# Decoding the transcriptome of calcified atherosclerotic plaque at single-cell resolution

**DOI:** 10.1038/s42003-022-04056-7

**Published:** 2022-10-12

**Authors:** Tom Alsaigh, Doug Evans, David Frankel, Ali Torkamani

**Affiliations:** 1 Scripps Research Translational Institute, La Jolla, CA USA; 2 Department of Molecular Medicine, Scripps Research, La Jolla, CA USA; 3 Department of Internal Medicine, Scripps Health, La Jolla, CA USA; 4 Department of Integrative Structural and Computational Biology, Scripps Research, La Jolla, CA USA; 5 Department of Vascular and Endovascular Surgery, Scripps Health, La Jolla, CA USA; 6 Present Address: Departments of General Internal Medicine and Vascular and Endovascular Surgery, University of California San Diego, La Jolla, CA USA

**Keywords:** Computational biology and bioinformatics, Cell biology

## Abstract

Atherogenesis involves an interplay of inflammation, tissue remodeling and cellular transdifferentiation (CTD), making it especially difficult to precisely delineate its pathophysiology. Here we use single-cell RNA sequencing and systems-biology approaches to analyze the transcriptional profiles of vascular smooth muscle cells (VSMCs) and endothelial cells (ECs) in calcified atherosclerotic core (AC) plaques and patient-matched proximal adjacent (PA) portions of carotid artery tissue from patients undergoing carotid endarterectomy. Our results reveal an anatomic distinction whereby PA cells express inflammatory mediators, while cells expressing matrix-secreting genes occupy a majority of the AC region. Systems biology analysis indicates that inflammation in PA ECs and VSMCs may be driven by TNFa signaling. Furthermore, we identify *POSTN, SPP1* and *IBSP* in AC VSMCs, and *ITLN1, SCX* and *S100A4* in AC ECs as possible candidate drivers of CTD in the atherosclerotic core. These results establish an anatomic framework for atherogenesis which forms the basis for exploration of a site-specific strategy for disruption of disease progression.

## Abstract (web summary)

Single-cell RNA sequencing and systems biology are used to profile the human vascular cell populations in calcified atherosclerotic core plaques from carotid endarterectomy samples, showing an anatomic distinction between gene expression of inflammatory versus matrix-secreting factors.

## Introduction

The pathophysiology of atherosclerosis is exceptionally complex and involves inflammation^1^, cellular transdifferentiation^2,3^, and dynamic interactions between a variety of cell types within the vascular wall^4,5^. For example, the phenotypic landscape of VSMCs is understood to be quite dynamic as these cells often participate in phenotype switching^6^ and contribute to the development of extracellular matrix producing cells in atherosclerosis^4^. Many of these pathogenic processes are mediated through molecular crosstalk between VSMCs^7^, however, disease progression in general involves coordinated genomic and molecular communication between the plethora of immune cell subtypes and other cellular components of the arterial wall^8,9^.

Evidence from genome-wide association studies (GWAS) suggests a plurality of genetic drivers of atherosclerosis acting through tissue-specific regulation^10^, underscoring the importance of uncovering cell-type specific genetic drivers of disease in order to expose therapeutic opportunities. Efforts made using microarrays to evaluate gene expression changes in patients with carotid artery plaque have yielded important information^11^, however, these bulk sequencing studies obscure the diverse plaque environment by assessing complete transcriptome profiles without distinction between predominant cell types contributing to gene expression.

More recent studies have advanced our understanding of the rich cellular heterogeneity within the plaque environment through single-cell transcriptomics approaches. For example, in murine atherosclerotic aortas three distinct macrophage subsets were identified, including inflammatory, Res-like, and TREM2hi macrophages^12^, enhancing our understanding of macrophage diversity within plaque. A predominance of T-cells in human carotid plaque lesions has been shown, enriching our understanding of CD4 and CD8 subpopulations in atherosclerosis^13^. In addition, immunophenotyping in carotid atherosclerosis revealed fundamental differences between T-cells and macrophages from symptomatic versus asymptomatic patients, including expansion of the CD4 + T-cell subset and macrophages with varied phenotypes in symptomatic patients^14^. Smooth muscle cell lineage tracing in concert with scRNAseq has identified *TCF21* as involved in a trajectory of phenotypic modulation whereby a group of contractile smooth muscle cells shift gene expression to comprise a distinct population of fibromyocytes^15^, raising the exciting possibility that other genes may also influence phenotypic modulation in atherosclerosis. Phenotypic plasticity in advanced human atherosclerotic lesions has recently been shown to be modulated by stem cell pluripotency genes (*KLF4* and *OCT4*), regulating cellular transitions to phenotypically diverse matrix-secreting cells such as osteogenic and chondrocyte-like cells^16^. These studies all highlight critical mechanisms involved with disease progression, however, no prior study has simultaneously performed single-cell analysis across the entire atherosclerotic plaque and proximal vascular wall within the same patient without cell selection in mouse or human tissue.

Here we developed a strategy to allow for single-cell RNA sequencing (scRNAseq) of atherosclerotic core (AC) plaques and patient-matched proximal adjacent (PA) portions of the carotid artery in their entirety, without preference for cell type, providing an unbiased view of the disease transcriptome. This sample pairing and preparation strategy, in concert with systems biology approaches, has allowed us to describe the transcriptome and identify possible key transcriptional drivers of disease processes in the vascular wall which may lay the groundwork for future therapeutic opportunities.

## Results

### Tissue source and processing

Paired sections of tissue, including both artery and plaque, were recovered from the atherosclerotic core (AC) and proximally adjacent (PA) region of three patients with asymptomatic type VII calcified plaques who underwent carotid endarterectomy (Fig. S1a, Table S1). Due to the rich cellular composition of carotid artery and plethora of debris in plaque (i.e., lipid, fibrinogen, etc.), dissociation and generation of single-cell suspensions amenable to single-cell RNA sequencing were difficult. After tissue collection, enzymatic digestion, RBC lysis, and filtration were the initial steps required to generate single cells (see “Methods” and Fig. S1b). However, despite efficient enzymatic dissociation and significant filtering of our sample, we were still challenged by abundant plaque debris, which ultimately resulted in poor single-cell capture rates. In order to overcome this issue without isolating specific cell types through cell-marker antibody labeling, we devised a strategy to label all cells in the sample with a far-red excitation-emission live/dead cell nuclear stain (DRAQ5). All cells in the sample were stained, with debris being left unstained by the dye. Previous studies have used nuclear staining in library preparation and sequencing experiments to discriminate single versus doublet cells during cell sorting without adverse effects for downstream applications such as single-cell and bulk RNA sequencing^17–20^. Subsequently, DRAQ5+ cells were manually gated and sorted from the remainder of the debris using FACS. Cells isolated from the entire filtered sample represented <1% of the total particles in the sample (Figs. S2a–S2f). Viability of remaining cells was assessed and was always >80% using this technique for cell separation (see “Methods”). The cells were then processed for single-cell sequencing.

### Cell-type detection and assignment

**Fig. 1:**
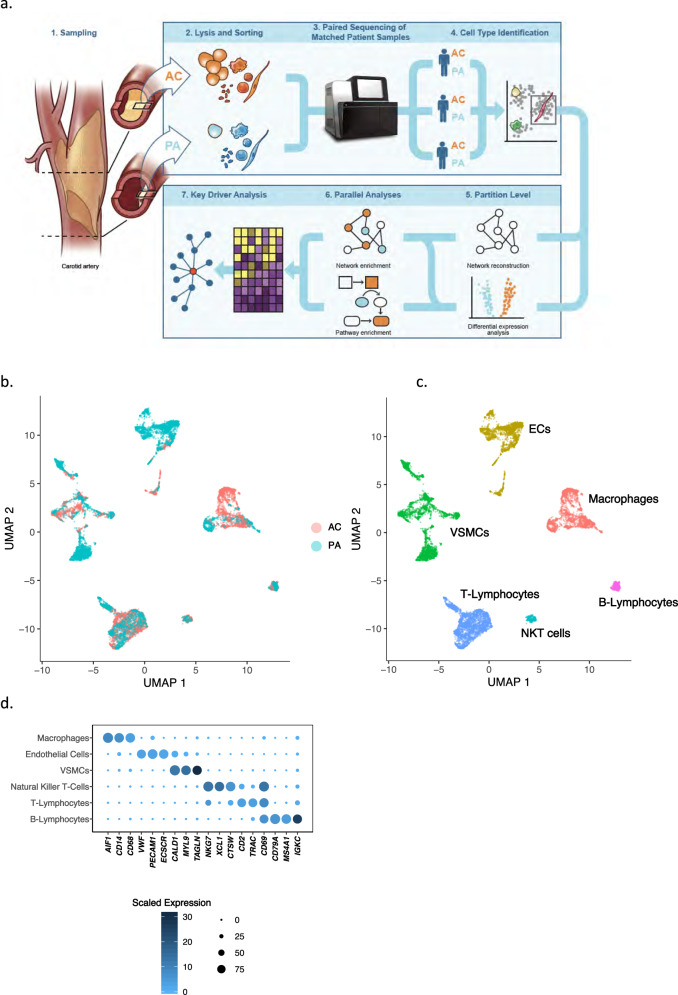
Data processing produces 6 main cell group partitions. **a** Schematic diagram of analytical steps from tissue dissociation to key driver analysis. **b**, **c** UMAP visualization of 6 major cell types following doublet removal via gene exclusion criteria (see Supplemental Methods), separated by anatomic location (**b**), and by cell type (**c**). **d** Dotplot depicting cell-type marker genes, resulting in the identification of macrophages, ECs, VSMCs, NKT cells, T- and B-Lymphocytes. Dot size depicts the fraction of cells expressing a gene. Dot color depicts the degree of expression of each gene. *n* = 3 for PA and AC groups.

The analytical approach in this manuscript is depicted in Fig. 1a. Generation of single cells from three patient-matched AC and PA samples (batched per patient on a single NextSeq flow cell) yielded 51,981 cells total, with an average of ~13,000 AC cells/patient and ~5000 PA cells/patient. Cell number disparities are due to the difference in size of the AC vs PA tissue itself. Given the abundance of AC versus PA cells, down-sampling was performed to equalize the contribution of each sample and condition to the unsupervised discovery of cell types and to mitigate bias due to class imbalance. UMAP-based clustering (see “Methods”) of this down-sampled dataset reveals 15 distinct cell partitions (Fig. S1c, d), representing 17,100 cells total. In order to assign partitions to major cell types we examined genes expressed in >80% of cells per partition and at a mean expression count >2. A dotplot representing three marker genes selected for each partition is presented in (Fig. S1e). A comparison of VSMC marker genes used in our study with those in the literature^15^ is provided (Fig. S1f). Cell-type labels assigned to these 15 initial partitions based on these marker genes include: T-lymphocytes (2 partitions), macrophages, VSMCs (2 partitions), ECs (2 partitions), B-lymphocytes, natural killer T-cells, B1-lymphocytes, mast cells, lymphoid progenitors, plasmacytoid dendritic cells, and an unidentified partition (Table S2). Following doublet filtering using a marker-gene exclusion method (see Supplemental Methods), removal of partitions with too few cells for differential gene expression analysis (mast cells, lymphoid progenitors, plasmacytoid dendritic cells, and the unidentified partition), and merging of partitions assigned to the same cell-type, we assessed differential gene expression between AC and PA regions across the 6 remaining major cell types: macrophages, ECs, VSMCs, NKT cells, T- and B-lymphocytes (Fig. 1b–d, Fig. S4, Supplementary Data 1–6). We performed a number of independent partitioning experiments using various algorithmic variations to confirm the reproducibility of these partitions and cell-label assignments (see Supplemental Methods).

### Differential gene expression—VSMC and EC

**Fig. 2:**
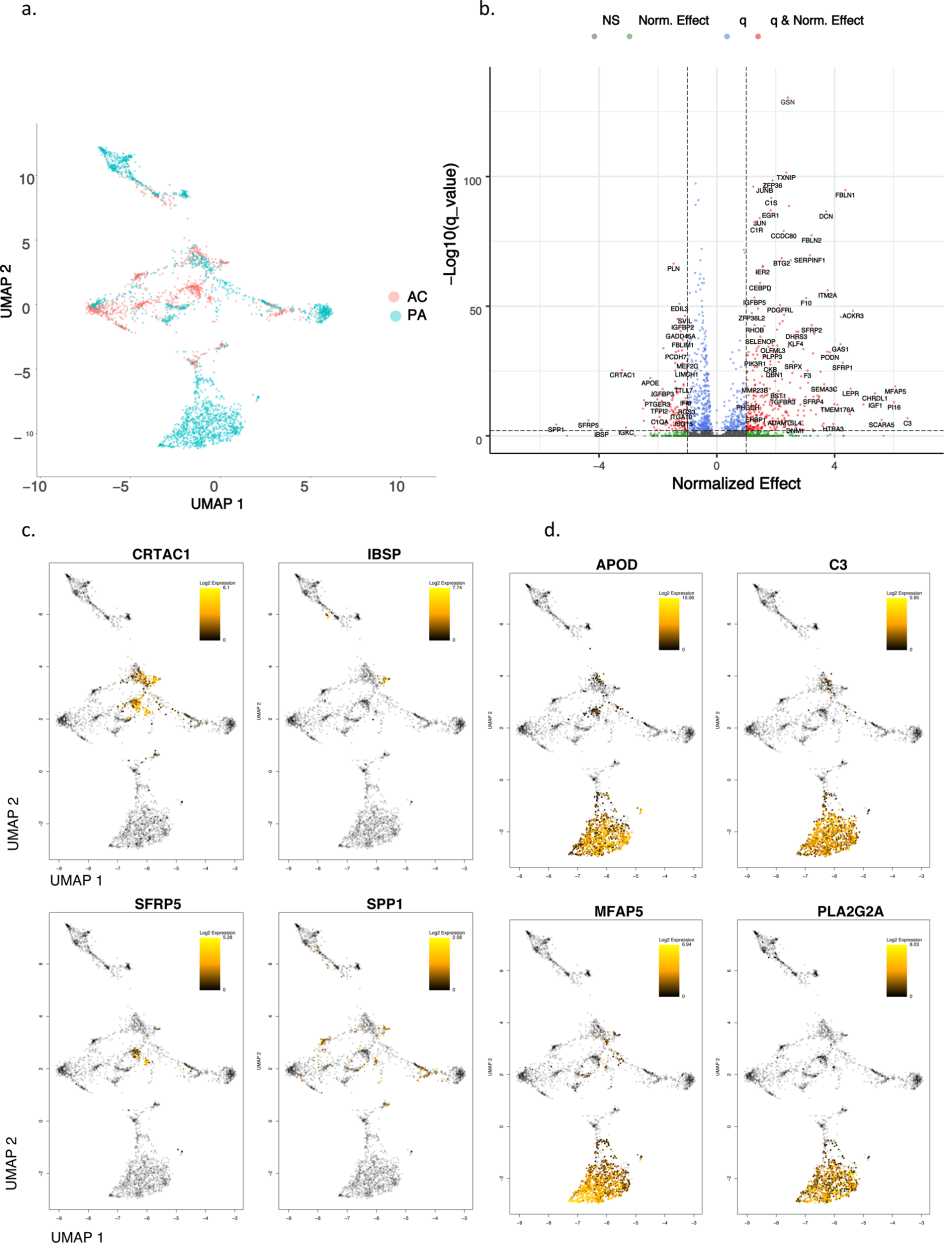

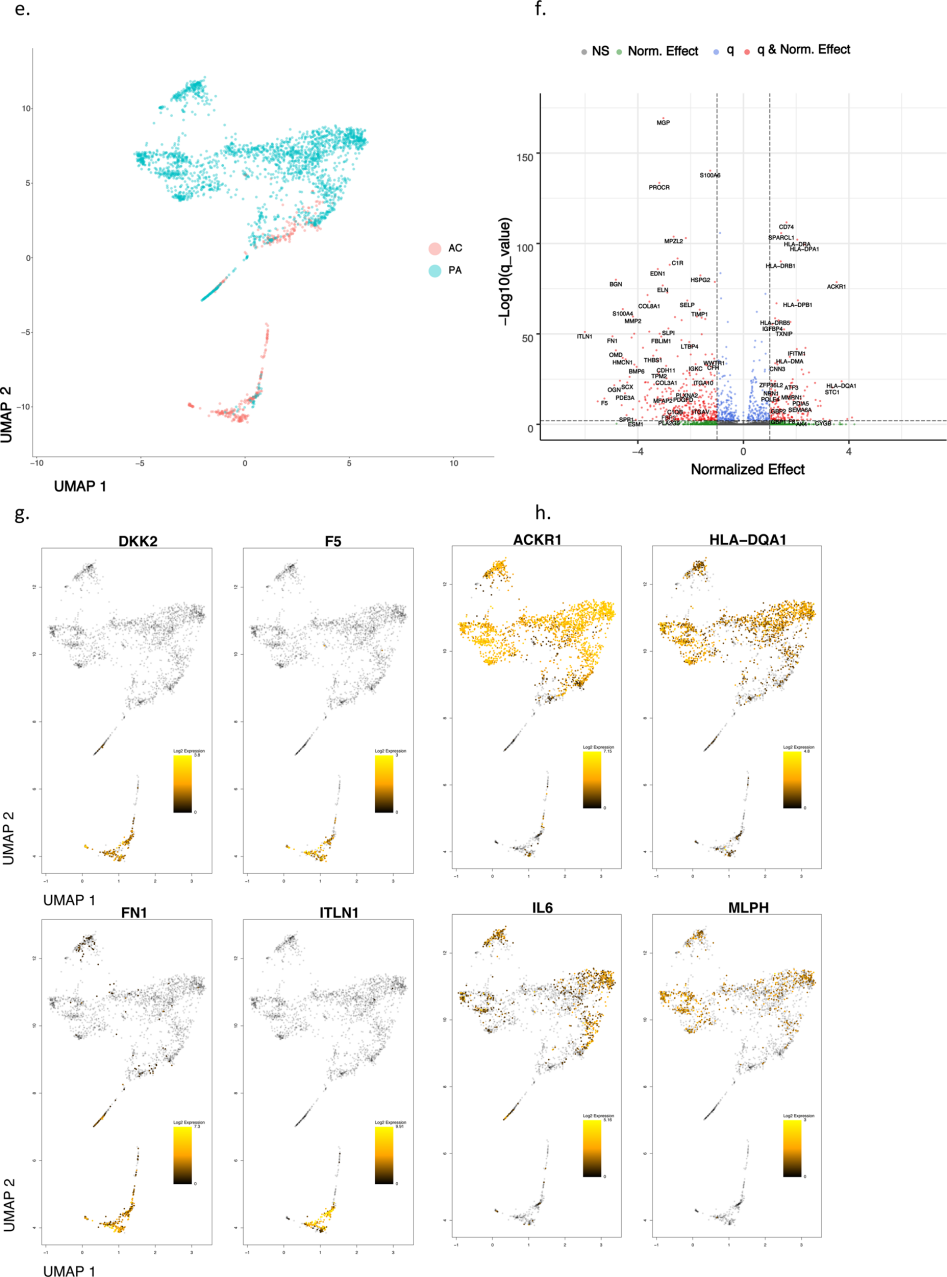
Differential gene expression in VSMCs and ECs. **a**, **e** UMAP visualization of VSMCs (**a**) and ECs (**e**), separated by anatomic location. **b**, **f** Volcano plots of the top differentially expressed genes in VSMCs (**b**) and ECs (**f**). Dotted lines represented *q*-value 0.5 and <−0.5 corresponding to PA and AC cells, respectively. **c**, **d** UMAP visualization of the top 4 upregulated genes in AC VSMCs (**c**), and PA VSMCs (**d**). Gray-colored cells indicate 0 expression of designated gene, while color bar gradient indicates lowest (black) to highest (yellow) gene expression level. **g**, **h** UMAP visualization of the top 4 upregulated genes in AC ECs (**g**), and PA ECs (**h**). Color scheme is similar to the above-described parameters. VSMCs = 3674 cells; ECs = 2764 cells. *n* = 3 for PA and AC groups.

**Fig. 3:**
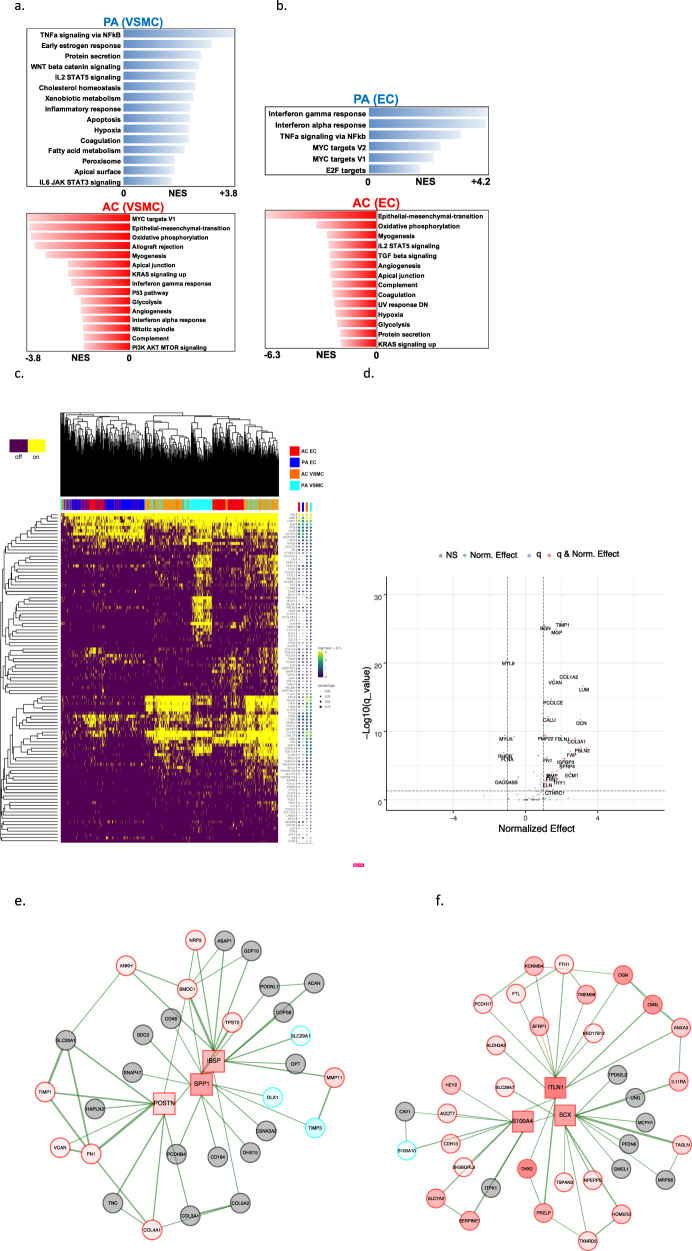
Gene set enrichment analysis and gene co-expression networks identify key gene drivers of EMT hallmark biologic process. **a**, **b** Normalized enrichment score (NES) ranking of all significant PA and AC Hallmarks generated from GSEA analysis of differentially expressed genes for VSMCs (**a**) and ECs (**b**) (FDR *q*-value < 0.05). **c** Fully clustered on/off heatmap visualization of overlap between leading edge EMT hallmark genes generated by GSEA. Heatmaps are downsampled and represent 448 cells from each cell type and anatomic location (1792 total cells). A dotplot corresponding to gene expression levels for each cell type in the heatmap is included. Dot size depicts the fraction of cells expressing a gene. Dot color depicts the degree of expression of each gene. **d** Volcano plot of differentially expressed genes between the two groups of VSMCs in (**c**). Dotted lines represented *q*-value<0.01 and normalized effect >0.5 and <−0.5. **e**, **f** Gene co-expression networks generated from VSMC Module 13 (**d**) and EC Module 1 (**e**) representing the EMT hallmark from GSEA analysis. Genes are separated by anatomic location (red = AC genes, cyan = PA genes), differential expression (darker shade = higher DE, gray = non-significantly DEGs), correlation with other connected genes (green line = positive correlation, orange line = negative correlation) and strength of correlation (connecting line thickness). Significantly DEGs (*q* < 0.05) with high connectivity scores (>0.3) are denoted by a box instead of a circle. *n* = 3 for PA and AC groups.

GWAS results have highlighted biological processes in the vessel wall as key drivers of coronary artery disease (CAD)^21^. Our prior work has demonstrated the vascular wall to be involved in the most impactful common genetic risk factor for CAD^22^. Our results here also demonstrate extensive differential expression in these cell types across anatomic locations compared to the remaining cell types. Therefore, we chose to focus our efforts on dissecting expression alterations in VSMCs and ECs in order to illuminate pathogenic genomic signatures within these cell-types. As above, each cell type is compared across anatomic location (Fig. 2a, e), and the top differentially expressed genes are shown (Fig. 3b, f), revealing interesting spatial and expression magnitude differences between AC and PA cells.

VSMCs generate three subclusters in the UMAP plot. A large fraction of PA VSMCs form a PA-specific VSMC subcluster. In contrast, AC VSMCs form 2 separate clusters both of which are intermingled with PA VSMCs. This suggests VSMCs occupy three major cell states, including one completely distinct PA subtype, and two that are predominantly AC VSMCs (Fig. 2a). The top four upregulated genes in the AC are sparsely expressed and include *SPP1, SFRP5, IBSP*, and *CRTAC1* (Fig. 2b, c), while *APOD, PLA2G2A, C3*, and *MFAP5* are upregulated in many PA VSMCs (Fig. 2b, d).

The spatial clustering of upregulated genes in AC VSMCs suggests the presence of separate subpopulations of matrix-secreting VSMCs involved with ECM remodeling (Fig. 2c). *SPP1* (osteopontin) is a secreted glycoprotein involved in bone remodeling^23^ and has been implicated in atherosclerosis for inhibiting vascular calcification and inflammation in the plaque milieu^24^. *IBSP* (bone sialoprotein) is a significant component of bone, cartilage, and other mineralized tissues^25^. *CRTAC1* is a marker to distinguish chondrocytes from osteoblasts and other mesenchymal stem cells^26,27^. These findings suggest the presence of cartilaginous and osseous matrix-secreting VSMCs in the AC region. *SFRP5*, an adipokine that is a direct *WNT* antagonist, reduces the secretion of inflammatory factors^28^ and is thought to exert favorable effects on the development of atherosclerosis^29^. The high expression of *SFRP5* in the AC suggests a deceleration of these inflammatory processes in the core of the plaque, and an overall shift in the AC to calcification and matrix remodeling.

Conversely, the upregulated genes in PA VSMCs are more ubiquitously expressed by VSMCs in a PA-specific region of the UMAP plot (Fig. 2d). *C3* is highly differentially expressed in many PA cells (Fig. 2d). Complement activation has long been appreciated for its role in atherosclerosis^30^, with maturation of plaque shown to be dependent, in part, on C3 opsonization for macrophage recruitment and stimulation of antibody responses^31^. Its predominance in our PA samples suggests complement activation in atherosclerosis is anatomically driven by VSMCs located adjacent to areas of maximal plaque build-up. *PLA2G2A* (phospholipase A2 group IIA), also selectively expressed by this group of cells, is pro-atherogenic, modulates LDL oxidation and cellular oxidative stress, and promotes inflammatory cytokine secretion^32^, further facilitating the inflammatory properties of this group of VSMCs. Full differential expression results for VSMCs are provided (Supplementary Data 5).

Overall, we identify increased calcification and ECM remodeling by VSMCs in the AC versus pro-inflammatory signaling by VSMCs in the PA. These differences in biological processes are strongly supported further in the systems analyses below.

In contrast to VSMCs, for ECs we observe a more complete separation of cells into two distinct subgroups (Fig. 2e). PA ECs significantly outnumber the AC ECs (2316 vs 448 cells, respectively), possibly due to intimal erosion and loss of endothelial cell layer integrity during advanced disease^5,33–35^ resulting in fewer captured ECs in the AC. Cellular transdifferentiation may also cause a subpopulation of ECs to lose common EC marker expression, resulting in lower numbers of ECs identified in AC compared to the PA counterpart. Histologic assessment of AC plaque collected from our patients supports the assertion of endothelial layer attenuation as the principal reason for lower AC EC capture (Fig. S3b, c). In contrast to VSMCs, there is a skew toward higher magnitude expression changes in AC ECs vs PA ECs. The top four upregulated genes are *ITLN1, DKK2, F5*, and *FN1* in the AC and *IL6, MLPH, HLA-DQA1*, and *ACKR1* in PA ECs (Fig. 2g, h).

The upregulated genes in AC ECs again suggest a synthetic profile. *ITLN* (omentin) is an adipokine enhancing insulin-sensitivity in adipocytes^36^. Interestingly, circulating plasma omentin levels were shown to negatively correlate with carotid intima-media thickness^37^, inhibit TNF-induced vascular inflammation in human ECs^38^, and promote revascularization^39^, suggesting an anti-inflammatory and intimal repair role in AC ECs. *DKK2* further indicates intimal repair as it stimulates angiogenesis in ECs^40^. The significant upregulation of *FN1* (fibronectin) in this group further suggests active ECM remodeling and may serve as a marker for mesenchymal cells and EMT-related processes^41^.

Similarly to PA VSMCs, the upregulated genes in PA ECs suggest an overall inflammatory profile. Central players in inflammation and antigen presentation are upregulated specifically in PA ECs (Fig. 2h). *IL6*, a key inflammatory cytokine associated with plaque^42^, is the most upregulated gene. Furthermore, *ACKR1*, highly upregulated in many PA ECs, binds and internalizes numerous chemokines and facilitates their presentation on the cell surface in order to boost leukocyte recruitment and augment inflammation^43^. Antigen presentation on ECs via *HLA-DQA1* (MHC class II molecule) may support activation and exhaustion of CD4+ T-cells^44,45^ as previously described. Full differential expression results for ECs are provided (Supplementary Data 6).

Overall, we identify two main EC subtypes: synthetic ECs in the AC that appear to participate in intimal repair, revascularization, and ECM modulation, and inflammatory ECs in the PA region that likely facilitate inflammation via antigen/chemokine presentation and recruitment of immune cells, including CD4+ T-cells. These differences in biological processes are strongly supported further in the systems analyses below.

### Hallmark processes—VSMCs and ECs

In order to explore the anatomic differences for these cell types further, gene set enrichment analysis (GSEA) was used to asses hallmark processes most significantly altered in VSMCs and ECs (Fig. 3a, b). Epithelial to mesenchymal transition (EMT), oxidative phosphorylation, and myogenesis gene upregulation were strongly enriched in both AC VSMCs and ECs, collectively suggesting an increase in cellular metabolic activity and proliferation. In contrast, a distinctly inflammatory profile was seen in PA VSMCs and ECs, with IFN gamma/alpha responses and TNFa signaling via NFkB dominating the enriched processes in these groups of cells. Because EMT and TNFa signaling were both shared and strongly enriched processes in the two cell types, the gene signatures associated with these hallmarks were further scrutinized through generation of heatmaps consisting of leading-edge differentially expressed genes from each hallmark process (EMT—Fig. 3c, TNFa signaling via NFkB—Fig. S5a).

While overlapping at the hallmark level, separation of cells by cell type as well as anatomic location in the EMT hallmark heatmap suggests the overlapping processes are mediated by distinct gene sets in each cell type. Moreover, analysis of EMT hallmark genes further supports the presence of 2 cellular subtypes of AC VSMCs as they appear to cluster into two distinct groups of cells with dichotomous expression of contractile *(MYL9, TPM2, TAGLN, FLNA*) versus synthetic/EMT (*POSTN, LUM, FBLN2, DCN, PCOLCE2, MGP, COL3A1*) gene signatures (Fig. 3c, d). These results indicate a group of VSMCs in the AC may perform the contractile functions of the blood vessel wall, while the other group of VSMCs may be involved with CTD and ECM remodeling. Furthermore, cells with an ACTA2 + Thy1− gene signature in Fig. 3c may be, in part, plaque-stabilizing myofibroblasts (orange line), indicating that these contractile cells may also have a large role in ECM remodeling.

In contrast to distinct subclustering of cells by EMT-related genes, there appears to be a common gradient of genes involved in inflammation and response to inflammation expressed throughout the atherosclerotic tissue, with higher levels of TNF-related inflammatory genes expressed in PA VSMCs and ECs compared to AC cells, indicating a predominance of inflammatory processes occurring in the PA region overall (Fig. S5a). Collectively these genes (*EIF1, FOS, JUN, JUNB, ZFP36, PNRC1, KLF2, IER2, CEBPD, NFKBIA, GADD45B, EGR1, PPP1R15A*, and *SOCS3*), in addition to IL6 expression in PA ECs, appear to coordinate the inflammatory response pathways in plaque and its adjacent structures. All cell types analyzed thus far are coordinated along this gradient of inflammation.

### Network analysis

To further dissect VSMC and EC anatomical gene expression differences in order to identify candidate key genes driving the significant hallmark processes, we reconstructed gene co-expression networks using a partial correlation-based approach (see “Methods”), defined modules by clustering, and overlaid differential expression analysis results on these modules to identify those enriched in genes differentially expressed between AC and PA tissues.

Using this strategy, 31 and 39 distinct gene network modules were generated in our VSMC and EC datasets, respectively (see Supplemental “Methods”, Supplementary Data 7, 8). Of these, 8 modules in VSMCs, and 5 modules in ECs were enriched with differentially expressed genes (*p*-value < 0.05, Fisher’s exact test, see “Methods”). Furthermore, differentially expressed EMT-related hallmark genes overlapped significantly and specifically with a single VSMC and EC module. Differentially expressed TNFa signaling via NFkB-related hallmark genes also overlapped significantly with one VSMCs and EC module (*p*-value < 0.05, Fisher’s exact test). No other hallmark processes overlapped with generated network modules.

### EMT hallmark in VSMCs and ECs from the atherosclerotic core

The EMT gene signature generated from GSEA analysis of network modules and the robust upregulation of genes found in matrix-secreting cells in this cohort suggests the possibility of CTD occurring and/or completing in the atherosclerotic core. Therefore, in order to further characterize genes which may stimulate CTD in AC VSMCs and ECs we examined gene co-expression networks in conjunction with differential expression data from the modules enriched with EMT hallmark genes. In VSMCs we identified 9 genes (*SPP1, IBSP, POSTN, MMP11, COL15A1, FN1, COL4A1, SMOC1, TIMP1*) whose expression was significantly upregulated in AC cells and with strong network connectivity (see “Methods”). Among these genes we identify *POSTN*, *SPP1*, and *IBSP* as possible key gene drivers of CTD processes in AC VSMCs due to their strong central connectivity and high degree of differential expression in the network module (Fig. 3e). *POSTN* (periostin) is expressed by osteoblasts and other connective tissue cell types involved with ECM maturation^46^ and stabilization during EMT in non-cardiac lineages^47,48^. *POSTN, SPP1*, and *IBSP* are highly interconnected in our network and likely serve as drivers of CTD by modulating other correlated genes such as *TIMP1, VCAN, TPST2, SMOC1, MMP11, FN1*, and *COL4A1* (Fig. 3e), all genes which are involved with cellular differentiation^49^ and extracellular matrix remodeling^50,51^.

In our EC network we identified 18 genes (*ITLN1, FN1, OMD, S100A4, SCX, PRELP, GDF7, TMP2, SERPINE2, SLPI, HEY2, IGFBP3, FOXC2, RARRES2, PTGDS, TAGLN, LINC01235,* and *COL6A2*) whose expression was significantly upregulated in AC cells and with strong network connectivity. Among these genes, we identify *ITLN1, S100A4*, and *SCX* as possible gene drivers of CTD in ECs associated with the AC (Fig. 3f). *ITLN1* (omentin) is highly upregulated in ECs associated with the atherosclerotic core, and network data indicate it is strongly correlated with genes involved with cellular proliferation and ECM modulation. *ITLN* is also strongly correlated to *OGN* (osteoglycin) which induces ectopic bone formation^52^, indicating that *ITLN1* may modulate ECs with osteoblast-like features in the atherosclerotic core. *SCX* (scleraxin), a transcription factor that plays a critical role in mesoderm formation, and the development of chondrocyte lineages^53^, as well as regulating gene expression involved with ECM synthesis and breakdown in tenocytes^54^, is co-expressed with *IL11RA*, an interleukin receptor implicated in chondrogenesis^55^, as well as with a variety of genes involved with cellular development and modulation of ECM structures. Thus, *SCX* may modulate chondrocyte-like ECs in the AC. *S100A4* is a calcium-binding protein that is highly expressed in smooth muscle cells of human coronary arteries during intimal thickening^56^, and silencing this gene in endothelial cells prevents endothelial tube formation and tumor angiogenesis in mice^57^. Co-expression with *HEY2*, a transcription factor involved with *NOTCH* signaling and critical for vascular development^58^, may indicate an important role in repair via re-endothelialization of plaque-denuded artery.

### TNFa signaling via NFKB in proximally adjacent VSMCs and ECs

Next, genes critical to stimulating TNFa signaling via NFkB in PA VSMCs and ECs were evaluated. In the VSMC module we identified 14 genes (*APOLD1, MT1A, ZFP36, EGR1, JUNB, FOSB, JUN, FOS, RERGL, MT1M, DNAJB1, CCNH, HSPA1B*, and *HSPA1A*) whose expression was significantly upregulated in PA cells and with strong network connectivity. Among these genes we identify immediate-early (IE) genes *ZFP36, EGR1, JUNB, FOSB*, and *FOS* as critical response genes in this hallmark process. Importantly, the paired-sample study design in which AC and PA samples from the same patient are processed identically at the same time ensures that these IE genes preferentially upregulated in the PA region are critical for the inflammatory response and not an artifact of tissue processing stressors.

In the EC module we identified two genes (*IER2* and *FOS*) whose expression was significantly increased in PA EC cells (Fig. S5e), and are highly correlated with other critical transcription factors in our network, including *FOSB, JUNB, EGR1*, and *ZFP36*, further supporting this group of gene’s importance in the TNFa signaling hallmark (Fig. S5d).

### Evaluation of cellular subpopulations

**Fig. 4:**
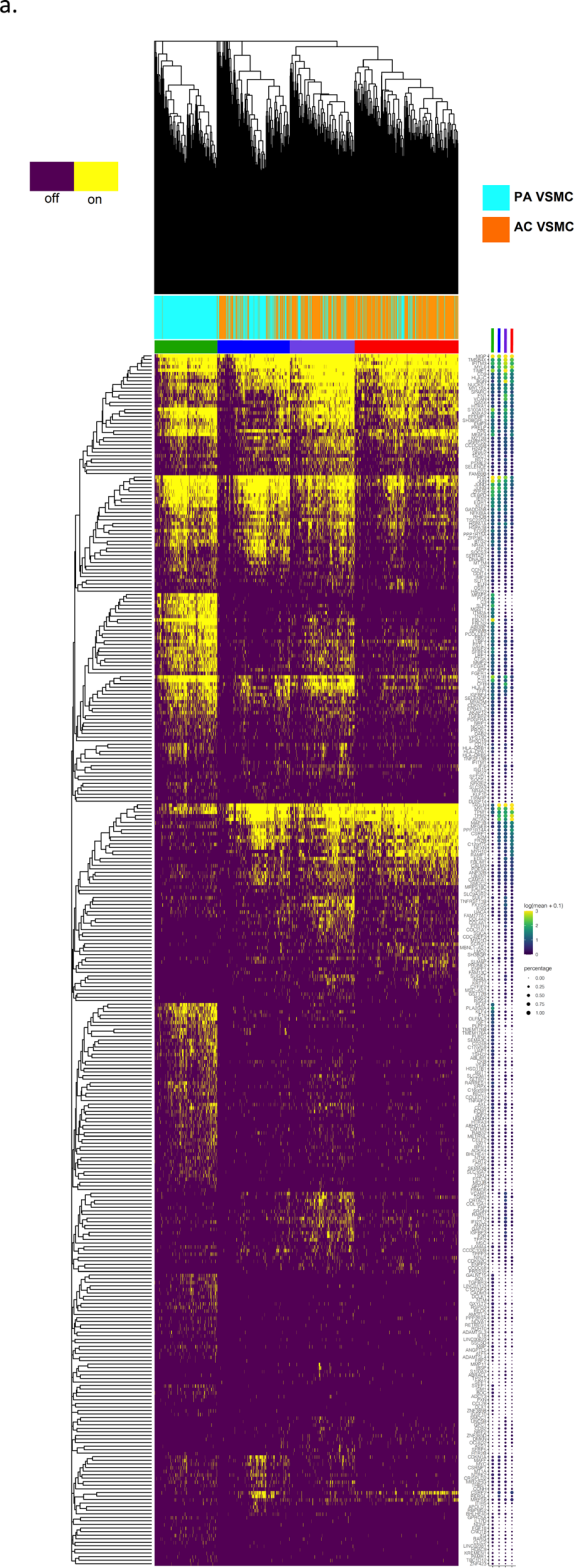

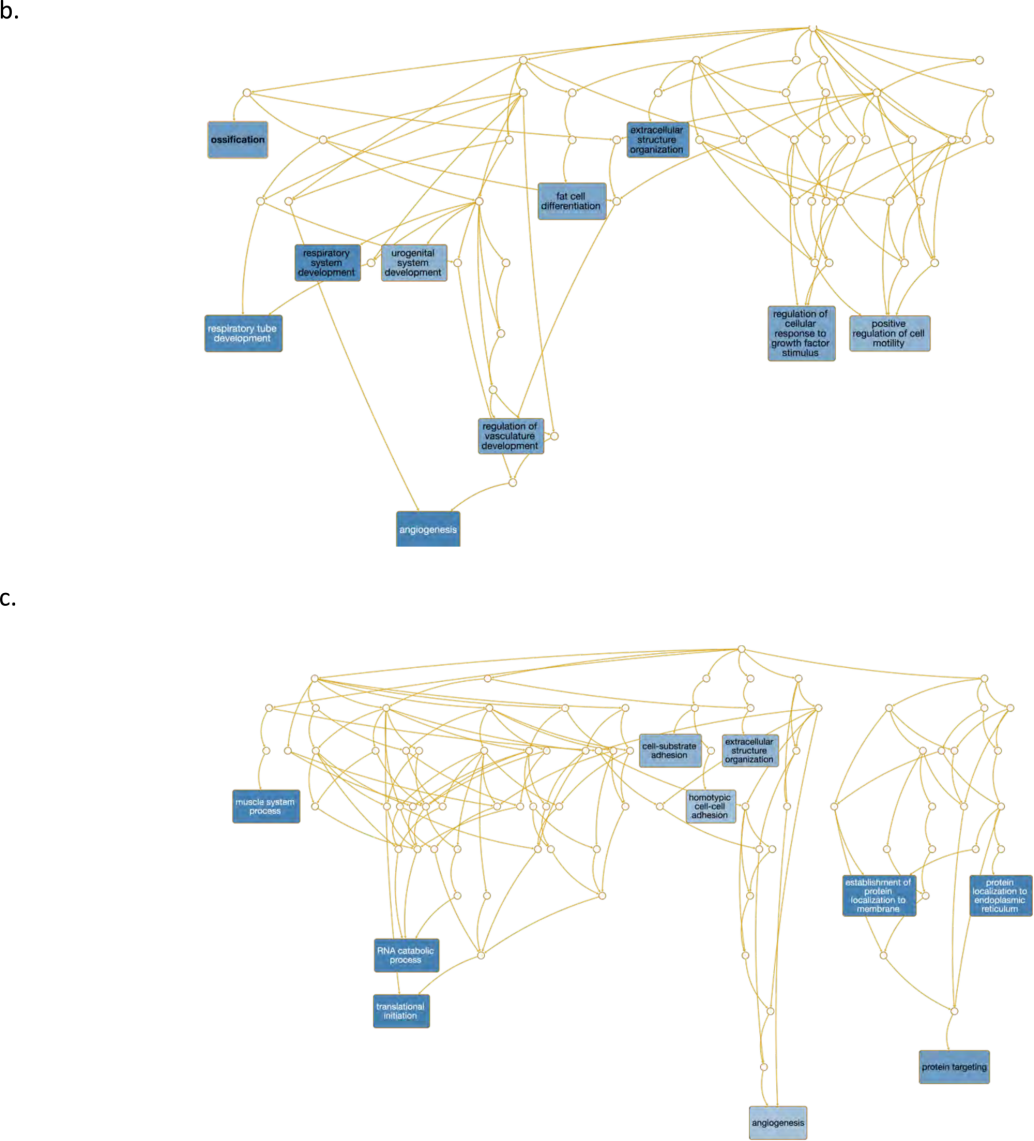

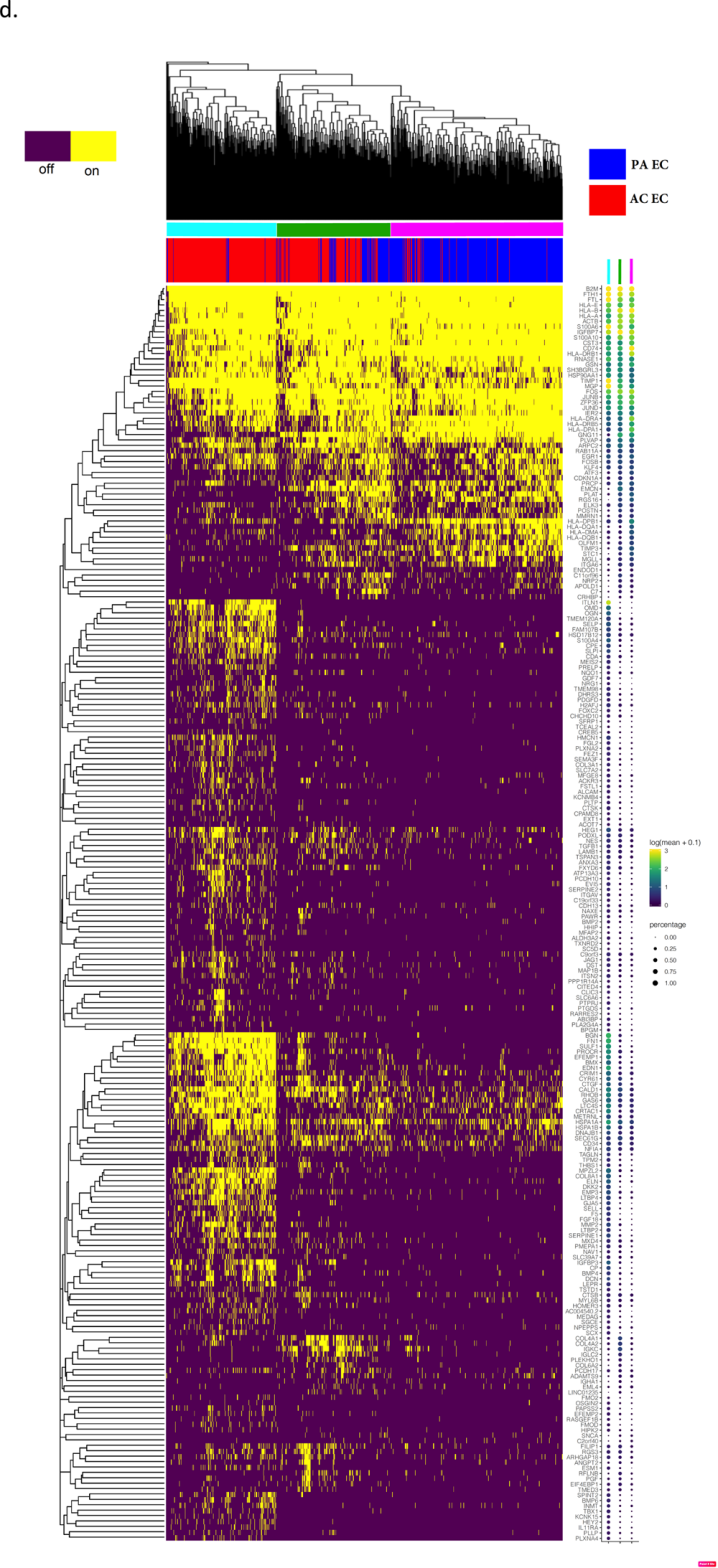

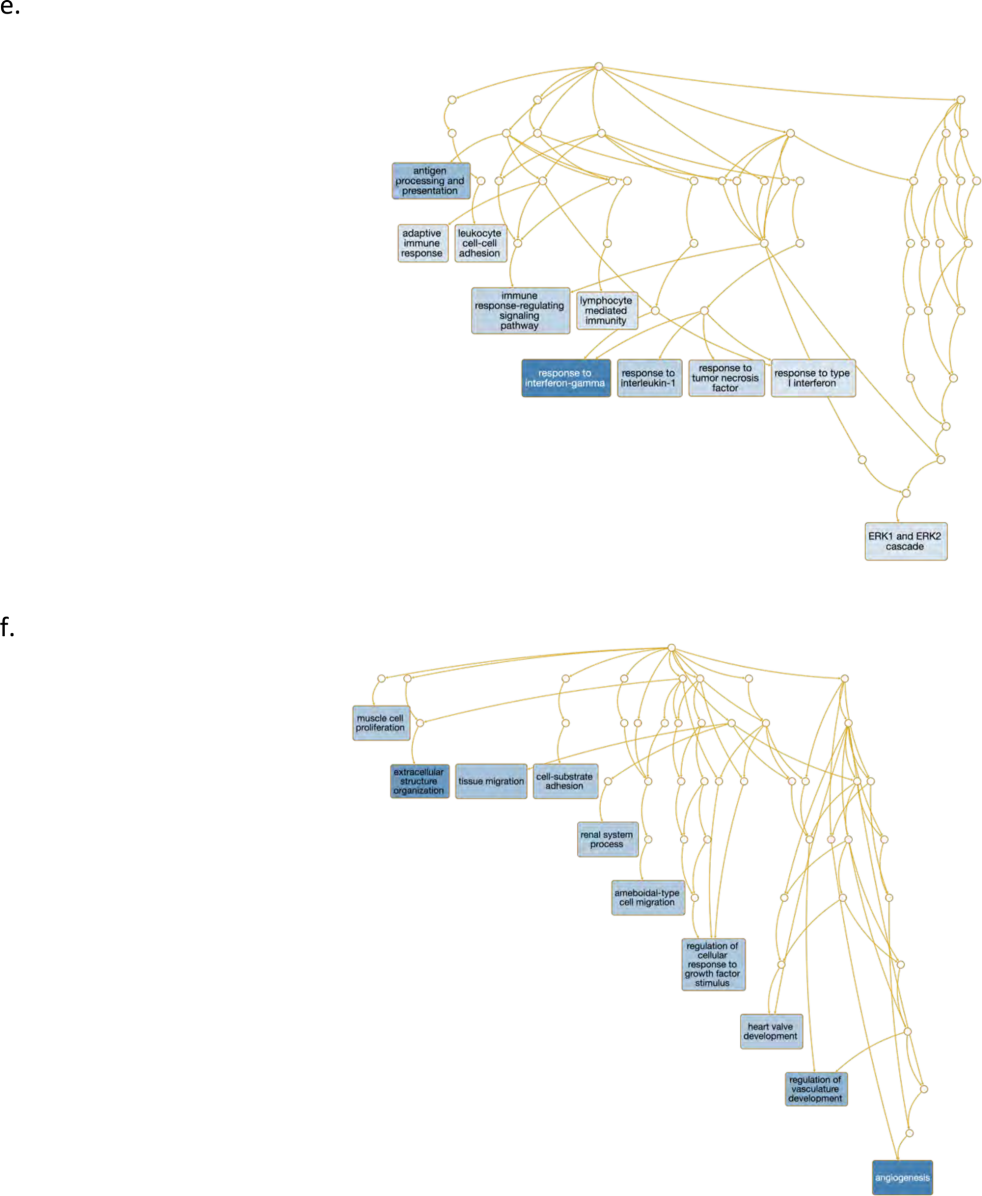
Evaluation of VSMC and EC subpopulations. **a**, **d** Biclustered heatmap visualization of all significant genes (*q* < 0.05) from VSMC (**a**) and EC (**d**) modules enriched with differentially expressed genes. **a** 1224 VSMCs from each anatomic location (2448 cells total). Large color bar denotes PA (cyan) and AC (orange) VSMCs. Small color bar above denotes distinct cell subpopulations (blue, forest green, lime green, brown, purple, magenta, red). **d** 448 ECs from each anatomic location (896 cells total) in. Large color bar denotes PA (blue) and AC (red) ECs. Small color bar above denotes distinct cell subpopulations (cyan, green, magenta). A dotplot corresponding to gene expression levels for each cell subpopulation on the heatmap is included. Colored dots next to specific genes correspond to critical genes related to the designated cell subpopulation. Continuous gene expression based gene ontology enrichment analysis of biological function performed based on the fraction of AC cells per subpopulation of VSMCs (**b**, **c**) and ECs (**e**, **f**). *n* = 3 for PA and AC groups.

Finally, in order to identify and characterize refined subpopulations from each anatomic region, we selected the 7 VSMC and 5 EC differentially expressed modules described above and biclustered cells and genes (Fig. 4a, d). The likely biological functions of these subpopulations were then inferred based on the genes differentially expressed and subsequent gene ontology enrichment analysis across these subpopulations. A continuous gene expression model, based on the fraction of AC cells per subpopulation, and subsequent gene ontology enrichment analysis was used to evaluate these cell subtype differences (Fig. 4b, c, e, f).

### VSMCs

We identified four cell subpopulations of VSMCs with some overlapping features in our analysis (Fig. 4a). The four subpopulations appear to form a continuum of cell states, starting with a population that consists exclusively of PA VSMCs (Fig. 4a, green bar), characterized by genes involved in recruitment of inflammatory mediators, with early signs of CTD. Specifically, *C3* (opsonization and macrophage recruitment; normalized effect = 6.5, *q* = 1.74e−07) is highly differentially expressed in this subpopulation and likely augments PA inflammation and macrophage recruitment. This group of VSMCs also shows evidence of early migratory and CTD-like qualities given the expression of *FBN1, SEMA3C, HTRA3*, and *C1QTNF3*, (normalized effect = 2.77, 3.65, 4.0, 3.58, respectively; *q* = 6.93e−41, 1.25e−20, 2.53e−05, 0.00012, respectively) genes that are both highly differentially expressed in this cohort and with high signal strength in our networks (Fig. 4a, Supplementary Data 7). *FBN1* (ECM component) is strongly correlated with *TGFBR3, SEMA3C*, and *CD248* (modulators of EMT-like processes)^59–61^. Interestingly, this group of cells co-expresses *IGSF10*, a marker of early osteochondroprogenitor cells^62^, *TMEM119* (bone formation and mineralization; promotes differentiation of myeloblasts into osteoblasts)^63,64^, and *WNT11* (bone formation)^65^ (Supplementary Data 7).

On the other end of this continuum, we identify a subpopulation of ~70% AC cells (Fig. 4a, red bar) that have elevated expression of *POSTN* (osteoblasts; normalized effect = −2.206, *q* = 3.60e−16)*, CRTAC1* (chrondrocytes; normalized effect = −3.22, *q* = 3.91e−26), *TNFRSF11B* (bone remodeling; normalized effect = −0.98, *q* = 7.31e−06)^66^, *ENG* (VSMC migration; normalized effect = 0.87, *q* = 1.41e−13)^67^, *COL4A2*, and *COL4A1* (cell proliferation, association with CAD; normalized effect = −0.98, −1.03 and *q* = 3.17e−15, 5.68e−11, respectively)^68,69^. Collectively, the differential gene expression data and the underlying biology behind our gene co-expression networks support this group of cells as likely representing synthetic osteoblast- and chondrocyte-like VSMCs which facilitate calcification and cartilaginous matrix-secretion and reside largely in the AC.

Furthermore, gene ontology enrichment analysis provides a clear progression from muscle system processes, extracellular structure reorganization, and catabolic processes enriched in the PA to processes involved with CTD such as ossification, fat cell differentiation, and regulation of cell motility, adhesion, and cellular transdifferentiation enriched in the AC (Fig. 4b, c). The shift in cell states supports a continuum of cell state changes leading to increased CTD in the atherosclerotic core.

### ECs

Overall, we observe three EC subpopulations. Like VSMCs, these cells display transitory properties as they move through a continuum of cell states (Fig. 4d). First, there is a group comprised near exclusively of inflammatory PA ECs that is involved in recruitment of inflammatory mediators (Fig. 4d, magenta bar). This group has a greater number of cells expressing immune genes such as the cluster of HLA genes, as well as *CD74* (normalized effect = 1.63, *q* = 2.07e−112), a gene which forms part of the invariant chain of the MHC II complex and is a receptor for the cytokine macrophage migration inhibitory factor (MIF)^70^. The upregulation of MHC class II complex in this subset of PA ECs complements our previous finding of CD4 + T-cell recruitment to this subpopulation of PA ECs, leading to over-activation and exhaustion via antigen-persistence.

The next group of cells is intermediate in its composition of AC (67.5%) and PA (~32.5%) ECs with a mixed gene expression profile with characteristics similar to each of the other two groups of cells (Fig. 4d, green bar), likely representing dysfunctional ECs that are in transition from the inflamed subtype to the CTD subtype described below.

The final group of cells is largely comprised of ECs from the AC (96.8%) (Fig. 4d, cyan bar) and is largely devoid of endothelial-marker gene *EMCN*^71^ (normalized effect = 0.86, *q* = 1.17e−09). Critical EMT genes identified earlier (*ITLN1*, *SCX*, and *S100A4*) are predominantly expressed in this large cluster of AC ECs alongside highly correlated genes *OMD, OGN*, and *CRTAC1*, again indicating that this population of ECs likely represents the main group of transdifferentiated ECs.

Gene ontology enrichment analysis further supports this shift in EC cell state from cells primarily involved with immune response (antigen processing and presentation, adaptive immune response, etc.) to cell states predominantly involved with proliferation, migration, vascular development, and angiogenesis (Fig. 4e, f).

## Discussion

**Fig. 5:**
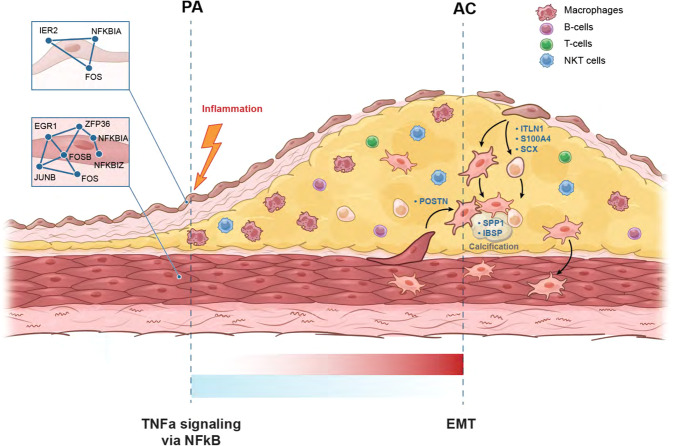
Schematic of atherosclerotic plaque by anatomic location. Illustrates key hallmark processes in PA and AC VSMCs and ECs. Inflammatory gene signatures shown in the PA portion, and matrix secreting genes up-regulated in the AC region. See main text for details.

In this study, unbiased single-cell transcriptomics of calcified atheromatous plaque in asymptomatic patients revealed 15 distinct cell types, including six major cell types; macrophages, ECs, VSMCs, NKT cells, T-, and B-lymphocytes. When expression signatures were contrasted across AC and PA plaque regions, VSMCs and ECs demonstrated the greatest degree of anatomical diversity. Our findings demonstrate that both VSMCs and ECs likely contribute to increased calcification and ECM remodeling activity in the AC. In the PA region, inflammatory signaling, including recruitment of immune cells, is enriched in both cell types (Fig.5). However, differing biological processes and CTD molecular networks underly this convergent biology.

VSMC plasticity in response to injury and environmental cues is central to vascular homeostasis and remodeling^72^. *KLF4*-dependent modulation of phenotype switching in VSMCs was shown to be pathogenic, and deletion of this gene in a murine model of atherosclerosis reduced lesion size^73^, suggesting that targeting modulators of CTD in atherosclerosis may be an effective treatment strategy. Subsequent analysis has found *KLF4*-dependent osteogenic SMC phenotypes contributing to plaque calcification and a *LGLSAL3* + SMC population with chondrocyte-like gene signature that was decreased in SMC-*KLF4* knockout^16^, underscoring the remarkable plasticity of cell types encountered in plaque. Recent single-cell analysis of atherosclerotic plaque has similarly found a predominance of endothelial cell transition to mesenchymal cells^13^. It is therefore critical to unmask modulators of CTD, and to explore this phenomenon in other cells of the vascular wall such as ECs. In our study we identify groups of VSMCs that express both the core VSMC marker genes and the transcription factors (i.e., *SCX*) and effectors (i.e., *IBSP, SPP1*) of matrix-secreting cells, supporting the notion of real CTD occurring and/or completing in the AC, albeit without true lineage tracing in this dataset. CTD in the AC to osteoblast- and chondrocyte-like cell states may be defined by different molecular networks with correlated driver genes: *POSTN, SPP1,* and *IBSP* in VSMCs, vs *ITLN1, SCX*, and *S100A4* in ECs. While these groups of genes may promote CTD in their respective cell types, their pathogenic versus protective role are unclear and scrutinization of these genes will be critical to fully appreciate their contribution to disease progression.

Similarly, while inflammatory signaling is enriched in the PA for both cell types, the molecular networks engaged in inflammatory signaling differs across VSMCs and ECs. Both cell types appear to respond to inflammatory signaling via IE-mediated expression of NFkB inhibitors; *NFKBIA* and *TNFAIP2*. However, VSMCs engage in immune cell recruitment through expression of *C3* (complement component 3), whereas ECs engage in immune cell recruitment through MHC class II presentation. A wide variety of other inflammatory processes are activated in both cell types, converging on TNFa signaling via NFkB in both cell types and IL6 signaling in ECs—both likely augmented by vascular wall driven recruitment of inflammatory mediators of early plaque development^74^.

Importantly, this study explores asymptomatic calcified atheromatous plaque, offering detailed analysis of plaque burden, but admittedly limits the conclusions that may be drawn regarding plaque vulnerability and rupture as seen in symptomatic patients. However, illuminating the anatomic landscape of calcified atheroma at single-cell resolution underscores the intriguing possibility of employing site-specific therapies to curtail disease progression. Given the recent focus on targeting inflammation to impede atherogenesis^75^, our results highlight the importance of targeting inflammatory mediators at the nidus of disease, thereby halting the development of plaque and progression of blood vessel cells into pathogenic CTD. Similar strategies may be contemplated for the CTD observed in our patients, though it is unclear whether AC calcification is pathogenic or stabilizing and protective.

The dataset in this study serves as a census for transcripts in calcified atherosclerotic plaque, and important limitations should be noted, including the inability to perform more thorough histologic validation of our sample due to the limited amount of tissue available, all of which must be processed to maximize single-cell yield. The limited amount of tissue obtained also posed challenges for spatial RNA identification of critically discussed genes. In addition, it is not possible to definitively prove the occurrence of CTD due to the lack of lineage tracing ability in this sample, or the addition of other validation studies in vitro, such as gene knock-down studies in vascular cell lines which may provide robust evidence of identified CTD-stimulating genes. Our gene co-expression networks, while implying correlation between genes and revealing important insight into underlying biology, do not allow us to identify key genes as definitive drivers of gene expression without requisite functional assays. Last, the final definition of subclusters was defined by re-clustering cells of the same cell-type by specific hallmark gene sets and defining subclusters by nested cuts of the hierarchical tree. This approach raises the possibility of other unidentified nested subclusters.

Overall, our study provides anatomic insight into atherogenesis by examining key cellular processes occurring in the atherosclerotic core and adjacent arterial tissue and lays the groundwork for continuing efforts to unravel the complexity of this disease.

## Methods

### Selection of patients

Identification and consenting of three patients with severe carotid plaque formation requiring carotid endarterectomy occurred in collaboration with the Scripps Health Biorepository under IRB# 19-7332 approved by the Scripps Institutional Review Board. Informed patient consent was obtained for all samples collected. Patient characteristics and comorbidities are presented in Table S1. Plaques were characterized by histopathology according to AHA classification scheme^76^. Briefly, near full thickness (except adventitia) sections of artery and plaque were recovered from the atherosclerotic core, based on surgeon’s determination of area of largest plaque burden, usually near the carotid bifurcation. A section of artery located ~1 cm proximal to the AC was then recovered from the same patient during the cut-down portion of the endarterectomy, resulting in 3 replicates of both PA and AC sections. These specimens were immediately transported for tissue processing.

### Tissue processing and generation of single-cell suspensions

AC and PA carotid artery from patients undergoing carotid endarterectomy was immediately collected from the operating room and transferred on ice for tissue dissociation (Fig. S1a, b). Samples were weighed, minced with surgical blades, then placed in conical tubes with pronase (5000 U/ml) and collagenase II (0.1% solution). Tissue was then incubated at 37 °C for 1 h with continuous gentle agitation. 30 min into the incubation period, DNase I solution was added, and the entire solution remained incubated for an additional 30 min. Pipetting mixture up and down every 10 min helped break tissue apart. After 1-h incubation was complete, 10% FBS + Complete EC media (Cell Applications, Inc) was immediately added to dissociated tissue to quench enzymatic activity. The sample was then inverted a few times and then set down for 30 s, allowing all debris to settle at the bottom. The entire solution was carefully transferred, without settled debris, to another conical tube (use pipettor to transfer solution near debris pellet carefully, removing all solution but leaving pellet at bottom of tube). Sample was then centrifuged at 500 × *g* for 5 min and supernatant discarded. Cell pellet was resuspended in 5 mL 1X RBC lysis buffer and incubated at RT for 5 min. 30 mL 1X PBS was added to stop the reaction and the sample was then spun immediately at 500 × *g* for 5 min at RT. The supernatant was discarded, and cell pellet resuspended in 10 mL FACS buffer composed of: 1X PBS, 2.5 mM EDTA, 25 mM HEPES, 1% FBS (heat-inactivated), and 1% Pen-Strep. To wash excess reagents, the sample is centrifuged again at 500 × *g* for 5 min, supernatant discarded, then cell pellet is again resuspended in 2 mL FACS buffer. 2 μl of 5 mM DRAQ5 is added to each tube, and solution is pipetted up and down a few times. Next, solution is triturated with P1000 and entire sample sieved through 100 μM cell strainer fitted onto 50 mL conical tube. This step is repeated next through a 40 μM cell strainer. The solution is placed on ice and immediately transported for FACS. Cells were sorted on a MoFLo Astrios EQ cell sorter (Beckman Coulter) with a 100 µm nozzle tip at a sheath pressure of 20 psi. Cells were distinguished from cellular debris by gating DRAQ5 positive events and doublets excluded using appropriate FSC and SSC gating (Fig. S2a–f). Cells isolated represented <1% of the total particles in the sample which was then transported for evaluation of viability and subsequent sequencing.

Cells were then processed on the 10X Genomics Chromium single cell 3’ v3 gene expression platform. Cells were counted and checked for viability using a Countess II Automated Cell Counter (Thermo Fisher). Sequencing libraries were prepared as recommended in the user manual with cell target numbers ranging between 6000 and 22,000 cells per sample. Completed sequencing libraries were then sequenced on an Illumina NextSeq500 using 28 × 91 paired-end reads with 8 base i7 index reads to demultiplex different samples. Aggregating our 6 samples (3 patient-matched PA and AC samples) resulted in 51,981 cells, a sequencing depth of 15,549 reads/cell, 1339 median genes/cell and 3776 UMI/cell.

### Single-cell transcriptomics

We used a combination of publicly available tools and custom scripts to process single-cell transcriptomic data. These methods have been developed and refined by our lab for the analysis of single-cell transcriptomics of human atherosclerotic plaque tissue, an extremely challenging tissue for single-cell sequencing given the variable degree of cellular viability and cellular and extracellular matrix heterogeneity present in plaque tissue from vascular wall to the fatty plaque tissue itself. A combination of 10X Cell Ranger^77^, Monocle^78^, Seurat^79^, and custom R and python scripts as well as pathway analysis tools are combined in a comprehensive single-cell quality control and analysis pipeline. Data has been deposited: https://www.ncbi.nlm.nih.gov/geo/query/acc.cgi?acc=GSE159677. Please see Supplemental Methods and Figs. S6–S14 for more detailed data analysis methods.

### Cell set preparations, aggregation, and initial cell-type identification

Raw single-cell sequencing data were processed independently per sample using the 10X Genomic Chromium platform^77^. 10X Genomic’s Cell Ranger with default parameters for read mapping^80^, sample quality control, unique molecular identifier filtration, normalization, and expression quantification. Further quality filtering is performed by Seurat^79^ to remove cells with >10% mitochondrial mRNA (total mRNA), in addition to cells with <200 or >4000 genes expressed. Quality control resulted in a reduction of 6145 cells, down to 45,836 cells total prior to downsampling. Cell sets were then down-sampled to 17,100 cells to account for imbalances in total cell counts across samples which may adversely influence clustering analysis.

Aggregated cell sets were then further processed by Monocle for cell-type sub-setting^78^. Dimensionality reduction was performed as standard via principal component analysis in Monocle and UMAP partitioning is applied^81^, which we have found to perform better than t-SNE in practice. Cell type assignment was a largely manual process facilitated by partition level differential gene expression analysis to identify 3 known marker genes per cell type that were expressed in >80% of cells and at a mean expression count >2. At this point partitions were assigned to cell types.

### Doublet analysis and filtering

In order to identify and remove distressed cells, or rare cell types and artifacts, we first estimated doublet-rates and attempted to filter doublets using the Scrublet package^82^. However, we found that Scrublet and other doublet detection packages were not effective at identifying doublets in highly heterogenous samples like atherosclerotic plaque. Therefore, as an alternative we identify doublets based on a combination of the inappropriate expression of cell-type marker genes coupled with elevated read counts. Marker genes that should be ubiquitously expressed (>90%) in one cell type (partition) and rarely expressed (<10%) in other cell types (partitions) were used to mark potential doublet cells. Cells with multiple inappropriate marker genes expressed (≥2) are tagged for removal. For example, *CD2* expression (cell adhesion molecule specific to T- and NKT-cells) was detected at higher-than-expected levels in VSMCs (3%) and ECs (2.1%), and thus these cells were excluded from analysis. An upward shift in read counts across all doublets relative to the population average was used to validate the doublet identification strategy. In addition, we perform gene co-expression network reconstruction using a custom partial correlations approach (described below), which is sensitive to rare outlying correlation events, and enables us to detect inappropriate co-expression of genes. Inappropriate networks reflective of unexpected cell-types were identified during the doublet detection process to validate our filtration strategy. Remaining cells were re-clustered producing six major partitions; macrophages, ECs, VSMCs, NKT cells, T- and B-lymphocytes (Fig. 1c, d). Patient-specific cells are shown in Fig. S3a.

### Differential gene expression analysis

Differential expression analysis was performed using Monocle^78^, which uses a generalized linear regression model approach, and was adjusted for patient-specific and other confounder effects as covariates. Modeling includes an adjustment for patient identification as a corrective term. In order to have commensurate measures of differential expression, each gene’s expression level was normalized prior to fitting the model, allowing the resulting model coefficient to be interpreted directly as that gene’s effect size (normalized effect). The sign of the coefficient determines up- or down-regulation. A corrected *p*-value was computed for each coefficient using Benjamini and Hochberg. Consistency of gene expression differences at a biological process level was evaluated by Gene Set Enrichment Analysis applied to single-cell gene differential expression data ranked by normalized effect.

### Gene networks

We reconstructed gene expression networks using a modified Weighted Gene Co-Expression Network Analysis^83^ approach we developed using partial correlations and applied to single-cell data. All pairwise gene-gene correlations are computed with partial correlations adjusted for the rest of the genome using a Penrose-Moore pseudo inverse with applied shrinkage parameter (uses the R package corpcor). The resulting matrix is linearized and then subjected to a false discovery analysis using R package fdrtool. Networks are reconstructed from the resulting top 20,000 most significant partial correlations by applying an FDR threshold to the pairwise co-expression edges (threshold estimated empirically from the distribution) with modules defined by Louvain clustering using default parameters^81^, where edge weights (distances) were set to the reciprocal of the absolute partial correlation. Because clustering was performed using weighted edges, FDR thresholds were relaxed and allowed to exceed 0.05 (mean FDR ~0.25).

### Gene module network plotting and selection of significant modules

Network plots were created using igraph’s built-in plotting functions. Cluster level plotting with colorization schemes aiding in visual interpretation of gene-to-gene relationships was used. For module plots containing a greater number of genes (over 100), additional filtering was used to help elucidate each module’s significant gene-to-gene relationships. For selected modules in the VSMC and EC cell types, module subnetworks were constructed by filtering and maintaining genes that were in the top 15 percent of those most connected within the network (higher ranked strength), along with all the modules’ differentially expressed genes. This resulted in modules on the order of 10 to 30 genes. Nodes were colorized gray for non-differentially expressed genes, and a cyan to red gradient was used for positive to negative normalized effect levels (qualified with *q* < 0.05). Dark red genes (nodes in the network) were indicative of genes significantly upregulated in AC cells, while dark cyan genes were lower expressing the AC cells.

In order to focus our analysis on key genetic drivers of calcified plaque build-up, we chose to concentrate on selected modules exhibiting significant interactions between DE genes. This was determined by first setting a threshold of *p* < 0.05 for differential expression overlap between modules (determined by one-tailed Fisher exact test). A contingency table was constructed for each module in the network based on the overlap of significant DE genes to the module genes. Modules do not overlap with each other due to the use of Louvain clustering. Modules with disproportionate numbers of DE genes rank as the most significant, while those with few or no DE genes rank as the least significant. This resulted in 8/31 modules being selected for further investigation in the VSMC dataset, and 7/36 modules in the EC dataset. Next, within each of these modules, genes were sorted by normalized effect and *q*-value < 0.05 in order to better interrogate significantly differentially expressed genes. After this, the gene list was sorted by strength of connections, with >0.3 being used as a cutoff for genes to explore further, given the greater correlations with other genes observed with higher strength score. Modules with genes present satisfying these criteria moved forward with analysis. Within the chosen modules, genes were again sorted by normalized effect and *q* < 0.05. Genes with NE > 0.5 or <−0.5 were chosen and plotted, and those with signal strength >0.3 were chosen for further examination.

### Heatmaps

All heatmaps were constructed from gene expression data, with individual cells plotted along the horizonal axis and genes plotted along the vertical axis. Prior to plotting, expression data was converted to binary form (on/off), with each gene plotted as on if the expression level of that gene had an RNA count of two or more. A binary distance method was then used to drive hierarchical clustering along both axes using complete-linkage clustering. All heatmaps are accompanied by dot plots on the right side the heatmap, showing gene expression levels for each of the cell subsets in the heatmap. These dot plots are scaled based on the raw RNA counts.

### Statistics and reproducibility

Wherever possible, commonly available tools and statistical methods in performing computational analysis were used. Greater detail may be found within the supplementary methods. For experimental reproducibility, we overcame the lack of biological and technical replicates by aggregating samples and then comparing the cell subtype transcriptional profiles of the 3 patient sample pairs, checking for consistency across samples, as well as confirming the transcriptional homogeneity within cell types. This is the preferred approach in detecting batch effects and is commonly used in single-cell pipelines. This method did not require exotic cell dataset integration for discovering and defining cell type clusters or phenotype identification.

The general approach starts with an examination of the sample level cell cluster counts (Fig. S8b). The table demonstrates that each cluster contains significant cells from all samples and are well represented. Further, there is spatial and graphical separation between clusters (Fig. 1c, d). Key marker gene expression homogeneity within clusters and across samples is shown by visually examining cell marker plots (Fig. S4). This is further confirmed by the gene exclusion results (Fig. S9).

This demonstrates that the expression of key cell marker genes is well represented across samples while remaining specific to cell types, confirming that sample level biological and technical artifacts have minimal effect on our analysis. Further details on partition level gene network and module level analysis may be found in Supplementary Data 9–15.

### Reporting summary

Further information on research design is available in the Nature Research Reporting Summary linked to this article.

## Supplementary information

Supplementary Information

Description of Additional Supplementary Files

Supplementary Data 1

Supplementary Data 2

Supplementary Data 3

Supplementary Data 4

Supplementary Data 5

Supplementary Data 6

Supplementary Data 7

Supplementary Data 8

Supplementary Data 9

Supplementary Data 10

Supplementary Data 11

Supplementary Data 12

Supplementary Data 13

Supplementary Data 14

Supplementary Data 15

Reporting Summary

## Data Availability

The scRNAseq datasets generated during and/or analyzed during the current study are available in the Gene Expression Omnibus (GEO) at the following address: https://www.ncbi.nlm.nih.gov/geo/query/acc.cgi?acc=GSE159677.

## Code availability

All analysis code related to the manuscript has been made publicly available at the following address: https://github.com/TorkamaniLab/Carotid.Art.Dis.scRNA_Seq, 10.5281/zenodo.7065390.
