# Supplementary material for: Decoding the transcriptome of calcified atherosclerotic plaque at single-cell resolution
Source: Commun Biol. 2022 Oct 12;5:1084. doi: 10.1038/s42003-022-04056-7 (PMC9556750; doi:10.1038/s42003-022-04056-7)
Supplement: Supplementary file 1 — Supplementary Information [file 42003_2022_4056_MOESM1_ESM.pdf]

Supplemental Methods

Selection of patients:

Identification and consenting of three patients with severe carotid plaque formation requiring carotid endarterectomy occurred in collaboration with the Scripps Health Biorepository under IRB# 19-7332 approved by the Scripps Institutional Review Board. Patient characteristics and comorbidities are presented in Supplementary Table 1. Plaques were characterized by histopathology according to AHA classification scheme. Briefly, near full thickness sections of artery and plaque were recovered from the atherosclerotic core (except adventitia), and full thickness proximally adjacent arterial sections were recovered from the same patient during the cut-down portion of the endarterectomy. These specimens were immediately transported for tissue processing.

Aggregated cell data set:

10X Genomics CellRanger v3.0.2 was used to count the RNA molecules and then aggregate the 6 samples into a single cell set. The 6 aggregated samples (3 patient-matched PA and AC samples) resulted in 51,981 cells with an average sequencing depth of 15,549 reads/cell, 1,339 median genes/cell and median 3,776 UMIs/cell. Aggregation was performed with default parameters (with normalization). The individual six sample Cell Ranger results are summarized in Fig. S6A. The aggregated six sample Cell Ranger results are summarized in Fig. S6B.

Processing Cell Sets Using the Monocle 3 (Beta) Tool Set:

Unless otherwise stated, each of the following analyses were performed with the Monocle R package (beta release version 3). Each cell dataset was processed using the standard Monocle pipeline. This includes principle component dimensionality reduction (100 PCAs are used), UMAP dimensionality reduction (2 dimensions are used), Louvain partitioning and clustering, and top marker analysis.

Five main subsets of the cell data sets were produced, with each set derived from the prior, adding further refinements in each step. These cell sets can be summarized as follows:

1. Original 51,981 Cells, As Aggregated from Cell Ranger, Unfiltered
2. QC Filtered 45,836 Cells, Filtered for Hi/Lo Gene Count and Hi mtRNA Content
3. Down-sampled 17,100 Cells, Down Sampled to Equalize Across Patient and Tissue Location
4. Remixed 16,287 Cells, Remove Small Cell Groups and Recombine Similar Cell
5. De-doubleted 13,070 Cells, Remove Contaminated and Doubleted Cells

Preliminary cell filtering:

Cell filtering was performed on the original cell set using standard methods, primarily to remove cells with very high mitochondrial RNA content. The Seurat pbmc3k tutorial was used as a rough guide for setting thresholds for this filtering. Cells with greater than 4000 genes were removed (the default Seurat threshold is 2500 genes). Cells which had total mitochondrial RNA abundance greater than 10% were
removed (the Seurat default threshold is 5%). Cells with less than 200 genes we removed (Seurat default). In total, this resulted in 11.8% of the cells being removed, with the overwhelming majority of those removed due to high mitochondrial RNA content (10.8% - i.e. 92% of removed cells were due to high mitochondrial

expression). Fig. S6B highlights the low quality cells removed from our dataset. Sample specific filtering results are summarized in Fig. S6A.

Sample level down-sampling:

Because of the limited PA tissue, there was roughly a 3-to-1 AC to PA cell count ratio in the aggregated cell set. To account for this imbalance, cells were down-sampled such that each of the six original groups would contribute an equal number of cells to the final set. Patient 3 established the target down sampling count as their PA tissue had the fewest number of cells (2850 cells). This down sampling resulted in a new aggregated cell set with 17,100 cells. See Fig.S6A for sample cell counts prior to down sampling.

Preliminary Monocle cell partitioning:

The down-sampled cell set were then processed using the Monocle R package (beta release version 3). Dimensionality reduction was performed using standard principal component analysis with 100 components. Two-dimension UMAP dimensionality reduction was used, and partitioning/clustering was performed using the Louvain algorithm as provided by Monocle. From this we identified 15 initial partitions. See Fig. S8A for details on resulting cell counts per sample per partition.

Initial cell type identification and partition assessment:

Initial cell type identification was largely a manual process facilitated by partition level differential gene expression analysis to identify 3 known marker genes per cell type that were expressed in greater than 80% of cells and at a mean expression count greater than 2. Using known marker genes, each of the 15 partitions were assigned to a cell type label except for partition 1, which had only 24 cells coming from a single sample (patient 3 AC). There is some indication from the expression that from these cells are progenitor cells, however we could not conclusively assign a cell type label to this partition. See Fig. S8A for the initial cell type assignments. See Fig. S1D for a cell plot of the partitions with cell types labels. See Fig. S1E for a dot plot of these top markers across all partitions.

Partition filtering and remix:

Besides partition 1, some of the partitions discovered in the prior step contained too few cells for differential expression analysis where ideally there would be a significant number of cells from each patient and each location (AC or PA) in each partition. Therefore, smaller partitions, where differential gene expression would be underpowered, were removed from further consideration. In addition, partitions with the same cell-type label were merged for downstream differential expression analysis. The threshold for keeping a partition was that at least 250 cells be present in that partition in both AC and PA samples. That resulted in the exclusion of partitions 1, 4, 11, 12, 13, 14, and 15, and the combining of partitions 2 and 5 and partitions 3 and 6 (VSMCs and ECs). The removal of the smaller partitions resulted in a total removal of 813 cells, going from 17,100 down to 16,287 cells. See Fig. S8A and Fig. S8B for details.

Final Monocle cell partitioning:

The new remixed cell subset consisting of 16,287 was not reprocessed through the Monocle 3 standard pipeline. However, to confirm that our final partitions are robust to

our data processing pipeline, we re-partitioned the final filtered dataset and confirmed that 99.93% of cells are re-assigned to the same cell identity. Furthermore, additional exploratory partitioning analyses with other tools (primarily Seurat as well as alternative embeddings with Monocle) confirmed that the two VSMC partitions and two EC partitions could be considered a single partition depending upon the nature of the algorithm used, and that similarly NK-cells and T-cells formed joint or separate partitions depending upon the algorithmic approach. All partitions were fully reproducible.

Doublet, Multiplet, and Contaminant Detection:
Doublets occur in single-cell expression data at an expected rate given the single-cell capture technology. Similarly, background RNA or contaminant cell fragments attached to cells can lead to unexpected gene expression profiles that confound differential expression results. We identified and eliminated these doublet / multiplet / contaminant cells using a gene exclusion approach, which we then validated with a standard doublet detection algorithm – Scrublet. Both approaches resulted in doublet numbers matching statistical expectations.

Gene exclusion analysis:
First, to investigate the extent of doublet and contamination issues, a gene exclusion analysis was undertaken to characterize the appearance of genes commonly expressed in one cell type (partition) but rarely expressed in other cell types (partitions). The principle concern is that the appearance of unexpected genes, especially marker genes for a known cell-type, in some small fraction of cells assigned to an alternative cell-type. To find unexpected genes, we first identified genes that are ubiquitously expressed within individual partitions. A 90% minimum threshold of cells expressing a gene was set for finding ubiquitously expressed genes for each partition. In turn, a rareness test was performed using those same ubiquitous genes, but now within the other partitions, looking instead for rare expression of genes ubiquitously expressed in other partitions. In this case, a 10% (maximum) threshold was chosen. This resulted in 2 to 14 genes per partition that met this 90/10 rule and could be used to tag suspicious cells, that is cells that may include contaminants or cells that might have been formed from two or more dissimilar cells. See Fig. S9 for the details of this analysis. Two exceptions were made to the 90/10 rule; The putative combined VSMC partition (VSMC.5.7) threshold was reduced from 90% to 87%. This was needed in order to capture more ubiquitously expressed genes with matching rare genes in other partitions, and the putative T Lymphocyte (CYTOT.9) cells threshold was reduced to 78% because only two ubiquitous genes were discovered that satisfied the 90/10 rule. To assess these potentially problematic cells, we first determined whether there is an increase in total RNA in the candidate doublets / multiplets / contaminants. The principle at work here is the contaminated cells and doublet cells should, on average, contain more total RNA than uncontaminated cells and singlets. That is characterized as ratio of mean total RNA expression for cells with the rare marker compared to the mean total RNA expression of cells within the same partition but without that marker. 93% of these candidate doublets show an increase in total RNA, and 65% show a significant (p-value < 0.05) increase in total RNA. Thus, we filtered and removed cells if the candidate doublet / multiplet / contaminant cell in question expressed 2 or

more inappropriate marker genes for its resident partition, conditioned further by filtering only those cells with a concomitant increase in total RNA (p-value < 0.05). Summary statistics for the number of tagged candidates, and the changes in total RNA levels are provided in Fig. S10A, Fig. S10B, and Fig. S10C. Noteworthy in Fig. S10A is the increased total RNA in each partition due to the tagged cells. Further, the number of filtered AC cells to PA cells is roughly 3-to-1 as expected. The absolute number of cells filtered is consistent with the predicted ratio of doublet rates. Fig. S11 presents a cell plot of the cell partition colorized by removed doublets / contaminants.

Doublet Validation using Scrublet:
Scrublet is a validated algorithmic approach for the identification of doublets. Scrublet generates synthetic doublets computationally, and then uses graphic methods to discover similar process-derived latent doublets based on geometric distance of UMAP embedding, that is the distance between the synthesized doublet and the process-derived doublets. Synthesized doublets are also used detect other synthesized doublets, and this is used as a check on the ability or this method to collocate doublets more generally. The Scrublet pipeline was run on the 16,287 cell set using default parameters. Scrublet has an input parameter for the estimated doublet rate, so three values were tried, 6% (the default), 10%, and 20%. The results were nearly identical for the three, reliably identifying only 1.3% of all cells (205 doublets).
Fig. S12 displays the Scrublet results. The first pair of plots are score histograms of the predicted doublets (left plot) and the synthesized doublets (right plot). These distributions are bimodal, with the distribution of the synthesized doublet plot serving as guide for selecting a cutoff for predicted doublets. Note that Scrublet is not able to detect all synthesized doublets. The cell plots below in Fig. S12 highlight the scored and predicted doublets. Note that high scoring doublets are spatially intermixed with the cores of the clustered cell group, suggesting that the method of uniquely identifying doublets from synthesized doublets is adversely affected in scenarios of high cellular heterogeneity in the true singlet cell populations. Regardless, doublets identified by Scrublet and our gene exclusion method were concordant 94% of the time, validating our doublet / contaminant detection and removal approach.

Partition level differential expression analysis:
Monocle 3 was used to perform differential expression analysis on each of the six cell partition cell types, where cells were regressed across AC and PA phenotypes. Patient ID was included in the models and was treated as a batch effect categorical covariate (and also corrects for sequencing batch). For each partition analyzed, on the order of 20,000 genes were successfully scored. Unless stated otherwise, differentially expressed genes were ranked as most significant based on having the highest absolute Normalized Effect but prequalified by having a q-values less than 0.05.

Partition level gene networks - partial correlation with gene level clustering:
We reconstructed gene expression networks using a modified Weighted Gene Co-Expression Network Analysis approach we developed using partial correlations and applied to single-cell data. All pairwise gene-gene correlations are computed with partial

correlations adjusted for the rest of the genome using Penrose-Moore pseudo inverse with applied shrinkage parameter (uses the R package corpcor).
The resulting partial correlation matrix is linearized and then subjected to a false discovery analysis using R package fdrtool. See Fig. S13A and Fig. S13B for the results of applying the FDR tool to the VSMC and EC cell sets, respectively.
Gene modules were then extracted by applying igraph weighted Louvain clustering to the high-density networks, with edge weights (distances) set to the reciprocal of the absolute partial correlation. Because clustering was performed using weighted edges, FDR thresholds were relaxed and allowed to exceed 0.05, with a typical FDR of 0.25.
Both the EC and VSMC high-density networks are provided as Supplementary Data. Supplementary Data 7 and 8 for the VSMC edge and node attributes, and Supplementary Data 9 and 10 for the EC edge and node attributes respectively. A dictionary of the relevant network attributes is provided in Supplementary Data 15.

Module enrichment analysis:
Module level significance is measured by the abundance of differentially expressed genes per module relative to expectations based on random chance. Supplementary Data 11 shows module level enrichment analysis for EC modules with Supplementary Data 12 cataloging the individual gene level changes for EC modules significantly enriched with differentially expressed genes. Supplementary Data 13 shows module level enrichment analysis for VSMC modules with Supplementary Data 14 cataloging the individual gene level changes for VSMC modules significantly enriched with differentially expressed genes. For module level enrichment, the first and second columns contain the module ID and the number of genes for that module, and then the columns labeled de_overlap_est and de_overlap_pval represent the overlap ratio and p-value as taken from the Fisher Exact test, where the total number of genes in the network, and the ratio of total differentially expressed genes in the network, are compared to the total number of genes in the module, and its ratio of differentially expressed genes. de_overlap_est is the ratio of increase in differentially expressed genes for that module, and the de_overlap_pval column is the p-value for that overlap ratio. This table has been sorted by de_overlap_pval, from smallest to largest. There is no distinction made as to whether the differentially expressed genes are positive or negative, only their significance is considered. Subnetwork plots were generated for the most significantly differentially expressed genes by again filtering based on q-value > 0.05 and plotting the top 15% of genes based on degree connectivity and normalized effect. Intermediate connecting genes were added automatically to completely connect the resultant network. In the resultant network diagrams, square nodes are used to highlight key genes included due to connectivity and normalized effect, those that that were added to complete the network are displayed as round nodes. Magenta colorization denotes genes upregulated in AC cells, gray indicate genes that are not significantly differentially expressed, and cyan colorization indicates genes upregulated PA cells.

VSMC and EC heatmaps
All heat maps in this paper are constructed from gene expression data, with individual cells organized and plotted along the horizonal axis and genes organized and plotted

along the vertical axis. Prior to plotting, expression data is converted to binary form (on/off), with each gene plotted in one of two colors, as a binary on or off. The threshold for considering a gene “on” was two or more reads. The binary distance measure was then used to derive hierarchical clustering along both axes using complete-linkage clustering. The dot plots shown on the right edge of heatmaps depict expression level for cell subpopulations and are computed from the continuous RNA counts. Sub-clusters are defined using the dendrogram cuts.

Sub-population Differential Expression Analysis

Differential expression across the defined sub-clusters was determined by defining for each sub-cluster a continuous independent variable defined as the fraction of AC cells per subcluster as displayed in Fig S14A for the EC heatmap and Figure S14B for the VSMC Heatmap. This regression is performed as described previously for global differential expression analyses, adjusted for patient ID, but with the outcome being a continuous outcome defined by the resident subcluster AC-cell abundance for each cell. The results of these regression analyses are tabulated in Supplementary Data 10 for the EC heatmap and Supplementary Data 9 for the VSMC heatmap. These tables represent all the genes in the respective cells set for this regression. The last column, with the heading ‘Corrected NE’, is the same as the Normalized Effect column, but only when the q-value column is less than 0.05, otherwise it is zero. The gene names, coupled with ranking provided with Corrected NE, is suitable for GSEA.

Gene Ontology Overlap and Gene Set Enrichment Analyses

For all differential expression analyses, genes were ranked based on their Normalized Effect as generated by the Monocle 3 differential expression process described above. Genes with non-significant q-values (q-value > 0.05) have their Normalized Effect set to 0. Gene set enrichment analysis is then executed using GSEA 4.0.0 using default parameters using these adjusted Normalized Effects as input ranks. Gene ontology overlap analysis was performed using only significant genes (unranked), where the significance of overlap with gene-ontology terms is calculated in the WEB-based Gene SeT AnaLysis Toolkit.

Extended Data:

Figure S1:

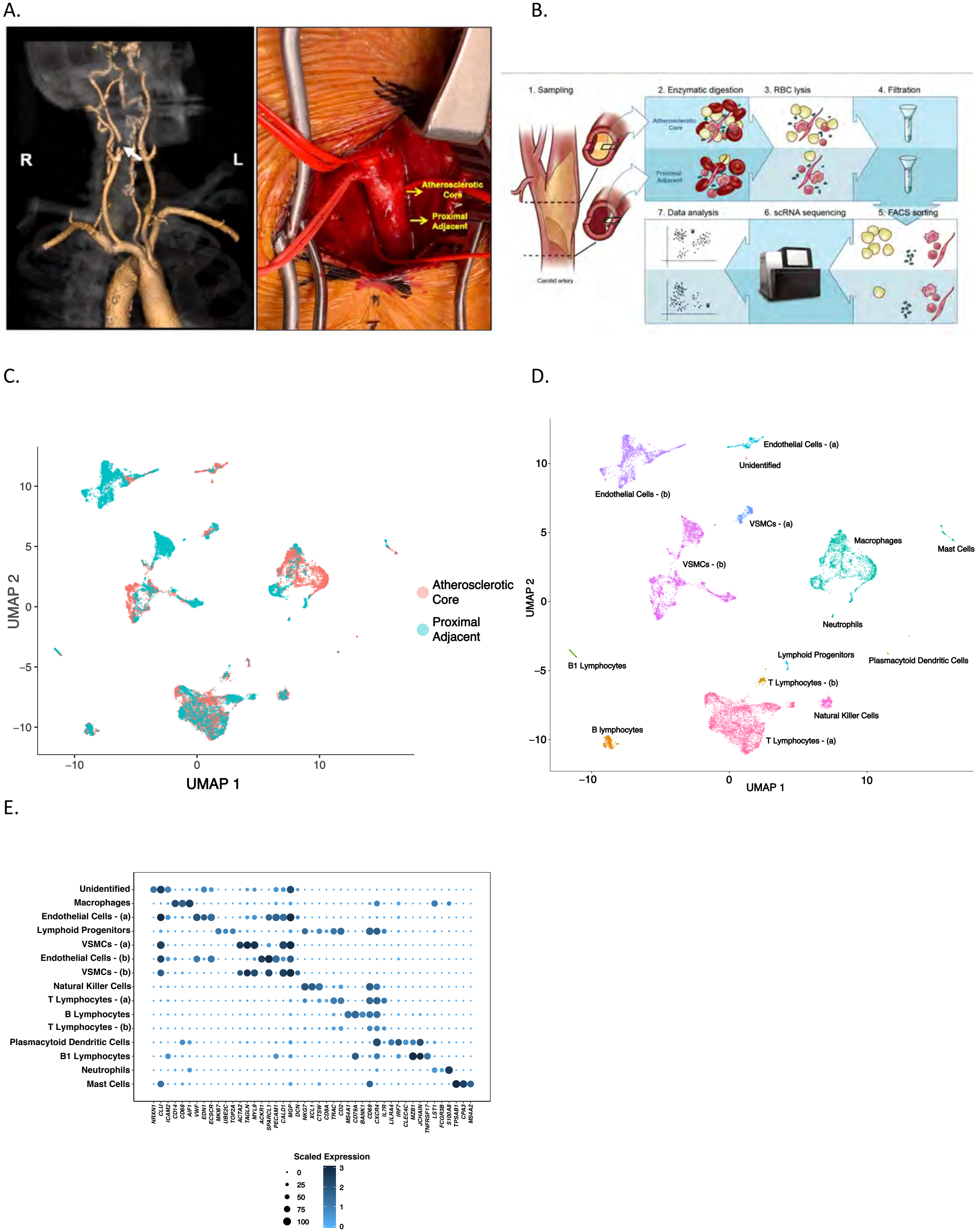

Fig. S1: Complete dissociation and scRNAseq profiling of 51,981 cells from the AC and PA region results in 15 distinct cell populations. a, Computed tomography angiogram demonstrating right internal carotid occlusion with >70% stenosis due to plaque (left panel)**;** anatomic location of dissected tissue for experiments presented (right panel). **b,** Schematic depicting steps necessary for complete dissociation of artery and plaque from their respective anatomic regions (details in Methods). **c-d,** UMAP visualization of down-sampled cells (n=17,100 cells) split by anatomic location **(c)**, and by cell-type **(d)**. **e**, cell-type marker genes for 15 distinct cell populations presented as a dotplot. Dot size depicts the fraction of cells expressing a gene. Dot color depicts the degree of expression of each gene.

Figure S2:

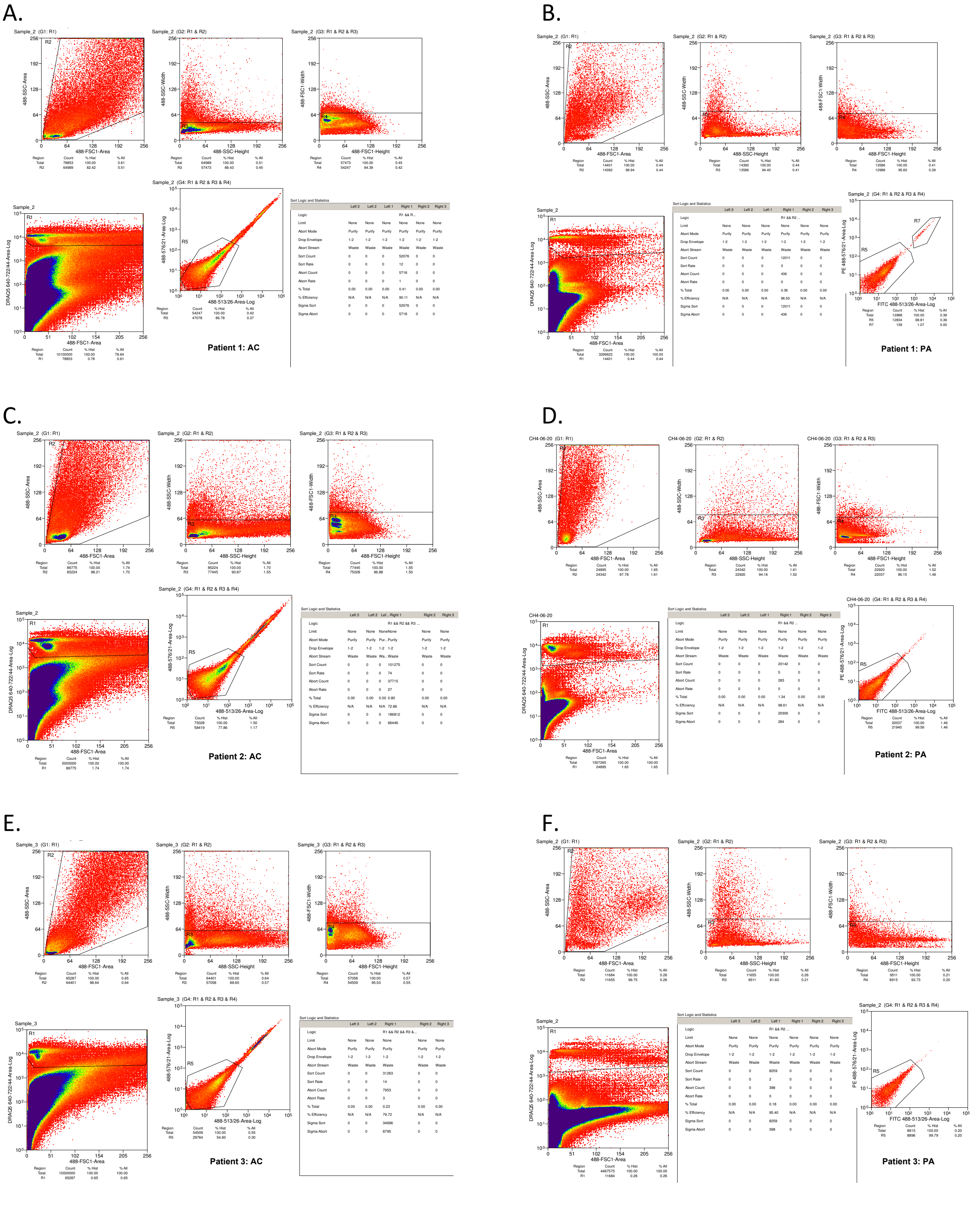

Fig. S2: Fluorescence Activated Cell Sorting (FACS) of samples. a-f, Cells from dissociated carotid artery and plaque from both AC and PA samples were labeled with DRAQ5, a far-red excitation/emission nuclear stain. Cells were distinguished from cellular debris by gating DRAQ5 positive events and doublets excluded using appropriate FSC and SSC gating.

Figure S3:

A.

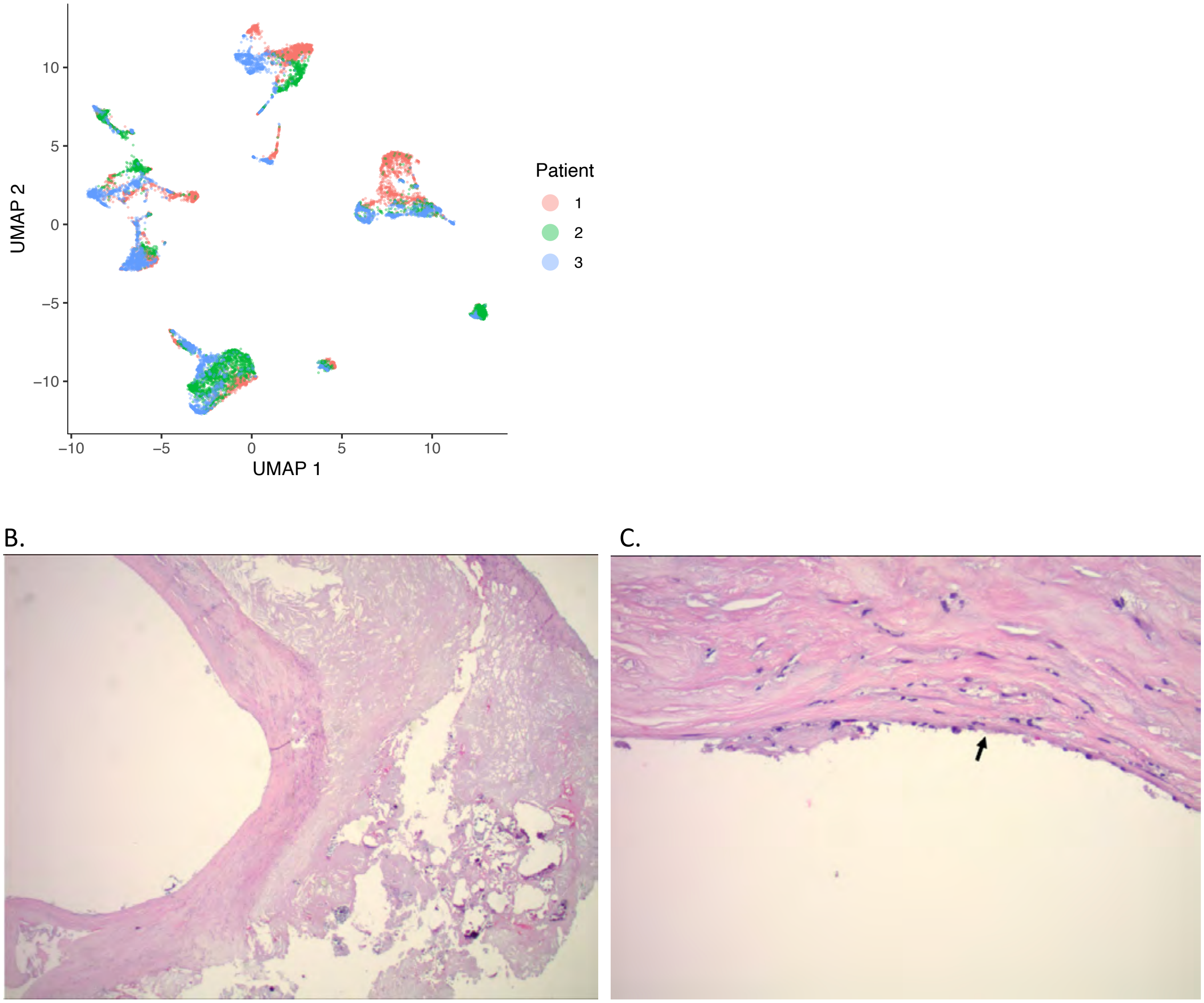

Fig. S3: Fig. S2: Aggregated patient data sets.
(A) UMAP of aggregated, down-sampled cell sets separated by patient.
(B) Low magnification H&E histologic staining of atherosclerotic plaque (AC) tissue from patient 3.
(C) High magnification histologic image of same tissue sample, arrow depicting disrupted EC layer.

Figure S4:

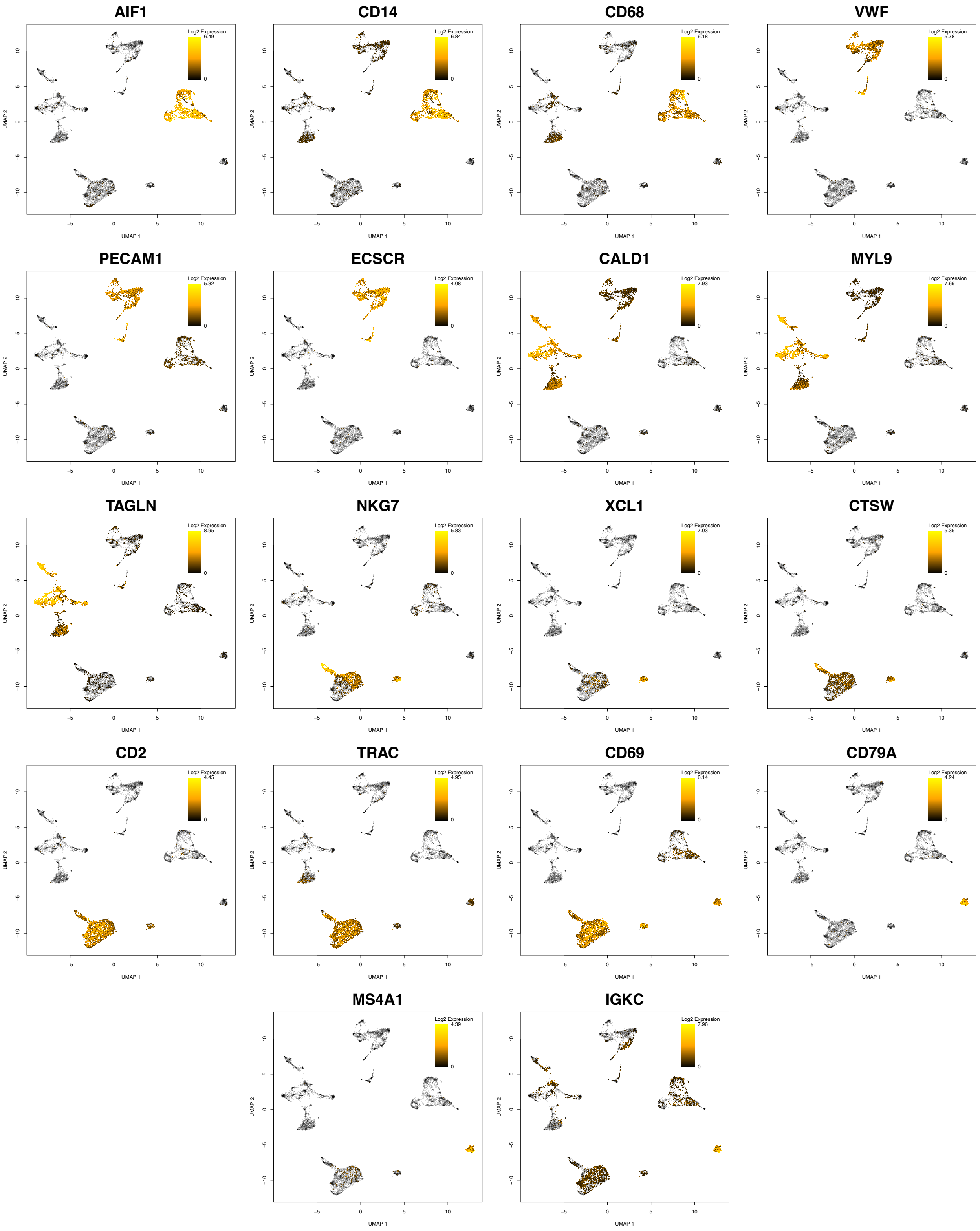

Fig. S4: Gene markers for identified cell types. UMAP plots of selected cell-marker genes from dotplot (Fig. 1D). Gray-colored cells indicate 0 expression of designated gene, while color bar gradient indicates lowest (black) to highest (yellow) gene expression level.

Figure S5:

A.

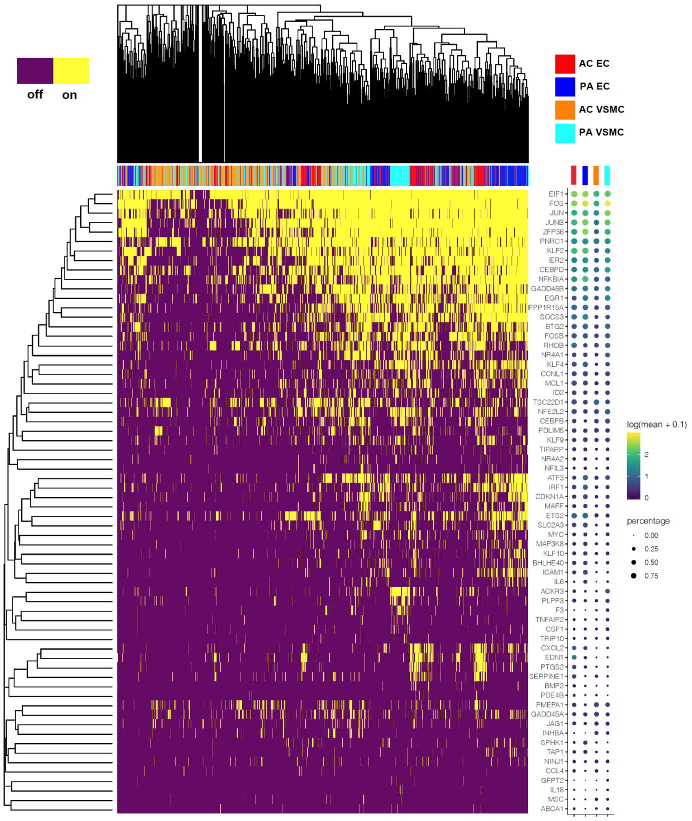

B.

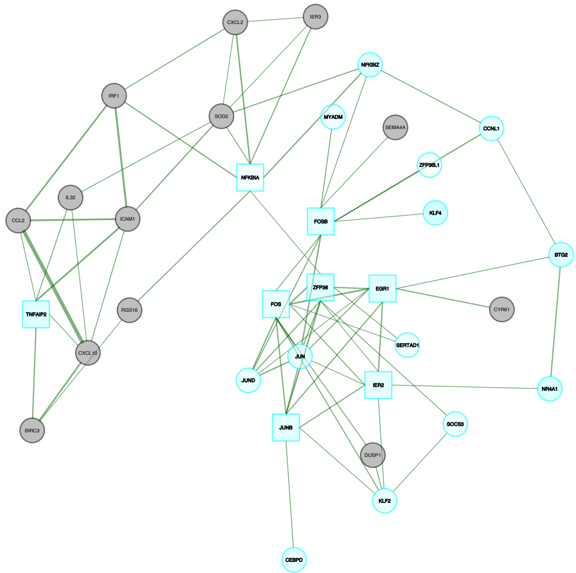

C.

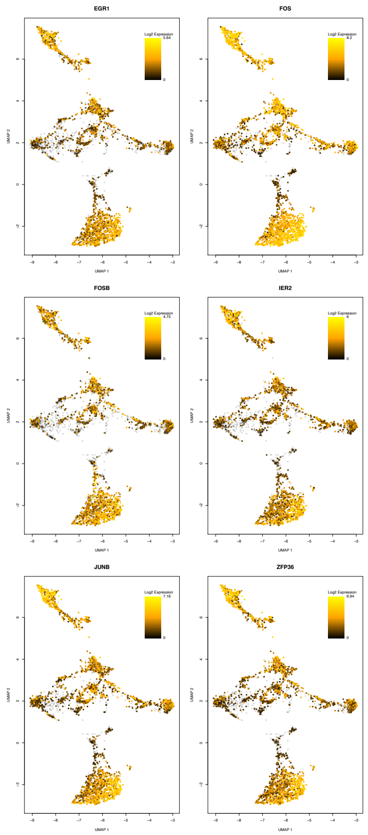

D.

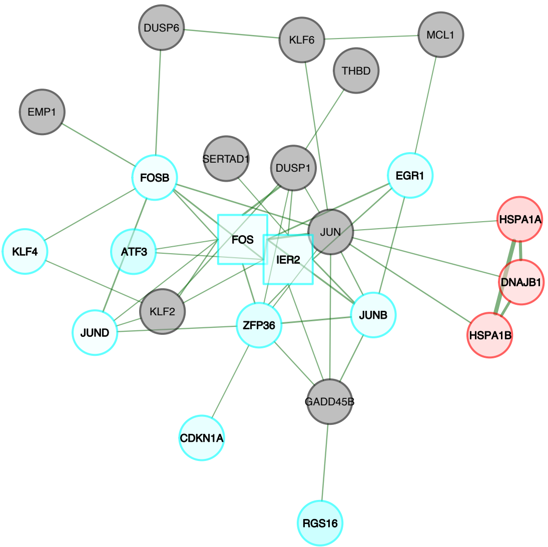

E.

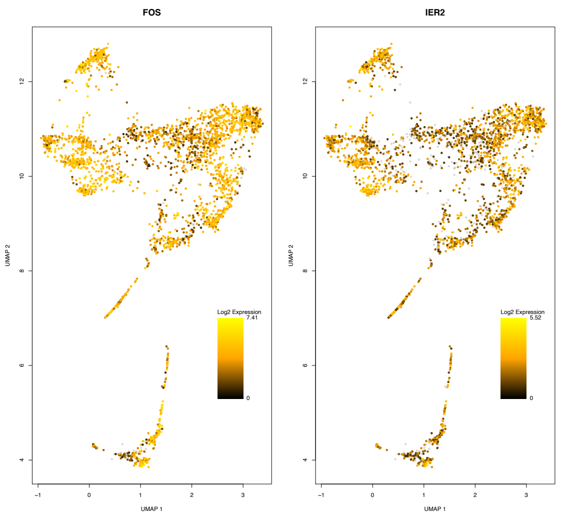

Fig. S5: Gene set enrichment analysis and gene co-expression networks identify key gene drivers of TNFa signaling via NFkB hallmark biologic process. **a**, Fully clustered on/off heatmap visualization of overlap between significantly differentially expressed genes in VSMCs and ECs and leading edge EMT Hallmark genes generated by GSEA. Heatmaps are downsampled and represent 448 cells from each cell type and anatomic location (1792 total cells). A dotplot corresponding to gene expression levels for each cell type in the heatmap is included. Dot size depicts the fraction of cells expressing a gene. Dot color depicts the degree of expression of each gene. **b,d** Gene co-expression networks generated from VSMC Module 31 (b) and EC Module 36 (d) representing the TNFa signaling via NFkB hallmark from GSEA analysis. Genes are separated by anatomic location (red=AC genes, cyan=PA genes), differential expression (darker shade=higher DE, gray=non-significantly DEGs), correlation with other connected genes (green line=positive correlation, orange line=negative correlation) and strength of correlation (connecting line thickness). Significantly DEGs ($q<0.05$) with high connectivity scores (>0.3) are denoted by a box instead of a circle. **c,e** UMAP distribution of boxed genes from (b), (d), respectively. VSMCs=3,674 cells; ECs=2,764 cells.

Figure S6:

Description	Patient 1 (AC)	Patient 1 (PA)	Patient 2 (AC)	Patient 2 (PA)	Patient 3 (AC)	Patient 3 (PA)
Estimated Number of Cells	11,015	3,716	15,960	5,523	12,388	3,379
Mean Reads per Cell	24,791	63,201	15,457	57,130	20,364	80,123
Median Genes per Cell	1,667	1,968	1,204	1,466	1,557	2,213
Number of Reads	273,078,128	234,857,523	246,707,399	315,532,869	252,278,050	270,736,818
Valid Barcodes	98.10%60.30%	97.90%	97.80%42.10%	97.90%	97.70%46.40%	98.00%
Sequencing Saturation	96.60%89.10%	83.40%	96.30%87.40%	81.20%	96.70%89.40%	84.10%
Q30 Bases in Barcode	96.50%95.80%	96.50%	95.60%95.70%	96.20%	96.10%96.30%	96.70%
Q30 Bases in RNA Read		89.40%		87.40%		88.60%
Q30 Bases in Sample Index	96.30%	96.30%	95.10%	95.80%	96.30%	96.30%
Q30 Bases in UMI	93.20%	95.80%	90.20%	95.70%	93.20%	96.20%
	2.60%		3.80%		4.20%	
Reads Mapped to Genome	11.00%	95.80%	14.40%	95.30%	21.70%	95.90%
Reads Mapped Confidently to Genome	79.70%	90.10%	72.00%	90.00%	67.30%	92.80%
Reads Mapped Confidently to Intergenic Regions	75.50%	3.90%	68.30%	3.60%	63.20%	3.90%
Reads Mapped Confidently to Intronic Regions	1.10%	12.20%	1.20%	13.00%	1.70%	20.30%
Reads Mapped Confidently to Exonic Regions		74.00%		73.50%		68.50%
Reads Mapped Confidently to Transcriptome	11,015	70.00%	15,960	69.30%	12,388	64.40%
Reads Mapped Antisense to Gene	93.60%	1.30%	93.70%	1.20%	91.70%	1.50%
	24,791		15,457		20,364	
Estimated Number of Cells	1,667	3,716	1,204	5,523	1,557	3,379
Fraction Reads in Cells	22,745	92.10%	23,696	94.20%	23,699	93.70%
Mean Reads per Cell	5,162	63,201	3,388	57,130	4,565	80,123
Median Genes per Cell		1,968		1,466		2,213
Total Genes Detected	02dat20190515ti	21,665	02dat20190620ti	22,583	01dat20190717ti	22,299
Median UMI Counts per Cell	sCARconDIS	6,016	sCARconDIS	4,805	sCARconDIS	6,994
Name	GRCh38	01dat20190515ti sCARconHEL	GRCh38	01dat20190620ti sCARconHEA	GRCh38	01dat20190717ti sCARconHEA
Description						
Transcriptome		GRCh38		GRCh38		GRCh38
Chemistry	Single Cell 3' v3	Single Cell 3' v3	Single Cell 3' v3	Single Cell 3' v3	Single Cell 3' v3	Single Cell 3' v3
Cell Ranger Version	3.0.2	3.0.2	3.0.2	3.0.2	3.0.2	3.0.2

Description	Patient 1-3 Aggreg
Estimated Number of Cells	51,981
Mean Reads per Cell	15,549
Median Genes per Cell	1,339
Pre-Normalization Number of Reads	1,593,190,787
Post-Normalization Number of Reads	808,276,523
01dat20190515tisCARconN? Fraction of Reads Kept	24.30%
02dat20190515tisCARcon?A Fraction of Reads Kept	56.40%
01dat20190620tisCARconN? Fraction of Reads Kept	26.50%
02dat20190620tisCARcon?A Fraction of Reads Kept	100.00%
01dat20190717tisCARconN? Fraction of Reads Kept	20.40%
02dat20190717tisCARcon?A Fraction of Reads Kept	83.90%
01dat20190515tisCARconN? Pre-Normalization Total Reads per Cell	63,201
02dat20190515tisCARcon?A Pre-Normalization Total Reads per Cell	24,791
01dat20190620tisCARconN? Pre-Normalization Total Reads per Cell	57,130
02dat20190620tisCARcon?A Pre-Normalization Total Reads per Cell	15,457
01dat20190717tisCARconN? Pre-Normalization Total Reads per Cell	80,123
02dat20190717tisCARcon?A Pre-Normalization Total Reads per Cell	20,364
01dat20190515tisCARconN? Pre-Normalization Confidently Mapped Barcoded Reads per Cell	40,071
02dat20190515tisCARcon?A Pre-Normalization Confidently Mapped Barcoded Reads per Cell	17,245
01dat20190620tisCARconN? Pre-Normalization Confidently Mapped Barcoded Reads per Cell	36,682
02dat20190620tisCARcon?A Pre-Normalization Confidently Mapped Barcoded Reads per Cell	9,723
01dat20190717tisCARconN? Pre-Normalization Confidently Mapped Barcoded Reads per Cell	47,615
02dat20190717tisCARcon?A Pre-Normalization Confidently Mapped Barcoded Reads per Cell	11,589
Estimated Number of Cells	51,981
Fraction Reads in Cells	93.10%
Pre-Normalization Mean Reads per Cell	30,649
Post-Normalization Mean Reads per Cell	15,549
Median Genes per Cell	1,339
Median UMI Counts per Cell	3,776
Name	AGGREGATEM APPED- tisCAR6samples
Description	
Transcriptome	GRCh38
Chemistry	Single Cell 3' v3
Cell Ranger Version	3.0.2
Low Post-Normalization Read Depth: Ideal > 50%. There may be large differences in sequencing depth across the input libraries. Application performance is likely to be affected.	20.40%

Fig. S6: Results of 10x Genomics CellRanger Count and Aggr
(A) Results from six runs of CellRanger Count side-by-side. The tissue samples were preprocessed separately and sequenced separately.
(B) Results of one run of CellRanger Aggr on the six samples. The default normalization mode was used (mapped).

Figure S7:

A.

Sample	N.Cells	Cells.MT.lt.10pct	Cells.Gene.gt.200	Cells.Gene.lt.4000	Cells.Kept	MT.lt.10.pct	pct.Genes.gt.200	pct.Genes.lt.4000	pct.kept
**Patient 1 AC**	11015	10331	10876	10967	10273	93.8%	98.7%	99.6%	93.3%
**Patient 1 PA**	3716	3112	3471	3713	3104	83.7%	93.4%	99.9%	83.5%
**Patient 2 AC**	15960	14296	15923	15822	14157	89.6%	99.8%	99.1%	88.7%
**Patient 2 PA**	5523	4931	5420	5512	4896	89.3%	98.1%	99.8%	88.6%
**Patient 3 AC**	12388	10806	12322	12138	10556	87.2%	99.5%	98.0%	85.2%
**Patient 3 PA**	3379	2882	3255	3372	2850	85.3%	96.3%	99.8%	84.3%
**Summary of Filtering**		46358	51267	51524	45836				
**As Percent of Total**		89.2%	98.6%	99.1%	88.2%				

Choice for filtering cutoffs were influenced by Seurat pipeline:
https://satijalab.org/seurat/v3.1/pbmc3k_tutorial.html

Downsample Threshold Cell Set

B.

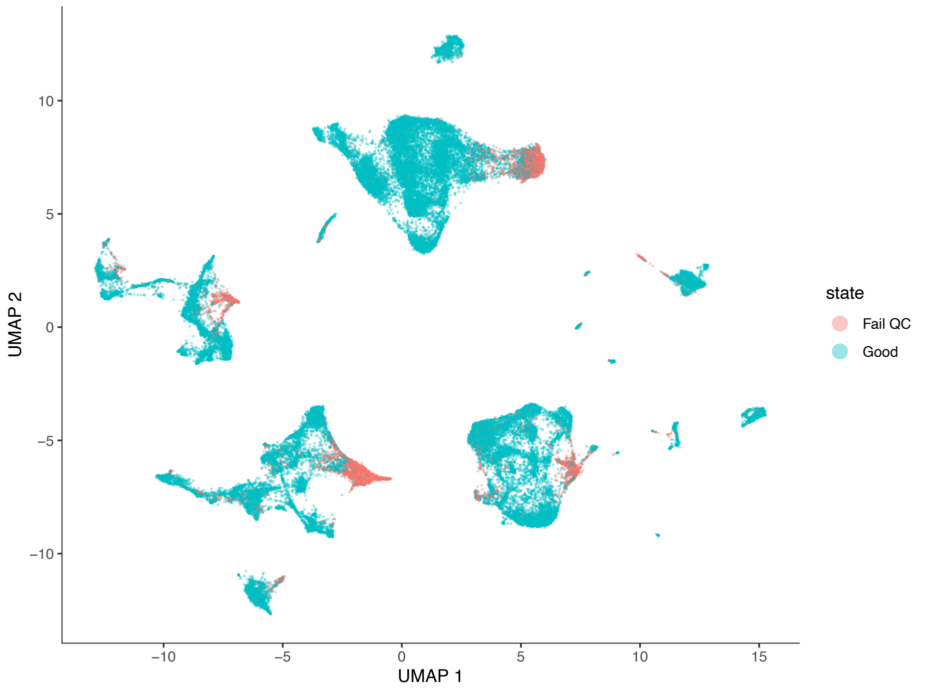

Fig. S7: Summary of Cell Filtering
(A) Summary of cell counts per sample, with the numbers and fractions of cells meeting three criteria. First, the column labeled Cell.MT.lt.10pct is the number of cells (per sample) with total mtRNA less than 10 percent. Second and third, the columns labeled Cell.Gene.gt.200 and Cell.Gene.lt.4000 are the number of cells with greater than 200 genes and less than 4000 genes, respectively. The Cells.Kept column is the number of cells that remain when all three filters are applied. The remaining columns recapitulate the cell count columns as percentages of total cells. For reference, a summation of the cell counts and percentages is shown at the bottom of the columns 2 through 5.
(B) Standard Monocle 3 pipeline cell plot with cells colorized by cells kept (cyan = Good), and cells removed (red = Fail QC).

Figure S8:

A.

Column1	Column2	Column3	Column4	Column5	Column6	Column7	Column8	Column9	Column11	Column12	Column13
Patient ID	ID Number 1	ID Number 1	ID Number 2	ID Number 2	ID Number 3	ID Number 3	Total	0	Remix Action	Temporary Cell/Partition Name	Final Cell/Partition Name
Condition	(PA)	(AC)	(PA)	(AC)	(PA)	(AC)		0		After Remix, Prior to Doublet Removal	After Doublet Removal
1-Unidentified	0	24	0	0	0	0	24	24	Remove	FAILQC2.1.4.11-15	
2-Macrophages	235	1323	162	339	498	537	3094	3094	As Is	MACRO.2	Macrophages
3-Endothelial Cells - (a)	31	184	0	10	3	192	420	420	Recombine 3+6	ENDO.3.6	EC or Endothelial Cells
4-Lymphoid Progenitors	1	21	8	13	4	40	87	87	Remove	FAILQC2.1.4.11-15	
5-VSMCs - Contractile	316	127	0	17	23	6	489	489	Recombine 5+7	VSMC.5.7	VSMC
6-Endothelial Cells - (b)	1273	4	409	268	750	6	2710	2710	Recombine 3+6	ENDO.3.6	EC or Endothelial Cells
7-VSMCs - Synthetic	688	266	601	395	959	828	3737	3737	Recombine 5+7	VSMC.5.7	VSMC
8-Natural Killer Cells	51	116	85	56	26	36	370	370	As Is	NKC.8	NKT Cells or Natural Killer T-Cells
9-Cytotoxic T Lymphocytes	242	698	1155	1343	481	1025	4944	4944	As Is	CYTOT.9	T-Cells or T Lymphocytes
10-B Lymphocytes	2	10	244	216	9	42	523	523	As Is	BLYMPH.10	B-Cells or B Lymphocytes
11-T lymphocytes	2	36	68	44	22	48	220	220	Remove	FAILQC2.1.4.11-15	
12-Plasmacytoid Dendritic Cells	0	6	4	9	3	14	36	36	Remove	FAILQC2.1.4.11-15	
13-B1 Lymphocytes	3	13	74	104	0	4	198	198	Remove	FAILQC2.1.4.11-15	
14-Neutrophils	1	6	37	0	14	0	58	58	Remove	FAILQC2.1.4.11-15	
15-Mast Cells	5	16	3	36	58	72	190	190	Remove	FAILQC2.1.4.11-15	
	2850	2850	2850	2850	2850	2850	17100				

B.

Column1	Column2	Column3	Column4	Column5	Column6	Column7	Column8	Column9	Column10	Column11	Column11	Column12
Patient ID	ID Number 1	ID Number 1	ID Number 2	ID Number 2	ID Number 3	ID Number 3	Total	Mean				
Condition	(PA)	(AC)	(PA)	(AC)	(PA)	(AC)	Cells	UMI		AC/Total	PA/Total	Both/Total
MACRO.2	235	1323	162	339	498	537	3094	7929	MACRO.2	5.5%	13.5%	19.0%
ENDO.3.6	1304	188	409	278	753	198	3130	5915	ENDO.3.6	15.1%	4.1%	19.2%
VSMC.5.7	1004	393	601	412	982	834	4226	5923	VSMC.5.7	15.9%	10.1%	25.9%
NKC.8	51	116	85	56	26	36	370	2522	NKC.8	1.0%	1.3%	2.3%
CYTOT.9	242	698	1155	1343	481	1025	4944	3090	CYTOT.9	11.5%	18.8%	30.4%
BLYMPH.10	2	10	244	216	9	42	523	3414	BLYMPH.10	1.6%	1.6%	3.2%
FAILQC2.1.4.11-15	12	122	194	206	101	178	813	9565		1.9%	3.1%	5.0%
Total Cells	2838	2728	2656	2644	2749	2672	16287			8243	8044	16287

NOTE: FailQC Cells Removed from Totals at Bottom of Each Column

Fig. S8: Summary of Partition Level Filtering and Remixing (Down-Sampled Cell Set)
(A) The 15 putative cell types are shown with a breakdown of the number of cells per patient sample (3 pairs). Totals at the bottom of these columns show 2850 each and is the result of the down-sampling used to balance the number of cells from each patient (1-3) and sample location (AC or PA). Column 11 shows the action that is taken to remove small partitions and to combine very similar cell types, specifically combining the two putative VSMC partitions (a) and (b) and the two putative EC partitions (a) and (b). Column 12 shows the temporary partition names used for tracking cell groups in the doublet analysis, and column 13 is a preview of the cell type assigned names for these partitions after doublet filtering is applied.
(B) Summary of the filtering actions taken in (A), including sample-by-sample cell counts (columns 2-7), total cell counts for cell type (column 8), the mean total UMI count for cells in each partition (column 9). Columns 11-13 show the factions of cells in each cell type associated with each tissue location type, AC and PA. The last row shows the cells that remain from each sample, after the removal of cells designated as FAILQC2.1.4.11-15 column 1. This filtering resulted in the removal of 813 cells from the original 17,100 shown in (A), with 16,287 cells retained.

Figure S9:

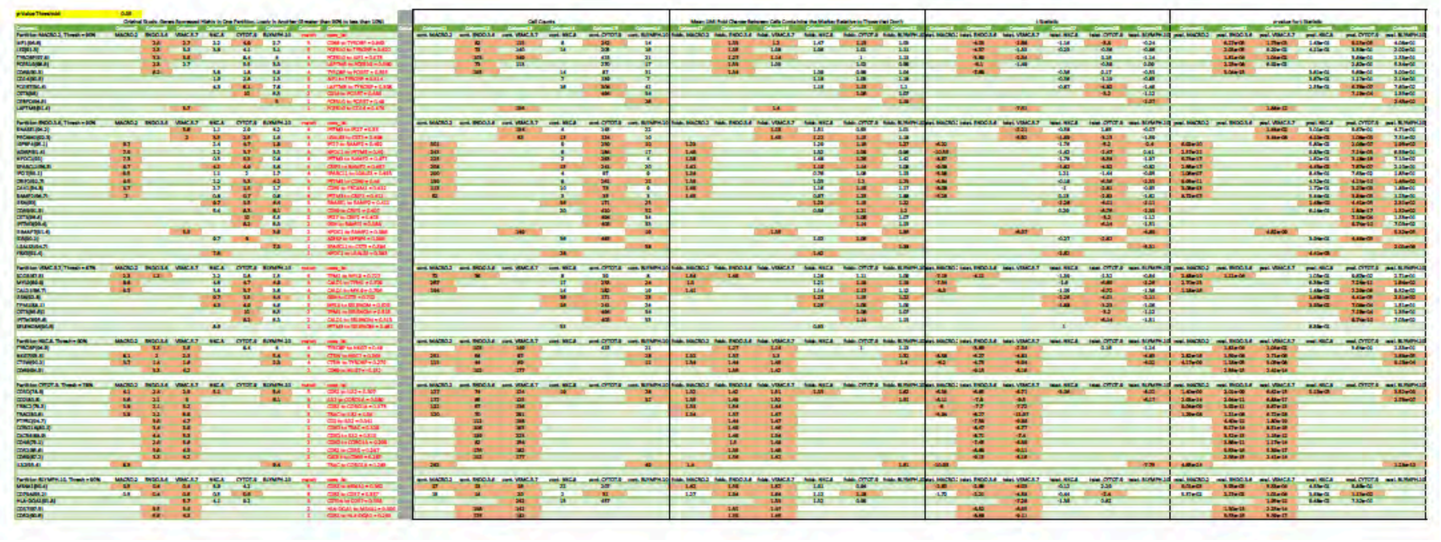

Fig. S9: Five Panel Gene Exclusion Analysis Worksheet

This worksheet shows the 6 remixed cell type partitions, temporarily named, with each partition represented by a select set of genes as shown in the left column of the first panel. These select genes are chosen because they highly represent their associated cells type, typically expressed in 90% or more of the cells (actual percentage shown inside parenthesis), while also being very lowly expressed in the cells of one or more other partitions. Continuing with the first panel, columns 2-7 show the fractional (percentage) of cells represented in the other partitions, but only if the fraction is less than 10%, otherwise they are left blank.The remaining panels and columns of the worksheet show statistics associated with shifts in mean expression, such as the mean expression shift t score and p-value for the fraction of cells containing these markers, compared to cells that do not contain these markers. Orange highlighted cases correspond to those instances were a uncommon marker is associated with cells that have a marked increase in total RNA with a p-value of 0.05 or less. Because these markers are rare, and because thy cells that contain them have a marked increase in total RNA, these are ideal candidates for further doublet / contaminant filtration.

Figure S10:

Summary of doublet cells removed (percentages)					
AC	PA				
29.2	10.6				
ID Number 1	ID Number 2	ID Number 3			
13.8	23.6	22.1			
BLYMPH.10	CYTOT.9	ENDO.3.6	MACRO.2	NKC.8	VSMC.5.7
14.3	31.2	11.7	20.6	11.9	13.1

Mean UMI Cell Counts in Filter Cells and Doublet Cells

	-Dblt Mean UMI	+Dblt Mean UMI	Fold Change
1-Macrophages	7289	10394	1.426
2-Endothelial Cells	5663	7819	1.381
3-VSMCs	5687	7492	1.317
4-Natural Kill Cells	2436	3161	1.298
5-Cytotoxic T Lymphocytes	2935	3429	1.168
6-B Lymphocytes	3220	4569	1.419

B.

Remixed and Doublet Filtered Partition Stats

Patient Condition	ID Number 1 PA	ID Number 1 AC	ID Number 2 PA	ID Number 2 AC	ID Number 3 PA	ID Number 3 AC	Total Cells	Mean UMI
1-Macrophages	204	1119	132	221	421	359	2456	7289
2-Endothelial Cells	1248	132	348	170	720	146	2764	5663
3-VSMCs	970	294	550	269	930	661	3674	5687
4-Natural Kill T-Cells	42	106	78	48	24	28	326	2436
5-T Lymphocytes	165	509	952	886	351	539	3402	2935
6-B Lymphocytes	1	8	229	164	8	38	448	3220

Doublet Stats

Patient Condition	ID Number 1 PA	ID Number 1 AC	ID Number 2 PA	ID Number 2 AC	ID Number 3 PA	ID Number 3 AC	Total Cells	Mean UMI
1-Macrophages	31	204	30	118	77	178	638	10394
2-Endothelial Cells	56	56	61	108	33	52	366	7819
3-VSMCs	34	99	51	143	52	173	552	7492
4-Natural Kill T-Cells	9	10	7	8	2	8	44	3161
5-T Lymphocytes	77	189	203	457	130	486	1542	3429
6-B Lymphocytes	1	2	15	52	1	4	75	4569

2630	2168	2289	1758	2454	1771	13070

Loss of cells = 3217 19.8%

Total Cells = 16287

C.

	PA Cells%	AC Cells%	PA Cells	AC Cells	DownSample
1-Macrophages	10%	30%	757	1699	757
2-Endothelial Cells	31%	8%	2316	448	448
3-VSMCs	33%	21%	2450	1224	1224
4-Natural Kill Cells	2%	3%	144	182	144
5-Cytotoxic T Lymphocy	20%	34%	1468	1934	1468
6-B Lymphocytes	3%	4%	238	210	210

Fig. S10: Prospective Summary and Statistics of Doublet / Contaminant Filtering
(A) The top panel shows the fraction of cells that would be removed from each cell subgroup (tissue location, patient, and putative cell type). The filtering criteria used for these data are that cells must contain at least two rare markers and that those markers have to each be associated with an upshift in mean total RNA with a p-value less than 0.05. The lower panel shows the shifts in mean total RNA for each of the cell types based this filtering, and the last column shows the fold change. The cell type assignments have been renamed but are the same cell sets as shown in the top panel.
(B) The top and bottom panels show a breakdown in cell counts for each cell type for each patient and tissue location. Total remaining cell counts are shown in the last full row. The loss of cells due to this filtering is 3,216, from the original 16,287, resulting in 13,070 cells. The mean UMI counts in the top and bottom panels reflect the shift in mean total RNA in the filtered (upper panel) to doublet cells (lower panel), from which the fold change Is computed in Fig. 13A.
(C) This is the final filtered cell sets, with cell counts and percentages of cells per sample that remain after filtering. The final column, labeled DownSample, is the minimum of the PA Cells and AC Cells columns, and will be used later in constructing cell sets used in the heatmap analyses.

Figure S11:

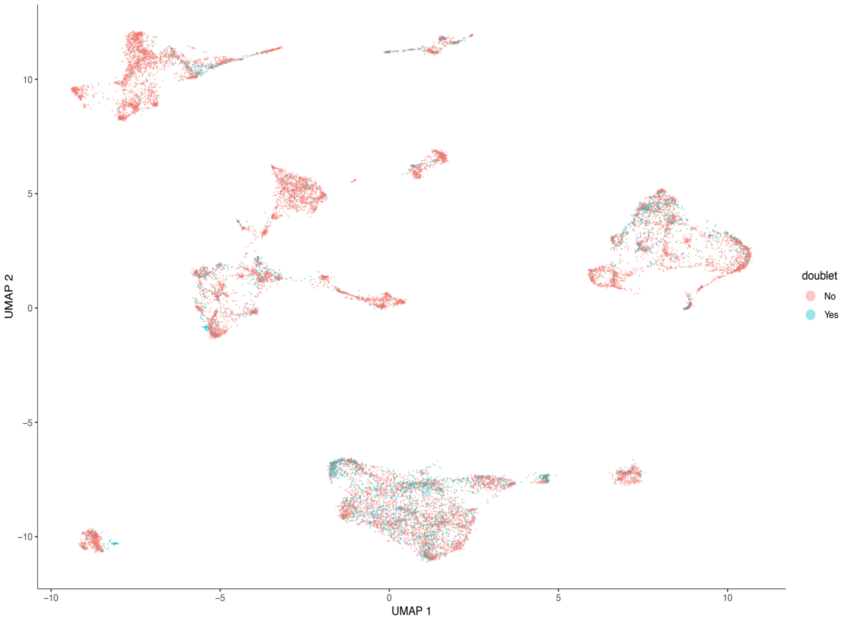

Fig. S11: Plot of Doublet / Contaminant Cells Contrasted with Cells Kept
A colorized cell plot from the Monocle pipeline, colorized by cells kept (doublet = No = red), and cells removed (doublet = Yes = cyan).

Figure S12:

```
## Scrublet case 2, expected rate = 10%
scrub = scr.Scrublet(counts_matrix_down, expected_doublet_rate = .10, n_neighbors = 100)
```

```
doublet_scores, predicted_doublets = scrub.scrub_doublets(min_counts=2,
                                                          min_cells=3,
                                                          min_gene_variability_pctl=85,
                                                          n_prin_comps=30)
```

```
Preprocessing...
Simulating doublets...
Embedding transcriptomes using PCA...
Calculating doublet scores...
Automatically set threshold at doublet score = 0.49
Detected doublet rate = 1.3%
Estimated detectable doublet fraction = 33.1%
Overall doublet rate:
        Expected   = 10.0%
        Estimated  = 3.8%
Elapsed time: 29.9 seconds
```

```
scrub.plot_histogram();
```

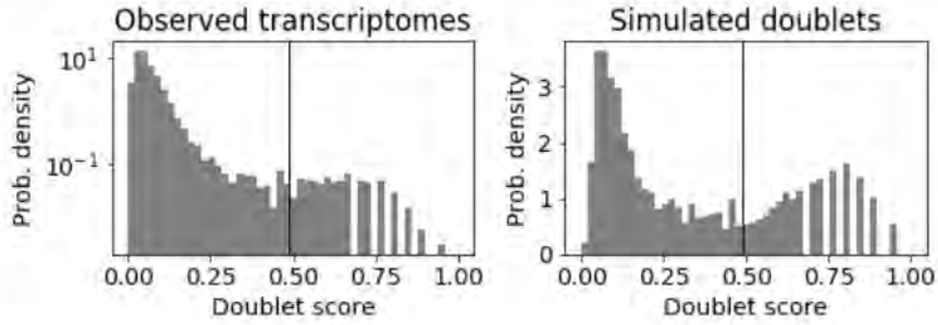


```
scrub.plot_embedding('UMAP', order_points='TRUE');

# scrub.plot_embedding('tSNE', order_points=True);
# scrub.plot_embedding('FA', order_points=True);
```

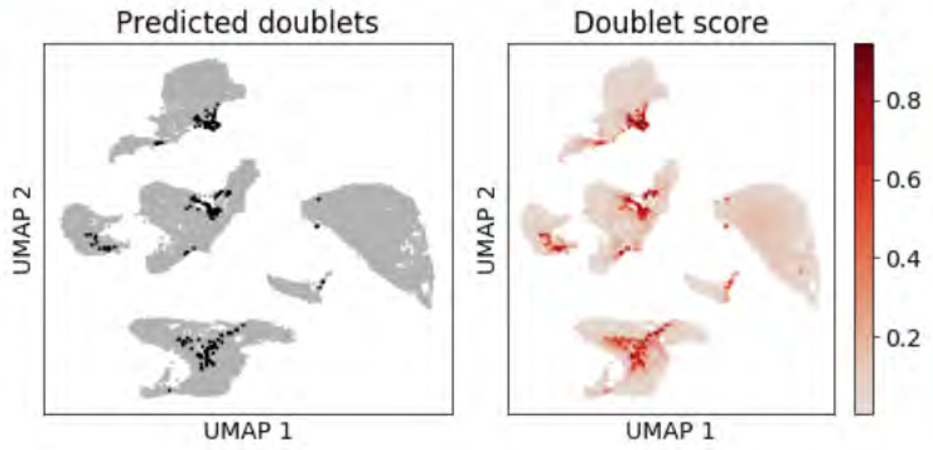

Fig. S12: Scrublet Analysis Results

The Srcublet analysis results are based on an expected doublet rate 0f 10%, roughly in line with the predicted doublet rate in Extended Data Fig. 11). The first pair of plots in this figure are score histograms of the predicted doublets (left plot) and the synthesized doublets (right plot). These distributions are bimodal, with the distribution of the synthesized doublet plot serving as guide for selecting a cutoff for predicted doublets. Note that Scrublet is not able to detect all synthesized doublets. The cell plots at the bottom highlight the scored and predicted doublets. Note that high scoring doublets are spatially intermixed with the cores of the clustered cell group, suggesting that the method of uniquely identifying doublets from synthesized doublets is adversely affected in scenarios of high cellular heterogeneity in the true singlet cell populations.

Figure S13:

A.

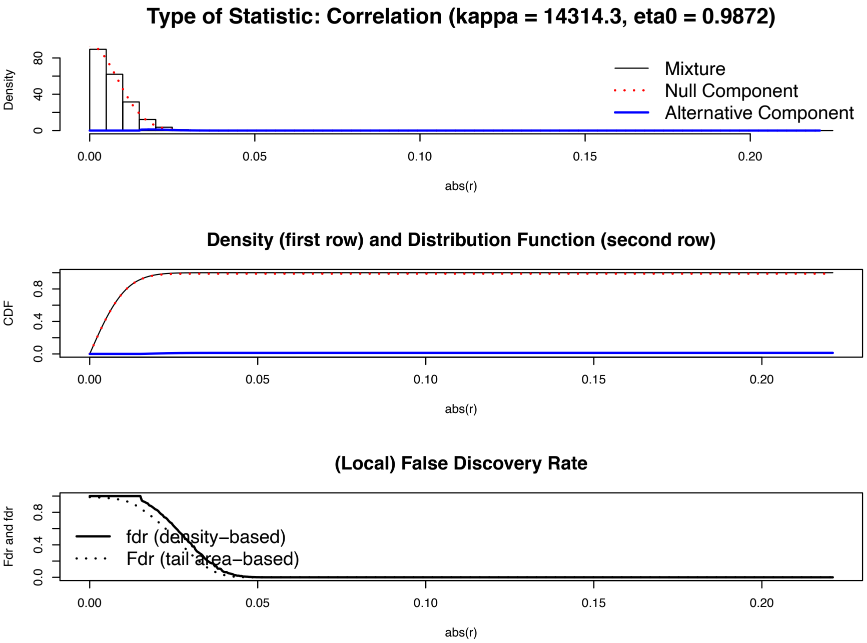

B.

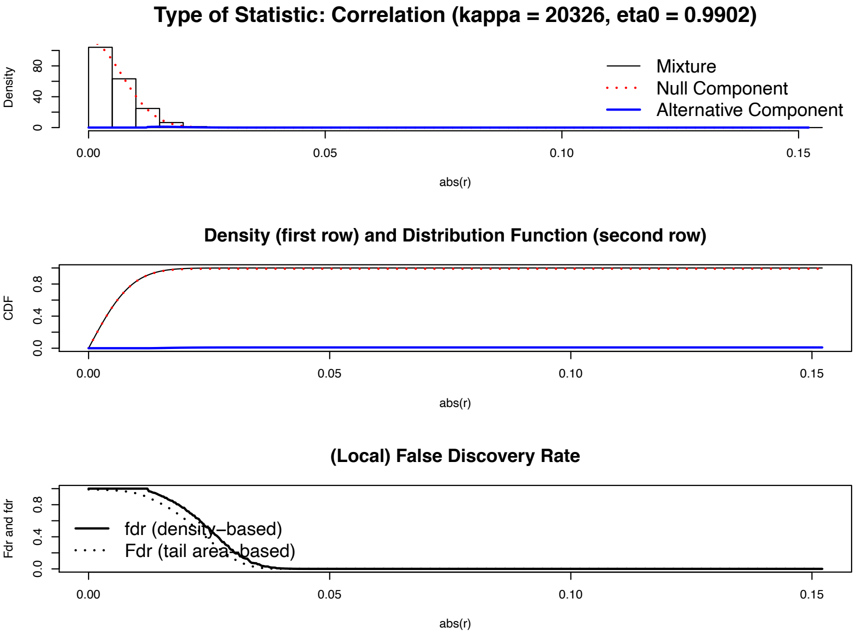

Fig. S13: Results of FDR Analysis On Partial Correlations

(A) Standard output of the fdrtool package used to compute false discovery rates of the partial correlations from the VSMC cell set. The null set is estimated to be 98.72% of all partial correlations. Note that more conservative tail based FDRs are used to guide construction of the VSMC network (top 20,000 best FDR partial correlations).

(B) Similar output as (A), except as run against the partial correlations from the EC cell set. In this case, the null set is estimated to be 99.02% of all EC partial correlations.

Figure S14:

A.

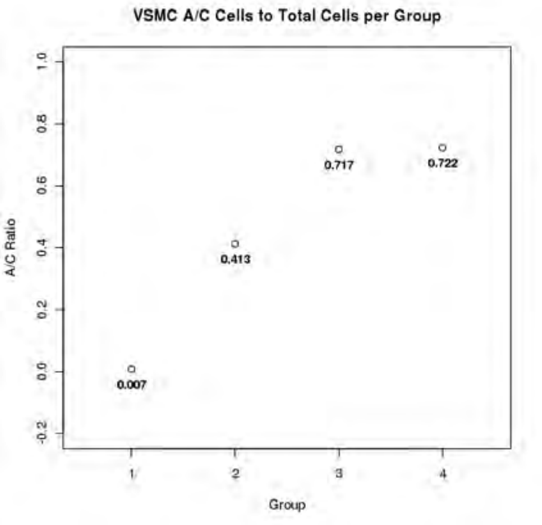

B.

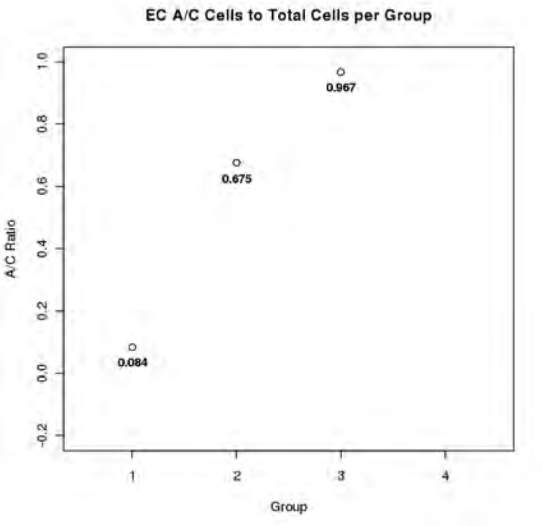

Fig. S14: AC Cell Abundance Plots By Heatmap Group
(A) Plots of the relative AC cell abundance of 4 cell group (clusters) shown in the VSMC heatmap. These groups follow the heatmap group order left to right.
(B) Similar plot to (A), but with 3 cell groups (clusters) shown in the EC heatmap, also grouped according to the heatmap group order left to right.

Patient	Age	Smoker	Diabetes (A1C)	Hypertension	Dyslipidemia	Statin	Symptomatic	AHA Classification
1	82	Yes	Yes (9.3%)	Yes	Yes	Yes	No	Type VII Calcified
2	87	No	Yes (6.4%)	Yes	Yes	Yes	No	Type VII Calcified
3	65	Yes	No (5.5%)	Yes	Yes	Yes	No	Type VII Calcified

Table S1. Patient comorbidities and carotid plaque classification.

Patient ID	ID Number 1	ID Number 1	ID Number 2	ID Number 2	ID Number 3	ID Number 3	Total
Condition	PA	AC	PA	AC	PA	AC	
1-Unidentified	0	24	0	0	0	0	24
2-Macrophages	235	1323	162	339	498	537	3094
3-Endothelial Cells (a)	31	184	0	10	3	192	420
4-Lymphoid Progenitors	1	21	8	13	4	40	87
5-VSMCs (a)	316	127	0	17	23	6	489
6-Endothelial Cells (b)	1273	4	409	268	750	6	2710
7-VSMCs (b)	688	266	601	395	959	828	3737
8-Natural Killer Cells	51	116	85	56	26	36	370
9-T-Lymphocytes (a)	242	698	1155	1343	481	1025	4944
10-B-Lymphocytes	2	10	244	216	9	42	523
11-T-lymphocytes (b)	2	36	68	44	22	48	220
12-Plasmacytoid Dendritic Cells	0	6	4	9	3	14	36
13-B1-Lymphocytes	3	13	74	104	0	4	198
14-Neutrophils	1	6	37	0	14	0	58
15-Mast Cells	5	16	3	36	58	72	190

Table S2. Breakdown of cell types identified by patient and anatomic region. Number of cells listed in each group.
