# Supplementary material for: Decoding the transcriptome of calcified atherosclerotic plaque at single-cell resolution
Source: Commun Biol. 2022 Oct 12;5:1084. doi: 10.1038/s42003-022-04056-7 (PMC9556750; doi:10.1038/s42003-022-04056-7)
Supplement: Supplementary file 2 — Description of Additional Supplementary Files [file 42003_2022_4056_MOESM2_ESM.pdf]

Description of Additional Supplementary Files

File name: Supplementary Data 1
Description: Full differential gene expression results for macrophages.

File name: Supplementary Data 2
Description: Full differential gene expression results for T-cells.

File name: Supplementary Data 3
Description: Full differential gene expression results for B-cells.

File name: Supplementary Data 4
Description: Full differential gene expression results for NKT cells.

File name: Supplementary Data 5
Description: Full differential gene expression results for VSMCs.

File name: Supplementary Data 6
Description: Full differential gene expression results for ECs.

File name: Supplementary Data 7
Description: Gene network modules generated in VSMC datasets

File name: Supplementary Data 8
Description: Gene network modules generated in EC datasets

File name: Supplementary Data 9
Description: Sub-population differential expression and regression analysis for VSMC heatmap.

File name: Supplementary Data 10
Description: Sub-population differential expression and regression analysis for EC heatmap.

File name: Supplementary Data 11
Description: Module level enrichment analysis for EC modules.

File name: Supplementary Data 12
Description: Individual gene level changes for EC modules significantly enriched with differentially expressed genes.

File name: Supplementary Data 13
Description: Module level enrichment analysis for VSMC modules.

File name: Supplementary Data 14

Description: Individual gene level changes for VSMC modules significantly enriched with differentially expressed genes.

File name: Supplementary Data 15
Description: Dictionary of the relevant network attributes.
