# Supplementary material for: Decoding the transcriptome of calcified atherosclerotic plaque at single-cell resolution
Source: Commun Biol. 2022 Oct 12;5:1084. doi: 10.1038/s42003-022-04056-7 (PMC9556750; doi:10.1038/s42003-022-04056-7)
Supplement: Supplementary file 3 — Supplementary Data 1 [file 42003_2022_4056_MOESM3_ESM.pdf]

Full differential gene expression results for macrophages.

gene_short_name	estimate	std_err	test_val	p_value	normalized_effect	model_component	q_value
FABP4	-7.853728	7.1228094	-1.1026	0.27	-9.726483553	count	1
MT1G	-6.0358186	5.3858463	-1.1207	0.263	-6.925075976	count	1
CCL7	-16.7579479	579.0145799	-0.0289	0.9769	-6.868106239	count	1
MT1H	-17.6708884	1377.056097	-0.0128	0.9898	-6.053447143	count	1
FN1	-4.182696	0.9226583	-4.5333	6.09E-06	-5.836693239	count	0.14450352
APOC1	-4.024983	0.6292108	-6.3969	1.89E-10	-5.715760418	count	4.55E-06
SLAMF9	-4.6222961	1.152633	-4.0102	6.25E-05	-5.065717032	count	1
JAKMIP2	-17.278782	473.843205	-0.0365	0.971	-4.238259241	count	1
FABP5	-2.901351	0.2718002	-10.6746	4.95E-26	-4.175069457	count	1.20E-21
SPINK1	-17.304822	1476.497968	-0.0117	0.991	-4.033058447	count	1
MMP7	-17.223127	2177.085365	-0.0079	0.994	-3.866270316	count	1
APOE	-2.665305	0.2952539	-9.0272	3.47E-19	-3.836529057	count	8.41E-15
CHI3L1	-17.337018	1283.111367	-0.0135	0.989	-3.61589264	count	1
SDS	-2.5194276	0.2993028	-8.4177	6.44E-17	-3.437768891	count	1.56E-12
GPNMB	-2.4480897	0.2232244	-10.9669	2.35E-27	-3.43735946	count	5.70E-23
C15orf48	-2.354126	0.2362872	-9.963	6.00E-23	-3.359733272	count	1.46E-18
MMP12	-17.037736	1556.760097	-0.0109	0.991	-3.205020923	count	1
CST6	-2.714291	0.8178351	-3.3189	0.000917	-3.092241901	count	1
SPP1	-2.119065	0.2607386	-8.1272	6.89E-16	-3.05517052	count	1.67E-11
ITGB8	-2.879657	0.8966875	-3.2114	0.00134	-2.869865874	count	1
ITLN1	-2.975454	1.8882932	-1.5757	0.115	-2.612679614	count	1
PPARG	-1.940231	0.3999775	-4.8509	1.31E-06	-2.550891807	count	0.0311911
OTOA	-2.3736584	0.3836663	-6.1868	7.17E-10	-2.505438762	count	1.72E-05
RAB42	-2.1979653	0.5379194	-4.086	4.53E-05	-2.463514849	count	1
NT5E	-17.775165	1208.754147	-0.0147	0.988	-2.45108539	count	1
ADAMDEC1	-2.1004575	0.3487197	-6.0233	1.97E-09	-2.418982449	count	4.73E-05
LPL	-2.3980836	0.8340443	-2.8752	0.00407	-2.410360467	count	1
PCOLCE2	-2.156078	0.835055	-2.582	0.00988	-2.384582188	count	1
LINC01705	-17.512754	1298.906837	-0.0135	0.989	-2.353553084	count	1
PHLDA1	-1.6462835	0.1789086	-9.2018	7.31E-20	-2.337190824	count	1.77E-15
CTSL	-1.6091682	0.1261635	-12.7546	4.02E-36	-2.312753575	count	9.76E-32
MT1X	-1.6161869	0.3940643	-4.1013	4.24E-05	-2.295441381	count	0.9993256
CXCL5	-1.759827	0.8557194	-2.0565	0.0398	-2.236971867	count	1
CSTB	-1.532215	0.1168619	-13.1113	5.22E-38	-2.207815419	count	1.27E-33
MIR210HG	-2.425064	1.044312	-2.3222	0.0203	-2.133934736	count	1
SDC2	-1.5357132	0.2350775	-6.5328	7.82E-11	-2.133776294	count	1.88E-06
TFPI	-1.671809	0.528581	-3.1628	0.00158	-2.122979266	count	1
NRIP3	-1.7205729	0.4538373	-3.7912	0.000154	-2.095764976	count	1
AC022092.1	-16.67632	824.0777138	-0.0202	0.984	-2.076270616	count	1
CD109	-1.571515	0.305002	-5.1525	2.78E-07	-2.070141728	count	0.006635304
FBP1	-1.4324131	0.1615803	-8.865	1.44E-18	-2.047087521	count	3.49E-14
LY6K	-16.539338	703.228147	-0.0235	0.981	-2.045369828	count	1
MATK	-2.236576	0.9355289	-2.3907	0.0169	-2.03645617	count	1
GSDME	-1.9767523	0.7259346	-2.723	0.00651	-2.033543787	count	1

SCD	-1.6483733	0.3961881	-4.1606	3.28E-05	-1.998529314	count	0.77408
SLC16A10	-1.449779	0.2241593	-6.4676	1.20E-10	-1.953399075	count	2.89E-06
ACP5	-1.386632	0.1680482	-8.2514	2.52E-16	-1.951021408	count	6.10E-12
ADSSL1	-2.007947	0.8483685	-2.3668	0.018	-1.90214026	count	1
TM4SF19	-2.284186	1.466638	-1.5574	0.119	-1.881787561	count	1
HS3ST1	-1.4221524	0.3601901	-3.9483	8.09E-05	-1.876560557	count	1
ENO2	-2.305484	1.5454954	-1.4917	0.136	-1.860555443	count	1
GOLGA7B	-17.072333	927.408354	-0.0184	0.985	-1.84462627	count	1
ANKRD29	-16.530588	812.1649817	-0.0204	0.984	-1.844626167	count	1
ANGPTL4	-1.538627	0.6087138	-2.5277	0.0115	-1.813084555	count	1
ST14	-1.4059535	0.307697	-4.5693	5.14E-06	-1.804720946	count	0.12201846
IGLC2	-1.8339188	0.2713414	-6.7587	1.73E-11	-1.796278529	count	4.17E-07
LINC01857	-2.5548807	1.0636969	-2.4019	0.0164	-1.786873548	count	1
MMP9	-1.2771939	0.4508488	-2.8329	0.00465	-1.776202945	count	1
MMP19	-1.2683375	0.2280245	-5.5623	2.95E-08	-1.753839381	count	0.000706496
SPOCD1	-16.964828	1089.96318	-0.0156	0.988	-1.73270474	count	1
PLIN2	-1.2021334	0.0963508	-12.4766	1.11E-34	-1.727069231	count	2.70E-30
ABCG1	-1.3563006	0.2345934	-5.7815	8.34E-09	-1.718116569	count	0.000200077
SLC39A8	-1.2654986	0.3213341	-3.9383	8.44E-05	-1.706575851	count	1
OLR1	-1.2096251	0.1179055	-10.2593	3.29E-24	-1.70565781	count	7.98E-20
CLLU1OS	-16.926011	969.5722077	-0.0175	0.986	-1.693380812	count	1
EFNA5	-16.92555	1978.134449	-0.0086	0.993	-1.693380811	count	1
TGM2	-1.7305661	0.7047839	-2.4555	0.0141	-1.68955912	count	1
ABCA1	-1.2046475	0.1528141	-7.8831	4.76E-15	-1.680926397	count	1.15E-10
AGRP	-1.717485	1.047495	-1.6396	0.101	-1.653708308	count	1
FHAD1	-1.7009516	0.6069456	-2.8025	0.00511	-1.633025045	count	1
KLHDC8B	-1.4082636	0.3456082	-4.0747	4.75E-05	-1.627591066	count	1
LINC01943	-1.435282	0.5224185	-2.7474	0.00605	-1.599156144	count	1
NCS1	-1.961491	0.7801747	-2.5142	0.012	-1.589687811	count	1
HSD3B7	-1.2676495	0.2986899	-4.244	2.28E-05	-1.584567328	count	0.5388096
ZFP62	-2.016306	0.8722942	-2.3115	0.0209	-1.581992538	count	1
C1orf21	-2.7006403	0.791083	-3.4139	0.000651	-1.574156486	count	1
ALDH1A2	-16.799531	1054.541453	-0.0159	0.987	-1.568538693	count	1
TIMP1	-1.0881445	0.1294858	-8.4036	7.23E-17	-1.568362846	count	1.75E-12
CSF1	-2.019728	1.0158972	-1.9881	0.0469	-1.565736102	count	1
SLC2A5	-1.228682	0.3656546	-3.3602	0.000791	-1.560425854	count	1
KIFC3	-1.3638477	0.3533685	-3.8596	0.000117	-1.546955999	count	1
FABP3	-1.4670846	0.7048435	-2.0814	0.0375	-1.542797065	count	1
CTSD	-1.0708559	0.086337	-12.4032	2.62E-34	-1.542075336	count	6.36E-30
MME	-1.66332	1.078463	-1.5423	0.123	-1.537846362	count	1
SERPINE1	-1.5977881	0.6015071	-2.6563	0.00795	-1.532807422	count	1
AC109826.1	-1.840701	0.4990994	-3.688	0.000231	-1.52806377	count	1
ERO1A	-1.0873079	0.1592828	-6.8263	1.09E-11	-1.526614929	count	2.63E-07
TMEM45A	-2.4855197	1.3745415	-1.8083	0.0707	-1.523414383	count	1
HIF1A-AS2	-1.324646	0.5038858	-2.6289	0.00862	-1.522287989	count	1

SLC6A8	-1.4933862	0.5774093	-2.5864	0.00976	-1.51897192	count	1
RARRES1	-1.861865	0.4333083	-4.2969	1.80E-05	-1.507589878	count	0.42579
RALA	-1.0615467	0.1108231	-9.5788	2.31E-21	-1.502950545	count	5.60E-17
AC004988.1	-16.916007	1059.996739	-0.016	0.987	-1.478873802	count	1
AC004130.1	-16.7831291	860.5236575	-0.0195	0.984	-1.478873416	count	1
SH3D21	-2.2358079	1.2248551	-1.8254	0.0681	-1.477760256	count	1
RAI14	-1.347254	0.60913	-2.2118	0.0271	-1.474047667	count	1
AK4	-1.6428907	0.701752	-2.3411	0.0193	-1.467843604	count	1
NR1H3	-1.3525284	0.4060548	-3.3309	0.000878	-1.449239245	count	1
AP001453.2	-2.021311	1.2369392	-1.6341	0.102	-1.447604081	count	1
SPHK1	-1.1355532	0.2912562	-3.8988	9.93E-05	-1.445250566	count	1
LINC01094	-1.1003868	0.1827093	-6.0226	1.97E-09	-1.440509866	count	4.73E-05
DUSP4	-1.1962393	0.3238198	-3.6942	0.000225	-1.438846978	count	1
AL138724.1	-17.094318	2021.187871	-0.0085	0.993	-1.431860382	count	1
ARL4C	-1.0043344	0.1033559	-9.7172	6.29E-22	-1.420111264	count	1.52E-17
IL4I1	-1.0508105	0.2460233	-4.2712	2.02E-05	-1.420037366	count	0.4777098
CXXC5	-1.030118	0.2077735	-4.9579	7.61E-07	-1.409203548	count	0.018133869
EDNRB	-1.3711431	0.429361	-3.1935	0.00142	-1.392265576	count	1
PLA2G7	-0.9909204	0.1678656	-5.9031	4.06E-09	-1.384614674	count	9.75E-05
GZMA	-18.0051722	1280.00079	-0.0141	0.989	-1.383264095	count	1
ASPHD1	-16.829472	1760.04905	-0.0096	0.992	-1.383264016	count	1
GCHFR	-0.9824161	0.1946862	-5.0462	4.84E-07	-1.378433619	count	0.01154098
CADM1	-2.0137475	0.5996036	-3.3585	0.000796	-1.376407662	count	1
SCIN	-2.1621509	0.8501532	-2.5432	0.011	-1.376245146	count	1
LHFPL2	-1.0168171	0.1805995	-5.6302	2.00E-08	-1.369119107	count	0.00047924
LXN	-1.105412	0.2945794	-3.7525	0.000179	-1.36033067	count	1
GK	-0.9591309	0.095601	-10.0326	3.05E-23	-1.354211048	count	7.40E-19
IL7R	-1.4445355	0.5139661	-2.8106	0.00498	-1.336230165	count	1
DNAH11	-16.544192	1663.931905	-0.0099	0.992	-1.332973615	count	1
B4GALT2	-17.3689868	1036.227709	-0.0168	0.987	-1.332973348	count	1
HAMP	-1.1266952	0.4951172	-2.2756	0.023	-1.330280544	count	1
TACSTD2	-2.8580353	1.6052903	-1.7804	0.0751	-1.329799358	count	1
CXCL10	-0.9668391	0.6342427	-1.5244	0.1275	-1.32498441	count	1
MT1M	-1.3997877	0.6859397	-2.0407	0.0414	-1.324320227	count	1
SMPDL3A	-1.0007018	0.2109923	-4.7428	2.23E-06	-1.319758783	count	0.05303386
CYSTM1	-0.9281053	0.1165121	-7.9657	2.49E-15	-1.308686022	count	6.02E-11
TKTL1	-1.699127	0.9763084	-1.7404	0.0819	-1.304582265	count	1
PROCR	-1.4996108	0.5197937	-2.885	0.00395	-1.302483119	count	1
VWA5A	-1.020542	0.3662675	-2.7863	0.00537	-1.301085324	count	1
SDSL	-0.9876354	0.1848225	-5.3437	9.95E-08	-1.29091291	count	0.002378448
MREG	-17.483181	2539.266946	-0.0069	0.995	-1.280866051	count	1
SIT1	-16.484338	2034.413778	-0.0081	0.994	-1.28086596	count	1
MITF	-0.9491907	0.2289925	-4.1451	3.51E-05	-1.277352402	count	0.8280441
MT1E	-1.1372166	0.3261537	-3.4868	0.000498	-1.270783822	count	1
CDCP1	-1.0492273	0.3332535	-3.1484	0.00166	-1.269786043	count	1

PLOD2	-2.001488	1.1120188	-1.7999	0.072	-1.269734898	count	1
KCNMA1	-1.008081	0.1878715	-5.3658	8.81E-08	-1.269497648	count	0.002106471
L3MBTL4-AS1	-1.5917621	0.6168447	-2.5805	0.00992	-1.266966974	count	1
CHCHD6	-1.3889267	0.5235452	-2.6529	0.00803	-1.265405685	count	1
GLA	-0.9214785	0.1246749	-7.3911	1.99E-13	-1.264408333	count	4.81E-09
CD81	-0.8797939	0.0683205	-12.8775	9.12E-37	-1.262964574	count	2.22E-32
SMOX	-1.2463681	0.440667	-2.8284	0.00472	-1.254618363	count	1
CCL20	-0.9079987	0.3131925	-2.8992	0.00377	-1.253348315	count	1
SYNJ2	-1.8128474	0.6402446	-2.8315	0.00467	-1.250971822	count	1
TREM2	-0.8883865	0.1649103	-5.3871	7.84E-08	-1.249688592	count	0.001874701
IGKC	-0.9173176	0.1819427	-5.0418	4.95E-07	-1.249466207	count	0.011802285
MAPK13	-0.9904833	0.386939	-2.5598	0.0105	-1.247588221	count	1
NPC1	-1.0909742	0.391379	-2.7875	0.00535	-1.24238785	count	1
TMEM158	-0.9172697	0.2772221	-3.3088	0.000951	-1.228688605	count	1
HACD1	-17.4486879	1151.250769	-0.0152	0.988	-1.226805978	count	1
CRTAC1	-17.4459171	2958.116184	-0.0059	0.995	-1.226805978	count	1
CDK14	-1.161713	0.4892491	-2.3745	0.0177	-1.225370238	count	1
DSE	-0.8756747	0.1121356	-7.8091	8.47E-15	-1.222130596	count	2.05E-10
CELSR1	-1.715199	1.729978	-0.9915	0.322	-1.209960335	count	1
CENPM	-1.828478	2.1197134	-0.8626	0.388	-1.208091232	count	1
CD9	-0.844591	0.0938596	-8.9985	4.47E-19	-1.20744478	count	1.08E-14
CXCL9	-2.200688	1.18052	-1.8642	0.0624	-1.204664269	count	1
NPL	-0.8507718	0.1164566	-7.3055	3.72E-13	-1.199849608	count	8.98E-09
KCNN4	-1.0171333	0.3626628	-2.8046	0.00508	-1.198899982	count	1
UCK2	-1.3830664	0.5324153	-2.5977	0.00944	-1.189262051	count	1
PCBD1	-0.8578062	0.1336486	-6.4184	1.65E-10	-1.188162963	count	3.97E-06
HIC1	-1.059388	0.3576203	-2.9623	0.00308	-1.186672628	count	1
RUNX3	-0.9912869	0.2797335	-3.5437	0.000402	-1.18627391	count	1
SLAMF8	-0.8883245	0.1467885	-6.0517	1.65E-09	-1.179604006	count	3.96E-05
RAB13	-0.8310595	0.1218035	-6.823	1.12E-11	-1.171459067	count	2.70E-07
ACOT4	-16.625003	1023.643639	-0.0162	0.987	-1.170641281	count	1
MYO10	-16.8595571	868.4515805	-0.0194	0.985	-1.170640983	count	1
TCTEX1D1	-16.7227473	767.1447327	-0.0218	0.983	-1.17064097	count	1
LRRC6	-1.861024	1.6694203	-1.1148	0.265	-1.166399953	count	1
LGALS3	-0.7974459	0.0837459	-9.5222	3.91E-21	-1.148275984	count	9.48E-17
VKORC1	-0.8102714	0.0941713	-8.6042	1.35E-17	-1.144202719	count	3.27E-13
P2RY11	-1.2257123	0.5099893	-2.4034	0.0163	-1.138092045	count	1
SATB1	-0.8961655	0.2435278	-3.6799	0.000238	-1.137225021	count	1
INO80B	-1.599936	0.774701	-2.0652	0.039	-1.136428035	count	1
FGGY	-1.3180357	0.5954587	-2.2135	0.027	-1.134219073	count	1
MGP	-0.8197388	0.2209141	-3.7107	0.000211	-1.131093363	count	1
LPIN1	-1.0279546	0.3366062	-3.0539	0.00228	-1.128896559	count	1
TCEAL9	-0.8591555	0.2087258	-4.1162	3.98E-05	-1.127395175	count	0.9385238
RGS1	-0.8024431	0.0972456	-8.2517	2.52E-16	-1.125573494	count	6.10E-12
FAM89A	-0.8695051	0.2645652	-3.2865	0.00103	-1.120099576	count	1

MGLL	-0.7930192	0.202118	-3.9235	8.97E-05	-1.118183594	count	1
SHB	-2.2847239	0.653327	-3.4971	0.000479	-1.113091291	count	1
CACNA1G	-17.274853	2074.937854	-0.0083	0.993	-1.112200952	count	1
FLRT2	-17.274155	1806.521748	-0.0096	0.992	-1.112200952	count	1
SHISAL2A	-17.273719	1603.070718	-0.0108	0.991	-1.112200952	count	1
PTPN13	-16.27785	2589.026385	-0.0063	0.995	-1.112200862	count	1
STX1A	-16.5683536	1979.724292	-0.0084	0.993	-1.112200588	count	1
NMB	-0.9179373	0.3847115	-2.386	0.0171	-1.106869573	count	1
IGHG3	-1.520553	0.8799274	-1.728	0.0841	-1.099203538	count	1
CTSB	-0.7624065	0.057848	-13.1795	2.25E-38	-1.09743385	count	5.47E-34
PRDM1	-0.7936339	0.1417148	-5.6002	2.38E-08	-1.096795382	count	0.000570177
ADAM9	-0.802952	0.1409372	-5.6972	1.36E-08	-1.096142245	count	0.000326142
MRO	-1.4323275	0.6305579	-2.2715	0.0232	-1.093626346	count	1
METTL1	-0.986038	0.4484316	-2.1989	0.028	-1.09181094	count	1
CD3E	-2.3834704	1.7727651	-1.3445	0.179	-1.090826839	count	1
SLC8B1	-0.8712614	0.2481989	-3.5103	0.000456	-1.090700957	count	1
CD82	-0.8000382	0.2384843	-3.3547	0.000807	-1.078956806	count	1
CRADD	-0.9059879	0.3111727	-2.9115	0.00363	-1.076074076	count	1
NUFIP1	-0.9668001	0.285575	-3.3855	0.000722	-1.072765504	count	1
LINC01588	-1.2819389	0.6826629	-1.8779	0.0605	-1.070494788	count	1
MINPP1	-1.3864067	0.5304488	-2.6136	0.00901	-1.06877437	count	1
PIF1	-1.1716922	0.9074694	-1.2912	0.197	-1.068056061	count	1
ARHGAP22	-0.9018207	0.2537268	-3.5543	0.000386	-1.062864235	count	1
TUBA1C	-0.7432159	0.0847839	-8.766	3.39E-18	-1.060933339	count	8.21E-14
LITAF	-0.7382417	0.0605779	-12.1866	3.28E-33	-1.057036261	count	7.97E-29
SNAPC1	-0.7554713	0.1624734	-4.6498	3.50E-06	-1.056443798	count	0.08316
HIST1H1B	-17.360758	1493.521027	-0.0116	0.991	-1.051292783	count	1
IL36RN	-17.196466	3241.051387	-0.0053	0.996	-1.051292775	count	1
TMEM145	-17.195329	2522.089442	-0.0068	0.995	-1.051292775	count	1
GAL	-17.195259	2287.746891	-0.0075	0.994	-1.051292775	count	1
AGAP2-AS1	-17.193122	1491.470793	-0.0115	0.991	-1.051292775	count	1
AC092484.1	-16.7072473	1605.911652	-0.0104	0.992	-1.051292667	count	1
OASL	-0.7854805	0.2787394	-2.818	0.00487	-1.049830024	count	1
HES4	-0.8245017	0.2617658	-3.1498	0.00165	-1.049686202	count	1
HMGB3	-0.8184147	0.2760004	-2.9653	0.00305	-1.047308874	count	1
GPR183	-0.7279719	0.0664538	-10.9546	2.68E-27	-1.045371818	count	6.51E-23
COL6A1	-1.3438851	0.8851295	-1.5183	0.129	-1.045150125	count	1
SEPHS2	-0.7555485	0.1299593	-5.8137	6.90E-09	-1.039810174	count	0.000165572
RASGRP3	-0.8959261	0.3199604	-2.8001	0.00515	-1.037230561	count	1
MIR155HG	-0.9167366	0.339947	-2.6967	0.00705	-1.035969975	count	1
UGCG	-0.759231	0.1337115	-5.6781	1.52E-08	-1.031095407	count	0.00036442
LAT	-0.9760307	0.5481256	-1.7807	0.0751	-1.024642289	count	1
WDR54	-0.8707796	0.3878811	-2.245	0.0249	-1.020105213	count	1
TMEM106C	-0.897037	0.4048071	-2.216	0.0268	-1.018783734	count	1
FAM20C	-0.7509287	0.2793043	-2.6886	0.00722	-1.017505181	count	1

FLVCR2	-0.9317996	0.3188277	-2.9226	0.0035	-1.015011885	count	1
DEFB1	-1.476104	0.9734934	-1.5163	0.13	-1.012426722	count	1
MSANTD3	-0.9331419	0.3917041	-2.3823	0.0173	-1.007915707	count	1
CSKMT	-0.9122844	0.4214025	-2.1649	0.0305	-1.00748972	count	1
FBXW8	-1.5804022	0.8256706	-1.9141	0.0557	-1.007059081	count	1
IARS	-1.0764441	0.3931108	-2.7383	0.00622	-1.006189455	count	1
P4HA2	-0.8422133	0.4708833	-1.7886	0.0738	-1.00366855	count	1
CREBL2	-0.766205	0.1693224	-4.5251	6.32E-06	-1.002534844	count	0.14992936
MAOA	-2.1366741	1.1261005	-1.8974	0.0579	-1.001517164	count	1
DNASE2	-0.7280688	0.132335	-5.5017	4.15E-08	-1.000565604	count	0.000993303
CALR	-0.6939774	0.0573437	-12.1021	8.69E-33	-0.99438649	count	2.11E-28
GAL3ST4	-0.857906	0.3044136	-2.8182	0.00487	-0.994201948	count	1
HES1	-0.7625194	0.2231578	-3.417	0.000644	-0.994086425	count	1
LIPA	-0.7020688	0.1150871	-6.1003	1.23E-09	-0.992166523	count	2.96E-05
NAV1	-0.8982149	0.4036689	-2.2251	0.0262	-0.989792101	count	1
IBSP	-17.108824	2749.882549	-0.0062	0.995	-0.98769989	count	1
PNCK	-16.821029	1254.276469	-0.0134	0.989	-0.987699872	count	1
PRR22	-16.746922	2789.731941	-0.006	0.995	-0.987699867	count	1
PAQR5	-16.745366	1214.810503	-0.0138	0.989	-0.987699867	count	1
ST18	-17.328618	1232.750567	-0.0141	0.989	-0.987699614	count	1
SIGLEC10	-0.8397394	0.1676396	-5.0092	5.85E-07	-0.986416455	count	0.01394406
CD63	-0.6821981	0.0422388	-16.151	7.68E-56	-0.982432332	count	1.87E-51
GLUL	-0.6821164	0.0556985	-12.2466	1.64E-33	-0.980328255	count	3.98E-29
CENPN	-1.0925444	0.5635552	-1.9387	0.0527	-0.978809989	count	1
AC078883.3	-1.571026	1.291903	-1.2161	0.224	-0.976090765	count	1
PPP1R14B	-0.6823748	0.0811017	-8.4138	6.65E-17	-0.97406006	count	1.61E-12
VASH1	-0.931179	0.3131311	-2.9738	0.00297	-0.967718047	count	1
VEGFB	-0.6818426	0.0983692	-6.9315	5.31E-12	-0.963225459	count	1.28E-07
HENMT1	-0.9903238	0.4646669	-2.1313	0.0332	-0.962158079	count	1
ABHD12	-0.7254523	0.1626618	-4.4599	8.57E-06	-0.960567732	count	0.20321184
MARCO	-0.6708014	0.1369583	-4.8979	1.03E-06	-0.959242692	count	0.02453151
OLFML2B	-0.7118996	0.1485857	-4.7912	1.76E-06	-0.956430246	count	0.04187568
RRP1	-0.7811031	0.293596	-2.6605	0.00785	-0.953108111	count	1
TDP1	-0.9522872	0.4928323	-1.9323	0.0534	-0.951031567	count	1
CHML	-0.7237003	0.1901472	-3.806	0.000145	-0.950103537	count	1
PEA15	-0.677791	0.1062911	-6.3767	2.15E-10	-0.948769766	count	5.17E-06
AC092069.1	-0.7669189	0.345068	-2.2225	0.0263	-0.948690491	count	1
MT1F	-0.6995657	0.2177797	-3.2123	0.00133	-0.948468383	count	1
JCHAIN	-2.3349178	0.8247546	-2.831	0.00468	-0.947444302	count	1
PLS3	-1.8966855	1.3553792	-1.3994	0.162	-0.946653379	count	1
TPM2	-0.9622675	0.3906324	-2.4634	0.0138	-0.94597073	count	1
GBE1	-0.719499	0.2998691	-2.3994	0.0165	-0.945221763	count	1
TNIP2	-0.7198587	0.1797192	-4.0055	6.37E-05	-0.944262246	count	1
CTNNAL1	-1.124678	0.5884242	-1.9113	0.0561	-0.941573439	count	1
MAPK6	-0.7100265	0.2125619	-3.3403	0.000849	-0.939920146	count	1

SLC7A1		-1.7178975	0.7083673	-2.4252	0.0154	-0.933583245	count	1
LLGL2		-1.841737	1.0024094	-1.8373	0.0663	-0.932515107	count	1
PRKAR1B		-1.0025396	0.6159648	-1.6276	0.104	-0.930706545	count	1
GPC4		-1.09504	1.4510891	-0.7546	0.451	-0.921855122	count	1
DNAJC5B		-17.492718	1394.467034	-0.0125	0.99	-0.921173529	count	1
EPS8L1		-17.3661787	1694.082308	-0.0103	0.992	-0.921173525	count	1
USP2		-17.0747218	1409.186883	-0.0121	0.99	-0.921173512	count	1
LINC01091		-17.015672	4751.378798	-0.0036	0.997	-0.921173237	count	1
FAM72D		-17.015054	4300.805328	-0.004	0.997	-0.921173237	count	1
TDO2		-17.015054	4300.805328	-0.004	0.997	-0.921173237	count	1
FBXO24		-17.015054	4300.805328	-0.004	0.997	-0.921173237	count	1
	2-Mar	-17.013721	3421.512797	-0.005	0.996	-0.921173237	count	1
GZMM		-17.012352	2415.638058	-0.007	0.994	-0.921173237	count	1
IL36B		-17.011761	2116.932588	-0.008	0.994	-0.921173237	count	1
DCAF4L1		-16.692191	2910.097897	-0.0057	0.995	-0.921173217	count	1
FGF11		-16.691382	1814.348904	-0.0092	0.993	-0.921173217	count	1
CYP27A1		-0.6513609	0.155761	-4.1818	2.99E-05	-0.914584189	count	0.7059091
EEPD1		-0.7805281	0.4503396	-1.7332	0.0832	-0.913057845	count	1
GZMK		-1.9137845	1.1319267	-1.6907	0.091	-0.911027937	count	1
LDHA		-0.6321425	0.0589773	-10.7184	3.15E-26	-0.908022085	count	7.64E-22
ALDOC		-0.9013767	0.6082176	-1.482	0.138	-0.906585961	count	1
IL32		-1.894525	1.0167521	-1.8633	0.0625	-0.906470521	count	1
TDP2		-0.668494	0.2144538	-3.1172	0.00185	-0.905515775	count	1
FAM162A		-0.6402098	0.125005	-5.1215	3.27E-07	-0.905463878	count	0.007803201
CLN8		-0.6750227	0.1676348	-4.0267	5.83E-05	-0.905290556	count	1
NKAPL		-0.9145043	0.5795784	-1.5779	0.115	-0.905212682	count	1
TTLL5		-1.0859505	0.5622679	-1.9314	0.0536	-0.902670497	count	1
P4HA1		-0.6389143	0.1272407	-5.0213	5.50E-07	-0.902658564	count	0.0131109
ABCC3		-0.7023401	0.2441787	-2.8763	0.00406	-0.901452102	count	1
ORC6		-1.3333106	0.7949283	-1.6773	0.0936	-0.900226344	count	1
ST20		-0.715495	0.3101569	-2.3069	0.0211	-0.899512196	count	1
SLC38A6		-0.695143	0.2509839	-2.7697	0.00565	-0.89902377	count	1
PPFIBP1		-0.8792907	0.5279552	-1.6655	0.0959	-0.892937663	count	1
NDUFA4L2		-2.0114793	1.8600398	-1.0814	0.28	-0.889817855	count	1
OPTN		-0.8890425	0.3996764	-2.2244	0.0262	-0.889539684	count	1
FMNL2		-0.6759146	0.1919293	-3.5217	0.000437	-0.885967489	count	1
MT2A		-0.6156809	0.1653644	-3.7232	0.000201	-0.88532863	count	1
CYTOR		-0.6529833	0.1594052	-4.0964	4.33E-05	-0.884282273	count	1
OCIAD2		-1.2990428	0.7049343	-1.8428	0.0655	-0.88399151	count	1
CERCAM		-1.982496	1.0441818	-1.8986	0.0577	-0.883839208	count	1
	10-Sep	-0.7032523	0.2682962	-2.6212	0.00882	-0.879886708	count	1
LBX2		-1.398592	1.563832	-0.8943	0.371	-0.879629998	count	1
CD72		-0.6757502	0.1629762	-4.1463	3.49E-05	-0.878707416	count	0.8233608
MSR1		-0.6146248	0.0810235	-7.5858	4.66E-14	-0.878292802	count	1.13E-09
C17orf58		-0.7639812	0.3869494	-1.9744	0.0485	-0.871095204	count	1

SPATS2	-0.8375774	0.4017226	-2.085	0.0372	-0.87103028	count	1
RGCC	-0.6042106	0.1044659	-5.7838	8.23E-09	-0.869426882	count	0.000197446
FNIP2	-0.6381362	0.1507838	-4.2321	2.40E-05	-0.86589631	count	0.567048
PRDX4	-0.6143705	0.1107712	-5.5463	3.23E-08	-0.864004963	count	0.000773488
CTPS1	-1.1780454	0.6245041	-1.8864	0.0594	-0.863156513	count	1
RAB11B-AS1	-0.9144319	0.5412507	-1.6895	0.0913	-0.860848677	count	1
TMEM52B	-0.8498302	0.4859456	-1.7488	0.0804	-0.860748386	count	1
BNIP3	-0.6271825	0.2012563	-3.1163	0.00185	-0.860502907	count	1
ARID5B	-0.6213069	0.1524735	-4.0749	4.75E-05	-0.858144986	count	1
RHOH	-0.8904691	0.3509773	-2.5371	0.0112	-0.856745304	count	1
SH2B2	-0.8202569	0.3740098	-2.1931	0.0284	-0.85487714	count	1
ALCAM	-0.6238069	0.1544337	-4.0393	5.53E-05	-0.854230481	count	1
NEK2	-16.909627	4506.010222	-0.0038	0.997	-0.851430906	count	1
ADGRF5	-16.909627	4506.010222	-0.0038	0.997	-0.851430906	count	1
GSEC	-16.905309	1931.085166	-0.0088	0.993	-0.851430906	count	1
ADAMTS2	-16.904738	1651.751937	-0.0102	0.992	-0.851430906	count	1
ZP1	-16.904738	1651.751937	-0.0102	0.992	-0.851430906	count	1
ICOS	-17.2911557	3767.668758	-0.0046	0.996	-0.851430858	count	1
AC011611.4	-17.2891264	1847.525059	-0.0094	0.993	-0.851430858	count	1
SCG5	-17.2888624	1695.56179	-0.0102	0.992	-0.851430858	count	1
LMNTD2	-16.8761149	1171.4986	-0.0144	0.989	-0.85143084	count	1
AL162231.1	-16.828495	1087.146117	-0.0155	0.988	-0.851430837	count	1
POLR3H	-0.7350984	0.3900315	-1.8847	0.0596	-0.850187969	count	1
PPIF	-0.5990136	0.10597	-5.6527	1.76E-08	-0.850176193	count	0.000421837
PRADC1	-0.7122934	0.2779828	-2.5624	0.0105	-0.849214685	count	1
KCNJ15	-0.9447545	0.610809	-1.5467	0.122	-0.848655668	count	1
DTYMK	-0.6586963	0.2474689	-2.6617	0.00782	-0.848558085	count	1
NECTIN2	-0.6214845	0.1370294	-4.5354	6.03E-06	-0.84711271	count	0.1430919
PLPP5	-0.6599637	0.2394906	-2.7557	0.0059	-0.846886723	count	1
INSIG1	-0.5899997	0.0873875	-6.7515	1.82E-11	-0.84520078	count	4.39E-07
AQP9	-0.6081214	0.1633183	-3.7235	0.000201	-0.844792151	count	1
CNBD2	-0.7341382	0.3313341	-2.2157	0.0268	-0.844401971	count	1
GNG10	-0.8939687	0.4100882	-2.1799	0.0294	-0.844257802	count	1
LMNB2	-0.8720196	0.3842225	-2.2696	0.0233	-0.841300471	count	1
FTL	-0.5814662	0.0431243	-13.4835	5.03E-40	-0.838823466	count	1.22E-35
MARCKSL1	-0.605079	0.1614675	-3.7474	0.000183	-0.837502525	count	1
GZF1	-0.6055055	0.1397564	-4.3326	1.53E-05	-0.831040084	count	0.3620898
CAPG	-0.5768877	0.0666353	-8.6574	8.59E-18	-0.826337437	count	2.08E-13
YDJC	-0.6373755	0.2282227	-2.7928	0.00527	-0.823131944	count	1
LGALS1	-0.5701068	0.052703	-10.8173	1.13E-26	-0.821237549	count	2.74E-22
HS3ST2	-2.1587871	0.9984418	-2.1622	0.0307	-0.820807348	count	1
CYB5A	-0.6093722	0.1843652	-3.3052	0.000963	-0.819432731	count	1
BOLA2B	-0.870434	0.7740215	-1.1246	0.261	-0.817113279	count	1
IL1RN	-0.5685577	0.1296798	-4.3843	1.21E-05	-0.816518018	count	0.2865764
TAF4	-1.564324	0.6759484	-2.3143	0.0207	-0.816465427	count	1

ADAM28	-0.6359048	0.161112	-3.947	8.14E-05	-0.815693806	count	1
SLC36A1	-0.9001864	0.5121538	-1.7576	0.0789	-0.814646138	count	1
ANKRD9	-0.6373996	0.2290774	-2.7825	0.00544	-0.814140297	count	1
TLCD2	-0.9877337	0.5923557	-1.6675	0.0955	-0.810879488	count	1
SIPA1L2	-0.674097	0.2301044	-2.9295	0.00343	-0.809273123	count	1
STX6	-0.5930187	0.1429822	-4.1475	3.48E-05	-0.809186336	count	0.8210364
EFEMP1	-2.0654245	1.4277557	-1.4466	0.148	-0.805642242	count	1
BAG3	-0.7160736	0.2509105	-2.8539	0.00435	-0.804236076	count	1
FRRS1	-1.2278794	0.8332108	-1.4737	0.141	-0.80409333	count	1
MRAS	-0.6364726	0.2661477	-2.3914	0.0169	-0.802978856	count	1
PTGR1	-0.6573467	0.2727397	-2.4102	0.016	-0.802785653	count	1
PDLIM4	-1.2712345	1.0538849	-1.2062	0.228	-0.799048614	count	1
UCHL3	-0.574048	0.1427755	-4.0206	5.98E-05	-0.798433185	count	1
ANG	-0.6010214	0.2075361	-2.896	0.00381	-0.798183396	count	1
IL18BP	-0.625454	0.3038813	-2.0582	0.0397	-0.797265573	count	1
METTL7B	-0.7419624	0.4696128	-1.5799	0.114	-0.797198111	count	1
ZNF891	-1.1639104	0.5114828	-2.2756	0.023	-0.795563048	count	1
UBE2C	-1.0062483	1.3778068	-0.7303	0.465	-0.795100418	count	1
CFAP36	-0.635123	0.2539237	-2.5012	0.0124	-0.791266918	count	1
TMEM163	-0.9966806	0.4148404	-2.4026	0.0164	-0.78902676	count	1
PPP1R3E	-1.1959692	0.5636003	-2.122	0.0339	-0.789024873	count	1
SCYL3	-1.7507142	0.8311042	-2.1065	0.0353	-0.788795988	count	1
ZNF569	-1.24758	0.7788465	-1.6018	0.109	-0.788621768	count	1
MTHFS	-0.6017667	0.2317597	-2.5965	0.00947	-0.788233091	count	1
IL18	-0.5634047	0.1010707	-5.5744	2.76E-08	-0.78799154	count	0.00066113
AL133453.1	-0.5865009	0.2181096	-2.689	0.00721	-0.787582131	count	1
PLPP1	-1.3767632	1.0905041	-1.2625	0.207	-0.785692259	count	1
LINC00243	-1.5790931	0.6820733	-2.3151	0.0207	-0.784203893	count	1
FCGR3A	-0.5448835	0.0734827	-7.4151	1.66E-13	-0.781882964	count	4.01E-09
MAFF	-0.5800269	0.1223127	-4.7422	2.24E-06	-0.781772292	count	0.05326944
PPA1	-0.5586182	0.0990161	-5.6417	1.88E-08	-0.780698043	count	0.000450523
C1orf122	-0.5606558	0.1363798	-4.111	4.07E-05	-0.780343078	count	0.9595839
IER3	-0.5418647	0.0640043	-8.4661	4.30E-17	-0.779611884	count	1.04E-12
2-Mar	-0.5577679	0.1008824	-5.5289	3.56E-08	-0.778683194	count	0.0008523
SLX4	-1.1733248	0.6701478	-1.7508	0.0801	-0.778146951	count	1
ZNF503-AS2	-16.954676	1156.125636	-0.0147	0.988	-0.778144731	count	1
ZKSCAN7	-16.790939	4246.386754	-0.004	0.997	-0.778144723	count	1
AP001330.1	-16.790939	4246.386754	-0.004	0.997	-0.778144723	count	1
REM2	-16.790939	4246.386754	-0.004	0.997	-0.778144723	count	1
ALG1L2	-16.790144	3751.349153	-0.0045	0.996	-0.778144723	count	1
CLEC19A	-16.790144	3751.349153	-0.0045	0.996	-0.778144723	count	1
AC079313.2	-16.789454	3091.540504	-0.0054	0.996	-0.778144723	count	1
SNX22	-16.78749	2116.689374	-0.0079	0.994	-0.778144723	count	1
DAGLA	-16.787081	1979.06912	-0.0085	0.993	-0.778144723	count	1
C4orf47	-16.786897	1831.466658	-0.0092	0.993	-0.778144723	count	1

MAFIP	-17.2058232	2553.016865	-0.0067	0.995	-0.77814462	count	1
CD3D	-17.1192645	2034.660897	-0.0084	0.993	-0.778144617	count	1
GOLGA8R	-17.119202	2216.819178	-0.0077	0.994	-0.778144617	count	1
AP001453.4	-16.5698146	1348.022162	-0.0123	0.99	-0.778144589	count	1
C6orf223	-16.5694229	1146.925907	-0.0144	0.988	-0.778144589	count	1
GPRIN3	-0.5711379	0.175157	-3.2607	0.00113	-0.777962504	count	1
BEX3	-0.6005175	0.2567892	-2.3386	0.0194	-0.777416325	count	1
ABLIM3	-1.2807803	1.1312524	-1.1322	0.258	-0.776677574	count	1
TMEM70	-0.5498421	0.0966033	-5.6918	1.41E-08	-0.776311851	count	0.00033809
HEY1	-1.1176338	1.0011033	-1.1164	0.264	-0.77191509	count	1
IFI6	-0.5405041	0.0971573	-5.5632	2.94E-08	-0.771149056	count	0.00070413
NOP16	-0.6098367	0.2511024	-2.4286	0.0152	-0.769714836	count	1
MIF	-0.5380335	0.0823196	-6.5359	7.66E-11	-0.768379032	count	1.85E-06
ATP6V1F	-0.5333918	0.0522432	-10.2098	5.38E-24	-0.766044595	count	1.30E-19
S100B	-0.671624	0.5828744	-1.1523	0.249	-0.765262438	count	1
TARBP2	-0.8356767	0.4225318	-1.9778	0.0481	-0.764260068	count	1
ENG	-0.5547204	0.1398012	-3.9679	7.46E-05	-0.764156748	count	1
RAB7B	-0.6116965	0.2903008	-2.1071	0.0352	-0.763165571	count	1
AC147651.4	-0.6261252	0.234145	-2.6741	0.00754	-0.762532438	count	1
DHRS3	-0.5689931	0.1968275	-2.8908	0.00388	-0.762054052	count	1
AL035413.1	-1.1387788	0.6390961	-1.7819	0.0749	-0.761254067	count	1
MMP2	-0.9747515	0.7260916	-1.3425	0.18	-0.760440992	count	1
SMIM12	-0.5554898	0.1398392	-3.9723	7.32E-05	-0.760384546	count	1
PRSS21	-0.6753983	0.5960038	-1.1332	0.257	-0.758885514	count	1
HIF1A	-0.5313767	0.071445	-7.4376	1.41E-13	-0.75833956	count	3.41E-09
CCR5	-0.6597282	0.3122934	-2.1125	0.0347	-0.757245311	count	1
ITGA6	-0.7801364	0.4000682	-1.95	0.0513	-0.756245722	count	1
CLEC5A	-0.5459785	0.1874684	-2.9124	0.00362	-0.755421007	count	1
PAICS	-0.5904274	0.2274824	-2.5955	0.0095	-0.755301791	count	1
EXOC6B	-0.888939	0.4733759	-1.8779	0.0605	-0.754885799	count	1
AC016831.1	-0.6402461	0.2170285	-2.9501	0.00321	-0.75421615	count	1
GPR137B	-0.5625537	0.1435228	-3.9196	9.11E-05	-0.754080388	count	1
PAM	-0.7625925	0.4462909	-1.7087	0.0876	-0.753592227	count	1
ME2	-0.5429005	0.13648	-3.9779	7.15E-05	-0.751752952	count	1
TMEM251	-0.5453904	0.1592833	-3.424	0.000627	-0.750450519	count	1
PRDX1	-0.5222921	0.0544067	-9.5998	1.90E-21	-0.749748576	count	4.61E-17
ENOSF1	-0.5908144	0.2322922	-2.5434	0.011	-0.74953606	count	1
MGAT4A	-0.5369143	0.1041207	-5.1567	2.71E-07	-0.749045674	count	0.00646877
RAB33A	-0.805897	0.4734591	-1.7021	0.0889	-0.748679551	count	1
FAM107B	-0.5267496	0.0939844	-5.6047	2.32E-08	-0.748642981	count	0.000555872
ACOT11	-0.9330762	0.7387347	-1.2631	0.207	-0.747884669	count	1
CCDC167	-0.5595676	0.2686964	-2.0825	0.0374	-0.747328869	count	1
TSC22D1	-0.5555	0.226795	-2.4493	0.0144	-0.747112257	count	1
SH3PXD2B	-1.0685241	0.3892566	-2.745	0.00609	-0.746094068	count	1
TYMS	-1.0028624	0.8114133	-1.2359	0.217	-0.745846426	count	1

PTTG1	-0.598691	0.2998352	-1.9967	0.046	-0.745636188	count	1
POLR3E	-0.928618	0.4011841	-2.3147	0.0207	-0.744951043	count	1
ZDHHC12	-0.5497688	0.1592425	-3.4524	0.000565	-0.744517388	count	1
COBLL1	-1.5752109	1.4781325	-1.0657	0.287	-0.744270221	count	1
TPI1	-0.5166967	0.0524912	-9.8435	1.89E-22	-0.743510603	count	4.58E-18
PGK1	-0.5170106	0.055292	-9.3506	1.90E-20	-0.743163502	count	4.60E-16
MGST3	-0.5180444	0.0582334	-8.896	1.10E-18	-0.742802412	count	2.66E-14
PKD1	-1.0974918	0.5138536	-2.1358	0.0328	-0.740589923	count	1
LILRB4	-0.5200094	0.0863071	-6.0251	1.94E-09	-0.740159075	count	4.66E-05
LINC00513	-1.1389843	0.4539716	-2.5089	0.0122	-0.738660073	count	1
PTPN1	-0.5289645	0.1022144	-5.1751	2.46E-07	-0.738487431	count	0.005873004
HMGA1	-0.5260502	0.108598	-4.844	1.35E-06	-0.737829426	count	0.03213945
RALGDS	-0.5891025	0.2290728	-2.5717	0.0102	-0.735969963	count	1
RBM4B	-0.9575417	0.4691662	-2.0409	0.0414	-0.734590406	count	1
RABEPK	-0.6688658	0.3301983	-2.0256	0.0429	-0.73446658	count	1
FRMD4A	-0.6460712	0.2685601	-2.4057	0.0162	-0.733072438	count	1
VOPP1	-0.5854443	0.1413312	-4.1424	3.55E-05	-0.731558248	count	0.837445
HLA-DQB2	-0.7181035	0.2991901	-2.4002	0.0165	-0.730044236	count	1
NACA2	-1.522714	0.9343986	-1.6296	0.103	-0.729718532	count	1
TPRG1	-1.1183671	0.8952443	-1.2492	0.212	-0.728781274	count	1
SOGA1	-0.7162529	0.347977	-2.0583	0.0397	-0.72833742	count	1
LPCAT3	-0.6481513	0.2847188	-2.2765	0.0229	-0.727562804	count	1
CISD2	-0.5177693	0.1024477	-5.054	4.65E-07	-0.725444178	count	0.011088855
ADAL	-0.9425533	0.4393679	-2.1452	0.032	-0.725279745	count	1
CBR3	-1.3771007	0.6591434	-2.0892	0.0368	-0.721293283	count	1
CEP128	-1.0220196	0.4668759	-2.1891	0.0287	-0.720949331	count	1
F3	-1.376013	0.8767621	-1.5694	0.117	-0.720928469	count	1
COL6A2	-0.9354631	0.5871464	-1.5932	0.111	-0.72084994	count	1
VAC14	-0.5936017	0.3096211	-1.9172	0.0553	-0.719523087	count	1
BATF	-0.5871347	0.2435359	-2.4109	0.016	-0.719301037	count	1
ENO1	-0.5005018	0.0578674	-8.6491	9.22E-18	-0.719223884	count	2.23E-13
FAM210A	-0.554516	0.1752133	-3.1648	0.00157	-0.716819506	count	1
FPGS	-0.6176446	0.3570094	-1.7301	0.0837	-0.715774827	count	1
STPG1	-1.8355219	1.1376912	-1.6134	0.107	-0.714622903	count	1
SLC26A6	-0.7638242	0.5500241	-1.3887	0.165	-0.714227867	count	1
TMEM51	-0.5571281	0.2524824	-2.2066	0.0274	-0.713274941	count	1
LY6G5C	-0.7813129	0.9452029	-0.8266	0.409	-0.712555686	count	1
ASB1	-0.6632707	0.4522083	-1.4667	0.143	-0.711897883	count	1
SPAG4	-0.640476	0.4821308	-1.3284	0.184	-0.711249616	count	1
ZNF184	-1.4573118	0.6158253	-2.3664	0.018	-0.710752095	count	1
NEU3	-0.7507992	0.4532136	-1.6566	0.0977	-0.710699745	count	1
POLR2H	-0.5444545	0.1842945	-2.9543	0.00316	-0.709852538	count	1
FERMT2	-0.8569657	0.527755	-1.6238	0.105	-0.70910402	count	1
ACOT13	-0.5163823	0.1693156	-3.0498	0.00231	-0.70838181	count	1
IGLC3	-0.5474263	0.3675457	-1.4894	0.137	-0.707883984	count	1

UBL3	-0.5397914	0.1402167	-3.8497	0.000121	-0.707659301	count	1
HNRNPA1P48	-0.8540344	0.5010629	-1.7044	0.0884	-0.707060169	count	1
CBLB	-0.6769899	0.4540913	-1.4909	0.136	-0.705498612	count	1
ADA	-0.6546537	0.3098421	-2.1129	0.0347	-0.703326746	count	1
DOK5	-1.7739079	0.9764354	-1.8167	0.0694	-0.702639133	count	1
RHEB	-0.4952776	0.0768983	-6.4407	1.43E-10	-0.702309077	count	3.44E-06
EIF4A3	-0.4943544	0.0912621	-5.4169	6.66E-08	-0.701120467	count	0.001592939
PNMA1	-0.6817547	0.4397788	-1.5502	0.121	-0.7010576	count	1
NAP1L5	-17.5423813	2192.864501	-0.008	0.994	-0.700935462	count	1
RASGEF1A	-17.5021155	1895.863086	-0.0092	0.993	-0.700935461	count	1
P2RY10	-17.4610944	1593.518801	-0.011	0.991	-0.70093546	count	1
PPY	-17.1130677	2066.428562	-0.0083	0.993	-0.700935451	count	1
ZCWPW2	-17.1128026	1891.210087	-0.009	0.993	-0.700935451	count	1
PCAT6	-17.1125277	1698.105733	-0.0101	0.992	-0.700935451	count	1
CIB2	-17.1116666	1273.686734	-0.0134	0.989	-0.700935451	count	1
AC007014.1	-16.5036533	2715.300031	-0.0061	0.995	-0.700935423	count	1
TJP1	-16.5034247	2375.585446	-0.0069	0.994	-0.700935423	count	1
ZNF284	-16.5025798	1478.524195	-0.0112	0.991	-0.700935423	count	1
LINC02245	-16.656299	3969.928468	-0.0042	0.997	-0.700935209	count	1
ITGA11	-16.656299	3969.928468	-0.0042	0.997	-0.700935209	count	1
BRICD5	-16.653784	2467.224088	-0.0068	0.995	-0.700935209	count	1
OSCP1	-16.653036	2038.963135	-0.0082	0.993	-0.700935209	count	1
PROC	-16.652841	1874.813839	-0.0089	0.993	-0.700935209	count	1
S100A16	-16.652394	1694.701228	-0.0098	0.992	-0.700935209	count	1
BIRC7	-16.652394	1694.701228	-0.0098	0.992	-0.700935209	count	1
FZD3	-16.651939	1493.20175	-0.0112	0.991	-0.700935209	count	1
MLEC	-0.4972781	0.0908296	-5.4748	4.82E-08	-0.700539956	count	0.001153474
FKTN	-1.7608398	0.9511118	-1.8513	0.0642	-0.700017489	count	1
AC058791.1	-0.5350142	0.1644493	-3.2534	0.00116	-0.699929504	count	1
TMEM217	-1.5588136	1.2047958	-1.2938	0.196	-0.698996854	count	1
AC139530.1	-0.7860688	0.5434843	-1.4464	0.148	-0.698814027	count	1
ST6GALNAC4	-0.6213544	0.2839936	-2.1879	0.0288	-0.696714298	count	1
INTS5	-0.8538985	0.5517602	-1.5476	0.122	-0.694813005	count	1
AKR1B1	-0.4988616	0.0962735	-5.1817	2.38E-07	-0.694514941	count	0.005682964
ETFBKMT	-1.4009796	0.8481895	-1.6517	0.0987	-0.69364884	count	1
ZNF331	-0.4818434	0.0676672	-7.1208	1.40E-12	-0.691884942	count	3.38E-08
PKIB	-0.5219057	0.2138583	-2.4404	0.0147	-0.691538861	count	1
CXorf40B	-0.5904623	0.3050454	-1.9357	0.053	-0.691459351	count	1
MACC1	-1.2092756	0.5418111	-2.2319	0.0257	-0.691345951	count	1
MT-ND6	-0.5419031	0.2121813	-2.554	0.0107	-0.691216971	count	1
KYAT3	-0.5902271	0.3010834	-1.9603	0.0501	-0.689377362	count	1
HAUS2	-0.5592766	0.2301981	-2.4295	0.0152	-0.687505872	count	1
IFI27L1	-0.6578852	0.343633	-1.9145	0.0557	-0.687171952	count	1
DNAJC9	-0.5801618	0.3232938	-1.7945	0.0729	-0.686724192	count	1
TPM4	-0.4789789	0.0580251	-8.2547	2.46E-16	-0.686136469	count	5.95E-12

P4HB	-0.4816277	0.0772123	-6.2377	5.21E-10	-0.685867336	count	1.25E-05
ACAA2	-0.491198	0.1118825	-4.3903	1.18E-05	-0.685343368	count	0.2795184
SBF2-AS1	-0.6849748	0.507157	-1.3506	0.177	-0.684082826	count	1
ATOX1	-0.4822369	0.0807611	-5.9712	2.70E-09	-0.683983795	count	6.48E-05
SLAMF7	-1.368365	0.4359724	-3.1387	0.00172	-0.683413697	count	1
GALE	-0.5700942	0.3325829	-1.7141	0.0866	-0.682850518	count	1
PXMP2	-1.1870698	0.6379093	-1.8609	0.0629	-0.68243732	count	1
LIMS1	-0.4778964	0.0697172	-6.8548	9.00E-12	-0.682352791	count	2.17E-07
EML4	-0.4849079	0.1302341	-3.7234	0.000201	-0.682317772	count	1
S100A13	-0.5088309	0.2311071	-2.2017	0.0278	-0.681858849	count	1
CYR61	-1.1191381	1.4085963	-0.7945	0.427	-0.680696982	count	1
HLA-DOB	-0.9805096	0.6831482	-1.4353	0.151	-0.679199346	count	1
HDAC11	-0.8912129	0.9253723	-0.9631	0.336	-0.678188048	count	1
FAH	-0.5122812	0.2499353	-2.0497	0.0405	-0.677156819	count	1
INAFM2	-0.5538229	0.2005088	-2.7621	0.00579	-0.676664839	count	1
FAM104A	-0.5313421	0.2088617	-2.544	0.011	-0.676193629	count	1
ABCB8	-0.6704661	0.3922701	-1.7092	0.0875	-0.676025937	count	1
CDKN3	-0.744469	0.6413332	-1.1608	0.246	-0.674833735	count	1
SLC41A1	-1.8997868	1.2003429	-1.5827	0.114	-0.674461196	count	1
CRNDE	-0.909889	0.5782433	-1.5735	0.116	-0.674068672	count	1
TNFAIP8L1	-0.6483641	0.3975452	-1.6309	0.103	-0.673815376	count	1
FNDC10	-0.6990059	0.4588933	-1.5232	0.128	-0.673224813	count	1
PPP1R14A	-1.3354382	0.6582044	-2.0289	0.0426	-0.672828651	count	1
SLC2A1	-1.4562367	1.0627362	-1.3703	0.171	-0.671587799	count	1
SLC28A3	-1.4562367	1.949165	-0.7471	0.455	-0.671587799	count	1
SMAD7	-1.1582586	0.5586101	-2.0735	0.0382	-0.670679006	count	1
DZIP1L	-1.1537647	0.7535038	-1.5312	0.126	-0.668824553	count	1
SERPINF2	-0.8316763	0.611821	-1.3593	0.174	-0.667149911	count	1
COG7	-1.148459	0.4641777	-2.4742	0.0134	-0.666627769	count	1
SMIM3	-0.4860564	0.1467113	-3.313	0.000936	-0.666040506	count	1
BYSL	-0.795241	0.6127304	-1.2979	0.194	-0.665473263	count	1
PTRH1	-0.5413781	0.2372213	-2.2822	0.0226	-0.665232517	count	1
ACTN1	-0.483563	0.1460087	-3.3119	0.00094	-0.665009934	count	1
PTCRA	-1.2154404	0.8384891	-1.4496	0.147	-0.663836796	count	1
PIGW	-1.2154404	0.8632775	-1.4079	0.159	-0.663836796	count	1
ITGAV	-0.5178761	0.1959032	-2.6435	0.00826	-0.663725439	count	1
ALG5	-0.4918621	0.1544931	-3.1837	0.00147	-0.663143992	count	1
DSN1	-0.8061444	0.5512573	-1.4624	0.144	-0.66181311	count	1
ENTPD5	-0.8624151	0.6562434	-1.3142	0.189	-0.660084875	count	1
ADORA2B	-0.6617377	0.4757135	-1.391	0.164	-0.657670677	count	1
MIPEP	-0.9094473	0.6237043	-1.4581	0.145	-0.657266583	count	1
HMOX1	-0.4557998	0.1161404	-3.9246	8.93E-05	-0.654775616	count	1
ZSCAN5A	-0.739533	0.5138218	-1.4393	0.15	-0.653746435	count	1
POLR2J3	-1.1886779	1.3457443	-0.8833	0.377	-0.653681409	count	1
TANC2	-0.601178	0.2821661	-2.1306	0.0332	-0.652878149	count	1

NOV	-1.5456067	1.2559018	-1.2307	0.219	-0.652556697	count	1
GADD45GIP1	-0.459734	0.0752095	-6.1127	1.14E-09	-0.651634625	count	2.74E-05
S100A2	-1.00598	0.7959112	-1.2639	0.206	-0.651304274	count	1
CXCL1	-0.4735311	0.3848467	-1.2304	0.219	-0.650584585	count	1
SOX15	-1.760755	1.5494086	-1.1364	0.256	-0.650247622	count	1
SRRD	-0.5792735	0.3920648	-1.4775	0.14	-0.649714265	count	1
RMDN3	-0.5142225	0.232408	-2.2126	0.027	-0.649652815	count	1
HSPD1	-0.4554413	0.0632008	-7.2063	7.62E-13	-0.648052682	count	1.84E-08
GAS2L3	-0.5083608	0.1965676	-2.5862	0.00976	-0.647705487	count	1
IFRD2	-0.5359352	0.2859526	-1.8742	0.061	-0.647407403	count	1
AC007038.2	-1.3677252	1.491324	-0.9171	0.359	-0.646142692	count	1
SIGLEC12	-1.5151266	1.1349802	-1.3349	0.182	-0.645147503	count	1
SDF2L1	-0.4581397	0.107711	-4.2534	2.18E-05	-0.644685723	count	0.5153738
APOO	-0.6876266	0.4871205	-1.4116	0.158	-0.643539817	count	1
SDC4	-0.5176083	0.3038499	-1.7035	0.0886	-0.642643867	count	1
CLCF1	-0.7952267	0.6872585	-1.1571	0.247	-0.642317426	count	1
WDR90	-1.7181811	1.5651052	-1.0978	0.272	-0.642247816	count	1
MRGBP	-0.5434819	0.258157	-2.1052	0.0354	-0.642073142	count	1
SLC25A35	-1.2423713	1.465501	-0.8477	0.397	-0.641506513	count	1
MAP3K20	-0.4800907	0.1997959	-2.4029	0.0163	-0.640716277	count	1
SLC37A2	-0.5379632	0.3607653	-1.4912	0.136	-0.640320974	count	1
CSTF1	-0.5779687	0.3291392	-1.756	0.0792	-0.637873589	count	1
IL21R	-0.8761196	0.50331	-1.7407	0.0819	-0.637644498	count	1
PHF19	-0.5209212	0.2066964	-2.5202	0.0118	-0.636780947	count	1
ZDHHC14	-0.7048087	0.3668739	-1.9211	0.0548	-0.634952217	count	1
YWHAH	-0.4466265	0.0769864	-5.8014	7.42E-09	-0.634904444	count	0.000178035
TUBB	-0.4448483	0.0681659	-6.526	8.18E-11	-0.634460557	count	1.97E-06
LRRC59	-0.4680776	0.1433669	-3.2649	0.00111	-0.634025456	count	1
MYO1E	-0.4817473	0.2371178	-2.0317	0.0423	-0.631590988	count	1
RIOX1	-0.7005193	0.4528905	-1.5468	0.122	-0.631522576	count	1
CASC15	-1.063438	2.022951	-0.5257	0.599	-0.63036523	count	1
C1orf198	-1.2104338	0.9167516	-1.3204	0.187	-0.630269311	count	1
CCT3	-0.4522202	0.0938392	-4.8191	1.53E-06	-0.629925606	count	0.03641553
CDC7	-1.127446	1.9923575	-0.5659	0.572	-0.629736986	count	1
SEC22A	-0.8370199	0.4938499	-1.6949	0.0902	-0.629424014	count	1
HSP90AA1	-0.4376797	0.0524754	-8.3407	1.22E-16	-0.629332928	count	2.95E-12
KRT18	-1.2074072	0.7243846	-1.6668	0.0957	-0.629191233	count	1
MYDGF	-0.4401941	0.0697973	-6.3067	3.37E-10	-0.628070099	count	8.11E-06
PRR3	-0.6464539	0.3777454	-1.7113	0.0871	-0.627273201	count	1
DDB1	-0.5150134	0.217043	-2.3729	0.0177	-0.626521571	count	1
IFITM10	-1.6386114	0.9337664	-1.7548	0.0794	-0.626516997	count	1
CCSER1	-1.9494991	0.8193446	-2.3793	0.0174	-0.626407734	count	1
GSTO1	-0.4360926	0.0555406	-7.8518	6.08E-15	-0.626094311	count	1.47E-10
FAM220A	-0.831592	0.6654745	-1.2496	0.212	-0.626030546	count	1
STIP1	-0.4677977	0.1515855	-3.086	0.00205	-0.625935755	count	1

IFT81	-0.6745181	0.6635699	-1.0165	0.309	-0.625515707	count	1
ARFGAP3	-0.4526251	0.1233719	-3.6688	0.000249	-0.624436989	count	1
AC016831.5	-0.4688432	0.1885424	-2.4867	0.013	-0.623964635	count	1
SPSB1	-0.6905229	0.6033892	-1.1444	0.253	-0.623507217	count	1
NIPAL3	-0.7231062	0.7833183	-0.9231	0.356	-0.622693857	count	1
ZNF267	-0.4454695	0.1087447	-4.0965	4.33E-05	-0.622358724	count	1
OGFOD1	-0.765072	0.4268762	-1.7923	0.0732	-0.621448313	count	1
FAM114A1	-0.4826791	0.2098064	-2.3006	0.0215	-0.621211785	count	1
FAM92A	-0.9454286	0.8208638	-1.1517	0.25	-0.620686951	count	1
SPIC	-17.865758	2552.47042	-0.007	0.994	-0.619359615	count	1
COLEC10	-16.500851	3673.056765	-0.0045	0.996	-0.619359576	count	1
FOXM1	-16.500851	3673.056765	-0.0045	0.996	-0.619359576	count	1
GNAO1	-16.500851	3673.056765	-0.0045	0.996	-0.619359576	count	1
FBXO17	-16.500851	3673.056765	-0.0045	0.996	-0.619359576	count	1
WDR78	-16.499953	3119.559889	-0.0053	0.996	-0.619359576	count	1
FHL2	-16.499953	3119.559889	-0.0053	0.996	-0.619359576	count	1
FOXP2	-16.499953	3119.559889	-0.0053	0.996	-0.619359576	count	1
TRDC	-16.499953	3119.559889	-0.0053	0.996	-0.619359576	count	1
RSPH9	-16.498302	2116.043982	-0.0078	0.994	-0.619359576	count	1
RTCA-AS1	-16.497624	1725.99618	-0.0096	0.992	-0.619359576	count	1
DAW1	-16.497624	1725.99618	-0.0096	0.992	-0.619359576	count	1
SEMA5A	-16.497624	1725.99618	-0.0096	0.992	-0.619359576	count	1
AC123595.1	-16.497624	1725.99618	-0.0096	0.992	-0.619359576	count	1
MS4A6E	-16.497624	1725.99618	-0.0096	0.992	-0.619359576	count	1
GREM1	-16.497624	1725.99618	-0.0096	0.992	-0.619359576	count	1
ACTC1	-16.497624	1725.99618	-0.0096	0.992	-0.619359576	count	1
ZNF507	-16.497624	1725.99618	-0.0096	0.992	-0.619359576	count	1
TMEM147-AS1	-16.497144	1493.39174	-0.011	0.991	-0.619359576	count	1
SLC7A11	-17.7492734	1369.763136	-0.013	0.99	-0.619359513	count	1
AC107871.1	-17.5446847	2332.581503	-0.0075	0.994	-0.61935951	count	1
DNAH14	-17.5335097	2290.361658	-0.0077	0.994	-0.619359509	count	1
HIST1H2BK	-17.4297257	2698.322878	-0.0065	0.995	-0.619359507	count	1
SPOCK2	-17.4297257	2698.322878	-0.0065	0.995	-0.619359507	count	1
AC083862.2	-17.4295648	2522.568986	-0.0069	0.994	-0.619359507	count	1
AKR1E2	-17.4293132	2128.232118	-0.0082	0.993	-0.619359507	count	1
RNASEH2A	-17.4293132	2128.232118	-0.0082	0.993	-0.619359507	count	1
AL358472.5	-17.4291255	1900.658589	-0.0092	0.993	-0.619359507	count	1
ARNTL2	-17.3648599	1150.929616	-0.0151	0.988	-0.619359506	count	1
AC026785.2	-17.0680346	1274.408965	-0.0134	0.989	-0.619359498	count	1
AL031775.1	-17.0110585	2304.995688	-0.0074	0.994	-0.619359497	count	1
AC008035.1	-17.0110585	2304.995688	-0.0074	0.994	-0.619359497	count	1
AL133415.1	-17.0104071	1698.961362	-0.01	0.992	-0.619359496	count	1
CYP27B1	-17.0104071	1698.961362	-0.01	0.992	-0.619359496	count	1
NRAV	-17.0104071	1698.961362	-0.01	0.992	-0.619359496	count	1
SPC25	-16.4303458	2528.147155	-0.0065	0.995	-0.619359472	count	1

E2F8	-16.4303458	2528.147155	-0.0065	0.995	-0.619359472	count	1
THUMPD3	-0.4746101	0.2083676	-2.2778	0.0228	-0.618635898	count	1
HDAC2-AS2	-1.408322	1.0250993	-1.3738	0.17	-0.617743277	count	1
ACER3	-0.4851305	0.2056774	-2.3587	0.0184	-0.617637957	count	1
HSD17B4	-0.4466584	0.1163603	-3.8386	0.000127	-0.617105853	count	1
ZNF200	-0.6486022	0.5314442	-1.2205	0.222	-0.617014223	count	1
CD2	-1.589567	0.7811804	-2.0348	0.042	-0.616296204	count	1
TMEM150A	-0.4735255	0.2419875	-1.9568	0.0505	-0.615614329	count	1
FAM229A	-1.4002645	1.7156686	-0.8162	0.414	-0.615582427	count	1
MRPL17	-0.4527196	0.1656593	-2.7328	0.00632	-0.615219147	count	1
AP002449.1	-1.8694655	1.2530649	-1.4919	0.136	-0.614648258	count	1
WIPI1	-0.4790414	0.1809487	-2.6474	0.00816	-0.614568123	count	1
CD84	-0.4371897	0.0977345	-4.4732	8.06E-06	-0.613757288	count	0.19115096
WSB2	-0.4662697	0.225626	-2.0666	0.0389	-0.6136904	count	1
BHLHE40	-0.452679	0.1265985	-3.5757	0.000356	-0.613566909	count	1
CCDC189	-1.5758719	0.736266	-2.1404	0.0324	-0.613368491	count	1
ZNF140	-1.0248988	0.5926673	-1.7293	0.0839	-0.613260734	count	1
HSPH1	-0.4475833	0.1082475	-4.1348	3.67E-05	-0.611350149	count	0.8656796
CAPN15	-0.5826848	0.4094672	-1.423	0.155	-0.610314902	count	1
ATF5	-0.4345181	0.1352737	-3.2121	0.00133	-0.608340024	count	1
DGCR8	-0.8852189	0.5443492	-1.6262	0.104	-0.608116505	count	1
TK1	-0.8025881	0.6492911	-1.2361	0.217	-0.607737969	count	1
TMUB1	-0.4537744	0.1811331	-2.5052	0.0123	-0.60721966	count	1
NRAS	-0.4721547	0.2170414	-2.1754	0.0297	-0.605930697	count	1
FBXO32	-1.0684587	0.7982479	-1.3385	0.181	-0.605722556	count	1
SRM	-0.4405916	0.1301704	-3.3847	0.000724	-0.604621114	count	1
FLOT1	-0.4293875	0.0901468	-4.7632	2.02E-06	-0.604146759	count	0.04804974
TM4SF1	-1.356592	1.0926016	-1.2416	0.214	-0.60363584	count	1
TMEM38B	-0.474787	0.2268925	-2.0926	0.0365	-0.603588131	count	1
SCARB2	-0.4306857	0.1121098	-3.8416	0.000125	-0.603574001	count	1
MRPL40	-0.4338329	0.1226788	-3.5363	0.000413	-0.603344209	count	1
SND1-IT1	-0.7745285	0.5330517	-1.453	0.146	-0.603185637	count	1
BCAP31	-0.4222774	0.0718063	-5.8808	4.64E-09	-0.601458021	count	0.000111369
DNAJC18	-2.2558225	1.0400205	-2.169	0.0302	-0.600556195	count	1
PRIM2	-0.6524934	0.4185577	-1.5589	0.119	-0.600074682	count	1
SUOX	-1.769145	1.1445987	-1.5456	0.122	-0.598715632	count	1
ID4	-1.7689513	1.3311055	-1.3289	0.184	-0.598683547	count	1
PTPN23	-0.6598063	0.3697037	-1.7847	0.0744	-0.598680569	count	1
FAM24B	-0.8680281	0.9403363	-0.9231	0.356	-0.598559827	count	1
AL035446.1	-1.2138863	1.0268503	-1.1821	0.237	-0.597729936	count	1
EPB41L3	-0.4285141	0.0986088	-4.3456	1.45E-05	-0.597518914	count	0.343215
MAGED2	-0.4586347	0.156617	-2.9284	0.00344	-0.597427585	count	1
ARID5A	-0.4326938	0.115083	-3.7598	0.000174	-0.596704185	count	1
HSPA5	-0.4158414	0.0694683	-5.9861	2.47E-09	-0.595690974	count	5.93E-05
IRAK1	-0.4339138	0.1374808	-3.1562	0.00162	-0.595349511	count	1

ZNF584	-0.8313193	1.4140591	-0.5879	0.557	-0.594732677	count	1
IRAK2	-0.6228346	0.4090737	-1.5225	0.128	-0.594711401	count	1
ZEB1	-1.2039726	0.7261669	-1.658	0.0974	-0.594420693	count	1
PDLIM1	-0.6726262	0.3438942	-1.9559	0.0506	-0.592875295	count	1
MANF	-0.4297424	0.1319089	-3.2579	0.00114	-0.592820081	count	1
VIM	-0.4105188	0.0430754	-9.5302	3.63E-21	-0.592042236	count	8.80E-17
S100A11	-0.4106421	0.0312581	-13.1371	3.80E-38	-0.591978423	count	9.23E-34
TAMM41	-0.6125561	0.4707814	-1.3011	0.193	-0.591585545	count	1
KCTD13	-0.5530929	0.5066106	-1.0918	0.275	-0.591514221	count	1
AHCY	-0.4629949	0.2052394	-2.2559	0.0242	-0.591236994	count	1
DDN-AS1	-0.9281618	0.7071901	-1.3125	0.189	-0.590746861	count	1
P2RX4	-0.4259836	0.1501991	-2.8361	0.0046	-0.590746544	count	1
MRM2	-0.4766047	0.2227285	-2.1398	0.0325	-0.5906744	count	1
CXorf40A	-0.5005026	0.3330817	-1.5026	0.133	-0.59034252	count	1
LGMN	-0.4124386	0.130006	-3.1725	0.00153	-0.589771632	count	1
TIMP4	-1.183971	1.4727691	-0.8039	0.422	-0.587672328	count	1
DLG5	-1.183971	2.4307803	-0.4871	0.626	-0.587672328	count	1
MRPS6	-0.4179604	0.1193465	-3.5021	0.00047	-0.58747669	count	1
MAST4	-1.4512938	0.8150417	-1.7806	0.0751	-0.585202865	count	1
PLEK	-0.4073799	0.0608905	-6.6904	2.75E-11	-0.585010624	count	6.63E-07
GEM	-0.4607699	0.3815068	-1.2078	0.227	-0.584993582	count	1
MAD2L1	-1.0846604	0.8463155	-1.2816	0.2	-0.583508732	count	1
RNF122	-0.6243647	0.5263917	-1.1861	0.236	-0.58340383	count	1
CREB3	-0.4407571	0.1699904	-2.5928	0.00958	-0.582719593	count	1
CTSZ	-0.404496	0.0454422	-8.9013	1.05E-18	-0.581606407	count	2.54E-14
OGN	-1.6664588	0.885477	-1.882	0.06	-0.58093886	count	1
AC092683.1	-1.6661413	0.9264884	-1.7983	0.0722	-0.580881471	count	1
THBD	-0.4064252	0.0732894	-5.5455	3.25E-08	-0.580766939	count	0.000778213
KANK1	-1.432447	0.8665312	-1.6531	0.0984	-0.580693794	count	1
PRDM8	-0.4667706	0.4443127	-1.0505	0.294	-0.57882698	count	1
ADAMTS6	-1.1528264	0.9220474	-1.2503	0.211	-0.576972069	count	1
NUPR1	-0.4204373	0.2405656	-1.7477	0.0806	-0.576551439	count	1
ELL2	-0.424028	0.1130162	-3.7519	0.00018	-0.576506596	count	1
FKBP2	-0.4034966	0.0685026	-5.8902	4.39E-09	-0.575921808	count	0.000105373
ELMO2	-0.8603155	0.615139	-1.3986	0.162	-0.575777241	count	1
SPINT2	-0.4079158	0.0800284	-5.0971	3.71E-07	-0.575651675	count	0.008850576
DNAJC25-GNG10	-0.993857	0.7052916	-1.4091	0.159	-0.573992486	count	1
BX284668.6	-1.2533247	0.762094	-1.6446	0.1	-0.573769804	count	1
MTFR1	-0.6479706	0.427302	-1.5164	0.13	-0.573455332	count	1
SIGLEC7	-0.4421714	0.2210339	-2.0005	0.0456	-0.572774175	count	1
AL512329.2	-1.2492507	1.0910947	-1.145	0.252	-0.572543798	count	1
SGPL1	-0.4631651	0.263772	-1.7559	0.0792	-0.571924579	count	1
FEN1	-0.7466256	0.4124001	-1.8104	0.0703	-0.571683922	count	1
NT5DC2	-0.6101688	0.4050463	-1.5064	0.132	-0.571343322	count	1
PAQR3	-1.613975	0.7221712	-2.2349	0.0255	-0.571245132	count	1

HINFP	-0.6672059	0.3989206	-1.6725	0.0945	-0.571036982	count	1
USP5	-0.6936825	0.4429921	-1.5659	0.118	-0.570874946	count	1
HGF	-0.4731463	0.214917	-2.2015	0.0278	-0.570681832	count	1
GPX4	-0.3967718	0.0408478	-9.7134	6.53E-22	-0.570333954	count	1.58E-17
HK1	-0.4277598	0.153466	-2.7873	0.00536	-0.569356637	count	1
HSPA2	-1.602959	0.7337243	-2.1847	0.029	-0.56915628	count	1
ZBED3	-0.5338107	0.3286081	-1.6245	0.104	-0.568932279	count	1
ITGA5	-0.4340645	0.2335774	-1.8583	0.0632	-0.568812536	count	1
MED18	-0.7224199	0.5707149	-1.2658	0.206	-0.568279286	count	1
TXNDC17	-0.4002197	0.0767086	-5.2174	1.97E-07	-0.568074993	count	0.004705542
SEMA6B	-0.4670327	0.297573	-1.5695	0.117	-0.567908709	count	1
STK17A	-0.4152879	0.1395155	-2.9766	0.00294	-0.567544084	count	1
MCOLN2	-2.771212	1.471312	-1.8835	0.0598	-0.566576661	count	1
RAD50	-0.6732576	0.5311093	-1.2676	0.205	-0.566098571	count	1
RABL2A	-1.1208052	0.7713518	-1.453	0.146	-0.565723686	count	1
RBM38	-0.4584994	0.2369616	-1.9349	0.0531	-0.565451561	count	1
GNL3	-0.4340389	0.1652606	-2.6264	0.00868	-0.564443322	count	1
ILKAP	-0.4786177	0.2676051	-1.7885	0.0738	-0.56404818	count	1
PDXK	-0.3960313	0.0800876	-4.945	8.13E-07	-0.563998643	count	0.019372164
NENF	-0.3969405	0.0820691	-4.8367	1.40E-06	-0.563442715	count	0.033327
PLSCR1	-0.3946426	0.0636482	-6.2004	6.59E-10	-0.563425049	count	1.58E-05
OPN3	-0.4249134	0.2013769	-2.11	0.035	-0.563149441	count	1
AC002091.2	-0.8054304	0.8828913	-0.9123	0.362	-0.562987385	count	1
NTAN1	-0.4002672	0.1177693	-3.3987	0.000688	-0.562666762	count	1
MAPKAP1	-0.4218171	0.1678641	-2.5128	0.012	-0.562244402	count	1
FBXO31	-1.3575707	1.1030463	-1.2307	0.219	-0.562107255	count	1
DYRK4	-0.4384292	0.2155664	-2.0338	0.0421	-0.56206707	count	1
ABHD15	-0.5331975	0.3273575	-1.6288	0.103	-0.56167988	count	1
C4orf3	-0.3912636	0.0604898	-6.4683	1.19E-10	-0.560379237	count	2.87E-06
SWAP70	-0.4125439	0.1281433	-3.2194	0.0013	-0.560010418	count	1
AL023806.1	-1.5529556	1.1599114	-1.3389	0.181	-0.559432793	count	1
NXT2	-0.6115917	0.414246	-1.4764	0.14	-0.559116223	count	1
SCYL1	-0.4629415	0.2782865	-1.6635	0.0963	-0.558767688	count	1
FAM50A	-0.3982724	0.105758	-3.7659	0.00017	-0.55815722	count	1
UTP25	-0.8276814	0.6338867	-1.3057	0.192	-0.557973447	count	1
SIGLEC9	-0.4326163	0.1801705	-2.4012	0.0164	-0.557600572	count	1
RANBP2	-0.4062528	0.1453556	-2.7949	0.00523	-0.557173086	count	1
DNMT3A	-0.4805913	0.2908862	-1.6522	0.0986	-0.55689615	count	1
C14orf119	-0.4023641	0.1262554	-3.1869	0.00146	-0.556151998	count	1
ALDH1B1	-1.3341759	0.8280205	-1.6113	0.107	-0.55607531	count	1
RCN1	-0.5045159	0.2914543	-1.731	0.0836	-0.555983559	count	1
BNIP3L	-0.3879014	0.0583871	-6.6436	3.76E-11	-0.555119203	count	9.06E-07
ABCB4	-2.5630781	1.308713	-1.9585	0.0503	-0.554741711	count	1
DCTN2	-0.4086168	0.1344848	-3.0384	0.0024	-0.554733806	count	1
MAN1B1	-0.4864147	0.233353	-2.0845	0.0372	-0.554293131	count	1

LRRC23	-0.5493983	0.3968238	-1.3845	0.166	-0.554080663	count	1
IL7	-1.0848476	0.6134402	-1.7685	0.0771	-0.552790462	count	1
CALM3	-0.3874029	0.0737864	-5.2503	1.65E-07	-0.552641279	count	0.003942015
GNPDA1	-0.3955456	0.1208836	-3.2721	0.00108	-0.551844487	count	1
HDHD3	-0.5557446	0.619667	-0.8968	0.37	-0.550854566	count	1
FAM129B	-0.4261633	0.2869423	-1.4852	0.138	-0.550751715	count	1
LY9	-0.941119	0.6494377	-1.4491	0.147	-0.550630908	count	1
MED19	-0.4223115	0.1901451	-2.221	0.0264	-0.550203829	count	1
FTH1	-0.3812218	0.0294814	-12.9309	4.76E-37	-0.549953203	count	1.16E-32
PDGFA	-0.5443495	0.2809123	-1.9378	0.0528	-0.549339411	count	1
TTC21A	-0.9374651	0.8641242	-1.0849	0.278	-0.548983495	count	1
ABRAXAS2	-0.4419251	0.2035848	-2.1707	0.03	-0.548821991	count	1
PDCD4-AS1	-0.7315952	0.4751806	-1.5396	0.124	-0.548481378	count	1
GPR35	-0.4550491	0.2824709	-1.611	0.107	-0.548425475	count	1
GOLIM4	-0.4024358	0.1392906	-2.8892	0.0039	-0.547313293	count	1
SLC39A11	-0.4662356	0.2235989	-2.0851	0.0372	-0.547096719	count	1
CHCHD1	-0.3968967	0.1555896	-2.5509	0.0108	-0.547053738	count	1
ZNF510	-0.7520236	1.0868586	-0.6919	0.489	-0.546998887	count	1
LINC00900	-0.7520236	0.6062194	-1.2405	0.215	-0.546998887	count	1
GPAA1	-0.4026202	0.1460288	-2.7571	0.00587	-0.546145542	count	1
CENPF	-0.5455752	0.5007969	-1.0894	0.276	-0.546077887	count	1
C1GALT1C1	-0.4255616	0.2185579	-1.9471	0.0516	-0.545899643	count	1
NQO1	-0.727225	0.517839	-1.4043	0.16	-0.545682254	count	1
LINC00526	-1.1627584	0.7403016	-1.5707	0.116	-0.545634237	count	1
LINC01480	-0.4382706	0.3677315	-1.1918	0.233	-0.54517168	count	1
BAMBI	-0.6450379	0.8341274	-0.7733	0.439	-0.545045464	count	1
NAF1	-0.4170198	0.2120314	-1.9668	0.0493	-0.54484004	count	1
POLR1C	-0.4861034	0.5196421	-0.9355	0.35	-0.543606714	count	1
LINC00884	-0.4683774	0.3071504	-1.5249	0.127	-0.543291166	count	1
CTTNBP2NL	-0.4112835	0.1449013	-2.8384	0.00457	-0.542974086	count	1
KPNA2	-0.4014472	0.1114537	-3.6019	0.000322	-0.542887176	count	1
MRPL15	-0.4025361	0.1615905	-2.4911	0.0128	-0.542154028	count	1
TIMM8A	-0.6086279	0.4906364	-1.2405	0.215	-0.542072092	count	1
EVA1B	-0.399374	0.1574454	-2.5366	0.0113	-0.542050185	count	1
TCP1	-0.3958429	0.1003116	-3.9461	8.17E-05	-0.541896404	count	1
PLEKHM2	-0.3933005	0.1417592	-2.7744	0.00557	-0.541837499	count	1
PPP1R3B	-0.4804186	0.3654918	-1.3144	0.189	-0.541790284	count	1
MFSD13A	-0.5010326	0.3279029	-1.528	0.127	-0.541730013	count	1
RUBCNL	-0.5194212	0.3397142	-1.529	0.126	-0.541130834	count	1
MKI67	-0.8312777	0.8359568	-0.9944	0.32	-0.541003279	count	1
ARHGAP18	-0.3793225	0.0724755	-5.2338	1.80E-07	-0.540988814	count	0.00430002
BRCA1	-0.6070162	0.5484353	-1.1068	0.268	-0.540776157	count	1
ALG2	-0.4076236	0.1472127	-2.7689	0.00567	-0.540553889	count	1
AMIGO2	-1.756703	0.9311149	-1.8867	0.0593	-0.540262014	count	1
FCHO2	-0.3904815	0.1304195	-2.994	0.00278	-0.540092973	count	1

TMEM86A	-0.5254527	0.3782625	-1.3891	0.165	-0.539554429	count	1
RRAGD	-0.399658	0.1882831	-2.1226	0.0339	-0.539282997	count	1
HIST1H2BN	-0.9742357	0.7421034	-1.3128	0.189	-0.539055049	count	1
ITGB1	-0.3773929	0.0718621	-5.2516	1.64E-07	-0.537924272	count	0.003918288
HAGHL	-0.4649437	0.3600732	-1.2912	0.197	-0.537820191	count	1
TRUB2	-0.4970463	0.284472	-1.7473	0.0807	-0.537644959	count	1
PPP1R17	-0.8628786	1.815733	-0.4752	0.635	-0.536698526	count	1
ADGRE2	-0.3966262	0.1391665	-2.85	0.00441	-0.536435536	count	1
ABCD1	-0.4481755	0.3041959	-1.4733	0.141	-0.535778183	count	1
AL161935.3	-0.4500474	0.2500625	-1.7997	0.072	-0.535467999	count	1
ENC1	-0.4976278	0.3534073	-1.4081	0.159	-0.535401169	count	1
RPP40	-0.7112339	0.4670061	-1.523	0.128	-0.535388511	count	1
SMYD3	-0.5491542	0.5429082	-1.0115	0.312	-0.535017751	count	1
TUBA1B	-0.3719139	0.0489715	-7.5945	4.36E-14	-0.534751631	count	1.05E-09
TMED3	-0.3953973	0.1478247	-2.6748	0.00753	-0.534351646	count	1
RTCB	-0.3923257	0.1330577	-2.9485	0.00322	-0.534165581	count	1
EGLN3	-0.4218784	0.3963168	-1.0645	0.287	-0.533826649	count	1
ADGRB1	-17.7988987	2638.253693	-0.0067	0.995	-0.532892935	count	1
FRMD6	-17.798853	1907.507903	-0.0093	0.993	-0.532892935	count	1
SULF1	-17.4788308	1741.348731	-0.01	0.992	-0.532892931	count	1
COL3A1	-17.4786412	1478.180055	-0.0118	0.991	-0.532892931	count	1
HIST1H2BH	-17.4726681	1583.935758	-0.011	0.991	-0.532892931	count	1
LINC02175	-17.3493969	2175.698639	-0.008	0.994	-0.532892929	count	1
ZFYVE9	-17.3492286	1905.069146	-0.0091	0.993	-0.532892929	count	1
AC064836.3	-17.3492286	1905.069146	-0.0091	0.993	-0.532892929	count	1
AL358852.1	-17.3492286	1905.069146	-0.0091	0.993	-0.532892929	count	1
KLHL11	-17.3492286	1905.069146	-0.0091	0.993	-0.532892929	count	1
AC099568.2	-17.2227894	2398.618802	-0.0072	0.994	-0.532892926	count	1
VCAM1	-17.2224215	1692.659517	-0.0102	0.992	-0.532892926	count	1
AC092587.1	-16.8969752	2951.194046	-0.0057	0.995	-0.532892918	count	1
AL133351.1	-16.8961981	1964.005507	-0.0086	0.993	-0.532892918	count	1
CA12	-16.8961981	1964.005507	-0.0086	0.993	-0.532892918	count	1
AC023043.1	-16.8959327	1699.25233	-0.0099	0.992	-0.532892918	count	1
A4GALT	-16.8959327	1699.25233	-0.0099	0.992	-0.532892918	count	1
UBOX5	-16.8378582	1260.669742	-0.0134	0.989	-0.532892916	count	1
MAFA	-18.18994	1486.581556	-0.0122	0.99	-0.53289285	count	1
HSD17B1	-17.462882	2386.718109	-0.0073	0.994	-0.532892842	count	1
COL7A1	-17.315103	3308.612874	-0.0052	0.996	-0.532892839	count	1
HIST1H2AK	-17.314607	2916.160831	-0.0059	0.995	-0.532892839	count	1
AL080317.1	-17.314607	2916.160831	-0.0059	0.995	-0.532892839	count	1
NOTCH3	-17.314607	2916.160831	-0.0059	0.995	-0.532892839	count	1
CPNE9	-17.314102	2462.404928	-0.007	0.994	-0.532892839	count	1
AC091808.1	-17.314102	2462.404928	-0.007	0.994	-0.532892839	count	1
C8orf88	-17.314102	2462.404928	-0.007	0.994	-0.532892839	count	1
BAALC	-17.314102	2462.404928	-0.007	0.994	-0.532892839	count	1

AL359182.2	-17.314102	2462.404928	-0.007	0.994	-0.532892839	count	1
AP001453.1	-17.314102	2462.404928	-0.007	0.994	-0.532892839	count	1
NTSR1	-17.314102	2462.404928	-0.007	0.994	-0.532892839	count	1
AP000695.2	-17.314102	2462.404928	-0.007	0.994	-0.532892839	count	1
SPATA18	-16.317066	3350.573769	-0.0049	0.996	-0.532892806	count	1
PHEX	-16.317066	3350.573769	-0.0049	0.996	-0.532892806	count	1
JAM3	-16.317066	3350.573769	-0.0049	0.996	-0.532892806	count	1
IGHV1-18	-16.317066	3350.573769	-0.0049	0.996	-0.532892806	count	1
AC116914.2	-16.317066	3350.573769	-0.0049	0.996	-0.532892806	count	1
AC010522.1	-16.317066	3350.573769	-0.0049	0.996	-0.532892806	count	1
OSBP2	-16.317066	3350.573769	-0.0049	0.996	-0.532892806	count	1
DPYSL4	-16.315322	2219.318368	-0.0074	0.994	-0.532892805	count	1
CCDC65	-16.315322	2219.318368	-0.0074	0.994	-0.532892805	count	1
AC015712.1	-16.315322	2219.318368	-0.0074	0.994	-0.532892805	count	1
SLC2A11	-1.4259893	0.8881854	-1.6055	0.109	-0.53288894	count	1
UTP18	-0.3946746	0.1610966	-2.4499	0.0144	-0.532319707	count	1
SCX	-1.1214646	2.5336977	-0.4426	0.658	-0.532181823	count	1
ELAC1	-0.640326	0.3494446	-1.8324	0.067	-0.532013935	count	1
CENPL	-1.4202543	1.2024463	-1.1811	0.238	-0.531624757	count	1
ZC4H2	-2.24237	1.3641039	-1.6438	0.1	-0.531336939	count	1
Z93241.1	-0.5164953	0.4512861	-1.1445	0.253	-0.530929585	count	1
SDC3	-0.4070427	0.1957263	-2.0797	0.0377	-0.530639719	count	1
IFT46	-1.1166274	0.7871221	-1.4186	0.156	-0.530579939	count	1
COPS4	-0.4107239	0.1811576	-2.2672	0.0235	-0.529034634	count	1
POLR2J2	-0.7473596	0.8053698	-0.928	0.354	-0.528900782	count	1
TFPT	-0.3899933	0.1869111	-2.0865	0.037	-0.528524703	count	1
ARL8B	-0.3761302	0.096702	-3.8896	0.000103	-0.527841807	count	1
AL136454.1	-1.0171332	0.7045629	-1.4436	0.149	-0.527553229	count	1
CMC4	-0.7722354	0.5931318	-1.302	0.193	-0.526978163	count	1
SNX12	-0.529017	0.4159788	-1.2717	0.204	-0.526195751	count	1
CENPK	-0.8872382	0.6978654	-1.2714	0.204	-0.525955825	count	1
TSPYL4	-0.5798359	0.4689065	-1.2366	0.216	-0.525888033	count	1
BHLHE41	-0.3939251	0.157858	-2.4954	0.0126	-0.52511049	count	1
SIPA1L3	-0.8393222	0.5897819	-1.4231	0.155	-0.524932014	count	1
CARMIL1	-0.5691459	0.392069	-1.4516	0.147	-0.523691996	count	1
RBM41	-0.5124319	0.3574568	-1.4335	0.152	-0.523175391	count	1
PDIA6	-0.3660451	0.065603	-5.5797	2.67E-08	-0.523100102	count	0.000639625
NPC2	-0.3628623	0.0338319	-10.7254	2.93E-26	-0.522436705	count	7.11E-22
VEZT	-0.4326926	0.2891797	-1.4963	0.135	-0.522283306	count	1
USF2	-0.3683991	0.0726315	-5.0722	4.23E-07	-0.522194887	count	0.010088127
ATP6V1D	-0.3759285	0.1077761	-3.4881	0.000495	-0.521702116	count	1
NIT2	-0.4125616	0.2354778	-1.752	0.0799	-0.521117753	count	1
RPS6KA2	-0.6884925	0.5890607	-1.1688	0.243	-0.520610662	count	1
GPI	-0.3758393	0.1427396	-2.633	0.00852	-0.520324535	count	1
ZNF738	-0.5815362	0.6030092	-0.9644	0.335	-0.520181507	count	1

MRPS14	-0.3973159	0.1976277	-2.0104	0.0445	-0.520144497	count	1
CAVIN3	-0.4194997	0.367096	-1.1428	0.253	-0.520142225	count	1
C19orf24	-0.3717063	0.0969435	-3.8343	0.000129	-0.520103105	count	1
ZFYVE16	-0.3686596	0.1114637	-3.3074	0.000955	-0.519987328	count	1
ZBED4	-0.5560064	0.4191985	-1.3264	0.185	-0.518820965	count	1
NAALADL1	-0.5205963	0.36349	-1.4322	0.152	-0.518384451	count	1
FBLN5	-1.616697	1.0669071	-1.5153	0.13	-0.518081095	count	1
CCRL2	-0.4025613	0.2094275	-1.9222	0.0547	-0.517839711	count	1
TOX	-0.9915858	2.3457591	-0.4227	0.673	-0.517728106	count	1
ZNF530	-0.9915858	1.1473285	-0.8643	0.388	-0.517728106	count	1
MCRIP2	-0.37495	0.1558857	-2.4053	0.0162	-0.516945553	count	1
KBTBD8	-0.4168565	0.1952842	-2.1346	0.0329	-0.516175206	count	1
VAV3-AS1	-1.1885926	2.3845318	-0.4985	0.618	-0.516030351	count	1
SIRT2	-0.3905096	0.1502895	-2.5984	0.00942	-0.515927667	count	1
SNCG	-1.3458762	1.2245581	-1.0991	0.272	-0.514698283	count	1
TARS	-0.4024041	0.1634584	-2.4618	0.0139	-0.514394274	count	1
BRPF1	-0.53758	0.3542475	-1.5175	0.129	-0.514218764	count	1
DDX39A	-0.3748748	0.1325685	-2.8278	0.00473	-0.512769177	count	1
STIMATE	-0.6578614	0.4924122	-1.336	0.182	-0.512456177	count	1
TNFRSF21	-0.4308701	0.254888	-1.6904	0.0911	-0.512149992	count	1
UBN1	-0.3758247	0.1551002	-2.4231	0.0155	-0.51178541	count	1
OTUD6B	-0.5045825	0.2913619	-1.7318	0.0834	-0.511743785	count	1
KLHL5	-0.3882699	0.1730963	-2.2431	0.025	-0.51027621	count	1
PSD4	-0.4816002	0.3776475	-1.2753	0.202	-0.510270468	count	1
PCBD2	-0.5212641	0.3343357	-1.5591	0.119	-0.509759803	count	1
CCDC71L	-0.3834421	0.1976375	-1.9401	0.0525	-0.508900002	count	1
KNOP1	-0.4186671	0.2209491	-1.8949	0.0582	-0.508818565	count	1
CHST2	-0.5094861	0.4143691	-1.2295	0.219	-0.508047462	count	1
CIITA	-0.4171252	0.1792677	-2.3268	0.0201	-0.506995865	count	1
PKD2L1	-0.6195134	0.6752816	-0.9174	0.359	-0.50696431	count	1
GPRC5D-AS1	-0.6195134	0.5731599	-1.0809	0.28	-0.50696431	count	1
ZFAND6	-0.3615586	0.1085474	-3.3309	0.000879	-0.506704745	count	1
PBDC1	-0.3702629	0.1419513	-2.6084	0.00915	-0.506321969	count	1
MTPAP	-0.4364186	0.3823389	-1.1414	0.254	-0.506011323	count	1
RWDD2A	-1.5464309	1.0728809	-1.4414	0.15	-0.505922498	count	1
TCEANC	-1.9759803	1.3867231	-1.4249	0.154	-0.505918806	count	1
IL1R1	-0.3979108	0.3137224	-1.2684	0.205	-0.505735003	count	1
JRK	-0.8949658	0.8532765	-1.0489	0.294	-0.505121345	count	1
ADAT3	-1.039923	0.8037608	-1.2938	0.196	-0.504436148	count	1
TTC19	-0.3833538	0.229353	-1.6715	0.0948	-0.503906922	count	1
PDIA3	-0.3515017	0.0532301	-6.6034	4.91E-11	-0.503503283	count	1.18E-06
AIFM2	-0.7049633	0.5685828	-1.2399	0.215	-0.503354108	count	1
MRNIP	-0.6622419	0.3511626	-1.8859	0.0594	-0.503350243	count	1
CDC42EP4	-0.4956899	0.3472859	-1.4273	0.154	-0.50327634	count	1
FPR3	-0.3711255	0.1182421	-3.1387	0.00172	-0.502807655	count	1

MICA	-0.5441871	0.3805128	-1.4301	0.153	-0.502605914	count	1
RENBP	-0.4007102	0.1833575	-2.1854	0.029	-0.502193744	count	1
MCU	-0.5992773	0.7185757	-0.834	0.404	-0.50150508	count	1
SEC61G	-0.3508489	0.0678511	-5.1709	2.52E-07	-0.501413713	count	0.006015492
MEX3D	-0.7272215	0.5900398	-1.2325	0.218	-0.501122328	count	1
SCOC-AS1	-1.5156768	1.274371	-1.1894	0.234	-0.500373229	count	1
ATP1B3	-0.3513097	0.0618912	-5.6762	1.54E-08	-0.500155655	count	0.000369184
DVL3	-0.4535645	0.3421885	-1.3255	0.185	-0.500064735	count	1
ARMCX6	-0.386349	0.1625096	-2.3774	0.0175	-0.499772575	count	1
ARSK	-1.2822843	0.8142992	-1.5747	0.115	-0.49942249	count	1
AKR1A1	-0.3509019	0.0643333	-5.4544	5.40E-08	-0.498981641	count	0.001292058
MARCKS	-0.3471321	0.0542609	-6.3975	1.89E-10	-0.498176333	count	4.55E-06
NMNAT1	-0.5254881	0.4122228	-1.2748	0.203	-0.498133965	count	1
PELO	-0.3919545	0.3105965	-1.2619	0.207	-0.497788656	count	1
SDCCAG8	-0.360535	0.1339409	-2.6917	0.00716	-0.497596104	count	1
SASH1	-0.4098529	0.2231241	-1.8369	0.0663	-0.497442084	count	1
PHLDA2	-0.3607555	0.1683361	-2.1431	0.0322	-0.497167092	count	1
CAPN10	-0.485034	0.4419853	-1.0974	0.273	-0.496848484	count	1
SDE2	-0.377942	0.1456507	-2.5949	0.00952	-0.496610224	count	1
POP4	-0.3689014	0.1657815	-2.2252	0.0262	-0.496388784	count	1
AC021016.3	-0.6711897	0.9128857	-0.7352	0.462	-0.496310574	count	1
TP53BP1	-0.4277009	0.4813226	-0.8886	0.374	-0.496255309	count	1
PTGS1	-0.3983096	0.1811806	-2.1984	0.028	-0.495902135	count	1
C12orf45	-0.3713755	0.225256	-1.6487	0.0993	-0.495601586	count	1
CCDC107	-0.3541346	0.0982929	-3.6028	0.000321	-0.495503191	count	1
NDUFAF6	-0.5222225	0.3555445	-1.4688	0.142	-0.49526789	count	1
PLEK2	-0.5219671	0.3014108	-1.7317	0.0834	-0.495043608	count	1
LRRC42	-0.4353824	0.3094657	-1.4069	0.16	-0.494833367	count	1
CENPW	-0.3866723	0.2083458	-1.8559	0.0636	-0.494651398	count	1
CTSH	-0.3454491	0.048704	-7.0928	1.71E-12	-0.494483325	count	4.13E-08
CCDC88A	-0.3482813	0.0954972	-3.647	0.000271	-0.494123712	count	1
MRPL20	-0.3484089	0.0806247	-4.3214	1.61E-05	-0.493870386	count	0.3809582
C1QB	-0.3426769	0.083663	-4.0959	4.34E-05	-0.493462352	count	1
ARL3	-0.3629832	0.1602857	-2.2646	0.0236	-0.493098565	count	1
AC005838.2	-0.7762649	1.067219	-0.7274	0.467	-0.492631654	count	1
PABPC4	-0.3523134	0.0890466	-3.9565	7.82E-05	-0.492331089	count	1
MRPL39	-0.4437351	0.3399789	-1.3052	0.192	-0.491913959	count	1
TEX2	-0.4693115	0.3947606	-1.1889	0.235	-0.49175018	count	1
FKBP14	-0.6857541	0.4631627	-1.4806	0.139	-0.491595857	count	1
SKIL	-0.3505934	0.0900647	-3.8927	0.000102	-0.491588579	count	1
NETO2	-0.45177	0.2468578	-1.8301	0.0674	-0.490965539	count	1
C11orf45	-0.6432168	0.6525553	-0.9857	0.324	-0.490705641	count	1
AC008736.1	-0.5969651	0.9098846	-0.6561	0.512	-0.490482448	count	1
NUDT9	-0.3886533	0.243034	-1.5992	0.11	-0.490393983	count	1
AC008741.2	-0.8123556	0.9515252	-0.8537	0.393	-0.490291795	count	1

DNAJB6	-0.3451935	0.0692536	-4.9845	6.65E-07	-0.489895886	count	0.015848945
SMIM4	-0.3645492	0.1598998	-2.2799	0.0227	-0.489629665	count	1
TIGD5	-0.5216162	0.4771811	-1.0931	0.274	-0.48917839	count	1
PFKP	-0.404491	0.2715953	-1.4893	0.137	-0.489173357	count	1
TXNRD1	-0.3558898	0.1325927	-2.6841	0.00732	-0.489145239	count	1
KCNMB4	-0.4938101	0.3537626	-1.3959	0.163	-0.489125707	count	1
AL355075.4	-0.5941173	0.411296	-1.4445	0.149	-0.488389501	count	1
AC005261.1	-0.5939994	0.4959199	-1.1978	0.231	-0.488302794	count	1
PIK3R5	-0.3883937	0.2473387	-1.5703	0.116	-0.487083404	count	1
IRF8	-0.3550617	0.0946098	-3.7529	0.000179	-0.486907445	count	1
SLC17A5	-0.3815199	0.228009	-1.6733	0.0944	-0.486768383	count	1
NANS	-0.3439715	0.0748049	-4.5982	4.48E-06	-0.486653613	count	0.10638208
ISG15	-0.3427521	0.1189704	-2.881	0.004	-0.48652502	count	1
IFI27	-0.3673755	0.5558429	-0.6609	0.509	-0.486470799	count	1
SERINC5	-0.3660123	0.2350385	-1.5572	0.12	-0.485826862	count	1
XCL1	-0.987404	1.3632525	-0.7243	0.469	-0.485716665	count	1
CENPX	-0.3490742	0.1186399	-2.9423	0.00329	-0.484918534	count	1
EID2	-0.388093	0.2799849	-1.3861	0.166	-0.484135056	count	1
PALB2	-0.697948	0.5759853	-1.2117	0.226	-0.483974602	count	1
MOAP1	-0.3785374	0.2733534	-1.3848	0.166	-0.483970295	count	1
KHDRBS3	-1.787629	1.0499596	-1.7026	0.0888	-0.483916938	count	1
TMEM138	-0.381507	0.2455802	-1.5535	0.12	-0.483732514	count	1
HTR7	-1.0807795	0.7941724	-1.3609	0.174	-0.483461713	count	1
HIST2H2BF	-0.7245659	0.5308491	-1.3649	0.172	-0.48295464	count	1
APOLD1	-0.5018082	0.7328313	-0.6848	0.494	-0.482392993	count	1
ATP6V1H	-0.3662296	0.1984827	-1.8451	0.0651	-0.481427771	count	1
EPAS1	-0.4689015	0.2871927	-1.6327	0.103	-0.48125073	count	1
CRELD1	-0.4020182	0.3413003	-1.1779	0.239	-0.481090207	count	1
APOC2	-1.7629108	1.0321437	-1.708	0.0878	-0.480749588	count	1
TRAF2	-0.5406415	0.7285612	-0.7421	0.458	-0.479812142	count	1
TLCD1	-1.4076315	1.1799757	-1.1929	0.233	-0.479727988	count	1
WDR35	-0.8957151	0.6814113	-1.3145	0.189	-0.479346004	count	1
ZNF674-AS1	-0.8943988	0.7984277	-1.1202	0.263	-0.47880223	count	1
MRTO4	-0.361365	0.2350037	-1.5377	0.124	-0.477906125	count	1
ENAH	-2.5479935	1.4681085	-1.7356	0.0828	-0.477725344	count	1
PGM2L1	-0.5470442	0.3246757	-1.6849	0.0921	-0.47768015	count	1
C19orf47	-0.8903851	0.5108531	-1.7429	0.0815	-0.477141242	count	1
LRRC29	-1.060533	0.5982277	-1.7728	0.0764	-0.477057566	count	1
ACRBP	-0.5289314	0.3489445	-1.5158	0.13	-0.47703248	count	1
HSPE1	-0.334615	0.0694597	-4.8174	1.54E-06	-0.476633105	count	0.036652
REXO1	-0.4113127	0.2619256	-1.5703	0.116	-0.476402833	count	1
GNPDA2	-0.8876785	0.6082341	-1.4594	0.145	-0.476018797	count	1
PVR	-0.7440302	0.6088014	-1.2221	0.222	-0.475666379	count	1
LYRM4	-0.3972765	0.2162653	-1.837	0.0663	-0.475574216	count	1
NUDT3	-0.3627375	0.1518708	-2.3885	0.017	-0.475502739	count	1

PLD3	-0.333475	0.08157	-4.0882	4.49E-05	-0.475281984	count	1
LY6G5B	-1.054179	1.3443664	-0.7841	0.433	-0.475028673	count	1
LCA5	-1.054179	1.1762207	-0.8962	0.37	-0.475028673	count	1
SLC44A1	-0.3860382	0.1915182	-2.0157	0.0439	-0.474349795	count	1
RCC1L	-0.5334017	0.305733	-1.7447	0.0812	-0.473929701	count	1
DIXDC1	-0.8822563	0.9424623	-0.9361	0.349	-0.473764253	count	1
CPNE2	-0.3706202	0.2046326	-1.8111	0.0702	-0.473568853	count	1
NDUFAF3	-0.3357155	0.0815717	-4.1156	3.99E-05	-0.473553031	count	0.940842
UAP1	-0.4143642	0.285725	-1.4502	0.147	-0.47349762	count	1
HSP90AB1	-0.3290625	0.0415496	-7.9197	3.58E-15	-0.473492172	count	8.66E-11
FNDC3B	-0.3420124	0.1508065	-2.2679	0.0234	-0.472947785	count	1
C10orf55	-1.0464725	0.7377831	-1.4184	0.156	-0.472555556	count	1
ZNF549	-1.0464725	1.4620126	-0.7158	0.474	-0.472555556	count	1
ZMYND19	-0.5998559	0.53456	-1.1221	0.262	-0.472410044	count	1
TUBB2A	-0.350031	0.1717497	-2.038	0.0417	-0.471972712	count	1
BLOC1S6	-0.3425794	0.1030938	-3.323	0.000904	-0.471880642	count	1
SCAANT1	-1.3683755	1.1666379	-1.1729	0.241	-0.471766992	count	1
PTPMT1	-0.3608212	0.2274362	-1.5865	0.113	-0.471551642	count	1
SLC25A19	-0.3481661	0.1879003	-1.8529	0.064	-0.471480661	count	1
VDR	-0.4281313	0.3005138	-1.4247	0.154	-0.470993355	count	1
NOL4L	-0.5468576	0.4102039	-1.3331	0.183	-0.46985093	count	1
AP1S1	-0.3531219	0.1682473	-2.0988	0.0359	-0.469544462	count	1
CD68	-0.3262385	0.0425461	-7.6679	2.50E-14	-0.469527959	count	6.04E-10
UBASH3B	-0.409139	0.349883	-1.1694	0.242	-0.467735542	count	1
ATP13A3	-0.334516	0.1312888	-2.5479	0.0109	-0.467536557	count	1
IDH2	-0.3374664	0.1177848	-2.8651	0.0042	-0.467237867	count	1
ZFYVE28	-0.4745886	0.4896532	-0.9692	0.333	-0.466988629	count	1
TMEM176A	-0.3284814	0.0846957	-3.8784	0.000108	-0.465593619	count	1
ISCA2	-0.3467695	0.1773788	-1.955	0.0507	-0.465187523	count	1
AFG1L	-0.930916	0.7963835	-1.1689	0.243	-0.464823484	count	1
ZNF618	-0.487194	0.3483521	-1.3986	0.162	-0.464328146	count	1
BET1L	-0.3861318	0.2280981	-1.6928	0.0906	-0.46362583	count	1
C15orf40	-0.3505799	0.1901473	-1.8437	0.0653	-0.463582221	count	1
GPR160	-0.4145484	0.2956427	-1.4022	0.161	-0.462832847	count	1
RANBP1	-0.3303079	0.0946703	-3.489	0.000493	-0.462385703	count	1
SPIRE1	-0.3741669	0.2628273	-1.4236	0.155	-0.462342283	count	1
TPX2	-1.0134535	0.8320724	-1.218	0.223	-0.46180551	count	1
IGHA1	-0.6178891	0.2462025	-2.5097	0.0121	-0.461771842	count	1
HPS5	-0.3687229	0.1729498	-2.132	0.0331	-0.461091071	count	1
MPP1	-0.3243011	0.0932655	-3.4772	0.000516	-0.460108672	count	1
EREG	-0.3215341	0.1354334	-2.3741	0.0177	-0.45999688	count	1
AC068025.2	-0.8483704	2.0996845	-0.404	0.686	-0.459497312	count	1
CKM	-0.8483704	1.0629205	-0.7982	0.425	-0.459497312	count	1
LRP12	-0.3974782	0.3867413	-1.0278	0.304	-0.459442971	count	1
TRAPPC2B	-0.3468884	0.209538	-1.6555	0.098	-0.459437694	count	1

SNX24	-0.3607888	0.2243069	-1.6085	0.108	-0.459385061	count	1
AC067852.2	-0.7498732	0.6320785	-1.1864	0.236	-0.459300905	count	1
HSPA4	-0.3355809	0.1307922	-2.5658	0.0104	-0.459235703	count	1
WASHC3	-0.3377255	0.1293261	-2.6114	0.00907	-0.459172106	count	1
RER1	-0.3231998	0.0696359	-4.6413	3.64E-06	-0.458877783	count	0.08647912
HMCN1	-0.9150718	2.311036	-0.396	0.692	-0.458820018	count	1
AC098487.1	-0.9150718	1.067018	-0.8576	0.391	-0.458820018	count	1
PEX11A	-0.9150718	1.0957561	-0.8351	0.404	-0.458820018	count	1
AC078850.1	-0.654661	0.8787866	-0.745	0.456	-0.458137228	count	1
BEND3	-1.1231883	1.173751	-0.9569	0.339	-0.457782135	count	1
TMEM234	-0.4000652	0.3046643	-1.3131	0.189	-0.457715299	count	1
MMP24OS	-0.3224312	0.0879336	-3.6668	0.000251	-0.457587601	count	1
DPP3	-0.4266069	0.2621099	-1.6276	0.104	-0.457360509	count	1
RBAK	-1.300036	0.7605444	-1.7093	0.0875	-0.457297286	count	1
ANKRD22	-0.5641619	0.283213	-1.992	0.0465	-0.456986567	count	1
CIB1	-0.3203965	0.0591092	-5.4204	6.53E-08	-0.456960518	count	0.001562041
OSER1	-0.3309245	0.113969	-2.9036	0.00372	-0.456732567	count	1
ANKS6	-0.652141	0.8275058	-0.7881	0.431	-0.456615396	count	1
GNA12	-0.3499208	0.3272689	-1.0692	0.285	-0.456294268	count	1
E2F6	-0.4724257	0.4383698	-1.0777	0.281	-0.45597384	count	1
POMGNT1	-0.4626356	0.4518177	-1.0239	0.306	-0.455936082	count	1
NUDT14	-0.338531	0.1713595	-1.9756	0.0483	-0.455759827	count	1
TFIP11	-0.4249058	0.3176617	-1.3376	0.181	-0.45561831	count	1
PRKCH	-0.495576	0.3965678	-1.2497	0.212	-0.455253359	count	1
PLIN3	-0.3221325	0.0856736	-3.76	0.000174	-0.454802595	count	1
NELFE	-0.3341461	0.1634603	-2.0442	0.041	-0.454762148	count	1
CEBPE	-0.4239487	0.425825	-0.9956	0.32	-0.454637763	count	1
PLA2G4C	-0.4709254	0.4533751	-1.0387	0.299	-0.454618184	count	1
CA2	-0.3521328	0.2479855	-1.42	0.156	-0.45436169	count	1
MED26	-0.5605517	0.3353924	-1.6713	0.0948	-0.454356208	count	1
GPR82	-0.6064368	0.4475632	-1.355	0.176	-0.454235582	count	1
DCTPP1	-0.3429907	0.1659829	-2.0664	0.0389	-0.453890205	count	1
RNASET2	-0.3158552	0.0461088	-6.8502	9.29E-12	-0.453846197	count	2.24E-07
MIR181A1HG	-0.3399782	0.1598349	-2.1271	0.0335	-0.453777777	count	1
MYL6B	-0.3540661	0.2221244	-1.594	0.111	-0.453624928	count	1
ZNF506	-0.3821119	0.3759532	-1.0164	0.31	-0.45358474	count	1
CCNYL1	-0.372495	0.2465241	-1.511	0.131	-0.452325068	count	1
KDELR2	-0.3179408	0.0729304	-4.3595	1.36E-05	-0.452216873	count	0.32198
FUOM	-0.3191923	0.0995194	-3.2073	0.00136	-0.452000109	count	1
GATC	-0.4987064	0.3831823	-1.3015	0.193	-0.451861158	count	1
CD36	-0.3142736	0.0967791	-3.2473	0.00118	-0.451774435	count	1
PPP1R3D	-0.4448509	0.4617438	-0.9634	0.335	-0.450906631	count	1
RPF1	-0.3778637	0.2711556	-1.3935	0.164	-0.450861953	count	1
RAP2B	-0.3213345	0.0985638	-3.2602	0.00113	-0.450832772	count	1
TMEM135	-0.5835535	0.5411657	-1.0783	0.281	-0.45031879	count	1

TMIGD3	-0.4027924	0.3403401	-1.1835	0.237	-0.450196573	count	1
ACP2	-0.3461049	0.1992265	-1.7372	0.0825	-0.45013453	count	1
HS3ST3B1	-0.9784295	0.4716139	-2.0746	0.0381	-0.450127581	count	1
CAMSAP1	-0.3724924	0.2673783	-1.3931	0.164	-0.44958897	count	1
MYLPF	-0.8878241	1.2348085	-0.719	0.472	-0.448347563	count	1
IQCG	-0.400772	0.3678426	-1.0895	0.276	-0.448021694	count	1
FAM129A	-0.32648	0.1091059	-2.9923	0.0028	-0.447970973	count	1
FCGR1A	-0.3140774	0.0756376	-4.1524	3.40E-05	-0.447868952	count	0.802298
COPB2	-0.3249452	0.1173889	-2.7681	0.00568	-0.447545044	count	1
NAA50	-0.3253798	0.139138	-2.3385	0.0194	-0.447406288	count	1
PIM3	-0.3134667	0.0739725	-4.2376	2.34E-05	-0.446857164	count	0.5529186
HSP90B1	-0.3109503	0.0604523	-5.1437	2.91E-07	-0.446825159	count	0.006945006
AC127070.1	-0.9682995	0.954605	-1.0143	0.311	-0.446696631	count	1
ZNF235	-0.9682995	1.2085645	-0.8012	0.423	-0.446696631	count	1
ANKRD54	-0.5269335	0.4722492	-1.1158	0.265	-0.446458292	count	1
KCNK13	-0.4921882	0.4678887	-1.0519	0.293	-0.446396939	count	1
NDUFAF4	-0.3560477	0.2456589	-1.4494	0.147	-0.44613848	count	1
SORD	-0.4435904	0.3397696	-1.3056	0.192	-0.446027138	count	1
AC245595.1	-0.484477	0.4774163	-1.0148	0.31	-0.445790574	count	1
ENPP4	-0.4290973	0.3434251	-1.2495	0.212	-0.445634865	count	1
SYCE1L	-1.2458785	1.1059772	-1.1265	0.26	-0.445261956	count	1
RAB20	-0.3145638	0.0768657	-4.0924	4.41E-05	-0.445172739	count	1
AC010864.1	-0.7216188	0.7691095	-0.9383	0.348	-0.444916223	count	1
SMARCAL1	-0.4653078	0.3465582	-1.3427	0.18	-0.444815636	count	1
AL391069.2	-1.0764158	0.6055058	-1.7777	0.0756	-0.444567521	count	1
FLT1	-0.3368817	0.2201265	-1.5304	0.126	-0.444531406	count	1
TTC3	-0.326014	0.1205575	-2.7042	0.00689	-0.444269659	count	1
ARMC6	-0.4499771	0.4252134	-1.0582	0.29	-0.44418751	count	1
PAQR8	-0.6309157	0.4472211	-1.4107	0.158	-0.443720577	count	1
PGBD5	-1.5068881	1.0095561	-1.4926	0.136	-0.443656888	count	1
NIPAL2	-0.4955629	0.4806682	-1.031	0.303	-0.44292682	count	1
RBFA	-0.3565565	0.2484007	-1.4354	0.151	-0.44239108	count	1
SPAG16	-0.6284412	0.7435475	-0.8452	0.398	-0.442208299	count	1
CD80	-1.231848	0.4862684	-2.5333	0.0114	-0.442059719	count	1
EMSY	-0.4048521	0.287508	-1.4081	0.159	-0.4420591	count	1
NFRKB	-0.4221967	0.4112286	-1.0267	0.305	-0.441817715	count	1
RBX1	-0.3101687	0.0629626	-4.9262	8.94E-07	-0.441395051	count	0.02129955
DNAAF5	-1.0651953	0.6649016	-1.602	0.109	-0.441329403	count	1
EMG1	-0.3505923	0.2000726	-1.7523	0.0798	-0.441114943	count	1
KCTD10	-0.387921	0.4389027	-0.8838	0.377	-0.441092621	count	1
UHRF1	-18.288248	1887.960737	-0.0097	0.992	-0.44091196	count	1
CCDC183	-18.120875	2241.094188	-0.0081	0.994	-0.440911958	count	1
TNFRSF11B	-17.89633	1724.961797	-0.0104	0.992	-0.440911957	count	1
AL645608.8	-17.627232	1501.103572	-0.0117	0.991	-0.440911954	count	1
AC118553.1	-17.261135	3456.82378	-0.005	0.996	-0.440911949	count	1

KLRD1	-17.260956	2792.052847	-0.0062	0.995	-0.440911949	count	1
HIST1H1A	-17.260782	2242.034839	-0.0077	0.994	-0.440911949	count	1
ZNF286A	-17.260782	2242.034839	-0.0077	0.994	-0.440911949	count	1
ADNP-AS1	-17.260782	2242.034839	-0.0077	0.994	-0.440911949	count	1
RAB11FIP5	-17.260647	1908.530744	-0.009	0.993	-0.440911949	count	1
AGBL3	-17.260647	1908.530744	-0.009	0.993	-0.440911949	count	1
PNMA6A	-17.260647	1908.530744	-0.009	0.993	-0.440911949	count	1
MMP8	-17.260647	1908.530744	-0.009	0.993	-0.440911949	count	1
ZNF670-ZNF695	-17.092317	4936.997261	-0.0035	0.997	-0.440911946	count	1
GRIA2	-17.092317	4936.997261	-0.0035	0.997	-0.440911946	count	1
UPK2	-17.092317	4936.997261	-0.0035	0.997	-0.440911946	count	1
RHOJ	-17.092317	4936.997261	-0.0035	0.997	-0.440911946	count	1
AL358472.3	-17.091438	3900.277613	-0.0044	0.997	-0.440911946	count	1
PXDN	-17.091438	3900.277613	-0.0044	0.997	-0.440911946	count	1
AOX1	-17.091438	3900.277613	-0.0044	0.997	-0.440911946	count	1
LINC02541	-17.091438	3900.277613	-0.0044	0.997	-0.440911946	count	1
AP000919.2	-17.091438	3900.277613	-0.0044	0.997	-0.440911946	count	1
FNDC5	-17.090688	3018.567076	-0.0057	0.995	-0.440911946	count	1
C1orf74	-17.090688	3018.567076	-0.0057	0.995	-0.440911946	count	1
AL109615.3	-17.090688	3018.567076	-0.0057	0.995	-0.440911946	count	1
AP003419.2	-17.090688	3018.567076	-0.0057	0.995	-0.440911946	count	1
NCAM1	-17.090688	3018.567076	-0.0057	0.995	-0.440911946	count	1
AC010186.1	-17.090688	3018.567076	-0.0057	0.995	-0.440911946	count	1
CEP170B	-17.090688	3018.567076	-0.0057	0.995	-0.440911946	count	1
RBFOX1	-17.090688	3018.567076	-0.0057	0.995	-0.440911946	count	1
LINC02139	-17.090688	3018.567076	-0.0057	0.995	-0.440911946	count	1
EPHA2	-17.090158	2462.524239	-0.0069	0.994	-0.440911946	count	1
WNT5A	-17.090158	2462.524239	-0.0069	0.994	-0.440911946	count	1
PDE5A	-17.090158	2462.524239	-0.0069	0.994	-0.440911946	count	1
TAB2-AS1	-17.090158	2462.524239	-0.0069	0.994	-0.440911946	count	1
IGHG4	-17.090158	2462.524239	-0.0069	0.994	-0.440911946	count	1
AC026471.2	-17.090158	2462.524239	-0.0069	0.994	-0.440911946	count	1
FAM83G	-17.090158	2462.524239	-0.0069	0.994	-0.440911946	count	1
KRT36	-17.090158	2462.524239	-0.0069	0.994	-0.440911946	count	1
GTSE1	-17.090158	2462.524239	-0.0069	0.994	-0.440911946	count	1
TMPRSS13	-17.7386298	2428.48399	-0.0073	0.994	-0.440911841	count	1
AL136961.1	-17.7386124	2125.354018	-0.0083	0.993	-0.440911841	count	1
MYL4	-17.7386124	2125.354018	-0.0083	0.993	-0.440911841	count	1
AL590428.1	-17.7380567	1905.111185	-0.0093	0.993	-0.440911841	count	1
TCTN2	-17.7380567	1905.111185	-0.0093	0.993	-0.440911841	count	1
SOX18	-17.7380567	1905.111185	-0.0093	0.993	-0.440911841	count	1
ATXN7L2	-17.7380498	2238.068342	-0.0079	0.994	-0.440911841	count	1
HCG25	-17.4435506	2042.538974	-0.0085	0.993	-0.440911838	count	1
AL031777.3	-17.4239365	1776.626022	-0.0098	0.992	-0.440911837	count	1
AGMAT	-16.9702505	1468.674279	-0.0116	0.991	-0.440911829	count	1

PANO1	-16.9702505	1468.674279	-0.0116	0.991	-0.440911829	count	1
AC018761.3	-16.7657804	2023.615166	-0.0083	0.993	-0.440911824	count	1
FCRLA	-16.7655341	1698.50873	-0.0099	0.992	-0.440911824	count	1
AC008040.1	-16.7655341	1698.50873	-0.0099	0.992	-0.440911824	count	1
KLF1	-16.7655341	1698.50873	-0.0099	0.992	-0.440911824	count	1
AL109811.2	-16.5853428	1797.066808	-0.0092	0.993	-0.440911819	count	1
TINAGL1	-16.5853285	1273.438624	-0.013	0.99	-0.440911819	count	1
CACNA2D3	-0.3874433	0.2459974	-1.575	0.115	-0.440567616	count	1
MICALL1	-0.5006242	0.6136456	-0.8158	0.415	-0.440421059	count	1
DDHD1	-0.342077	0.214055	-1.5981	0.11	-0.440085097	count	1
RPS27L	-0.3074478	0.0651096	-4.722	2.47E-06	-0.439690566	count	0.05872672
ARSG	-0.4107471	0.2712138	-1.5145	0.13	-0.438548401	count	1
IFIT5	-0.498303	0.4066685	-1.2253	0.221	-0.438540789	count	1
PMM2	-0.353372	0.2367686	-1.4925	0.136	-0.438519565	count	1
MYL5	-0.4898781	0.3537614	-1.3848	0.166	-0.438231789	count	1
PPAT	-0.4898781	0.3909387	-1.2531	0.21	-0.438231789	count	1
KHDC4	-0.4314786	0.3058455	-1.4108	0.158	-0.438061536	count	1
GTF3C6	-0.3096714	0.0842582	-3.6753	0.000243	-0.437364332	count	1
FKBP4	-0.3348933	0.1597404	-2.0965	0.0361	-0.437247447	count	1
PHLDB1	-0.5992612	0.5744951	-1.0431	0.297	-0.437239297	count	1
ELK3	-0.3327325	0.2031018	-1.6383	0.101	-0.437172924	count	1
NME1	-0.3158516	0.1289804	-2.4488	0.0144	-0.436964569	count	1
TIGAR	-0.3287676	0.1667959	-1.9711	0.0488	-0.4369193	count	1
AVPI1	-0.3150025	0.150406	-2.0943	0.0363	-0.436677431	count	1
PDZD11	-0.3411541	0.2099008	-1.6253	0.104	-0.436515872	count	1
HM13	-0.3072091	0.0671126	-4.5775	4.94E-06	-0.436028703	count	0.11728548
SLC1A3	-0.3352624	0.1534565	-2.1847	0.029	-0.435910641	count	1
CCNJ	-0.561446	0.5184263	-1.083	0.279	-0.43507319	count	1
DOCK10	-0.3182658	0.1480976	-2.149	0.0317	-0.435037304	count	1
AVEN	-0.4359183	0.391682	-1.1129	0.266	-0.434983049	count	1
AKR7A2	-0.3152518	0.1206967	-2.6119	0.00906	-0.434934491	count	1
B4GALT5	-0.3492536	0.2508867	-1.3921	0.164	-0.434786878	count	1
MRPS7	-0.3152466	0.1259275	-2.5034	0.0124	-0.434691435	count	1
CYTH3	-0.5767241	0.854729	-0.6747	0.5	-0.434493811	count	1
XPO4	-0.5332319	0.4259129	-1.252	0.211	-0.434320544	count	1
ETFB	-0.305693	0.0832232	-3.6732	0.000245	-0.434048503	count	1
PAFAH1B3	-0.3466488	0.2215426	-1.5647	0.118	-0.434005954	count	1
GSPT1	-0.3083961	0.0899652	-3.4279	0.000618	-0.43389978	count	1
RASGRF1	-1.931878	1.6332837	-1.1828	0.237	-0.433147169	count	1
HSD17B8	-0.4833361	0.3818609	-1.2657	0.206	-0.432816824	count	1
LSS	-1.035677	0.6745309	-1.5354	0.125	-0.432683587	count	1
ZBTB5	-0.6365315	0.9236355	-0.6892	0.491	-0.432479829	count	1
MAK16	-0.3914766	0.3770962	-1.0381	0.299	-0.432196138	count	1
DPH5	-0.6360445	0.372557	-1.7072	0.0879	-0.432194111	count	1
SYNC	-0.4904512	0.4070184	-1.205	0.228	-0.43216844	count	1

EFL1	-0.3695727	0.346542	-1.0665	0.286	-0.432072169	count	1
SMURF1	-0.4622233	0.4522129	-1.0221	0.307	-0.432051706	count	1
URGCP	-1.188834	0.7530256	-1.5787	0.115	-0.432023022	count	1
BOLA3	-0.3233149	0.1903676	-1.6984	0.0896	-0.43123008	count	1
NSMAF	-0.344551	0.2500862	-1.3777	0.168	-0.430257731	count	1
LRSAM1	-0.4875898	0.3831088	-1.2727	0.203	-0.429841587	count	1
SMUG1	-0.3537815	0.2958208	-1.1959	0.232	-0.429275539	count	1
B3GNTL1	-0.4428011	0.402934	-1.0989	0.272	-0.429087013	count	1
SOCS7	-1.0223455	0.6442698	-1.5868	0.113	-0.428717828	count	1
FAIM	-0.3523647	0.38652	-0.9116	0.362	-0.428434621	count	1
ZDHHC19	-1.1719536	0.7625277	-1.5369	0.124	-0.427992404	count	1
IGLON5	-0.9133185	0.9455847	-0.9659	0.334	-0.427652813	count	1
TAF4B	-1.170077	0.6607761	-1.7708	0.0767	-0.427541171	count	1
RBBP9	-0.5495663	0.5229543	-1.0509	0.293	-0.426818449	count	1
PPARD	-0.4159141	0.3003574	-1.3847	0.166	-0.42634982	count	1
MAN1B1-DT	-0.5640159	0.5395771	-1.0453	0.296	-0.425967034	count	1
LEAP2	-0.7234447	0.586425	-1.2337	0.217	-0.425750916	count	1
TBC1D2	-0.3504934	0.2958438	-1.1847	0.236	-0.42537594	count	1
AARSD1	-1.010323	0.6803496	-1.485	0.138	-0.425108742	count	1
BCKDK	-0.3081033	0.1280074	-2.4069	0.0162	-0.424953972	count	1
BTG1	-0.2949836	0.0531914	-5.5457	3.24E-08	-0.424902061	count	0.00077585
UBE2T	-0.8262431	0.8290268	-0.9966	0.319	-0.423982595	count	1
PLEKHB2	-0.3034769	0.0858722	-3.5341	0.000417	-0.42376596	count	1
NUDT22	-0.3115014	0.1536485	-2.0274	0.0427	-0.422966789	count	1
NDUFB2	-0.2951532	0.0476208	-6.198	6.69E-10	-0.422628601	count	1.61E-05
PKM	-0.2940801	0.0544733	-5.3986	7.36E-08	-0.421926775	count	0.00176007
ELOC	-0.2960305	0.0677429	-4.3699	1.29E-05	-0.421857268	count	0.3054978
NSMCE1	-0.3144812	0.1451258	-2.167	0.0303	-0.421664419	count	1
STK39	-0.5283764	0.4303579	-1.2278	0.22	-0.421620055	count	1
IFNAR2	-0.310547	0.1525867	-2.0352	0.0419	-0.421470526	count	1
SLC30A1	-0.3288985	0.2092109	-1.5721	0.116	-0.421469232	count	1
PLAU	-0.3072008	0.1703437	-1.8034	0.0714	-0.420092708	count	1
RAN	-0.2934386	0.0527355	-5.5643	2.92E-08	-0.419896471	count	0.000699369
IDE	-0.4481141	0.4049306	-1.1066	0.269	-0.419707578	count	1
AC008608.2	-0.6141334	0.7560387	-0.8123	0.417	-0.419264556	count	1
DRAM1	-0.3053526	0.1497147	-2.0396	0.0415	-0.418873644	count	1
ST3GAL6	-0.3271078	0.1933478	-1.6918	0.0908	-0.418812411	count	1
PLTP	-0.2935455	0.0947136	-3.0993	0.00196	-0.418638637	count	1
PDLIM7	-0.3247582	0.2114164	-1.5361	0.125	-0.417756285	count	1
AC116407.1	-0.7066058	0.9574016	-0.738	0.461	-0.417513623	count	1
FAM49B	-0.2931266	0.0537478	-5.4537	5.43E-08	-0.417102442	count	0.001299182
CHI3L2	-0.5676119	0.5957974	-0.9527	0.341	-0.416773827	count	1
LRFN4	-0.4247576	0.4172049	-1.0181	0.309	-0.416701741	count	1
AC093525.7	-1.7701184	1.1387037	-1.5545	0.12	-0.416675759	count	1
KCNQ1OT1	-0.3356798	0.2182993	-1.5377	0.124	-0.416353197	count	1

TSEN54	-0.4501709	0.4374392	-1.0291	0.304	-0.416312576	count	1
USP54	-0.9812521	2.0542391	-0.4777	0.633	-0.416252541	count	1
TRIM27	-0.3143788	0.1839686	-1.7089	0.0876	-0.416220529	count	1
NDN	-0.3315466	0.2616157	-1.2673	0.205	-0.415979416	count	1
CCT6A	-0.2946154	0.075642	-3.8949	0.000101	-0.415520269	count	1
PLB1	-0.3560685	0.2464386	-1.4449	0.149	-0.415492154	count	1
HLA-DMB	-0.29143	0.0573472	-5.0819	4.02E-07	-0.415157708	count	0.009588906
CDV3	-0.2897107	0.0554342	-5.2262	1.88E-07	-0.41465108	count	0.004490944
EHHADH	-1.1151941	1.0310038	-1.0817	0.28	-0.41405193	count	1
BIK	-1.1151941	1.0310038	-1.0817	0.28	-0.41405193	count	1
GM2A	-0.3050284	0.1558029	-1.9578	0.0504	-0.413826329	count	1
MEGF8	-0.8007412	0.5608848	-1.4276	0.154	-0.413606921	count	1
SLC35B1	-0.3125089	0.165162	-1.8921	0.0586	-0.413356219	count	1
SFXN1	-0.3611059	0.3637235	-0.9928	0.321	-0.413038708	count	1
CGAS	-0.3177572	0.1513349	-2.0997	0.0359	-0.412839153	count	1
ZNF501	-0.6958246	1.4253284	-0.4882	0.625	-0.412197488	count	1
AL391834.2	-0.6958246	1.2200215	-0.5703	0.569	-0.412197488	count	1
AC009118.2	-0.6958246	1.4874802	-0.4678	0.64	-0.412197488	count	1
ZNF234	-0.6589406	0.9894285	-0.666	0.505	-0.412178209	count	1
FCHO1	-0.3805033	0.4218072	-0.9021	0.367	-0.412154548	count	1
NRP2	-0.2933284	0.1368291	-2.1438	0.0322	-0.41197781	count	1
PSMD1	-0.3009999	0.126658	-2.3765	0.0176	-0.410759817	count	1
SERPINB9	-0.2950519	0.0977656	-3.018	0.00257	-0.410539661	count	1
PDLIM3	-0.7929067	0.8316436	-0.9534	0.34	-0.410385538	count	1
RAP1GDS1	-0.3187583	0.1991236	-1.6008	0.11	-0.410145829	count	1
SNRNP35	-0.3093532	0.1699253	-1.8205	0.0688	-0.409836743	count	1
TMEM161B-AS1	-0.4309617	0.4206958	-1.0244	0.306	-0.409372529	count	1
SLC25A43	-0.4549502	0.4359429	-1.0436	0.297	-0.40917367	count	1
TADA3	-0.2996565	0.1305194	-2.2959	0.0218	-0.409107958	count	1
TIPARP	-0.2883003	0.0829611	-3.4751	0.000519	-0.40901533	count	1
SEL1L3	-0.622183	0.5734188	-1.085	0.278	-0.40875367	count	1
MERTK	-0.3045583	0.1897773	-1.6048	0.109	-0.408620756	count	1
B3GALNT2	-0.3543021	0.3139675	-1.1285	0.259	-0.408295511	count	1
AC009948.1	-0.5378486	0.3890436	-1.3825	0.167	-0.408253353	count	1
CXCL3	-0.2837624	0.129887	-2.1847	0.029	-0.408071816	count	1
PSMD14	-0.2997037	0.1387737	-2.1597	0.0309	-0.407837662	count	1
PEX11B	-0.4071331	0.3441972	-1.1828	0.237	-0.407718203	count	1
AC112907.3	-2.847028	2.205989	-1.2906	0.197	-0.407623293	count	1
LRRC47	-0.3936551	0.3104563	-1.268	0.205	-0.407580461	count	1
RNF220	-0.3334197	0.2671996	-1.2478	0.212	-0.407418469	count	1
MEA1	-0.292816	0.1211726	-2.4165	0.0157	-0.406762905	count	1
MCUB	-0.2878208	0.0920258	-3.1276	0.00178	-0.406735294	count	1
GNL2	-0.3062292	0.1601137	-1.9126	0.0559	-0.406704052	count	1
GMFB	-0.3048872	0.1775894	-1.7168	0.0861	-0.406025064	count	1
AL645728.1	-0.6167473	0.492663	-1.2519	0.211	-0.405665936	count	1

NPM3	-0.3493519	0.3092655	-1.1296	0.259	-0.405375456	count	1
RNF115	-0.3049047	0.1554456	-1.9615	0.0499	-0.405147952	count	1
SELENOI	-0.5679816	0.4818514	-1.1787	0.239	-0.404679158	count	1
VSIG10L	-0.5325185	0.6651922	-0.8005	0.423	-0.404619468	count	1
NAA40	-0.4560986	0.8049838	-0.5666	0.571	-0.404071454	count	1
GNGT2	-0.4069042	0.3062917	-1.3285	0.184	-0.403878338	count	1
REEP6	-1.6585726	1.0499966	-1.5796	0.114	-0.403865166	count	1
PSMC3	-0.2898021	0.1018304	-2.8459	0.00446	-0.403300861	count	1
MLLT1	-0.4190175	0.3204758	-1.3075	0.191	-0.403094502	count	1
MS4A4A	-0.2822435	0.0776709	-3.6338	0.000285	-0.403026441	count	1
NFAT5	-0.3231355	0.194264	-1.6634	0.0964	-0.402853011	count	1
SLC30A4	-1.0711153	0.7040519	-1.5214	0.128	-0.402805656	count	1
HAUS6	-0.3638779	0.2717221	-1.3392	0.181	-0.402777191	count	1
AL136987.1	-0.5298085	0.432358	-1.2254	0.221	-0.402768555	count	1
TSPOAP1-AS1	-0.5862519	0.6221713	-0.9423	0.346	-0.402602784	count	1
AL355488.1	-0.6751085	1.1438944	-0.5902	0.555	-0.401889995	count	1
POP7	-0.3056251	0.1918064	-1.5934	0.111	-0.401714814	count	1
AC008083.2	-0.8407186	0.8868602	-0.948	0.343	-0.401397144	count	1
PSMB1	-0.280511	0.0461303	-6.0808	1.38E-09	-0.401316096	count	3.32E-05
WDR83	-0.4217771	0.3413684	-1.2355	0.217	-0.401154949	count	1
MRPL41	-0.2833793	0.0840757	-3.3705	0.000762	-0.40095479	count	1
TPST1	-0.4600007	0.5328063	-0.8634	0.388	-0.400856207	count	1
TRMT5	-0.4315707	0.4002959	-1.0781	0.281	-0.400186593	count	1
DCAF17	-0.4573297	0.4738106	-0.9652	0.335	-0.398698447	count	1
MON1A	-0.7112707	0.7847277	-0.9064	0.365	-0.398614133	count	1
AKAP6	-0.7112707	1.6467914	-0.4319	0.666	-0.398614133	count	1
PDHA1	-0.2940352	0.1629068	-1.8049	0.0712	-0.39860574	count	1
YARS	-0.3433256	0.2341586	-1.4662	0.143	-0.398571813	count	1
NCEH1	-0.3055191	0.2148387	-1.4221	0.155	-0.398541456	count	1
CTPS2	-0.7644167	0.9243425	-0.827	0.408	-0.398536274	count	1
OTUD3	-0.6681726	0.5603074	-1.1925	0.233	-0.398411765	count	1
ZFAND2A	-0.319003	0.1875607	-1.7008	0.0891	-0.398353145	count	1
ADSL	-0.3814159	0.3228177	-1.1815	0.238	-0.398251914	count	1
ADCY3	-0.5230522	0.5946684	-0.8796	0.379	-0.398144242	count	1
AC007114.1	-0.5576143	0.6016085	-0.9269	0.354	-0.398132232	count	1
GAPDH	-0.2760971	0.0407128	-6.7816	1.48E-11	-0.398132197	count	3.57E-07
LINC01006	-0.8313193	1.4053735	-0.5915	0.554	-0.397904304	count	1
AC073611.1	-0.3195402	0.3143291	-1.0166	0.309	-0.397875257	count	1
AF001548.2	-1.0521369	1.3343025	-0.7885	0.43	-0.397848177	count	1
NAGS	-1.0521369	1.0450717	-1.0068	0.314	-0.397848177	count	1
AC025048.4	-1.0521369	1.0450717	-1.0068	0.314	-0.397848177	count	1
HPRT1	-0.2883409	0.1368894	-2.1064	0.0353	-0.397672767	count	1
HSD17B7	-0.3645909	0.3124895	-1.1667	0.243	-0.397642707	count	1
NUDT19	-0.3729466	0.3500606	-1.0654	0.287	-0.39739851	count	1
GPR107	-0.3893021	0.2807115	-1.3868	0.166	-0.397234611	count	1

ACKR3	-0.3831072	0.5349936	-0.7161	0.474	-0.3971351	count	1
TMEM134	-0.2854813	0.1187019	-2.405	0.0162	-0.39691785	count	1
OSTC	-0.2791598	0.0731918	-3.8141	0.00014	-0.396753816	count	1
TMEM156	-0.5553679	0.4775005	-1.1631	0.245	-0.396709327	count	1
MAPRE1	-0.2851824	0.0997275	-2.8596	0.00428	-0.396685681	count	1
SLC43A3	-0.2945671	0.1500861	-1.9627	0.0498	-0.396534655	count	1
LRPAP1	-0.2778486	0.0628696	-4.4194	1.03E-05	-0.396487881	count	0.2440997
TMEM147	-0.2811804	0.0859387	-3.2719	0.00108	-0.39627854	count	1
CHPF	-1.0400692	0.863091	-1.2051	0.228	-0.394659324	count	1
LINC00476	-0.5521283	0.5780322	-0.9552	0.34	-0.394654653	count	1
MR1	-0.2967568	0.1730487	-1.7149	0.0865	-0.394423118	count	1
IFNGR2	-0.2775722	0.0747184	-3.7149	0.000208	-0.394005157	count	1
DTNBP1	-0.2992668	0.1458618	-2.0517	0.0403	-0.393900265	count	1
LAIR1	-0.2794121	0.0731031	-3.8222	0.000136	-0.393836602	count	1
DDX55	-0.3366789	0.2858712	-1.1777	0.239	-0.393452131	count	1
RDX	-0.2887738	0.1359156	-2.1247	0.0337	-0.393329817	count	1
MCFD2	-0.2852364	0.1213992	-2.3496	0.0189	-0.393249066	count	1
NDUFA11	-0.2763286	0.0643159	-4.2964	1.80E-05	-0.393236117	count	0.42579
H2AFV	-0.275681	0.0648838	-4.2488	2.23E-05	-0.393100227	count	0.5270605
BZW1	-0.2745211	0.0600371	-4.5725	5.06E-06	-0.393019383	count	0.1201244
LUCAT1	-0.3059275	0.2367588	-1.2921	0.196	-0.392799623	count	1
CCT2	-0.2789563	0.1019314	-2.7367	0.00625	-0.392527958	count	1
MTOR	-0.4493773	0.4677492	-0.9607	0.337	-0.392261525	count	1
RARA-AS1	-0.3149096	0.2628975	-1.1978	0.231	-0.392209243	count	1
AGAP6	-0.5930984	0.9007882	-0.6584	0.51	-0.392130036	count	1
CCNB1	-0.5686222	0.7420174	-0.7663	0.444	-0.391946715	count	1
NQO2	-0.2792051	0.1082013	-2.5804	0.00993	-0.391744329	count	1
TOMM70	-0.332936	0.2157902	-1.5429	0.123	-0.391436563	count	1
CXCL16	-0.2743	0.0660195	-4.1548	3.37E-05	-0.391305418	count	0.7952526
SNX8	-0.2892193	0.1594409	-1.814	0.0698	-0.390940543	count	1
NPAT	-0.3622142	0.2828449	-1.2806	0.2	-0.390914995	count	1
C2orf74	-0.2980838	0.183785	-1.6219	0.105	-0.390730055	count	1
PBX3	-0.3219333	0.2387399	-1.3485	0.178	-0.390649248	count	1
AC093827.4	-0.5904306	0.4582576	-1.2884	0.198	-0.390592647	count	1
CYFIP1	-0.2805104	0.0979196	-2.8647	0.00421	-0.390590728	count	1
CNOT9	-0.2986884	0.1866308	-1.6004	0.11	-0.390515693	count	1
ACOX2	-0.5454987	0.7774154	-0.7017	0.483	-0.390439983	count	1
SMIM37	-0.2896645	0.1486146	-1.9491	0.0514	-0.390360308	count	1
CCDC126	-0.3473482	0.3072917	-1.1304	0.258	-0.39017941	count	1
MZT2A	-0.2787696	0.1311718	-2.1252	0.0337	-0.38952622	count	1
GAN	-0.3879625	0.458158	-0.8468	0.397	-0.389435914	count	1
GRAMD1B	-0.3953978	0.3583662	-1.1033	0.27	-0.389365094	count	1
CRELD2	-0.2871896	0.1363044	-2.107	0.0352	-0.389184616	count	1
SEC61B	-0.2712527	0.0475711	-5.7021	1.33E-08	-0.389171734	count	0.000318961
FLCN	-0.3527788	0.290149	-1.2159	0.224	-0.389058526	count	1

NCBP2	-0.2900874	0.1518501	-1.9104	0.0562	-0.388802564	count	1
MX1	-0.2763373	0.131619	-2.0995	0.0359	-0.388736007	count	1
CKS2	-0.2788392	0.116181	-2.4	0.0165	-0.388722756	count	1
LACC1	-0.3114897	0.2139822	-1.4557	0.146	-0.388022561	count	1
SNAI1	-0.3935676	0.2910817	-1.3521	0.176	-0.387653202	count	1
WDHD1	-0.8040328	0.8351732	-0.9627	0.336	-0.387641614	count	1
CD247	-1.5298992	1.1530973	-1.3268	0.185	-0.387460282	count	1
ANXA2	-0.2694851	0.0468147	-5.7564	9.66E-09	-0.387104718	count	0.000231724
TCAIM	-0.3204545	0.2781554	-1.1521	0.249	-0.386471376	count	1
MGAT4B	-0.2853148	0.1706888	-1.6715	0.0947	-0.386029171	count	1
ANKRD11	-0.2771268	0.1337581	-2.0719	0.0384	-0.38524122	count	1
ABCF1	-0.2789396	0.1132364	-2.4633	0.0138	-0.38497887	count	1
WDR5	-0.3945587	0.4007362	-0.9846	0.325	-0.384775305	count	1
SLU7	-0.2862794	0.1261185	-2.2699	0.0233	-0.384694106	count	1
REPS1	-0.3792728	0.3131745	-1.2111	0.226	-0.384348581	count	1
ZNF268	-0.7308559	0.5499218	-1.329	0.184	-0.384305606	count	1
BLOC1S1	-0.2684238	0.0556177	-4.8262	1.48E-06	-0.384044358	count	0.03522696
TMEM51-AS1	-1.1838259	1.1094918	-1.067	0.286	-0.384032492	count	1
DERL3	-1.1838259	1.1945095	-0.9911	0.322	-0.384032492	count	1
RAB5C	-0.2681611	0.0615401	-4.3575	1.37E-05	-0.382789068	count	0.3243201
HLA-DQA1	-0.2654073	0.0684047	-3.88	0.000107	-0.382138611	count	1
MYL6	-0.2651645	0.0301124	-8.8058	2.40E-18	-0.38212708	count	5.81E-14
CCDC25	-0.2781026	0.2011236	-1.3827	0.167	-0.38207784	count	1
ANXA5	-0.2656687	0.0381168	-6.9699	4.06E-12	-0.381869485	count	9.79E-08
NDUFC2	-0.2673979	0.0620893	-4.3067	1.72E-05	-0.381447816	count	0.4069176
FAM27C	-0.5742553	0.6782186	-0.8467	0.397	-0.381226072	count	1
ZDHHC24	-0.2874817	0.1630383	-1.7633	0.078	-0.380569248	count	1
DNAJC21	-0.2733591	0.1365344	-2.0021	0.0454	-0.380506526	count	1
HOMER3	-0.2720351	0.1006907	-2.7017	0.00695	-0.380226882	count	1
ZNF131	-0.300689	0.186897	-1.6088	0.108	-0.380202823	count	1
DDX10	-0.3312328	0.3084891	-1.0737	0.283	-0.379808729	count	1
WDYHV1	-0.3580048	0.4648168	-0.7702	0.441	-0.379726249	count	1
ZNF697	-0.7831385	0.7375667	-1.0618	0.288	-0.37965892	count	1
AC093895.1	-0.3513863	0.3887389	-0.9039	0.366	-0.379643295	count	1
CUL7	-0.5979911	0.6982114	-0.8565	0.392	-0.37924616	count	1
C8orf76	-0.2929479	0.2239679	-1.308	0.191	-0.379244594	count	1
MPI	-0.3574426	0.3765535	-0.9492	0.343	-0.379152516	count	1
HIST2H2AC	-0.3306313	0.2432779	-1.3591	0.174	-0.379137732	count	1
DST	-0.3146994	0.2002475	-1.5716	0.116	-0.378847778	count	1
AC100810.1	-0.2708012	0.1286473	-2.105	0.0354	-0.378078701	count	1
ACSL3	-0.2697657	0.1161934	-2.3217	0.0203	-0.377701455	count	1
SMIM25	-0.2655812	0.106703	-2.489	0.0129	-0.37749147	count	1
LSR	-0.4171092	0.3726503	-1.1193	0.263	-0.377285951	count	1
ZNF141	-0.312648	0.270942	-1.1539	0.249	-0.377249614	count	1
ZC3HAV1	-0.2907867	0.1433555	-2.0284	0.0426	-0.37648008	count	1

APBB1IP	-0.2640656	0.0691297	-3.8199	0.000137	-0.375805163	count	1
AF117829.1	-0.3768075	0.3527814	-1.0681	0.286	-0.37541251	count	1
AC068580.4	-0.4768872	0.829399	-0.575	0.565	-0.375374492	count	1
XCR1	-0.6227073	0.7142917	-0.8718	0.383	-0.375272983	count	1
BCAT1	-0.2692847	0.1409202	-1.9109	0.0561	-0.375207282	count	1
B3GNT5	-0.2760939	0.1439838	-1.9175	0.0553	-0.374734526	count	1
BTBD8	-0.8520679	0.5499214	-1.5494	0.121	-0.374635374	count	1
MCM7	-0.3421923	0.5101218	-0.6708	0.502	-0.374029334	count	1
OLMALINC	-0.9641032	0.7855529	-1.2273	0.22	-0.373922731	count	1
ZNF438	-0.31482	0.3611373	-0.8717	0.383	-0.373617443	count	1
REV3L	-0.2747974	0.1498331	-1.834	0.0668	-0.373541025	count	1
PDPN	-0.5610291	0.5159263	-1.0874	0.277	-0.373509561	count	1
EXOSC2	-0.5610291	0.5134066	-1.0928	0.275	-0.373509561	count	1
CHEK2	-0.406596	0.5652566	-0.7193	0.472	-0.373482954	count	1
ZNF22	-0.2775233	0.1691304	-1.6409	0.101	-0.373269639	count	1
GLRX2	-0.2713048	0.1418004	-1.9133	0.0558	-0.373258573	count	1
GDF11	-0.9616379	0.5723545	-1.6801	0.0931	-0.373230359	count	1
RASA1	-0.3054491	0.2634374	-1.1595	0.246	-0.372485749	count	1
TAGLN	-0.2876266	0.2405466	-1.1957	0.232	-0.372436829	count	1
ABI3	-0.2780273	0.1334163	-2.0839	0.0373	-0.37206671	count	1
JPT1	-0.2610032	0.072142	-3.6179	0.000303	-0.372064606	count	1
FTO	-0.5168014	0.5698086	-0.907	0.365	-0.372042821	count	1
PSMD8	-0.2607202	0.0574558	-4.5378	5.96E-06	-0.37195232	count	0.14143676
COPRS	-0.2745273	0.1796924	-1.5278	0.127	-0.371940514	count	1
AC073111.5	-1.4197031	1.0763782	-1.319	0.187	-0.37190441	count	1
ENTR1	-0.3102342	0.2428097	-1.2777	0.201	-0.371900062	count	1
MAP3K8	-0.2607852	0.0692647	-3.7651	2.00E-04	-0.371889032	count	1
RGS12	-0.3438861	0.3620685	-0.9498	0.342	-0.371819365	count	1
NASP	-0.2691925	0.0994289	-2.7074	0.00683	-0.371489292	count	1
LSM2	-0.2676685	0.1206254	-2.219	0.0266	-0.371294827	count	1
ARL10	-1.41473	0.768596	-1.8407	0.0658	-0.371167819	count	1
EMC9	-0.2999207	0.3141348	-0.9548	0.34	-0.371014441	count	1
SOD3	-2.0739768	1.5745769	-1.3172	0.188	-0.370363927	count	1
DNAJC15	-0.2603089	0.0778476	-3.3438	0.000839	-0.370219148	count	1
METRNL	-0.2582888	0.0528181	-4.8902	1.07E-06	-0.37019982	count	0.02548205
VAMP8	-0.2576089	0.0388184	-6.6363	3.95E-11	-0.370090382	count	9.52E-07
TNPO1	-0.2855808	0.1897056	-1.5054	0.132	-0.369523247	count	1
SPCS3	-0.2598864	0.0676998	-3.8388	0.000127	-0.369431641	count	1
HMGN1	-0.2582749	0.063478	-4.0687	4.88E-05	-0.369273319	count	1
SERPINB9P1	-0.4955887	0.5276377	-0.9393	0.348	-0.369061533	count	1
KIAA0753	-0.4955887	0.6289053	-0.788	0.431	-0.369061533	count	1
UBE2D4	-0.3122324	0.2571302	-1.2143	0.225	-0.368666152	count	1
THBS3	-0.4456475	0.5100928	-0.8737	0.382	-0.368549124	count	1
GPR146	-0.6470663	0.5168926	-1.2518	0.211	-0.368336365	count	1
MPHOSPH6	-0.2805983	0.1638486	-1.7125	0.0869	-0.36827	count	1

AL590096.1	-0.5779312	1.8171946	-0.318	0.75	-0.36817065	count	1
DNAJC8	-0.2594454	0.07832	-3.3126	0.000938	-0.368092773	count	1
DYRK3	-1.1091468	0.8811981	-1.2587	0.208	-0.367920611	count	1
DOCK4	-0.2768948	0.2222109	-1.2461	0.213	-0.367917304	count	1
MAP4K3	-0.3025809	0.2707529	-1.1176	0.264	-0.367616135	count	1
CAMTA1	-0.2617615	0.0893923	-2.9282	0.00344	-0.367584278	count	1
C8orf58	-0.3996593	0.6002802	-0.6658	0.506	-0.367480498	count	1
VAT1	-0.2717437	0.178615	-1.5214	0.128	-0.367479723	count	1
ANKRD36	-0.3832715	0.3610381	-1.0616	0.289	-0.366444786	count	1
ZNF146	-0.3281633	0.3323237	-0.9875	0.324	-0.366099304	count	1
SLC15A3	-0.2687447	0.1380016	-1.9474	0.0516	-0.365625402	count	1
SEC11C	-0.2610367	0.1045494	-2.4968	0.0126	-0.36535862	count	1
VDAC1	-0.256799	0.0687563	-3.7349	0.000192	-0.365203664	count	1
ERP27	-0.6029909	0.9959818	-0.6054	0.545	-0.365055702	count	1
CCT6B	-0.6029909	0.6616995	-0.9113	0.362	-0.365055702	count	1
DEPP1	-1.9919099	1.1928867	-1.6698	0.0951	-0.364592773	count	1
SPATA5L1	-0.3350684	0.367562	-0.9116	0.362	-0.364581222	count	1
HNRNPF	-0.2564666	0.0581237	-4.4124	1.07E-05	-0.364443196	count	0.2535472
FUT7	-0.396112	0.4728381	-0.8377	0.402	-0.364405632	count	1
PKP4	-0.8216658	0.668685	-1.2288	0.219	-0.364292086	count	1
SBNO1	-0.2735824	0.1930073	-1.4175	0.156	-0.363553823	count	1
CEP68	-0.2926784	0.2320727	-1.2611	0.207	-0.363338524	count	1
TIMM8B	-0.2605582	0.1099909	-2.3689	0.0179	-0.363326584	count	1
ACE	-0.9241125	0.8127839	-1.137	0.256	-0.362538129	count	1
JPT2	-0.3453977	0.5399081	-0.6397	0.522	-0.362075451	count	1
UCP2	-0.2542531	0.0731119	-3.4776	0.000515	-0.361239284	count	1
NTMT1	-0.2667107	0.1718359	-1.5521	0.121	-0.361222794	count	1
USP31	-0.6774785	0.4365973	-1.5517	0.121	-0.361060689	count	1
ACAD9	-0.3396445	0.3149917	-1.0783	0.281	-0.3609495	count	1
HMGCL	-0.2901295	0.2176994	-1.3327	0.183	-0.36077126	count	1
PSMB2	-0.2550544	0.0775117	-3.2905	0.00101	-0.36068912	count	1
POLR3A	-0.4180477	0.44648	-0.9363	0.349	-0.360596628	count	1
ANTXR1	-1.93456	1.1720526	-1.6506	0.099	-0.360295038	count	1
AP2M1	-0.2536007	0.063989	-3.9632	7.61E-05	-0.360249231	count	1
MZT2B	-0.252957	0.0641116	-3.9456	8.18E-05	-0.359957332	count	1
ATP9B	-0.2852246	0.2448719	-1.1648	0.244	-0.359624022	count	1
GLRX3	-0.2623672	0.1229816	-2.1334	0.033	-0.358979314	count	1
TDG	-0.2620688	0.143249	-1.8295	0.0675	-0.358978792	count	1
MCCC1	-0.4540415	0.3549379	-1.2792	0.201	-0.358872028	count	1
ABHD14A	-0.3021139	0.2350541	-1.2853	0.199	-0.358859426	count	1
THEM4	-0.4084275	0.3972556	-1.0281	0.304	-0.358819082	count	1
CENPV	-0.5146784	0.7191086	-0.7157	0.474	-0.358761977	count	1
ATP6V0D1	-0.2517844	0.064226	-3.9203	9.09E-05	-0.357904509	count	1
FAM98A	-0.2783865	0.2395417	-1.1622	0.245	-0.3578682	count	1
MAPKAPK5-AS1	-0.2974919	0.2175268	-1.3676	0.172	-0.357743132	count	1

CXCL11	-0.9062384	0.9155529	-0.9898	0.322	-0.357343305	count	1
HYOU1	-0.3089369	0.2388422	-1.2935	0.196	-0.357310703	count	1
FAM122B	-0.3731081	0.3836113	-0.9726	0.331	-0.357214186	count	1
AHSA1	-0.271904	0.136472	-1.9924	0.0464	-0.356970294	count	1
ITPR1	-0.3167694	0.3229764	-0.9808	0.327	-0.35678819	count	1
TESK1	-0.2821066	0.2414296	-1.1685	0.243	-0.35661101	count	1
EIF3C	-0.4300547	0.4817401	-0.8927	0.372	-0.356596774	count	1
MPV17	-0.2622272	0.1595449	-1.6436	0.1	-0.356556585	count	1
FAM213B	-0.6670663	0.3781726	-1.7639	0.0779	-0.356438375	count	1
MYH10	-0.9031264	0.6307741	-1.4318	0.152	-0.35643208	count	1
SPCS2	-0.2489297	0.0511818	-4.8636	1.23E-06	-0.356050557	count	0.02928876
PGF	-0.9015515	1.2732045	-0.7081	0.479	-0.355970175	count	1
PKIG	-0.7214654	0.5966942	-1.2091	0.227	-0.355462814	count	1
RMND5B	-0.331865	0.4572134	-0.7258	0.468	-0.355135308	count	1
DDO	-0.7204472	0.7746975	-0.93	0.352	-0.355055352	count	1
AC020928.1	-0.6195134	0.8360911	-0.741	0.459	-0.354993992	count	1
TMEM173	-0.2619182	0.1099367	-2.3824	0.0173	-0.354965171	count	1
FCGR2B	-0.2500617	0.1075868	-2.3243	0.0202	-0.35472576	count	1
PPIB	-0.2464104	0.0472142	-5.219	1.95E-07	-0.354112835	count	0.004657965
SEC22C	-0.3057877	0.2972443	-1.0287	0.304	-0.353756	count	1
CCT5	-0.2494626	0.0725361	-3.4392	0.000593	-0.353601505	count	1
AL645568.1	-1.0458446	1.0662451	-0.9809	0.327	-0.353517344	count	1
QPCTL	-1.0458446	1.0384332	-1.0071	0.314	-0.353517344	count	1
TBC1D8B	-1.3012396	1.1129762	-1.1692	0.242	-0.353499542	count	1
PRDM10	-1.3012396	1.1519097	-1.1296	0.259	-0.353499542	count	1
SIRPA	-0.2555723	0.1230159	-2.0776	0.0379	-0.353412209	count	1
DUSP14	-0.2848757	0.2852962	-0.9985	0.318	-0.353258966	count	1
ARL1	-0.2616754	0.1633605	-1.6018	0.109	-0.353224977	count	1
CCDC152	-0.3948906	0.319742	-1.235	0.217	-0.353144818	count	1
PYM1	-0.2588301	0.1596571	-1.6212	0.105	-0.352699644	count	1
TBCEL	-1.0417787	0.8198526	-1.2707	0.204	-0.35256824	count	1
CASP5	-0.3637834	0.3533965	-1.0294	0.303	-0.35252693	count	1
EIF2S1	-0.256824	0.1232284	-2.0841	0.0373	-0.352457185	count	1
EDEM2	-0.2662845	0.1511773	-1.7614	0.0783	-0.351660792	count	1
AC231981.1	-0.5027917	0.9274848	-0.5421	0.588	-0.351332585	count	1
PC	-0.655479	1.259875	-0.5203	0.603	-0.351260507	count	1
AL049840.1	-0.3301654	0.4954741	-0.6664	0.505	-0.351223354	count	1
DHX35	-0.4832291	0.639636	-0.7555	0.45	-0.350205485	count	1
SLC25A39	-0.2548571	0.1189065	-2.1433	0.0322	-0.350007864	count	1
IFT43	-0.2850767	0.2645495	-1.0776	0.281	-0.349995468	count	1
BTF3L4	-0.2485782	0.0832765	-2.985	0.00286	-0.349987147	count	1
LINC00342	-0.8797508	0.9712129	-0.9058	0.365	-0.349522857	count	1
TMEM64	-0.573165	0.5338868	-1.0736	0.283	-0.34938901	count	1
SOCS3	-0.2454231	0.0613233	-4.0021	6.46E-05	-0.34923718	count	1
METTL2A	-0.3153701	0.3256841	-0.9683	0.333	-0.34901974	count	1

DPCD	-0.2986375	0.3317742	-0.9001	0.368	-0.348958783	count	1
SSTR2	-1.7958416	0.828459	-2.1677	0.0303	-0.348927413	count	1
HCFC1R1	-0.2499929	0.1272078	-1.9652	0.0495	-0.34891264	count	1
UBE2F	-0.2568525	0.1242421	-2.0674	0.0388	-0.3483269	count	1
RTN4	-0.2431827	0.0501496	-4.8491	1.32E-06	-0.347711618	count	0.03142788
HOMER2	-0.4386289	0.5084795	-0.8626	0.388	-0.347650045	count	1
ALDH7A1	-0.4013496	0.365538	-1.098	0.272	-0.347116537	count	1
HIST1H1C	-0.2710715	0.2128568	-1.2735	0.203	-0.346936331	count	1
MRPS25	-0.2612823	0.1771586	-1.4748	0.14	-0.346855563	count	1
BCL7A	-0.4780334	0.9092174	-0.5258	0.599	-0.346795675	count	1
CHMP6	-0.29669	0.2477194	-1.1977	0.231	-0.346733885	count	1
INO80C	-0.3023463	0.2476446	-1.2209	0.222	-0.346267028	count	1
GALNS	-0.2919991	0.2810343	-1.039	0.299	-0.346194571	count	1
LBX2-AS1	-0.4617469	0.5454666	-0.8465	0.397	-0.346100197	count	1
LINC01311	-1.255553	1.2408185	-1.0119	0.312	-0.345907278	count	1
DHDH	-0.6005144	0.8300484	-0.7235	0.469	-0.345671393	count	1
DECR2	-0.4358769	0.4893726	-0.8907	0.373	-0.345638799	count	1
GPR42	-0.4252443	0.5851467	-0.7267	0.467	-0.345582581	count	1
IPP	-1.2533247	1.0224833	-1.2258	0.22	-0.345529703	count	1
CEP55	-1.2533247	1.1339506	-1.1053	0.269	-0.345529703	count	1
DDAH2	-0.242871	0.0724997	-3.35	0.00082	-0.345349184	count	1
UPP1	-0.2429252	0.0817911	-2.9701	0.00301	-0.345106617	count	1
YBX3	-0.2410776	0.0586463	-4.1107	4.07E-05	-0.345054269	count	0.9595839
TSC1	-0.3387053	0.3005029	-1.1271	0.26	-0.344883368	count	1
MSRB2	-0.2472226	0.0967288	-2.5558	0.0107	-0.344654042	count	1
HAUS8	-0.2809376	0.2335747	-1.2028	0.229	-0.344380993	count	1
CLTA	-0.2405801	0.0504001	-4.7734	1.92E-06	-0.344379641	count	0.04567488
SLC3A2	-0.241798	0.0610661	-3.9596	7.72E-05	-0.344371659	count	1
KANSL3	-0.3195225	0.4573694	-0.6986	0.485	-0.344369264	count	1
MAGI3	-1.742858	0.9896315	-1.7611	0.0783	-0.344197634	count	1
SGSM3	-1.741014	0.9322438	-1.8676	0.0619	-0.344028982	count	1
TUBGCP2	-0.2458045	0.0961906	-2.5554	0.0107	-0.343744481	count	1
PGAP2	-0.3188495	0.4595344	-0.6939	0.488	-0.343667069	count	1
C2	-0.2939444	0.1453013	-2.023	0.0432	-0.343595974	count	1
ADRM1	-0.2442771	0.0939627	-2.5997	0.00939	-0.343501536	count	1
SMIM1	-0.3620919	0.4782076	-0.7572	0.449	-0.34321717	count	1
ANKRD6	-0.5613728	0.7872076	-0.7131	0.476	-0.343124953	count	1
XPO5	-0.4723561	0.4504312	-1.0487	0.294	-0.343060563	count	1
CXCR4	-0.2405585	0.0587806	-4.0925	4.41E-05	-0.342745757	count	1
AC009570.1	-18.000696	2441.15086	-0.0074	0.994	-0.342664675	count	1
ZFP28	-18.000031	1888.739609	-0.0095	0.992	-0.342664675	count	1
ANOS1	-17.932205	1498.325741	-0.012	0.99	-0.342664675	count	1
FAM111B	-17.859823	1752.025593	-0.0102	0.992	-0.342664674	count	1
DOCK6	-17.69616	2316.64302	-0.0076	0.994	-0.342664673	count	1
DEGS2	-17.413596	1257.526218	-0.0138	0.989	-0.34266467	count	1

LINC02081	-17.313202	1262.415737	-0.0137	0.989	-0.342664669	count	1
AL121936.1	-17.160748	3031.025473	-0.0057	0.995	-0.342664667	count	1
AC004466.1	-17.160653	2343.912326	-0.0073	0.994	-0.342664667	count	1
WDR62	-17.160653	2343.912326	-0.0073	0.994	-0.342664667	count	1
ZNF229	-17.160653	2343.912326	-0.0073	0.994	-0.342664667	count	1
HIST3H2BB	-17.160572	1909.836305	-0.009	0.993	-0.342664667	count	1
TCF7L1	-17.160572	1909.836305	-0.009	0.993	-0.342664667	count	1
TNIP3	-17.160572	1909.836305	-0.009	0.993	-0.342664667	count	1
AC021097.1	-17.160572	1909.836305	-0.009	0.993	-0.342664667	count	1
CA3-AS1	-17.160572	1909.836305	-0.009	0.993	-0.342664667	count	1
AC136475.5	-17.160572	1909.836305	-0.009	0.993	-0.342664667	count	1
KIF11	-17.160572	1909.836305	-0.009	0.993	-0.342664667	count	1
SPESP1	-17.160572	1909.836305	-0.009	0.993	-0.342664667	count	1
PRC1	-17.160572	1909.836305	-0.009	0.993	-0.342664667	count	1
AC145207.2	-17.160572	1909.836305	-0.009	0.993	-0.342664667	count	1
AC002116.2	-17.160572	1909.836305	-0.009	0.993	-0.342664667	count	1
ZNF527	-17.160572	1909.836305	-0.009	0.993	-0.342664667	count	1
CADM4	-17.160572	1909.836305	-0.009	0.993	-0.342664667	count	1
CD27	-17.01934	2209.976639	-0.0077	0.994	-0.342664665	count	1
DPYD-AS2	-16.802934	4271.931304	-0.0039	0.997	-0.342664662	count	1
AC110769.1	-16.802934	4271.931304	-0.0039	0.997	-0.342664662	count	1
LINC02037	-16.802934	4271.931304	-0.0039	0.997	-0.342664662	count	1
ANKRD31	-16.802934	4271.931304	-0.0039	0.997	-0.342664662	count	1
AL031123.2	-16.802934	4271.931304	-0.0039	0.997	-0.342664662	count	1
HIST1H3E	-16.802934	4271.931304	-0.0039	0.997	-0.342664662	count	1
AL157371.2	-16.802934	4271.931304	-0.0039	0.997	-0.342664662	count	1
TRGC2	-16.802934	4271.931304	-0.0039	0.997	-0.342664662	count	1
AC004922.1	-16.802934	4271.931304	-0.0039	0.997	-0.342664662	count	1
AC104958.2	-16.802934	4271.931304	-0.0039	0.997	-0.342664662	count	1
ST8SIA6	-16.802934	4271.931304	-0.0039	0.997	-0.342664662	count	1
TMEM26-AS1	-16.802934	4271.931304	-0.0039	0.997	-0.342664662	count	1
TMEM254-AS1	-16.802934	4271.931304	-0.0039	0.997	-0.342664662	count	1
AC010201.2	-16.802934	4271.931304	-0.0039	0.997	-0.342664662	count	1
USP12-AS2	-16.802934	4271.931304	-0.0039	0.997	-0.342664662	count	1
CTRB2	-16.802934	4271.931304	-0.0039	0.997	-0.342664662	count	1
AC087741.2	-16.802934	4271.931304	-0.0039	0.997	-0.342664662	count	1
AC139530.3	-16.802934	4271.931304	-0.0039	0.997	-0.342664662	count	1
SBSN	-16.802934	4271.931304	-0.0039	0.997	-0.342664662	count	1
ZNF497	-16.802934	4271.931304	-0.0039	0.997	-0.342664662	count	1
DEPDC1	-16.802147	3181.716909	-0.0053	0.996	-0.342664662	count	1
AL139412.1	-16.802147	3181.716909	-0.0053	0.996	-0.342664662	count	1
ELF3	-16.802147	3181.716909	-0.0053	0.996	-0.342664662	count	1
AC009495.3	-16.802147	3181.716909	-0.0053	0.996	-0.342664662	count	1
DUBR	-16.802147	3181.716909	-0.0053	0.996	-0.342664662	count	1
PPBP	-16.802147	3181.716909	-0.0053	0.996	-0.342664662	count	1

C9	-16.802147	3181.716909	-0.0053	0.996	-0.342664662	count	1
HIST1H3G	-16.802147	3181.716909	-0.0053	0.996	-0.342664662	count	1
HIST1H2BL	-16.802147	3181.716909	-0.0053	0.996	-0.342664662	count	1
SH3BGRL2	-16.802147	3181.716909	-0.0053	0.996	-0.342664662	count	1
AC009902.2	-16.802147	3181.716909	-0.0053	0.996	-0.342664662	count	1
RSPO2	-16.802147	3181.716909	-0.0053	0.996	-0.342664662	count	1
CD81-AS1	-16.802147	3181.716909	-0.0053	0.996	-0.342664662	count	1
AP000769.2	-16.802147	3181.716909	-0.0053	0.996	-0.342664662	count	1
AL163636.1	-16.802147	3181.716909	-0.0053	0.996	-0.342664662	count	1
KTN1-AS1	-16.802147	3181.716909	-0.0053	0.996	-0.342664662	count	1
ISL2	-16.802147	3181.716909	-0.0053	0.996	-0.342664662	count	1
AC103810.2	-16.802147	3181.716909	-0.0053	0.996	-0.342664662	count	1
MYBL2	-16.802147	3181.716909	-0.0053	0.996	-0.342664662	count	1
PRR19	-16.802147	3181.716909	-0.0053	0.996	-0.342664662	count	1
SEC14L2	-16.802147	3181.716909	-0.0053	0.996	-0.342664662	count	1
PTPRU	-16.801591	2462.538409	-0.0068	0.995	-0.342664662	count	1
AL591845.1	-16.801591	2462.538409	-0.0068	0.995	-0.342664662	count	1
AL162431.2	-16.801591	2462.538409	-0.0068	0.995	-0.342664662	count	1
LAMC2	-16.801591	2462.538409	-0.0068	0.995	-0.342664662	count	1
LINC01412	-16.801591	2462.538409	-0.0068	0.995	-0.342664662	count	1
AC078802.1	-16.801591	2462.538409	-0.0068	0.995	-0.342664662	count	1
AC007126.1	-16.801591	2462.538409	-0.0068	0.995	-0.342664662	count	1
CXCL6	-16.801591	2462.538409	-0.0068	0.995	-0.342664662	count	1
SEC24B-AS1	-16.801591	2462.538409	-0.0068	0.995	-0.342664662	count	1
CCNA2	-16.801591	2462.538409	-0.0068	0.995	-0.342664662	count	1
AL603910.1	-16.801591	2462.538409	-0.0068	0.995	-0.342664662	count	1
AL080250.1	-16.801591	2462.538409	-0.0068	0.995	-0.342664662	count	1
AC090192.2	-16.801591	2462.538409	-0.0068	0.995	-0.342664662	count	1
LY6E-DT	-16.801591	2462.538409	-0.0068	0.995	-0.342664662	count	1
FRRS1L	-16.801591	2462.538409	-0.0068	0.995	-0.342664662	count	1
AP003486.1	-16.801591	2462.538409	-0.0068	0.995	-0.342664662	count	1
AC008014.1	-16.801591	2462.538409	-0.0068	0.995	-0.342664662	count	1
AHNAK2	-16.801591	2462.538409	-0.0068	0.995	-0.342664662	count	1
TPSB2	-16.801591	2462.538409	-0.0068	0.995	-0.342664662	count	1
RPGRIP1L	-16.801591	2462.538409	-0.0068	0.995	-0.342664662	count	1
PLLP	-16.801591	2462.538409	-0.0068	0.995	-0.342664662	count	1
BCAR1	-16.801591	2462.538409	-0.0068	0.995	-0.342664662	count	1
ARHGAP23	-16.801591	2462.538409	-0.0068	0.995	-0.342664662	count	1
PCGF2	-16.801591	2462.538409	-0.0068	0.995	-0.342664662	count	1
ITGB3	-16.801591	2462.538409	-0.0068	0.995	-0.342664662	count	1
LPO	-16.801591	2462.538409	-0.0068	0.995	-0.342664662	count	1
SMIM24	-16.801591	2462.538409	-0.0068	0.995	-0.342664662	count	1
ANKLE1	-16.801591	2462.538409	-0.0068	0.995	-0.342664662	count	1
AC011476.3	-16.801591	2462.538409	-0.0068	0.995	-0.342664662	count	1
C22orf15	-16.801591	2462.538409	-0.0068	0.995	-0.342664662	count	1

SGSM1	-16.801591	2462.538409	-0.0068	0.995	-0.342664662	count	1
LINC00649	-16.801591	2462.538409	-0.0068	0.995	-0.342664662	count	1
TRBC2	-17.9534219	1936.980662	-0.0093	0.993	-0.342664553	count	1
BCL9	-17.6706094	1899.742585	-0.0093	0.993	-0.342664551	count	1
AC233280.1	-17.6706094	1899.742585	-0.0093	0.993	-0.342664551	count	1
HIST1H2BJ	-17.6706094	1899.742585	-0.0093	0.993	-0.342664551	count	1
CIT	-17.6706094	1899.742585	-0.0093	0.993	-0.342664551	count	1
IL19	-17.6136785	2795.79997	-0.0063	0.995	-0.34266455	count	1
CDS1	-17.6136785	2795.79997	-0.0063	0.995	-0.34266455	count	1
AC245140.1	-17.6136785	2795.79997	-0.0063	0.995	-0.34266455	count	1
AC092123.1	-17.6136785	2795.79997	-0.0063	0.995	-0.34266455	count	1
KCNB1	-17.6136785	2795.79997	-0.0063	0.995	-0.34266455	count	1
GK3P	-17.4040624	1254.260295	-0.0139	0.989	-0.342664548	count	1
TRIM59	-17.1917669	1348.544978	-0.0127	0.99	-0.342664546	count	1
CHKB-DT	-17.1915305	1981.156612	-0.0087	0.993	-0.342664546	count	1
CFI	-17.0646334	1265.127397	-0.0135	0.989	-0.342664544	count	1
LAMP3	-16.9142748	1633.113308	-0.0104	0.992	-0.342664542	count	1
PAWR	-16.9140974	1270.230187	-0.0133	0.989	-0.342664542	count	1
STON1	-16.6141737	2774.217094	-0.006	0.995	-0.342664536	count	1
AC145343.1	-16.6138894	2117.102124	-0.0078	0.994	-0.342664536	count	1
CDCA3	-16.5090353	1273.464108	-0.013	0.99	-0.342664533	count	1
AP002761.3	-16.5089923	1528.32454	-0.0108	0.991	-0.342664533	count	1
CEBPB-AS1	-16.5089923	1528.32454	-0.0108	0.991	-0.342664533	count	1
AC023908.3	-16.508909	1940.033991	-0.0085	0.993	-0.342664533	count	1
SYNM	-16.508909	1940.033991	-0.0085	0.993	-0.342664533	count	1
RNASEH1-AS1	-0.3764664	0.4389402	-0.8577	0.391	-0.342574378	count	1
RTN4IP1	-0.5322936	0.6912459	-0.77	0.441	-0.342536521	count	1
H2AFY	-0.2387197	0.0496857	-4.8046	1.64E-06	-0.342527359	count	0.03902708
DSTN	-0.2422499	0.0906873	-2.6713	0.00761	-0.342482217	count	1
GPN1	-0.2768255	0.2710684	-1.0212	0.307	-0.34234065	count	1
3-Mar	-0.4304562	0.457716	-0.9404	0.347	-0.341670575	count	1
CARNMT1	-0.3273888	0.5728222	-0.5715	0.568	-0.341489205	count	1
AL592295.4	-1.713096	1.4968727	-1.1445	0.253	-0.341441089	count	1
DNAL1	-0.454939	0.4887583	-0.9308	0.352	-0.341439497	count	1
PLBD2	-0.2676884	0.2532605	-1.057	0.291	-0.341272545	count	1
PHLPP2	-1.228414	0.8339999	-1.4729	0.141	-0.34126186	count	1
IMMT	-0.2509519	0.1207501	-2.0783	0.0378	-0.341230473	count	1
AC016831.7	-0.2698916	0.2076272	-1.2999	0.194	-0.340965894	count	1
MOV10	-0.4409195	0.4125815	-1.0687	0.285	-0.340820857	count	1
CD59	-0.2495121	0.1267394	-1.9687	0.0491	-0.340730984	count	1
ZCCHC14	-0.350927	0.5117272	-0.6858	0.493	-0.340636552	count	1
FCGR2A	-0.2382556	0.0570038	-4.1796	3.02E-05	-0.340452651	count	0.7129314
CFH	-0.8494768	0.4385329	-1.9371	0.0528	-0.340404008	count	1
ISY1	-0.2876549	0.2285455	-1.2586	0.208	-0.340245559	count	1
PPP1R35	-0.2692409	0.2300018	-1.1706	0.242	-0.339740981	count	1

CCR7	-1.6888435	1.2393833	-1.3626	0.173	-0.339140558	count	1
MRPL51	-0.2397242	0.0819663	-2.9247	0.00348	-0.338346353	count	1
TRAPPC4	-0.2496294	0.1374852	-1.8157	0.0695	-0.337662644	count	1
SPATC1L	-0.3006044	0.2955975	-1.0169	0.309	-0.337640336	count	1
KRR1	-0.255972	0.1786489	-1.4328	0.152	-0.337419635	count	1
CALU	-0.2496529	0.1682604	-1.4837	0.138	-0.336996701	count	1
SPATA6	-0.2832179	0.2238082	-1.2654	0.206	-0.336857425	count	1
EMC6	-0.2412191	0.104906	-2.2994	0.0216	-0.336755516	count	1
B3GNT2	-0.2455718	0.139114	-1.7653	0.0776	-0.336614314	count	1
CD3G	-0.9742357	1.1455224	-0.8505	0.395	-0.33636914	count	1
MADCAM1	-0.9742357	1.1230558	-0.8675	0.386	-0.33636914	count	1
MTFP1	-0.9742357	1.1455224	-0.8505	0.395	-0.33636914	count	1
RNF19B	-0.2787125	0.2502829	-1.1136	0.266	-0.336314817	count	1
CPVL	-0.2341695	0.0652501	-3.5888	0.000339	-0.336258047	count	1
AP2S1	-0.2344765	0.0480904	-4.8757	1.15E-06	-0.336220719	count	0.0273861
CCT4	-0.2394092	0.091183	-2.6256	0.0087	-0.33527649	count	1
KRTCAP3	-0.7387022	2.3282174	-0.3173	0.751	-0.334973988	count	1
STXBP5-AS1	-0.7387022	1.0881501	-0.6789	0.497	-0.334973988	count	1
DOPEY1	-0.3208331	0.4530291	-0.7082	0.479	-0.334890985	count	1
GNB4	-0.2445952	0.1247304	-1.961	0.05	-0.334719666	count	1
ZBTB46	-0.9673715	0.5728217	-1.6888	0.0914	-0.334676592	count	1
FICD	-0.3524392	0.3950356	-0.8922	0.372	-0.334504576	count	1
SLCO4A1	-0.459181	0.440434	-1.0426	0.297	-0.334355493	count	1
PDCD6	-0.2362487	0.075082	-3.1465	0.00167	-0.334032647	count	1
FRG1	-0.2388784	0.1003669	-2.3801	0.0174	-0.33395102	count	1
AC108863.1	-1.633834	0.783197	-2.0861	0.0371	-0.333735779	count	1
GCLM	-0.2765288	0.2903765	-0.9523	0.341	-0.333726742	count	1
ATP6AP1	-0.2376314	0.0846746	-2.8064	0.00505	-0.333683213	count	1
YIF1A	-0.2393631	0.1129704	-2.1188	0.0342	-0.333166868	count	1
PIK3R6	-1.6237262	0.741685	-2.1892	0.0287	-0.332713778	count	1
ISOC2	-0.2499943	0.1812344	-1.3794	0.168	-0.332451805	count	1
LYRM2	-0.2422165	0.1349253	-1.7952	0.0727	-0.332446456	count	1
DAPK2	-1.6181471	1.1024994	-1.4677	0.142	-0.332145822	count	1
UROD	-0.2435205	0.1569506	-1.5516	0.121	-0.33164876	count	1
ETF1	-0.2362284	0.0788302	-2.9967	0.00276	-0.331423448	count	1
ACADS	-0.2700623	0.22584	-1.1958	0.232	-0.33126275	count	1
NPM1	-0.2308469	0.0406093	-5.6846	1.47E-08	-0.331125634	count	0.000352462
TRIM47	-0.3484253	0.3354767	-1.0386	0.299	-0.330874044	count	1
GEMIN8	-0.3887018	0.5655416	-0.6873	0.492	-0.330864913	count	1
FBXL22	-1.605533	0.8223709	-1.9523	0.051	-0.330851442	count	1
SERF2	-0.2292694	0.0284717	-8.0525	1.25E-15	-0.330393881	count	3.02E-11
CHCHD3	-0.2479169	0.1551604	-1.5978	0.11	-0.330300177	count	1
GPALPP1	-0.2775644	0.2308897	-1.2022	0.229	-0.330262125	count	1
FXYD1	-0.6085358	0.7463092	-0.8154	0.415	-0.329917147	count	1
S100A10	-0.2286811	0.0508199	-4.4998	7.12E-06	-0.329685349	count	0.16887928

C1orf54	-0.238433	0.1294401	-1.842	0.0656	-0.329514661	count	1
SSB	-0.231877	0.0707513	-3.2774	0.00106	-0.329443819	count	1
HSPA9	-0.2363887	0.0898459	-2.631	0.00857	-0.329325648	count	1
USO1	-0.2509553	0.1735974	-1.4456	0.148	-0.329309205	count	1
PIR	-0.4861564	0.5534734	-0.8784	0.38	-0.328851893	count	1
SETDB2	-0.2597508	0.2354066	-1.1034	0.27	-0.32870848	count	1
TUSC2	-0.2420836	0.1418345	-1.7068	0.088	-0.328667159	count	1
JHY	-0.7212917	0.5551137	-1.2994	0.194	-0.328615834	count	1
ZNF502	-1.583218	1.195781	-1.324	0.186	-0.328526222	count	1
UQCC3	-0.2490749	0.1734837	-1.4357	0.151	-0.328033286	count	1
TMEM50A	-0.229428	0.0508998	-4.5074	6.87E-06	-0.327924338	count	0.16296327
IQCB1	-0.2890862	0.2806876	-1.0299	0.303	-0.327703921	count	1
LMAN2	-0.2314515	0.0719491	-3.2169	0.00131	-0.327449794	count	1
ZNF14	-1.1497734	0.8403381	-1.3682	0.171	-0.327212055	count	1
SLBP	-0.2365867	0.1118316	-2.1156	0.0345	-0.327178906	count	1
LMAN1	-0.2339552	0.1045073	-2.2387	0.0253	-0.327154673	count	1
PEX1	-0.2785487	0.3603518	-0.773	0.44	-0.326937495	count	1
NUP93	-0.2946183	0.3598132	-0.8188	0.413	-0.326674184	count	1
POMP	-0.2276199	0.0436802	-5.2111	2.03E-07	-0.32638834	count	0.004848655
MIEF1	-0.2972302	0.399739	-0.7436	0.457	-0.326280391	count	1
GUSB	-0.2329138	0.1146417	-2.0317	0.0423	-0.325959735	count	1
AC004951.1	-0.4086157	0.4585644	-0.8911	0.373	-0.325593352	count	1
GYS1	-0.5026457	0.5821077	-0.8635	0.388	-0.325558987	count	1
ANKRD10	-0.2348692	0.1190113	-1.9735	0.0486	-0.325538282	count	1
BMP1	-0.8012332	0.6467285	-1.2389	0.216	-0.325469561	count	1
MTHFD2	-0.2373969	0.1114153	-2.1307	0.0332	-0.325460715	count	1
TNS3	-0.2520388	0.235785	-1.0689	0.285	-0.325219176	count	1
PPIH	-0.2445969	0.1626458	-1.5039	0.133	-0.325178724	count	1
LENG9	-0.3982426	0.410168	-0.9709	0.332	-0.325146648	count	1
IL10RB	-0.39778	0.3583758	-1.11	0.267	-0.324794661	count	1
ARV1	-0.2734469	0.2693521	-1.0152	0.31	-0.32462034	count	1
EHMT1	-0.2488986	0.2051948	-1.213	0.225	-0.324247686	count	1
SUMO3	-0.2289502	0.0764727	-2.9939	0.00278	-0.324235179	count	1
NAA20	-0.2300217	0.0896184	-2.5667	0.0103	-0.324233262	count	1
ZWILCH	-0.459941	0.4669025	-0.9851	0.325	-0.324201186	count	1
EXD3	-0.4297363	0.4128709	-1.0408	0.298	-0.324065021	count	1
SKA2	-0.2613993	0.2449427	-1.0672	0.286	-0.324060125	count	1
PSMD11	-0.2332519	0.1167734	-1.9975	0.0459	-0.323048228	count	1
ATP6AP2	-0.2259009	0.0498913	-4.5279	6.24E-06	-0.322957423	count	0.148044
TEX10	-0.3355557	0.4134807	-0.8115	0.417	-0.322862192	count	1
ESCO2	-0.7923214	0.9263855	-0.8553	0.392	-0.322656039	count	1
GOLGA8B	-0.7923214	1.26686	-0.6254	0.532	-0.322656039	count	1
TCF7	-0.4276083	0.8461539	-0.5054	0.613	-0.322589321	count	1
ERCC1	-0.2309688	0.1046148	-2.2078	0.0274	-0.322558834	count	1
DDOST	-0.2319424	0.1057251	-2.1938	0.0283	-0.322173601	count	1

GK-AS1	-0.5531437	0.5134485	-1.0773	0.281	-0.321991032	count	1
RNF145	-0.2360348	0.1348156	-1.7508	0.0801	-0.32186773	count	1
LUZP1	-0.2413898	0.1616892	-1.4929	0.136	-0.321667038	count	1
SRCAP	-0.78874	0.5936238	-1.3287	0.184	-0.321520536	count	1
CNDP2	-0.2308352	0.1044036	-2.211	0.0271	-0.321391444	count	1
TBC1D22B	-0.6380343	0.7460643	-0.8552	0.393	-0.321205147	count	1
PMEPA1	-0.274261	0.3143228	-0.8725	0.383	-0.321057997	count	1
L2HGDH	-1.1166274	1.4018576	-0.7965	0.426	-0.321020988	count	1
ZNF324B	-1.1166274	1.1222908	-0.995	0.32	-0.321020988	count	1
AP001636.3	-0.7006285	0.9262466	-0.7564	0.449	-0.320976316	count	1
RAD51AP1	-0.2890259	0.3380664	-0.8549	0.393	-0.320636102	count	1
DEXI	-0.2777998	0.3466394	-0.8014	0.423	-0.319914176	count	1
GPR18	-0.4374515	0.4278658	-1.0224	0.307	-0.3198853	count	1
CALM1	-0.2222611	0.0363272	-6.1183	1.10E-09	-0.319752005	count	2.64E-05
HLA-DPB1	-0.2216375	0.0512129	-4.3278	1.57E-05	-0.319491635	count	0.3715248
CATSPER1	-0.23752	0.1653832	-1.4362	0.151	-0.319263981	count	1
DNAJC5	-0.2455234	0.1904926	-1.2889	0.198	-0.319189093	count	1
FCMR	-0.5167732	1.0502659	-0.492	0.623	-0.319074919	count	1
CCNB2	-0.5167732	0.7823459	-0.6605	0.509	-0.319074919	count	1
ELP5	-0.2573111	0.2348371	-1.0957	0.273	-0.3190638	count	1
TSPAN3	-0.233183	0.1345138	-1.7335	0.0831	-0.31892736	count	1
ATP5MD	-0.2240274	0.0625578	-3.5811	0.000349	-0.318806822	count	1
ASPH	-0.227531	0.1070718	-2.125	0.0337	-0.31870461	count	1
ZNF484	-0.5152335	0.6272097	-0.8215	0.411	-0.318234524	count	1
ORMDL3	-0.9023188	0.717635	-1.2574	0.209	-0.318203127	count	1
DAXX	-0.2538034	0.2802436	-0.9057	0.365	-0.318027322	count	1
CFAP44	-2.8685432	1.4334524	-2.0011	0.0455	-0.317961253	count	1
AL139089.1	-1.4826891	1.1352802	-1.306	0.192	-0.317472702	count	1
NPIPB6	-1.4826891	1.507243	-0.9837	0.325	-0.317472702	count	1
AC027097.2	-1.4826891	0.998978	-1.4842	0.138	-0.317472702	count	1
KATNA1	-0.2515303	0.2353345	-1.0688	0.285	-0.317277814	count	1
ATP2C1	-0.2664287	0.2013939	-1.3229	0.186	-0.317254659	count	1
EMC8	-0.2456234	0.1772286	-1.3859	0.166	-0.317025061	count	1
FLNB	-0.269894	0.3999209	-0.6749	0.5	-0.316979605	count	1
ASPM	-0.7739051	0.7424906	-1.0423	0.297	-0.316787343	count	1
GOLGA8A	-0.5125736	0.6942686	-0.7383	0.46	-0.31678112	count	1
KAT2A	-0.6275299	0.3845909	-1.6317	0.103	-0.316766375	count	1
FYTTD1	-0.2316255	0.1263928	-1.8326	0.067	-0.31674439	count	1
IL10RB-DT	-0.2355344	0.1584596	-1.4864	0.137	-0.316390397	count	1
DUSP8	-0.3636223	0.4162288	-0.8736	0.382	-0.31635992	count	1
SERPINF1	-0.2371758	0.1442882	-1.6438	0.1	-0.316091371	count	1
PINLYP	-0.4315372	0.6526118	-0.6612	0.509	-0.315922525	count	1
REXO4	-0.2682211	0.2880927	-0.931	0.352	-0.315053201	count	1
PTPN2	-0.2238569	0.0894775	-2.5018	0.0124	-0.314892562	count	1
SYNGR2	-0.2221454	0.0749303	-2.9647	0.00306	-0.31478832	count	1

MFSD14B	-0.2581698	0.2450045	-1.0537	0.292	-0.31452216	count	1
CARF	-0.6824657	0.6365057	-1.0722	0.284	-0.314177035	count	1
TMBIM6	-0.2184649	0.037152	-5.8803	4.65E-09	-0.314049073	count	0.000111605
ORAI2	-0.251235	0.1848114	-1.3594	0.174	-0.313973707	count	1
SLC16A3	-0.2225341	0.0825482	-2.6958	0.00707	-0.313915971	count	1
ATP5PF	-0.2193235	0.0518981	-4.226	2.47E-05	-0.313864854	count	0.5835622
MORC2-AS1	-0.367296	0.8864543	-0.4143	0.679	-0.31372318	count	1
RPN2	-0.2218367	0.0741157	-2.9931	0.00279	-0.313574485	count	1
PSMG1	-0.2472843	0.1973342	-1.2531	0.21	-0.31348479	count	1
TXNL4B	-0.3669031	0.3955737	-0.9275	0.354	-0.313407294	count	1
SEMA4C	-0.8832827	0.588166	-1.5018	0.133	-0.313232406	count	1
MCF2L2	-0.4428421	0.6041747	-0.733	0.464	-0.313222846	count	1
DPP7	-0.2198545	0.0630896	-3.4848	0.000501	-0.313092271	count	1
ERRFI1	-0.5049647	0.3809142	-1.3257	0.185	-0.312612377	count	1
SLC35E2A	-0.8809166	0.6544173	-1.3461	0.178	-0.31260976	count	1
PDPK1	-0.2388057	0.1840083	-1.2978	0.194	-0.31250336	count	1
SLC49A3	-0.2562827	0.2221101	-1.1539	0.249	-0.312258969	count	1
PUS7	-0.3812851	0.4072117	-0.9363	0.349	-0.312202269	count	1
RAB38	-0.4410695	0.5351287	-0.8242	0.41	-0.312079813	count	1
PAPSS1	-0.2267565	0.129983	-1.7445	0.0812	-0.312078304	count	1
PIK3R1	-0.2247087	0.0967371	-2.3229	0.0203	-0.31203313	count	1
HSD17B14	-0.2566057	0.274634	-0.9344	0.35	-0.312024884	count	1
CHN2	-0.3134524	0.310912	-1.0082	0.313	-0.311835648	count	1
DERL1	-0.225074	0.128237	-1.7551	0.0794	-0.311689574	count	1
ZNF846	-0.2780794	0.3105433	-0.8955	0.371	-0.311608481	count	1
ZPR1	-0.2360468	0.1873474	-1.2599	0.208	-0.311535688	count	1
MMAA	-0.4577127	0.8089046	-0.5658	0.572	-0.311453941	count	1
STK38L	-0.2283575	0.1339801	-1.7044	0.0884	-0.311381159	count	1
PTMS	-0.2215591	0.0914288	-2.4233	0.0155	-0.310974363	count	1
RAB8A	-0.2207959	0.0857482	-2.5749	0.0101	-0.310926829	count	1
PSMC2	-0.2289967	0.129284	-1.7713	0.0766	-0.310913954	count	1
MPG	-0.2219039	0.1081616	-2.0516	0.0403	-0.310842538	count	1
DACT3	-0.6736103	0.6598874	-1.0208	0.307	-0.310833316	count	1
ZNF444	-0.4232547	0.5059399	-0.8366	0.403	-0.310355551	count	1
IMPA1	-0.2568214	0.2133011	-1.204	0.229	-0.31033356	count	1
CDKN1A	-0.218624	0.0748561	-2.9206	0.00353	-0.310032294	count	1
FAM96B	-0.217798	0.0604707	-3.6017	0.000322	-0.309952185	count	1
MRPS17	-0.3057663	0.765441	-0.3995	0.69	-0.309950911	count	1
RHOC	-0.2196666	0.0901143	-2.4376	0.0149	-0.309745612	count	1
AMD1	-0.2211629	0.0772089	-2.8645	0.00421	-0.309712507	count	1
FOCAD	-0.866934	0.6280996	-1.3802	0.168	-0.308908353	count	1
C19orf70	-0.2174034	0.074772	-2.9075	0.00368	-0.308825317	count	1
FRMD6-AS1	-1.4069228	1.2225224	-1.1508	0.25	-0.308485372	count	1
FZD5	-0.4355031	0.4404627	-0.9887	0.323	-0.308484382	count	1
ZNF485	-0.5265983	1.3767879	-0.3825	0.702	-0.308448673	count	1

AC010542.2	-0.5620354	0.7345217	-0.7652	0.444	-0.308193408	count	1
CTSA	-0.2166262	0.0692806	-3.1268	0.00179	-0.308162583	count	1
SLC4A2	-0.290278	0.3226468	-0.8997	0.368	-0.308112812	count	1
VEGFA	-0.2193179	0.0990476	-2.2143	0.0269	-0.307803453	count	1
SNRPD1	-0.2180086	0.0870154	-2.5054	0.0123	-0.307597701	count	1
CAMK1D	-0.2388177	0.1735503	-1.3761	0.169	-0.307519506	count	1
NDUFAF5	-0.3527915	0.4419455	-0.7983	0.425	-0.307454126	count	1
JOSD2	-0.2203063	0.1090969	-2.0194	0.0436	-0.307384211	count	1
STMN1	-0.2179348	0.1205209	-1.8083	0.0707	-0.307041049	count	1
YBX1	-0.2129675	0.0285875	-7.4497	1.29E-13	-0.306815116	count	3.12E-09
MAP1LC3B2	-0.4322764	0.4938208	-0.8754	0.381	-0.306395996	count	1
GNG5	-0.2129471	0.0395159	-5.3889	7.77E-08	-0.30602368	count	0.00185804
AC074327.1	-0.4488525	0.5872015	-0.7644	0.445	-0.305986028	count	1
ZNF471	-1.0385505	1.0370026	-1.0015	0.317	-0.305783463	count	1
RAC1	-0.2126408	0.0347254	-6.1235	1.06E-09	-0.30574518	count	2.55E-05
TOE1	-0.3818992	0.4711034	-0.8106	0.418	-0.305734338	count	1
PALD1	-0.3040261	0.3254848	-0.9341	0.35	-0.305582281	count	1
ELANE	-0.4161692	0.6952728	-0.5986	0.55	-0.305576992	count	1
DDHD2	-0.3723299	0.4486033	-0.83	0.407	-0.305332264	count	1
ANAPC11	-0.2143256	0.0604183	-3.5474	0.000396	-0.305191	count	1
AL591846.2	-0.7366855	0.7665078	-0.9611	0.337	-0.304700633	count	1
TAGAP	-0.2181666	0.0949809	-2.297	0.0217	-0.304638764	count	1
CACYBP	-0.216488	0.0854038	-2.5349	0.0113	-0.304629036	count	1
MOB4	-0.2330113	0.1564658	-1.4892	0.137	-0.304578417	count	1
DBR1	-0.3431788	0.5937863	-0.5779	0.563	-0.304522622	count	1
PPM1J	-0.4660971	0.8843244	-0.5271	0.598	-0.304278885	count	1
GINS2	-0.4660971	0.6883675	-0.6771	0.498	-0.304278885	count	1
HIST1H1E	-0.2262842	0.2371567	-0.9542	0.34	-0.304040169	count	1
EFHD2	-0.2117469	0.0471556	-4.4904	7.44E-06	-0.303738193	count	0.17645448
ZNF670	-1.3670652	1.2088991	-1.1308	0.258	-0.303519151	count	1
AC091564.6	-1.3670652	1.2088991	-1.1308	0.258	-0.303519151	count	1
TYW1	-0.3180264	0.3110605	-1.0224	0.307	-0.303235596	count	1
MET	-0.5954091	0.7620487	-0.7813	0.435	-0.303017909	count	1
USP14	-0.2209034	0.1299135	-1.7004	0.0892	-0.302725583	count	1
AL121832.2	-0.8435065	0.768013	-1.0983	0.272	-0.302622644	count	1
COL1A2	-0.6518772	0.5151435	-1.2654	0.206	-0.302547147	count	1
HDAC4	-0.3604061	0.3967545	-0.9084	0.364	-0.302329335	count	1
RCAN3	-0.2825771	0.3105587	-0.9099	0.363	-0.302070893	count	1
ARHGAP10	-0.2222974	0.1813485	-1.2258	0.22	-0.302017432	count	1
SLC39A1	-0.2175931	0.1177454	-1.848	0.0647	-0.301771731	count	1
DDX11	-0.3396323	0.3859933	-0.8799	0.379	-0.301536425	count	1
MED12	-0.3199244	0.5292775	-0.6045	0.546	-0.301324774	count	1
PHB	-0.2147653	0.0873777	-2.4579	0.014	-0.301131309	count	1
FAM71A	-2.328968	1.2131164	-1.9198	0.055	-0.300786627	count	1
CBFA2T2	-0.3152467	0.449176	-0.7018	0.483	-0.300695787	count	1

TPD52L2	-0.2179614	0.1453951	-1.4991	0.134	-0.30064008	count	1
ACYP1	-0.2539834	0.3252963	-0.7808	0.435	-0.300319198	count	1
FAM96A	-0.2124706	0.0798299	-2.6615	0.00783	-0.300037335	count	1
B4GALT7	-0.2351781	0.2291364	-1.0264	0.305	-0.299954042	count	1
POP1	-0.3183672	0.3807301	-0.8362	0.403	-0.299921378	count	1
GTF2IRD2B	-2.300766	1.2168823	-1.8907	0.0588	-0.299623192	count	1
FKBP3	-0.2165021	0.1333315	-1.6238	0.105	-0.299388979	count	1
SLC2A8	-0.2634989	0.2335376	-1.1283	0.259	-0.299337421	count	1
RAB9A	-0.2204012	0.1417475	-1.5549	0.12	-0.299302542	count	1
AC073508.3	-0.8312541	1.0515493	-0.7905	0.429	-0.299293002	count	1
PPP4C	-0.2104738	0.0764283	-2.7539	0.00593	-0.299120373	count	1
MCM4	-0.3939787	0.4622786	-0.8523	0.394	-0.299089875	count	1
GGA1	-0.216179	0.1465096	-1.4755	0.14	-0.299018219	count	1
GMPR	-0.2851126	0.403013	-0.7075	0.479	-0.298754062	count	1
ZNF611	-0.3058038	0.7330817	-0.4171	0.677	-0.298552271	count	1
C14orf93	-0.6412916	0.4914333	-1.3049	0.192	-0.298469908	count	1
VMA21	-0.2131082	0.0958172	-2.2241	0.0262	-0.297852228	count	1
KIF13B	-0.2305265	0.2180868	-1.057	0.291	-0.297723629	count	1
RGS13	-1.32131	1.0681256	-1.237	0.216	-0.297607862	count	1
HDGFL3	-0.295659	0.3343177	-0.8844	0.377	-0.297466451	count	1
HSPB1	-0.2094262	0.0824989	-2.5385	0.0112	-0.297228642	count	1
MFSD5	-0.2584357	0.37853	-0.6827	0.495	-0.297023511	count	1
ZNF329	-0.637143	1.0120277	-0.6296	0.529	-0.296864605	count	1
IFT52	-0.2507937	0.2866178	-0.875	0.382	-0.296613946	count	1
ARFGAP1	-0.2829069	0.4186326	-0.6758	0.499	-0.296512518	count	1
TRPV2	-0.2467976	0.2301702	-1.0722	0.284	-0.296412091	count	1
KCTD9	-0.3181332	0.3555074	-0.8949	0.371	-0.295914891	count	1
NDUFB8	-0.2075449	0.057813	-3.5899	0.000337	-0.295545653	count	1
OLFML3	-0.3323905	0.4422027	-0.7517	0.452	-0.295427656	count	1
APOBEC3F	-0.53526	0.676734	-0.7909	0.429	-0.295421105	count	1
RNF7	-0.2070133	0.0625108	-3.3116	0.000941	-0.295350386	count	1
ATP6V1B2	-0.2095764	0.0890316	-2.354	0.0187	-0.294986305	count	1
COA7	-0.3020018	0.4319529	-0.6992	0.485	-0.294981628	count	1
SLC5A3	-0.2325237	0.2493523	-0.9325	0.351	-0.294977744	count	1
GPSM1	-0.8135455	1.0741615	-0.7574	0.449	-0.294429009	count	1
MYO9B	-0.2110608	0.1279847	-1.6491	0.0993	-0.294261057	count	1
MPC1	-0.2129323	0.0971886	-2.1909	0.0286	-0.294128871	count	1
M6PR	-0.2062128	0.0647845	-3.1831	0.00148	-0.293991473	count	1
SUGP2	-0.2331309	0.2442452	-0.9545	0.34	-0.293966359	count	1
DGUOK	-0.21069	0.1036927	-2.0319	0.0423	-0.293448924	count	1
FIZ1	-0.4701432	0.6839644	-0.6874	0.492	-0.293324211	count	1
AC083798.2	-0.8092649	0.6694993	-1.2088	0.227	-0.293244063	count	1
CHORDC1	-0.2159107	0.1400274	-1.5419	0.123	-0.293083803	count	1
MRRF	-0.258713	0.2759517	-0.9375	0.349	-0.292845101	count	1
CREB3L4	-0.3973051	0.5232652	-0.7593	0.448	-0.292782553	count	1

AC124283.1	-0.6262519	0.4643419	-1.3487	0.178	-0.292630454	count	1
LAMC1	-0.3288339	0.6749112	-0.4872	0.626	-0.292422079	count	1
RABAC1	-0.2049789	0.0619048	-3.3112	0.000942	-0.292377789	count	1
LINC01011	-0.8053582	0.6952678	-1.1583	0.247	-0.292159483	count	1
HTRA4	-2.125894	1.0193844	-2.0855	0.0371	-0.291678205	count	1
POLR3G	-0.9702326	1.0880352	-0.8917	0.373	-0.291672105	count	1
CLMP	-0.9702326	1.1566616	-0.8388	0.402	-0.291672105	count	1
UBE2L3	-0.2054738	0.0711708	-2.8871	0.00392	-0.291554271	count	1
RPL35	-0.2025852	0.0304438	-6.6544	3.50E-11	-0.29135654	count	8.43E-07
MKL2	-0.3007552	0.3879961	-0.7752	0.438	-0.290686948	count	1
SPAG9	-0.2048274	0.0796915	-2.5703	0.0102	-0.290592921	count	1
GMPPB	-0.2430084	0.3062946	-0.7934	0.428	-0.290545203	count	1
ACTR1B	-0.2363114	0.2753424	-0.8582	0.391	-0.290432979	count	1
MINOS1	-0.2048232	0.0700592	-2.9236	0.00349	-0.29042138	count	1
EIF5	-0.2044863	0.0623446	-3.2799	0.00105	-0.290353822	count	1
GMNN	-0.2409026	0.2404101	-1.002	0.316	-0.290106816	count	1
UTP11	-0.2152574	0.1592885	-1.3514	0.177	-0.290090969	count	1
INTS7	-0.3811481	0.7837781	-0.4863	0.627	-0.290036111	count	1
CENPP	-0.3811481	0.5172115	-0.7369	0.461	-0.290036111	count	1
MRPL55	-0.2106424	0.1282015	-1.6431	0.1	-0.289849424	count	1
SPATA2L	-0.2324864	0.2369742	-0.9811	0.327	-0.289557985	count	1
POLD4	-0.3030628	0.3494834	-0.8672	0.386	-0.289538873	count	1
VBP1	-0.2133231	0.1466256	-1.4549	0.146	-0.289283517	count	1
IP6K2	-0.234457	0.2424792	-0.9669	0.334	-0.288692947	count	1
TUFT1	-0.6159605	0.7642925	-0.8059	0.42	-0.288603005	count	1
NAT10	-0.2951122	0.3704591	-0.7966	0.426	-0.288501594	count	1
C8orf33	-0.2182703	0.2059962	-1.0596	0.289	-0.287924786	count	1
PHPT1	-0.2023486	0.0737677	-2.743	0.00613	-0.287498711	count	1
NCL	-0.2003487	0.0470744	-4.256	2.16E-05	-0.28746845	count	0.5106672
ID3	-0.206916	0.1839001	-1.1252	0.261	-0.287071762	count	1
SGK1	-0.1997938	0.0612971	-3.2594	0.00113	-0.287025502	count	1
LINC01534	-1.242375	1.0535413	-1.1792	0.238	-0.286861715	count	1
TRIP10	-0.2614234	0.3607293	-0.7247	0.469	-0.286458377	count	1
NDUFB7	-0.2014086	0.0720514	-2.7953	0.00522	-0.28582627	count	1
RBM8A	-0.2002332	0.0560929	-3.5697	0.000364	-0.285824957	count	1
ZNHIT2	-0.3549949	0.5342104	-0.6645	0.506	-0.28552344	count	1
C10orf95	-0.6075687	0.9148075	-0.6641	0.507	-0.285299893	count	1
SNHG15	-0.204358	0.1028364	-1.9872	0.047	-0.285234027	count	1
FSD1L	-0.3637261	0.5458566	-0.6663	0.505	-0.285103219	count	1
GGCT	-0.2147452	0.1587242	-1.3529	0.176	-0.28508102	count	1
MBTD1	-0.3386068	0.3968015	-0.8533	0.394	-0.285052693	count	1
CLUAP1	-0.2756017	0.3905953	-0.7056	0.481	-0.284882539	count	1
DERL2	-0.208019	0.1244066	-1.6721	0.0946	-0.284654288	count	1
6-Sep	-0.2079352	0.1082622	-1.9207	0.0549	-0.28454009	count	1
RNF185	-0.3094708	0.4065907	-0.7611	0.447	-0.284332746	count	1

CCSER2	-0.2236813	0.2026605	-1.1037	0.27	-0.284199969	count	1
LMAN2L	-0.7754903	0.6713209	-1.1552	0.248	-0.283768157	count	1
ANKRD36B	-0.3721305	0.4930114	-0.7548	0.45	-0.283643989	count	1
TOR3A	-0.2169812	0.1738077	-1.2484	0.212	-0.283598223	count	1
NIP7	-0.2298586	0.2567829	-0.8951	0.371	-0.283592402	count	1
ERMAP	-0.3041972	0.3925053	-0.775	0.438	-0.283496673	count	1
CD47	-0.2012296	0.0846236	-2.3779	0.0175	-0.283481533	count	1
FHL1	-0.3081048	0.4303068	-0.716	0.474	-0.283132231	count	1
AP006623.1	-1.9575034	1.0809182	-1.811	0.0703	-0.282701461	count	1
RNF6	-0.2475715	0.2281385	-1.0852	0.278	-0.282699135	count	1
C12orf49	-0.2193446	0.2064504	-1.0625	0.288	-0.282673236	count	1
MROH1	-0.2633928	0.3033	-0.8684	0.385	-0.282106007	count	1
ACBD6	-0.2076305	0.1519583	-1.3664	0.172	-0.281969834	count	1
LETM2	-0.4085125	0.5368102	-0.761	0.447	-0.28080114	count	1
ELAC2	-0.2760486	0.3595007	-0.7679	0.443	-0.280787057	count	1
BLMH	-0.2439884	0.2960705	-0.8241	0.41	-0.280738088	count	1
DAD1	-0.1960925	0.0545886	-3.5922	0.000334	-0.280638962	count	1
GRB2	-0.1957493	0.0460265	-4.253	2.19E-05	-0.28043244	count	0.517716
STOM	-0.2081926	0.1250461	-1.6649	0.0961	-0.280344271	count	1
MRPL18	-0.200784	0.0925364	-2.1698	0.0301	-0.280322154	count	1
DFFA	-0.233737	0.2731527	-0.8557	0.392	-0.280305458	count	1
CD226	-0.3197504	0.577085	-0.5541	0.58	-0.280078192	count	1
TMEM165	-0.1990215	0.0868983	-2.2903	0.0221	-0.279911563	count	1
AFTPH	-0.2137615	0.170341	-1.2549	0.21	-0.279798044	count	1
CWC15	-0.2010027	0.123373	-1.6292	0.103	-0.279321802	count	1
GGA2	-0.2079861	0.1608709	-1.2929	0.196	-0.279292497	count	1
FAHD2B	-1.1887693	1.030052	-1.1541	0.249	-0.279152473	count	1
DBN1	-1.1887693	1.1701911	-1.0159	0.31	-0.279152473	count	1
IPO4	-1.1887693	1.030052	-1.1541	0.249	-0.279152473	count	1
SLFN13	-1.1887693	1.1701911	-1.0159	0.31	-0.279152473	count	1
SUGP1	-0.2768445	0.3269885	-0.8466	0.397	-0.279152077	count	1
RAB18	-0.2051859	0.1264757	-1.6223	0.105	-0.279014868	count	1
NARS2	-0.3656003	0.4327462	-0.8448	0.398	-0.279000174	count	1
MAST2	-0.4696575	0.5551198	-0.846	0.398	-0.278739356	count	1
NFKBIE	-0.2077934	0.16413	-1.266	0.206	-0.278306546	count	1
MDN1	-0.2874193	0.4558314	-0.6305	0.528	-0.27827165	count	1
GPR132	-0.2383651	0.2041047	-1.1679	0.243	-0.278070821	count	1
PARD6G-AS1	-0.7552477	0.4638415	-1.6282	0.104	-0.277980405	count	1
BOLA2-SMG1P6	-0.6572207	0.7005612	-0.9381	0.348	-0.277869427	count	1
UQCRFS1	-0.1966049	0.0832221	-2.3624	0.0182	-0.277755959	count	1
TRAM2	-0.4036829	0.6310262	-0.6397	0.522	-0.277754184	count	1
COX14	-0.1974196	0.0821058	-2.4045	0.0163	-0.277400997	count	1
RPL27A	-0.1933973	0.0379991	-5.0895	3.86E-07	-0.277343864	count	0.00920803
TCTN3	-0.2863155	0.2657445	-1.0774	0.281	-0.277241953	count	1
B3GALT6	-0.2271294	0.2429071	-0.935	0.35	-0.277223377	count	1

SMARCC1	-0.2080672	0.1721047	-1.209	0.227	-0.277046492	count	1
UQCC2	-0.2029873	0.1248029	-1.6265	0.104	-0.276751745	count	1
IGF2R	-0.2018316	0.1681225	-1.2005	0.23	-0.276434077	count	1
AP3D1	-0.2157952	0.1775887	-1.2151	0.224	-0.276315802	count	1
PODXL	-1.1693888	1.3125035	-0.891	0.373	-0.276280686	count	1
AL136040.1	-1.1686596	0.7384459	-1.5826	0.114	-0.27617174	count	1
TMED10	-0.1943491	0.0684254	-2.8403	0.00454	-0.276001123	count	1
TMEM17	-0.4951539	1.5116908	-0.3275	0.743	-0.275928726	count	1
SRSF2	-0.1953902	0.0702219	-2.7825	0.00544	-0.275592344	count	1
EZH2	-0.2952894	0.3649868	-0.809	0.419	-0.275531283	count	1
POLR2D	-0.2375187	0.269863	-0.8801	0.379	-0.275316366	count	1
CLPP	-0.2016773	0.133753	-1.5078	0.132	-0.275216185	count	1
KLHL21	-0.2701513	0.318082	-0.8493	0.396	-0.27497342	count	1
COPS8	-0.2097706	0.1944713	-1.0787	0.281	-0.2749719	count	1
SETD5	-0.2140373	0.2429805	-0.8809	0.378	-0.274884462	count	1
LPAR5	-0.3408739	0.3870489	-0.8807	0.379	-0.27483097	count	1
DUSP16	-0.4367559	0.4628561	-0.9436	0.345	-0.274506973	count	1
PLGRKT	-0.2052482	0.1507222	-1.3618	0.173	-0.274371605	count	1
PSMD3	-0.2020453	0.1232547	-1.6393	0.101	-0.274349122	count	1
SH3BP4	-0.5795896	0.5404497	-1.0724	0.284	-0.274162945	count	1
KLHDC1	-0.5795896	0.5896615	-0.9829	0.326	-0.274162945	count	1
ARL2	-0.1985954	0.1155357	-1.7189	0.0858	-0.273940793	count	1
AL020996.1	-0.8878241	1.0519419	-0.844	0.399	-0.273644832	count	1
LINC01526	-0.8878241	1.3772968	-0.6446	0.519	-0.273644832	count	1
VLDLR	-0.8878241	1.0611704	-0.8366	0.403	-0.273644832	count	1
DLGAP5	-0.8878241	1.0611704	-0.8366	0.403	-0.273644832	count	1
ACOT2	-0.8878241	1.0611704	-0.8366	0.403	-0.273644832	count	1
RAC3	-0.8878241	1.0519419	-0.844	0.399	-0.273644832	count	1
ASIP	-0.8878241	1.0611704	-0.8366	0.403	-0.273644832	count	1
AC006116.8	-0.8878241	1.0611704	-0.8366	0.403	-0.273644832	count	1
BACE2	-0.8873977	0.744934	-1.1912	0.234	-0.273548617	count	1
SMARCAD1	-0.2384713	0.2438014	-0.9781	0.328	-0.273521949	count	1
EIF5B	-0.1935697	0.082194	-2.355	0.0186	-0.273087084	count	1
GALC	-0.2197763	0.2348422	-0.9358	0.349	-0.273076382	count	1
EIF5A	-0.1909945	0.0538903	-3.5441	0.000401	-0.272760963	count	1
UQCRHL	-0.4130553	0.5351415	-0.7719	0.44	-0.272709085	count	1
INF2	-0.2228754	0.3353805	-0.6645	0.506	-0.272623547	count	1
KIF5B	-0.1931041	0.0685758	-2.8159	0.0049	-0.272382971	count	1
CDK10	-0.277902	0.2840474	-0.9784	0.328	-0.272260332	count	1
SLC26A11	-0.316103	0.4041113	-0.7822	0.434	-0.272190707	count	1
HEXA	-0.1933332	0.1062641	-1.8194	0.069	-0.272164898	count	1
AURKC	-1.787629	1.3340195	-1.34	0.18	-0.272120886	count	1
COQ2	-0.2059132	0.1797757	-1.1454	0.252	-0.272034315	count	1
RHOQ	-0.1949536	0.0725338	-2.6878	0.00724	-0.272013247	count	1
CD70	-1.1405897	0.8961243	-1.2728	0.203	-0.271928491	count	1

UBE2N	-0.193242	0.0918572	-2.1037	0.0355	-0.27191956	count	1
BCORL1	-0.3944336	0.5825558	-0.6771	0.498	-0.271899981	count	1
MPP6	-0.6397697	0.8880501	-0.7204	0.471	-0.271787691	count	1
PELP1	-0.3155058	0.3761585	-0.8388	0.402	-0.271701788	count	1
COMMD7	-0.1956001	0.1105041	-1.7701	0.0768	-0.271616444	count	1
NACC1	-0.237636	0.3287433	-0.7229	0.47	-0.271570836	count	1
COMMD10	-0.2006072	0.1609613	-1.2463	0.213	-0.271440819	count	1
TIMM50	-0.2101687	0.2174357	-0.9666	0.334	-0.271423497	count	1
PPT1	-0.1917305	0.0608123	-3.1528	0.00164	-0.271210967	count	1
POLR2K	-0.1942921	0.102193	-1.9012	0.0574	-0.270874382	count	1
C5orf34	-0.4099575	0.9967237	-0.4113	0.681	-0.270840295	count	1
VPS35	-0.1907185	0.0638591	-2.9866	0.00285	-0.270739452	count	1
CRYZ	-0.2633845	0.607487	-0.4336	0.665	-0.270498022	count	1
C6orf52	-0.5702237	0.8785008	-0.6491	0.516	-0.27039215	count	1
AC006027.1	-0.5702237	1.95502	-0.2917	0.771	-0.27039215	count	1
CNOT6	-0.2631776	0.350235	-0.7514	0.452	-0.270291755	count	1
ARID4B	-0.1942745	0.0991587	-1.9592	0.0502	-0.270154569	count	1
SLC22A18AS	-0.2372658	0.2896507	-0.8191	0.413	-0.270116945	count	1
LINC02542	-0.634954	1.7243502	-0.3682	0.713	-0.270097371	count	1
FAM86B1	-0.634954	1.1671709	-0.544	0.586	-0.270097371	count	1
ADPRHL1	-0.634954	1.1671709	-0.544	0.586	-0.270097371	count	1
C19orf44	-0.634954	1.2393887	-0.5123	0.608	-0.270097371	count	1
HOMER3-AS1	-0.634954	1.0877855	-0.5837	0.559	-0.270097371	count	1
SHANK3	-0.634954	1.1671709	-0.544	0.586	-0.270097371	count	1
SMCR5	-0.8714095	0.79357	-1.0981	0.272	-0.269919138	count	1
SYT1	-0.8705282	0.7329205	-1.1878	0.235	-0.269717813	count	1
TBC1D8-AS1	-0.5670617	0.69006	-0.8218	0.411	-0.269114251	count	1
CLMN	-0.22077	0.2919256	-0.7563	0.45	-0.269033321	count	1
MAPK9	-0.2392408	0.2500878	-0.9566	0.339	-0.268985696	count	1
DLC1	-0.5662224	0.6699043	-0.8452	0.398	-0.268774657	count	1
ACADVL	-0.1899113	0.0873915	-2.1731	0.0299	-0.268559238	count	1
PITPNA	-0.1941179	0.1367498	-1.4195	0.156	-0.268361692	count	1
UBAC1	-0.1921489	0.1109746	-1.7315	0.0835	-0.268274844	count	1
INTS2	-0.3244599	0.4332876	-0.7488	0.454	-0.268212294	count	1
BCLAF3	-0.2354845	0.3981828	-0.5914	0.554	-0.268127803	count	1
AC005726.5	-0.3243011	0.4706397	-0.6891	0.491	-0.268088051	count	1
NUP98	-0.215934	0.2009339	-1.0747	0.283	-0.267935577	count	1
NUDT1	-0.1908568	0.1036354	-1.8416	0.0657	-0.267428559	count	1
AL035587.1	-0.8603871	0.7235617	-1.1891	0.235	-0.267391754	count	1
NIPSNAP1	-0.2541073	0.3905257	-0.6507	0.515	-0.267138531	count	1
NSMCE3	-0.1968391	0.1569911	-1.2538	0.21	-0.266887997	count	1
MIF4GD	-0.1932599	0.121262	-1.5937	0.111	-0.266826864	count	1
SSR3	-0.1869186	0.0565355	-3.3062	0.001	-0.266659325	count	1
ARPC5L	-0.1916763	0.1248494	-1.5353	0.125	-0.266072455	count	1
FAM173A	-0.1925939	0.111782	-1.7229	0.085	-0.266017793	count	1

DHRS11	-0.284374	0.5225966	-0.5442	0.586	-0.26574135	count	1
CHUK	-0.2582862	0.3223133	-0.8014	0.423	-0.265412221	count	1
TTC1	-0.1907877	0.1011095	-1.8869	0.0593	-0.265293399	count	1
MRPL36	-0.1919512	0.1363102	-1.4082	0.159	-0.265132858	count	1
MAP1LC3B	-0.1853935	0.0576119	-3.218	0.00131	-0.265085198	count	1
B4GALT1-AS1	-0.3693321	0.5750937	-0.6422	0.521	-0.265046552	count	1
BRD3OS	-0.4197973	0.6826282	-0.615	0.539	-0.264828003	count	1
CORO1C	-0.1921124	0.1531817	-1.2541	0.21	-0.264641044	count	1
LSM7	-0.1860534	0.0694904	-2.6774	0.00747	-0.264476363	count	1
RNF181	-0.1855847	0.0683664	-2.7146	0.00668	-0.264402993	count	1
ACBD3	-0.1885418	0.1109128	-1.6999	0.0893	-0.26419153	count	1
SFXN4	-0.2253526	0.2766965	-0.8144	0.415	-0.263953666	count	1
ABL2	-0.2010638	0.1990593	-1.0101	0.313	-0.263639402	count	1
PIGZ	-0.3117014	0.9255702	-0.3368	0.736	-0.263540716	count	1
TMEM8A	-0.2033261	0.196589	-1.0343	0.301	-0.263533607	count	1
MICAL2	-0.2543367	0.2722678	-0.9341	0.35	-0.263512832	count	1
GOSR2	-0.216577	0.2232466	-0.9701	0.332	-0.263459298	count	1
PFDN2	-0.1851572	0.0678887	-2.7274	0.00643	-0.263333514	count	1
HIST1H4C	-0.1889925	0.1604974	-1.1775	0.239	-0.263327103	count	1
MRS2	-0.2684392	0.3471932	-0.7732	0.439	-0.263297571	count	1
FCRLB	-0.2854692	0.516629	-0.5526	0.581	-0.2631642	count	1
CST7	-0.2177116	0.2735461	-0.7959	0.426	-0.263148827	count	1
NOMO1	-0.304858	0.6112772	-0.4987	0.618	-0.262967468	count	1
RPF2	-0.1917671	0.1234935	-1.5529	0.121	-0.262944712	count	1
COX8A	-0.1834254	0.0445135	-4.1207	3.90E-05	-0.262901212	count	0.919737
HES6	-0.298997	0.3992483	-0.7489	0.454	-0.26272498	count	1
GTF2H1	-0.219119	0.206435	-1.0614	0.289	-0.262395122	count	1
RDH10	-0.3100258	0.501146	-0.6186	0.536	-0.262194158	count	1
SSR4	-0.1826836	0.0422986	-4.3189	1.63E-05	-0.262024914	count	0.385658
SLAMF1	-1.0764158	0.8531133	-1.2618	0.207	-0.261857505	count	1
ZMIZ2	-0.2437137	0.3459577	-0.7045	0.481	-0.261538521	count	1
GAS8	-0.3781414	0.832711	-0.4541	0.65	-0.261527843	count	1
BAG2	-0.4373147	0.5335871	-0.8196	0.413	-0.261463326	count	1
DHX32	-0.251859	0.4407393	-0.5714	0.568	-0.261015839	count	1
MAGED1	-0.3309552	0.5488423	-0.603	0.547	-0.260919703	count	1
AL021068.1	-0.8320738	0.8023904	-1.037	0.3	-0.26080461	count	1
NXT1	-0.185013	0.0926287	-1.9974	0.0459	-0.260612082	count	1
CDC20	-0.3302738	0.9012916	-0.3664	0.714	-0.260413539	count	1
ILK	-0.1879642	0.1069867	-1.7569	0.0791	-0.260356959	count	1
LAPTM5	-0.1812031	0.0339074	-5.3441	9.93E-08	-0.260211828	count	0.002373767
GPATCH4	-0.2054603	0.2203283	-0.9325	0.351	-0.260058314	count	1
SMIM30	-0.203682	0.159745	-1.275	0.202	-0.259874149	count	1
ZCCHC17	-0.1909126	0.1408273	-1.3557	0.175	-0.259623787	count	1
COX20	-0.1844954	0.1085796	-1.6992	0.0894	-0.259506031	count	1
DACH1	-1.612705	1.0499575	-1.536	0.125	-0.259383454	count	1

SLC17A9	-0.2615904	0.5216478	-0.5015	0.616	-0.25938339	count	1
TGIF1	-0.185098	0.0888917	-2.0823	0.0374	-0.259344579	count	1
NKIRAS1	-0.2274964	0.2762892	-0.8234	0.41	-0.259199921	count	1
GGCX	-0.2003286	0.188756	-1.0613	0.289	-0.258817803	count	1
GLOD4	-0.1910277	0.1326766	-1.4398	0.15	-0.25853777	count	1
NPRL2	-0.2555703	0.3670792	-0.6962	0.486	-0.258336319	count	1
RPLP0	-0.1793645	0.0322284	-5.5654	2.90E-08	-0.258317129	count	0.000694608
AC008915.2	-0.2798842	0.5047779	-0.5545	0.579	-0.258215881	count	1
NFS1	-0.2417025	0.4114101	-0.5875	0.557	-0.257823211	count	1
HLA-DRA	-0.178681	0.0472362	-3.7827	0.000159	-0.257694456	count	1
USP9X	-0.1982857	0.1704672	-1.1632	0.245	-0.257459004	count	1
SAT1	-0.1785758	0.0351745	-5.0769	4.13E-07	-0.257384571	count	0.009850463
G3BP2	-0.1834489	0.0909373	-2.0173	0.0438	-0.257205624	count	1
PGM3	-0.2237401	0.3501099	-0.6391	0.523	-0.256923146	count	1
CYP51A1	-0.3708058	0.5244934	-0.707	0.48	-0.256832714	count	1
TMEM201	-1.0432195	1.1229209	-0.929	0.353	-0.256440756	count	1
SPIN4	-1.0432195	1.1229209	-0.929	0.353	-0.256440756	count	1
ABHD8	-0.2207916	0.2917786	-0.7567	0.449	-0.256250461	count	1
NR4A3	-0.1818403	0.1135237	-1.6018	0.109	-0.256119166	count	1
MORF4L2	-0.1886995	0.1287197	-1.466	0.143	-0.256036906	count	1
ZBTB8A	-1.04024	0.8139698	-1.278	0.201	-0.255947471	count	1
PIM1	-0.1898324	0.1562073	-1.2153	0.224	-0.255740939	count	1
ERN1	-0.1874359	0.1380431	-1.3578	0.175	-0.255689515	count	1
TMEM126A	-0.186698	0.1383571	-1.3494	0.177	-0.25533318	count	1
BRWD1-AS2	-0.6786178	0.7751184	-0.8755	0.381	-0.255323181	count	1
SLC22A4	-0.354371	0.5423266	-0.6534	0.514	-0.255048347	count	1
SPARC	-0.2521755	0.458442	-0.5501	0.582	-0.255004258	count	1
ZNF808	-0.2286216	0.506019	-0.4518	0.651	-0.254989906	count	1
CFDP1	-0.1831357	0.122786	-1.4915	0.136	-0.254940104	count	1
CBWD6	-0.4873153	0.7642389	-0.6376	0.524	-0.254797619	count	1
PI4K2A	-0.2021931	0.2379086	-0.8499	0.395	-0.254356364	count	1
PPME1	-0.2589481	0.3850595	-0.6725	0.501	-0.25428486	count	1
BRI3	-0.1766324	0.0372313	-4.7442	2.21E-06	-0.254101396	count	0.05256043
CDC37L1-DT	-0.5302677	0.7724588	-0.6865	0.492	-0.254063787	count	1
BBOF1	-0.5302677	0.9339045	-0.5678	0.57	-0.254063787	count	1
CABLES2	-0.5302677	0.8335721	-0.6361	0.525	-0.254063787	count	1
PTTG1IP	-0.1788035	0.0711379	-2.5135	0.012	-0.254042824	count	1
RCC1	-0.2584926	0.4617299	-0.5598	0.576	-0.253851731	count	1
OAZ3	-0.8027612	0.6709982	-1.1964	0.232	-0.253839432	count	1
LINC00484	-0.8027612	0.8198446	-0.9792	0.328	-0.253839432	count	1
ZNF410	-0.8027612	0.9389105	-0.855	0.393	-0.253839432	count	1
B3GAT3	-0.1857023	0.1378126	-1.3475	0.178	-0.253715693	count	1
FBXL8	-0.381597	0.513193	-0.7436	0.457	-0.253603411	count	1
CHMP2B	-0.180935	0.0926562	-1.9528	0.051	-0.253477184	count	1
ARIH2	-0.1934989	0.2034409	-0.9511	0.342	-0.253290334	count	1

SNCA	-0.192143	0.1528093	-1.2574	0.209	-0.253247321	count	1
CHMP3	-0.1848247	0.1405681	-1.3148	0.189	-0.252881756	count	1
GRWD1	-0.2436055	0.3451336	-0.7058	0.48	-0.252687507	count	1
LYNX1	-1.02052	0.639651	-1.5954	0.111	-0.252653646	count	1
AC090527.3	-0.3983872	0.6543273	-0.6089	0.543	-0.252492271	count	1
ESF1	-0.1874007	0.1682076	-1.1141	0.265	-0.252480504	count	1
LINS1	-0.379683	0.420589	-0.9027	0.367	-0.25243184	count	1
ATP6V0B	-0.175553	0.0352143	-4.9853	6.62E-07	-0.252353361	count	0.015778108
EID2B	-0.3114088	0.4618763	-0.6742	0.5	-0.252334003	count	1
TMEM260	-0.2595197	0.5180352	-0.501	0.616	-0.252147255	count	1
SMAD6	-0.6681726	1.0126583	-0.6598	0.509	-0.25214219	count	1
XKR6	-0.6681725	0.9500819	-0.7033	0.482	-0.252142161	count	1
AP005329.3	-0.2656115	0.3474269	-0.7645	0.445	-0.25198987	count	1
KLHL42	-0.3278598	0.3845041	-0.8527	0.394	-0.251918236	count	1
CLDN23	-0.2512119	0.3756569	-0.6687	0.504	-0.251779247	count	1
LSM10	-0.1801396	0.091129	-1.9768	0.0482	-0.251620015	count	1
HSDL1	-0.2463602	0.3395105	-0.7256	0.468	-0.25143276	count	1
PRRC2C	-0.1767948	0.0657872	-2.6874	0.00725	-0.251422587	count	1
SECISBP2	-0.1879148	0.1701083	-1.1047	0.269	-0.251322953	count	1
TRIM21	-0.2506735	0.3128987	-0.8011	0.423	-0.251255465	count	1
AC079922.2	-0.377653	0.6079131	-0.6212	0.535	-0.251188113	count	1
CHTOP	-0.1910297	0.1655104	-1.1542	0.249	-0.251041591	count	1
UMPS	-0.2400638	0.3245979	-0.7396	0.46	-0.250961004	count	1
ACVR1	-0.2437691	0.4538476	-0.5371	0.591	-0.250896187	count	1
NDUFC1	-0.1774245	0.0791117	-2.2427	0.025	-0.250831718	count	1
PLVAP	-1.5028693	1.5410051	-0.9753	0.33	-0.250308571	count	1
TESK2	-0.2326636	0.3981105	-0.5844	0.559	-0.249951147	count	1
UPF3B	-0.2004397	0.2222481	-0.9019	0.367	-0.249688346	count	1
BAHD1	-0.3245969	0.498407	-0.6513	0.515	-0.249557422	count	1
TBK1	-0.1997155	0.1690197	-1.1816	0.237	-0.248795606	count	1
OPA3	-0.217208	0.3011081	-0.7214	0.471	-0.248629521	count	1
PDE12	-0.2313888	0.2918662	-0.7928	0.428	-0.248612601	count	1
TAB1	-0.5171306	0.6096479	-0.8482	0.396	-0.248609405	count	1
ZNF428	-0.1827051	0.1365187	-1.3383	0.181	-0.248445751	count	1
CCDC115	-0.183416	0.1361885	-1.3468	0.178	-0.248351664	count	1
PSMD7	-0.1754467	0.0781102	-2.2461	0.0248	-0.248325534	count	1
NHP2	-0.1763523	0.0955552	-1.8456	0.0651	-0.248235688	count	1
SELENOW	-0.1741821	0.0712542	-2.4445	0.0146	-0.24820379	count	1
LRRC3	-0.7793508	0.8405471	-0.9272	0.354	-0.248169107	count	1
ACSM3	-0.4124039	0.485478	-0.8495	0.396	-0.247959444	count	1
TTLL1	-0.4722815	0.7159683	-0.6596	0.51	-0.247851355	count	1
PGAM1	-0.1731685	0.0603486	-2.8695	0.00415	-0.247839535	count	1
RSPRY1	-0.2133192	0.2698609	-0.7905	0.429	-0.247716366	count	1
NIPA2	-0.1869563	0.1407896	-1.3279	0.184	-0.247662382	count	1
GSTM3	-0.4383976	0.8466258	-0.5178	0.605	-0.247603106	count	1

AL512353.1	-1.46916	1.1353105	-1.2941	0.196	-0.247342779	count	1
SMYD5	-1.46916	1.1353105	-1.2941	0.196	-0.247342779	count	1
CACNA1A	-0.2639147	0.7108074	-0.3713	0.71	-0.247305232	count	1
DUSP23	-0.1760301	0.0986585	-1.7842	0.0745	-0.247149143	count	1
UBE2Z	-0.1902885	0.167269	-1.1376	0.255	-0.246956826	count	1
TRIM3	-0.5131312	0.7258816	-0.7069	0.48	-0.246940445	count	1
SLC35F6	-0.1861728	0.192516	-0.9671	0.334	-0.246502972	count	1
NDUFV2	-0.1728691	0.0594232	-2.9091	0.0037	-0.246391962	count	1
SMPD3	-0.9831256	1.2483993	-0.7875	0.431	-0.246264716	count	1
NOP14	-0.1918921	0.2483456	-0.7727	0.44	-0.245468252	count	1
LAIR2	-0.6461852	0.9064143	-0.7129	0.476	-0.24537265	count	1
NOP56	-0.183796	0.1439763	-1.2766	0.202	-0.245331927	count	1
RAB7A	-0.1723826	0.0588775	-2.9278	0.00344	-0.245189258	count	1
MED8	-0.1802289	0.144081	-1.2509	0.211	-0.244907741	count	1
WDR1	-0.1767597	0.1134005	-1.5587	0.119	-0.244821495	count	1
AC025164.1	-0.1762141	0.2795359	-0.6304	0.529	-0.244481621	count	1
EIF4EBP1	-0.1724703	0.0775774	-2.2232	0.0263	-0.244369007	count	1
EIF5AL1	-0.5064527	0.7824255	-0.6473	0.518	-0.244144687	count	1
C5orf63	-1.432447	1.004187	-1.4265	0.154	-0.244011602	count	1
SNRPE	-0.1736905	0.0916291	-1.8956	0.0581	-0.244007596	count	1
ATP6V0A2	-0.2816977	0.3412315	-0.8255	0.409	-0.243858897	count	1
MILR1	-0.1819152	0.1383631	-1.3148	0.189	-0.2437252	count	1
NUTF2	-0.1743089	0.1046197	-1.6661	0.0958	-0.24363409	count	1
PDAP1	-0.1735219	0.0986002	-1.7599	0.0786	-0.243271105	count	1
WDR43	-0.1789955	0.1484885	-1.2054	0.228	-0.243176414	count	1
NDUFV3	-0.1743894	0.1073771	-1.6241	0.104	-0.242405564	count	1
BTRC	-0.2620747	0.4553817	-0.5755	0.565	-0.2423803	count	1
ACOX3	-0.2548147	0.4291855	-0.5937	0.553	-0.242088963	count	1
SNRNP25	-0.1813419	0.1470326	-1.2333	0.218	-0.242073099	count	1
HELLS	-0.4598217	0.7201245	-0.6385	0.523	-0.2420499	count	1
TMX2	-0.189562	0.1761748	-1.076	0.282	-0.242009311	count	1
PHETA2	-0.4014461	0.6902425	-0.5816	0.561	-0.241964958	count	1
SRR	-0.3801963	0.5648011	-0.6732	0.501	-0.241909711	count	1
CD58	-0.1741235	0.1233135	-1.412	0.158	-0.241892106	count	1
PSMB6	-0.1692563	0.0530598	-3.1899	0.00144	-0.241703073	count	1
ZNF706	-0.1689871	0.0539788	-3.1306	0.00176	-0.241692848	count	1
TBCD	-0.2612851	0.2964227	-0.8815	0.378	-0.241676245	count	1
MALSU1	-0.1810908	0.1675041	-1.0811	0.28	-0.241635899	count	1
PRRG1	-0.7527666	0.8204925	-0.9175	0.359	-0.241612885	count	1
ADAM8	-0.1787566	0.1745534	-1.0241	0.306	-0.241572751	count	1
LAPTM4A	-0.1686045	0.0470014	-3.5872	0.000341	-0.24145643	count	1
TMEM68	-0.2474638	0.4359527	-0.5676	0.57	-0.240796022	count	1
ORMDL2	-0.1722697	0.1056434	-1.6307	0.103	-0.240789695	count	1
SH3BGRL3	-0.1668481	0.0354664	-4.7044	2.69E-06	-0.240519028	count	0.06394399
AC092140.1	-1.392884	1.1118937	-1.2527	0.21	-0.240301008	count	1

RBL1	-0.6298345	0.5790746	-1.0877	0.277	-0.240273671	count	1
GSN	-0.1685282	0.0734752	-2.2937	0.0219	-0.239711886	count	1
IL1A	-0.4956513	1.0425607	-0.4754	0.635	-0.239599664	count	1
SNAPC2	-0.190002	0.1942281	-0.9782	0.328	-0.239473755	count	1
CD22	-0.3104948	0.5943036	-0.5225	0.601	-0.239318815	count	1
SAP130	-0.2759649	0.501487	-0.5503	0.582	-0.239105814	count	1
LRP5L	-0.3193578	0.5566232	-0.5737	0.566	-0.238811306	count	1
MYEF2	-0.93914	0.8669407	-1.0833	0.279	-0.238506097	count	1
MPLKIP	-0.1723153	0.1164102	-1.4802	0.139	-0.238452013	count	1
APAF1	-0.1809522	0.1508595	-1.1995	0.23	-0.238447757	count	1
NDUFS5	-0.1664843	0.0524901	-3.1717	0.00153	-0.238183967	count	1
C5orf22	-0.2444133	0.5684471	-0.43	0.667	-0.237917906	count	1
UQCR11	-0.1664574	0.0532886	-3.1237	0.0018	-0.237843548	count	1
GUK1	-0.1660384	0.0491118	-3.3808	0.000734	-0.237831556	count	1
SPDL1	-0.2999801	0.4872917	-0.6156	0.538	-0.237775681	count	1
MIATNB	-0.3729953	0.5967774	-0.625	0.532	-0.237694787	count	1
TOM1L2	-0.2691491	0.4570426	-0.5889	0.556	-0.237556199	count	1
NUCB2	-0.1721297	0.1222339	-1.4082	0.159	-0.237539214	count	1
SLC38A1	-0.7355889	0.4661188	-1.5781	0.115	-0.237309646	count	1
AL606760.3	-17.4310003	3773.429215	-0.0046	0.996	-0.237234527	count	1
AL139339.1	-17.4310003	3773.429215	-0.0046	0.996	-0.237234527	count	1
AC134312.1	-17.4310003	3773.429215	-0.0046	0.996	-0.237234527	count	1
ZNF610	-17.4310003	3773.429215	-0.0046	0.996	-0.237234527	count	1
AL357079.1	-17.4308585	2783.921146	-0.0063	0.995	-0.237234527	count	1
LINC01160	-17.4308585	2783.921146	-0.0063	0.995	-0.237234527	count	1
AL162258.2	-17.4308585	2783.921146	-0.0063	0.995	-0.237234527	count	1
AL590723.1	-17.4308585	2783.921146	-0.0063	0.995	-0.237234527	count	1
GPC1	-17.4308585	2783.921146	-0.0063	0.995	-0.237234527	count	1
C3orf33	-17.4308585	2783.921146	-0.0063	0.995	-0.237234527	count	1
AC092535.1	-17.4308585	2783.921146	-0.0063	0.995	-0.237234527	count	1
LIFR	-17.4308585	2783.921146	-0.0063	0.995	-0.237234527	count	1
AC008875.1	-17.4308585	2783.921146	-0.0063	0.995	-0.237234527	count	1
MRPS30-DT	-17.4308585	2783.921146	-0.0063	0.995	-0.237234527	count	1
AC109322.1	-17.4308585	2783.921146	-0.0063	0.995	-0.237234527	count	1
ASS1	-17.4308585	2783.921146	-0.0063	0.995	-0.237234527	count	1
ADAM12	-17.4308585	2783.921146	-0.0063	0.995	-0.237234527	count	1
AC022509.4	-17.4308585	2783.921146	-0.0063	0.995	-0.237234527	count	1
IKZF3	-17.4308585	2783.921146	-0.0063	0.995	-0.237234527	count	1
AC130371.2	-17.4308585	2783.921146	-0.0063	0.995	-0.237234527	count	1
CPXM1	-17.4308585	2783.921146	-0.0063	0.995	-0.237234527	count	1
GRIN3B	-17.4308585	2783.921146	-0.0063	0.995	-0.237234527	count	1
NEURL3	-17.421322	2094.167041	-0.0083	0.993	-0.237234527	count	1
EVC2	-17.421322	2094.167041	-0.0083	0.993	-0.237234527	count	1
AL441883.1	-17.421322	2094.167041	-0.0083	0.993	-0.237234527	count	1
LAMB1	-17.421322	2094.167041	-0.0083	0.993	-0.237234527	count	1

DNAJC12	-17.421322	2094.167041	-0.0083	0.993	-0.237234527	count	1
FGF5	-17.421196	2698.119368	-0.0065	0.995	-0.237234527	count	1
E2F1	-17.421196	2698.119368	-0.0065	0.995	-0.237234527	count	1
AL031663.3	-17.3619141	1596.679103	-0.0109	0.991	-0.237234527	count	1
ANXA3	-17.3610703	1312.10275	-0.0132	0.989	-0.237234527	count	1
ADTRP	-17.3610703	1312.10275	-0.0132	0.989	-0.237234527	count	1
LPAR3	-17.0439327	1906.12048	-0.0089	0.993	-0.237234524	count	1
DENND2C	-17.0439327	1906.12048	-0.0089	0.993	-0.237234524	count	1
MEX3A	-17.0439327	1906.12048	-0.0089	0.993	-0.237234524	count	1
KIF14	-17.0439327	1906.12048	-0.0089	0.993	-0.237234524	count	1
CCDC150	-17.0439327	1906.12048	-0.0089	0.993	-0.237234524	count	1
AC012510.1	-17.0439327	1906.12048	-0.0089	0.993	-0.237234524	count	1
AC092053.3	-17.0439327	1906.12048	-0.0089	0.993	-0.237234524	count	1
PSMD6-AS1	-17.0439327	1906.12048	-0.0089	0.993	-0.237234524	count	1
HCG20	-17.0439327	1906.12048	-0.0089	0.993	-0.237234524	count	1
THBS2	-17.0439327	1906.12048	-0.0089	0.993	-0.237234524	count	1
SLC38A5	-17.0439327	1906.12048	-0.0089	0.993	-0.237234524	count	1
AC087273.2	-17.0439327	1906.12048	-0.0089	0.993	-0.237234524	count	1
ADRB3	-17.0439327	1906.12048	-0.0089	0.993	-0.237234524	count	1
MELK	-17.0439327	1906.12048	-0.0089	0.993	-0.237234524	count	1
AC090587.1	-17.0439327	1906.12048	-0.0089	0.993	-0.237234524	count	1
CCDC81	-17.0439327	1906.12048	-0.0089	0.993	-0.237234524	count	1
TEAD4	-17.0439327	1906.12048	-0.0089	0.993	-0.237234524	count	1
LINC01481	-17.0439327	1906.12048	-0.0089	0.993	-0.237234524	count	1
SPATA13-AS1	-17.0439327	1906.12048	-0.0089	0.993	-0.237234524	count	1
CPB2-AS1	-17.0439327	1906.12048	-0.0089	0.993	-0.237234524	count	1
RBM26-AS1	-17.0439327	1906.12048	-0.0089	0.993	-0.237234524	count	1
AC091057.2	-17.0439327	1906.12048	-0.0089	0.993	-0.237234524	count	1
AC051619.5	-17.0439327	1906.12048	-0.0089	0.993	-0.237234524	count	1
BCAS1	-17.0439327	1906.12048	-0.0089	0.993	-0.237234524	count	1
ZNF625	-17.0439327	1906.12048	-0.0089	0.993	-0.237234524	count	1
PIK3R2	-17.0439327	1906.12048	-0.0089	0.993	-0.237234524	count	1
AC011479.1	-17.0439327	1906.12048	-0.0089	0.993	-0.237234524	count	1
CRYBB2	-17.0439327	1906.12048	-0.0089	0.993	-0.237234524	count	1
AP001434.1	-17.0439327	1906.12048	-0.0089	0.993	-0.237234524	count	1
SELP	-17.0439248	2527.770254	-0.0067	0.995	-0.237234524	count	1
MOGAT1	-17.0439248	2527.770254	-0.0067	0.995	-0.237234524	count	1
ITK	-17.0439248	2527.770254	-0.0067	0.995	-0.237234524	count	1
CPXM2	-17.0439248	2527.770254	-0.0067	0.995	-0.237234524	count	1
CALB2	-17.0439248	2527.770254	-0.0067	0.995	-0.237234524	count	1
RND2	-17.0439248	2527.770254	-0.0067	0.995	-0.237234524	count	1
ERG	-17.0439248	2527.770254	-0.0067	0.995	-0.237234524	count	1
C12orf60	-16.8506404	1391.939263	-0.0121	0.99	-0.237234522	count	1
CCNT2-AS1	-16.85023	1264.134854	-0.0133	0.989	-0.237234522	count	1
CAV2	-16.85023	1264.134854	-0.0133	0.989	-0.237234522	count	1

CXCR3	-16.85023	1264.134854	-0.0133	0.989	-0.237234522	count	1
SLC16A13	-16.85023	1264.134854	-0.0133	0.989	-0.237234522	count	1
KAZN	-16.8134966	1289.369266	-0.013	0.99	-0.237234521	count	1
SLC25A34	-18.566613	2265.505006	-0.0082	0.993	-0.237234423	count	1
ACTN2	-18.40018	2346.293979	-0.0078	0.994	-0.237234422	count	1
B3GNT9	-18.399538	2117.899843	-0.0087	0.993	-0.237234422	count	1
SLC47A1	-18.196182	1868.179119	-0.0097	0.992	-0.237234422	count	1
ICA1L	-17.940905	1876.912456	-0.0096	0.992	-0.237234421	count	1
MIAT	-17.940905	1876.912456	-0.0096	0.992	-0.237234421	count	1
ZNF749	-17.932512	1912.902376	-0.0094	0.993	-0.237234421	count	1
DIAPH3	-17.783356	2672.845456	-0.0067	0.995	-0.23723442	count	1
NAT2	-17.706694	1762.003592	-0.01	0.992	-0.23723442	count	1
AC114956.1	-17.594487	2674.601446	-0.0066	0.995	-0.237234419	count	1
AL359643.3	-17.593663	1889.168622	-0.0093	0.993	-0.237234419	count	1
BACH2	-17.593663	1889.168622	-0.0093	0.993	-0.237234419	count	1
AL590708.1	-17.593663	1889.168622	-0.0093	0.993	-0.237234419	count	1
AC044839.1	-17.593663	1889.168622	-0.0093	0.993	-0.237234419	count	1
ZNF582-AS1	-17.593663	1889.168622	-0.0093	0.993	-0.237234419	count	1
AL590787.1	-17.593544	2505.517522	-0.007	0.994	-0.237234419	count	1
AC010999.2	-17.566309	1558.078956	-0.0113	0.991	-0.237234419	count	1
ACSM1	-17.440746	2020.827098	-0.0086	0.993	-0.237234418	count	1
FOXD2-AS1	-17.371153	1538.27023	-0.0113	0.991	-0.237234418	count	1
AL160006.1	-17.20582	1915.40763	-0.009	0.993	-0.237234416	count	1
ZKSCAN2	-17.202784	1657.673019	-0.0104	0.992	-0.237234416	count	1
AL663074.1	-16.395751	3485.021594	-0.0047	0.996	-0.237234405	count	1
NEGR1	-16.395751	3485.021594	-0.0047	0.996	-0.237234405	count	1
PALMD	-16.395751	3485.021594	-0.0047	0.996	-0.237234405	count	1
AC244453.2	-16.395751	3485.021594	-0.0047	0.996	-0.237234405	count	1
ERVMER61-1	-16.395751	3485.021594	-0.0047	0.996	-0.237234405	count	1
HSD11B1	-16.395751	3485.021594	-0.0047	0.996	-0.237234405	count	1
AL353593.2	-16.395751	3485.021594	-0.0047	0.996	-0.237234405	count	1
AC016738.1	-16.395751	3485.021594	-0.0047	0.996	-0.237234405	count	1
GALNT5	-16.395751	3485.021594	-0.0047	0.996	-0.237234405	count	1
FSIP2	-16.395751	3485.021594	-0.0047	0.996	-0.237234405	count	1
AC131235.4	-16.395751	3485.021594	-0.0047	0.996	-0.237234405	count	1
SLC26A1	-16.395751	3485.021594	-0.0047	0.996	-0.237234405	count	1
DTHD1	-16.395751	3485.021594	-0.0047	0.996	-0.237234405	count	1
PRDM5	-16.395751	3485.021594	-0.0047	0.996	-0.237234405	count	1
AC020703.1	-16.395751	3485.021594	-0.0047	0.996	-0.237234405	count	1
LINC01948	-16.395751	3485.021594	-0.0047	0.996	-0.237234405	count	1
KLHL3	-16.395751	3485.021594	-0.0047	0.996	-0.237234405	count	1
CXXC5-AS1	-16.395751	3485.021594	-0.0047	0.996	-0.237234405	count	1
AL157373.2	-16.395751	3485.021594	-0.0047	0.996	-0.237234405	count	1
AC003092.1	-16.395751	3485.021594	-0.0047	0.996	-0.237234405	count	1
STRIP2	-16.395751	3485.021594	-0.0047	0.996	-0.237234405	count	1

PFKFB1	-16.395751	3485.021594	-0.0047	0.996	-0.237234405	count	1
AF131216.4	-16.395751	3485.021594	-0.0047	0.996	-0.237234405	count	1
CHRNA6	-16.395751	3485.021594	-0.0047	0.996	-0.237234405	count	1
PMP2	-16.395751	3485.021594	-0.0047	0.996	-0.237234405	count	1
AP002907.1	-16.395751	3485.021594	-0.0047	0.996	-0.237234405	count	1
PSCA	-16.395751	3485.021594	-0.0047	0.996	-0.237234405	count	1
AC233992.3	-16.395751	3485.021594	-0.0047	0.996	-0.237234405	count	1
FBXO10	-16.395751	3485.021594	-0.0047	0.996	-0.237234405	count	1
WNK2	-16.395751	3485.021594	-0.0047	0.996	-0.237234405	count	1
AL158152.2	-16.395751	3485.021594	-0.0047	0.996	-0.237234405	count	1
PTGES	-16.395751	3485.021594	-0.0047	0.996	-0.237234405	count	1
SARDH	-16.395751	3485.021594	-0.0047	0.996	-0.237234405	count	1
AL138921.1	-16.395751	3485.021594	-0.0047	0.996	-0.237234405	count	1
AL121820.1	-16.395751	3485.021594	-0.0047	0.996	-0.237234405	count	1
IGHV3-33	-16.395751	3485.021594	-0.0047	0.996	-0.237234405	count	1
SLC27A2	-16.395751	3485.021594	-0.0047	0.996	-0.237234405	count	1
AC104590.1	-16.395751	3485.021594	-0.0047	0.996	-0.237234405	count	1
UBAP1L	-16.395751	3485.021594	-0.0047	0.996	-0.237234405	count	1
AC130650.2	-16.395751	3485.021594	-0.0047	0.996	-0.237234405	count	1
PLA2G10	-16.395751	3485.021594	-0.0047	0.996	-0.237234405	count	1
LINC02195	-16.395751	3485.021594	-0.0047	0.996	-0.237234405	count	1
AC009093.5	-16.395751	3485.021594	-0.0047	0.996	-0.237234405	count	1
MT3	-16.395751	3485.021594	-0.0047	0.996	-0.237234405	count	1
AC010542.5	-16.395751	3485.021594	-0.0047	0.996	-0.237234405	count	1
AC009120.5	-16.395751	3485.021594	-0.0047	0.996	-0.237234405	count	1
AC135782.3	-16.395751	3485.021594	-0.0047	0.996	-0.237234405	count	1
AC004771.4	-16.395751	3485.021594	-0.0047	0.996	-0.237234405	count	1
AC005899.6	-16.395751	3485.021594	-0.0047	0.996	-0.237234405	count	1
RAPGEFL1	-16.395751	3485.021594	-0.0047	0.996	-0.237234405	count	1
SDK2	-16.395751	3485.021594	-0.0047	0.996	-0.237234405	count	1
AC087289.4	-16.395751	3485.021594	-0.0047	0.996	-0.237234405	count	1
AC027601.1	-16.395751	3485.021594	-0.0047	0.996	-0.237234405	count	1
AC090772.3	-16.395751	3485.021594	-0.0047	0.996	-0.237234405	count	1
AC017100.1	-16.395751	3485.021594	-0.0047	0.996	-0.237234405	count	1
AC011444.1	-16.395751	3485.021594	-0.0047	0.996	-0.237234405	count	1
AC011511.3	-16.395751	3485.021594	-0.0047	0.996	-0.237234405	count	1
AC008764.3	-16.395751	3485.021594	-0.0047	0.996	-0.237234405	count	1
GNG8	-16.395751	3485.021594	-0.0047	0.996	-0.237234405	count	1
AC016027.1	-16.395751	3485.021594	-0.0047	0.996	-0.237234405	count	1
RFPL3S	-16.395751	3485.021594	-0.0047	0.996	-0.237234405	count	1
FP236383.3	-16.395751	3485.021594	-0.0047	0.996	-0.237234405	count	1
VWA1	-16.395168	2462.462616	-0.0067	0.995	-0.237234405	count	1
AL109917.1	-16.395168	2462.462616	-0.0067	0.995	-0.237234405	count	1
CDCA8	-16.395168	2462.462616	-0.0067	0.995	-0.237234405	count	1
RAD54L	-16.395168	2462.462616	-0.0067	0.995	-0.237234405	count	1

MROH7	-16.395168	2462.462616	-0.0067	0.995	-0.237234405	count	1
AC119674.2	-16.395168	2462.462616	-0.0067	0.995	-0.237234405	count	1
ADGRL4	-16.395168	2462.462616	-0.0067	0.995	-0.237234405	count	1
AC093157.2	-16.395168	2462.462616	-0.0067	0.995	-0.237234405	count	1
HIST2H4A	-16.395168	2462.462616	-0.0067	0.995	-0.237234405	count	1
AL391832.1	-16.395168	2462.462616	-0.0067	0.995	-0.237234405	count	1
SDC1	-16.395168	2462.462616	-0.0067	0.995	-0.237234405	count	1
IGKV3-20	-16.395168	2462.462616	-0.0067	0.995	-0.237234405	count	1
DUSP19	-16.395168	2462.462616	-0.0067	0.995	-0.237234405	count	1
LINC01792	-16.395168	2462.462616	-0.0067	0.995	-0.237234405	count	1
HJURP	-16.395168	2462.462616	-0.0067	0.995	-0.237234405	count	1
SYN2	-16.395168	2462.462616	-0.0067	0.995	-0.237234405	count	1
CCR4	-16.395168	2462.462616	-0.0067	0.995	-0.237234405	count	1
AC112220.4	-16.395168	2462.462616	-0.0067	0.995	-0.237234405	count	1
CCR3	-16.395168	2462.462616	-0.0067	0.995	-0.237234405	count	1
CCDC36	-16.395168	2462.462616	-0.0067	0.995	-0.237234405	count	1
AC006252.1	-16.395168	2462.462616	-0.0067	0.995	-0.237234405	count	1
SPATA12	-16.395168	2462.462616	-0.0067	0.995	-0.237234405	count	1
AC073352.1	-16.395168	2462.462616	-0.0067	0.995	-0.237234405	count	1
SLC12A8	-16.395168	2462.462616	-0.0067	0.995	-0.237234405	count	1
LINC02029	-16.395168	2462.462616	-0.0067	0.995	-0.237234405	count	1
LINC02069	-16.395168	2462.462616	-0.0067	0.995	-0.237234405	count	1
ATP13A4	-16.395168	2462.462616	-0.0067	0.995	-0.237234405	count	1
AC019077.1	-16.395168	2462.462616	-0.0067	0.995	-0.237234405	count	1
BBS12	-16.395168	2462.462616	-0.0067	0.995	-0.237234405	count	1
AC109927.2	-16.395168	2462.462616	-0.0067	0.995	-0.237234405	count	1
ANKDD1B	-16.395168	2462.462616	-0.0067	0.995	-0.237234405	count	1
DMGDH	-16.395168	2462.462616	-0.0067	0.995	-0.237234405	count	1
MZB1	-16.395168	2462.462616	-0.0067	0.995	-0.237234405	count	1
SPINK6	-16.395168	2462.462616	-0.0067	0.995	-0.237234405	count	1
FGF18	-16.395168	2462.462616	-0.0067	0.995	-0.237234405	count	1
GPRIN1	-16.395168	2462.462616	-0.0067	0.995	-0.237234405	count	1
AC145098.1	-16.395168	2462.462616	-0.0067	0.995	-0.237234405	count	1
AC136604.3	-16.395168	2462.462616	-0.0067	0.995	-0.237234405	count	1
HIST1H3I	-16.395168	2462.462616	-0.0067	0.995	-0.237234405	count	1
PPT2-EGFL8	-16.395168	2462.462616	-0.0067	0.995	-0.237234405	count	1
Z97832.2	-16.395168	2462.462616	-0.0067	0.995	-0.237234405	count	1
TDRD6	-16.395168	2462.462616	-0.0067	0.995	-0.237234405	count	1
AL080317.3	-16.395168	2462.462616	-0.0067	0.995	-0.237234405	count	1
KLHL7-DT	-16.395168	2462.462616	-0.0067	0.995	-0.237234405	count	1
HOXA4	-16.395168	2462.462616	-0.0067	0.995	-0.237234405	count	1
TRGV5	-16.395168	2462.462616	-0.0067	0.995	-0.237234405	count	1
TRGV3	-16.395168	2462.462616	-0.0067	0.995	-0.237234405	count	1
SUN3	-16.395168	2462.462616	-0.0067	0.995	-0.237234405	count	1
CDHR3	-16.395168	2462.462616	-0.0067	0.995	-0.237234405	count	1

LEP	-16.395168	2462.462616	-0.0067	0.995	-0.237234405	count	1
DGKI	-16.395168	2462.462616	-0.0067	0.995	-0.237234405	count	1
WDR86	-16.395168	2462.462616	-0.0067	0.995	-0.237234405	count	1
LINC00630	-16.395168	2462.462616	-0.0067	0.995	-0.237234405	count	1
APLN	-16.395168	2462.462616	-0.0067	0.995	-0.237234405	count	1
XPNPEP2	-16.395168	2462.462616	-0.0067	0.995	-0.237234405	count	1
MAMLD1	-16.395168	2462.462616	-0.0067	0.995	-0.237234405	count	1
HCFC1-AS1	-16.395168	2462.462616	-0.0067	0.995	-0.237234405	count	1
AC100810.3	-16.395168	2462.462616	-0.0067	0.995	-0.237234405	count	1
CPA6	-16.395168	2462.462616	-0.0067	0.995	-0.237234405	count	1
ATP6V0D2	-16.395168	2462.462616	-0.0067	0.995	-0.237234405	count	1
AL928970.1	-16.395168	2462.462616	-0.0067	0.995	-0.237234405	count	1
IL33	-16.395168	2462.462616	-0.0067	0.995	-0.237234405	count	1
AL158071.3	-16.395168	2462.462616	-0.0067	0.995	-0.237234405	count	1
ROR2	-16.395168	2462.462616	-0.0067	0.995	-0.237234405	count	1
CCDC180	-16.395168	2462.462616	-0.0067	0.995	-0.237234405	count	1
LHX2	-16.395168	2462.462616	-0.0067	0.995	-0.237234405	count	1
AC107884.1	-16.395168	2462.462616	-0.0067	0.995	-0.237234405	count	1
TMEM9B-AS1	-16.395168	2462.462616	-0.0067	0.995	-0.237234405	count	1
GNG3	-16.395168	2462.462616	-0.0067	0.995	-0.237234405	count	1
MYEOV	-16.395168	2462.462616	-0.0067	0.995	-0.237234405	count	1
UCP3	-16.395168	2462.462616	-0.0067	0.995	-0.237234405	count	1
TECTA	-16.395168	2462.462616	-0.0067	0.995	-0.237234405	count	1
OR8G1	-16.395168	2462.462616	-0.0067	0.995	-0.237234405	count	1
OR8G5	-16.395168	2462.462616	-0.0067	0.995	-0.237234405	count	1
CDNF	-16.395168	2462.462616	-0.0067	0.995	-0.237234405	count	1
TMEM26	-16.395168	2462.462616	-0.0067	0.995	-0.237234405	count	1
AC024597.1	-16.395168	2462.462616	-0.0067	0.995	-0.237234405	count	1
AC073370.1	-16.395168	2462.462616	-0.0067	0.995	-0.237234405	count	1
MORN4	-16.395168	2462.462616	-0.0067	0.995	-0.237234405	count	1
WDR11-AS1	-16.395168	2462.462616	-0.0067	0.995	-0.237234405	count	1
AC005342.2	-16.395168	2462.462616	-0.0067	0.995	-0.237234405	count	1
AC024257.4	-16.395168	2462.462616	-0.0067	0.995	-0.237234405	count	1
TROAP	-16.395168	2462.462616	-0.0067	0.995	-0.237234405	count	1
AC023055.1	-16.395168	2462.462616	-0.0067	0.995	-0.237234405	count	1
IFNG	-16.395168	2462.462616	-0.0067	0.995	-0.237234405	count	1
DAO	-16.395168	2462.462616	-0.0067	0.995	-0.237234405	count	1
CABP1	-16.395168	2462.462616	-0.0067	0.995	-0.237234405	count	1
LINC00544	-16.395168	2462.462616	-0.0067	0.995	-0.237234405	count	1
LRRC63	-16.395168	2462.462616	-0.0067	0.995	-0.237234405	count	1
PTGDR	-16.395168	2462.462616	-0.0067	0.995	-0.237234405	count	1
AKAP5	-16.395168	2462.462616	-0.0067	0.995	-0.237234405	count	1
ZC2HC1C	-16.395168	2462.462616	-0.0067	0.995	-0.237234405	count	1
AL133279.1	-16.395168	2462.462616	-0.0067	0.995	-0.237234405	count	1
AC124312.1	-16.395168	2462.462616	-0.0067	0.995	-0.237234405	count	1

OIP5	-16.395168	2462.462616	-0.0067	0.995	-0.237234405	count	1
AC090970.2	-16.395168	2462.462616	-0.0067	0.995	-0.237234405	count	1
AC023906.5	-16.395168	2462.462616	-0.0067	0.995	-0.237234405	count	1
RORA-AS1	-16.395168	2462.462616	-0.0067	0.995	-0.237234405	count	1
AC107241.1	-16.395168	2462.462616	-0.0067	0.995	-0.237234405	count	1
IQCH	-16.395168	2462.462616	-0.0067	0.995	-0.237234405	count	1
SENP8	-16.395168	2462.462616	-0.0067	0.995	-0.237234405	count	1
CEMIP	-16.395168	2462.462616	-0.0067	0.995	-0.237234405	count	1
AC068870.2	-16.395168	2462.462616	-0.0067	0.995	-0.237234405	count	1
BICDL2	-16.395168	2462.462616	-0.0067	0.995	-0.237234405	count	1
NPIPB2	-16.395168	2462.462616	-0.0067	0.995	-0.237234405	count	1
PRRT2	-16.395168	2462.462616	-0.0067	0.995	-0.237234405	count	1
AC026461.3	-16.395168	2462.462616	-0.0067	0.995	-0.237234405	count	1
ARHGEF15	-16.395168	2462.462616	-0.0067	0.995	-0.237234405	count	1
AC015922.4	-16.395168	2462.462616	-0.0067	0.995	-0.237234405	count	1
AC011815.1	-16.395168	2462.462616	-0.0067	0.995	-0.237234405	count	1
CCDC68	-16.395168	2462.462616	-0.0067	0.995	-0.237234405	count	1
ATP8B1	-16.395168	2462.462616	-0.0067	0.995	-0.237234405	count	1
LINC01909	-16.395168	2462.462616	-0.0067	0.995	-0.237234405	count	1
AL049634.2	-16.395168	2462.462616	-0.0067	0.995	-0.237234405	count	1
AL121890.4	-16.395168	2462.462616	-0.0067	0.995	-0.237234405	count	1
LINC00237	-16.395168	2462.462616	-0.0067	0.995	-0.237234405	count	1
ZNF337-AS1	-16.395168	2462.462616	-0.0067	0.995	-0.237234405	count	1
ZNF341-AS1	-16.395168	2462.462616	-0.0067	0.995	-0.237234405	count	1
LINC01270	-16.395168	2462.462616	-0.0067	0.995	-0.237234405	count	1
BX640514.2	-16.395168	2462.462616	-0.0067	0.995	-0.237234405	count	1
OGFR-AS1	-16.395168	2462.462616	-0.0067	0.995	-0.237234405	count	1
FNDC11	-16.395168	2462.462616	-0.0067	0.995	-0.237234405	count	1
PLPPR3	-16.395168	2462.462616	-0.0067	0.995	-0.237234405	count	1
PCSK4	-16.395168	2462.462616	-0.0067	0.995	-0.237234405	count	1
AC010336.4	-16.395168	2462.462616	-0.0067	0.995	-0.237234405	count	1
AC136469.1	-16.395168	2462.462616	-0.0067	0.995	-0.237234405	count	1
NANOS3	-16.395168	2462.462616	-0.0067	0.995	-0.237234405	count	1
PPP1R13L	-16.395168	2462.462616	-0.0067	0.995	-0.237234405	count	1
KIR3DL2	-16.395168	2462.462616	-0.0067	0.995	-0.237234405	count	1
RFPL4A	-16.395168	2462.462616	-0.0067	0.995	-0.237234405	count	1
IGLV2-14	-16.395168	2462.462616	-0.0067	0.995	-0.237234405	count	1
IGLC7	-16.395168	2462.462616	-0.0067	0.995	-0.237234405	count	1
Z95115.1	-16.395168	2462.462616	-0.0067	0.995	-0.237234405	count	1
AL022326.1	-16.395168	2462.462616	-0.0067	0.995	-0.237234405	count	1
ENTHD1	-16.395168	2462.462616	-0.0067	0.995	-0.237234405	count	1
AP001062.1	-16.395168	2462.462616	-0.0067	0.995	-0.237234405	count	1
DHRS12	-0.2121274	0.2697772	-0.7863	0.432	-0.236931168	count	1
CYP2S1	-0.2433426	0.4636795	-0.5248	0.6	-0.236907148	count	1
AL355338.1	-0.3165695	0.5078567	-0.6233	0.533	-0.236848045	count	1

EXOSC8	-0.1740521	0.1535697	-1.1334	0.257	-0.23666531	count	1
LACTB	-0.1666724	0.0748841	-2.2257	0.0261	-0.236638861	count	1
COX5A	-0.1652577	0.0491317	-3.3636	0.000781	-0.236532807	count	1
METTL17	-0.2259176	0.3793833	-0.5955	0.552	-0.236523889	count	1
PITRM1	-0.2454886	0.3363752	-0.7298	0.466	-0.236284714	count	1
CREG1	-0.1662414	0.0719201	-2.3115	0.0209	-0.236223444	count	1
MRPL13	-0.1700563	0.1074904	-1.5821	0.114	-0.236183228	count	1
FAHD1	-0.1887411	0.2321741	-0.8129	0.416	-0.235955755	count	1
ISOC1	-0.2307551	0.3269333	-0.7058	0.48	-0.235916926	count	1
HIVEP3	-0.2051261	0.2634865	-0.7785	0.436	-0.235889624	count	1
DCAF4	-0.5403833	0.8814715	-0.613	0.54	-0.235829875	count	1
COG6	-0.2477552	0.4140832	-0.5983	0.55	-0.235598686	count	1
GTF2B	-0.1669914	0.0816394	-2.0455	0.0409	-0.23551706	count	1
MLF2	-0.1683841	0.0960001	-1.754	0.0796	-0.235318938	count	1
PEX3	-0.2473338	0.3359469	-0.7362	0.462	-0.235210853	count	1
M1AP	-0.9207933	0.9516048	-0.9676	0.333	-0.235190681	count	1
TYSND1	-0.2471336	0.5717018	-0.4323	0.666	-0.235026587	count	1
ALKBH5	-0.1859638	0.2124917	-0.8752	0.382	-0.235022614	count	1
CCDC134	-0.2762244	0.5382308	-0.5132	0.608	-0.234860697	count	1
RCHY1	-0.1912937	0.2178999	-0.8779	0.38	-0.234857442	count	1
TMEM87B	-0.2019045	0.2477677	-0.8149	0.415	-0.234659903	count	1
OSTM1	-0.1744421	0.1457426	-1.1969	0.231	-0.234620622	count	1
PTGER2	-0.1691342	0.1438346	-1.1759	0.24	-0.234502271	count	1
TMBIM1	-0.1715737	0.1131698	-1.5161	0.13	-0.234380748	count	1
FBXW11	-0.1805938	0.1966455	-0.9184	0.359	-0.234275706	count	1
PLK2	-0.2124962	0.2193093	-0.9689	0.333	-0.233893166	count	1
SLF2	-0.1862224	0.2688471	-0.6927	0.489	-0.233821011	count	1
DNAJC3	-0.1658064	0.0835255	-1.9851	0.0472	-0.233680672	count	1
CAV1	-0.2872128	0.7765339	-0.3699	0.712	-0.233673757	count	1
CD14	-0.162118	0.0449689	-3.6051	0.000318	-0.233546795	count	1
RHEBL1	-0.3860784	0.4411916	-0.8751	0.382	-0.233502126	count	1
HMGN4	-0.2485996	0.4236143	-0.5869	0.557	-0.233431585	count	1
PIM2	-0.2143795	0.3208921	-0.6681	0.504	-0.233411326	count	1
N4BP1	-0.1759916	0.1453653	-1.2107	0.226	-0.233335579	count	1
AC027097.1	-0.2556774	0.3810046	-0.6711	0.502	-0.233330679	count	1
SORBS1	-1.320787	1.621479	-0.8146	0.415	-0.233204028	count	1
ZNF426	-0.3850934	0.4039064	-0.9534	0.34	-0.232957479	count	1
RIT1	-0.1653701	0.0929436	-1.7793	0.0753	-0.232657703	count	1
CTGF	-0.3211836	0.4724504	-0.6798	0.497	-0.232639066	count	1
RAB32	-0.1631327	0.0619921	-2.6315	0.00855	-0.23240394	count	1
WDR73	-0.2731948	0.3295272	-0.8291	0.407	-0.232395107	count	1
GPR65	-0.1675274	0.0987589	-1.6963	0.09	-0.232383088	count	1
RFX3	-0.3470858	0.4772299	-0.7273	0.467	-0.232318421	count	1
ZNF266	-0.3470408	0.5380972	-0.6449	0.519	-0.232290438	count	1
DYNC1I2	-0.1665583	0.1020072	-1.6328	0.103	-0.232175959	count	1

TREML1	-0.7150826	1.2791884	-0.559	0.576	-0.232103241	count	1
UBAC2-AS1	-0.7150826	1.109106	-0.6447	0.519	-0.232103241	count	1
AL359397.2	-0.7150826	1.0987975	-0.6508	0.515	-0.232103241	count	1
AC016876.1	-0.7150826	1.0987975	-0.6508	0.515	-0.232103241	count	1
ZNF396	-0.7150826	1.1757129	-0.6082	0.543	-0.232103241	count	1
CEBPB	-0.1613208	0.0426624	-3.7813	0.00016	-0.232030099	count	1
PPHLN1	-0.1733055	0.1423665	-1.2173	0.224	-0.231970885	count	1
MXD1	-0.1648513	0.0993814	-1.6588	0.0973	-0.231855053	count	1
WDR44	-0.2265524	0.2872288	-0.7888	0.43	-0.23172822	count	1
BCAS2	-0.1656226	0.1377327	-1.2025	0.229	-0.231598138	count	1
GEMIN2	-0.2777521	0.418969	-0.6629	0.507	-0.231358607	count	1
ABCC5	-0.209067	0.3127159	-0.6686	0.504	-0.231354776	count	1
ATL3	-0.1772306	0.1593617	-1.1121	0.266	-0.231332772	count	1
RPL7L1	-0.1696075	0.1315865	-1.2889	0.198	-0.231299836	count	1
AC243960.1	-0.19622	0.4056363	-0.4837	0.629	-0.23099373	count	1
ZSWIM9	-0.4058716	0.6219839	-0.6525	0.514	-0.230978891	count	1
ZNF821	-0.2661314	0.4353066	-0.6114	0.541	-0.230931509	count	1
GNL1	-0.1849527	0.2955133	-0.6259	0.531	-0.230925919	count	1
UGDH	-0.3448234	0.4488449	-0.7682	0.442	-0.230911237	count	1
THOC6	-0.1824317	0.2125813	-0.8582	0.391	-0.230880069	count	1
THOC1	-0.1854063	0.2496766	-0.7426	0.458	-0.23078918	count	1
KIF2A	-0.1705803	0.1418792	-1.2023	0.229	-0.230774507	count	1
ZFPM1	-0.1748647	0.177544	-0.9849	0.325	-0.230736206	count	1
MKKS	-0.2035394	0.2993637	-0.6799	0.497	-0.230499931	count	1
MRPL27	-0.1657373	0.1125683	-1.4723	0.141	-0.230473624	count	1
STRBP	-0.3298114	0.5917947	-0.5573	0.577	-0.23030948	count	1
ZFP64	-0.473767	0.7343212	-0.6452	0.519	-0.230302733	count	1
MYBBP1A	-0.2390316	0.5934412	-0.4028	0.687	-0.230258198	count	1
PDCD5	-0.1629259	0.0824772	-1.9754	0.0483	-0.23023663	count	1
LY86	-0.1616555	0.0641909	-2.5184	0.0119	-0.23011263	count	1
COMMD5	-0.1714622	0.149673	-1.1456	0.252	-0.23004781	count	1
NT5C2	-0.1902963	0.31808	-0.5983	0.55	-0.229894955	count	1
USB1	-0.1790768	0.2428037	-0.7375	0.461	-0.22988366	count	1
PRELID3A	-0.4726159	0.6467597	-0.7307	0.465	-0.229810438	count	1
SMC2	-0.1781483	0.2231939	-0.7982	0.425	-0.229763831	count	1
DYNLL1	-0.1601684	0.0432229	-3.7056	0.000216	-0.229730391	count	1
LSM12	-0.1640605	0.1055654	-1.5541	0.12	-0.229693454	count	1
TRIM25	-0.1702629	0.1576411	-1.0801	0.28	-0.229564898	count	1
ZC3H3	-0.2474002	0.386316	-0.6404	0.522	-0.22926825	count	1
BANF1	-0.1619498	0.0753464	-2.1494	0.0317	-0.229135425	count	1
KLHL7	-0.1904982	0.2995905	-0.6359	0.525	-0.229086984	count	1
CLN6	-0.2812705	0.6400529	-0.4394	0.66	-0.229065497	count	1
AP003392.4	-0.8869572	1.1896615	-0.7456	0.456	-0.228951788	count	1
ZNF10	-0.8869572	1.1161411	-0.7947	0.427	-0.228951788	count	1
CCL2	-0.1585173	0.127383	-1.2444	0.213	-0.22846015	count	1

PRKCI	-0.2135364	0.2912675	-0.7331	0.464	-0.228415627	count	1
TLR1	-0.1706068	0.1432189	-1.1912	0.234	-0.228375516	count	1
TIMP2	-0.1596891	0.0526222	-3.0346	0.00243	-0.228368008	count	1
KIAA1522	-0.5202529	0.8341689	-0.6237	0.533	-0.228270389	count	1
RRM2	-0.5202529	0.834169	-0.6237	0.533	-0.228270389	count	1
APTR	-0.2951766	0.5503561	-0.5363	0.592	-0.228132554	count	1
IER3IP1	-0.1693753	0.1685075	-1.0051	0.315	-0.227914529	count	1
ANKRD37	-0.1714455	0.2133786	-0.8035	0.422	-0.227898341	count	1
GNAI1	-0.6985051	0.6105416	-1.1441	0.253	-0.227838816	count	1
ARHGAP20	-0.3398425	0.7416659	-0.4582	0.647	-0.227808068	count	1
SEC13	-0.1665822	0.1281488	-1.2999	0.194	-0.227632781	count	1
CIC	-0.1976494	0.2867188	-0.6893	0.491	-0.227422546	count	1
RAB11FIP2	-0.2075647	0.2386341	-0.8698	0.384	-0.227371317	count	1
TCAF1	-0.2528428	0.3750124	-0.6742	0.5	-0.22736138	count	1
MAML2	-0.1674067	0.1789893	-0.9353	0.35	-0.227254926	count	1
ENDOV	-0.1884528	0.2953824	-0.638	0.524	-0.227181137	count	1
G3BP1	-0.1663705	0.1181329	-1.4083	0.159	-0.227101672	count	1
KIAA1324L	-0.6956549	1.1976917	-0.5808	0.561	-0.227100589	count	1
MRPL21	-0.1638115	0.1107366	-1.4793	0.139	-0.226984966	count	1
EIF3D	-0.1612242	0.0799442	-2.0167	0.0438	-0.226453846	count	1
TNFSF14	-0.1691768	0.3995041	-0.4235	0.672	-0.226379477	count	1
DEAF1	-0.2089253	0.3212493	-0.6504	0.516	-0.226300411	count	1
PLXNA3	-0.3013089	0.4432149	-0.6798	0.497	-0.226063382	count	1
GLRX5	-0.163643	0.1188668	-1.3767	0.169	-0.226014498	count	1
AC007950.2	-0.8713057	0.9112177	-0.9562	0.339	-0.226010656	count	1
RABGAP1	-0.1907445	0.2338953	-0.8155	0.415	-0.225910023	count	1
TEX30	-0.1855506	0.2760971	-0.672	0.502	-0.22567316	count	1
AURKA	-0.2186133	0.3380375	-0.6467	0.518	-0.225622508	count	1
FOXQ1	-0.3217728	0.883725	-0.3641	0.716	-0.22505211	count	1
LINC01426	-0.3706799	0.733043	-0.5057	0.613	-0.224956966	count	1
C7orf50	-0.1617457	0.1170081	-1.3823	0.167	-0.224913504	count	1
ZNF8	-0.86543	0.8917178	-0.9705	0.332	-0.22489742	count	1
GTF2H3	-0.1820524	0.2316066	-0.786	0.432	-0.224431342	count	1
ZNF619	-0.4224651	0.6638436	-0.6364	0.525	-0.22441431	count	1
SERBP1	-0.1570876	0.0551415	-2.8488	0.0044	-0.224307785	count	1
URB1	-0.3693471	0.7647109	-0.483	0.629	-0.224214271	count	1
AC004540.1	-0.6837588	0.9189191	-0.7441	0.457	-0.224003455	count	1
NCBP2-AS2	-0.1677404	0.1844355	-0.9095	0.363	-0.223810274	count	1
CD2BP2	-0.1634136	0.1324833	-1.2335	0.218	-0.223767635	count	1
MTCH2	-0.16148	0.1134155	-1.4238	0.155	-0.223709989	count	1
SSU72	-0.1585574	0.0816864	-1.941	0.0524	-0.22357206	count	1
TYW1B	-0.3326908	0.7755591	-0.429	0.668	-0.223340158	count	1
AP002360.1	-0.3911023	0.8152983	-0.4797	0.631	-0.223336196	count	1
AC009119.1	-0.5767241	0.8376987	-0.6885	0.491	-0.223325971	count	1
AC015917.2	-0.5767241	0.8376987	-0.6885	0.491	-0.223325971	count	1

AC005264.1	-0.5767241	0.8367429	-0.6892	0.491	-0.223325971	count	1
MEX3C	-0.2219628	0.3316083	-0.6694	0.503	-0.223220362	count	1
SEM1	-0.1565209	0.0566825	-2.7614	0.0058	-0.223205448	count	1
COA5	-0.1642415	0.1706339	-0.9625	0.336	-0.223090356	count	1
PPP1R8	-0.2124863	0.4568517	-0.4651	0.642	-0.222773312	count	1
PPID	-0.1687712	0.1858796	-0.908	0.364	-0.222739592	count	1
CTNNB1	-0.1574777	0.0924492	-1.7034	0.0886	-0.222735632	count	1
LRIG2	-0.3064291	0.4216385	-0.7268	0.467	-0.222574708	count	1
CAMSAP2	-0.191081	0.3055843	-0.6253	0.532	-0.222257618	count	1
AC078846.1	-0.2428875	0.5047706	-0.4812	0.63	-0.222054918	count	1
ZBTB21	-0.1832521	0.2543738	-0.7204	0.471	-0.221967457	count	1
GYG1	-0.1677099	0.1441228	-1.1637	0.245	-0.221952278	count	1
SSNA1	-0.157274	0.0822664	-1.9118	0.056	-0.221917723	count	1
APRT	-0.155094	0.0518958	-2.9886	0.00283	-0.221790223	count	1
AC008686.1	-1.212082	1.1495653	-1.0544	0.292	-0.22164227	count	1
COMMD2	-0.1627746	0.1487615	-1.0942	0.274	-0.221494008	count	1
ARL5A	-0.1832056	0.2307069	-0.7941	0.427	-0.221425383	count	1
TMEM37	-0.1683541	0.1827837	-0.9211	0.357	-0.221411739	count	1
PDXDC1	-0.1725871	0.2360311	-0.7312	0.465	-0.221397295	count	1
ATG4A	-0.241982	0.5057687	-0.4784	0.632	-0.221254954	count	1
PSMB5	-0.1586385	0.0996852	-1.5914	0.112	-0.221176899	count	1
MXRA7	-0.3285879	0.6188471	-0.531	0.595	-0.220770414	count	1
CBY1	-0.3148591	0.4565468	-0.6897	0.49	-0.2205157	count	1
B3GAT2	-0.4141527	0.6064804	-0.6829	0.495	-0.220440765	count	1
CEP170	-0.1571596	0.0944092	-1.6647	0.0961	-0.220408285	count	1
CTU1	-0.1848998	0.291384	-0.6346	0.526	-0.220249176	count	1
PPIL1	-0.2208987	0.3589889	-0.6153	0.538	-0.22009629	count	1
CMAS	-0.1687731	0.1825187	-0.9247	0.355	-0.21988242	count	1
MFSD1	-0.1551938	0.062597	-2.4793	0.0132	-0.219822858	count	1
EMILIN1	-0.4128466	0.6950351	-0.594	0.553	-0.219814787	count	1
ZNHIT6	-0.1964321	0.2994455	-0.656	0.512	-0.219695416	count	1
RAD23A	-0.1548929	0.0814735	-1.9011	0.0574	-0.219423371	count	1
PRRC1	-0.1735034	0.2776306	-0.6249	0.532	-0.219403217	count	1
ZNF330	-0.1632374	0.1499976	-1.0883	0.277	-0.2193811	count	1
MRPS2	-0.1735943	0.2075883	-0.8362	0.403	-0.219243839	count	1
NAGK	-0.1538438	0.0614782	-2.5024	0.0124	-0.219165405	count	1
RPS19	-0.1521503	0.0278484	-5.4635	5.14E-08	-0.219155531	count	0.001229951
B3GNT7	-0.2826276	0.3935364	-0.7182	0.473	-0.218918555	count	1
NME4	-0.1601253	0.1622336	-0.987	0.324	-0.218791747	count	1
PHKA2	-0.232476	0.374441	-0.6209	0.535	-0.218758636	count	1
DGCR6L	-0.1629153	0.1646893	-0.9892	0.323	-0.218629999	count	1
TMED2	-0.1573135	0.0943055	-1.6681	0.0954	-0.218411289	count	1
SIRT3	-0.274167	0.525235	-0.522	0.602	-0.218278835	count	1
CSRP1	-0.1616675	0.1584899	-1.02	0.308	-0.218231373	count	1
KLHL6	-0.1830265	0.2044566	-0.8952	0.371	-0.218044654	count	1

DHX38	-0.1778659	0.280718	-0.6336	0.526	-0.217719564	count	1
ENTPD6	-0.2059666	0.3010745	-0.6841	0.494	-0.217604749	count	1
EFNB1	-0.2990747	0.4873995	-0.6136	0.54	-0.217534895	count	1
SGO1	-0.3790662	0.7893328	-0.4802	0.631	-0.217064475	count	1
WDR34	-0.3790662	0.7607917	-0.4983	0.618	-0.217064475	count	1
EIF4E	-0.1552993	0.1301213	-1.1935	0.233	-0.216858131	count	1
DLGAP1-AS2	-0.8226393	0.6454925	-1.2744	0.203	-0.216638704	count	1
CATIP	-0.2793063	0.6197431	-0.4507	0.652	-0.216472411	count	1
GMEB2	-0.2093482	0.5437306	-0.385	0.7	-0.216276186	count	1
ATG7	-0.168879	0.2051336	-0.8233	0.41	-0.216242957	count	1
SBNO2	-0.1796699	0.2163181	-0.8306	0.406	-0.216212444	count	1
GALNT11	-0.1814265	0.2342157	-0.7746	0.439	-0.216161288	count	1
CCDC163	-0.820161	0.8081269	-1.0149	0.31	-0.216152134	count	1
CLDND2	-0.2585798	0.6955781	-0.3717	0.71	-0.216052516	count	1
UBXN7	-0.1939644	0.2658757	-0.7295	0.466	-0.21597097	count	1
AC106782.2	-0.3536881	0.6228208	-0.5679	0.57	-0.215451827	count	1
AC005332.7	-0.3758174	0.5212443	-0.721	0.471	-0.215364925	count	1
KHDRBS1	-0.1522106	0.067099	-2.2684	0.0234	-0.215294259	count	1
CLTB	-0.1526826	0.081451	-1.8745	0.061	-0.215107162	count	1
LCORL	-0.1639679	0.2114107	-0.7756	0.438	-0.214568974	count	1
STX17	-0.1733541	0.2997977	-0.5782	0.563	-0.214542527	count	1
UBE2V2	-0.1542462	0.1102212	-1.3994	0.162	-0.214527713	count	1
JMJD6	-0.1579873	0.1350353	-1.17	0.242	-0.214513065	count	1
KDSR	-0.1629336	0.1500265	-1.086	0.278	-0.214451257	count	1
ATP6V0E1	-0.1491664	0.0377821	-3.9481	8.10E-05	-0.214125396	count	1
YWHAG	-0.1532151	0.1076756	-1.4229	0.155	-0.214001617	count	1
MDC1	-0.3048108	0.4633926	-0.6578	0.511	-0.21389831	count	1
SYNGAP1	-0.3506186	1.3851026	-0.2531	0.8	-0.21372629	count	1
OST4	-0.1492328	0.0476152	-3.1341	0.00174	-0.213676799	count	1
ACTA2	-0.1965112	0.2323351	-0.8458	0.398	-0.213102965	count	1
NARF	-0.1611836	0.1894771	-0.8507	0.395	-0.213014121	count	1
SLC12A2	-0.2401201	0.3823354	-0.628	0.53	-0.212842249	count	1
D2HGDH	-0.2041642	0.4197489	-0.4864	0.627	-0.2126664	count	1
C1QBP	-0.1504906	0.0820727	-1.8336	0.0668	-0.21246728	count	1
FZR1	-0.2023545	0.3152218	-0.6419	0.521	-0.212373181	count	1
PDGFRB	-2.455543	1.4655018	-1.6756	0.094	-0.212236509	count	1
FBXO4	-0.2037267	0.3097003	-0.6578	0.511	-0.212220428	count	1
TULP4	-0.1705518	0.2143507	-0.7957	0.426	-0.211455232	count	1
SRP68	-0.1973416	0.2736468	-0.7212	0.471	-0.211426664	count	1
EBNA1BP2	-0.1591681	0.1745061	-0.9121	0.362	-0.21134894	count	1
OMD	-1.1182229	1.0403023	-1.0749	0.283	-0.210772649	count	1
AC145285.2	-1.1182229	1.0403023	-1.0749	0.283	-0.210772649	count	1
GSDMB	-1.1182229	1.3670667	-0.818	0.413	-0.210772649	count	1
ZNF417	-1.1182229	1.3670667	-0.818	0.413	-0.210772649	count	1
PSAP	-0.1461831	0.0335014	-4.3635	1.33E-05	-0.210519752	count	0.3149174

BGN	-0.1951558	0.6101733	-0.3198	0.749	-0.210417142	count	1
ANKRD65	-1.1146395	0.9632633	-1.1571	0.247	-0.210340612	count	1
CDH13	-1.1146395	0.9153164	-1.2178	0.223	-0.210340612	count	1
ITPKC	-0.5371738	0.5411927	-0.9926	0.321	-0.21031913	count	1
ATP8B2	-0.7905058	1.075667	-0.7349	0.462	-0.210259472	count	1
F2R	-0.7905058	1.2294638	-0.643	0.52	-0.210259472	count	1
MIR181A2HG	-0.7905058	1.075667	-0.7349	0.462	-0.210259472	count	1
SLFN12L	-0.7905058	1.075667	-0.7349	0.462	-0.210259472	count	1
AC145207.5	-0.7905058	1.075667	-0.7349	0.462	-0.210259472	count	1
CNN1	-0.7905058	1.075667	-0.7349	0.462	-0.210259472	count	1
PBX4	-0.7905058	1.075667	-0.7349	0.462	-0.210259472	count	1
AC243960.3	-0.7905058	1.7811212	-0.4438	0.657	-0.210259472	count	1
PSMC1	-0.1498516	0.0965529	-1.552	0.121	-0.20996448	count	1
RITA1	-0.2631175	0.3630719	-0.7247	0.469	-0.209875284	count	1
MSANTD4	-0.2630341	0.3404544	-0.7726	0.44	-0.209811719	count	1
SLC25A3	-0.1463823	0.0408094	-3.587	0.000341	-0.209807885	count	1
PLEKHM3	-0.4718217	0.4518071	-1.0443	0.296	-0.209698038	count	1
ZNF500	-0.7872301	0.7743809	-1.0166	0.309	-0.209600514	count	1
DYNC1LI2	-0.1555502	0.1511708	-1.029	0.304	-0.209526979	count	1
PLEKHN1	-0.4254854	0.8904156	-0.4779	0.633	-0.209371737	count	1
NECTIN4	-0.4254854	0.926407	-0.4593	0.646	-0.209371737	count	1
ZNF236-DT	-0.4254854	0.8777367	-0.4848	0.628	-0.209371737	count	1
ZNF552	-0.4254854	0.8262043	-0.515	0.607	-0.209371737	count	1
CEP290	-0.1780701	0.3303938	-0.539	0.59	-0.209274265	count	1
GNS	-0.1506735	0.0996137	-1.5126	0.131	-0.208821156	count	1
FAM149B1	-0.2552426	0.5830005	-0.4378	0.662	-0.208763322	count	1
DKC1	-0.1581951	0.186563	-0.8479	0.397	-0.208734274	count	1
LAMTOR5	-0.1467938	0.0681563	-2.1538	0.0314	-0.208477603	count	1
ZNF816-ZNF321P	-0.6253006	0.7323882	-0.8538	0.393	-0.208405979	count	1
ZIK1	-0.4232547	0.7219378	-0.5863	0.558	-0.208390694	count	1
AC006547.3	-0.5305698	1.0378485	-0.5112	0.609	-0.208114898	count	1
DNMT1	-0.1518194	0.1157342	-1.3118	0.19	-0.207897101	count	1
ZNF260	-0.3614827	0.590374	-0.6123	0.54	-0.207832177	count	1
NABP2	-0.1773337	0.2791257	-0.6353	0.525	-0.207788434	count	1
MGME1	-0.1737054	0.2336156	-0.7436	0.457	-0.207596976	count	1
LILRB1	-0.1522114	0.1275032	-1.1938	0.233	-0.207556869	count	1
TRMU	-0.2123717	0.4360265	-0.4871	0.626	-0.207544025	count	1
CDKAL1	-0.2427302	0.3381999	-0.7177	0.473	-0.207459894	count	1
EIF4G2	-0.145662	0.0596673	-2.4412	0.0147	-0.207292437	count	1
AC004846.1	-0.3071455	0.6238709	-0.4923	0.623	-0.207263159	count	1
HIVEP2	-0.307063	0.5204255	-0.59	0.555	-0.207210934	count	1
PPP1R15B	-0.1486774	0.1336178	-1.1127	0.266	-0.207047066	count	1
TSC22D2	-0.1525588	0.1673993	-0.9113	0.362	-0.207002162	count	1
ANXA2R	-0.2073949	0.3686325	-0.5626	0.574	-0.206967736	count	1
DYNC2H1	-0.359806	0.6660077	-0.5402	0.589	-0.206947483	count	1

LAP3	-0.1451445	0.0562799	-2.579	0.00997	-0.206939717	count	1
AC099778.1	-0.3380704	0.4752342	-0.7114	0.477	-0.206645345	count	1
DENND5B	-0.2464925	1.1811284	-0.2087	0.835	-0.206349873	count	1
NAGLU	-0.1611218	0.2068037	-0.7791	0.436	-0.206167708	count	1
CRIP2	-0.5244815	0.5889508	-0.8905	0.373	-0.206074562	count	1
PLEKHM1	-0.163468	0.2295986	-0.712	0.477	-0.206025393	count	1
KLHL15	-0.2514651	0.3490361	-0.7205	0.471	-0.205800995	count	1
GSTZ1	-0.1606885	0.219025	-0.7337	0.463	-0.205617055	count	1
SMS	-0.1451607	0.0807577	-1.7975	0.0724	-0.205476864	count	1
GUCY1B1	-0.6139554	0.6812132	-0.9013	0.368	-0.205305469	count	1
VAMP1	-0.7657715	0.6771702	-1.1308	0.258	-0.205243855	count	1
DNPH1	-0.1455461	0.09089	-1.6013	0.109	-0.205146859	count	1
RNASEH1	-0.161984	0.2420177	-0.6693	0.503	-0.204946024	count	1
FANCM	-0.3557699	0.3787386	-0.9394	0.348	-0.204814787	count	1
MIER2	-0.1887299	0.324255	-0.582	0.561	-0.204813724	count	1
TCAF2	-0.4590063	0.7883327	-0.5822	0.56	-0.204692191	count	1
GIMAP5	-0.7625188	0.6402552	-1.191	0.234	-0.204577392	count	1
RAB34	-0.1467789	0.1157224	-1.2684	0.205	-0.204498575	count	1
TCEA3	-1.0661981	0.9507749	-1.1214	0.262	-0.204373409	count	1
SSR2	-0.1429357	0.0545593	-2.6198	0.00885	-0.204313975	count	1
OXLD1	-0.1590989	0.2050374	-0.776	0.438	-0.204008278	count	1
MRPL11	-0.1462803	0.1103816	-1.3252	0.185	-0.203892993	count	1
HLA-DMA	-0.1417711	0.0496556	-2.8551	0.00434	-0.203874737	count	1
CRYL1	-0.1488995	0.1019561	-1.4604	0.144	-0.203830128	count	1
ZDHHC23	-2.153456	1.2629127	-1.7051	0.0883	-0.203675744	count	1
CTSV	-2.153456	1.849958	-1.1641	0.245	-0.203675744	count	1
LRRC39	-0.5167732	1.5811474	-0.3268	0.744	-0.203479971	count	1
TOLLIP-AS1	-0.5167732	1.1246937	-0.4595	0.646	-0.203479971	count	1
MRVI1-AS1	-0.5167732	1.186984	-0.4354	0.663	-0.203479971	count	1
NLRX1	-0.5167732	1.0911974	-0.4736	0.636	-0.203479971	count	1
AC025580.3	-0.5167732	1.0911974	-0.4736	0.636	-0.203479971	count	1
ZNF223	-0.5167732	1.3043674	-0.3962	0.692	-0.203479971	count	1
BBS4	-0.2616737	0.3728041	-0.7019	0.483	-0.203433612	count	1
TUBA1A	-0.1414545	0.066652	-2.1223	0.0339	-0.203359301	count	1
ARPC2	-0.1412589	0.0298315	-4.7352	2.31E-06	-0.203284158	count	0.05492718
SIVA1	-0.1429713	0.078048	-1.8318	0.0671	-0.203197319	count	1
YWHAQ	-0.1439899	0.0801272	-1.797	0.0725	-0.203188635	count	1
PDCL3	-0.1456838	0.1133132	-1.2857	0.199	-0.202922213	count	1
NUDCD1	-0.1868415	0.3336445	-0.56	0.576	-0.202800103	count	1
AC017002.3	-0.3773708	0.7764503	-0.486	0.627	-0.202642487	count	1
VTI1B	-0.1440666	0.098764	-1.4587	0.145	-0.202499622	count	1
PRPF38B	-0.1448604	0.0921889	-1.5713	0.116	-0.202382587	count	1
SRSF9	-0.1415813	0.0538353	-2.6299	0.00859	-0.202314681	count	1
POLR2J	-0.1431066	0.0863028	-1.6582	0.0974	-0.202296548	count	1
PCNX4	-0.1651302	0.1869914	-0.8831	0.377	-0.202276945	count	1

TMED4	-0.1447082	0.1042747	-1.3878	0.165	-0.202185247	count	1
GBGT1	-0.1643546	0.1977948	-0.8309	0.406	-0.202088419	count	1
DNAJB11	-0.1494655	0.1404794	-1.064	0.287	-0.202068547	count	1
TNFRSF8	-0.2275262	0.4004359	-0.5682	0.57	-0.202049009	count	1
CCR6	-2.101768	1.0367371	-2.0273	0.0427	-0.201949725	count	1
MRPS18C	-0.1440099	0.1045427	-1.3775	0.168	-0.201900898	count	1
QDPR	-0.1525364	0.1892968	-0.8058	0.42	-0.201859276	count	1
CDC123	-0.1469382	0.1177866	-1.2475	0.212	-0.201823066	count	1
SUSD6	-0.1534293	0.1518202	-1.0106	0.312	-0.201760139	count	1
UEVLD	-0.2020446	0.429853	-0.47	0.638	-0.20175376	count	1
CORO7	-0.1601735	0.2464047	-0.65	0.516	-0.201639632	count	1
TUFM	-0.1428097	0.0682834	-2.0914	0.0366	-0.201635577	count	1
TRAF3IP2	-0.2669768	0.5389298	-0.4954	0.62	-0.201557958	count	1
TMC4	-0.4077676	0.599408	-0.6803	0.496	-0.201545495	count	1
NUP50-DT	-0.1917837	0.3977839	-0.4821	0.63	-0.20149745	count	1
OAZ2	-0.1464228	0.1306669	-1.1206	0.263	-0.201453474	count	1
SRP54	-0.1473309	0.1325975	-1.1111	0.267	-0.201318505	count	1
CCDC30	-0.2666409	0.7286363	-0.3659	0.714	-0.201316544	count	1
SDCBP	-0.1399364	0.0413754	-3.3821	0.000731	-0.201011231	count	1
HAVCR2	-0.14314	0.0864516	-1.6557	0.0979	-0.200931492	count	1
TLN2	-0.4062946	0.471386	-0.8619	0.389	-0.200891334	count	1
TNFAIP1	-0.1838967	0.3999476	-0.4598	0.646	-0.200787089	count	1
AL928654.1	-1.0370172	1.3115291	-0.7907	0.429	-0.200662779	count	1
PWP1	-0.1491713	0.1662543	-0.8972	0.37	-0.200620754	count	1
NEK6	-0.1556434	0.1871844	-0.8315	0.406	-0.200373417	count	1
ZW10	-0.3723841	0.4725592	-0.788	0.431	-0.200202422	count	1
COPE	-0.1400052	0.049618	-2.8217	0.00482	-0.200172623	count	1
AC083973.1	-0.4460252	0.7020644	-0.6353	0.525	-0.199582506	count	1
ETV6	-0.1460376	0.1213306	-1.2036	0.229	-0.199118108	count	1
THAP11	-0.1504482	0.2212308	-0.6801	0.497	-0.198893862	count	1
RASGEF1B	-0.1395759	0.0790835	-1.7649	0.0777	-0.198406949	count	1
MICAL1	-0.1675152	0.245254	-0.683	0.495	-0.19815833	count	1
PSMA7	-0.1378141	0.0398477	-3.4585	0.000552	-0.197991572	count	1
EXT2	-0.2000955	0.3588716	-0.5576	0.577	-0.197889834	count	1
SMC3	-0.146071	0.1386863	-1.0532	0.292	-0.197613418	count	1
FZD2	-0.1701231	0.2854962	-0.5959	0.551	-0.19752701	count	1
ZNF622	-0.1460759	0.1465067	-0.9971	0.319	-0.197501783	count	1
CEP250	-0.3053122	0.4346408	-0.7024	0.482	-0.197368357	count	1
TXN	-0.1374779	0.0598318	-2.2977	0.0217	-0.197190755	count	1
REEP5	-0.1379302	0.0547857	-2.5176	0.0119	-0.197185137	count	1
ADH5	-0.1423336	0.1189938	-1.1961	0.232	-0.197053421	count	1
WDR46	-0.1543158	0.1843501	-0.8371	0.403	-0.196893267	count	1
RTL5	-0.2297452	0.5024641	-0.4572	0.648	-0.196753726	count	1
SYS1	-0.1481172	0.1588862	-0.9322	0.351	-0.196643177	count	1
AP005329.1	-0.7234447	0.8292863	-0.8724	0.383	-0.196445184	count	1

STARD3NL	-0.1443067	0.1587775	-0.9089	0.364	-0.19614866	count	1
TMSB4X	-0.1360294	0.0269309	-5.051	4.72E-07	-0.196138507	count	0.011255312
CIAPIN1	-0.1694211	0.2257756	-0.7504	0.453	-0.196051053	count	1
MRPL47	-0.1447539	0.1264397	-1.1448	0.252	-0.195779282	count	1
MRPS24	-0.9978423	1.0758831	-0.9275	0.354	-0.195540784	count	1
TMEM167A	-0.1375439	0.0763825	-1.8007	0.0719	-0.19544489	count	1
AURKAIP1	-0.1372758	0.0643086	-2.1346	0.0329	-0.195374121	count	1
XPC	-0.1566959	0.3382242	-0.4633	0.643	-0.195339727	count	1
SPOUT1	-0.2235067	0.3583753	-0.6237	0.533	-0.195190694	count	1
SH3BP5L	-0.2232611	0.5002369	-0.4463	0.655	-0.194983325	count	1
TRIM39	-0.2498634	0.6359111	-0.3929	0.694	-0.194651159	count	1
DHRS9	-0.1410915	0.2195794	-0.6426	0.521	-0.194583459	count	1
STRAP	-0.1412007	0.0965548	-1.4624	0.144	-0.19455995	count	1
ACIN1	-0.142915	0.1255179	-1.1386	0.255	-0.194552454	count	1
TTYH3	-0.1451096	0.1551383	-0.9354	0.35	-0.194223597	count	1
GNL3L	-0.1888955	0.3995768	-0.4727	0.636	-0.194008967	count	1
COX7A2	-0.1355664	0.0492846	-2.7507	0.00599	-0.194005845	count	1
ALDH2	-0.1371469	0.0639548	-2.1444	0.0321	-0.193992568	count	1
TBPL1	-0.157203	0.2271096	-0.6922	0.489	-0.193719472	count	1
C5orf51	-0.1618105	0.2578569	-0.6275	0.53	-0.193529281	count	1
TGFA	-0.9808895	0.9528925	-1.0294	0.303	-0.19327349	count	1
WWC2	-0.1878576	0.3311811	-0.5672	0.571	-0.192964615	count	1
PSMD12	-0.1383532	0.0952304	-1.4528	0.146	-0.192879468	count	1
IRGQ	-0.1763605	0.3343666	-0.5274	0.598	-0.192691093	count	1
RHOA	-0.1339478	0.0348717	-3.8412	0.000126	-0.192541452	count	1
KCTD5	-0.1608596	0.2841802	-0.566	0.571	-0.192403692	count	1
TMED9	-0.1359351	0.0826319	-1.6451	0.1	-0.192379961	count	1
SLC35E1	-0.1512438	0.2274857	-0.6648	0.506	-0.192358622	count	1
SLC12A7	-0.2061344	0.316915	-0.6504	0.515	-0.192090668	count	1
PDE8A	-0.1576643	0.3284127	-0.4801	0.631	-0.192071822	count	1
SNRPG	-0.1353536	0.0704134	-1.9223	0.0547	-0.191912413	count	1
DENND6A-DT	-1.838001	1.2705592	-1.4466	0.148	-0.191718166	count	1
PCBP1	-0.1337813	0.0450539	-2.9694	0.00301	-0.19162513	count	1
RPL3	-0.1330276	0.0293523	-4.5321	6.12E-06	-0.191420855	count	0.14520312
MTX2	-0.153412	0.2314114	-0.6629	0.507	-0.191277894	count	1
MAP9	-0.6988948	0.7888952	-0.8859	0.376	-0.191215471	count	1
YEATS2	-0.1729465	0.4472342	-0.3867	0.699	-0.191032286	count	1
SPTY2D1	-0.1445214	0.1717615	-0.8414	0.4	-0.19088296	count	1
ASCC1	-0.1789861	0.3547192	-0.5046	0.614	-0.190880001	count	1
ADGRA2	-0.9621425	1.1344572	-0.8481	0.396	-0.190729952	count	1
BSCL2	-0.9621425	1.1344572	-0.8481	0.396	-0.190729952	count	1
AL844908.1	-0.9621425	1.4152142	-0.6799	0.497	-0.190729952	count	1
SOD1	-0.1343632	0.0705646	-1.9041	0.057	-0.190593989	count	1
PYURF	-0.1354806	0.0889146	-1.5237	0.128	-0.190519762	count	1
MMADHC	-0.1387014	0.1127567	-1.2301	0.219	-0.190336304	count	1

LINC01503	-0.1511163	0.2343893	-0.6447	0.519	-0.190319816	count	1
STAM2	-0.1598411	0.2663096	-0.6002	0.548	-0.190213298	count	1
CD44	-0.132953	0.0623106	-2.1337	0.033	-0.190212709	count	1
ANAPC15	-0.1390524	0.1368717	-1.0159	0.31	-0.190195362	count	1
PIGF	-0.1460709	0.1891592	-0.7722	0.44	-0.190178575	count	1
ZSCAN29	-0.3821647	0.6362103	-0.6007	0.548	-0.190098373	count	1
SUGT1	-0.1351957	0.1032202	-1.3098	0.19	-0.190046903	count	1
TFCP2L1	-0.6930213	0.5345555	-1.2964	0.195	-0.189950371	count	1
C18orf25	-0.1863458	0.2658111	-0.701	0.483	-0.189836512	count	1
ILF3	-0.1369872	0.1193304	-1.148	0.251	-0.189780696	count	1
SLC35A4	-0.153136	0.2326056	-0.6584	0.51	-0.189724687	count	1
TMEM256	-0.1365451	0.1094486	-1.2476	0.212	-0.189592441	count	1
NOL8	-0.14838	0.2098401	-0.7071	0.48	-0.189570763	count	1
PINX1	-0.1607266	0.345219	-0.4656	0.642	-0.189119094	count	1
ACTR3	-0.1324207	0.0609619	-2.1722	0.0299	-0.189096286	count	1
TPGS2	-0.1439198	0.2025689	-0.7105	0.477	-0.189079742	count	1
PAFAH1B2	-0.1377625	0.1315456	-1.0473	0.295	-0.18894791	count	1
GNB1L	-0.2354845	0.4480955	-0.5255	0.599	-0.18870911	count	1
OPLAH	-0.6861336	0.5944093	-1.1543	0.248	-0.188459942	count	1
AL445472.1	-0.5530705	0.8298299	-0.6665	0.505	-0.188252733	count	1
GPKOW	-0.1670597	0.3026239	-0.552	0.581	-0.188145346	count	1
HTATIP2	-0.1362785	0.1200296	-1.1354	0.256	-0.188095319	count	1
AC090630.1	-0.3243011	0.9850727	-0.3292	0.742	-0.188036812	count	1
MAIP1	-0.173992	0.3639374	-0.4781	0.633	-0.187974531	count	1
PARK7	-0.1312691	0.0506195	-2.5933	0.00956	-0.187780554	count	1
AATK	-0.3048579	0.6820854	-0.4469	0.655	-0.18769532	count	1
AC009275.1	-0.4701432	0.7183625	-0.6545	0.513	-0.187512728	count	1
TBX6	-0.4701432	0.9600081	-0.4897	0.624	-0.187512728	count	1
MDH1	-0.1328358	0.0791857	-1.6775	0.0936	-0.187498338	count	1
NOL9	-0.1757239	0.3281058	-0.5356	0.592	-0.187460701	count	1
AL356417.3	-0.4689184	0.7403453	-0.6334	0.527	-0.187087025	count	1
MRPS23	-0.1389822	0.1484028	-0.9365	0.349	-0.18696562	count	1
C19orf12	-0.1728561	0.3853382	-0.4486	0.654	-0.186767274	count	1
AL358472.4	-0.5475907	0.7541999	-0.7261	0.468	-0.186683466	count	1
MRPL14	-0.131997	0.0906372	-1.4563	0.145	-0.186289819	count	1
GNB1	-0.1327911	0.0927843	-1.4312	0.153	-0.186280004	count	1
RBMX2	-0.1457212	0.1848763	-0.7882	0.431	-0.186195015	count	1
BTBD7	-0.1414769	0.1900737	-0.7443	0.457	-0.185767823	count	1
DHX8	-0.159298	0.2941043	-0.5416	0.588	-0.185716521	count	1
COL18A1	-0.4108171	0.5024198	-0.8177	0.414	-0.18552509	count	1
ZNF879	-0.9243565	0.66156	-1.3972	0.162	-0.185485883	count	1
TBC1D32	-0.9243565	0.9030643	-1.0236	0.306	-0.185485883	count	1
TIAM1	-0.1490713	0.22263	-0.6696	0.503	-0.185329213	count	1
GORAB	-0.2159271	0.3842492	-0.5619	0.574	-0.185309963	count	1
WIPF2	-0.1582572	0.2367633	-0.6684	0.504	-0.18510853	count	1

NDUFS6	-0.1310494	0.0743329	-1.763	0.078	-0.185078833	count	1
POC1B	-0.170225	0.4094373	-0.4158	0.678	-0.185049011	count	1
SEH1L	-0.1550188	0.2500089	-0.6201	0.535	-0.185015664	count	1
TUBG1	-0.1534531	0.3627061	-0.4231	0.672	-0.184964012	count	1
CCDC12	-0.1348334	0.1282175	-1.0516	0.293	-0.184660575	count	1
GPR89A	-0.3179566	0.6120765	-0.5195	0.603	-0.184622072	count	1
YEATS4	-0.1433992	0.2040301	-0.7028	0.482	-0.184529231	count	1
HECTD3	-0.1878965	0.616723	-0.3047	0.761	-0.184169201	count	1
ZNF195	-0.1569482	0.2895383	-0.5421	0.588	-0.18416536	count	1
SSBP3	-0.1476333	0.2640356	-0.5591	0.576	-0.184126762	count	1
LRRC14	-0.2600346	0.4721127	-0.5508	0.582	-0.184063463	count	1
PDSS1	-0.206334	0.4464272	-0.4622	0.644	-0.18379044	count	1
ZNF275	-0.911584	0.8834586	-1.0318	0.302	-0.183677313	count	1
SUV39H2	-0.911584	0.9800878	-0.9301	0.352	-0.183677313	count	1
LINC00672	-0.911584	0.8527045	-1.0691	0.285	-0.183677313	count	1
IL6R-AS1	-0.4585346	0.6524557	-0.7028	0.482	-0.183464881	count	1
PIAS4	-0.1667857	0.2976301	-0.5604	0.575	-0.183373709	count	1
XCL2	-0.5358108	1.8109382	-0.2959	0.767	-0.183290613	count	1
PHF23	-0.1383069	0.1666029	-0.8302	0.407	-0.183205686	count	1
PATL1	-0.1592557	0.2686634	-0.5928	0.553	-0.183113106	count	1
FAF2	-0.1425373	0.1935835	-0.7363	0.462	-0.183076554	count	1
CCDC15	-0.5345483	0.7001432	-0.7635	0.445	-0.182925423	count	1
ANXA11	-0.1289749	0.0705214	-1.8289	0.0675	-0.182831622	count	1
TCTN1	-0.227763	0.4676083	-0.4871	0.626	-0.182756513	count	1
ICMT	-0.1659685	0.388794	-0.4269	0.67	-0.182488478	count	1
TAF9	-0.1303643	0.1169309	-1.1149	0.265	-0.182305355	count	1
CANX	-0.1277787	0.0571416	-2.2362	0.0254	-0.182274555	count	1
ST13	-0.1279264	0.0634782	-2.0153	0.044	-0.182269762	count	1
NIFK	-0.1304289	0.1076387	-1.2117	0.226	-0.182183628	count	1
SLC15A4	-0.1445425	0.2299542	-0.6286	0.53	-0.182097286	count	1
AL138885.3	-1.633834	1.579376	-1.0345	0.301	-0.181900469	count	1
BRSK1	-1.633834	1.0274314	-1.5902	0.112	-0.181900469	count	1
ZNF91	-0.1512681	0.2562348	-0.5903	0.555	-0.181490138	count	1
STAG3	-0.6529123	0.6477095	-1.008	0.314	-0.181166356	count	1
PDCD2L	-0.6529123	0.6477095	-1.008	0.314	-0.181166356	count	1
PTGES3	-0.1271681	0.0586346	-2.1688	0.0302	-0.181116375	count	1
HLA-DQB1	-0.1259502	0.0518798	-2.4277	0.0153	-0.18103573	count	1
UBA2	-0.1350557	0.160301	-0.8425	0.4	-0.181030581	count	1
CARD19	-0.12899	0.1150954	-1.1207	0.263	-0.180509881	count	1
OGG1	-0.2101422	0.3733334	-0.5629	0.574	-0.180503639	count	1
BRK1	-0.1265868	0.0512406	-2.4704	0.0136	-0.180421433	count	1
EDF1	-0.1260899	0.0477367	-2.6414	0.00831	-0.180395855	count	1
HYLS1	-0.3100162	0.6705753	-0.4623	0.644	-0.180333246	count	1
BTG3	-0.1294676	0.112815	-1.1476	0.251	-0.180292005	count	1
HERPUD1	-0.1255399	0.0596995	-2.1029	0.0356	-0.180254959	count	1

TM9SF3	-0.1292098	0.0994379	-1.2994	0.194	-0.18012276	count	1
ABI1	-0.1319511	0.1241125	-1.0632	0.288	-0.180004213	count	1
RBSN	-0.1877135	0.3455469	-0.5432	0.587	-0.179877586	count	1
CNPY2	-0.1303663	0.1571385	-0.8296	0.407	-0.179663811	count	1
TPMT	-0.1355359	0.1343103	-1.0091	0.313	-0.179643822	count	1
AL683813.1	-0.8832827	0.7417659	-1.1908	0.234	-0.179604153	count	1
ABHD17A	-0.1295498	0.1119353	-1.1574	0.247	-0.179580417	count	1
ASAH1	-0.1251411	0.046043	-2.7179	0.00662	-0.179513127	count	1
ZNF519	-0.3584134	0.8671808	-0.4133	0.679	-0.179333267	count	1
MAPK12	-0.5207014	0.8284659	-0.6285	0.53	-0.178899897	count	1
ZNF263	-0.235557	0.3808775	-0.6185	0.536	-0.178839288	count	1
C3	-0.1321182	0.2194955	-0.6019	0.547	-0.178742085	count	1
UBXN2B	-0.1376776	0.2193208	-0.6277	0.53	-0.178605032	count	1
DUS3L	-0.1719256	0.349869	-0.4914	0.623	-0.178322058	count	1
MSC	-1.561725	1.0907918	-1.4317	0.152	-0.177974221	count	1
RANBP9	-0.1420253	0.2333685	-0.6086	0.543	-0.177967482	count	1
C16orf87	-0.1326499	0.1479247	-0.8967	0.37	-0.177954095	count	1
NSMF	-0.2029647	0.3573074	-0.568	0.57	-0.177789668	count	1
SDHB	-0.1272421	0.0899525	-1.4145	0.157	-0.17775234	count	1
RASSF4	-0.1259083	0.0822795	-1.5303	0.126	-0.177693073	count	1
PPP2CB	-0.1324219	0.1538321	-0.8608	0.389	-0.177581249	count	1
HEXB	-0.1253513	0.0719646	-1.7418	0.0817	-0.177575882	count	1
DLGAP1-AS1	-0.1472426	0.2624415	-0.561	0.575	-0.177545749	count	1
DOT1L	-0.1521097	0.344218	-0.4419	0.659	-0.177427236	count	1
NDUFV1	-0.1291764	0.1136101	-1.137	0.256	-0.177134082	count	1
P2RY1	-0.1751085	0.4179416	-0.419	0.675	-0.17703779	count	1
CETP	-0.2863251	0.7182286	-0.3987	0.69	-0.176990141	count	1
ZNF7	-0.1592022	0.3743	-0.4253	0.671	-0.176940469	count	1
SIN3A	-0.1436207	0.2391391	-0.6006	0.548	-0.176795884	count	1
MAP7D1	-0.1280689	0.1097534	-1.1669	0.243	-0.176787566	count	1
CDC37	-0.1246336	0.0674959	-1.8465	0.0649	-0.176638655	count	1
TXLNA	-0.1448873	0.2392606	-0.6056	0.545	-0.176637166	count	1
STOML2	-0.1286121	0.1602363	-0.8026	0.422	-0.176586259	count	1
AL161421.1	-0.149069	0.3092122	-0.4821	0.63	-0.176556186	count	1
COPS7A	-0.1367288	0.1766224	-0.7741	0.439	-0.176476387	count	1
CHAF1A	-0.2095551	0.3058673	-0.6851	0.493	-0.176449345	count	1
STX8	-0.1299368	0.1220416	-1.0647	0.287	-0.176395427	count	1
MT-ATP6	-0.1223769	0.0307059	-3.9854	6.93E-05	-0.176330064	count	1
MAP4K5	-0.1410475	0.2177619	-0.6477	0.517	-0.17623618	count	1
SNX7	-0.231472	0.3674731	-0.6299	0.529	-0.175865228	count	1
AKAP2	-1.52113	1.1378164	-1.3369	0.181	-0.175649266	count	1
CD151	-0.1287868	0.0982914	-1.3103	0.19	-0.175537742	count	1
APP	-0.1289672	0.1435022	-0.8987	0.369	-0.175311686	count	1
RASL10A	-0.2571495	0.5250376	-0.4898	0.624	-0.175267917	count	1
TMEM222	-0.1501185	0.3085393	-0.4865	0.627	-0.175129457	count	1

SH3BP5-AS1	-0.5077973	0.8487363	-0.5983	0.55	-0.175115288	count	1
FRS3	-0.5077973	0.8538619	-0.5947	0.552	-0.175115288	count	1
AJM1	-0.5077973	1.0347724	-0.4907	0.624	-0.175115288	count	1
NCOR2	-0.1307936	0.1750181	-0.7473	0.455	-0.175058825	count	1
DCXR	-0.1249834	0.0975841	-1.2808	0.2	-0.174934105	count	1
CCDC90B	-0.1275411	0.1343723	-0.9492	0.343	-0.174896924	count	1
EMILIN2	-0.1240913	0.0750546	-1.6533	0.0984	-0.174813326	count	1
CCL19	-1.500214	0.8732205	-1.718	0.0859	-0.174418036	count	1
ASB7	-0.2990747	0.470579	-0.6355	0.525	-0.174395885	count	1
CHCHD10	-0.1221724	0.0587284	-2.0803	0.0376	-0.17426026	count	1
TAF5L	-0.172102	0.3873692	-0.4443	0.657	-0.174056496	count	1
PCNX1	-0.1437971	0.2793068	-0.5148	0.607	-0.173823514	count	1
AL031848.2	-1.489796	1.481218	-1.0058	0.315	-0.173796062	count	1
TCEAL4	-0.1234054	0.11139	-1.1079	0.268	-0.173300249	count	1
APMAP	-0.1272869	0.1534548	-0.8295	0.407	-0.173292538	count	1
CDH26	-0.3806309	0.6627915	-0.5743	0.566	-0.173241088	count	1
SMTN	-0.3806309	0.8626004	-0.4413	0.659	-0.173241088	count	1
RPSA	-0.1204973	0.0364188	-3.3087	0.000951	-0.173228844	count	1
RELB	-0.1265615	0.1385951	-0.9132	0.361	-0.172972537	count	1
PAPOLA	-0.1229677	0.0799273	-1.5385	0.124	-0.172943775	count	1
RBL2	-0.1420576	0.1923211	-0.7386	0.46	-0.172493981	count	1
GMDS-DT	-0.2202113	0.4209725	-0.5231	0.601	-0.172428902	count	1
ITGB3BP	-0.1574827	0.306672	-0.5135	0.608	-0.17235901	count	1
AL132656.2	-1.464829	1.298091	-1.1284	0.259	-0.172281956	count	1
TOR1A	-0.128763	0.1646254	-0.7822	0.434	-0.172064756	count	1
STAM	-0.1352258	0.2195984	-0.6158	0.538	-0.171910917	count	1
ACBD5	-0.1375509	0.189245	-0.7268	0.467	-0.171897134	count	1
G2E3	-0.1419904	0.2584238	-0.5494	0.583	-0.171658228	count	1
RTL8B	-0.251118	0.7452227	-0.337	0.736	-0.17136092	count	1
PLXDC2	-0.1214721	0.082562	-1.4713	0.141	-0.17132113	count	1
PARM1	-0.6091694	0.7633704	-0.798	0.425	-0.171294916	count	1
AC012360.3	-0.2251625	0.9741886	-0.2311	0.817	-0.171262498	count	1
ACOT7	-0.2249738	0.5036273	-0.4467	0.655	-0.171124678	count	1
USP12	-0.1266765	0.1778503	-0.7123	0.476	-0.170978827	count	1
DMAC1	-0.125829	0.1453923	-0.8654	0.387	-0.170742362	count	1
KHK	-0.2501576	0.4332494	-0.5774	0.564	-0.17073787	count	1
PRMT1	-0.1218838	0.102339	-1.191	0.234	-0.170426465	count	1
AC008429.1	-0.8212991	0.8675742	-0.9467	0.344	-0.170360982	count	1
LINC01268	-0.8212991	0.8786115	-0.9348	0.35	-0.170360982	count	1
PRR14	-0.1522383	0.335318	-0.454	0.65	-0.170113107	count	1
AL137186.2	-0.1636744	0.4167024	-0.3928	0.695	-0.169910389	count	1
LINC01759	-0.6029909	1.1849751	-0.5089	0.611	-0.169875821	count	1
NOS1AP	-0.6029909	1.1849751	-0.5089	0.611	-0.169875821	count	1
DOCK3	-0.6029909	1.1304458	-0.5334	0.594	-0.169875821	count	1
POT1-AS1	-0.6029909	1.550106	-0.389	0.697	-0.169875821	count	1

AL391988.1	-0.6029909	1.2691712	-0.4751	0.635	-0.169875821	count	1
FBXL14	-0.6029909	1.1304458	-0.5334	0.594	-0.169875821	count	1
WNK1	-0.1232456	0.1089277	-1.1314	0.258	-0.16935212	count	1
UBA1	-0.1310391	0.2006236	-0.6532	0.514	-0.169319871	count	1
DNASE1L1	-0.1449617	0.1600311	-0.9058	0.365	-0.169175467	count	1
CA11	-0.1834503	0.3353827	-0.547	0.584	-0.169084857	count	1
OXSM	-0.1784259	0.3689601	-0.4836	0.629	-0.169079787	count	1
SRGAP1	-0.1272601	0.2207562	-0.5765	0.564	-0.169057267	count	1
SPIB	-0.1581832	0.3302073	-0.479	0.632	-0.169035548	count	1
SLC6A6	-0.1241594	0.1709343	-0.7264	0.468	-0.168836464	count	1
MPZL2	-0.2887062	0.6479002	-0.4456	0.656	-0.168740038	count	1
NUCKS1	-0.1186632	0.0690837	-1.7177	0.086	-0.168698799	count	1
TRIM16	-1.406195	0.9297599	-1.5124	0.131	-0.168591356	count	1
SCAMP3	-0.134041	0.2356523	-0.5688	0.57	-0.168497921	count	1
HCCS	-0.1270233	0.1611438	-0.7883	0.431	-0.168490547	count	1
PGPEP1	-0.140854	0.271342	-0.5191	0.604	-0.168262694	count	1
PEX19	-0.1478856	0.3069125	-0.4818	0.63	-0.168222586	count	1
ZDHHC4	-0.1303907	0.1827215	-0.7136	0.476	-0.168186345	count	1
ZNF714	-0.1882527	0.3707741	-0.5077	0.612	-0.168117469	count	1
NUTM2B-AS1	-0.1255384	0.17382	-0.7222	0.47	-0.168048726	count	1
F8A1	-0.129084	0.1839286	-0.7018	0.483	-0.168038338	count	1
SRP9	-0.1190789	0.0805308	-1.4787	0.139	-0.167973933	count	1
SNRPB	-0.1180539	0.054899	-2.1504	0.0316	-0.167851353	count	1
FOXC1	-0.8048576	0.6922496	-1.1627	0.245	-0.167833447	count	1
SNX1	-0.1229429	0.1333532	-0.9219	0.357	-0.167820872	count	1
USP8	-0.1215114	0.1192458	-1.019	0.308	-0.16760922	count	1
C19orf48	-0.1520989	0.4143723	-0.3671	0.714	-0.167443437	count	1
NDUFAB1	-0.117495	0.0690349	-1.702	0.0889	-0.16701003	count	1
CAMKMT	-0.2556742	0.4434319	-0.5766	0.564	-0.166984193	count	1
POGK	-0.1516038	0.2526488	-0.6001	0.549	-0.166905652	count	1
CLIC1	-0.1158975	0.0309112	-3.7494	0.000181	-0.16687557	count	1
PJA1	-0.2127557	0.4702196	-0.4525	0.651	-0.166802957	count	1
FAM53A	-1.378688	1.4503264	-0.9506	0.342	-0.166793044	count	1
COL4A2	-1.378688	1.010867	-1.3639	0.173	-0.166793044	count	1
CGRRF1	-0.1371972	0.2451141	-0.5597	0.576	-0.166639763	count	1
TMEM189	-0.13405	0.2059585	-0.6509	0.515	-0.166516421	count	1
TCEA1	-0.1175578	0.0827858	-1.42	0.156	-0.166487185	count	1
DALRD3	-0.1325741	0.2143393	-0.6185	0.536	-0.166435463	count	1
BEX4	-0.1183455	0.1217485	-0.972	0.331	-0.166167671	count	1
LINC02246	-0.794049	1.0790934	-0.7358	0.462	-0.166154294	count	1
FUT8	-0.2335237	0.4467958	-0.5227	0.601	-0.16613395	count	1
MNT	-0.1339556	0.2348289	-0.5704	0.568	-0.166128404	count	1
AC093627.4	-0.22515	0.4764457	-0.4726	0.637	-0.166026155	count	1
KDELR1	-0.1164586	0.0633576	-1.8381	0.0662	-0.165839863	count	1
PITHD1	-0.1209145	0.1535999	-0.7872	0.431	-0.165768008	count	1

PIGS	-0.1364517	0.2765432	-0.4934	0.622	-0.165741515	count	1
MYO1D	-0.4761374	0.743485	-0.6404	0.522	-0.165693558	count	1
FANCD2	-0.4080108	0.7969516	-0.512	0.609	-0.165507458	count	1
MTDH	-0.1156482	0.0495467	-2.3341	0.0197	-0.165453112	count	1
CHD3	-0.1333846	0.230697	-0.5782	0.563	-0.165425173	count	1
TPD52	-0.1883492	0.3255977	-0.5785	0.563	-0.165340012	count	1
RILPL1	-0.474111	0.5689957	-0.8332	0.405	-0.165083903	count	1
PARN	-0.1531362	0.259824	-0.5894	0.556	-0.164761922	count	1
BOP1	-0.1714918	0.3063638	-0.5598	0.576	-0.164666159	count	1
TMEM230	-0.1168726	0.0778114	-1.502	0.133	-0.164620396	count	1
AF129075.2	-1.3426727	1.3553721	-0.9906	0.322	-0.164371854	count	1
PDE7A	-0.1375483	0.2856571	-0.4815	0.63	-0.164348348	count	1
GNPTG	-0.1166691	0.0904532	-1.2898	0.197	-0.164347674	count	1
AC010680.3	-0.3004457	1.180064	-0.2546	0.799	-0.164283987	count	1
BIRC5	-0.3004457	0.7828468	-0.3838	0.701	-0.164283987	count	1
DNAJB1	-0.1155065	0.0989703	-1.1671	0.243	-0.164150721	count	1
TXNDC11	-0.1377161	0.252626	-0.5451	0.586	-0.164119952	count	1
CDCA7L	-0.1708485	0.3427607	-0.4984	0.618	-0.164061567	count	1
LRRC57	-0.1446215	0.263075	-0.5497	0.583	-0.163872873	count	1
MARF1	-0.1286477	0.2322502	-0.5539	0.58	-0.163782258	count	1
GPR108	-0.1183506	0.1199489	-0.9867	0.324	-0.163576415	count	1
CREB1	-0.1250573	0.1556475	-0.8035	0.422	-0.163534369	count	1
RTL10	-0.2144998	0.6031871	-0.3556	0.722	-0.163459003	count	1
DDX19A	-0.1527755	0.3713035	-0.4115	0.681	-0.163341759	count	1
TYMP	-0.1134445	0.037939	-2.9902	0.00282	-0.163123858	count	1
ZNF317	-0.2381024	0.3804316	-0.6259	0.531	-0.162895537	count	1
AP002478.1	-1.320787	0.911143	-1.4496	0.147	-0.162862716	count	1
SPATA13	-0.1328513	0.2141142	-0.6205	0.535	-0.162401081	count	1
PSMC5	-0.1151487	0.0736418	-1.5636	0.118	-0.162325034	count	1
MPC2	-0.115591	0.1221008	-0.9467	0.344	-0.162161813	count	1
HRAS	-0.121839	0.1849326	-0.6588	0.51	-0.162038514	count	1
ATF3	-0.1126889	0.0707467	-1.5929	0.111	-0.161904008	count	1
RAD18	-0.2006478	0.3905785	-0.5137	0.607	-0.16172242	count	1
NECAP2	-0.1171149	0.1243557	-0.9418	0.346	-0.161649242	count	1
SLAMF6	-0.7648622	0.9209919	-0.8305	0.406	-0.161549682	count	1
PLEKHA8	-0.1623645	0.4043179	-0.4016	0.688	-0.161284374	count	1
POLH	-0.3961786	0.7484676	-0.5293	0.597	-0.16122178	count	1
PROS1	-0.2114304	0.3410799	-0.6199	0.535	-0.161206827	count	1
PACSIN2	-0.1208302	0.1563501	-0.7728	0.44	-0.161076065	count	1
ZNF540	-0.2746838	1.4526059	-0.1891	0.85	-0.161045558	count	1
MIF-AS1	-0.2746838	0.8283578	-0.3316	0.74	-0.161045558	count	1
LRMDA	-0.1152876	0.1090653	-1.0571	0.291	-0.160926619	count	1
SUMO1	-0.1134791	0.0682268	-1.6633	0.0964	-0.160898903	count	1
SRD5A3	-0.1171829	0.129288	-0.9064	0.365	-0.160811613	count	1
PIDD1	-0.2583932	0.7162619	-0.3608	0.718	-0.160679387	count	1

TFB2M	-0.1616661	0.380125	-0.4253	0.671	-0.160603571	count	1
AP3S1	-0.1150804	0.1058918	-1.0868	0.277	-0.160467698	count	1
CLSPN	-0.1941908	0.4803392	-0.4043	0.686	-0.160401672	count	1
TRAF3	-0.1274262	0.2367696	-0.5382	0.59	-0.160233118	count	1
LINC01137	-0.3486295	0.8389572	-0.4156	0.678	-0.159984333	count	1
SNHG10	-0.3486295	0.8494975	-0.4104	0.682	-0.159984333	count	1
AC009126.1	-0.3927477	0.7653549	-0.5132	0.608	-0.159973378	count	1
TNKS1BP1	-0.3927477	0.7063908	-0.556	0.578	-0.159973378	count	1
EFTUD2	-0.1243209	0.1930558	-0.644	0.52	-0.159950293	count	1
RRP15	-0.1226231	0.2432308	-0.5041	0.614	-0.159542499	count	1
THRAP3	-0.1125261	0.0677358	-1.6612	0.0968	-0.159509747	count	1
TIMM9	-0.1263117	0.1889651	-0.6684	0.504	-0.159473028	count	1
PNO1	-0.1237703	0.1940464	-0.6378	0.524	-0.159395327	count	1
MFSD4B	-0.1603323	0.5061066	-0.3168	0.751	-0.159303048	count	1
FBXO45	-0.1586588	0.3307369	-0.4797	0.631	-0.159218268	count	1
PAIP1	-0.1189426	0.1523095	-0.7809	0.435	-0.159182689	count	1
RHBDD3	-0.158304	0.620536	-0.2551	0.799	-0.15886857	count	1
RPS10	-0.1111503	0.0464834	-2.3912	0.0169	-0.158839612	count	1
UNC45A	-0.1381999	0.2657904	-0.52	0.603	-0.158562513	count	1
CSNK1A1	-0.1114724	0.0614822	-1.8131	0.0699	-0.158528062	count	1
DCTN5	-0.1356441	0.3826078	-0.3545	0.723	-0.157887203	count	1
MYO6	-1.2498252	1.0957026	-1.1407	0.254	-0.157767286	count	1
TTLL11	-1.2498252	1.0957026	-1.1407	0.254	-0.157767286	count	1
AL137077.2	-1.2498252	1.3252282	-0.9431	0.346	-0.157767286	count	1
C6orf120	-0.1280435	0.2337857	-0.5477	0.584	-0.157753745	count	1
ATP1A1	-0.1123739	0.0873591	-1.2863	0.198	-0.157662648	count	1
ZNF790	-0.2685371	0.5558993	-0.4831	0.629	-0.157656243	count	1
PPP4R3A	-0.1152119	0.1262461	-0.9126	0.362	-0.157594128	count	1
ARPC5	-0.1094689	0.0350219	-3.1257	0.00179	-0.157370792	count	1
UBE2Q1	-0.1181465	0.1893986	-0.6238	0.533	-0.15736642	count	1
RMDN1	-0.1172671	0.172704	-0.679	0.497	-0.157320306	count	1
MAP4	-0.1159704	0.1271269	-0.9122	0.362	-0.157312998	count	1
PEBP1	-0.1107847	0.0756936	-1.4636	0.143	-0.157257836	count	1
VPS41	-0.1207217	0.1517386	-0.7956	0.426	-0.156957257	count	1
HLA-DRB5	-0.108927	0.0449479	-2.4234	0.0154	-0.156942377	count	1
ENPP2	-0.1305688	0.1613028	-0.8095	0.418	-0.156857342	count	1
FIBP	-0.1115889	0.0944165	-1.1819	0.237	-0.156666106	count	1
FAM207A	-0.1176241	0.1683929	-0.6985	0.485	-0.156600093	count	1
DCST1-AS1	-0.5460932	0.799359	-0.6832	0.495	-0.156514987	count	1
NHLRC1	-0.5460932	0.799359	-0.6832	0.495	-0.156514987	count	1
ANKRD16	-0.2663769	0.5504649	-0.4839	0.628	-0.156462717	count	1
BCOR	-0.1849942	0.3521771	-0.5253	0.599	-0.156361193	count	1
TSR1	-0.1261119	0.3520734	-0.3582	0.72	-0.156204101	count	1
IFNAR1	-0.1113421	0.0881107	-1.2637	0.206	-0.156154044	count	1
SARS	-0.1147665	0.1436885	-0.7987	0.425	-0.156094721	count	1

PITPNB	-0.1159972	0.1450042	-0.8	0.424	-0.156079841	count	1
ZNF250	-0.2505355	0.4394696	-0.5701	0.569	-0.156052836	count	1
ATP5MC3	-0.1086115	0.0453547	-2.3947	0.0167	-0.155865917	count	1
ABHD3	-0.1361857	0.2203766	-0.618	0.537	-0.155674524	count	1
MIR3945HG	-0.1217046	0.2101233	-0.5792	0.563	-0.155665719	count	1
SIK2	-0.1283538	0.307416	-0.4175	0.676	-0.155642987	count	1
EMC3	-0.1111491	0.0984213	-1.1293	0.259	-0.15561493	count	1
FOXK2	-0.1285696	0.2626822	-0.4894	0.625	-0.15555816	count	1
TNPO2	-0.1768119	0.4919287	-0.3594	0.719	-0.155472285	count	1
FSTL1	-0.3801963	1.0133333	-0.3752	0.708	-0.155384427	count	1
AC138356.1	-0.3801963	1.3877242	-0.274	0.784	-0.155384427	count	1
ZBTB3	-0.3801963	1.0748319	-0.3537	0.724	-0.155384427	count	1
AC022098.3	-0.3801963	0.8770262	-0.4335	0.665	-0.155384427	count	1
AC010331.1	-0.3801963	0.8009912	-0.4747	0.635	-0.155384427	count	1
RBM34	-0.2035028	0.5001724	-0.4069	0.684	-0.155377887	count	1
BBS7	-0.151883	0.4008346	-0.3789	0.705	-0.155309919	count	1
LAG3	-0.1656879	0.6295225	-0.2632	0.792	-0.155222351	count	1
ZNF778	-0.5401754	0.6090091	-0.887	0.375	-0.155094836	count	1
RB1	-0.110817	0.0903478	-1.2266	0.22	-0.154742839	count	1
COPB1	-0.1115682	0.1090147	-1.0234	0.306	-0.154676969	count	1
VCP	-0.1119288	0.0975068	-1.1479	0.251	-0.154674606	count	1
LRP1	-0.1097756	0.0905182	-1.2127	0.225	-0.154614172	count	1
IQSEC2	-0.138845	0.321772	-0.4315	0.666	-0.154582048	count	1
EMC2	-0.116482	0.1699321	-0.6855	0.493	-0.154556409	count	1
RALGAPB	-0.1672786	0.3586358	-0.4664	0.641	-0.154513451	count	1
U62317.5	-0.1274824	0.2669425	-0.4776	0.633	-0.154252725	count	1
TTN-AS1	-0.1646182	0.331336	-0.4968	0.619	-0.154241622	count	1
NCKAP5	-0.2350237	0.33865	-0.694	0.488	-0.154144055	count	1
INO80	-0.1181686	0.1867543	-0.6327	0.527	-0.154120185	count	1
UTP4	-0.1863314	0.4098604	-0.4546	0.649	-0.154101628	count	1
PSMD2	-0.1115144	0.112394	-0.9922	0.321	-0.154061196	count	1
CD300A	-0.109281	0.0952011	-1.1479	0.251	-0.154030862	count	1
C11orf49	-0.1493003	0.5186822	-0.2878	0.773	-0.153991287	count	1
TSPAN14	-0.1142085	0.1401032	-0.8152	0.415	-0.153787115	count	1
HIST1H2AC	-0.1400067	0.4261808	-0.3285	0.743	-0.15347127	count	1
SRP72	-0.1087207	0.0829711	-1.3103	0.19	-0.153430661	count	1
CD52	-0.1068382	0.0908416	-1.1761	0.24	-0.153364511	count	1
CBWD1	-0.1152015	0.1976944	-0.5827	0.56	-0.153313225	count	1
MRPS16	-0.1107791	0.1193015	-0.9286	0.353	-0.153088564	count	1
NAMPT	-0.1065117	0.0461626	-2.3073	0.0211	-0.153080016	count	1
ACTR2	-0.1068355	0.0462586	-2.3095	0.021	-0.15264833	count	1
DNAJB9	-0.1125055	0.1180937	-0.9527	0.341	-0.152453672	count	1
UBE2M	-0.1075067	0.0801516	-1.3413	0.18	-0.152325727	count	1
EIF5A2	-0.1991126	0.4716592	-0.4222	0.673	-0.152142436	count	1
AL163051.1	-0.5273375	0.5701648	-0.9249	0.355	-0.151994059	count	1

MYOF	-0.1140386	0.1973448	-0.5779	0.563	-0.15198225	count	1
SURF4	-0.1117835	0.1378639	-0.8108	0.418	-0.151969835	count	1
LRCH1	-0.1439421	0.382493	-0.3763	0.707	-0.151962485	count	1
STRN	-0.1228279	0.2236859	-0.5491	0.583	-0.151903956	count	1
FAM177A1	-0.1089016	0.1064481	-1.023	0.306	-0.151888934	count	1
HLA-DRB1	-0.1052337	0.0482625	-2.1804	0.0293	-0.151770546	count	1
THAP9	-1.1697709	0.7334864	-1.5948	0.111	-0.151631428	count	1
RPA3	-0.1101177	0.1343932	-0.8194	0.413	-0.151499831	count	1
TTC38	-0.1615649	0.4694846	-0.3441	0.731	-0.151440685	count	1
PHKG1	-0.1395424	0.2932382	-0.4759	0.634	-0.151239531	count	1
IPO9	-0.1612737	0.3453465	-0.467	0.641	-0.151173431	count	1
PCLAF	-0.2041804	0.4858474	-0.4203	0.674	-0.151137002	count	1
SRP14	-0.1050878	0.0334888	-3.138	0.00172	-0.151106611	count	1
MINK1	-0.1588011	0.3760103	-0.4223	0.673	-0.150858266	count	1
LEPROT	-0.1062667	0.0723727	-1.4683	0.142	-0.150822444	count	1
PAK1IP1	-0.1158706	0.196804	-0.5888	0.556	-0.150793758	count	1
HAUS1	-0.1154301	0.180605	-0.6391	0.523	-0.150782118	count	1
NUBP1	-0.11006	0.1193897	-0.9219	0.357	-0.150713647	count	1
LAMP1	-0.1059856	0.0682838	-1.5521	0.121	-0.150650083	count	1
LINC00667	-0.1122691	0.2002754	-0.5606	0.575	-0.150278148	count	1
LSM3	-0.1060288	0.0754997	-1.4044	0.16	-0.150189803	count	1
SPTAN1	-0.1175748	0.2080595	-0.5651	0.572	-0.1500887	count	1
PSMA3	-0.1065266	0.0815689	-1.306	0.192	-0.150079667	count	1
ISG20L2	-0.1111509	0.1603779	-0.6931	0.488	-0.150078779	count	1
MSX1	-0.519201	0.8914363	-0.5824	0.56	-0.150014653	count	1
TRAC	-0.519201	0.9626847	-0.5393	0.59	-0.150014653	count	1
R3HCC1	-0.1183408	0.2063923	-0.5734	0.566	-0.149841251	count	1
EFCAB13	-0.1853342	0.5147598	-0.36	0.719	-0.149754007	count	1
H2AFJ	-0.1050923	0.0776558	-1.3533	0.176	-0.149669383	count	1
PARP3	-0.2538777	0.5144047	-0.4935	0.622	-0.149532559	count	1
GMIP	-0.1205252	0.2058924	-0.5854	0.558	-0.149327775	count	1
MIS12	-0.1516847	0.3178693	-0.4772	0.633	-0.149315566	count	1
LZTFL1	-0.1569825	0.3909255	-0.4016	0.688	-0.149164828	count	1
ZNF880	-0.1195763	0.2747583	-0.4352	0.663	-0.149116101	count	1
RASAL2	-0.125879	0.2722142	-0.4624	0.644	-0.148898539	count	1
AC015982.1	-0.2926817	0.6363065	-0.46	0.646	-0.148809203	count	1
KCNAB2	-0.1100823	0.149499	-0.7363	0.462	-0.14859207	count	1
EIF4A1	-0.1051513	0.0766146	-1.3725	0.17	-0.148418455	count	1
KREMEN1	-0.1561429	0.499308	-0.3127	0.755	-0.148382737	count	1
C12orf65	-0.1269534	0.3038124	-0.4179	0.676	-0.148347192	count	1
HNRNPM	-0.1047903	0.0710751	-1.4744	0.141	-0.148217428	count	1
TRIM66	-0.1833383	0.5631112	-0.3256	0.745	-0.148189392	count	1
EPSTI1	-0.1046347	0.091266	-1.1465	0.252	-0.148075608	count	1
CRYAB	-0.1935974	0.8131395	-0.2381	0.812	-0.148070392	count	1
TWF2	-0.1040916	0.0730841	-1.4243	0.154	-0.147893389	count	1

MCRIP1	-0.1059793	0.1198925	-0.884	0.377	-0.147743661	count	1
ARFIP2	-0.1338701	0.27645	-0.4842	0.628	-0.147610523	count	1
NINJ1	-0.1029597	0.0598219	-1.7211	0.0854	-0.147598115	count	1
DHCR7	-0.1825463	0.4868659	-0.3749	0.708	-0.147568233	count	1
AC239798.2	-0.6786178	1.1283119	-0.6014	0.548	-0.147331055	count	1
AC108047.1	-0.6786178	1.0975105	-0.6183	0.536	-0.147331055	count	1
AC009950.1	-0.6786178	1.0975105	-0.6183	0.536	-0.147331055	count	1
AC011899.3	-0.6786178	1.2958427	-0.5237	0.601	-0.147331055	count	1
AC016394.1	-0.6786178	1.0975105	-0.6183	0.536	-0.147331055	count	1
ENTPD7	-0.6786178	1.0975105	-0.6183	0.536	-0.147331055	count	1
AC025580.2	-0.6786178	1.0975105	-0.6183	0.536	-0.147331055	count	1
AC099524.1	-0.6786178	1.0975105	-0.6183	0.536	-0.147331055	count	1
SNX25	-0.4161575	0.7132723	-0.5834	0.56	-0.147308819	count	1
ELK4	-0.1241317	0.2231813	-0.5562	0.578	-0.147262813	count	1
FH	-0.1161442	0.1830738	-0.6344	0.526	-0.147254498	count	1
KPNA1	-0.1159429	0.192023	-0.6038	0.546	-0.147177157	count	1
ATP5ME	-0.1036102	0.0663987	-1.5604	0.119	-0.147155914	count	1
MFSD12	-0.1057019	0.1119007	-0.9446	0.345	-0.146978599	count	1
GMCL1	-0.1310888	0.2833874	-0.4626	0.644	-0.146738105	count	1
GHRL	-0.2346353	0.5025484	-0.4669	0.641	-0.146639977	count	1
ZBTB17	-0.1472264	0.3191486	-0.4613	0.645	-0.146501603	count	1
AC073957.3	-0.5042471	0.7909641	-0.6375	0.524	-0.146347959	count	1
AP001257.1	-0.5042471	1.1293358	-0.4465	0.655	-0.146347959	count	1
PSMC3IP	-0.5042471	0.7099183	-0.7103	0.478	-0.146347959	count	1
ETS1	-0.1971808	0.8786778	-0.2244	0.822	-0.146140031	count	1
BRI3BP	-0.1112219	0.163813	-0.679	0.497	-0.145808835	count	1
CARS	-0.1212843	0.2936404	-0.413	0.68	-0.145786802	count	1
PRKAG1	-0.1158734	0.1819801	-0.6367	0.524	-0.145785473	count	1
TMEM176B	-0.1015844	0.0684683	-1.4837	0.138	-0.145551464	count	1
RNH1	-0.1016558	0.0467667	-2.1737	0.0298	-0.145482271	count	1
EIF6	-0.1029377	0.0826798	-1.245	0.213	-0.145361016	count	1
CLOCK	-0.1192684	0.2979929	-0.4002	0.689	-0.145319224	count	1
PLS1	-1.087759	0.6464321	-1.6827	0.0926	-0.144895513	count	1
LMBR1L	-0.1154577	0.1802079	-0.6407	0.522	-0.14486256	count	1
FGFR1OP2	-0.1041711	0.1052673	-0.9896	0.322	-0.144809692	count	1
MFSD10	-0.1035725	0.1254034	-0.8259	0.409	-0.144726739	count	1
AL133264.2	-0.6627556	0.8414392	-0.7876	0.431	-0.144614204	count	1
AC090559.1	-0.6627556	0.8414392	-0.7876	0.431	-0.144614204	count	1
C16orf89	-0.6627556	0.8414392	-0.7876	0.431	-0.144614204	count	1
AC008764.6	-0.6627556	1.0045702	-0.6597	0.509	-0.144614204	count	1
ZNF682	-0.6627556	0.8414392	-0.7876	0.431	-0.144614204	count	1
UBA6-AS1	-0.4957279	0.8111603	-0.6111	0.541	-0.144242336	count	1
NEFH	-0.2821298	0.504191	-0.5596	0.576	-0.143809164	count	1
AL024508.2	-0.6572003	0.9105502	-0.7218	0.471	-0.143655099	count	1
ZNF486	-0.6572003	0.7382075	-0.8903	0.373	-0.143655099	count	1

UBR7	-0.1379862	0.3043139	-0.4534	0.65	-0.143624348	count	1
CREB3L2	-0.1109436	0.2186543	-0.5074	0.612	-0.143588436	count	1
PSMB7	-0.1026256	0.090456	-1.1345	0.257	-0.143509297	count	1
CDC34	-0.1068528	0.1560967	-0.6845	0.494	-0.143163916	count	1
MMP2-AS1	-0.1504559	0.4621575	-0.3256	0.745	-0.143080651	count	1
HSD17B10	-0.1046015	0.1188532	-0.8801	0.379	-0.143020418	count	1
POLDIP2	-0.1112039	0.210037	-0.5294	0.597	-0.143012351	count	1
SAP30L	-0.116003	0.2923052	-0.3969	0.692	-0.143011833	count	1
DDX52	-0.1111558	0.1856901	-0.5986	0.549	-0.142950768	count	1
DDX56	-0.1130022	0.2681318	-0.4214	0.673	-0.1429378	count	1
AP1S3	-0.1686052	1.0626595	-0.1587	0.874	-0.14286635	count	1
SLC29A3	-0.1301834	0.2805707	-0.464	0.643	-0.142827169	count	1
MAP3K6	-0.4008214	0.4570061	-0.8771	0.381	-0.142494743	count	1
ALG6	-0.1430927	0.3579001	-0.3998	0.689	-0.142455484	count	1
HDGFL2	-0.1084797	0.2387568	-0.4544	0.65	-0.142413855	count	1
FBXO5	-0.1919161	0.469655	-0.4086	0.683	-0.142372721	count	1
CINP	-0.1052831	0.1683476	-0.6254	0.532	-0.1423094	count	1
TBCB	-0.1005132	0.0744735	-1.3496	0.177	-0.142155893	count	1
TMEM14B	-0.0999515	0.0696861	-1.4343	0.152	-0.142031181	count	1
NRSN2-AS1	-0.3992359	0.8916878	-0.4477	0.654	-0.141994407	count	1
KTN1	-0.1000787	0.0670743	-1.4921	0.136	-0.141932274	count	1
PGAP1	-0.3056787	0.6617473	-0.4619	0.644	-0.141812512	count	1
PRMT2	-0.1009475	0.1058309	-0.9539	0.34	-0.141679139	count	1
UQCRQ	-0.0994724	0.0625309	-1.5908	0.112	-0.141674576	count	1
SNRPA1	-0.104203	0.1388228	-0.7506	0.453	-0.141650484	count	1
NUDT8	-0.1671046	0.4351742	-0.384	0.701	-0.141627154	count	1
KCNC4	-0.397491	0.8451837	-0.4703	0.638	-0.141443189	count	1
GPR89B	-0.397491	0.853321	-0.4658	0.641	-0.141443189	count	1
TMEM177	-0.397491	1.0867763	-0.3658	0.715	-0.141443189	count	1
TSTD3	-0.397491	0.853321	-0.4658	0.641	-0.141443189	count	1
AC025259.3	-0.397491	0.853321	-0.4658	0.641	-0.141443189	count	1
AC005523.2	-0.2257381	0.3831864	-0.5891	0.556	-0.141343186	count	1
TRAPPC3	-0.1014002	0.1217027	-0.8332	0.405	-0.141167005	count	1
TSEN15	-0.1176984	0.2736974	-0.43	0.667	-0.141161668	count	1
BECN1	-0.1047096	0.1499247	-0.6984	0.485	-0.141029147	count	1
MSRA	-0.1032072	0.1299777	-0.794	0.427	-0.140841867	count	1
AC118553.2	-1.04024	1.0542749	-0.9867	0.324	-0.140773946	count	1
AC109454.2	-1.04024	1.0542749	-0.9867	0.324	-0.140773946	count	1
SEPT7-AS1	-1.04024	1.0542749	-0.9867	0.324	-0.140773946	count	1
ABCB1	-1.04024	1.2426744	-0.8371	0.403	-0.140773946	count	1
PNMA5	-1.04024	1.0542749	-0.9867	0.324	-0.140773946	count	1
THRIL	-1.04024	1.0542749	-0.9867	0.324	-0.140773946	count	1
CCDC168	-1.04024	1.1398628	-0.9126	0.362	-0.140773946	count	1
ZNF594	-1.04024	1.0542749	-0.9867	0.324	-0.140773946	count	1
MIR4435-2HG	-0.1077198	0.2053183	-0.5246	0.6	-0.140748078	count	1

LRBA	-0.196191	0.4353497	-0.4507	0.652	-0.140555258	count	1
PDCD10	-0.100584	0.092788	-1.084	0.278	-0.140359734	count	1
HDHD5	-0.1087263	0.1850611	-0.5875	0.557	-0.140233317	count	1
PRR11	-0.1618151	0.3920107	-0.4128	0.68	-0.139999957	count	1
GTPBP4	-0.1043175	0.1564476	-0.6668	0.505	-0.139831661	count	1
TENT4B	-0.110149	0.1831597	-0.6014	0.548	-0.139520203	count	1
SLC52A2	-0.1043216	0.1605827	-0.6496	0.516	-0.139437288	count	1
DUS4L	-0.1644105	0.4448225	-0.3696	0.712	-0.139400856	count	1
DYNC2LI1	-0.1642567	0.4117746	-0.3989	0.69	-0.139273704	count	1
OXCT2	-0.2995834	0.7141781	-0.4195	0.675	-0.139198457	count	1
TXNL4A	-0.0983147	0.0767351	-1.2812	0.2	-0.139163906	count	1
SOD2	-0.0971678	0.0614439	-1.5814	0.114	-0.139097399	count	1
CAPRIN1	-0.1020207	0.1483733	-0.6876	0.492	-0.139033221	count	1
BUD31	-0.0995554	0.104445	-0.9532	0.341	-0.138970583	count	1
TIMM17A	-0.1010883	0.1278492	-0.7907	0.429	-0.138911751	count	1
LDLRAD3	-0.2511285	0.4894709	-0.5131	0.608	-0.138886932	count	1
SPNS1	-0.1030964	0.1473064	-0.6999	0.484	-0.138861842	count	1
ATG3	-0.0977363	0.0617863	-1.5818	0.114	-0.138839982	count	1
GTF2A1	-0.1085953	0.2056096	-0.5282	0.597	-0.138827583	count	1
MIRLET7BHG	-0.2339408	0.7542167	-0.3102	0.756	-0.138393116	count	1
PHC1	-0.1630585	0.415961	-0.392	0.695	-0.138282891	count	1
UBE2E3	-0.0985525	0.0995082	-0.9904	0.322	-0.138235064	count	1
GLG1	-0.1027866	0.1486808	-0.6913	0.489	-0.138100609	count	1
SLC22A16	-0.1564689	0.4708994	-0.3323	0.74	-0.137987668	count	1
PIP5K1A	-0.1263967	0.2960767	-0.4269	0.669	-0.137951412	count	1
GOLGA2	-0.1008941	0.1615715	-0.6245	0.532	-0.13793209	count	1
CENPH	-0.1593459	0.4747581	-0.3356	0.737	-0.137913799	count	1
GPS1	-0.1078785	0.1867577	-0.5776	0.564	-0.137767388	count	1
CCZ1B	-0.2085874	0.3449452	-0.6047	0.545	-0.137536618	count	1
MYPOP	-0.1481555	0.3279422	-0.4518	0.651	-0.137196511	count	1
SUMF2	-0.1012951	0.1401712	-0.7227	0.47	-0.1371784	count	1
ERC1	-0.1068867	0.2227892	-0.4798	0.631	-0.137078537	count	1
TRMT10C	-0.0990787	0.1201097	-0.8249	0.41	-0.13705232	count	1
RAMMET	-0.1006219	0.1541877	-0.6526	0.514	-0.13686266	count	1
SYN1	-0.1983744	0.6793785	-0.292	0.77	-0.136767924	count	1
ZDHHC9	-0.1983744	0.6911635	-0.287	0.774	-0.136767924	count	1
ATP5MC1	-0.097111	0.0897069	-1.0825	0.279	-0.136703769	count	1
SFR1	-0.1154841	0.2433478	-0.4746	0.635	-0.136696527	count	1
RBM48	-0.1417965	0.4178684	-0.3393	0.734	-0.136651264	count	1
TRAF4	-0.1200325	0.3169874	-0.3787	0.705	-0.136274466	count	1
TMEM120A	-0.1024419	0.1581322	-0.6478	0.517	-0.135773201	count	1
SLC9B1	-0.3790662	1.0538974	-0.3597	0.719	-0.13558603	count	1
AC109347.1	-0.3790662	1.9962709	-0.1899	0.849	-0.13558603	count	1
USP27X-AS1	-0.3790662	1.1781752	-0.3217	0.748	-0.13558603	count	1
SCARA3	-0.3790662	1.9962709	-0.1899	0.849	-0.13558603	count	1

RND1	-0.3790662	1.429447	-0.2652	0.791	-0.13558603	count	1
AC027319.1	-0.3790662	1.2200511	-0.3107	0.756	-0.13558603	count	1
FAM3B	-0.3790662	1.0538974	-0.3597	0.719	-0.13558603	count	1
GNAS	-0.0941819	0.0315094	-2.989	0.00283	-0.135503439	count	1
ORAOV1	-0.1320566	0.3775012	-0.3498	0.727	-0.135322852	count	1
PTDSS2	-0.2152998	0.4124652	-0.522	0.602	-0.135101848	count	1
RAB3GAP2	-0.1153824	0.25621	-0.4503	0.653	-0.134934995	count	1
REPIN1	-0.1149338	0.2607462	-0.4408	0.659	-0.134840057	count	1
DGKE	-0.1156231	0.3194594	-0.3619	0.717	-0.13477418	count	1
IFNLR1	-0.3247558	1.4367243	-0.226	0.821	-0.134702159	count	1
IGFBP3	-0.3247558	0.9144356	-0.3551	0.723	-0.134702159	count	1
BEX2	-0.3247558	1.0120595	-0.3209	0.748	-0.134702159	count	1
AC011043.1	-0.3247558	0.9034833	-0.3594	0.719	-0.134702159	count	1
PRPF40A	-0.0951605	0.0813029	-1.1704	0.242	-0.134609966	count	1
DEDD	-0.1115362	0.2561214	-0.4355	0.663	-0.13446867	count	1
HTT	-0.1058823	0.2326719	-0.4551	0.649	-0.134465778	count	1
DOLPP1	-0.3750385	0.6188214	-0.6061	0.545	-0.134296698	count	1
ZNF720	-0.1187277	0.2906739	-0.4085	0.683	-0.134235162	count	1
PLXNC1	-0.1072475	0.1707941	-0.6279	0.53	-0.134226437	count	1
CDK5	-0.1051157	0.2452695	-0.4286	0.668	-0.134108211	count	1
C19orf54	-0.1469207	0.4170234	-0.3523	0.725	-0.134075923	count	1
CYCS	-0.0936141	0.0783786	-1.1944	0.232	-0.133934773	count	1
PPP1R18	-0.0949659	0.0846121	-1.1224	0.262	-0.133733019	count	1
MCAM	-0.9584358	1.2057457	-0.7949	0.427	-0.133285023	count	1
PRDX6	-0.0937103	0.0655907	-1.4287	0.153	-0.133078774	count	1
TIMM23	-0.1114602	0.2369372	-0.4704	0.638	-0.1330527	count	1
HSPB11	-0.0961008	0.1232121	-0.78	0.435	-0.132816431	count	1
NXN	-0.9527278	0.8521011	-1.1181	0.264	-0.132743332	count	1
NOL7	-0.0932279	0.0701869	-1.3283	0.184	-0.132633303	count	1
SQSTM1	-0.0924847	0.0570958	-1.6198	0.105	-0.132590665	count	1
MRPS34	-0.0936679	0.0874199	-1.0715	0.284	-0.132562316	count	1
NDFIP1	-0.0940781	0.0776619	-1.2114	0.226	-0.132550368	count	1
UTP14A	-0.1144685	0.2711959	-0.4221	0.673	-0.132530646	count	1
RANBP10	-0.2108966	0.4684165	-0.4502	0.653	-0.132460315	count	1
DARS	-0.0975971	0.1288872	-0.7572	0.449	-0.131943742	count	1
FN3KRP	-0.1061341	0.2475937	-0.4287	0.668	-0.13159537	count	1
RPL15	-0.0913192	0.0245759	-3.7158	0.000207	-0.131520882	count	1
CLIC2	-0.1162789	0.1937848	-0.6	0.549	-0.131491996	count	1
RFTN1	-0.1242882	0.2784867	-0.4463	0.655	-0.131467321	count	1
CFL1	-0.0912733	0.0281805	-3.2389	0.00122	-0.131353746	count	1
URI1	-0.0969644	0.1520725	-0.6376	0.524	-0.131326306	count	1
PNP	-0.0942599	0.1107348	-0.8512	0.395	-0.131298298	count	1
NSMCE2	-0.0986987	0.1557157	-0.6338	0.526	-0.131094484	count	1
COX6C	-0.0916539	0.0519324	-1.7649	0.0777	-0.131041692	count	1
ANXA4	-0.0936525	0.1046831	-0.8946	0.371	-0.130974704	count	1

AGAP5	-0.5856951	0.7252447	-0.8076	0.419	-0.130953164	count	1
GTF3C4	-0.1357488	0.4306811	-0.3152	0.753	-0.130919329	count	1
CBARP	-0.3143545	0.7681402	-0.4092	0.682	-0.130746814	count	1
PPP1R26	-0.363402	0.639538	-0.5682	0.57	-0.130553576	count	1
ZSCAN21	-0.2076815	0.6436459	-0.3227	0.747	-0.130528259	count	1
DENND2D	-0.1103394	0.2553236	-0.4322	0.666	-0.130274356	count	1
MECR	-0.1270167	0.3700645	-0.3432	0.731	-0.130227898	count	1
TRMT44	-0.312983	0.4977623	-0.6288	0.53	-0.130223497	count	1
MAPK1IP1L	-0.0932843	0.1002888	-0.9302	0.352	-0.129998697	count	1
DEGS1	-0.0931575	0.0954823	-0.9757	0.329	-0.129997473	count	1
VPS25	-0.0956636	0.1427438	-0.6702	0.503	-0.129788895	count	1
CREM	-0.0911269	0.0777542	-1.172	0.241	-0.129720126	count	1
BCAM	-0.5778824	0.675503	-0.8555	0.392	-0.129524729	count	1
NT5C	-0.0930858	0.1142851	-0.8145	0.415	-0.129470181	count	1
KIAA1958	-0.2181029	0.6543584	-0.3333	0.739	-0.129469277	count	1
AHDC1	-0.3108316	0.5048486	-0.6157	0.538	-0.129401751	count	1
TSHZ3	-0.1556932	0.3236124	-0.4811	0.63	-0.129383151	count	1
C1D	-0.093277	0.1014126	-0.9198	0.358	-0.129261666	count	1
CHCHD2	-0.0900014	0.0382335	-2.354	0.0187	-0.129252497	count	1
LINC01970	-0.3103741	0.5276563	-0.5882	0.556	-0.129226878	count	1
ARF4	-0.0913901	0.0846706	-1.0794	0.281	-0.129196863	count	1
ANKLE2	-0.0955522	0.1735518	-0.5506	0.582	-0.129104892	count	1
PPP4R2	-0.0932063	0.1231052	-0.7571	0.449	-0.128973634	count	1
GABARAP	-0.1032469	0.1957312	-0.5275	0.598	-0.128857007	count	1
SSBP1	-0.0904318	0.0618808	-1.4614	0.144	-0.128788202	count	1
TIMM13	-0.0914217	0.0891698	-1.0253	0.305	-0.128655507	count	1
RAB10	-0.090684	0.0735374	-1.2332	0.218	-0.128457556	count	1
ZFP14	-0.1314806	0.3032276	-0.4336	0.665	-0.128333691	count	1
BANP	-0.101606	0.2532721	-0.4012	0.688	-0.128260519	count	1
RAB1A	-0.0913166	0.0881013	-1.0365	0.3	-0.128065192	count	1
FP565260.1	-0.4316682	0.6006971	-0.7186	0.472	-0.128017011	count	1
BEND5	-0.4315999	0.8733869	-0.4942	0.621	-0.127999343	count	1
LINC01560	-0.4315999	0.8733869	-0.4942	0.621	-0.127999343	count	1
AC142472.1	-0.4315999	0.8739748	-0.4938	0.621	-0.127999343	count	1
UNKL	-0.2302628	0.4132604	-0.5572	0.577	-0.127953273	count	1
EML3	-0.1534072	0.3937399	-0.3896	0.697	-0.127528761	count	1
POLR3D	-0.108986	0.2621623	-0.4157	0.678	-0.127511079	count	1
ATMIN	-0.1095378	0.2708376	-0.4044	0.686	-0.127307384	count	1
ASF1A	-0.0999063	0.2143606	-0.4661	0.641	-0.127204807	count	1
CLSTN1	-0.1093693	0.2449186	-0.4466	0.655	-0.127113071	count	1
DAPK1	-0.0984058	0.1882496	-0.5227	0.601	-0.12708753	count	1
NELFCD	-0.0980535	0.2275062	-0.431	0.667	-0.127074522	count	1
TTLL4	-0.1350501	0.4579026	-0.2949	0.768	-0.127019123	count	1
TNFRSF12A	-0.0940196	0.1582251	-0.5942	0.552	-0.126958112	count	1
XRRA1	-0.1332066	0.4215922	-0.316	0.752	-0.126949982	count	1

SLC31A1	-0.0935669	0.1548338	-0.6043	0.546	-0.126879428	count	1
ARAP3	-0.427194	0.739719	-0.5775	0.564	-0.126857714	count	1
HAUS7	-0.2710026	0.6339845	-0.4275	0.669	-0.126824341	count	1
P3H2	-0.1601908	0.3230609	-0.4959	0.62	-0.126704899	count	1
SMIM15	-0.091572	0.1415747	-0.6468	0.518	-0.126633439	count	1
CLYBL	-0.2011079	0.7273131	-0.2765	0.782	-0.126569382	count	1
KIAA0895L	-0.5603454	0.6980625	-0.8027	0.422	-0.126288813	count	1
CIPC	-0.1381784	0.3524541	-0.392	0.695	-0.126246331	count	1
TRIM62	-0.5564266	0.8461405	-0.6576	0.511	-0.125560105	count	1
AC004520.1	-0.5564266	1.0110891	-0.5503	0.582	-0.125560105	count	1
HDAC3	-0.0937243	0.1510747	-0.6204	0.535	-0.125465119	count	1
PTN	-0.2438394	1.5006961	-0.1625	0.871	-0.125433316	count	1
RAB40C	-0.1682492	0.5800122	-0.2901	0.772	-0.125343576	count	1
TOMM40	-0.0912749	0.1614591	-0.5653	0.572	-0.125224161	count	1
LYSMD2	-0.0892629	0.1000761	-0.892	0.373	-0.124850677	count	1
WBP1L	-0.1056895	0.2241414	-0.4715	0.637	-0.124822811	count	1
DCP1A	-0.0959844	0.2066118	-0.4646	0.642	-0.124808443	count	1
DHRS1	-0.0957894	0.2331152	-0.4109	0.681	-0.124749637	count	1
HARS2	-0.2986764	0.516833	-0.5779	0.563	-0.124739904	count	1
BUB1	-0.8713056	1.1511046	-0.7569	0.449	-0.124734813	count	1
KCNMB3	-0.8713056	1.1511046	-0.7569	0.449	-0.124734813	count	1
RAD9B	-0.8713056	1.4123739	-0.6169	0.537	-0.124734813	count	1
ARHGAP11B	-0.8713056	1.1511046	-0.7569	0.449	-0.124734813	count	1
ZNF433	-0.8713056	1.1511046	-0.7569	0.449	-0.124734813	count	1
PIN4	-0.0904808	0.1262854	-0.7165	0.474	-0.124663325	count	1
NEDD8	-0.086987	0.0514383	-1.6911	0.0909	-0.124460828	count	1
ZNF614	-0.1669861	0.5493668	-0.304	0.761	-0.124430447	count	1
NCKAP5L	-0.10047	0.2003154	-0.5016	0.616	-0.124398355	count	1
DISP1	-0.1728561	0.6489743	-0.2664	0.79	-0.124373051	count	1
ANKAR	-0.1407043	0.4151404	-0.3389	0.735	-0.12436384	count	1
ARL6IP1	-0.0886868	0.0879846	-1.008	0.314	-0.124290777	count	1
UNK	-0.1150387	0.3681627	-0.3125	0.755	-0.124211677	count	1
UBA5	-0.1082991	0.2701973	-0.4008	0.689	-0.124059517	count	1
AC104695.3	-0.1873319	0.5566124	-0.3366	0.736	-0.124046414	count	1
EXOSC3	-0.0958324	0.1972694	-0.4858	0.627	-0.123886193	count	1
ZFP30	-0.8623482	0.819061	-1.0528	0.293	-0.123821013	count	1
ZNF280B	-0.8623482	0.9049316	-0.9529	0.341	-0.123821013	count	1
MTG1	-0.2405043	0.6310063	-0.3811	0.703	-0.123815591	count	1
AC093249.6	-0.2405043	0.605307	-0.3973	0.691	-0.123815591	count	1
PTMA	-0.0858358	0.0229437	-3.7411	0.000187	-0.123744637	count	1
GPR85	-0.8591631	0.8995776	-0.9551	0.34	-0.123494482	count	1
PCDH17	-18.525121	2745.083433	-0.0067	0.995	-0.123488054	count	1
ZDBF2	-18.524312	2053.985462	-0.009	0.993	-0.123488054	count	1
ASPN	-18.352372	2325.654407	-0.0079	0.994	-0.123488054	count	1
SIGLEC8	-18.351541	2099.172962	-0.0087	0.993	-0.123488054	count	1

KRT17	-18.161614	1965.489598	-0.0092	0.993	-0.123488053	count	1
HLX-AS1	-18.142466	2438.068072	-0.0074	0.994	-0.123488053	count	1
ZNF215	-18.142466	2438.068072	-0.0074	0.994	-0.123488053	count	1
ALDH1L1	-18.141339	2164.167784	-0.0084	0.993	-0.123488053	count	1
AC104984.3	-18.092035	1649.029008	-0.011	0.991	-0.123488053	count	1
FAM110D	-18.09112	3117.481017	-0.0058	0.995	-0.123488053	count	1
GPX8	-18.09112	3117.481017	-0.0058	0.995	-0.123488053	count	1
IGHA2	-18.087598	2697.592818	-0.0067	0.995	-0.123488053	count	1
GALNT4	-17.973237	2194.691266	-0.0082	0.993	-0.123488053	count	1
SLC8A1-AS1	-17.940974	2055.784912	-0.0087	0.993	-0.123488053	count	1
ZNF665	-17.940974	2055.784912	-0.0087	0.993	-0.123488053	count	1
ADGRV1	-17.906361	1557.664006	-0.0115	0.991	-0.123488053	count	1
PIK3CD-AS2	-17.899091	3114.605105	-0.0057	0.995	-0.123488053	count	1
AL139424.3	-17.899091	3114.605105	-0.0057	0.995	-0.123488053	count	1
Z98257.1	-17.899091	3114.605105	-0.0057	0.995	-0.123488053	count	1
AL606760.2	-17.899091	3114.605105	-0.0057	0.995	-0.123488053	count	1
AL356488.2	-17.899091	3114.605105	-0.0057	0.995	-0.123488053	count	1
PGLYRP4	-17.899091	3114.605105	-0.0057	0.995	-0.123488053	count	1
KCNT2	-17.899091	3114.605105	-0.0057	0.995	-0.123488053	count	1
SOX13	-17.899091	3114.605105	-0.0057	0.995	-0.123488053	count	1
DTL	-17.899091	3114.605105	-0.0057	0.995	-0.123488053	count	1
SPATA45	-17.899091	3114.605105	-0.0057	0.995	-0.123488053	count	1
AL513314.2	-17.899091	3114.605105	-0.0057	0.995	-0.123488053	count	1
LGALS8-AS1	-17.899091	3114.605105	-0.0057	0.995	-0.123488053	count	1
AC012074.1	-17.899091	3114.605105	-0.0057	0.995	-0.123488053	count	1
AC006369.1	-17.899091	3114.605105	-0.0057	0.995	-0.123488053	count	1
AC019171.1	-17.899091	3114.605105	-0.0057	0.995	-0.123488053	count	1
ADD2	-17.899091	3114.605105	-0.0057	0.995	-0.123488053	count	1
TMEM182	-17.899091	3114.605105	-0.0057	0.995	-0.123488053	count	1
LINC01963	-17.899091	3114.605105	-0.0057	0.995	-0.123488053	count	1
GRIP2	-17.899091	3114.605105	-0.0057	0.995	-0.123488053	count	1
CXCR6	-17.899091	3114.605105	-0.0057	0.995	-0.123488053	count	1
AC084035.1	-17.899091	3114.605105	-0.0057	0.995	-0.123488053	count	1
AC092953.2	-17.899091	3114.605105	-0.0057	0.995	-0.123488053	count	1
NSG1	-17.899091	3114.605105	-0.0057	0.995	-0.123488053	count	1
AC098829.1	-17.899091	3114.605105	-0.0057	0.995	-0.123488053	count	1
AC124016.2	-17.899091	3114.605105	-0.0057	0.995	-0.123488053	count	1
NPNT	-17.899091	3114.605105	-0.0057	0.995	-0.123488053	count	1
FAT4	-17.899091	3114.605105	-0.0057	0.995	-0.123488053	count	1
SPOCK3	-17.899091	3114.605105	-0.0057	0.995	-0.123488053	count	1
TRIP13	-17.899091	3114.605105	-0.0057	0.995	-0.123488053	count	1
CCNO	-17.899091	3114.605105	-0.0057	0.995	-0.123488053	count	1
KCNIP1	-17.899091	3114.605105	-0.0057	0.995	-0.123488053	count	1
AC136604.2	-17.899091	3114.605105	-0.0057	0.995	-0.123488053	count	1
HIST1H2AH	-17.899091	3114.605105	-0.0057	0.995	-0.123488053	count	1

C6orf163	-17.899091	3114.605105	-0.0057	0.995	-0.123488053	count	1
AL353135.1	-17.899091	3114.605105	-0.0057	0.995	-0.123488053	count	1
AL023581.2	-17.899091	3114.605105	-0.0057	0.995	-0.123488053	count	1
AL355297.3	-17.899091	3114.605105	-0.0057	0.995	-0.123488053	count	1
AL139393.2	-17.899091	3114.605105	-0.0057	0.995	-0.123488053	count	1
TFR2	-17.899091	3114.605105	-0.0057	0.995	-0.123488053	count	1
ATP6V1FNB	-17.899091	3114.605105	-0.0057	0.995	-0.123488053	count	1
AC073320.1	-17.899091	3114.605105	-0.0057	0.995	-0.123488053	count	1
GHET1	-17.899091	3114.605105	-0.0057	0.995	-0.123488053	count	1
BMX	-17.899091	3114.605105	-0.0057	0.995	-0.123488053	count	1
INE2	-17.899091	3114.605105	-0.0057	0.995	-0.123488053	count	1
AFF2	-17.899091	3114.605105	-0.0057	0.995	-0.123488053	count	1
PRRG3	-17.899091	3114.605105	-0.0057	0.995	-0.123488053	count	1
MSC-AS1	-17.899091	3114.605105	-0.0057	0.995	-0.123488053	count	1
AP001330.5	-17.899091	3114.605105	-0.0057	0.995	-0.123488053	count	1
ARHGEF39	-17.899091	3114.605105	-0.0057	0.995	-0.123488053	count	1
COL27A1	-17.899091	3114.605105	-0.0057	0.995	-0.123488053	count	1
PPP1R26-AS1	-17.899091	3114.605105	-0.0057	0.995	-0.123488053	count	1
SLC1A2	-17.899091	3114.605105	-0.0057	0.995	-0.123488053	count	1
POU2AF1	-17.899091	3114.605105	-0.0057	0.995	-0.123488053	count	1
CYP17A1	-17.899091	3114.605105	-0.0057	0.995	-0.123488053	count	1
AC016957.2	-17.899091	3114.605105	-0.0057	0.995	-0.123488053	count	1
C12orf42	-17.899091	3114.605105	-0.0057	0.995	-0.123488053	count	1
ASCL4	-17.899091	3114.605105	-0.0057	0.995	-0.123488053	count	1
AC026367.1	-17.899091	3114.605105	-0.0057	0.995	-0.123488053	count	1
AC027290.1	-17.899091	3114.605105	-0.0057	0.995	-0.123488053	count	1
DNAH10	-17.899091	3114.605105	-0.0057	0.995	-0.123488053	count	1
MAB21L1	-17.899091	3114.605105	-0.0057	0.995	-0.123488053	count	1
AC001226.1	-17.899091	3114.605105	-0.0057	0.995	-0.123488053	count	1
AC005480.1	-17.899091	3114.605105	-0.0057	0.995	-0.123488053	count	1
LINC01550	-17.899091	3114.605105	-0.0057	0.995	-0.123488053	count	1
JAG2	-17.899091	3114.605105	-0.0057	0.995	-0.123488053	count	1
SCG3	-17.899091	3114.605105	-0.0057	0.995	-0.123488053	count	1
THAP10	-17.899091	3114.605105	-0.0057	0.995	-0.123488053	count	1
ACAN	-17.899091	3114.605105	-0.0057	0.995	-0.123488053	count	1
SRRM2-AS1	-17.899091	3114.605105	-0.0057	0.995	-0.123488053	count	1
AC007216.4	-17.899091	3114.605105	-0.0057	0.995	-0.123488053	count	1
AC130466.1	-17.899091	3114.605105	-0.0057	0.995	-0.123488053	count	1
AC009090.2	-17.899091	3114.605105	-0.0057	0.995	-0.123488053	count	1
AC027682.1	-17.899091	3114.605105	-0.0057	0.995	-0.123488053	count	1
HASPIN	-17.899091	3114.605105	-0.0057	0.995	-0.123488053	count	1
MYH3	-17.899091	3114.605105	-0.0057	0.995	-0.123488053	count	1
MYH7B	-17.899091	3114.605105	-0.0057	0.995	-0.123488053	count	1
BCAS4	-17.899091	3114.605105	-0.0057	0.995	-0.123488053	count	1
PLIN4	-17.899091	3114.605105	-0.0057	0.995	-0.123488053	count	1

ADGRL1	-17.899091	3114.605105	-0.0057	0.995	-0.123488053	count	1
LIPE	-17.899091	3114.605105	-0.0057	0.995	-0.123488053	count	1
NLGN4Y	-17.899091	3114.605105	-0.0057	0.995	-0.123488053	count	1
AC253536.6	-17.899091	3114.605105	-0.0057	0.995	-0.123488053	count	1
PVALB	-17.899091	3114.605105	-0.0057	0.995	-0.123488053	count	1
SLC16A8	-17.899091	3114.605105	-0.0057	0.995	-0.123488053	count	1
GRAP2	-17.899091	3114.605105	-0.0057	0.995	-0.123488053	count	1
EFCAB6	-17.899091	3114.605105	-0.0057	0.995	-0.123488053	count	1
GCSAM	-17.889086	1996.687505	-0.009	0.993	-0.123488053	count	1
CASZ1	-17.870915	1857.103643	-0.0096	0.992	-0.123488053	count	1
LMOD1	-17.870915	1857.103643	-0.0096	0.992	-0.123488053	count	1
CYB561D2	-17.794111	1827.1213	-0.0097	0.992	-0.123488053	count	1
ZNF365	-17.794111	1827.1213	-0.0097	0.992	-0.123488053	count	1
RAD51	-17.794111	1827.1213	-0.0097	0.992	-0.123488053	count	1
RN7SL832P	-17.7737	2064.251785	-0.0086	0.993	-0.123488053	count	1
AC005540.1	-17.7737	2064.251785	-0.0086	0.993	-0.123488053	count	1
CACNA1C-AS2	-17.7737	2064.251785	-0.0086	0.993	-0.123488053	count	1
AC008758.4	-17.7737	2064.251785	-0.0086	0.993	-0.123488053	count	1
UNC80	-17.742531	2935.421747	-0.006	0.995	-0.123488053	count	1
SPINK2	-17.742531	2935.421747	-0.006	0.995	-0.123488053	count	1
ITPR3	-17.742531	2935.421747	-0.006	0.995	-0.123488053	count	1
THEMIS	-17.742531	2935.421747	-0.006	0.995	-0.123488053	count	1
AL136320.1	-17.742531	2935.421747	-0.006	0.995	-0.123488053	count	1
AC006064.2	-17.742531	2935.421747	-0.006	0.995	-0.123488053	count	1
GZMH	-17.742531	2935.421747	-0.006	0.995	-0.123488053	count	1
IGLV3-1	-17.742531	2935.421747	-0.006	0.995	-0.123488053	count	1
AL513550.1	-17.740777	2716.887115	-0.0065	0.995	-0.123488053	count	1
TRIM6	-17.740777	2716.887115	-0.0065	0.995	-0.123488053	count	1
DNM1P35	-17.740777	2716.887115	-0.0065	0.995	-0.123488053	count	1
ADAM32	-17.670684	1594.044523	-0.0111	0.991	-0.123488052	count	1
AL158151.3	-17.670684	1594.044523	-0.0111	0.991	-0.123488052	count	1
AC093827.5	-17.553023	2175.93826	-0.0081	0.994	-0.123488052	count	1
HIST1H2BF	-17.552096	2045.655339	-0.0086	0.993	-0.123488052	count	1
AC147651.1	-17.552096	2045.655339	-0.0086	0.993	-0.123488052	count	1
ZNF608	-17.502184	2084.392974	-0.0084	0.993	-0.123488052	count	1
RNF217-AS1	-17.502184	2084.392974	-0.0084	0.993	-0.123488052	count	1
RNFT2	-17.502184	2084.392974	-0.0084	0.993	-0.123488052	count	1
AL137786.1	-17.502184	2084.392974	-0.0084	0.993	-0.123488052	count	1
ARNT2	-17.502184	2084.392974	-0.0084	0.993	-0.123488052	count	1
ZNF23	-17.502184	2084.392974	-0.0084	0.993	-0.123488052	count	1
ZNF556	-17.502184	2084.392974	-0.0084	0.993	-0.123488052	count	1
AC007663.3	-17.502184	2084.392974	-0.0084	0.993	-0.123488052	count	1
RAVER2	-17.500989	1868.169847	-0.0094	0.993	-0.123488052	count	1
AC135507.1	-17.500989	1868.169847	-0.0094	0.993	-0.123488052	count	1
LSAMP	-17.500989	1868.169847	-0.0094	0.993	-0.123488052	count	1

TRPC1	-17.500989	1868.169847	-0.0094	0.993	-0.123488052	count	1
SEMA3A	-17.500989	1868.169847	-0.0094	0.993	-0.123488052	count	1
AL683807.1	-17.500989	1868.169847	-0.0094	0.993	-0.123488052	count	1
AZIN1-AS1	-17.500989	1868.169847	-0.0094	0.993	-0.123488052	count	1
ABO	-17.500989	1868.169847	-0.0094	0.993	-0.123488052	count	1
AC087241.3	-17.500989	1868.169847	-0.0094	0.993	-0.123488052	count	1
TSC22D1-AS1	-17.500989	1868.169847	-0.0094	0.993	-0.123488052	count	1
MSLN	-17.500989	1868.169847	-0.0094	0.993	-0.123488052	count	1
AC009163.7	-17.500989	1868.169847	-0.0094	0.993	-0.123488052	count	1
ARHGAP44	-17.500989	1868.169847	-0.0094	0.993	-0.123488052	count	1
MOCOS	-17.500989	1868.169847	-0.0094	0.993	-0.123488052	count	1
LINC00663	-17.500989	1868.169847	-0.0094	0.993	-0.123488052	count	1
OSBPL10	-17.4966	1674.47573	-0.0104	0.992	-0.123488052	count	1
ADAMTS4	-17.314235	2076.210816	-0.0083	0.993	-0.123488051	count	1
ABCG8	-17.314235	2076.210816	-0.0083	0.993	-0.123488051	count	1
LINC01305	-17.314235	2076.210816	-0.0083	0.993	-0.123488051	count	1
CP	-17.314235	2076.210816	-0.0083	0.993	-0.123488051	count	1
AC096564.1	-17.314235	2076.210816	-0.0083	0.993	-0.123488051	count	1
MIR583HG	-17.314235	2076.210816	-0.0083	0.993	-0.123488051	count	1
LINC02201	-17.314235	2076.210816	-0.0083	0.993	-0.123488051	count	1
ADAMTSL2	-17.314235	2076.210816	-0.0083	0.993	-0.123488051	count	1
AL137145.2	-17.314235	2076.210816	-0.0083	0.993	-0.123488051	count	1
AL133417.1	-17.314235	2076.210816	-0.0083	0.993	-0.123488051	count	1
AC090617.5	-17.229564	1473.714778	-0.0117	0.991	-0.123488051	count	1
PHGDH	-17.211639	1244.530295	-0.0138	0.989	-0.123488051	count	1
AL096855.1	-17.197067	2752.218623	-0.0062	0.995	-0.123488051	count	1
PINK1-AS	-17.197067	2752.218623	-0.0062	0.995	-0.123488051	count	1
LINC01355	-17.197067	2752.218623	-0.0062	0.995	-0.123488051	count	1
LINC02574	-17.197067	2752.218623	-0.0062	0.995	-0.123488051	count	1
LINC02586	-17.197067	2752.218623	-0.0062	0.995	-0.123488051	count	1
S100A3	-17.197067	2752.218623	-0.0062	0.995	-0.123488051	count	1
NR1I3	-17.197067	2752.218623	-0.0062	0.995	-0.123488051	count	1
CCDC181	-17.197067	2752.218623	-0.0062	0.995	-0.123488051	count	1
PLEKHA6	-17.197067	2752.218623	-0.0062	0.995	-0.123488051	count	1
SERTAD4-AS1	-17.197067	2752.218623	-0.0062	0.995	-0.123488051	count	1
FIGLA	-17.197067	2752.218623	-0.0062	0.995	-0.123488051	count	1
SULT1C2	-17.197067	2752.218623	-0.0062	0.995	-0.123488051	count	1
SGPP2	-17.197067	2752.218623	-0.0062	0.995	-0.123488051	count	1
AC099541.1	-17.197067	2752.218623	-0.0062	0.995	-0.123488051	count	1
AC092910.3	-17.197067	2752.218623	-0.0062	0.995	-0.123488051	count	1
IL20RB	-17.197067	2752.218623	-0.0062	0.995	-0.123488051	count	1
CAMK2N2	-17.197067	2752.218623	-0.0062	0.995	-0.123488051	count	1
AC109347.2	-17.197067	2752.218623	-0.0062	0.995	-0.123488051	count	1
MGARP	-17.197067	2752.218623	-0.0062	0.995	-0.123488051	count	1
EDA2R	-17.197067	2752.218623	-0.0062	0.995	-0.123488051	count	1

PDGFRL	-17.197067	2752.218623	-0.0062	0.995	-0.123488051	count	1
AC100814.1	-17.197067	2752.218623	-0.0062	0.995	-0.123488051	count	1
AL358113.1	-17.197067	2752.218623	-0.0062	0.995	-0.123488051	count	1
CXCR5	-17.197067	2752.218623	-0.0062	0.995	-0.123488051	count	1
AL158163.1	-17.197067	2752.218623	-0.0062	0.995	-0.123488051	count	1
CLEC1B	-17.197067	2752.218623	-0.0062	0.995	-0.123488051	count	1
AC025031.4	-17.197067	2752.218623	-0.0062	0.995	-0.123488051	count	1
PTPRB	-17.197067	2752.218623	-0.0062	0.995	-0.123488051	count	1
LINC00426	-17.197067	2752.218623	-0.0062	0.995	-0.123488051	count	1
ITGBL1	-17.197067	2752.218623	-0.0062	0.995	-0.123488051	count	1
PPP1R36	-17.197067	2752.218623	-0.0062	0.995	-0.123488051	count	1
LINC00239	-17.197067	2752.218623	-0.0062	0.995	-0.123488051	count	1
BUB1B	-17.197067	2752.218623	-0.0062	0.995	-0.123488051	count	1
AC018362.2	-17.197067	2752.218623	-0.0062	0.995	-0.123488051	count	1
DUOX1	-17.197067	2752.218623	-0.0062	0.995	-0.123488051	count	1
CSPG4	-17.197067	2752.218623	-0.0062	0.995	-0.123488051	count	1
AC124068.2	-17.197067	2752.218623	-0.0062	0.995	-0.123488051	count	1
NKD1	-17.197067	2752.218623	-0.0062	0.995	-0.123488051	count	1
CDH5	-17.197067	2752.218623	-0.0062	0.995	-0.123488051	count	1
AC092718.8	-17.197067	2752.218623	-0.0062	0.995	-0.123488051	count	1
CORO6	-17.197067	2752.218623	-0.0062	0.995	-0.123488051	count	1
SMIM5	-17.197067	2752.218623	-0.0062	0.995	-0.123488051	count	1
NDC80	-17.197067	2752.218623	-0.0062	0.995	-0.123488051	count	1
ABALON	-17.197067	2752.218623	-0.0062	0.995	-0.123488051	count	1
SLA2	-17.197067	2752.218623	-0.0062	0.995	-0.123488051	count	1
KISS1R	-17.197067	2752.218623	-0.0062	0.995	-0.123488051	count	1
PRR36	-17.197067	2752.218623	-0.0062	0.995	-0.123488051	count	1
F2RL3	-17.197067	2752.218623	-0.0062	0.995	-0.123488051	count	1
ZNF547	-17.197067	2752.218623	-0.0062	0.995	-0.123488051	count	1
AP001065.1	-17.197067	2752.218623	-0.0062	0.995	-0.123488051	count	1
AL627309.1	-16.700967	4059.590711	-0.0041	0.997	-0.123488048	count	1
AL390719.2	-16.700967	4059.590711	-0.0041	0.997	-0.123488048	count	1
TTLL10	-16.700967	4059.590711	-0.0041	0.997	-0.123488048	count	1
AL139246.3	-16.700967	4059.590711	-0.0041	0.997	-0.123488048	count	1
FBXO2	-16.700967	4059.590711	-0.0041	0.997	-0.123488048	count	1
AL121992.1	-16.700967	4059.590711	-0.0041	0.997	-0.123488048	count	1
AL450998.3	-16.700967	4059.590711	-0.0041	0.997	-0.123488048	count	1
MFAP2	-16.700967	4059.590711	-0.0041	0.997	-0.123488048	count	1
GPR3	-16.700967	4059.590711	-0.0041	0.997	-0.123488048	count	1
LINC01778	-16.700967	4059.590711	-0.0041	0.997	-0.123488048	count	1
AC114488.2	-16.700967	4059.590711	-0.0041	0.997	-0.123488048	count	1
TMEM35B	-16.700967	4059.590711	-0.0041	0.997	-0.123488048	count	1
TMCO2	-16.700967	4059.590711	-0.0041	0.997	-0.123488048	count	1
PTPRF	-16.700967	4059.590711	-0.0041	0.997	-0.123488048	count	1
KIF2C	-16.700967	4059.590711	-0.0041	0.997	-0.123488048	count	1

AL358075.2	-16.700967	4059.590711	-0.0041	0.997	-0.123488048	count	1
AC093424.1	-16.700967	4059.590711	-0.0041	0.997	-0.123488048	count	1
AC099794.1	-16.700967	4059.590711	-0.0041	0.997	-0.123488048	count	1
AL031429.1	-16.700967	4059.590711	-0.0041	0.997	-0.123488048	count	1
LINC01725	-16.700967	4059.590711	-0.0041	0.997	-0.123488048	count	1
AL121989.1	-16.700967	4059.590711	-0.0041	0.997	-0.123488048	count	1
HFM1	-16.700967	4059.590711	-0.0041	0.997	-0.123488048	count	1
FAM19A3	-16.700967	4059.590711	-0.0041	0.997	-0.123488048	count	1
SYT6	-16.700967	4059.590711	-0.0041	0.997	-0.123488048	count	1
LINC01762	-16.700967	4059.590711	-0.0041	0.997	-0.123488048	count	1
AC245014.1	-16.700967	4059.590711	-0.0041	0.997	-0.123488048	count	1
NUDT4B	-16.700967	4059.590711	-0.0041	0.997	-0.123488048	count	1
LYSMD1	-16.700967	4059.590711	-0.0041	0.997	-0.123488048	count	1
TDRKH-AS1	-16.700967	4059.590711	-0.0041	0.997	-0.123488048	count	1
AL365181.3	-16.700967	4059.590711	-0.0041	0.997	-0.123488048	count	1
KIRREL1	-16.700967	4059.590711	-0.0041	0.997	-0.123488048	count	1
LINC01704	-16.700967	4059.590711	-0.0041	0.997	-0.123488048	count	1
OR6N1	-16.700967	4059.590711	-0.0041	0.997	-0.123488048	count	1
AL139011.2	-16.700967	4059.590711	-0.0041	0.997	-0.123488048	count	1
AL591806.3	-16.700967	4059.590711	-0.0041	0.997	-0.123488048	count	1
ILDR2	-16.700967	4059.590711	-0.0041	0.997	-0.123488048	count	1
AL022310.1	-16.700967	4059.590711	-0.0041	0.997	-0.123488048	count	1
AXDND1	-16.700967	4059.590711	-0.0041	0.997	-0.123488048	count	1
NMNAT2	-16.700967	4059.590711	-0.0041	0.997	-0.123488048	count	1
AL445228.2	-16.700967	4059.590711	-0.0041	0.997	-0.123488048	count	1
LINC00862	-16.700967	4059.590711	-0.0041	0.997	-0.123488048	count	1
AC104463.2	-16.700967	4059.590711	-0.0041	0.997	-0.123488048	count	1
ADORA1	-16.700967	4059.590711	-0.0041	0.997	-0.123488048	count	1
AL031316.1	-16.700967	4059.590711	-0.0041	0.997	-0.123488048	count	1
AL136379.1	-16.700967	4059.590711	-0.0041	0.997	-0.123488048	count	1
OBSCN-AS1	-16.700967	4059.590711	-0.0041	0.997	-0.123488048	count	1
AL121990.1	-16.700967	4059.590711	-0.0041	0.997	-0.123488048	count	1
LINC01348	-16.700967	4059.590711	-0.0041	0.997	-0.123488048	count	1
AL357556.4	-16.700967	4059.590711	-0.0041	0.997	-0.123488048	count	1
EDARADD	-16.700967	4059.590711	-0.0041	0.997	-0.123488048	count	1
CATSPERE	-16.700967	4059.590711	-0.0041	0.997	-0.123488048	count	1
AL591848.4	-16.700967	4059.590711	-0.0041	0.997	-0.123488048	count	1
ZNF695	-16.700967	4059.590711	-0.0041	0.997	-0.123488048	count	1
OR2L2	-16.700967	4059.590711	-0.0041	0.997	-0.123488048	count	1
LINC00299	-16.700967	4059.590711	-0.0041	0.997	-0.123488048	count	1
LINC01814	-16.700967	4059.590711	-0.0041	0.997	-0.123488048	count	1
AC012065.3	-16.700967	4059.590711	-0.0041	0.997	-0.123488048	count	1
LINC02580	-16.700967	4059.590711	-0.0041	0.997	-0.123488048	count	1
AC009501.1	-16.700967	4059.590711	-0.0041	0.997	-0.123488048	count	1
AC016700.3	-16.700967	4059.590711	-0.0041	0.997	-0.123488048	count	1

LINC01293	-16.700967	4059.590711	-0.0041	0.997	-0.123488048	count	1
AC012511.1	-16.700967	4059.590711	-0.0041	0.997	-0.123488048	count	1
CD8A	-16.700967	4059.590711	-0.0041	0.997	-0.123488048	count	1
AC133644.2	-16.700967	4059.590711	-0.0041	0.997	-0.123488048	count	1
ZAP70	-16.700967	4059.590711	-0.0041	0.997	-0.123488048	count	1
CHST10	-16.700967	4059.590711	-0.0041	0.997	-0.123488048	count	1
NT5DC4	-16.700967	4059.590711	-0.0041	0.997	-0.123488048	count	1
AC016745.1	-16.700967	4059.590711	-0.0041	0.997	-0.123488048	count	1
DPP10	-16.700967	4059.590711	-0.0041	0.997	-0.123488048	count	1
POTEE	-16.700967	4059.590711	-0.0041	0.997	-0.123488048	count	1
CCDC74A	-16.700967	4059.590711	-0.0041	0.997	-0.123488048	count	1
LINC01876	-16.700967	4059.590711	-0.0041	0.997	-0.123488048	count	1
LINC02478	-16.700967	4059.590711	-0.0041	0.997	-0.123488048	count	1
AC008063.2	-16.700967	4059.590711	-0.0041	0.997	-0.123488048	count	1
GRB14	-16.700967	4059.590711	-0.0041	0.997	-0.123488048	count	1
CCDC173	-16.700967	4059.590711	-0.0041	0.997	-0.123488048	count	1
AC016737.2	-16.700967	4059.590711	-0.0041	0.997	-0.123488048	count	1
AC010894.3	-16.700967	4059.590711	-0.0041	0.997	-0.123488048	count	1
CHN1	-16.700967	4059.590711	-0.0041	0.997	-0.123488048	count	1
AC019080.4	-16.700967	4059.590711	-0.0041	0.997	-0.123488048	count	1
NCKAP1	-16.700967	4059.590711	-0.0041	0.997	-0.123488048	count	1
LINC01827	-16.700967	4059.590711	-0.0041	0.997	-0.123488048	count	1
SATB2-AS1	-16.700967	4059.590711	-0.0041	0.997	-0.123488048	count	1
AC005037.1	-16.700967	4059.590711	-0.0041	0.997	-0.123488048	count	1
AC012668.3	-16.700967	4059.590711	-0.0041	0.997	-0.123488048	count	1
AC013448.2	-16.700967	4059.590711	-0.0041	0.997	-0.123488048	count	1
SCYGR4	-16.700967	4059.590711	-0.0041	0.997	-0.123488048	count	1
SLC16A14	-16.700967	4059.590711	-0.0041	0.997	-0.123488048	count	1
SNORC	-16.700967	4059.590711	-0.0041	0.997	-0.123488048	count	1
SAG	-16.700967	4059.590711	-0.0041	0.997	-0.123488048	count	1
AC112715.1	-16.700967	4059.590711	-0.0041	0.997	-0.123488048	count	1
AC068313.1	-16.700967	4059.590711	-0.0041	0.997	-0.123488048	count	1
AC018809.2	-16.700967	4059.590711	-0.0041	0.997	-0.123488048	count	1
COLQ	-16.700967	4059.590711	-0.0041	0.997	-0.123488048	count	1
ZNF385D	-16.700967	4059.590711	-0.0041	0.997	-0.123488048	count	1
LINC01980	-16.700967	4059.590711	-0.0041	0.997	-0.123488048	count	1
CSPG5	-16.700967	4059.590711	-0.0041	0.997	-0.123488048	count	1
PLXNB1	-16.700967	4059.590711	-0.0041	0.997	-0.123488048	count	1
SEMA3F	-16.700967	4059.590711	-0.0041	0.997	-0.123488048	count	1
ITIH3	-16.700967	4059.590711	-0.0041	0.997	-0.123488048	count	1
AC116036.2	-16.700967	4059.590711	-0.0041	0.997	-0.123488048	count	1
FAM3D	-16.700967	4059.590711	-0.0041	0.997	-0.123488048	count	1
LINC00882	-16.700967	4059.590711	-0.0041	0.997	-0.123488048	count	1
BTLA	-16.700967	4059.590711	-0.0041	0.997	-0.123488048	count	1
AC074044.1	-16.700967	4059.590711	-0.0041	0.997	-0.123488048	count	1

AC128687.2	-16.700967	4059.590711	-0.0041	0.997	-0.123488048	count	1
ARHGAP31-AS1	-16.700967	4059.590711	-0.0041	0.997	-0.123488048	count	1
ALG1L	-16.700967	4059.590711	-0.0041	0.997	-0.123488048	count	1
PODXL2	-16.700967	4059.590711	-0.0041	0.997	-0.123488048	count	1
KBTBD12	-16.700967	4059.590711	-0.0041	0.997	-0.123488048	count	1
EFCC1	-16.700967	4059.590711	-0.0041	0.997	-0.123488048	count	1
LINC02014	-16.700967	4059.590711	-0.0041	0.997	-0.123488048	count	1
LINC02021	-16.700967	4059.590711	-0.0041	0.997	-0.123488048	count	1
BFSP2	-16.700967	4059.590711	-0.0041	0.997	-0.123488048	count	1
EPHB1	-16.700967	4059.590711	-0.0041	0.997	-0.123488048	count	1
FOXL2	-16.700967	4059.590711	-0.0041	0.997	-0.123488048	count	1
CLSTN2	-16.700967	4059.590711	-0.0041	0.997	-0.123488048	count	1
TM4SF18	-16.700967	4059.590711	-0.0041	0.997	-0.123488048	count	1
AC104472.3	-16.700967	4059.590711	-0.0041	0.997	-0.123488048	count	1
LINC00881	-16.700967	4059.590711	-0.0041	0.997	-0.123488048	count	1
AC080013.6	-16.700967	4059.590711	-0.0041	0.997	-0.123488048	count	1
IL12A	-16.700967	4059.590711	-0.0041	0.997	-0.123488048	count	1
PEX5L-AS1	-16.700967	4059.590711	-0.0041	0.997	-0.123488048	count	1
KLHL6-AS1	-16.700967	4059.590711	-0.0041	0.997	-0.123488048	count	1
ABCC5-AS1	-16.700967	4059.590711	-0.0041	0.997	-0.123488048	count	1
CHRD	-16.700967	4059.590711	-0.0041	0.997	-0.123488048	count	1
AC139887.1	-16.700967	4059.590711	-0.0041	0.997	-0.123488048	count	1
ADRA2C	-16.700967	4059.590711	-0.0041	0.997	-0.123488048	count	1
C1QTNF7	-16.700967	4059.590711	-0.0041	0.997	-0.123488048	count	1
AC099550.1	-16.700967	4059.590711	-0.0041	0.997	-0.123488048	count	1
LINC02357	-16.700967	4059.590711	-0.0041	0.997	-0.123488048	count	1
UCHL1	-16.700967	4059.590711	-0.0041	0.997	-0.123488048	count	1
AC110792.3	-16.700967	4059.590711	-0.0041	0.997	-0.123488048	count	1
AC069307.1	-16.700967	4059.590711	-0.0041	0.997	-0.123488048	count	1
AC104806.2	-16.700967	4059.590711	-0.0041	0.997	-0.123488048	count	1
AC053527.1	-16.700967	4059.590711	-0.0041	0.997	-0.123488048	count	1
AC110615.1	-16.700967	4059.590711	-0.0041	0.997	-0.123488048	count	1
PPEF2	-16.700967	4059.590711	-0.0041	0.997	-0.123488048	count	1
AC104785.1	-16.700967	4059.590711	-0.0041	0.997	-0.123488048	count	1
FGF2	-16.700967	4059.590711	-0.0041	0.997	-0.123488048	count	1
GYPE	-16.700967	4059.590711	-0.0041	0.997	-0.123488048	count	1
REELD1	-16.700967	4059.590711	-0.0041	0.997	-0.123488048	count	1
FAM160A1	-16.700967	4059.590711	-0.0041	0.997	-0.123488048	count	1
AC107214.2	-16.700967	4059.590711	-0.0041	0.997	-0.123488048	count	1
AHRR	-16.700967	4059.590711	-0.0041	0.997	-0.123488048	count	1
AC026740.1	-16.700967	4059.590711	-0.0041	0.997	-0.123488048	count	1
AC091891.2	-16.700967	4059.590711	-0.0041	0.997	-0.123488048	count	1
AC034229.1	-16.700967	4059.590711	-0.0041	0.997	-0.123488048	count	1
EGFLAM	-16.700967	4059.590711	-0.0041	0.997	-0.123488048	count	1
LINC01033	-16.700967	4059.590711	-0.0041	0.997	-0.123488048	count	1

AC010359.1	-16.700967	4059.590711	-0.0041	0.997	-0.123488048	count	1
AC010273.1	-16.700967	4059.590711	-0.0041	0.997	-0.123488048	count	1
SERF1B	-16.700967	4059.590711	-0.0041	0.997	-0.123488048	count	1
AC008972.1	-16.700967	4059.590711	-0.0041	0.997	-0.123488048	count	1
AC113404.1	-16.700967	4059.590711	-0.0041	0.997	-0.123488048	count	1
BHMT2	-16.700967	4059.590711	-0.0041	0.997	-0.123488048	count	1
CMYA5	-16.700967	4059.590711	-0.0041	0.997	-0.123488048	count	1
AC104118.1	-16.700967	4059.590711	-0.0041	0.997	-0.123488048	count	1
SPATA9	-16.700967	4059.590711	-0.0041	0.997	-0.123488048	count	1
AC104123.1	-16.700967	4059.590711	-0.0041	0.997	-0.123488048	count	1
AC012603.1	-16.700967	4059.590711	-0.0041	0.997	-0.123488048	count	1
TMEM232	-16.700967	4059.590711	-0.0041	0.997	-0.123488048	count	1
LOX	-16.700967	4059.590711	-0.0041	0.997	-0.123488048	count	1
KIAA1024L	-16.700967	4059.590711	-0.0041	0.997	-0.123488048	count	1
ACSL6	-16.700967	4059.590711	-0.0041	0.997	-0.123488048	count	1
SMAD5-AS1	-16.700967	4059.590711	-0.0041	0.997	-0.123488048	count	1
AC104116.1	-16.700967	4059.590711	-0.0041	0.997	-0.123488048	count	1
PCDHB4	-16.700967	4059.590711	-0.0041	0.997	-0.123488048	count	1
PLAC8L1	-16.700967	4059.590711	-0.0041	0.997	-0.123488048	count	1
POU4F3	-16.700967	4059.590711	-0.0041	0.997	-0.123488048	count	1
SPINK5	-16.700967	4059.590711	-0.0041	0.997	-0.123488048	count	1
HTR4	-16.700967	4059.590711	-0.0041	0.997	-0.123488048	count	1
CARMN	-16.700967	4059.590711	-0.0041	0.997	-0.123488048	count	1
SLC6A7	-16.700967	4059.590711	-0.0041	0.997	-0.123488048	count	1
C5orf58	-16.700967	4059.590711	-0.0041	0.997	-0.123488048	count	1
NEURL1B	-16.700967	4059.590711	-0.0041	0.997	-0.123488048	count	1
STC2	-16.700967	4059.590711	-0.0041	0.997	-0.123488048	count	1
FAM153A	-16.700967	4059.590711	-0.0041	0.997	-0.123488048	count	1
COL23A1	-16.700967	4059.590711	-0.0041	0.997	-0.123488048	count	1
ZFP2	-16.700967	4059.590711	-0.0041	0.997	-0.123488048	count	1
LINC01962	-16.700967	4059.590711	-0.0041	0.997	-0.123488048	count	1
AL138831.1	-16.700967	4059.590711	-0.0041	0.997	-0.123488048	count	1
AL136309.2	-16.700967	4059.590711	-0.0041	0.997	-0.123488048	count	1
ERVFRD-1	-16.700967	4059.590711	-0.0041	0.997	-0.123488048	count	1
CAP2	-16.700967	4059.590711	-0.0041	0.997	-0.123488048	count	1
AL031775.2	-16.700967	4059.590711	-0.0041	0.997	-0.123488048	count	1
HIST1H3B	-16.700967	4059.590711	-0.0041	0.997	-0.123488048	count	1
GTF2H4	-16.700967	4059.590711	-0.0041	0.997	-0.123488048	count	1
NCR3	-16.700967	4059.590711	-0.0041	0.997	-0.123488048	count	1
C6orf47-AS1	-16.700967	4059.590711	-0.0041	0.997	-0.123488048	count	1
AL662796.1	-16.700967	4059.590711	-0.0041	0.997	-0.123488048	count	1
TCP11	-16.700967	4059.590711	-0.0041	0.997	-0.123488048	count	1
POLH-AS1	-16.700967	4059.590711	-0.0041	0.997	-0.123488048	count	1
LRRC1	-16.700967	4059.590711	-0.0041	0.997	-0.123488048	count	1
AL121972.1	-16.700967	4059.590711	-0.0041	0.997	-0.123488048	count	1

AL080317.2	-16.700967	4059.590711	-0.0041	0.997	-0.123488048	count	1
PTPRK	-16.700967	4059.590711	-0.0041	0.997	-0.123488048	count	1
AL356124.2	-16.700967	4059.590711	-0.0041	0.997	-0.123488048	count	1
ENPP3	-16.700967	4059.590711	-0.0041	0.997	-0.123488048	count	1
AL596188.1	-16.700967	4059.590711	-0.0041	0.997	-0.123488048	count	1
AL353596.1	-16.700967	4059.590711	-0.0041	0.997	-0.123488048	count	1
AL138737.1	-16.700967	4059.590711	-0.0041	0.997	-0.123488048	count	1
AL031056.2	-16.700967	4059.590711	-0.0041	0.997	-0.123488048	count	1
SOD2	-16.700967	4059.590711	-0.0041	0.997	-0.123488048	count	1
SMOC2	-16.700967	4059.590711	-0.0041	0.997	-0.123488048	count	1
SLC29A4	-16.700967	4059.590711	-0.0041	0.997	-0.123488048	count	1
AC007029.1	-16.700967	4059.590711	-0.0041	0.997	-0.123488048	count	1
AC005014.2	-16.700967	4059.590711	-0.0041	0.997	-0.123488048	count	1
ABCB5	-16.700967	4059.590711	-0.0041	0.997	-0.123488048	count	1
KIAA0895	-16.700967	4059.590711	-0.0041	0.997	-0.123488048	count	1
GLI3	-16.700967	4059.590711	-0.0041	0.997	-0.123488048	count	1
MLXIPL	-16.700967	4059.590711	-0.0041	0.997	-0.123488048	count	1
RUNDC3B	-16.700967	4059.590711	-0.0041	0.997	-0.123488048	count	1
DLX5	-16.700967	4059.590711	-0.0041	0.997	-0.123488048	count	1
TMEM130	-16.700967	4059.590711	-0.0041	0.997	-0.123488048	count	1
AC093668.3	-16.700967	4059.590711	-0.0041	0.997	-0.123488048	count	1
NFE4	-16.700967	4059.590711	-0.0041	0.997	-0.123488048	count	1
AC005046.1	-16.700967	4059.590711	-0.0041	0.997	-0.123488048	count	1
TSPAN12	-16.700967	4059.590711	-0.0041	0.997	-0.123488048	count	1
FSCN3	-16.700967	4059.590711	-0.0041	0.997	-0.123488048	count	1
SMKR1	-16.700967	4059.590711	-0.0041	0.997	-0.123488048	count	1
ZC3HAV1L	-16.700967	4059.590711	-0.0041	0.997	-0.123488048	count	1
DENND2A	-16.700967	4059.590711	-0.0041	0.997	-0.123488048	count	1
CTAGE6	-16.700967	4059.590711	-0.0041	0.997	-0.123488048	count	1
AC073314.1	-16.700967	4059.590711	-0.0041	0.997	-0.123488048	count	1
AC008060.1	-16.700967	4059.590711	-0.0041	0.997	-0.123488048	count	1
AC004554.2	-16.700967	4059.590711	-0.0041	0.997	-0.123488048	count	1
LINC02154	-16.700967	4059.590711	-0.0041	0.997	-0.123488048	count	1
CLTRN	-16.700967	4059.590711	-0.0041	0.997	-0.123488048	count	1
LINC01281	-16.700967	4059.590711	-0.0041	0.997	-0.123488048	count	1
RGN	-16.700967	4059.590711	-0.0041	0.997	-0.123488048	count	1
ZNF157	-16.700967	4059.590711	-0.0041	0.997	-0.123488048	count	1
FAM156B	-16.700967	4059.590711	-0.0041	0.997	-0.123488048	count	1
ZNF711	-16.700967	4059.590711	-0.0041	0.997	-0.123488048	count	1
PABPC5	-16.700967	4059.590711	-0.0041	0.997	-0.123488048	count	1
TSPAN6	-16.700967	4059.590711	-0.0041	0.997	-0.123488048	count	1
BHLHB9	-16.700967	4059.590711	-0.0041	0.997	-0.123488048	count	1
TCEAL5	-16.700967	4059.590711	-0.0041	0.997	-0.123488048	count	1
TMEM31	-16.700967	4059.590711	-0.0041	0.997	-0.123488048	count	1
TMSB15B-AS1	-16.700967	4059.590711	-0.0041	0.997	-0.123488048	count	1

ALG13-AS1	-16.700967	4059.590711	-0.0041	0.997	-0.123488048	count	1
SH2D1A	-16.700967	4059.590711	-0.0041	0.997	-0.123488048	count	1
INTS6L-AS1	-16.700967	4059.590711	-0.0041	0.997	-0.123488048	count	1
FMR1NB	-16.700967	4059.590711	-0.0041	0.997	-0.123488048	count	1
AC004908.3	-16.700967	4059.590711	-0.0041	0.997	-0.123488048	count	1
TDRP	-16.700967	4059.590711	-0.0041	0.997	-0.123488048	count	1
AC104964.4	-16.700967	4059.590711	-0.0041	0.997	-0.123488048	count	1
FAM66D	-16.700967	4059.590711	-0.0041	0.997	-0.123488048	count	1
FAM86B2	-16.700967	4059.590711	-0.0041	0.997	-0.123488048	count	1
TRMT9B	-16.700967	4059.590711	-0.0041	0.997	-0.123488048	count	1
LZTS1	-16.700967	4059.590711	-0.0041	0.997	-0.123488048	count	1
AC100861.2	-16.700967	4059.590711	-0.0041	0.997	-0.123488048	count	1
PBK	-16.700967	4059.590711	-0.0041	0.997	-0.123488048	count	1
MBOAT4	-16.700967	4059.590711	-0.0041	0.997	-0.123488048	count	1
AC026979.3	-16.700967	4059.590711	-0.0041	0.997	-0.123488048	count	1
AC103724.4	-16.700967	4059.590711	-0.0041	0.997	-0.123488048	count	1
AC012103.1	-16.700967	4059.590711	-0.0041	0.997	-0.123488048	count	1
LACTB2-AS1	-16.700967	4059.590711	-0.0041	0.997	-0.123488048	count	1
AC022274.1	-16.700967	4059.590711	-0.0041	0.997	-0.123488048	count	1
AC036214.1	-16.700967	4059.590711	-0.0041	0.997	-0.123488048	count	1
AC009686.2	-16.700967	4059.590711	-0.0041	0.997	-0.123488048	count	1
AC018616.1	-16.700967	4059.590711	-0.0041	0.997	-0.123488048	count	1
AC010834.2	-16.700967	4059.590711	-0.0041	0.997	-0.123488048	count	1
AC023632.2	-16.700967	4059.590711	-0.0041	0.997	-0.123488048	count	1
AC083836.1	-16.700967	4059.590711	-0.0041	0.997	-0.123488048	count	1
AC012213.3	-16.700967	4059.590711	-0.0041	0.997	-0.123488048	count	1
DPYS	-16.700967	4059.590711	-0.0041	0.997	-0.123488048	count	1
ZFPM2	-16.700967	4059.590711	-0.0041	0.997	-0.123488048	count	1
RAD21-AS1	-16.700967	4059.590711	-0.0041	0.997	-0.123488048	count	1
LINC00861	-16.700967	4059.590711	-0.0041	0.997	-0.123488048	count	1
CCDC26	-16.700967	4059.590711	-0.0041	0.997	-0.123488048	count	1
AC087045.2	-16.700967	4059.590711	-0.0041	0.997	-0.123488048	count	1
AC134682.1	-16.700967	4059.590711	-0.0041	0.997	-0.123488048	count	1
LY6H	-16.700967	4059.590711	-0.0041	0.997	-0.123488048	count	1
RHPN1-AS1	-16.700967	4059.590711	-0.0041	0.997	-0.123488048	count	1
AC084125.1	-16.700967	4059.590711	-0.0041	0.997	-0.123488048	count	1
VLDLR-AS1	-16.700967	4059.590711	-0.0041	0.997	-0.123488048	count	1
AL135786.2	-16.700967	4059.590711	-0.0041	0.997	-0.123488048	count	1
AL360014.1	-16.700967	4059.590711	-0.0041	0.997	-0.123488048	count	1
AL137847.2	-16.700967	4059.590711	-0.0041	0.997	-0.123488048	count	1
SHC3	-16.700967	4059.590711	-0.0041	0.997	-0.123488048	count	1
BARX1	-16.700967	4059.590711	-0.0041	0.997	-0.123488048	count	1
AL359182.1	-16.700967	4059.590711	-0.0041	0.997	-0.123488048	count	1
FAM225B	-16.700967	4059.590711	-0.0041	0.997	-0.123488048	count	1
RNF183	-16.700967	4059.590711	-0.0041	0.997	-0.123488048	count	1

WDR31	-16.700967	4059.590711	-0.0041	0.997	-0.123488048	count	1
BSPRY	-16.700967	4059.590711	-0.0041	0.997	-0.123488048	count	1
C9orf43	-16.700967	4059.590711	-0.0041	0.997	-0.123488048	count	1
AL157935.2	-16.700967	4059.590711	-0.0041	0.997	-0.123488048	count	1
QRFP	-16.700967	4059.590711	-0.0041	0.997	-0.123488048	count	1
AIF1L	-16.700967	4059.590711	-0.0041	0.997	-0.123488048	count	1
AK8	-16.700967	4059.590711	-0.0041	0.997	-0.123488048	count	1
GFI1B	-16.700967	4059.590711	-0.0041	0.997	-0.123488048	count	1
DRD4	-16.700967	4059.590711	-0.0041	0.997	-0.123488048	count	1
TRIM34	-16.700967	4059.590711	-0.0041	0.997	-0.123488048	count	1
AC091564.2	-16.700967	4059.590711	-0.0041	0.997	-0.123488048	count	1
OR10A2	-16.700967	4059.590711	-0.0041	0.997	-0.123488048	count	1
AC069360.1	-16.700967	4059.590711	-0.0041	0.997	-0.123488048	count	1
GALNT18	-16.700967	4059.590711	-0.0041	0.997	-0.123488048	count	1
LINC00958	-16.700967	4059.590711	-0.0041	0.997	-0.123488048	count	1
CALCB	-16.700967	4059.590711	-0.0041	0.997	-0.123488048	count	1
KCNC1	-16.700967	4059.590711	-0.0041	0.997	-0.123488048	count	1
AC009549.1	-16.700967	4059.590711	-0.0041	0.997	-0.123488048	count	1
CSTF3-DT	-16.700967	4059.590711	-0.0041	0.997	-0.123488048	count	1
AL356215.1	-16.700967	4059.590711	-0.0041	0.997	-0.123488048	count	1
FJX1	-16.700967	4059.590711	-0.0041	0.997	-0.123488048	count	1
AC087521.1	-16.700967	4059.590711	-0.0041	0.997	-0.123488048	count	1
C11orf94	-16.700967	4059.590711	-0.0041	0.997	-0.123488048	count	1
AGBL2	-16.700967	4059.590711	-0.0041	0.997	-0.123488048	count	1
OR5B21	-16.700967	4059.590711	-0.0041	0.997	-0.123488048	count	1
AP000442.2	-16.700967	4059.590711	-0.0041	0.997	-0.123488048	count	1
MS4A2	-16.700967	4059.590711	-0.0041	0.997	-0.123488048	count	1
AP000777.2	-16.700967	4059.590711	-0.0041	0.997	-0.123488048	count	1
AP003721.4	-16.700967	4059.590711	-0.0041	0.997	-0.123488048	count	1
C11orf95	-16.700967	4059.590711	-0.0041	0.997	-0.123488048	count	1
OVOL1	-16.700967	4059.590711	-0.0041	0.997	-0.123488048	count	1
SNX32	-16.700967	4059.590711	-0.0041	0.997	-0.123488048	count	1
SLC29A2	-16.700967	4059.590711	-0.0041	0.997	-0.123488048	count	1
AP003716.1	-16.700967	4059.590711	-0.0041	0.997	-0.123488048	count	1
ARAP1-AS2	-16.700967	4059.590711	-0.0041	0.997	-0.123488048	count	1
AP001922.5	-16.700967	4059.590711	-0.0041	0.997	-0.123488048	count	1
AP002360.3	-16.700967	4059.590711	-0.0041	0.997	-0.123488048	count	1
AP001189.6	-16.700967	4059.590711	-0.0041	0.997	-0.123488048	count	1
KCTD14	-16.700967	4059.590711	-0.0041	0.997	-0.123488048	count	1
AP003063.1	-16.700967	4059.590711	-0.0041	0.997	-0.123488048	count	1
DSCAML1	-16.700967	4059.590711	-0.0041	0.997	-0.123488048	count	1
USP2-AS1	-16.700967	4059.590711	-0.0041	0.997	-0.123488048	count	1
AP003501.2	-16.700967	4059.590711	-0.0041	0.997	-0.123488048	count	1
AP001318.2	-16.700967	4059.590711	-0.0041	0.997	-0.123488048	count	1
AL590068.1	-16.700967	4059.590711	-0.0041	0.997	-0.123488048	count	1

ZNF239	-16.700967	4059.590711	-0.0041	0.997	-0.123488048	count	1
AL137026.1	-16.700967	4059.590711	-0.0041	0.997	-0.123488048	count	1
OR13A1	-16.700967	4059.590711	-0.0041	0.997	-0.123488048	count	1
PRF1	-16.700967	4059.590711	-0.0041	0.997	-0.123488048	count	1
DNAJC9-AS1	-16.700967	4059.590711	-0.0041	0.997	-0.123488048	count	1
AL139241.1	-16.700967	4059.590711	-0.0041	0.997	-0.123488048	count	1
NKX2-3	-16.700967	4059.590711	-0.0041	0.997	-0.123488048	count	1
INA	-16.700967	4059.590711	-0.0041	0.997	-0.123488048	count	1
TDRD1	-16.700967	4059.590711	-0.0041	0.997	-0.123488048	count	1
AL390763.1	-16.700967	4059.590711	-0.0041	0.997	-0.123488048	count	1
AL162274.2	-16.700967	4059.590711	-0.0041	0.997	-0.123488048	count	1
AL162274.1	-16.700967	4059.590711	-0.0041	0.997	-0.123488048	count	1
LINC00942	-16.700967	4059.590711	-0.0041	0.997	-0.123488048	count	1
AC005842.1	-16.700967	4059.590711	-0.0041	0.997	-0.123488048	count	1
GALNT8	-16.700967	4059.590711	-0.0041	0.997	-0.123488048	count	1
AC006064.1	-16.700967	4059.590711	-0.0041	0.997	-0.123488048	count	1
A2ML1	-16.700967	4059.590711	-0.0041	0.997	-0.123488048	count	1
AC008115.4	-16.700967	4059.590711	-0.0041	0.997	-0.123488048	count	1
AC125611.3	-16.700967	4059.590711	-0.0041	0.997	-0.123488048	count	1
FIGNL2	-16.700967	4059.590711	-0.0041	0.997	-0.123488048	count	1
AC034102.6	-16.700967	4059.590711	-0.0041	0.997	-0.123488048	count	1
LRP1-AS	-16.700967	4059.590711	-0.0041	0.997	-0.123488048	count	1
DTX3	-16.700967	4059.590711	-0.0041	0.997	-0.123488048	count	1
DPY19L2	-16.700967	4059.590711	-0.0041	0.997	-0.123488048	count	1
GLIPR1L1	-16.700967	4059.590711	-0.0041	0.997	-0.123488048	count	1
AC011611.3	-16.700967	4059.590711	-0.0041	0.997	-0.123488048	count	1
AC107032.2	-16.700967	4059.590711	-0.0041	0.997	-0.123488048	count	1
C12orf50	-16.700967	4059.590711	-0.0041	0.997	-0.123488048	count	1
AC024909.1	-16.700967	4059.590711	-0.0041	0.997	-0.123488048	count	1
AC138123.1	-16.700967	4059.590711	-0.0041	0.997	-0.123488048	count	1
AC073655.2	-16.700967	4059.590711	-0.0041	0.997	-0.123488048	count	1
AC089983.1	-16.700967	4059.590711	-0.0041	0.997	-0.123488048	count	1
AC079385.2	-16.700967	4059.590711	-0.0041	0.997	-0.123488048	count	1
TBX3	-16.700967	4059.590711	-0.0041	0.997	-0.123488048	count	1
KSR2	-16.700967	4059.590711	-0.0041	0.997	-0.123488048	count	1
AC131159.2	-16.700967	4059.590711	-0.0041	0.997	-0.123488048	count	1
TMEM233	-16.700967	4059.590711	-0.0041	0.997	-0.123488048	count	1
PITPNM2	-16.700967	4059.590711	-0.0041	0.997	-0.123488048	count	1
AC068790.8	-16.700967	4059.590711	-0.0041	0.997	-0.123488048	count	1
PABPC3	-16.700967	4059.590711	-0.0041	0.997	-0.123488048	count	1
LINC02340	-16.700967	4059.590711	-0.0041	0.997	-0.123488048	count	1
UBE2L5	-16.700967	4059.590711	-0.0041	0.997	-0.123488048	count	1
LINC00545	-16.700967	4059.590711	-0.0041	0.997	-0.123488048	count	1
AL138820.1	-16.700967	4059.590711	-0.0041	0.997	-0.123488048	count	1
AL354696.2	-16.700967	4059.590711	-0.0041	0.997	-0.123488048	count	1

TUSC8	-16.700967	4059.590711	-0.0041	0.997	-0.123488048	count	1
LINC00330	-16.700967	4059.590711	-0.0041	0.997	-0.123488048	count	1
TEX29	-16.700967	4059.590711	-0.0041	0.997	-0.123488048	count	1
ATP11A-AS1	-16.700967	4059.590711	-0.0041	0.997	-0.123488048	count	1
GRTP1	-16.700967	4059.590711	-0.0041	0.997	-0.123488048	count	1
AL136295.6	-16.700967	4059.590711	-0.0041	0.997	-0.123488048	count	1
AL049830.3	-16.700967	4059.590711	-0.0041	0.997	-0.123488048	count	1
AL139353.2	-16.700967	4059.590711	-0.0041	0.997	-0.123488048	count	1
AL133163.2	-16.700967	4059.590711	-0.0041	0.997	-0.123488048	count	1
AL132639.3	-16.700967	4059.590711	-0.0041	0.997	-0.123488048	count	1
AL049875.1	-16.700967	4059.590711	-0.0041	0.997	-0.123488048	count	1
LINC02315	-16.700967	4059.590711	-0.0041	0.997	-0.123488048	count	1
AL139099.1	-16.700967	4059.590711	-0.0041	0.997	-0.123488048	count	1
AL139317.3	-16.700967	4059.590711	-0.0041	0.997	-0.123488048	count	1
LINC00520	-16.700967	4059.590711	-0.0041	0.997	-0.123488048	count	1
SIX1	-16.700967	4059.590711	-0.0041	0.997	-0.123488048	count	1
FAM71D	-16.700967	4059.590711	-0.0041	0.997	-0.123488048	count	1
PLEKHH1	-16.700967	4059.590711	-0.0041	0.997	-0.123488048	count	1
ACTN1-AS1	-16.700967	4059.590711	-0.0041	0.997	-0.123488048	count	1
PLEKHD1	-16.700967	4059.590711	-0.0041	0.997	-0.123488048	count	1
KCNK10	-16.700967	4059.590711	-0.0041	0.997	-0.123488048	count	1
AL162171.1	-16.700967	4059.590711	-0.0041	0.997	-0.123488048	count	1
BCL11B	-16.700967	4059.590711	-0.0041	0.997	-0.123488048	count	1
BEGAIN	-16.700967	4059.590711	-0.0041	0.997	-0.123488048	count	1
AL138976.2	-16.700967	4059.590711	-0.0041	0.997	-0.123488048	count	1
AL583722.4	-16.700967	4059.590711	-0.0041	0.997	-0.123488048	count	1
GOLGA8M	-16.700967	4059.590711	-0.0041	0.997	-0.123488048	count	1
AC055876.5	-16.700967	4059.590711	-0.0041	0.997	-0.123488048	count	1
AC091057.6	-16.700967	4059.590711	-0.0041	0.997	-0.123488048	count	1
AC010809.2	-16.700967	4059.590711	-0.0041	0.997	-0.123488048	count	1
RASGRP1	-16.700967	4059.590711	-0.0041	0.997	-0.123488048	count	1
AC022929.2	-16.700967	4059.590711	-0.0041	0.997	-0.123488048	count	1
AC020661.1	-16.700967	4059.590711	-0.0041	0.997	-0.123488048	count	1
CHAC1	-16.700967	4059.590711	-0.0041	0.997	-0.123488048	count	1
AC022306.2	-16.700967	4059.590711	-0.0041	0.997	-0.123488048	count	1
AC022087.1	-16.700967	4059.590711	-0.0041	0.997	-0.123488048	count	1
AC092755.2	-16.700967	4059.590711	-0.0041	0.997	-0.123488048	count	1
AC100827.4	-16.700967	4059.590711	-0.0041	0.997	-0.123488048	count	1
C15orf59	-16.700967	4059.590711	-0.0041	0.997	-0.123488048	count	1
AC090260.1	-16.700967	4059.590711	-0.0041	0.997	-0.123488048	count	1
AC016705.1	-16.700967	4059.590711	-0.0041	0.997	-0.123488048	count	1
TMC3-AS1	-16.700967	4059.590711	-0.0041	0.997	-0.123488048	count	1
PCSK6-AS1	-16.700967	4059.590711	-0.0041	0.997	-0.123488048	count	1
AC004754.1	-16.700967	4059.590711	-0.0041	0.997	-0.123488048	count	1
CCDC78	-16.700967	4059.590711	-0.0041	0.997	-0.123488048	count	1

TPSAB1	-16.700967	4059.590711	-0.0041	0.997	-0.123488048	count	1
AC120498.9	-16.700967	4059.590711	-0.0041	0.997	-0.123488048	count	1
BAIAP3	-16.700967	4059.590711	-0.0041	0.997	-0.123488048	count	1
SYNGR3	-16.700967	4059.590711	-0.0041	0.997	-0.123488048	count	1
NPW	-16.700967	4059.590711	-0.0041	0.997	-0.123488048	count	1
TEKT5	-16.700967	4059.590711	-0.0041	0.997	-0.123488048	count	1
AC130456.5	-16.700967	4059.590711	-0.0041	0.997	-0.123488048	count	1
AC092375.2	-16.700967	4059.590711	-0.0041	0.997	-0.123488048	count	1
AC092338.2	-16.700967	4059.590711	-0.0041	0.997	-0.123488048	count	1
AC109460.2	-16.700967	4059.590711	-0.0041	0.997	-0.123488048	count	1
AC009133.2	-16.700967	4059.590711	-0.0041	0.997	-0.123488048	count	1
AC106886.3	-16.700967	4059.590711	-0.0041	0.997	-0.123488048	count	1
PYDC1	-16.700967	4059.590711	-0.0041	0.997	-0.123488048	count	1
ITGAD	-16.700967	4059.590711	-0.0041	0.997	-0.123488048	count	1
AC018845.3	-16.700967	4059.590711	-0.0041	0.997	-0.123488048	count	1
ZNF423	-16.700967	4059.590711	-0.0041	0.997	-0.123488048	count	1
AC007610.1	-16.700967	4059.590711	-0.0041	0.997	-0.123488048	count	1
AC007493.2	-16.700967	4059.590711	-0.0041	0.997	-0.123488048	count	1
DOK4	-16.700967	4059.590711	-0.0041	0.997	-0.123488048	count	1
AC092118.2	-16.700967	4059.590711	-0.0041	0.997	-0.123488048	count	1
NDRG4	-16.700967	4059.590711	-0.0041	0.997	-0.123488048	count	1
CDH11	-16.700967	4059.590711	-0.0041	0.997	-0.123488048	count	1
LINC01572	-16.700967	4059.590711	-0.0041	0.997	-0.123488048	count	1
AC092718.3	-16.700967	4059.590711	-0.0041	0.997	-0.123488048	count	1
AC126696.1	-16.700967	4059.590711	-0.0041	0.997	-0.123488048	count	1
LINC02166	-16.700967	4059.590711	-0.0041	0.997	-0.123488048	count	1
AC087392.1	-16.700967	4059.590711	-0.0041	0.997	-0.123488048	count	1
AC027796.3	-16.700967	4059.590711	-0.0041	0.997	-0.123488048	count	1
BCL6B	-16.700967	4059.590711	-0.0041	0.997	-0.123488048	count	1
SLC35G6	-16.700967	4059.590711	-0.0041	0.997	-0.123488048	count	1
GUCY2D	-16.700967	4059.590711	-0.0041	0.997	-0.123488048	count	1
MFSD6L	-16.700967	4059.590711	-0.0041	0.997	-0.123488048	count	1
AC002091.1	-16.700967	4059.590711	-0.0041	0.997	-0.123488048	count	1
HS3ST3A1	-16.700967	4059.590711	-0.0041	0.997	-0.123488048	count	1
DRC3	-16.700967	4059.590711	-0.0041	0.997	-0.123488048	count	1
AC007952.7	-16.700967	4059.590711	-0.0041	0.997	-0.123488048	count	1
VTN	-16.700967	4059.590711	-0.0041	0.997	-0.123488048	count	1
AC104984.1	-16.700967	4059.590711	-0.0041	0.997	-0.123488048	count	1
AC005899.5	-16.700967	4059.590711	-0.0041	0.997	-0.123488048	count	1
AC060766.4	-16.700967	4059.590711	-0.0041	0.997	-0.123488048	count	1
RASL10B	-16.700967	4059.590711	-0.0041	0.997	-0.123488048	count	1
SRCIN1	-16.700967	4059.590711	-0.0041	0.997	-0.123488048	count	1
AC004231.1	-16.700967	4059.590711	-0.0041	0.997	-0.123488048	count	1
ZNF385C	-16.700967	4059.590711	-0.0041	0.997	-0.123488048	count	1
C17orf113	-16.700967	4059.590711	-0.0041	0.997	-0.123488048	count	1

AC099811.3	-16.700967	4059.590711	-0.0041	0.997	-0.123488048	count	1
SOST	-16.700967	4059.590711	-0.0041	0.997	-0.123488048	count	1
ITGA2B	-16.700967	4059.590711	-0.0041	0.997	-0.123488048	count	1
AC005670.1	-16.700967	4059.590711	-0.0041	0.997	-0.123488048	count	1
TMEM92	-16.700967	4059.590711	-0.0041	0.997	-0.123488048	count	1
AC015813.4	-16.700967	4059.590711	-0.0041	0.997	-0.123488048	count	1
AC015813.3	-16.700967	4059.590711	-0.0041	0.997	-0.123488048	count	1
AC005972.1	-16.700967	4059.590711	-0.0041	0.997	-0.123488048	count	1
AC005332.3	-16.700967	4059.590711	-0.0041	0.997	-0.123488048	count	1
KIF19	-16.700967	4059.590711	-0.0041	0.997	-0.123488048	count	1
AC021683.1	-16.700967	4059.590711	-0.0041	0.997	-0.123488048	count	1
C1QTNF1	-16.700967	4059.590711	-0.0041	0.997	-0.123488048	count	1
AC145207.3	-16.700967	4059.590711	-0.0041	0.997	-0.123488048	count	1
PYCR1	-16.700967	4059.590711	-0.0041	0.997	-0.123488048	count	1
AC139099.3	-16.700967	4059.590711	-0.0041	0.997	-0.123488048	count	1
AC139099.1	-16.700967	4059.590711	-0.0041	0.997	-0.123488048	count	1
AP001496.2	-16.700967	4059.590711	-0.0041	0.997	-0.123488048	count	1
AP005059.2	-16.700967	4059.590711	-0.0041	0.997	-0.123488048	count	1
AP005482.1	-16.700967	4059.590711	-0.0041	0.997	-0.123488048	count	1
GATA6-AS1	-16.700967	4059.590711	-0.0041	0.997	-0.123488048	count	1
AC090912.2	-16.700967	4059.590711	-0.0041	0.997	-0.123488048	count	1
LAMA3	-16.700967	4059.590711	-0.0041	0.997	-0.123488048	count	1
AC012417.1	-16.700967	4059.590711	-0.0041	0.997	-0.123488048	count	1
SLC14A1	-16.700967	4059.590711	-0.0041	0.997	-0.123488048	count	1
SIRPG	-16.700967	4059.590711	-0.0041	0.997	-0.123488048	count	1
AL049712.1	-16.700967	4059.590711	-0.0041	0.997	-0.123488048	count	1
PRND	-16.700967	4059.590711	-0.0041	0.997	-0.123488048	count	1
AL133396.2	-16.700967	4059.590711	-0.0041	0.997	-0.123488048	count	1
AL121890.2	-16.700967	4059.590711	-0.0041	0.997	-0.123488048	count	1
CHGB	-16.700967	4059.590711	-0.0041	0.997	-0.123488048	count	1
AL021396.1	-16.700967	4059.590711	-0.0041	0.997	-0.123488048	count	1
PARAL1	-16.700967	4059.590711	-0.0041	0.997	-0.123488048	count	1
SNAP25	-16.700967	4059.590711	-0.0041	0.997	-0.123488048	count	1
LINC01752	-16.700967	4059.590711	-0.0041	0.997	-0.123488048	count	1
AL161938.1	-16.700967	4059.590711	-0.0041	0.997	-0.123488048	count	1
AL121892.1	-16.700967	4059.590711	-0.0041	0.997	-0.123488048	count	1
AL121761.2	-16.700967	4059.590711	-0.0041	0.997	-0.123488048	count	1
AL035252.3	-16.700967	4059.590711	-0.0041	0.997	-0.123488048	count	1
NINL	-16.700967	4059.590711	-0.0041	0.997	-0.123488048	count	1
FOXS1	-16.700967	4059.590711	-0.0041	0.997	-0.123488048	count	1
TTLL9	-16.700967	4059.590711	-0.0041	0.997	-0.123488048	count	1
AL133227.1	-16.700967	4059.590711	-0.0041	0.997	-0.123488048	count	1
AL031666.1	-16.700967	4059.590711	-0.0041	0.997	-0.123488048	count	1
AL157838.1	-16.700967	4059.590711	-0.0041	0.997	-0.123488048	count	1
EEF1A2	-16.700967	4059.590711	-0.0041	0.997	-0.123488048	count	1

LINC00266-1	-16.700967	4059.590711	-0.0041	0.997	-0.123488048	count	1
FGF22	-16.700967	4059.590711	-0.0041	0.997	-0.123488048	count	1
ANKRD24	-16.700967	4059.590711	-0.0041	0.997	-0.123488048	count	1
AC005339.1	-16.700967	4059.590711	-0.0041	0.997	-0.123488048	count	1
TEX45	-16.700967	4059.590711	-0.0041	0.997	-0.123488048	count	1
CAMSAP3	-16.700967	4059.590711	-0.0041	0.997	-0.123488048	count	1
AC011451.1	-16.700967	4059.590711	-0.0041	0.997	-0.123488048	count	1
ZNF559-ZNF177	-16.700967	4059.590711	-0.0041	0.997	-0.123488048	count	1
COL5A3	-16.700967	4059.590711	-0.0041	0.997	-0.123488048	count	1
AC011472.1	-16.700967	4059.590711	-0.0041	0.997	-0.123488048	count	1
ELAVL3	-16.700967	4059.590711	-0.0041	0.997	-0.123488048	count	1
MAST1	-16.700967	4059.590711	-0.0041	0.997	-0.123488048	count	1
AC011446.1	-16.700967	4059.590711	-0.0041	0.997	-0.123488048	count	1
AC010319.4	-16.700967	4059.590711	-0.0041	0.997	-0.123488048	count	1
UNC13A	-16.700967	4059.590711	-0.0041	0.997	-0.123488048	count	1
TSSK6	-16.700967	4059.590711	-0.0041	0.997	-0.123488048	count	1
ZNF730	-16.700967	4059.590711	-0.0041	0.997	-0.123488048	count	1
ZNF724	-16.700967	4059.590711	-0.0041	0.997	-0.123488048	count	1
RGS9BP	-16.700967	4059.590711	-0.0041	0.997	-0.123488048	count	1
AC008747.1	-16.700967	4059.590711	-0.0041	0.997	-0.123488048	count	1
WTIP	-16.700967	4059.590711	-0.0041	0.997	-0.123488048	count	1
FXYD7	-16.700967	4059.590711	-0.0041	0.997	-0.123488048	count	1
AD000090.1	-16.700967	4059.590711	-0.0041	0.997	-0.123488048	count	1
AD000671.3	-16.700967	4059.590711	-0.0041	0.997	-0.123488048	count	1
NPHS1	-16.700967	4059.590711	-0.0041	0.997	-0.123488048	count	1
GGN	-16.700967	4059.590711	-0.0041	0.997	-0.123488048	count	1
LGALS4	-16.700967	4059.590711	-0.0041	0.997	-0.123488048	count	1
ZNF285	-16.700967	4059.590711	-0.0041	0.997	-0.123488048	count	1
GIPR	-16.700967	4059.590711	-0.0041	0.997	-0.123488048	count	1
DACT3-AS1	-16.700967	4059.590711	-0.0041	0.997	-0.123488048	count	1
KCNA7	-16.700967	4059.590711	-0.0041	0.997	-0.123488048	count	1
AC063977.6	-16.700967	4059.590711	-0.0041	0.997	-0.123488048	count	1
AC008750.2	-16.700967	4059.590711	-0.0041	0.997	-0.123488048	count	1
AC092070.3	-16.700967	4059.590711	-0.0041	0.997	-0.123488048	count	1
KIR2DL4	-16.700967	4059.590711	-0.0041	0.997	-0.123488048	count	1
AC020922.4	-16.700967	4059.590711	-0.0041	0.997	-0.123488048	count	1
ZNF667	-16.700967	4059.590711	-0.0041	0.997	-0.123488048	count	1
AC005498.2	-16.700967	4059.590711	-0.0041	0.997	-0.123488048	count	1
ZNF154	-16.700967	4059.590711	-0.0041	0.997	-0.123488048	count	1
AC012313.8	-16.700967	4059.590711	-0.0041	0.997	-0.123488048	count	1
AC012313.7	-16.700967	4059.590711	-0.0041	0.997	-0.123488048	count	1
PCDH11Y	-16.700967	4059.590711	-0.0041	0.997	-0.123488048	count	1
HDHD5-AS1	-16.700967	4059.590711	-0.0041	0.997	-0.123488048	count	1
RTN4R	-16.700967	4059.590711	-0.0041	0.997	-0.123488048	count	1
AC007731.3	-16.700967	4059.590711	-0.0041	0.997	-0.123488048	count	1

LRRC74B	-16.700967	4059.590711	-0.0041	0.997	-0.123488048	count	1
AP000550.1	-16.700967	4059.590711	-0.0041	0.997	-0.123488048	count	1
RAB36	-16.700967	4059.590711	-0.0041	0.997	-0.123488048	count	1
Z97353.2	-16.700967	4059.590711	-0.0041	0.997	-0.123488048	count	1
C22orf42	-16.700967	4059.590711	-0.0041	0.997	-0.123488048	count	1
PDXP	-16.700967	4059.590711	-0.0041	0.997	-0.123488048	count	1
KDELR3	-16.700967	4059.590711	-0.0041	0.997	-0.123488048	count	1
DMC1	-16.700967	4059.590711	-0.0041	0.997	-0.123488048	count	1
FAM83F	-16.700967	4059.590711	-0.0041	0.997	-0.123488048	count	1
PNPLA3	-16.700967	4059.590711	-0.0041	0.997	-0.123488048	count	1
AL021392.1	-16.700967	4059.590711	-0.0041	0.997	-0.123488048	count	1
CU638689.4	-16.700967	4059.590711	-0.0041	0.997	-0.123488048	count	1
CU638689.5	-16.700967	4059.590711	-0.0041	0.997	-0.123488048	count	1
FP236241.1	-16.700967	4059.590711	-0.0041	0.997	-0.123488048	count	1
SMIM11B	-16.700967	4059.590711	-0.0041	0.997	-0.123488048	count	1
AF127577.1	-16.700967	4059.590711	-0.0041	0.997	-0.123488048	count	1
AF124730.1	-16.700967	4059.590711	-0.0041	0.997	-0.123488048	count	1
AP000311.1	-16.700967	4059.590711	-0.0041	0.997	-0.123488048	count	1
AP000704.1	-16.700967	4059.590711	-0.0041	0.997	-0.123488048	count	1
LCA5L	-16.700967	4059.590711	-0.0041	0.997	-0.123488048	count	1
ZNF295-AS1	-16.700967	4059.590711	-0.0041	0.997	-0.123488048	count	1
AC145212.1	-16.700967	4059.590711	-0.0041	0.997	-0.123488048	count	1
WDR4	-0.1655162	0.4500587	-0.3678	0.713	-0.123367269	count	1
RGS10	-0.0861327	0.0548783	-1.5695	0.117	-0.12330862	count	1
PEX13	-0.103728	0.2712479	-0.3824	0.702	-0.123219986	count	1
FKBP7	-0.1600179	0.5895013	-0.2714	0.786	-0.123098213	count	1
PUF60	-0.0886744	0.1005412	-0.882	0.378	-0.123073701	count	1
SEC14L1	-0.088985	0.0995422	-0.8939	0.371	-0.122928472	count	1
CD320	-0.147706	0.4535686	-0.3257	0.745	-0.122897919	count	1
CYP2U1	-0.1596917	0.3982073	-0.401	0.688	-0.122854123	count	1
PAFAH1B1	-0.0873948	0.0890038	-0.9819	0.326	-0.122785582	count	1
SERTAD3	-0.0879854	0.1064506	-0.8265	0.409	-0.122747472	count	1
RAP2C	-0.0975516	0.2035035	-0.4794	0.632	-0.12267123	count	1
PIK3C2B	-0.2933049	0.7979251	-0.3676	0.713	-0.122669436	count	1
SLC20A1	-0.0916254	0.1521486	-0.6022	0.547	-0.122662159	count	1
NFE2L2	-0.0857365	0.0570717	-1.5023	0.133	-0.122608584	count	1
GPD1L	-0.3389565	0.4977247	-0.681	0.496	-0.122602553	count	1
SUB1	-0.0855709	0.050616	-1.6906	0.091	-0.122563147	count	1
EPHB2	-0.1097607	0.3595828	-0.3052	0.76	-0.12249808	count	1
C1QC	-0.0850731	0.0774125	-1.099	0.272	-0.122495109	count	1
CDC42BPA	-0.2604079	0.3756152	-0.6933	0.488	-0.122188442	count	1
RAP1B	-0.085875	0.0565792	-1.5178	0.129	-0.122170042	count	1
MGAT1	-0.0862405	0.062577	-1.3782	0.168	-0.122163746	count	1
RPS5	-0.0848548	0.0308845	-2.7475	0.00605	-0.122115552	count	1
MAGIX	-0.1936039	0.4523615	-0.428	0.669	-0.122036102	count	1

PRRC2A	-0.0993993	0.2219079	-0.4479	0.654	-0.121971491	count	1
KCTD7	-0.1143623	0.386752	-0.2957	0.767	-0.121916705	count	1
TMC6	-0.0927409	0.1598649	-0.5801	0.562	-0.121892553	count	1
HIVEP1	-0.0981702	0.1996574	-0.4917	0.623	-0.121770605	count	1
GRN	-0.0847782	0.0407941	-2.0782	0.0378	-0.121746418	count	1
MSH3	-0.1311705	0.272289	-0.4817	0.63	-0.121738314	count	1
UBE2A	-0.0862576	0.0808411	-1.067	0.286	-0.121699422	count	1
AC091271.1	-0.0989243	0.2473893	-0.3999	0.689	-0.121621783	count	1
ICE2	-0.1001279	0.2581765	-0.3878	0.698	-0.121615648	count	1
NDUFA4	-0.0847723	0.0440019	-1.9266	0.0541	-0.121449689	count	1
ATP6V1G1	-0.0845959	0.0427544	-1.9786	0.048	-0.121433616	count	1
GALNT1	-0.0897549	0.1240387	-0.7236	0.469	-0.121400644	count	1
CISD1	-0.0924578	0.2075885	-0.4454	0.656	-0.121365107	count	1
AC108134.2	-0.3351571	0.9189327	-0.3647	0.715	-0.121356099	count	1
C11orf58	-0.0850549	0.0570399	-1.4911	0.136	-0.121304199	count	1
PEX7	-0.1241632	0.3983215	-0.3117	0.755	-0.121296191	count	1
SARAF	-0.0848312	0.043997	-1.9281	0.054	-0.121279504	count	1
KDELC2	-0.4055057	0.4643691	-0.8732	0.383	-0.12118957	count	1
QKI	-0.0850214	0.0622693	-1.3654	0.172	-0.121122236	count	1
GFPT1	-0.104009	0.2696138	-0.3858	0.7	-0.120929029	count	1
TFDP1	-0.0929242	0.1796559	-0.5172	0.605	-0.120842546	count	1
CAMLG	-0.0879157	0.1415735	-0.621	0.535	-0.1202941	count	1
KIAA0556	-0.1811666	0.4649758	-0.3896	0.697	-0.12011071	count	1
EIF3J	-0.085169	0.0985962	-0.8638	0.388	-0.119796608	count	1
TCEAL3	-0.086913	0.1380777	-0.6294	0.529	-0.119754641	count	1
SLC4A7	-0.0854821	0.1186049	-0.7207	0.471	-0.119575915	count	1
GATAD2A	-0.0956775	0.2233624	-0.4284	0.668	-0.119454427	count	1
POLD1	-0.1373015	0.4877629	-0.2815	0.778	-0.119218187	count	1
MTRF1L	-0.0972397	0.2151872	-0.4519	0.651	-0.119104496	count	1
SNX3	-0.0831218	0.051563	-1.612	0.107	-0.119080877	count	1
CCDC32	-0.0962732	0.2003714	-0.4805	0.631	-0.119022344	count	1
IPO13	-0.1462068	0.4929025	-0.2966	0.767	-0.118885293	count	1
MAGOH	-0.0843574	0.0825746	-1.0216	0.307	-0.118726919	count	1
CCNE2	-0.2521934	0.5996658	-0.4206	0.674	-0.118575844	count	1
IFNGR1	-0.0830182	0.0531336	-1.5624	0.118	-0.1185697	count	1
FOXO4	-0.1877494	0.5659285	-0.3318	0.74	-0.118488898	count	1
FAM133B	-0.0837312	0.080303	-1.0427	0.297	-0.118372642	count	1
STARD9	-0.5161099	0.6877238	-0.7505	0.453	-0.117943318	count	1
NOP2	-0.2506261	0.6972381	-0.3595	0.719	-0.11788477	count	1
ARHGEF7	-0.1076597	0.2793471	-0.3854	0.7	-0.117697079	count	1
IL2RA	-0.1053719	0.2572275	-0.4096	0.682	-0.117642512	count	1
NDUFAF7	-0.1232469	0.3355075	-0.3673	0.713	-0.117602971	count	1
SLIRP	-0.0844184	0.1019303	-0.8282	0.408	-0.117528993	count	1
AAED1	-0.0842647	0.1155538	-0.7292	0.466	-0.117472898	count	1
RRAGA	-0.0862773	0.1319111	-0.6541	0.513	-0.117296795	count	1

HLA-DPA1	-0.0813201	0.0547698	-1.4848	0.138	-0.117269016	count	1
MESD	-0.0831484	0.0861082	-0.9656	0.334	-0.117240965	count	1
NOL11	-0.1065367	0.3015743	-0.3533	0.724	-0.117125395	count	1
SCOC	-0.0904733	0.17304	-0.5228	0.601	-0.116981957	count	1
IGHM	-0.7972658	1.2709058	-0.6273	0.531	-0.116980432	count	1
ARPC1A	-0.0848885	0.1119937	-0.758	0.449	-0.116560425	count	1
MTR	-0.1012044	0.2804551	-0.3609	0.718	-0.115993981	count	1
SNAPC5	-0.092986	0.2013712	-0.4618	0.644	-0.115930149	count	1
FLNA	-0.0813855	0.0927384	-0.8776	0.38	-0.115773979	count	1
MRPL50	-0.0869631	0.1679141	-0.5179	0.605	-0.115770514	count	1
SMAP1	-0.0827151	0.1076416	-0.7684	0.442	-0.115740876	count	1
NCKAP1L	-0.0832126	0.0982123	-0.8473	0.397	-0.115718616	count	1
PCK2	-0.0924295	0.2134879	-0.4329	0.665	-0.115592208	count	1
CDC42EP2	-0.0891384	0.2030633	-0.439	0.661	-0.115558864	count	1
UBP1	-0.0952661	0.2750436	-0.3464	0.729	-0.11548962	count	1
SPR	-0.0989213	0.3164824	-0.3126	0.755	-0.115440642	count	1
PRUNE2	-0.3169656	0.4351131	-0.7285	0.466	-0.115348177	count	1
COPZ1	-0.0824282	0.0945635	-0.8717	0.383	-0.115315907	count	1
SNF8	-0.0817397	0.0882066	-0.9267	0.354	-0.115303934	count	1
HCG18	-0.0944781	0.282763	-0.3341	0.738	-0.115035952	count	1
ZNF799	-0.2058661	0.5255293	-0.3917	0.695	-0.115027968	count	1
PSMD4	-0.0817381	0.082099	-0.9956	0.32	-0.115027538	count	1
SERPINB6	-0.0809978	0.0701475	-1.1547	0.248	-0.114718457	count	1
TINF2	-0.0866493	0.1734597	-0.4995	0.617	-0.114650125	count	1
AC069544.1	-0.1199463	0.6114173	-0.1962	0.844	-0.114500089	count	1
REEP4	-0.0894758	0.1771958	-0.505	0.614	-0.114229653	count	1
HTATSF1	-0.0880909	0.1799152	-0.4896	0.624	-0.114205288	count	1
PSMD10	-0.092276	0.2365309	-0.3901	0.696	-0.114104302	count	1
PROX1	-0.4958732	0.5649119	-0.8778	0.38	-0.11403707	count	1
VPS26A	-0.081563	0.0955245	-0.8538	0.393	-0.11394934	count	1
ZBTB20-AS2	-0.7675392	0.9636642	-0.7965	0.426	-0.113735958	count	1
7-Sep	-0.0794682	0.0489763	-1.6226	0.105	-0.113694548	count	1
ZFP36L1	-0.0790488	0.0577053	-1.3699	0.171	-0.113548155	count	1
SRA1	-0.0799676	0.0860121	-0.9297	0.353	-0.113480747	count	1
SEPSECS-AS1	-0.1305649	0.6654966	-0.1962	0.844	-0.113479678	count	1
CISD3	-0.0811516	0.1025125	-0.7916	0.429	-0.113417609	count	1
CCT8	-0.0801936	0.0742163	-1.0805	0.28	-0.113201704	count	1
SUCLA2	-0.0896398	0.2075373	-0.4319	0.666	-0.112917494	count	1
SLC39A4	-0.0836204	0.1569302	-0.5329	0.594	-0.112833362	count	1
ABI2	-0.1013468	0.2516774	-0.4027	0.687	-0.112633111	count	1
RALGPS2	-0.1048525	0.2926938	-0.3582	0.72	-0.112612589	count	1
NOP10	-0.0784745	0.0516568	-1.5192	0.129	-0.112406188	count	1
FBXW5	-0.0813251	0.1650729	-0.4927	0.622	-0.112313167	count	1
C1RL-AS1	-0.2379981	0.8989356	-0.2648	0.791	-0.112295551	count	1
SNRNP200	-0.0845488	0.1623208	-0.5209	0.603	-0.111999126	count	1

RFC1	-0.0818455	0.1437396	-0.5694	0.569	-0.111762355	count	1
BMF	-0.0914829	0.3922252	-0.2332	0.816	-0.111639914	count	1
TLK2	-0.085893	0.2048582	-0.4193	0.675	-0.111638401	count	1
NEK1	-0.0924671	0.4482715	-0.2063	0.837	-0.111605832	count	1
UQCR10	-0.0780526	0.0574377	-1.3589	0.174	-0.111604847	count	1
TMEM126B	-0.0826428	0.1443865	-0.5724	0.567	-0.111589326	count	1
FOSL2	-0.0789157	0.0901621	-0.8753	0.382	-0.111531165	count	1
RALY	-0.0797867	0.0890171	-0.8963	0.37	-0.111519982	count	1
ZNF446	-0.2152377	0.6657898	-0.3233	0.747	-0.11147052	count	1
USP37	-0.0893665	0.1945403	-0.4594	0.646	-0.111437407	count	1
ATRAID	-0.0787129	0.0749675	-1.05	0.294	-0.11123427	count	1
AC023509.4	-0.1278438	0.5817367	-0.2198	0.826	-0.111158402	count	1
ZDHHC6	-0.0844897	0.1808171	-0.4673	0.64	-0.11114722	count	1
PPM1G	-0.0790723	0.0903952	-0.8747	0.382	-0.111055279	count	1
DDX41	-0.0879855	0.1953552	-0.4504	0.652	-0.110990156	count	1
GSC	-0.2632872	1.0550026	-0.2496	0.803	-0.110982118	count	1
RAMP2	-0.2632872	0.9263742	-0.2842	0.776	-0.110982118	count	1
TBKBP1	-0.2632872	1.004232	-0.2622	0.793	-0.110982118	count	1
ZNF155	-0.2632872	0.9317084	-0.2826	0.778	-0.110982118	count	1
RPL36AL	-0.07716	0.034288	-2.2504	0.0245	-0.110863648	count	1
DCSTAMP	-0.1360503	0.7156665	-0.1901	0.849	-0.110805494	count	1
OAS3	-0.0919662	0.3084109	-0.2982	0.766	-0.110742388	count	1
UBALD2	-0.0782196	0.0717624	-1.09	0.276	-0.110719985	count	1
CBLL1	-0.0919198	0.2579324	-0.3564	0.722	-0.110686821	count	1
LAMP2	-0.0784003	0.0797694	-0.9828	0.326	-0.110596271	count	1
LAMB3	-0.2133296	0.9454468	-0.2256	0.822	-0.110531836	count	1
PACSIN3	-0.2133296	0.7908747	-0.2697	0.787	-0.110531836	count	1
CMC1	-0.0799633	0.1283816	-0.6229	0.533	-0.109996937	count	1
AIDA	-0.0841998	0.1810797	-0.465	0.642	-0.109946862	count	1
FO393401.1	-0.0981596	0.2755802	-0.3562	0.722	-0.109655208	count	1
SKP1	-0.0764414	0.0449005	-1.7025	0.0888	-0.109560801	count	1
ANO7	-0.4722815	1.1726611	-0.4027	0.687	-0.109412393	count	1
ZBED8	-0.4722815	1.2025025	-0.3927	0.695	-0.109412393	count	1
HIST1H2BG	-0.4722815	1.1726611	-0.4027	0.687	-0.109412393	count	1
KIF24	-0.4722815	1.3851632	-0.341	0.733	-0.109412393	count	1
P3H4	-0.4722815	1.3851632	-0.341	0.733	-0.109412393	count	1
ZNF671	-0.4722815	1.1726611	-0.4027	0.687	-0.109412393	count	1
EAF1	-0.0808756	0.1904632	-0.4246	0.671	-0.109277243	count	1
EXOSC7	-0.0827823	0.1852937	-0.4468	0.655	-0.10923664	count	1
HMG20B	-0.0802498	0.1439705	-0.5574	0.577	-0.109199324	count	1
ATAD5	-0.1052021	0.3341397	-0.3148	0.753	-0.109005267	count	1
SCRN3	-0.1229365	0.4288751	-0.2866	0.774	-0.108931926	count	1
LIX1L-AS1	-0.4689184	0.8167814	-0.5741	0.566	-0.108746885	count	1
HECW2	-0.4689184	0.9684151	-0.4842	0.628	-0.108746885	count	1
AP000763.3	-0.4689184	0.8472218	-0.5535	0.58	-0.108746885	count	1

RNFT1-DT	-0.4689184	0.8766384	-0.5349	0.593	-0.108746885	count	1
XRN2	-0.0764221	0.0615849	-1.2409	0.215	-0.108574818	count	1
EHBP1	-0.1017138	0.4260837	-0.2387	0.811	-0.108562737	count	1
ZBTB25	-0.1002997	0.3274617	-0.3063	0.759	-0.108443284	count	1
COX5B	-0.0755624	0.0415772	-1.8174	0.0693	-0.1084105	count	1
VAV3	-0.0824941	0.2086401	-0.3954	0.693	-0.108321449	count	1
DPH3	-0.078211	0.1108648	-0.7055	0.481	-0.107971886	count	1
APIP	-0.078945	0.1306336	-0.6043	0.546	-0.10780769	count	1
PSMA1	-0.0758729	0.0671673	-1.1296	0.259	-0.107601044	count	1
APPL1	-0.0766727	0.0935602	-0.8195	0.413	-0.107486539	count	1
CHID1	-0.0810919	0.1404753	-0.5773	0.564	-0.107373151	count	1
ACSS2	-0.1019838	0.3557747	-0.2867	0.774	-0.10733871	count	1
NUDT5	-0.079839	0.1369799	-0.5829	0.56	-0.107330314	count	1
HS1BP3	-0.0943444	0.2539242	-0.3715	0.71	-0.107323449	count	1
ZFYVE21	-0.0817938	0.2235191	-0.3659	0.714	-0.107190399	count	1
STX7	-0.0765012	0.0896517	-0.8533	0.394	-0.107186731	count	1
GPAT4	-0.092755	0.2652518	-0.3497	0.727	-0.107175829	count	1
STARD5	-0.1107534	0.4626649	-0.2394	0.811	-0.107135363	count	1
TRIM13	-0.0838811	0.2228872	-0.3763	0.707	-0.107112119	count	1
MSH6	-0.0822839	0.1609647	-0.5112	0.609	-0.107046111	count	1
DPY30	-0.0769514	0.1170433	-0.6575	0.511	-0.106966661	count	1
SELENOS	-0.0756666	0.0773719	-0.978	0.328	-0.106948425	count	1
AL139289.2	-0.2918139	0.9118215	-0.32	0.749	-0.106932637	count	1
MAPK10	-0.2918139	1.5153948	-0.1926	0.847	-0.106932637	count	1
AL359644.1	-0.2918139	0.8196777	-0.356	0.722	-0.106932637	count	1
AC136475.1	-0.2918139	1.0756275	-0.2713	0.786	-0.106932637	count	1
RPL36A	-0.0761394	0.0757849	-1.0047	0.315	-0.106845623	count	1
LY96	-0.0750776	0.0692347	-1.0844	0.278	-0.106772519	count	1
BCCIP	-0.0805471	0.1722456	-0.4676	0.64	-0.106710838	count	1
NEK11	-0.7046919	0.7905014	-0.8914	0.373	-0.106621377	count	1
TBC1D16	-0.7046919	0.87603	-0.8044	0.421	-0.106621377	count	1
CCNE1	-0.7046919	0.7905014	-0.8914	0.373	-0.106621377	count	1
C21orf58	-0.7046919	0.8846898	-0.7965	0.426	-0.106621377	count	1
QSER1	-0.0825863	0.2702158	-0.3056	0.76	-0.106429237	count	1
GPM6B	-0.456261	0.5933897	-0.7689	0.442	-0.10622814	count	1
TIMP3	-0.0861578	0.7290518	-0.1182	0.906	-0.106193946	count	1
SCAF1	-0.1110012	0.3889039	-0.2854	0.775	-0.106077585	count	1
PSMG4	-0.0810454	0.1965197	-0.4124	0.68	-0.105915994	count	1
TBC1D19	-0.4544735	0.8229044	-0.5523	0.581	-0.105870644	count	1
POLR2L	-0.0741454	0.0621712	-1.1926	0.233	-0.105699921	count	1
TXNL1	-0.0752812	0.0874186	-0.8612	0.389	-0.105659987	count	1
C1orf35	-0.0788754	0.1627877	-0.4845	0.628	-0.105585109	count	1
PSMA2	-0.0758649	0.1071241	-0.7082	0.479	-0.105436334	count	1
PIGG	-0.1188696	0.3853999	-0.3084	0.758	-0.105388318	count	1
EPG5	-0.1131547	0.369259	-0.3064	0.759	-0.105263343	count	1

HIC2	-0.0990745	0.5353442	-0.1851	0.853	-0.10505337	count	1
AC007325.4	-0.2018882	0.7009565	-0.288	0.773	-0.104884489	count	1
PNPO	-0.1027908	0.2724351	-0.3773	0.706	-0.104745592	count	1
AGFG2	-0.6885987	1.2044369	-0.5717	0.568	-0.104742747	count	1
SKAP1	-0.6885987	1.1036482	-0.6239	0.533	-0.104742747	count	1
ZSCAN22	-0.6885987	1.2682727	-0.5429	0.587	-0.104742747	count	1
NAA10	-0.0749851	0.1070446	-0.7005	0.484	-0.104669572	count	1
USP13	-0.6874395	0.6390759	-1.0757	0.282	-0.10460652	count	1
NSUN4	-0.0919113	0.417952	-0.2199	0.826	-0.104575099	count	1
NBPF12	-0.2843787	0.5627354	-0.5054	0.613	-0.104420629	count	1
GALM	-0.0831711	0.234376	-0.3549	0.723	-0.10421378	count	1
PRKCA	-0.0975587	0.2943058	-0.3315	0.74	-0.10416862	count	1
PPIA	-0.0724519	0.032909	-2.2016	0.0278	-0.104163384	count	1
C19orf53	-0.072871	0.0555019	-1.3129	0.189	-0.10396233	count	1
PTGR2	-0.130512	0.3095477	-0.4216	0.673	-0.103734799	count	1
PIAS2	-0.1008198	0.2361377	-0.427	0.669	-0.103653325	count	1
CD207	-1.9948141	2.088497	-0.9551	0.3396	-0.103607161	count	1
OSBPL8	-0.0731247	0.0875153	-0.8356	0.403	-0.103583327	count	1
TP53BP2	-0.0792475	0.1959556	-0.4044	0.686	-0.103572701	count	1
AC006333.2	-0.0822831	0.305976	-0.2689	0.788	-0.103544485	count	1
TMCC1	-0.0880322	0.3437974	-0.2561	0.798	-0.103467422	count	1
LGALS9	-0.072779	0.073461	-0.9907	0.322	-0.10344793	count	1
FAAP24	-0.2179424	0.7122987	-0.306	0.76	-0.103341709	count	1
DCLK2	-1.9800126	2.2275407	-0.8889	0.3742	-0.103319416	count	1
USPL1	-0.0943509	0.2612239	-0.3612	0.718	-0.103267961	count	1
COL8A2	-0.1425927	0.4353475	-0.3275	0.743	-0.103166217	count	1
ATP6V1E2	-0.1162289	0.3755637	-0.3095	0.757	-0.103085143	count	1
AC026304.1	-0.1833383	1.312477	-0.1397	0.889	-0.102957975	count	1
IL10	-0.0734713	0.2135542	-0.344	0.731	-0.102566515	count	1
MAPK3	-0.0793977	0.1746488	-0.4546	0.649	-0.102521126	count	1
ARHGEF26	-1.921258	1.0348711	-1.8565	0.0635	-0.102136903	count	1
TSPAN13	-0.1609382	0.7247094	-0.2221	0.824	-0.102128091	count	1
YAE1D1	-0.1320738	0.4264353	-0.3097	0.757	-0.102084432	count	1
PSMA5	-0.0724559	0.075768	-0.9563	0.339	-0.102083729	count	1
TMEM9B	-0.0731841	0.098463	-0.7433	0.457	-0.102012791	count	1
ULK4	-0.1170916	0.3578275	-0.3272	0.744	-0.101967377	count	1
GALK1	-0.0735473	0.124262	-0.5919	0.554	-0.101888419	count	1
GDF15	-0.0929729	0.4616051	-0.2014	0.84	-0.101771963	count	1
NDUFB6	-0.0724462	0.0957179	-0.7569	0.449	-0.101541065	count	1
SRSF10	-0.0722392	0.1226584	-0.5889	0.556	-0.101404691	count	1
C4orf48	-0.0716945	0.0771581	-0.9292	0.353	-0.10134649	count	1
EIF3B	-0.0757761	0.2290768	-0.3308	0.741	-0.101317143	count	1
IFT20	-0.0741822	0.1177616	-0.6299	0.529	-0.101198516	count	1
LIN7B	-0.1085052	0.3441432	-0.3153	0.753	-0.100998476	count	1
SPATA20	-0.097387	0.2836041	-0.3434	0.731	-0.100988079	count	1

TIMMDC1	-0.0745424	0.1273942	-0.5851	0.559	-0.100859145	count	1
ZNF551	-0.1935974	0.7468105	-0.2592	0.795	-0.100772112	count	1
HIST1H4H	-0.2374638	0.4999104	-0.475	0.635	-0.100769119	count	1
CHMP2A	-0.0706394	0.0651281	-1.0846	0.2782	-0.100687324	count	1
EIF1B	-0.0707468	0.0693858	-1.0196	0.308	-0.100514908	count	1
ECI2	-0.0773746	0.1799151	-0.4301	0.667	-0.100267119	count	1
VKORC1L1	-0.085398	0.2552832	-0.3345	0.738	-0.100074986	count	1
TMTC3	-0.1171485	0.4428446	-0.2645	0.791	-0.100034031	count	1
GPRC5C	-0.1920423	0.3129849	-0.6136	0.54	-0.099998877	count	1
NF2	-0.0907674	0.4271887	-0.2125	0.832	-0.099924319	count	1
SLC9A1	-0.112595	0.43028	-0.2617	0.794	-0.099912798	count	1
HPS1	-0.0732177	0.1366125	-0.536	0.592	-0.099907577	count	1
MTMR1	-0.1255007	0.4745285	-0.2645	0.791	-0.099833091	count	1
RPL13A	-0.0693768	0.0306549	-2.2632	0.0237	-0.099782167	count	1
SPATA7	-0.100575	0.3250568	-0.3094	0.757	-0.099589345	count	1
AL078604.2	-1.804128	2.070401	-0.8714	0.3836	-0.099576068	count	1
BBS1	-1.804128	1.276397	-1.4135	0.158	-0.099576068	count	1
MCF2L	-1.804128	2.1522761	-0.8382	0.402	-0.099576068	count	1
ISLR	-1.804128	2.1522761	-0.8382	0.402	-0.099576068	count	1
TRPV4	-0.3253277	0.4385241	-0.7419	0.458	-0.099530063	count	1
MEI1	-0.0931496	0.3879029	-0.2401	0.81	-0.099501983	count	1
TANK	-0.0721054	0.1039948	-0.6934	0.488	-0.099421097	count	1
BANK1	-0.1655015	0.6230942	-0.2656	0.791	-0.099359768	count	1
STAU2	-0.0871954	0.3020579	-0.2887	0.773	-0.099245146	count	1
ZNF649	-0.0991946	0.3767816	-0.2633	0.792	-0.099241412	count	1
ZMYND11	-0.0851762	0.2676397	-0.3182	0.75	-0.099163497	count	1
MICALL2	-0.1757854	0.6782698	-0.2592	0.796	-0.09888239	count	1
LDLRAD4	-0.0726933	0.1943883	-0.374	0.708	-0.098805535	count	1
C22orf34	-0.1753417	1.0001479	-0.1753	0.861	-0.098642515	count	1
UBE2J1	-0.0703248	0.1051877	-0.6686	0.504	-0.098482453	count	1
DUSP3	-0.0720947	0.13379	-0.5389	0.59	-0.098262674	count	1
ARL6IP5	-0.0685651	0.0469154	-1.4615	0.144	-0.09818248	count	1
AL031280.1	-1.7382	1.121868	-1.5494	0.121	-0.098006921	count	1
CPQ	-0.0705696	0.1083011	-0.6516	0.515	-0.097937523	count	1
TMEM141	-0.0703424	0.1131067	-0.6219	0.534	-0.097903566	count	1
SRRM1	-0.0685154	0.0563997	-1.2148	0.2246	-0.097855555	count	1
DYNLRB1	-0.0687715	0.0700609	-0.9816	0.326	-0.097575306	count	1
ZCCHC10	-0.0730323	0.1621004	-0.4505	0.652	-0.097528943	count	1
NECTIN1	-0.1460686	0.5860206	-0.2493	0.803	-0.097511527	count	1
RBM28	-0.0764933	0.234363	-0.3264	0.744	-0.097491204	count	1
DRAM2	-0.0692762	0.0800856	-0.865	0.387	-0.097447003	count	1
CFL2	-0.1295171	0.3429727	-0.3776	0.706	-0.097147286	count	1
DARS-AS1	-0.1527064	0.5577615	-0.2738	0.784	-0.097066775	count	1
ELP1	-0.1527064	0.529296	-0.2885	0.773	-0.097066775	count	1
RNASE6	-0.0683555	0.0718169	-0.9518	0.341	-0.096965167	count	1

PPDPF	-0.0677762	0.0734998	-0.9221	0.357	-0.096634782	count	1
BLVRB	-0.0672344	0.0525882	-1.2785	0.201	-0.096629765	count	1
HIST1H2AG	-0.6210416	0.8368133	-0.7422	0.458	-0.096597153	count	1
ATG16L1	-0.4088184	0.524926	-0.7788	0.436	-0.096588702	count	1
RARS	-0.0771198	0.2020411	-0.3817	0.703	-0.096516885	count	1
CEP104	-0.0937756	0.2905324	-0.3228	0.747	-0.096481658	count	1
AC026250.1	-0.3143545	0.9668854	-0.3251	0.745	-0.096478658	count	1
MS4A7	-0.0671801	0.0575148	-1.168	0.243	-0.096466465	count	1
ZNF213-AS1	-0.1849294	0.6301497	-0.2935	0.769	-0.096454579	count	1
AC010654.1	-0.1179845	0.4338157	-0.272	0.786	-0.096366125	count	1
RNF187	-0.0717927	0.1501791	-0.478	0.633	-0.096351508	count	1
C1orf159	-0.1710122	0.4656247	-0.3673	0.713	-0.096299285	count	1
HMMR	-0.1514051	0.7033762	-0.2153	0.83	-0.096265039	count	1
C6orf203	-0.0900398	0.3274864	-0.2749	0.783	-0.09620813	count	1
CORO1B	-0.0684042	0.0934126	-0.7323	0.464	-0.096193596	count	1
LINC00920	-0.3127241	0.5694975	-0.5491	0.583	-0.096023482	count	1
TRDMT1	-0.0979844	0.3817496	-0.2567	0.797	-0.096015026	count	1
ORC3	-0.0807006	0.3153897	-0.2559	0.798	-0.096006302	count	1
ZFYVE19	-0.084238	0.3918071	-0.215	0.83	-0.095900638	count	1
SAP18	-0.0670125	0.0493353	-1.3583	0.174	-0.095864412	count	1
LRR1	-0.0807825	0.2863999	-0.2821	0.778	-0.095836446	count	1
KHDRBS2	-0.1699822	0.573863	-0.2962	0.767	-0.095741127	count	1
FOXRED1	-0.0895923	0.4640591	-0.1931	0.847	-0.095733982	count	1
FAM89B	-0.0684173	0.1096182	-0.6241	0.533	-0.095306065	count	1
ATP5IF1	-0.0669679	0.0631664	-1.0602	0.289	-0.095047898	count	1
LPXN	-0.0703245	0.1454397	-0.4835	0.629	-0.095013727	count	1
CD1B	-1.619621	1.339221	-1.2094	0.227	-0.094932434	count	1
TMEM219	-0.0663171	0.0591816	-1.1206	0.263	-0.094624925	count	1
URB2	-0.1982408	0.620742	-0.3194	0.749	-0.094453946	count	1
WDR5B	-0.1982408	0.6525112	-0.3038	0.761	-0.094453946	count	1
MAGI2	-0.1982408	0.6525112	-0.3038	0.761	-0.094453946	count	1
FAM222A	-0.6034094	0.8691944	-0.6942	0.488	-0.094401067	count	1
AL365361.1	-0.306546	0.6694488	-0.4579	0.647	-0.094294457	count	1
LAMTOR1	-0.065956	0.0549704	-1.1998	0.23	-0.094293393	count	1
FGFRL1	-0.1410492	0.643055	-0.2193	0.826	-0.094252782	count	1
TRAPPC2L	-0.0674894	0.1125003	-0.5999	0.549	-0.094145525	count	1
AL590560.2	-1.590985	1.229861	-1.2936	0.196	-0.094138296	count	1
RAPGEF5	-1.590985	1.229861	-1.2936	0.196	-0.094138296	count	1
AC037487.2	-0.253151	0.901589	-0.2808	0.779	-0.093749134	count	1
TRMT12	-0.1096088	0.4234117	-0.2589	0.796	-0.093699957	count	1
CHMP1A	-0.0734965	0.2025923	-0.3628	0.717	-0.093683544	count	1
AL356056.2	-0.2194962	0.6961817	-0.3153	0.753	-0.093576429	count	1
CLNK	-0.5962322	0.9236078	-0.6455	0.519	-0.09349872	count	1
ZNF367	-0.5962322	0.8566748	-0.696	0.487	-0.09349872	count	1
HNRNPA3	-0.0653578	0.05447	-1.1999	0.23	-0.093190451	count	1

SNRNP40	-0.0705378	0.1592995	-0.4428	0.658	-0.093168131	count	1
SNAP29	-0.0686337	0.1306087	-0.5255	0.599	-0.093014024	count	1
ABHD14B	-0.0692648	0.1524954	-0.4542	0.65	-0.092928786	count	1
FBXO46	-0.1195817	0.5223556	-0.2289	0.819	-0.092622943	count	1
ELK1	-0.0754501	0.2430582	-0.3104	0.756	-0.092156515	count	1
PSMA4	-0.0649869	0.0643044	-1.0106	0.312	-0.092144739	count	1
OSBPL7	-0.3871673	0.567326	-0.6824	0.495	-0.092084474	count	1
CD180	-0.0768796	0.2911618	-0.264	0.792	-0.091970893	count	1
SEC24D	-0.0707607	0.2086794	-0.3391	0.735	-0.091871545	count	1
PCYT1A	-0.0724556	0.1779357	-0.4072	0.684	-0.091815386	count	1
OCIAD1	-0.0647928	0.0726954	-0.8913	0.373	-0.091734745	count	1
C1orf56	-0.066042	0.1439001	-0.4589	0.646	-0.091363242	count	1
ZMAT3	-0.0865974	0.2990797	-0.2895	0.772	-0.091281258	count	1
SMIM29	-0.0664443	0.1343999	-0.4944	0.621	-0.091181136	count	1
NPHP4	-0.3820781	0.6221892	-0.6141	0.539	-0.091016099	count	1
EXOSC6	-0.0683687	0.2125652	-0.3216	0.748	-0.09097685	count	1
TKTL2	-1.48291	1.217315	-1.2182	0.223	-0.090947264	count	1
AL512625.1	-1.48291	1.066513	-1.3904	0.165	-0.090947264	count	1
GINS3	-1.48291	1.066513	-1.3904	0.165	-0.090947264	count	1
GLO1	-0.0687306	0.168392	-0.4082	0.683	-0.090939659	count	1
CD274	-0.381608	0.7090838	-0.5382	0.591	-0.090917189	count	1
DNAJB12	-0.0654431	0.109405	-0.5982	0.55	-0.090298193	count	1
DBI	-0.0628183	0.0462537	-1.3581	0.175	-0.090240279	count	1
ARCN1	-0.0656788	0.1211639	-0.5421	0.588	-0.089867748	count	1
RAB4A	-0.0660586	0.1201039	-0.55	0.582	-0.089823326	count	1
MTMR2	-0.1027907	0.3532646	-0.291	0.771	-0.08969696	count	1
IGHG2	-1.443018	0.6758587	-2.1351	0.0329	-0.08968815	count	1
ZBTB41	-0.0946305	0.2941167	-0.3217	0.748	-0.089456707	count	1
RIDA	-0.0911981	0.2923257	-0.312	0.755	-0.089435227	count	1
HMGN5	-0.104377	0.312374	-0.3341	0.738	-0.08929613	count	1
MRPS21	-0.06304	0.0743956	-0.8474	0.397	-0.089254147	count	1
TSEN34	-0.0631627	0.1007997	-0.6266	0.531	-0.088885372	count	1
GRAMD4	-0.0820007	0.2921026	-0.2807	0.779	-0.088805086	count	1
MEIKIN	-0.3709514	0.7170702	-0.5173	0.605	-0.08866744	count	1
ANO5	-0.3709514	0.8120055	-0.4568	0.648	-0.08866744	count	1
ITGA1	-0.5581938	0.8188871	-0.6816	0.496	-0.088634017	count	1
C6orf47	-0.0723646	0.2272886	-0.3184	0.75	-0.088581428	count	1
ATP2A3	-0.0913449	0.4535282	-0.2014	0.84	-0.088564829	count	1
MTERF2	-0.1849105	0.6107387	-0.3028	0.762	-0.088388753	count	1
CD2AP	-0.0675484	0.1766293	-0.3824	0.702	-0.088380225	count	1
AC002310.1	-0.369381	0.6742376	-0.5478	0.584	-0.088334536	count	1
ATP5F1C	-0.0618903	0.0504038	-1.2279	0.2196	-0.088311645	count	1
IDNK	-0.0733112	0.2862163	-0.2561	0.798	-0.088163595	count	1
SYNCRIP	-0.0627514	0.0796212	-0.7881	0.431	-0.088029731	count	1
TM2D1	-0.0653388	0.1554778	-0.4202	0.674	-0.087836196	count	1

PALLD	-0.0728189	0.195391	-0.3727	0.709	-0.087785826	count	1
DRAP1	-0.0615575	0.0660845	-0.9315	0.352	-0.087701579	count	1
CLEC2B	-0.0613764	0.0676145	-0.9077	0.3641	-0.08763607	count	1
PRELID1	-0.0611667	0.0495465	-1.2345	0.217	-0.087618867	count	1
AC009088.1	-1.378087	1.40928	-0.9779	0.328	-0.087539315	count	1
VASH2	-1.3780868	1.5622851	-0.8821	0.3778	-0.087539293	count	1
GNPTAB	-0.0653274	0.1707289	-0.3826	0.702	-0.087333351	count	1
LIG4	-0.0815273	0.2679127	-0.3043	0.761	-0.087181669	count	1
PYROXD1	-0.0721083	0.2611891	-0.2761	0.783	-0.087137394	count	1
SUCLG2	-0.0678943	0.1596993	-0.4251	0.671	-0.087120404	count	1
NUF2	-1.365715	1.0895615	-1.2535	0.21	-0.08711547	count	1
MANEAL	-0.5460932	1.1315662	-0.4826	0.629	-0.087057184	count	1
CD160	-0.5460932	1.1315662	-0.4826	0.629	-0.087057184	count	1
C2orf81	-0.5460932	1.1315662	-0.4826	0.629	-0.087057184	count	1
CDCA7	-0.5460932	1.1933187	-0.4576	0.647	-0.087057184	count	1
PLK4	-0.5460932	1.1315662	-0.4826	0.629	-0.087057184	count	1
AC091982.3	-0.5460932	1.1933187	-0.4576	0.647	-0.087057184	count	1
GET4	-0.5460932	1.1315662	-0.4826	0.629	-0.087057184	count	1
AC068389.4	-0.5460932	1.1315662	-0.4826	0.629	-0.087057184	count	1
PPP1R32	-0.5460932	1.4322371	-0.3813	0.703	-0.087057184	count	1
TESPA1	-0.5460932	1.1315662	-0.4826	0.629	-0.087057184	count	1
PCDH9	-0.5460932	1.1315662	-0.4826	0.629	-0.087057184	count	1
TOMM20L	-0.5460932	1.1315662	-0.4826	0.629	-0.087057184	count	1
CARD14	-0.5460932	1.1933187	-0.4576	0.647	-0.087057184	count	1
FBXO15	-0.5460932	1.1315662	-0.4826	0.629	-0.087057184	count	1
SH2D3A	-0.5460932	1.1315662	-0.4826	0.629	-0.087057184	count	1
EPHX3	-0.5460932	1.1315662	-0.4826	0.629	-0.087057184	count	1
MRPS11	-0.0652455	0.177181	-0.3682	0.713	-0.087031747	count	1
GCSH	-0.074214	0.277706	-0.2672	0.789	-0.086466859	count	1
C1R	-0.2017519	0.6328498	-0.3188	0.75	-0.086403382	count	1
PKP2	-0.2314736	0.3713621	-0.6233	0.533	-0.086226063	count	1
NUP62	-0.0641704	0.2155615	-0.2977	0.766	-0.085968316	count	1
FMN1	-0.0721875	0.2062238	-0.35	0.726	-0.085924673	count	1
CX3CL1	-1.331068	1.710024	-0.7784	0.436	-0.085903338	count	1
NUCB1	-0.0613091	0.0861658	-0.7115	0.477	-0.085729775	count	1
ZC3H13	-0.0616545	0.1109223	-0.5558	0.578	-0.085603575	count	1
LGALSL	-1.320787	1.0194426	-1.2956	0.195	-0.085536371	count	1
ZNF662	-1.320787	1.822703	-0.7246	0.469	-0.085536371	count	1
DLL1	-1.320787	1.716973	-0.7693	0.442	-0.085536371	count	1
AC007541.1	-1.320787	1.0194426	-1.2956	0.195	-0.085536371	count	1
SIGIRR	-0.0612985	0.1023183	-0.5991	0.549	-0.085341986	count	1
FAM136A	-0.0633473	0.163671	-0.387	0.699	-0.085316121	count	1
RAB31	-0.0599875	0.0710028	-0.8449	0.398	-0.08488848	count	1
KRAS	-0.0604158	0.0977117	-0.6183	0.536	-0.084845144	count	1
PTOV1-AS1	-0.197832	0.7810773	-0.2533	0.8	-0.084809438	count	1

ADARB1	-0.1035814	0.4238302	-0.2444	0.807	-0.08479282	count	1
HYAL3	-0.2723828	0.7771218	-0.3505	0.726	-0.08461228	count	1
2-Sep	-0.060372	0.0885524	-0.6818	0.495	-0.084547943	count	1
TFB1M	-0.0860579	0.3654558	-0.2355	0.814	-0.084444332	count	1
AC068768.1	-1.290037	1.056757	-1.2208	0.222	-0.084418626	count	1
BRD9	-0.0684636	0.2180997	-0.3139	0.754	-0.084308489	count	1
TMEM223	-0.0653775	0.2283064	-0.2864	0.775	-0.084228665	count	1
NBDY	-0.0602895	0.0957609	-0.6296	0.529	-0.084087897	count	1
DVL2	-0.0901072	0.5343651	-0.1686	0.866	-0.084070579	count	1
CERS4	-0.0901072	0.365826	-0.2463	0.805	-0.084070579	count	1
ZNF653	-0.3461478	0.9146036	-0.3785	0.705	-0.083368245	count	1
FAM50B	-0.0905648	0.4286088	-0.2113	0.833	-0.083266956	count	1
GPATCH11	-0.0621057	0.1603124	-0.3874	0.698	-0.083206657	count	1
MRPL28	-0.0600531	0.1197238	-0.5016	0.616	-0.08310689	count	1
SHTN1	-0.0592481	0.0858847	-0.6899	0.49	-0.083083847	count	1
CDK2	-0.0990745	0.5193012	-0.1908	0.849	-0.083048992	count	1
RNF13	-0.058233	0.0683012	-0.8526	0.394	-0.082875124	count	1
TWF1	-0.0637232	0.1781407	-0.3577	0.721	-0.082822572	count	1
ST3GAL4	-0.0790666	0.3412339	-0.2317	0.817	-0.082785729	count	1
TEPSIN	-0.0982485	0.3632878	-0.2704	0.787	-0.082366845	count	1
IQCN	-0.1175369	0.914481	-0.1285	0.898	-0.082282734	count	1
DCUN1D5	-0.0622431	0.1999432	-0.3113	0.756	-0.082185504	count	1
TMEM11	-0.0658455	0.2494134	-0.264	0.792	-0.082073011	count	1
MAPKAPK2	-0.0613993	0.1604836	-0.3826	0.702	-0.081796393	count	1
GABPB1	-0.0701671	0.2574876	-0.2725	0.785	-0.08177463	count	1
SNRPN	-0.0617814	0.1690335	-0.3655	0.715	-0.081481103	count	1
ZNF226	-0.0673661	0.2586521	-0.2605	0.795	-0.081429272	count	1
SLC16A1-AS1	-0.099342	0.4233398	-0.2347	0.814	-0.081376032	count	1
MMEL1	-0.5011557	0.6648347	-0.7538	0.451	-0.0810758	count	1
CMPK1	-0.0589505	0.120122	-0.4908	0.624	-0.081009878	count	1
PARP4	-0.0616409	0.1907738	-0.3231	0.747	-0.08099391	count	1
GART	-0.0646646	0.2184128	-0.2961	0.767	-0.080976861	count	1
HNRNPAB	-0.056701	0.0699466	-0.8106	0.418	-0.080676245	count	1
NMRK1	-0.0624536	0.2030389	-0.3076	0.758	-0.080620552	count	1
UBE2E2	-0.0592482	0.1489699	-0.3977	0.691	-0.080594893	count	1
UBA6	-0.0613999	0.2248689	-0.273	0.785	-0.08057215	count	1
AC090198.1	-1.18366	1.182579	-1.0009	0.317	-0.080308989	count	1
RMRP	-1.18366	1.1067293	-1.0695	0.285	-0.080308989	count	1
KDELC1	-1.18366	1.365757	-0.8667	0.386	-0.080308989	count	1
AC022098.4	-1.18366	1.1067293	-1.0695	0.285	-0.080308989	count	1
DAP3	-0.0595611	0.1398995	-0.4257	0.67	-0.080224093	count	1
EFCAB11	-0.0800158	0.403291	-0.1984	0.843	-0.080222747	count	1
AC244090.1	-0.0609018	0.1744813	-0.349	0.727	-0.080176823	count	1
ALKBH7	-0.0564498	0.0673943	-0.8376	0.402	-0.080093783	count	1
VRK3	-0.064136	0.2408837	-0.2663	0.79	-0.080073989	count	1

GPATCH2L	-0.0600167	0.1592022	-0.377	0.706	-0.080031777	count	1
LINC01637	-0.067949	0.277053	-0.2453	0.806	-0.079969605	count	1
URB1-AS1	-0.0692315	0.3300137	-0.2098	0.834	-0.079837309	count	1
USP40	-0.0929935	0.5684897	-0.1636	0.87	-0.079689908	count	1
MOK	-0.0970797	0.5840669	-0.1662	0.868	-0.079550799	count	1
C7orf31	-0.139634	0.4736242	-0.2948	0.768	-0.079175019	count	1
EEF2KMT	-0.084775	0.3958246	-0.2142	0.83	-0.079149074	count	1
MVB12A	-0.0589263	0.1530536	-0.385	0.7	-0.079109189	count	1
TSTD1	-0.0572103	0.1494192	-0.3829	0.702	-0.078983076	count	1
PSENEN	-0.0560039	0.0793314	-0.7059	0.48	-0.078959632	count	1
SRP19	-0.0567036	0.1268124	-0.4471	0.655	-0.078926991	count	1
RSRC1	-0.0576904	0.1284896	-0.449	0.653	-0.078865805	count	1
AGPAT2	-0.0562897	0.1095422	-0.5139	0.607	-0.078808248	count	1
ZNF407	-0.0725898	0.3600949	-0.2016	0.84	-0.078679406	count	1
IRAK1BP1	-0.4830433	0.8684696	-0.5562	0.578	-0.0786084	count	1
NLGN2	-0.4830433	1.022004	-0.4726	0.637	-0.0786084	count	1
ZDHHC16	-0.0674364	0.4371769	-0.1543	0.877	-0.078606964	count	1
HERC5	-0.0582924	0.1981301	-0.2942	0.769	-0.078571928	count	1
HDDC2	-0.0593642	0.1744301	-0.3403	0.734	-0.078436248	count	1
GAA	-0.0559252	0.0816092	-0.6853	0.493	-0.078422053	count	1
NEAT1	-0.0542909	0.0679141	-0.7994	0.424	-0.078223551	count	1
SOWAHD	-0.0674565	0.3265111	-0.2066	0.836	-0.078085037	count	1
VPS29	-0.0547236	0.0583813	-0.9373	0.349	-0.078066912	count	1
SLC22A15	-0.0784981	0.4445643	-0.1766	0.86	-0.077922201	count	1
RPS6KA1	-0.0625033	0.192261	-0.3251	0.745	-0.077919801	count	1
CLCN2	-0.4776356	0.9064438	-0.5269	0.598	-0.077865364	count	1
SLC9B2	-0.4776356	0.8144097	-0.5865	0.558	-0.077865364	count	1
NANOS1	-0.4776356	0.7706077	-0.6198	0.535	-0.077865364	count	1
COG8	-0.4776356	0.7396663	-0.6457	0.519	-0.077865364	count	1
LAMTOR2	-0.0547192	0.0628864	-0.8701	0.384	-0.077802554	count	1
SCAMP1	-0.059267	0.1709094	-0.3468	0.729	-0.077673864	count	1
RRM1	-0.0753364	0.3071167	-0.2453	0.806	-0.077657531	count	1
B3GALT4	-0.0612879	0.2338202	-0.2621	0.793	-0.077505773	count	1
P3H3	-1.115414	1.260005	-0.8852	0.376	-0.077463594	count	1
TSPAN31	-0.0608507	0.1856542	-0.3278	0.743	-0.077148675	count	1
PLA2G2D	-0.3173819	0.6336122	-0.5009	0.616	-0.077111928	count	1
ASTN2	-1.1016767	1.373904	-0.8019	0.423	-0.076870124	count	1
RNF212	-0.1054756	0.6498092	-0.1623	0.871	-0.076821947	count	1
COMMD4	-0.0555652	0.1350911	-0.4113	0.681	-0.076799678	count	1
ACTR6	-0.0591205	0.2127625	-0.2779	0.781	-0.076727371	count	1
GLB1L	-0.066646	0.3319539	-0.2008	0.841	-0.076580278	count	1
SF3B6	-0.05394	0.0730916	-0.738	0.461	-0.076549436	count	1
ABCD4	-0.0612947	0.2721051	-0.2253	0.822	-0.076295663	count	1
CAPN10-DT	-0.1087336	0.5908926	-0.184	0.854	-0.076243964	count	1
HSD11B1L	-0.0888635	0.4496601	-0.1976	0.843	-0.076196537	count	1

MTFR1L	-0.0611058	0.2782897	-0.2196	0.826	-0.076182777	count	1
SNHG25	-0.0738771	0.4402542	-0.1678	0.867	-0.076164626	count	1
GTF2IRD2	-0.1339656	0.5181295	-0.2586	0.796	-0.076055105	count	1
ANPEP	-0.0538947	0.1178957	-0.4571	0.648	-0.075990153	count	1
RIC8B	-0.1006713	0.8193734	-0.1229	0.902	-0.075887757	count	1
METTL6	-0.0676234	0.3263532	-0.2072	0.836	-0.075729755	count	1
EEF1B2	-0.0525599	0.034231	-1.5354	0.125	-0.075592959	count	1
SLC20A2	-0.076097	0.3328644	-0.2286	0.819	-0.075558971	count	1
YWHAE	-0.0529567	0.0595503	-0.8893	0.374	-0.07547222	count	1
ARHGEF6	-0.060209	0.1721484	-0.3498	0.727	-0.07506797	count	1
AC073332.1	-0.0655018	0.3572786	-0.1833	0.855	-0.074978429	count	1
POLR1E	-0.0874084	0.4223709	-0.2069	0.836	-0.074964701	count	1
AC005229.4	-0.099347	0.55664	-0.1785	0.858	-0.074906464	count	1
EXOSC9	-0.0593923	0.2336841	-0.2542	0.799	-0.074714124	count	1
NUP160	-0.0705725	0.2977661	-0.237	0.813	-0.074504655	count	1
ALOX5	-0.0530891	0.1016781	-0.5221	0.602	-0.074382983	count	1
HGS	-0.0601913	0.2572421	-0.234	0.815	-0.074153778	count	1
ZNF160	-0.0666761	0.2923637	-0.2281	0.82	-0.07393947	count	1
ASMTL-AS1	-1.035722	0.916086	-1.1306	0.258	-0.073920981	count	1
COL14A1	-1.035198	0.8560716	-1.2092	0.227	-0.073896865	count	1
SEC23IP	-0.0677225	0.2558133	-0.2647	0.791	-0.073872385	count	1
AL158850.1	-1.03373	1.200626	-0.861	0.389	-0.073829297	count	1
AL158071.4	-1.03373	1.139308	-0.9073	0.364	-0.073829297	count	1
NHLRC4	-1.03373	1.200626	-0.861	0.389	-0.073829297	count	1
TMEM179B	-0.0520359	0.0769044	-0.6766	0.499	-0.073750503	count	1
CTDNEP1	-0.05223	0.0808003	-0.6464	0.518	-0.07371289	count	1
TNRC6B	-0.053762	0.1160832	-0.4631	0.643	-0.073623259	count	1
NDUFA13	-0.0527737	0.0997498	-0.5291	0.597	-0.073567675	count	1
PRKRA	-0.0576829	0.1917119	-0.3009	0.764	-0.073491402	count	1
STUB1	-0.0522028	0.0918933	-0.5681	0.57	-0.073431586	count	1
H1F0	-0.0528162	0.1538042	-0.3434	0.731	-0.073395395	count	1
SNHG12	-0.0558748	0.1893675	-0.2951	0.768	-0.073383245	count	1
MOCS2	-0.0583517	0.2032717	-0.2871	0.774	-0.073305461	count	1
PON2	-0.0642692	0.2113806	-0.304	0.761	-0.073277509	count	1
AP001462.1	-0.2331677	0.7354827	-0.317	0.751	-0.073244522	count	1
PIGP	-0.0541405	0.1465847	-0.3693	0.712	-0.073243149	count	1
ELAVL1	-0.0545761	0.139597	-0.391	0.696	-0.073191362	count	1
NDUFB11	-0.0511562	0.0491204	-1.0414	0.298	-0.07317893	count	1
FAN1	-0.2329326	0.6040109	-0.3856	0.7	-0.073175548	count	1
SLC25A33	-0.057771	0.1860483	-0.3105	0.756	-0.073162555	count	1
SYVN1	-0.0806816	0.3586048	-0.225	0.822	-0.073125036	count	1
AC005332.5	-0.11397	0.5619345	-0.2028	0.839	-0.073011387	count	1
MORF4L1	-0.0510145	0.0506111	-1.008	0.3136	-0.072909704	count	1
KNL1	-1.013863	0.7079191	-1.4322	0.152	-0.072906185	count	1
NDUFA8	-0.0529455	0.1174457	-0.4508	0.652	-0.072899925	count	1

NUDCD2	-0.0539112	0.1484791	-0.3631	0.717	-0.072888146	count	1
POLD2	-0.0589516	0.2728585	-0.2161	0.829	-0.072763998	count	1
ABCA5	-0.1133412	0.5156607	-0.2198	0.826	-0.072617675	count	1
AAMP	-0.0536487	0.1338669	-0.4008	0.689	-0.072510884	count	1
HSBP1	-0.0507387	0.0591936	-0.8572	0.391	-0.072446455	count	1
ERGIC2	-0.0538786	0.1348956	-0.3994	0.69	-0.072423505	count	1
CYLD	-0.0535029	0.1509777	-0.3544	0.723	-0.072244587	count	1
LINC01303	-0.0809847	0.4502139	-0.1799	0.857	-0.072177164	count	1
CNOT6L	-0.0547841	0.1522818	-0.3598	0.719	-0.072136684	count	1
SLC25A29	-0.0639419	0.38556	-0.1658	0.868	-0.071963107	count	1
PAIP2B	-0.2933049	0.7416491	-0.3955	0.693	-0.071783494	count	1
ALDH18A1	-0.2933049	0.7416491	-0.3955	0.693	-0.071783494	count	1
AC018362.1	-0.2933049	0.7567711	-0.3876	0.698	-0.071783494	count	1
MAD2L1BP	-0.0551911	0.2684155	-0.2056	0.837	-0.071637826	count	1
COL4A3BP	-0.0515795	0.1101619	-0.4682	0.64	-0.071460425	count	1
SCP2	-0.0505707	0.0786089	-0.6433	0.52	-0.071143537	count	1
METTL23	-0.052463	0.1563345	-0.3356	0.737	-0.071040815	count	1
FNBP1	-0.0513537	0.0991536	-0.5179	0.605	-0.071030753	count	1
FUBP1	-0.0535267	0.1455326	-0.3678	0.713	-0.070969503	count	1
MTIF3	-0.0508518	0.1047012	-0.4857	0.627	-0.070896319	count	1
TCEANC2	-0.1053226	0.4420618	-0.2383	0.812	-0.070865955	count	1
ITGAX	-0.0501078	0.0951875	-0.5264	0.599	-0.070791545	count	1
DCTN3	-0.0504039	0.0838404	-0.6012	0.548	-0.070768617	count	1
PIP4K2C	-0.134062	0.5386796	-0.2489	0.803	-0.070746796	count	1
ACTG1	-0.0490956	0.0344615	-1.4247	0.1544	-0.070718921	count	1
MTMR9	-0.0608724	0.2682633	-0.2269	0.821	-0.070239687	count	1
NAPA	-0.0502823	0.1023287	-0.4914	0.623	-0.070235296	count	1
PCNT	-0.0612662	0.281617	-0.2176	0.828	-0.070151929	count	1
MCMBP	-0.0571274	0.2004577	-0.285	0.776	-0.070124595	count	1
ACOT9	-0.0508894	0.1353652	-0.3759	0.707	-0.070115951	count	1
CEP152	-0.0760513	0.4787194	-0.1589	0.874	-0.070054739	count	1
HORMAD1	-0.9527278	1.0736556	-0.8874	0.375	-0.069965593	count	1
AC073046.1	-0.9527278	1.0736556	-0.8874	0.375	-0.069965593	count	1
AC137630.2	-0.9527278	1.0736556	-0.8874	0.375	-0.069965593	count	1
DEPDC1B	-0.9527278	1.0736556	-0.8874	0.375	-0.069965593	count	1
MTBP	-0.9527278	1.0736556	-0.8874	0.375	-0.069965593	count	1
AL354733.3	-0.9527278	1.0736556	-0.8874	0.375	-0.069965593	count	1
EXPH5	-0.9527278	1.515811	-0.6285	0.53	-0.069965593	count	1
PSD	-0.9527278	1.1635679	-0.8188	0.413	-0.069965593	count	1
AC069209.1	-0.9527278	1.0736556	-0.8874	0.375	-0.069965593	count	1
AC015967.1	-0.9527278	1.0736556	-0.8874	0.375	-0.069965593	count	1
LPIN3	-0.9527278	1.643165	-0.5798	0.562	-0.069965593	count	1
AC005944.1	-0.9527278	1.0736556	-0.8874	0.375	-0.069965593	count	1
LINC01694	-0.9527278	1.515811	-0.6285	0.53	-0.069965593	count	1
METAP2	-0.0504305	0.1147499	-0.4395	0.66	-0.069936136	count	1

FAM199X	-0.0555805	0.2295604	-0.2421	0.809	-0.069931136	count	1
SLC2A14	-0.9508769	0.8186868	-1.1615	0.246	-0.069874168	count	1
WASL	-0.0521038	0.1958422	-0.266	0.79	-0.069852354	count	1
PTAFR	-0.0496599	0.1021832	-0.486	0.627	-0.069776548	count	1
MRPL22	-0.0515297	0.1546689	-0.3332	0.739	-0.069672065	count	1
UBE3A	-0.050885	0.1352709	-0.3762	0.707	-0.069556339	count	1
FAM228B	-0.0584575	0.2437412	-0.2398	0.81	-0.069449488	count	1
AC127496.5	-0.1438359	0.8856267	-0.1624	0.871	-0.069438691	count	1
DYNC1LI1	-0.0499316	0.1011277	-0.4937	0.622	-0.069425091	count	1
CMSS1	-0.0596531	0.2733682	-0.2182	0.827	-0.069334263	count	1
CUL3	-0.0518851	0.1665956	-0.3114	0.755	-0.069296951	count	1
SMPD1	-0.0662997	0.3425961	-0.1935	0.847	-0.068966594	count	1
ATP5MF	-0.048322	0.0504331	-0.9581	0.3381	-0.06894338	count	1
AC078883.1	-0.0820204	0.5605313	-0.1463	0.884	-0.068929417	count	1
ID2	-0.0488373	0.0963844	-0.5067	0.612	-0.068731519	count	1
CDC73	-0.050505	0.1349349	-0.3743	0.708	-0.068731283	count	1
SIGLEC11	-0.2177667	0.550637	-0.3955	0.693	-0.068705546	count	1
CD164	-0.0482772	0.0657317	-0.7345	0.463	-0.068627755	count	1
ABHD4	-0.129747	0.5845503	-0.222	0.824	-0.068537014	count	1
FBXO28	-0.0578154	0.2467979	-0.2343	0.815	-0.068494462	count	1
ZNF554	-0.410404	0.7278428	-0.5639	0.573	-0.068380186	count	1
ZNF17	-0.410404	0.6369471	-0.6443	0.519	-0.068380186	count	1
EHD4	-0.0499285	0.1254379	-0.398	0.691	-0.068322403	count	1
NOCT	-0.0534432	0.2386187	-0.224	0.823	-0.067943174	count	1
VDAC2	-0.0476273	0.0588041	-0.8099	0.418	-0.067876987	count	1
ARF1	-0.0474845	0.04618	-1.0282	0.3039	-0.067859607	count	1
NDUFA6	-0.0478952	0.0752584	-0.6364	0.525	-0.067849388	count	1
RPS20	-0.0472734	0.0340892	-1.3868	0.166	-0.067835526	count	1
NABP1	-0.0481454	0.1042795	-0.4617	0.644	-0.067817917	count	1
RIOK3	-0.048433	0.1041471	-0.465	0.642	-0.067719711	count	1
BRIX1	-0.0536148	0.2412477	-0.2222	0.824	-0.067647889	count	1
MRPS12	-0.0488736	0.1291029	-0.3786	0.705	-0.067602823	count	1
IFIH1	-0.0561007	0.1884354	-0.2977	0.766	-0.067535323	count	1
AC005162.3	-0.9044556	0.7673943	-1.1786	0.239	-0.067533963	count	1
PAK3	-0.9044556	0.9356897	-0.9666	0.334	-0.067533963	count	1
TRIM58	-0.2133296	1.872474	-0.1139	0.909	-0.067390035	count	1
AC008443.5	-0.2133296	1.119772	-0.1905	0.849	-0.067390035	count	1
WASF1	-0.2133296	1.119772	-0.1905	0.849	-0.067390035	count	1
AC087672.2	-0.2133296	1.119772	-0.1905	0.849	-0.067390035	count	1
ZNF202	-0.2133296	1.119772	-0.1905	0.849	-0.067390035	count	1
LINC00167	-0.2133296	1.872474	-0.1139	0.909	-0.067390035	count	1
NUAK1	-0.2133296	1.0960392	-0.1946	0.846	-0.067390035	count	1
AL161772.1	-0.2133296	1.0960392	-0.1946	0.846	-0.067390035	count	1
KDM8	-0.2133296	1.2764278	-0.1671	0.867	-0.067390035	count	1
AC004771.1	-0.2133296	1.2764278	-0.1671	0.867	-0.067390035	count	1

AC011498.4	-0.2133296	1.0960392	-0.1946	0.846	-0.067390035	count	1
ARHGEF18	-0.2133296	1.2764278	-0.1671	0.867	-0.067390035	count	1
LINC00665	-0.2133296	1.0960392	-0.1946	0.846	-0.067390035	count	1
AC008440.2	-0.2133296	1.2764278	-0.1671	0.867	-0.067390035	count	1
AC002470.1	-0.2133296	1.119772	-0.1905	0.849	-0.067390035	count	1
RNF227	-0.2734408	0.4926659	-0.555	0.579	-0.067323988	count	1
TRIM33	-0.050403	0.2027009	-0.2487	0.804	-0.067071263	count	1
UFM1	-0.0479097	0.1120572	-0.4275	0.669	-0.067063756	count	1
CCDC124	-0.0482307	0.1099188	-0.4388	0.661	-0.06699189	count	1
BUB3	-0.0486376	0.1304579	-0.3728	0.709	-0.066777998	count	1
SLC35A5	-0.0736017	0.2666267	-0.276	0.783	-0.066770934	count	1
LIMK1	-0.0615055	0.2643112	-0.2327	0.816	-0.066730804	count	1
ARHGEF35	-0.8875695	0.8301569	-1.0692	0.285	-0.066659842	count	1
AEBP1	-0.1531528	0.611845	-0.2503	0.802	-0.066402962	count	1
SLC35B4	-0.0564702	0.3042386	-0.1856	0.853	-0.066303906	count	1
SF3B4	-0.0490245	0.1428014	-0.3433	0.731	-0.066204191	count	1
BBS10	-0.3946516	0.8212829	-0.4805	0.631	-0.066090828	count	1
RSPH3	-0.0550785	0.2815313	-0.1956	0.845	-0.065977847	count	1
MRPL12	-0.0482082	0.1504839	-0.3204	0.749	-0.065914804	count	1
ITFG2-AS1	-0.1518701	0.6441771	-0.2358	0.814	-0.065868055	count	1
AC022211.2	-0.8711115	1.25031	-0.6967	0.486	-0.065795931	count	1
CD79A	-0.8711115	1.210499	-0.7196	0.472	-0.065795931	count	1
AC009061.2	-0.0702082	0.5008951	-0.1402	0.889	-0.06566933	count	1
NDUFS7	-0.0463182	0.0671084	-0.6902	0.49	-0.065529137	count	1
SGTA	-0.0530736	0.2214633	-0.2396	0.811	-0.06552858	count	1
MED4	-0.0481851	0.1427461	-0.3376	0.736	-0.065422062	count	1
ENTPD3-AS1	-0.0672349	0.3712764	-0.1811	0.856	-0.065372921	count	1
DNHD1	-0.8624539	0.8221627	-1.049	0.294	-0.065336708	count	1
SC5D	-0.058285	0.2756359	-0.2115	0.833	-0.065320521	count	1
ZNF469	-0.2639355	0.6994664	-0.3773	0.706	-0.065169674	count	1
CBWD2	-0.0621382	0.2479238	-0.2506	0.802	-0.065168865	count	1
AC004803.1	-0.1719718	0.9216989	-0.1866	0.852	-0.06509001	count	1
CDKL1	-0.1719718	0.925533	-0.1858	0.853	-0.06509001	count	1
ZNF354C	-0.1499134	0.8436426	-0.1777	0.859	-0.065051383	count	1
SRI	-0.0456176	0.0824667	-0.5532	0.58	-0.064674282	count	1
ANP32B	-0.0453258	0.0543916	-0.8333	0.405	-0.064594723	count	1
RB1-DT	-0.2604903	0.9635663	-0.2703	0.787	-0.064385578	count	1
MRPL52	-0.045736	0.0833728	-0.5486	0.583	-0.064356781	count	1
MAX	-0.0473068	0.121797	-0.3884	0.698	-0.06428284	count	1
PJVK	-0.381608	0.839866	-0.4544	0.65	-0.064175659	count	1
SPEG	-0.381608	0.8832674	-0.432	0.666	-0.064175659	count	1
KIF18A	-0.381608	0.839866	-0.4544	0.65	-0.064175659	count	1
AL133467.1	-0.381608	0.839866	-0.4544	0.65	-0.064175659	count	1
NDUFA1	-0.0449489	0.0531884	-0.8451	0.3981	-0.064128709	count	1
ZNF782	-0.8350737	0.6615495	-1.2623	0.207	-0.063862455	count	1

CDC26	-0.0457292	0.105602	-0.433	0.665	-0.063828064	count	1
RRP7A	-0.0452385	0.1058229	-0.4275	0.669	-0.063816397	count	1
ALG3	-0.0476643	0.1595081	-0.2988	0.765	-0.063776768	count	1
IDH1	-0.0475585	0.148692	-0.3198	0.749	-0.063581118	count	1
NR6A1	-0.073967	0.5237176	-0.1412	0.888	-0.063560338	count	1
PDCD11	-0.0688081	0.3796895	-0.1812	0.856	-0.06344183	count	1
NAA15	-0.0499354	0.1777584	-0.2809	0.779	-0.063417049	count	1
SUV39H1	-0.1671047	0.8781318	-0.1903	0.849	-0.063329575	count	1
AC093512.1	-0.1671047	0.8716661	-0.1917	0.848	-0.063329575	count	1
SPAG5	-0.1671047	0.8781318	-0.1903	0.849	-0.063329575	count	1
TPM3	-0.0440391	0.0347292	-1.2681	0.205	-0.063314607	count	1
C9orf3	-0.1668824	0.5801303	-0.2877	0.774	-0.063249056	count	1
AF127577.4	-0.8235864	0.8869078	-0.9286	0.353	-0.063233922	count	1
HADHB	-0.0445703	0.0787606	-0.5659	0.572	-0.062737196	count	1
UQCC1	-0.3710332	0.5412337	-0.6855	0.493	-0.06261	count	1
MOSPD1	-0.0596816	0.3552516	-0.168	0.867	-0.062607387	count	1
YRDC	-0.0474702	0.166637	-0.2849	0.776	-0.062558701	count	1
SLC16A4	-0.1649425	0.8160432	-0.2021	0.84	-0.062545977	count	1
ITGA10	-0.1649425	0.81986	-0.2012	0.841	-0.062545977	count	1
AC010997.5	-0.1649425	0.8744404	-0.1886	0.85	-0.062545977	count	1
PFKM	-0.1649425	0.7452985	-0.2213	0.825	-0.062545977	count	1
MCM8	-0.1649425	0.8326648	-0.1981	0.843	-0.062545977	count	1
EGLN2	-0.1649425	0.7431347	-0.222	0.824	-0.062545977	count	1
TFG	-0.0459176	0.1299726	-0.3533	0.724	-0.062540954	count	1
RNF43	-0.8093501	0.8686358	-0.9317	0.352	-0.062446661	count	1
PSMD6	-0.0456684	0.1186469	-0.3849	0.7	-0.062418156	count	1
MDFIC	-0.0456532	0.1849809	-0.2468	0.805	-0.062370046	count	1
BORA	-0.8068162	0.8991809	-0.8973	0.37	-0.062305562	count	1
TNFRSF10C	-0.0921014	0.6536356	-0.1409	0.888	-0.062126496	count	1
DBF4B	-0.1637199	0.7474789	-0.219	0.827	-0.062102478	count	1
AL031595.2	-0.1637199	0.6571979	-0.2491	0.803	-0.062102478	count	1
LIN54	-0.0695213	0.390591	-0.178	0.859	-0.062057297	count	1
POLE	-0.3668619	0.6445339	-0.5692	0.569	-0.061989189	count	1
VSIG8	-0.3661425	0.9046994	-0.4047	0.686	-0.061881938	count	1
ANKRD39	-0.0532027	0.2731736	-0.1948	0.846	-0.061864591	count	1
PNKP	-0.0473008	0.2132023	-0.2219	0.824	-0.061751363	count	1
LRRC37A	-0.1274093	1.5132551	-0.0842	0.933	-0.061749998	count	1
AC104532.2	-0.1274093	0.7476824	-0.1704	0.865	-0.061749998	count	1
DDX24	-0.0433067	0.0667831	-0.6485	0.517	-0.06166471	count	1
GRASP	-0.0448149	0.101497	-0.4415	0.659	-0.061610109	count	1
HAT1	-0.0458065	0.1487042	-0.308	0.758	-0.061515637	count	1
GGNBP2	-0.0440996	0.0983508	-0.4484	0.654	-0.06141019	count	1
TAF1A	-0.0871251	0.4621788	-0.1885	0.85	-0.06133492	count	1
HEATR6	-0.0549476	0.2980776	-0.1843	0.854	-0.061299449	count	1
PELI3	-0.0560616	0.4160604	-0.1347	0.893	-0.061214469	count	1

NFYB	-0.0502516	0.2640884	-0.1903	0.849	-0.061207771	count	1
PHF6	-0.0479587	0.1766011	-0.2716	0.786	-0.061196123	count	1
SNX6	-0.0427319	0.0620941	-0.6882	0.4914	-0.060998951	count	1
CSTF2	-0.0723832	0.4433401	-0.1633	0.87	-0.060917635	count	1
TMEM208	-0.0431922	0.0942805	-0.4581	0.647	-0.060726291	count	1
AL353764.1	-0.1908455	1.1661608	-0.1637	0.87	-0.060670148	count	1
COL15A1	-0.1908455	0.9645036	-0.1979	0.843	-0.060670148	count	1
LINC01146	-0.1908455	0.8440173	-0.2261	0.821	-0.060670148	count	1
MAMSTR	-0.1908455	1.1661608	-0.1637	0.87	-0.060670148	count	1
AC084018.2	-0.1144542	0.7136733	-0.1604	0.873	-0.060668912	count	1
DIABLO	-0.1144542	0.7166078	-0.1597	0.873	-0.060668912	count	1
CD99	-0.042241	0.050271	-0.8403	0.401	-0.060629804	count	1
TMEM160	-0.0429982	0.0909766	-0.4726	0.637	-0.060590424	count	1
MDH2	-0.0426332	0.0673202	-0.6333	0.527	-0.060492527	count	1
RXRB	-0.0618249	0.3716221	-0.1664	0.868	-0.060150466	count	1
GANAB	-0.0459991	0.1910603	-0.2408	0.81	-0.060142577	count	1
ALDH4A1	-0.7675392	1.1770254	-0.6521	0.514	-0.060080603	count	1
AL512408.1	-0.7675392	1.1755742	-0.6529	0.514	-0.060080603	count	1
DLEC1	-0.7675392	1.1755742	-0.6529	0.514	-0.060080603	count	1
TERC	-0.7675392	1.1755742	-0.6529	0.514	-0.060080603	count	1
WWC2-AS2	-0.7675392	1.1755742	-0.6529	0.514	-0.060080603	count	1
FANCC	-0.7675392	1.1755742	-0.6529	0.514	-0.060080603	count	1
SYCP3	-0.7675392	1.1755742	-0.6529	0.514	-0.060080603	count	1
AL138963.3	-0.7675392	1.1755742	-0.6529	0.514	-0.060080603	count	1
AC090825.1	-0.7675392	1.1755742	-0.6529	0.514	-0.060080603	count	1
AC005224.4	-0.7675392	1.528769	-0.5021	0.616	-0.060080603	count	1
TEX14	-0.7675392	1.1755742	-0.6529	0.514	-0.060080603	count	1
TMEM87A	-0.0458179	0.1687008	-0.2716	0.786	-0.060074092	count	1
EPRS	-0.044148	0.1199125	-0.3682	0.713	-0.059994413	count	1
PGGHG	-0.0766517	0.3467615	-0.2211	0.825	-0.059794365	count	1
DYNLT1	-0.0418808	0.0676327	-0.6192	0.536	-0.059612808	count	1
OSTF1	-0.0417892	0.0579537	-0.7211	0.471	-0.059523643	count	1
LTBP3	-0.0491716	0.3249347	-0.1513	0.88	-0.059498917	count	1
SRSF8	-0.0482224	0.2842268	-0.1697	0.865	-0.059445383	count	1
R3HCC1L	-0.0484487	0.3083354	-0.1571	0.875	-0.059263639	count	1
PFKFB3	-0.0435776	0.115438	-0.3775	0.706	-0.05923567	count	1
HNRNPR	-0.0420453	0.0890327	-0.4722	0.637	-0.059233312	count	1
SH3YL1	-0.0516277	0.3631266	-0.1422	0.887	-0.059157293	count	1
HINT1	-0.0412678	0.0468005	-0.8818	0.378	-0.059157108	count	1
FBXL15	-0.0429874	0.1514381	-0.2839	0.777	-0.059139853	count	1
PGAM5	-0.0607223	0.378009	-0.1606	0.872	-0.059085265	count	1
OSBPL2	-0.0500885	0.3353978	-0.1493	0.881	-0.05901847	count	1
DNAJA1	-0.0412559	0.0504518	-0.8177	0.414	-0.059016411	count	1
ZNF580	-0.0440982	0.16759	-0.2631	0.792	-0.058908971	count	1
RBM47	-0.0424263	0.1238846	-0.3425	0.732	-0.058735783	count	1

LINC02019	-0.0774861	0.9657969	-0.0802	0.936	-0.058641392	count	1
HIBADH	-0.0505365	0.2473264	-0.2043	0.838	-0.058568674	count	1
ZNF334	-0.0959888	1.100857	-0.0872	0.931	-0.058471577	count	1
SETD3	-0.0439364	0.1401123	-0.3136	0.754	-0.05823913	count	1
CSTF3	-0.0512936	0.3182044	-0.1612	0.872	-0.05804477	count	1
PRDM2	-0.0437597	0.1761707	-0.2484	0.804	-0.058005204	count	1
BATF2	-0.7317518	0.7167178	-1.021	0.307	-0.057990272	count	1
CYB5RL	-0.1816061	0.7594619	-0.2391	0.811	-0.057882662	count	1
AP003354.2	-0.1816061	0.7594619	-0.2391	0.811	-0.057882662	count	1
BAK1	-0.0463782	0.2172871	-0.2134	0.831	-0.057770548	count	1
UQCRC1	-0.0410674	0.090257	-0.455	0.649	-0.057619637	count	1
TMEM191C	-0.066844	0.4859739	-0.1375	0.891	-0.057498357	count	1
B2M	-0.0398585	0.0201103	-1.982	0.0476	-0.057491961	count	1
TMCO6	-0.0605245	0.4174912	-0.145	0.885	-0.057455542	count	1
GTF2A2	-0.0406756	0.0786964	-0.5169	0.605	-0.057318656	count	1
MINCR	-0.0615522	0.5162871	-0.1192	0.905	-0.056804638	count	1
PIGM	-0.0674389	0.3872195	-0.1742	0.862	-0.056798092	count	1
ALG11	-0.0840312	0.622705	-0.1349	0.893	-0.056769603	count	1
ZC3H15	-0.0399889	0.0716658	-0.558	0.577	-0.056728377	count	1
FBXO41	-0.0929935	0.5259191	-0.1768	0.86	-0.056681934	count	1
RAB2A	-0.0398426	0.0673838	-0.5913	0.554	-0.05666763	count	1
UBC	-0.0393393	0.0315589	-1.2465	0.213	-0.056646576	count	1
PRMT3	-0.067131	0.4112648	-0.1632	0.87	-0.056541347	count	1
SEC62	-0.0394277	0.0535031	-0.7369	0.461	-0.056520775	count	1
ZBTB8OS	-0.0405525	0.1161503	-0.3491	0.727	-0.056424753	count	1
ABRACL	-0.0395745	0.0634665	-0.6235	0.533	-0.05640296	count	1
MAD1L1	-0.0438651	0.2435241	-0.1801	0.857	-0.056396091	count	1
IL24	-0.1765595	0.8162251	-0.2163	0.829	-0.056353702	count	1
IDH1-AS1	-0.1154859	0.8696872	-0.1328	0.894	-0.056129801	count	1
CTNNBL1	-0.0412544	0.1478748	-0.279	0.78	-0.056065526	count	1
IGSF21	-0.0428297	0.1445405	-0.2963	0.767	-0.056004567	count	1
SNRPD3	-0.0395224	0.0726354	-0.5441	0.586	-0.055936029	count	1
C5	-0.6969145	0.6019978	-1.1577	0.247	-0.055896409	count	1
KATNAL1	-0.0614985	0.6897191	-0.0892	0.929	-0.055880135	count	1
SYT11	-0.046872	0.2274446	-0.2061	0.837	-0.055725938	count	1
TLN1	-0.0387331	0.0520419	-0.7443	0.457	-0.055444469	count	1
PRCP	-0.0400123	0.1115431	-0.3587	0.72	-0.055443757	count	1
THAP4	-0.0513307	0.3217007	-0.1596	0.873	-0.055398996	count	1
FRA10AC1	-0.0431452	0.1856419	-0.2324	0.816	-0.055303352	count	1
MAF1	-0.0396753	0.0985377	-0.4026	0.687	-0.055287569	count	1
DCUN1D4	-0.0706701	0.410362	-0.1722	0.863	-0.055182103	count	1
RALBP1	-0.039201	0.093642	-0.4186	0.676	-0.055174419	count	1
CLK3	-0.043426	0.2072635	-0.2095	0.834	-0.055165462	count	1
AL158835.1	-0.2204687	1.025811	-0.2149	0.83	-0.0551494	count	1
WHRN	-0.1133529	0.544516	-0.2082	0.835	-0.055120914	count	1

OAZ1	-0.0382314	0.0231612	-1.6507	0.0989	-0.055085761	count	1
AC245452.1	-0.1260721	0.5792719	-0.2176	0.828	-0.055033306	count	1
RINT1	-0.0615344	0.4047923	-0.152	0.879	-0.054987324	count	1
KTI12	-0.0477821	0.2974283	-0.1607	0.872	-0.0549794	count	1
RNPS1	-0.0395595	0.1129952	-0.3501	0.726	-0.054816674	count	1
SLC25A1	-0.0421543	0.2042465	-0.2064	0.837	-0.054786336	count	1
SDHC	-0.0388995	0.0828522	-0.4695	0.639	-0.054575953	count	1
RPL27	-0.0379393	0.0333365	-1.1381	0.255	-0.054491887	count	1
SOWAHC	-0.0571165	0.427075	-0.1337	0.894	-0.054242769	count	1
RPL41	-0.0375718	0.020377	-1.8438	0.0653	-0.054167858	count	1
LRRIQ3	-0.3143545	1.2345272	-0.2546	0.799	-0.054017783	count	1
CFB	-0.3143545	1.2345272	-0.2546	0.799	-0.054017783	count	1
PIP5KL1	-0.3143545	1.5161908	-0.2073	0.836	-0.054017783	count	1
PHYHD1	-0.3143545	1.202161	-0.2615	0.794	-0.054017783	count	1
BACE1-AS	-0.3143545	1.2345272	-0.2546	0.799	-0.054017783	count	1
AC090061.1	-0.3143545	1.2345272	-0.2546	0.799	-0.054017783	count	1
LINC02391	-0.3143545	1.202161	-0.2615	0.794	-0.054017783	count	1
PLSCR3	-0.3143545	1.2345272	-0.2546	0.799	-0.054017783	count	1
ZNF382	-0.3143545	1.2345272	-0.2546	0.799	-0.054017783	count	1
AC021092.1	-0.3143545	1.202161	-0.2615	0.794	-0.054017783	count	1
ZNF112	-0.3143545	1.2345272	-0.2546	0.799	-0.054017783	count	1
CDK4	-0.0400747	0.1666175	-0.2405	0.81	-0.053925732	count	1
PPP1R2	-0.0382343	0.0840372	-0.455	0.649	-0.053861546	count	1
IBTK	-0.0410408	0.2027667	-0.2024	0.84	-0.053782087	count	1
RAB5A	-0.0393693	0.120736	-0.3261	0.744	-0.053767601	count	1
EIF2B1	-0.0422669	0.1759039	-0.2403	0.81	-0.053759856	count	1
PPM1A	-0.0432572	0.2183064	-0.1981	0.843	-0.053714423	count	1
NRBP1	-0.0386325	0.1343601	-0.2875	0.774	-0.053545745	count	1
DTX3L	-0.0416673	0.1847282	-0.2256	0.822	-0.053521573	count	1
SAMD13	-0.3096509	0.7652368	-0.4046	0.686	-0.053289429	count	1
TMEM161A	-0.0474993	0.2850244	-0.1666	0.868	-0.053021855	count	1
TBRG1	-0.0384763	0.1293657	-0.2974	0.766	-0.052964022	count	1
MTRF1	-0.0581027	0.4677546	-0.1242	0.901	-0.052818104	count	1
PRPF31	-0.0385208	0.1407104	-0.2738	0.784	-0.052755361	count	1
PLCE1	-0.0989341	0.8767853	-0.1128	0.91	-0.052625982	count	1
ITGA7	-0.0989341	0.8260253	-0.1198	0.905	-0.052625982	count	1
ZNF599	-0.0989341	1.117569	-0.0885	0.929	-0.052625982	count	1
GINM1	-0.0378765	0.10256	-0.3693	0.712	-0.052559522	count	1
SLC39A6	-0.0427446	0.1885582	-0.2267	0.821	-0.052509429	count	1
TDRD3	-0.0422781	0.248842	-0.1699	0.865	-0.052412162	count	1
MRPL19	-0.0408279	0.162707	-0.2509	0.802	-0.052391893	count	1
HUWE1	-0.0410197	0.217349	-0.1887	0.85	-0.052356696	count	1
ZNF32	-0.0419229	0.2119892	-0.1978	0.843	-0.052315069	count	1
XRCC3	-0.6385284	0.8062399	-0.792	0.428	-0.052253208	count	1
LINC01678	-0.0380821	0.139668	-0.2727	0.785	-0.052216774	count	1

TAF11	-0.038961	0.1438048	-0.2709	0.786	-0.052098715	count	1
PSEN2	-0.0592802	0.3758727	-0.1577	0.875	-0.052046334	count	1
ARHGAP32	-0.0710121	0.7545953	-0.0941	0.925	-0.052035913	count	1
RRP1B	-0.0398757	0.1997759	-0.1996	0.842	-0.052032013	count	1
CYFIP2	-0.084282	0.4798698	-0.1756	0.861	-0.05146405	count	1
AP002954.1	-0.0702032	0.4796986	-0.1463	0.884	-0.051450437	count	1
CHRNB1	-0.0506045	0.2946472	-0.1717	0.864	-0.051383474	count	1
SNRPC	-0.0363497	0.0787235	-0.4617	0.644	-0.051299713	count	1
QTRT2	-0.0398889	0.2560204	-0.1558	0.876	-0.051188812	count	1
CDK2AP1	-0.0955952	0.5020192	-0.1904	0.849	-0.050888071	count	1
ATAD3A	-0.0519027	0.2928039	-0.1773	0.859	-0.05055483	count	1
TMEM258	-0.0353347	0.0621976	-0.5681	0.57	-0.050236136	count	1
NLE1	-0.0874972	0.588863	-0.1486	0.882	-0.050175605	count	1
GINS1	-0.2896891	0.9783271	-0.2961	0.767	-0.050172006	count	1
PARL	-0.0365121	0.1089958	-0.335	0.738	-0.05015852	count	1
APBB2	-0.131084	0.5365325	-0.2443	0.807	-0.050152523	count	1
SUZ12	-0.0365485	0.1300099	-0.2811	0.779	-0.049776944	count	1
ZNF805	-0.0933353	0.5240413	-0.1781	0.859	-0.049710261	count	1
TAPBP	-0.0353768	0.091013	-0.3887	0.698	-0.049698515	count	1
C1QA	-0.0344403	0.0743804	-0.463	0.643	-0.049599805	count	1
SNRNP27	-0.0369123	0.201261	-0.1834	0.854	-0.04958199	count	1
KAT6B	-0.0411298	0.2992465	-0.1374	0.891	-0.049434845	count	1
SCMH1	-0.0651637	0.5658219	-0.1152	0.908	-0.049418408	count	1
DBT	-0.0599038	0.4108918	-0.1458	0.884	-0.049368344	count	1
SMARCB1	-0.035922	0.1261842	-0.2847	0.776	-0.049247256	count	1
DEF8	-0.0364266	0.1539139	-0.2367	0.813	-0.049056058	count	1
SELENOK	-0.03401	0.0757709	-0.4489	0.654	-0.048848784	count	1
MMP23B	-0.2805033	0.7799094	-0.3597	0.719	-0.048723094	count	1
MAPK8IP1	-0.2805033	0.7745144	-0.3622	0.717	-0.048723094	count	1
AC008764.8	-0.2805033	0.6156575	-0.4556	0.649	-0.048723094	count	1
LILRA4	-0.0995176	0.5308937	-0.1875	0.851	-0.048551365	count	1
C12orf75	-0.0542106	0.3940845	-0.1376	0.891	-0.048490584	count	1
PPP2R1B	-0.0454045	0.4422375	-0.1027	0.918	-0.048388447	count	1
LRRC27	-0.0714489	0.4594049	-0.1555	0.876	-0.048384013	count	1
NKILA	-0.277634	0.8304401	-0.3343	0.738	-0.048268669	count	1
AL590764.1	-0.0839337	0.4670278	-0.1797	0.857	-0.048168808	count	1
TMEM65	-0.0386677	0.2228547	-0.1735	0.862	-0.048105934	count	1
DNAJC30	-0.0464294	0.3740354	-0.1241	0.901	-0.048000101	count	1
GLMN	-0.0502907	0.3662214	-0.1373	0.891	-0.047799829	count	1
HMGXB3	-0.0353362	0.1726594	-0.2047	0.838	-0.047779486	count	1
LYAR	-0.0350229	0.1359079	-0.2577	0.797	-0.047747058	count	1
PRDM4	-0.0447935	0.3836737	-0.1167	0.907	-0.047739985	count	1
CCDC58	-0.0404707	0.3277247	-0.1235	0.902	-0.047715696	count	1
FBXO34	-0.0366025	0.1646808	-0.2223	0.824	-0.047693404	count	1
OSGEP	-0.0350467	0.1385388	-0.253	0.8	-0.047596758	count	1

SNRPD2	-0.0332991	0.0578311	-0.5758	0.565	-0.047523301	count	1
PKD2L2	-0.5660077	1.1391677	-0.4969	0.619	-0.047486923	count	1
ACHE	-0.5660077	1.1391677	-0.4969	0.619	-0.047486923	count	1
C20orf197	-0.5660077	1.223277	-0.4627	0.644	-0.047486923	count	1
AC011498.1	-0.5660077	1.1391677	-0.4969	0.619	-0.047486923	count	1
NEK8	-0.0735265	0.6955165	-0.1057	0.916	-0.047480596	count	1
AHI1	-0.0362043	0.2158251	-0.1677	0.867	-0.047451503	count	1
AC009133.1	-0.5652309	0.7092245	-0.797	0.426	-0.047434387	count	1
MZT1	-0.0357013	0.1713643	-0.2083	0.835	-0.047309042	count	1
RNF25	-0.057195	0.4154393	-0.1377	0.891	-0.047155325	count	1
LIPE-AS1	-0.0468556	0.355052	-0.132	0.895	-0.04714554	count	1
DYRK1B	-0.0641486	0.4904315	-0.1308	0.896	-0.047062785	count	1
SPCS1	-0.0329128	0.0528624	-0.6226	0.534	-0.046990289	count	1
CHST12	-0.0378698	0.2571932	-0.1472	0.883	-0.046876158	count	1
MTHFD1L	-0.0377255	0.2943224	-0.1282	0.898	-0.046858208	count	1
RAB14	-0.0332344	0.0873103	-0.3806	0.703	-0.046766087	count	1
ZNF395	-0.0452191	0.4338008	-0.1042	0.917	-0.046754522	count	1
ZNHIT1	-0.0325753	0.0718524	-0.4534	0.65	-0.046332422	count	1
NUTM2A-AS1	-0.0367155	0.2116696	-0.1735	0.862	-0.04629799	count	1
TIMM17B	-0.0331652	0.1125536	-0.2947	0.768	-0.046211041	count	1
LARP4	-0.0350055	0.178916	-0.1957	0.845	-0.046204959	count	1
FIS1	-0.0324783	0.0590291	-0.5502	0.582	-0.046179477	count	1
CCDC153	-0.0803856	0.6055012	-0.1328	0.894	-0.046167561	count	1
ITGB1BP1	-0.0331402	0.1169763	-0.2833	0.777	-0.046078844	count	1
CDK1	-0.0863188	0.6447306	-0.1339	0.894	-0.046045572	count	1
DNAJB5	-0.051437	0.5019555	-0.1025	0.918	-0.046026785	count	1
FUCA1	-0.0337893	0.1121996	-0.3012	0.763	-0.045975255	count	1
TRIM24	-0.0393416	0.2519981	-0.1561	0.876	-0.045946029	count	1
STK32C	-0.036334	0.2266105	-0.1603	0.873	-0.045879162	count	1
YIF1B	-0.0339911	0.16675	-0.2038	0.838	-0.04585774	count	1
EIF1AX	-0.0323868	0.0741896	-0.4365	0.662	-0.045823915	count	1
USP46-AS1	-0.074795	0.6179232	-0.121	0.904	-0.045759806	count	1
UTP3	-0.034779	0.1943023	-0.179	0.858	-0.045707598	count	1
AC062017.1	-0.0449568	0.5185101	-0.0867	0.931	-0.045675821	count	1
HS2ST1	-0.0405089	0.31446	-0.1288	0.898	-0.045673734	count	1
CCDC191	-0.093055	0.4256099	-0.2186	0.827	-0.045467492	count	1
BNIP1	-0.0455874	0.3472804	-0.1313	0.896	-0.045417811	count	1
ATP6V0E2	-0.0451208	0.3141119	-0.1436	0.886	-0.045408422	count	1
TMEM237	-0.0702199	0.6909294	-0.1016	0.919	-0.045374666	count	1
EVL	-0.0334636	0.1463697	-0.2286	0.819	-0.045358779	count	1
AL355922.1	-0.0926224	0.5443497	-0.1702	0.865	-0.045260713	count	1
SSH1	-0.0381032	0.273839	-0.1391	0.889	-0.04506616	count	1
OTUD4	-0.0394876	0.2476329	-0.1595	0.873	-0.044725081	count	1
ACAA1	-0.0320529	0.0951584	-0.3368	0.736	-0.044716398	count	1
TMEM53	-0.0378049	0.2745883	-0.1377	0.891	-0.04458033	count	1

AQP1	-0.5224034	0.8761359	-0.5963	0.551	-0.044488356	count	1
STIM2	-0.0358074	0.2026211	-0.1767	0.86	-0.044479953	count	1
KPNA6	-0.0348898	0.2288467	-0.1525	0.879	-0.044442463	count	1
ADAP2	-0.0310277	0.0811368	-0.3824	0.702	-0.044122104	count	1
LEO1	-0.0356107	0.2147409	-0.1658	0.868	-0.043926965	count	1
BBS5	-0.0545504	0.9985305	-0.0546	0.956	-0.043887343	count	1
LRRC75B	-0.0896888	0.7912999	-0.1133	0.91	-0.04385736	count	1
LARS2	-0.0896057	0.5743641	-0.156	0.876	-0.043817577	count	1
LCP2	-0.0318472	0.099599	-0.3198	0.749	-0.043769076	count	1
RBP1	-0.064541	0.6143353	-0.1051	0.916	-0.043762827	count	1
AP2A2	-0.0334576	0.1380067	-0.2424	0.808	-0.043699315	count	1
KDM1A	-0.0348081	0.2878262	-0.1209	0.904	-0.04364956	count	1
RPS6KL1	-0.2484519	0.9384224	-0.2648	0.791	-0.043596123	count	1
TMEM204	-0.2484519	0.9334979	-0.2662	0.79	-0.043596123	count	1
AL008635.1	-0.2484519	0.9334979	-0.2662	0.79	-0.043596123	count	1
AP001059.2	-0.2484519	0.9169425	-0.271	0.786	-0.043596123	count	1
PRPF6	-0.0317542	0.1399495	-0.2269	0.821	-0.043493566	count	1
AGER	-0.1130232	0.6526207	-0.1732	0.863	-0.043447229	count	1
NMT1	-0.0323819	0.1678021	-0.193	0.847	-0.04327089	count	1
ANKHD1-EIF4EBP3	-0.5050637	0.7352596	-0.6869	0.492	-0.043267585	count	1
DENND1B	-0.03353	0.2087311	-0.1606	0.872	-0.042712752	count	1
ARF6	-0.0305062	0.0879294	-0.3469	0.729	-0.042674774	count	1
PAXIP1	-0.0529788	0.5092974	-0.104	0.917	-0.04263341	count	1
PCNA	-0.0332559	0.2003087	-0.166	0.868	-0.042599187	count	1
C3AR1	-0.029808	0.0630713	-0.4726	0.637	-0.042366764	count	1
MAP3K14	-0.0444841	0.3530601	-0.126	0.9	-0.042310397	count	1
PA2G4	-0.0297311	0.0727003	-0.409	0.683	-0.042173806	count	1
MFSD2A	-0.0427112	0.3737931	-0.1143	0.909	-0.042116614	count	1
LYRM9	-0.0430602	0.444854	-0.0968	0.923	-0.041984501	count	1
ANO6	-0.0315566	0.1998202	-0.1579	0.875	-0.041973055	count	1
FOXO1	-0.0343576	0.2430832	-0.1413	0.888	-0.041972932	count	1
GCNT2	-0.0388034	0.3697436	-0.1049	0.916	-0.041925487	count	1
RAB1B	-0.0311295	0.1557621	-0.1999	0.842	-0.041867158	count	1
UBL5	-0.0292461	0.0486528	-0.6011	0.548	-0.041854778	count	1
LRRC40	-0.0398218	0.2961788	-0.1345	0.893	-0.041854017	count	1
ASMTL	-0.033464	0.248594	-0.1346	0.893	-0.041642594	count	1
TOP1MT	-0.0398121	0.4422811	-0.09	0.928	-0.041521804	count	1
NDUFA5	-0.0298903	0.1051489	-0.2843	0.776	-0.041486538	count	1
ZNF90	-0.084632	0.4909416	-0.1724	0.863	-0.041433664	count	1
UBAP1	-0.0316318	0.1868627	-0.1693	0.866	-0.041377364	count	1
NUBP2	-0.0316892	0.1777721	-0.1783	0.859	-0.041097929	count	1
BEX1	-0.066844	0.67691	-0.0987	0.921	-0.040961645	count	1
MGRN1	-0.0314794	0.3730794	-0.0844	0.933	-0.040927837	count	1
CSNK2A1	-0.0306351	0.1593824	-0.1922	0.848	-0.040768418	count	1
NFE2L3	-0.0384725	0.190251	-0.2022	0.84	-0.040739887	count	1

JMY	-0.0331169	0.2202263	-0.1504	0.88	-0.040623674	count	1
CASTOR3	-0.1047798	0.5954432	-0.176	0.86	-0.04036495	count	1
SLC37A3	-0.1047798	0.5933502	-0.1766	0.86	-0.04036495	count	1
XYLT1	-0.0442328	0.3957254	-0.1118	0.911	-0.040282577	count	1
IDO1	-0.039622	0.5419704	-0.0731	0.942	-0.040278091	count	1
FDXR	-0.0418919	0.5331285	-0.0786	0.937	-0.039857267	count	1
NNMT	-0.0904831	0.5177454	-0.1748	0.861	-0.039847575	count	1
DESI1	-0.0294828	0.1629814	-0.1809	0.856	-0.039766225	count	1
ZDHHC2	-0.0306975	0.1848101	-0.1661	0.868	-0.039709446	count	1
TRAF1	-0.0352872	0.3294898	-0.1071	0.915	-0.039618367	count	1
ACTR10	-0.0289687	0.1636922	-0.177	0.86	-0.039614319	count	1
COX7B	-0.0277157	0.053578	-0.5173	0.605	-0.039578603	count	1
TGFB2	-0.0739196	0.5808146	-0.1273	0.899	-0.039540593	count	1
EPOP	-0.0739196	0.6108063	-0.121	0.904	-0.039540593	count	1
ROCK2	-0.0301131	0.155728	-0.1934	0.847	-0.039529252	count	1
HIRA	-0.0476723	0.5028482	-0.0948	0.924	-0.039360808	count	1
CHMP5	-0.027795	0.0775417	-0.3585	0.72	-0.039301795	count	1
CD1A	-0.2220366	0.7612231	-0.2917	0.771	-0.039286766	count	1
AC106047.1	-0.2220366	0.7612231	-0.2917	0.771	-0.039286766	count	1
TRIM60	-0.2220366	1.021223	-0.2174	0.828	-0.039286766	count	1
AC006064.4	-0.2220366	0.7685758	-0.2889	0.773	-0.039286766	count	1
CXCL12	-0.0302808	0.1907858	-0.1587	0.874	-0.039273332	count	1
XRCC5	-0.0275905	0.0604083	-0.4567	0.648	-0.03921347	count	1
CD163	-0.0274114	0.0668367	-0.4101	0.682	-0.039091459	count	1
FCER1G	-0.0270419	0.0255655	-1.0578	0.29	-0.038960502	count	1
ARHGAP35	-0.0350073	0.3242759	-0.108	0.914	-0.038921371	count	1
MGMT	-0.0281738	0.1108299	-0.2542	0.799	-0.038856061	count	1
FAM13A-AS1	-0.0674568	0.6035584	-0.1118	0.911	-0.038848962	count	1
SNAP47	-0.0358777	0.4335577	-0.0828	0.934	-0.038774412	count	1
PINK1	-0.0309524	0.1832185	-0.1689	0.866	-0.03864527	count	1
FAM168A	-0.0390366	0.3249656	-0.1201	0.904	-0.038508937	count	1
MEF2C-AS1	-0.0565368	0.5184183	-0.1091	0.913	-0.038392926	count	1
CATSPER2	-0.0870559	0.6514532	-0.1336	0.894	-0.038370612	count	1
SUMO2	-0.026713	0.0365716	-0.7304	0.465	-0.038353947	count	1
COMMD8	-0.0278638	0.1151028	-0.2421	0.809	-0.038310424	count	1
GLIPR2	-0.0267439	0.0798314	-0.335	0.738	-0.038163837	count	1
MED21	-0.0313602	0.2041188	-0.1536	0.878	-0.037985082	count	1
CDC42	-0.026431	0.0371888	-0.7107	0.477	-0.037941631	count	1
CCDC171	-0.0614339	0.6292253	-0.0976	0.922	-0.037687748	count	1
GABARAPL2	-0.0264505	0.0643672	-0.4109	0.681	-0.037677654	count	1
NUFIP2	-0.0269116	0.1002432	-0.2685	0.788	-0.037658373	count	1
TMED5	-0.0265289	0.09262	-0.2864	0.775	-0.037434424	count	1
POLR3GL	-0.0271488	0.1194962	-0.2272	0.82	-0.037321306	count	1
FAM120AOS	-0.0278488	0.1479464	-0.1882	0.851	-0.037234059	count	1
TRERF1	-0.0365929	0.4862273	-0.0753	0.94	-0.037210546	count	1

BRCA2	-0.0305651	0.2488964	-0.1228	0.902	-0.037189361	count	1
TMEM123	-0.026122	0.0633423	-0.4124	0.68	-0.037149876	count	1
MRPS9	-0.0300687	0.2392378	-0.1257	0.9	-0.037101208	count	1
ARF4-AS1	-0.0840312	0.7697284	-0.1092	0.913	-0.037064985	count	1
ZBED3-AS1	-0.0840312	0.7687341	-0.1093	0.913	-0.037064985	count	1
LRRC49	-0.4199852	0.9264474	-0.4533	0.65	-0.037038164	count	1
ABCE1	-0.027306	0.1465599	-0.1863	0.852	-0.036962273	count	1
PAN3-AS1	-0.2077307	0.6674987	-0.3112	0.756	-0.036921051	count	1
ADAMTS1	-0.2077307	0.8516569	-0.2439	0.807	-0.036921051	count	1
GNA13	-0.0263841	0.1002635	-0.2631	0.792	-0.036738427	count	1
IGFBP7	-0.0263657	0.1385188	-0.1903	0.849	-0.036726496	count	1
NCOR1	-0.0260096	0.0870085	-0.2989	0.765	-0.036718969	count	1
ILVBL	-0.0288197	0.2146052	-0.1343	0.893	-0.036588692	count	1
SELENOT	-0.0259089	0.0678335	-0.3819	0.703	-0.036574249	count	1
ARMCX3	-0.0264315	0.1317883	-0.2006	0.841	-0.036390987	count	1
EIF1AD	-0.0285746	0.2323459	-0.123	0.902	-0.036365164	count	1
MT-ND3	-0.0251996	0.0355565	-0.7087	0.479	-0.036255464	count	1
AC037459.2	-0.4068395	0.7682875	-0.5295	0.596	-0.036039469	count	1
SLC25A25	-0.032064	0.2846904	-0.1126	0.91	-0.036008597	count	1
UBE2D3	-0.0251579	0.0477855	-0.5265	0.599	-0.035991536	count	1
CLUH	-0.0407956	0.543347	-0.0751	0.94	-0.035908826	count	1
PLCD1	-0.0729676	0.5547836	-0.1315	0.895	-0.035820488	count	1
UBE2B	-0.0251112	0.0557423	-0.4505	0.652	-0.035813256	count	1
ZNF710	-0.0325911	0.2927468	-0.1113	0.911	-0.035658305	count	1
STX12	-0.0255918	0.0952686	-0.2686	0.788	-0.035615096	count	1
NDUFA2	-0.0250078	0.0647197	-0.3864	0.699	-0.035589474	count	1
PPP1R16B	-0.4008463	0.8888874	-0.451	0.652	-0.035580892	count	1
AC027307.3	-0.4008463	0.8888874	-0.451	0.652	-0.035580892	count	1
IPO7	-0.0271684	0.1575432	-0.1725	0.863	-0.035569315	count	1
WDR81	-0.0382714	0.3898834	-0.0982	0.922	-0.03542407	count	1
DUSP2	-0.0247202	0.0993283	-0.2489	0.803	-0.035386427	count	1
UBXN4	-0.0248513	0.0667713	-0.3722	0.71	-0.035359258	count	1
DNAJC14	-0.0338279	0.3383142	-0.1	0.92	-0.035301195	count	1
DCAF13	-0.0262074	0.1571082	-0.1668	0.868	-0.035114854	count	1
CDK8	-0.0377396	0.5334135	-0.0708	0.944	-0.034934177	count	1
SGK3	-0.0254276	0.134923	-0.1885	0.851	-0.034824938	count	1
C2orf68	-0.0280544	0.2888518	-0.0971	0.923	-0.034803528	count	1
NEURL2	-0.3890643	0.5592986	-0.6956	0.487	-0.03467339	count	1
RBMXL1	-0.032182	0.2967211	-0.1085	0.914	-0.034569494	count	1
UBE2J2	-0.0255415	0.1457171	-0.1753	0.861	-0.034487816	count	1
TRIM69	-0.0273237	0.2333314	-0.1171	0.907	-0.034374446	count	1
ARHGAP1	-0.0300604	0.288934	-0.104	0.917	-0.034218445	count	1
PATJ	-0.381608	1.1886192	-0.3211	0.748	-0.034094948	count	1
TEX41	-0.381608	1.1886192	-0.3211	0.748	-0.034094948	count	1
DLX2	-0.381608	1.1886192	-0.3211	0.748	-0.034094948	count	1

FAM171B	-0.381608	1.1886192	-0.3211	0.748	-0.034094948	count	1
AC114730.2	-0.381608	1.1886192	-0.3211	0.748	-0.034094948	count	1
FBLN2	-0.381608	1.308419	-0.2917	0.771	-0.034094948	count	1
TNK2-AS1	-0.381608	1.1886192	-0.3211	0.748	-0.034094948	count	1
STBD1	-0.381608	1.1886192	-0.3211	0.748	-0.034094948	count	1
AC126283.1	-0.381608	1.1886192	-0.3211	0.748	-0.034094948	count	1
AC105285.1	-0.381608	1.1886192	-0.3211	0.748	-0.034094948	count	1
PLN	-0.381608	1.308419	-0.2917	0.771	-0.034094948	count	1
AL591468.1	-0.381608	1.1886192	-0.3211	0.748	-0.034094948	count	1
KCND1	-0.381608	1.1886192	-0.3211	0.748	-0.034094948	count	1
NEFM	-0.381608	1.308419	-0.2917	0.771	-0.034094948	count	1
PKIA	-0.381608	1.1886192	-0.3211	0.748	-0.034094948	count	1
ERICD	-0.381608	1.1886192	-0.3211	0.748	-0.034094948	count	1
AL357874.1	-0.381608	1.1886192	-0.3211	0.748	-0.034094948	count	1
AL358781.1	-0.381608	1.1886192	-0.3211	0.748	-0.034094948	count	1
NPFFR1	-0.381608	1.1886192	-0.3211	0.748	-0.034094948	count	1
SYCE1	-0.381608	1.1886192	-0.3211	0.748	-0.034094948	count	1
ADCY6	-0.381608	1.1886192	-0.3211	0.748	-0.034094948	count	1
AC073592.1	-0.381608	1.1886192	-0.3211	0.748	-0.034094948	count	1
AC091057.3	-0.381608	1.1886192	-0.3211	0.748	-0.034094948	count	1
KLHL25	-0.381608	1.1886192	-0.3211	0.748	-0.034094948	count	1
AC018557.1	-0.381608	1.1886192	-0.3211	0.748	-0.034094948	count	1
AC127024.5	-0.381608	1.1886192	-0.3211	0.748	-0.034094948	count	1
SYDE1	-0.381608	1.1886192	-0.3211	0.748	-0.034094948	count	1
CYP4F22	-0.381608	1.1886192	-0.3211	0.748	-0.034094948	count	1
BCKDHA	-0.381608	1.1886192	-0.3211	0.748	-0.034094948	count	1
ARVCF	-0.381608	1.1886192	-0.3211	0.748	-0.034094948	count	1
PLA2G4A	-0.0362743	0.5090031	-0.0713	0.943	-0.034072256	count	1
IFI27L2	-0.0242076	0.1072145	-0.2258	0.821	-0.034048883	count	1
TUBB4B	-0.0239817	0.0771506	-0.3108	0.756	-0.034012778	count	1
PI4K2B	-0.0385804	0.4306665	-0.0896	0.929	-0.03396929	count	1
B4GAT1	-0.1900327	0.5200806	-0.3654	0.715	-0.033963261	count	1
EDEM1	-0.025364	0.1617872	-0.1568	0.875	-0.033725345	count	1
SNHG21	-0.0398001	0.472683	-0.0842	0.933	-0.033656427	count	1
CFAP20	-0.0273646	0.2391493	-0.1144	0.909	-0.033642934	count	1
EXO5	-0.1027908	0.6686335	-0.1537	0.878	-0.033485471	count	1
RNASE3	-0.1027908	0.7692206	-0.1336	0.894	-0.033485471	count	1
HLA-DQA2	-0.0414656	0.071022	-0.5838	0.559	-0.033428278	count	1
TMEM59	-0.0233789	0.0461625	-0.5064	0.6126	-0.03342431	count	1
AC027307.2	-0.0404357	0.9481758	-0.0426	0.966	-0.033422246	count	1
NUDT21	-0.0252219	0.1466464	-0.172	0.863	-0.033261248	count	1
TES	-0.0257414	0.1610998	-0.1598	0.873	-0.033244169	count	1
WDR19	-0.129056	0.7171531	-0.18	0.857	-0.033164618	count	1
PTGES2	-0.0248052	0.1561595	-0.1588	0.874	-0.033138293	count	1
SOX12	-0.0573648	0.5806912	-0.0988	0.921	-0.033107566	count	1

RSU1	-0.0242469	0.1247935	-0.1943	0.846	-0.033047023	count	1
ABAT	-0.0485221	0.4361594	-0.1112	0.911	-0.032999558	count	1
CYB561	-0.0746182	0.4982688	-0.1498	0.881	-0.032989075	count	1
PRR5L	-0.1282531	0.5210643	-0.2461	0.806	-0.032966004	count	1
SLC35D1	-0.0614305	0.491198	-0.1251	0.9	-0.032951196	count	1
AEBP2	-0.0283037	0.2642395	-0.1071	0.915	-0.03285273	count	1
ME3	-0.0534252	0.7556717	-0.0707	0.944	-0.032827844	count	1
AP002433.1	-0.0534252	1.600515	-0.0334	0.973	-0.032827844	count	1
EPN1	-0.0241584	0.1414789	-0.1708	0.864	-0.032816766	count	1
C14orf28	-0.0481235	0.51481	-0.0935	0.926	-0.032730899	count	1
AIG1	-0.0245022	0.1638724	-0.1495	0.881	-0.032674434	count	1
MRPL54	-0.0230234	0.0875137	-0.2631	0.793	-0.032491133	count	1
AC021054.1	-0.0391055	0.6059524	-0.0645	0.949	-0.032329218	count	1
PPARGC1B	-0.0471376	0.3673814	-0.1283	0.898	-0.032066222	count	1
NRL	-0.0724924	0.6170179	-0.1175	0.906	-0.03206592	count	1
WBP2NL	-0.0724924	0.6773708	-0.107	0.915	-0.03206592	count	1
ARSA	-0.0241245	0.1803236	-0.1338	0.894	-0.032045946	count	1
DUSP5	-0.0233499	0.1472752	-0.1585	0.874	-0.032036944	count	1
SCO1	-0.0275028	0.2326868	-0.1182	0.906	-0.032036647	count	1
TIMM29	-0.0297849	0.3333314	-0.0894	0.929	-0.032001436	count	1
AL606760.1	-0.3534516	1.0886027	-0.3247	0.745	-0.031881683	count	1
AKIP1	-0.0243736	0.2009209	-0.1213	0.903	-0.03186754	count	1
SP140	-0.0315416	0.2796445	-0.1128	0.91	-0.031788504	count	1
CEP19	-0.0447857	0.4525461	-0.099	0.921	-0.031770585	count	1
AQP3	-0.0641486	0.7468562	-0.0859	0.932	-0.031555809	count	1
CASTOR1	-0.0641486	0.6508201	-0.0986	0.921	-0.031555809	count	1
CCDC122	-0.0513307	0.5781847	-0.0888	0.929	-0.031554194	count	1
AC048382.5	-0.0513307	0.5767825	-0.089	0.929	-0.031554194	count	1
AC020912.1	-0.0513307	0.6636232	-0.0773	0.938	-0.031554194	count	1
BSG	-0.0221264	0.062183	-0.3558	0.722	-0.031466634	count	1
CD300LB	-0.0237946	0.1750606	-0.1359	0.892	-0.031220288	count	1
BMP2K	-0.0224728	0.1004399	-0.2237	0.823	-0.031190932	count	1
ERGIC3	-0.0223059	0.1096122	-0.2035	0.839	-0.031122054	count	1
ASL	-0.0256714	0.2126533	-0.1207	0.904	-0.031032443	count	1
8-Sep	-0.0628938	0.4571038	-0.1376	0.891	-0.030947563	count	1
TMX1	-0.0222269	0.1092657	-0.2034	0.839	-0.030776284	count	1
DNAJC7	-0.0218441	0.0910653	-0.2399	0.81	-0.03070697	count	1
TAX1BP1	-0.0214323	0.0617736	-0.347	0.729	-0.030479638	count	1
KCTD17	-0.0281968	0.3264824	-0.0864	0.931	-0.03029946	count	1
CLP1	-0.0254616	0.3477305	-0.0732	0.942	-0.030226049	count	1
RECK	-0.02959	0.359519	-0.0823	0.934	-0.030111276	count	1
HDAC2	-0.0218182	0.1351507	-0.1614	0.872	-0.029759604	count	1
KLF12	-0.3266344	0.598754	-0.5455	0.585	-0.029730685	count	1
AMDHD2	-0.0229373	0.1727838	-0.1328	0.894	-0.029705589	count	1
POLR2I	-0.0216049	0.1449221	-0.1491	0.882	-0.029692432	count	1

SLC25A24	-0.0212998	0.1083702	-0.1965	0.844	-0.029685167	count	1
FAM120C	-0.043477	0.4714073	-0.0922	0.927	-0.02959613	count	1
LAGE3	-0.0210847	0.1193525	-0.1767	0.86	-0.029393302	count	1
ERLIN1	-0.0243643	0.2001349	-0.1217	0.903	-0.029384544	count	1
C22orf39	-0.0220505	0.1804521	-0.1222	0.903	-0.029336736	count	1
SIL1	-0.0216267	0.1421885	-0.1521	0.879	-0.029287034	count	1
EMP2	-0.0236126	0.1482506	-0.1593	0.873	-0.029249019	count	1
GLS	-0.0208697	0.1032725	-0.2021	0.84	-0.029180517	count	1
ZNF436-AS1	-0.074795	0.739756	-0.1011	0.919	-0.029038224	count	1
ATL1	-0.074795	0.820605	-0.0911	0.927	-0.029038224	count	1
SOCS1	-0.0241025	0.2669399	-0.0903	0.928	-0.028998249	count	1
TSPAN10	-0.1121364	0.8572018	-0.1308	0.896	-0.028958912	count	1
RWDD2B	-0.0537838	0.5024399	-0.107	0.915	-0.02889831	count	1
TMEM184B	-0.029922	0.402636	-0.0743	0.941	-0.028872593	count	1
FAM20A	-0.0252243	0.2584414	-0.0976	0.922	-0.028842826	count	1
ATF7	-0.0230425	0.2205963	-0.1045	0.917	-0.02878019	count	1
TNFSF13	-0.0494352	0.5125449	-0.0965	0.923	-0.028578826	count	1
WIZ	-0.0277386	0.4097817	-0.0677	0.946	-0.028486883	count	1
GON4L	-0.0216525	0.1760384	-0.123	0.902	-0.028291635	count	1
ACP6	-0.0863188	1.0228455	-0.0844	0.933	-0.028246419	count	1
TNNI2	-0.0209966	0.1639822	-0.128	0.898	-0.028245861	count	1
CAMKK1	-0.0318096	0.4065224	-0.0782	0.938	-0.028033664	count	1
NME7	-0.027757	0.4432171	-0.0626	0.95	-0.027985465	count	1
RNF10	-0.0210434	0.1884996	-0.1116	0.911	-0.027787476	count	1
C5orf24	-0.0210254	0.1973048	-0.1066	0.915	-0.027763722	count	1
MTHFD2L	-0.0207862	0.2197329	-0.0946	0.925	-0.027495586	count	1
GSKIP	-0.0266123	0.2518034	-0.1057	0.916	-0.027089442	count	1
EEF1E1	-0.0203745	0.1839837	-0.1107	0.912	-0.027025201	count	1
ASB6	-0.0271279	0.4660601	-0.0582	0.954	-0.026796563	count	1
SAE1	-0.0224551	0.2151881	-0.1044	0.917	-0.02673705	count	1
MGAT2	-0.0203414	0.1616382	-0.1258	0.9	-0.026710247	count	1
CYB5R3	-0.0191094	0.1036584	-0.1843	0.854	-0.026684966	count	1
CLIP1	-0.0196668	0.1405472	-0.1399	0.889	-0.026657725	count	1
ARMC5	-0.0684699	0.4333411	-0.158	0.874	-0.02662588	count	1
POLR1A	-0.029541	0.6845204	-0.0432	0.966	-0.026511096	count	1
ANKZF1	-0.0289927	0.3559068	-0.0815	0.935	-0.026455591	count	1
GTPBP2	-0.0248515	0.3385498	-0.0734	0.941	-0.026349571	count	1
FKBP1B	-0.0258275	0.4026025	-0.0642	0.949	-0.026292698	count	1
VSIG4	-0.0184543	0.0712688	-0.2589	0.796	-0.026253618	count	1
RTN2	-0.0283103	0.3144348	-0.09	0.928	-0.02623687	count	1
SBDS	-0.0185409	0.0801712	-0.2313	0.817	-0.026195207	count	1
11-Sep	-0.0198047	0.1386855	-0.1428	0.886	-0.026183123	count	1
NUDC	-0.0186281	0.0864005	-0.2156	0.829	-0.026116991	count	1
MRPS10	-0.0197716	0.1508159	-0.1311	0.896	-0.026046055	count	1
LAMA4	-0.2805033	0.9408038	-0.2982	0.766	-0.025930991	count	1

OMG	-0.2805033	0.944513	-0.297	0.767	-0.025930991	count	1
NSMCE4A	-0.020122	0.2108439	-0.0954	0.924	-0.025815537	count	1
AL583839.1	-0.0993269	1.1103791	-0.0895	0.929	-0.025746366	count	1
APBB1	-0.0993269	0.8636628	-0.115	0.908	-0.025746366	count	1
AC032044.1	-0.0993269	0.7801571	-0.1273	0.899	-0.025746366	count	1
SFT2D1	-0.0182797	0.0862946	-0.2118	0.832	-0.025722176	count	1
NCLN	-0.021072	0.2393906	-0.088	0.93	-0.02570721	count	1
NOXA1	-0.0348177	0.4224553	-0.0824	0.934	-0.025673774	count	1
SCCPDH	-0.0191506	0.1659952	-0.1154	0.908	-0.025480622	count	1
DEPDC7	-0.2750998	0.9383558	-0.2932	0.769	-0.025477598	count	1
VASN	-0.2750998	1.056868	-0.2603	0.795	-0.025477598	count	1
AC015726.1	-0.2750998	1.010125	-0.2723	0.785	-0.025477598	count	1
LYPD5	-0.2750998	0.9383558	-0.2932	0.769	-0.025477598	count	1
RBFOX2	-0.2750998	0.8732483	-0.315	0.753	-0.025477598	count	1
AL353194.1	-0.0254939	0.3950396	-0.0645	0.949	-0.025454495	count	1
LY75	-0.0332729	0.4867947	-0.0684	0.946	-0.025367718	count	1
MCUR1	-0.018493	0.1427297	-0.1296	0.897	-0.02532029	count	1
C12orf73	-0.0223755	0.3101121	-0.0722	0.942	-0.0252634	count	1
ACVR1B	-0.0245726	0.4673289	-0.0526	0.958	-0.025243535	count	1
CHD9	-0.0180591	0.1065944	-0.1694	0.865	-0.025162814	count	1
IMP4	-0.0182622	0.1687739	-0.1082	0.914	-0.025125864	count	1
RABL6	-0.0188623	0.1706376	-0.1105	0.912	-0.025059521	count	1
CPOX	-0.0464429	0.4428733	-0.1049	0.916	-0.024994388	count	1
TMEM19	-0.0197518	0.233369	-0.0846	0.933	-0.024958797	count	1
TRMT112	-0.0174531	0.0586484	-0.2976	0.766	-0.024910585	count	1
CCDC28B	-0.0239739	0.387207	-0.0619	0.951	-0.024629973	count	1
RPS21	-0.0170653	0.0336813	-0.5067	0.612	-0.024499618	count	1
TAF6	-0.0234492	0.3335291	-0.0703	0.944	-0.024495087	count	1
ETV7	-0.0453593	0.4918975	-0.0922	0.927	-0.024417039	count	1
TAF3	-0.0196808	0.1975906	-0.0996	0.921	-0.024383422	count	1
AGAP3	-0.0200172	0.1975639	-0.1013	0.919	-0.024369539	count	1
RAB30	-0.1336637	0.6726742	-0.1987	0.843	-0.02431144	count	1
APEX1	-0.0172194	0.0754324	-0.2283	0.819	-0.024261574	count	1
ARF3	-0.0173788	0.1181641	-0.1471	0.883	-0.023902647	count	1
CLDN1	-0.0179453	0.2441523	-0.0735	0.941	-0.02388938	count	1
CAB39L	-0.0267968	0.5411919	-0.0495	0.961	-0.02363203	count	1
PPP6C	-0.0176203	0.1561547	-0.1128	0.91	-0.023554414	count	1
GPRC5A	-0.0316509	0.6867439	-0.0461	0.963	-0.023351299	count	1
HIST1H2BD	-0.1271926	0.7326831	-0.1736	0.862	-0.02318078	count	1
EPCAM	-0.1260721	0.983862	-0.1281	0.898	-0.022984527	count	1
CLEC4F	-0.1260721	0.7855534	-0.1605	0.873	-0.022984527	count	1
DDX42	-0.0171275	0.1849735	-0.0926	0.926	-0.022778788	count	1
LINC01315	-0.0288868	0.5722713	-0.0505	0.96	-0.022708315	count	1
GDE1	-0.0174881	0.1477941	-0.1183	0.906	-0.022652864	count	1
PID1	-0.0162732	0.1235756	-0.1317	0.895	-0.022631445	count	1

NDRG2	-0.0243217	0.2588876	-0.0939	0.925	-0.022551639	count	1
PSD3	-0.0203391	0.3299905	-0.0616	0.951	-0.02240177	count	1
REX1BD	-0.0157211	0.0692439	-0.227	0.82	-0.022395664	count	1
DYNC1H1	-0.0158342	0.1268424	-0.1248	0.901	-0.022132735	count	1
PEX26	-0.0221436	0.360051	-0.0615	0.951	-0.022117347	count	1
APBB3	-0.0173892	0.2214364	-0.0785	0.937	-0.021823545	count	1
TM6SF1	-0.0159575	0.1503269	-0.1062	0.915	-0.021579194	count	1
WFS1	-0.0432701	0.6256901	-0.0692	0.945	-0.021388302	count	1
SEC61A2	-0.0476723	0.8900487	-0.0536	0.957	-0.021214911	count	1
PAPLN	-0.0476723	0.7610162	-0.0626	0.95	-0.021214911	count	1
NOL10	-0.019679	0.2735561	-0.0719	0.943	-0.021162549	count	1
PTOV1	-0.0159826	0.2163023	-0.0739	0.941	-0.02100344	count	1
CDK19	-0.018637	0.2901403	-0.0642	0.949	-0.020951554	count	1
AC069185.1	-0.035986	0.7114562	-0.0506	0.96	-0.020862563	count	1
AL021707.6	-0.0628679	0.6113293	-0.1028	0.918	-0.020704046	count	1
SULT1B1	-0.2181436	0.5966145	-0.3656	0.715	-0.020590943	count	1
CPT2	-0.0188864	0.3045988	-0.062	0.951	-0.020567004	count	1
RFC3	-0.0237273	0.625168	-0.038	0.97	-0.020534926	count	1
SHLD1	-0.0168126	0.2756576	-0.061	0.951	-0.020426859	count	1
FTCDNL1	-0.0295667	0.4971945	-0.0595	0.953	-0.020178775	count	1
ENKD1	-0.0294338	0.5672956	-0.0519	0.959	-0.020088565	count	1
NDUFS8	-0.0141766	0.0772392	-0.1835	0.854	-0.020045621	count	1
MIR99AHG	-0.2107859	0.7048752	-0.299	0.765	-0.019945219	count	1
RPS19BP1	-0.0140755	0.0958601	-0.1468	0.883	-0.019894037	count	1
GIGYF2	-0.0152505	0.1753501	-0.087	0.931	-0.01985487	count	1
MED27	-0.0166489	0.2613167	-0.0637	0.949	-0.019830689	count	1
F8	-0.0340239	0.528005	-0.0644	0.949	-0.01973315	count	1
AC098613.1	-0.0213235	0.4292379	-0.0497	0.96	-0.019476678	count	1
AC024257.3	-0.1041948	0.735075	-0.1417	0.887	-0.019124562	count	1
ARHGDIA	-0.0134587	0.0689492	-0.1952	0.845	-0.0190935	count	1
ZNF658	-0.0385804	0.7851412	-0.0491	0.961	-0.019090777	count	1
ABHD2	-0.0141842	0.1487442	-0.0954	0.924	-0.019048628	count	1
MRPL35	-0.0143826	0.1813841	-0.0793	0.937	-0.018973146	count	1
TTLL7	-0.0724924	0.9737966	-0.0744	0.941	-0.018936709	count	1
BX323046.1	-0.0724924	1.0410207	-0.0696	0.944	-0.018936709	count	1
AC018638.7	-0.0724924	1.3091132	-0.0554	0.956	-0.018936709	count	1
AL365203.2	-0.0724924	1.125101	-0.0644	0.949	-0.018936709	count	1
ODF3L1	-0.0724924	0.8723876	-0.0831	0.934	-0.018936709	count	1
KCNG2	-0.0724924	0.8723876	-0.0831	0.934	-0.018936709	count	1
ZNF737	-0.0724924	0.8723876	-0.0831	0.934	-0.018936709	count	1
KYNU	-0.0137993	0.1200212	-0.115	0.908	-0.018850822	count	1
REL	-0.0131557	0.0601545	-0.2187	0.827	-0.018817994	count	1
C16orf91	-0.0146488	0.2603399	-0.0563	0.955	-0.018758169	count	1
APEX2	-0.0160641	0.3353043	-0.0479	0.962	-0.018726672	count	1
OXSR1	-0.0136744	0.1482718	-0.0922	0.927	-0.01865451	count	1

LCK	-0.0565368	1.0144026	-0.0557	0.956	-0.01865097	count	1
AC013400.1	-0.0565368	1.0142335	-0.0557	0.956	-0.01865097	count	1
AP003469.4	-0.0565368	1.0139229	-0.0558	0.956	-0.01865097	count	1
ZBTB26	-0.0565368	1.2100903	-0.0467	0.963	-0.01865097	count	1
DTX4	-0.0565368	0.9001992	-0.0628	0.95	-0.01865097	count	1
AC000403.1	-0.0565368	1.0144026	-0.0557	0.956	-0.01865097	count	1
SULT1A3	-0.0565368	0.9007395	-0.0628	0.95	-0.01865097	count	1
PLD6	-0.0565368	0.9012795	-0.0627	0.95	-0.01865097	count	1
RIPOR3	-0.0565368	0.9009299	-0.0628	0.95	-0.01865097	count	1
SH3GLB1	-0.0129548	0.072309	-0.1792	0.858	-0.018430363	count	1
ENY2	-0.0128983	0.0521871	-0.2472	0.8048	-0.018422559	count	1
PIK3IP1	-0.0164025	0.180042	-0.0911	0.927	-0.018354685	count	1
TECPR1	-0.0168154	0.3539796	-0.0475	0.962	-0.018203446	count	1
FUBP3	-0.0159463	0.2658895	-0.06	0.952	-0.018170925	count	1
HELZ2	-0.0176618	0.3039154	-0.0581	0.954	-0.018156645	count	1
SMARCD1	-0.0144493	0.2095989	-0.0689	0.945	-0.018109624	count	1
MXD4	-0.0129074	0.0996114	-0.1296	0.897	-0.017987379	count	1
ZNF764	-0.021639	0.6821725	-0.0317	0.975	-0.017936034	count	1
SIAH1	-0.0139288	0.1872051	-0.0744	0.941	-0.017928276	count	1
VPS28	-0.0124521	0.0506623	-0.2458	0.8059	-0.017794965	count	1
HACL1	-0.0144907	0.2479091	-0.0585	0.953	-0.017486375	count	1
CHAC2	-0.0525326	0.6056301	-0.0867	0.931	-0.017348779	count	1
TBL1XR1	-0.0126988	0.1548614	-0.082	0.935	-0.017146041	count	1
RPL9	-0.0117897	0.0249189	-0.4731	0.636	-0.016974775	count	1
COX6A1	-0.0118293	0.0458183	-0.2582	0.796	-0.016954297	count	1
KLF16	-0.013063	0.1779658	-0.0734	0.941	-0.016862677	count	1
IDI1	-0.0121836	0.1083395	-0.1125	0.91	-0.016822151	count	1
RAD54L2	-0.0170092	0.3906656	-0.0435	0.965	-0.016820166	count	1
SMIM19	-0.0120157	0.1295055	-0.0928	0.926	-0.016618182	count	1
PHF13	-0.0138227	0.253473	-0.0545	0.957	-0.016598662	count	1
SVBP	-0.0118886	0.1253629	-0.0948	0.924	-0.016580968	count	1
MYO7A	-0.0267699	0.3365705	-0.0795	0.937	-0.016537406	count	1
PSMB4	-0.011658	0.0790112	-0.1475	0.883	-0.016360445	count	1
SERPING1	-0.0117558	0.1394859	-0.0843	0.933	-0.016236263	count	1
MACO1	-0.0126202	0.2194539	-0.0575	0.954	-0.015993343	count	1
HSPA8	-0.0111344	0.0470068	-0.2369	0.813	-0.015985088	count	1
PSMG2	-0.0113304	0.0783017	-0.1447	0.885	-0.015956718	count	1
PEX10	-0.0153507	0.2958503	-0.0519	0.959	-0.015918945	count	1
SLMAP	-0.0131747	0.2382609	-0.0553	0.956	-0.015899509	count	1
GTPBP3	-0.0176184	0.3554724	-0.0496	0.96	-0.015836226	count	1
USE1	-0.0119313	0.1717095	-0.0695	0.945	-0.01579539	count	1
TAF15	-0.0120843	0.1370106	-0.0882	0.93	-0.015709125	count	1
ZNF605	-0.0204961	0.4780347	-0.0429	0.966	-0.015659399	count	1
DTD2	-0.0240002	0.6451428	-0.0372	0.97	-0.01564763	count	1
ICAM4	-0.0211453	0.505142	-0.0419	0.967	-0.015628481	count	1

EIF2B2	-0.0134035	0.2588133	-0.0518	0.959	-0.015627867	count	1
MRPS30	-0.0128116	0.2264117	-0.0566	0.955	-0.015603343	count	1
UCKL1	-0.0126354	0.2624087	-0.0482	0.962	-0.015575565	count	1
CCM2	-0.0114824	0.1355631	-0.0847	0.933	-0.01552907	count	1
SOAT1	-0.0113258	0.1518257	-0.0746	0.941	-0.015479428	count	1
OGFOD3	-0.0118488	0.1941622	-0.061	0.951	-0.015377541	count	1
RPS16	-0.0106162	0.0268453	-0.3955	0.693	-0.015281429	count	1
CYP20A1	-0.0111393	0.1628329	-0.0684	0.945	-0.015151738	count	1
ZC3H18	-0.0118174	0.1743732	-0.0678	0.946	-0.015101233	count	1
CDC5L	-0.0108518	0.1076655	-0.1008	0.92	-0.015044847	count	1
FDX2	-0.0157441	0.422913	-0.0372	0.97	-0.015026081	count	1
BPGM	-0.0138948	0.4024136	-0.0345	0.972	-0.014747313	count	1
ATXN7L1	-0.019264	0.3701775	-0.052	0.959	-0.014721025	count	1
CSDE1	-0.010373	0.0787261	-0.1318	0.895	-0.014646274	count	1
GBP7	-0.0784495	0.9799505	-0.0801	0.936	-0.014513267	count	1
UGDH-AS1	-0.0784495	0.9799505	-0.0801	0.936	-0.014513267	count	1
BEND6	-0.0784495	0.9799505	-0.0801	0.936	-0.014513267	count	1
AC069503.2	-0.0784495	0.9799505	-0.0801	0.936	-0.014513267	count	1
CPNE3	-0.0103901	0.1224102	-0.0849	0.932	-0.014503449	count	1
RBM12B	-0.0146357	0.3373067	-0.0434	0.965	-0.014476808	count	1
SDF2	-0.0105891	0.1355523	-0.0781	0.938	-0.01447592	count	1
NDUFB10	-0.0101145	0.0575249	-0.1758	0.86	-0.014426595	count	1
C17orf75	-0.0132468	0.3091791	-0.0428	0.966	-0.014344721	count	1
AL031590.1	-0.1469191	0.9794685	-0.15	0.881	-0.014199145	count	1
NDUFB3	-0.0100435	0.0828919	-0.1212	0.904	-0.014159336	count	1
DNAJC24	-0.0125493	0.3225985	-0.0389	0.969	-0.014114419	count	1
STEAP3	-0.0172672	0.4006271	-0.0431	0.966	-0.013972253	count	1
AC004918.1	-0.018076	0.5090394	-0.0355	0.972	-0.013815875	count	1
TXLNG	-0.0125268	0.2626809	-0.0477	0.962	-0.013806077	count	1
MISP3	-0.0235154	0.5429098	-0.0433	0.965	-0.013668365	count	1
ACSL1	-0.0098827	0.0989038	-0.0999	0.92	-0.013587181	count	1
COX6B1	-0.0094493	0.0375592	-0.2516	0.801	-0.01356311	count	1
SUPT6H	-0.0100399	0.1483739	-0.0677	0.946	-0.013374733	count	1
KIF21B	-0.0337554	0.5688031	-0.0593	0.953	-0.013243408	count	1
RTF2	-0.0093803	0.0937161	-0.1001	0.92	-0.013224425	count	1
MT-ND4	-0.009154	0.030785	-0.2974	0.766	-0.013191465	count	1
HGH1	-0.0201299	0.423416	-0.0475	0.962	-0.013133977	count	1
DNMBP	-0.013904	0.2995757	-0.0464	0.963	-0.013095557	count	1
ATG12	-0.0094561	0.1315941	-0.0719	0.943	-0.013039218	count	1
BMP8B	-0.1336637	0.9509513	-0.1406	0.888	-0.012974586	count	1
PASK	-0.1336637	0.9509513	-0.1406	0.888	-0.012974586	count	1
MEG8	-0.1336637	0.9509513	-0.1406	0.888	-0.012974586	count	1
ZNF726	-0.1336637	0.9509513	-0.1406	0.888	-0.012974586	count	1
AL117339.5	-0.0281243	0.6543517	-0.043	0.966	-0.012574952	count	1
AC002467.1	-0.0200135	0.4079213	-0.0491	0.961	-0.012380215	count	1

ZCRB1	-0.008834	0.1140657	-0.0774	0.938	-0.012292874	count	1
BLVRA	-0.0086177	0.0791809	-0.1088	0.913	-0.012193015	count	1
ATAD3B	-0.0129954	0.3567003	-0.0364	0.971	-0.012066626	count	1
LRCH3	-0.0110338	0.4430569	-0.0249	0.98	-0.012023695	count	1
HLA-A	-0.0083394	0.0296317	-0.2814	0.778	-0.012011713	count	1
PDE6B	-0.0152062	0.4556386	-0.0334	0.973	-0.01197976	count	1
UTP14C	-0.01611	0.4490502	-0.0359	0.971	-0.011917072	count	1
ESD	-0.0083413	0.0835916	-0.0998	0.921	-0.011792126	count	1
NAPG	-0.0086616	0.1769311	-0.049	0.961	-0.011610587	count	1
LINC02345	-0.0210579	0.2940691	-0.0716	0.943	-0.011395924	count	1
RAB4B	-0.0229377	0.6788291	-0.0338	0.973	-0.011391026	count	1
POU5F2	-0.0148738	0.6955894	-0.0214	0.983	-0.011374322	count	1
SAMHD1	-0.0079009	0.0592148	-0.1334	0.894	-0.011268035	count	1
RAB11FIP1	-0.0077944	0.0857425	-0.0909	0.928	-0.011116485	count	1
UBXN10-AS1	-0.1121364	1.283587	-0.0874	0.93	-0.0109622	count	1
AL357055.3	-0.1121364	1.296036	-0.0865	0.931	-0.0109622	count	1
TTC30B	-0.1121364	1.296036	-0.0865	0.931	-0.0109622	count	1
ATG9A	-0.1121364	1.283587	-0.0874	0.93	-0.0109622	count	1
CD96	-0.1121364	1.296036	-0.0865	0.931	-0.0109622	count	1
ACTRT3	-0.1121364	1.296036	-0.0865	0.931	-0.0109622	count	1
AC008957.1	-0.1121364	1.296036	-0.0865	0.931	-0.0109622	count	1
PRR16	-0.1121364	1.296036	-0.0865	0.931	-0.0109622	count	1
CHSY3	-0.1121364	1.296036	-0.0865	0.931	-0.0109622	count	1
TTC26	-0.1121364	1.296036	-0.0865	0.931	-0.0109622	count	1
LINC01546	-0.1121364	1.296036	-0.0865	0.931	-0.0109622	count	1
CTHRC1	-0.1121364	1.296036	-0.0865	0.931	-0.0109622	count	1
SPATC1	-0.1121364	1.283587	-0.0874	0.93	-0.0109622	count	1
AQP11	-0.1121364	1.283587	-0.0874	0.93	-0.0109622	count	1
AP002986.1	-0.1121364	1.283587	-0.0874	0.93	-0.0109622	count	1
AL158211.3	-0.1121364	1.296036	-0.0865	0.931	-0.0109622	count	1
OXGR1	-0.1121364	1.313611	-0.0854	0.932	-0.0109622	count	1
AC123768.4	-0.1121364	1.296036	-0.0865	0.931	-0.0109622	count	1
EFCAB5	-0.1121364	1.313611	-0.0854	0.932	-0.0109622	count	1
CDK5R1	-0.1121364	1.296036	-0.0865	0.931	-0.0109622	count	1
AL121760.1	-0.1121364	1.296036	-0.0865	0.931	-0.0109622	count	1
LMTK3	-0.1121364	1.296036	-0.0865	0.931	-0.0109622	count	1
C19orf84	-0.1121364	1.296036	-0.0865	0.931	-0.0109622	count	1
AC008735.2	-0.1121364	1.283587	-0.0874	0.93	-0.0109622	count	1
SCO2	-0.1121364	1.283587	-0.0874	0.93	-0.0109622	count	1
NRDC	-0.0077917	0.107479	-0.0725	0.942	-0.010960884	count	1
TRIM38	-0.0079105	0.101506	-0.0779	0.938	-0.010942638	count	1
PPP2R2B	-0.0586752	0.678121	-0.0865	0.931	-0.010920709	count	1
PIWIL4	-0.0586752	0.7053292	-0.0832	0.934	-0.010920709	count	1
SERTAD1	-0.0076772	0.0834978	-0.0919	0.927	-0.010864841	count	1
NTPCR	-0.0079504	0.1909507	-0.0416	0.967	-0.010721395	count	1

NEPRO	-0.0089905	0.2612978	-0.0344	0.973	-0.010683676	count	1
AAK1	-0.007679	0.1205068	-0.0637	0.949	-0.010604801	count	1
TRAPPC1	-0.0071901	0.0714085	-0.1007	0.92	-0.010221814	count	1
CENPU	-0.0092679	0.3120676	-0.0297	0.976	-0.010217099	count	1
OXER1	-0.0181484	0.6418595	-0.0283	0.977	-0.009827601	count	1
SARS2	-0.0181484	0.578512	-0.0314	0.975	-0.009827601	count	1
UBE2D2	-0.0067321	0.0603373	-0.1116	0.911	-0.009584119	count	1
CHTF18	-0.0284633	0.6695821	-0.0425	0.966	-0.009460999	count	1
AL355816.2	-0.0355042	0.6879268	-0.0516	0.959	-0.009373214	count	1
SCLY	-0.0355042	0.6879268	-0.0516	0.959	-0.009373214	count	1
ZFR	-0.0067953	0.1127243	-0.0603	0.952	-0.009332952	count	1
RPS2	-0.0064118	0.0255213	-0.2512	0.802	-0.009241763	count	1
HADHA	-0.0064678	0.0532957	-0.1214	0.903	-0.009216634	count	1
PPOX	-0.0108867	0.3709525	-0.0293	0.977	-0.009038084	count	1
GOT1	-0.0086555	0.4737062	-0.0183	0.985	-0.008906018	count	1
CKS1B	-0.006824	0.2476313	-0.0276	0.978	-0.008459897	count	1
SPRYD7	-0.006793	0.255385	-0.0266	0.979	-0.008406695	count	1
VAMP7	-0.0065347	0.2356402	-0.0277	0.978	-0.00840509	count	1
RBBP5	-0.0073755	0.3063728	-0.0241	0.981	-0.008336869	count	1
WTAP	-0.0058649	0.0878465	-0.0668	0.947	-0.008269602	count	1
STRIP1	-0.0090681	0.3444029	-0.0263	0.979	-0.008159958	count	1
DUSP10	-0.0063025	0.1943798	-0.0324	0.974	-0.008106499	count	1
ZCCHC7	-0.0063542	0.1977003	-0.0321	0.974	-0.00791743	count	1
TAF2	-0.0076595	0.294797	-0.026	0.979	-0.007881972	count	1
INAFM1	-0.0058417	0.2248613	-0.026	0.979	-0.007513932	count	1
MYL12B	-0.0050916	0.0366228	-0.139	0.889	-0.007313741	count	1
GDPD1	-0.0070711	0.5017169	-0.0141	0.989	-0.007144763	count	1
FAM49A	-0.005212	0.1191563	-0.0437	0.965	-0.007131926	count	1
RABIF	-0.0054175	0.2169615	-0.025	0.98	-0.007107467	count	1
HNRNPDL	-0.0048228	0.0446583	-0.108	0.914	-0.006911617	count	1
HSPA1L	-0.0673866	0.6909164	-0.0975	0.922	-0.006684588	count	1
CXorf38	-0.0048802	0.137054	-0.0356	0.972	-0.006670752	count	1
RCN2	-0.0051452	0.1831305	-0.0281	0.978	-0.006597983	count	1
PFDN6	-0.0048416	0.1618714	-0.0299	0.976	-0.006456765	count	1
KEAP1	-0.0055732	0.2574273	-0.0216	0.983	-0.006407152	count	1
GALR2	-0.0069628	0.5324364	-0.0131	0.99	-0.00626722	count	1
EIF2S2	-0.0042408	0.076337	-0.0556	0.956	-0.006033675	count	1
PLCB3	-0.0055212	0.3278971	-0.0168	0.987	-0.005777379	count	1
NUP35	-0.0075239	0.4300673	-0.0175	0.986	-0.005760594	count	1
AL118506.1	-0.006633	0.4161379	-0.0159	0.987	-0.005754224	count	1
CDK5RAP1	-0.0048043	0.2799522	-0.0172	0.986	-0.005604801	count	1
ELOB	-0.0038522	0.0458275	-0.0841	0.933	-0.005524374	count	1
ERLEC1	-0.0038967	0.1450108	-0.0269	0.979	-0.00539135	count	1
CDK13	-0.0039838	0.1637869	-0.0243	0.981	-0.005263186	count	1
NPIPB5	-0.0044793	0.3055298	-0.0147	0.988	-0.005188989	count	1

ZNF557	-0.0047318	0.3944118	-0.012	0.99	-0.004912047	count	1
MRPL57	-0.003367	0.0765229	-0.044	0.965	-0.004764205	count	1
GBA	-0.0037644	0.209615	-0.018	0.986	-0.004760055	count	1
DNAAF1	-0.0468521	0.257251	-0.1821	0.855	-0.004678728	count	1
BRD8	-0.0038952	0.2679398	-0.0145	0.988	-0.004630223	count	1
YIPF5	-0.0033708	0.1797796	-0.0187	0.985	-0.004470861	count	1
VPS4A	-0.0034246	0.1699323	-0.0202	0.984	-0.004456776	count	1
ODC1	-0.003139	0.1697673	-0.0185	0.985	-0.004294551	count	1
GPR84	-0.003276	0.1883719	-0.0174	0.986	-0.004209963	count	1
CPM	-0.0029951	0.1050084	-0.0285	0.977	-0.004106881	count	1
ANAPC7	-0.0037314	0.3659705	-0.0102	0.992	-0.004068682	count	1
BLM	-0.0052815	0.6103518	-0.0087	0.993	-0.004045199	count	1
RPS11	-0.0028005	0.0342927	-0.0817	0.935	-0.004025222	count	1
JTB	-0.002797	0.0645858	-0.0433	0.965	-0.003972294	count	1
ATP6V0A1	-0.0028183	0.1649079	-0.0171	0.986	-0.003849043	count	1
SARM1	-0.0384007	0.7922533	-0.0485	0.961	-0.003845275	count	1
MAP1S	-0.0030891	0.2271987	-0.0136	0.989	-0.003843353	count	1
KMO	-0.0032052	0.288143	-0.0111	0.991	-0.003685428	count	1
FCGRT	-0.0024861	0.0401544	-0.0619	0.951	-0.003576062	count	1
EMC4	-0.002506	0.0987141	-0.0254	0.98	-0.003516532	count	1
AC005921.2	-0.002914	0.2832423	-0.0103	0.992	-0.003510184	count	1
NUS1	-0.0026336	0.2025388	-0.013	0.99	-0.003450672	count	1
TCEAL8	-0.0024771	0.1365505	-0.0181	0.986	-0.003383841	count	1
BABAM1	-0.0023684	0.1061025	-0.0223	0.982	-0.00326364	count	1
CCDC43	-0.0024844	0.2043693	-0.0122	0.99	-0.003259698	count	1
RBM17	-0.0022513	0.0908495	-0.0248	0.98	-0.003155307	count	1
SDHA	-0.0023977	0.186094	-0.0129	0.99	-0.00306848	count	1
AP002748.3	-0.0061033	0.5288838	-0.0115	0.991	-0.003042574	count	1
BEST1	-0.0023321	0.267353	-0.0087	0.993	-0.003039909	count	1
INCENP	-0.0040455	0.4572476	-0.0088	0.993	-0.00299869	count	1
EAF2	-0.0021404	0.12118	-0.0177	0.986	-0.002911903	count	1
SLC35D2	-0.0021368	0.1593755	-0.0134	0.989	-0.002826439	count	1
SRGAP2B	-0.0025941	0.1948539	-0.0133	0.989	-0.002775363	count	1
ATXN10	-0.0018687	0.1450038	-0.0129	0.99	-0.002530145	count	1
ZNF562	-0.0018515	0.2531099	-0.0073	0.994	-0.002174761	count	1
NAXE	-0.0016002	0.1408446	-0.0114	0.991	-0.002172318	count	1
PTPRO	-0.0019829	0.4015014	-0.0049	0.996	-0.002149338	count	1
AC097103.2	-0.0036429	1.0868929	-0.0034	0.997	-0.002126175	count	1
ENOPH1	-0.0017005	0.1941401	-0.0088	0.993	-0.002112301	count	1
RPL23	-0.001434	0.0328897	-0.0436	0.965	-0.002060217	count	1
ZNF829	-0.0026774	1.1598884	-0.0023	0.998	-0.001913506	count	1
AC004148.2	-0.0097583	0.6592889	-0.0148	0.988	-0.001843335	count	1
DNAJC1	-0.0013242	0.1009917	-0.0131	0.99	-0.001843208	count	1
PIP4K2A	-0.0012882	0.1196325	-0.0108	0.991	-0.001769752	count	1
MMP25-AS1	-0.0044057	0.631265	-0.007	0.994	-0.001741375	count	1

GTF2H5	-0.0012222	0.1106227	-0.011	0.991	-0.001696052	count	1
AC016727.1	-0.0036429	0.8179104	-0.0045	0.996	-0.001638389	count	1
ABHD14A-ACY1	-0.0036429	0.8177902	-0.0045	0.996	-0.001638389	count	1
ZNF596	-0.0036429	0.8179104	-0.0045	0.996	-0.001638389	count	1
AP003352.1	-0.0036429	1.0445515	-0.0035	0.997	-0.001638389	count	1
AL354920.1	-0.0036429	1.0444573	-0.0035	0.997	-0.001638389	count	1
CTSW	-0.0036429	0.9747757	-0.0037	0.997	-0.001638389	count	1
AC010336.1	-0.0036429	0.8177902	-0.0045	0.996	-0.001638389	count	1
PARVB	-0.0011315	0.11215	-0.0101	0.992	-0.001576034	count	1
P2RX7	-0.0012029	0.1659438	-0.0072	0.994	-0.001569258	count	1
TUBB6	-0.0011268	0.1265092	-0.0089	0.993	-0.001555827	count	1
SART3	-0.001351	0.2783522	-0.0049	0.996	-0.001455201	count	1
SZRD1	-0.001045	0.1361276	-0.0077	0.994	-0.001427237	count	1
PPP6R1	-0.0011113	0.2041008	-0.0054	0.996	-0.001380457	count	1
NECAB3	-0.0021586	0.418333	-0.0052	0.996	-0.00126025	count	1
SET	-0.0008694	0.0511673	-0.017	0.9864	-0.001242873	count	1
SLC25A22	-0.0014825	0.4824966	-0.0031	0.998	-0.001232467	count	1
PAQR6	-0.0036429	1.37961	-0.0026	0.998	-0.000970462	count	1
AL353708.3	-0.0036429	1.1574636	-0.0031	0.997	-0.000970462	count	1
PPFIA4	-0.0036429	1.1578035	-0.0031	0.997	-0.000970462	count	1
ATP6V1C2	-0.0036429	1.1578035	-0.0031	0.997	-0.000970462	count	1
AC007038.1	-0.0036429	1.1578035	-0.0031	0.997	-0.000970462	count	1
AC009974.1	-0.0036429	1.1578035	-0.0031	0.997	-0.000970462	count	1
ZNF620	-0.0036429	1.37961	-0.0026	0.998	-0.000970462	count	1
LNP1	-0.0036429	1.1578035	-0.0031	0.997	-0.000970462	count	1
KIAA1257	-0.0036429	1.37961	-0.0026	0.998	-0.000970462	count	1
PXYLP1	-0.0036429	1.1574636	-0.0031	0.997	-0.000970462	count	1
AC068620.1	-0.0036429	1.1578035	-0.0031	0.997	-0.000970462	count	1
NEIL3	-0.0036429	1.1578035	-0.0031	0.997	-0.000970462	count	1
AC026691.1	-0.0036429	1.37961	-0.0026	0.998	-0.000970462	count	1
ZNF165	-0.0036429	1.1574636	-0.0031	0.997	-0.000970462	count	1
AC105446.1	-0.0036429	1.1578035	-0.0031	0.997	-0.000970462	count	1
C8orf37	-0.0036429	1.1574636	-0.0031	0.997	-0.000970462	count	1
AL356489.3	-0.0036429	1.1574636	-0.0031	0.997	-0.000970462	count	1
STKLD1	-0.0036429	1.1578035	-0.0031	0.997	-0.000970462	count	1
SCUBE2	-0.0036429	1.1578035	-0.0031	0.997	-0.000970462	count	1
LIPT2	-0.0036429	1.1578035	-0.0031	0.997	-0.000970462	count	1
LINC02550	-0.0036429	1.37961	-0.0026	0.998	-0.000970462	count	1
ABLIM1	-0.0036429	1.740388	-0.0021	0.998	-0.000970462	count	1
AC026333.3	-0.0036429	1.1574636	-0.0031	0.997	-0.000970462	count	1
AL133371.2	-0.0036429	1.1578035	-0.0031	0.997	-0.000970462	count	1
CLBA1	-0.0036429	1.37961	-0.0026	0.998	-0.000970462	count	1
MAP1A	-0.0036429	1.1574636	-0.0031	0.997	-0.000970462	count	1
MESP1	-0.0036429	1.1578035	-0.0031	0.997	-0.000970462	count	1
PKMYT1	-0.0036429	1.1578035	-0.0031	0.997	-0.000970462	count	1

ATP2A1-AS1	-0.0036429	1.1574636	-0.0031	0.997	-0.000970462	count	1
MC1R	-0.0036429	1.1578035	-0.0031	0.997	-0.000970462	count	1
CEP112	-0.0036429	1.37961	-0.0026	0.998	-0.000970462	count	1
AC005332.8	-0.0036429	1.37961	-0.0026	0.998	-0.000970462	count	1
AL121899.1	-0.0036429	1.1578035	-0.0031	0.997	-0.000970462	count	1
SPC24	-0.0036429	1.1578035	-0.0031	0.997	-0.000970462	count	1
AC010487.2	-0.0036429	1.1578035	-0.0031	0.997	-0.000970462	count	1
MLC1	-0.0036429	1.1578035	-0.0031	0.997	-0.000970462	count	1
LINC01547	-0.0036429	1.1578035	-0.0031	0.997	-0.000970462	count	1
EFNA3	-0.00473	0.808442	-0.0059	0.995	-0.000894846	count	1
CNIH3	-0.00473	0.8085966	-0.0058	0.995	-0.000894846	count	1
ZNF786	-0.00473	0.7541279	-0.0063	0.995	-0.000894846	count	1
GARNL3	-0.00473	0.754559	-0.0063	0.995	-0.000894846	count	1
ZBTB2	-0.0009365	0.3720413	-0.0025	0.998	-0.000828782	count	1
SOX4	-0.0005065	0.1456336	-0.0035	0.997	-0.000700052	count	1
PPP2CA	-2.17E-05	0.1024337	-2.00E-04	1	-3.02E-05	count	1
CD5	-18.169022	1691.610154	-0.0107	0.991	-3.72E-08	count	1
CCDC85C	-1.8994837	0.7977577	-2.381	0.0173	-2.99E-08	count	1
GTSF1	-1.2107129	0.4152493	-2.9156	0.0036	-2.37E-08	count	1
AC093525.4	-1.0334775	0.8601001	-1.2016	0.23	-2.15E-08	count	1
AL954642.1	-0.8243504	0.5095365	-1.6178	0.106	-1.84E-08	count	1
AL731563.3	-0.6638257	0.6282464	-1.0566	0.291	-1.57E-08	count	1
ANGPT2	-0.6203618	0.5119998	-1.2116	0.226	-1.49E-08	count	1
NOX4	-18.7498142	2522.961576	-0.0074	0.994	-1.36E-08	count	1
GPR75	-18.7498142	2522.961576	-0.0074	0.994	-1.36E-08	count	1
AC015727.1	-18.7498142	2522.961576	-0.0074	0.994	-1.36E-08	count	1
PTPRS	-18.7498142	2522.961576	-0.0074	0.994	-1.36E-08	count	1
ANKRD33B	-18.7360435	2220.130283	-0.0084	0.993	-1.36E-08	count	1
AL022323.3	-18.6211908	1754.161086	-0.0106	0.992	-1.36E-08	count	1
AP001267.2	-18.5986116	2038.513951	-0.0091	0.993	-1.36E-08	count	1
DIO2	-18.2412221	1979.151666	-0.0092	0.993	-1.36E-08	count	1
AKNAD1	-18.4688718	2192.324165	-0.0084	0.993	-1.36E-08	count	1
CAMK4	-18.4688718	2192.324165	-0.0084	0.993	-1.36E-08	count	1
AC211476.2	-18.4688718	2192.324165	-0.0084	0.993	-1.36E-08	count	1
TFPI2	-18.4688718	2192.324165	-0.0084	0.993	-1.36E-08	count	1
CD40LG	-18.4688718	2192.324165	-0.0084	0.993	-1.36E-08	count	1
AC083809.1	-18.4688718	2192.324165	-0.0084	0.993	-1.36E-08	count	1
AC100835.2	-18.4688718	2192.324165	-0.0084	0.993	-1.36E-08	count	1
ZNF114	-18.4688718	2192.324165	-0.0084	0.993	-1.36E-08	count	1
SCHIP1	-18.1588298	2023.268447	-0.009	0.993	-1.35E-08	count	1
SMO	-0.4957827	0.6750333	-0.7345	0.463	-1.24E-08	count	1
FAM129C	-2.238752	1.227489	-1.8238	0.0683	-1.17E-08	count	1
ECM2	-2.1052206	0.9153687	-2.2999	0.0215	-1.14E-08	count	1
PREX2	-1.9510699	0.9680762	-2.0154	0.044	-1.11E-08	count	1
SPRY3	-1.8732101	1.4009598	-1.3371	0.181	-1.09E-08	count	1

AC012645.4	-1.7687484	0.8946825	-1.977	0.0482	-1.06E-08	count	1
AC007620.3	-1.7687484	1.0399837	-1.7007	0.0891	-1.06E-08	count	1
AL121652.1	-1.6763339	1.1734298	-1.4286	0.153	-1.04E-08	count	1
GJA1	-1.6763339	1.3122069	-1.2775	0.202	-1.04E-08	count	1
AC012467.2	-1.5456048	0.9716621	-1.5907	0.112	-9.97E-09	count	1
AC245297.2	-1.5456048	0.9716621	-1.5907	0.112	-9.97E-09	count	1
ITPRIPL1	-0.373768	0.4406468	-0.8482	0.396	-9.76E-09	count	1
CYS1	-1.4305494	0.8825188	-1.621	0.105	-9.57E-09	count	1
AUNIP	-1.4120734	0.8757092	-1.6125	0.107	-9.52E-09	count	1
TPBG	-1.1736256	0.8191836	-1.4327	0.152	-8.56E-09	count	1
CDCA2	-1.1109527	0.8452878	-1.3143	0.189	-8.28E-09	count	1
CLIC3	-1.1109527	0.7770778	-1.4297	0.153	-8.28E-09	count	1
SPIRE2	-1.1109527	0.947253	-1.1728	0.241	-8.28E-09	count	1
CAPN3	-0.9918519	0.9447676	-1.0498	0.294	-7.69E-09	count	1
STAC	-0.9490996	0.8173814	-1.1611	0.246	-7.47E-09	count	1
JPH4	-0.9035882	0.5670456	-1.5935	0.111	-7.22E-09	count	1
TNFSF4	-0.6456441	0.8399452	-0.7687	0.442	-5.64E-09	count	1
INSL3	-0.6456441	0.8399452	-0.7687	0.442	-5.64E-09	count	1
TUBG2	-0.6456441	0.8399452	-0.7687	0.442	-5.64E-09	count	1
ACTG2	-0.6456441	0.8399452	-0.7687	0.442	-5.64E-09	count	1
AL355304.1	-0.6456441	0.8399452	-0.7687	0.442	-5.64E-09	count	1
LINC01063	-0.6456441	0.8399452	-0.7687	0.442	-5.64E-09	count	1
AC004846.2	-0.6456441	0.8399452	-0.7687	0.442	-5.64E-09	count	1
CFAP97D1	-0.6456441	0.84006	-0.7686	0.442	-5.64E-09	count	1
SGIP1	-0.6456441	0.8399452	-0.7687	0.442	-5.64E-09	count	1
PDE6A	-0.6456441	0.8399452	-0.7687	0.442	-5.64E-09	count	1
ZNF473	-0.6456441	0.8399452	-0.7687	0.442	-5.64E-09	count	1
ABCA10	-0.6456441	1.0332868	-0.6248	0.532	-5.64E-09	count	1
ZFP57	-0.6124899	0.5322154	-1.1508	0.25	-5.41E-09	count	1
AC107959.1	-0.5647756	0.7123264	-0.7929	0.428	-5.07E-09	count	1
AC106795.2	-18.1492175	1932.519667	-0.0094	0.993	-5.00E-09	count	1
NTM	-18.1492175	1932.519667	-0.0094	0.993	-5.00E-09	count	1
TC2N	-18.1492175	1932.519667	-0.0094	0.993	-5.00E-09	count	1
WWC1	-18.1492175	1932.519667	-0.0094	0.993	-5.00E-09	count	1
EFCAB9	-18.1492175	1932.519667	-0.0094	0.993	-5.00E-09	count	1
THY1	-18.1492175	1932.519667	-0.0094	0.993	-5.00E-09	count	1
POSTN	-18.1492175	1932.519667	-0.0094	0.993	-5.00E-09	count	1
AL031666.2	-18.1492175	1932.519667	-0.0094	0.993	-5.00E-09	count	1
TMIGD2	-18.1492175	1932.519667	-0.0094	0.993	-5.00E-09	count	1
WFDC3	-18.1492175	1932.519667	-0.0094	0.993	-5.00E-09	count	1
FAM184A	-18.1492175	1932.519667	-0.0094	0.993	-5.00E-09	count	1
AC011471.2	-18.1492175	1932.519667	-0.0094	0.993	-5.00E-09	count	1
AC073263.2	-18.1492175	1932.519667	-0.0094	0.993	-5.00E-09	count	1
RHCG	-18.1492175	1932.519667	-0.0094	0.993	-5.00E-09	count	1
LINC01356	-18.1492175	1932.519667	-0.0094	0.993	-5.00E-09	count	1

AC006213.1	-18.1492175	1932.519667	-0.0094	0.993	-5.00E-09	count	1
RARB	-18.1492175	1932.519667	-0.0094	0.993	-5.00E-09	count	1
LIN37	-18.1492175	1932.519667	-0.0094	0.993	-5.00E-09	count	1
Z68871.1	-18.7709922	1468.020922	-0.0128	0.99	-5.00E-09	count	1
GAD1	-18.7709922	1468.020922	-0.0128	0.99	-5.00E-09	count	1
ZNF773	-18.7709922	1468.020922	-0.0128	0.99	-5.00E-09	count	1
CNKSR3	-18.66134	1934.491173	-0.0096	0.992	-5.00E-09	count	1
CLRN3	-18.66134	1934.491173	-0.0096	0.992	-5.00E-09	count	1
SCML4	-18.66134	1934.491173	-0.0096	0.992	-5.00E-09	count	1
AP001107.2	-18.77231	1899.649652	-0.0099	0.992	-5.00E-09	count	1
TRAT1	-18.993572	2295.917823	-0.0083	0.993	-5.00E-09	count	1
FUT1	-19.0610187	1800.902468	-0.0106	0.992	-5.00E-09	count	1
COL4A1	-19.2395746	2203.399068	-0.0087	0.993	-5.00E-09	count	1
RBM15-AS1	-18.7740488	2553.719074	-0.0074	0.994	-4.99E-09	count	1
PCDHGB2	-18.7740488	2553.719074	-0.0074	0.994	-4.99E-09	count	1
RSPH4A	-18.7740488	2553.719074	-0.0074	0.994	-4.99E-09	count	1
AC012560.1	-18.7740488	2553.719074	-0.0074	0.994	-4.99E-09	count	1
AC087286.2	-18.7740488	2553.719074	-0.0074	0.994	-4.99E-09	count	1
TMEM78	-18.7740488	2553.719074	-0.0074	0.994	-4.99E-09	count	1
GABRD	-18.9945996	2893.869995	-0.0066	0.995	-4.99E-09	count	1
AC106886.5	-18.4203338	1911.9697	-0.0096	0.992	-4.99E-09	count	1
TPTEP2-CSNK1E	-18.4203338	1911.9697	-0.0096	0.992	-4.99E-09	count	1
OTUB2	-18.4203338	1911.9697	-0.0096	0.992	-4.99E-09	count	1
MCOLN3	-19.4681798	2551.404622	-0.0076	0.994	-4.99E-09	count	1
DTX1	-19.2866875	2719.318142	-0.0071	0.994	-4.99E-09	count	1
LINC00844	-19.2866875	2719.318142	-0.0071	0.994	-4.99E-09	count	1
ATP9A	-19.2866875	2719.318142	-0.0071	0.994	-4.99E-09	count	1
CRISPLD1	-19.4686813	3069.818202	-0.0063	0.995	-4.99E-09	count	1
BDH1	-19.4686813	3069.818202	-0.0063	0.995	-4.99E-09	count	1
HIST1H4I	-19.4686813	3069.818202	-0.0063	0.995	-4.99E-09	count	1
CCNB3	-18.421466	2249.781849	-0.0082	0.993	-4.99E-09	count	1
PCDHB14	-19.0638415	2951.894243	-0.0065	0.995	-4.99E-09	count	1
OLFML1	-19.0638415	2951.894243	-0.0065	0.995	-4.99E-09	count	1
AL121757.1	-19.0638415	2951.894243	-0.0065	0.995	-4.99E-09	count	1
KLHL13	-19.0638415	2951.894243	-0.0065	0.995	-4.99E-09	count	1
KRTAP5-8	-19.0638415	2951.894243	-0.0065	0.995	-4.99E-09	count	1
AC138028.1	-19.0638415	2951.894243	-0.0065	0.995	-4.99E-09	count	1
AL590648.2	-19.287568	3301.280262	-0.0058	0.995	-4.99E-09	count	1
TRAPPC12-AS1	-19.287568	3301.280262	-0.0058	0.995	-4.99E-09	count	1
CA9	-19.287568	3301.280262	-0.0058	0.995	-4.99E-09	count	1
TOM1L1	-19.287568	3301.280262	-0.0058	0.995	-4.99E-09	count	1
CDC42EP5	-18.6286142	2518.48108	-0.0074	0.994	-4.99E-09	count	1
AL157888.1	-17.9254142	1835.131859	-0.0098	0.992	-4.97E-09	count	1
AC007383.3	-18.2157226	2121.812261	-0.0086	0.993	-4.97E-09	count	1
LINC00683	-18.7688917	2797.860757	-0.0067	0.995	-4.97E-09	count	1

FANK1	-18.7688917	2797.860757	-0.0067	0.995	-4.97E-09	count	1
AL157402.2	-18.6194605	2596.435361	-0.0072	0.994	-4.97E-09	count	1
AC005670.2	-0.1593104	0.6931143	-0.2298	0.818	-4.48E-09	count	1
MCMDC2	-0.4391085	0.6176914	-0.7109	0.477	-4.12E-09	count	1
KRBA1	-0.4317173	0.5029861	-0.8583	0.391	-4.06E-09	count	1
AC005840.2	-0.4182652	0.9946649	-0.4205	0.674	-3.96E-09	count	1
PLEKHG6	-0.4182652	0.9946649	-0.4205	0.674	-3.96E-09	count	1
C11orf80	-0.382454	0.7289795	-0.5246	0.6	-3.66E-09	count	1
NBPF3	-1.2579227	0.649591	-1.9365	0.0529	-3.28E-09	count	1
TMEM98	-1.2579227	0.649591	-1.9365	0.0529	-3.28E-09	count	1
SGCA	-1.2579227	0.649591	-1.9365	0.0529	-3.28E-09	count	1
AL731577.1	-0.1125293	0.3975903	-0.283	0.777	-3.22E-09	count	1
CENPO	-0.8524576	0.6894391	-1.2365	0.216	-2.55E-09	count	1
AC015987.1	-0.8524576	0.6894391	-1.2365	0.216	-2.55E-09	count	1
RNF207	-0.8524576	0.6894391	-1.2365	0.216	-2.55E-09	count	1
SEMA4F	-0.8524576	0.6894391	-1.2365	0.216	-2.55E-09	count	1
GPR174	-0.8524576	0.6894391	-1.2365	0.216	-2.55E-09	count	1
ZNF462	-0.8524576	0.6894391	-1.2365	0.216	-2.55E-09	count	1
ZNF461	-0.8524576	0.8336323	-1.0226	0.307	-2.55E-09	count	1
AC138150.2	-0.8524576	0.8336323	-1.0226	0.307	-2.55E-09	count	1
LINC01134	-18.6677302	2421.510289	-0.0077	0.994	-1.84E-09	count	1
AL050343.1	-18.6677302	2421.510289	-0.0077	0.994	-1.84E-09	count	1
AC099791.2	-18.6677302	2421.510289	-0.0077	0.994	-1.84E-09	count	1
AL391335.1	-18.6677302	2421.510289	-0.0077	0.994	-1.84E-09	count	1
AL596325.1	-18.6677302	2421.510289	-0.0077	0.994	-1.84E-09	count	1
AC138969.1	-18.6677302	2421.510289	-0.0077	0.994	-1.84E-09	count	1
EME1	-18.6677302	2421.510289	-0.0077	0.994	-1.84E-09	count	1
SAMD11	-18.6677302	2421.510289	-0.0077	0.994	-1.84E-09	count	1
PLA2G2C	-18.6677302	2421.510289	-0.0077	0.994	-1.84E-09	count	1
LINC01635	-18.6677302	2421.510289	-0.0077	0.994	-1.84E-09	count	1
EPHA8	-18.6677302	2421.510289	-0.0077	0.994	-1.84E-09	count	1
SMPDL3B	-18.6677302	2421.510289	-0.0077	0.994	-1.84E-09	count	1
TMEM269	-18.6677302	2421.510289	-0.0077	0.994	-1.84E-09	count	1
ZSWIM5	-18.6677302	2421.510289	-0.0077	0.994	-1.84E-09	count	1
AC104170.2	-18.6677302	2421.510289	-0.0077	0.994	-1.84E-09	count	1
AL603832.1	-18.6677302	2421.510289	-0.0077	0.994	-1.84E-09	count	1
AL157904.1	-18.6677302	2421.510289	-0.0077	0.994	-1.84E-09	count	1
SRGAP2-AS1	-18.6677302	2421.510289	-0.0077	0.994	-1.84E-09	count	1
PDZK1	-18.6677302	2421.510289	-0.0077	0.994	-1.84E-09	count	1
BGLAP	-18.6677302	2421.510289	-0.0077	0.994	-1.84E-09	count	1
ETV3L	-18.6677302	2421.510289	-0.0077	0.994	-1.84E-09	count	1
ATP1A2	-18.6677302	2421.510289	-0.0077	0.994	-1.84E-09	count	1
MAEL	-18.6677302	2421.510289	-0.0077	0.994	-1.84E-09	count	1
CNTN2	-18.6677302	2421.510289	-0.0077	0.994	-1.84E-09	count	1
TMEM81	-18.6677302	2421.510289	-0.0077	0.994	-1.84E-09	count	1

PIGR	-18.6677302	2421.510289	-0.0077	0.994	-1.84E-09	count	1
IRF6	-18.6677302	2421.510289	-0.0077	0.994	-1.84E-09	count	1
AC096637.2	-18.6677302	2421.510289	-0.0077	0.994	-1.84E-09	count	1
AC096642.1	-18.6677302	2421.510289	-0.0077	0.994	-1.84E-09	count	1
LINC01354	-18.6677302	2421.510289	-0.0077	0.994	-1.84E-09	count	1
LINC01132	-18.6677302	2421.510289	-0.0077	0.994	-1.84E-09	count	1
RNF144A	-18.6677302	2421.510289	-0.0077	0.994	-1.84E-09	count	1
EFR3B	-18.6677302	2421.510289	-0.0077	0.994	-1.84E-09	count	1
AC092567.1	-18.6677302	2421.510289	-0.0077	0.994	-1.84E-09	count	1
LINC02576	-18.6677302	2421.510289	-0.0077	0.994	-1.84E-09	count	1
AC073263.1	-18.6677302	2421.510289	-0.0077	0.994	-1.84E-09	count	1
ADRA2B	-18.6677302	2421.510289	-0.0077	0.994	-1.84E-09	count	1
AC108868.1	-18.6677302	2421.510289	-0.0077	0.994	-1.84E-09	count	1
CCDC148	-18.6677302	2421.510289	-0.0077	0.994	-1.84E-09	count	1
AC104076.1	-18.6677302	2421.510289	-0.0077	0.994	-1.84E-09	count	1
PDE1A	-18.6677302	2421.510289	-0.0077	0.994	-1.84E-09	count	1
ASIC4	-18.6677302	2421.510289	-0.0077	0.994	-1.84E-09	count	1
AC013476.1	-18.6677302	2421.510289	-0.0077	0.994	-1.84E-09	count	1
AC104667.1	-18.6677302	2421.510289	-0.0077	0.994	-1.84E-09	count	1
BOK	-18.6677302	2421.510289	-0.0077	0.994	-1.84E-09	count	1
AC098614.4	-18.6677302	2421.510289	-0.0077	0.994	-1.84E-09	count	1
VIPR1-AS1	-18.6677302	2421.510289	-0.0077	0.994	-1.84E-09	count	1
UCN2	-18.6677302	2421.510289	-0.0077	0.994	-1.84E-09	count	1
SUCLG2-AS1	-18.6677302	2421.510289	-0.0077	0.994	-1.84E-09	count	1
FOXP1-AS1	-18.6677302	2421.510289	-0.0077	0.994	-1.84E-09	count	1
CADM2	-18.6677302	2421.510289	-0.0077	0.994	-1.84E-09	count	1
CD200	-18.6677302	2421.510289	-0.0077	0.994	-1.84E-09	count	1
GAP43	-18.6677302	2421.510289	-0.0077	0.994	-1.84E-09	count	1
NPHP3-AS1	-18.6677302	2421.510289	-0.0077	0.994	-1.84E-09	count	1
PLCH1	-18.6677302	2421.510289	-0.0077	0.994	-1.84E-09	count	1
SPTSSB	-18.6677302	2421.510289	-0.0077	0.994	-1.84E-09	count	1
AC090425.2	-18.6677302	2421.510289	-0.0077	0.994	-1.84E-09	count	1
CTBP1-AS	-18.6677302	2421.510289	-0.0077	0.994	-1.84E-09	count	1
AC092546.1	-18.6677302	2421.510289	-0.0077	0.994	-1.84E-09	count	1
AC006160.1	-18.6677302	2421.510289	-0.0077	0.994	-1.84E-09	count	1
PDGFRA	-18.6677302	2421.510289	-0.0077	0.994	-1.84E-09	count	1
TMPRSS11D	-18.6677302	2421.510289	-0.0077	0.994	-1.84E-09	count	1
LINC02435	-18.6677302	2421.510289	-0.0077	0.994	-1.84E-09	count	1
AC131956.2	-18.6677302	2421.510289	-0.0077	0.994	-1.84E-09	count	1
AC098679.1	-18.6677302	2421.510289	-0.0077	0.994	-1.84E-09	count	1
AC021087.1	-18.6677302	2421.510289	-0.0077	0.994	-1.84E-09	count	1
C5orf38	-18.6677302	2421.510289	-0.0077	0.994	-1.84E-09	count	1
AC112187.3	-18.6677302	2421.510289	-0.0077	0.994	-1.84E-09	count	1
AC035140.1	-18.6677302	2421.510289	-0.0077	0.994	-1.84E-09	count	1
FAM169A	-18.6677302	2421.510289	-0.0077	0.994	-1.84E-09	count	1

AC113383.1	-18.6677302	2421.510289	-0.0077	0.994	-1.84E-09	count	1
AC099509.1	-18.6677302	2421.510289	-0.0077	0.994	-1.84E-09	count	1
PRDM6	-18.6677302	2421.510289	-0.0077	0.994	-1.84E-09	count	1
AC135457.1	-18.6677302	2421.510289	-0.0077	0.994	-1.84E-09	count	1
AC008781.2	-18.6677302	2421.510289	-0.0077	0.994	-1.84E-09	count	1
AFAP1L1	-18.6677302	2421.510289	-0.0077	0.994	-1.84E-09	count	1
AC106801.1	-18.6677302	2421.510289	-0.0077	0.994	-1.84E-09	count	1
IL12B	-18.6677302	2421.510289	-0.0077	0.994	-1.84E-09	count	1
AC008378.1	-18.6677302	2421.510289	-0.0077	0.994	-1.84E-09	count	1
AL138720.1	-18.6677302	2421.510289	-0.0077	0.994	-1.84E-09	count	1
HIST1H2BO	-18.6677302	2421.510289	-0.0077	0.994	-1.84E-09	count	1
AL662890.1	-18.6677302	2421.510289	-0.0077	0.994	-1.84E-09	count	1
TRIM10	-18.6677302	2421.510289	-0.0077	0.994	-1.84E-09	count	1
HCG27	-18.6677302	2421.510289	-0.0077	0.994	-1.84E-09	count	1
ZBTB12	-18.6677302	2421.510289	-0.0077	0.994	-1.84E-09	count	1
AL645940.1	-18.6677302	2421.510289	-0.0077	0.994	-1.84E-09	count	1
AL035588.1	-18.6677302	2421.510289	-0.0077	0.994	-1.84E-09	count	1
AL583856.2	-18.6677302	2421.510289	-0.0077	0.994	-1.84E-09	count	1
OOEP	-18.6677302	2421.510289	-0.0077	0.994	-1.84E-09	count	1
FHL5	-18.6677302	2421.510289	-0.0077	0.994	-1.84E-09	count	1
ALDH8A1	-18.6677302	2421.510289	-0.0077	0.994	-1.84E-09	count	1
PERP	-18.6677302	2421.510289	-0.0077	0.994	-1.84E-09	count	1
GRM1	-18.6677302	2421.510289	-0.0077	0.994	-1.84E-09	count	1
ISPD	-18.6677302	2421.510289	-0.0077	0.994	-1.84E-09	count	1
HOXA9	-18.6677302	2421.510289	-0.0077	0.994	-1.84E-09	count	1
HOXA13	-18.6677302	2421.510289	-0.0077	0.994	-1.84E-09	count	1
AC005091.1	-18.6677302	2421.510289	-0.0077	0.994	-1.84E-09	count	1
WIPF3	-18.6677302	2421.510289	-0.0077	0.994	-1.84E-09	count	1
TRGC1	-18.6677302	2421.510289	-0.0077	0.994	-1.84E-09	count	1
C7orf25	-18.6677302	2421.510289	-0.0077	0.994	-1.84E-09	count	1
EGFR	-18.6677302	2421.510289	-0.0077	0.994	-1.84E-09	count	1
PEG10	-18.6677302	2421.510289	-0.0077	0.994	-1.84E-09	count	1
FBXL13	-18.6677302	2421.510289	-0.0077	0.994	-1.84E-09	count	1
STRA8	-18.6677302	2421.510289	-0.0077	0.994	-1.84E-09	count	1
TRBVB	-18.6677302	2421.510289	-0.0077	0.994	-1.84E-09	count	1
C7orf33	-18.6677302	2421.510289	-0.0077	0.994	-1.84E-09	count	1
AOC1	-18.6677302	2421.510289	-0.0077	0.994	-1.84E-09	count	1
AC099552.1	-18.6677302	2421.510289	-0.0077	0.994	-1.84E-09	count	1
CRLF2	-18.6677302	2421.510289	-0.0077	0.994	-1.84E-09	count	1
RAI2	-18.6677302	2421.510289	-0.0077	0.994	-1.84E-09	count	1
EFHC2	-18.6677302	2421.510289	-0.0077	0.994	-1.84E-09	count	1
JADE3	-18.6677302	2421.510289	-0.0077	0.994	-1.84E-09	count	1
CCDC120	-18.6677302	2421.510289	-0.0077	0.994	-1.84E-09	count	1
DLG3	-18.6677302	2421.510289	-0.0077	0.994	-1.84E-09	count	1
PHKA1	-18.6677302	2421.510289	-0.0077	0.994	-1.84E-09	count	1

AMOT	-18.6677302	2421.510289	-0.0077	0.994	-1.84E-09	count	1
AC087269.1	-18.6677302	2421.510289	-0.0077	0.994	-1.84E-09	count	1
SOX7	-18.6677302	2421.510289	-0.0077	0.994	-1.84E-09	count	1
RBPMS	-18.6677302	2421.510289	-0.0077	0.994	-1.84E-09	count	1
AC103724.3	-18.6677302	2421.510289	-0.0077	0.994	-1.84E-09	count	1
AC090739.1	-18.6677302	2421.510289	-0.0077	0.994	-1.84E-09	count	1
CLVS1	-18.6677302	2421.510289	-0.0077	0.994	-1.84E-09	count	1
SLCO5A1	-18.6677302	2421.510289	-0.0077	0.994	-1.84E-09	count	1
AC010834.3	-18.6677302	2421.510289	-0.0077	0.994	-1.84E-09	count	1
AC087752.4	-18.6677302	2421.510289	-0.0077	0.994	-1.84E-09	count	1
AARD	-18.6677302	2421.510289	-0.0077	0.994	-1.84E-09	count	1
ARHGAP39	-18.6677302	2421.510289	-0.0077	0.994	-1.84E-09	count	1
AL161725.1	-18.6677302	2421.510289	-0.0077	0.994	-1.84E-09	count	1
TRBV26OR9-2	-18.6677302	2421.510289	-0.0077	0.994	-1.84E-09	count	1
FRMPD1	-18.6677302	2421.510289	-0.0077	0.994	-1.84E-09	count	1
FOXE1	-18.6677302	2421.510289	-0.0077	0.994	-1.84E-09	count	1
CAVIN4	-18.6677302	2421.510289	-0.0077	0.994	-1.84E-09	count	1
AL356309.2	-18.6677302	2421.510289	-0.0077	0.994	-1.84E-09	count	1
AL592211.1	-18.6677302	2421.510289	-0.0077	0.994	-1.84E-09	count	1
PRDM12	-18.6677302	2421.510289	-0.0077	0.994	-1.84E-09	count	1
AL590226.1	-18.6677302	2421.510289	-0.0077	0.994	-1.84E-09	count	1
AC069287.2	-18.6677302	2421.510289	-0.0077	0.994	-1.84E-09	count	1
AC104389.4	-18.6677302	2421.510289	-0.0077	0.994	-1.84E-09	count	1
UBQLNL	-18.6677302	2421.510289	-0.0077	0.994	-1.84E-09	count	1
OR56B1	-18.6677302	2421.510289	-0.0077	0.994	-1.84E-09	count	1
AC080023.1	-18.6677302	2421.510289	-0.0077	0.994	-1.84E-09	count	1
AC079329.1	-18.6677302	2421.510289	-0.0077	0.994	-1.84E-09	count	1
SAA2	-18.6677302	2421.510289	-0.0077	0.994	-1.84E-09	count	1
SAA1	-18.6677302	2421.510289	-0.0077	0.994	-1.84E-09	count	1
LDHC	-18.6677302	2421.510289	-0.0077	0.994	-1.84E-09	count	1
AP003721.3	-18.6677302	2421.510289	-0.0077	0.994	-1.84E-09	count	1
AP003721.1	-18.6677302	2421.510289	-0.0077	0.994	-1.84E-09	count	1
AC004923.4	-18.6677302	2421.510289	-0.0077	0.994	-1.84E-09	count	1
AP002490.1	-18.6677302	2421.510289	-0.0077	0.994	-1.84E-09	count	1
WNT11	-18.6677302	2421.510289	-0.0077	0.994	-1.84E-09	count	1
THRSP	-18.6677302	2421.510289	-0.0077	0.994	-1.84E-09	count	1
CCDC89	-18.6677302	2421.510289	-0.0077	0.994	-1.84E-09	count	1
AP000880.1	-18.6677302	2421.510289	-0.0077	0.994	-1.84E-09	count	1
AP004147.1	-18.6677302	2421.510289	-0.0077	0.994	-1.84E-09	count	1
AP000646.1	-18.6677302	2421.510289	-0.0077	0.994	-1.84E-09	count	1
CASC10	-18.6677302	2421.510289	-0.0077	0.994	-1.84E-09	count	1
UNC5B-AS1	-18.6677302	2421.510289	-0.0077	0.994	-1.84E-09	count	1
AC067750.1	-18.6677302	2421.510289	-0.0077	0.994	-1.84E-09	count	1
SLIT1	-18.6677302	2421.510289	-0.0077	0.994	-1.84E-09	count	1
AL157786.1	-18.6677302	2421.510289	-0.0077	0.994	-1.84E-09	count	1

AL513190.1	-18.6677302	2421.510289	-0.0077	0.994	-1.84E-09	count	1
EDRF1-AS1	-18.6677302	2421.510289	-0.0077	0.994	-1.84E-09	count	1
AL451069.2	-18.6677302	2421.510289	-0.0077	0.994	-1.84E-09	count	1
PRMT8	-18.6677302	2421.510289	-0.0077	0.994	-1.84E-09	count	1
AC018630.2	-18.6677302	2421.510289	-0.0077	0.994	-1.84E-09	count	1
RERGL	-18.6677302	2421.510289	-0.0077	0.994	-1.84E-09	count	1
AC053513.1	-18.6677302	2421.510289	-0.0077	0.994	-1.84E-09	count	1
LINC00477	-18.6677302	2421.510289	-0.0077	0.994	-1.84E-09	count	1
PTHLH	-18.6677302	2421.510289	-0.0077	0.994	-1.84E-09	count	1
AC121338.1	-18.6677302	2421.510289	-0.0077	0.994	-1.84E-09	count	1
AC121338.2	-18.6677302	2421.510289	-0.0077	0.994	-1.84E-09	count	1
FAIM2	-18.6677302	2421.510289	-0.0077	0.994	-1.84E-09	count	1
KRT7	-18.6677302	2421.510289	-0.0077	0.994	-1.84E-09	count	1
NEUROD4	-18.6677302	2421.510289	-0.0077	0.994	-1.84E-09	count	1
ERBB3	-18.6677302	2421.510289	-0.0077	0.994	-1.84E-09	count	1
AC083805.3	-18.6677302	2421.510289	-0.0077	0.994	-1.84E-09	count	1
AC020611.2	-18.6677302	2421.510289	-0.0077	0.994	-1.84E-09	count	1
AC089998.1	-18.6677302	2421.510289	-0.0077	0.994	-1.84E-09	count	1
LINC02458	-18.6677302	2421.510289	-0.0077	0.994	-1.84E-09	count	1
AC079907.2	-18.6677302	2421.510289	-0.0077	0.994	-1.84E-09	count	1
CCDC63	-18.6677302	2421.510289	-0.0077	0.994	-1.84E-09	count	1
RASAL1	-18.6677302	2421.510289	-0.0077	0.994	-1.84E-09	count	1
AC127164.1	-18.6677302	2421.510289	-0.0077	0.994	-1.84E-09	count	1
AC026333.4	-18.6677302	2421.510289	-0.0077	0.994	-1.84E-09	count	1
LINC02405	-18.6677302	2421.510289	-0.0077	0.994	-1.84E-09	count	1
GJA3	-18.6677302	2421.510289	-0.0077	0.994	-1.84E-09	count	1
AL160153.1	-18.6677302	2421.510289	-0.0077	0.994	-1.84E-09	count	1
FGF14-AS2	-18.6677302	2421.510289	-0.0077	0.994	-1.84E-09	count	1
NFATC4	-18.6677302	2421.510289	-0.0077	0.994	-1.84E-09	count	1
FOXA1	-18.6677302	2421.510289	-0.0077	0.994	-1.84E-09	count	1
BMP4	-18.6677302	2421.510289	-0.0077	0.994	-1.84E-09	count	1
AL139300.1	-18.6677302	2421.510289	-0.0077	0.994	-1.84E-09	count	1
LINC00638	-18.6677302	2421.510289	-0.0077	0.994	-1.84E-09	count	1
AC021755.3	-18.6677302	2421.510289	-0.0077	0.994	-1.84E-09	count	1
TYRO3	-18.6677302	2421.510289	-0.0077	0.994	-1.84E-09	count	1
SLC28A2	-18.6677302	2421.510289	-0.0077	0.994	-1.84E-09	count	1
AC084757.2	-18.6677302	2421.510289	-0.0077	0.994	-1.84E-09	count	1
LINC01169	-18.6677302	2421.510289	-0.0077	0.994	-1.84E-09	count	1
SH2D7	-18.6677302	2421.510289	-0.0077	0.994	-1.84E-09	count	1
AC013565.3	-18.6677302	2421.510289	-0.0077	0.994	-1.84E-09	count	1
WDR93	-18.6677302	2421.510289	-0.0077	0.994	-1.84E-09	count	1
AC068831.1	-18.6677302	2421.510289	-0.0077	0.994	-1.84E-09	count	1
AL031705.1	-18.6677302	2421.510289	-0.0077	0.994	-1.84E-09	count	1
KREMEN2	-18.6677302	2421.510289	-0.0077	0.994	-1.84E-09	count	1
AC007601.2	-18.6677302	2421.510289	-0.0077	0.994	-1.84E-09	count	1

MIR193BHG	-18.6677302	2421.510289	-0.0077	0.994	-1.84E-09	count	1
AC092287.1	-18.6677302	2421.510289	-0.0077	0.994	-1.84E-09	count	1
AC012645.1	-18.6677302	2421.510289	-0.0077	0.994	-1.84E-09	count	1
AC116348.1	-18.6677302	2421.510289	-0.0077	0.994	-1.84E-09	count	1
SLC5A2	-18.6677302	2421.510289	-0.0077	0.994	-1.84E-09	count	1
SLC12A3	-18.6677302	2421.510289	-0.0077	0.994	-1.84E-09	count	1
ADGRG5	-18.6677302	2421.510289	-0.0077	0.994	-1.84E-09	count	1
MMP15	-18.6677302	2421.510289	-0.0077	0.994	-1.84E-09	count	1
AC092115.2	-18.6677302	2421.510289	-0.0077	0.994	-1.84E-09	count	1
MTSS1L	-18.6677302	2421.510289	-0.0077	0.994	-1.84E-09	count	1
HYDIN	-18.6677302	2421.510289	-0.0077	0.994	-1.84E-09	count	1
TMEM231	-18.6677302	2421.510289	-0.0077	0.994	-1.84E-09	count	1
CA5A	-18.6677302	2421.510289	-0.0077	0.994	-1.84E-09	count	1
AC134312.5	-18.6677302	2421.510289	-0.0077	0.994	-1.84E-09	count	1
AC138028.3	-18.6677302	2421.510289	-0.0077	0.994	-1.84E-09	count	1
LINC00304	-18.6677302	2421.510289	-0.0077	0.994	-1.84E-09	count	1
SLC22A31	-18.6677302	2421.510289	-0.0077	0.994	-1.84E-09	count	1
TRPV3	-18.6677302	2421.510289	-0.0077	0.994	-1.84E-09	count	1
AC124066.1	-18.6677302	2421.510289	-0.0077	0.994	-1.84E-09	count	1
AC104982.1	-18.6677302	2421.510289	-0.0077	0.994	-1.84E-09	count	1
ARL5C	-18.6677302	2421.510289	-0.0077	0.994	-1.84E-09	count	1
AC040934.1	-18.6677302	2421.510289	-0.0077	0.994	-1.84E-09	count	1
AC103702.2	-18.6677302	2421.510289	-0.0077	0.994	-1.84E-09	count	1
LINC02073	-18.6677302	2421.510289	-0.0077	0.994	-1.84E-09	count	1
HLF	-18.6677302	2421.510289	-0.0077	0.994	-1.84E-09	count	1
C17orf77	-18.6677302	2421.510289	-0.0077	0.994	-1.84E-09	count	1
OTOP2	-18.6677302	2421.510289	-0.0077	0.994	-1.84E-09	count	1
MAFG-DT	-18.6677302	2421.510289	-0.0077	0.994	-1.84E-09	count	1
AP005329.2	-18.6677302	2421.510289	-0.0077	0.994	-1.84E-09	count	1
AP005059.1	-18.6677302	2421.510289	-0.0077	0.994	-1.84E-09	count	1
DSG2	-18.6677302	2421.510289	-0.0077	0.994	-1.84E-09	count	1
MYO5B	-18.6677302	2421.510289	-0.0077	0.994	-1.84E-09	count	1
ONECUT2	-18.6677302	2421.510289	-0.0077	0.994	-1.84E-09	count	1
CDH7	-18.6677302	2421.510289	-0.0077	0.994	-1.84E-09	count	1
AC104423.1	-18.6677302	2421.510289	-0.0077	0.994	-1.84E-09	count	1
AC021594.1	-18.6677302	2421.510289	-0.0077	0.994	-1.84E-09	count	1
LINC01754	-18.6677302	2421.510289	-0.0077	0.994	-1.84E-09	count	1
AL354813.1	-18.6677302	2421.510289	-0.0077	0.994	-1.84E-09	count	1
LAMA5	-18.6677302	2421.510289	-0.0077	0.994	-1.84E-09	count	1
AL121829.2	-18.6677302	2421.510289	-0.0077	0.994	-1.84E-09	count	1
AC020916.2	-18.6677302	2421.510289	-0.0077	0.994	-1.84E-09	count	1
CLEC17A	-18.6677302	2421.510289	-0.0077	0.994	-1.84E-09	count	1
AC020911.1	-18.6677302	2421.510289	-0.0077	0.994	-1.84E-09	count	1
AC020913.1	-18.6677302	2421.510289	-0.0077	0.994	-1.84E-09	count	1
USHBP1	-18.6677302	2421.510289	-0.0077	0.994	-1.84E-09	count	1

PLEKHF1	-18.6677302	2421.510289	-0.0077	0.994	-1.84E-09	count	1
AC020907.3	-18.6677302	2421.510289	-0.0077	0.994	-1.84E-09	count	1
CEACAM1	-18.6677302	2421.510289	-0.0077	0.994	-1.84E-09	count	1
GPR4	-18.6677302	2421.510289	-0.0077	0.994	-1.84E-09	count	1
NOVA2	-18.6677302	2421.510289	-0.0077	0.994	-1.84E-09	count	1
MEIS3	-18.6677302	2421.510289	-0.0077	0.994	-1.84E-09	count	1
SLC6A16	-18.6677302	2421.510289	-0.0077	0.994	-1.84E-09	count	1
NAPSA	-18.6677302	2421.510289	-0.0077	0.994	-1.84E-09	count	1
AC010320.1	-18.6677302	2421.510289	-0.0077	0.994	-1.84E-09	count	1
COX6B2	-18.6677302	2421.510289	-0.0077	0.994	-1.84E-09	count	1
ZNF460-AS1	-18.6677302	2421.510289	-0.0077	0.994	-1.84E-09	count	1
AC004076.1	-18.6677302	2421.510289	-0.0077	0.994	-1.84E-09	count	1
LINC01658	-18.6677302	2421.510289	-0.0077	0.994	-1.84E-09	count	1
AP000350.7	-18.6677302	2421.510289	-0.0077	0.994	-1.84E-09	count	1
SUSD2	-18.6677302	2421.510289	-0.0077	0.994	-1.84E-09	count	1
LINC01422	-18.6677302	2421.510289	-0.0077	0.994	-1.84E-09	count	1
PIK3IP1-AS1	-18.6677302	2421.510289	-0.0077	0.994	-1.84E-09	count	1
BAIAP2L2	-18.6677302	2421.510289	-0.0077	0.994	-1.84E-09	count	1
ARHGAP8	-18.6677302	2421.510289	-0.0077	0.994	-1.84E-09	count	1
Z97192.1	-18.6677302	2421.510289	-0.0077	0.994	-1.84E-09	count	1
MIOX	-18.6677302	2421.510289	-0.0077	0.994	-1.84E-09	count	1
AP000223.1	-18.6677302	2421.510289	-0.0077	0.994	-1.84E-09	count	1
AP000317.1	-18.6677302	2421.510289	-0.0077	0.994	-1.84E-09	count	1
TSPEAR-AS1	-18.6677302	2421.510289	-0.0077	0.994	-1.84E-09	count	1
AP001469.2	-18.6677302	2421.510289	-0.0077	0.994	-1.84E-09	count	1
AL109811.3	-18.6677302	2421.510289	-0.0077	0.994	-1.84E-09	count	1
AL590434.1	-18.6677302	2421.510289	-0.0077	0.994	-1.84E-09	count	1
NUP210L	-18.6677302	2421.510289	-0.0077	0.994	-1.84E-09	count	1
LINC01460	-18.6677302	2421.510289	-0.0077	0.994	-1.84E-09	count	1
MEIS1	-18.6677302	2421.510289	-0.0077	0.994	-1.84E-09	count	1
AC012447.1	-18.6677302	2421.510289	-0.0077	0.994	-1.84E-09	count	1
RFTN2	-18.6677302	2421.510289	-0.0077	0.994	-1.84E-09	count	1
KIT	-18.6677302	2421.510289	-0.0077	0.994	-1.84E-09	count	1
CRYGN	-18.6677302	2421.510289	-0.0077	0.994	-1.84E-09	count	1
LINC00102	-18.6677302	2421.510289	-0.0077	0.994	-1.84E-09	count	1
HDC	-18.6677302	2421.510289	-0.0077	0.994	-1.84E-09	count	1
CHRNA3	-18.6677302	2421.510289	-0.0077	0.994	-1.84E-09	count	1
AC100793.2	-18.6677302	2421.510289	-0.0077	0.994	-1.84E-09	count	1
LINC02078	-18.6677302	2421.510289	-0.0077	0.994	-1.84E-09	count	1
SNPH	-18.6677302	2421.510289	-0.0077	0.994	-1.84E-09	count	1
AC004156.1	-18.6677302	2421.510289	-0.0077	0.994	-1.84E-09	count	1
BX322562.1	-18.6677302	2421.510289	-0.0077	0.994	-1.84E-09	count	1
EBF1	-19.363641	2421.469698	-0.008	0.994	-1.84E-09	count	1
C6orf132	-19.363641	2421.469698	-0.008	0.994	-1.84E-09	count	1
WASF3	-19.363641	2421.469698	-0.008	0.994	-1.84E-09	count	1

TENT5B	-19.363641	2421.469698	-0.008	0.994	-1.84E-09	count	1
AC074099.1	-19.363641	2421.469698	-0.008	0.994	-1.84E-09	count	1
SYNPO2	-19.363641	2421.469698	-0.008	0.994	-1.84E-09	count	1
PNMA2	-19.363641	2421.469698	-0.008	0.994	-1.84E-09	count	1
EHF	-19.363641	2421.469698	-0.008	0.994	-1.84E-09	count	1
CBX2	-19.363641	2421.469698	-0.008	0.994	-1.84E-09	count	1
DRAXIN	-19.363641	2421.469698	-0.008	0.994	-1.84E-09	count	1
AL451074.6	-19.363641	2421.469698	-0.008	0.994	-1.84E-09	count	1
LAD1	-19.363641	2421.469698	-0.008	0.994	-1.84E-09	count	1
RAB17	-19.363641	2421.469698	-0.008	0.994	-1.84E-09	count	1
TBX18	-19.363641	2421.469698	-0.008	0.994	-1.84E-09	count	1
AL732314.4	-19.363641	2421.469698	-0.008	0.994	-1.84E-09	count	1
HSPA12A	-19.363641	2421.469698	-0.008	0.994	-1.84E-09	count	1
COL4A2-AS1	-19.363641	2421.469698	-0.008	0.994	-1.84E-09	count	1
C17orf99	-19.363641	2421.469698	-0.008	0.994	-1.84E-09	count	1
CLUL1	-19.363641	2421.469698	-0.008	0.994	-1.84E-09	count	1
NPR1	-19.363641	2421.469698	-0.008	0.994	-1.84E-09	count	1
NCAPG	-19.363641	2421.469698	-0.008	0.994	-1.84E-09	count	1
AC067735.1	-19.363641	2421.469698	-0.008	0.994	-1.84E-09	count	1
DACT1	-19.363641	2421.469698	-0.008	0.994	-1.84E-09	count	1
BRIP1	-19.363641	2421.469698	-0.008	0.994	-1.84E-09	count	1
AC008752.2	-19.363641	2421.469698	-0.008	0.994	-1.84E-09	count	1
CHMP1B-AS1	-19.365217	3431.971417	-0.0056	0.995	-1.84E-09	count	1
ACOD1	-19.365217	3431.971417	-0.0056	0.995	-1.84E-09	count	1
UNC5C	-19.365217	3431.971417	-0.0056	0.995	-1.84E-09	count	1
AP006284.1	-19.365217	3431.971417	-0.0056	0.995	-1.84E-09	count	1
AL359220.1	-19.365217	3431.971417	-0.0056	0.995	-1.84E-09	count	1
AP001542.3	-19.365217	3431.971417	-0.0056	0.995	-1.84E-09	count	1
TGFBR3L	-19.365217	3431.971417	-0.0056	0.995	-1.84E-09	count	1
SLC8A2	-19.365217	3431.971417	-0.0056	0.995	-1.84E-09	count	1
LINC01122	-19.365217	3431.971417	-0.0056	0.995	-1.84E-09	count	1
CPA3	-19.365217	3431.971417	-0.0056	0.995	-1.84E-09	count	1
AP001610.1	-19.365217	3431.971417	-0.0056	0.995	-1.84E-09	count	1
UPK1B	-19.365217	3431.971417	-0.0056	0.995	-1.84E-09	count	1
AC067930.2	-19.365217	3431.971417	-0.0056	0.995	-1.84E-09	count	1
RCVRN	-19.365217	3431.971417	-0.0056	0.995	-1.84E-09	count	1
DDIT4L	-18.513372	1737.146346	-0.0107	0.991	-1.83E-09	count	1
ACBD7	-18.513372	1737.146346	-0.0107	0.991	-1.83E-09	count	1
C9orf153	-18.5154838	2464.899725	-0.0075	0.994	-1.83E-09	count	1
BEAN1-AS1	-18.5154838	2464.899725	-0.0075	0.994	-1.83E-09	count	1
CES3	-18.5154838	2464.899725	-0.0075	0.994	-1.83E-09	count	1
AC107952.2	-18.5154838	2464.899725	-0.0075	0.994	-1.83E-09	count	1
CRYGS	-18.5154838	2464.899725	-0.0075	0.994	-1.83E-09	count	1
FAM218A	-18.5154838	2464.899725	-0.0075	0.994	-1.83E-09	count	1
RHBDL1	-18.5154838	2464.899725	-0.0075	0.994	-1.83E-09	count	1

AC008753.2	-18.5154838	2464.899725	-0.0075	0.994	-1.83E-09	count	1
CYP2D6	-18.5154838	2464.899725	-0.0075	0.994	-1.83E-09	count	1
EMCN	-18.5154838	2464.899725	-0.0075	0.994	-1.83E-09	count	1
EGFL6	-18.5154838	2464.899725	-0.0075	0.994	-1.83E-09	count	1
AC105020.5	-18.5154838	2464.899725	-0.0075	0.994	-1.83E-09	count	1
AL022329.2	-18.5154838	2464.899725	-0.0075	0.994	-1.83E-09	count	1
AC104184.1	-18.9230629	2248.415785	-0.0084	0.993	-1.83E-09	count	1
AC011374.1	-18.9230629	2248.415785	-0.0084	0.993	-1.83E-09	count	1
AL590560.1	-0.1593104	0.6986937	-0.228	0.82	-1.65E-09	count	1
PNMA8A	-0.1593104	0.5931455	-0.2686	0.788	-1.65E-09	count	1
FAM71F2	-0.1593104	0.6803583	-0.2342	0.815	-1.65E-09	count	1
ITPR1-DT	-0.1593104	0.6803583	-0.2342	0.815	-1.65E-09	count	1
POC1B-GALNT4	-0.1593104	0.6803583	-0.2342	0.815	-1.65E-09	count	1
AC013652.2	-0.1593104	0.6803583	-0.2342	0.815	-1.65E-09	count	1
PPT2	-0.4182652	0.8924533	-0.4687	0.639	-1.46E-09	count	1
KRT31	-0.4182652	0.8924533	-0.4687	0.639	-1.46E-09	count	1
ZNF257	-0.077028	0.527679	-0.146	0.884	-8.20E-10	count	1
AC011374.2	-0.077028	0.5243187	-0.1469	0.883	-8.20E-10	count	1
AL136985.3	-18.8167105	2865.561954	-0.0066	0.995	-6.74E-10	count	1
AC093155.3	-18.8167105	2865.561954	-0.0066	0.995	-6.74E-10	count	1
FAM72C	-18.8167105	2865.561954	-0.0066	0.995	-6.74E-10	count	1
AL031733.2	-18.8167105	2865.561954	-0.0066	0.995	-6.74E-10	count	1
Z99127.1	-18.8167105	2865.561954	-0.0066	0.995	-6.74E-10	count	1
LINC01115	-18.8167105	2865.561954	-0.0066	0.995	-6.74E-10	count	1
NRIR	-18.8167105	2865.561954	-0.0066	0.995	-6.74E-10	count	1
SLC5A7	-18.8167105	2865.561954	-0.0066	0.995	-6.74E-10	count	1
RGPD8	-18.8167105	2865.561954	-0.0066	0.995	-6.74E-10	count	1
INHBB	-18.8167105	2865.561954	-0.0066	0.995	-6.74E-10	count	1
PLA2R1	-18.8167105	2865.561954	-0.0066	0.995	-6.74E-10	count	1
MYO1B	-18.8167105	2865.561954	-0.0066	0.995	-6.74E-10	count	1
YEATS2-AS1	-18.8167105	2865.561954	-0.0066	0.995	-6.74E-10	count	1
MEPE	-18.8167105	2865.561954	-0.0066	0.995	-6.74E-10	count	1
SNCA-AS1	-18.8167105	2865.561954	-0.0066	0.995	-6.74E-10	count	1
AC020900.1	-18.8167105	2865.561954	-0.0066	0.995	-6.74E-10	count	1
AL024498.1	-18.8167105	2865.561954	-0.0066	0.995	-6.74E-10	count	1
KCNK5	-18.8167105	2865.561954	-0.0066	0.995	-6.74E-10	count	1
GNMT	-18.8167105	2865.561954	-0.0066	0.995	-6.74E-10	count	1
RIMS1	-18.8167105	2865.561954	-0.0066	0.995	-6.74E-10	count	1
AL390208.1	-18.8167105	2865.561954	-0.0066	0.995	-6.74E-10	count	1
AC006042.2	-18.8167105	2865.561954	-0.0066	0.995	-6.74E-10	count	1
AC005154.4	-18.8167105	2865.561954	-0.0066	0.995	-6.74E-10	count	1
TRGV10	-18.8167105	2865.561954	-0.0066	0.995	-6.74E-10	count	1
TRGV7	-18.8167105	2865.561954	-0.0066	0.995	-6.74E-10	count	1
SEMA3D	-18.8167105	2865.561954	-0.0066	0.995	-6.74E-10	count	1
SPDYE3	-18.8167105	2865.561954	-0.0066	0.995	-6.74E-10	count	1

AC016831.6	-18.8167105	2865.561954	-0.0066	0.995	-6.74E-10	count	1
NUDT11	-18.8167105	2865.561954	-0.0066	0.995	-6.74E-10	count	1
FAM167A	-18.8167105	2865.561954	-0.0066	0.995	-6.74E-10	count	1
AC079209.2	-18.8167105	2865.561954	-0.0066	0.995	-6.74E-10	count	1
DSCC1	-18.8167105	2865.561954	-0.0066	0.995	-6.74E-10	count	1
AC100803.3	-18.8167105	2865.561954	-0.0066	0.995	-6.74E-10	count	1
KYAT1	-18.8167105	2865.561954	-0.0066	0.995	-6.74E-10	count	1
CNIH2	-18.8167105	2865.561954	-0.0066	0.995	-6.74E-10	count	1
NUTM2D	-18.8167105	2865.561954	-0.0066	0.995	-6.74E-10	count	1
KLRC4	-18.8167105	2865.561954	-0.0066	0.995	-6.74E-10	count	1
EIF2S3B	-18.8167105	2865.561954	-0.0066	0.995	-6.74E-10	count	1
ARHGEF25	-18.8167105	2865.561954	-0.0066	0.995	-6.74E-10	count	1
TRAV8-2	-18.8167105	2865.561954	-0.0066	0.995	-6.74E-10	count	1
SLC10A1	-18.8167105	2865.561954	-0.0066	0.995	-6.74E-10	count	1
DPF3	-18.8167105	2865.561954	-0.0066	0.995	-6.74E-10	count	1
AL138478.1	-18.8167105	2865.561954	-0.0066	0.995	-6.74E-10	count	1
AL137779.2	-18.8167105	2865.561954	-0.0066	0.995	-6.74E-10	count	1
DISP2	-18.8167105	2865.561954	-0.0066	0.995	-6.74E-10	count	1
AC021752.1	-18.8167105	2865.561954	-0.0066	0.995	-6.74E-10	count	1
LINC00926	-18.8167105	2865.561954	-0.0066	0.995	-6.74E-10	count	1
AC027237.2	-18.8167105	2865.561954	-0.0066	0.995	-6.74E-10	count	1
AC104938.1	-18.8167105	2865.561954	-0.0066	0.995	-6.74E-10	count	1
AC008731.1	-18.8167105	2865.561954	-0.0066	0.995	-6.74E-10	count	1
AC044802.2	-18.8167105	2865.561954	-0.0066	0.995	-6.74E-10	count	1
CARMIL2	-18.8167105	2865.561954	-0.0066	0.995	-6.74E-10	count	1
CLEC18A	-18.8167105	2865.561954	-0.0066	0.995	-6.74E-10	count	1
MAFTRR	-18.8167105	2865.561954	-0.0066	0.995	-6.74E-10	count	1
C16orf46	-18.8167105	2865.561954	-0.0066	0.995	-6.74E-10	count	1
AC087393.2	-18.8167105	2865.561954	-0.0066	0.995	-6.74E-10	count	1
FLJ45513	-18.8167105	2865.561954	-0.0066	0.995	-6.74E-10	count	1
SOX9	-18.8167105	2865.561954	-0.0066	0.995	-6.74E-10	count	1
AP001029.2	-18.8167105	2865.561954	-0.0066	0.995	-6.74E-10	count	1
TCF4-AS2	-18.8167105	2865.561954	-0.0066	0.995	-6.74E-10	count	1
XKR7	-18.8167105	2865.561954	-0.0066	0.995	-6.74E-10	count	1
ZNF831	-18.8167105	2865.561954	-0.0066	0.995	-6.74E-10	count	1
AC007292.2	-18.8167105	2865.561954	-0.0066	0.995	-6.74E-10	count	1
AC003956.1	-18.8167105	2865.561954	-0.0066	0.995	-6.74E-10	count	1
AC012313.6	-18.8167105	2865.561954	-0.0066	0.995	-6.74E-10	count	1
Z98885.3	-18.8167105	2865.561954	-0.0066	0.995	-6.74E-10	count	1
BX004987.1	-18.8167105	2865.561954	-0.0066	0.995	-6.74E-10	count	1
AC254633.1	-18.8167105	2865.561954	-0.0066	0.995	-6.74E-10	count	1
AL031283.1	-18.8167105	2865.561954	-0.0066	0.995	-6.74E-10	count	1
AL050343.2	-18.8167105	2865.561954	-0.0066	0.995	-6.74E-10	count	1
DAB1	-18.8167105	2865.561954	-0.0066	0.995	-6.74E-10	count	1
TNNI3K	-18.8167105	2865.561954	-0.0066	0.995	-6.74E-10	count	1

TSACC	-18.8167105	2865.561954	-0.0066	0.995	-6.74E-10	count	1
AL513217.1	-18.8167105	2865.561954	-0.0066	0.995	-6.74E-10	count	1
ALK	-18.8167105	2865.561954	-0.0066	0.995	-6.74E-10	count	1
GCC2-AS1	-18.8167105	2865.561954	-0.0066	0.995	-6.74E-10	count	1
AC074286.1	-18.8167105	2865.561954	-0.0066	0.995	-6.74E-10	count	1
KCNH8	-18.8167105	2865.561954	-0.0066	0.995	-6.74E-10	count	1
CCK	-18.8167105	2865.561954	-0.0066	0.995	-6.74E-10	count	1
STX19	-18.8167105	2865.561954	-0.0066	0.995	-6.74E-10	count	1
PLCXD2	-18.8167105	2865.561954	-0.0066	0.995	-6.74E-10	count	1
AC093788.1	-18.8167105	2865.561954	-0.0066	0.995	-6.74E-10	count	1
AC019163.1	-18.8167105	2865.561954	-0.0066	0.995	-6.74E-10	count	1
LINC01262	-18.8167105	2865.561954	-0.0066	0.995	-6.74E-10	count	1
C7	-18.8167105	2865.561954	-0.0066	0.995	-6.74E-10	count	1
CDO1	-18.8167105	2865.561954	-0.0066	0.995	-6.74E-10	count	1
HIST1H2AI	-18.8167105	2865.561954	-0.0066	0.995	-6.74E-10	count	1
UBD	-18.8167105	2865.561954	-0.0066	0.995	-6.74E-10	count	1
AL135905.1	-18.8167105	2865.561954	-0.0066	0.995	-6.74E-10	count	1
AL451064.1	-18.8167105	2865.561954	-0.0066	0.995	-6.74E-10	count	1
AC002464.1	-18.8167105	2865.561954	-0.0066	0.995	-6.74E-10	count	1
AL078590.3	-18.8167105	2865.561954	-0.0066	0.995	-6.74E-10	count	1
AL390955.2	-18.8167105	2865.561954	-0.0066	0.995	-6.74E-10	count	1
FAM180A	-18.8167105	2865.561954	-0.0066	0.995	-6.74E-10	count	1
PIP	-18.8167105	2865.561954	-0.0066	0.995	-6.74E-10	count	1
AC004889.1	-18.8167105	2865.561954	-0.0066	0.995	-6.74E-10	count	1
FAM239B	-18.8167105	2865.561954	-0.0066	0.995	-6.74E-10	count	1
HR	-18.8167105	2865.561954	-0.0066	0.995	-6.74E-10	count	1
RIMS2	-18.8167105	2865.561954	-0.0066	0.995	-6.74E-10	count	1
AC104316.2	-18.8167105	2865.561954	-0.0066	0.995	-6.74E-10	count	1
LINC01219	-18.8167105	2865.561954	-0.0066	0.995	-6.74E-10	count	1
TSKU	-18.8167105	2865.561954	-0.0066	0.995	-6.74E-10	count	1
LAYN	-18.8167105	2865.561954	-0.0066	0.995	-6.74E-10	count	1
SCN4B	-18.8167105	2865.561954	-0.0066	0.995	-6.74E-10	count	1
DKK1	-18.8167105	2865.561954	-0.0066	0.995	-6.74E-10	count	1
AC022509.1	-18.8167105	2865.561954	-0.0066	0.995	-6.74E-10	count	1
BCDIN3D-AS1	-18.8167105	2865.561954	-0.0066	0.995	-6.74E-10	count	1
LINC02389	-18.8167105	2865.561954	-0.0066	0.995	-6.74E-10	count	1
MYL2	-18.8167105	2865.561954	-0.0066	0.995	-6.74E-10	count	1
LINC00642	-18.8167105	2865.561954	-0.0066	0.995	-6.74E-10	count	1
IGHV4-34	-18.8167105	2865.561954	-0.0066	0.995	-6.74E-10	count	1
IGHV1-69-2	-18.8167105	2865.561954	-0.0066	0.995	-6.74E-10	count	1
TGM5	-18.8167105	2865.561954	-0.0066	0.995	-6.74E-10	count	1
AC068722.2	-18.8167105	2865.561954	-0.0066	0.995	-6.74E-10	count	1
UNC13C	-18.8167105	2865.561954	-0.0066	0.995	-6.74E-10	count	1
TMC7	-18.8167105	2865.561954	-0.0066	0.995	-6.74E-10	count	1
PRSS8	-18.8167105	2865.561954	-0.0066	0.995	-6.74E-10	count	1

AC044802.1	-18.8167105	2865.561954	-0.0066	0.995	-6.74E-10	count	1
TERB1	-18.8167105	2865.561954	-0.0066	0.995	-6.74E-10	count	1
AC091153.3	-18.8167105	2865.561954	-0.0066	0.995	-6.74E-10	count	1
WSCD1	-18.8167105	2865.561954	-0.0066	0.995	-6.74E-10	count	1
AC016876.3	-18.8167105	2865.561954	-0.0066	0.995	-6.74E-10	count	1
AC135178.2	-18.8167105	2865.561954	-0.0066	0.995	-6.74E-10	count	1
AC005224.1	-18.8167105	2865.561954	-0.0066	0.995	-6.74E-10	count	1
ANGPT4	-18.8167105	2865.561954	-0.0066	0.995	-6.74E-10	count	1
HAR1A	-18.8167105	2865.561954	-0.0066	0.995	-6.74E-10	count	1
DIRAS1	-18.8167105	2865.561954	-0.0066	0.995	-6.74E-10	count	1
AC011446.2	-18.8167105	2865.561954	-0.0066	0.995	-6.74E-10	count	1
OR1I1	-18.8167105	2865.561954	-0.0066	0.995	-6.74E-10	count	1
LINC01764	-18.8167105	2865.561954	-0.0066	0.995	-6.74E-10	count	1
MYBPC2	-18.8167105	2865.561954	-0.0066	0.995	-6.74E-10	count	1
FAM71E1	-18.8167105	2865.561954	-0.0066	0.995	-6.74E-10	count	1
VPREB3	-18.8167105	2865.561954	-0.0066	0.995	-6.74E-10	count	1
SHISA8	-18.8167105	2865.561954	-0.0066	0.995	-6.74E-10	count	1
TNFRSF13C	-18.8167105	2865.561954	-0.0066	0.995	-6.74E-10	count	1
AC233755.1	-18.8167105	2865.561954	-0.0066	0.995	-6.74E-10	count	1
HEYL	-18.8167105	2865.561954	-0.0066	0.995	-6.74E-10	count	1
TBR1	-18.8167105	2865.561954	-0.0066	0.995	-6.74E-10	count	1
LINC01013	-18.8167105	2865.561954	-0.0066	0.995	-6.74E-10	count	1
LINC00894	-18.8167105	2865.561954	-0.0066	0.995	-6.74E-10	count	1
AP003110.1	-18.8167105	2865.561954	-0.0066	0.995	-6.74E-10	count	1
AP002884.4	-18.8167105	2865.561954	-0.0066	0.995	-6.74E-10	count	1
SPAG6	-18.8167105	2865.561954	-0.0066	0.995	-6.74E-10	count	1
TMEM72-AS1	-18.8167105	2865.561954	-0.0066	0.995	-6.74E-10	count	1
AC123768.2	-18.8167105	2865.561954	-0.0066	0.995	-6.74E-10	count	1
AC018521.2	-18.8167105	2865.561954	-0.0066	0.995	-6.74E-10	count	1
AC007638.1	-18.8167105	2865.561954	-0.0066	0.995	-6.74E-10	count	1
AL133230.1	-18.8167105	2865.561954	-0.0066	0.995	-6.74E-10	count	1
S1PR5	-18.8167105	2865.561954	-0.0066	0.995	-6.74E-10	count	1
ANGPT1	-0.1777864	0.4627212	-0.3842	0.701	-6.73E-10	count	1
RASL11B	-0.1593104	0.7966078	-0.2	0.842	-6.07E-10	count	1
AC108866.1	-0.1593104	0.7966078	-0.2	0.842	-6.07E-10	count	1
MYOZ3	-0.1593104	0.7966078	-0.2	0.842	-6.07E-10	count	1
EPDR1	-0.1593104	0.7966078	-0.2	0.842	-6.07E-10	count	1
ACTR3C	-0.1593104	0.7966078	-0.2	0.842	-6.07E-10	count	1
AL162586.1	-0.1593104	0.7966078	-0.2	0.842	-6.07E-10	count	1
PIH1D2	-0.1593104	0.7966078	-0.2	0.842	-6.07E-10	count	1
AL133353.1	-0.1593104	0.7966078	-0.2	0.842	-6.07E-10	count	1
SLC4A1	-0.1593104	0.7966078	-0.2	0.842	-6.07E-10	count	1
CCBE1	-0.1593104	0.7966078	-0.2	0.842	-6.07E-10	count	1
PLCG1	-0.1593104	0.7966078	-0.2	0.842	-6.07E-10	count	1
AC022144.1	-0.1593104	0.7966078	-0.2	0.842	-6.07E-10	count	1

MUC1	-0.1593104	0.7966078	-0.2	0.842	-6.07E-10	count	1
PRELP	-0.1593104	0.7966078	-0.2	0.842	-6.07E-10	count	1
KIF26B	-0.1593104	0.7966078	-0.2	0.842	-6.07E-10	count	1
PDE8B	-0.1593104	0.7966078	-0.2	0.842	-6.07E-10	count	1
LAMA2	-0.1593104	0.7966078	-0.2	0.842	-6.07E-10	count	1
MID1	-0.1593104	0.7966078	-0.2	0.842	-6.07E-10	count	1
AC103957.2	-0.1593104	0.7966078	-0.2	0.842	-6.07E-10	count	1
AC018523.2	-0.1593104	0.7966078	-0.2	0.842	-6.07E-10	count	1
TPH1	-0.1593104	0.7966078	-0.2	0.842	-6.07E-10	count	1
RPS6KB2-AS1	-0.1593104	0.7966078	-0.2	0.842	-6.07E-10	count	1
NAALAD2	-0.1593104	0.7966078	-0.2	0.842	-6.07E-10	count	1
MAT1A	-0.1593104	0.7966078	-0.2	0.842	-6.07E-10	count	1
PPP1R3C	-0.1593104	0.7966078	-0.2	0.842	-6.07E-10	count	1
AL157832.3	-0.1593104	0.7966078	-0.2	0.842	-6.07E-10	count	1
LINC00562	-0.1593104	0.7966078	-0.2	0.842	-6.07E-10	count	1
AC009120.4	-0.1593104	0.7966078	-0.2	0.842	-6.07E-10	count	1
LINC02132	-0.1593104	0.7966078	-0.2	0.842	-6.07E-10	count	1
P2RX5-TAX1BP3	-0.1593104	0.7966078	-0.2	0.842	-6.07E-10	count	1
RHBDL3	-0.1593104	0.7966078	-0.2	0.842	-6.07E-10	count	1
ABCA8	-0.1593104	0.7966078	-0.2	0.842	-6.07E-10	count	1
ITGB4	-0.1593104	0.7966078	-0.2	0.842	-6.07E-10	count	1
RNF157	-0.1593104	0.7966078	-0.2	0.842	-6.07E-10	count	1
PCP2	-0.1593104	0.7966078	-0.2	0.842	-6.07E-10	count	1
ZNRF3-AS1	-0.1593104	0.7966078	-0.2	0.842	-6.07E-10	count	1
AL035681.1	-0.1593104	0.7966078	-0.2	0.842	-6.07E-10	count	1
SFTPD	-0.1593104	0.7966078	-0.2	0.842	-6.07E-10	count	1
AC079949.2	-0.1593104	0.7966078	-0.2	0.842	-6.07E-10	count	1
LRRC46	-0.1593104	0.7966078	-0.2	0.842	-6.07E-10	count	1
OR7A17	-0.1593104	0.7966078	-0.2	0.842	-6.07E-10	count	1
Z83847.1	-0.1593104	0.7966078	-0.2	0.842	-6.07E-10	count	1
ABI3BP	-0.1593104	0.5625623	-0.2832	0.777	-6.07E-10	count	1
ZNF366	-0.1593104	0.5625623	-0.2832	0.777	-6.07E-10	count	1
RECQL4	-0.1593104	0.5625623	-0.2832	0.777	-6.07E-10	count	1
AKR1C1	0.0069755	0.6857454	0.0102	0.992	7.65E-11	count	1
HTR2B	0.0069755	0.6855492	0.0102	0.992	7.65E-11	count	1
BVES	0.0069755	0.6857454	0.0102	0.992	7.65E-11	count	1
IGHEP2	0.0069755	0.6857454	0.0102	0.992	7.65E-11	count	1
AL031599.1	0.0636845	0.5602511	0.1137	0.91	7.12E-10	count	1
AXIN2	0.1780252	0.9599292	0.1855	0.853	7.62E-10	count	1
GNAL	0.1780252	0.9599292	0.1855	0.853	7.62E-10	count	1
HNRNPA1L2	0.1780252	0.9599292	0.1855	0.853	7.62E-10	count	1
AC012640.1	0.1780252	0.9599292	0.1855	0.853	7.62E-10	count	1
AC078795.3	0.1780252	0.9599292	0.1855	0.853	7.62E-10	count	1
FNDC4	0.1780252	0.9599292	0.1855	0.853	7.62E-10	count	1
KRBOX1	0.1780252	0.9599292	0.1855	0.853	7.62E-10	count	1

SPTA1	0.1780252	0.9599292	0.1855	0.853	7.62E-10	count	1
DYNC1I1	0.1780252	0.9599292	0.1855	0.853	7.62E-10	count	1
HCN2	0.1780252	0.9599292	0.1855	0.853	7.62E-10	count	1
HIST2H2AA4	0.1780252	0.9599292	0.1855	0.853	7.62E-10	count	1
AC007364.1	0.1780252	0.9599292	0.1855	0.853	7.62E-10	count	1
AC004067.1	0.1780252	0.9937802	0.1791	0.858	7.62E-10	count	1
Z94721.1	0.1780252	0.9599292	0.1855	0.853	7.62E-10	count	1
HPN	0.1780252	0.9937802	0.1791	0.858	7.62E-10	count	1
SMAD9	0.1780252	0.9599292	0.1855	0.853	7.62E-10	count	1
ALPK2	0.1780252	0.9599292	0.1855	0.853	7.62E-10	count	1
AC016722.2	0.1780252	0.9937802	0.1791	0.858	7.62E-10	count	1
APOBEC3D	0.1780252	0.9599292	0.1855	0.853	7.62E-10	count	1
FAM95B1	0.1780252	0.9599292	0.1855	0.853	7.62E-10	count	1
AC087203.3	0.1780252	0.9599292	0.1855	0.853	7.62E-10	count	1
SOX8	0.1780252	0.9599292	0.1855	0.853	7.62E-10	count	1
AC026254.2	0.1780252	0.9937802	0.1791	0.858	7.62E-10	count	1
AC010175.1	0.1780252	0.9599292	0.1855	0.853	7.62E-10	count	1
AC097359.2	0.1780252	0.9599292	0.1855	0.853	7.62E-10	count	1
C2orf15	0.1780252	0.9599292	0.1855	0.853	7.62E-10	count	1
FAM66C	0.1780252	0.9599292	0.1855	0.853	7.62E-10	count	1
INPP5J	0.1780252	0.9599292	0.1855	0.853	7.62E-10	count	1
LINC00671	0.1780252	0.9599292	0.1855	0.853	7.62E-10	count	1
DLEU7-AS1	0.1780252	0.9599292	0.1855	0.853	7.62E-10	count	1
AL137789.1	0.1780252	0.9599292	0.1855	0.853	7.62E-10	count	1
AC007220.1	0.1780252	0.9937802	0.1791	0.858	7.62E-10	count	1
POLQ	0.1780252	0.9937802	0.1791	0.858	7.62E-10	count	1
SLC26A4	0.1780252	0.9599292	0.1855	0.853	7.62E-10	count	1
AP003392.1	0.1780252	0.9599292	0.1855	0.853	7.62E-10	count	1
MFAP3L	0.1780252	0.9937802	0.1791	0.858	7.62E-10	count	1
GPR153	0.1780252	0.9599292	0.1855	0.853	7.62E-10	count	1
GRIN3A	0.1780252	0.9937802	0.1791	0.858	7.62E-10	count	1
MCM3AP-AS1	0.1780252	0.9599292	0.1855	0.853	7.62E-10	count	1
RHPN2	0.1780252	0.9599292	0.1855	0.853	7.62E-10	count	1
ZNF74	0.1393456	0.5271788	0.2643	0.792	1.60E-09	count	1
ZNF883	0.1780252	0.6902367	0.2579	0.796	2.07E-09	count	1
ROBO1	0.5153608	0.3772702	1.366	0.172	2.47E-09	count	1
AC007878.1	0.5153608	0.5350672	0.9632	0.336	2.47E-09	count	1
SCGB3A2	0.5153608	0.5350672	0.9632	0.336	2.47E-09	count	1
TBX21	0.5153608	0.5350672	0.9632	0.336	2.47E-09	count	1
AC105105.1	0.5153608	0.5350672	0.9632	0.336	2.47E-09	count	1
AC006504.1	0.5153608	0.5350672	0.9632	0.336	2.47E-09	count	1
AC092747.4	0.2157162	0.4556486	0.4734	0.636	2.54E-09	count	1
SDK1	0.5338367	0.8560789	0.6236	0.533	2.57E-09	count	1
AC139749.1	0.5338367	0.8560789	0.6236	0.533	2.57E-09	count	1
MORC1	0.5338367	0.8560789	0.6236	0.533	2.57E-09	count	1

AL355297.4	0.5338367	0.8560789	0.6236	0.533	2.57E-09	count	1
AL356019.2	0.5338367	0.8560789	0.6236	0.533	2.57E-09	count	1
GPR135	0.5338367	0.8560789	0.6236	0.533	2.57E-09	count	1
GOLGA8H	0.5338367	0.8560789	0.6236	0.533	2.57E-09	count	1
AC239799.2	0.5338367	0.6894391	0.7743	0.439	2.57E-09	count	1
CAPN12	0.5338367	0.6894391	0.7743	0.439	2.57E-09	count	1
TTC34	0.5338367	0.6894391	0.7743	0.439	2.57E-09	count	1
TOGARAM2	0.5338367	0.6894391	0.7743	0.439	2.57E-09	count	1
CYP26B1	0.5338367	0.6894391	0.7743	0.439	2.57E-09	count	1
LINC01238	0.5338367	0.6894391	0.7743	0.439	2.57E-09	count	1
PART1	0.5338367	0.6894391	0.7743	0.439	2.57E-09	count	1
MOCS1	0.5338367	0.6894391	0.7743	0.439	2.57E-09	count	1
LBHD1	0.5338367	0.6894391	0.7743	0.439	2.57E-09	count	1
CDCA5	0.5338367	0.6894391	0.7743	0.439	2.57E-09	count	1
ELOVL3	0.5338367	0.6894391	0.7743	0.439	2.57E-09	count	1
CCDC184	0.5338367	0.6894391	0.7743	0.439	2.57E-09	count	1
SEMA6D	0.5338367	0.6894391	0.7743	0.439	2.57E-09	count	1
AC087286.3	0.5338367	0.6894391	0.7743	0.439	2.57E-09	count	1
AC027130.1	0.5338367	0.6894391	0.7743	0.439	2.57E-09	count	1
SMG8	0.5338367	0.6894391	0.7743	0.439	2.57E-09	count	1
ZNF792	0.5338367	0.6894391	0.7743	0.439	2.57E-09	count	1
FAM198A	0.2265442	0.5775302	0.3923	0.695	2.68E-09	count	1
AC095055.1	0.2265442	0.5259894	0.4307	0.667	2.68E-09	count	1
C2orf80	0.2265442	0.7860802	0.2882	0.773	2.68E-09	count	1
AC055811.3	0.2461547	0.5132158	0.4796	0.632	2.93E-09	count	1
MYLK	0.1463533	0.4943841	0.296	0.767	4.58E-09	count	1
PCDHGA12	0.3860913	0.7039261	0.5485	0.583	4.82E-09	count	1
DUSP15	0.3860913	0.6319212	0.611	0.541	4.82E-09	count	1
MSANTD1	18.4303267	2731.527168	0.0067	0.995	4.82E-09	count	1
STEAP2	18.4303269	2731.527089	0.0067	0.995	4.82E-09	count	1
FHDC1	18.4303267	2731.526931	0.0067	0.995	4.82E-09	count	1
AC092354.1	18.4303267	2731.526931	0.0067	0.995	4.82E-09	count	1
INMT	18.4303266	2731.527036	0.0067	0.995	4.82E-09	count	1
ARSE	18.4303266	2731.527182	0.0067	0.995	4.82E-09	count	1
AC108860.2	18.4303266	2731.527182	0.0067	0.995	4.82E-09	count	1
AC100830.2	18.4303268	2731.527063	0.0067	0.995	4.82E-09	count	1
AC093525.3	18.4303266	2731.527182	0.0067	0.995	4.82E-09	count	1
ADM5	18.4303266	2731.527182	0.0067	0.995	4.82E-09	count	1
AL031283.2	18.4303265	2731.526957	0.0067	0.995	4.82E-09	count	1
AL139220.2	18.4303266	2731.527049	0.0067	0.995	4.82E-09	count	1
MAB21L3	18.4303266	2731.527089	0.0067	0.995	4.82E-09	count	1
AL078644.2	18.4303266	2731.526878	0.0067	0.995	4.82E-09	count	1
NR5A2	18.4303266	2731.527049	0.0067	0.995	4.82E-09	count	1
OR2C3	18.4303265	2731.526997	0.0067	0.995	4.82E-09	count	1
AC009237.15	18.4303264	2731.527195	0.0067	0.995	4.82E-09	count	1

AC012087.1	18.4303264	2731.527195	0.0067	0.995	4.82E-09	count	1
RBMS3	18.4303266	2731.527049	0.0067	0.995	4.82E-09	count	1
CDC25A	18.4303266	2731.527089	0.0067	0.995	4.82E-09	count	1
AC072039.2	18.4303266	2731.527049	0.0067	0.995	4.82E-09	count	1
KLB	18.4303264	2731.526944	0.0067	0.995	4.82E-09	count	1
NIM1K	18.4303268	2731.526904	0.0067	0.995	4.82E-09	count	1
AC008897.2	18.4303266	2731.527049	0.0067	0.995	4.82E-09	count	1
HCG9	18.4303266	2731.527049	0.0067	0.995	4.82E-09	count	1
GABRR2	18.4303266	2731.527049	0.0067	0.995	4.82E-09	count	1
STK31	18.4303266	2731.526878	0.0067	0.995	4.82E-09	count	1
TRPV6	18.4303266	2731.52701	0.0067	0.995	4.82E-09	count	1
WWC3-AS1	18.4303266	2731.527049	0.0067	0.995	4.82E-09	count	1
SHROOM4	18.4303264	2731.527089	0.0067	0.995	4.82E-09	count	1
SBSPON	18.4303266	2731.526878	0.0067	0.995	4.82E-09	count	1
AC100771.2	18.4303266	2731.527049	0.0067	0.995	4.82E-09	count	1
AP001107.7	18.4303266	2731.52701	0.0067	0.995	4.82E-09	count	1
AP001972.1	18.4303264	2731.527195	0.0067	0.995	4.82E-09	count	1
OLAH	18.4303265	2731.526997	0.0067	0.995	4.82E-09	count	1
CYP17A1-AS1	18.4303264	2731.526944	0.0067	0.995	4.82E-09	count	1
PRAP1	18.4303264	2731.527089	0.0067	0.995	4.82E-09	count	1
IQSEC3	18.4303266	2731.52701	0.0067	0.995	4.82E-09	count	1
THSD1	18.4303266	2731.527049	0.0067	0.995	4.82E-09	count	1
AC001226.2	18.4303264	2731.527089	0.0067	0.995	4.82E-09	count	1
WASIR2	18.4303264	2731.527195	0.0067	0.995	4.82E-09	count	1
AC010536.1	18.4303266	2731.526957	0.0067	0.995	4.82E-09	count	1
AC002553.2	18.4303268	2731.526904	0.0067	0.995	4.82E-09	count	1
AP005899.1	18.4303267	2731.526891	0.0067	0.995	4.82E-09	count	1
AP001010.1	18.4303264	2731.527195	0.0067	0.995	4.82E-09	count	1
PCAT18	18.4303266	2731.527049	0.0067	0.995	4.82E-09	count	1
AL391095.1	18.4303266	2731.527049	0.0067	0.995	4.82E-09	count	1
APOBEC3B-AS1	18.4303266	2731.526957	0.0067	0.995	4.82E-09	count	1
Z83851.1	18.4303265	2731.526997	0.0067	0.995	4.82E-09	count	1
AL591163.1	18.4303265	2731.526983	0.0067	0.995	4.82E-09	count	1
AK5	18.4303267	2731.527023	0.0067	0.995	4.82E-09	count	1
AL390066.1	18.4303267	2731.527023	0.0067	0.995	4.82E-09	count	1
C1orf220	18.4303264	2731.527116	0.0067	0.995	4.82E-09	count	1
GREB1	18.4303264	2731.52697	0.0067	0.995	4.82E-09	count	1
AC073869.5	18.4303265	2731.526983	0.0067	0.995	4.82E-09	count	1
AC131097.3	18.4303264	2731.526957	0.0067	0.995	4.82E-09	count	1
UBE2E2-AS1	18.4303266	2731.527155	0.0067	0.995	4.82E-09	count	1
TMEM110-MUSTN1	18.4303265	2731.526983	0.0067	0.995	4.82E-09	count	1
AC131235.3	18.4303266	2731.52697	0.0067	0.995	4.82E-09	count	1
AC109927.1	18.4303267	2731.527023	0.0067	0.995	4.82E-09	count	1
AC034229.4	18.4303263	2731.52697	0.0067	0.995	4.82E-09	count	1
PCDHB15	18.4303264	2731.526904	0.0067	0.995	4.82E-09	count	1

MAS1	18.4303263	2731.52697	0.0067	0.995	4.82E-09	count	1
AC079760.2	18.4303265	2731.526983	0.0067	0.995	4.82E-09	count	1
AL161909.2	18.4303265	2731.527155	0.0067	0.995	4.82E-09	count	1
LINC01474	18.4303265	2731.527155	0.0067	0.995	4.82E-09	count	1
AC138230.1	18.4303265	2731.526983	0.0067	0.995	4.82E-09	count	1
CATSPERZ	18.4303266	2731.52697	0.0067	0.995	4.82E-09	count	1
AC005520.5	18.4303265	2731.527023	0.0067	0.995	4.82E-09	count	1
AC002550.2	18.4303264	2731.526904	0.0067	0.995	4.82E-09	count	1
AC002550.1	18.4303264	2731.526997	0.0067	0.995	4.82E-09	count	1
BOLA2	18.4303264	2731.52697	0.0067	0.995	4.82E-09	count	1
NRN1L	18.4303267	2731.52701	0.0067	0.995	4.82E-09	count	1
FKBP10	18.4303266	2731.527234	0.0067	0.995	4.82E-09	count	1
AC023983.2	18.4303264	2731.526997	0.0067	0.995	4.82E-09	count	1
ATCAY	18.4303266	2731.527234	0.0067	0.995	4.82E-09	count	1
BORCS8-MEF2B	18.4303264	2731.526904	0.0067	0.995	4.82E-09	count	1
AC007773.1	18.4303265	2731.527023	0.0067	0.995	4.82E-09	count	1
AC005392.2	18.4303263	2731.52697	0.0067	0.995	4.82E-09	count	1
ZNF320	18.4303264	2731.526957	0.0067	0.995	4.82E-09	count	1
AC008753.3	18.4303263	2731.52697	0.0067	0.995	4.82E-09	count	1
AC007666.1	18.4303265	2731.527023	0.0067	0.995	4.82E-09	count	1
PARS2	18.4303263	2731.526997	0.0067	0.995	4.82E-09	count	1
AC119674.1	18.4303265	2731.527182	0.0067	0.995	4.82E-09	count	1
AC244453.3	18.4303264	2731.527116	0.0067	0.995	4.82E-09	count	1
AL590714.1	18.4303266	2731.527063	0.0067	0.995	4.82E-09	count	1
LINC01934	18.4303267	2731.527168	0.0067	0.995	4.82E-09	count	1
AMOTL2	18.4303265	2731.527116	0.0067	0.995	4.82E-09	count	1
SHOX2	18.4303264	2731.526944	0.0067	0.995	4.82E-09	count	1
LINC01206	18.4303266	2731.527063	0.0067	0.995	4.82E-09	count	1
LINC00887	18.4303265	2731.526957	0.0067	0.995	4.82E-09	count	1
AC046143.1	18.4303264	2731.526931	0.0067	0.995	4.82E-09	count	1
AC139887.4	18.4303264	2731.527023	0.0067	0.995	4.82E-09	count	1
AC096564.2	18.4303264	2731.526931	0.0067	0.995	4.82E-09	count	1
AC008592.5	18.4303265	2731.52701	0.0067	0.995	4.82E-09	count	1
C1QTNF2	18.4303265	2731.526983	0.0067	0.995	4.82E-09	count	1
AL353759.1	18.4303264	2731.527116	0.0067	0.995	4.82E-09	count	1
ZNF391	18.4303262	2731.52697	0.0067	0.995	4.82E-09	count	1
AL078581.1	18.4303264	2731.526997	0.0067	0.995	4.82E-09	count	1
PNLDC1	18.4303265	2731.526983	0.0067	0.995	4.82E-09	count	1
AMPH	18.4303265	2731.527063	0.0067	0.995	4.82E-09	count	1
AC007566.1	18.4303264	2731.527023	0.0067	0.995	4.82E-09	count	1
LRGUK	18.4303264	2731.527116	0.0067	0.995	4.82E-09	count	1
AC026904.2	18.4303265	2731.527182	0.0067	0.995	4.82E-09	count	1
AC100801.1	18.4303265	2731.526957	0.0067	0.995	4.82E-09	count	1
AL353743.2	18.4303264	2731.527076	0.0067	0.995	4.82E-09	count	1
C9orf170	18.4303264	2731.526931	0.0067	0.995	4.82E-09	count	1

SYT9	18.4303265	2731.527208	0.0067	0.995	4.82E-09	count	1
STK33	18.4303264	2731.527023	0.0067	0.995	4.82E-09	count	1
AP002770.1	18.4303267	2731.527142	0.0067	0.995	4.82E-09	count	1
AL121749.1	18.4303263	2731.527049	0.0067	0.995	4.82E-09	count	1
GFRA1	18.4303264	2731.527023	0.0067	0.995	4.82E-09	count	1
AC036103.1	18.4303266	2731.526931	0.0067	0.995	4.82E-09	count	1
AP3B2	18.4303264	2731.527116	0.0067	0.995	4.82E-09	count	1
AC099518.4	18.4303264	2731.527023	0.0067	0.995	4.82E-09	count	1
KCNJ12	18.4303264	2731.526931	0.0067	0.995	4.82E-09	count	1
LINC01415	18.4303266	2731.527063	0.0067	0.995	4.82E-09	count	1
AC011450.1	18.4303265	2731.526983	0.0067	0.995	4.82E-09	count	1
AC003002.3	18.4303265	2731.526878	0.0067	0.995	4.82E-09	count	1
SMIM34B	18.4303265	2731.526878	0.0067	0.995	4.82E-09	count	1
SRARP	18.4303263	2731.526983	0.0067	0.995	4.82E-09	count	1
IQCC	18.4303265	2731.52701	0.0067	0.995	4.82E-09	count	1
AL592166.1	18.4303264	2731.527076	0.0067	0.995	4.82E-09	count	1
PODN	18.4303264	2731.526838	0.0067	0.995	4.82E-09	count	1
BCAN	18.4303266	2731.527089	0.0067	0.995	4.82E-09	count	1
MYBPH	18.4303263	2731.526983	0.0067	0.995	4.82E-09	count	1
MAP1LC3C	18.4303264	2731.526957	0.0067	0.995	4.82E-09	count	1
AC092687.3	18.4303264	2731.527076	0.0067	0.995	4.82E-09	count	1
KLHL29	18.4303262	2731.527023	0.0067	0.995	4.82E-09	count	1
AGBL5	18.4303264	2731.526957	0.0067	0.995	4.82E-09	count	1
GCKR	18.4303263	2731.526983	0.0067	0.995	4.82E-09	count	1
AC007250.1	18.4303262	2731.52697	0.0067	0.995	4.82E-09	count	1
AC107081.2	18.4303264	2731.527076	0.0067	0.995	4.82E-09	count	1
AC073869.3	18.4303264	2731.526957	0.0067	0.995	4.82E-09	count	1
AC007750.1	18.4303264	2731.526957	0.0067	0.995	4.82E-09	count	1
NOSTRIN	18.4303266	2731.527089	0.0067	0.995	4.82E-09	count	1
KIAA2012	18.4303263	2731.526983	0.0067	0.995	4.82E-09	count	1
AC090948.3	18.4303263	2731.526983	0.0067	0.995	4.82E-09	count	1
MAGI1	18.4303262	2731.526931	0.0067	0.995	4.82E-09	count	1
TMEM212	18.4303263	2731.526904	0.0067	0.995	4.82E-09	count	1
AC116651.1	18.4303266	2731.526997	0.0067	0.995	4.82E-09	count	1
AC097504.2	18.4303266	2731.526997	0.0067	0.995	4.82E-09	count	1
LRAT	18.4303264	2731.527076	0.0067	0.995	4.82E-09	count	1
LINC02427	18.4303264	2731.527076	0.0067	0.995	4.82E-09	count	1
ROPN1L	18.4303265	2731.527076	0.0067	0.995	4.82E-09	count	1
RNF180	18.4303264	2731.526957	0.0067	0.995	4.82E-09	count	1
SNCAIP	18.4303264	2731.526957	0.0067	0.995	4.82E-09	count	1
DND1	18.4303264	2731.526997	0.0067	0.995	4.82E-09	count	1
SLC36A3	18.4303263	2731.52701	0.0067	0.995	4.82E-09	count	1
SLIT3	18.4303264	2731.526957	0.0067	0.995	4.82E-09	count	1
AL139095.4	18.4303265	2731.526997	0.0067	0.995	4.82E-09	count	1
DDR1-DT	18.4303262	2731.526997	0.0067	0.995	4.82E-09	count	1

POU5F1	18.4303264	2731.526957	0.0067	0.995	4.82E-09	count	1
LY6G6C	18.4303264	2731.526838	0.0067	0.995	4.82E-09	count	1
RHAG	18.4303263	2731.526957	0.0067	0.995	4.82E-09	count	1
AL354892.2	18.4303263	2731.526983	0.0067	0.995	4.82E-09	count	1
ZNF853	18.4303265	2731.527129	0.0067	0.995	4.82E-09	count	1
HOXA3	18.4303266	2731.527089	0.0067	0.995	4.82E-09	count	1
SPDYE1	18.4303264	2731.526957	0.0067	0.995	4.82E-09	count	1
ZPBP	18.4303264	2731.526904	0.0067	0.995	4.82E-09	count	1
CFAP69	18.4303262	2731.526931	0.0067	0.995	4.82E-09	count	1
CHRDL1	18.4303264	2731.526838	0.0067	0.995	4.82E-09	count	1
FGF20	18.4303265	2731.526997	0.0067	0.995	4.82E-09	count	1
PENK	18.4303264	2731.526891	0.0067	0.995	4.82E-09	count	1
AC067930.5	18.4303262	2731.527023	0.0067	0.995	4.82E-09	count	1
LMO1	18.4303263	2731.526904	0.0067	0.995	4.82E-09	count	1
AC018410.1	18.4303263	2731.526904	0.0067	0.995	4.82E-09	count	1
AP003064.2	18.4303264	2731.527063	0.0067	0.995	4.82E-09	count	1
KDM4D	18.4303265	2731.526997	0.0067	0.995	4.82E-09	count	1
AP000757.1	18.4303263	2731.526904	0.0067	0.995	4.82E-09	count	1
PARD3	18.4303264	2731.526838	0.0067	0.995	4.82E-09	count	1
ASAH2	18.4303264	2731.526957	0.0067	0.995	4.82E-09	count	1
EBF3	18.4303264	2731.526838	0.0067	0.995	4.82E-09	count	1
B4GALNT3	18.4303262	2731.52697	0.0067	0.995	4.82E-09	count	1
GUCY2C	18.4303264	2731.527063	0.0067	0.995	4.82E-09	count	1
ATP7B	18.4303262	2731.526997	0.0067	0.995	4.82E-09	count	1
AC007182.1	18.4303263	2731.526904	0.0067	0.995	4.82E-09	count	1
TMEM63C	18.4303262	2731.527023	0.0067	0.995	4.82E-09	count	1
TEPP	18.4303264	2731.526865	0.0067	0.995	4.82E-09	count	1
NECAB2	18.4303264	2731.526838	0.0067	0.995	4.82E-09	count	1
AC107982.3	18.4303262	2731.526931	0.0067	0.995	4.82E-09	count	1
AC087294.1	18.4303264	2731.526957	0.0067	0.995	4.82E-09	count	1
CCL1	18.4303262	2731.527023	0.0067	0.995	4.82E-09	count	1
AC091132.1	18.4303263	2731.526983	0.0067	0.995	4.82E-09	count	1
LINC01483	18.4303264	2731.526957	0.0067	0.995	4.82E-09	count	1
QRICH2	18.4303263	2731.526983	0.0067	0.995	4.82E-09	count	1
AC022966.1	18.4303264	2731.526957	0.0067	0.995	4.82E-09	count	1
AC090229.1	18.4303264	2731.526904	0.0067	0.995	4.82E-09	count	1
TMEM74B	18.4303262	2731.526931	0.0067	0.995	4.82E-09	count	1
ADAM33	18.4303263	2731.526983	0.0067	0.995	4.82E-09	count	1
ITCH-AS1	18.4303264	2731.526957	0.0067	0.995	4.82E-09	count	1
AC007292.1	18.4303264	2731.526957	0.0067	0.995	4.82E-09	count	1
CYP4F12	18.4303264	2731.526838	0.0067	0.995	4.82E-09	count	1
ZNF221	18.4303264	2731.526997	0.0067	0.995	4.82E-09	count	1
EHD2	18.4303264	2731.526997	0.0067	0.995	4.82E-09	count	1
XKR3	18.4303266	2731.527089	0.0067	0.995	4.82E-09	count	1
AC004832.6	18.4303263	2731.526983	0.0067	0.995	4.82E-09	count	1

AC254562.2	18.4303264	2731.526904	0.0067	0.995	4.82E-09	count	1
GRIK1	18.4303263	2731.526997	0.0067	0.995	4.82E-09	count	1
AP000238.1	18.4303263	2731.526957	0.0067	0.995	4.82E-09	count	1
AP000253.1	18.4303264	2731.526904	0.0067	0.995	4.82E-09	count	1
TMPRSS3	18.4303265	2731.527129	0.0067	0.995	4.82E-09	count	1
SCNN1D	18.4303263	2731.526891	0.0067	0.995	4.82E-09	count	1
PRDM16	18.4303265	2731.527036	0.0067	0.995	4.82E-09	count	1
DPYD-AS1	18.4303261	2731.527076	0.0067	0.995	4.82E-09	count	1
FCRL6	18.4303266	2731.527129	0.0067	0.995	4.82E-09	count	1
LINC01645	18.4303263	2731.526997	0.0067	0.995	4.82E-09	count	1
CFHR3	18.4303262	2731.526825	0.0067	0.995	4.82E-09	count	1
MARK1	18.4303263	2731.526997	0.0067	0.995	4.82E-09	count	1
CAPN8	18.430326	2731.527049	0.0067	0.995	4.82E-09	count	1
AC011747.2	18.4303263	2731.526957	0.0067	0.995	4.82E-09	count	1
MIR3681HG	18.4303262	2731.52701	0.0067	0.995	4.82E-09	count	1
AC093690.1	18.4303263	2731.527129	0.0067	0.995	4.82E-09	count	1
PTH2R	18.4303265	2731.527049	0.0067	0.995	4.82E-09	count	1
CPS1	18.4303263	2731.526957	0.0067	0.995	4.82E-09	count	1
AC131097.4	18.4303263	2731.527129	0.0067	0.995	4.82E-09	count	1
AC104187.1	18.4303263	2731.526957	0.0067	0.995	4.82E-09	count	1
AC137630.1	18.4303263	2731.526957	0.0067	0.995	4.82E-09	count	1
HTR1F	18.4303263	2731.526891	0.0067	0.995	4.82E-09	count	1
PTTG2	18.4303263	2731.526931	0.0067	0.995	4.82E-09	count	1
TXK	18.4303263	2731.527129	0.0067	0.995	4.82E-09	count	1
LNX1	18.4303262	2731.52701	0.0067	0.995	4.82E-09	count	1
BTC	18.4303266	2731.526983	0.0067	0.995	4.82E-09	count	1
GALNTL6	18.4303264	2731.526944	0.0067	0.995	4.82E-09	count	1
PLEKHG4B	18.4303263	2731.526878	0.0067	0.995	4.82E-09	count	1
AC034236.3	18.4303261	2731.527076	0.0067	0.995	4.82E-09	count	1
SFTA2	18.4303261	2731.52697	0.0067	0.995	4.82E-09	count	1
PI16	18.4303263	2731.52697	0.0067	0.995	4.82E-09	count	1
BVES-AS1	18.4303263	2731.526891	0.0067	0.995	4.82E-09	count	1
FAM131B	18.4303264	2731.527023	0.0067	0.995	4.82E-09	count	1
NUDT10	18.4303264	2731.526838	0.0067	0.995	4.82E-09	count	1
GLUD2	18.430326	2731.527049	0.0067	0.995	4.82E-09	count	1
TMLHE-AS1	18.4303263	2731.526997	0.0067	0.995	4.82E-09	count	1
AC107959.5	18.4303264	2731.52701	0.0067	0.995	4.82E-09	count	1
DUSP26	18.4303265	2731.527036	0.0067	0.995	4.82E-09	count	1
AC087362.1	18.4303264	2731.527049	0.0067	0.995	4.82E-09	count	1
RAD54B	18.4303262	2731.526825	0.0067	0.995	4.82E-09	count	1
AC090922.1	18.4303262	2731.526931	0.0067	0.995	4.82E-09	count	1
PTPRD-AS1	18.4303263	2731.527129	0.0067	0.995	4.82E-09	count	1
TRPM3	18.430326	2731.527049	0.0067	0.995	4.82E-09	count	1
OR52I1	18.4303263	2731.526865	0.0067	0.995	4.82E-09	count	1
AC023946.1	18.4303263	2731.526957	0.0067	0.995	4.82E-09	count	1

ABTB2	18.4303263	2731.527063	0.0067	0.995	4.82E-09	count	1
RERG	18.4303262	2731.526931	0.0067	0.995	4.82E-09	count	1
ASIC1	18.4303263	2731.526865	0.0067	0.995	4.82E-09	count	1
MYBPC1	18.4303263	2731.526983	0.0067	0.995	4.82E-09	count	1
AL158063.1	18.4303264	2731.527116	0.0067	0.995	4.82E-09	count	1
AL138694.1	18.4303262	2731.526865	0.0067	0.995	4.82E-09	count	1
AF111167.1	18.4303262	2731.526851	0.0067	0.995	4.82E-09	count	1
C14orf132	18.4303263	2731.526891	0.0067	0.995	4.82E-09	count	1
SNURF	18.4303263	2731.527129	0.0067	0.995	4.82E-09	count	1
DPH6-DT	18.4303263	2731.526957	0.0067	0.995	4.82E-09	count	1
AC013391.1	18.430326	2731.527049	0.0067	0.995	4.82E-09	count	1
CRTC3-AS1	18.4303263	2731.527129	0.0067	0.995	4.82E-09	count	1
AC109597.2	18.430326	2731.527049	0.0067	0.995	4.82E-09	count	1
NPIPA3	18.4303265	2731.527036	0.0067	0.995	4.82E-09	count	1
AC133919.2	18.4303263	2731.526865	0.0067	0.995	4.82E-09	count	1
AC240565.2	18.4303264	2731.52701	0.0067	0.995	4.82E-09	count	1
TEKT3	18.4303263	2731.526957	0.0067	0.995	4.82E-09	count	1
AC005549.1	18.4303263	2731.526957	0.0067	0.995	4.82E-09	count	1
AC060766.6	18.4303264	2731.52701	0.0067	0.995	4.82E-09	count	1
LINC00511	18.4303264	2731.526944	0.0067	0.995	4.82E-09	count	1
AC120049.1	18.4303263	2731.526957	0.0067	0.995	4.82E-09	count	1
AC100778.3	18.4303263	2731.526957	0.0067	0.995	4.82E-09	count	1
AL049794.1	18.4303263	2731.526891	0.0067	0.995	4.82E-09	count	1
CCM2L	18.4303262	2731.526931	0.0067	0.995	4.82E-09	count	1
AL023803.2	18.4303262	2731.52701	0.0067	0.995	4.82E-09	count	1
AL031055.1	18.4303261	2731.527076	0.0067	0.995	4.82E-09	count	1
CSE1L-AS1	18.4303263	2731.526931	0.0067	0.995	4.82E-09	count	1
AC011498.6	18.4303263	2731.52697	0.0067	0.995	4.82E-09	count	1
TM6SF2	18.4303263	2731.526957	0.0067	0.995	4.82E-09	count	1
ZNF418	18.4303263	2731.526957	0.0067	0.995	4.82E-09	count	1
LINC00896	18.4303263	2731.526983	0.0067	0.995	4.82E-09	count	1
SCARF2	18.4303261	2731.52697	0.0067	0.995	4.82E-09	count	1
SLC5A4	18.4303262	2731.52701	0.0067	0.995	4.82E-09	count	1
AL121672.3	18.4303263	2731.526957	0.0067	0.995	4.82E-09	count	1
LINC00158	18.4303263	2731.526931	0.0067	0.995	4.82E-09	count	1
LINC00310	18.4303264	2731.527142	0.0067	0.995	4.82E-09	count	1
AP001412.1	18.4303263	2731.526878	0.0067	0.995	4.82E-09	count	1
LINC01786	18.4303263	2731.526931	0.0067	0.995	4.82E-09	count	1
AL513327.1	18.4303264	2731.527049	0.0067	0.995	4.82E-09	count	1
AL596275.2	18.4303263	2731.526931	0.0067	0.995	4.82E-09	count	1
GNG12-AS1	18.4303264	2731.526997	0.0067	0.995	4.82E-09	count	1
DNASE2B	18.4303264	2731.526904	0.0067	0.995	4.82E-09	count	1
CD5L	18.4303263	2731.527116	0.0067	0.995	4.82E-09	count	1
C1orf143	18.4303261	2731.526904	0.0067	0.995	4.82E-09	count	1
AC009242.1	18.4303263	2731.527116	0.0067	0.995	4.82E-09	count	1

FAM228A	18.4303261	2731.526957	0.0067	0.995	4.82E-09	count	1
AC012073.1	18.4303264	2731.526957	0.0067	0.995	4.82E-09	count	1
AC008280.3	18.4303264	2731.526904	0.0067	0.995	4.82E-09	count	1
AC017002.1	18.4303263	2731.526997	0.0067	0.995	4.82E-09	count	1
KIF5C	18.4303262	2731.52701	0.0067	0.995	4.82E-09	count	1
AC022001.3	18.4303263	2731.526944	0.0067	0.995	4.82E-09	count	1
LINC02026	18.4303263	2731.526997	0.0067	0.995	4.82E-09	count	1
AC024560.3	18.430326	2731.526772	0.0067	0.995	4.82E-09	count	1
AC108477.1	18.4303263	2731.527116	0.0067	0.995	4.82E-09	count	1
PCDHB3	18.4303261	2731.526917	0.0067	0.995	4.82E-09	count	1
LINC02569	18.4303261	2731.526904	0.0067	0.995	4.82E-09	count	1
TEAD3	18.430326	2731.526772	0.0067	0.995	4.82E-09	count	1
AL136304.1	18.4303263	2731.527116	0.0067	0.995	4.82E-09	count	1
LINC02518	18.4303262	2731.526825	0.0067	0.995	4.82E-09	count	1
AL627422.2	18.4303262	2731.527036	0.0067	0.995	4.82E-09	count	1
AC019117.2	18.4303262	2731.527036	0.0067	0.995	4.82E-09	count	1
AC004837.2	18.4303262	2731.527023	0.0067	0.995	4.82E-09	count	1
ABCA13	18.430326	2731.526838	0.0067	0.995	4.82E-09	count	1
ZNF727	18.4303263	2731.526931	0.0067	0.995	4.82E-09	count	1
AC004990.1	18.4303262	2731.527036	0.0067	0.995	4.82E-09	count	1
KIAA1549	18.4303263	2731.526931	0.0067	0.995	4.82E-09	count	1
RNF32	18.4303263	2731.526931	0.0067	0.995	4.82E-09	count	1
SRPX	18.4303263	2731.526944	0.0067	0.995	4.82E-09	count	1
TMSB15B	18.4303263	2731.527142	0.0067	0.995	4.82E-09	count	1
PIWIL2	18.4303263	2731.526931	0.0067	0.995	4.82E-09	count	1
GPR20	18.4303262	2731.52701	0.0067	0.995	4.82E-09	count	1
AL359636.1	18.4303264	2731.527049	0.0067	0.995	4.82E-09	count	1
TUBB8	18.4303262	2731.526825	0.0067	0.995	4.82E-09	count	1
AC073651.1	18.4303261	2731.527089	0.0067	0.995	4.82E-09	count	1
SCN8A	18.4303263	2731.52701	0.0067	0.995	4.82E-09	count	1
GLS2	18.4303264	2731.527049	0.0067	0.995	4.82E-09	count	1
AC026367.3	18.4303261	2731.527089	0.0067	0.995	4.82E-09	count	1
LINC00462	18.4303263	2731.526931	0.0067	0.995	4.82E-09	count	1
AL132780.1	18.4303261	2731.526917	0.0067	0.995	4.82E-09	count	1
AL157756.1	18.4303263	2731.526944	0.0067	0.995	4.82E-09	count	1
AC122108.2	18.4303262	2731.527036	0.0067	0.995	4.82E-09	count	1
TPM1-AS	18.430326	2731.526772	0.0067	0.995	4.82E-09	count	1
RPL3L	18.4303262	2731.527036	0.0067	0.995	4.82E-09	count	1
IRX5	18.430326	2731.526772	0.0067	0.995	4.82E-09	count	1
BEAN1	18.4303261	2731.526904	0.0067	0.995	4.82E-09	count	1
AC138627.1	18.4303261	2731.527089	0.0067	0.995	4.82E-09	count	1
AC091153.4	18.4303263	2731.526944	0.0067	0.995	4.82E-09	count	1
LINC02210-CRHR1	18.4303263	2731.526983	0.0067	0.995	4.82E-09	count	1
HOXB3	18.4303264	2731.526838	0.0067	0.995	4.82E-09	count	1
C17orf82	18.4303264	2731.527049	0.0067	0.995	4.82E-09	count	1

GRIN2C	18.4303262	2731.526798	0.0067	0.995	4.82E-09	count	1
TRERNA1	18.4303264	2731.526931	0.0067	0.995	4.82E-09	count	1
PALM	18.430326	2731.526772	0.0067	0.995	4.82E-09	count	1
AC008770.2	18.4303263	2731.526944	0.0067	0.995	4.82E-09	count	1
AC010422.3	18.4303264	2731.526997	0.0067	0.995	4.82E-09	count	1
AC016590.3	18.4303263	2731.527116	0.0067	0.995	4.82E-09	count	1
ZNF233	18.4303261	2731.527089	0.0067	0.995	4.82E-09	count	1
ZNF135	18.4303264	2731.526838	0.0067	0.995	4.82E-09	count	1
AL022322.1	18.4303264	2731.526904	0.0067	0.995	4.82E-09	count	1
AL121992.2	18.4303261	2731.526838	0.0067	0.995	4.82E-09	count	1
UBXN10	18.4303262	2731.526983	0.0067	0.995	4.82E-09	count	1
AL136115.1	18.4303261	2731.526865	0.0067	0.995	4.82E-09	count	1
PDZK1IP1	18.4303262	2731.526983	0.0067	0.995	4.82E-09	count	1
AL445231.1	18.4303261	2731.526865	0.0067	0.995	4.82E-09	count	1
MYOC	18.4303259	2731.526838	0.0067	0.995	4.82E-09	count	1
SUSD4	18.4303262	2731.527049	0.0067	0.995	4.82E-09	count	1
PLEKHH2	18.4303261	2731.52701	0.0067	0.995	4.82E-09	count	1
PARD3B	18.4303261	2731.527049	0.0067	0.995	4.82E-09	count	1
ACKR2	18.430326	2731.526838	0.0067	0.995	4.82E-09	count	1
HYAL1	18.4303263	2731.527076	0.0067	0.995	4.82E-09	count	1
TM4SF4	18.4303261	2731.527116	0.0067	0.995	4.82E-09	count	1
AC146944.4	18.4303261	2731.527049	0.0067	0.995	4.82E-09	count	1
AC008438.1	18.4303262	2731.526865	0.0067	0.995	4.82E-09	count	1
TTK	18.430326	2731.526838	0.0067	0.995	4.82E-09	count	1
AC024084.1	18.4303261	2731.52697	0.0067	0.995	4.82E-09	count	1
AC107959.3	18.4303262	2731.526838	0.0067	0.995	4.82E-09	count	1
PGM5-AS1	18.4303261	2731.527089	0.0067	0.995	4.82E-09	count	1
ALDH1L2	18.4303261	2731.526838	0.0067	0.995	4.82E-09	count	1
AL365394.1	18.4303261	2731.527063	0.0067	0.995	4.82E-09	count	1
STXBP6	18.430326	2731.526838	0.0067	0.995	4.82E-09	count	1
AC012236.1	18.4303261	2731.527116	0.0067	0.995	4.82E-09	count	1
PRSS27	18.4303261	2731.527089	0.0067	0.995	4.82E-09	count	1
AC009119.2	18.4303261	2731.526891	0.0067	0.995	4.82E-09	count	1
AC009123.1	18.4303262	2731.527049	0.0067	0.995	4.82E-09	count	1
AL035461.2	18.4303261	2731.526865	0.0067	0.995	4.82E-09	count	1
ZNF99	18.4303261	2731.526865	0.0067	0.995	4.82E-09	count	1
AC011466.1	18.4303263	2731.527076	0.0067	0.995	4.82E-09	count	1
TULP2	18.4303262	2731.526983	0.0067	0.995	4.82E-09	count	1
AC245884.8	18.4303261	2731.526798	0.0067	0.995	4.82E-09	count	1
GRM7-AS1	18.430326	2731.527049	0.0067	0.995	4.82E-09	count	1
RPL34-AS1	18.4303261	2731.526957	0.0067	0.995	4.82E-09	count	1
IDO2	18.4303261	2731.527102	0.0067	0.995	4.82E-09	count	1
CDKN2B-AS1	18.430326	2731.526983	0.0067	0.995	4.82E-09	count	1
AP000936.1	18.4303262	2731.527102	0.0067	0.995	4.82E-09	count	1
LINC02551	18.4303261	2731.526865	0.0067	0.995	4.82E-09	count	1

KRT80	18.4303262	2731.527102	0.0067	0.995	4.82E-09	count	1
AC011939.3	18.4303261	2731.526865	0.0067	0.995	4.82E-09	count	1
AC027277.1	18.430326	2731.526983	0.0067	0.995	4.82E-09	count	1
AL117328.2	18.4303263	2731.526983	0.0067	0.995	4.82E-09	count	1
AL445248.1	18.4303262	2731.526851	0.0067	0.995	4.82E-09	count	1
AC096586.1	18.4303262	2731.526891	0.0067	0.995	4.82E-09	count	1
FIRRE	18.4303262	2731.526851	0.0067	0.995	4.82E-09	count	1
CYP7A1	18.4303262	2731.526851	0.0067	0.995	4.82E-09	count	1
CDH3	18.4303262	2731.526851	0.0067	0.995	4.82E-09	count	1
LRRC7	18.430326	2731.526931	0.0067	0.995	4.82E-09	count	1
KCND3	18.430326	2731.526983	0.0067	0.995	4.82E-09	count	1
KCNS3	18.430326	2731.526983	0.0067	0.995	4.82E-09	count	1
LIMS3	18.430326	2731.526944	0.0067	0.995	4.82E-09	count	1
ZFP37	18.430326	2731.526931	0.0067	0.995	4.82E-09	count	1
AL731533.2	18.430326	2731.526825	0.0067	0.995	4.82E-09	count	1
SFTPD-AS1	18.4303259	2731.526917	0.0067	0.995	4.82E-09	count	1
ABCC2	18.430326	2731.526944	0.0067	0.995	4.82E-09	count	1
LINC01993	18.430326	2731.526931	0.0067	0.995	4.82E-09	count	1
AC010271.1	18.4303262	2731.526917	0.0067	0.995	4.82E-09	count	1
Z82217.1	18.4303262	2731.527063	0.0067	0.995	4.82E-09	count	1
CREB3L1	18.4303259	2731.526825	0.0067	0.995	4.82E-09	count	1
CSMD1	19.7400066	1476.028717	0.0134	0.989	4.88E-09	count	1
TRIM71	19.7400062	1476.029	0.0134	0.989	4.88E-09	count	1
AC022217.3	19.7400057	1476.028582	0.0134	0.989	4.88E-09	count	1
FAM209B	19.7400056	1476.028582	0.0134	0.989	4.88E-09	count	1
AL049795.1	19.0442689	1479.888577	0.0129	0.99	4.89E-09	count	1
LINC01960	19.0442686	1479.888712	0.0129	0.99	4.89E-09	count	1
AC079354.3	19.0442686	1479.888712	0.0129	0.99	4.89E-09	count	1
AC078881.1	19.0442686	1479.888712	0.0129	0.99	4.89E-09	count	1
EGFL8	19.0442689	1479.888577	0.0129	0.99	4.89E-09	count	1
AC131011.1	19.0442686	1479.888712	0.0129	0.99	4.89E-09	count	1
AL354710.2	19.0442686	1479.888712	0.0129	0.99	4.89E-09	count	1
AC027682.2	19.0442686	1479.888712	0.0129	0.99	4.89E-09	count	1
CHMP4C	19.0442687	1479.888651	0.0129	0.99	4.89E-09	count	1
NHLH1	19.0442687	1479.888725	0.0129	0.99	4.89E-09	count	1
AC010746.1	19.0442688	1479.888759	0.0129	0.99	4.89E-09	count	1
DNAH12	19.0442686	1479.888698	0.0129	0.99	4.89E-09	count	1
LINC02102	19.0442686	1479.888685	0.0129	0.99	4.89E-09	count	1
EPB41L4A	19.0442686	1479.888698	0.0129	0.99	4.89E-09	count	1
GRID2IP	19.0442688	1479.888759	0.0129	0.99	4.89E-09	count	1
AC092849.1	19.0442686	1479.888671	0.0129	0.99	4.89E-09	count	1
TRBV9	19.0442686	1479.888685	0.0129	0.99	4.89E-09	count	1
AC084026.2	19.0442687	1479.888691	0.0129	0.99	4.89E-09	count	1
SFTA1P	19.0442686	1479.888644	0.0129	0.99	4.89E-09	count	1
MANSC4	19.0442687	1479.888725	0.0129	0.99	4.89E-09	count	1

AC007496.2	19.0442686	1479.888563	0.0129	0.99	4.89E-09	count	1
CKLF-CMTM1	19.0442686	1479.888698	0.0129	0.99	4.89E-09	count	1
AC010531.6	19.0442686	1479.888698	0.0129	0.99	4.89E-09	count	1
AC021491.4	19.0442686	1479.888671	0.0129	0.99	4.89E-09	count	1
FAM83D	19.0442686	1479.888671	0.0129	0.99	4.89E-09	count	1
LGI4	19.0442686	1479.888644	0.0129	0.99	4.89E-09	count	1
Z95114.2	19.0442686	1479.888671	0.0129	0.99	4.89E-09	count	1
CACHD1	19.0442686	1479.888631	0.0129	0.99	4.89E-09	count	1
WDR63	19.0442687	1479.888678	0.0129	0.99	4.89E-09	count	1
TMPRSS7	19.0442686	1479.888631	0.0129	0.99	4.89E-09	count	1
MARVELD2	19.0442687	1479.888685	0.0129	0.99	4.89E-09	count	1
LRRC73	19.0442686	1479.888671	0.0129	0.99	4.89E-09	count	1
RAB40A	19.0442686	1479.888651	0.0129	0.99	4.89E-09	count	1
AC136624.2	19.0442686	1479.888671	0.0129	0.99	4.89E-09	count	1
TMED6	19.0442685	1479.888624	0.0129	0.99	4.89E-09	count	1
AC011921.1	19.0442686	1479.888631	0.0129	0.99	4.89E-09	count	1
PARD6G	19.0442687	1479.888664	0.0129	0.99	4.89E-09	count	1
LYPD8	19.0442686	1479.888604	0.0129	0.99	4.89E-09	count	1
DES	19.0442686	1479.888617	0.0129	0.99	4.89E-09	count	1
NPY1R	19.0442686	1479.888617	0.0129	0.99	4.89E-09	count	1
C9orf163	19.0442686	1479.888631	0.0129	0.99	4.89E-09	count	1
AP001267.3	19.0442686	1479.888664	0.0129	0.99	4.89E-09	count	1
AC079315.1	19.0442686	1479.888638	0.0129	0.99	4.89E-09	count	1
AC023034.1	19.0442687	1479.888638	0.0129	0.99	4.89E-09	count	1
FMOD	19.0442684	1479.888597	0.0129	0.99	4.89E-09	count	1
SPINK9	19.0442686	1479.888691	0.0129	0.99	4.89E-09	count	1
TMEM200A	19.0442685	1479.888631	0.0129	0.99	4.89E-09	count	1
FAM171A1	19.0442684	1479.888597	0.0129	0.99	4.89E-09	count	1
MUCL1	19.0442684	1479.888644	0.0129	0.99	4.89E-09	count	1
AC090181.1	19.0442686	1479.888691	0.0129	0.99	4.89E-09	count	1
AC020978.5	19.0442684	1479.888631	0.0129	0.99	4.89E-09	count	1
PSMA8	19.0442686	1479.888691	0.0129	0.99	4.89E-09	count	1
3-Sep	19.0442684	1479.888597	0.0129	0.99	4.89E-09	count	1
MAPK11	19.0442684	1479.888597	0.0129	0.99	4.89E-09	count	1
AP000569.1	19.0442684	1479.888631	0.0129	0.99	4.89E-09	count	1
AP000322.2	19.0442684	1479.888597	0.0129	0.99	4.89E-09	count	1
AC018682.1	19.0442683	1479.888604	0.0129	0.99	4.89E-09	count	1
LINC01513	19.0442683	1479.888691	0.0129	0.99	4.89E-09	count	1
PCDHB9	19.0442683	1479.888604	0.0129	0.99	4.89E-09	count	1
ELFN1-AS1	19.0442683	1479.888671	0.0129	0.99	4.89E-09	count	1
OR5AN1	19.0442684	1479.888638	0.0129	0.99	4.89E-09	count	1
MS4A3	19.0442683	1479.888651	0.0129	0.99	4.89E-09	count	1
AL512506.3	19.0442684	1479.888597	0.0129	0.99	4.89E-09	count	1
SLITRK5	19.0442683	1479.888651	0.0129	0.99	4.89E-09	count	1
TBC1D3D	19.0442685	1479.888604	0.0129	0.99	4.89E-09	count	1

AC097724.1	19.0442682	1479.888617	0.0129	0.99	4.89E-09	count	1
AC007391.1	19.0442682	1479.888617	0.0129	0.99	4.89E-09	count	1
PCDHB2	19.0442684	1479.88859	0.0129	0.99	4.89E-09	count	1
AL021997.3	19.0442682	1479.888617	0.0129	0.99	4.89E-09	count	1
SFRP1	19.0442682	1479.888624	0.0129	0.99	4.89E-09	count	1
AL391839.2	19.0442682	1479.888617	0.0129	0.99	4.89E-09	count	1
BX248123.1	19.0442682	1479.888617	0.0129	0.99	4.89E-09	count	1
ADRA2A	19.0442684	1479.888651	0.0129	0.99	4.89E-09	count	1
AC121761.1	19.0442682	1479.888611	0.0129	0.99	4.89E-09	count	1
APBA2	19.0442682	1479.888624	0.0129	0.99	4.89E-09	count	1
AC009041.2	19.0442682	1479.888617	0.0129	0.99	4.89E-09	count	1
AP005432.2	19.0442684	1479.88859	0.0129	0.99	4.89E-09	count	1
C20orf144	19.0442682	1479.888604	0.0129	0.99	4.89E-09	count	1
AL354760.1	19.0442683	1479.888624	0.0129	0.99	4.89E-09	count	1
AC112220.3	19.0442682	1479.888631	0.0129	0.99	4.89E-09	count	1
OPRK1	19.0442682	1479.888631	0.0129	0.99	4.89E-09	count	1
SAMD12	19.0442682	1479.888631	0.0129	0.99	4.89E-09	count	1
AL512625.3	19.0442683	1479.888624	0.0129	0.99	4.89E-09	count	1
BICDL1	19.0442682	1479.888631	0.0129	0.99	4.89E-09	count	1
C1orf167	19.0442681	1479.888644	0.0129	0.99	4.89E-09	count	1
DMP1	19.0442683	1479.888631	0.0129	0.99	4.89E-09	count	1
LINC01393	19.0442683	1479.888698	0.0129	0.99	4.89E-09	count	1
TTC16	19.0442683	1479.888698	0.0129	0.99	4.89E-09	count	1
AL136526.1	19.0442683	1479.888698	0.0129	0.99	4.89E-09	count	1
AC087286.1	19.0442683	1479.888631	0.0129	0.99	4.89E-09	count	1
AC134407.1	19.0442683	1479.888698	0.0129	0.99	4.89E-09	count	1
FOXD3-AS1	19.0442681	1479.88851	0.0129	0.99	4.89E-09	count	1
PRL	19.0442681	1479.88851	0.0129	0.99	4.89E-09	count	1
LINC00504	0.9393018	0.7580156	1.2392	0.215	5.13E-09	count	1
AL021707.5	0.9393018	0.649591	1.446	0.148	5.13E-09	count	1
CFAP70	0.9393018	0.649591	1.446	0.148	5.13E-09	count	1
KAT14	0.4246942	0.5208916	0.8153	0.415	5.37E-09	count	1
KIZ-AS1	1.0016945	0.7688079	1.3029	0.193	5.58E-09	count	1
YAP1	1.0016945	0.7688079	1.3029	0.193	5.58E-09	count	1
AC007620.2	1.0016945	0.7688079	1.3029	0.193	5.58E-09	count	1
COL24A1	1.0016945	0.7688079	1.3029	0.193	5.58E-09	count	1
AL590399.1	1.0016945	0.7688079	1.3029	0.193	5.58E-09	count	1
MYB	1.0016945	0.7688079	1.3029	0.193	5.58E-09	count	1
DLX4	1.0016945	0.7688079	1.3029	0.193	5.58E-09	count	1
B9D1	1.0016945	0.7688079	1.3029	0.193	5.58E-09	count	1
ZNF710-AS1	0.5259245	0.6118511	0.8596	0.39	6.87E-09	count	1
PYGM	0.5338367	0.6556708	0.8142	0.416	6.99E-09	count	1
AC005034.3	0.5338367	0.486565	1.0972	0.273	6.99E-09	count	1
BCL2L14	1.208508	0.5430113	2.2256	0.0261	7.12E-09	count	1
NEBL	1.208508	0.4627212	2.6117	0.0091	7.12E-09	count	1

FADS2	0.6950648	0.4840703	1.4359	0.151	9.58E-09	count	1
LURAP1	0.7031844	0.5238275	1.3424	0.18	9.72E-09	count	1
SRXN1	1.6139731	0.5773503	2.7955	0.0052	1.05E-08	count	1
LRRC17	0.7569803	0.642633	1.1779	0.239	1.06E-08	count	1
AC013652.1	0.7743155	0.6601244	1.173	0.241	1.09E-08	count	1
AC068491.3	0.7743155	0.8266783	0.9367	0.349	1.09E-08	count	1
SH2D6	0.7743155	0.8266783	0.9367	0.349	1.09E-08	count	1
HSPB2	0.7743155	0.8266783	0.9367	0.349	1.09E-08	count	1
AL353708.1	0.7743155	0.8266783	0.9367	0.349	1.09E-08	count	1
TRPV1	0.7743155	0.8266783	0.9367	0.349	1.09E-08	count	1
CLDN16	0.7743155	0.8266783	0.9367	0.349	1.09E-08	count	1
AC137723.1	0.7743155	0.8266783	0.9367	0.349	1.09E-08	count	1
AC093227.1	0.7743155	0.8525727	0.9082	0.364	1.09E-08	count	1
GZMB	0.7743155	0.8525727	0.9082	0.364	1.09E-08	count	1
AC011337.1	0.7743155	0.8525727	0.9082	0.364	1.09E-08	count	1
TMCC2	0.7743155	0.8525727	0.9082	0.364	1.09E-08	count	1
KCNH3	0.7743155	0.8525727	0.9082	0.364	1.09E-08	count	1
AC007326.4	0.7743155	0.8525727	0.9082	0.364	1.09E-08	count	1
NPIPB15	0.7951589	0.5728693	1.388	0.165	1.13E-08	count	1
NOMO2	0.7951589	0.6455904	1.2317	0.218	1.13E-08	count	1
SEMA6C	0.8902569	0.6414993	1.3878	0.165	1.30E-08	count	1
VAT1L	18.5321042	2874.128727	0.0064	0.995	1.31E-08	count	1
OSR1	18.5321038	2874.128809	0.0064	0.995	1.31E-08	count	1
SGCD	18.532104	2874.128665	0.0064	0.995	1.31E-08	count	1
AC012150.1	18.532104	2874.128665	0.0064	0.995	1.31E-08	count	1
AC141586.2	18.5321038	2874.128809	0.0064	0.995	1.31E-08	count	1
FCRL1	18.5321038	2874.128583	0.0064	0.995	1.31E-08	count	1
AC018638.6	18.5321035	2874.128665	0.0064	0.995	1.31E-08	count	1
AC008121.2	18.5321038	2874.128583	0.0064	0.995	1.31E-08	count	1
AL121845.1	18.5321039	2874.128768	0.0064	0.995	1.31E-08	count	1
MATN1	18.532104	2874.128799	0.0064	0.995	1.31E-08	count	1
LIPH	18.532104	2874.128799	0.0064	0.995	1.31E-08	count	1
AC004982.1	18.532104	2874.128799	0.0064	0.995	1.31E-08	count	1
ZNF709	18.532104	2874.128799	0.0064	0.995	1.31E-08	count	1
CCDC33	18.5321036	2874.128635	0.0064	0.995	1.31E-08	count	1
HBB	18.5321033	2874.128573	0.0064	0.995	1.31E-08	count	1
TDRD10	18.8196994	2028.19145	0.0093	0.993	1.31E-08	count	1
AC110285.2	18.8196994	2028.191421	0.0093	0.993	1.31E-08	count	1
LLGL1	18.8192076	1652.963425	0.0114	0.991	1.31E-08	count	1
RAPGEF3	18.8192072	1652.963401	0.0114	0.991	1.31E-08	count	1
INSM1	18.5313554	2139.407022	0.0087	0.993	1.31E-08	count	1
PARVA	18.5313554	2139.407091	0.0087	0.993	1.31E-08	count	1
KCNJ11	18.5313556	2139.407022	0.0087	0.993	1.31E-08	count	1
AIRE	18.5313557	2139.407205	0.0087	0.993	1.31E-08	count	1
MAP2	18.5313552	2139.406908	0.0087	0.993	1.31E-08	count	1

CXCR1	18.5313553	2139.406976	0.0087	0.993	1.31E-08	count	1
SKIDA1	18.5313554	2139.407045	0.0087	0.993	1.31E-08	count	1
AC139491.7	18.5313549	2139.406931	0.0087	0.993	1.31E-08	count	1
RASGEF1C	18.5313554	2139.407007	0.0087	0.993	1.31E-08	count	1
ZNF79	18.5313557	2139.407137	0.0087	0.993	1.31E-08	count	1
CCDC154	18.5313552	2139.406954	0.0087	0.993	1.31E-08	count	1
AC027702.1	18.5313551	2139.406992	0.0087	0.993	1.31E-08	count	1
AC009159.3	18.5313555	2139.407091	0.0087	0.993	1.31E-08	count	1
PIFO	18.5308183	1654.35969	0.0112	0.991	1.31E-08	count	1
NDFIP2	18.5308184	1654.359743	0.0112	0.991	1.31E-08	count	1
LINC01238	18.5308184	1654.359661	0.0112	0.991	1.31E-08	count	1
SEMA3B	18.5308183	1654.359666	0.0112	0.991	1.31E-08	count	1
PGA4	18.5308185	1654.35969	0.0112	0.991	1.31E-08	count	1
AC022916.1	18.5308181	1654.35969	0.0112	0.991	1.31E-08	count	1
LOXHD1	18.5308183	1654.359655	0.0112	0.991	1.31E-08	count	1
KCTD21-AS1	18.5308181	1654.359548	0.0112	0.991	1.31E-08	count	1
HOXB-AS3	18.5308185	1654.359619	0.0112	0.991	1.31E-08	count	1
AC091173.1	18.5308182	1654.359637	0.0112	0.991	1.31E-08	count	1
DMPK	18.5308182	1654.359719	0.0112	0.991	1.31E-08	count	1
AC008467.1	18.5308179	1654.359637	0.0112	0.991	1.31E-08	count	1
AC084855.2	18.1250804	2344.888095	0.0077	0.994	1.31E-08	count	1
SCGB1B2P	18.1250804	2344.888229	0.0077	0.994	1.31E-08	count	1
AC226118.1	18.1250802	2344.88812	0.0077	0.994	1.31E-08	count	1
GPRASP2	18.1250801	2344.888078	0.0077	0.994	1.31E-08	count	1
LINC02172	18.1250801	2344.888154	0.0077	0.994	1.31E-08	count	1
AL355802.2	18.1250803	2344.888279	0.0077	0.994	1.31E-08	count	1
AC126177.8	18.1250801	2344.888154	0.0077	0.994	1.31E-08	count	1
MTRNR2L1	18.1250801	2344.88817	0.0077	0.994	1.31E-08	count	1
AC007998.4	18.1250802	2344.888104	0.0077	0.994	1.31E-08	count	1
SLC2A10	18.1250802	2344.888129	0.0077	0.994	1.31E-08	count	1
AC104170.1	18.1250801	2344.888062	0.0077	0.994	1.31E-08	count	1
FAXC	18.1250802	2344.888129	0.0077	0.994	1.31E-08	count	1
LINC00629	18.1250801	2344.888162	0.0077	0.994	1.31E-08	count	1
PHYHIP	18.1250801	2344.888162	0.0077	0.994	1.31E-08	count	1
COL5A1	18.1250801	2344.888162	0.0077	0.994	1.31E-08	count	1
ARMC4	18.1250802	2344.888154	0.0077	0.994	1.31E-08	count	1
OLFM4	18.1250802	2344.888129	0.0077	0.994	1.31E-08	count	1
CELF4	18.1250802	2344.888129	0.0077	0.994	1.31E-08	count	1
LINC01273	18.1250801	2344.888104	0.0077	0.994	1.31E-08	count	1
FAM43B	18.1250803	2344.888129	0.0077	0.994	1.31E-08	count	1
AGTR1	18.1250801	2344.88812	0.0077	0.994	1.31E-08	count	1
AC106881.1	18.1250803	2344.888129	0.0077	0.994	1.31E-08	count	1
FRG1-DT	18.1250803	2344.888129	0.0077	0.994	1.31E-08	count	1
BMP6	18.1250801	2344.88812	0.0077	0.994	1.31E-08	count	1
VAX1	18.1250799	2344.888137	0.0077	0.994	1.31E-08	count	1

AC009093.1	18.1250801	2344.88812	0.0077	0.994	1.31E-08	count	1
LINC01624	18.1250801	2344.888087	0.0077	0.994	1.31E-08	count	1
LANCL3	18.1250801	2344.888087	0.0077	0.994	1.31E-08	count	1
AL356275.1	18.1250799	2344.888078	0.0077	0.994	1.31E-08	count	1
AL160408.4	18.1250799	2344.888078	0.0077	0.994	1.31E-08	count	1
ALS2CR12	18.12508	2344.888095	0.0077	0.994	1.31E-08	count	1
OR5H14	18.1250799	2344.888078	0.0077	0.994	1.31E-08	count	1
OR2A1-AS1	18.1250799	2344.888078	0.0077	0.994	1.31E-08	count	1
ARMS2	18.1250799	2344.888078	0.0077	0.994	1.31E-08	count	1
LINC00470	18.1250799	2344.888078	0.0077	0.994	1.31E-08	count	1
TLE2	18.1250799	2344.888078	0.0077	0.994	1.31E-08	count	1
OR2B6	18.1250799	2344.888129	0.0077	0.994	1.31E-08	count	1
AL139384.2	18.1244968	1655.628137	0.0109	0.991	1.31E-08	count	1
AC024267.4	18.1244967	1655.628214	0.0109	0.991	1.31E-08	count	1
SPATA25	18.1244966	1655.628102	0.0109	0.991	1.31E-08	count	1
AL162742.1	18.1244967	1655.628179	0.0109	0.991	1.31E-08	count	1
UBE2Q2L	18.1244964	1655.628137	0.0109	0.991	1.31E-08	count	1
AC239803.1	18.1244965	1655.628137	0.0109	0.991	1.31E-08	count	1
AL353593.1	18.1244966	1655.628102	0.0109	0.991	1.31E-08	count	1
CD8B2	18.1244967	1655.628155	0.0109	0.991	1.31E-08	count	1
GALNT15	18.1244965	1655.628126	0.0109	0.991	1.31E-08	count	1
LRRTM2	18.1244966	1655.628126	0.0109	0.991	1.31E-08	count	1
PCDHGB7	18.1244965	1655.628096	0.0109	0.991	1.31E-08	count	1
GPT	18.1244964	1655.628137	0.0109	0.991	1.31E-08	count	1
AL356056.1	18.1244965	1655.628078	0.0109	0.991	1.31E-08	count	1
ENO4	18.1244966	1655.628161	0.0109	0.991	1.31E-08	count	1
AC138207.7	18.1244965	1655.62812	0.0109	0.991	1.31E-08	count	1
PSPN	18.1244966	1655.628102	0.0109	0.991	1.31E-08	count	1
GSTM5	18.1244966	1655.628096	0.0109	0.991	1.31E-08	count	1
AC137630.3	18.1244963	1655.628055	0.0109	0.991	1.31E-08	count	1
LINC02150	18.1244965	1655.628137	0.0109	0.991	1.31E-08	count	1
ITGA2	18.1244965	1655.628078	0.0109	0.991	1.31E-08	count	1
JAZF1-AS1	18.1244964	1655.628102	0.0109	0.991	1.31E-08	count	1
TMEM225B	18.1244964	1655.628067	0.0109	0.991	1.31E-08	count	1
AC007938.3	18.1244964	1655.628043	0.0109	0.991	1.31E-08	count	1
AC124947.1	18.1244964	1655.628031	0.0109	0.991	1.31E-08	count	1
AC012085.2	18.1244966	1655.628126	0.0109	0.991	1.31E-08	count	1
AC055713.1	18.1244966	1655.628108	0.0109	0.991	1.31E-08	count	1
AL031600.3	18.1244965	1655.628084	0.0109	0.991	1.31E-08	count	1
HOXB8	18.1244964	1655.628108	0.0109	0.991	1.31E-08	count	1
AC092835.1	18.1244965	1655.628043	0.0109	0.991	1.31E-08	count	1
AC009226.1	18.1244965	1655.628132	0.0109	0.991	1.31E-08	count	1
ZDHHC11B	18.1244964	1655.628078	0.0109	0.991	1.31E-08	count	1
AC026412.3	18.1244965	1655.628179	0.0109	0.991	1.31E-08	count	1
AC104113.1	18.1244963	1655.628049	0.0109	0.991	1.31E-08	count	1

RGMB-AS1	18.1244966	1655.628143	0.0109	0.991	1.31E-08	count	1
FIBCD1	18.1244964	1655.628161	0.0109	0.991	1.31E-08	count	1
AC022400.6	18.1244964	1655.628143	0.0109	0.991	1.31E-08	count	1
AL121929.2	18.1244964	1655.628078	0.0109	0.991	1.31E-08	count	1
NPIPB13	18.1244964	1655.628155	0.0109	0.991	1.31E-08	count	1
HAS3	18.1244965	1655.628179	0.0109	0.991	1.31E-08	count	1
SLC5A10	18.1244965	1655.628155	0.0109	0.991	1.31E-08	count	1
PADI6	18.1244965	1655.628114	0.0109	0.991	1.31E-08	count	1
PM20D1	18.1244964	1655.62812	0.0109	0.991	1.31E-08	count	1
GPR150	18.1244964	1655.628126	0.0109	0.991	1.31E-08	count	1
AC091948.1	18.1244964	1655.628126	0.0109	0.991	1.31E-08	count	1
AL662797.1	18.1244963	1655.628049	0.0109	0.991	1.31E-08	count	1
AL356124.1	18.1244963	1655.628108	0.0109	0.991	1.31E-08	count	1
ZFP92	18.1244965	1655.628084	0.0109	0.991	1.31E-08	count	1
ORM1	18.1244964	1655.628126	0.0109	0.991	1.31E-08	count	1
AL121748.2	18.1244963	1655.628078	0.0109	0.991	1.31E-08	count	1
AL157832.1	18.1244964	1655.628049	0.0109	0.991	1.31E-08	count	1
MYO1A	18.1244964	1655.628084	0.0109	0.991	1.31E-08	count	1
PKN2-AS1	18.1244963	1655.628149	0.0109	0.991	1.31E-08	count	1
CCDC85A	18.1244963	1655.628078	0.0109	0.991	1.31E-08	count	1
AC005740.4	18.1244963	1655.628037	0.0109	0.991	1.31E-08	count	1
TMEM45B	18.1244961	1655.628043	0.0109	0.991	1.31E-08	count	1
AC016394.2	18.1244964	1655.628137	0.0109	0.991	1.31E-08	count	1
AC008763.1	18.1244962	1655.628067	0.0109	0.991	1.31E-08	count	1
PCDHGA6	18.1244963	1655.628137	0.0109	0.991	1.31E-08	count	1
FAM161B	0.3860913	0.5295237	0.7291	0.466	1.31E-08	count	1
TMEM198	19.4392274	1802.949017	0.0108	0.991	1.32E-08	count	1
LINC00211	18.8628971	2589.943635	0.0073	0.994	1.32E-08	count	1
AL360181.2	18.8627186	1949.026634	0.0097	0.992	1.32E-08	count	1
MYO7B	18.8626715	1949.016967	0.0097	0.992	1.32E-08	count	1
DMTN	19.1497917	1560.031092	0.0123	0.99	1.32E-08	count	1
LINC00885	19.1497916	1560.031092	0.0123	0.99	1.32E-08	count	1
HDAC11-AS1	19.1497912	1560.031155	0.0123	0.99	1.32E-08	count	1
GPR137C	19.1497914	1560.031071	0.0123	0.99	1.32E-08	count	1
AC108463.3	19.1497911	1560.030976	0.0123	0.99	1.32E-08	count	1
TENM1	19.1497911	1560.030976	0.0123	0.99	1.32E-08	count	1
AL845472.1	19.1497911	1560.030976	0.0123	0.99	1.32E-08	count	1
AC116366.2	18.7413273	1271.851491	0.0147	0.988	1.32E-08	count	1
AC139795.2	18.7413271	1271.851375	0.0147	0.988	1.32E-08	count	1
SLIT2	18.7413271	1271.851452	0.0147	0.988	1.32E-08	count	1
AC079807.1	18.7413269	1271.851529	0.0147	0.988	1.32E-08	count	1
ASPG	18.741327	1271.851469	0.0147	0.988	1.32E-08	count	1
CSMD2	18.7413267	1271.851512	0.0147	0.988	1.32E-08	count	1
AC092384.1	19.0511414	2138.991388	0.0089	0.993	1.33E-08	count	1
LINC02471	19.0511855	2139.002037	0.0089	0.993	1.33E-08	count	1

ZNF132	18.5593643	1938.565008	0.0096	0.992	1.33E-08	count	1
FZD9	18.559371	1938.580507	0.0096	0.992	1.33E-08	count	1
AC135012.1	18.5593575	1938.565196	0.0096	0.992	1.33E-08	count	1
AL355490.2	18.5593627	1938.573078	0.0096	0.992	1.33E-08	count	1
PLGLB1	18.5593673	1938.568881	0.0096	0.992	1.33E-08	count	1
FOXC2	18.5593442	1938.574036	0.0096	0.992	1.33E-08	count	1
AL512770.1	18.5593568	1938.577419	0.0096	0.992	1.33E-08	count	1
AC004593.3	18.5593564	1938.577405	0.0096	0.992	1.33E-08	count	1
CRX	18.5593426	1938.569983	0.0096	0.992	1.33E-08	count	1
LINC00691	18.5593548	1938.561272	0.0096	0.992	1.33E-08	count	1
F12	18.559341	1938.570177	0.0096	0.992	1.33E-08	count	1
SLC4A3	18.5593349	1938.570271	0.0096	0.992	1.33E-08	count	1
FBXL16	18.5593314	1938.56647	0.0096	0.992	1.33E-08	count	1
OCLN	19.0500971	1919.879614	0.0099	0.992	1.33E-08	count	1
LYPD2	0.4003053	0.698917	0.5728	0.567	1.36E-08	count	1
KALRN	0.9393018	0.9379904	1.0014	0.317	1.39E-08	count	1
PDE3A	0.9393018	0.9379904	1.0014	0.317	1.39E-08	count	1
AL691403.1	0.9393018	0.9379904	1.0014	0.317	1.39E-08	count	1
IKZF2	1.035242	0.5922974	1.7478	0.0806	1.58E-08	count	1
TCIM	1.1285195	1.1129849	1.014	0.311	1.77E-08	count	1
AF230666.1	1.1285195	0.9176323	1.2298	0.219	1.77E-08	count	1
ZNF578	1.1285195	0.7844669	1.4386	0.15	1.77E-08	count	1
MORF4L2-AS1	1.2269839	0.7087989	1.7311	0.0836	1.97E-08	count	1
PRRX1	1.2269839	0.7087989	1.7311	0.0836	1.97E-08	count	1
AC103996.2	1.2269839	0.6285584	1.9521	0.051	1.97E-08	count	1
AC006238.1	1.2269839	0.6285584	1.9521	0.051	1.97E-08	count	1
AC087627.1	1.30515	0.8825956	1.4788	0.139	2.14E-08	count	1
AC107214.1	1.30515	0.8825956	1.4788	0.139	2.14E-08	count	1
EHD3	1.3836902	0.8834411	1.5663	0.117	2.32E-08	count	1
GFPT2	1.4670031	1.2263035	1.1963	0.232	2.51E-08	count	1
FRY-AS1	1.5296759	0.8691379	1.76	0.0785	2.65E-08	count	1
ACKR1	1.5296759	0.8314648	1.8397	0.0659	2.65E-08	count	1
SULT1A2	1.632449	0.606461	2.6918	0.0072	2.89E-08	count	1
PGA5	1.632449	0.4268866	3.8241	1.00E-04	2.89E-08	count	1
ZNF550	1.6643649	0.9709579	1.7141	0.0866	2.97E-08	count	1
TIMD4	0.8306861	0.5295785	1.5686	0.117	3.25E-08	count	1
TBX1	1.7865997	0.9594085	1.8622	0.0627	3.25E-08	count	1
GIPC2	0.8359784	0.5021302	1.6649	0.0961	3.27E-08	count	1
MED12L	1.8908357	0.9606564	1.9683	0.0491	3.51E-08	count	1
OVCH1-AS1	18.5170796	2852.619212	0.0065	0.995	3.53E-08	count	1
AL731571.1	18.3833226	2668.079438	0.0069	0.995	3.53E-08	count	1
SPATA8	18.634236	2252.338292	0.0083	0.993	3.54E-08	count	1
NPIPA5	18.6338913	1984.814992	0.0094	0.993	3.54E-08	count	1
FBN1	18.8339124	1941.025214	0.0097	0.992	3.54E-08	count	1
GPR12	18.9206606	1967.766782	0.0096	0.992	3.54E-08	count	1

AC108102.1	18.045449	2253.359221	0.008	0.994	3.54E-08	count	1
AC087289.5	18.045449	2253.359221	0.008	0.994	3.54E-08	count	1
AC008443.3	18.0454486	2253.359138	0.008	0.994	3.54E-08	count	1
LINC02080	18.0454486	2253.359138	0.008	0.994	3.54E-08	count	1
GABRB3	18.0454491	2253.35915	0.008	0.994	3.54E-08	count	1
C4BPB	18.8329046	1166.252364	0.0161	0.987	3.54E-08	count	1
AASS	18.3809023	1257.41354	0.0146	0.988	3.54E-08	count	1
NXPH3	18.3809024	1257.413543	0.0146	0.988	3.54E-08	count	1
RRAS2	18.2266691	1420.969497	0.0128	0.99	3.54E-08	count	1
FGF13	18.3805488	1135.820261	0.0162	0.987	3.55E-08	count	1
ARHGAP42	18.0439994	1491.361787	0.0121	0.99	3.55E-08	count	1
MIR497HG	17.820388	1590.701359	0.0112	0.991	3.55E-08	count	1
RBM44	17.8203879	1590.701346	0.0112	0.991	3.55E-08	count	1
ADAD2	17.8203878	1590.701334	0.0112	0.991	3.55E-08	count	1
CAMK2B	17.8203878	1590.701296	0.0112	0.991	3.55E-08	count	1
BEX5	17.8203878	1590.701317	0.0112	0.991	3.55E-08	count	1
FAM72B	18.0433773	1187.479445	0.0152	0.988	3.55E-08	count	1
ZNF280C	18.0433774	1187.479411	0.0152	0.988	3.55E-08	count	1
TRIM50	17.820181	1421.940772	0.0125	0.99	3.55E-08	count	1
AL590867.1	17.8201809	1421.940809	0.0125	0.99	3.55E-08	count	1
LINC02376	18.0429284	1001.659618	0.018	0.986	3.55E-08	count	1
AC011825.4	18.7859724	2647.3234	0.0071	0.994	3.55E-08	count	1
ANK2	18.4303895	1764.694729	0.0104	0.992	3.57E-08	count	1
ZC3H11A	18.4297618	1350.589038	0.0136	0.989	3.57E-08	count	1
AC025423.1	18.4294722	1180.810853	0.0156	0.988	3.57E-08	count	1
AC127521.1	18.2756677	1734.994634	0.0105	0.992	3.57E-08	count	1
AC013264.1	19.1237262	1539.851798	0.0124	0.99	3.58E-08	count	1
AC079601.1	19.1237262	1539.851798	0.0124	0.99	3.58E-08	count	1
AC013553.4	19.1237262	1539.851798	0.0124	0.99	3.58E-08	count	1
RHEX	18.8435991	1338.597618	0.0141	0.989	3.58E-08	count	1
GPRC5B	18.0912116	1454.470078	0.0124	0.99	3.58E-08	count	1
TRPC6	18.6625951	1222.772234	0.0153	0.988	3.58E-08	count	1
C17orf97	18.0909473	1182.990657	0.0153	0.988	3.58E-08	count	1
MYLK-AS1	18.0909514	1182.992108	0.0153	0.988	3.58E-08	count	1
ZCCHC12	18.8140271	1609.707141	0.0117	0.991	3.59E-08	count	1
CCNA1	18.2547523	1308.636746	0.0139	0.989	3.59E-08	count	1
AC012368.2	18.2547519	1308.640878	0.0139	0.989	3.59E-08	count	1
AC092746.1	18.2539205	1174.505072	0.0155	0.988	3.60E-08	count	1
CA8	0.9031511	0.550742	1.6399	0.101	3.61E-08	count	1
CYP4F3	0.9214451	0.7388707	1.2471	0.212	3.71E-08	count	1
LOXL1	1.0901897	0.563536	1.9346	0.0532	4.60E-08	count	1
ABCA9	1.367888	0.5587916	2.4479	0.0144	6.22E-08	count	1
AMY2B	1.3836902	0.7057044	1.9607	0.05	6.29E-08	count	1
ZNF585B	1.4437825	0.8071989	1.7886	0.0738	6.67E-08	count	1
AL121985.1	1.5454376	0.8876171	1.7411	0.0818	7.30E-08	count	1

HOXA5	1.7865997	0.409011	4.3681	1.31E-05	8.89E-08	count	0.3102211
RGPD2	1.8506953	0.8069838	2.2933	0.0219	9.36E-08	count	1
CNTN4	17.8342277	2027.516223	0.0088	0.993	9.61E-08	count	1
FOXL2NB	17.8342277	2027.51618	0.0088	0.993	9.61E-08	count	1
ARHGEF5	17.9987579	2201.362747	0.0082	0.993	9.63E-08	count	1
CACNB4	18.5198799	810.2949949	0.0229	0.982	9.68E-08	count	1
SGCB	2.0449251	0.8808434	2.3216	0.0203	1.06E-07	count	1
GCOM1	2.2385848	1.1821203	1.8937	0.0584	1.19E-07	count	1
AL078639.1	2.3255962	1.4296555	1.6267	0.104	1.25E-07	count	1
AC132192.1	2.4451378	0.8537101	2.8641	0.0042	1.33E-07	count	1
KCNAB1	2.5387496	1.3754229	1.8458	0.065	1.39E-07	count	1
IL12RB2	2.6491411	0.7773905	3.4077	7.00E-04	1.46E-07	count	1
CXCL13	3.33674	0.6779655	4.9217	9.15E-07	6.01E-07	count	0.021798045
PANX1	0.000118	0.351208	3.00E-04	1	0.000102462	count	1
EMC7	8.47E-05	0.0957679	9.00E-04	0.999	0.000118365	count	1
PSMB8-AS1	9.78E-05	0.1559696	6.00E-04	0.999	0.00013091	count	1
ANAPC13	0.0001531	0.1439231	0.0011	0.999	0.000206499	count	1
ZNF100	0.0001904	0.2323082	8.00E-04	0.999	0.000229953	count	1
CYC1	0.0001713	0.0761019	0.0023	0.998	0.000242458	count	1
TNFSF12	0.0002249	0.0789157	0.0028	0.998	0.000316745	count	1
APH1B	0.0002807	0.1408409	0.002	0.998	0.000380858	count	1
CD93	0.0002964	0.1002478	0.003	0.998	0.000414558	count	1
CRACR2A	0.0009625	0.7053461	0.0014	0.999	0.000433358	count	1
NIPA1	0.0009625	0.7053461	0.0014	0.999	0.000433358	count	1
RMND1	0.0006346	0.3002257	0.0021	0.998	0.000659117	count	1
AL160408.2	0.0027806	0.9677966	0.0029	0.998	0.000742089	count	1
HARS	0.0006144	0.2136912	0.0029	0.998	0.000757872	count	1
JMJD8	0.0006724	0.2117414	0.0032	0.997	0.000824636	count	1
TMCC3	0.0008418	0.2941972	0.0029	0.998	0.000842865	count	1
MRPL32	0.0006634	0.1433376	0.0046	0.996	0.000884005	count	1
BAZ1A	0.0007011	0.0868441	0.0081	0.994	0.000980805	count	1
GADD45A	0.0008193	0.2274288	0.0036	0.997	0.001055132	count	1
TTC27	0.0014422	0.4013179	0.0036	0.997	0.001105813	count	1
AXIN1	0.0014422	0.419368	0.0034	0.997	0.001105813	count	1
MCTS1	0.0008146	0.1129333	0.0072	0.994	0.001115488	count	1
RPS29	0.0008588	0.0315157	0.0273	0.978	0.001234432	count	1
MAPKAPK3	0.0009686	0.1248846	0.0078	0.994	0.001322941	count	1
CISH	0.0013994	0.2817125	0.005	0.996	0.001386625	count	1
ABCA7	0.0015504	0.5109643	0.003	0.998	0.001442179	count	1
WDCP	0.0168136	1.094296	0.0154	0.988	0.001713855	count	1
TMEM255A	0.0168136	0.9553703	0.0176	0.986	0.001713855	count	1
KITLG	0.0168136	1.020771	0.0165	0.987	0.001713855	count	1
ADGRD1	0.0168136	1.021003	0.0165	0.987	0.001713855	count	1
CORO2A	0.0024969	0.6071296	0.0041	0.997	0.001786116	count	1
PDE4A	0.0014835	0.2279283	0.0065	0.995	0.001846122	count	1

MCRS1	0.0016393	0.2062643	0.0079	0.994	0.002133751	count	1
IFT22	0.0024163	0.4543452	0.0053	0.996	0.002213693	count	1
GJC2	0.0049771	1.0217766	0.0049	0.996	0.002487376	count	1
TAPT1-AS1	0.0049771	0.9074413	0.0055	0.996	0.002487376	count	1
NFYC-AS1	0.0137794	0.5843113	0.0236	0.981	0.002621377	count	1
CCT7	0.0019558	0.1021135	0.0192	0.985	0.00271571	count	1
PTPN7	0.0022155	0.2153928	0.0103	0.992	0.002823083	count	1
MIR34AHG	0.0084473	0.6053633	0.014	0.989	0.002835567	count	1
AC006059.1	0.0058	0.6471915	0.009	0.993	0.00289917	count	1
AL031708.1	0.0058	0.7832307	0.0074	0.994	0.00289917	count	1
LINC00910	0.0058	0.7418924	0.0078	0.994	0.00289917	count	1
VPS36	0.0021342	0.1263009	0.0169	0.987	0.002940651	count	1
OAS2	0.0023272	0.2103175	0.0111	0.991	0.002978806	count	1
SNX5	0.0021483	0.087483	0.0246	0.98	0.003005841	count	1
ST8SIA4	0.0021629	0.1172112	0.0185	0.985	0.003005886	count	1
TTC39C	0.0024879	0.2147661	0.0116	0.991	0.003025921	count	1
CYB5D2	0.0026962	0.3128998	0.0086	0.993	0.003113354	count	1
MED30	0.0023397	0.1610678	0.0145	0.988	0.003151492	count	1
ERLIN2	0.002877	0.2705993	0.0106	0.992	0.003506884	count	1
STX4	0.0026038	0.112609	0.0231	0.982	0.003636035	count	1
ZBTB1	0.0027584	0.1290489	0.0214	0.983	0.003675871	count	1
AC083880.1	0.0049771	0.6824694	0.0073	0.994	0.003694826	count	1
LSM1	0.0027132	0.126927	0.0214	0.983	0.003706719	count	1
PPFIA1	0.0027591	0.1318738	0.0209	0.983	0.003722911	count	1
DDRGK1	0.002737	0.1087845	0.0252	0.98	0.003778218	count	1
CMTM3	0.0028037	0.086193	0.0325	0.974	0.003941918	count	1
MS4A1	0.01224	1.1184248	0.0109	0.991	0.004112816	count	1
CIRBP-AS1	0.01224	1.118357	0.0109	0.991	0.004112816	count	1
HIST3H2A	0.0045238	0.3207518	0.0141	0.989	0.004145579	count	1
WDR20	0.00362	0.2522313	0.0144	0.989	0.004164369	count	1
LRRC34	0.0247206	0.9450064	0.0262	0.979	0.004718227	count	1
SLC39A14	0.0247206	0.902012	0.0274	0.978	0.004718227	count	1
PIH1D1	0.0034774	0.1372965	0.0253	0.98	0.00475082	count	1
HAX1	0.0034628	0.1001777	0.0346	0.972	0.004833651	count	1
TSPAN17	0.0051935	0.2882488	0.018	0.986	0.005031581	count	1
GMPPA	0.0040765	0.2138661	0.0191	0.985	0.005113465	count	1
TMED8	0.0047145	0.3089099	0.0153	0.988	0.005144307	count	1
LAT2	0.0037585	0.09376	0.0401	0.968	0.005175499	count	1
TAF10	0.0037652	0.0584871	0.0644	0.949	0.005365407	count	1
GPAT3	0.0042095	0.1638102	0.0257	0.98	0.005612494	count	1
ACYP2	0.0046086	0.186295	0.0247	0.98	0.005745351	count	1
CLPTM1L	0.0043256	0.1442609	0.03	0.976	0.005756186	count	1
LRP10	0.0042387	0.1396112	0.0304	0.976	0.005765751	count	1
PEAK3	0.0220747	0.465619	0.0474	0.962	0.005923328	count	1
AC092279.1	0.0149408	0.7323174	0.0204	0.984	0.005934111	count	1

SERAC1	0.0067046	0.3943952	0.017	0.986	0.005939476	count	1
HPS3	0.0044693	0.1619809	0.0276	0.978	0.005953246	count	1
APBA3	0.0064196	0.6296286	0.0102	0.992	0.006061096	count	1
FUCA2	0.0045844	0.0960104	0.0477	0.962	0.006359343	count	1
MTCH1	0.0045783	0.0871845	0.0525	0.958	0.006431364	count	1
ATAT1	0.0073589	0.4593389	0.016	0.987	0.006636078	count	1
ARHGEF37	0.0107836	0.5196903	0.0208	0.983	0.006711336	count	1
FBXO7	0.0049274	0.1481976	0.0332	0.973	0.006756046	count	1
IQGAP3	0.0654732	0.8110503	0.0807	0.936	0.00677821	count	1
MAPK1	0.0049706	0.1132664	0.0439	0.965	0.006836639	count	1
NRSN2	0.0661193	0.8199917	0.0806	0.936	0.006846501	count	1
SMG1	0.0051525	0.1488138	0.0346	0.972	0.006943141	count	1
SLC27A4	0.0206574	0.5489226	0.0376	0.97	0.00695663	count	1
HNRNPLL	0.0058514	0.2348014	0.0249	0.98	0.006979127	count	1
UFD1	0.0050347	0.1206853	0.0417	0.967	0.007044623	count	1
APLF	0.0122027	0.72636	0.0168	0.987	0.00714531	count	1
ZKSCAN1	0.0056571	0.2836824	0.0199	0.984	0.007201029	count	1
JADE2	0.0102524	0.3622848	0.0283	0.977	0.007343779	count	1
CDC42SE1	0.005357	0.0869355	0.0616	0.951	0.00750578	count	1
PAPSS2	0.0059325	0.2031823	0.0292	0.977	0.007602796	count	1
POLRMT	0.0116033	0.3613818	0.0321	0.974	0.007616299	count	1
NHSL1	0.0098998	0.5249886	0.0189	0.985	0.007830024	count	1
SMARCA5	0.0056935	0.0927203	0.0614	0.951	0.007940242	count	1
TSPOAP1	0.0146748	0.6141614	0.0239	0.981	0.008003051	count	1
SCRIB	0.0087658	0.3985926	0.022	0.982	0.008037203	count	1
AC036176.1	0.0129141	0.6874596	0.0188	0.985	0.008040638	count	1
BNIP2	0.005749	0.0832395	0.0691	0.945	0.008075554	count	1
MIR22HG	0.0057959	0.10012	0.0579	0.954	0.008140997	count	1
GPR137	0.0071106	0.3432646	0.0207	0.983	0.008149527	count	1
LETM1	0.0069256	0.2369544	0.0292	0.977	0.008367454	count	1
NOP58	0.0062277	0.141642	0.044	0.965	0.008428335	count	1
TM2D2	0.0061477	0.130083	0.0473	0.962	0.008449877	count	1
AACS	0.0168869	0.4905659	0.0344	0.973	0.008462121	count	1
SLC25A53	0.0093597	0.3788929	0.0247	0.98	0.008582379	count	1
TBC1D9	0.0067171	0.1663905	0.0404	0.968	0.008660839	count	1
ZNF775	0.0847634	0.6028954	0.1406	0.888	0.008829004	count	1
TNKS2	0.0066949	0.1642176	0.0408	0.967	0.00887751	count	1
PFDN1	0.0063408	0.0969256	0.0654	0.948	0.008881964	count	1
THOC7	0.0066152	0.0854186	0.0774	0.938	0.009297285	count	1
PGP	0.0066497	0.0834005	0.0797	0.936	0.009363752	count	1
ISCU	0.0066371	0.0747187	0.0888	0.929	0.009395829	count	1
H2AFZ	0.006659	0.0515361	0.1292	0.897	0.009555321	count	1
NRF1	0.0146328	0.5267317	0.0278	0.978	0.009610294	count	1
ALDH6A1	0.0087681	0.3329102	0.0263	0.979	0.009626505	count	1
SEL1L	0.0069536	0.1380713	0.0504	0.96	0.009636921	count	1

BTBD19	0.0089995	0.4524977	0.0199	0.984	0.009638291	count	1
TIMM10	0.0071319	0.1266841	0.0563	0.955	0.00971908	count	1
ORC2	0.0111852	0.4835314	0.0231	0.982	0.009727122	count	1
SORBS3	0.0089688	0.2549054	0.0352	0.972	0.009730744	count	1
ZNF33B	0.0116978	0.3148555	0.0372	0.97	0.009965303	count	1
PQLC2	0.0104551	0.4278616	0.0244	0.981	0.010135168	count	1
TAX1BP3	0.0079851	0.2304755	0.0346	0.972	0.010177311	count	1
SRRT	0.0077834	0.1675872	0.0464	0.963	0.010242604	count	1
CREBZF	0.0082352	0.208836	0.0394	0.969	0.01025146	count	1
AC107375.1	0.0380749	0.844895	0.0451	0.964	0.010262454	count	1
RRAGC	0.0077031	0.1657563	0.0465	0.963	0.010299451	count	1
PWWP2B	0.0086879	0.2759389	0.0315	0.975	0.010497685	count	1
SMPD4	0.0106061	0.3838811	0.0276	0.978	0.010837217	count	1
CEP89	0.0115146	0.4150517	0.0277	0.978	0.010878154	count	1
SLC37A1	0.0120985	0.3619101	0.0334	0.973	0.01091681	count	1
POLR2A	0.0085519	0.1990666	0.043	0.966	0.010983706	count	1
DHX58	0.0097698	0.3241696	0.0301	0.976	0.011006931	count	1
SMYD4	0.0120492	0.2952765	0.0408	0.967	0.011052243	count	1
PRPF38A	0.0084656	0.1818902	0.0465	0.963	0.011080226	count	1
RFFL	0.0117284	0.3372463	0.0348	0.972	0.011371138	count	1
FKBP15	0.0084472	0.1302996	0.0648	0.948	0.01156409	count	1
AL359915.2	0.0430924	0.734965	0.0586	0.953	0.011631079	count	1
NAIF1	0.029688	0.491057	0.0605	0.952	0.01183469	count	1
MEX3B	0.023582	0.4529099	0.0521	0.958	0.011834825	count	1
E2F3	0.0098111	0.2174663	0.0451	0.964	0.011855605	count	1
RAPGEF2	0.0100084	0.2530501	0.0396	0.968	0.011872888	count	1
TMEM14C	0.0084567	0.0666871	0.1268	0.899	0.012072218	count	1
FBXO33	0.0097395	0.2283184	0.0427	0.966	0.012104689	count	1
ARL6IP6	0.0093481	0.1720509	0.0543	0.957	0.01215111	count	1
RGS5	0.0135198	0.751709	0.018	0.986	0.012201521	count	1
H3F3B	0.0085027	0.0304477	0.2793	0.78	0.012255952	count	1
PDE6D	0.0098741	0.2247066	0.0439	0.965	0.012272055	count	1
TCEAL1	0.0102111	0.2139787	0.0477	0.962	0.012339235	count	1
CHCHD7	0.0090079	0.1235952	0.0729	0.942	0.012390406	count	1
FBXO30	0.0113419	0.3528534	0.0321	0.974	0.012719067	count	1
ETFA	0.0091735	0.0989765	0.0927	0.926	0.012775474	count	1
YWHAZ	0.0089216	0.0492709	0.1811	0.856	0.01278356	count	1
RWDD1	0.0090407	0.0745099	0.1213	0.903	0.01281787	count	1
CYTH4	0.0094261	0.1443949	0.0653	0.948	0.013028211	count	1
RAB22A	0.009781	0.1662847	0.0588	0.953	0.013089733	count	1
ATF6	0.0097041	0.117943	0.0823	0.934	0.013095969	count	1
SORT1	0.0103547	0.187249	0.0553	0.956	0.013258102	count	1
TCTEX1D2	0.0174477	0.5649289	0.0309	0.975	0.013412661	count	1
PTPRJ	0.0100435	0.1522798	0.066	0.947	0.013576464	count	1
TFAP2E	0.0716501	0.9205733	0.0778	0.938	0.013867062	count	1

IFT172	0.0716501	1.0246684	0.0699	0.944	0.013867062	count	1
AGAP1	0.0716501	0.9205733	0.0778	0.938	0.013867062	count	1
AC107464.3	0.0716501	0.9205733	0.0778	0.938	0.013867062	count	1
AC120053.1	0.0716501	0.9145319	0.0783	0.938	0.013867062	count	1
VSTM4	0.0716501	0.9205733	0.0778	0.938	0.013867062	count	1
CCDC9B	0.0716501	1.0246684	0.0699	0.944	0.013867062	count	1
AC048382.6	0.0716501	0.9205733	0.0778	0.938	0.013867062	count	1
AC011481.2	0.0716501	1.1771429	0.0609	0.951	0.013867062	count	1
RFT1	0.0133748	0.3042158	0.044	0.965	0.014020416	count	1
CCAR2	0.0141369	0.2884874	0.049	0.961	0.014027086	count	1
COA6	0.0100931	0.1077825	0.0936	0.925	0.01402827	count	1
TNFRSF10D	0.0178929	0.3573642	0.0501	0.96	0.014169557	count	1
SORL1	0.0104265	0.1504596	0.0693	0.945	0.01425905	count	1
C1orf53	0.0207958	0.4956829	0.042	0.967	0.014323291	count	1
CMTM6	0.0101655	0.0618382	0.1644	0.869	0.014436136	count	1
TAB3	0.013402	0.3548434	0.0378	0.97	0.014634467	count	1
CDIPT	0.0110031	0.1659287	0.0663	0.947	0.014651898	count	1
TNFRSF10A	0.0138933	0.343631	0.0404	0.968	0.014676093	count	1
ATP6V1C1	0.0109011	0.14855	0.0734	0.942	0.014745397	count	1
PIN1	0.0106196	0.1011138	0.105	0.916	0.014849189	count	1
CC2D1B	0.0167461	0.4652864	0.036	0.971	0.01485482	count	1
AL133342.1	0.012633	0.2371588	0.0533	0.958	0.015502759	count	1
DIP2A	0.0124503	0.2863145	0.0435	0.965	0.01571218	count	1
POU3F1	0.1488244	0.5685668	0.2618	0.794	0.015816156	count	1
IFI30	0.0110795	0.0915191	0.1211	0.904	0.015860649	count	1
SLC11A2	0.0125213	0.2210412	0.0566	0.955	0.016132474	count	1
SLC2A13	0.0164717	0.3956107	0.0416	0.967	0.016167649	count	1
STARD3	0.0126166	0.1841317	0.0685	0.945	0.016345734	count	1
SLC39A3	0.0124261	0.1623827	0.0765	0.939	0.01660901	count	1
MYH11	0.0241758	0.4716737	0.0513	0.959	0.016661383	count	1
TARBP1	0.0167971	0.4505415	0.0373	0.97	0.016671374	count	1
RILP	0.0124879	0.1485792	0.084	0.933	0.016938301	count	1
CENPS	0.0423637	0.7164213	0.0591	0.953	0.016940767	count	1
CBLN3	0.0423637	0.8029478	0.0528	0.958	0.016940767	count	1
SNRK-AS1	0.1593638	0.9958372	0.16	0.873	0.016991781	count	1
SOX5	0.1593638	1.1113606	0.1434	0.886	0.016991781	count	1
ZNF20	0.1593638	1.0504143	0.1517	0.879	0.016991781	count	1
BLCAP	0.0143224	0.2060568	0.0695	0.945	0.017043823	count	1
UBE2R2	0.0122346	0.071641	0.1708	0.864	0.017325193	count	1
UBE2E1	0.0128472	0.1340951	0.0958	0.924	0.017425776	count	1
PRKDC	0.0126507	0.1053938	0.12	0.904	0.017444817	count	1
P3H1	0.0157856	0.3300832	0.0478	0.962	0.017708347	count	1
MCAT	0.0147022	0.316268	0.0465	0.963	0.017727878	count	1
ZNF136	0.0224913	0.388537	0.0579	0.954	0.017823745	count	1
AC007448.3	0.0201119	0.4805198	0.0419	0.967	0.017848424	count	1

AC010280.2	0.1674885	0.9433476	0.1775	0.859	0.017903098	count	1
TMCO1	0.0127639	0.0849166	0.1503	0.881	0.018017548	count	1
PLRG1	0.0140634	0.2240605	0.0628	0.95	0.018028574	count	1
SLC19A2	0.016426	0.342103	0.048	0.962	0.018045454	count	1
COA4	0.0129945	0.110336	0.1178	0.906	0.018181	count	1
GRAMD2B	0.0673354	0.5435545	0.1239	0.901	0.018297083	count	1
MYZAP	0.0673354	0.5888245	0.1144	0.909	0.018297083	count	1
GRPEL1	0.0131589	0.0959573	0.1371	0.891	0.018348356	count	1
CAHM	0.0189984	0.4065832	0.0467	0.963	0.018434758	count	1
MEGF6	0.0682723	0.6172925	0.1106	0.912	0.018556467	count	1
KLF8	0.1733584	0.799501	0.2168	0.828	0.018564235	count	1
VMP1	0.0131147	0.0727393	0.1803	0.857	0.018691933	count	1
EIF4G1	0.0138209	0.1326521	0.1042	0.917	0.018846168	count	1
DCLRE1B	0.0216872	0.4050625	0.0535	0.957	0.018887108	count	1
SAR1A	0.0134905	0.0970506	0.139	0.889	0.018923295	count	1
ACP1	0.013606	0.1014869	0.1341	0.893	0.018996036	count	1
DCPS	0.0145471	0.1780954	0.0817	0.935	0.019096884	count	1
TNRC6C	0.038085	0.4750346	0.0802	0.936	0.019175241	count	1
AKIRIN1	0.0138815	0.1081391	0.1284	0.898	0.019176614	count	1
LINC00482	0.0328567	0.8409777	0.0391	0.969	0.019320366	count	1
WIPF1	0.0138344	0.0950693	0.1455	0.884	0.019347894	count	1
ZFP1	0.0353946	0.4572039	0.0774	0.938	0.019388206	count	1
TPRKB	0.014233	0.1325742	0.1074	0.915	0.019514382	count	1
JAK1	0.0138038	0.0622146	0.2219	0.824	0.019635764	count	1
WDTC1	0.0205877	0.3430282	0.06	0.952	0.019732076	count	1
FAM72A	0.0210862	0.3058149	0.069	0.945	0.019943282	count	1
RAPGEF1	0.0148075	0.1554647	0.0952	0.924	0.020130291	count	1
PCDHGC3	0.0741486	1.2090624	0.0613	0.951	0.020186347	count	1
AC008393.1	0.0741486	1.0916312	0.0679	0.946	0.020186347	count	1
ANLN	0.0741486	0.9355963	0.0793	0.937	0.020186347	count	1
CYP3A5	0.0741486	1.0920987	0.0679	0.946	0.020186347	count	1
FAM111A-DT	0.0741486	0.9344635	0.0793	0.937	0.020186347	count	1
CR381653.1	0.0741486	0.9350096	0.0793	0.937	0.020186347	count	1
SLX4IP	0.0256783	0.3992853	0.0643	0.949	0.020359365	count	1
NREP	0.0172472	0.2908904	0.0593	0.953	0.02040892	count	1
VPS33B	0.0373662	0.5958285	0.0627	0.95	0.020476751	count	1
MED16	0.0177849	0.2486472	0.0715	0.943	0.020633765	count	1
RASSF8	0.0206229	0.3288346	0.0627	0.95	0.020691939	count	1
AC108471.2	0.1060709	0.8189803	0.1295	0.897	0.020737256	count	1
ZNF564	0.1060709	0.8189803	0.1295	0.897	0.020737256	count	1
SYCE3	0.1060709	0.8111691	0.1308	0.896	0.020737256	count	1
PPP4R3B	0.0157067	0.1631499	0.0963	0.923	0.020774688	count	1
IPO8	0.0186298	0.2693785	0.0692	0.945	0.020800776	count	1
FARSA	0.0163721	0.1754983	0.0933	0.926	0.020990062	count	1
CDCA4	0.0186037	0.3411475	0.0545	0.957	0.021069301	count	1

MFAP1	0.016091	0.1615104	0.0996	0.921	0.021258487	count	1
COX17	0.0150021	0.0760903	0.1972	0.844	0.021261677	count	1
CCDC112	0.0155424	0.1271067	0.1223	0.903	0.021275512	count	1
NADK2	0.0216863	0.3913692	0.0554	0.956	0.021298149	count	1
ERH	0.0150287	0.0740709	0.2029	0.839	0.021366561	count	1
RPS18	0.0148376	0.0261541	0.5673	0.571	0.021382925	count	1
DSC2	0.0156303	0.1188581	0.1315	0.895	0.02143906	count	1
USP34	0.0157801	0.1501694	0.1051	0.916	0.021558735	count	1
PTPN11	0.016073	0.157586	0.102	0.919	0.021625129	count	1
CTNND1	0.0164736	0.2506159	0.0657	0.948	0.021684191	count	1
CHD4	0.0157191	0.1343882	0.117	0.907	0.021699985	count	1
BAD	0.0161404	0.1434239	0.1125	0.91	0.021707759	count	1
THUMPD1	0.0160672	0.125909	0.1276	0.898	0.021762588	count	1
MRPL43	0.015589	0.0939046	0.166	0.868	0.021849803	count	1
CLEC11A	0.0158696	0.1383647	0.1147	0.909	0.021911021	count	1
ZNF830	0.0207332	0.3147334	0.0659	0.947	0.021915122	count	1
ZSCAN18	0.0335694	0.4187251	0.0802	0.936	0.022125157	count	1
ZNF134	0.0321813	0.4424887	0.0727	0.942	0.022210316	count	1
ADORA3	0.0273335	0.3293124	0.083	0.934	0.02226703	count	1
RAD1	0.0184505	0.2707972	0.0681	0.946	0.022358563	count	1
COPS3	0.0165052	0.1279951	0.129	0.897	0.022528982	count	1
SLC41A2	0.0239355	0.4139449	0.0578	0.954	0.022645747	count	1
GPX7	0.030423	0.5476332	0.0556	0.956	0.022680768	count	1
AL353719.1	0.030423	0.7657124	0.0397	0.968	0.022680768	count	1
CYB561A3	0.0177721	0.1752322	0.1014	0.919	0.022945774	count	1
PTPN9	0.0214117	0.4115456	0.052	0.959	0.02295666	count	1
SURF2	0.0175414	0.2285203	0.0768	0.939	0.02296574	count	1
TMEM80	0.0182442	0.2130176	0.0856	0.932	0.02323518	count	1
ETFRF1	0.0173531	0.1636287	0.1061	0.916	0.023237567	count	1
RUVBL1	0.0185299	0.2284673	0.0811	0.935	0.023453074	count	1
SGSH	0.0195963	0.3935018	0.0498	0.96	0.02345539	count	1
BACH1-IT2	0.2161909	0.792011	0.273	0.785	0.023457972	count	1
SMIM7	0.016898	0.1053542	0.1604	0.873	0.023648483	count	1
ANAPC2	0.0402195	0.5559121	0.0723	0.942	0.023685073	count	1
TOP2B	0.0185237	0.1860794	0.0995	0.921	0.023750496	count	1
RPL36	0.0166527	0.0325595	0.5115	0.609	0.023939426	count	1
ELOVL1	0.0177696	0.1362378	0.1304	0.896	0.024076549	count	1
BRD7	0.0174792	0.1079397	0.1619	0.871	0.024218744	count	1
WNT2B	0.1236455	0.7938892	0.1557	0.876	0.024297244	count	1
AC025682.1	0.1236455	0.7027443	0.1759	0.86	0.024297244	count	1
RAB35	0.0193479	0.1850707	0.1045	0.917	0.024321645	count	1
MMACHC	0.0716501	0.6480358	0.1106	0.912	0.02445283	count	1
GRB10	0.0716501	0.6501708	0.1102	0.912	0.02445283	count	1
NFKB1	0.0178976	0.138538	0.1292	0.897	0.024490134	count	1
CNOT11	0.0209525	0.2363494	0.0887	0.929	0.024646482	count	1

NUP43	0.0262753	0.356279	0.0737	0.941	0.02486631	count	1
CSNK2B	0.0174515	0.0588678	0.2965	0.767	0.024869235	count	1
MMAB	0.0210399	0.3605596	0.0584	0.953	0.024902698	count	1
LRP11	0.2298273	0.8674098	0.265	0.791	0.025041612	count	1
CNPY4	0.0324975	0.4370386	0.0744	0.941	0.025042095	count	1
AC010618.3	0.0324975	0.5747553	0.0565	0.955	0.025042095	count	1
SMC4	0.0198196	0.191951	0.1033	0.918	0.025119218	count	1
LRFN1	0.0921138	0.6002377	0.1535	0.878	0.025201337	count	1
IGFBP5	0.0744784	0.4660103	0.1598	0.873	0.025436703	count	1
CLDN15	0.0463537	0.5083738	0.0912	0.927	0.025450229	count	1
DCHS1	0.0463537	0.475745	0.0974	0.922	0.025450229	count	1
UPF1	0.0233142	0.252105	0.0925	0.926	0.025627164	count	1
GLT8D1	0.0217806	0.276499	0.0788	0.937	0.025702337	count	1
RFK	0.0197191	0.1836883	0.1074	0.915	0.02570272	count	1
C1orf174	0.0200265	0.1772376	0.113	0.91	0.025809281	count	1
GNAI2	0.0180724	0.0341209	0.5297	0.596	0.025922551	count	1
TXNDC12	0.0185673	0.0950506	0.1953	0.845	0.025992324	count	1
C10orf25	0.0761402	0.889873	0.0856	0.932	0.026015436	count	1
MCM3	0.0268699	0.3344374	0.0803	0.936	0.026095706	count	1
WDR92	0.0397294	0.4549312	0.0873	0.93	0.026215031	count	1
SLC23A2	0.0262409	0.4168861	0.0629	0.95	0.026344144	count	1
LSM5	0.0188147	0.0970018	0.194	0.846	0.026498839	count	1
SAP30BP	0.0196245	0.1509357	0.13	0.897	0.02650733	count	1
PRDX5	0.018586	0.0580708	0.3201	0.749	0.02652217	count	1
GPR157	0.0322297	0.4613333	0.0699	0.944	0.02692536	count	1
ZMAT2	0.0196842	0.1194692	0.1648	0.869	0.027056619	count	1
TMED1	0.0203182	0.2093603	0.097	0.923	0.027126547	count	1
PLAGL2	0.0251403	0.2925664	0.0859	0.932	0.02731354	count	1
GPER1	0.2494877	0.6337559	0.3937	0.694	0.027346639	count	1
SHCBP1	0.0367166	0.5476003	0.0671	0.947	0.027401148	count	1
RASA2	0.021606	0.1801652	0.1199	0.905	0.02748751	count	1
FAM192A	0.0206288	0.1387435	0.1487	0.882	0.027528769	count	1
GSTA4	0.2514173	0.676166	0.3718	0.71	0.027574255	count	1
TRAPPC6A	0.0203673	0.1329788	0.1532	0.878	0.02762219	count	1
TMEM109	0.0201561	0.1100428	0.1832	0.855	0.027632667	count	1
TRAPPC9	0.0296891	0.3739164	0.0794	0.937	0.0277114	count	1
EIF3I	0.0197081	0.0723649	0.2723	0.785	0.027888245	count	1
TBC1D20	0.0226382	0.2866565	0.079	0.937	0.02790294	count	1
SPPL3	0.0223688	0.2178905	0.1027	0.918	0.027953755	count	1
PPIL4	0.0203196	0.1120442	0.1814	0.856	0.027973686	count	1
EIF3A	0.0198018	0.0763303	0.2594	0.795	0.028029487	count	1
VAMP4	0.0223772	0.2611453	0.0857	0.932	0.02813343	count	1
GRINA	0.0201456	0.0973095	0.207	0.836	0.028152891	count	1
SLC7A6OS	0.0259628	0.2626656	0.0988	0.921	0.028209096	count	1
NDUFA10	0.0204123	0.0988262	0.2065	0.836	0.028324514	count	1

MTMR8	0.2586483	1.0014526	0.2583	0.796	0.028429435	count	1
AC091563.1	0.2586483	0.942174	0.2745	0.784	0.028429435	count	1
SPAG8	0.2586483	1.0014526	0.2583	0.796	0.028429435	count	1
DKK3	0.2586483	1.104727	0.2341	0.815	0.028429435	count	1
PRDM11	0.2586483	1.0014526	0.2583	0.796	0.028429435	count	1
CLEC6A	0.2586483	1.0014526	0.2583	0.796	0.028429435	count	1
AC135050.1	0.2586483	1.0014526	0.2583	0.796	0.028429435	count	1
MARVELD3	0.2586483	0.942174	0.2745	0.784	0.028429435	count	1
ZFP3	0.2586483	1.0014526	0.2583	0.796	0.028429435	count	1
AC008763.2	0.2586483	1.104727	0.2341	0.815	0.028429435	count	1
LRFN3	0.2586483	0.942174	0.2745	0.784	0.028429435	count	1
MED7	0.0236614	0.2896602	0.0817	0.935	0.028474548	count	1
HMGB1	0.0200341	0.037588	0.533	0.594	0.028810339	count	1
LINC01353	0.0459979	0.4968464	0.0926	0.926	0.028823897	count	1
RRAS	0.0206339	0.1052392	0.1961	0.845	0.028837648	count	1
OBSCN	0.1050536	0.8350864	0.1258	0.9	0.028843396	count	1
CD86	0.02034	0.0665966	0.3054	0.76	0.028936516	count	1
CEBPZOS	0.0218118	0.1914684	0.1139	0.909	0.029010111	count	1
HELZ	0.0218071	0.1563298	0.1395	0.889	0.029102068	count	1
HPGD	0.0391285	0.306734	0.1276	0.899	0.029212703	count	1
RCC2	0.022094	0.1725952	0.128	0.898	0.029277523	count	1
YBEY	0.0235581	0.237249	0.0993	0.921	0.029532392	count	1
SNW1	0.0211699	0.1010272	0.2095	0.834	0.029642567	count	1
CEP72	0.0502729	1.1858875	0.0424	0.966	0.029665466	count	1
HIST1H2AE	0.0502729	0.909805	0.0553	0.956	0.029665466	count	1
PLPP4	0.0502729	0.8139014	0.0618	0.951	0.029665466	count	1
ZNF256	0.0502729	1.5216874	0.033	0.974	0.029665466	count	1
SNX4	0.0228383	0.1931256	0.1183	0.906	0.030345236	count	1
SESN2	0.0248008	0.2106796	0.1177	0.906	0.030392409	count	1
CLEC2D	0.0330623	0.3766575	0.0878	0.93	0.03040608	count	1
HMGN3-AS1	0.0552914	0.6357135	0.087	0.931	0.030414592	count	1
NAA25	0.0294734	0.2641531	0.1116	0.911	0.030440233	count	1
BDH2	0.0262856	0.3342185	0.0786	0.937	0.030513481	count	1
KLHL24	0.0235611	0.1406116	0.1676	0.867	0.030640585	count	1
ZNF678	0.042647	0.426996	0.0999	0.92	0.030718552	count	1
ZMIZ1	0.0239524	0.21508	0.1114	0.911	0.03093209	count	1
AP003068.2	0.2797728	0.661931	0.4227	0.673	0.030947669	count	1
AL133245.1	0.2828022	0.6452812	0.4383	0.661	0.031311235	count	1
PYCARD-AS1	0.0308966	0.3848487	0.0803	0.936	0.031340257	count	1
RPA2	0.0236016	0.1575886	0.1498	0.881	0.031392076	count	1
RPL10	0.0220161	0.0216498	1.0169	0.309	0.031751058	count	1
MRPL42	0.0240696	0.1535228	0.1568	0.875	0.03178783	count	1
TCIRG1	0.0227522	0.0933167	0.2438	0.807	0.031919501	count	1
EIPR1	0.0328567	0.3750918	0.0876	0.93	0.031931293	count	1
VCPIP1	0.0271636	0.1874724	0.1449	0.885	0.032065296	count	1

PNKD	0.0230862	0.1020023	0.2263	0.821	0.032078792	count	1
RUNX1	0.0230404	0.1164261	0.1979	0.843	0.032184168	count	1
C17orf80	0.0304848	0.4413424	0.0691	0.945	0.032251284	count	1
PMVK	0.0236251	0.1271548	0.1858	0.853	0.032273844	count	1
SH3BGRL	0.0225499	0.038678	0.583	0.56	0.032356882	count	1
ALDOA	0.023748	0.1678275	0.1415	0.887	0.032357053	count	1
CFAP298	0.0248984	0.1823794	0.1365	0.891	0.032433557	count	1
SLFN5	0.0271542	0.2668905	0.1017	0.919	0.032515939	count	1
GPBP1L1	0.02495	0.1568173	0.1591	0.874	0.032551798	count	1
PPP3CC	0.0294687	0.3107874	0.0948	0.924	0.032588576	count	1
MECP2	0.0239065	0.1540097	0.1552	0.877	0.032643484	count	1
PUM3	0.0241351	0.1513945	0.1594	0.873	0.032645309	count	1
PPP1CA	0.0229967	0.0605851	0.3796	0.704	0.032698213	count	1
SRSF1	0.0250641	0.1571231	0.1595	0.873	0.032725828	count	1
DARS2	0.043817	0.4983386	0.0879	0.93	0.032738233	count	1
GATD1	0.0341115	0.3295883	0.1035	0.918	0.032744463	count	1
MYO1G	0.0243173	0.1538293	0.1581	0.874	0.032805178	count	1
ENSA	0.0231504	0.0663214	0.3491	0.727	0.032805874	count	1
TM7SF3	0.0269006	0.1855353	0.145	0.885	0.032969184	count	1
TMBIM4	0.0231305	0.0490061	0.472	0.637	0.033095156	count	1
WDR18	0.0251447	0.1964892	0.128	0.898	0.033188801	count	1
GMPS	0.0253143	0.1934968	0.1308	0.896	0.033510486	count	1
LYN	0.0236672	0.067685	0.3497	0.727	0.033636599	count	1
SPACA9	0.0569745	0.617187	0.0923	0.926	0.033665215	count	1
MFGE8	0.1699024	0.614658	0.2764	0.782	0.033835772	count	1
AL096865.1	0.0741486	0.7073423	0.1048	0.917	0.033962266	count	1
GTF3A	0.0238953	0.0703394	0.3397	0.734	0.0339733	count	1
CASP9	0.0293983	0.347313	0.0846	0.933	0.034008657	count	1
CAPZB	0.0238021	0.0459066	0.5185	0.604	0.034060866	count	1
LZIC	0.0251481	0.127113	0.1978	0.843	0.034204046	count	1
PRIM1	0.0334741	0.3956706	0.0846	0.933	0.03428148	count	1
RPL37A	0.0238895	0.0313775	0.7614	0.447	0.034333378	count	1
FBXL19	0.0476726	0.5988032	0.0796	0.937	0.034367845	count	1
RORA	0.1247	0.565333	0.2206	0.825	0.034420917	count	1
MUTYH	0.0387336	0.5273482	0.0734	0.941	0.034458392	count	1
TRAM1	0.0244283	0.0751746	0.325	0.745	0.034582177	count	1
RABL2B	0.0360891	0.4018686	0.0898	0.928	0.034650608	count	1
AP000692.2	0.0436441	0.7851174	0.0556	0.956	0.034699215	count	1
AC005332.4	0.0860064	0.7538602	0.1141	0.909	0.034762635	count	1
HNRNPC	0.0245914	0.0598559	0.4108	0.681	0.034950863	count	1
ZEB2	0.0244826	0.0606322	0.4038	0.686	0.035038049	count	1
PTGER4	0.0248769	0.1014121	0.2453	0.806	0.035058177	count	1
TRPM4	0.0356447	0.3488577	0.1022	0.919	0.035059891	count	1
TRIAP1	0.0273853	0.1953849	0.1402	0.889	0.035402489	count	1
AC079209.1	0.0511536	0.4434841	0.1153	0.908	0.035423233	count	1

RXYLT1	0.0301638	0.2594544	0.1163	0.907	0.035502323	count	1
SMCO4	0.0252963	0.0828685	0.3053	0.76	0.035714155	count	1
CTU2	0.0353274	0.3612329	0.0978	0.922	0.035850719	count	1
DAZAP1	0.0259422	0.1162928	0.2231	0.823	0.035859302	count	1
PTCD3	0.0341818	0.2922838	0.1169	0.907	0.035901342	count	1
STAG2	0.0258821	0.1053491	0.2457	0.806	0.035976506	count	1
DNAAF4	0.0358775	0.3831054	0.0936	0.925	0.036054601	count	1
ARPC3	0.0250562	0.0242568	1.033	0.302	0.036089402	count	1
RTRAF	0.0254385	0.0598458	0.4251	0.671	0.036096168	count	1
RPUSD2	0.0501512	0.5370413	0.0934	0.926	0.036169914	count	1
MS4A6A	0.0253048	0.0466744	0.5422	0.588	0.036369879	count	1
IMP3	0.0259341	0.0882789	0.2938	0.769	0.036575064	count	1
CAPZA1	0.0258399	0.0634373	0.4073	0.684	0.036643494	count	1
KPNA3	0.0277289	0.1443639	0.1921	0.848	0.036646042	count	1
OS9	0.0260708	0.0737287	0.3536	0.724	0.036695716	count	1
MCM9	0.0394504	0.3784136	0.1043	0.917	0.036865634	count	1
PRKAR2A	0.0274834	0.1519316	0.1809	0.856	0.036872862	count	1
EPS15L1	0.0361587	0.3179894	0.1137	0.909	0.037040635	count	1
PFKFB4	0.0444362	0.3658262	0.1215	0.903	0.037187796	count	1
BROX	0.0269666	0.106843	0.2524	0.801	0.037249419	count	1
ERV3-1	0.0293701	0.2362152	0.1243	0.901	0.037331427	count	1
RAMP1	0.0922591	0.644895	0.1431	0.886	0.037346486	count	1
PDCD1LG2	0.0539715	0.4148853	0.1301	0.897	0.037393158	count	1
LINC00623	0.030262	0.2401527	0.126	0.9	0.037518302	count	1
CC2D1A	0.0373298	0.4500101	0.083	0.934	0.037519685	count	1
LRRC28	0.0322273	0.3267481	0.0986	0.921	0.037688174	count	1
HSD17B12	0.0279125	0.1541498	0.1811	0.856	0.037781359	count	1
PLEC	0.0280551	0.1578502	0.1777	0.859	0.037824542	count	1
SLC35B2	0.0314287	0.2775641	0.1132	0.91	0.037838437	count	1
YTHDF2	0.0275987	0.122927	0.2245	0.822	0.037870221	count	1
IFT27	0.030793	0.2035227	0.1513	0.88	0.037897107	count	1
ZNF385A	0.0271779	0.1142396	0.2379	0.812	0.038006145	count	1
SF3B5	0.0267945	0.0631431	0.4243	0.671	0.038114383	count	1
THAP2	0.0319543	0.2589069	0.1234	0.902	0.038171609	count	1
PPRC1	0.0388071	0.4058406	0.0956	0.924	0.038183454	count	1
GNG7	0.0304057	0.2513766	0.121	0.904	0.03824058	count	1
SLC25A38	0.0342116	0.3695483	0.0926	0.926	0.038244195	count	1
ZNF77	0.13938	0.5631823	0.2475	0.805	0.03862601	count	1
AC007365.1	0.0516828	0.7320253	0.0706	0.944	0.038664917	count	1
TDRKH	0.0704017	0.5781614	0.1218	0.903	0.038849117	count	1
XYLT2	0.0704017	0.6042496	0.1165	0.907	0.038849117	count	1
SAMD4B	0.0300055	0.2235908	0.1342	0.893	0.038897825	count	1
RHOT2	0.031835	0.2203463	0.1445	0.885	0.03894643	count	1
EIF4H	0.0280825	0.0896627	0.3132	0.754	0.0389627	count	1
LINC01473	0.1943158	0.7360607	0.264	0.792	0.038968307	count	1

RBM3	0.0273216	0.0505801	0.5402	0.589	0.039012529	count	1
ZCCHC2	0.0294747	0.1572469	0.1874	0.851	0.039087264	count	1
BAZ1B	0.0307404	0.1686674	0.1823	0.855	0.039432596	count	1
FAM151B	0.049671	0.5973899	0.0831	0.934	0.039527137	count	1
TBCK	0.0716501	0.4571321	0.1567	0.875	0.039548304	count	1
PMPCA	0.0315071	0.2185882	0.1441	0.885	0.039627688	count	1
GSTM2	0.3522197	1.070735	0.329	0.742	0.039808988	count	1
ACTR3B	0.3522197	0.8681808	0.4057	0.685	0.039808988	count	1
USP20	0.3522197	0.8681808	0.4057	0.685	0.039808988	count	1
PCCA	0.0426006	0.2982335	0.1428	0.886	0.039824425	count	1
FTX	0.0314203	0.2258973	0.1391	0.889	0.039940682	count	1
CASP7	0.0379365	0.2334931	0.1625	0.871	0.040161881	count	1
GOLPH3	0.0310096	0.1843799	0.1682	0.866	0.040200887	count	1
ATXN1	0.0301216	0.1544297	0.1951	0.845	0.040224935	count	1
PHACTR1	0.0281924	0.0745165	0.3783	0.705	0.040297761	count	1
ARPC4	0.028309	0.06102	0.4639	0.643	0.04029915	count	1
TYW5	0.0471363	0.4537186	0.1039	0.917	0.040353973	count	1
TYROBP	0.0281037	0.0212268	1.324	0.186	0.040517195	count	1
ZNF544	0.0733808	0.5490549	0.1336	0.894	0.040518195	count	1
TMEM206	0.0324641	0.265207	0.1224	0.903	0.040523894	count	1
TTL	0.0337285	0.2861719	0.1179	0.906	0.040811027	count	1
A2M-AS1	0.0528241	0.5091471	0.1038	0.917	0.040836458	count	1
TRIO	0.0331114	0.2486932	0.1331	0.894	0.040909116	count	1
IAH1	0.0292938	0.088935	0.3294	0.742	0.041082008	count	1
DUSP11	0.0331405	0.2222756	0.1491	0.881	0.041302454	count	1
PSMC6	0.0299605	0.1098857	0.2727	0.785	0.041344024	count	1
BLOC1S5	0.0368718	0.3071383	0.12	0.904	0.041428969	count	1
RUVBL2	0.0338758	0.2925237	0.1158	0.908	0.041447477	count	1
DPM2	0.0306971	0.1551797	0.1978	0.843	0.041591049	count	1
C11orf54	0.0354069	0.2437108	0.1453	0.884	0.041687035	count	1
AC008074.3	0.0498114	0.4004772	0.1244	0.901	0.041718081	count	1
TOR1B	0.0354655	0.3076304	0.1153	0.908	0.041756181	count	1
ATP5S	0.0308291	0.1561445	0.1974	0.843	0.041855328	count	1
RUSC1-AS1	0.1034483	0.7161855	0.1444	0.885	0.041989271	count	1
JAGN1	0.0318489	0.1660128	0.1918	0.848	0.042095908	count	1
GAS6-AS1	0.0502729	0.577303	0.0871	0.931	0.042107354	count	1
AC009005.1	0.0828496	0.5357787	0.1546	0.877	0.042126872	count	1
AC090114.2	0.0492047	0.4673225	0.1053	0.916	0.042136752	count	1
RPL8	0.0292809	0.0251006	1.1665	0.244	0.042204927	count	1
THTPA	0.0399892	0.2815676	0.142	0.887	0.042342838	count	1
NEK3	0.0361206	0.2265747	0.1594	0.873	0.042392468	count	1
SCAND1	0.0297794	0.0661644	0.4501	0.653	0.042443133	count	1
LRIF1	0.032041	0.2699204	0.1187	0.906	0.0424935	count	1
CHIT1	0.0926328	0.8028294	0.1154	0.908	0.042609221	count	1
ST3GAL6-AS1	0.0926328	1.1409191	0.0812	0.935	0.042609221	count	1

POLN	0.0926328	1.1409191	0.0812	0.935	0.042609221	count	1
MND1	0.0926328	0.8028294	0.1154	0.908	0.042609221	count	1
CCNF	0.0926328	0.8028294	0.1154	0.908	0.042609221	count	1
ANKEF1	0.0926328	0.8028294	0.1154	0.908	0.042609221	count	1
RHOG	0.0299141	0.0510107	0.5864	0.558	0.04266382	count	1
CALCOCO1	0.0339234	0.2424953	0.1399	0.889	0.042732073	count	1
ZSWIM4	0.1236455	0.6700241	0.1845	0.854	0.042764709	count	1
ZNF503	0.0318377	0.1635031	0.1947	0.846	0.042819032	count	1
THYN1	0.0315263	0.1339323	0.2354	0.814	0.042861499	count	1
AL139260.1	0.0617861	0.5163915	0.1196	0.905	0.04286618	count	1
MAP3K10	0.0617861	0.5768871	0.1071	0.915	0.04286618	count	1
SDHAF2	0.0322074	0.1748696	0.1842	0.854	0.04286967	count	1
DIS3	0.0331484	0.1596088	0.2077	0.835	0.042900524	count	1
H3F3A	0.0297761	0.0251779	1.1826	0.237	0.042905549	count	1
PPP1R7	0.0308147	0.087559	0.3519	0.725	0.04307085	count	1
VHL	0.0340361	0.1963045	0.1734	0.862	0.043216907	count	1
PNMA8B	0.3797529	0.7542869	0.5035	0.615	0.04326768	count	1
TMEM41A	0.0385476	0.264613	0.1457	0.884	0.043317332	count	1
TMEM203	0.0349998	0.257009	0.1362	0.892	0.04332493	count	1
PPCS	0.0308619	0.0904452	0.3412	0.733	0.043370336	count	1
SH2B1	0.0445907	0.3468664	0.1286	0.898	0.043391118	count	1
YIPF2	0.03419	0.2158618	0.1584	0.874	0.043519061	count	1
CHTF8	0.0343809	0.2388926	0.1439	0.886	0.043600309	count	1
COPS9	0.030864	0.0719829	0.4288	0.668	0.043662617	count	1
IRF2BPL	0.0349444	0.2667617	0.131	0.896	0.043694144	count	1
FOXD2	0.216874	0.8013055	0.2707	0.787	0.043771031	count	1
TAF1A-AS1	0.216874	0.8919104	0.2432	0.808	0.043771031	count	1
FZD7	0.216874	0.8046245	0.2695	0.788	0.043771031	count	1
KLHL2	0.0389696	0.2812073	0.1386	0.89	0.043792933	count	1
AL590617.2	0.0500974	0.4148241	0.1208	0.904	0.043796268	count	1
DDX49	0.03457	0.2040259	0.1694	0.865	0.043840457	count	1
ZCCHC9	0.0333563	0.204054	0.1635	0.87	0.043843417	count	1
UBAP2	0.0350389	0.2208912	0.1586	0.874	0.043880503	count	1
SIMC1	0.0698662	0.4843824	0.1442	0.885	0.043980917	count	1
CALCR	0.1580818	0.7184522	0.22	0.826	0.044029594	count	1
SEC61A1	0.0331685	0.1877225	0.1767	0.86	0.044083185	count	1
PRDX3	0.0310986	0.0643463	0.4833	0.629	0.044106136	count	1
DNAJC19	0.0323376	0.1278281	0.253	0.8	0.044131756	count	1
NKRF	0.0392989	0.3720545	0.1056	0.916	0.04416408	count	1
YTHDC1	0.0318275	0.1149548	0.2769	0.782	0.044171011	count	1
ZSWIM7	0.0321212	0.1095103	0.2933	0.769	0.044440709	count	1
GOLGA4	0.0321317	0.0942359	0.341	0.733	0.044562077	count	1
TATDN1	0.0336446	0.1676985	0.2006	0.841	0.044693368	count	1
AP000547.3	0.0561738	0.4595268	0.1222	0.903	0.044746019	count	1
TTLL12	0.1295615	0.4877671	0.2656	0.791	0.044878184	count	1

AFF1	0.0343241	0.1744968	0.1967	0.844	0.044961876	count	1
GPCPD1	0.031715	0.0922384	0.3438	0.731	0.045014063	count	1
UBE2H	0.0329296	0.1513614	0.2176	0.828	0.045022281	count	1
PHF10	0.0342783	0.1928409	0.1778	0.859	0.045056543	count	1
SRGAP3	0.0403373	0.2581227	0.1563	0.876	0.045113026	count	1
WRAP53	0.0453353	0.4581719	0.0989	0.921	0.04513224	count	1
ZNF707	0.1621478	0.731904	0.2215	0.825	0.045211264	count	1
N6AMT1	0.062765	0.485969	0.1292	0.897	0.045363797	count	1
SLC19A1	0.0473042	0.314887	0.1502	0.881	0.045476422	count	1
PLPP3	0.0377914	0.298999	0.1264	0.899	0.045515024	count	1
POM121	0.0369125	0.2389533	0.1545	0.877	0.045528988	count	1
WDR13	0.0351237	0.1943958	0.1807	0.857	0.045540853	count	1
PADI2	0.0686603	0.4801784	0.143	0.886	0.045545956	count	1
ZNF566	0.0495045	0.7732454	0.064	0.949	0.045619349	count	1
COMMD1	0.032706	0.1043255	0.3135	0.754	0.045664642	count	1
LMNA	0.031964	0.0779856	0.4099	0.682	0.045791099	count	1
ZNF248	0.0830208	0.4909069	0.1691	0.866	0.045932961	count	1
KXD1	0.0330523	0.120208	0.275	0.783	0.045937542	count	1
HPCAL1	0.0330584	0.1290878	0.2561	0.798	0.045950903	count	1
CLN5	0.0345813	0.1624582	0.2129	0.831	0.045962626	count	1
NET1	0.0507478	0.2746302	0.1848	0.853	0.046016792	count	1
SPEN	0.0340286	0.1329548	0.2559	0.798	0.046162518	count	1
PARP12	0.0400036	0.2350243	0.1702	0.865	0.0463101	count	1
ITGA4	0.0331704	0.0912272	0.3636	0.716	0.046320834	count	1
ZNF600	0.2289966	0.532762	0.4298	0.667	0.046375816	count	1
UBB	0.032282	0.0367694	0.878	0.38	0.046376446	count	1
CDC23	0.0472108	0.3218019	0.1467	0.883	0.046494137	count	1
GHITM	0.0328565	0.0633546	0.5186	0.604	0.046539542	count	1
LY6E	0.0326038	0.0843823	0.3864	0.699	0.046549737	count	1
SAT2	0.0328049	0.0732993	0.4475	0.655	0.046599125	count	1
ECHS1	0.0333616	0.0970931	0.3436	0.731	0.046964976	count	1
TP53TG1	0.0365352	0.2042268	0.1789	0.858	0.046973308	count	1
UBE2I	0.033119	0.0751073	0.441	0.659	0.047016619	count	1
LSP1	0.0328657	0.0616518	0.5331	0.594	0.047111431	count	1
FASTKD2	0.0411897	0.3440325	0.1197	0.905	0.047128463	count	1
AGAP2	0.0748607	0.5465504	0.137	0.891	0.047169658	count	1
NDST2	0.0484545	0.4026754	0.1203	0.904	0.047171029	count	1
DESI2	0.0352509	0.1549792	0.2275	0.82	0.047706836	count	1
TRIM68	0.2352118	0.9899699	0.2376	0.812	0.047717724	count	1
AC091814.1	0.2352118	0.9899699	0.2376	0.812	0.047717724	count	1
PWAR6	0.2352118	0.977298	0.2407	0.81	0.047717724	count	1
FSIP1	0.2352118	0.977298	0.2407	0.81	0.047717724	count	1
NT5M	0.2352118	0.9875452	0.2382	0.812	0.047717724	count	1
ADORA2A-AS1	0.2352118	0.9875452	0.2382	0.812	0.047717724	count	1
APOBEC3H	0.2352118	0.9899699	0.2376	0.812	0.047717724	count	1

NT5C3B	0.0438685	0.2777264	0.158	0.875	0.047735461	count	1
SPAG1	0.0637509	0.4861401	0.1311	0.896	0.047786931	count	1
UNG	0.0637509	0.4713992	0.1352	0.892	0.047786931	count	1
CRHBP	0.0557707	0.3308054	0.1686	0.866	0.047802655	count	1
MYO15B	0.0557707	0.3968567	0.1405	0.888	0.047802655	count	1
ZNF319	0.0637776	1.0224862	0.0624	0.95	0.04780715	count	1
HCLS1	0.0336695	0.0556945	0.6045	0.546	0.047872136	count	1
EXD2	0.086793	0.6814946	0.1274	0.899	0.048057535	count	1
XRCC6	0.03406	0.0749747	0.4543	0.65	0.048093502	count	1
RABL3	0.0461689	0.382926	0.1206	0.904	0.04816089	count	1
RRN3	0.0522816	0.3021758	0.173	0.863	0.048194827	count	1
APH1A	0.0344763	0.0863744	0.3991	0.69	0.048201644	count	1
SURF6	0.0411116	0.3281693	0.1253	0.9	0.048265292	count	1
RNF217	0.0403427	0.2556187	0.1578	0.875	0.048345291	count	1
MFAP3	0.1050536	0.9415496	0.1116	0.911	0.04845976	count	1
NRARP	0.0362728	0.2618026	0.1386	0.89	0.048468799	count	1
AC009065.4	0.0819223	0.7541799	0.1086	0.914	0.04864727	count	1
TSKS	0.0819223	0.7092161	0.1155	0.908	0.04864727	count	1
CCDC51	0.0583147	0.4833984	0.1206	0.904	0.048898617	count	1
LINC00294	0.1060709	0.6021006	0.1762	0.86	0.048940357	count	1
ZNF593	0.0356359	0.1442322	0.2471	0.805	0.048995309	count	1
LINC00893	0.0777384	0.934018	0.0832	0.934	0.049009601	count	1
AGFG1	0.0353273	0.1074075	0.3289	0.742	0.049442313	count	1
DDIT3	0.0363789	0.138358	0.2629	0.793	0.049578663	count	1
TOP1	0.034864	0.0669006	0.5211	0.602	0.049581887	count	1
NMRAL1	0.0393328	0.2090352	0.1882	0.851	0.049826165	count	1
PIGBOS1	0.0379942	0.1980308	0.1919	0.848	0.049845063	count	1
HRH2	0.0367033	0.1336066	0.2747	0.784	0.049918275	count	1
PNPT1	0.0408849	0.2141574	0.1909	0.849	0.050041228	count	1
ADI1	0.0361699	0.0927189	0.3901	0.696	0.050429529	count	1
GTPBP6	0.0387766	0.1484056	0.2613	0.794	0.050453573	count	1
CCNC	0.0383897	0.1716157	0.2237	0.823	0.050466945	count	1
GCLC	0.0481045	0.2896054	0.1661	0.868	0.050589184	count	1
GLMP	0.0374713	0.1513307	0.2476	0.804	0.050698249	count	1
PTPRM	0.1243402	0.4931286	0.2521	0.801	0.050723008	count	1
KCMF1	0.0380085	0.1430718	0.2657	0.791	0.050767322	count	1
DUSP12	0.0423929	0.242541	0.1748	0.861	0.050808141	count	1
AL138966.2	0.1461253	0.8552665	0.1709	0.864	0.050827982	count	1
BIVM	0.1461253	0.7409142	0.1972	0.844	0.050827982	count	1
AC116913.1	0.1461253	0.9173933	0.1593	0.873	0.050827982	count	1
NR1H2	0.0368441	0.1157223	0.3184	0.75	0.050878452	count	1
VAPA	0.0356567	0.0508622	0.701	0.4833	0.050948862	count	1
PDCL	0.0408913	0.182385	0.2242	0.823	0.051062913	count	1
CLIC4	0.0395073	0.2198472	0.1797	0.857	0.051095056	count	1
UFL1	0.0380826	0.1349118	0.2823	0.778	0.051244383	count	1

UBFD1	0.0565203	0.3307792	0.1709	0.864	0.051288229	count	1
SACS	0.0527167	0.3729833	0.1413	0.888	0.051344341	count	1
MRPL23	0.0367532	0.1016539	0.3616	0.718	0.051350632	count	1
ACTN4	0.0372827	0.1229049	0.3033	0.762	0.051751425	count	1
TOP2A	0.1017308	0.8093468	0.1257	0.9	0.05193991	count	1
KCNK7	0.1272842	0.9468374	0.1344	0.893	0.051960508	count	1
BTBD11	0.1272842	0.8407863	0.1514	0.88	0.051960508	count	1
AC004943.2	0.1272842	0.8407863	0.1514	0.88	0.051960508	count	1
AC006157.1	0.1272842	1.5945048	0.0798	0.936	0.051960508	count	1
RARS2	0.0439387	0.2564176	0.1714	0.864	0.052078067	count	1
PRR15	0.4482087	1.211345	0.37	0.711	0.052082244	count	1
AMER1	0.4482087	1.085943	0.4127	0.68	0.052082244	count	1
LINC02099	0.4482087	1.085943	0.4127	0.68	0.052082244	count	1
FAM84B	0.4482087	1.085943	0.4127	0.68	0.052082244	count	1
ZFP41	0.4482087	1.085943	0.4127	0.68	0.052082244	count	1
AC023355.1	0.4482087	1.211345	0.37	0.711	0.052082244	count	1
GP1BA	0.4482087	1.0116915	0.443	0.658	0.052082244	count	1
AC010761.2	0.4482087	1.079644	0.4151	0.678	0.052082244	count	1
AC015819.1	0.4482087	1.085943	0.4127	0.68	0.052082244	count	1
ARHGAP33	0.4482087	1.079644	0.4151	0.678	0.052082244	count	1
CCDC13	0.0826486	0.7546693	0.1095	0.913	0.052153584	count	1
MFHAS1	0.0468751	0.286598	0.1636	0.87	0.052184757	count	1
NEMP2	0.0721097	0.5041355	0.143	0.886	0.052199582	count	1
SYTL3	0.0422758	0.2237907	0.1889	0.85	0.052254978	count	1
SLC39A7	0.0410364	0.1732095	0.2369	0.813	0.05248962	count	1
HCST	0.0367236	0.0464835	0.79	0.43	0.052733484	count	1
CDAN1	0.2586483	0.7449146	0.3472	0.728	0.052817098	count	1
PIK3AP1	0.0388094	0.1372299	0.2828	0.777	0.052880279	count	1
SNX33	0.0761419	0.5103072	0.1492	0.881	0.052958585	count	1
PCIF1	0.0424095	0.2205155	0.1923	0.848	0.053046269	count	1
RFESD	0.1537275	0.7033645	0.2186	0.827	0.053574673	count	1
PSMD13	0.0386725	0.1028271	0.3761	0.707	0.053735198	count	1
IFI16	0.0377446	0.0568634	0.6638	0.507	0.053742799	count	1
PFN1	0.0374057	0.0272845	1.371	0.171	0.0538946	count	1
SINHCAF	0.0398428	0.1506785	0.2644	0.791	0.053959837	count	1
CUTA	0.038169	0.0682182	0.5595	0.576	0.054011942	count	1
COG1	0.0569075	0.3527631	0.1613	0.872	0.054048081	count	1
TMEM170A	0.0404924	0.1471983	0.2751	0.783	0.054063656	count	1
ISCA1	0.0392574	0.1362221	0.2882	0.773	0.054139223	count	1
FARS2	0.0511536	0.3071715	0.1665	0.868	0.054218667	count	1
ECH1	0.0382492	0.0662403	0.5774	0.564	0.054449681	count	1
CRTAM	0.0497184	0.2973941	0.1672	0.867	0.054453161	count	1
PDCD6IP	0.0394568	0.1219299	0.3236	0.746	0.054456056	count	1
SCRG1	0.0785694	0.3993153	0.1968	0.844	0.054670032	count	1
RPL10A	0.0380212	0.0312563	1.2164	0.224	0.054722708	count	1

PHTF1	0.0480362	0.2837682	0.1693	0.866	0.054757577	count	1
USP42	0.0577766	0.3563648	0.1621	0.871	0.054878998	count	1
AC106707.1	0.0654851	0.3237103	0.2023	0.84	0.054966715	count	1
SQOR	0.0393498	0.0875228	0.4496	0.653	0.05507811	count	1
HMGN2	0.0385588	0.0590326	0.6532	0.5137	0.055117468	count	1
ZNF813	0.1584011	0.6296061	0.2516	0.801	0.055268222	count	1
CENPT	0.0529637	0.2863088	0.185	0.853	0.055283914	count	1
AP001189.5	0.4725612	0.7909152	0.5975	0.55	0.055291322	count	1
RRBP1	0.0392994	0.0832781	0.4719	0.637	0.055487845	count	1
HSPBP1	0.0468306	0.2655165	0.1764	0.86	0.055515333	count	1
CAMK1	0.0403603	0.1102167	0.3662	0.714	0.055589849	count	1
PTP4A2	0.039169	0.0677075	0.5785	0.563	0.055680124	count	1
SLC8A1	0.0397455	0.0940756	0.4225	0.673	0.055692397	count	1
TMOD3	0.0395932	0.092536	0.4279	0.669	0.055724876	count	1
MUL1	0.0614228	0.3838675	0.16	0.873	0.05577103	count	1
GID8	0.0415668	0.1441368	0.2884	0.773	0.05615344	count	1
IK	0.0398317	0.0778702	0.5115	0.609	0.056191783	count	1
CEP41	0.0669842	0.488917	0.137	0.891	0.05623687	count	1
AMDHD1	0.1012677	0.6965495	0.1454	0.884	0.056239799	count	1
L3MBTL1	0.1012677	1.2869119	0.0787	0.937	0.056239799	count	1
BLOC1S2	0.0402117	0.1151322	0.3493	0.727	0.056285003	count	1
EIF4E2	0.0399263	0.0800237	0.4989	0.618	0.056350757	count	1
SLAIN2	0.0408683	0.1352466	0.3022	0.763	0.056379574	count	1
ZNF350	0.0481624	0.3950849	0.1219	0.903	0.056380884	count	1
ORAI1	0.0417796	0.1478143	0.2826	0.777	0.056441173	count	1
NCK1-DT	0.0456089	0.2545309	0.1792	0.858	0.056486245	count	1
ALKBH2	0.0484304	0.335784	0.1442	0.885	0.05669558	count	1
AAMDC	0.0431348	0.1706475	0.2528	0.8	0.056859016	count	1
SELENBP1	0.4853224	0.8120123	0.5977	0.55	0.056988182	count	1
AC008914.1	0.4853224	0.8120123	0.5977	0.55	0.056988182	count	1
FP565260.6	0.4853224	0.7463248	0.6503	0.516	0.056988182	count	1
ERI3	0.0456666	0.2122196	0.2152	0.83	0.057038403	count	1
DR1	0.0418631	0.1461687	0.2864	0.775	0.057194231	count	1
RHOBTB1	0.0698183	0.3121243	0.2237	0.823	0.057233008	count	1
MAMDC4	0.1238797	1.0707655	0.1157	0.908	0.057388407	count	1
AC079174.2	0.1238797	1.2483789	0.0992	0.921	0.057388407	count	1
PTPN21	0.1238797	1.6715811	0.0741	0.941	0.057388407	count	1
STX1B	0.1238797	0.8696794	0.1424	0.887	0.057388407	count	1
RPP14	0.0584668	0.4701522	0.1244	0.901	0.057648574	count	1
PAF1	0.0450111	0.1867013	0.2411	0.81	0.057708097	count	1
UNC5B	0.0970351	0.4872094	0.1992	0.842	0.057793326	count	1
CAPRIN2	0.0724355	0.3718746	0.1948	0.846	0.057840925	count	1
PRKAG2-AS1	0.042794	0.1661828	0.2575	0.797	0.057924426	count	1
MTREX	0.0456623	0.2485588	0.1837	0.854	0.057933545	count	1
TCTE3	0.0772454	0.7021497	0.11	0.912	0.058028409	count	1

CREB5	0.0435325	0.1358829	0.3204	0.749	0.058100229	count	1
AL022069.1	0.1418817	0.7687729	0.1846	0.854	0.058121155	count	1
AC011944.1	0.2063004	0.8732296	0.2362	0.813	0.058200237	count	1
UBAP2L	0.0465079	0.2017829	0.2305	0.818	0.058447974	count	1
FAHD2A	0.0555397	0.391841	0.1417	0.887	0.058448062	count	1
HMBS	0.0544011	0.2668578	0.2039	0.838	0.058493602	count	1
SNX9	0.0429252	0.122184	0.3513	0.725	0.058578335	count	1
GDPD5	0.1264034	0.5913577	0.2138	0.831	0.05859089	count	1
SGK494	0.0756871	0.4733268	0.1599	0.873	0.058720113	count	1
ADCK2	0.0462385	0.2047856	0.2258	0.821	0.058812153	count	1
AL034417.3	0.2858546	1.2553215	0.2277	0.82	0.058814282	count	1
RIMKLA	0.2858546	1.2388281	0.2307	0.818	0.058814282	count	1
AL356356.1	0.2858546	1.2553215	0.2277	0.82	0.058814282	count	1
TRIM46	0.2858546	1.2388281	0.2307	0.818	0.058814282	count	1
AC013271.1	0.2858546	1.2388281	0.2307	0.818	0.058814282	count	1
NPHP1	0.2858546	1.2388281	0.2307	0.818	0.058814282	count	1
COL8A1	0.2858546	1.2388281	0.2307	0.818	0.058814282	count	1
AC016596.1	0.2858546	1.2553215	0.2277	0.82	0.058814282	count	1
AC010245.2	0.2858546	1.2388281	0.2307	0.818	0.058814282	count	1
NRN1	0.2858546	1.2388281	0.2307	0.818	0.058814282	count	1
AL354719.2	0.2858546	1.2553215	0.2277	0.82	0.058814282	count	1
SRSF12	0.2858546	1.2553215	0.2277	0.82	0.058814282	count	1
Z97989.1	0.2858546	1.2388281	0.2307	0.818	0.058814282	count	1
THSD7A	0.2858546	1.2388281	0.2307	0.818	0.058814282	count	1
SLC12A9-AS1	0.2858546	1.2553215	0.2277	0.82	0.058814282	count	1
VGF	0.2858546	1.2553215	0.2277	0.82	0.058814282	count	1
RASA4B	0.2858546	1.2388281	0.2307	0.818	0.058814282	count	1
AC246817.2	0.2858546	1.2388281	0.2307	0.818	0.058814282	count	1
AC138696.2	0.2858546	1.2388281	0.2307	0.818	0.058814282	count	1
ZNF252P-AS1	0.2858546	1.6004755	0.1786	0.858	0.058814282	count	1
GLIDR	0.2858546	1.2388281	0.2307	0.818	0.058814282	count	1
TNC	0.2858546	1.2388281	0.2307	0.818	0.058814282	count	1
RNF208	0.2858546	1.2388281	0.2307	0.818	0.058814282	count	1
AP003774.1	0.2858546	1.2388281	0.2307	0.818	0.058814282	count	1
AP002761.1	0.2858546	1.2388281	0.2307	0.818	0.058814282	count	1
SYTL2	0.2858546	1.2388281	0.2307	0.818	0.058814282	count	1
MMP3	0.2858546	1.2553215	0.2277	0.82	0.058814282	count	1
MCM10	0.2858546	1.2553215	0.2277	0.82	0.058814282	count	1
AL512598.1	0.2858546	1.2388281	0.2307	0.818	0.058814282	count	1
BICC1	0.2858546	1.2388281	0.2307	0.818	0.058814282	count	1
AC092794.1	0.2858546	1.2388281	0.2307	0.818	0.058814282	count	1
AC009318.1	0.2858546	1.2553215	0.2277	0.82	0.058814282	count	1
AC073610.3	0.2858546	1.2388281	0.2307	0.818	0.058814282	count	1
PMEL	0.2858546	1.2388281	0.2307	0.818	0.058814282	count	1
AC089984.1	0.2858546	1.2553215	0.2277	0.82	0.058814282	count	1

WDR66	0.2858546	1.2388281	0.2307	0.818	0.058814282	count	1
AL136295.2	0.2858546	1.2388281	0.2307	0.818	0.058814282	count	1
POLE2	0.2858546	1.2388281	0.2307	0.818	0.058814282	count	1
AL355916.1	0.2858546	1.2388281	0.2307	0.818	0.058814282	count	1
AC090515.2	0.2858546	1.2388281	0.2307	0.818	0.058814282	count	1
AC068338.3	0.2858546	1.2388281	0.2307	0.818	0.058814282	count	1
ZNF774	0.2858546	1.2388281	0.2307	0.818	0.058814282	count	1
ADGRG3	0.2858546	1.2388281	0.2307	0.818	0.058814282	count	1
ZNF19	0.2858546	1.2388281	0.2307	0.818	0.058814282	count	1
AC092718.1	0.2858546	1.6004755	0.1786	0.858	0.058814282	count	1
TIAF1	0.2858546	1.2388281	0.2307	0.818	0.058814282	count	1
C17orf53	0.2858546	1.2388281	0.2307	0.818	0.058814282	count	1
CA4	0.2858546	1.6004755	0.1786	0.858	0.058814282	count	1
AC132938.3	0.2858546	1.6004755	0.1786	0.858	0.058814282	count	1
AL121894.1	0.2858546	1.2388281	0.2307	0.818	0.058814282	count	1
SLC25A23	0.2858546	1.2388281	0.2307	0.818	0.058814282	count	1
AC011445.2	0.2858546	1.2553215	0.2277	0.82	0.058814282	count	1
CLTCL1	0.2858546	1.2388281	0.2307	0.818	0.058814282	count	1
AF127936.1	0.2858546	1.2388281	0.2307	0.818	0.058814282	count	1
AP001505.1	0.2858546	1.2388281	0.2307	0.818	0.058814282	count	1
AL354822.1	0.2858546	1.2388281	0.2307	0.818	0.058814282	count	1
COA3	0.0420848	0.1061624	0.3964	0.692	0.0588169	count	1
SPAG7	0.0425071	0.100226	0.4241	0.672	0.058909243	count	1
AC099518.5	0.0538724	0.3530666	0.1526	0.879	0.059022619	count	1
CYB5R4	0.0424262	0.1080707	0.3926	0.695	0.059107921	count	1
CTCF	0.0446053	0.1612508	0.2766	0.782	0.059112134	count	1
BMPR2	0.056734	0.2762936	0.2053	0.837	0.059240145	count	1
LTBP4	0.0765599	0.3490511	0.2193	0.826	0.059405287	count	1
DBNL	0.0424893	0.0897594	0.4734	0.636	0.059542529	count	1
CHMP1B	0.0415215	0.0782116	0.5309	0.596	0.059573735	count	1
KMT5C	0.0671271	0.4953763	0.1355	0.892	0.059937245	count	1
YIPF3	0.0433268	0.111218	0.3896	0.697	0.060096863	count	1
AC092164.1	0.1008545	0.834058	0.1209	0.904	0.060113102	count	1
FAM219A	0.0624985	0.5020116	0.1245	0.901	0.060186177	count	1
SLC25A5	0.0420279	0.0424657	0.9897	0.322	0.060299091	count	1
IGF1	0.044683	0.1589551	0.2811	0.779	0.060502457	count	1
TRAK1	0.0518889	0.2506594	0.207	0.836	0.060550253	count	1
APOPT1	0.0450368	0.157259	0.2864	0.775	0.06063396	count	1
ZC3H10	0.0723324	0.4543641	0.1592	0.874	0.060772524	count	1
FASN	0.0761415	0.495645	0.1536	0.878	0.060833944	count	1
PPIE	0.0472751	0.1651519	0.2863	0.775	0.06098595	count	1
TOMM22	0.0435581	0.0887548	0.4908	0.624	0.061145273	count	1
CRK	0.046202	0.1523395	0.3033	0.762	0.061230802	count	1
AMOTL1	0.1499405	0.6755973	0.2219	0.824	0.061539767	count	1
PLXDC1	0.0689353	0.4089017	0.1686	0.866	0.061565996	count	1

ZNF287	0.2179409	0.9143607	0.2384	0.812	0.061672455	count	1
CCR10	0.2179409	0.9143607	0.2384	0.812	0.061672455	count	1
PRR34	0.2179409	0.9143607	0.2384	0.812	0.061672455	count	1
SBF1	0.0679323	0.4046486	0.1679	0.867	0.061731514	count	1
TSTA3	0.046953	0.1576523	0.2978	0.766	0.061859991	count	1
AC007613.1	0.0803045	0.4745272	0.1692	0.866	0.062346941	count	1
MPZL1	0.0507967	0.2152944	0.2359	0.813	0.062577726	count	1
DNAJC25	0.0572637	0.3423907	0.1672	0.867	0.062755324	count	1
RGL2	0.0527936	0.1902737	0.2775	0.781	0.062789781	count	1
AC104794.2	0.0584074	0.2801711	0.2085	0.835	0.062822813	count	1
ARHGAP25	0.0503237	0.2286553	0.2201	0.826	0.062967733	count	1
TFEC	0.0460194	0.1188163	0.3873	0.699	0.063066546	count	1
SNHG9	0.0489076	0.2629765	0.186	0.852	0.063095575	count	1
SPART	0.0472119	0.1452596	0.325	0.745	0.063213792	count	1
DCAF5	0.0506957	0.2099008	0.2415	0.809	0.063334215	count	1
RUFY1	0.0477031	0.1376033	0.3467	0.729	0.063359311	count	1
DNM2	0.0488252	0.1372298	0.3558	0.722	0.063390241	count	1
MUS81	0.0573238	0.2608311	0.2198	0.826	0.06353176	count	1
ACVR2A	0.0844974	0.398164	0.2122	0.832	0.0635501	count	1
GATD3B	0.1144438	0.7048654	0.1624	0.871	0.063728952	count	1
PHF11	0.0468112	0.1257771	0.3722	0.71	0.063839939	count	1
PPP2R5B	0.0570455	0.4995736	0.1142	0.909	0.06388004	count	1
MUT	0.0641257	0.3715045	0.1726	0.863	0.06396354	count	1
LCP1	0.0446304	0.0422356	1.0567	0.291	0.06399194	count	1
VRK1	0.0544891	0.2579326	0.2113	0.833	0.064024194	count	1
TM9SF2	0.0458773	0.0967023	0.4744	0.635	0.064149101	count	1
CD19	0.1382858	0.7998261	0.1729	0.863	0.064270178	count	1
TOMM7	0.0450381	0.0452383	0.9956	0.32	0.06432003	count	1
C17orf51	0.0966457	0.5406286	0.1788	0.858	0.064435129	count	1
ZNF583	0.0786631	0.4959414	0.1586	0.874	0.064565948	count	1
LINC02470	0.0926328	0.734747	0.1261	0.9	0.064612588	count	1
TOMM20	0.0453771	0.0547071	0.8295	0.407	0.064619299	count	1
TP53I13	0.0480939	0.1575175	0.3053	0.76	0.064632394	count	1
BCL3	0.0471392	0.1315334	0.3584	0.72	0.064653964	count	1
STK11IP	0.0725307	0.586268	0.1237	0.902	0.064806751	count	1
CCR1	0.0468702	0.119483	0.3923	0.695	0.06517392	count	1
CCDC174	0.0489831	0.1843702	0.2657	0.791	0.065322355	count	1
IMMP1L	0.0616002	0.3458178	0.1781	0.859	0.065351986	count	1
GABARAPL1	0.0463108	0.0843855	0.5488	0.583	0.065409055	count	1
AC109587.1	0.3157495	0.7642442	0.4132	0.68	0.065499718	count	1
TEN1	0.0521253	0.2128261	0.2449	0.807	0.065523477	count	1
SCAMP2	0.0463271	0.0698932	0.6628	0.508	0.065529039	count	1
MAP2K6	0.0942484	0.3581792	0.2631	0.792	0.06575778	count	1
ABCD3	0.0646409	0.2911826	0.222	0.824	0.065790515	count	1
NDUFS3	0.0474932	0.1127921	0.4211	0.674	0.065847867	count	1

ERCC6	0.057982	0.299041	0.1939	0.846	0.065854206	count	1
MT-CO2	0.045704	0.0268105	1.7047	0.0884	0.065892341	count	1
TRNP1	0.2321488	0.641994	0.3616	0.718	0.065937375	count	1
TSSC4	0.0490529	0.1461629	0.3356	0.737	0.066022534	count	1
LRRC45	0.0878434	0.4628119	0.1898	0.849	0.066101935	count	1
CLEC14A	0.0850962	1.1679758	0.0729	0.942	0.066115978	count	1
MRPS15	0.0477153	0.1080758	0.4415	0.659	0.06622638	count	1
TECR	0.0476048	0.1005655	0.4734	0.636	0.066236693	count	1
NBPF1	0.0807856	0.4439874	0.182	0.856	0.066328347	count	1
TASP1	0.0807856	0.3694289	0.2187	0.827	0.066328347	count	1
ZUP1	0.0575276	0.2401332	0.2396	0.811	0.066423088	count	1
C9orf16	0.0470908	0.0682255	0.6902	0.49	0.066838925	count	1
VAMP3	0.047683	0.0838858	0.5684	0.57	0.066930452	count	1
NTHL1	0.0622807	0.4594818	0.1355	0.892	0.067011051	count	1
PLEKHJ1	0.0490826	0.1348815	0.3639	0.716	0.067081445	count	1
ITPA	0.0492212	0.1401222	0.3513	0.725	0.067255831	count	1
NDC1	0.0965618	0.4653962	0.2075	0.836	0.06739867	count	1
CHPT1	0.0500084	0.1216156	0.4112	0.681	0.067407742	count	1
UGP2	0.0488928	0.1160196	0.4214	0.673	0.067488371	count	1
ZMYM2	0.0499313	0.1354085	0.3687	0.712	0.067531189	count	1
DMAP1	0.0540084	0.1925208	0.2805	0.779	0.067589315	count	1
ERO1B	0.0548727	0.2012144	0.2727	0.785	0.067612558	count	1
PCSK7	0.055447	0.2048485	0.2707	0.787	0.06762729	count	1
EZR	0.0475198	0.0799168	0.5946	0.552	0.067713082	count	1
CCNG2	0.0615373	0.215501	0.2856	0.775	0.067849868	count	1
THAP7	0.0567531	0.2323859	0.2442	0.807	0.067892799	count	1
BTBD3	0.0826883	0.5784471	0.1429	0.886	0.067909123	count	1
YIPF6	0.0513653	0.1970317	0.2607	0.794	0.068300877	count	1
FERMT3	0.0482832	0.0669343	0.7214	0.471	0.068312948	count	1
ENDOD1	0.0607354	0.4995617	0.1216	0.903	0.06836312	count	1
NEK10	0.5698047	0.7619191	0.7479	0.455	0.068482648	count	1
ACSL4	0.0498162	0.1338194	0.3723	0.71	0.068711433	count	1
LMF2	0.0550802	0.1923636	0.2863	0.775	0.069040289	count	1
ADPGK	0.050102	0.117468	0.4265	0.67	0.069062422	count	1
DDX43	0.5740714	0.6197715	0.9263	0.354	0.069075036	count	1
ZNF668	0.0599213	0.2563214	0.2338	0.815	0.069198002	count	1
EXOC8	0.0613175	0.3351106	0.183	0.855	0.069345518	count	1
FRMD8	0.0616453	0.2575569	0.2393	0.811	0.069391939	count	1
SFT2D2	0.0518177	0.1834898	0.2824	0.778	0.069475914	count	1
SLC35E4	0.0633654	0.3368187	0.1881	0.851	0.069476297	count	1
TAP1	0.0541464	0.1241251	0.4362	0.663	0.069660863	count	1
GTF2F2	0.0514425	0.146404	0.3514	0.725	0.069664783	count	1
CASP3	0.0545815	0.204754	0.2666	0.79	0.069773133	count	1
TBL2	0.0556353	0.2352954	0.2364	0.813	0.069946267	count	1
KIAA1614	0.2456156	0.9812744	0.2503	0.802	0.070007011	count	1

TAS2R4	0.2456156	0.9857669	0.2492	0.803	0.070007011	count	1
EIF2AK2	0.0501796	0.1219396	0.4115	0.681	0.070040036	count	1
ELP6	0.0602259	0.2375813	0.2535	0.8	0.070068951	count	1
ABHD16A	0.2459551	0.7652815	0.3214	0.748	0.070109947	count	1
CNN2	0.0509937	0.1216823	0.4191	0.675	0.070139874	count	1
NDUFA3	0.0497339	0.0793337	0.6269	0.531	0.070238193	count	1
HNRNPUL2	0.0606693	0.2765462	0.2194	0.826	0.070330027	count	1
DDX5	0.0490371	0.034899	1.4051	0.16	0.070339197	count	1
STXBP5	0.1008217	0.4096792	0.2461	0.806	0.070423497	count	1
CEP76	0.1520492	0.5962727	0.255	0.799	0.070884691	count	1
GSDMD	0.0529124	0.1122835	0.4712	0.638	0.07091604	count	1
ROMO1	0.0507128	0.0903475	0.5613	0.575	0.071117145	count	1
METTL26	0.0504989	0.0880704	0.5734	0.566	0.071168699	count	1
AL355388.2	0.589203	0.977433	0.6028	0.547	0.071184989	count	1
SLC25A26	0.0583673	0.2218067	0.2631	0.792	0.071199866	count	1
TTC23	0.5894214	0.7813198	0.7544	0.451	0.071215541	count	1
AC005224.3	0.2019966	0.546039	0.3699	0.711	0.071246995	count	1
SS18L2	0.0517718	0.1439852	0.3596	0.719	0.071388533	count	1
DHX33	0.0869085	0.421417	0.2062	0.837	0.071418286	count	1
EHD1	0.0538278	0.1701172	0.3164	0.752	0.071429848	count	1
C6orf62	0.0514811	0.0985964	0.5221	0.602	0.071488988	count	1
SNIP1	0.0610351	0.2677227	0.228	0.82	0.071508342	count	1
CACNA2D4	0.0549273	0.2171966	0.2529	0.8	0.071620469	count	1
L3MBTL2	0.0642486	0.2933567	0.219	0.827	0.071621351	count	1
RGS9	0.0725966	0.4418647	0.1643	0.87	0.071687805	count	1
TLE1	0.1130626	0.3787313	0.2985	0.765	0.071753103	count	1
UBE2K	0.0521052	0.1188868	0.4383	0.661	0.071814355	count	1
DPYSL2	0.0518853	0.0863959	0.6006	0.548	0.071906195	count	1
RPL12	0.05005	0.0234715	2.1324	0.0331	0.072152653	count	1
LMOD3	0.0992327	0.5548045	0.1789	0.858	0.072158099	count	1
MAZ	0.0523811	0.1166864	0.4489	0.654	0.072194914	count	1
AL121658.1	0.2046005	0.7131707	0.2869	0.774	0.072211679	count	1
TRAPPC10	0.0528002	0.142601	0.3703	0.711	0.072246558	count	1
ETV5	0.0544226	0.1476249	0.3687	0.712	0.072371998	count	1
IKBKB	0.0629866	0.2320446	0.2714	0.786	0.072469437	count	1
NPRL3	0.0613493	0.4026648	0.1524	0.879	0.072571195	count	1
VPS33A	0.0737065	0.3251873	0.2267	0.821	0.072792317	count	1
ZSCAN32	0.0787569	0.4973173	0.1584	0.874	0.07283313	count	1
EBAG9	0.0554612	0.2124024	0.2611	0.794	0.073087518	count	1
RBM14	0.0675149	0.2972041	0.2272	0.82	0.073151267	count	1
CTNNA1	0.0528591	0.1058394	0.4994	0.618	0.073153146	count	1
MED10	0.0529907	0.113112	0.4685	0.639	0.073234624	count	1
LIMA1	0.059182	0.2195585	0.2696	0.788	0.073343613	count	1
SPRED2	0.0892962	0.4287975	0.2082	0.835	0.073405512	count	1
ZYX	0.0518022	0.0793629	0.6527	0.514	0.073423758	count	1

TPR	0.0520952	0.0849838	0.613	0.54	0.073464478	count	1
EPC2	0.0646693	0.2094839	0.3087	0.758	0.07348439	count	1
C12orf10	0.0536068	0.1377449	0.3892	0.697	0.073762731	count	1
PPA2	0.0553119	0.1476845	0.3745	0.708	0.073774073	count	1
CDK2AP2	0.0527815	0.1016335	0.5193	0.604	0.073791622	count	1
PTPN12	0.0525119	0.1078608	0.4868	0.626	0.073816753	count	1
PRMT5-AS1	0.3527055	0.6864161	0.5138	0.607	0.073901592	count	1
MAP2K2	0.0522998	0.0826801	0.6326	0.527	0.073903473	count	1
TEF	0.2586483	0.5771103	0.4482	0.654	0.073970557	count	1
TCF25	0.0520139	0.0773268	0.6726	0.501	0.073975384	count	1
TBL3	0.0777434	0.3962668	0.1962	0.844	0.074012949	count	1
RFPL1S	0.3539828	0.809975	0.437	0.662	0.074194684	count	1
ATP5F1A	0.0526603	0.0675108	0.78	0.435	0.074279306	count	1
C10orf143	0.0883203	0.6254893	0.1412	0.888	0.074370657	count	1
PPAN	0.0598275	0.2497721	0.2395	0.811	0.074404794	count	1
PDIA4	0.0540432	0.1228948	0.4398	0.66	0.074439067	count	1
RNF5	0.0543437	0.1316938	0.4127	0.68	0.07461589	count	1
USP6NL	0.0637279	0.3152865	0.2021	0.84	0.074675833	count	1
GLTP	0.0541188	0.1252598	0.4321	0.666	0.074718801	count	1
TLR10	0.1118554	0.6001455	0.1864	0.852	0.074778424	count	1
UFSP1	0.1118554	0.6432129	0.1739	0.862	0.074778424	count	1
TSR3	0.0545584	0.1251022	0.4361	0.663	0.074938988	count	1
SLC43A2	0.0532429	0.1122491	0.4743	0.635	0.075046874	count	1
TMEM248	0.0549942	0.1231059	0.4467	0.655	0.075082507	count	1
MGST2	0.0534803	0.0846912	0.6315	0.528	0.075209384	count	1
THOP1	0.0780292	0.4086841	0.1909	0.849	0.075271921	count	1
FAM3C	0.0587042	0.1818827	0.3228	0.747	0.075302257	count	1
AK9	0.1827887	0.4915549	0.3719	0.71	0.075602379	count	1
PGAP3	0.1191889	0.4922964	0.2421	0.809	0.075726998	count	1
ATG2B	0.0795583	0.4031865	0.1973	0.844	0.075756335	count	1
MFN1	0.0659017	0.2723384	0.242	0.809	0.075838359	count	1
SLC15A2	0.1833832	0.5892808	0.3112	0.756	0.075858775	count	1
C9orf64	0.0786482	0.3080564	0.2553	0.799	0.075874232	count	1
FIP1L1	0.0582048	0.1718935	0.3386	0.735	0.075902047	count	1
EI24	0.0605594	0.2320117	0.261	0.794	0.075926434	count	1
C3orf14	0.0693645	0.2657548	0.261	0.794	0.076090504	count	1
AL359541.1	0.6245294	0.9328551	0.6695	0.503	0.076165527	count	1
NDUFB2-AS1	0.6245294	0.9328551	0.6695	0.503	0.076165527	count	1
OR52K1	0.6245294	0.9946242	0.6279	0.53	0.076165527	count	1
FAM241B	0.6245294	0.9328551	0.6695	0.503	0.076165527	count	1
NUDT13	0.6245294	0.9328551	0.6695	0.503	0.076165527	count	1
TSPAN9	0.6245294	0.9963512	0.6268	0.531	0.076165527	count	1
ARHGAP11A	0.6245294	0.9328551	0.6695	0.503	0.076165527	count	1
ZNF517	0.1841472	0.5397034	0.3412	0.733	0.076188372	count	1
RBBP7	0.0570462	0.1518127	0.3758	0.707	0.076231729	count	1

LINC02001	0.0613939	0.2060373	0.298	0.766	0.076486992	count	1
HSPA13	0.0607984	0.2595226	0.2343	0.815	0.076564844	count	1
BRMS1	0.0556902	0.1545651	0.3603	0.719	0.076591888	count	1
COQ5	0.0645663	0.2991085	0.2159	0.829	0.076621147	count	1
AL356599.1	0.1851653	0.6525907	0.2837	0.777	0.076627763	count	1
IL2RG	0.0577189	0.1607706	0.359	0.72	0.076762084	count	1
GAB1	0.1208945	0.397293	0.3043	0.761	0.076834894	count	1
LPCAT2	0.0550447	0.0907384	0.6066	0.544	0.076853936	count	1
ZNF841	0.0987788	0.7386827	0.1337	0.894	0.076907976	count	1
TRNAU1AP	0.0566553	0.1666029	0.3401	0.734	0.076914276	count	1
TRIP4	0.0634692	0.2644674	0.24	0.81	0.076920457	count	1
KLHDC2	0.0603022	0.2015277	0.2992	0.765	0.076926115	count	1
AC011815.2	0.6300557	0.9206596	0.6844	0.494	0.076951517	count	1
MCEMP1	0.0552956	0.1594405	0.3468	0.729	0.076959993	count	1
TBCA	0.0539487	0.0551927	0.9775	0.3284	0.077027864	count	1
AP001056.2	0.0962678	0.56854	0.1693	0.866	0.077144227	count	1
RUFY4	0.269181	0.7812011	0.3446	0.73	0.077191769	count	1
VASP	0.0545746	0.062275	0.8763	0.381	0.07753295	count	1
NFKBIA	0.0539953	0.0605423	0.8919	0.373	0.077804931	count	1
PCCB	0.0687915	0.3371919	0.204	0.838	0.077839997	count	1
DSTYK	0.0683239	0.3444896	0.1983	0.843	0.077994941	count	1
DNAJB14	0.0583959	0.1589009	0.3675	0.713	0.078107728	count	1
DYNLT3	0.0567516	0.1459104	0.3889	0.697	0.078172817	count	1
AL451085.2	0.1118127	0.9356027	0.1195	0.905	0.07824751	count	1
SPRYD4	0.0859192	0.451762	0.1902	0.849	0.078249953	count	1
SP100	0.0554715	0.0772023	0.7185	0.473	0.078293853	count	1
CDC40	0.0559603	0.0956064	0.5853	0.558	0.078378532	count	1
C18orf21	0.0666727	0.2358474	0.2827	0.777	0.078398988	count	1
RAD17	0.0769369	0.3174954	0.2423	0.809	0.078399799	count	1
POLR2E	0.0556923	0.0768351	0.7248	0.469	0.078766571	count	1
GNE	0.0960432	0.4459759	0.2154	0.83	0.079027966	count	1
MPP5	0.0800772	0.3583333	0.2235	0.823	0.079136919	count	1
CSNK2A2	0.0587893	0.1693343	0.3472	0.728	0.079173553	count	1
DLEU2	0.0613254	0.2063628	0.2972	0.766	0.079299709	count	1
ZDHHC3	0.0590024	0.1474161	0.4002	0.689	0.079372443	count	1
RIPK3	0.074741	0.4589182	0.1629	0.871	0.079385134	count	1
RAB23	0.2242968	0.9246107	0.2426	0.808	0.07954621	count	1
ARL6IP4	0.0556574	0.0509379	1.0927	0.2747	0.079548318	count	1
NBAS	0.0683441	0.2356563	0.29	0.772	0.079555644	count	1
RFNG	0.0612508	0.2145179	0.2855	0.775	0.079622852	count	1
CDKN2C	0.0825103	0.3478984	0.2372	0.813	0.079633992	count	1
COPG2	0.1097632	0.448347	0.2448	0.807	0.079953661	count	1
IDUA	0.0785453	0.298987	0.2627	0.793	0.080051356	count	1
MMP14	0.0771866	0.2413619	0.3198	0.749	0.080081257	count	1
ADHFE1	0.3797529	0.5416499	0.7011	0.483	0.080146202	count	1

CDKN2AIPNL	0.0723008	0.2755385	0.2624	0.793	0.080223565	count	1
TRG-AS1	0.0895952	0.3785361	0.2367	0.813	0.080227205	count	1
FLYWCH2	0.0596573	0.1953389	0.3054	0.76	0.080254625	count	1
CLCN5	0.1263332	0.3657619	0.3454	0.73	0.080372133	count	1
RPP25L	0.0626885	0.2213974	0.2831	0.777	0.08042493	count	1
EPB41L2	0.0611277	0.1418465	0.4309	0.667	0.080861265	count	1
ZGLP1	0.0635872	0.2189005	0.2905	0.771	0.080934005	count	1
MTFMT	0.0720206	0.320061	0.225	0.822	0.081132676	count	1
PPFIBP2	0.0796054	0.3261804	0.2441	0.807	0.081140176	count	1
SNU13	0.0569988	0.0560283	1.0173	0.309	0.081148546	count	1
ZNF639	0.0679931	0.2673783	0.2543	0.799	0.081168229	count	1
HDAC10	0.0804346	0.3503941	0.2296	0.818	0.081197881	count	1
RPTOR	0.1012677	0.5870004	0.1725	0.863	0.081210686	count	1
PAIP2	0.0572346	0.0668753	0.8558	0.392	0.081215837	count	1
NOP53	0.0576042	0.0712926	0.808	0.419	0.08144904	count	1
RAB11A	0.0584634	0.1001462	0.5838	0.559	0.081450602	count	1
DHODH	0.0896635	0.3931625	0.2281	0.82	0.081697461	count	1
LINC00847	0.0635108	0.2182571	0.291	0.771	0.081977397	count	1
MBOAT2	0.2848397	0.4894117	0.582	0.561	0.082010247	count	1
SERINC2	0.1054094	0.3305203	0.3189	0.75	0.082153452	count	1
MORC4	0.198217	0.4705715	0.4212	0.674	0.082277906	count	1
THAP3	0.087558	0.3366175	0.2601	0.795	0.082285645	count	1
APTX	0.0691427	0.2586849	0.2673	0.789	0.082313299	count	1
AIMP1	0.0595867	0.1029959	0.5785	0.563	0.082415549	count	1
COX7A2L	0.0584304	0.0714016	0.8183	0.413	0.082486993	count	1
MYLIP	0.0635131	0.1540908	0.4122	0.68	0.082838427	count	1
AC009779.2	0.0830004	0.346916	0.2393	0.811	0.082951178	count	1
ARMCX5	0.0984054	0.4109111	0.2395	0.811	0.082978307	count	1
SLC16A5	0.0822549	0.3459015	0.2378	0.812	0.083050529	count	1
RNF103	0.0884065	0.3476537	0.2543	0.799	0.083091202	count	1
MTPN	0.0582386	0.0535355	1.0879	0.2768	0.083125018	count	1
BAG6	0.0632632	0.178158	0.3551	0.723	0.083226972	count	1
GCN1	0.138788	0.4653248	0.2983	0.766	0.083333339	count	1
UCKL1-AS1	0.6746252	0.6264392	1.0769	0.282	0.083357271	count	1
NFYA	0.1013653	0.3566477	0.2842	0.776	0.083470362	count	1
MCTP1	0.0655042	0.1404406	0.4664	0.641	0.08348049	count	1
PPIAL4G	0.3940779	1.0300171	0.3826	0.702	0.083485917	count	1
ANKRD33	0.3940779	0.9313036	0.4231	0.672	0.083485917	count	1
SMU1	0.060781	0.1276875	0.476	0.634	0.083585235	count	1
EPB41L4A-AS1	0.0669937	0.1843673	0.3634	0.716	0.083621936	count	1
ZRANB3	0.2352118	0.6948293	0.3385	0.735	0.083639079	count	1
AC096992.2	0.2352118	0.6948293	0.3385	0.735	0.083639079	count	1
AC020917.3	0.2352118	0.6956914	0.3381	0.735	0.083639079	count	1
ECT2	0.0846131	0.4263763	0.1984	0.843	0.083659233	count	1
FHOD1	0.0793302	0.2878079	0.2756	0.783	0.083663613	count	1

PET100	0.0598062	0.0973659	0.6142	0.539	0.083912079	count	1
PLCL1	0.1198165	0.4720766	0.2538	0.8	0.083962776	count	1
CD74	0.0582529	0.0436891	1.3333	0.183	0.084019559	count	1
MBNL3	0.1047243	0.3491503	0.2999	0.764	0.084025328	count	1
SLC35C1	0.0775605	0.3619598	0.2143	0.83	0.08410545	count	1
RBM22	0.0612155	0.1209213	0.5062	0.613	0.084107358	count	1
IRF7	0.0599918	0.1056859	0.5676	0.57	0.084157424	count	1
AC118549.1	0.0701237	0.2348776	0.2986	0.765	0.08417549	count	1
RAB8B	0.0597973	0.09951	0.6009	0.548	0.084257615	count	1
FAM241A	0.0659346	0.2247765	0.2933	0.769	0.084322481	count	1
ZNF35	0.0958783	0.4184449	0.2291	0.819	0.084324181	count	1
PHF5A	0.0611313	0.1340714	0.456	0.648	0.084345329	count	1
SGO2	0.0960177	0.3578252	0.2683	0.788	0.084448304	count	1
FGF7	0.682572	0.7952283	0.8583	0.391	0.084511741	count	1
TMEM159	0.0685601	0.2212593	0.3099	0.757	0.08453403	count	1
ATXN7L3	0.0679256	0.2639015	0.2574	0.797	0.084649561	count	1
AC005726.1	0.1805807	0.7527307	0.2399	0.81	0.084719352	count	1
ZNF570	0.1805807	0.5848386	0.3088	0.758	0.084719352	count	1
RBAK-RBAKDN	0.0752769	0.2796964	0.2691	0.788	0.084821079	count	1
TAF1B	0.0692293	0.2258276	0.3066	0.759	0.084855147	count	1
ZNF785	0.108874	0.9980141	0.1091	0.913	0.084898348	count	1
PRICKLE3	0.1809854	0.7499493	0.2413	0.809	0.084916775	count	1
RAB30-AS1	0.0645785	0.1970188	0.3278	0.743	0.084960614	count	1
RPS25	0.0590522	0.0249632	2.3656	0.0181	0.085042062	count	1
VPS37C	0.0716894	0.307463	0.2332	0.816	0.085109947	count	1
AGAP9	0.239239	0.5586199	0.4283	0.668	0.085154269	count	1
TSN	0.0674133	0.1939095	0.3477	0.728	0.085156325	count	1
TIGD6	0.1815423	0.6545851	0.2773	0.782	0.085188493	count	1
RPL4	0.0596052	0.040404	1.4752	0.14	0.085320492	count	1
PACS2	0.0719131	0.3016495	0.2384	0.812	0.085376648	count	1
TPT1	0.0592207	0.0251986	2.3502	0.0188	0.085394479	count	1
METTL18	0.1823572	0.6378222	0.2859	0.775	0.085586209	count	1
AL645933.2	0.1424743	0.4864943	0.2929	0.77	0.085607194	count	1
WDFY4	0.0702192	0.2752415	0.2551	0.799	0.085709736	count	1
AKT1S1	0.0768237	0.3013991	0.2549	0.799	0.085720947	count	1
MEG3	0.6909624	0.9187214	0.7521	0.452	0.085734717	count	1
CYP1B1-AS1	0.2062835	0.5725001	0.3603	0.719	0.085785951	count	1
CUTC	0.0661227	0.2379201	0.2779	0.781	0.085823667	count	1
TBC1D23	0.0689663	0.2058332	0.3351	0.738	0.086091759	count	1
NUP58	0.0665375	0.1451411	0.4584	0.647	0.086133852	count	1
MOB1A	0.0606184	0.0587857	1.0312	0.303	0.086190201	count	1
RPL7	0.0599744	0.0317866	1.8868	0.0593	0.08623282	count	1
NDUFB4	0.0606339	0.0605974	1.0006	0.317	0.086374394	count	1
UBL4A	0.0671776	0.3313868	0.2027	0.839	0.086378622	count	1
ZNF34	0.1024347	0.4408896	0.2323	0.816	0.086423759	count	1

TACC1	0.0615156	0.0798527	0.7704	0.441	0.086570257	count	1
ZNRF1	0.0775831	0.2990737	0.2594	0.795	0.08657324	count	1
FMNL3	0.0685657	0.2357953	0.2908	0.771	0.086616162	count	1
SMARCA4	0.0665503	0.2393171	0.2781	0.781	0.086669715	count	1
ARNTL	0.0859147	0.3429047	0.2505	0.802	0.086777327	count	1
TMEM191B	0.1239355	0.4523828	0.274	0.784	0.086909821	count	1
IDH3G	0.0622202	0.0914711	0.6802	0.496	0.086921464	count	1
WEE1	0.1446039	0.5848581	0.2472	0.805	0.0869222	count	1
WDR25	0.0823995	0.3240016	0.2543	0.799	0.086924376	count	1
FCGR1B	0.0629166	0.1109755	0.5669	0.571	0.087129527	count	1
FKRP	0.1450165	0.434364	0.3339	0.739	0.087177095	count	1
RECQL	0.0659221	0.1321668	0.4988	0.618	0.08741231	count	1
MANEA	0.0994595	0.2990426	0.3326	0.739	0.087514264	count	1
PEF1	0.0673198	0.1731131	0.3889	0.697	0.08760242	count	1
SPTLC3	0.1306714	0.5150224	0.2537	0.8	0.087648524	count	1
HSCB	0.0688252	0.1903779	0.3615	0.718	0.087724163	count	1
MCOLN1	0.0627882	0.1174532	0.5346	0.593	0.087829976	count	1
CCDC86	0.0816083	0.3955829	0.2063	0.837	0.087950144	count	1
CBX5	0.0682309	0.1905411	0.3581	0.72	0.087999584	count	1
ALAD	0.0871491	0.3453134	0.2524	0.801	0.088034914	count	1
NUP88	0.0871908	0.3064614	0.2845	0.776	0.088077403	count	1
SLC25A14	0.1256347	0.5375873	0.2337	0.815	0.088126696	count	1
ZNF677	0.1256347	0.5419107	0.2318	0.817	0.088126696	count	1
LINC02384	0.7081621	0.6754559	1.0484	0.295	0.088254431	count	1
JARID2	0.0657575	0.1602482	0.4103	0.682	0.08837157	count	1
HIST1H2BC	0.1260585	0.5531777	0.2279	0.82	0.088430304	count	1
AC003102.1	0.1212748	0.4409994	0.275	0.783	0.088505045	count	1
PDE6G	0.1212748	0.4154993	0.2919	0.77	0.088505045	count	1
AC074117.1	0.1175284	0.3941619	0.2982	0.766	0.088856047	count	1
AC243772.2	0.1478756	0.6310231	0.2343	0.815	0.088944424	count	1
DEK	0.0621988	0.0514693	1.2085	0.227	0.088992079	count	1
TIMM10B	0.0721724	0.2258862	0.3195	0.749	0.089003481	count	1
AC010226.1	0.1081048	0.5023703	0.2152	0.83	0.089105124	count	1
AGPS	0.070437	0.1712148	0.4114	0.681	0.089107369	count	1
AL022328.3	0.2499428	0.5941901	0.4206	0.674	0.089194725	count	1
CLTC	0.0636403	0.0846473	0.7518	0.452	0.089206313	count	1
MPPED2	0.7152709	0.4889227	1.463	0.144	0.089300835	count	1
COPS5	0.0646029	0.1075297	0.6008	0.548	0.089384034	count	1
NCF4	0.062962	0.070161	0.8974	0.37	0.089407257	count	1
PDK3	0.0765413	0.2926552	0.2615	0.794	0.089457844	count	1
MT-ND5	0.0623231	0.0357047	1.7455	0.081	0.089627063	count	1
MINDY2	0.0700793	0.2121923	0.3303	0.741	0.089736838	count	1
KLHDC3	0.0716286	0.1848596	0.3875	0.698	0.08984774	count	1
LINC00467	0.0823316	0.3035995	0.2712	0.786	0.089871532	count	1
CTIF	0.1339239	0.5739824	0.2333	0.816	0.089881499	count	1

FAM198B-AS1	0.1042156	0.3568224	0.2921	0.77	0.089911451	count	1
RUNX2	0.0942882	0.4127438	0.2284	0.819	0.089931361	count	1
FOXO6	0.4215622	0.837236	0.5035	0.615	0.089955942	count	1
C1orf115	0.4215622	0.837236	0.5035	0.615	0.089955942	count	1
GXYLT2	0.4215622	1.1268636	0.3741	0.708	0.089955942	count	1
ZSCAN12	0.4215622	0.837236	0.5035	0.615	0.089955942	count	1
EPHB4	0.4215622	0.837236	0.5035	0.615	0.089955942	count	1
KCP	0.4215622	0.8057521	0.5232	0.601	0.089955942	count	1
POMK	0.4215622	0.8691609	0.485	0.628	0.089955942	count	1
PPP1R13B	0.4215622	0.837236	0.5035	0.615	0.089955942	count	1
TRIM16L	0.4215622	0.837236	0.5035	0.615	0.089955942	count	1
LZTS3	0.4215622	0.8984256	0.4692	0.639	0.089955942	count	1
CUL1	0.073494	0.2284296	0.3217	0.748	0.090101617	count	1
PGRMC1	0.0666788	0.1454457	0.4584	0.647	0.090155216	count	1
RASD1	0.1192197	0.4231382	0.2818	0.778	0.090158617	count	1
ATP5PO	0.0631701	0.0510111	1.2384	0.2157	0.090216734	count	1
EIF2B4	0.0758017	0.2600438	0.2915	0.771	0.090275603	count	1
DNM3	0.2528183	0.8993785	0.2811	0.779	0.090283449	count	1
AC016773.1	0.2528183	0.9961672	0.2538	0.8	0.090283449	count	1
HOPX	0.2528183	0.7974075	0.3171	0.751	0.090283449	count	1
ZNF674	0.2528183	0.7957776	0.3177	0.751	0.090283449	count	1
AF196972.1	0.2528183	0.8696151	0.2907	0.771	0.090283449	count	1
PYCR3	0.2528183	0.9008369	0.2806	0.779	0.090283449	count	1
ANKRD42	0.0887188	0.4021644	0.2206	0.825	0.090509427	count	1
KCNK6	0.0722823	0.1760203	0.4106	0.681	0.09053127	count	1
DCAF7	0.0686901	0.152472	0.4505	0.652	0.090555221	count	1
COX7C	0.0631802	0.0404607	1.5615	0.119	0.090592187	count	1
ASB9	0.1611598	0.4494776	0.3585	0.72	0.090592569	count	1
RPL13	0.0628356	0.0223179	2.8155	0.00491	0.090593702	count	1
MRPS26	0.0672025	0.1512954	0.4442	0.657	0.090743693	count	1
SUPT4H1	0.0639997	0.069868	0.916	0.36	0.09078157	count	1
PTBP1	0.0676531	0.128619	0.526	0.599	0.090958156	count	1
ILF3-DT	0.0672407	0.156134	0.4307	0.667	0.091032086	count	1
RPS27	0.0632879	0.0257145	2.4612	0.0139	0.091128196	count	1
ACVR2B	0.1517705	0.5150673	0.2947	0.768	0.091354972	count	1
FASTKD1	0.0923789	0.6338214	0.1457	0.884	0.091411358	count	1
ULBP1	0.1764908	0.8696482	0.2029	0.839	0.091550775	count	1
TMEM246	0.1764908	1.4740086	0.1197	0.905	0.091550775	count	1
AC005363.2	0.1764908	0.6965141	0.2534	0.8	0.091550775	count	1
IPO9-AS1	0.1768234	0.8916918	0.1983	0.843	0.091729654	count	1
FUNDC2	0.0655849	0.1023483	0.6408	0.522	0.091778021	count	1
ATP2B1	0.064243	0.0660142	0.9732	0.3306	0.091835718	count	1
UBE3D	0.1770375	0.7437847	0.238	0.812	0.091844821	count	1
HKR1	0.1771868	0.5565172	0.3184	0.75	0.091925133	count	1
CAND1	0.0706935	0.217831	0.3245	0.746	0.091926389	count	1

TMEM192	0.0762515	0.2448809	0.3114	0.756	0.092030034	count	1
CRBN	0.0689423	0.1447064	0.4764	0.634	0.092071508	count	1
CENPB	0.072159	0.2154715	0.3349	0.738	0.092199896	count	1
SNRNP48	0.0722588	0.1825813	0.3958	0.692	0.092221258	count	1
IDS	0.0657217	0.1115356	0.5892	0.556	0.092381961	count	1
PLAUR	0.0642074	0.0600964	1.0684	0.285	0.092452211	count	1
RNF130	0.0645354	0.0485388	1.3296	0.184	0.092471603	count	1
MOSPD2	0.0733586	0.1918901	0.3823	0.702	0.092561421	count	1
RPS6	0.064313	0.0277288	2.3194	0.0205	0.092599549	count	1
REXO2	0.0687726	0.1419144	0.4846	0.628	0.092638834	count	1
MAFB	0.0646863	0.053436	1.2105	0.226	0.092859801	count	1
RPS12	0.0644544	0.0239296	2.6935	0.00712	0.092930314	count	1
XPNPEP1	0.0758557	0.3316161	0.2287	0.819	0.093007929	count	1
RAB6A	0.070228	0.1684111	0.417	0.677	0.093029011	count	1
TENT4A	0.0888781	0.3550379	0.2503	0.802	0.093078164	count	1
ARRDC2	0.0767639	0.1897114	0.4046	0.686	0.09309842	count	1
PHC2	0.0686239	0.1174174	0.5844	0.559	0.093184501	count	1
C1orf43	0.0659166	0.0766499	0.86	0.39	0.093258286	count	1
TGOLN2	0.0654402	0.0648348	1.0093	0.313	0.093272704	count	1
PANK4	0.1464183	0.5065849	0.289	0.773	0.093493678	count	1
NOL6	0.1464183	0.5757946	0.2543	0.799	0.093493678	count	1
HAPLN3	0.1989499	0.3783581	0.5258	0.599	0.093713313	count	1
ZNF703	0.0839866	0.2153678	0.39	0.697	0.093763769	count	1
IFT57	0.0769407	0.2011621	0.3825	0.702	0.093946224	count	1
TSPAN4	0.0670658	0.0981703	0.6832	0.495	0.094022949	count	1
UBE2W	0.0698674	0.1300623	0.5372	0.591	0.094046563	count	1
ADPRM	0.1400126	0.5304199	0.264	0.792	0.094068148	count	1
TLNRD1	0.0694633	0.149865	0.4635	0.643	0.094132281	count	1
CYBA	0.0653405	0.0246733	2.6482	0.00814	0.094144382	count	1
EXOC1	0.0703618	0.162572	0.4328	0.665	0.094181224	count	1
HIGD1A	0.0697662	0.1345183	0.5186	0.604	0.0943357	count	1
FNBP4	0.0690429	0.1213909	0.5688	0.57	0.094538002	count	1
ST20-AS1	0.1036237	0.4240578	0.2444	0.807	0.094577829	count	1
LMO4	0.067719	0.1077407	0.6285	0.53	0.094732035	count	1
LAPTM4B	0.1075945	0.4121605	0.2611	0.794	0.094771306	count	1
OTUB1	0.0687719	0.1388198	0.4954	0.62	0.094897872	count	1
LAMP5	0.2654623	0.6317792	0.4202	0.674	0.095087144	count	1
PRPS2	0.0748175	0.2275858	0.3287	0.742	0.095153406	count	1
RABGGTB	0.0725069	0.1670081	0.4342	0.664	0.095156176	count	1
CCDC146	0.1259548	0.4802674	0.2623	0.793	0.095352243	count	1
NSF	0.0765037	0.2159348	0.3543	0.723	0.095376588	count	1
RNF126	0.0713451	0.1766015	0.404	0.686	0.095499644	count	1
NFX1	0.0754053	0.3554165	0.2122	0.832	0.095538214	count	1
FNDC3A	0.0691329	0.1188056	0.5819	0.561	0.095591978	count	1
COX10	0.1065604	0.4604805	0.2314	0.817	0.095621363	count	1

CAP1	0.0668736	0.049275	1.3572	0.175	0.095636186	count	1
STK19	0.078534	0.2586835	0.3036	0.761	0.095689509	count	1
DDX46	0.0676401	0.0717966	0.9421	0.346	0.095843451	count	1
ZNF598	0.0922645	0.2977078	0.3099	0.757	0.095858757	count	1
GNB2	0.0674067	0.0620757	1.0859	0.278	0.095872839	count	1
ACTB	0.0664772	0.0279914	2.3749	0.0176	0.095874926	count	1
OTUD6B-AS1	0.0712144	0.1331364	0.5349	0.593	0.095933161	count	1
AC022098.1	0.0923485	0.3380713	0.2732	0.785	0.095946774	count	1
DOCK7	0.0760642	0.2386853	0.3187	0.75	0.096118389	count	1
ZC3H8	0.0835649	0.2571501	0.325	0.745	0.096278746	count	1
GPR161	0.4482087	0.7123416	0.6292	0.529	0.096306355	count	1
S100PBP	0.0899349	0.3158189	0.2848	0.776	0.096334041	count	1
USP36	0.0733596	0.152348	0.4815	0.63	0.096537927	count	1
KLHDC4	0.0956175	0.3140458	0.3045	0.761	0.09667037	count	1
AMZ1	0.1173063	0.7560703	0.1552	0.877	0.096814925	count	1
KIF3C	0.2055997	0.7369228	0.279	0.78	0.096985843	count	1
EFCAB7	0.3330084	0.7264342	0.4584	0.647	0.097052591	count	1
AC104653.1	0.1611662	0.6581805	0.2449	0.807	0.097183858	count	1
MEF2D	0.0734631	0.1908146	0.385	0.7	0.097267779	count	1
LSM4	0.0690426	0.0817793	0.8443	0.399	0.097283334	count	1
TRBC1	0.2063004	0.7251943	0.2845	0.776	0.097331183	count	1
SNX2	0.0684442	0.0585951	1.1681	0.243	0.097351995	count	1
MAN2C1	0.0887701	0.3555654	0.2497	0.803	0.097527765	count	1
ORAI3	0.0718058	0.134485	0.5339	0.593	0.097619904	count	1
C1QTNF12	0.7713801	1.468606	0.5252	0.599	0.097660211	count	1
AC114490.2	0.7713801	1.468606	0.5252	0.599	0.097660211	count	1
AL109741.1	0.7713801	1.468606	0.5252	0.599	0.097660211	count	1
TRIM45	0.7713801	1.468606	0.5252	0.599	0.097660211	count	1
ANXA9	0.7713801	1.468606	0.5252	0.599	0.097660211	count	1
AL358472.2	0.7713801	1.468606	0.5252	0.599	0.097660211	count	1
SH2D2A	0.7713801	1.4140999	0.5455	0.585	0.097660211	count	1
AL512306.2	0.7713801	1.468606	0.5252	0.599	0.097660211	count	1
AL512343.2	0.7713801	1.4140999	0.5455	0.585	0.097660211	count	1
BOLA3-AS1	0.7713801	1.468606	0.5252	0.599	0.097660211	count	1
CD8B	0.7713801	1.468606	0.5252	0.599	0.097660211	count	1
NCAPH	0.7713801	1.468606	0.5252	0.599	0.097660211	count	1
AC012360.2	0.7713801	1.468606	0.5252	0.599	0.097660211	count	1
LINC01191	0.7713801	1.468606	0.5252	0.599	0.097660211	count	1
AC068282.1	0.7713801	1.4140999	0.5455	0.585	0.097660211	count	1
AC097468.3	0.7713801	1.468606	0.5252	0.599	0.097660211	count	1
AC012485.3	0.7713801	1.468606	0.5252	0.599	0.097660211	count	1
KIF15	0.7713801	1.4140999	0.5455	0.585	0.097660211	count	1
TRAIP	0.7713801	1.468606	0.5252	0.599	0.097660211	count	1
PCBP4	0.7713801	1.4140999	0.5455	0.585	0.097660211	count	1
AC108693.2	0.7713801	1.468606	0.5252	0.599	0.097660211	count	1

ILDR1	0.7713801	1.468606	0.5252	0.599	0.097660211	count	1
EVC	0.7713801	1.468606	0.5252	0.599	0.097660211	count	1
AC024243.1	0.7713801	1.4140999	0.5455	0.585	0.097660211	count	1
TMEM150C	0.7713801	1.4140999	0.5455	0.585	0.097660211	count	1
DKK2	0.7713801	1.468606	0.5252	0.599	0.097660211	count	1
AC096711.2	0.7713801	1.468606	0.5252	0.599	0.097660211	count	1
TPPP	0.7713801	1.468606	0.5252	0.599	0.097660211	count	1
EIF4EBP3	0.7713801	1.468606	0.5252	0.599	0.097660211	count	1
AC008641.1	0.7713801	1.468606	0.5252	0.599	0.097660211	count	1
ZNF311	0.7713801	1.468606	0.5252	0.599	0.097660211	count	1
SLC44A4	0.7713801	1.468606	0.5252	0.599	0.097660211	count	1
AL662884.4	0.7713801	1.468606	0.5252	0.599	0.097660211	count	1
RCAN2	0.7713801	1.4140999	0.5455	0.585	0.097660211	count	1
EYS	0.7713801	1.4140999	0.5455	0.585	0.097660211	count	1
AL138828.1	0.7713801	1.468606	0.5252	0.599	0.097660211	count	1
AL356234.3	0.7713801	1.468606	0.5252	0.599	0.097660211	count	1
AL121956.1	0.7713801	1.4140999	0.5455	0.585	0.097660211	count	1
LINC01615	0.7713801	1.4140999	0.5455	0.585	0.097660211	count	1
MINDY4	0.7713801	1.468606	0.5252	0.599	0.097660211	count	1
AC073335.2	0.7713801	1.468606	0.5252	0.599	0.097660211	count	1
NPTX2	0.7713801	1.468606	0.5252	0.599	0.097660211	count	1
AC008264.2	0.7713801	1.468606	0.5252	0.599	0.097660211	count	1
AC009542.1	0.7713801	1.468606	0.5252	0.599	0.097660211	count	1
ZNF425	0.7713801	1.4140999	0.5455	0.585	0.097660211	count	1
BX890604.1	0.7713801	1.468606	0.5252	0.599	0.097660211	count	1
YY2	0.7713801	1.468606	0.5252	0.599	0.097660211	count	1
AC115618.1	0.7713801	1.468606	0.5252	0.599	0.097660211	count	1
FAM156A	0.7713801	1.4140999	0.5455	0.585	0.097660211	count	1
AL121601.1	0.7713801	1.468606	0.5252	0.599	0.097660211	count	1
FAM85B	0.7713801	1.468606	0.5252	0.599	0.097660211	count	1
LINC01301	0.7713801	1.468606	0.5252	0.599	0.097660211	count	1
CYP7B1	0.7713801	1.468606	0.5252	0.599	0.097660211	count	1
NCALD	0.7713801	1.468606	0.5252	0.599	0.097660211	count	1
PCA3	0.7713801	1.468606	0.5252	0.599	0.097660211	count	1
PTCH1	0.7713801	1.4140999	0.5455	0.585	0.097660211	count	1
AL359091.4	0.7713801	1.468606	0.5252	0.599	0.097660211	count	1
AL356481.1	0.7713801	1.468606	0.5252	0.599	0.097660211	count	1
AL590226.2	0.7713801	1.468606	0.5252	0.599	0.097660211	count	1
PNPLA7	0.7713801	1.468606	0.5252	0.599	0.097660211	count	1
MIR194-2HG	0.7713801	1.468606	0.5252	0.599	0.097660211	count	1
P4HA3	0.7713801	1.468606	0.5252	0.599	0.097660211	count	1
AP001528.1	0.7713801	1.468606	0.5252	0.599	0.097660211	count	1
TTC36	0.7713801	1.468606	0.5252	0.599	0.097660211	count	1
AL157395.1	0.7713801	1.468606	0.5252	0.599	0.097660211	count	1
AL137145.1	0.7713801	1.468606	0.5252	0.599	0.097660211	count	1

AC067747.1	0.7713801	1.468606	0.5252	0.599	0.097660211	count	1
AL136982.7	0.7713801	1.4140999	0.5455	0.585	0.097660211	count	1
AL592071.1	0.7713801	1.468606	0.5252	0.599	0.097660211	count	1
AKAP3	0.7713801	1.468606	0.5252	0.599	0.097660211	count	1
FAM90A1	0.7713801	1.468606	0.5252	0.599	0.097660211	count	1
TAS2R30	0.7713801	1.468606	0.5252	0.599	0.097660211	count	1
PDE6H	0.7713801	1.468606	0.5252	0.599	0.097660211	count	1
DDN	0.7713801	1.468606	0.5252	0.599	0.097660211	count	1
GLT8D2	0.7713801	1.4140999	0.5455	0.585	0.097660211	count	1
SLAIN1	0.7713801	1.468606	0.5252	0.599	0.097660211	count	1
LINC00379	0.7713801	1.468606	0.5252	0.599	0.097660211	count	1
AL358334.2	0.7713801	1.468606	0.5252	0.599	0.097660211	count	1
ARMH4	0.7713801	1.4140999	0.5455	0.585	0.097660211	count	1
AL391262.1	0.7713801	1.4140999	0.5455	0.585	0.097660211	count	1
KIF26A	0.7713801	1.468606	0.5252	0.599	0.097660211	count	1
DLL4	0.7713801	1.468606	0.5252	0.599	0.097660211	count	1
AC090510.1	0.7713801	1.468606	0.5252	0.599	0.097660211	count	1
SERINC4	0.7713801	1.468606	0.5252	0.599	0.097660211	count	1
AC016355.1	0.7713801	1.468606	0.5252	0.599	0.097660211	count	1
GOLGA6L10	0.7713801	1.4140999	0.5455	0.585	0.097660211	count	1
AC003965.1	0.7713801	1.468606	0.5252	0.599	0.097660211	count	1
AC109460.1	0.7713801	1.468606	0.5252	0.599	0.097660211	count	1
MYLK3	0.7713801	1.468606	0.5252	0.599	0.097660211	count	1
AC023813.3	0.7713801	1.468606	0.5252	0.599	0.097660211	count	1
MT1A	0.7713801	1.468606	0.5252	0.599	0.097660211	count	1
AC004771.3	0.7713801	1.468606	0.5252	0.599	0.097660211	count	1
PITPNM3	0.7713801	1.468606	0.5252	0.599	0.097660211	count	1
ATP1B2	0.7713801	1.4140999	0.5455	0.585	0.097660211	count	1
TMEM220-AS1	0.7713801	1.468606	0.5252	0.599	0.097660211	count	1
AC020558.1	0.7713801	1.468606	0.5252	0.599	0.097660211	count	1
AC122129.1	0.7713801	1.468606	0.5252	0.599	0.097660211	count	1
GRAPL	0.7713801	1.468606	0.5252	0.599	0.097660211	count	1
AC130324.3	0.7713801	1.468606	0.5252	0.599	0.097660211	count	1
AC006441.1	0.7713801	1.4140999	0.5455	0.585	0.097660211	count	1
LINC00854	0.7713801	1.468606	0.5252	0.599	0.097660211	count	1
MEIOC	0.7713801	1.468606	0.5252	0.599	0.097660211	count	1
SMIM6	0.7713801	1.468606	0.5252	0.599	0.097660211	count	1
DSCAS	0.7713801	1.468606	0.5252	0.599	0.097660211	count	1
AL031665.2	0.7713801	1.468606	0.5252	0.599	0.097660211	count	1
SDCBP2	0.7713801	1.4140999	0.5455	0.585	0.097660211	count	1
PDYN-AS1	0.7713801	1.468606	0.5252	0.599	0.097660211	count	1
MACROD2	0.7713801	1.4140999	0.5455	0.585	0.097660211	count	1
AL121772.3	0.7713801	1.468606	0.5252	0.599	0.097660211	count	1
DLGAP4-AS1	0.7713801	1.468606	0.5252	0.599	0.097660211	count	1
TLDC2	0.7713801	1.468606	0.5252	0.599	0.097660211	count	1

AL008726.1	0.7713801	1.4140999	0.5455	0.585	0.097660211	count	1
AL357033.1	0.7713801	1.468606	0.5252	0.599	0.097660211	count	1
COL9A3	0.7713801	1.468606	0.5252	0.599	0.097660211	count	1
PTK6	0.7713801	1.468606	0.5252	0.599	0.097660211	count	1
SHC2	0.7713801	1.468606	0.5252	0.599	0.097660211	count	1
AMH	0.7713801	1.468606	0.5252	0.599	0.097660211	count	1
ZNF426-DT	0.7713801	1.468606	0.5252	0.599	0.097660211	count	1
ANGPTL8	0.7713801	1.468606	0.5252	0.599	0.097660211	count	1
ZNF491	0.7713801	1.468606	0.5252	0.599	0.097660211	count	1
AC005614.1	0.7713801	1.4140999	0.5455	0.585	0.097660211	count	1
NTN5	0.7713801	1.468606	0.5252	0.599	0.097660211	count	1
LHB	0.7713801	1.4140999	0.5455	0.585	0.097660211	count	1
SIGLECL1	0.7713801	1.468606	0.5252	0.599	0.097660211	count	1
ZNF835	0.7713801	1.468606	0.5252	0.599	0.097660211	count	1
AC012313.1	0.7713801	1.468606	0.5252	0.599	0.097660211	count	1
CECR2	0.7713801	1.468606	0.5252	0.599	0.097660211	count	1
ZNRF3	0.7713801	1.468606	0.5252	0.599	0.097660211	count	1
LINC01521	0.7713801	1.468606	0.5252	0.599	0.097660211	count	1
LSM14A	0.0713841	0.1199403	0.5952	0.552	0.097684956	count	1
FNTA	0.0731074	0.1310649	0.5578	0.577	0.097688699	count	1
DHX9	0.0732709	0.1287285	0.5692	0.569	0.097725013	count	1
AC009948.4	0.7731887	1.002598	0.7712	0.441	0.097932574	count	1
STX18-AS1	0.7731887	1.214623	0.6366	0.524	0.097932574	count	1
LINC02104	0.7731887	1.083255	0.7138	0.475	0.097932574	count	1
CLSTN3	0.7731887	1.002598	0.7712	0.441	0.097932574	count	1
AC018904.1	0.7731887	1.075238	0.7191	0.472	0.097932574	count	1
BX539320.1	0.7731887	0.9680519	0.7987	0.425	0.097932574	count	1
PARP16	0.174104	0.5473484	0.3181	0.75	0.098120711	count	1
LGALS8	0.0737741	0.1458144	0.5059	0.613	0.098155208	count	1
SCML1	0.0743919	0.1717978	0.433	0.665	0.098443428	count	1
TBC1D22A	0.0723386	0.1563432	0.4627	0.644	0.098527654	count	1
WDR89	0.1748224	0.5488538	0.3185	0.75	0.098539587	count	1
FUS	0.0691467	0.0641969	1.0771	0.2815	0.098637398	count	1
IL1B	0.0686	0.0954437	0.7187	0.472	0.098676661	count	1
LINC00891	0.1547089	0.3470404	0.4458	0.656	0.098936575	count	1
ZNF430	0.0840889	0.2172608	0.387	0.699	0.098983779	count	1
ZNF559	0.1472083	0.4079093	0.3609	0.718	0.099026893	count	1
BZW2	0.075282	0.1878655	0.4007	0.689	0.099137724	count	1
CCDC82	0.072198	0.1320053	0.5469	0.584	0.099214019	count	1
CRIPT	0.0743737	0.1586194	0.4689	0.639	0.099473323	count	1
AUP1	0.0709039	0.0782219	0.9064	0.365	0.099628433	count	1
IGLL5	0.2111309	0.9957263	0.212	0.832	0.099714586	count	1
UBA52	0.0692611	0.0244936	2.8277	0.00473	0.099741375	count	1
ANKIB1	0.0789633	0.2374873	0.3325	0.74	0.099793695	count	1
AC009309.1	0.4630788	0.6665965	0.6947	0.487	0.099883113	count	1

SMIM26	0.0714341	0.08603	0.8303	0.406	0.099903188	count	1
GNPNAT1	0.090411	0.3340761	0.2706	0.787	0.099910339	count	1
CHD7	0.0841092	0.2974405	0.2828	0.777	0.100217355	count	1
FAM160A2	0.0852094	0.3229785	0.2638	0.792	0.100309581	count	1
RAB21	0.0724914	0.1028011	0.7052	0.481	0.100465623	count	1
BST2	0.0702846	0.051735	1.3585	0.174	0.100665731	count	1
AOAH	0.0718579	0.0774023	0.9284	0.353	0.100739349	count	1
PIK3CB	0.077126	0.1978578	0.3898	0.697	0.100792199	count	1
BASP1	0.0704781	0.077647	0.9077	0.364	0.100909781	count	1
AMFR	0.0828278	0.2074481	0.3993	0.69	0.100943516	count	1
EEFSEC	0.0863659	0.2762814	0.3126	0.755	0.10100257	count	1
NCK2	0.0822117	0.2325935	0.3535	0.724	0.101037489	count	1
TMEM102	0.0889647	0.3576504	0.2487	0.804	0.101264165	count	1
TIAL1	0.0735864	0.1231513	0.5975	0.55	0.101268158	count	1
ZFP90	0.0974328	0.3262334	0.2987	0.765	0.101276617	count	1
SRPRA	0.0728814	0.098586	0.7393	0.46	0.101277919	count	1
BFAR	0.0745749	0.1289885	0.5782	0.563	0.101471801	count	1
FKBP8	0.0716345	0.0798713	0.8969	0.37	0.101588233	count	1
SSSCA1	0.0742521	0.1397387	0.5314	0.595	0.101614602	count	1
MRPS35	0.0741053	0.1247064	0.5942	0.552	0.101624174	count	1
ZNF12	0.1027071	0.3696994	0.2778	0.781	0.101739976	count	1
GPATCH3	0.1203262	0.4594971	0.2619	0.793	0.101766759	count	1
IRS1	0.7992824	0.9064383	0.8818	0.378	0.101881934	count	1
ATP5F1D	0.0712018	0.0459736	1.5488	0.122	0.101908523	count	1
SCFD1	0.0789414	0.1449556	0.5446	0.586	0.102045808	count	1
RAD21	0.0740941	0.1124887	0.6587	0.51	0.102087856	count	1
GPBP1	0.0733769	0.0941914	0.779	0.436	0.102356521	count	1
IMPDH2	0.0761679	0.1510877	0.5041	0.614	0.102503511	count	1
CBR1	0.0737409	0.1075323	0.6858	0.493	0.102528334	count	1
NOC2L	0.0798097	0.168201	0.4745	0.635	0.102562083	count	1
STK40	0.0809842	0.2035634	0.3978	0.691	0.102629675	count	1
SLC25A16	0.0909877	0.5943099	0.1531	0.878	0.102640288	count	1
RBCK1	0.0749958	0.1147621	0.6535	0.514	0.102846855	count	1
MTFR2	0.2858546	0.881206	0.3244	0.746	0.102890702	count	1
TRIM32	0.2858546	0.8753512	0.3266	0.744	0.102890702	count	1
LINC01569	0.2858546	1.0114082	0.2826	0.777	0.102890702	count	1
REXO5	0.2858546	1.2394998	0.2306	0.818	0.102890702	count	1
UTRN	0.0765764	0.114945	0.6662	0.505	0.103015306	count	1
EXOC4	0.081026	0.2215227	0.3658	0.715	0.103074438	count	1
KIAA0825	0.2179409	0.7061131	0.3086	0.758	0.103082556	count	1
PCNP	0.0748251	0.1120355	0.6679	0.504	0.103096157	count	1
RC3H2	0.0955635	0.2680147	0.3566	0.721	0.103109737	count	1
UBR5	0.078846	0.1933384	0.4078	0.683	0.103199646	count	1
NDUFB9	0.0726237	0.0712057	1.0199	0.308	0.103336368	count	1
ZKSCAN4	0.35321	0.5608994	0.6297	0.529	0.103459016	count	1

CBX6	0.0772142	0.1415471	0.5455	0.585	0.103502622	count	1
COMMD9	0.0749948	0.1142235	0.6566	0.512	0.10350985	count	1
EID1	0.0728069	0.0538797	1.3513	0.177	0.103802068	count	1
BUD23	0.0760787	0.1363968	0.5578	0.577	0.103834207	count	1
ATP2A2	0.0771863	0.1507757	0.5119	0.609	0.103991513	count	1
UXT	0.0732488	0.0593822	1.2335	0.218	0.104212119	count	1
ZNF518A	0.0824635	0.2109916	0.3908	0.696	0.104232158	count	1
ABHD1	0.3557799	0.7963302	0.4468	0.655	0.104278136	count	1
ATP5F1E	0.0724285	0.0280734	2.58	0.00994	0.104306295	count	1
ZNF777	0.289564	0.7612099	0.3804	0.704	0.104317592	count	1
CHST11	0.0786089	0.1388104	0.5663	0.571	0.10443859	count	1
RPS3	0.072499	0.0256751	2.8237	0.00479	0.104461332	count	1
TPP1	0.0742619	0.0865632	0.8579	0.391	0.104637197	count	1
RUSC1	0.0859079	0.3107036	0.2765	0.782	0.104713777	count	1
SERPINH1	0.1268053	0.4831209	0.2625	0.793	0.104793966	count	1
NUDT7	0.1166838	0.4541261	0.2569	0.797	0.104837037	count	1
SMARCA2	0.0802667	0.1440325	0.5573	0.577	0.104906856	count	1
ZNF626	0.358129	0.4106182	0.8722	0.383	0.105027673	count	1
PNPLA8	0.0746471	0.0881923	0.8464	0.397	0.105076143	count	1
PSMB3	0.073573	0.045787	1.6069	0.108	0.105318674	count	1
NDUFA7	0.1102567	0.3567511	0.3091	0.757	0.105349156	count	1
ECI1	0.0769821	0.1132583	0.6797	0.497	0.105355508	count	1
RAP1A	0.074261	0.0731635	1.015	0.31	0.10539045	count	1
UBLCP1	0.0901262	0.2436516	0.3699	0.711	0.105424848	count	1
NSRP1	0.0752894	0.1245209	0.6046	0.545	0.105442161	count	1
AK6	0.077637	0.1365443	0.5686	0.57	0.105471563	count	1
ATP23	0.0971251	0.2879883	0.3373	0.736	0.105489801	count	1
RCL1	0.0908944	0.4049859	0.2244	0.822	0.105575029	count	1
EXOC5	0.0849403	0.2013264	0.4219	0.673	0.10575751	count	1
SFMBT1	0.0996077	0.4273101	0.2331	0.816	0.106025942	count	1
METTL15	0.0889626	0.2561172	0.3474	0.728	0.106029746	count	1
UBE2G2	0.078911	0.1772446	0.4452	0.656	0.106121012	count	1
RABEP1	0.0812973	0.1836671	0.4426	0.658	0.106493785	count	1
TERF2IP	0.0752743	0.0771664	0.9755	0.329	0.106670933	count	1
CCDC6	0.0819837	0.1786104	0.459	0.646	0.107156639	count	1
MED20	0.254892	0.6368543	0.4002	0.689	0.10718175	count	1
SIGLEC5	0.254892	0.6439012	0.3959	0.692	0.10718175	count	1
ZMPSTE24	0.0878207	0.1953149	0.4496	0.653	0.107290012	count	1
TMEM183A	0.0790286	0.1446371	0.5464	0.585	0.107364954	count	1
SLK	0.0820864	0.1482626	0.5537	0.58	0.107371971	count	1
MED14OS	0.1095842	0.2904438	0.3773	0.706	0.107384864	count	1
SAYSD1	0.0902322	0.2361672	0.3821	0.702	0.10755072	count	1
MRPS28	0.0831527	0.1928866	0.4311	0.666	0.107604941	count	1
FAM234B	0.2274423	0.5486907	0.4145	0.679	0.107796916	count	1
TTC7B	0.1688311	0.5288699	0.3192	0.75	0.108243565	count	1

ACTR1A	0.0794863	0.1408768	0.5642	0.573	0.108277329	count	1
TENT5A	0.0793564	0.1223952	0.6484	0.517	0.108314011	count	1
STAU1	0.0774062	0.0929323	0.8329	0.405	0.108317593	count	1
SS18	0.0881142	0.2152563	0.4093	0.682	0.108322773	count	1
LYPLAL1	0.0791983	0.1440427	0.5498	0.582	0.108438997	count	1
RNF34	0.0900853	0.2799841	0.3218	0.748	0.108535593	count	1
ATAD1	0.0981951	0.225542	0.4354	0.663	0.108577266	count	1
PCDHGA10	0.8433795	0.6946773	1.2141	0.225	0.108638566	count	1
TPRG1L	0.090658	0.2444873	0.3708	0.711	0.108660327	count	1
GPS2	0.0841894	0.1961958	0.4291	0.668	0.10904971	count	1
PRPF18	0.0877522	0.2312304	0.3795	0.704	0.109085775	count	1
AC072061.1	0.3713953	0.5985682	0.6205	0.535	0.109274947	count	1
TSHZ1	0.1035782	0.3863646	0.2681	0.789	0.109470788	count	1
PLAC9	0.1707807	0.4956634	0.3445	0.73	0.109531921	count	1
SELENOH	0.0770199	0.0551042	1.3977	0.162	0.109725459	count	1
RPGR	0.0923367	0.2426525	0.3805	0.704	0.109754629	count	1
WDPCP	0.1150105	0.5609445	0.205	0.838	0.10994911	count	1
ELP4	0.0961371	0.3224761	0.2981	0.766	0.109955179	count	1
CHST14	0.1087304	0.3378503	0.3218	0.748	0.11006923	count	1
EML1	0.2321488	0.5534786	0.4194	0.675	0.110138755	count	1
C1orf52	0.0804775	0.1275342	0.631	0.528	0.110145724	count	1
TMEM199	0.0874306	0.2002507	0.4366	0.662	0.11053283	count	1
JMJD1C	0.0777783	0.0695907	1.1177	0.264	0.110705423	count	1
LINC02062	0.2632183	0.978266	0.2691	0.788	0.110890404	count	1
HNRNPK	0.0779285	0.0492976	1.5808	0.114	0.111087915	count	1
ZNF347	0.1463344	0.5695257	0.2569	0.797	0.111130579	count	1
DHRSX	0.0839613	0.1869748	0.4491	0.653	0.11119938	count	1
CLK4	0.0931542	0.2143921	0.4345	0.664	0.11136399	count	1
C22orf46	0.1198892	0.3543324	0.3384	0.735	0.111410792	count	1
MRPL24	0.083437	0.200175	0.4168	0.677	0.111619585	count	1
VAV1	0.0866987	0.1957214	0.443	0.658	0.111670157	count	1
KPNA4	0.0805129	0.1041211	0.7733	0.439	0.111702842	count	1
CA5B	0.1065938	0.2962763	0.3598	0.719	0.111809084	count	1
KLHDC7B	0.1006218	0.3843961	0.2618	0.794	0.11188851	count	1
LINC02185	0.1155256	0.4635808	0.2492	0.803	0.111899224	count	1
HDLBP	0.0802164	0.1045639	0.7672	0.443	0.111925073	count	1
CLDND1	0.082345	0.1328336	0.6199	0.535	0.112030799	count	1
TP53I3	0.0876328	0.1911558	0.4584	0.647	0.112036509	count	1
ATR	0.1120419	0.3126675	0.3583	0.72	0.112304029	count	1
ZC3H7B	0.1433387	0.3632176	0.3946	0.693	0.112353089	count	1
ZBTB47	0.159468	0.582259	0.2739	0.784	0.112493963	count	1
DPAGT1	0.1018142	0.2750036	0.3702	0.711	0.112610202	count	1
NDUFB5	0.0796667	0.0647399	1.2306	0.219	0.112621399	count	1
SLC24A1	0.1536291	0.9470192	0.1622	0.871	0.112702462	count	1
SIKE1	0.0887049	0.2032368	0.4365	0.663	0.112736569	count	1

YY1	0.0791859	0.0640927	1.2355	0.217	0.112737786	count	1
SEC16A	0.0900896	0.2946915	0.3057	0.76	0.112745497	count	1
NOL3	0.1151619	0.419974	0.2742	0.784	0.11291665	count	1
PWWP2A	0.0886299	0.2290185	0.387	0.699	0.113052972	count	1
CAVIN1	0.1489389	0.6553157	0.2273	0.82	0.113153823	count	1
HIPK2	0.0861049	0.2138042	0.4027	0.687	0.113351915	count	1
MDP1	0.2386502	0.4919751	0.4851	0.628	0.113380843	count	1
PLEKHA5	0.1050105	0.3853809	0.2725	0.785	0.113391192	count	1
ZNF804A	0.140619	0.4506404	0.312	0.755	0.113414264	count	1
RTL6	0.2688822	0.5248422	0.5123	0.608	0.113420421	count	1
WIPI2	0.082498	0.1277217	0.6459	0.518	0.113488183	count	1
C2orf49	0.0865737	0.1984093	0.4363	0.663	0.113505066	count	1
PRIMPOL	0.117175	0.433892	0.2701	0.787	0.113516941	count	1
TEX264	0.0816684	0.0973519	0.8389	0.402	0.113537276	count	1
FAM174A	0.0914953	0.2691177	0.34	0.734	0.113559563	count	1
INPP5E	0.1263623	0.6173848	0.2047	0.838	0.113668255	count	1
TRPS1	0.0834364	0.1419839	0.5876	0.557	0.113695365	count	1
FBXO36	0.385126	0.838375	0.4594	0.646	0.113696655	count	1
RAB43	0.385126	0.8224141	0.4683	0.64	0.113696655	count	1
ULBP2	0.385126	0.9779565	0.3938	0.694	0.113696655	count	1
CENPI	0.385126	1.226056	0.3141	0.753	0.113696655	count	1
AC124798.1	0.385126	0.8224141	0.4683	0.64	0.113696655	count	1
TMTC4	0.385126	0.8255833	0.4665	0.641	0.113696655	count	1
AGPAT4	0.1023281	0.3015929	0.3393	0.734	0.113800438	count	1
SGMS1-AS1	0.3855594	0.6111712	0.6309	0.528	0.113836648	count	1
BCL2L1	0.0901643	0.2280698	0.3953	0.693	0.113845181	count	1
CCNDBP1	0.0829726	0.1137567	0.7294	0.466	0.11386786	count	1
MARK2	0.0895856	0.1744455	0.5135	0.608	0.114139905	count	1
FILNC1	0.161761	0.5266518	0.3071	0.759	0.114154812	count	1
ENO1-AS1	0.315505	0.7747077	0.4073	0.684	0.114359599	count	1
SERPINI1	0.315505	0.7235179	0.4361	0.663	0.114359599	count	1
LINC01852	0.315505	0.6879055	0.4586	0.647	0.114359599	count	1
DTWD2	0.1558494	0.3965922	0.393	0.694	0.114371719	count	1
LINC00106	0.880927	0.7337471	1.2006	0.23	0.114470592	count	1
AC023590.1	0.880927	0.7857269	1.1212	0.262	0.114470592	count	1
SEZ6	0.880927	0.8997634	0.9791	0.328	0.114470592	count	1
SHMT2	0.0915462	0.1967984	0.4652	0.642	0.114575659	count	1
SELENOF	0.0811462	0.0685003	1.1846	0.236	0.114577749	count	1
ZNF701	0.1076282	0.3499892	0.3075	0.758	0.114642127	count	1
TPGS1	0.0839433	0.1356766	0.6187	0.536	0.114871064	count	1
AC003681.1	0.2725379	0.480908	0.5667	0.571	0.115056497	count	1
CTNNBIP1	0.0854692	0.1488703	0.5741	0.566	0.115087269	count	1
MED9	0.189875	0.4728785	0.4015	0.688	0.115115054	count	1
C6orf48	0.0874118	0.13379	0.6534	0.514	0.115297541	count	1
CCDC138	0.2037139	0.6118322	0.333	0.739	0.115477289	count	1

AL159169.2	0.2738394	1.3252515	0.2066	0.836	0.115639552	count	1
AC073896.2	0.1912313	0.4823707	0.3964	0.692	0.115966653	count	1
NUDT18	0.1044123	0.3983284	0.2621	0.793	0.116136442	count	1
NUPL2	0.0933567	0.1776222	0.5256	0.599	0.116280566	count	1
FASTK	0.0917609	0.2088255	0.4394	0.66	0.116336796	count	1
N4BP2	0.0953151	0.2989926	0.3188	0.75	0.116489707	count	1
ERGIC1	0.0864672	0.131721	0.6564	0.512	0.116882168	count	1
YWHAB	0.0814065	0.0351076	2.3188	0.0205	0.116943159	count	1
CTBS	0.0839068	0.105221	0.7974	0.425	0.117006646	count	1
PARP15	0.2459551	0.5502695	0.447	0.655	0.117033487	count	1
PHACTR4	0.0908427	0.1823406	0.4982	0.618	0.117255141	count	1
RPL21	0.0814513	0.0250633	3.2498	0.00117	0.117306383	count	1
PI4KA	0.1079914	0.2567898	0.4205	0.674	0.117395032	count	1
RPP30	0.0897901	0.1729443	0.5192	0.604	0.11756328	count	1
AIF1	0.081636	0.0293002	2.7862	0.00537	0.117637438	count	1
UBQLN1	0.0910986	0.1892269	0.4814	0.63	0.117699158	count	1
EIF4B	0.0842694	0.0696338	1.2102	0.226	0.117806507	count	1
SYF2	0.0836968	0.0760767	1.1002	0.271	0.118347994	count	1
SPARCL1	0.1168758	0.6035004	0.1937	0.846	0.118408786	count	1
POC1B-AS1	0.5386618	0.6478088	0.8315	0.406	0.118421328	count	1
C5AR1	0.0823455	0.043848	1.878	0.0605	0.118431069	count	1
IMPACT	0.0923286	0.1966241	0.4696	0.639	0.118452807	count	1
TADA2A	0.1030987	0.2898897	0.3556	0.722	0.11846307	count	1
ELOA	0.0879915	0.1403538	0.6269	0.531	0.11861967	count	1
ZNF511	0.0857861	0.1219224	0.7036	0.482	0.118662216	count	1
LYSMD3	0.101041	0.2183605	0.4627	0.644	0.118672064	count	1
ZNF274	0.1099801	0.2980345	0.369	0.712	0.118805892	count	1
EGR4	0.4014803	0.7883167	0.5093	0.611	0.118997172	count	1
CCS	0.0867728	0.1291417	0.6719	0.502	0.119020205	count	1
SNRPB2	0.0842347	0.0775759	1.0858	0.278	0.119059457	count	1
NOB1	0.0931084	0.2676406	0.3479	0.728	0.119195352	count	1
SNRPA	0.0876418	0.1501687	0.5836	0.56	0.119280622	count	1
AGPAT5	0.0938952	0.2884115	0.3256	0.745	0.119357161	count	1
TFDP2	0.1113004	0.2621758	0.4245	0.671	0.119434617	count	1
MRPL58	0.0912773	0.1766822	0.5166	0.605	0.119515868	count	1
9-Mar	0.0980351	0.2542118	0.3856	0.7	0.119569364	count	1
CMC2	0.0859499	0.108304	0.7936	0.428	0.119595187	count	1
FAM104B	0.0951102	0.2456759	0.3871	0.699	0.119600406	count	1
CFAP45	0.9142266	0.8176887	1.1181	0.264	0.119701215	count	1
AC117382.2	0.9142266	0.9879596	0.9254	0.355	0.119701215	count	1
AC112236.2	0.9142266	0.8753444	1.0444	0.296	0.119701215	count	1
MSS51	0.9142266	1.0758	0.8498	0.396	0.119701215	count	1
SND1	0.0887945	0.1421934	0.6245	0.532	0.119704244	count	1
CEP95	0.1034136	0.2282396	0.4531	0.651	0.119764827	count	1
RPP25	0.102432	0.2491364	0.4111	0.681	0.119910848	count	1

XYLB	0.5446572	0.7410641	0.735	0.462	0.119917007	count	1
ADAMTS10	0.5446572	0.7547209	0.7217	0.471	0.119917007	count	1
MSTO1	0.135816	0.5446834	0.2493	0.803	0.120058728	count	1
COIL	0.0996678	0.2880486	0.346	0.729	0.120142786	count	1
ZRANB1	0.0998185	0.273399	0.3651	0.715	0.120325418	count	1
AL450998.2	0.0970138	0.2691669	0.3604	0.719	0.120439233	count	1
C8orf82	0.0935265	0.1872817	0.4994	0.618	0.120493417	count	1
PAK2	0.0844797	0.0577564	1.4627	0.1437	0.120571179	count	1
ZNF512	0.2849553	0.5038873	0.5655	0.572	0.120631885	count	1
ING5	0.0964178	0.2191904	0.4399	0.66	0.120698244	count	1
THAP5	0.0977903	0.2211456	0.4422	0.658	0.120742942	count	1
PSMF1	0.086674	0.1048227	0.8269	0.408	0.120964526	count	1
PARP1	0.0873558	0.1098214	0.7954	0.426	0.121221348	count	1
ATP5PD	0.0847728	0.0485099	1.7475	0.0807	0.121266651	count	1
SPIN3	0.5502916	0.8727479	0.6305	0.528	0.121325961	count	1
LRP5	0.5502916	0.8727479	0.6305	0.528	0.121325961	count	1
SYCP2	0.5502916	1.1323299	0.486	0.627	0.121325961	count	1
ZNF837	0.5502916	0.9011822	0.6106	0.541	0.121325961	count	1
C3orf70	0.9245585	1.015012	0.9109	0.362	0.121334944	count	1
LINC01554	0.9245585	0.7819196	1.1824	0.237	0.121334944	count	1
IFT122	0.1268493	0.6718097	0.1888	0.85	0.121424695	count	1
ZNF684	0.1348479	0.4291348	0.3142	0.753	0.121427386	count	1
NRBF2	0.086009	0.0787632	1.092	0.275	0.121524297	count	1
KCTD12	0.087145	0.0914434	0.953	0.341	0.121612038	count	1
IFITM3	0.0847198	0.0610541	1.3876	0.165	0.121637523	count	1
TOPBP1	0.1060375	0.228321	0.4644	0.642	0.121863614	count	1
NDUFAF2	0.0897118	0.1644197	0.5456	0.585	0.121903591	count	1
NT5C3A	0.0927926	0.2178493	0.4259	0.67	0.12201781	count	1
DOCK1	0.1660042	0.42505	0.3906	0.696	0.12202038	count	1
IFIT3	0.0906685	0.2588276	0.3503	0.726	0.122056783	count	1
CD83	0.0849885	0.0482941	1.7598	0.0786	0.122095777	count	1
SSBP4	0.0878753	0.1122523	0.7828	0.434	0.122264555	count	1
VPS9D1	0.1184886	0.3296462	0.3594	0.719	0.122336847	count	1
ESRRA	0.0956622	0.1982983	0.4824	0.63	0.122338488	count	1
SMIM10L1	0.0894748	0.1422413	0.629	0.529	0.122478426	count	1
ZNF717	0.1479795	0.5574102	0.2655	0.791	0.122652214	count	1
SP110	0.0870126	0.0856318	1.0161	0.31	0.12272692	count	1
CDK12	0.0917294	0.1434263	0.6396	0.523	0.122737147	count	1
ATP5PB	0.0864931	0.0591885	1.4613	0.144	0.122790851	count	1
IL6R	0.0903499	0.1390665	0.6497	0.516	0.12284014	count	1
CAPNS1	0.0868207	0.0666974	1.3017	0.193	0.122842333	count	1
CDK20	0.3375423	0.6969216	0.4843	0.628	0.122976749	count	1
HPSE	0.0912223	0.1654728	0.5513	0.581	0.123027377	count	1
G0S2	0.08566	0.1448631	0.5913	0.554	0.123046557	count	1
RPL23A	0.0855505	0.031843	2.6866	0.00727	0.123057032	count	1

SEPHS1	0.108949	0.2563153	0.4251	0.671	0.123059281	count	1
RAB24	0.1675639	0.4101649	0.4085	0.683	0.123197195	count	1
ZNF787	0.0926909	0.1433145	0.6468	0.518	0.123320453	count	1
LAS1L	0.1015528	0.2852202	0.3561	0.722	0.123321613	count	1
TRIM36	0.2033611	0.5446122	0.3734	0.709	0.12360053	count	1
ZNF24	0.0905108	0.1405102	0.6442	0.52	0.123845016	count	1
PNN	0.0880219	0.075124	1.1717	0.241	0.123961843	count	1
FBXO11	0.095186	0.159121	0.5982	0.55	0.123975404	count	1
EIF2AK1	0.0938678	0.1678512	0.5592	0.576	0.124062972	count	1
ZNF672	0.1026853	0.2454032	0.4184	0.676	0.124109185	count	1
SEC11A	0.0867333	0.0430716	2.0137	0.0442	0.124171992	count	1
CBL	0.0927082	0.143412	0.6464	0.518	0.12421151	count	1
PARP11	0.108571	0.3625835	0.2994	0.765	0.124280721	count	1
IQCK	0.3409784	0.5891933	0.5787	0.563	0.124327442	count	1
LINC02352	0.1931097	0.6459019	0.299	0.765	0.124347642	count	1
SCAF4	0.1017689	0.2304573	0.4416	0.659	0.124417311	count	1
C11orf1	0.1000324	0.2724828	0.3671	0.714	0.124420513	count	1
EDA	0.1692681	0.3648134	0.464	0.643	0.124483665	count	1
CCDC127	0.1030771	0.2396904	0.43	0.667	0.124585312	count	1
LAMTOR4	0.0870328	0.0432616	2.0118	0.0444	0.124710852	count	1
MAGOHB	0.0975413	0.199159	0.4898	0.624	0.124750283	count	1
GRK4	0.261348	0.804682	0.3248	0.745	0.12476436	count	1
PFDN5	0.0866658	0.0274458	3.1577	0.00161	0.124773345	count	1
SLC35E2B	0.1321969	0.6199522	0.2132	0.831	0.124869336	count	1
SETD4	0.138618	0.4083751	0.3394	0.734	0.124879598	count	1
TESMIN	0.2378602	0.6009633	0.3958	0.692	0.124947206	count	1
4-Sep	0.1846313	0.9206811	0.2005	0.841	0.125004402	count	1
CHMP4A	0.0890263	0.0972038	0.9159	0.36	0.125145125	count	1
RASSF7	0.1201719	0.3085682	0.3894	0.697	0.125171978	count	1
ELAVL4	0.1944606	0.4625466	0.4204	0.674	0.12524752	count	1
TXN2	0.0894089	0.0869105	1.0287	0.304	0.125427287	count	1
PHF20L1	0.0902647	0.1013646	0.8905	0.373	0.125433182	count	1
TERF1	0.1037828	0.1929682	0.5378	0.591	0.125442945	count	1
PTRH2	0.0953477	0.1614237	0.5907	0.555	0.125470371	count	1
ZNF276	0.1250437	0.2894875	0.4319	0.666	0.125498153	count	1
NFKBIB	0.10366	0.2763998	0.375	0.708	0.125597712	count	1
AC104596.1	0.5680355	1.0693511	0.5312	0.595	0.125783925	count	1
AL035530.2	0.5680355	1.0693511	0.5312	0.595	0.125783925	count	1
DNMBP-AS1	0.5680355	1.0693511	0.5312	0.595	0.125783925	count	1
ESPL1	0.5680355	1.0304698	0.5512	0.582	0.125783925	count	1
SGCG	0.5680355	1.0304698	0.5512	0.582	0.125783925	count	1
ASB2	0.5680355	1.0693511	0.5312	0.595	0.125783925	count	1
USP3-AS1	0.5680355	1.0304698	0.5512	0.582	0.125783925	count	1
AC005696.4	0.5680355	1.0693511	0.5312	0.595	0.125783925	count	1
PLCD3	0.5680355	1.0693511	0.5312	0.595	0.125783925	count	1

C20orf204	0.5680355	1.0304698	0.5512	0.582	0.125783925	count	1
GRIN2D	0.5680355	1.0304698	0.5512	0.582	0.125783925	count	1
IQCH-AS1	0.2966489	0.5561782	0.5334	0.594	0.125907729	count	1
AP3S2	0.123245	0.3576265	0.3446	0.73	0.126148143	count	1
SLC7A8	0.1006158	0.2410556	0.4174	0.676	0.126172365	count	1
CNBP	0.0883728	0.0486028	1.8183	0.0691	0.126204561	count	1
DCTN6	0.0923434	0.1640201	0.563	0.573	0.126300804	count	1
U62317.3	0.196096	1.1633922	0.1686	0.866	0.126337439	count	1
KIDINS220	0.0947101	0.1401225	0.6759	0.499	0.126563452	count	1
ZCCHC4	0.1431002	0.4774654	0.2997	0.764	0.126613397	count	1
BARD1	0.1023599	0.2571098	0.3981	0.691	0.126649031	count	1
PHF2	0.1138685	0.2855779	0.3987	0.69	0.126743805	count	1
ICAM1	0.0941049	0.1446777	0.6504	0.515	0.126785943	count	1
ERP44	0.0897072	0.0708404	1.2663	0.206	0.126807273	count	1
WASF2	0.088924	0.0572492	1.5533	0.12	0.126815268	count	1
BPTF	0.0911017	0.1157588	0.787	0.431	0.126872067	count	1
KRT10	0.0893612	0.0782416	1.1421	0.254	0.126926431	count	1
ASB3	0.2231785	0.5781512	0.386	0.7	0.126988736	count	1
NDEL1	0.0931487	0.1255085	0.7422	0.458	0.127015852	count	1
RPL26L1	0.0913323	0.1281213	0.7129	0.476	0.12707953	count	1
MACF1	0.0911045	0.1165797	0.7815	0.435	0.127164698	count	1
ATG14	0.1079249	0.2589083	0.4168	0.677	0.127224154	count	1
BCL11A	0.100751	0.2828701	0.3562	0.722	0.127441505	count	1
RNF114	0.0917148	0.0985583	0.9306	0.352	0.127546206	count	1
CKLF	0.0893699	0.0642917	1.3901	0.1646	0.127566443	count	1
DTNB	0.135124	0.4447642	0.3038	0.761	0.127675975	count	1
ARL8A	0.091975	0.0980642	0.9379	0.348	0.127697938	count	1
GNAI3	0.0906335	0.0841627	1.0769	0.282	0.1279068	count	1
EXOC3	0.1002458	0.2571477	0.3898	0.697	0.128075318	count	1
PTS	0.0967828	0.1747348	0.5539	0.58	0.128077041	count	1
CYB561D2	0.0940002	0.1270424	0.7399	0.459	0.128146962	count	1
SNX17	0.091706	0.0882611	1.039	0.299	0.128192522	count	1
SPECC1L	0.1812399	0.5356874	0.3383	0.735	0.128311147	count	1
MIEN1	0.0912181	0.0903685	1.0094	0.313	0.128345475	count	1
CAMTA2	0.1153955	0.313646	0.3679	0.713	0.128458028	count	1
FXYD5	0.0895045	0.0375253	2.3852	0.0171	0.128643526	count	1
RPLP2	0.0893626	0.0250003	3.5745	0.000358	0.128721223	count	1
CRYBB1	0.1169118	0.3601136	0.3247	0.745	0.1287273	count	1
PEX14	0.1819074	0.4587316	0.3965	0.692	0.128797754	count	1
ITPK1	0.1043047	0.1789904	0.5827	0.56	0.128825772	count	1
ANKS1A	0.1052145	0.203	0.5183	0.604	0.128924693	count	1
FAM53C	0.0959149	0.1446039	0.6633	0.507	0.128937382	count	1
ALG14	0.1147946	0.4299257	0.267	0.789	0.129093749	count	1
AL669831.5	0.1521045	0.5578507	0.2727	0.785	0.129192869	count	1
PFKL	0.0945673	0.1242781	0.7609	0.447	0.129230673	count	1

TK2	0.1023021	0.1979414	0.5168	0.605	0.129234717	count	1
SLC1A5	0.1161224	0.2530417	0.4589	0.646	0.129274181	count	1
STAM-AS1	0.2005492	0.7003578	0.2864	0.775	0.129308262	count	1
AC006449.2	0.2005492	0.6587622	0.3044	0.761	0.129308262	count	1
PPP1R12A	0.0924038	0.095454	0.968	0.333	0.129423106	count	1
SAMM50	0.1015846	0.2019174	0.5031	0.615	0.129486907	count	1
BRD2	0.0924538	0.1070718	0.8635	0.388	0.129519549	count	1
POLR2C	0.101261	0.1794874	0.5642	0.573	0.1295254	count	1
SOCS2	0.3048503	0.4411315	0.6911	0.49	0.129622626	count	1
AKAP13	0.0913754	0.0804146	1.1363	0.256	0.129643996	count	1
PBX2	0.1076112	0.2195233	0.4902	0.624	0.129773206	count	1
P4HTM	0.108774	0.2795481	0.3891	0.697	0.129787631	count	1
RIPK2	0.0933945	0.0939312	0.9943	0.32	0.129938672	count	1
NARS	0.0941982	0.1000102	0.9419	0.346	0.130089274	count	1
CD1C	0.0993123	0.2611738	0.3803	0.704	0.130162638	count	1
ORC5	0.1271958	0.5036755	0.2525	0.801	0.130240453	count	1
SP4	0.1534648	0.392041	0.3915	0.695	0.130371775	count	1
TSFM	0.1011682	0.2139351	0.4729	0.636	0.130373218	count	1
AL392172.1	0.0995028	0.2370523	0.4198	0.675	0.130413055	count	1
TRIP12	0.0952238	0.1279658	0.7441	0.457	0.130497681	count	1
RYK	0.1044181	0.238961	0.437	0.662	0.130550688	count	1
S1PR1	0.2291804	0.4792088	0.4782	0.633	0.130554385	count	1
TENT5C	0.2291804	0.4819546	0.4755	0.634	0.130554385	count	1
PQLC1	0.0940686	0.0995454	0.945	0.345	0.130637821	count	1
QRSL1	0.1208384	0.2791947	0.4328	0.665	0.13065129	count	1
NAPA-AS1	0.2483207	0.5244798	0.4735	0.636	0.130716816	count	1
LARP4B	0.1036646	0.2923185	0.3546	0.723	0.13078047	count	1
RPS4Y1	0.0911659	0.0483874	1.8841	0.0597	0.130862751	count	1
SAAL1	0.1385305	0.3647534	0.3798	0.704	0.13094447	count	1
PLEKHA7	0.9849361	0.6592556	1.494	0.135	0.130980424	count	1
TP53	0.1176513	0.2752759	0.4274	0.669	0.130991082	count	1
MRPL2	0.1055053	0.2212295	0.4769	0.633	0.131031658	count	1
RRP36	0.0981029	0.1673617	0.5862	0.558	0.131108018	count	1
AP006621.3	0.1934154	0.7269428	0.2661	0.79	0.131147175	count	1
EML2	0.120565	0.3228538	0.3734	0.709	0.131195555	count	1
WRAP73	0.1102953	0.2092564	0.5271	0.598	0.131236007	count	1
ABCF2	0.1336073	0.3110036	0.4296	0.668	0.13125379	count	1
FAM3A	0.1061969	0.2393113	0.4438	0.657	0.131419871	count	1
MTHFD1	0.133849	0.4256396	0.3145	0.753	0.131494511	count	1
PBRM1	0.0959539	0.1234904	0.777	0.437	0.131499799	count	1
MTHFR	0.1026289	0.2003002	0.5124	0.608	0.131856452	count	1
RNF41	0.1042989	0.1784556	0.5845	0.559	0.131948065	count	1
ZNF771	0.2505939	0.6595197	0.38	0.704	0.131973554	count	1
KRBA2	0.2505939	0.6596397	0.3799	0.704	0.131973554	count	1
NUP85	0.1267992	0.3319488	0.382	0.703	0.132153914	count	1

MTG2	0.115901	0.323914	0.3578	0.721	0.132169003	count	1
MALAT1	0.0916432	0.0418049	2.1922	0.02846	0.132206698	count	1
FAM32A	0.0949317	0.1230213	0.7717	0.44	0.132262393	count	1
MED23	0.1468191	0.3146548	0.4666	0.641	0.132399497	count	1
AC103706.1	0.2173371	0.4905586	0.443	0.658	0.132435692	count	1
LSG1	0.1075044	0.2581196	0.4165	0.677	0.132543512	count	1
AP3B1	0.0966698	0.124349	0.7774	0.437	0.132869201	count	1
CHAMP1	0.1068124	0.2711583	0.3939	0.694	0.132893886	count	1
SREBF1	0.104509	0.229922	0.4545	0.649	0.132903742	count	1
RDH11	0.0997663	0.1437542	0.694	0.488	0.132957487	count	1
AC020909.2	0.1450528	0.4168832	0.3479	0.728	0.133046026	count	1
ILF2	0.0946138	0.0777773	1.2165	0.224	0.133100727	count	1
CCDC97	0.1209853	0.2888818	0.4188	0.675	0.133254145	count	1
AATF	0.0998581	0.1578412	0.6326	0.527	0.133273065	count	1
WDR45B	0.0983996	0.1306472	0.7532	0.451	0.133501011	count	1
MMGT1	0.1057199	0.2082633	0.5076	0.612	0.133570761	count	1
RNASEH2C	0.0945103	0.0815774	1.1585	0.247	0.13357922	count	1
SCRN1	0.1236172	0.2877323	0.4296	0.668	0.133685893	count	1
RBM4	0.1189442	0.3122597	0.3809	0.703	0.133799928	count	1
DPM3	0.0970022	0.1184959	0.8186	0.413	0.133802995	count	1
DYNLL2	0.1049806	0.2224929	0.4718	0.637	0.133991962	count	1
PTP4A1	0.0979368	0.1257877	0.7786	0.436	0.134022653	count	1
ZBTB33	0.1285941	0.3834231	0.3354	0.737	0.134046217	count	1
DDX50	0.1034428	0.2069739	0.4998	0.617	0.134074407	count	1
ERMN	1.004202	0.8263364	1.2152	0.224	0.134092081	count	1
AC021321.1	1.004202	0.9831385	1.0214	0.307	0.134092081	count	1
SOX6	1.004202	1.119051	0.8974	0.37	0.134092081	count	1
GOLGA6L4	1.004202	1.030436	0.9745	0.33	0.134092081	count	1
RTN4RL2	0.1979933	0.5718298	0.3462	0.729	0.134355256	count	1
FAM102B	0.1020866	0.2039829	0.5005	0.617	0.134452123	count	1
COMT	0.094414	0.0623946	1.5132	0.13	0.13447313	count	1
LINC02076	0.3157764	1.2359528	0.2555	0.798	0.134590365	count	1
WNT5B	0.602915	0.6027943	1.0002	0.317	0.134638385	count	1
FER	0.1186569	0.2474691	0.4795	0.632	0.134736833	count	1
ZCCHC8	0.1224895	0.2156654	0.568	0.57	0.134926428	count	1
HAGH	0.0979642	0.1017869	0.9624	0.336	0.13493449	count	1
MRPS36	0.0963747	0.0986051	0.9774	0.328	0.134943373	count	1
PPP1R21	0.1169254	0.2071294	0.5645	0.572	0.135010027	count	1
RIPK1	0.1055861	0.2132165	0.4952	0.62	0.135079263	count	1
LRRC8B	0.1771326	0.5500553	0.322	0.747	0.135152416	count	1
MANBAL	0.1307978	0.2997199	0.4364	0.663	0.135198957	count	1
ATXN7	0.2564532	0.6631295	0.3867	0.699	0.13521767	count	1
PHB2	0.0957806	0.0778885	1.2297	0.219	0.135259966	count	1
CCDC34	0.1774988	0.4752402	0.3735	0.709	0.135439306	count	1
NMT2	0.1677903	0.6134835	0.2735	0.784	0.135852171	count	1

TMEM267	0.1535016	0.3967163	0.3869	0.699	0.135992553	count	1
TPCN2	0.1242412	0.3278514	0.379	0.705	0.136070434	count	1
CFLAR	0.0967364	0.0962984	1.0045	0.315	0.136413174	count	1
FAAP20	0.0972987	0.097336	0.9996	0.318	0.136454603	count	1
UPF2	0.0985671	0.1348321	0.731	0.465	0.136510743	count	1
TXNDC15	0.1015918	0.148106	0.6859	0.493	0.136742891	count	1
SMDT1	0.0962369	0.0638472	1.5073	0.132	0.136863969	count	1
C3orf38	0.1065451	0.2015474	0.5286	0.597	0.136906991	count	1
PPP6R3	0.1065287	0.1824603	0.5838	0.559	0.137028504	count	1
RTN1	0.126733	0.205911	0.6155	0.538	0.137090058	count	1
YIPF1	0.109459	0.2464352	0.4442	0.657	0.137100393	count	1
SLC27A5	0.1578528	0.3958364	0.3988	0.69	0.137141147	count	1
AC073389.1	0.2858546	0.9257076	0.3088	0.758	0.137166803	count	1
LATS2-AS1	0.2858546	0.8506572	0.336	0.737	0.137166803	count	1
DPH1	0.2858546	0.7777886	0.3675	0.713	0.137166803	count	1
ZNF681	0.2858546	0.7142045	0.4002	0.689	0.137166803	count	1
AL080276.2	0.4568268	0.6908814	0.6612	0.509	0.137205112	count	1
MTF1	0.1121992	0.3090685	0.363	0.717	0.137239737	count	1
CMTM8	0.2125296	0.5396016	0.3939	0.694	0.137322073	count	1
BCAP29	0.0987554	0.1151713	0.8575	0.391	0.137334342	count	1
CD276	0.1297174	0.3191022	0.4065	0.684	0.137408234	count	1
RANBP6	0.1233648	0.2932584	0.4207	0.674	0.137410391	count	1
TNIK	0.2025371	0.4336166	0.4671	0.64	0.13754398	count	1
ZNF281	0.1059359	0.1585866	0.668	0.504	0.137559913	count	1
SFPQ	0.0966232	0.0591836	1.6326	0.103	0.137727335	count	1
ZMYM1	0.1418695	0.378769	0.3746	0.708	0.137801602	count	1
TBC1D25	0.1704117	0.5288375	0.3222	0.747	0.138025463	count	1
NEU1	0.0997901	0.1166691	0.8553	0.392	0.138115252	count	1
MIR503HG	0.323733	0.5428122	0.5964	0.551	0.138221273	count	1
PHRF1	0.1152021	0.3062925	0.3761	0.707	0.138264802	count	1
HEMK1	0.1757144	0.3675569	0.4781	0.633	0.138386508	count	1
CLPTM1	0.1064025	0.1940617	0.5483	0.584	0.138404273	count	1
RUFY3	0.1113428	0.2256285	0.4935	0.622	0.138793973	count	1
METTL5	0.1000427	0.1135064	0.8814	0.378	0.138880844	count	1
CSNK1G2	0.1044895	0.1366754	0.7645	0.445	0.138915767	count	1
B3GNT8	0.1540163	0.4122421	0.3736	0.709	0.139010433	count	1
RPIA	0.1079582	0.1859784	0.5805	0.562	0.139016346	count	1
METTL21A	0.1055729	0.1709863	0.6174	0.537	0.139057418	count	1
RPL18A	0.0964945	0.0260076	3.7102	0.000212	0.139063861	count	1
DNAH17	0.2047126	0.4796888	0.4268	0.67	0.139072285	count	1
WDR83OS	0.0973319	0.0517468	1.8809	0.0601	0.139170529	count	1
CUEDC2	0.1001393	0.1057079	0.9473	0.344	0.13937599	count	1
FBXO38	0.1161645	0.275575	0.4215	0.673	0.139427167	count	1
CD40	0.103718	0.1510535	0.6866	0.492	0.139715189	count	1
UQCRC2	0.0995949	0.0822848	1.2104	0.226	0.139746444	count	1

AC127002.1	0.3799147	0.7356077	0.5165	0.606	0.139764933	count	1
AC027682.6	0.3799147	0.809365	0.4694	0.639	0.139764933	count	1
MRPL3	0.101637	0.1131943	0.8979	0.369	0.139953683	count	1
KMT2E	0.0990094	0.0868096	1.1405	0.254	0.13998309	count	1
SLF1	0.1459491	0.2796546	0.5219	0.602	0.139997724	count	1
UFC1	0.0985991	0.0638589	1.544	0.123	0.140002804	count	1
TNFAIP8	0.101114	0.1084306	0.9325	0.351	0.14013252	count	1
TTC5	0.1223306	0.2784092	0.4394	0.66	0.140160219	count	1
CDR2L	0.6245294	0.6159681	1.0139	0.311	0.140185234	count	1
TPST2	0.1029543	0.1260463	0.8168	0.414	0.140305552	count	1
NME6	0.1157642	0.2188459	0.529	0.597	0.140351712	count	1
ANGEL1	0.1733437	0.4669518	0.3712	0.711	0.14045803	count	1
MUC20-OT1	0.1056411	0.174529	0.6053	0.545	0.140596027	count	1
SLC25A42	0.6270848	0.6975737	0.899	0.369	0.140844003	count	1
ZC2HC1A	0.129386	0.2342222	0.5524	0.581	0.14089296	count	1
CRIP3	1.0460113	0.9772792	1.0703	0.285	0.140898207	count	1
AL021707.1	1.0460113	0.9772792	1.0703	0.285	0.140898207	count	1
PIGN	0.1340047	0.4121763	0.3251	0.745	0.140901984	count	1
NBEAL1	0.1065903	0.1751967	0.6084	0.543	0.140926839	count	1
POLR2M	0.1470136	0.2887735	0.5091	0.611	0.141034988	count	1
TREM1	0.0991821	0.0824195	1.2034	0.229	0.141175662	count	1
ATG5	0.1050796	0.1664254	0.6314	0.528	0.141230845	count	1
RPS9	0.0980838	0.0238177	4.1181	3.95E-05	0.14127388	count	0.931489
KDM5B	0.1112102	0.2401417	0.4631	0.643	0.141284248	count	1
C10orf88	0.2081026	0.6045838	0.3442	0.731	0.141455847	count	1
ZNF69	0.2081026	0.6222527	0.3344	0.738	0.141455847	count	1
DAPL1	0.1316542	0.3609363	0.3648	0.715	0.141514438	count	1
HIKESHI	0.1029627	0.1322075	0.7788	0.436	0.141532234	count	1
NOLC1	0.1094838	0.1582766	0.6917	0.489	0.141541037	count	1
CDC42SE2	0.1041955	0.1225026	0.8506	0.395	0.141583707	count	1
PGS1	0.1114796	0.2108725	0.5287	0.597	0.141627975	count	1
ALDH3A2	0.1237582	0.2118904	0.5841	0.559	0.141809346	count	1
C19orf66	0.1066138	0.1895894	0.5623	0.574	0.141821278	count	1
GHDC	0.1273488	0.2386348	0.5337	0.594	0.141889575	count	1
AC239800.2	1.052649	0.7681175	1.3704	0.171	0.141985279	count	1
AL158212.2	1.052649	0.6214817	1.6938	0.0904	0.141985279	count	1
GNA15	0.104303	0.1340044	0.7784	0.436	0.142000063	count	1
SLC7A5	0.1180146	0.2169188	0.544	0.586	0.142034695	count	1
CCDC28A	0.1062228	0.1745331	0.6086	0.543	0.142051688	count	1
MOB3B	0.1262925	0.280198	0.4507	0.652	0.142140313	count	1
RBM26	0.1101118	0.1591048	0.6921	0.489	0.142489259	count	1
PYCR2	0.1117738	0.2146636	0.5207	0.603	0.142529579	count	1
PLOD3	0.1148361	0.2080216	0.552	0.581	0.142679959	count	1
MARK3	0.1072134	0.1561193	0.6867	0.492	0.142694207	count	1
MIR762HG	0.1676569	0.5258479	0.3188	0.75	0.142695051	count	1

AC019069.1	0.2701007	0.7489171	0.3607	0.718	0.142800584	count	1
USP35	0.2701007	0.7397177	0.3651	0.715	0.142800584	count	1
GADD45B	0.0991695	0.0479766	2.067	0.0388	0.14280373	count	1
ATP5F1B	0.1008149	0.0560436	1.7989	0.0722	0.142867771	count	1
NOL12	0.1394392	0.3714386	0.3754	0.707	0.142940592	count	1
AL121987.2	0.3340584	0.7401298	0.4514	0.652	0.142949885	count	1
RPL31	0.0998261	0.0378167	2.6397	0.00835	0.143082669	count	1
PRKX	0.1324338	0.2170909	0.61	0.542	0.143322646	count	1
LONP1	0.1208198	0.2883594	0.419	0.675	0.143419719	count	1
NBPF10	0.2509853	0.7008664	0.3581	0.72	0.143571632	count	1
HOMER1	0.1826188	0.3491741	0.523	0.601	0.143968176	count	1
SLC4A1AP	0.1077969	0.1533315	0.703	0.482	0.144030641	count	1
MIDN	0.1016852	0.0856736	1.1869	0.235	0.144090972	count	1
COMMD3	0.1044568	0.1034994	1.0092	0.313	0.14416816	count	1
DNM1L	0.1076335	0.1753997	0.6136	0.54	0.144319377	count	1
FEZ2	0.1027537	0.0974186	1.0548	0.292	0.144373383	count	1
MRPL4	0.1045168	0.1355214	0.7712	0.441	0.144527102	count	1
PXK	0.1119184	0.1676393	0.6676	0.504	0.144700332	count	1
TTC8	0.1959791	0.4399599	0.4454	0.656	0.144731261	count	1
ZDHHC18	0.1455054	0.3512788	0.4142	0.679	0.144761772	count	1
SIGMAR1	0.1253763	0.2972729	0.4218	0.673	0.144846889	count	1
CAMK2G	0.1293493	0.3007279	0.4301	0.667	0.144888933	count	1
UBXN6	0.118971	0.1650153	0.721	0.471	0.144933394	count	1
DNPEP	0.1085492	0.1558397	0.6965	0.486	0.144971089	count	1
REST	0.1051805	0.1196887	0.8788	0.38	0.144985051	count	1
SNX20	0.1169034	0.1999546	0.5846	0.559	0.145004142	count	1
NOD2	0.1323164	0.3042041	0.435	0.664	0.145005378	count	1
POLDIP3	0.1255306	0.273535	0.4589	0.646	0.145026589	count	1
ZNF326	0.1086769	0.154261	0.7045	0.481	0.145073887	count	1
RPLP1	0.1006103	0.0220759	4.5575	5.43E-06	0.1450824	count	0.12888105
NFATC2	0.1637104	0.3199838	0.5116	0.609	0.145220122	count	1
COX18	0.1417564	0.3804146	0.3726	0.709	0.145347313	count	1
WDR47	0.1638514	0.2752451	0.5953	0.552	0.145347723	count	1
HNRNPA0	0.1031929	0.0736555	1.401	0.161	0.145439979	count	1
DHX15	0.1143468	0.1658533	0.6894	0.491	0.145469309	count	1
PILRA	0.1020326	0.0601233	1.6971	0.0898	0.145540473	count	1
TSPYL2	0.1142887	0.2311969	0.4943	0.621	0.145574287	count	1
TRAP1	0.1214416	0.2292239	0.5298	0.596	0.145802771	count	1
ACAT2	0.1163593	0.1862551	0.6247	0.532	0.146012023	count	1
HOOK3	0.1047067	0.1022025	1.0245	0.306	0.146031653	count	1
ZNF442	1.078448	0.9147384	1.179	0.239	0.146226283	count	1
ZNF512B	0.2764643	0.6357011	0.4349	0.664	0.146349078	count	1
PLA2G12A	0.2059508	0.4321775	0.4765	0.634	0.146390656	count	1
CNOT7	0.1071053	0.1387653	0.7718	0.44	0.146462213	count	1
TRPT1	0.1176878	0.2441561	0.482	0.63	0.146495698	count	1

TMSB10	0.1016242	0.0230286	4.413	1.06E-05	0.146520736	count	0.2511882
KPNA5	0.1429001	0.4003985	0.3569	0.721	0.146535553	count	1
CLNS1A	0.1049422	0.086449	1.2139	0.225	0.146574213	count	1
CDC42EP1	0.159924	0.4630697	0.3454	0.73	0.146941329	count	1
SRSF7	0.1032319	0.0687193	1.5022	0.133	0.147050862	count	1
KHNYN	0.1513404	0.2765805	0.5472	0.584	0.147147027	count	1
ACO1	0.1334772	0.4554506	0.2931	0.769	0.147152909	count	1
SIPA1	0.1098287	0.1498969	0.7327	0.464	0.14720798	count	1
TBC1D12	0.1150505	0.195331	0.589	0.556	0.147238339	count	1
TTLL3	0.172978	0.4353771	0.3973	0.691	0.147326539	count	1
CCNI	0.1029066	0.0447659	2.2988	0.0216	0.147484555	count	1
DOCK5	0.10893	0.1830282	0.5952	0.552	0.14750336	count	1
ASNA1	0.1068203	0.1117355	0.956	0.339	0.147568211	count	1
FBXO6	0.122662	0.2004202	0.612	0.541	0.147664687	count	1
TAOK1	0.1084426	0.153575	0.7061	0.48	0.147685573	count	1
CCND2	0.1112727	0.1725152	0.645	0.519	0.147723192	count	1
VIL1	0.4882519	0.9350717	0.5222	0.602	0.147725316	count	1
AMT	0.4882519	0.9383899	0.5203	0.603	0.147725316	count	1
MELTF-AS1	0.4882519	0.9317417	0.524	0.6	0.147725316	count	1
HIST1H3A	0.4882519	0.9383899	0.5203	0.603	0.147725316	count	1
ACSS3	0.4882519	1.1271663	0.4332	0.665	0.147725316	count	1
ABHD12B	0.4882519	0.9350717	0.5222	0.602	0.147725316	count	1
CHRFAM7A	0.4882519	0.9350717	0.5222	0.602	0.147725316	count	1
TCAP	0.4882519	0.9317417	0.524	0.6	0.147725316	count	1
SDCBP2-AS1	0.4882519	0.9383899	0.5203	0.603	0.147725316	count	1
WBP4	0.1078051	0.1275289	0.8453	0.398	0.147739183	count	1
BTN2A2	0.1185685	0.1968474	0.6023	0.547	0.147848031	count	1
SGF29	0.1241995	0.2451556	0.5066	0.612	0.147896379	count	1
ELMSAN1	0.107942	0.1283801	0.8408	0.401	0.148249966	count	1
RBM6	0.1102614	0.1631267	0.6759	0.499	0.148327427	count	1
RTCA	0.1102892	0.1509849	0.7305	0.465	0.148421557	count	1
RPS27A	0.1030094	0.0222155	4.6368	3.72E-06	0.148484346	count	0.08837232
HNMT	0.1045612	0.0753663	1.3874	0.165	0.148538596	count	1
BTBD1	0.1194059	0.2031277	0.5878	0.557	0.148645653	count	1
AC091729.3	0.2183254	0.6062725	0.3601	0.719	0.148658747	count	1
SMG6	0.1205182	0.2853677	0.4223	0.673	0.148679231	count	1
TBC1D9B	0.1137354	0.1881143	0.6046	0.545	0.1486891	count	1
MAPKAPK5	0.1314817	0.2811861	0.4676	0.64	0.148744925	count	1
ELMOD3	0.188664	0.3149479	0.599	0.549	0.148863737	count	1
MED24	0.1711459	0.3931385	0.4353	0.663	0.148941671	count	1
CSPP1	0.1368526	0.285627	0.4791	0.632	0.149111374	count	1
AC093157.1	0.3092951	0.6092191	0.5077	0.612	0.149136802	count	1
NKAPD1	0.1175046	0.2154545	0.5454	0.586	0.149317025	count	1
OSBP	0.1162364	0.2436119	0.4771	0.633	0.149411117	count	1
NDUFAF8	0.1092739	0.1297013	0.8425	0.4	0.14987764	count	1

NDRG1	0.1117596	0.1902143	0.5875	0.557	0.149928466	count	1
ABHD11	0.1259115	0.2235507	0.5632	0.573	0.14994945	count	1
THAP9-AS1	0.1184021	0.1705512	0.6942	0.488	0.150073972	count	1
CTBP2	0.1149081	0.1460675	0.7867	0.432	0.150112128	count	1
PIGT	0.1115559	0.1436483	0.7766	0.437	0.150187014	count	1
ZNF358	0.1156135	0.2784202	0.4152	0.678	0.150429594	count	1
DDI2	0.1195355	0.294614	0.4057	0.685	0.150467584	count	1
C6orf136	0.1381962	0.3243548	0.4261	0.67	0.150591223	count	1
STEAP4	0.2627436	0.3370964	0.7794	0.436	0.150631979	count	1
ANAPC5	0.111585	0.1144695	0.9748	0.33	0.150659248	count	1
CEP131	0.2213757	0.5672288	0.3903	0.696	0.150812344	count	1
LRP8	0.4974013	0.8015641	0.6205	0.535	0.150812567	count	1
CNN3	0.4974013	0.8043062	0.6184	0.536	0.150812567	count	1
VANGL1	0.4974013	0.8192486	0.6071	0.544	0.150812567	count	1
PLPP7	0.4974013	0.8015641	0.6205	0.535	0.150812567	count	1
NTRK3	0.4974013	0.807039	0.6163	0.538	0.150812567	count	1
AL035563.1	0.4974013	0.8192486	0.6071	0.544	0.150812567	count	1
ELMOD2	0.1222401	0.2221345	0.5503	0.582	0.150815594	count	1
BEND7	0.6655643	0.7463856	0.8917	0.373	0.150838868	count	1
ZFHX2	0.6655643	0.6967689	0.9552	0.34	0.150838868	count	1
AC123768.3	0.6655643	0.7929035	0.8394	0.401	0.150838868	count	1
ZNF48	0.6655643	0.7463856	0.8917	0.373	0.150838868	count	1
DDX21	0.1054751	0.061377	1.7185	0.0858	0.150937439	count	1
PMFBP1	0.1487465	0.4115697	0.3614	0.718	0.151158575	count	1
FO704657.1	1.108716	1.029867	1.0766	0.282	0.151233663	count	1
AL354956.1	1.108716	1.313742	0.8439	0.399	0.151233663	count	1
TIE1	1.108716	1.313742	0.8439	0.399	0.151233663	count	1
HIST2H2AA3	1.108716	1.313742	0.8439	0.399	0.151233663	count	1
LINC01133	1.108716	1.313742	0.8439	0.399	0.151233663	count	1
AL451074.2	1.108716	1.313742	0.8439	0.399	0.151233663	count	1
AC009237.14	1.108716	1.029867	1.0766	0.282	0.151233663	count	1
DNER	1.108716	1.313742	0.8439	0.399	0.151233663	count	1
CAMP	1.108716	1.313742	0.8439	0.399	0.151233663	count	1
LDB2	1.108716	1.029867	1.0766	0.282	0.151233663	count	1
FAM47E	1.108716	1.313742	0.8439	0.399	0.151233663	count	1
SORBS2	1.108716	1.313742	0.8439	0.399	0.151233663	count	1
PLCXD3	1.108716	1.029867	1.0766	0.282	0.151233663	count	1
AC025175.1	1.108716	1.313742	0.8439	0.399	0.151233663	count	1
IRGM	1.108716	1.313742	0.8439	0.399	0.151233663	count	1
HIST1H4F	1.108716	1.313742	0.8439	0.399	0.151233663	count	1
HIST1H3H	1.108716	1.313742	0.8439	0.399	0.151233663	count	1
CPNE5	1.108716	1.313742	0.8439	0.399	0.151233663	count	1
FILIP1	1.108716	1.313742	0.8439	0.399	0.151233663	count	1
AR	1.108716	1.313742	0.8439	0.399	0.151233663	count	1
NXF3	1.108716	1.029867	1.0766	0.282	0.151233663	count	1

AC090186.1	1.108716	1.313742	0.8439	0.399	0.151233663	count	1
E2F5	1.108716	1.029867	1.0766	0.282	0.151233663	count	1
PRRX2	1.108716	1.313742	0.8439	0.399	0.151233663	count	1
CCDC183-AS1	1.108716	1.313742	0.8439	0.399	0.151233663	count	1
AP000781.1	1.108716	1.313742	0.8439	0.399	0.151233663	count	1
AP001830.1	1.108716	1.029867	1.0766	0.282	0.151233663	count	1
AP000866.2	1.108716	1.029867	1.0766	0.282	0.151233663	count	1
CUBN	1.108716	1.313742	0.8439	0.399	0.151233663	count	1
KCNIP2	1.108716	1.313742	0.8439	0.399	0.151233663	count	1
CLEC4C	1.108716	1.313742	0.8439	0.399	0.151233663	count	1
AC087386.1	1.108716	1.029867	1.0766	0.282	0.151233663	count	1
AC025043.1	1.108716	1.029867	1.0766	0.282	0.151233663	count	1
AC106782.1	1.108716	1.313742	0.8439	0.399	0.151233663	count	1
MYO15A	1.108716	1.313742	0.8439	0.399	0.151233663	count	1
AC005899.7	1.108716	1.029867	1.0766	0.282	0.151233663	count	1
CSF3	1.108716	1.029867	1.0766	0.282	0.151233663	count	1
HID1	1.108716	1.029867	1.0766	0.282	0.151233663	count	1
MTRNR2L3	1.108716	1.029867	1.0766	0.282	0.151233663	count	1
IGFL3	1.108716	1.029867	1.0766	0.282	0.151233663	count	1
AC008440.1	1.108716	1.029867	1.0766	0.282	0.151233663	count	1
AP000357.2	1.108716	1.029867	1.0766	0.282	0.151233663	count	1
Z99774.1	1.108716	1.313742	0.8439	0.399	0.151233663	count	1
CXADR	1.108716	1.029867	1.0766	0.282	0.151233663	count	1
PMM1	0.1202011	0.2690004	0.4468	0.655	0.151309554	count	1
SAMD1	0.1159051	0.1748546	0.6629	0.507	0.151535547	count	1
AK2	0.111678	0.1651198	0.6763	0.499	0.15164311	count	1
AHCTF1	0.1155011	0.1515551	0.7621	0.446	0.151667022	count	1
MTIF2	0.1212748	0.1889643	0.6418	0.521	0.151736177	count	1
IARS2	0.1148869	0.1653364	0.6949	0.487	0.151746744	count	1
PIGQ	0.165074	0.3903972	0.4228	0.672	0.151763861	count	1
KMT5A	0.112795	0.1673441	0.674	0.5	0.151915945	count	1
HUS1	0.1152707	0.1979485	0.5823	0.56	0.151970077	count	1
TNFRSF4	0.2649915	0.5457251	0.4856	0.627	0.151984973	count	1
SSR1	0.1081726	0.088832	1.2177	0.223	0.152010649	count	1
HERC3	0.1331529	0.2870765	0.4638	0.643	0.15201929	count	1
RPS6KB1	0.1315425	0.2162504	0.6083	0.543	0.152030717	count	1
CDK11B	0.1183747	0.1945224	0.6085	0.543	0.152171129	count	1
ARAF	0.1229179	0.2219012	0.5539	0.58	0.152229052	count	1
CEPT1	0.1226989	0.259912	0.4721	0.637	0.152232578	count	1
DHX37	0.1928302	0.541905	0.3558	0.722	0.15224223	count	1
ZNF613	0.501931	0.7129042	0.7041	0.481	0.152344978	count	1
AXL	0.1116874	0.1990392	0.5611	0.575	0.152492779	count	1
VIRMA	0.1212842	0.1992353	0.6087	0.543	0.152679747	count	1
DNAJA2	0.1098079	0.0976955	1.124	0.261	0.15270809	count	1
NBPF11	0.2663787	0.6536397	0.4075	0.684	0.152820432	count	1

MPRIP-AS1	0.2663787	0.704405	0.3782	0.705	0.152820432	count	1
OSR2	0.6738629	0.9393992	0.7173	0.473	0.15301262	count	1
PDZD7	0.6738629	0.8176064	0.8242	0.41	0.15301262	count	1
CAPZA2	0.107502	0.0580107	1.8531	0.064	0.153017189	count	1
CBX7	0.1537275	0.4721603	0.3256	0.745	0.153066854	count	1
NANP	0.1594592	0.4156728	0.3836	0.701	0.153178693	count	1
AC133919.1	1.120929	0.8015909	1.3984	0.162	0.153263224	count	1
SPACA6P-AS	1.120929	1.019292	1.0997	0.272	0.153263224	count	1
SAC3D1	0.1303143	0.2488439	0.5237	0.601	0.153322429	count	1
ZMYND15	0.1273672	0.2061829	0.6177	0.537	0.15336738	count	1
ING1	0.1133745	0.1276041	0.8885	0.374	0.153486705	count	1
FXR1	0.1109077	0.1095834	1.0121	0.312	0.153586239	count	1
PLA2G15	0.1173648	0.1905674	0.6159	0.538	0.153681192	count	1
WBP1	0.3574899	0.8295939	0.4309	0.667	0.153749763	count	1
MARS2	0.3574899	0.7088695	0.5043	0.614	0.153749763	count	1
ZNF816	0.3574899	0.7033447	0.5083	0.611	0.153749763	count	1
METTL25	0.1600574	0.3565128	0.449	0.654	0.153763143	count	1
SNN	0.1113109	0.1175918	0.9466	0.344	0.153781258	count	1
SNHG18	0.5067329	1.1191883	0.4528	0.651	0.153972478	count	1
ZKSCAN3	0.5067329	0.7311985	0.693	0.488	0.153972478	count	1
SNAI3-AS1	0.5067329	0.7311985	0.693	0.488	0.153972478	count	1
TMEM143	0.5067329	0.7125979	0.7111	0.477	0.153972478	count	1
DDX19B	0.1327603	0.2652399	0.5005	0.617	0.154038981	count	1
FLII	0.1141926	0.1257509	0.9081	0.364	0.15418827	count	1
PCYT2	0.1399942	0.4096852	0.3417	0.733	0.154413724	count	1
NUBPL	0.226547	0.5182682	0.4371	0.662	0.154467986	count	1
TPCN1	0.1280093	0.2334422	0.5484	0.583	0.15453881	count	1
LCMT1	0.1169419	0.1874298	0.6239	0.533	0.154656182	count	1
PJA2	0.1098762	0.089893	1.2223	0.222	0.154806808	count	1
RALGAPA1	0.13349	0.3378124	0.3952	0.693	0.154892733	count	1
UQCRB	0.1079856	0.0396044	2.7266	0.00644	0.154910523	count	1
H1FX	0.1085861	0.0794245	1.3672	0.172	0.1549696	count	1
C18orf54	0.5099814	0.8177831	0.6236	0.533	0.155075132	count	1
PSMA6	0.1164311	0.2506677	0.4645	0.642	0.15507634	count	1
CWC27	0.1194791	0.1887725	0.6329	0.527	0.155078338	count	1
CELF2	0.1111461	0.1173576	0.9471	0.344	0.155132002	count	1
KANSL1-AS1	0.1173765	0.1406821	0.8343	0.404	0.155232751	count	1
UBXN1	0.1090814	0.056579	1.9279	0.054	0.155303207	count	1
WDR45	0.1153432	0.15341	0.7519	0.452	0.155526616	count	1
CBX3	0.1097088	0.0664134	1.6519	0.0987	0.15558752	count	1
HLA-B	0.1079857	0.0263132	4.1039	4.20E-05	0.155678837	count	0.98994
POLR3K	0.1219423	0.144291	0.8451	0.398	0.155739168	count	1
AC096733.2	0.1504313	0.4133649	0.3639	0.716	0.155770884	count	1
RACK1	0.108155	0.025444	4.2507	2.21E-05	0.15580275	count	0.5223998
OXCT1	0.2540796	0.511175	0.4971	0.619	0.155861378	count	1

THAP7-AS1	0.2540796	0.5418039	0.469	0.639	0.155861378	count	1
PPP5D1	1.137133	1.009722	1.1262	0.26	0.155964033	count	1
PARPBP	0.2190137	0.9421382	0.2325	0.816	0.156001783	count	1
AC087164.1	0.2719969	0.5669775	0.4797	0.631	0.156208087	count	1
ENTPD4	0.1551927	0.3470414	0.4472	0.655	0.156215917	count	1
BBIP1	0.1172395	0.2289541	0.5121	0.609	0.156234054	count	1
FEM1C	0.1336823	0.2668978	0.5009	0.617	0.156250058	count	1
C1orf216	0.1649822	0.3403499	0.4847	0.628	0.156403446	count	1
SERINC1	0.1120608	0.0936896	1.1961	0.232	0.156410192	count	1
CABP4	0.4215622	0.6026032	0.6996	0.484	0.156541868	count	1
FAM177B	0.2411619	0.5513387	0.4374	0.662	0.156599711	count	1
BAG1	0.1109285	0.0782259	1.4181	0.156	0.156727881	count	1
ATG10	0.1398541	0.2793157	0.5007	0.617	0.156773792	count	1
NIF3L1	0.1367598	0.2808932	0.4869	0.626	0.156841977	count	1
ZNF236	0.1400126	0.4085203	0.3427	0.732	0.156953242	count	1
TOM1	0.1156938	0.1469457	0.7873	0.431	0.156970354	count	1
UTP23	0.1150974	0.2207558	0.5214	0.602	0.157082437	count	1
MSN	0.1102084	0.0530107	2.079	0.0377	0.157189653	count	1
SCAMP1-AS1	0.2051549	0.4390841	0.4672	0.64	0.157190457	count	1
RPS15	0.109117	0.0234269	4.6578	3.37E-06	0.15723335	count	0.08008131
HLA-E	0.1094593	0.0349714	3.13	0.00177	0.157388653	count	1
STAMBP	0.1188295	0.1962366	0.6055	0.545	0.157435468	count	1
GAPVD1	0.1219238	0.1727755	0.7057	0.48	0.157689095	count	1
WBP2	0.1135099	0.1156042	0.9819	0.326	0.157709238	count	1
LYPLA2	0.1191984	0.1735507	0.6868	0.492	0.157743071	count	1
QSOX1	0.1174344	0.1585668	0.7406	0.459	0.157816962	count	1
SMYD2	0.138854	0.2696207	0.515	0.607	0.157888894	count	1
ARRDC4	0.1234914	0.2453861	0.5033	0.615	0.157909527	count	1
NDUFA12	0.111144	0.0622312	1.786	0.0742	0.158018084	count	1
NAIP	0.1150354	0.0929833	1.2372	0.216	0.158068922	count	1
LRRFIP1	0.1102485	0.0513183	2.1483	0.0318	0.158154623	count	1
GLIS3	0.146072	0.277252	0.5269	0.598	0.158254841	count	1
PUS10	0.2138453	0.4524044	0.4727	0.636	0.158361286	count	1
SNAPC4	0.1857284	0.5375149	0.3455	0.73	0.158448797	count	1
JAK3	0.1312325	0.2112644	0.6212	0.535	0.158456735	count	1
DGUOK-AS1	0.3275174	0.6689755	0.4896	0.624	0.158513458	count	1
SPTLC1	0.1213467	0.1496091	0.8111	0.417	0.158554307	count	1
DXO	0.1785141	0.3907891	0.4568	0.648	0.158639371	count	1
FAM174B	0.3680842	0.613477	0.6	0.549	0.158664082	count	1
PTPRE	0.1107807	0.0562382	1.9698	0.049	0.158751851	count	1
ALYREF	0.1133669	0.1017894	1.1137	0.266	0.158869153	count	1
ACVRL1	0.1562257	0.4493109	0.3477	0.728	0.158871162	count	1
CNPPD1	0.1214003	0.1692949	0.7171	0.473	0.15898444	count	1
LINC01374	0.4277602	0.6003987	0.7125	0.476	0.159061591	count	1
WHAMM	0.1228758	0.1779076	0.6907	0.49	0.159073686	count	1

PTGDS	1.1561213	0.4756691	2.4305	0.0151	0.15913996	count	1
NOP14-AS1	0.2233177	0.5797081	0.3852	0.7	0.159176546	count	1
ATF1	0.1216426	0.1744824	0.6972	0.486	0.159184476	count	1
RUBCN	0.1460741	0.3507853	0.4164	0.677	0.159273885	count	1
PAG1	0.118831	0.1541497	0.7709	0.441	0.159506009	count	1
PCTP	0.1361823	0.3039219	0.4481	0.654	0.159747189	count	1
BAZ2A	0.1229572	0.1733864	0.7092	0.478	0.159749842	count	1
MYO19	0.2021827	0.4337853	0.4661	0.641	0.159840013	count	1
MTERF4	0.1300223	0.2103247	0.6182	0.537	0.159842275	count	1
TLR2	0.1138472	0.0769482	1.4795	0.139	0.159948816	count	1
C12orf43	0.1297215	0.2573681	0.504	0.614	0.160101525	count	1
GAS6	0.1156569	0.1030297	1.1226	0.262	0.16010908	count	1
GFER	0.1202198	0.1576931	0.7624	0.446	0.16021732	count	1
PTPN22	0.1371448	0.2744967	0.4996	0.617	0.160330816	count	1
ADAT1	0.1609417	0.3083959	0.5219	0.602	0.16036427	count	1
MTMR6	0.1219297	0.1523876	0.8001	0.424	0.160465151	count	1
CEACAM4	0.1280818	0.1916711	0.6682	0.504	0.160552616	count	1
PUSL1	0.1327129	0.2838896	0.4675	0.64	0.160653305	count	1
TCOF1	0.1395278	0.2322332	0.6008	0.548	0.160703973	count	1
TLR7	0.1302709	0.2087333	0.6241	0.533	0.16078368	count	1
DPP8	0.1313483	0.194903	0.6739	0.5	0.160812989	count	1
Z98884.1	0.4321562	1.0378484	0.4164	0.677	0.160852295	count	1
AL049597.2	0.4321562	0.9088362	0.4755	0.634	0.160852295	count	1
S100A5	0.4321562	1.2567506	0.3439	0.731	0.160852295	count	1
AC073896.3	0.4321562	0.9132932	0.4732	0.636	0.160852295	count	1
DMWD	0.4321562	0.9517671	0.4541	0.65	0.160852295	count	1
PRKRIP1	0.1256815	0.204262	0.6153	0.538	0.160905616	count	1
RELT	0.1196603	0.1758789	0.6804	0.496	0.160942059	count	1
UNC50	0.1195382	0.1587874	0.7528	0.452	0.160961124	count	1
EPM2A	0.2799267	0.4519258	0.6194	0.536	0.161000488	count	1
SPPL2A	0.1153148	0.0971233	1.1873	0.235	0.161012124	count	1
ATXN7L3B	0.1209492	0.1691621	0.715	0.475	0.161030778	count	1
PLK3	0.1165659	0.1314948	0.8865	0.375	0.1610764	count	1
FKBP1A	0.1127847	0.052652	2.1421	0.0323	0.161129808	count	1
DPH2	0.2479665	0.4602835	0.5387	0.59	0.161206785	count	1
TRMT1L	0.1585674	0.3316338	0.4781	0.633	0.161288017	count	1
CSNK1E	0.1406714	0.3036477	0.4633	0.643	0.161369352	count	1
SNHG19	0.2178234	0.5229761	0.4165	0.677	0.161405524	count	1
DDX23	0.1333521	0.2306243	0.5782	0.563	0.161432391	count	1
AC105277.1	0.3035135	0.8553695	0.3548	0.723	0.1615216	count	1
FAM229B	0.3035135	0.7817397	0.3883	0.698	0.1615216	count	1
AL691447.2	0.3035135	0.7817397	0.3883	0.698	0.1615216	count	1
FOXN3	0.1186497	0.1163235	1.02	0.308	0.161616101	count	1
ZFYVE1	0.1937952	0.4168845	0.4649	0.642	0.16162665	count	1
MBP	0.1148792	0.0906669	1.267	0.205	0.161651819	count	1

MAP3K2	0.1152587	0.0974121	1.1832	0.237	0.161668347	count	1
SPATA2	0.2813777	0.440604	0.6386	0.523	0.161878786	count	1
PTPRA	0.1185853	0.1451741	0.8168	0.414	0.161930087	count	1
FHL3	0.1198278	0.1424997	0.8409	0.4	0.162034153	count	1
CASS4	0.1431368	0.2375556	0.6025	0.547	0.16206071	count	1
CUX1	0.1184644	0.1240889	0.9547	0.34	0.162204012	count	1
AC137767.1	0.2277393	0.5017519	0.4539	0.65	0.162442179	count	1
SF3B2	0.1149952	0.0911893	1.2611	0.207	0.162483544	count	1
ZNF71	0.2644816	0.5501657	0.4807	0.631	0.162544741	count	1
THRA	0.1287595	0.2211521	0.5822	0.56	0.162604183	count	1
KCNE5	0.2825816	0.7284233	0.3879	0.698	0.162607836	count	1
MYOZ2	0.2646759	0.7898003	0.3351	0.738	0.162669793	count	1
TRMT61B	0.1615788	0.331734	0.4871	0.626	0.162744126	count	1
HHAT	0.2122167	0.8772616	0.2419	0.809	0.162770944	count	1
TBC1D14	0.133249	0.1989283	0.6698	0.503	0.162803296	count	1
BCS1L	0.1693105	0.4230953	0.4002	0.689	0.162812376	count	1
STX2	0.1316865	0.215027	0.6124	0.54	0.162850948	count	1
PLEKHA3	0.1191628	0.154169	0.7729	0.44	0.163019648	count	1
ZNF148	0.1223325	0.1500537	0.8153	0.415	0.16312152	count	1
CIRBP	0.1145463	0.0597635	1.9167	0.0554	0.163300961	count	1
ST3GAL2	0.1605636	0.3134114	0.5123	0.608	0.163349064	count	1
RNPEPL1	0.1187282	0.1085925	1.0933	0.274	0.163388417	count	1
CACUL1	0.1189753	0.1216274	0.9782	0.328	0.163400138	count	1
HYAL2	0.1384435	0.2526036	0.5481	0.584	0.163492962	count	1
NSFL1C	0.119201	0.1297964	0.9184	0.359	0.163616004	count	1
AGO2	0.1234521	0.1734458	0.7118	0.477	0.163763866	count	1
ALS2CL	1.184257	1.006275	1.1769	0.239	0.163866674	count	1
OSBPL11	0.1222181	0.1809871	0.6753	0.5	0.16392922	count	1
CSNK1D	0.1195534	0.1233103	0.9695	0.332	0.164003461	count	1
RHOBTB2	0.2139015	0.4864366	0.4397	0.66	0.164103901	count	1
NDUFS1	0.1294899	0.1717634	0.7539	0.451	0.164198555	count	1
GEMIN6	0.1325449	0.2465838	0.5375	0.591	0.164223386	count	1
FTSJ1	0.1432802	0.3262933	0.4391	0.661	0.164390043	count	1
C2orf69	0.1481198	0.2742642	0.5401	0.589	0.164395605	count	1
PUDP	0.1517984	0.4769567	0.3183	0.75	0.16453363	count	1
CNP	0.1285278	0.2598059	0.4947	0.621	0.164566514	count	1
ANKRD35	0.2858546	0.6201478	0.4609	0.645	0.164591341	count	1
MPZ	0.2858546	0.6201478	0.4609	0.645	0.164591341	count	1
PIAS3	0.1929376	0.4531412	0.4258	0.67	0.164752492	count	1
TRABD	0.1202222	0.1251219	0.9608	0.337	0.164788847	count	1
APOM	0.1638579	0.4499305	0.3642	0.716	0.165075744	count	1
IP6K1	0.1364881	0.24301	0.5617	0.574	0.165255391	count	1
TMEM127	0.1223745	0.176723	0.6925	0.489	0.165263647	count	1
MAP3K7CL	0.1353864	0.2682332	0.5047	0.614	0.165432065	count	1
BORCS6	0.1335777	0.2289289	0.5835	0.56	0.16551074	count	1

RABEP2	0.1702281	0.3895646	0.437	0.662	0.165836019	count	1
CNIH4	0.1181888	0.1138106	1.0385	0.299	0.165980106	count	1
MKRN1	0.1238937	0.1512175	0.8193	0.413	0.166113065	count	1
ZP3	0.2700982	0.376101	0.7182	0.473	0.166162761	count	1
PPP1CC	0.1204713	0.1054361	1.1426	0.253	0.166333311	count	1
MTMR14	0.1229401	0.1322492	0.9296	0.353	0.166468097	count	1
SMNDC1	0.1224884	0.1303969	0.9394	0.348	0.166543744	count	1
GTF2I	0.1225522	0.1166324	1.0508	0.293	0.166676577	count	1
TST	0.1313099	0.2062864	0.6365	0.524	0.166735019	count	1
TULP3	0.2439146	0.4321814	0.5644	0.573	0.166787112	count	1
FDXACB1	0.3436074	0.4972469	0.691	0.49	0.166844147	count	1
SLC16A7	0.156274	0.2559225	0.6106	0.542	0.167138729	count	1
GAS2L1	0.1464917	0.2450311	0.5978	0.55	0.167396726	count	1
RPL28	0.1163785	0.0231616	5.0246	5.41E-07	0.167782596	count	0.012896899
ZNF761	0.2010152	0.4413128	0.4555	0.649	0.167809255	count	1
CNNM4	0.2119758	0.4646965	0.4562	0.648	0.167815719	count	1
KRIT1	0.131868	0.1908398	0.691	0.49	0.16787071	count	1
PPIG	0.1189777	0.0725479	1.64	0.101	0.168268292	count	1
PLXND1	0.1269596	0.1684549	0.7537	0.451	0.168337892	count	1
POLR2F	0.1203923	0.1146397	1.0502	0.294	0.168353268	count	1
ZWINT	0.3157566	0.5733186	0.5508	0.582	0.168436035	count	1
TMEM259	0.1243739	0.1334608	0.9319	0.351	0.168467343	count	1
MICU2	0.1300007	0.1781076	0.7299	0.466	0.168490986	count	1
DCLRE1A	0.2737726	0.643768	0.4253	0.671	0.168533204	count	1
AC040169.1	0.2737726	0.643768	0.4253	0.671	0.168533204	count	1
KIF3B	0.1587603	0.3317093	0.4786	0.632	0.168587885	count	1
ZNF76	0.2588609	0.459536	0.5633	0.573	0.168603149	count	1
PLEKHO2	0.1267344	0.1508404	0.8402	0.401	0.169009154	count	1
ZC3HC1	0.1901111	0.3864311	0.492	0.623	0.169183205	count	1
MRPL33	0.1206646	0.1000096	1.2065	0.228	0.169184359	count	1
ZNF449	0.25983	0.5157678	0.5038	0.614	0.16926229	count	1
DDT	0.1187916	0.0688437	1.7255	0.0846	0.169269684	count	1
TMEM14A	0.1680939	0.3465426	0.4851	0.628	0.169411871	count	1
MEN1	0.2282954	0.4804397	0.4752	0.635	0.169435378	count	1
COQ10A	0.220668	0.4391243	0.5025	0.615	0.169463432	count	1
RNF152	0.391421	0.7938654	0.4931	0.622	0.169557052	count	1
RPL39L	0.1400171	0.2671891	0.524	0.6	0.169558859	count	1
PIGK	0.1609362	0.2775473	0.5799	0.562	0.16961412	count	1
CLN3	0.1683249	0.5358692	0.3141	0.753	0.169648424	count	1
TWSG1	0.1485403	0.3806548	0.3902	0.696	0.169760682	count	1
AC240565.1	1.22275	0.8453773	1.4464	0.148	0.170371242	count	1
AC004817.3	0.2040668	0.3408063	0.5988	0.549	0.170425654	count	1
MAP2K7	0.1394958	0.302757	0.4608	0.645	0.170487624	count	1
RAD51B	0.1882972	0.425206	0.4428	0.658	0.170644723	count	1
SNRPF	0.12174	0.0853543	1.4263	0.154	0.170704744	count	1

DAP	0.1260647	0.1359586	0.9272	0.354	0.170709461	count	1
AGRN	0.2494551	0.5436286	0.4589	0.646	0.170730522	count	1
IGFLR1	0.1394059	0.2054384	0.6786	0.497	0.170744534	count	1
HBEGF	0.1195814	0.0759127	1.5752	0.115	0.171055998	count	1
HUS1B	0.4570962	0.8540455	0.5352	0.593	0.171067195	count	1
CDK16	0.1496858	0.2544817	0.5882	0.556	0.171082775	count	1
ST3GAL1	0.1299086	0.1623815	0.8	0.424	0.171116699	count	1
CMIP	0.1267767	0.1352333	0.9375	0.349	0.171510701	count	1
REPS2	0.1832449	0.378458	0.4842	0.628	0.171515325	count	1
AC034199.1	1.2305199	1.092564	1.1263	0.26	0.171689141	count	1
RREB1	0.1316169	0.209049	0.6296	0.529	0.171757652	count	1
CMTR1	0.18352	0.3888716	0.4719	0.637	0.171778055	count	1
EDC3	0.197178	0.4312115	0.4573	0.648	0.172157457	count	1
SMARCA1	0.2515028	0.6779245	0.371	0.711	0.172189601	count	1
TMEM273	0.1589216	0.2726698	0.5828	0.56	0.17235136	count	1
RFC4	0.1841676	0.3604053	0.511	0.609	0.172396589	count	1
DDB2	0.1400269	0.2465797	0.5679	0.57	0.172566481	count	1
NSD2	0.1546805	0.3060111	0.5055	0.613	0.172684433	count	1
TGS1	0.12776	0.1480477	0.863	0.388	0.172787734	count	1
TBCC	0.1362313	0.2033055	0.6701	0.503	0.172791899	count	1
ELOF1	0.1279397	0.1942158	0.6588	0.51	0.17279894	count	1
WDR27	0.561966	0.7929839	0.7087	0.479	0.172902884	count	1
TTC4	0.3553566	0.5444094	0.6527	0.514	0.172957391	count	1
PROSER3	0.2023613	0.8690219	0.2329	0.816	0.173008789	count	1
LRRC25	0.1245736	0.0916579	1.3591	0.174	0.17316074	count	1
DDR1	1.239247	0.8992237	1.3781	0.168	0.173171284	count	1
MAP2K3	0.12456	0.1155592	1.0779	0.281	0.173216418	count	1
COP1	0.1375188	0.1744882	0.7881	0.431	0.17323123	count	1
FYB1	0.1212215	0.0510861	2.3729	0.0177	0.173305886	count	1
RAD51C	0.1286592	0.2045219	0.6291	0.529	0.173340802	count	1
HINT3	0.1600863	0.224014	0.7146	0.475	0.173630397	count	1
RPL7A	0.1204987	0.0244374	4.9309	8.73E-07	0.17368438	count	0.020800971
STK11	0.1368029	0.2182659	0.6268	0.531	0.173746386	count	1
LNPK	0.1434532	0.2457237	0.5838	0.559	0.173750436	count	1
XPO1	0.1279772	0.1500787	0.8527	0.394	0.173828434	count	1
CCDC50	0.127992	0.1287411	0.9942	0.32	0.173848584	count	1
EEF2	0.1220456	0.0454394	2.6859	0.00728	0.174132426	count	1
NFIC	0.1279664	0.1266479	1.0104	0.312	0.174151776	count	1
ANXA7	0.1244618	0.0963288	1.2921	0.196	0.174280612	count	1
ZNF211	0.3579328	0.5983242	0.5982	0.55	0.174301183	count	1
LINC00278	0.3579328	0.8006264	0.4471	0.655	0.174301183	count	1
ZC3H4	0.1713988	0.3102204	0.5525	0.581	0.174548721	count	1
STK32B	0.4020939	0.678255	0.5928	0.553	0.174569663	count	1
TIAM2	0.4020939	0.6347733	0.6334	0.527	0.174569663	count	1
HLCS	0.4020939	0.6239782	0.6444	0.519	0.174569663	count	1

SMC6	0.1452859	0.2546194	0.5706	0.568	0.174653105	count	1
NAA38	0.1243174	0.0832024	1.4942	0.135	0.174693292	count	1
DENND1C	0.1791753	0.3538777	0.5063	0.613	0.174712723	count	1
ZC3H12A	0.1313512	0.1494604	0.8788	0.38	0.174745538	count	1
FKBP9	0.2554591	0.4419632	0.578	0.563	0.175011121	count	1
LTB	0.1431788	0.2748649	0.5209	0.602	0.175020212	count	1
MUM1	0.1512665	0.2728583	0.5544	0.579	0.175045342	count	1
RAB40B	0.2840866	0.4346337	0.6536	0.513	0.175201778	count	1
SIGLEC14	0.1649527	0.2463178	0.6697	0.503	0.175254317	count	1
GARS	0.1361162	0.1886658	0.7215	0.471	0.175266863	count	1
ARID1B	0.1320531	0.152348	0.8668	0.386	0.175308544	count	1
UQCRH	0.1224353	0.0412524	2.968	0.00303	0.175399055	count	1
CLEC3B	1.252874	0.7822929	1.6015	0.109	0.175489321	count	1
GLB1	0.1308123	0.1237665	1.0569	0.291	0.17556314	count	1
HPF1	0.1332657	0.1563454	0.8524	0.394	0.175668935	count	1
PEX11G	0.1851211	0.4339563	0.4266	0.67	0.175878676	count	1
NDUFAF1	0.1427321	0.2641691	0.5403	0.589	0.175922373	count	1
PRKD3	0.1442669	0.2580513	0.5591	0.576	0.175970365	count	1
BCL7C	0.1255459	0.1055115	1.1899	0.234	0.176013536	count	1
HSDL2	0.1343527	0.1858319	0.723	0.47	0.176013853	count	1
AL356441.1	1.2569057	1.1205265	1.1217	0.262	0.176176121	count	1
LINC01703	1.2569057	1.319048	0.9529	0.341	0.176176121	count	1
OR2B11	1.2569057	1.1205265	1.1217	0.262	0.176176121	count	1
AC098828.2	1.2569057	1.319048	0.9529	0.341	0.176176121	count	1
LRRC2	1.2569057	1.319048	0.9529	0.341	0.176176121	count	1
ITIH4	1.2569057	1.319048	0.9529	0.341	0.176176121	count	1
ATP1B3-AS1	1.2569057	1.1205265	1.1217	0.262	0.176176121	count	1
HTRA3	1.2569057	1.319048	0.9529	0.341	0.176176121	count	1
AC027607.1	1.2569057	1.319048	0.9529	0.341	0.176176121	count	1
AC020741.1	1.2569057	1.319048	0.9529	0.341	0.176176121	count	1
GPR63	1.2569057	1.458152	0.862	0.389	0.176176121	count	1
FRK	1.2569057	1.458152	0.862	0.389	0.176176121	count	1
AC007285.1	1.2569057	1.319048	0.9529	0.341	0.176176121	count	1
AL022157.1	1.2569057	1.319048	0.9529	0.341	0.176176121	count	1
AL162231.2	1.2569057	1.319048	0.9529	0.341	0.176176121	count	1
SMC5-AS1	1.2569057	1.319048	0.9529	0.341	0.176176121	count	1
B3GNT10	1.2569057	1.319048	0.9529	0.341	0.176176121	count	1
CTTN	1.2569057	1.319048	0.9529	0.341	0.176176121	count	1
SYT15	1.2569057	1.458152	0.862	0.389	0.176176121	count	1
RNASE4	1.2569057	1.319048	0.9529	0.341	0.176176121	count	1
AC005479.2	1.2569057	1.458152	0.862	0.389	0.176176121	count	1
AL049840.2	1.2569057	1.319048	0.9529	0.341	0.176176121	count	1
MYO5C	1.2569057	1.458152	0.862	0.389	0.176176121	count	1
AC010761.1	1.2569057	1.319048	0.9529	0.341	0.176176121	count	1
ITGA3	1.2569057	1.1205265	1.1217	0.262	0.176176121	count	1

CFAP61	1.2569057	1.319048	0.9529	0.341	0.176176121	count	1
Z98752.4	1.2569057	1.319048	0.9529	0.341	0.176176121	count	1
STAP2	1.2569057	1.319048	0.9529	0.341	0.176176121	count	1
AC008622.2	1.2569057	1.1205265	1.1217	0.262	0.176176121	count	1
PTOV1-AS2	1.2569057	1.458152	0.862	0.389	0.176176121	count	1
ADPRHL2	0.1339328	0.1706834	0.7847	0.433	0.176321043	count	1
ZFYVE26	0.1980182	0.3843935	0.5151	0.606	0.176387863	count	1
PPP1CB	0.1238105	0.0595665	2.0785	0.0378	0.176500703	count	1
TARDBP	0.1414601	0.1966745	0.7193	0.472	0.176858189	count	1
ADM	0.1305371	0.1312483	0.9946	0.32	0.176946353	count	1
RFWD3	0.2118569	0.4131286	0.5128	0.608	0.177113566	count	1
SUPT16H	0.1295967	0.1097011	1.1814	0.238	0.1773242	count	1
SEC63	0.1315226	0.1336767	0.9839	0.325	0.177465597	count	1
ABCC4	0.199222	0.6607406	0.3015	0.763	0.177485824	count	1
RPL26	0.1233514	0.0264893	4.6567	3.39E-06	0.177665341	count	0.08055318
CUL5	0.1333577	0.15524	0.859	0.39	0.177694833	count	1
PDPR	0.1554802	0.3103187	0.501	0.616	0.177773307	count	1
UBTD2	0.1542087	0.2855552	0.54	0.589	0.177780111	count	1
VPS51	0.1388775	0.1568021	0.8857	0.376	0.177884163	count	1
CNOT1	0.1375174	0.1703217	0.8074	0.42	0.177948496	count	1
CASC4	0.1333929	0.1759051	0.7583	0.448	0.178088515	count	1
UBE2Q2	0.1333807	0.1744158	0.7647	0.445	0.178483097	count	1
CHRAC1	0.1318141	0.1396294	0.944	0.345	0.178515027	count	1
KIAA0586	0.1548921	0.2406752	0.6436	0.52	0.178575737	count	1
ZNF664	0.162725	0.3173795	0.5127	0.608	0.17874498	count	1
RAPH1	0.142387	0.2017947	0.7056	0.481	0.178865816	count	1
PSIP1	0.1296635	0.1144751	1.1327	0.257	0.178909519	count	1
TAF12	0.1304187	0.1167272	1.1173	0.264	0.178974436	count	1
PNPLA4	0.1725383	0.3079022	0.5604	0.575	0.179016011	count	1
AL136115.2	0.7713801	1.0188793	0.7571	0.449	0.17902115	count	1
AL603839.3	0.7713801	1.3644481	0.5653	0.572	0.17902115	count	1
ORC1	0.7713801	1.0188793	0.7571	0.449	0.17902115	count	1
FALEC	0.7713801	1.1715293	0.6584	0.51	0.17902115	count	1
RGS5	0.7713801	1.037977	0.7432	0.457	0.17902115	count	1
AC012358.3	0.7713801	1.037977	0.7432	0.457	0.17902115	count	1
LINC01116	0.7713801	0.9994168	0.7718	0.44	0.17902115	count	1
AC008966.1	0.7713801	1.0188793	0.7571	0.449	0.17902115	count	1
SYCP2L	0.7713801	1.0188793	0.7571	0.449	0.17902115	count	1
PCDH11X	0.7713801	1.037977	0.7432	0.457	0.17902115	count	1
GPRASP1	0.7713801	1.037977	0.7432	0.457	0.17902115	count	1
EPS8L2	0.7713801	0.9994168	0.7718	0.44	0.17902115	count	1
FIBIN	0.7713801	1.468606	0.5252	0.599	0.17902115	count	1
AP001160.2	0.7713801	1.037977	0.7432	0.457	0.17902115	count	1
LRTOMT	0.7713801	1.037977	0.7432	0.457	0.17902115	count	1
ZEB1-AS1	0.7713801	1.0188793	0.7571	0.449	0.17902115	count	1

NEK5	0.7713801	1.0188793	0.7571	0.449	0.17902115	count	1
EDC4	0.7713801	1.037977	0.7432	0.457	0.17902115	count	1
DHRS13	0.7713801	1.0188793	0.7571	0.449	0.17902115	count	1
MPP3	0.7713801	1.0188793	0.7571	0.449	0.17902115	count	1
AC068234.2	0.7713801	1.037977	0.7432	0.457	0.17902115	count	1
AC011825.2	0.7713801	1.3644481	0.5653	0.572	0.17902115	count	1
AC005523.1	0.7713801	1.0188793	0.7571	0.449	0.17902115	count	1
ZNF788P	0.7713801	1.1715293	0.6584	0.51	0.17902115	count	1
PGLYRP2	0.7713801	1.0188793	0.7571	0.449	0.17902115	count	1
ZNF850	0.7713801	1.0188793	0.7571	0.449	0.17902115	count	1
ZNF793	0.7713801	1.4141	0.5455	0.585	0.17902115	count	1
EDDM13	0.7713801	1.1715293	0.6584	0.51	0.17902115	count	1
C1QTNF6	0.7713801	1.037977	0.7432	0.457	0.17902115	count	1
TOX4	0.1276252	0.0949627	1.344	0.179	0.179033903	count	1
HIBCH	0.1536377	0.3244807	0.4735	0.636	0.179152365	count	1
DCK	0.1386176	0.1416717	0.9784	0.328	0.179209255	count	1
TIPRL	0.1302964	0.1359693	0.9583	0.338	0.179307109	count	1
GSTK1	0.1257762	0.054675	2.3004	0.0215	0.179364901	count	1
TADA1	0.7726871	0.8592072	0.8993	0.369	0.179375355	count	1
OSBPL6	0.7726871	0.8177903	0.9448	0.345	0.179375355	count	1
C7orf61	0.7726871	0.8592072	0.8993	0.369	0.179375355	count	1
ACTL10	0.7726871	0.8592072	0.8993	0.369	0.179375355	count	1
TYW3	0.1341951	0.1529054	0.8776	0.38	0.179413791	count	1
SMURF2	0.1475588	0.2377436	0.6207	0.535	0.179607022	count	1
SMAD1	0.3354815	0.5849101	0.5736	0.566	0.179636682	count	1
POLA2	0.3354815	0.5106453	0.657	0.511	0.179636682	count	1
NAPB	0.3354815	0.4821486	0.6958	0.487	0.179636682	count	1
ZNF616	0.2620427	0.5426644	0.4829	0.629	0.179713614	count	1
CHM	0.1339032	0.1497083	0.8944	0.371	0.179797427	count	1
TMF1	0.1292431	0.1123355	1.1505	0.25	0.17994234	count	1
SKAP2	0.1279366	0.0758942	1.6857	0.092	0.180078764	count	1
RECQL5	0.4790831	0.6581414	0.7279	0.467	0.180150044	count	1
TAF1D	0.1280737	0.0881483	1.4529	0.146	0.180245859	count	1
CMTM4	0.4143394	1.0044357	0.4125	0.68	0.180344398	count	1
GCC2	0.1296584	0.1090609	1.1889	0.235	0.180355068	count	1
XBP1	0.1277159	0.0742615	1.7198	0.0856	0.180697111	count	1
ECSIT	0.1617804	0.2679638	0.6037	0.546	0.180702512	count	1
TOPORS	0.1381128	0.1398811	0.9874	0.324	0.180959784	count	1
ARL13B	0.151968	0.2274615	0.6681	0.504	0.181242406	count	1
CRYBG1	0.137445	0.1300854	1.0566	0.291	0.181314551	count	1
ZNF862	0.371577	0.6007818	0.6185	0.536	0.181438297	count	1
TMEM86B	0.371577	0.6926517	0.5365	0.592	0.181438297	count	1
FBXW2	0.1495371	0.2340136	0.639	0.523	0.181609459	count	1
RING1	0.1431781	0.1899449	0.7538	0.451	0.181651428	count	1
SCFD2	0.2941531	0.4088984	0.7194	0.472	0.181731223	count	1

KAT6A	0.1429485	0.1766021	0.8094	0.418	0.181825032	count	1
KATNB1	0.1912996	0.3775958	0.5066	0.612	0.181869238	count	1
NAGPA	0.138611	0.1885147	0.7353	0.462	0.181878088	count	1
MTMR10	0.150574	0.283814	0.5305	0.596	0.181992899	count	1
NRIP1	0.1332834	0.1612185	0.8267	0.408	0.182002895	count	1
TMA7	0.1269692	0.0419836	3.0243	0.00252	0.182127801	count	1
FBXW7	0.1397833	0.1992509	0.7015	0.483	0.182313958	count	1
HBP1	0.1348508	0.1199774	1.124	0.261	0.182343118	count	1
UNC93B1	0.1303488	0.0875202	1.4894	0.137	0.182403186	count	1
RPN1	0.1302887	0.093292	1.3966	0.163	0.182574722	count	1
MED29	0.1328513	0.1289498	1.0303	0.303	0.182666087	count	1
PANK2	0.1385877	0.1827552	0.7583	0.448	0.182710968	count	1
INTS6	0.1357689	0.1562397	0.869	0.385	0.182741401	count	1
AC243829.1	0.4197244	0.5783199	0.7258	0.468	0.182891772	count	1
ZNF443	0.4864555	0.8999408	0.5405	0.589	0.183211653	count	1
TANGO2	0.1571409	0.2752411	0.5709	0.568	0.183276268	count	1
GUF1	0.1928797	0.3846397	0.5015	0.616	0.183402445	count	1
RBM10	0.1679706	0.3202784	0.5245	0.6	0.183459305	count	1
GTF2F1	0.1332563	0.1236539	1.0777	0.281	0.183484045	count	1
RBM7	0.1470361	0.1869324	0.7866	0.432	0.183571026	count	1
CLEC10A	0.1317264	0.1072139	1.2286	0.219	0.183651487	count	1
IL12RB1	0.2100932	0.3513197	0.598	0.55	0.183726903	count	1
LINC01003	0.1408116	0.1940274	0.7257	0.468	0.183807789	count	1
UBQLN4	0.2150927	0.3688082	0.5832	0.56	0.184191985	count	1
AC005225.2	0.790874	0.9377149	0.8434	0.399	0.184318993	count	1
C19orf33	0.790874	0.8046216	0.9829	0.326	0.184318993	count	1
CNTROB	0.3184024	0.5002542	0.6365	0.525	0.184432466	count	1
C2orf42	0.343899	0.5358375	0.6418	0.521	0.184439089	count	1
FAM20B	0.1847198	0.3766169	0.4905	0.624	0.184484538	count	1
GDI2	0.1305979	0.0647936	2.0156	0.044	0.184529566	count	1
PGLS	0.1289469	0.0479013	2.6919	0.00715	0.184544869	count	1
HMGXB4	0.1361789	0.194956	0.6985	0.485	0.184613938	count	1
ZNF181	0.2263466	0.4195505	0.5395	0.59	0.184748606	count	1
CCDC47	0.1312174	0.0797876	1.6446	0.1	0.184821967	count	1
PPP6R2	0.1509155	0.2971922	0.5078	0.612	0.184943361	count	1
NEDD4	0.2209719	0.3435926	0.6431	0.52	0.184954812	count	1
AC240274.1	0.5967236	0.4090127	1.4589	0.145	0.185010004	count	1
GALNT6	0.1664786	0.2648839	0.6285	0.53	0.185019369	count	1
PPP1R3G	0.2330231	0.5712307	0.4079	0.683	0.185025408	count	1
SREK1IP1	0.1321167	0.1004432	1.3153	0.189	0.185038591	count	1
CPSF3	0.1640556	0.2327682	0.7048	0.481	0.185129528	count	1
ZNF513	0.1874749	0.3731787	0.5024	0.615	0.185177958	count	1
SH3KBP1	0.1324027	0.0828654	1.5978	0.11	0.185426755	count	1
AC010642.2	0.1458698	0.2147436	0.6793	0.497	0.185561126	count	1
MYCBP2	0.1325622	0.0971954	1.3639	0.173	0.185584979	count	1

ZNF92	0.167906	0.2938946	0.5713	0.568	0.185586269	count	1
COG2	0.1598066	0.27189	0.5878	0.557	0.185731817	count	1
ASXL1	0.14065	0.1621254	0.8675	0.386	0.185790625	count	1
LBR	0.1335917	0.1179884	1.1322	0.258	0.18583461	count	1
AC004241.1	0.3207578	0.5922552	0.5416	0.588	0.185876448	count	1
BAX	0.1308778	0.0667737	1.96	0.0501	0.185909247	count	1
CYBC1	0.1361239	0.1086132	1.2533	0.21	0.185934807	count	1
TBC1D7	0.1433431	0.184547	0.7767	0.437	0.186030389	count	1
CD53	0.1301354	0.0477477	2.7255	0.0065	0.186119269	count	1
PATZ1	0.3469252	0.4251156	0.8161	0.415	0.186168898	count	1
UCHL5	0.1420861	0.1622587	0.8757	0.381	0.186322849	count	1
ARHGDIB	0.1298313	0.0357697	3.6296	0.00029	0.18641352	count	1
AC107959.4	0.4272165	0.4925502	0.8674	0.386	0.186443914	count	1
MRPL16	0.1358311	0.115196	1.1791	0.238	0.186454149	count	1
RPL38	0.1300123	0.0354234	3.6702	0.000247	0.186461423	count	1
HNRNPA2B1	0.1300188	0.0388072	3.3504	0.000819	0.186655368	count	1
ZNF316	0.213476	0.3297725	0.6473	0.517	0.186762785	count	1
DDX18	0.132772	0.0819979	1.6192	0.106	0.187064166	count	1
PLOD1	0.1628684	0.2648056	0.615	0.539	0.187101436	count	1
FDPS	0.1359064	0.1171021	1.1606	0.246	0.187204077	count	1
MRPL48	0.1518535	0.2709175	0.5605	0.575	0.187243533	count	1
AC084346.2	0.6035605	0.7763349	0.7774	0.437	0.187408724	count	1
AL157938.3	0.6035605	0.7889991	0.765	0.444	0.187408724	count	1
AP003392.6	0.6035605	0.7584041	0.7958	0.426	0.187408724	count	1
SIAE	0.6035605	0.7738528	0.7799	0.436	0.187408724	count	1
EMID1	1.322619	0.7634871	1.7323	0.0833	0.187420524	count	1
GUCD1	0.1475331	0.1918418	0.769	0.442	0.187450503	count	1
PIP5K1C	0.1596654	0.2691764	0.5932	0.553	0.187554344	count	1
DUT	0.1331853	0.0932163	1.4288	0.153	0.187688267	count	1
CCDC9	0.1778897	0.355808	0.5	0.617	0.187751871	count	1
POLG2	0.1729947	0.468823	0.369	0.712	0.187820493	count	1
AL353622.1	0.604777	0.8045314	0.7517	0.452	0.187836116	count	1
MIR3142HG	0.604777	0.8421133	0.7182	0.473	0.187836116	count	1
RPS6KB2	0.1402889	0.1772342	0.7915	0.429	0.187840415	count	1
HIF1AN	0.1636516	0.3999483	0.4092	0.682	0.188010619	count	1
PRR7	0.1795987	0.3680124	0.488	0.626	0.188054688	count	1
ALKBH4	0.1732348	0.4320854	0.4009	0.689	0.188084682	count	1
TPP2	0.1449967	0.1764453	0.8218	0.411	0.188186315	count	1
CHIC1	0.3844623	0.7706398	0.4989	0.618	0.188209172	count	1
AP000845.1	0.3844623	0.8323994	0.4619	0.644	0.188209172	count	1
AC018413.1	0.3844623	0.7581035	0.5071	0.612	0.188209172	count	1
ZNF543	0.3844623	0.8405036	0.4574	0.647	0.188209172	count	1
AL022322.2	0.3844623	0.8323994	0.4619	0.644	0.188209172	count	1
MYL9	0.1770248	0.3624943	0.4884	0.625	0.188268842	count	1
COG3	0.1593126	0.3497309	0.4555	0.649	0.188363651	count	1

COX4I1	0.1309207	0.0280538	4.6668	3.22E-06	0.188391001	count	0.0765233
TVP23B	0.1747266	0.3172319	0.5508	0.582	0.188467058	count	1
AC018797.2	0.385126	0.5902951	0.6524	0.514	0.188558734	count	1
LINC00957	0.385126	0.6671663	0.5773	0.564	0.188558734	count	1
PORCN	0.2881092	0.4841314	0.5951	0.552	0.188582134	count	1
NAA30	0.190921	0.4196791	0.4549	0.649	0.18864606	count	1
TMEM18	0.1361106	0.1314398	1.0355	0.301	0.188716733	count	1
WDR59	0.2115492	0.3449638	0.6133	0.54	0.188745876	count	1
BIRC2	0.1401181	0.1432989	0.9778	0.328	0.188824602	count	1
ZNF789	0.2377416	0.3563323	0.6672	0.505	0.188896235	count	1
CALHM6	0.1570586	0.1538576	1.0208	0.307	0.188922645	count	1
ZNF253	0.4326078	0.5322095	0.8129	0.416	0.189005744	count	1
CXCR2	1.3322248	0.9509183	1.401	0.161	0.189071541	count	1
ELP3	0.1742493	0.3058775	0.5697	0.569	0.189201072	count	1
ZSCAN26	0.2083103	0.4770866	0.4366	0.662	0.189221883	count	1
KLF11	0.1523257	0.2451583	0.6213	0.534	0.189239545	count	1
GDI1	0.1472665	0.1804609	0.8161	0.415	0.189302472	count	1
RPAIN	0.1364986	0.1090047	1.2522	0.211	0.189394778	count	1
NEURL4	0.2319688	0.9217573	0.2517	0.801	0.189481139	count	1
REEP3	0.1357632	0.1166173	1.1642	0.244	0.189539584	count	1
CMTM7	0.1368665	0.0995875	1.3743	0.169	0.189694833	count	1
SUCLG1	0.1374314	0.0991805	1.3857	0.166	0.190177138	count	1
SLC26A2	0.1635989	0.2711662	0.6033	0.546	0.190183501	count	1
GFM2	0.2270848	0.4201444	0.5405	0.589	0.190223001	count	1
LTBP2	0.2905308	0.347233	0.8367	0.403	0.190244149	count	1
LRRC37A3	1.3390987	1.045264	1.2811	0.2	0.190254038	count	1
CNNM3	0.3279315	0.6844555	0.4791	0.632	0.190280978	count	1
GNG12	0.3075903	0.3839719	0.8011	0.423	0.190478876	count	1
SMIM20	0.1402712	0.1390776	1.0086	0.313	0.190624747	count	1
LCLAT1	0.2486387	0.4324058	0.575	0.565	0.191720336	count	1
FIGNL1	0.2672769	0.6461276	0.4137	0.679	0.19182682	count	1
PXN-AS1	0.2672769	0.5912551	0.4521	0.651	0.19182682	count	1
DNAJC27	0.2015693	0.6714709	0.3002	0.764	0.191842622	count	1
PRSS23	1.348495	0.6065373	2.2233	0.0263	0.191871816	count	1
TRABD2A	0.4390606	0.7794505	0.5633	0.573	0.192078247	count	1
TTC12	0.4390606	0.7794505	0.5633	0.573	0.192078247	count	1
LKAAEAR1	0.4390606	0.6946211	0.6321	0.527	0.192078247	count	1
FANCA	0.392235	0.4346439	0.9024	0.367	0.192307836	count	1
ZNF624	0.4395947	0.5476351	0.8027	0.422	0.19233286	count	1
KCNAB3	1.351332	1.132474	1.1933	0.233	0.192360425	count	1
AHR	0.1416336	0.1286978	1.1005	0.271	0.192481022	count	1
ZMIZ1-AS1	0.2357336	0.5356727	0.4401	0.66	0.192653805	count	1
IQGAP1	0.1353658	0.0602493	2.2468	0.0247	0.19287349	count	1
FAM160B2	0.2428082	0.418429	0.5803	0.562	0.193057731	count	1
COPS6	0.1378574	0.0942757	1.4623	0.144	0.193075684	count	1

CFAP97	0.1456782	0.1600574	0.9102	0.363	0.193357264	count	1
CCZ1	0.1536645	0.2028219	0.7576	0.449	0.193411207	count	1
SRRM2	0.1352643	0.0542732	2.4923	0.0128	0.193707058	count	1
AC003101.2	0.360186	0.8057035	0.447	0.655	0.193769245	count	1
LACTB2	0.149288	0.22066	0.6766	0.499	0.193782191	count	1
KMT2A	0.141957	0.1405508	1.01	0.313	0.193782307	count	1
LIX1L	0.1604682	0.2380574	0.6741	0.5	0.194049764	count	1
EIF2A	0.1405334	0.0908716	1.5465	0.122	0.194085875	count	1
FMR1	0.1516013	0.2041407	0.7426	0.458	0.194269274	count	1
NEURL1	0.2177799	0.3634201	0.5993	0.549	0.194448663	count	1
LINC00921	0.2608893	0.474441	0.5499	0.582	0.194575949	count	1
SYK	0.1395098	0.1050238	1.3284	0.184	0.194659527	count	1
INSIG2	0.1575687	0.2219189	0.71	0.478	0.194718116	count	1
CFLAR-AS1	0.283068	0.4665572	0.6067	0.544	0.194791578	count	1
THAP12	0.1461062	0.1753482	0.8332	0.405	0.194838059	count	1
MAFG	0.1396521	0.1132605	1.233	0.218	0.19507469	count	1
PPP2R3C	0.141992	0.1330981	1.0668	0.286	0.195116324	count	1
PSMC4	0.1395394	0.0944026	1.4781	0.14	0.195134558	count	1
GPATCH1	0.2275942	0.3667125	0.6206	0.535	0.195205267	count	1
AC116366.1	0.6261912	0.5654067	1.1075	0.268	0.195388354	count	1
SECTM1	0.1468928	0.1208731	1.2153	0.224	0.195596773	count	1
DDX58	0.1634821	0.2239046	0.7301	0.465	0.195651204	count	1
DHFR2	0.31558	0.7159299	0.4408	0.659	0.19569731	count	1
BMI1	0.1815795	0.3357992	0.5407	0.589	0.195965027	count	1
RBM12	0.1625754	0.2791079	0.5825	0.56	0.19612362	count	1
APOBR	0.1486132	0.2282726	0.651	0.515	0.196229724	count	1
FAM221A	0.2340832	0.3918636	0.5974	0.55	0.196263659	count	1
LANCL2	0.2851424	0.5324648	0.5355	0.592	0.196284118	count	1
AL590822.2	1.37497	0.6288247	2.1866	0.0289	0.196437539	count	1
MTCP1	1.37497	1.145237	1.2006	0.23	0.196437539	count	1
GXYLT1	0.1777461	0.2817192	0.6309	0.528	0.1966044	count	1
IKBIP	0.1412679	0.0965714	1.4628	0.144	0.196670994	count	1
ATP5MG	0.1371669	0.0390372	3.5137	0.00045	0.196794911	count	1
GTF2E2	0.1521729	0.1915557	0.7944	0.427	0.196821431	count	1
RMC1	0.1771912	0.2529619	0.7005	0.484	0.197077098	count	1
RIOK1	0.152842	0.2356211	0.6487	0.517	0.197114094	count	1
IRF4	0.2250153	0.3912235	0.5752	0.565	0.197135892	count	1
KLHL12	0.2132711	0.3683438	0.579	0.563	0.197151912	count	1
RANGRF	0.1479854	0.1865821	0.7931	0.428	0.197255401	count	1
CCAR1	0.1513088	0.1553429	0.974	0.33	0.197415089	count	1
AL031714.1	0.4507117	1.0299973	0.4376	0.662	0.197643117	count	1
YJEFN3	0.4507117	0.7316632	0.616	0.538	0.197643117	count	1
NKAP	0.1452791	0.1216225	1.1945	0.232	0.197774299	count	1
ANAPC16	0.1394108	0.0748703	1.862	0.0627	0.197777847	count	1
MBNL1-AS1	0.2753601	0.4816819	0.5717	0.568	0.197874367	count	1

PDLIM5	0.1473346	0.1365427	1.079	0.281	0.197890477	count	1
DHX30	0.1581787	0.2162165	0.7316	0.464	0.197899462	count	1
BCL2L2	0.18887	0.471234	0.4008	0.689	0.197919837	count	1
H6PD	0.1657133	0.2726464	0.6078	0.543	0.198345123	count	1
CLHC1	0.3023778	0.6021317	0.5022	0.616	0.198392243	count	1
ARHGEF3	0.1865437	0.2850602	0.6544	0.513	0.198547705	count	1
RPS8	0.1377132	0.0240875	5.7172	1.21E-08	0.19855089	count	0.000290219
DHX40	0.1627842	0.2077868	0.7834	0.433	0.198735994	count	1
VPS16	0.1621622	0.2175037	0.7456	0.456	0.198831814	count	1
TUBGCP3	0.1709953	0.2541365	0.6728	0.501	0.198871421	count	1
TRIQK	0.1577711	0.2196178	0.7184	0.473	0.19890398	count	1
FBXO9	0.1463017	0.1417001	1.0325	0.302	0.198953315	count	1
ZNF644	0.1463096	0.1369212	1.0686	0.285	0.198964086	count	1
NMD3	0.1591087	0.1961448	0.8112	0.417	0.199070718	count	1
DHRS7	0.1400625	0.0680268	2.0589	0.0396	0.199282984	count	1
USP38	0.1757165	0.234685	0.7487	0.454	0.19938743	count	1
DNTTIP2	0.1412544	0.0914402	1.5448	0.123	0.199459814	count	1
RPE	0.16715	0.226839	0.7369	0.461	0.199513999	count	1
RPL14	0.1385705	0.0248397	5.5786	2.69E-08	0.199648549	count	0.00064439
FAM204A	0.1422315	0.0865364	1.6436	0.1	0.199728082	count	1
AL136162.1	0.3045749	0.5968112	0.5103	0.61	0.199906472	count	1
NATD1	0.3710512	0.4503596	0.8239	0.41	0.20002099	count	1
SETD7	0.1610393	0.22647	0.7111	0.477	0.200140878	count	1
IL13RA1	0.1430041	0.0851578	1.6793	0.0932	0.200150982	count	1
BAP1	0.2003305	0.3738731	0.5358	0.592	0.200375531	count	1
DNAJC2	0.1462506	0.1333887	1.0964	0.273	0.200381695	count	1
TNFRSF1A	0.143878	0.0821449	1.7515	0.08	0.20046809	count	1
RPL22L1	0.1415177	0.0824535	1.7163	0.0862	0.20049826	count	1
HSPA1A	0.1393774	0.0775314	1.7977	0.0723	0.200540009	count	1
C1orf123	0.1456193	0.1317333	1.1054	0.269	0.200711444	count	1
CEP57L1	0.2031374	0.3243375	0.6263	0.531	0.200957816	count	1
C2CD3	0.2031374	0.4989547	0.4071	0.684	0.200957816	count	1
PIGA	0.1902624	0.3698675	0.5144	0.607	0.201019039	count	1
CES2	0.1670748	0.3410063	0.4899	0.624	0.201076006	count	1
ASF1B	0.8524591	0.6809776	1.2518	0.211	0.201258327	count	1
CLASP1	0.1841105	0.370384	0.4971	0.619	0.201334513	count	1
SEMA6A-AS1	0.8529447	1.069174	0.7978	0.425	0.201393071	count	1
MAP3K13	0.1469026	0.1331596	1.1032	0.27	0.201485574	count	1
MFF	0.1446466	0.1170918	1.2353	0.217	0.201710837	count	1
HDGF	0.1447186	0.1089695	1.3281	0.184	0.201755552	count	1
DPH6	0.1692963	0.2551448	0.6635	0.507	0.202099325	count	1
VPS35L	0.1827521	0.2740883	0.6668	0.505	0.202215305	count	1
ZDHHC13	0.2152907	0.7502037	0.287	0.774	0.20221818	count	1
SLC30A7	0.1676348	0.219826	0.7626	0.446	0.202279599	count	1
DBF4	0.1561052	0.201698	0.774	0.439	0.202310026	count	1

H2AFX	0.1609824	0.1829905	0.8797	0.379	0.202378311	count	1
AL034549.1	0.2070521	0.4766972	0.4343	0.664	0.20246531	count	1
FGFR1OP	0.2156755	0.3954397	0.5454	0.586	0.202588041	count	1
CPE	0.6469752	0.5646762	1.1457	0.252	0.202769686	count	1
ADD1	0.1508989	0.1621219	0.9308	0.352	0.20277523	count	1
NUP54	0.1623464	0.2321116	0.6994	0.484	0.202816945	count	1
ZCWPW1	0.1988404	0.4633776	0.4291	0.668	0.203006125	count	1
FAM98C	0.1659306	0.2282828	0.7269	0.467	0.203053556	count	1
UCN	0.6480194	0.9605233	0.6747	0.5	0.20314184	count	1
AC009962.1	0.6480194	1.454252	0.4456	0.656	0.20314184	count	1
ZSCAN31	0.6480194	1.0230415	0.6334	0.527	0.20314184	count	1
AL512791.2	0.6480194	0.9634124	0.6726	0.501	0.20314184	count	1
AC010542.4	0.6480194	0.9605233	0.6747	0.5	0.20314184	count	1
AP005131.1	0.6480194	1.119601	0.5788	0.563	0.20314184	count	1
CHAF1B	0.6480194	0.9634124	0.6726	0.501	0.20314184	count	1
SHPK	0.6483378	0.745956	0.8691	0.385	0.203255345	count	1
FADD	0.1858582	0.3063915	0.6066	0.544	0.203272531	count	1
POLI	0.1858582	0.4388065	0.4236	0.672	0.203272531	count	1
USP21	0.4625327	0.6009292	0.7697	0.442	0.203311596	count	1
WDR76	0.4625327	0.6096328	0.7587	0.448	0.203311596	count	1
ARHGAP9	0.1573379	0.1537641	1.0232	0.306	0.203341523	count	1
PEMT	0.1768595	0.2564823	0.6896	0.491	0.203355747	count	1
TMEM30A	0.1484294	0.1322702	1.1222	0.262	0.203459155	count	1
NRDE2	0.1769558	0.2697839	0.6559	0.512	0.203467718	count	1
PDK2	0.1743463	0.2496727	0.6983	0.485	0.203553529	count	1
MHENCR	0.1821278	0.3883796	0.4689	0.639	0.203722732	count	1
MKRN2	0.1803607	0.3053945	0.5906	0.555	0.203755574	count	1
IFT140	0.2955499	1.1401469	0.2592	0.795	0.203785583	count	1
KCNA3	0.4139213	0.6632132	0.6241	0.533	0.203799651	count	1
MYBPC3	0.4139213	1.0065425	0.4112	0.681	0.203799651	count	1
HOTAIRM1	0.1504489	0.1269617	1.185	0.236	0.203825384	count	1
STK16	0.1629021	0.2060697	0.7905	0.429	0.203849059	count	1
AC007388.1	0.1596233	0.2589542	0.6164	0.538	0.203903524	count	1
SYPL1	0.1460551	0.1089077	1.3411	0.18	0.203998085	count	1
RNF38	0.1729829	0.361139	0.479	0.632	0.20403001	count	1
ARHGAP31	0.1877522	0.3009806	0.6238	0.533	0.204075463	count	1
IKZF4	0.2641509	0.4942432	0.5345	0.593	0.204133561	count	1
FAU	0.1416396	0.0226274	6.2597	4.54E-10	0.204142846	count	1.09E-05
DOHH	0.1807528	0.4200151	0.4303	0.667	0.204203954	count	1
ETNK1	0.1483036	0.1259954	1.1771	0.239	0.20445262	count	1
RBM25	0.1437639	0.0726437	1.979	0.0479	0.204514805	count	1
INPP4B	0.6519234	0.723877	0.9006	0.368	0.204534363	count	1
BTBD10	0.1661317	0.2302676	0.7215	0.471	0.204577199	count	1
CDK9	0.1563199	0.2047006	0.7637	0.445	0.204619248	count	1
DENR	0.1491232	0.1298252	1.1486	0.251	0.204741679	count	1

ESCO1	0.1574423	0.2003732	0.7857	0.432	0.204775497	count	1
PCYOX1L	0.4160626	0.3892076	1.069	0.285	0.204938806	count	1
RNF2	0.1605427	0.2174861	0.7382	0.46	0.20508474	count	1
ZNF205	0.2339583	0.4872028	0.4802	0.631	0.20519324	count	1
RPGRIP1	0.3800354	0.6393702	0.5944	0.552	0.205206944	count	1
SEC23A	0.1688669	0.2098862	0.8046	0.421	0.205282014	count	1
MT-ATP8	0.1525489	0.1516325	1.006	0.314	0.20532132	count	1
TMEM216	0.17016	0.2605351	0.6531	0.514	0.205353212	count	1
STMP1	0.1443391	0.065529	2.2027	0.0277	0.205374724	count	1
CDK6	0.1580762	0.1782951	0.8866	0.375	0.205426624	count	1
NAP1L1	0.1442209	0.0610586	2.362	0.0183	0.205467076	count	1
CD163L1	0.1856696	0.3630475	0.5114	0.609	0.20548707	count	1
PARP9	0.1561123	0.1492766	1.0458	0.296	0.205509378	count	1
CCDC66	0.1565415	0.1799852	0.8697	0.385	0.205510434	count	1
ARHGAP30	0.1492189	0.1087343	1.3723	0.17	0.205882709	count	1
KLHDC10	0.1851733	0.2786764	0.6645	0.506	0.206072549	count	1
TNS2	1.431815	0.7682421	1.8638	0.0625	0.206273245	count	1
SPEF2	0.3319184	1.0844571	0.3061	0.76	0.206407992	count	1
NONO	0.1519357	0.1213666	1.2519	0.211	0.206461916	count	1
RBMX	0.1486155	0.0989422	1.502	0.133	0.20654639	count	1
ATG101	0.1512216	0.1379824	1.0959	0.273	0.206609337	count	1
PCID2	0.1616688	0.1946199	0.8307	0.406	0.206773162	count	1
SDHD	0.1470306	0.0862106	1.7055	0.0882	0.206843188	count	1
PPM1B	0.170221	0.1842509	0.9239	0.356	0.206941901	count	1
BNC2	0.355036	0.5034792	0.7052	0.481	0.207012634	count	1
BX255925.3	0.3330084	0.5872588	0.5671	0.571	0.207124413	count	1
SAV1	0.1685598	0.2541239	0.6633	0.507	0.207169292	count	1
NUP155	0.2205318	0.4434252	0.4973	0.619	0.207258228	count	1
WBP11	0.1519547	0.1412227	1.076	0.282	0.207312687	count	1
BCL2L11	0.1640237	0.1715311	0.9562	0.339	0.207424634	count	1
LIN9	0.8752097	0.8365327	1.0462	0.296	0.20759053	count	1
AC090152.1	0.8752097	0.8025996	1.0905	0.276	0.20759053	count	1
DNALI1	0.3157764	0.6371674	0.4956	0.62	0.207641495	count	1
AL136962.1	0.3157764	0.6767427	0.4666	0.641	0.207641495	count	1
BICD1	0.288489	0.438158	0.6584	0.51	0.207725364	count	1
LHFPL6	0.288489	0.5422228	0.532	0.595	0.207725364	count	1
DIMT1	0.1701241	0.2395704	0.7101	0.478	0.207770296	count	1
NR2C1	0.2075851	0.3562483	0.5827	0.56	0.20777514	count	1
GLRX	0.1455065	0.0570386	2.551	0.0108	0.207831647	count	1
HINT2	0.1509314	0.1251621	1.2059	0.228	0.207909497	count	1
DMAC2	0.1904586	0.3058145	0.6228	0.533	0.208376131	count	1
ZNF302	0.1756245	0.3032293	0.5792	0.563	0.20848717	count	1
CDC16	0.1756757	0.2519729	0.6972	0.486	0.208548542	count	1
CEBPA	0.1606111	0.1636091	0.9817	0.326	0.208554301	count	1
BOD1	0.1693521	0.2265374	0.7476	0.455	0.20857359	count	1

LSM8	0.1493278	0.0865451	1.7254	0.0846	0.208575958	count	1
RAPGEF6	0.2291724	0.4628937	0.4951	0.621	0.208670554	count	1
FNIP1	0.150812	0.1228253	1.2279	0.22	0.208813911	count	1
SAR1B	0.151887	0.1292361	1.1753	0.24	0.208936063	count	1
NOP9	0.2066048	0.4442431	0.4651	0.642	0.208980218	count	1
AAAS	0.1783315	0.3069824	0.5809	0.561	0.208992877	count	1
MPND	0.1732729	0.2431205	0.7127	0.476	0.209143164	count	1
ATP8A1	0.2554188	0.5397607	0.4732	0.636	0.209289796	count	1
NCAPH2	0.1812903	0.3062158	0.592	0.554	0.209357543	count	1
PIBF1	0.1730292	0.2627528	0.6585	0.51	0.209372478	count	1
RPS15A	0.1454097	0.0258076	5.6344	1.96E-08	0.209508783	count	0.000469675
RNASEH2B	0.1508401	0.0983718	1.5334	0.125	0.209643509	count	1
NSA2	0.1473949	0.0626907	2.3511	0.0188	0.209670347	count	1
SLC31A2	0.1484631	0.0889927	1.6683	0.0954	0.209724069	count	1
C16orf74	0.1796364	0.3788	0.4742	0.635	0.209795848	count	1
YME1L1	0.1515171	0.1140882	1.3281	0.184	0.209820121	count	1
TUT1	0.2632183	0.657731	0.4002	0.689	0.20987482	count	1
AC018529.2	0.2267436	0.3249821	0.6977	0.485	0.209919861	count	1
BCL2L13	0.1634248	0.1990495	0.821	0.412	0.209965749	count	1
C6orf89	0.1578282	0.1502672	1.0503	0.294	0.209997038	count	1
TCF19	0.5502916	0.6241421	0.8817	0.378	0.210050291	count	1
BRF2	0.5502916	0.6163436	0.8928	0.372	0.210050291	count	1
ANKRD52	0.4766136	0.560493	0.8503	0.395	0.210092946	count	1
SCAMP4	0.1661567	0.1907297	0.8712	0.384	0.210139319	count	1
MYOM2	0.3601964	0.7677059	0.4692	0.639	0.210214096	count	1
AC138207.5	0.3601964	0.7132659	0.505	0.614	0.210214096	count	1
AKR1C3	0.3601965	0.6671938	0.5399	0.589	0.210214161	count	1
FAM214B	0.1629912	0.2272539	0.7172	0.473	0.210273993	count	1
EWSR1	0.1520032	0.1187916	1.2796	0.201	0.210409564	count	1
TUBGCP4	0.2239607	0.5222479	0.4288	0.668	0.21055838	count	1
CDK3	0.4266459	0.6985671	0.6107	0.541	0.210580723	count	1
DVL1	0.2208979	0.4499617	0.4909	0.624	0.210667208	count	1
UMAD1	0.1914452	0.4178131	0.4582	0.647	0.210743193	count	1
CENPE	0.4781087	0.410369	1.1651	0.244	0.210814828	count	1
CIART	1.4580827	0.6830038	2.1348	0.0329	0.210829706	count	1
LRP2BP	1.4580827	0.9933214	1.4679	0.142	0.210829706	count	1
STAG1	0.174329	0.2062804	0.8451	0.398	0.210959076	count	1
PTP4A3	0.5526914	0.5913221	0.9347	0.35	0.211070519	count	1
ZNF133	0.5526914	0.5778526	0.9565	0.339	0.211070519	count	1
CNOT2	0.1535687	0.1329223	1.1553	0.248	0.211100076	count	1
DGKH	0.2456842	0.3795052	0.6474	0.517	0.211197113	count	1
TRAM2-AS1	0.2458107	0.4300645	0.5716	0.568	0.21130917	count	1
ABHD13	0.1613576	0.2179246	0.7404	0.459	0.211402103	count	1
FAM131A	0.171983	0.2394801	0.7182	0.473	0.211410033	count	1
IGSF6	0.1473078	0.0554756	2.6554	0.00797	0.211423733	count	1

IKZF5	0.2071992	0.4163522	0.4977	0.619	0.211700292	count	1
AP1B1	0.1568934	0.1296121	1.2105	0.226	0.21173342	count	1
RASA4	0.1687168	0.2605756	0.6475	0.517	0.211843924	count	1
AC025171.3	0.273803	0.550161	0.4977	0.619	0.211882133	count	1
ZNF420	0.273803	0.4975383	0.5503	0.582	0.211882133	count	1
LYPLA1	0.1528478	0.1149848	1.3293	0.184	0.212196759	count	1
TRIM8	0.1538214	0.1316187	1.1687	0.243	0.212246297	count	1
MBLAC2	0.55607	0.7257722	0.7662	0.444	0.212508196	count	1
RGS3	0.3077559	0.4914814	0.6262	0.531	0.212611221	count	1
IFT88	0.1820976	0.2459976	0.7402	0.459	0.212701287	count	1
PPP2R2D	0.1637112	0.1984203	0.8251	0.409	0.212786729	count	1
AL451050.2	1.4693778	1.405249	1.0456	0.296	0.212790689	count	1
NEB	1.4693778	1.369788	1.0727	0.284	0.212790689	count	1
LINC02447	1.4693778	1.1071362	1.3272	0.185	0.212790689	count	1
HIST1H4B	1.4693778	1.369788	1.0727	0.284	0.212790689	count	1
TREML2	1.4693778	1.369788	1.0727	0.284	0.212790689	count	1
ESR1	1.4693778	1.369788	1.0727	0.284	0.212790689	count	1
TMEM184A	1.4693778	1.405249	1.0456	0.296	0.212790689	count	1
SUGCT	1.4693778	1.1071362	1.3272	0.185	0.212790689	count	1
NOS3	1.4693778	1.369788	1.0727	0.284	0.212790689	count	1
AL136141.1	1.4693778	1.369788	1.0727	0.284	0.212790689	count	1
AC078927.1	1.4693778	1.1071362	1.3272	0.185	0.212790689	count	1
RNF31	1.4693778	1.1071362	1.3272	0.185	0.212790689	count	1
AC092295.2	1.4693778	1.405249	1.0456	0.296	0.212790689	count	1
KCNJ14	1.4693778	1.369788	1.0727	0.284	0.212790689	count	1
PRCC	0.1653264	0.2212621	0.7472	0.455	0.213088595	count	1
AC027031.2	0.3236487	0.5583094	0.5797	0.562	0.213092531	count	1
METAP1	0.1774576	0.2353307	0.7541	0.451	0.213118336	count	1
LHPP	0.1775281	0.2263499	0.7843	0.433	0.213203787	count	1
SVIP	0.1636915	0.2027995	0.8072	0.42	0.213302727	count	1
ARMH3	0.1781213	0.2773942	0.6421	0.521	0.213337111	count	1
LARP1B	0.1802761	0.251829	0.7159	0.474	0.213401889	count	1
FBH1	0.1898885	0.3052444	0.6221	0.534	0.213606906	count	1
TRIM28	0.1599582	0.1603313	0.9977	0.319	0.213687926	count	1
WDR70	0.1732012	0.202863	0.8538	0.393	0.213773727	count	1
CD4	0.153261	0.0876738	1.7481	0.0806	0.213781765	count	1
AGAP4	0.3431815	0.8237594	0.4166	0.677	0.213821997	count	1
LRRC20	0.3431815	0.7168143	0.4788	0.632	0.213821997	count	1
SLC46A2	0.2273641	0.3876894	0.5865	0.558	0.213836161	count	1
DDX27	0.1571748	0.1365731	1.1508	0.25	0.213840073	count	1
PREX1	0.1558251	0.1352907	1.1518	0.25	0.213881017	count	1
SRGN	0.1483991	0.0307085	4.8325	1.43E-06	0.213942418	count	0.03403972
PGRMC2	0.1644703	0.1743681	0.9432	0.346	0.213962646	count	1
ITGA9	0.3952181	0.3810779	1.0371	0.3	0.21400447	count	1
AL008729.2	0.6787945	0.5884782	1.1535	0.249	0.214165887	count	1

ZBED5-AS1	0.1733314	0.2898538	0.598	0.55	0.214348467	count	1
TOR1AIP2	0.1550495	0.1220917	1.2699	0.204	0.214395292	count	1
JMJD4	0.2120372	0.3382103	0.6269	0.531	0.214582726	count	1
ARFGEF1	0.1608558	0.1658248	0.97	0.332	0.214891488	count	1
HLTF	0.1727512	0.2203613	0.7839	0.433	0.215181378	count	1
AC009093.2	0.298427	0.4862455	0.6137	0.539	0.215205329	count	1
COQ10B	0.1581204	0.1358485	1.1639	0.245	0.215529728	count	1
SCAF11	0.151766	0.0700882	2.1654	0.0305	0.215716355	count	1
SRF	0.1860536	0.3416443	0.5446	0.586	0.21576458	count	1
ZNF496	0.2992123	0.4799478	0.6234	0.533	0.215797233	count	1
AMN1	0.2112691	0.3611954	0.5849	0.559	0.215937799	count	1
GSTP1	0.1500549	0.0369062	4.0658	4.94E-05	0.215980947	count	1
LSM6	0.1532884	0.0961673	1.594	0.111	0.216096009	count	1
PCM1	0.1545485	0.1179822	1.3099	0.19	0.216268678	count	1
LRPPRC	0.1684933	0.1781148	0.946	0.344	0.216281129	count	1
ZNF597	0.3129071	0.502084	0.6232	0.533	0.216344773	count	1
ZNF490	0.3002538	0.6951001	0.432	0.666	0.216582447	count	1
SAFB2	0.1647488	0.1401283	1.1757	0.24	0.216636634	count	1
CEP164	0.1830958	0.219674	0.8335	0.405	0.216773791	count	1
CTSK	0.4000223	0.6550894	0.6106	0.541	0.216796961	count	1
MPST	0.1599742	0.1330445	1.2024	0.229	0.21697017	count	1
PRPF39	0.2523757	0.3323484	0.7594	0.448	0.21712887	count	1
ELF2	0.1569511	0.1069269	1.4678	0.142	0.217182934	count	1
BIRC6	0.1622245	0.1590964	1.0197	0.308	0.217413216	count	1
ARPC1B	0.1516312	0.0404194	3.7514	0.00018	0.217456811	count	1
EIF1	0.1509537	0.0220282	6.8527	9.13E-12	0.217506514	count	2.20E-07
TNS1	0.1722143	0.2593496	0.664	0.507	0.217546696	count	1
PLEKHB1	0.5680355	0.7279728	0.7803	0.435	0.217612532	count	1
RGS16	0.1916324	0.3318179	0.5775	0.564	0.217677096	count	1
GIT2	0.1683817	0.1639306	1.0272	0.304	0.217692541	count	1
LEPROTL1	0.1566212	0.0971149	1.6127	0.107	0.217764248	count	1
PAXBP1	0.162032	0.174742	0.9273	0.354	0.217874183	count	1
CCNT2	0.1909523	0.2812535	0.6789	0.497	0.217880295	count	1
MTA2	0.2003646	0.291131	0.6882	0.491	0.217994413	count	1
PEX16	0.1692966	0.1929977	0.8772	0.38	0.218011365	count	1
RELA	0.1712255	0.1862847	0.9192	0.358	0.218020456	count	1
SMIM10	0.2392557	0.4578316	0.5226	0.601	0.218100669	count	1
NFATC3	0.1897446	0.2286244	0.8299	0.407	0.218348583	count	1
U2AF1L4	0.1972023	0.2797921	0.7048	0.481	0.218432507	count	1
TEDC1	0.2159809	0.3242899	0.666	0.505	0.218653098	count	1
KIAA1324	0.5706602	1.123	0.5082	0.611	0.218734815	count	1
CCL28	0.5706602	0.9891124	0.5769	0.564	0.218734815	count	1
LINC00265	0.5706602	0.8566439	0.6662	0.505	0.218734815	count	1
LINC00539	0.5706602	0.7825141	0.7293	0.466	0.218734815	count	1
FUT8-AS1	0.5706602	0.9748212	0.5854	0.558	0.218734815	count	1

LINC01679	0.5706602	0.7825141	0.7293	0.466	0.218734815	count	1
MYL12A	0.1522359	0.0356025	4.276	1.98E-05	0.218777151	count	0.46827
DDX6	0.1577864	0.1148964	1.3733	0.17	0.218828016	count	1
NDUFS4	0.1585271	0.1056365	1.5007	0.134	0.219245109	count	1
ZFAND5	0.1540267	0.057404	2.6832	0.00734	0.219273704	count	1
DCUN1D1	0.178531	0.1758231	1.0154	0.31	0.219525267	count	1
SF3A1	0.177089	0.1865344	0.9494	0.343	0.219846314	count	1
DLD	0.1692915	0.1781218	0.9504	0.342	0.219885277	count	1
OAF	0.1997264	0.3356636	0.595	0.552	0.219992725	count	1
ZHX1	0.1797857	0.192296	0.9349	0.35	0.220152418	count	1
TMSB4Y	0.2937652	0.5896471	0.4982	0.618	0.220155505	count	1
UBTD1	0.1843197	0.2734243	0.6741	0.5	0.2202111	count	1
RPL11	0.1527697	0.0220046	6.9426	4.91E-12	0.220261093	count	1.18E-07
STXBP1	0.4060442	0.6180302	0.657	0.511	0.220303139	count	1
AC137932.3	0.4060442	0.6671781	0.6086	0.543	0.220303139	count	1
RAB11FIP3	0.2842982	1.1374054	0.25	0.803	0.220328794	count	1
PGM1	0.1785652	0.2503596	0.7132	0.476	0.220447325	count	1
AARS	0.2117476	0.2969757	0.713	0.476	0.220449613	count	1
RPS4X	0.1530021	0.0258758	5.9129	3.83E-09	0.220467602	count	9.20E-05
CALD1	0.2845038	0.4459284	0.638	0.524	0.220494493	count	1
SLC29A1	0.1793519	0.2680643	0.6691	0.504	0.220542959	count	1
ZNF121	0.2101972	0.3110312	0.6758	0.499	0.220667091	count	1
SLC9A3	0.696956	0.7815038	0.8918	0.373	0.220721179	count	1
SPG21	0.1584652	0.1217787	1.3013	0.193	0.220805996	count	1
AP3M2	0.228275	0.3716361	0.6142	0.539	0.220857288	count	1
SNAPIN	0.1602952	0.1261912	1.2703	0.204	0.220948402	count	1
WDR12	0.187792	0.2725412	0.689	0.491	0.2209531	count	1
DOLK	0.3350269	0.5153323	0.6501	0.516	0.22099275	count	1
TRAPPC11	0.1984822	0.2566667	0.7733	0.439	0.221091812	count	1
FDX1	0.1571898	0.1034349	1.5197	0.129	0.221107884	count	1
ADIPOR1	0.1583584	0.0977986	1.6192	0.106	0.221154017	count	1
SURF1	0.1594257	0.1000376	1.5937	0.111	0.221240843	count	1
AL391121.1	0.2144277	0.406834	0.5271	0.598	0.221306618	count	1
RNF214	0.1746478	0.1804667	0.9678	0.333	0.221549234	count	1
RPL24	0.1539291	0.0269663	5.7082	1.28E-08	0.221616058	count	0.000306995
SPRY2	0.1969924	0.2510094	0.7848	0.433	0.221705443	count	1
KDM5D	0.2391689	0.3914074	0.611	0.541	0.221725777	count	1
ODF2	0.2214223	0.2770656	0.7992	0.424	0.221914492	count	1
KIAA1586	0.1764876	0.2239011	0.7882	0.431	0.222008383	count	1
PIGH	0.1829979	0.2547226	0.7184	0.473	0.222086631	count	1
INTS4	0.2360888	0.370747	0.6368	0.524	0.222248642	count	1
HNRNPD	0.1566668	0.0612131	2.5594	0.0105	0.222561264	count	1
TMEM255B	0.1838436	0.261731	0.7024	0.482	0.222578822	count	1
TCEA2	0.1686267	0.1792973	0.9405	0.347	0.222773558	count	1
TTC17	0.1791588	0.2197921	0.8151	0.415	0.222833953	count	1

CCDC18	0.1856143	0.3051478	0.6083	0.543	0.223008654	count	1
C12orf76	0.1796841	0.1930056	0.931	0.352	0.223093036	count	1
ARHGEF9	0.1954647	0.3291327	0.5939	0.553	0.223094111	count	1
MTHFSD	0.3380969	0.4833838	0.6994	0.484	0.223128678	count	1
NAP1L4	0.1628789	0.1511607	1.0775	0.281	0.223222832	count	1
EEF1A1	0.1551317	0.0229441	6.7613	1.70E-11	0.223707354	count	4.10E-07
OTULINL	0.1629864	0.1033279	1.5774	0.115	0.223734668	count	1
SAFB	0.1671389	0.1406069	1.1887	0.235	0.223827812	count	1
PCBP2	0.1575637	0.0629998	2.501	0.0124	0.223832114	count	1
GIT1	0.1998832	0.2823878	0.7078	0.479	0.223859702	count	1
CCDC137	0.1821579	0.2200001	0.828	0.408	0.224022166	count	1
GORASP2	0.171134	0.1967864	0.8696	0.385	0.224273273	count	1
LENG1	0.1756882	0.1913139	0.9183	0.359	0.224287028	count	1
PIGC	0.1713304	0.181992	0.9414	0.347	0.224364828	count	1
RNF11	0.1692278	0.1699059	0.996	0.319	0.224497054	count	1
RGL4	1.537195	1.010429	1.5213	0.128	0.22457772	count	1
JPX	0.1734644	0.2089056	0.8303	0.406	0.224724173	count	1
MAT2A	0.1608762	0.0989477	1.6259	0.104	0.224842143	count	1
P2RY2	0.3602039	0.5645064	0.6381	0.523	0.225073623	count	1
LSM14B	0.1889095	0.2888316	0.654	0.513	0.225095027	count	1
IGHG1	0.2020689	0.4447401	0.4544	0.65	0.225143841	count	1
CIAO1	0.1713521	0.1445951	1.185	0.236	0.225205125	count	1
PMAIP1	0.1633862	0.1263713	1.2929	0.196	0.225255792	count	1
KLF5	0.5080484	0.4766417	1.0659	0.287	0.22534461	count	1
SRSF11	0.158871	0.0661452	2.4019	0.0164	0.225383082	count	1
MT-CO3	0.1565056	0.0261869	5.9765	2.61E-09	0.225631618	count	6.27E-05
OTUD1	0.1584904	0.0841708	1.883	0.0598	0.225712841	count	1
LINC00685	0.2364245	0.383426	0.6166	0.538	0.225838869	count	1
PEX6	0.2519403	0.3337226	0.7549	0.45	0.225849069	count	1
ZBTB7A	0.1621548	0.1101635	1.4719	0.141	0.225934178	count	1
KRTCAP2	0.1608435	0.0897368	1.7924	0.0732	0.225950394	count	1
CMTR2	0.2209534	0.3524457	0.6269	0.531	0.226032176	count	1
RBMS1	0.161357	0.0886515	1.8201	0.0689	0.226082945	count	1
RHCE	1.5464032	1.281003	1.2072	0.227	0.226178966	count	1
AC007383.2	1.5464032	1.281003	1.2072	0.227	0.226178966	count	1
AC097634.3	1.5464032	1.281003	1.2072	0.227	0.226178966	count	1
PHLDB2	1.5464032	1.508711	1.025	0.305	0.226178966	count	1
STARD4-AS1	1.5464032	1.361144	1.1361	0.256	0.226178966	count	1
PRR7-AS1	1.5464032	1.281003	1.2072	0.227	0.226178966	count	1
AL353803.4	1.5464032	1.361144	1.1361	0.256	0.226178966	count	1
C11orf65	1.5464032	1.095628	1.4114	0.158	0.226178966	count	1
AC022021.1	1.5464032	1.281003	1.2072	0.227	0.226178966	count	1
AL121928.1	1.5464032	1.361144	1.1361	0.256	0.226178966	count	1
HOXC4	1.5464032	1.508711	1.025	0.305	0.226178966	count	1
AL589745.1	1.5464032	1.361144	1.1361	0.256	0.226178966	count	1

LOXL1-AS1	1.5464032	1.281003	1.2072	0.227	0.226178966	count	1
AC027020.2	1.5464032	1.361144	1.1361	0.256	0.226178966	count	1
CASKIN2	1.5464032	0.9943113	1.5553	0.12	0.226178966	count	1
SLC35E3	0.1763062	0.2111313	0.8351	0.404	0.226370428	count	1
TMEM60	0.172277	0.1803748	0.9551	0.34	0.226582755	count	1
MBTPS1	0.1741289	0.1820584	0.9564	0.339	0.226594501	count	1
SASH3	0.1775546	0.192061	0.9245	0.355	0.226684803	count	1
GPR171	0.3627118	0.6782736	0.5348	0.593	0.226736003	count	1
LYRM7	0.2177781	0.3013639	0.7226	0.47	0.226844931	count	1
CCNK	0.169788	0.1494871	1.1358	0.256	0.226870855	count	1
ME1	0.190511	0.2004279	0.9505	0.342	0.227022845	count	1
SPATA24	0.4175912	0.5771797	0.7235	0.469	0.227044391	count	1
CES4A	0.4175912	0.6018047	0.6939	0.488	0.227044391	count	1
AMZ2	0.1730489	0.1766033	0.9799	0.327	0.22712518	count	1
HDAC1	0.170787	0.159968	1.0676	0.286	0.227191586	count	1
LNX2	0.3875344	0.4985329	0.7773	0.437	0.227257962	count	1
EVI5L	0.3875344	0.5396602	0.7181	0.473	0.227257962	count	1
TOX2	0.9451946	0.844897	1.1187	0.263	0.227304263	count	1
GTF2E1	0.2768811	0.3919949	0.7063	0.48	0.227515901	count	1
NCBP1	0.2103683	0.4615476	0.4558	0.649	0.227544212	count	1
HIP1	0.1829872	0.2224187	0.8227	0.411	0.227632822	count	1
TAOK3	0.1614417	0.0851573	1.8958	0.0581	0.227669833	count	1
RFXANK	0.1685521	0.1627172	1.0359	0.3	0.227758174	count	1
DNAJC13	0.1807894	0.1858329	0.9729	0.331	0.227797279	count	1
MOSPD3	0.1767298	0.2329955	0.7585	0.448	0.22787236	count	1
AP001160.3	0.4590482	0.5757712	0.7973	0.425	0.227973318	count	1
CACNB3	0.3455088	0.6565223	0.5263	0.599	0.228293005	count	1
MLXIP	0.1763932	0.1683626	1.0477	0.295	0.228326116	count	1
INTS10	0.1765695	0.142282	1.241	0.215	0.228338367	count	1
CRYBG3	0.1775222	0.204521	0.868	0.385	0.228428902	count	1
IFITM1	0.254892	0.4132924	0.6167	0.537	0.228572779	count	1
RPS23	0.1586152	0.024344	6.5156	8.76E-11	0.228635411	count	2.11E-06
VPS50	0.2160009	0.2736736	0.7893	0.43	0.2286981	count	1
AL137802.2	0.9503231	1.0349031	0.9183	0.359	0.228762142	count	1
AZIN2	0.9503231	1.1525823	0.8245	0.41	0.228762142	count	1
LINC00607	0.9503231	1.07092	0.8874	0.375	0.228762142	count	1
NRG2	0.9503231	1.0349031	0.9183	0.359	0.228762142	count	1
HLA-G	0.9503231	1.0349031	0.9183	0.359	0.228762142	count	1
AC114271.1	0.9503231	1.07092	0.8874	0.375	0.228762142	count	1
FAM227A	0.9503231	1.388264	0.6845	0.494	0.228762142	count	1
AC097376.2	0.2196678	0.3256346	0.6746	0.5	0.228850175	count	1
PTPRC	0.1594003	0.0365096	4.366	1.32E-05	0.228878359	count	0.3125628
ITPR2	0.1665952	0.1024221	1.6266	0.104	0.22887838	count	1
TRIM22	0.1683332	0.1220007	1.3798	0.168	0.228940417	count	1
ANKRD17	0.1791928	0.1777859	1.0079	0.314	0.229061151	count	1

GLI4	0.1998543	0.2931074	0.6818	0.495	0.229164297	count	1
DLEU7	0.2337706	0.4989148	0.4686	0.639	0.22919712	count	1
C11orf24	0.1942599	0.2919045	0.6655	0.506	0.229401367	count	1
RPL5	0.1592351	0.0273317	5.826	6.42E-09	0.229417353	count	0.000154061
AC110079.2	0.9527416	0.5567392	1.7113	0.0872	0.229450253	count	1
TJAP1	0.2435706	0.4342157	0.5609	0.575	0.229473847	count	1
ERCC4	0.2109881	0.3048302	0.6921	0.489	0.229737247	count	1
TIFAB	0.4223858	0.6943607	0.6083	0.543	0.229850494	count	1
AL606469.1	0.4223858	0.7731558	0.5463	0.585	0.229850494	count	1
RIOX2	0.2206108	0.3239784	0.6809	0.496	0.229851048	count	1
PAFAH2	0.3479099	0.4760824	0.7308	0.465	0.229968282	count	1
DNAJC3-DT	0.2962645	0.4651436	0.6369	0.524	0.22998618	count	1
IFI35	0.1656183	0.1137358	1.4562	0.145	0.230118943	count	1
NGLY1	0.1701433	0.1730002	0.9835	0.325	0.230224101	count	1
DDX28	0.2410076	0.391039	0.6163	0.538	0.230325574	count	1
USP32	0.1921876	0.2114577	0.9089	0.364	0.230354486	count	1
KIN	0.1701816	0.1361271	1.2502	0.211	0.230425885	count	1
FBXW9	0.3324989	0.5704402	0.5829	0.56	0.230592718	count	1
TRIR	0.1617864	0.0502969	3.2166	0.00131	0.230626357	count	1
LINC00954	0.5189151	0.7856725	0.6605	0.509	0.230652564	count	1
ST6GALNAC6	0.5189151	0.8072089	0.6429	0.52	0.230652564	count	1
ARHGEF17	0.5189151	0.7856725	0.6605	0.509	0.230652564	count	1
SEMA7A	0.5189151	0.9665659	0.5369	0.591	0.230652564	count	1
ZNF784	0.5189151	0.7756938	0.669	0.504	0.230652564	count	1
AC005261.3	0.7243656	0.6669055	1.0862	0.278	0.230682355	count	1
ARPIN	0.1818163	0.1828573	0.9943	0.32	0.230704593	count	1
ZNF180	0.5191432	0.4974671	1.0436	0.297	0.230764174	count	1
STAT4	0.3073413	0.52317	0.5875	0.557	0.230781842	count	1
AC016065.1	0.3073413	0.5134464	0.5986	0.55	0.230781842	count	1
KLHL23	0.4643119	1.250548	0.3713	0.71	0.230815422	count	1
AC010894.2	0.4643119	0.8905093	0.5214	0.602	0.230815422	count	1
LINC02018	0.4643119	0.799271	0.5809	0.561	0.230815422	count	1
AC019205.1	0.4643119	1.4766725	0.3144	0.753	0.230815422	count	1
C9orf116	0.4643119	0.9107924	0.5098	0.61	0.230815422	count	1
AL022238.2	0.4643119	0.9918243	0.4681	0.64	0.230815422	count	1
MAGT1	0.1715651	0.1386725	1.2372	0.216	0.230829553	count	1
RFX7	0.2739359	0.352645	0.7768	0.437	0.230848751	count	1
GALNT2	0.1813136	0.2091107	0.8671	0.386	0.230950267	count	1
THOC5	0.2327913	0.2619768	0.8886	0.374	0.230953794	count	1
TXNDC9	0.1720294	0.1582577	1.087	0.277	0.230993144	count	1
STAC3	0.1751688	0.179764	0.9744	0.33	0.231017616	count	1
OXNAD1	0.3494658	0.5511984	0.634	0.526	0.23105444	count	1
SF3A3	0.1723891	0.1391752	1.2386	0.216	0.231087574	count	1
ISG20	0.1752553	0.1869856	0.9373	0.349	0.231132225	count	1
AC060780.1	0.2577147	0.4526883	0.5693	0.569	0.231179001	count	1

VPS52	0.4650529	0.4340468	1.0714	0.284	0.231215883	count	1
TRIM11	0.1978761	0.2296307	0.8617	0.389	0.231346031	count	1
TMEM120B	0.242283	0.3486206	0.695	0.487	0.23157482	count	1
NOC3L	0.1876805	0.2183136	0.8597	0.39	0.231794789	count	1
TFAM	0.1672685	0.1371428	1.2197	0.223	0.231888604	count	1
DCLRE1C	0.2005762	0.3320344	0.6041	0.546	0.231904669	count	1
RARRES2	0.4260902	0.8077924	0.5275	0.598	0.232021339	count	1
SIRT5	0.2692388	0.3516913	0.7656	0.444	0.232115998	count	1
SLC37A4	0.2313978	0.3829052	0.6043	0.546	0.23212837	count	1
ATP11C	0.2247244	0.2651079	0.8477	0.397	0.232142039	count	1
CCDC102B	0.3208809	0.5815569	0.5518	0.581	0.232178035	count	1
SAMD8	0.1854883	0.2091189	0.887	0.375	0.232328141	count	1
ZADH2	0.1901032	0.2086051	0.9113	0.362	0.232390926	count	1
PCDHGB6	1.5831444	0.9436053	1.6778	0.0935	0.232567419	count	1
AC025171.2	0.2995914	0.5390664	0.5558	0.578	0.232676158	count	1
COQ3	0.2995914	0.6202061	0.4831	0.629	0.232676158	count	1
AP002807.1	0.3961879	0.4904996	0.8077	0.419	0.232681939	count	1
PTGER3	0.5233191	0.705991	0.7413	0.459	0.232808864	count	1
AC080013.1	0.6034584	0.8468988	0.7126	0.476	0.232837609	count	1
STAP1	0.6034584	0.8468988	0.7126	0.476	0.232837609	count	1
HHIP	0.6034584	1.3907564	0.4339	0.664	0.232837609	count	1
Z84485.1	0.6034584	0.8468988	0.7126	0.476	0.232837609	count	1
AC093799.1	0.6034584	1.0596664	0.5695	0.569	0.232837609	count	1
UXT-AS1	0.6034584	1.3907564	0.4339	0.664	0.232837609	count	1
PEBP4	0.6034584	0.8468988	0.7126	0.476	0.232837609	count	1
CSNK2A3	0.6034584	0.8468988	0.7126	0.476	0.232837609	count	1
RTKN2	0.6034584	0.8468988	0.7126	0.476	0.232837609	count	1
SLC25A15	0.6034584	0.8468988	0.7126	0.476	0.232837609	count	1
COG8	0.6034584	0.8468988	0.7126	0.476	0.232837609	count	1
DYNLRB2	0.6034584	0.8468988	0.7126	0.476	0.232837609	count	1
CDC6	0.6034584	1.0596664	0.5695	0.569	0.232837609	count	1
AC007993.3	0.6034584	1.3907564	0.4339	0.664	0.232837609	count	1
AC132872.2	0.6034584	0.8468988	0.7126	0.476	0.232837609	count	1
C21orf62-AS1	0.6034584	0.8468988	0.7126	0.476	0.232837609	count	1
IRF1	0.1635139	0.0805634	2.0296	0.0425	0.232880963	count	1
FAM213A	0.1880349	0.1936859	0.9708	0.332	0.23311871	count	1
EIF3F	0.1634123	0.05034	3.2462	0.00119	0.233184702	count	1
ZFAND4	0.5242059	0.5314823	0.9863	0.324	0.233243439	count	1
TSPAN15	0.217032	0.2423076	0.8957	0.371	0.233254192	count	1
AGGF1	0.226099	0.3239924	0.6979	0.485	0.233589877	count	1
KBTBD2	0.180996	0.1887306	0.959	0.338	0.233639715	count	1
LENG8-AS1	0.277188	0.6650063	0.4168	0.677	0.233684745	count	1
RNMT	0.166038	0.0980442	1.6935	0.0905	0.23378762	count	1
PRR13	0.1637304	0.0516753	3.1684	0.0016	0.233831382	count	1
CCL3	0.1622584	0.0781855	2.0753	0.0381	0.233860007	count	1

ITGB7	0.2007764	0.2951073	0.6804	0.496	0.233924684	count	1
SLC22A5	0.5257136	0.5916099	0.8886	0.374	0.233982524	count	1
ABCC10	0.3233354	0.4154428	0.7783	0.436	0.234039395	count	1
ASTE1	0.2924587	0.4146862	0.7053	0.481	0.234113226	count	1
PRKAR1A	0.1667129	0.0856763	1.9458	0.0518	0.234126556	count	1
METTL2B	0.1948585	0.2427313	0.8028	0.422	0.234226804	count	1
RPS7	0.1626091	0.0242958	6.6929	2.70E-11	0.234355268	count	6.51E-07
NUSAP1	0.2524839	0.5018447	0.5031	0.615	0.234408947	count	1
TAF13	0.1826719	0.1959457	0.9323	0.351	0.234594492	count	1
PPP3R1	0.1795198	0.1567735	1.1451	0.252	0.234604218	count	1
GON7	0.1816801	0.2021819	0.8986	0.369	0.234758809	count	1
AP1AR	0.1900957	0.2269372	0.8377	0.402	0.234802832	count	1
MEMO1	0.2530199	0.3182814	0.795	0.427	0.234920196	count	1
SLCO2B1	0.1722281	0.1163416	1.4804	0.139	0.234993961	count	1
EMD	0.168797	0.1112449	1.5173	0.129	0.235019903	count	1
AC002456.1	0.9725464	0.8259221	1.1775	0.239	0.235099361	count	1
LRRC24	0.9725464	0.9118858	1.0665	0.286	0.235099361	count	1
TET2	0.1701366	0.1239096	1.3731	0.17	0.235132186	count	1
AP002884.1	0.4001732	0.7146593	0.5599	0.576	0.23518453	count	1
MAGEF1	0.2060153	0.2800763	0.7356	0.462	0.235295439	count	1
SDF4	0.1688701	0.1012428	1.668	0.0954	0.235485238	count	1
BIRC3	0.1697275	0.1101437	1.541	0.123	0.235508893	count	1
PRNP	0.1693996	0.0925733	1.8299	0.0674	0.235539481	count	1
WASHC5	0.2081206	0.2686541	0.7747	0.439	0.235553265	count	1
RAB5IF	0.1680418	0.0833534	2.016	0.0439	0.235573544	count	1
SLC40A1	0.167858	0.1501601	1.1179	0.264	0.235667998	count	1
FMO4	0.4323875	0.5503523	0.7857	0.432	0.235717196	count	1
FMC1	0.1916483	0.2657969	0.721	0.471	0.23579548	count	1
EIF3G	0.1658801	0.0578089	2.8695	0.00415	0.235821756	count	1
CDK5RAP3	0.1756098	0.1591205	1.1036	0.27	0.236010934	count	1
TRMT61A	0.2106984	0.4177282	0.5044	0.614	0.236147584	count	1
VPS8	0.1945015	0.2242272	0.8674	0.386	0.236180368	count	1
UPF3A	0.17059	0.1843733	0.9252	0.355	0.236225257	count	1
HNRNPH3	0.1713701	0.0964478	1.7768	0.0757	0.236288879	count	1
TMCO4	0.219874	0.2895299	0.7594	0.448	0.236361012	count	1
NUDT17	0.6116357	0.8471102	0.722	0.47	0.23637609	count	1
ROM1	0.6116357	0.7279803	0.8402	0.401	0.23637609	count	1
LARP1	0.1743836	0.1459188	1.1951	0.232	0.236647904	count	1
AC116667.1	0.3574899	0.6437048	0.5554	0.579	0.236663403	count	1
PMF1	0.1739287	0.1384384	1.2564	0.209	0.23670415	count	1
TDRD7	0.2549678	0.4186411	0.609	0.543	0.236778591	count	1
COG4	0.2174292	0.2613642	0.8319	0.406	0.236865274	count	1
LATS2	0.2056707	0.2346888	0.8764	0.381	0.236910316	count	1
TGFBRAP1	0.2219565	0.3515208	0.6314	0.528	0.23691527	count	1
NMI	0.1734637	0.1167669	1.4856	0.138	0.236916208	count	1

LIG1	0.2880472	0.3964547	0.7266	0.468	0.237034093	count	1
CSRNP2	0.435189	0.4560269	0.9543	0.34	0.237363615	count	1
ZDHHC5	0.1837456	0.2010286	0.914	0.361	0.23744353	count	1
MLH1	0.2088917	0.293237	0.7124	0.476	0.237549977	count	1
FAM219B	0.2108764	0.262859	0.8022	0.422	0.237553822	count	1
DUSP18	0.2054142	0.3022912	0.6795	0.497	0.237568361	count	1
TMEM161B	0.2421884	0.3348941	0.7232	0.47	0.237645299	count	1
SHARPIN	0.1722196	0.1219424	1.4123	0.158	0.237654185	count	1
UTY	0.1977765	0.2770564	0.7138	0.475	0.237769905	count	1
CENPC	0.1796279	0.1467232	1.2243	0.221	0.23780645	count	1
ZNF770	0.1973073	0.2562462	0.77	0.441	0.23782918	count	1
ZNF669	0.2488481	0.3265865	0.762	0.446	0.238009917	count	1
LINC00115	0.2347794	0.4620888	0.5081	0.611	0.238091792	count	1
RSL1D1	0.1674565	0.0594477	2.8169	0.00489	0.238183508	count	1
ARMC7	0.2268198	0.3947478	0.5746	0.566	0.23844772	count	1
C1orf50	0.2826926	0.6946511	0.407	0.684	0.238489666	count	1
C17orf67	0.1913261	0.29032	0.659	0.51	0.238505256	count	1
TCTEX1D4	0.4786786	0.9655368	0.4958	0.62	0.238596017	count	1
RPS28	0.1656523	0.024768	6.6882	2.79E-11	0.238660646	count	6.72E-07
SERP1	0.1662899	0.03945	4.2152	2.59E-05	0.23869782	count	0.611758
GNB5	0.3070738	0.4370864	0.7025	0.482	0.238734005	count	1
ATP6V1E1	0.1710262	0.0969699	1.7637	0.0779	0.238954415	count	1
ASCC2	0.1837981	0.2315843	0.7937	0.427	0.239042097	count	1
SMIM8	0.1891244	0.2867254	0.6596	0.51	0.239063236	count	1
MTRNR2L12	0.1679134	0.0667619	2.5151	0.012	0.23921298	count	1
RETREG3	0.2169433	0.3104714	0.6988	0.485	0.239255214	count	1
NORAD	0.1713214	0.1040398	1.6467	0.0997	0.239483768	count	1
ERI2	0.361628	0.5302331	0.682	0.495	0.239560803	count	1
DRG2	0.1883688	0.2067023	0.9113	0.362	0.239696829	count	1
ERI1	0.2073269	0.2400288	0.8638	0.388	0.239808332	count	1
SF3B1	0.1696526	0.0641107	2.6462	0.00819	0.239937064	count	1
ACACA	0.2781611	0.4419509	0.6294	0.529	0.240067817	count	1
LINC01010	0.3627219	0.5625876	0.6447	0.519	0.240327273	count	1
PCGF6	0.242033	0.4119078	0.5876	0.557	0.240333482	count	1
TICAM1	0.1945384	0.2337251	0.8323	0.405	0.24033757	count	1
ENDOG	0.1899615	0.2451178	0.775	0.438	0.240463399	count	1
MTSS1	0.1720017	0.1023235	1.681	0.0929	0.240549532	count	1
SENP5	0.187322	0.1935225	0.968	0.333	0.240604156	count	1
TBCE	0.7521517	0.5919597	1.2706	0.204	0.240861371	count	1
AL731661.1	0.3839777	0.6890344	0.5573	0.577	0.240880096	count	1
MS4A4E	0.4829621	0.5378116	0.898	0.369	0.240922419	count	1
PROSER2	0.4093228	0.6732547	0.608	0.543	0.240941019	count	1
PIK3R4	0.4836217	0.5573384	0.8677	0.386	0.241280917	count	1
KLC1	0.193698	0.2274907	0.8515	0.395	0.241485951	count	1
TRIP6	0.1853919	0.1976005	0.9382	0.348	0.241536565	count	1

SLC25A45	0.2065621	0.330181	0.6256	0.532	0.241623211	count	1
BCAT2	0.2320096	0.3058515	0.7586	0.448	0.241960796	count	1
PAQR4	0.2410422	0.3112361	0.7745	0.439	0.242019384	count	1
GTPBP8	0.1962893	0.2454025	0.7999	0.424	0.242043438	count	1
TOR2A	0.1791989	0.1346733	1.3306	0.183	0.242195219	count	1
ZNF766	0.2303318	0.3059292	0.7529	0.452	0.242210027	count	1
INTS8	0.2568605	0.2902909	0.8848	0.376	0.242333081	count	1
IPMK	0.1811832	0.1464351	1.2373	0.216	0.242494431	count	1
HEG1	0.348907	0.419155	0.8324	0.405	0.242582968	count	1
SMAP2	0.1705945	0.0505404	3.3754	0.000749	0.24266958	count	1
SPINDOC	0.3350587	0.4047944	0.8277	0.408	0.242945896	count	1
CHMP4B	0.1739885	0.1073319	1.621	0.105	0.242978727	count	1
FUNDC1	0.2085081	0.2173056	0.9595	0.337	0.243043595	count	1
AREL1	0.2757544	0.5248469	0.5254	0.599	0.243055759	count	1
FARSB	0.2330924	0.3781228	0.6164	0.538	0.243112218	count	1
MPRIP	0.2051184	0.3174221	0.6462	0.518	0.243142167	count	1
LDHB	0.1726371	0.0807457	2.138	0.0326	0.243147995	count	1
ANKRD12	0.1711515	0.0815554	2.0986	0.036	0.243204744	count	1
CPT1A	0.1880782	0.2277823	0.8257	0.409	0.243310658	count	1
PRKAB2	0.4131182	0.4258399	0.9701	0.332	0.24333336	count	1
GALNT3	0.2579581	0.3121129	0.8265	0.409	0.243396541	count	1
ZNF765	0.2333672	0.6277577	0.3717	0.71	0.243404464	count	1
ZNF814	0.2510103	0.3822223	0.6567	0.511	0.243407351	count	1
ABR	0.1956592	0.2099148	0.9321	0.351	0.243527194	count	1
AL121983.1	0.4459096	0.5682673	0.7847	0.433	0.24367668	count	1
SERPINB1	0.1701758	0.0526785	3.2305	0.00125	0.243700915	count	1
FAP	0.4882519	1.356081	0.36	0.719	0.243799488	count	1
AC092171.5	0.4882519	0.7747852	0.6302	0.529	0.243799488	count	1
ADCY4	0.4882519	0.7090909	0.6886	0.491	0.243799488	count	1
AC007114.2	0.4882519	0.7056005	0.692	0.489	0.243799488	count	1
RPS6KC1	0.2084403	0.2847394	0.732	0.464	0.243846703	count	1
PIK3CA	0.1913776	0.2204645	0.8681	0.385	0.243857756	count	1
CRABP2	0.4462261	1.1152982	0.4001	0.689	0.243863354	count	1
ADIPOR2	0.1864613	0.1760465	1.0592	0.29	0.243914084	count	1
ALDH3B1	0.1855676	0.1804054	1.0286	0.304	0.243983427	count	1
EPHB3	1.649396	0.7946876	2.0755	0.038	0.244073228	count	1
AC010491.1	1.649396	1.291076	1.2775	0.202	0.244073228	count	1
AC022364.1	1.649396	0.7946876	2.0755	0.038	0.244073228	count	1
PUS3	0.2118872	0.3697402	0.5731	0.567	0.244164583	count	1
SPATA33	0.2383807	0.4148576	0.5746	0.566	0.244236884	count	1
MRFAP1	0.1748228	0.0772756	2.2623	0.0238	0.244267966	count	1
AC093673.1	0.1849897	0.2079521	0.8896	0.374	0.244342917	count	1
LINC02361	1.005158	0.820863	1.2245	0.221	0.244455086	count	1
CXorf21	0.1743471	0.1310683	1.3302	0.184	0.244598521	count	1
PDCD4	0.1794328	0.114007	1.5739	0.116	0.244792721	count	1

RINL	0.2437592	0.3832865	0.636	0.525	0.244808702	count	1
C1orf112	0.2973093	0.4559114	0.6521	0.514	0.244947581	count	1
TRMO	0.2220408	0.303085	0.7326	0.464	0.244966639	count	1
CTR9	0.1861847	0.1736545	1.0722	0.284	0.24529671	count	1
PDHB	0.1787357	0.1292907	1.3824	0.167	0.245785581	count	1
PLAGL1	0.1950608	0.1915607	1.0183	0.309	0.245915971	count	1
ABCF3	0.2081365	0.2813049	0.7399	0.459	0.2459779	count	1
STX5	0.1894476	0.1576325	1.2018	0.23	0.245999807	count	1
HADH	0.1903914	0.2621001	0.7264	0.468	0.246083873	count	1
ZNF207	0.176784	0.1077472	1.6407	0.101	0.246210819	count	1
SPG7	0.1857917	0.1684128	1.1032	0.27	0.246292129	count	1
OLA1	0.1795415	0.1176459	1.5261	0.127	0.246521232	count	1
ARL16	0.2176947	0.2308089	0.9432	0.346	0.246544584	count	1
AC087500.1	0.4937564	0.6357882	0.7766	0.437	0.246798145	count	1
SEC24C	0.2236771	0.3161585	0.7075	0.479	0.246800804	count	1
INTS12	0.2117117	0.2760395	0.767	0.443	0.246824167	count	1
PANK1	0.6362018	0.6749432	0.9426	0.346	0.24705879	count	1
ZNF470	0.6362018	0.7966112	0.7986	0.425	0.24705879	count	1
ANKS3	0.2701007	0.4898162	0.5514	0.581	0.247066046	count	1
U2AF1	1.014261	0.4501258	2.2533	0.0243	0.247078026	count	1
EFEMP2	0.2802742	0.5012149	0.5592	0.576	0.247170062	count	1
MSL1	0.193966	0.1810645	1.0713	0.284	0.24717885	count	1
ZNRF2	0.1857484	0.1430703	1.2983	0.194	0.247182887	count	1
DENND6B	0.1934363	0.1931167	1.0017	0.317	0.247391874	count	1
SYNGR1	0.187914	0.1650231	1.1387	0.255	0.247421393	count	1
GPN3	0.1804508	0.1270683	1.4201	0.156	0.247525323	count	1
CERS6	0.2003592	0.1685419	1.1888	0.235	0.247592117	count	1
PGM2	0.1845375	0.1775831	1.0392	0.299	0.247648601	count	1
ABCB7	0.2020435	0.2255745	0.8957	0.371	0.247663566	count	1
BTD	0.2116862	0.2539986	0.8334	0.405	0.247690364	count	1
NOD1	0.3413359	0.7879504	0.4332	0.665	0.247725758	count	1
RBBP6	0.1788813	0.1149265	1.5565	0.12	0.247830683	count	1
ZBED6	0.7713801	0.836741	0.9219	0.357	0.247951716	count	1
SLC23A3	0.7713801	1.0942259	0.705	0.481	0.247951716	count	1
FAM83H	0.7713801	0.836741	0.9219	0.357	0.247951716	count	1
KLLN	0.7713801	0.836741	0.9219	0.357	0.247951716	count	1
ITPK1-AS1	0.7713801	1.0540588	0.7318	0.464	0.247951716	count	1
OMA1	0.2086744	0.331712	0.6291	0.529	0.24816737	count	1
RANBP3	0.2277159	0.2456382	0.927	0.354	0.248261829	count	1
COMMD6	0.1733434	0.0482725	3.5909	0.000336	0.2482947	count	1
RFC2	0.1961276	0.1865158	1.0515	0.293	0.248326282	count	1
PTCD2	0.7724033	0.8550775	0.9033	0.366	0.248330045	count	1
AP001157.1	0.3741418	0.6162987	0.6071	0.544	0.248342429	count	1
KLK1	0.7728975	0.6260772	1.2345	0.217	0.248512813	count	1
AC093525.6	0.6396553	0.7389513	0.8656	0.387	0.24856679	count	1

ZNF699	0.2878776	0.5522446	0.5213	0.602	0.248744626	count	1
ALKBH3	0.2106261	0.3060657	0.6882	0.491	0.248954327	count	1
NFYC	0.1867293	0.1835804	1.0172	0.309	0.249002264	count	1
NCOA7	0.1890359	0.1778549	1.0629	0.288	0.249072037	count	1
MIOS	0.1967802	0.2275091	0.8649	0.387	0.249158657	count	1
TRIM41	0.264038	0.4242607	0.6223	0.534	0.249291206	count	1
RTL8A	0.1935259	0.1975878	0.9794	0.327	0.249412194	count	1
CABIN1	0.2030956	0.276303	0.735	0.462	0.24949319	count	1
RRP12	0.2149024	0.2358957	0.911	0.362	0.24965274	count	1
CTBP1	0.1922655	0.1751485	1.0977	0.272	0.249680421	count	1
RPS14	0.173221	0.0226129	7.6603	2.65E-14	0.249705336	count	6.40E-10
THADA	0.2374508	0.3759193	0.6317	0.528	0.249842384	count	1
LRRC8C	0.2142803	0.2322159	0.9228	0.356	0.2498563	count	1
PITPNM1	0.2323315	0.3248707	0.7152	0.475	0.249993848	count	1
AC020910.4	0.4998558	0.5652931	0.8842	0.377	0.25012662	count	1
AC024909.2	0.5586201	0.6012222	0.9291	0.353	0.25019844	count	1
PAAF1	0.2181687	0.3261293	0.669	0.504	0.250451906	count	1
ZNF362	0.2421872	0.2943207	0.8229	0.411	0.250558141	count	1
DERA	0.1835645	0.1354136	1.3556	0.175	0.250632259	count	1
BACE1	0.3040104	0.4569405	0.6653	0.506	0.250683213	count	1
CARM1	0.2175679	0.2554588	0.8517	0.394	0.250797967	count	1
TBC1D4	0.2969838	0.3063038	0.9696	0.332	0.250991359	count	1
HMOX2	0.1906135	0.1836109	1.0381	0.299	0.25116119	count	1
ZBTB43	0.189111	0.2067592	0.9146	0.36	0.251274791	count	1
RCBTB1	0.6458578	0.5720957	1.1289	0.259	0.25127897	count	1
RBM18	0.1896184	0.2267657	0.8362	0.403	0.251388854	count	1
CSK	0.180746	0.0971257	1.861	0.0629	0.251401878	count	1
RPS3A	0.1745275	0.0244169	7.1478	1.16E-12	0.251570633	count	2.80E-08
TTC31	0.253141	0.3823461	0.6621	0.508	0.251626668	count	1
OGFOD2	0.400127	0.4111063	0.9733	0.331	0.251677466	count	1
RPL29	0.1746835	0.0250141	6.9834	3.70E-12	0.251750746	count	8.93E-08
CITED2	0.1771284	0.1110548	1.595	0.111	0.251842744	count	1
UBE2S	0.1780306	0.084403	2.1093	0.035	0.252260303	count	1
NES	0.7831002	0.8008273	0.9779	0.328	0.252291529	count	1
CAD	0.7831002	0.609526	1.2848	0.199	0.252291529	count	1
GCNA	0.6484533	0.5981101	1.0842	0.278	0.252415351	count	1
RHOBTB3	0.2043032	0.2088088	0.9784	0.328	0.252509483	count	1
SUSD1	0.226318	0.2702555	0.8374	0.402	0.252586952	count	1
LLPH	0.1839432	0.1454953	1.2643	0.206	0.252630785	count	1
VIPAS39	0.3242169	0.372601	0.8701	0.384	0.252654206	count	1
SF1	0.1786793	0.0781266	2.287	0.0223	0.252668941	count	1
GTF3C2	0.2637766	0.3075373	0.8577	0.391	0.2526713	count	1
BOLA1	0.2638304	0.3812662	0.692	0.489	0.252724202	count	1
SNRNP70	0.1828156	0.1018889	1.7943	0.0729	0.252878883	count	1
SECISBP2L	0.193568	0.1826515	1.0598	0.289	0.253065626	count	1

IL6ST	0.1917205	0.1625111	1.1797	0.238	0.253118155	count	1
NDRG3	0.2130375	0.2949628	0.7223	0.47	0.253415358	count	1
GEMIN5	0.3488494	0.452374	0.7712	0.441	0.253456964	count	1
KCTD2	0.3637369	0.499755	0.7278	0.467	0.253464378	count	1
AC109630.1	0.3815175	0.565399	0.6748	0.5	0.253532177	count	1
DOCK11	0.196983	0.146345	1.346	0.178	0.253633862	count	1
LUC7L3	0.1815522	0.0964034	1.8833	0.0598	0.253645244	count	1
7-Mar	0.1883973	0.1225759	1.537	0.124	0.253759046	count	1
ALDH16A1	0.2009848	0.2028423	0.9908	0.322	0.253809567	count	1
IST1	0.1886073	0.1490921	1.265	0.206	0.253848244	count	1
MRPS31	0.2013326	0.1863633	1.0803	0.28	0.253884261	count	1
MVK	0.5067329	0.5095357	0.9945	0.32	0.253886643	count	1
PIK3CD-AS1	1.7065036	1.337144	1.2762	0.202	0.253960544	count	1
WNT6	1.7065036	1.064872	1.6025	0.109	0.253960544	count	1
AC024060.1	1.7065036	1.337144	1.2762	0.202	0.253960544	count	1
ZNF713	1.7065036	1.153889	1.4789	0.139	0.253960544	count	1
RAB39B	1.7065036	1.064872	1.6025	0.109	0.253960544	count	1
SPATA41	1.7065036	1.337144	1.2762	0.202	0.253960544	count	1
ZSWIM3	1.7065036	1.064872	1.6025	0.109	0.253960544	count	1
SSC5D	1.7065036	1.337144	1.2762	0.202	0.253960544	count	1
VPS54	0.2277968	0.3506297	0.6497	0.516	0.254263207	count	1
ROCK1	0.1797879	0.0733703	2.4504	0.0143	0.254419522	count	1
CCL5	0.1968243	0.3016872	0.6524	0.514	0.254450582	count	1
DHRS4L2	0.1838729	0.0976301	1.8834	0.0598	0.254861064	count	1
EFR3A	0.1860949	0.1655783	1.1239	0.261	0.254969326	count	1
FAM120A	0.1891592	0.1269515	1.49	0.136	0.254980376	count	1
PRKCSH	0.19342	0.1555294	1.2436	0.214	0.255045416	count	1
CRYZL1	0.2014013	0.2313534	0.8705	0.384	0.255053799	count	1
SRC	0.2443737	0.3391491	0.7205	0.471	0.255119559	count	1
ADCK5	0.31775	0.4369732	0.7272	0.467	0.255212741	count	1
ZNF324	0.2489074	0.5321389	0.4677	0.64	0.255257342	count	1
NEMF	0.1884294	0.1260992	1.4943	0.135	0.255302591	count	1
TBC1D15	0.1968239	0.136169	1.4454	0.148	0.25540618	count	1
DCTN1	0.2029744	0.2287193	0.8874	0.375	0.255592381	count	1
WSB1	0.1804151	0.0702896	2.5667	0.0103	0.255608935	count	1
CCDC130	0.1991623	0.2013281	0.9892	0.323	0.255634508	count	1
CEP78	0.3845983	0.3314519	1.1603	0.246	0.255702918	count	1
ATL2	0.2543868	0.426126	0.597	0.551	0.255731054	count	1
NAE1	0.2061172	0.2234076	0.9226	0.356	0.255735217	count	1
NRROS	0.2042842	0.204775	0.9976	0.319	0.256064353	count	1
TTC9C	0.2134142	0.2256356	0.9458	0.344	0.256077149	count	1
AC055822.1	0.7934483	0.9979214	0.7951	0.427	0.256134536	count	1
AC024940.1	0.7934483	0.7452926	1.0646	0.287	0.256134536	count	1
STXBP3	0.1885384	0.150776	1.2505	0.211	0.256222563	count	1
KLHL17	0.3286089	1.0991073	0.299	0.765	0.256229566	count	1

POLA1	0.2962468	0.428051	0.6921	0.489	0.256232509	count	1
SLC30A5	0.2222306	0.2440931	0.9104	0.363	0.256245709	count	1
GABPA	0.2234519	0.2815777	0.7936	0.428	0.256600913	count	1
AKAP8	0.2061738	0.2229841	0.9246	0.355	0.256727206	count	1
MAPK8	0.2090992	0.2821729	0.741	0.459	0.256938075	count	1
CHST13	0.2257603	0.2555776	0.8833	0.377	0.257011776	count	1
EIF3E	0.180415	0.0495319	3.6424	0.000276	0.257111553	count	1
RCOR1	0.1876814	0.1220432	1.5378	0.124	0.257204075	count	1
MIR222HG	0.2270185	0.4382658	0.518	0.605	0.257260438	count	1
RPL6	0.1785511	0.0245495	7.2731	4.70E-13	0.257313977	count	1.13E-08
AL159163.1	0.3042496	0.5663457	0.5372	0.591	0.257362151	count	1
ACADM	0.1909436	0.1632046	1.17	0.242	0.257394901	count	1
HHLA3	0.256026	0.3664569	0.6987	0.485	0.257417385	count	1
PUM1	0.1967532	0.1663798	1.1826	0.237	0.25745267	count	1
TMEM167B	0.194302	0.1706682	1.1385	0.255	0.257628058	count	1
LINC00656	1.7283342	0.9429855	1.8328	0.0669	0.257729281	count	1
VPS37A	0.2018276	0.18597	1.0853	0.278	0.257894647	count	1
COPZ2	0.2656227	0.3635087	0.7307	0.465	0.257948589	count	1
AL356512.1	0.3699008	0.5424604	0.6819	0.495	0.25799938	count	1
INTS13	0.2982924	0.3958626	0.7535	0.451	0.258064681	count	1
ABRAXAS1	0.2175708	0.2705705	0.8041	0.421	0.258077243	count	1
TRIM52	0.2247712	0.3164477	0.7103	0.478	0.258136982	count	1
TOMM5	0.1926737	0.1844178	1.0448	0.296	0.258168416	count	1
KIAA0040	0.1976514	0.1935462	1.0212	0.307	0.258231089	count	1
AC245060.5	0.2626623	0.5296445	0.4959	0.62	0.258244279	count	1
CLCN3	0.2023605	0.1861595	1.087	0.277	0.258269797	count	1
SLC35A2	0.2141056	0.2685162	0.7974	0.425	0.258294821	count	1
IL16	0.1947028	0.1524935	1.2768	0.202	0.258454537	count	1
CTSG	0.4103283	0.9478798	0.4329	0.665	0.258522707	count	1
TEX22	0.4103283	0.6535561	0.6278	0.53	0.258522707	count	1
HDAC8	0.2160414	0.2732592	0.7906	0.429	0.258540067	count	1
LINC01410	0.2699101	0.4052905	0.666	0.505	0.258706399	count	1
RIMS3	1.054508	0.9979126	1.0567	0.291	0.258731422	count	1
PRKAR2A-AS1	1.054508	1.041727	1.0123	0.312	0.258731422	count	1
TF	1.054508	1.2816545	0.8228	0.411	0.258731422	count	1
ADCY2	1.054508	1.1781147	0.8951	0.371	0.258731422	count	1
TSLP	1.054508	0.9663408	1.0912	0.275	0.258731422	count	1
AC091959.3	1.054508	0.9189394	1.1475	0.251	0.258731422	count	1
AL137003.2	1.054508	0.9663408	1.0912	0.275	0.258731422	count	1
AC004839.1	1.054508	0.9979126	1.0567	0.291	0.258731422	count	1
MYBL1	1.054508	1.001006	1.0534	0.292	0.258731422	count	1
RFX3-AS1	1.054508	1.041727	1.0123	0.312	0.258731422	count	1
FZD8	1.054508	0.8689562	1.2135	0.225	0.258731422	count	1
CAPS2	1.054508	1.041727	1.0123	0.312	0.258731422	count	1
DDTL	1.054508	0.9663408	1.0912	0.275	0.258731422	count	1

MRPL49	0.2330724	0.2614263	0.8915	0.373	0.258819914	count	1
CDK18	0.4715553	0.5265296	0.8956	0.371	0.258858905	count	1
LMBR1	0.3891381	0.3685154	1.056	0.291	0.258904889	count	1
TMEM43	0.2015324	0.2385246	0.8449	0.398	0.258979833	count	1
AMMECR1L	0.411106	0.5370285	0.7655	0.444	0.259045332	count	1
RCE1	0.2023184	0.2528311	0.8002	0.424	0.259130377	count	1
KMT2C	0.1883969	0.1153326	1.6335	0.102	0.259154348	count	1
CSF2RA	0.1856743	0.1089947	1.7035	0.0886	0.259217092	count	1
FURIN	0.2136152	0.2248559	0.95	0.342	0.259629187	count	1
TMOD2	0.2175668	0.2053845	1.0593	0.29	0.259636475	count	1
C11orf71	0.2210431	0.2972452	0.7436	0.457	0.259683675	count	1
FBXO21	0.2128863	0.2305972	0.9232	0.356	0.259938359	count	1
AKT1	0.1914089	0.1348784	1.4191	0.156	0.259986091	count	1
TDRD9	1.741681	0.7671703	2.2703	0.0233	0.26002984	count	1
MDK	0.3154734	0.5050167	0.6247	0.532	0.260514372	count	1
WDSUB1	0.2347381	0.286004	0.8208	0.412	0.260699822	count	1
CSAD	0.2100932	0.3044658	0.69	0.49	0.260712446	count	1
ATP7A	0.2235651	0.2856412	0.7827	0.434	0.260823414	count	1
WDR61	0.1941106	0.1417651	1.3692	0.171	0.260976191	count	1
TMEM214	0.2315556	0.3911433	0.592	0.554	0.261207988	count	1
AC100786.1	0.4758569	0.5874035	0.8101	0.418	0.261416371	count	1
TMCO3	0.2028216	0.2383816	0.8508	0.395	0.261471295	count	1
RPL22	0.1816216	0.0280561	6.4735	1.15E-10	0.261502932	count	2.77E-06
TCP11L1	0.2446438	0.2963352	0.8256	0.409	0.261598176	count	1
PUS1	0.2693892	0.3553834	0.758	0.449	0.261702711	count	1
DDX20	0.2466142	0.289727	0.8512	0.395	0.261755879	count	1
SKI	0.2260599	0.2518934	0.8974	0.37	0.261771175	count	1
UBL7-AS1	0.2169651	0.2769908	0.7833	0.434	0.261781668	count	1
PYCARD	0.1825363	0.0421312	4.3326	1.53E-05	0.261873714	count	0.3620898
ZNF480	0.2106485	0.2776956	0.7586	0.448	0.261883057	count	1
ARL2BP	0.1919684	0.14521	1.322	0.186	0.261949075	count	1
PIP4P1	0.192752	0.1208937	1.5944	0.111	0.261968352	count	1
POR	0.1922429	0.1778673	1.0808	0.28	0.262058302	count	1
PSRC1	0.3259354	0.5962629	0.5466	0.585	0.26206765	count	1
SHQ1	0.3259354	0.5569402	0.5852	0.558	0.26206765	count	1
RP9	0.2027002	0.2053767	0.987	0.324	0.262095538	count	1
FBXO25	0.2146417	0.2583478	0.8308	0.406	0.262103176	count	1
SLC25A6	0.1824321	0.0379316	4.8095	1.60E-06	0.262167091	count	0.0380768
LINC01772	0.443002	0.6296983	0.7035	0.482	0.262259879	count	1
GRPEL2	0.443002	0.6356669	0.6969	0.486	0.262259879	count	1
DIRC3	1.75609	1.12861	1.556	0.12	0.262510164	count	1
FAM124B	1.75609	1.274602	1.3778	0.168	0.262510164	count	1
AC116563.1	1.75609	1.328848	1.3215	0.186	0.262510164	count	1
SCNN1A	1.75609	1.328848	1.3215	0.186	0.262510164	count	1
CGNL1	1.75609	0.9956429	1.7638	0.0779	0.262510164	count	1

AC012173.1	1.75609	1.572253	1.1169	0.264	0.262510164	count	1
AC099518.1	1.75609	0.921996	1.9047	0.0569	0.262510164	count	1
PRDX2	0.1881675	0.1288194	1.4607	0.144	0.262542176	count	1
PMP22	0.1875707	0.1061795	1.7665	0.0774	0.262558557	count	1
RAB12	0.2155052	0.2422558	0.8896	0.374	0.262566745	count	1
RHOU	0.2025135	0.1708576	1.1853	0.236	0.262595396	count	1
CLINT1	0.1865697	0.0891514	2.0927	0.0365	0.26262386	count	1
IL3RA	0.25594	0.3731235	0.6859	0.493	0.262629805	count	1
EXOSC1	0.1954978	0.1593214	1.2271	0.22	0.262636057	count	1
CSNK1G3	0.2065348	0.2229961	0.9262	0.354	0.262649537	count	1
GRIPAP1	0.1958746	0.1892041	1.0353	0.301	0.262703576	count	1
MRPL34	0.1871603	0.0861251	2.1731	0.0299	0.262916126	count	1
NFATC2IP	0.2060477	0.1985725	1.0376	0.3	0.263009711	count	1
ARMC9	0.8120867	0.6655467	1.2202	0.223	0.263082318	count	1
COPS7B	0.2321067	0.3291255	0.7052	0.481	0.263113207	count	1
VCPKMT	0.2211307	0.4427569	0.4994	0.618	0.26315575	count	1
MYO9A	0.2332667	0.2588023	0.9013	0.368	0.263167842	count	1
POLR1D	0.1863952	0.0695941	2.6783	0.00745	0.263240933	count	1
TMEM128	0.2384229	0.2493421	0.9562	0.339	0.26334693	count	1
FAM57A	0.3620696	0.6813235	0.5314	0.595	0.263567165	count	1
APBA1	0.6738629	0.6249822	1.0782	0.281	0.263584861	count	1
OGA	0.194071	0.1696668	1.1438	0.253	0.263990881	count	1
NMNAT3	0.3494825	0.3790146	0.9221	0.357	0.263995313	count	1
MT-CO1	0.1830787	0.0238961	7.6615	2.63E-14	0.264064015	count	6.36E-10
IRF9	0.2082156	0.2537717	0.8205	0.412	0.264107948	count	1
TIFA	0.2226914	0.2278587	0.9773	0.329	0.264223979	count	1
TMEM9	0.2008326	0.1884514	1.0657	0.287	0.264343612	count	1
LTV1	0.2043469	0.2283173	0.895	0.371	0.264492346	count	1
INPP5A	0.3127483	0.4467046	0.7001	0.484	0.264826511	count	1
FCF1	0.2364115	0.2549332	0.9273	0.354	0.265427264	count	1
PLEKHO1	0.1865721	0.0744877	2.5047	0.0123	0.265457289	count	1
AC007384.1	0.2639612	0.4687308	0.5631	0.573	0.26558703	count	1
BORCS8	0.1995431	0.1520901	1.312	0.19	0.265764537	count	1
TCF4	0.1988024	0.1547474	1.2847	0.199	0.265937145	count	1
CNOT8	0.2088485	0.1763446	1.1843	0.236	0.265952571	count	1
RSL24D1	0.1887087	0.0802815	2.3506	0.0188	0.266258556	count	1
LPP-AS2	0.4493565	0.689203	0.652	0.514	0.266304741	count	1
FIG4	0.225864	0.2438973	0.9261	0.355	0.266316597	count	1
SELENOP	0.1854146	0.0896147	2.069	0.0386	0.266387437	count	1
SERPINA1	0.1859714	0.053697	3.4634	0.000543	0.266464701	count	1
BCL2L12	0.216477	0.2289781	0.9454	0.345	0.266639794	count	1
BCL2A1	0.1857251	0.0658728	2.8195	0.00485	0.266640397	count	1
OSCAR	0.1957044	0.1319391	1.4833	0.138	0.266861042	count	1
NAGA	0.1961044	0.1114272	1.7599	0.0785	0.267058056	count	1
ARID4A	0.1923836	0.1054145	1.825	0.0681	0.267112118	count	1

MRPS33	0.1968011	0.1623545	1.2122	0.226	0.267178199	count	1
SART1	0.2258631	0.26973	0.8374	0.402	0.267186715	count	1
MEF2A	0.1908894	0.0940269	2.0302	0.0424	0.267327724	count	1
SHKBP1	0.191065	0.083706	2.2826	0.0225	0.267359244	count	1
RPL19	0.1854672	0.0219877	8.435	5.57E-17	0.267400616	count	1.35E-12
AC016745.2	0.682621	0.815455	0.8371	0.403	0.267453152	count	1
STX17-AS1	0.682621	0.8420904	0.8106	0.418	0.267453152	count	1
CRAT	0.2381977	0.3311048	0.7194	0.472	0.26746458	count	1
NDUFA9	0.1968518	0.1377043	1.4295	0.153	0.267559699	count	1
RHBDD1	0.3158654	0.3930875	0.8035	0.422	0.267567597	count	1
TMC8	0.2383639	0.260934	0.9135	0.361	0.267654162	count	1
LONRF1	0.2206772	0.2374493	0.9294	0.353	0.267655452	count	1
VARS2	0.5938164	0.6072571	0.9779	0.328	0.267718058	count	1
UNC13B	0.5938164	0.5912772	1.0043	0.315	0.267718058	count	1
ZNF213	0.5939776	0.7288098	0.815	0.415	0.267798726	count	1
C16orf45	0.5939776	0.7668984	0.7745	0.439	0.267798726	count	1
TBP	0.2487776	0.3957927	0.6286	0.53	0.26802729	count	1
TRMT13	0.2636884	0.3930154	0.6709	0.502	0.268100164	count	1
HEBP1	0.1911113	0.0935763	2.0423	0.0412	0.268105155	count	1
BET1	0.2260156	0.2331482	0.9694	0.332	0.268215939	count	1
SPAST	0.2101602	0.2020417	1.0402	0.298	0.268297644	count	1
NBPF9	0.2879709	0.5754194	0.5005	0.617	0.268369393	count	1
GNAQ	0.1983221	0.1115788	1.7774	0.0756	0.26849683	count	1
PAK1	0.1947653	0.1029897	1.8911	0.0587	0.268587764	count	1
FAM78A	0.2295943	0.2795388	0.8213	0.412	0.268919444	count	1
DGKA	0.2157711	0.2919234	0.7391	0.46	0.269249894	count	1
SAMD9	0.1985072	0.1546353	1.2837	0.199	0.269258486	count	1
WDR82	0.2040212	0.1789259	1.1403	0.254	0.269272373	count	1
RICTOR	0.2177938	0.2670533	0.8155	0.415	0.269340982	count	1
SNX30	0.2170533	0.2300905	0.9433	0.346	0.269428847	count	1
CHURC1	0.1930162	0.0950305	2.0311	0.0424	0.269509691	count	1
FNTB	0.3111376	0.4527239	0.6873	0.492	0.269587326	count	1
DENND5A	0.2105493	0.1928112	1.092	0.275	0.269748068	count	1
MT-CYB	0.1871744	0.0287892	6.5016	9.60E-11	0.269828974	count	2.31E-06
RRAGB	0.2457004	0.340027	0.7226	0.47	0.269906293	count	1
RDH13	0.3570867	0.4898459	0.729	0.466	0.270024539	count	1
NFKB2	0.2137512	0.1881652	1.136	0.256	0.270061642	count	1
GCA	0.1906211	0.066324	2.8741	0.00409	0.270090779	count	1
PPP2R2A	0.1963519	0.1203771	1.6311	0.103	0.270321209	count	1
EIF2AK3	0.2588107	0.258943	0.9995	0.318	0.270514897	count	1
DENND4A	0.2126853	0.2337379	0.9099	0.363	0.270530809	count	1
LARS	0.1961417	0.126455	1.5511	0.121	0.270535044	count	1
TACO1	0.2421505	0.3287936	0.7365	0.462	0.270549119	count	1
ARMC10	0.2056325	0.1922562	1.0696	0.285	0.271058099	count	1
PRRG2	0.3198436	0.6662	0.4801	0.631	0.271068509	count	1

CLASP2	0.2247807	0.2503363	0.8979	0.369	0.271316433	count	1
KBTBD7	0.3131197	0.6301459	0.4969	0.619	0.271368049	count	1
YPEL1	0.3589253	0.5175829	0.6935	0.488	0.271483952	count	1
KDM2B	0.2274462	0.2650581	0.8581	0.391	0.271566111	count	1
MCM5	0.2104618	0.2035678	1.0339	0.301	0.271662298	count	1
MAP3K7	0.2298066	0.2440365	0.9417	0.346	0.271909859	count	1
MOB2	0.2109625	0.2033954	1.0372	0.3	0.272037653	count	1
DNAJA3	0.2731731	0.3236316	0.8441	0.399	0.272045192	count	1
ALG10B	0.3289471	0.5697953	0.5773	0.564	0.272101476	count	1
ETHE1	0.1949587	0.0914837	2.1311	0.0332	0.272180156	count	1
MKLN1	0.2201424	0.2335839	0.9425	0.346	0.272273001	count	1
AFF3	0.2968201	0.4217273	0.7038	0.482	0.272297833	count	1
RHBDD2	0.2080524	0.1928629	1.0788	0.281	0.272324512	count	1
EIF3K	0.1897102	0.0364012	5.2116	2.03E-07	0.272337319	count	0.004848655
TMEM140	0.2369611	0.2757903	0.8592	0.39	0.272340249	count	1
RPA1	0.3143235	0.336348	0.9345	0.35	0.272449886	count	1
ZMYM5	0.2159823	0.175862	1.2281	0.22	0.272510032	count	1
SPA17	0.3215712	0.4200861	0.7655	0.444	0.272589745	count	1
FYCO1	0.2531018	0.4916018	0.5149	0.607	0.272775501	count	1
DGKQ	0.6040284	0.540951	1.1166	0.264	0.272834259	count	1
TRADD	0.2031498	0.1374562	1.4779	0.14	0.272847351	count	1
CNST	0.2204485	0.260205	0.8472	0.397	0.273174692	count	1
MRM3	0.2488703	0.3421705	0.7273	0.467	0.273450431	count	1
HNRNPUL1	0.2031437	0.1219982	1.6651	0.096	0.273598929	count	1
TBC1D2B	0.21278	0.1643406	1.2948	0.196	0.273827705	count	1
BACH1	0.1941441	0.1000777	1.9399	0.0525	0.273926682	count	1
TRAPPC13	0.2694916	0.3236794	0.8326	0.405	0.274140443	count	1
OSBPL1A	0.2042211	0.172259	1.1855	0.236	0.274292337	count	1
ACSL5	0.2563784	0.3824103	0.6704	0.503	0.274395464	count	1
PIGV	0.2754961	0.3615372	0.762	0.446	0.274417307	count	1
EMP3	0.190807	0.0454646	4.1968	2.80E-05	0.274462176	count	0.66122
STT3B	0.2033047	0.133573	1.522	0.128	0.274521992	count	1
NCDN	1.108716	0.8341143	1.3292	0.184	0.274562565	count	1
SPTY2D1OS	1.108716	0.9763295	1.1356	0.256	0.274562565	count	1
ACAP1	0.2306507	0.2136324	1.0797	0.28	0.274622866	count	1
DNTTIP1	0.2061752	0.1402255	1.4703	0.142	0.274780399	count	1
SACM1L	0.2362566	0.2133134	1.1076	0.268	0.274804435	count	1
HNRNPA1	0.192159	0.0456203	4.2121	2.62E-05	0.274846226	count	0.6188178
POLR1B	0.3048022	0.5580038	0.5462	0.585	0.274872578	count	1
AFG3L2	0.2081308	0.2214754	0.9397	0.347	0.274899884	count	1
AC116407.2	0.2996779	0.5132922	0.5838	0.559	0.275004064	count	1
CREBRF	0.2019203	0.1299317	1.554	0.12	0.275005408	count	1
EBI3	0.2266828	0.1873211	1.2101	0.226	0.275018778	count	1
MPDU1	0.2131929	0.1647	1.2944	0.196	0.275211472	count	1
RGS19	0.1943109	0.0707796	2.7453	0.00609	0.275288104	count	1

ATP5MC2	0.1914455	0.0337196	5.6776	1.53E-08	0.275315308	count	0.000366802
C8orf59	0.194219	0.0720409	2.696	0.00707	0.275347475	count	1
RNFT1	0.2035582	0.1745165	1.1664	0.244	0.275431474	count	1
UBE2D1	0.1941955	0.0674226	2.8803	0.00401	0.275452069	count	1
PHLDB3	0.3424635	0.4304444	0.7956	0.426	0.275947439	count	1
UHMK1	0.2119291	0.1657124	1.2789	0.201	0.276095088	count	1
TNFSF8	0.2223804	0.216383	1.0277	0.304	0.276103263	count	1
RPUSD3	0.213957	0.2543125	0.8413	0.4	0.276478826	count	1
PARP14	0.1977408	0.0823997	2.3998	0.0165	0.276548661	count	1
PNPLA6	0.2241651	0.191214	1.1723	0.241	0.276756002	count	1
ABCA3	0.7036679	0.8025588	0.8768	0.381	0.276786926	count	1
LARP7	0.1978879	0.1003776	1.9714	0.0488	0.276818937	count	1
TTC32	0.2261473	0.2254225	1.0032	0.316	0.276914284	count	1
PHF1	0.2219324	0.2801308	0.7922	0.428	0.277007728	count	1
PCMT1	0.1965391	0.0709245	2.7711	0.00563	0.277033414	count	1
ZFP69B	1.1175489	0.768622	1.454	0.146	0.277155566	count	1
AC239868.2	1.1175489	0.7881798	1.4179	0.156	0.277155566	count	1
MLYCD	0.2848947	0.3472763	0.8204	0.412	0.277182701	count	1
WDR55	0.2225186	0.2075933	1.0719	0.284	0.277266998	count	1
IMMP2L	0.2887512	0.3310222	0.8723	0.383	0.277285884	count	1
BID	0.1954363	0.0746701	2.6173	0.00892	0.277338827	count	1
TNFSF15	0.2297821	0.331982	0.6922	0.489	0.277421399	count	1
UBE3C	0.2212055	0.2601937	0.8502	0.395	0.27746068	count	1
RNF213	0.1967034	0.0871434	2.2572	0.0241	0.277693486	count	1
PHETA1	0.3667951	0.4081696	0.8986	0.369	0.277737792	count	1
CD300C	0.2107518	0.1906626	1.1054	0.269	0.277843272	count	1
OTULIN	0.2140534	0.222416	0.9624	0.336	0.277909471	count	1
CAGE1	1.8472188	1.349633	1.3687	0.171	0.278101065	count	1
LINC01127	1.120929	0.6890357	1.6268	0.104	0.278148686	count	1
AC025181.2	0.3031918	0.4605824	0.6583	0.51	0.278333574	count	1
MBD4	0.1999529	0.1090274	1.834	0.0668	0.278406964	count	1
ZFP69	0.852997	0.7285691	1.1708	0.242	0.278445628	count	1
C2orf92	0.852997	0.7582168	1.125	0.261	0.278445628	count	1
VTA1	0.2140208	0.1749761	1.2231	0.221	0.278600338	count	1
TRMT10A	0.2738517	0.3354462	0.8164	0.414	0.27868222	count	1
NKX3-1	0.6157406	0.8194007	0.7515	0.452	0.278719919	count	1
BCO2	0.2688335	0.4508791	0.5962	0.551	0.278753022	count	1
PRKACB	0.2249816	0.1720634	1.3075	0.191	0.278845642	count	1
GRHPR	0.2027768	0.1191598	1.7017	0.0889	0.278889438	count	1
ETS2	0.1954818	0.0595059	3.2851	0.00103	0.2789473	count	1
APEH	0.2268159	0.2189466	1.0359	0.3	0.279501635	count	1
AC090204.1	0.2234017	0.272745	0.8191	0.413	0.279788995	count	1
GGT5	0.6179656	0.9346691	0.6612	0.509	0.279840165	count	1
TUT4	0.205465	0.1905808	1.0781	0.281	0.279849883	count	1
RPL18	0.1942713	0.0244872	7.9336	3.21E-15	0.279966346	count	7.76E-11

RPS26	0.1945526	0.0277487	7.0112	3.04E-12	0.280100619	count	7.33E-08
TENT2	0.219413	0.1803848	1.2164	0.224	0.280200013	count	1
CLCC1	0.2958128	0.2959293	0.9996	0.318	0.280203823	count	1
IREB2	0.2215417	0.1943146	1.1401	0.254	0.280380971	count	1
ZNF641	0.2349684	0.2840894	0.8271	0.408	0.280656661	count	1
CDC42BPB	0.2387554	0.2501434	0.9545	0.34	0.280769595	count	1
EME2	0.5555304	0.557003	0.9974	0.319	0.280780035	count	1
KLHL9	0.252656	0.4216484	0.5992	0.549	0.280946175	count	1
DHRS4	0.2250673	0.1889535	1.1911	0.234	0.280956281	count	1
DOK2	0.1978977	0.0665996	2.9715	0.00299	0.281052425	count	1
GEN1	0.5088106	0.638852	0.7964	0.426	0.281110524	count	1
UCK1	0.2575155	0.3562437	0.7229	0.47	0.281363568	count	1
C1orf109	0.3317565	0.3750035	0.8847	0.376	0.28156949	count	1
ANXA1	0.1955073	0.0446896	4.3748	1.27E-05	0.281680753	count	0.3007741
TMEM33	0.2061915	0.1347585	1.5301	0.126	0.281697722	count	1
USP16	0.2029938	0.1105844	1.8356	0.0665	0.281743161	count	1
VPS37B	0.2119061	0.1864354	1.1366	0.256	0.28186805	count	1
RB1CC1	0.2087565	0.1498214	1.3934	0.164	0.282110361	count	1
RAD9A	0.2363251	0.3124645	0.7563	0.45	0.282296892	count	1
PPIL3	0.2288122	0.2913312	0.7854	0.432	0.282550186	count	1
PVT1	0.2941015	0.4152805	0.7082	0.479	0.282572931	count	1
TECPR2	0.2602823	0.4663408	0.5581	0.577	0.28259314	count	1
KIF1B	0.2087596	0.1507838	1.3845	0.166	0.282773798	count	1
C21orf2	0.2144722	0.1897076	1.1305	0.258	0.282775339	count	1
PTPN18	0.2051354	0.1090812	1.8806	0.0601	0.282787303	count	1
BABAM2	0.2131962	0.2059133	1.0354	0.301	0.282810863	count	1
RAD23B	0.2068752	0.1151714	1.7962	0.0726	0.282901466	count	1
DHDDS	0.2517672	0.2690746	0.9357	0.35	0.282955711	count	1
CGGBP1	0.2053006	0.1164632	1.7628	0.0781	0.2830628	count	1
SMARCE1	0.2270181	0.200909	1.13	0.259	0.283413851	count	1
DPH7	0.2758426	0.3328032	0.8288	0.407	0.283536925	count	1
ELF4	0.2785889	0.3622062	0.7691	0.442	0.283620224	count	1
RIOK2	0.2244299	0.2157566	1.0402	0.298	0.283666704	count	1
B3GLCT	0.560924	0.6415068	0.8744	0.382	0.283775035	count	1
MFAP4	0.560924	0.6016629	0.9323	0.351	0.283775035	count	1
TNFAIP3	0.208185	0.0999158	2.0836	0.0373	0.283791925	count	1
PPP1R16A	0.3272109	0.3182715	1.0281	0.304	0.284048077	count	1
NFIL3	0.2013184	0.0717269	2.8067	0.00504	0.28427049	count	1
CKMT2-AS1	0.4054687	0.8536978	0.475	0.635	0.28430569	count	1
IFRD1	0.2036342	0.111988	1.8184	0.0691	0.284556007	count	1
IL18RAP	1.886343	1.134695	1.6624	0.0966	0.284734242	count	1
AL355353.1	1.886343	1.0411	1.8119	0.0701	0.284734242	count	1
AC023509.2	1.886343	1.0411	1.8119	0.0701	0.284734242	count	1
AC005520.2	0.4493077	0.5869413	0.7655	0.444	0.284850595	count	1
HPS4	0.2498679	0.2937415	0.8506	0.395	0.284890185	count	1

S100A6	0.1977623	0.0414469	4.7715	1.94E-06	0.285087204	count	0.04614872
CHD1	0.2007974	0.0759212	2.6448	0.00823	0.28511696	count	1
LAMTOR3	0.2149422	0.1587318	1.3541	0.176	0.285139034	count	1
AMMECR1	0.3158567	0.598416	0.5278	0.598	0.285188333	count	1
RAB2B	0.2490329	0.2978045	0.8362	0.403	0.285194956	count	1
DYRK1A	0.2214567	0.2415875	0.9167	0.359	0.285372469	count	1
TRIM35	0.4792095	0.5105934	0.9385	0.348	0.285400294	count	1
ZNF169	0.4792095	0.4905191	0.9769	0.329	0.285400294	count	1
NCOA4	0.2033658	0.0771415	2.6363	0.00844	0.285431914	count	1
PHAX	0.2136555	0.1629547	1.3111	0.19	0.285767374	count	1
RETREG2	0.2177438	0.1901658	1.145	0.252	0.285932802	count	1
DICER1	0.2068628	0.1208585	1.7116	0.0871	0.285938527	count	1
POLR2G	0.2064425	0.1026148	2.0118	0.0443	0.286257331	count	1
POLE4	0.2024455	0.0791219	2.5587	0.0106	0.286371689	count	1
HTRA2	0.230599	0.2290515	1.0068	0.314	0.286405682	count	1
TRAF7	0.2212657	0.2068204	1.0698	0.285	0.286540927	count	1
VMO1	0.2016046	0.1109051	1.8178	0.0692	0.286698097	count	1
SLC50A1	0.2169947	0.1652347	1.3133	0.189	0.287027581	count	1
MVP	0.2102935	0.1231333	1.7079	0.0878	0.287040475	count	1
QARS	0.2178396	0.1280811	1.7008	0.0891	0.287427432	count	1
INTS11	0.211921	0.1448963	1.4626	0.144	0.287522702	count	1
HACD3	0.226836	0.2062693	1.0997	0.272	0.2875332	count	1
PNRC2	0.203966	0.0816398	2.4984	0.0125	0.287615231	count	1
FN3K	0.4534438	0.5688303	0.7972	0.425	0.287659948	count	1
MTRNR2L10	1.153801	0.7385584	1.5622	0.118	0.287834149	count	1
CCDC77	0.2689513	0.3275736	0.821	0.412	0.288129562	count	1
CACTIN	0.303931	0.4320794	0.7034	0.482	0.288129708	count	1
PPIP5K2	0.2154908	0.1530127	1.4083	0.159	0.288233697	count	1
TNFSF13B	0.2023649	0.0600139	3.372	0.000758	0.288323819	count	1
ZNF680	0.4544746	0.4293243	1.0586	0.29	0.288360556	count	1
SLC25A13	0.2235641	0.1893816	1.1805	0.238	0.288403416	count	1
FBN2	0.8793611	0.5882641	1.4948	0.135	0.288425194	count	1
R3HDM2	0.2096513	0.1169076	1.7933	0.073	0.288477539	count	1
AC034111.1	0.5698545	0.8866596	0.6427	0.52	0.288743639	count	1
ZNF525	0.5698545	0.6898782	0.826	0.409	0.288743639	count	1
IMPDH1	0.2155022	0.1292765	1.667	0.0956	0.28877167	count	1
GPR34	0.2067566	0.1020634	2.0258	0.0429	0.288856006	count	1
ATF7IP2	0.2394471	0.2865184	0.8357	0.403	0.289226487	count	1
ATP5MPL	0.2034035	0.0594516	3.4213	0.000633	0.289310994	count	1
MRPS18B	0.2141541	0.1718231	1.2464	0.213	0.289533724	count	1
NUDT6	0.3208167	0.4517014	0.7102	0.478	0.28982386	count	1
KLF3	0.2040055	0.0784291	2.6011	0.00935	0.289928129	count	1
GMPR2	0.2159087	0.1523797	1.4169	0.157	0.289940351	count	1
TBXAS1	0.2056371	0.0894686	2.2984	0.0216	0.290035465	count	1
TNFRSF1B	0.2037104	0.0672957	3.0271	0.00249	0.290037495	count	1

KANSL1	0.2138728	0.1390247	1.5384	0.124	0.290091909	count	1
TBC1D10A	0.2483387	0.2894382	0.858	0.391	0.290136852	count	1
TUBGCP5	0.2773996	0.3195502	0.8681	0.385	0.290385086	count	1
BAG5	0.2184283	0.1584586	1.3785	0.168	0.29043204	count	1
TLE3	0.2140832	0.1575708	1.3586	0.174	0.290555731	count	1
MYO5A	0.2177449	0.1606113	1.3557	0.175	0.290567196	count	1
PDK1	0.2462422	0.2876097	0.8562	0.392	0.290667973	count	1
TNXB	1.921826	1.360505	1.4126	0.158	0.290712989	count	1
TG	1.921826	1.638869	1.1727	0.241	0.290712989	count	1
LINC02334	1.921826	1.638869	1.1727	0.241	0.290712989	count	1
GSDMA	1.921826	0.8744343	2.1978	0.0281	0.290712989	count	1
AC011484.1	1.921826	1.038138	1.8512	0.0643	0.290712989	count	1
ZNF772	1.921826	1.281501	1.4997	0.134	0.290712989	count	1
AC002451.1	0.4140922	0.5722958	0.7236	0.469	0.290718402	count	1
SRFBP1	0.2133699	0.1413003	1.51	0.131	0.290808962	count	1
CKB	0.3507777	0.3181133	1.1027	0.27	0.290946195	count	1
TNFRSF9	1.1643777	0.7420391	1.5692	0.117	0.290959866	count	1
JKAMP	0.2143877	0.1318304	1.6262	0.104	0.291144783	count	1
GKAP1	0.2665763	0.2757234	0.9668	0.334	0.291453482	count	1
ARMC1	0.2360053	0.215055	1.0974	0.273	0.291522875	count	1
MAP4K1	0.3718313	0.4618114	0.8052	0.421	0.291608019	count	1
HAAO	0.2835644	0.3139866	0.9031	0.367	0.291665184	count	1
NFU1	0.2244091	0.1881632	1.1926	0.233	0.291704843	count	1
DTD1	0.2224621	0.1857886	1.1974	0.231	0.291740536	count	1
MID1IP1	0.209962	0.1290985	1.6264	0.104	0.29181183	count	1
SNX11	0.2509903	0.3212571	0.7813	0.435	0.292190642	count	1
IPCEF1	0.3234494	0.3792464	0.8529	0.394	0.29228608	count	1
ATXN2	0.221967	0.1568405	1.4152	0.157	0.292324307	count	1
RTTN	0.4604697	0.5698769	0.808	0.419	0.292438909	count	1
SEC22B	0.2148634	0.1559858	1.3775	0.168	0.292459546	count	1
CCDC142	0.5768137	0.5663634	1.0185	0.309	0.292623713	count	1
NUP205	0.3086193	0.4381667	0.7043	0.481	0.292712038	count	1
ATG4B	0.2384146	0.2601428	0.9165	0.36	0.292727522	count	1
METTL16	0.2591371	0.28938	0.8955	0.371	0.29284643	count	1
MARVELD1	0.2497428	0.260531	0.9586	0.338	0.292850141	count	1
TAB2	0.2391711	0.2466421	0.9697	0.332	0.293034162	count	1
ZEB2-AS1	0.2255791	0.1810231	1.2461	0.213	0.293235854	count	1
VPS39	0.2718965	0.3036972	0.8953	0.371	0.293444678	count	1
SLC35F3	0.8929953	1.1426336	0.7815	0.435	0.293609303	count	1
C19orf57	0.8929953	0.8806131	1.0141	0.311	0.293609303	count	1
ZNF45	0.8929953	0.8967149	0.9959	0.319	0.293609303	count	1
SETDB1	0.2531721	0.2522931	1.0035	0.316	0.293635135	count	1
PCNX3	0.3014706	0.3384851	0.8906	0.373	0.293775858	count	1
SLC25A46	0.2533096	0.2270908	1.1155	0.265	0.293796959	count	1
GSS	0.2208515	0.211132	1.046	0.296	0.293828276	count	1

HIGD2A	0.2054562	0.0453053	4.5349	6.04E-06	0.293971457	count	0.14332316
KPTN	0.3457952	0.4335859	0.7975	0.425	0.293977248	count	1
IBA57	0.2785448	0.375617	0.7416	0.458	0.294052401	count	1
MICU1	0.2212962	0.1550105	1.4276	0.154	0.294105248	count	1
ASH1L	0.2155835	0.1448354	1.4885	0.137	0.294139027	count	1
RNF135	0.2144452	0.1341829	1.5982	0.11	0.294244478	count	1
AREG	0.2043763	0.115666	1.767	0.0774	0.294309256	count	1
MTX3	0.3642463	0.5436802	0.67	0.503	0.294316745	count	1
SPIN2B	0.3202356	0.4447726	0.72	0.472	0.294513512	count	1
CDKN2AIP	0.2288167	0.1879441	1.2175	0.224	0.294925971	count	1
ATP6V1A	0.2167148	0.1363512	1.5894	0.112	0.294988809	count	1
DDIT4	0.207388	0.0814988	2.5447	0.011	0.295058714	count	1
CDKN1C	0.2131332	0.1887624	1.1291	0.259	0.295171056	count	1
KIF20B	0.2555286	0.2259214	1.1311	0.258	0.2952292	count	1
AC012615.1	0.269976	0.3541202	0.7624	0.446	0.29524227	count	1
PRAF2	0.2929455	0.3353414	0.8736	0.382	0.295514493	count	1
DPY19L4	0.2929773	0.4028776	0.7272	0.467	0.295547405	count	1
RNF121	0.2929773	0.3802405	0.7705	0.441	0.295547405	count	1
FOXN3-AS1	0.3116024	0.3683143	0.846	0.398	0.295629635	count	1
RPS6KA4	0.243076	0.1894726	1.2829	0.2	0.29584669	count	1
ACAT1	0.2277804	0.1790804	1.2719	0.204	0.295853131	count	1
ZBTB11-AS1	0.2875539	0.3247262	0.8855	0.376	0.295868303	count	1
STOML1	0.4040293	0.444599	0.9087	0.364	0.295870697	count	1
MAFK	0.2361957	0.29281	0.8067	0.42	0.295961363	count	1
CROT	0.3036644	0.3519163	0.8629	0.388	0.295975344	count	1
AMPD3	0.270688	0.2672619	1.0128	0.311	0.29603596	count	1
EPB41	0.2396492	0.1826183	1.3123	0.19	0.296070151	count	1
AC079305.1	0.327651	0.3949352	0.8296	0.407	0.296218096	count	1
TAPT1	0.2447287	0.2473825	0.9893	0.323	0.296434049	count	1
KIAA0930	0.2152842	0.1158326	1.8586	0.0632	0.296514651	count	1
TSPYL1	0.2154862	0.1288609	1.6722	0.0946	0.296530259	count	1
EIF4A2	0.2132366	0.0912061	2.338	0.0195	0.296538083	count	1
RLF	0.227223	0.1664562	1.3651	0.172	0.296640655	count	1
AGA	0.2392537	0.1702945	1.4049	0.16	0.296712725	count	1
LILRA6	0.2416289	0.2345253	1.0303	0.303	0.296716331	count	1
BTF3	0.206324	0.0308257	6.6932	2.70E-11	0.29674352	count	6.51E-07
AFDN	0.2427261	0.2613803	0.9286	0.353	0.296780239	count	1
MYD88	0.2133913	0.1073031	1.9887	0.0468	0.296850167	count	1
CYREN	0.240784	0.3056508	0.7878	0.431	0.296894664	count	1
ZNF514	0.3576553	0.4709012	0.7595	0.448	0.296900962	count	1
DNAJA4	0.3415091	0.4868384	0.7015	0.483	0.296950706	count	1
DDX1	0.2248094	0.158011	1.4227	0.155	0.297054443	count	1
PHYKPL	0.2219647	0.1484764	1.4949	0.135	0.297071606	count	1
PARVG	0.2127194	0.0965735	2.2027	0.0277	0.297128509	count	1
AC010969.2	0.4978255	0.7156596	0.6956	0.487	0.297384242	count	1

AIP	0.2149974	0.115264	1.8653	0.0623	0.297637494	count	1
MLX	0.2142701	0.110617	1.937	0.0529	0.297711948	count	1
CYB561D1	0.5365475	0.6336728	0.8467	0.397	0.297823373	count	1
ZNF222	0.5365475	0.5985531	0.8964	0.37	0.297823373	count	1
5-Mar	0.2285297	0.2028937	1.1264	0.26	0.297865363	count	1
ARID2	0.254873	0.2373899	1.0736	0.283	0.297880726	count	1
PISD	0.2781101	0.2211905	1.2573	0.209	0.298148721	count	1
DHX36	0.212689	0.09416	2.2588	0.024	0.298179271	count	1
EIF3M	0.210481	0.0667778	3.152	0.00164	0.298331067	count	1
TWNK	0.5376023	0.6905407	0.7785	0.436	0.298461343	count	1
AC007728.2	0.5376023	0.6905407	0.7785	0.436	0.298461343	count	1
LY86-AS1	0.5873935	0.6963836	0.8435	0.399	0.298536111	count	1
FAM124A	0.5873935	0.6963836	0.8435	0.399	0.298536111	count	1
KIF17	1.1900084	1.1003261	1.0815	0.28	0.298552578	count	1
STIL	1.1900084	1.1036536	1.0782	0.281	0.298552578	count	1
AL360270.3	1.1900084	1.0729548	1.1091	0.267	0.298552578	count	1
PRPF40B	1.1900084	1.072572	1.1095	0.267	0.298552578	count	1
PAGR1	1.1900084	1.0029742	1.1865	0.236	0.298552578	count	1
USP6	1.1900084	1.0029742	1.1865	0.236	0.298552578	count	1
MRPL38	1.1900084	0.9690911	1.228	0.22	0.298552578	count	1
RPL17-C18orf32	1.1900084	1.0029742	1.1865	0.236	0.298552578	count	1
BACH1-AS1	1.1900084	1.0029742	1.1865	0.236	0.298552578	count	1
EXOSC4	0.2195865	0.1561778	1.406	0.16	0.2986631	count	1
AC005076.1	0.445141	0.4828858	0.9218	0.357	0.298712075	count	1
C16orf95	1.971816	1.284072	1.5356	0.125	0.29906966	count	1
KLHL22	0.3601965	0.5549076	0.6491	0.516	0.299103322	count	1
RAB11B	0.2217655	0.1568966	1.4135	0.158	0.29912513	count	1
ARRDC1-AS1	0.2718084	0.3699875	0.7346	0.463	0.299138302	count	1
G6PC3	0.2441838	0.2128922	1.147	0.252	0.299242948	count	1
MX2	0.2123701	0.1036679	2.0486	0.0406	0.299354802	count	1
NBN	0.2217822	0.1359323	1.6316	0.103	0.299578367	count	1
USP30	0.4091245	0.5280956	0.7747	0.439	0.299815063	count	1
BRAF	0.2392712	0.2019038	1.1851	0.236	0.299851114	count	1
AL008729.1	0.9103129	0.7160616	1.2713	0.204	0.300215999	count	1
AC007228.2	0.9103129	1.0578546	0.8605	0.39	0.300215999	count	1
FAM189B	0.3263725	0.416994	0.7827	0.434	0.300351624	count	1
DHFR	0.3263725	0.4179895	0.7808	0.435	0.300351624	count	1
PRKCE	0.427032	0.4331534	0.9859	0.324	0.30036573	count	1
CSGALNACT2	0.216194	0.1032349	2.0942	0.0363	0.300425758	count	1
MCM6	0.3322022	0.4480002	0.7415	0.458	0.300480722	count	1
AC004812.2	0.3322022	0.4113458	0.8076	0.419	0.300480722	count	1
AK3	0.2470068	0.2447727	1.0091	0.313	0.300685684	count	1
ZMYND8	0.2424631	0.2129056	1.1388	0.255	0.300733131	count	1
PEPD	0.2134245	0.0801969	2.6613	0.00784	0.300803672	count	1
SOCS5	0.3086295	0.3668563	0.8413	0.4	0.300956204	count	1

PICALM	0.2163433	0.1039408	2.0814	0.0375	0.301125338	count	1
SLC25A17	0.3463067	0.4393756	0.7882	0.431	0.301288124	count	1
KRI1	0.3090116	0.3726672	0.8292	0.407	0.301339682	count	1
FUT11	0.2738483	0.3796666	0.7213	0.471	0.30142624	count	1
ZRANB2	0.217812	0.1128907	1.9294	0.0538	0.301460538	count	1
AP3M1	0.2501737	0.3247355	0.7704	0.441	0.301550763	count	1
RWDD4	0.2234104	0.1546408	1.4447	0.149	0.30168033	count	1
HHEX	0.2348378	0.1762005	1.3328	0.183	0.301781449	count	1
ZNF3	0.3732027	0.5712434	0.6533	0.514	0.301894468	count	1
SEMA4B	0.3732027	0.5160564	0.7232	0.47	0.301894468	count	1
RPL35A	0.2096118	0.0252984	8.2856	1.91E-16	0.301967832	count	4.62E-12
SPOP	0.2256593	0.1455458	1.5504	0.121	0.302040417	count	1
MORN1	0.6618333	0.9444299	0.7008	0.484	0.302063036	count	1
AL133551.1	0.6618333	1.145342	0.5778	0.563	0.302063036	count	1
AC116667.2	0.6618333	1.0189528	0.6495	0.516	0.302063036	count	1
LINC01431	0.6618333	1.145342	0.5778	0.563	0.302063036	count	1
IER5L	0.2181666	0.140432	1.5535	0.12	0.302119127	count	1
ZHX1-C8orf76	0.4747123	0.6142892	0.7728	0.44	0.302152532	count	1
ZNF468	0.3974653	0.5562475	0.7145	0.475	0.302218221	count	1
HDAC5	0.2455665	0.1968886	1.2472	0.212	0.302236584	count	1
FANCB	0.373697	0.4407096	0.8479	0.397	0.302313096	count	1
NIPSNAP2	0.2164193	0.1052806	2.0556	0.0399	0.302383231	count	1
CTSC	0.2110961	0.0697035	3.0285	0.00248	0.302446187	count	1
RBM45	0.3287284	0.362024	0.908	0.364	0.302594531	count	1
POLR2J3	0.2180542	0.2078912	1.0489	0.294	0.302734201	count	1
LTBR	0.2221516	0.144525	1.5371	0.124	0.302978602	count	1
CRLS1	0.2348343	0.1853771	1.2668	0.205	0.303049902	count	1
PHKG2	0.2302944	0.1976352	1.1653	0.244	0.303150996	count	1
HOXB4	0.9181805	0.6279281	1.4622	0.144	0.303225419	count	1
PLAA	0.3072222	0.3435198	0.8943	0.371	0.30331958	count	1
UBR1	0.3195572	0.3099861	1.0309	0.303	0.30341703	count	1
UNC119B	0.5072168	0.6716764	0.7552	0.45	0.303451565	count	1
STMN3	0.5072168	0.6098504	0.8317	0.406	0.303451565	count	1
TGDS	0.2791175	0.3061801	0.9116	0.362	0.303459712	count	1
LRRC41	0.252425	0.2253439	1.1202	0.263	0.30348037	count	1
IKBKG	0.240343	0.1895081	1.2682	0.205	0.303523065	count	1
TPRA1	0.230307	0.2428993	0.9482	0.343	0.30357651	count	1
CUL4A	0.2355317	0.2339861	1.0066	0.314	0.303645692	count	1
BCL7B	0.2218188	0.1279791	1.7332	0.0832	0.303682736	count	1
CERS2	0.230408	0.1746263	1.3194	0.187	0.303710431	count	1
AC007336.1	0.3299167	0.3771702	0.8747	0.382	0.303726198	count	1
ING3	0.2415825	0.1927639	1.2533	0.21	0.30373519	count	1
QTRT1	0.2673722	0.2567131	1.0415	0.298	0.303770513	count	1
BRWD3	0.2813099	0.2471484	1.1382	0.255	0.303815916	count	1
TCTA	0.2550262	0.3514831	0.7256	0.468	0.304029875	count	1

C12orf57	0.2178853	0.1406166	1.5495	0.121	0.304255332	count	1
FAM69A	2.00335	1.349064	1.485	0.138	0.304297228	count	1
AL450384.2	2.00335	1.382159	1.4494	0.147	0.304297228	count	1
DELE1	0.2485577	0.1921704	1.2934	0.196	0.304662503	count	1
NIT1	0.2337558	0.1783404	1.3107	0.19	0.304722773	count	1
A1BG	0.2288817	0.1551307	1.4754	0.14	0.304729519	count	1
PAPOLG	0.3124917	0.2826835	1.1054	0.269	0.304833425	count	1
AC243829.4	0.4537019	0.4376502	1.0367	0.3	0.304846093	count	1
TSPYL5	0.7663487	0.5266192	1.4552	0.146	0.304887486	count	1
CDS2	0.2299536	0.1934828	1.1885	0.235	0.305162869	count	1
DECR1	0.2179865	0.0873081	2.4967	0.0126	0.305172473	count	1
ZNF43	0.3056033	0.4666803	0.6548	0.513	0.305240161	count	1
DIP2C	1.2126473	0.6715856	1.8056	0.0711	0.305278533	count	1
AASDH	0.2616181	0.3015631	0.8675	0.386	0.305879642	count	1
SUMO4	0.4346305	0.4618423	0.9411	0.347	0.306044571	count	1
YTHDF3	0.229243	0.1525212	1.503	0.133	0.306138983	count	1
ITM2B	0.2125824	0.0374216	5.6807	1.50E-08	0.30627076	count	0.00035964
TRMT10B	0.2922937	0.2738515	1.0673	0.286	0.306343426	count	1
SPRYD3	0.2922937	0.2769084	1.0556	0.291	0.306343426	count	1
AC018647.2	0.3787011	0.5227992	0.7244	0.469	0.306553557	count	1
ARID3A	0.2352764	0.1773547	1.3266	0.185	0.306718364	count	1
INTS14	0.2952276	0.3369432	0.8762	0.381	0.306789484	count	1
HEXIM2	0.5127064	0.5599305	0.9157	0.36	0.307004825	count	1
DDX3X	0.2155315	0.0576805	3.7366	0.000191	0.307090507	count	1
ITFG1	0.2391993	0.2005996	1.1924	0.233	0.307091327	count	1
GOLGB1	0.2195204	0.1058429	2.074	0.0382	0.307116869	count	1
SPDYA	0.7713801	0.7265483	1.0617	0.288	0.307161992	count	1
MSX2	0.7713801	0.7265483	1.0617	0.288	0.307161992	count	1
KIFC1	0.7713801	0.7129038	1.082	0.279	0.307161992	count	1
SCML2	0.7713801	0.8986348	0.8584	0.391	0.307161992	count	1
C19orf73	0.4820775	0.9426843	0.5114	0.609	0.307189134	count	1
RNLS	0.5130606	0.5174062	0.9916	0.321	0.307234251	count	1
WDR24	0.5130606	0.6516971	0.7873	0.431	0.307234251	count	1
CDK5RAP2	0.2407127	0.2141349	1.1241	0.261	0.307251475	count	1
LTN1	0.2423101	0.2167825	1.1178	0.264	0.307319347	count	1
FBXL6	0.27047	0.2670066	1.013	0.311	0.307349529	count	1
TRIM65	0.3698243	0.3312766	1.1164	0.264	0.307457737	count	1
AL078459.1	0.7722208	0.6758913	1.1425	0.253	0.30754231	count	1
C9orf106	0.7726871	0.609279	1.2682	0.205	0.307753301	count	1
KDM4A	0.3241803	0.3304705	0.981	0.327	0.307947657	count	1
SRPK2	0.2271746	0.1254546	1.8108	0.0703	0.30820724	count	1
SPSB2	0.3158787	0.3347862	0.9435	0.346	0.308235593	count	1
TLDC1	0.4049909	0.4772685	0.8486	0.396	0.308250812	count	1
C9orf85	0.2451741	0.2730318	0.898	0.369	0.30829293	count	1
GOLT1B	0.2400916	0.2180189	1.1012	0.271	0.308585421	count	1

TET3	0.2648756	0.239086	1.1079	0.268	0.30859932	count	1
PLPBP	0.2335557	0.1741197	1.3414	0.18	0.308870825	count	1
EHBP1L1	0.228343	0.1444847	1.5804	0.114	0.308903546	count	1
RBM39	0.2171795	0.0509685	4.2611	2.11E-05	0.308958007	count	0.4989095
MED17	0.3354815	0.401668	0.8352	0.404	0.309028942	count	1
TMEM79	2.0324637	1.33726	1.5199	0.129	0.309091337	count	1
TMTC1	2.0324637	0.9738479	2.087	0.037	0.309091337	count	1
ARRB2	0.2167282	0.0568914	3.8095	0.000143	0.309238546	count	1
NEDD4L	0.2722637	0.3172202	0.8583	0.391	0.309422409	count	1
APOBEC3G	0.2380114	0.1527886	1.5578	0.119	0.309509982	count	1
TAF7	0.2225755	0.0989333	2.2498	0.0246	0.309787784	count	1
UROS	0.232588	0.2042236	1.1389	0.255	0.309851624	count	1
SLC25A37	0.2187952	0.1213423	1.8031	0.0715	0.310025013	count	1
MED25	0.2572908	0.2524366	1.0192	0.308	0.310236632	count	1
N4BP2L1	0.2446526	0.1597442	1.5315	0.126	0.310316299	count	1
RSAD2	0.2653685	0.3803542	0.6977	0.485	0.310329461	count	1
CALM2	0.2159884	0.0347945	6.2075	6.30E-10	0.310526814	count	1.52E-05
ZNF567	0.3644607	0.351702	1.0363	0.3	0.310528481	count	1
OPRL1	0.4406788	0.4474295	0.9849	0.325	0.310572029	count	1
SYNRG	0.2398398	0.167538	1.4316	0.152	0.310770079	count	1
TGFBR1	0.2334064	0.1728585	1.3503	0.177	0.310785817	count	1
NUDT2	0.2564417	0.2085313	1.2298	0.219	0.310794174	count	1
CNOT4	0.2289726	0.138077	1.6583	0.0974	0.311030736	count	1
CENPBD1	0.2824815	0.3499459	0.8072	0.42	0.311115434	count	1
AIFM3	0.3842024	0.6309063	0.609	0.543	0.311220444	count	1
CLEC1A	0.4882519	0.5385786	0.9066	0.365	0.31141843	count	1
PPP1R15A	0.2173263	0.05753	3.7776	0.000162	0.311438486	count	1
XIAP	0.2287002	0.156736	1.4591	0.145	0.311534494	count	1
RPH3A	0.7814065	0.6171011	1.2663	0.206	0.31170263	count	1
AL136295.5	0.4633872	0.4147041	1.1174	0.264	0.311800943	count	1
PDS5A	0.2407155	0.1951721	1.2333	0.218	0.311912986	count	1
IER3-AS1	0.385126	0.4525982	0.8509	0.395	0.312004492	count	1
MIS18A	0.3001769	0.3348241	0.8965	0.37	0.312058457	count	1
YKT6	0.2507648	0.2415883	1.038	0.299	0.312271648	count	1
PXN	0.2512185	0.2432532	1.0327	0.302	0.312280506	count	1
PRR34-AS1	0.2488586	0.2313113	1.0759	0.282	0.312481116	count	1
LIPT1	0.2980822	0.3589518	0.8304	0.406	0.312554402	count	1
BAHCC1	0.6825945	0.5651148	1.2079	0.227	0.312668191	count	1
LINC02453	0.7835436	0.6485857	1.2081	0.227	0.31267184	count	1
TRIM14	0.2415388	0.2007397	1.2032	0.229	0.312690144	count	1
GOLGA7	0.2261588	0.1048049	2.1579	0.031	0.312731826	count	1
EEA1	0.224746	0.0954136	2.3555	0.0186	0.31288089	count	1
NCOA6	0.2766682	0.2763762	1.0011	0.317	0.313007402	count	1
TOB1	0.2259512	0.1136677	1.9878	0.0469	0.31301273	count	1
ZXDB	1.2387731	0.7454011	1.6619	0.0967	0.31306114	count	1

ZBTB38	0.2326979	0.1534753	1.5162	0.13	0.313224754	count	1
FGD5	2.059534	1.326399	1.5527	0.121	0.313519798	count	1
GLIPR1L2	2.059534	1.473036	1.3982	0.162	0.313519798	count	1
TMEM38A	2.059534	1.326399	1.5527	0.121	0.313519798	count	1
CAPS	0.3985148	0.5334379	0.7471	0.455	0.313618642	count	1
EXOC3-AS1	0.3402962	0.4247	0.8013	0.423	0.31362111	count	1
P2RY6	0.2552875	0.2330209	1.0956	0.273	0.31367711	count	1
CALHM2	0.2427686	0.1838914	1.3202	0.187	0.313990048	count	1
ASNSD1	0.2428159	0.2198679	1.1044	0.27	0.314051679	count	1
AF064858.1	0.4921884	0.5398051	0.9118	0.362	0.314118116	count	1
ARHGAP15	0.2452763	0.1788476	1.3714	0.17	0.314243437	count	1
PDIK1L	0.4456637	0.5000275	0.8913	0.373	0.314308257	count	1
SUFU	0.3687592	0.3511024	1.0503	0.294	0.314348708	count	1
SCAF8	0.2708093	0.3210244	0.8436	0.399	0.314411012	count	1
RNF8	0.2472895	0.2493434	0.9918	0.321	0.314524561	count	1
NAAA	0.2259853	0.103315	2.1873	0.0288	0.314577647	count	1
RPL32	0.2182488	0.0226869	9.62	1.57E-21	0.314605921	count	3.81E-17
IQCE	0.2781262	0.3105438	0.8956	0.371	0.314685876	count	1
AC124242.1	1.244387	0.7472563	1.6653	0.096	0.314736063	count	1
TMEM187	0.2768777	0.3416976	0.8103	0.418	0.314756396	count	1
GOPC	0.2352998	0.135026	1.7426	0.0815	0.315009045	count	1
NHS	0.5251707	0.5302905	0.9903	0.322	0.315090621	count	1
MRPL37	0.23347	0.1510753	1.5454	0.122	0.315098485	count	1
SEC31A	0.2283402	0.1217112	1.8761	0.0608	0.315229813	count	1
KLF6	0.2192138	0.0604299	3.6276	0.000292	0.315279783	count	1
POLR2B	0.2383842	0.1504333	1.5847	0.113	0.315293904	count	1
NR3C1	0.2250111	0.0884948	2.5426	0.0111	0.315316656	count	1
RNF168	0.2286159	0.1699868	1.3449	0.179	0.315561555	count	1
ASRGL1	0.2526149	0.2110775	1.1968	0.232	0.315691464	count	1
AP5Z1	0.2681922	0.2679332	1.001	0.317	0.315893459	count	1
MOSMO	0.2648897	0.3234953	0.8188	0.413	0.315947762	count	1
SLC25A10	0.790874	0.8946774	0.884	0.377	0.315999798	count	1
CRLF3	0.2382239	0.1783756	1.3355	0.182	0.316019652	count	1
CSNK1G1	0.2967522	0.338812	0.8759	0.381	0.316202821	count	1
THOC3	0.2781586	0.2807377	0.9908	0.322	0.316237644	count	1
PHLPP1	0.6189683	0.5772932	1.0722	0.284	0.316277358	count	1
COQ4	0.2404352	0.218536	1.1002	0.271	0.316365681	count	1
ZNF283	0.4487246	0.5951566	0.754	0.451	0.316604536	count	1
MIER1	0.2274669	0.1120181	2.0306	0.0424	0.316611978	count	1
MFN2	0.3244478	0.3299037	0.9835	0.325	0.316851279	count	1
AC138150.1	0.3205384	0.6070424	0.528	0.598	0.316852292	count	1
KIFAP3	0.2585121	0.2016139	1.2822	0.2	0.317003527	count	1
ALPK1	0.2591587	0.2357876	1.0991	0.272	0.317106501	count	1
NACA	0.220094	0.0256585	8.5778	1.69E-17	0.317127361	count	4.09E-13
ITM2A	0.6205107	0.6899061	0.8994	0.369	0.317147625	count	1

AC111182.1	0.6205107	0.661338	0.9383	0.348	0.317147625	count	1
JAML	0.2265601	0.0778296	2.911	0.00364	0.317199076	count	1
SPIDR	0.2460199	0.2166105	1.1358	0.256	0.317271571	count	1
RMI2	0.6917276	0.7162816	0.9657	0.334	0.317350808	count	1
SRSF4	0.2306275	0.1144416	2.0152	0.044	0.317432038	count	1
NDUFS2	0.2301031	0.1088312	2.1143	0.0346	0.317467401	count	1
TIA1	0.2632527	0.222051	1.1856	0.236	0.317516681	count	1
USP30-AS1	0.955506	0.5162273	1.8509	0.0643	0.317567757	count	1
MEST	0.4321562	0.5259573	0.8217	0.411	0.317701778	count	1
DCTN4	0.2466507	0.2117794	1.1647	0.244	0.317760996	count	1
RFC5	0.4503002	0.4515342	0.9973	0.319	0.317787172	count	1
SYNE2	0.3342108	0.681531	0.4904	0.624	0.317789617	count	1
SMC1A	0.2314353	0.1213462	1.9072	0.0566	0.317826291	count	1
L3HYPDH	0.3178574	0.3655466	0.8695	0.385	0.317826762	count	1
PRKAA1	0.2444482	0.21703	1.1263	0.26	0.317939967	count	1
IMPAD1	0.246334	0.1906985	1.2917	0.197	0.318004019	count	1
TANGO6	0.4041603	0.3941585	1.0254	0.305	0.318291578	count	1
MFSD14A	1.2569057	0.8647305	1.4535	0.146	0.318474428	count	1
VWCE	1.2569057	0.8647305	1.4535	0.146	0.318474428	count	1
AC011511.1	1.2569057	0.9321136	1.3484	0.178	0.318474428	count	1
AL021707.7	1.2569057	0.8647305	1.4535	0.146	0.318474428	count	1
SLC36A4	0.2417773	0.1551933	1.5579	0.119	0.318576796	count	1
LPCAT4	0.3222508	0.4654666	0.6923	0.489	0.318594557	count	1
FGD6	0.2714183	0.2608258	1.0406	0.298	0.318646998	count	1
LINC02158	0.38288	0.5194319	0.7371	0.461	0.318812612	count	1
COPA	0.2314312	0.1196278	1.9346	0.0532	0.31882341	count	1
MTO1	0.3586291	0.3762406	0.9532	0.341	0.319082734	count	1
GEMIN7	0.2737984	0.287004	0.954	0.34	0.31915552	count	1
MCCC2	0.3228526	0.3411632	0.9463	0.344	0.319206966	count	1
NAXD	0.3521788	0.3677298	0.9577	0.338	0.319232502	count	1
CHST15	0.2408389	0.1831286	1.3151	0.189	0.319323734	count	1
TOMM40L	0.2934132	0.3686567	0.7959	0.426	0.319330157	count	1
ZNF660	0.9606819	0.7463346	1.2872	0.198	0.319564819	count	1
DEPDC4	2.0968086	1.2495184	1.6781	0.0935	0.319568373	count	1
KDM6B	0.2298069	0.1157292	1.9857	0.0472	0.319603446	count	1
C7orf26	0.2978481	0.3205645	0.9291	0.353	0.319781584	count	1
L3MBTL4	0.500746	0.4993817	1.0027	0.316	0.31999584	count	1
LUC7L	0.2438268	0.170945	1.4263	0.154	0.320406261	count	1
WDFY2	0.2483145	0.1950168	1.2733	0.203	0.320580387	count	1
DCAF8	0.2668109	0.2732976	0.9763	0.329	0.32100188	count	1
TMED7	0.2420008	0.1617062	1.4965	0.135	0.321241868	count	1
MYH9	0.230879	0.0999745	2.3094	0.021	0.321538015	count	1
DHCR24	2.1090641	1.429207	1.4757	0.14	0.321544289	count	1
RBM5-AS1	2.1090641	1.429207	1.4757	0.14	0.321544289	count	1
RPUSD4	0.3253194	0.3994619	0.8144	0.415	0.321717844	count	1

SEC23B	0.2547336	0.2164396	1.1769	0.239	0.321866561	count	1
PIGO	0.3434721	0.3456007	0.9938	0.32	0.321922876	count	1
BAIAP2-DT	0.3184173	0.3120799	1.0203	0.308	0.321924604	count	1
TBC1D10B	0.2676205	0.215287	1.2431	0.214	0.321988574	count	1
GABPB2	0.2844712	0.3585931	0.7933	0.428	0.321993424	count	1
PRUNE1	0.5038476	0.685332	0.7352	0.462	0.322129103	count	1
GID4	0.3222205	0.3192965	1.0092	0.313	0.322313894	count	1
FKBPL	0.3048347	0.3235083	0.9423	0.346	0.322467776	count	1
SLC51A	0.7020186	0.7275822	0.9649	0.335	0.322639501	count	1
AC008494.3	0.7020186	0.8081481	0.8687	0.385	0.322639501	count	1
EBPL	0.2420957	0.1812869	1.3354	0.182	0.322752428	count	1
UBR3	0.2930758	0.2978291	0.984	0.325	0.32301923	count	1
CEP192	0.3133183	0.3061126	1.0235	0.306	0.323070462	count	1
CARD11	0.47904	0.6839192	0.7004	0.484	0.323074561	count	1
POC1A	0.4574524	0.5148233	0.8886	0.374	0.323160905	count	1
NOSIP	0.2336911	0.1243919	1.8787	0.0604	0.323224308	count	1
NBR1	0.2545126	0.1606244	1.5845	0.113	0.323368526	count	1
DUS1L	0.2419083	0.1591205	1.5203	0.129	0.323753409	count	1
SREK1	0.2413189	0.1507483	1.6008	0.11	0.323822687	count	1
ZDHHC17	0.2758232	0.2656961	1.0381	0.299	0.323895945	count	1
FEM1B	0.2574655	0.1995349	1.2903	0.197	0.323898904	count	1
SLC25A51	0.3141378	0.3826962	0.8209	0.412	0.323937319	count	1
RNF4	0.2614844	0.2057008	1.2712	0.204	0.323969522	count	1
CYB5R2	0.9721001	0.803167	1.2103	0.226	0.323977111	count	1
LINC01537	0.9721001	1.0243929	0.949	0.343	0.323977111	count	1
GAS6-DT	0.9721001	0.8323758	1.1679	0.243	0.323977111	count	1
SAMD15	0.9721001	0.803167	1.2103	0.226	0.323977111	count	1
OTUD7A	0.9721001	0.9429096	1.031	0.303	0.323977111	count	1
COL1A1	0.9721001	0.9249416	1.051	0.293	0.323977111	count	1
LINC01597	0.9721001	0.8522975	1.1406	0.254	0.323977111	count	1
HMG20A	0.3088776	0.3061646	1.0089	0.313	0.324150783	count	1
MT-ND4L	0.22755	0.0676733	3.3625	0.000784	0.324362829	count	1
AIFM1	0.2792496	0.2246583	1.243	0.214	0.324366241	count	1
TTI1	0.3999823	0.3988286	1.0029	0.316	0.324636211	count	1
NSL1	0.2363329	0.1099306	2.1498	0.0317	0.324701966	count	1
PDZD8	0.2765569	0.2896744	0.9547	0.34	0.324770447	count	1
HLA-DOA	0.2429093	0.1508369	1.6104	0.107	0.324797015	count	1
LMNB1	0.2768806	0.1939289	1.4277	0.153	0.325156281	count	1
POT1	0.3586607	0.435453	0.8236	0.41	0.32533143	count	1
ZNF576	0.2752033	0.278169	0.9893	0.323	0.325356548	count	1
RNPEP	0.2379939	0.1266127	1.8797	0.0603	0.325385684	count	1
KL	2.1333285	1.388395	1.5365	0.125	0.325436972	count	1
ZNF439	0.9762866	0.5603373	1.7423	0.0816	0.325597178	count	1
RPAP2	0.2939738	0.2707962	1.0856	0.278	0.325949466	count	1
ENOX2	0.3036817	0.2655122	1.1438	0.253	0.32618569	count	1

ZER1	0.3821633	0.3921685	0.9745	0.33	0.326281803	count	1
PFDN4	0.2457866	0.1477153	1.6639	0.0963	0.326296302	count	1
ACO2	0.2468273	0.1496587	1.6493	0.0992	0.32632635	count	1
ARHGEF2	0.2429	0.1479516	1.6418	0.101	0.326894778	count	1
COX19	0.2400288	0.1561034	1.5376	0.124	0.327027208	count	1
AP4S1	0.3307039	0.3651423	0.9057	0.365	0.327201835	count	1
NOA1	0.2700039	0.2855828	0.9454	0.345	0.327438651	count	1
BORCS7	0.2412383	0.1118093	2.1576	0.0311	0.327468035	count	1
CTTNBP2	0.2989802	0.2733131	1.0939	0.274	0.327630265	count	1
ZNF225	0.981557	0.8053248	1.2188	0.223	0.32763843	count	1
NHLRC3	0.2654788	0.1846886	1.4374	0.151	0.327689445	count	1
ZBTB14	0.3208948	0.3545682	0.905	0.366	0.327874038	count	1
TMEM115	0.2748943	0.2430031	1.1312	0.258	0.328046727	count	1
RALB	0.2426443	0.1307877	1.8553	0.0637	0.328114143	count	1
NSDHL	0.3126607	0.305688	1.0228	0.306	0.328218561	count	1
NARFL	0.3401351	0.3607111	0.943	0.346	0.328258015	count	1
PPP2R5D	0.4299581	0.4416297	0.9736	0.33	0.328335604	count	1
EEF1AKMT2	0.2639979	0.2439063	1.0824	0.279	0.328336158	count	1
TEX261	0.310759	0.2806	1.1075	0.268	0.328884778	count	1
CWC22	0.2563451	0.1849236	1.3862	0.166	0.328918472	count	1
RSBN1L	0.235208	0.1093145	2.1517	0.0315	0.328957676	count	1
PRKCZ	2.156153	0.9282949	2.3227	0.0203	0.329074545	count	1
CLEC12B	2.156153	1.058677	2.0366	0.0418	0.329074545	count	1
GAR1	0.2562113	0.2061023	1.2431	0.214	0.329112915	count	1
RNF26	0.3694965	0.3723815	0.9923	0.321	0.329140985	count	1
RIN2	0.243646	0.1349522	1.8054	0.0711	0.329246793	count	1
HLA-C	0.2289656	0.0306289	7.4755	1.06E-13	0.329392762	count	2.56E-09
HARBI1	0.5884739	0.6264733	0.9393	0.348	0.329432287	count	1
ABCB10	0.4176609	0.3815601	1.0946	0.274	0.329488757	count	1
AC006480.2	0.5886211	0.5631463	1.0452	0.296	0.329522464	count	1
HMGA1P4	0.5886211	0.6770245	0.8694	0.385	0.329522464	count	1
VPS4B	0.2371023	0.1059592	2.2377	0.0253	0.329666023	count	1
KARS	0.2492877	0.1600473	1.5576	0.119	0.329803103	count	1
NDUFV2-AS1	0.3295443	0.3488771	0.9446	0.345	0.329852445	count	1
GOLGA5	0.2654264	0.2539809	1.0451	0.296	0.330131776	count	1
PPIC	0.3576158	0.4256631	0.8401	0.401	0.33017205	count	1
ALG9	0.4486165	0.4850795	0.9248	0.355	0.330541302	count	1
SIRT6	0.2700427	0.311165	0.8678	0.386	0.330583734	count	1
EFCAB14	0.2451236	0.1420893	1.7251	0.0846	0.330779558	count	1
ANKMY1	0.4900531	0.4997058	0.9807	0.327	0.331031024	count	1
MAP4K4	0.2453452	0.155306	1.5798	0.114	0.331079984	count	1
PSMB8	0.2324961	0.0594787	3.9089	9.52E-05	0.33114487	count	1
SPI1	0.2310622	0.0430943	5.3618	9.01E-08	0.331220633	count	0.002154111
APLP2	0.2310117	0.0507571	4.5513	5.59E-06	0.331266593	count	0.13266188
PABPC1L	0.468248	0.8337971	0.5616	0.574	0.331288434	count	1

RIMKLB	0.371842	0.4277493	0.8693	0.385	0.331314427	count	1
RBM15	0.330964	0.4700974	0.704	0.481	0.331314727	count	1
MBOAT7	0.2419272	0.1174157	2.0604	0.0395	0.331385651	count	1
ZBTB34	0.4079208	0.4051023	1.007	0.314	0.331401573	count	1
TIPARP-AS1	0.3482483	0.3739609	0.9312	0.352	0.331590651	count	1
1-Sep	0.2652388	0.2483099	1.0682	0.286	0.331631246	count	1
RTKN	0.8254797	1.0123034	0.8154	0.415	0.331785105	count	1
FAM126A	0.2796799	0.3156366	0.8861	0.376	0.331794187	count	1
EPHX1	0.2621233	0.1680073	1.5602	0.119	0.332227922	count	1
CDC25B	0.4917894	0.3851248	1.277	0.202	0.332287242	count	1
ELF1	0.2334007	0.0576098	4.0514	5.25E-05	0.332352706	count	1
DHPS	0.245936	0.1432015	1.7174	0.086	0.332355474	count	1
ZFAT	0.7208856	0.6301524	1.144	0.253	0.332369088	count	1
AC061992.1	0.7208856	0.6862815	1.0504	0.294	0.332369088	count	1
SCYL2	0.284959	0.2176885	1.309	0.191	0.332372007	count	1
TNIP1	0.2634013	0.1890232	1.3935	0.164	0.332438964	count	1
BRWD1	0.2557504	0.1888226	1.3544	0.176	0.332454698	count	1
LCOR	0.2516218	0.171339	1.4686	0.142	0.332495482	count	1
ZC3H12D	0.3492419	0.3797756	0.9196	0.358	0.332568712	count	1
ZFAS1	0.2334891	0.0599409	3.8953	0.000101	0.33260891	count	1
EIF3L	0.2356124	0.0621047	3.7938	0.000152	0.332654894	count	1
ZNF823	0.6480194	0.7682315	0.8435	0.399	0.332723828	count	1
POLK	0.2556058	0.1816442	1.4072	0.16	0.3328481	count	1
TBC1D5	0.2541899	0.1737392	1.4631	0.144	0.332881942	count	1
FRMD4B	0.236792	0.0887706	2.6675	0.00769	0.332894999	count	1
WWOX	0.3076603	0.4046591	0.7603	0.447	0.332913243	count	1
AC080013.5	0.5527507	0.5215758	1.0598	0.289	0.333069567	count	1
UBL7	0.2609711	0.2123594	1.2289	0.219	0.333337873	count	1
SRSF6	0.244868	0.1468987	1.6669	0.0957	0.333466922	count	1
AL035071.1	0.3371645	0.3536246	0.9535	0.34	0.333787729	count	1
PRELID3B	0.2458101	0.1533395	1.603	0.109	0.333800915	count	1
FBXO8	0.297767	0.2828442	1.0528	0.293	0.333911744	count	1
ETV2	0.5540787	0.4810258	1.1519	0.249	0.333938248	count	1
TAGLN2	0.2324637	0.0443029	5.2471	1.68E-07	0.334078334	count	0.00401352
CTDP1	0.3067913	0.2997373	1.0235	0.306	0.334207099	count	1
ZNF85	0.3067994	0.2955434	1.0381	0.299	0.334216114	count	1
PLPP6	0.6507284	0.5585757	1.165	0.244	0.334263311	count	1
AP000919.4	0.6507284	0.5928816	1.0976	0.273	0.334263311	count	1
GDPD3	1.3096647	0.7977364	1.6417	0.101	0.334271513	count	1
SWI5	0.2605739	0.212212	1.2279	0.22	0.334390138	count	1
UBE2L6	0.2384237	0.0922178	2.5854	0.00978	0.334524198	count	1
SFXN2	1.3105833	0.7836832	1.6723	0.0946	0.334547096	count	1
LRRFIP2	0.245623	0.1335272	1.8395	0.066	0.334768484	count	1
IRF2BP2	0.2392536	0.0926085	2.5835	0.00984	0.334808659	count	1
TBCE	0.4541515	0.546637	0.8308	0.406	0.334869094	count	1

GOLGA8N	0.6519234	0.6457713	1.0095	0.313	0.33494272	count	1
IL18R1	0.4382137	0.5467074	0.8016	0.423	0.335000359	count	1
AHCYL2	0.7259875	0.6362447	1.1411	0.254	0.335007412	count	1
RNF215	0.4732686	0.4722892	1.0021	0.316	0.335074869	count	1
SYNPO	1.3127844	0.9099471	1.4427	0.149	0.335207488	count	1
CEP83-DT	1.3127844	0.9958201	1.3183	0.188	0.335207488	count	1
PODNL1	1.3127844	0.9958201	1.3183	0.188	0.335207488	count	1
TMEM121B	1.3127844	0.9099471	1.4427	0.149	0.335207488	count	1
TMEM243	0.2450973	0.1177711	2.0811	0.0375	0.335367462	count	1
RNF149	0.2356945	0.0646616	3.645	0.000273	0.335385833	count	1
BTK	0.2523377	0.1474119	1.7118	0.0871	0.335418115	count	1
C6orf106	0.2562162	0.187669	1.3653	0.172	0.33555386	count	1
RPL37	0.2332172	0.0257273	9.065	2.48E-19	0.33591385	count	6.01E-15
UBTF	0.2555727	0.1697315	1.5057	0.132	0.336414828	count	1
TXNDC16	0.3016554	0.254801	1.1839	0.237	0.336532546	count	1
SIRT7	0.2545364	0.168476	1.5108	0.131	0.336581849	count	1
PLEKHA1	0.2828691	0.3128809	0.9041	0.366	0.336680584	count	1
BRMS1L	0.3535875	0.4365035	0.81	0.418	0.336848184	count	1
LINC01358	1.0053459	0.8372559	1.2008	0.23	0.336875286	count	1
AL360270.1	1.0053459	0.8372559	1.2008	0.23	0.336875286	count	1
NBPF19	0.3209719	0.4497163	0.7137	0.475	0.337162365	count	1
WDR6	0.2890505	0.2868473	1.0077	0.314	0.337220733	count	1
PIGX	0.2727682	0.2145905	1.2711	0.204	0.337456055	count	1
C9orf78	0.2398587	0.0901497	2.6607	0.00785	0.337498697	count	1
ZNF57	0.8386331	0.8635476	0.9711	0.332	0.337816028	count	1
NUMBL	0.8386331	0.7172574	1.1692	0.242	0.337816028	count	1
DNASE1L3	0.2772725	0.4321346	0.6416	0.521	0.337995109	count	1
AL451165.2	0.3192653	0.3381951	0.944	0.345	0.338107167	count	1
ELMO1	0.263165	0.1707451	1.5413	0.123	0.338120334	count	1
MAPRE2	0.2580049	0.2029376	1.2714	0.204	0.338170175	count	1
PQLC3	0.2435547	0.1047845	2.3243	0.0202	0.338193022	count	1
C1orf127	1.008924	0.8986123	1.1228	0.262	0.338267744	count	1
ZMYND12	1.008924	0.8986123	1.1228	0.262	0.338267744	count	1
AC239868.3	1.008924	1.2214938	0.826	0.409	0.338267744	count	1
EFNA1	1.008924	0.8986123	1.1228	0.262	0.338267744	count	1
AL121983.2	1.008924	1.2214938	0.826	0.409	0.338267744	count	1
KIAA1614-AS1	1.008924	0.8986123	1.1228	0.262	0.338267744	count	1
CD34	1.008924	0.8986123	1.1228	0.262	0.338267744	count	1
AL390728.5	1.008924	0.8986123	1.1228	0.262	0.338267744	count	1
EML6	1.008924	0.8986123	1.1228	0.262	0.338267744	count	1
AC099522.2	1.008924	0.8986123	1.1228	0.262	0.338267744	count	1
CHRNA10	1.008924	0.8986123	1.1228	0.262	0.338267744	count	1
LINC00702	1.008924	1.2214938	0.826	0.409	0.338267744	count	1
LINC01465	1.008924	0.8986123	1.1228	0.262	0.338267744	count	1
SYNJ2BP-COX16	1.008924	0.8986123	1.1228	0.262	0.338267744	count	1

PLK1	1.008924	0.8986123	1.1228	0.262	0.338267744	count	1
AC009118.3	1.008924	0.8986123	1.1228	0.262	0.338267744	count	1
AC130343.2	1.008924	0.8986123	1.1228	0.262	0.338267744	count	1
AC099811.4	1.008924	1.2214938	0.826	0.409	0.338267744	count	1
ANGPTL6	1.008924	0.8986123	1.1228	0.262	0.338267744	count	1
CU634019.6	1.008924	1.2214938	0.826	0.409	0.338267744	count	1
PRKAG2	0.2481599	0.1383746	1.7934	0.073	0.338328967	count	1
CNIH1	0.2433121	0.109043	2.2313	0.0257	0.338507424	count	1
ERCC3	0.3017901	0.3115217	0.9688	0.333	0.338510591	count	1
CD38	0.366456	0.3604383	1.0167	0.309	0.338638848	count	1
IGIP	0.603529	0.6511259	0.9269	0.354	0.338671817	count	1
NT5DC3	0.603529	0.6364031	0.9483	0.343	0.338671817	count	1
DPY19L3	0.603529	0.7362616	0.8197	0.412	0.338671817	count	1
C4orf33	0.2761422	0.2339567	1.1803	0.238	0.338883534	count	1
ZNF408	0.4293454	0.4569994	0.9395	0.348	0.339204806	count	1
ATAD2	0.3178534	0.3076202	1.0333	0.302	0.339225639	count	1
CEP63	0.2568475	0.1562505	1.6438	0.1	0.339444275	count	1
RPS24	0.2354647	0.0221199	10.6449	6.71E-26	0.33947886	count	1.63E-21
SLC25A44	0.3738616	0.3045679	1.2275	0.22	0.339661412	count	1
METTL8	0.3357032	0.3183489	1.0545	0.292	0.33990368	count	1
USP28	0.3209288	0.3027036	1.0602	0.289	0.339911896	count	1
SNX18	0.2681988	0.1827659	1.4674	0.142	0.340001283	count	1
C1orf131	0.2598532	0.1748736	1.4859	0.137	0.340087139	count	1
CBR4	0.3265745	0.3069639	1.0639	0.287	0.340221799	count	1
ZNF264	0.2977134	0.2538513	1.1728	0.241	0.340435769	count	1
KRCC1	0.2525719	0.1484181	1.7018	0.0889	0.340499938	count	1
MRE11	0.2955642	0.2157328	1.37	0.171	0.340878312	count	1
IL15RA	0.3985401	0.3039605	1.3112	0.19	0.34090271	count	1
NPEPPS	0.2575101	0.1611698	1.5978	0.11	0.340956497	count	1
ARSB	0.3072809	0.2870935	1.0703	0.285	0.341006492	count	1
KCNJ5	0.3632178	0.3641565	0.9974	0.319	0.341098941	count	1
ZNF84	0.3056736	0.3166824	0.9652	0.335	0.341104679	count	1
FDFT1	0.2570256	0.1669877	1.5392	0.124	0.341112321	count	1
STAT3	0.2412729	0.0862682	2.7968	0.0052	0.341198334	count	1
ZNF675	0.2894134	0.2602559	1.112	0.266	0.341274595	count	1
FLT3	0.3903657	0.4170587	0.936	0.349	0.341305931	count	1
PCED1B	0.3991782	0.4331158	0.9216	0.357	0.341473303	count	1
ZNF516	0.3017774	0.3317662	0.9096	0.363	0.341950282	count	1
PSTK	0.3913072	0.4499037	0.8698	0.385	0.34216463	count	1
ERCC2	0.4096721	0.3976376	1.0303	0.303	0.342205923	count	1
BAIAP2	0.2771102	0.2776918	0.9979	0.318	0.342213325	count	1
CYHR1	0.2548227	0.1859981	1.37	0.171	0.342326328	count	1
CETN3	0.3207721	0.2906969	1.1035	0.27	0.342415001	count	1
CUEDC1	0.2791183	0.3450836	0.8088	0.419	0.342579903	count	1
MZF1	0.4476721	0.4793808	0.9339	0.35	0.342650246	count	1

CCNL2	0.2638212	0.2352481	1.1215	0.262	0.342718276	count	1
ZNF844	0.4477998	0.4117977	1.0874	0.277	0.342753622	count	1
TRMT2A	0.3147563	0.2454981	1.2821	0.2	0.34307584	count	1
ARMCX1	0.2820793	0.2014434	1.4003	0.162	0.343111932	count	1
AC025159.1	0.2779984	0.2523465	1.1017	0.271	0.343322899	count	1
FAM41C	0.4485458	0.4742519	0.9458	0.344	0.343357639	count	1
ELP2	0.2797511	0.2571308	1.088	0.277	0.343365958	count	1
HDHD2	0.3656797	0.2931008	1.2476	0.212	0.343494203	count	1
DCP2	0.2493677	0.1072015	2.3262	0.0201	0.343790385	count	1
CHFR	0.2946001	0.2995624	0.9834	0.325	0.343800406	count	1
UFSP2	0.2981757	0.2205227	1.3521	0.176	0.343942039	count	1
GFOD2	0.3397648	0.3588768	0.9467	0.344	0.344134622	count	1
KDM2A	0.2527403	0.1351018	1.8707	0.0615	0.344138042	count	1
ARHGAP21	0.2536755	0.1607741	1.5778	0.115	0.344320273	count	1
CROCC	0.8529447	0.5209548	1.6373	0.102	0.344396739	count	1
AC018816.1	0.8530348	0.8176262	1.0433	0.297	0.344438228	count	1
USP22	0.259561	0.2230431	1.1637	0.245	0.344497525	count	1
AL355472.1	0.4123095	0.3225511	1.2783	0.201	0.344515295	count	1
DNAJC17	0.2725468	0.236695	1.1515	0.25	0.344595919	count	1
MITD1	0.260945	0.1713269	1.5231	0.128	0.34467506	count	1
PYGO2	0.5368001	0.4843488	1.1083	0.268	0.344889158	count	1
PNRC1	0.2397485	0.0374505	6.4017	1.83E-10	0.344962155	count	4.41E-06
EP400	0.2866442	0.2309239	1.2413	0.215	0.345192797	count	1
ARPP19	0.2533426	0.1372321	1.8461	0.065	0.345241227	count	1
EIF4G3	0.2565897	0.1337101	1.919	0.0551	0.345411006	count	1
TSPAN33	0.2649744	0.1291702	2.0514	0.0403	0.345433603	count	1
MEAF6	0.2472067	0.0992308	2.4912	0.0128	0.345440905	count	1
MUC12	0.537619	0.564923	0.9517	0.341	0.345456937	count	1
PKD2	0.3573802	0.3595129	0.9941	0.32	0.345460074	count	1
ZC3H14	0.2828435	0.1902732	1.4865	0.137	0.345670458	count	1
HERC2	0.2816189	0.2152195	1.3085	0.191	0.345686323	count	1
SEC31B	2.2637447	1.393512	1.6245	0.104	0.345884129	count	1
TOR1AIP1	0.2665411	0.1526096	1.7466	0.0808	0.345963156	count	1
SRSF3	0.2433014	0.0539228	4.512	6.72E-06	0.346077828	count	0.15941184
RRP8	0.2900837	0.2323097	1.2487	0.212	0.34643588	count	1
MFNG	0.2623025	0.1519189	1.7266	0.0844	0.34647967	count	1
RASSF3	0.2586053	0.1287364	2.0088	0.0447	0.346535264	count	1
MPHOSPH8	0.2447888	0.0840781	2.9114	0.00363	0.346737069	count	1
PTPN6	0.2454264	0.0774029	3.1708	0.00154	0.347023426	count	1
PPP2R3A	0.5401934	0.6596342	0.8189	0.413	0.347242615	count	1
DUSP28	0.2801191	0.295524	0.9479	0.343	0.347322195	count	1
IRAK4	0.2670177	0.1643401	1.6248	0.104	0.347519743	count	1
NBPF14	0.2803225	0.1962872	1.4281	0.153	0.347577257	count	1
AP002495.2	0.674101	0.6747668	0.999	0.318	0.347585876	count	1
ACLY	0.2814796	0.2256803	1.2472	0.212	0.347672436	count	1

ST6GAL1	0.2784184	0.1991325	1.3982	0.162	0.347695098	count	1
PRRC2B	0.2705937	0.1939435	1.3952	0.163	0.347746837	count	1
AL138995.1	0.3550915	0.4439684	0.7998	0.424	0.347756049	count	1
BCL10	0.2522911	0.1164106	2.1673	0.0303	0.347774995	count	1
PRPS1	0.2675665	0.2122241	1.2608	0.208	0.347932819	count	1
DZANK1	0.427274	0.863253	0.495	0.621	0.347939425	count	1
POLR3B	0.5413507	0.5602288	0.9663	0.334	0.34804569	count	1
AGO3	0.2787127	0.2269835	1.2279	0.22	0.348066601	count	1
LONRF3	0.2760993	0.3550338	0.7777	0.437	0.348108925	count	1
MMP25	2.278581	0.919512	2.478	0.0133	0.348156029	count	1
GPATCH8	0.2646409	0.182479	1.4503	0.147	0.348190454	count	1
OPHN1	0.4545385	0.4890119	0.9295	0.353	0.348213089	count	1
NCAPD3	0.2942574	0.2298339	1.2803	0.201	0.348228868	count	1
KLHL20	0.3475108	0.3320701	1.0465	0.295	0.348380199	count	1
CAPN1	0.259945	0.1617346	1.6072	0.108	0.348495026	count	1
ZNF219	0.3406643	0.3959532	0.8604	0.39	0.348646162	count	1
CASD1	0.3022231	0.2351947	1.285	0.199	0.348691855	count	1
TRA2B	0.2478208	0.1037132	2.3895	0.0169	0.348757295	count	1
ATPAF1	0.2904392	0.2173004	1.3366	0.181	0.348866936	count	1
GALNT7	0.286199	0.2042465	1.4012	0.161	0.349015913	count	1
HESX1	0.514851	0.6310611	0.8158	0.415	0.349018831	count	1
AP001059.3	0.514851	0.7019195	0.7335	0.463	0.349018831	count	1
RPAP1	0.676741	0.5366857	1.261	0.207	0.349095305	count	1
CA13	0.8634587	0.7740665	1.1155	0.265	0.349243382	count	1
ZNF66	0.8634587	0.7767831	1.1116	0.266	0.349243382	count	1
ZFP82	0.8634587	0.7814765	1.1049	0.269	0.349243382	count	1
LRRC75A	0.263582	0.1822156	1.4465	0.148	0.349255801	count	1
ZBTB44	0.2915885	0.2203071	1.3236	0.186	0.349277011	count	1
MASTL	0.3614755	0.2971026	1.2167	0.224	0.349551935	count	1
HP1BP3	0.246894	0.0774069	3.1896	0.00144	0.349565436	count	1
ZNF224	0.2926983	0.3174632	0.922	0.357	0.349603655	count	1
MED15	0.2786146	0.2638813	1.0558	0.291	0.349681827	count	1
EEF1D	0.2434355	0.0330636	7.3626	2.45E-13	0.349954208	count	5.92E-09
USP19	0.3491768	0.3895212	0.8964	0.37	0.350100696	count	1
KAT8	0.2636637	0.1537384	1.715	0.0865	0.350174537	count	1
TMA16	0.2611448	0.1630736	1.6014	0.109	0.35041794	count	1
OGFR	0.2559168	0.1258462	2.0336	0.0421	0.350543384	count	1
ZNF277	0.2676066	0.1884767	1.4198	0.156	0.35057813	count	1
CEP295	0.3065049	0.3062821	1.0007	0.317	0.350672189	count	1
OSER1-DT	0.8668858	0.6095091	1.4223	0.155	0.350825365	count	1
PKNOX1	0.2835167	0.2358323	1.2022	0.229	0.350908739	count	1
GLIPR1	0.245608	0.0498243	4.9295	8.80E-07	0.350940894	count	0.02096688
AZIN1	0.2586653	0.1399053	1.8489	0.0646	0.35112315	count	1
SNX16	0.3055782	0.2443399	1.2506	0.211	0.35113742	count	1
ATP1B1	0.249038	0.0955052	2.6076	0.00917	0.351188341	count	1

ITFG2	0.3860988	0.2705712	1.427	0.154	0.351224808	count	1
ZMAT5	0.2647036	0.1806791	1.465	0.143	0.351364851	count	1
MPHOSPH10	0.2712075	0.1656254	1.6375	0.102	0.351409935	count	1
SH3GL1	0.2729455	0.2230925	1.2235	0.221	0.351554648	count	1
FAAH	0.4105366	0.4418839	0.9291	0.353	0.35164125	count	1
RBM19	0.3167169	0.2405195	1.3168	0.188	0.351697028	count	1
TNKS	0.3168097	0.3231555	0.9804	0.327	0.35180222	count	1
PYROXD2	1.04388	0.6343577	1.6456	0.1	0.351914125	count	1
PHLDA3	0.5817354	0.4715194	1.2337	0.217	0.35208997	count	1
MINDY1	0.4762702	0.4391484	1.0845	0.278	0.352214522	count	1
SBF2	0.2734923	0.1776469	1.5395	0.124	0.352264889	count	1
PTBP3	0.253967	0.1049245	2.4205	0.0156	0.352434544	count	1
CAT	0.2498834	0.0750907	3.3278	0.000888	0.352808285	count	1
CTF1	0.4221352	0.7862477	0.5369	0.591	0.353128967	count	1
LIMCH1	1.047202	0.5547435	1.8877	0.0592	0.353214675	count	1
ACCS	0.5207953	0.4767039	1.0925	0.275	0.353345339	count	1
LPIN2	0.2593071	0.1485675	1.7454	0.081	0.353403196	count	1
CARD6	0.2878351	0.258674	1.1127	0.266	0.353410995	count	1
SLC38A9	0.3564027	0.354666	1.0049	0.315	0.3534367	count	1
AC009053.2	0.4976393	0.606954	0.8199	0.412	0.353513618	count	1
SULF2	0.2644085	0.1808242	1.4622	0.144	0.353705639	count	1
PPP2R5C	0.2565001	0.1099608	2.3327	0.0197	0.353832731	count	1
PANK3	0.2876268	0.2561033	1.1231	0.262	0.353904474	count	1
MED28	0.2555344	0.1163404	2.1964	0.0282	0.354209761	count	1
AL138762.1	0.47881	0.9453646	0.5065	0.613	0.354211368	count	1
POLG	0.339655	0.292174	1.1625	0.245	0.354214346	count	1
MAP7	0.3893631	0.4084721	0.9532	0.341	0.354313433	count	1
CASP6	0.3208789	0.30957	1.0365	0.3	0.35432874	count	1
HSF2	0.3533865	0.3061694	1.1542	0.249	0.354449912	count	1
PER2	0.284355	0.2249197	1.2643	0.206	0.354573019	count	1
POP5	0.2631803	0.150969	1.7433	0.0814	0.354603585	count	1
EXOC2	0.3463426	0.3153342	1.0983	0.272	0.354622961	count	1
RC3H1	0.273203	0.2026054	1.3484	0.178	0.354678583	count	1
STK4	0.2513393	0.077953	3.2242	0.00128	0.354729423	count	1
MT-ND2	0.2462983	0.0381837	6.4503	1.34E-10	0.354756236	count	3.23E-06
COASY	0.2871804	0.206212	1.3926	0.164	0.354797528	count	1
CAPN7	0.279141	0.2230748	1.2513	0.211	0.355424801	count	1
RNF170	0.3108384	0.2733665	1.1371	0.256	0.35572124	count	1
NUP188	0.3382938	0.348375	0.9711	0.332	0.355833981	count	1
HLX	0.2737759	0.1684521	1.6252	0.104	0.35607023	count	1
KLF13	0.2545614	0.112176	2.2693	0.0233	0.356114095	count	1
SMARCD2	0.2883939	0.1958841	1.4723	0.141	0.35631458	count	1
F11R	0.2950626	0.3290828	0.8966	0.37	0.356419964	count	1
HNRNPU	0.2485706	0.0592272	4.1969	2.80E-05	0.356479807	count	0.66122
AC009403.1	0.3289606	0.336951	0.9763	0.329	0.356502827	count	1

MFSD6	0.3790529	0.3966527	0.9556	0.339	0.356522076	count	1
EPB41L5	0.6898156	0.7142085	0.9658	0.334	0.356583179	count	1
JMJD1C-AS1	0.6898156	0.8001977	0.8621	0.389	0.356583179	count	1
SMAGP	0.5019783	0.5240888	0.9578	0.338	0.356806575	count	1
IMPA2	0.2615242	0.1389849	1.8817	0.06	0.356809614	count	1
ZG16B	0.416352	0.3937403	1.0574	0.29	0.356855329	count	1
E2F4	0.2682154	0.1509193	1.7772	0.0757	0.357037898	count	1
PIK3R3	0.8806706	0.5605825	1.571	0.116	0.357199168	count	1
RCBTB2	0.2644939	0.1411793	1.8735	0.0611	0.357304952	count	1
POMC	0.2880814	0.1954805	1.4737	0.141	0.357309671	count	1
HDX	0.3743665	0.3596815	1.0408	0.298	0.357351957	count	1
SON	0.250068	0.0535376	4.6709	3.16E-06	0.357582923	count	0.07510056
RNF123	0.5266782	0.4329492	1.2165	0.224	0.357632613	count	1
SFRP2	2.3415585	1.128019	2.0758	0.038	0.357668879	count	1
LASP1	0.262593	0.1658081	1.5837	0.113	0.357708363	count	1
KMT5B	0.2817006	0.2071036	1.3602	0.174	0.357772043	count	1
WDR60	0.2813478	0.2615533	1.0757	0.282	0.357795109	count	1
ABHD6	0.3114272	0.2963389	1.0509	0.293	0.357980038	count	1
ZNF341	2.343685	1.44718	1.6195	0.105	0.357986409	count	1
AC026369.3	0.4277126	0.406449	1.0523	0.293	0.35802544	count	1
ZSCAN2	0.483695	0.5162378	0.937	0.349	0.358055012	count	1
ZNF568	0.483695	0.5377494	0.8995	0.368	0.358055012	count	1
ZNF574	0.483695	0.5126819	0.9435	0.346	0.358055012	count	1
CDPF1	0.4522411	0.5048517	0.8958	0.37	0.358309445	count	1
ACOX1	0.2874417	0.2145128	1.34	0.18	0.358465032	count	1
AC007262.2	0.7713801	0.6906269	1.1169	0.264	0.358613578	count	1
LZTR1	0.484463	0.4797329	1.0099	0.313	0.358659631	count	1
TFRC	0.2547368	0.1211613	2.1025	0.0356	0.358753752	count	1
TGFBR3	2.34888	1.199749	1.9578	0.0504	0.358760576	count	1
PCF11	0.2609887	0.1318439	1.9795	0.0479	0.358768593	count	1
CLECL1	0.304035	0.3043262	0.999	0.318	0.358790743	count	1
ZBTB22	0.3085416	0.2572233	1.1995	0.23	0.358978792	count	1
CRAMP1	0.7720936	0.8561641	0.9018	0.367	0.358986481	count	1
BRD3	0.290569	0.1677458	1.7322	0.0834	0.359034091	count	1
WDR53	0.5049163	0.4891075	1.0323	0.302	0.359037985	count	1
MAK	0.7725279	0.5517204	1.4002	0.162	0.359213487	count	1
TRAPPC6B	0.29189	0.206185	1.4157	0.157	0.35921463	count	1
GSE1	0.5051548	0.3196903	1.5801	0.114	0.359219192	count	1
AC147067.1	0.358002	0.3280655	1.0913	0.275	0.359221362	count	1
FBXO44	0.7728203	0.5215657	1.4817	0.139	0.35936635	count	1
ARHGAP26	0.2602013	0.130077	2.0004	0.0456	0.359414594	count	1
ZBED1	0.2835144	0.2196859	1.2905	0.197	0.359609619	count	1
PPM1M	0.2752531	0.1652851	1.6653	0.096	0.359822352	count	1
BPNT1	0.4108005	0.4189783	0.9805	0.327	0.359976166	count	1
NUDT4	0.2745504	0.1829838	1.5004	0.134	0.360013966	count	1

PTK2B	0.282044	0.2434102	1.1587	0.247	0.360064333	count	1
CEBPD	0.2499446	0.0423809	5.8976	4.20E-09	0.360172422	count	0.000100821
LIMK2	0.3322962	0.3119894	1.0651	0.287	0.360202352	count	1
ARRDC5	0.8871806	0.6635098	1.3371	0.181	0.360215085	count	1
AC084033.3	0.3243848	0.3111714	1.0425	0.297	0.360392621	count	1
NGDN	0.2863329	0.2532706	1.1305	0.258	0.360599524	count	1
U73166.1	0.696956	0.6002407	1.1611	0.246	0.360681698	count	1
PRDM15	0.696956	0.6056021	1.1508	0.25	0.360681698	count	1
VARS	0.3067075	0.2487113	1.2332	0.218	0.360758022	count	1
COPS2	0.2630537	0.1158196	2.2712	0.0232	0.360821503	count	1
NUDT16L1	0.2830497	0.1940696	1.4585	0.145	0.360909088	count	1
CAAP1	0.2824903	0.2155423	1.3106	0.19	0.361081237	count	1
TMEM263	0.3040296	0.3037194	1.001	0.317	0.361129251	count	1
ST3GAL3	0.6401288	0.5638048	1.1354	0.256	0.361267968	count	1
TMEM202-AS1	0.6401288	0.5891734	1.0865	0.277	0.361267968	count	1
DGCR6	0.6401288	0.5891734	1.0865	0.277	0.361267968	count	1
ZNF526	0.3291121	0.5940959	0.554	0.58	0.361395771	count	1
TRPC4AP	0.2888173	0.2066118	1.3979	0.162	0.361439022	count	1
WAPL	0.2663467	0.1410492	1.8883	0.0591	0.36159759	count	1
AC006504.5	0.4882519	0.5785125	0.844	0.399	0.36164394	count	1
TSPO	0.2514772	0.0368407	6.8261	1.10E-11	0.361795623	count	2.65E-07
MGA	0.3098638	0.2671726	1.1598	0.246	0.361914776	count	1
AL135925.1	0.3020093	0.2523318	1.1969	0.231	0.361942541	count	1
SP2	0.3320512	0.3796611	0.8746	0.382	0.362361708	count	1
TRAPPC12	0.2985427	0.1765304	1.6912	0.0909	0.362522161	count	1
KIF16B	0.3507464	0.3263714	1.0747	0.283	0.362761246	count	1
TXNRD3	1.07179	1.0145409	1.0564	0.291	0.362861216	count	1
NSD3	0.2591763	0.0996772	2.6002	0.00937	0.363197358	count	1
USP7	0.2713375	0.1469316	1.8467	0.0649	0.363363491	count	1
DPYSL3	0.5106891	0.4942625	1.0332	0.302	0.363426407	count	1
TRIP11	0.2700748	0.1617605	1.6696	0.0951	0.363515468	count	1
FRYL	0.2797405	0.2118521	1.3205	0.187	0.363564279	count	1
EIF1AY	0.2610574	0.1239356	2.1064	0.0353	0.363749804	count	1
ZFAND3	0.2730948	0.1570274	1.7392	0.0821	0.363764916	count	1
ST5	0.7816186	0.7870789	0.9931	0.321	0.363969873	count	1
ECHDC1	0.2629959	0.1080171	2.4348	0.015	0.364039625	count	1
SLC25A28	0.2862134	0.1629892	1.756	0.0792	0.364042955	count	1
TMEM175	0.3106253	0.2763096	1.1242	0.261	0.364150784	count	1
HBS1L	0.2914548	0.2754648	1.058	0.29	0.364158193	count	1
NOC4L	0.3246753	0.2899009	1.12	0.263	0.364708863	count	1
FECH	0.3718711	0.3389623	1.0971	0.273	0.364739542	count	1
AIRN	2.389403	1.446081	1.6523	0.0986	0.364748537	count	1
STX18	0.2996396	0.2149037	1.3943	0.163	0.364759423	count	1
MYSM1	0.2924489	0.2798724	1.0449	0.296	0.364780295	count	1
METTL14	0.3111916	0.2832466	1.0987	0.272	0.364825666	count	1

AMPD2	0.2797464	0.201458	1.3886	0.165	0.364840586	count	1
TOR4A	0.3249469	0.2591564	1.2539	0.21	0.365020165	count	1
BRAP	0.3391012	0.3391251	0.9999	0.317	0.365168567	count	1
SAMSN1	0.2569637	0.0687273	3.7389	0.000189	0.365188898	count	1
TXLNB	0.4083058	0.315041	1.296	0.195	0.36521907	count	1
AC233723.1	0.7840997	0.7328914	1.0699	0.285	0.36526957	count	1
TMEM106B	0.2914245	0.2108777	1.382	0.167	0.365342353	count	1
HMGN3	0.2586905	0.0783829	3.3003	0.00098	0.365569936	count	1
MFSD3	0.3884037	0.382672	1.015	0.31	0.365647949	count	1
KCNMB1	0.3329171	0.2773623	1.2003	0.23	0.365667916	count	1
TMEM185B	0.3946135	0.3263782	1.2091	0.227	0.36569017	count	1
WDR11	0.2758468	0.2417478	1.1411	0.254	0.366040305	count	1
AC099063.4	1.4154761	0.8315954	1.7021	0.0889	0.366092192	count	1
LINC01736	1.4154761	0.8315954	1.7021	0.0889	0.366092192	count	1
CXCL14	1.4154761	1.307151	1.0829	0.279	0.366092192	count	1
ZNF300	1.4154761	1.021294	1.386	0.166	0.366092192	count	1
SPDYE16	1.4154761	1.0545611	1.3422	0.18	0.366092192	count	1
TMEM266	1.4154761	1.38413	1.0226	0.307	0.366092192	count	1
AIMP2	0.2797826	0.1725237	1.6217	0.105	0.366365257	count	1
RWDD3	0.6034584	0.5979392	1.0092	0.313	0.366426622	count	1
PDCD1	0.6034584	0.5979392	1.0092	0.313	0.366426622	count	1
KIFC2	0.6035734	0.4840204	1.247	0.213	0.36650269	count	1
TGFB1I1	0.6038933	0.4563939	1.3232	0.186	0.366714329	count	1
TSR2	0.2766005	0.1617714	1.7098	0.0874	0.367046807	count	1
VPS26B	0.2994878	0.2449215	1.2228	0.222	0.367099827	count	1
RAVER1	0.4627628	1.011448	0.4575	0.647	0.367117727	count	1
AP005019.1	1.083071	0.6707652	1.6147	0.107	0.367298035	count	1
IER5	0.2605396	0.0871665	2.989	0.00283	0.367324742	count	1
CCDC59	0.2679557	0.1097867	2.4407	0.0147	0.36733744	count	1
VDAC3	0.2629143	0.1020577	2.5761	0.01	0.367412169	count	1
PRPSAP1	0.282692	0.2225889	1.27	0.204	0.36743099	count	1
AC062029.1	0.7883215	0.5855004	1.3464	0.178	0.367482622	count	1
CNOT10	0.3157838	0.3143292	1.0046	0.315	0.367551185	count	1
INPP5B	0.4504353	0.3404092	1.3232	0.186	0.367812699	count	1
ADA2	0.2646419	0.1035834	2.5549	0.0107	0.367815341	count	1
ANKRD40	0.2920085	0.2107386	1.3856	0.166	0.36782303	count	1
RELL1	0.3060248	0.2576634	1.1877	0.235	0.367861767	count	1
SDHAF1	0.3137443	0.3203546	0.9794	0.327	0.367868266	count	1
AURKB	0.7891219	0.7227912	1.0918	0.275	0.367902394	count	1
C3orf62	0.3556614	0.2827605	1.2578	0.209	0.367988184	count	1
RPS17	0.2820519	0.1402519	2.011	0.0444	0.368177717	count	1
STK25	0.2762025	0.1850832	1.4923	0.136	0.368310941	count	1
HIRIP3	0.3231378	0.3349699	0.9647	0.335	0.368377816	count	1
RFX2	0.3091027	0.271734	1.1375	0.255	0.368387925	count	1
NEDD1	0.3131163	0.3480279	0.8997	0.368	0.36842011	count	1

NPTN	0.2654411	0.112447	2.3606	0.0183	0.368538812	count	1
ANKMY2	0.3711739	0.3704126	1.0021	0.316	0.368560814	count	1
AC100858.3	1.423983	0.9639486	1.4772	0.14	0.368654477	count	1
CPNE7	1.423983	0.9639486	1.4772	0.14	0.368654477	count	1
SOCS4	0.2871229	0.2052525	1.3989	0.162	0.368771117	count	1
ORMDL1	0.2632472	0.0914761	2.8778	0.00404	0.368837275	count	1
SLC18B1	0.3283799	0.3139788	1.0459	0.296	0.36895575	count	1
LDB1	0.3105645	0.283065	1.0971	0.273	0.369012551	count	1
THEMIS2	0.26273	0.0876621	2.9971	0.00275	0.369035077	count	1
WAC	0.2687158	0.1178103	2.2809	0.0226	0.369142311	count	1
THOC2	0.270575	0.1340603	2.0183	0.0437	0.369210239	count	1
DNAJB4	0.315016	0.2260559	1.3935	0.164	0.369384287	count	1
SAMD9L	0.2756258	0.1511849	1.8231	0.0684	0.369471804	count	1
ALG1	0.3766743	0.3471128	1.0852	0.278	0.36960884	count	1
ERAL1	0.3387082	0.2565419	1.3203	0.187	0.369795333	count	1
MIA3	0.2695277	0.1206592	2.2338	0.0256	0.36981253	count	1
TLR4	0.2736298	0.116415	2.3505	0.0188	0.369967771	count	1
ANKRD28	0.2636237	0.1111915	2.3709	0.0178	0.370093173	count	1
MT-ND1	0.2570013	0.0362828	7.0833	1.83E-12	0.370126236	count	4.42E-08
SDR39U1	0.3218367	0.2541793	1.2662	0.206	0.370167513	count	1
FAM53B	0.3821723	0.2838106	1.3466	0.178	0.370270452	count	1
ACD	0.3648393	0.249983	1.4595	0.145	0.370307864	count	1
RFX5	0.3463474	0.2947273	1.1751	0.24	0.370411323	count	1
CYB5R1	0.2779435	0.1405637	1.9773	0.0481	0.370646518	count	1
ZC3H6	0.2851921	0.1884604	1.5133	0.13	0.370706759	count	1
YAF2	0.2840079	0.1933769	1.4687	0.142	0.370750724	count	1
SYAP1	0.2615814	0.0945347	2.767	0.0057	0.370837912	count	1
NDST1	0.355187	0.3538056	1.0039	0.316	0.370860382	count	1
PSME3	0.3069105	0.2219536	1.3828	0.167	0.370935164	count	1
HOMEZ	0.432128	0.4305726	1.0036	0.316	0.371027495	count	1
PTPRN2	0.3338093	0.2981942	1.1194	0.263	0.3710902	count	1
CASP4	0.262884	0.0761588	3.4518	0.000566	0.371193431	count	1
MAP3K4	0.2939013	0.241614	1.2164	0.224	0.371339921	count	1
FXN	0.3135851	0.2293498	1.3673	0.172	0.37146874	count	1
PAOX	0.3559021	0.4025963	0.884	0.377	0.371627575	count	1
MRPS5	0.2684488	0.1332705	2.0143	0.0441	0.371867985	count	1
KBTBD11	0.3207282	0.2795746	1.1472	0.251	0.371943951	count	1
CD6	1.095187	0.6945091	1.5769	0.115	0.372070459	count	1
MRPL1	0.2936884	0.2130227	1.3787	0.168	0.372130029	count	1
RPL39	0.258262	0.024985	10.3367	1.52E-24	0.372156825	count	3.69E-20
HCFC1	0.3430847	0.330161	1.0391	0.299	0.372177801	count	1
UBR5-AS1	0.3330588	0.4622656	0.7205	0.471	0.372318453	count	1
VPS26C	0.2872329	0.1821042	1.5773	0.115	0.372698432	count	1
CCHCR1	0.5023254	0.406941	1.2344	0.217	0.372748894	count	1
ZNF839	0.5768602	0.5179593	1.1137	0.266	0.372785428	count	1

KSR1	0.5474561	0.4128052	1.3262	0.185	0.37281788	count	1
CEP162	0.3513507	0.2833175	1.2401	0.215	0.372983447	count	1
DCAF12	0.3085941	0.2247992	1.3728	0.17	0.372998881	count	1
TIGD7	0.4251925	0.6765284	0.6285	0.53	0.373165619	count	1
IQCD	0.3515357	0.4581748	0.7673	0.443	0.373184939	count	1
GMFG	0.2602429	0.0401026	6.4894	1.04E-10	0.373244977	count	2.50E-06
RBM42	0.2742047	0.1195388	2.2939	0.0219	0.373288803	count	1
STT3A	0.3131733	0.2665	1.1751	0.24	0.373314141	count	1
TOB2	0.27412	0.142608	1.9222	0.0547	0.373378313	count	1
DROSHA	0.503287	0.5051312	0.9963	0.319	0.373508805	count	1
RPUSD1	0.3342157	0.2741589	1.2191	0.223	0.373639107	count	1
GMEB1	0.2945503	0.1974609	1.4917	0.136	0.373749737	count	1
EMC10	0.2723934	0.1303121	2.0903	0.0367	0.373759512	count	1
ITGB2	0.260484	0.0385296	6.7606	1.71E-11	0.373813137	count	4.12E-07
SLC11A1	0.2620993	0.0956191	2.7411	0.00617	0.373882988	count	1
RABGAP1L	0.2833437	0.2080806	1.3617	0.173	0.373981416	count	1
UBE4A	0.2885353	0.201755	1.4301	0.153	0.374050972	count	1
CST3	0.2594175	0.0415559	6.2426	5.05E-10	0.374068127	count	1.21E-05
CEACAM21	0.4863616	0.432983	1.1233	0.261	0.374095095	count	1
BOD1L1	0.2643197	0.0821167	3.2188	0.0013	0.374096464	count	1
STAT2	0.2761684	0.1382908	1.997	0.0459	0.37417051	count	1
GGPS1	0.2966372	0.2385129	1.2437	0.214	0.374280264	count	1
STYXL1	0.3040273	0.22386	1.3581	0.175	0.374341434	count	1
ZSCAN20	0.9182308	0.8689214	1.0567	0.291	0.374649488	count	1
KDM4A-AS1	0.9182308	1.05643	0.8692	0.385	0.374649488	count	1
LTA	0.9182308	1.0351217	0.8871	0.375	0.374649488	count	1
GTF2IRD1	0.9182308	1.05643	0.8692	0.385	0.374649488	count	1
GNRH1	0.9182308	0.8689214	1.0567	0.291	0.374649488	count	1
PRNCR1	0.9182308	1.0351217	0.8871	0.375	0.374649488	count	1
CDT1	0.9182308	0.9948409	0.923	0.356	0.374649488	count	1
C19orf81	0.9182308	0.9948409	0.923	0.356	0.374649488	count	1
IL17RC	0.3690377	0.336217	1.0976	0.272	0.374699047	count	1
LRCH4	0.2890704	0.2115301	1.3666	0.172	0.37475046	count	1
JAG1	0.4113893	0.3536268	1.1633	0.245	0.375198064	count	1
TMEM229B	0.9196146	0.6806243	1.3511	0.177	0.37529464	count	1
PPP1R11	0.2710722	0.1158444	2.34	0.0194	0.375462125	count	1
FAM98B	0.3596499	0.3459829	1.0395	0.299	0.375649514	count	1
IL1RL2	0.8040595	0.5543067	1.4506	0.147	0.375748982	count	1
GALK2	0.3228271	0.2456324	1.3143	0.189	0.375893609	count	1
TIGD2	0.66389	0.6505901	1.0204	0.308	0.376035988	count	1
BX537318.1	0.66389	0.5710723	1.1625	0.245	0.376035988	count	1
CCDC85B	0.265268	0.0801429	3.3099	0.000947	0.376052281	count	1
FARP1	0.3255628	0.2352261	1.384	0.166	0.376118258	count	1
THG1L	0.3106402	0.2305423	1.3474	0.178	0.376475	count	1
GPR180	0.3938069	0.3814925	1.0323	0.302	0.37659488	count	1

MAML3	0.2928543	0.2166923	1.3515	0.177	0.376616332	count	1
SLC25A4	0.3835882	0.6266085	0.6122	0.54	0.376623839	count	1
PDHX	0.3426933	0.288711	1.187	0.235	0.37665268	count	1
CYB5D1	0.5073384	0.5140907	0.9869	0.324	0.376712036	count	1
TSC22D4	0.2761203	0.1488045	1.8556	0.0636	0.376712862	count	1
ZNFX1	0.2942902	0.1893306	1.5544	0.12	0.376758801	count	1
CBX4	0.2951277	0.1578668	1.8695	0.0617	0.376929142	count	1
SLC35F5	0.3102688	0.2633563	1.1781	0.239	0.376959767	count	1
TRIM44	0.2715925	0.1226962	2.2135	0.027	0.376966518	count	1
CPLANE1	0.3305385	0.419559	0.7878	0.431	0.376979532	count	1
CHMP7	0.3305527	0.3285077	1.0062	0.314	0.376996041	count	1
RDH14	0.3203455	0.2271317	1.4104	0.159	0.377068212	count	1
EXOG	0.375288	0.3565573	1.0525	0.293	0.377118932	count	1
AKAP12	2.4754134	1.53595	1.6117	0.107	0.377140717	count	1
AC021739.2	2.4754134	1.53595	1.6117	0.107	0.377140717	count	1
NID1	0.6656665	0.6054523	1.0995	0.272	0.377143114	count	1
USP49	0.4495247	0.4604788	0.9762	0.329	0.37722263	count	1
C17orf100	1.108716	0.7072473	1.5676	0.117	0.377408124	count	1
AL121761.1	1.108716	0.7353296	1.5078	0.132	0.377408124	count	1
KIAA1671	1.108716	0.8340581	1.3293	0.184	0.377408124	count	1
ALS2	0.5542553	0.4348667	1.2745	0.203	0.377801305	count	1
DMD	0.9252908	0.6073921	1.5234	0.128	0.377942686	count	1
MBTPS2	0.4758569	0.5787854	0.8222	0.411	0.37810425	count	1
SENP2	0.3463021	0.2601819	1.331	0.183	0.378282022	count	1
AL033527.5	0.7278808	0.6549281	1.1114	0.267	0.378504653	count	1
SDAD1	0.2871783	0.1698876	1.6904	0.0911	0.37857514	count	1
EIF2D	0.315758	0.2871659	1.0996	0.272	0.378669273	count	1
LMLN	0.8096116	0.647549	1.2503	0.211	0.378671341	count	1
SHLD3	0.6681611	0.6342226	1.0535	0.292	0.378698472	count	1
AC004982.2	0.9270196	0.7265572	1.2759	0.202	0.378749576	count	1
OSBPL5	0.9270196	0.8236669	1.1255	0.26	0.378749576	count	1
AP000487.1	0.9270196	0.6906885	1.3422	0.18	0.378749576	count	1
PTGIS	0.9270196	0.8040362	1.153	0.249	0.378749576	count	1
PAXX	0.2746062	0.1136672	2.4159	0.0158	0.378770615	count	1
UBQLN2	0.3292172	0.2535641	1.2984	0.194	0.37881601	count	1
UAP1L1	0.3490854	0.2971854	1.1746	0.24	0.378845164	count	1
GPN2	0.3514954	0.3164146	1.1109	0.267	0.378848957	count	1
TTC13	0.4515052	0.4739349	0.9527	0.341	0.378969445	count	1
EIF2S3	0.2727228	0.0836871	3.2588	0.00113	0.378991705	count	1
SYP	0.6689242	0.5919851	1.13	0.259	0.379174428	count	1
KCTD18	0.4233279	0.3971617	1.0659	0.287	0.379248442	count	1
CEP350	0.2767923	0.1314999	2.1049	0.0354	0.379335436	count	1
IRF5	0.3450902	0.230603	1.4965	0.135	0.379347715	count	1
TSC2	0.3698622	0.3069568	1.2049	0.228	0.379427778	count	1
ZNF577	0.3546935	0.3432958	1.0332	0.302	0.379566288	count	1

PDCD7	0.2872229	0.1441516	1.9925	0.0464	0.379626054	count	1
ZNF106	0.2699665	0.0801283	3.3692	0.000766	0.379667103	count	1
POU2F1	0.3150493	0.2850339	1.1053	0.269	0.379906548	count	1
ARMT1	0.289247	0.1600551	1.8072	0.0709	0.379990705	count	1
FBXO27	1.115635	0.7908176	1.4107	0.158	0.380141173	count	1
VIPR1	1.462908	0.5459126	2.6797	0.00742	0.380380825	count	1
AKT2	0.2960825	0.2424787	1.2211	0.222	0.380384875	count	1
SLC25A36	0.278214	0.1283298	2.168	0.0303	0.380426488	count	1
PDE7B	2.500038	1.028617	2.4305	0.0151	0.380606922	count	1
PGGT1B	0.2896372	0.1657144	1.7478	0.0806	0.380781429	count	1
NAPEPLD	0.4790564	0.4364894	1.0975	0.273	0.380792885	count	1
RPL30	0.2642172	0.0229975	11.489	8.55E-30	0.380878536	count	2.08E-25
EED	0.3216129	0.2590356	1.2416	0.215	0.381132761	count	1
MED22	0.4107039	0.3282312	1.2513	0.211	0.381203848	count	1
NR2C2	0.3648695	0.3205227	1.1384	0.255	0.381254086	count	1
ABHD10	0.3792885	0.3140456	1.2077	0.227	0.381267064	count	1
RTN3	0.2704162	0.0713147	3.7919	0.000153	0.381323276	count	1
ZNF25	0.3985781	0.4176942	0.9542	0.34	0.381326289	count	1
MRPL53	0.443775	0.4482704	0.99	0.322	0.38151573	count	1
RNASEK	0.2785452	0.1361081	2.0465	0.0408	0.381663911	count	1
NFE2L1	0.301154	0.2787519	1.0804	0.28	0.381688013	count	1
SHOC2	0.2748339	0.1233967	2.2272	0.026	0.381845101	count	1
TNFAIP8L2	0.2820983	0.1519521	1.8565	0.0635	0.381865755	count	1
SPRTN	0.3183893	0.2271235	1.4018	0.161	0.381872625	count	1
DYSF	0.4548329	0.338452	1.3439	0.179	0.381905879	count	1
C2orf76	0.309015	0.2283918	1.353	0.176	0.382113461	count	1
UBXN2A	0.3130609	0.2406485	1.3009	0.193	0.382224708	count	1
MAP1LC3A	0.3308373	0.2805389	1.1793	0.238	0.382324687	count	1
BRD4	0.2745092	0.0957259	2.8677	0.00417	0.382351454	count	1
ELOA-AS1	0.6274949	0.6538075	0.9598	0.337	0.382368593	count	1
SLC5A6	0.6274949	0.6275693	0.9999	0.317	0.382368593	count	1
ANO8	0.8177014	0.6321214	1.2936	0.196	0.382935041	count	1
STK3	0.3233692	0.2307408	1.4014	0.161	0.383247895	count	1
SPSB3	0.2862314	0.146383	1.9554	0.0507	0.383250127	count	1
TRIB3	0.3486478	0.3761261	0.9269	0.354	0.383349122	count	1
TMEM236	0.6757741	0.4081451	1.6557	0.0979	0.383450099	count	1
FBL	0.2846063	0.1644294	1.7309	0.0836	0.383480496	count	1
GAK	0.3043795	0.2151206	1.4149	0.157	0.38357665	count	1
AF213884.3	0.6295076	0.6817279	0.9234	0.356	0.383707143	count	1
PI4KB	0.3270673	0.2502232	1.3071	0.191	0.383759314	count	1
JUN	0.2667864	0.0859553	3.1038	0.00193	0.383817302	count	1
PPP5C	0.4690165	0.4510689	1.0398	0.299	0.383818278	count	1
DCP1B	0.7374505	0.5808306	1.2696	0.204	0.384043213	count	1
REV1	0.2991226	0.2084847	1.4347	0.151	0.384751713	count	1
KHSRP	0.3181952	0.2057858	1.5462	0.122	0.384772763	count	1

SUPT3H	0.3917076	0.3970421	0.9866	0.324	0.38487087	count	1
ZFHX3	0.2823851	0.1225153	2.3049	0.0213	0.384894019	count	1
TRAK2	0.335846	0.3475231	0.9664	0.334	0.384900147	count	1
DLAT	0.3716678	0.3496616	1.0629	0.288	0.385033595	count	1
LILRB2	0.2738186	0.0863271	3.1719	0.00153	0.385095159	count	1
L1TD1	1.1284209	0.7847236	1.438	0.151	0.385197617	count	1
FOXD1	1.1284209	0.8274372	1.3638	0.173	0.385197617	count	1
ZNF251	0.4843064	0.5548058	0.8729	0.383	0.385208085	count	1
LINC02207	0.5644556	0.3077486	1.8341	0.0668	0.385290511	count	1
MAL	2.534346	2.0175867	1.2561	0.2092	0.385373714	count	1
PPP2R5A	0.3067364	0.1938539	1.5823	0.114	0.385377378	count	1
CCP110	0.3297743	0.265648	1.2414	0.215	0.385581628	count	1
CAMK2D	0.3031077	0.2008054	1.5095	0.131	0.385750157	count	1
ICE1	0.2875179	0.1693713	1.6976	0.0897	0.385843794	count	1
RHOT1	0.2977307	0.1914743	1.5549	0.12	0.386073796	count	1
EFNB2	0.5958498	0.7726819	0.7711	0.441	0.386092361	count	1
LPAR6	0.276488	0.108896	2.539	0.0112	0.386232238	count	1
KCTD11	0.5960685	0.5916206	1.0075	0.314	0.386245932	count	1
ASCC3	0.2941586	0.1718312	1.7119	0.087	0.386491303	count	1
MKNK1	0.2793777	0.1374689	2.0323	0.0422	0.38673434	count	1
FUT10	0.9451946	0.735308	1.2854	0.199	0.387247685	count	1
SNUPN	0.2980964	0.2020078	1.4757	0.14	0.387270494	count	1
EXOC7	0.3037553	0.2147223	1.4146	0.157	0.387574808	count	1
RPH3AL	0.4614504	0.5160029	0.8943	0.371	0.387750421	count	1
PTER	0.3816421	0.3037985	1.2562	0.209	0.3878971	count	1
IL1RAP	0.323347	0.1945145	1.6623	0.0966	0.387909955	count	1
CCSAP	0.2947235	0.1914443	1.5395	0.124	0.388063389	count	1
SLC35B3	0.357396	0.3291978	1.0857	0.278	0.388086581	count	1
MDM2	0.294593	0.1645031	1.7908	0.0734	0.388157087	count	1
NUDT12	0.6362018	0.4832065	1.3166	0.188	0.38816314	count	1
YPEL5	0.2733962	0.0749991	3.6453	0.000273	0.388330767	count	1
ADCY7	0.3135821	0.1708831	1.8351	0.0666	0.388591251	count	1
OSGEPL1	0.5436655	0.4477961	1.2141	0.225	0.388594086	count	1
ATP13A2	0.3629818	0.2810765	1.2914	0.197	0.388666806	count	1
CCNY	0.2830164	0.1085087	2.6082	0.00916	0.388786373	count	1
PRRG4	0.3350603	0.2840652	1.1795	0.238	0.388871347	count	1
VPS45	0.3496514	0.2642181	1.3233	0.186	0.389096612	count	1
RAB29	0.3038649	0.1670778	1.8187	0.0691	0.389135253	count	1
ACAD8	0.4752127	0.390869	1.2158	0.224	0.389167647	count	1
B9D2	0.36086	0.2749886	1.3123	0.19	0.389198457	count	1
CASK	0.387025	0.3170367	1.2208	0.222	0.38929547	count	1
METRN	0.310821	0.2317966	1.3409	0.18	0.389296863	count	1
TRAPPC2	0.3587948	0.3276653	1.095	0.274	0.389642909	count	1
ZNF2	0.9503231	0.7566291	1.256	0.209	0.389650204	count	1
SCD5	0.9503231	0.8761219	1.0847	0.278	0.389650204	count	1

AC012306.2	0.33328	0.2675562	1.2456	0.213	0.389753055	count	1
ZNF398	0.5451966	0.6983322	0.7807	0.435	0.389766647	count	1
CCPG1	0.2781605	0.0982972	2.8298	0.0047	0.389780185	count	1
CEP85	0.4529898	0.488777	0.9268	0.354	0.389828724	count	1
TRMT6	0.2829052	0.1514084	1.8685	0.0618	0.389928241	count	1
STK26	0.3567704	0.3075046	1.1602	0.246	0.389992871	count	1
SLC22A18	0.3030179	0.2042613	1.4835	0.138	0.39023099	count	1
AC104986.2	0.4764606	1.0664414	0.4468	0.655	0.390245707	count	1
C12orf66	0.4764606	0.4951158	0.9623	0.336	0.390245707	count	1
ZNF587B	0.5061233	0.6967169	0.7264	0.468	0.390248281	count	1
C3orf58	0.2859631	0.1336583	2.1395	0.0325	0.390497311	count	1
EPM2AIP1	0.3193197	0.2040725	1.5647	0.118	0.390866184	count	1
CHCHD5	0.2833354	0.1137367	2.4912	0.0128	0.390918221	count	1
CYTIP	0.2743734	0.0652564	4.2045	2.71E-05	0.390942285	count	0.6399936
CD300E	0.2878029	0.1488851	1.9331	0.0533	0.390985311	count	1
KIAA2013	0.3041873	0.1525218	1.9944	0.0462	0.391326913	count	1
AC007686.3	0.5472907	0.5261833	1.0401	0.298	0.39137094	count	1
PARD6A	0.3323263	0.2992695	1.1105	0.267	0.391412421	count	1
CD46	0.2765947	0.0815605	3.3913	0.000707	0.391424199	count	1
CPSF6	0.3055583	0.1898209	1.6097	0.108	0.391782765	count	1
VPS72	0.3207994	0.246137	1.3033	0.193	0.39180379	count	1
DDX39B	0.3024816	0.1647417	1.8361	0.0665	0.391914219	count	1
SRPRB	0.3104749	0.1840463	1.6869	0.0917	0.391931877	count	1
SQLE	0.3025484	0.2812824	1.0756	0.282	0.392001523	count	1
ANKRD36C	0.3940352	0.3193734	1.2338	0.217	0.392030787	count	1
ADAM10	0.2837569	0.1090228	2.6027	0.0093	0.392063895	count	1
SLC45A4	0.42221	0.3355788	1.2582	0.208	0.392321563	count	1
ATF4	0.2819284	0.0951145	2.9641	0.00306	0.392713911	count	1
TSNAX	0.2921424	0.1297385	2.2518	0.0244	0.392910406	count	1
RABGGTA	0.3198426	0.1986227	1.6103	0.107	0.393240264	count	1
RPL34	0.2730195	0.0238611	11.442	1.43E-29	0.393489575	count	3.47E-25
HOXB-AS1	0.6063892	0.4642043	1.3063	0.192	0.393500164	count	1
ZNF589	0.5285831	0.5456342	0.9687	0.333	0.393550541	count	1
THNSL2	1.1495826	0.7441422	1.5448	0.123	0.393581881	count	1
RUFY2	0.3194515	0.3051509	1.0469	0.295	0.393583211	count	1
ZNF700	0.5286609	0.460391	1.1483	0.251	0.393612327	count	1
SPATA5	0.4573718	0.4848187	0.9434	0.346	0.393786459	count	1
CHD6	0.3154608	0.1666053	1.8935	0.0584	0.393830111	count	1
USP18	0.480745	0.489619	0.9819	0.326	0.393948829	count	1
PIKFYVE	0.333525	0.215947	1.5445	0.123	0.394186537	count	1
MFSD9	0.5294653	0.5819238	0.9099	0.363	0.394251251	count	1
LFNG	0.2859006	0.1372668	2.0828	0.0374	0.39427301	count	1
LOXL3	0.3962397	0.3879942	1.0213	0.307	0.394297904	count	1
AZU1	0.4579513	0.451027	1.0154	0.31	0.394310073	count	1
PHF7	0.5111872	0.5037493	1.0148	0.31	0.394397204	count	1

ESYT1	0.3252639	0.2830578	1.1491	0.251	0.39445697	count	1
FAM216A	0.4062611	0.3972084	1.0228	0.307	0.394464663	count	1
ARGLU1	0.277329	0.0726595	3.8168	0.000139	0.394533506	count	1
TMEM181	0.3772522	0.3268697	1.1541	0.249	0.394564685	count	1
MKS1	1.152821	0.7179008	1.6058	0.108	0.394866693	count	1
CDC27	0.2955251	0.1479424	1.9976	0.0459	0.395023393	count	1
NAA35	0.3431449	0.3520906	0.9746	0.33	0.395152922	count	1
UNC119	0.3046887	0.145708	2.0911	0.0366	0.395174799	count	1
ICAM5	0.5524204	0.5244114	1.0534	0.292	0.395303588	count	1
SH2B3	0.2934565	0.1479675	1.9832	0.0475	0.395622209	count	1
CDIP1	0.3574224	0.3041136	1.1753	0.24	0.39562892	count	1
FADS3	0.323203	0.2264329	1.4274	0.154	0.395684778	count	1
AL024507.2	0.5789258	0.8039123	0.7201	0.472	0.395941071	count	1
IDH3B	0.2947614	0.1300147	2.2671	0.0235	0.396125637	count	1
ADIRF	0.367197	0.5421833	0.6773	0.498	0.396208242	count	1
SYT17	0.3652193	0.413381	0.8835	0.377	0.396794019	count	1
AKAP9	0.283756	0.0836542	3.392	0.000705	0.396994028	count	1
ZNF518B	0.3708499	0.3008403	1.2327	0.218	0.397314093	count	1
BCL2	0.3047076	0.1629812	1.8696	0.0617	0.397328957	count	1
NEU4	0.4041203	0.4644827	0.87	0.384	0.397496972	count	1
STX10	0.2832212	0.0948353	2.9865	0.00285	0.397713287	count	1
WARS2	0.4044714	0.3236073	1.2499	0.211	0.397854417	count	1
MED31	0.3019413	0.2165627	1.3942	0.163	0.397910678	count	1
LETMD1	0.3134541	0.1816846	1.7253	0.0846	0.397991753	count	1
HDAC6	0.3806786	0.4687654	0.8121	0.417	0.398251431	count	1
RMND5A	0.319593	0.2610163	1.2244	0.221	0.398346679	count	1
ATG4D	0.4218617	0.4875544	0.8653	0.387	0.39840951	count	1
USP48	0.2977023	0.1589632	1.8728	0.0612	0.398517741	count	1
SNX19	0.3719701	0.3164088	1.1756	0.24	0.398545865	count	1
ATPAF2	0.3209941	0.2092058	1.5343	0.125	0.398650507	count	1
TRIM5	0.3420848	0.3156098	1.0839	0.279	0.398730403	count	1
AC002553.1	0.7007662	0.5675575	1.2347	0.217	0.399100681	count	1
PTEN	0.2877542	0.0786598	3.6582	0.000259	0.399117791	count	1
AEN	0.3887909	0.4139955	0.9391	0.348	0.399446511	count	1
STK10	0.3024394	0.1366812	2.2127	0.027	0.399627191	count	1
AC106791.1	1.1648231	0.7983742	1.459	0.145	0.399631357	count	1
AL136038.3	1.1648231	0.7983742	1.459	0.145	0.399631357	count	1
MXI1	0.2940044	0.1324893	2.2191	0.0266	0.399687224	count	1
AP003108.2	0.6153914	0.6392913	0.9626	0.336	0.399839883	count	1
SSRP1	0.3141694	0.2070274	1.5175	0.129	0.399974286	count	1
THAP8	0.4753496	0.491026	0.9681	0.333	0.400048052	count	1
TOLLIP	0.2913047	0.1426273	2.0424	0.0412	0.400217007	count	1
FOXP4	0.423799	0.438984	0.9654	0.334	0.400311556	count	1
N4BP2L2	0.2836926	0.0792837	3.5782	0.000353	0.400360584	count	1
FAM173B	0.4239803	0.5158314	0.8219	0.411	0.400489584	count	1

RTF1	0.2857572	0.109906	2.6	0.00938	0.400588219	count	1
KIAA1147	0.3198338	0.3234712	0.9888	0.323	0.400718597	count	1
HEATR3	0.3226394	0.2526148	1.2772	0.202	0.400719479	count	1
OXR1	0.2991333	0.139099	2.1505	0.0316	0.400812899	count	1
AL117379.1	0.6553562	0.6978189	0.9391	0.348	0.40094662	count	1
YPEL2	0.3051791	0.1584047	1.9266	0.0541	0.401082077	count	1
MRPL9	0.3093545	0.1844406	1.6773	0.0936	0.401279308	count	1
HDDC3	0.314432	0.1781976	1.7645	0.0778	0.401336671	count	1
BMT2	0.4390607	0.3796787	1.1564	0.248	0.401540776	count	1
GSK3A	0.3153894	0.1796573	1.7555	0.0793	0.401543602	count	1
KATNAL2	0.8529731	0.6532852	1.3057	0.192	0.401600276	count	1
ICOSLG	0.3649246	0.2914363	1.2522	0.211	0.401676138	count	1
SHC1	0.348805	0.3391736	1.0284	0.304	0.401798138	count	1
NPEPL1	0.3473709	0.2492342	1.3938	0.164	0.40179906	count	1
ADPRH	0.3488172	0.2614862	1.334	0.182	0.401812465	count	1
TNFRSF14	0.2931509	0.105367	2.7822	0.00544	0.401847465	count	1
BCAR3	0.4036477	0.3663743	1.1017	0.271	0.401921317	count	1
HNRNPH2	0.2939622	0.1271638	2.3117	0.0209	0.401958436	count	1
MBD1	0.346378	0.2687931	1.2886	0.198	0.402254004	count	1
GTDC1	0.3702054	0.2922862	1.2666	0.205	0.402347564	count	1
IRF2	0.2879883	0.1061215	2.7138	0.0067	0.402541895	count	1
SPTSSA	0.2926416	0.1176642	2.4871	0.0129	0.402677676	count	1
CLPX	0.3226914	0.1850004	1.7443	0.0812	0.402966654	count	1
ITGB5	0.3284464	0.2175231	1.5099	0.131	0.403088513	count	1
SH2D4A	0.7076719	0.4760112	1.4867	0.137	0.403438897	count	1
SLC38A10	0.3143645	0.1767535	1.7785	0.0754	0.40364626	count	1
ESS2	0.4211529	0.4887859	0.8616	0.389	0.403758062	count	1
SIGLEC15	0.7713801	0.6880124	1.1212	0.262	0.403764885	count	1
KIAA1551	0.2939357	0.1186166	2.478	0.0133	0.404313951	count	1
SOS1	0.331695	0.2148606	1.5438	0.123	0.404348957	count	1
MIER3	0.450349	0.5040599	0.8934	0.372	0.40457086	count	1
AES	0.2890869	0.086859	3.3282	0.000887	0.404656746	count	1
NUB1	0.2964908	0.1008786	2.9391	0.00332	0.404734129	count	1
ACAD10	0.5076035	0.4175391	1.2157	0.224	0.404851843	count	1
ZNF800	0.3115487	0.1582294	1.969	0.0491	0.404899108	count	1
PLXNB2	0.3124255	0.1647915	1.8959	0.0581	0.404913206	count	1
GGACT	0.4427325	0.3869026	1.1443	0.253	0.405044861	count	1
YOD1	0.4811558	0.3408496	1.4116	0.158	0.405193914	count	1
RFLNB	0.2927617	0.1551781	1.8866	0.0593	0.405492629	count	1
KCNRG	1.5464032	1.0153588	1.523	0.128	0.405507789	count	1
AC009404.1	0.8605168	0.5962275	1.4433	0.149	0.405607624	count	1
AL117335.1	0.4292063	0.3831728	1.1201	0.263	0.40562332	count	1
SUPT5H	0.3022862	0.1613972	1.8729	0.0612	0.405780337	count	1
FOSL1	0.3294349	0.224619	1.4666	0.143	0.406048352	count	1
TMEM185A	0.3645984	0.3672069	0.9929	0.321	0.406112992	count	1

POFUT2	0.4439026	0.3196172	1.3889	0.165	0.406161931	count	1
ANKRD44	0.3049242	0.1476552	2.0651	0.039	0.406229282	count	1
NME3	0.2886822	0.0928993	3.1075	0.00191	0.406274893	count	1
GPR162	0.3713117	0.3270607	1.1353	0.256	0.406282522	count	1
FCER1A	0.2874366	0.2238013	1.2843	0.199	0.406344561	count	1
PLPPR2	0.3822252	0.3309855	1.1548	0.248	0.406672651	count	1
CRIM1	0.5097858	0.4810396	1.0598	0.289	0.406696157	count	1
UGGT2	0.3594609	0.2923764	1.2294	0.219	0.406724491	count	1
METAP1D	0.9868529	0.7699899	1.2816	0.2	0.406818414	count	1
B4GALT1	0.2875651	0.0842764	3.4122	0.000655	0.406923029	count	1
ACAP2	0.2885411	0.0851901	3.387	0.000718	0.406997863	count	1
COA1	0.3152948	0.176775	1.7836	0.0746	0.407044874	count	1
TRAF6	0.4620181	0.4014357	1.1509	0.25	0.407060371	count	1
USP53	0.3170593	0.1800096	1.7613	0.0783	0.407140096	count	1
SLC7A6	0.5942468	0.377247	1.5752	0.115	0.407250778	count	1
MCTP2	0.5460521	0.3347981	1.631	0.103	0.407447507	count	1
MAP2K4	0.3472794	0.2396508	1.4491	0.147	0.40790244	count	1
KPNB1	0.2931341	0.1117614	2.6229	0.00877	0.40794132	count	1
HGSNAT	0.3144707	0.1810242	1.7372	0.0825	0.408729708	count	1
MORC3	0.3023742	0.1291864	2.3406	0.0193	0.408756156	count	1
RSRC2	0.2898072	0.0878569	3.2986	0.000985	0.408922459	count	1
MICB	0.4742657	0.3519462	1.3476	0.178	0.409071852	count	1
AL139246.5	0.317518	0.2235664	1.4202	0.156	0.40908262	count	1
ASPHD2	0.4553286	0.498925	0.9126	0.362	0.409249314	count	1
TTI2	0.3847195	0.3382599	1.1373	0.256	0.409399689	count	1
PAXIP1-AS2	0.4475174	0.3294041	1.3586	0.174	0.409614154	count	1
WWP2	0.3947697	0.2708516	1.4575	0.145	0.409696065	count	1
TMEM129	0.3556925	0.3409203	1.0433	0.297	0.409889001	count	1
AC079015.1	0.7818753	0.669054	1.1686	0.243	0.40989109	count	1
LINC02328	0.7818753	0.6449851	1.2122	0.226	0.40989109	count	1
ZNF548	0.7818753	0.6454322	1.2114	0.226	0.40989109	count	1
ENGASE	0.416492	0.3241989	1.2847	0.199	0.410103	count	1
CXCL2	0.2851979	0.0962787	2.9622	0.00308	0.410150063	count	1
SLA	0.2917288	0.0832433	3.5045	0.000466	0.410247855	count	1
KATNBL1	0.3003719	0.1254499	2.3944	0.0167	0.410262129	count	1
TMEM268	0.3990358	0.3153003	1.2656	0.206	0.410301536	count	1
ATF2	0.3285539	0.1982094	1.6576	0.0975	0.410377491	count	1
MXRA8	1.5630278	1.188027	1.3156	0.188	0.410501041	count	1
TRIM61	1.5630278	0.7261906	2.1524	0.0315	0.410501041	count	1
GGH	0.3829499	0.3768167	1.0163	0.31	0.410627624	count	1
MMS22L	0.4875467	0.6621578	0.7363	0.462	0.410863801	count	1
HTRA1	0.2978572	0.1631478	1.8257	0.068	0.410891411	count	1
LUC7L2	0.3355188	0.1863402	1.8006	0.0719	0.410975496	count	1
ZNF16	0.8706889	0.6222925	1.3992	0.162	0.411019497	count	1
ALAS1	0.3414519	0.2279247	1.4981	0.134	0.411125227	count	1

CEP83	0.3733949	0.3130971	1.1926	0.233	0.411225939	count	1
ERBIN	0.3074073	0.1419144	2.1661	0.0304	0.411397888	count	1
FCGR3B	0.7848177	0.7604354	1.0321	0.302	0.411610747	count	1
AZI2	0.2998079	0.1289271	2.3254	0.0201	0.411782298	count	1
LINC02193	2.735886	1.19696	2.2857	0.0224	0.411865263	count	1
DBP	0.3257824	0.2057358	1.5835	0.113	0.412078216	count	1
BAZ2B	0.2962465	0.1145751	2.5856	0.00978	0.412106186	count	1
TBC1D13	0.4776676	0.3973238	1.2022	0.229	0.412155017	count	1
LIN52	1.197267	0.705666	1.6966	0.0899	0.412537447	count	1
NT5DC1	0.3347399	0.1984166	1.6871	0.0917	0.412675498	count	1
LPCAT1	0.3114779	0.2089412	1.4907	0.136	0.41268319	count	1
FOXP1	0.3020258	0.1270364	2.3775	0.0175	0.412734671	count	1
FAM208A	0.3170481	0.1779735	1.7814	0.075	0.412845059	count	1
OGDH	0.3301152	0.1992547	1.6567	0.0977	0.413061423	count	1
DNAJC28	0.7230893	0.8632381	0.8376	0.402	0.413145019	count	1
PLD2	0.3799856	0.3488443	1.0893	0.276	0.413249625	count	1
KNTC1	0.6740269	0.5008143	1.3459	0.178	0.413453747	count	1
DONSON	0.6740269	0.4994653	1.3495	0.177	0.413453747	count	1
AP001437.1	0.5762177	0.6255552	0.9211	0.357	0.413597976	count	1
PHACTR2	0.3027313	0.1304398	2.3209	0.0204	0.413703081	count	1
BRAT1	0.3214448	0.2324802	1.3827	0.167	0.41374397	count	1
CEP57	0.3060454	0.1347915	2.2705	0.0233	0.413745701	count	1
RNF138	0.3301408	0.1721313	1.918	0.0552	0.413788125	count	1
HECTD4	0.3713332	0.3503362	1.0599	0.289	0.413788723	count	1
STARD7	0.3167024	0.1724959	1.836	0.0665	0.413792256	count	1
LINC02482	0.3986262	0.4359253	0.9144	0.361	0.413819924	count	1
PIPOX	0.6035419	0.6161165	0.9796	0.327	0.414128421	count	1
UTP6	0.3083418	0.1407491	2.1907	0.0286	0.414135308	count	1
SNAI3	0.4204956	0.3704276	1.1352	0.256	0.414186979	count	1
BBX	0.3027329	0.112648	2.6874	0.00725	0.414199573	count	1
IQCJ-SCHIP1	0.8771959	0.8338283	1.052	0.293	0.414486293	count	1
AL117336.3	0.8771959	0.7482159	1.1724	0.241	0.414486293	count	1
SLX1A	0.8771959	0.7482159	1.1724	0.241	0.414486293	count	1
AC026471.4	0.8771959	0.8610528	1.0187	0.308	0.414486293	count	1
GOSR1	0.3092884	0.1424932	2.1705	0.0301	0.414503703	count	1
MTM1	0.3924976	0.2724093	1.4408	0.15	0.414523517	count	1
GLYR1	0.3175485	0.2051984	1.5475	0.122	0.414564138	count	1
ZNF687	0.4322522	0.4112012	1.0512	0.293	0.41481387	count	1
DUSP22	0.3110655	0.1538745	2.0216	0.0433	0.415108205	count	1
ECM1	0.5197891	0.321726	1.6156	0.106	0.415159138	count	1
CCNQ	0.3165035	0.1765982	1.7922	0.0732	0.415161428	count	1
TMPPE	1.004805	0.6887817	1.4588	0.145	0.415289212	count	1
AC007611.1	1.004805	0.6542149	1.5359	0.125	0.415289212	count	1
ZNF852	0.6769846	0.5297304	1.278	0.201	0.415439158	count	1
AL132639.2	0.6769846	0.5646385	1.199	0.231	0.415439158	count	1

SCRN2	0.4271019	0.3201662	1.334	0.182	0.415463908	count	1
GATM	0.3061106	0.1498861	2.0423	0.0412	0.415731163	count	1
ZNF692	1.0059036	0.7286817	1.3804	0.168	0.415808283	count	1
ZBTB4	0.3297204	0.2045456	1.612	0.107	0.415882763	count	1
IPO11	0.5570259	0.6218027	0.8958	0.37	0.416200341	count	1
FAM76A	0.3207747	0.2193487	1.4624	0.144	0.41622659	count	1
GATD3A	0.6389375	0.7141309	0.8947	0.371	0.416474614	count	1
PSMG3	0.3121013	0.1888338	1.6528	0.0985	0.416709083	count	1
MRPS22	0.315716	0.1579555	1.9988	0.0457	0.417031446	count	1
ABL1	0.4094828	0.3641141	1.1246	0.261	0.417126247	count	1
YIPF4	0.3062385	0.1301464	2.353	0.0187	0.417337442	count	1
MACROD1	0.4188103	0.3931382	1.0653	0.287	0.417548436	count	1
NVL	0.4483483	0.3306119	1.3561	0.175	0.417649982	count	1
AC048341.1	0.7303392	0.7717066	0.9464	0.344	0.417719015	count	1
ZBTB18	0.3841517	0.3651326	1.0521	0.293	0.417897117	count	1
DCAF16	0.3841517	0.3152026	1.2187	0.223	0.417897117	count	1
SLC35C2	0.3382479	0.2055985	1.6452	0.1	0.41791361	count	1
FAM120B	0.3926256	0.3824512	1.0266	0.305	0.418048632	count	1
TMEM119	1.2121041	0.7125142	1.7012	0.089	0.418450978	count	1
KLF10	0.3008716	0.1118759	2.6893	0.00721	0.418513341	count	1
BCKDHB	0.5827137	0.5205743	1.1194	0.263	0.41860599	count	1
HLA-F	0.2958679	0.075751	3.9058	9.65E-05	0.418703575	count	1
DNAH1	1.2136261	0.7905475	1.5352	0.125	0.419057957	count	1
AC011416.3	1.2136261	0.7978174	1.5212	0.128	0.419057957	count	1
STXBP4	1.2136261	0.832662	1.4575	0.145	0.419057957	count	1
ZNF304	1.2136261	0.9329251	1.3009	0.193	0.419057957	count	1
RSF1	0.3006935	0.1142932	2.6309	0.00857	0.419096499	count	1
LRRC61	0.682621	0.5788251	1.1793	0.238	0.419225763	count	1
ATP10A	0.682621	0.5916456	1.1538	0.249	0.419225763	count	1
SDR42E2	0.682621	0.5949877	1.1473	0.251	0.419225763	count	1
KRBOX4	0.5420088	0.3686748	1.4702	0.142	0.419732631	count	1
TELO2	0.3884506	0.3374794	1.151	0.25	0.419754674	count	1
IRF3	0.3218378	0.1730557	1.8597	0.063	0.419858776	count	1
RBM15B	0.3747516	0.2253876	1.6627	0.0965	0.420011943	count	1
ZNHIT3	0.3049801	0.1243192	2.4532	0.0142	0.420028331	count	1
RNF144B	0.3249504	0.1115472	2.9131	0.00361	0.420055969	count	1
ECHDC2	0.4212518	0.4099647	1.0275	0.304	0.420067657	count	1
HMBOX1	0.3710496	0.2919215	1.2711	0.204	0.420124395	count	1
DCUN1D3	0.3488186	0.2288762	1.524	0.128	0.420136084	count	1
WDR33	0.3081453	0.1219187	2.5275	0.0116	0.420173007	count	1
ADNP2	0.3554301	0.3117236	1.1402	0.254	0.420535031	count	1
USF1	0.3566634	0.2778614	1.2836	0.199	0.42059501	count	1
TMEM238	0.3680309	0.3856655	0.9543	0.34	0.420650562	count	1
DOCK2	0.3027838	0.1165492	2.5979	0.00944	0.420750869	count	1
NUAK2	0.4269377	0.368944	1.1572	0.247	0.42076304	count	1

TAF8	0.3568137	0.2509911	1.4216	0.155	0.420775421	count	1
CACNB1	1.5975089	0.9181603	1.7399	0.082	0.42084161	count	1
MYOM1	1.5975089	0.9489505	1.6834	0.0924	0.42084161	count	1
LZTS2	0.3444949	0.3388704	1.0166	0.309	0.421167842	count	1
ZNF654	0.3539364	0.3016756	1.1732	0.241	0.421441293	count	1
ZBTB10	0.3671171	0.204018	1.7994	0.0721	0.421484824	count	1
TGFBR2	0.306621	0.1098767	2.7906	0.0053	0.421591921	count	1
LINC00309	2.815831	0.9341503	3.0143	0.0026	0.421638379	count	1
HCK	0.2994135	0.0846371	3.5376	0.000411	0.421659228	count	1
RIPOR1	0.3426401	0.2304801	1.4866	0.137	0.421662698	count	1
ZNF354A	0.3804893	0.265355	1.4339	0.152	0.421780869	count	1
PDSS2	0.3827827	0.3133725	1.2215	0.222	0.421820053	count	1
SMAD4	0.3532219	0.2445401	1.4444	0.149	0.421867055	count	1
SPPL2B	0.4065272	0.3106176	1.3088	0.191	0.422274696	count	1
AC114760.2	0.410371	0.3521979	1.1652	0.244	0.422327904	count	1
MAT2B	0.3378838	0.1831678	1.8447	0.0652	0.422902336	count	1
SYNJ1	0.3840742	0.2706358	1.4192	0.156	0.423278318	count	1
C15orf39	0.3412708	0.236862	1.4408	0.15	0.423361397	count	1
SERINC3	0.3072488	0.1332978	2.305	0.0213	0.4233899	count	1
KCTD21	0.7393218	0.5494157	1.3457	0.179	0.423394661	count	1
NEK7	0.3479794	0.2047856	1.6992	0.0894	0.423475114	count	1
CLEC7A	0.2965536	0.0547053	5.4209	6.51E-08	0.423618692	count	0.001557322
FKBP5	0.3110796	0.0791263	3.9314	8.68E-05	0.42385154	count	1
RNF141	0.3089542	0.1351753	2.2856	0.0224	0.423874321	count	1
DICER1-AS1	0.6168055	0.6200905	0.9947	0.32	0.423963176	count	1
AHCYL1	0.3167692	0.1382842	2.2907	0.0221	0.424006582	count	1
CDC14B	2.8357771	1.343483	2.1108	0.0349	0.424010405	count	1
AF111167.2	0.8954696	0.5172438	1.7312	0.0835	0.42424194	count	1
ATM	0.3215652	0.1562552	2.0579	0.0397	0.424257419	count	1
ARFGEF2	0.3827419	0.2606628	1.4683	0.142	0.42433805	count	1
SPIN1	0.3334613	0.2183875	1.5269	0.127	0.424802039	count	1
SLTM	0.3021549	0.0859269	3.5164	0.000445	0.424848328	count	1
AC004865.2	0.4054068	0.3218895	1.2595	0.208	0.424903676	count	1
TRIM72	2.8433975	1.700759	1.6718	0.0947	0.424909694	count	1
APOL2	0.3629118	0.2608635	1.3912	0.164	0.425062344	count	1
CYTH2	0.3168839	0.1464267	2.1641	0.0306	0.425125732	count	1
ZNF621	0.6512179	0.4765068	1.3666	0.172	0.425179877	count	1
LDLRAP1	0.4638539	0.4186031	1.1081	0.268	0.425239482	count	1
ZNF655	0.3150261	0.1490523	2.1135	0.0347	0.425343599	count	1
SMG9	0.421695	0.2970231	1.4197	0.156	0.425374765	count	1
Z93930.2	0.3963463	0.3703401	1.0702	0.285	0.425388736	count	1
FITM2	0.5915252	0.5190017	1.1397	0.255	0.42540858	count	1
TOGARAM1	0.3777862	0.3391606	1.1139	0.265	0.425746989	count	1
ATXN7	0.5494381	0.9557184	0.5749	0.565	0.42586033	count	1
LOH12CR2	0.8985259	0.6444169	1.3943	0.163	0.425876375	count	1

PROCA1	0.5044515	0.4832796	1.0438	0.297	0.42589044	count	1
CPNE1	0.3156873	0.1045579	3.0193	0.00256	0.426241348	count	1
PPP1R10	0.3010307	0.0834338	3.608	0.000315	0.426443823	count	1
CCDC71	0.5698545	0.5169591	1.1023	0.27	0.426454582	count	1
MRPL44	0.3458779	0.2098415	1.6483	0.0994	0.426596918	count	1
ANKRD13A	0.3242577	0.1912486	1.6955	0.0901	0.426661914	count	1
TMUB2	0.324035	0.1776988	1.8235	0.0683	0.426962246	count	1
MED13L	0.3214332	0.1354554	2.373	0.0177	0.426969036	count	1
ING4	0.3596343	0.2375945	1.5136	0.13	0.426990445	count	1
C9orf139	0.5708744	0.4106811	1.3901	0.165	0.427270815	count	1
DPM1	0.3287034	0.1817212	1.8088	0.0706	0.427395367	count	1
STK35	0.4286738	0.3848026	1.114	0.265	0.427730836	count	1
IL4R	0.3358017	0.2100022	1.599	0.11	0.427815759	count	1
GALNT12	0.5349593	1.0117967	0.5287	0.597	0.428021333	count	1
KIF3A	0.4083878	0.3354193	1.2175	0.224	0.428121888	count	1
KDM5A	0.3104422	0.1348292	2.3025	0.0214	0.428246657	count	1
NRP1	0.306244	0.1105009	2.7714	0.00562	0.428335508	count	1
CYTH1	0.3098498	0.1589999	1.9487	0.0514	0.428506961	count	1
IL10RA	0.3049549	0.0947849	3.2173	0.00131	0.428602176	count	1
RRS1	0.4023887	0.318236	1.2644	0.206	0.42873976	count	1
PPP2R1A	0.3145658	0.1284848	2.4483	0.0144	0.428740634	count	1
BTBD6	0.3436911	0.219008	1.5693	0.117	0.428771119	count	1
CARHSP1	0.3141469	0.1404392	2.2369	0.0254	0.429270098	count	1
SUPT7L	0.3512115	0.3016601	1.1643	0.244	0.429499976	count	1
ADAR	0.3129964	0.1166128	2.6841	0.00732	0.429533529	count	1
SERGEF	0.3667402	0.2380178	1.5408	0.123	0.429630703	count	1
BLOC1S4	0.3226542	0.1634219	1.9744	0.0485	0.429784285	count	1
TMEM184C	0.4173974	0.3263861	1.2788	0.201	0.42979112	count	1
CCDC159	0.3429608	0.2537674	1.3515	0.177	0.430055432	count	1
SMIM13	0.3494576	0.2261687	1.5451	0.122	0.430170697	count	1
YJU2	0.355187	0.3055446	1.1625	0.245	0.430214981	count	1
TTC9	1.6290862	0.9405895	1.732	0.0834	0.430288411	count	1
ZMAT1	0.4779409	0.2992858	1.5969	0.11	0.430539469	count	1
AC096667.1	0.3159579	0.1415727	2.2318	0.0257	0.430647175	count	1
TRIM23	0.4875865	0.330599	1.4749	0.14	0.430712658	count	1
ICK	0.4044993	0.2618423	1.5448	0.123	0.431052518	count	1
TMEM42	0.4014939	0.2879376	1.3944	0.163	0.431066542	count	1
RNF169	0.3236514	0.2093731	1.5458	0.122	0.431121681	count	1
ARMC8	0.3375728	0.1955571	1.7262	0.0844	0.431191112	count	1
ZNF768	0.4486642	0.4321945	1.0381	0.299	0.431193148	count	1
CAB39	0.3164215	0.1323893	2.3901	0.0169	0.431397743	count	1
NEK4	0.3669309	0.287563	1.276	0.202	0.431415251	count	1
MFSD11	0.3498842	0.2348607	1.4898	0.136	0.431606865	count	1
ARL17A	0.4705098	0.4287368	1.0974	0.273	0.43161653	count	1
XPA	0.3161863	0.1622109	1.9492	0.0514	0.431637775	count	1

SLC25A12	0.5242603	0.4139089	1.2666	0.205	0.431716183	count	1
C7orf43	0.3965826	0.3208716	1.236	0.217	0.431776686	count	1
PIK3C3	0.3920345	0.2460996	1.593	0.111	0.432270692	count	1
RAB3IL1	0.4335006	0.2119664	2.0451	0.0409	0.432718416	count	1
ING2	0.308307	0.1250178	2.4661	0.0137	0.432863105	count	1
ZNF563	0.8211393	0.7840943	1.0472	0.295	0.432912572	count	1
OGFRL1	0.3070554	0.0863466	3.5561	0.000384	0.432921351	count	1
HSPA14	0.4443836	0.3734834	1.1898	0.234	0.432922723	count	1
PCMTD1	0.3128536	0.1162825	2.6905	0.00718	0.433054418	count	1
DDX3Y	0.3105075	0.1117967	2.7774	0.00552	0.433148745	count	1
ITCH	0.350426	0.2078292	1.6861	0.0919	0.433167638	count	1
UBIAD1	0.4066013	0.3612047	1.1257	0.26	0.433356392	count	1
PRPF8	0.3223128	0.1482923	2.1735	0.0298	0.433378726	count	1
TTYH2	0.5261885	0.2852385	1.8447	0.0652	0.433396082	count	1
ZNF652	0.3293591	0.1569195	2.0989	0.0359	0.433427954	count	1
MLF1	0.3552725	0.256466	1.3853	0.166	0.433525871	count	1
C12orf4	0.3775845	0.2656353	1.4214	0.155	0.433754143	count	1
COLGALT1	0.3160854	0.1298503	2.4342	0.015	0.433880656	count	1
WASHC4	0.3176244	0.1145558	2.7727	0.0056	0.434255209	count	1
C1orf162	0.3029971	0.049916	6.0701	1.48E-09	0.434333906	count	3.56E-05
LMF1	0.4142456	0.3488919	1.1873	0.235	0.434449043	count	1
TAF1C	0.5799147	0.4853658	1.1948	0.232	0.434512197	count	1
ACADSB	0.6644849	0.4860965	1.367	0.172	0.434606554	count	1
U2SURP	0.3088013	0.0882444	3.4994	0.000475	0.434670963	count	1
AP2B1	0.3300751	0.1980257	1.6668	0.0957	0.434682872	count	1
LDHD	0.9150507	0.509546	1.7958	0.0726	0.434726812	count	1
SLC25A11	0.3150706	0.1108083	2.8434	0.0045	0.434871186	count	1
ARHGAP29	1.2539922	0.7461076	1.6807	0.0929	0.435178357	count	1
HECTD1	0.3191273	0.148347	2.1512	0.0316	0.43556028	count	1
VEZF1	0.331745	0.1510824	2.1958	0.0282	0.435645411	count	1
CUL2	0.3362913	0.1777929	1.8915	0.0587	0.435717047	count	1
KIAA2026	0.3243219	0.1782152	1.8198	0.0689	0.435731134	count	1
SIRT1	0.3420405	0.1968619	1.7375	0.0824	0.435851235	count	1
SZT2	0.419259	0.343033	1.2222	0.222	0.435915606	count	1
POLM	0.4274262	0.4329046	0.9873	0.324	0.436019065	count	1
CNPY3	0.3061165	0.0573895	5.334	1.05E-07	0.436042783	count	0.00250971
PDRG1	0.4004268	0.2701731	1.4821	0.138	0.436072561	count	1
DISC1	0.4475978	0.3546972	1.2619	0.207	0.43617434	count	1
ARG2	0.5160831	0.4224764	1.2216	0.222	0.436253772	count	1
GPT2	1.049421	0.7656643	1.3706	0.171	0.436428456	count	1
LINC02381	0.6672064	0.5382957	1.2395	0.215	0.436543061	count	1
MIA2	0.3265663	0.1677082	1.9472	0.0516	0.436581288	count	1
RPS6KA3	0.3228309	0.1433975	2.2513	0.0245	0.436717641	count	1
ZSCAN16-AS1	0.3320954	0.1865452	1.7802	0.0752	0.436746254	count	1
HK2	0.3819752	0.323396	1.1811	0.238	0.436931659	count	1

TAOK2	0.5174454	0.3959157	1.307	0.191	0.437468772	count	1
JDP2	0.3118406	0.0885922	3.52	0.000439	0.437699713	count	1
ELL	0.3385078	0.1638224	2.0663	0.0389	0.437750904	count	1
PLD1	0.4249234	0.2950942	1.44	0.15	0.437791997	count	1
FAM161A	0.5841067	0.6602378	0.8847	0.376	0.4378739	count	1
DDA1	0.3252764	0.1745516	1.8635	0.0625	0.437922062	count	1
CEP85L	0.3454971	0.2206843	1.5656	0.118	0.437930955	count	1
FLVCR1-DT	0.4077196	0.450846	0.9043	0.366	0.43793774	count	1
RBBP4	0.3190366	0.1332015	2.3951	0.0167	0.438128221	count	1
C20orf27	0.3170026	0.0924533	3.4288	0.000616	0.438170481	count	1
PPM1L	0.4861072	0.3112974	1.5616	0.119	0.438246177	count	1
TIGD3	0.6361067	0.6640073	0.958	0.338	0.438317175	count	1
COL4A2-AS2	0.6361067	0.6383046	0.9966	0.319	0.438317175	count	1
TMEM218	0.3380033	0.1809295	1.8681	0.0619	0.438377105	count	1
UGGT1	0.3609301	0.2195438	1.644	0.1	0.438394282	count	1
NCAPG2	0.6083191	0.4442138	1.3694	0.171	0.438403375	count	1
COQ9	0.372771	0.2540548	1.4673	0.142	0.438410141	count	1
ZKSCAN5	0.9222498	0.6215741	1.4837	0.138	0.438589455	count	1
TFAP4	0.9222498	0.5851685	1.576	0.115	0.438589455	count	1
NR4A2	0.305886	0.0574241	5.3268	1.09E-07	0.438606229	count	0.002605209
FOXO3	0.3189462	0.1178216	2.707	0.00684	0.438608723	count	1
WDR74	0.3578102	0.2296138	1.5583	0.119	0.438684132	count	1
PSMD9	0.3731088	0.2029369	1.8385	0.0661	0.438814837	count	1
MORN2	0.3579444	0.2976683	1.2025	0.229	0.438851074	count	1
HMGCS1	0.3773838	0.3360723	1.1229	0.262	0.438984875	count	1
AC008906.1	1.658246	1.305714	1.27	0.204	0.438988329	count	1
AC012150.2	1.658246	1.379544	1.202	0.229	0.438988329	count	1
BTN3A1	0.4347836	0.3174905	1.3694	0.171	0.439037129	count	1
MBNL1	0.3108142	0.0836842	3.7141	0.000208	0.439267988	count	1
CEACAM3	0.7126829	0.4481576	1.5903	0.112	0.43948796	count	1
SCAP	0.4509853	0.3056207	1.4756	0.14	0.439602778	count	1
AC104506.1	0.5662181	0.4382004	1.2921	0.196	0.439729363	count	1
EIF4EBP2	0.3307873	0.1675028	1.9748	0.0484	0.439732207	count	1
ASPSCR1	0.3507595	0.2133398	1.6441	0.1	0.439957179	count	1
JOSD1	0.3209541	0.1505289	2.1322	0.0331	0.440033766	count	1
ZHX2	0.8333407	0.6737656	1.2368	0.216	0.440098113	count	1
CRTC3	0.3425474	0.2967295	1.1544	0.248	0.440208995	count	1
SNHG8	0.3127429	0.0843518	3.7076	0.000214	0.440296991	count	1
TNFRSF10B	0.3371056	0.1484693	2.2705	0.0233	0.44032149	count	1
PKN1	0.320513	0.1172712	2.7331	0.00632	0.440341489	count	1
ZNF432	0.9257819	0.5551172	1.6677	0.0955	0.440486079	count	1
TMEM205	0.3145898	0.0985423	3.1924	0.00143	0.440546159	count	1
CYP4V2	0.4412364	0.3348346	1.3178	0.188	0.440718283	count	1
CARD8	0.326434	0.1375774	2.3727	0.0177	0.440835176	count	1
RBPJ	0.3104508	0.0700289	4.4332	9.69E-06	0.440863426	count	0.22967238

DRAIC	0.6732904	1.2052639	0.5586	0.576	0.440875551	count	1
TARSL2	0.3629426	0.324965	1.1169	0.264	0.440876792	count	1
ABCG2	0.4365815	0.5148055	0.8481	0.396	0.440915582	count	1
ITPRIPL2	0.3230862	0.1340031	2.411	0.016	0.440989486	count	1
TSG101	0.317444	0.1090758	2.9103	0.00364	0.441085303	count	1
ABHD17B	0.4806453	0.3566328	1.3477	0.178	0.441339272	count	1
FOXK1	0.3956128	0.2619469	1.5103	0.131	0.441503147	count	1
TTC33	0.4809857	0.3653404	1.3165	0.188	0.441666058	count	1
RPL17	0.3193291	0.1130417	2.8249	0.00477	0.44172955	count	1
SHISA5	0.3196242	0.1252809	2.5513	0.0108	0.441739744	count	1
PHF20	0.3164353	0.0916151	3.454	0.000562	0.441845858	count	1
ANP32E	0.3176755	0.1027017	3.0932	0.002	0.441961174	count	1
ODF3B	0.3150366	0.0886634	3.5532	0.000388	0.442084074	count	1
PPP2R5E	0.327176	0.1502588	2.1774	0.0295	0.442162162	count	1
OSGIN1	0.4332522	0.2798297	1.5483	0.122	0.442162272	count	1
SETD2	0.339187	0.1694821	2.0013	0.0455	0.442317647	count	1
BRCC3	0.3571982	0.2091891	1.7075	0.0878	0.442533022	count	1
STAMBPL1	0.5007768	0.4612827	1.0856	0.278	0.442951005	count	1
ARMCX2	0.5703929	0.5344173	1.0673	0.286	0.443186043	count	1
ACTL6A	0.3511405	0.2064993	1.7004	0.0892	0.443227047	count	1
NDUFB1	0.3120392	0.0700153	4.4567	8.70E-06	0.443319065	count	0.2062857
CDKL3	0.5909268	0.4770488	1.2387	0.216	0.443348314	count	1
PSME1	0.3096498	0.0437995	7.0697	2.02E-12	0.44351076	count	4.87E-08
GBP2	0.3218632	0.0905394	3.555	0.000385	0.443608743	count	1
RRNAD1	0.3915441	0.3054845	1.2817	0.2	0.443857799	count	1
VPS11	0.4395082	0.3008126	1.4611	0.144	0.443974295	count	1
NINJ2	0.367328	0.2017545	1.8207	0.0688	0.443993141	count	1
ZNF282	0.7719278	0.5339581	1.4457	0.148	0.444073233	count	1
ZNF573	0.7719278	0.5751573	1.3421	0.18	0.444073233	count	1
CD1E	0.3776934	0.4054799	0.9315	0.352	0.44430841	count	1
FBXW4	0.4685826	0.4559957	1.0276	0.304	0.444428415	count	1
FUT4	0.3920828	0.3395064	1.1549	0.248	0.444482233	count	1
DCAF11	0.3822161	0.2440053	1.5664	0.117	0.444718156	count	1
IQGAP2	0.3134094	0.0758563	4.1316	3.72E-05	0.445079779	count	0.8774364
RAP2A	0.3295129	0.1523216	2.1633	0.0306	0.445338394	count	1
NUMB	0.324754	0.1203271	2.6989	0.007	0.445362636	count	1
ZNF217	0.3320571	0.1551604	2.1401	0.0324	0.44541528	count	1
INPP4A	0.3657555	0.2189103	1.6708	0.0949	0.445444683	count	1
TNPO3	0.3738653	0.2702168	1.3836	0.167	0.445591919	count	1
KCNQ3	0.6459964	0.5871705	1.1002	0.271	0.445691003	count	1
PMS1	0.4040846	0.2500131	1.6163	0.106	0.445896871	count	1
AL691432.2	0.4635695	0.3978258	1.1653	0.244	0.446100579	count	1
NR2C2AP	0.3935339	0.285389	1.3789	0.168	0.446164446	count	1
OXA1L	0.327862	0.1145209	2.8629	0.00423	0.446338772	count	1
GDPGP1	0.6186027	0.4641445	1.3328	0.183	0.446379468	count	1

ASAP1	0.3210252	0.1079938	2.9726	0.00298	0.446437228	count	1
GPX3	0.3838378	0.2578783	1.4884	0.137	0.446642732	count	1
PPP1R12B	0.4951904	0.3727725	1.3284	0.184	0.446829071	count	1
FAM215B	0.3855503	0.3090343	1.2476	0.212	0.44687865	count	1
ATF6B	0.339569	0.2259461	1.5029	0.133	0.44697353	count	1
ID2-AS1	1.284112	0.9484273	1.3539	0.176	0.447229986	count	1
LRIG1	1.284112	1.2155904	1.0564	0.291	0.447229986	count	1
AC104825.1	1.284112	1.2188783	1.0535	0.292	0.447229986	count	1
AMACR	1.284112	0.9484273	1.3539	0.176	0.447229986	count	1
AC011405.1	1.284112	0.9484273	1.3539	0.176	0.447229986	count	1
PXT1	1.284112	0.9484273	1.3539	0.176	0.447229986	count	1
RAMP3	1.284112	0.9484273	1.3539	0.176	0.447229986	count	1
RPA4	1.284112	0.9484273	1.3539	0.176	0.447229986	count	1
TACR2	1.284112	0.9484273	1.3539	0.176	0.447229986	count	1
AL731569.1	1.284112	1.1513374	1.1153	0.265	0.447229986	count	1
MSRB3	1.284112	1.1513374	1.1153	0.265	0.447229986	count	1
KIF23	1.284112	0.9484273	1.3539	0.176	0.447229986	count	1
LARP6	1.284112	0.9484273	1.3539	0.176	0.447229986	count	1
AC092119.2	1.284112	0.9484273	1.3539	0.176	0.447229986	count	1
RTN4RL1	1.284112	0.9484273	1.3539	0.176	0.447229986	count	1
ZNF416	1.284112	0.9484273	1.3539	0.176	0.447229986	count	1
AL441992.1	0.422564	0.3473337	1.2166	0.224	0.44724034	count	1
CCNT1	0.3690552	0.3006214	1.2276	0.22	0.447282416	count	1
UBE2G1	0.3459208	0.1764191	1.9608	0.05	0.447430951	count	1
LDAH	0.6826029	0.5334784	1.2795	0.201	0.447516052	count	1
SKP2	0.5757488	0.4817024	1.1952	0.232	0.447624162	count	1
SMAD2	0.3435228	0.1658527	2.0713	0.0384	0.447635835	count	1
OARD1	0.3482805	0.1817468	1.9163	0.0554	0.447652952	count	1
TRMT2B	0.7778097	0.4892869	1.5897	0.112	0.447815894	count	1
SFXN5	0.4231133	0.3112575	1.3594	0.174	0.447839085	count	1
NFXL1	1.2857405	0.5270663	2.4394	0.0148	0.447881911	count	1
TNRC6A	0.3432169	0.2175622	1.5776	0.115	0.448000401	count	1
UTF1	0.3991743	0.29291	1.3628	0.173	0.448041159	count	1
RGMA	0.7783745	0.5118264	1.5208	0.128	0.448175465	count	1
AC015819.2	1.074157	0.5753207	1.8671	0.062	0.448197188	count	1
CLK2	0.4800107	0.3493108	1.3742	0.17	0.448461094	count	1
EVI5	0.3290682	0.1372357	2.3978	0.0166	0.448484551	count	1
MPO	1.0749195	0.7817256	1.3751	0.169	0.448560434	count	1
CRTAP	0.3209997	0.0840216	3.8204	0.000137	0.448625391	count	1
TMEM39A	0.4274086	0.2483242	1.7212	0.0853	0.448682184	count	1
ZNF587	0.4663461	0.3648893	1.278	0.201	0.448880858	count	1
RMDN2	0.4977094	0.3902209	1.2755	0.202	0.449211349	count	1
CCDC91	0.3338191	0.1328835	2.5121	0.0121	0.449313004	count	1
HEATR1	0.4358353	0.2980247	1.4624	0.144	0.449405084	count	1
PANX2	0.780313	0.7073724	1.1031	0.27	0.449409853	count	1

YES1	1.6942875	0.9317109	1.8185	0.0691	0.449705593	count	1
LINC00324	0.347802	0.213062	1.6324	0.103	0.449887981	count	1
RASSF1	0.3404517	0.1517392	2.2437	0.0249	0.449962626	count	1
MRPL10	0.3677203	0.2265291	1.6233	0.105	0.449995586	count	1
AP5M1	0.4102672	0.2878705	1.4252	0.154	0.450045369	count	1
TMEM41B	0.3486488	0.214833	1.6229	0.105	0.45007357	count	1
DHX29	0.3256986	0.1259504	2.5859	0.00977	0.450097004	count	1
BRF1	0.4453907	0.3579729	1.2442	0.214	0.45012545	count	1
MTRNR2L8	0.3509013	0.1452717	2.4155	0.0158	0.451056526	count	1
JRKL	0.4111859	0.3292504	1.2489	0.212	0.451079546	count	1
AL357054.4	1.2937393	0.7978871	1.6215	0.105	0.451085186	count	1
MPZL3	1.2937393	0.7758252	1.6676	0.0955	0.451085186	count	1
CEMIP2	0.3494541	0.1805989	1.935	0.0531	0.451123512	count	1
ZBTB39	1.0807261	0.7156995	1.51	0.131	0.451327789	count	1
ZNF581	0.3519261	0.1762138	1.9972	0.0459	0.451352982	count	1
APOL3	0.4201208	0.3333733	1.2602	0.208	0.451638411	count	1
ZNF689	0.852997	0.5001643	1.7054	0.0882	0.451703678	count	1
PMS2	0.4762115	0.3873982	1.2293	0.219	0.451971229	count	1
AL928921.2	1.702071	1.0380211	1.6397	0.101	0.452014113	count	1
DCDC2B	1.702071	1.0380211	1.6397	0.101	0.452014113	count	1
CCDC17	1.702071	1.0380211	1.6397	0.101	0.452014113	count	1
S100A1	1.702071	1.0380211	1.6397	0.101	0.452014113	count	1
KLHDC9	1.702071	1.0380211	1.6397	0.101	0.452014113	count	1
LINC01740	1.702071	1.0380211	1.6397	0.101	0.452014113	count	1
AL117350.1	1.702071	1.0380211	1.6397	0.101	0.452014113	count	1
LINC01914	1.702071	1.0380211	1.6397	0.101	0.452014113	count	1
ST3GAL5-AS1	1.702071	1.0380211	1.6397	0.101	0.452014113	count	1
LINC00471	1.702071	1.0380211	1.6397	0.101	0.452014113	count	1
SNED1	1.702071	1.0380211	1.6397	0.101	0.452014113	count	1
LINC00852	1.702071	1.0380211	1.6397	0.101	0.452014113	count	1
AC112220.2	1.702071	1.0380211	1.6397	0.101	0.452014113	count	1
SLC35G2	1.702071	1.0380211	1.6397	0.101	0.452014113	count	1
FGFBP2	1.702071	1.0380211	1.6397	0.101	0.452014113	count	1
LINC01093	1.702071	1.0380211	1.6397	0.101	0.452014113	count	1
AC008799.2	1.702071	1.0380211	1.6397	0.101	0.452014113	count	1
AL137003.1	1.702071	1.0380211	1.6397	0.101	0.452014113	count	1
HIST1H4J	1.702071	1.0380211	1.6397	0.101	0.452014113	count	1
SYNGAP1-AS1	1.702071	1.0380211	1.6397	0.101	0.452014113	count	1
CAPN11	1.702071	1.0380211	1.6397	0.101	0.452014113	count	1
ADGB	1.702071	1.0380211	1.6397	0.101	0.452014113	count	1
HOXA1	1.702071	1.0380211	1.6397	0.101	0.452014113	count	1
AC018645.2	1.702071	1.0380211	1.6397	0.101	0.452014113	count	1
ATP6V0E2-AS1	1.702071	1.0380211	1.6397	0.101	0.452014113	count	1
ATG9B	1.702071	1.0380211	1.6397	0.101	0.452014113	count	1
EXTL3-AS1	1.702071	1.0380211	1.6397	0.101	0.452014113	count	1

RUNX1T1	1.702071	1.0380211	1.6397	0.101	0.452014113	count	1
PKN3	1.702071	1.0380211	1.6397	0.101	0.452014113	count	1
AC068580.3	1.702071	1.0380211	1.6397	0.101	0.452014113	count	1
AC006299.1	1.702071	1.0380211	1.6397	0.101	0.452014113	count	1
AP003419.3	1.702071	1.0380211	1.6397	0.101	0.452014113	count	1
IDI2-AS1	1.702071	1.0380211	1.6397	0.101	0.452014113	count	1
MEIG1	1.702071	1.0380211	1.6397	0.101	0.452014113	count	1
THNSL1	1.702071	1.0380211	1.6397	0.101	0.452014113	count	1
AC021028.1	1.702071	1.0380211	1.6397	0.101	0.452014113	count	1
KAZALD1	1.702071	1.0380211	1.6397	0.101	0.452014113	count	1
AC022075.1	1.702071	1.0380211	1.6397	0.101	0.452014113	count	1
HIGD1C	1.702071	1.0380211	1.6397	0.101	0.452014113	count	1
CCDC62	1.702071	1.0380211	1.6397	0.101	0.452014113	count	1
SEC23A-AS1	1.702071	1.0380211	1.6397	0.101	0.452014113	count	1
AL121820.2	1.702071	1.0380211	1.6397	0.101	0.452014113	count	1
CNGB1	1.702071	1.0380211	1.6397	0.101	0.452014113	count	1
AC027682.4	1.702071	1.0380211	1.6397	0.101	0.452014113	count	1
P2RX5	1.702071	1.0380211	1.6397	0.101	0.452014113	count	1
PIMREG	1.702071	1.0380211	1.6397	0.101	0.452014113	count	1
AC005288.1	1.702071	1.0380211	1.6397	0.101	0.452014113	count	1
KRT15	1.702071	1.0380211	1.6397	0.101	0.452014113	count	1
AP005671.1	1.702071	1.0380211	1.6397	0.101	0.452014113	count	1
GATA6	1.702071	1.0380211	1.6397	0.101	0.452014113	count	1
DBNDD2	1.702071	1.0380211	1.6397	0.101	0.452014113	count	1
AC092068.1	1.702071	1.0380211	1.6397	0.101	0.452014113	count	1
TINCR	1.702071	1.0380211	1.6397	0.101	0.452014113	count	1
TDRD12	1.702071	1.0380211	1.6397	0.101	0.452014113	count	1
SIX5	1.702071	1.0380211	1.6397	0.101	0.452014113	count	1
MMP11	1.702071	1.0380211	1.6397	0.101	0.452014113	count	1
AP001043.1	1.702071	1.0380211	1.6397	0.101	0.452014113	count	1
CRNKL1	0.3551006	0.2046949	1.7348	0.0829	0.452090305	count	1
KMT2B	0.3510791	0.1944929	1.8051	0.0712	0.452282483	count	1
SLC45A3	0.8539844	0.727081	1.1745	0.24	0.452287625	count	1
NADSYN1	0.3582999	0.2601031	1.3775	0.168	0.452373771	count	1
PECAM1	0.3271362	0.1279983	2.5558	0.0107	0.452502119	count	1
MTERF1	0.5344457	0.5418198	0.9864	0.324	0.452652915	count	1
TNFRSF18	0.78578	0.6587347	1.1929	0.233	0.452893149	count	1
ZFY-AS1	0.78578	0.8712276	0.9019	0.367	0.452893149	count	1
FAM200B	0.3254119	0.106555	3.0539	0.00228	0.453051672	count	1
ECPAS	0.3643454	0.2427702	1.5008	0.134	0.453236269	count	1
TRIM7	0.6035605	0.3853511	1.5663	0.117	0.45350585	count	1
TGFBI	0.3208168	0.0745511	4.3033	1.75E-05	0.453602361	count	0.4139975
FXR2	0.4713114	0.4168014	1.1308	0.258	0.453855292	count	1
TMEM116	0.502832	0.4582958	1.0972	0.273	0.454058543	count	1
APOL4	1.0864724	0.6599989	1.6462	0.0999	0.454068062	count	1

KCTD20	0.3295862	0.1482079	2.2238	0.0263	0.454216449	count	1
GDAP1	0.5503103	0.4481838	1.2279	0.22	0.454455671	count	1
STARD10	0.3779645	0.2427296	1.5571	0.12	0.454572627	count	1
C8orf44	0.6582346	0.5144515	1.2795	0.201	0.454833275	count	1
AC132192.2	0.4791201	0.4839405	0.99	0.322	0.454849034	count	1
PITPNC1	0.371657	0.2197389	1.6914	0.0909	0.454886277	count	1
ITGAE	0.3316823	0.1404525	2.3615	0.0183	0.455003676	count	1
ARRDC3-AS1	0.7359726	0.6268399	1.1741	0.24	0.455259481	count	1
ADAT2	0.693514	0.5802895	1.1951	0.232	0.455310004	count	1
COPG1	0.3834258	0.2339545	1.6389	0.101	0.455755417	count	1
PIGL	0.4877636	0.3882509	1.2563	0.209	0.456026431	count	1
ADRB1	0.7910887	0.4407551	1.7948	0.0728	0.456278665	count	1
MED13	0.3370612	0.1684893	2.0005	0.0456	0.456389758	count	1
VAV2	0.5158168	0.4267908	1.2086	0.227	0.456934999	count	1
SELENOM	0.3417144	0.1856895	1.8402	0.0659	0.456970928	count	1
ANGEL2	0.3898372	0.2340528	1.6656	0.0959	0.457222152	count	1
BMP2	0.9577114	0.639241	1.4982	0.134	0.457674183	count	1
UHRF1BP1L	0.3456089	0.167271	2.0662	0.0389	0.45769671	count	1
FRZB	0.5402134	0.7539365	0.7165	0.474	0.457813493	count	1
BCL9L	0.517111	0.3801199	1.3604	0.174	0.458139787	count	1
PPIL2	0.3726688	0.2303659	1.6177	0.106	0.458183396	count	1
HEBP2	0.322453	0.0731319	4.4092	1.08E-05	0.458213813	count	0.255906
GPR141	0.5172189	0.3704575	1.3962	0.163	0.458240236	count	1
CD28	0.4532708	0.2957457	1.5326	0.125	0.458372275	count	1
SNX14	0.3746311	0.1984525	1.8878	0.0592	0.458581938	count	1
DNAL4	0.5076646	0.3798589	1.3365	0.182	0.458634605	count	1
CXorf56	0.429766	0.3662829	1.1733	0.241	0.458780815	count	1
DIDO1	0.3886351	0.2537439	1.5316	0.126	0.45901953	count	1
EIF4ENIF1	0.5181651	0.3219174	1.6096	0.108	0.459121204	count	1
MSL3	0.3354529	0.1206251	2.781	0.00546	0.459179911	count	1
RAB3IP	0.6106474	0.4794784	1.2736	0.203	0.459213075	count	1
SGPP1	0.4708002	0.2466766	1.9086	0.0564	0.45968738	count	1
CCNH	0.3272732	0.0831471	3.9361	8.51E-05	0.459749497	count	1
TM2D3	0.3408097	0.1416847	2.4054	0.0162	0.460025557	count	1
PLP2	0.322716	0.083352	3.8717	0.000111	0.460073541	count	1
PFKFB2	0.477893	0.5991754	0.7976	0.425	0.460453962	count	1
SLC48A1	0.368989	0.2783712	1.3255	0.185	0.460757977	count	1
HIST1H2BE	1.7320997	1.010385	1.7143	0.0866	0.460899428	count	1
AC099489.1	3.1811996	2.2894634	1.3895	0.1648	0.460911674	count	1
USP27X	0.9637973	0.6772854	1.423	0.155	0.460958752	count	1
ZNF623	0.9637973	0.6804307	1.4165	0.157	0.460958752	count	1
SUN1	0.4351565	0.2721077	1.5992	0.11	0.460975445	count	1
PRPF19	0.3742236	0.1950793	1.9183	0.0552	0.461109259	count	1
PIGB	0.8689982	0.5584645	1.556	0.12	0.461177337	count	1
MFSD14C	0.3736063	0.308013	1.213	0.225	0.461297892	count	1

DGAT2	0.4321413	0.3367429	1.2833	0.2	0.461391391	count	1
NELFB	0.3807113	0.1983966	1.9189	0.0551	0.461640112	count	1
HERC1	0.3635561	0.2095074	1.7353	0.0828	0.461735661	count	1
MAN1A2	0.3543188	0.159027	2.228	0.026	0.461834397	count	1
ZNF529	0.6385563	0.502118	1.2717	0.204	0.46189538	count	1
USP4	0.3483651	0.1909963	1.8239	0.0683	0.46193574	count	1
ZFYVE27	0.4237185	0.4274579	0.9913	0.322	0.462137263	count	1
MCM3AP	0.3927273	0.2810008	1.3976	0.162	0.462336983	count	1
ATP5MGL	0.871462	0.6917267	1.2598	0.208	0.462638089	count	1
SENP6	0.3341336	0.1380319	2.4207	0.0156	0.462749779	count	1
TXNRD2	0.4100586	0.2513876	1.6312	0.103	0.462989478	count	1
TBC1D30	0.4080528	0.3586368	1.1378	0.255	0.463007807	count	1
INHBA	0.3683713	0.349379	1.0544	0.292	0.463086363	count	1
RGP1	0.5124008	0.3563693	1.4378	0.151	0.463122403	count	1
TMEM220	0.3679136	0.2131676	1.7259	0.0845	0.463238473	count	1
RNF20	0.3737013	0.2329869	1.604	0.109	0.463269384	count	1
ZNF37A	0.4411037	0.2478591	1.7797	0.0753	0.463512942	count	1
MAPRE3	0.5037473	0.5365932	0.9388	0.348	0.463552886	count	1
AC013394.1	0.3593789	0.2476869	1.4509	0.147	0.463580559	count	1
ANP32A	0.3284703	0.0786576	4.176	3.07E-05	0.46364076	count	0.7247042
BCR	0.705215	0.6706567	1.0515	0.293	0.463684104	count	1
PCGF3	0.4049098	0.2458301	1.6471	0.0997	0.463753486	count	1
ZFPL1	0.3856204	0.2324025	1.6593	0.0972	0.463938222	count	1
STN1	0.3856204	0.2207173	1.7471	0.0807	0.463938222	count	1
CEP135	0.3659608	0.2484522	1.473	0.141	0.46418092	count	1
NPLOC4	0.3840222	0.2918642	1.3158	0.188	0.464508746	count	1
AC103691.1	0.5618584	0.4624295	1.215	0.224	0.464566044	count	1
RNF175	1.108716	0.6233494	1.7786	0.0754	0.464689949	count	1
ZNF763	1.108716	0.7529074	1.4726	0.141	0.464689949	count	1
CNFN	0.8043754	0.5486275	1.4662	0.143	0.464764512	count	1
ATP13A1	0.464465	0.3212492	1.4458	0.148	0.46478617	count	1
ZYG11B	0.4008281	0.2118589	1.892	0.0586	0.46495864	count	1
PRPF4	0.3621203	0.1927704	1.8785	0.0604	0.465105002	count	1
AL139384.1	0.3952389	0.2925966	1.3508	0.177	0.46535093	count	1
IWS1	0.3458657	0.1610273	2.1479	0.0318	0.465440961	count	1
MADD	0.3942932	0.3525044	1.1185	0.263	0.465829543	count	1
PRPF4B	0.3309903	0.0879613	3.7629	0.000172	0.465926118	count	1
POGZ	0.3732085	0.1883106	1.9819	0.0476	0.466097733	count	1
AC005837.1	1.3317222	0.8895824	1.497	0.135	0.466306564	count	1
INSR	0.39097	0.1895092	2.0631	0.0392	0.466348867	count	1
CCDC106	0.4305246	0.2659715	1.6187	0.106	0.466526578	count	1
MINDY3	0.4839783	0.3877426	1.2482	0.212	0.466560054	count	1
SMIM14	0.3401925	0.124711	2.7278	0.00642	0.46673093	count	1
DPP4	0.6741706	0.5450017	1.237	0.216	0.466766073	count	1
FAM193A	0.4114357	0.2952093	1.3937	0.164	0.466935425	count	1

MAD2L2	0.3482361	0.1582847	2.2001	0.0279	0.467053432	count	1
ZNF451	0.3662658	0.17219	2.1271	0.0335	0.467077427	count	1
AL450326.1	0.4916937	0.4217199	1.1659	0.244	0.46730234	count	1
RFX1	0.3983045	0.2584024	1.5414	0.123	0.467349814	count	1
HSPA1B	0.3268762	0.0842697	3.8789	0.000108	0.467393268	count	1
MBLAC1	0.9758215	0.7714671	1.2649	0.206	0.467455748	count	1
CACFD1	0.9758215	0.7969647	1.2244	0.221	0.467455748	count	1
LINC00865	0.9758215	0.9091252	1.0734	0.283	0.467455748	count	1
ABCB9	0.9758215	1.0023479	0.9735	0.33	0.467455748	count	1
DOK3	0.3535654	0.1753056	2.0169	0.0438	0.467731817	count	1
FPGT	0.4287535	0.2812588	1.5244	0.128	0.46777974	count	1
DOK1	0.3643068	0.1773045	2.0547	0.04	0.467943457	count	1
MIR646HG	1.75609	0.8503709	2.0651	0.039	0.467971775	count	1
KLHL36	0.3699786	0.2593552	1.4265	0.154	0.467994754	count	1
RBBP8	0.3830841	0.2451751	1.5625	0.118	0.46800243	count	1
6-Mar	0.352133	0.1550136	2.2716	0.0232	0.468320898	count	1
TBC1D1	0.3398056	0.1278361	2.6581	0.00791	0.468358293	count	1
CNOT3	0.4322936	0.2846542	1.5187	0.129	0.468497551	count	1
FAM91A1	0.3526722	0.1828152	1.9291	0.0538	0.46904333	count	1
CHKB	0.7127746	0.5747549	1.2401	0.215	0.469102871	count	1
FUK	0.8826469	0.5771502	1.5293	0.126	0.46927596	count	1
PAX8	0.4014382	0.2920599	1.3745	0.169	0.469351959	count	1
CCDC186	0.3462352	0.1458167	2.3745	0.0177	0.469501846	count	1
MED14	0.3951452	0.3111529	1.2699	0.204	0.469943628	count	1
FAM198B	0.3602284	0.1745022	2.0643	0.0391	0.470013335	count	1
PIGU	0.4945579	0.4324471	1.1436	0.253	0.470142017	count	1
KNSTRN	0.4752457	0.355436	1.3371	0.181	0.470251141	count	1
ZMYM3	0.4697997	0.3816656	1.2309	0.218	0.470323231	count	1
YTHDF1	0.3581959	0.2343695	1.5283	0.127	0.470333669	count	1
FAM234A	0.3898902	0.259495	1.5025	0.133	0.470462465	count	1
CHPF2	0.3933089	0.2522304	1.5593	0.119	0.470615994	count	1
ASNS	1.1211981	0.8009676	1.3998	0.162	0.470659923	count	1
ZNF696	1.1211981	0.9611637	1.1665	0.244	0.470659923	count	1
DDX47	1.1211981	0.9007285	1.2448	0.213	0.470659923	count	1
AL139353.1	1.1211981	0.8523089	1.3155	0.188	0.470659923	count	1
DPEP3	1.1211981	0.9611637	1.1665	0.244	0.470659923	count	1
AC015813.1	1.1211981	0.9007285	1.2448	0.213	0.470659923	count	1
CLDN5	1.1211981	1.042734	1.0752	0.282	0.470659923	count	1
TRAF3IP2-AS1	0.4602992	0.4543576	1.0131	0.311	0.470737528	count	1
NUDT15	0.3853915	0.246136	1.5658	0.118	0.47086561	count	1
RTL8C	0.3449048	0.1418576	2.4313	0.0151	0.470920589	count	1
ZFP91	0.3837588	0.1995679	1.9229	0.0546	0.470989876	count	1
KBTBD4	0.6798976	0.485636	1.4	0.162	0.471062029	count	1
ARHGEF11	0.4316925	0.300197	1.438	0.151	0.471074641	count	1
ZNF394	0.3499272	0.1565914	2.2347	0.0255	0.471131715	count	1

PURA	0.3435367	0.1422796	2.4145	0.0158	0.471138426	count	1
TMEM97	0.4608968	0.363957	1.2663	0.206	0.471369904	count	1
MXD3	0.625726	0.3882198	1.6118	0.107	0.471377926	count	1
TIMM44	0.4065007	0.2591517	1.5686	0.117	0.471677441	count	1
FAM102A	0.8154156	0.7369254	1.1065	0.269	0.471829012	count	1
DSEL	1.1238996	0.7487911	1.501	0.133	0.471952871	count	1
KCTD15	1.1238996	0.7007901	1.6038	0.109	0.471952871	count	1
PARP6	0.4531043	0.3818578	1.1866	0.236	0.472275052	count	1
ARHGEF12	0.6055593	0.3485538	1.7373	0.0825	0.472396824	count	1
NLK	0.5567919	0.4311042	1.2915	0.197	0.472671851	count	1
TLR6	0.414635	0.270013	1.5356	0.125	0.472937274	count	1
CYBB	0.329969	0.0456405	7.2297	6.44E-13	0.473025728	count	1.55E-08
APPBP2	0.4130738	0.243412	1.697	0.0898	0.473314012	count	1
TMEM91	0.3571259	0.1877878	1.9018	0.0573	0.473358031	count	1
ATAD2B	0.3712443	0.2190285	1.695	0.0902	0.47349937	count	1
C9orf72	0.3428747	0.0949613	3.6107	0.000312	0.473629996	count	1
PTRHD1	0.3412922	0.1006786	3.3899	0.00071	0.473703267	count	1
ZCCHC24	0.4153824	0.5713036	0.7271	0.467	0.473809263	count	1
RASSF5	0.3483491	0.1382284	2.5201	0.0118	0.474377355	count	1
SCAPER	0.3891825	0.2853661	1.3638	0.173	0.474438387	count	1
AC005280.2	0.3699253	0.1963216	1.8843	0.0596	0.474691922	count	1
SH3BP1	0.3569654	0.1767657	2.0194	0.0436	0.474795906	count	1
ZNF565	0.6300008	0.4980627	1.2649	0.206	0.474831977	count	1
CLPB	0.3935549	0.2155457	1.8259	0.068	0.47495997	count	1
DNAJC10	0.3639156	0.1828626	1.9901	0.0467	0.475270458	count	1
IZUMO4	0.8929953	0.6611426	1.3507	0.177	0.475426851	count	1
ZNF441	0.8929953	0.6554522	1.3624	0.173	0.475426851	count	1
SETD9	0.3939557	0.2342355	1.6819	0.0927	0.475451918	count	1
SPRED1	0.3524072	0.1401766	2.514	0.012	0.475598155	count	1
CPEB4	0.3461315	0.1095217	3.1604	0.00159	0.475695418	count	1
ADAMTSL4	0.3867139	0.2379691	1.6251	0.104	0.47571444	count	1
JUP	0.7663487	0.4044452	1.8948	0.0582	0.475921408	count	1
AC008040.5	1.7839572	0.7558647	2.3602	0.0183	0.476155257	count	1
SEC24A	0.4456637	0.3702137	1.2038	0.229	0.476265594	count	1
KLHL26	1.1329583	0.7053438	1.6062	0.108	0.47629042	count	1
ZBTB16	0.9922293	0.2485041	3.9928	6.72E-05	0.476336939	count	1
ATIC	0.3921682	0.2386881	1.643	0.101	0.476965951	count	1
ATXN2L	0.37301	0.2231317	1.6717	0.0947	0.476977604	count	1
MRPL46	0.3812495	0.2106507	1.8099	0.0704	0.477106787	count	1
ARAP1	0.3613865	0.1795461	2.0128	0.0442	0.477548476	count	1
TRAPPC5	0.6118758	0.4468988	1.3692	0.171	0.477660826	count	1
GPAM	0.8246293	0.6460713	1.2764	0.202	0.477733802	count	1
MBOAT1	0.6889688	0.4271554	1.6129	0.107	0.477874612	count	1
FAM8A1	0.528229	0.3801744	1.3894	0.165	0.478141992	count	1
CD3EAP	0.4410373	0.3671866	1.2011	0.23	0.478244676	count	1

BCLAF1	0.3409259	0.095361	3.5751	0.000357	0.478289518	count	1
RNF111	0.3668756	0.2113995	1.7355	0.0828	0.478356744	count	1
ADCK1	0.6897918	0.5710315	1.208	0.227	0.478493177	count	1
FCER2	3.381364	1.473398	2.2949	0.0218	0.478782397	count	1
AP5B1	0.3891933	0.2541168	1.5316	0.126	0.478810803	count	1
P2RX1	0.4896412	0.3017984	1.6224	0.105	0.478831672	count	1
ODR4	0.3878012	0.2218647	1.7479	0.0806	0.479085307	count	1
PPM1D	0.4027592	0.2295111	1.7549	0.0794	0.479168114	count	1
EBLN3P	0.3643404	0.1930504	1.8873	0.0592	0.479176607	count	1
CPSF2	0.3761369	0.2186141	1.7206	0.0855	0.479189302	count	1
AC239800.3	0.5512456	0.4232859	1.3023	0.193	0.47924068	count	1
FPR1	0.3363643	0.064762	5.1939	2.23E-07	0.479316557	count	0.005325686
ZNF83	0.4165127	0.3270405	1.2736	0.203	0.479481479	count	1
TPRG1-AS1	0.5954182	0.4348563	1.3692	0.171	0.47960088	count	1
ERMP1	0.5119007	0.6177594	0.8286	0.407	0.479630224	count	1
RAP2C-AS1	0.7718708	0.640259	1.2056	0.228	0.479688242	count	1
ALOX15B	0.7718708	0.5164394	1.4946	0.135	0.479688242	count	1
CXCL8	0.3332173	0.0756194	4.4065	1.10E-05	0.479889616	count	0.260623
PRR5	0.4368224	0.3515801	1.2425	0.214	0.479976085	count	1
KCTD3	0.5123378	0.4510064	1.136	0.256	0.480058353	count	1
TTF1	0.3698572	0.2107866	1.7547	0.0794	0.48010577	count	1
NBPF20	1.366269	1.170204	1.1675	0.243	0.480159885	count	1
FLYWCH1	0.5306929	0.3443795	1.541	0.123	0.480482956	count	1
MANBA	0.3582386	0.1403593	2.5523	0.0108	0.480772828	count	1
SLC9A7	0.4982121	0.3449869	1.4441	0.149	0.48086091	count	1
GGA3	0.4498914	0.3447614	1.3049	0.192	0.480920182	count	1
FILIP1L	0.3435034	0.1156022	2.9714	0.00299	0.481075945	count	1
NLRC4	0.4805395	0.2925436	1.6426	0.101	0.481481048	count	1
DLG4	0.5138351	0.4057936	1.2662	0.206	0.481525134	count	1
PYGL	0.3507509	0.1203923	2.9134	0.00361	0.481585364	count	1
ST6GALNAC3	0.9036593	0.5563254	1.6243	0.104	0.481774531	count	1
HMCES	0.3907791	0.2952221	1.3237	0.186	0.481817663	count	1
SERTAD2	0.3610548	0.1517436	2.3794	0.0174	0.481818952	count	1
ASH1L-AS1	0.5322796	0.3987408	1.3349	0.182	0.481990889	count	1
CASC3	0.3972376	0.243615	1.6306	0.103	0.482016111	count	1
ZNF292	0.3789676	0.2357968	1.6072	0.108	0.48219838	count	1
UBA3	0.374491	0.1587547	2.3589	0.0184	0.48224363	count	1
NUP107	0.4079303	0.306835	1.3295	0.184	0.482254875	count	1
KANTR	0.6647679	0.6040204	1.1006	0.271	0.482354275	count	1
CPTP	0.4292391	0.4105579	1.0455	0.296	0.48263331	count	1
ZBTB11	0.369325	0.1759382	2.0992	0.0359	0.482801392	count	1
TNK2	0.4422286	0.273158	1.6189	0.106	0.482894346	count	1
PUS7L	0.3763685	0.2390819	1.5742	0.116	0.483051406	count	1
DEDD2	0.360802	0.1467515	2.4586	0.014	0.48313615	count	1
SRP14-AS1	0.5686558	0.3789541	1.5006	0.134	0.483327013	count	1

FLAD1	0.4026197	0.3069435	1.3117	0.19	0.483375247	count	1
PCGF5	0.3558172	0.1255912	2.8331	0.00465	0.483474699	count	1
ZNF780B	0.6191645	0.5990546	1.0336	0.301	0.483741338	count	1
CDR2	0.5563465	0.6238762	0.8918	0.373	0.483918625	count	1
PCDH12	0.7334539	0.442228	1.6585	0.0973	0.483959352	count	1
SETD6	0.5693641	0.5248683	1.0848	0.278	0.483963714	count	1
RPS13	0.3358524	0.0257417	13.047	1.15E-37	0.484106305	count	2.79E-33
TGFB1	0.3401187	0.0639223	5.3208	1.13E-07	0.484140442	count	0.002700587
C15orf61	0.365411	0.1545786	2.3639	0.0182	0.484426703	count	1
CHP1	0.3481155	0.0979711	3.5532	0.000388	0.484452546	count	1
IL17RE	0.7343328	0.5444714	1.3487	0.178	0.484591813	count	1
GSR	0.3767535	0.1822663	2.067	0.0388	0.484652625	count	1
AC026803.2	0.8355129	0.6720962	1.2431	0.214	0.4847192	count	1
GDAP2	0.3920563	0.2045906	1.9163	0.0554	0.485400174	count	1
MMS19	0.5711949	0.4551288	1.255	0.21	0.485609774	count	1
AC108673.3	0.9103129	0.53276	1.7087	0.0876	0.485739659	count	1
CKAP2	0.3715368	0.173261	2.1444	0.0321	0.486115828	count	1
ASB13	0.6442343	0.437174	1.4736	0.141	0.486349243	count	1
ARRB1	0.3656266	0.1707894	2.1408	0.0324	0.486403784	count	1
PPM1N	0.4853198	0.2957075	1.6412	0.101	0.486451922	count	1
PCOLCE	0.603529	0.6339348	0.952	0.341	0.486557373	count	1
HSPA6	0.3575444	0.2039396	1.7532	0.0797	0.486691654	count	1
SYMPK	0.4021905	0.2967591	1.3553	0.175	0.486862353	count	1
ZNF747	0.6229248	0.5540277	1.1244	0.261	0.486880924	count	1
ADK	0.3588884	0.1570713	2.2849	0.0224	0.486914711	count	1
FGD3	0.4589177	0.2481788	1.8491	0.0646	0.486942952	count	1
AC092490.1	0.7825938	0.6552482	1.1943	0.232	0.487011834	count	1
ABCC6	0.8390924	0.8575423	0.9785	0.328	0.487019009	count	1
TLK1	0.3587852	0.1639552	2.1883	0.0287	0.487232045	count	1
ZNF667-AS1	0.6043429	0.6524433	0.9263	0.354	0.487255911	count	1
SNHG7	0.3503621	0.10431	3.3589	0.000795	0.48726435	count	1
SF3B3	0.4069747	0.2593476	1.5692	0.117	0.487266953	count	1
ZNF524	0.3536462	0.1246574	2.8369	0.00459	0.487430401	count	1
S1PR2	0.4864886	0.3920274	1.241	0.215	0.487667735	count	1
FANCL	0.4868616	0.389719	1.2493	0.212	0.488055776	count	1
AC110769.2	0.5886732	0.4373344	1.346	0.178	0.488110341	count	1
CSF2RB	0.4241024	0.1869186	2.2689	0.0234	0.488414113	count	1
SOCS6	0.4092565	0.2701555	1.5149	0.13	0.488568381	count	1
PITPNA-AS1	0.4346025	0.3043268	1.4281	0.153	0.488814154	count	1
STK36	0.9155404	0.6239541	1.4673	0.142	0.488857365	count	1
CNEP1R1	0.390523	0.1910575	2.044	0.0411	0.488870741	count	1
CTSO	0.4324421	0.2401801	1.8005	0.0719	0.488883906	count	1
CSF1R	0.3452147	0.0693473	4.9781	6.87E-07	0.488894233	count	0.016371897
HELQ	0.413482	0.2492017	1.6592	0.0972	0.48894642	count	1
SMAD5	0.3899873	0.2084776	1.8706	0.0615	0.489019338	count	1

TIRAP	0.5750949	0.6742037	0.853	0.394	0.489117663	count	1
CELF1	0.3636919	0.166771	2.1808	0.0293	0.489387436	count	1
SLC25A20	0.47409	0.5133814	0.9235	0.356	0.49023258	count	1
IPPK	0.787613	0.5564973	1.4153	0.157	0.490443883	count	1
SLC4A8	0.5518791	0.4979327	1.1083	0.268	0.490588259	count	1
ZNF185	0.5011975	0.3227873	1.5527	0.121	0.490595892	count	1
C2CD2L	0.4660805	0.2712631	1.7182	0.0859	0.49061758	count	1
CD7	0.4701731	0.402208	1.169	0.243	0.490663469	count	1
AC087645.2	0.84512	0.5104106	1.6558	0.0979	0.490894367	count	1
IFI44L	0.3590554	0.1603723	2.2389	0.0253	0.490950223	count	1
FAM126B	0.484426	0.2489437	1.9459	0.0518	0.491052213	count	1
SCNM1	0.3555354	0.1213303	2.9303	0.00342	0.491111652	count	1
GPR155	0.389847	0.1636305	2.3825	0.0173	0.491218168	count	1
PDE3B	0.4346321	0.2269642	1.915	0.0556	0.491420124	count	1
WDR26	0.3713835	0.1790918	2.0737	0.0382	0.491497122	count	1
HIPK3	0.3611637	0.1198892	3.0125	0.00262	0.491504304	count	1
ZNF273	0.6507284	0.4873097	1.3353	0.182	0.491612327	count	1
GSTM4	0.5778738	0.3129484	1.8465	0.0649	0.49161834	count	1
FAM76B	0.3885274	0.2090585	1.8585	0.0632	0.49175527	count	1
ANAPC4	0.4120263	0.2573013	1.6013	0.109	0.491936403	count	1
SHLD2	0.3972699	0.2617763	1.5176	0.129	0.491950722	count	1
ARID3B	0.4907853	0.3442663	1.4256	0.154	0.492138673	count	1
COMTD1	0.3862273	0.1879418	2.055	0.04	0.492197768	count	1
INPP1	0.4045133	0.2750774	1.4705	0.142	0.492231114	count	1
TMEM131	0.3757258	0.2006807	1.8723	0.0613	0.492429521	count	1
AMBRA1	0.6778979	0.5755167	1.1779	0.239	0.492634103	count	1
ZNF232	0.5939216	0.5311191	1.1182	0.264	0.492729424	count	1
KIAA1143	0.3718653	0.2651572	1.4024	0.161	0.492751938	count	1
AP001269.4	0.5101532	0.4602501	1.1084	0.268	0.492877799	count	1
SLFN11	0.3761407	0.1836594	2.048	0.0407	0.492978246	count	1
YTHDF3-AS1	0.4435258	0.3149233	1.4084	0.159	0.493551936	count	1
ARL14EP	0.372261	0.1689921	2.2028	0.0277	0.493580484	count	1
SETX	0.3650168	0.1376153	2.6524	0.00804	0.493619211	count	1
C9orf40	0.446251	0.4949912	0.9015	0.367	0.493703741	count	1
ARF5	0.3482461	0.0692553	5.0284	5.30E-07	0.493717948	count	0.0126352
LINC00909	0.4148456	0.2903434	1.4288	0.153	0.493821331	count	1
TP53RK	0.3901518	0.2136151	1.8264	0.0679	0.493837155	count	1
ANKHD1	0.4205084	0.2274424	1.8489	0.0646	0.493938997	count	1
EGLN1	0.3981146	0.2147276	1.854	0.0639	0.493982382	count	1
ECD	0.3952769	0.2203317	1.794	0.0729	0.494045874	count	1
SPHK2	0.6538835	0.3669443	1.782	0.0749	0.494171211	count	1
MYC	0.4985784	0.3046308	1.6367	0.102	0.494259764	count	1
EIF3H	0.3458673	0.0461007	7.5024	8.71E-14	0.494432074	count	2.10E-09
MED1	0.4026128	0.321972	1.2505	0.211	0.494495985	count	1
WAS	0.3518941	0.0789628	4.4565	8.71E-06	0.494538004	count	0.2065141

POGLUT1	0.41296	0.2442286	1.6909	0.091	0.494564656	count	1
LINC01252	1.0263421	0.7267568	1.4122	0.158	0.494854942	count	1
CBWD3	0.5053946	0.4277344	1.1816	0.237	0.494872549	count	1
BDP1	0.3561861	0.1315425	2.7078	0.00682	0.494932658	count	1
MAPKBP1	0.7487256	0.5520095	1.3564	0.175	0.494961347	count	1
ZZEF1	0.3734897	0.2474725	1.5092	0.131	0.495222642	count	1
NIN	0.3701611	0.1419014	2.6086	0.00915	0.495757006	count	1
ZNF555	0.7128824	0.5714194	1.2476	0.212	0.495880166	count	1
NAT1	0.6144251	0.5063534	1.2134	0.225	0.495915815	count	1
ATRX	0.3540877	0.0900581	3.9318	8.67E-05	0.496049246	count	1
GTF3C3	0.4485664	0.2743808	1.6348	0.102	0.496334316	count	1
C5orf15	0.3743482	0.1556449	2.4051	0.0162	0.496370069	count	1
RRM2B	0.3875972	0.2058297	1.8831	0.0598	0.497044187	count	1
GYPC	0.3496993	0.0793953	4.4045	1.11E-05	0.497179259	count	0.262959
RBM33	0.3780601	0.1545235	2.4466	0.0145	0.497380327	count	1
KLHL28	0.4206547	0.2419289	1.7388	0.0822	0.497595767	count	1
AL359711.2	0.9302852	0.5454641	1.7055	0.0882	0.497662438	count	1
POLD3	0.3818952	0.2021124	1.8895	0.0589	0.498130724	count	1
PREB	0.3978405	0.214223	1.8571	0.0634	0.49815759	count	1
ANXA6	0.3798059	0.1647539	2.3053	0.0212	0.4982118	count	1
ATRN	0.4226133	0.2315799	1.8249	0.0681	0.498237561	count	1
TFE3	0.4343705	0.2286305	1.8999	0.0576	0.498284313	count	1
DHTKD1	0.6589919	0.391187	1.6846	0.0922	0.498316813	count	1
FBXL3	0.4167289	0.1938942	2.1493	0.0317	0.499161497	count	1
CAST	0.3483655	0.0441348	7.8932	4.40E-15	0.499162876	count	1.06E-10
FKBP11	0.5164034	0.3587521	1.4394	0.15	0.499174644	count	1
TRAF3IP1	0.4874985	0.3172446	1.5367	0.125	0.499562778	count	1
AL445686.2	1.0360274	0.7432668	1.3939	0.163	0.50012482	count	1
LINC01816	1.0360274	0.8663135	1.1959	0.232	0.50012482	count	1
AC064801.1	1.0360274	0.9984929	1.0376	0.3	0.50012482	count	1
SH3BGR	1.0360274	0.7241734	1.4306	0.153	0.50012482	count	1
PHF21A	0.3981013	0.2057527	1.9349	0.0531	0.500158422	count	1
PIAS1	0.3728795	0.1695246	2.1996	0.0279	0.500335746	count	1
DYM	0.3907608	0.2039517	1.9159	0.0555	0.500550413	count	1
AFMID	0.4218784	0.231609	1.8215	0.0687	0.500737589	count	1
GVQW3	1.4181491	0.845284	1.6777	0.0935	0.500962129	count	1
LRRC32	1.4181491	0.8668396	1.636	0.102	0.500962129	count	1
AC135279.3	1.4181491	0.8668396	1.636	0.102	0.500962129	count	1
ANKFY1	0.4475174	0.2636601	1.6973	0.0898	0.500964218	count	1
ACTR8	0.4250207	0.3140527	1.3533	0.176	0.501132909	count	1
TBC1D24	0.6035605	0.3904512	1.5458	0.122	0.501221568	count	1
DAAM2	3.6827805	2.2409033	1.6434	0.1004	0.501237385	count	1
STX3	0.3867107	0.2055587	1.8813	0.0601	0.501245555	count	1
TRPM2	0.4149572	0.2425525	1.7108	0.0872	0.501248002	count	1
CKAP5	0.4224611	0.2366677	1.785	0.0744	0.501442643	count	1

TMEM250	0.425444	0.2708494	1.5708	0.116	0.50164205	count	1
DCBLD2	0.6631626	0.4393553	1.5094	0.131	0.501703745	count	1
ARL5B	0.3754247	0.1573011	2.3867	0.0171	0.502380697	count	1
NSUN6	0.4291585	0.2319989	1.8498	0.0645	0.502449994	count	1
HYPK	0.6418342	0.4930083	1.3019	0.193	0.502695503	count	1
RBM43	0.495624	0.3106336	1.5955	0.111	0.502826531	count	1
PTPA	0.4045034	0.232581	1.7392	0.0821	0.50298819	count	1
ERVK3-1	0.4818412	0.3611109	1.3343	0.182	0.503252836	count	1
NDOR1	0.8643347	0.5104324	1.6933	0.0905	0.503269768	count	1
SMG7	0.4214515	0.1999617	2.1077	0.0352	0.503402091	count	1
CEBPG	0.373186	0.1536235	2.4292	0.0152	0.503439711	count	1
TCN2	0.3945205	0.19167	2.0583	0.0397	0.503544675	count	1
DENND4C	0.3961953	0.2030051	1.9517	0.0511	0.503708583	count	1
TAF9B	0.5552514	0.4652917	1.1933	0.233	0.503858095	count	1
VAMP2	0.3598114	0.0955469	3.7658	0.00017	0.503866939	count	1
ZNF460	0.8072474	0.4921789	1.6402	0.101	0.50389368	count	1
CENPQ	0.4676153	0.3182085	1.4695	0.142	0.504271458	count	1
MBD2	0.3563081	0.0772015	4.6153	4.13E-06	0.504363151	count	0.0980875
ABHD18	0.4532007	0.4389896	1.0324	0.302	0.504604566	count	1
MROH6	0.4713788	0.3997127	1.1793	0.238	0.504607873	count	1
DAPK3	0.4831036	0.3868384	1.2489	0.212	0.504615816	count	1
ZSCAN30	0.693208	0.8427524	0.8226	0.411	0.504646461	count	1
CTDSPL2	0.3964253	0.1884503	2.1036	0.0355	0.504684713	count	1
SLC1A4	0.4190078	0.3299143	1.2701	0.204	0.504839198	count	1
DAZAP2	0.3553519	0.0674389	5.2692	1.49E-07	0.505021194	count	0.003560504
USP51	0.5222737	0.3267425	1.5984	0.11	0.505093023	count	1
ATXN3	0.3802437	0.1648611	2.3064	0.0212	0.50514357	count	1
TRAFD1	0.5033992	0.2592878	1.9415	0.0523	0.505276657	count	1
ST7L	0.4467043	0.3545684	1.2599	0.208	0.505409255	count	1
C11orf74	0.4164399	0.3137194	1.3274	0.184	0.505719035	count	1
PTK2	0.430363	0.2898367	1.4848	0.138	0.505754024	count	1
NSUN2	0.4494074	0.2684524	1.6741	0.0942	0.505890426	count	1
DNAJB2	0.4210671	0.2215951	1.9002	0.0575	0.505930396	count	1
EIF2B5	0.4140333	0.2368639	1.748	0.0806	0.506440989	count	1
SCIMP	0.373029	0.1122243	3.324	0.000901	0.50668718	count	1
PYGB	0.4604982	0.3671515	1.2542	0.21	0.506725116	count	1
SATB2	1.4332175	0.7080976	2.024	0.0431	0.507000635	count	1
SPATS2L	0.3738864	0.1422545	2.6283	0.00863	0.507220984	count	1
PDS5B	0.3916807	0.1827183	2.1436	0.0322	0.507255645	count	1
RASL11A	0.4488035	0.3748426	1.1973	0.231	0.507843234	count	1
SERPINB8	0.3734117	0.1387874	2.6905	0.00718	0.508119207	count	1
PDE4DIP	0.3888263	0.184872	2.1032	0.0355	0.508119765	count	1
RARA	0.3691202	0.133672	2.7614	0.0058	0.508211147	count	1
STX16	0.3922298	0.2401125	1.6335	0.102	0.508473858	count	1
ATXN1L	0.4750174	0.3403368	1.3957	0.163	0.50862403	count	1

EYA3	0.4030709	0.2297027	1.7548	0.0794	0.508885202	count	1
BMS1	0.3905141	0.2010868	1.942	0.0522	0.50904089	count	1
MAP3K12	0.3955947	0.2547896	1.5526	0.121	0.509162469	count	1
ZFAND2B	0.3840154	0.1699372	2.2597	0.0239	0.509897798	count	1
SLC25A32	0.423349	0.2626601	1.6118	0.107	0.510164043	count	1
LPP	0.3769221	0.142461	2.6458	0.0082	0.510174933	count	1
ZNF18	0.8753228	0.5033916	1.7389	0.0822	0.510361079	count	1
RIC8A	0.3855167	0.1950358	1.9766	0.0482	0.510676473	count	1
BAG4	0.3820394	0.1805823	2.1156	0.0345	0.510800992	count	1
HSF1	0.391912	0.1736367	2.2571	0.0241	0.510881061	count	1
RASSF1-AS1	0.5088455	0.4597156	1.1069	0.268	0.51095497	count	1
FANCE	1.443179	0.7157604	2.0163	0.0439	0.510991376	count	1
TMEM63A	0.4849596	0.3639572	1.3325	0.183	0.511151885	count	1
NKG7	0.4349255	0.3241207	1.3419	0.18	0.511227045	count	1
DDX31	0.8180648	0.5984478	1.367	0.172	0.511319603	count	1
PEX2	0.3888625	0.150448	2.5847	0.0098	0.511348768	count	1
GPSM2	0.5742845	0.4276958	1.3427	0.179	0.511580402	count	1
MBIP	0.4322963	0.3076759	1.405	0.16	0.511643455	count	1
FEZ1	1.4450717	0.5598171	2.5813	0.0099	0.511749234	count	1
RYBP	0.3822058	0.1711143	2.2336	0.0256	0.511764207	count	1
C5AR2	0.3944756	0.1930732	2.0431	0.0411	0.511909278	count	1
OAT	0.3882315	0.1885026	2.0596	0.0395	0.511960575	count	1
CHIC2	0.3732731	0.1303189	2.8643	0.00421	0.512228453	count	1
EPOR	0.3989289	0.2507677	1.5908	0.112	0.512340906	count	1
FAAP100	0.5756498	0.386235	1.4904	0.136	0.512861591	count	1
LSMEM1	1.4485997	0.657377	2.2036	0.0276	0.513162077	count	1
WDR3	0.4534268	0.2905279	1.5607	0.119	0.513205345	count	1
KIAA1191	0.4295869	0.2429414	1.7683	0.0771	0.51330467	count	1
CWC25	0.3757102	0.1435051	2.6181	0.0089	0.513334873	count	1
GPHN	0.6348254	0.5263172	1.2062	0.228	0.513476412	count	1
GTF2H2C	0.6549299	0.3734791	1.7536	0.0796	0.513673123	count	1
CARD9	0.4132508	0.1832594	2.255	0.0242	0.514026359	count	1
AP1M1	0.4458635	0.2154637	2.0693	0.0386	0.514055676	count	1
CPD	0.3849989	0.1791681	2.1488	0.0317	0.514275493	count	1
ERICH1	0.366302	0.098163	3.7316	0.000195	0.514376637	count	1
TCF3	0.4673854	0.3037268	1.5388	0.124	0.514517169	count	1
MPPE1	0.4153912	0.1871166	2.22	0.0265	0.514734173	count	1
SH2D3C	0.5395951	0.3758919	1.4355	0.151	0.514928465	count	1
GLUD1	0.371001	0.1226392	3.0251	0.00251	0.514962782	count	1
LMTK2	0.679679	0.4759962	1.4279	0.153	0.51513635	count	1
DAB2	0.3609699	0.084695	4.262	2.10E-05	0.515209924	count	0.496566
PSTPIP2	0.4016037	0.1577999	2.545	0.011	0.51521484	count	1
GATAD1	0.3858953	0.1419732	2.7181	0.00661	0.515220598	count	1
ITSN2	0.3702673	0.1101099	3.3627	0.000784	0.515330615	count	1
C1GALT1	0.3907702	0.1683566	2.3211	0.0204	0.515337677	count	1

GCDH	0.4744804	0.3364517	1.4102	0.159	0.51560198	count	1
MED6	0.4049138	0.2005921	2.0186	0.0436	0.515625081	count	1
CCDC84	0.4814778	0.3280153	1.4679	0.142	0.515758263	count	1
TROVE2	0.3733554	0.1243027	3.0036	0.0027	0.515911497	count	1
PABPC1	0.3592362	0.0347358	10.342	1.44E-24	0.515938293	count	3.49E-20
PRTFDC1	1.216067	0.6202923	1.9605	0.0501	0.516211064	count	1
PIP4P2	0.3761551	0.1431155	2.6283	0.00863	0.516419414	count	1
ZNF431	0.4821852	0.2986093	1.6148	0.106	0.516539702	count	1
AUH	0.4859793	0.3400121	1.4293	0.153	0.516594252	count	1
AP2A1	0.3980519	0.187391	2.1242	0.0338	0.516598511	count	1
NKIRAS2	0.381863	0.1437759	2.656	0.00796	0.516724807	count	1
ATAD3C	1.9243683	1.1474879	1.677	0.0937	0.516772594	count	1
AL031432.3	1.9243683	1.1474879	1.677	0.0937	0.516772594	count	1
LINC00853	1.9243683	1.654342	1.1632	0.245	0.516772594	count	1
AMIGO1	1.9243683	1.1474879	1.677	0.0937	0.516772594	count	1
PLSCR2	1.9243683	1.1474879	1.677	0.0937	0.516772594	count	1
TMEM44	1.9243683	1.576046	1.221	0.222	0.516772594	count	1
MPIG6B	1.9243683	1.1474879	1.677	0.0937	0.516772594	count	1
AL133338.1	1.9243683	1.1474879	1.677	0.0937	0.516772594	count	1
BX322234.1	1.9243683	1.1474879	1.677	0.0937	0.516772594	count	1
HOXA-AS2	1.9243683	1.1474879	1.677	0.0937	0.516772594	count	1
AC067930.1	1.9243683	1.1474879	1.677	0.0937	0.516772594	count	1
FAM225A	1.9243683	1.1474879	1.677	0.0937	0.516772594	count	1
LGR4	1.9243683	1.1474879	1.677	0.0937	0.516772594	count	1
BDNF-AS	1.9243683	1.1474879	1.677	0.0937	0.516772594	count	1
NRXN2	1.9243683	1.1474879	1.677	0.0937	0.516772594	count	1
AP000866.1	1.9243683	1.1474879	1.677	0.0937	0.516772594	count	1
AC068987.5	1.9243683	1.1474879	1.677	0.0937	0.516772594	count	1
AC126614.1	1.9243683	1.1474879	1.677	0.0937	0.516772594	count	1
MEIS2	1.9243683	1.654342	1.1632	0.245	0.516772594	count	1
AC079322.1	1.9243683	1.1474879	1.677	0.0937	0.516772594	count	1
NPIPA1	1.9243683	1.1474879	1.677	0.0937	0.516772594	count	1
AC135782.1	1.9243683	1.1474879	1.677	0.0937	0.516772594	count	1
AC008946.1	1.9243683	1.1474879	1.677	0.0937	0.516772594	count	1
SPTBN4	1.9243683	1.1474879	1.677	0.0937	0.516772594	count	1
AC010247.2	1.9243683	1.654342	1.1632	0.245	0.516772594	count	1
AC011468.5	1.9243683	1.1474879	1.677	0.0937	0.516772594	count	1
OLIG2	1.9243683	1.1474879	1.677	0.0937	0.516772594	count	1
AC004854.2	0.5148753	0.337321	1.5264	0.127	0.517245598	count	1
ZNF780A	0.4200446	0.251307	1.6714	0.0948	0.517377462	count	1
ARIH2OS	0.682621	0.540044	1.264	0.206	0.517532331	count	1
LSM11	0.8273478	0.5909658	1.4	0.162	0.517700899	count	1
NLN	0.4308993	0.2161163	1.9938	0.0463	0.517963257	count	1
POMT2	0.7805606	0.6473182	1.2058	0.228	0.517974824	count	1
PXMP4	0.6401288	0.4082512	1.568	0.117	0.518049735	count	1

AL391069.3	0.5352059	0.3465868	1.5442	0.123	0.518145578	count	1
PARP2	0.5352059	0.3369071	1.5886	0.112	0.518145578	count	1
EXOC6	0.4091749	0.1979339	2.0672	0.0388	0.51823027	count	1
CLCN7	0.4246434	0.2279446	1.8629	0.0626	0.518408234	count	1
IFI44	0.3771152	0.1289248	2.9251	0.00348	0.518478012	count	1
WWTR1	0.8287373	0.6405519	1.2938	0.196	0.518656742	count	1
HACE1	0.8287373	0.5652389	1.4662	0.143	0.518656742	count	1
B4GALT4	0.4427742	0.3035089	1.4589	0.145	0.5187323	count	1
AKNA	0.3957782	0.2368965	1.6707	0.0949	0.518959416	count	1
CUL9	0.7435944	0.5213811	1.4262	0.154	0.519098222	count	1
GLE1	0.5112356	0.3667669	1.3939	0.163	0.519265627	count	1
IL27	0.8891662	0.5405992	1.6448	0.1	0.519309238	count	1
AC093484.2	0.8891662	0.6049134	1.4699	0.142	0.519309238	count	1
SIK1	0.8891754	0.5031566	1.7672	0.0773	0.519315198	count	1
LPGAT1	0.3749218	0.1333889	2.8107	0.00498	0.519318358	count	1
HACD2	0.4588146	0.2220387	2.0664	0.0389	0.519456679	count	1
CC2D2A	1.2236579	1.0256861	1.193	0.233	0.519866384	count	1
SUPT20H	0.447356	0.2149407	2.0813	0.0375	0.520156235	count	1
ARIH1	0.3859515	0.1521432	2.5368	0.0112	0.520169332	count	1
PPP3CB	0.4262125	0.1898634	2.2448	0.0249	0.520356052	count	1
NHLRC2	0.4976921	0.3397467	1.4649	0.143	0.520379485	count	1
THAP6	0.4726366	0.2892579	1.634	0.102	0.520461488	count	1
GSK3B	0.3972578	0.1813475	2.1906	0.0286	0.520917747	count	1
SUCNR1	0.5310958	0.2648331	2.0054	0.045	0.521106509	count	1
MIR4458HG	0.5734991	0.3904172	1.4689	0.142	0.521274598	count	1
DIRC2	0.4088175	0.1599542	2.5558	0.0107	0.521343531	count	1
DDX51	0.5544917	0.3813101	1.4542	0.146	0.521458784	count	1
RNF44	0.4707033	0.2426311	1.94	0.0525	0.521511998	count	1
ZNF836	0.6111662	0.4048186	1.5097	0.131	0.521651859	count	1
KIAA0232	0.3903887	0.159928	2.441	0.0147	0.521790038	count	1
SKIV2L	0.5742193	0.3721828	1.5428	0.123	0.521962805	count	1
AKAP8L	0.396029	0.1929935	2.052	0.0403	0.521973387	count	1
CTSF	0.4237404	0.2325604	1.8221	0.0686	0.522002176	count	1
PHIP	0.3810664	0.1296638	2.9389	0.00332	0.522058742	count	1
AC026202.2	1.4711102	0.8947039	1.6442	0.1	0.522172375	count	1
H1FX-AS1	1.4711102	0.8947039	1.6442	0.1	0.522172375	count	1
FAM149A	1.4711102	1.0902625	1.3493	0.177	0.522172375	count	1
UNC5CL	1.4711102	1.0278079	1.4313	0.152	0.522172375	count	1
AL136131.3	1.4711102	0.8947039	1.6442	0.1	0.522172375	count	1
TMEM67	1.4711102	0.8947039	1.6442	0.1	0.522172375	count	1
INCA1	1.4711102	0.8947039	1.6442	0.1	0.522172375	count	1
PRSS57	1.4711102	0.8947039	1.6442	0.1	0.522172375	count	1
TPK1	0.4491451	0.221894	2.0241	0.0431	0.522282613	count	1
SYNE1	0.4300697	0.1921119	2.2386	0.0253	0.522560796	count	1
TMEM101	0.4178011	0.213808	1.9541	0.0508	0.522605921	count	1

API5	0.4110438	0.1885564	2.18	0.0294	0.522825306	count	1
ARFRP1	0.4020899	0.2214696	1.8156	0.0696	0.522873303	count	1
LILRB3	0.3738939	0.1081125	3.4584	0.000553	0.522951472	count	1
SMN1	0.6281667	0.4910986	1.2791	0.201	0.522951555	count	1
EBP	0.3999421	0.1763268	2.2682	0.0234	0.523218862	count	1
PSME2	0.3655996	0.0572564	6.3853	2.04E-10	0.523289707	count	4.91E-06
ZNF791	0.406554	0.1811269	2.2446	0.0249	0.52342832	count	1
CPSF4	0.4725211	0.2721407	1.7363	0.0826	0.523581667	count	1
FAM168B	0.4187666	0.2423612	1.7279	0.0841	0.523830908	count	1
ATP8B4	0.450474	0.2147523	2.0976	0.036	0.523862264	count	1
SLC46A3	0.4091807	0.2022049	2.0236	0.0431	0.524426547	count	1
PAN2	0.557642	0.4328641	1.2883	0.198	0.524561344	count	1
NSD1	0.3829304	0.1301451	2.9423	0.00329	0.524861153	count	1
ALKBH1	0.5496655	0.3851869	1.427	0.154	0.52497625	count	1
AC017083.1	0.6918166	0.6043939	1.1446	0.252	0.525027577	count	1
SH3GLB2	0.4093003	0.2516446	1.6265	0.104	0.525204462	count	1
SAP30	0.3765272	0.0754152	4.9927	6.37E-07	0.52535313	count	0.015182895
GAB2	0.447083	0.21165	2.1124	0.0348	0.525819501	count	1
DOCK8	0.3718373	0.0837864	4.4379	9.48E-06	0.526021398	count	0.22474236
AP001056.1	0.9777295	0.5536051	1.7661	0.0775	0.526099322	count	1
SNX29	0.3816557	0.1190259	3.2065	0.00136	0.526116533	count	1
SRGAP2C	0.4344275	0.218832	1.9852	0.0472	0.526590573	count	1
CIZ1	0.4447043	0.3319915	1.3395	0.181	0.526628519	count	1
LINC01954	0.7212415	0.4952191	1.4564	0.145	0.526709931	count	1
FAM86C1	0.8407802	0.6072885	1.3845	0.166	0.526948387	count	1
ZBTB49	0.5601913	0.5030944	1.1135	0.266	0.52707285	count	1
FTSJ3	0.4609611	0.2878984	1.6011	0.109	0.527087795	count	1
LMO2	0.3746532	0.0788921	4.7489	2.16E-06	0.527240359	count	0.0513756
AC098850.3	0.5138938	0.502735	1.0222	0.307	0.527617124	count	1
EIF2B3	0.4148376	0.2348113	1.7667	0.0774	0.527711779	count	1
ANKRD13D	0.385574	0.142646	2.703	0.00692	0.527901539	count	1
BICD2	0.3989215	0.152673	2.6129	0.00903	0.527921904	count	1
PHF14	0.3900016	0.165431	2.3575	0.0185	0.528700993	count	1
TTTY14	0.6190777	0.7617127	0.8127	0.416	0.528808499	count	1
SRSF5	0.3718909	0.0604002	6.1571	8.63E-10	0.528858953	count	2.07E-05
RRP9	0.4864237	0.3317408	1.4663	0.143	0.528971422	count	1
LMO7	0.9044362	0.7620394	1.1869	0.235	0.529197191	count	1
TAF6L	0.5106299	0.3482098	1.4664	0.143	0.529387769	count	1
MIS18BP1	0.3773242	0.0785729	4.8022	1.66E-06	0.529730312	count	0.0394997
LTBP1	0.983973	0.7500073	1.312	0.19	0.52985268	count	1
LRRC8A	0.5110809	0.3483944	1.467	0.143	0.529871966	count	1
CPAMD8	0.757843	0.567882	1.3345	0.182	0.529903993	count	1
PKN2	0.3829029	0.1208486	3.1685	0.00155	0.529962047	count	1
BTAF1	0.4069463	0.2170505	1.8749	0.0609	0.530211294	count	1
ZSWIM8	0.4323272	0.2663936	1.6229	0.105	0.530411426	count	1

GRK5	0.6985362	0.4131271	1.6909	0.091	0.530510585	count	1
ARHGEF1	0.3971309	0.1235902	3.2133	0.00133	0.530604941	count	1
ANKRD26	0.4759575	0.2686729	1.7715	0.0766	0.530638619	count	1
PTAR1	0.4273396	0.2424038	1.7629	0.078	0.530804687	count	1
EID3	0.5640283	0.4463674	1.2636	0.206	0.530854388	count	1
FASTKD5	0.5224809	0.3482987	1.5001	0.134	0.531123899	count	1
GPSM3	0.3718625	0.0485777	7.655	2.76E-14	0.531279434	count	6.67E-10
FAR2	0.4667226	0.2795802	1.6694	0.0952	0.531284351	count	1
SMC5	0.3934015	0.1471749	2.673	0.00757	0.531291261	count	1
CLCN6	0.5284745	0.3491883	1.5134	0.13	0.531448086	count	1
STAT1	0.3799836	0.086257	4.4052	1.10E-05	0.531537688	count	0.260623
SOS2	0.4099515	0.1530494	2.6786	0.00744	0.531694725	count	1
THUMPD3-AS1	0.388205	0.1499038	2.5897	0.00966	0.532127112	count	1
UBAC2	0.3807917	0.0912683	4.1722	3.12E-05	0.532138567	count	0.7364448
PELI1	0.395386	0.1297039	3.0484	0.00233	0.532331648	count	1
ZFY	0.43489	0.2365673	1.8383	0.0661	0.532385646	count	1
IFITM2	0.3716466	0.0565094	6.5767	5.86E-11	0.532387768	count	1.41E-06
GOT2	0.5493308	0.3119111	1.7612	0.0783	0.532424111	count	1
ALG10	0.9882533	0.6076132	1.6265	0.104	0.532426952	count	1
INTS6-AS1	0.5294261	0.3425209	1.5457	0.122	0.532442679	count	1
POLE3	0.388809	0.1323074	2.9387	0.00333	0.532598861	count	1
GOLGA3	0.4133588	0.1873643	2.2062	0.0275	0.532872873	count	1
ANKRD50	0.5243363	0.3172277	1.6529	0.0985	0.533081764	count	1
SFXN3	0.4115413	0.2020047	2.0373	0.0417	0.533256572	count	1
APPL2	0.4602377	0.2016902	2.2819	0.0226	0.533282702	count	1
FBXO48	0.5304696	0.484354	1.0952	0.274	0.533533422	count	1
NELFA	0.4646735	0.3828252	1.2138	0.225	0.533887812	count	1
TMX3	0.4133317	0.2420344	1.7077	0.0878	0.533972802	count	1
PML	0.4235433	0.1907173	2.2208	0.0265	0.534265095	count	1
IL15	0.4431315	0.1790195	2.4753	0.0134	0.534447638	count	1
GFM1	0.4484951	0.2242432	2	0.0456	0.534681562	count	1
KIF1BP	0.4377742	0.2385228	1.8354	0.0666	0.534713907	count	1
STX11	0.3790109	0.0747064	5.0733	4.20E-07	0.535618996	count	0.010017
SCPEP1	0.382466	0.0805646	4.7473	2.18E-06	0.535644638	count	0.05184912
DNAAF2	0.4215651	0.2482734	1.698	0.0896	0.535644726	count	1
MAP7D3	0.4312922	0.216113	1.9957	0.0461	0.53578931	count	1
MAP3K11	0.4046872	0.1439646	2.811	0.00498	0.535955427	count	1
EPHB6	0.4510558	0.26559	1.6983	0.0896	0.536076007	count	1
TPRN	0.5608321	0.3686326	1.5214	0.128	0.536131413	count	1
LEMD3	0.4928205	0.3883435	1.269	0.205	0.536137967	count	1
CCNG1	0.4379645	0.2237672	1.9572	0.0504	0.536212845	count	1
KLC2	0.7334721	0.597318	1.2279	0.22	0.536362531	count	1
DCAKD	0.6274949	0.4429592	1.4166	0.157	0.536430534	count	1
METTL4	0.4965317	0.3489574	1.4229	0.155	0.536435191	count	1
LRRC4	1.1025546	0.6500649	1.6961	0.09	0.536448133	count	1

THUMPD2	0.4227938	0.2829241	1.4944	0.135	0.53647458	count	1
BTN2A1	0.4577207	0.2309025	1.9823	0.0476	0.536624404	count	1
AFF4	0.3875499	0.1193328	3.2476	0.00118	0.536804834	count	1
PTGIR	0.5467325	0.3350524	1.6318	0.103	0.537104594	count	1
UVSSA	0.4130236	0.2038502	2.0261	0.0429	0.537240284	count	1
WDR41	0.4224248	0.2052217	2.0584	0.0397	0.537486761	count	1
USP1	0.4046256	0.1525447	2.6525	0.00804	0.537492287	count	1
TATDN3	0.4184767	0.1846969	2.2657	0.0236	0.537742797	count	1
EHMT2	0.494416	0.2546913	1.9412	0.0523	0.537926083	count	1
FGD5-AS1	0.3962742	0.1177215	3.3662	0.000774	0.537949682	count	1
USP10	0.4060789	0.1644442	2.4694	0.0136	0.538477549	count	1
CNNM2	1.1062894	0.5471617	2.0219	0.0433	0.538492954	count	1
SFSWAP	0.404827	0.1602097	2.5269	0.0116	0.538682398	count	1
XAF1	0.3862834	0.1244568	3.1038	0.00193	0.538752143	count	1
CH25H	0.3828476	0.1293752	2.9592	0.00311	0.538884153	count	1
MON2	0.4295814	0.2451711	1.7522	0.0799	0.539387609	count	1
NNT	0.4486538	0.3207022	1.399	0.162	0.539710894	count	1
TRAPPC8	0.4658276	0.2281225	2.042	0.0413	0.539908996	count	1
FAM214A	0.4930722	0.3802405	1.2967	0.195	0.54009593	count	1
TMEM242	0.4026723	0.1676959	2.4012	0.0164	0.540363929	count	1
MBNL2	0.4431892	0.1827333	2.4253	0.0154	0.541441843	count	1
ZBTB48	0.4671866	0.242088	1.9298	0.0537	0.541520373	count	1
MLST8	0.4619273	0.2906561	1.5893	0.112	0.541663413	count	1
LRRC37B	0.5088418	0.3508134	1.4505	0.147	0.541705447	count	1
GIN1	0.7405844	0.4425841	1.6733	0.0944	0.541982859	count	1
AL359258.2	0.8630574	0.6791471	1.2708	0.204	0.542319511	count	1
PRSS36	0.5591606	0.3653173	1.5306	0.126	0.542374125	count	1
FBXO22	0.4609311	0.2255986	2.0431	0.0411	0.542456279	count	1
HEXIM1	0.4293055	0.2186834	1.9631	0.0497	0.542462248	count	1
ASB8	0.4152397	0.1791909	2.3173	0.0206	0.542531876	count	1
ITSN1	0.4066193	0.1722642	2.3604	0.0183	0.542547705	count	1
HAUS5	0.7413272	0.7213629	1.0277	0.304	0.542570135	count	1
TSNARE1	0.8634587	0.5873376	1.4701	0.142	0.542596788	count	1
ZNF440	0.8634587	0.5683117	1.5193	0.129	0.542596788	count	1
CHD2	0.3998285	0.1390868	2.8747	0.00408	0.542632022	count	1
MRM1	1.0053459	0.7007634	1.4346	0.152	0.542718169	count	1
MRPS18A	0.4291404	0.2063051	2.0801	0.0376	0.543061822	count	1
ZNF575	0.9260384	0.6022729	1.5376	0.124	0.543215472	count	1
XPO6	0.4502775	0.2218758	2.0294	0.0425	0.543226954	count	1
NFKBIZ	0.3886987	0.1109033	3.5048	0.000465	0.543315918	count	1
IKZF1	0.4040312	0.1486705	2.7176	0.00662	0.543377774	count	1
WDR75	0.492954	0.4097467	1.2031	0.229	0.543486289	count	1
FAM208B	0.4357864	0.191616	2.2743	0.023	0.543492915	count	1
AL078590.2	1.2735793	0.8301379	1.5342	0.125	0.543927223	count	1
LINC00968	1.2735793	0.7629577	1.6693	0.0952	0.543927223	count	1

ZSWIM1	1.2735793	0.7629577	1.6693	0.0952	0.543927223	count	1
AAGAB	0.4998742	0.3107353	1.6087	0.108	0.544045156	count	1
DDX60L	0.4032829	0.1554439	2.5944	0.00953	0.544109342	count	1
SGTB	0.4085579	0.1469731	2.7798	0.00548	0.544285752	count	1
MTUS1	0.6362018	0.3662476	1.7371	0.0825	0.544323414	count	1
RIN1	0.4908474	0.2964803	1.6556	0.0979	0.544465066	count	1
CHSY1	0.4037261	0.1303808	3.0965	0.00198	0.544711316	count	1
HECA	0.4050687	0.1576531	2.5694	0.0102	0.544782911	count	1
TRIB2	1.1178689	0.7797609	1.4336	0.152	0.544836193	count	1
PNOC	1.1178689	0.7295322	1.5323	0.126	0.544836193	count	1
EFHB	1.008924	0.6346924	1.5896	0.112	0.544874756	count	1
AC008443.6	1.008924	0.6820795	1.4792	0.139	0.544874756	count	1
KIF21A	1.008924	0.7576082	1.3317	0.183	0.544874756	count	1
AC004233.3	1.008924	0.6346924	1.5896	0.112	0.544874756	count	1
TM9SF1	0.7775549	0.5083483	1.5296	0.126	0.544886659	count	1
ZNRD1	0.3989021	0.1288311	3.0963	0.00198	0.544920841	count	1
DNLZ	0.9287073	0.6041033	1.5373	0.124	0.544949706	count	1
MAP2K1	0.4223422	0.1369084	3.0849	0.00206	0.545170657	count	1
SUDS3	0.3997607	0.1447575	2.7616	0.00579	0.545244744	count	1
PPWD1	0.4299101	0.2581863	1.6651	0.096	0.545623215	count	1
STK24	0.4095871	0.1482889	2.7621	0.00579	0.545667853	count	1
ULK3	0.4606784	0.2853088	1.6147	0.107	0.545938949	count	1
MCL1	0.3807214	0.0450233	8.4561	4.68E-17	0.546122954	count	1.13E-12
DEF6	0.4148648	0.1699207	2.4415	0.0147	0.546258086	count	1
FLI1	0.4170411	0.1858125	2.2444	0.0249	0.546685205	count	1
THAP1	0.4464121	0.2447267	1.8241	0.0683	0.546732049	count	1
SLC27A3	0.422321	0.1532655	2.7555	0.0059	0.546833738	count	1
RAB3A	0.4646394	0.3103191	1.4973	0.134	0.546914027	count	1
IGBP1	0.3940917	0.0959687	4.1065	4.15E-05	0.547341426	count	0.978238
ZNF865	0.6396731	0.4952189	1.2917	0.197	0.547472528	count	1
CAPN2	0.3947727	0.1145929	3.445	0.000581	0.547521772	count	1
SLC38A2	0.3889045	0.1009337	3.8531	0.00012	0.547542288	count	1
IGHMBP2	0.6744771	0.5432867	1.2415	0.215	0.547747714	count	1
RAF1	0.4264891	0.1806835	2.3604	0.0183	0.548160824	count	1
PHKB	0.4111671	0.1562883	2.6308	0.00857	0.548663671	count	1
STARD4	0.5113149	0.3231234	1.5824	0.114	0.54876351	count	1
SIRPD	0.7500837	0.4932872	1.5206	0.128	0.549497436	count	1
A2M	0.3940371	0.1228671	3.207	0.00136	0.549813669	count	1
EYA2	0.8740955	0.5143221	1.6995	0.0893	0.549950974	count	1
PQBP1	0.39983	0.1147216	3.4852	5.00E-04	0.549966115	count	1
GBP1	0.4079489	0.1427505	2.8578	0.0043	0.55065675	count	1
CAVIN2	0.7853429	0.3203107	2.4518	0.0143	0.550816548	count	1
PRMT6	1.0188416	0.5555016	1.8341	0.0668	0.550855309	count	1
NUP50	0.4131351	0.1675976	2.465	0.0138	0.551021714	count	1
PSPH	0.6995052	0.4650687	1.5041	0.133	0.551185473	count	1

GCC1	0.6600513	0.3780904	1.7457	0.081	0.551213875	count	1
HNRNPL	0.4055164	0.1441958	2.8123	0.00496	0.551252657	count	1
TARS2	0.644069	0.4294235	1.4998	0.134	0.551462336	count	1
GPR27	1.2896541	0.614361	2.0992	0.0359	0.551680528	count	1
AC005082.1	0.5365883	0.4333634	1.2382	0.216	0.551798781	count	1
PRLR	0.786709	0.5159267	1.5248	0.127	0.551857303	count	1
TTBK2	0.462851	0.2952813	1.5675	0.117	0.552141493	count	1
AC108673.2	1.5464032	0.8414758	1.8377	0.0662	0.55222842	count	1
AL162414.1	1.5464032	0.651042	2.3753	0.0176	0.55222842	count	1
AC016588.2	1.5464032	0.8414758	1.8377	0.0662	0.55222842	count	1
GBP5	0.4470106	0.1971904	2.2669	0.0235	0.552304512	count	1
DZIP3	0.8775458	0.6012258	1.4596	0.145	0.552338417	count	1
NUP133	0.577269	0.3384948	1.7054	0.0882	0.552576951	count	1
MSL2	0.4928085	0.3254272	1.5143	0.13	0.553069083	count	1
AC027644.3	0.4257016	0.1886539	2.2565	0.0241	0.553395313	count	1
COQ8B	0.5438655	0.3453489	1.5748	0.115	0.553711932	count	1
ETFDH	0.4774903	0.2556308	1.8679	0.0619	0.553742613	count	1
FBXL5	0.404057	0.1165772	3.466	0.000537	0.553845069	count	1
LINC02035	0.5562371	0.4536443	1.2262	0.22	0.553861548	count	1
TBC1D10C	0.4738795	0.2520005	1.8805	0.0602	0.553883775	count	1
EAPP	0.4034796	0.1286217	3.1369	0.00173	0.553890271	count	1
RAE1	0.4376255	0.1737582	2.5186	0.0118	0.553945291	count	1
ZNF189	0.6323715	0.3815738	1.6573	0.0976	0.554017596	count	1
AC004687.1	0.407697	0.1495843	2.7255	0.00647	0.55406654	count	1
SLC5A9	2.0580676	1.0459481	1.9677	0.0492	0.554272538	count	1
TRH	2.0580676	1.1225812	1.8333	0.0669	0.554272538	count	1
AL139041.1	2.0580676	1.0459481	1.9677	0.0492	0.554272538	count	1
ABCA2	2.0580676	1.017587	2.0225	0.0432	0.554272538	count	1
AL133215.2	2.0580676	1.0459481	1.9677	0.0492	0.554272538	count	1
AC005476.2	2.0580676	1.0459481	1.9677	0.0492	0.554272538	count	1
CCDC102A	2.0580676	1.017587	2.0225	0.0432	0.554272538	count	1
LINC01664	2.0580676	1.0459481	1.9677	0.0492	0.554272538	count	1
C16orf72	0.408669	0.1409978	2.8984	0.00378	0.554704254	count	1
ITGB2-AS1	0.4310532	0.1972158	2.1857	0.0289	0.554725664	count	1
PIK3CD	0.4892503	0.247483	1.9769	0.0482	0.554820996	count	1
ZNF606	0.8312527	0.9320784	0.8918	0.373	0.554823526	count	1
KIF13A	0.4199404	0.1727992	2.4302	0.0152	0.554906839	count	1
APOBEC3C	0.4158395	0.1378815	3.0159	0.00259	0.554948042	count	1
MTA1	0.4974865	0.2982822	1.6678	0.0955	0.555313638	count	1
NCK1	0.418097	0.1648183	2.5367	0.0113	0.555858553	count	1
OCEL1	0.4301164	0.1563056	2.7518	0.00597	0.55590417	count	1
FLT3LG	0.7295944	0.3980658	1.8328	0.0669	0.55591597	count	1
PNISR	0.3946713	0.1036864	3.8064	0.000144	0.556598847	count	1
BICRA	0.9466917	0.5223693	1.8123	0.0701	0.556648708	count	1
CHD1L	0.480417	0.2433557	1.9741	0.0485	0.557215891	count	1

ATP11B	0.4616856	0.2842111	1.6244	0.104	0.557250447	count	1
MVD	0.482447	0.2591237	1.8618	0.0627	0.557258745	count	1
MOB3A	0.4194045	0.1880591	2.2302	0.0258	0.557290285	count	1
MBD5	0.4452223	0.2287995	1.9459	0.0518	0.557420213	count	1
DAAM1	0.4403668	0.1967887	2.2378	0.0253	0.557462346	count	1
CCL3L1	0.3873807	0.1013461	3.8224	0.000135	0.557589823	count	1
NR1D2	0.4461835	0.1646454	2.71	0.00678	0.557660722	count	1
TMEM245	0.4893486	0.2571187	1.9032	0.0571	0.557701152	count	1
TIMM23B	0.7318292	0.4947129	1.4793	0.139	0.557747871	count	1
SS18L1	0.6234964	0.3398403	1.8347	0.0667	0.5578973	count	1
FARP2	0.5377207	0.3696065	1.4548	0.146	0.558510574	count	1
MANEA-DT	0.795517	0.527191	1.509	0.131	0.558571696	count	1
NBEA	0.795517	1.0377297	0.7666	0.443	0.558571696	count	1
PLXNA1	0.5610018	0.3641829	1.5404	0.124	0.558803192	count	1
ARFIP1	0.4217968	0.1514089	2.7858	0.00538	0.55881435	count	1
C20orf194	0.4463442	0.2410365	1.8518	0.0642	0.558845607	count	1
EXOSC5	0.4206891	0.1682666	2.5001	0.0125	0.559011566	count	1
CEBPZ	0.4032518	0.1314527	3.0677	0.00218	0.559254398	count	1
DDX54	0.4355576	0.2079239	2.0948	0.0363	0.559309317	count	1
PMPCB	0.4145633	0.1378405	3.0076	0.00266	0.559432943	count	1
CASP1	0.3947206	0.0688922	5.7295	1.13E-08	0.559680202	count	0.000271053
RGS2	0.38982	0.0565663	6.8914	7.00E-12	0.559847228	count	1.69E-07
ZNF688	0.4356453	0.1946682	2.2379	0.0253	0.560070289	count	1
KMT2D	0.5502994	0.3121304	1.763	0.078	0.560517337	count	1
CEP70	0.9531598	0.8242428	1.1564	0.248	0.560861489	count	1
CALML4	0.9531598	0.6745584	1.413	0.158	0.560861489	count	1
ZNF629	0.6715984	0.4302206	1.5611	0.119	0.561476826	count	1
GNLY	0.5456987	0.6145309	0.888	0.375	0.561521342	count	1
PSPC1	0.4323376	0.1787607	2.4185	0.0157	0.561587273	count	1
SFI1	0.6552266	0.5591251	1.1719	0.241	0.561598703	count	1
RETREG1	1.3108243	0.6378167	2.0552	0.04	0.561893268	count	1
DYRK2	0.4865405	0.314289	1.5481	0.122	0.562100105	count	1
ZNF384	0.6412533	0.3683993	1.7406	0.0819	0.562250436	count	1
TRNT1	0.4633975	0.2336581	1.9832	0.0475	0.562359799	count	1
TRIM73	0.8005398	0.4633183	1.7278	0.0841	0.562403994	count	1
CBX1	0.4175136	0.1665064	2.5075	0.0122	0.562561384	count	1
CAMK2N1	2.0888534	1.1207677	1.8638	0.0625	0.562716452	count	1
INKA2	2.0888534	1.1207677	1.8638	0.0625	0.562716452	count	1
HCN3	2.0888534	1.1207677	1.8638	0.0625	0.562716452	count	1
LINC00886	2.0888534	1.1207677	1.8638	0.0625	0.562716452	count	1
FLNC	2.0888534	1.1207677	1.8638	0.0625	0.562716452	count	1
NALT1	2.0888534	1.1207677	1.8638	0.0625	0.562716452	count	1
IGSF22	2.0888534	1.1207677	1.8638	0.0625	0.562716452	count	1
AL392046.1	2.0888534	1.319267	1.5833	0.113	0.562716452	count	1
TEX9	2.0888534	1.1207677	1.8638	0.0625	0.562716452	count	1

ERBB2	2.0888534	1.1207677	1.8638	0.0625	0.562716452	count	1
AL118508.1	2.0888534	1.1207677	1.8638	0.0625	0.562716452	count	1
CLEC4M	2.0888534	1.319267	1.5833	0.113	0.562716452	count	1
SLC10A7	0.9560654	0.5561936	1.7189	0.0858	0.562754825	count	1
RNF146	0.4285475	0.191345	2.2397	0.0252	0.562786215	count	1
LINC02397	1.3127844	0.8695903	1.5097	0.131	0.562838878	count	1
HIST1H2AL	0.7381878	0.5856315	1.2605	0.208	0.562962847	count	1
ZBED5	0.4529522	0.2089893	2.1673	0.0303	0.563123022	count	1
CPSF1	0.5420894	0.3485598	1.5552	0.12	0.563214018	count	1
MAN2B1	0.4586445	0.2325235	1.9725	0.0487	0.563256255	count	1
GINS4	0.6927232	0.3425121	2.0225	0.0432	0.563576159	count	1
VAPB	0.4769376	0.3224929	1.4789	0.139	0.563690953	count	1
AC242426.2	0.6073547	0.6399468	0.9491	0.343	0.564000141	count	1
PDP1	0.4554353	0.2145624	2.1226	0.0339	0.564036256	count	1
XAB2	0.5537676	0.3039751	1.8218	0.0686	0.564187552	count	1
AKAP1	0.5430191	0.3600267	1.5083	0.132	0.564215215	count	1
NCOA2	0.4438125	0.2464102	1.8011	0.0718	0.564290001	count	1
FAM110A	0.4227057	0.1667855	2.5344	0.0113	0.564475702	count	1
RNF219	0.4776547	0.2361758	2.0225	0.0432	0.564556563	count	1
LINC02363	1.5774313	1.2144657	1.2989	0.194	0.564565094	count	1
AC008771.1	1.5774313	0.9213426	1.7121	0.087	0.564565094	count	1
UNC79	1.5774313	0.9213426	1.7121	0.087	0.564565094	count	1
AC091132.5	1.5774313	1.1678557	1.3507	0.177	0.564565094	count	1
PPP1R27	1.5774313	1.0039341	1.5712	0.116	0.564565094	count	1
CEACAM19	1.5774313	0.9470184	1.6657	0.0959	0.564565094	count	1
CRCP	0.4258596	0.177693	2.3966	0.0166	0.564936707	count	1
MICU3	0.8041217	0.5375111	1.496	0.135	0.56513819	count	1
SPG11	0.4444789	0.1820675	2.4413	0.0147	0.56514848	count	1
IFIT1	0.4154597	0.2764193	1.503	0.133	0.56535498	count	1
PBLD	0.9600792	0.5864644	1.6371	0.102	0.565371181	count	1
DLST	0.4937801	0.2795619	1.7663	0.0775	0.565568928	count	1
LMBRD1	0.4213193	0.1497794	2.8129	0.00495	0.565574128	count	1
XRCC4	0.4347229	0.1911203	2.2746	0.023	0.566283619	count	1
ZNF81	0.7713801	0.5613596	1.3741	0.17	0.566376576	count	1
PHC3	0.4508033	0.3113349	1.448	0.148	0.566425493	count	1
AP1G1	0.4278997	0.2406024	1.7785	0.0755	0.566615807	count	1
VGLL4	0.454959	0.1946133	2.3378	0.0195	0.566727758	count	1
RNF14	0.4995465	0.262591	1.9024	0.0572	0.566803301	count	1
SNTB1	0.4577295	0.428809	1.0674	0.286	0.566923317	count	1
MSH2	0.6103691	0.3478643	1.7546	0.0794	0.566941074	count	1
SHROOM1	0.6103691	0.3386941	1.8021	0.0716	0.566941074	count	1
ZNF28	0.9627013	0.5209963	1.8478	0.0648	0.567080882	count	1
NFKBIL1	0.4508231	0.2778921	1.6223	0.105	0.567375996	count	1
DCAF1	0.5838533	0.3463089	1.6859	0.0919	0.567415359	count	1
SPRY1	0.6470235	0.3764341	1.7188	0.0858	0.567603577	count	1

FAM43A	0.6111097	0.3787926	1.6133	0.107	0.567663787	count	1
DDX17	0.3995109	0.0840935	4.7508	2.14E-06	0.567734711	count	0.05090204
BCDIN3D	0.7191226	0.4534637	1.5858	0.113	0.567762159	count	1
EIF2AK4	0.4166042	0.1133769	3.6745	0.000243	0.568043619	count	1
TOMM34	0.4876833	0.3003886	1.6235	0.105	0.568152381	count	1
RNF139	0.4254874	0.1982478	2.1462	0.032	0.568219947	count	1
SENCR	0.5215727	0.2756347	1.8923	0.0586	0.568399268	count	1
TMPO	0.4137846	0.1226388	3.374	0.000752	0.568462314	count	1
ADNP	0.4270277	0.1581349	2.7004	0.00697	0.568471999	count	1
CASP8AP2	0.4809183	0.217775	2.2083	0.0273	0.568496526	count	1
SLC27A1	0.488099	0.2984458	1.6355	0.102	0.568647833	count	1
BPHL	0.5254873	0.3110894	1.6892	0.0913	0.5687264	count	1
ADAM17	0.4108288	0.1134797	3.6203	3.00E-04	0.568860352	count	1
NAB1	0.4506152	0.2393477	1.8827	0.0599	0.568900486	count	1
HCAR2	0.4131977	0.1477482	2.7966	0.0052	0.568981983	count	1
ENTPD1	0.4300707	0.1376355	3.1247	0.0018	0.569154264	count	1
SPINT1	0.4769761	0.2442673	1.9527	0.051	0.569336211	count	1
WDFY1	0.4605996	0.2812975	1.6374	0.102	0.569371197	count	1
SETMAR	1.1628456	0.6766504	1.7185	0.0858	0.569515469	count	1
ZNF483	1.1628456	0.8874462	1.3103	0.19	0.569515469	count	1
KIAA0391	1.1628456	0.8045857	1.4453	0.149	0.569515469	count	1
SP2-AS1	1.1628456	0.6766504	1.7185	0.0858	0.569515469	count	1
1-Mar	0.4064634	0.0892954	4.5519	5.58E-06	0.569522695	count	0.13243014
TLR5	0.4354489	0.1546943	2.8149	0.00492	0.569682951	count	1
RBM23	0.4505576	0.2078755	2.1674	0.0303	0.56969294	count	1
AC105020.6	0.8101706	0.4954381	1.6353	0.102	0.569758171	count	1
HYI	0.4574987	0.2035537	2.2476	0.0247	0.569940492	count	1
LINC02285	0.9673181	0.5141522	1.8814	0.06	0.570092249	count	1
TMTC2	0.721949	0.4285637	1.6846	0.0922	0.570153673	count	1
LTA4H	0.4038919	0.0801724	5.0378	5.05E-07	0.570205681	count	0.01204021
RHBDF2	0.4301514	0.184441	2.3322	0.0198	0.570332621	count	1
THEM6	0.776365	0.4511449	1.7209	0.0854	0.570333746	count	1
OAS1	0.4042147	0.089293	4.5268	6.27E-06	0.570753139	count	0.14874948
SERPINB2	0.4073723	0.2764603	1.4735	0.141	0.57088273	count	1
NIPBL	0.4100254	0.0997855	4.1091	4.10E-05	0.570993129	count	0.966575
FCHSD2	0.4218734	0.1411557	2.9887	0.00283	0.57102925	count	1
AC244021.1	2.120691	1.009649	2.1004	0.0358	0.571367296	count	1
SHE	2.120691	0.9205696	2.3037	0.0213	0.571367296	count	1
CLCN4	2.120691	1.365817	1.5527	0.121	0.571367296	count	1
PRR14L	0.4470266	0.1803824	2.4782	0.0133	0.571438015	count	1
WDR48	0.4787826	0.245146	1.9531	0.0509	0.571536379	count	1
CDKN1B	0.4103708	0.0963261	4.2602	2.12E-05	0.571536724	count	0.5012316
AC008972.2	0.7781164	0.8221186	0.9465	0.344	0.571724599	count	1
SLC38A7	0.5609069	0.3445262	1.6281	0.104	0.57174657	count	1
BSDC1	0.4559244	0.2941427	1.55	0.121	0.57199826	count	1

ALKBH6	0.5323933	0.3210712	1.6582	0.0974	0.572133428	count	1
DPY19L1	0.5675771	0.3560442	1.5941	0.111	0.572396767	count	1
ERP29	0.4016042	0.0489382	8.2064	3.64E-16	0.573077422	count	8.80E-12
TXNIP	0.3993225	0.0624333	6.396	1.90E-10	0.573114818	count	4.57E-06
AC004069.1	0.7255943	0.419637	1.7291	0.0839	0.573239279	count	1
AKAP7	0.4939209	0.2315183	2.1334	0.033	0.573250868	count	1
DNAJC16	0.5980984	0.3340754	1.7903	0.0735	0.573459336	count	1
VPS13D	0.4775269	0.3073771	1.5536	0.12	0.573471223	count	1
NIFK-AS1	0.6172257	0.3998491	1.5436	0.123	0.57363418	count	1
POMGNT2	0.8152915	0.5503788	1.4813	0.139	0.573671847	count	1
BLZF1	0.4906099	0.2462956	1.992	0.0465	0.573886931	count	1
CCDC7	0.7517323	0.5835178	1.2883	0.198	0.574084557	count	1
MFAP5	0.6080711	0.4443616	1.3684	0.171	0.574377948	count	1
AC008105.3	0.4878387	0.2679117	1.8209	0.0687	0.574826845	count	1
MYO1C	0.4760347	0.3104199	1.5335	0.125	0.574902668	count	1
PTPN4	0.5996627	0.5569843	1.0766	0.282	0.575029445	count	1
LIMD1	0.5435157	0.3172446	1.7132	0.0868	0.57508143	count	1
FBXL4	0.55848	0.3112617	1.7942	0.0729	0.575175626	count	1
LCMT2	18.0441746	1167.694012	0.0155	0.988	0.575502158	count	1
HOXB5	18.072149	758.9498303	0.0238	0.981	0.575502159	count	1
GABRB2	18.2145087	931.3120318	0.0196	0.984	0.57550216	count	1
CACNA1D	18.2429163	1059.82375	0.0172	0.9863	0.57550216	count	1
AC025569.1	18.2865714	1421.948345	0.0129	0.99	0.57550216	count	1
CCL14	18.3662078	874.6311153	0.021	0.9832	0.575502161	count	1
CLDN4	18.3665489	1033.614715	0.0178	0.9858	0.575502161	count	1
STX16-NPEPL1	18.4129917	1180.629386	0.0156	0.988	0.575502161	count	1
AL391244.3	18.6617547	1369.961069	0.0136	0.989	0.575502163	count	1
UTS2	18.6617547	1369.96108	0.0136	0.989	0.575502163	count	1
AL358473.1	18.6617547	1369.961062	0.0136	0.989	0.575502163	count	1
AC013468.1	18.6617547	1369.961067	0.0136	0.989	0.575502163	count	1
ADGRA3	18.6617547	1369.961071	0.0136	0.989	0.575502163	count	1
PCDH1	18.6617548	1369.961085	0.0136	0.989	0.575502163	count	1
SLC26A8	18.6617547	1369.961069	0.0136	0.989	0.575502163	count	1
U47924.2	18.6617547	1369.961067	0.0136	0.989	0.575502163	count	1
AC023509.3	18.6617547	1369.961089	0.0136	0.989	0.575502163	count	1
AL137779.1	18.6617547	1369.961078	0.0136	0.989	0.575502163	count	1
FAM81A	18.6617547	1369.961085	0.0136	0.989	0.575502163	count	1
LINC02206	18.6617547	1369.961074	0.0136	0.989	0.575502163	count	1
AC005696.1	18.6617547	1369.961078	0.0136	0.989	0.575502163	count	1
AC092117.1	18.6617584	1369.96379	0.0136	0.989	0.575502163	count	1
AC138207.2	18.6617584	1369.963779	0.0136	0.989	0.575502163	count	1
LINC02085	18.6617585	1369.963799	0.0136	0.989	0.575502163	count	1
SLC35G1	18.6617585	1369.963795	0.0136	0.989	0.575502163	count	1
AL357078.1	18.6617591	1369.964174	0.0136	0.989	0.575502163	count	1
AC015971.1	18.6617591	1369.964177	0.0136	0.989	0.575502163	count	1

AC009948.3	18.6617591	1369.964157	0.0136	0.989	0.575502163	count	1
ZMYND10	18.6617591	1369.964168	0.0136	0.989	0.575502163	count	1
TM4SF19-AS1	18.6617591	1369.96417	0.0136	0.989	0.575502163	count	1
SLCO4C1	18.6617591	1369.964174	0.0136	0.989	0.575502163	count	1
STC1	18.6617591	1369.964161	0.0136	0.989	0.575502163	count	1
SCARA5	18.6617591	1369.964166	0.0136	0.989	0.575502163	count	1
OLFML2A	18.6617591	1369.964166	0.0136	0.989	0.575502163	count	1
OR52K2	18.6617591	1369.964161	0.0136	0.989	0.575502163	count	1
GSTO2	18.6617591	1369.964163	0.0136	0.989	0.575502163	count	1
AICDA	18.6617591	1369.964168	0.0136	0.989	0.575502163	count	1
TGFB3	18.661759	1369.964168	0.0136	0.989	0.575502163	count	1
AC007614.1	18.6617591	1369.964161	0.0136	0.989	0.575502163	count	1
PLEKHG4	18.6617591	1369.964181	0.0136	0.989	0.575502163	count	1
AC009802.1	18.661759	1369.964154	0.0136	0.989	0.575502163	count	1
RTEL1-TNFRSF6B	18.6617591	1369.96417	0.0136	0.989	0.575502163	count	1
AC119396.1	18.6617591	1369.964179	0.0136	0.989	0.575502163	count	1
AC007786.1	18.6617591	1369.964174	0.0136	0.989	0.575502163	count	1
AC006213.2	18.6617591	1369.964159	0.0136	0.989	0.575502163	count	1
ADORA2A	18.6617591	1369.964168	0.0136	0.989	0.575502163	count	1
HOXA10	18.6617592	1369.964181	0.0136	0.989	0.575502163	count	1
PLA2G2A	18.6643422	1530.662854	0.0122	0.9903	0.575502163	count	1
IL31RA	18.7976097	1375.468012	0.0137	0.989	0.575502164	count	1
AC003991.1	18.7976097	1375.468022	0.0137	0.989	0.575502164	count	1
MATN2	18.7976097	1375.46801	0.0137	0.989	0.575502164	count	1
DAB2IP	18.7976097	1375.468022	0.0137	0.989	0.575502164	count	1
AC104031.1	18.7976097	1375.468017	0.0137	0.989	0.575502164	count	1
LTB4R2	18.7976096	1375.467994	0.0137	0.989	0.575502164	count	1
AP000915.1	18.7976114	1375.469634	0.0137	0.989	0.575502164	count	1
ITPKB-AS1	18.7976116	1375.469634	0.0137	0.989	0.575502164	count	1
AC078845.1	18.7976115	1375.469634	0.0137	0.989	0.575502164	count	1
ABCF2	18.7976156	1375.469242	0.0137	0.989	0.575502164	count	1
AC005253.1	18.7976198	1375.470764	0.0137	0.989	0.575502164	count	1
AP001528.2	18.79769	1781.21132	0.0106	0.992	0.575502164	count	1
NPAS3	18.79769	1781.21132	0.0106	0.992	0.575502164	count	1
PAK6	18.7976901	1781.211332	0.0106	0.992	0.575502164	count	1
FPGT-TNNI3K	18.9083814	1654.998096	0.0114	0.991	0.575502164	count	1
ZC3H11B	18.9083814	1654.998074	0.0114	0.991	0.575502164	count	1
ZNF625-ZNF20	18.9083913	1654.998857	0.0114	0.991	0.575502164	count	1
NEO1	18.9083926	1655.000483	0.0114	0.991	0.575502164	count	1
AC009480.1	18.9085841	2108.340833	0.009	0.993	0.575502164	count	1
AC105345.1	18.9085841	2108.340833	0.009	0.993	0.575502164	count	1
CUZD1	18.9085843	2108.340854	0.009	0.993	0.575502164	count	1
EMG1	18.9085998	2108.343581	0.009	0.993	0.575502164	count	1
ANKRD34B	18.9340587	1535.959232	0.0123	0.99	0.575502164	count	1
SAP30L-AS1	18.9340586	1535.959224	0.0123	0.99	0.575502164	count	1

PDP2	18.9340587	1535.959219	0.0123	0.99	0.575502164	count	1
SAPCD2	18.9340661	1535.957492	0.0123	0.99	0.575502164	count	1
AC092368.3	18.9560243	982.850499	0.0193	0.9846	0.575502164	count	1
AC022098.2	18.956836	1161.899285	0.0163	0.987	0.575502164	count	1
DOC2A	19.0442047	1541.87875	0.0124	0.99	0.575502165	count	1
PKDCC	19.1030569	1519.093327	0.0126	0.99	0.575502165	count	1
AL049780.2	19.2493127	1431.949063	0.0134	0.9893	0.575502165	count	1
AC091488.1	19.7601536	1634.485681	0.0121	0.99	0.575502167	count	1
TNKS2-AS1	19.7601572	1634.485603	0.0121	0.99	0.575502167	count	1
GCAT	19.7601573	1634.485596	0.0121	0.99	0.575502167	count	1
PPIL6	19.7601601	1634.484834	0.0121	0.99	0.575502167	count	1
LRRC36	19.7601604	1634.484813	0.0121	0.99	0.575502167	count	1
SPIN2A	19.7601602	1634.484791	0.0121	0.99	0.575502167	count	1
AC136944.2	19.7601602	1634.484827	0.0121	0.99	0.575502167	count	1
AP000640.1	19.760168	1634.491681	0.0121	0.99	0.575502167	count	1
AL450468.2	19.7601745	1634.48724	0.0121	0.99	0.575502167	count	1
AC098934.4	20.4390722	2044.230305	0.01	0.992	0.575502168	count	1
LINC00628	20.4390722	2044.230294	0.01	0.992	0.575502168	count	1
ADGRF3	20.4390722	2044.230213	0.01	0.992	0.575502168	count	1
TMEM169	20.4390722	2044.230282	0.01	0.992	0.575502168	count	1
DOK7	20.4390722	2044.230202	0.01	0.992	0.575502168	count	1
ENPP6	20.4390722	2044.230305	0.01	0.992	0.575502168	count	1
BTNL9	20.4390722	2044.230328	0.01	0.992	0.575502168	count	1
FZD6	20.4390722	2044.230294	0.01	0.992	0.575502168	count	1
AC090579.1	20.4390722	2044.230213	0.01	0.992	0.575502168	count	1
LINC01230	20.4390722	2044.230294	0.01	0.992	0.575502168	count	1
FOCAD-AS1	20.4390722	2044.230294	0.01	0.992	0.575502168	count	1
AL162426.1	20.4390722	2044.230236	0.01	0.992	0.575502168	count	1
AC025263.1	20.4390722	2044.230294	0.01	0.992	0.575502168	count	1
AL158196.1	20.4390722	2044.230282	0.01	0.992	0.575502168	count	1
LINC00449	20.4390722	2044.230305	0.01	0.992	0.575502168	count	1
AL110504.1	20.4390722	2044.230271	0.01	0.992	0.575502168	count	1
LIPC	20.4390722	2044.230236	0.01	0.992	0.575502168	count	1
MEGF11	20.4390722	2044.230294	0.01	0.992	0.575502168	count	1
SHBG	20.4390722	2044.230213	0.01	0.992	0.575502168	count	1
C17orf64	20.4390721	2044.23019	0.01	0.992	0.575502168	count	1
TBX2	20.4390722	2044.230202	0.01	0.992	0.575502168	count	1
AL357033.3	20.4390722	2044.230282	0.01	0.992	0.575502168	count	1
TMPRSS9	20.4390722	2044.230202	0.01	0.992	0.575502168	count	1
SULT2B1	20.4390722	2044.230282	0.01	0.992	0.575502168	count	1
TMEM52	20.4390724	2044.230316	0.01	0.992	0.575502168	count	1
TMEM54	20.4390723	2044.230374	0.01	0.992	0.575502168	count	1
CLDN19	20.4390724	2044.23042	0.01	0.992	0.575502168	count	1
DUSP27	20.4390723	2044.230271	0.01	0.992	0.575502168	count	1
SELE	20.4390724	2044.230339	0.01	0.992	0.575502168	count	1

AL449106.1	20.4390723	2044.230316	0.01	0.992	0.575502168	count	1
COLGALT2	20.4390724	2044.230248	0.01	0.992	0.575502168	count	1
AL596220.1	20.4390723	2044.230259	0.01	0.992	0.575502168	count	1
AL121655.1	20.4390723	2044.230339	0.01	0.992	0.575502168	count	1
RMDN2-AS1	20.4390724	2044.230236	0.01	0.992	0.575502168	count	1
AC007389.3	20.4390724	2044.230236	0.01	0.992	0.575502168	count	1
AC008277.1	20.4390724	2044.230351	0.01	0.992	0.575502168	count	1
LINC01806	20.4390726	2044.230282	0.01	0.992	0.575502168	count	1
AC064836.2	20.4390724	2044.230294	0.01	0.992	0.575502168	count	1
AC073052.2	20.4390724	2044.230248	0.01	0.992	0.575502168	count	1
COL4A4	20.4390724	2044.230282	0.01	0.992	0.575502168	count	1
ERICH6	20.4390724	2044.230339	0.01	0.992	0.575502168	count	1
AC096577.1	20.4390724	2044.230248	0.01	0.992	0.575502168	count	1
AC021151.1	20.4390724	2044.230236	0.01	0.992	0.575502168	count	1
LINC02218	20.4390726	2044.230248	0.01	0.992	0.575502168	count	1
ELOVL7	20.4390724	2044.230248	0.01	0.992	0.575502168	count	1
LINC02242	20.4390723	2044.230236	0.01	0.992	0.575502168	count	1
SERF1A	20.4390723	2044.230351	0.01	0.992	0.575502168	count	1
AC008522.1	20.4390723	2044.230374	0.01	0.992	0.575502168	count	1
SEMA6A	20.4390724	2044.230294	0.01	0.992	0.575502168	count	1
AC113361.1	20.4390723	2044.230236	0.01	0.992	0.575502168	count	1
PCDHGA11	20.4390724	2044.230305	0.01	0.992	0.575502168	count	1
RANBP17	20.4390724	2044.230225	0.01	0.992	0.575502168	count	1
MYLK4	20.4390724	2044.230362	0.01	0.992	0.575502168	count	1
PNPLA1	20.4390726	2044.230248	0.01	0.992	0.575502168	count	1
AL365275.1	20.4390724	2044.230294	0.01	0.992	0.575502168	count	1
PNMA3	20.4390723	2044.230316	0.01	0.992	0.575502168	count	1
AVPR2	20.4390723	2044.230328	0.01	0.992	0.575502168	count	1
AC011773.3	20.4390724	2044.230271	0.01	0.992	0.575502168	count	1
SYBU	20.4390724	2044.230316	0.01	0.992	0.575502168	count	1
AC090921.1	20.4390724	2044.230294	0.01	0.992	0.575502168	count	1
C9orf24	20.4390724	2044.230236	0.01	0.992	0.575502168	count	1
AL133410.1	20.4390726	2044.230248	0.01	0.992	0.575502168	count	1
PCAT7	20.4390724	2044.230339	0.01	0.992	0.575502168	count	1
AL049629.2	20.4390724	2044.230225	0.01	0.992	0.575502168	count	1
ANGPTL5	20.4390723	2044.230282	0.01	0.992	0.575502168	count	1
CARD17	20.4390723	2044.230339	0.01	0.992	0.575502168	count	1
SMIM35	20.4390724	2044.230305	0.01	0.992	0.575502168	count	1
AP003392.5	20.4390724	2044.230351	0.01	0.992	0.575502168	count	1
C10orf67	20.4390724	2044.230339	0.01	0.992	0.575502168	count	1
AC010998.2	20.4390723	2044.230351	0.01	0.992	0.575502168	count	1
NRIP2	20.4390724	2044.230294	0.01	0.992	0.575502168	count	1
MIR200CHG	20.4390723	2044.230305	0.01	0.992	0.575502168	count	1
KLRK1	20.4390724	2044.230282	0.01	0.992	0.575502168	count	1
H3F3C	20.4390724	2044.23042	0.01	0.992	0.575502168	count	1

C12orf54	20.4390724	2044.230282	0.01	0.992	0.575502168	count	1
E2F7	20.4390723	2044.230374	0.01	0.992	0.575502168	count	1
USP44	20.4390723	2044.230271	0.01	0.992	0.575502168	count	1
AC069234.4	20.4390723	2044.230374	0.01	0.992	0.575502168	count	1
HNF1A	20.4390724	2044.230271	0.01	0.992	0.575502168	count	1
HPD	20.4390724	2044.230339	0.01	0.992	0.575502168	count	1
CLIP1-AS1	20.4390724	2044.230339	0.01	0.992	0.575502168	count	1
SERP2	20.4390723	2044.230225	0.01	0.992	0.575502168	count	1
G2E3-AS1	20.4390724	2044.230305	0.01	0.992	0.575502168	count	1
SERPINA5	20.4390723	2044.230339	0.01	0.992	0.575502168	count	1
AL133367.1	20.4390723	2044.230328	0.01	0.992	0.575502168	count	1
GOLGA8S	20.4390723	2044.230374	0.01	0.992	0.575502168	count	1
USP50	20.4390723	2044.230339	0.01	0.992	0.575502168	count	1
AC073167.1	20.4390724	2044.230282	0.01	0.992	0.575502168	count	1
LINC00924	20.4390724	2044.230259	0.01	0.992	0.575502168	count	1
AC004233.4	20.4390727	2044.230294	0.01	0.992	0.575502168	count	1
AC109597.1	20.4390726	2044.230271	0.01	0.992	0.575502168	count	1
AC138904.3	20.4390727	2044.230294	0.01	0.992	0.575502168	count	1
AC092723.1	20.4390724	2044.230339	0.01	0.992	0.575502168	count	1
ZNF286B	20.4390723	2044.230305	0.01	0.992	0.575502168	count	1
KRT27	20.4390724	2044.230362	0.01	0.992	0.575502168	count	1
HAP1	20.4390726	2044.230248	0.01	0.992	0.575502168	count	1
RUNDC3A	20.4390724	2044.230316	0.01	0.992	0.575502168	count	1
AC005920.1	20.4390723	2044.230259	0.01	0.992	0.575502168	count	1
AC011195.2	20.4390724	2044.230248	0.01	0.992	0.575502168	count	1
AC005332.6	20.4390724	2044.230305	0.01	0.992	0.575502168	count	1
GPR142	20.4390726	2044.230271	0.01	0.992	0.575502168	count	1
AP005530.1	20.4390723	2044.230271	0.01	0.992	0.575502168	count	1
CABYR	20.4390723	2044.230374	0.01	0.992	0.575502168	count	1
LINC01915	20.4390724	2044.230248	0.01	0.992	0.575502168	count	1
AC104365.3	20.4390723	2044.230374	0.01	0.992	0.575502168	count	1
DOK6	20.4390724	2044.230282	0.01	0.992	0.575502168	count	1
AC090360.1	20.4390723	2044.230328	0.01	0.992	0.575502168	count	1
TCF15	20.4390724	2044.230248	0.01	0.992	0.575502168	count	1
LINC01729	20.4390724	2044.230236	0.01	0.992	0.575502168	count	1
AL050403.2	20.4390726	2044.230305	0.01	0.992	0.575502168	count	1
SLC24A3	20.4390724	2044.230282	0.01	0.992	0.575502168	count	1
SGK2	20.4390724	2044.230339	0.01	0.992	0.575502168	count	1
AL139352.1	20.4390724	2044.230339	0.01	0.992	0.575502168	count	1
SLC12A5-AS1	20.4390726	2044.230271	0.01	0.992	0.575502168	count	1
BMP7	20.4390727	2044.230294	0.01	0.992	0.575502168	count	1
JSRP1	20.4390723	2044.230259	0.01	0.992	0.575502168	count	1
AC024575.1	20.4390724	2044.230248	0.01	0.992	0.575502168	count	1
AC011448.1	20.4390723	2044.230259	0.01	0.992	0.575502168	count	1
AC092329.3	20.4390723	2044.230248	0.01	0.992	0.575502168	count	1

AD000671.2	20.4390723	2044.230236	0.01	0.992	0.575502168	count	1
NKPD1	20.4390724	2044.230225	0.01	0.992	0.575502168	count	1
AC020922.2	20.4390726	2044.230248	0.01	0.992	0.575502168	count	1
RFPL4AL1	20.4390724	2044.230385	0.01	0.992	0.575502168	count	1
CDC45	20.4390724	2044.230282	0.01	0.992	0.575502168	count	1
HSF2BP	20.4390724	2044.230316	0.01	0.992	0.575502168	count	1
DIRAS3	20.4390725	2044.230328	0.01	0.992	0.575502168	count	1
LHX4	20.4390725	2044.230305	0.01	0.992	0.575502168	count	1
AC015969.1	20.4390725	2044.230305	0.01	0.992	0.575502168	count	1
AC010894.4	20.4390725	2044.230271	0.01	0.992	0.575502168	count	1
AC007163.1	20.4390725	2044.230305	0.01	0.992	0.575502168	count	1
AC098820.3	20.4390725	2044.230282	0.01	0.992	0.575502168	count	1
CHDH	20.4390725	2044.230328	0.01	0.992	0.575502168	count	1
LINC02024	20.4390725	2044.230294	0.01	0.992	0.575502168	count	1
GATA2-AS1	20.4390725	2044.230305	0.01	0.992	0.575502168	count	1
AC019131.2	20.4390725	2044.230351	0.01	0.992	0.575502168	count	1
PCDHAC2	20.4390725	2044.230316	0.01	0.992	0.575502168	count	1
AC139795.3	20.4390725	2044.230305	0.01	0.992	0.575502168	count	1
AL023583.1	20.4390725	2044.230305	0.01	0.992	0.575502168	count	1
SCUBE3	20.4390725	2044.230305	0.01	0.992	0.575502168	count	1
AL365226.1	20.4390725	2044.230316	0.01	0.992	0.575502168	count	1
TRGV6	20.4390725	2044.230305	0.01	0.992	0.575502168	count	1
AC027644.4	20.4390725	2044.230305	0.01	0.992	0.575502168	count	1
AC093673.2	20.4390725	2044.230305	0.01	0.992	0.575502168	count	1
XIAP-AS1	20.4390725	2044.230282	0.01	0.992	0.575502168	count	1
MCPH1-AS1	20.4390725	2044.230282	0.01	0.992	0.575502168	count	1
AL391834.1	20.4390725	2044.230316	0.01	0.992	0.575502168	count	1
HRCT1	20.4390725	2044.230305	0.01	0.992	0.575502168	count	1
AL390067.1	20.4390725	2044.230305	0.01	0.992	0.575502168	count	1
TNNT3	20.4390725	2044.230339	0.01	0.992	0.575502168	count	1
OR2AT4	20.4390725	2044.230282	0.01	0.992	0.575502168	count	1
MALRD1	20.4390725	2044.230294	0.01	0.992	0.575502168	count	1
RBP4	20.4390725	2044.230351	0.01	0.992	0.575502168	count	1
CFAP43	20.4390725	2044.230305	0.01	0.992	0.575502168	count	1
AC068896.1	20.4390725	2044.230316	0.01	0.992	0.575502168	count	1
AC079907.1	20.4390725	2044.230282	0.01	0.992	0.575502168	count	1
AL627171.2	20.4390725	2044.230282	0.01	0.992	0.575502168	count	1
AL136038.5	20.4390725	2044.230305	0.01	0.992	0.575502168	count	1
SMOC1	20.4390725	2044.230282	0.01	0.992	0.575502168	count	1
AL583722.2	20.4390725	2044.230305	0.01	0.992	0.575502168	count	1
AC100830.3	20.4390725	2044.230305	0.01	0.992	0.575502168	count	1
AC120114.1	20.4390725	2044.230316	0.01	0.992	0.575502168	count	1
AC092118.1	20.4390725	2044.230305	0.01	0.992	0.575502168	count	1
RNASEK-C17orf49	20.4390725	2044.230316	0.01	0.992	0.575502168	count	1
AC080112.1	20.4390725	2044.230271	0.01	0.992	0.575502168	count	1

HIGD1B	20.4390725	2044.230305	0.01	0.992	0.575502168	count	1
EFCAB3	20.4390725	2044.230282	0.01	0.992	0.575502168	count	1
AL121890.5	20.4390725	2044.230351	0.01	0.992	0.575502168	count	1
AL035661.1	20.4390725	2044.230316	0.01	0.992	0.575502168	count	1
AC027307.1	20.4390725	2044.230282	0.01	0.992	0.575502168	count	1
AC005786.2	20.4390725	2044.230305	0.01	0.992	0.575502168	count	1
AP000552.1	20.4390725	2044.230282	0.01	0.992	0.575502168	count	1
TSHZ2	18.6617548	1369.961096	0.0136	0.989	0.57550221	count	1
CFAP99	18.6617585	1369.963786	0.0136	0.989	0.57550221	count	1
DNA2	18.6617591	1369.964161	0.0136	0.989	0.57550221	count	1
AC022509.3	20.4390724	2044.230316	0.01	0.992	0.575502215	count	1
PRTN3	20.4390725	2044.230339	0.01	0.992	0.575502215	count	1
AC016737.1	20.4390727	2044.230385	0.01	0.992	0.575502215	count	1
AC117395.1	20.4390727	2044.230385	0.01	0.992	0.575502215	count	1
AF186192.1	20.4390727	2044.230385	0.01	0.992	0.575502215	count	1
CFAP161	20.4390727	2044.230385	0.01	0.992	0.575502215	count	1
CCDC113	20.4390727	2044.230385	0.01	0.992	0.575502215	count	1
KRTAP19-8	20.4390727	2044.230385	0.01	0.992	0.575502215	count	1
RBM27	0.5919344	0.3013938	1.964	0.0496	0.575624529	count	1
USP39	0.4597961	0.1980169	2.322	0.0203	0.575942484	count	1
ADO	0.454835	0.2115696	2.1498	0.0317	0.576032076	count	1
GTPBP10	0.5490795	0.3884368	1.4136	0.158	0.576084666	count	1
SHISA4	0.5776737	0.2936097	1.9675	0.0492	0.576112835	count	1
MRPL45	0.4679944	0.2113069	2.2148	0.0269	0.576210515	count	1
ZBTB40	0.6202691	0.3294847	1.8825	0.0599	0.576606582	count	1
SPATA1	0.7844344	0.5732725	1.3683	0.171	0.576744356	count	1
MYCL	0.6319412	0.3376922	1.8714	0.0614	0.577311697	count	1
VPS13C	0.4103239	0.1049093	3.9112	9.43E-05	0.577416578	count	1
ANAPC10	0.4440837	0.1946212	2.2818	0.0226	0.577554248	count	1
SIN3B	0.4486028	0.1734799	2.5859	0.00977	0.5775838	count	1
AP001816.1	0.5230272	0.3065012	1.7064	0.0881	0.577638715	count	1
RMI1	0.6114272	0.3378647	1.8097	0.0705	0.577703004	count	1
FBXL12	0.4663538	0.2120524	2.1992	0.028	0.577779303	count	1
ID1	0.5727477	0.4408943	1.2991	0.194	0.577823346	count	1
TBCCD1	0.6731008	0.5585789	1.205	0.228	0.577864439	count	1
HPS6	0.5794941	0.4082946	1.4193	0.156	0.578004582	count	1
PGM5	1.611489	0.6327587	2.5468	0.0109	0.578063871	count	1
STARD13	0.6737926	0.3024481	2.2278	0.026	0.578494671	count	1
DNAJC11	0.5242387	0.2921029	1.7947	0.0728	0.579016278	count	1
CS	0.4896794	0.2488266	1.968	0.0492	0.579077356	count	1
NUDT16	0.4257685	0.0967225	4.402	1.12E-05	0.579126959	count	0.2653168
PDCD2	0.4201975	0.1227898	3.4221	0.000632	0.57929615	count	1
EBLN2	0.9816805	0.5479651	1.7915	0.0733	0.579468353	count	1
NBPF15	1.6152527	0.8840099	1.8272	0.0678	0.579552574	count	1
TTC30A	1.6152527	0.9407268	1.717	0.0861	0.579552574	count	1

GPC2	1.6152527	0.9407268	1.717	0.0861	0.579552574	count	1
ARHGEF10	1.6152527	1.0524766	1.5347	0.125	0.579552574	count	1
LINC00654	1.6152527	0.9407268	1.717	0.0861	0.579552574	count	1
AL031846.2	1.6152527	1.0022209	1.6117	0.107	0.579552574	count	1
ZNF638	0.4337898	0.147117	2.9486	0.00322	0.579691169	count	1
WASHC2C	0.5217768	0.1761559	2.962	0.00309	0.579781229	count	1
INTS9	0.6346463	0.4304677	1.4743	0.141	0.579914426	count	1
AGL	0.4860515	0.2703202	1.7981	0.0723	0.580391727	count	1
ZNF571	0.9182308	0.717165	1.2804	0.201	0.580557891	count	1
CBFB	0.4565758	0.2632809	1.7342	0.083	0.580736428	count	1
FOLR2	0.4063434	0.0880366	4.6156	4.12E-06	0.581027489	count	0.09785412
SSPN	0.5697392	0.4009015	1.4211	0.155	0.581105187	count	1
STRADB	0.5640631	0.3231619	1.7455	0.081	0.581145141	count	1
FAM193B	0.6481932	0.4033393	1.6071	0.108	0.581242227	count	1
NPIPB4	0.8680187	0.7722683	1.124	0.261	0.581689287	count	1
BTN3A2	0.4678152	0.1522371	3.0729	0.00214	0.581896695	count	1
C11orf68	0.5179812	0.3692051	1.403	0.161	0.582110412	count	1
DPEP2	0.4380976	0.1653802	2.649	0.00812	0.58234431	count	1
CTBP1-DT	0.6629361	0.4535968	1.4615	0.144	0.582384116	count	1
SMCR8	0.4894342	0.2515041	1.946	0.0518	0.582661334	count	1
DCBLD1	0.7919272	0.4136517	1.9145	0.0557	0.582702163	count	1
EFNA4	0.5552183	0.5779876	0.9606	0.337	0.582756912	count	1
ZNF75A	0.5084411	0.2869885	1.7716	0.0766	0.582789188	count	1
TATDN2	0.6500936	0.3332089	1.951	0.0512	0.583041229	count	1
UXS1	0.4725629	0.2303661	2.0514	0.0403	0.583184162	count	1
FOXJ2	0.5115335	0.2846192	1.7973	0.0724	0.58364614	count	1
NCAPD2	0.6083191	0.3587486	1.6957	0.0901	0.583722611	count	1
SMN2	0.9229123	0.7444833	1.2397	0.215	0.583812581	count	1
ZNF740	0.7160154	0.5781054	1.2386	0.216	0.583832023	count	1
H2AFY2	0.6390256	0.3369439	1.8965	0.058	0.584129576	count	1
APOBEC3B	0.794077	0.4078736	1.9469	0.0517	0.584412475	count	1
IL27RA	0.4595307	0.1952611	2.3534	0.0187	0.584545302	count	1
FOXJ3	0.4580383	0.1991134	2.3004	0.0215	0.584950421	count	1
CRKL	0.5154403	0.4030162	1.279	0.201	0.585317757	count	1
FAM172A	0.4664445	0.2530358	1.8434	0.0654	0.585394656	count	1
ZNF124	0.4887418	0.2340669	2.088	0.0369	0.585465712	count	1
WASHC2A	0.4657712	0.2269822	2.052	0.0403	0.585511777	count	1
SNX10	0.4157299	0.0896737	4.636	3.74E-06	0.585666046	count	0.0888437
ZNF107	0.6410943	0.4292478	1.4935	0.135	0.586121419	count	1
ZBTB7B	0.4761296	0.2408284	1.977	0.0481	0.586398347	count	1
SCAI	0.9270196	0.5455666	1.6992	0.0894	0.586669246	count	1
POPDC2	1.194129	0.7129172	1.675	0.0941	0.586711536	count	1
AC012640.2	0.6115434	0.4413529	1.3856	0.166	0.586962546	count	1
R3HDM1	0.4701887	0.1984823	2.3689	0.0179	0.587077034	count	1
MKNK2	0.4292159	0.1210327	3.5463	0.000398	0.58743296	count	1

RNGTT	0.528717	0.2524453	2.0944	0.0363	0.587717536	count	1
ERG28	0.5126939	0.2895378	1.7707	0.0767	0.587787678	count	1
INO80E	0.4969136	0.2874832	1.7285	0.084	0.587818518	count	1
IGFBP4	0.5713554	0.2796355	2.0432	0.0411	0.588946726	count	1
LST1	0.4108275	0.0496618	8.2725	2.13E-16	0.589050928	count	5.15E-12
CCDC125	0.476389	0.2373427	2.0072	0.0448	0.589216431	count	1
SLC22A23	1.367596	0.7176104	1.9058	0.0568	0.589275507	count	1
LAMB2	0.6443879	0.351724	1.8321	0.0671	0.589293546	count	1
SENP3	0.8358911	0.4886195	1.7107	0.0873	0.589437749	count	1
AASDHPPT	0.4413646	0.162815	2.7108	0.00676	0.58959657	count	1
HELB	0.5568791	0.3106583	1.7926	0.0732	0.589718741	count	1
ST3GAL5	0.4600802	0.2166646	2.1235	0.0338	0.589795022	count	1
PSMD5	0.5981052	0.2979319	2.0075	0.0448	0.589850358	count	1
INPP5D	0.46698	0.2076995	2.2483	0.0246	0.589858256	count	1
ABHD17C	1.0834679	0.5448903	1.9884	0.0469	0.589943385	count	1
SH3BP2	0.4267794	0.1364406	3.128	0.00178	0.590162977	count	1
SRPK1	0.4565605	0.1850176	2.4677	0.0137	0.590485817	count	1
AC119428.2	1.370149	0.6718179	2.0395	0.0415	0.590506422	count	1
UTP20	0.5987609	0.3588298	1.6686	0.0953	0.590525334	count	1
AL031963.3	0.7238377	0.5033399	1.4381	0.151	0.590646664	count	1
ATG4C	0.4650079	0.2137392	2.1756	0.0297	0.590787097	count	1
RNF113A	0.4633762	0.1917592	2.4164	0.0157	0.591087663	count	1
BBS2	0.6592081	0.392245	1.6806	0.093	0.591674539	count	1
MED11	0.4341984	0.1528994	2.8398	0.00455	0.591851376	count	1
C2CD5	0.5637517	0.3514436	1.6041	0.109	0.592037785	count	1
AC073283.1	2.198335	1.1461401	1.918	0.0552	0.592097941	count	1
TXNDC5	2.198335	1.1618592	1.8921	0.0586	0.592097941	count	1
AC005616.1	2.198335	1.1461401	1.918	0.0552	0.592097941	count	1
CCDC57	0.4796931	0.246385	1.9469	0.0517	0.592132532	count	1
IL23A	1.2043538	0.7119782	1.6916	0.0909	0.592336061	count	1
CSE1L	0.5594714	0.3085618	1.8132	0.0699	0.592560099	count	1
PDE4D	0.5026671	0.252856	1.988	0.0469	0.592689848	count	1
GPD2	0.521773	0.2799654	1.8637	0.0625	0.592700526	count	1
KIAA1328	0.6177347	0.2952853	2.092	0.0365	0.593186775	count	1
RALGAPA2	0.5556317	0.3342813	1.6622	0.0966	0.59325981	count	1
VPS18	0.5225657	0.2941253	1.7767	0.0757	0.593624907	count	1
ALPK3	0.7503727	0.4622775	1.6232	0.105	0.594247293	count	1
CEBPA-DT	0.9381435	0.6199208	1.5133	0.13	0.594411561	count	1
MSMO1	0.6190492	0.3235435	1.9133	0.0558	0.594508762	count	1
EDN1	0.93839	0.7291132	1.287	0.198	0.594583197	count	1
CATSPERG	0.93839	0.561326	1.6717	0.0947	0.594583197	count	1
XRN1	0.4473023	0.1349481	3.3146	0.000931	0.594686173	count	1
TRIM26	0.5080997	0.237371	2.1405	0.0324	0.594821607	count	1
CLIP2	0.9387452	0.4364767	2.1507	0.0316	0.594830558	count	1
VSIG10	0.7286864	0.4489474	1.6231	0.105	0.594873678	count	1

ASAP2	0.9388092	0.5805872	1.617	0.106	0.594875143	count	1
NAA16	0.523701	0.2740797	1.9108	0.0562	0.594948879	count	1
RALGPS1	1.0920486	0.6142259	1.7779	0.0755	0.595146234	count	1
SLC12A9	0.456736	0.1462818	3.1223	0.00182	0.595273425	count	1
NR2F2	0.7518425	0.858978	0.8753	0.382	0.595495263	count	1
IFFO2	0.6396682	0.6041354	1.0588	0.29	0.59557537	count	1
DPF2	0.5624062	0.3189656	1.7632	0.078	0.595777618	count	1
PLEKHA4	1.0067039	0.6472954	1.5552	0.12	0.595831651	count	1
TNFRSF11A	0.6297574	0.3725372	1.6905	0.0911	0.595883895	count	1
RP2	0.4690431	0.1579194	2.9701	0.00301	0.595983979	count	1
AC044849.1	0.730138	0.3601452	2.0273	0.0427	0.59613961	count	1
ZNF227	0.6515411	0.4079766	1.597	0.11	0.596186656	count	1
OSM	0.4223243	0.1137891	3.7115	0.000211	0.596263314	count	1
ZNF628	0.7303392	0.4941789	1.4779	0.14	0.596315098	count	1
GAS7	0.4437874	0.1380653	3.2143	0.00132	0.596488313	count	1
METTL3	0.5905962	0.4520739	1.3064	0.192	0.596575731	count	1
TRAF5	0.5844654	0.4485514	1.303	0.193	0.596725872	count	1
C6orf226	0.4644607	0.2166451	2.1439	0.0321	0.596893104	count	1
SCARB1	0.6410932	0.3214969	1.9941	0.0463	0.596970249	count	1
CNTLN	0.4486918	0.1773944	2.5293	0.0115	0.597227649	count	1
TMEM144	0.5102986	0.2420208	2.1085	0.0351	0.59745527	count	1
CHKA	0.4709147	0.171403	2.7474	0.00605	0.597548298	count	1
FAM122C	0.8106819	0.5828805	1.3908	0.164	0.597636285	count	1
CR1	0.4745545	0.1817057	2.6117	0.00907	0.597687421	count	1
KCNC3	0.7319457	0.4269739	1.7143	0.0866	0.597716364	count	1
SNAP23	0.4354373	0.1058155	4.1151	4.00E-05	0.597867546	count	0.94316
USP33	0.4543447	0.1509858	3.0092	0.00265	0.597894453	count	1
TACC3	0.5053701	0.1840094	2.7464	0.00607	0.598041537	count	1
RHNO1	0.4948888	0.2665826	1.8564	0.0635	0.598119029	count	1
GULP1	2.221378	1.1738719	1.8924	0.0586	0.598146208	count	1
AC105760.2	2.221378	1.1738719	1.8924	0.0586	0.598146208	count	1
TRIM2	2.221378	1.0901705	2.0376	0.0417	0.598146208	count	1
MTRNR2L6	2.221378	1.1105563	2.0002	0.0456	0.598146208	count	1
AC100849.1	2.221378	1.188244	1.8695	0.0617	0.598146208	count	1
AL139383.1	2.221378	1.1105563	2.0002	0.0456	0.598146208	count	1
DMKN	2.221378	1.330075	1.6701	0.095	0.598146208	count	1
ZNF415	2.221378	1.1105563	2.0002	0.0456	0.598146208	count	1
AC132872.3	2.2215355	1.522	1.4596	0.145	0.598187356	count	1
ZNF704	1.3865624	0.6235244	2.2238	0.0263	0.598416811	count	1
EPB41L1	1.3865624	0.535603	2.5888	0.00969	0.598416811	count	1
XPR1	0.4916084	0.3636413	1.3519	0.177	0.598770044	count	1
PRKACA	0.4473377	0.1358051	3.294	0.001	0.598828593	count	1
ARFGAP2	0.4555507	0.1951036	2.3349	0.0196	0.599497065	count	1
PSME4	0.4837631	0.2055515	2.3535	0.0187	0.599707811	count	1
PPCDC	0.5183385	0.2499783	2.0735	0.0382	0.599753967	count	1

RAB5B	0.4978311	0.2919319	1.7053	0.0883	0.600071211	count	1
AC135050.3	0.8501721	0.5527144	1.5382	0.124	0.600387995	count	1
LGALS3BP	0.4463815	0.1609689	2.7731	0.00559	0.600765102	count	1
CRY1	0.601448	0.5214927	1.1533	0.249	0.60084484	count	1
GTF3C1	0.4899745	0.2364301	2.0724	0.0383	0.601054738	count	1
MYO1F	0.431742	0.1032094	4.1832	2.98E-05	0.601154881	count	0.703578
ARL17B	1.3922829	0.7876077	1.7677	0.0772	0.601172902	count	1
KCTD6	0.5346136	0.3216876	1.6619	0.0967	0.601328899	count	1
AC020915.3	0.7162991	0.5789212	1.2373	0.216	0.601335867	count	1
UBXN11	0.4678787	0.1679791	2.7853	0.00539	0.60134047	count	1
CCDC61	0.6101454	0.3628033	1.6818	0.0927	0.60225117	count	1
FMO2	1.6729951	0.7546593	2.2169	0.0267	0.602307326	count	1
AC019254.1	1.6729951	0.8188923	2.043	0.0412	0.602307326	count	1
KLF7	0.4797536	0.1776694	2.7003	0.00698	0.602351852	count	1
USP24	0.4799291	0.2588826	1.8538	0.0639	0.60257552	count	1
ALOX5AP	0.421618	0.0758185	5.5609	2.97E-08	0.602596825	count	0.000711256
ALG13	0.4323632	0.1274194	3.3932	0.000702	0.602655546	count	1
LIAS	0.5487565	0.3428702	1.6005	0.11	0.603030822	count	1
TRIM37	0.5968975	0.3381185	1.7653	0.0776	0.603203506	count	1
TMEM63B	0.7183942	0.3878823	1.8521	0.0641	0.603208832	count	1
GNA11	0.6192202	0.4324723	1.4318	0.152	0.603391489	count	1
STARD8	0.6040169	0.4344572	1.3903	0.165	0.603520504	count	1
NHSL2	0.6280816	0.3365256	1.8664	0.0621	0.603597163	count	1
SYNJ2BP	0.4832653	0.2199808	2.1969	0.0281	0.603662655	count	1
OCRL	0.8183378	0.5517827	1.4831	0.138	0.603741106	count	1
DFFB	1.1065884	0.8116057	1.3635	0.173	0.603968103	count	1
ZNF419	0.7391464	0.585847	1.2617	0.207	0.60400009	count	1
DMXL1	0.4589958	0.1799399	2.5508	0.0108	0.604075474	count	1
COX11	0.5265804	0.2091584	2.5176	0.0119	0.604119192	count	1
A1BG-AS1	0.6203107	0.3586297	1.7297	0.0838	0.604502751	count	1
MSI2	0.5982104	0.3627951	1.6489	0.0993	0.604584893	count	1
PDGFB	0.518266	0.1919969	2.6993	0.007	0.60463883	count	1
UHRF2	0.6124935	0.3025576	2.0244	0.043	0.604671226	count	1
TMEM69	0.506124	0.1926728	2.6269	0.00867	0.604864929	count	1
TMEM44-AS1	0.6732748	0.5260558	1.2799	0.201	0.605014753	count	1
PSEN1	0.4625926	0.1955469	2.3656	0.0181	0.605071731	count	1
TTC37	0.4730502	0.1651444	2.8645	0.00421	0.605144109	count	1
AGPAT3	0.4627411	0.1637064	2.8267	0.00474	0.605268012	count	1
CD48	0.4256105	0.0627893	6.7784	1.52E-11	0.605854151	count	3.66E-07
USP3	0.4333132	0.0986915	4.3906	1.18E-05	0.606670402	count	0.2795184
ZNF41	2.254578	0.743894	3.0308	0.00246	0.606772621	count	1
HSPA14	0.4925009	0.2031275	2.4246	0.0154	0.60691314	count	1
SLC10A3	0.4915263	0.2587528	1.8996	0.0576	0.606990167	count	1
GRK6	0.4553466	0.1599271	2.8472	0.00445	0.607159037	count	1
ITPKB	0.8230129	0.4599016	1.7895	0.0737	0.607471423	count	1

WRB	0.5002522	0.2325098	2.1515	0.0315	0.607949893	count	1
HERC4	0.4747051	0.1943626	2.4424	0.0147	0.608044106	count	1
FAM118B	0.6421347	0.3383199	1.898	0.0578	0.608179176	count	1
AP001107.9	1.2334513	0.7584171	1.6263	0.104	0.608349631	count	1
AC011603.2	1.2334513	0.7351431	1.6778	0.0935	0.608349631	count	1
AL135999.1	1.6884231	0.7675189	2.1998	0.0279	0.608357529	count	1
INIP	0.4601108	0.1786756	2.5751	0.0101	0.609282145	count	1
ZBTB6	0.6651937	0.4707682	1.413	0.158	0.609356801	count	1
MARS	0.5477207	0.2408171	2.2744	0.023	0.609469926	count	1
CBWD5	0.497901	0.2324929	2.1416	0.0323	0.609532719	count	1
RASA3	0.4981301	0.2496263	1.9955	0.0461	0.609818254	count	1
FAM180B	1.692227	1.550471	1.0914	0.2752	0.609847127	count	1
VAMP5	0.4334564	0.0833074	5.2031	2.12E-07	0.610214303	count	0.005063196
ATP10D	0.4868523	0.2102478	2.3156	0.0207	0.610362544	count	1
RGPD5	0.6037098	0.4102605	1.4715	0.141	0.610372936	count	1
FES	0.4649856	0.1470714	3.1616	0.00159	0.610666491	count	1
CMKLR1	0.5427187	0.295575	1.8361	0.0665	0.61070255	count	1
AC020765.2	0.7095213	0.5174821	1.3711	0.17	0.611106968	count	1
PRPSAP2	0.4969247	0.2109902	2.3552	0.0186	0.611113878	count	1
CLK1	0.4382755	0.1119079	3.9164	9.23E-05	0.61147178	count	1
CERS5	0.5165412	0.2585323	1.998	0.0458	0.611554174	count	1
FMNL1	0.4416714	0.110396	4.0008	6.50E-05	0.611579083	count	1
MCC	1.1191256	0.9356071	1.1961	0.232	0.611580183	count	1
GAPLINC	0.6361721	0.3803487	1.6726	0.0945	0.611744501	count	1
HSD17B11	0.4321241	0.0667103	6.4776	1.12E-10	0.612275426	count	2.70E-06
AKAP10	0.5781268	0.3257599	1.7747	0.0761	0.613025875	count	1
ALKBH8	0.867083	0.5189974	1.6707	0.0949	0.613374995	count	1
LARGE1	2.281462	0.9170953	2.4877	0.0129	0.613681107	count	1
KIAA0100	0.4692144	0.1880824	2.4947	0.0127	0.613825209	count	1
YARS2	0.5705356	0.2881834	1.9798	0.0478	0.614527526	count	1
ARL6	1.7045946	0.8952256	1.9041	0.057	0.614684643	count	1
AL109615.2	1.7045946	0.926769	1.8393	0.066	0.614684643	count	1
LINC01684	1.7045946	1.1117616	1.5332	0.125	0.614684643	count	1
RASGRP2	0.4699349	0.1753108	2.6806	0.0074	0.614777761	count	1
MTA3	0.640066	0.4875242	1.3129	0.189	0.615667881	count	1
MS4A14	0.6157096	0.3125223	1.9701	0.0489	0.615707024	count	1
UBALD1	0.5676719	0.3143727	1.8057	0.0711	0.615910184	count	1
BTBD9	0.7758983	0.4804488	1.6149	0.106	0.615947878	count	1
ZNF174	0.7758983	0.4182852	1.855	0.0637	0.615947878	count	1
ZNF646	0.5857705	0.4399083	1.3316	0.183	0.616016349	count	1
GOLPH3L	0.8026454	0.4710497	1.704	0.0885	0.616041457	count	1
ZFC3H1	0.447283	0.1362466	3.2829	0.00104	0.616154617	count	1
CCDC92	0.4932132	0.2685524	1.8366	0.0664	0.616286682	count	1
PICK1	0.6503349	0.2519606	2.5811	0.00991	0.616333133	count	1
CDYL	0.4805214	0.2133486	2.2523	0.0244	0.61634011	count	1

BBC3	0.530226	0.2344755	2.2613	0.0238	0.616432424	count	1
ATE1	0.5140915	0.2357548	2.1806	0.0293	0.616467643	count	1
UACA	0.5142956	0.2210431	2.3267	0.0201	0.616717429	count	1
LINC01506	1.0388519	0.4922516	2.1104	0.0349	0.616899369	count	1
GAREM1	2.295881	1.0430137	2.2012	0.0278	0.617357368	count	1
AC040970.1	0.6418771	0.3106092	2.0665	0.0389	0.617493303	count	1
RPP21	0.7344894	0.4205095	1.7467	0.0808	0.617611093	count	1
GOLGA1	0.5121543	0.2716288	1.8855	0.0595	0.617683422	count	1
U2AF1L5	0.6624227	0.3885235	1.705	0.0883	0.617872372	count	1
TAZ	0.5012691	0.2340126	2.1421	0.0323	0.617907357	count	1
C18orf65	0.5875673	0.5091494	1.154	0.249	0.617975005	count	1
EXOSC10	0.5202167	0.2449182	2.124	0.0338	0.618085384	count	1
CPEB3	0.9722574	0.5454672	1.7824	0.0748	0.618201934	count	1
UPK3A	0.5522594	0.5585711	0.9887	0.323	0.618276571	count	1
SLC25A40	0.5467311	0.2642337	2.0691	0.0386	0.618648612	count	1
UBE3B	0.5833493	0.3075634	1.8967	0.058	0.618760741	count	1
CTDSP1	0.4533091	0.1126926	4.0225	5.93E-05	0.619055217	count	1
NNT-AS1	0.5417646	0.2767483	1.9576	0.0504	0.619066094	count	1
WAC-AS1	0.4635106	0.1428954	3.2437	0.0012	0.619112001	count	1
ASCL2	0.4726361	0.178219	2.652	0.00805	0.61935982	count	1
GIPC1	0.5084396	0.263997	1.9259	0.0542	0.619658605	count	1
TMEM39B	0.5232881	0.2212495	2.3651	0.0181	0.619719583	count	1
PLEKHG2	0.5600692	0.2865819	1.9543	0.0508	0.619815665	count	1
DIS3L	0.6445605	0.3632986	1.7742	0.0762	0.620198341	count	1
P2RY12	1.044166	0.3657982	2.8545	0.00435	0.620386165	count	1
SLC39A13	0.6281425	0.4133138	1.5198	0.129	0.620813047	count	1
ZNF561	0.5644603	0.3758308	1.5019	0.133	0.620828949	count	1
ORC4	0.5106589	0.2205954	2.3149	0.0207	0.620839598	count	1
PREP	0.6072365	0.3111965	1.9513	0.0511	0.620920349	count	1
BCAS3	0.8769251	0.4578907	1.9151	0.0556	0.620943025	count	1
SP3	0.4543682	0.1409163	3.2244	0.00128	0.620987968	count	1
SEMA4A	0.510861	0.193582	2.639	0.00837	0.621089987	count	1
FGD2	0.4714746	0.1497423	3.1486	0.00166	0.621111957	count	1
NEK9	0.5905277	0.3229562	1.8285	0.0676	0.621202668	count	1
ZDHHC21	0.568978	0.3069751	1.8535	0.0639	0.621755791	count	1
PRMT7	0.7211688	0.3563663	2.0237	0.0431	0.621764696	count	1
AP003774.3	1.2578277	0.4892508	2.5709	0.0102	0.621770437	count	1
CX3CR1	0.4541019	0.1362606	3.3326	0.000873	0.621834038	count	1
PFN2	2.3150489	1.146531	2.0192	0.0436	0.622212598	count	1
DEPDC5	0.6466986	0.3902111	1.6573	0.0976	0.622354168	count	1
TVP23A	0.6220849	0.316261	1.967	0.0493	0.622356884	count	1
MOGS	0.5192504	0.2732059	1.9006	0.0575	0.622782218	count	1
ZDHHC7	0.4923003	0.1548548	3.1791	0.0015	0.62325329	count	1
C11orf98	0.7852612	0.5346291	1.4688	0.142	0.623921915	count	1
YPEL3	0.4405396	0.0827568	5.3233	1.11E-07	0.624593189	count	0.0026529

PACERR	1.4421768	1.051599	1.3714	0.17	0.625183153	count	1
OSBPL9	0.4841078	0.1737351	2.7865	0.00537	0.625235216	count	1
SGSM2	0.6052463	0.4030794	1.5016	0.133	0.625269873	count	1
LINC02432	0.9273824	0.4130969	2.245	0.0249	0.625280381	count	1
HMGCR	0.4978071	0.23464	2.1216	0.034	0.625369919	count	1
AL359198.1	1.265273	0.6295408	2.0098	0.0446	0.62586999	count	1
PDE4B	0.4518783	0.0928137	4.8687	1.20E-06	0.625954914	count	0.0285756
ZNF630	1.7336057	0.8365598	2.0723	0.0383	0.625995309	count	1
AC124319.2	1.7336057	0.8842021	1.9606	0.05	0.625995309	count	1
ARHGAP24	0.4672934	0.1937206	2.4122	0.0159	0.626060411	count	1
ATP8B3	0.8151296	0.4812755	1.6937	0.0905	0.626363279	count	1
CERK	0.5141131	0.2557442	2.0103	0.0445	0.626703832	count	1
PGBD4	2.3332661	1.1644131	2.0038	0.0452	0.626793063	count	1
ZNF781	2.3332661	1.0055139	2.3205	0.0204	0.626793063	count	1
DENND4B	0.5258615	0.2257638	2.3293	0.0199	0.626978633	count	1
B3GALNT1	0.7108298	0.5990499	1.1866	0.236	0.627021701	count	1
TLE4	0.4562082	0.1280608	3.5624	0.000374	0.627056894	count	1
METTL22	0.5393846	0.2753594	1.9588	0.0502	0.627341484	count	1
COL6A3	0.9858814	0.7308566	1.3489	0.177	0.627721153	count	1
MANSC1	1.2689208	0.7647187	1.6593	0.0972	0.627878553	count	1
SP140L	0.4685896	0.162891	2.8767	0.00405	0.628107342	count	1
TNFRSF14-AS1	0.7464903	0.5394955	1.3837	0.167	0.628364882	count	1
AKAP11	0.4922687	0.2061532	2.3879	0.017	0.628449965	count	1
CDK11A	0.4842127	0.2109491	2.2954	0.0218	0.628564008	count	1
TALDO1	0.4381025	0.0457692	9.572	2.46E-21	0.628620624	count	5.96E-17
NCBP3	0.4651641	0.1624064	2.8642	0.00422	0.628690392	count	1
ERCC8	0.6530137	0.394267	1.6563	0.0978	0.628724049	count	1
AP4M1	0.6085898	0.3484724	1.7465	0.0809	0.628858936	count	1
BRPF3	0.7471978	0.5232815	1.4279	0.153	0.628999242	count	1
CD99L2	0.5716726	0.3479913	1.6428	0.101	0.629009981	count	1
RPAP3	0.4855173	0.1656816	2.9304	0.00342	0.62903152	count	1
RSBN1	0.4829364	0.1738501	2.7779	0.00551	0.629242069	count	1
BIN3	0.5126307	0.2479846	2.0672	0.0388	0.629355676	count	1
SIPA1L1	0.4931051	0.1701549	2.898	0.00379	0.629532182	count	1
ERCC5	0.7135297	0.3992463	1.7872	0.074	0.62954441	count	1
VMAC	0.7135297	0.4229488	1.687	0.0917	0.62954441	count	1
CCL4	0.4371255	0.1025749	4.2615	2.11E-05	0.630148502	count	0.4989095
HCFC2	0.7143448	0.3272216	2.1831	0.0291	0.630306149	count	1
AC009506.1	0.6229248	0.365589	1.7039	0.0885	0.630618203	count	1
COTL1	0.438875	0.043398	10.1128	1.40E-23	0.63101856	count	3.40E-19
PTPDC1	0.8210082	0.5446708	1.5073	0.132	0.631227927	count	1
SLC12A4	0.8533229	0.4287365	1.9903	0.0467	0.631697302	count	1
ASGR2	0.4853037	0.1855975	2.6148	0.00898	0.631785128	count	1
ARL4A	0.4432215	0.0677918	6.538	7.56E-11	0.631843465	count	1.82E-06
NUP153	0.5013789	0.274223	1.8284	0.0676	0.631987509	count	1

GCFC2	0.567421	0.2956314	1.9194	0.0551	0.632051179	count	1
TUBE1	0.6565217	0.3772068	1.7405	0.0819	0.63226404	count	1
AC093323.1	0.6666858	0.443298	1.5039	0.133	0.63261044	count	1
ZNF318	0.5358382	0.3375104	1.5876	0.113	0.63270774	count	1
TNFSF9	0.4624675	0.1697716	2.7241	0.00649	0.632902908	count	1
HERC6	0.6251302	0.5505645	1.1354	0.256	0.632943964	count	1
AP000787.1	2.3587404	1.118571	2.1087	0.0351	0.633141974	count	1
KDM5C	0.5930935	0.2893019	2.0501	0.0405	0.634681199	count	1
TCERG1	0.4673408	0.1522419	3.0697	0.00217	0.634683243	count	1
TM9SF4	0.510643	0.1951179	2.6171	0.00892	0.634849403	count	1
PRPF3	0.5033195	0.294603	1.7085	0.0877	0.635470897	count	1
CYSLTR2	0.7199264	0.3322163	2.167	0.0303	0.635523885	count	1
AL391832.3	0.9413995	0.5269176	1.7866	0.0741	0.635606586	count	1
URM1	0.4867059	0.1426963	3.4108	0.000658	0.635884454	count	1
FMO5	0.7554351	0.3915552	1.9293	0.0538	0.636388241	count	1
DCTD	0.5038293	0.1918843	2.6257	0.0087	0.637103862	count	1
TMEM62	0.8009389	0.4046518	1.9793	0.0479	0.637290197	count	1
NSUN3	0.5957201	0.356899	1.6692	0.0952	0.637589928	count	1
ADSS	0.4659652	0.1348993	3.4542	0.000561	0.637717183	count	1
KDM7A	0.4839735	0.1448203	3.3419	0.000845	0.63774347	count	1
KDM4C	0.5193812	0.2853859	1.8199	0.0689	0.63779409	count	1
PCGF1	0.5420106	0.295171	1.8363	0.0664	0.637857905	count	1
GAPT	0.4688628	0.1207936	3.8815	0.000107	0.637937762	count	1
SLC30A9	0.4977632	0.189811	2.6224	0.00878	0.637973019	count	1
PDE1B	0.5961388	0.5777985	1.0317	0.302	0.638053651	count	1
MMP17	1.764941	0.5810897	3.0373	0.00241	0.63815051	count	1
AL606491.1	1.163007	0.944256	1.2317	0.218	0.638254479	count	1
AC092171.4	1.163007	0.8050232	1.4447	0.149	0.638254479	count	1
LRRCC1	0.6453511	0.3425759	1.8838	0.0597	0.638589367	count	1
AC093297.2	1.4704768	0.9303936	1.5805	0.114	0.63877286	count	1
EP300	0.4738588	0.1508603	3.141	0.0017	0.638808657	count	1
PCED1A	0.740072	0.4121161	1.7958	0.0727	0.639087281	count	1
ZFX	0.4791431	0.1811918	2.6444	0.00824	0.639166649	count	1
UVRAG	0.4667031	0.1115568	4.1835	2.97E-05	0.639188383	count	0.7012467
SLC2A3	0.4470856	0.0787675	5.676	1.54E-08	0.639227342	count	0.000369184
PLEKHA2	0.4861664	0.1924339	2.5264	0.0116	0.639257121	count	1
LRMP	0.5230587	0.1641241	3.187	0.00146	0.639382571	count	1
UBE4B	0.5739627	0.2449977	2.3427	0.0192	0.639556376	count	1
ZNF746	0.6464273	0.331743	1.9486	0.0515	0.639701943	count	1
AC087741.1	0.6186916	0.5045854	1.2261	0.22	0.639708574	count	1
ZNF138	0.6387826	0.3402537	1.8774	0.0606	0.639790984	count	1
MAP3K3	0.4793771	0.1560376	3.0722	0.00215	0.639820565	count	1
PHF3	0.4612	0.105696	4.3635	1.33E-05	0.640056694	count	0.3149174
EVI2A	0.4583968	0.0906587	5.0563	4.59E-07	0.640069152	count	0.010946232
PPP1R37	0.619043	0.3893609	1.5899	0.112	0.64008614	count	1

RDH5	0.7250444	0.5538871	1.309	0.191	0.640310618	count	1
SETD1B	0.6973691	0.3512428	1.9854	0.0472	0.64046419	count	1
MYO18A	0.6973691	0.3782952	1.8435	0.0654	0.64046419	count	1
AP1G2	0.6085267	0.3156529	1.9278	0.054	0.640842936	count	1
AC245140.2	0.8055633	0.475365	1.6946	0.0903	0.641237137	count	1
CUL4B	0.5826245	0.2292136	2.5418	0.0111	0.641440917	count	1
LRP3	0.5992855	0.3479944	1.7221	0.0852	0.6415392	count	1
KIF27	1.0059036	0.5197998	1.9352	0.0531	0.641727621	count	1
TADA2B	0.6867673	0.3453648	1.9885	0.0469	0.641780784	count	1
MLLT3	1.0768722	0.6017653	1.7895	0.0737	0.641870685	count	1
AKIRIN2	0.456261	0.0738337	6.1796	7.50E-10	0.641894149	count	1.80E-05
DUS2	0.641042	0.4639257	1.3818	0.167	0.642151913	count	1
IRAK3	0.4757424	0.0930233	5.1142	3.39E-07	0.642611535	count	0.008089218
TCF12	0.4920402	0.1924605	2.5566	0.0106	0.642930563	count	1
CAMTA1-DT	1.296606	1.1304284	1.147	0.251	0.643122041	count	1
CPLANE2	1.296606	0.7192112	1.8028	0.0715	0.643122041	count	1
CASTOR2	1.296606	0.6692785	1.9373	0.0528	0.643122041	count	1
CLCN1	1.296606	0.6692785	1.9373	0.0528	0.643122041	count	1
AC080038.1	1.296606	0.837537	1.5481	0.122	0.643122041	count	1
TMCC1-AS1	1.4799132	0.7024672	2.1067	0.0352	0.643298458	count	1
SPNS2	2.4000815	1.195162	2.0082	0.0447	0.643303518	count	1
MICAL3	0.9522109	0.7556711	1.2601	0.208	0.643578933	count	1
MIGA1	0.5384428	0.304062	1.7708	0.0767	0.644327723	count	1
MKL1	0.5781252	0.2983722	1.9376	0.0528	0.644333669	count	1
TIMM22	0.5661057	0.312333	1.8125	0.07	0.644473706	count	1
RIF1	0.482623	0.1784921	2.7039	0.0069	0.644526803	count	1
SIAH2	0.4760263	0.1362196	3.4945	0.000483	0.644629818	count	1
ZNF691	1.2998517	0.6955317	1.8689	0.0618	0.644908622	count	1
TCHH	0.8698914	0.428951	2.028	0.0427	0.64496852	count	1
CCDC40	1.4837489	1.119428	1.3255	0.185	0.645137131	count	1
BRD1	0.4943465	0.189001	2.6156	0.00896	0.645420254	count	1
ARRDC3	0.4643362	0.1243697	3.7335	0.000193	0.645549716	count	1
RSRP1	0.4548817	0.0820742	5.5423	3.30E-08	0.645673683	count	0.000790152
MNS1	1.3013094	0.6485878	2.0064	0.0449	0.645711071	count	1
HEXA-AS1	1.3013094	0.6560559	1.9835	0.0474	0.645711071	count	1
SMAD3	0.5943707	0.3375258	1.761	0.0784	0.645852183	count	1
ALMS1	1.0119967	0.6763765	1.4962	0.135	0.645993697	count	1
ZNF345	1.3022926	0.695405	1.8727	0.0612	0.646252313	count	1
AL365205.1	1.786376	1.1766098	1.5182	0.129	0.6464262	count	1
ZMYM4	0.4871594	0.1861425	2.6171	0.00892	0.646641631	count	1
MTX1	0.4703095	0.1366447	3.4418	0.000587	0.646801646	count	1
ZNF335	0.7675857	0.3934329	1.951	0.0512	0.647297899	count	1
MEF2C	0.4551537	0.0758804	5.9983	2.29E-09	0.647355138	count	5.50E-05
AL662844.4	1.1782002	0.5490503	2.1459	0.032	0.647498824	count	1
PUM2	0.5335799	0.1913934	2.7879	0.00535	0.647568804	count	1

JUNB	0.4497032	0.036234	12.4111	2.39E-34	0.647734221	count	5.81E-30
ENTPD1-AS1	0.9579695	0.558889	1.7141	0.0866	0.647827934	count	1
AL390036.1	0.5588435	0.2796984	1.998	0.0458	0.647830864	count	1
ZNF397	0.5723534	0.2813797	2.0341	0.042	0.648465408	count	1
CEP120	0.6269118	0.2692466	2.3284	0.02	0.648543722	count	1
CD37	0.4532192	0.047432	9.5551	2.88E-21	0.648656692	count	6.98E-17
SLC35A1	0.6938576	0.2944237	2.3567	0.0185	0.648753909	count	1
MLH3	0.615914	0.3337819	1.8453	0.0651	0.648911739	count	1
STXBP2	0.4567813	0.0757453	6.0305	1.88E-09	0.649105154	count	4.52E-05
AC092803.2	0.4908781	0.1644073	2.9857	0.00286	0.649583958	count	1
DCAF15	0.5464184	0.2766624	1.975	0.0484	0.649915636	count	1
ZNF230	0.6117253	0.4670853	1.3097	0.19	0.649961298	count	1
TTC21B	0.6740459	0.335552	2.0088	0.0447	0.64996433	count	1
ZNF528	0.6565044	0.424906	1.5451	0.122	0.650124494	count	1
FAM217B	0.5526384	0.3127043	1.7673	0.0773	0.650658841	count	1
SULT1C4	2.430584	1.0305385	2.3586	0.0184	0.650686355	count	1
DHX34	0.6848539	0.3687043	1.8575	0.0634	0.650724738	count	1
ACOT8	0.510342	0.2242156	2.2761	0.0229	0.650986418	count	1
MMD	0.5048589	0.279053	1.8092	0.0705	0.651671473	count	1
GAB3	0.5812427	0.2567613	2.2637	0.0237	0.651693202	count	1
BCL6	0.4854109	0.1325722	3.6615	0.000256	0.651735372	count	1
AP4B1	0.8181026	0.4120345	1.9855	0.0472	0.651947724	count	1
ANAPC1	0.6670252	0.3580928	1.8627	0.0626	0.652208724	count	1
TCF7L2	0.5884708	0.2306197	2.5517	0.0108	0.652230942	count	1
C16orf70	0.538906	0.3535356	1.5243	0.128	0.652412381	count	1
SLC46A1	0.6512774	0.3694881	1.7626	0.0781	0.652852679	count	1
LAX1	1.5003966	1.2518776	1.1985	0.231	0.653110981	count	1
CCL24	1.5003966	1.1278147	1.3304	0.184	0.653110981	count	1
AL357033.4	1.5003966	0.7232451	2.0745	0.0381	0.653110981	count	1
FGL2	0.4552762	0.0559691	8.1344	6.50E-16	0.653161069	count	1.57E-11
TMEM50B	0.4919409	0.1476217	3.3324	0.000874	0.65343914	count	1
CCDC69	0.496145	0.1583279	3.1337	0.00175	0.653475342	count	1
NCOA3	0.5038837	0.1463366	3.4433	0.000584	0.653765451	count	1
DIP2B	0.5900168	0.2330769	2.5314	0.0114	0.653997219	count	1
AL445524.1	0.5418471	0.2092025	2.5901	0.00965	0.654237034	count	1
ARHGAP19	0.7753446	0.4061621	1.909	0.0564	0.654270625	count	1
RNF216	0.4969014	0.1563185	3.1788	0.0015	0.654481732	count	1
CANT1	0.5582153	0.2786178	2.0035	0.0452	0.654933167	count	1
MDM4	0.4794039	0.1444324	3.3192	0.000916	0.655245426	count	1
TKFC	0.6395771	0.3645735	1.7543	0.0795	0.655361682	count	1
NXF1	0.5373512	0.2612212	2.0571	0.0398	0.655580977	count	1
ZXDA	1.8112827	1.0435147	1.7358	0.0827	0.655999658	count	1
CHEK1	1.8112827	0.9084316	1.9939	0.0463	0.655999658	count	1
AL691403.2	1.8112827	0.8792659	2.06	0.0395	0.655999658	count	1
SERPINB10	1.8112827	0.9880133	1.8333	0.0669	0.655999658	count	1

MYNN	0.6036806	0.3266097	1.8483	0.0647	0.656306506	count	1
DCAF6	0.5499835	0.2591697	2.1221	0.0339	0.656375029	count	1
TRRAP	0.595892	0.2718566	2.1919	0.0285	0.656512773	count	1
DPYD	0.4845546	0.1252286	3.8694	0.000112	0.656933499	count	1
EEF1AKMT3	1.0282293	0.6612378	1.555	0.12	0.657366677	count	1
CARD16	0.4624259	0.0608916	7.5943	4.37E-14	0.657453821	count	1.06E-09
TRIOBP	0.5014289	0.1754907	2.8573	0.00431	0.657502158	count	1
ADH1B	1.5098389	0.7848612	1.9237	0.0545	0.657628713	count	1
AC068888.1	1.5098389	0.8703662	1.7347	0.0829	0.657628713	count	1
AC108134.3	0.8003964	0.3643295	2.1969	0.0281	0.657632371	count	1
CIR1	0.4696084	0.0905833	5.1843	2.35E-07	0.65775655	count	0.0056118
FAM19A2	0.8533201	0.4510863	1.8917	0.0586	0.658012655	count	1
TUT7	0.4738353	0.1167337	4.0591	5.08E-05	0.658169738	count	1
GASAL1	1.0293873	0.5388148	1.9105	0.0562	0.658178386	count	1
FAM135A	0.6644404	0.3577737	1.8572	0.0634	0.658338615	count	1
TOP3A	0.5870049	0.2602383	2.2556	0.0242	0.658344404	count	1
PM20D2	1.1021476	0.5844008	1.8859	0.0594	0.658497909	count	1
COX7A1	1.1021476	0.9087477	1.2128	0.225	0.658497909	count	1
UIMC1	0.525766	0.2591284	2.029	0.0426	0.658800425	count	1
SPTBN1	0.692967	0.4384309	1.5806	0.114	0.658822952	count	1
CSTF2T	0.6251723	0.2799343	2.2333	0.0256	0.659030441	count	1
IPO5	0.5521684	0.2665554	2.0715	0.0384	0.659039527	count	1
IVNS1ABP	0.4709488	0.0959989	4.9058	9.92E-07	0.659283629	count	0.023628448
PPP1R12C	0.5524078	0.2611474	2.1153	0.0345	0.659331492	count	1
GSTCD	0.8879874	0.4621592	1.9214	0.0548	0.659484468	count	1
GMDS	0.6206104	0.3097523	2.0036	0.0452	0.659744428	count	1
ATG13	0.6373278	0.3480765	1.831	0.0672	0.65974697	count	1
MBD3	0.5719254	0.2377119	2.406	0.0162	0.660518774	count	1
AC022706.1	1.5171663	0.6579902	2.3058	0.0212	0.661132013	count	1
FAF1	0.5381774	0.2238388	2.4043	0.0163	0.661304193	count	1
SNTB2	0.575429	0.3386495	1.6992	0.0894	0.661685387	count	1
ANK3	1.3313855	0.8239337	1.6159	0.106	0.662263049	count	1
PIP4K2B	0.7339088	0.340131	2.1577	0.031	0.662725739	count	1
DDX59	0.5148067	0.2195868	2.3444	0.0191	0.663210364	count	1
AL356488.3	0.9791576	0.4242525	2.308	0.0211	0.66347635	count	1
AL591895.1	0.9791714	0.856298	1.1435	0.253	0.663486552	count	1
SEMA3C	0.5188033	0.3624234	1.4315	0.152	0.663651068	count	1
LINC01184	0.5616172	0.3860254	1.4549	0.146	0.663860197	count	1
THBS1	0.4748736	0.1158327	4.0997	4.27E-05	0.664315102	count	1
ACY1	1.833216	1.089773	1.6822	0.0927	0.664390804	count	1
TMEM106A	0.5189463	0.2020151	2.5688	0.0103	0.664671565	count	1
MN1	2.491399	1.29162	1.9289	0.0539	0.665110572	count	1
AC139887.2	0.7365683	0.5823499	1.2648	0.206	0.665264202	count	1
DLGAP4	0.5123537	0.1862365	2.7511	0.00598	0.665535093	count	1
KAT5	0.5841085	0.2585405	2.2593	0.024	0.665540124	count	1

COQ8A	0.6119891	0.2663975	2.2973	0.0217	0.665642	count	1
LMBRD2	0.689801	0.386574	1.7844	0.0745	0.665900063	count	1
LRRC8C-DT	0.8344891	0.5091418	1.639	0.101	0.665961931	count	1
SH3BP5	0.4824964	0.1194745	4.0385	5.54E-05	0.666084329	count	1
KLC4	1.2090445	0.6866911	1.7607	0.0784	0.666274364	count	1
RNASEH2B-AS1	1.2090445	0.6338101	1.9076	0.0566	0.666274364	count	1
ZFP36L2	0.4651124	0.0565907	8.2189	3.29E-16	0.666287779	count	7.96E-12
AP5S1	0.8635793	0.512222	1.6859	0.0919	0.666532294	count	1
LINC01504	0.6500936	0.3338989	1.947	0.0517	0.666580165	count	1
HIST1H4A	1.1146845	0.8327714	1.3385	0.181	0.66675154	count	1
8-Mar	0.5854167	0.2985279	1.961	0.05	0.667071841	count	1
SCLT1	0.5214568	0.1860556	2.8027	0.00511	0.667091821	count	1
ADGRE1	0.7537961	0.4160878	1.8116	0.0702	0.667242094	count	1
SLC39A10	0.4908935	0.1563554	3.1396	0.00171	0.667303433	count	1
WDR77	0.5364767	0.222207	2.4143	0.0158	0.667519559	count	1
POMZP3	0.8117141	0.4596745	1.7658	0.0775	0.667575259	count	1
SAP25	0.7018405	0.3750075	1.8715	0.0614	0.667686408	count	1
BISPR	0.5560425	0.2508748	2.2164	0.0268	0.667866324	count	1
KDM3A	0.5669263	0.2541545	2.2306	0.0258	0.667880097	count	1
ACSF2	0.8372677	0.3354564	2.4959	0.0126	0.668340156	count	1
MYCBP	0.5738597	0.238038	2.4108	0.016	0.668458983	count	1
ATN1	0.6189023	0.3975092	1.557	0.12	0.668464467	count	1
UBR2	0.5093571	0.1710985	2.977	0.00294	0.668538291	count	1
PDLIM2	0.4878993	0.1368164	3.5661	0.000369	0.668543338	count	1
CDC14A	0.5679953	0.3648393	1.5568	0.12	0.6691691	count	1
AP001160.1	0.7408048	0.4389	1.6879	0.0916	0.669309092	count	1
TP53I11	0.5933984	0.3422637	1.7337	0.0831	0.669430251	count	1
AC012368.1	0.6037077	0.2737328	2.2055	0.0275	0.669646637	count	1
MGAM	0.9006827	0.6139605	1.467	0.143	0.669680557	count	1
USP47	0.5095817	0.1698536	3.0001	0.00273	0.669867633	count	1
ZNF579	0.6201911	0.4048928	1.5317	0.126	0.669904226	count	1
MIR29B2CHG	0.5647203	0.2823038	2.0004	0.0456	0.669930316	count	1
XKR8	0.5970566	0.3085211	1.9352	0.0531	0.66995272	count	1
ARRDC1	0.4948078	0.1551154	3.1899	0.00144	0.670476401	count	1
PSMB9	0.4715249	0.0664152	7.0996	1.63E-12	0.670688145	count	3.93E-08
RAP1GAP2	0.8407504	0.5097157	1.6494	0.0992	0.671321753	count	1
AC040977.1	0.9900689	0.5605769	1.7662	0.0775	0.671543277	count	1
ASXL2	0.5530142	0.228919	2.4158	0.0158	0.671629518	count	1
MAVS	0.528747	0.252221	2.0964	0.0362	0.672033405	count	1
CREBBP	0.5129546	0.1654585	3.1002	0.00196	0.672239829	count	1
ZNF383	0.8425131	0.4601999	1.8308	0.0673	0.672831174	count	1
TMEM107	0.5010247	0.1364738	3.6712	0.000247	0.672869731	count	1
ECHDC3	0.6496159	0.3061101	2.1222	0.0339	0.672975048	count	1
IRF2BP1	0.871625	0.488895	1.7828	0.0747	0.673218615	count	1
GRAMD1A	0.5275858	0.1791575	2.9448	0.00326	0.673293574	count	1

CDK17	0.6634977	0.3072908	2.1592	0.0309	0.673471344	count	1
PRKCB	0.4932491	0.131846	3.7411	0.000187	0.673588702	count	1
FGD4	0.5120412	0.1325184	3.8639	0.000114	0.673640771	count	1
THRB	1.125169	0.6144677	1.8311	0.0672	0.673656843	count	1
KIF1C	0.6282525	0.291366	2.1562	0.0312	0.673662609	count	1
C1RL	0.6037586	0.3679593	1.6408	0.101	0.67378176	count	1
ARAP2	0.5609844	0.2619245	2.1418	0.0323	0.673928417	count	1
AL391422.3	1.0526448	0.5547477	1.8975	0.0579	0.674491878	count	1
NCR3LG1	1.0526448	0.6121247	1.7197	0.0856	0.674491878	count	1
FFAR4	0.8734787	0.4523774	1.9309	0.0536	0.674759689	count	1
SMG5	0.6513343	0.3486733	1.868	0.0619	0.674825846	count	1
METTL9	0.4783245	0.0772446	6.1923	6.93E-10	0.675138701	count	1.67E-05
NEDD9	0.4972481	0.1261412	3.942	8.31E-05	0.67536511	count	1
SELENOO	0.5532674	0.2438173	2.2692	0.0233	0.675377843	count	1
INTS1	0.5955646	0.3052677	1.951	0.0512	0.675518155	count	1
ARHGAP12	0.5483911	0.2238368	2.45	0.0144	0.675597822	count	1
DTWD1	0.5309349	0.1902048	2.7914	0.00529	0.675798057	count	1
MAP10	1.054508	0.4895367	2.1541	0.0313	0.675799621	count	1
CTDSP2	0.5042323	0.1504409	3.3517	0.000815	0.675969122	count	1
FRMD3	0.9961409	0.425936	2.3387	0.0194	0.676034726	count	1
ABCC1	0.6130915	0.3040439	2.0165	0.0439	0.676071187	count	1
HEATR5A	0.722482	0.3810529	1.896	0.0581	0.676947862	count	1
ZNF44	0.6669801	0.3765459	1.7713	0.0766	0.677155719	count	1
HAUS4	0.6103617	0.3134927	1.947	0.0517	0.677257459	count	1
WDR7	0.7497755	0.3432842	2.1841	0.029	0.677878679	count	1
TIGD1	1.0579513	0.5822441	1.817	0.0693	0.67821658	count	1
PCSK5	0.6838895	0.4489774	1.5232	0.128	0.678491058	count	1
SLC9A6	0.9123403	0.4054719	2.2501	0.0245	0.679051697	count	1
RIN3	0.4970527	0.1247367	3.9848	6.95E-05	0.679168407	count	1
FAM210B	0.5197652	0.1975679	2.6308	0.00857	0.679570921	count	1
TERF2	0.5420856	0.2237841	2.4224	0.0155	0.67958335	count	1
TAF1	0.5767187	0.2364478	2.4391	0.0148	0.679690507	count	1
KBTBD3	0.624824	0.315243	1.982	0.0476	0.680073582	count	1
NAA60	0.5329518	0.2600891	2.0491	0.0406	0.680237984	count	1
PCNX2	0.6856629	0.5199454	1.3187	0.187	0.680330091	count	1
DMXL2	0.4905068	0.1285147	3.8167	0.000139	0.68036876	count	1
NME8	0.9143439	0.5204451	1.7568	0.0791	0.680663104	count	1
ERAP2	0.60323	0.2103293	2.868	0.00417	0.680844961	count	1
SENP1	0.7150253	0.3752153	1.9056	0.0568	0.680867936	count	1
PPP3CA	0.4950853	0.12421	3.9859	6.92E-05	0.681327108	count	1
SELENON	0.567038	0.2587276	2.1916	0.0285	0.681356179	count	1
GEMIN4	0.7156586	0.3951683	1.811	0.0703	0.681501417	count	1
MAN1C1	0.8276229	0.4224064	1.9593	0.0502	0.681567067	count	1
TGIF2	0.7399926	0.2998819	2.4676	0.0137	0.681810126	count	1
FGFR1	0.604162	0.3154446	1.9153	0.0556	0.681927373	count	1

CLEC9A	0.5573809	0.3910353	1.4254	0.154	0.682155895	count	1
LGI2	1.0047675	0.4026123	2.4956	0.0126	0.682418495	count	1
TONSL	1.8814576	0.7038898	2.6729	0.00757	0.682707218	count	1
NF1	0.5281915	0.2419702	2.1829	0.0291	0.682904785	count	1
CCDC170	0.5403442	0.1866074	2.8956	0.00382	0.682945685	count	1
ARID1A	0.511307	0.1481347	3.4516	0.000567	0.683172923	count	1
TNRC18	0.5669614	0.210822	2.6893	0.00721	0.683258044	count	1
ARHGEF10L	0.6234467	0.2326901	2.6793	0.00743	0.683291542	count	1
ENO3	1.0060677	0.6048861	1.6632	0.0964	0.683380915	count	1
NOM1	0.546015	0.2383918	2.2904	0.0221	0.683383655	count	1
CRTC1	0.7420541	0.3649211	2.0335	0.0421	0.683813516	count	1
CCDC117	0.5941858	0.2918372	2.036	0.0419	0.683834613	count	1
PPP2R3B	0.7421138	0.4778099	1.5532	0.121	0.68387152	count	1
MPV17L2	0.5782125	0.272264	2.1237	0.0338	0.683937392	count	1
TMEM117	1.1413424	0.5678795	2.0098	0.0446	0.684312719	count	1
PES1	0.5541648	0.2098207	2.6411	0.00832	0.684326769	count	1
LINC01004	1.5658337	0.9094394	1.7218	0.0852	0.684337385	count	1
LCAT	1.5658337	0.7887229	1.9853	0.0472	0.684337385	count	1
FAM111A	0.5144233	0.156224	3.2929	0.00101	0.684384133	count	1
RASAL3	0.5921318	0.2525492	2.3446	0.0191	0.68447161	count	1
FAM45A	0.492918	0.1262894	3.9031	9.75E-05	0.68470453	count	1
PSMG3-AS1	1.0673959	0.5245499	2.0349	0.042	0.684848067	count	1
ZNF609	0.5512466	0.2987134	1.8454	0.0651	0.684876231	count	1
GPANK1	0.5373511	0.1916742	2.8035	0.0051	0.685017022	count	1
FOXN2	0.4991664	0.123189	4.052	5.24E-05	0.685143569	count	1
EPC1	0.4952701	0.1355813	3.6529	0.000265	0.685234007	count	1
COX15	0.5813699	0.2716125	2.1404	0.0324	0.685302335	count	1
MTMR3	0.5984501	0.2788498	2.1461	0.032	0.685666139	count	1
VCAN	0.4769104	0.0815393	5.8488	5.61E-09	0.68623819	count	0.00013464
MAP3K5	0.5710497	0.2681691	2.1294	0.0333	0.686279728	count	1
AC025171.4	2.5840835	1.1334279	2.2799	0.0227	0.686317766	count	1
CCNL1	0.4823302	0.0771439	6.2523	4.75E-10	0.686337595	count	1.14E-05
CCNB1IP1	0.6183172	0.2658189	2.3261	0.0201	0.68636109	count	1
AL118516.1	0.5031863	0.1526259	3.2969	0.000992	0.686508805	count	1
FBLN1	1.892861	0.6683165	2.8323	0.00466	0.687007646	count	1
NAA80	0.6842389	0.6186179	1.1061	0.269	0.687371054	count	1
LINC01220	0.7930239	0.5559205	1.4265	0.154	0.687767216	count	1
ZNF721	0.5679625	0.247363	2.2961	0.0218	0.688317643	count	1
KDM4B	0.5458701	0.2843868	1.9195	0.055	0.68896029	count	1
SRD5A1	0.7231591	0.3622676	1.9962	0.046	0.689006449	count	1
AC006449.6	0.5804806	0.356465	1.6284	0.104	0.68905874	count	1
NLRC5	0.8614586	0.3249956	2.6507	0.00808	0.689067199	count	1
PRX	1.898538	0.9471539	2.0045	0.0451	0.68914445	count	1
PHTF2	0.5768759	0.2199102	2.6232	0.00876	0.689190851	count	1
CORO1A	0.4813953	0.0515026	9.347	1.96E-20	0.689301754	count	4.75E-16

ASGR1	0.4907158	0.0931637	5.2672	1.51E-07	0.689389977	count	0.003608145
AGO4	0.5409737	0.1926785	2.8077	0.00503	0.689701085	count	1
ACTR5	0.8371127	0.4476836	1.8699	0.0616	0.689921645	count	1
SHPRH	0.5335938	0.194071	2.7495	0.00601	0.689977636	count	1
TMEM150B	0.7782939	0.5614086	1.3863	0.166	0.690240191	count	1
RNF167	0.5141053	0.150045	3.4263	0.000622	0.690270254	count	1
PACRGL	0.8628723	0.4701763	1.8352	0.0666	0.690279643	count	1
KLRG1	1.1506806	0.6828581	1.6851	0.0921	0.69046722	count	1
RND3	0.5401735	0.385445	1.4014	0.161	0.690487129	count	1
CHRNE	0.5416637	0.214147	2.5294	0.0115	0.690593328	count	1
RBMS2	0.634415	0.312658	2.0291	0.0426	0.690865585	count	1
ERMARD	0.7254295	0.3947424	1.8377	0.0662	0.691279081	count	1
ERCC6L2	0.588601	0.2696339	2.183	0.0291	0.691462597	count	1
TMEM186	0.6971479	0.4459594	1.5633	0.118	0.692245939	count	1
SP1	0.5571003	0.2248993	2.4771	0.0133	0.692275556	count	1
COQ6	0.6814759	0.3216987	2.1184	0.0342	0.692502221	count	1
XPOT	0.6214443	0.2871176	2.1644	0.0305	0.694126939	count	1
HNRNPH1	0.48869	0.0772576	6.3255	2.99E-10	0.694232573	count	7.19E-06
SIRPB1	0.5094013	0.1363163	3.7369	0.000191	0.694466016	count	1
HVCN1	0.523576	0.1417264	3.6943	0.000225	0.694602116	count	1
ZNF70	1.3905271	0.7354572	1.8907	0.0588	0.694765228	count	1
EZH1	0.5703419	0.2499157	2.2821	0.0226	0.694890242	count	1
SUCO	0.5267003	0.1890141	2.7866	0.00537	0.695129423	count	1
VTI1A	0.543784	0.2234556	2.4335	0.015	0.695166953	count	1
HAS1	0.6340065	0.3623174	1.7499	0.0803	0.695247072	count	1
AQR	0.5679663	0.2159278	2.6304	0.00858	0.69536103	count	1
AL022328.4	0.8210316	0.4029234	2.0377	0.0417	0.695420452	count	1
SRGAP2	0.5167997	0.142519	3.6262	0.000294	0.695440545	count	1
VPS13B	0.6014011	0.2998901	2.0054	0.045	0.695468468	count	1
RAB3GAP1	0.5530278	0.2033442	2.7197	0.00658	0.695882369	count	1
GORASP1	0.6588996	0.408494	1.613	0.107	0.695948426	count	1
NIPSNAP3A	0.5280875	0.1682305	3.1391	0.00172	0.695992758	count	1
NR3C2	1.5904326	0.8768598	1.8138	0.0698	0.696019905	count	1
GRK3	0.5222386	0.1393076	3.7488	0.000182	0.696052302	count	1
AL118558.3	0.6779946	0.3012104	2.2509	0.0245	0.696385566	count	1
SUMF1	0.5715465	0.2950807	1.9369	0.0529	0.696386924	count	1
AC234772.3	0.9758215	0.6653079	1.4667	0.143	0.697322475	count	1
AC010173.1	0.9355214	0.4510381	2.0742	0.0382	0.697708438	count	1
KCNJ2	0.7214288	0.3312159	2.1781	0.0295	0.697950294	count	1
EPHA1-AS1	1.08608	0.4168293	2.6056	0.00923	0.697974418	count	1
ARC	2.638107	1.095782	2.4075	0.0161	0.698238069	count	1
FBRSL1	0.5865752	0.3493936	1.6788	0.0933	0.698784036	count	1
SLC9A9	0.5419925	0.1609874	3.3667	0.000773	0.699470565	count	1
HCG11	0.7454659	0.3724373	2.0016	0.0454	0.699632639	count	1
C12orf29	0.6052722	0.3218843	1.8804	0.0602	0.700062653	count	1

TTPAL	0.7236704	0.489407	1.4787	0.139	0.700224715	count	1
TMEM241	1.400569	0.7665934	1.827	0.0678	0.700275352	count	1
HEATR5B	0.6574427	0.2918198	2.2529	0.0244	0.700363987	count	1
CD33	0.5028268	0.099033	5.0774	4.11E-07	0.700910651	count	0.009803172
MIEF2	0.9397292	0.5089668	1.8463	0.065	0.701097981	count	1
NME2	0.7353816	0.4273057	1.721	0.0854	0.701245374	count	1
MORC2	0.850622	0.4691454	1.8131	0.0699	0.701825022	count	1
AC026471.1	0.6644007	0.510067	1.3026	0.193	0.701977934	count	1
HIST1H1D	0.5824335	0.273437	2.13	0.0333	0.702301269	count	1
NUP37	0.5986961	0.2700497	2.217	0.0267	0.703611621	count	1
FAM114A2	0.5991859	0.2465213	2.4306	0.0151	0.704201221	count	1
HHIPL1	2.6659486	0.9442763	2.8233	0.00479	0.704252843	count	1
TNFAIP6	0.5634542	0.2984576	1.8879	0.0592	0.70430565	count	1
UBR4	0.5274502	0.1594268	3.3084	0.000952	0.704569243	count	1
RAB28	0.5843385	0.2659522	2.1972	0.0281	0.704646892	count	1
RAC2	0.4967386	0.0718085	6.9175	5.84E-12	0.705085217	count	1.41E-07
ARHGEF19	0.9100716	0.5096772	1.7856	0.0743	0.705223491	count	1
CD300LF	0.5849425	0.1999331	2.9257	0.00347	0.705390635	count	1
AL160272.1	0.8322058	0.5916514	1.4066	0.16	0.705507081	count	1
ZNF212	1.0968996	0.5561913	1.9722	0.0487	0.70557948	count	1
PIEZO1	0.6158155	0.2946068	2.0903	0.0367	0.706112846	count	1
CCDC80	1.2751054	0.5837271	2.1844	0.029	0.706495117	count	1
OPA1	0.5694891	0.2467666	2.3078	0.0211	0.706529233	count	1
C4orf46	0.730925	0.3825289	1.9108	0.0562	0.707587994	count	1
SLC16A1	0.6817548	0.2895135	2.3548	0.0186	0.707626837	count	1
AVL9	0.6451176	0.287479	2.2441	0.0249	0.70783513	count	1
PTCD1	1.0393072	0.4869859	2.1342	0.0329	0.708006427	count	1
KLRB1	1.277851	1.132028	1.1288	0.259	0.708166301	count	1
POM121C	0.589105	0.2143929	2.7478	0.00604	0.708450301	count	1
ZGRF1	1.4166503	0.7530396	1.8812	0.0601	0.7090924	count	1
DENND6A	0.5880298	0.3151243	1.866	0.0622	0.709192548	count	1
TRUB1	0.8364313	0.4393761	1.9037	0.0571	0.709323392	count	1
EEF2K	0.6281295	0.3028276	2.0742	0.0382	0.709783126	count	1
AL162377.1	0.8600844	0.4722561	1.8212	0.0687	0.710169329	count	1
ATG2A	0.5473651	0.1960764	2.7916	0.00529	0.710189403	count	1
ADPGK-AS1	0.9517376	0.6240387	1.5251	0.127	0.710775851	count	1
GRSF1	0.5245095	0.1209707	4.3358	1.51E-05	0.711016699	count	0.3573868
PDE2A	0.8182443	0.393592	2.0789	0.0377	0.711025195	count	1
MTF2	0.5409685	0.1930455	2.8023	0.00511	0.711037924	count	1
DENND3	0.548046	0.1852849	2.9579	0.00313	0.711083749	count	1
NUDCD3	0.6073209	0.2824901	2.1499	0.0317	0.711265972	count	1
ZNF337	0.9954104	0.6388115	1.5582	0.119	0.712509939	count	1
KIF22	0.5296587	0.1676009	3.1602	0.0016	0.712580147	count	1
UPRT	0.7589197	0.3262802	2.326	0.0201	0.712929221	count	1
AC144652.1	0.5682744	0.2407842	2.3601	0.0183	0.712961906	count	1

UHRF1BP1	0.6588173	0.3269904	2.0148	0.044	0.713110834	count	1
PTGS2	0.5113094	0.1581239	3.2336	0.00124	0.713143503	count	1
MFSD8	0.7092519	0.3512987	2.0189	0.0436	0.713620823	count	1
HIST2H2BE	0.955431	0.3990156	2.3945	0.0167	0.713753813	count	1
NR1D1	0.86429	0.4780651	1.8079	0.0707	0.713879722	count	1
SPOPL	0.5411801	0.1936859	2.7941	0.00524	0.713937884	count	1
ZDHHC20	0.5381319	0.1796905	2.9948	0.00277	0.71410134	count	1
ADCY9	0.8908582	0.5945969	1.4983	0.134	0.714305932	count	1
AC098818.2	0.6600863	0.50545	1.3059	0.192	0.714532075	count	1
GADD45G	0.5315591	0.2358331	2.254	0.0243	0.714536608	count	1
PET117	0.6950095	0.3221131	2.1577	0.0311	0.714590079	count	1
SLC2A4RG	0.5468527	0.1782161	3.0685	0.00218	0.714786797	count	1
PLBD1-AS1	1.1876494	0.6866195	1.7297	0.0838	0.714841252	count	1
SAXO2	1.1876494	0.6114972	1.9422	0.0522	0.714841252	count	1
ZNF888	1.1876494	0.6458305	1.8389	0.066	0.714841252	count	1
USP46	0.6706352	0.3284535	2.0418	0.0413	0.714936883	count	1
ZNF558	1.9682431	0.870036	2.2623	0.0238	0.715133138	count	1
PAN3	0.5594404	0.1814055	3.0839	0.00207	0.715466733	count	1
GOLM1	0.6562975	0.2979922	2.2024	0.0277	0.715512326	count	1
BATF3	0.5815845	0.1871759	3.1072	0.00191	0.715671616	count	1
RNF19A	0.525918	0.1349261	3.8978	9.97E-05	0.715679249	count	1
CCND3	0.5267369	0.1155134	4.56	5.37E-06	0.715923891	count	0.12746769
EDRF1	0.6243919	0.364458	1.7132	0.0868	0.71621807	count	1
UBXN8	0.6971172	0.3175377	2.1954	0.0282	0.716846572	count	1
GIMAP1	0.5399765	0.1366137	3.9526	7.95E-05	0.717010838	count	1
CYSLTR1	0.541435	0.1408197	3.8449	0.000124	0.717171972	count	1
GBA2	0.653383	0.3562137	1.8342	0.0667	0.717204582	count	1
MAEA	0.5769547	0.2080385	2.7733	0.00559	0.717384989	count	1
MAN2B2	0.6454449	0.2352224	2.744	0.00611	0.717436382	count	1
RTP4	0.8072106	0.4354718	1.8536	0.0639	0.717441809	count	1
APC	0.5484018	0.1738202	3.155	0.00162	0.717446442	count	1
DNAJC4	0.5184212	0.1066925	4.859	1.25E-06	0.717744024	count	0.02976375
RARG	0.7639815	0.3869733	1.9742	0.0485	0.717935129	count	1
FANCI	1.1926301	0.631952	1.8872	0.0592	0.718125726	count	1
NFKBID	0.510694	0.0952155	5.3636	8.92E-08	0.718423246	count	0.002132683
AHNAK	0.5041592	0.0705599	7.1451	1.18E-12	0.718947499	count	2.85E-08
HIPK1	0.5423687	0.160551	3.3782	0.000741	0.719332261	count	1
TRPM7	0.5708333	0.200815	2.8426	0.00451	0.719822006	count	1
CMPK2	0.6304489	0.3293964	1.914	0.0557	0.719868034	count	1
FEM1A	0.6931656	0.3072483	2.256	0.0242	0.719947703	count	1
GLCE	0.6197063	0.2564102	2.4169	0.0157	0.720283015	count	1
SLCO3A1	0.5377455	0.1476731	3.6415	0.000277	0.720945103	count	1
E4F1	0.7011466	0.3937998	1.7805	0.0751	0.72116124	count	1
STAT6	0.5436636	0.156354	3.4771	0.000516	0.721954044	count	1
PCF11-AS1	1.1984517	0.5854594	2.047	0.0408	0.721964785	count	1

SPACA6	0.6951342	0.3411339	2.0377	0.0417	0.722074206	count	1
STRN3	0.5497775	0.1827684	3.0081	0.00266	0.722186079	count	1
AC097634.1	1.1211981	0.6751406	1.6607	0.0969	0.722667295	count	1
MAN2A1	0.5642704	0.2145661	2.6298	0.0086	0.723550335	count	1
WDR91	0.6228201	0.2817541	2.2105	0.0272	0.723995935	count	1
YPEL4	0.9326696	0.5385274	1.7319	0.0834	0.724072732	count	1
LONP2	0.5407333	0.1788009	3.0242	0.00252	0.724289692	count	1
GIMAP6	0.7121388	0.2362916	3.0138	0.00261	0.725015156	count	1
RELL2	1.0624843	0.4477795	2.3728	0.0177	0.72519806	count	1
ZNF582	1.1253241	0.5900906	1.907	0.0566	0.725569832	count	1
CCDC93	0.5607696	0.2043418	2.7443	0.00611	0.72557442	count	1
SH3RF1	0.6194316	0.2964619	2.0894	0.0368	0.725805278	count	1
CDC37L1	0.5952525	0.2514855	2.3669	0.018	0.725856622	count	1
TSGA10	0.6611274	0.4796557	1.3783	0.168	0.725987514	count	1
LGALS12	1.4475793	0.6835032	2.1179	0.0343	0.726024187	count	1
ARSD	0.6001985	0.2975115	2.0174	0.0438	0.726223964	count	1
ZNF445	0.7602959	0.3527667	2.1552	0.0312	0.726226048	count	1
TM7SF2	0.9051438	0.3775833	2.3972	0.0166	0.726587142	count	1
NOTCH1	0.658734	0.2857064	2.3056	0.0212	0.728074383	count	1
STAT5A	0.5747019	0.1770397	3.2462	0.00119	0.728161867	count	1
CCDC14	0.6215129	0.2637279	2.3566	0.0185	0.72830481	count	1
AC008124.1	0.6551306	0.4561227	1.4363	0.151	0.728543184	count	1
DLG1	0.6057068	0.2971142	2.0386	0.0416	0.728851921	count	1
ENPP1	0.8582171	0.6608837	1.2986	0.194	0.72901676	count	1
GSPT2	0.8196087	0.4200416	1.9513	0.0511	0.729121351	count	1
TIPIN	0.6448601	0.3100381	2.0799	0.0376	0.729249813	count	1
SLC12A6	0.5832856	0.251357	2.3205	0.0204	0.729518727	count	1
ZNF93	0.7427371	0.3871836	1.9183	0.0552	0.729907453	count	1
ZMYM6	0.6603687	0.319185	2.0689	0.0387	0.729939439	count	1
TMLHE	0.6306042	0.2315396	2.7235	0.0065	0.730149031	count	1
KBTBD6	2.792011	1.407034	1.9843	0.0473	0.730384327	count	1
ATP6V0C	1.3150665	0.6115327	2.1504	0.0316	0.730806423	count	1
LYL1	0.527688	0.1150243	4.5876	4.71E-06	0.73106289	count	0.11183424
AP004609.3	0.8054519	0.5103598	1.5782	0.115	0.73119575	count	1
COL9A2	0.9771458	0.4506401	2.1684	0.0302	0.731274037	count	1
RPP38	0.5783519	0.2170346	2.6648	0.00775	0.73174788	count	1
BIN1	0.5455374	0.1528597	3.5689	0.000365	0.731825009	count	1
GIMAP4	0.5200391	0.0722714	7.1956	8.22E-13	0.73192762	count	1.98E-08
EXTL3	0.7043429	0.3433805	2.0512	0.0404	0.732024996	count	1
ZNF75D	1.07179	0.4809009	2.2287	0.0259	0.732104442	count	1
POLB	0.5938034	0.1879895	3.1587	0.0016	0.732615872	count	1
SLC16A6	0.5699144	0.2502821	2.2771	0.0229	0.732652612	count	1
AC106739.1	1.2148554	0.6462805	1.8798	0.0603	0.732782217	count	1
CXXC1	0.6024883	0.2409689	2.5003	0.0125	0.732976688	count	1
SLC7A7	0.5245468	0.0760993	6.8929	6.93E-12	0.733252181	count	1.67E-07

AC005070.3	1.6701696	0.7347701	2.2731	0.0231	0.733630613	count	1
ARL15	0.6993986	0.3672227	1.9046	0.057	0.733704832	count	1
MLLT10	0.5647277	0.1818709	3.1051	0.00192	0.733719838	count	1
AC004921.1	0.7208366	0.3785458	1.9042	0.057	0.734249518	count	1
DIS3L2	0.6427117	0.3489091	1.8421	0.0656	0.734267222	count	1
JUND	0.5100163	0.0323646	15.7585	2.28E-53	0.73448932	count	5.54E-49
FAM185A	1.2182144	0.6471248	1.8825	0.0599	0.734997221	count	1
CSRNP1	0.5271743	0.10171	5.1831	2.36E-07	0.735136141	count	0.005635444
YY1AP1	0.6188587	0.2011379	3.0768	0.00212	0.735699442	count	1
PDIA5	0.7154945	0.4774168	1.4987	0.134	0.736533787	count	1
EEF1AKMT4	0.7024664	0.3426251	2.0502	0.0404	0.737048446	count	1
WDR36	0.6107092	0.2579567	2.3675	0.018	0.737137059	count	1
XPNPEP3	0.6452653	0.3376868	1.9108	0.0561	0.737266824	count	1
GTF3C5	0.6908684	0.3123843	2.2116	0.0271	0.737309856	count	1
SLC9A8	0.7324442	0.3330266	2.1994	0.0279	0.737998935	count	1
DAG1	1.3269379	0.4977284	2.666	0.00773	0.738022952	count	1
UBN2	0.6098334	0.266793	2.2858	0.0224	0.738128114	count	1
SSBP2	0.5842264	0.205749	2.8395	0.00456	0.738151838	count	1
KIZ	0.7721919	0.3133428	2.4644	0.0138	0.738168419	count	1
REC8	1.028512	0.4324274	2.3785	0.0175	0.738205983	count	1
PHF12	0.6298878	0.2703166	2.3302	0.0199	0.738365018	count	1
ZNF718	1.4701825	0.6654115	2.2094	0.0272	0.73837301	count	1
MPV17L	1.4701825	0.7060506	2.0823	0.0374	0.73837301	count	1
TRA2A	0.5359889	0.1268379	4.2258	2.47E-05	0.738629025	count	0.5835622
LTC4S	0.5293839	0.1245572	4.2501	2.22E-05	0.73881492	count	0.5247414
FAM200A	0.8300626	0.440252	1.8854	0.0595	0.738976695	count	1
FFAR2	0.5955647	0.3008812	1.9794	0.0479	0.739467528	count	1
HABP4	1.0303931	0.5060429	2.0362	0.0418	0.739667304	count	1
MTMR4	0.7184734	0.3525971	2.0377	0.0417	0.73972711	count	1
ZC3H7A	0.5496374	0.1743744	3.1521	0.00164	0.739994361	count	1
AC108488.1	0.8149534	0.5603796	1.4543	0.146	0.740314989	count	1
FRAT1	0.5639601	0.173156	3.2569	0.00114	0.740444782	count	1
FAS	0.7121635	0.2500786	2.8478	0.00444	0.740480175	count	1
NRG1	0.5803658	0.2311087	2.5112	0.0121	0.740671635	count	1
NOMO3	1.3317222	0.5389163	2.4711	0.0135	0.740930359	count	1
POLL	0.7000486	0.2961794	2.3636	0.0182	0.74110027	count	1
CCR2	0.7062885	0.296333	2.3834	0.0172	0.741215014	count	1
NEIL1	0.9537661	0.5800434	1.6443	0.1	0.741691168	count	1
NKTR	0.5381446	0.1348341	3.9912	6.77E-05	0.741752119	count	1
CWF19L2	0.562902	0.1631605	3.45	0.00057	0.741838398	count	1
NSUN5	0.6261048	0.2778839	2.2531	0.0243	0.74196979	count	1
JAZF1	0.5791806	0.1546154	3.7459	0.000184	0.742016094	count	1
RHOF	0.6283286	0.2716064	2.3134	0.0208	0.742031172	count	1
NAPRT	0.5407604	0.1219432	4.4345	9.63E-06	0.742572313	count	0.22827915
PAK4	1.0859047	0.6433308	1.6879	0.0915	0.742583535	count	1

CD79B	1.0869043	0.4715262	2.3051	0.0212	0.743325819	count	1
C19orf25	0.5508923	0.1894777	2.9074	0.00368	0.743511228	count	1
TMEM254	0.672262	0.350645	1.9172	0.0553	0.743513651	count	1
PAXIP1-AS1	0.7155206	0.3864852	1.8514	0.0642	0.744110883	count	1
RASSF2	0.5486437	0.1477356	3.7137	0.000209	0.744224429	count	1
KDM1B	0.6070981	0.2319994	2.6168	0.00893	0.744227609	count	1
MTAP	0.5911157	0.2850058	2.074	0.0382	0.744602195	count	1
CHCHD4	0.6692327	0.2976203	2.2486	0.0246	0.744724767	count	1
CDC42EP3	0.537813	0.121421	4.4293	9.87E-06	0.744831408	count	0.23392887
S1PR3	0.6517492	0.382628	1.7033	0.0886	0.744884879	count	1
ASB16-AS1	0.7480361	0.5183019	1.4432	0.149	0.745156751	count	1
HIST1H4E	0.8999892	0.4779876	1.8829	0.0598	0.745414688	count	1
INO80D	0.662502	0.2365149	2.8011	0.00513	0.745700033	count	1
ZBTB20	0.5844159	0.1848505	3.1616	0.00159	0.745915751	count	1
FBRS	0.575296	0.1868529	3.0789	0.0021	0.746144515	count	1
FANCF	0.8569338	0.5450911	1.5721	0.116	0.746782101	count	1
INTU	1.0915997	0.5410254	2.0176	0.0437	0.746812697	count	1
ULK2	0.7496567	0.3515321	2.1325	0.0331	0.74684459	count	1
RUNDC1	0.7594081	0.3300869	2.3006	0.0215	0.747070294	count	1
CTSS	0.5191505	0.0367871	14.1123	1.54E-43	0.747634562	count	3.74E-39
ASH2L	0.6194507	0.3062651	2.0226	0.0432	0.747914989	count	1
FBF1	2.0582029	0.9356486	2.1998	0.0279	0.747950447	count	1
LRRC58	0.5700617	0.1978176	2.8818	0.00399	0.747951892	count	1
PABPN1	0.5457724	0.1249594	4.3676	1.31E-05	0.747989695	count	0.3102211
AC010240.3	1.702071	1.0380211	1.6397	0.101	0.748551582	count	1
CAPN5	1.702071	0.7881152	2.1597	0.0309	0.748551582	count	1
AL359232.1	1.702071	0.7334359	2.3207	0.0204	0.748551582	count	1
PIK3C2A	0.6067143	0.1807665	3.3563	0.000802	0.748844724	count	1
CHD8	0.5804765	0.1977551	2.9353	0.00336	0.748981434	count	1
CPEB2	0.6864738	0.386476	1.7762	0.0758	0.749552043	count	1
HOXB2	0.6864738	0.3525539	1.9471	0.0516	0.749552043	count	1
ZBTB24	0.5819127	0.234875	2.4775	0.0133	0.750018681	count	1
AC011978.2	1.7052872	0.7403583	2.3033	0.0213	0.750051657	count	1
WWC3	0.6738944	0.2755951	2.4452	0.0145	0.750076487	count	1
RASSF8-AS1	1.4923464	0.6896534	2.1639	0.0306	0.750458484	count	1
PLEKHF2	0.5809774	0.1993844	2.9139	0.0036	0.750459881	count	1
LEKR1	0.8108002	0.7815349	1.0374	0.3	0.75079139	count	1
PLA1A	0.9652346	0.5164931	1.8688	0.0618	0.751276748	count	1
DGKZ	0.5557824	0.157816	3.5217	0.000437	0.751313735	count	1
TLR8	0.5964854	0.1937572	3.0785	0.0021	0.751475833	count	1
SNX21	0.8840819	0.3852801	2.2946	0.0218	0.752432053	count	1
SLC41A3	0.646702	0.4062565	1.5919	0.112	0.752491227	count	1
SSH2	0.5554519	0.1252793	4.4337	9.67E-06	0.752757715	count	0.22920801
ZNF493	0.7562368	0.4369903	1.7306	0.0837	0.753699339	count	1
FAR1	0.5577493	0.169991	3.2811	0.00105	0.75370921	count	1

LNPEP	0.5623772	0.1573531	3.574	0.000358	0.753899229	count	1
MCM2	1.3535414	0.7184791	1.8839	0.0597	0.754182088	count	1
ZNF595	1.3535414	0.7649977	1.7693	0.077	0.754182088	count	1
MALINC1	1.3535414	0.8118608	1.6672	0.0956	0.754182088	count	1
SCAMP5	1.3535414	0.8915718	1.5182	0.129	0.754182088	count	1
DDX60	0.6384618	0.2508116	2.5456	0.011	0.754288717	count	1
PPP1R9B	0.5960033	0.2403189	2.4801	0.0132	0.754433498	count	1
SMCHD1	0.5394164	0.0914712	5.8971	4.21E-09	0.754566638	count	0.000101057
GIMAP2	0.620051	0.1706155	3.6342	0.000285	0.754784493	count	1
PLD4	0.5675585	0.1850949	3.0663	0.00219	0.75489592	count	1
TMOD1	0.8872205	0.4187832	2.1186	0.0342	0.755275716	count	1
ISYNA1	1.0071864	0.4973162	2.0252	0.043	0.755541263	count	1
OGT	0.5810395	0.1866364	3.1132	0.00187	0.755903864	count	1
IKBKE	0.7082166	0.2942751	2.4066	0.0162	0.756513419	count	1
SLC2A9	0.6796978	0.3296436	2.0619	0.0393	0.756740679	count	1
OFD1	0.5802287	0.1699287	3.4145	0.000649	0.756936156	count	1
IER2	0.5283648	0.0604868	8.7352	4.42E-18	0.757308417	count	1.07E-13
DHRS7B	0.5754134	0.2307788	2.4934	0.0127	0.758504749	count	1
SLC35A3	0.6573038	0.3844562	1.7097	0.0874	0.758530675	count	1
FAM117A	1.0548473	0.5739346	1.8379	0.0662	0.758672668	count	1
MLLT6	0.5843678	0.2420962	2.4138	0.0159	0.75881553	count	1
RAB39A	0.7522945	0.3241683	2.3207	0.0204	0.758891694	count	1
RGS18	0.5757799	0.1368809	4.2064	2.69E-05	0.758993027	count	0.6352973
ZFAND1	0.5750774	0.165732	3.4699	0.00053	0.759137737	count	1
SNX13	0.5792099	0.1644982	3.5211	0.000438	0.759479062	count	1
TESC	0.5712806	0.144996	3.94	8.38E-05	0.759894276	count	1
TMEM8B	1.056597	0.4856196	2.1758	0.0297	0.760033076	count	1
IFFO1	0.6096935	0.1986585	3.0691	0.00217	0.760294288	count	1
VILL	1.174892	0.5764675	2.0381	0.0416	0.760451831	count	1
WDFY3	0.5877159	0.2273576	2.585	0.0098	0.760897645	count	1
TUBD1	0.7460991	0.4431119	1.6838	0.0924	0.761097727	count	1
LYSMD4	0.893704	0.4691302	1.905	0.0569	0.761151553	count	1
SPNS3	0.7184855	0.3762791	1.9094	0.0563	0.761366049	count	1
ODF2L	0.6470481	0.2980956	2.1706	0.0301	0.761882024	count	1
CC2D2B	2.0996406	0.9322178	2.2523	0.0244	0.762769768	count	1
IGSF8	0.653017	0.3290956	1.9843	0.0473	0.763152475	count	1
FBXL18	2.100796	1.020723	2.0581	0.0397	0.763180003	count	1
ALG8	0.6531624	0.3172057	2.0591	0.0396	0.763326741	count	1
AC064807.1	1.0171789	0.479237	2.1225	0.0339	0.76361977	count	1
ARHGAP27	0.6079755	0.2790624	2.1786	0.0295	0.76362147	count	1
ZNF142	0.8756144	0.4766914	1.8369	0.0664	0.764076565	count	1
B4GALT3	0.6009805	0.2321719	2.5885	0.0097	0.764205241	count	1
CLASRP	0.6682607	0.2167145	3.0836	0.00207	0.764295199	count	1
MLKL	0.6403513	0.2130051	3.0063	0.00267	0.764370625	count	1
AC092171.3	1.1808337	0.5823427	2.0277	0.0427	0.76463393	count	1

PHF8	0.7102455	0.3002703	2.3654	0.0181	0.764915232	count	1
CITED4	0.6266544	0.223204	2.8075	0.00503	0.764936474	count	1
CCDC149	0.6495947	0.2610446	2.4884	0.0129	0.764954481	count	1
CNTRL	0.5726313	0.1338289	4.2788	1.95E-05	0.76508712	count	0.4611945
RANGAP1	0.7353547	0.3235904	2.2725	0.0231	0.765575507	count	1
SPON2	1.264612	0.5920308	2.1361	0.0328	0.765583308	count	1
CCDC136	1.264612	0.7147442	1.7693	0.077	0.765583308	count	1
MALT1	0.5646693	0.139741	4.0408	5.49E-05	0.765604855	count	1
LINC01136	0.899677	0.5143323	1.7492	0.0804	0.766566495	count	1
GPATCH2	0.5870292	0.1761129	3.3333	0.000871	0.766597872	count	1
SLC25A30	0.983973	0.5643668	1.7435	0.0814	0.766949237	count	1
SIK3	0.5985679	0.2035887	2.9401	0.00331	0.76721009	count	1
PURB	0.5641136	0.1304874	4.3231	1.60E-05	0.768249268	count	0.378608
LANCL1	0.8612625	0.5289709	1.6282	0.104	0.768426728	count	1
RCAN1	0.5883485	0.1737953	3.3853	0.000722	0.769014577	count	1
PIP5K1B	1.0243813	0.5167798	1.9822	0.0476	0.769444337	count	1
SLFN12	0.660912	0.248597	2.6586	0.0079	0.769460946	count	1
IFIT2	0.5684142	0.2380202	2.3881	0.017	0.769917855	count	1
HERPUD2	0.5789254	0.1725854	3.3544	0.000807	0.770161555	count	1
HILPDA	0.7150543	0.4024957	1.7766	0.0758	0.770280254	count	1
FSTL3	0.8634571	0.3705821	2.33	0.0199	0.770500158	count	1
AC245297.3	0.6018591	0.2574745	2.3375	0.0195	0.770515091	count	1
KIAA1841	0.7333622	0.3795097	1.9324	0.0534	0.770754404	count	1
KLHL18	0.6157753	0.2127281	2.8947	0.00383	0.770862785	count	1
CARD8-AS1	0.6504253	0.2237527	2.9069	0.00368	0.7714872	count	1
NCSTN	0.6035938	0.1978064	3.0514	0.0023	0.771772069	count	1
TIMM21	0.7415726	0.3061463	2.4223	0.0155	0.772309356	count	1
CRTC2	0.6934568	0.2751174	2.5206	0.0118	0.772548102	count	1
MAP4K2	0.9065336	0.3205496	2.8281	0.00472	0.77278448	count	1
CMBL	1.3842299	0.5810202	2.3824	0.0173	0.772795344	count	1
TSEN2	1.3843884	0.6541268	2.1164	0.0344	0.772891374	count	1
AL390957.1	1.3846785	0.7945203	1.7428	0.0815	0.773067169	count	1
STS	1.3846785	0.7993034	1.7324	0.0833	0.773067169	count	1
FFAR3	0.5765784	0.2579754	2.235	0.0255	0.773469718	count	1
FP700111.1	1.0745123	0.596072	1.8027	0.0716	0.773965573	count	1
NECAP1	0.5949264	0.2030472	2.93	0.00342	0.774194856	count	1
TTC7A	0.6065022	0.2136073	2.8393	0.00456	0.774524286	count	1
NR2F6	0.7244841	0.4198257	1.7257	0.0845	0.774536942	count	1
ZNF776	1.7582702	0.8465213	2.0771	0.0379	0.774635589	count	1
NISCH	0.583872	0.1781861	3.2768	0.00106	0.774940051	count	1
DCUN1D2	0.9937742	0.554485	1.7922	0.0732	0.775151313	count	1
CEP44	0.7093657	0.2661369	2.6654	0.00774	0.775411324	count	1
ERF	0.6021911	0.1805999	3.3344	0.000868	0.775628636	count	1
F13A1	0.5439255	0.0858858	6.3331	2.85E-10	0.776040906	count	6.86E-06
PRELID2	0.7450446	0.4817896	1.5464	0.122	0.776070405	count	1

MNAT1	0.6086641	0.2246875	2.7089	0.0068	0.776280609	count	1
AC009113.1	1.0776733	0.7225228	1.4915	0.136	0.77642446	count	1
ANKRD46	0.7988785	0.4074788	1.9605	0.05	0.77672795	count	1
NAT14	0.9109997	0.5126349	1.7771	0.0757	0.776835715	count	1
EEF1G	1.5413892	0.6904155	2.2326	0.0257	0.777105692	count	1
STAT5B	0.6674321	0.3039615	2.1958	0.0282	0.777250789	count	1
NOTCH2	0.5901929	0.1613243	3.6584	0.000259	0.777632614	count	1
AC012651.1	3.050161	1.363085	2.2377	0.0253	0.778250909	count	1
SDHAF3	0.5875165	0.1897198	3.0968	0.00198	0.778346604	count	1
PPP1R3F	1.2842954	0.5926122	2.1672	0.0303	0.778549635	count	1
DDIAS	1.2842954	0.6893337	1.8631	0.0626	0.778549635	count	1
CCL4L2	0.5406231	0.1240281	4.3589	1.36E-05	0.779153994	count	0.32198
CASP2	0.717838	0.3264457	2.199	0.028	0.779319485	count	1
JAM2	1.2019794	0.6453633	1.8625	0.0627	0.779516108	count	1
ZNF429	0.7349947	0.5513637	1.333	0.183	0.779529856	count	1
GIGYF1	0.6179082	0.228328	2.7062	0.00685	0.780168687	count	1
COG5	0.7357346	0.2721621	2.7033	0.00691	0.780344263	count	1
AL592494.3	1.54749	0.8631647	1.7928	0.0731	0.780410241	count	1
NAT8L	1.54749	1.0627122	1.4562	0.145	0.780410241	count	1
AC006033.2	1.54749	0.8248743	1.876	0.0608	0.780410241	count	1
TAPBPL	0.6522617	0.2047542	3.1856	0.00146	0.781397168	count	1
SNRK	0.6230513	0.1816008	3.4309	0.000612	0.781517784	count	1
HOOK2	0.6450504	0.250642	2.5736	0.0101	0.781677983	count	1
MAP2K5	0.7199673	0.5182405	1.3893	0.165	0.781711915	count	1
LYRM1	0.6316342	0.2343809	2.6949	0.00709	0.781861531	count	1
S100Z	0.6871628	0.2501372	2.7471	0.00606	0.782615436	count	1
NSUN7	1.1402658	0.4841304	2.3553	0.0186	0.782969657	count	1
COX16	0.6462805	0.3455396	1.8704	0.0616	0.783200187	count	1
XPO7	0.7027759	0.2551262	2.7546	0.00592	0.78326048	count	1
CCDC121	1.0868545	0.5225215	2.08	0.0376	0.783567135	count	1
MAU2	0.7124019	0.3055261	2.3317	0.0198	0.784229586	count	1
ITPRIP	0.5957785	0.1861923	3.1998	0.00139	0.784492814	count	1
AL117332.1	1.1425216	0.5831261	1.9593	0.0502	0.784646081	count	1
FAM110B	0.9197049	0.3527236	2.6074	0.00918	0.784734677	count	1
CRACR2B	1.005545	0.420391	2.3919	0.0168	0.785005569	count	1
SARNP	0.6638552	0.3274524	2.0273	0.0427	0.78502847	count	1
IL1R2	0.5971656	0.2046285	2.9183	0.00355	0.785150415	count	1
NCF2	0.5524216	0.0590833	9.3499	1.91E-20	0.785402644	count	4.63E-16
BIN2	0.5679949	0.1050053	5.4092	6.94E-08	0.786011439	count	0.00165977
EAF1-AS1	1.044862	0.5539567	1.8862	0.0594	0.786013928	count	1
STK17B	0.552449	0.0633068	8.7265	4.76E-18	0.786469725	count	1.15E-13
MOB1B	0.6219452	0.1988655	3.1275	0.00178	0.786585666	count	1
NPHP3	0.9750481	0.3988011	2.4449	0.0146	0.786817203	count	1
INPPL1	0.6732087	0.2070678	3.2512	0.00116	0.787362553	count	1
ACACB	1.5605415	0.6413932	2.433	0.015	0.78747222	count	1

DTX2	0.6913286	0.2507831	2.7567	0.00588	0.787497451	count	1
AC015912.3	0.6311555	0.384579	1.6412	0.101	0.787534572	count	1
RAB27A	0.5861524	0.1901791	3.0821	0.00208	0.787864789	count	1
CMTM2	1.41146	0.5649639	2.4983	0.0125	0.789281092	count	1
SPECC1	0.5823601	0.1506435	3.8658	0.000114	0.789499816	count	1
MTERF3	0.7269727	0.3045158	2.3873	0.017	0.789584696	count	1
NAALADL2	1.7915484	0.8875443	2.0185	0.0436	0.789948467	count	1
AFAP1	1.7915484	0.8148933	2.1985	0.028	0.789948467	count	1
POLR3C	0.6937583	0.2511074	2.7628	0.00577	0.790345263	count	1
USP15	0.5579913	0.0732172	7.621	3.57E-14	0.791675813	count	8.63E-10
HDAC7	0.7927031	0.2957959	2.6799	0.00741	0.791733273	count	1
ACPP	0.852861	0.272086	3.1345	0.00174	0.791910508	count	1
RETN	0.5946818	0.1375955	4.322	1.61E-05	0.792682801	count	0.3809582
ZNF143	0.6322103	0.1958111	3.2287	0.00126	0.793201688	count	1
POMT1	1.0539836	0.461142	2.2856	0.0224	0.793396444	count	1
AC068473.5	2.1872353	1.0138218	2.1574	0.0311	0.793432357	count	1
HS6ST1	0.7611369	0.3274201	2.3246	0.0202	0.793511051	count	1
PRRT3	1.2236579	0.6661091	1.837	0.0663	0.794769924	count	1
POFUT1	1.223747	0.5020623	2.4374	0.0149	0.794832647	count	1
SASS6	0.6825142	0.3316753	2.0578	0.0397	0.795278078	count	1
QRICH1	0.6799267	0.2638154	2.5773	0.01	0.795421908	count	1
LEPR	1.1570387	0.5956139	1.9426	0.0522	0.795434738	count	1
RHPN1	1.1570387	0.5997601	1.9292	0.0538	0.795434738	count	1
C2CD2	0.9094711	0.4592426	1.9804	0.0478	0.795463258	count	1
CCDC22	0.6374728	0.1947816	3.2728	0.00108	0.795556187	count	1
ADD3	0.6246286	0.2018323	3.0948	0.00199	0.795855328	count	1
GBF1	0.6729252	0.2989646	2.2509	0.0245	0.796015589	count	1
ZNF736	0.7636238	0.4465574	1.71	0.0874	0.796207555	count	1
MRFAP1L1	0.6988932	0.3816618	1.8312	0.0672	0.79636479	count	1
CASP8	0.5971341	0.1680956	3.5523	0.000389	0.796858906	count	1
CYP2R1	0.807733	0.4111966	1.9643	0.0496	0.796913686	count	1
AL121603.2	0.7644086	0.372445	2.0524	0.0402	0.797058556	count	1
ANKH	0.6035551	0.3218922	1.875	0.0609	0.798231797	count	1
SGMS2	0.6249472	0.212897	2.9354	0.00336	0.798429719	count	1
CBFA2T3	0.8454148	0.5143712	1.6436	0.1	0.798683546	count	1
IVD	0.630552	0.2139813	2.9468	0.00324	0.798870703	count	1
IL11RA	1.4285226	0.6533992	2.1863	0.0289	0.799594646	count	1
GRAP	1.1627811	0.564237	2.0608	0.0394	0.799702292	count	1
KDM3B	0.6294795	0.2406219	2.6161	0.00895	0.799859542	count	1
TUSC1	0.7475433	0.3046499	2.4538	0.0142	0.800110302	count	1
CYP1B1	0.5789458	0.1766086	3.2781	0.00106	0.80035838	count	1
SF3A2	0.7836008	0.3234272	2.4228	0.0155	0.801021653	count	1
SIGLEC1	0.6056956	0.1837383	3.2965	0.000993	0.801092342	count	1
C9orf66	0.7068841	0.303974	2.3255	0.0201	0.801557561	count	1
BORCS5	0.631959	0.2071914	3.0501	0.00231	0.801882867	count	1

Z82244.2	0.8496198	0.4471609	1.9	0.0575	0.802862719	count	1
MYADM	0.5644314	0.0748684	7.539	6.63E-14	0.803517251	count	1.60E-09
ZNF322	0.6414622	0.2423498	2.6468	0.00818	0.803580399	count	1
OSBPL3	0.7346707	0.4141916	1.7737	0.0762	0.804029772	count	1
WARS	0.6043102	0.125799	4.8038	1.65E-06	0.804265277	count	0.0392634
ZNF529-AS1	1.1135436	0.5619489	1.9816	0.0476	0.804335754	count	1
EVA1C	0.9665992	0.5747415	1.6818	0.0927	0.80441049	count	1
AC009414.2	1.823263	1.2788352	1.4257	0.154	0.80444234	count	1
MST1	1.823263	1.2788352	1.4257	0.154	0.80444234	count	1
LDOC1	1.823263	1.2788352	1.4257	0.154	0.80444234	count	1
HYKK	1.823263	0.8288207	2.1998	0.0279	0.80444234	count	1
AC011447.3	1.823263	0.8288207	2.1998	0.0279	0.80444234	count	1
ADM2	1.823263	1.22194	1.4921	0.136	0.80444234	count	1
ATP2B4	0.6475145	0.2116435	3.0595	0.00224	0.805178844	count	1
ZBTB45	1.0298657	0.4911396	2.0969	0.0361	0.805377567	count	1
MAPK14	0.624477	0.1700512	3.6723	0.000246	0.805643654	count	1
AP1S2	0.5629992	0.0570082	9.8758	1.39E-22	0.80573661	count	3.37E-18
CLDN7	0.7735868	0.3270741	2.3652	0.0181	0.807013395	count	1
PARP8	0.6355601	0.1970996	3.2246	0.00128	0.807705274	count	1
EVI2B	0.5667155	0.0526714	10.7595	2.06E-26	0.80771311	count	5.00E-22
SSFA2	0.5979752	0.1339764	4.4633	8.44E-06	0.808198639	count	0.20013772
SUPV3L1	0.790428	0.3002786	2.6323	0.00853	0.808297773	count	1
MRC2	1.175007	0.5912891	1.9872	0.047	0.808787536	count	1
SDHAF4	0.7293729	0.3729299	1.9558	0.0506	0.808808137	count	1
MAN1A1	0.6001241	0.1334651	4.4965	7.23E-06	0.808881283	count	0.17148114
FUZ	0.709608	0.2924377	2.4265	0.0153	0.808929624	count	1
C18orf32	0.6560351	0.1993491	3.2909	0.00101	0.809132225	count	1
WRN	0.8308513	0.35276	2.3553	0.0186	0.809349986	count	1
AC092053.2	1.035164	0.6015597	1.7208	0.0854	0.809817223	count	1
ZHX3	1.445641	0.7869428	1.837	0.0663	0.809927596	count	1
AP002387.2	0.7626345	0.3387682	2.2512	0.0245	0.809972745	count	1
CYBRD1	0.6517426	0.2317261	2.8126	0.00495	0.810531726	count	1
MB21D2	0.7771673	0.2729491	2.8473	0.00445	0.810898002	count	1
AC083843.3	1.4477054	0.533191	2.7152	0.00667	0.811172624	count	1
TMEM99	1.4477054	0.6164079	2.3486	0.0189	0.811172624	count	1
PRMT9	0.6044499	0.1926171	3.1381	0.00172	0.811190819	count	1
SLC39A9	0.8584827	0.3866761	2.2202	0.0265	0.811673855	count	1
SLC2A6	0.7275682	0.3158251	2.3037	0.0213	0.811781132	count	1
LEMD2	0.684311	0.280523	2.4394	0.0148	0.812661147	count	1
ZBTB37	0.7531468	0.3991434	1.8869	0.0593	0.812822827	count	1
PLCL2	0.6523022	0.224284	2.9084	0.00367	0.812835633	count	1
DRG1	0.648932	0.2115175	3.068	0.00218	0.813099397	count	1
RILPL2	0.5693921	0.0567101	10.0404	2.83E-23	0.813130393	count	6.86E-19
U2AF2	0.6479724	0.238334	2.7188	0.0066	0.813316307	count	1
GATB	0.813762	0.3860205	2.1081	0.0351	0.813729376	count	1

ZKSCAN8	0.9295573	0.5443652	1.7076	0.0878	0.814106263	count	1
PRH1	0.7665565	0.3768713	2.034	0.0421	0.814295611	count	1
RNF166	0.6004598	0.1303151	4.6078	4.28E-06	0.81450024	count	0.10163716
KLRF1	0.8147675	0.3716036	2.1926	0.0284	0.814780148	count	1
IGFBP2	0.7349569	0.5408986	1.3588	0.174	0.815201308	count	1
RNASE2	0.6126857	0.1514814	4.0446	5.40E-05	0.816454934	count	1
TRMT11	0.8069484	0.3459036	2.3329	0.0197	0.816530949	count	1
PEAK1	0.7061994	0.3402117	2.0758	0.038	0.816544915	count	1
IGF2BP2	0.6718024	0.516245	1.3013	0.193	0.816992311	count	1
ZSCAN16	0.8383468	0.3808882	2.201	0.0278	0.817004788	count	1
PRMT5	0.7832002	0.3490477	2.2438	0.0249	0.817444691	count	1
CFD	0.569217	0.0544269	10.4584	4.48E-25	0.817526857	count	1.09E-20
LIN7A	0.9134327	0.3336176	2.738	0.00623	0.817775301	count	1
PXDC1	0.6311288	0.1732372	3.6431	0.000275	0.817857619	count	1
GRHL1	1.6172993	0.8336982	1.9399	0.0525	0.818042233	count	1
ZNF175	1.8532947	0.7480513	2.4775	0.0133	0.818073263	count	1
QPRT	0.7999716	0.3194886	2.5039	0.0123	0.818472664	count	1
CYTL1	0.6996147	0.2929386	2.3883	0.017	0.819052872	count	1
FBXO3	0.7587804	0.3087279	2.4578	0.014	0.819120745	count	1
NUP214	0.5791332	0.082031	7.0599	2.16E-12	0.81979852	count	5.21E-08
AL442128.2	0.8012269	0.3677809	2.1785	0.0295	0.819811308	count	1
FAM206A	0.7784874	0.3326489	2.3403	0.0193	0.820079579	count	1
DET1	0.9844716	0.6603081	1.4909	0.136	0.820266145	count	1
HIST4H4	1.6226872	0.6348192	2.5561	0.0106	0.82093143	count	1
AC022182.2	1.1919308	0.5844595	2.0394	0.0415	0.821362885	count	1
SESTD1	0.6427127	0.1718363	3.7403	0.000188	0.821465332	count	1
LUM	0.9180473	0.7685478	1.1945	0.232	0.822145574	count	1
ZNF182	0.9382814	0.4348693	2.1576	0.0311	0.82220798	count	1
PPTC7	0.6436147	0.2498839	2.5757	0.0101	0.822635178	count	1
TBC1D8	0.8131222	0.337538	2.409	0.0161	0.823051373	count	1
KLF9	0.635888	0.1343874	4.7318	2.35E-06	0.823245083	count	0.05587595
RAD51D	0.7965524	0.4140209	1.9239	0.0545	0.823609071	count	1
SENP7	0.6652081	0.2133332	3.1182	0.00184	0.824211979	count	1
ARHGAP5	0.6431722	0.1750665	3.6739	0.000244	0.825223497	count	1
MTURN	0.666282	0.2283938	2.9173	0.00356	0.825567311	count	1
PGBD1	1.1410505	0.5789372	1.9709	0.0488	0.8257449	count	1
AC145124.1	1.1410505	0.582848	1.9577	0.0504	0.8257449	count	1
AC108718.1	1.356171	0.5795324	2.3401	0.0194	0.825810518	count	1
BBS9	1.1980447	0.5178489	2.3135	0.0208	0.825905121	count	1
PGD	0.6102642	0.1295411	4.711	2.60E-06	0.827332591	count	0.0618098
PECR	0.8005502	0.3794989	2.1095	0.035	0.827912377	count	1
TRIM56	0.6572129	0.3974579	1.6535	0.0983	0.827928886	count	1
USP25	0.6485672	0.1966363	3.2983	0.000987	0.827952711	count	1
SUSD3	0.8006872	0.2490438	3.215	0.00132	0.828059872	count	1
EFCAB2	0.751653	0.3024433	2.4853	0.013	0.828909761	count	1

S100A4	0.575116	0.0383341	15.0027	9.59E-49	0.828997979	count	2.33E-44
PHYH	0.614089	0.1741518	3.5262	0.000429	0.829191266	count	1
OTUD5	0.6628461	0.2573664	2.5755	0.0101	0.829331115	count	1
YTHDC2	0.6205578	0.1896141	3.2727	0.00108	0.829731352	count	1
AC068338.2	1.2735793	0.6255164	2.036	0.0419	0.829868597	count	1
G6PD	0.6197139	0.1481886	4.1819	2.99E-05	0.830266188	count	0.7059091
AC099850.1	1.6420872	0.8613206	1.9065	0.0567	0.831314887	count	1
LRG1	0.6228455	0.2188392	2.8461	0.00446	0.831944299	count	1
ADAM19	0.8216487	0.3767027	2.1812	0.0293	0.832059391	count	1
GLYCTK	0.7897103	0.3252071	2.4283	0.0152	0.832362538	count	1
FOPNL	0.6703821	0.2653888	2.526	0.0116	0.832461323	count	1
VRK2	0.6410296	0.1701267	3.768	0.000168	0.832542477	count	1
AC074032.1	0.8221474	0.3786038	2.1715	0.03	0.832586349	count	1
ANKRD27	1.0285096	0.4292602	2.396	0.0166	0.832988542	count	1
DUSP1	0.5787847	0.0414216	13.973	9.50E-43	0.833270702	count	2.31E-38
TEFM	0.699392	0.2666664	2.6227	0.00878	0.833814497	count	1
CASP10	0.7120129	0.2248087	3.1672	0.00156	0.833942758	count	1
FLVCR1	0.7921529	0.3765341	2.1038	0.0355	0.835036588	count	1
GFRA2	0.8686696	0.2255791	3.8508	0.000121	0.835313577	count	1
MGAT5	0.6778015	0.2156944	3.1424	0.0017	0.836499486	count	1
TFCP2	0.8175699	0.3436735	2.3789	0.0174	0.837245906	count	1
GRK2	0.6102367	0.1242314	4.9121	9.60E-07	0.837286049	count	0.02286912
CTH	1.2136261	0.472226	2.57	0.0102	0.837478752	count	1
GALNT10	0.8269439	0.3683462	2.245	0.0249	0.837655214	count	1
BLNK	0.661686	0.1985425	3.3327	0.000873	0.837672318	count	1
AGTPBP1	0.6233013	0.1479597	4.2126	2.62E-05	0.837861277	count	0.6188178
EMB	0.6083188	0.108436	5.6099	2.25E-08	0.838097765	count	0.000539123
NXPE3	0.8590748	0.3072876	2.7957	0.00522	0.838185849	count	1
RETSAT	1.1101534	0.4317859	2.5711	0.0102	0.838881208	count	1
NDE1	0.6687123	0.2063091	3.2413	0.00121	0.839796582	count	1
AL033528.2	1.9025903	0.7932282	2.3985	0.0165	0.840236219	count	1
PLCB2	0.6862184	0.1790584	3.8324	0.00013	0.841239915	count	1
CTNS	0.745403	0.2992654	2.4908	0.0128	0.842001156	count	1
ANTXR2	0.6491059	0.1890031	3.4344	0.000604	0.842321559	count	1
GLTPD2	1.291343	0.626451	2.0614	0.0394	0.84234377	count	1
RIC3	1.3824127	0.8009553	1.726	0.0845	0.843018731	count	1
ADGRE3	1.3826652	0.5641347	2.4509	0.0143	0.843184134	count	1
VCL	0.6751031	0.2352342	2.8699	0.00414	0.843368978	count	1
ZSWIM6	0.6236289	0.1462471	4.2642	2.08E-05	0.843466034	count	0.4918576
RPRD1A	0.7865904	0.4194008	1.8755	0.0608	0.843474622	count	1
WDR37	0.7600674	0.3063285	2.4812	0.0132	0.843968127	count	1
IFT80	2.3387476	0.8593187	2.7216	0.00654	0.844176327	count	1
S100P	1.0770979	0.334014	3.2247	0.00128	0.844971308	count	1
COCH	3.5107143	1.515855	2.316	0.0206	0.845690671	count	1
ZSCAN25	1.043765	0.5424651	1.9241	0.0545	0.846174251	count	1

ALDH9A1	0.6386149	0.1666822	3.8313	0.000131	0.846217237	count	1
GNPAT	0.7349111	0.2552892	2.8787	0.00403	0.846797118	count	1
TBC1D31	2.3470186	1.0545273	2.2257	0.0261	0.846858153	count	1
SMARCC2	0.6300167	0.1518281	4.1495	3.44E-05	0.846966059	count	0.811668
AL021368.2	1.2979629	0.5603506	2.3163	0.0206	0.84699036	count	1
TUBA4A	0.6075405	0.1298547	4.6786	3.05E-06	0.847080663	count	0.07248935
CDKN2B	0.8818973	0.4749324	1.8569	0.0634	0.84866577	count	1
FPR2	0.6991695	0.1744903	4.0069	6.33E-05	0.848707321	count	1
GBP3	0.7094794	0.2644293	2.6831	0.00734	0.848884294	count	1
KIF9	0.8379429	0.3456881	2.424	0.0154	0.849282268	count	1
RAB11FIP4	1.0474737	0.4427665	2.3657	0.0181	0.849380288	count	1
LINC00662	0.7058704	0.3111362	2.2687	0.0234	0.849713508	count	1
LILRA2	0.6101592	0.104641	5.831	6.24E-09	0.849928036	count	0.000149754
AC083843.2	1.9243683	0.8107598	2.3735	0.0177	0.849939601	count	1
FXYD2	1.9243683	0.8488598	2.267	0.0235	0.849939601	count	1
PPFIA3	1.9243683	0.8488598	2.267	0.0235	0.849939601	count	1
CALCOCO2	0.6059852	0.0993431	6.0999	1.23E-09	0.850173489	count	2.96E-05
HSBP1L1	0.6478628	0.2301015	2.8156	0.00491	0.850586944	count	1
EOGT	1.3941511	0.682142	2.0438	0.0411	0.850705972	count	1
AL390728.6	0.8714796	0.3960128	2.2006	0.0279	0.850869899	count	1
RCOR3	0.6852151	0.2171825	3.155	0.00162	0.851221827	count	1
RNF40	0.7583881	0.3269116	2.3199	0.0204	0.852295202	count	1
SWSAP1	0.7402419	0.3353715	2.2072	0.0274	0.853109867	count	1
KIAA0355	0.8087585	0.3738352	2.1634	0.0306	0.853222395	count	1
TTC14	0.6321327	0.1694469	3.7306	0.000195	0.853417509	count	1
METTL13	0.779741	0.4275764	1.8236	0.0683	0.85507765	count	1
SETD1A	0.779741	0.335459	2.3244	0.0202	0.85507765	count	1
SESN3	0.6884737	0.1547553	4.4488	9.02E-06	0.855344234	count	0.21384616
DGAT1	0.653352	0.1924093	3.3956	0.000696	0.855766409	count	1
EEF1AKMT1	0.8041693	0.3333516	2.4124	0.0159	0.855788076	count	1
SEMA4D	0.6720569	0.2130114	3.155	0.00162	0.856037939	count	1
NEIL2	0.9988462	0.4164468	2.3985	0.0165	0.856662778	count	1
STRN4	0.7762065	0.2861054	2.713	0.00671	0.856895111	count	1
AL021453.1	0.9991144	0.4528922	2.2061	0.0275	0.856906808	count	1
AC069224.1	1.6912183	0.6255744	2.7035	0.00691	0.857464692	count	1
AL390729.1	1.525741	0.7300826	2.0898	0.0367	0.858050701	count	1
SVIL	0.7354315	0.2274036	3.234	0.00124	0.858609403	count	1
AC106739.2	0.8793418	0.3611494	2.4348	0.015	0.85891196	count	1
MPHOSPH9	0.8472224	0.3268625	2.592	0.0096	0.859095284	count	1
SLPI	1.9465945	0.6257213	3.111	0.00189	0.859784899	count	1
TLR3	1.1359836	0.4910731	2.3133	0.0208	0.859802717	count	1
AKAP17A	0.6376138	0.1463207	4.3576	1.37E-05	0.859838735	count	0.3243201
TFEB	0.7032106	0.1997032	3.5213	0.000437	0.860388781	count	1
LRRC8D	0.7697784	0.3152827	2.4415	0.0147	0.860404189	count	1
KAT7	0.7086699	0.2043637	3.4677	0.000534	0.860485551	count	1

AC097534.2	0.8489756	0.3167523	2.6803	0.00741	0.860949644	count	1
PCYOX1	0.7293814	0.263591	2.7671	0.0057	0.86136717	count	1
RPRD1B	0.7712669	0.2663392	2.8958	0.00382	0.86212017	count	1
HIST2H3D	2.395218	0.9655569	2.4807	0.0132	0.862297952	count	1
LINC01888	2.395218	0.8986123	2.6655	0.00774	0.862297952	count	1
MALL	2.395218	0.8986123	2.6655	0.00774	0.862297952	count	1
AC053503.5	2.395218	0.8986123	2.6655	0.00774	0.862297952	count	1
ACAD11	2.395218	0.8986123	2.6655	0.00774	0.862297952	count	1
LINC00472	2.395218	0.8986123	2.6655	0.00774	0.862297952	count	1
LINC00271	2.395218	0.8986123	2.6655	0.00774	0.862297952	count	1
AL355312.2	2.395218	0.9655569	2.4807	0.0132	0.862297952	count	1
GPC3	2.395218	0.9655569	2.4807	0.0132	0.862297952	count	1
AP001372.3	2.395218	0.8986123	2.6655	0.00774	0.862297952	count	1
AL391684.1	2.395218	0.9655569	2.4807	0.0132	0.862297952	count	1
C10orf91	2.395218	0.9655569	2.4807	0.0132	0.862297952	count	1
PEX12	2.395218	0.9655569	2.4807	0.0132	0.862297952	count	1
AC004528.2	2.395218	0.9655569	2.4807	0.0132	0.862297952	count	1
GCH1	0.7141147	0.1793007	3.9828	7.01E-05	0.862391995	count	1
PFAS	1.098852	0.5297981	2.0741	0.0382	0.863214113	count	1
AC015712.2	1.414389	0.6917644	2.0446	0.041	0.863943222	count	1
SIK1B	1.414389	0.5595335	2.5278	0.0115	0.863943222	count	1
PPM1F	0.6831793	0.3037553	2.2491	0.0246	0.863976859	count	1
CCDC24	1.7038352	0.8657374	1.9681	0.0492	0.864143907	count	1
GHRLOS	1.7038352	1.2578695	1.3545	0.176	0.864143907	count	1
ARMCX4	1.7055723	0.5792966	2.9442	0.00327	0.86506229	count	1
LENG8	0.7012172	0.2404818	2.9159	0.00358	0.865959039	count	1
C11orf96	0.8994743	0.4453055	2.0199	0.0435	0.866417566	count	1
CALCRL	0.9649567	0.3518811	2.7423	0.00615	0.866608732	count	1
ARHGAP6	0.6846573	0.2062786	3.3191	0.000916	0.867225063	count	1
FAM222B	1.3271965	0.5576893	2.3798	0.0174	0.867491563	count	1
DOPEY2	0.7407287	0.2790011	2.6549	0.00798	0.868453135	count	1
XRCC1	0.7470793	0.2419773	3.0874	0.00204	0.868913994	count	1
FASTKD3	0.9314877	0.3808158	2.446	0.0145	0.868984319	count	1
FGFBP3	0.9676435	0.472563	2.0476	0.0407	0.86915717	count	1
HFE	0.9321351	0.3579576	2.604	0.00927	0.869619799	count	1
VIM-AS1	0.6872548	0.2462655	2.7907	0.0053	0.870567741	count	1
NCOA5	1.1493872	0.5067941	2.268	0.0234	0.870658073	count	1
AARS2	1.547166	0.7106814	2.177	0.0296	0.870849121	count	1
CCDC88B	0.7299119	0.2421336	3.0145	0.0026	0.871069022	count	1
ZXDC	0.7888972	0.2836812	2.7809	0.00546	0.871369069	count	1
ETAA1	0.9045741	0.5966395	1.5161	0.13	0.871569909	count	1
NAB2	0.7215589	0.2719729	2.6531	0.00803	0.871579197	count	1
TCP11L2	0.7411959	0.4073504	1.8196	0.0689	0.872396345	count	1
SGMS1	0.6497051	0.156522	4.1509	3.42E-05	0.872474255	count	0.8069832
AL627171.1	0.8598752	0.3487725	2.4654	0.0138	0.872480572	count	1

PLBD1	0.6173109	0.0744762	8.2887	1.86E-16	0.873363925	count	4.50E-12
SIDT2	0.7482779	0.2118378	3.5323	0.00042	0.874000768	count	1
EXTL2	0.9540292	0.4508507	2.1161	0.0344	0.874291783	count	1
LIN7C	0.6970062	0.2436815	2.8603	0.00427	0.874406957	count	1
GTF2H2	0.8210484	0.3831822	2.1427	0.0322	0.874426978	count	1
ARHGAP4	0.6541939	0.1506333	4.343	1.46E-05	0.874677144	count	0.3455674
CCND1	0.6894684	0.233274	2.9556	0.00315	0.874744143	count	1
PARG	0.8289005	0.3560009	2.3284	0.02	0.87529568	count	1
CD24	1.4320491	1.0138574	1.4125	0.158	0.875475564	count	1
MIIP	0.6575647	0.1559463	4.2166	2.57E-05	0.876360931	count	0.6070854
RNF125	0.7052005	0.229896	3.0675	0.00218	0.876510312	count	1
MTMR12	0.8101048	0.2900284	2.7932	0.00526	0.8765608	count	1
AGO1	0.7163343	0.2879419	2.4878	0.0129	0.876773359	count	1
SLC30A6	1.0802138	0.4266697	2.5317	0.0114	0.877687917	count	1
CLEC16A	1.049805	0.4472251	2.3474	0.019	0.878284426	count	1
BTBD2	0.7465316	0.260733	2.8632	0.00423	0.878834117	count	1
MSRB1	0.6566388	0.1409544	4.6585	3.36E-06	0.878880501	count	0.07984704
PELI2	0.8015681	0.282891	2.8335	0.00464	0.879829801	count	1
PACS1	0.7327363	0.2180447	3.3605	0.00079	0.880114322	count	1
ACAP3	0.7744148	0.3460073	2.2381	0.0253	0.880494193	count	1
KLHL35	1.9938216	0.9266302	2.1517	0.0315	0.880502826	count	1
DDIT4-AS1	1.9938216	1.0182547	1.9581	0.0503	0.880502826	count	1
KCNJ2-AS1	1.9938216	0.8392115	2.3758	0.0176	0.880502826	count	1
GGT1	1.9938216	0.8392115	2.3758	0.0176	0.880502826	count	1
RCN3	0.9432649	0.3735982	2.5248	0.0116	0.880546036	count	1
CYYR1	1.735108	0.821909	2.1111	0.0349	0.880631396	count	1
LPAR1	1.1201803	0.5927385	1.8898	0.0589	0.881100524	count	1
TBC1D17	1.0529996	0.3979152	2.6463	0.00819	0.881122789	count	1
TMEM164	0.7879922	0.3040169	2.5919	0.0096	0.881407261	count	1
FAXDC2	1.0263421	0.4171191	2.4605	0.0139	0.88168644	count	1
PHOSPHO2	1.3476398	0.7144048	1.8864	0.0594	0.881808042	count	1
SPICE1	0.9620257	0.4088129	2.3532	0.0187	0.882015234	count	1
TPM1	0.6718218	0.1612373	4.1667	3.20E-05	0.882410851	count	0.755296
RAD52	1.2156191	0.5822647	2.0877	0.0369	0.883758736	count	1
GSTT2B	0.7661794	0.3231724	2.3708	0.0178	0.88384036	count	1
C21orf91	0.7276148	0.214873	3.3863	0.00072	0.883981807	count	1
PER3	0.7072143	0.2364572	2.9909	0.00281	0.884206353	count	1
ETV3	0.6591309	0.1833318	3.5953	0.00033	0.884414191	count	1
USF3	0.6895736	0.2888062	2.3877	0.017	0.88453928	count	1
DAGLB	0.7740746	0.2357661	3.2832	0.00104	0.884631563	count	1
FRS2	0.8241311	0.3479749	2.3684	0.0179	0.885226292	count	1
FAM157C	1.4478401	0.4126426	3.5087	0.000458	0.885771836	count	1
PPIP5K1	1.030909	0.7547686	1.3659	0.172	0.88584365	count	1
IQSEC1	0.6798073	0.1599675	4.2497	2.22E-05	0.886117429	count	0.5247414
POLR3F	0.7754909	0.3178665	2.4397	0.0148	0.886296473	count	1

ACSM5	1.2195757	0.4754727	2.565	0.0104	0.886834461	count	1
TRIB1	0.6529091	0.1192419	5.4755	4.81E-08	0.887075293	count	0.001151129
LIPN	0.9068667	0.2771519	3.2721	0.00108	0.887082159	count	1
SH3RF3	1.1704493	0.5840253	2.0041	0.0452	0.887712348	count	1
TNNT1	0.7290118	0.2615966	2.7868	0.00536	0.888077349	count	1
AC026401.3	1.0925695	0.4131649	2.6444	0.00824	0.888372062	count	1
POU2F2	0.6350603	0.0898783	7.0658	2.07E-12	0.888667355	count	4.99E-08
USP11	0.8102612	0.3088913	2.6231	0.00877	0.889692635	count	1
AL139274.2	1.284112	0.7694217	1.6689	0.0953	0.889769459	count	1
HSF4	2.486537	1.220077	2.038	0.0417	0.890650311	count	1
UBE2O	0.8605713	0.3835232	2.2439	0.0249	0.892597078	count	1
MON1B	0.7194212	0.27196	2.6453	0.00821	0.892680507	count	1
GATA2	2.495488	1.0863664	2.2971	0.0217	0.89336464	count	1
F2RL1	2.495488	1.0324181	2.4171	0.0157	0.89336464	count	1
AC116366.3	2.495488	1.0324181	2.4171	0.0157	0.89336464	count	1
CASC8	2.495488	1.0324181	2.4171	0.0157	0.89336464	count	1
CEP295NL	2.495488	1.0324181	2.4171	0.0157	0.89336464	count	1
ALG12	0.8251658	0.3317245	2.4875	0.0129	0.893435444	count	1
MPP7	1.4614765	0.4273894	3.4195	0.000638	0.894650535	count	1
ERAP1	0.7873035	0.2594813	3.0341	0.00244	0.895579706	count	1
MIB2	0.8928207	0.3609655	2.4734	0.0134	0.896470996	count	1
FAM13B	0.6924202	0.2041346	3.392	0.000705	0.896492921	count	1
FAM184B	0.9595912	0.4789147	2.0037	0.0452	0.896578888	count	1
AL050341.2	0.9443789	0.4923917	1.9179	0.0552	0.897216793	count	1
DIAPH2	0.6581124	0.1615631	4.0734	4.78E-05	0.897980876	count	1
CBX8	1.1836335	0.6048583	1.9569	0.0505	0.898384578	count	1
SRBD1	0.7243214	0.2229563	3.2487	0.00117	0.89887366	count	1
MSANTD2	0.8240312	0.4933214	1.6704	0.095	0.898891941	count	1
VASH1-AS1	1.4707226	0.666173	2.2077	0.0274	0.900663599	count	1
ZNF280D	0.7165769	0.2473211	2.8974	0.0038	0.900960485	count	1
AC009831.1	1.376384	0.7791669	1.7665	0.0774	0.901904446	count	1
EDEM3	0.6789524	0.1785509	3.8026	0.000147	0.901943934	count	1
TMEM209	0.8880337	0.358415	2.4777	0.0133	0.90228766	count	1
ZNF615	1.188803	0.7306557	1.627	0.104	0.902568292	count	1
ALOX12-AS1	0.8698331	0.3400719	2.5578	0.0106	0.902589473	count	1
PRKAR2B	0.7703503	0.2814326	2.7372	0.00624	0.904082812	count	1
FAM227B	1.7799913	0.7849761	2.2676	0.0234	0.904115331	count	1
TCHP	0.7299797	0.2437701	2.9945	0.00278	0.904122407	count	1
TRMT1	0.6577805	0.1163543	5.6533	1.76E-08	0.90429944	count	0.000421837
ABTB1	0.6626378	0.1320883	5.0166	5.64E-07	0.904449926	count	0.013444068
R3HDM4	0.697081	0.1872491	3.7227	0.000202	0.904492568	count	1
MAF	0.6482914	0.101762	6.3707	2.24E-10	0.904510115	count	5.39E-06
MDM1	1.3810448	0.4053633	3.4069	0.000668	0.905159173	count	1
AL161785.1	1.3829686	0.4459814	3.101	0.00195	0.906502184	count	1
ABT1	0.6965866	0.2005386	3.4736	0.000522	0.906542568	count	1

MAP3K21	1.0819442	0.52816	2.0485	0.0406	0.906841526	count	1
DCAF10	0.7389755	0.2473421	2.9877	0.00284	0.907250869	count	1
TANC1	2.0580676	0.7732558	2.6616	0.00783	0.908223724	count	1
RAB3D	0.7874691	0.2775875	2.8368	0.00459	0.909081387	count	1
LINC01089	0.9572235	0.4018115	2.3823	0.0173	0.910025835	count	1
RNASE1	0.6319663	0.0866031	7.2973	3.95E-13	0.910871193	count	9.54E-09
AC007881.3	1.6149319	0.6270114	2.5756	0.0101	0.9110856	count	1
RNF139-AS1	1.1198484	0.5695453	1.9662	0.0494	0.911959132	count	1
VWA8	0.8702122	0.6722595	1.2945	0.196	0.912031305	count	1
ZC3H12C	0.9450145	0.4887233	1.9336	0.0533	0.912451014	count	1
CIDEB	0.7836622	0.2730464	2.8701	0.00414	0.912612308	count	1
ROGDI	0.6932708	0.1480041	4.6841	2.97E-06	0.913030524	count	0.07059393
FBXL19-AS1	1.7974257	0.9389931	1.9142	0.0557	0.91317724	count	1
MAML1	0.8430478	0.3174975	2.6553	0.00798	0.91348017	count	1
CTC1	1.6200274	0.6374851	2.5413	0.0111	0.914094737	count	1
EPS8	0.6777512	0.1363118	4.9721	7.08E-07	0.914423008	count	0.01687164
NUMA1	0.686708	0.1734824	3.9584	7.76E-05	0.914537205	count	1
TMEM131L	0.8906909	0.3078922	2.8929	0.00385	0.915373166	count	1
GBP4	0.7100762	0.1842662	3.8535	0.000119	0.91560415	count	1
MEPCE	0.749447	0.2194434	3.4152	0.000648	0.915848324	count	1
GFOD1	0.7869096	0.2646207	2.9737	0.00297	0.916493347	count	1
MGST1	0.6563257	0.1206976	5.4378	5.93E-08	0.917341206	count	0.001418753
INPP5F	0.7910312	0.3125369	2.531	0.0114	0.917436442	count	1
PILRB	0.8406581	0.3124081	2.6909	0.00717	0.917652134	count	1
ARNT	0.7327292	0.2435867	3.0081	0.00266	0.918361851	count	1
FAM160B1	0.8682178	0.4264509	2.0359	0.0419	0.918421842	count	1
RPS6KA5	0.7515396	0.3163302	2.3758	0.0176	0.918457634	count	1
ZNF333	0.8031341	0.4689803	1.7125	0.0869	0.918804919	count	1
NECTIN3	2.5830007	1.426052	1.8113	0.0702	0.919285835	count	1
CNGA1	2.5830007	1.0795869	2.3926	0.0168	0.919285835	count	1
TAS2R14	2.5830007	1.0795869	2.3926	0.0168	0.919285835	count	1
SNX27	0.6833053	0.1337282	5.1097	3.48E-07	0.919648984	count	0.008302932
LATS1	0.8263546	0.2629258	3.1429	0.00169	0.920024719	count	1
SLC33A1	0.8218398	0.3449569	2.3824	0.0173	0.920468011	count	1
METTL7A	0.6929097	0.115484	6	2.26E-09	0.920674237	count	5.43E-05
TMEM168	0.8784141	0.2912424	3.0161	0.00259	0.920959967	count	1
AL359513.1	2.0888534	0.7917158	2.6384	0.00838	0.921308642	count	1
UBE2V1	0.7474887	0.2445606	3.0565	0.00226	0.92217489	count	1
C16orf86	0.9414227	0.3178326	2.962	0.00309	0.92247634	count	1
P2RY13	0.6572367	0.0921056	7.1357	1.26E-12	0.922582057	count	3.04E-08
TBRG4	0.7636253	0.2282139	3.3461	0.000832	0.923527668	count	1
FAM13A	0.7089358	0.1935359	3.6631	0.000254	0.923700289	count	1
SMPD2	0.9564575	0.3907285	2.4479	0.0144	0.924025018	count	1
MIB1	0.8993633	0.3173279	2.8342	0.00463	0.924649813	count	1
LRWD1	0.97213	0.4433041	2.1929	0.0284	0.924894625	count	1

TSTD2	0.8669917	0.3823029	2.2678	0.0234	0.925208117	count	1
AKTIP	1.0492924	0.5020987	2.0898	0.0367	0.925450549	count	1
EPS15	0.6778603	0.1237098	5.4794	4.70E-08	0.925624286	count	0.001124851
CD302	0.6596154	0.0717497	9.1933	7.90E-20	0.926954027	count	1.91E-15
NGRN	1.0285989	0.465052	2.2118	0.0271	0.927004707	count	1
DGLUCY	0.8142964	0.2268405	3.5897	0.000338	0.927190229	count	1
UBA7	1.0523411	0.3188932	3.3	0.000981	0.928287967	count	1
SLC44A2	1.0524448	0.3781748	2.783	0.00543	0.92838447	count	1
KLHL8	0.7586963	0.2116604	3.5845	0.000344	0.92969622	count	1
PLEKHH3	1.4174745	0.540178	2.6241	0.00874	0.930554652	count	1
DGKG	1.0133085	0.4482299	2.2607	0.0239	0.93157216	count	1
SH3PXD2A	0.9791844	0.3317832	2.9513	0.00319	0.931932298	count	1
PER1	0.6862211	0.1089283	6.2998	3.52E-10	0.932371209	count	8.47E-06
SCARF1	0.8815244	0.26726	3.2984	0.000986	0.933027254	count	1
CLEC4D	0.7813471	0.2048758	3.8138	0.00014	0.933923509	count	1
PPM1K	0.7796915	0.2438559	3.1973	0.0014	0.934903908	count	1
DHRS4-AS1	0.766728	0.2426677	3.1596	0.0016	0.934997498	count	1
IGSF10	1.4241887	0.529656	2.6889	0.00722	0.935226261	count	1
DHX57	1.1146049	0.3258855	3.4202	0.000636	0.93586045	count	1
PPARA	0.8307218	0.350609	2.3694	0.0179	0.936174645	count	1
MAPK7	0.8313673	0.2558763	3.2491	0.00117	0.936923874	count	1
ZSCAN9	1.2842545	0.5004461	2.5662	0.0103	0.937048406	count	1
SYTL1	1.3484133	0.4031059	3.3451	0.000835	0.937319769	count	1
MATR3	0.8857431	0.3036933	2.9166	0.00357	0.937658602	count	1
SEC16B	2.6506688	1.276468	2.0766	0.0379	0.938551708	count	1
AL512306.3	2.6506688	1.991831	1.3308	0.183	0.938551708	count	1
AC084036.1	2.6506688	1.0708119	2.4754	0.0134	0.938551708	count	1
ZRSR2	0.7562501	0.2053139	3.6834	0.000235	0.939242336	count	1
MATN1-AS1	2.131814	0.8437107	2.5267	0.0116	0.939343718	count	1
DMTF1	0.8205954	0.2583052	3.1768	0.00151	0.939350846	count	1
LINC00996	1.8482106	0.764774	2.4167	0.0157	0.939369307	count	1
PLCG2	0.6695395	0.1210028	5.5333	3.48E-08	0.940036253	count	0.000833216
SPTLC2	0.7056916	0.1132079	6.2336	5.35E-10	0.94006954	count	1.29E-05
RAB33B	0.849162	0.2594267	3.2732	0.00108	0.940175232	count	1
TNFRSF25	1.6649858	0.6388197	2.6063	0.00921	0.940536178	count	1
HEXDC	0.7162942	0.2045011	3.5026	0.000469	0.940763016	count	1
CPNE8	0.7006053	0.1518093	4.615	4.13E-06	0.941011322	count	0.0980875
KLF4	0.6578609	0.0581316	11.3168	5.60E-29	0.941179043	count	1.36E-24
ZNF354B	0.9734707	0.3678373	2.6465	0.00819	0.941236754	count	1
ANKRD23	2.6609832	1.2401129	2.1458	0.032	0.941428235	count	1
SEZ6L	2.6609832	1.0771069	2.4705	0.0136	0.941428235	count	1
CLEC4A	0.676044	0.0947601	7.1343	1.28E-12	0.942214692	count	3.09E-08
SEC24B	0.8118695	0.270048	3.0064	0.00267	0.942255427	count	1
TBL1X	0.746097	0.2063634	3.6155	0.000306	0.943391476	count	1
STYX	0.7565627	0.2516146	3.0068	0.00267	0.943421143	count	1

INPP5K	0.7845348	0.2750093	2.8528	0.00437	0.943771571	count	1
AKT3	0.8373261	0.2858561	2.9292	0.00343	0.94384083	count	1
LINC00346	0.8209251	0.3775931	2.1741	0.0298	0.944341293	count	1
MCPH1	0.8647357	0.2530103	3.4178	0.000642	0.944832257	count	1
NADK	0.7564631	0.1887663	4.0074	6.32E-05	0.945116353	count	1
MRI1	0.8180495	0.401097	2.0395	0.0415	0.945360616	count	1
TTF2	0.7814885	0.2546121	3.0693	0.00217	0.945601996	count	1
MTRR	1.1590531	0.3904568	2.9685	0.00302	0.945845296	count	1
ZNF586	1.5407545	0.49638	3.104	0.00193	0.946001962	count	1
FAM122A	0.7496184	0.2534709	2.9574	0.00313	0.946386002	count	1
VSIR	0.6673467	0.0622976	10.7122	3.35E-26	0.94674374	count	8.13E-22
ROBO4	2.152247	0.692947	3.1059	0.00192	0.947827794	count	1
AGPAT1	0.7347372	0.2595088	2.8313	0.00467	0.94784712	count	1
STAB1	0.6827758	0.1083064	6.3041	3.43E-10	0.947994167	count	8.25E-06
AC103591.3	0.7041485	0.2967657	2.3727	0.0177	0.948353767	count	1
ANKRA2	0.904547	0.3348253	2.7015	0.00695	0.949418967	count	1
ARL11	0.7275529	0.1981022	3.6726	0.000245	0.950021162	count	1
RLIM	0.7537012	0.1992335	3.783	0.000159	0.950051648	count	1
VPS13A	0.8101929	0.3334649	2.4296	0.0152	0.952047553	count	1
LINC01176	1.450646	0.6232521	2.3275	0.02	0.953605031	count	1
BX284668.5	0.7451471	0.1964833	3.7924	0.000153	0.954459148	count	1
KIAA1109	0.7326246	0.158143	4.6327	3.80E-06	0.954955876	count	0.0902576
ACSF3	0.7719775	0.2307825	3.345	0.000835	0.95508138	count	1
ZNF101	0.9109344	0.5424205	1.6794	0.0932	0.956377002	count	1
CENPJ	0.9397001	0.3770125	2.4925	0.0128	0.957029583	count	1
RGL1	0.7159806	0.1568544	4.5646	5.25E-06	0.957084969	count	0.1246245
PIK3CG	0.8896299	0.2596961	3.4257	0.000623	0.958177913	count	1
S1PR4	0.75309	0.2014257	3.7388	0.000189	0.958188943	count	1
CDADC1	0.8770361	0.2623826	3.3426	0.000842	0.958722804	count	1
CWF19L1	0.8502736	0.3065777	2.7734	0.00559	0.958873057	count	1
SMIM27	0.7977989	0.222902	3.5791	0.000351	0.96008355	count	1
AC114490.3	2.7315296	1.0318309	2.6473	0.00817	0.960674909	count	1
AP003774.4	2.7315296	1.0318309	2.6473	0.00817	0.960674909	count	1
AC020656.2	1.564376	0.6481014	2.4138	0.0159	0.961203284	count	1
HECTD2	0.8734278	0.2747793	3.1787	0.0015	0.96142513	count	1
BTN3A3	1.0449742	0.3218873	3.2464	0.00118	0.962183523	count	1
CLU	0.7977271	0.4397537	1.814	0.0698	0.962878182	count	1
TMPO-AS1	1.1787881	0.6119663	1.9262	0.0542	0.962892739	count	1
RPRD2	0.8258537	0.3324927	2.4838	0.0131	0.963057894	count	1
EPN2	1.0902477	0.4418608	2.4674	0.0137	0.963565395	count	1
GALT	0.766942	0.2076753	3.693	0.000226	0.963709576	count	1
ZNF343	1.8963808	0.7412267	2.5584	0.0106	0.963915062	count	1
ADRB2	0.7326466	0.1765735	4.1492	3.45E-05	0.964008903	count	0.813993
RBM5	0.7357479	0.1697032	4.3355	1.51E-05	0.964225364	count	0.3573868
SIRPB2	0.7929397	0.3312872	2.3935	0.0168	0.965079209	count	1

DIAPH1	0.7151563	0.1359929	5.2588	1.58E-07	0.965701043	count	0.003775252
PRKAB1	1.0926743	0.4366523	2.5024	0.0124	0.965823382	count	1
ZBP1	1.2674475	1.0359523	1.2235	0.221	0.966127991	count	1
DPP9	0.7902751	0.2530338	3.1232	0.00181	0.966777294	count	1
AC090517.4	1.2236579	0.6328326	1.9336	0.0533	0.967796897	count	1
MAPK8IP3	1.1223093	0.4515017	2.4857	0.013	0.969052171	count	1
FZD1	0.7461737	0.2301382	3.2423	0.0012	0.970018329	count	1
TSPAN2	1.3931463	0.5520871	2.5234	0.0117	0.970274561	count	1
ANKRD49	0.8113783	0.2678033	3.0298	0.00247	0.970654516	count	1
ACBD4	1.1244226	0.4051563	2.7753	0.00556	0.970975331	count	1
BLOC1S3	0.9342633	0.3914169	2.3869	0.0171	0.972158279	count	1
AC010883.1	1.395767	0.6417283	2.175	0.0297	0.972201285	count	1
AC124016.1	1.395767	0.5545322	2.517	0.0119	0.972201285	count	1
EFHC1	1.1558507	0.4438251	2.6043	0.00926	0.972490038	count	1
NTNG2	0.878156	0.3118583	2.8159	0.0049	0.973312264	count	1
PRKCD	0.7591399	0.1508331	5.033	5.18E-07	0.973891647	count	0.012349638
PRAG1	1.1576412	0.4328909	2.6742	0.00754	0.974079455	count	1
AC023157.3	0.7963802	0.2440848	3.2627	0.00112	0.974395965	count	1
NUP210	0.9045381	0.3208389	2.8193	0.00485	0.974795947	count	1
CFAP58-DT	1.586088	0.8089229	1.9607	0.05	0.975130008	count	1
CYB5B	0.7421178	0.1794403	4.1357	3.66E-05	0.976611793	count	0.8633574
RIC1	0.8655757	0.2507139	3.4524	0.000565	0.976643541	count	1
KMT2E-AS1	0.8277344	0.2485542	3.3302	0.000881	0.976912189	count	1
BUD13	0.866415	0.2930113	2.9569	0.00314	0.977618352	count	1
PRKN	2.796085	1.1210477	2.4942	0.0127	0.977631994	count	1
RGL3	2.796085	1.120051	2.4964	0.0126	0.977631994	count	1
ZNF30	2.796085	1.1990069	2.332	0.0198	0.977631994	count	1
9-Sep	0.7238496	0.1311673	5.5185	3.78E-08	0.978305327	count	0.000904894
WRNIP1	0.8262907	0.287164	2.8774	0.00404	0.978771983	count	1
POC5	0.9404359	0.4020312	2.3392	0.0194	0.978826939	count	1
TMEM104	1.2371225	0.4353318	2.8418	0.00452	0.979057876	count	1
TRIT1	1.0436216	0.3736988	2.7927	0.00527	0.979153703	count	1
USP45	1.0267393	0.4576141	2.2437	0.0249	0.979385192	count	1
SWT1	0.9720573	0.6001552	1.6197	0.105	0.979567088	count	1
PLIN5	1.3396769	0.5926468	2.2605	0.0239	0.979941246	count	1
CNRIP1	0.7974975	0.2148947	3.7111	0.000211	0.980563264	count	1
TRAF3IP3	0.7264982	0.1370722	5.3001	1.26E-07	0.981140902	count	0.003011148
AC144831.1	1.065223	0.4280483	2.4886	0.0129	0.981757805	count	1
PAXBP1-AS1	1.1383517	0.4059758	2.804	0.00509	0.983649378	count	1
AAR2	0.9759689	0.3290878	2.9657	0.00305	0.983671412	count	1
LIG3	0.9361605	0.3536264	2.6473	0.00817	0.983862938	count	1
AC005332.1	0.9058415	0.3253173	2.7845	0.0054	0.983931158	count	1
GGT7	1.2037457	0.492181	2.4457	0.0145	0.984437418	count	1
RERE	0.7385522	0.1437119	5.1391	2.98E-07	0.985396583	count	0.00711177
MEDAG	18.6516772	1259.393233	0.0148	0.988	0.985786068	count	1

AC093388.1	19.2691062	1564.758214	0.0123	0.9902	0.985786073	count	1
TNN	19.3561681	1938.641643	0.01	0.992	0.985786073	count	1
HIP1R	19.559496	1320.282742	0.0148	0.9882	0.985786074	count	1
ADGRG1	20.1345959	1238.268074	0.0163	0.987	0.985786076	count	1
CELSR3	20.1358452	1756.601605	0.0115	0.991	0.985786076	count	1
OR7G2	20.1358452	1756.601605	0.0115	0.991	0.985786076	count	1
AC007249.2	20.1358453	1756.601655	0.0115	0.991	0.985786076	count	1
NCBP2-AS1	20.1358453	1756.601677	0.0115	0.991	0.985786076	count	1
ECT2L	20.1358453	1756.601677	0.0115	0.991	0.985786076	count	1
HEPH	20.1358453	1756.601655	0.0115	0.991	0.985786076	count	1
GRIK5	20.1358453	1756.601677	0.0115	0.991	0.985786076	count	1
SH3TC2	18.601927	920.1423476	0.0202	0.984	0.985786138	count	1
AC009318.3	19.2683063	1357.302788	0.0142	0.9887	0.985786144	count	1
ADAMTS5	19.2683104	1357.302344	0.0142	0.9887	0.985786144	count	1
AC010680.4	19.3305558	1258.052218	0.0154	0.988	0.985786145	count	1
UST	19.355698	1368.299796	0.0141	0.989	0.985786145	count	1
PRICKLE1	19.355698	1368.299796	0.0141	0.989	0.985786145	count	1
PTPRG	19.4277769	2106.811277	0.0092	0.993	0.985786145	count	1
LINC01126	19.4448558	1526.726132	0.0127	0.9898	0.985786145	count	1
SCGB3A1	19.4452758	1721.520012	0.0113	0.991	0.985786145	count	1
LINC02217	20.1345958	1238.268145	0.0163	0.987	0.985786148	count	1
CDC20B	20.1345958	1238.268156	0.0163	0.987	0.985786148	count	1
AC007216.2	20.1345957	1238.26813	0.0163	0.987	0.985786148	count	1
AC110285.6	20.1345958	1238.268094	0.0163	0.987	0.985786148	count	1
IL11	20.1345957	1238.268074	0.0163	0.987	0.985786148	count	1
FAM87B	20.1345961	1238.268115	0.0163	0.987	0.985786148	count	1
TAS1R3	20.134596	1238.268115	0.0163	0.987	0.985786148	count	1
ROR1	20.134596	1238.26813	0.0163	0.987	0.985786148	count	1
KCNN1	20.134596	1238.268125	0.0163	0.987	0.985786148	count	1
PLA2G6	20.1345956	1238.268104	0.0163	0.987	0.985786148	count	1
CNTNAP2	20.1345959	1238.268084	0.0163	0.987	0.985786148	count	1
AL590399.3	20.1345962	1238.268161	0.0163	0.987	0.985786148	count	1
LINC02257	20.135845	1756.601663	0.0115	0.991	0.985786148	count	1
HOOK1	20.1358452	1756.601576	0.0115	0.991	0.985786148	count	1
AL449266.1	20.1358452	1756.601648	0.0115	0.991	0.985786148	count	1
DNM3OS	20.1358452	1756.601612	0.0115	0.991	0.985786148	count	1
AC018693.1	20.1358453	1756.60167	0.0115	0.991	0.985786148	count	1
AC007389.5	20.1358453	1756.601714	0.0115	0.991	0.985786148	count	1
AC007879.3	20.1358452	1756.601612	0.0115	0.991	0.985786148	count	1
APOA1	20.1358452	1756.601626	0.0115	0.991	0.985786148	count	1
CYP46A1	20.1358453	1756.601655	0.0115	0.991	0.985786148	count	1
MKRN3	20.1358452	1756.60175	0.0115	0.991	0.985786148	count	1
HSPB6	20.1358452	1756.601648	0.0115	0.991	0.985786148	count	1
FP565260.3	20.1358455	1756.601743	0.0115	0.991	0.985786148	count	1
AC097382.2	20.1358451	1756.601619	0.0115	0.991	0.985786148	count	1

DUOXA1	20.1358451	1756.601648	0.0115	0.991	0.985786148	count	1
AC015674.1	20.1358451	1756.601619	0.0115	0.991	0.985786148	count	1
AC013403.2	20.1358454	1756.601721	0.0115	0.991	0.985786148	count	1
AC023128.1	20.1358454	1756.601655	0.0115	0.991	0.985786148	count	1
RGS7BP	20.1358454	1756.601721	0.0115	0.991	0.985786148	count	1
AL031123.1	20.1358454	1756.601655	0.0115	0.991	0.985786148	count	1
AC110285.5	20.1358454	1756.601721	0.0115	0.991	0.985786148	count	1
AC004221.1	20.1358454	1756.601655	0.0115	0.991	0.985786148	count	1
WNT7B	20.1358454	1756.601721	0.0115	0.991	0.985786148	count	1
MVB12B	0.9671209	0.2876906	3.3617	0.000787	0.986099888	count	1
PRKD2	0.9150475	0.3789269	2.4148	0.0158	0.986512784	count	1
HMGB2	0.6986995	0.0853264	8.1886	4.20E-16	0.986778266	count	1.02E-11
CKAP4	0.7384122	0.1507486	4.8983	1.03E-06	0.986827745	count	0.02453151
CPED1	0.8128832	0.2415033	3.3659	0.000775	0.987176383	count	1
RXRA	0.7217197	0.1267368	5.6946	1.38E-08	0.987236024	count	0.000330924
YLPM1	0.8334656	0.271326	3.0718	0.00215	0.987473407	count	1
GANC	0.9487399	0.297508	3.189	0.00145	0.987798966	count	1
PBXIP1	0.8281837	0.2199615	3.7651	0.00017	0.987943126	count	1
ATOH8	1.5003966	0.5222435	2.873	0.0041	0.988023038	count	1
ZBTB42	1.5003966	0.5400968	2.778	0.00551	0.988023038	count	1
RABGEF1	0.8231632	0.2200604	3.7406	0.000188	0.988228548	count	1
MEFV	1.1182756	0.2718512	4.1136	4.02E-05	0.989641601	count	0.9478356
FOSB	0.6916567	0.0643136	10.7544	2.17E-26	0.990928374	count	5.27E-22
TNFAIP2	0.706715	0.0967853	7.3019	3.82E-13	0.991023488	count	9.22E-09
ANKDD1A	0.9832846	0.4382046	2.2439	0.0249	0.991347795	count	1
NFATC1	0.7964842	0.3363051	2.3683	0.0179	0.992150139	count	1
TMX4	0.7155344	0.13385	5.3458	9.83E-08	0.992332536	count	0.00234996
PLA2G16	0.7612496	0.1409185	5.4021	7.22E-08	0.992737139	count	0.001726663
NCKIPSD	1.0408045	0.4952213	2.1017	0.0357	0.99342125	count	1
ACSS1	1.301886	0.3674092	3.5434	0.000402	0.99388802	count	1
LBH	1.0415537	0.4011327	2.5965	0.00947	0.994168892	count	1
APOOL	0.8173289	0.2298028	3.5567	0.000383	0.995382076	count	1
PPP4R1	0.7949627	0.2166376	3.6696	0.000248	0.995935736	count	1
AC106028.4	0.754962	0.2195719	3.4383	0.000595	0.995954594	count	1
ZNF414	0.9104647	0.4065992	2.2392	0.0252	0.99648678	count	1
PREPL	0.9393778	0.3148421	2.9836	0.00288	0.996567334	count	1
SELPLG	0.7778525	0.1453544	5.3514	9.54E-08	0.996969678	count	0.002280728
KANK2	1.258574	0.394523	3.1901	0.00144	0.996984433	count	1
SYNE3	0.9788659	0.2659826	3.6802	0.000238	0.998553359	count	1
HSPA4L	2.2794458	0.9740702	2.3401	0.0194	0.999212697	count	1
TREML4	2.2794458	0.8339961	2.7332	0.00632	0.999212697	count	1
TAF5	1.0323034	0.3685612	2.8009	0.00514	1.000773691	count	1
SEPSECS	1.1308939	0.3811723	2.9669	0.00304	1.001377239	count	1
CD69	0.7140088	0.1619584	4.4086	1.09E-05	1.001505653	count	0.2582646
PSKH1	1.2648599	0.4841584	2.6125	0.00904	1.002233668	count	1

CAPNS2	1.4367189	0.8057791	1.783	0.0747	1.002250038	count	1
SH3TC1	0.7829999	0.1880509	4.1638	3.24E-05	1.003661835	count	0.7647048
PSMA3-AS1	0.7487097	0.158748	4.7163	2.54E-06	1.005262334	count	0.0603885
KAT2B	0.8691592	0.4198987	2.0699	0.0386	1.006042207	count	1
ATG16L2	0.7637158	0.1556044	4.9081	9.80E-07	1.006125651	count	0.02334458
FOS	0.6988844	0.0512621	13.6335	7.51E-41	1.007517252	count	1.83E-36
FBXO42	0.9084315	0.3568504	2.5457	0.011	1.007929704	count	1
AC145285.6	1.2310191	0.5854546	2.1027	0.0356	1.007958363	count	1
PARD6B	1.637896	0.8372648	1.9562	0.0505	1.008172463	count	1
LINC02256	1.0008223	0.417625	2.3965	0.0166	1.009750959	count	1
TPD52L1	1.78553	0.873738	2.0436	0.0411	1.010343805	count	1
SESN1	0.7946333	0.1640769	4.8431	1.36E-06	1.010397799	count	0.03237616
CDKN2A	1.3224414	0.5842751	2.2634	0.0237	1.010428689	count	1
RCCD1	1.3225016	0.5894815	2.2435	0.025	1.010477077	count	1
SREBF2	1.0760239	0.3048159	3.5301	0.000423	1.010997263	count	1
APOBEC3A	0.7151821	0.1719362	4.1596	3.30E-05	1.01114513	count	0.778767
MCEE	0.8535367	0.253929	3.3613	0.000788	1.011819424	count	1
MOCS3	1.2011256	0.5129032	2.3418	0.0193	1.012654422	count	1
CLIP4	0.8574361	0.2385799	3.5939	0.000332	1.012823188	count	1
ELOVL5	0.7758028	0.1558231	4.9787	6.84E-07	1.012888755	count	0.016301088
AL355001.2	1.0791611	0.3577086	3.0169	0.00258	1.014079873	count	1
GSAP	0.8013412	0.2093053	3.8286	0.000132	1.014399366	count	1
JADE1	0.8767997	0.2744494	3.1948	0.00142	1.015117435	count	1
MNDA	0.708079	0.0474252	14.9304	2.60E-48	1.017588073	count	6.32E-44
AL451085.1	1.7990467	0.7993425	2.2507	0.0245	1.018061613	count	1
FANCG	1.284112	0.5292654	2.4262	0.0153	1.018299604	count	1
PCMTD2	0.8427283	0.3145332	2.6793	0.00743	1.018391297	count	1
PSMB10	0.7363242	0.1032088	7.1343	1.28E-12	1.018524797	count	3.09E-08
TTC28	2.9630146	1.1092562	2.6712	0.00761	1.018586315	count	1
MELTF	1.1263232	0.5562999	2.0247	0.043	1.019759756	count	1
TIMELESS	1.656285	0.6073616	2.727	0.00644	1.01983178	count	1
LINGO3	2.0086149	0.7586202	2.6477	0.00816	1.019871947	count	1
CD101	1.1518683	0.4233468	2.7209	0.00656	1.020877119	count	1
NPDC1	1.0530081	0.3670866	2.8686	0.00416	1.021725922	count	1
ZFP36	0.7098621	0.0414323	17.1331	3.06E-62	1.021992445	count	7.44E-58
RACGAP1	1.1070533	0.4770029	2.3209	0.0204	1.022183164	count	1
CD244	1.0874968	0.3426966	3.1734	0.00153	1.022269888	count	1
CETN2	0.797484	0.2114852	3.7709	0.000167	1.022494656	count	1
GLCCI1	1.0545427	0.3577151	2.948	0.00323	1.023278758	count	1
CD200R1	1.1845204	0.3066569	3.8627	0.000115	1.025626547	count	1
MAP1B	1.3418685	0.7669034	1.7497	0.0803	1.026039714	count	1
FADS1	0.7887091	0.2118457	3.723	0.000201	1.02703443	count	1
RAI1	1.2182145	0.5299607	2.2987	0.0216	1.027797457	count	1
TTTY15	0.880509	0.2750488	3.2013	0.00139	1.028459827	count	1
STON2	1.1602304	0.3997924	2.9021	0.00374	1.028648306	count	1

C15orf41	1.559922	0.7584452	2.0567	0.0398	1.028923118	count	1
AC007032.1	0.7994759	0.1892156	4.2252	2.47E-05	1.028923803	count	0.5835622
EGR2	0.7424082	0.1460161	5.0844	3.97E-07	1.029192709	count	0.009470038
KDM6A	1.0460354	0.33325	3.1389	0.00172	1.02971424	count	1
ZNF436	2.3585458	0.8512441	2.7707	0.00564	1.029867675	count	1
HOXB7	1.3468564	0.477331	2.8216	0.00482	1.030044151	count	1
XXYLT1	0.9985787	0.3134892	3.1854	0.00146	1.030858592	count	1
CPPED1	0.7418551	0.0986253	7.522	7.53E-14	1.031217896	count	1.82E-09
RNASEL	0.9041131	0.3130581	2.888	0.00391	1.032490713	count	1
ZDHHC8	1.0806374	0.3761799	2.8727	0.00411	1.033164717	count	1
U62317.2	1.141195	0.4016443	2.8413	0.00453	1.033863761	count	1
GRIP1	1.6806965	0.7760653	2.1657	0.0304	1.035250365	count	1
HAL	1.5694206	0.5076449	3.0916	0.00201	1.035418404	count	1
ST7-AS1	1.263767	0.4697678	2.6902	0.00719	1.036161304	count	1
DAPP1	0.7692524	0.139909	5.4982	4.23E-08	1.036354335	count	0.001012408
SCN1B	0.8028261	0.2331769	3.443	0.000585	1.038087312	count	1
PTGFRN	1.4859826	0.4424658	3.3584	0.000796	1.038226374	count	1
OSGIN2	0.8457667	0.290099	2.9154	0.00358	1.038599941	count	1
RSAD1	0.9354118	0.3003701	3.1142	0.00187	1.038788217	count	1
AC093627.5	1.6867588	0.7648212	2.2054	0.0275	1.039068489	count	1
TICAM2	1.5765157	0.6118173	2.5768	0.01	1.040263979	count	1
FHIT	0.9034234	0.3084264	2.9291	0.00343	1.041914053	count	1
FRAT2	0.7663265	0.122314	6.2652	4.38E-10	1.042708393	count	1.05E-05
RNF24	0.7931689	0.1800632	4.4049	1.10E-05	1.04293244	count	0.260623
TNF	0.7728524	0.1825443	4.2338	2.38E-05	1.043842038	count	0.5623464
P2RY8	1.3155048	0.4665905	2.8194	0.00485	1.044456152	count	1
ZNF33A	0.7862736	0.1835857	4.2829	1.92E-05	1.044753767	count	0.4541184
PTBP2	1.0356285	0.3367509	3.0754	0.00213	1.046273723	count	1
MAGEH1	0.9367361	0.3093672	3.0279	0.00249	1.046914684	count	1
ZNF117	0.9168848	0.238846	3.8388	0.000127	1.047473423	count	1
ANO10	0.9858875	0.3047687	3.2349	0.00123	1.047673686	count	1
EGFL7	0.7794519	0.1757871	4.4341	9.65E-06	1.047877401	count	0.2287436
EIF4E3	0.949532	0.2661163	3.5681	0.000366	1.047948231	count	1
ADAM15	0.8196767	0.1737953	4.7163	2.54E-06	1.048601181	count	0.0603885
C20orf96	1.037983	0.4060252	2.5564	0.0106	1.048743962	count	1
FGR	0.7443506	0.0858327	8.6721	7.58E-18	1.048994134	count	1.84E-13
CTLA4	1.5904326	0.537381	2.9596	0.00311	1.049753228	count	1
ARHGAP45	0.7788613	0.1308019	5.9545	2.98E-09	1.050299632	count	7.15E-05
CIP2A	1.2804049	0.4700573	2.7239	0.0065	1.050471117	count	1
MPEG1	0.7499001	0.0804239	9.3243	2.41E-20	1.050516992	count	5.84E-16
ADGRE5	0.7627247	0.1018727	7.487	9.77E-14	1.050536145	count	2.36E-09
STRADA	0.997363	0.3290168	3.0313	0.00246	1.050570359	count	1
ICA1	0.8461301	0.2518348	3.3599	0.000792	1.050818577	count	1
EARS2	1.858809	0.8767815	2.12	0.0341	1.051889554	count	1
VSTM1	1.504776	0.2864497	5.2532	1.62E-07	1.051895505	count	0.003870666

SIDT1	1.7079803	0.440988	3.8731	0.00011	1.052399104	count	1
ABHD5	0.7553763	0.0984768	7.6706	2.45E-14	1.052582859	count	5.92E-10
KRT8	1.435472	0.6471919	2.218	0.0266	1.053645035	count	1
TTC39B	1.1195538	0.4187999	2.6732	0.00756	1.053755646	count	1
ROBO3	1.1018419	0.3665619	3.0059	0.00267	1.054313245	count	1
SLC6A12	1.1881049	0.7027863	1.6906	0.091	1.054537853	count	1
HOXB6	2.0837792	0.5460214	3.8163	0.000139	1.056295313	count	1
MIGA2	1.5109276	0.6982682	2.1638	0.0306	1.056362854	count	1
BST1	0.7629018	0.1090107	6.9984	3.33E-12	1.057741009	count	8.03E-08
KANSL2	0.8711367	0.2831322	3.0768	0.00212	1.059415696	count	1
ZNF546	1.7193198	0.8236226	2.0875	0.0369	1.059499269	count	1
MBD6	0.851721	0.2162751	3.9381	8.44E-05	1.060069947	count	1
MRC1	0.7455928	0.0804036	9.2731	3.84E-20	1.060458272	count	9.30E-16
CCDC144A	0.8444594	0.3420215	2.469	0.0136	1.060933927	count	1
HAUS3	0.9920959	0.311791	3.1819	0.00148	1.063688386	count	1
LILRA5	0.7778162	0.1183469	6.5723	6.03E-11	1.063929546	count	1.45E-06
ZCCHC3	0.964337	0.3383159	2.8504	0.0044	1.064785155	count	1
LINC01023	1.5227987	0.7171112	2.1235	0.0338	1.064973302	count	1
CD55	0.7472263	0.0668869	11.1715	2.68E-28	1.066269293	count	6.51E-24
FBXL20	0.9376968	0.2631159	3.5638	0.000372	1.066281455	count	1
ZNF346	1.6164434	0.622386	2.5972	0.00946	1.067432478	count	1
AC048341.2	1.6164434	0.6966101	2.3204	0.0204	1.067432478	count	1
PPM1H	2.107536	0.6692785	3.149	0.00166	1.067619903	count	1
BTG2	0.7451396	0.0718378	10.3725	1.06E-24	1.06884884	count	2.57E-20
AL158152.1	1.5289411	0.610053	2.5062	0.0123	1.069423148	count	1
AC087190.1	0.9147519	0.2888062	3.1674	0.00156	1.069454535	count	1
SUN2	0.8039473	0.1678202	4.7905	1.76E-06	1.071057307	count	0.04187568
TRIM4	0.8475038	0.2127304	3.9839	6.98E-05	1.072060293	count	1
ARMH1	0.9335754	0.3253005	2.8699	0.00414	1.072426524	count	1
HIPK1-AS1	1.6250474	0.6284831	2.5857	0.00978	1.073263789	count	1
UTP15	1.0886916	0.4048693	2.689	0.00722	1.073430412	count	1
DENND1A	0.9146972	0.2083923	4.3893	1.19E-05	1.073769753	count	0.2818634
HACD4	0.7711585	0.102473	7.5255	7.33E-14	1.074307874	count	1.77E-09
AC073352.2	0.9412236	0.4619111	2.0377	0.0417	1.076028274	count	1
GABPB1-AS1	0.7924491	0.1748259	4.5328	6.10E-06	1.07676743	count	0.1447347
INHBA-AS1	3.2535754	0.9659721	3.3682	0.000768	1.080183808	count	1
SLC25A25-AS1	1.910005	0.6950483	2.748	0.00604	1.080464489	count	1
AC087482.1	1.910005	1.0192552	1.8739	0.0611	1.080464489	count	1
SFT2D3	0.9360809	0.3148929	2.9727	0.00298	1.080560214	count	1
TMEM71	0.8932078	0.2050724	4.3556	1.38E-05	1.080694609	count	0.3266598
GJB2	0.9453784	0.5132411	1.842	0.0656	1.080902964	count	1
USP9Y	1.2785216	0.4146537	3.0833	0.00207	1.081137732	count	1
GTPBP1	0.8942656	0.2757287	3.2433	0.0012	1.082000408	count	1
HCAR3	0.7872845	0.154314	5.1018	3.62E-07	1.082095273	count	0.008636596
PNPLA2	0.8041102	0.1569588	5.1231	3.24E-07	1.0827664	count	0.007731936

SSX2IP	1.2486633	0.5181356	2.4099	0.016	1.083825672	count	1
AC026979.2	1.1144985	0.3252263	3.4268	0.000621	1.083920097	count	1
IGF1R	1.0381272	0.4112973	2.524	0.0117	1.084389871	count	1
RASGRP4	0.9697569	0.2787107	3.4794	0.000511	1.084897771	count	1
APOL6	0.8076094	0.1477519	5.466	5.07E-08	1.085555708	count	0.001213251
NEXN	1.0122937	0.3600397	2.8116	0.00497	1.086052224	count	1
ZNF467	0.8077732	0.1460059	5.5325	3.49E-08	1.086277306	count	0.000835576
ARHGEF40	0.8667657	0.1870163	4.6347	3.76E-06	1.087458275	count	0.08931128
PTCH2	1.224094	0.4598191	2.6621	0.00782	1.087922212	count	1
SPN	0.8430218	0.2207258	3.8193	0.000137	1.088297016	count	1
GLDN	1.9243683	1.18623	1.6223	0.105	1.088410976	count	1
L3MBTL3	0.9306587	0.2583396	3.6025	0.000321	1.088500067	count	1
RHOB	0.764639	0.0651362	11.7391	5.34E-31	1.089578581	count	1.30E-26
QSOX2	1.6494253	0.7309259	2.2566	0.0241	1.089738905	count	1
MRPL30	0.9329708	0.2400271	3.8869	0.000104	1.091268445	count	1
CARS2	0.825904	0.1741839	4.7416	2.24E-06	1.092147308	count	0.05326944
FYN	0.8394301	0.1809238	4.6397	3.67E-06	1.09284626	count	0.08718819
AC025423.4	2.5392994	0.8853024	2.8683	0.00416	1.095956779	count	1
CHERP	0.9450285	0.2955689	3.1973	0.0014	1.096181252	count	1
RYR1	1.1128836	0.4192406	2.6545	0.00799	1.098209184	count	1
AC011899.2	0.9875228	0.3351527	2.9465	0.00324	1.098397506	count	1
ZNF708	0.9391959	0.3159291	2.9728	0.00298	1.098722039	count	1
TAP2	0.9011415	0.2740673	3.288	0.00102	1.099578236	count	1
DGKD	0.9062308	0.2047748	4.4255	1.00E-05	1.099907187	count	0.237
LINC00987	1.9456781	0.7753305	2.5095	0.0122	1.100141643	count	1
ANKRD13C	1.0878293	0.352311	3.0877	0.00204	1.101026589	count	1
TP53INP1	1.0030542	0.3745333	2.6781	0.00745	1.101131172	count	1
HSPBAP1	0.8898122	0.2229024	3.9919	6.75E-05	1.101309671	count	1
MSH5	2.181289	1.312691	1.6617	0.0967	1.102178091	count	1
TNFAIP8L3	1.1176311	0.2745838	4.0703	4.84E-05	1.103070192	count	1
AC067930.4	1.3040154	0.4563563	2.8574	0.00431	1.103629371	count	1
AP003481.1	1.3041994	0.5371453	2.428	0.0153	1.103791531	count	1
ZNF627	1.343035	0.5282887	2.5422	0.0111	1.104195542	count	1
C5orf30	1.5017782	0.5694854	2.6371	0.00842	1.104234951	count	1
RNPC3	0.8393347	0.2236788	3.7524	0.000179	1.105183502	count	1
LDLR	0.9943523	0.2026612	4.9065	9.88E-07	1.10620926	count	0.02353416
TKT	0.7719776	0.047954	16.0983	1.66E-55	1.106264244	count	4.04E-51
JAK2	0.8835455	0.1632821	5.4112	6.87E-08	1.106810811	count	0.001643098
AL357060.1	1.7965408	0.5111128	3.515	0.000448	1.107400005	count	1
LYZ	0.7684416	0.0491822	15.6244	1.56E-52	1.107999944	count	3.79E-48
VENTX	0.9491632	0.2694591	3.5225	0.000435	1.110656358	count	1
AC016575.1	1.6814393	0.6611863	2.5431	0.011	1.111264662	count	1
PLAG1	2.5840834	0.8687699	2.9744	0.00296	1.111452942	count	1
LRRC37A2	1.3521962	0.493464	2.7402	0.00618	1.112032596	count	1
FAM118A	0.8072251	0.1557961	5.1813	2.38E-07	1.112392357	count	0.005682964

TRANK1	1.3141708	0.3944864	3.3313	0.000877	1.112577824	count	1
CEP97	1.315039	0.4271102	3.0789	0.0021	1.113342552	count	1
FLOT2	0.9800289	0.2465982	3.9742	7.27E-05	1.115719086	count	1
KIAA1468	1.0309503	0.348372	2.9593	0.00311	1.115769831	count	1
SNTA1	1.284112	0.6907258	1.8591	0.0631	1.115906312	count	1
ECE1	1.2044917	0.3063434	3.9318	8.66E-05	1.116192815	count	1
AC093635.1	1.4567539	0.6154515	2.367	0.018	1.117815204	count	1
ATP11A	0.890788	0.1914547	4.6527	3.45E-06	1.118087428	count	0.08197545
SNAPC3	0.9326927	0.3565312	2.616	0.00895	1.119170909	count	1
NCOA1	0.8505966	0.1463663	5.8114	7.00E-09	1.120170753	count	0.000167965
NLRP3	0.8171364	0.1005054	8.1303	6.72E-16	1.120298818	count	1.63E-11
AC012645.3	1.2091007	0.4331219	2.7916	0.00529	1.120631188	count	1
TBX19	1.9838468	0.6546713	3.0303	0.00247	1.120972554	count	1
IDH3A	0.8824126	0.2370236	3.7229	0.000201	1.122095889	count	1
MRPS27	0.9920118	0.3223849	3.0771	0.00211	1.123568904	count	1
LINC00937	0.8757066	0.2076609	4.217	2.57E-05	1.125655225	count	0.6070854
IGFBP6	1.3682875	0.4081303	3.3526	0.000813	1.125783442	count	1
GOLGA8Q	2.2358403	0.9870627	2.2651	0.0236	1.127134628	count	1
CHST7	0.9801593	0.2819017	3.477	0.000516	1.127322478	count	1
NBPF26	2.6419074	0.8453515	3.1252	0.0018	1.130935852	count	1
KIF9-AS1	2.247452	0.8495899	2.6453	0.00821	1.132378462	count	1
AP003680.1	2.247452	0.7610957	2.9529	0.00318	1.132378462	count	1
C2orf40	1.4756367	0.7699335	1.9166	0.0554	1.132793623	count	1
MOB3C	0.980359	0.3138047	3.1241	0.0018	1.132959721	count	1
RGS14	0.9191305	0.2394426	3.8386	0.000127	1.133103382	count	1
PLXNA2	1.2463104	0.4215384	2.9566	0.00314	1.13334212	count	1
QPCT	0.8431877	0.151408	5.569	2.84E-08	1.13378193	count	0.000680265
LIMD2	0.8054131	0.0769787	10.4628	4.29E-25	1.136841841	count	1.04E-20
NR4A1	0.7955869	0.0726746	10.9473	2.90E-27	1.136960366	count	7.04E-23
GK5	0.9680543	0.3146873	3.0762	0.00212	1.137885409	count	1
AC084064.1	3.6042785	1.0572812	3.409	0.000663	1.139420531	count	1
DGCR2	1.0705729	0.2949024	3.6303	0.000289	1.140705106	count	1
AP000766.1	2.2673549	0.7544898	3.0051	0.00268	1.141310221	count	1
LTB4R	0.9159365	0.2184396	4.1931	2.85E-05	1.141394433	count	0.6729705
LGALS2	0.8205544	0.1514456	5.4181	6.61E-08	1.143156345	count	0.001581046
ZNF296	1.0648986	0.3120048	3.4131	0.000653	1.144278329	count	1
STK38	0.8794172	0.2059512	4.27	2.03E-05	1.144406853	count	0.4800544
PRAM1	0.8792073	0.1841586	4.7742	1.91E-06	1.145257819	count	0.0454389
AP4E1	1.0574134	0.3656936	2.8915	0.00387	1.145270221	count	1
FOXRED2	1.7330627	0.5681577	3.0503	0.00231	1.14569483	count	1
SLC9A3R1	0.9502398	0.2202732	4.3139	1.67E-05	1.147727037	count	0.3951053
CD1D	0.8766122	0.1470001	5.9633	2.83E-09	1.149171417	count	6.80E-05
PRR12	1.6402057	0.530601	3.0912	0.00202	1.14931996	count	1
PLSCR4	2.0383947	0.8582764	2.375	0.0176	1.150325326	count	1
SFRP4	2.0383947	1.0538632	1.9342	0.0532	1.150325326	count	1

NAT9	0.9614056	0.2366097	4.0633	4.99E-05	1.150587972	count	1
DUSP7	1.2649423	0.4263531	2.9669	0.00304	1.150922221	count	1
ICAM2	0.8855351	0.149792	5.9118	3.86E-09	1.151305833	count	9.27E-05
LINC00528	1.398772	0.5733818	2.4395	0.0148	1.151778881	count	1
METTL27	1.092201	0.3758181	2.9062	0.00369	1.153896087	count	1
ARHGAP5-AS1	1.5024176	0.5405278	2.7795	0.00549	1.153977118	count	1
AL354707.1	1.5690148	0.8485747	1.849	0.0646	1.155089903	count	1
NEMP1	1.4511918	0.4516171	3.2133	0.00133	1.156737784	count	1
KANSL1L	1.1057307	0.3102289	3.5642	0.000372	1.157387509	count	1
EMC1	1.0066825	0.322425	3.1222	0.00182	1.158573311	count	1
RUSC2	1.3315095	0.5266951	2.528	0.0115	1.158681412	count	1
LINC00960	2.3076225	1.1077126	2.0832	0.0373	1.15915949	count	1
ADGRB3	2.3076225	0.9714691	2.3754	0.0176	1.15915949	count	1
WASHC1	0.8934984	0.1884961	4.7401	2.26E-06	1.159385217	count	0.05374054
PROSER1	1.1582382	0.3837943	3.0179	0.00257	1.160149465	count	1
ZNF487	0.9617156	0.3812639	2.5224	0.0117	1.161856573	count	1
PLAC8	0.8818447	0.154863	5.6944	1.39E-08	1.162646566	count	0.000333308
CCDC151	1.0102737	0.3281611	3.0786	0.0021	1.162804081	count	1
INKA1	1.4586078	0.5717695	2.551	0.0108	1.162830105	count	1
ARL4D	1.1251543	0.3438342	3.2724	0.00108	1.166303052	count	1
AC133550.2	1.765742	0.9035165	1.9543	0.0508	1.167299167	count	1
AC110995.1	1.6667446	0.5864602	2.842	0.00452	1.168157527	count	1
BICRAL	1.2842724	0.3902996	3.2905	0.00101	1.169139719	count	1
MAST3	1.1291748	0.3259457	3.4643	0.000541	1.170599453	count	1
AC084871.2	1.901457	0.6576384	2.8913	0.00387	1.171094458	count	1
AUTS2	1.5910983	0.5517372	2.8838	0.00396	1.171682331	count	1
AC009961.1	1.3461757	0.6574973	2.0474	0.0407	1.17188587	count	1
C17orf49	1.1330173	0.3235618	3.5017	0.000471	1.174705136	count	1
FNBP1L	1.4762833	0.518625	2.8465	0.00446	1.177329058	count	1
PCSK6	1.321072	0.7345749	1.7984	0.0722	1.177553268	count	1
GCNT1	1.0287765	0.2865913	3.5897	0.000338	1.178734946	count	1
AC132872.1	1.323245	0.4889047	2.7065	0.00685	1.17955467	count	1
TWIST2	2.800683	0.9690703	2.8901	0.00389	1.181366273	count	1
AC069277.1	2.800683	0.9690703	2.8901	0.00389	1.181366273	count	1
FBXL2	2.800683	0.8894891	3.1486	0.00166	1.181366273	count	1
AC073349.1	2.800683	0.9690703	2.8901	0.00389	1.181366273	count	1
AC084082.1	2.800683	0.9690703	2.8901	0.00389	1.181366273	count	1
MESP2	2.800683	0.9690703	2.8901	0.00389	1.181366273	count	1
ZNF607	2.800683	0.9690703	2.8901	0.00389	1.181366273	count	1
DEPTOR	1.1082312	0.2925574	3.7881	0.000155	1.182035073	count	1
ZNF592	1.0382265	0.3243075	3.2014	0.00139	1.18366547	count	1
IFT74	0.9173395	0.2296742	3.9941	6.69E-05	1.184132538	count	1
KIAA1211L	1.0152531	0.3423094	2.9659	0.00305	1.184787527	count	1
LYST	0.8512671	0.108455	7.849	6.21E-15	1.187032821	count	1.50E-10
PEX5	1.3316421	0.3833725	3.4735	0.000523	1.187286311	count	1

LYPD3	1.2562794	0.4063483	3.0916	0.00201	1.187672296	count	1
KCNQ1	0.9281657	0.1486931	6.2422	5.07E-10	1.189408484	count	1.22E-05
CDH23	1.2389071	0.3770983	3.2854	0.00103	1.190690178	count	1
AC002070.1	2.3977766	0.8338548	2.8755	0.00407	1.198023307	count	1
AIM2	1.5031838	0.3260537	4.6102	4.23E-06	1.199333737	count	0.10045404
AGTRAP	0.8521582	0.086563	9.8444	1.88E-22	1.199461435	count	4.56E-18
DLGAP3	1.1451764	0.3659873	3.129	0.00177	1.199926364	count	1
RAB44	2.863548	1.0145737	2.8224	0.0048	1.200091352	count	1
POU6F1	2.863548	1.0821904	2.6461	0.0082	1.200091352	count	1
TLE6	2.863548	1.0821904	2.6461	0.0082	1.200091352	count	1
AATBC	1.7135258	0.4922625	3.4809	0.000508	1.20113236	count	1
WDR17	1.379294	0.6853553	2.0125	0.0443	1.201642143	count	1
OLIG1	1.5080932	0.4638957	3.2509	0.00117	1.203341365	count	1
AC037459.3	2.4141698	0.806943	2.9917	0.0028	1.204923819	count	1
SLC43A1	1.2740524	0.5234474	2.434	0.015	1.205013527	count	1
SULT1A1	0.8762829	0.1108716	7.9036	4.06E-15	1.205394038	count	9.82E-11
PATL2	1.636812	0.5033077	3.2521	0.00116	1.205838397	count	1
ARMC2	1.3530657	0.4234692	3.1952	0.00142	1.206987776	count	1
DTNA	1.2200702	0.3131646	3.8959	1.00E-04	1.207766779	count	1
DUSP6	0.8566308	0.0985679	8.6908	6.46E-18	1.208274127	count	1.56E-13
VPS53	0.935638	0.2331776	4.0126	6.19E-05	1.210786977	count	1
AC040162.1	1.328617	0.3828775	3.4701	0.000529	1.210839308	count	1
STIM1	1.1177059	0.3456109	3.234	0.00124	1.212426755	count	1
MAP3K1	0.9221087	0.1546769	5.9615	2.86E-09	1.213447868	count	6.87E-05
MLLT11	1.1953375	0.3335724	3.5834	0.000346	1.21356897	count	1
LILRB5	0.9640919	0.1631036	5.9109	3.88E-09	1.213708677	count	9.31E-05
INTS3	1.1111069	0.310236	3.5815	0.000348	1.214329254	count	1
CAMKK2	0.913742	0.1995705	4.5785	4.92E-06	1.215087844	count	0.11681556
OBSL1	2.921448	1.1811404	2.4734	0.0135	1.216717882	count	1
SPINT1-AS1	1.524824	0.706553	2.1581	0.031	1.216978698	count	1
INTS6L	1.2152958	0.3558932	3.4148	0.000649	1.219123433	count	1
GAMT	1.0698078	0.2977608	3.5928	0.000334	1.220514839	count	1
PTDSS1	0.9713121	0.2300978	4.2213	2.52E-05	1.220758325	count	0.5952996
MARK4	1.2344644	0.3735589	3.3046	0.000965	1.222440222	count	1
ALDH1A1	0.9765145	0.1471813	6.6348	3.99E-11	1.2228668	count	9.61E-07
APOL1	1.1201646	0.2767898	4.047	5.35E-05	1.224479742	count	1
ZNF26	1.2940609	0.3814736	3.3923	0.000704	1.224511841	count	1
DNASE1	2.4617209	0.745336	3.3028	0.000971	1.224645489	count	1
AL359265.3	2.9693264	1.0541607	2.8168	0.00489	1.230021501	count	1
C15orf65	2.9693264	1.115671	2.6615	0.00783	1.230021501	count	1
PSTPIP1	0.9110878	0.1275638	7.1422	1.21E-12	1.230593539	count	2.92E-08
TNFSF10	0.8687016	0.105734	8.2159	3.37E-16	1.230953049	count	8.15E-12
KCNE1	0.9980117	0.3033229	3.2903	0.00102	1.232044033	count	1
CLDN12	2.198335	0.8432658	2.6069	0.00919	1.233350597	count	1
IL17RA	0.9135285	0.1658026	5.5097	3.97E-08	1.233913541	count	0.000950259

SLC9A3R2	1.4169572	0.4170462	3.3976	0.000691	1.235368225	count	1
AC015802.6	1.7633467	0.5631748	3.1311	0.00176	1.235903676	count	1
WWP1	0.9144012	0.1603457	5.7027	1.32E-08	1.23609411	count	0.000316576
VNN1	1.029383	0.3062422	3.3613	0.000788	1.2375504	count	1
IRS2	0.9386498	0.1186221	7.9129	3.77E-15	1.241910752	count	9.11E-11
ZNF197	1.5571271	0.4156337	3.7464	0.000184	1.243218106	count	1
AL121944.1	1.0662674	0.3487057	3.0578	0.00225	1.245593454	count	1
ITM2C	1.012051	0.2386489	4.2408	2.31E-05	1.246732826	count	0.5458761
CCL8	0.8746723	0.1981212	4.4148	1.05E-05	1.246828893	count	0.248829
CCDC144NL	1.280227	0.3649107	3.5083	0.000459	1.250912692	count	1
PTX3	0.9041965	0.230777	3.9181	9.17E-05	1.255133993	count	1
WDR49	3.0726595	1.1886592	2.585	0.0098	1.257378231	count	1
GATAD2B	1.0559511	0.2209173	4.7798	1.86E-06	1.257422421	count	0.04425126
TSC22D3	0.8879633	0.0643959	13.7891	1.02E-41	1.258376213	count	2.48E-37
UNC13D	1.031647	0.2845039	3.6261	0.000294	1.258512831	count	1
NFIX	1.1782118	0.4061562	2.9009	0.00375	1.258718282	count	1
AC008267.5	1.2882032	0.4326943	2.9772	0.00294	1.258912768	count	1
CRISPLD2	1.4112017	0.3594796	3.9257	8.89E-05	1.260258612	count	1
NAV2	1.7122073	0.4150552	4.1253	3.83E-05	1.261557094	count	0.9033055
ITGAM	0.9223956	0.1477153	6.2444	5.00E-10	1.266655742	count	1.20E-05
RBKS	1.0298905	0.265315	3.8818	0.000106	1.269050848	count	1
CABLES1	1.9232134	0.5522814	3.4823	0.000506	1.269077287	count	1
TEP1	1.3644635	0.3066197	4.45	8.97E-06	1.269518847	count	0.21266973
DNAJC27-AS1	1.9243683	0.6452832	2.9822	0.00289	1.269808335	count	1
DHX16	0.9758457	0.2720757	3.5867	0.000341	1.270089951	count	1
KCNE3	0.991257	0.1798584	5.5113	3.93E-08	1.271286684	count	0.000940724
TNFSF18	1.6531397	0.6794652	2.433	0.015	1.271664992	count	1
MRVI1	1.4271647	0.4070441	3.5062	0.000463	1.274831766	count	1
RGMB	2.2848678	0.7230662	3.16	0.0016	1.276243287	count	1
RCSD1	0.9463752	0.1009948	9.3705	1.58E-20	1.277008154	count	3.83E-16
NLRP1	0.9615572	0.1984468	4.8454	1.34E-06	1.279228648	count	0.03190272
NLRP12	1.2083142	0.3319355	3.6402	0.000278	1.280071087	count	1
AC064805.1	1.5101658	0.5622466	2.6859	0.00728	1.283635024	count	1
CDK7	1.2956907	0.3501981	3.6999	0.00022	1.284715294	count	1
HSPG2	1.671013	0.7709987	2.1673	0.0303	1.285424328	count	1
C17orf107	1.0036184	0.2201518	4.5588	5.40E-06	1.285620909	count	0.1281744
APCDD1	3.1879088	1.1075954	2.8782	0.00403	1.28575114	count	1
EXOC3L1	2.6167045	0.7819235	3.3465	0.000831	1.28583213	count	1
ATP6AP1L	3.1918479	1.221527	2.613	0.00903	1.286681899	count	1
AK1	2.100954	0.6857788	3.0636	0.00221	1.287090201	count	1
AL360012.1	1.8379658	0.4948502	3.7142	0.000208	1.287256601	count	1
CTDSPL	1.3836066	0.3575784	3.8694	0.000112	1.287740682	count	1
AC093677.2	1.9564527	1.1279263	1.7346	0.0829	1.290021603	count	1
NRGN	0.9468935	0.1565918	6.0469	1.70E-09	1.291354714	count	4.08E-05
AC079630.1	1.6193755	0.3924319	4.1265	3.81E-05	1.293414882	count	0.8986266

CDKL5	1.366421	0.4344297	3.1453	0.00168	1.294782638	count	1
ULK1	1.201399	0.3022594	3.9747	7.25E-05	1.295023782	count	1
AC008759.3	3.2343073	1.2295619	2.6305	0.00858	1.296553539	count	1
AC009812.1	2.3272928	0.8321498	2.7967	0.0052	1.296724739	count	1
AC087239.1	1.1863275	0.2889992	4.105	4.18E-05	1.298531949	count	0.9852678
AC093495.1	2.1225421	1.0081009	2.1055	0.0354	1.299197333	count	1
NBL1	1.2940556	0.4047788	3.1969	0.00141	1.300240544	count	1
AC021188.1	19.641114	1738.531958	0.0113	0.991	1.304854494	count	1
STK24-AS1	18.7620173	1072.223078	0.0175	0.986	1.304854571	count	1
ETV1	18.8111616	917.287194	0.0205	0.984	1.304854572	count	1
ZNF845	18.8111616	917.2871985	0.0205	0.984	1.304854572	count	1
BMPR1A	18.9693158	931.3556514	0.0204	0.984	1.304854574	count	1
OVGP1	18.9955792	1008.526166	0.0188	0.985	1.304854574	count	1
PEAR1	19.5439008	1306.594202	0.015	0.988	1.304854579	count	1
LYG2	19.5439008	1306.59422	0.015	0.988	1.304854579	count	1
AL138895.1	19.5439008	1306.59422	0.015	0.988	1.304854579	count	1
NANOG	19.5439008	1306.59422	0.015	0.988	1.304854579	count	1
ANKS1B	19.5439008	1306.59422	0.015	0.988	1.304854579	count	1
MMRN1	19.5439009	1306.594232	0.015	0.988	1.304854579	count	1
NIPAL4	19.5439009	1306.594244	0.015	0.988	1.304854579	count	1
FKBP1C	19.5439009	1306.594236	0.015	0.988	1.304854579	count	1
AC025034.1	19.5439009	1306.594244	0.015	0.988	1.304854579	count	1
FAM209A	19.5439009	1306.594244	0.015	0.988	1.304854579	count	1
PKD1L3	19.6400881	1490.389196	0.0132	0.9895	1.30485458	count	1
AL590648.3	19.6411142	1738.532008	0.0113	0.991	1.30485458	count	1
AL450306.1	19.6411141	1738.531947	0.0113	0.991	1.30485458	count	1
TVP23C	19.641114	1738.53193	0.0113	0.991	1.30485458	count	1
TMEM221	19.6411142	1738.531947	0.0113	0.991	1.30485458	count	1
AC022762.2	19.6877804	1474.522805	0.0134	0.989	1.30485458	count	1
ALDH5A1	20.5425082	1601.337881	0.0128	0.99	1.304854583	count	1
KIAA0319L	1.0544221	0.2458844	4.2883	1.87E-05	1.305700687	count	0.4423111
NYNRIN	1.4625919	0.4812151	3.0394	0.0024	1.307082671	count	1
KIAA0513	1.0585	0.2576102	4.1089	4.11E-05	1.307866712	count	0.9688914
VNN3	2.1432755	0.5830793	3.6758	0.000242	1.310738491	count	1
SAMD4A	1.2540602	0.2990684	4.1932	2.85E-05	1.317003705	count	0.6729705
PARP10	1.1475019	0.2707398	4.2384	2.33E-05	1.317791365	count	0.550579
CDYL2	2.3726003	0.7905038	3.0014	0.00271	1.318189565	count	1
LILRA1	0.9759701	0.1594252	6.1218	1.07E-09	1.318842372	count	2.57E-05
ZNF585A	2.0028646	0.6019582	3.3272	0.00089	1.318924967	count	1
RAB37	2.1613105	0.6191315	3.4909	0.00049	1.320707355	count	1
TEC	1.2461308	0.3257813	3.8251	0.000134	1.32102999	count	1
EMP1	0.9420169	0.1563847	6.0237	1.96E-09	1.322480225	count	4.71E-05
AL450311.2	2.0116787	0.9060727	2.2202	0.0265	1.324367874	count	1
CSTA	0.9315963	0.0667151	13.9638	1.07E-42	1.324961565	count	2.60E-38
CD209	1.156022	0.2232774	5.1775	2.43E-07	1.327753115	count	0.005801625

GIMAP8	1.0862967	0.1965501	5.5268	3.61E-08	1.32971234	count	0.000864234
LINC00877	1.2156561	0.2597988	4.6792	3.04E-06	1.331294616	count	0.07225472
NRM	1.0569392	0.2776751	3.8064	0.000144	1.334501678	count	1
C16orf54	1.0137177	0.1982888	5.1123	3.43E-07	1.335169518	count	0.00818398
PGBD2	1.364726	0.3925995	3.4761	0.000518	1.335421729	count	1
RARRES3	0.9774075	0.1141311	8.5639	1.90E-17	1.336967963	count	4.60E-13
NRBP2	1.6751864	0.5332479	3.1415	0.0017	1.337969642	count	1
MROH8	2.0350165	0.7266144	2.8007	0.00514	1.338706778	count	1
KLF2	0.9365665	0.0864133	10.8382	9.08E-27	1.341696085	count	2.20E-22
ITGAL	1.0917527	0.2043603	5.3423	1.00E-07	1.343184851	count	0.0023903
LIF	1.442538	0.4342607	3.3218	0.000907	1.343619854	count	1
COLEC12	1.0398194	0.1838152	5.6569	1.72E-08	1.344107638	count	0.000412284
CCL23	1.475108	0.4247479	3.4729	0.000524	1.347420884	count	1
ZNF783	1.6908038	0.5791421	2.9195	0.00354	1.350354994	count	1
GIMAP7	0.9830907	0.1242329	7.9133	3.76E-15	1.351927228	count	9.09E-11
LRRK1	1.0996713	0.2341481	4.6965	2.79E-06	1.353057342	count	0.0663183
C9orf47	2.222336	0.5860262	3.7922	0.000153	1.353945426	count	1
ADAP1	1.1044441	0.1928757	5.7262	1.15E-08	1.355669871	count	0.000275839
TMEM170B	1.0464304	0.1697177	6.1657	8.18E-10	1.358699728	count	1.97E-05
AL161457.2	1.9441971	0.7290474	2.6668	0.00771	1.358711782	count	1
CDKN2D	0.9825776	0.1175474	8.359	1.05E-16	1.362015175	count	2.54E-12
ZNF532	1.2857392	0.4014602	3.2027	0.00138	1.36383571	count	1
CLEC12A	0.9949858	0.0944573	10.5337	2.09E-25	1.363935262	count	5.07E-21
ESYT2	1.0339199	0.2249297	4.5966	4.51E-06	1.366138855	count	0.10708995
CSF3R	0.9764305	0.1082084	9.0236	3.58E-19	1.372999395	count	8.67E-15
ATF7IP	1.0407443	0.1645562	6.3246	3.01E-10	1.375226226	count	7.24E-06
FAM117B	1.4064798	0.3501737	4.0165	6.08E-05	1.376957565	count	1
CLEC4E	0.9864555	0.0893599	11.0391	1.10E-27	1.377808988	count	2.67E-23
NPPA-AS1	2.2742489	0.7308302	3.1119	0.00188	1.381605487	count	1
TWISTNB	0.9821644	0.1530445	6.4175	1.66E-10	1.382718462	count	4.00E-06
AC006942.1	2.1104642	0.6063233	3.4808	0.000509	1.384319283	count	1
TSPAN32	1.3051852	0.2773511	4.7059	2.67E-06	1.384811985	count	0.06347124
GPBAR1	1.1150671	0.1855703	6.0089	2.15E-09	1.384816064	count	5.16E-05
GNG2	1.062797	0.1538053	6.91	6.15E-12	1.388400814	count	1.48E-07
ATRIP	1.4922783	0.6032057	2.4739	0.0134	1.390500923	count	1
HDAC9	1.0685894	0.1889265	5.6561	1.73E-08	1.397327186	count	0.000414664
PLCXD1	1.1863188	0.3511508	3.3784	0.000741	1.399696558	count	1
LRRC69	2.1452636	0.8113399	2.6441	0.00824	1.404962587	count	1
CDA	1.0383842	0.200049	5.1906	2.27E-07	1.405367099	count	0.005420987
TYK2	1.2359291	0.2969976	4.1614	3.27E-05	1.406302208	count	0.7717527
TMEM88	1.9168273	0.5501735	3.484	0.000503	1.408217326	count	1
LINC01001	1.7106976	0.4343377	3.9386	8.42E-05	1.411959935	count	1
FCHSD1	1.3623975	0.4188853	3.2524	0.00116	1.418412038	count	1
INVS	1.671536	0.4977698	3.3581	0.000797	1.421211263	count	1
FSCN1	1.160555	0.2366133	4.9049	9.96E-07	1.421813632	count	0.023722728

MAN2A2	1.4346516	0.2646114	5.4217	6.48E-08	1.424973882	count	0.00155021
EXT1	1.369675	0.3976752	3.4442	0.000582	1.426083138	count	1
EIF3J-DT	1.1801657	0.3081245	3.8302	0.000131	1.426149182	count	1
TCF20	1.3212458	0.4355579	3.0335	0.00244	1.426613362	count	1
RFXAP	1.418758	0.4063091	3.4918	0.000488	1.427721142	count	1
HIST2H2AB	2.050494	1.041888	1.9681	0.0492	1.428062753	count	1
AC034236.2	2.050494	0.9105335	2.252	0.0244	1.428062753	count	1
AC087623.3	1.562909	0.681727	2.2926	0.022	1.428175249	count	1
GLT1D1	1.3765007	0.3422693	4.0217	5.95E-05	1.433273639	count	1
SHF	2.05887	0.8396041	2.4522	0.0143	1.433429917	count	1
TSPAN7	3.088365	0.8196954	3.7677	0.000169	1.442490991	count	1
PLAT	2.6609832	0.8505095	3.1287	0.00178	1.444557211	count	1
AC093462.1	2.6609832	0.9579936	2.7777	0.00552	1.444557211	count	1
F5	1.2154821	0.3097504	3.9241	8.95E-05	1.445323104	count	1
MZF1-AS1	1.4190395	0.4295681	3.3034	0.000969	1.445628238	count	1
CCL17	2.216321	0.722285	3.0685	0.00218	1.446309317	count	1
RHBDF1	1.702071	1.0380211	1.6397	0.101	1.446818185	count	1
SMARCD3	1.193974	0.2188808	5.4549	5.39E-08	1.455490429	count	0.001289719
PDGFC	1.1799143	0.1964481	6.0062	2.18E-09	1.469320002	count	5.24E-05
NOTCH2NL	1.3864716	0.3563078	3.8912	0.000102	1.472169736	count	1
CPSF7	1.1954789	0.2678329	4.4635	8.43E-06	1.472342685	count	0.19990902
SLITRK4	1.4827087	0.3898835	3.803	0.000146	1.473038533	count	1
CENPA	3.213349	1.130143	2.8433	0.0045	1.476736963	count	1
ST7	1.4198944	0.3927411	3.6153	0.000306	1.478883678	count	1
LPAR2	1.31861	0.3576196	3.6872	0.000232	1.484554566	count	1
PDE9A	2.7644835	0.7802309	3.5432	0.000403	1.485482065	count	1
NFAM1	1.1402963	0.181151	6.2947	3.64E-10	1.489024262	count	8.76E-06
SFMBT2	1.2893796	0.3574931	3.6067	0.000316	1.490420151	count	1
ARHGAP17	1.1608761	0.2072115	5.6024	2.35E-08	1.494880677	count	0.000563013
WLS	1.1406074	0.1779512	6.4097	1.74E-10	1.498742748	count	4.19E-06
CRY2	1.334615	0.3824617	3.4895	0.000492	1.50273833	count	1
CCDC96	2.1693521	0.5886981	3.685	0.000234	1.502817998	count	1
TFAP2C	2.8350539	0.622127	4.557	5.44E-06	1.512035791	count	0.12911296
VNN2	1.303659	0.229714	5.6751	1.55E-08	1.514087317	count	0.000371551
FCAR	1.1637459	0.2144747	5.426	6.33E-08	1.516903354	count	0.001514389
AC245128.3	1.1212654	0.1673644	6.6995	2.58E-11	1.52038726	count	6.22E-07
LRRK2	1.1824077	0.1630074	7.2537	5.41E-13	1.520897343	count	1.31E-08
AC020916.1	1.0765453	0.1286864	8.3657	9.90E-17	1.523013981	count	2.40E-12
COQ7	1.2368814	0.2207186	5.6039	2.33E-08	1.523776209	count	0.000558245
IGF2BP3	3.420262	1.4192335	2.4099	0.016	1.527212094	count	1
AC011444.2	1.999021	0.6780431	2.9482	0.00323	1.528381438	count	1
HP	1.9249737	0.4975057	3.8692	0.000112	1.531076289	count	1
ICAM3	1.123451	0.1181127	9.5117	4.31E-21	1.531077589	count	1.04E-16
TCFL5	1.6162375	0.6675161	2.4213	0.0155	1.533282942	count	1
SETBP1	1.8636135	0.6464379	2.8829	0.00397	1.534204506	count	1

FXYD6	1.2916393	0.2288428	5.6442	1.85E-08	1.536762654	count	0.000443353
JARID2-AS1	1.8169294	0.5532367	3.2842	0.00104	1.541727059	count	1
TP53INP2	1.7705958	0.4242825	4.1732	3.11E-05	1.543660378	count	0.7341155
NACC2	1.3514587	0.3046032	4.4368	9.53E-06	1.547359472	count	0.22591818
AGK	1.4399888	0.3654825	3.94	8.38E-05	1.555887295	count	1
FCN1	1.0876625	0.0659892	16.4824	5.74E-58	1.56433071	count	1.40E-53
PTPN20	19.9089016	1450.810584	0.0137	0.9891	1.565979327	count	1
AC114811.2	19.832348	1191.443737	0.0166	0.987	1.565979422	count	1
DLX6-AS1	19.832348	1191.443755	0.0166	0.987	1.565979422	count	1
CCDC194	19.832348	1191.443737	0.0166	0.987	1.565979422	count	1
AC008649.1	19.8323481	1191.443763	0.0166	0.987	1.565979422	count	1
AC079834.2	19.8336562	1510.289992	0.0131	0.99	1.565979422	count	1
GADL1	19.8336562	1510.289992	0.0131	0.99	1.565979422	count	1
SERPINI2	19.8336562	1510.289992	0.0131	0.99	1.565979422	count	1
AC004771.5	19.8336562	1510.289992	0.0131	0.99	1.565979422	count	1
TSPAN5	1.9060016	0.6797819	2.8038	0.00509	1.567314141	count	1
AL135791.1	1.723724	0.6329402	2.7234	0.00651	1.57328023	count	1
HRH1	1.3091056	0.2143742	6.1066	1.18E-09	1.573597354	count	2.84E-05
TMEM94	1.4516761	0.3520457	4.1235	3.85E-05	1.580946929	count	0.907984
AC009630.2	2.4631526	0.9628673	2.5581	0.0106	1.581030671	count	1
CCDC157	2.695846	0.7524217	3.5829	0.000346	1.583857038	count	1
ENHO	1.6234918	0.5252066	3.0911	0.00202	1.589673453	count	1
PLCB1	1.5468435	0.3596261	4.3013	1.76E-05	1.59410915	count	0.4163456
AC020911.2	1.6821196	0.4775023	3.5227	0.000435	1.5947897	count	1
AC245014.3	1.1932945	0.1874893	6.3646	2.33E-10	1.595237162	count	5.61E-06
AL592183.1	1.6372323	0.3849656	4.2529	2.19E-05	1.602928757	count	0.517716
NFIB	2.209022	0.889859	2.4824	0.0131	1.603374045	count	1
RNF150	1.6976141	0.4157066	4.0837	4.57E-05	1.609154319	count	1
HSH2D	1.4385097	0.2852425	5.0431	4.92E-07	1.610450444	count	0.011731248
AL356020.1	2.761422	1.460094	1.8913	0.0587	1.611598386	count	1
CRIP1	1.1731544	0.1191951	9.8423	1.92E-22	1.611813185	count	4.66E-18
C16orf58	1.414212	0.4363093	3.2413	0.00121	1.619471979	count	1
MTMR11	1.3187749	0.2159401	6.1071	1.18E-09	1.621313918	count	2.84E-05
IL6	1.9186142	0.7749137	2.4759	0.0134	1.623689516	count	1
ZNF254	1.5060698	0.3876676	3.885	0.000105	1.627214644	count	1
AC073195.1	2.247452	0.5644209	3.9819	7.04E-05	1.627604855	count	1
SSH3	1.7193706	0.5608355	3.0657	0.0022	1.629256292	count	1
PAQR7	1.7253691	0.3716543	4.6424	3.63E-06	1.634784375	count	0.08624517
AC020656.1	1.1430648	0.0771137	14.8231	1.13E-47	1.635228868	count	2.75E-43
NCF1	1.1578583	0.0861522	13.4397	8.74E-40	1.637949849	count	2.12E-35
SLC35F2	2.1753673	0.8254976	2.6352	0.00846	1.649987052	count	1
CES1	1.322602	0.2396677	5.5185	3.78E-08	1.651512038	count	0.000904894
AL391832.2	1.4752262	0.3381157	4.3631	1.34E-05	1.661531459	count	0.3172584
CCDC88C	1.366585	0.2837685	4.8158	1.56E-06	1.662717977	count	0.03712644
ANK1	3.311509	1.0217057	3.2412	0.00121	1.663518305	count	1

PDGFD	3.311509	1.0217057	3.2412	0.00121	1.663518305	count	1
C19orf38	1.2184285	0.1046324	11.6448	1.53E-30	1.668009093	count	3.72E-26
LMCD1	2.458549	0.8230181	2.9872	0.00284	1.67106708	count	1
EGR1	1.214468	0.1616252	7.5141	7.98E-14	1.673692293	count	1.93E-09
C3orf18	3.380762	1.1211255	3.0155	0.00259	1.681717093	count	1
NIPSNAP3B	1.894962	0.6853052	2.7651	0.00573	1.686489439	count	1
ATP2B1-AS1	1.1783762	0.1068832	11.0249	1.28E-27	1.686833422	count	3.11E-23
AL034397.3	1.3702423	0.2661531	5.1483	2.84E-07	1.704053539	count	0.006778228
AC106712.1	3.501326	1.155831	3.0293	0.00248	1.711268124	count	1
C1S	3.501326	1.1986065	2.9212	0.00352	1.711268124	count	1
RRAD	1.5544225	0.4336703	3.5843	0.000345	1.716986782	count	1
AC007952.4	1.3087595	0.179762	7.2805	4.46E-13	1.717683199	count	1.08E-08
PLEKHG3	2.189104	0.6555858	3.3392	0.000853	1.721441957	count	1
GUCY1A1	1.7989257	0.4669663	3.8524	0.00012	1.730196396	count	1
NBEAL2	1.617155	0.2804887	5.7655	9.16E-09	1.731486125	count	0.000219739
HK3	1.4609185	0.2587574	5.6459	1.83E-08	1.732268182	count	0.000438578
FBLIM1	1.83677	0.6576164	2.7931	0.00526	1.736262945	count	1
CSGALNACT1	1.4872394	0.2806305	5.2996	1.26E-07	1.742716464	count	0.003011148
TMEM154	1.430765	0.1869007	7.6552	2.76E-14	1.750175065	count	6.67E-10
CLEC4G	1.683517	0.290942	5.7864	8.11E-09	1.751173913	count	0.000194575
TOP3B	1.7495546	0.6496767	2.693	0.00713	1.756999264	count	1
GABBR1	1.5323588	0.4929372	3.1086	0.0019	1.763036566	count	1
GAS1	1.8681482	0.7406039	2.5225	0.0117	1.76441616	count	1
DNM1	3.8004711	1.00355	3.787	0.000156	1.773797461	count	1
BPI	2.6819986	0.6942061	3.8634	0.000115	1.786770493	count	1
PCBP3	19.2736161	1022.653449	0.0188	0.985	1.787006243	count	1
LRRC4C	19.3037483	1436.559676	0.0134	0.989	1.787006243	count	1
AL355574.1	20.0579197	1689.495603	0.0119	0.991	1.787006251	count	1
ADAMTSL3	20.0579197	1689.495603	0.0119	0.991	1.787006251	count	1
LRP6	19.2414793	1319.095282	0.0146	0.988	1.787006345	count	1
SLC25A30-AS1	20.0579201	1689.49566	0.0119	0.991	1.787006353	count	1
AL158071.5	3.9227822	1.0292147	3.8114	0.000142	1.795352271	count	1
VWF	1.876525	0.7364708	2.548	0.0109	1.80116975	count	1
FRY	1.9893276	0.4950336	4.0186	6.03E-05	1.802770187	count	1
ADGRG6	1.9742846	0.3829769	5.1551	2.74E-07	1.825315061	count	0.006540106
LIMS2	2.072464	0.9325173	2.2224	0.0263	1.832016605	count	1
SLC24A4	1.8825097	0.3045078	6.1821	7.38E-10	1.834115504	count	1.77E-05
MEGF9	1.4048906	0.167717	8.3766	9.05E-17	1.842178909	count	2.19E-12
AL596094.1	2.633267	1.1022024	2.3891	0.017	1.850180827	count	1
AVIL	2.633267	0.8031521	3.2787	0.00106	1.850180827	count	1
CELSR2	3.49383	0.8504229	4.1083	4.12E-05	1.855137782	count	0.9712076
AC012360.1	3.49383	0.974526	3.5852	0.000343	1.855137782	count	1
ACY3	3.522773	1.075975	3.274	0.00107	1.862445791	count	1
SLC2A12	3.5227728	2.7723594	1.2707	0.204	1.86244596	count	1
SHMT1	1.516151	0.2443411	6.2051	6.40E-10	1.872295819	count	1.54E-05

NOTCH4	1.667667	0.3700666	4.5064	6.90E-06	1.875293453	count	0.163668
FBXL17	1.8400754	0.383266	4.801	1.67E-06	1.887750897	count	0.03973598
AC004825.2	2.728563	0.872659	3.1267	0.00179	1.899107384	count	1
OR11G2	2.289857	0.6557038	3.4922	0.000488	1.90320557	count	1
ELN	2.954834	0.8157232	3.6223	0.000298	1.910519022	count	1
SLC12A5	1.6024063	0.276674	5.7917	7.86E-09	1.930581609	count	0.000188585
ITGA8	3.00759	1.309182	2.2973	0.0217	1.932238499	count	1
ZDHHC1	1.6872498	0.2821841	5.9793	2.57E-09	1.956362157	count	6.17E-05
EGR3	1.5416374	0.2444522	6.3065	3.37E-10	1.95810153	count	8.11E-06
CHADL	2.102565	0.4952096	4.2458	2.26E-05	1.96796042	count	0.5341284
DLG2	2.707702	0.7800743	3.4711	0.000527	1.969645889	count	1
PAM16	19.3759879	1254.038813	0.0155	0.9877	1.978626359	count	1
HRNR	20.2403474	1850.848111	0.0109	0.991	1.978626369	count	1
ANKUB1	20.2403474	1850.848111	0.0109	0.991	1.978626369	count	1
CFP	1.42835	0.1007001	14.1842	5.98E-44	1.991888326	count	1.45E-39
PDK4	1.482914	0.1579319	9.3896	1.33E-20	2.01456059	count	3.22E-16
S100A9	1.3993004	0.088826	15.7533	2.46E-53	2.015941292	count	5.98E-49
AP003086.1	1.7434558	0.3540996	4.9236	9.06E-07	2.019654929	count	0.021584544
1-Mar	2.1681572	0.3498942	6.1966	6.75E-10	2.02257125	count	1.62E-05
F10	3.647981	1.087845	3.3534	0.00081	2.024265392	count	1
CSRP2	2.269222	0.8233409	2.7561	0.00589	2.027287202	count	1
GNG11	1.981472	0.5417902	3.6573	0.00026	2.065739397	count	1
TUBGCP6	2.2736714	0.497148	4.5734	5.04E-06	2.070687296	count	0.11965464
AL391807.1	2.640445	0.6452279	4.0923	4.41E-05	2.07250778	count	1
RBP7	1.5153009	0.1155212	13.1171	4.86E-38	2.100571473	count	1.18E-33
PLEKHG1	3.195165	1.225583	2.6071	0.00919	2.103668818	count	1
PADI4	2.601758	0.3851246	6.7556	1.77E-11	2.110052209	count	4.27E-07
NFIA	1.6825377	0.2656477	6.3337	2.84E-10	2.113060106	count	6.83E-06
HPGDS	1.6538334	0.1958672	8.4436	5.19E-17	2.116872517	count	1.26E-12
CCL18	1.5173915	0.3273324	4.6356	3.75E-06	2.130014946	count	0.0890775
TPPP3	1.996815	0.3424672	5.8307	6.25E-09	2.136530697	count	0.000149988
C11orf21	2.0530702	0.4249524	4.8313	1.44E-06	2.154546319	count	0.03427632
ECSCR	2.677261	1.186324	2.2568	0.0241	2.155920769	count	1
NFE2	2.5317425	0.4966563	5.0976	3.70E-07	2.171459315	count	0.00882709
RIPOR2	1.7539825	0.1698711	10.3254	1.70E-24	2.18391855	count	4.12E-20
FOLR3	2.2475161	0.2300708	9.7688	3.86E-22	2.241096104	count	9.36E-18
AC027575.2	19.6012669	1479.659141	0.0132	0.9894	2.29911814	count	1
PRG4	20.5255869	2134.567182	0.0096	0.992	2.299118265	count	1
PROK2	1.871709	0.361707	5.1747	2.47E-07	2.334865348	count	0.005896384
CCL13	2.805191	0.5417123	5.1784	2.42E-07	2.344661281	count	0.005777992
S100A8	1.6351444	0.117021	13.9731	9.49E-43	2.355834048	count	2.31E-38
ABCA6	3.6903438	0.6868052	5.3732	8.46E-08	2.360306683	count	0.002022871
PSMD6-AS2	4.144489	1.8736603	2.212	0.0271	2.362392407	count	1
SELL	1.7689094	0.1431586	12.3563	4.55E-34	2.383422588	count	1.11E-29
MAMDC2	2.4546039	0.3481193	7.051	2.30E-12	2.415037528	count	5.55E-08

OLFM1	2.390315	0.4673466	5.1146	3.39E-07	2.415846907	count	0.008089218
ESAM	19.7823562	1613.827298	0.0123	0.9902	2.436099118	count	1
TMEM47	3.118916	1.485135	2.1001	0.0358	2.455088148	count	1
AL138899.1	2.831778	0.7115857	3.9795	7.11E-05	2.460951083	count	1
FCGBP	1.8179726	0.3354744	5.4191	6.57E-08	2.513494204	count	0.001571544
CEP126	18.9063107	1075.301432	0.0176	0.986	2.561194292	count	1
EFHD1	4.2007252	3.7157673	1.1305	0.2584	2.662203626	count	1
RPS6KA6	4.2134584	2.2983444	1.8333	0.0669	2.664758991	count	1
DCN	2.8343462	0.5476636	5.1753	2.46E-07	2.821461805	count	0.005873004
C2CD4B	4.341128	0.9894658	4.3873	1.20E-05	2.851544607	count	0.28422
WISP2	4.4193557	1.9752643	2.2373	0.0254	2.866257468	count	1
APOD	19.3426438	940.276298	0.0206	0.9836	2.882163519	count	1
P2RY14	2.6053393	0.261826	9.9507	6.76E-23	2.895391933	count	1.64E-18
DPT	3.513249	0.8275233	4.2455	2.26E-05	2.913515209	count	0.5341284
CCDC141	4.9545502	2.1733552	2.2797	0.0227	2.943925601	count	1
PLEKHG5	19.4730636	955.7473798	0.0204	0.9837	2.975033131	count	1
S100A12	2.1201061	0.161932	13.0926	6.58E-38	3.002639027	count	1.60E-33
SCN9A	3.4574168	0.4835528	7.15	1.14E-12	3.067226029	count	2.75E-08
TTN	3.7357891	0.8368362	4.4642	8.40E-06	3.648857664	count	0.199206
LYVE1	3.3549124	0.3295958	10.1789	7.29E-24	3.842679041	count	1.77E-19
