# Supplementary material for: Decoding the transcriptome of calcified atherosclerotic plaque at single-cell resolution
Source: Commun Biol. 2022 Oct 12;5:1084. doi: 10.1038/s42003-022-04056-7 (PMC9556750; doi:10.1038/s42003-022-04056-7)
Supplement: Supplementary file 4 — Supplementary Data 2 [file 42003_2022_4056_MOESM4_ESM.pdf]

**Full differential gene expression results for T-cells.**

| gene_short_name | estimate | std_err | test_val | p_value | normalized_effect | model_component | q_value |
|---|---|---|---|---|---|---|---|
| SPP1 | -4.5821068 | 1.0604845 | -4.3208 | 1.60E-05 | -5.673479533 | count | 0.384256 |
| APOC1 | -17.932354 | 592.099898 | -0.0303 | 0.976 | -4.866909656 | count | 1 |
| APOE | -3.3841943 | 0.736581 | -4.5945 | 4.50E-06 | -4.190406145 | count | 0.1082745 |
| ITLN1 | -17.882229 | 738.0153187 | -0.0242 | 0.981 | -4.074785791 | count | 1 |
| IGKC | -2.3024627 | 0.0819754 | -28.0872 | 2.89E-156 | -3.152885805 | count | 7.03E-152 |
| CPVL | -17.737501 | 993.6453293 | -0.0179 | 0.986 | -2.738799102 | count | 1 |
| FN1 | -3.3586466 | 0.8451283 | -3.9741 | 7.21E-05 | -2.733847537 | count | 1 |
| MGP | -1.9954048 | 0.1472577 | -13.5504 | 8.88E-41 | -2.722991675 | count | 2.16E-36 |
| CCL20 | -3.2935327 | 1.4148698 | -2.3278 | 0.02 | -2.687842463 | count | 1 |
| MS4A7 | -3.448449 | 1.5521173 | -2.2218 | 0.0264 | -2.55627894 | count | 1 |
| IGLC2 | -2.1453451 | 0.1460838 | -14.6857 | 2.19E-47 | -2.480280904 | count | 5.32E-43 |
| CXCL3 | -2.467837 | 0.9346565 | -2.6404 | 0.00832 | -2.470832249 | count | 1 |
| CCL2 | -1.9264816 | 0.3479804 | -5.5362 | 3.33E-08 | -2.421125137 | count | 0.000804628 |
| C1QA | -2.6012081 | 0.5068639 | -5.132 | 3.03E-07 | -2.383301853 | count | 0.00731139 |
| CD86 | -17.054126 | 985.9503087 | -0.0173 | 0.986 | -2.238235802 | count | 1 |
| LYZ | -1.7338212 | 0.3401844 | -5.0967 | 3.64E-07 | -2.192290142 | count | 0.0087815 |
| IL1B | -2.7205517 | 0.9111841 | -2.9857 | 0.00285 | -2.138192154 | count | 1 |
| CD68 | -1.8843182 | 0.4556866 | -4.1351 | 3.63E-05 | -2.08581289 | count | 0.8705466 |
| PPP1R14A | -2.7708666 | 0.8479046 | -3.2679 | 0.00109 | -2.084237857 | count | 1 |
| RNASE1 | -1.644742 | 0.3933158 | -4.1817 | 2.97E-05 | -2.070823791 | count | 0.7124733 |
| CD14 | -2.0341351 | 0.4673415 | -4.3526 | 1.39E-05 | -2.057903156 | count | 0.3338919 |
| MT1H | -17.29334 | 1377.623198 | -0.0126 | 0.99 | -2.046531099 | count | 1 |
| MS4A4A | -2.810909 | 1.3978831 | -2.0108 | 0.0444 | -1.930251686 | count | 1 |
| TAGLN | -1.5681707 | 0.2073233 | -7.5639 | 5.01E-14 | -1.870962623 | count | 1.22E-09 |
| CTSL | -1.504728 | 0.2510194 | -5.9945 | 2.26E-09 | -1.832083729 | count | 5.47E-05 |
| C5orf17 | -18.08898 | 1633.908086 | -0.0111 | 0.991 | -1.82539154 | count | 1 |
| TPSB2 | -16.859728 | 1046.945944 | -0.0161 | 0.987 | -1.825391416 | count | 1 |
| AL117379.1 | -17.736597 | 1527.376081 | -0.0116 | 0.991 | -1.825391311 | count | 1 |
| ABCA1 | -2.1875461 | 0.7974038 | -2.7433 | 0.00611 | -1.813894545 | count | 1 |
| C1QB | -1.8547707 | 0.4031784 | -4.6004 | 4.37E-06 | -1.813411053 | count | 0.10515094 |
| TPM2 | -1.9453667 | 0.354798 | -5.483 | 4.49E-08 | -1.813077784 | count | 0.001084739 |
| C15orf48 | -1.557815 | 0.8565712 | -1.8187 | 0.0691 | -1.801361253 | count | 1 |
| CXCL2 | -2.0474655 | 0.6433057 | -3.1827 | 0.00147 | -1.800064544 | count | 1 |
| HMOX1 | -1.723795 | 0.5487265 | -3.1414 | 0.0017 | -1.780548524 | count | 1 |
| SDS | -17.6049717 | 1289.109583 | -0.0137 | 0.989 | -1.743444528 | count | 1 |
| MUC20 | -17.650153 | 2071.551051 | -0.0085 | 0.993 | -1.74344443 | count | 1 |
| MARCO | -16.982873 | 1094.333334 | -0.0155 | 0.988 | -1.743444361 | count | 1 |
| C1QC | -1.7603227 | 0.5223729 | -3.3699 | 0.00076 | -1.728136552 | count | 1 |
| FABP4 | -1.353858 | 0.3940721 | -3.4356 | 0.000598 | -1.726690304 | count | 1 |
| RAB32 | -2.4464493 | 1.1668549 | -2.0966 | 0.0361 | -1.726365162 | count | 1 |
| AQP9 | -17.923285 | 1963.847383 | -0.0091 | 0.993 | -1.656561559 | count | 1 |
| AC008040.1 | -17.601944 | 2075.727912 | -0.0085 | 0.993 | -1.65656154 | count | 1 |
| JCHAIN | -1.72997 | 0.2513857 | -6.8817 | 7.01E-12 | -1.649141537 | count | 1.70E-07 |
| VCAN | -2.4938554 | 0.7209934 | -3.4589 | 0.000549 | -1.642447094 | count | 1 |

| | | | | | | | |
|---|---|---|---|---|---|---|---|
| BGN | -2.2625395 | 0.5735536 | -3.9448 | 8.15E-05 | -1.61513035 | count | 1 |
| CXCL8 | -1.312535 | 0.440274 | -2.9812 | 0.00289 | -1.610440858 | count | 1 |
| ZNF69 | -2.226386 | 1.1451594 | -1.9442 | 0.052 | -1.601581578 | count | 1 |
| MYL9 | -2.058107 | 0.5959088 | -3.4537 | 0.00056 | -1.575051817 | count | 1 |
| OGN | -2.4164333 | 1.0031641 | -2.4088 | 0.0161 | -1.565426476 | count | 1 |
| CD300LB | -17.827886 | 1892.75613 | -0.0094 | 0.992 | -1.564108888 | count | 1 |
| SOX18 | -17.061447 | 1184.45748 | -0.0144 | 0.989 | -1.564108829 | count | 1 |
| CRTAC1 | -17.9887682 | 1161.631679 | -0.0155 | 0.988 | -1.564108418 | count | 1 |
| IGHG3 | -2.123499 | 0.3171155 | -6.6963 | 2.49E-11 | -1.561049749 | count | 6.03E-07 |
| IFIT1 | -1.5388017 | 0.4255106 | -3.6164 | 0.000303 | -1.55998709 | count | 1 |
| GBP7 | -1.4859533 | 0.4205999 | -3.5329 | 0.000416 | -1.469761129 | count | 1 |
| CXCL1 | -17.209579 | 1396.416572 | -0.0123 | 0.99 | -1.465322718 | count | 1 |
| MMP19 | -17.161166 | 1174.398452 | -0.0146 | 0.988 | -1.465322714 | count | 1 |
| HLA-DRA | -1.001033 | 0.1038747 | -9.6369 | 1.06E-21 | -1.417685587 | count | 2.58E-17 |
| LY86 | -2.450199 | 1.403975 | -1.7452 | 0.081 | -1.389983551 | count | 1 |
| IL1RN | -2.8069147 | 1.1848269 | -2.3691 | 0.0179 | -1.387493864 | count | 1 |
| SELENOP | -2.4351697 | 0.629901 | -3.866 | 0.000113 | -1.386335459 | count | 1 |
| RGS5 | -1.8891609 | 0.5234164 | -3.6093 | 0.000311 | -1.372651668 | count | 1 |
| CYBB | -18.0330142 | 1132.409351 | -0.0159 | 0.987 | -1.359272499 | count | 1 |
| PLA2G7 | -17.917365 | 1842.387199 | -0.0097 | 0.992 | -1.359272495 | count | 1 |
| LILRA5 | -17.663611 | 2425.008907 | -0.0073 | 0.994 | -1.359272484 | count | 1 |
| LINC02531 | -17.604846 | 2173.454666 | -0.0081 | 0.994 | -1.359272481 | count | 1 |
| CTSG | -17.43701 | 1712.375113 | -0.0102 | 0.992 | -1.359272472 | count | 1 |
| ADAP2 | -17.286983 | 1361.775733 | -0.0127 | 0.99 | -1.359272462 | count | 1 |
| TREM2 | -17.133198 | 1402.418875 | -0.0122 | 0.99 | -1.35927245 | count | 1 |
| PTN | -17.122678 | 1398.180684 | -0.0122 | 0.99 | -1.359272449 | count | 1 |
| GPNMB | -17.088925 | 1075.066532 | -0.0159 | 0.987 | -1.359272446 | count | 1 |
| SMPDL3A | -16.367591 | 1130.567107 | -0.0145 | 0.988 | -1.359272356 | count | 1 |
| NXPH4 | -2.328868 | 1.2181899 | -1.9117 | 0.056 | -1.359226048 | count | 1 |
| NOV | -1.457871 | 1.0785852 | -1.3517 | 0.177 | -1.351670484 | count | 1 |
| TLCD1 | -2.2346282 | 0.8978295 | -2.4889 | 0.0129 | -1.333196937 | count | 1 |
| MFSD3 | -1.501594 | 0.6884256 | -2.1812 | 0.0292 | -1.30718004 | count | 1 |
| RNASE6 | -1.1115875 | 0.4029766 | -2.7584 | 0.00584 | -1.302495063 | count | 1 |
| EFEMP1 | -3.4644802 | 1.373906 | -2.5216 | 0.0117 | -1.28211978 | count | 1 |
| IGHA1 | -2.038261 | 0.1994091 | -10.2215 | 3.54E-24 | -1.272466044 | count | 8.60E-20 |
| C1orf54 | -1.8827253 | 1.0450418 | -1.8016 | 0.0717 | -1.272358393 | count | 1 |
| IGHG1 | -1.396669 | 0.2087909 | -6.6893 | 2.61E-11 | -1.262570639 | count | 6.32E-07 |
| A2M | -1.4636862 | 0.4960582 | -2.9506 | 0.00319 | -1.254791931 | count | 1 |
| HPCA | -17.471782 | 2573.782375 | -0.0068 | 0.995 | -1.244803986 | count | 1 |
| PRELID3A | -17.468752 | 1510.90541 | -0.0116 | 0.991 | -1.244803985 | count | 1 |
| SLAMF9 | -17.467929 | 1319.551628 | -0.0132 | 0.989 | -1.244803985 | count | 1 |
| AL391262.1 | -17.373874 | 1757.193764 | -0.0099 | 0.992 | -1.24480398 | count | 1 |
| CCL8 | -17.054399 | 1041.363891 | -0.0164 | 0.987 | -1.244803959 | count | 1 |
| NES | -16.57759 | 1221.959741 | -0.0136 | 0.989 | -1.244803912 | count | 1 |
| NAV2 | -17.5395548 | 1453.390464 | -0.0121 | 0.99 | -1.244803655 | count | 1 |

| | | | | | | | |
|---|---|---|---|---|---|---|---|
| MAMSTR | -17.3277737 | 1202.578042 | -0.0144 | 0.989 | -1.244803644 | count | 1 |
| LGMN | -1.7958648 | 0.8934042 | -2.0101 | 0.0445 | -1.236901696 | count | 1 |
| IFI27 | -1.047613 | 0.374199 | -2.7996 | 0.00515 | -1.23411651 | count | 1 |
| CYR61 | -1.7647186 | 0.5477999 | -3.2215 | 0.00129 | -1.223667987 | count | 1 |
| ZNF354C | -2.0825736 | 1.2016867 | -1.733 | 0.0832 | -1.222861703 | count | 1 |
| HLA-DMB | -0.9985252 | 0.2120851 | -4.7081 | 2.60E-06 | -1.217843568 | count | 0.0625976 |
| RBAK | -1.7490289 | 0.5923809 | -2.9525 | 0.00317 | -1.216896077 | count | 1 |
| PHF13 | -1.0426202 | 0.1877074 | -5.5545 | 3.00E-08 | -1.206950836 | count | 0.00072498 |
| FCGR2A | -1.8216998 | 0.6477028 | -2.8126 | 0.00494 | -1.194406778 | count | 1 |
| KIAA1841 | -1.1952298 | 0.4447452 | -2.6874 | 0.00724 | -1.18650904 | count | 1 |
| AIM2 | -1.0901732 | 0.2558224 | -4.2614 | 2.09E-05 | -1.158876677 | count | 0.5017881 |
| CAV1 | -1.8590385 | 0.7034577 | -2.6427 | 0.00826 | -1.14989071 | count | 1 |
| SPI1 | -1.6826394 | 0.9762765 | -1.7235 | 0.0849 | -1.137643308 | count | 1 |
| EDN1 | -1.7937683 | 0.9780406 | -1.834 | 0.0667 | -1.126233931 | count | 1 |
| AC020922.3 | -17.703399 | 2051.768299 | -0.0086 | 0.993 | -1.12046368 | count | 1 |
| TMEM244 | -17.557869 | 1960.676393 | -0.009 | 0.993 | -1.120463675 | count | 1 |
| AC135803.1 | -17.31629 | 2381.260388 | -0.0073 | 0.994 | -1.120463664 | count | 1 |
| LINC00877 | -17.31629 | 2381.260388 | -0.0073 | 0.994 | -1.120463664 | count | 1 |
| RADIL | -17.31629 | 2381.260388 | -0.0073 | 0.994 | -1.120463664 | count | 1 |
| AC026250.1 | -17.31629 | 2381.260388 | -0.0073 | 0.994 | -1.120463664 | count | 1 |
| ISLR2 | -17.31629 | 2381.260388 | -0.0073 | 0.994 | -1.120463664 | count | 1 |
| AC120114.1 | -17.31454 | 1771.946915 | -0.0098 | 0.992 | -1.120463664 | count | 1 |
| LINC02327 | -17.31437 | 1680.538855 | -0.0103 | 0.992 | -1.120463663 | count | 1 |
| CHI3L1 | -17.30579 | 1734.178152 | -0.01 | 0.992 | -1.120463663 | count | 1 |
| AC026202.3 | -17.227703 | 1670.815274 | -0.0103 | 0.992 | -1.120463659 | count | 1 |
| NMU | -17.227703 | 1670.815274 | -0.0103 | 0.992 | -1.120463659 | count | 1 |
| TMEM255B | -16.958251 | 1465.720232 | -0.0116 | 0.991 | -1.120463642 | count | 1 |
| PCDH7 | -18.29519 | 1899.77651 | -0.0096 | 0.992 | -1.120463618 | count | 1 |
| DPT | -17.3988395 | 1300.22781 | -0.0134 | 0.989 | -1.12046359 | count | 1 |
| CTGF | -1.1299424 | 0.3395347 | -3.3279 | 0.000884 | -1.116398816 | count | 1 |
| EIF5AL1 | -1.9323984 | 0.9841606 | -1.9635 | 0.0497 | -1.109055019 | count | 1 |
| TMEM176B | -1.9266093 | 0.8860229 | -2.1744 | 0.0297 | -1.107253995 | count | 1 |
| RAD18 | -1.0447658 | 0.2896608 | -3.6069 | 0.000314 | -1.105904919 | count | 1 |
| MAFB | -1.4926847 | 0.5366472 | -2.7815 | 0.00544 | -1.09594118 | count | 1 |
| HLA-DQA1 | -0.8308973 | 0.1432508 | -5.8003 | 7.23E-09 | -1.094549427 | count | 0.00017485 |
| ABCG1 | -0.8550204 | 0.1899082 | -4.5023 | 6.95E-06 | -1.08950763 | count | 0.1671336 |
| PLAC9 | -2.5372582 | 1.0168599 | -2.4952 | 0.0126 | -1.079012603 | count | 1 |
| FGD4 | -2.5144511 | 1.7474924 | -1.4389 | 0.15 | -1.0754433 | count | 1 |
| MYLK | -1.6491174 | 0.9973522 | -1.6535 | 0.0983 | -1.06978274 | count | 1 |
| PDIK1L | -1.0517774 | 0.3209777 | -3.2768 | 0.00106 | -1.064016965 | count | 1 |
| IFI44L | -0.838496 | 0.2423432 | -3.46 | 0.000547 | -1.063758457 | count | 1 |
| CDKN3 | -0.9114399 | 0.3893155 | -2.3411 | 0.0193 | -1.056425245 | count | 1 |
| RSAD2 | -0.9058868 | 0.3512831 | -2.5788 | 0.00996 | -1.050476526 | count | 1 |
| AIF1 | -0.8157064 | 0.2957685 | -2.7579 | 0.00585 | -1.049554148 | count | 1 |
| TMEM176A | -1.336655 | 0.9164345 | -1.4585 | 0.145 | -1.049520619 | count | 1 |

| | | | | | | | |
|---|---|---|---|---|---|---|---|
| CCL18 | -1.5950185 | 0.7750186 | -2.058 | 0.0397 | -1.047198825 | count | 1 |
| TYMP | -0.747502 | 0.1135517 | -6.5829 | 5.32E-11 | -1.042915365 | count | 1.29E-06 |
| FOLR2 | -2.3214483 | 0.8948897 | -2.5941 | 0.00952 | -1.042258119 | count | 1 |
| GM2A | -1.4680792 | 0.7646558 | -1.9199 | 0.055 | -1.039180299 | count | 1 |
| AC092747.4 | -2.2652082 | 1.2714422 | -1.7816 | 0.0749 | -1.031518378 | count | 1 |
| WDR90 | -1.913838 | 1.2868614 | -1.4872 | 0.137 | -1.031278342 | count | 1 |
| ALDH2 | -1.2418945 | 0.7006902 | -1.7724 | 0.0764 | -1.024214195 | count | 1 |
| OLR1 | -2.2178529 | 1.4451257 | -1.5347 | 0.125 | -1.022074964 | count | 1 |
| ITPRIPL2 | -1.0085712 | 0.5149166 | -1.9587 | 0.0502 | -1.012529583 | count | 1 |
| FBP1 | -0.8995193 | 0.3877218 | -2.32 | 0.0204 | -1.007114993 | count | 1 |
| MZB1 | -0.9376484 | 0.3104862 | -3.0199 | 0.00255 | -1.004427914 | count | 1 |
| PXDC1 | -1.492285 | 1.3426091 | -1.1115 | 0.266 | -1.002034526 | count | 1 |
| MKI67 | -1.3791499 | 0.8115621 | -1.6994 | 0.0893 | -0.994345851 | count | 1 |
| RAB13 | -1.294778 | 0.7265296 | -1.7821 | 0.0748 | -0.988786063 | count | 1 |
| IGFBP7 | -0.7077141 | 0.1302767 | -5.4324 | 5.95E-08 | -0.984812116 | count | 0.001437163 |
| TPX2 | -1.2290887 | 0.5866172 | -2.0952 | 0.0362 | -0.984620912 | count | 1 |
| CLN6 | -2.6016936 | 1.3598533 | -1.9132 | 0.0558 | -0.984399567 | count | 1 |
| UNC13A | -18.0532991 | 2480.187721 | -0.0073 | 0.994 | -0.984387765 | count | 1 |
| CHST1 | -17.8443294 | 1967.830633 | -0.0091 | 0.993 | -0.98438776 | count | 1 |
| IL18 | -17.6426509 | 1132.647391 | -0.0156 | 0.988 | -0.984387754 | count | 1 |
| SDC2 | -17.4161454 | 1194.529163 | -0.0146 | 0.988 | -0.984387747 | count | 1 |
| IBSP | -17.3488453 | 1490.673626 | -0.0116 | 0.991 | -0.984387744 | count | 1 |
| PILRA | -17.068264 | 1416.756103 | -0.012 | 0.99 | -0.984387731 | count | 1 |
| GAPT | -17.855923 | 2247.013102 | -0.0079 | 0.994 | -0.984387546 | count | 1 |
| TREM1 | -17.57578 | 2516.324379 | -0.007 | 0.994 | -0.984387538 | count | 1 |
| HCK | -17.498673 | 1456.31628 | -0.012 | 0.99 | -0.984387536 | count | 1 |
| IGLC7 | -17.232002 | 1750.293945 | -0.0098 | 0.992 | -0.984387525 | count | 1 |
| AL390036.1 | -17.132223 | 2171.888485 | -0.0079 | 0.994 | -0.98438752 | count | 1 |
| TBC1D8-AS1 | -17.132223 | 2171.888485 | -0.0079 | 0.994 | -0.98438752 | count | 1 |
| ST3GAL6 | -17.132223 | 2171.888485 | -0.0079 | 0.994 | -0.98438752 | count | 1 |
| LINC01511 | -17.132223 | 2171.888485 | -0.0079 | 0.994 | -0.98438752 | count | 1 |
| AC005082.1 | -17.132223 | 2171.888485 | -0.0079 | 0.994 | -0.98438752 | count | 1 |
| CST6 | -17.132223 | 2171.888485 | -0.0079 | 0.994 | -0.98438752 | count | 1 |
| METTL7B | -17.132223 | 2171.888485 | -0.0079 | 0.994 | -0.98438752 | count | 1 |
| DHRS9 | -17.130459 | 1563.372944 | -0.011 | 0.991 | -0.98438752 | count | 1 |
| ANXA3 | -17.130459 | 1563.372944 | -0.011 | 0.991 | -0.98438752 | count | 1 |
| SOX30 | -17.130459 | 1563.372944 | -0.011 | 0.991 | -0.98438752 | count | 1 |
| IGF2BP3 | -17.130459 | 1563.372944 | -0.011 | 0.991 | -0.98438752 | count | 1 |
| AL450311.2 | -17.130459 | 1563.372944 | -0.011 | 0.991 | -0.98438752 | count | 1 |
| LGALS4 | -16.872097 | 1193.125469 | -0.0141 | 0.989 | -0.984387505 | count | 1 |
| MX1 | -0.7235415 | 0.1486423 | -4.8677 | 1.18E-06 | -0.982275048 | count | 0.02843918 |
| NR1H3 | -2.0114597 | 1.1245031 | -1.7888 | 0.0737 | -0.976343008 | count | 1 |
| AC051619.5 | -1.34402 | 1.1304028 | -1.189 | 0.235 | -0.975970087 | count | 1 |
| SPATS2L | -0.7529896 | 0.1769435 | -4.2555 | 2.14E-05 | -0.975589701 | count | 0.5137284 |
| DIXDC1 | -0.945589 | 0.5875871 | -1.6093 | 0.108 | -0.969235578 | count | 1 |

| | | | | | | | |
|---|---|---|---|---|---|---|---|
| QPCT | -1.3290676 | 0.5968113 | -2.2269 | 0.026 | -0.968033972 | count | 1 |
| TIMP2 | -0.7945367 | 0.3685455 | -2.1559 | 0.0312 | -0.961019864 | count | 1 |
| HELZ2 | -0.837184 | 0.2593595 | -3.2279 | 0.00126 | -0.960634029 | count | 1 |
| EFHD1 | -1.2442298 | 0.8892111 | -1.3993 | 0.162 | -0.959443107 | count | 1 |
| CLEC7A | -1.6792404 | 0.7027581 | -2.3895 | 0.0169 | -0.957411208 | count | 1 |
| CCNE1 | -2.3942722 | 1.2238204 | -1.9564 | 0.0505 | -0.95516852 | count | 1 |
| TRAF3IP2 | -0.8748712 | 0.3157144 | -2.7711 | 0.00562 | -0.951926404 | count | 1 |
| HLA-DRB5 | -0.6834472 | 0.1080348 | -6.3262 | 2.84E-10 | -0.948609101 | count | 6.88E-06 |
| TM4SF1 | -1.3683516 | 0.4809195 | -2.8453 | 0.00446 | -0.943479608 | count | 1 |
| DYNLT1 | -0.6628817 | 0.0714101 | -9.2827 | 2.86E-20 | -0.940155772 | count | 6.95E-16 |
| ZNF584 | -1.4520047 | 0.5469864 | -2.6546 | 0.00798 | -0.931008288 | count | 1 |
| NR2F6 | -1.4457714 | 0.4768701 | -3.0318 | 0.00245 | -0.928293592 | count | 1 |
| CEP112 | -1.5911543 | 0.7710294 | -2.0637 | 0.0391 | -0.926259781 | count | 1 |
| LMO2 | -1.4336394 | 0.9933881 | -1.4432 | 0.149 | -0.922978675 | count | 1 |
| BMF | -1.423667 | 0.658588 | -2.1617 | 0.0307 | -0.918578553 | count | 1 |
| SERPINE1 | -2.1684371 | 1.5129911 | -1.4332 | 0.152 | -0.91677427 | count | 1 |
| GPR35 | -0.9283951 | 0.3726306 | -2.4915 | 0.0128 | -0.914677163 | count | 1 |
| G0S2 | -0.7614564 | 0.5645723 | -1.3487 | 0.178 | -0.912918506 | count | 1 |
| IGHM | -1.2256855 | 0.7061338 | -1.7358 | 0.0827 | -0.911272635 | count | 1 |
| SNHG21 | -0.9031137 | 0.450055 | -2.0067 | 0.0449 | -0.90606538 | count | 1 |
| FHL2 | -1.3947201 | 0.6260825 | -2.2277 | 0.026 | -0.905647811 | count | 1 |
| FAM171A2 | -1.746922 | 0.9524706 | -1.8341 | 0.0667 | -0.905643223 | count | 1 |
| RBM11 | -3.0000187 | 1.3090408 | -2.2918 | 0.022 | -0.905493636 | count | 1 |
| ING2 | -0.6543636 | 0.1020994 | -6.4091 | 1.67E-10 | -0.904807022 | count | 4.04E-06 |
| MAP1B | -1.52152 | 0.6467162 | -2.3527 | 0.0187 | -0.900240906 | count | 1 |
| ZFAND4 | -1.3789553 | 0.5206998 | -2.6483 | 0.00813 | -0.898505314 | count | 1 |
| SMYD5 | -1.2018388 | 0.4970677 | -2.4179 | 0.0157 | -0.89770924 | count | 1 |
| ACTA2 | -0.6761603 | 0.1624924 | -4.1612 | 3.24E-05 | -0.897101259 | count | 0.7770492 |
| NUPR1 | -1.2723411 | 0.704716 | -1.8055 | 0.0711 | -0.894985599 | count | 1 |
| SAPCD2 | -2.0538289 | 1.3004758 | -1.5793 | 0.114 | -0.894313105 | count | 1 |
| SHTN1 | -1.0471353 | 0.6402985 | -1.6354 | 0.102 | -0.893025093 | count | 1 |
| AK1 | -1.5001273 | 0.6889371 | -2.1775 | 0.0295 | -0.8919951 | count | 1 |
| HSPG2 | -2.0313518 | 0.9194028 | -2.2094 | 0.0272 | -0.889653488 | count | 1 |
| RCBTB1 | -0.8104988 | 0.3519887 | -2.3026 | 0.0214 | -0.887556874 | count | 1 |
| HNRNPA1P48 | -0.9324999 | 0.2831702 | -3.2931 | 0.001 | -0.887445464 | count | 1 |
| NRAV | -1.351691 | 0.9024074 | -1.4979 | 0.134 | -0.885984953 | count | 1 |
| TOP2B | -0.6704262 | 0.1356785 | -4.9413 | 8.14E-07 | -0.884993442 | count | 0.019626354 |
| VSIG10 | -1.1738092 | 0.6626722 | -1.7713 | 0.0766 | -0.8815405 | count | 1 |
| DISP2 | -1.9926124 | 1.7343755 | -1.1489 | 0.251 | -0.881419223 | count | 1 |
| PLTP | -1.6661577 | 0.6257457 | -2.6627 | 0.00779 | -0.881068643 | count | 1 |
| KNL1 | -1.6601021 | 0.7102207 | -2.3374 | 0.0195 | -0.879166369 | count | 1 |
| CDK1 | -0.9385666 | 0.4668925 | -2.0102 | 0.0445 | -0.875214376 | count | 1 |
| TEP1 | -1.3273047 | 0.572898 | -2.3168 | 0.0206 | -0.874605592 | count | 1 |
| ZNF45 | -1.161899 | 0.620106 | -1.8737 | 0.0611 | -0.874596115 | count | 1 |
| CD36 | -0.7440519 | 0.5467116 | -1.361 | 0.174 | -0.873935444 | count | 1 |

| | | | | | | | |
|---|---|---|---|---|---|---|---|
| THBS2 | -1.9526945 | 1.5940518 | -1.225 | 0.221 | -0.87265962 | count | 1 |
| ISG15 | -0.6129801 | 0.0887128 | -6.9097 | 5.77E-12 | -0.870838013 | count | 1.40E-07 |
| CBR3 | -0.8081111 | 0.3068613 | -2.6335 | 0.00849 | -0.869216166 | count | 1 |
| FCGR2B | -1.0511072 | 0.5030743 | -2.0894 | 0.0367 | -0.869081144 | count | 1 |
| ARMCX5 | -0.7785182 | 0.3285606 | -2.3695 | 0.0179 | -0.868877152 | count | 1 |
| SYNC | -0.7322473 | 0.1909614 | -3.8345 | 0.000128 | -0.868818812 | count | 1 |
| OAS3 | -0.9518972 | 0.4286252 | -2.2208 | 0.0264 | -0.867058391 | count | 1 |
| C2orf40 | -1.4319908 | 0.6496203 | -2.2044 | 0.0276 | -0.864927072 | count | 1 |
| ZBTB11 | -0.6662968 | 0.1332896 | -4.9989 | 6.06E-07 | -0.862897587 | count | 0.014613084 |
| MTUS1 | -0.9458505 | 0.537276 | -1.7605 | 0.0784 | -0.862323367 | count | 1 |
| FANCI | -1.2091083 | 0.6007735 | -2.0126 | 0.0442 | -0.861531443 | count | 1 |
| SLC43A2 | -1.899075 | 1.2906573 | -1.4714 | 0.141 | -0.860442437 | count | 1 |
| AL163051.1 | -1.888868 | 0.8828685 | -2.1395 | 0.0325 | -0.858056909 | count | 1 |
| WHRN | -0.8768326 | 0.4031188 | -2.1751 | 0.0297 | -0.855707388 | count | 1 |
| VPS33B | -0.9884161 | 0.5287253 | -1.8694 | 0.0616 | -0.851084384 | count | 1 |
| PTTG1 | -0.6027437 | 0.1067314 | -5.6473 | 1.76E-08 | -0.848746769 | count | 0.000425445 |
| LEAP2 | -1.2729907 | 0.588281 | -2.1639 | 0.0305 | -0.84864283 | count | 1 |
| TP53TG5 | -1.558161 | 1.5370068 | -1.0138 | 0.311 | -0.845858353 | count | 1 |
| RGS17 | -1.012032 | 0.853825 | -1.1853 | 0.236 | -0.842362087 | count | 1 |
| TPPP | -1.1727159 | 0.7593576 | -1.5444 | 0.123 | -0.841727826 | count | 1 |
| EZR | -0.5862506 | 0.0484502 | -12.1001 | 5.00E-33 | -0.840340507 | count | 1.22E-28 |
| ANG | -0.9170114 | 0.5513781 | -1.6631 | 0.0964 | -0.839583798 | count | 1 |
| SCX | -1.247599 | 0.9558297 | -1.3053 | 0.192 | -0.836210664 | count | 1 |
| AC068338.2 | -0.8886741 | 0.4688674 | -1.8954 | 0.0581 | -0.834542082 | count | 1 |
| DEPDC1 | -17.906992 | 3199.457207 | -0.0056 | 0.996 | -0.834127889 | count | 1 |
| LINC01954 | -17.906992 | 3199.457207 | -0.0056 | 0.996 | -0.834127889 | count | 1 |
| HR | -17.906992 | 3199.457207 | -0.0056 | 0.996 | -0.834127889 | count | 1 |
| CNTNAP3 | -17.906992 | 3199.457207 | -0.0056 | 0.996 | -0.834127889 | count | 1 |
| ITGA7 | -17.906992 | 3199.457207 | -0.0056 | 0.996 | -0.834127889 | count | 1 |
| PCDH17 | -17.906992 | 3199.457207 | -0.0056 | 0.996 | -0.834127889 | count | 1 |
| AC087292.1 | -17.906992 | 3199.457207 | -0.0056 | 0.996 | -0.834127889 | count | 1 |
| AC116407.1 | -17.906992 | 3199.457207 | -0.0056 | 0.996 | -0.834127889 | count | 1 |
| FSCN2 | -17.906992 | 3199.457207 | -0.0056 | 0.996 | -0.834127889 | count | 1 |
| AC137723.1 | -17.906992 | 3199.457207 | -0.0056 | 0.996 | -0.834127889 | count | 1 |
| SYT4 | -17.906992 | 3199.457207 | -0.0056 | 0.996 | -0.834127889 | count | 1 |
| AL451062.3 | -17.90538 | 2258.318553 | -0.0079 | 0.994 | -0.834127889 | count | 1 |
| KLLN | -17.90538 | 2258.318553 | -0.0079 | 0.994 | -0.834127889 | count | 1 |
| SYCE1 | -17.90538 | 2258.318553 | -0.0079 | 0.994 | -0.834127889 | count | 1 |
| AL031055.1 | -17.90538 | 2258.318553 | -0.0079 | 0.994 | -0.834127889 | count | 1 |
| KREMEN1 | -17.90538 | 2258.318553 | -0.0079 | 0.994 | -0.834127889 | count | 1 |
| AC234772.3 | -17.74201 | 2604.201066 | -0.0068 | 0.995 | -0.834127886 | count | 1 |
| ADGRG3 | -17.74201 | 2604.201066 | -0.0068 | 0.995 | -0.834127886 | count | 1 |
| GPR25 | -17.740928 | 2099.017963 | -0.0085 | 0.993 | -0.834127886 | count | 1 |
| AP000759.1 | -17.740928 | 2099.017963 | -0.0085 | 0.993 | -0.834127886 | count | 1 |
| TVP23C-CDRT4 | -17.740928 | 2099.017963 | -0.0085 | 0.993 | -0.834127886 | count | 1 |

| | | | | | | | |
|---|---|---|---|---|---|---|---|
| GML | -17.425789 | 1930.306399 | -0.009 | 0.993 | -0.834127877 | count | 1 |
| RASAL1 | -17.42554 | 1788.621693 | -0.0097 | 0.992 | -0.834127877 | count | 1 |
| SCARB1 | -17.42554 | 1788.621693 | -0.0097 | 0.992 | -0.834127877 | count | 1 |
| CLEC5A | -17.395777 | 1774.355811 | -0.0098 | 0.992 | -0.834127877 | count | 1 |
| PTPRD | -17.06209 | 1785.142008 | -0.0096 | 0.992 | -0.834127864 | count | 1 |
| CXCL12 | -18.3003207 | 1274.529984 | -0.0144 | 0.989 | -0.834127769 | count | 1 |
| FAP | -17.9593581 | 2481.239715 | -0.0072 | 0.994 | -0.834127763 | count | 1 |
| PAX8 | -17.9586844 | 2001.967993 | -0.009 | 0.993 | -0.834127763 | count | 1 |
| NEURL2 | -17.9586844 | 2001.967993 | -0.009 | 0.993 | -0.834127763 | count | 1 |
| HSPA4L | -17.8345694 | 2414.237031 | -0.0074 | 0.994 | -0.834127761 | count | 1 |
| LTBP1 | -17.8340544 | 1078.001627 | -0.0165 | 0.987 | -0.834127761 | count | 1 |
| RDM1 | -17.8268047 | 1658.684437 | -0.0107 | 0.991 | -0.834127761 | count | 1 |
| HIST1H2AJ | -17.7309109 | 2551.036632 | -0.007 | 0.994 | -0.834127759 | count | 1 |
| AC093627.5 | -17.7295431 | 1800.883421 | -0.0098 | 0.992 | -0.834127759 | count | 1 |
| RAB42 | -17.6554693 | 1954.921792 | -0.009 | 0.993 | -0.834127757 | count | 1 |
| ZBTB20-AS1 | -17.6371027 | 1966.760234 | -0.009 | 0.993 | -0.834127757 | count | 1 |
| COX7A1 | -17.6188597 | 1081.037422 | -0.0163 | 0.987 | -0.834127756 | count | 1 |
| FMO2 | -17.6187673 | 1285.658273 | -0.0137 | 0.989 | -0.834127756 | count | 1 |
| CLIC4 | -17.465848 | 1110.648536 | -0.0157 | 0.987 | -0.834127752 | count | 1 |
| PLXNB2 | -17.4361127 | 1772.411554 | -0.0098 | 0.992 | -0.834127751 | count | 1 |
| CDCP1 | -17.2562243 | 1916.878584 | -0.009 | 0.993 | -0.834127745 | count | 1 |
| COPS4 | -0.6215198 | 0.1456873 | -4.2661 | 2.04E-05 | -0.832153244 | count | 0.489804 |
| CCR5 | -0.664613 | 0.243438 | -2.7301 | 0.00636 | -0.831527188 | count | 1 |
| RBSN | -0.7262059 | 0.3434446 | -2.1145 | 0.0345 | -0.831013046 | count | 1 |
| CENPF | -0.6361993 | 0.2708248 | -2.3491 | 0.0189 | -0.827120478 | count | 1 |
| KIAA0355 | -0.668136 | 0.2858481 | -2.3374 | 0.0195 | -0.824865147 | count | 1 |
| FGFR1 | -1.2241721 | 0.3632849 | -3.3697 | 0.000761 | -0.824573575 | count | 1 |
| ZNF90 | -1.0767859 | 0.3548125 | -3.0348 | 0.00243 | -0.823684006 | count | 1 |
| EIF4EBP1 | -0.6079209 | 0.1607576 | -3.7816 | 0.000159 | -0.819719013 | count | 1 |
| ALG1L | -1.7348708 | 1.7859395 | -0.9714 | 0.331 | -0.819647996 | count | 1 |
| TIMP1 | -0.5771438 | 0.0813724 | -7.0926 | 1.59E-12 | -0.818773769 | count | 3.86E-08 |
| STAU2 | -0.661912 | 0.2023088 | -3.2718 | 0.00108 | -0.817470733 | count | 1 |
| SLC11A1 | -0.9742595 | 0.5895858 | -1.6524 | 0.0985 | -0.816075812 | count | 1 |
| AKAP2 | -1.063709 | 0.844346 | -1.2598 | 0.208 | -0.815662075 | count | 1 |
| LTBR | -2.1850131 | 1.2607046 | -1.7332 | 0.0832 | -0.813553911 | count | 1 |
| MLKL | -0.7187845 | 0.2375274 | -3.0261 | 0.0025 | -0.81228715 | count | 1 |
| NNMT | -1.7006875 | 0.5385684 | -3.1578 | 0.0016 | -0.810484348 | count | 1 |
| CEBPD | -0.5722384 | 0.1564307 | -3.6581 | 3.00E-04 | -0.808324727 | count | 1 |
| BIVM | -1.0030064 | 0.4794191 | -2.0921 | 0.0365 | -0.808278622 | count | 1 |
| IGSF6 | -0.7546119 | 0.5571263 | -1.3545 | 0.176 | -0.808041244 | count | 1 |
| TNK2 | -0.6879207 | 0.2793031 | -2.463 | 0.0138 | -0.807383737 | count | 1 |
| TIGD5 | -0.7931896 | 0.3181955 | -2.4928 | 0.0127 | -0.806386885 | count | 1 |
| HMGN5 | -0.7602749 | 0.3303907 | -2.3011 | 0.0214 | -0.805055247 | count | 1 |
| LYSMD4 | -0.850448 | 0.5065261 | -1.679 | 0.0932 | -0.80286667 | count | 1 |
| MIR3142HG | -2.1063486 | 0.9362225 | -2.2498 | 0.0245 | -0.800683058 | count | 1 |

| | | | | | | | |
|---|---|---|---|---|---|---|---|
| DVL3 | -0.7993335 | 0.379219 | -2.1078 | 0.0351 | -0.800345185 | count | 1 |
| PFKP | -0.6300873 | 0.1436456 | -4.3864 | 1.19E-05 | -0.800035292 | count | 0.2859451 |
| ATP13A3 | -0.6578963 | 0.2457752 | -2.6768 | 0.00747 | -0.799979583 | count | 1 |
| FAR2 | -0.7988219 | 0.37993 | -2.1026 | 0.0356 | -0.799882314 | count | 1 |
| IFI6 | -0.5730984 | 0.1458878 | -3.9284 | 8.72E-05 | -0.799548806 | count | 1 |
| SARDH | -0.7117299 | 0.405528 | -1.7551 | 0.0793 | -0.799082099 | count | 1 |
| AMIGO2 | -0.683861 | 0.2407915 | -2.8401 | 0.00454 | -0.798750481 | count | 1 |
| TMEM140 | -0.6973741 | 0.2469074 | -2.8244 | 0.00476 | -0.794837705 | count | 1 |
| CXCL10 | -1.4110822 | 0.9396631 | -1.5017 | 0.133 | -0.793376743 | count | 1 |
| VWF | -1.0262921 | 0.655993 | -1.5645 | 0.118 | -0.792414901 | count | 1 |
| CPSF2 | -0.6487247 | 0.2268072 | -2.8602 | 0.00426 | -0.791957132 | count | 1 |
| MIR22HG | -0.5934428 | 0.1508386 | -3.9343 | 8.51E-05 | -0.791252185 | count | 1 |
| MAT2A | -0.6578509 | 0.1495366 | -4.3993 | 1.12E-05 | -0.791090908 | count | 0.2691696 |
| ALDH3A2 | -0.7158887 | 0.3152779 | -2.2707 | 0.0232 | -0.791009209 | count | 1 |
| HCFC1R1 | -0.5749828 | 0.1463879 | -3.9278 | 8.74E-05 | -0.788834214 | count | 1 |
| SPATA33 | -0.7864952 | 0.2690372 | -2.9234 | 0.00349 | -0.788707335 | count | 1 |
| KCNK1 | -1.622237 | 1.7935053 | -0.9045 | 0.366 | -0.788536431 | count | 1 |
| CORO1C | -1.076543 | 0.5352604 | -2.0113 | 0.0444 | -0.78745208 | count | 1 |
| SLC43A1 | -0.7976948 | 0.4670539 | -1.7079 | 0.0877 | -0.786350002 | count | 1 |
| AC133644.2 | -0.6645238 | 0.2272471 | -2.9242 | 0.00348 | -0.78505701 | count | 1 |
| AC004585.1 | -0.5937493 | 0.1873956 | -3.1684 | 0.00155 | -0.785055117 | count | 1 |
| USP37 | -0.6671756 | 0.190121 | -3.5092 | 0.000455 | -0.784220935 | count | 1 |
| HECTD2 | -1.0120967 | 0.6043915 | -1.6746 | 0.0941 | -0.783481681 | count | 1 |
| CLEC14A | -0.8231661 | 0.6667808 | -1.2345 | 0.217 | -0.779991342 | count | 1 |
| VMO1 | -1.063613 | 1.1178312 | -0.9515 | 0.341 | -0.779939906 | count | 1 |
| PAK1 | -0.7098796 | 0.281903 | -2.5182 | 0.0118 | -0.778118188 | count | 1 |
| FAM174B | -1.5848072 | 1.1177198 | -1.4179 | 0.156 | -0.777603422 | count | 1 |
| SPRED1 | -1.5805723 | 0.9499206 | -1.6639 | 0.0962 | -0.776347275 | count | 1 |
| ATP9A | -1.9687536 | 1.2273941 | -1.604 | 0.109 | -0.776017906 | count | 1 |
| B3GNT9 | -0.8344886 | 0.6137207 | -1.3597 | 0.174 | -0.773102805 | count | 1 |
| SOD3 | -0.9133195 | 0.5430014 | -1.682 | 0.0927 | -0.772724666 | count | 1 |
| MRPL38 | -0.9467194 | 0.6489505 | -1.4588 | 0.145 | -0.770371096 | count | 1 |
| KRBA1 | -1.1175402 | 0.5919042 | -1.888 | 0.0591 | -0.769562568 | count | 1 |
| MS4A6A | -0.7933468 | 0.4111392 | -1.9296 | 0.0537 | -0.769126025 | count | 1 |
| MMEL1 | -1.3450086 | 0.7887883 | -1.7052 | 0.0883 | -0.768031548 | count | 1 |
| ACTN1 | -0.7769199 | 0.2804862 | -2.7699 | 0.00564 | -0.767840712 | count | 1 |
| TPRN | -0.7765466 | 0.2751056 | -2.8227 | 0.00479 | -0.767506984 | count | 1 |
| NDC80 | -0.8273202 | 0.314941 | -2.6269 | 0.00866 | -0.767230428 | count | 1 |
| GK | -0.7744092 | 0.3334803 | -2.3222 | 0.0203 | -0.765595456 | count | 1 |
| USP6NL | -1.5412139 | 0.6872182 | -2.2427 | 0.025 | -0.764484964 | count | 1 |
| LINC01759 | -1.2018737 | 0.7887229 | -1.5238 | 0.128 | -0.764177986 | count | 1 |
| NASP | -0.5511493 | 0.0747872 | -7.3696 | 2.14E-13 | -0.763974195 | count | 5.19E-09 |
| IL1R1 | -1.9050521 | 0.9145638 | -2.083 | 0.0373 | -0.763612366 | count | 1 |
| TPSAB1 | -1.9030629 | 1.2548386 | -1.5166 | 0.129 | -0.76321448 | count | 1 |
| PPP1R14B | -0.5388771 | 0.0875995 | -6.1516 | 8.56E-10 | -0.76296175 | count | 2.07E-05 |

| | | | | | | | |
|---|---|---|---|---|---|---|---|
| NUP43 | -0.9354524 | 0.3709109 | -2.522 | 0.0117 | -0.762664735 | count | 1 |
| LACTB | -0.5612683 | 0.1277278 | -4.3943 | 1.15E-05 | -0.762559476 | count | 0.2763565 |
| NADK2 | -0.9344214 | 0.3209692 | -2.9112 | 0.00362 | -0.761957588 | count | 1 |
| PPIF | -0.59728 | 0.1912565 | -3.1229 | 0.00181 | -0.760983751 | count | 1 |
| SERPINA1 | -0.8195344 | 0.7935614 | -1.0327 | 0.302 | -0.760834817 | count | 1 |
| CENPU | -1.098883 | 0.4603952 | -2.3868 | 0.017 | -0.759591609 | count | 1 |
| GPRC5D-AS1 | -0.7541144 | 0.4718851 | -1.5981 | 0.11 | -0.759147367 | count | 1 |
| OASL | -0.5360414 | 0.1108314 | -4.8366 | 1.38E-06 | -0.758656402 | count | 0.03325386 |
| CCDC146 | -0.7810689 | 0.340765 | -2.2921 | 0.022 | -0.758411202 | count | 1 |
| METTL6 | -0.7214331 | 0.2848261 | -2.5329 | 0.0114 | -0.758409176 | count | 1 |
| CLN8 | -0.7414379 | 0.2885137 | -2.5699 | 0.0102 | -0.758270565 | count | 1 |
| TCTEX1D2 | -0.643604 | 0.2266078 | -2.8402 | 0.00454 | -0.757794127 | count | 1 |
| CALD1 | -0.9283383 | 0.4105113 | -2.2614 | 0.0238 | -0.757778578 | count | 1 |
| NACA2 | -0.9280377 | 0.5471755 | -1.6961 | 0.09 | -0.757571781 | count | 1 |
| TRIP6 | -0.8356709 | 0.4567787 | -1.8295 | 0.0674 | -0.756269989 | count | 1 |
| AL109741.1 | -0.8092548 | 0.5115983 | -1.5818 | 0.114 | -0.752362951 | count | 1 |
| RBAK-RBAKDN | -0.5990653 | 0.1709387 | -3.5046 | 0.000463 | -0.750243745 | count | 1 |
| C17orf51 | -0.9580533 | 0.5311077 | -1.8039 | 0.0713 | -0.748901483 | count | 1 |
| C8orf88 | -0.8258143 | 0.6331159 | -1.3044 | 0.192 | -0.748417446 | count | 1 |
| OTULIN | -0.5636947 | 0.1369962 | -4.1147 | 3.97E-05 | -0.747375496 | count | 0.9518472 |
| AC007566.1 | -1.481718 | 1.4795026 | -1.0015 | 0.317 | -0.745898424 | count | 1 |
| THBD | -1.2865239 | 0.589371 | -2.1829 | 0.0291 | -0.744656108 | count | 1 |
| RMI1 | -0.7649056 | 0.3675596 | -2.081 | 0.0375 | -0.744239208 | count | 1 |
| KIF21B | -0.7807824 | 0.2670322 | -2.9239 | 0.00348 | -0.744015817 | count | 1 |
| MYLIP | -0.5858848 | 0.1236927 | -4.7366 | 2.26E-06 | -0.743968838 | count | 0.05442758 |
| HIF1A-AS2 | -1.8101515 | 1.3744315 | -1.317 | 0.188 | -0.743898715 | count | 1 |
| AC005332.8 | -0.9049197 | 0.770988 | -1.1737 | 0.241 | -0.741583579 | count | 1 |
| R3HCC1L | -0.6163886 | 0.1990568 | -3.0965 | 0.00197 | -0.740251363 | count | 1 |
| ITGA1 | -0.5923224 | 0.2180702 | -2.7162 | 0.00664 | -0.740220114 | count | 1 |
| HAUS3 | -0.561577 | 0.1337812 | -4.1977 | 2.77E-05 | -0.739697016 | count | 0.6645784 |
| ARHGEF5 | -0.9951027 | 0.4252596 | -2.34 | 0.0193 | -0.739285193 | count | 1 |
| TTF2 | -0.6445738 | 0.241377 | -2.6704 | 0.00761 | -0.737802671 | count | 1 |
| ATAD2 | -0.5628106 | 0.1674011 | -3.362 | 0.000782 | -0.737289486 | count | 1 |
| BASP1 | -0.8984884 | 0.5802344 | -1.5485 | 0.122 | -0.737106456 | count | 1 |
| ISOC2 | -0.5562654 | 0.2092963 | -2.6578 | 0.0079 | -0.73704188 | count | 1 |
| AC137932.3 | -1.7785153 | 0.7447118 | -2.3882 | 0.017 | -0.736986403 | count | 1 |
| OVCH1-AS1 | -0.8979082 | 0.5597456 | -1.6041 | 0.109 | -0.736701938 | count | 1 |
| PLEKHO2 | -0.6629899 | 0.3568222 | -1.858 | 0.0632 | -0.736064113 | count | 1 |
| MALSU1 | -0.5479519 | 0.1415959 | -3.8698 | 0.000111 | -0.73416473 | count | 1 |
| KSR1 | -0.8942324 | 0.5714468 | -1.5649 | 0.118 | -0.734136688 | count | 1 |
| HSPA4 | -0.5416951 | 0.1207037 | -4.4878 | 7.43E-06 | -0.731630572 | count | 0.17866921 |
| NARS2 | -0.6710734 | 0.3070366 | -2.1856 | 0.0289 | -0.7315941 | count | 1 |
| CAVIN3 | -1.2546318 | 0.660595 | -1.8992 | 0.0576 | -0.731531527 | count | 1 |
| AC245060.5 | -0.7237862 | 0.3461271 | -2.0911 | 0.0366 | -0.731195772 | count | 1 |
| EMG1 | -1.747952 | 1.3699856 | -1.2759 | 0.202 | -0.730141701 | count | 1 |

| | | | | | | | |
|---|---|---|---|---|---|---|---|
| FBXO31 | -1.4293683 | 0.5066104 | -2.8214 | 0.00481 | -0.728878424 | count | 1 |
| SPARCL1 | -1.742137 | 0.4771462 | -3.6512 | 0.000265 | -0.72882084 | count | 1 |
| SAC3D1 | -0.611706 | 0.2568124 | -2.3819 | 0.0173 | -0.72862499 | count | 1 |
| KCTD10 | -0.7099114 | 0.3368888 | -2.1073 | 0.0352 | -0.72859809 | count | 1 |
| VANGL1 | -0.7801584 | 0.3638277 | -2.1443 | 0.0321 | -0.728213296 | count | 1 |
| HBP1 | -0.5311627 | 0.0895063 | -5.9344 | 3.25E-09 | -0.724538618 | count | 7.86E-05 |
| RANBP9 | -0.6061711 | 0.2001452 | -3.0287 | 0.00247 | -0.722298063 | count | 1 |
| NIPSNAP3A | -0.59595 | 0.2752383 | -2.1652 | 0.0304 | -0.722210164 | count | 1 |
| VSIG4 | -0.8433313 | 0.7960157 | -1.0594 | 0.289 | -0.721518589 | count | 1 |
| SMIM11B | -1.401168 | 1.2725673 | -1.1011 | 0.271 | -0.719446769 | count | 1 |
| LINC01094 | -1.401168 | 1.321098 | -1.0606 | 0.289 | -0.719446769 | count | 1 |
| MSL3 | -0.531952 | 0.1196074 | -4.4475 | 8.97E-06 | -0.71825066 | count | 0.21565674 |
| LGALS9 | -0.5470227 | 0.2165119 | -2.5265 | 0.0116 | -0.716174493 | count | 1 |
| FAM227B | -0.7327959 | 0.3336042 | -2.1966 | 0.0281 | -0.715863662 | count | 1 |
| DHX8 | -0.6031162 | 0.1781516 | -3.3854 | 0.000719 | -0.715543483 | count | 1 |
| AL158835.1 | -0.9556784 | 0.7731583 | -1.2361 | 0.217 | -0.715242901 | count | 1 |
| TNFAIP2 | -1.6832969 | 0.8242554 | -2.0422 | 0.0412 | -0.7151093 | count | 1 |
| RBMXL1 | -0.6195821 | 0.1802941 | -3.4365 | 0.000596 | -0.715012333 | count | 1 |
| ODF3B | -0.6851913 | 0.3176328 | -2.1572 | 0.0311 | -0.714381962 | count | 1 |
| FHL1 | -0.6599269 | 0.2304388 | -2.8638 | 0.00421 | -0.713291021 | count | 1 |
| KBTBD6 | -1.2104798 | 0.5415383 | -2.2353 | 0.0255 | -0.712916645 | count | 1 |
| TEPP | -1.3808701 | 0.708106 | -1.9501 | 0.0512 | -0.712542595 | count | 1 |
| CEP57L1 | -0.7035157 | 0.3553953 | -1.9795 | 0.0478 | -0.712372691 | count | 1 |
| LMNA | -0.5022183 | 0.0918374 | -5.4686 | 4.87E-08 | -0.712106105 | count | 0.001176495 |
| POU3F1 | -0.5999586 | 0.2917689 | -2.0563 | 0.0398 | -0.711949009 | count | 1 |
| CYSTM1 | -0.5324697 | 0.1586188 | -3.3569 | 0.000797 | -0.711723732 | count | 1 |
| KIAA0513 | -0.6824391 | 0.323774 | -2.1078 | 0.0351 | -0.711720017 | count | 1 |
| C1RL-AS1 | -0.9011857 | 0.4210087 | -2.1405 | 0.0324 | -0.711543345 | count | 1 |
| JAML | -0.5293043 | 0.1523629 | -3.474 | 0.000519 | -0.711382849 | count | 1 |
| FAM129B | -1.3768282 | 0.6901023 | -1.9951 | 0.0461 | -0.711156207 | count | 1 |
| ANPEP | -0.9005497 | 1.0299589 | -0.8744 | 0.382 | -0.711119951 | count | 1 |
| CNNM4 | -1.3764363 | 0.5679167 | -2.4237 | 0.0154 | -0.711021545 | count | 1 |
| AC091982.3 | -1.205865 | 1.1099855 | -1.0864 | 0.277 | -0.71094103 | count | 1 |
| B3GNT5 | -1.0919369 | 0.86322 | -1.265 | 0.206 | -0.710807505 | count | 1 |
| EPHA4 | -0.7133664 | 0.3570147 | -1.9981 | 0.0458 | -0.710458435 | count | 1 |
| PLEKHO1 | -0.509768 | 0.103988 | -4.9022 | 9.92E-07 | -0.708485186 | count | 0.023915136 |
| ITGA5 | -0.7556543 | 0.3126834 | -2.4167 | 0.0157 | -0.707681944 | count | 1 |
| SSFA2 | -0.7096837 | 0.2829302 | -2.5083 | 0.0122 | -0.707098753 | count | 1 |
| IFI44 | -0.5288464 | 0.1653177 | -3.199 | 0.00139 | -0.704821387 | count | 1 |
| IL4I1 | -0.6386723 | 0.4142032 | -1.5419 | 0.123 | -0.704648038 | count | 1 |
| TMEM191C | -0.57939 | 0.3029122 | -1.9127 | 0.0559 | -0.702846453 | count | 1 |
| CPNE7 | -0.5786417 | 0.2381053 | -2.4302 | 0.0151 | -0.701970308 | count | 1 |
| ABCF3 | -0.6057469 | 0.2053785 | -2.9494 | 0.00321 | -0.699779868 | count | 1 |
| CTSH | -0.541309 | 0.1977013 | -2.738 | 0.00621 | -0.698427769 | count | 1 |
| AC092119.2 | -1.6146303 | 1.4702806 | -1.0982 | 0.272 | -0.698296815 | count | 1 |

| | | | | | | | |
|---|---|---|---|---|---|---|---|
| CAPG | -0.4927906 | 0.1147451 | -4.2947 | 1.80E-05 | -0.697936058 | count | 0.432252 |
| FDX2 | -0.8114259 | 0.4423248 | -1.8345 | 0.0667 | -0.697678997 | count | 1 |
| FAAP100 | -0.8389033 | 0.3415775 | -2.456 | 0.0141 | -0.695023918 | count | 1 |
| VASH1 | -2.1865625 | 0.9807625 | -2.2295 | 0.0258 | -0.694318099 | count | 1 |
| CEP126 | -0.7588066 | 0.3858747 | -1.9665 | 0.0493 | -0.69426366 | count | 1 |
| GINS2 | -0.8372891 | 0.5830264 | -1.4361 | 0.151 | -0.693868822 | count | 1 |
| LGALS3 | -0.4843361 | 0.0868297 | -5.578 | 2.62E-08 | -0.693089243 | count | 0.000633228 |
| ZNF547 | -1.163921 | 0.7655296 | -1.5204 | 0.129 | -0.692722381 | count | 1 |
| SEC22C | -0.5338581 | 0.1704696 | -3.1317 | 0.00175 | -0.692093269 | count | 1 |
| GEM | -0.7764267 | 0.3987676 | -1.9471 | 0.0516 | -0.690725194 | count | 1 |
| TYMS | -1.5808912 | 0.9977042 | -1.5845 | 0.113 | -0.689706792 | count | 1 |
| PGLYRP2 | -0.5568158 | 0.2956577 | -1.8833 | 0.0597 | -0.68967679 | count | 1 |
| MT-ND6 | -0.5407976 | 0.1241512 | -4.356 | 1.36E-05 | -0.689445016 | count | 0.3267128 |
| XRCC2 | -1.5726235 | 1.1313824 | -1.39 | 0.165 | -0.687568078 | count | 1 |
| GALNT2 | -0.538174 | 0.2583255 | -2.0833 | 0.0373 | -0.687464392 | count | 1 |
| GLA | -0.6274471 | 0.2580708 | -2.4313 | 0.0151 | -0.686798946 | count | 1 |
| CSKMT | -0.5436497 | 0.1795397 | -3.028 | 0.00248 | -0.685883315 | count | 1 |
| C11orf96 | -0.7475193 | 0.3158556 | -2.3666 | 0.018 | -0.685010857 | count | 1 |
| PMP22 | -0.964956 | 0.7215727 | -1.3373 | 0.181 | -0.684980566 | count | 1 |
| ZNF217 | -0.4959294 | 0.1048477 | -4.73 | 2.34E-06 | -0.684195104 | count | 0.0563472 |
| CDH13 | -1.2985269 | 0.8185295 | -1.5864 | 0.113 | -0.68352757 | count | 1 |
| TAPT1 | -0.5685466 | 0.224662 | -2.5307 | 0.0114 | -0.682058077 | count | 1 |
| GOLGA6L9 | -1.550776 | 1.3306175 | -1.1655 | 0.244 | -0.681851822 | count | 1 |
| LINC01963 | -1.550776 | 1.5923987 | -0.9739 | 0.33 | -0.681851822 | count | 1 |
| CTHRC1 | -1.5470748 | 0.9217114 | -1.6785 | 0.0933 | -0.680874321 | count | 1 |
| HELLS | -0.5666018 | 0.2370771 | -2.3899 | 0.0169 | -0.679808512 | count | 1 |
| INO80B | -0.6922562 | 0.2516139 | -2.7513 | 0.00597 | -0.679623145 | count | 1 |
| EVA1B | -0.549323 | 0.1974209 | -2.7825 | 0.00542 | -0.67867725 | count | 1 |
| WEE1 | -0.5764897 | 0.2436303 | -2.3662 | 0.018 | -0.678559295 | count | 1 |
| ANKS1B | -0.8126933 | 0.6136398 | -1.3244 | 0.185 | -0.676170805 | count | 1 |
| LRRC61 | -0.5438275 | 0.3598374 | -1.5113 | 0.131 | -0.675951138 | count | 1 |
| HIST1H1E | -0.4775336 | 0.1127791 | -4.2342 | 2.35E-05 | -0.673932102 | count | 0.5640235 |
| PITPNM1 | -0.5432958 | 0.2561194 | -2.1213 | 0.034 | -0.673412043 | count | 1 |
| PSMA2 | -0.4864714 | 0.1088499 | -4.4692 | 8.11E-06 | -0.672850498 | count | 0.19499684 |
| PHLDB3 | -0.6513101 | 0.310915 | -2.0948 | 0.0363 | -0.672736757 | count | 1 |
| EAF2 | -0.7317904 | 0.3683626 | -1.9866 | 0.047 | -0.67205499 | count | 1 |
| MT1E | -0.5171055 | 0.1772454 | -2.9175 | 0.00355 | -0.67173864 | count | 1 |
| MCAT | -0.6397276 | 0.2655542 | -2.409 | 0.016 | -0.670157783 | count | 1 |
| MAFG | -0.5092636 | 0.1469206 | -3.4663 | 0.000534 | -0.669842446 | count | 1 |
| BRI3 | -0.4752509 | 0.0902499 | -5.2659 | 1.48E-07 | -0.667689318 | count | 0.003572276 |
| ZNF165 | -1.0069131 | 0.4724143 | -2.1314 | 0.0331 | -0.667160313 | count | 1 |
| AC005332.4 | -0.7711409 | 0.5529378 | -1.3946 | 0.163 | -0.667140144 | count | 1 |
| LARP4 | -0.5259416 | 0.1684215 | -3.1228 | 0.00181 | -0.666954215 | count | 1 |
| MSR1 | -1.2524535 | 0.981102 | -1.2766 | 0.202 | -0.666576497 | count | 1 |
| AL645608.3 | -17.616962 | 2767.561006 | -0.0064 | 0.995 | -0.666378765 | count | 1 |

| | | | | | | | |
|---|---|---|---|---|---|---|---|
| AL391845.1 | -17.616962 | 2767.561006 | -0.0064 | 0.995 | -0.666378765 | count | 1 |
| AC092807.3 | -17.616962 | 2767.561006 | -0.0064 | 0.995 | -0.666378765 | count | 1 |
| C1orf226 | -17.616962 | 2767.561006 | -0.0064 | 0.995 | -0.666378765 | count | 1 |
| AL445483.1 | -17.616962 | 2767.561006 | -0.0064 | 0.995 | -0.666378765 | count | 1 |
| CAPN8 | -17.616962 | 2767.561006 | -0.0064 | 0.995 | -0.666378765 | count | 1 |
| FAM178B | -17.616962 | 2767.561006 | -0.0064 | 0.995 | -0.666378765 | count | 1 |
| EVC | -17.616962 | 2767.561006 | -0.0064 | 0.995 | -0.666378765 | count | 1 |
| AC096734.2 | -17.616962 | 2767.561006 | -0.0064 | 0.995 | -0.666378765 | count | 1 |
| AC079341.1 | -17.616962 | 2767.561006 | -0.0064 | 0.995 | -0.666378765 | count | 1 |
| AC092354.1 | -17.616962 | 2767.561006 | -0.0064 | 0.995 | -0.666378765 | count | 1 |
| AL133268.3 | -17.616962 | 2767.561006 | -0.0064 | 0.995 | -0.666378765 | count | 1 |
| HIST1H3J | -17.616962 | 2767.561006 | -0.0064 | 0.995 | -0.666378765 | count | 1 |
| AL034345.2 | -17.616962 | 2767.561006 | -0.0064 | 0.995 | -0.666378765 | count | 1 |
| SNAP91 | -17.616962 | 2767.561006 | -0.0064 | 0.995 | -0.666378765 | count | 1 |
| HOXA3 | -17.616962 | 2767.561006 | -0.0064 | 0.995 | -0.666378765 | count | 1 |
| FAM156A | -17.616962 | 2767.561006 | -0.0064 | 0.995 | -0.666378765 | count | 1 |
| AP005717.1 | -17.616962 | 2767.561006 | -0.0064 | 0.995 | -0.666378765 | count | 1 |
| PAPPA-AS1 | -17.616962 | 2767.561006 | -0.0064 | 0.995 | -0.666378765 | count | 1 |
| IFITM3 | -17.616962 | 2767.561006 | -0.0064 | 0.995 | -0.666378765 | count | 1 |
| SOX6 | -17.616962 | 2767.561006 | -0.0064 | 0.995 | -0.666378765 | count | 1 |
| LDLRAD3 | -17.616962 | 2767.561006 | -0.0064 | 0.995 | -0.666378765 | count | 1 |
| AP003721.4 | -17.616962 | 2767.561006 | -0.0064 | 0.995 | -0.666378765 | count | 1 |
| GNG3 | -17.616962 | 2767.561006 | -0.0064 | 0.995 | -0.666378765 | count | 1 |
| AP002770.1 | -17.616962 | 2767.561006 | -0.0064 | 0.995 | -0.666378765 | count | 1 |
| AP003086.1 | -17.616962 | 2767.561006 | -0.0064 | 0.995 | -0.666378765 | count | 1 |
| HPSE2 | -17.616962 | 2767.561006 | -0.0064 | 0.995 | -0.666378765 | count | 1 |
| HMX2 | -17.616962 | 2767.561006 | -0.0064 | 0.995 | -0.666378765 | count | 1 |
| IGHV3-43 | -17.616962 | 2767.561006 | -0.0064 | 0.995 | -0.666378765 | count | 1 |
| LARP6 | -17.616962 | 2767.561006 | -0.0064 | 0.995 | -0.666378765 | count | 1 |
| AC087286.2 | -17.616962 | 2767.561006 | -0.0064 | 0.995 | -0.666378765 | count | 1 |
| AC027682.3 | -17.616962 | 2767.561006 | -0.0064 | 0.995 | -0.666378765 | count | 1 |
| AC092718.8 | -17.616962 | 2767.561006 | -0.0064 | 0.995 | -0.666378765 | count | 1 |
| EFCAB5 | -17.616962 | 2767.561006 | -0.0064 | 0.995 | -0.666378765 | count | 1 |
| DNMT3B | -17.616962 | 2767.561006 | -0.0064 | 0.995 | -0.666378765 | count | 1 |
| AC005786.3 | -17.616962 | 2767.561006 | -0.0064 | 0.995 | -0.666378765 | count | 1 |
| PODNL1 | -17.616962 | 2767.561006 | -0.0064 | 0.995 | -0.666378765 | count | 1 |
| IGLON5 | -17.616962 | 2767.561006 | -0.0064 | 0.995 | -0.666378765 | count | 1 |
| AURKC | -17.616962 | 2767.561006 | -0.0064 | 0.995 | -0.666378765 | count | 1 |
| GSTT2B | -17.616962 | 2767.561006 | -0.0064 | 0.995 | -0.666378765 | count | 1 |
| AL008719.1 | -17.616962 | 2767.561006 | -0.0064 | 0.995 | -0.666378765 | count | 1 |
| CARD10 | -17.616962 | 2767.561006 | -0.0064 | 0.995 | -0.666378765 | count | 1 |
| ENTHD1 | -17.616962 | 2767.561006 | -0.0064 | 0.995 | -0.666378765 | count | 1 |
| AL359643.3 | -17.615786 | 2059.98771 | -0.0086 | 0.993 | -0.666378765 | count | 1 |
| MMP7 | -17.615786 | 2059.98771 | -0.0086 | 0.993 | -0.666378765 | count | 1 |
| AC069503.2 | -17.615786 | 2059.98771 | -0.0086 | 0.993 | -0.666378765 | count | 1 |

| | | | | | | | |
|---|---|---|---|---|---|---|---|
| CCL7 | -17.615786 | 2059.98771 | -0.0086 | 0.993 | -0.666378765 | count | 1 |
| C1orf194 | -17.610329 | 2383.803337 | -0.0074 | 0.994 | -0.666378765 | count | 1 |
| DGKG | -17.610329 | 2383.803337 | -0.0074 | 0.994 | -0.666378765 | count | 1 |
| DNAAF4 | -17.610329 | 2383.803337 | -0.0074 | 0.994 | -0.666378765 | count | 1 |
| TRPM4 | -17.610329 | 2383.803337 | -0.0074 | 0.994 | -0.666378765 | count | 1 |
| COL3A1 | -17.559749 | 702.0748184 | -0.025 | 0.98 | -0.666378764 | count | 1 |
| FABP3 | -17.250298 | 2068.808989 | -0.0083 | 0.993 | -0.666378757 | count | 1 |
| PLPP3 | -17.250298 | 2068.808989 | -0.0083 | 0.993 | -0.666378757 | count | 1 |
| LYSMD1 | -17.250298 | 2068.808989 | -0.0083 | 0.993 | -0.666378757 | count | 1 |
| HAAO | -17.250298 | 2068.808989 | -0.0083 | 0.993 | -0.666378757 | count | 1 |
| LHFPL2 | -17.250298 | 2068.808989 | -0.0083 | 0.993 | -0.666378757 | count | 1 |
| ADAMDEC1 | -17.250298 | 2068.808989 | -0.0083 | 0.993 | -0.666378757 | count | 1 |
| AC134682.1 | -17.250298 | 2068.808989 | -0.0083 | 0.993 | -0.666378757 | count | 1 |
| AC123768.2 | -17.250298 | 2068.808989 | -0.0083 | 0.993 | -0.666378757 | count | 1 |
| ACTC1 | -17.250298 | 2068.808989 | -0.0083 | 0.993 | -0.666378757 | count | 1 |
| AC007608.3 | -17.250298 | 2068.808989 | -0.0083 | 0.993 | -0.666378757 | count | 1 |
| AP001267.3 | -16.964511 | 1456.579625 | -0.0116 | 0.991 | -0.666378748 | count | 1 |
| RAB31 | -18.1555158 | 1300.185101 | -0.014 | 0.989 | -0.666378613 | count | 1 |
| EMCN | -18.076246 | 1689.861381 | -0.0107 | 0.991 | -0.666378612 | count | 1 |
| CCDC149 | -18.0618239 | 2826.077675 | -0.0064 | 0.995 | -0.666378612 | count | 1 |
| ROR2 | -18.0618239 | 2826.077675 | -0.0064 | 0.995 | -0.666378612 | count | 1 |
| WDR31 | -18.0618239 | 2826.077675 | -0.0064 | 0.995 | -0.666378612 | count | 1 |
| RHOJ | -18.0618239 | 2826.077675 | -0.0064 | 0.995 | -0.666378612 | count | 1 |
| AC090515.2 | -18.0618239 | 2826.077675 | -0.0064 | 0.995 | -0.666378612 | count | 1 |
| PTGES3L | -18.0618239 | 2826.077675 | -0.0064 | 0.995 | -0.666378612 | count | 1 |
| PLA2G4C | -18.0618239 | 2826.077675 | -0.0064 | 0.995 | -0.666378612 | count | 1 |
| ASGR1 | -18.0611922 | 2047.198923 | -0.0088 | 0.993 | -0.666378612 | count | 1 |
| UNC5CL | -17.8543291 | 2254.609924 | -0.0079 | 0.994 | -0.666378609 | count | 1 |
| SIGLEC10 | -17.8537168 | 2080.684509 | -0.0086 | 0.993 | -0.666378609 | count | 1 |
| KRT81 | -17.7846263 | 1579.103653 | -0.0113 | 0.991 | -0.666378608 | count | 1 |
| FRZB | -17.7756241 | 1237.182684 | -0.0144 | 0.989 | -0.666378608 | count | 1 |
| SULF1 | -17.6209989 | 1131.81353 | -0.0156 | 0.988 | -0.666378605 | count | 1 |
| AL139424.1 | -17.5999307 | 2334.626781 | -0.0075 | 0.994 | -0.666378605 | count | 1 |
| BARX1 | -17.5999307 | 2334.626781 | -0.0075 | 0.994 | -0.666378605 | count | 1 |
| PAPPA | -17.5999307 | 2334.626781 | -0.0075 | 0.994 | -0.666378605 | count | 1 |
| BMP2 | -17.5993748 | 2079.232889 | -0.0085 | 0.993 | -0.666378605 | count | 1 |
| RIN2 | -17.5981871 | 1535.113962 | -0.0115 | 0.991 | -0.666378605 | count | 1 |
| PKIG | -17.4890988 | 1221.108192 | -0.0143 | 0.989 | -0.666378603 | count | 1 |
| CSRP2 | -17.4849185 | 1211.849064 | -0.0144 | 0.988 | -0.666378603 | count | 1 |
| SFTPB | -17.3671627 | 1938.831036 | -0.009 | 0.993 | -0.6663786 | count | 1 |
| NKPD1 | -17.3668473 | 1642.55546 | -0.0106 | 0.992 | -0.6663786 | count | 1 |
| FPR3 | -17.3666115 | 1472.253801 | -0.0118 | 0.991 | -0.6663786 | count | 1 |
| LINC01562 | -17.3248165 | 1916.868141 | -0.009 | 0.993 | -0.666378599 | count | 1 |
| CFAP70 | -17.2129586 | 1187.16684 | -0.0145 | 0.988 | -0.666378596 | count | 1 |
| BHLHB9 | -17.1747133 | 1614.228741 | -0.0106 | 0.992 | -0.666378595 | count | 1 |

| | | | | | | | |
|---|---|---|---|---|---|---|---|
| MXRA8 | -16.9889389 | 1162.390107 | -0.0146 | 0.988 | -0.666378589 | count | 1 |
| AC009088.1 | -16.9888772 | 1286.369931 | -0.0132 | 0.989 | -0.666378589 | count | 1 |
| DGCR6 | -0.7455783 | 0.6132658 | -1.2158 | 0.224 | -0.666254492 | count | 1 |
| TMEM86B | -1.9847611 | 0.9144967 | -2.1703 | 0.0301 | -0.665535586 | count | 1 |
| PGLYRP1 | -1.099222 | 1.1707174 | -0.9389 | 0.348 | -0.663687165 | count | 1 |
| AL450326.1 | -1.476972 | 0.6598022 | -2.2385 | 0.0253 | -0.661837907 | count | 1 |
| AC098850.3 | -1.474385 | 1.1919432 | -1.237 | 0.216 | -0.661116192 | count | 1 |
| PACSIN1 | -1.474385 | 1.3411262 | -1.0994 | 0.272 | -0.661116192 | count | 1 |
| ZNF235 | -0.826294 | 0.5244502 | -1.5755 | 0.115 | -0.660839951 | count | 1 |
| SLC30A4 | -0.8258548 | 0.5702399 | -1.4483 | 0.148 | -0.660537597 | count | 1 |
| RBBP9 | -0.6846257 | 0.3419092 | -2.0024 | 0.0453 | -0.660470021 | count | 1 |
| AC114490.3 | -0.9935544 | 0.7212316 | -1.3776 | 0.168 | -0.660113593 | count | 1 |
| LMNB1 | -0.5370483 | 0.1797745 | -2.9873 | 0.00283 | -0.65985577 | count | 1 |
| SPTY2D1 | -0.4800958 | 0.1094779 | -4.3853 | 1.19E-05 | -0.658568519 | count | 0.2859451 |
| SNAI3 | -0.5530596 | 0.1881397 | -2.9396 | 0.00331 | -0.658337388 | count | 1 |
| PLXNA1 | -1.4633626 | 0.9666983 | -1.5138 | 0.13 | -0.658026616 | count | 1 |
| RABL3 | -0.8216194 | 0.3160223 | -2.5999 | 0.00937 | -0.657618912 | count | 1 |
| CCHCR1 | -0.6672765 | 0.4627067 | -1.4421 | 0.149 | -0.65706559 | count | 1 |
| HSD3B7 | -0.8617174 | 0.6863965 | -1.2554 | 0.209 | -0.656045407 | count | 1 |
| PHF23 | -0.479283 | 0.0989603 | -4.8432 | 1.33E-06 | -0.655674024 | count | 0.03205167 |
| N4BP3 | -0.7112405 | 0.434267 | -1.6378 | 0.102 | -0.655019912 | count | 1 |
| ZNF426 | -0.5965217 | 0.2987447 | -1.9968 | 0.0459 | -0.654771032 | count | 1 |
| TNFAIP8L1 | -0.6931889 | 0.2976482 | -2.3289 | 0.0199 | -0.654557555 | count | 1 |
| MIATNB | -0.6081648 | 0.2706991 | -2.2466 | 0.0247 | -0.653857849 | count | 1 |
| NRP2 | -1.907113 | 1.2593508 | -1.5144 | 0.13 | -0.65307878 | count | 1 |
| ARHGAP44 | -1.907113 | 1.3951417 | -1.367 | 0.172 | -0.65307878 | count | 1 |
| LINC02361 | -0.5509694 | 0.2898916 | -1.9006 | 0.0574 | -0.652865536 | count | 1 |
| ZC3HAV1 | -0.4689813 | 0.072344 | -6.4827 | 1.03E-10 | -0.651700158 | count | 2.49E-06 |
| KIF1B | -0.6380184 | 0.2678562 | -2.3819 | 0.0173 | -0.650796415 | count | 1 |
| ZDHHC20 | -0.4961344 | 0.1633411 | -3.0374 | 0.0024 | -0.649928285 | count | 1 |
| RNF170 | -0.5606803 | 0.2820036 | -1.9882 | 0.0469 | -0.649886608 | count | 1 |
| ACOT2 | -1.8870617 | 0.9212246 | -2.0484 | 0.0406 | -0.649728543 | count | 1 |
| AC036176.1 | -0.9733759 | 0.5759509 | -1.69 | 0.0911 | -0.649372007 | count | 1 |
| SPSB2 | -0.5338361 | 0.2657756 | -2.0086 | 0.0447 | -0.649332406 | count | 1 |
| TMEM199 | -0.5501003 | 0.2393927 | -2.2979 | 0.0216 | -0.648666973 | count | 1 |
| LPIN2 | -0.4651237 | 0.0980392 | -4.7443 | 2.18E-06 | -0.648393104 | count | 0.05250312 |
| PHACTR1 | -0.8496944 | 0.6824128 | -1.2451 | 0.213 | -0.648278926 | count | 1 |
| ATG2A | -0.5438662 | 0.1550511 | -3.5077 | 0.000458 | -0.647779819 | count | 1 |
| AC016831.5 | -0.5547708 | 0.1304873 | -4.2515 | 2.18E-05 | -0.647033112 | count | 0.523309 |
| AL359198.1 | -1.2008322 | 1.0191162 | -1.1783 | 0.239 | -0.646962672 | count | 1 |
| THAP8 | -0.9676028 | 0.4709141 | -2.0547 | 0.04 | -0.646277244 | count | 1 |
| TAF1 | -0.6435542 | 0.2587243 | -2.4874 | 0.0129 | -0.646140301 | count | 1 |
| KDSR | -0.4663554 | 0.1128476 | -4.1326 | 3.67E-05 | -0.646064892 | count | 0.8801027 |
| APOBEC3G | -0.4506986 | 0.0635761 | -7.0891 | 1.63E-12 | -0.645717231 | count | 3.95E-08 |
| CCDC6 | -0.5307667 | 0.1425381 | -3.7237 | 2.00E-04 | -0.645713863 | count | 1 |

| | | | | | | | |
|---|---|---|---|---|---|---|---|
| ZC3H12A | -0.4723923 | 0.1121894 | -4.2107 | 2.61E-05 | -0.64513505 | count | 0.6262434 |
| RELL1 | -0.5717629 | 0.2694418 | -2.122 | 0.0339 | -0.644738956 | count | 1 |
| ABCB9 | -1.8523456 | 1.0940491 | -1.6931 | 0.0905 | -0.643794783 | count | 1 |
| NUP88 | -0.5623924 | 0.2105753 | -2.6707 | 0.0076 | -0.643658121 | count | 1 |
| GATD3B | -0.8943414 | 0.7566743 | -1.1819 | 0.237 | -0.643495798 | count | 1 |
| UTP18 | -0.4952072 | 0.1385253 | -3.5749 | 0.000355 | -0.642915885 | count | 1 |
| TRIM27 | -0.4971497 | 0.1614829 | -3.0787 | 0.0021 | -0.642557479 | count | 1 |
| GCHFR | -0.4545887 | 0.1004265 | -4.5266 | 6.20E-06 | -0.642049999 | count | 0.1491162 |
| PANO1 | -1.4043568 | 0.6050062 | -2.3212 | 0.0203 | -0.641057739 | count | 1 |
| SNX30 | -0.8385366 | 0.3572546 | -2.3472 | 0.019 | -0.641032675 | count | 1 |
| CACYBP | -0.4524497 | 0.0730192 | -6.1963 | 6.47E-10 | -0.64089302 | count | 1.57E-05 |
| CKAP2 | -0.469366 | 0.1183958 | -3.9644 | 7.51E-05 | -0.640792672 | count | 1 |
| OSGIN2 | -0.562471 | 0.2952009 | -1.9054 | 0.0568 | -0.639358479 | count | 1 |
| IL6 | -1.397954 | 1.1821509 | -1.1826 | 0.237 | -0.63917238 | count | 1 |
| PANK3 | -0.5440149 | 0.2538787 | -2.1428 | 0.0322 | -0.638441575 | count | 1 |
| B3GNT10 | -1.3942755 | 1.4882041 | -0.9369 | 0.349 | -0.638085473 | count | 1 |
| FASTKD3 | -0.688863 | 0.3260848 | -2.1125 | 0.0347 | -0.636331645 | count | 1 |
| FBXO45 | -0.5518554 | 0.2047903 | -2.6947 | 0.00708 | -0.636186309 | count | 1 |
| SERPINB8 | -0.6201304 | 0.241617 | -2.5666 | 0.0103 | -0.63378265 | count | 1 |
| AC097534.2 | -0.6024963 | 0.2625254 | -2.295 | 0.0218 | -0.63354997 | count | 1 |
| TMEM14C | -0.4503016 | 0.098203 | -4.5854 | 4.69E-06 | -0.633437333 | count | 0.11283202 |
| ZFYVE9 | -1.1658408 | 0.7144718 | -1.6318 | 0.103 | -0.633289441 | count | 1 |
| CENPN | -0.7030159 | 0.3074086 | -2.2869 | 0.0223 | -0.632033289 | count | 1 |
| RPUSD4 | -0.5557004 | 0.2757734 | -2.0151 | 0.044 | -0.631997133 | count | 1 |
| CRIP2 | -0.5297934 | 0.2610971 | -2.0291 | 0.0425 | -0.631588763 | count | 1 |
| IRF4 | -0.555276 | 0.1744788 | -3.1825 | 0.00147 | -0.631535439 | count | 1 |
| GPAT4 | -0.7840222 | 0.3377046 | -2.3216 | 0.0203 | -0.63147432 | count | 1 |
| 4-Sep | -0.6383193 | 0.6618859 | -0.9644 | 0.335 | -0.630703275 | count | 1 |
| EMP1 | -0.5179905 | 0.3384645 | -1.5304 | 0.126 | -0.630634832 | count | 1 |
| CLEC4A | -1.7779715 | 1.2474064 | -1.4253 | 0.154 | -0.630497667 | count | 1 |
| AEN | -0.5103696 | 0.1575931 | -3.2385 | 0.00121 | -0.629955643 | count | 1 |
| ACSL4 | -0.6160416 | 0.2093493 | -2.9426 | 0.00328 | -0.629882044 | count | 1 |
| FKBP4 | -0.4817129 | 0.1438993 | -3.3476 | 0.000824 | -0.629828276 | count | 1 |
| FIG4 | -0.6805809 | 0.3533627 | -1.926 | 0.0542 | -0.629378705 | count | 1 |
| PLIN2 | -0.4460144 | 0.0820048 | -5.4389 | 5.74E-08 | -0.629293588 | count | 0.001386497 |
| WBP1L | -0.541295 | 0.3001065 | -1.8037 | 0.0714 | -0.628298388 | count | 1 |
| EXOC3-AS1 | -0.6135494 | 0.3881535 | -1.5807 | 0.114 | -0.627502433 | count | 1 |
| MCUR1 | -0.4726802 | 0.1552512 | -3.0446 | 0.00235 | -0.627315302 | count | 1 |
| NCK1-DT | -0.5282447 | 0.258734 | -2.0417 | 0.0413 | -0.626873682 | count | 1 |
| MAPK13 | -0.4730286 | 0.17164 | -2.7559 | 0.00588 | -0.626632095 | count | 1 |
| SDHAF4 | -0.5746468 | 0.3313528 | -1.7342 | 0.083 | -0.626060119 | count | 1 |
| TK2 | -0.5941542 | 0.3282206 | -1.8102 | 0.0703 | -0.625300163 | count | 1 |
| AC019205.1 | -0.928894 | 0.4681663 | -1.9841 | 0.0473 | -0.62527874 | count | 1 |
| YWHAH | -0.4442286 | 0.0836161 | -5.3127 | 1.15E-07 | -0.625030582 | count | 0.0027761 |
| DERA | -0.5194137 | 0.2546076 | -2.0401 | 0.0414 | -0.625017351 | count | 1 |

| | | | | | | | |
|---|---|---|---|---|---|---|---|
| SLC6A6 | -0.5380823 | 0.2077747 | -2.5897 | 0.00965 | -0.624713366 | count | 1 |
| CHAC2 | -0.644292 | 0.3541927 | -1.819 | 0.069 | -0.624651887 | count | 1 |
| TMEM158 | -0.9262165 | 0.775334 | -1.1946 | 0.232 | -0.623810317 | count | 1 |
| IL10 | -0.4862984 | 0.4635079 | -1.0492 | 0.294 | -0.623667139 | count | 1 |
| SIRT5 | -0.6305354 | 0.3468935 | -1.8177 | 0.0692 | -0.623578357 | count | 1 |
| AC006504.5 | -0.6729089 | 0.3431202 | -1.9611 | 0.0499 | -0.6229206 | count | 1 |
| UGCG | -0.4690191 | 0.1328632 | -3.5301 | 0.000421 | -0.622522995 | count | 1 |
| ATG9A | -1.0107157 | 0.693638 | -1.4571 | 0.145 | -0.622118067 | count | 1 |
| MFAP4 | -1.730076 | 1.3086919 | -1.322 | 0.186 | -0.621498398 | count | 1 |
| ZNF575 | -0.9205799 | 0.6616542 | -1.3913 | 0.164 | -0.620712268 | count | 1 |
| DEDD | -0.5134164 | 0.2303017 | -2.2293 | 0.0259 | -0.620524828 | count | 1 |
| HK1 | -0.4766579 | 0.1607017 | -2.9661 | 0.00304 | -0.6201086 | count | 1 |
| ASB3 | -0.7665735 | 0.4684872 | -1.6363 | 0.102 | -0.619197473 | count | 1 |
| MCOLN2 | -0.5434875 | 0.238697 | -2.2769 | 0.0229 | -0.618694272 | count | 1 |
| PTGIS | -0.8531664 | 0.8154537 | -1.0462 | 0.296 | -0.618625565 | count | 1 |
| CTSB | -0.4446317 | 0.1176872 | -3.7781 | 0.000161 | -0.618602131 | count | 1 |
| TOPORS | -0.4550278 | 0.0991182 | -4.5908 | 4.58E-06 | -0.618532148 | count | 0.1101948 |
| CFH | -0.5564581 | 0.2568384 | -2.1666 | 0.0303 | -0.61834199 | count | 1 |
| SFXN3 | -0.5510191 | 0.2142238 | -2.5722 | 0.0101 | -0.617617061 | count | 1 |
| GNA15 | -0.503356 | 0.2488261 | -2.0229 | 0.0432 | -0.617583647 | count | 1 |
| COLGALT2 | -0.7997386 | 0.4968668 | -1.6096 | 0.108 | -0.615547057 | count | 1 |
| PDCD1 | -0.4519371 | 0.1206981 | -3.7444 | 0.000184 | -0.614920478 | count | 1 |
| RPP25L | -0.5632628 | 0.2306328 | -2.4422 | 0.0146 | -0.614285965 | count | 1 |
| LTC4S | -0.4758381 | 0.2073403 | -2.295 | 0.0218 | -0.613591114 | count | 1 |
| MGLL | -0.907064 | 0.6349592 | -1.4285 | 0.153 | -0.613246262 | count | 1 |
| MESP1 | -1.311106 | 1.4538817 | -0.9018 | 0.367 | -0.612733678 | count | 1 |
| GCFC2 | -0.4915897 | 0.2189521 | -2.2452 | 0.0248 | -0.612640737 | count | 1 |
| STX6 | -0.4819843 | 0.1908008 | -2.5261 | 0.0116 | -0.612399606 | count | 1 |
| DUSP7 | -0.53358 | 0.2494415 | -2.1391 | 0.0325 | -0.612016395 | count | 1 |
| HTT | -0.7561856 | 0.2336019 | -3.2371 | 0.00122 | -0.611845718 | count | 1 |
| PCNX3 | -0.6996026 | 0.3340508 | -2.0943 | 0.0363 | -0.611723172 | count | 1 |
| KIF5B | -0.433323 | 0.0778435 | -5.5666 | 2.80E-08 | -0.610358644 | count | 0.000676676 |
| ZNF382 | -0.9862268 | 0.4042023 | -2.4399 | 0.0147 | -0.610236019 | count | 1 |
| EXOSC10 | -0.5084771 | 0.1950341 | -2.6071 | 0.00917 | -0.609685076 | count | 1 |
| SLC7A6OS | -0.4717265 | 0.199457 | -2.3651 | 0.0181 | -0.609348383 | count | 1 |
| MID1IP1 | -0.4495499 | 0.1561373 | -2.8792 | 0.00401 | -0.609108558 | count | 1 |
| NBEAL1 | -0.453746 | 0.1305779 | -3.4749 | 0.000517 | -0.609035324 | count | 1 |
| PLEKHN1 | -0.8991006 | 0.5594943 | -1.607 | 0.108 | -0.608822774 | count | 1 |
| CCNG2 | -0.5515993 | 0.2132145 | -2.5871 | 0.00972 | -0.607853886 | count | 1 |
| DYNC1H1 | -0.4468465 | 0.1013158 | -4.4104 | 1.06E-05 | -0.607237492 | count | 0.2547816 |
| POGZ | -0.4844064 | 0.1872919 | -2.5864 | 0.00974 | -0.607068105 | count | 1 |
| PSMD12 | -0.4365028 | 0.0892225 | -4.8923 | 1.04E-06 | -0.606502904 | count | 0.02507024 |
| AP003108.2 | -0.8933706 | 0.6322768 | -1.4129 | 0.158 | -0.605628591 | count | 1 |
| RBM15 | -0.610874 | 0.3020864 | -2.0222 | 0.0432 | -0.605509558 | count | 1 |
| AC139795.3 | -1.097011 | 0.9611719 | -1.1413 | 0.254 | -0.6054912 | count | 1 |

| | | | | | | | |
|---|---|---|---|---|---|---|---|
| BET1L | -0.5146545 | 0.2277164 | -2.2601 | 0.0239 | -0.605193421 | count | 1 |
| CCNE2 | -0.5900027 | 0.3146671 | -1.875 | 0.0609 | -0.604941074 | count | 1 |
| SMG7 | -0.5234187 | 0.2005859 | -2.6094 | 0.00911 | -0.604664402 | count | 1 |
| COPRS | -0.5138498 | 0.247664 | -2.0748 | 0.0381 | -0.604279875 | count | 1 |
| ACOT9 | -0.4938113 | 0.2215263 | -2.2291 | 0.0259 | -0.604145422 | count | 1 |
| CLEC2B | -0.4212698 | 0.05571 | -7.5618 | 5.08E-14 | -0.603973311 | count | 1.23E-09 |
| TTN | -0.5192024 | 0.2899669 | -1.7906 | 0.0735 | -0.603604497 | count | 1 |
| TMPO | -0.4303195 | 0.0871768 | -4.9362 | 8.35E-07 | -0.603303088 | count | 0.02013185 |
| SAFB2 | -0.4501918 | 0.1109557 | -4.0574 | 5.07E-05 | -0.603074341 | count | 1 |
| TGFBI | -0.7411627 | 0.3953091 | -1.8749 | 0.0609 | -0.601157263 | count | 1 |
| IFT27 | -0.4791023 | 0.2140442 | -2.2383 | 0.0253 | -0.600572383 | count | 1 |
| LRRC8D | -0.6307342 | 0.2670916 | -2.3615 | 0.0183 | -0.60033922 | count | 1 |
| SLC39A1 | -0.4646042 | 0.15442 | -3.0087 | 0.00264 | -0.598391182 | count | 1 |
| SENP5 | -0.4566577 | 0.1673315 | -2.7291 | 0.00638 | -0.598297668 | count | 1 |
| MORC4 | -0.6137143 | 0.3254556 | -1.8857 | 0.0594 | -0.597201333 | count | 1 |
| MRPL36 | -0.4310188 | 0.1158941 | -3.7191 | 0.000203 | -0.597036319 | count | 1 |
| AMN1 | -0.6420977 | 0.3352435 | -1.9153 | 0.0555 | -0.596817487 | count | 1 |
| AL606491.1 | -0.7053384 | 0.6541975 | -1.0782 | 0.281 | -0.596806324 | count | 1 |
| CDH20 | -1.6070959 | 1.0762789 | -1.4932 | 0.135 | -0.59674663 | count | 1 |
| AL592183.1 | -0.6122221 | 0.3291321 | -1.8601 | 0.063 | -0.595855301 | count | 1 |
| NFE2L3 | -0.4922535 | 0.2285937 | -2.1534 | 0.0314 | -0.595688283 | count | 1 |
| DLAT | -0.6582899 | 0.3609421 | -1.8238 | 0.0683 | -0.595509036 | count | 1 |
| TRIM25 | -0.5182643 | 0.2775225 | -1.8675 | 0.0619 | -0.595127772 | count | 1 |
| TPP1 | -0.4525263 | 0.1795585 | -2.5202 | 0.0118 | -0.593663096 | count | 1 |
| PDGFRB | -1.249455 | 0.8040779 | -1.5539 | 0.12 | -0.592965933 | count | 1 |
| NEFH | -1.5884141 | 0.8665275 | -1.8331 | 0.0669 | -0.592771837 | count | 1 |
| TIMELESS | -1.2484621 | 0.8512061 | -1.4667 | 0.143 | -0.592640675 | count | 1 |
| MAP4K2 | -0.5195515 | 0.2099847 | -2.4742 | 0.0134 | -0.592532264 | count | 1 |
| IL13RA1 | -1.2474224 | 1.0371808 | -1.2027 | 0.229 | -0.592299829 | count | 1 |
| AC004771.1 | -1.2474224 | 1.0936401 | -1.1406 | 0.254 | -0.592299829 | count | 1 |
| CD70 | -0.4278363 | 0.1764774 | -2.4243 | 0.0154 | -0.592268521 | count | 1 |
| PDE4D | -0.4319385 | 0.1033725 | -4.1785 | 3.01E-05 | -0.592242195 | count | 0.7220388 |
| IFIT3 | -0.4671764 | 0.2879397 | -1.6225 | 0.105 | -0.591460578 | count | 1 |
| ID3 | -0.4467947 | 0.1835481 | -2.4342 | 0.015 | -0.591224319 | count | 1 |
| AKIP1 | -0.4811397 | 0.2244901 | -2.1433 | 0.0322 | -0.591035616 | count | 1 |
| TNFRSF9 | -0.5259874 | 0.1957785 | -2.6866 | 0.00725 | -0.590757731 | count | 1 |
| RBBP8 | -0.6523223 | 0.3707429 | -1.7595 | 0.0786 | -0.590592696 | count | 1 |
| PFKFB3 | -0.4654293 | 0.1458075 | -3.1921 | 0.00143 | -0.590559668 | count | 1 |
| VAT1 | -0.5211276 | 0.2747214 | -1.8969 | 0.0579 | -0.590010889 | count | 1 |
| AL031708.1 | -0.7612773 | 0.5945593 | -1.2804 | 0.2 | -0.589842431 | count | 1 |
| AL049840.1 | -0.5927805 | 0.254001 | -2.3338 | 0.0197 | -0.588791447 | count | 1 |
| SVBP | -0.4277717 | 0.1390845 | -3.0756 | 0.00212 | -0.588588212 | count | 1 |
| ARHGAP18 | -0.4236218 | 0.1071348 | -3.9541 | 7.84E-05 | -0.588379917 | count | 1 |
| PINK1 | -0.489056 | 0.199973 | -2.4456 | 0.0145 | -0.587093952 | count | 1 |
| MPZL1 | -0.6683381 | 0.4161487 | -1.606 | 0.108 | -0.587035994 | count | 1 |

| | | | | | | | |
|---|---|---|---|---|---|---|---|
| AMD1 | -0.4148021 | 0.0630049 | -6.5837 | 5.30E-11 | -0.586545249 | count | 1.28E-06 |
| ZNF467 | -0.8588302 | 0.4397092 | -1.9532 | 0.0509 | -0.586174277 | count | 1 |
| LRIG1 | -0.6136652 | 0.2480687 | -2.4738 | 0.0134 | -0.585333848 | count | 1 |
| OTUD7A | -1.5539358 | 0.90545 | -1.7162 | 0.0862 | -0.585282512 | count | 1 |
| FOXN3-AS1 | -0.9349615 | 0.6540715 | -1.4294 | 0.153 | -0.584826129 | count | 1 |
| ZXDC | -0.5997748 | 0.2815116 | -2.1306 | 0.0332 | -0.584603989 | count | 1 |
| BLVRB | -0.4396857 | 0.1661525 | -2.6463 | 0.00818 | -0.584589847 | count | 1 |
| FAM43A | -0.5601215 | 0.2414721 | -2.3196 | 0.0204 | -0.584051401 | count | 1 |
| IFT22 | -0.5078687 | 0.2947209 | -1.7232 | 0.0849 | -0.583637724 | count | 1 |
| TARS | -0.436933 | 0.149998 | -2.9129 | 0.0036 | -0.583352613 | count | 1 |
| ADGRF3 | -1.2199 | 1.2425763 | -0.9818 | 0.326 | -0.583188713 | count | 1 |
| SMAD9 | -1.2199 | 1.665085 | -0.7326 | 0.464 | -0.583188713 | count | 1 |
| TFB2M | -0.5388991 | 0.2622534 | -2.0549 | 0.04 | -0.583124241 | count | 1 |
| UBTD1 | -0.9315283 | 0.5002846 | -1.862 | 0.0627 | -0.583098483 | count | 1 |
| ANXA2 | -0.4091641 | 0.0632142 | -6.4727 | 1.10E-10 | -0.582924296 | count | 2.66E-06 |
| AC020915.1 | -1.5431734 | 1.062122 | -1.4529 | 0.146 | -0.582903392 | count | 1 |
| TMEM185A | -0.5863571 | 0.2824458 | -2.076 | 0.038 | -0.582835694 | count | 1 |
| NDUFAF4 | -0.4224606 | 0.1189475 | -3.5517 | 0.000388 | -0.582772559 | count | 1 |
| NR1D1 | -0.5384671 | 0.3568939 | -1.5088 | 0.131 | -0.582679575 | count | 1 |
| TNKS | -0.4715735 | 0.2021906 | -2.3323 | 0.0197 | -0.581448046 | count | 1 |
| GGH | -0.7929484 | 0.5721662 | -1.3859 | 0.166 | -0.581355936 | count | 1 |
| PNMA1 | -0.5056513 | 0.2130824 | -2.373 | 0.0177 | -0.581184088 | count | 1 |
| GNL1 | -0.4466224 | 0.1281522 | -3.4851 | 0.000498 | -0.580855721 | count | 1 |
| AL391069.3 | -0.7116438 | 0.7324496 | -0.9716 | 0.331 | -0.57996214 | count | 1 |
| IFNGR1 | -0.4106314 | 0.0761351 | -5.3935 | 7.38E-08 | -0.579783001 | count | 0.001782344 |
| INMT | -1.0355727 | 1.0015206 | -1.034 | 0.301 | -0.579655681 | count | 1 |
| FO704657.1 | -1.0355727 | 1.0448922 | -0.9911 | 0.322 | -0.579655681 | count | 1 |
| ARPC5L | -0.405048 | 0.0541738 | -7.4768 | 9.64E-14 | -0.579619818 | count | 2.34E-09 |
| KCNQ5 | -2.4120083 | 1.4189931 | -1.6998 | 0.0893 | -0.579329343 | count | 1 |
| SGMS1 | -0.4778796 | 0.2056622 | -2.3236 | 0.0202 | -0.578776365 | count | 1 |
| NFATC2 | -0.4663297 | 0.1599656 | -2.9152 | 0.00358 | -0.578630812 | count | 1 |
| TCAIM | -0.5922228 | 0.2772659 | -2.1359 | 0.0328 | -0.577757517 | count | 1 |
| CKLF | -0.4029496 | 0.0614149 | -6.5611 | 6.15E-11 | -0.576775078 | count | 1.49E-06 |
| AC004687.1 | -0.4262304 | 0.1237821 | -3.4434 | 0.000581 | -0.576606444 | count | 1 |
| RIN1 | -1.200224 | 0.9188882 | -1.3062 | 0.192 | -0.57657029 | count | 1 |
| GTF2I | -0.4193971 | 0.1239516 | -3.3836 | 0.000724 | -0.57536458 | count | 1 |
| MT1F | -0.4152715 | 0.1247997 | -3.3275 | 0.000886 | -0.575229136 | count | 1 |
| ATP8B2 | -0.4646908 | 0.1937731 | -2.3981 | 0.0165 | -0.574937528 | count | 1 |
| HLA-DRB1 | -0.3999532 | 0.0660147 | -6.0585 | 1.52E-09 | -0.574199324 | count | 3.68E-05 |
| BICD1 | -0.5184889 | 0.2180866 | -2.3774 | 0.0175 | -0.572983223 | count | 1 |
| PMVK | -0.4144127 | 0.1252012 | -3.31 | 0.000943 | -0.572465097 | count | 1 |
| TNFRSF10B | -0.4806028 | 0.2274534 | -2.113 | 0.0347 | -0.572076169 | count | 1 |
| LIN54 | -0.5275311 | 0.2645294 | -1.9942 | 0.0462 | -0.5714088 | count | 1 |
| AC016957.2 | -0.734083 | 0.5228917 | -1.4039 | 0.16 | -0.571405415 | count | 1 |
| HIST2H2AC | -0.4404513 | 0.14787 | -2.9786 | 0.00292 | -0.571328275 | count | 1 |

| | | | | | | | |
|---|---|---|---|---|---|---|---|
| TMEM223 | -0.4172648 | 0.1482372 | -2.8148 | 0.00491 | -0.571285805 | count | 1 |
| P2RY11 | -0.6987732 | 0.3847506 | -1.8162 | 0.0694 | -0.570641144 | count | 1 |
| ITM2A | -0.3977033 | 0.0588316 | -6.76 | 1.62E-11 | -0.569973314 | count | 3.93E-07 |
| CCDC80 | -1.48578 | 0.8367751 | -1.7756 | 0.0759 | -0.569878568 | count | 1 |
| DNAJA1 | -0.3968374 | 0.044521 | -8.9135 | 7.90E-19 | -0.569419284 | count | 1.92E-14 |
| XYLT1 | -0.6267008 | 0.433521 | -1.4456 | 0.148 | -0.569370796 | count | 1 |
| GON7 | -0.4290415 | 0.1684426 | -2.5471 | 0.0109 | -0.569093141 | count | 1 |
| BNIP1 | -0.4461041 | 0.1948621 | -2.2893 | 0.0221 | -0.568815286 | count | 1 |
| SMARCAL1 | -0.7299625 | 0.303012 | -2.409 | 0.016 | -0.568592961 | count | 1 |
| WIPI2 | -0.4204839 | 0.1226041 | -3.4296 | 0.000612 | -0.568285514 | count | 1 |
| TAF5L | -0.551958 | 0.2744133 | -2.0114 | 0.0444 | -0.568192628 | count | 1 |
| RAB20 | -0.7719134 | 0.3054075 | -2.5275 | 0.0115 | -0.56808748 | count | 1 |
| GGCT | -0.453623 | 0.1839502 | -2.466 | 0.0137 | -0.567868194 | count | 1 |
| PLAUR | -0.4260026 | 0.2096083 | -2.0324 | 0.0422 | -0.567565506 | count | 1 |
| PGRMC1 | -0.4298913 | 0.1559418 | -2.7567 | 0.00587 | -0.566866876 | count | 1 |
| ANKRD52 | -0.6421729 | 0.4457047 | -1.4408 | 0.15 | -0.566160201 | count | 1 |
| OGFRL1 | -0.495499 | 0.2378632 | -2.0831 | 0.0373 | -0.566125153 | count | 1 |
| AUTS2 | -0.5580091 | 0.2469009 | -2.2601 | 0.0239 | -0.565567889 | count | 1 |
| AP3D1 | -0.4877495 | 0.20018 | -2.4366 | 0.0149 | -0.564899183 | count | 1 |
| CELSR3 | -1.4639282 | 0.9359071 | -1.5642 | 0.118 | -0.56476772 | count | 1 |
| TIGAR | -0.4310209 | 0.1684507 | -2.5587 | 0.0105 | -0.564507781 | count | 1 |
| AC100786.1 | -1.1629002 | 0.7621154 | -1.5259 | 0.127 | -0.563773311 | count | 1 |
| LINC01678 | -0.6619117 | 0.3888812 | -1.7021 | 0.0888 | -0.563741874 | count | 1 |
| PAXIP1-AS2 | -0.4867025 | 0.2613632 | -1.8622 | 0.0627 | -0.563728225 | count | 1 |
| NUSAP1 | -0.576529 | 0.3117861 | -1.8491 | 0.0645 | -0.56348167 | count | 1 |
| H2AFZ | -0.3924619 | 0.0413313 | -9.4955 | 4.00E-21 | -0.563446318 | count | 9.72E-17 |
| WDR25 | -0.4965495 | 0.3111365 | -1.5959 | 0.111 | -0.563252746 | count | 1 |
| CPE | -1.4571131 | 0.791254 | -1.8415 | 0.0656 | -0.56315637 | count | 1 |
| ADNP2 | -0.5553906 | 0.2648522 | -2.097 | 0.0361 | -0.563072016 | count | 1 |
| EDARADD | -0.5465139 | 0.2344866 | -2.3307 | 0.0198 | -0.56290458 | count | 1 |
| ESS2 | -0.4698712 | 0.2352137 | -1.9976 | 0.0458 | -0.562250321 | count | 1 |
| APOBEC3H | -0.4076948 | 0.1272129 | -3.2048 | 0.00136 | -0.562087272 | count | 1 |
| RNF145 | -0.4181804 | 0.1306845 | -3.1999 | 0.00139 | -0.561792879 | count | 1 |
| WNK1 | -0.4021997 | 0.0897937 | -4.4792 | 7.74E-06 | -0.561344777 | count | 0.18611604 |
| SCRG1 | -0.8155519 | 0.3929876 | -2.0753 | 0.038 | -0.561315636 | count | 1 |
| TSC22D1 | -0.4760727 | 0.2097719 | -2.2695 | 0.0233 | -0.561257777 | count | 1 |
| ZNF582-AS1 | -0.68525 | 0.5852201 | -1.1709 | 0.242 | -0.560795739 | count | 1 |
| TNFRSF11B | -2.2030219 | 1.3221944 | -1.6662 | 0.0958 | -0.560253212 | count | 1 |
| HTRA1 | -0.8135872 | 0.4439311 | -1.8327 | 0.0669 | -0.560174349 | count | 1 |
| DESI1 | -0.4224807 | 0.1617795 | -2.6115 | 0.00906 | -0.559450724 | count | 1 |
| ZFP1 | -0.5350965 | 0.2700364 | -1.9816 | 0.0476 | -0.559352641 | count | 1 |
| IDNK | -0.4304378 | 0.1962165 | -2.1937 | 0.0283 | -0.55934211 | count | 1 |
| RPP14 | -0.5611301 | 0.2976308 | -1.8853 | 0.0595 | -0.559341913 | count | 1 |
| FAM8A1 | -0.5349932 | 0.2871987 | -1.8628 | 0.0626 | -0.559250379 | count | 1 |
| SYTL2 | -0.4208574 | 0.1559718 | -2.6983 | 0.007 | -0.5588722 | count | 1 |

| | | | | | | | |
|---|---|---|---|---|---|---|---|
| MC1R | -0.987525 | 1.4216483 | -0.6946 | 0.487 | -0.558772404 | count | 1 |
| NMB | -0.5274555 | 0.2372603 | -2.2231 | 0.0263 | -0.558731424 | count | 1 |
| PTCD3 | -0.4624959 | 0.1959393 | -2.3604 | 0.0183 | -0.558413601 | count | 1 |
| LDLRAD4 | -0.4034526 | 0.1427836 | -2.8256 | 0.00475 | -0.558080063 | count | 1 |
| AKAP1 | -0.5199826 | 0.3230884 | -1.6094 | 0.108 | -0.557610129 | count | 1 |
| GMEB2 | -0.5958213 | 0.3348625 | -1.7793 | 0.0753 | -0.55711702 | count | 1 |
| AP002852.1 | -1.1432704 | 0.867784 | -1.3175 | 0.188 | -0.556914671 | count | 1 |
| PHLDA3 | -0.4746471 | 0.2895362 | -1.6393 | 0.101 | -0.556645717 | count | 1 |
| ARL17A | -0.8796143 | 0.4404413 | -1.9971 | 0.0459 | -0.556577371 | count | 1 |
| S100A11 | -0.3866316 | 0.0433005 | -8.929 | 6.89E-19 | -0.556314971 | count | 1.67E-14 |
| SLBP | -0.3962464 | 0.0799324 | -4.9573 | 7.50E-07 | -0.55628401 | count | 0.018084 |
| TRIM52 | -0.4374142 | 0.1519102 | -2.8794 | 0.00401 | -0.55567971 | count | 1 |
| HDAC11 | -0.7106488 | 0.4444693 | -1.5989 | 0.11 | -0.555344577 | count | 1 |
| MFSD14A | -0.9788074 | 0.8117584 | -1.2058 | 0.228 | -0.554919209 | count | 1 |
| CHPF | -0.5011764 | 0.2720744 | -1.8421 | 0.0656 | -0.554654788 | count | 1 |
| NXF1 | -0.5162923 | 0.2423701 | -2.1302 | 0.0332 | -0.553840565 | count | 1 |
| VPS39 | -0.5367927 | 0.3320503 | -1.6166 | 0.106 | -0.553443928 | count | 1 |
| GPN3 | -0.4121569 | 0.1606752 | -2.5652 | 0.0104 | -0.552266239 | count | 1 |
| AURKA | -0.972708 | 0.4786055 | -2.0324 | 0.0422 | -0.552211495 | count | 1 |
| IQCE | -0.6058685 | 0.3511804 | -1.7252 | 0.0846 | -0.551980712 | count | 1 |
| MDGA1 | -0.7050037 | 0.788155 | -0.8945 | 0.371 | -0.551451813 | count | 1 |
| BARD1 | -0.4424944 | 0.2175986 | -2.0335 | 0.0421 | -0.551265957 | count | 1 |
| UQCRHL | -0.7981293 | 0.5598818 | -1.4255 | 0.154 | -0.55115696 | count | 1 |
| BCL6 | -0.5024092 | 0.1831223 | -2.7436 | 0.00611 | -0.550848619 | count | 1 |
| KRT18 | -0.5193938 | 0.3042909 | -1.7069 | 0.0879 | -0.550613666 | count | 1 |
| DGUOK-AS1 | -1.1245133 | 0.9350157 | -1.2027 | 0.229 | -0.550277715 | count | 1 |
| CRYBG3 | -0.703268 | 0.6223892 | -1.1299 | 0.259 | -0.550253055 | count | 1 |
| STRBP | -0.428709 | 0.2025665 | -2.1164 | 0.0344 | -0.55009754 | count | 1 |
| HMG20A | -0.4924379 | 0.2854277 | -1.7253 | 0.0846 | -0.550096051 | count | 1 |
| RBPJ | -0.3958539 | 0.0936096 | -4.2288 | 2.41E-05 | -0.550013301 | count | 0.5783759 |
| MYH10 | -2.1018885 | 0.8203315 | -2.5622 | 0.0104 | -0.54966744 | count | 1 |
| TSPAN10 | -0.7947761 | 1.0604463 | -0.7495 | 0.454 | -0.549191865 | count | 1 |
| PAQR4 | -0.9654044 | 0.7292754 | -1.3238 | 0.186 | -0.548956458 | count | 1 |
| DNAJB6 | -0.3860093 | 0.0593886 | -6.4997 | 9.22E-11 | -0.548928051 | count | 2.23E-06 |
| CSTA | -0.6426278 | 0.9122833 | -0.7044 | 0.481 | -0.548885669 | count | 1 |
| SRRD | -0.4765387 | 0.2192499 | -2.1735 | 0.0298 | -0.548880159 | count | 1 |
| CBX8 | -0.6421595 | 0.4699901 | -1.3663 | 0.172 | -0.548523574 | count | 1 |
| IL15 | -0.5313522 | 0.2987254 | -1.7787 | 0.0754 | -0.548139165 | count | 1 |
| FAM110C | -0.792343 | 0.7598005 | -1.0428 | 0.297 | -0.547763985 | count | 1 |
| IGLC3 | -0.4055799 | 0.1771234 | -2.2898 | 0.0221 | -0.547710247 | count | 1 |
| SAXO2 | -1.115831 | 1.1115637 | -1.0038 | 0.316 | -0.547177919 | count | 1 |
| AC002116.2 | -1.115831 | 1.3734425 | -0.8124 | 0.417 | -0.547177919 | count | 1 |
| ZNF718 | -0.503128 | 0.3758583 | -1.3386 | 0.181 | -0.546161084 | count | 1 |
| BCOR | -0.4976908 | 0.2732931 | -1.8211 | 0.0687 | -0.54589552 | count | 1 |
| PLEKHG2 | -0.6965416 | 0.4287443 | -1.6246 | 0.104 | -0.54559925 | count | 1 |

| | | | | | | | |
|---|---|---|---|---|---|---|---|
| ZNF596 | -0.6645135 | 0.7771539 | -0.8551 | 0.393 | -0.545596193 | count | 1 |
| ATP1B3 | -0.3936353 | 0.0889212 | -4.4268 | 9.87E-06 | -0.545540571 | count | 0.23725506 |
| GDF15 | -0.8582663 | 1.1958156 | -0.7177 | 0.473 | -0.545455102 | count | 1 |
| METTL15 | -0.4620081 | 0.1837826 | -2.5139 | 0.012 | -0.545173999 | count | 1 |
| B3GALT2 | -1.1081901 | 0.8582416 | -1.2912 | 0.197 | -0.54443546 | count | 1 |
| ADCK2 | -0.5013706 | 0.2199684 | -2.2793 | 0.0227 | -0.544337719 | count | 1 |
| ETAA1 | -0.4792413 | 0.2850285 | -1.6814 | 0.0928 | -0.544335438 | count | 1 |
| ZNF581 | -0.4331711 | 0.1633525 | -2.6518 | 0.00804 | -0.544157786 | count | 1 |
| MKNK2 | -0.3983267 | 0.1180836 | -3.3733 | 0.000751 | -0.544124571 | count | 1 |
| PINLYP | -2.0442712 | 1.0643706 | -1.9206 | 0.0549 | -0.543203078 | count | 1 |
| CHD4 | -0.393755 | 0.0991566 | -3.971 | 7.30E-05 | -0.542692692 | count | 1 |
| ASTN2 | -0.9503931 | 0.5984377 | -1.5881 | 0.112 | -0.542222662 | count | 1 |
| ZNF189 | -0.6594906 | 0.4181297 | -1.5772 | 0.115 | -0.541895904 | count | 1 |
| RAD51AP1 | -2.0325534 | 1.125568 | -1.8058 | 0.071 | -0.541848289 | count | 1 |
| DHRS1 | -0.4118285 | 0.1824626 | -2.2571 | 0.0241 | -0.541589945 | count | 1 |
| RAB15 | -1.1000036 | 0.8950089 | -1.229 | 0.219 | -0.541482032 | count | 1 |
| ERV3-1 | -0.4584254 | 0.303701 | -1.5095 | 0.131 | -0.541071279 | count | 1 |
| AC025181.2 | -0.5519761 | 0.5002971 | -1.1033 | 0.27 | -0.541017862 | count | 1 |
| PEX7 | -0.5518953 | 0.3128411 | -1.7641 | 0.0778 | -0.540943673 | count | 1 |
| GFPT1 | -0.4634177 | 0.1871965 | -2.4756 | 0.0134 | -0.540833573 | count | 1 |
| MRTO4 | -0.4112477 | 0.1882742 | -2.1843 | 0.029 | -0.540224112 | count | 1 |
| RNF114 | -0.4008111 | 0.1093826 | -3.6643 | 0.000252 | -0.54005223 | count | 1 |
| MIDN | -0.385626 | 0.0799499 | -4.8233 | 1.47E-06 | -0.539987337 | count | 0.03542112 |
| IFI30 | -0.7280039 | 0.4232749 | -1.7199 | 0.0855 | -0.539976871 | count | 1 |
| TRBV28 | -0.6875311 | 0.2382572 | -2.8857 | 0.00393 | -0.539344693 | count | 1 |
| C1orf109 | -0.471114 | 0.2779165 | -1.6952 | 0.0901 | -0.53923496 | count | 1 |
| MSH6 | -0.4121768 | 0.123092 | -3.3485 | 0.000821 | -0.538825373 | count | 1 |
| TRIM24 | -0.4614414 | 0.2116388 | -2.1803 | 0.0293 | -0.538599058 | count | 1 |
| CHN1 | -0.4811411 | 0.2797292 | -1.72 | 0.0855 | -0.537963054 | count | 1 |
| C4orf48 | -0.3872075 | 0.10364 | -3.7361 | 0.00019 | -0.537959885 | count | 1 |
| PTPN11 | -0.3953788 | 0.141998 | -2.7844 | 0.00539 | -0.537862607 | count | 1 |
| BDH2 | -0.4144971 | 0.1860314 | -2.2281 | 0.0259 | -0.537368787 | count | 1 |
| CUL5 | -0.4012995 | 0.1381158 | -2.9055 | 0.00369 | -0.537086764 | count | 1 |
| TRIB2 | -0.419136 | 0.1975584 | -2.1216 | 0.0339 | -0.537045085 | count | 1 |
| HIP1R | -0.5125924 | 0.4226241 | -1.2129 | 0.225 | -0.537013493 | count | 1 |
| AC243829.1 | -0.6521126 | 0.4093108 | -1.5932 | 0.111 | -0.536447542 | count | 1 |
| UPF1 | -0.5585451 | 0.2491985 | -2.2414 | 0.0251 | -0.536341754 | count | 1 |
| RIDA | -0.5272654 | 0.3383717 | -1.5582 | 0.119 | -0.536155968 | count | 1 |
| FAM84B | -0.5460323 | 0.2404698 | -2.2707 | 0.0232 | -0.535556298 | count | 1 |
| ZNF267 | -0.3899132 | 0.1068666 | -3.6486 | 0.000268 | -0.535532457 | count | 1 |
| TACO1 | -0.4270393 | 0.2439976 | -1.7502 | 0.0802 | -0.535252927 | count | 1 |
| PDE3B | -0.4508649 | 0.1466275 | -3.0749 | 0.00212 | -0.535097007 | count | 1 |
| MORC3 | -0.3938584 | 0.104392 | -3.7729 | 0.000164 | -0.535066793 | count | 1 |
| SKP2 | -1.0817909 | 0.5278987 | -2.0492 | 0.0405 | -0.534855271 | count | 1 |
| KLC1 | -0.4505435 | 0.1836871 | -2.4528 | 0.0142 | -0.534726315 | count | 1 |

| | | | | | | | |
|---|---|---|---|---|---|---|---|
| SPATA2L | -0.4222453 | 0.1709199 | -2.4704 | 0.0135 | -0.534445247 | count | 1 |
| ZNF823 | -0.7191119 | 0.4474359 | -1.6072 | 0.108 | -0.534216538 | count | 1 |
| GAK | -0.4914678 | 0.2160168 | -2.2751 | 0.023 | -0.534050338 | count | 1 |
| TMEM44-AS1 | -1.9657553 | 1.6128944 | -1.2188 | 0.223 | -0.533857075 | count | 1 |
| UFSP1 | -1.3385742 | 0.8975687 | -1.4913 | 0.136 | -0.533776411 | count | 1 |
| PSMD2 | -0.3902567 | 0.1150234 | -3.3928 | 7.00E-04 | -0.533590343 | count | 1 |
| SMC4 | -0.4134226 | 0.1447116 | -2.8569 | 0.0043 | -0.533504173 | count | 1 |
| PREPL | -0.4412232 | 0.1788314 | -2.4673 | 0.0137 | -0.533381884 | count | 1 |
| TCAF1 | -0.5434539 | 0.2793665 | -1.9453 | 0.0518 | -0.533184264 | count | 1 |
| ST6GALNAC4 | -0.4389199 | 0.2650657 | -1.6559 | 0.0978 | -0.532767304 | count | 1 |
| MYH11 | -0.9294596 | 0.3539276 | -2.6261 | 0.00867 | -0.532734103 | count | 1 |
| ITGB5 | -1.9539496 | 1.3324324 | -1.4665 | 0.143 | -0.532396319 | count | 1 |
| SLFN11 | -0.4462398 | 0.1602544 | -2.7846 | 0.00539 | -0.532302929 | count | 1 |
| HIST1H1B | -0.5822033 | 0.3885455 | -1.4984 | 0.134 | -0.532080589 | count | 1 |
| HMGA1 | -0.3823495 | 0.0929504 | -4.1135 | 3.99E-05 | -0.532006453 | count | 0.9566025 |
| RCCD1 | -1.072161 | 0.6275741 | -1.7084 | 0.0876 | -0.531319933 | count | 1 |
| TUFT1 | -0.8313342 | 0.8638233 | -0.9624 | 0.336 | -0.531243805 | count | 1 |
| TACC1 | -0.3855933 | 0.09806 | -3.9322 | 8.58E-05 | -0.531068181 | count | 1 |
| AC073896.2 | -0.5309804 | 0.3777233 | -1.4057 | 0.16 | -0.53105015 | count | 1 |
| ITGA11 | -1.942332 | 1.2972424 | -1.4973 | 0.134 | -0.530944328 | count | 1 |
| VPS13D | -0.5218066 | 0.2085409 | -2.5022 | 0.0124 | -0.530909142 | count | 1 |
| CCDC88A | -0.4334391 | 0.255904 | -1.6938 | 0.0904 | -0.530173244 | count | 1 |
| RUFY1 | -0.4384733 | 0.1869908 | -2.3449 | 0.0191 | -0.530140684 | count | 1 |
| CAMKK2 | -0.6175344 | 0.3272935 | -1.8868 | 0.0593 | -0.529395737 | count | 1 |
| PELP1 | -0.5392047 | 0.2492553 | -2.1633 | 0.0306 | -0.529271427 | count | 1 |
| ZFP28 | -0.7601546 | 0.8599849 | -0.8839 | 0.377 | -0.528715438 | count | 1 |
| KRT86 | -0.7601546 | 0.8829365 | -0.8609 | 0.389 | -0.528715438 | count | 1 |
| ZNF791 | -0.3958801 | 0.1091136 | -3.6281 | 0.00029 | -0.528702072 | count | 1 |
| HIST1H2BH | -0.8265321 | 0.4849725 | -1.7043 | 0.0884 | -0.528688839 | count | 1 |
| TRIQK | -0.8259354 | 0.6330998 | -1.3046 | 0.192 | -0.528370914 | count | 1 |
| ISG20 | -0.3688132 | 0.0541821 | -6.8069 | 1.17E-11 | -0.528004035 | count | 2.84E-07 |
| SBNO1 | -0.406471 | 0.1302912 | -3.1197 | 0.00183 | -0.527886219 | count | 1 |
| ZFYVE1 | -0.5771535 | 0.3245654 | -1.7782 | 0.0755 | -0.527814374 | count | 1 |
| LYPLAL1 | -0.4095837 | 0.18719 | -2.1881 | 0.0287 | -0.527757234 | count | 1 |
| KLHL18 | -0.5374794 | 0.2117872 | -2.5378 | 0.0112 | -0.527681383 | count | 1 |
| SLC1A5 | -0.3985892 | 0.1529867 | -2.6054 | 0.00922 | -0.527671119 | count | 1 |
| GLRX | -0.3705051 | 0.0778586 | -4.7587 | 2.03E-06 | -0.526482108 | count | 0.04889458 |
| AP001816.1 | -0.4561432 | 0.2979593 | -1.5309 | 0.126 | -0.526151081 | count | 1 |
| HSF2 | -0.4046956 | 0.1802904 | -2.2447 | 0.0249 | -0.525613036 | count | 1 |
| S1PR2 | -0.5745187 | 0.3154606 | -1.8212 | 0.0687 | -0.525585676 | count | 1 |
| MRC1 | -1.8996399 | 1.2866097 | -1.4765 | 0.14 | -0.525482808 | count | 1 |
| AC090825.1 | -1.305146 | 1.2025786 | -1.0853 | 0.278 | -0.525017208 | count | 1 |
| AC087482.1 | -1.305146 | 1.2433322 | -1.0497 | 0.294 | -0.525017208 | count | 1 |
| AL353596.1 | -1.305146 | 1.3634536 | -0.9572 | 0.339 | -0.525017208 | count | 1 |
| TSTD3 | -0.6112522 | 0.6269355 | -0.975 | 0.33 | -0.524488597 | count | 1 |

| | | | | | | | |
|---|---|---|---|---|---|---|---|
| ZNF41 | -0.5451936 | 0.3977752 | -1.3706 | 0.171 | -0.524353476 | count | 1 |
| TNFSF13B | -0.4285093 | 0.2680272 | -1.5988 | 0.11 | -0.524281052 | count | 1 |
| CLEC12A | -1.0501077 | 0.825587 | -1.272 | 0.203 | -0.523141399 | count | 1 |
| DTX2 | -0.4714732 | 0.3200188 | -1.4733 | 0.141 | -0.523059017 | count | 1 |
| CD9 | -0.3927553 | 0.2062522 | -1.9042 | 0.057 | -0.522867088 | count | 1 |
| AL136040.1 | -1.0490968 | 0.7032083 | -1.4919 | 0.136 | -0.522763774 | count | 1 |
| SPOPL | -0.4525656 | 0.2760254 | -1.6396 | 0.101 | -0.52215597 | count | 1 |
| ATG101 | -0.3750952 | 0.1036931 | -3.6174 | 0.000302 | -0.521869272 | count | 1 |
| HDGF | -0.3725931 | 0.0950163 | -3.9214 | 8.98E-05 | -0.52186763 | count | 1 |
| IGIP | -1.2921809 | 0.9759714 | -1.324 | 0.186 | -0.521562656 | count | 1 |
| FERMT2 | -1.2921809 | 0.9843383 | -1.3127 | 0.189 | -0.521562656 | count | 1 |
| ECPAS | -0.4584264 | 0.2695666 | -1.7006 | 0.0891 | -0.521506 | count | 1 |
| BCL9 | -0.8127452 | 0.5227364 | -1.5548 | 0.12 | -0.521318099 | count | 1 |
| CISD1 | -0.4017913 | 0.1723924 | -2.3307 | 0.0198 | -0.52113088 | count | 1 |
| GFM2 | -0.6068634 | 0.3592981 | -1.689 | 0.0913 | -0.521053882 | count | 1 |
| AP001453.1 | -1.8657801 | 1.2014461 | -1.5529 | 0.121 | -0.521007923 | count | 1 |
| ATG13 | -0.4433376 | 0.2550959 | -1.7379 | 0.0823 | -0.521000359 | count | 1 |
| ADIRF | -0.5300564 | 0.3115243 | -1.7015 | 0.0889 | -0.52083162 | count | 1 |
| HLA-DQB1 | -0.3862591 | 0.1070922 | -3.6068 | 0.000314 | -0.520619221 | count | 1 |
| UBL3 | -0.3880146 | 0.1229752 | -3.1552 | 0.00162 | -0.52059295 | count | 1 |
| LYSMD3 | -0.4025893 | 0.1649514 | -2.4407 | 0.0147 | -0.520559998 | count | 1 |
| ARIH2 | -0.3792944 | 0.1224343 | -3.0979 | 0.00196 | -0.520536001 | count | 1 |
| CMBL | -1.287861 | 0.8225107 | -1.5658 | 0.117 | -0.5204044 | count | 1 |
| NETO2 | -0.5853028 | 0.412077 | -1.4204 | 0.156 | -0.520121907 | count | 1 |
| NCAPG | -1.0412017 | 0.9507756 | -1.0951 | 0.274 | -0.519806025 | count | 1 |
| FRMD4A | -1.2854226 | 0.7699431 | -1.6695 | 0.0951 | -0.519749005 | count | 1 |
| MSMP | -0.9009514 | 1.340905 | -0.6719 | 0.502 | -0.519627914 | count | 1 |
| POM121 | -0.4726544 | 0.2037351 | -2.3199 | 0.0204 | -0.51953304 | count | 1 |
| LRRIQ3 | -0.8079219 | 0.6277039 | -1.2871 | 0.198 | -0.518727069 | count | 1 |
| ANKZF1 | -0.4146734 | 0.1511566 | -2.7433 | 0.00611 | -0.518694655 | count | 1 |
| ZBTB4 | -0.4412324 | 0.20492 | -2.1532 | 0.0314 | -0.518597289 | count | 1 |
| SDHA | -0.3825507 | 0.1198438 | -3.1921 | 0.00143 | -0.518548037 | count | 1 |
| BCL2L1 | -0.4019335 | 0.2003423 | -2.0062 | 0.0449 | -0.518047397 | count | 1 |
| MAP7D3 | -0.3993341 | 0.1797879 | -2.2211 | 0.0264 | -0.517988592 | count | 1 |
| TBRG1 | -0.3756448 | 0.1162864 | -3.2303 | 0.00125 | -0.517877976 | count | 1 |
| CIITA | -1.0354854 | 0.6784275 | -1.5263 | 0.127 | -0.517655337 | count | 1 |
| GMDS-DT | -0.5373524 | 0.2924994 | -1.8371 | 0.0663 | -0.517291221 | count | 1 |
| LTBP3 | -0.408659 | 0.1832472 | -2.2301 | 0.0258 | -0.516383441 | count | 1 |
| MED25 | -0.4302122 | 0.2656945 | -1.6192 | 0.105 | -0.516018335 | count | 1 |
| CDKN1C | -0.3881873 | 0.1689666 | -2.2974 | 0.0217 | -0.515955882 | count | 1 |
| YIF1A | -0.3736504 | 0.1404007 | -2.6613 | 0.00782 | -0.515557453 | count | 1 |
| ZNF101 | -0.3967664 | 0.1364436 | -2.9079 | 0.00366 | -0.515456562 | count | 1 |
| NOP9 | -0.8912775 | 0.5191263 | -1.7169 | 0.0861 | -0.515132144 | count | 1 |
| COL1A2 | -1.820602 | 0.6522814 | -2.7911 | 0.00528 | -0.514834285 | count | 1 |
| CHEK1 | -0.5989232 | 0.4216734 | -1.4203 | 0.156 | -0.514826072 | count | 1 |

| | | | | | | | |
|---|---|---|---|---|---|---|---|
| AC127521.1 | -0.504799 | 0.33643 | -1.5005 | 0.134 | -0.514515202 | count | 1 |
| SIX5 | -0.6517397 | 1.0050612 | -0.6485 | 0.517 | -0.514270305 | count | 1 |
| MPP7 | -0.4517701 | 0.2294647 | -1.9688 | 0.0491 | -0.514187417 | count | 1 |
| SUSD3 | -0.3716913 | 0.0986351 | -3.7683 | 0.000167 | -0.514141904 | count | 1 |
| VAMP1 | -0.5336643 | 0.4170286 | -1.2797 | 0.201 | -0.513963971 | count | 1 |
| PKD1 | -0.6218344 | 0.3226625 | -1.9272 | 0.054 | -0.513925972 | count | 1 |
| MIOS | -0.4513873 | 0.2512452 | -1.7966 | 0.0725 | -0.513766263 | count | 1 |
| ELAC2 | -0.4546561 | 0.2508448 | -1.8125 | 0.07 | -0.513498753 | count | 1 |
| EMB | -0.3618772 | 0.063006 | -5.7435 | 1.01E-08 | -0.513493568 | count | 0.000244218 |
| TP53BP2 | -0.3952271 | 0.1240215 | -3.1868 | 0.00145 | -0.513484109 | count | 1 |
| NSD2 | -0.4474971 | 0.2320689 | -1.9283 | 0.0539 | -0.51308089 | count | 1 |
| CASZ1 | -1.022764 | 0.4005099 | -2.5537 | 0.0107 | -0.512841257 | count | 1 |
| AL136295.2 | -1.805836 | 1.2591132 | -1.4342 | 0.152 | -0.512764962 | count | 1 |
| OIP5 | -1.805836 | 1.5499094 | -1.1651 | 0.244 | -0.512764962 | count | 1 |
| ZNF324B | -0.7961743 | 0.5952403 | -1.3376 | 0.181 | -0.512389585 | count | 1 |
| CXorf21 | -0.8847485 | 0.5292915 | -1.6716 | 0.0947 | -0.512084067 | count | 1 |
| EREG | -0.8844015 | 0.9325827 | -0.9483 | 0.343 | -0.511921732 | count | 1 |
| IPO8 | -0.4433596 | 0.2565491 | -1.7282 | 0.084 | -0.511864502 | count | 1 |
| NUP58 | -0.4285641 | 0.2043267 | -2.0974 | 0.036 | -0.511765553 | count | 1 |
| AC137630.1 | -1.2550916 | 0.8087515 | -1.5519 | 0.121 | -0.511500436 | count | 1 |
| CREB3 | -0.39043 | 0.1420477 | -2.7486 | 0.00602 | -0.511393991 | count | 1 |
| MEMO1 | -0.5576088 | 0.4396205 | -1.2684 | 0.205 | -0.51123732 | count | 1 |
| UXS1 | -0.3857822 | 0.1507657 | -2.5588 | 0.0105 | -0.510903681 | count | 1 |
| OAS2 | -0.3905196 | 0.1857405 | -2.1025 | 0.0356 | -0.510874716 | count | 1 |
| DNAJC13 | -0.4454479 | 0.2667096 | -1.6702 | 0.095 | -0.510806477 | count | 1 |
| KMT2D | -0.5934013 | 0.3200509 | -1.8541 | 0.0638 | -0.510484682 | count | 1 |
| KLF16 | -0.3948519 | 0.1238944 | -3.187 | 0.00145 | -0.509886831 | count | 1 |
| TOX2 | -0.4997334 | 0.264784 | -1.8873 | 0.0592 | -0.509618766 | count | 1 |
| ALOX12-AS1 | -0.6158398 | 0.3783483 | -1.6277 | 0.104 | -0.509436272 | count | 1 |
| ZNF57 | -0.5920096 | 0.5378075 | -1.1008 | 0.271 | -0.509389169 | count | 1 |
| MFGE8 | -0.7274581 | 0.3755689 | -1.9369 | 0.0528 | -0.509065463 | count | 1 |
| CEP76 | -0.4843138 | 0.3219521 | -1.5043 | 0.133 | -0.508772942 | count | 1 |
| RBM8A | -0.355368 | 0.0437618 | -8.1205 | 6.43E-16 | -0.508745601 | count | 1.56E-11 |
| CASP7 | -0.4302644 | 0.1972798 | -2.181 | 0.0293 | -0.508743943 | count | 1 |
| TBCE | -1.0094897 | 0.7035661 | -1.4348 | 0.151 | -0.507776816 | count | 1 |
| LAYN | -0.8753208 | 0.6210181 | -1.4095 | 0.159 | -0.507662973 | count | 1 |
| SLFN5 | -0.3627689 | 0.0749218 | -4.842 | 1.34E-06 | -0.507024877 | count | 0.03229132 |
| LMOD3 | -0.5256582 | 0.3715999 | -1.4146 | 0.157 | -0.506729099 | count | 1 |
| ATF7 | -0.4081962 | 0.2027157 | -2.0136 | 0.0441 | -0.506462526 | count | 1 |
| TMEM99 | -0.406766 | 0.2486299 | -1.636 | 0.102 | -0.506196345 | count | 1 |
| SNX7 | -1.0038031 | 0.9611891 | -1.0443 | 0.296 | -0.505594465 | count | 1 |
| RAB33A | -0.3771302 | 0.1863557 | -2.0237 | 0.0431 | -0.505411135 | count | 1 |
| CARMIL1 | -0.5673267 | 0.3924845 | -1.4455 | 0.148 | -0.505383247 | count | 1 |
| SEC23B | -0.4026909 | 0.2203926 | -1.8272 | 0.0678 | -0.505295745 | count | 1 |
| MXD4 | -0.3594851 | 0.0881534 | -4.0779 | 4.65E-05 | -0.505133725 | count | 1 |

| | | | | | | | |
|---|---|---|---|---|---|---|---|
| MRPL1 | -0.3783148 | 0.1128933 | -3.3511 | 0.000814 | -0.505075198 | count | 1 |
| UBE2K | -0.3673023 | 0.1015923 | -3.6155 | 0.000304 | -0.504824634 | count | 1 |
| GNG11 | -1.2303827 | 0.5295983 | -2.3232 | 0.0202 | -0.504647941 | count | 1 |
| FTSJ3 | -0.42858 | 0.1956369 | -2.1907 | 0.0285 | -0.504138153 | count | 1 |
| XAF1 | -0.364766 | 0.1233825 | -2.9564 | 0.00313 | -0.503881377 | count | 1 |
| SAMD9L | -0.3623386 | 0.1200271 | -3.0188 | 0.00256 | -0.503568794 | count | 1 |
| CLASP1 | -0.4787115 | 0.3036837 | -1.5763 | 0.115 | -0.503156124 | count | 1 |
| TRIM41 | -0.4528422 | 0.3073694 | -1.4733 | 0.141 | -0.503146258 | count | 1 |
| DENND6B | -0.5014647 | 0.323045 | -1.5523 | 0.121 | -0.503132342 | count | 1 |
| DMKN | -0.5213452 | 0.4757243 | -1.0959 | 0.273 | -0.502824724 | count | 1 |
| FARP1 | -0.7771435 | 0.5141333 | -1.5116 | 0.131 | -0.502042365 | count | 1 |
| ADA | -0.385174 | 0.1774992 | -2.17 | 0.0301 | -0.501996437 | count | 1 |
| TRBV19 | -1.22076 | 0.885499 | -1.3786 | 0.168 | -0.501946619 | count | 1 |
| ATP6V0B | -0.3521172 | 0.0661956 | -5.3193 | 1.11E-07 | -0.501554639 | count | 0.002679762 |
| INTS6-AS1 | -0.4142441 | 0.3149001 | -1.3155 | 0.188 | -0.50153037 | count | 1 |
| EOGT | -1.7249414 | 0.7073734 | -2.4385 | 0.0148 | -0.500965145 | count | 1 |
| CEP104 | -0.4024489 | 0.2017391 | -1.9949 | 0.0461 | -0.500926892 | count | 1 |
| AGO2 | -0.3980841 | 0.1528528 | -2.6044 | 0.00924 | -0.500869759 | count | 1 |
| POP1 | -0.4831227 | 0.3209937 | -1.5051 | 0.132 | -0.500801352 | count | 1 |
| PCSK7 | -0.3803059 | 0.1065778 | -3.5683 | 0.000364 | -0.500057597 | count | 1 |
| PTPN22 | -0.360812 | 0.0924592 | -3.9024 | 9.71E-05 | -0.49995498 | count | 1 |
| ACOX3 | -0.8588954 | 0.4125957 | -2.0817 | 0.0374 | -0.499904726 | count | 1 |
| AREL1 | -0.5177811 | 0.4186562 | -1.2368 | 0.216 | -0.499594697 | count | 1 |
| PARP16 | -0.4537244 | 0.318166 | -1.4261 | 0.154 | -0.499511399 | count | 1 |
| ECSCR | -1.211219 | 0.7209651 | -1.68 | 0.093 | -0.499250361 | count | 1 |
| AC004951.1 | -0.771942 | 0.5147224 | -1.4997 | 0.134 | -0.499196822 | count | 1 |
| IQCH | -1.210713 | 1.2389059 | -0.9772 | 0.329 | -0.499107031 | count | 1 |
| AL031775.1 | -1.210713 | 1.2786351 | -0.9469 | 0.344 | -0.499107031 | count | 1 |
| SLC14A2 | -1.210713 | 1.408388 | -0.8596 | 0.39 | -0.499107031 | count | 1 |
| CKS1B | -0.4285235 | 0.2014461 | -2.1272 | 0.0335 | -0.49833631 | count | 1 |
| PLEKHM2 | -0.3898581 | 0.1857945 | -2.0983 | 0.0359 | -0.498228822 | count | 1 |
| GALC | -0.4737973 | 0.2775218 | -1.7072 | 0.0879 | -0.498223251 | count | 1 |
| EIF2AK2 | -0.3590441 | 0.118115 | -3.0398 | 0.00239 | -0.497834504 | count | 1 |
| SMC3 | -0.3544275 | 0.0838928 | -4.2248 | 2.45E-05 | -0.497324002 | count | 0.587951 |
| GZF1 | -0.3993979 | 0.2286607 | -1.7467 | 0.0808 | -0.497201283 | count | 1 |
| PPP6R2 | -0.3976712 | 0.1790334 | -2.2212 | 0.0264 | -0.496478692 | count | 1 |
| COQ10A | -0.3987501 | 0.185426 | -2.1505 | 0.0316 | -0.496410087 | count | 1 |
| SMARCD2 | -0.4553923 | 0.1956483 | -2.3276 | 0.02 | -0.496392171 | count | 1 |
| NIN | -0.3728872 | 0.1273845 | -2.9273 | 0.00344 | -0.496257219 | count | 1 |
| ACAD10 | -0.9792175 | 0.5357459 | -1.8278 | 0.0677 | -0.496070196 | count | 1 |
| PIM3 | -0.3482592 | 0.0647382 | -5.3795 | 7.97E-08 | -0.495936095 | count | 0.001924596 |
| MSRB2 | -0.3862302 | 0.2112268 | -1.8285 | 0.0676 | -0.495513539 | count | 1 |
| LMO7 | -0.4207295 | 0.2414929 | -1.7422 | 0.0816 | -0.495152461 | count | 1 |
| XPA | -0.3569488 | 0.1157051 | -3.085 | 0.00205 | -0.495046 | count | 1 |
| PLBD2 | -0.6243232 | 0.4531713 | -1.3777 | 0.168 | -0.494816377 | count | 1 |

| | | | | | | | |
|---|---|---|---|---|---|---|---|
| SMIM25 | -0.512467 | 0.7788971 | -0.6579 | 0.511 | -0.494772666 | count | 1 |
| SEPHS2 | -0.3607259 | 0.1144895 | -3.1507 | 0.00164 | -0.494370263 | count | 1 |
| DDX58 | -0.3918153 | 0.1665456 | -2.3526 | 0.0187 | -0.494307484 | count | 1 |
| ZNHIT2 | -0.388768 | 0.2387685 | -1.6282 | 0.104 | -0.493841457 | count | 1 |
| IFI16 | -0.3474678 | 0.0576939 | -6.0226 | 1.90E-09 | -0.493760317 | count | 4.60E-05 |
| GTPBP1 | -0.500852 | 0.2236506 | -2.2394 | 0.0252 | -0.49374707 | count | 1 |
| COL1A1 | -1.6771751 | 1.3291602 | -1.2618 | 0.207 | -0.493617743 | count | 1 |
| SAT1 | -0.3461061 | 0.0612168 | -5.6538 | 1.70E-08 | -0.49354211 | count | 0.000410975 |
| N4BP1 | -0.3554681 | 0.0923898 | -3.8475 | 0.000122 | -0.493401519 | count | 1 |
| ZNF419 | -0.5106095 | 0.3924368 | -1.3011 | 0.193 | -0.493085458 | count | 1 |
| TBC1D2 | -0.7004292 | 0.5598293 | -1.2511 | 0.211 | -0.492593906 | count | 1 |
| RFX2 | -0.8423404 | 0.8261341 | -1.0196 | 0.308 | -0.492013664 | count | 1 |
| METTL23 | -0.3510099 | 0.0953679 | -3.6806 | 0.000236 | -0.491911094 | count | 1 |
| AL078639.1 | -1.6656325 | 1.0370777 | -1.6061 | 0.108 | -0.491798693 | count | 1 |
| MACO1 | -0.3887159 | 0.1615115 | -2.4067 | 0.0161 | -0.491603284 | count | 1 |
| PDP1 | -0.4198263 | 0.1896969 | -2.2131 | 0.027 | -0.491381411 | count | 1 |
| PBLD | -0.9665415 | 1.0186289 | -0.9489 | 0.343 | -0.491103045 | count | 1 |
| USP36 | -0.3891089 | 0.12909 | -3.0142 | 0.0026 | -0.490950572 | count | 1 |
| HSP90AA1 | -0.3415216 | 0.0375771 | -9.0886 | 1.67E-19 | -0.490727283 | count | 4.06E-15 |
| PHRF1 | -0.4661795 | 0.2266375 | -2.0569 | 0.0398 | -0.490565573 | count | 1 |
| DNPH1 | -0.3578853 | 0.1054451 | -3.394 | 0.000697 | -0.49050115 | count | 1 |
| NUP98 | -0.3917089 | 0.1366949 | -2.8656 | 0.00419 | -0.490486618 | count | 1 |
| STMN1 | -0.3484869 | 0.0834554 | -4.1757 | 3.04E-05 | -0.49007786 | count | 0.7292048 |
| DCAF11 | -0.3792492 | 0.1905705 | -1.9901 | 0.0467 | -0.490014648 | count | 1 |
| ATP6V1B2 | -0.444769 | 0.2056727 | -2.1625 | 0.0306 | -0.490013262 | count | 1 |
| CAPRIN1 | -0.3780438 | 0.1450923 | -2.6055 | 0.00921 | -0.490003271 | count | 1 |
| UBTF | -0.3521622 | 0.1020022 | -3.4525 | 0.000562 | -0.489213433 | count | 1 |
| FAM110A | -0.3594386 | 0.1582131 | -2.2719 | 0.0232 | -0.489095389 | count | 1 |
| MFSD5 | -0.3949358 | 0.3004053 | -1.3147 | 0.189 | -0.488844461 | count | 1 |
| FCN1 | -0.6158796 | 0.8229391 | -0.7484 | 0.454 | -0.488782293 | count | 1 |
| NAA50 | -0.3573391 | 0.1082983 | -3.2996 | 0.000978 | -0.488687674 | count | 1 |
| TVP23C | -0.5651334 | 0.4981334 | -1.1345 | 0.257 | -0.4881277 | count | 1 |
| CSNK1D | -0.3683413 | 0.114699 | -3.2114 | 0.00133 | -0.487572887 | count | 1 |
| AC008124.1 | -0.4058449 | 0.2626572 | -1.5452 | 0.122 | -0.487483122 | count | 1 |
| LYRM9 | -0.4269646 | 0.2221933 | -1.9216 | 0.0547 | -0.486837994 | count | 1 |
| UBR5 | -0.3958428 | 0.1631083 | -2.4269 | 0.0153 | -0.486806844 | count | 1 |
| FAM160B1 | -0.3864077 | 0.1987528 | -1.9442 | 0.052 | -0.486428389 | count | 1 |
| VIM | -0.3372707 | 0.0301851 | -11.1734 | 1.72E-28 | -0.486278218 | count | 4.18E-24 |
| RTL10 | -0.4927739 | 0.4228202 | -1.1654 | 0.244 | -0.486217546 | count | 1 |
| STIP1 | -0.3602845 | 0.126942 | -2.8382 | 0.00456 | -0.48589422 | count | 1 |
| ORAI2 | -0.3726829 | 0.1669327 | -2.2325 | 0.0256 | -0.485251623 | count | 1 |
| RAB4B | -0.7461244 | 0.668428 | -1.1162 | 0.264 | -0.484963584 | count | 1 |
| CAB39 | -0.3595802 | 0.1317662 | -2.7289 | 0.00639 | -0.484951985 | count | 1 |
| FAM161A | -0.6438054 | 0.4286121 | -1.5021 | 0.133 | -0.484527276 | count | 1 |
| TMEM102 | -0.38784 | 0.2186183 | -1.7741 | 0.0761 | -0.484425212 | count | 1 |

| | | | | | | | |
|---|---|---|---|---|---|---|---|
| CTSZ | -0.3526883 | 0.1289798 | -2.7344 | 0.00628 | -0.484237561 | count | 1 |
| AC017002.3 | -0.4391186 | 0.6224414 | -0.7055 | 0.481 | -0.484011828 | count | 1 |
| GNRH1 | -1.6169995 | 1.3044366 | -1.2396 | 0.215 | -0.483943794 | count | 1 |
| EIF2B4 | -0.3768131 | 0.1654333 | -2.2777 | 0.0228 | -0.483604898 | count | 1 |
| SNCG | -1.1563862 | 0.7166069 | -1.6137 | 0.107 | -0.483401384 | count | 1 |
| ZNF225 | -0.946999 | 0.7501396 | -1.2624 | 0.207 | -0.483369623 | count | 1 |
| FLAD1 | -0.3647238 | 0.1597437 | -2.2832 | 0.0225 | -0.483301755 | count | 1 |
| DZIP3 | -0.3772375 | 0.1843943 | -2.0458 | 0.0409 | -0.483253475 | count | 1 |
| PELO | -0.3559829 | 0.1369993 | -2.5984 | 0.00941 | -0.483044141 | count | 1 |
| COL8A1 | -1.6081377 | 1.3936713 | -1.1539 | 0.249 | -0.482478847 | count | 1 |
| MFAP1 | -0.3496099 | 0.1087907 | -3.2136 | 0.00132 | -0.482305512 | count | 1 |
| NAA38 | -0.3422012 | 0.0971901 | -3.5209 | 0.000436 | -0.481860685 | count | 1 |
| ALG9 | -0.7397613 | 0.4966137 | -1.4896 | 0.136 | -0.481427488 | count | 1 |
| CAMK2N2 | -0.682203 | 0.7852833 | -0.8687 | 0.385 | -0.481371044 | count | 1 |
| FURIN | -0.6388945 | 0.3339968 | -1.9129 | 0.0558 | -0.481231167 | count | 1 |
| DAPK3 | -0.4131023 | 0.2592806 | -1.5933 | 0.111 | -0.480898694 | count | 1 |
| GRK5 | -0.4563951 | 0.2635627 | -1.7316 | 0.0834 | -0.480710571 | count | 1 |
| AC022390.1 | -1.1463146 | 0.5666058 | -2.0231 | 0.0431 | -0.480424356 | count | 1 |
| POLH | -0.5768401 | 0.3946513 | -1.4616 | 0.144 | -0.479981406 | count | 1 |
| AGO1 | -0.5548916 | 0.3313333 | -1.6747 | 0.0941 | -0.479973249 | count | 1 |
| INTS2 | -0.4553672 | 0.3404037 | -1.3377 | 0.181 | -0.479674002 | count | 1 |
| NDUFC2 | -0.3370622 | 0.0753143 | -4.4754 | 7.88E-06 | -0.479673462 | count | 0.1894746 |
| LSM11 | -0.602892 | 0.6192058 | -0.9737 | 0.33 | -0.479461828 | count | 1 |
| STIM1 | -0.417131 | 0.212616 | -1.9619 | 0.0499 | -0.479296001 | count | 1 |
| GAS2L1 | -0.7352259 | 0.6632727 | -1.1085 | 0.268 | -0.478900307 | count | 1 |
| GMNN | -0.3605191 | 0.1306871 | -2.7586 | 0.00584 | -0.478680842 | count | 1 |
| ZNF699 | -0.4298353 | 0.277243 | -1.5504 | 0.121 | -0.478457919 | count | 1 |
| PORCN | -0.467414 | 0.3390157 | -1.3787 | 0.168 | -0.478233708 | count | 1 |
| RB1CC1 | -0.3661838 | 0.1197353 | -3.0583 | 0.00224 | -0.478183772 | count | 1 |
| UBXN2B | -0.5188922 | 0.3067 | -1.6919 | 0.0908 | -0.478097229 | count | 1 |
| NDUFB10 | -0.3351632 | 0.0561394 | -5.9702 | 2.61E-09 | -0.4780348 | count | 6.31E-05 |
| SNX9 | -0.4189364 | 0.178331 | -2.3492 | 0.0189 | -0.477961152 | count | 1 |
| MRPS16 | -0.3480356 | 0.1278809 | -2.7216 | 0.00653 | -0.477570579 | count | 1 |
| SERPINH1 | -0.7323947 | 0.3991234 | -1.835 | 0.0666 | -0.477319898 | count | 1 |
| CARMIL2 | -0.4180107 | 0.2637901 | -1.5846 | 0.113 | -0.476936813 | count | 1 |
| MTRNR2L8 | -0.3815645 | 0.1524539 | -2.5028 | 0.0124 | -0.476724398 | count | 1 |
| KIF4A | -18.290806 | 2172.949896 | -0.0084 | 0.993 | -0.476526428 | count | 1 |
| ATP5MGL | -17.774184 | 1172.703042 | -0.0152 | 0.988 | -0.476526424 | count | 1 |
| CA6 | -17.455882 | 2135.394602 | -0.0082 | 0.993 | -0.47652642 | count | 1 |
| LINC01765 | -17.455882 | 2135.394602 | -0.0082 | 0.993 | -0.47652642 | count | 1 |
| ECM1 | -17.455882 | 2135.394602 | -0.0082 | 0.993 | -0.47652642 | count | 1 |
| CD1C | -17.455882 | 2135.394602 | -0.0082 | 0.993 | -0.47652642 | count | 1 |
| RHEX | -17.455882 | 2135.394602 | -0.0082 | 0.993 | -0.47652642 | count | 1 |
| 2-Mar | -17.455882 | 2135.394602 | -0.0082 | 0.993 | -0.47652642 | count | 1 |
| AC012442.1 | -17.455882 | 2135.394602 | -0.0082 | 0.993 | -0.47652642 | count | 1 |

| | | | | | | | |
|---|---|---|---|---|---|---|---|
| TMEM37 | -17.455882 | 2135.394602 | -0.0082 | 0.993 | -0.47652642 | count | 1 |
| LHFPL4 | -17.455882 | 2135.394602 | -0.0082 | 0.993 | -0.47652642 | count | 1 |
| AC135507.1 | -17.455882 | 2135.394602 | -0.0082 | 0.993 | -0.47652642 | count | 1 |
| AC097634.1 | -17.455882 | 2135.394602 | -0.0082 | 0.993 | -0.47652642 | count | 1 |
| STBD1 | -17.455882 | 2135.394602 | -0.0082 | 0.993 | -0.47652642 | count | 1 |
| MAPK10 | -17.455882 | 2135.394602 | -0.0082 | 0.993 | -0.47652642 | count | 1 |
| RTL8B | -17.455882 | 2135.394602 | -0.0082 | 0.993 | -0.47652642 | count | 1 |
| SAXO1 | -17.455882 | 2135.394602 | -0.0082 | 0.993 | -0.47652642 | count | 1 |
| PARVA | -17.455882 | 2135.394602 | -0.0082 | 0.993 | -0.47652642 | count | 1 |
| WNT11 | -17.455882 | 2135.394602 | -0.0082 | 0.993 | -0.47652642 | count | 1 |
| NDN | -17.455882 | 2135.394602 | -0.0082 | 0.993 | -0.47652642 | count | 1 |
| SPTLC3 | -17.455882 | 2135.394602 | -0.0082 | 0.993 | -0.47652642 | count | 1 |
| SMC1B | -17.455882 | 2135.394602 | -0.0082 | 0.993 | -0.47652642 | count | 1 |
| C1orf167 | -17.208987 | 2256.870203 | -0.0076 | 0.994 | -0.476526416 | count | 1 |
| EPHA2 | -17.208987 | 2256.870203 | -0.0076 | 0.994 | -0.476526416 | count | 1 |
| SPOCD1 | -17.208987 | 2256.870203 | -0.0076 | 0.994 | -0.476526416 | count | 1 |
| TMEM35B | -17.208987 | 2256.870203 | -0.0076 | 0.994 | -0.476526416 | count | 1 |
| RAD54L | -17.208987 | 2256.870203 | -0.0076 | 0.994 | -0.476526416 | count | 1 |
| AC105942.1 | -17.208987 | 2256.870203 | -0.0076 | 0.994 | -0.476526416 | count | 1 |
| AL356441.1 | -17.208987 | 2256.870203 | -0.0076 | 0.994 | -0.476526416 | count | 1 |
| HLX | -17.208987 | 2256.870203 | -0.0076 | 0.994 | -0.476526416 | count | 1 |
| AC098828.2 | -17.208987 | 2256.870203 | -0.0076 | 0.994 | -0.476526416 | count | 1 |
| PFN4 | -17.208987 | 2256.870203 | -0.0076 | 0.994 | -0.476526416 | count | 1 |
| AC104695.2 | -17.208987 | 2256.870203 | -0.0076 | 0.994 | -0.476526416 | count | 1 |
| AC017083.2 | -17.208987 | 2256.870203 | -0.0076 | 0.994 | -0.476526416 | count | 1 |
| LIMS3 | -17.208987 | 2256.870203 | -0.0076 | 0.994 | -0.476526416 | count | 1 |
| AC012447.1 | -17.208987 | 2256.870203 | -0.0076 | 0.994 | -0.476526416 | count | 1 |
| CCDC148 | -17.208987 | 2256.870203 | -0.0076 | 0.994 | -0.476526416 | count | 1 |
| AC011997.1 | -17.208987 | 2256.870203 | -0.0076 | 0.994 | -0.476526416 | count | 1 |
| LINC01792 | -17.208987 | 2256.870203 | -0.0076 | 0.994 | -0.476526416 | count | 1 |
| MLPH | -17.208987 | 2256.870203 | -0.0076 | 0.994 | -0.476526416 | count | 1 |
| LINC01990 | -17.208987 | 2256.870203 | -0.0076 | 0.994 | -0.476526416 | count | 1 |
| NR1I2 | -17.208987 | 2256.870203 | -0.0076 | 0.994 | -0.476526416 | count | 1 |
| TMCC1-AS1 | -17.208987 | 2256.870203 | -0.0076 | 0.994 | -0.476526416 | count | 1 |
| MRAS | -17.208987 | 2256.870203 | -0.0076 | 0.994 | -0.476526416 | count | 1 |
| PLOD2 | -17.208987 | 2256.870203 | -0.0076 | 0.994 | -0.476526416 | count | 1 |
| AC147067.2 | -17.208987 | 2256.870203 | -0.0076 | 0.994 | -0.476526416 | count | 1 |
| AC069307.1 | -17.208987 | 2256.870203 | -0.0076 | 0.994 | -0.476526416 | count | 1 |
| BTC | -17.208987 | 2256.870203 | -0.0076 | 0.994 | -0.476526416 | count | 1 |
| HHIP-AS1 | -17.208987 | 2256.870203 | -0.0076 | 0.994 | -0.476526416 | count | 1 |
| AC025176.1 | -17.208987 | 2256.870203 | -0.0076 | 0.994 | -0.476526416 | count | 1 |
| AC005618.1 | -17.208987 | 2256.870203 | -0.0076 | 0.994 | -0.476526416 | count | 1 |
| AC011337.1 | -17.208987 | 2256.870203 | -0.0076 | 0.994 | -0.476526416 | count | 1 |
| WWC1 | -17.208987 | 2256.870203 | -0.0076 | 0.994 | -0.476526416 | count | 1 |
| LINC01962 | -17.208987 | 2256.870203 | -0.0076 | 0.994 | -0.476526416 | count | 1 |

| | | | | | | | |
|---|---|---|---|---|---|---|---|
| HLA-DQB1-AS1 | -17.208987 | 2256.870203 | -0.0076 | 0.994 | -0.476526416 | count | 1 |
| TREML1 | -17.208987 | 2256.870203 | -0.0076 | 0.994 | -0.476526416 | count | 1 |
| AC002480.2 | -17.208987 | 2256.870203 | -0.0076 | 0.994 | -0.476526416 | count | 1 |
| AC073115.2 | -17.208987 | 2256.870203 | -0.0076 | 0.994 | -0.476526416 | count | 1 |
| AC073349.1 | -17.208987 | 2256.870203 | -0.0076 | 0.994 | -0.476526416 | count | 1 |
| TRBV10-3 | -17.208987 | 2256.870203 | -0.0076 | 0.994 | -0.476526416 | count | 1 |
| CRYGN | -17.208987 | 2256.870203 | -0.0076 | 0.994 | -0.476526416 | count | 1 |
| BMX | -17.208987 | 2256.870203 | -0.0076 | 0.994 | -0.476526416 | count | 1 |
| COL4A5 | -17.208987 | 2256.870203 | -0.0076 | 0.994 | -0.476526416 | count | 1 |
| KLHL13 | -17.208987 | 2256.870203 | -0.0076 | 0.994 | -0.476526416 | count | 1 |
| HTRA4 | -17.208987 | 2256.870203 | -0.0076 | 0.994 | -0.476526416 | count | 1 |
| AC090579.1 | -17.208987 | 2256.870203 | -0.0076 | 0.994 | -0.476526416 | count | 1 |
| LY6E-DT | -17.208987 | 2256.870203 | -0.0076 | 0.994 | -0.476526416 | count | 1 |
| AC067930.2 | -17.208987 | 2256.870203 | -0.0076 | 0.994 | -0.476526416 | count | 1 |
| AL157884.2 | -17.208987 | 2256.870203 | -0.0076 | 0.994 | -0.476526416 | count | 1 |
| PRSS3 | -17.208987 | 2256.870203 | -0.0076 | 0.994 | -0.476526416 | count | 1 |
| TAL2 | -17.208987 | 2256.870203 | -0.0076 | 0.994 | -0.476526416 | count | 1 |
| PRRX2 | -17.208987 | 2256.870203 | -0.0076 | 0.994 | -0.476526416 | count | 1 |
| FIBIN | -17.208987 | 2256.870203 | -0.0076 | 0.994 | -0.476526416 | count | 1 |
| DEPDC7 | -17.208987 | 2256.870203 | -0.0076 | 0.994 | -0.476526416 | count | 1 |
| SLC37A2 | -17.208987 | 2256.870203 | -0.0076 | 0.994 | -0.476526416 | count | 1 |
| CYP17A1-AS1 | -17.208987 | 2256.870203 | -0.0076 | 0.994 | -0.476526416 | count | 1 |
| DDX11-AS1 | -17.208987 | 2256.870203 | -0.0076 | 0.994 | -0.476526416 | count | 1 |
| AC008083.3 | -17.208987 | 2256.870203 | -0.0076 | 0.994 | -0.476526416 | count | 1 |
| ADCY6 | -17.208987 | 2256.870203 | -0.0076 | 0.994 | -0.476526416 | count | 1 |
| ASIC1 | -17.208987 | 2256.870203 | -0.0076 | 0.994 | -0.476526416 | count | 1 |
| TRPV4 | -17.208987 | 2256.870203 | -0.0076 | 0.994 | -0.476526416 | count | 1 |
| TRDV2 | -17.208987 | 2256.870203 | -0.0076 | 0.994 | -0.476526416 | count | 1 |
| SLC7A8 | -17.208987 | 2256.870203 | -0.0076 | 0.994 | -0.476526416 | count | 1 |
| AL157912.1 | -17.208987 | 2256.870203 | -0.0076 | 0.994 | -0.476526416 | count | 1 |
| JMJD7-PLA2G4B | -17.208987 | 2256.870203 | -0.0076 | 0.994 | -0.476526416 | count | 1 |
| C2CD4A | -17.208987 | 2256.870203 | -0.0076 | 0.994 | -0.476526416 | count | 1 |
| AC055855.1 | -17.208987 | 2256.870203 | -0.0076 | 0.994 | -0.476526416 | count | 1 |
| AC090260.1 | -17.208987 | 2256.870203 | -0.0076 | 0.994 | -0.476526416 | count | 1 |
| CMTM4 | -17.208987 | 2256.870203 | -0.0076 | 0.994 | -0.476526416 | count | 1 |
| DYNLRB2 | -17.208987 | 2256.870203 | -0.0076 | 0.994 | -0.476526416 | count | 1 |
| FGF11 | -17.208987 | 2256.870203 | -0.0076 | 0.994 | -0.476526416 | count | 1 |
| RASL10B | -17.208987 | 2256.870203 | -0.0076 | 0.994 | -0.476526416 | count | 1 |
| AC006441.1 | -17.208987 | 2256.870203 | -0.0076 | 0.994 | -0.476526416 | count | 1 |
| GRB7 | -17.208987 | 2256.870203 | -0.0076 | 0.994 | -0.476526416 | count | 1 |
| ZPBP2 | -17.208987 | 2256.870203 | -0.0076 | 0.994 | -0.476526416 | count | 1 |
| HCRT | -17.208987 | 2256.870203 | -0.0076 | 0.994 | -0.476526416 | count | 1 |
| LRRC37A | -17.208987 | 2256.870203 | -0.0076 | 0.994 | -0.476526416 | count | 1 |
| ITGB3 | -17.208987 | 2256.870203 | -0.0076 | 0.994 | -0.476526416 | count | 1 |
| GH1 | -17.208987 | 2256.870203 | -0.0076 | 0.994 | -0.476526416 | count | 1 |

| | | | | | | | |
|---|---|---|---|---|---|---|---|
| ST6GALNAC2 | -17.208987 | 2256.870203 | -0.0076 | 0.994 | -0.476526416 | count | 1 |
| PPP4R1-AS1 | -17.208987 | 2256.870203 | -0.0076 | 0.994 | -0.476526416 | count | 1 |
| KCNK15-AS1 | -17.208987 | 2256.870203 | -0.0076 | 0.994 | -0.476526416 | count | 1 |
| AZU1 | -17.208987 | 2256.870203 | -0.0076 | 0.994 | -0.476526416 | count | 1 |
| GRIN3B | -17.208987 | 2256.870203 | -0.0076 | 0.994 | -0.476526416 | count | 1 |
| AC123912.4 | -17.208987 | 2256.870203 | -0.0076 | 0.994 | -0.476526416 | count | 1 |
| FBXO17 | -17.208987 | 2256.870203 | -0.0076 | 0.994 | -0.476526416 | count | 1 |
| ZNF534 | -17.208987 | 2256.870203 | -0.0076 | 0.994 | -0.476526416 | count | 1 |
| LILRA6 | -17.208987 | 2256.870203 | -0.0076 | 0.994 | -0.476526416 | count | 1 |
| AC245884.10 | -17.208987 | 2256.870203 | -0.0076 | 0.994 | -0.476526416 | count | 1 |
| IGLV1-47 | -17.208987 | 2256.870203 | -0.0076 | 0.994 | -0.476526416 | count | 1 |
| BAIAP2L2 | -17.208987 | 2256.870203 | -0.0076 | 0.994 | -0.476526416 | count | 1 |
| CU638689.5 | -17.208987 | 2256.870203 | -0.0076 | 0.994 | -0.476526416 | count | 1 |
| KCNE1 | -17.208987 | 2256.870203 | -0.0076 | 0.994 | -0.476526416 | count | 1 |
| AC008972.2 | -17.2081 | 1593.575898 | -0.0108 | 0.991 | -0.476526416 | count | 1 |
| HIST1H3C | -18.2899845 | 2999.432559 | -0.0061 | 0.995 | -0.476526266 | count | 1 |
| SLC26A4-AS1 | -18.2896011 | 2470.647166 | -0.0074 | 0.994 | -0.476526266 | count | 1 |
| UNC5B-AS1 | -18.2896011 | 2470.647166 | -0.0074 | 0.994 | -0.476526266 | count | 1 |
| AC018529.2 | -18.2896011 | 2470.647166 | -0.0074 | 0.994 | -0.476526266 | count | 1 |
| KCNE4 | -18.0342021 | 1685.739 | -0.0107 | 0.991 | -0.476526264 | count | 1 |
| ELAVL4 | -18.033758 | 2968.103815 | -0.0061 | 0.995 | -0.476526264 | count | 1 |
| GREB1 | -18.033758 | 2968.103815 | -0.0061 | 0.995 | -0.476526264 | count | 1 |
| RAB6C | -18.033758 | 2968.103815 | -0.0061 | 0.995 | -0.476526264 | count | 1 |
| TANC1 | -18.033758 | 2968.103815 | -0.0061 | 0.995 | -0.476526264 | count | 1 |
| CYP27A1 | -18.033758 | 2968.103815 | -0.0061 | 0.995 | -0.476526264 | count | 1 |
| CADM2 | -18.033758 | 2968.103815 | -0.0061 | 0.995 | -0.476526264 | count | 1 |
| SLIT2 | -18.033758 | 2968.103815 | -0.0061 | 0.995 | -0.476526264 | count | 1 |
| AC026412.3 | -18.033758 | 2968.103815 | -0.0061 | 0.995 | -0.476526264 | count | 1 |
| HEY2 | -18.033758 | 2968.103815 | -0.0061 | 0.995 | -0.476526264 | count | 1 |
| RPS6KA2 | -18.033758 | 2968.103815 | -0.0061 | 0.995 | -0.476526264 | count | 1 |
| AC022893.1 | -18.033758 | 2968.103815 | -0.0061 | 0.995 | -0.476526264 | count | 1 |
| PLEKHA7 | -18.033758 | 2968.103815 | -0.0061 | 0.995 | -0.476526264 | count | 1 |
| AL355075.3 | -18.033758 | 2968.103815 | -0.0061 | 0.995 | -0.476526264 | count | 1 |
| AC005696.4 | -18.033758 | 2968.103815 | -0.0061 | 0.995 | -0.476526264 | count | 1 |
| SOST | -18.033758 | 2968.103815 | -0.0061 | 0.995 | -0.476526264 | count | 1 |
| DTNA | -18.033758 | 2968.103815 | -0.0061 | 0.995 | -0.476526264 | count | 1 |
| AF038458.2 | -18.033758 | 2968.103815 | -0.0061 | 0.995 | -0.476526264 | count | 1 |
| HMGN3-AS1 | -18.0331952 | 2431.701348 | -0.0074 | 0.994 | -0.476526264 | count | 1 |
| LINC01762 | -17.9604619 | 2608.394193 | -0.0069 | 0.995 | -0.476526264 | count | 1 |
| TRPC3 | -17.9604619 | 2608.394193 | -0.0069 | 0.995 | -0.476526264 | count | 1 |
| RUNDC3B | -17.9604619 | 2608.394193 | -0.0069 | 0.995 | -0.476526264 | count | 1 |
| SMIM10L2A | -17.9604619 | 2608.394193 | -0.0069 | 0.995 | -0.476526264 | count | 1 |
| AC103957.2 | -17.9604619 | 2608.394193 | -0.0069 | 0.995 | -0.476526264 | count | 1 |
| ACTN3 | -17.9604619 | 2608.394193 | -0.0069 | 0.995 | -0.476526264 | count | 1 |
| MMP12 | -17.9604619 | 2608.394193 | -0.0069 | 0.995 | -0.476526264 | count | 1 |

| | | | | | | | |
|---|---|---|---|---|---|---|---|
| SDSL | -17.9604619 | 2608.394193 | -0.0069 | 0.995 | -0.476526264 | count | 1 |
| AJUBA | -17.9604619 | 2608.394193 | -0.0069 | 0.995 | -0.476526264 | count | 1 |
| LINC02274 | -17.9604619 | 2608.394193 | -0.0069 | 0.995 | -0.476526264 | count | 1 |
| SNX22 | -17.9604619 | 2608.394193 | -0.0069 | 0.995 | -0.476526264 | count | 1 |
| HOXB6 | -17.9604619 | 2608.394193 | -0.0069 | 0.995 | -0.476526264 | count | 1 |
| MRC2 | -17.9604619 | 2608.394193 | -0.0069 | 0.995 | -0.476526264 | count | 1 |
| MYO5B | -17.9604619 | 2608.394193 | -0.0069 | 0.995 | -0.476526264 | count | 1 |
| LRRC25 | -17.9604619 | 2608.394193 | -0.0069 | 0.995 | -0.476526264 | count | 1 |
| HSPB6 | -17.9604619 | 2608.394193 | -0.0069 | 0.995 | -0.476526264 | count | 1 |
| AC096887.1 | -17.9602304 | 2154.952354 | -0.0083 | 0.993 | -0.476526264 | count | 1 |
| RSPH4A | -17.9602304 | 2154.952354 | -0.0083 | 0.993 | -0.476526264 | count | 1 |
| LINC01481 | -17.9602304 | 2154.952354 | -0.0083 | 0.993 | -0.476526264 | count | 1 |
| ST14 | -17.9584573 | 2573.277729 | -0.007 | 0.994 | -0.476526264 | count | 1 |
| CLDN7 | -17.8938972 | 1496.211734 | -0.012 | 0.99 | -0.476526263 | count | 1 |
| CD93 | -17.7726457 | 1572.747648 | -0.0113 | 0.991 | -0.476526262 | count | 1 |
| KCNN3 | -17.7356936 | 2346.357241 | -0.0076 | 0.994 | -0.476526261 | count | 1 |
| TACC2 | -17.7356936 | 2346.357241 | -0.0076 | 0.994 | -0.476526261 | count | 1 |
| GPR63 | -17.7347442 | 2000.411082 | -0.0089 | 0.993 | -0.476526261 | count | 1 |
| AL080317.2 | -17.7347442 | 2000.411082 | -0.0089 | 0.993 | -0.476526261 | count | 1 |
| GPRC5A | -17.7347442 | 2000.411082 | -0.0089 | 0.993 | -0.476526261 | count | 1 |
| AC091057.4 | -17.7347442 | 2000.411082 | -0.0089 | 0.993 | -0.476526261 | count | 1 |
| RARRES2 | -17.7340553 | 1802.815199 | -0.0098 | 0.992 | -0.476526261 | count | 1 |
| WDR66 | -17.7312903 | 1728.356342 | -0.0103 | 0.992 | -0.476526261 | count | 1 |
| FAM110B | -17.7238733 | 1787.550737 | -0.0099 | 0.992 | -0.476526261 | count | 1 |
| AL135818.2 | -17.6561447 | 1715.590053 | -0.0103 | 0.992 | -0.47652626 | count | 1 |
| AL928654.1 | -17.6561337 | 2147.630097 | -0.0082 | 0.993 | -0.47652626 | count | 1 |
| PRNCR1 | -17.6290664 | 1284.943216 | -0.0137 | 0.989 | -0.47652626 | count | 1 |
| COL4A2 | -17.5907085 | 1081.498989 | -0.0163 | 0.987 | -0.47652626 | count | 1 |
| P4HA2 | -17.5545281 | 1832.787258 | -0.0096 | 0.992 | -0.476526259 | count | 1 |
| CR381653.1 | -17.5540267 | 1875.665939 | -0.0094 | 0.993 | -0.476526259 | count | 1 |
| CELF5 | -17.5357224 | 2026.974894 | -0.0087 | 0.993 | -0.476526259 | count | 1 |
| PALMD | -17.535143 | 1584.732784 | -0.0111 | 0.991 | -0.476526259 | count | 1 |
| IGKV3-20 | -17.535143 | 1584.732784 | -0.0111 | 0.991 | -0.476526259 | count | 1 |
| ASS1 | -17.534855 | 1453.290165 | -0.0121 | 0.99 | -0.476526259 | count | 1 |
| HSPB7 | -17.5153356 | 1710.707466 | -0.0102 | 0.992 | -0.476526259 | count | 1 |
| AL031772.1 | -17.5153356 | 1710.707466 | -0.0102 | 0.992 | -0.476526259 | count | 1 |
| DDIAS | -17.5152688 | 1559.554801 | -0.0112 | 0.991 | -0.476526259 | count | 1 |
| EIF2S3B | -17.5152688 | 1559.554801 | -0.0112 | 0.991 | -0.476526259 | count | 1 |
| AC137630.3 | -17.4473446 | 1063.109957 | -0.0164 | 0.987 | -0.476526258 | count | 1 |
| AC092343.1 | -17.4470039 | 2093.443689 | -0.0083 | 0.993 | -0.476526258 | count | 1 |
| ARMC12 | -17.4470039 | 2093.443689 | -0.0083 | 0.993 | -0.476526258 | count | 1 |
| MPDZ | -17.4470039 | 2093.443689 | -0.0083 | 0.993 | -0.476526258 | count | 1 |
| GNAZ | -17.4470039 | 2093.443689 | -0.0083 | 0.993 | -0.476526258 | count | 1 |
| SLC1A3 | -17.4463538 | 1804.282201 | -0.0097 | 0.992 | -0.476526258 | count | 1 |
| AP001318.2 | -17.4463538 | 1804.282201 | -0.0097 | 0.992 | -0.476526258 | count | 1 |

| | | | | | | | |
|---|---|---|---|---|---|---|---|
| AC005695.3 | -17.4169546 | 1931.241301 | -0.009 | 0.993 | -0.476526257 | count | 1 |
| COL5A1 | -17.4169445 | 1534.903232 | -0.0113 | 0.991 | -0.476526257 | count | 1 |
| AC104170.1 | -17.4168557 | 1798.71277 | -0.0097 | 0.992 | -0.476526257 | count | 1 |
| ASAP2 | -17.4168074 | 1791.669529 | -0.0097 | 0.992 | -0.476526257 | count | 1 |
| LINC01355 | -17.2612851 | 1605.696561 | -0.0108 | 0.991 | -0.476526255 | count | 1 |
| STAB1 | -17.250311 | 1062.758199 | -0.0162 | 0.987 | -0.476526254 | count | 1 |
| KRT17 | -17.2450489 | 1536.332846 | -0.0112 | 0.991 | -0.476526254 | count | 1 |
| AL034550.2 | -17.2450489 | 1536.332846 | -0.0112 | 0.991 | -0.476526254 | count | 1 |
| ADGRF5 | -17.2450268 | 1793.343421 | -0.0096 | 0.992 | -0.476526254 | count | 1 |
| ARHGAP20 | -17.2450268 | 1793.343421 | -0.0096 | 0.992 | -0.476526254 | count | 1 |
| SERTAD4-AS1 | -17.1634631 | 1054.720438 | -0.0163 | 0.987 | -0.476526253 | count | 1 |
| CD163 | -17.133763 | 1085.861567 | -0.0158 | 0.987 | -0.476526252 | count | 1 |
| PAPSS2 | -17.0880719 | 1118.433472 | -0.0153 | 0.988 | -0.476526251 | count | 1 |
| GAB1 | -17.083985 | 1671.601949 | -0.0102 | 0.992 | -0.476526251 | count | 1 |
| TTK | -17.083985 | 1671.601949 | -0.0102 | 0.992 | -0.476526251 | count | 1 |
| STS | -17.083985 | 1671.601949 | -0.0102 | 0.992 | -0.476526251 | count | 1 |
| PIWIL4 | -17.083985 | 1671.601949 | -0.0102 | 0.992 | -0.476526251 | count | 1 |
| CENPA | -17.0836571 | 1512.986422 | -0.0113 | 0.991 | -0.476526251 | count | 1 |
| AC109454.2 | -17.0836571 | 1512.986422 | -0.0113 | 0.991 | -0.476526251 | count | 1 |
| LILRB4 | -17.0004108 | 1058.595231 | -0.0161 | 0.987 | -0.476526249 | count | 1 |
| CCR9 | -16.854745 | 1523.98713 | -0.0111 | 0.991 | -0.476526246 | count | 1 |
| TRIM36 | -16.854745 | 1523.98713 | -0.0111 | 0.991 | -0.476526246 | count | 1 |
| LY6G5B | -16.854745 | 1523.98713 | -0.0111 | 0.991 | -0.476526246 | count | 1 |
| IGHEP2 | -16.854745 | 1523.98713 | -0.0111 | 0.991 | -0.476526246 | count | 1 |
| NRP1 | -16.854745 | 1523.98713 | -0.0111 | 0.991 | -0.476526246 | count | 1 |
| AL133523.1 | -16.854745 | 1523.98713 | -0.0111 | 0.991 | -0.476526246 | count | 1 |
| TPPP3 | -16.854745 | 1523.98713 | -0.0111 | 0.991 | -0.476526246 | count | 1 |
| MEIS2 | -16.7733854 | 1258.483533 | -0.0133 | 0.989 | -0.476526243 | count | 1 |
| CBY1 | -0.4002403 | 0.3194876 | -1.2528 | 0.21 | -0.47649003 | count | 1 |
| MARCKS | -0.9296126 | 0.4762738 | -1.9518 | 0.051 | -0.47641212 | count | 1 |
| TRIM68 | -1.5690488 | 1.0308195 | -1.5221 | 0.128 | -0.475890869 | count | 1 |
| DUS4L | -0.4170179 | 0.3112601 | -1.3398 | 0.18 | -0.475838033 | count | 1 |
| UBASH3B | -0.5024676 | 0.3293585 | -1.5256 | 0.127 | -0.475273694 | count | 1 |
| PRIMPOL | -0.4907598 | 0.2854517 | -1.7192 | 0.0857 | -0.475000852 | count | 1 |
| PAG1 | -0.3541252 | 0.1235001 | -2.8674 | 0.00416 | -0.474846126 | count | 1 |
| ZNF700 | -0.4505628 | 0.3291229 | -1.369 | 0.171 | -0.47482589 | count | 1 |
| OPA3 | -0.4716834 | 0.289061 | -1.6318 | 0.103 | -0.474746222 | count | 1 |
| LILRB3 | -0.9247954 | 0.8040768 | -1.1501 | 0.25 | -0.474471523 | count | 1 |
| ZNF580 | -0.3546029 | 0.1547537 | -2.2914 | 0.022 | -0.474435673 | count | 1 |
| FILIP1L | -0.5296932 | 0.4465791 | -1.1861 | 0.236 | -0.47424294 | count | 1 |
| TRAF4 | -0.3624632 | 0.1477636 | -2.453 | 0.0142 | -0.473996721 | count | 1 |
| MCM6 | -0.462849 | 0.2378521 | -1.946 | 0.0517 | -0.473780675 | count | 1 |
| ANKLE2 | -0.3497467 | 0.1206721 | -2.8983 | 0.00378 | -0.47366506 | count | 1 |
| EXD2 | -0.9226136 | 0.5464354 | -1.6884 | 0.0914 | -0.473590755 | count | 1 |
| OGFOD1 | -0.414627 | 0.2403311 | -1.7252 | 0.0846 | -0.473191163 | count | 1 |

| | | | | | | | |
|---|---|---|---|---|---|---|---|
| DNAAF5 | -0.5464003 | 0.4558963 | -1.1985 | 0.231 | -0.473190802 | count | 1 |
| PPP2CA | -0.3398848 | 0.0845583 | -4.0195 | 5.96E-05 | -0.473001964 | count | 1 |
| LIMS1 | -0.3407423 | 0.1040023 | -3.2763 | 0.00106 | -0.472862787 | count | 1 |
| ABHD13 | -0.3610213 | 0.1530938 | -2.3582 | 0.0184 | -0.472729724 | count | 1 |
| DDTL | -1.1183177 | 0.6883286 | -1.6247 | 0.104 | -0.472040323 | count | 1 |
| FRMD4B | -0.3448454 | 0.1087401 | -3.1713 | 0.00153 | -0.471883217 | count | 1 |
| WDR41 | -0.3925159 | 0.3273135 | -1.1992 | 0.231 | -0.471833685 | count | 1 |
| FAM129A | -0.3487571 | 0.1125494 | -3.0987 | 0.00196 | -0.471823416 | count | 1 |
| CASP6 | -0.3554374 | 0.1716831 | -2.0703 | 0.0385 | -0.471562804 | count | 1 |
| NAGA | -0.6244834 | 0.3330336 | -1.8751 | 0.0609 | -0.471519588 | count | 1 |
| POLR1E | -0.3708137 | 0.1607944 | -2.3061 | 0.0212 | -0.471380185 | count | 1 |
| TNFRSF10D | -0.7217848 | 0.4762675 | -1.5155 | 0.13 | -0.471377742 | count | 1 |
| USP51 | -0.7217848 | 0.5795222 | -1.2455 | 0.213 | -0.471377742 | count | 1 |
| CAMLG | -0.3346781 | 0.0756856 | -4.422 | 1.01E-05 | -0.471221732 | count | 0.2427737 |
| RTP4 | -0.3609785 | 0.2654215 | -1.36 | 0.174 | -0.470838274 | count | 1 |
| DENND3 | -0.426194 | 0.3216051 | -1.3252 | 0.185 | -0.470259406 | count | 1 |
| ZNF639 | -0.356395 | 0.1488371 | -2.3945 | 0.0167 | -0.46998858 | count | 1 |
| IPO5 | -0.4140373 | 0.2033499 | -2.0361 | 0.0418 | -0.469046942 | count | 1 |
| B3GNTL1 | -0.4077115 | 0.3195028 | -1.2761 | 0.202 | -0.468780911 | count | 1 |
| RFFL | -0.3999411 | 0.2215947 | -1.8048 | 0.0712 | -0.468710035 | count | 1 |
| PLCG1 | -0.3880773 | 0.2699626 | -1.4375 | 0.151 | -0.468614206 | count | 1 |
| SETD7 | -0.4644456 | 0.2897893 | -1.6027 | 0.109 | -0.467815087 | count | 1 |
| PMEPA1 | -0.3584285 | 0.169794 | -2.111 | 0.0348 | -0.467551062 | count | 1 |
| COA7 | -0.6184956 | 0.4323407 | -1.4306 | 0.153 | -0.46746733 | count | 1 |
| HIF1A | -0.3375232 | 0.0892782 | -3.7806 | 0.000159 | -0.46742173 | count | 1 |
| ACO1 | -0.6596988 | 0.4291882 | -1.5371 | 0.124 | -0.467386021 | count | 1 |
| FSCN1 | -0.4726281 | 0.2751616 | -1.7176 | 0.086 | -0.467369503 | count | 1 |
| TCOF1 | -0.3962976 | 0.2007038 | -1.9745 | 0.0484 | -0.467119269 | count | 1 |
| CASK | -0.3525043 | 0.1244606 | -2.8323 | 0.00465 | -0.466816831 | count | 1 |
| AL358075.2 | -0.9054059 | 1.0879237 | -0.8322 | 0.405 | -0.466603784 | count | 1 |
| GPM6B | -0.6158056 | 0.281382 | -2.1885 | 0.0287 | -0.465643631 | count | 1 |
| RYBP | -0.3889659 | 0.1309414 | -2.9705 | 0.00299 | -0.465568118 | count | 1 |
| EPM2A | -0.6564357 | 0.5696274 | -1.1524 | 0.249 | -0.46534649 | count | 1 |
| ELOA | -0.3393097 | 0.1124164 | -3.0183 | 0.00256 | -0.465188892 | count | 1 |
| KTI12 | -0.3721647 | 0.2334908 | -1.5939 | 0.111 | -0.465179891 | count | 1 |
| HOOK3 | -0.345894 | 0.1391548 | -2.4857 | 0.013 | -0.46517079 | count | 1 |
| USP22 | -0.3558127 | 0.1488413 | -2.3906 | 0.0169 | -0.464795917 | count | 1 |
| ZSCAN25 | -0.4905978 | 0.3966687 | -1.2368 | 0.216 | -0.464704132 | count | 1 |
| TRDMT1 | -0.4793456 | 0.2813411 | -1.7038 | 0.0885 | -0.464556658 | count | 1 |
| ZNF131 | -0.3406578 | 0.1083064 | -3.1453 | 0.00167 | -0.464520923 | count | 1 |
| PCGF2 | -1.5036777 | 1.1974102 | -1.2558 | 0.209 | -0.464403332 | count | 1 |
| WAPL | -0.345869 | 0.1076681 | -3.2124 | 0.00133 | -0.464193461 | count | 1 |
| IGF2BP2 | -0.7848819 | 1.0778633 | -0.7282 | 0.467 | -0.464067862 | count | 1 |
| OSGEPL1-AS1 | -0.7848819 | 1.098329 | -0.7146 | 0.475 | -0.464067862 | count | 1 |
| NRSN2 | -0.7848819 | 1.098329 | -0.7146 | 0.475 | -0.464067862 | count | 1 |

| | | | | | | | |
|---|---|---|---|---|---|---|---|
| ICOS | -0.3302763 | 0.093664 | -3.5262 | 0.000427 | -0.464038408 | count | 1 |
| CEP85 | -0.5811768 | 0.4270238 | -1.361 | 0.174 | -0.463772873 | count | 1 |
| USP14 | -0.3421638 | 0.1085825 | -3.1512 | 0.00164 | -0.463712566 | count | 1 |
| SETX | -0.3471955 | 0.1141497 | -3.0416 | 0.00237 | -0.463527306 | count | 1 |
| FAM220A | -0.6123138 | 0.3669135 | -1.6688 | 0.0952 | -0.463273315 | count | 1 |
| PDE4DIP | -0.3523556 | 0.1805253 | -1.9518 | 0.051 | -0.463169069 | count | 1 |
| EIF1AD | -0.3633686 | 0.1642588 | -2.2122 | 0.027 | -0.463017163 | count | 1 |
| NAGK | -0.3431364 | 0.1371965 | -2.5011 | 0.0124 | -0.462943123 | count | 1 |
| ATXN3 | -0.3683085 | 0.1702718 | -2.1631 | 0.0306 | -0.462859951 | count | 1 |
| TTC7A | -0.4330926 | 0.2487759 | -1.7409 | 0.0818 | -0.462781245 | count | 1 |
| AGPAT3 | -0.4022353 | 0.2119891 | -1.8974 | 0.0579 | -0.462660244 | count | 1 |
| EXOC8 | -0.4279115 | 0.2634936 | -1.624 | 0.104 | -0.462650527 | count | 1 |
| FDXR | -0.3846921 | 0.2479234 | -1.5517 | 0.121 | -0.462634684 | count | 1 |
| SMARCD3 | -0.553946 | 0.3566151 | -1.5533 | 0.12 | -0.462493833 | count | 1 |
| SMAD5 | -0.3881936 | 0.2724293 | -1.4249 | 0.154 | -0.462480629 | count | 1 |
| EBPL | -0.3452205 | 0.1310319 | -2.6346 | 0.00846 | -0.462334104 | count | 1 |
| PPP1R35 | -0.3317971 | 0.0991265 | -3.3472 | 0.000825 | -0.462257223 | count | 1 |
| RALA | -0.3305934 | 0.094858 | -3.4851 | 0.000498 | -0.462150953 | count | 1 |
| FBXL22 | -0.418452 | 0.4549403 | -0.9198 | 0.358 | -0.462005115 | count | 1 |
| ABAT | -0.8940902 | 0.4689856 | -1.9064 | 0.0567 | -0.461970101 | count | 1 |
| EBNA1BP2 | -0.3496398 | 0.1530921 | -2.2839 | 0.0224 | -0.461659309 | count | 1 |
| SHLD2 | -0.3536979 | 0.1770977 | -1.9972 | 0.0459 | -0.461451142 | count | 1 |
| IMPA2 | -0.4577957 | 0.5128016 | -0.8927 | 0.372 | -0.461435873 | count | 1 |
| DCAF12 | -0.4068968 | 0.2709076 | -1.502 | 0.133 | -0.461199525 | count | 1 |
| ATG3 | -0.3308603 | 0.1015289 | -3.2588 | 0.00113 | -0.46117982 | count | 1 |
| F8 | -0.4431264 | 0.5236589 | -0.8462 | 0.397 | -0.46112949 | count | 1 |
| BRD8 | -0.3548156 | 0.1932688 | -1.8359 | 0.0665 | -0.460947851 | count | 1 |
| FKBP7 | -0.7782143 | 0.6069951 | -1.2821 | 0.2 | -0.460768887 | count | 1 |
| PCAT1 | -0.4567751 | 0.335204 | -1.3627 | 0.173 | -0.460455885 | count | 1 |
| AC006449.2 | -0.7771443 | 1.2263519 | -0.6337 | 0.526 | -0.460238404 | count | 1 |
| KLHL21 | -0.4648209 | 0.3482415 | -1.3348 | 0.182 | -0.460038443 | count | 1 |
| IL2RA | -0.3658846 | 0.2089391 | -1.7512 | 0.08 | -0.459862551 | count | 1 |
| PPARD | -0.8889241 | 0.5784307 | -1.5368 | 0.124 | -0.459844322 | count | 1 |
| ZNF275 | -0.7759654 | 0.4234404 | -1.8325 | 0.067 | -0.459653561 | count | 1 |
| MAD1L1 | -0.3304794 | 0.1011523 | -3.2671 | 0.0011 | -0.45858847 | count | 1 |
| AL160313.1 | -1.471458 | 1.0623752 | -1.3851 | 0.166 | -0.458519269 | count | 1 |
| ANKRD39 | -0.3468505 | 0.1477335 | -2.3478 | 0.0189 | -0.458486215 | count | 1 |
| SLC16A1-AS1 | -0.3779077 | 0.2410077 | -1.568 | 0.117 | -0.458451294 | count | 1 |
| AP1AR | -0.3736345 | 0.1927615 | -1.9383 | 0.0527 | -0.458447719 | count | 1 |
| TMSB4X | -0.3178931 | 0.0168764 | -18.8365 | 2.32E-75 | -0.458385856 | count | 5.64E-71 |
| E2F3 | -0.4012696 | 0.2449891 | -1.6379 | 0.102 | -0.458383919 | count | 1 |
| LAP3 | -0.3344228 | 0.1152566 | -2.9016 | 0.00374 | -0.458366483 | count | 1 |
| YIPF2 | -0.4076056 | 0.226435 | -1.8001 | 0.0719 | -0.458348191 | count | 1 |
| C9orf3 | -0.6448422 | 0.4468449 | -1.4431 | 0.149 | -0.458076368 | count | 1 |
| TRIOBP | -0.4396538 | 0.2739143 | -1.6051 | 0.109 | -0.457667725 | count | 1 |

| | | | | | | | |
|---|---|---|---|---|---|---|---|
| SPINK2 | -0.4717703 | 0.3748432 | -1.2586 | 0.208 | -0.457607149 | count | 1 |
| SPART | -0.3740957 | 0.162583 | -2.301 | 0.0215 | -0.457376934 | count | 1 |
| FAHD1 | -0.4610106 | 0.2714913 | -1.6981 | 0.0896 | -0.456455134 | count | 1 |
| C21orf62-AS1 | -0.6953319 | 0.5828508 | -1.193 | 0.233 | -0.456428766 | count | 1 |
| PYCARD | -0.3224352 | 0.0890464 | -3.621 | 0.000298 | -0.456357665 | count | 1 |
| FRS2 | -0.470301 | 0.3405288 | -1.3811 | 0.167 | -0.456257576 | count | 1 |
| AC016876.1 | -0.5079284 | 0.4047561 | -1.2549 | 0.21 | -0.456060088 | count | 1 |
| GNG12 | -1.4574136 | 0.9782187 | -1.4899 | 0.136 | -0.455907603 | count | 1 |
| TMEM186 | -0.412704 | 0.3626533 | -1.138 | 0.255 | -0.455868867 | count | 1 |
| SKI | -0.3737015 | 0.1890814 | -1.9764 | 0.0482 | -0.455213421 | count | 1 |
| USP45 | -0.4916716 | 0.3251183 | -1.5123 | 0.131 | -0.45456081 | count | 1 |
| FMR1 | -0.3411974 | 0.1416075 | -2.4095 | 0.016 | -0.454461003 | count | 1 |
| MPC1 | -0.3248927 | 0.1011562 | -3.2118 | 0.00133 | -0.454268807 | count | 1 |
| MTIF2 | -0.350955 | 0.186504 | -1.8818 | 0.06 | -0.453911233 | count | 1 |
| SRF | -0.4676612 | 0.3050668 | -1.533 | 0.125 | -0.453831528 | count | 1 |
| MIR222HG | -0.403203 | 0.2447925 | -1.6471 | 0.0996 | -0.453547543 | count | 1 |
| AL359220.1 | -0.5217579 | 0.2501559 | -2.0857 | 0.0371 | -0.453397004 | count | 1 |
| CDKN2C | -0.3448095 | 0.2083976 | -1.6546 | 0.0981 | -0.453352392 | count | 1 |
| DAXX | -0.3337766 | 0.1193816 | -2.7959 | 0.0052 | -0.453336605 | count | 1 |
| ATP1B1 | -0.3260934 | 0.132461 | -2.4618 | 0.0139 | -0.453147334 | count | 1 |
| RAB28 | -0.4141884 | 0.2020687 | -2.0497 | 0.0405 | -0.453040254 | count | 1 |
| MRS2 | -0.3964462 | 0.2927718 | -1.3541 | 0.176 | -0.453028781 | count | 1 |
| CLEC16A | -0.4489212 | 0.4141738 | -1.0839 | 0.278 | -0.452906237 | count | 1 |
| SLC35A3 | -0.4346248 | 0.2241049 | -1.9394 | 0.0525 | -0.452649564 | count | 1 |
| USP13 | -0.6876031 | 0.5153525 | -1.3342 | 0.182 | -0.452025115 | count | 1 |
| BAK1 | -0.3310037 | 0.1357356 | -2.4386 | 0.0148 | -0.451813874 | count | 1 |
| ZNF587B | -1.0525925 | 0.5450052 | -1.9313 | 0.0535 | -0.451723657 | count | 1 |
| HERC2 | -0.3655677 | 0.1691254 | -2.1615 | 0.0307 | -0.451723486 | count | 1 |
| PCED1A | -0.4335405 | 0.3969958 | -1.0921 | 0.275 | -0.451566848 | count | 1 |
| TRAPPC13 | -0.4169617 | 0.2680829 | -1.5553 | 0.12 | -0.451234345 | count | 1 |
| EDC3 | -0.4331747 | 0.3963222 | -1.093 | 0.274 | -0.451201526 | count | 1 |
| CAND1 | -0.3271743 | 0.1099063 | -2.9768 | 0.00293 | -0.451138765 | count | 1 |
| TCEAL3 | -0.3450948 | 0.1976804 | -1.7457 | 0.0809 | -0.450949396 | count | 1 |
| ZMYND19 | -0.4468872 | 0.2715577 | -1.6456 | 0.0999 | -0.450948643 | count | 1 |
| FAM126B | -0.4550252 | 0.2090895 | -2.1762 | 0.0296 | -0.450819181 | count | 1 |
| ATP11A | -0.5015351 | 0.3999104 | -1.2541 | 0.21 | -0.450695131 | count | 1 |
| BRPF1 | -0.386477 | 0.287934 | -1.3422 | 0.18 | -0.450692144 | count | 1 |
| PUS3 | -0.3634779 | 0.2142468 | -1.6965 | 0.0899 | -0.450589556 | count | 1 |
| ZNF431 | -0.3887541 | 0.1886783 | -2.0604 | 0.0394 | -0.450498652 | count | 1 |
| AL392172.1 | -0.4210984 | 0.3150386 | -1.3367 | 0.181 | -0.450443221 | count | 1 |
| GTDC1 | -0.4864586 | 0.2314391 | -2.1019 | 0.0356 | -0.450031398 | count | 1 |
| SLC35E4 | -0.3908883 | 0.335515 | -1.165 | 0.244 | -0.449960402 | count | 1 |
| CLPP | -0.3186528 | 0.0704562 | -4.5227 | 6.31E-06 | -0.449773499 | count | 0.1517555 |
| UBE4B | -0.4316075 | 0.2413847 | -1.788 | 0.0739 | -0.449636022 | count | 1 |
| ERCC1 | -0.3346754 | 0.1435228 | -2.3319 | 0.0198 | -0.449597589 | count | 1 |

| | | | | | | | |
|---|---|---|---|---|---|---|---|
| MFHAS1 | -0.3675715 | 0.1925649 | -1.9088 | 0.0564 | -0.449551686 | count | 1 |
| NFE2L2 | -0.316856 | 0.0680859 | -4.6538 | 3.38E-06 | -0.449402068 | count | 0.08135322 |
| MT2A | -0.3139661 | 0.0620161 | -5.0627 | 4.35E-07 | -0.449380246 | count | 0.010493505 |
| KDM5B | -0.3468048 | 0.1691451 | -2.0503 | 0.0404 | -0.449311615 | count | 1 |
| ANKRD13C | -0.3673093 | 0.2048601 | -1.793 | 0.0731 | -0.44923707 | count | 1 |
| TEX30 | -0.4024295 | 0.2138853 | -1.8815 | 0.06 | -0.44890981 | count | 1 |
| ID4 | -1.4190593 | 0.9680918 | -1.4658 | 0.143 | -0.448628072 | count | 1 |
| DCP1B | -0.3740652 | 0.3054975 | -1.2244 | 0.221 | -0.448118328 | count | 1 |
| EMP2 | -1.0409924 | 0.8241668 | -1.2631 | 0.207 | -0.448044576 | count | 1 |
| FKBPL | -0.3722746 | 0.2188631 | -1.7009 | 0.089 | -0.448014793 | count | 1 |
| DYNC1LI2 | -0.3505766 | 0.1529278 | -2.2924 | 0.0219 | -0.447839604 | count | 1 |
| LUC7L3 | -0.3207144 | 0.0834072 | -3.8452 | 0.000123 | -0.447701077 | count | 1 |
| ESD | -0.3186583 | 0.0894822 | -3.5611 | 0.000374 | -0.447676306 | count | 1 |
| ACP5 | -0.3346485 | 0.1789203 | -1.8704 | 0.0615 | -0.447616913 | count | 1 |
| LIX1L | -0.3309831 | 0.1210099 | -2.7352 | 0.00627 | -0.447445505 | count | 1 |
| TAP1 | -0.3189756 | 0.0861418 | -3.7029 | 0.000217 | -0.447351259 | count | 1 |
| CHM | -0.3330173 | 0.1482151 | -2.2469 | 0.0247 | -0.447079861 | count | 1 |
| ZNF446 | -0.44275 | 0.4061969 | -1.09 | 0.276 | -0.446963849 | count | 1 |
| MNT | -0.513506 | 0.2893949 | -1.7744 | 0.0761 | -0.446732219 | count | 1 |
| CBWD3 | -0.459935 | 0.2965119 | -1.5512 | 0.121 | -0.446721053 | count | 1 |
| TNIP3 | -0.5577673 | 0.3144332 | -1.7739 | 0.0762 | -0.446713471 | count | 1 |
| OTUD3 | -0.4423173 | 0.305806 | -1.4464 | 0.148 | -0.446546847 | count | 1 |
| KLHL7 | -0.3826904 | 0.2097762 | -1.8243 | 0.0682 | -0.446386114 | count | 1 |
| PCBD1 | -0.331161 | 0.1628244 | -2.0339 | 0.042 | -0.446359459 | count | 1 |
| AL139089.1 | -0.6260688 | 0.589277 | -1.0624 | 0.288 | -0.446224947 | count | 1 |
| RSPH3 | -0.6259735 | 0.4267353 | -1.4669 | 0.142 | -0.446164545 | count | 1 |
| ATXN1L | -0.4345796 | 0.2568085 | -1.6922 | 0.0907 | -0.446096221 | count | 1 |
| ZDHHC14 | -0.411998 | 0.3841113 | -1.0726 | 0.284 | -0.446050875 | count | 1 |
| PIK3R3 | -0.4119439 | 0.4313809 | -0.9549 | 0.34 | -0.445994355 | count | 1 |
| PSMD14 | -0.3245775 | 0.1113998 | -2.9136 | 0.0036 | -0.445988807 | count | 1 |
| MCM8 | -1.034084 | 0.6042143 | -1.7115 | 0.0871 | -0.445840073 | count | 1 |
| ZMIZ1 | -0.4693265 | 0.4442421 | -1.0565 | 0.291 | -0.445673183 | count | 1 |
| STX10 | -0.3265908 | 0.1143391 | -2.8563 | 0.00431 | -0.44564158 | count | 1 |
| KDM5A | -0.3242142 | 0.1003587 | -3.2306 | 0.00125 | -0.445633459 | count | 1 |
| WWP2 | -0.4271641 | 0.2705306 | -1.579 | 0.114 | -0.44519442 | count | 1 |
| MMP9 | -0.7467406 | 0.6835011 | -1.0925 | 0.275 | -0.445039305 | count | 1 |
| WBP4 | -0.326421 | 0.1307662 | -2.4962 | 0.0126 | -0.445034681 | count | 1 |
| HNRNPAB | -0.3130723 | 0.0654309 | -4.7848 | 1.78E-06 | -0.444974172 | count | 0.04288198 |
| G3BP2 | -0.3124016 | 0.0583655 | -5.3525 | 9.25E-08 | -0.444672726 | count | 0.002233413 |
| FAM126A | -0.432756 | 0.2718635 | -1.5918 | 0.112 | -0.444303999 | count | 1 |
| DNAJA2 | -0.3153609 | 0.0788347 | -4.0003 | 6.46E-05 | -0.444230982 | count | 1 |
| FGFR1OP2 | -0.3127106 | 0.0653785 | -4.7831 | 1.80E-06 | -0.444153499 | count | 0.043362 |
| SAMSN1 | -0.3125368 | 0.0527537 | -5.9245 | 3.45E-09 | -0.444146815 | count | 8.34E-05 |
| SPNS3 | -0.3390765 | 0.1798888 | -1.8849 | 0.0595 | -0.443740788 | count | 1 |
| CEP350 | -0.3251705 | 0.1110493 | -2.9282 | 0.00343 | -0.443339985 | count | 1 |

| | | | | | | | |
|---|---|---|---|---|---|---|---|
| HES4 | -0.5527291 | 0.5201252 | -1.0627 | 0.288 | -0.443022221 | count | 1 |
| KCNK6 | -0.6208518 | 0.2846626 | -2.181 | 0.0293 | -0.442914286 | count | 1 |
| PDZD7 | -1.0242152 | 0.9820781 | -1.0429 | 0.297 | -0.442673517 | count | 1 |
| MRPL17 | -0.3219585 | 0.143733 | -2.24 | 0.0252 | -0.442548781 | count | 1 |
| DVL2 | -0.5520128 | 0.2495918 | -2.2117 | 0.0271 | -0.442496869 | count | 1 |
| CDR2 | -0.3602205 | 0.1582456 | -2.2763 | 0.0229 | -0.442288042 | count | 1 |
| TRMT2B | -0.7412642 | 0.3144077 | -2.3577 | 0.0184 | -0.442275889 | count | 1 |
| MCOLN1 | -0.3859773 | 0.2830655 | -1.3636 | 0.173 | -0.441390933 | count | 1 |
| XPO5 | -0.5265288 | 0.3952413 | -1.3322 | 0.183 | -0.441362498 | count | 1 |
| USP4 | -0.355889 | 0.1760954 | -2.021 | 0.0434 | -0.441340613 | count | 1 |
| SLC7A5 | -0.3736301 | 0.1656401 | -2.2557 | 0.0242 | -0.441019673 | count | 1 |
| ALG6 | -0.371643 | 0.2620315 | -1.4183 | 0.156 | -0.441013712 | count | 1 |
| AL139260.1 | -0.8426016 | 1.137205 | -0.7409 | 0.459 | -0.440493554 | count | 1 |
| AL138762.1 | -0.8423272 | 0.7420172 | -1.1352 | 0.256 | -0.440377371 | count | 1 |
| POLR1B | -0.4064151 | 0.3538731 | -1.1485 | 0.251 | -0.440214615 | count | 1 |
| SAFB | -0.3293383 | 0.1201812 | -2.7403 | 0.00617 | -0.439883512 | count | 1 |
| MED17 | -0.3846098 | 0.2625501 | -1.4649 | 0.143 | -0.439869246 | count | 1 |
| APP | -0.4885474 | 0.2307982 | -2.1168 | 0.0344 | -0.439763406 | count | 1 |
| PAQR3 | -0.4431154 | 0.3457743 | -1.2815 | 0.2 | -0.439579017 | count | 1 |
| TJP3 | -1.0142676 | 0.6429141 | -1.5776 | 0.115 | -0.43946088 | count | 1 |
| ETF1 | -0.3229941 | 0.1219834 | -2.6479 | 0.00814 | -0.439204557 | count | 1 |
| CDKN1B | -0.3093966 | 0.0604215 | -5.1206 | 3.21E-07 | -0.439199449 | count | 0.007745088 |
| TBC1D4 | -0.3489997 | 0.1337443 | -2.6095 | 0.00911 | -0.438962009 | count | 1 |
| STEAP4 | -1.0124435 | 0.8461409 | -1.1965 | 0.232 | -0.438869534 | count | 1 |
| CNOT7 | -0.318224 | 0.0975062 | -3.2636 | 0.00111 | -0.438734449 | count | 1 |
| TESK1 | -0.3513424 | 0.2291223 | -1.5334 | 0.125 | -0.438354104 | count | 1 |
| RPL27A | -0.3052322 | 0.0244661 | -12.4757 | 5.85E-35 | -0.438316463 | count | 1.42E-30 |
| DYNLL2 | -0.3282984 | 0.1110425 | -2.9565 | 0.00313 | -0.437792691 | count | 1 |
| FBXW8 | -1.0090005 | 0.5130709 | -1.9666 | 0.0493 | -0.437751396 | count | 1 |
| KBTBD2 | -0.3335415 | 0.1357783 | -2.4565 | 0.0141 | -0.437653945 | count | 1 |
| LUM | -1.362957 | 0.4131139 | -3.2992 | 0.00098 | -0.437584329 | count | 1 |
| PSMD8 | -0.3065711 | 0.0569904 | -5.3793 | 7.98E-08 | -0.4375409 | count | 0.001926931 |
| SLC3A2 | -0.3062592 | 0.0534449 | -5.7304 | 1.09E-08 | -0.43737465 | count | 0.000263551 |
| NOP10 | -0.3071465 | 0.0726902 | -4.2254 | 2.45E-05 | -0.43735868 | count | 0.587951 |
| TMEM131 | -0.3495179 | 0.1898314 | -1.8412 | 0.0657 | -0.437318365 | count | 1 |
| ETFDH | -0.3630799 | 0.2069037 | -1.7548 | 0.0794 | -0.437173846 | count | 1 |
| GSPT1 | -0.3103334 | 0.0663254 | -4.679 | 3.00E-06 | -0.437064707 | count | 0.072219 |
| FAM20C | -0.6613354 | 0.7996531 | -0.827 | 0.408 | -0.436937316 | count | 1 |
| AC092650.1 | -0.6613354 | 1.052415 | -0.6284 | 0.53 | -0.436937316 | count | 1 |
| IL6ST | -0.3415739 | 0.1093012 | -3.1251 | 0.00179 | -0.435617979 | count | 1 |
| SCML1 | -0.5718883 | 0.1959549 | -2.9185 | 0.00354 | -0.435585425 | count | 1 |
| HDAC5 | -0.411318 | 0.277481 | -1.4823 | 0.138 | -0.43503149 | count | 1 |
| STARD7 | -0.3250662 | 0.1293744 | -2.5126 | 0.012 | -0.434901393 | count | 1 |
| DIAPH2 | -0.3400995 | 0.1612145 | -2.1096 | 0.035 | -0.434630591 | count | 1 |
| RC3H1 | -0.3421244 | 0.1799972 | -1.9007 | 0.0574 | -0.434479404 | count | 1 |

| | | | | | | | |
|---|---|---|---|---|---|---|---|
| LIMA1 | -0.3191218 | 0.1382878 | -2.3077 | 0.0211 | -0.434173744 | count | 1 |
| LINC00309 | -0.9967218 | 0.8980377 | -1.1099 | 0.267 | -0.433743389 | count | 1 |
| DR1 | -0.3125879 | 0.1023271 | -3.0548 | 0.00227 | -0.433683437 | count | 1 |
| AL136454.1 | -0.9964684 | 0.4171427 | -2.3888 | 0.017 | -0.433660334 | count | 1 |
| PIAS4 | -0.3762731 | 0.2470259 | -1.5232 | 0.128 | -0.433568341 | count | 1 |
| PDE4B | -0.3065729 | 0.067214 | -4.5611 | 5.27E-06 | -0.433455198 | count | 0.12675931 |
| PPIL4 | -0.3108556 | 0.0799993 | -3.8857 | 0.000104 | -0.433393596 | count | 1 |
| CEP19 | -0.3996693 | 0.3274823 | -1.2204 | 0.222 | -0.433153999 | count | 1 |
| FAM193A | -0.3876134 | 0.2595547 | -1.4934 | 0.135 | -0.432873668 | count | 1 |
| RFX1 | -0.3730888 | 0.2166823 | -1.7218 | 0.0852 | -0.432794812 | count | 1 |
| IRF7 | -0.3159258 | 0.1277587 | -2.4728 | 0.0135 | -0.432684219 | count | 1 |
| CKS2 | -0.3124485 | 0.1012015 | -3.0874 | 0.00204 | -0.432549125 | count | 1 |
| SMC2 | -0.3359067 | 0.1890822 | -1.7765 | 0.0757 | -0.432520722 | count | 1 |
| KMT2B | -0.3257327 | 0.1207433 | -2.6977 | 0.00702 | -0.432499334 | count | 1 |
| MDP1 | -0.4356102 | 0.4052737 | -1.0749 | 0.283 | -0.432478339 | count | 1 |
| CTNNBL1 | -0.327641 | 0.1327834 | -2.4675 | 0.0137 | -0.432427892 | count | 1 |
| TEDC1 | -0.3496343 | 0.2178424 | -1.605 | 0.109 | -0.432366784 | count | 1 |
| RASA1 | -0.4660892 | 0.2707592 | -1.7214 | 0.0853 | -0.432266127 | count | 1 |
| KCNN4 | -0.4034295 | 0.3291016 | -1.2259 | 0.22 | -0.432211434 | count | 1 |
| CRTAM | -0.3156245 | 0.120515 | -2.619 | 0.00886 | -0.432115143 | count | 1 |
| HSP90AB1 | -0.3002297 | 0.030515 | -9.8388 | 1.53E-22 | -0.432103805 | count | 3.72E-18 |
| PRKAR1B | -0.3986125 | 0.3579165 | -1.1137 | 0.265 | -0.432047029 | count | 1 |
| LRRC20 | -1.335103 | 0.9790489 | -1.3637 | 0.173 | -0.431923211 | count | 1 |
| TNRC6C | -0.3450845 | 0.1432366 | -2.4092 | 0.016 | -0.431856362 | count | 1 |
| NLRX1 | -1.3342865 | 0.9793417 | -1.3624 | 0.173 | -0.431755438 | count | 1 |
| MYL5 | -0.4949742 | 0.4150038 | -1.1927 | 0.233 | -0.431698515 | count | 1 |
| NINJ1 | -0.3123855 | 0.1326161 | -2.3556 | 0.0186 | -0.431631835 | count | 1 |
| ZFR2 | -0.9901281 | 1.1290181 | -0.877 | 0.381 | -0.431577888 | count | 1 |
| PVRIG | -0.9901281 | 1.1529627 | -0.8588 | 0.391 | -0.431577888 | count | 1 |
| SYNE4 | -0.9901281 | 1.2419473 | -0.7972 | 0.425 | -0.431577888 | count | 1 |
| PPIL6 | -0.9901281 | 1.304186 | -0.7592 | 0.448 | -0.431577888 | count | 1 |
| UBR7 | -0.4026404 | 0.236624 | -1.7016 | 0.0889 | -0.431395637 | count | 1 |
| BCR | -0.602453 | 0.2967683 | -2.03 | 0.0424 | -0.431178734 | count | 1 |
| ARID1B | -0.3201607 | 0.1191994 | -2.6859 | 0.00727 | -0.430815164 | count | 1 |
| AEBP1 | -2.689675 | 0.7692554 | -3.4965 | 0.000478 | -0.430753343 | count | 1 |
| C17orf58 | -0.4936353 | 0.4964793 | -0.9943 | 0.32 | -0.430608824 | count | 1 |
| ZNF780A | -0.3404537 | 0.2268148 | -1.501 | 0.133 | -0.430444665 | count | 1 |
| DCLRE1B | -0.8189741 | 0.5613685 | -1.4589 | 0.145 | -0.430422294 | count | 1 |
| GAS6 | -0.4523528 | 0.3976496 | -1.1376 | 0.255 | -0.430405448 | count | 1 |
| PI4KB | -0.364386 | 0.2609489 | -1.3964 | 0.163 | -0.430351287 | count | 1 |
| GPR155 | -0.3447815 | 0.1699194 | -2.0291 | 0.0425 | -0.430296113 | count | 1 |
| AC093673.1 | -0.3359504 | 0.1919065 | -1.7506 | 0.0801 | -0.430227073 | count | 1 |
| LARP1 | -0.3318788 | 0.1295815 | -2.5612 | 0.0105 | -0.43019281 | count | 1 |
| MAST4 | -0.4930295 | 0.2643595 | -1.865 | 0.0623 | -0.430115635 | count | 1 |
| SORBS3 | -0.366089 | 0.2123173 | -1.7243 | 0.0848 | -0.429957547 | count | 1 |

| | | | | | | | |
|---|---|---|---|---|---|---|---|
| SUDS3 | -0.3114151 | 0.1193914 | -2.6084 | 0.00914 | -0.429854102 | count | 1 |
| ANAPC7 | -0.3679706 | 0.218152 | -1.6868 | 0.0917 | -0.42962353 | count | 1 |
| RABGAP1 | -0.3599657 | 0.2216357 | -1.6241 | 0.104 | -0.429561451 | count | 1 |
| CANT1 | -0.4177649 | 0.2869052 | -1.4561 | 0.145 | -0.429542007 | count | 1 |
| DPEP2 | -0.4176015 | 0.3093131 | -1.3501 | 0.177 | -0.429380829 | count | 1 |
| SDC4 | -0.4410693 | 0.3085727 | -1.4294 | 0.153 | -0.429297193 | count | 1 |
| SOD2 | -0.3154058 | 0.1044944 | -3.0184 | 0.00256 | -0.429148242 | count | 1 |
| FUOM | -0.321203 | 0.1651992 | -1.9443 | 0.0519 | -0.428759153 | count | 1 |
| PDLIM7 | -0.3912142 | 0.3412462 | -1.1464 | 0.252 | -0.428715281 | count | 1 |
| LRFN4 | -0.4238243 | 0.3157036 | -1.3425 | 0.18 | -0.428684125 | count | 1 |
| LRRC37B | -0.39519 | 0.256105 | -1.5431 | 0.123 | -0.428460442 | count | 1 |
| AL118558.3 | -0.5613294 | 0.5019416 | -1.1183 | 0.264 | -0.428279355 | count | 1 |
| TNFSF10 | -0.3006144 | 0.1090923 | -2.7556 | 0.00589 | -0.428098893 | count | 1 |
| EEF1AKMT1 | -0.4161584 | 0.3183878 | -1.3071 | 0.191 | -0.427957022 | count | 1 |
| RNF227 | -0.4743426 | 0.4839312 | -0.9802 | 0.327 | -0.42775683 | count | 1 |
| C21orf2 | -0.3254539 | 0.1714366 | -1.8984 | 0.0577 | -0.427653642 | count | 1 |
| SH2D1A | -0.3054411 | 0.0826998 | -3.6934 | 0.000225 | -0.42748398 | count | 1 |
| KCTD21 | -0.811078 | 0.5211764 | -1.5562 | 0.12 | -0.427026223 | count | 1 |
| SMAP2 | -0.3013638 | 0.0519416 | -5.802 | 7.16E-09 | -0.426892325 | count | 0.000173165 |
| ABCB10 | -0.4383906 | 0.3659049 | -1.1981 | 0.231 | -0.426816171 | count | 1 |
| LEPROTL1 | -0.2979476 | 0.0402991 | -7.3934 | 1.79E-13 | -0.426810319 | count | 4.34E-09 |
| TXLNA | -0.3460666 | 0.2215905 | -1.5617 | 0.118 | -0.426645928 | count | 1 |
| SWAP70 | -0.5075328 | 0.3689014 | -1.3758 | 0.169 | -0.426602309 | count | 1 |
| STYXL1 | -0.3497515 | 0.2602758 | -1.3438 | 0.179 | -0.426572703 | count | 1 |
| EPB41L2 | -0.530382 | 0.3599806 | -1.4734 | 0.141 | -0.426565982 | count | 1 |
| BBS7 | -0.4145186 | 0.2889913 | -1.4344 | 0.152 | -0.426338583 | count | 1 |
| OXSR1 | -0.3348667 | 0.1613025 | -2.076 | 0.038 | -0.426294533 | count | 1 |
| SDHAF1 | -0.3298553 | 0.1777091 | -1.8562 | 0.0635 | -0.426248865 | count | 1 |
| NDFIP1 | -0.3006239 | 0.0615885 | -4.8812 | 1.10E-06 | -0.426143213 | count | 0.0265144 |
| FABP5 | -0.2997255 | 0.0973739 | -3.0781 | 0.0021 | -0.425993664 | count | 1 |
| INKA1 | -0.6421787 | 0.5001026 | -1.2841 | 0.199 | -0.425816225 | count | 1 |
| LAGE3 | -0.3061552 | 0.1075732 | -2.846 | 0.00445 | -0.425780277 | count | 1 |
| GZMK | -0.2953964 | 0.0529266 | -5.5812 | 2.58E-08 | -0.425519873 | count | 0.000623586 |
| TRAV8-2 | -0.4873217 | 0.3520115 | -1.3844 | 0.166 | -0.425463986 | count | 1 |
| DCP2 | -0.3076474 | 0.1012806 | -3.0376 | 0.0024 | -0.425428142 | count | 1 |
| NPLOC4 | -0.3439023 | 0.2239755 | -1.5354 | 0.125 | -0.425394786 | count | 1 |
| HDDC3 | -0.318117 | 0.1638036 | -1.9421 | 0.0522 | -0.425347796 | count | 1 |
| KLHL5 | -0.3560654 | 0.2591776 | -1.3738 | 0.17 | -0.425002941 | count | 1 |
| SETD3 | -0.3292082 | 0.1403665 | -2.3453 | 0.0191 | -0.424719847 | count | 1 |
| ACTR5 | -0.4010706 | 0.3195644 | -1.2551 | 0.21 | -0.424584929 | count | 1 |
| TRMU | -0.3592777 | 0.2229249 | -1.6117 | 0.107 | -0.424449878 | count | 1 |
| AC018809.2 | -1.2986164 | 1.3861522 | -0.9368 | 0.349 | -0.424324873 | count | 1 |
| TMEM225B | -1.2986164 | 1.4076721 | -0.9225 | 0.356 | -0.424324873 | count | 1 |
| PRKACA | -0.5272041 | 0.2918324 | -1.8065 | 0.0709 | -0.424214758 | count | 1 |
| NUFIP2 | -0.3074371 | 0.0869123 | -3.5373 | 0.00041 | -0.424046324 | count | 1 |

| | | | | | | | |
|---|---|---|---|---|---|---|---|
| LINC00662 | -0.3519312 | 0.2228065 | -1.5795 | 0.114 | -0.424011623 | count | 1 |
| SEPHS1 | -0.3608815 | 0.1913381 | -1.8861 | 0.0594 | -0.423978982 | count | 1 |
| TERF2IP | -0.2956116 | 0.0435533 | -6.7874 | 1.34E-11 | -0.42329249 | count | 3.25E-07 |
| TXNDC15 | -0.3115904 | 0.1266331 | -2.4606 | 0.0139 | -0.42317958 | count | 1 |
| PHTF2 | -0.3107385 | 0.1300245 | -2.3898 | 0.0169 | -0.423028758 | count | 1 |
| TET3 | -0.7034673 | 0.5119581 | -1.3741 | 0.17 | -0.42298966 | count | 1 |
| SKA2 | -0.3135051 | 0.1427533 | -2.1961 | 0.0281 | -0.422983192 | count | 1 |
| CCDC93 | -0.3543161 | 0.172241 | -2.0571 | 0.0398 | -0.422957647 | count | 1 |
| MARF1 | -0.3408207 | 0.2001649 | -1.7027 | 0.0887 | -0.422953209 | count | 1 |
| NCL | -0.2941409 | 0.0354339 | -8.3011 | 1.47E-16 | -0.42271759 | count | 3.57E-12 |
| TENT4A | -0.6367108 | 0.3055469 | -2.0838 | 0.0372 | -0.422623825 | count | 1 |
| ERRFI1 | -0.4251521 | 0.2545606 | -1.6701 | 0.095 | -0.422561558 | count | 1 |
| AC005520.2 | -0.5022688 | 0.6113954 | -0.8215 | 0.411 | -0.422494956 | count | 1 |
| WWTR1 | -0.8002037 | 0.6326567 | -1.2648 | 0.206 | -0.422324325 | count | 1 |
| MRPS6 | -0.2966596 | 0.0804967 | -3.6854 | 0.000232 | -0.422295014 | count | 1 |
| FLVCR1 | -0.8000272 | 0.437996 | -1.8266 | 0.0679 | -0.422247788 | count | 1 |
| S100Z | -1.2887388 | 0.9693034 | -1.3296 | 0.184 | -0.422231862 | count | 1 |
| USP30-AS1 | -0.3572848 | 0.2192406 | -1.6296 | 0.103 | -0.422146405 | count | 1 |
| RECK | -0.3712284 | 0.2585694 | -1.4357 | 0.151 | -0.42185552 | count | 1 |
| ATP11B | -0.3432697 | 0.1552942 | -2.2104 | 0.0271 | -0.421831193 | count | 1 |
| APOBEC3F | -0.3773441 | 0.3552015 | -1.0623 | 0.288 | -0.421733674 | count | 1 |
| ELP6 | -0.3203879 | 0.1583863 | -2.0228 | 0.0432 | -0.421547179 | count | 1 |
| MTERF4 | -0.3059443 | 0.1063165 | -2.8777 | 0.00403 | -0.421403403 | count | 1 |
| PSMC1 | -0.3026678 | 0.1007173 | -3.0051 | 0.00267 | -0.421099188 | count | 1 |
| SP100 | -0.2956693 | 0.0566753 | -5.2169 | 1.93E-07 | -0.421017135 | count | 0.004658055 |
| C14orf119 | -0.3025287 | 0.100558 | -3.0085 | 0.00264 | -0.420831023 | count | 1 |
| FAM117B | -0.4089241 | 0.2590101 | -1.5788 | 0.114 | -0.420812379 | count | 1 |
| PGM2L1 | -0.322183 | 0.153937 | -2.093 | 0.0364 | -0.42075849 | count | 1 |
| BAP1 | -0.4416082 | 0.3802587 | -1.1613 | 0.246 | -0.420703662 | count | 1 |
| FOXO1 | -0.3069911 | 0.0950756 | -3.2289 | 0.00125 | -0.420200672 | count | 1 |
| ZNF691 | -0.5495857 | 0.6257729 | -0.8783 | 0.38 | -0.420117697 | count | 1 |
| DDX60 | -0.3299309 | 0.1735356 | -1.9012 | 0.0574 | -0.420092213 | count | 1 |
| AL139384.1 | -0.4224202 | 0.4319278 | -0.978 | 0.328 | -0.419966793 | count | 1 |
| ELMO1 | -0.3416712 | 0.1764533 | -1.9363 | 0.0529 | -0.419899992 | count | 1 |
| SF3A1 | -0.3183243 | 0.1375547 | -2.3142 | 0.0207 | -0.419791249 | count | 1 |
| EHD4 | -0.326901 | 0.1369119 | -2.3877 | 0.017 | -0.419562443 | count | 1 |
| RAD51D | -0.4514368 | 0.3574129 | -1.2631 | 0.207 | -0.41942161 | count | 1 |
| PPP1R3B | -0.5838873 | 0.336813 | -1.7336 | 0.0831 | -0.419243006 | count | 1 |
| AP2A2 | -0.3660995 | 0.2354071 | -1.5552 | 0.12 | -0.419238368 | count | 1 |
| PARVG | -0.3070841 | 0.1194596 | -2.5706 | 0.0102 | -0.418996372 | count | 1 |
| PNPT1 | -0.3421447 | 0.1942595 | -1.7613 | 0.0783 | -0.418995776 | count | 1 |
| S100A16 | -2.4515283 | 1.2251327 | -2.001 | 0.0455 | -0.418924466 | count | 1 |
| PATL1 | -0.3458307 | 0.2262575 | -1.5285 | 0.126 | -0.418565589 | count | 1 |
| C9orf85 | -0.3341185 | 0.2025051 | -1.6499 | 0.0991 | -0.418335368 | count | 1 |
| PTTG1IP | -0.307783 | 0.1260455 | -2.4418 | 0.0147 | -0.418243486 | count | 1 |

| | | | | | | | |
|---|---|---|---|---|---|---|---|
| SCAF8 | -0.4202597 | 0.2188987 | -1.9199 | 0.055 | -0.417913505 | count | 1 |
| CCDC88B | -0.3399099 | 0.1847308 | -1.84 | 0.0659 | -0.417771696 | count | 1 |
| OTUD6B | -0.3770192 | 0.248938 | -1.5145 | 0.13 | -0.417625348 | count | 1 |
| KTN1 | -0.2930708 | 0.0574987 | -5.097 | 3.64E-07 | -0.417526578 | count | 0.0087815 |
| CCDC34 | -0.3479621 | 0.3197729 | -1.0882 | 0.277 | -0.417465397 | count | 1 |
| ZNF34 | -0.38886 | 0.3221539 | -1.2071 | 0.227 | -0.417127894 | count | 1 |
| GNL3 | -0.3289282 | 0.135587 | -2.426 | 0.0153 | -0.417020362 | count | 1 |
| NDUFS4 | -0.3040023 | 0.1260875 | -2.411 | 0.016 | -0.416437366 | count | 1 |
| PDCD2L | -0.5166522 | 0.309825 | -1.6676 | 0.0955 | -0.416388207 | count | 1 |
| TASP1 | -0.3692234 | 0.2176892 | -1.6961 | 0.09 | -0.416370707 | count | 1 |
| CD28 | -0.2996137 | 0.1014816 | -2.9524 | 0.00317 | -0.416003422 | count | 1 |
| INTS13 | -0.3371939 | 0.1757222 | -1.9189 | 0.0551 | -0.415885824 | count | 1 |
| OPTN | -0.2925879 | 0.0710411 | -4.1186 | 3.90E-05 | -0.415735418 | count | 0.935142 |
| EPHB6 | -0.3498547 | 0.2121422 | -1.6492 | 0.0992 | -0.41569512 | count | 1 |
| KNSTRN | -0.5780872 | 0.4383376 | -1.3188 | 0.187 | -0.415494883 | count | 1 |
| FAM104B | -0.3164415 | 0.2055813 | -1.5393 | 0.124 | -0.415429609 | count | 1 |
| KHNYN | -0.47458 | 0.2845399 | -1.6679 | 0.0954 | -0.415049066 | count | 1 |
| STIM2 | -0.3031148 | 0.1047653 | -2.8933 | 0.00384 | -0.414766096 | count | 1 |
| AC109446.3 | -0.4458631 | 0.3103717 | -1.4365 | 0.151 | -0.414521382 | count | 1 |
| PAF1 | -0.336013 | 0.142025 | -2.3659 | 0.018 | -0.414452912 | count | 1 |
| BLM | -0.3777374 | 0.3125635 | -1.2085 | 0.227 | -0.414396276 | count | 1 |
| WDR73 | -0.3817705 | 0.2211892 | -1.726 | 0.0844 | -0.414374245 | count | 1 |
| COG4 | -0.385816 | 0.201251 | -1.9171 | 0.0553 | -0.413970858 | count | 1 |
| ZNF564 | -0.6211674 | 0.6873233 | -0.9037 | 0.366 | -0.413504837 | count | 1 |
| POLR2B | -0.302319 | 0.1026482 | -2.9452 | 0.00325 | -0.413365011 | count | 1 |
| CACTIN | -0.51251 | 0.4101531 | -1.2496 | 0.212 | -0.413307634 | count | 1 |
| KIF5C | -0.3496297 | 0.265388 | -1.3174 | 0.188 | -0.413292302 | count | 1 |
| DUSP4 | -0.2928408 | 0.1021163 | -2.8677 | 0.00416 | -0.413228024 | count | 1 |
| MRPL14 | -0.2946519 | 0.1065837 | -2.7645 | 0.00573 | -0.412966797 | count | 1 |
| ZNF549 | -0.4436308 | 0.4014935 | -1.105 | 0.269 | -0.412556622 | count | 1 |
| STAG2 | -0.297383 | 0.0918481 | -3.2378 | 0.00122 | -0.412409817 | count | 1 |
| PPP1R3E | -0.5729552 | 0.4078795 | -1.4047 | 0.16 | -0.412170928 | count | 1 |
| BTBD10 | -0.315538 | 0.1994002 | -1.5824 | 0.114 | -0.412166245 | count | 1 |
| PLSCR1 | -0.3016515 | 0.1514262 | -1.9921 | 0.0464 | -0.412130992 | count | 1 |
| CPNE2 | -0.3362472 | 0.3006314 | -1.1185 | 0.263 | -0.411895497 | count | 1 |
| ADRB1 | -1.2400535 | 1.1671278 | -1.0625 | 0.288 | -0.411687929 | count | 1 |
| RABGAP1L | -0.3008354 | 0.0995821 | -3.021 | 0.00254 | -0.411506107 | count | 1 |
| LGALS1 | -0.2860483 | 0.0616851 | -4.6372 | 3.66E-06 | -0.411270435 | count | 0.08808156 |
| MRPL21 | -0.2954731 | 0.1091924 | -2.706 | 0.00684 | -0.410976448 | count | 1 |
| IPO13 | -0.9277918 | 0.642383 | -1.4443 | 0.149 | -0.410646877 | count | 1 |
| GTSCR1 | -0.773157 | 0.7270959 | -1.0633 | 0.288 | -0.410504567 | count | 1 |
| NCOA3 | -0.3138304 | 0.141698 | -2.2148 | 0.0268 | -0.410496212 | count | 1 |
| PEX10 | -0.4120183 | 0.4154712 | -0.9917 | 0.321 | -0.410070984 | count | 1 |
| STK35 | -0.569282 | 0.3829877 | -1.4864 | 0.137 | -0.409787409 | count | 1 |
| ARMCX3 | -0.3193976 | 0.1188316 | -2.6878 | 0.00723 | -0.409281168 | count | 1 |

| | | | | | | | |
|---|---|---|---|---|---|---|---|
| SLC25A44 | -0.3913104 | 0.293423 | -1.3336 | 0.182 | -0.409194369 | count | 1 |
| HIVEP3 | -0.3479612 | 0.2352048 | -1.4794 | 0.139 | -0.409126095 | count | 1 |
| BLOC1S1 | -0.2883121 | 0.0750349 | -3.8424 | 0.000124 | -0.408886499 | count | 1 |
| BISPR | -0.3178166 | 0.2334746 | -1.3612 | 0.174 | -0.408775367 | count | 1 |
| TIFA | -0.2990614 | 0.1176107 | -2.5428 | 0.011 | -0.408773396 | count | 1 |
| SRGAP3 | -0.3803131 | 0.3021687 | -1.2586 | 0.208 | -0.40825871 | count | 1 |
| AL365203.2 | -1.2221889 | 1.067398 | -1.145 | 0.252 | -0.407722913 | count | 1 |
| TRAPPC4 | -0.2893747 | 0.080672 | -3.5871 | 0.000339 | -0.407286734 | count | 1 |
| DOK4 | -1.2199175 | 0.7858582 | -1.5523 | 0.121 | -0.407215047 | count | 1 |
| ZC3H10 | -0.4827379 | 0.4724016 | -1.0219 | 0.307 | -0.407191047 | count | 1 |
| TPM4 | -0.2861213 | 0.0620809 | -4.6088 | 4.20E-06 | -0.407181086 | count | 0.1010688 |
| BNIP3L | -0.2942191 | 0.0898863 | -3.2732 | 0.00107 | -0.407127928 | count | 1 |
| VPS26A | -0.2928261 | 0.0924419 | -3.1677 | 0.00155 | -0.407082867 | count | 1 |
| MCF2L2 | -0.5649195 | 0.808712 | -0.6985 | 0.485 | -0.406951869 | count | 1 |
| PTPRK | -2.2496763 | 1.28256 | -1.7541 | 0.0795 | -0.406648687 | count | 1 |
| AL031846.2 | -0.9159532 | 0.8157464 | -1.1228 | 0.262 | -0.406577507 | count | 1 |
| ARMCX2 | -0.9159532 | 0.8548736 | -1.0714 | 0.284 | -0.406577507 | count | 1 |
| SLC36A1 | -0.9159532 | 0.8669901 | -1.0565 | 0.291 | -0.406577507 | count | 1 |
| TUBA4A | -0.2836369 | 0.0439005 | -6.4609 | 1.19E-10 | -0.406552043 | count | 2.88E-06 |
| CNPY2 | -0.294091 | 0.1101164 | -2.6707 | 0.0076 | -0.406457343 | count | 1 |
| GCA | -0.3154038 | 0.2101251 | -1.501 | 0.133 | -0.406417045 | count | 1 |
| ZCCHC9 | -0.310899 | 0.1622382 | -1.9163 | 0.0554 | -0.406165382 | count | 1 |
| TPM1 | -0.3495309 | 0.2560213 | -1.3652 | 0.172 | -0.406090639 | count | 1 |
| ACAT1 | -0.3063336 | 0.1367703 | -2.2398 | 0.0252 | -0.405788672 | count | 1 |
| VPS13A | -0.3393238 | 0.1422934 | -2.3847 | 0.0171 | -0.405408879 | count | 1 |
| NDFIP2 | -0.3106116 | 0.1752066 | -1.7728 | 0.0763 | -0.405247138 | count | 1 |
| HNRNPD | -0.286123 | 0.0609632 | -4.6934 | 2.79E-06 | -0.405152596 | count | 0.06716925 |
| NMT2 | -0.3389274 | 0.1969201 | -1.7211 | 0.0853 | -0.404944412 | count | 1 |
| D2HGDH | -0.3817484 | 0.2498485 | -1.5279 | 0.127 | -0.404825727 | count | 1 |
| AFTPH | -0.3175032 | 0.1716287 | -1.8499 | 0.0644 | -0.404463313 | count | 1 |
| TAF1B | -0.2998538 | 0.1648565 | -1.8189 | 0.069 | -0.40443657 | count | 1 |
| SURF4 | -0.2901567 | 0.1026854 | -2.8257 | 0.00475 | -0.404236746 | count | 1 |
| LGALS8 | -0.3264819 | 0.1479296 | -2.207 | 0.0274 | -0.404178726 | count | 1 |
| DIRC2 | -0.5266247 | 0.5178264 | -1.017 | 0.309 | -0.404051909 | count | 1 |
| Z84485.1 | -0.9070641 | 0.8217746 | -1.1038 | 0.27 | -0.40350211 | count | 1 |
| MAP3K7 | -0.3672482 | 0.2529259 | -1.452 | 0.147 | -0.403226432 | count | 1 |
| CLTA | -0.284265 | 0.0747046 | -3.8052 | 0.000144 | -0.403203732 | count | 1 |
| AC117382.2 | -0.5249952 | 0.8214519 | -0.6391 | 0.523 | -0.402906343 | count | 1 |
| KLHL15 | -0.6648558 | 0.3866462 | -1.7195 | 0.0856 | -0.4029033 | count | 1 |
| LNX2 | -0.4323872 | 0.3418877 | -1.2647 | 0.206 | -0.402641604 | count | 1 |
| ZNF277 | -0.3092945 | 0.1428988 | -2.1644 | 0.0305 | -0.402414422 | count | 1 |
| GPSM2 | -0.5242421 | 0.3446193 | -1.5212 | 0.128 | -0.402376658 | count | 1 |
| SNTA1 | -0.3164322 | 0.2180294 | -1.4513 | 0.147 | -0.402260858 | count | 1 |
| DHX16 | -0.3132254 | 0.1924719 | -1.6274 | 0.104 | -0.402210677 | count | 1 |
| LY6G5C | -0.3626281 | 0.3053878 | -1.1874 | 0.235 | -0.402131274 | count | 1 |

| | | | | | | | |
|---|---|---|---|---|---|---|---|
| NCOR2 | -0.3317159 | 0.1799728 | -1.8431 | 0.0654 | -0.401784396 | count | 1 |
| IFT43 | -0.3503024 | 0.2016779 | -1.7369 | 0.0825 | -0.40158266 | count | 1 |
| DUSP22 | -0.3081832 | 0.1779954 | -1.7314 | 0.0835 | -0.401553623 | count | 1 |
| MGA | -0.3158454 | 0.1766606 | -1.7879 | 0.0739 | -0.401524113 | count | 1 |
| EIF4A1 | -0.2874462 | 0.0729674 | -3.9394 | 8.33E-05 | -0.40140287 | count | 1 |
| ANKRD9 | -0.4309688 | 0.3226191 | -1.3358 | 0.182 | -0.401388579 | count | 1 |
| RIMKLB | -0.4751128 | 0.3352018 | -1.4174 | 0.156 | -0.401188805 | count | 1 |
| ZCCHC18 | -0.6609237 | 0.5320864 | -1.2421 | 0.214 | -0.400836016 | count | 1 |
| IFIT2 | -0.3017717 | 0.2859842 | -1.0552 | 0.291 | -0.400564294 | count | 1 |
| ZDHHC6 | -0.306109 | 0.1386642 | -2.2076 | 0.0273 | -0.400491402 | count | 1 |
| AC007325.4 | -1.1898777 | 0.8754176 | -1.3592 | 0.174 | -0.400418649 | count | 1 |
| RASSF2 | -0.3684678 | 0.2284501 | -1.6129 | 0.107 | -0.400374334 | count | 1 |
| USP47 | -0.3004724 | 0.1258407 | -2.3877 | 0.017 | -0.400289944 | count | 1 |
| MINOS1 | -0.2826583 | 0.0773421 | -3.6546 | 0.000261 | -0.400139433 | count | 1 |
| ODC1 | -0.284984 | 0.086896 | -3.2796 | 0.00105 | -0.400053844 | count | 1 |
| CEP89 | -0.3877956 | 0.2767237 | -1.4014 | 0.161 | -0.3998785 | count | 1 |
| PCM1 | -0.2873344 | 0.0766622 | -3.7481 | 0.000181 | -0.39976641 | count | 1 |
| OAZ3 | -0.7485959 | 1.266165 | -0.5912 | 0.554 | -0.399616258 | count | 1 |
| AC008946.1 | -0.7485959 | 1.266165 | -0.5912 | 0.554 | -0.399616258 | count | 1 |
| AC093673.2 | -0.7485959 | 1.4576124 | -0.5136 | 0.608 | -0.399616258 | count | 1 |
| MAPK11 | -0.5201225 | 0.3106587 | -1.6743 | 0.0942 | -0.39947645 | count | 1 |
| NANOS1 | -0.6580817 | 0.6445865 | -1.0209 | 0.307 | -0.399339339 | count | 1 |
| CSNK1E | -0.3509078 | 0.2292046 | -1.531 | 0.126 | -0.399335485 | count | 1 |
| USP3-AS1 | -2.1435722 | 1.2591123 | -1.7024 | 0.0888 | -0.399234506 | count | 1 |
| VEGFB | -0.2851176 | 0.0972958 | -2.9304 | 0.00341 | -0.39916874 | count | 1 |
| CELF1 | -0.3080972 | 0.1512331 | -2.0372 | 0.0417 | -0.399066036 | count | 1 |
| EEF1AKMT4 | -0.5967315 | 0.4275099 | -1.3958 | 0.163 | -0.399037954 | count | 1 |
| USP39 | -0.3167071 | 0.1762097 | -1.7973 | 0.0724 | -0.398891045 | count | 1 |
| TNFAIP8 | -0.2796541 | 0.0539277 | -5.1857 | 2.28E-07 | -0.398783026 | count | 0.005502324 |
| PPP1R3D | -0.3866514 | 0.3085963 | -1.2529 | 0.21 | -0.398742007 | count | 1 |
| OAZ2 | -0.3808845 | 0.220032 | -1.731 | 0.0835 | -0.398672952 | count | 1 |
| MAN2C1 | -0.4927665 | 0.2806445 | -1.7558 | 0.0792 | -0.398561103 | count | 1 |
| UBAC1 | -0.3001157 | 0.1597522 | -1.8786 | 0.0604 | -0.398383452 | count | 1 |
| NFKB2 | -0.3558518 | 0.1372268 | -2.5932 | 0.00955 | -0.39835369 | count | 1 |
| MAPK6 | -0.3176202 | 0.1955009 | -1.6246 | 0.104 | -0.397964607 | count | 1 |
| CP | -0.8910042 | 1.02778 | -0.8669 | 0.386 | -0.397902464 | count | 1 |
| EMILIN1 | -0.8910042 | 1.0797149 | -0.8252 | 0.409 | -0.397902464 | count | 1 |
| RNF157 | -0.3399148 | 0.2480407 | -1.3704 | 0.171 | -0.397572017 | count | 1 |
| LRRC41 | -0.3490295 | 0.2372721 | -1.471 | 0.141 | -0.397250093 | count | 1 |
| NEDD4 | -0.3847435 | 0.3432084 | -1.121 | 0.262 | -0.396846307 | count | 1 |
| PSEN1 | -0.3484794 | 0.1993278 | -1.7483 | 0.0805 | -0.396639227 | count | 1 |
| MIR646HG | -2.108397 | 1.82836 | -1.1532 | 0.249 | -0.396616499 | count | 1 |
| UBE2E2 | -0.5926221 | 0.678219 | -0.8738 | 0.382 | -0.39658939 | count | 1 |
| OMD | -0.6527318 | 0.780944 | -0.8358 | 0.403 | -0.396516295 | count | 1 |
| SSH1 | -0.4518681 | 0.3157245 | -1.4312 | 0.152 | -0.396379599 | count | 1 |

| | | | | | | | |
|---|---|---|---|---|---|---|---|
| FBXO22 | -0.3406751 | 0.2129571 | -1.5997 | 0.11 | -0.396027459 | count | 1 |
| FHOD1 | -0.4142604 | 0.3313634 | -1.2502 | 0.211 | -0.395882026 | count | 1 |
| SNAPC2 | -0.2953946 | 0.1434333 | -2.0595 | 0.0395 | -0.395781178 | count | 1 |
| BCL7A | -2.0971801 | 1.3608175 | -1.5411 | 0.123 | -0.395764075 | count | 1 |
| RAB2A | -0.2813256 | 0.0803412 | -3.5016 | 0.000468 | -0.395644083 | count | 1 |
| CCDC91 | -0.2846594 | 0.0952187 | -2.9895 | 0.00281 | -0.395619302 | count | 1 |
| AL022069.1 | -0.7391377 | 0.7588836 | -0.974 | 0.33 | -0.395383989 | count | 1 |
| SESN2 | -0.3636878 | 0.16764 | -2.1695 | 0.0301 | -0.395335061 | count | 1 |
| NIPAL3 | -0.3152179 | 0.1698176 | -1.8562 | 0.0635 | -0.39499562 | count | 1 |
| IER3 | -0.2853449 | 0.1306235 | -2.1845 | 0.029 | -0.394987447 | count | 1 |
| DDAH2 | -0.2871096 | 0.1259344 | -2.2798 | 0.0227 | -0.394982897 | count | 1 |
| TCFL5 | -0.4358017 | 0.4243729 | -1.0269 | 0.305 | -0.394918486 | count | 1 |
| AC015819.1 | -0.6496196 | 0.7027366 | -0.9244 | 0.355 | -0.394870621 | count | 1 |
| EPS8L1 | -0.4666182 | 0.5676713 | -0.822 | 0.411 | -0.394484096 | count | 1 |
| HERC3 | -0.328174 | 0.1880525 | -1.7451 | 0.0811 | -0.394158174 | count | 1 |
| CLDND2 | -0.4490872 | 0.420214 | -1.0687 | 0.285 | -0.394084492 | count | 1 |
| CD4 | -0.2928591 | 0.1306685 | -2.2412 | 0.0251 | -0.394057783 | count | 1 |
| CHTOP | -0.3003663 | 0.1414392 | -2.1236 | 0.0338 | -0.394036458 | count | 1 |
| FBXO25 | -0.3762473 | 0.2173817 | -1.7308 | 0.0836 | -0.393985706 | count | 1 |
| SLFN12 | -0.3517383 | 0.3384908 | -1.0391 | 0.299 | -0.393868911 | count | 1 |
| NCBP1 | -0.3366594 | 0.2822017 | -1.193 | 0.233 | -0.393844441 | count | 1 |
| BTG3 | -0.2780053 | 0.0718303 | -3.8703 | 0.000111 | -0.393805245 | count | 1 |
| DHODH | -0.3709951 | 0.3281884 | -1.1304 | 0.258 | -0.393794833 | count | 1 |
| DICER1 | -0.3035039 | 0.1867196 | -1.6255 | 0.104 | -0.393785474 | count | 1 |
| TMCO3 | -0.3814855 | 0.2943877 | -1.2959 | 0.195 | -0.393607284 | count | 1 |
| EIF1AX | -0.2793944 | 0.0748011 | -3.7352 | 0.000191 | -0.393586072 | count | 1 |
| PFDN2 | -0.2778643 | 0.0687114 | -4.0439 | 5.37E-05 | -0.393569718 | count | 1 |
| ANO9 | -0.3513421 | 0.2750532 | -1.2774 | 0.202 | -0.393436788 | count | 1 |
| NUP205 | -0.4856821 | 0.3424325 | -1.4183 | 0.156 | -0.393244361 | count | 1 |
| KCTD13 | -0.3305624 | 0.2581244 | -1.2806 | 0.2 | -0.393213726 | count | 1 |
| ZNF33B | -0.3358083 | 0.1910444 | -1.7578 | 0.0789 | -0.3928696 | count | 1 |
| EXOC1 | -0.3062778 | 0.174608 | -1.7541 | 0.0795 | -0.392658264 | count | 1 |
| ZBTB20-AS2 | -0.5426705 | 0.4889352 | -1.1099 | 0.267 | -0.392410611 | count | 1 |
| ARHGAP21 | -0.6449658 | 0.3024434 | -2.1325 | 0.033 | -0.392405097 | count | 1 |
| NRIP1 | -0.3746698 | 0.2492657 | -1.5031 | 0.133 | -0.392390128 | count | 1 |
| PPFIA1 | -0.3167534 | 0.1774682 | -1.7848 | 0.0744 | -0.392312986 | count | 1 |
| METTL22 | -0.7322527 | 0.5016983 | -1.4595 | 0.145 | -0.392289397 | count | 1 |
| SNX2 | -0.28456 | 0.1016466 | -2.7995 | 0.00515 | -0.392185493 | count | 1 |
| HAUS7 | -0.4839761 | 0.7094267 | -0.6822 | 0.495 | -0.391962032 | count | 1 |
| RPA4 | -0.87313 | 1.3818564 | -0.6319 | 0.528 | -0.391604528 | count | 1 |
| ARNT2 | -0.87313 | 1.4930402 | -0.5848 | 0.559 | -0.391604528 | count | 1 |
| ZNF23 | -0.87313 | 1.4930402 | -0.5848 | 0.559 | -0.391604528 | count | 1 |
| KIFC3 | -0.3495085 | 0.7025994 | -0.4975 | 0.619 | -0.391436529 | count | 1 |
| VPS35L | -0.5087172 | 0.3668329 | -1.3868 | 0.166 | -0.391423346 | count | 1 |
| AGPAT2 | -0.2866473 | 0.1287235 | -2.2268 | 0.026 | -0.391253643 | count | 1 |

| | | | | | | | |
|---|---|---|---|---|---|---|---|
| PAOX | -0.3852761 | 0.3164674 | -1.2174 | 0.224 | -0.391195975 | count | 1 |
| MOCS2 | -0.2979616 | 0.1889068 | -1.5773 | 0.115 | -0.390909714 | count | 1 |
| EXOC6B | -0.3997563 | 0.3698825 | -1.0808 | 0.28 | -0.390841152 | count | 1 |
| CEPT1 | -0.3360292 | 0.2438452 | -1.378 | 0.168 | -0.390742829 | count | 1 |
| MORN2 | -0.481924 | 0.462944 | -1.041 | 0.298 | -0.390418539 | count | 1 |
| RRAD | -0.8697032 | 0.5265912 | -1.6516 | 0.0987 | -0.390389086 | count | 1 |
| TBCEL | -0.4444892 | 0.5523898 | -0.8047 | 0.421 | -0.39028535 | count | 1 |
| MTDH | -0.2728297 | 0.054185 | -5.0351 | 5.02E-07 | -0.389894543 | count | 0.012107236 |
| CD82 | -0.2794883 | 0.1076394 | -2.5965 | 0.00946 | -0.38979599 | count | 1 |
| LUZP1 | -0.2843217 | 0.1033187 | -2.7519 | 0.00596 | -0.389744134 | count | 1 |
| TMEM120B | -0.2800029 | 0.1081632 | -2.5887 | 0.00967 | -0.389599307 | count | 1 |
| CPA5 | -0.5057557 | 0.5460475 | -0.9262 | 0.354 | -0.389326554 | count | 1 |
| CRACR2B | -0.7256204 | 0.7771216 | -0.9337 | 0.351 | -0.389297476 | count | 1 |
| POLR3E | -0.3344534 | 0.1983694 | -1.686 | 0.0919 | -0.38894956 | count | 1 |
| AKAP8 | -0.3057136 | 0.1504521 | -2.032 | 0.0422 | -0.388797194 | count | 1 |
| MTHFR | -0.3974602 | 0.3385024 | -1.1742 | 0.24 | -0.388691925 | count | 1 |
| TM9SF4 | -0.3342033 | 0.2164482 | -1.544 | 0.123 | -0.38866491 | count | 1 |
| EP400 | -0.3763014 | 0.1992881 | -1.8882 | 0.0591 | -0.388448571 | count | 1 |
| TRAM2-AS1 | -0.8641209 | 0.8866342 | -0.9746 | 0.33 | -0.388403761 | count | 1 |
| AC008764.6 | -0.5040627 | 0.4629891 | -1.0887 | 0.276 | -0.388126846 | count | 1 |
| TRG-AS1 | -0.2811223 | 0.1320566 | -2.1288 | 0.0333 | -0.388104596 | count | 1 |
| GGACT | -0.3886262 | 0.2717721 | -1.43 | 0.153 | -0.387724878 | count | 1 |
| KPNA2 | -0.2801855 | 0.0846683 | -3.3092 | 0.000945 | -0.387689666 | count | 1 |
| WDR43 | -0.2830816 | 0.1140523 | -2.482 | 0.0131 | -0.387629242 | count | 1 |
| ZNF367 | -0.5353509 | 0.5193324 | -1.0308 | 0.303 | -0.387597662 | count | 1 |
| LXN | -0.3160473 | 0.2429453 | -1.3009 | 0.193 | -0.387539153 | count | 1 |
| BBS2 | -0.5030561 | 0.3402379 | -1.4785 | 0.139 | -0.387413186 | count | 1 |
| UBE3C | -0.3601572 | 0.2040801 | -1.7648 | 0.0777 | -0.387282821 | count | 1 |
| GRIPAP1 | -0.2975466 | 0.1430942 | -2.0794 | 0.0377 | -0.387275581 | count | 1 |
| USP3 | -0.2794384 | 0.0912748 | -3.0615 | 0.00222 | -0.387193297 | count | 1 |
| PTMS | -0.277516 | 0.1382309 | -2.0076 | 0.0448 | -0.386992822 | count | 1 |
| KCNMB1 | -1.9870778 | 1.176794 | -1.6886 | 0.0914 | -0.386931218 | count | 1 |
| CHERP | -0.3808079 | 0.222963 | -1.7079 | 0.0877 | -0.386828826 | count | 1 |
| PGM2 | -0.3054549 | 0.1912389 | -1.5972 | 0.11 | -0.386744597 | count | 1 |
| SECISBP2 | -0.2822208 | 0.1029149 | -2.7423 | 0.00613 | -0.386738884 | count | 1 |
| HACD3 | -0.2943088 | 0.1661985 | -1.7708 | 0.0767 | -0.386625537 | count | 1 |
| KLHDC2 | -0.283474 | 0.1197217 | -2.3678 | 0.018 | -0.386616107 | count | 1 |
| LAS1L | -0.3091811 | 0.1982978 | -1.5592 | 0.119 | -0.386476245 | count | 1 |
| SRRT | -0.2862692 | 0.1139162 | -2.513 | 0.012 | -0.386459415 | count | 1 |
| ALCAM | -0.6334922 | 0.4575949 | -1.3844 | 0.166 | -0.386302619 | count | 1 |
| HBEGF | -0.3591799 | 0.3156942 | -1.1377 | 0.255 | -0.386263645 | count | 1 |
| YAE1D1 | -0.3869211 | 0.3642655 | -1.0622 | 0.288 | -0.386091076 | count | 1 |
| CLSTN3 | -0.31983 | 0.2091038 | -1.5295 | 0.126 | -0.386008027 | count | 1 |
| REXO2 | -0.2786275 | 0.1038029 | -2.6842 | 0.00731 | -0.385987489 | count | 1 |
| APOLD1 | -0.3135674 | 0.1960956 | -1.5991 | 0.11 | -0.385889446 | count | 1 |

| | | | | | | | |
|---|---|---|---|---|---|---|---|
| PRDX4 | -0.2877472 | 0.1415642 | -2.0326 | 0.0422 | -0.385886253 | count | 1 |
| LPIN1 | -0.3040618 | 0.1211093 | -2.5106 | 0.0121 | -0.385875692 | count | 1 |
| STK17A | -0.2691081 | 0.0451485 | -5.9605 | 2.77E-09 | -0.385784408 | count | 6.70E-05 |
| MNAT1 | -0.2850134 | 0.1277059 | -2.2318 | 0.0257 | -0.385666602 | count | 1 |
| C6orf48 | -0.2820622 | 0.0861669 | -3.2734 | 0.00107 | -0.385650353 | count | 1 |
| DAP3 | -0.2840512 | 0.1039059 | -2.7337 | 0.00629 | -0.38559856 | count | 1 |
| CAMK1 | -0.3734141 | 0.3667302 | -1.0182 | 0.309 | -0.385572847 | count | 1 |
| MIR762HG | -0.4029464 | 0.3084485 | -1.3064 | 0.192 | -0.38555998 | count | 1 |
| KTN1-AS1 | -1.9702262 | 1.1360471 | -1.7343 | 0.083 | -0.385501883 | count | 1 |
| NUDT22 | -0.2810943 | 0.1317132 | -2.1341 | 0.0329 | -0.385476305 | count | 1 |
| DCUN1D4 | -0.3857046 | 0.2746879 | -1.4042 | 0.16 | -0.384925039 | count | 1 |
| DRAP1 | -0.2695546 | 0.0614729 | -4.3849 | 1.20E-05 | -0.384848394 | count | 0.288324 |
| NT5M | -0.6306514 | 0.5547663 | -1.1368 | 0.256 | -0.384786429 | count | 1 |
| USP1 | -0.2749295 | 0.100368 | -2.7392 | 0.00619 | -0.384603767 | count | 1 |
| CCDC107 | -0.2701949 | 0.0643727 | -4.1974 | 2.77E-05 | -0.384581786 | count | 0.6645784 |
| AP1G2 | -0.3135973 | 0.1870787 | -1.6763 | 0.0938 | -0.384581201 | count | 1 |
| TOP2A | -0.8533686 | 0.4884833 | -1.747 | 0.0807 | -0.384560645 | count | 1 |
| ASL | -0.4236705 | 0.3043466 | -1.3921 | 0.164 | -0.38450416 | count | 1 |
| PRELID3B | -0.2790293 | 0.108495 | -2.5718 | 0.0102 | -0.384483962 | count | 1 |
| IL4R | -0.312407 | 0.1665927 | -1.8753 | 0.0608 | -0.384482848 | count | 1 |
| GALK1 | -0.3029226 | 0.2208423 | -1.3717 | 0.17 | -0.384447169 | count | 1 |
| ST3GAL1 | -0.3093084 | 0.1186694 | -2.6065 | 0.00919 | -0.384402958 | count | 1 |
| FMC1 | -0.3082504 | 0.1818033 | -1.6955 | 0.0901 | -0.384237888 | count | 1 |
| ZEB1 | -0.3000859 | 0.1439646 | -2.0844 | 0.0372 | -0.384071125 | count | 1 |
| GLG1 | -0.2857545 | 0.1129534 | -2.5298 | 0.0115 | -0.384042226 | count | 1 |
| ATG5 | -0.2767494 | 0.0972642 | -2.8453 | 0.00446 | -0.383649113 | count | 1 |
| LHFPL6 | -1.1183962 | 0.6404904 | -1.7462 | 0.0809 | -0.383641276 | count | 1 |
| SARM1 | -0.472876 | 0.3796509 | -1.2456 | 0.213 | -0.383599782 | count | 1 |
| SFMBT1 | -0.3488301 | 0.2067705 | -1.687 | 0.0917 | -0.383560665 | count | 1 |
| FAM89B | -0.269531 | 0.062241 | -4.3304 | 1.53E-05 | -0.383530616 | count | 0.3674601 |
| RPA1 | -0.3221882 | 0.2231377 | -1.4439 | 0.149 | -0.383437077 | count | 1 |
| LIME1 | -0.7124674 | 0.8511466 | -0.8371 | 0.403 | -0.383332157 | count | 1 |
| WDR61 | -0.2949209 | 0.1572592 | -1.8754 | 0.0608 | -0.383331301 | count | 1 |
| HIC1 | -0.384007 | 0.2354919 | -1.6307 | 0.103 | -0.383297345 | count | 1 |
| UQCC3 | -0.286009 | 0.1671876 | -1.7107 | 0.0872 | -0.3832895 | count | 1 |
| CSRP1 | -0.2815993 | 0.1313334 | -2.1442 | 0.0321 | -0.383214559 | count | 1 |
| LIN7B | -0.4103865 | 0.3064589 | -1.3391 | 0.181 | -0.383149845 | count | 1 |
| CORO1B | -0.2702338 | 0.0760933 | -3.5513 | 0.000388 | -0.382934099 | count | 1 |
| SPRYD7 | -0.3133619 | 0.2890536 | -1.0841 | 0.278 | -0.382898092 | count | 1 |
| GPRIN3 | -0.2778277 | 0.0924183 | -3.0062 | 0.00266 | -0.382834938 | count | 1 |
| PRPF6 | -0.2794391 | 0.1064741 | -2.6245 | 0.00872 | -0.3828054 | count | 1 |
| TMEM164 | -0.569547 | 0.62492 | -0.9114 | 0.362 | -0.382756804 | count | 1 |
| MAF | -0.2760859 | 0.0997046 | -2.769 | 0.00565 | -0.382648973 | count | 1 |
| NUDT7 | -0.3555368 | 0.3358029 | -1.0588 | 0.29 | -0.382462714 | count | 1 |
| FAR1 | -0.3267208 | 0.193396 | -1.6894 | 0.0912 | -0.382453472 | count | 1 |

| | | | | | | | |
|---|---|---|---|---|---|---|---|
| NDEL1 | -0.2976401 | 0.138612 | -2.1473 | 0.0318 | -0.38241167 | count | 1 |
| HIST1H4C | -0.2681043 | 0.0643563 | -4.1659 | 3.18E-05 | -0.382364718 | count | 0.762723 |
| ANP32E | -0.2691893 | 0.0644723 | -4.1753 | 3.05E-05 | -0.382164003 | count | 0.731573 |
| NDUFB8 | -0.2673807 | 0.0584982 | -4.5708 | 5.03E-06 | -0.381889909 | count | 0.12100671 |
| NIT1 | -0.3001784 | 0.2467811 | -1.2164 | 0.224 | -0.38183936 | count | 1 |
| CMPK2 | -0.3471294 | 0.3453455 | -1.0052 | 0.315 | -0.38174141 | count | 1 |
| NUTM2B-AS1 | -0.2806616 | 0.1153658 | -2.4328 | 0.015 | -0.381399969 | count | 1 |
| Z98885.3 | -0.8431866 | 0.8240664 | -1.0232 | 0.306 | -0.380898097 | count | 1 |
| ANKRD13B | -1.1070006 | 1.1276858 | -0.9817 | 0.326 | -0.380886686 | count | 1 |
| KLHL24 | -0.2860308 | 0.1261788 | -2.2669 | 0.0235 | -0.380858249 | count | 1 |
| RGS10 | -0.2665662 | 0.0558275 | -4.7748 | 1.87E-06 | -0.380711728 | count | 0.04504456 |
| AP4B1-AS1 | -1.9154028 | 1.2088074 | -1.5845 | 0.113 | -0.380703052 | count | 1 |
| KIF1C | -0.3581509 | 0.3532627 | -1.0138 | 0.311 | -0.380587242 | count | 1 |
| AL359915.2 | -0.8420839 | 0.618563 | -1.3614 | 0.173 | -0.380500088 | count | 1 |
| CHMP2B | -0.2755459 | 0.0934132 | -2.9498 | 0.0032 | -0.380334791 | count | 1 |
| ASB7 | -0.4187842 | 0.3058549 | -1.3692 | 0.171 | -0.380298973 | count | 1 |
| PRKAB2 | -0.3493613 | 0.3723011 | -0.9384 | 0.348 | -0.380204062 | count | 1 |
| SNRNP48 | -0.3040524 | 0.165947 | -1.8322 | 0.067 | -0.380150304 | count | 1 |
| ZBTB2 | -0.2994959 | 0.177235 | -1.6898 | 0.0912 | -0.380149306 | count | 1 |
| UBA2 | -0.2770623 | 0.0990645 | -2.7968 | 0.00519 | -0.380103816 | count | 1 |
| PLK3 | -0.2753144 | 0.1020178 | -2.6987 | 0.007 | -0.380016485 | count | 1 |
| CINP | -0.3000691 | 0.1885224 | -1.5917 | 0.112 | -0.380007116 | count | 1 |
| SERBP1 | -0.2660406 | 0.0477527 | -5.5712 | 2.73E-08 | -0.379988022 | count | 0.000659786 |
| FAM228B | -0.3575027 | 0.283632 | -1.2604 | 0.208 | -0.37991979 | count | 1 |
| SGTB | -0.2788099 | 0.1227319 | -2.2717 | 0.0232 | -0.379785164 | count | 1 |
| EIF5 | -0.2659035 | 0.0465768 | -5.7089 | 1.23E-08 | -0.379717817 | count | 0.000297377 |
| AK9 | -0.3262682 | 0.3049349 | -1.07 | 0.285 | -0.379628048 | count | 1 |
| OCIAD2 | -0.2663709 | 0.0605268 | -4.4009 | 1.11E-05 | -0.379577438 | count | 0.2667774 |
| HSD17B1 | -0.6208505 | 0.6138736 | -1.0114 | 0.312 | -0.379539502 | count | 1 |
| DRAM1 | -0.8393785 | 0.5056437 | -1.66 | 0.097 | -0.379522471 | count | 1 |
| UBE2B | -0.2655254 | 0.0538449 | -4.9313 | 8.56E-07 | -0.378842749 | count | 0.020637304 |
| SLC4A10 | -0.7023684 | 0.7255783 | -0.968 | 0.333 | -0.378723266 | count | 1 |
| SLC40A1 | -0.3323513 | 0.285575 | -1.1638 | 0.245 | -0.378705286 | count | 1 |
| ZNF394 | -0.2725175 | 0.0835966 | -3.2599 | 0.00113 | -0.378580919 | count | 1 |
| DCUN1D1 | -0.3018798 | 0.1647851 | -1.832 | 0.067 | -0.378498266 | count | 1 |
| SCRN1 | -1.097011 | 1.0804397 | -1.0153 | 0.31 | -0.378453555 | count | 1 |
| HLA-DOA | -1.097011 | 1.2688286 | -0.8646 | 0.387 | -0.378453555 | count | 1 |
| SUN3 | -1.097011 | 1.3369066 | -0.8206 | 0.412 | -0.378453555 | count | 1 |
| TMEM45A | -1.097011 | 1.493283 | -0.7346 | 0.463 | -0.378453555 | count | 1 |
| RTN4RL2 | -1.097011 | 1.493283 | -0.7346 | 0.463 | -0.378453555 | count | 1 |
| SPNS2 | -1.097011 | 1.493283 | -0.7346 | 0.463 | -0.378453555 | count | 1 |
| ARHGEF35 | -0.5214055 | 0.4384933 | -1.1891 | 0.234 | -0.378388362 | count | 1 |
| AC106028.4 | -0.3559655 | 0.6091264 | -0.5844 | 0.559 | -0.378336587 | count | 1 |
| SIL1 | -0.3119033 | 0.2426099 | -1.2856 | 0.199 | -0.378179994 | count | 1 |
| WRNIP1 | -0.3105696 | 0.2063607 | -1.505 | 0.132 | -0.378095148 | count | 1 |

| | | | | | | | |
|---|---|---|---|---|---|---|---|
| STK24 | -0.2765189 | 0.1029458 | -2.6861 | 0.00727 | -0.377957358 | count | 1 |
| LYL1 | -0.346846 | 0.2822034 | -1.2291 | 0.219 | -0.377543298 | count | 1 |
| ELOA-AS1 | -0.4154718 | 0.4108376 | -1.0113 | 0.312 | -0.377444889 | count | 1 |
| SEC24B-AS1 | -0.4645605 | 0.6276197 | -0.7402 | 0.459 | -0.377313998 | count | 1 |
| AL591846.2 | -0.4645605 | 0.7736612 | -0.6005 | 0.548 | -0.377313998 | count | 1 |
| HMGXB4 | -0.2952913 | 0.1316936 | -2.2423 | 0.025 | -0.377255804 | count | 1 |
| TLE3 | -0.288108 | 0.1402532 | -2.0542 | 0.04 | -0.377145971 | count | 1 |
| CREBBP | -0.3023869 | 0.1518828 | -1.9909 | 0.0466 | -0.377026873 | count | 1 |
| C17orf80 | -0.3393357 | 0.2875943 | -1.1799 | 0.238 | -0.376969092 | count | 1 |
| GPR137 | -0.3166252 | 0.2477997 | -1.2777 | 0.201 | -0.376936426 | count | 1 |
| DNAJC7 | -0.2692531 | 0.0901788 | -2.9858 | 0.00285 | -0.376828397 | count | 1 |
| PAFAH1B1 | -0.2669125 | 0.0680425 | -3.9227 | 8.93E-05 | -0.376427248 | count | 1 |
| DKC1 | -0.2907192 | 0.1484796 | -1.958 | 0.0503 | -0.376177619 | count | 1 |
| UBE3A | -0.2763135 | 0.1073188 | -2.5747 | 0.0101 | -0.375703365 | count | 1 |
| UCHL5 | -0.274933 | 0.1251555 | -2.1967 | 0.0281 | -0.375649174 | count | 1 |
| TRIB3 | -0.3295196 | 0.2945447 | -1.1187 | 0.263 | -0.375551744 | count | 1 |
| PSAT1 | -0.5170834 | 0.3759925 | -1.3752 | 0.169 | -0.375523601 | count | 1 |
| STX18-AS1 | -1.8580744 | 1.3031001 | -1.4259 | 0.154 | -0.37543356 | count | 1 |
| HSPD1 | -0.2642533 | 0.0564854 | -4.6783 | 3.01E-06 | -0.375395385 | count | 0.07245672 |
| BYSL | -0.4128504 | 0.4164124 | -0.9914 | 0.322 | -0.375184268 | count | 1 |
| LIG1 | -0.3135373 | 0.2500826 | -1.2537 | 0.21 | -0.375144144 | count | 1 |
| SMARCD1 | -0.2757062 | 0.1309985 | -2.1047 | 0.0354 | -0.375060077 | count | 1 |
| CAVIN2 | -0.61158 | 0.9446441 | -0.6474 | 0.517 | -0.374553772 | count | 1 |
| MGRN1 | -0.3129233 | 0.2301612 | -1.3596 | 0.174 | -0.374422284 | count | 1 |
| EAF1-AS1 | -1.0791436 | 0.9899787 | -1.0901 | 0.276 | -0.374059208 | count | 1 |
| DDB1 | -0.2957926 | 0.1838298 | -1.6091 | 0.108 | -0.373778371 | count | 1 |
| BRICD5 | -1.0770306 | 0.8875676 | -1.2135 | 0.225 | -0.373535858 | count | 1 |
| AC027097.1 | -0.4594523 | 0.3649501 | -1.2589 | 0.208 | -0.373443648 | count | 1 |
| PLPP2 | -1.83679 | 0.6226155 | -2.9501 | 0.0032 | -0.373409604 | count | 1 |
| IL17RE | -0.4593604 | 0.8069742 | -0.5692 | 0.569 | -0.37337395 | count | 1 |
| SPTAN1 | -0.2682841 | 0.0924721 | -2.9012 | 0.00374 | -0.373209768 | count | 1 |
| PPP1R21 | -0.3358185 | 0.2123961 | -1.5811 | 0.114 | -0.373160593 | count | 1 |
| FBXO34 | -0.2745247 | 0.1144442 | -2.3988 | 0.0165 | -0.372920255 | count | 1 |
| PPP5D1 | -0.6895243 | 0.5912873 | -1.1661 | 0.244 | -0.372825655 | count | 1 |
| TRIM5 | -0.3036761 | 0.2316293 | -1.311 | 0.19 | -0.372594706 | count | 1 |
| AC002310.1 | -0.4582034 | 0.3905836 | -1.1731 | 0.241 | -0.372496344 | count | 1 |
| SLC5A3 | -0.2809036 | 0.1636141 | -1.7169 | 0.0861 | -0.372354198 | count | 1 |
| SLC35A5 | -0.3318491 | 0.3009366 | -1.1027 | 0.27 | -0.372140005 | count | 1 |
| SPIDR | -0.3010094 | 0.211691 | -1.4219 | 0.155 | -0.371894364 | count | 1 |
| RPGR | -0.3414786 | 0.2447958 | -1.395 | 0.163 | -0.371861324 | count | 1 |
| MARVELD1 | -0.6872544 | 0.9607709 | -0.7153 | 0.474 | -0.371779209 | count | 1 |
| ROBO3 | -0.5113994 | 0.6553112 | -0.7804 | 0.435 | -0.371748579 | count | 1 |
| JAG2 | -0.551225 | 0.5294629 | -1.0411 | 0.298 | -0.371673141 | count | 1 |
| CARHSP1 | -0.2679849 | 0.1067201 | -2.5111 | 0.0121 | -0.371540295 | count | 1 |
| SPATA13 | -0.290713 | 0.15676 | -1.8545 | 0.0638 | -0.371469804 | count | 1 |

| | | | | | | | |
|---|---|---|---|---|---|---|---|
| ANXA5 | -0.261184 | 0.065386 | -3.9945 | 6.62E-05 | -0.371382548 | count | 1 |
| RFC1 | -0.2635628 | 0.0835273 | -3.1554 | 0.00162 | -0.371357198 | count | 1 |
| KIAA0100 | -0.3538535 | 0.1929965 | -1.8335 | 0.0668 | -0.371285488 | count | 1 |
| MTHFD1L | -0.3646494 | 0.3843708 | -0.9487 | 0.343 | -0.370998556 | count | 1 |
| SCYL1 | -0.3206655 | 0.1936821 | -1.6556 | 0.0979 | -0.370855985 | count | 1 |
| KLHDC7B | -0.3306678 | 0.3057864 | -1.0814 | 0.28 | -0.370847128 | count | 1 |
| TRIM26 | -0.3146801 | 0.2011779 | -1.5642 | 0.118 | -0.370741219 | count | 1 |
| EPN2 | -0.3783052 | 0.3350572 | -1.1291 | 0.259 | -0.370714077 | count | 1 |
| AKAP9 | -0.2619794 | 0.070281 | -3.7276 | 0.000196 | -0.370552838 | count | 1 |
| RAC1 | -0.2596155 | 0.0548404 | -4.734 | 2.29E-06 | -0.370551373 | count | 0.05514778 |
| PPP1R16B | -0.3127413 | 0.1834751 | -1.7045 | 0.0884 | -0.370494334 | count | 1 |
| ZBTB6 | -0.3274916 | 0.3719555 | -0.8805 | 0.379 | -0.370416918 | count | 1 |
| BRD1 | -0.2698147 | 0.0897954 | -3.0048 | 0.00268 | -0.370330033 | count | 1 |
| NBL1 | -0.2968795 | 0.2030601 | -1.462 | 0.144 | -0.370249883 | count | 1 |
| FAM102A | -0.2764179 | 0.122142 | -2.2631 | 0.0237 | -0.370241737 | count | 1 |
| HPF1 | -0.2709459 | 0.1384869 | -1.9565 | 0.0505 | -0.369768663 | count | 1 |
| IDH1 | -0.3295783 | 0.2233911 | -1.4753 | 0.14 | -0.369654502 | count | 1 |
| TRAV3 | -0.6825866 | 0.9445964 | -0.7226 | 0.47 | -0.369623341 | count | 1 |
| SPATS2 | -0.2981118 | 0.2524449 | -1.1809 | 0.238 | -0.369541545 | count | 1 |
| RAB27A | -0.2668572 | 0.1016149 | -2.6262 | 0.00867 | -0.369485398 | count | 1 |
| NFIX | -1.795873 | 0.8937021 | -2.0095 | 0.0446 | -0.369412813 | count | 1 |
| TDRKH | -0.4192607 | 0.3980518 | -1.0533 | 0.292 | -0.36934387 | count | 1 |
| AQP3 | -0.2622532 | 0.0926801 | -2.8297 | 0.00469 | -0.369342564 | count | 1 |
| SPNS1 | -0.2908464 | 0.1900185 | -1.5306 | 0.126 | -0.369294975 | count | 1 |
| ASNSD1 | -0.272779 | 0.1365464 | -1.9977 | 0.0458 | -0.369202212 | count | 1 |
| ZMYM3 | -0.4773645 | 0.4580734 | -1.0421 | 0.297 | -0.369106837 | count | 1 |
| ABHD3 | -0.3042284 | 0.1659937 | -1.8328 | 0.0669 | -0.369021097 | count | 1 |
| GLIPR2 | -0.2595735 | 0.0862199 | -3.0106 | 0.00263 | -0.368942026 | count | 1 |
| AFF1 | -0.2918827 | 0.1870085 | -1.5608 | 0.119 | -0.36889561 | count | 1 |
| HIBCH | -0.2898653 | 0.1989728 | -1.4568 | 0.145 | -0.368866535 | count | 1 |
| HLA-DPA1 | -0.2569111 | 0.0589189 | -4.3604 | 1.34E-05 | -0.368834664 | count | 0.321935 |
| EMC3 | -0.2639727 | 0.1022549 | -2.5815 | 0.00988 | -0.368765741 | count | 1 |
| HIST1H2BF | -0.6007213 | 0.5884885 | -1.0208 | 0.307 | -0.368685734 | count | 1 |
| CMTR1 | -0.4340924 | 0.2721825 | -1.5949 | 0.111 | -0.368637812 | count | 1 |
| CAPS2 | -0.5460736 | 0.6625498 | -0.8242 | 0.41 | -0.368540924 | count | 1 |
| LRCH4 | -0.2938266 | 0.166771 | -1.7619 | 0.0782 | -0.368527116 | count | 1 |
| PDE7A | -0.2703408 | 0.1024078 | -2.6398 | 0.00833 | -0.368305042 | count | 1 |
| CALU | -0.2887139 | 0.1558164 | -1.8529 | 0.064 | -0.368192091 | count | 1 |
| SMYD2 | -0.2859409 | 0.1746553 | -1.6372 | 0.102 | -0.368190662 | count | 1 |
| FAM98A | -0.2899117 | 0.1723778 | -1.6818 | 0.0927 | -0.368121512 | count | 1 |
| MICB | -0.3340359 | 0.2394539 | -1.395 | 0.163 | -0.36771647 | count | 1 |
| NATD1 | -0.5987791 | 0.6033749 | -0.9924 | 0.321 | -0.367632987 | count | 1 |
| CLSPN | -0.4169387 | 0.3309094 | -1.26 | 0.208 | -0.367408329 | count | 1 |
| STRIP1 | -0.3452728 | 0.2685611 | -1.2856 | 0.199 | -0.367310392 | count | 1 |
| LRBA | -0.2867673 | 0.1631184 | -1.758 | 0.0788 | -0.367203837 | count | 1 |

| | | | | | | | |
|---|---|---|---|---|---|---|---|
| CCDC90B | -0.2659255 | 0.1146925 | -2.3186 | 0.0205 | -0.367202163 | count | 1 |
| STK40 | -0.3078108 | 0.2547036 | -1.2085 | 0.227 | -0.366626613 | count | 1 |
| ITGB1BP1 | -0.2610978 | 0.0917801 | -2.8448 | 0.00447 | -0.366599241 | count | 1 |
| SLC12A4 | -0.5425701 | 0.5192191 | -1.045 | 0.296 | -0.366406703 | count | 1 |
| TSPAN15 | -0.5961965 | 0.4869144 | -1.2244 | 0.221 | -0.366231611 | count | 1 |
| EPB41L4A-AS1 | -0.2838744 | 0.107203 | -2.648 | 0.00813 | -0.366189935 | count | 1 |
| CTNNA1 | -0.2925649 | 0.2312999 | -1.2649 | 0.206 | -0.365969416 | count | 1 |
| VKORC1 | -0.2625387 | 0.1025064 | -2.5612 | 0.0105 | -0.365932229 | count | 1 |
| DTYMK | -0.2942323 | 0.1871968 | -1.5718 | 0.116 | -0.365914568 | count | 1 |
| NAMPT | -0.2627371 | 0.099181 | -2.6491 | 0.00811 | -0.365839904 | count | 1 |
| LONRF3 | -0.6738726 | 0.6328152 | -1.0649 | 0.287 | -0.365584511 | count | 1 |
| VWA8 | -0.4145453 | 0.3069948 | -1.3503 | 0.177 | -0.365411838 | count | 1 |
| NBPF9 | -0.8005508 | 0.714555 | -1.1203 | 0.263 | -0.36531542 | count | 1 |
| AC015849.1 | -0.4486783 | 0.8860178 | -0.5064 | 0.613 | -0.365258089 | count | 1 |
| PIK3CB | -0.3065632 | 0.2102363 | -1.4582 | 0.145 | -0.365166394 | count | 1 |
| FANCE | -0.5013417 | 0.4050467 | -1.2377 | 0.216 | -0.36504783 | count | 1 |
| TP53 | -0.3253319 | 0.2337946 | -1.3915 | 0.164 | -0.365004071 | count | 1 |
| FLVCR1-DT | -0.364897 | 0.3480756 | -1.0483 | 0.295 | -0.364928397 | count | 1 |
| TMEM204 | -0.2873294 | 0.1905421 | -1.508 | 0.132 | -0.364879073 | count | 1 |
| TBCD | -0.2878049 | 0.1603734 | -1.7946 | 0.0728 | -0.364652609 | count | 1 |
| GBE1 | -0.4710533 | 0.4661441 | -1.0105 | 0.312 | -0.364583119 | count | 1 |
| GALNT11 | -0.293112 | 0.1912238 | -1.5328 | 0.125 | -0.36453956 | count | 1 |
| SLC16A3 | -0.2726424 | 0.1520076 | -1.7936 | 0.073 | -0.364367376 | count | 1 |
| PLXNA2 | -1.7458705 | 1.1905681 | -1.4664 | 0.143 | -0.364334597 | count | 1 |
| DDR1 | -1.7458705 | 1.249596 | -1.3971 | 0.162 | -0.364334597 | count | 1 |
| CENPI | -1.7458705 | 1.4803065 | -1.1794 | 0.238 | -0.364334597 | count | 1 |
| APPL1 | -0.2645168 | 0.1073292 | -2.4645 | 0.0138 | -0.364244149 | count | 1 |
| PDCD6 | -0.2574498 | 0.0728589 | -3.5335 | 0.000415 | -0.364233612 | count | 1 |
| AC124319.2 | -1.0397928 | 1.0328147 | -1.0068 | 0.314 | -0.364185587 | count | 1 |
| LRRC14 | -0.3380067 | 0.2759946 | -1.2247 | 0.221 | -0.364135611 | count | 1 |
| NCOA2 | -0.2955937 | 0.1823627 | -1.6209 | 0.105 | -0.364082197 | count | 1 |
| PRKCE | -0.797195 | 0.4990286 | -1.5975 | 0.11 | -0.364072021 | count | 1 |
| MRPS33 | -0.2638376 | 0.1186757 | -2.2232 | 0.0263 | -0.363937417 | count | 1 |
| PDP2 | -0.4701499 | 0.4249853 | -1.1063 | 0.269 | -0.36393473 | count | 1 |
| ZNF813 | -0.3792988 | 0.6003572 | -0.6318 | 0.528 | -0.363886756 | count | 1 |
| CYTOR | -0.2563563 | 0.0724468 | -3.5385 | 0.000408 | -0.363824675 | count | 1 |
| CTU1 | -0.3118941 | 0.27259 | -1.1442 | 0.253 | -0.363231482 | count | 1 |
| HGF | -1.7352618 | 1.343054 | -1.292 | 0.196 | -0.36322908 | count | 1 |
| JMJD6 | -0.2601883 | 0.0833127 | -3.123 | 0.00181 | -0.363120643 | count | 1 |
| RIOX2 | -0.2992261 | 0.2520417 | -1.1872 | 0.235 | -0.363047061 | count | 1 |
| TNFSF9 | -0.2547126 | 0.1131369 | -2.2514 | 0.0244 | -0.362808733 | count | 1 |
| MINK1 | -0.3366526 | 0.3093509 | -1.0883 | 0.277 | -0.362717378 | count | 1 |
| ZNF92 | -0.2788509 | 0.1566239 | -1.7804 | 0.0751 | -0.362630376 | count | 1 |
| AGL | -0.2814436 | 0.1734744 | -1.6224 | 0.105 | -0.362456228 | count | 1 |
| AC245297.3 | -0.2621357 | 0.1024084 | -2.5597 | 0.0105 | -0.362268201 | count | 1 |

| | | | | | | | |
|---|---|---|---|---|---|---|---|
| TFPT | -0.2621864 | 0.136325 | -1.9232 | 0.0545 | -0.362245658 | count | 1 |
| HSPB11 | -0.2573838 | 0.0891519 | -2.887 | 0.00391 | -0.362169441 | count | 1 |
| MITD1 | -0.264847 | 0.1126672 | -2.3507 | 0.0188 | -0.362075237 | count | 1 |
| ZNRF1 | -0.2632352 | 0.121731 | -2.1624 | 0.0307 | -0.362043976 | count | 1 |
| APOOL | -0.3401409 | 0.2532181 | -1.3433 | 0.179 | -0.362010087 | count | 1 |
| ETS2 | -0.2763996 | 0.1606416 | -1.7206 | 0.0854 | -0.361945794 | count | 1 |
| AC114760.2 | -0.3149603 | 0.1547401 | -2.0354 | 0.0419 | -0.361923247 | count | 1 |
| ZNF48 | -0.3688639 | 0.2924424 | -1.2613 | 0.207 | -0.361821628 | count | 1 |
| TAOK2 | -0.6653256 | 0.3615021 | -1.8404 | 0.0658 | -0.361605099 | count | 1 |
| DLG3 | -0.3442589 | 0.2487359 | -1.384 | 0.166 | -0.361527127 | count | 1 |
| RAPGEF5 | -1.0293729 | 0.9586703 | -1.0738 | 0.283 | -0.361525772 | count | 1 |
| DECR2 | -0.4437727 | 0.4490311 | -0.9883 | 0.323 | -0.361521026 | count | 1 |
| NEK11 | -0.4666915 | 0.6281389 | -0.743 | 0.458 | -0.36145055 | count | 1 |
| ATP6V1G2 | -1.7155119 | 1.085353 | -1.5806 | 0.114 | -0.361144295 | count | 1 |
| RPS19BP1 | -0.2547243 | 0.0708701 | -3.5942 | 0.00033 | -0.36109717 | count | 1 |
| PRPF3 | -0.2798552 | 0.1607116 | -1.7414 | 0.0817 | -0.361054777 | count | 1 |
| TTC33 | -0.3140488 | 0.1740419 | -1.8044 | 0.0713 | -0.360897543 | count | 1 |
| RTN2 | -0.6637599 | 0.9184952 | -0.7227 | 0.47 | -0.360874209 | count | 1 |
| YEATS2 | -0.3603421 | 0.4089928 | -0.881 | 0.378 | -0.360537951 | count | 1 |
| DCUN1D5 | -0.2716526 | 0.1501543 | -1.8092 | 0.0705 | -0.360525901 | count | 1 |
| ST6GALNAC6 | -0.3537962 | 0.3462973 | -1.0217 | 0.307 | -0.360333356 | count | 1 |
| ADAT3 | -0.4236765 | 0.4714619 | -0.8986 | 0.369 | -0.360303185 | count | 1 |
| XYLT2 | -0.442095 | 0.3967996 | -1.1142 | 0.265 | -0.360241532 | count | 1 |
| CCDC144NL | -0.5848305 | 0.5498442 | -1.0636 | 0.288 | -0.360043851 | count | 1 |
| LACC1 | -0.4645476 | 0.4961687 | -0.9363 | 0.349 | -0.359909017 | count | 1 |
| CCDC106 | -1.0225452 | 0.9285523 | -1.1012 | 0.271 | -0.35977255 | count | 1 |
| ECI1 | -0.2665314 | 0.1398779 | -1.9055 | 0.0568 | -0.359523467 | count | 1 |
| SDCBP2 | -1.0212157 | 0.6750972 | -1.5127 | 0.13 | -0.359430212 | count | 1 |
| MRPL41 | -0.2531157 | 0.0704812 | -3.5913 | 0.000334 | -0.359424267 | count | 1 |
| TMEM238 | -0.2569252 | 0.0893575 | -2.8753 | 0.00406 | -0.359291843 | count | 1 |
| AL354822.1 | -0.4920668 | 0.7505307 | -0.6556 | 0.512 | -0.358844882 | count | 1 |
| PRKD2 | -0.2875987 | 0.1719539 | -1.6725 | 0.0945 | -0.358821122 | count | 1 |
| PHACTR4 | -0.2812214 | 0.1633789 | -1.7213 | 0.0853 | -0.358737531 | count | 1 |
| MAGED2 | -0.2570195 | 0.1010233 | -2.5442 | 0.011 | -0.358655743 | count | 1 |
| SLC9A6 | -0.6589473 | 0.443096 | -1.4871 | 0.137 | -0.358623822 | count | 1 |
| MANF | -0.2597374 | 0.094109 | -2.76 | 0.00581 | -0.358593175 | count | 1 |
| ZNF592 | -0.4395839 | 0.3200899 | -1.3733 | 0.17 | -0.358325078 | count | 1 |
| FBXL20 | -0.3823787 | 0.2852477 | -1.3405 | 0.18 | -0.358164716 | count | 1 |
| DMXL1 | -0.3094227 | 0.1870492 | -1.6542 | 0.0982 | -0.358112318 | count | 1 |
| DNAJC1 | -0.252515 | 0.0754688 | -3.346 | 0.000829 | -0.3580885 | count | 1 |
| APH1B | -0.2845748 | 0.2132428 | -1.3345 | 0.182 | -0.357994484 | count | 1 |
| RCAN3 | -0.2574057 | 0.0856374 | -3.0058 | 0.00267 | -0.357990686 | count | 1 |
| PRMT1 | -0.2534166 | 0.0795599 | -3.1852 | 0.00146 | -0.357990303 | count | 1 |
| TRAPPC10 | -0.2549349 | 0.0884965 | -2.8807 | 0.00399 | -0.357818507 | count | 1 |
| PGD | -0.351098 | 0.2728221 | -1.2869 | 0.198 | -0.35767789 | count | 1 |

| | | | | | | | |
|---|---|---|---|---|---|---|---|
| BEX4 | -0.2679511 | 0.126438 | -2.1192 | 0.0341 | -0.357553329 | count | 1 |
| DCAF16 | -0.2766451 | 0.1692639 | -1.6344 | 0.102 | -0.357551415 | count | 1 |
| RUVBL1 | -0.2618345 | 0.1310505 | -1.998 | 0.0458 | -0.357539828 | count | 1 |
| S100A2 | -0.7793496 | 0.943687 | -0.8259 | 0.409 | -0.357418341 | count | 1 |
| SMIM1 | -1.6799046 | 0.702206 | -2.3923 | 0.0168 | -0.357296503 | count | 1 |
| ARID4B | -0.2519414 | 0.0572245 | -4.4027 | 1.10E-05 | -0.357290708 | count | 0.264385 |
| PIK3R2 | -0.6559934 | 0.755895 | -0.8678 | 0.386 | -0.357239811 | count | 1 |
| SMOX | -1.6792404 | 1.1564726 | -1.452 | 0.147 | -0.357223631 | count | 1 |
| TFPI | -1.6787092 | 1.092224 | -1.537 | 0.124 | -0.357165312 | count | 1 |
| DDX60L | -0.28607 | 0.1651325 | -1.7324 | 0.0833 | -0.356937603 | count | 1 |
| PTK2B | -0.2750797 | 0.1825604 | -1.5068 | 0.132 | -0.356688569 | count | 1 |
| AKIRIN1 | -0.2539467 | 0.0706083 | -3.5966 | 0.000327 | -0.356687111 | count | 1 |
| TCP1 | -0.2557593 | 0.0861182 | -2.9699 | 0.003 | -0.356332921 | count | 1 |
| NAPSA | -0.7757316 | 0.9743685 | -0.7961 | 0.426 | -0.356060822 | count | 1 |
| NAB2 | -0.3231176 | 0.2609318 | -1.2383 | 0.216 | -0.355996325 | count | 1 |
| CCDC85B | -0.2484957 | 0.0493669 | -5.0336 | 5.06E-07 | -0.355946881 | count | 0.012203202 |
| PRRC2C | -0.2495149 | 0.0458204 | -5.4455 | 5.53E-08 | -0.355730942 | count | 0.001335882 |
| PIAS1 | -0.2770108 | 0.1413753 | -1.9594 | 0.0501 | -0.355511017 | count | 1 |
| ENDOG | -0.2916927 | 0.1649197 | -1.7687 | 0.077 | -0.355452448 | count | 1 |
| B4GALT2 | -0.4026042 | 0.5869368 | -0.6859 | 0.493 | -0.355429491 | count | 1 |
| TRAM2 | -1.0048651 | 0.5861219 | -1.7144 | 0.0865 | -0.355194409 | count | 1 |
| ABHD2 | -0.2950136 | 0.2397802 | -1.2304 | 0.219 | -0.354969007 | count | 1 |
| SPRY2 | -0.4170113 | 0.5081241 | -0.8207 | 0.412 | -0.354955241 | count | 1 |
| GALM | -0.2666817 | 0.1366539 | -1.9515 | 0.0511 | -0.354952259 | count | 1 |
| IST1 | -0.2586297 | 0.1223 | -2.1147 | 0.0345 | -0.35479629 | count | 1 |
| ZSWIM8 | -0.3480404 | 0.2799483 | -1.2432 | 0.214 | -0.354666783 | count | 1 |
| RNF4 | -0.2667044 | 0.1234715 | -2.16 | 0.0308 | -0.354663112 | count | 1 |
| INTS10 | -0.2627186 | 0.122424 | -2.146 | 0.0319 | -0.354625716 | count | 1 |
| CCR6 | -0.2722353 | 0.1666926 | -1.6332 | 0.103 | -0.354614226 | count | 1 |
| ZNF688 | -0.2658276 | 0.1511238 | -1.759 | 0.0787 | -0.354439215 | count | 1 |
| STAP1 | -1.0018437 | 0.7270247 | -1.378 | 0.168 | -0.354406482 | count | 1 |
| NLRP1 | -0.270428 | 0.1426105 | -1.8963 | 0.058 | -0.354188628 | count | 1 |
| YES1 | -0.3606786 | 0.2019796 | -1.7857 | 0.0742 | -0.354095582 | count | 1 |
| ELK3 | -0.2584599 | 0.1257211 | -2.0558 | 0.0399 | -0.353925367 | count | 1 |
| CBL | -0.2724759 | 0.1789885 | -1.5223 | 0.128 | -0.35388415 | count | 1 |
| DLEU2 | -0.3282034 | 0.2393282 | -1.3714 | 0.17 | -0.353859755 | count | 1 |
| SNX10 | -0.2590698 | 0.1514166 | -1.711 | 0.0872 | -0.353781316 | count | 1 |
| NPL | -0.4154235 | 0.4115889 | -1.0093 | 0.313 | -0.353679569 | count | 1 |
| HIC2 | -0.5215198 | 0.3371429 | -1.5469 | 0.122 | -0.353515793 | count | 1 |
| LRP1 | -0.5725678 | 0.7700777 | -0.7435 | 0.457 | -0.35333085 | count | 1 |
| PWWP2A | -0.2637107 | 0.141107 | -1.8689 | 0.0617 | -0.353324444 | count | 1 |
| SPIN1 | -0.3032043 | 0.1860945 | -1.6293 | 0.103 | -0.353302518 | count | 1 |
| AC013264.1 | -0.2836443 | 0.1774125 | -1.5988 | 0.11 | -0.352913016 | count | 1 |
| PARVB | -0.2817607 | 0.1996185 | -1.4115 | 0.158 | -0.352617376 | count | 1 |
| CHCHD10 | -0.2488112 | 0.0745313 | -3.3383 | 0.000852 | -0.352604823 | count | 1 |

| | | | | | | | |
|---|---|---|---|---|---|---|---|
| LINC01215 | -0.6459182 | 0.4027022 | -1.604 | 0.109 | -0.35250317 | count | 1 |
| BRMS1L | -0.3266205 | 0.2778343 | -1.1756 | 0.24 | -0.352198767 | count | 1 |
| C1R | -0.9932916 | 0.4539639 | -2.188 | 0.0287 | -0.352167505 | count | 1 |
| EED | -0.2605636 | 0.1332201 | -1.9559 | 0.0506 | -0.352151784 | count | 1 |
| TMEM171 | -0.7645795 | 0.6374743 | -1.1994 | 0.23 | -0.351858295 | count | 1 |
| DBP | -0.2613123 | 0.151141 | -1.7289 | 0.0839 | -0.351850189 | count | 1 |
| DOCK8 | -0.2522671 | 0.0728648 | -3.4621 | 0.000543 | -0.351711375 | count | 1 |
| MINDY1 | -1.627816 | 1.144063 | -1.4228 | 0.155 | -0.351456159 | count | 1 |
| NECTIN2 | -0.9893621 | 0.8253995 | -1.1986 | 0.231 | -0.351134398 | count | 1 |
| BATF | -0.2470623 | 0.0787513 | -3.1372 | 0.0017 | -0.351093324 | count | 1 |
| PPP2CB | -0.2820665 | 0.2021377 | -1.3954 | 0.163 | -0.350974359 | count | 1 |
| CERK | -0.2675698 | 0.1488124 | -1.798 | 0.0723 | -0.350917141 | count | 1 |
| CIDEB | -0.3651157 | 0.2531487 | -1.4423 | 0.149 | -0.350824424 | count | 1 |
| ALDH6A1 | -0.3846946 | 0.2758074 | -1.3948 | 0.163 | -0.350796339 | count | 1 |
| AKAP8L | -0.2681326 | 0.1166546 | -2.2985 | 0.0216 | -0.350751292 | count | 1 |
| AC010175.1 | -0.987525 | 1.5250491 | -0.6475 | 0.517 | -0.350650448 | count | 1 |
| SCD | -0.7612567 | 0.7758332 | -0.9812 | 0.327 | -0.350600857 | count | 1 |
| PPCDC | -0.3844452 | 0.3827147 | -1.0045 | 0.315 | -0.35057945 | count | 1 |
| CLCN7 | -0.3968054 | 0.3692072 | -1.0747 | 0.283 | -0.350569032 | count | 1 |
| ZNF148 | -0.2540168 | 0.1073849 | -2.3655 | 0.0181 | -0.350534675 | count | 1 |
| CYYR1 | -1.618242 | 1.1674969 | -1.3861 | 0.166 | -0.350354895 | count | 1 |
| PPM1F | -0.4794201 | 0.4273173 | -1.1219 | 0.262 | -0.350350492 | count | 1 |
| SNAPC4 | -0.5670463 | 0.4830797 | -1.1738 | 0.241 | -0.350295667 | count | 1 |
| KPNA1 | -0.279782 | 0.1763764 | -1.5863 | 0.113 | -0.350170536 | count | 1 |
| SNTB2 | -0.2616639 | 0.1335625 | -1.9591 | 0.0502 | -0.350062165 | count | 1 |
| DGCR6L | -0.2506291 | 0.1136874 | -2.2045 | 0.0276 | -0.349970588 | count | 1 |
| DBF4 | -0.2537988 | 0.1052446 | -2.4115 | 0.0159 | -0.349944241 | count | 1 |
| NCKAP5L | -0.3173236 | 0.3478918 | -0.9121 | 0.362 | -0.349767631 | count | 1 |
| PSMA5 | -0.246135 | 0.0711624 | -3.4588 | 0.000549 | -0.349755047 | count | 1 |
| ID1 | -0.3203961 | 0.2388705 | -1.3413 | 0.18 | -0.349488615 | count | 1 |
| OTUD4 | -0.3203164 | 0.2508509 | -1.2769 | 0.202 | -0.349403868 | count | 1 |
| CACNB3 | -0.4274866 | 0.4446193 | -0.9615 | 0.336 | -0.34906965 | count | 1 |
| SEPSECS-AS1 | -0.3483963 | 0.4607947 | -0.7561 | 0.45 | -0.349001408 | count | 1 |
| TMTC2 | -0.4273699 | 0.5834744 | -0.7325 | 0.464 | -0.348980181 | count | 1 |
| LINC01550 | -0.2604423 | 0.1581312 | -1.647 | 0.0997 | -0.348971362 | count | 1 |
| GADD45A | -0.2517358 | 0.1025548 | -2.4546 | 0.0142 | -0.348930778 | count | 1 |
| FAM91A1 | -0.2957029 | 0.2487194 | -1.1889 | 0.235 | -0.348774602 | count | 1 |
| POT1 | -0.3946624 | 0.3216837 | -1.2269 | 0.22 | -0.348770676 | count | 1 |
| CDV3 | -0.2456439 | 0.0594695 | -4.1306 | 3.71E-05 | -0.348666448 | count | 0.889658 |
| CFAP44 | -0.4268419 | 0.6278678 | -0.6798 | 0.497 | -0.348575339 | count | 1 |
| SEC14L2 | -0.4764368 | 0.475308 | -1.0024 | 0.316 | -0.348340587 | count | 1 |
| UBE2J2 | -0.2554329 | 0.1201416 | -2.1261 | 0.0336 | -0.348241653 | count | 1 |
| TYK2 | -0.3544667 | 0.3206159 | -1.1056 | 0.269 | -0.348221984 | count | 1 |
| CD44 | -0.243391 | 0.0457719 | -5.3175 | 1.12E-07 | -0.348196851 | count | 0.002703792 |
| TUBB | -0.2438136 | 0.0554692 | -4.3955 | 1.14E-05 | -0.347988384 | count | 0.2739648 |

| | | | | | | | |
|---|---|---|---|---|---|---|---|
| SERAC1 | -0.3709993 | 0.3699065 | -1.003 | 0.316 | -0.347959403 | count | 1 |
| SPINT2 | -0.2627005 | 0.143758 | -1.8274 | 0.0677 | -0.347696892 | count | 1 |
| DUSP23 | -0.2506664 | 0.1213417 | -2.0658 | 0.0389 | -0.347529249 | count | 1 |
| AL365361.1 | -0.3261204 | 0.2743836 | -1.1886 | 0.235 | -0.347502141 | count | 1 |
| TRAV39 | -0.6352333 | 0.7566054 | -0.8396 | 0.401 | -0.347452938 | count | 1 |
| TIMM17B | -0.2476974 | 0.1021722 | -2.4243 | 0.0154 | -0.347268518 | count | 1 |
| SMG6 | -0.2619423 | 0.12837 | -2.0405 | 0.0414 | -0.347049609 | count | 1 |
| CEP95 | -0.2648536 | 0.1530767 | -1.7302 | 0.0837 | -0.346944618 | count | 1 |
| JPT1 | -0.2430326 | 0.0629534 | -3.8605 | 0.000115 | -0.346923676 | count | 1 |
| ANKRD18B | -0.7514511 | 1.1129476 | -0.6752 | 0.5 | -0.346875935 | count | 1 |
| ZNF330 | -0.2625164 | 0.1390793 | -1.8875 | 0.0592 | -0.346730337 | count | 1 |
| MPP5 | -0.4739779 | 0.4618646 | -1.0262 | 0.305 | -0.346682239 | count | 1 |
| APOBEC3C | -0.2440372 | 0.0858917 | -2.8412 | 0.00452 | -0.346607649 | count | 1 |
| ZNF160 | -0.2784278 | 0.2037777 | -1.3663 | 0.172 | -0.34650228 | count | 1 |
| FAM214B | -0.4730037 | 0.4068282 | -1.1627 | 0.245 | -0.346024777 | count | 1 |
| DNAJC3-DT | -0.3285721 | 0.4463111 | -0.7362 | 0.462 | -0.345531199 | count | 1 |
| MASTL | -0.4719709 | 0.2821896 | -1.6725 | 0.0945 | -0.345327507 | count | 1 |
| DNM2 | -0.2684699 | 0.1366706 | -1.9644 | 0.0496 | -0.345287277 | count | 1 |
| RNASEK | -0.2495949 | 0.0960707 | -2.598 | 0.00942 | -0.345167227 | count | 1 |
| NDUFB2 | -0.2414308 | 0.0564646 | -4.2758 | 1.96E-05 | -0.344982813 | count | 0.4706352 |
| CHCHD5 | -0.2483241 | 0.1165222 | -2.1311 | 0.0331 | -0.344935356 | count | 1 |
| RRP36 | -0.2486764 | 0.119429 | -2.0822 | 0.0374 | -0.344927328 | count | 1 |
| CIB1 | -0.2407072 | 0.0446893 | -5.3862 | 7.68E-08 | -0.344890044 | count | 0.001854643 |
| SPARC | -1.571669 | 0.6414771 | -2.4501 | 0.0143 | -0.344870566 | count | 1 |
| TSTA3 | -0.2504059 | 0.1241718 | -2.0166 | 0.0438 | -0.34467697 | count | 1 |
| RBMS1 | -0.2467314 | 0.0866109 | -2.8487 | 0.00442 | -0.344491 | count | 1 |
| AC011825.4 | -0.9642905 | 1.0916606 | -0.8833 | 0.377 | -0.344477781 | count | 1 |
| EGFL7 | -0.4428177 | 0.6431208 | -0.6885 | 0.491 | -0.344216689 | count | 1 |
| CCDC57 | -0.2552267 | 0.1391756 | -1.8338 | 0.0668 | -0.344140985 | count | 1 |
| DHX9 | -0.2558018 | 0.108079 | -2.3668 | 0.018 | -0.344012819 | count | 1 |
| AC123768.3 | -0.4424184 | 0.7818164 | -0.5659 | 0.572 | -0.343927181 | count | 1 |
| LINC01943 | -0.2595424 | 0.1657467 | -1.5659 | 0.117 | -0.343891435 | count | 1 |
| RHBDD1 | -0.4031455 | 0.3363854 | -1.1985 | 0.231 | -0.343793614 | count | 1 |
| CXCR3 | -0.2414073 | 0.0839719 | -2.8749 | 0.00407 | -0.343740825 | count | 1 |
| MCM7 | -0.2896766 | 0.1992541 | -1.4538 | 0.146 | -0.343626393 | count | 1 |
| OAS1 | -0.260898 | 0.2113299 | -1.2346 | 0.217 | -0.343463287 | count | 1 |
| RAB5B | -0.2965006 | 0.2321033 | -1.2775 | 0.202 | -0.343439147 | count | 1 |
| PBDC1 | -0.2472312 | 0.1074795 | -2.3003 | 0.0215 | -0.343421693 | count | 1 |
| YJU2 | -0.271471 | 0.192095 | -1.4132 | 0.158 | -0.343376747 | count | 1 |
| FLVCR2 | -0.5050737 | 0.9168484 | -0.5509 | 0.582 | -0.343363932 | count | 1 |
| FAM135A | -0.5541674 | 0.5807755 | -0.9542 | 0.34 | -0.343185907 | count | 1 |
| PTPRJ | -0.2611839 | 0.1862873 | -1.402 | 0.161 | -0.343026066 | count | 1 |
| PRDM4 | -0.3142154 | 0.3118559 | -1.0076 | 0.314 | -0.34291334 | count | 1 |
| EEF2K | -0.4407721 | 0.3738223 | -1.1791 | 0.238 | -0.342733129 | count | 1 |
| ADSSL1 | -0.5529873 | 0.6740164 | -0.8204 | 0.412 | -0.34253232 | count | 1 |

| | | | | | | | |
|---|---|---|---|---|---|---|---|
| HPRT1 | -0.249403 | 0.1111358 | -2.2441 | 0.0249 | -0.342422692 | count | 1 |
| ZNF891 | -0.3355644 | 0.2696147 | -1.2446 | 0.213 | -0.34235945 | count | 1 |
| GRASP | -0.2694043 | 0.1847011 | -1.4586 | 0.145 | -0.342351276 | count | 1 |
| C15orf61 | -0.2474909 | 0.1075037 | -2.3022 | 0.0214 | -0.342267064 | count | 1 |
| CAMK1D | -0.2656144 | 0.1837515 | -1.4455 | 0.148 | -0.34225655 | count | 1 |
| MSN | -0.2407326 | 0.0624838 | -3.8527 | 0.000119 | -0.342234283 | count | 1 |
| AL592494.3 | -0.3480054 | 0.3318091 | -1.0488 | 0.294 | -0.342103267 | count | 1 |
| RAB11FIP3 | -0.4010301 | 0.5281085 | -0.7594 | 0.448 | -0.342086502 | count | 1 |
| NAPRT | -0.2592276 | 0.1909505 | -1.3576 | 0.175 | -0.342046805 | count | 1 |
| PPP1CC | -0.2458378 | 0.0909335 | -2.7035 | 0.0069 | -0.342010335 | count | 1 |
| LSM1 | -0.2453555 | 0.0978224 | -2.5082 | 0.0122 | -0.341819503 | count | 1 |
| ZNF268 | -0.4005762 | 0.3835592 | -1.0444 | 0.296 | -0.341720061 | count | 1 |
| DGKH | -0.4005304 | 0.3145933 | -1.2732 | 0.203 | -0.341683075 | count | 1 |
| SLC16A4 | -0.9537455 | 0.7014384 | -1.3597 | 0.174 | -0.341644379 | count | 1 |
| IFT57 | -0.2545641 | 0.1370173 | -1.8579 | 0.0633 | -0.341641292 | count | 1 |
| LINC00324 | -0.2530015 | 0.133807 | -1.8908 | 0.0587 | -0.341577302 | count | 1 |
| AC055713.1 | -0.9533273 | 0.9502211 | -1.0033 | 0.316 | -0.341531593 | count | 1 |
| NDUFB4 | -0.240077 | 0.0650298 | -3.6918 | 0.000226 | -0.341272552 | count | 1 |
| INPP5F | -0.3997738 | 0.4489979 | -0.8904 | 0.373 | -0.341072134 | count | 1 |
| BCAT1 | -1.5392412 | 0.9637001 | -1.5972 | 0.11 | -0.340925406 | count | 1 |
| CTSA | -0.242631 | 0.0962491 | -2.5209 | 0.0118 | -0.340620382 | count | 1 |
| CRYBG1 | -0.2465974 | 0.0654704 | -3.7665 | 0.000168 | -0.340602927 | count | 1 |
| ZDHHC3 | -0.2547763 | 0.1589776 | -1.6026 | 0.109 | -0.340359623 | count | 1 |
| ENPP1 | -0.6201387 | 1.0191971 | -0.6085 | 0.543 | -0.340271232 | count | 1 |
| HSBP1 | -0.2452231 | 0.125453 | -1.9547 | 0.0507 | -0.340079016 | count | 1 |
| RRAGC | -0.2722672 | 0.1573386 | -1.7305 | 0.0836 | -0.339918181 | count | 1 |
| CDKL2 | -0.5482483 | 0.5417514 | -1.012 | 0.312 | -0.339904139 | count | 1 |
| STARD5 | -0.4638649 | 0.4926508 | -0.9416 | 0.346 | -0.339845173 | count | 1 |
| AC124016.1 | -0.276537 | 0.3579596 | -0.7725 | 0.44 | -0.339738613 | count | 1 |
| KANK3 | -0.6186789 | 0.4517703 | -1.3695 | 0.171 | -0.339573747 | count | 1 |
| RNF7 | -0.2383922 | 0.0632135 | -3.7712 | 0.000165 | -0.33954634 | count | 1 |
| FYCO1 | -0.2949186 | 0.2377612 | -1.2404 | 0.215 | -0.339337945 | count | 1 |
| NOP14-AS1 | -0.3178003 | 0.2798066 | -1.1358 | 0.256 | -0.338874011 | count | 1 |
| TRMT10A | -0.292425 | 0.3162253 | -0.9247 | 0.355 | -0.338805554 | count | 1 |
| LPXN | -0.247986 | 0.1151982 | -2.1527 | 0.0314 | -0.338709242 | count | 1 |
| FAM133B | -0.2388471 | 0.0660817 | -3.6144 | 0.000305 | -0.33864773 | count | 1 |
| ASMTL | -0.2755388 | 0.1726605 | -1.5958 | 0.111 | -0.338528283 | count | 1 |
| PNN | -0.2384269 | 0.0584678 | -4.0779 | 4.65E-05 | -0.338508191 | count | 1 |
| AC018816.1 | -0.3823276 | 0.3518251 | -1.0867 | 0.277 | -0.338397491 | count | 1 |
| PHF10 | -0.2592029 | 0.1407441 | -1.8417 | 0.0656 | -0.338248386 | count | 1 |
| MT1X | -0.2409797 | 0.0964437 | -2.4987 | 0.0125 | -0.338236957 | count | 1 |
| PCLAF | -0.6158796 | 0.874048 | -0.7046 | 0.481 | -0.33823484 | count | 1 |
| APOL3 | -0.2619113 | 0.1688381 | -1.5513 | 0.121 | -0.338109175 | count | 1 |
| PSMA7 | -0.2353581 | 0.0396364 | -5.9379 | 3.18E-09 | -0.338085099 | count | 7.69E-05 |
| ARHGAP35 | -0.2741135 | 0.1696545 | -1.6157 | 0.106 | -0.337964847 | count | 1 |

| | | | | | | | |
|---|---|---|---|---|---|---|---|
| MFSD2A | -0.9400039 | 0.6660396 | -1.4113 | 0.158 | -0.337922002 | count | 1 |
| SULF2 | -0.9400039 | 0.7249427 | -1.2967 | 0.195 | -0.337922002 | count | 1 |
| HPGD | -0.3368901 | 0.2774007 | -1.2145 | 0.225 | -0.337859432 | count | 1 |
| PCCA | -0.3308882 | 0.3111441 | -1.0635 | 0.288 | -0.337737807 | count | 1 |
| AL031316.1 | -0.614241 | 0.7841255 | -0.7833 | 0.433 | -0.337450215 | count | 1 |
| TPGS1 | -0.2436696 | 0.1045742 | -2.3301 | 0.0199 | -0.337323741 | count | 1 |
| RDX | -0.2708005 | 0.1706637 | -1.5867 | 0.113 | -0.337122804 | count | 1 |
| MIEF1 | -0.3587501 | 0.3297174 | -1.0881 | 0.277 | -0.336939591 | count | 1 |
| POGK | -0.3120376 | 0.2352131 | -1.3266 | 0.185 | -0.336873209 | count | 1 |
| NDUFAB1 | -0.237323 | 0.0725402 | -3.2716 | 0.00108 | -0.336820575 | count | 1 |
| OSGEPL1 | -0.280919 | 0.3072027 | -0.9144 | 0.361 | -0.336718051 | count | 1 |
| TRIM33 | -0.2630623 | 0.1359472 | -1.935 | 0.0531 | -0.336477844 | count | 1 |
| ADGRL4 | -1.5032605 | 1.0760283 | -1.397 | 0.162 | -0.336423597 | count | 1 |
| PIN4 | -0.2452369 | 0.1366477 | -1.7947 | 0.0728 | -0.336376404 | count | 1 |
| POLD3 | -0.2768668 | 0.2130618 | -1.2995 | 0.194 | -0.336301473 | count | 1 |
| NELFCD | -0.246719 | 0.1224135 | -2.0155 | 0.0439 | -0.336264084 | count | 1 |
| ARL8B | -0.2535962 | 0.1288943 | -1.9675 | 0.0492 | -0.336064543 | count | 1 |
| LYG1 | -1.4998284 | 1.149523 | -1.3047 | 0.192 | -0.335987249 | count | 1 |
| PIK3C3 | -0.2815794 | 0.1671904 | -1.6842 | 0.0922 | -0.335875387 | count | 1 |
| CD79B | -0.2511714 | 0.1691133 | -1.4852 | 0.138 | -0.335841982 | count | 1 |
| POLR3A | -0.3190665 | 0.2387978 | -1.3361 | 0.182 | -0.335813563 | count | 1 |
| SLC35E1 | -0.2739815 | 0.1469848 | -1.864 | 0.0624 | -0.335427212 | count | 1 |
| MDM1 | -0.293533 | 0.2124413 | -1.3817 | 0.167 | -0.335352966 | count | 1 |
| AP2B1 | -0.2501809 | 0.1364917 | -1.8329 | 0.0669 | -0.33529975 | count | 1 |
| TRIP12 | -0.2525033 | 0.1230044 | -2.0528 | 0.0402 | -0.335275371 | count | 1 |
| ARID5A | -0.2388194 | 0.0717112 | -3.3303 | 0.000877 | -0.335175936 | count | 1 |
| ZNF44 | -0.2543903 | 0.1702363 | -1.4943 | 0.135 | -0.33495668 | count | 1 |
| ST8SIA6 | -0.4300696 | 0.4559124 | -0.9433 | 0.346 | -0.33495352 | count | 1 |
| NCBP2 | -0.2442531 | 0.1181434 | -2.0674 | 0.0388 | -0.334793274 | count | 1 |
| SLK | -0.2458087 | 0.1228854 | -2.0003 | 0.0455 | -0.334725561 | count | 1 |
| CGAS | -0.2459895 | 0.1406517 | -1.7489 | 0.0804 | -0.334657741 | count | 1 |
| SLC10A7 | -0.3661643 | 0.3698463 | -0.99 | 0.322 | -0.334640092 | count | 1 |
| NIT2 | -0.2571278 | 0.1651285 | -1.5571 | 0.12 | -0.334608994 | count | 1 |
| FAM104A | -0.2440566 | 0.1448893 | -1.6844 | 0.0922 | -0.33440317 | count | 1 |
| HMBOX1 | -0.5371554 | 0.294605 | -1.8233 | 0.0683 | -0.333729908 | count | 1 |
| ZCRB1 | -0.2391462 | 0.1116763 | -2.1414 | 0.0323 | -0.333622795 | count | 1 |
| ATP5MC1 | -0.2406652 | 0.0926918 | -2.5964 | 0.00946 | -0.333332182 | count | 1 |
| SPOP | -0.24353 | 0.1164607 | -2.0911 | 0.0366 | -0.333309533 | count | 1 |
| RAB11B-AS1 | -0.406992 | 0.4082817 | -0.9968 | 0.319 | -0.333303794 | count | 1 |
| ZNF568 | -0.2807496 | 0.2783987 | -1.0084 | 0.313 | -0.333205593 | count | 1 |
| CENPE | -0.5360474 | 0.3941009 | -1.3602 | 0.174 | -0.333111484 | count | 1 |
| H3F3A | -0.2313577 | 0.0283067 | -8.1733 | 4.19E-16 | -0.332934297 | count | 1.02E-11 |
| PPP1R2 | -0.2332651 | 0.0501112 | -4.6549 | 3.36E-06 | -0.332874601 | count | 0.0808752 |
| SIVA1 | -0.2343938 | 0.0672272 | -3.4866 | 0.000495 | -0.332870223 | count | 1 |
| ZNF10 | -0.3641257 | 0.2941803 | -1.2378 | 0.216 | -0.332857599 | count | 1 |

| | | | | | | | |
|---|---|---|---|---|---|---|---|
| HADH | -0.2818764 | 0.1851098 | -1.5228 | 0.128 | -0.332734716 | count | 1 |
| CXCR6 | -0.240167 | 0.1131987 | -2.1216 | 0.0339 | -0.332720193 | count | 1 |
| ZNF736 | -0.389341 | 0.3704744 | -1.0509 | 0.293 | -0.332633161 | count | 1 |
| VDAC1 | -0.2359807 | 0.070657 | -3.3398 | 0.000847 | -0.332485837 | count | 1 |
| DLGAP1-AS1 | -0.2534109 | 0.1635732 | -1.5492 | 0.121 | -0.332483755 | count | 1 |
| GOLPH3L | -0.3313161 | 0.2853565 | -1.1611 | 0.246 | -0.3324514 | count | 1 |
| CSF2RB | -0.7139376 | 0.9261316 | -0.7709 | 0.441 | -0.33242977 | count | 1 |
| PLEC | -0.2533007 | 0.1585617 | -1.5975 | 0.11 | -0.332340219 | count | 1 |
| UBA6 | -0.2866421 | 0.185443 | -1.5457 | 0.122 | -0.332226243 | count | 1 |
| ACSL3 | -0.2534788 | 0.1424756 | -1.7791 | 0.0753 | -0.33215456 | count | 1 |
| ZNF207 | -0.2416835 | 0.0942073 | -2.5654 | 0.0103 | -0.331974359 | count | 1 |
| HSD17B10 | -0.240664 | 0.1120049 | -2.1487 | 0.0317 | -0.331894757 | count | 1 |
| SNHG9 | -0.2454868 | 0.1415854 | -1.7338 | 0.083 | -0.331875776 | count | 1 |
| SDE2 | -0.2950658 | 0.1710577 | -1.7249 | 0.0846 | -0.331763637 | count | 1 |
| MFSD4B | -0.3530062 | 0.3217157 | -1.0973 | 0.273 | -0.331759976 | count | 1 |
| PLEKHB2 | -0.2490846 | 0.1194606 | -2.0851 | 0.0371 | -0.331674047 | count | 1 |
| AP003469.4 | -0.9170687 | 0.7377822 | -1.243 | 0.214 | -0.331633184 | count | 1 |
| SLC25A36 | -0.244231 | 0.1032229 | -2.3661 | 0.018 | -0.331624571 | count | 1 |
| WDR82 | -0.2452671 | 0.1114033 | -2.2016 | 0.0278 | -0.331580209 | count | 1 |
| 6-Sep | -0.2335439 | 0.062433 | -3.7407 | 0.000187 | -0.331536229 | count | 1 |
| BPGM | -0.266989 | 0.1683116 | -1.5863 | 0.113 | -0.331425133 | count | 1 |
| PTPN14 | -1.4639282 | 1.2962028 | -1.1294 | 0.259 | -0.331349337 | count | 1 |
| IKZF3 | -0.2359266 | 0.08393 | -2.811 | 0.00497 | -0.331327951 | count | 1 |
| INPP5D | -0.2548863 | 0.1559684 | -1.6342 | 0.102 | -0.331223954 | count | 1 |
| ZNF239 | -1.4622056 | 0.8772069 | -1.6669 | 0.0956 | -0.331123396 | count | 1 |
| HNMT | -1.4622056 | 0.9335767 | -1.5662 | 0.117 | -0.331123396 | count | 1 |
| AC009403.1 | -0.2713195 | 0.3359341 | -0.8077 | 0.419 | -0.330960013 | count | 1 |
| MCC | -1.459667 | 0.7216597 | -2.0227 | 0.0432 | -0.330789935 | count | 1 |
| RSPRY1 | -0.264043 | 0.1833887 | -1.4398 | 0.15 | -0.330691239 | count | 1 |
| ZNF175 | -0.6000193 | 0.6952591 | -0.863 | 0.388 | -0.330612953 | count | 1 |
| LMCD1 | -0.3862959 | 0.4963609 | -0.7783 | 0.436 | -0.330164868 | count | 1 |
| NHS | -0.5986469 | 0.7286346 | -0.8216 | 0.411 | -0.329950569 | count | 1 |
| ZMAT1 | -0.350914 | 0.2579659 | -1.3603 | 0.174 | -0.329871388 | count | 1 |
| ANTXR2 | -0.2502052 | 0.1677203 | -1.4918 | 0.136 | -0.329857755 | count | 1 |
| ZNF540 | -0.3227668 | 0.4640085 | -0.6956 | 0.487 | -0.329700022 | count | 1 |
| MTRR | -0.3422002 | 0.2488579 | -1.3751 | 0.169 | -0.329620859 | count | 1 |
| TRAPPC2L | -0.236598 | 0.1041807 | -2.271 | 0.0232 | -0.329481837 | count | 1 |
| PTGES3 | -0.2305013 | 0.0451745 | -5.1025 | 3.54E-07 | -0.329445838 | count | 0.008540604 |
| BCL3 | -0.2400906 | 0.1165 | -2.0609 | 0.0394 | -0.329109396 | count | 1 |
| MRPL58 | -0.2468516 | 0.1679327 | -1.4699 | 0.142 | -0.329006193 | count | 1 |
| IDH3B | -0.2422928 | 0.1195008 | -2.0275 | 0.0427 | -0.328836605 | count | 1 |
| SFT2D3 | -0.52835 | 0.3131447 | -1.6872 | 0.0916 | -0.328806722 | count | 1 |
| MTCP1 | -0.9067164 | 0.8919797 | -1.0165 | 0.309 | -0.328763282 | count | 1 |
| NDUFS3 | -0.23726 | 0.0993849 | -2.3873 | 0.017 | -0.328704895 | count | 1 |
| RAMMET | -0.2374002 | 0.1261185 | -1.8824 | 0.0599 | -0.328671001 | count | 1 |

| | | | | | | | |
|---|---|---|---|---|---|---|---|
| HLA-DMA | -0.2360852 | 0.1228183 | -1.9222 | 0.0547 | -0.328478582 | count | 1 |
| SFI1 | -0.297531 | 0.1876836 | -1.5853 | 0.113 | -0.328442565 | count | 1 |
| IL7R | -0.2282861 | 0.0402239 | -5.6754 | 1.50E-08 | -0.32829815 | count | 0.00036264 |
| CEP192 | -0.2851053 | 0.2090578 | -1.3638 | 0.173 | -0.328254599 | count | 1 |
| RAB11FIP1 | -0.2328582 | 0.0859413 | -2.7095 | 0.00677 | -0.328218815 | count | 1 |
| SLCO3A1 | -0.2563283 | 0.1744211 | -1.4696 | 0.142 | -0.327943862 | count | 1 |
| KATNA1 | -0.2446238 | 0.1392861 | -1.7563 | 0.0791 | -0.327892848 | count | 1 |
| FAM118A | -0.2358042 | 0.0906857 | -2.6002 | 0.00936 | -0.32772046 | count | 1 |
| MAD2L1 | -0.2968168 | 0.2119009 | -1.4007 | 0.161 | -0.327671712 | count | 1 |
| DTNBP1 | -0.2425629 | 0.1367085 | -1.7743 | 0.0761 | -0.327553512 | count | 1 |
| EIF2S2 | -0.2304954 | 0.0647217 | -3.5613 | 0.000374 | -0.327503518 | count | 1 |
| PARD6A | -0.2403273 | 0.1415018 | -1.6984 | 0.0895 | -0.327442248 | count | 1 |
| BUB1 | -0.3693098 | 0.5084075 | -0.7264 | 0.468 | -0.327409258 | count | 1 |
| ZFYVE16 | -0.2682816 | 0.1819165 | -1.4748 | 0.14 | -0.32730308 | count | 1 |
| CAV2 | -1.4333449 | 0.9123602 | -1.571 | 0.116 | -0.327290859 | count | 1 |
| MLX | -0.2370276 | 0.1205032 | -1.967 | 0.0493 | -0.327166538 | count | 1 |
| INAFM1 | -0.3064673 | 0.3493134 | -0.8773 | 0.38 | -0.327099189 | count | 1 |
| PLP2 | -0.2281004 | 0.0536586 | -4.251 | 2.19E-05 | -0.327077758 | count | 0.5256876 |
| PAICS | -0.259694 | 0.1894869 | -1.3705 | 0.171 | -0.32702572 | count | 1 |
| SENP7 | -0.2374203 | 0.1007382 | -2.3568 | 0.0185 | -0.326962503 | count | 1 |
| PCBD2 | -0.3199859 | 0.3213872 | -0.9956 | 0.319 | -0.326944517 | count | 1 |
| THOC2 | -0.2421009 | 0.0998345 | -2.425 | 0.0154 | -0.326932681 | count | 1 |
| BAZ1B | -0.2455431 | 0.1214191 | -2.0223 | 0.0432 | -0.326694257 | count | 1 |
| ADM | -0.4444826 | 0.4171978 | -1.0654 | 0.287 | -0.326667241 | count | 1 |
| SNRNP25 | -0.2448672 | 0.1746557 | -1.402 | 0.161 | -0.326656227 | count | 1 |
| DOCK9 | -0.3100728 | 0.2837308 | -1.0928 | 0.275 | -0.326602254 | count | 1 |
| ATOX1 | -0.2436391 | 0.1403741 | -1.7356 | 0.0827 | -0.326580152 | count | 1 |
| AMPD3 | -0.6988926 | 0.3492762 | -2.001 | 0.0455 | -0.326548777 | count | 1 |
| MRPL28 | -0.2402828 | 0.139061 | -1.7279 | 0.0841 | -0.326287477 | count | 1 |
| CEP152 | -0.367977 | 0.2942188 | -1.2507 | 0.211 | -0.326281911 | count | 1 |
| MBD3 | -0.2684895 | 0.2081596 | -1.2898 | 0.197 | -0.326262995 | count | 1 |
| ZFY-AS1 | -0.5909583 | 0.9518885 | -0.6208 | 0.535 | -0.326231266 | count | 1 |
| CDC20 | -0.897328 | 1.0158322 | -0.8833 | 0.377 | -0.326143721 | count | 1 |
| MYO1D | -0.4773105 | 0.4456861 | -1.071 | 0.284 | -0.326067354 | count | 1 |
| TBC1D5 | -0.2562808 | 0.1833083 | -1.3981 | 0.162 | -0.325835812 | count | 1 |
| GSDME | -1.422289 | 0.798282 | -1.7817 | 0.0749 | -0.325798991 | count | 1 |
| UCK1 | -0.3014803 | 0.2873787 | -1.0491 | 0.294 | -0.325752358 | count | 1 |
| HLTF | -0.2488087 | 0.1614581 | -1.541 | 0.123 | -0.325659152 | count | 1 |
| C5orf30 | -0.3135959 | 0.3371017 | -0.9303 | 0.352 | -0.325591347 | count | 1 |
| ENG | -0.3049679 | 0.3728437 | -0.818 | 0.413 | -0.325539425 | count | 1 |
| RMI2 | -1.4199411 | 0.9370074 | -1.5154 | 0.13 | -0.325480471 | count | 1 |
| MRPS34 | -0.2287563 | 0.0689674 | -3.3169 | 0.00092 | -0.325447646 | count | 1 |
| CDK5 | -0.2771453 | 0.3121299 | -0.8879 | 0.375 | -0.325395029 | count | 1 |
| TMF1 | -0.2326111 | 0.0762685 | -3.0499 | 0.00231 | -0.32528012 | count | 1 |
| BCL2L11 | -0.3373153 | 0.185341 | -1.82 | 0.0689 | -0.325085269 | count | 1 |

| | | | | | | | |
|---|---|---|---|---|---|---|---|
| MTHFD2L | -0.2752445 | 0.2664938 | -1.0328 | 0.302 | -0.325030748 | count | 1 |
| CLN3 | -0.5882684 | 0.5228245 | -1.1252 | 0.261 | -0.324926676 | count | 1 |
| RAP2C-AS1 | -0.3961547 | 0.4256411 | -0.9307 | 0.352 | -0.324923747 | count | 1 |
| LTB4R | -0.2821455 | 0.2433539 | -1.1594 | 0.246 | -0.324908605 | count | 1 |
| KHK | -0.3369977 | 0.3634435 | -0.9272 | 0.354 | -0.324790201 | count | 1 |
| KPNB1 | -0.2376318 | 0.1023549 | -2.3216 | 0.0203 | -0.324756167 | count | 1 |
| SLC9A9 | -0.2565354 | 0.1839912 | -1.3943 | 0.163 | -0.324674509 | count | 1 |
| AP1M1 | -0.2605583 | 0.1824712 | -1.4279 | 0.153 | -0.324516563 | count | 1 |
| FAM207A | -0.2455359 | 0.175253 | -1.401 | 0.161 | -0.324449821 | count | 1 |
| HOMER3 | -0.8906713 | 0.9581478 | -0.9296 | 0.353 | -0.324276597 | count | 1 |
| UCHL3 | -0.2372488 | 0.144523 | -1.6416 | 0.101 | -0.324234806 | count | 1 |
| BCAP29 | -0.2363736 | 0.1286703 | -1.837 | 0.0663 | -0.324149242 | count | 1 |
| C10orf95 | -0.3445416 | 0.3738506 | -0.9216 | 0.357 | -0.324112835 | count | 1 |
| ZC3H6 | -0.2335822 | 0.1170486 | -1.9956 | 0.0461 | -0.324050365 | count | 1 |
| CNOT6L | -0.2306053 | 0.0584521 | -3.9452 | 8.13E-05 | -0.323997046 | count | 1 |
| LONP1 | -0.3119827 | 0.3166306 | -0.9853 | 0.325 | -0.323963292 | count | 1 |
| FBXW7 | -0.2398299 | 0.1223453 | -1.9603 | 0.05 | -0.323880743 | count | 1 |
| SSH2 | -0.2426938 | 0.1214175 | -1.9988 | 0.0457 | -0.323773584 | count | 1 |
| AMFR | -0.2876284 | 0.2212511 | -1.3 | 0.194 | -0.323570309 | count | 1 |
| C3AR1 | -0.2808637 | 0.2855765 | -0.9835 | 0.325 | -0.32345911 | count | 1 |
| THOC6 | -0.2696462 | 0.2072624 | -1.301 | 0.193 | -0.323401896 | count | 1 |
| TFB1M | -0.2588854 | 0.2011524 | -1.287 | 0.198 | -0.323395954 | count | 1 |
| NDUFS7 | -0.2291408 | 0.0768532 | -2.9815 | 0.00289 | -0.32312998 | count | 1 |
| CCNO | -0.6897764 | 1.1933995 | -0.578 | 0.563 | -0.322960994 | count | 1 |
| NT5DC1 | -0.242196 | 0.1270566 | -1.9062 | 0.0567 | -0.322837236 | count | 1 |
| HSD17B14 | -0.51727 | 0.660057 | -0.7837 | 0.433 | -0.322583922 | count | 1 |
| EFNA4 | -0.249784 | 0.2141868 | -1.1662 | 0.244 | -0.322583745 | count | 1 |
| SNRK | -0.2369199 | 0.1005865 | -2.3554 | 0.0186 | -0.322522946 | count | 1 |
| KCTD17 | -0.3105547 | 0.2800454 | -1.1089 | 0.268 | -0.322521689 | count | 1 |
| ABCD4 | -0.2488798 | 0.1859346 | -1.3385 | 0.181 | -0.322481326 | count | 1 |
| BRD9 | -0.2483857 | 0.1383178 | -1.7958 | 0.0726 | -0.322351092 | count | 1 |
| NFIC | -0.2514206 | 0.1504535 | -1.6711 | 0.0948 | -0.322349327 | count | 1 |
| TEC | -0.5828838 | 0.5373521 | -1.0847 | 0.278 | -0.322309926 | count | 1 |
| MIR4435-2HG | -0.2334299 | 0.1398785 | -1.6688 | 0.0952 | -0.322302921 | count | 1 |
| BUD31 | -0.2288701 | 0.076232 | -3.0023 | 0.0027 | -0.322173109 | count | 1 |
| MRPL4 | -0.2333234 | 0.110615 | -2.1093 | 0.035 | -0.322156336 | count | 1 |
| PEX12 | -0.8829301 | 0.78415 | -1.126 | 0.26 | -0.32209508 | count | 1 |
| RBM14 | -0.3518279 | 0.2420466 | -1.4536 | 0.146 | -0.322083674 | count | 1 |
| ANAPC4 | -0.2510777 | 0.1962768 | -1.2792 | 0.201 | -0.321913563 | count | 1 |
| GFOD2 | -0.4121977 | 0.2653545 | -1.5534 | 0.12 | -0.321896792 | count | 1 |
| MYO5A | -0.3014313 | 0.204792 | -1.4719 | 0.141 | -0.321858691 | count | 1 |
| ZNF605 | -0.2943 | 0.318386 | -0.9243 | 0.355 | -0.321677021 | count | 1 |
| RXRA | -0.3145696 | 0.3250012 | -0.9679 | 0.333 | -0.321572968 | count | 1 |
| CCDC97 | -0.2615663 | 0.2211545 | -1.1827 | 0.237 | -0.321572843 | count | 1 |
| PLCE1 | -0.8803305 | 0.5895668 | -1.4932 | 0.135 | -0.321360037 | count | 1 |

| | | | | | | | |
|---|---|---|---|---|---|---|---|
| RCN1 | -0.3198956 | 0.3610625 | -0.886 | 0.376 | -0.321349736 | count | 1 |
| PPM1B | -0.251114 | 0.1533767 | -1.6372 | 0.102 | -0.321332592 | count | 1 |
| ZKSCAN1 | -0.2654033 | 0.1805674 | -1.4698 | 0.142 | -0.321231444 | count | 1 |
| ZSWIM3 | -1.388651 | 0.5629175 | -2.4669 | 0.0137 | -0.321178326 | count | 1 |
| USP25 | -0.2455955 | 0.1491011 | -1.6472 | 0.0996 | -0.321058804 | count | 1 |
| BEX2 | -0.2419029 | 0.1451545 | -1.6665 | 0.0957 | -0.320978695 | count | 1 |
| OXNAD1 | -0.2295699 | 0.0769541 | -2.9832 | 0.00287 | -0.320949621 | count | 1 |
| ARCN1 | -0.2326261 | 0.1102374 | -2.1102 | 0.0349 | -0.320663637 | count | 1 |
| UPF3A | -0.2284146 | 0.0928395 | -2.4603 | 0.0139 | -0.320324943 | count | 1 |
| TNPO2 | -0.4351517 | 0.3284739 | -1.3248 | 0.185 | -0.320288608 | count | 1 |
| GPX4 | -0.2235771 | 0.0465752 | -4.8003 | 1.65E-06 | -0.320177912 | count | 0.0397518 |
| LSR | -0.2523079 | 0.130391 | -1.935 | 0.0531 | -0.320116476 | count | 1 |
| DDX17 | -0.2264037 | 0.0600293 | -3.7716 | 0.000165 | -0.320111139 | count | 1 |
| CHEK2 | -0.409752 | 0.6087054 | -0.6732 | 0.501 | -0.320103663 | count | 1 |
| FNDC4 | -1.380316 | 1.26998 | -1.0869 | 0.277 | -0.320013862 | count | 1 |
| RGS13 | -1.380316 | 1.326389 | -1.0407 | 0.298 | -0.320013862 | count | 1 |
| RAB11FIP4 | -0.2995533 | 0.2279784 | -1.314 | 0.189 | -0.319903149 | count | 1 |
| NCF1 | -0.2274084 | 0.1004072 | -2.2649 | 0.0236 | -0.319754942 | count | 1 |
| RBBP7 | -0.2300194 | 0.0986419 | -2.3319 | 0.0198 | -0.319640148 | count | 1 |
| CTCF | -0.2310027 | 0.0976331 | -2.366 | 0.018 | -0.319611325 | count | 1 |
| ERBB3 | -0.4090416 | 0.7700637 | -0.5312 | 0.595 | -0.319582535 | count | 1 |
| C8orf76 | -0.242822 | 0.1730377 | -1.4033 | 0.161 | -0.319455916 | count | 1 |
| PDCD5 | -0.225124 | 0.0750804 | -2.9984 | 0.00273 | -0.319430833 | count | 1 |
| LZIC | -0.2356237 | 0.1392574 | -1.692 | 0.0907 | -0.319319325 | count | 1 |
| MTIF3 | -0.2267022 | 0.0940671 | -2.41 | 0.016 | -0.319309269 | count | 1 |
| BCAS1 | -0.8729146 | 1.070679 | -0.8153 | 0.415 | -0.319256363 | count | 1 |
| HCFC1 | -0.295284 | 0.2502232 | -1.1801 | 0.238 | -0.319215274 | count | 1 |
| PAIP2B | -0.4335362 | 0.4426596 | -0.9794 | 0.327 | -0.319181965 | count | 1 |
| KLHL25 | -0.6800993 | 0.483794 | -1.4058 | 0.16 | -0.319132373 | count | 1 |
| MISP3 | -0.3725891 | 0.4197908 | -0.8876 | 0.375 | -0.319025861 | count | 1 |
| ARFGAP2 | -0.2392658 | 0.1433758 | -1.6688 | 0.0952 | -0.318953746 | count | 1 |
| SAT2 | -0.2274439 | 0.1104116 | -2.06 | 0.0395 | -0.318673338 | count | 1 |
| SQLE | -0.2510692 | 0.202672 | -1.2388 | 0.216 | -0.318559857 | count | 1 |
| ZDHHC13 | -0.2667245 | 0.2826253 | -0.9437 | 0.345 | -0.31841561 | count | 1 |
| TRNAU1AP | -0.2374485 | 0.1372216 | -1.7304 | 0.0836 | -0.31832602 | count | 1 |
| SBF1 | -0.2943239 | 0.2461892 | -1.1955 | 0.232 | -0.31820171 | count | 1 |
| YBX3 | -0.2350045 | 0.1209202 | -1.9435 | 0.052 | -0.318133328 | count | 1 |
| PLCB1 | -0.2850686 | 0.2197783 | -1.2971 | 0.195 | -0.317952207 | count | 1 |
| TCHP | -0.2399904 | 0.1628391 | -1.4738 | 0.141 | -0.317827035 | count | 1 |
| TAB1 | -0.3579496 | 0.3997557 | -0.8954 | 0.371 | -0.317786357 | count | 1 |
| TOLLIP-AS1 | -0.3160842 | 0.4595944 | -0.6877 | 0.492 | -0.317638443 | count | 1 |
| TXNRD1 | -0.2351855 | 0.1277767 | -1.8406 | 0.0658 | -0.317638278 | count | 1 |
| FBLN7 | -0.2502234 | 0.2044492 | -1.2239 | 0.221 | -0.317496882 | count | 1 |
| FYTTD1 | -0.2283805 | 0.0933442 | -2.4466 | 0.0145 | -0.31749091 | count | 1 |
| AKAP13 | -0.2238167 | 0.0550784 | -4.0636 | 4.94E-05 | -0.317453483 | count | 1 |

| | | | | | | | |
|---|---|---|---|---|---|---|---|
| DEXI | -0.2460398 | 0.162518 | -1.5139 | 0.13 | -0.317243175 | count | 1 |
| WASHC4 | -0.2436408 | 0.1780518 | -1.3684 | 0.171 | -0.317189979 | count | 1 |
| XPOT | -0.3007642 | 0.258696 | -1.1626 | 0.245 | -0.317051188 | count | 1 |
| TRAPPC2B | -0.2323171 | 0.1330738 | -1.7458 | 0.0809 | -0.316991251 | count | 1 |
| CRK | -0.2698673 | 0.1680496 | -1.6059 | 0.108 | -0.31698588 | count | 1 |
| SUPT16H | -0.2292606 | 0.1008925 | -2.2723 | 0.0231 | -0.31697353 | count | 1 |
| LRP12 | -0.674624 | 0.5695258 | -1.1845 | 0.236 | -0.316956975 | count | 1 |
| MAX | -0.2282957 | 0.0888593 | -2.5692 | 0.0102 | -0.316934549 | count | 1 |
| PCK2 | -0.2792644 | 0.2559042 | -1.0913 | 0.275 | -0.316921571 | count | 1 |
| NELFB | -0.2541435 | 0.1920258 | -1.3235 | 0.186 | -0.31661469 | count | 1 |
| NPEPPS | -0.2467244 | 0.1674558 | -1.4734 | 0.141 | -0.316380307 | count | 1 |
| SLC2A3 | -0.2219876 | 0.0560833 | -3.9582 | 7.71E-05 | -0.316352981 | count | 1 |
| PTPN7 | -0.2227948 | 0.0769452 | -2.8955 | 0.00381 | -0.316293994 | count | 1 |
| TERF1 | -0.2357186 | 0.120321 | -1.9591 | 0.0502 | -0.316251945 | count | 1 |
| BRAF | -0.2530229 | 0.1596886 | -1.5845 | 0.113 | -0.316150939 | count | 1 |
| NFYA | -0.3276558 | 0.3162473 | -1.0361 | 0.3 | -0.316100517 | count | 1 |
| PHPT1 | -0.2230784 | 0.0857138 | -2.6026 | 0.00929 | -0.315982979 | count | 1 |
| RTF2 | -0.2273009 | 0.0930009 | -2.4441 | 0.0146 | -0.315933459 | count | 1 |
| FAM50A | -0.2264381 | 0.0985219 | -2.2984 | 0.0216 | -0.315786775 | count | 1 |
| ROMO1 | -0.2278733 | 0.0880097 | -2.5892 | 0.00966 | -0.315665453 | count | 1 |
| ATG16L1 | -0.2856132 | 0.2415462 | -1.1824 | 0.237 | -0.315567029 | count | 1 |
| KPNA6 | -0.2433396 | 0.1721369 | -1.4136 | 0.158 | -0.315358661 | count | 1 |
| CDC5L | -0.2260028 | 0.0945902 | -2.3893 | 0.0169 | -0.315280777 | count | 1 |
| AATF | -0.2309841 | 0.1096607 | -2.1064 | 0.0352 | -0.315042978 | count | 1 |
| CCDC186 | -0.2260859 | 0.0829005 | -2.7272 | 0.00642 | -0.315039321 | count | 1 |
| SYMPK | -0.3545681 | 0.2603903 | -1.3617 | 0.173 | -0.314915937 | count | 1 |
| RGCC | -0.2185263 | 0.0457042 | -4.7813 | 1.82E-06 | -0.314758344 | count | 0.04384198 |
| EXOC2 | -0.2511479 | 0.203958 | -1.2314 | 0.218 | -0.314709958 | count | 1 |
| FAM89A | -0.2663144 | 0.1857884 | -1.4334 | 0.152 | -0.314646507 | count | 1 |
| WDR76 | -0.2729859 | 0.2388136 | -1.1431 | 0.253 | -0.314544728 | count | 1 |
| SRA1 | -0.2255366 | 0.1095138 | -2.0594 | 0.0395 | -0.314482115 | count | 1 |
| RPA3 | -0.2258103 | 0.0890785 | -2.535 | 0.0113 | -0.314386641 | count | 1 |
| WBP1 | -0.855506 | 0.6144985 | -1.3922 | 0.164 | -0.314278301 | count | 1 |
| RERE | -0.2507735 | 0.1635442 | -1.5334 | 0.125 | -0.314245663 | count | 1 |
| RARA | -0.232384 | 0.143771 | -1.6163 | 0.106 | -0.314059385 | count | 1 |
| RPAIN | -0.224691 | 0.0909089 | -2.4716 | 0.0135 | -0.314056885 | count | 1 |
| NIPA1 | -0.4015048 | 0.3080511 | -1.3034 | 0.193 | -0.314045818 | count | 1 |
| COPB2 | -0.2285042 | 0.1146299 | -1.9934 | 0.0463 | -0.314036852 | count | 1 |
| OSBPL8 | -0.2244603 | 0.0856401 | -2.621 | 0.00881 | -0.313948748 | count | 1 |
| GPS2 | -0.2273159 | 0.1045201 | -2.1749 | 0.0297 | -0.31380289 | count | 1 |
| ARHGAP17 | -0.2936451 | 0.2301836 | -1.2757 | 0.202 | -0.313746496 | count | 1 |
| AC233280.1 | -0.8532229 | 0.9511482 | -0.897 | 0.37 | -0.313621301 | count | 1 |
| ST3GAL5 | -0.239497 | 0.1526736 | -1.5687 | 0.117 | -0.31355638 | count | 1 |
| ESR1 | -0.6657737 | 0.9424287 | -0.7064 | 0.48 | -0.313426634 | count | 1 |
| KRAS | -0.2221651 | 0.0760377 | -2.9218 | 0.0035 | -0.313240217 | count | 1 |

| | | | | | | | |
|---|---|---|---|---|---|---|---|
| COMMD8 | -0.2262973 | 0.1111528 | -2.0359 | 0.0418 | -0.313195267 | count | 1 |
| TFG | -0.2309563 | 0.1059322 | -2.1802 | 0.0293 | -0.313189563 | count | 1 |
| ERMAP | -1.332442 | 0.9434884 | -1.4123 | 0.158 | -0.313176281 | count | 1 |
| PRC1 | -1.332442 | 0.9745706 | -1.3672 | 0.172 | -0.313176281 | count | 1 |
| TAF5 | -0.2664494 | 0.1880482 | -1.4169 | 0.157 | -0.313033958 | count | 1 |
| MAPRE3 | -0.6646745 | 0.4942553 | -1.3448 | 0.179 | -0.312986963 | count | 1 |
| ITSN2 | -0.2242546 | 0.0962432 | -2.3301 | 0.0199 | -0.31289636 | count | 1 |
| PPP1R12A | -0.2217096 | 0.0662245 | -3.3478 | 0.000823 | -0.312842314 | count | 1 |
| ZFAND2A | -0.2305943 | 0.1389845 | -1.6591 | 0.0972 | -0.312700797 | count | 1 |
| ETHE1 | -0.2228068 | 0.0967957 | -2.3018 | 0.0214 | -0.312679559 | count | 1 |
| ATF4 | -0.2217725 | 0.0651486 | -3.4041 | 0.000672 | -0.312594799 | count | 1 |
| ST8SIA1 | -0.2889809 | 0.2594988 | -1.1136 | 0.266 | -0.312557954 | count | 1 |
| KHDC1 | -0.6634537 | 0.5252842 | -1.263 | 0.207 | -0.312498337 | count | 1 |
| SNHG7 | -0.220437 | 0.071188 | -3.0965 | 0.00197 | -0.312456936 | count | 1 |
| RNF103 | -0.3105816 | 0.2819775 | -1.1014 | 0.271 | -0.312274841 | count | 1 |
| SPHK2 | -0.3514531 | 0.3257245 | -1.079 | 0.281 | -0.31226929 | count | 1 |
| KDM3A | -0.2708581 | 0.1708272 | -1.5856 | 0.113 | -0.3121352 | count | 1 |
| MAP3K8 | -0.2229756 | 0.1104507 | -2.0188 | 0.0436 | -0.312039863 | count | 1 |
| AC091057.2 | -0.3403428 | 1.0410888 | -0.3269 | 0.744 | -0.311989228 | count | 1 |
| ATP2B4 | -0.2300573 | 0.1191481 | -1.9309 | 0.0536 | -0.311975735 | count | 1 |
| ZNF143 | -0.2475959 | 0.1603879 | -1.5437 | 0.123 | -0.311941862 | count | 1 |
| LRRC8C | -0.2565433 | 0.1582835 | -1.6208 | 0.105 | -0.311931449 | count | 1 |
| ERBIN | -0.2283772 | 0.1097328 | -2.0812 | 0.0375 | -0.311900024 | count | 1 |
| PSMB4 | -0.2208635 | 0.0784054 | -2.8169 | 0.00488 | -0.311796162 | count | 1 |
| AC006157.1 | -0.6617001 | 0.9021818 | -0.7334 | 0.463 | -0.311795893 | count | 1 |
| Z83851.1 | -0.6617001 | 1.0452832 | -0.633 | 0.527 | -0.311795893 | count | 1 |
| TCEAL8 | -0.2332043 | 0.1317919 | -1.7695 | 0.0769 | -0.311697634 | count | 1 |
| TMEM64 | -0.3044717 | 0.2694135 | -1.1301 | 0.259 | -0.311541954 | count | 1 |
| SLC27A4 | -0.6608511 | 0.4693959 | -1.4079 | 0.159 | -0.311455556 | count | 1 |
| GCH1 | -0.2507101 | 0.1633251 | -1.535 | 0.125 | -0.311440316 | count | 1 |
| GTPBP6 | -0.2280315 | 0.120399 | -1.894 | 0.0583 | -0.311166257 | count | 1 |
| MRFAP1 | -0.2206124 | 0.0625836 | -3.5251 | 0.000429 | -0.311122076 | count | 1 |
| KCMF1 | -0.2270635 | 0.1143629 | -1.9855 | 0.0472 | -0.310972438 | count | 1 |
| AC069544.1 | -0.4967314 | 0.5991437 | -0.8291 | 0.407 | -0.310967322 | count | 1 |
| IFFO2 | -0.2557379 | 0.1984022 | -1.289 | 0.197 | -0.310964537 | count | 1 |
| RNGTT | -0.2602377 | 0.1777501 | -1.4641 | 0.143 | -0.310781228 | count | 1 |
| BMPR2 | -0.3969345 | 0.3072233 | -1.292 | 0.196 | -0.310681339 | count | 1 |
| ASRGL1 | -0.8423404 | 1.2193653 | -0.6908 | 0.49 | -0.310476419 | count | 1 |
| ISL2 | -0.8423404 | 1.2193653 | -0.6908 | 0.49 | -0.310476419 | count | 1 |
| AL138895.1 | -0.8423404 | 1.232232 | -0.6836 | 0.494 | -0.310476419 | count | 1 |
| AL139317.3 | -0.8423404 | 1.232232 | -0.6836 | 0.494 | -0.310476419 | count | 1 |
| LIPA | -0.2361146 | 0.1585342 | -1.4894 | 0.136 | -0.310320614 | count | 1 |
| KHDC4 | -0.2655631 | 0.2069541 | -1.2832 | 0.2 | -0.310153409 | count | 1 |
| MAPK8IP3 | -0.5579998 | 0.4435159 | -1.2581 | 0.208 | -0.310126272 | count | 1 |
| PHAX | -0.2243492 | 0.102359 | -2.1918 | 0.0285 | -0.310124722 | count | 1 |

| | | | | | | | |
|---|---|---|---|---|---|---|---|
| MUC20-OT1 | -0.2404061 | 0.1524154 | -1.5773 | 0.115 | -0.310037277 | count | 1 |
| ASF1B | -0.4518137 | 0.4980472 | -0.9072 | 0.364 | -0.310008379 | count | 1 |
| RPS27L | -0.2166131 | 0.0605462 | -3.5776 | 0.000352 | -0.30998291 | count | 1 |
| CEP83 | -0.3141451 | 0.246551 | -1.2742 | 0.203 | -0.309885161 | count | 1 |
| DENND1A | -0.4511326 | 0.4077859 | -1.1063 | 0.269 | -0.30957711 | count | 1 |
| BBC3 | -0.2209386 | 0.1005527 | -2.1972 | 0.0281 | -0.309576834 | count | 1 |
| PTCH1 | -0.3953735 | 0.397087 | -0.9957 | 0.319 | -0.309530969 | count | 1 |
| AVPI1 | -0.2704503 | 0.2017844 | -1.3403 | 0.18 | -0.309451919 | count | 1 |
| EIF5B | -0.2175964 | 0.0659782 | -3.298 | 0.001 | -0.309430075 | count | 1 |
| DPM1 | -0.2407126 | 0.149858 | -1.6063 | 0.108 | -0.309317431 | count | 1 |
| H3F3C | -1.306106 | 0.6348798 | -2.0572 | 0.0397 | -0.309303731 | count | 1 |
| LINC02001 | -0.2388335 | 0.1854866 | -1.2876 | 0.198 | -0.309065265 | count | 1 |
| AC104653.1 | -0.5556951 | 0.7443134 | -0.7466 | 0.455 | -0.308990323 | count | 1 |
| ZFAND6 | -0.2218561 | 0.0912977 | -2.43 | 0.0151 | -0.308894686 | count | 1 |
| CKB | -0.327747 | 0.2704558 | -1.2118 | 0.226 | -0.308890812 | count | 1 |
| ZNF283 | -0.492857 | 0.6076723 | -0.8111 | 0.417 | -0.308764131 | count | 1 |
| LDOC1 | -0.3469947 | 0.3316534 | -1.0463 | 0.296 | -0.308477164 | count | 1 |
| IL23R | -0.3748938 | 0.6847199 | -0.5475 | 0.584 | -0.308397512 | count | 1 |
| SLC9B2 | -0.417839 | 0.3472341 | -1.2033 | 0.229 | -0.308394506 | count | 1 |
| ZNF174 | -0.284994 | 0.3260853 | -0.874 | 0.382 | -0.308343092 | count | 1 |
| PPWD1 | -0.2249216 | 0.1288933 | -1.745 | 0.0811 | -0.308277472 | count | 1 |
| CHAMP1 | -0.2497153 | 0.1961282 | -1.2732 | 0.203 | -0.308227811 | count | 1 |
| PSMD5 | -0.2760648 | 0.2181776 | -1.2653 | 0.206 | -0.308108614 | count | 1 |
| IMMT | -0.2266151 | 0.1157039 | -1.9586 | 0.0502 | -0.307958988 | count | 1 |
| HNRNPU | -0.2161199 | 0.0487612 | -4.4322 | 9.62E-06 | -0.307743864 | count | 0.23125518 |
| C19orf25 | -0.2272495 | 0.114828 | -1.979 | 0.0479 | -0.307681099 | count | 1 |
| SIT1 | -0.2174223 | 0.0812889 | -2.6747 | 0.00752 | -0.307548276 | count | 1 |
| PLAGL2 | -0.3004243 | 0.2643151 | -1.1366 | 0.256 | -0.307515349 | count | 1 |
| EME2 | -0.5523715 | 0.607858 | -0.9087 | 0.364 | -0.307349908 | count | 1 |
| CRYGS | -1.2921373 | 1.3034496 | -0.9913 | 0.322 | -0.30721736 | count | 1 |
| TBC1D8B | -1.2921373 | 1.3933121 | -0.9274 | 0.354 | -0.30721736 | count | 1 |
| BMI1 | -0.2545593 | 0.1873218 | -1.3589 | 0.174 | -0.306944536 | count | 1 |
| CCDC167 | -0.216923 | 0.09309 | -2.3302 | 0.0199 | -0.306908606 | count | 1 |
| FLYWCH2 | -0.228005 | 0.1570697 | -1.4516 | 0.147 | -0.306811998 | count | 1 |
| EMC7 | -0.2186335 | 0.0876936 | -2.4932 | 0.0127 | -0.306801137 | count | 1 |
| SKIL | -0.222781 | 0.0986534 | -2.2582 | 0.024 | -0.306773939 | count | 1 |
| UBL4A | -0.2377297 | 0.1617456 | -1.4698 | 0.142 | -0.30661291 | count | 1 |
| ABHD14A | -0.2191999 | 0.1102673 | -1.9879 | 0.0469 | -0.306312299 | count | 1 |
| MIA2 | -0.2232978 | 0.1050644 | -2.1253 | 0.0336 | -0.306279705 | count | 1 |
| GGNBP2 | -0.2170177 | 0.0672909 | -3.2251 | 0.00127 | -0.306252476 | count | 1 |
| L3MBTL3 | -0.2619855 | 0.1761058 | -1.4877 | 0.137 | -0.306040666 | count | 1 |
| ADAR | -0.2221451 | 0.0992598 | -2.238 | 0.0253 | -0.305992656 | count | 1 |
| MBTD1 | -0.3167694 | 0.3504559 | -0.9039 | 0.366 | -0.305949286 | count | 1 |
| RABIF | -0.2249584 | 0.1220703 | -1.8429 | 0.0654 | -0.305867128 | count | 1 |
| RAMP2 | -1.2831516 | 0.8074787 | -1.5891 | 0.112 | -0.305863208 | count | 1 |

| | | | | | | | |
|---|---|---|---|---|---|---|---|
| TNIK | -0.2315967 | 0.1410367 | -1.6421 | 0.101 | -0.305814704 | count | 1 |
| GNB4 | -0.8261752 | 1.1585767 | -0.7131 | 0.476 | -0.305764484 | count | 1 |
| KDF1 | -0.8261752 | 1.1970013 | -0.6902 | 0.49 | -0.305764484 | count | 1 |
| RAB3IP | -0.2599014 | 0.2035825 | -1.2766 | 0.202 | -0.305457877 | count | 1 |
| H2AFV | -0.2143632 | 0.0578738 | -3.704 | 2.00E-04 | -0.305398181 | count | 1 |
| CCDC141 | -0.4443948 | 0.3025355 | -1.4689 | 0.142 | -0.305304546 | count | 1 |
| XRN1 | -0.2200713 | 0.0925023 | -2.3791 | 0.0174 | -0.304746397 | count | 1 |
| SUB1 | -0.2121741 | 0.0360897 | -5.8791 | 4.52E-09 | -0.304693994 | count | 0.000109321 |
| SPAG9 | -0.219166 | 0.0909604 | -2.4095 | 0.016 | -0.304595926 | count | 1 |
| U47924.2 | -0.6437216 | 0.7577817 | -0.8495 | 0.396 | -0.30455492 | count | 1 |
| NOC4L | -0.2926512 | 0.2151118 | -1.3605 | 0.174 | -0.30441213 | count | 1 |
| ZBTB7A | -0.2167 | 0.081803 | -2.649 | 0.00811 | -0.304314122 | count | 1 |
| SMARCA5 | -0.2175226 | 0.0760031 | -2.862 | 0.00424 | -0.304305425 | count | 1 |
| TRDV1 | -0.8208271 | 1.088034 | -0.7544 | 0.451 | -0.304194926 | count | 1 |
| IGFBP5 | -0.442606 | 0.3707258 | -1.1939 | 0.233 | -0.304168253 | count | 1 |
| OCEL1 | -0.2425997 | 0.1997893 | -1.2143 | 0.225 | -0.304105302 | count | 1 |
| SBF2-AS1 | -0.4422918 | 0.5324528 | -0.8307 | 0.406 | -0.303968601 | count | 1 |
| RAD9A | -0.2305569 | 0.1415976 | -1.6283 | 0.104 | -0.303773193 | count | 1 |
| PDXK | -0.2264814 | 0.1536905 | -1.4736 | 0.141 | -0.303696965 | count | 1 |
| MESD | -0.2151839 | 0.0866576 | -2.4832 | 0.0131 | -0.30366842 | count | 1 |
| EMC8 | -0.2307386 | 0.1692584 | -1.3632 | 0.173 | -0.303660036 | count | 1 |
| ANKH | -0.2362378 | 0.179148 | -1.3187 | 0.187 | -0.303613668 | count | 1 |
| RNF19A | -0.2137214 | 0.0562848 | -3.7971 | 0.000149 | -0.303426475 | count | 1 |
| RAD17 | -0.2474766 | 0.1922145 | -1.2875 | 0.198 | -0.303360785 | count | 1 |
| ZC3H7B | -0.274208 | 0.3111893 | -0.8812 | 0.378 | -0.303221018 | count | 1 |
| PPM1D | -0.2564036 | 0.1861023 | -1.3778 | 0.168 | -0.303107841 | count | 1 |
| ZNF121 | -0.2610447 | 0.2225819 | -1.1728 | 0.241 | -0.303038779 | count | 1 |
| MIGA1 | -0.234914 | 0.1854568 | -1.2667 | 0.205 | -0.303009569 | count | 1 |
| GPANK1 | -0.2410315 | 0.1562216 | -1.5429 | 0.123 | -0.302968871 | count | 1 |
| KDM1B | -0.3679035 | 0.3900443 | -0.9432 | 0.346 | -0.302939213 | count | 1 |
| ZNF597 | -0.5433645 | 0.3934626 | -1.381 | 0.167 | -0.302891059 | count | 1 |
| SAYSD1 | -0.2403411 | 0.2245893 | -1.0701 | 0.285 | -0.302888742 | count | 1 |
| ZADH2 | -0.2479498 | 0.2051625 | -1.2086 | 0.227 | -0.302796716 | count | 1 |
| ZNF638 | -0.2251551 | 0.1149468 | -1.9588 | 0.0502 | -0.30278962 | count | 1 |
| ERGIC3 | -0.2191596 | 0.1107498 | -1.9789 | 0.0479 | -0.302657361 | count | 1 |
| IRF2BP1 | -0.255934 | 0.2295347 | -1.115 | 0.265 | -0.302560744 | count | 1 |
| NHP2 | -0.2156344 | 0.0940548 | -2.2926 | 0.0219 | -0.302535267 | count | 1 |
| PGM3 | -0.286635 | 0.2523445 | -1.1359 | 0.256 | -0.302520782 | count | 1 |
| PAFAH1B2 | -0.2216226 | 0.126881 | -1.7467 | 0.0808 | -0.302322814 | count | 1 |
| PSMA8 | -0.3855778 | 0.5501583 | -0.7008 | 0.483 | -0.302298091 | count | 1 |
| MRPL52 | -0.2159055 | 0.0976236 | -2.2116 | 0.0271 | -0.302242593 | count | 1 |
| BICDL1 | -0.2298126 | 0.1217679 | -1.8873 | 0.0592 | -0.302090865 | count | 1 |
| TMEM8A | -0.2267832 | 0.1706732 | -1.3288 | 0.184 | -0.301871869 | count | 1 |
| CDC37L1 | -0.2256217 | 0.1372975 | -1.6433 | 0.1 | -0.301615244 | count | 1 |
| DHFR2 | -0.5407818 | 0.5670142 | -0.9537 | 0.34 | -0.301608926 | count | 1 |

| | | | | | | | |
|---|---|---|---|---|---|---|---|
| SLC35E2B | -0.3285068 | 0.2706323 | -1.2138 | 0.225 | -0.301553806 | count | 1 |
| AP001160.1 | -0.3196144 | 0.2808811 | -1.1379 | 0.255 | -0.301496339 | count | 1 |
| SLC33A1 | -0.3387303 | 0.3437987 | -0.9853 | 0.325 | -0.301435185 | count | 1 |
| DYNC2H1 | -0.6354636 | 0.3777453 | -1.6823 | 0.0926 | -0.301205014 | count | 1 |
| PLEKHA6 | -0.4790138 | 0.9792908 | -0.4891 | 0.625 | -0.300861464 | count | 1 |
| OAZ1 | -0.209019 | 0.029113 | -7.1796 | 8.55E-13 | -0.300691125 | count | 2.07E-08 |
| SP140 | -0.2167488 | 0.1060075 | -2.0447 | 0.041 | -0.300629515 | count | 1 |
| ATP1A1 | -0.2175493 | 0.0955201 | -2.2775 | 0.0228 | -0.300519265 | count | 1 |
| CIC | -0.280961 | 0.2739201 | -1.0257 | 0.305 | -0.300506017 | count | 1 |
| SLAMF1 | -0.2155256 | 0.1069498 | -2.0152 | 0.044 | -0.30045698 | count | 1 |
| CENPW | -0.5380764 | 0.4871883 | -1.1045 | 0.269 | -0.300264177 | count | 1 |
| MIER1 | -0.2109526 | 0.0602957 | -3.4986 | 5.00E-04 | -0.300251721 | count | 1 |
| IGLL5 | -0.8074175 | 1.0561083 | -0.7645 | 0.445 | -0.30023609 | count | 1 |
| SLC38A6 | -0.2772443 | 0.3116401 | -0.8896 | 0.374 | -0.300141659 | count | 1 |
| DCTN3 | -0.2118724 | 0.0735296 | -2.8815 | 0.00398 | -0.299926195 | count | 1 |
| CCNG1 | -0.2261492 | 0.1314924 | -1.7199 | 0.0855 | -0.299901533 | count | 1 |
| MORF4L2 | -0.2333063 | 0.1122026 | -2.0793 | 0.0377 | -0.299875995 | count | 1 |
| PCNA | -0.2180602 | 0.1246197 | -1.7498 | 0.0802 | -0.299729647 | count | 1 |
| TWF1 | -0.2282588 | 0.1483184 | -1.539 | 0.124 | -0.299696788 | count | 1 |
| PARP11 | -0.2520958 | 0.1981942 | -1.272 | 0.203 | -0.29967671 | count | 1 |
| SERTAD3 | -0.2116227 | 0.0840612 | -2.5175 | 0.0119 | -0.299617811 | count | 1 |
| ZFAND5 | -0.214403 | 0.0756298 | -2.8349 | 0.00461 | -0.299317647 | count | 1 |
| AC067852.2 | -0.4762198 | 0.4460641 | -1.0676 | 0.286 | -0.299260666 | count | 1 |
| TLDC1 | -0.4347507 | 0.5060473 | -0.8591 | 0.39 | -0.299168944 | count | 1 |
| NCOR1 | -0.2097381 | 0.0605756 | -3.4624 | 5.00E-04 | -0.299110509 | count | 1 |
| BTG1 | -0.2074602 | 0.0252091 | -8.2296 | 2.65E-16 | -0.299015649 | count | 6.43E-12 |
| DPY30 | -0.2133577 | 0.1032596 | -2.0662 | 0.0389 | -0.298939342 | count | 1 |
| PVT1 | -0.234598 | 0.1604918 | -1.4617 | 0.144 | -0.298508567 | count | 1 |
| A1BG-AS1 | -0.2756684 | 0.3158747 | -0.8727 | 0.383 | -0.298472515 | count | 1 |
| PPFIBP1 | -0.4747902 | 0.5586051 | -0.85 | 0.395 | -0.298440832 | count | 1 |
| NPIPB5 | -0.4747655 | 0.261101 | -1.8183 | 0.0691 | -0.298426786 | count | 1 |
| COX7B | -0.209403 | 0.0549844 | -3.8084 | 1.00E-04 | -0.298256956 | count | 1 |
| SMUG1 | -0.2625206 | 0.2785742 | -0.9424 | 0.346 | -0.298254779 | count | 1 |
| SDR42E1 | -0.5340239 | 0.6579794 | -0.8116 | 0.417 | -0.29824653 | count | 1 |
| NUFIP1 | -0.2313623 | 0.2079194 | -1.1127 | 0.266 | -0.297938036 | count | 1 |
| CHAF1A | -0.4324028 | 0.326342 | -1.325 | 0.185 | -0.297671695 | count | 1 |
| SMCHD1 | -0.2103609 | 0.0552597 | -3.8068 | 0.000143 | -0.297523919 | count | 1 |
| MRPS23 | -0.2191101 | 0.1424901 | -1.5377 | 0.124 | -0.297500754 | count | 1 |
| AP002807.1 | -0.5324823 | 0.7904334 | -0.6737 | 0.501 | -0.297477982 | count | 1 |
| ELF1 | -0.2081007 | 0.0426288 | -4.8817 | 1.10E-06 | -0.297459599 | count | 0.0265144 |
| LYPLA2 | -0.2166869 | 0.1221394 | -1.7741 | 0.0761 | -0.297449521 | count | 1 |
| CTDSP2 | -0.2313645 | 0.1566513 | -1.4769 | 0.14 | -0.297399735 | count | 1 |
| CWC22 | -0.2282658 | 0.1214937 | -1.8788 | 0.0604 | -0.297312737 | count | 1 |
| NARF | -0.2239553 | 0.1354766 | -1.6531 | 0.0984 | -0.297008503 | count | 1 |
| CD74 | -0.2064129 | 0.0478395 | -4.3147 | 1.64E-05 | -0.297004879 | count | 0.393846 |

| | | | | | | | |
|---|---|---|---|---|---|---|---|
| BAZ1A | -0.2107063 | 0.0720429 | -2.9247 | 0.00347 | -0.296997798 | count | 1 |
| MCAM | -0.4722721 | 0.4438679 | -1.064 | 0.287 | -0.296995551 | count | 1 |
| KDM7A | -0.2593571 | 0.2150299 | -1.2061 | 0.228 | -0.296972675 | count | 1 |
| ARHGEF2 | -0.2356005 | 0.1798263 | -1.3102 | 0.19 | -0.296969888 | count | 1 |
| DICER1-AS1 | -0.3455545 | 0.5469016 | -0.6318 | 0.528 | -0.296920054 | count | 1 |
| DRAXIN | -0.3455545 | 0.6064427 | -0.5698 | 0.569 | -0.296920054 | count | 1 |
| RABGEF1 | -0.3005391 | 0.2931819 | -1.0251 | 0.305 | -0.296867732 | count | 1 |
| SEC16A | -0.2774783 | 0.2444582 | -1.1351 | 0.256 | -0.296865109 | count | 1 |
| DHX40 | -0.2509893 | 0.2030702 | -1.236 | 0.217 | -0.296798046 | count | 1 |
| NDUFC1 | -0.2128105 | 0.0955756 | -2.2266 | 0.026 | -0.296776457 | count | 1 |
| TPI1 | -0.2068109 | 0.0429061 | -4.8201 | 1.50E-06 | -0.296737835 | count | 0.036141 |
| FAM13A-AS1 | -0.3597288 | 0.5474093 | -0.6571 | 0.511 | -0.296540702 | count | 1 |
| LEMD3 | -0.2589667 | 0.1942921 | -1.3329 | 0.183 | -0.296533126 | count | 1 |
| LINC01771 | -0.4306115 | 0.7038008 | -0.6118 | 0.541 | -0.296528384 | count | 1 |
| ATP5IF1 | -0.2076377 | 0.0477786 | -4.3458 | 1.43E-05 | -0.296499489 | count | 0.3434717 |
| CLIC5 | -0.3065822 | 0.306101 | -1.0016 | 0.317 | -0.296425976 | count | 1 |
| KIAA0040 | -0.2142672 | 0.1116177 | -1.9197 | 0.055 | -0.296374887 | count | 1 |
| ATP2B1 | -0.2103826 | 0.0779054 | -2.7005 | 0.00696 | -0.296140497 | count | 1 |
| HBS1L | -0.2258149 | 0.1442662 | -1.5653 | 0.118 | -0.296138807 | count | 1 |
| AL117332.1 | -0.4299533 | 0.5172559 | -0.8312 | 0.406 | -0.296108098 | count | 1 |
| PITPNB | -0.2155551 | 0.1152988 | -1.8695 | 0.0616 | -0.296101687 | count | 1 |
| ATP5MF | -0.2091095 | 0.070319 | -2.9737 | 0.00296 | -0.296092899 | count | 1 |
| EFR3A | -0.217735 | 0.1212086 | -1.7964 | 0.0725 | -0.296083951 | count | 1 |
| C12orf29 | -0.2246772 | 0.1348976 | -1.6655 | 0.0959 | -0.296073236 | count | 1 |
| DHX58 | -0.2675086 | 0.3285451 | -0.8142 | 0.416 | -0.295958039 | count | 1 |
| TIMM9 | -0.2248226 | 0.1597425 | -1.4074 | 0.159 | -0.29592213 | count | 1 |
| MOB1B | -0.529213 | 0.3551046 | -1.4903 | 0.136 | -0.295846171 | count | 1 |
| RPRD1A | -0.2411689 | 0.2407744 | -1.0016 | 0.317 | -0.295716158 | count | 1 |
| FEM1B | -0.2528635 | 0.2209318 | -1.1445 | 0.252 | -0.295545209 | count | 1 |
| SLC16A11 | -0.2883838 | 0.4223244 | -0.6828 | 0.495 | -0.295516569 | count | 1 |
| ZSCAN18 | -0.2438574 | 0.1967902 | -1.2392 | 0.215 | -0.29547413 | count | 1 |
| TNFRSF12A | -0.2184319 | 0.1401705 | -1.5583 | 0.119 | -0.295456774 | count | 1 |
| NPHP1 | -1.2154405 | 1.031695 | -1.1781 | 0.239 | -0.295351779 | count | 1 |
| HERPUD1 | -0.2058324 | 0.0468841 | -4.3902 | 1.17E-05 | -0.295300383 | count | 0.281151 |
| ZSCAN2 | -0.2987418 | 0.3965633 | -0.7533 | 0.451 | -0.295145159 | count | 1 |
| RRNAD1 | -0.2834485 | 0.3140305 | -0.9026 | 0.367 | -0.295078111 | count | 1 |
| ENO1 | -0.2061343 | 0.0519505 | -3.9679 | 7.40E-05 | -0.295060407 | count | 1 |
| KIF9-AS1 | -0.4282398 | 0.6821121 | -0.6278 | 0.53 | -0.295013434 | count | 1 |
| NR2F2 | -1.2127462 | 0.604272 | -2.007 | 0.0448 | -0.29492216 | count | 1 |
| NUBP2 | -0.2189823 | 0.1196758 | -1.8298 | 0.0674 | -0.294922125 | count | 1 |
| TSC2 | -0.3310148 | 0.2356681 | -1.4046 | 0.16 | -0.294846178 | count | 1 |
| ATG4A | -0.259422 | 0.2440825 | -1.0628 | 0.288 | -0.294795129 | count | 1 |
| CEBPG | -0.2170684 | 0.1241334 | -1.7487 | 0.0804 | -0.294739569 | count | 1 |
| FBLIM1 | -0.3308443 | 0.4560865 | -0.7254 | 0.468 | -0.294700412 | count | 1 |
| DEK | -0.2058165 | 0.050468 | -4.0782 | 4.64E-05 | -0.294587817 | count | 1 |

| | | | | | | | |
|---|---|---|---|---|---|---|---|
| PIN1 | -0.2088804 | 0.0807295 | -2.5874 | 0.00971 | -0.294558486 | count | 1 |
| GTF2A1 | -0.2336409 | 0.1635765 | -1.4283 | 0.153 | -0.294522516 | count | 1 |
| WDR26 | -0.2450599 | 0.1732131 | -1.4148 | 0.157 | -0.294296155 | count | 1 |
| SAMD12 | -0.4270873 | 0.4527203 | -0.9434 | 0.346 | -0.294276757 | count | 1 |
| YY1 | -0.206249 | 0.054008 | -3.8189 | 1.00E-04 | -0.294192164 | count | 1 |
| HCCS | -0.241732 | 0.2334251 | -1.0356 | 0.3 | -0.294136184 | count | 1 |
| MLLT10 | -0.2264477 | 0.147776 | -1.5324 | 0.126 | -0.294082248 | count | 1 |
| MAP3K5 | -0.2473165 | 0.188117 | -1.3147 | 0.189 | -0.294072551 | count | 1 |
| SCAF1 | -0.3420464 | 0.3628046 | -0.9428 | 0.346 | -0.29403846 | count | 1 |
| NACC1 | -0.2714572 | 0.3270946 | -0.8299 | 0.407 | -0.294009849 | count | 1 |
| WDR46 | -0.2278171 | 0.1533616 | -1.4855 | 0.138 | -0.293924132 | count | 1 |
| IZUMO4 | -0.303722 | 0.4084751 | -0.7436 | 0.457 | -0.29374802 | count | 1 |
| ZNF787 | -0.2395139 | 0.1578004 | -1.5178 | 0.129 | -0.293709539 | count | 1 |
| ZNF93 | -0.3561006 | 0.3455207 | -1.0306 | 0.303 | -0.29369554 | count | 1 |
| RRAGB | -0.2564096 | 0.2852269 | -0.899 | 0.369 | -0.293653493 | count | 1 |
| DSTYK | -0.2743649 | 0.2329606 | -1.1777 | 0.239 | -0.293608306 | count | 1 |
| PMAIP1 | -0.2106599 | 0.0958106 | -2.1987 | 0.028 | -0.293591905 | count | 1 |
| HAUS2 | -0.227157 | 0.1754743 | -1.2945 | 0.196 | -0.293579131 | count | 1 |
| NOC2L | -0.2229791 | 0.1356591 | -1.6437 | 0.1 | -0.293510329 | count | 1 |
| GPR18 | -0.2240012 | 0.2040295 | -1.0979 | 0.272 | -0.293399627 | count | 1 |
| DIDO1 | -0.2253471 | 0.1622773 | -1.3887 | 0.165 | -0.293105919 | count | 1 |
| FGF18 | -0.3956605 | 0.6536065 | -0.6053 | 0.545 | -0.293046175 | count | 1 |
| ABHD11 | -0.2463708 | 0.2677256 | -0.9202 | 0.358 | -0.292963242 | count | 1 |
| PQLC2 | -0.4247445 | 0.5069063 | -0.8379 | 0.402 | -0.292778192 | count | 1 |
| MPLKIP | -0.2120547 | 0.114632 | -1.8499 | 0.0644 | -0.292715983 | count | 1 |
| LPCAT4 | -0.2240152 | 0.1770953 | -1.2649 | 0.206 | -0.292636854 | count | 1 |
| MAP4K1 | -0.2178309 | 0.1526247 | -1.4272 | 0.154 | -0.292576782 | count | 1 |
| AP4S1 | -0.4645355 | 0.3243141 | -1.4324 | 0.152 | -0.292545239 | count | 1 |
| ORC4 | -0.2345646 | 0.1802264 | -1.3015 | 0.193 | -0.292466755 | count | 1 |
| GAPDH | -0.2028353 | 0.0266036 | -7.6244 | 3.16E-14 | -0.292381727 | count | 7.67E-10 |
| MXI1 | -0.2131486 | 0.1064139 | -2.003 | 0.0453 | -0.292300127 | count | 1 |
| TRAPPC6A | -0.2076193 | 0.0838852 | -2.475 | 0.0134 | -0.292232132 | count | 1 |
| BACE2 | -0.7804087 | 0.7143438 | -1.0925 | 0.275 | -0.292160719 | count | 1 |
| KANK1 | -0.7804087 | 0.7333699 | -1.0641 | 0.287 | -0.292160719 | count | 1 |
| SAMD9 | -0.2080472 | 0.0875332 | -2.3768 | 0.0175 | -0.292033533 | count | 1 |
| CWC15 | -0.208639 | 0.0976886 | -2.1358 | 0.0328 | -0.291824549 | count | 1 |
| GRIP2 | -0.4632394 | 1.0195658 | -0.4543 | 0.65 | -0.291798225 | count | 1 |
| STAM | -0.2220876 | 0.1469162 | -1.5117 | 0.131 | -0.291644363 | count | 1 |
| COMMD5 | -0.2153764 | 0.1444328 | -1.4912 | 0.136 | -0.291507791 | count | 1 |
| VIM-AS1 | -0.242604 | 0.2178951 | -1.1134 | 0.266 | -0.291384155 | count | 1 |
| RND1 | -0.4225307 | 0.3716289 | -1.137 | 0.256 | -0.291360881 | count | 1 |
| ITGA3 | -0.3708196 | 0.6014356 | -0.6166 | 0.538 | -0.291355537 | count | 1 |
| ILF3 | -0.209373 | 0.0912026 | -2.2957 | 0.0218 | -0.291292421 | count | 1 |
| SLTM | -0.2056174 | 0.0672931 | -3.0556 | 0.00226 | -0.291278695 | count | 1 |
| LTK | -1.1896158 | 0.8952199 | -1.3289 | 0.184 | -0.291197425 | count | 1 |

| | | | | | | | |
|---|---|---|---|---|---|---|---|
| TM9SF3 | -0.2120795 | 0.1030686 | -2.0577 | 0.0397 | -0.291144985 | count | 1 |
| PDCD2 | -0.2082703 | 0.0914768 | -2.2768 | 0.0229 | -0.291110881 | count | 1 |
| RRS1-AS1 | -0.3704372 | 0.8854208 | -0.4184 | 0.676 | -0.29107128 | count | 1 |
| ZC3H3 | -0.2837313 | 0.2728971 | -1.0397 | 0.299 | -0.29087216 | count | 1 |
| PDLIM5 | -0.2290442 | 0.1958478 | -1.1695 | 0.242 | -0.290854691 | count | 1 |
| FAM76A | -0.2232342 | 0.1602685 | -1.3929 | 0.164 | -0.290803227 | count | 1 |
| IRAK1BP1 | -0.3921486 | 0.507097 | -0.7733 | 0.439 | -0.290604428 | count | 1 |
| ZNF200 | -0.2883571 | 0.2913881 | -0.9896 | 0.322 | -0.2905459 | count | 1 |
| TARSL2 | -0.2214998 | 0.1160683 | -1.9084 | 0.0564 | -0.290515214 | count | 1 |
| ATG4B | -0.2352058 | 0.1995625 | -1.1786 | 0.239 | -0.290508031 | count | 1 |
| NAIP | -0.460701 | 0.3687294 | -1.2494 | 0.212 | -0.290334016 | count | 1 |
| HDHD3 | -0.2234993 | 0.1954939 | -1.1433 | 0.253 | -0.290279695 | count | 1 |
| ZNF583 | -0.5178884 | 0.3360256 | -1.5412 | 0.123 | -0.290174169 | count | 1 |
| C12orf42 | -0.3254967 | 0.4767265 | -0.6828 | 0.495 | -0.290125065 | count | 1 |
| GAPVD1 | -0.2356902 | 0.1598776 | -1.4742 | 0.141 | -0.290106665 | count | 1 |
| RFC5 | -0.4601809 | 0.3289053 | -1.3991 | 0.162 | -0.290033819 | count | 1 |
| CHCHD7 | -0.2122249 | 0.1078835 | -1.9672 | 0.0492 | -0.289917855 | count | 1 |
| OSER1 | -0.2075366 | 0.0866462 | -2.3952 | 0.0167 | -0.289880356 | count | 1 |
| MYL6 | -0.2012001 | 0.0277792 | -7.2428 | 5.41E-13 | -0.289793622 | count | 1.31E-08 |
| AFAP1 | -0.2510786 | 0.30079 | -0.8347 | 0.404 | -0.289701824 | count | 1 |
| FIBP | -0.2084435 | 0.1114389 | -1.8705 | 0.0615 | -0.289615858 | count | 1 |
| CMSS1 | -0.2642998 | 0.1797318 | -1.4705 | 0.142 | -0.289546904 | count | 1 |
| POLR3K | -0.2098833 | 0.1194543 | -1.757 | 0.079 | -0.289486742 | count | 1 |
| DGCR2 | -0.3246643 | 0.2785076 | -1.1657 | 0.244 | -0.289412259 | count | 1 |
| BOLA2B | -0.6065951 | 0.446287 | -1.3592 | 0.174 | -0.28937606 | count | 1 |
| TCEAL4 | -0.2181901 | 0.1530603 | -1.4255 | 0.154 | -0.289116741 | count | 1 |
| RNF10 | -0.2317255 | 0.1413391 | -1.6395 | 0.101 | -0.288961369 | count | 1 |
| MED4 | -0.2077989 | 0.0896995 | -2.3166 | 0.0206 | -0.288945735 | count | 1 |
| RFTN1 | -0.2249924 | 0.1576966 | -1.4267 | 0.154 | -0.288729701 | count | 1 |
| INPPL1 | -0.2732403 | 0.328679 | -0.8313 | 0.406 | -0.288709248 | count | 1 |
| SETD2 | -0.2127162 | 0.114096 | -1.8644 | 0.0624 | -0.288704144 | count | 1 |
| ZNF281 | -0.2294298 | 0.1517587 | -1.5118 | 0.131 | -0.288519714 | count | 1 |
| EIF4E | -0.2111446 | 0.1119941 | -1.8853 | 0.0595 | -0.288447175 | count | 1 |
| MT-ATP6 | -0.2001376 | 0.0225664 | -8.8688 | 1.17E-18 | -0.288355581 | count | 2.84E-14 |
| CMC2 | -0.204354 | 0.0770459 | -2.6524 | 0.00803 | -0.288307337 | count | 1 |
| PEX16 | -0.2138576 | 0.1426449 | -1.4992 | 0.134 | -0.288238114 | count | 1 |
| EZH2 | -0.2766254 | 0.2182374 | -1.2675 | 0.205 | -0.288146627 | count | 1 |
| TNIP1 | -0.2178497 | 0.1465939 | -1.4861 | 0.137 | -0.288073783 | count | 1 |
| API5 | -0.2207344 | 0.1610524 | -1.3706 | 0.171 | -0.287978924 | count | 1 |
| TRIP4 | -0.2576885 | 0.1966759 | -1.3102 | 0.19 | -0.28797396 | count | 1 |
| NSUN5 | -0.2478201 | 0.1983974 | -1.2491 | 0.212 | -0.287918651 | count | 1 |
| CEMIP2 | -0.2141338 | 0.088119 | -2.4301 | 0.0151 | -0.28783615 | count | 1 |
| CRTC1 | -1.169002 | 0.4565907 | -2.5603 | 0.0105 | -0.287822677 | count | 1 |
| PPIAL4G | -0.5131462 | 0.8456736 | -0.6068 | 0.544 | -0.287789907 | count | 1 |
| AC020571.1 | -0.387592 | 0.2795068 | -1.3867 | 0.166 | -0.28743171 | count | 1 |

| | | | | | | | |
|---|---|---|---|---|---|---|---|
| NDUFA13 | -0.2060259 | 0.0974712 | -2.1137 | 0.0346 | -0.287426144 | count | 1 |
| ABHD17B | -0.2428876 | 0.2062271 | -1.1778 | 0.239 | -0.287348297 | count | 1 |
| HSPA6 | -0.3650952 | 0.474828 | -0.7689 | 0.442 | -0.287096509 | count | 1 |
| BCL11B | -0.2143605 | 0.1138938 | -1.8821 | 0.0599 | -0.287085824 | count | 1 |
| PRR11 | -0.7633933 | 0.7363781 | -1.0367 | 0.3 | -0.287003246 | count | 1 |
| ELP2 | -0.2306315 | 0.1536181 | -1.5013 | 0.133 | -0.28674673 | count | 1 |
| ERF | -0.2199595 | 0.1371213 | -1.6041 | 0.109 | -0.28656548 | count | 1 |
| HYAL2 | -0.3212766 | 0.4375926 | -0.7342 | 0.463 | -0.286509613 | count | 1 |
| ZW10 | -0.3642414 | 0.3635498 | -1.0019 | 0.316 | -0.286460568 | count | 1 |
| GEMIN2 | -0.2562181 | 0.2972371 | -0.862 | 0.389 | -0.286360311 | count | 1 |
| SGPP2 | -0.7609228 | 0.4609396 | -1.6508 | 0.0989 | -0.286249913 | count | 1 |
| DESI2 | -0.2105714 | 0.1361889 | -1.5462 | 0.122 | -0.286096338 | count | 1 |
| RIC1 | -0.3323784 | 0.3418629 | -0.9723 | 0.331 | -0.286081621 | count | 1 |
| INSIG1 | -0.2075047 | 0.1127269 | -1.8408 | 0.0657 | -0.286052049 | count | 1 |
| EIF3J | -0.2037047 | 0.0885831 | -2.2996 | 0.0215 | -0.286037118 | count | 1 |
| NOL8 | -0.2137145 | 0.1588766 | -1.3452 | 0.179 | -0.286001976 | count | 1 |
| AMT | -0.7599661 | 0.9207176 | -0.8254 | 0.409 | -0.28595787 | count | 1 |
| ZFP90 | -0.2246231 | 0.1688427 | -1.3304 | 0.183 | -0.285920512 | count | 1 |
| TIMMDC1 | -0.2113751 | 0.1344662 | -1.572 | 0.116 | -0.285784073 | count | 1 |
| PKD2L2 | -1.1555086 | 0.6924475 | -1.6687 | 0.0953 | -0.285585091 | count | 1 |
| CEP250 | -0.2474528 | 0.241485 | -1.0247 | 0.306 | -0.28558274 | count | 1 |
| MEGF9 | -0.247426 | 0.2664018 | -0.9288 | 0.353 | -0.285552285 | count | 1 |
| NAA30 | -0.2376411 | 0.2350795 | -1.0109 | 0.312 | -0.285497013 | count | 1 |
| DHX30 | -0.2262655 | 0.1679906 | -1.3469 | 0.178 | -0.285307499 | count | 1 |
| GPRASP1 | -0.4130824 | 0.4124585 | -1.0015 | 0.317 | -0.285298097 | count | 1 |
| SNED1 | -1.153702 | 0.7005792 | -1.6468 | 0.0997 | -0.285283783 | count | 1 |
| SLC27A5 | -0.2439191 | 0.239062 | -1.0203 | 0.308 | -0.285241675 | count | 1 |
| PEX14 | -0.4518862 | 0.281008 | -1.6081 | 0.108 | -0.285237052 | count | 1 |
| NME3 | -0.2023732 | 0.0802523 | -2.5217 | 0.0117 | -0.285137657 | count | 1 |
| CCDC32 | -0.2141074 | 0.1421991 | -1.5057 | 0.132 | -0.285085467 | count | 1 |
| PSMB7 | -0.2056332 | 0.0967021 | -2.1265 | 0.0335 | -0.285063032 | count | 1 |
| NDUFA11 | -0.2004492 | 0.0690754 | -2.9019 | 0.00373 | -0.284923381 | count | 1 |
| YBEY | -0.2215832 | 0.1809414 | -1.2246 | 0.221 | -0.284920696 | count | 1 |
| FCGRT | -0.2169004 | 0.1996975 | -1.0861 | 0.277 | -0.284873514 | count | 1 |
| RGS16 | -0.213857 | 0.164076 | -1.3034 | 0.193 | -0.284753751 | count | 1 |
| AL031280.1 | -0.7555964 | 0.5695981 | -1.3265 | 0.185 | -0.284621818 | count | 1 |
| TMEM160 | -0.2012364 | 0.076384 | -2.6345 | 0.00846 | -0.284555427 | count | 1 |
| PLA2G6 | -0.4118716 | 0.3846333 | -1.0708 | 0.284 | -0.284519541 | count | 1 |
| SIDT2 | -0.4117845 | 0.4765633 | -0.8641 | 0.388 | -0.284463513 | count | 1 |
| TMEM205 | -0.2079581 | 0.1704061 | -1.2204 | 0.222 | -0.284225683 | count | 1 |
| PSME4 | -0.2566658 | 0.2008632 | -1.2778 | 0.201 | -0.284186113 | count | 1 |
| INE2 | -0.5939798 | 0.9055518 | -0.6559 | 0.512 | -0.284149178 | count | 1 |
| BCAS3 | -0.2479241 | 0.3287959 | -0.754 | 0.451 | -0.28409007 | count | 1 |
| ZNF230 | -0.2817005 | 0.3153803 | -0.8932 | 0.372 | -0.284017395 | count | 1 |
| PPIL3 | -0.2258141 | 0.2072232 | -1.0897 | 0.276 | -0.284013533 | count | 1 |

| | | | | | | | |
|---|---|---|---|---|---|---|---|
| SCAMP4 | -0.225222 | 0.1835045 | -1.2273 | 0.22 | -0.284003243 | count | 1 |
| NUB1 | -0.202775 | 0.0818733 | -2.4767 | 0.0133 | -0.283966335 | count | 1 |
| AHCTF1 | -0.2341373 | 0.1719199 | -1.3619 | 0.173 | -0.283833625 | count | 1 |
| TC2N | -0.2060756 | 0.0792279 | -2.601 | 0.00933 | -0.283751216 | count | 1 |
| DHX15 | -0.2254885 | 0.1497357 | -1.5059 | 0.132 | -0.283607674 | count | 1 |
| USP33 | -0.2130646 | 0.1229066 | -1.7335 | 0.0831 | -0.283447138 | count | 1 |
| TTI2 | -0.2337959 | 0.2501976 | -0.9344 | 0.35 | -0.283424545 | count | 1 |
| MAP2K4 | -0.2408858 | 0.218987 | -1.1 | 0.271 | -0.28341992 | count | 1 |
| TENT5A | -0.2192048 | 0.1593342 | -1.3758 | 0.169 | -0.283374178 | count | 1 |
| SYTL3 | -0.2081463 | 0.0840178 | -2.4774 | 0.0133 | -0.283092941 | count | 1 |
| NBEAL2 | -0.2366757 | 0.2468975 | -0.9586 | 0.338 | -0.283000186 | count | 1 |
| PLIN3 | -0.2181272 | 0.17084 | -1.2768 | 0.202 | -0.282909208 | count | 1 |
| MADD | -0.3074197 | 0.2567703 | -1.1973 | 0.231 | -0.282881004 | count | 1 |
| LRRC42 | -0.24871 | 0.2387404 | -1.0418 | 0.298 | -0.282822637 | count | 1 |
| POMP | -0.1984753 | 0.0616926 | -3.2172 | 0.00131 | -0.282792679 | count | 1 |
| ZNF430 | -0.2163796 | 0.1258436 | -1.7194 | 0.0856 | -0.28272525 | count | 1 |
| AL033527.5 | -0.5029871 | 0.8793076 | -0.572 | 0.567 | -0.282664134 | count | 1 |
| NEK7 | -0.2126624 | 0.1679869 | -1.2659 | 0.206 | -0.28265284 | count | 1 |
| PHF20L1 | -0.2038123 | 0.0909253 | -2.2415 | 0.0251 | -0.282481867 | count | 1 |
| GCLC | -0.3588213 | 0.2539928 | -1.4127 | 0.158 | -0.282419282 | count | 1 |
| COX8A | -0.1971011 | 0.0485046 | -4.0636 | 4.94E-05 | -0.282357114 | count | 1 |
| CCDC191 | -0.3162913 | 0.353056 | -0.8959 | 0.37 | -0.282233195 | count | 1 |
| TLK2 | -0.213154 | 0.1387822 | -1.5359 | 0.125 | -0.282192207 | count | 1 |
| NBN | -0.213531 | 0.1196925 | -1.784 | 0.0745 | -0.282093071 | count | 1 |
| PGAP1 | -0.3583769 | 0.3117941 | -1.1494 | 0.25 | -0.282087609 | count | 1 |
| C17orf67 | -0.2241544 | 0.2098828 | -1.068 | 0.286 | -0.281944598 | count | 1 |
| ULK4 | -0.2478827 | 0.2447137 | -1.0129 | 0.311 | -0.281897195 | count | 1 |
| RILPL2 | -0.1989575 | 0.0682337 | -2.9158 | 0.00357 | -0.281893606 | count | 1 |
| FYN | -0.1987737 | 0.0640135 | -3.1052 | 0.00192 | -0.281607608 | count | 1 |
| ZNF836 | -0.2846388 | 0.3350507 | -0.8495 | 0.396 | -0.281604384 | count | 1 |
| ADAM28 | -1.1315317 | 0.9456975 | -1.1965 | 0.232 | -0.281552981 | count | 1 |
| AC064836.3 | -1.1315317 | 0.9590909 | -1.1798 | 0.238 | -0.281552981 | count | 1 |
| CDC42EP5 | -1.1315317 | 0.9923854 | -1.1402 | 0.254 | -0.281552981 | count | 1 |
| AC096677.1 | -1.1315317 | 1.128105 | -1.003 | 0.316 | -0.281552981 | count | 1 |
| BAMBI | -0.4071106 | 0.4268564 | -0.9537 | 0.34 | -0.281454591 | count | 1 |
| XRCC1 | -0.2206046 | 0.2101899 | -1.0495 | 0.294 | -0.281447768 | count | 1 |
| ITM2C | -0.1977788 | 0.0949115 | -2.0838 | 0.0373 | -0.281398706 | count | 1 |
| IPO9 | -0.2437658 | 0.2453559 | -0.9935 | 0.321 | -0.28139199 | count | 1 |
| ORC2 | -0.2341409 | 0.2489804 | -0.9404 | 0.347 | -0.281342925 | count | 1 |
| MPV17L | -0.744904 | 0.6814712 | -1.0931 | 0.274 | -0.28133746 | count | 1 |
| PCNX4 | -0.2741811 | 0.1703521 | -1.6095 | 0.108 | -0.281324653 | count | 1 |
| FBXO28 | -0.2300258 | 0.189949 | -1.211 | 0.226 | -0.281147758 | count | 1 |
| RBM27 | -0.5867026 | 0.32511 | -1.8046 | 0.0712 | -0.281118093 | count | 1 |
| ETS1 | -0.1970617 | 0.0442461 | -4.4538 | 8.71E-06 | -0.28108629 | count | 0.20941453 |
| TCAF2 | -0.3785006 | 0.3167826 | -1.1948 | 0.232 | -0.281085912 | count | 1 |

| | | | | | | | |
|---|---|---|---|---|---|---|---|
| SLC25A10 | -0.4446311 | 0.719521 | -0.618 | 0.537 | -0.28102759 | count | 1 |
| SPINT1 | -0.2785824 | 0.4213835 | -0.6611 | 0.509 | -0.280956117 | count | 1 |
| CEP55 | -1.1270746 | 0.8089353 | -1.3933 | 0.164 | -0.280795472 | count | 1 |
| AC092164.1 | -1.1270746 | 0.8489114 | -1.3277 | 0.184 | -0.280795472 | count | 1 |
| EEF1A2 | -1.1270746 | 0.987075 | -1.1418 | 0.254 | -0.280795472 | count | 1 |
| CALHM6 | -1.1270746 | 1.0284756 | -1.0959 | 0.273 | -0.280795472 | count | 1 |
| CEBPZ | -0.1995518 | 0.0854886 | -2.3343 | 0.0196 | -0.280772772 | count | 1 |
| C1QTNF6 | -1.126836 | 0.6579821 | -1.7126 | 0.0869 | -0.28075485 | count | 1 |
| ZBED4 | -0.4058386 | 0.2333183 | -1.7394 | 0.0821 | -0.280634804 | count | 1 |
| TUBGCP5 | -0.2466995 | 0.2096597 | -1.1767 | 0.239 | -0.280573437 | count | 1 |
| HERC4 | -0.2133466 | 0.1483198 | -1.4384 | 0.15 | -0.280572886 | count | 1 |
| BRK1 | -0.1975982 | 0.0609672 | -3.2411 | 0.0012 | -0.280527825 | count | 1 |
| NET1 | -0.4055934 | 0.3848861 | -1.0538 | 0.292 | -0.280476726 | count | 1 |
| PDXDC1 | -0.2312081 | 0.1694421 | -1.3645 | 0.172 | -0.280323237 | count | 1 |
| C4orf33 | -0.2688584 | 0.2525064 | -1.0648 | 0.287 | -0.280244933 | count | 1 |
| EIF3C | -0.7413257 | 0.418397 | -1.7718 | 0.0765 | -0.280233518 | count | 1 |
| ACTR6 | -0.202026 | 0.1092077 | -1.8499 | 0.0644 | -0.280074611 | count | 1 |
| PTRHD1 | -0.2010345 | 0.1057498 | -1.901 | 0.0574 | -0.280063768 | count | 1 |
| PHF3 | -0.2005119 | 0.073867 | -2.7145 | 0.00667 | -0.280005366 | count | 1 |
| ZNF511 | -0.2059618 | 0.1369614 | -1.5038 | 0.133 | -0.279995692 | count | 1 |
| ZBED1 | -0.2582436 | 0.2316328 | -1.1149 | 0.265 | -0.279985749 | count | 1 |
| GOLGA4 | -0.2011739 | 0.079294 | -2.5371 | 0.0112 | -0.279961528 | count | 1 |
| ARID4A | -0.2001599 | 0.0813444 | -2.4606 | 0.0139 | -0.279911689 | count | 1 |
| UTP14C | -0.3554315 | 0.4826542 | -0.7364 | 0.462 | -0.279888101 | count | 1 |
| KANSL1 | -0.2002683 | 0.0802434 | -2.4958 | 0.0126 | -0.279828909 | count | 1 |
| AC016727.1 | -0.3384454 | 0.5250643 | -0.6446 | 0.519 | -0.27980453 | count | 1 |
| RBM25 | -0.1964106 | 0.0485984 | -4.0415 | 5.43E-05 | -0.279720595 | count | 1 |
| TRIM3 | -0.4968764 | 0.5205421 | -0.9545 | 0.34 | -0.279569172 | count | 1 |
| ICA1 | -0.2955353 | 0.3290831 | -0.8981 | 0.369 | -0.279514462 | count | 1 |
| ALG12 | -0.2438178 | 0.2608111 | -0.9348 | 0.35 | -0.279457948 | count | 1 |
| GTF2B | -0.1955893 | 0.0542112 | -3.6079 | 0.000313 | -0.279305338 | count | 1 |
| LRRFIP2 | -0.208167 | 0.1243822 | -1.6736 | 0.0943 | -0.279249769 | count | 1 |
| SNRPN | -0.1974041 | 0.0756899 | -2.6081 | 0.00915 | -0.279235529 | count | 1 |
| RIPK1 | -0.2171082 | 0.1548708 | -1.4019 | 0.161 | -0.279208408 | count | 1 |
| HECA | -0.2051644 | 0.0981024 | -2.0913 | 0.0366 | -0.279186219 | count | 1 |
| DEF8 | -0.2219367 | 0.1807117 | -1.2281 | 0.219 | -0.279179602 | count | 1 |
| ANKRD34A | -0.3757456 | 0.4764917 | -0.7886 | 0.43 | -0.279158832 | count | 1 |
| CIP2A | -0.3239012 | 0.5160301 | -0.6277 | 0.53 | -0.279086324 | count | 1 |
| NAA10 | -0.1976564 | 0.0816157 | -2.4218 | 0.0155 | -0.279077201 | count | 1 |
| IFIH1 | -0.2257836 | 0.2046558 | -1.1032 | 0.27 | -0.278987311 | count | 1 |
| NSMCE2 | -0.2186006 | 0.1446197 | -1.5116 | 0.131 | -0.278910976 | count | 1 |
| AC026401.3 | -0.4953697 | 0.4402542 | -1.1252 | 0.261 | -0.278804684 | count | 1 |
| APTR | -0.4027164 | 0.3786858 | -1.0635 | 0.288 | -0.278620765 | count | 1 |
| C16orf58 | -0.2875493 | 0.3303225 | -0.8705 | 0.384 | -0.278571815 | count | 1 |
| ANKRD35 | -0.2713902 | 0.3899149 | -0.696 | 0.486 | -0.278531024 | count | 1 |

| | | | | | | | |
|---|---|---|---|---|---|---|---|
| AGAP2 | -0.2254094 | 0.2003105 | -1.1253 | 0.261 | -0.278529546 | count | 1 |
| SMARCC1 | -0.2117504 | 0.1174017 | -1.8036 | 0.0714 | -0.278485803 | count | 1 |
| FBXO3 | -0.2113813 | 0.1465998 | -1.4419 | 0.149 | -0.278331091 | count | 1 |
| MRPL35 | -0.2201342 | 0.1964152 | -1.1208 | 0.262 | -0.278328489 | count | 1 |
| NAA20 | -0.199288 | 0.1064726 | -1.8717 | 0.0613 | -0.278216289 | count | 1 |
| TRMT1L | -0.2445072 | 0.2648391 | -0.9232 | 0.356 | -0.278120102 | count | 1 |
| LONP2 | -0.2068205 | 0.1457345 | -1.4192 | 0.156 | -0.278048372 | count | 1 |
| RIF1 | -0.2113351 | 0.1343189 | -1.5734 | 0.116 | -0.27794275 | count | 1 |
| ZNF408 | -0.3111697 | 0.291397 | -1.0679 | 0.286 | -0.277833779 | count | 1 |
| FAM111A-DT | -0.2507429 | 0.4313564 | -0.5813 | 0.561 | -0.277746871 | count | 1 |
| IFT122 | -1.108754 | 0.6520338 | -1.7005 | 0.0891 | -0.277655611 | count | 1 |
| STX4 | -0.2011967 | 0.1146022 | -1.7556 | 0.0792 | -0.277380138 | count | 1 |
| ABI2 | -0.2438107 | 0.2613207 | -0.933 | 0.351 | -0.277340504 | count | 1 |
| C1S | -0.5773476 | 0.7029736 | -0.8213 | 0.412 | -0.277204412 | count | 1 |
| IREB2 | -0.2235709 | 0.1748712 | -1.2785 | 0.201 | -0.277183373 | count | 1 |
| INTS8 | -0.2620653 | 0.2567086 | -1.0209 | 0.307 | -0.277159661 | count | 1 |
| NOTCH2NL | -0.4377575 | 0.5369331 | -0.8153 | 0.415 | -0.277027511 | count | 1 |
| AQP1 | -0.5758965 | 0.7770895 | -0.7411 | 0.459 | -0.276595619 | count | 1 |
| C5AR1 | -0.2793757 | 0.6122724 | -0.4563 | 0.648 | -0.276540171 | count | 1 |
| PCYT2 | -0.2519483 | 0.2264141 | -1.1128 | 0.266 | -0.276270589 | count | 1 |
| ZNF507 | -0.2408735 | 0.3083932 | -0.7811 | 0.435 | -0.276134957 | count | 1 |
| CREBRF | -0.1981028 | 0.0835276 | -2.3717 | 0.0178 | -0.27589289 | count | 1 |
| ABHD8 | -0.4357794 | 0.5476603 | -0.7957 | 0.426 | -0.275874204 | count | 1 |
| TUBA1C | -0.1945745 | 0.0774709 | -2.5116 | 0.0121 | -0.275836715 | count | 1 |
| ZDHHC7 | -0.2686947 | 0.2701481 | -0.9946 | 0.32 | -0.275831391 | count | 1 |
| HOXB-AS1 | -0.2733303 | 0.2883015 | -0.9481 | 0.343 | -0.275795129 | count | 1 |
| BMP2K | -0.2994467 | 0.4036292 | -0.7419 | 0.458 | -0.275794117 | count | 1 |
| TMUB1 | -0.1986463 | 0.0968298 | -2.0515 | 0.0403 | -0.275639412 | count | 1 |
| RBM41 | -0.2783389 | 0.2724348 | -1.0217 | 0.307 | -0.275541852 | count | 1 |
| LSM14A | -0.1967421 | 0.0853556 | -2.305 | 0.0212 | -0.27539444 | count | 1 |
| RNF111 | -0.212274 | 0.161975 | -1.3105 | 0.19 | -0.275367683 | count | 1 |
| MPC2 | -0.1949109 | 0.0747324 | -2.6081 | 0.00914 | -0.275345344 | count | 1 |
| SLC22A18 | -0.2602676 | 0.2460625 | -1.0577 | 0.29 | -0.275299445 | count | 1 |
| AC015813.1 | -0.725022 | 0.7526514 | -0.9633 | 0.335 | -0.275173151 | count | 1 |
| NFRKB | -0.2324563 | 0.2299026 | -1.0111 | 0.312 | -0.275167185 | count | 1 |
| CBR1 | -0.2030589 | 0.1573623 | -1.2904 | 0.197 | -0.275054637 | count | 1 |
| FOXO3 | -0.2277667 | 0.1989043 | -1.1451 | 0.252 | -0.275012997 | count | 1 |
| PRRC2B | -0.2126886 | 0.1341414 | -1.5856 | 0.113 | -0.275007698 | count | 1 |
| CLUH | -0.3323307 | 0.4223663 | -0.7868 | 0.431 | -0.274975772 | count | 1 |
| NPPA-AS1 | -1.0926945 | 1.1121037 | -0.9825 | 0.326 | -0.274867732 | count | 1 |
| CLEC10A | -1.0926945 | 1.3941292 | -0.7838 | 0.433 | -0.274867732 | count | 1 |
| AC007878.1 | -1.0926945 | 1.4496743 | -0.7538 | 0.451 | -0.274867732 | count | 1 |
| DLL1 | -1.0926945 | 1.4496743 | -0.7538 | 0.451 | -0.274867732 | count | 1 |
| AC092910.3 | -1.0926945 | 1.554823 | -0.7028 | 0.482 | -0.274867732 | count | 1 |
| TNFRSF18 | -0.2144765 | 0.1744356 | -1.2295 | 0.219 | -0.274802295 | count | 1 |

| | | | | | | | |
|---|---|---|---|---|---|---|---|
| PRDX1 | -0.1921592 | 0.0540692 | -3.5539 | 0.000385 | -0.274736365 | count | 1 |
| MAML3 | -1.091901 | 0.7179456 | -1.5209 | 0.128 | -0.274729148 | count | 1 |
| C2orf76 | -0.2347515 | 0.2374417 | -0.9887 | 0.323 | -0.274668402 | count | 1 |
| DUSP8 | -0.2151521 | 0.1648063 | -1.3055 | 0.192 | -0.274544695 | count | 1 |
| QDPR | -0.2283038 | 0.1821782 | -1.2532 | 0.21 | -0.274411634 | count | 1 |
| IRF2 | -0.1956939 | 0.085843 | -2.2797 | 0.0227 | -0.274200492 | count | 1 |
| G3BP1 | -0.1981909 | 0.0943911 | -2.0997 | 0.0358 | -0.274057576 | count | 1 |
| MBLAC1 | -0.3475733 | 0.4250915 | -0.8176 | 0.414 | -0.274009469 | count | 1 |
| MPG | -0.1954345 | 0.0937041 | -2.0857 | 0.0371 | -0.273771724 | count | 1 |
| CRNDE | -0.5690164 | 0.6925226 | -0.8217 | 0.411 | -0.273702824 | count | 1 |
| GIMAP1 | -0.1939024 | 0.0781569 | -2.4809 | 0.0132 | -0.273656409 | count | 1 |
| SYNGAP1 | -0.3168918 | 0.294602 | -1.0757 | 0.282 | -0.273289258 | count | 1 |
| PGP | -0.1938004 | 0.0862176 | -2.2478 | 0.0247 | -0.273241596 | count | 1 |
| TGIF2 | -0.225241 | 0.191175 | -1.1782 | 0.239 | -0.273168695 | count | 1 |
| AC027097.2 | -1.0829527 | 0.9660221 | -1.121 | 0.262 | -0.273160606 | count | 1 |
| CCZ1B | -0.2758287 | 0.2762563 | -0.9985 | 0.318 | -0.273123878 | count | 1 |
| AP002387.2 | -0.1970733 | 0.1094828 | -1.8 | 0.0719 | -0.273102258 | count | 1 |
| C3orf14 | -0.2817308 | 0.3505388 | -0.8037 | 0.422 | -0.273097812 | count | 1 |
| GOLGB1 | -0.1953655 | 0.0780196 | -2.5041 | 0.0123 | -0.273068596 | count | 1 |
| FSD2 | -0.4839761 | 1.0631324 | -0.4552 | 0.649 | -0.273006308 | count | 1 |
| AC027290.1 | -1.0812927 | 1.0952498 | -0.9873 | 0.324 | -0.2728685 | count | 1 |
| AC104695.3 | -1.0812927 | 1.2283936 | -0.8802 | 0.379 | -0.2728685 | count | 1 |
| RASA4B | -1.0812927 | 1.2283936 | -0.8802 | 0.379 | -0.2728685 | count | 1 |
| AC099063.4 | -0.2814149 | 0.4258245 | -0.6609 | 0.509 | -0.272800404 | count | 1 |
| TAF1D | -0.1919962 | 0.0505704 | -3.7966 | 0.000149 | -0.27277056 | count | 1 |
| SEC14L1 | -0.2078401 | 0.1317539 | -1.5775 | 0.115 | -0.272703397 | count | 1 |
| TNKS2 | -0.2012959 | 0.1237932 | -1.6261 | 0.104 | -0.272675539 | count | 1 |
| CCDC50 | -0.2302802 | 0.2510288 | -0.9173 | 0.359 | -0.272624058 | count | 1 |
| FAM208A | -0.2058797 | 0.1297911 | -1.5862 | 0.113 | -0.272611918 | count | 1 |
| NF1 | -0.237706 | 0.2267577 | -1.0483 | 0.295 | -0.272558498 | count | 1 |
| KCTD6 | -0.2459557 | 0.2413222 | -1.0192 | 0.308 | -0.272537834 | count | 1 |
| RAP1GDS1 | -0.2044489 | 0.1298125 | -1.575 | 0.115 | -0.272527964 | count | 1 |
| MAP4K4 | -0.2118355 | 0.1269698 | -1.6684 | 0.0953 | -0.272475421 | count | 1 |
| NFKBIL1 | -0.2277474 | 0.2094757 | -1.0872 | 0.277 | -0.272452727 | count | 1 |
| RSBN1 | -0.1933126 | 0.0705468 | -2.7402 | 0.00617 | -0.272431391 | count | 1 |
| DCTN4 | -0.2358446 | 0.23497 | -1.0037 | 0.316 | -0.272381282 | count | 1 |
| NTMT1 | -0.2008409 | 0.11611 | -1.7297 | 0.0838 | -0.272358391 | count | 1 |
| REEP5 | -0.1905799 | 0.0591831 | -3.2202 | 0.00129 | -0.272341693 | count | 1 |
| PAPSS1 | -0.2153027 | 0.1992889 | -1.0804 | 0.28 | -0.27226968 | count | 1 |
| AC004816.1 | -0.4823563 | 0.7190785 | -0.6708 | 0.502 | -0.272179477 | count | 1 |
| MCM3AP | -0.2391712 | 0.2426709 | -0.9856 | 0.324 | -0.272145438 | count | 1 |
| TRAV23DV6 | -1.0770306 | 0.886075 | -1.2155 | 0.224 | -0.272116866 | count | 1 |
| AC007249.2 | -1.0770306 | 1.267231 | -0.8499 | 0.395 | -0.272116866 | count | 1 |
| ZNF300 | -0.5649195 | 1.0867827 | -0.5198 | 0.603 | -0.271975313 | count | 1 |
| PDE2A | -0.5649195 | 1.0867827 | -0.5198 | 0.603 | -0.271975313 | count | 1 |

| | | | | | | | |
|---|---|---|---|---|---|---|---|
| HS3ST1 | -0.5649195 | 1.1995168 | -0.471 | 0.638 | -0.271975313 | count | 1 |
| AL731661.1 | -0.5649195 | 1.1995168 | -0.471 | 0.638 | -0.271975313 | count | 1 |
| AC109322.1 | -0.5649195 | 1.2474214 | -0.4529 | 0.651 | -0.271975313 | count | 1 |
| RAMP3 | -0.5649195 | 1.381202 | -0.409 | 0.683 | -0.271975313 | count | 1 |
| ANXA1 | -0.1886591 | 0.0406693 | -4.6389 | 3.64E-06 | -0.271864711 | count | 0.08760388 |
| ADCK1 | -0.4286332 | 0.7225794 | -0.5932 | 0.553 | -0.271699738 | count | 1 |
| SPINT1-AS1 | -0.5642318 | 0.4294598 | -1.3138 | 0.189 | -0.271684969 | count | 1 |
| ATXN7L3 | -0.2197386 | 0.2211423 | -0.9937 | 0.32 | -0.271590304 | count | 1 |
| ASB2 | -0.2142291 | 0.2533163 | -0.8457 | 0.398 | -0.271567308 | count | 1 |
| UPF2 | -0.1932934 | 0.0866455 | -2.2309 | 0.0258 | -0.271549993 | count | 1 |
| RBM28 | -0.2175537 | 0.163692 | -1.329 | 0.184 | -0.271449759 | count | 1 |
| PBXIP1 | -0.192806 | 0.0704813 | -2.7356 | 0.00626 | -0.271434697 | count | 1 |
| H2AFJ | -0.1921974 | 0.0861501 | -2.231 | 0.0257 | -0.271431486 | count | 1 |
| TPD52L2 | -0.2195675 | 0.1951685 | -1.125 | 0.261 | -0.271380873 | count | 1 |
| TNIP2 | -0.1979177 | 0.1154365 | -1.7145 | 0.0865 | -0.271179325 | count | 1 |
| CTNS | -0.3912104 | 0.4445229 | -0.8801 | 0.379 | -0.271177781 | count | 1 |
| PSMB5 | -0.1971573 | 0.1133281 | -1.7397 | 0.082 | -0.271167393 | count | 1 |
| DENND2D | -0.1953005 | 0.0933199 | -2.0928 | 0.0364 | -0.271064534 | count | 1 |
| NDUFB1 | -0.1920564 | 0.0792516 | -2.4234 | 0.0154 | -0.271054451 | count | 1 |
| IFT74 | -0.2216278 | 0.2294948 | -0.9657 | 0.334 | -0.270990335 | count | 1 |
| MGME1 | -0.2106671 | 0.1858537 | -1.1335 | 0.257 | -0.270983072 | count | 1 |
| SP110 | -0.1909413 | 0.0652384 | -2.9268 | 0.00345 | -0.270944513 | count | 1 |
| ZFYVE26 | -0.7114085 | 0.6498282 | -1.0948 | 0.274 | -0.270909394 | count | 1 |
| KLC4 | -0.4797918 | 0.5393988 | -0.8895 | 0.374 | -0.270869142 | count | 1 |
| MEI1 | -0.1995125 | 0.1267632 | -1.5739 | 0.116 | -0.270848637 | count | 1 |
| MOK | -0.5619417 | 0.5120079 | -1.0975 | 0.272 | -0.27071736 | count | 1 |
| ZNF530 | -0.5618055 | 0.6861772 | -0.8187 | 0.413 | -0.270659772 | count | 1 |
| TOPBP1 | -0.225145 | 0.1794257 | -1.2548 | 0.21 | -0.270658765 | count | 1 |
| AL450992.2 | -0.7104964 | 1.1181017 | -0.6354 | 0.525 | -0.270622485 | count | 1 |
| COCH | -0.7104964 | 1.3077537 | -0.5433 | 0.587 | -0.270622485 | count | 1 |
| AC093627.4 | -0.7104964 | 1.3259389 | -0.5358 | 0.592 | -0.270622485 | count | 1 |
| NPAS1 | -0.7104964 | 1.4191967 | -0.5006 | 0.617 | -0.270622485 | count | 1 |
| POLR2J2 | -0.3135691 | 0.4661769 | -0.6726 | 0.501 | -0.270537153 | count | 1 |
| FOCAD | -1.067373 | 0.4899755 | -2.1784 | 0.0294 | -0.270405187 | count | 1 |
| CBX4 | -0.1974234 | 0.1063776 | -1.8559 | 0.0636 | -0.270402683 | count | 1 |
| DUSP19 | -1.0670954 | 0.8458117 | -1.2616 | 0.207 | -0.27035566 | count | 1 |
| RBMX2 | -0.2000063 | 0.1375735 | -1.4538 | 0.146 | -0.270312653 | count | 1 |
| GPR157 | -0.7094132 | 0.5071434 | -1.3988 | 0.162 | -0.270281538 | count | 1 |
| AC136475.1 | -0.4785766 | 0.3453108 | -1.3859 | 0.166 | -0.270247693 | count | 1 |
| FAM122C | -0.246165 | 0.2601122 | -0.9464 | 0.344 | -0.270044833 | count | 1 |
| TUBA1A | -0.1882766 | 0.0547424 | -3.4393 | 0.00059 | -0.270006007 | count | 1 |
| TDP2 | -0.2026777 | 0.1710249 | -1.1851 | 0.236 | -0.269939592 | count | 1 |
| SAMD1 | -0.2175889 | 0.1532046 | -1.4203 | 0.156 | -0.269836593 | count | 1 |
| COG8 | -0.7077146 | 0.4709613 | -1.5027 | 0.133 | -0.269746441 | count | 1 |
| AGPAT4 | -0.2547446 | 0.3300957 | -0.7717 | 0.44 | -0.26958048 | count | 1 |

| | | | | | | | |
|---|---|---|---|---|---|---|---|
| WDR59 | -0.2409343 | 0.2579617 | -0.934 | 0.35 | -0.269565514 | count | 1 |
| NOTCH2 | -0.2718193 | 0.3179394 | -0.8549 | 0.393 | -0.269259018 | count | 1 |
| PSME2 | -0.1876221 | 0.0470387 | -3.9887 | 6.78E-05 | -0.269249796 | count | 1 |
| NOC3L | -0.2058481 | 0.1686017 | -1.2209 | 0.222 | -0.269046508 | count | 1 |
| ANKRD37 | -0.2079712 | 0.1418622 | -1.466 | 0.143 | -0.268948373 | count | 1 |
| ZNF793 | -0.4238041 | 0.53259 | -0.7957 | 0.426 | -0.268871733 | count | 1 |
| DERL2 | -0.1930548 | 0.0945851 | -2.0411 | 0.0413 | -0.268830023 | count | 1 |
| TIMM23 | -0.2153596 | 0.2217001 | -0.9714 | 0.331 | -0.268736525 | count | 1 |
| ABCF1 | -0.1916762 | 0.0873429 | -2.1945 | 0.0283 | -0.268580479 | count | 1 |
| UBLCP1 | -0.1998345 | 0.1460035 | -1.3687 | 0.171 | -0.268507335 | count | 1 |
| HIST1H2BC | -0.3401143 | 0.4824215 | -0.705 | 0.481 | -0.268415422 | count | 1 |
| GGT7 | -0.5562329 | 0.3407122 | -1.6326 | 0.103 | -0.268300288 | count | 1 |
| RPS6KB2 | -0.2008667 | 0.1378512 | -1.4571 | 0.145 | -0.268233008 | count | 1 |
| EPS15 | -0.1931406 | 0.0995437 | -1.9403 | 0.0524 | -0.268184595 | count | 1 |
| FAM96B | -0.1885718 | 0.0603689 | -3.1237 | 0.0018 | -0.268182481 | count | 1 |
| ADAM17 | -0.2066911 | 0.1667432 | -1.2396 | 0.215 | -0.268171591 | count | 1 |
| EIF2AK4 | -0.1992471 | 0.1953168 | -1.0201 | 0.308 | -0.268093061 | count | 1 |
| CCT6A | -0.1903497 | 0.079405 | -2.3972 | 0.0166 | -0.268070252 | count | 1 |
| ZNF569 | -0.2304277 | 0.2221396 | -1.0373 | 0.3 | -0.267991964 | count | 1 |
| DNMBP | -0.4741332 | 0.8365322 | -0.5668 | 0.571 | -0.267972384 | count | 1 |
| SMYD4 | -0.2607719 | 0.3396908 | -0.7677 | 0.443 | -0.267887935 | count | 1 |
| ZNF7 | -0.2110625 | 0.2146824 | -0.9831 | 0.326 | -0.267584644 | count | 1 |
| TPR | -0.1876431 | 0.0583635 | -3.2151 | 0.0013 | -0.26757608 | count | 1 |
| YIF1B | -0.2110114 | 0.2068358 | -1.0202 | 0.308 | -0.267520366 | count | 1 |
| NOTCH1 | -0.2648619 | 0.2552882 | -1.0375 | 0.3 | -0.267461604 | count | 1 |
| ANAPC11 | -0.1890157 | 0.07669 | -2.4647 | 0.0138 | -0.26742631 | count | 1 |
| HLCS | -1.050439 | 0.5991234 | -1.7533 | 0.0796 | -0.267374268 | count | 1 |
| NAP1L1 | -0.1877823 | 0.0496101 | -3.7852 | 0.000156 | -0.267371489 | count | 1 |
| RFC3 | -0.3585337 | 0.369187 | -0.9711 | 0.332 | -0.267076815 | count | 1 |
| PARP8 | -0.1905511 | 0.0828758 | -2.2992 | 0.0216 | -0.266942403 | count | 1 |
| NT5DC3 | -0.4711186 | 0.6478653 | -0.7272 | 0.467 | -0.266426062 | count | 1 |
| CBX3 | -0.1872421 | 0.0599036 | -3.1257 | 0.001789 | -0.266375427 | count | 1 |
| SOS1 | -0.2032218 | 0.138039 | -1.4722 | 0.141 | -0.266338794 | count | 1 |
| IKZF1 | -0.188521 | 0.0605156 | -3.1152 | 0.00185 | -0.266159725 | count | 1 |
| CENPQ | -0.2549989 | 0.3881514 | -0.657 | 0.511 | -0.266115403 | count | 1 |
| ZBTB14 | -0.2286686 | 0.2182956 | -1.0475 | 0.295 | -0.265973956 | count | 1 |
| PHLDA2 | -0.2164342 | 0.1982564 | -1.0917 | 0.275 | -0.265690412 | count | 1 |
| ZBTB32 | -0.3364694 | 0.4817232 | -0.6985 | 0.485 | -0.265676892 | count | 1 |
| CA13 | -1.040811 | 0.7074382 | -1.4712 | 0.141 | -0.265634461 | count | 1 |
| HSPE1 | -0.1859377 | 0.0483583 | -3.845 | 0.000123 | -0.265627755 | count | 1 |
| INIP | -0.2020643 | 0.1548176 | -1.3052 | 0.192 | -0.265496055 | count | 1 |
| FRA10AC1 | -0.1988007 | 0.1587899 | -1.252 | 0.211 | -0.265486557 | count | 1 |
| C8orf82 | -0.2114472 | 0.1934675 | -1.0929 | 0.275 | -0.265388333 | count | 1 |
| GSTO1 | -0.1885157 | 0.0850432 | -2.2167 | 0.0267 | -0.265380808 | count | 1 |
| PIP5K1C | -0.2504627 | 0.2914217 | -0.8595 | 0.39 | -0.265142631 | count | 1 |

| | | | | | | | |
|---|---|---|---|---|---|---|---|
| SERPING1 | -0.5485933 | 0.5795735 | -0.9465 | 0.344 | -0.265054512 | count | 1 |
| DTX3L | -0.2049088 | 0.1636809 | -1.2519 | 0.211 | -0.265013758 | count | 1 |
| CHCHD1 | -0.1916649 | 0.1197668 | -1.6003 | 0.11 | -0.264988377 | count | 1 |
| EIF4G2 | -0.1877874 | 0.0648893 | -2.894 | 0.00383 | -0.26496438 | count | 1 |
| EXOSC9 | -0.1922639 | 0.1115951 | -1.7229 | 0.085 | -0.264942635 | count | 1 |
| VCAM1 | -0.4170567 | 0.4483227 | -0.9303 | 0.352 | -0.264910814 | count | 1 |
| LCP1 | -0.1846396 | 0.0431222 | -4.2818 | 1.91E-05 | -0.264876187 | count | 0.4586483 |
| UBN2 | -0.2345622 | 0.2416808 | -0.9705 | 0.332 | -0.264832977 | count | 1 |
| CABIN1 | -0.1966609 | 0.1255628 | -1.5662 | 0.117 | -0.26480547 | count | 1 |
| PPA1 | -0.1883865 | 0.0705481 | -2.6703 | 0.00761 | -0.264692908 | count | 1 |
| CTTNBP2NL | -0.6913552 | 0.7415695 | -0.9323 | 0.351 | -0.26456495 | count | 1 |
| SP4 | -0.2221611 | 0.1975535 | -1.1246 | 0.261 | -0.264521621 | count | 1 |
| CARD19 | -0.1936312 | 0.1287826 | -1.5036 | 0.133 | -0.264487087 | count | 1 |
| DDX54 | -0.2044902 | 0.1536236 | -1.3311 | 0.183 | -0.264475869 | count | 1 |
| TIMM50 | -0.2153942 | 0.2097738 | -1.0268 | 0.305 | -0.264426275 | count | 1 |
| LIN7C | -0.2667394 | 0.2025073 | -1.3172 | 0.188 | -0.264357374 | count | 1 |
| KDM6B | -0.2054714 | 0.1305539 | -1.5738 | 0.116 | -0.264345273 | count | 1 |
| COTL1 | -0.1840804 | 0.0483855 | -3.8045 | 0.000145 | -0.264282619 | count | 1 |
| SBDS | -0.1850304 | 0.0497715 | -3.7176 | 0.000204 | -0.264250993 | count | 1 |
| MTA1 | -0.2407686 | 0.2116319 | -1.1377 | 0.255 | -0.264230189 | count | 1 |
| AP2A1 | -0.2302237 | 0.234172 | -0.9831 | 0.326 | -0.264103802 | count | 1 |
| NPC2 | -0.1880468 | 0.0909461 | -2.0677 | 0.0387 | -0.264002769 | count | 1 |
| PAXIP1 | -0.2860872 | 0.3676164 | -0.7782 | 0.436 | -0.263886881 | count | 1 |
| LRRFIP1 | -0.1842144 | 0.0472455 | -3.8991 | 9.84E-05 | -0.263724849 | count | 1 |
| ODR4 | -0.2298388 | 0.2168827 | -1.0597 | 0.289 | -0.263668639 | count | 1 |
| SNRNP200 | -0.1982434 | 0.1216653 | -1.6294 | 0.103 | -0.263579729 | count | 1 |
| SIAH1 | -0.2033116 | 0.1750714 | -1.1613 | 0.246 | -0.263393856 | count | 1 |
| MAPRE2 | -0.2011177 | 0.1276465 | -1.5756 | 0.115 | -0.262899469 | count | 1 |
| AIFM2 | -0.3325651 | 0.6985389 | -0.4761 | 0.634 | -0.262739867 | count | 1 |
| UQCRFS1 | -0.1855996 | 0.0761887 | -2.4361 | 0.0149 | -0.26268096 | count | 1 |
| TMCO6 | -0.3324769 | 0.3721506 | -0.8934 | 0.372 | -0.262673485 | count | 1 |
| EFCAB11 | -0.2706536 | 0.4464092 | -0.6063 | 0.544 | -0.262656142 | count | 1 |
| RWDD1 | -0.1837374 | 0.0559956 | -3.2813 | 0.001 | -0.26260498 | count | 1 |
| PUM2 | -0.2065994 | 0.1675785 | -1.2329 | 0.218 | -0.26256924 | count | 1 |
| PWP1 | -0.1911802 | 0.1099675 | -1.7385 | 0.0822 | -0.262535353 | count | 1 |
| FBXO21 | -0.1983629 | 0.1441074 | -1.3765 | 0.169 | -0.262435799 | count | 1 |
| MED28 | -0.1926817 | 0.1029063 | -1.8724 | 0.0612 | -0.262380158 | count | 1 |
| IL18R1 | -0.2074019 | 0.1589526 | -1.3048 | 0.192 | -0.262356598 | count | 1 |
| STAT1 | -0.1872867 | 0.087737 | -2.1346 | 0.0329 | -0.26234495 | count | 1 |
| ZSWIM4 | -0.5420362 | 0.6833976 | -0.7931 | 0.428 | -0.262258438 | count | 1 |
| CD27 | -0.1851624 | 0.0828707 | -2.2344 | 0.0255 | -0.262207277 | count | 1 |
| CDKN2D | -0.1833314 | 0.0567356 | -3.2313 | 0.00124 | -0.262202001 | count | 1 |
| ZNF280D | -0.2213411 | 0.2224797 | -0.9949 | 0.32 | -0.262170148 | count | 1 |
| BOD1 | -0.2053412 | 0.1854901 | -1.107 | 0.268 | -0.262116248 | count | 1 |
| RNF122 | -0.4626707 | 0.4608927 | -1.0039 | 0.316 | -0.262081339 | count | 1 |

| | | | | | | | |
|---|---|---|---|---|---|---|---|
| AC012368.1 | -1.0209062 | 0.8948351 | -1.1409 | 0.254 | -0.261998789 | count | 1 |
| MECOM | -1.0209062 | 1.142582 | -0.8935 | 0.372 | -0.261998789 | count | 1 |
| CNN2 | -0.184621 | 0.0729691 | -2.5301 | 0.0114 | -0.261994346 | count | 1 |
| PCOLCE2 | -0.6828818 | 0.7020184 | -0.9727 | 0.331 | -0.26186128 | count | 1 |
| MBD4 | -0.1871229 | 0.0925467 | -2.0219 | 0.0433 | -0.26185122 | count | 1 |
| MON2 | -0.2222054 | 0.2185234 | -1.0168 | 0.309 | -0.261718066 | count | 1 |
| FAM184A | -0.2410406 | 0.2060036 | -1.1701 | 0.242 | -0.261679329 | count | 1 |
| ZNF224 | -0.1951117 | 0.1331018 | -1.4659 | 0.143 | -0.261615096 | count | 1 |
| GSKIP | -0.1995966 | 0.1663917 | -1.1996 | 0.23 | -0.261614125 | count | 1 |
| PURA | -0.186245 | 0.0857746 | -2.1713 | 0.03 | -0.261610618 | count | 1 |
| ZSCAN9 | -0.3154378 | 0.4890705 | -0.645 | 0.519 | -0.261588494 | count | 1 |
| STX8 | -0.1912525 | 0.1093512 | -1.749 | 0.0804 | -0.261568896 | count | 1 |
| ZFAND2B | -0.1894028 | 0.1102823 | -1.7174 | 0.086 | -0.261525858 | count | 1 |
| KIAA0391 | -0.6814169 | 0.6102682 | -1.1166 | 0.264 | -0.261392498 | count | 1 |
| UQCR11 | -0.1829871 | 0.0539481 | -3.3919 | 7.00E-04 | -0.26133825 | count | 1 |
| GOT1 | -0.2081613 | 0.1970225 | -1.0565 | 0.291 | -0.261298254 | count | 1 |
| NEIL2 | -0.2541747 | 0.3151738 | -0.8065 | 0.42 | -0.261263915 | count | 1 |
| FAM206A | -0.246699 | 0.3961319 | -0.6228 | 0.533 | -0.261238968 | count | 1 |
| CENPX | -0.1870807 | 0.0964172 | -1.9403 | 0.0524 | -0.261205635 | count | 1 |
| PICALM | -0.2036274 | 0.1531021 | -1.33 | 0.184 | -0.260998398 | count | 1 |
| PLEKHA8 | -0.2498555 | 0.3193299 | -0.7824 | 0.434 | -0.260862202 | count | 1 |
| SLC26A2 | -0.2687093 | 0.2679265 | -1.0029 | 0.316 | -0.26082066 | count | 1 |
| ZBTB8OS | -0.1885475 | 0.116752 | -1.6149 | 0.106 | -0.260819513 | count | 1 |
| MAP4 | -0.1885534 | 0.0990859 | -1.9029 | 0.0571 | -0.260762611 | count | 1 |
| GNAQ | -0.3016149 | 0.3043538 | -0.991 | 0.322 | -0.260614211 | count | 1 |
| UBE2D2 | -0.1820079 | 0.045972 | -3.9591 | 7.68E-05 | -0.260554197 | count | 1 |
| CFAP36 | -0.188717 | 0.108838 | -1.7339 | 0.083 | -0.260438929 | count | 1 |
| CARD9 | -1.0124435 | 0.9426976 | -1.074 | 0.283 | -0.260437265 | count | 1 |
| AC100810.1 | -0.2092483 | 0.1956296 | -1.0696 | 0.285 | -0.260396462 | count | 1 |
| SHISAL2A | -0.1937201 | 0.1515873 | -1.2779 | 0.201 | -0.260324971 | count | 1 |
| TCP11L2 | -0.2028945 | 0.1753759 | -1.1569 | 0.247 | -0.260065483 | count | 1 |
| PARP9 | -0.1943682 | 0.1418327 | -1.3704 | 0.171 | -0.260017447 | count | 1 |
| AL139289.2 | -0.6771034 | 0.8817953 | -0.7679 | 0.443 | -0.260009725 | count | 1 |
| HIST2H4B | -0.6771034 | 0.9629748 | -0.7031 | 0.482 | -0.260009725 | count | 1 |
| REL | -0.1827865 | 0.0557144 | -3.2808 | 0.00105 | -0.259961247 | count | 1 |
| MAT2B | -0.1927383 | 0.1141916 | -1.6879 | 0.0915 | -0.259883583 | count | 1 |
| ZFYVE19 | -0.2421859 | 0.2798928 | -0.8653 | 0.387 | -0.259839409 | count | 1 |
| FMNL1 | -0.1894927 | 0.0985222 | -1.9233 | 0.0545 | -0.259764923 | count | 1 |
| PKP4 | -0.4579122 | 0.5017199 | -0.9127 | 0.361 | -0.259626676 | count | 1 |
| PPP1R12C | -0.2390912 | 0.2455968 | -0.9735 | 0.33 | -0.259601494 | count | 1 |
| METTL16 | -0.2073715 | 0.1877471 | -1.1045 | 0.269 | -0.259597869 | count | 1 |
| ZFP69 | -0.6757982 | 0.5659425 | -1.1941 | 0.233 | -0.259590639 | count | 1 |
| AFDN | -0.199382 | 0.1873017 | -1.0645 | 0.287 | -0.259536337 | count | 1 |
| YIPF1 | -0.2450139 | 0.2631988 | -0.9309 | 0.352 | -0.259490324 | count | 1 |
| FBXL16 | -0.2363586 | 0.2505518 | -0.9434 | 0.346 | -0.259474555 | count | 1 |

| | | | | | | | |
|---|---|---|---|---|---|---|---|
| OLA1 | -0.1883968 | 0.1053067 | -1.789 | 0.0737 | -0.259472463 | count | 1 |
| ST7 | -0.3476875 | 0.4757311 | -0.7308 | 0.465 | -0.259425646 | count | 1 |
| MZT2A | -0.1815632 | 0.0595496 | -3.0489 | 0.00231 | -0.259360515 | count | 1 |
| PIAS3 | -0.2295987 | 0.2935479 | -0.7822 | 0.434 | -0.259314728 | count | 1 |
| FRG1 | -0.1854336 | 0.0932064 | -1.9895 | 0.0467 | -0.259246953 | count | 1 |
| LINC01137 | -0.2242593 | 0.300734 | -0.7457 | 0.456 | -0.259184847 | count | 1 |
| ETFB | -0.183511 | 0.0843344 | -2.176 | 0.0296 | -0.258955718 | count | 1 |
| ATXN7L1 | -0.2443046 | 0.2387265 | -1.0234 | 0.306 | -0.25875412 | count | 1 |
| ZNF775 | -0.2889838 | 0.4177374 | -0.6918 | 0.489 | -0.258705679 | count | 1 |
| ZNF33A | -0.2209194 | 0.1468328 | -1.5046 | 0.133 | -0.258691535 | count | 1 |
| ZSWIM9 | -0.2476685 | 0.3907824 | -0.6338 | 0.526 | -0.258626952 | count | 1 |
| MAP9 | -0.1926771 | 0.178059 | -1.0821 | 0.279 | -0.258556114 | count | 1 |
| TMEM200A | -0.228894 | 0.2621962 | -0.873 | 0.383 | -0.258530935 | count | 1 |
| PRKACB | -0.1898399 | 0.1075261 | -1.7655 | 0.0776 | -0.258522908 | count | 1 |
| ZNF706 | -0.181359 | 0.0627086 | -2.8921 | 0.00385 | -0.258251005 | count | 1 |
| SEC23IP | -0.2305819 | 0.251077 | -0.9184 | 0.358 | -0.258166998 | count | 1 |
| OTULINL | -0.1904215 | 0.1241339 | -1.534 | 0.125 | -0.258138688 | count | 1 |
| MRPS15 | -0.186799 | 0.1093957 | -1.7076 | 0.0878 | -0.258076727 | count | 1 |
| ADD1 | -0.1945445 | 0.1103617 | -1.7628 | 0.078 | -0.257936463 | count | 1 |
| TMEM144 | -18.2606301 | 2992.971879 | -0.0061 | 0.995 | -0.257849083 | count | 1 |
| PLA2G2C | -18.2598327 | 2431.556712 | -0.0075 | 0.994 | -0.257849083 | count | 1 |
| PKP3 | -18.2598327 | 2431.556712 | -0.0075 | 0.994 | -0.257849083 | count | 1 |
| NTRK2 | -18.1992227 | 2199.433283 | -0.0083 | 0.993 | -0.257849083 | count | 1 |
| TCL1A | -18.1988517 | 1842.997102 | -0.0099 | 0.992 | -0.257849083 | count | 1 |
| PODXL | -18.0879611 | 1641.425466 | -0.011 | 0.991 | -0.257849083 | count | 1 |
| ZNRF3 | -17.9491926 | 1540.052544 | -0.0117 | 0.991 | -0.257849082 | count | 1 |
| SERTAD4 | -17.84058 | 2360.735678 | -0.0076 | 0.994 | -0.257849082 | count | 1 |
| AC096667.1 | -17.84058 | 2360.735678 | -0.0076 | 0.994 | -0.257849082 | count | 1 |
| CPB2-AS1 | -17.84058 | 2360.735678 | -0.0076 | 0.994 | -0.257849082 | count | 1 |
| LINC00926 | -17.84058 | 2360.735678 | -0.0076 | 0.994 | -0.257849082 | count | 1 |
| RGMA | -17.84058 | 2360.735678 | -0.0076 | 0.994 | -0.257849082 | count | 1 |
| AC113615.1 | -17.7510294 | 2437.134916 | -0.0073 | 0.994 | -0.257849081 | count | 1 |
| DDX25 | -17.7510294 | 2437.134916 | -0.0073 | 0.994 | -0.257849081 | count | 1 |
| AC132872.3 | -17.7510294 | 2437.134916 | -0.0073 | 0.994 | -0.257849081 | count | 1 |
| CRYBB2 | -17.7510294 | 2437.134916 | -0.0073 | 0.994 | -0.257849081 | count | 1 |
| MN1 | -17.7510294 | 2437.134916 | -0.0073 | 0.994 | -0.257849081 | count | 1 |
| SUCNR1 | -17.6053651 | 1844.745799 | -0.0095 | 0.992 | -0.25784908 | count | 1 |
| PTGR1 | -17.4405459 | 1889.474313 | -0.0092 | 0.993 | -0.257849079 | count | 1 |
| AC067750.1 | -17.4403623 | 1559.114424 | -0.0112 | 0.991 | -0.257849079 | count | 1 |
| C3orf52 | -17.3357695 | 1799.70082 | -0.0096 | 0.992 | -0.257849078 | count | 1 |
| ANLN | -17.2659414 | 1843.147255 | -0.0094 | 0.993 | -0.257849078 | count | 1 |
| AC079313.2 | -17.2659414 | 1843.147255 | -0.0094 | 0.993 | -0.257849078 | count | 1 |
| AC025423.4 | -17.2659414 | 1843.147255 | -0.0094 | 0.993 | -0.257849078 | count | 1 |
| CCDC68 | -17.2659414 | 1843.147255 | -0.0094 | 0.993 | -0.257849078 | count | 1 |
| SLC2A9 | -17.1513385 | 1653.652796 | -0.0104 | 0.992 | -0.257849077 | count | 1 |

| | | | | | | | |
|---|---|---|---|---|---|---|---|
| 1-Mar | -16.9038573 | 1150.803768 | -0.0147 | 0.988 | -0.257849074 | count | 1 |
| PNMA3 | -17.839412 | 1513.825686 | -0.0118 | 0.991 | -0.257848987 | count | 1 |
| FNBP1L | -17.66867 | 1583.767542 | -0.0112 | 0.991 | -0.257848986 | count | 1 |
| USP43 | -17.594392 | 2096.898876 | -0.0084 | 0.993 | -0.257848986 | count | 1 |
| AC004156.1 | -17.594392 | 2096.898876 | -0.0084 | 0.993 | -0.257848986 | count | 1 |
| AL157786.1 | -17.593277 | 1705.086262 | -0.0103 | 0.992 | -0.257848986 | count | 1 |
| PDPN | -17.51339 | 2627.890078 | -0.0067 | 0.995 | -0.257848985 | count | 1 |
| TMEM51 | -17.51339 | 2627.890078 | -0.0067 | 0.995 | -0.257848985 | count | 1 |
| PIFO | -17.51339 | 2627.890078 | -0.0067 | 0.995 | -0.257848985 | count | 1 |
| KIAA1211L | -17.51339 | 2627.890078 | -0.0067 | 0.995 | -0.257848985 | count | 1 |
| AC009506.1 | -17.51339 | 2627.890078 | -0.0067 | 0.995 | -0.257848985 | count | 1 |
| AC068196.1 | -17.51339 | 2627.890078 | -0.0067 | 0.995 | -0.257848985 | count | 1 |
| AC098820.3 | -17.51339 | 2627.890078 | -0.0067 | 0.995 | -0.257848985 | count | 1 |
| OXTR | -17.51339 | 2627.890078 | -0.0067 | 0.995 | -0.257848985 | count | 1 |
| HDAC11-AS1 | -17.51339 | 2627.890078 | -0.0067 | 0.995 | -0.257848985 | count | 1 |
| XCR1 | -17.51339 | 2627.890078 | -0.0067 | 0.995 | -0.257848985 | count | 1 |
| RASSF6 | -17.51339 | 2627.890078 | -0.0067 | 0.995 | -0.257848985 | count | 1 |
| LINC02100 | -17.51339 | 2627.890078 | -0.0067 | 0.995 | -0.257848985 | count | 1 |
| AC012636.1 | -17.51339 | 2627.890078 | -0.0067 | 0.995 | -0.257848985 | count | 1 |
| AL035696.3 | -17.51339 | 2627.890078 | -0.0067 | 0.995 | -0.257848985 | count | 1 |
| AL023581.2 | -17.51339 | 2627.890078 | -0.0067 | 0.995 | -0.257848985 | count | 1 |
| AC236972.3 | -17.51339 | 2627.890078 | -0.0067 | 0.995 | -0.257848985 | count | 1 |
| GLDC | -17.51339 | 2627.890078 | -0.0067 | 0.995 | -0.257848985 | count | 1 |
| LCN15 | -17.51339 | 2627.890078 | -0.0067 | 0.995 | -0.257848985 | count | 1 |
| AC084337.2 | -17.51339 | 2627.890078 | -0.0067 | 0.995 | -0.257848985 | count | 1 |
| AP002990.1 | -17.51339 | 2627.890078 | -0.0067 | 0.995 | -0.257848985 | count | 1 |
| AP001453.2 | -17.51339 | 2627.890078 | -0.0067 | 0.995 | -0.257848985 | count | 1 |
| AP001767.3 | -17.51339 | 2627.890078 | -0.0067 | 0.995 | -0.257848985 | count | 1 |
| CLLU1OS | -17.51339 | 2627.890078 | -0.0067 | 0.995 | -0.257848985 | count | 1 |
| SKA3 | -17.51339 | 2627.890078 | -0.0067 | 0.995 | -0.257848985 | count | 1 |
| AC004816.2 | -17.51339 | 2627.890078 | -0.0067 | 0.995 | -0.257848985 | count | 1 |
| SAMD15 | -17.51339 | 2627.890078 | -0.0067 | 0.995 | -0.257848985 | count | 1 |
| AC087564.1 | -17.51339 | 2627.890078 | -0.0067 | 0.995 | -0.257848985 | count | 1 |
| AC092118.2 | -17.51339 | 2627.890078 | -0.0067 | 0.995 | -0.257848985 | count | 1 |
| KCNJ2 | -17.51339 | 2627.890078 | -0.0067 | 0.995 | -0.257848985 | count | 1 |
| AC027601.4 | -17.51339 | 2627.890078 | -0.0067 | 0.995 | -0.257848985 | count | 1 |
| FCER2 | -17.51339 | 2627.890078 | -0.0067 | 0.995 | -0.257848985 | count | 1 |
| C19orf33 | -17.51339 | 2627.890078 | -0.0067 | 0.995 | -0.257848985 | count | 1 |
| CABP7 | -17.51339 | 2627.890078 | -0.0067 | 0.995 | -0.257848985 | count | 1 |
| RAB43 | -17.306285 | 1150.418347 | -0.015 | 0.988 | -0.257848984 | count | 1 |
| AC007114.1 | -0.4050378 | 0.6867152 | -0.5898 | 0.555 | -0.257827917 | count | 1 |
| SS18 | -0.198585 | 0.1414224 | -1.4042 | 0.16 | -0.257718338 | count | 1 |
| RPL23 | -0.1794424 | 0.0290505 | -6.1769 | 7.31E-10 | -0.257709513 | count | 1.77E-05 |
| ARID3A | -0.5313144 | 0.4095277 | -1.2974 | 0.195 | -0.25766614 | count | 1 |
| UBE2Q2 | -0.1864656 | 0.1217496 | -1.5316 | 0.126 | -0.257617212 | count | 1 |

| | | | | | | | |
|---|---|---|---|---|---|---|---|
| IRAK1 | -0.2036152 | 0.180391 | -1.1287 | 0.259 | -0.257603127 | count | 1 |
| ZC2HC1A | -0.2716595 | 0.3710906 | -0.7321 | 0.464 | -0.257589972 | count | 1 |
| CDK4 | -0.1917491 | 0.16594 | -1.1555 | 0.248 | -0.257503168 | count | 1 |
| MALT1 | -0.1893589 | 0.0966293 | -1.9596 | 0.0501 | -0.257499859 | count | 1 |
| ST3GAL3 | -0.5308577 | 0.3982193 | -1.3331 | 0.183 | -0.257469982 | count | 1 |
| AC073332.1 | -0.2259917 | 0.2648011 | -0.8534 | 0.393 | -0.257368633 | count | 1 |
| EPRS | -0.1858142 | 0.1071829 | -1.7336 | 0.0831 | -0.257356749 | count | 1 |
| TOR1A | -0.19177 | 0.1543822 | -1.2422 | 0.214 | -0.25734381 | count | 1 |
| TMEM63A | -0.218439 | 0.1885287 | -1.1587 | 0.247 | -0.257336288 | count | 1 |
| DCAF15 | -0.2002627 | 0.1507165 | -1.3287 | 0.184 | -0.257209136 | count | 1 |
| EIF6 | -0.1834602 | 0.0968341 | -1.8946 | 0.0582 | -0.257147159 | count | 1 |
| EMC9 | -0.2079293 | 0.1930746 | -1.0769 | 0.282 | -0.257127164 | count | 1 |
| MYSM1 | -0.2059762 | 0.1586759 | -1.2981 | 0.194 | -0.257126803 | count | 1 |
| SURF2 | -0.1844723 | 0.1100448 | -1.6763 | 0.0938 | -0.257035209 | count | 1 |
| GSDMD | -0.184068 | 0.0937004 | -1.9644 | 0.0496 | -0.256928791 | count | 1 |
| TPK1 | -0.2046158 | 0.2324122 | -0.8804 | 0.379 | -0.256883722 | count | 1 |
| STRADA | -0.2025472 | 0.1915627 | -1.0573 | 0.29 | -0.256869726 | count | 1 |
| HARBI1 | -0.3690763 | 0.6259767 | -0.5896 | 0.555 | -0.256767686 | count | 1 |
| RCC2 | -0.2124913 | 0.2086838 | -1.0182 | 0.309 | -0.256764501 | count | 1 |
| NABP1 | -0.1909859 | 0.1052296 | -1.8149 | 0.0696 | -0.256665284 | count | 1 |
| ADSS | -0.1860249 | 0.0912537 | -2.0385 | 0.0416 | -0.25666141 | count | 1 |
| PPP2R2D | -0.1962747 | 0.1372658 | -1.4299 | 0.153 | -0.2566042 | count | 1 |
| CASP3 | -0.1879263 | 0.1110983 | -1.6915 | 0.0908 | -0.256275563 | count | 1 |
| OFD1 | -0.1836739 | 0.0873299 | -2.1032 | 0.0355 | -0.256095715 | count | 1 |
| SMIM19 | -0.1829084 | 0.1032257 | -1.7719 | 0.0765 | -0.25609479 | count | 1 |
| ALDH1B1 | -0.6647725 | 0.9004193 | -0.7383 | 0.46 | -0.256037433 | count | 1 |
| AP000866.2 | -0.6647725 | 0.9870086 | -0.6735 | 0.501 | -0.256037433 | count | 1 |
| AC015967.1 | -0.6647725 | 1.2198765 | -0.545 | 0.586 | -0.256037433 | count | 1 |
| ANAPC1 | -0.2023655 | 0.1902086 | -1.0639 | 0.287 | -0.256034046 | count | 1 |
| UBALD1 | -0.2184982 | 0.2306303 | -0.9474 | 0.344 | -0.255891986 | count | 1 |
| SUMO4 | -0.4015545 | 0.2745802 | -1.4624 | 0.144 | -0.255768613 | count | 1 |
| APBB3 | -0.4015472 | 0.4423279 | -0.9078 | 0.364 | -0.255764293 | count | 1 |
| MAGT1 | -0.200746 | 0.1637385 | -1.226 | 0.22 | -0.25574703 | count | 1 |
| TXN | -0.178519 | 0.0501033 | -3.563 | 0.000372 | -0.25569644 | count | 1 |
| PARP1 | -0.182533 | 0.0842266 | -2.1672 | 0.0303 | -0.255665513 | count | 1 |
| WDR47 | -0.2695643 | 0.2618659 | -1.0294 | 0.303 | -0.255659994 | count | 1 |
| CAPN1 | -0.1881167 | 0.1350791 | -1.3926 | 0.164 | -0.255561092 | count | 1 |
| GLYR1 | -0.2262007 | 0.1870993 | -1.209 | 0.227 | -0.255534601 | count | 1 |
| RPS20 | -0.1777027 | 0.0232342 | -7.6483 | 2.63E-14 | -0.25551421 | count | 6.38E-10 |
| S100A10 | -0.1773573 | 0.0375379 | -4.7247 | 2.40E-06 | -0.255481732 | count | 0.0577872 |
| CDK2 | -0.342005 | 0.4055561 | -0.8433 | 0.399 | -0.255405566 | count | 1 |
| LRRC34 | -0.9850194 | 0.9308941 | -1.0581 | 0.29 | -0.255311617 | count | 1 |
| SETD1B | -0.2409323 | 0.2812488 | -0.8567 | 0.392 | -0.255252589 | count | 1 |
| TMPO-AS1 | -0.34177 | 0.4717413 | -0.7245 | 0.469 | -0.255239139 | count | 1 |
| MAPKAPK2 | -0.2011755 | 0.1300896 | -1.5464 | 0.122 | -0.255143 | count | 1 |

| | | | | | | | |
|---|---|---|---|---|---|---|---|
| CPOX | -0.2689963 | 0.2759951 | -0.9746 | 0.33 | -0.25513662 | count | 1 |
| N4BP2 | -0.2101607 | 0.1620304 | -1.297 | 0.195 | -0.2550667 | count | 1 |
| NDUFS6 | -0.1805808 | 0.0778349 | -2.32 | 0.0204 | -0.25502859 | count | 1 |
| HAGH | -0.1828785 | 0.0941939 | -1.9415 | 0.0523 | -0.254818682 | count | 1 |
| DTNB | -0.2686215 | 0.2773499 | -0.9685 | 0.333 | -0.254791236 | count | 1 |
| IDH3A | -0.2067294 | 0.1969435 | -1.0497 | 0.294 | -0.254790763 | count | 1 |
| TEX10 | -0.2567955 | 0.3150479 | -0.8151 | 0.415 | -0.254746719 | count | 1 |
| ZFX | -0.1898077 | 0.1263019 | -1.5028 | 0.133 | -0.254721095 | count | 1 |
| ALOX5AP | -0.1776444 | 0.0519311 | -3.4208 | 0.000632 | -0.254709223 | count | 1 |
| S100A6 | -0.1768147 | 0.0314073 | -5.6297 | 1.95E-08 | -0.254685228 | count | 0.000471335 |
| SCMH1 | -0.3996726 | 0.3569795 | -1.1196 | 0.263 | -0.25465482 | count | 1 |
| DDX59 | -0.1932296 | 0.1598621 | -1.2087 | 0.227 | -0.254553051 | count | 1 |
| GPR89A | -0.365662 | 0.29762 | -1.2286 | 0.219 | -0.254534122 | count | 1 |
| ZC3H14 | -0.2001461 | 0.1667394 | -1.2004 | 0.23 | -0.254427105 | count | 1 |
| GPBP1 | -0.1791649 | 0.052895 | -3.3872 | 0.000714 | -0.254334712 | count | 1 |
| NMRAL1 | -0.1876035 | 0.1278557 | -1.4673 | 0.142 | -0.254331655 | count | 1 |
| JARID2 | -0.2250375 | 0.1521525 | -1.479 | 0.139 | -0.254240158 | count | 1 |
| ARF6 | -0.1784145 | 0.0542726 | -3.2874 | 0.001 | -0.254024108 | count | 1 |
| TMEM50A | -0.1771066 | 0.0487709 | -3.6314 | 0.000286 | -0.25376098 | count | 1 |
| CCT2 | -0.17943 | 0.073781 | -2.4319 | 0.0151 | -0.253739538 | count | 1 |
| GCN1 | -0.3395965 | 0.3778284 | -0.8988 | 0.369 | -0.253699289 | count | 1 |
| MYCBP2 | -0.178547 | 0.0619273 | -2.8832 | 0.00396 | -0.253522562 | count | 1 |
| TIPIN | -0.2088656 | 0.2472899 | -0.8446 | 0.398 | -0.253510726 | count | 1 |
| PER1 | -0.2079219 | 0.12455 | -1.6694 | 0.0951 | -0.253407183 | count | 1 |
| HDGFL2 | -0.190208 | 0.1509196 | -1.2603 | 0.208 | -0.253405298 | count | 1 |
| TMEM192 | -0.2284008 | 0.2643899 | -0.8639 | 0.388 | -0.253401908 | count | 1 |
| NFAT5 | -0.2137902 | 0.1611162 | -1.3269 | 0.185 | -0.253330792 | count | 1 |
| SLC38A7 | -0.5211537 | 0.6038526 | -0.863 | 0.388 | -0.253290956 | count | 1 |
| AP1S3 | -0.3047358 | 0.4976037 | -0.6124 | 0.54 | -0.253071925 | count | 1 |
| PSMD13 | -0.1813212 | 0.0875871 | -2.0702 | 0.0385 | -0.252983689 | count | 1 |
| HLA-DPB1 | -0.1763313 | 0.0593974 | -2.9687 | 0.00301 | -0.252853758 | count | 1 |
| MRPS11 | -0.1883688 | 0.1449599 | -1.2995 | 0.194 | -0.252797778 | count | 1 |
| NUPL2 | -0.193071 | 0.1558844 | -1.2386 | 0.216 | -0.252777083 | count | 1 |
| FUT8 | -0.2030724 | 0.1636828 | -1.2406 | 0.215 | -0.252776151 | count | 1 |
| NDUFS5 | -0.1764765 | 0.0457798 | -3.8549 | 0.000118 | -0.252678624 | count | 1 |
| RAB40B | -0.3192197 | 0.3766682 | -0.8475 | 0.397 | -0.252672784 | count | 1 |
| TSPO | -0.1762466 | 0.0486302 | -3.6242 | 0.000294 | -0.252494976 | count | 1 |
| ATP6V0D1 | -0.1823933 | 0.1162663 | -1.5688 | 0.117 | -0.252325678 | count | 1 |
| NFKB1 | -0.1946911 | 0.1157123 | -1.6825 | 0.0926 | -0.252293172 | count | 1 |
| PLD2 | -0.9688151 | 0.6778389 | -1.4293 | 0.153 | -0.252235687 | count | 1 |
| DGLUCY | -0.225144 | 0.2032808 | -1.1076 | 0.268 | -0.252172351 | count | 1 |
| MB21D2 | -0.3373064 | 0.54391 | -0.6202 | 0.535 | -0.252075568 | count | 1 |
| PRDM15 | -0.253919 | 0.4777897 | -0.5314 | 0.595 | -0.251962763 | count | 1 |
| OPA1 | -0.2150513 | 0.2046929 | -1.0506 | 0.294 | -0.251904965 | count | 1 |
| BST1 | -0.3615008 | 0.9131163 | -0.3959 | 0.692 | -0.251808135 | count | 1 |

| | | | | | | | |
|---|---|---|---|---|---|---|---|
| CARD11 | -0.2177105 | 0.2008966 | -1.0837 | 0.279 | -0.251716146 | count | 1 |
| ADTRP | -0.2227479 | 0.2640076 | -0.8437 | 0.399 | -0.251691577 | count | 1 |
| KLHDC10 | -0.2446109 | 0.3095506 | -0.7902 | 0.429 | -0.25164582 | count | 1 |
| MMRN2 | -0.9654044 | 1.250416 | -0.7721 | 0.44 | -0.251583768 | count | 1 |
| TRIM45 | -0.9654044 | 1.4393098 | -0.6707 | 0.502 | -0.251583768 | count | 1 |
| MNX1 | -0.9654044 | 1.4393098 | -0.6707 | 0.502 | -0.251583768 | count | 1 |
| DNAH10 | -0.9654044 | 1.4393098 | -0.6707 | 0.502 | -0.251583768 | count | 1 |
| USP16 | -0.1791222 | 0.0741932 | -2.4143 | 0.0158 | -0.2515176 | count | 1 |
| CEP57 | -0.1846393 | 0.0954869 | -1.9337 | 0.0532 | -0.251462933 | count | 1 |
| HMGN1 | -0.1754786 | 0.0449844 | -3.9009 | 9.77E-05 | -0.251403771 | count | 1 |
| RHNO1 | -0.1922378 | 0.1800563 | -1.0677 | 0.286 | -0.251355291 | count | 1 |
| GPD2 | -0.4419316 | 0.4703472 | -0.9396 | 0.347 | -0.251344203 | count | 1 |
| ATP6V1D | -0.1853405 | 0.1196997 | -1.5484 | 0.122 | -0.251274097 | count | 1 |
| KISS1R | -0.4417121 | 0.5517588 | -0.8006 | 0.423 | -0.251230028 | count | 1 |
| GZMA | -0.174309 | 0.042538 | -4.0977 | 4.27E-05 | -0.251107534 | count | 1 |
| FKBP3 | -0.1820331 | 0.1181294 | -1.541 | 0.123 | -0.251024946 | count | 1 |
| PKD2 | -0.3356784 | 0.3782106 | -0.8875 | 0.375 | -0.250920519 | count | 1 |
| LINC00665 | -0.2796447 | 0.4088136 | -0.684 | 0.494 | -0.25061979 | count | 1 |
| DLG4 | -0.6477936 | 0.8519447 | -0.7604 | 0.447 | -0.250520644 | count | 1 |
| AC114490.2 | -0.6477936 | 0.9351257 | -0.6927 | 0.489 | -0.250520644 | count | 1 |
| ZBED5 | -0.2063417 | 0.1762943 | -1.1704 | 0.242 | -0.250477808 | count | 1 |
| MBOAT1 | -0.3593729 | 0.3141711 | -1.1439 | 0.253 | -0.250412538 | count | 1 |
| OSBPL2 | -0.2124529 | 0.2179455 | -0.9748 | 0.33 | -0.250367919 | count | 1 |
| ACAT2 | -0.1846698 | 0.1161255 | -1.5903 | 0.112 | -0.2502308 | count | 1 |
| STN1 | -0.181166 | 0.1124264 | -1.6114 | 0.107 | -0.249903242 | count | 1 |
| QPRT | -0.6457324 | 0.6090547 | -1.0602 | 0.289 | -0.249847205 | count | 1 |
| CCDC74A | -0.5130146 | 0.7921003 | -0.6477 | 0.517 | -0.249769963 | count | 1 |
| PPP6C | -0.1818849 | 0.1214363 | -1.4978 | 0.134 | -0.249544058 | count | 1 |
| CISD2 | -0.1804428 | 0.114517 | -1.5757 | 0.115 | -0.249508418 | count | 1 |
| UBA1 | -0.2248176 | 0.2507386 | -0.8966 | 0.37 | -0.24948947 | count | 1 |
| TIMM17A | -0.18454 | 0.1283838 | -1.4374 | 0.151 | -0.24948348 | count | 1 |
| SLC4A7 | -0.1820617 | 0.1184509 | -1.537 | 0.124 | -0.249326633 | count | 1 |
| HEATR6 | -0.2053693 | 0.2560067 | -0.8022 | 0.422 | -0.249309077 | count | 1 |
| STK17B | -0.1738436 | 0.0380543 | -4.5683 | 5.09E-06 | -0.249287601 | count | 0.12244504 |
| GATAD2A | -0.1943716 | 0.1398429 | -1.3899 | 0.165 | -0.249212822 | count | 1 |
| EFNA1 | -0.2998472 | 0.5734598 | -0.5229 | 0.601 | -0.249172539 | count | 1 |
| RUFY3 | -0.2462872 | 0.3040176 | -0.8101 | 0.418 | -0.249131205 | count | 1 |
| TPO | -0.9523077 | 1.0634043 | -0.8955 | 0.371 | -0.249065866 | count | 1 |
| ESAM | -0.9523077 | 1.221902 | -0.7794 | 0.436 | -0.249065866 | count | 1 |
| GINS1 | -0.9523077 | 1.221902 | -0.7794 | 0.436 | -0.249065866 | count | 1 |
| TESC | -0.1820128 | 0.1634492 | -1.1136 | 0.266 | -0.248868197 | count | 1 |
| RPS10 | -0.1733752 | 0.0326924 | -5.3032 | 1.21E-07 | -0.248810237 | count | 0.002920819 |
| DDX56 | -0.1991665 | 0.1892751 | -1.0523 | 0.293 | -0.248695218 | count | 1 |
| B3GAT2 | -0.2414606 | 0.2927196 | -0.8249 | 0.409 | -0.248473665 | count | 1 |
| HEMK1 | -0.2377235 | 0.2753265 | -0.8634 | 0.388 | -0.248450854 | count | 1 |

| | | | | | | | |
|---|---|---|---|---|---|---|---|
| ENC1 | -0.2094683 | 0.2121927 | -0.9872 | 0.324 | -0.248267789 | count | 1 |
| FGFBP3 | -0.2554059 | 0.3411154 | -0.7487 | 0.454 | -0.248240125 | count | 1 |
| TPM3 | -0.1728992 | 0.0354157 | -4.882 | 1.10E-06 | -0.248236395 | count | 0.0265144 |
| SLC23A3 | -0.5091635 | 0.9512103 | -0.5353 | 0.592 | -0.248098943 | count | 1 |
| ZMPSTE24 | -0.1934374 | 0.1749855 | -1.1054 | 0.269 | -0.248022836 | count | 1 |
| LTA | -0.1805411 | 0.2052874 | -0.8795 | 0.379 | -0.24796373 | count | 1 |
| TMC8 | -0.1871537 | 0.1286858 | -1.4543 | 0.146 | -0.247931859 | count | 1 |
| PTPN2 | -0.1757825 | 0.0821916 | -2.1387 | 0.0325 | -0.247922832 | count | 1 |
| COX14 | -0.1753633 | 0.0694607 | -2.5246 | 0.0116 | -0.247821765 | count | 1 |
| ITPR2 | -0.1953464 | 0.1451177 | -1.3461 | 0.178 | -0.247803059 | count | 1 |
| NCKAP1L | -0.1874896 | 0.1341684 | -1.3974 | 0.162 | -0.247586281 | count | 1 |
| TPCN1 | -0.3124358 | 0.3655163 | -0.8548 | 0.393 | -0.247538842 | count | 1 |
| EPN1 | -0.1994475 | 0.2004568 | -0.995 | 0.32 | -0.247530643 | count | 1 |
| RPP30 | -0.1865919 | 0.1526204 | -1.2226 | 0.222 | -0.24744069 | count | 1 |
| ARRDC1 | -0.1840944 | 0.1253584 | -1.4685 | 0.142 | -0.247439082 | count | 1 |
| TNFAIP1 | -0.2545572 | 0.3192136 | -0.7975 | 0.425 | -0.247436258 | count | 1 |
| ISCA1 | -0.1762655 | 0.0810428 | -2.175 | 0.0297 | -0.247247068 | count | 1 |
| MYNN | -0.1901486 | 0.1651098 | -1.1516 | 0.25 | -0.247214135 | count | 1 |
| USP9X | -0.1958095 | 0.1659479 | -1.1799 | 0.238 | -0.247192934 | count | 1 |
| CBWD1 | -0.1973715 | 0.1858532 | -1.062 | 0.288 | -0.24717849 | count | 1 |
| VEGFA | -0.4338088 | 0.5133907 | -0.845 | 0.398 | -0.247111397 | count | 1 |
| TMEM70 | -0.1794993 | 0.1036524 | -1.7317 | 0.0834 | -0.247097313 | count | 1 |
| MDN1 | -0.2541516 | 0.2491598 | -1.02 | 0.308 | -0.247052033 | count | 1 |
| RBBP6 | -0.1768884 | 0.0995037 | -1.7777 | 0.0755 | -0.247036314 | count | 1 |
| RAB5IF | -0.1766171 | 0.0942745 | -1.8734 | 0.0611 | -0.246975304 | count | 1 |
| STK38L | -0.2051849 | 0.2265214 | -0.9058 | 0.365 | -0.246913694 | count | 1 |
| POP7 | -0.1790394 | 0.1256192 | -1.4253 | 0.154 | -0.246906442 | count | 1 |
| NUP153 | -0.1914673 | 0.1620568 | -1.1815 | 0.237 | -0.2468853 | count | 1 |
| ARPC2 | -0.1715125 | 0.0311392 | -5.5079 | 3.90E-08 | -0.246848515 | count | 0.00094224 |
| TMEM246 | -0.9404613 | 1.0040286 | -0.9367 | 0.349 | -0.246768306 | count | 1 |
| CD2 | -0.1714145 | 0.0335074 | -5.1157 | 3.30E-07 | -0.246706287 | count | 0.00796191 |
| SIRPG | -0.1827962 | 0.1411159 | -1.2954 | 0.195 | -0.246682399 | count | 1 |
| CETN2 | -0.214722 | 0.2473188 | -0.8682 | 0.385 | -0.24655935 | count | 1 |
| PPM1G | -0.173386 | 0.0702908 | -2.4667 | 0.0137 | -0.246484848 | count | 1 |
| FAM3C | -0.1854488 | 0.1621323 | -1.1438 | 0.253 | -0.246174149 | count | 1 |
| POLR2I | -0.1777932 | 0.1036267 | -1.7157 | 0.0863 | -0.246096039 | count | 1 |
| CD151 | -0.1866831 | 0.1724141 | -1.0828 | 0.279 | -0.24597119 | count | 1 |
| AC020928.1 | -0.2742852 | 0.4200147 | -0.653 | 0.514 | -0.245970486 | count | 1 |
| PSMC2 | -0.1773998 | 0.1078069 | -1.6455 | 0.1 | -0.245784509 | count | 1 |
| MGAT5 | -0.1973542 | 0.1787061 | -1.1044 | 0.27 | -0.245716718 | count | 1 |
| FAM120A | -0.184548 | 0.1476382 | -1.25 | 0.211 | -0.24545031 | count | 1 |
| AL121832.2 | -0.9332744 | 0.7825626 | -1.1926 | 0.233 | -0.24536514 | count | 1 |
| CUL1 | -0.1881174 | 0.1474123 | -1.2761 | 0.202 | -0.245311297 | count | 1 |
| TTL | -0.2312692 | 0.3149351 | -0.7343 | 0.463 | -0.245207332 | count | 1 |
| SQOR | -0.1776632 | 0.114746 | -1.5483 | 0.122 | -0.245151606 | count | 1 |

| | | | | | | | |
|---|---|---|---|---|---|---|---|
| TXNDC17 | -0.1746109 | 0.0934487 | -1.8685 | 0.0618 | -0.245036891 | count | 1 |
| CENPH | -0.2280538 | 0.2635844 | -0.8652 | 0.387 | -0.244948076 | count | 1 |
| KCNK12 | -0.327154 | 0.4432525 | -0.7381 | 0.461 | -0.244862019 | count | 1 |
| ATP6V0E2 | -0.1791007 | 0.1093073 | -1.6385 | 0.101 | -0.24479783 | count | 1 |
| SOX12 | -0.2576352 | 0.4185987 | -0.6155 | 0.538 | -0.244653369 | count | 1 |
| ABCC10 | -0.2129372 | 0.3149831 | -0.676 | 0.499 | -0.244536943 | count | 1 |
| KLHL42 | -0.2224914 | 0.2785379 | -0.7988 | 0.424 | -0.244498217 | count | 1 |
| NUDT1 | -0.174967 | 0.1105614 | -1.5825 | 0.114 | -0.24442945 | count | 1 |
| JMJD1C | -0.1744595 | 0.0725783 | -2.4037 | 0.0163 | -0.244374638 | count | 1 |
| TRMT11 | -0.1981942 | 0.1625819 | -1.219 | 0.223 | -0.24436327 | count | 1 |
| CCDC12 | -0.1723299 | 0.0768766 | -2.2416 | 0.025 | -0.244277553 | count | 1 |
| NELL2 | -0.2049115 | 0.1761288 | -1.1634 | 0.245 | -0.244206485 | count | 1 |
| ZNF565 | -0.5001554 | 0.5218183 | -0.9585 | 0.338 | -0.244177584 | count | 1 |
| EXOC6 | -0.1929024 | 0.1792518 | -1.0762 | 0.282 | -0.244147225 | count | 1 |
| LINC00672 | -0.5000849 | 0.4973941 | -1.0054 | 0.315 | -0.244146831 | count | 1 |
| EHBP1 | -0.2245867 | 0.248387 | -0.9042 | 0.366 | -0.244119876 | count | 1 |
| AC090061.1 | -0.2245056 | 0.5219222 | -0.4302 | 0.667 | -0.244033208 | count | 1 |
| HDAC1 | -0.175381 | 0.0966869 | -1.8139 | 0.0698 | -0.244000588 | count | 1 |
| BCCIP | -0.1837945 | 0.1390105 | -1.3222 | 0.186 | -0.243987857 | count | 1 |
| CLIP1 | -0.1786786 | 0.1121967 | -1.5925 | 0.111 | -0.243914718 | count | 1 |
| FOXP1 | -0.1740352 | 0.0746491 | -2.3314 | 0.0198 | -0.243810977 | count | 1 |
| LINC00342 | -0.6270976 | 0.557249 | -1.1253 | 0.261 | -0.243722109 | count | 1 |
| RIOK2 | -0.1934369 | 0.1475441 | -1.311 | 0.19 | -0.243598968 | count | 1 |
| AC079922.2 | -0.2240361 | 0.353277 | -0.6342 | 0.526 | -0.243531444 | count | 1 |
| CYP2R1 | -0.2172759 | 0.2789362 | -0.7789 | 0.436 | -0.243489945 | count | 1 |
| SCAF11 | -0.1721872 | 0.0631823 | -2.7252 | 0.00646 | -0.243466352 | count | 1 |
| CCDC71L | -0.1863788 | 0.1463302 | -1.2737 | 0.203 | -0.243400121 | count | 1 |
| ZBTB18 | -0.3069705 | 0.3908831 | -0.7853 | 0.432 | -0.243394755 | count | 1 |
| RIPK2 | -0.194258 | 0.1510459 | -1.2861 | 0.199 | -0.243309477 | count | 1 |
| LANCL3 | -0.6258476 | 0.7378683 | -0.8482 | 0.396 | -0.243308879 | count | 1 |
| ENTPD1-AS1 | -0.2449855 | 0.3375407 | -0.7258 | 0.468 | -0.243305749 | count | 1 |
| AAK1 | -0.1727654 | 0.0719959 | -2.3997 | 0.0165 | -0.243215962 | count | 1 |
| GYPC | -0.1696171 | 0.049887 | -3.4 | 0.000682 | -0.243035268 | count | 1 |
| KIZ | -0.2130015 | 0.1910144 | -1.1151 | 0.265 | -0.242776718 | count | 1 |
| TRABD | -0.1734837 | 0.0864367 | -2.0071 | 0.0448 | -0.24263378 | count | 1 |
| KIAA1143 | -0.1817189 | 0.1448301 | -1.2547 | 0.21 | -0.242564011 | count | 1 |
| LUC7L2 | -0.1802723 | 0.1135085 | -1.5882 | 0.112 | -0.242489798 | count | 1 |
| SLC38A5 | -0.2394443 | 0.3522207 | -0.6798 | 0.497 | -0.242360667 | count | 1 |
| SNRNP27 | -0.1780847 | 0.124244 | -1.4333 | 0.152 | -0.242333394 | count | 1 |
| FARSB | -0.2067253 | 0.2337679 | -0.8843 | 0.377 | -0.242267115 | count | 1 |
| NUDT15 | -0.1896795 | 0.1751058 | -1.0832 | 0.279 | -0.24225638 | count | 1 |
| MROH6 | -0.4956784 | 0.7910285 | -0.6266 | 0.531 | -0.2422221 | count | 1 |
| C1orf198 | -0.4956784 | 0.8277804 | -0.5988 | 0.549 | -0.2422221 | count | 1 |
| AL445524.1 | -0.3785023 | 0.5160901 | -0.7334 | 0.463 | -0.242066462 | count | 1 |
| TNS4 | -0.6220634 | 0.7901959 | -0.7872 | 0.431 | -0.242056081 | count | 1 |

| | | | | | | | |
|---|---|---|---|---|---|---|---|
| QTRT2 | -0.2282177 | 0.229764 | -0.9933 | 0.321 | -0.242031512 | count | 1 |
| VPS8 | -0.2390619 | 0.2386378 | -1.0018 | 0.317 | -0.241982033 | count | 1 |
| ETFBKMT | -0.4946998 | 0.5332329 | -0.9277 | 0.354 | -0.241794084 | count | 1 |
| EPSTI1 | -0.1751894 | 0.1496603 | -1.1706 | 0.242 | -0.241678064 | count | 1 |
| DHPS | -0.1751811 | 0.1035345 | -1.692 | 0.0907 | -0.241666641 | count | 1 |
| VGLL4 | -0.1834981 | 0.1448769 | -1.2666 | 0.205 | -0.241512066 | count | 1 |
| DDX3X | -0.1702676 | 0.0552709 | -3.0806 | 0.00208 | -0.241499726 | count | 1 |
| ELOVL4 | -0.268935 | 0.4080287 | -0.6591 | 0.51 | -0.241322733 | count | 1 |
| ALAS1 | -0.1893317 | 0.1948184 | -0.9718 | 0.331 | -0.24130265 | count | 1 |
| TMOD1 | -0.6197802 | 0.7467501 | -0.83 | 0.407 | -0.241298899 | count | 1 |
| IMPDH2 | -0.1783485 | 0.1201501 | -1.4844 | 0.138 | -0.241282925 | count | 1 |
| CAMSAP2 | -0.6193775 | 0.839424 | -0.7379 | 0.461 | -0.24116525 | count | 1 |
| DGUOK | -0.1718204 | 0.0777867 | -2.2089 | 0.0273 | -0.241100321 | count | 1 |
| AP3B1 | -0.1804408 | 0.12773 | -1.4127 | 0.158 | -0.241067282 | count | 1 |
| VPS4B | -0.1730431 | 0.0844956 | -2.048 | 0.0406 | -0.241056057 | count | 1 |
| SUMO1 | -0.1694335 | 0.0612074 | -2.7682 | 0.00567 | -0.241031797 | count | 1 |
| SPICE1 | -0.2168965 | 0.3399179 | -0.6381 | 0.523 | -0.240832791 | count | 1 |
| KPNA4 | -0.1760937 | 0.1067024 | -1.6503 | 0.099 | -0.240797322 | count | 1 |
| UBQLN4 | -0.2893531 | 0.3603386 | -0.803 | 0.422 | -0.240782814 | count | 1 |
| PHF6 | -0.176638 | 0.1562889 | -1.1302 | 0.258 | -0.240709376 | count | 1 |
| CPNE3 | -0.1717201 | 0.0896828 | -1.9147 | 0.0556 | -0.240659183 | count | 1 |
| CD79A | -0.9090093 | 0.6080443 | -1.495 | 0.135 | -0.240575468 | count | 1 |
| MRPS2 | -0.181192 | 0.1498981 | -1.2088 | 0.227 | -0.240548053 | count | 1 |
| HIST1H2BG | -0.9084613 | 0.7691366 | -1.1811 | 0.238 | -0.240466366 | count | 1 |
| RPS11 | -0.1671862 | 0.0242803 | -6.8857 | 6.82E-12 | -0.240415953 | count | 1.65E-07 |
| FKBP5 | -0.1764372 | 0.0729165 | -2.4197 | 0.0156 | -0.240325554 | count | 1 |
| ITPR3 | -0.2596326 | 0.2937621 | -0.8838 | 0.377 | -0.240189535 | count | 1 |
| TET1 | -0.4908279 | 0.5484448 | -0.8949 | 0.371 | -0.240098563 | count | 1 |
| NELFE | -0.1772351 | 0.1358667 | -1.3045 | 0.192 | -0.240055731 | count | 1 |
| SOD1 | -0.1675581 | 0.0419101 | -3.998 | 6.52E-05 | -0.23999908 | count | 1 |
| SLC25A32 | -0.1873614 | 0.1694956 | -1.1054 | 0.269 | -0.239805696 | count | 1 |
| RBBP4 | -0.1737793 | 0.1004012 | -1.7308 | 0.0836 | -0.239804509 | count | 1 |
| RNF141 | -0.2411737 | 0.2813467 | -0.8572 | 0.391 | -0.239606909 | count | 1 |
| COG3 | -0.2179481 | 0.2725994 | -0.7995 | 0.424 | -0.239584231 | count | 1 |
| FBXW9 | -0.6145603 | 0.6665146 | -0.9221 | 0.357 | -0.239564075 | count | 1 |
| CLCF1 | -0.2020266 | 0.2196918 | -0.9196 | 0.358 | -0.239543874 | count | 1 |
| ZBTB45 | -0.2118121 | 0.4098851 | -0.5168 | 0.605 | -0.239507006 | count | 1 |
| MAP3K1 | -0.206991 | 0.1870251 | -1.1068 | 0.268 | -0.239476727 | count | 1 |
| SFPQ | -0.1679425 | 0.0468088 | -3.5878 | 0.000338 | -0.239439536 | count | 1 |
| ZNF853 | -0.2761772 | 0.5658621 | -0.4881 | 0.626 | -0.239387422 | count | 1 |
| LPP | -0.1882271 | 0.1584454 | -1.188 | 0.235 | -0.239377886 | count | 1 |
| GALNS | -0.2518546 | 0.2706481 | -0.9306 | 0.352 | -0.239308678 | count | 1 |
| GAN | -0.2458294 | 0.2611446 | -0.9414 | 0.347 | -0.239160706 | count | 1 |
| SPPL3 | -0.1879407 | 0.1741198 | -1.0794 | 0.28 | -0.239016098 | count | 1 |
| GMPS | -0.1746042 | 0.1187511 | -1.4703 | 0.142 | -0.238861877 | count | 1 |

| | | | | | | | |
|---|---|---|---|---|---|---|---|
| RASGRP1 | -0.1800981 | 0.10806 | -1.6666 | 0.0957 | -0.23862633 | count | 1 |
| ZNF318 | -0.2076021 | 0.2876871 | -0.7216 | 0.471 | -0.238488649 | count | 1 |
| TIMM21 | -0.2749906 | 0.3318447 | -0.8287 | 0.407 | -0.238393584 | count | 1 |
| SLC19A1 | -0.417086 | 0.4786391 | -0.8714 | 0.384 | -0.238348679 | count | 1 |
| TTC39B | -0.1841609 | 0.162253 | -1.135 | 0.256 | -0.238334498 | count | 1 |
| XPC | -0.1765676 | 0.1345675 | -1.3121 | 0.19 | -0.238305729 | count | 1 |
| CCDC88C | -0.1723442 | 0.0931907 | -1.8494 | 0.0645 | -0.238273504 | count | 1 |
| UNC119B | -0.3721495 | 0.4968385 | -0.749 | 0.454 | -0.238267933 | count | 1 |
| DCST1-AS1 | -0.89705 | 0.8990631 | -0.9978 | 0.318 | -0.238185079 | count | 1 |
| WDR36 | -0.195885 | 0.2052615 | -0.9543 | 0.34 | -0.237903554 | count | 1 |
| TWF2 | -0.1687766 | 0.0826536 | -2.042 | 0.0412 | -0.237782812 | count | 1 |
| TIAM2 | -0.2443729 | 0.3782436 | -0.6461 | 0.518 | -0.237778116 | count | 1 |
| HELB | -0.1775416 | 0.0999496 | -1.7763 | 0.0758 | -0.237595071 | count | 1 |
| SSRP1 | -0.1863761 | 0.1677063 | -1.1113 | 0.267 | -0.237560361 | count | 1 |
| KIF3B | -0.1896056 | 0.2119945 | -0.8944 | 0.371 | -0.237526168 | count | 1 |
| ATXN10 | -0.1717228 | 0.1037167 | -1.6557 | 0.0979 | -0.237476589 | count | 1 |
| LARS | -0.1721557 | 0.0992392 | -1.7348 | 0.0829 | -0.237435008 | count | 1 |
| UGP2 | -0.1702698 | 0.0680272 | -2.503 | 0.0124 | -0.237399603 | count | 1 |
| CACNA2D4 | -0.1918358 | 0.2720106 | -0.7053 | 0.481 | -0.237390578 | count | 1 |
| ABL1 | -0.6078762 | 0.3993406 | -1.5222 | 0.128 | -0.237335075 | count | 1 |
| EVI5L | -0.607239 | 0.6095212 | -0.9963 | 0.319 | -0.23712214 | count | 1 |
| GGCX | -0.2134527 | 0.2064365 | -1.034 | 0.301 | -0.237065868 | count | 1 |
| EHBP1L1 | -0.1929014 | 0.1938366 | -0.9952 | 0.32 | -0.237053576 | count | 1 |
| GFM1 | -0.1951508 | 0.2003871 | -0.9739 | 0.33 | -0.237020155 | count | 1 |
| CSTB | -0.1651617 | 0.0544448 | -3.0336 | 0.00244 | -0.23687979 | count | 1 |
| SETDB1 | -0.2094529 | 0.3392661 | -0.6174 | 0.537 | -0.236875869 | count | 1 |
| TOB2 | -0.1782335 | 0.1271615 | -1.4016 | 0.161 | -0.236864608 | count | 1 |
| GYG1 | -0.1676674 | 0.1012221 | -1.6564 | 0.0977 | -0.236840717 | count | 1 |
| DIP2C | -0.8903462 | 0.9652758 | -0.9224 | 0.356 | -0.236836507 | count | 1 |
| CLDN15 | -0.8901061 | 0.47117 | -1.8891 | 0.059 | -0.236788188 | count | 1 |
| AC016575.1 | -0.3386496 | 0.8204444 | -0.4128 | 0.68 | -0.236763227 | count | 1 |
| INTS9 | -0.2981918 | 0.4102332 | -0.7269 | 0.467 | -0.236723295 | count | 1 |
| AC008105.3 | -0.184154 | 0.1645507 | -1.1191 | 0.263 | -0.236645845 | count | 1 |
| IGHMBP2 | -0.369373 | 0.3676448 | -1.0047 | 0.315 | -0.236604752 | count | 1 |
| NKRF | -0.2296178 | 0.1991282 | -1.1531 | 0.249 | -0.236531279 | count | 1 |
| RTL5 | -0.3691788 | 0.6617571 | -0.5579 | 0.577 | -0.236488345 | count | 1 |
| LRRC58 | -0.2027462 | 0.2353357 | -0.8615 | 0.389 | -0.236182163 | count | 1 |
| PDK4 | -0.886914 | 0.6672692 | -1.3292 | 0.184 | -0.236143759 | count | 1 |
| TMEM87A | -0.1735285 | 0.1133309 | -1.5312 | 0.126 | -0.236037342 | count | 1 |
| SLC25A19 | -0.2195287 | 0.2595838 | -0.8457 | 0.398 | -0.235947317 | count | 1 |
| TMEM165 | -0.1687258 | 0.0928916 | -1.8164 | 0.0694 | -0.235925083 | count | 1 |
| DTHD1 | -0.1773235 | 0.1452724 | -1.2206 | 0.222 | -0.235881611 | count | 1 |
| IQCN | -0.3372999 | 0.6033301 | -0.5591 | 0.576 | -0.235870656 | count | 1 |
| AC087645.2 | -0.3680373 | 0.8663821 | -0.4248 | 0.671 | -0.235803989 | count | 1 |
| GRN | -0.1770188 | 0.1905487 | -0.929 | 0.353 | -0.235477963 | count | 1 |

| | | | | | | | |
|---|---|---|---|---|---|---|---|
| TMED3 | -0.1747708 | 0.1335624 | -1.3085 | 0.191 | -0.235433666 | count | 1 |
| PSMB1 | -0.1646132 | 0.0534108 | -3.082 | 0.00207 | -0.23542244 | count | 1 |
| CEP41 | -0.2249906 | 0.3688183 | -0.61 | 0.542 | -0.235394355 | count | 1 |
| SPATA2 | -0.2417517 | 0.268361 | -0.9008 | 0.368 | -0.235288806 | count | 1 |
| MRPL51 | -0.1668811 | 0.0852068 | -1.9585 | 0.0502 | -0.235261021 | count | 1 |
| CUL3 | -0.1778532 | 0.1288776 | -1.38 | 0.168 | -0.235173205 | count | 1 |
| RIT1 | -0.171728 | 0.1160676 | -1.4796 | 0.139 | -0.23512187 | count | 1 |
| AHNAK | -0.16499 | 0.0636427 | -2.5924 | 0.0096 | -0.234973617 | count | 1 |
| COPB1 | -0.1686561 | 0.091218 | -1.8489 | 0.0646 | -0.234955398 | count | 1 |
| TTC19 | -0.182649 | 0.1246167 | -1.4657 | 0.143 | -0.234723432 | count | 1 |
| IGFBP3 | -0.2074904 | 0.2463758 | -0.8422 | 0.4 | -0.234686473 | count | 1 |
| NAGPA | -0.1872213 | 0.2032446 | -0.9212 | 0.357 | -0.234561381 | count | 1 |
| FAM114A2 | -0.1988149 | 0.2128541 | -0.934 | 0.35 | -0.234472864 | count | 1 |
| LAPTM5 | -0.1634373 | 0.0415098 | -3.9373 | 8.41E-05 | -0.234362325 | count | 1 |
| SLC29A1 | -0.4776673 | 0.7821577 | -0.6107 | 0.541 | -0.234311069 | count | 1 |
| NANP | -0.2700972 | 0.4208568 | -0.6418 | 0.521 | -0.234291689 | count | 1 |
| RNF13 | -0.1687849 | 0.1029263 | -1.6399 | 0.101 | -0.234083051 | count | 1 |
| UBAP2L | -0.1811249 | 0.2059417 | -0.8795 | 0.379 | -0.23403282 | count | 1 |
| EAPP | -0.1655008 | 0.0803987 | -2.0585 | 0.0396 | -0.233812639 | count | 1 |
| GALNT8 | -0.8753956 | 1.0031949 | -0.8726 | 0.383 | -0.233806551 | count | 1 |
| BX537318.1 | -0.8753956 | 1.0031949 | -0.8726 | 0.383 | -0.233806551 | count | 1 |
| ECHDC3 | -0.8753956 | 1.1590281 | -0.7553 | 0.45 | -0.233806551 | count | 1 |
| SSSCA1 | -0.1676664 | 0.129509 | -1.2946 | 0.196 | -0.233619154 | count | 1 |
| FMNL3 | -0.2065201 | 0.2618574 | -0.7887 | 0.43 | -0.23360376 | count | 1 |
| ERP27 | -0.3113667 | 0.457333 | -0.6808 | 0.496 | -0.233595194 | count | 1 |
| MAP1LC3B2 | -0.3110463 | 0.4175082 | -0.745 | 0.456 | -0.233365926 | count | 1 |
| H2AFY | -0.1657638 | 0.0850941 | -1.948 | 0.0515 | -0.23305953 | count | 1 |
| TMEM189 | -0.180004 | 0.2205001 | -0.8163 | 0.414 | -0.232984668 | count | 1 |
| PCNX1 | -0.1763404 | 0.1364196 | -1.2926 | 0.196 | -0.232929345 | count | 1 |
| ATAD2B | -0.180533 | 0.1500609 | -1.2031 | 0.229 | -0.232450208 | count | 1 |
| CISD3 | -0.1646516 | 0.1030668 | -1.5975 | 0.11 | -0.232323453 | count | 1 |
| RAB35 | -0.1948032 | 0.1563282 | -1.2461 | 0.213 | -0.232282744 | count | 1 |
| HIST1H2AL | -0.4728371 | 0.4543643 | -1.0407 | 0.298 | -0.232177499 | count | 1 |
| RPGRIP1L | -0.4726535 | 0.519656 | -0.9096 | 0.363 | -0.23209631 | count | 1 |
| RRS1 | -0.1773513 | 0.1425474 | -1.2442 | 0.214 | -0.231987975 | count | 1 |
| ACAA1 | -0.1705274 | 0.1185609 | -1.4383 | 0.15 | -0.231967068 | count | 1 |
| ZNF25 | -0.2581511 | 0.3502965 | -0.737 | 0.461 | -0.231935063 | count | 1 |
| BRCC3 | -0.1934587 | 0.1919552 | -1.0078 | 0.314 | -0.231844082 | count | 1 |
| TMEM263 | -0.1688306 | 0.121795 | -1.3862 | 0.166 | -0.231839776 | count | 1 |
| CTDP1 | -0.2156182 | 0.191594 | -1.1254 | 0.261 | -0.231814224 | count | 1 |
| NDUFA2 | -0.164018 | 0.0757414 | -2.1655 | 0.0304 | -0.23180477 | count | 1 |
| DUSP16 | -0.1860738 | 0.2069934 | -0.8989 | 0.369 | -0.231779646 | count | 1 |
| CCND2 | -0.1636182 | 0.0675501 | -2.4222 | 0.0155 | -0.231587524 | count | 1 |
| USP31 | -0.8643989 | 0.4944889 | -1.7481 | 0.0805 | -0.231558173 | count | 1 |
| RPIA | -0.1703947 | 0.1286976 | -1.324 | 0.186 | -0.231557105 | count | 1 |

| | | | | | | | |
|---|---|---|---|---|---|---|---|
| S100PBP | -0.1847908 | 0.1925803 | -0.9596 | 0.337 | -0.231538512 | count | 1 |
| GNG10 | -0.4713851 | 0.5878147 | -0.8019 | 0.423 | -0.231535141 | count | 1 |
| GEMIN8 | -0.1986787 | 0.2546742 | -0.7801 | 0.435 | -0.231498436 | count | 1 |
| ZNF577 | -0.2245668 | 0.4864122 | -0.4617 | 0.644 | -0.231429477 | count | 1 |
| SLC30A6 | -0.2327473 | 0.3307513 | -0.7037 | 0.482 | -0.231419658 | count | 1 |
| FAM222B | -0.3604623 | 0.3607895 | -0.9991 | 0.318 | -0.231254626 | count | 1 |
| EIF2S1 | -0.168689 | 0.1063426 | -1.5863 | 0.113 | -0.231147388 | count | 1 |
| NCBP2-AS2 | -0.1672718 | 0.1291832 | -1.2948 | 0.195 | -0.230971017 | count | 1 |
| CLK4 | -0.1731404 | 0.1329639 | -1.3022 | 0.193 | -0.23095956 | count | 1 |
| RHOG | -0.162342 | 0.0649732 | -2.4986 | 0.0125 | -0.230943995 | count | 1 |
| TRIP10 | -0.3297912 | 0.4128923 | -0.7987 | 0.425 | -0.230897039 | count | 1 |
| HTATSF1 | -0.1681186 | 0.1030578 | -1.6313 | 0.103 | -0.230864412 | count | 1 |
| DIS3L2 | -0.1899984 | 0.2334893 | -0.8137 | 0.416 | -0.230818789 | count | 1 |
| CWF19L2 | -0.1664939 | 0.099264 | -1.6773 | 0.0936 | -0.230809255 | count | 1 |
| BOLA3 | -0.1669192 | 0.1170869 | -1.4256 | 0.154 | -0.230788785 | count | 1 |
| SLU7 | -0.1670168 | 0.0994879 | -1.6788 | 0.0933 | -0.230743359 | count | 1 |
| KCTD9 | -0.2238779 | 0.2704104 | -0.8279 | 0.408 | -0.230733266 | count | 1 |
| DCTN6 | -0.1719983 | 0.1027608 | -1.6738 | 0.0943 | -0.230562711 | count | 1 |
| SPAG1 | -0.2020659 | 0.2327351 | -0.8682 | 0.385 | -0.230471996 | count | 1 |
| RBM4 | -0.1733283 | 0.131488 | -1.3182 | 0.188 | -0.230372442 | count | 1 |
| IDI1 | -0.1633475 | 0.072421 | -2.2555 | 0.0242 | -0.23037036 | count | 1 |
| FNBP1 | -0.1636972 | 0.0736968 | -2.2212 | 0.0264 | -0.230325544 | count | 1 |
| STYK1 | -0.3067736 | 0.507126 | -0.6049 | 0.545 | -0.230306018 | count | 1 |
| COL4A3BP | -0.1781867 | 0.1589964 | -1.1207 | 0.262 | -0.230257476 | count | 1 |
| CALM3 | -0.1618488 | 0.0700997 | -2.3088 | 0.021 | -0.230177358 | count | 1 |
| MRPS22 | -0.1723097 | 0.1302543 | -1.3229 | 0.186 | -0.230052733 | count | 1 |
| PCOLCE | -0.2417371 | 0.4491452 | -0.5382 | 0.59 | -0.229936808 | count | 1 |
| AKT3 | -0.1722602 | 0.1539072 | -1.1192 | 0.263 | -0.229789975 | count | 1 |
| ANO10 | -0.3281218 | 0.3391914 | -0.9674 | 0.333 | -0.229789443 | count | 1 |
| SLAIN1 | -0.2556751 | 0.2726219 | -0.9378 | 0.348 | -0.22977595 | count | 1 |
| NCK2 | -0.1660148 | 0.078963 | -2.1024 | 0.0356 | -0.229769923 | count | 1 |
| POLR3G | -0.8555476 | 0.7219962 | -1.185 | 0.236 | -0.229736039 | count | 1 |
| TPH1 | -0.8555476 | 0.7498446 | -1.141 | 0.254 | -0.229736039 | count | 1 |
| AC231981.1 | -0.2646572 | 0.4996731 | -0.5297 | 0.596 | -0.229725159 | count | 1 |
| MBD2 | -0.1631461 | 0.0752804 | -2.1672 | 0.0303 | -0.229709359 | count | 1 |
| PLEKHB1 | -0.1849258 | 0.2292758 | -0.8066 | 0.42 | -0.229648019 | count | 1 |
| PDE12 | -0.1906576 | 0.1997038 | -0.9547 | 0.34 | -0.229598386 | count | 1 |
| LMTK2 | -0.2225347 | 0.2980752 | -0.7466 | 0.455 | -0.229375547 | count | 1 |
| ARNTL2 | -0.8533108 | 0.9076949 | -0.9401 | 0.347 | -0.22927386 | count | 1 |
| DGKE | -0.1779909 | 0.1534522 | -1.1599 | 0.246 | -0.22919579 | count | 1 |
| RMND1 | -0.1870891 | 0.2417806 | -0.7738 | 0.439 | -0.229123026 | count | 1 |
| ZNF140 | -0.1942216 | 0.2321773 | -0.8365 | 0.403 | -0.229113444 | count | 1 |
| WWP1 | -0.1710073 | 0.1201955 | -1.4227 | 0.155 | -0.229062781 | count | 1 |
| ADAT2 | -0.4655383 | 0.3575272 | -1.3021 | 0.193 | -0.228943913 | count | 1 |
| KNOP1 | -0.1721942 | 0.1864357 | -0.9236 | 0.356 | -0.228871195 | count | 1 |

| | | | | | | | |
|---|---|---|---|---|---|---|---|
| HIVEP1 | -0.2348711 | 0.1884468 | -1.2464 | 0.213 | -0.228747568 | count | 1 |
| TKFC | -0.2469195 | 0.3281621 | -0.7524 | 0.452 | -0.228746264 | count | 1 |
| POU2F1 | -0.2634749 | 0.31524 | -0.8358 | 0.403 | -0.2287318 | count | 1 |
| FZD7 | -0.3562651 | 0.8137765 | -0.4378 | 0.662 | -0.228728019 | count | 1 |
| MLH1 | -0.1860599 | 0.2100887 | -0.8856 | 0.376 | -0.228715695 | count | 1 |
| ZRSR2 | -0.1702258 | 0.1378875 | -1.2345 | 0.217 | -0.228701193 | count | 1 |
| UBR1 | -0.1961144 | 0.1740969 | -1.1265 | 0.26 | -0.228544394 | count | 1 |
| WBP2NL | -0.5816623 | 0.8485259 | -0.6855 | 0.493 | -0.228511323 | count | 1 |
| SPAG5 | -2.4670905 | 1.277334 | -1.9314 | 0.0535 | -0.22841465 | count | 1 |
| EMC6 | -0.1622845 | 0.0927969 | -1.7488 | 0.0804 | -0.228360374 | count | 1 |
| ZNF317 | -0.2627477 | 0.3602098 | -0.7294 | 0.466 | -0.228120644 | count | 1 |
| NOL10 | -0.1862316 | 0.2139351 | -0.8705 | 0.384 | -0.228081718 | count | 1 |
| ALKBH8 | -0.234101 | 0.2875352 | -0.8142 | 0.416 | -0.228014835 | count | 1 |
| UBE2M | -0.1606897 | 0.0683586 | -2.3507 | 0.0188 | -0.227761352 | count | 1 |
| PARP3 | -0.2393692 | 0.3112147 | -0.7691 | 0.442 | -0.227740254 | count | 1 |
| VPS29 | -0.1605975 | 0.0709743 | -2.2628 | 0.0237 | -0.227690361 | count | 1 |
| AMZ2 | -0.1705389 | 0.1223598 | -1.3938 | 0.163 | -0.227502609 | count | 1 |
| ZMYND8 | -0.1735473 | 0.1427579 | -1.2157 | 0.224 | -0.227338813 | count | 1 |
| DERL1 | -0.1637913 | 0.0968355 | -1.6914 | 0.0908 | -0.227317831 | count | 1 |
| RRAGD | -0.5780958 | 0.429559 | -1.3458 | 0.178 | -0.227300724 | count | 1 |
| AC104532.2 | -0.4617901 | 0.455001 | -1.0149 | 0.31 | -0.22727887 | count | 1 |
| NFYC | -0.1729701 | 0.1624046 | -1.0651 | 0.287 | -0.227166685 | count | 1 |
| ZNF32 | -0.1674675 | 0.1466145 | -1.1422 | 0.253 | -0.227116068 | count | 1 |
| IGHG4 | -0.4614003 | 0.719455 | -0.6413 | 0.521 | -0.227105539 | count | 1 |
| CBLL1 | -0.1765744 | 0.1433837 | -1.2315 | 0.218 | -0.226961933 | count | 1 |
| SLC35A1 | -0.2084805 | 0.2705013 | -0.7707 | 0.441 | -0.226884816 | count | 1 |
| GID4 | -0.2167013 | 0.3417396 | -0.6341 | 0.526 | -0.226877824 | count | 1 |
| MRPS5 | -0.1626046 | 0.1043443 | -1.5583 | 0.119 | -0.226875895 | count | 1 |
| AC008915.2 | -0.8417519 | 0.8212499 | -1.025 | 0.305 | -0.22687433 | count | 1 |
| AC130343.2 | -0.8415051 | 0.7245483 | -1.1614 | 0.246 | -0.226822883 | count | 1 |
| UBE2S | -0.1584742 | 0.0530585 | -2.9868 | 0.00284 | -0.226786695 | count | 1 |
| CHD1 | -0.1607106 | 0.0729353 | -2.2035 | 0.0276 | -0.226655507 | count | 1 |
| CMTM3 | -0.1605624 | 0.0836491 | -1.9195 | 0.055 | -0.226637345 | count | 1 |
| PGLS | -0.1593798 | 0.0703679 | -2.2649 | 0.0236 | -0.226517034 | count | 1 |
| C1orf35 | -0.1628961 | 0.1060134 | -1.5366 | 0.124 | -0.226401516 | count | 1 |
| DYNC1I2 | -0.1648235 | 0.1138354 | -1.4479 | 0.148 | -0.226350194 | count | 1 |
| ARHGAP11B | -0.3520447 | 0.6264069 | -0.562 | 0.574 | -0.226183236 | count | 1 |
| AC008608.2 | -0.5747887 | 0.5316848 | -1.0811 | 0.28 | -0.226176007 | count | 1 |
| MCRIP1 | -0.16437 | 0.1026764 | -1.6009 | 0.11 | -0.22617594 | count | 1 |
| PPP6R1 | -0.1699807 | 0.1387234 | -1.2253 | 0.221 | -0.22594086 | count | 1 |
| DHRS13 | -0.227096 | 0.3091101 | -0.7347 | 0.463 | -0.225920643 | count | 1 |
| TTC31 | -0.2708205 | 0.3441426 | -0.7869 | 0.431 | -0.225903406 | count | 1 |
| SCART1 | -0.5738186 | 1.0302694 | -0.557 | 0.578 | -0.225845698 | count | 1 |
| TRMT2A | -0.1807092 | 0.1908127 | -0.947 | 0.344 | -0.225816041 | count | 1 |
| CNOT6 | -0.209945 | 0.2524505 | -0.8316 | 0.406 | -0.225813215 | count | 1 |

| | | | | | | | |
|---|---|---|---|---|---|---|---|
| PPP2R2A | -0.1638819 | 0.0994194 | -1.6484 | 0.0994 | -0.225784025 | count | 1 |
| RNASEH1-AS1 | -0.1979009 | 0.297332 | -0.6656 | 0.506 | -0.225780611 | count | 1 |
| ORMDL2 | -0.1611776 | 0.1066369 | -1.5115 | 0.131 | -0.225740334 | count | 1 |
| ELMSAN1 | -0.1689128 | 0.1296895 | -1.3024 | 0.193 | -0.225723432 | count | 1 |
| GMPR | -0.3927534 | 0.9962528 | -0.3942 | 0.693 | -0.225482612 | count | 1 |
| TUSC2 | -0.1638048 | 0.122552 | -1.3366 | 0.181 | -0.225399965 | count | 1 |
| LRRC59 | -0.1889624 | 0.1562875 | -1.2091 | 0.227 | -0.225386621 | count | 1 |
| NUS1 | -0.177133 | 0.1953631 | -0.9067 | 0.365 | -0.225357555 | count | 1 |
| PSPN | -0.5720769 | 0.8148975 | -0.702 | 0.483 | -0.2252522 | count | 1 |
| LSM4 | -0.1597448 | 0.0898859 | -1.7772 | 0.0756 | -0.225228402 | count | 1 |
| CREB1 | -0.1697542 | 0.1351355 | -1.2562 | 0.209 | -0.225203707 | count | 1 |
| TUBG2 | -0.8334557 | 0.7532771 | -1.1064 | 0.269 | -0.225140529 | count | 1 |
| TCF19 | -0.3919769 | 0.4674807 | -0.8385 | 0.402 | -0.225069786 | count | 1 |
| STOML2 | -0.1631529 | 0.1167255 | -1.3977 | 0.162 | -0.225045019 | count | 1 |
| SLC25A33 | -0.1918091 | 0.1708156 | -1.1229 | 0.262 | -0.224975564 | count | 1 |
| STX11 | -0.1641053 | 0.1213405 | -1.3524 | 0.176 | -0.22496549 | count | 1 |
| CCDC134 | -0.2695268 | 0.4139534 | -0.6511 | 0.515 | -0.224861733 | count | 1 |
| LDLR | -0.1850035 | 0.1880626 | -0.9837 | 0.325 | -0.224803813 | count | 1 |
| IDH3G | -0.1608576 | 0.0962803 | -1.6707 | 0.0949 | -0.224682581 | count | 1 |
| GABBR1 | -0.3494913 | 0.4514915 | -0.7741 | 0.439 | -0.224641554 | count | 1 |
| FAM192A | -0.1686368 | 0.1469723 | -1.1474 | 0.251 | -0.224577 | count | 1 |
| NUP133 | -0.320258 | 0.3486073 | -0.9187 | 0.358 | -0.224562965 | count | 1 |
| RLF | -0.1777525 | 0.1290731 | -1.3771 | 0.169 | -0.22454924 | count | 1 |
| IFIT5 | -0.1953045 | 0.2332355 | -0.8374 | 0.402 | -0.224530413 | count | 1 |
| NDUFV1 | -0.1645199 | 0.1136261 | -1.4479 | 0.148 | -0.224342176 | count | 1 |
| AC007950.2 | -0.2688416 | 0.7895393 | -0.3405 | 0.733 | -0.22430986 | count | 1 |
| NUDT3 | -0.2983887 | 0.2723411 | -1.0956 | 0.273 | -0.224288474 | count | 1 |
| F2RL1 | -0.281878 | 0.578002 | -0.4877 | 0.626 | -0.224276834 | count | 1 |
| ABHD15 | -0.2687848 | 0.2875395 | -0.9348 | 0.35 | -0.224264111 | count | 1 |
| RIC3 | -0.1645459 | 0.1504894 | -1.0934 | 0.274 | -0.224067947 | count | 1 |
| RFC4 | -0.1787422 | 0.2382637 | -0.7502 | 0.453 | -0.224012979 | count | 1 |
| ECHDC1 | -0.1630039 | 0.1139601 | -1.4304 | 0.153 | -0.224008874 | count | 1 |
| TMEM147 | -0.161805 | 0.1004138 | -1.6114 | 0.107 | -0.223900043 | count | 1 |
| TRIM47 | -0.2683008 | 0.330109 | -0.8128 | 0.416 | -0.22387421 | count | 1 |
| MTMR8 | -0.389641 | 0.6424477 | -0.6065 | 0.544 | -0.223827053 | count | 1 |
| CPSF7 | -0.1885985 | 0.2269655 | -0.831 | 0.406 | -0.22378263 | count | 1 |
| CEP290 | -0.1713992 | 0.1577505 | -1.0865 | 0.277 | -0.223617586 | count | 1 |
| MYH9 | -0.1584354 | 0.0737546 | -2.1481 | 0.0318 | -0.223530142 | count | 1 |
| AC010864.1 | -0.3890344 | 0.6588932 | -0.5904 | 0.555 | -0.223504139 | count | 1 |
| SLC25A5 | -0.1558477 | 0.0433613 | -3.5942 | 0.00033 | -0.22346094 | count | 1 |
| RGS1 | -0.1569775 | 0.0668651 | -2.3477 | 0.0189 | -0.223442931 | count | 1 |
| GALNT6 | -0.1915799 | 0.2335646 | -0.8202 | 0.412 | -0.223318357 | count | 1 |
| TNFRSF1B | -0.1600828 | 0.0817152 | -1.959 | 0.0502 | -0.223219033 | count | 1 |
| TRBV27 | -0.824098 | 0.9054 | -0.9102 | 0.363 | -0.223173276 | count | 1 |
| TTC39C-AS1 | -0.29674 | 0.2643685 | -1.1224 | 0.262 | -0.223103309 | count | 1 |

| | | | | | | | |
|---|---|---|---|---|---|---|---|
| RANBP2 | -0.1725222 | 0.126299 | -1.366 | 0.172 | -0.222976925 | count | 1 |
| NEO1 | -0.24788 | 0.4874393 | -0.5085 | 0.611 | -0.222969549 | count | 1 |
| YWHAB | -0.1549335 | 0.0348815 | -4.4417 | 9.21E-06 | -0.222799373 | count | 0.2214084 |
| PLOD1 | -0.2476284 | 0.5668415 | -0.4369 | 0.662 | -0.22274964 | count | 1 |
| NSF | -0.2069457 | 0.1963517 | -1.054 | 0.292 | -0.222638276 | count | 1 |
| XPNPEP1 | -0.1797641 | 0.2083825 | -0.8627 | 0.388 | -0.222566514 | count | 1 |
| H6PD | -0.1983512 | 0.3397357 | -0.5838 | 0.559 | -0.222564776 | count | 1 |
| DNAJC30 | -0.1950077 | 0.2720092 | -0.7169 | 0.473 | -0.222520172 | count | 1 |
| PPP2R1B | -0.1965619 | 0.2494149 | -0.7881 | 0.431 | -0.22248319 | count | 1 |
| BCL9L | -0.1759476 | 0.1851677 | -0.9502 | 0.342 | -0.222284031 | count | 1 |
| TOP1MT | -0.2041299 | 0.3181438 | -0.6416 | 0.521 | -0.222221477 | count | 1 |
| TBCCD1 | -0.2191405 | 0.3704919 | -0.5915 | 0.554 | -0.22221648 | count | 1 |
| ZSCAN22 | -0.4503192 | 0.5608783 | -0.8029 | 0.422 | -0.222164308 | count | 1 |
| HELZ | -0.1636975 | 0.1204442 | -1.3591 | 0.174 | -0.22213698 | count | 1 |
| 11-Sep | -0.1626805 | 0.111756 | -1.4557 | 0.146 | -0.22213261 | count | 1 |
| C6orf47 | -0.1771779 | 0.1746167 | -1.0147 | 0.31 | -0.222066075 | count | 1 |
| 2-Sep | -0.1599057 | 0.0953139 | -1.6777 | 0.0935 | -0.222028083 | count | 1 |
| LINC01934 | -0.1771364 | 0.1773986 | -0.9985 | 0.318 | -0.222014422 | count | 1 |
| HSPH1 | -0.1632516 | 0.103688 | -1.5744 | 0.115 | -0.221877374 | count | 1 |
| KMT5B | -0.1710758 | 0.160661 | -1.0648 | 0.287 | -0.221850421 | count | 1 |
| MIR194-2HG | -2.254688 | 1.268113 | -1.778 | 0.0755 | -0.221725261 | count | 1 |
| C16orf91 | -0.1651211 | 0.1506997 | -1.0957 | 0.273 | -0.221705906 | count | 1 |
| ANKRD17 | -0.177913 | 0.1785382 | -0.9965 | 0.319 | -0.221688043 | count | 1 |
| AZI2 | -0.1667757 | 0.1535168 | -1.0864 | 0.277 | -0.221485102 | count | 1 |
| CEP97 | -0.2326326 | 0.2618103 | -0.8886 | 0.374 | -0.221484607 | count | 1 |
| MPHOSPH6 | -0.1699943 | 0.1599554 | -1.0628 | 0.288 | -0.22147266 | count | 1 |
| SND1 | -0.1937498 | 0.1782378 | -1.087 | 0.277 | -0.221102198 | count | 1 |
| GPR137B | -0.174063 | 0.1760875 | -0.9885 | 0.323 | -0.220974159 | count | 1 |
| PAXBP1 | -0.1606662 | 0.1051094 | -1.5286 | 0.126 | -0.22094901 | count | 1 |
| PEF1 | -0.164886 | 0.135819 | -1.214 | 0.225 | -0.220892104 | count | 1 |
| CCDC15 | -0.3430247 | 0.5220418 | -0.6571 | 0.511 | -0.220730267 | count | 1 |
| EIF3A | -0.1593891 | 0.0803356 | -1.984 | 0.0473 | -0.220670683 | count | 1 |
| PHACTR2 | -0.1597448 | 0.0990254 | -1.6132 | 0.107 | -0.220658181 | count | 1 |
| PDHA1 | -0.164074 | 0.1305337 | -1.2569 | 0.209 | -0.220463845 | count | 1 |
| MRM1 | -0.2767669 | 0.6636412 | -0.417 | 0.677 | -0.220364445 | count | 1 |
| A1BG | -0.1592295 | 0.1153506 | -1.3804 | 0.168 | -0.219888622 | count | 1 |
| PSENEN | -0.1567558 | 0.0888242 | -1.7648 | 0.0777 | -0.219867188 | count | 1 |
| RASA2 | -0.1592513 | 0.0916367 | -1.7379 | 0.0823 | -0.219858741 | count | 1 |
| GGA2 | -0.1695047 | 0.1607868 | -1.0542 | 0.292 | -0.219823403 | count | 1 |
| NLN | -0.8081706 | 0.9736325 | -0.8301 | 0.407 | -0.219796465 | count | 1 |
| Z99774.1 | -0.8081706 | 1.1144122 | -0.7252 | 0.468 | -0.219796465 | count | 1 |
| OLFM2 | -0.2205425 | 0.4360245 | -0.5058 | 0.613 | -0.219535649 | count | 1 |
| LEPR | -0.8064097 | 0.5996101 | -1.3449 | 0.179 | -0.219421015 | count | 1 |
| CD83 | -0.16585 | 0.1491636 | -1.1119 | 0.266 | -0.219366906 | count | 1 |
| GCLM | -0.1606912 | 0.1281035 | -1.2544 | 0.21 | -0.219328604 | count | 1 |

| | | | | | | | |
|---|---|---|---|---|---|---|---|
| TSHZ3 | -0.5546705 | 0.696616 | -0.7962 | 0.426 | -0.219289206 | count | 1 |
| SET | -0.1529247 | 0.0401576 | -3.8081 | 0.000143 | -0.219265078 | count | 1 |
| ELOB | -0.1530117 | 0.0449984 | -3.4004 | 0.000681 | -0.219059174 | count | 1 |
| LINC00685 | -0.2361102 | 0.3116934 | -0.7575 | 0.449 | -0.218988849 | count | 1 |
| LINC01806 | -0.3401086 | 0.5548465 | -0.613 | 0.54 | -0.218963263 | count | 1 |
| ZBTB5 | -0.3401086 | 0.566062 | -0.6008 | 0.548 | -0.218963263 | count | 1 |
| MT-ND1 | -0.1521446 | 0.0289991 | -5.2465 | 1.64E-07 | -0.218932694 | count | 0.003958304 |
| SOCS4 | -0.1670936 | 0.1488153 | -1.1228 | 0.262 | -0.218923404 | count | 1 |
| ATAT1 | -0.2199073 | 0.4498831 | -0.4888 | 0.625 | -0.218916322 | count | 1 |
| CASC3 | -0.1903228 | 0.1868089 | -1.0188 | 0.308 | -0.218869432 | count | 1 |
| ETV2 | -0.2620505 | 0.4519197 | -0.5799 | 0.562 | -0.218834322 | count | 1 |
| IFI27L2 | -0.1557847 | 0.0966309 | -1.6122 | 0.107 | -0.218457325 | count | 1 |
| FSD1L | -0.2901811 | 0.4575 | -0.6343 | 0.526 | -0.218381967 | count | 1 |
| NDUFA6 | -0.1541632 | 0.0672619 | -2.292 | 0.022 | -0.218314884 | count | 1 |
| ADGRE2 | -0.2900118 | 0.5610865 | -0.5169 | 0.605 | -0.218259969 | count | 1 |
| TUBB2A | -0.156333 | 0.0924298 | -1.6914 | 0.0909 | -0.2182069 | count | 1 |
| GTF3C6 | -0.1543419 | 0.0821593 | -1.8786 | 0.0604 | -0.21806278 | count | 1 |
| SARAF | -0.1517437 | 0.0302917 | -5.0094 | 5.74E-07 | -0.218039615 | count | 0.01384201 |
| SIRT3 | -0.202497 | 0.2960921 | -0.6839 | 0.494 | -0.217926114 | count | 1 |
| NUMA1 | -0.1642404 | 0.1452102 | -1.1311 | 0.258 | -0.217917531 | count | 1 |
| TXNDC11 | -0.1846044 | 0.194253 | -0.9503 | 0.342 | -0.21788261 | count | 1 |
| GPSM1 | -0.7990498 | 0.8015255 | -0.9969 | 0.319 | -0.217846563 | count | 1 |
| ZNF586 | -0.1908538 | 0.2798715 | -0.6819 | 0.495 | -0.21783674 | count | 1 |
| REV3L | -0.1769767 | 0.1441602 | -1.2276 | 0.22 | -0.217637244 | count | 1 |
| GLRX3 | -0.1602288 | 0.1131331 | -1.4163 | 0.157 | -0.217557479 | count | 1 |
| ZBTB11-AS1 | -0.2888997 | 0.342132 | -0.8444 | 0.398 | -0.217458397 | count | 1 |
| KIAA0232 | -0.1612709 | 0.1444601 | -1.1164 | 0.264 | -0.217447809 | count | 1 |
| PSMB6 | -0.1526344 | 0.062527 | -2.4411 | 0.0147 | -0.217230869 | count | 1 |
| CEP162 | -0.210454 | 0.2742791 | -0.7673 | 0.443 | -0.217148643 | count | 1 |
| NBPF20 | -0.7953264 | 0.8394074 | -0.9475 | 0.343 | -0.217047162 | count | 1 |
| GATM | -0.7953264 | 0.8943079 | -0.8893 | 0.374 | -0.217047162 | count | 1 |
| CD34 | -0.7953264 | 0.91445 | -0.8697 | 0.385 | -0.217047162 | count | 1 |
| IL11RA | -0.22775 | 0.4742418 | -0.4802 | 0.631 | -0.21694458 | count | 1 |
| MNDA | -0.2015206 | 0.5026709 | -0.4009 | 0.689 | -0.21689142 | count | 1 |
| ATP9B | -0.1871567 | 0.198413 | -0.9433 | 0.346 | -0.216784486 | count | 1 |
| SCRN3 | -0.1930842 | 0.3136766 | -0.6156 | 0.538 | -0.216730629 | count | 1 |
| ARFGAP3 | -0.1577885 | 0.105048 | -1.5021 | 0.133 | -0.216710839 | count | 1 |
| AHDC1 | -0.2490005 | 0.3401527 | -0.732 | 0.464 | -0.216544732 | count | 1 |
| ROCK1 | -0.1534769 | 0.0761926 | -2.0143 | 0.0441 | -0.216301503 | count | 1 |
| SSU72 | -0.1525882 | 0.0659634 | -2.3132 | 0.0208 | -0.216229198 | count | 1 |
| IFNAR1 | -0.1594024 | 0.1177786 | -1.3534 | 0.176 | -0.216209334 | count | 1 |
| GTPBP2 | -0.2869893 | 0.4367387 | -0.6571 | 0.511 | -0.216080739 | count | 1 |
| STK3 | -0.2869893 | 0.4414241 | -0.6501 | 0.516 | -0.216080728 | count | 1 |
| KCNAB3 | -0.5452441 | 0.9706553 | -0.5617 | 0.574 | -0.216035911 | count | 1 |
| SUMF2 | -0.1564223 | 0.1072084 | -1.459 | 0.145 | -0.21590133 | count | 1 |

| | | | | | | | |
|---|---|---|---|---|---|---|---|
| AC007686.3 | -0.1817167 | 0.2687815 | -0.6761 | 0.499 | -0.215695515 | count | 1 |
| DOPEY2 | -0.1920729 | 0.2833159 | -0.6779 | 0.498 | -0.215609925 | count | 1 |
| CHD7 | -0.2003034 | 0.1927315 | -1.0393 | 0.299 | -0.215601313 | count | 1 |
| TUT7 | -0.1582031 | 0.1251782 | -1.2638 | 0.206 | -0.215351216 | count | 1 |
| COQ6 | -0.183467 | 0.2475015 | -0.7413 | 0.459 | -0.215291132 | count | 1 |
| MSC-AS1 | -0.5430689 | 0.4544922 | -1.1949 | 0.232 | -0.215282793 | count | 1 |
| MGMT | -0.154147 | 0.0981417 | -1.5707 | 0.116 | -0.215024563 | count | 1 |
| SUPT3H | -0.1708826 | 0.2167221 | -0.7885 | 0.43 | -0.21481465 | count | 1 |
| MADCAM1 | -0.7849562 | 0.5189064 | -1.5127 | 0.13 | -0.214810375 | count | 1 |
| MRPS12 | -0.1536481 | 0.1048335 | -1.4656 | 0.143 | -0.214751632 | count | 1 |
| GPBP1L1 | -0.1650462 | 0.1323341 | -1.2472 | 0.212 | -0.214736938 | count | 1 |
| SMURF2 | -0.163201 | 0.1602186 | -1.0186 | 0.308 | -0.214655272 | count | 1 |
| GSK3A | -0.1599572 | 0.1366336 | -1.1707 | 0.242 | -0.214637132 | count | 1 |
| C7orf57 | -2.065723 | 1.576489 | -1.3103 | 0.19 | -0.2145681 | count | 1 |
| ZNHIT3 | -0.153863 | 0.0854996 | -1.7996 | 0.072 | -0.214559368 | count | 1 |
| POSTN | -2.06357 | 0.8298213 | -2.4868 | 0.0129 | -0.214479197 | count | 1 |
| C12orf45 | -0.1627159 | 0.1475077 | -1.1031 | 0.27 | -0.214275593 | count | 1 |
| UBE3B | -0.2248457 | 0.3410136 | -0.6593 | 0.51 | -0.214241672 | count | 1 |
| PRSS35 | -0.332245 | 0.9754935 | -0.3406 | 0.733 | -0.214188359 | count | 1 |
| SMC5 | -0.1592545 | 0.0962679 | -1.6543 | 0.0982 | -0.214160199 | count | 1 |
| MT-ND5 | -0.1489866 | 0.0320562 | -4.6477 | 3.48E-06 | -0.21412216 | count | 0.08375664 |
| EDEM3 | -0.1776363 | 0.1988095 | -0.8935 | 0.372 | -0.214054774 | count | 1 |
| PMM1 | -0.1605968 | 0.1576636 | -1.0186 | 0.308 | -0.213909157 | count | 1 |
| ZNF784 | -0.4318915 | 0.6143639 | -0.703 | 0.482 | -0.213888438 | count | 1 |
| MAP10 | -0.4318915 | 0.7125287 | -0.6061 | 0.544 | -0.213888438 | count | 1 |
| SNRPF | -0.1522406 | 0.0700134 | -2.1745 | 0.0297 | -0.21388181 | count | 1 |
| ZNF846 | -0.1904675 | 0.2559094 | -0.7443 | 0.457 | -0.213830504 | count | 1 |
| SENP1 | -0.1661607 | 0.2257784 | -0.7359 | 0.462 | -0.213648571 | count | 1 |
| CYB5A | -0.1570072 | 0.1304695 | -1.2034 | 0.229 | -0.213624529 | count | 1 |
| SCAND1 | -0.1499424 | 0.0645916 | -2.3214 | 0.0203 | -0.213565506 | count | 1 |
| KANSL3 | -0.1938654 | 0.2279166 | -0.8506 | 0.395 | -0.213477462 | count | 1 |
| UTP3 | -0.1619165 | 0.1476779 | -1.0964 | 0.273 | -0.213475433 | count | 1 |
| TRMT1 | -0.1770985 | 0.2278182 | -0.7774 | 0.437 | -0.21341233 | count | 1 |
| BORCS6 | -0.1569158 | 0.1264564 | -1.2409 | 0.215 | -0.213395835 | count | 1 |
| GBP1 | -0.1515486 | 0.1081095 | -1.4018 | 0.161 | -0.213325435 | count | 1 |
| DHX38 | -0.1683501 | 0.1772393 | -0.9498 | 0.342 | -0.213263967 | count | 1 |
| HEXDC | -0.1574705 | 0.1475773 | -1.067 | 0.286 | -0.213242068 | count | 1 |
| CFLAR | -0.1520625 | 0.0809277 | -1.879 | 0.0603 | -0.213170327 | count | 1 |
| ING1 | -0.155216 | 0.1031255 | -1.5051 | 0.132 | -0.213111922 | count | 1 |
| 7-Mar | -0.1546203 | 0.0954606 | -1.6197 | 0.105 | -0.213050375 | count | 1 |
| CS | -0.2003779 | 0.2503217 | -0.8005 | 0.423 | -0.212978101 | count | 1 |
| STAMBPL1 | -0.1649212 | 0.1688847 | -0.9765 | 0.329 | -0.212834427 | count | 1 |
| RTF1 | -0.1508838 | 0.0709261 | -2.1273 | 0.0335 | -0.212825709 | count | 1 |
| SCYL2 | -0.1671972 | 0.2153499 | -0.7764 | 0.438 | -0.212790844 | count | 1 |
| PDSS2 | -0.1849172 | 0.2594865 | -0.7126 | 0.476 | -0.212722514 | count | 1 |

| | | | | | | | |
|---|---|---|---|---|---|---|---|
| BZW1 | -0.1488461 | 0.0502427 | -2.9625 | 0.0031 | -0.212659355 | count | 1 |
| SUFU | -0.3023516 | 0.3729217 | -0.8108 | 0.418 | -0.212606978 | count | 1 |
| NSFL1C | -0.155521 | 0.1246411 | -1.2478 | 0.212 | -0.212559551 | count | 1 |
| ZNF696 | -0.5348426 | 0.589065 | -0.908 | 0.364 | -0.212426496 | count | 1 |
| SUGCT | -0.5348426 | 0.7405729 | -0.7222 | 0.47 | -0.212426496 | count | 1 |
| CHD6 | -0.1607455 | 0.1207601 | -1.3311 | 0.183 | -0.212412862 | count | 1 |
| GPR183 | -0.1483898 | 0.0575924 | -2.5766 | 0.01 | -0.212368194 | count | 1 |
| HNRNPA1L2 | -0.7735684 | 0.6875724 | -1.1251 | 0.261 | -0.212336296 | count | 1 |
| POLR1A | -0.2538519 | 0.4883357 | -0.5198 | 0.603 | -0.212209781 | count | 1 |
| HNRNPLL | -0.1597373 | 0.1301626 | -1.2272 | 0.22 | -0.212172926 | count | 1 |
| NAP1L4 | -0.1511699 | 0.0740646 | -2.0411 | 0.0413 | -0.212089316 | count | 1 |
| MKRN2 | -0.1776773 | 0.1995094 | -0.8906 | 0.373 | -0.212049551 | count | 1 |
| ATF7IP2 | -0.1585522 | 0.1305193 | -1.2148 | 0.225 | -0.211926152 | count | 1 |
| AL354733.3 | -0.3012406 | 0.4101192 | -0.7345 | 0.463 | -0.211862638 | count | 1 |
| C11orf24 | -0.2656539 | 0.3979619 | -0.6675 | 0.504 | -0.211836679 | count | 1 |
| NUDT5 | -0.1511141 | 0.1020784 | -1.4804 | 0.139 | -0.21181761 | count | 1 |
| NRBP1 | -0.1521489 | 0.0915007 | -1.6628 | 0.0964 | -0.211691438 | count | 1 |
| ZCCHC2 | -0.1608882 | 0.1486555 | -1.0823 | 0.279 | -0.211626062 | count | 1 |
| NOP58 | -0.1505396 | 0.073162 | -2.0576 | 0.0397 | -0.211557584 | count | 1 |
| PREX1 | -0.1565182 | 0.127325 | -1.2293 | 0.219 | -0.2114573 | count | 1 |
| SPG11 | -0.1790551 | 0.1901468 | -0.9417 | 0.346 | -0.21139636 | count | 1 |
| PQBP1 | -0.1533665 | 0.0986445 | -1.5547 | 0.12 | -0.21132628 | count | 1 |
| SSBP2 | -0.1671836 | 0.1603997 | -1.0423 | 0.297 | -0.211280237 | count | 1 |
| FTX | -0.1685146 | 0.1687312 | -0.9987 | 0.318 | -0.211279157 | count | 1 |
| SOWAHD | -0.4260123 | 0.7227132 | -0.5895 | 0.556 | -0.211232696 | count | 1 |
| SRP68 | -0.2344709 | 0.1970652 | -1.1898 | 0.234 | -0.211229691 | count | 1 |
| PMF1 | -0.1504469 | 0.0894004 | -1.6828 | 0.0925 | -0.21119059 | count | 1 |
| RABEP1 | -0.1613539 | 0.142935 | -1.1289 | 0.259 | -0.211155715 | count | 1 |
| AP5M1 | -0.16573 | 0.1873273 | -0.8847 | 0.376 | -0.210934334 | count | 1 |
| MCM9 | -0.2161543 | 0.3202472 | -0.675 | 0.5 | -0.210904268 | count | 1 |
| TAX1BP1 | -0.1478362 | 0.0536269 | -2.7568 | 0.0059 | -0.210837008 | count | 1 |
| NOL7 | -0.148205 | 0.0637358 | -2.3253 | 0.0201 | -0.210791645 | count | 1 |
| PUM3 | -0.1524445 | 0.1035422 | -1.4723 | 0.141 | -0.210704575 | count | 1 |
| ARMT1 | -0.1706661 | 0.2033855 | -0.8391 | 0.401 | -0.210673711 | count | 1 |
| EDF1 | -0.1470777 | 0.0434752 | -3.383 | 0.000725 | -0.210642122 | count | 1 |
| ZXDB | -0.263974 | 0.3959432 | -0.6667 | 0.505 | -0.210545079 | count | 1 |
| TYW1B | -0.3647259 | 0.4171749 | -0.8743 | 0.382 | -0.210494034 | count | 1 |
| DSN1 | -0.2266941 | 0.3615824 | -0.6269 | 0.531 | -0.210468469 | count | 1 |
| AC010331.1 | -0.7646957 | 0.8576002 | -0.8917 | 0.373 | -0.210395842 | count | 1 |
| DMAP1 | -0.1569006 | 0.1453745 | -1.0793 | 0.281 | -0.210390945 | count | 1 |
| CYTIP | -0.1466102 | 0.0344304 | -4.2582 | 2.12E-05 | -0.210381757 | count | 0.5089484 |
| ZNF789 | -0.1930852 | 0.2954278 | -0.6536 | 0.513 | -0.210368086 | count | 1 |
| PEMT | -0.1857161 | 0.2498435 | -0.7433 | 0.457 | -0.21035353 | count | 1 |
| BUD13 | -0.1628215 | 0.1924408 | -0.8461 | 0.398 | -0.21013847 | count | 1 |
| ATP5ME | -0.1480999 | 0.0677455 | -2.1861 | 0.0289 | -0.210098304 | count | 1 |

| | | | | | | | |
|---|---|---|---|---|---|---|---|
| CABP4 | -0.7631373 | 0.8794723 | -0.8677 | 0.386 | -0.210053868 | count | 1 |
| CENPK | -0.1540474 | 0.1863557 | -0.8266 | 0.409 | -0.21000248 | count | 1 |
| ZNF222 | -0.1975327 | 0.2780001 | -0.7105 | 0.477 | -0.210000906 | count | 1 |
| MROH1 | -0.2631673 | 0.3251367 | -0.8094 | 0.418 | -0.209924602 | count | 1 |
| ASPSCR1 | -0.1593791 | 0.1821899 | -0.8748 | 0.382 | -0.2098995 | count | 1 |
| AC105446.1 | -0.2260467 | 0.4154094 | -0.5442 | 0.586 | -0.20988195 | count | 1 |
| KLHL22 | -0.1885124 | 0.3187206 | -0.5915 | 0.554 | -0.20972633 | count | 1 |
| FSTL1 | -0.7613355 | 0.896571 | -0.8492 | 0.396 | -0.209658027 | count | 1 |
| AC004889.1 | -0.7613355 | 0.99025 | -0.7688 | 0.442 | -0.209658027 | count | 1 |
| DNAJC21 | -0.1483748 | 0.0818579 | -1.8126 | 0.07 | -0.209639752 | count | 1 |
| ARL6 | -0.2197324 | 0.4925703 | -0.4461 | 0.656 | -0.209478636 | count | 1 |
| NUDT6 | -0.2777786 | 0.4966744 | -0.5593 | 0.576 | -0.209426447 | count | 1 |
| CRIM1 | -0.1773459 | 0.2544642 | -0.6969 | 0.486 | -0.20939772 | count | 1 |
| AMBRA1 | -0.262469 | 0.4163357 | -0.6304 | 0.528 | -0.209387392 | count | 1 |
| WASHC3 | -0.1500928 | 0.0988908 | -1.5178 | 0.129 | -0.20934374 | count | 1 |
| USP20 | -0.2100746 | 0.24053 | -0.8734 | 0.383 | -0.209319009 | count | 1 |
| KDELC2 | -0.1941925 | 0.3694052 | -0.5257 | 0.599 | -0.209120427 | count | 1 |
| CNOT8 | -0.1535037 | 0.1283726 | -1.1958 | 0.232 | -0.209069651 | count | 1 |
| DCAF17 | -0.180321 | 0.2627101 | -0.6864 | 0.493 | -0.208950297 | count | 1 |
| UBE2E1 | -0.1620894 | 0.1545667 | -1.0487 | 0.294 | -0.20882541 | count | 1 |
| ECT2 | -0.3616155 | 0.5907327 | -0.6121 | 0.54 | -0.208819582 | count | 1 |
| SUMO2 | -0.1453393 | 0.0348982 | -4.1647 | 3.20E-05 | -0.208798514 | count | 0.767488 |
| DSCC1 | -0.3615012 | 0.9183426 | -0.3936 | 0.694 | -0.208758019 | count | 1 |
| SPINDOC | -0.2094962 | 0.2903691 | -0.7215 | 0.471 | -0.208753855 | count | 1 |
| TLCD2 | -0.3232966 | 0.4537125 | -0.7126 | 0.476 | -0.20873704 | count | 1 |
| DUSP5 | -0.1472163 | 0.0725967 | -2.0279 | 0.0427 | -0.208715168 | count | 1 |
| FKBP2 | -0.1474498 | 0.0787906 | -1.8714 | 0.0614 | -0.208554118 | count | 1 |
| ETV5 | -0.2962313 | 0.6201917 | -0.4776 | 0.633 | -0.208503044 | count | 1 |
| CPM | -0.7560159 | 0.5088091 | -1.4859 | 0.137 | -0.208486739 | count | 1 |
| MAML2 | -0.1798787 | 0.1391362 | -1.2928 | 0.196 | -0.208443156 | count | 1 |
| SUPT5H | -0.1573222 | 0.1230683 | -1.2783 | 0.201 | -0.208349781 | count | 1 |
| RHEB | -0.1484368 | 0.0799311 | -1.8571 | 0.0634 | -0.208305069 | count | 1 |
| NDUFV2 | -0.1458022 | 0.0542487 | -2.6877 | 0.00723 | -0.208263539 | count | 1 |
| IQCB1 | -0.1579372 | 0.1509345 | -1.0464 | 0.295 | -0.208250064 | count | 1 |
| BECN1 | -0.151369 | 0.1284831 | -1.1781 | 0.239 | -0.208122253 | count | 1 |
| PROSER1 | -0.753427 | 0.3507299 | -2.1482 | 0.0318 | -0.207915156 | count | 1 |
| AC073195.1 | -0.5217482 | 0.763541 | -0.6833 | 0.494 | -0.207853393 | count | 1 |
| BRAP | -0.1834397 | 0.2546131 | -0.7205 | 0.471 | -0.207805319 | count | 1 |
| DNAJC10 | -0.1573763 | 0.1697326 | -0.9272 | 0.354 | -0.207748559 | count | 1 |
| VAMP2 | -0.1451755 | 0.0491173 | -2.9557 | 0.00314 | -0.207736635 | count | 1 |
| XRRA1 | -0.1708376 | 0.2676459 | -0.6383 | 0.523 | -0.207728061 | count | 1 |
| KIAA1324 | -0.2602043 | 0.4507096 | -0.5773 | 0.564 | -0.207644314 | count | 1 |
| RBM47 | -1.9078006 | 1.557642 | -1.2248 | 0.221 | -0.207571798 | count | 1 |
| NR2C2AP | -0.158795 | 0.1825803 | -0.8697 | 0.385 | -0.207538255 | count | 1 |
| PDZD11 | -0.1546151 | 0.1583987 | -0.9761 | 0.329 | -0.207491723 | count | 1 |

| | | | | | | | |
|---|---|---|---|---|---|---|---|
| TCF3 | -0.1778557 | 0.240291 | -0.7402 | 0.459 | -0.20748297 | count | 1 |
| SUGP1 | -0.1778087 | 0.2393159 | -0.743 | 0.458 | -0.207428693 | count | 1 |
| DYRK1B | -0.1978161 | 0.2207244 | -0.8962 | 0.37 | -0.207426892 | count | 1 |
| STX1A | -0.2232312 | 0.4063048 | -0.5494 | 0.583 | -0.207330188 | count | 1 |
| KIAA2013 | -0.1585773 | 0.1773553 | -0.8941 | 0.371 | -0.20725498 | count | 1 |
| AC006333.2 | -0.2943094 | 0.5712927 | -0.5152 | 0.606 | -0.207212515 | count | 1 |
| HABP4 | -0.1616425 | 0.1530949 | -1.0558 | 0.291 | -0.20706612 | count | 1 |
| CDK2AP2 | -0.145477 | 0.0668507 | -2.1761 | 0.0296 | -0.207028428 | count | 1 |
| AHSA1 | -0.1503187 | 0.1066796 | -1.4091 | 0.159 | -0.207009515 | count | 1 |
| ABLIM1 | -0.1670804 | 0.1566914 | -1.0663 | 0.286 | -0.206973051 | count | 1 |
| THYN1 | -0.148004 | 0.1036041 | -1.4286 | 0.153 | -0.206960786 | count | 1 |
| BAG3 | -0.1645749 | 0.1571889 | -1.047 | 0.295 | -0.206934939 | count | 1 |
| FAM215B | -0.2002851 | 0.2182811 | -0.9176 | 0.359 | -0.206835075 | count | 1 |
| TRIM13 | -0.1571464 | 0.1502304 | -1.046 | 0.296 | -0.206724368 | count | 1 |
| LINC00265 | -0.7478676 | 0.8252209 | -0.9063 | 0.365 | -0.206684575 | count | 1 |
| SLC39A8 | -0.170614 | 0.2206202 | -0.7733 | 0.439 | -0.206581116 | count | 1 |
| CKMT2-AS1 | -0.2735607 | 0.4381596 | -0.6243 | 0.532 | -0.206372556 | count | 1 |
| RABAC1 | -0.1441376 | 0.0529993 | -2.7196 | 0.00657 | -0.206353504 | count | 1 |
| E2F1 | -1.881407 | 0.957499 | -1.9649 | 0.0495 | -0.206303073 | count | 1 |
| DPP4 | -0.1578428 | 0.1573708 | -1.003 | 0.316 | -0.206299213 | count | 1 |
| HID1 | -0.51727 | 0.9425819 | -0.5488 | 0.583 | -0.206281957 | count | 1 |
| HFE | -0.51727 | 0.9650341 | -0.536 | 0.592 | -0.206281957 | count | 1 |
| DYNLL1 | -0.1439081 | 0.0476394 | -3.0208 | 0.00254 | -0.206268595 | count | 1 |
| ATP6AP2 | -0.1455514 | 0.0716671 | -2.0309 | 0.0423 | -0.206236759 | count | 1 |
| LINC00513 | -0.5169031 | 0.1978385 | -2.6128 | 0.00902 | -0.206153045 | count | 1 |
| DDX39A | -0.1484097 | 0.1113332 | -1.333 | 0.183 | -0.206134589 | count | 1 |
| NUBP1 | -0.153598 | 0.1627738 | -0.9436 | 0.345 | -0.206131092 | count | 1 |
| ENTPD3-AS1 | -0.2110924 | 0.2998766 | -0.7039 | 0.482 | -0.206066255 | count | 1 |
| GLT8D1 | -0.1560885 | 0.1601722 | -0.9745 | 0.33 | -0.206055206 | count | 1 |
| DNAJC27 | -0.1789857 | 0.2313165 | -0.7738 | 0.439 | -0.205972526 | count | 1 |
| SLC30A9 | -0.1662174 | 0.1931347 | -0.8606 | 0.39 | -0.205911414 | count | 1 |
| RAI1 | -0.3561262 | 0.4649187 | -0.766 | 0.444 | -0.205859115 | count | 1 |
| TTC9C | -0.161356 | 0.1767539 | -0.9129 | 0.361 | -0.205846907 | count | 1 |
| C5orf24 | -0.1546459 | 0.1517655 | -1.019 | 0.308 | -0.205822762 | count | 1 |
| TXNL1 | -0.1461586 | 0.0802068 | -1.8223 | 0.0685 | -0.205763769 | count | 1 |
| NECTIN3 | -0.1866448 | 0.2140436 | -0.872 | 0.383 | -0.205630855 | count | 1 |
| UBE2D3 | -0.1437444 | 0.0459856 | -3.1259 | 0.00179 | -0.205618103 | count | 1 |
| PARP4 | -0.1665258 | 0.1588362 | -1.0484 | 0.295 | -0.205599141 | count | 1 |
| ABCA7 | -0.4134968 | 0.3545797 | -1.1662 | 0.244 | -0.205554531 | count | 1 |
| POLR2J | -0.1452299 | 0.0746049 | -1.9467 | 0.0517 | -0.205416899 | count | 1 |
| TSPAN13 | -1.8619366 | 1.183482 | -1.5733 | 0.116 | -0.20534791 | count | 1 |
| PLEKHF1 | -0.1468955 | 0.1141099 | -1.2873 | 0.198 | -0.205267435 | count | 1 |
| IRGQ | -0.1986064 | 0.3545995 | -0.5601 | 0.575 | -0.205130608 | count | 1 |
| VPS37B | -0.1563066 | 0.1202871 | -1.2994 | 0.194 | -0.205114853 | count | 1 |
| HNRNPH3 | -0.1471554 | 0.0753381 | -1.9533 | 0.0509 | -0.204754455 | count | 1 |

| | | | | | | | |
|---|---|---|---|---|---|---|---|
| ZCCHC8 | -0.161208 | 0.1670392 | -0.9651 | 0.335 | -0.204748013 | count | 1 |
| SULT1A3 | -1.849092 | 0.8606436 | -2.1485 | 0.0317 | -0.204708715 | count | 1 |
| RIC8B | -0.1981905 | 0.318483 | -0.6223 | 0.534 | -0.204708241 | count | 1 |
| DNAH14 | -0.7388963 | 1.1521464 | -0.6413 | 0.521 | -0.204689414 | count | 1 |
| RHBDF2 | -0.1857597 | 0.1997247 | -0.9301 | 0.352 | -0.204668419 | count | 1 |
| TUBGCP2 | -0.1529538 | 0.1152238 | -1.3274 | 0.184 | -0.204634787 | count | 1 |
| ADGRE1 | -0.3164253 | 0.4729601 | -0.669 | 0.504 | -0.204538392 | count | 1 |
| CHMP2A | -0.1442276 | 0.0831884 | -1.7337 | 0.0831 | -0.204427264 | count | 1 |
| CNOT3 | -0.173088 | 0.2670042 | -0.6483 | 0.517 | -0.204417037 | count | 1 |
| NUDT14 | -0.1532888 | 0.1322252 | -1.1593 | 0.246 | -0.204390913 | count | 1 |
| NR1D2 | -0.1565712 | 0.1290947 | -1.2128 | 0.225 | -0.204357693 | count | 1 |
| LRRC63 | -0.4103243 | 0.6389586 | -0.6422 | 0.521 | -0.204109859 | count | 1 |
| ZNRD1 | -0.1441242 | 0.0792011 | -1.8197 | 0.0689 | -0.203964629 | count | 1 |
| HECTD4 | -0.2006603 | 0.2573171 | -0.7798 | 0.436 | -0.20381105 | count | 1 |
| MAP1LC3B | -0.1425676 | 0.0514442 | -2.7713 | 0.0056 | -0.203653725 | count | 1 |
| UBASH3A | -0.158032 | 0.1893876 | -0.8344 | 0.404 | -0.203624152 | count | 1 |
| 9-Mar | -0.1467635 | 0.1047788 | -1.4007 | 0.161 | -0.203364729 | count | 1 |
| HCFC2 | -0.1910837 | 0.2274942 | -0.8399 | 0.401 | -0.20324733 | count | 1 |
| HP1BP3 | -0.1427986 | 0.0594816 | -2.4007 | 0.01642 | -0.203202676 | count | 1 |
| HKDC1 | -0.2880987 | 0.5028829 | -0.5729 | 0.567 | -0.20303618 | count | 1 |
| C7orf61 | -0.7312851 | 0.8593498 | -0.851 | 0.395 | -0.202987637 | count | 1 |
| EXOSC7 | -0.1510201 | 0.1489755 | -1.0137 | 0.311 | -0.20297707 | count | 1 |
| MED10 | -0.1436446 | 0.0759266 | -1.8919 | 0.0586 | -0.202924312 | count | 1 |
| SERPINE2 | -0.2419226 | 0.4645473 | -0.5208 | 0.603 | -0.202543333 | count | 1 |
| SHC1 | -0.175912 | 0.2767546 | -0.6356 | 0.525 | -0.202472621 | count | 1 |
| C7 | -1.8031659 | 1.116411 | -1.6151 | 0.106 | -0.202363096 | count | 1 |
| BIN3 | -0.1694378 | 0.2196479 | -0.7714 | 0.441 | -0.20230119 | count | 1 |
| N4BP2L1 | -0.1504835 | 0.1327144 | -1.1339 | 0.257 | -0.202258035 | count | 1 |
| SLC27A1 | -0.3492928 | 0.5635998 | -0.6198 | 0.535 | -0.202164178 | count | 1 |
| TP53RK | -0.152874 | 0.1611463 | -0.9487 | 0.343 | -0.202050605 | count | 1 |
| NONO | -0.1462986 | 0.1015642 | -1.4405 | 0.15 | -0.202009664 | count | 1 |
| PRPF8 | -0.1531022 | 0.1227813 | -1.247 | 0.213 | -0.201899504 | count | 1 |
| KDM2A | -0.1490879 | 0.1013964 | -1.4703 | 0.142 | -0.201805666 | count | 1 |
| FZD6 | -0.217122 | 0.5680253 | -0.3822 | 0.702 | -0.201787403 | count | 1 |
| MOB4 | -0.1452331 | 0.0977717 | -1.4854 | 0.138 | -0.201729428 | count | 1 |
| RBM12 | -0.1697668 | 0.220236 | -0.7708 | 0.441 | -0.201637412 | count | 1 |
| ZNF107 | -0.1644081 | 0.1942301 | -0.8465 | 0.397 | -0.201550698 | count | 1 |
| SCML4 | -0.1458419 | 0.0973639 | -1.4979 | 0.134 | -0.201542289 | count | 1 |
| P2RX5 | -0.2111979 | 0.3178045 | -0.6646 | 0.506 | -0.201516585 | count | 1 |
| CCDC92 | -0.1522992 | 0.1690527 | -0.9009 | 0.368 | -0.201510389 | count | 1 |
| GAA | -0.1777731 | 0.2914119 | -0.61 | 0.542 | -0.201458595 | count | 1 |
| PJA2 | -0.1439023 | 0.078919 | -1.8234 | 0.0683 | -0.201447759 | count | 1 |
| CSNK1G2 | -0.1478082 | 0.1033546 | -1.4301 | 0.153 | -0.201425044 | count | 1 |
| PSMG3-AS1 | -0.3113377 | 0.8310573 | -0.3746 | 0.708 | -0.201422579 | count | 1 |
| ZNF850 | -0.3113377 | 0.968247 | -0.3215 | 0.748 | -0.201422579 | count | 1 |

| | | | | | | | |
|---|---|---|---|---|---|---|---|
| COPS6 | -0.1423495 | 0.0818367 | -1.7394 | 0.082 | -0.201412203 | count | 1 |
| RORA | -0.1418465 | 0.0588638 | -2.4097 | 0.016 | -0.201327446 | count | 1 |
| PSMB2 | -0.1424585 | 0.0849645 | -1.6767 | 0.0937 | -0.201287779 | count | 1 |
| ZNF563 | -0.3110909 | 0.3994277 | -0.7788 | 0.436 | -0.201271279 | count | 1 |
| APIP | -0.1496995 | 0.1504904 | -0.9947 | 0.32 | -0.201207461 | count | 1 |
| IPO7 | -0.1635064 | 0.1702847 | -0.9602 | 0.337 | -0.201190245 | count | 1 |
| ARHGAP19 | -0.4032292 | 0.5362566 | -0.7519 | 0.452 | -0.200871155 | count | 1 |
| THUMPD3-AS1 | -0.1443101 | 0.1019686 | -1.4152 | 0.157 | -0.200838554 | count | 1 |
| CNBD2 | -0.1592738 | 0.2120225 | -0.7512 | 0.453 | -0.200835131 | count | 1 |
| NEDD9 | -0.1458892 | 0.1253507 | -1.1638 | 0.245 | -0.200668383 | count | 1 |
| ERCC4 | -0.1559288 | 0.2080806 | -0.7494 | 0.454 | -0.200558146 | count | 1 |
| HTRA2 | -0.1598709 | 0.1735421 | -0.9212 | 0.357 | -0.200508771 | count | 1 |
| PCID2 | -0.1492562 | 0.1212272 | -1.2312 | 0.218 | -0.200469199 | count | 1 |
| CLDND1 | -0.1423445 | 0.0769491 | -1.8499 | 0.0644 | -0.200399645 | count | 1 |
| PLLP | -0.4021342 | 0.6119954 | -0.6571 | 0.511 | -0.200370349 | count | 1 |
| ANP32B | -0.1400719 | 0.0462646 | -3.0276 | 0.0025 | -0.200265789 | count | 1 |
| LARP4B | -0.173963 | 0.2166492 | -0.803 | 0.422 | -0.20025264 | count | 1 |
| COX6A1 | -0.1395972 | 0.0410982 | -3.3967 | 0.00069 | -0.200251738 | count | 1 |
| DOT1L | -0.2649901 | 0.4489338 | -0.5903 | 0.555 | -0.200154383 | count | 1 |
| H2AFX | -0.1493102 | 0.1137835 | -1.3122 | 0.19 | -0.200091227 | count | 1 |
| SLC35B4 | -0.163745 | 0.2723596 | -0.6012 | 0.548 | -0.199973576 | count | 1 |
| KIF11 | -0.499079 | 0.9041937 | -0.552 | 0.581 | -0.199859462 | count | 1 |
| TYW1 | -0.1583933 | 0.2013366 | -0.7867 | 0.432 | -0.19973138 | count | 1 |
| SAP30 | -0.144934 | 0.0934525 | -1.5509 | 0.121 | -0.199608356 | count | 1 |
| HECTD1 | -0.1505148 | 0.1337942 | -1.125 | 0.261 | -0.19957131 | count | 1 |
| CYLD | -0.1409581 | 0.0736241 | -1.9146 | 0.0556 | -0.199518707 | count | 1 |
| ITPRIPL1 | -0.1605007 | 0.1826155 | -0.8789 | 0.38 | -0.199516535 | count | 1 |
| WDR48 | -0.1578091 | 0.1702938 | -0.9267 | 0.354 | -0.199501381 | count | 1 |
| EFTUD2 | -0.1791343 | 0.2219463 | -0.8071 | 0.42 | -0.199419555 | count | 1 |
| LINC00526 | -0.4977486 | 0.5264321 | -0.9455 | 0.344 | -0.199387286 | count | 1 |
| TSR2 | -0.1440326 | 0.1161301 | -1.2403 | 0.215 | -0.19934976 | count | 1 |
| AP1G1 | -0.15291 | 0.153276 | -0.9976 | 0.319 | -0.19931205 | count | 1 |
| RRM1 | -0.1677594 | 0.1893703 | -0.8859 | 0.376 | -0.199273985 | count | 1 |
| NAXD | -0.2086097 | 0.3034245 | -0.6875 | 0.492 | -0.199099001 | count | 1 |
| RDH10 | -0.2635011 | 0.3628703 | -0.7262 | 0.468 | -0.199072342 | count | 1 |
| MX2 | -0.146962 | 0.1244155 | -1.1812 | 0.238 | -0.199049432 | count | 1 |
| AC009005.1 | -0.3989942 | 0.8806251 | -0.4531 | 0.651 | -0.198932869 | count | 1 |
| GIMAP5 | -0.1549233 | 0.179425 | -0.8634 | 0.388 | -0.198892943 | count | 1 |
| JAGN1 | -0.1487338 | 0.1375604 | -1.0812 | 0.28 | -0.198844692 | count | 1 |
| ZNF274 | -0.1664234 | 0.1937174 | -0.8591 | 0.39 | -0.198732548 | count | 1 |
| MAN1B1 | -0.1588932 | 0.1760665 | -0.9025 | 0.367 | -0.198725835 | count | 1 |
| EXOC5 | -0.1550215 | 0.1583055 | -0.9793 | 0.328 | -0.198627838 | count | 1 |
| MRPS9 | -0.1491891 | 0.1498573 | -0.9955 | 0.32 | -0.198584766 | count | 1 |
| MRFAP1L1 | -0.1494537 | 0.1275613 | -1.1716 | 0.241 | -0.198561175 | count | 1 |
| AL691403.2 | -0.3423214 | 1.0901043 | -0.314 | 0.754 | -0.198383723 | count | 1 |

| | | | | | | | |
|---|---|---|---|---|---|---|---|
| AL139220.2 | -0.3423214 | 1.133989 | -0.3019 | 0.763 | -0.198383723 | count | 1 |
| C16orf72 | -0.1576756 | 0.1283006 | -1.229 | 0.219 | -0.198311494 | count | 1 |
| HMCN1 | -1.7275496 | 1.432603 | -1.2059 | 0.228 | -0.198289044 | count | 1 |
| TRADD | -0.1406652 | 0.0854567 | -1.646 | 0.0998 | -0.198277919 | count | 1 |
| MED1 | -0.1668623 | 0.2374796 | -0.7026 | 0.482 | -0.198217604 | count | 1 |
| ECI2 | -0.1447794 | 0.1262236 | -1.147 | 0.251 | -0.198072341 | count | 1 |
| AIMP1 | -0.14221 | 0.1024621 | -1.3879 | 0.165 | -0.197992188 | count | 1 |
| MIGA2 | -0.1815118 | 0.3026793 | -0.5997 | 0.549 | -0.19792481 | count | 1 |
| ZNF766 | -0.1538221 | 0.1727898 | -0.8902 | 0.373 | -0.197861736 | count | 1 |
| ZNF354B | -0.1664324 | 0.3494978 | -0.4762 | 0.634 | -0.19771134 | count | 1 |
| AVL9 | -0.202329 | 0.2691316 | -0.7518 | 0.452 | -0.197678151 | count | 1 |
| SELENOM | -0.1404305 | 0.0839818 | -1.6722 | 0.0946 | -0.19767385 | count | 1 |
| ECD | -0.1556053 | 0.1898334 | -0.8197 | 0.412 | -0.197671016 | count | 1 |
| LYRM2 | -0.1439895 | 0.1211377 | -1.1886 | 0.235 | -0.197660384 | count | 1 |
| CALM2 | -0.13761 | 0.0348321 | -3.9507 | 7.95E-05 | -0.197560811 | count | 1 |
| IRF2BPL | -0.1487523 | 0.1392309 | -1.0684 | 0.285 | -0.197439777 | count | 1 |
| LIX1L-AS1 | -0.3404356 | 0.3250825 | -1.0472 | 0.295 | -0.197359207 | count | 1 |
| FOSL1 | -0.2468575 | 0.335053 | -0.7368 | 0.461 | -0.197347657 | count | 1 |
| RGL4 | -0.1411085 | 0.0973965 | -1.4488 | 0.147 | -0.197219712 | count | 1 |
| PACSIN2 | -0.1686931 | 0.2394274 | -0.7046 | 0.481 | -0.196895869 | count | 1 |
| C20orf194 | -0.3395232 | 0.4124591 | -0.8232 | 0.41 | -0.196863231 | count | 1 |
| FAM24B | -0.3394643 | 0.4113018 | -0.8253 | 0.409 | -0.196831208 | count | 1 |
| DDX1 | -0.1507656 | 0.1616221 | -0.9328 | 0.351 | -0.196812116 | count | 1 |
| LINC00426 | -0.1526911 | 0.2068569 | -0.7381 | 0.46 | -0.196775353 | count | 1 |
| ZNF579 | -0.20132 | 0.3602489 | -0.5588 | 0.576 | -0.196711371 | count | 1 |
| PSMA3-AS1 | -0.1474889 | 0.1087009 | -1.3568 | 0.175 | -0.196682814 | count | 1 |
| CBLB | -0.1496257 | 0.124069 | -1.206 | 0.228 | -0.196628979 | count | 1 |
| NCOA5 | -0.303384 | 0.3717612 | -0.8161 | 0.415 | -0.196539432 | count | 1 |
| GCNT1 | -0.1782866 | 0.3076737 | -0.5795 | 0.562 | -0.19653716 | count | 1 |
| FRMD8 | -0.1823375 | 0.2187788 | -0.8334 | 0.405 | -0.196528989 | count | 1 |
| PCF11-AS1 | -1.69618 | 0.8830278 | -1.9209 | 0.0548 | -0.196518528 | count | 1 |
| EMG1 | -0.1493512 | 0.1489735 | -1.0025 | 0.316 | -0.19650947 | count | 1 |
| NCDN | -0.3935757 | 0.8345935 | -0.4716 | 0.637 | -0.19644734 | count | 1 |
| PRKX | -0.1583763 | 0.1708601 | -0.9269 | 0.354 | -0.196261713 | count | 1 |
| NKAP | -0.1402516 | 0.0887814 | -1.5797 | 0.114 | -0.196187644 | count | 1 |
| POLK | -0.1496199 | 0.144229 | -1.0374 | 0.3 | -0.196122148 | count | 1 |
| PEX6 | -0.171587 | 0.2492421 | -0.6884 | 0.491 | -0.196079239 | count | 1 |
| TMEM38B | -0.1729379 | 0.2184342 | -0.7917 | 0.429 | -0.196039124 | count | 1 |
| HDDC2 | -0.1448763 | 0.1143758 | -1.2667 | 0.205 | -0.195889626 | count | 1 |
| DHX36 | -0.1385189 | 0.0658827 | -2.1025 | 0.0356 | -0.195888183 | count | 1 |
| MICAL2 | -0.1713813 | 0.2466214 | -0.6949 | 0.487 | -0.195846642 | count | 1 |
| KRTAP5-AS1 | -0.6994189 | 0.7931442 | -0.8818 | 0.378 | -0.195771842 | count | 1 |
| DBH | -0.6994189 | 0.9313915 | -0.7509 | 0.453 | -0.195771842 | count | 1 |
| FAM177A1 | -0.1378461 | 0.0604815 | -2.2791 | 0.0227 | -0.195697104 | count | 1 |
| RAPGEF6 | -0.3919007 | 0.2249475 | -1.7422 | 0.0816 | -0.195677735 | count | 1 |

| | | | | | | | |
|---|---|---|---|---|---|---|---|
| RAB12 | -0.2446965 | 0.3022534 | -0.8096 | 0.418 | -0.195676654 | count | 1 |
| NUP214 | -0.1518033 | 0.1564218 | -0.9705 | 0.332 | -0.19563666 | count | 1 |
| MED13L | -0.1502825 | 0.1595901 | -0.9417 | 0.346 | -0.195611566 | count | 1 |
| CALCOCO1 | -0.1646132 | 0.1802898 | -0.913 | 0.361 | -0.195568706 | count | 1 |
| ATIC | -0.1465116 | 0.138773 | -1.0558 | 0.291 | -0.195553761 | count | 1 |
| GNB2 | -0.1376715 | 0.0675847 | -2.037 | 0.0417 | -0.195540312 | count | 1 |
| SLC30A7 | -0.1600884 | 0.1962934 | -0.8156 | 0.415 | -0.19554018 | count | 1 |
| SH2D3A | -0.1635598 | 0.209492 | -0.7807 | 0.435 | -0.195341333 | count | 1 |
| TAF11 | -0.1447927 | 0.1272883 | -1.1375 | 0.255 | -0.19529136 | count | 1 |
| CSNK1A1 | -0.1378495 | 0.0624873 | -2.206 | 0.0274 | -0.195267437 | count | 1 |
| NAPG | -0.1439075 | 0.1418039 | -1.0148 | 0.31 | -0.195245527 | count | 1 |
| IPCEF1 | -0.1495854 | 0.1082087 | -1.3824 | 0.167 | -0.194996882 | count | 1 |
| RALB | -0.1589854 | 0.2078638 | -0.7649 | 0.444 | -0.194949556 | count | 1 |
| AP3S1 | -0.1396186 | 0.1058679 | -1.3188 | 0.187 | -0.194938162 | count | 1 |
| BCAS4 | -0.1549371 | 0.1802518 | -0.8596 | 0.39 | -0.194887289 | count | 1 |
| XPO7 | -0.2040405 | 0.3032308 | -0.6729 | 0.501 | -0.194827622 | count | 1 |
| TMEM208 | -0.1409003 | 0.1170955 | -1.2033 | 0.229 | -0.194776104 | count | 1 |
| BCAS2 | -0.1378332 | 0.0681936 | -2.0212 | 0.0433 | -0.194709264 | count | 1 |
| UBE2I | -0.1366519 | 0.0578368 | -2.3627 | 0.0182 | -0.194676528 | count | 1 |
| CALM1 | -0.1350841 | 0.0223673 | -6.0394 | 1.71E-09 | -0.194660614 | count | 4.14E-05 |
| SNU13 | -0.1362494 | 0.0483192 | -2.8198 | 0.0048 | -0.194648038 | count | 1 |
| BANP | -0.1527744 | 0.1859366 | -0.8216 | 0.411 | -0.194532141 | count | 1 |
| DEDD2 | -0.1417775 | 0.1156733 | -1.2257 | 0.22 | -0.194491385 | count | 1 |
| ORMDL1 | -0.1369638 | 0.0664414 | -2.0614 | 0.0393 | -0.194473712 | count | 1 |
| TMEM260 | -0.194899 | 0.2606713 | -0.7477 | 0.455 | -0.194469027 | count | 1 |
| CCT3 | -0.1381418 | 0.0751812 | -1.8375 | 0.0662 | -0.194455368 | count | 1 |
| GLOD4 | -0.1404091 | 0.1027631 | -1.3663 | 0.172 | -0.194342973 | count | 1 |
| AP001107.9 | -0.6926364 | 0.6238223 | -1.1103 | 0.267 | -0.194217024 | count | 1 |
| PRR14L | -0.1572324 | 0.1902972 | -0.8262 | 0.409 | -0.194201584 | count | 1 |
| LINC00539 | -0.2994161 | 0.5236075 | -0.5718 | 0.567 | -0.194097874 | count | 1 |
| STK10 | -0.1407782 | 0.1012587 | -1.3903 | 0.165 | -0.194070239 | count | 1 |
| SERPINB9P1 | -0.6919179 | 0.6316528 | -1.0954 | 0.273 | -0.194051977 | count | 1 |
| ITPR1 | -0.2147754 | 0.1998936 | -1.0744 | 0.283 | -0.193914536 | count | 1 |
| MAPKAPK5-AS1 | -0.1460814 | 0.129924 | -1.1244 | 0.261 | -0.193906781 | count | 1 |
| BCLAF3 | -0.175774 | 0.2364721 | -0.7433 | 0.457 | -0.193801201 | count | 1 |
| MICAL3 | -0.3334725 | 0.2873653 | -1.1604 | 0.246 | -0.193569339 | count | 1 |
| TMEM168 | -0.2218286 | 0.2762449 | -0.803 | 0.422 | -0.193539544 | count | 1 |
| BTBD1 | -0.1551511 | 0.1803024 | -0.8605 | 0.39 | -0.193502351 | count | 1 |
| AKAP11 | -0.1469962 | 0.1578531 | -0.9312 | 0.352 | -0.193422582 | count | 1 |
| TALDO1 | -0.1366307 | 0.0736346 | -1.8555 | 0.0636 | -0.193408079 | count | 1 |
| NAXE | -0.1394808 | 0.1081072 | -1.2902 | 0.197 | -0.193289611 | count | 1 |
| EXOC3 | -0.1654951 | 0.2120539 | -0.7804 | 0.435 | -0.193197879 | count | 1 |
| ATF2 | -0.1573828 | 0.147935 | -1.0639 | 0.287 | -0.19299803 | count | 1 |
| NFATC1 | -0.1687367 | 0.1880071 | -0.8975 | 0.37 | -0.192855685 | count | 1 |
| SAP30L | -0.161297 | 0.1674055 | -0.9635 | 0.335 | -0.192660862 | count | 1 |

| | | | | | | | |
|---|---|---|---|---|---|---|---|
| NDUFA10 | -0.1386164 | 0.1055026 | -1.3139 | 0.189 | -0.192591727 | count | 1 |
| SPRTN | -0.1491422 | 0.1680442 | -0.8875 | 0.375 | -0.192565285 | count | 1 |
| ANKRD55 | -0.2069008 | 0.3308702 | -0.6253 | 0.532 | -0.192496247 | count | 1 |
| DDX18 | -0.1358031 | 0.0622001 | -2.1833 | 0.0291 | -0.192398056 | count | 1 |
| CORO7 | -0.1507537 | 0.1389711 | -1.0848 | 0.278 | -0.192390393 | count | 1 |
| HMGB3 | -0.1966543 | 0.3833664 | -0.513 | 0.608 | -0.192238213 | count | 1 |
| MLYCD | -0.1742154 | 0.2859872 | -0.6092 | 0.542 | -0.192103523 | count | 1 |
| SGK3 | -0.1498705 | 0.1619062 | -0.9257 | 0.355 | -0.192060278 | count | 1 |
| FEM1C | -0.1760617 | 0.2103558 | -0.837 | 0.403 | -0.192057192 | count | 1 |
| BID | -0.1409093 | 0.1294749 | -1.0883 | 0.277 | -0.19204503 | count | 1 |
| C6orf120 | -0.1553228 | 0.1947712 | -0.7975 | 0.425 | -0.191858419 | count | 1 |
| DYNLRB1 | -0.1348878 | 0.0634433 | -2.1261 | 0.0336 | -0.191810726 | count | 1 |
| PSMB9 | -0.1335645 | 0.0434303 | -3.0754 | 0.00212 | -0.191808067 | count | 1 |
| EMD | -0.1362044 | 0.0823046 | -1.6549 | 0.098 | -0.191779821 | count | 1 |
| IRF1 | -0.1332185 | 0.0409886 | -3.2501 | 0.00116 | -0.191778949 | count | 1 |
| CCAR1 | -0.1422631 | 0.1207589 | -1.1781 | 0.239 | -0.191763988 | count | 1 |
| DYNC1LI1 | -0.1382763 | 0.1013764 | -1.364 | 0.173 | -0.191753469 | count | 1 |
| PANK2 | -0.1458395 | 0.1385111 | -1.0529 | 0.292 | -0.191672271 | count | 1 |
| SLC31A2 | -0.166409 | 0.2866331 | -0.5806 | 0.562 | -0.191643068 | count | 1 |
| NIPSNAP2 | -0.1420375 | 0.131362 | -1.0813 | 0.28 | -0.191585265 | count | 1 |
| MIEF2 | -0.2710804 | 0.487558 | -0.556 | 0.578 | -0.191546159 | count | 1 |
| HAPLN3 | -0.1650808 | 0.2033592 | -0.8118 | 0.417 | -0.191459444 | count | 1 |
| BX890604.1 | -0.2708049 | 0.4440284 | -0.6099 | 0.542 | -0.191359593 | count | 1 |
| ZNF799 | -0.2117318 | 0.2968255 | -0.7133 | 0.476 | -0.191231278 | count | 1 |
| RPL13A | -0.1327251 | 0.0173634 | -7.6439 | 2.72E-14 | -0.191205604 | count | 6.60E-10 |
| BSCL2 | -0.4747902 | 0.7622693 | -0.6229 | 0.533 | -0.191186333 | count | 1 |
| GMCL1 | -0.1470798 | 0.1411277 | -1.0422 | 0.297 | -0.191168581 | count | 1 |
| TRIM46 | -0.3819579 | 0.6357881 | -0.6008 | 0.548 | -0.191097061 | count | 1 |
| SRP54 | -0.1382601 | 0.0966388 | -1.4307 | 0.153 | -0.191082623 | count | 1 |
| TIAL1 | -0.1362722 | 0.0839814 | -1.6226 | 0.105 | -0.191059488 | count | 1 |
| PHYKPL | -0.1397648 | 0.1184798 | -1.1797 | 0.238 | -0.190991549 | count | 1 |
| AC011416.3 | -0.6785498 | 0.8452845 | -0.8027 | 0.422 | -0.190966401 | count | 1 |
| GLS | -0.1346524 | 0.0620757 | -2.1692 | 0.0301 | -0.190931233 | count | 1 |
| ULK1 | -0.1996477 | 0.4007634 | -0.4982 | 0.618 | -0.190717095 | count | 1 |
| VPS35 | -0.1367007 | 0.0937065 | -1.4588 | 0.145 | -0.190627559 | count | 1 |
| TAF6 | -0.1604182 | 0.2120455 | -0.7565 | 0.449 | -0.190626212 | count | 1 |
| TATDN3 | -0.1519223 | 0.221508 | -0.6859 | 0.493 | -0.190597643 | count | 1 |
| TIRAP | -0.2109307 | 0.4426048 | -0.4766 | 0.634 | -0.190524691 | count | 1 |
| TRMT6 | -0.1423574 | 0.1398345 | -1.018 | 0.309 | -0.190500853 | count | 1 |
| E4F1 | -0.1666332 | 0.2357161 | -0.7069 | 0.48 | -0.190475948 | count | 1 |
| SCNM1 | -0.1382118 | 0.1245696 | -1.1095 | 0.267 | -0.190425099 | count | 1 |
| EIF4A2 | -0.1341421 | 0.0491384 | -2.7299 | 0.00637 | -0.190420718 | count | 1 |
| AC026471.2 | -0.3804929 | 0.7411352 | -0.5134 | 0.608 | -0.190420358 | count | 1 |
| TOP1 | -0.1355466 | 0.0724709 | -1.8704 | 0.0615 | -0.19041466 | count | 1 |
| ADCY7 | -0.1665602 | 0.228106 | -0.7302 | 0.465 | -0.190393348 | count | 1 |

| | | | | | | | |
|---|---|---|---|---|---|---|---|
| ACTR3 | -0.1334016 | 0.0515084 | -2.5899 | 0.0096 | -0.190391494 | count | 1 |
| R3HDM4 | -0.1376036 | 0.108192 | -1.2718 | 0.204 | -0.190324041 | count | 1 |
| PLOD3 | -0.1786895 | 0.279989 | -0.6382 | 0.523 | -0.190246929 | count | 1 |
| FGFRL1 | -0.2691351 | 0.4682001 | -0.5748 | 0.565 | -0.190228481 | count | 1 |
| RSRC1 | -0.1396133 | 0.1176971 | -1.1862 | 0.236 | -0.190104546 | count | 1 |
| UTP14A | -0.1599543 | 0.2284241 | -0.7003 | 0.484 | -0.190079507 | count | 1 |
| TRA2B | -0.134531 | 0.0602942 | -2.2312 | 0.0257 | -0.190060182 | count | 1 |
| TAF15 | -0.14244 | 0.1035815 | -1.3751 | 0.169 | -0.189971053 | count | 1 |
| SLC25A30 | -0.2102497 | 0.4494134 | -0.4678 | 0.64 | -0.189923926 | count | 1 |
| H3F3B | -0.1317042 | 0.0246617 | -5.3404 | 9.88E-08 | -0.18986689 | count | 0.002385328 |
| CD52 | -0.1318601 | 0.0311753 | -4.2296 | 2.40E-05 | -0.189813091 | count | 0.576 |
| RBM5 | -0.1406797 | 0.1351963 | -1.0406 | 0.298 | -0.189758697 | count | 1 |
| GSDMB | -1.583538 | 0.5456313 | -2.9022 | 0.00373 | -0.189751071 | count | 1 |
| ZNF837 | -0.1985858 | 0.5399164 | -0.3678 | 0.713 | -0.189722834 | count | 1 |
| NUDT4B | -0.4705211 | 1.1683206 | -0.4027 | 0.687 | -0.189650371 | count | 1 |
| E2F6 | -0.3787919 | 0.3356828 | -1.1284 | 0.259 | -0.189634063 | count | 1 |
| MORN3 | -0.2504728 | 0.2766611 | -0.9053 | 0.365 | -0.189582795 | count | 1 |
| BAZ2A | -0.1491841 | 0.1390346 | -1.073 | 0.283 | -0.189556433 | count | 1 |
| ZNF16 | -0.209787 | 0.3662875 | -0.5727 | 0.567 | -0.189515683 | count | 1 |
| IP6K1 | -0.1514676 | 0.1633938 | -0.927 | 0.354 | -0.189493502 | count | 1 |
| CCNC | -0.1480963 | 0.1487708 | -0.9955 | 0.32 | -0.189413084 | count | 1 |
| NOP56 | -0.1365525 | 0.0920536 | -1.4834 | 0.138 | -0.189408641 | count | 1 |
| LEO1 | -0.1375048 | 0.1338395 | -1.0274 | 0.304 | -0.189395195 | count | 1 |
| TRIB1 | -0.3258102 | 0.3525741 | -0.9241 | 0.356 | -0.189386309 | count | 1 |
| PDCL3 | -0.1348796 | 0.083903 | -1.6076 | 0.108 | -0.189361134 | count | 1 |
| FASTK | -0.1482975 | 0.1597816 | -0.9281 | 0.353 | -0.189271476 | count | 1 |
| JTB | -0.1327901 | 0.0553133 | -2.4007 | 0.0164 | -0.189156003 | count | 1 |
| CCT5 | -0.1340601 | 0.0816055 | -1.6428 | 0.101 | -0.18899032 | count | 1 |
| AC073111.5 | -0.1510339 | 0.2135044 | -0.7074 | 0.479 | -0.1889541 | count | 1 |
| TAF12 | -0.1356692 | 0.1100415 | -1.2329 | 0.218 | -0.188932133 | count | 1 |
| BEST1 | -0.4683043 | 0.7052354 | -0.664 | 0.507 | -0.188851443 | count | 1 |
| HAUS1 | -0.1433278 | 0.1491569 | -0.9609 | 0.337 | -0.188837456 | count | 1 |
| IER3IP1 | -0.1367595 | 0.1117339 | -1.224 | 0.221 | -0.188756362 | count | 1 |
| DONSON | -0.2667934 | 0.4434195 | -0.6017 | 0.547 | -0.188641142 | count | 1 |
| SETD5 | -0.1491436 | 0.1699264 | -0.8777 | 0.38 | -0.188605611 | count | 1 |
| ZMYM6 | -0.2247905 | 0.3230502 | -0.6958 | 0.487 | -0.18860462 | count | 1 |
| DHRS12 | -0.1595201 | 0.1885004 | -0.8463 | 0.397 | -0.188529488 | count | 1 |
| LSM7 | -0.1322289 | 0.0619866 | -2.1332 | 0.033 | -0.188456828 | count | 1 |
| NBEA | -0.6677431 | 1.298476 | -0.5143 | 0.607 | -0.188453072 | count | 1 |
| HEBP1 | -0.1473014 | 0.1932663 | -0.7622 | 0.446 | -0.188401373 | count | 1 |
| TIMM10B | -0.1413557 | 0.1580097 | -0.8946 | 0.371 | -0.188362064 | count | 1 |
| ELK4 | -0.1438319 | 0.1288304 | -1.1164 | 0.264 | -0.188315238 | count | 1 |
| HNRNPM | -0.1327464 | 0.0633043 | -2.097 | 0.0361 | -0.188301061 | count | 1 |
| MATR3 | -0.323801 | 0.2230691 | -1.4516 | 0.147 | -0.188287285 | count | 1 |
| WDR24 | -0.6670224 | 0.7265565 | -0.9181 | 0.359 | -0.188284915 | count | 1 |

| | | | | | | | |
|---|---|---|---|---|---|---|---|
| TMED8 | -0.3757908 | 0.3937346 | -0.9544 | 0.34 | -0.188245308 | count | 1 |
| SCRN2 | -0.1545913 | 0.2102983 | -0.7351 | 0.462 | -0.188114391 | count | 1 |
| BEX5 | -0.1600926 | 0.1670904 | -0.9581 | 0.338 | -0.188103501 | count | 1 |
| WAC | -0.1361037 | 0.0880199 | -1.5463 | 0.122 | -0.18805764 | count | 1 |
| KBTBD3 | -0.1456164 | 0.2039566 | -0.714 | 0.475 | -0.188033245 | count | 1 |
| PSMB3 | -0.131741 | 0.0536938 | -2.4536 | 0.0142 | -0.18802245 | count | 1 |
| NOL9 | -0.1502391 | 0.193687 | -0.7757 | 0.438 | -0.187965538 | count | 1 |
| NEDD4L | -0.6656283 | 0.6019103 | -1.1059 | 0.269 | -0.18795924 | count | 1 |
| RHOBTB2 | -0.6656283 | 0.6791188 | -0.9801 | 0.327 | -0.18795924 | count | 1 |
| MED30 | -0.1355861 | 0.0999422 | -1.3566 | 0.175 | -0.18794387 | count | 1 |
| TCF20 | -0.1845552 | 0.2347006 | -0.7863 | 0.432 | -0.187717341 | count | 1 |
| CUTC | -0.1374969 | 0.1371417 | -1.0026 | 0.316 | -0.187657464 | count | 1 |
| GOLGA2 | -0.1406973 | 0.1381942 | -1.0181 | 0.309 | -0.187654018 | count | 1 |
| ALG2 | -0.1421703 | 0.1681135 | -0.8457 | 0.398 | -0.187534753 | count | 1 |
| BRWD3 | -0.1738373 | 0.2492722 | -0.6974 | 0.486 | -0.187485742 | count | 1 |
| ZWILCH | -0.3740994 | 0.3648133 | -1.0255 | 0.305 | -0.187461772 | count | 1 |
| CNOT1 | -0.1417075 | 0.1367393 | -1.0363 | 0.3 | -0.187343254 | count | 1 |
| RBFA | -0.1506332 | 0.1684966 | -0.894 | 0.371 | -0.187325127 | count | 1 |
| ZNF585B | -0.2010895 | 0.5091343 | -0.395 | 0.693 | -0.187203893 | count | 1 |
| RSF1 | -0.1320885 | 0.07231 | -1.8267 | 0.0678 | -0.187182399 | count | 1 |
| ANKRD20A8P | -1.54314 | 1.388933 | -1.111 | 0.267 | -0.187160905 | count | 1 |
| STPG1 | -0.191309 | 0.4502497 | -0.4249 | 0.671 | -0.187108145 | count | 1 |
| RSL24D1 | -0.1317974 | 0.0576964 | -2.2843 | 0.0224 | -0.187059066 | count | 1 |
| TRBV29-1 | -0.6617001 | 1.1584252 | -0.5712 | 0.568 | -0.187040206 | count | 1 |
| OAF | -0.6617001 | 1.1584252 | -0.5712 | 0.568 | -0.187040206 | count | 1 |
| PRAF2 | -0.1411347 | 0.1650605 | -0.855 | 0.393 | -0.186984367 | count | 1 |
| KDM3B | -0.1695056 | 0.2155616 | -0.7863 | 0.432 | -0.186971032 | count | 1 |
| SLC9A3R2 | -0.2068932 | 0.3953111 | -0.5234 | 0.601 | -0.18696143 | count | 1 |
| TRAP1 | -0.1518361 | 0.2307536 | -0.658 | 0.511 | -0.186924121 | count | 1 |
| GIMAP8 | -0.1549102 | 0.2202469 | -0.7033 | 0.482 | -0.186874788 | count | 1 |
| TNFRSF10A | -0.145751 | 0.1559094 | -0.9348 | 0.35 | -0.186806167 | count | 1 |
| UBE2Z | -0.13687 | 0.1415043 | -0.9672 | 0.333 | -0.186803699 | count | 1 |
| SARNP | -0.1711802 | 0.2253666 | -0.7596 | 0.448 | -0.186797488 | count | 1 |
| STK11 | -0.1484562 | 0.2005313 | -0.7403 | 0.459 | -0.186780572 | count | 1 |
| MRPL3 | -0.1336183 | 0.1035367 | -1.2905 | 0.197 | -0.186763766 | count | 1 |
| C9orf78 | -0.1304342 | 0.0492235 | -2.6498 | 0.00809 | -0.186687299 | count | 1 |
| SERTAD1 | -0.1318077 | 0.0691915 | -1.905 | 0.0569 | -0.186651587 | count | 1 |
| DENND4B | -0.1540274 | 0.2170742 | -0.7096 | 0.478 | -0.186643686 | count | 1 |
| LYRM4 | -0.1645499 | 0.1906678 | -0.863 | 0.388 | -0.186629008 | count | 1 |
| XRCC5 | -0.130893 | 0.0537291 | -2.4362 | 0.0149 | -0.186517658 | count | 1 |
| XPO1 | -0.1387102 | 0.120004 | -1.1559 | 0.248 | -0.186477831 | count | 1 |
| BRD2 | -0.1316427 | 0.0601037 | -2.1903 | 0.0286 | -0.186454575 | count | 1 |
| ENAH | -0.4615438 | 0.9878067 | -0.4672 | 0.64 | -0.186409225 | count | 1 |
| AC011815.2 | -0.4615438 | 1.0201399 | -0.4524 | 0.651 | -0.186409225 | count | 1 |
| ITSN1 | -1.5316045 | 1.100897 | -1.3912 | 0.164 | -0.186404938 | count | 1 |

| | | | | | | | |
|---|---|---|---|---|---|---|---|
| DNAJC8 | -0.1311387 | 0.0637729 | -2.0563 | 0.0398 | -0.186402515 | count | 1 |
| ENY2 | -0.1311702 | 0.0642427 | -2.0418 | 0.0412 | -0.186292196 | count | 1 |
| MTMR2 | -0.1865471 | 0.3073783 | -0.6069 | 0.544 | -0.186277046 | count | 1 |
| C9orf40 | -0.1566883 | 0.2196465 | -0.7134 | 0.476 | -0.186229739 | count | 1 |
| B9D2 | -0.1575165 | 0.194861 | -0.8084 | 0.419 | -0.186181235 | count | 1 |
| PRKCSH | -0.1471973 | 0.1412906 | -1.0418 | 0.298 | -0.18615737 | count | 1 |
| GDE1 | -0.1435942 | 0.1654729 | -0.8678 | 0.386 | -0.186062898 | count | 1 |
| MAPK3 | -0.1457318 | 0.1906248 | -0.7645 | 0.445 | -0.186012917 | count | 1 |
| ADH1B | -1.5255102 | 0.8435241 | -1.8085 | 0.0706 | -0.186002539 | count | 1 |
| TOX | -0.1385147 | 0.1271938 | -1.089 | 0.276 | -0.185946133 | count | 1 |
| MPHOSPH9 | -0.1533951 | 0.1847403 | -0.8303 | 0.406 | -0.185882982 | count | 1 |
| C19orf70 | -0.1313073 | 0.0752197 | -1.7456 | 0.081 | -0.185858742 | count | 1 |
| DYRK4 | -0.144088 | 0.1949298 | -0.7392 | 0.46 | -0.185738132 | count | 1 |
| EFNB2 | -0.6550797 | 0.5815345 | -1.1265 | 0.26 | -0.18548629 | count | 1 |
| TPRG1 | -0.1486799 | 0.1862338 | -0.7984 | 0.425 | -0.185479012 | count | 1 |
| PNKP | -0.1365145 | 0.1301436 | -1.049 | 0.294 | -0.185433972 | count | 1 |
| ARFGEF2 | -0.1680802 | 0.2533224 | -0.6635 | 0.507 | -0.185416994 | count | 1 |
| RCL1 | -0.158704 | 0.2278699 | -0.6965 | 0.486 | -0.185340257 | count | 1 |
| PRDM2 | -0.1376619 | 0.1048744 | -1.3126 | 0.189 | -0.185202444 | count | 1 |
| AL441992.1 | -0.1585282 | 0.2417231 | -0.6558 | 0.512 | -0.185136761 | count | 1 |
| OCIAD1 | -0.1302654 | 0.0677306 | -1.9233 | 0.0545 | -0.185065283 | count | 1 |
| ATXN2 | -0.1492239 | 0.1602966 | -0.9309 | 0.352 | -0.184989664 | count | 1 |
| ARMC1 | -0.1367927 | 0.1416699 | -0.9656 | 0.334 | -0.184986965 | count | 1 |
| SLC35D1 | -0.1629535 | 0.2703442 | -0.6028 | 0.547 | -0.18483686 | count | 1 |
| SMS | -0.134444 | 0.1187578 | -1.1321 | 0.258 | -0.184771821 | count | 1 |
| PEA15 | -0.1511587 | 0.2312129 | -0.6538 | 0.513 | -0.184706847 | count | 1 |
| RBM45 | -0.1784914 | 0.3214425 | -0.5553 | 0.579 | -0.184665868 | count | 1 |
| AC009948.1 | -0.2042082 | 0.5023053 | -0.4065 | 0.684 | -0.184589877 | count | 1 |
| MAEA | -0.1416086 | 0.1674164 | -0.8458 | 0.398 | -0.18436693 | count | 1 |
| YPEL2 | -0.1671153 | 0.1956363 | -0.8542 | 0.393 | -0.184364823 | count | 1 |
| ZNF622 | -0.1365412 | 0.1117737 | -1.2216 | 0.222 | -0.184308164 | count | 1 |
| KIAA1109 | -0.1381634 | 0.1182527 | -1.1684 | 0.243 | -0.18428469 | count | 1 |
| SINHCAF | -0.1319396 | 0.0836004 | -1.5782 | 0.115 | -0.184173177 | count | 1 |
| TAS2R14 | -1.496004 | 1.042709 | -1.4347 | 0.151 | -0.184024872 | count | 1 |
| ATP5MC3 | -0.1284799 | 0.0479201 | -2.6811 | 0.00737 | -0.184009747 | count | 1 |
| PHLPP1 | -0.3665824 | 0.6992359 | -0.5243 | 0.6 | -0.183972204 | count | 1 |
| KLHL35 | -0.3159114 | 0.7183558 | -0.4398 | 0.66 | -0.183962835 | count | 1 |
| PLS3 | -1.494101 | 0.7272708 | -2.0544 | 0.04 | -0.183895542 | count | 1 |
| ZHX1 | -0.1436938 | 0.2040886 | -0.7041 | 0.481 | -0.183809067 | count | 1 |
| ZRANB2 | -0.1307228 | 0.0825846 | -1.5829 | 0.114 | -0.183718008 | count | 1 |
| TMEM169 | -0.2595275 | 0.7799894 | -0.3327 | 0.739 | -0.183707828 | count | 1 |
| EIF3B | -0.1435247 | 0.1696296 | -0.8461 | 0.398 | -0.183593783 | count | 1 |
| EID2 | -0.1440166 | 0.1544952 | -0.9322 | 0.351 | -0.183435511 | count | 1 |
| ZNHIT6 | -0.1474768 | 0.1667598 | -0.8844 | 0.377 | -0.183423142 | count | 1 |
| ATL2 | -0.1680175 | 0.2450653 | -0.6856 | 0.493 | -0.183387621 | count | 1 |

| | | | | | | | |
|---|---|---|---|---|---|---|---|
| ELF2 | -0.1318578 | 0.0899281 | -1.4663 | 0.143 | -0.183359341 | count | 1 |
| RRP1 | -0.1424942 | 0.1961896 | -0.7263 | 0.468 | -0.18335623 | count | 1 |
| NPAT | -0.1359561 | 0.1299798 | -1.046 | 0.296 | -0.183284495 | count | 1 |
| UPP1 | -0.1299222 | 0.0859882 | -1.5109 | 0.131 | -0.183257283 | count | 1 |
| MAP3K13 | -0.1438177 | 0.1673376 | -0.8594 | 0.39 | -0.183183412 | count | 1 |
| PRPSAP1 | -0.1428738 | 0.1520753 | -0.9395 | 0.348 | -0.183135614 | count | 1 |
| SUGT1 | -0.1300991 | 0.0927269 | -1.403 | 0.161 | -0.182860146 | count | 1 |
| BTF3L4 | -0.1319242 | 0.0999084 | -1.3205 | 0.187 | -0.182661417 | count | 1 |
| PRKCI | -0.1513319 | 0.2452862 | -0.617 | 0.537 | -0.182589323 | count | 1 |
| UNC13D | -0.1512946 | 0.191722 | -0.7891 | 0.43 | -0.182544644 | count | 1 |
| STX16 | -0.1458603 | 0.157593 | -0.9256 | 0.355 | -0.182518066 | count | 1 |
| STRN | -0.149945 | 0.1780532 | -0.8421 | 0.4 | -0.182499216 | count | 1 |
| IRF2BP2 | -0.1299906 | 0.0775713 | -1.6758 | 0.0939 | -0.182484927 | count | 1 |
| SRRM1 | -0.1274809 | 0.0438402 | -2.9079 | 0.00366 | -0.18244324 | count | 1 |
| UQCC1 | -0.6418546 | 0.522351 | -1.2288 | 0.219 | -0.182362795 | count | 1 |
| CDKL5 | -0.6418546 | 0.5325624 | -1.2052 | 0.228 | -0.182362795 | count | 1 |
| EXOC7 | -0.1550776 | 0.1653561 | -0.9378 | 0.348 | -0.182260531 | count | 1 |
| PRSS21 | -0.2169878 | 0.5773797 | -0.3758 | 0.707 | -0.182234506 | count | 1 |
| NDRG1 | -0.1688987 | 0.1585427 | -1.0653 | 0.287 | -0.182225929 | count | 1 |
| AC022706.1 | -0.1401459 | 0.1819853 | -0.7701 | 0.441 | -0.182192552 | count | 1 |
| CNIH4 | -0.1299051 | 0.1052654 | -1.2341 | 0.217 | -0.182184011 | count | 1 |
| RIOK3 | -0.1314913 | 0.1008109 | -1.3043 | 0.192 | -0.182149742 | count | 1 |
| ARL2 | -0.1318339 | 0.1211167 | -1.0885 | 0.276 | -0.182118832 | count | 1 |
| CRYAB | -1.467658 | 0.6194771 | -2.3692 | 0.0179 | -0.182078183 | count | 1 |
| PDE4A | -0.1822206 | 0.302047 | -0.6033 | 0.546 | -0.182028053 | count | 1 |
| SIN3A | -0.138428 | 0.1476498 | -0.9375 | 0.349 | -0.181966454 | count | 1 |
| RNMT | -0.1287808 | 0.0662447 | -1.944 | 0.052 | -0.181919902 | count | 1 |
| TRIM2 | -0.6398117 | 0.6084844 | -1.0515 | 0.293 | -0.181877965 | count | 1 |
| RETREG3 | -0.1666037 | 0.2102597 | -0.7924 | 0.428 | -0.181862788 | count | 1 |
| TMOD3 | -0.1289812 | 0.0868077 | -1.4858 | 0.137 | -0.181808654 | count | 1 |
| PACS1 | -0.1355785 | 0.1389729 | -0.9756 | 0.329 | -0.181740925 | count | 1 |
| TRNP1 | -1.4613144 | 1.041361 | -1.4033 | 0.161 | -0.181636188 | count | 1 |
| DLG1 | -0.1383005 | 0.1700746 | -0.8132 | 0.416 | -0.18157228 | count | 1 |
| PHKA2 | -0.2563046 | 0.3836088 | -0.6681 | 0.504 | -0.18151569 | count | 1 |
| ATP8A2 | -1.458402 | 1.001524 | -1.4562 | 0.145 | -0.181432464 | count | 1 |
| STAT5B | -0.1484347 | 0.170948 | -0.8683 | 0.385 | -0.181400283 | count | 1 |
| SF3B6 | -0.127902 | 0.0657337 | -1.9458 | 0.0518 | -0.181396126 | count | 1 |
| AC005261.3 | -0.6376504 | 0.6295002 | -1.0129 | 0.311 | -0.181364486 | count | 1 |
| NRIP3 | -0.6374136 | 0.4187714 | -1.5221 | 0.128 | -0.181308169 | count | 1 |
| AC001226.1 | -1.4565052 | 1.208771 | -1.2049 | 0.228 | -0.181299549 | count | 1 |
| SNHG18 | -0.6370931 | 0.9193534 | -0.693 | 0.488 | -0.181231909 | count | 1 |
| AC002553.2 | -0.6370931 | 0.9974207 | -0.6387 | 0.523 | -0.181231909 | count | 1 |
| AC092687.3 | -0.278556 | 0.7017523 | -0.3969 | 0.691 | -0.18120251 | count | 1 |
| AHCY | -0.1572142 | 0.2232387 | -0.7042 | 0.481 | -0.181152126 | count | 1 |
| TRMT10C | -0.1293402 | 0.0820416 | -1.5765 | 0.115 | -0.181108261 | count | 1 |

| | | | | | | | |
|---|---|---|---|---|---|---|---|
| IFI35 | -0.1326508 | 0.1287881 | -1.03 | 0.303 | -0.181057173 | count | 1 |
| STYX | -0.1386108 | 0.1420867 | -0.9755 | 0.329 | -0.181005905 | count | 1 |
| ZCCHC10 | -0.1334312 | 0.139534 | -0.9563 | 0.339 | -0.180966959 | count | 1 |
| GSR | -0.1570418 | 0.1813224 | -0.8661 | 0.387 | -0.18095531 | count | 1 |
| RINL | -0.1335482 | 0.1349724 | -0.9894 | 0.323 | -0.180924543 | count | 1 |
| TSEN2 | -0.4463613 | 0.418602 | -1.0663 | 0.286 | -0.180893149 | count | 1 |
| AC016773.1 | -0.2069218 | 0.4436943 | -0.4664 | 0.641 | -0.180849274 | count | 1 |
| TNRC6A | -0.1377013 | 0.1219678 | -1.129 | 0.259 | -0.180788427 | count | 1 |
| RFX3 | -0.2382586 | 0.3791318 | -0.6284 | 0.53 | -0.180650707 | count | 1 |
| RNF216 | -0.1335461 | 0.1144958 | -1.1664 | 0.244 | -0.180607176 | count | 1 |
| RPL26 | -0.1253432 | 0.0177015 | -7.0809 | 1.73E-12 | -0.180599025 | count | 4.19E-08 |
| IFT80 | -0.2775093 | 0.3435905 | -0.8077 | 0.419 | -0.180552827 | count | 1 |
| UBE2D1 | -0.1317174 | 0.1229689 | -1.0711 | 0.284 | -0.180517279 | count | 1 |
| SH3GLB1 | -0.1275799 | 0.0819968 | -1.5559 | 0.12 | -0.180490289 | count | 1 |
| MFAP3 | -0.4451571 | 0.5219704 | -0.8528 | 0.394 | -0.180453783 | count | 1 |
| TMEM184B | -0.1715959 | 0.3857554 | -0.4448 | 0.656 | -0.180312589 | count | 1 |
| HHEX | -0.3586404 | 0.4565978 | -0.7855 | 0.432 | -0.180272372 | count | 1 |
| ZC3H8 | -0.135106 | 0.1371752 | -0.9849 | 0.325 | -0.180218726 | count | 1 |
| NUDT2 | -0.1400414 | 0.153233 | -0.9139 | 0.361 | -0.180213918 | count | 1 |
| ZRANB1 | -0.1587545 | 0.2081016 | -0.7629 | 0.446 | -0.18012114 | count | 1 |
| SEMA4D | -0.1320758 | 0.0981598 | -1.3455 | 0.179 | -0.180113498 | count | 1 |
| CASC4 | -0.137536 | 0.1326821 | -1.0366 | 0.3 | -0.180102614 | count | 1 |
| MRPS26 | -0.1300618 | 0.1043918 | -1.2459 | 0.213 | -0.180086656 | count | 1 |
| SGTA | -0.1472641 | 0.2179277 | -0.6757 | 0.499 | -0.179979068 | count | 1 |
| MLF2 | -0.1286837 | 0.1028325 | -1.2514 | 0.211 | -0.179950483 | count | 1 |
| PATJ | -0.1472014 | 0.2003113 | -0.7349 | 0.462 | -0.179902942 | count | 1 |
| IQGAP2 | -0.1264971 | 0.0648343 | -1.9511 | 0.0511 | -0.179791348 | count | 1 |
| SAR1A | -0.1271385 | 0.0712951 | -1.7833 | 0.0746 | -0.179601711 | count | 1 |
| RPS6KC1 | -0.1582572 | 0.3002135 | -0.5271 | 0.598 | -0.179562463 | count | 1 |
| TEX2 | -0.2053716 | 0.5153672 | -0.3985 | 0.69 | -0.179526789 | count | 1 |
| KIAA0895L | -0.2757135 | 0.5391347 | -0.5114 | 0.609 | -0.17943761 | count | 1 |
| FAF1 | -0.1388394 | 0.1757218 | -0.7901 | 0.43 | -0.179319176 | count | 1 |
| AC103702.2 | -0.1580269 | 0.3557668 | -0.4442 | 0.657 | -0.179303731 | count | 1 |
| CREM | -0.1261491 | 0.0554851 | -2.2736 | 0.0231 | -0.179279744 | count | 1 |
| NMRK1 | -0.1275765 | 0.0964409 | -1.3228 | 0.186 | -0.179247198 | count | 1 |
| GSEC | -0.3072561 | 0.7395769 | -0.4154 | 0.678 | -0.179202726 | count | 1 |
| RNF25 | -0.1435885 | 0.2258632 | -0.6357 | 0.525 | -0.179163308 | count | 1 |
| GPCPD1 | -0.129058 | 0.0951514 | -1.3563 | 0.175 | -0.179144328 | count | 1 |
| AFAP1L2 | -0.2361497 | 0.4437012 | -0.5322 | 0.595 | -0.179105039 | count | 1 |
| ZNF331 | -0.1256388 | 0.0692092 | -1.8153 | 0.0696 | -0.179060947 | count | 1 |
| ZFP36L1 | -0.1247867 | 0.055237 | -2.2591 | 0.0239 | -0.178989458 | count | 1 |
| ADH5 | -0.1263872 | 0.0783908 | -1.6123 | 0.107 | -0.178908642 | count | 1 |
| RPP40 | -0.1789747 | 0.3193568 | -0.5604 | 0.575 | -0.178837925 | count | 1 |
| ACTN4 | -0.1268201 | 0.0874354 | -1.4504 | 0.147 | -0.17868075 | count | 1 |
| AC006064.4 | -0.4398887 | 0.6994375 | -0.6289 | 0.529 | -0.178528323 | count | 1 |

| | | | | | | | |
|---|---|---|---|---|---|---|---|
| PYURF | -0.1255542 | 0.0661045 | -1.8993 | 0.0576 | -0.178387862 | count | 1 |
| ANKRD46 | -0.186494 | 0.2634875 | -0.7078 | 0.479 | -0.178385027 | count | 1 |
| RAD50 | -0.1570068 | 0.3046497 | -0.5154 | 0.606 | -0.178157581 | count | 1 |
| TLR5 | -0.3539744 | 0.7021255 | -0.5041 | 0.614 | -0.178092489 | count | 1 |
| DNAJC9 | -0.1314295 | 0.1111583 | -1.1824 | 0.237 | -0.178060881 | count | 1 |
| SELENON | -0.305158 | 0.3639066 | -0.8386 | 0.402 | -0.178046331 | count | 1 |
| WNT2B | -0.4384535 | 0.6766139 | -0.648 | 0.517 | -0.178002882 | count | 1 |
| EML4 | -0.1251718 | 0.0540754 | -2.3148 | 0.0207 | -0.177980426 | count | 1 |
| HSPA8 | -0.1234542 | 0.0303633 | -4.0659 | 4.89E-05 | -0.177730811 | count | 1 |
| SNW1 | -0.1257124 | 0.0744838 | -1.6878 | 0.0915 | -0.177676471 | count | 1 |
| RBM38 | -0.1256964 | 0.0652321 | -1.9269 | 0.0541 | -0.177599484 | count | 1 |
| CDK5RAP1 | -0.1356166 | 0.1635348 | -0.8293 | 0.407 | -0.177598279 | count | 1 |
| SLC29A2 | -0.1777128 | 0.5173091 | -0.3435 | 0.731 | -0.177597166 | count | 1 |
| PUM1 | -0.138533 | 0.1422662 | -0.9738 | 0.33 | -0.177596494 | count | 1 |
| CCSER2 | -0.1258718 | 0.0680762 | -1.849 | 0.0645 | -0.177587051 | count | 1 |
| PTPN1 | -0.1283626 | 0.0889144 | -1.4437 | 0.149 | -0.177471334 | count | 1 |
| RB1 | -0.1341534 | 0.1336977 | -1.0034 | 0.316 | -0.177388881 | count | 1 |
| RPL35 | -0.1231953 | 0.02072 | -5.9457 | 3.03E-09 | -0.177351544 | count | 7.33E-05 |
| CORO1A | -0.1231499 | 0.0330119 | -3.7305 | 0.000194 | -0.177177627 | count | 1 |
| NPM1 | -0.123237 | 0.0277441 | -4.4419 | 9.20E-06 | -0.177168845 | count | 0.2211772 |
| AC098487.1 | -0.3519527 | 0.6639798 | -0.5301 | 0.596 | -0.177146549 | count | 1 |
| SCAMP1-AS1 | -0.1685396 | 0.2750547 | -0.6127 | 0.54 | -0.177143997 | count | 1 |
| SH3GLB2 | -0.1320275 | 0.1454166 | -0.9079 | 0.364 | -0.177128532 | count | 1 |
| AP1S2 | -0.1295979 | 0.117801 | -1.1001 | 0.271 | -0.176975508 | count | 1 |
| SMIM13 | -0.1523963 | 0.2196441 | -0.6938 | 0.488 | -0.176876266 | count | 1 |
| ZMYM2 | -0.1308486 | 0.1125484 | -1.1626 | 0.245 | -0.176861369 | count | 1 |
| ZC3H18 | -0.1379544 | 0.1446197 | -0.9539 | 0.34 | -0.176858039 | count | 1 |
| LSM8 | -0.1246693 | 0.0659967 | -1.889 | 0.059 | -0.176667297 | count | 1 |
| SRSF4 | -0.1261438 | 0.0926454 | -1.3616 | 0.173 | -0.176659687 | count | 1 |
| EIF4EBP2 | -0.1308266 | 0.1351855 | -0.9678 | 0.333 | -0.176613179 | count | 1 |
| COX18 | -0.1734417 | 0.2910026 | -0.596 | 0.551 | -0.17658298 | count | 1 |
| MMGT1 | -0.1391935 | 0.171655 | -0.8109 | 0.417 | -0.176511672 | count | 1 |
| SYP | -0.2487751 | 0.518234 | -0.48 | 0.631 | -0.176385013 | count | 1 |
| DIAPH1 | -0.128028 | 0.095758 | -1.337 | 0.181 | -0.176364008 | count | 1 |
| AC022098.1 | -0.1700028 | 0.4701347 | -0.3616 | 0.718 | -0.176007211 | count | 1 |
| TCF4 | -1.3823551 | 0.81747 | -1.691 | 0.0909 | -0.175935471 | count | 1 |
| IWS1 | -0.1268464 | 0.1087833 | -1.166 | 0.244 | -0.175846663 | count | 1 |
| UFD1 | -0.1247371 | 0.0900879 | -1.3846 | 0.166 | -0.175803983 | count | 1 |
| IP6K2 | -0.1308988 | 0.127266 | -1.0285 | 0.304 | -0.175749232 | count | 1 |
| DPM3 | -0.1271438 | 0.1119288 | -1.1359 | 0.256 | -0.175743071 | count | 1 |
| DNAJB4 | -0.1589618 | 0.1906948 | -0.8336 | 0.405 | -0.175467845 | count | 1 |
| EYA3 | -0.1573848 | 0.236976 | -0.6641 | 0.507 | -0.175461766 | count | 1 |
| FOSL2 | -0.1273681 | 0.0946914 | -1.3451 | 0.179 | -0.175456476 | count | 1 |
| HNRNPR | -0.1237608 | 0.0633404 | -1.9539 | 0.0508 | -0.17540463 | count | 1 |
| OBSCN | -0.3480867 | 0.5502926 | -0.6325 | 0.527 | -0.17533529 | count | 1 |

| | | | | | | | |
|---|---|---|---|---|---|---|---|
| REEP3 | -0.1288055 | 0.1397004 | -0.922 | 0.357 | -0.175334976 | count | 1 |
| KDELR2 | -0.124577 | 0.0874785 | -1.4241 | 0.155 | -0.175237792 | count | 1 |
| NAB1 | -0.1603922 | 0.2402537 | -0.6676 | 0.504 | -0.175159549 | count | 1 |
| CENPB | -0.1422208 | 0.1830994 | -0.7767 | 0.437 | -0.175158496 | count | 1 |
| NOD1 | -0.2686947 | 0.482902 | -0.5564 | 0.578 | -0.17507178 | count | 1 |
| GAMT | -0.1407234 | 0.1933115 | -0.728 | 0.467 | -0.175070915 | count | 1 |
| FAM13B | -0.1379126 | 0.1449021 | -0.9518 | 0.341 | -0.174895201 | count | 1 |
| KIF18A | -0.6105298 | 0.4468587 | -1.3663 | 0.172 | -0.174862158 | count | 1 |
| ENGASE | -0.2464049 | 0.5582773 | -0.4414 | 0.659 | -0.174767249 | count | 1 |
| AIFM1 | -0.1396229 | 0.2142376 | -0.6517 | 0.515 | -0.174754963 | count | 1 |
| EI24 | -0.1408635 | 0.1748645 | -0.8056 | 0.421 | -0.174685044 | count | 1 |
| RP9 | -0.1280079 | 0.1495987 | -0.8557 | 0.392 | -0.174655551 | count | 1 |
| STK25 | -0.1287483 | 0.1299589 | -0.9907 | 0.322 | -0.174534081 | count | 1 |
| SHARPIN | -0.1260563 | 0.1137961 | -1.1077 | 0.268 | -0.174376616 | count | 1 |
| RNF19B | -0.1925969 | 0.2829248 | -0.6807 | 0.496 | -0.174316385 | count | 1 |
| UBE2T | -0.1491617 | 0.2525018 | -0.5907 | 0.555 | -0.174288504 | count | 1 |
| FOXN3 | -0.1275533 | 0.1149249 | -1.1099 | 0.267 | -0.174263422 | count | 1 |
| RAB5A | -0.1287123 | 0.0986374 | -1.3049 | 0.192 | -0.174187879 | count | 1 |
| PSTPIP1 | -0.1254271 | 0.0906975 | -1.3829 | 0.167 | -0.174147987 | count | 1 |
| AC020656.1 | -0.6070147 | 0.6631841 | -0.9153 | 0.36 | -0.174011486 | count | 1 |
| KPTN | -0.6070147 | 0.7271578 | -0.8348 | 0.404 | -0.174011486 | count | 1 |
| PAPOLA | -0.1234178 | 0.0719812 | -1.7146 | 0.0865 | -0.173999618 | count | 1 |
| CBFA2T2 | -0.1775565 | 0.2286446 | -0.7766 | 0.437 | -0.173883321 | count | 1 |
| ME2 | -0.132077 | 0.1296693 | -1.0186 | 0.308 | -0.173857459 | count | 1 |
| RAF1 | -0.170635 | 0.1685362 | -1.0125 | 0.311 | -0.173767337 | count | 1 |
| AL020996.1 | -0.2664821 | 0.8517353 | -0.3129 | 0.754 | -0.173693178 | count | 1 |
| WASL | -0.138367 | 0.1678267 | -0.8245 | 0.41 | -0.173680557 | count | 1 |
| ABRACL | -0.121172 | 0.050431 | -2.4027 | 0.0163 | -0.173500979 | count | 1 |
| ABT1 | -0.1249302 | 0.1020729 | -1.2239 | 0.221 | -0.173459004 | count | 1 |
| LSM2 | -0.1226161 | 0.0895472 | -1.3693 | 0.171 | -0.173453613 | count | 1 |
| CIB2 | -0.1770936 | 0.3147988 | -0.5626 | 0.574 | -0.17343753 | count | 1 |
| FAM131B | -0.4260123 | 0.8297797 | -0.5134 | 0.608 | -0.173431861 | count | 1 |
| KRT8 | -0.4260123 | 0.8856134 | -0.481 | 0.631 | -0.173431861 | count | 1 |
| JAKMIP1 | -0.1371843 | 0.175588 | -0.7813 | 0.435 | -0.173121478 | count | 1 |
| LNPK | -0.1387158 | 0.1967002 | -0.7052 | 0.481 | -0.173116351 | count | 1 |
| TGIF1 | -0.1236724 | 0.0768429 | -1.6094 | 0.108 | -0.173113351 | count | 1 |
| CAPZA2 | -0.1237077 | 0.0877605 | -1.4096 | 0.159 | -0.17309405 | count | 1 |
| MIR210HG | -0.2654857 | 0.8067287 | -0.3291 | 0.742 | -0.173071988 | count | 1 |
| CSTF3 | -0.1369242 | 0.2228228 | -0.6145 | 0.539 | -0.172794867 | count | 1 |
| TDRD3 | -0.1376364 | 0.1838165 | -0.7488 | 0.454 | -0.172768235 | count | 1 |
| RHOD | -0.2273794 | 0.5885812 | -0.3863 | 0.699 | -0.172666187 | count | 1 |
| SNAP29 | -0.1295074 | 0.1588345 | -0.8154 | 0.415 | -0.172618876 | count | 1 |
| BDP1 | -0.1221124 | 0.0738504 | -1.6535 | 0.0983 | -0.172602397 | count | 1 |
| SPG21 | -0.1293569 | 0.1541854 | -0.839 | 0.402 | -0.172571555 | count | 1 |
| PCYOX1 | -0.1598393 | 0.2748052 | -0.5816 | 0.561 | -0.172566496 | count | 1 |

| | | | | | | | |
|---|---|---|---|---|---|---|---|
| RNF115 | -0.1238306 | 0.0938232 | -1.3198 | 0.187 | -0.172404396 | count | 1 |
| GIMAP4 | -0.1203513 | 0.0525935 | -2.2883 | 0.0222 | -0.172309113 | count | 1 |
| LNX1 | -0.5997992 | 0.7368925 | -0.814 | 0.416 | -0.172259602 | count | 1 |
| TRIR | -0.120138 | 0.0403509 | -2.9773 | 0.00293 | -0.172256868 | count | 1 |
| CCDC30 | -0.3409775 | 0.4397765 | -0.7753 | 0.438 | -0.17199637 | count | 1 |
| IGFBP2 | -0.1318313 | 0.3741894 | -0.3523 | 0.725 | -0.171938198 | count | 1 |
| SYNGR2 | -0.1237783 | 0.1041166 | -1.1888 | 0.235 | -0.171825509 | count | 1 |
| ST6GALNAC1 | -0.2262338 | 0.4973638 | -0.4549 | 0.649 | -0.171823838 | count | 1 |
| AC104986.2 | -0.2262338 | 0.5535149 | -0.4087 | 0.683 | -0.171823838 | count | 1 |
| RRP1B | -0.1239917 | 0.1068428 | -1.1605 | 0.246 | -0.17181577 | count | 1 |
| TMEM159 | -0.1339866 | 0.1715714 | -0.7809 | 0.435 | -0.171793226 | count | 1 |
| EIF4A3 | -0.1215823 | 0.0737837 | -1.6478 | 0.0995 | -0.171790769 | count | 1 |
| GADD45GIP1 | -0.1211888 | 0.0706988 | -1.7142 | 0.0866 | -0.171698326 | count | 1 |
| FUBP1 | -0.1293884 | 0.1016331 | -1.2731 | 0.203 | -0.171644307 | count | 1 |
| SERPINB1 | -0.1206499 | 0.0663657 | -1.818 | 0.0692 | -0.171609602 | count | 1 |
| RBCK1 | -0.1218135 | 0.0835945 | -1.4572 | 0.145 | -0.171519672 | count | 1 |
| USO1 | -0.1364678 | 0.1621833 | -0.8414 | 0.4 | -0.171308855 | count | 1 |
| MIS18BP1 | -0.1227677 | 0.121033 | -1.0143 | 0.31 | -0.171276767 | count | 1 |
| KDM5D | -0.1508787 | 0.2127124 | -0.7093 | 0.478 | -0.171268969 | count | 1 |
| NAPB | -0.2928508 | 0.4479861 | -0.6537 | 0.513 | -0.171243402 | count | 1 |
| DNMT1 | -0.122776 | 0.0878039 | -1.3983 | 0.162 | -0.171204505 | count | 1 |
| SGCA | -0.5949467 | 0.9083704 | -0.655 | 0.513 | -0.171077126 | count | 1 |
| PISD | -0.1473343 | 0.2762162 | -0.5334 | 0.594 | -0.171050198 | count | 1 |
| THAP7 | -0.1254727 | 0.1233048 | -1.0176 | 0.309 | -0.170969574 | count | 1 |
| RAE1 | -0.1303494 | 0.1607588 | -0.8108 | 0.418 | -0.170949514 | count | 1 |
| MRPL39 | -0.1283411 | 0.1622119 | -0.7912 | 0.429 | -0.170913899 | count | 1 |
| TTC39C | -0.1211497 | 0.0761977 | -1.5899 | 0.112 | -0.17090551 | count | 1 |
| HERC1 | -0.1368288 | 0.1664309 | -0.8221 | 0.411 | -0.170773944 | count | 1 |
| TSPOAP1 | -0.1707438 | 0.5646177 | -0.3024 | 0.762 | -0.170739384 | count | 1 |
| FLII | -0.1367702 | 0.1671598 | -0.8182 | 0.413 | -0.170701195 | count | 1 |
| EP300 | -0.1427703 | 0.1422781 | -1.0035 | 0.316 | -0.170689659 | count | 1 |
| NTNG2 | -0.188334 | 0.5735575 | -0.3284 | 0.743 | -0.170537472 | count | 1 |
| BACH2 | -0.1740021 | 0.2793586 | -0.6229 | 0.533 | -0.170459234 | count | 1 |
| ANK3 | -0.1528506 | 0.1524913 | -1.0024 | 0.316 | -0.170457733 | count | 1 |
| ZBTB44 | -0.1337594 | 0.1596726 | -0.8377 | 0.402 | -0.170429982 | count | 1 |
| BRF1 | -0.1880923 | 0.2873295 | -0.6546 | 0.513 | -0.170323099 | count | 1 |
| SLC25A39 | -0.125472 | 0.1260259 | -0.9956 | 0.32 | -0.170287338 | count | 1 |
| SYNRG | -0.1212519 | 0.071392 | -1.6984 | 0.0895 | -0.17028242 | count | 1 |
| NT5C3A | -0.1229071 | 0.1109891 | -1.1074 | 0.268 | -0.170275146 | count | 1 |
| GCC2 | -0.1199309 | 0.0569803 | -2.1048 | 0.0354 | -0.170271073 | count | 1 |
| ATXN1 | -0.1297536 | 0.1116993 | -1.1616 | 0.245 | -0.170170794 | count | 1 |
| USF3 | -0.1324081 | 0.1456191 | -0.9093 | 0.363 | -0.170109807 | count | 1 |
| CASD1 | -0.1362898 | 0.1900187 | -0.7172 | 0.473 | -0.170104793 | count | 1 |
| DNAJB1 | -0.11848 | 0.0589449 | -2.01 | 0.0445 | -0.170024448 | count | 1 |
| CLK2 | -0.1667347 | 0.3084573 | -0.5405 | 0.589 | -0.169852174 | count | 1 |

| | | | | | | | |
|---|---|---|---|---|---|---|---|
| CD81 | -0.1181559 | 0.040335 | -2.9294 | 0.00342 | -0.169825254 | count | 1 |
| NKX3-1 | -0.4162197 | 0.4995656 | -0.8332 | 0.405 | -0.169813491 | count | 1 |
| SPTBN1 | -0.1318526 | 0.1611852 | -0.818 | 0.413 | -0.169719393 | count | 1 |
| NENF | -0.1204048 | 0.0876755 | -1.3733 | 0.17 | -0.169689673 | count | 1 |
| OXR1 | -0.1288984 | 0.1636205 | -0.7878 | 0.431 | -0.169686964 | count | 1 |
| RING1 | -0.1308359 | 0.1732708 | -0.7551 | 0.45 | -0.169594929 | count | 1 |
| HIST1H2AC | -0.1470773 | 0.2602426 | -0.5652 | 0.572 | -0.169572086 | count | 1 |
| AC009133.1 | -0.1817022 | 0.3059343 | -0.5939 | 0.553 | -0.169496959 | count | 1 |
| MTPAP | -0.1480863 | 0.2920167 | -0.5071 | 0.612 | -0.169464918 | count | 1 |
| NPTXR | -0.4149159 | 0.6038847 | -0.6871 | 0.492 | -0.169330383 | count | 1 |
| GYS1 | -0.5877497 | 0.517897 | -1.1349 | 0.257 | -0.169316993 | count | 1 |
| PDCD4 | -0.1188613 | 0.0545986 | -2.177 | 0.0295 | -0.169271631 | count | 1 |
| SMCR8 | -0.2226014 | 0.3286958 | -0.6772 | 0.498 | -0.169151017 | count | 1 |
| CRELD2 | -0.1236522 | 0.1162745 | -1.0635 | 0.288 | -0.169084808 | count | 1 |
| ZGRF1 | -0.5864761 | 0.6406251 | -0.9155 | 0.36 | -0.169004715 | count | 1 |
| METAP2 | -0.1210116 | 0.0887957 | -1.3628 | 0.173 | -0.168987938 | count | 1 |
| RECQL | -0.1220357 | 0.1003861 | -1.2157 | 0.224 | -0.168822854 | count | 1 |
| RPF2 | -0.1266288 | 0.1575617 | -0.8037 | 0.422 | -0.168792663 | count | 1 |
| USP10 | -0.1262423 | 0.1330238 | -0.949 | 0.343 | -0.168716246 | count | 1 |
| MGAT4B | -0.131661 | 0.1753858 | -0.7507 | 0.453 | -0.168483399 | count | 1 |
| F12 | -0.1924465 | 0.5462869 | -0.3523 | 0.725 | -0.168480079 | count | 1 |
| NSD3 | -0.1179731 | 0.0529997 | -2.2259 | 0.0261 | -0.168452125 | count | 1 |
| MOCS3 | -0.4124347 | 0.5320815 | -0.7751 | 0.438 | -0.168410107 | count | 1 |
| MAMLD1 | -1.2852263 | 1.0664815 | -1.2051 | 0.228 | -0.168397118 | count | 1 |
| AL132656.2 | -1.2852263 | 1.0664815 | -1.2051 | 0.228 | -0.168397118 | count | 1 |
| CEBPB-AS1 | -1.2852263 | 1.0664815 | -1.2051 | 0.228 | -0.168397118 | count | 1 |
| OLFM1 | -1.2852263 | 1.354476 | -0.9489 | 0.343 | -0.168397118 | count | 1 |
| KCNAB2 | -0.129266 | 0.1604668 | -0.8056 | 0.421 | -0.168356015 | count | 1 |
| ARPC1A | -0.1296626 | 0.1750837 | -0.7406 | 0.459 | -0.168350961 | count | 1 |
| C2orf49 | -0.1244198 | 0.1574451 | -0.7902 | 0.429 | -0.168292486 | count | 1 |
| LY6E | -0.1173278 | 0.0514118 | -2.2821 | 0.0225 | -0.168232015 | count | 1 |
| UBC | -0.1165535 | 0.0227378 | -5.126 | 3.13E-07 | -0.167911037 | count | 0.007552377 |
| PRPSAP2 | -0.1352645 | 0.1580936 | -0.8556 | 0.392 | -0.167779771 | count | 1 |
| FOXO4 | -0.4107098 | 0.50757 | -0.8092 | 0.418 | -0.167769667 | count | 1 |
| UFL1 | -0.1213415 | 0.1260829 | -0.9624 | 0.336 | -0.167734626 | count | 1 |
| AC011978.2 | -0.2863763 | 0.6529747 | -0.4386 | 0.661 | -0.167651108 | count | 1 |
| KHSRP | -0.1750338 | 0.2360831 | -0.7414 | 0.458 | -0.167612121 | count | 1 |
| NDUFS8 | -0.1183224 | 0.0830358 | -1.425 | 0.154 | -0.167599843 | count | 1 |
| HOXA-AS2 | -0.4100137 | 0.8301507 | -0.4939 | 0.621 | -0.167511054 | count | 1 |
| LYST | -0.1205899 | 0.1270351 | -0.9493 | 0.343 | -0.167510084 | count | 1 |
| FTL | -0.1162152 | 0.0230111 | -5.0504 | 4.64E-07 | -0.167485569 | count | 0.01119168 |
| STXBP5 | -0.1794826 | 0.3049641 | -0.5885 | 0.556 | -0.167464786 | count | 1 |
| ABHD4 | -0.2564901 | 0.5016866 | -0.5113 | 0.609 | -0.167453685 | count | 1 |
| BAG1 | -0.119098 | 0.0934914 | -1.2739 | 0.203 | -0.167443438 | count | 1 |
| PAXIP1-AS1 | -0.1550333 | 0.2310388 | -0.671 | 0.502 | -0.167436541 | count | 1 |

| | | | | | | | |
|---|---|---|---|---|---|---|---|
| SAE1 | -0.1263268 | 0.1467495 | -0.8608 | 0.389 | -0.167424305 | count | 1 |
| IFRD1 | -0.1213315 | 0.08657 | -1.4015 | 0.161 | -0.167254339 | count | 1 |
| DPP8 | -0.1275771 | 0.16474 | -0.7744 | 0.439 | -0.167105758 | count | 1 |
| UBE2J1 | -0.1240813 | 0.1369844 | -0.9058 | 0.365 | -0.166975964 | count | 1 |
| LANCL1 | -0.1496588 | 0.2646125 | -0.5656 | 0.572 | -0.166933265 | count | 1 |
| FAM173B | -0.2194883 | 0.3808001 | -0.5764 | 0.564 | -0.166857927 | count | 1 |
| SIRT6 | -0.1381385 | 0.2358958 | -0.5856 | 0.558 | -0.166774969 | count | 1 |
| AGPAT5 | -0.1401929 | 0.2359527 | -0.5942 | 0.552 | -0.166764654 | count | 1 |
| PFN2 | -0.2552816 | 0.7530181 | -0.339 | 0.735 | -0.16669751 | count | 1 |
| SVIP | -0.1176273 | 0.073466 | -1.6011 | 0.109 | -0.166507774 | count | 1 |
| ACVR1 | -0.3292216 | 0.3363058 | -0.9789 | 0.328 | -0.16645182 | count | 1 |
| STK32C | -0.1899186 | 0.2937715 | -0.6465 | 0.518 | -0.166315346 | count | 1 |
| SLIRP | -0.1249808 | 0.1204195 | -1.0379 | 0.299 | -0.166297322 | count | 1 |
| WDR34 | -0.5754302 | 0.8993585 | -0.6398 | 0.522 | -0.166286204 | count | 1 |
| CCDC47 | -0.1192473 | 0.0988596 | -1.2062 | 0.228 | -0.16620584 | count | 1 |
| RHOF | -0.1172784 | 0.0696095 | -1.6848 | 0.0921 | -0.166121954 | count | 1 |
| ZFYVE21 | -0.1430355 | 0.1992764 | -0.7178 | 0.473 | -0.166099707 | count | 1 |
| HNRNPA3 | -0.1163966 | 0.0500276 | -2.3266 | 0.02 | -0.166091281 | count | 1 |
| RSBN1L | -0.1168591 | 0.0648077 | -1.8032 | 0.0715 | -0.166062072 | count | 1 |
| PPP2R5A | -0.1237512 | 0.1188345 | -1.0414 | 0.298 | -0.16605271 | count | 1 |
| AGO3 | -0.133414 | 0.1784729 | -0.7475 | 0.455 | -0.166025605 | count | 1 |
| AC016831.7 | -0.128933 | 0.1014362 | -1.2711 | 0.204 | -0.165976337 | count | 1 |
| ATP5F1C | -0.1165985 | 0.064838 | -1.7983 | 0.0722 | -0.165954569 | count | 1 |
| TM2D3 | -0.1195949 | 0.1018943 | -1.1737 | 0.241 | -0.165844831 | count | 1 |
| ZCCHC4 | -0.1656888 | 0.3481853 | -0.4759 | 0.634 | -0.165759251 | count | 1 |
| CDH23 | -0.4050379 | 0.9977802 | -0.4059 | 0.685 | -0.165659807 | count | 1 |
| LZTFL1 | -0.1689723 | 0.3466946 | -0.4874 | 0.626 | -0.165609628 | count | 1 |
| SPAG16 | -0.1424963 | 0.2217123 | -0.6427 | 0.52 | -0.165478583 | count | 1 |
| DNAJC14 | -0.2533332 | 0.3759987 | -0.6738 | 0.501 | -0.165477674 | count | 1 |
| PGAM5 | -0.1433895 | 0.2629571 | -0.5453 | 0.586 | -0.165355614 | count | 1 |
| SH3KBP1 | -0.1161526 | 0.0574743 | -2.0209 | 0.0434 | -0.165289406 | count | 1 |
| JAK1 | -0.1153094 | 0.0420973 | -2.7391 | 0.00619 | -0.16527795 | count | 1 |
| BBIP1 | -0.1225609 | 0.1615365 | -0.7587 | 0.448 | -0.165267983 | count | 1 |
| BRD7 | -0.1179591 | 0.0893906 | -1.3196 | 0.187 | -0.165249929 | count | 1 |
| SNRPC | -0.118232 | 0.0902379 | -1.3102 | 0.19 | -0.165233027 | count | 1 |
| MYL12B | -0.1146932 | 0.0289014 | -3.9684 | 7.38E-05 | -0.165092794 | count | 1 |
| ELAVL1 | -0.1217953 | 0.1237475 | -0.9842 | 0.325 | -0.165036289 | count | 1 |
| ZNF385D | -0.2815007 | 0.9237204 | -0.3047 | 0.761 | -0.164939847 | count | 1 |
| CHN2 | -0.2524114 | 0.4135751 | -0.6103 | 0.542 | -0.164900267 | count | 1 |
| HNRNPC | -0.1157626 | 0.0501597 | -2.3079 | 0.0211 | -0.164832498 | count | 1 |
| DYRK1A | -0.1392526 | 0.1558907 | -0.8933 | 0.372 | -0.164751 | count | 1 |
| SLC31A1 | -0.3256192 | 0.4725008 | -0.6891 | 0.491 | -0.164746995 | count | 1 |
| SLC35C2 | -0.12243 | 0.1456603 | -0.8405 | 0.401 | -0.16464381 | count | 1 |
| IKBKB | -0.148973 | 0.198684 | -0.7498 | 0.453 | -0.164553603 | count | 1 |
| RAB3D | -0.3249538 | 0.4964266 | -0.6546 | 0.513 | -0.164431806 | count | 1 |

| | | | | | | | |
|---|---|---|---|---|---|---|---|
| ARHGEF1 | -0.116352 | 0.0706293 | -1.6474 | 0.0996 | -0.164395426 | count | 1 |
| IKBIP | -0.1208054 | 0.1223416 | -0.9874 | 0.323 | -0.164386426 | count | 1 |
| TSR3 | -0.1184387 | 0.1049842 | -1.1282 | 0.259 | -0.164352264 | count | 1 |
| VCPKMT | -0.128924 | 0.1431794 | -0.9004 | 0.368 | -0.164295622 | count | 1 |
| PNPLA8 | -0.1185281 | 0.0858592 | -1.3805 | 0.168 | -0.164177912 | count | 1 |
| PQLC1 | -0.1279564 | 0.1477779 | -0.8659 | 0.387 | -0.164093151 | count | 1 |
| KANSL1L | -0.5664687 | 0.5369592 | -1.055 | 0.292 | -0.164067555 | count | 1 |
| AKTIP | -0.1209392 | 0.140576 | -0.8603 | 0.39 | -0.164059891 | count | 1 |
| PIGM | -0.1518566 | 0.3839387 | -0.3955 | 0.692 | -0.164043585 | count | 1 |
| TM9SF2 | -0.1202654 | 0.1284461 | -0.9363 | 0.349 | -0.164037972 | count | 1 |
| TPMT | -0.1283653 | 0.1792336 | -0.7162 | 0.474 | -0.163940834 | count | 1 |
| PGK1 | -0.1144729 | 0.046168 | -2.4795 | 0.0132 | -0.163852619 | count | 1 |
| ARNTL | -0.1253911 | 0.1612079 | -0.7778 | 0.437 | -0.163801969 | count | 1 |
| PSMG2 | -0.1162959 | 0.0821325 | -1.416 | 0.157 | -0.163770598 | count | 1 |
| ULK2 | -0.2036281 | 0.4389949 | -0.4639 | 0.643 | -0.163719052 | count | 1 |
| RALBP1 | -0.1164829 | 0.0855976 | -1.3608 | 0.174 | -0.163657714 | count | 1 |
| GBP4 | -0.1173918 | 0.113864 | -1.031 | 0.303 | -0.163594613 | count | 1 |
| DCN | -0.3994795 | 0.3809388 | -1.0487 | 0.294 | -0.16358632 | count | 1 |
| IK | -0.1152053 | 0.0584027 | -1.9726 | 0.0486 | -0.163565454 | count | 1 |
| ATRX | -0.1159431 | 0.0665205 | -1.743 | 0.0814 | -0.163554185 | count | 1 |
| ANKRD11 | -0.1161749 | 0.0748618 | -1.5519 | 0.121 | -0.163544716 | count | 1 |
| C2CD5 | -0.1941808 | 0.2869124 | -0.6768 | 0.499 | -0.163537667 | count | 1 |
| PAQR8 | -0.1577916 | 0.3786492 | -0.4167 | 0.677 | -0.16352834 | count | 1 |
| COMMD1 | -0.1175825 | 0.1140208 | -1.0312 | 0.303 | -0.163468979 | count | 1 |
| HMG20B | -0.1222675 | 0.1356585 | -0.9013 | 0.367 | -0.16341785 | count | 1 |
| STK4 | -0.1140068 | 0.0369824 | -3.0827 | 0.00207 | -0.163397222 | count | 1 |
| GPD1L | -0.1666154 | 0.3151433 | -0.5287 | 0.597 | -0.163335465 | count | 1 |
| DDX43 | -0.5635003 | 0.8149973 | -0.6914 | 0.489 | -0.163329964 | count | 1 |
| PDK1 | -0.1631847 | 0.2605987 | -0.6262 | 0.531 | -0.163290444 | count | 1 |
| CITED4 | -0.1365076 | 0.1956835 | -0.6976 | 0.485 | -0.163252715 | count | 1 |
| PLEKHA2 | -0.1296487 | 0.1747506 | -0.7419 | 0.458 | -0.163231134 | count | 1 |
| MRPL18 | -0.1165259 | 0.0976033 | -1.1939 | 0.233 | -0.163223803 | count | 1 |
| NME6 | -0.1269953 | 0.179116 | -0.709 | 0.478 | -0.16318355 | count | 1 |
| MRPL55 | -0.1189016 | 0.1178896 | -1.0086 | 0.313 | -0.163103888 | count | 1 |
| FAM160B2 | -0.1600068 | 0.2680189 | -0.597 | 0.551 | -0.163091997 | count | 1 |
| LMBRD2 | -0.1491618 | 0.3070619 | -0.4858 | 0.627 | -0.163023926 | count | 1 |
| AC037459.3 | -0.2493842 | 0.9799539 | -0.2545 | 0.799 | -0.163002706 | count | 1 |
| PITPNA | -0.1233457 | 0.1684218 | -0.7324 | 0.464 | -0.162961882 | count | 1 |
| HNRNPF | -0.1140509 | 0.0490552 | -2.3249 | 0.0201 | -0.162863413 | count | 1 |
| RPF1 | -0.1233061 | 0.137964 | -0.8938 | 0.372 | -0.162726991 | count | 1 |
| TFE3 | -0.1659538 | 0.3328839 | -0.4985 | 0.618 | -0.162696894 | count | 1 |
| SKAP1 | -0.1137612 | 0.0545472 | -2.0856 | 0.0371 | -0.162632835 | count | 1 |
| PDHX | -0.144417 | 0.2510488 | -0.5753 | 0.565 | -0.162615071 | count | 1 |
| SLC25A37 | -0.1243003 | 0.1536433 | -0.809 | 0.419 | -0.162607765 | count | 1 |
| TMEM121 | -0.1794021 | 0.3401909 | -0.5274 | 0.598 | -0.162607273 | count | 1 |

| | | | | | | | |
|---|---|---|---|---|---|---|---|
| MTERF2 | -0.1568449 | 0.2514988 | -0.6236 | 0.533 | -0.162559763 | count | 1 |
| RTL8C | -0.1267017 | 0.2072761 | -0.6113 | 0.541 | -0.162490601 | count | 1 |
| LRRCC1 | -0.156764 | 0.3497313 | -0.4482 | 0.654 | -0.162476986 | count | 1 |
| KIAA0586 | -0.1223861 | 0.1384693 | -0.8839 | 0.377 | -0.162381436 | count | 1 |
| FNBP4 | -0.1157709 | 0.0733894 | -1.5775 | 0.115 | -0.162304849 | count | 1 |
| ISCU | -0.1138419 | 0.056722 | -2.007 | 0.0448 | -0.162301619 | count | 1 |
| NIFK | -0.1156572 | 0.092255 | -1.2537 | 0.21 | -0.162220701 | count | 1 |
| RAB34 | -0.128091 | 0.3014634 | -0.4249 | 0.671 | -0.162104167 | count | 1 |
| NFKBID | -0.1242137 | 0.1360411 | -0.9131 | 0.361 | -0.162037107 | count | 1 |
| PRIM2 | -0.1376836 | 0.199098 | -0.6915 | 0.489 | -0.161968405 | count | 1 |
| NDUFA7 | -0.158822 | 0.2753891 | -0.5767 | 0.564 | -0.161900645 | count | 1 |
| ACOX1 | -0.1785667 | 0.3031331 | -0.5891 | 0.556 | -0.161864708 | count | 1 |
| PHC3 | -0.1198657 | 0.1406091 | -0.8525 | 0.394 | -0.161848904 | count | 1 |
| HEXB | -0.1186957 | 0.143806 | -0.8254 | 0.409 | -0.161827366 | count | 1 |
| GOLPH3 | -0.1203965 | 0.1321121 | -0.9113 | 0.362 | -0.161800058 | count | 1 |
| VOPP1 | -0.1245553 | 0.1236057 | -1.0077 | 0.314 | -0.16174346 | count | 1 |
| C17orf100 | -0.3192282 | 0.5106045 | -0.6252 | 0.532 | -0.161715828 | count | 1 |
| AHI1 | -0.1289884 | 0.1654066 | -0.7798 | 0.436 | -0.161510017 | count | 1 |
| PPT1 | -0.1184957 | 0.1280014 | -0.9257 | 0.355 | -0.161480604 | count | 1 |
| TRMT61B | -0.1292973 | 0.2199936 | -0.5877 | 0.557 | -0.161421118 | count | 1 |
| AL024508.2 | -0.2468559 | 0.516375 | -0.4781 | 0.633 | -0.161416311 | count | 1 |
| ZNF587 | -0.2750211 | 0.3588349 | -0.7664 | 0.443 | -0.161328533 | count | 1 |
| MPP6 | -0.1582475 | 0.2337084 | -0.6771 | 0.498 | -0.161322875 | count | 1 |
| QARS | -0.1233869 | 0.1503656 | -0.8206 | 0.412 | -0.161192572 | count | 1 |
| TMEM219 | -0.1138852 | 0.0745725 | -1.5272 | 0.127 | -0.161163114 | count | 1 |
| RAD1 | -0.1221791 | 0.1643353 | -0.7435 | 0.457 | -0.160868527 | count | 1 |
| FAM96A | -0.1161464 | 0.1281036 | -0.9067 | 0.365 | -0.160844897 | count | 1 |
| BTBD11 | -0.5533397 | 0.5384521 | -1.0276 | 0.304 | -0.160795625 | count | 1 |
| CNFN | -0.2740643 | 1.0755018 | -0.2548 | 0.799 | -0.160794496 | count | 1 |
| ADAT1 | -0.163969 | 0.2060407 | -0.7958 | 0.426 | -0.16078068 | count | 1 |
| RRP7A | -0.1160535 | 0.1295602 | -0.8957 | 0.37 | -0.160677606 | count | 1 |
| CHIC2 | -0.115195 | 0.0927743 | -1.2417 | 0.214 | -0.16059127 | count | 1 |
| SIRT1 | -0.1249465 | 0.1564639 | -0.7986 | 0.425 | -0.160561233 | count | 1 |
| VCP | -0.1165432 | 0.0925445 | -1.2593 | 0.208 | -0.160517916 | count | 1 |
| ARL13B | -0.1602671 | 0.2092647 | -0.7659 | 0.444 | -0.160412466 | count | 1 |
| RBM33 | -0.118356 | 0.1123425 | -1.0535 | 0.292 | -0.160385521 | count | 1 |
| CRLF1 | -1.1896158 | 1.194433 | -0.996 | 0.319 | -0.160378154 | count | 1 |
| RPS16 | -0.1112734 | 0.0187554 | -5.9329 | 3.28E-09 | -0.160279056 | count | 7.93E-05 |
| ADAMTS6 | -0.1990879 | 0.4375527 | -0.455 | 0.649 | -0.160162886 | count | 1 |
| RFNG | -0.1222475 | 0.1771016 | -0.6903 | 0.49 | -0.160147343 | count | 1 |
| RAB10 | -0.1163825 | 0.1086852 | -1.0708 | 0.284 | -0.160145754 | count | 1 |
| SPAST | -0.1378119 | 0.1742494 | -0.7909 | 0.429 | -0.160080747 | count | 1 |
| OST4 | -0.1116933 | 0.0393841 | -2.836 | 0.0046 | -0.160043989 | count | 1 |
| CRIPT | -0.1206101 | 0.154712 | -0.7796 | 0.436 | -0.160031576 | count | 1 |
| ALDOC | -0.131921 | 0.2539179 | -0.5195 | 0.603 | -0.160019445 | count | 1 |

| | | | | | | | |
|---|---|---|---|---|---|---|---|
| GPATCH4 | -0.1196541 | 0.1562573 | -0.7658 | 0.444 | -0.159933679 | count | 1 |
| MYBL2 | -1.184393 | 1.173914 | -1.0089 | 0.313 | -0.159922351 | count | 1 |
| VWA7 | -1.184393 | 1.236326 | -0.958 | 0.338 | -0.159922351 | count | 1 |
| ERICH1 | -0.1141288 | 0.0929793 | -1.2275 | 0.22 | -0.159908782 | count | 1 |
| TBC1D31 | -0.1350596 | 0.268839 | -0.5024 | 0.615 | -0.159824879 | count | 1 |
| CCS | -0.1162085 | 0.1325047 | -0.877 | 0.381 | -0.159749646 | count | 1 |
| NDUFS1 | -0.1227918 | 0.1546147 | -0.7942 | 0.427 | -0.159710637 | count | 1 |
| AL031963.3 | -0.1761158 | 0.4755404 | -0.3703 | 0.711 | -0.15968533 | count | 1 |
| STRADB | -0.2440314 | 0.390948 | -0.6242 | 0.533 | -0.159642353 | count | 1 |
| CDC42EP2 | -0.5484987 | 0.5455434 | -1.0054 | 0.315 | -0.15958269 | count | 1 |
| PET117 | -0.1264045 | 0.2585506 | -0.4889 | 0.625 | -0.159579652 | count | 1 |
| AC147651.4 | -0.159408 | 0.3769131 | -0.4229 | 0.672 | -0.159564726 | count | 1 |
| APOBEC3A | -0.5482483 | 0.8412686 | -0.6517 | 0.515 | -0.159519856 | count | 1 |
| CDC42BPG | -0.5482483 | 0.8670399 | -0.6323 | 0.527 | -0.159519856 | count | 1 |
| SLC16A13 | -0.2717203 | 0.549713 | -0.4943 | 0.621 | -0.159485339 | count | 1 |
| LTA4H | -0.1218477 | 0.149717 | -0.8139 | 0.416 | -0.15940974 | count | 1 |
| SWT1 | -0.1818332 | 0.3368895 | -0.5397 | 0.589 | -0.159382358 | count | 1 |
| WDR55 | -0.1265549 | 0.1638106 | -0.7726 | 0.44 | -0.159353943 | count | 1 |
| LRRC75A | -0.1183687 | 0.1364985 | -0.8672 | 0.386 | -0.159306033 | count | 1 |
| THAP12 | -0.1152946 | 0.1088776 | -1.0589 | 0.29 | -0.159303087 | count | 1 |
| ABHD16A | -1.177254 | 0.7415603 | -1.5875 | 0.112 | -0.159296247 | count | 1 |
| UBXN4 | -0.1116874 | 0.057651 | -1.9373 | 0.0528 | -0.159059402 | count | 1 |
| FAM241B | -0.1328657 | 0.2387086 | -0.5566 | 0.578 | -0.158925702 | count | 1 |
| FOXJ1 | -0.2086931 | 0.4527623 | -0.4609 | 0.645 | -0.158889383 | count | 1 |
| 7-Sep | -0.1105231 | 0.0346527 | -3.1894 | 0.00144 | -0.158877164 | count | 1 |
| CD72 | -0.2706087 | 0.4875793 | -0.555 | 0.579 | -0.158864078 | count | 1 |
| INPP1 | -0.1811423 | 0.3116433 | -0.5812 | 0.561 | -0.158789285 | count | 1 |
| ZNF195 | -0.1193154 | 0.1478744 | -0.8069 | 0.42 | -0.158779035 | count | 1 |
| PER3 | -0.1971403 | 0.3865546 | -0.51 | 0.61 | -0.158636011 | count | 1 |
| GNB1 | -0.1152455 | 0.1039814 | -1.1083 | 0.268 | -0.158583586 | count | 1 |
| ARID1A | -0.1210374 | 0.1280764 | -0.945 | 0.345 | -0.158567124 | count | 1 |
| CRCP | -0.1197149 | 0.1420519 | -0.8428 | 0.399 | -0.158520769 | count | 1 |
| PRPF4 | -0.1272625 | 0.1775064 | -0.7169 | 0.473 | -0.158408834 | count | 1 |
| TBC1D1 | -0.1172019 | 0.122169 | -0.9593 | 0.337 | -0.158358256 | count | 1 |
| ZNF516 | -0.3855212 | 0.7330542 | -0.5259 | 0.599 | -0.1583539 | count | 1 |
| LINC02352 | -0.3855212 | 0.7462841 | -0.5166 | 0.605 | -0.1583539 | count | 1 |
| NOL3 | -0.543519 | 0.7215662 | -0.7532 | 0.451 | -0.158331451 | count | 1 |
| EXOSC3 | -0.1187425 | 0.1477973 | -0.8034 | 0.422 | -0.158307615 | count | 1 |
| WRN | -0.1371863 | 0.2636782 | -0.5203 | 0.603 | -0.158258776 | count | 1 |
| SDHB | -0.1138013 | 0.0994403 | -1.1444 | 0.253 | -0.158187157 | count | 1 |
| MVP | -0.1162883 | 0.11641 | -0.999 | 0.318 | -0.158173343 | count | 1 |
| KXD1 | -0.1140266 | 0.114164 | -0.9988 | 0.318 | -0.158132904 | count | 1 |
| PHF7 | -0.1649207 | 0.3503196 | -0.4708 | 0.638 | -0.158083705 | count | 1 |
| CHSY1 | -0.1335627 | 0.2187393 | -0.6106 | 0.542 | -0.158065699 | count | 1 |
| POLR2H | -0.116758 | 0.1249326 | -0.9346 | 0.35 | -0.158043495 | count | 1 |

| | | | | | | | |
|---|---|---|---|---|---|---|---|
| RALGAPA1 | -0.1218486 | 0.1395729 | -0.873 | 0.383 | -0.157986505 | count | 1 |
| DDX21 | -0.1105558 | 0.0512312 | -2.158 | 0.031 | -0.157874052 | count | 1 |
| AL021368.2 | -0.5412742 | 0.502806 | -1.0765 | 0.282 | -0.157766251 | count | 1 |
| BCKDHB | -0.1545243 | 0.2419812 | -0.6386 | 0.523 | -0.157577018 | count | 1 |
| EMSY | -0.1475943 | 0.2051394 | -0.7195 | 0.472 | -0.15751244 | count | 1 |
| NDUFAF8 | -0.1131494 | 0.1072241 | -1.0553 | 0.291 | -0.157492724 | count | 1 |
| CLPB | -0.1867583 | 0.361961 | -0.516 | 0.606 | -0.157428254 | count | 1 |
| FASLG | -0.1145567 | 0.1559438 | -0.7346 | 0.463 | -0.157375746 | count | 1 |
| KIF3A | -0.1207447 | 0.1275471 | -0.9467 | 0.344 | -0.157057095 | count | 1 |
| CDKN1A | -0.1142085 | 0.1094664 | -1.0433 | 0.297 | -0.157056616 | count | 1 |
| MRPL15 | -0.1173887 | 0.1774735 | -0.6614 | 0.508 | -0.157042761 | count | 1 |
| CHPT1 | -0.1265602 | 0.1493427 | -0.8474 | 0.397 | -0.15703806 | count | 1 |
| EIF1AY | -0.1122044 | 0.0964554 | -1.1633 | 0.245 | -0.15701398 | count | 1 |
| PTMA | -0.1088575 | 0.0167318 | -6.506 | 8.85E-11 | -0.156943698 | count | 2.14E-06 |
| UBE2Q1 | -0.115933 | 0.1307919 | -0.8864 | 0.375 | -0.156836836 | count | 1 |
| GDAP1 | -0.2058513 | 0.4775358 | -0.4311 | 0.666 | -0.156787361 | count | 1 |
| TMEM250 | -0.1159365 | 0.150542 | -0.7701 | 0.441 | -0.156747679 | count | 1 |
| KIF20B | -0.1196097 | 0.1390691 | -0.8601 | 0.39 | -0.156702615 | count | 1 |
| UBN1 | -0.1136885 | 0.106166 | -1.0709 | 0.284 | -0.156687058 | count | 1 |
| EGLN3 | -0.5365711 | 0.6444053 | -0.8327 | 0.405 | -0.156579562 | count | 1 |
| CD5 | -0.1126138 | 0.1152023 | -0.9775 | 0.328 | -0.156507221 | count | 1 |
| CDC16 | -0.1222354 | 0.1958781 | -0.624 | 0.533 | -0.156469404 | count | 1 |
| ALKBH5 | -0.1195282 | 0.1294378 | -0.9234 | 0.356 | -0.156384858 | count | 1 |
| FH | -0.1232416 | 0.1747467 | -0.7053 | 0.481 | -0.156369628 | count | 1 |
| ZNF740 | -0.1532517 | 0.3957704 | -0.3872 | 0.699 | -0.156296085 | count | 1 |
| PPHLN1 | -0.1135149 | 0.0916122 | -1.2391 | 0.215 | -0.156255507 | count | 1 |
| AC108863.1 | -0.2657646 | 0.3535033 | -0.7518 | 0.452 | -0.156153607 | count | 1 |
| C6orf89 | -0.1176478 | 0.1457378 | -0.8073 | 0.42 | -0.156111646 | count | 1 |
| ZSWIM6 | -0.1851335 | 0.3279523 | -0.5645 | 0.572 | -0.156089298 | count | 1 |
| PLEKHG1 | -0.2383544 | 0.4422511 | -0.539 | 0.59 | -0.156071469 | count | 1 |
| FBH1 | -0.1411221 | 0.2373587 | -0.5946 | 0.552 | -0.155964159 | count | 1 |
| KCTD18 | -0.1426005 | 0.2792253 | -0.5107 | 0.61 | -0.155924226 | count | 1 |
| ABL2 | -0.1588419 | 0.3330579 | -0.4769 | 0.633 | -0.155827223 | count | 1 |
| GRAMD1B | -0.1527765 | 0.2713002 | -0.5631 | 0.573 | -0.155817699 | count | 1 |
| THOP1 | -0.2044713 | 0.365058 | -0.5601 | 0.575 | -0.155765956 | count | 1 |
| EFNB1 | -0.2650687 | 0.5891849 | -0.4499 | 0.653 | -0.155763804 | count | 1 |
| SSB | -0.1094334 | 0.0607883 | -1.8002 | 0.0719 | -0.155748447 | count | 1 |
| ADSL | -0.1301724 | 0.260403 | -0.4999 | 0.617 | -0.155724671 | count | 1 |
| GBP5 | -0.1094589 | 0.0712188 | -1.5369 | 0.124 | -0.155615297 | count | 1 |
| PER2 | -0.1357531 | 0.2508073 | -0.5413 | 0.588 | -0.155465212 | count | 1 |
| ZNF546 | -0.3778497 | 0.5880367 | -0.6426 | 0.521 | -0.155462657 | count | 1 |
| UBQLN2 | -0.1367637 | 0.181284 | -0.7544 | 0.451 | -0.155380922 | count | 1 |
| IFFO1 | -0.1240353 | 0.2239972 | -0.5537 | 0.58 | -0.155337169 | count | 1 |
| METTL5 | -0.1100998 | 0.0917985 | -1.1994 | 0.23 | -0.15526011 | count | 1 |
| UBXN1 | -0.1085387 | 0.0468315 | -2.3176 | 0.0205 | -0.155234094 | count | 1 |

| | | | | | | | |
|---|---|---|---|---|---|---|---|
| PSMD1 | -0.1158378 | 0.1272911 | -0.91 | 0.363 | -0.155220862 | count | 1 |
| MMD | -0.123929 | 0.1828184 | -0.6779 | 0.498 | -0.155204664 | count | 1 |
| KLF13 | -0.1091111 | 0.0603544 | -1.8078 | 0.0707 | -0.155031329 | count | 1 |
| ARFIP2 | -0.1324877 | 0.1961354 | -0.6755 | 0.499 | -0.154946893 | count | 1 |
| OMA1 | -0.1212353 | 0.1717143 | -0.706 | 0.48 | -0.154871031 | count | 1 |
| INPP5B | -0.1545885 | 0.357356 | -0.4326 | 0.665 | -0.154806376 | count | 1 |
| PEBP1 | -0.1082102 | 0.0497007 | -2.1772 | 0.0295 | -0.154762713 | count | 1 |
| PRELID1 | -0.1082722 | 0.0566363 | -1.9117 | 0.056 | -0.15473041 | count | 1 |
| AC008549.2 | -0.3758861 | 0.4867963 | -0.7722 | 0.44 | -0.154720848 | count | 1 |
| MICU1 | -0.125519 | 0.1750183 | -0.7172 | 0.473 | -0.154697106 | count | 1 |
| CAMTA1 | -0.1126117 | 0.1059208 | -1.0632 | 0.288 | -0.154654334 | count | 1 |
| HMCES | -0.1130521 | 0.1212831 | -0.9321 | 0.351 | -0.154550593 | count | 1 |
| ZNF839 | -0.1919107 | 0.300165 | -0.6394 | 0.523 | -0.15453198 | count | 1 |
| ILKAP | -0.1177299 | 0.1368027 | -0.8606 | 0.39 | -0.1544501 | count | 1 |
| LANCL2 | -0.2357628 | 0.5418072 | -0.4351 | 0.663 | -0.154438945 | count | 1 |
| PCMT1 | -0.1092134 | 0.0720154 | -1.5165 | 0.129 | -0.154381538 | count | 1 |
| USP19 | -0.2625733 | 0.4202413 | -0.6248 | 0.532 | -0.154365151 | count | 1 |
| SLX4 | -0.2356182 | 0.4219729 | -0.5584 | 0.577 | -0.154347812 | count | 1 |
| RTN4R | -0.1572653 | 0.3749437 | -0.4194 | 0.675 | -0.154303001 | count | 1 |
| KCNK5 | -1.1209403 | 0.7433336 | -1.508 | 0.132 | -0.15423317 | count | 1 |
| WARS2 | -0.1443324 | 0.2806705 | -0.5142 | 0.607 | -0.154068943 | count | 1 |
| METRNL | -0.1086813 | 0.0859108 | -1.265 | 0.206 | -0.153882995 | count | 1 |
| ARPP19 | -0.1104281 | 0.0965061 | -1.1443 | 0.253 | -0.153765192 | count | 1 |
| MYBBP1A | -0.1752648 | 0.3125109 | -0.5608 | 0.575 | -0.153739916 | count | 1 |
| FP565260.1 | -0.3732196 | 0.5371254 | -0.6948 | 0.487 | -0.153712346 | count | 1 |
| LENG1 | -0.1115913 | 0.1220046 | -0.9146 | 0.36 | -0.153707359 | count | 1 |
| ZHX1-C8orf76 | -0.2015536 | 0.6954608 | -0.2898 | 0.772 | -0.15360501 | count | 1 |
| TRAPPC3 | -0.110773 | 0.0982268 | -1.1277 | 0.26 | -0.153591738 | count | 1 |
| MPV17L2 | -0.1458687 | 0.2875529 | -0.5073 | 0.612 | -0.15358856 | count | 1 |
| AC006369.1 | -0.1271345 | 0.1865777 | -0.6814 | 0.496 | -0.153568803 | count | 1 |
| AC083843.3 | -0.3726806 | 0.7789691 | -0.4784 | 0.632 | -0.153508336 | count | 1 |
| CLTC | -0.121572 | 0.1446392 | -0.8405 | 0.401 | -0.153505318 | count | 1 |
| SNX13 | -0.1374871 | 0.2151716 | -0.639 | 0.523 | -0.153478421 | count | 1 |
| SCAMP3 | -0.1177745 | 0.1646164 | -0.7154 | 0.474 | -0.153438487 | count | 1 |
| BIRC6 | -0.113795 | 0.1091634 | -1.0424 | 0.297 | -0.153371666 | count | 1 |
| HDAC4 | -0.1690086 | 0.3364418 | -0.5023 | 0.615 | -0.153358447 | count | 1 |
| NECTIN1 | -0.5238693 | 0.7553885 | -0.6935 | 0.488 | -0.153358317 | count | 1 |
| AGBL5 | -0.5237636 | 0.6220381 | -0.842 | 0.4 | -0.153331407 | count | 1 |
| PAQR6 | -0.5237636 | 0.6481091 | -0.8081 | 0.419 | -0.153331358 | count | 1 |
| FAM210A | -0.1196241 | 0.1857484 | -0.644 | 0.52 | -0.153139624 | count | 1 |
| ATP5F1B | -0.1077256 | 0.0573894 | -1.8771 | 0.0606 | -0.153103843 | count | 1 |
| OAT | -0.116994 | 0.1375976 | -0.8503 | 0.395 | -0.152867226 | count | 1 |
| SLC38A2 | -0.1087348 | 0.0731293 | -1.4869 | 0.137 | -0.152840876 | count | 1 |
| TMEM161B | -0.1525848 | 0.2128384 | -0.7169 | 0.473 | -0.15282682 | count | 1 |
| SDCBP | -0.1073611 | 0.0574686 | -1.8682 | 0.0618 | -0.152763224 | count | 1 |

| | | | | | | | |
|---|---|---|---|---|---|---|---|
| NPPC | -0.5214055 | 0.6878183 | -0.7581 | 0.448 | -0.152730669 | count | 1 |
| ANKRD31 | -0.5214055 | 0.8626555 | -0.6044 | 0.546 | -0.152730669 | count | 1 |
| RNF32 | -0.5214055 | 0.8644036 | -0.6032 | 0.546 | -0.152730669 | count | 1 |
| BICD2 | -0.13441 | 0.3006602 | -0.447 | 0.655 | -0.152728696 | count | 1 |
| SCARB2 | -0.1215477 | 0.2176502 | -0.5585 | 0.577 | -0.152664246 | count | 1 |
| ZNF641 | -0.1471749 | 0.2729974 | -0.5391 | 0.59 | -0.152657097 | count | 1 |
| ANKIB1 | -0.1163568 | 0.1560061 | -0.7458 | 0.456 | -0.152654139 | count | 1 |
| CDC40 | -0.1090856 | 0.0865558 | -1.2603 | 0.208 | -0.152653732 | count | 1 |
| ZNF485 | -0.2329008 | 0.54954 | -0.4238 | 0.672 | -0.15263434 | count | 1 |
| GOPC | -0.1099717 | 0.1009227 | -1.0897 | 0.276 | -0.152617014 | count | 1 |
| CDK11A | -0.1119152 | 0.1193709 | -0.9375 | 0.349 | -0.152528834 | count | 1 |
| FNDC3A | -0.1143992 | 0.1434339 | -0.7976 | 0.425 | -0.152393922 | count | 1 |
| PUS7 | -0.1493616 | 0.2951286 | -0.5061 | 0.613 | -0.152378689 | count | 1 |
| ZC3H13 | -0.108945 | 0.0927902 | -1.1741 | 0.24 | -0.152353241 | count | 1 |
| CKAP2L | -0.2134873 | 0.9537331 | -0.2238 | 0.823 | -0.152167933 | count | 1 |
| AC092140.1 | -1.098365 | 0.6055948 | -1.8137 | 0.0698 | -0.152140456 | count | 1 |
| RMDN1 | -0.1126468 | 0.1835942 | -0.6136 | 0.54 | -0.151927301 | count | 1 |
| UVSSA | -0.1198954 | 0.2358093 | -0.5084 | 0.611 | -0.151775928 | count | 1 |
| GAS1 | -0.2129029 | 0.5215866 | -0.4082 | 0.683 | -0.151764519 | count | 1 |
| PDS5B | -0.1158839 | 0.168992 | -0.6857 | 0.493 | -0.151631489 | count | 1 |
| GALK2 | -0.1217551 | 0.1873429 | -0.6499 | 0.516 | -0.151586242 | count | 1 |
| CCDC136 | -0.2125768 | 0.4080655 | -0.5209 | 0.602 | -0.151539384 | count | 1 |
| COX5A | -0.1059907 | 0.0623723 | -1.6993 | 0.0894 | -0.151440967 | count | 1 |
| BPTF | -0.1072943 | 0.0672936 | -1.5944 | 0.111 | -0.151436924 | count | 1 |
| C19orf24 | -0.1074987 | 0.0898789 | -1.196 | 0.232 | -0.151260128 | count | 1 |
| TRGV9 | -0.2570143 | 0.3806926 | -0.6751 | 0.5 | -0.1512445 | count | 1 |
| MARK3 | -0.1166277 | 0.1263107 | -0.9233 | 0.356 | -0.151239974 | count | 1 |
| POC1B | -0.1210579 | 0.18438 | -0.6566 | 0.512 | -0.151182531 | count | 1 |
| NEXN | -0.5151054 | 0.6488924 | -0.7938 | 0.427 | -0.151121812 | count | 1 |
| GTF2F2 | -0.1150396 | 0.1308995 | -0.8788 | 0.38 | -0.150931165 | count | 1 |
| NDUFB6 | -0.1073531 | 0.0890036 | -1.2062 | 0.228 | -0.150731935 | count | 1 |
| FLCN | -0.1785634 | 0.3421046 | -0.522 | 0.602 | -0.150669231 | count | 1 |
| LINC00528 | -0.2959919 | 0.5023346 | -0.5892 | 0.556 | -0.150623675 | count | 1 |
| TRPM7 | -0.1130688 | 0.1440171 | -0.7851 | 0.432 | -0.150487129 | count | 1 |
| ZBTB43 | -0.1161887 | 0.1618618 | -0.7178 | 0.473 | -0.150423232 | count | 1 |
| IDH2 | -0.1065742 | 0.0809034 | -1.3173 | 0.188 | -0.150421229 | count | 1 |
| SNHG12 | -0.1076411 | 0.0895541 | -1.202 | 0.229 | -0.150402222 | count | 1 |
| PLCB2 | -0.1408304 | 0.2251764 | -0.6254 | 0.532 | -0.150369969 | count | 1 |
| HIST1H2AH | -0.2554059 | 0.578735 | -0.4413 | 0.659 | -0.150340341 | count | 1 |
| CYTH2 | -0.1122564 | 0.1285642 | -0.8732 | 0.383 | -0.150313963 | count | 1 |
| FXR2 | -0.1530995 | 0.316086 | -0.4844 | 0.628 | -0.150273299 | count | 1 |
| ZMYM4 | -0.1136893 | 0.1618862 | -0.7023 | 0.483 | -0.150238853 | count | 1 |
| GLTP | -0.1095825 | 0.1099974 | -0.9962 | 0.319 | -0.150229171 | count | 1 |
| REM2 | -0.295136 | 0.839114 | -0.3517 | 0.725 | -0.150212968 | count | 1 |
| ESCO2 | -0.295136 | 0.8600989 | -0.3431 | 0.732 | -0.150212968 | count | 1 |

| | | | | | | | |
|---|---|---|---|---|---|---|---|
| FBXO10 | -0.295136 | 0.9697836 | -0.3043 | 0.761 | -0.150212968 | count | 1 |
| CDC27 | -0.1130459 | 0.1328447 | -0.851 | 0.395 | -0.150169513 | count | 1 |
| PSMC5 | -0.1061067 | 0.0688927 | -1.5402 | 0.124 | -0.150161813 | count | 1 |
| CD6 | -0.1063477 | 0.0825039 | -1.289 | 0.197 | -0.150027606 | count | 1 |
| NEK6 | -0.1562559 | 0.3573726 | -0.4372 | 0.662 | -0.149903747 | count | 1 |
| NCOA1 | -0.1131743 | 0.1359732 | -0.8323 | 0.405 | -0.149882941 | count | 1 |
| CHRNA5 | -0.363001 | 0.5994343 | -0.6056 | 0.545 | -0.149835326 | count | 1 |
| CREG1 | -0.1280781 | 0.2178903 | -0.5878 | 0.557 | -0.149825516 | count | 1 |
| GABARAPL2 | -0.1051382 | 0.0588797 | -1.7856 | 0.0742 | -0.149771214 | count | 1 |
| DOCK2 | -0.11413 | 0.1206403 | -0.946 | 0.344 | -0.149741282 | count | 1 |
| HYI | -0.1214101 | 0.2356192 | -0.5153 | 0.606 | -0.149658596 | count | 1 |
| AP000866.5 | -0.5091635 | 1.2716612 | -0.4004 | 0.689 | -0.149598942 | count | 1 |
| GRHL1 | -0.5091635 | 1.3458378 | -0.3783 | 0.705 | -0.149598942 | count | 1 |
| RAI14 | -0.5091635 | 1.3458378 | -0.3783 | 0.705 | -0.149598942 | count | 1 |
| VGF | -0.5091635 | 1.3458378 | -0.3783 | 0.705 | -0.149598942 | count | 1 |
| C9orf116 | -0.5091635 | 1.3458378 | -0.3783 | 0.705 | -0.149598942 | count | 1 |
| SCARF1 | -0.5091635 | 1.3458378 | -0.3783 | 0.705 | -0.149598942 | count | 1 |
| STX16-NPEPL1 | -0.5091635 | 1.3458378 | -0.3783 | 0.705 | -0.149598942 | count | 1 |
| FUT11 | -0.1201184 | 0.2259474 | -0.5316 | 0.595 | -0.149558084 | count | 1 |
| AL359644.1 | -0.508914 | 0.8630528 | -0.5897 | 0.555 | -0.149534878 | count | 1 |
| ZNF260 | -0.1464557 | 0.3183453 | -0.4601 | 0.646 | -0.149450598 | count | 1 |
| AC107959.1 | -0.5083894 | 0.5980377 | -0.8501 | 0.395 | -0.14940016 | count | 1 |
| DUSP14 | -0.112041 | 0.1499414 | -0.7472 | 0.455 | -0.149394781 | count | 1 |
| FAM149A | -1.0686116 | 1.064636 | -1.0037 | 0.316 | -0.149326195 | count | 1 |
| RNASEH2B | -0.1061019 | 0.0862059 | -1.2308 | 0.218 | -0.149309512 | count | 1 |
| SART3 | -0.1149327 | 0.1666418 | -0.6897 | 0.49 | -0.14928875 | count | 1 |
| ISY1 | -0.1165825 | 0.1508189 | -0.773 | 0.44 | -0.149260397 | count | 1 |
| SDF2L1 | -0.1055756 | 0.0878604 | -1.2016 | 0.23 | -0.149141182 | count | 1 |
| MGAT1 | -0.1079469 | 0.1046633 | -1.0314 | 0.302 | -0.149123827 | count | 1 |
| CBR4 | -0.1346578 | 0.3474727 | -0.3875 | 0.698 | -0.148884446 | count | 1 |
| AP003716.1 | -1.063351 | 0.6026754 | -1.7644 | 0.0778 | -0.148821853 | count | 1 |
| CTBP2 | -0.1695116 | 0.4287857 | -0.3953 | 0.693 | -0.148790261 | count | 1 |
| SMIM12 | -0.1076666 | 0.1156 | -0.9314 | 0.352 | -0.148777073 | count | 1 |
| AC233723.1 | -0.5059046 | 0.7950011 | -0.6364 | 0.525 | -0.148761508 | count | 1 |
| ESPN | -0.2266205 | 0.7442986 | -0.3045 | 0.761 | -0.148667999 | count | 1 |
| SLC25A4 | -0.1128206 | 0.1801991 | -0.6261 | 0.531 | -0.148581123 | count | 1 |
| STMN3 | -0.1108676 | 0.1261781 | -0.8787 | 0.38 | -0.148575806 | count | 1 |
| DNAJC2 | -0.1071714 | 0.1104648 | -0.9702 | 0.332 | -0.148535923 | count | 1 |
| UVRAG | -0.1091207 | 0.1222415 | -0.8927 | 0.372 | -0.148441386 | count | 1 |
| TAOK3 | -0.1063747 | 0.0813732 | -1.3072 | 0.191 | -0.14840538 | count | 1 |
| NBPF19 | -0.1945206 | 0.2691949 | -0.7226 | 0.47 | -0.148388388 | count | 1 |
| NFATC2IP | -0.1150001 | 0.1384031 | -0.8309 | 0.406 | -0.148373648 | count | 1 |
| TGFBRAP1 | -0.1543889 | 0.2946809 | -0.5239 | 0.6 | -0.148139286 | count | 1 |
| FRAT1 | -0.1266196 | 0.2136446 | -0.5927 | 0.553 | -0.148131019 | count | 1 |
| SRPK1 | -0.1082645 | 0.1059404 | -1.0219 | 0.307 | -0.148016131 | count | 1 |

| | | | | | | | |
|---|---|---|---|---|---|---|---|
| PAK2 | -0.103844 | 0.0581012 | -1.7873 | 0.074 | -0.147951917 | count | 1 |
| C16orf87 | -0.1080609 | 0.1337807 | -0.8077 | 0.419 | -0.14791828 | count | 1 |
| IARS | -0.1229919 | 0.216358 | -0.5685 | 0.57 | -0.147906529 | count | 1 |
| TRIM8 | -0.112577 | 0.1142063 | -0.9857 | 0.324 | -0.147898188 | count | 1 |
| CCZ1 | -0.1120357 | 0.1434647 | -0.7809 | 0.435 | -0.147893895 | count | 1 |
| EPHA1-AS1 | -0.1281241 | 0.2352668 | -0.5446 | 0.586 | -0.147881312 | count | 1 |
| EFHC1 | -0.1750841 | 0.3120565 | -0.5611 | 0.575 | -0.14779519 | count | 1 |
| SNX24 | -0.1217856 | 0.2576771 | -0.4726 | 0.637 | -0.147793304 | count | 1 |
| COPE | -0.1035343 | 0.0542218 | -1.9095 | 0.0563 | -0.147785524 | count | 1 |
| MFSD11 | -0.1141398 | 0.1568311 | -0.7278 | 0.467 | -0.147779457 | count | 1 |
| HIP1 | -0.1334968 | 0.2921178 | -0.457 | 0.648 | -0.14761222 | count | 1 |
| TRAV30 | -0.2894783 | 0.9593818 | -0.3017 | 0.763 | -0.147494293 | count | 1 |
| ACVR1C | -0.5009497 | 0.5003333 | -1.0012 | 0.317 | -0.147485288 | count | 1 |
| COMMD4 | -0.1084563 | 0.1274099 | -0.8512 | 0.395 | -0.147465338 | count | 1 |
| YLPM1 | -0.1252554 | 0.2169301 | -0.5774 | 0.564 | -0.147444747 | count | 1 |
| AC074044.1 | -0.2893289 | 0.5897485 | -0.4906 | 0.624 | -0.147422412 | count | 1 |
| TECPR2 | -0.1746101 | 0.3582058 | -0.4875 | 0.626 | -0.147403446 | count | 1 |
| METTL18 | -0.1276412 | 0.2414185 | -0.5287 | 0.597 | -0.147328004 | count | 1 |
| RAB14 | -0.1053153 | 0.0795748 | -1.3235 | 0.186 | -0.147261359 | count | 1 |
| CKAP5 | -0.1147616 | 0.2074632 | -0.5532 | 0.58 | -0.147233439 | count | 1 |
| MRNIP | -0.1212394 | 0.2153826 | -0.5629 | 0.574 | -0.147134094 | count | 1 |
| MEF2C | -0.2061533 | 0.5120124 | -0.4026 | 0.687 | -0.147099702 | count | 1 |
| AL139246.3 | -0.1619236 | 0.4338319 | -0.3732 | 0.709 | -0.147040937 | count | 1 |
| LFNG | -0.1077247 | 0.1333829 | -0.8076 | 0.419 | -0.146961468 | count | 1 |
| PYM1 | -0.1073604 | 0.1297757 | -0.8273 | 0.408 | -0.1469609 | count | 1 |
| FBXW5 | -0.1056578 | 0.0943952 | -1.1193 | 0.263 | -0.146699734 | count | 1 |
| AL133453.1 | -0.1086911 | 0.1184182 | -0.9179 | 0.359 | -0.146697464 | count | 1 |
| MARS | -0.1326069 | 0.2064022 | -0.6425 | 0.521 | -0.146636925 | count | 1 |
| PCNP | -0.1047038 | 0.0813856 | -1.2865 | 0.198 | -0.146622877 | count | 1 |
| ATP6V0C | -0.205081 | 0.5736484 | -0.3575 | 0.721 | -0.146357678 | count | 1 |
| RPP38 | -0.1077438 | 0.1366772 | -0.7883 | 0.431 | -0.146347921 | count | 1 |
| B3GNT2 | -0.1078 | 0.109261 | -0.9866 | 0.324 | -0.146346994 | count | 1 |
| THADA | -0.1814424 | 0.2493311 | -0.7277 | 0.467 | -0.14629876 | count | 1 |
| SNUPN | -0.1171049 | 0.1876909 | -0.6239 | 0.533 | -0.146267932 | count | 1 |
| MRPS36 | -0.1048802 | 0.0945112 | -1.1097 | 0.267 | -0.146250464 | count | 1 |
| ARHGEF12 | -0.1321351 | 0.3256699 | -0.4057 | 0.685 | -0.146119801 | count | 1 |
| CCDC85C | -0.2046 | 0.5387874 | -0.3797 | 0.704 | -0.146029869 | count | 1 |
| PSIP1 | -0.1026504 | 0.059302 | -1.731 | 0.0835 | -0.146013862 | count | 1 |
| REPS1 | -0.1319291 | 0.2672686 | -0.4936 | 0.622 | -0.145893998 | count | 1 |
| DRAIC | -0.3525543 | 0.5819336 | -0.6058 | 0.545 | -0.145851703 | count | 1 |
| CEP164 | -0.1109481 | 0.1718411 | -0.6456 | 0.519 | -0.145764235 | count | 1 |
| SYNCRIP | -0.1034525 | 0.0799386 | -1.2941 | 0.196 | -0.145684699 | count | 1 |
| COG6 | -0.172414 | 0.2845156 | -0.606 | 0.545 | -0.145587819 | count | 1 |
| MON1A | -0.1656491 | 0.3932384 | -0.4212 | 0.674 | -0.145463328 | count | 1 |
| TUBA1B | -0.1013761 | 0.0414692 | -2.4446 | 0.0146 | -0.145331915 | count | 1 |

| | | | | | | | |
|---|---|---|---|---|---|---|---|
| ALS2 | -0.2212282 | 0.4409258 | -0.5017 | 0.616 | -0.145255517 | count | 1 |
| H2AFY2 | -0.2033885 | 0.4477362 | -0.4543 | 0.65 | -0.145185959 | count | 1 |
| IBTK | -0.1233042 | 0.160922 | -0.7662 | 0.444 | -0.145162718 | count | 1 |
| AC020915.3 | -0.1299491 | 0.2657162 | -0.4891 | 0.625 | -0.145134318 | count | 1 |
| SNRPG | -0.1020569 | 0.0587088 | -1.7384 | 0.0822 | -0.145073838 | count | 1 |
| NIPA2 | -0.1119702 | 0.1378415 | -0.8123 | 0.417 | -0.144979573 | count | 1 |
| NUP50 | -0.1080492 | 0.1242881 | -0.8693 | 0.385 | -0.14491894 | count | 1 |
| TMC4 | -0.4908279 | 0.7056292 | -0.6956 | 0.487 | -0.144866812 | count | 1 |
| AC005332.6 | -0.4908279 | 0.7241577 | -0.6778 | 0.498 | -0.144866812 | count | 1 |
| LINC01281 | -0.4906408 | 0.8495164 | -0.5776 | 0.564 | -0.144818265 | count | 1 |
| AL627422.2 | -0.4906408 | 0.9347484 | -0.5249 | 0.6 | -0.144818265 | count | 1 |
| GALNT7 | -0.1273874 | 0.2416533 | -0.5271 | 0.598 | -0.144810581 | count | 1 |
| MYO1C | -0.1714564 | 0.3483233 | -0.4922 | 0.623 | -0.144795801 | count | 1 |
| UQCRQ | -0.1019721 | 0.0688176 | -1.4818 | 0.138 | -0.144764523 | count | 1 |
| RAP1A | -0.1019507 | 0.0596027 | -1.7105 | 0.0873 | -0.144760859 | count | 1 |
| WWC3 | -0.1140028 | 0.2215363 | -0.5146 | 0.607 | -0.144693184 | count | 1 |
| WDR13 | -0.1177783 | 0.1905449 | -0.6181 | 0.537 | -0.144677595 | count | 1 |
| METTL25 | -0.1262593 | 0.258985 | -0.4875 | 0.626 | -0.144673643 | count | 1 |
| ZMAT3 | -0.1214963 | 0.221337 | -0.5489 | 0.583 | -0.144659854 | count | 1 |
| ZSCAN12 | -0.2025535 | 0.5750316 | -0.3522 | 0.725 | -0.144607653 | count | 1 |
| AL009179.1 | -1.019297 | 0.905684 | -1.1254 | 0.26 | -0.144518828 | count | 1 |
| KBTBD7 | -0.4893488 | 0.5305058 | -0.9224 | 0.356 | -0.144482947 | count | 1 |
| HSPA9 | -0.1024555 | 0.0743777 | -1.3775 | 0.168 | -0.144465687 | count | 1 |
| RPL15 | -0.1002122 | 0.0185734 | -5.3955 | 7.30E-08 | -0.144352667 | count | 0.001763096 |
| TFAM | -0.105047 | 0.1050351 | -1.0001 | 0.317 | -0.144330094 | count | 1 |
| PIGW | -0.2020493 | 0.4299021 | -0.47 | 0.638 | -0.144258382 | count | 1 |
| SLC37A3 | -0.1257638 | 0.3002296 | -0.4189 | 0.675 | -0.144110056 | count | 1 |
| AC023157.3 | -0.1640351 | 0.32239 | -0.5088 | 0.611 | -0.144072194 | count | 1 |
| CA11 | -0.2442744 | 0.3469916 | -0.704 | 0.481 | -0.144067432 | count | 1 |
| HAUS8 | -0.1229424 | 0.2045716 | -0.601 | 0.548 | -0.143857551 | count | 1 |
| SLC7A6 | -0.1214751 | 0.2186247 | -0.5556 | 0.578 | -0.143849572 | count | 1 |
| TRANK1 | -0.1099009 | 0.1627281 | -0.6754 | 0.499 | -0.143825565 | count | 1 |
| ADAM9 | -0.1782626 | 0.7494041 | -0.2379 | 0.812 | -0.143793147 | count | 1 |
| NSMAF | -0.1287371 | 0.2084961 | -0.6175 | 0.537 | -0.143791898 | count | 1 |
| STAG1 | -0.1138394 | 0.1537368 | -0.7405 | 0.459 | -0.143780996 | count | 1 |
| ELP3 | -0.1312993 | 0.2345274 | -0.5598 | 0.576 | -0.143679392 | count | 1 |
| CCT4 | -0.1018404 | 0.0780025 | -1.3056 | 0.192 | -0.143652245 | count | 1 |
| RANBP1 | -0.1018473 | 0.0761613 | -1.3373 | 0.181 | -0.143542355 | count | 1 |
| MEX3C | -0.1168039 | 0.1742637 | -0.6703 | 0.503 | -0.143486544 | count | 1 |
| AC003102.1 | -0.1460138 | 0.4549855 | -0.3209 | 0.748 | -0.143411474 | count | 1 |
| DHDDS | -0.118656 | 0.2264191 | -0.5241 | 0.6 | -0.143383681 | count | 1 |
| COMMD3 | -0.1036277 | 0.1224892 | -0.846 | 0.398 | -0.143240858 | count | 1 |
| C16orf74 | -0.2427411 | 0.4572583 | -0.5309 | 0.596 | -0.143201278 | count | 1 |
| FBXO32 | -0.1049637 | 0.1040231 | -1.009 | 0.313 | -0.143003328 | count | 1 |
| AP2M1 | -0.1015441 | 0.0793669 | -1.2794 | 0.201 | -0.142848597 | count | 1 |

| | | | | | | | |
|---|---|---|---|---|---|---|---|
| ZNF253 | -0.1424796 | 0.2486809 | -0.5729 | 0.567 | -0.142831964 | count | 1 |
| ATP5MPL | -0.1010853 | 0.0745201 | -1.3565 | 0.175 | -0.1428198 | count | 1 |
| GUCD1 | -0.1133521 | 0.2112107 | -0.5367 | 0.592 | -0.142797566 | count | 1 |
| ANK1 | -1.0017508 | 0.9365309 | -1.0696 | 0.285 | -0.142764634 | count | 1 |
| PRRC2A | -0.1265905 | 0.195359 | -0.648 | 0.517 | -0.142701715 | count | 1 |
| SRSF11 | -0.1000142 | 0.0547161 | -1.8279 | 0.0677 | -0.142691755 | count | 1 |
| TFAP4 | -0.1687415 | 0.3823618 | -0.4413 | 0.659 | -0.142549278 | count | 1 |
| ACAP1 | -0.1002193 | 0.0511681 | -1.9586 | 0.0502 | -0.142548915 | count | 1 |
| RNFT1 | -0.1133533 | 0.1692369 | -0.6698 | 0.503 | -0.142414919 | count | 1 |
| SLC2A13 | -0.4811469 | 0.4597846 | -1.0465 | 0.295 | -0.142348234 | count | 1 |
| UQCC2 | -0.1036424 | 0.1346334 | -0.7698 | 0.441 | -0.142253479 | count | 1 |
| DNAJC5 | -0.1144912 | 0.211948 | -0.5402 | 0.589 | -0.142130851 | count | 1 |
| ADCY3 | -0.1329354 | 0.4192129 | -0.3171 | 0.751 | -0.142023339 | count | 1 |
| HINT1 | -0.09871 | 0.0298601 | -3.3057 | 0.000957 | -0.141952007 | count | 1 |
| APOL1 | -0.1678732 | 0.3253642 | -0.516 | 0.606 | -0.141830449 | count | 1 |
| CEP170 | -0.115002 | 0.2088151 | -0.5507 | 0.582 | -0.141797057 | count | 1 |
| DIRC3 | -0.4785559 | 0.7285645 | -0.6568 | 0.511 | -0.141671759 | count | 1 |
| TPBG | -0.4785559 | 0.8284391 | -0.5777 | 0.564 | -0.141671759 | count | 1 |
| LMBR1L | -0.1161606 | 0.1741875 | -0.6669 | 0.505 | -0.141593745 | count | 1 |
| MIIP | -0.1077628 | 0.1512105 | -0.7127 | 0.476 | -0.141590767 | count | 1 |
| TMED5 | -0.1026969 | 0.1010618 | -1.0162 | 0.31 | -0.14155797 | count | 1 |
| TLN1 | -0.0993711 | 0.0571806 | -1.7378 | 0.0823 | -0.141545618 | count | 1 |
| ATP5PD | -0.0991876 | 0.0609976 | -1.6261 | 0.104 | -0.141512798 | count | 1 |
| KRI1 | -0.1123084 | 0.1934577 | -0.5805 | 0.562 | -0.141488045 | count | 1 |
| ACOT11 | -0.4776673 | 1.020033 | -0.4683 | 0.64 | -0.141439544 | count | 1 |
| GRAPL | -0.4776673 | 1.1027796 | -0.4331 | 0.665 | -0.141439544 | count | 1 |
| MUM1 | -0.1208153 | 0.216251 | -0.5587 | 0.576 | -0.141384717 | count | 1 |
| TRIM28 | -0.1084731 | 0.1388993 | -0.7809 | 0.435 | -0.141356601 | count | 1 |
| RAD51C | -0.1050547 | 0.157671 | -0.6663 | 0.505 | -0.141324795 | count | 1 |
| GNG5 | -0.0988817 | 0.0546627 | -1.8089 | 0.0705 | -0.141274795 | count | 1 |
| CUL7 | -0.1848653 | 0.6766759 | -0.2732 | 0.785 | -0.141208921 | count | 1 |
| CD40LG | -0.1015599 | 0.1022948 | -0.9928 | 0.321 | -0.141132684 | count | 1 |
| C5orf34 | -0.1320885 | 0.4738347 | -0.2788 | 0.78 | -0.141127372 | count | 1 |
| CAPN7 | -0.1095328 | 0.1509938 | -0.7254 | 0.468 | -0.141086109 | count | 1 |
| DDX46 | -0.0988139 | 0.0609144 | -1.6222 | 0.1049 | -0.140742272 | count | 1 |
| RPP21 | -0.1316898 | 0.2196931 | -0.5994 | 0.549 | -0.140705529 | count | 1 |
| RAD21 | -0.100447 | 0.0720826 | -1.3935 | 0.164 | -0.140630632 | count | 1 |
| PPP1R12B | -0.2751898 | 0.3851438 | -0.7145 | 0.475 | -0.140598997 | count | 1 |
| NFYB | -0.1047058 | 0.174083 | -0.6015 | 0.548 | -0.140550186 | count | 1 |
| PERP | -0.1048666 | 0.1367062 | -0.7671 | 0.443 | -0.140326864 | count | 1 |
| GINM1 | -0.1040143 | 0.1153812 | -0.9015 | 0.367 | -0.140306924 | count | 1 |
| PLK2 | -0.1596124 | 0.3163017 | -0.5046 | 0.614 | -0.14025741 | count | 1 |
| EMILIN2 | -0.119108 | 0.2171266 | -0.5486 | 0.583 | -0.140253367 | count | 1 |
| MPHOSPH10 | -0.1005428 | 0.0977203 | -1.0289 | 0.304 | -0.140184493 | count | 1 |
| OXLD1 | -0.1008612 | 0.1248044 | -0.8082 | 0.419 | -0.14013429 | count | 1 |

| | | | | | | | |
|---|---|---|---|---|---|---|---|
| LCMT1 | -0.1018523 | 0.1283372 | -0.7936 | 0.427 | -0.139946722 | count | 1 |
| COMTD1 | -0.1021749 | 0.1302316 | -0.7846 | 0.433 | -0.139928191 | count | 1 |
| SLC12A6 | -0.1456739 | 0.3026694 | -0.4813 | 0.63 | -0.139893919 | count | 1 |
| NOP14 | -0.1042922 | 0.1435829 | -0.7264 | 0.468 | -0.139890374 | count | 1 |
| MGST3 | -0.0983744 | 0.0670191 | -1.4679 | 0.142 | -0.139819042 | count | 1 |
| METRN | -0.1040954 | 0.1608277 | -0.6472 | 0.518 | -0.139732498 | count | 1 |
| STAT5A | -0.1367175 | 0.1840069 | -0.743 | 0.458 | -0.139626873 | count | 1 |
| CD96 | -0.0983474 | 0.066362 | -1.482 | 0.138 | -0.13955124 | count | 1 |
| ZC4H2 | -0.1952265 | 0.442351 | -0.4413 | 0.659 | -0.13952651 | count | 1 |
| GPR132 | -0.1084711 | 0.1397759 | -0.776 | 0.438 | -0.139460924 | count | 1 |
| TES | -0.0994051 | 0.0965794 | -1.0293 | 0.303 | -0.139395334 | count | 1 |
| SMARCAD1 | -0.1134268 | 0.1660524 | -0.6831 | 0.495 | -0.139357767 | count | 1 |
| TRIM22 | -0.0993063 | 0.0858223 | -1.1571 | 0.247 | -0.139273011 | count | 1 |
| PIGBOS1 | -0.1091963 | 0.2017726 | -0.5412 | 0.588 | -0.13924645 | count | 1 |
| ANKRD13A | -0.1070823 | 0.16511 | -0.6486 | 0.517 | -0.139122473 | count | 1 |
| PHC1 | -0.1362161 | 0.2569436 | -0.5301 | 0.596 | -0.139120607 | count | 1 |
| TRMT12 | -0.1270898 | 0.2987301 | -0.4254 | 0.671 | -0.139113185 | count | 1 |
| MRPS24 | -0.3349588 | 0.6738165 | -0.4971 | 0.619 | -0.139096343 | count | 1 |
| CRB3 | -0.3349588 | 0.7010602 | -0.4778 | 0.633 | -0.139096343 | count | 1 |
| DNAJA3 | -0.1244987 | 0.2140462 | -0.5816 | 0.561 | -0.139095665 | count | 1 |
| TMCC2 | -0.9631994 | 1.0348657 | -0.9307 | 0.352 | -0.138828658 | count | 1 |
| TUT4 | -0.0988402 | 0.0733414 | -1.3477 | 0.178 | -0.1387881 | count | 1 |
| MAP7D1 | -0.1017573 | 0.1080419 | -0.9418 | 0.346 | -0.138641881 | count | 1 |
| ZNF502 | -0.4666016 | 0.6265905 | -0.7447 | 0.457 | -0.138537999 | count | 1 |
| MAGIX | -0.4665158 | 0.8016576 | -0.5819 | 0.561 | -0.138515391 | count | 1 |
| DBF4B | -0.4665158 | 0.8695977 | -0.5365 | 0.592 | -0.138515391 | count | 1 |
| AL031733.2 | -0.4665158 | 0.8835441 | -0.528 | 0.598 | -0.138515391 | count | 1 |
| TMEM198 | -0.4665158 | 1.015752 | -0.4593 | 0.646 | -0.138515391 | count | 1 |
| NDUFA4 | -0.0965773 | 0.0408647 | -2.3633 | 0.0182 | -0.138482126 | count | 1 |
| PAIP2 | -0.0970051 | 0.053617 | -1.8092 | 0.0705 | -0.138457031 | count | 1 |
| SHROOM1 | -0.9594881 | 0.8084821 | -1.1868 | 0.235 | -0.138443751 | count | 1 |
| GBP2 | -0.0988102 | 0.0856421 | -1.1538 | 0.249 | -0.138411428 | count | 1 |
| AL451085.2 | -0.1478597 | 0.3305381 | -0.4473 | 0.655 | -0.138403663 | count | 1 |
| SEC24B | -0.1292958 | 0.2239901 | -0.5772 | 0.564 | -0.138172022 | count | 1 |
| DPH6 | -0.1137737 | 0.2261294 | -0.5031 | 0.615 | -0.138120235 | count | 1 |
| RTP5 | -0.332245 | 1.0616461 | -0.313 | 0.754 | -0.138049393 | count | 1 |
| TRAV1-2 | -0.332245 | 1.162242 | -0.2859 | 0.775 | -0.138049393 | count | 1 |
| SLC7A11 | -0.332245 | 1.3045372 | -0.2547 | 0.799 | -0.138049393 | count | 1 |
| AC079171.1 | -0.332245 | 1.3045372 | -0.2547 | 0.799 | -0.138049393 | count | 1 |
| AC244197.2 | -0.332245 | 1.3045372 | -0.2547 | 0.799 | -0.138049393 | count | 1 |
| AL731569.1 | -0.332245 | 1.3045372 | -0.2547 | 0.799 | -0.138049393 | count | 1 |
| DIAPH3 | -0.332245 | 1.3045372 | -0.2547 | 0.799 | -0.138049393 | count | 1 |
| CDRT4 | -0.332245 | 1.3045372 | -0.2547 | 0.799 | -0.138049393 | count | 1 |
| ZGLP1 | -0.332245 | 1.3045372 | -0.2547 | 0.799 | -0.138049393 | count | 1 |
| TMEM38A | -0.332245 | 1.3045372 | -0.2547 | 0.799 | -0.138049393 | count | 1 |

| | | | | | | | |
|---|---|---|---|---|---|---|---|
| CYP2S1 | -0.332245 | 1.3045372 | -0.2547 | 0.799 | -0.138049393 | count | 1 |
| LHB | -0.332245 | 1.3045372 | -0.2547 | 0.799 | -0.138049393 | count | 1 |
| FAM71E1 | -0.332245 | 1.3045372 | -0.2547 | 0.799 | -0.138049393 | count | 1 |
| MTHFD2 | -0.100656 | 0.0869875 | -1.1571 | 0.247 | -0.137904442 | count | 1 |
| KLF12 | -0.1107058 | 0.1588811 | -0.6968 | 0.486 | -0.137888898 | count | 1 |
| MIER3 | -0.1472263 | 0.2638619 | -0.558 | 0.577 | -0.137819528 | count | 1 |
| AURKAIP1 | -0.0969721 | 0.069922 | -1.3869 | 0.166 | -0.137799823 | count | 1 |
| CAVIN1 | -0.3315378 | 0.4777705 | -0.6939 | 0.488 | -0.137776285 | count | 1 |
| POLR3GL | -0.0970717 | 0.0725671 | -1.3377 | 0.181 | -0.137719842 | count | 1 |
| TRGV10 | -0.1514809 | 0.3301222 | -0.4589 | 0.646 | -0.137710706 | count | 1 |
| COPS2 | -0.1000519 | 0.0973288 | -1.028 | 0.304 | -0.137703758 | count | 1 |
| POLD4 | -0.1325582 | 0.3025875 | -0.4381 | 0.661 | -0.13765721 | count | 1 |
| PTPRN2 | -0.1076644 | 0.2409149 | -0.4469 | 0.655 | -0.137595568 | count | 1 |
| RAB6B | -0.9513097 | 0.8509126 | -1.118 | 0.264 | -0.137591816 | count | 1 |
| MRPL12 | -0.1026682 | 0.1556217 | -0.6597 | 0.509 | -0.137501897 | count | 1 |
| MTF1 | -0.1270516 | 0.2869211 | -0.4428 | 0.658 | -0.137492448 | count | 1 |
| AC004854.2 | -0.1702444 | 0.4526232 | -0.3761 | 0.707 | -0.137465247 | count | 1 |
| MAPK9 | -0.1127545 | 0.1759118 | -0.641 | 0.522 | -0.137462422 | count | 1 |
| FANCD2 | -0.3306139 | 0.4244756 | -0.7789 | 0.436 | -0.137419414 | count | 1 |
| AIP | -0.0967629 | 0.0677772 | -1.4277 | 0.153 | -0.137392802 | count | 1 |
| PTDSS1 | -0.122934 | 0.1985328 | -0.6192 | 0.536 | -0.137361256 | count | 1 |
| FRAT2 | -0.1043974 | 0.1374605 | -0.7595 | 0.448 | -0.137350683 | count | 1 |
| ZNHIT1 | -0.0968615 | 0.0774749 | -1.2502 | 0.211 | -0.137329412 | count | 1 |
| ICE2 | -0.1031194 | 0.150919 | -0.6833 | 0.494 | -0.137275023 | count | 1 |
| AL022238.2 | -0.9471826 | 0.7487988 | -1.2649 | 0.206 | -0.137159941 | count | 1 |
| GLB1 | -0.107779 | 0.2163659 | -0.4981 | 0.618 | -0.137139441 | count | 1 |
| CDC6 | -0.4612428 | 0.8098479 | -0.5695 | 0.569 | -0.137126278 | count | 1 |
| AKT1 | -0.1009384 | 0.1284027 | -0.7861 | 0.432 | -0.137119575 | count | 1 |
| ZNF526 | -0.3296526 | 0.3979215 | -0.8284 | 0.407 | -0.137047927 | count | 1 |
| RTL8A | -0.1074592 | 0.2354753 | -0.4564 | 0.648 | -0.137039109 | count | 1 |
| CCT6B | -0.2674432 | 0.6814625 | -0.3925 | 0.695 | -0.136843189 | count | 1 |
| TXNL4A | -0.0966999 | 0.0754434 | -1.2818 | 0.2 | -0.136841646 | count | 1 |
| LYRM1 | -0.102427 | 0.1516757 | -0.6753 | 0.5 | -0.136841343 | count | 1 |
| VEZF1 | -0.1011545 | 0.1295094 | -0.7811 | 0.435 | -0.13671505 | count | 1 |
| XRN2 | -0.0965932 | 0.0702991 | -1.374 | 0.17 | -0.136698051 | count | 1 |
| NQO2 | -0.101467 | 0.165423 | -0.6134 | 0.54 | -0.136696005 | count | 1 |
| SMARCE1 | -0.1069505 | 0.1462783 | -0.7311 | 0.465 | -0.136686342 | count | 1 |
| AL596223.1 | -0.9404613 | 1.458832 | -0.6447 | 0.519 | -0.136453792 | count | 1 |
| MICU2 | -0.1015571 | 0.1121335 | -0.9057 | 0.365 | -0.136432867 | count | 1 |
| TARS2 | -0.1499659 | 0.3340074 | -0.449 | 0.653 | -0.136355261 | count | 1 |
| COX7A2 | -0.0951198 | 0.0450165 | -2.113 | 0.0347 | -0.136225991 | count | 1 |
| ZNF3 | -0.1274 | 0.2830486 | -0.4501 | 0.653 | -0.136165066 | count | 1 |
| COX20 | -0.0974816 | 0.0852009 | -1.1441 | 0.253 | -0.136137642 | count | 1 |
| FBL | -0.0983349 | 0.0892785 | -1.1014 | 0.271 | -0.136106858 | count | 1 |
| CUL4A | -0.1178108 | 0.2021051 | -0.5829 | 0.56 | -0.13605723 | count | 1 |

| | | | | | | | |
|---|---|---|---|---|---|---|---|
| TTC22 | -0.1496189 | 0.3795719 | -0.3942 | 0.693 | -0.136044739 | count | 1 |
| CD46 | -0.0970144 | 0.0804617 | -1.2057 | 0.228 | -0.136013576 | count | 1 |
| NCEH1 | -0.4564453 | 0.627776 | -0.7271 | 0.467 | -0.135858896 | count | 1 |
| PSMA3 | -0.0961043 | 0.0692957 | -1.3869 | 0.166 | -0.135743003 | count | 1 |
| NRBF2 | -0.0966467 | 0.0865933 | -1.1161 | 0.264 | -0.135681441 | count | 1 |
| SMIM7 | -0.0972939 | 0.1084567 | -0.8971 | 0.37 | -0.135681422 | count | 1 |
| CRKL | -0.1092383 | 0.2433869 | -0.4488 | 0.654 | -0.135637908 | count | 1 |
| RAB21 | -0.0976089 | 0.0966915 | -1.0095 | 0.313 | -0.135564275 | count | 1 |
| SWI5 | -0.1084651 | 0.2076869 | -0.5223 | 0.602 | -0.135520953 | count | 1 |
| SMARCA2 | -0.0992437 | 0.0903216 | -1.0988 | 0.272 | -0.135409021 | count | 1 |
| UBE2V2 | -0.0976035 | 0.1072448 | -0.9101 | 0.363 | -0.135381553 | count | 1 |
| EPOR | -0.1068232 | 0.1941556 | -0.5502 | 0.582 | -0.135289081 | count | 1 |
| DXO | -0.1376078 | 0.2929592 | -0.4697 | 0.639 | -0.135258709 | count | 1 |
| COMMD7 | -0.0963596 | 0.0885784 | -1.0878 | 0.277 | -0.13523468 | count | 1 |
| A2M-AS1 | -0.1537545 | 0.3557685 | -0.4322 | 0.666 | -0.135198437 | count | 1 |
| TROVE2 | -0.0979924 | 0.0955519 | -1.0255 | 0.305 | -0.135121319 | count | 1 |
| IL6R | -0.1106595 | 0.2099158 | -0.5272 | 0.598 | -0.134920707 | count | 1 |
| APOBEC3D | -0.1371884 | 0.3807168 | -0.3603 | 0.719 | -0.134851595 | count | 1 |
| MAP1S | -0.1138184 | 0.2656029 | -0.4285 | 0.668 | -0.13483492 | count | 1 |
| AKNA | -0.0997341 | 0.107336 | -0.9292 | 0.353 | -0.134798715 | count | 1 |
| TEX264 | -0.0952438 | 0.0779937 | -1.2212 | 0.222 | -0.134707756 | count | 1 |
| ACSF3 | -0.1164938 | 0.2118167 | -0.55 | 0.582 | -0.134546245 | count | 1 |
| DAPP1 | -0.1082545 | 0.1850896 | -0.5849 | 0.559 | -0.134421548 | count | 1 |
| TYW3 | -0.0987374 | 0.1394977 | -0.7078 | 0.479 | -0.13440495 | count | 1 |
| WASF2 | -0.0948646 | 0.0709933 | -1.3362 | 0.182 | -0.134329495 | count | 1 |
| SRFBP1 | -0.099384 | 0.1427174 | -0.6964 | 0.486 | -0.134326361 | count | 1 |
| TAB3 | -0.1662728 | 0.3675077 | -0.4524 | 0.651 | -0.13432575 | count | 1 |
| METTL2A | -0.1127639 | 0.2334967 | -0.4829 | 0.629 | -0.134320559 | count | 1 |
| AC064801.1 | -0.2622363 | 0.7238507 | -0.3623 | 0.717 | -0.134311837 | count | 1 |
| TMEM173 | -0.0964448 | 0.0788923 | -1.2225 | 0.222 | -0.134214086 | count | 1 |
| NDUFB7 | -0.0944824 | 0.0697153 | -1.3553 | 0.175 | -0.134156098 | count | 1 |
| TBC1D19 | -0.1223456 | 0.2268851 | -0.5392 | 0.59 | -0.133963602 | count | 1 |
| PDE1B | -0.3214832 | 0.6131733 | -0.5243 | 0.6 | -0.133884071 | count | 1 |
| PSMB8 | -0.0935638 | 0.0532863 | -1.7559 | 0.0792 | -0.133881841 | count | 1 |
| AC016596.1 | -0.2611684 | 0.8350705 | -0.3128 | 0.754 | -0.13379199 | count | 1 |
| TP53BP1 | -0.1235817 | 0.2154572 | -0.5736 | 0.566 | -0.13377026 | count | 1 |
| METTL9 | -0.0948561 | 0.0775075 | -1.2238 | 0.221 | -0.133470626 | count | 1 |
| ZKSCAN5 | -0.2605029 | 0.389918 | -0.6681 | 0.504 | -0.13346791 | count | 1 |
| CCAR2 | -0.1081931 | 0.215287 | -0.5026 | 0.615 | -0.133438975 | count | 1 |
| BCL10 | -0.0983293 | 0.1166978 | -0.8426 | 0.4 | -0.133366776 | count | 1 |
| TTF1 | -0.0968396 | 0.1297978 | -0.7461 | 0.456 | -0.133068116 | count | 1 |
| SLC25A24 | -0.1001972 | 0.1388801 | -0.7215 | 0.471 | -0.133008318 | count | 1 |
| UNC93B1 | -0.1089703 | 0.2330948 | -0.4675 | 0.64 | -0.132870961 | count | 1 |
| RAB8B | -0.0940755 | 0.074391 | -1.2646 | 0.206 | -0.132781244 | count | 1 |
| EIPR1 | -0.1114254 | 0.2419427 | -0.4605 | 0.645 | -0.132734918 | count | 1 |

| | | | | | | | |
|---|---|---|---|---|---|---|---|
| PKM | -0.0932637 | 0.0662866 | -1.407 | 0.16 | -0.132674359 | count | 1 |
| PDGFB | -0.1853535 | 0.3774763 | -0.491 | 0.623 | -0.132661088 | count | 1 |
| LRMP | -0.1078839 | 0.1663441 | -0.6486 | 0.517 | -0.13257843 | count | 1 |
| AP002495.2 | -0.1379269 | 0.3284554 | -0.4199 | 0.675 | -0.132552026 | count | 1 |
| RNF41 | -0.1046145 | 0.200211 | -0.5225 | 0.601 | -0.132501874 | count | 1 |
| HMGN2 | -0.0926912 | 0.0520762 | -1.7799 | 0.0752 | -0.132497564 | count | 1 |
| MCUB | -0.0936578 | 0.067902 | -1.3793 | 0.168 | -0.132427622 | count | 1 |
| ZYG11B | -0.1730857 | 0.2867512 | -0.6036 | 0.546 | -0.13242222 | count | 1 |
| ACD | -0.1003567 | 0.1647623 | -0.6091 | 0.542 | -0.132207041 | count | 1 |
| PYROXD1 | -0.1002221 | 0.1832981 | -0.5468 | 0.585 | -0.132185312 | count | 1 |
| ACBD6 | -0.0958809 | 0.1159922 | -0.8266 | 0.409 | -0.13217404 | count | 1 |
| PSAP | -0.0941247 | 0.0869158 | -1.0829 | 0.279 | -0.13211482 | count | 1 |
| SLC43A3 | -0.1845309 | 0.4902299 | -0.3764 | 0.707 | -0.132088092 | count | 1 |
| VIRMA | -0.1003639 | 0.1416538 | -0.7085 | 0.479 | -0.132057187 | count | 1 |
| PRKDC | -0.0948733 | 0.0867056 | -1.0942 | 0.274 | -0.132029223 | count | 1 |
| EMC2 | -0.0994036 | 0.1564444 | -0.6354 | 0.525 | -0.131957189 | count | 1 |
| ZNF552 | -0.1558908 | 0.3627692 | -0.4297 | 0.667 | -0.131894359 | count | 1 |
| RUBCN | -0.144957 | 0.2598237 | -0.5579 | 0.577 | -0.13187056 | count | 1 |
| ZSCAN16 | -0.1141409 | 0.2481755 | -0.4599 | 0.646 | -0.131846188 | count | 1 |
| YOD1 | -0.1268485 | 0.3073793 | -0.4127 | 0.68 | -0.131787649 | count | 1 |
| ADRM1 | -0.0931013 | 0.0778568 | -1.1958 | 0.232 | -0.131758797 | count | 1 |
| SLC4A1AP | -0.0968065 | 0.1296214 | -0.7468 | 0.455 | -0.131715936 | count | 1 |
| CLCC1 | -0.1370304 | 0.3188617 | -0.4297 | 0.667 | -0.131701658 | count | 1 |
| DNAJC25 | -0.1202574 | 0.2599505 | -0.4626 | 0.644 | -0.131695844 | count | 1 |
| SYPL1 | -0.0948701 | 0.0822755 | -1.1531 | 0.249 | -0.131652046 | count | 1 |
| UBXN7 | -0.1067197 | 0.215418 | -0.4954 | 0.62 | -0.131629693 | count | 1 |
| EMC1 | -0.1555196 | 0.3317064 | -0.4688 | 0.639 | -0.131586065 | count | 1 |
| INO80D | -0.1031434 | 0.1506253 | -0.6848 | 0.494 | -0.131553853 | count | 1 |
| SLPI | -0.8945215 | 0.539377 | -1.6584 | 0.0973 | -0.131532913 | count | 1 |
| RPAP3 | -0.0979679 | 0.1296916 | -0.7554 | 0.45 | -0.131523001 | count | 1 |
| SAAL1 | -0.1087868 | 0.2369608 | -0.4591 | 0.646 | -0.131517255 | count | 1 |
| ERN1 | -0.0982656 | 0.1065309 | -0.9224 | 0.356 | -0.131511445 | count | 1 |
| ADAL | -0.126498 | 0.3519848 | -0.3594 | 0.719 | -0.131427152 | count | 1 |
| TBC1D2B | -0.0981557 | 0.1238955 | -0.7922 | 0.428 | -0.131364656 | count | 1 |
| LINS1 | -0.1019604 | 0.1113217 | -0.9159 | 0.36 | -0.131362325 | count | 1 |
| RAB11FIP2 | -0.1047776 | 0.2030575 | -0.516 | 0.606 | -0.131313833 | count | 1 |
| PRRT3 | -0.136543 | 0.425679 | -0.3208 | 0.748 | -0.131239274 | count | 1 |
| VAV1 | -0.1026626 | 0.1789975 | -0.5735 | 0.566 | -0.131224326 | count | 1 |
| SELENOO | -0.1184699 | 0.2491208 | -0.4756 | 0.634 | -0.13112683 | count | 1 |
| HPCAL1 | -0.0954843 | 0.1051964 | -0.9077 | 0.364 | -0.131070759 | count | 1 |
| ROM1 | -0.2555762 | 0.7468946 | -0.3422 | 0.732 | -0.131065965 | count | 1 |
| ZNF558 | -0.1620901 | 0.4930534 | -0.3287 | 0.742 | -0.131015722 | count | 1 |
| CDC14A | -0.0984335 | 0.1021515 | -0.9636 | 0.335 | -0.130927158 | count | 1 |
| DPP7 | -0.0922961 | 0.0686743 | -1.344 | 0.179 | -0.130926068 | count | 1 |
| SLC20A2 | -0.1150851 | 0.230491 | -0.4993 | 0.618 | -0.130922263 | count | 1 |

| | | | | | | | |
|---|---|---|---|---|---|---|---|
| ORAOV1 | -0.1396995 | 0.2852948 | -0.4897 | 0.624 | -0.130872064 | count | 1 |
| ORMDL3 | -0.0998249 | 0.1347152 | -0.741 | 0.459 | -0.130849371 | count | 1 |
| TMEM41A | -0.1008216 | 0.1926504 | -0.5233 | 0.601 | -0.130801974 | count | 1 |
| PNP | -0.0926149 | 0.0927341 | -0.9987 | 0.318 | -0.130791468 | count | 1 |
| ZFYVE28 | -0.0971809 | 0.1325912 | -0.7329 | 0.464 | -0.130752113 | count | 1 |
| AC138150.1 | -0.1360148 | 0.3335165 | -0.4078 | 0.683 | -0.130738134 | count | 1 |
| ESYT1 | -0.1012264 | 0.1654784 | -0.6117 | 0.541 | -0.130656369 | count | 1 |
| ATF7IP | -0.0952385 | 0.0863648 | -1.1027 | 0.27 | -0.130639189 | count | 1 |
| RETREG1 | -0.0996405 | 0.1368768 | -0.728 | 0.467 | -0.13060828 | count | 1 |
| ARHGAP31 | -0.3129279 | 0.4478767 | -0.6987 | 0.485 | -0.130557594 | count | 1 |
| PHIP | -0.0950026 | 0.1034999 | -0.9179 | 0.359 | -0.130547074 | count | 1 |
| PPA2 | -0.0974281 | 0.1316029 | -0.7403 | 0.459 | -0.130497679 | count | 1 |
| FKBP14 | -0.2539372 | 0.4110605 | -0.6178 | 0.537 | -0.130265799 | count | 1 |
| C7orf26 | -0.1070748 | 0.2067902 | -0.5178 | 0.605 | -0.130026728 | count | 1 |
| STAT4 | -0.0942301 | 0.0935403 | -1.0074 | 0.314 | -0.129901047 | count | 1 |
| TSEN34 | -0.0943677 | 0.1162017 | -0.8121 | 0.417 | -0.12984828 | count | 1 |
| GRSF1 | -0.0948019 | 0.122439 | -0.7743 | 0.439 | -0.129793579 | count | 1 |
| SLC41A2 | -0.2528658 | 0.7169454 | -0.3527 | 0.724 | -0.129742444 | count | 1 |
| HECW2 | -0.1966415 | 0.6840149 | -0.2875 | 0.774 | -0.129615004 | count | 1 |
| RNF213 | -0.0909448 | 0.0570191 | -1.595 | 0.111 | -0.129423352 | count | 1 |
| AC068870.2 | -0.4322193 | 0.6247271 | -0.6919 | 0.489 | -0.129406613 | count | 1 |
| SRSF2 | -0.0913618 | 0.0506011 | -1.8055 | 0.0711 | -0.129403882 | count | 1 |
| AC027644.3 | -0.0996939 | 0.1914945 | -0.5206 | 0.603 | -0.129343062 | count | 1 |
| GLIPR1L2 | -0.4318916 | 0.8432626 | -0.5122 | 0.609 | -0.129318738 | count | 1 |
| DLG2 | -0.4318916 | 0.9237608 | -0.4675 | 0.64 | -0.129318738 | count | 1 |
| PLK4 | -0.4318916 | 0.970437 | -0.445 | 0.656 | -0.129318738 | count | 1 |
| BFSP2 | -0.4318916 | 1.041005 | -0.4149 | 0.678 | -0.129318738 | count | 1 |
| FBXL3 | -0.0967536 | 0.1342347 | -0.7208 | 0.471 | -0.129276967 | count | 1 |
| MT-ND3 | -0.0898735 | 0.0249469 | -3.6026 | 0.00032 | -0.12927453 | count | 1 |
| PPTC7 | -0.106001 | 0.197207 | -0.5375 | 0.591 | -0.129267111 | count | 1 |
| WHAMM | -0.0946225 | 0.107718 | -0.8784 | 0.38 | -0.12917027 | count | 1 |
| TMEM60 | -0.0974756 | 0.1879616 | -0.5186 | 0.604 | -0.129139106 | count | 1 |
| PTPRE | -0.0933614 | 0.1065822 | -0.876 | 0.381 | -0.12899706 | count | 1 |
| GPS1 | -0.0969063 | 0.1530138 | -0.6333 | 0.527 | -0.128900123 | count | 1 |
| C12orf65 | -0.0918526 | 0.1005549 | -0.9135 | 0.361 | -0.128841767 | count | 1 |
| MED26 | -0.1260337 | 0.2200663 | -0.5727 | 0.567 | -0.128829649 | count | 1 |
| YTHDC1 | -0.0914875 | 0.0761919 | -1.2008 | 0.23 | -0.128779407 | count | 1 |
| RFX7 | -0.1308748 | 0.2803879 | -0.4668 | 0.641 | -0.128718956 | count | 1 |
| STT3B | -0.0945829 | 0.1015418 | -0.9315 | 0.352 | -0.128695044 | count | 1 |
| TMEM9 | -0.1015854 | 0.2010825 | -0.5052 | 0.613 | -0.128678659 | count | 1 |
| GEN1 | -0.1679662 | 0.4565334 | -0.3679 | 0.713 | -0.128594035 | count | 1 |
| LMNTD2 | -0.159011 | 0.5548657 | -0.2866 | 0.774 | -0.128576648 | count | 1 |
| BAD | -0.0943714 | 0.1248513 | -0.7559 | 0.45 | -0.128470667 | count | 1 |
| SMIM27 | -0.0991633 | 0.138425 | -0.7164 | 0.474 | -0.128444574 | count | 1 |
| PIDD1 | -0.2167311 | 0.416995 | -0.5197 | 0.603 | -0.128431509 | count | 1 |

| | | | | | | | |
|---|---|---|---|---|---|---|---|
| PA2G4 | -0.0901073 | 0.0591246 | -1.524 | 0.1276 | -0.12839243 | count | 1 |
| ABCD2 | -0.1215803 | 0.2445591 | -0.4971 | 0.619 | -0.128253585 | count | 1 |
| CCDC126 | -0.113665 | 0.2411116 | -0.4714 | 0.637 | -0.12823354 | count | 1 |
| DHRS4 | -0.104328 | 0.2371206 | -0.44 | 0.66 | -0.128227576 | count | 1 |
| IFT20 | -0.0962145 | 0.1468915 | -0.655 | 0.513 | -0.128219952 | count | 1 |
| RPL36A | -0.0908684 | 0.0548909 | -1.6554 | 0.0979 | -0.128202886 | count | 1 |
| ARHGAP25 | -0.095017 | 0.1308232 | -0.7263 | 0.468 | -0.128107422 | count | 1 |
| DNAJB12 | -0.0959162 | 0.1373431 | -0.6984 | 0.485 | -0.128050363 | count | 1 |
| CTLA4 | -0.0946017 | 0.1454175 | -0.6506 | 0.515 | -0.128027274 | count | 1 |
| PSMG3 | -0.0989647 | 0.1540895 | -0.6423 | 0.521 | -0.127970159 | count | 1 |
| ZNF654 | -0.1181657 | 0.2392717 | -0.4939 | 0.621 | -0.127956595 | count | 1 |
| ZNF182 | -0.1404727 | 0.3157106 | -0.4449 | 0.656 | -0.127851266 | count | 1 |
| TRIM65 | -0.1123301 | 0.2635318 | -0.4262 | 0.67 | -0.127809108 | count | 1 |
| FYB1 | -0.0892742 | 0.0403588 | -2.212 | 0.027 | -0.127778579 | count | 1 |
| UGT8 | -0.4260123 | 0.8571594 | -0.497 | 0.619 | -0.127739484 | count | 1 |
| AC008610.1 | -0.4260123 | 0.9544872 | -0.4463 | 0.655 | -0.127739484 | count | 1 |
| KLK1 | -0.4260123 | 1.0023465 | -0.425 | 0.671 | -0.127739484 | count | 1 |
| AC124798.1 | -0.4260123 | 1.071287 | -0.3977 | 0.691 | -0.127739484 | count | 1 |
| ADGRB1 | -0.4260123 | 1.0867484 | -0.392 | 0.695 | -0.127739484 | count | 1 |
| UBE2Q2L | -0.4260123 | 1.0867484 | -0.392 | 0.695 | -0.127739484 | count | 1 |
| RGS9BP | -0.4260123 | 1.0867484 | -0.392 | 0.695 | -0.127739484 | count | 1 |
| SNX6 | -0.0895397 | 0.0596451 | -1.5012 | 0.133 | -0.127687575 | count | 1 |
| NANS | -0.0936797 | 0.1068073 | -0.8771 | 0.38 | -0.127652421 | count | 1 |
| INTS6L | -0.1209695 | 0.2516574 | -0.4807 | 0.631 | -0.127615174 | count | 1 |
| CDYL | -0.1021042 | 0.18645 | -0.5476 | 0.584 | -0.12760392 | count | 1 |
| COMMD2 | -0.0919586 | 0.110946 | -0.8289 | 0.407 | -0.127530984 | count | 1 |
| ITGAE | -0.0929656 | 0.1263607 | -0.7357 | 0.462 | -0.127478298 | count | 1 |
| OSBPL3 | -0.1505759 | 0.2517638 | -0.5981 | 0.55 | -0.12747742 | count | 1 |
| KLHL11 | -0.1504736 | 0.3966466 | -0.3794 | 0.704 | -0.127392348 | count | 1 |
| GRAMD4 | -0.1323712 | 0.3656348 | -0.362 | 0.717 | -0.127279732 | count | 1 |
| TSC22D2 | -0.0995667 | 0.1478692 | -0.6733 | 0.501 | -0.127279715 | count | 1 |
| ERO1B | -0.0989837 | 0.1311376 | -0.7548 | 0.45 | -0.127053632 | count | 1 |
| PARP2 | -0.1320983 | 0.3447336 | -0.3832 | 0.702 | -0.127020604 | count | 1 |
| TSPAN9 | -0.21418 | 0.8233839 | -0.2601 | 0.795 | -0.126975097 | count | 1 |
| SLC41A1 | -0.1924632 | 0.2619744 | -0.7347 | 0.463 | -0.126943973 | count | 1 |
| CARD17 | -0.3035145 | 0.9241657 | -0.3284 | 0.743 | -0.126881958 | count | 1 |
| ARFGEF1 | -0.0921074 | 0.1086898 | -0.8474 | 0.397 | -0.126822691 | count | 1 |
| FAM219B | -0.1043953 | 0.1990037 | -0.5246 | 0.6 | -0.126787963 | count | 1 |
| WDR33 | -0.0908785 | 0.0890425 | -1.0206 | 0.308 | -0.1267213 | count | 1 |
| CCDC115 | -0.0912337 | 0.1079832 | -0.8449 | 0.398 | -0.126718509 | count | 1 |
| SLC14A1 | -0.8487932 | 0.5831956 | -1.4554 | 0.146 | -0.126469064 | count | 1 |
| AC127070.1 | -0.2132831 | 0.620258 | -0.3439 | 0.731 | -0.126462733 | count | 1 |
| SNRNP35 | -0.0912781 | 0.1343571 | -0.6794 | 0.497 | -0.126318161 | count | 1 |
| SELENOK | -0.0881653 | 0.0462009 | -1.9083 | 0.0564 | -0.126211906 | count | 1 |
| ZNF571 | -0.143321 | 0.3013693 | -0.4756 | 0.634 | -0.126170342 | count | 1 |

| | | | | | | | |
|---|---|---|---|---|---|---|---|
| ACSL1 | -0.2126509 | 0.4367567 | -0.4869 | 0.626 | -0.126101481 | count | 1 |
| LYPLA1 | -0.0920363 | 0.1187195 | -0.7752 | 0.438 | -0.126012158 | count | 1 |
| FAM69A | -0.1178079 | 0.2639056 | -0.4464 | 0.655 | -0.12600146 | count | 1 |
| SH3GL1 | -0.1082801 | 0.2143277 | -0.5052 | 0.613 | -0.12598204 | count | 1 |
| ARRDC3 | -0.0911369 | 0.1113029 | -0.8188 | 0.413 | -0.125960474 | count | 1 |
| KMT2A | -0.0889523 | 0.0641659 | -1.3863 | 0.166 | -0.125948745 | count | 1 |
| LAT | -0.0882865 | 0.0582985 | -1.5144 | 0.13 | -0.12590341 | count | 1 |
| BZW2 | -0.0970232 | 0.1533849 | -0.6325 | 0.527 | -0.125887572 | count | 1 |
| CHRAC1 | -0.090927 | 0.1069262 | -0.8504 | 0.395 | -0.125864065 | count | 1 |
| ZC3H15 | -0.0884256 | 0.0607235 | -1.4562 | 0.145 | -0.125801307 | count | 1 |
| DUSP3 | -0.4188161 | 0.409321 | -1.0232 | 0.306 | -0.125799553 | count | 1 |
| AC074032.1 | -0.1176132 | 0.2966448 | -0.3965 | 0.692 | -0.125795003 | count | 1 |
| DYNLT3 | -0.0895284 | 0.08736 | -1.0248 | 0.306 | -0.125721534 | count | 1 |
| FAM111B | -0.8416244 | 0.68906 | -1.2214 | 0.222 | -0.125660019 | count | 1 |
| ORAI3 | -0.1004899 | 0.2486907 | -0.4041 | 0.686 | -0.125594059 | count | 1 |
| MRPL22 | -0.0899744 | 0.1047717 | -0.8588 | 0.391 | -0.125564166 | count | 1 |
| AC073389.1 | -0.300133 | 0.6211229 | -0.4832 | 0.629 | -0.125557628 | count | 1 |
| TRMT44 | -0.300066 | 0.427479 | -0.7019 | 0.483 | -0.125531334 | count | 1 |
| SIAH2 | -0.0892959 | 0.0820429 | -1.0884 | 0.276 | -0.125382007 | count | 1 |
| PSMG4 | -0.092377 | 0.1293578 | -0.7141 | 0.475 | -0.125374123 | count | 1 |
| FEZ2 | -0.0940277 | 0.15894 | -0.5916 | 0.554 | -0.125311566 | count | 1 |
| NBPF15 | -0.1302958 | 0.4356781 | -0.2991 | 0.765 | -0.125308687 | count | 1 |
| ARG2 | -0.1747964 | 0.3672521 | -0.476 | 0.634 | -0.125296358 | count | 1 |
| LIMK2 | -0.1336308 | 0.3285299 | -0.4068 | 0.684 | -0.125262357 | count | 1 |
| TRMT10B | -0.0981078 | 0.2240291 | -0.4379 | 0.661 | -0.12515169 | count | 1 |
| CNOT11 | -0.0986572 | 0.1676126 | -0.5886 | 0.556 | -0.124982 | count | 1 |
| MLEC | -0.0899267 | 0.1025527 | -0.8769 | 0.381 | -0.12495628 | count | 1 |
| SBF2 | -0.2102965 | 0.2808846 | -0.7487 | 0.454 | -0.124755371 | count | 1 |
| ADNP | -0.0916247 | 0.1184281 | -0.7737 | 0.439 | -0.1247369 | count | 1 |
| GPR174 | -0.0893883 | 0.0980373 | -0.9118 | 0.362 | -0.124665848 | count | 1 |
| LMO4 | -0.0885422 | 0.0844509 | -1.0484 | 0.295 | -0.124636043 | count | 1 |
| NDUFB3 | -0.0887016 | 0.0930489 | -0.9533 | 0.341 | -0.124439015 | count | 1 |
| WIPF1 | -0.0883357 | 0.0769988 | -1.1472 | 0.251 | -0.124312594 | count | 1 |
| FUS | -0.0869267 | 0.0445458 | -1.9514 | 0.0511 | -0.12424546 | count | 1 |
| RPAP1 | -0.2093826 | 0.4189327 | -0.4998 | 0.617 | -0.124232542 | count | 1 |
| RNF34 | -0.0918327 | 0.1314139 | -0.6988 | 0.485 | -0.124136665 | count | 1 |
| MAPKAP1 | -0.0964392 | 0.1423311 | -0.6776 | 0.498 | -0.124037106 | count | 1 |
| HIKESHI | -0.0893643 | 0.1102248 | -0.8107 | 0.418 | -0.124018871 | count | 1 |
| RSRC2 | -0.0868328 | 0.0478058 | -1.8164 | 0.0694 | -0.123833295 | count | 1 |
| ANKRD10 | -0.0910465 | 0.1196395 | -0.761 | 0.447 | -0.123828464 | count | 1 |
| SLC9A8 | -0.1615862 | 0.2912206 | -0.5549 | 0.579 | -0.123815379 | count | 1 |
| RNF168 | -0.0888681 | 0.0878757 | -1.0113 | 0.312 | -0.12381411 | count | 1 |
| NDUFB2-AS1 | -0.8243669 | 0.8086319 | -1.0195 | 0.308 | -0.123695385 | count | 1 |
| WBP11 | -0.0888878 | 0.0876508 | -1.0141 | 0.311 | -0.123683653 | count | 1 |
| MYDGF | -0.0875641 | 0.0809699 | -1.0814 | 0.28 | -0.123662374 | count | 1 |

| | | | | | | | |
|---|---|---|---|---|---|---|---|
| AL133342.1 | -0.0955903 | 0.226553 | -0.4219 | 0.673 | -0.123618858 | count | 1 |
| FAM168A | -0.1459022 | 0.4086768 | -0.357 | 0.721 | -0.123588464 | count | 1 |
| SASS6 | -0.0991308 | 0.221303 | -0.4479 | 0.654 | -0.123526561 | count | 1 |
| UQCRB | -0.0860411 | 0.0326024 | -2.6391 | 0.00835 | -0.123508371 | count | 1 |
| C12orf76 | -0.0938356 | 0.1534515 | -0.6115 | 0.541 | -0.123487197 | count | 1 |
| VPS50 | -0.112623 | 0.2124714 | -0.5301 | 0.596 | -0.123399164 | count | 1 |
| POLB | -0.0946133 | 0.1596574 | -0.5926 | 0.553 | -0.123341587 | count | 1 |
| VMAC | -0.0978423 | 0.2672699 | -0.3661 | 0.714 | -0.123326888 | count | 1 |
| EVL | -0.0861618 | 0.0465792 | -1.8498 | 0.0644 | -0.12329822 | count | 1 |
| ZNRF2 | -0.0932214 | 0.128011 | -0.7282 | 0.467 | -0.123250326 | count | 1 |
| LSM10 | -0.0876502 | 0.0963414 | -0.9098 | 0.363 | -0.123234336 | count | 1 |
| COX5B | -0.0860436 | 0.0482494 | -1.7833 | 0.0746 | -0.123229779 | count | 1 |
| PSMD4 | -0.0873593 | 0.0798562 | -1.094 | 0.274 | -0.123200554 | count | 1 |
| LNPEP | -0.0900452 | 0.1030516 | -0.8738 | 0.382 | -0.123190689 | count | 1 |
| AC008763.2 | -0.2074866 | 0.7520108 | -0.2759 | 0.783 | -0.123147304 | count | 1 |
| AC025171.4 | -0.2939485 | 0.7178803 | -0.4095 | 0.682 | -0.123130133 | count | 1 |
| PPP1R13B | -0.1278906 | 0.304378 | -0.4202 | 0.674 | -0.123023403 | count | 1 |
| FUT10 | -0.1350668 | 0.4166833 | -0.3241 | 0.746 | -0.123000574 | count | 1 |
| COL18A1 | -0.1519712 | 0.2927368 | -0.5191 | 0.604 | -0.122992559 | count | 1 |
| APBB1IP | -0.0861436 | 0.0501862 | -1.7165 | 0.0862 | -0.122984066 | count | 1 |
| POLE3 | -0.088754 | 0.0921338 | -0.9633 | 0.335 | -0.122919123 | count | 1 |
| UBAP1 | -0.0963268 | 0.1810834 | -0.5319 | 0.595 | -0.122886832 | count | 1 |
| SREBF1 | -0.171348 | 0.3136696 | -0.5463 | 0.585 | -0.122885487 | count | 1 |
| BAG6 | -0.1036464 | 0.1861901 | -0.5567 | 0.578 | -0.122847424 | count | 1 |
| SREBF2 | -0.1601019 | 0.3001756 | -0.5334 | 0.594 | -0.122702369 | count | 1 |
| PSMC6 | -0.0889946 | 0.095886 | -0.9281 | 0.353 | -0.122691613 | count | 1 |
| SNX1 | -0.0921412 | 0.1522468 | -0.6052 | 0.545 | -0.12268971 | count | 1 |
| LIPT2 | -0.1308151 | 0.6700356 | -0.1952 | 0.845 | -0.122657165 | count | 1 |
| TAB2 | -0.1013746 | 0.1733601 | -0.5848 | 0.559 | -0.122597595 | count | 1 |
| HIST1H2BK | -0.2382348 | 0.6332179 | -0.3762 | 0.707 | -0.122572298 | count | 1 |
| MED19 | -0.0884967 | 0.111682 | -0.7924 | 0.428 | -0.122182039 | count | 1 |
| RPS18 | -0.0846226 | 0.0163359 | -5.1801 | 2.35E-07 | -0.12201084 | count | 0.00567102 |
| SFSWAP | -0.0885624 | 0.1175794 | -0.7532 | 0.451 | -0.121985145 | count | 1 |
| TSTD1 | -0.0857244 | 0.0659539 | -1.2998 | 0.194 | -0.121973274 | count | 1 |
| LDHA | -0.0848947 | 0.0354902 | -2.3921 | 0.0168 | -0.121945806 | count | 1 |
| TNFSF12 | -0.0860388 | 0.0744819 | -1.1552 | 0.248 | -0.121914347 | count | 1 |
| ANKRD27 | -0.1383884 | 0.3269202 | -0.4233 | 0.672 | -0.121894387 | count | 1 |
| ZNF20 | -0.8081706 | 1.176089 | -0.6872 | 0.492 | -0.121829577 | count | 1 |
| PCDH10 | -0.8081706 | 1.371422 | -0.5893 | 0.556 | -0.121829577 | count | 1 |
| BGLAP | -0.8081706 | 1.391391 | -0.5808 | 0.561 | -0.121829577 | count | 1 |
| IGLV3-9 | -0.8081706 | 1.391391 | -0.5808 | 0.561 | -0.121829577 | count | 1 |
| BLVRA | -0.0920866 | 0.1493535 | -0.6166 | 0.538 | -0.121753201 | count | 1 |
| THTPA | -0.1098757 | 0.3454234 | -0.3181 | 0.75 | -0.121682881 | count | 1 |
| NSD1 | -0.087367 | 0.095166 | -0.918 | 0.359 | -0.121637175 | count | 1 |
| ESCO1 | -0.0870835 | 0.0977088 | -0.8913 | 0.373 | -0.121590214 | count | 1 |

| | | | | | | | |
|---|---|---|---|---|---|---|---|
| TIGD3 | -0.8061024 | 0.6180344 | -1.3043 | 0.192 | -0.121589772 | count | 1 |
| PSMD7 | -0.0856082 | 0.0757849 | -1.1296 | 0.259 | -0.121422641 | count | 1 |
| COPS3 | -0.0870029 | 0.0971496 | -0.8956 | 0.371 | -0.121321536 | count | 1 |
| CD58 | -0.086689 | 0.1086919 | -0.7976 | 0.425 | -0.121201635 | count | 1 |
| TCF25 | -0.0854947 | 0.0607272 | -1.4078 | 0.159 | -0.121160577 | count | 1 |
| DNPEP | -0.0891791 | 0.128306 | -0.6951 | 0.487 | -0.121104924 | count | 1 |
| MINDY2 | -0.0974696 | 0.1659981 | -0.5872 | 0.557 | -0.121080703 | count | 1 |
| ORAI1 | -0.085233 | 0.0699829 | -1.2179 | 0.223 | -0.12100543 | count | 1 |
| SP1 | -0.1229099 | 0.2772259 | -0.4434 | 0.658 | -0.120971741 | count | 1 |
| PLEKHJ1 | -0.0872439 | 0.1186416 | -0.7354 | 0.462 | -0.120915645 | count | 1 |
| TMEM256 | -0.0870134 | 0.1075985 | -0.8087 | 0.419 | -0.120911764 | count | 1 |
| VDAC2 | -0.0849203 | 0.059365 | -1.4305 | 0.153 | -0.120852655 | count | 1 |
| CDKN2AIP | -0.0876387 | 0.0913297 | -0.9596 | 0.337 | -0.120788118 | count | 1 |
| CLIC1 | -0.0838954 | 0.0320168 | -2.6204 | 0.00882 | -0.120767971 | count | 1 |
| C9orf72 | -0.1226027 | 0.2160431 | -0.5675 | 0.57 | -0.120672699 | count | 1 |
| INO80E | -0.0957248 | 0.1922899 | -0.4978 | 0.619 | -0.120666864 | count | 1 |
| SENP2 | -0.0988968 | 0.2117392 | -0.4671 | 0.64 | -0.120640729 | count | 1 |
| JADE1 | -0.0934197 | 0.1666887 | -0.5604 | 0.575 | -0.120607715 | count | 1 |
| FLI1 | -0.0935894 | 0.1351625 | -0.6924 | 0.489 | -0.120607604 | count | 1 |
| PDLIM3 | -0.1421558 | 0.6138428 | -0.2316 | 0.817 | -0.120467817 | count | 1 |
| 1-Sep | -0.0843506 | 0.0600328 | -1.4051 | 0.16 | -0.120458344 | count | 1 |
| NFU1 | -0.0910938 | 0.1434086 | -0.6352 | 0.525 | -0.120443344 | count | 1 |
| B3GALT6 | -0.0917198 | 0.2095903 | -0.4376 | 0.662 | -0.120407125 | count | 1 |
| LYSMD2 | -0.085251 | 0.0833234 | -1.0231 | 0.306 | -0.120381418 | count | 1 |
| BUB3 | -0.0846867 | 0.0610644 | -1.3868 | 0.166 | -0.120225641 | count | 1 |
| CYB561D1 | -0.1247815 | 0.3920379 | -0.3183 | 0.75 | -0.120067678 | count | 1 |
| ZC3H4 | -0.0917757 | 0.1893761 | -0.4846 | 0.628 | -0.119996492 | count | 1 |
| ATP5MD | -0.0842575 | 0.0642102 | -1.3122 | 0.19 | -0.119893382 | count | 1 |
| NCLN | -0.1044447 | 0.2806119 | -0.3722 | 0.71 | -0.119828791 | count | 1 |
| SHKBP1 | -0.0866047 | 0.1078085 | -0.8033 | 0.422 | -0.119796497 | count | 1 |
| GNG7 | -0.2325685 | 0.4610243 | -0.5045 | 0.614 | -0.119783843 | count | 1 |
| TTC1 | -0.0853521 | 0.090827 | -0.9397 | 0.347 | -0.119631326 | count | 1 |
| CDK5RAP3 | -0.0909768 | 0.1398125 | -0.6507 | 0.515 | -0.119585608 | count | 1 |
| ADIPOR2 | -0.0956417 | 0.1732518 | -0.552 | 0.581 | -0.119556339 | count | 1 |
| C16orf46 | -0.7886236 | 0.9936339 | -0.7937 | 0.427 | -0.119549219 | count | 1 |
| ELN | -0.2848447 | 0.6463293 | -0.4407 | 0.659 | -0.119544075 | count | 1 |
| AC019077.1 | -0.3954772 | 1.1714899 | -0.3376 | 0.736 | -0.119455069 | count | 1 |
| HIST1H4I | -0.2315652 | 0.898855 | -0.2576 | 0.797 | -0.119289436 | count | 1 |
| SMPD4 | -0.1047706 | 0.261389 | -0.4008 | 0.689 | -0.119261314 | count | 1 |
| KIAA2026 | -0.0868913 | 0.1005902 | -0.8638 | 0.388 | -0.119246306 | count | 1 |
| MPP1 | -0.0920361 | 0.2537005 | -0.3628 | 0.717 | -0.119237071 | count | 1 |
| ANAPC5 | -0.0869093 | 0.0961934 | -0.9035 | 0.366 | -0.119227604 | count | 1 |
| ARL6IP4 | -0.0833666 | 0.0485511 | -1.7171 | 0.0861 | -0.119181462 | count | 1 |
| UHRF1BP1L | -0.097641 | 0.2293277 | -0.4258 | 0.67 | -0.119115273 | count | 1 |
| NMD3 | -0.1029798 | 0.1751767 | -0.5879 | 0.557 | -0.119028042 | count | 1 |

| | | | | | | | |
|---|---|---|---|---|---|---|---|
| SLC25A11 | -0.0875709 | 0.1366105 | -0.641 | 0.522 | -0.11898646 | count | 1 |
| MORF4L1 | -0.0832296 | 0.0454576 | -1.8309 | 0.0672 | -0.11898278 | count | 1 |
| ETV6 | -0.0960027 | 0.1658193 | -0.579 | 0.563 | -0.118872166 | count | 1 |
| SLC35E2A | -0.2830577 | 0.4283384 | -0.6608 | 0.509 | -0.118838384 | count | 1 |
| PRKCA | -0.1096217 | 0.1791809 | -0.6118 | 0.541 | -0.118775706 | count | 1 |
| HMGCS1 | -0.094155 | 0.1657277 | -0.5681 | 0.57 | -0.118694596 | count | 1 |
| TNFSF14 | -0.0853808 | 0.1227137 | -0.6958 | 0.487 | -0.118521209 | count | 1 |
| ATP6V0E1 | -0.0826204 | 0.041683 | -1.9821 | 0.0475 | -0.118499097 | count | 1 |
| DFFB | -0.2821373 | 0.3586236 | -0.7867 | 0.431 | -0.11847469 | count | 1 |
| MUT | -0.1121541 | 0.2656146 | -0.4222 | 0.673 | -0.118394271 | count | 1 |
| GTSF1 | -0.0988144 | 0.2949031 | -0.3351 | 0.738 | -0.118389665 | count | 1 |
| CHKB | -0.1341553 | 0.4603219 | -0.2914 | 0.771 | -0.118220861 | count | 1 |
| ASB13 | -0.3904227 | 0.7183376 | -0.5435 | 0.587 | -0.118070444 | count | 1 |
| SLC39A3 | -0.0901577 | 0.1828819 | -0.493 | 0.622 | -0.117885938 | count | 1 |
| KMT2E | -0.0824302 | 0.0460746 | -1.7891 | 0.0737 | -0.117848689 | count | 1 |
| CARNMT1 | -0.0950813 | 0.2260451 | -0.4206 | 0.674 | -0.117735558 | count | 1 |
| PRKAR2A | -0.0893281 | 0.1456228 | -0.6134 | 0.54 | -0.117568311 | count | 1 |
| C19orf47 | -0.3885605 | 0.6436823 | -0.6037 | 0.546 | -0.117559403 | count | 1 |
| YTHDF2 | -0.0841724 | 0.0891976 | -0.9437 | 0.345 | -0.117397137 | count | 1 |
| RHOU | -0.1383205 | 0.3752206 | -0.3686 | 0.712 | -0.117270098 | count | 1 |
| ACBD3 | -0.084538 | 0.1105813 | -0.7645 | 0.445 | -0.117195965 | count | 1 |
| AHR | -0.1126027 | 0.1953628 | -0.5764 | 0.564 | -0.117118407 | count | 1 |
| RASGEF1A | -0.0977382 | 0.2922604 | -0.3344 | 0.738 | -0.11710623 | count | 1 |
| ZNF778 | -0.3865972 | 0.4347996 | -0.8891 | 0.374 | -0.117020014 | count | 1 |
| KRBOX4 | -0.1036357 | 0.2375773 | -0.4362 | 0.663 | -0.116990417 | count | 1 |
| ACAP2 | -0.0832135 | 0.0854759 | -0.9735 | 0.33 | -0.116966057 | count | 1 |
| NLRP3 | -0.1019085 | 0.2429479 | -0.4195 | 0.675 | -0.116935991 | count | 1 |
| ZNF296 | -0.0895248 | 0.1691134 | -0.5294 | 0.597 | -0.116894446 | count | 1 |
| TMLHE | -0.0980235 | 0.2703217 | -0.3626 | 0.717 | -0.116846355 | count | 1 |
| NUDT21 | -0.0851025 | 0.114261 | -0.7448 | 0.456 | -0.1167518 | count | 1 |
| NUP37 | -0.0912247 | 0.1986545 | -0.4592 | 0.646 | -0.11664675 | count | 1 |
| SNX25 | -0.1439521 | 0.3776868 | -0.3811 | 0.703 | -0.116618867 | count | 1 |
| SOCS5 | -0.1160432 | 0.2876184 | -0.4035 | 0.687 | -0.116595458 | count | 1 |
| AC002470.1 | -0.7626673 | 0.6444572 | -1.1834 | 0.237 | -0.116472567 | count | 1 |
| URB2 | -0.2257411 | 0.5211113 | -0.4332 | 0.665 | -0.116415443 | count | 1 |
| ARL5A | -0.1022286 | 0.2494044 | -0.4099 | 0.682 | -0.11638517 | count | 1 |
| MSRB1 | -0.0980985 | 0.2474476 | -0.3964 | 0.692 | -0.116303894 | count | 1 |
| HEBP2 | -0.082084 | 0.0715474 | -1.1473 | 0.251 | -0.116301862 | count | 1 |
| CBFB | -0.0904063 | 0.142589 | -0.634 | 0.526 | -0.116298984 | count | 1 |
| BABAM1 | -0.0842928 | 0.1130449 | -0.7457 | 0.456 | -0.116284557 | count | 1 |
| WDR18 | -0.0929704 | 0.1921176 | -0.4839 | 0.628 | -0.116228643 | count | 1 |
| SEC13 | -0.0848651 | 0.1333126 | -0.6366 | 0.524 | -0.116159307 | count | 1 |
| ATP13A2 | -0.1020111 | 0.2745465 | -0.3716 | 0.71 | -0.116139037 | count | 1 |
| GPR65 | -0.0821392 | 0.0694998 | -1.1819 | 0.237 | -0.116092904 | count | 1 |
| CDK19 | -0.1115958 | 0.2799517 | -0.3986 | 0.69 | -0.116080262 | count | 1 |

| | | | | | | | |
|---|---|---|---|---|---|---|---|
| ASH1L | -0.083947 | 0.0895909 | -0.937 | 0.349 | -0.116063644 | count | 1 |
| CNP | -0.089399 | 0.1692589 | -0.5282 | 0.597 | -0.116019977 | count | 1 |
| APRT | -0.080913 | 0.0416355 | -1.9434 | 0.0521 | -0.115969155 | count | 1 |
| SHPRH | -0.0887917 | 0.1317457 | -0.674 | 0.5 | -0.115939464 | count | 1 |
| LDB1 | -0.0967179 | 0.1853393 | -0.5218 | 0.602 | -0.11588933 | count | 1 |
| TMED4 | -0.0819159 | 0.0797707 | -1.0269 | 0.305 | -0.115859359 | count | 1 |
| ZBTB48 | -0.1113777 | 0.2173152 | -0.5125 | 0.608 | -0.11585537 | count | 1 |
| PLAA | -0.127079 | 0.2635303 | -0.4822 | 0.63 | -0.115822493 | count | 1 |
| EID3 | -0.1025598 | 0.2680295 | -0.3826 | 0.702 | -0.11578344 | count | 1 |
| KLHL36 | -0.0900884 | 0.1391834 | -0.6473 | 0.518 | -0.115667418 | count | 1 |
| MRPL54 | -0.0817861 | 0.0763754 | -1.0708 | 0.284 | -0.115601109 | count | 1 |
| UGDH | -0.2747754 | 0.3690733 | -0.7445 | 0.457 | -0.115560132 | count | 1 |
| USP9Y | -0.1267437 | 0.2629342 | -0.482 | 0.63 | -0.115520903 | count | 1 |
| PLEKHA5 | -0.0873545 | 0.1546293 | -0.5649 | 0.572 | -0.115509309 | count | 1 |
| ZNF397 | -0.0979742 | 0.2364949 | -0.4143 | 0.679 | -0.11549338 | count | 1 |
| RPLP1 | -0.0800275 | 0.0155551 | -5.1448 | 2.83E-07 | -0.115421263 | count | 0.006829073 |
| RPAP2 | -0.0865876 | 0.1270173 | -0.6817 | 0.495 | -0.115414294 | count | 1 |
| FAM222A | -0.22358 | 0.5487156 | -0.4075 | 0.684 | -0.115347288 | count | 1 |
| SUZ12 | -0.0832737 | 0.0898591 | -0.9267 | 0.354 | -0.115336256 | count | 1 |
| DOK1 | -0.0888644 | 0.2011065 | -0.4419 | 0.659 | -0.115327907 | count | 1 |
| MED21 | -0.084511 | 0.1231941 | -0.686 | 0.493 | -0.115175015 | count | 1 |
| CRNKL1 | -0.0871009 | 0.1580476 | -0.5511 | 0.582 | -0.11517465 | count | 1 |
| TTLL5 | -0.1500106 | 0.4558918 | -0.329 | 0.742 | -0.11512285 | count | 1 |
| INTS3 | -0.1357228 | 0.3180173 | -0.4268 | 0.67 | -0.115102511 | count | 1 |
| AC058791.1 | -0.0847859 | 0.0752448 | -1.1268 | 0.26 | -0.115085873 | count | 1 |
| ELL | -0.0893403 | 0.197691 | -0.4519 | 0.651 | -0.114931367 | count | 1 |
| DNAJC11 | -0.1303236 | 0.3909917 | -0.3333 | 0.739 | -0.114892533 | count | 1 |
| HCG27 | -0.3788649 | 0.6870725 | -0.5514 | 0.581 | -0.114890141 | count | 1 |
| MSMO1 | -0.0875067 | 0.1527423 | -0.5729 | 0.567 | -0.114888407 | count | 1 |
| MAP2K1 | -0.0915902 | 0.152695 | -0.5998 | 0.549 | -0.114842055 | count | 1 |
| NMT1 | -0.0878878 | 0.1544989 | -0.5689 | 0.569 | -0.114761935 | count | 1 |
| HIVEP2 | -0.0978998 | 0.1828909 | -0.5353 | 0.592 | -0.11470685 | count | 1 |
| TAMM41 | -0.0943921 | 0.2039681 | -0.4628 | 0.644 | -0.114689725 | count | 1 |
| GPN2 | -0.1057458 | 0.2247551 | -0.4705 | 0.638 | -0.114607062 | count | 1 |
| PSD4 | -0.0919408 | 0.2046152 | -0.4493 | 0.653 | -0.114598408 | count | 1 |
| ZNF410 | -0.2220595 | 0.4296238 | -0.5169 | 0.605 | -0.1145952 | count | 1 |
| HNRNPH2 | -0.0870463 | 0.1280417 | -0.6798 | 0.497 | -0.114571513 | count | 1 |
| PML | -0.0922082 | 0.2062292 | -0.4471 | 0.655 | -0.1145681 | count | 1 |
| CHD3 | -0.0828729 | 0.1061238 | -0.7809 | 0.435 | -0.114488324 | count | 1 |
| ZNF432 | -0.1923278 | 0.326414 | -0.5892 | 0.556 | -0.114443436 | count | 1 |
| SEC62 | -0.0800484 | 0.0501618 | -1.5958 | 0.1106 | -0.114442801 | count | 1 |
| CENPS | -0.3768897 | 0.671812 | -0.561 | 0.575 | -0.114344671 | count | 1 |
| HIST2H2BE | -0.3768897 | 0.7838741 | -0.4808 | 0.631 | -0.114344671 | count | 1 |
| AL157392.5 | -0.3768897 | 0.8391786 | -0.4491 | 0.653 | -0.114344671 | count | 1 |
| CEP44 | -0.0988824 | 0.2077812 | -0.4759 | 0.634 | -0.114318086 | count | 1 |

| | | | | | | | |
|---|---|---|---|---|---|---|---|
| SH3BGRL3 | -0.0792941 | 0.0247564 | -3.203 | 0.00137 | -0.114306803 | count | 1 |
| CHD9 | -0.0825549 | 0.1031086 | -0.8007 | 0.423 | -0.114228891 | count | 1 |
| APBB1 | -0.1053814 | 0.2071712 | -0.5087 | 0.611 | -0.114215018 | count | 1 |
| GLI4 | -0.0914253 | 0.1886613 | -0.4846 | 0.628 | -0.113958092 | count | 1 |
| HDHD5 | -0.0914182 | 0.2187295 | -0.418 | 0.676 | -0.113949273 | count | 1 |
| GJA1 | -0.7406361 | 1.175611 | -0.63 | 0.529 | -0.113817344 | count | 1 |
| BIRC3 | -0.0800979 | 0.0598554 | -1.3382 | 0.181 | -0.113734501 | count | 1 |
| PTP4A2 | -0.0797261 | 0.0541207 | -1.4731 | 0.1408 | -0.113709633 | count | 1 |
| PDS5A | -0.0856568 | 0.1531155 | -0.5594 | 0.576 | -0.113628791 | count | 1 |
| ATP6V1F | -0.079601 | 0.0630644 | -1.2622 | 0.207 | -0.113481945 | count | 1 |
| ETNK1 | -0.0827249 | 0.1159391 | -0.7135 | 0.476 | -0.11332265 | count | 1 |
| MAGOHB | -0.0843825 | 0.156608 | -0.5388 | 0.59 | -0.113314206 | count | 1 |
| RNF126 | -0.0807144 | 0.0894428 | -0.9024 | 0.367 | -0.11329104 | count | 1 |
| FGGY | -0.1335043 | 0.4021365 | -0.332 | 0.74 | -0.11325024 | count | 1 |
| UTRN | -0.0807294 | 0.0790278 | -1.0215 | 0.307 | -0.113183225 | count | 1 |
| PTAR1 | -0.0871833 | 0.1717476 | -0.5076 | 0.612 | -0.113151483 | count | 1 |
| HSF1 | -0.0870279 | 0.1397747 | -0.6226 | 0.534 | -0.113130548 | count | 1 |
| SETD4 | -0.1056803 | 0.2930156 | -0.3607 | 0.718 | -0.113129711 | count | 1 |
| PRIM1 | -0.0872829 | 0.2053423 | -0.4251 | 0.671 | -0.113094311 | count | 1 |
| SOCS2 | -0.0857763 | 0.1686269 | -0.5087 | 0.611 | -0.113039114 | count | 1 |
| LINC02086 | -0.3721495 | 0.9525648 | -0.3907 | 0.696 | -0.113033248 | count | 1 |
| SLA | -0.0797653 | 0.053673 | -1.4861 | 0.137 | -0.112799519 | count | 1 |
| GUK1 | -0.0785373 | 0.0418736 | -1.8756 | 0.0608 | -0.112693329 | count | 1 |
| MOSPD3 | -0.0834691 | 0.1582693 | -0.5274 | 0.598 | -0.112561529 | count | 1 |
| MYLK-AS1 | -0.1326032 | 0.5978171 | -0.2218 | 0.824 | -0.112497601 | count | 1 |
| GFI1 | -0.1003087 | 0.1803103 | -0.5563 | 0.578 | -0.112241103 | count | 1 |
| SCYL3 | -0.1077925 | 0.2763767 | -0.39 | 0.697 | -0.112157382 | count | 1 |
| MSL1 | -0.0863814 | 0.1542419 | -0.56 | 0.575 | -0.112113234 | count | 1 |
| SERF1A | -0.2659359 | 0.7750093 | -0.3431 | 0.732 | -0.112047606 | count | 1 |
| ENTPD1 | -0.2658642 | 0.4246102 | -0.6261 | 0.531 | -0.112019059 | count | 1 |
| MIA3 | -0.0810728 | 0.0978245 | -0.8288 | 0.407 | -0.112003867 | count | 1 |
| MDM4 | -0.0815204 | 0.1036549 | -0.7865 | 0.432 | -0.11188361 | count | 1 |
| CTR9 | -0.0837823 | 0.1349923 | -0.6206 | 0.535 | -0.111781652 | count | 1 |
| TXLNG | -0.089027 | 0.1529423 | -0.5821 | 0.561 | -0.111638538 | count | 1 |
| FAM107B | -0.0781775 | 0.0499318 | -1.5657 | 0.118 | -0.111588466 | count | 1 |
| TESK2 | -0.1872368 | 0.3700453 | -0.506 | 0.613 | -0.111509476 | count | 1 |
| SEMA4C | -0.1070678 | 0.2667331 | -0.4014 | 0.688 | -0.111409618 | count | 1 |
| SLC26A6 | -0.1870625 | 0.5287912 | -0.3538 | 0.724 | -0.111408931 | count | 1 |
| BCL7B | -0.080236 | 0.0909532 | -0.8822 | 0.378 | -0.11136144 | count | 1 |
| RUNX3 | -0.0787631 | 0.0642872 | -1.2252 | 0.221 | -0.111350189 | count | 1 |
| SCG5 | -0.1868871 | 1.1239753 | -0.1663 | 0.868 | -0.111307742 | count | 1 |
| AC009159.3 | -0.1868871 | 1.267742 | -0.1474 | 0.883 | -0.111307742 | count | 1 |
| MTF2 | -0.0822684 | 0.1198291 | -0.6865 | 0.492 | -0.111293819 | count | 1 |
| U2AF1L4 | -0.0821716 | 0.1239852 | -0.6628 | 0.508 | -0.111229046 | count | 1 |
| UTP20 | -0.1447131 | 0.3172736 | -0.4561 | 0.648 | -0.111135238 | count | 1 |

| | | | | | | | |
|---|---|---|---|---|---|---|---|
| FXYD2 | -0.1677779 | 0.647716 | -0.259 | 0.796 | -0.111086562 | count | 1 |
| PSMD11 | -0.0796931 | 0.1006195 | -0.792 | 0.428 | -0.111079435 | count | 1 |
| CCNY | -0.0840564 | 0.1366299 | -0.6152 | 0.538 | -0.110906782 | count | 1 |
| SMIM26 | -0.0787721 | 0.0841541 | -0.936 | 0.349 | -0.11080068 | count | 1 |
| GALNT1 | -0.0835026 | 0.1275163 | -0.6548 | 0.513 | -0.110776439 | count | 1 |
| PCNX2 | -0.0915376 | 0.2143454 | -0.4271 | 0.669 | -0.110750235 | count | 1 |
| SGSM2 | -0.1081571 | 0.3369382 | -0.321 | 0.748 | -0.11071811 | count | 1 |
| ATP5PO | -0.0776531 | 0.059235 | -1.3109 | 0.19 | -0.110703385 | count | 1 |
| PRPF40B | -0.185839 | 0.5814135 | -0.3196 | 0.749 | -0.110702971 | count | 1 |
| DNTTIP1 | -0.0869184 | 0.1840863 | -0.4722 | 0.637 | -0.110672105 | count | 1 |
| CES4A | -0.214109 | 0.403665 | -0.5304 | 0.596 | -0.11065511 | count | 1 |
| PUS1 | -0.0936597 | 0.2773265 | -0.3377 | 0.736 | -0.110431616 | count | 1 |
| CMTM7 | -0.0791751 | 0.088261 | -0.8971 | 0.37 | -0.110300209 | count | 1 |
| CLTB | -0.0778072 | 0.073827 | -1.0539 | 0.292 | -0.110280319 | count | 1 |
| ANKRD18A | -0.1663956 | 0.5708497 | -0.2915 | 0.771 | -0.110194746 | count | 1 |
| HLA-A | -0.0764187 | 0.0187839 | -4.0683 | 4.84E-05 | -0.110164696 | count | 1 |
| WDR91 | -0.1530998 | 0.4053868 | -0.3777 | 0.706 | -0.110084982 | count | 1 |
| KIAA1671 | -0.1530998 | 0.4226765 | -0.3622 | 0.717 | -0.110084982 | count | 1 |
| TAF3 | -0.083063 | 0.1165793 | -0.7125 | 0.476 | -0.110080806 | count | 1 |
| TANK | -0.0782835 | 0.0753288 | -1.0392 | 0.299 | -0.110040238 | count | 1 |
| SLC52A2 | -0.081266 | 0.1306367 | -0.6221 | 0.534 | -0.110004909 | count | 1 |
| CNTRL | -0.080184 | 0.0962336 | -0.8332 | 0.405 | -0.109759329 | count | 1 |
| RHOT1 | -0.0919447 | 0.2068614 | -0.4445 | 0.657 | -0.109632461 | count | 1 |
| IMPA1 | -0.0860673 | 0.1542906 | -0.5578 | 0.577 | -0.109591443 | count | 1 |
| DNAL1 | -0.2597496 | 0.3978239 | -0.6529 | 0.514 | -0.109580964 | count | 1 |
| SGF29 | -0.0810982 | 0.1321568 | -0.6137 | 0.539 | -0.109440331 | count | 1 |
| DHRS3 | -0.0830143 | 0.1343312 | -0.618 | 0.537 | -0.10940649 | count | 1 |
| ZNF257 | -0.7047512 | 0.3929031 | -1.7937 | 0.0729 | -0.109405566 | count | 1 |
| FARS2 | -0.0872172 | 0.1897178 | -0.4597 | 0.646 | -0.109376251 | count | 1 |
| ZNF609 | -0.0939341 | 0.2333805 | -0.4025 | 0.687 | -0.109375338 | count | 1 |
| GNAS | -0.0761216 | 0.0322879 | -2.3576 | 0.0185 | -0.10937302 | count | 1 |
| FAM131A | -0.1834943 | 0.3660231 | -0.5013 | 0.616 | -0.109349204 | count | 1 |
| PRPF38A | -0.080013 | 0.114828 | -0.6968 | 0.486 | -0.109344974 | count | 1 |
| EGR4 | -0.358847 | 0.6941204 | -0.517 | 0.605 | -0.10933531 | count | 1 |
| SCOC-AS1 | -0.358847 | 0.7691146 | -0.4666 | 0.641 | -0.10933531 | count | 1 |
| AC245452.1 | -0.358847 | 0.817204 | -0.4391 | 0.661 | -0.10933531 | count | 1 |
| SGO1 | -0.358847 | 1.013917 | -0.3539 | 0.723 | -0.10933531 | count | 1 |
| DDX49 | -0.0833636 | 0.1652071 | -0.5046 | 0.614 | -0.109317822 | count | 1 |
| BMS1 | -0.0832039 | 0.1397418 | -0.5954 | 0.552 | -0.109250931 | count | 1 |
| SEMA7A | -0.7034277 | 0.786226 | -0.8947 | 0.371 | -0.109240775 | count | 1 |
| CD47 | -0.076711 | 0.0601086 | -1.2762 | 0.202 | -0.109215907 | count | 1 |
| ZBTB20 | -0.0808437 | 0.1099083 | -0.7356 | 0.462 | -0.109097385 | count | 1 |
| BACH1 | -0.0880665 | 0.1480667 | -0.5948 | 0.552 | -0.109079497 | count | 1 |
| C4orf46 | -0.1418795 | 0.2997832 | -0.4733 | 0.636 | -0.108999842 | count | 1 |
| GMFG | -0.0758876 | 0.0378167 | -2.0067 | 0.0449 | -0.108975789 | count | 1 |

| | | | | | | | |
|---|---|---|---|---|---|---|---|
| HINT2 | -0.0781901 | 0.1099221 | -0.7113 | 0.477 | -0.108967207 | count | 1 |
| NUDC | -0.0774109 | 0.0790587 | -0.9792 | 0.328 | -0.108781995 | count | 1 |
| PLXNC1 | -0.1128356 | 0.3162784 | -0.3568 | 0.721 | -0.108694109 | count | 1 |
| RBX1 | -0.0764232 | 0.0684021 | -1.1173 | 0.264 | -0.108590603 | count | 1 |
| OSBPL9 | -0.0821096 | 0.1409654 | -0.5825 | 0.56 | -0.108587109 | count | 1 |
| NSMCE1 | -0.07921 | 0.1146234 | -0.691 | 0.49 | -0.108554792 | count | 1 |
| TBCB | -0.0761585 | 0.0660988 | -1.1522 | 0.249 | -0.108492649 | count | 1 |
| DBT | -0.0987589 | 0.2934751 | -0.3365 | 0.737 | -0.108309223 | count | 1 |
| SF3B2 | -0.0763134 | 0.0647726 | -1.1782 | 0.239 | -0.108248541 | count | 1 |
| FAM92A | -0.3546952 | 0.9861485 | -0.3597 | 0.719 | -0.108175819 | count | 1 |
| FAM98C | -0.0817063 | 0.1450773 | -0.5632 | 0.573 | -0.10817182 | count | 1 |
| CD109 | -0.1407344 | 0.579437 | -0.2429 | 0.808 | -0.108136412 | count | 1 |
| ZNF785 | -0.1407344 | 0.5823544 | -0.2417 | 0.809 | -0.108136412 | count | 1 |
| ZNF266 | -0.1225545 | 0.3113096 | -0.3937 | 0.694 | -0.108134972 | count | 1 |
| ZKSCAN8 | -0.1813643 | 0.5724637 | -0.3168 | 0.751 | -0.108118417 | count | 1 |
| S1PR1 | -0.0875644 | 0.2084308 | -0.4201 | 0.674 | -0.108086861 | count | 1 |
| HSPB1 | -0.0771777 | 0.0798087 | -0.967 | 0.334 | -0.108073157 | count | 1 |
| CPD | -0.0892995 | 0.1694575 | -0.527 | 0.598 | -0.108053203 | count | 1 |
| MRPL20 | -0.0760466 | 0.0704902 | -1.0788 | 0.281 | -0.107918247 | count | 1 |
| TUBG1 | -0.100761 | 0.3237362 | -0.3112 | 0.756 | -0.107901773 | count | 1 |
| CRTC3 | -0.0857063 | 0.1441154 | -0.5947 | 0.552 | -0.107787075 | count | 1 |
| NOP2 | -0.2552401 | 0.387946 | -0.6579 | 0.511 | -0.107778557 | count | 1 |
| PIP4K2B | -0.093166 | 0.3082323 | -0.3023 | 0.762 | -0.107743333 | count | 1 |
| ICK | -0.0863868 | 0.2221911 | -0.3888 | 0.697 | -0.10769825 | count | 1 |
| CCDC28A | -0.080318 | 0.1331028 | -0.6034 | 0.546 | -0.107616566 | count | 1 |
| DSEL | -0.352092 | 0.3863258 | -0.9114 | 0.362 | -0.107447529 | count | 1 |
| TKT | -0.0760541 | 0.0757282 | -1.0043 | 0.315 | -0.107240479 | count | 1 |
| AGAP1 | -0.3512516 | 0.5184729 | -0.6775 | 0.498 | -0.10721222 | count | 1 |
| TRIM35 | -0.1012834 | 0.31527 | -0.3213 | 0.748 | -0.107005714 | count | 1 |
| TMEM156 | -0.0828116 | 0.1705166 | -0.4857 | 0.627 | -0.106945388 | count | 1 |
| CCDC189 | -0.3502327 | 0.7234914 | -0.4841 | 0.628 | -0.106926735 | count | 1 |
| MTM1 | -0.0974642 | 0.2228117 | -0.4374 | 0.662 | -0.106898538 | count | 1 |
| AC008403.3 | -0.3499905 | 0.5693277 | -0.6147 | 0.539 | -0.106858857 | count | 1 |
| BIK | -0.0945867 | 0.2956523 | -0.3199 | 0.749 | -0.106833811 | count | 1 |
| HMGB2 | -0.0749405 | 0.0600799 | -1.2473 | 0.2124 | -0.106821662 | count | 1 |
| BLOC1S6 | -0.0798531 | 0.1255833 | -0.6359 | 0.525 | -0.106821645 | count | 1 |
| SIGMAR1 | -0.0828625 | 0.201456 | -0.4113 | 0.681 | -0.106817873 | count | 1 |
| BOLA2-SMG1P6 | -0.2526239 | 0.5331722 | -0.4738 | 0.636 | -0.1067312 | count | 1 |
| DCP1A | -0.0795289 | 0.1284332 | -0.6192 | 0.536 | -0.106644417 | count | 1 |
| CCDC130 | -0.0793369 | 0.1320882 | -0.6006 | 0.548 | -0.106627322 | count | 1 |
| ILK | -0.0772059 | 0.1136132 | -0.6796 | 0.497 | -0.106422822 | count | 1 |
| CCDC171 | -0.3481863 | 0.4415073 | -0.7886 | 0.43 | -0.106352985 | count | 1 |
| TPP2 | -0.0858217 | 0.1653753 | -0.519 | 0.604 | -0.106308421 | count | 1 |
| AC073896.3 | -0.6799715 | 0.8446916 | -0.805 | 0.421 | -0.106295715 | count | 1 |
| PPP4R3A | -0.0781729 | 0.1028978 | -0.7597 | 0.447 | -0.10628765 | count | 1 |

| | | | | | | | |
|---|---|---|---|---|---|---|---|
| ALKBH3 | -0.0824085 | 0.1998255 | -0.4124 | 0.68 | -0.106234041 | count | 1 |
| ATG2B | -0.0938867 | 0.2841638 | -0.3304 | 0.741 | -0.106047643 | count | 1 |
| VPS13B | -0.0883716 | 0.2066094 | -0.4277 | 0.669 | -0.105930176 | count | 1 |
| UBE2G2 | -0.0764797 | 0.1064555 | -0.7184 | 0.473 | -0.105776066 | count | 1 |
| PRKAR2B | -0.2501676 | 0.6187883 | -0.4043 | 0.686 | -0.105746741 | count | 1 |
| NBDY | -0.0757595 | 0.0932695 | -0.8123 | 0.417 | -0.105654117 | count | 1 |
| DOCK10 | -0.0773743 | 0.0947982 | -0.8162 | 0.414 | -0.105604992 | count | 1 |
| CALY | -0.084924 | 0.2791782 | -0.3042 | 0.761 | -0.105547073 | count | 1 |
| ZNF777 | -0.1155487 | 0.4576105 | -0.2525 | 0.801 | -0.105438769 | count | 1 |
| STXBP3 | -0.0768391 | 0.1059222 | -0.7254 | 0.468 | -0.105427086 | count | 1 |
| DNAJC18 | -0.1370737 | 0.3656976 | -0.3748 | 0.708 | -0.105374321 | count | 1 |
| SYAP1 | -0.0760628 | 0.0900021 | -0.8451 | 0.398 | -0.105357356 | count | 1 |
| PI4K2A | -0.0950425 | 0.3180038 | -0.2989 | 0.765 | -0.105356766 | count | 1 |
| REC8 | -0.1154187 | 0.3293651 | -0.3504 | 0.726 | -0.105321549 | count | 1 |
| POLI | -0.1092747 | 0.280989 | -0.3889 | 0.697 | -0.105298635 | count | 1 |
| NLE1 | -0.1046337 | 0.3301582 | -0.3169 | 0.751 | -0.105233279 | count | 1 |
| TRAV27 | -0.671555 | 0.9103404 | -0.7377 | 0.461 | -0.105227586 | count | 1 |
| TDRKH-AS1 | -0.203127 | 0.6190112 | -0.3281 | 0.743 | -0.105192003 | count | 1 |
| KAT7 | -0.0795689 | 0.1204733 | -0.6605 | 0.509 | -0.10511655 | count | 1 |
| PHF11 | -0.0752248 | 0.0920841 | -0.8169 | 0.414 | -0.105075722 | count | 1 |
| USP8 | -0.0759867 | 0.0986222 | -0.7705 | 0.441 | -0.105039855 | count | 1 |
| INTS14 | -0.0837004 | 0.2025321 | -0.4133 | 0.679 | -0.10497929 | count | 1 |
| ZNF384 | -0.1235838 | 0.2807461 | -0.4402 | 0.66 | -0.104955012 | count | 1 |
| SLC15A3 | -0.2481415 | 0.9538888 | -0.2601 | 0.795 | -0.104933883 | count | 1 |
| COQ9 | -0.1007753 | 0.2460102 | -0.4096 | 0.682 | -0.104913122 | count | 1 |
| TCTA | -0.0821882 | 0.2051974 | -0.4005 | 0.689 | -0.104897392 | count | 1 |
| TMEM120A | -0.08129 | 0.1797579 | -0.4522 | 0.651 | -0.10479561 | count | 1 |
| FIP1L1 | -0.0763041 | 0.1013029 | -0.7532 | 0.451 | -0.104731777 | count | 1 |
| NLK | -0.1454981 | 0.3987189 | -0.3649 | 0.715 | -0.10473165 | count | 1 |
| DEAF1 | -0.0892569 | 0.2068484 | -0.4315 | 0.666 | -0.104627274 | count | 1 |
| TRIM73 | -0.0882011 | 0.2317882 | -0.3805 | 0.704 | -0.104620877 | count | 1 |
| CYP20A1 | -0.0756758 | 0.1040882 | -0.727 | 0.467 | -0.104610459 | count | 1 |
| TOMM20 | -0.0732037 | 0.0462818 | -1.5817 | 0.114 | -0.10459236 | count | 1 |
| BTBD2 | -0.0897855 | 0.2537107 | -0.3539 | 0.723 | -0.104567954 | count | 1 |
| KLHL20 | -0.0903697 | 0.227192 | -0.3978 | 0.691 | -0.104525565 | count | 1 |
| USP7 | -0.0775622 | 0.1207911 | -0.6421 | 0.521 | -0.10446661 | count | 1 |
| SMIM10L1 | -0.0749931 | 0.10098 | -0.7427 | 0.458 | -0.104459753 | count | 1 |
| RRBP1 | -0.0781543 | 0.1102105 | -0.7091 | 0.478 | -0.104376151 | count | 1 |
| ARHGAP30 | -0.074822 | 0.0955233 | -0.7833 | 0.434 | -0.10425848 | count | 1 |
| GOLGA7B | -0.0972684 | 0.3681629 | -0.2642 | 0.792 | -0.104187687 | count | 1 |
| TTC9 | -0.0817158 | 0.1865331 | -0.4381 | 0.661 | -0.104065265 | count | 1 |
| NELFA | -0.0831776 | 0.2125676 | -0.3913 | 0.696 | -0.104023623 | count | 1 |
| ALG8 | -0.0831752 | 0.2236179 | -0.372 | 0.71 | -0.10402063 | count | 1 |
| PPP1R15B | -0.081679 | 0.1285517 | -0.6354 | 0.525 | -0.104018523 | count | 1 |
| FXR1 | -0.0745325 | 0.0831499 | -0.8964 | 0.37 | -0.104012082 | count | 1 |

| | | | | | | | |
|---|---|---|---|---|---|---|---|
| RGPD5 | -0.1224488 | 0.3298653 | -0.3712 | 0.711 | -0.104004675 | count | 1 |
| FAM72A | -0.3395965 | 0.7651805 | -0.4438 | 0.657 | -0.103937787 | count | 1 |
| LMBR1 | -0.1740421 | 0.2837334 | -0.6134 | 0.54 | -0.103880192 | count | 1 |
| ZNF346 | -0.3392399 | 0.4497267 | -0.7543 | 0.451 | -0.103837319 | count | 1 |
| AC069185.1 | -0.0997179 | 0.6132575 | -0.1626 | 0.871 | -0.103820789 | count | 1 |
| TMC6 | -0.0745059 | 0.0886023 | -0.8409 | 0.4 | -0.103818328 | count | 1 |
| RCC1L | -0.086555 | 0.1946714 | -0.4446 | 0.657 | -0.103761447 | count | 1 |
| MIB2 | -0.0755154 | 0.1278004 | -0.5909 | 0.555 | -0.103759969 | count | 1 |
| TMEM218 | -0.080705 | 0.2026689 | -0.3982 | 0.69 | -0.103649361 | count | 1 |
| MVK | -0.2447566 | 0.3858296 | -0.6344 | 0.526 | -0.103574237 | count | 1 |
| UQCRH | -0.0722863 | 0.0442229 | -1.6346 | 0.102 | -0.103515924 | count | 1 |
| ELL2 | -0.1733907 | 0.1614014 | -1.0743 | 0.283 | -0.103502608 | count | 1 |
| TAX1BP3 | -0.0882752 | 0.2861468 | -0.3085 | 0.758 | -0.103481788 | count | 1 |
| ZNF133 | -0.337938 | 0.5575316 | -0.6061 | 0.544 | -0.103470251 | count | 1 |
| ENOX2 | -0.0888229 | 0.2275537 | -0.3903 | 0.696 | -0.103452176 | count | 1 |
| RALGAPA2 | -0.096359 | 0.2375804 | -0.4056 | 0.685 | -0.103220294 | count | 1 |
| CCDC152 | -0.0835654 | 0.2609788 | -0.3202 | 0.749 | -0.103167074 | count | 1 |
| SHISA5 | -0.0729809 | 0.0810363 | -0.9006 | 0.368 | -0.102984651 | count | 1 |
| STARD10 | -0.086806 | 0.2094 | -0.4145 | 0.679 | -0.102973119 | count | 1 |
| INO80 | -0.0760173 | 0.1190149 | -0.6387 | 0.523 | -0.10290925 | count | 1 |
| ZNF83 | -0.0809759 | 0.1658518 | -0.4882 | 0.625 | -0.10288893 | count | 1 |
| U2AF1L5 | -0.1550913 | 0.4635929 | -0.3345 | 0.738 | -0.102886359 | count | 1 |
| ZNF341 | -0.1984746 | 0.7660987 | -0.2591 | 0.796 | -0.102870413 | count | 1 |
| GNPDA1 | -0.0771482 | 0.1518272 | -0.5081 | 0.611 | -0.102852732 | count | 1 |
| MTBP | -0.3353095 | 0.6905861 | -0.4855 | 0.627 | -0.102728394 | count | 1 |
| RAP2B | -0.0738283 | 0.0930034 | -0.7938 | 0.427 | -0.102705111 | count | 1 |
| RNF40 | -0.1020942 | 0.2640667 | -0.3866 | 0.699 | -0.102701166 | count | 1 |
| CXCL16 | -0.1124494 | 0.4083936 | -0.2753 | 0.783 | -0.102643221 | count | 1 |
| IGSF22 | -0.1018856 | 0.5570805 | -0.1829 | 0.855 | -0.102493119 | count | 1 |
| C19orf57 | -0.3344536 | 1.0993492 | -0.3042 | 0.761 | -0.10248658 | count | 1 |
| PROS1 | -0.3344536 | 1.1316905 | -0.2955 | 0.768 | -0.10248658 | count | 1 |
| RPL4 | -0.0714259 | 0.0302898 | -2.3581 | 0.0184 | -0.102464315 | count | 1 |
| GTF3C2 | -0.0932749 | 0.3084847 | -0.3024 | 0.762 | -0.102332196 | count | 1 |
| PIKFYVE | -0.1259995 | 0.2489324 | -0.5062 | 0.613 | -0.102300967 | count | 1 |
| CSDE1 | -0.072385 | 0.0658036 | -1.1 | 0.271 | -0.10229337 | count | 1 |
| PIK3R1 | -0.072064 | 0.0551836 | -1.3059 | 0.192 | -0.102210063 | count | 1 |
| AMMECR1L | -0.3333064 | 0.4300409 | -0.7751 | 0.438 | -0.102162375 | count | 1 |
| TBC1D24 | -0.1418457 | 0.4895343 | -0.2898 | 0.772 | -0.10215515 | count | 1 |
| HERC5 | -0.0769836 | 0.1638936 | -0.4697 | 0.639 | -0.102142959 | count | 1 |
| PSMD6 | -0.0733477 | 0.0921604 | -0.7959 | 0.426 | -0.102132552 | count | 1 |
| CD248 | -0.241172 | 0.9024234 | -0.2672 | 0.789 | -0.102132138 | count | 1 |
| CRBN | -0.0726682 | 0.0861427 | -0.8436 | 0.399 | -0.101946775 | count | 1 |
| IQGAP1 | -0.0720199 | 0.066683 | -1.08 | 0.28 | -0.101929883 | count | 1 |
| SCO1 | -0.0849597 | 0.1931598 | -0.4398 | 0.66 | -0.101856597 | count | 1 |
| MUTYH | -0.0863364 | 0.2500055 | -0.3453 | 0.73 | -0.101834603 | count | 1 |

| | | | | | | | |
|---|---|---|---|---|---|---|---|
| ID2 | -0.0712618 | 0.0609774 | -1.1687 | 0.243 | -0.101822466 | count | 1 |
| PABPC4 | -0.0753662 | 0.1192018 | -0.6323 | 0.527 | -0.101715496 | count | 1 |
| TDG | -0.0743528 | 0.12373 | -0.6009 | 0.548 | -0.101618157 | count | 1 |
| GRAMD2B | -0.1079484 | 0.3333814 | -0.3238 | 0.746 | -0.101443289 | count | 1 |
| ECHS1 | -0.0728985 | 0.1084708 | -0.6721 | 0.502 | -0.10141256 | count | 1 |
| APPBP2 | -0.0844637 | 0.1963495 | -0.4302 | 0.667 | -0.101264294 | count | 1 |
| NEXMIF | -0.6407124 | 0.935917 | -0.6846 | 0.494 | -0.101261725 | count | 1 |
| RYR1 | -0.1108391 | 0.3298104 | -0.3361 | 0.737 | -0.101190003 | count | 1 |
| DPY19L1 | -0.1189538 | 0.3171154 | -0.3751 | 0.708 | -0.101076663 | count | 1 |
| TIPARP | -0.0715359 | 0.0772862 | -0.9256 | 0.355 | -0.10091741 | count | 1 |
| NFKBIZ | -0.0756259 | 0.1099653 | -0.6877 | 0.492 | -0.100916515 | count | 1 |
| UPF3B | -0.0748276 | 0.1519766 | -0.4924 | 0.622 | -0.100856784 | count | 1 |
| AMDHD2 | -0.0891786 | 0.2389023 | -0.3733 | 0.709 | -0.100758185 | count | 1 |
| C12orf60 | -0.1941902 | 0.6606454 | -0.2939 | 0.769 | -0.100728684 | count | 1 |
| MXD3 | -0.1941902 | 0.685968 | -0.2831 | 0.777 | -0.100728684 | count | 1 |
| NXT1 | -0.0708709 | 0.0665092 | -1.0656 | 0.287 | -0.100633031 | count | 1 |
| CNPY4 | -0.0858154 | 0.2403893 | -0.357 | 0.721 | -0.100611059 | count | 1 |
| EXOSC8 | -0.0721931 | 0.1047437 | -0.6892 | 0.491 | -0.100562233 | count | 1 |
| IRF9 | -0.0739248 | 0.1277247 | -0.5788 | 0.563 | -0.100518491 | count | 1 |
| PON2 | -0.0906306 | 0.3994978 | -0.2269 | 0.821 | -0.10049448 | count | 1 |
| DNAJB9 | -0.0724233 | 0.0775054 | -0.9344 | 0.35 | -0.100414328 | count | 1 |
| SPTLC1 | -0.0775764 | 0.1613031 | -0.4809 | 0.631 | -0.100374813 | count | 1 |
| TRAPPC6B | -0.0828888 | 0.1757463 | -0.4716 | 0.637 | -0.100324834 | count | 1 |
| TMEM65 | -0.0812392 | 0.1730768 | -0.4694 | 0.639 | -0.100304501 | count | 1 |
| TULP4 | -0.0795227 | 0.1583276 | -0.5023 | 0.616 | -0.100299878 | count | 1 |
| TP53I13 | -0.0738222 | 0.1483494 | -0.4976 | 0.619 | -0.10027526 | count | 1 |
| THEMIS | -0.0723603 | 0.0940941 | -0.769 | 0.442 | -0.100256887 | count | 1 |
| AC108134.3 | -0.1677038 | 0.3373304 | -0.4971 | 0.619 | -0.100202504 | count | 1 |
| BSDC1 | -0.0823993 | 0.1514504 | -0.5441 | 0.586 | -0.100170451 | count | 1 |
| RPS19 | -0.069474 | 0.0204652 | -3.3947 | 0.000695 | -0.100162829 | count | 1 |
| CHMP7 | -0.081383 | 0.1646881 | -0.4942 | 0.621 | -0.100120592 | count | 1 |
| NLGN4Y | -0.6310403 | 0.7305468 | -0.8638 | 0.388 | -0.100001265 | count | 1 |
| KLRB1 | -0.0698709 | 0.069304 | -1.0082 | 0.3134 | -0.099991352 | count | 1 |
| RNF215 | -0.1013753 | 0.455682 | -0.2225 | 0.824 | -0.099967258 | count | 1 |
| GDI2 | -0.0712169 | 0.0755516 | -0.9426 | 0.346 | -0.099877416 | count | 1 |
| VWA5A | -0.325178 | 0.4755649 | -0.6838 | 0.494 | -0.099859455 | count | 1 |
| ZFP69B | -0.192446 | 0.4918322 | -0.3913 | 0.696 | -0.09985574 | count | 1 |
| SLC22A5 | -0.3249538 | 0.6019611 | -0.5398 | 0.589 | -0.099795799 | count | 1 |
| HMOX2 | -0.0702792 | 0.077244 | -0.9098 | 0.363 | -0.099793103 | count | 1 |
| KANSL1-AS1 | -0.0728956 | 0.095723 | -0.7615 | 0.446 | -0.099792899 | count | 1 |
| MRPL33 | -0.0715616 | 0.091678 | -0.7806 | 0.435 | -0.09978687 | count | 1 |
| TSPAN4 | -0.1011517 | 0.3601926 | -0.2808 | 0.779 | -0.099748721 | count | 1 |
| F2R | -0.0732134 | 0.150997 | -0.4849 | 0.628 | -0.099748336 | count | 1 |
| SSR4 | -0.0692819 | 0.0369016 | -1.8775 | 0.0605 | -0.099473364 | count | 1 |
| INVS | -0.1169973 | 0.4027047 | -0.2905 | 0.771 | -0.099436475 | count | 1 |

| | | | | | | | |
|---|---|---|---|---|---|---|---|
| FAM214A | -0.0808169 | 0.166851 | -0.4844 | 0.628 | -0.099426435 | count | 1 |
| CCT8 | -0.0700948 | 0.0714029 | -0.9817 | 0.326 | -0.099426339 | count | 1 |
| TBC1D22A | -0.0754253 | 0.1630811 | -0.4625 | 0.644 | -0.09942274 | count | 1 |
| PPAT | -0.08571 | 0.2221142 | -0.3859 | 0.7 | -0.099161238 | count | 1 |
| PTER | -0.0985306 | 0.2976351 | -0.331 | 0.741 | -0.099146013 | count | 1 |
| MID2 | -0.1907443 | 0.5126947 | -0.372 | 0.71 | -0.099003488 | count | 1 |
| AP5S1 | -0.1051615 | 0.4530616 | -0.2321 | 0.816 | -0.09885099 | count | 1 |
| HNRNPUL1 | -0.0723341 | 0.0966268 | -0.7486 | 0.454 | -0.098776834 | count | 1 |
| C9orf16 | -0.0687835 | 0.0519507 | -1.324 | 0.186 | -0.098476134 | count | 1 |
| MYO18A | -0.3197496 | 0.3996699 | -0.8 | 0.424 | -0.098316103 | count | 1 |
| INPP4B | -0.0711145 | 0.0870807 | -0.8166 | 0.414 | -0.098310296 | count | 1 |
| PDGFA | -0.0976884 | 0.3844048 | -0.2541 | 0.799 | -0.098305484 | count | 1 |
| CRTAP | -0.0729281 | 0.1459349 | -0.4997 | 0.617 | -0.098299903 | count | 1 |
| DDB2 | -0.0723828 | 0.1325586 | -0.546 | 0.585 | -0.098162963 | count | 1 |
| TRAV17 | -0.3190674 | 0.3306923 | -0.9648 | 0.335 | -0.098121844 | count | 1 |
| AC004148.2 | -0.1640722 | 0.4032785 | -0.4068 | 0.684 | -0.0980916 | count | 1 |
| POLD2 | -0.0753026 | 0.1731223 | -0.435 | 0.664 | -0.09807092 | count | 1 |
| RNF181 | -0.0695255 | 0.0923488 | -0.7529 | 0.452 | -0.098038769 | count | 1 |
| ARHGAP11A | -0.1637348 | 0.515853 | -0.3174 | 0.751 | -0.097895344 | count | 1 |
| SMARCC2 | -0.0716863 | 0.1072171 | -0.6686 | 0.504 | -0.097849925 | count | 1 |
| C1orf43 | -0.0697865 | 0.0879 | -0.7939 | 0.427 | -0.097802491 | count | 1 |
| USP15 | -0.0693114 | 0.0621616 | -1.115 | 0.265 | -0.097780576 | count | 1 |
| DDX11 | -0.0952995 | 0.2570763 | -0.3707 | 0.711 | -0.097657155 | count | 1 |
| LARP7 | -0.0692674 | 0.0952746 | -0.727 | 0.467 | -0.097629672 | count | 1 |
| GRB2 | -0.0686831 | 0.066173 | -1.0379 | 0.299 | -0.097494549 | count | 1 |
| SMIM20 | -0.0719178 | 0.1465301 | -0.4908 | 0.624 | -0.09747846 | count | 1 |
| DTWD1 | -0.0762628 | 0.2087552 | -0.3653 | 0.715 | -0.097353062 | count | 1 |
| ACTR3C | -0.3160235 | 0.5232831 | -0.6039 | 0.546 | -0.097254239 | count | 1 |
| DBI | -0.0678949 | 0.0551346 | -1.2314 | 0.218 | -0.097097048 | count | 1 |
| UBL7-AS1 | -0.0746552 | 0.1972426 | -0.3785 | 0.705 | -0.09707959 | count | 1 |
| MAP2K3 | -0.0715518 | 0.1269369 | -0.5637 | 0.573 | -0.097037317 | count | 1 |
| KRR1 | -0.0695098 | 0.0863226 | -0.8052 | 0.421 | -0.096991541 | count | 1 |
| NCF4 | -0.0717873 | 0.1405919 | -0.5106 | 0.61 | -0.096764205 | count | 1 |
| CCDC112 | -0.0740678 | 0.202406 | -0.3659 | 0.714 | -0.096751044 | count | 1 |
| LAMTOR2 | -0.0696567 | 0.1013688 | -0.6872 | 0.492 | -0.096750585 | count | 1 |
| SETD9 | -0.1058767 | 0.285786 | -0.3705 | 0.711 | -0.096708505 | count | 1 |
| TMEM106A | -0.1340628 | 0.473253 | -0.2833 | 0.777 | -0.096655522 | count | 1 |
| NDUFAF2 | -0.0703997 | 0.1263444 | -0.5572 | 0.577 | -0.096635261 | count | 1 |
| JMY | -0.075365 | 0.1328322 | -0.5674 | 0.571 | -0.096614203 | count | 1 |
| RAB1A | -0.0713593 | 0.1163525 | -0.6133 | 0.54 | -0.096611046 | count | 1 |
| RREB1 | -0.3136795 | 0.2929782 | -1.0707 | 0.284 | -0.096585201 | count | 1 |
| ATP6V1E2 | -0.0870314 | 0.3363055 | -0.2588 | 0.796 | -0.096525727 | count | 1 |
| RAB30 | -0.0823113 | 0.2518867 | -0.3268 | 0.744 | -0.096520253 | count | 1 |
| ARID5B | -0.068021 | 0.0658068 | -1.0336 | 0.301 | -0.096473518 | count | 1 |
| MBIP | -0.0747964 | 0.1559073 | -0.4797 | 0.631 | -0.096442658 | count | 1 |

| | | | | | | | |
|---|---|---|---|---|---|---|---|
| IER5 | -0.0698948 | 0.0997326 | -0.7008 | 0.483 | -0.096438793 | count | 1 |
| CTF1 | -0.2266205 | 1.0200094 | -0.2222 | 0.824 | -0.096254365 | count | 1 |
| LCAT | -0.3122929 | 0.7824649 | -0.3991 | 0.69 | -0.096189027 | count | 1 |
| ADI1 | -0.0693389 | 0.1066663 | -0.6501 | 0.516 | -0.096050908 | count | 1 |
| STON1 | -0.3115919 | 1.074942 | -0.2899 | 0.772 | -0.09598864 | count | 1 |
| AC025283.2 | -0.3111895 | 0.8350616 | -0.3727 | 0.709 | -0.095873586 | count | 1 |
| S100A13 | -0.3111359 | 0.4860429 | -0.6401 | 0.522 | -0.095858285 | count | 1 |
| ADPRH | -0.3109284 | 0.7466022 | -0.4165 | 0.677 | -0.095798913 | count | 1 |
| FCGR1A | -0.3109284 | 0.7751216 | -0.4011 | 0.688 | -0.095798913 | count | 1 |
| NUP85 | -0.0970933 | 0.2893611 | -0.3355 | 0.737 | -0.095780632 | count | 1 |
| POLR3H | -0.0769922 | 0.2083859 | -0.3695 | 0.712 | -0.095718036 | count | 1 |
| CWC27 | -0.0718934 | 0.1403968 | -0.5121 | 0.609 | -0.09568071 | count | 1 |
| MEF2A | -0.0721405 | 0.1599573 | -0.451 | 0.652 | -0.095628931 | count | 1 |
| MTCH1 | -0.0682488 | 0.0967162 | -0.7057 | 0.48 | -0.095576757 | count | 1 |
| PHF21A | -0.0810082 | 0.2055937 | -0.394 | 0.694 | -0.09557551 | count | 1 |
| HCG18 | -0.0725172 | 0.1328431 | -0.5459 | 0.585 | -0.095481592 | count | 1 |
| RNF139-AS1 | -0.0967814 | 0.3179309 | -0.3044 | 0.761 | -0.09547555 | count | 1 |
| MAP3K11 | -0.079864 | 0.1987881 | -0.4018 | 0.688 | -0.095282794 | count | 1 |
| PHF14 | -0.0690411 | 0.1054506 | -0.6547 | 0.513 | -0.095206129 | count | 1 |
| L2HGDH | -0.1430676 | 0.4945354 | -0.2893 | 0.772 | -0.095083326 | count | 1 |
| NEK9 | -0.1117542 | 0.353523 | -0.3161 | 0.752 | -0.095037229 | count | 1 |
| SLC27A2 | -0.1587537 | 0.4628505 | -0.343 | 0.732 | -0.094995279 | count | 1 |
| SMAP1 | -0.0676773 | 0.0838449 | -0.8072 | 0.42 | -0.094980485 | count | 1 |
| MTMR10 | -0.0984086 | 0.4713011 | -0.2088 | 0.835 | -0.094922786 | count | 1 |
| BRD3 | -0.0754265 | 0.1737898 | -0.434 | 0.664 | -0.094893277 | count | 1 |
| KRIT1 | -0.0699002 | 0.1409393 | -0.496 | 0.62 | -0.094852176 | count | 1 |
| ZBTB25 | -0.0739744 | 0.1491455 | -0.496 | 0.62 | -0.094835552 | count | 1 |
| KLF5 | -0.2227431 | 0.3297709 | -0.6754 | 0.499 | -0.0946818 | count | 1 |
| FBXW2 | -0.0717221 | 0.1499933 | -0.4782 | 0.633 | -0.094659923 | count | 1 |
| RBBP5 | -0.0760391 | 0.1704001 | -0.4462 | 0.655 | -0.094536542 | count | 1 |
| DHRS7B | -0.0861042 | 0.2984656 | -0.2885 | 0.773 | -0.094509951 | count | 1 |
| TNRC18 | -0.1225985 | 0.3006148 | -0.4078 | 0.683 | -0.094424852 | count | 1 |
| HMGB1 | -0.0655763 | 0.027822 | -2.357 | 0.0185 | -0.094361494 | count | 1 |
| EHMT2 | -0.0738725 | 0.1667795 | -0.4429 | 0.658 | -0.09430885 | count | 1 |
| SFR1 | -0.0778954 | 0.2626317 | -0.2966 | 0.767 | -0.094301887 | count | 1 |
| FAM221A | -0.1002573 | 0.3201827 | -0.3131 | 0.754 | -0.094285664 | count | 1 |
| EBAG9 | -0.0688802 | 0.1202149 | -0.573 | 0.567 | -0.094224845 | count | 1 |
| C2orf69 | -0.0757449 | 0.2077017 | -0.3647 | 0.715 | -0.094171824 | count | 1 |
| PCMTD2 | -0.0744591 | 0.1919709 | -0.3879 | 0.698 | -0.094171079 | count | 1 |
| RFXANK | -0.0695816 | 0.1476532 | -0.4713 | 0.637 | -0.094094658 | count | 1 |
| DGKA | -0.0706259 | 0.1145423 | -0.6166 | 0.538 | -0.094084194 | count | 1 |
| UBE3D | -0.1571644 | 0.4922574 | -0.3193 | 0.75 | -0.094068894 | count | 1 |
| MTRNR2L12 | -0.0659721 | 0.047111 | -1.4004 | 0.161 | -0.09406768 | count | 1 |
| GRPEL2 | -0.122081 | 0.3238573 | -0.377 | 0.706 | -0.094032576 | count | 1 |
| AP1S1 | -0.0703151 | 0.1614363 | -0.4356 | 0.663 | -0.094001905 | count | 1 |

| | | | | | | | |
|---|---|---|---|---|---|---|---|
| ERP44 | -0.0676067 | 0.0935755 | -0.7225 | 0.47 | -0.093963144 | count | 1 |
| MRPS25 | -0.0704605 | 0.1633563 | -0.4313 | 0.666 | -0.093776559 | count | 1 |
| ZNF85 | -0.0761806 | 0.266114 | -0.2863 | 0.775 | -0.093740112 | count | 1 |
| TBC1D9B | -0.0863758 | 0.2308445 | -0.3742 | 0.708 | -0.093738769 | count | 1 |
| AL136987.1 | -0.1800305 | 0.4786104 | -0.3762 | 0.707 | -0.09362471 | count | 1 |
| TSTD2 | -0.0995061 | 0.2635318 | -0.3776 | 0.706 | -0.09358597 | count | 1 |
| GGPS1 | -0.0714362 | 0.1502404 | -0.4755 | 0.634 | -0.093580008 | count | 1 |
| LCMT2 | -0.3030819 | 0.7127608 | -0.4252 | 0.671 | -0.093550358 | count | 1 |
| AC109347.1 | -0.1562422 | 0.9910553 | -0.1577 | 0.875 | -0.093531118 | count | 1 |
| PRPF38B | -0.0656766 | 0.0570202 | -1.1518 | 0.2495 | -0.093528238 | count | 1 |
| ODF2 | -0.0737604 | 0.2344044 | -0.3147 | 0.753 | -0.093520279 | count | 1 |
| MAP2K2 | -0.0655212 | 0.061479 | -1.0658 | 0.287 | -0.093380168 | count | 1 |
| UBE2H | -0.067363 | 0.1065477 | -0.6322 | 0.527 | -0.093315824 | count | 1 |
| MANEA-DT | -0.0859797 | 0.3189224 | -0.2696 | 0.787 | -0.093311423 | count | 1 |
| INTS1 | -0.0992105 | 0.2980701 | -0.3328 | 0.739 | -0.093310611 | count | 1 |
| CRLS1 | -0.0735831 | 0.1862114 | -0.3952 | 0.693 | -0.093296031 | count | 1 |
| IPMK | -0.0966002 | 0.2812519 | -0.3435 | 0.731 | -0.093193862 | count | 1 |
| DLK2 | -0.5791451 | 0.4896818 | -1.1827 | 0.237 | -0.093100405 | count | 1 |
| CREBZF | -0.0693579 | 0.1152276 | -0.6019 | 0.547 | -0.093095077 | count | 1 |
| TUBB4B | -0.065342 | 0.0567707 | -1.151 | 0.25 | -0.093054133 | count | 1 |
| PASK | -0.0830677 | 0.1304143 | -0.637 | 0.524 | -0.093048511 | count | 1 |
| AC068491.3 | -0.5786955 | 0.8937434 | -0.6475 | 0.517 | -0.093039598 | count | 1 |
| MAFK | -0.1289191 | 0.2642138 | -0.4879 | 0.626 | -0.093013854 | count | 1 |
| RRAS | -0.0702276 | 0.1622833 | -0.4327 | 0.665 | -0.092999177 | count | 1 |
| YBX1 | -0.0646426 | 0.0299596 | -2.1577 | 0.031 | -0.092981802 | count | 1 |
| THAP4 | -0.0725203 | 0.2114636 | -0.3429 | 0.732 | -0.092975512 | count | 1 |
| ZBED6CL | -0.3004197 | 0.8364673 | -0.3592 | 0.72 | -0.092785381 | count | 1 |
| FBXL15 | -0.0675147 | 0.1188128 | -0.5682 | 0.57 | -0.092711735 | count | 1 |
| ZNF544 | -0.0835576 | 0.2550556 | -0.3276 | 0.743 | -0.092693439 | count | 1 |
| COL4A1 | -0.575817 | 0.7368612 | -0.7814 | 0.435 | -0.092649889 | count | 1 |
| TSSC4 | -0.0668756 | 0.1183358 | -0.5651 | 0.572 | -0.092526771 | count | 1 |
| PRDM10 | -0.5745129 | 0.6829792 | -0.8412 | 0.4 | -0.092473079 | count | 1 |
| ERCC6L2 | -0.0798864 | 0.2100595 | -0.3803 | 0.704 | -0.092452992 | count | 1 |
| MAN2B2 | -0.0862133 | 0.2554133 | -0.3375 | 0.736 | -0.092418715 | count | 1 |
| MBTPS2 | -0.1775826 | 0.6097954 | -0.2912 | 0.771 | -0.092392612 | count | 1 |
| TCEANC | -0.0886415 | 0.3825135 | -0.2317 | 0.817 | -0.092367214 | count | 1 |
| AC124312.1 | -0.5735897 | 0.5805181 | -0.9881 | 0.323 | -0.092347826 | count | 1 |
| FIGNL1 | -0.0840815 | 0.2515566 | -0.3342 | 0.738 | -0.092302063 | count | 1 |
| CLK3 | -0.0681701 | 0.128806 | -0.5292 | 0.597 | -0.092298249 | count | 1 |
| SLC48A1 | -0.1278906 | 0.434027 | -0.2947 | 0.768 | -0.092285019 | count | 1 |
| HEATR5B | -0.0839823 | 0.2533476 | -0.3315 | 0.74 | -0.092193765 | count | 1 |
| TOR1AIP1 | -0.0674573 | 0.1153259 | -0.5849 | 0.559 | -0.092123823 | count | 1 |
| RLIM | -0.0745784 | 0.1742317 | -0.428 | 0.669 | -0.092104805 | count | 1 |
| AL606760.3 | -0.1769682 | 0.6766596 | -0.2615 | 0.794 | -0.092083182 | count | 1 |
| ZNF326 | -0.0681534 | 0.1303718 | -0.5228 | 0.601 | -0.092051392 | count | 1 |

| | | | | | | | |
|---|---|---|---|---|---|---|---|
| RBM34 | -0.0756928 | 0.1931678 | -0.3918 | 0.695 | -0.092044211 | count | 1 |
| UBQLN1 | -0.067863 | 0.1257242 | -0.5398 | 0.589 | -0.09193628 | count | 1 |
| LINC00957 | -0.1130025 | 0.4044287 | -0.2794 | 0.78 | -0.091893648 | count | 1 |
| CHMP4B | -0.0682444 | 0.133283 | -0.512 | 0.609 | -0.091671044 | count | 1 |
| OXCT1 | -0.070229 | 0.1500003 | -0.4682 | 0.64 | -0.091612562 | count | 1 |
| ASCC2 | -0.0712883 | 0.1678344 | -0.4248 | 0.671 | -0.091581467 | count | 1 |
| PPID | -0.0678375 | 0.1441194 | -0.4707 | 0.638 | -0.091507294 | count | 1 |
| TRAT1 | -0.0647502 | 0.0651539 | -0.9938 | 0.32 | -0.09150222 | count | 1 |
| CEP128 | -0.0948163 | 0.3260762 | -0.2908 | 0.771 | -0.091487774 | count | 1 |
| EGLN1 | -0.0697013 | 0.1784772 | -0.3905 | 0.696 | -0.09143315 | count | 1 |
| PIGP | -0.0687478 | 0.1396917 | -0.4921 | 0.623 | -0.091413025 | count | 1 |
| BRD4 | -0.0656079 | 0.0874313 | -0.7504 | 0.453 | -0.09141119 | count | 1 |
| HAUS5 | -0.0971465 | 0.3059544 | -0.3175 | 0.751 | -0.091387468 | count | 1 |
| TOMM5 | -0.0686465 | 0.1373058 | -0.5 | 0.617 | -0.091365799 | count | 1 |
| LTB | -0.0634904 | 0.0557929 | -1.138 | 0.255 | -0.091301783 | count | 1 |
| ZNF675 | -0.0689935 | 0.1520016 | -0.4539 | 0.65 | -0.091268887 | count | 1 |
| ISG20L2 | -0.0671403 | 0.1127359 | -0.5956 | 0.552 | -0.091208754 | count | 1 |
| PSMA6 | -0.0749553 | 0.169162 | -0.4431 | 0.658 | -0.091150286 | count | 1 |
| DDX39B | -0.0668769 | 0.1010898 | -0.6616 | 0.508 | -0.091124068 | count | 1 |
| GMEB1 | -0.0692606 | 0.1683184 | -0.4115 | 0.681 | -0.091088833 | count | 1 |
| ATG9B | -0.1180575 | 0.5921302 | -0.1994 | 0.842 | -0.090980898 | count | 1 |
| FOXJ3 | -0.0684465 | 0.1247455 | -0.5487 | 0.583 | -0.090923959 | count | 1 |
| PSMD10 | -0.073869 | 0.2098316 | -0.352 | 0.725 | -0.090904154 | count | 1 |
| OSBP | -0.0685629 | 0.1446928 | -0.4739 | 0.636 | -0.090893707 | count | 1 |
| RILPL1 | -0.2132054 | 0.4556455 | -0.4679 | 0.64 | -0.090802174 | count | 1 |
| IBA57 | -0.213137 | 0.3629833 | -0.5872 | 0.557 | -0.090774283 | count | 1 |
| WSB2 | -0.0689512 | 0.1848757 | -0.373 | 0.709 | -0.0906826 | count | 1 |
| CCDC18 | -0.0963813 | 0.2419308 | -0.3984 | 0.69 | -0.090674288 | count | 1 |
| SPEN | -0.0722117 | 0.1360308 | -0.5308 | 0.596 | -0.090607165 | count | 1 |
| MYB | -0.2926774 | 0.4490446 | -0.6518 | 0.515 | -0.090554871 | count | 1 |
| FAS | -0.0664514 | 0.114567 | -0.58 | 0.562 | -0.090544882 | count | 1 |
| FAM76B | -0.0682754 | 0.1309046 | -0.5216 | 0.602 | -0.090513146 | count | 1 |
| UBE2N | -0.0636589 | 0.0659521 | -0.9652 | 0.334 | -0.090401757 | count | 1 |
| ZCWPW1 | -0.1358533 | 0.361926 | -0.3754 | 0.707 | -0.090386937 | count | 1 |
| TMEM248 | -0.064851 | 0.0972881 | -0.6666 | 0.505 | -0.090373109 | count | 1 |
| ZNF714 | -0.0842632 | 0.3083166 | -0.2733 | 0.785 | -0.090340685 | count | 1 |
| BLOC1S2 | -0.0643577 | 0.0898283 | -0.7165 | 0.474 | -0.090219932 | count | 1 |
| SERPINF1 | -0.5579859 | 0.5607438 | -0.9951 | 0.32 | -0.090219621 | count | 1 |
| CAPRIN2 | -0.0698893 | 0.2070373 | -0.3376 | 0.736 | -0.089960798 | count | 1 |
| NAA25 | -0.0788313 | 0.2390065 | -0.3298 | 0.742 | -0.089870055 | count | 1 |
| SCLT1 | -0.0709897 | 0.1655821 | -0.4287 | 0.668 | -0.089793715 | count | 1 |
| KMT5A | -0.0681035 | 0.1491397 | -0.4566 | 0.648 | -0.089786949 | count | 1 |
| DDA1 | -0.0670964 | 0.1412436 | -0.475 | 0.635 | -0.089779493 | count | 1 |
| HPS1 | -0.0658884 | 0.1367374 | -0.4819 | 0.63 | -0.089778519 | count | 1 |
| CHAF1B | -0.5546129 | 0.5051154 | -1.098 | 0.272 | -0.089756791 | count | 1 |

| | | | | | | | |
|---|---|---|---|---|---|---|---|
| TADA2A | -0.0709517 | 0.2445856 | -0.2901 | 0.772 | -0.089745764 | count | 1 |
| GNA12 | -0.0908864 | 0.3581925 | -0.2537 | 0.8 | -0.089706146 | count | 1 |
| AC011603.2 | -0.5539626 | 0.6686567 | -0.8285 | 0.407 | -0.08966744 | count | 1 |
| 9-Sep | -0.0637074 | 0.074741 | -0.8524 | 0.394 | -0.089611623 | count | 1 |
| PPP3R1 | -0.0690512 | 0.1532953 | -0.4504 | 0.652 | -0.089516754 | count | 1 |
| GBP3 | -0.067495 | 0.203181 | -0.3322 | 0.74 | -0.089480109 | count | 1 |
| CRYZ | -0.0717284 | 0.2397654 | -0.2992 | 0.765 | -0.089472551 | count | 1 |
| SEC63 | -0.0679025 | 0.1325564 | -0.5123 | 0.609 | -0.089415382 | count | 1 |
| JHY | -0.0734084 | 0.2433096 | -0.3017 | 0.763 | -0.089275094 | count | 1 |
| SUPT7L | -0.0704016 | 0.1851998 | -0.3801 | 0.704 | -0.089271613 | count | 1 |
| RBM17 | -0.0635048 | 0.0796276 | -0.7975 | 0.425 | -0.089231251 | count | 1 |
| ACAD8 | -0.0811418 | 0.3172335 | -0.2558 | 0.798 | -0.089092126 | count | 1 |
| ARL4C | -0.0619429 | 0.0367386 | -1.686 | 0.0919 | -0.089026732 | count | 1 |
| DEPDC5 | -0.1336759 | 0.3858875 | -0.3464 | 0.729 | -0.088967361 | count | 1 |
| RBMX | -0.0636488 | 0.0713838 | -0.8916 | 0.373 | -0.088874222 | count | 1 |
| PRKD3 | -0.0692795 | 0.1711888 | -0.4047 | 0.686 | -0.088829363 | count | 1 |
| EIF1B | -0.0623071 | 0.0661491 | -0.9419 | 0.346 | -0.08866172 | count | 1 |
| TICAM1 | -0.0732022 | 0.2959685 | -0.2473 | 0.805 | -0.088638508 | count | 1 |
| P4HTM | -0.0660496 | 0.1620038 | -0.4077 | 0.684 | -0.088523257 | count | 1 |
| TMX2 | -0.0666874 | 0.142234 | -0.4689 | 0.639 | -0.088501834 | count | 1 |
| VPS33A | -0.0694464 | 0.2052149 | -0.3384 | 0.735 | -0.088475029 | count | 1 |
| MRPL57 | -0.0623757 | 0.0747241 | -0.8347 | 0.404 | -0.08843085 | count | 1 |
| AFF4 | -0.0664994 | 0.1162981 | -0.5718 | 0.567 | -0.088341159 | count | 1 |
| NHSL2 | -0.0813237 | 0.2966537 | -0.2741 | 0.784 | -0.088286332 | count | 1 |
| DCAF7 | -0.0658699 | 0.1428389 | -0.4611 | 0.645 | -0.088212347 | count | 1 |
| BAG4 | -0.0663654 | 0.153953 | -0.4311 | 0.666 | -0.088163402 | count | 1 |
| ADPRM | -0.077293 | 0.2078228 | -0.3719 | 0.71 | -0.08812413 | count | 1 |
| STAP2 | -0.2065693 | 0.7390721 | -0.2795 | 0.78 | -0.088093307 | count | 1 |
| ZNF383 | -0.0785945 | 0.2430641 | -0.3233 | 0.746 | -0.088061979 | count | 1 |
| ABCD3 | -0.0821061 | 0.2371294 | -0.3463 | 0.729 | -0.08804138 | count | 1 |
| EIF2B2 | -0.0676487 | 0.19691 | -0.3436 | 0.731 | -0.087985148 | count | 1 |
| ATP6V1E1 | -0.0634089 | 0.1132473 | -0.5599 | 0.576 | -0.087964119 | count | 1 |
| TMEM87B | -0.0726166 | 0.198373 | -0.3661 | 0.714 | -0.087931682 | count | 1 |
| JPT2 | -0.0872251 | 0.3464649 | -0.2518 | 0.801 | -0.0878526 | count | 1 |
| NAT14 | -0.1319576 | 0.5505708 | -0.2397 | 0.811 | -0.087846407 | count | 1 |
| P4HA1 | -0.0677756 | 0.1755278 | -0.3861 | 0.699 | -0.087717975 | count | 1 |
| ABRAXAS1 | -0.0701795 | 0.1656029 | -0.4238 | 0.672 | -0.087545497 | count | 1 |
| CETN3 | -0.0805332 | 0.2614616 | -0.308 | 0.758 | -0.087432837 | count | 1 |
| CDC26 | -0.0622436 | 0.0823268 | -0.7561 | 0.45 | -0.0872577 | count | 1 |
| PPP4R2 | -0.0627584 | 0.0854446 | -0.7345 | 0.463 | -0.087193319 | count | 1 |
| ZNF496 | -0.5359914 | 0.5787383 | -0.9261 | 0.354 | -0.087183662 | count | 1 |
| AC097376.2 | -0.071592 | 0.1759652 | -0.4069 | 0.684 | -0.087072875 | count | 1 |
| PRKAG1 | -0.0653367 | 0.168661 | -0.3874 | 0.698 | -0.087047819 | count | 1 |
| RNF113A | -0.062547 | 0.0980048 | -0.6382 | 0.523 | -0.08703747 | count | 1 |
| C14orf28 | -0.16695 | 0.4075504 | -0.4096 | 0.682 | -0.087027357 | count | 1 |

| | | | | | | | |
|---|---|---|---|---|---|---|---|
| MYO9B | -0.0658765 | 0.1424171 | -0.4626 | 0.644 | -0.086956627 | count | 1 |
| TCERG1 | -0.0638593 | 0.1166801 | -0.5473 | 0.584 | -0.086932463 | count | 1 |
| VTA1 | -0.0663256 | 0.1466873 | -0.4522 | 0.651 | -0.086777381 | count | 1 |
| EPC2 | -0.0690572 | 0.1427825 | -0.4837 | 0.629 | -0.086658795 | count | 1 |
| RNF5 | -0.0623672 | 0.1040702 | -0.5993 | 0.549 | -0.086519961 | count | 1 |
| MRPL24 | -0.0647469 | 0.1558085 | -0.4156 | 0.678 | -0.086493939 | count | 1 |
| YRDC | -0.0646572 | 0.1305001 | -0.4955 | 0.62 | -0.086447834 | count | 1 |
| TMEM181 | -0.101524 | 0.2045496 | -0.4963 | 0.62 | -0.086437598 | count | 1 |
| PITPNM2 | -0.1297652 | 0.7163182 | -0.1812 | 0.856 | -0.086415276 | count | 1 |
| LMAN2L | -0.2779079 | 0.5702968 | -0.4873 | 0.626 | -0.086275599 | count | 1 |
| ZNF19 | -0.1295351 | 0.9452751 | -0.137 | 0.891 | -0.086265019 | count | 1 |
| TECPR1 | -0.0762346 | 0.2388354 | -0.3192 | 0.75 | -0.0861998 | count | 1 |
| LAMTOR5 | -0.0609836 | 0.0753789 | -0.809 | 0.419 | -0.086175857 | count | 1 |
| SLC26A11 | -0.1192787 | 0.316037 | -0.3774 | 0.706 | -0.086173701 | count | 1 |
| TMEM126A | -0.0631567 | 0.130183 | -0.4851 | 0.628 | -0.086018754 | count | 1 |
| CLUAP1 | -0.0747992 | 0.2185114 | -0.3423 | 0.732 | -0.085960345 | count | 1 |
| ABCB7 | -0.0716282 | 0.1990693 | -0.3598 | 0.719 | -0.085926732 | count | 1 |
| DDX27 | -0.0627972 | 0.1118418 | -0.5615 | 0.575 | -0.085765518 | count | 1 |
| SSNA1 | -0.060351 | 0.0745348 | -0.8097 | 0.418 | -0.085741697 | count | 1 |
| PARP10 | -0.0654945 | 0.1802298 | -0.3634 | 0.716 | -0.085691813 | count | 1 |
| BIRC2 | -0.062634 | 0.1125999 | -0.5563 | 0.578 | -0.085616471 | count | 1 |
| ITGB1 | -0.0598988 | 0.0569763 | -1.0513 | 0.2932 | -0.085601459 | count | 1 |
| GOLGA3 | -0.0684488 | 0.1978595 | -0.3459 | 0.729 | -0.085391986 | count | 1 |
| NINJ2 | -0.0626747 | 0.1698474 | -0.369 | 0.712 | -0.085321403 | count | 1 |
| SLC25A16 | -0.0964092 | 0.2980502 | -0.3235 | 0.746 | -0.085305424 | count | 1 |
| TNS1 | -0.5217482 | 0.9784174 | -0.5333 | 0.594 | -0.085195017 | count | 1 |
| GOSR1 | -0.061769 | 0.1121198 | -0.5509 | 0.582 | -0.085108431 | count | 1 |
| BAG5 | -0.0621825 | 0.1139612 | -0.5456 | 0.585 | -0.085070752 | count | 1 |
| PADI4 | -0.5208085 | 0.8387893 | -0.6209 | 0.535 | -0.085063195 | count | 1 |
| PITRM1-AS1 | -0.5208085 | 0.9494692 | -0.5485 | 0.583 | -0.085063195 | count | 1 |
| MED6 | -0.0623757 | 0.1259983 | -0.4951 | 0.621 | -0.08499668 | count | 1 |
| RALY | -0.0601134 | 0.0776524 | -0.7741 | 0.439 | -0.084993144 | count | 1 |
| FAM172A | -0.0637605 | 0.1388743 | -0.4591 | 0.646 | -0.084950642 | count | 1 |
| SP3 | -0.0617985 | 0.1114245 | -0.5546 | 0.579 | -0.084927759 | count | 1 |
| SERF2 | -0.058882 | 0.0254257 | -2.3158 | 0.0206 | -0.084781165 | count | 1 |
| ZNF24 | -0.0607345 | 0.0923303 | -0.6578 | 0.511 | -0.084669039 | count | 1 |
| JOSD2 | -0.0618303 | 0.1195263 | -0.5173 | 0.605 | -0.084657736 | count | 1 |
| PPP2R5D | -0.0899297 | 0.3132838 | -0.2871 | 0.774 | -0.084656915 | count | 1 |
| AP003392.4 | -0.1039624 | 0.4518843 | -0.2301 | 0.818 | -0.084634415 | count | 1 |
| HIST1H4E | -0.141007 | 0.3328754 | -0.4236 | 0.672 | -0.084621684 | count | 1 |
| MTA2 | -0.0695228 | 0.212337 | -0.3274 | 0.743 | -0.084563725 | count | 1 |
| PIK3CA | -0.0673536 | 0.1940617 | -0.3471 | 0.729 | -0.084526082 | count | 1 |
| LEMD2 | -0.0666787 | 0.204985 | -0.3253 | 0.745 | -0.084353017 | count | 1 |
| C18orf54 | -0.2712097 | 0.7650372 | -0.3545 | 0.723 | -0.084324419 | count | 1 |
| RPL28 | -0.0584476 | 0.0123686 | -4.7255 | 2.39E-06 | -0.084295346 | count | 0.05754881 |

| | | | | | | | |
|---|---|---|---|---|---|---|---|
| BTN2A2 | -0.1034942 | 0.2708785 | -0.3821 | 0.702 | -0.084257999 | count | 1 |
| KIF22 | -0.0632024 | 0.1414174 | -0.4469 | 0.655 | -0.084049323 | count | 1 |
| RNASET2 | -0.0592608 | 0.0602678 | -0.9833 | 0.326 | -0.084000986 | count | 1 |
| GRAP | -0.0833119 | 0.2382386 | -0.3497 | 0.727 | -0.083938411 | count | 1 |
| EXOC4 | -0.064021 | 0.1595176 | -0.4013 | 0.688 | -0.083881836 | count | 1 |
| BLMH | -0.0702177 | 0.2075755 | -0.3383 | 0.735 | -0.083812369 | count | 1 |
| LINC00623 | -0.0598939 | 0.0884818 | -0.6769 | 0.499 | -0.083721855 | count | 1 |
| ZNF681 | -0.1603498 | 0.3924442 | -0.4086 | 0.683 | -0.083685821 | count | 1 |
| RPS17 | -0.0627915 | 0.1105678 | -0.5679 | 0.57 | -0.083583343 | count | 1 |
| TMEM43 | -0.0628947 | 0.1545878 | -0.4069 | 0.684 | -0.083558983 | count | 1 |
| RXRB | -0.0789476 | 0.2325736 | -0.3395 | 0.734 | -0.083545239 | count | 1 |
| ZNF208 | -0.0980006 | 0.445433 | -0.22 | 0.826 | -0.083470941 | count | 1 |
| VPS45 | -0.0744662 | 0.2696721 | -0.2761 | 0.782 | -0.083457402 | count | 1 |
| TRAF3 | -0.0663089 | 0.160514 | -0.4131 | 0.68 | -0.083449081 | count | 1 |
| CCDC14 | -0.0641167 | 0.1543293 | -0.4155 | 0.678 | -0.083399258 | count | 1 |
| SEC61A1 | -0.064855 | 0.2034095 | -0.3188 | 0.75 | -0.083332841 | count | 1 |
| PDK3 | -0.0730577 | 0.2136341 | -0.342 | 0.732 | -0.083315537 | count | 1 |
| AC114811.2 | -0.1387772 | 0.8926159 | -0.1555 | 0.876 | -0.083313758 | count | 1 |
| ZDHHC17 | -0.0671462 | 0.215998 | -0.3109 | 0.756 | -0.083234977 | count | 1 |
| TCP11L1 | -0.0785981 | 0.3359742 | -0.2339 | 0.815 | -0.083177503 | count | 1 |
| TSG101 | -0.0601061 | 0.1094913 | -0.549 | 0.583 | -0.083080402 | count | 1 |
| DNASE1 | -0.1591492 | 0.3858132 | -0.4125 | 0.68 | -0.083077079 | count | 1 |
| FAM19A2 | -0.1941696 | 0.4158907 | -0.4669 | 0.641 | -0.083010868 | count | 1 |
| ZNF365 | -0.0747691 | 0.2717777 | -0.2751 | 0.783 | -0.082990123 | count | 1 |
| AL162231.1 | -0.1147349 | 0.5269144 | -0.2177 | 0.828 | -0.082943041 | count | 1 |
| SLF1 | -0.0591246 | 0.0943877 | -0.6264 | 0.531 | -0.082897129 | count | 1 |
| RXYLT1 | -0.0656461 | 0.2237096 | -0.2934 | 0.769 | -0.08283716 | count | 1 |
| FANCM | -0.0770698 | 0.3148205 | -0.2448 | 0.807 | -0.082670233 | count | 1 |
| DELE1 | -0.068187 | 0.2103404 | -0.3242 | 0.746 | -0.082583879 | count | 1 |
| FAM234A | -0.137446 | 0.3425464 | -0.4012 | 0.688 | -0.082532423 | count | 1 |
| GATAD2B | -0.0640756 | 0.1717872 | -0.373 | 0.709 | -0.082491534 | count | 1 |
| MMS22L | -0.0768436 | 0.3938936 | -0.1951 | 0.845 | -0.0824289 | count | 1 |
| TMEM39B | -0.0742325 | 0.2547325 | -0.2914 | 0.771 | -0.082397302 | count | 1 |
| DNAJA4 | -0.1925406 | 0.3960869 | -0.4861 | 0.627 | -0.082341154 | count | 1 |
| STOML1 | -0.0832973 | 0.3901184 | -0.2135 | 0.831 | -0.082269627 | count | 1 |
| CCM2 | -0.0587799 | 0.0885812 | -0.6636 | 0.507 | -0.082246416 | count | 1 |
| LTB4R2 | -0.2640672 | 0.729583 | -0.3619 | 0.717 | -0.082236666 | count | 1 |
| EXO5 | -0.1009609 | 0.4940731 | -0.2043 | 0.838 | -0.08222053 | count | 1 |
| TMEM123 | -0.0577684 | 0.0634453 | -0.9105 | 0.363 | -0.08211953 | count | 1 |
| SGPL1 | -0.0719429 | 0.2548319 | -0.2823 | 0.778 | -0.082049433 | count | 1 |
| POLR2J3 | -0.0608496 | 0.1137067 | -0.5351 | 0.593 | -0.08203701 | count | 1 |
| TANGO2 | -0.0774658 | 0.309962 | -0.2499 | 0.803 | -0.081985984 | count | 1 |
| AC147067.1 | -0.0690355 | 0.2610587 | -0.2644 | 0.791 | -0.081963778 | count | 1 |
| RAPGEF2 | -0.0785452 | 0.3074556 | -0.2555 | 0.798 | -0.081909413 | count | 1 |
| ZNF620 | -0.1567494 | 0.5026682 | -0.3118 | 0.755 | -0.081859472 | count | 1 |

| | | | | | | | |
|---|---|---|---|---|---|---|---|
| NDUFA8 | -0.0593343 | 0.1295361 | -0.4581 | 0.647 | -0.081854254 | count | 1 |
| ARF3 | -0.0629997 | 0.1767391 | -0.3565 | 0.722 | -0.081685335 | count | 1 |
| MUL1 | -0.0810453 | 0.3113364 | -0.2603 | 0.795 | -0.081670029 | count | 1 |
| DDX42 | -0.0607531 | 0.1244429 | -0.4882 | 0.625 | -0.081619622 | count | 1 |
| PPP1R7 | -0.0578998 | 0.0871287 | -0.6645 | 0.506 | -0.081613303 | count | 1 |
| YIPF6 | -0.0622493 | 0.1695736 | -0.3671 | 0.714 | -0.081564116 | count | 1 |
| LRPAP1 | -0.0576432 | 0.0812474 | -0.7095 | 0.478 | -0.081482132 | count | 1 |
| TCTN2 | -0.4953697 | 0.8776496 | -0.5644 | 0.573 | -0.081464933 | count | 1 |
| MOGS | -0.0645039 | 0.1634829 | -0.3946 | 0.693 | -0.081399013 | count | 1 |
| AC138696.2 | -0.2611684 | 0.9629934 | -0.2712 | 0.786 | -0.081387234 | count | 1 |
| BRWD1-AS2 | -0.2611684 | 0.963444 | -0.2711 | 0.786 | -0.081387234 | count | 1 |
| ANKRD50 | -0.2611684 | 1.087233 | -0.2402 | 0.81 | -0.081387234 | count | 1 |
| PIGL | -0.0792486 | 0.2547798 | -0.311 | 0.756 | -0.081312797 | count | 1 |
| FADS3 | -0.0671133 | 0.2531333 | -0.2651 | 0.791 | -0.081287289 | count | 1 |
| CCNQ | -0.0592702 | 0.1233341 | -0.4806 | 0.631 | -0.081280731 | count | 1 |
| RPL7 | -0.0564438 | 0.0237656 | -2.375 | 0.0176 | -0.081228304 | count | 1 |
| TARBP1 | -0.0687653 | 0.2621034 | -0.2624 | 0.793 | -0.081180458 | count | 1 |
| RBM26 | -0.0621 | 0.1176409 | -0.5279 | 0.598 | -0.081025584 | count | 1 |
| EVC2 | -0.4913335 | 0.4724217 | -1.04 | 0.298 | -0.080888781 | count | 1 |
| TADA1 | -0.121021 | 0.3429862 | -0.3528 | 0.724 | -0.080697468 | count | 1 |
| DHRS4L2 | -0.0594285 | 0.1435688 | -0.4139 | 0.679 | -0.080656089 | count | 1 |
| ENTR1 | -0.0633765 | 0.15621 | -0.4057 | 0.685 | -0.080573084 | count | 1 |
| NUCKS1 | -0.0564513 | 0.0532376 | -1.0604 | 0.2891 | -0.08049788 | count | 1 |
| PDLIM2 | -0.0570826 | 0.082588 | -0.6912 | 0.49 | -0.080461819 | count | 1 |
| DENND4C | -0.0637385 | 0.1837898 | -0.3468 | 0.729 | -0.080435231 | count | 1 |
| MRPL49 | -0.0609442 | 0.1583877 | -0.3848 | 0.7 | -0.080265795 | count | 1 |
| C5orf63 | -0.0852135 | 0.318443 | -0.2676 | 0.789 | -0.080253236 | count | 1 |
| HGSNAT | -0.0715868 | 0.2322719 | -0.3082 | 0.758 | -0.080244383 | count | 1 |
| JAK2 | -0.0671926 | 0.2667845 | -0.2519 | 0.801 | -0.080212983 | count | 1 |
| SLC35G2 | -0.2570562 | 0.4860853 | -0.5288 | 0.597 | -0.080180181 | count | 1 |
| GPALPP1 | -0.0646745 | 0.2015154 | -0.3209 | 0.748 | -0.080178618 | count | 1 |
| KIAA1551 | -0.0560977 | 0.0513551 | -1.0923 | 0.275 | -0.08013624 | count | 1 |
| GIMAP6 | -0.0634337 | 0.177736 | -0.3569 | 0.721 | -0.080051416 | count | 1 |
| SH2D2A | -0.0571823 | 0.09292 | -0.6154 | 0.538 | -0.08004434 | count | 1 |
| VAPB | -0.0663863 | 0.203099 | -0.3269 | 0.744 | -0.080042411 | count | 1 |
| STX17 | -0.0594789 | 0.1428865 | -0.4163 | 0.677 | -0.079909679 | count | 1 |
| TMBIM4 | -0.0559928 | 0.0592999 | -0.9442 | 0.345 | -0.079890789 | count | 1 |
| TMED2 | -0.0575067 | 0.0863874 | -0.6657 | 0.506 | -0.079850825 | count | 1 |
| ZNF677 | -0.0726474 | 0.3238008 | -0.2244 | 0.822 | -0.079809634 | count | 1 |
| UBE2E3 | -0.0575456 | 0.1022946 | -0.5625 | 0.574 | -0.079743514 | count | 1 |
| SFT2D2 | -0.0664092 | 0.1828255 | -0.3632 | 0.716 | -0.079684968 | count | 1 |
| RPL27 | -0.0553976 | 0.0239856 | -2.3096 | 0.021 | -0.07963022 | count | 1 |
| TBC1D22B | -0.1324685 | 0.4299973 | -0.3081 | 0.758 | -0.07960785 | count | 1 |
| GNL2 | -0.0599652 | 0.1651012 | -0.3632 | 0.716 | -0.079510817 | count | 1 |
| CDCA4 | -0.0608561 | 0.1594962 | -0.3816 | 0.703 | -0.079405154 | count | 1 |

| | | | | | | | |
|---|---|---|---|---|---|---|---|
| MLLT6 | -0.0594062 | 0.1102525 | -0.5388 | 0.59 | -0.079367934 | count | 1 |
| CACNB1 | -0.4806902 | 0.4549543 | -1.0566 | 0.291 | -0.079362602 | count | 1 |
| SCAMP1 | -0.0641352 | 0.1536628 | -0.4174 | 0.676 | -0.079239813 | count | 1 |
| STAT3 | -0.0561627 | 0.0627537 | -0.895 | 0.371 | -0.079140377 | count | 1 |
| AGAP6 | -0.1513294 | 0.4537847 | -0.3335 | 0.739 | -0.079105384 | count | 1 |
| NUP54 | -0.0611831 | 0.1369491 | -0.4468 | 0.655 | -0.079062618 | count | 1 |
| LRRC23 | -0.0799398 | 0.2528095 | -0.3162 | 0.752 | -0.078976384 | count | 1 |
| C1D | -0.0567668 | 0.1048575 | -0.5414 | 0.588 | -0.078921662 | count | 1 |
| LATS2 | -0.1180143 | 0.4239362 | -0.2784 | 0.781 | -0.078727765 | count | 1 |
| CACNA1C-AS2 | -0.2520521 | 0.5293395 | -0.4762 | 0.634 | -0.078708012 | count | 1 |
| TXNDC9 | -0.0570026 | 0.1215211 | -0.4691 | 0.639 | -0.078685038 | count | 1 |
| COLQ | -0.1018856 | 0.7134094 | -0.1428 | 0.886 | -0.078681387 | count | 1 |
| POLDIP2 | -0.0634553 | 0.188127 | -0.3373 | 0.736 | -0.078670807 | count | 1 |
| R3HDM1 | -0.0596932 | 0.1453549 | -0.4107 | 0.681 | -0.078425403 | count | 1 |
| BTAF1 | -0.0628442 | 0.1945541 | -0.323 | 0.747 | -0.078416221 | count | 1 |
| TUBGCP3 | -0.0720794 | 0.2554431 | -0.2822 | 0.778 | -0.078299414 | count | 1 |
| DCBLD2 | -0.2504216 | 0.7830966 | -0.3198 | 0.749 | -0.078227524 | count | 1 |
| FIS1 | -0.0549629 | 0.0650517 | -0.8449 | 0.398 | -0.078219616 | count | 1 |
| RPS6KA3 | -0.058324 | 0.1146464 | -0.5087 | 0.611 | -0.078118425 | count | 1 |
| MRPL11 | -0.0561874 | 0.1058207 | -0.531 | 0.595 | -0.07811658 | count | 1 |
| SIPA1 | -0.0571462 | 0.1191817 | -0.4795 | 0.632 | -0.078019682 | count | 1 |
| YIPF3 | -0.0566693 | 0.1229844 | -0.4608 | 0.645 | -0.077988049 | count | 1 |
| ZNF566 | -0.0716903 | 0.2611601 | -0.2745 | 0.784 | -0.077878773 | count | 1 |
| INPP4A | -0.0620432 | 0.1437324 | -0.4317 | 0.666 | -0.077876349 | count | 1 |
| ASCC3 | -0.0567358 | 0.1108919 | -0.5116 | 0.609 | -0.07777846 | count | 1 |
| NTPCR | -0.0584815 | 0.1711435 | -0.3417 | 0.733 | -0.077625511 | count | 1 |
| NRGN | -0.468519 | 0.6522275 | -0.7183 | 0.473 | -0.077605054 | count | 1 |
| CFL1 | -0.0538858 | 0.02577 | -2.091 | 0.0366 | -0.077585909 | count | 1 |
| DDX5 | -0.0540167 | 0.0269062 | -2.0076 | 0.0448 | -0.077541535 | count | 1 |
| PPIG | -0.0544424 | 0.060424 | -0.901 | 0.368 | -0.077367397 | count | 1 |
| SCFD1 | -0.0593857 | 0.1416721 | -0.4192 | 0.675 | -0.077255198 | count | 1 |
| SLC25A25-AS1 | -0.4659854 | 0.4811734 | -0.9684 | 0.333 | -0.077237552 | count | 1 |
| CHKA | -0.1801279 | 0.4228639 | -0.426 | 0.67 | -0.077222748 | count | 1 |
| PCBP4 | -0.0637151 | 0.1971364 | -0.3232 | 0.747 | -0.077182827 | count | 1 |
| PDZD8 | -0.0626706 | 0.1682741 | -0.3724 | 0.71 | -0.07715779 | count | 1 |
| GALNT10 | -0.0652893 | 0.2053888 | -0.3179 | 0.751 | -0.077090108 | count | 1 |
| IL12RB2 | -0.4645311 | 0.6323838 | -0.7346 | 0.463 | -0.077026351 | count | 1 |
| TMEM59 | -0.0538413 | 0.0469945 | -1.1457 | 0.252 | -0.077025382 | count | 1 |
| MAP3K3 | -0.0620853 | 0.1992051 | -0.3117 | 0.755 | -0.076976323 | count | 1 |
| STRAP | -0.054919 | 0.0849134 | -0.6468 | 0.518 | -0.076897766 | count | 1 |
| MTMR4 | -0.0725334 | 0.2756952 | -0.2631 | 0.792 | -0.076793242 | count | 1 |
| ARAP2 | -0.0548097 | 0.0758726 | -0.7224 | 0.47 | -0.076754749 | count | 1 |
| AMPD2 | -0.0671548 | 0.2994067 | -0.2243 | 0.823 | -0.076609577 | count | 1 |
| TNFRSF13B | -0.4612428 | 0.7563305 | -0.6098 | 0.542 | -0.076548104 | count | 1 |
| MYO7A | -0.4612428 | 0.7760968 | -0.5943 | 0.552 | -0.076548104 | count | 1 |

| | | | | | | | |
|---|---|---|---|---|---|---|---|
| COX15 | -0.0676518 | 0.2629409 | -0.2573 | 0.797 | -0.076533716 | count | 1 |
| CLSTN1 | -0.0596702 | 0.156383 | -0.3816 | 0.703 | -0.07653064 | count | 1 |
| PHETA1 | -0.1784122 | 0.8195545 | -0.2177 | 0.828 | -0.076513149 | count | 1 |
| LRP8 | -0.4607229 | 0.497069 | -0.9269 | 0.354 | -0.0764724 | count | 1 |
| ZBTB41 | -0.0647435 | 0.2314006 | -0.2798 | 0.78 | -0.07644771 | count | 1 |
| PIAS2 | -0.0639277 | 0.215533 | -0.2966 | 0.767 | -0.076327083 | count | 1 |
| HRAS | -0.055825 | 0.1339798 | -0.4167 | 0.677 | -0.076316417 | count | 1 |
| DDX51 | -0.073082 | 0.3491416 | -0.2093 | 0.834 | -0.076243644 | count | 1 |
| KMT2C | -0.0571426 | 0.1210184 | -0.4722 | 0.637 | -0.07621308 | count | 1 |
| IFRD2 | -0.0596626 | 0.1768292 | -0.3374 | 0.736 | -0.076201744 | count | 1 |
| ATP5MG | -0.0530254 | 0.0325352 | -1.6298 | 0.103 | -0.076162434 | count | 1 |
| ZP3 | -0.145345 | 0.3758876 | -0.3867 | 0.699 | -0.076057953 | count | 1 |
| ZMAT2 | -0.0550329 | 0.1000944 | -0.5498 | 0.582 | -0.075809852 | count | 1 |
| RASA4 | -0.0766293 | 0.3085947 | -0.2483 | 0.804 | -0.0757273 | count | 1 |
| CLCN6 | -0.1445362 | 0.5748414 | -0.2514 | 0.801 | -0.075645561 | count | 1 |
| SUCLA2 | -0.0582349 | 0.1599048 | -0.3642 | 0.716 | -0.075517168 | count | 1 |
| INSR | -0.241172 | 0.956865 | -0.252 | 0.801 | -0.075494666 | count | 1 |
| ENPP2 | -0.241172 | 0.9703299 | -0.2485 | 0.804 | -0.075494666 | count | 1 |
| EFHB | -0.241172 | 0.9836104 | -0.2452 | 0.806 | -0.075494666 | count | 1 |
| ASB16 | -0.241172 | 1.238934 | -0.1947 | 0.846 | -0.075494666 | count | 1 |
| HLA-DOB | -0.241172 | 1.280139 | -0.1884 | 0.851 | -0.075494666 | count | 1 |
| PAK4 | -0.113021 | 0.4097667 | -0.2758 | 0.783 | -0.07545254 | count | 1 |
| SFRP4 | -0.4536533 | 0.6594873 | -0.6879 | 0.492 | -0.075440647 | count | 1 |
| HARS2 | -0.0763041 | 0.3203315 | -0.2382 | 0.812 | -0.075408029 | count | 1 |
| EVI2B | -0.0529551 | 0.057054 | -0.9282 | 0.3534 | -0.075398372 | count | 1 |
| MCM5 | -0.0655263 | 0.226715 | -0.289 | 0.773 | -0.075342412 | count | 1 |
| SDCBP2-AS1 | -0.0974759 | 0.3732633 | -0.2611 | 0.794 | -0.075318309 | count | 1 |
| RTRAF | -0.0529653 | 0.0586756 | -0.9027 | 0.367 | -0.075314342 | count | 1 |
| HLA-G | -0.4526559 | 0.547247 | -0.8272 | 0.408 | -0.075294724 | count | 1 |
| YIPF5 | -0.054255 | 0.1159946 | -0.4677 | 0.64 | -0.075289209 | count | 1 |
| YWHAZ | -0.0524255 | 0.0353824 | -1.4817 | 0.139 | -0.07526994 | count | 1 |
| FAM50B | -0.0599093 | 0.2851791 | -0.2101 | 0.834 | -0.075203532 | count | 1 |
| HADHA | -0.0529235 | 0.0693642 | -0.763 | 0.446 | -0.075105911 | count | 1 |
| TBC1D10A | -0.0572282 | 0.1404108 | -0.4076 | 0.684 | -0.075094177 | count | 1 |
| IPPK | -0.1247298 | 0.406797 | -0.3066 | 0.759 | -0.075050983 | count | 1 |
| SNX3 | -0.0526665 | 0.0641953 | -0.8204 | 0.412 | -0.075007981 | count | 1 |
| TRMT112 | -0.0524808 | 0.0479479 | -1.0945 | 0.2738 | -0.074963726 | count | 1 |
| BAX | -0.0524172 | 0.0668849 | -0.7837 | 0.433 | -0.074962501 | count | 1 |
| SYNJ2 | -0.0774402 | 0.2458552 | -0.315 | 0.753 | -0.074839323 | count | 1 |
| SLC50A1 | -0.0563119 | 0.1442415 | -0.3904 | 0.696 | -0.074749193 | count | 1 |
| MLLT1 | -0.0741184 | 0.4241264 | -0.1748 | 0.861 | -0.074732223 | count | 1 |
| MRPL37 | -0.0554196 | 0.155615 | -0.3561 | 0.722 | -0.074673154 | count | 1 |
| SMG1 | -0.0579576 | 0.1204106 | -0.4813 | 0.63 | -0.074628469 | count | 1 |
| NDUFS2 | -0.0552642 | 0.1260115 | -0.4386 | 0.661 | -0.074563451 | count | 1 |
| POGLUT1 | -0.0605302 | 0.2087955 | -0.2899 | 0.772 | -0.074528938 | count | 1 |

| | | | | | | | |
|---|---|---|---|---|---|---|---|
| SUPT4H1 | -0.0527591 | 0.0825891 | -0.6388 | 0.523 | -0.074499317 | count | 1 |
| LYRM7 | -0.0592663 | 0.2416925 | -0.2452 | 0.806 | -0.07439806 | count | 1 |
| PPP1CA | -0.0520549 | 0.0594015 | -0.8763 | 0.381 | -0.074363957 | count | 1 |
| SMNDC1 | -0.0537098 | 0.0899818 | -0.5969 | 0.551 | -0.074340636 | count | 1 |
| PROCA1 | -0.0811654 | 0.4636136 | -0.1751 | 0.861 | -0.074321563 | count | 1 |
| RFLNB | -0.0535569 | 0.0945822 | -0.5662 | 0.571 | -0.074219351 | count | 1 |
| VAMP8 | -0.0518102 | 0.0538311 | -0.9625 | 0.336 | -0.074122319 | count | 1 |
| UBL5 | -0.0516769 | 0.0472638 | -1.0934 | 0.274 | -0.073948599 | count | 1 |
| MAGI3 | -0.141181 | 0.3857345 | -0.366 | 0.714 | -0.073933469 | count | 1 |
| TPGS2 | -0.0551852 | 0.1392251 | -0.3964 | 0.692 | -0.073918845 | count | 1 |
| NOP16 | -0.0586936 | 0.166692 | -0.3521 | 0.725 | -0.07388476 | count | 1 |
| CLN5 | -0.0633452 | 0.209743 | -0.302 | 0.763 | -0.07387713 | count | 1 |
| SMURF1 | -0.122737 | 0.3508228 | -0.3499 | 0.726 | -0.073875582 | count | 1 |
| CHMP4A | -0.052621 | 0.0802849 | -0.6554 | 0.512 | -0.073799603 | count | 1 |
| CSPP1 | -0.0593113 | 0.168253 | -0.3525 | 0.724 | -0.073785848 | count | 1 |
| SLMAP | -0.0594663 | 0.191373 | -0.3107 | 0.756 | -0.073736506 | count | 1 |
| PRDM8 | -0.0686808 | 0.2484859 | -0.2764 | 0.782 | -0.07371473 | count | 1 |
| LPAR2 | -0.0566949 | 0.1367839 | -0.4145 | 0.679 | -0.073643334 | count | 1 |
| B3GALNT2 | -0.0627346 | 0.2461459 | -0.2549 | 0.799 | -0.07363756 | count | 1 |
| ALDH9A1 | -0.0542057 | 0.1095483 | -0.4948 | 0.621 | -0.073573883 | count | 1 |
| SEMA4F | -0.4398077 | 0.7918777 | -0.5554 | 0.579 | -0.073407149 | count | 1 |
| OSBPL11 | -0.0757655 | 0.2812718 | -0.2694 | 0.788 | -0.073231874 | count | 1 |
| SGSH | -0.0652934 | 0.2952879 | -0.2211 | 0.825 | -0.073217744 | count | 1 |
| SLC45A4 | -0.2333767 | 0.4338984 | -0.5379 | 0.591 | -0.073181936 | count | 1 |
| HACL1 | -0.0548975 | 0.1605824 | -0.3419 | 0.732 | -0.073155535 | count | 1 |
| NLRC3 | -0.0619352 | 0.2125222 | -0.2914 | 0.771 | -0.073141823 | count | 1 |
| CSRNP2 | -0.0775826 | 0.3830135 | -0.2026 | 0.839 | -0.073119298 | count | 1 |
| STUB1 | -0.051699 | 0.070922 | -0.729 | 0.466 | -0.073090815 | count | 1 |
| AL135905.1 | -0.4375821 | 1.005326 | -0.4353 | 0.663 | -0.073078694 | count | 1 |
| CXCR4 | -0.050807 | 0.0279831 | -1.8156 | 0.0695 | -0.073032164 | count | 1 |
| FECH | -0.0724081 | 0.3656367 | -0.198 | 0.843 | -0.073017968 | count | 1 |
| FOXD2 | -0.1393418 | 0.4903185 | -0.2842 | 0.776 | -0.072994052 | count | 1 |
| MTMR3 | -0.109071 | 0.2818424 | -0.387 | 0.699 | -0.072858044 | count | 1 |
| NDRG2 | -0.0942081 | 0.3573084 | -0.2637 | 0.792 | -0.072823559 | count | 1 |
| ZNF337 | -0.0853758 | 0.2874265 | -0.297 | 0.766 | -0.072820826 | count | 1 |
| LMF1 | -0.0662337 | 0.3061671 | -0.2163 | 0.829 | -0.072793823 | count | 1 |
| AL591895.1 | -0.0794702 | 0.4013717 | -0.198 | 0.843 | -0.072781566 | count | 1 |
| UBAP2 | -0.0643157 | 0.1792271 | -0.3589 | 0.72 | -0.072773818 | count | 1 |
| RPL39L | -0.0550791 | 0.1907716 | -0.2887 | 0.773 | -0.072722194 | count | 1 |
| TCF12 | -0.060569 | 0.1770465 | -0.3421 | 0.732 | -0.072696605 | count | 1 |
| ZFP62 | -0.0594424 | 0.2392182 | -0.2485 | 0.804 | -0.072632654 | count | 1 |
| LIG3 | -0.0675907 | 0.3233658 | -0.209 | 0.834 | -0.072550217 | count | 1 |
| PEG10 | -0.2311292 | 0.7854501 | -0.2943 | 0.769 | -0.072513494 | count | 1 |
| FAM161B | -0.1382937 | 0.4302213 | -0.3214 | 0.748 | -0.072458416 | count | 1 |
| SRBD1 | -0.0590662 | 0.1910874 | -0.3091 | 0.757 | -0.072458403 | count | 1 |

| | | | | | | | |
|---|---|---|---|---|---|---|---|
| ZNF284 | -0.0816497 | 0.4846871 | -0.1685 | 0.866 | -0.072358594 | count | 1 |
| DDX24 | -0.0504564 | 0.0379477 | -1.3296 | 0.184 | -0.072319133 | count | 1 |
| EIF2AK3 | -0.0766385 | 0.3112333 | -0.2462 | 0.806 | -0.072235938 | count | 1 |
| POLE4 | -0.0514744 | 0.0927652 | -0.5549 | 0.579 | -0.072233401 | count | 1 |
| ELOC | -0.0510352 | 0.0671344 | -0.7602 | 0.447 | -0.072126247 | count | 1 |
| RPP25 | -0.0932796 | 0.5352187 | -0.1743 | 0.862 | -0.072114311 | count | 1 |
| RGS5 | -0.0932796 | 0.6059409 | -0.1539 | 0.878 | -0.072114311 | count | 1 |
| RAB3A | -0.0642222 | 0.26305 | -0.2441 | 0.807 | -0.072021188 | count | 1 |
| RER1 | -0.0507506 | 0.0793769 | -0.6394 | 0.523 | -0.071921036 | count | 1 |
| METTL14 | -0.0561627 | 0.1806263 | -0.3109 | 0.756 | -0.071891987 | count | 1 |
| AL583810.1 | -0.1668967 | 0.8847559 | -0.1886 | 0.85 | -0.071737163 | count | 1 |
| AC090527.3 | -0.1668967 | 0.8847559 | -0.1886 | 0.85 | -0.071737163 | count | 1 |
| AL354732.1 | -0.1668967 | 1.0840987 | -0.1539 | 0.878 | -0.071737163 | count | 1 |
| MND1 | -0.1668967 | 1.0840987 | -0.1539 | 0.878 | -0.071737163 | count | 1 |
| FOXM1 | -0.1668967 | 1.0840987 | -0.1539 | 0.878 | -0.071737163 | count | 1 |
| HSDL2 | -0.0606413 | 0.200914 | -0.3018 | 0.763 | -0.071618348 | count | 1 |
| CASTOR3 | -0.0724083 | 0.4481761 | -0.1616 | 0.872 | -0.07158183 | count | 1 |
| PHF20 | -0.050952 | 0.0719503 | -0.7082 | 0.479 | -0.07150857 | count | 1 |
| AC027307.2 | -0.1662935 | 0.7767594 | -0.2141 | 0.83 | -0.071486357 | count | 1 |
| SHISA2 | -0.4260123 | 1.0712871 | -0.3977 | 0.691 | -0.071364118 | count | 1 |
| IL26 | -0.4260123 | 1.0867484 | -0.392 | 0.695 | -0.071364118 | count | 1 |
| SPESP1 | -0.4260123 | 1.0867484 | -0.392 | 0.695 | -0.071364118 | count | 1 |
| SRP14 | -0.0496154 | 0.0303387 | -1.6354 | 0.102 | -0.071344642 | count | 1 |
| EIF4ENIF1 | -0.0577269 | 0.247358 | -0.2334 | 0.815 | -0.071340031 | count | 1 |
| C8orf44 | -0.0874011 | 0.4861266 | -0.1798 | 0.857 | -0.071292783 | count | 1 |
| MOAP1 | -0.054438 | 0.1535308 | -0.3546 | 0.723 | -0.071145442 | count | 1 |
| KHDRBS1 | -0.0500754 | 0.0590652 | -0.8478 | 0.397 | -0.071143544 | count | 1 |
| PRDX3 | -0.051269 | 0.1046046 | -0.4901 | 0.624 | -0.07113199 | count | 1 |
| DOCK7 | -0.0801774 | 0.3255459 | -0.2463 | 0.805 | -0.071064805 | count | 1 |
| PRDX2 | -0.0496121 | 0.0543541 | -0.9128 | 0.361 | -0.070949768 | count | 1 |
| IFT81 | -0.1352192 | 0.720572 | -0.1877 | 0.851 | -0.070885978 | count | 1 |
| MEGF8 | -0.1352192 | 0.8106589 | -0.1668 | 0.868 | -0.070885978 | count | 1 |
| AL359397.2 | -0.1350923 | 0.7415461 | -0.1822 | 0.855 | -0.070821038 | count | 1 |
| UBE2R2 | -0.0507594 | 0.0937123 | -0.5417 | 0.588 | -0.070816688 | count | 1 |
| AC090948.3 | -0.4220147 | 0.5606051 | -0.7528 | 0.452 | -0.070768933 | count | 1 |
| DUS2 | -0.0650906 | 0.4144688 | -0.157 | 0.875 | -0.070740616 | count | 1 |
| HADHB | -0.0508475 | 0.108138 | -0.4702 | 0.638 | -0.070696286 | count | 1 |
| EPAS1 | -0.1173005 | 0.4059605 | -0.2889 | 0.773 | -0.070665029 | count | 1 |
| OGFR | -0.0518615 | 0.1190499 | -0.4356 | 0.663 | -0.070646874 | count | 1 |
| WASHC2A | -0.0599952 | 0.205644 | -0.2917 | 0.771 | -0.070431781 | count | 1 |
| TRAV38-2DV8 | -0.4179993 | 0.6192137 | -0.675 | 0.5 | -0.070169671 | count | 1 |
| AKR1B1 | -0.0508008 | 0.1085639 | -0.4679 | 0.64 | -0.070168586 | count | 1 |
| SERINC5 | -0.0529925 | 0.1455929 | -0.364 | 0.716 | -0.070126995 | count | 1 |
| DENND4A | -0.0561528 | 0.1386948 | -0.4049 | 0.686 | -0.070083887 | count | 1 |
| CAMK2D | -0.0548466 | 0.1615267 | -0.3396 | 0.734 | -0.070061137 | count | 1 |

| | | | | | | | |
|---|---|---|---|---|---|---|---|
| RBM39 | -0.0490006 | 0.039894 | -1.2283 | 0.219 | -0.069948027 | count | 1 |
| LCK | -0.0487467 | 0.0457664 | -1.0651 | 0.287 | -0.069871427 | count | 1 |
| COA4 | -0.0502042 | 0.1102253 | -0.4555 | 0.649 | -0.069774187 | count | 1 |
| IRS2 | -0.0786545 | 0.1856684 | -0.4236 | 0.672 | -0.069726118 | count | 1 |
| YWHAE | -0.0496253 | 0.0817085 | -0.6073 | 0.544 | -0.069692592 | count | 1 |
| CSNK2A1 | -0.053885 | 0.1374058 | -0.3922 | 0.695 | -0.069646104 | count | 1 |
| NAA16 | -0.0545861 | 0.1580427 | -0.3454 | 0.73 | -0.069575651 | count | 1 |
| SLC41A3 | -0.0529684 | 0.1776045 | -0.2982 | 0.766 | -0.069511737 | count | 1 |
| UBIAD1 | -0.0588474 | 0.2203185 | -0.2671 | 0.789 | -0.069505826 | count | 1 |
| CTPS1 | -0.0898598 | 0.3086173 | -0.2912 | 0.771 | -0.069500564 | count | 1 |
| BX255925.3 | -0.115294 | 0.688434 | -0.1675 | 0.867 | -0.069478585 | count | 1 |
| PLCL2 | -0.0547508 | 0.1528819 | -0.3581 | 0.72 | -0.069461489 | count | 1 |
| TMEM177 | -0.1036443 | 0.4101585 | -0.2527 | 0.801 | -0.06928841 | count | 1 |
| FNTB | -0.0955369 | 0.5181815 | -0.1844 | 0.854 | -0.069246359 | count | 1 |
| ZNF720 | -0.0525227 | 0.160217 | -0.3278 | 0.743 | -0.069188058 | count | 1 |
| TBC1D20 | -0.0533084 | 0.1694638 | -0.3146 | 0.753 | -0.069021711 | count | 1 |
| GLB1L | -0.1145121 | 0.6737449 | -0.17 | 0.865 | -0.06901603 | count | 1 |
| DCTPP1 | -0.0512573 | 0.1355293 | -0.3782 | 0.705 | -0.068974127 | count | 1 |
| MEX3D | -0.0807776 | 0.3867029 | -0.2089 | 0.835 | -0.068934056 | count | 1 |
| SPIRE1 | -0.4094764 | 0.4866801 | -0.8414 | 0.4 | -0.068892962 | count | 1 |
| ALKBH6 | -0.0553554 | 0.2451381 | -0.2258 | 0.821 | -0.068874651 | count | 1 |
| HIRIP3 | -0.0542771 | 0.1611963 | -0.3367 | 0.736 | -0.068861573 | count | 1 |
| FAM200B | -0.0495556 | 0.1176478 | -0.4212 | 0.674 | -0.068830439 | count | 1 |
| DYRK3 | -0.1597137 | 0.3630995 | -0.4399 | 0.66 | -0.068746379 | count | 1 |
| PCBP1 | -0.0480501 | 0.0474549 | -1.0125 | 0.311 | -0.068741388 | count | 1 |
| PDPK1 | -0.0535371 | 0.1694887 | -0.3159 | 0.752 | -0.068677174 | count | 1 |
| PJA1 | -0.058125 | 0.2203787 | -0.2638 | 0.792 | -0.06865501 | count | 1 |
| ARRDC3-AS1 | -0.2181695 | 0.7806106 | -0.2795 | 0.78 | -0.068645068 | count | 1 |
| WDR70 | -0.0574691 | 0.1736665 | -0.3309 | 0.741 | -0.068636413 | count | 1 |
| CYFIP2 | -0.0513854 | 0.1111502 | -0.4623 | 0.644 | -0.068603399 | count | 1 |
| WNT10A | -0.0571062 | 0.2482851 | -0.23 | 0.818 | -0.068551216 | count | 1 |
| ACTR2 | -0.0480766 | 0.0538429 | -0.8929 | 0.372 | -0.068480321 | count | 1 |
| TIMM8B | -0.0501588 | 0.1370807 | -0.3659 | 0.714 | -0.06845639 | count | 1 |
| PEX26 | -0.0801867 | 0.2874396 | -0.279 | 0.78 | -0.068434281 | count | 1 |
| PFDN4 | -0.0508489 | 0.1343457 | -0.3785 | 0.705 | -0.068326237 | count | 1 |
| HINFP | -0.0628044 | 0.2185311 | -0.2874 | 0.774 | -0.06826637 | count | 1 |
| PCF11 | -0.049177 | 0.0898976 | -0.547 | 0.584 | -0.068246067 | count | 1 |
| NUDT16L1 | -0.0491361 | 0.1023795 | -0.4799 | 0.631 | -0.06821902 | count | 1 |
| SNHG19 | -0.1127702 | 0.3509236 | -0.3214 | 0.748 | -0.067985126 | count | 1 |
| POMT1 | -0.0830249 | 0.3967859 | -0.2092 | 0.834 | -0.067758186 | count | 1 |
| RPL3 | -0.0469402 | 0.0177402 | -2.646 | 0.00818 | -0.067639706 | count | 1 |
| AL139246.5 | -0.0524433 | 0.1893706 | -0.2769 | 0.782 | -0.067538823 | count | 1 |
| GFER | -0.0496906 | 0.1306591 | -0.3803 | 0.704 | -0.067486615 | count | 1 |
| TRIM56 | -0.0581687 | 0.1947407 | -0.2987 | 0.765 | -0.067397466 | count | 1 |
| TXNIP | -0.0468587 | 0.0329458 | -1.4223 | 0.155 | -0.067379082 | count | 1 |

| | | | | | | | |
|---|---|---|---|---|---|---|---|
| SLC12A9-AS1 | -0.3989188 | 0.8583407 | -0.4648 | 0.642 | -0.067302503 | count | 1 |
| ST20 | -0.0788322 | 0.3267487 | -0.2413 | 0.809 | -0.067288403 | count | 1 |
| CEP72 | -0.1560659 | 0.6199946 | -0.2517 | 0.801 | -0.067224123 | count | 1 |
| TSPAN5 | -0.0500889 | 0.1375346 | -0.3642 | 0.716 | -0.067204641 | count | 1 |
| TOM1L2 | -0.0867467 | 0.3293569 | -0.2634 | 0.792 | -0.067119174 | count | 1 |
| PRPF40A | -0.0473747 | 0.0730417 | -0.6486 | 0.517 | -0.067078956 | count | 1 |
| CFP | -0.2122534 | 0.3592398 | -0.5908 | 0.555 | -0.066871132 | count | 1 |
| CRYL1 | -0.0501447 | 0.1415398 | -0.3543 | 0.723 | -0.06682855 | count | 1 |
| ITGA2 | -0.1550913 | 0.6615601 | -0.2344 | 0.815 | -0.066817026 | count | 1 |
| AP3M2 | -0.04997 | 0.1066308 | -0.4686 | 0.639 | -0.066773524 | count | 1 |
| ARHGAP4 | -0.0502905 | 0.1255356 | -0.4006 | 0.689 | -0.066764949 | count | 1 |
| SELENOH | -0.0468935 | 0.0636014 | -0.7373 | 0.461 | -0.066671783 | count | 1 |
| C6orf203 | -0.0543193 | 0.2307346 | -0.2354 | 0.814 | -0.066647985 | count | 1 |
| ANKRD12 | -0.0465186 | 0.0435408 | -1.0684 | 0.285 | -0.066598943 | count | 1 |
| GNA13 | -0.0495129 | 0.1313618 | -0.3769 | 0.706 | -0.066532677 | count | 1 |
| CHST10 | -0.1543419 | 0.5695813 | -0.271 | 0.786 | -0.066503882 | count | 1 |
| SREK1 | -0.0494109 | 0.1254124 | -0.394 | 0.694 | -0.066491746 | count | 1 |
| USP24 | -0.0513424 | 0.1866677 | -0.275 | 0.783 | -0.06647981 | count | 1 |
| TOX4 | -0.0472646 | 0.0787135 | -0.6005 | 0.548 | -0.066412829 | count | 1 |
| GRK2 | -0.0477739 | 0.1020365 | -0.4682 | 0.64 | -0.066356922 | count | 1 |
| USP5 | -0.0747583 | 0.303698 | -0.2462 | 0.806 | -0.066299186 | count | 1 |
| C12orf49 | -0.0503354 | 0.1648215 | -0.3054 | 0.76 | -0.066229086 | count | 1 |
| TRPS1 | -0.0541862 | 0.1763107 | -0.3073 | 0.759 | -0.066224453 | count | 1 |
| APTX | -0.0584327 | 0.2170495 | -0.2692 | 0.788 | -0.066139787 | count | 1 |
| REST | -0.0467912 | 0.06967 | -0.6716 | 0.502 | -0.066125781 | count | 1 |
| C1orf52 | -0.0473286 | 0.0927882 | -0.5101 | 0.61 | -0.066010386 | count | 1 |
| VPS4A | -0.0487843 | 0.1428852 | -0.3414 | 0.733 | -0.06599387 | count | 1 |
| GRINA | -0.0599819 | 0.2312814 | -0.2593 | 0.795 | -0.065949317 | count | 1 |
| KIF2A | -0.0462436 | 0.0636379 | -0.7267 | 0.467 | -0.065944102 | count | 1 |
| RFWD3 | -0.0680995 | 0.3919103 | -0.1738 | 0.862 | -0.065867335 | count | 1 |
| PARK7 | -0.0459241 | 0.048604 | -0.9449 | 0.345 | -0.065820208 | count | 1 |
| TTBK2 | -0.0621069 | 0.2940669 | -0.2112 | 0.833 | -0.065803767 | count | 1 |
| SUCO | -0.0493555 | 0.1328225 | -0.3716 | 0.71 | -0.065777835 | count | 1 |
| EIF3D | -0.046797 | 0.0702305 | -0.6663 | 0.505 | -0.065775842 | count | 1 |
| GRHPR | -0.0479057 | 0.1056001 | -0.4537 | 0.65 | -0.065578573 | count | 1 |
| GLRX2 | -0.0496065 | 0.1816561 | -0.2731 | 0.785 | -0.065428849 | count | 1 |
| RASAL3 | -0.0467155 | 0.0851962 | -0.5483 | 0.584 | -0.065415448 | count | 1 |
| AGGF1 | -0.0713391 | 0.3274018 | -0.2179 | 0.828 | -0.065387427 | count | 1 |
| ADGRE5 | -0.0464311 | 0.0736125 | -0.6307 | 0.528 | -0.065336182 | count | 1 |
| IFI27L1 | -0.0616021 | 0.278462 | -0.2212 | 0.825 | -0.065271277 | count | 1 |
| PES1 | -0.0659807 | 0.3173432 | -0.2079 | 0.835 | -0.065263289 | count | 1 |
| CTBP1 | -0.0479361 | 0.130439 | -0.3675 | 0.713 | -0.065173743 | count | 1 |
| ENOPH1 | -0.0474824 | 0.1256235 | -0.378 | 0.705 | -0.065149791 | count | 1 |
| RPL9 | -0.045203 | 0.0200455 | -2.255 | 0.0242 | -0.065121512 | count | 1 |
| ZNF484 | -0.0762214 | 0.3050502 | -0.2499 | 0.803 | -0.065078719 | count | 1 |

| | | | | | | | |
|---|---|---|---|---|---|---|---|
| OGFOD3 | -0.0511009 | 0.1938442 | -0.2636 | 0.792 | -0.06499216 | count | 1 |
| SLC25A22 | -0.055266 | 0.2444636 | -0.2261 | 0.821 | -0.064895295 | count | 1 |
| RWDD2A | -0.2056227 | 0.4431847 | -0.464 | 0.643 | -0.064876974 | count | 1 |
| EIF3J-DT | -0.0555412 | 0.2126791 | -0.2612 | 0.794 | -0.064801729 | count | 1 |
| C1orf112 | -0.3819763 | 0.5686975 | -0.6717 | 0.502 | -0.06472947 | count | 1 |
| IL2RB | -0.0473257 | 0.129163 | -0.3664 | 0.714 | -0.064503203 | count | 1 |
| VCPIP1 | -0.0500732 | 0.1447288 | -0.346 | 0.729 | -0.064490908 | count | 1 |
| GLMN | -0.0518193 | 0.1768884 | -0.2929 | 0.77 | -0.064483373 | count | 1 |
| EPB41 | -0.0460323 | 0.0855507 | -0.5381 | 0.591 | -0.064425004 | count | 1 |
| MKRN1 | -0.0482241 | 0.1147287 | -0.4203 | 0.674 | -0.0643869 | count | 1 |
| TXNDC16 | -0.0606793 | 0.2623053 | -0.2313 | 0.817 | -0.064297757 | count | 1 |
| CUX1 | -0.0518233 | 0.1994651 | -0.2598 | 0.795 | -0.064277982 | count | 1 |
| LINC00467 | -0.0583203 | 0.4021027 | -0.145 | 0.885 | -0.064129233 | count | 1 |
| AC011447.3 | -0.1484128 | 0.5774563 | -0.257 | 0.797 | -0.064022945 | count | 1 |
| MRPL34 | -0.0451771 | 0.0753062 | -0.5999 | 0.549 | -0.063868723 | count | 1 |
| DHRSX | -0.0501862 | 0.2227581 | -0.2253 | 0.822 | -0.063830663 | count | 1 |
| MAP7 | -0.201854 | 0.8609595 | -0.2345 | 0.815 | -0.063740742 | count | 1 |
| TMCO4 | -0.051927 | 0.2080457 | -0.2496 | 0.803 | -0.063718834 | count | 1 |
| SLC35B1 | -0.0477083 | 0.1453625 | -0.3282 | 0.743 | -0.063403551 | count | 1 |
| SSBP1 | -0.0443722 | 0.0610436 | -0.7269 | 0.467 | -0.063263109 | count | 1 |
| NEMP2 | -0.2001345 | 0.3597735 | -0.5563 | 0.578 | -0.063221684 | count | 1 |
| GSK3B | -0.0478059 | 0.1469273 | -0.3254 | 0.745 | -0.06320103 | count | 1 |
| RNF31 | -0.1463302 | 0.6453358 | -0.2268 | 0.821 | -0.06315008 | count | 1 |
| ANKRD13D | -0.0455292 | 0.0926364 | -0.4915 | 0.623 | -0.063143747 | count | 1 |
| NBAS | -0.0480928 | 0.1878748 | -0.256 | 0.798 | -0.063120831 | count | 1 |
| SEC22B | -0.0469306 | 0.1268796 | -0.3699 | 0.711 | -0.06311174 | count | 1 |
| NUP107 | -0.048208 | 0.1782546 | -0.2704 | 0.787 | -0.063013328 | count | 1 |
| CARD8 | -0.0460254 | 0.112415 | -0.4094 | 0.682 | -0.062954745 | count | 1 |
| TRIM62 | -0.062362 | 0.4370062 | -0.1427 | 0.887 | -0.06293864 | count | 1 |
| PRMT6 | -0.1456482 | 0.4655693 | -0.3128 | 0.754 | -0.062864074 | count | 1 |
| MAD2L2 | -0.0454134 | 0.1222541 | -0.3715 | 0.71 | -0.06279872 | count | 1 |
| DDX23 | -0.0494311 | 0.1493395 | -0.331 | 0.741 | -0.062723274 | count | 1 |
| DNAJC15 | -0.0443414 | 0.0829961 | -0.5343 | 0.593 | -0.062559117 | count | 1 |
| SEC61B | -0.0436685 | 0.0485497 | -0.8995 | 0.368 | -0.062530477 | count | 1 |
| PHKB | -0.04785 | 0.1656068 | -0.2889 | 0.773 | -0.062362816 | count | 1 |
| KIN | -0.0449024 | 0.1236276 | -0.3632 | 0.716 | -0.062343747 | count | 1 |
| NUBPL | -0.0729355 | 0.3299558 | -0.221 | 0.825 | -0.062295773 | count | 1 |
| ATP5F1E | -0.0432526 | 0.0268455 | -1.6112 | 0.107 | -0.062261428 | count | 1 |
| MLXIP | -0.0579218 | 0.23354 | -0.248 | 0.804 | -0.062213355 | count | 1 |
| ADA2 | -0.0548828 | 0.2100685 | -0.2613 | 0.794 | -0.062134437 | count | 1 |
| MFSD14B | -0.0528988 | 0.2541275 | -0.2082 | 0.835 | -0.062122992 | count | 1 |
| VAPA | -0.0437328 | 0.0563444 | -0.7762 | 0.438 | -0.06211 | count | 1 |
| EIF3M | -0.0436948 | 0.0599019 | -0.7294 | 0.466 | -0.062090594 | count | 1 |
| MIR155HG | -0.0477552 | 0.1998681 | -0.2389 | 0.811 | -0.062046035 | count | 1 |
| GAB2 | -0.3643197 | 0.7647943 | -0.4764 | 0.634 | -0.062020815 | count | 1 |

| | | | | | | | |
|---|---|---|---|---|---|---|---|
| APOA1 | -0.3643197 | 0.7770295 | -0.4689 | 0.639 | -0.062020815 | count | 1 |
| ZNF644 | -0.0444978 | 0.0930827 | -0.478 | 0.633 | -0.06201066 | count | 1 |
| PPAN | -0.0460085 | 0.1515346 | -0.3036 | 0.761 | -0.062002851 | count | 1 |
| TEX9 | -0.1433874 | 0.7306164 | -0.1963 | 0.844 | -0.061915408 | count | 1 |
| ARSA | -0.0506484 | 0.1848131 | -0.2741 | 0.784 | -0.061909663 | count | 1 |
| LRCH3 | -0.0556582 | 0.2097173 | -0.2654 | 0.791 | -0.061851536 | count | 1 |
| DNHD1 | -0.1427669 | 0.7701796 | -0.1854 | 0.853 | -0.061654882 | count | 1 |
| NIPSNAP1 | -0.049859 | 0.1540461 | -0.3237 | 0.746 | -0.061635405 | count | 1 |
| TRIM38 | -0.0450824 | 0.1082762 | -0.4164 | 0.677 | -0.061613714 | count | 1 |
| CLK1 | -0.043928 | 0.0725824 | -0.6052 | 0.545 | -0.061575301 | count | 1 |
| SPCS1 | -0.0429698 | 0.0460702 | -0.9327 | 0.351 | -0.061539938 | count | 1 |
| AC093157.2 | -0.1942473 | 0.7604612 | -0.2554 | 0.798 | -0.06144126 | count | 1 |
| PRSS16 | -0.1941902 | 0.8174911 | -0.2375 | 0.812 | -0.061423968 | count | 1 |
| LDB2 | -0.1941902 | 0.8977014 | -0.2163 | 0.829 | -0.061423968 | count | 1 |
| SMIM29 | -0.0442434 | 0.1257173 | -0.3519 | 0.725 | -0.06130418 | count | 1 |
| ZNF668 | -0.0496196 | 0.2400939 | -0.2067 | 0.836 | -0.061121326 | count | 1 |
| SRGAP2B | -0.0909721 | 0.3725317 | -0.2442 | 0.807 | -0.0609296 | count | 1 |
| RALGPS1 | -0.0712727 | 0.4126477 | -0.1727 | 0.863 | -0.060886693 | count | 1 |
| MYD88 | -0.046148 | 0.1668735 | -0.2765 | 0.782 | -0.060872072 | count | 1 |
| BTBD6 | -0.0447456 | 0.1216198 | -0.3679 | 0.713 | -0.060869946 | count | 1 |
| BBS12 | -0.1407876 | 0.4306661 | -0.3269 | 0.744 | -0.060823407 | count | 1 |
| ZSCAN32 | -0.0541734 | 0.2986008 | -0.1814 | 0.856 | -0.06078868 | count | 1 |
| COQ2 | -0.0601654 | 0.2916976 | -0.2063 | 0.837 | -0.06073251 | count | 1 |
| SUSD6 | -0.0460524 | 0.1784227 | -0.2581 | 0.796 | -0.060523826 | count | 1 |
| MRE11 | -0.0475712 | 0.1853679 | -0.2566 | 0.797 | -0.060509723 | count | 1 |
| ZNF559 | -0.0586897 | 0.3338899 | -0.1758 | 0.86 | -0.060315115 | count | 1 |
| GRPEL1 | -0.0429376 | 0.0819481 | -0.524 | 0.6 | -0.060290573 | count | 1 |
| EIF3I | -0.0425597 | 0.0682174 | -0.6239 | 0.533 | -0.060277944 | count | 1 |
| PGAP2 | -0.0494678 | 0.2240048 | -0.2208 | 0.825 | -0.060220966 | count | 1 |
| CCDC125 | -0.0567215 | 0.2935015 | -0.1933 | 0.847 | -0.060120922 | count | 1 |
| SAMD8 | -0.0535528 | 0.2275984 | -0.2353 | 0.814 | -0.060094519 | count | 1 |
| NSRP1 | -0.0429555 | 0.0989671 | -0.434 | 0.664 | -0.060070465 | count | 1 |
| SNF8 | -0.0428802 | 0.096671 | -0.4436 | 0.657 | -0.060054477 | count | 1 |
| PPP2R3B | -0.0574058 | 0.2726761 | -0.2105 | 0.833 | -0.059959424 | count | 1 |
| UNC119 | -0.0447865 | 0.1402019 | -0.3194 | 0.749 | -0.059903809 | count | 1 |
| CNBP | -0.0418161 | 0.0407567 | -1.026 | 0.305 | -0.059892889 | count | 1 |
| MGST2 | -0.0482643 | 0.3132929 | -0.1541 | 0.878 | -0.059871655 | count | 1 |
| KMT5C | -0.0573137 | 0.2521288 | -0.2273 | 0.82 | -0.059863636 | count | 1 |
| QRSL1 | -0.0519062 | 0.2210825 | -0.2348 | 0.814 | -0.059726388 | count | 1 |
| GIT2 | -0.0474105 | 0.1442056 | -0.3288 | 0.742 | -0.059704401 | count | 1 |
| GOLT1B | -0.0446129 | 0.1546975 | -0.2884 | 0.773 | -0.059569659 | count | 1 |
| APAF1 | -0.0487271 | 0.223699 | -0.2178 | 0.828 | -0.059565858 | count | 1 |
| SLC35B3 | -0.0579467 | 0.2378888 | -0.2436 | 0.808 | -0.059554955 | count | 1 |
| N4BP2L2 | -0.0417333 | 0.0499714 | -0.8351 | 0.404 | -0.059498784 | count | 1 |
| SAP18 | -0.0415007 | 0.0472394 | -0.8785 | 0.38 | -0.059492892 | count | 1 |

| | | | | | | | |
|---|---|---|---|---|---|---|---|
| WARS | -0.0516245 | 0.2622784 | -0.1968 | 0.844 | -0.059403153 | count | 1 |
| ERGIC2 | -0.0425353 | 0.0966957 | -0.4399 | 0.66 | -0.059366426 | count | 1 |
| HGS | -0.0469209 | 0.2159699 | -0.2173 | 0.828 | -0.059245703 | count | 1 |
| CHST15 | -0.3456162 | 1.201812 | -0.2876 | 0.774 | -0.059121292 | count | 1 |
| ATP10A | -0.0665418 | 0.3001128 | -0.2217 | 0.825 | -0.059062843 | count | 1 |
| USP12 | -0.0446153 | 0.135333 | -0.3297 | 0.742 | -0.058920618 | count | 1 |
| AC132872.1 | -0.1118434 | 0.6145664 | -0.182 | 0.856 | -0.058871931 | count | 1 |
| SUPT6H | -0.0461863 | 0.1450151 | -0.3185 | 0.75 | -0.058750729 | count | 1 |
| PSMA1 | -0.0414444 | 0.0675686 | -0.6134 | 0.54 | -0.058746189 | count | 1 |
| CCNH | -0.0417072 | 0.0662812 | -0.6292 | 0.529 | -0.058732128 | count | 1 |
| TRBC2 | -0.0407508 | 0.0364432 | -1.1182 | 0.264 | -0.058679455 | count | 1 |
| MTHFS | -0.0441259 | 0.1707999 | -0.2583 | 0.796 | -0.058467643 | count | 1 |
| WASHC2C | -0.0503668 | 0.2122846 | -0.2373 | 0.812 | -0.058381809 | count | 1 |
| C19orf53 | -0.0407664 | 0.0438678 | -0.9293 | 0.353 | -0.05836492 | count | 1 |
| FANCL | -0.0577377 | 0.4905737 | -0.1177 | 0.906 | -0.058293337 | count | 1 |
| GTF2E2 | -0.0430724 | 0.1422897 | -0.3027 | 0.762 | -0.058272539 | count | 1 |
| DEF6 | -0.0412171 | 0.0773278 | -0.533 | 0.594 | -0.058271545 | count | 1 |
| BRF2 | -0.0541114 | 0.3227303 | -0.1677 | 0.867 | -0.058135814 | count | 1 |
| TTC14 | -0.0420447 | 0.1151236 | -0.3652 | 0.715 | -0.058111682 | count | 1 |
| GTF2H5 | -0.0422915 | 0.1112397 | -0.3802 | 0.704 | -0.05807344 | count | 1 |
| FKBP15 | -0.0653303 | 0.2397328 | -0.2725 | 0.785 | -0.057994784 | count | 1 |
| PLCL1 | -0.0614253 | 0.2654129 | -0.2314 | 0.817 | -0.057979116 | count | 1 |
| SBNO2 | -0.0631398 | 0.2003112 | -0.3152 | 0.753 | -0.057918816 | count | 1 |
| THOC7 | -0.0410655 | 0.0861834 | -0.4765 | 0.634 | -0.057908226 | count | 1 |
| TRPV2 | -0.0450799 | 0.1940675 | -0.2323 | 0.816 | -0.057842832 | count | 1 |
| TOR2A | -0.0441443 | 0.2014909 | -0.2191 | 0.827 | -0.057788478 | count | 1 |
| RTN4 | -0.0409458 | 0.0747827 | -0.5475 | 0.584 | -0.057603686 | count | 1 |
| ITGBL1 | -0.3353095 | 0.8060836 | -0.416 | 0.677 | -0.057510188 | count | 1 |
| RGL1 | -0.3353095 | 0.9344718 | -0.3588 | 0.72 | -0.057510188 | count | 1 |
| NAPEPLD | -0.0499545 | 0.3528111 | -0.1416 | 0.887 | -0.057486723 | count | 1 |
| AC016394.1 | -0.0741189 | 0.5795864 | -0.1279 | 0.898 | -0.057439527 | count | 1 |
| PCNT | -0.0462896 | 0.1923658 | -0.2406 | 0.81 | -0.057426296 | count | 1 |
| RELL2 | -0.0483016 | 0.2740644 | -0.1762 | 0.86 | -0.05740372 | count | 1 |
| NDUFB11 | -0.0400846 | 0.0468564 | -0.8555 | 0.392 | -0.057397558 | count | 1 |
| LRRC45 | -0.0527677 | 0.2870614 | -0.1838 | 0.854 | -0.057394934 | count | 1 |
| SNAPC3 | -0.0502527 | 0.2618661 | -0.1919 | 0.848 | -0.057382375 | count | 1 |
| ZNF234 | -0.0568243 | 0.4507221 | -0.1261 | 0.9 | -0.057375366 | count | 1 |
| CHMP5 | -0.0408821 | 0.0873242 | -0.4682 | 0.64 | -0.057347762 | count | 1 |
| ATP2C1 | -0.0464443 | 0.2396798 | -0.1938 | 0.846 | -0.057217098 | count | 1 |
| RPS9 | -0.039669 | 0.0190521 | -2.0821 | 0.0374 | -0.057139209 | count | 1 |
| HIGD1A | -0.0413173 | 0.1203832 | -0.3432 | 0.731 | -0.057106778 | count | 1 |
| INAFM2 | -0.331015 | 0.4154762 | -0.7967 | 0.426 | -0.056836103 | count | 1 |
| EXOG | -0.0428327 | 0.177421 | -0.2414 | 0.809 | -0.05681512 | count | 1 |
| SLC25A26 | -0.0444723 | 0.1416241 | -0.314 | 0.754 | -0.056702452 | count | 1 |
| AC010226.1 | -0.3296064 | 0.7436355 | -0.4432 | 0.658 | -0.056614633 | count | 1 |

| | | | | | | | |
|---|---|---|---|---|---|---|---|
| AC068338.3 | -0.3296064 | 0.782491 | -0.4212 | 0.674 | -0.056614633 | count | 1 |
| FKBP1A | -0.0400076 | 0.0767779 | -0.5211 | 0.602 | -0.056585235 | count | 1 |
| CAST | -0.0394374 | 0.0488587 | -0.8072 | 0.42 | -0.056536936 | count | 1 |
| VRK1 | -0.042186 | 0.1399014 | -0.3015 | 0.763 | -0.056380634 | count | 1 |
| ZEB1-AS1 | -0.1301723 | 0.5122716 | -0.2541 | 0.799 | -0.056352616 | count | 1 |
| GPATCH2 | -0.0432941 | 0.1411685 | -0.3067 | 0.759 | -0.056345436 | count | 1 |
| AC012645.3 | -0.0506464 | 0.2296858 | -0.2205 | 0.825 | -0.056299409 | count | 1 |
| GLCCI1 | -0.0426328 | 0.1294705 | -0.3293 | 0.742 | -0.056239827 | count | 1 |
| PSMD3 | -0.0419484 | 0.1298927 | -0.3229 | 0.747 | -0.05611083 | count | 1 |
| C1orf122 | -0.0413954 | 0.1188774 | -0.3482 | 0.728 | -0.05610408 | count | 1 |
| TOM1 | -0.0458832 | 0.2001357 | -0.2293 | 0.819 | -0.056095874 | count | 1 |
| PIPOX | -0.1295351 | 1.0604773 | -0.1221 | 0.903 | -0.056083635 | count | 1 |
| AATK | -0.1295351 | 1.0602493 | -0.1222 | 0.903 | -0.056083635 | count | 1 |
| EBI3 | -0.1295351 | 1.0602493 | -0.1222 | 0.903 | -0.056083635 | count | 1 |
| HIST1H4F | -0.1295351 | 1.1413925 | -0.1135 | 0.91 | -0.056083635 | count | 1 |
| SPTY2D1OS | -0.1295351 | 1.2596605 | -0.1028 | 0.918 | -0.056083635 | count | 1 |
| FANCG | -0.0578796 | 0.3922372 | -0.1476 | 0.883 | -0.056033146 | count | 1 |
| WTAP | -0.0396057 | 0.0674729 | -0.587 | 0.557 | -0.056007159 | count | 1 |
| CDS2 | -0.0421268 | 0.133947 | -0.3145 | 0.753 | -0.055992407 | count | 1 |
| HEXIM2 | -0.0684563 | 0.5437843 | -0.1259 | 0.9 | -0.055964043 | count | 1 |
| STAU1 | -0.0398992 | 0.0807138 | -0.4943 | 0.621 | -0.055949796 | count | 1 |
| CCNK | -0.0409551 | 0.1022716 | -0.4005 | 0.689 | -0.055851671 | count | 1 |
| WDR53 | -0.0460638 | 0.246 | -0.1873 | 0.851 | -0.055842912 | count | 1 |
| EMP3 | -0.0388056 | 0.0373428 | -1.0392 | 0.299 | -0.055840804 | count | 1 |
| SP140L | -0.0404227 | 0.0965706 | -0.4186 | 0.676 | -0.055824939 | count | 1 |
| QSOX1 | -0.0462168 | 0.2692725 | -0.1716 | 0.864 | -0.055773536 | count | 1 |
| KPNA3 | -0.0413024 | 0.1286277 | -0.3211 | 0.748 | -0.055739439 | count | 1 |
| TRIM11 | -0.0436499 | 0.1665803 | -0.262 | 0.793 | -0.055655306 | count | 1 |
| LINC00402 | -0.0920018 | 0.3143413 | -0.2927 | 0.77 | -0.055647654 | count | 1 |
| PRADC1 | -0.0431869 | 0.2074686 | -0.2082 | 0.835 | -0.055632799 | count | 1 |
| CD63 | -0.0389067 | 0.0610938 | -0.6368 | 0.524 | -0.055595765 | count | 1 |
| AZIN1 | -0.0406089 | 0.1120341 | -0.3625 | 0.717 | -0.055595454 | count | 1 |
| AP006284.1 | -0.0764977 | 0.5912216 | -0.1294 | 0.897 | -0.055589089 | count | 1 |
| ADAM10 | -0.0414346 | 0.1278746 | -0.324 | 0.746 | -0.055514967 | count | 1 |
| RPL37A | -0.038518 | 0.0221587 | -1.7383 | 0.0823 | -0.055397629 | count | 1 |
| ZSCAN20 | -0.0825977 | 0.8569439 | -0.0964 | 0.923 | -0.055388024 | count | 1 |
| AGA | -0.0408139 | 0.1347397 | -0.3029 | 0.762 | -0.055316479 | count | 1 |
| MOSPD2 | -0.0458363 | 0.2030771 | -0.2257 | 0.821 | -0.055315275 | count | 1 |
| SLC25A53 | -0.057117 | 0.2987739 | -0.1912 | 0.848 | -0.055298591 | count | 1 |
| SLC38A10 | -0.0448789 | 0.1831273 | -0.2451 | 0.806 | -0.055291977 | count | 1 |
| MANBAL | -0.0528851 | 0.3693311 | -0.1432 | 0.886 | -0.055256092 | count | 1 |
| XIAP | -0.0409397 | 0.1330214 | -0.3078 | 0.758 | -0.055213929 | count | 1 |
| AC010642.2 | -0.0487094 | 0.1958515 | -0.2487 | 0.804 | -0.055164965 | count | 1 |
| PARP14 | -0.0398663 | 0.1047122 | -0.3807 | 0.703 | -0.055116896 | count | 1 |
| FAM111A | -0.0400731 | 0.1061727 | -0.3774 | 0.706 | -0.055105141 | count | 1 |

| | | | | | | | |
|---|---|---|---|---|---|---|---|
| CCR7 | -0.0387786 | 0.0760267 | -0.5101 | 0.61 | -0.055054801 | count | 1 |
| TOMM7 | -0.0382554 | 0.0288753 | -1.3248 | 0.185 | -0.054919152 | count | 1 |
| ACP1 | -0.0391429 | 0.0872175 | -0.4488 | 0.654 | -0.054896022 | count | 1 |
| AL133467.1 | -0.0907313 | 1.0801357 | -0.084 | 0.933 | -0.054890165 | count | 1 |
| SS18L1 | -0.075397 | 0.4597656 | -0.164 | 0.87 | -0.054797306 | count | 1 |
| MDH2 | -0.03845 | 0.062152 | -0.6186 | 0.536 | -0.054744378 | count | 1 |
| SPOUT1 | -0.0468509 | 0.2335063 | -0.2006 | 0.841 | -0.054686795 | count | 1 |
| ATP7A | -0.0443391 | 0.2607532 | -0.17 | 0.865 | -0.054628077 | count | 1 |
| CAPN10 | -0.0522065 | 0.2153469 | -0.2424 | 0.808 | -0.054549799 | count | 1 |
| ARPC5 | -0.038024 | 0.0464067 | -0.8194 | 0.413 | -0.054518041 | count | 1 |
| CCNI | -0.0379712 | 0.0356523 | -1.065 | 0.287 | -0.05447798 | count | 1 |
| AC008686.1 | -0.3160235 | 0.8035765 | -0.3933 | 0.694 | -0.054470073 | count | 1 |
| POLR3D | -0.0426122 | 0.1724257 | -0.2471 | 0.805 | -0.054453311 | count | 1 |
| MAP3K2 | -0.0404254 | 0.1111454 | -0.3637 | 0.716 | -0.054446877 | count | 1 |
| RANGRF | -0.0397638 | 0.1223735 | -0.3249 | 0.745 | -0.054417056 | count | 1 |
| CTBS | -0.0400296 | 0.1407558 | -0.2844 | 0.776 | -0.05434473 | count | 1 |
| KIF21A | -0.0403455 | 0.1223963 | -0.3296 | 0.742 | -0.054301368 | count | 1 |
| AC099518.5 | -0.0809093 | 0.8364814 | -0.0967 | 0.923 | -0.054269069 | count | 1 |
| USP27X | -0.0896694 | 0.4226285 | -0.2122 | 0.832 | -0.054256802 | count | 1 |
| SACS | -0.0573752 | 0.2159286 | -0.2657 | 0.79 | -0.054176579 | count | 1 |
| AC004865.2 | -0.0411467 | 0.1943835 | -0.2117 | 0.832 | -0.054150695 | count | 1 |
| EIF3G | -0.0378008 | 0.0445938 | -0.8477 | 0.397 | -0.054093809 | count | 1 |
| C12orf57 | -0.0376576 | 0.0382612 | -0.9842 | 0.325 | -0.054062722 | count | 1 |
| ZBP1 | -0.0408153 | 0.1669887 | -0.2444 | 0.807 | -0.053967638 | count | 1 |
| OXA1L | -0.0398677 | 0.1278101 | -0.3119 | 0.755 | -0.053939776 | count | 1 |
| AC243960.1 | -0.0380929 | 0.093613 | -0.4069 | 0.684 | -0.05385876 | count | 1 |
| RNF24 | -0.0447765 | 0.2158189 | -0.2075 | 0.836 | -0.053780238 | count | 1 |
| RBM15B | -0.0439849 | 0.1863259 | -0.2361 | 0.813 | -0.053779204 | count | 1 |
| FAM241A | -0.0424686 | 0.190927 | -0.2224 | 0.824 | -0.053768637 | count | 1 |
| GPHN | -0.0656581 | 0.3729073 | -0.1761 | 0.86 | -0.053693969 | count | 1 |
| RPN1 | -0.0390166 | 0.1140913 | -0.342 | 0.732 | -0.053437981 | count | 1 |
| PPIC | -0.1679662 | 0.8255543 | -0.2035 | 0.839 | -0.053433413 | count | 1 |
| MS4A1 | -0.0418433 | 0.1795234 | -0.2331 | 0.816 | -0.053354806 | count | 1 |
| NT5C3B | -0.0437313 | 0.1789827 | -0.2443 | 0.807 | -0.053250215 | count | 1 |
| TCF7 | -0.0385528 | 0.0869198 | -0.4435 | 0.657 | -0.053228723 | count | 1 |
| FGFR1OP | -0.0517112 | 0.281465 | -0.1837 | 0.854 | -0.053171912 | count | 1 |
| ARPC3 | -0.0369555 | 0.0318149 | -1.1616 | 0.245 | -0.053164446 | count | 1 |
| SMCR5 | -0.3076281 | 0.6606776 | -0.4656 | 0.642 | -0.053136343 | count | 1 |
| SUPV3L1 | -0.0426765 | 0.150137 | -0.2843 | 0.776 | -0.053124002 | count | 1 |
| B4GALT3 | -0.0391679 | 0.1316015 | -0.2976 | 0.766 | -0.053087007 | count | 1 |
| RTN3 | -0.0386588 | 0.1236606 | -0.3126 | 0.755 | -0.05304919 | count | 1 |
| CBX1 | -0.0387731 | 0.1293887 | -0.2997 | 0.764 | -0.05304018 | count | 1 |
| MCTS1 | -0.0390673 | 0.1173748 | -0.3328 | 0.739 | -0.053039099 | count | 1 |
| RACGAP1 | -0.0790002 | 0.512056 | -0.1543 | 0.877 | -0.053003168 | count | 1 |
| PTEN | -0.038226 | 0.1014895 | -0.3766 | 0.706 | -0.052969759 | count | 1 |

| | | | | | | | |
|---|---|---|---|---|---|---|---|
| PBRM1 | -0.0385621 | 0.1051637 | -0.3667 | 0.714 | -0.052954871 | count | 1 |
| THEMIS2 | -0.0434847 | 0.2604735 | -0.1669 | 0.867 | -0.05295048 | count | 1 |
| HDLBP | -0.0405592 | 0.143138 | -0.2834 | 0.777 | -0.052789842 | count | 1 |
| PTGR2 | -0.165851 | 0.5591523 | -0.2966 | 0.767 | -0.052784684 | count | 1 |
| TENT4B | -0.0512024 | 0.1846051 | -0.2774 | 0.782 | -0.052650796 | count | 1 |
| PLEKHM1 | -0.0448128 | 0.2400688 | -0.1867 | 0.852 | -0.052648147 | count | 1 |
| AC009318.3 | -0.0997665 | 0.8329721 | -0.1198 | 0.905 | -0.05262472 | count | 1 |
| NCOA4 | -0.0421256 | 0.150496 | -0.2799 | 0.78 | -0.052603254 | count | 1 |
| MCF2L-AS1 | -0.0997163 | 0.4955932 | -0.2012 | 0.841 | -0.052598694 | count | 1 |
| AC005332.7 | -0.0591266 | 0.4164566 | -0.142 | 0.887 | -0.052521268 | count | 1 |
| PTP4A1 | -0.0384657 | 0.1017743 | -0.378 | 0.705 | -0.052332043 | count | 1 |
| SFMBT2 | -0.0426268 | 0.2095526 | -0.2034 | 0.839 | -0.052326115 | count | 1 |
| AJM1 | -0.0553263 | 0.4496088 | -0.1231 | 0.902 | -0.052251799 | count | 1 |
| WDR83OS | -0.0365658 | 0.0571632 | -0.6397 | 0.522 | -0.052236628 | count | 1 |
| SEC23A | -0.0422279 | 0.1623035 | -0.2602 | 0.795 | -0.0522171 | count | 1 |
| HMGCR | -0.0449887 | 0.1951887 | -0.2305 | 0.818 | -0.052162589 | count | 1 |
| BORCS7 | -0.0388306 | 0.1374683 | -0.2825 | 0.778 | -0.052149823 | count | 1 |
| AC104506.1 | -0.1202049 | 0.3906602 | -0.3077 | 0.758 | -0.052137187 | count | 1 |
| CLYBL | -0.0715151 | 0.273353 | -0.2616 | 0.794 | -0.052002968 | count | 1 |
| ATF6 | -0.0390422 | 0.167424 | -0.2332 | 0.816 | -0.051946334 | count | 1 |
| AC008083.2 | -0.0564899 | 0.388103 | -0.1456 | 0.884 | -0.051852429 | count | 1 |
| CALR | -0.0361161 | 0.0496179 | -0.7279 | 0.4667 | -0.051588336 | count | 1 |
| NPEPL1 | -0.0431219 | 0.2237824 | -0.1927 | 0.847 | -0.051535312 | count | 1 |
| DDHD2 | -0.0428726 | 0.2260952 | -0.1896 | 0.85 | -0.05149787 | count | 1 |
| GIGYF2 | -0.0406566 | 0.1549857 | -0.2623 | 0.793 | -0.051477538 | count | 1 |
| RPUSD2 | -0.0428439 | 0.2323818 | -0.1844 | 0.854 | -0.051463462 | count | 1 |
| TBK1 | -0.0391493 | 0.1584844 | -0.247 | 0.805 | -0.051325747 | count | 1 |
| NCAPD3 | -0.04337 | 0.2045819 | -0.212 | 0.832 | -0.051263628 | count | 1 |
| HIST3H2A | -0.0407071 | 0.1927739 | -0.2112 | 0.833 | -0.051133323 | count | 1 |
| UBE4A | -0.0399493 | 0.1546626 | -0.2583 | 0.796 | -0.051054574 | count | 1 |
| CD99L2 | -0.1601438 | 0.4224048 | -0.3791 | 0.705 | -0.051031115 | count | 1 |
| C22orf39 | -0.0378882 | 0.1443329 | -0.2625 | 0.793 | -0.050959953 | count | 1 |
| SRI | -0.0357715 | 0.0650933 | -0.5495 | 0.583 | -0.05089573 | count | 1 |
| GORASP2 | -0.0372848 | 0.1204641 | -0.3095 | 0.757 | -0.050872469 | count | 1 |
| FXYD1 | -0.0407775 | 0.2339885 | -0.1743 | 0.862 | -0.05076363 | count | 1 |
| AGAP3 | -0.0619833 | 0.3841391 | -0.1614 | 0.872 | -0.050710423 | count | 1 |
| METTL26 | -0.0358128 | 0.0827265 | -0.4329 | 0.665 | -0.050561184 | count | 1 |
| ACBD5 | -0.0381528 | 0.150985 | -0.2527 | 0.801 | -0.050505602 | count | 1 |
| S1PR4 | -0.0362125 | 0.0992463 | -0.3649 | 0.715 | -0.050485835 | count | 1 |
| WNT10B | -0.0833221 | 0.6701582 | -0.1243 | 0.901 | -0.050466435 | count | 1 |
| DMPK | -0.0832882 | 0.6360096 | -0.131 | 0.896 | -0.050446169 | count | 1 |
| AC027307.3 | -0.0832552 | 0.5981002 | -0.1392 | 0.889 | -0.050426444 | count | 1 |
| SMIM37 | -0.0363695 | 0.123174 | -0.2953 | 0.768 | -0.050361588 | count | 1 |
| MZT2B | -0.0351228 | 0.0427088 | -0.8224 | 0.411 | -0.050282998 | count | 1 |
| TIGD7 | -0.1572776 | 0.5372684 | -0.2927 | 0.77 | -0.050148732 | count | 1 |

| | | | | | | | |
|---|---|---|---|---|---|---|---|
| RANGAP1 | -0.0487538 | 0.2117533 | -0.2302 | 0.818 | -0.050142336 | count | 1 |
| MRPL42 | -0.0367089 | 0.1126651 | -0.3258 | 0.745 | -0.050132055 | count | 1 |
| JSRP1 | -0.2887116 | 0.6930589 | -0.4166 | 0.677 | -0.050108226 | count | 1 |
| HRH2 | -0.074457 | 0.2076028 | -0.3587 | 0.72 | -0.049987746 | count | 1 |
| LRCH1 | -0.0494765 | 0.3152639 | -0.1569 | 0.875 | -0.049985751 | count | 1 |
| BOD1L1 | -0.0351443 | 0.0655213 | -0.5364 | 0.592 | -0.049921377 | count | 1 |
| ARHGEF26 | -0.0945541 | 0.5692918 | -0.1661 | 0.868 | -0.049920018 | count | 1 |
| SELENOS | -0.0351398 | 0.0772285 | -0.455 | 0.649 | -0.049704515 | count | 1 |
| B4GALNT3 | -0.0639938 | 0.5544153 | -0.1154 | 0.908 | -0.049655406 | count | 1 |
| GIPR | -0.0580068 | 0.6417208 | -0.0904 | 0.928 | -0.049625923 | count | 1 |
| CSNK2A2 | -0.0367192 | 0.1279332 | -0.287 | 0.774 | -0.049621473 | count | 1 |
| PSMA4 | -0.0350275 | 0.0743985 | -0.4708 | 0.638 | -0.049596749 | count | 1 |
| ALKBH7 | -0.0347951 | 0.066288 | -0.5249 | 0.6 | -0.049465176 | count | 1 |
| RPS21 | -0.0343531 | 0.021202 | -1.6203 | 0.105 | -0.049451143 | count | 1 |
| LAG3 | -0.0351739 | 0.1098474 | -0.3202 | 0.749 | -0.049447734 | count | 1 |
| TTC3 | -0.0359488 | 0.0939535 | -0.3826 | 0.702 | -0.049419532 | count | 1 |
| RNASEL | -0.0459713 | 0.2966129 | -0.155 | 0.877 | -0.049417711 | count | 1 |
| AC024909.2 | -0.1137061 | 0.7993004 | -0.1423 | 0.887 | -0.049379613 | count | 1 |
| DARS | -0.035871 | 0.1069596 | -0.3354 | 0.737 | -0.04936211 | count | 1 |
| ULK3 | -0.0412749 | 0.1969478 | -0.2096 | 0.834 | -0.049332105 | count | 1 |
| TESPA1 | -0.0380597 | 0.1365725 | -0.2787 | 0.781 | -0.049298828 | count | 1 |
| GABPB1 | -0.0380245 | 0.1603911 | -0.2371 | 0.813 | -0.049168142 | count | 1 |
| ILF2 | -0.0347372 | 0.0718221 | -0.4837 | 0.629 | -0.049151721 | count | 1 |
| FLOT1 | -0.0364554 | 0.1132506 | -0.3219 | 0.748 | -0.04913695 | count | 1 |
| SEL1L | -0.0376833 | 0.1548385 | -0.2434 | 0.808 | -0.049125423 | count | 1 |
| JADE2 | -0.0379585 | 0.1513976 | -0.2507 | 0.802 | -0.049082889 | count | 1 |
| SNX20 | -0.0372044 | 0.1801502 | -0.2065 | 0.836 | -0.049026694 | count | 1 |
| IFT140 | -0.2816719 | 0.5966659 | -0.4721 | 0.637 | -0.048973209 | count | 1 |
| DOPEY1 | -0.05981 | 0.263693 | -0.2268 | 0.821 | -0.048944702 | count | 1 |
| YKT6 | -0.0406477 | 0.221734 | -0.1833 | 0.855 | -0.04883019 | count | 1 |
| ENKD1 | -0.0804907 | 0.5121738 | -0.1572 | 0.875 | -0.048773107 | count | 1 |
| CTNNAL1 | -0.0503381 | 0.3589025 | -0.1403 | 0.888 | -0.048764536 | count | 1 |
| MAP3K14-AS1 | -0.1524619 | 0.5237535 | -0.2911 | 0.771 | -0.048663585 | count | 1 |
| MAP3K14 | -0.0429405 | 0.2776328 | -0.1547 | 0.877 | -0.048647563 | count | 1 |
| RBPMS | -0.2793306 | 0.9680185 | -0.2886 | 0.773 | -0.048594744 | count | 1 |
| ING3 | -0.0368744 | 0.1553003 | -0.2374 | 0.812 | -0.0485922 | count | 1 |
| ATP5PF | -0.0339554 | 0.0508659 | -0.6675 | 0.504 | -0.048586607 | count | 1 |
| MFSD14C | -0.0415918 | 0.214109 | -0.1943 | 0.846 | -0.048561074 | count | 1 |
| RENBP | -0.0801259 | 0.4330317 | -0.185 | 0.853 | -0.048554822 | count | 1 |
| ABCB8 | -0.0432382 | 0.231532 | -0.1867 | 0.852 | -0.048549607 | count | 1 |
| AC142472.1 | -0.1519126 | 0.4847811 | -0.3134 | 0.754 | -0.048493975 | count | 1 |
| DNAJB2 | -0.0385478 | 0.1693163 | -0.2277 | 0.82 | -0.048424557 | count | 1 |
| NCOA6 | -0.0420057 | 0.2338848 | -0.1796 | 0.857 | -0.048360101 | count | 1 |
| ZNF782 | -0.1513932 | 0.5137336 | -0.2947 | 0.768 | -0.048333556 | count | 1 |
| LINC00294 | -0.1509863 | 0.6787135 | -0.2225 | 0.824 | -0.048207864 | count | 1 |

| | | | | | | | |
|---|---|---|---|---|---|---|---|
| ASAP1 | -0.0365985 | 0.1386185 | -0.264 | 0.792 | -0.048109919 | count | 1 |
| AKAP7 | -0.0371181 | 0.1583582 | -0.2344 | 0.815 | -0.048080398 | count | 1 |
| EEF2 | -0.0336252 | 0.0399947 | -0.8407 | 0.401 | -0.048042209 | count | 1 |
| PRRX1 | -0.1501168 | 0.896947 | -0.1674 | 0.867 | -0.047939172 | count | 1 |
| MTX3 | -0.0616732 | 0.3333171 | -0.185 | 0.853 | -0.047868498 | count | 1 |
| UQCR10 | -0.0335422 | 0.0584301 | -0.5741 | 0.566 | -0.047857902 | count | 1 |
| DLGAP3 | -0.0904643 | 0.6850869 | -0.132 | 0.895 | -0.047794303 | count | 1 |
| TUBB6 | -0.0904643 | 0.7794189 | -0.1161 | 0.908 | -0.047794303 | count | 1 |
| CAMK4 | -0.0357715 | 0.1013309 | -0.353 | 0.724 | -0.047772484 | count | 1 |
| SS18L2 | -0.0335023 | 0.0696596 | -0.4809 | 0.631 | -0.047546634 | count | 1 |
| ZNF800 | -0.0347976 | 0.0993687 | -0.3502 | 0.726 | -0.047543943 | count | 1 |
| TBCA | -0.0332515 | 0.0545219 | -0.6099 | 0.542 | -0.047537839 | count | 1 |
| USP32 | -0.0517278 | 0.3755961 | -0.1377 | 0.89 | -0.047503224 | count | 1 |
| RMND5A | -0.0374214 | 0.1668355 | -0.2243 | 0.823 | -0.047502421 | count | 1 |
| TECR | -0.0334931 | 0.0706544 | -0.474 | 0.636 | -0.047466287 | count | 1 |
| CCDC24 | -0.2721593 | 0.9193495 | -0.296 | 0.767 | -0.047432495 | count | 1 |
| AP001453.4 | -0.2721593 | 0.9193495 | -0.296 | 0.767 | -0.047432495 | count | 1 |
| FAM110D | -0.2721593 | 1.056395 | -0.2576 | 0.797 | -0.047432495 | count | 1 |
| UPK2 | -0.2721593 | 1.056395 | -0.2576 | 0.797 | -0.047432495 | count | 1 |
| ZFYVE27 | -0.0421665 | 0.2383226 | -0.1769 | 0.86 | -0.047349247 | count | 1 |
| TPT1 | -0.0328257 | 0.016087 | -2.0405 | 0.0414 | -0.047334933 | count | 1 |
| ARMH4 | -0.2706087 | 0.8308964 | -0.3257 | 0.745 | -0.047180589 | count | 1 |
| PRKAR1A | -0.0349067 | 0.1141075 | -0.3059 | 0.76 | -0.04714371 | count | 1 |
| UBALD2 | -0.0329651 | 0.0476843 | -0.6913 | 0.4894 | -0.04712497 | count | 1 |
| GIPC1 | -0.0347692 | 0.1319712 | -0.2635 | 0.792 | -0.047073733 | count | 1 |
| CNTROB | -0.1470928 | 0.4631785 | -0.3176 | 0.751 | -0.04700394 | count | 1 |
| PRR29 | -0.1469555 | 0.9506081 | -0.1546 | 0.877 | -0.046961433 | count | 1 |
| SAMD3 | -0.0329623 | 0.0827749 | -0.3982 | 0.69 | -0.046907129 | count | 1 |
| TCEAL1 | -0.0448526 | 0.3126917 | -0.1434 | 0.886 | -0.04689114 | count | 1 |
| ARRDC1-AS1 | -0.0547324 | 0.2385598 | -0.2294 | 0.819 | -0.046841274 | count | 1 |
| C7orf50 | -0.0334348 | 0.1041751 | -0.3209 | 0.748 | -0.046823419 | count | 1 |
| CDC123 | -0.0336496 | 0.0994355 | -0.3384 | 0.735 | -0.046705049 | count | 1 |
| COX19 | -0.0341203 | 0.1226278 | -0.2782 | 0.781 | -0.046697478 | count | 1 |
| PLCB3 | -0.1069971 | 0.8412568 | -0.1272 | 0.899 | -0.046525357 | count | 1 |
| SYDE2 | -0.0691165 | 0.354933 | -0.1947 | 0.846 | -0.046437879 | count | 1 |
| HERC6 | -0.0468471 | 0.3083006 | -0.152 | 0.879 | -0.046412336 | count | 1 |
| METTL21A | -0.0371438 | 0.1881607 | -0.1974 | 0.844 | -0.046390606 | count | 1 |
| HYPK | -0.0636428 | 0.3996635 | -0.1592 | 0.873 | -0.046326995 | count | 1 |
| EBLN2 | -0.0635757 | 0.415223 | -0.1531 | 0.878 | -0.046278563 | count | 1 |
| PARP6 | -0.0407814 | 0.2739888 | -0.1488 | 0.882 | -0.046207192 | count | 1 |
| BBX | -0.0327636 | 0.079918 | -0.41 | 0.682 | -0.046017905 | count | 1 |
| CCNL2 | -0.0342509 | 0.1270351 | -0.2696 | 0.787 | -0.046003106 | count | 1 |
| SEC24C | -0.0412705 | 0.273082 | -0.1511 | 0.88 | -0.045903272 | count | 1 |
| SYNE2 | -0.0321881 | 0.0566076 | -0.5686 | 0.57 | -0.045851194 | count | 1 |
| FOXK2 | -0.0420875 | 0.2297676 | -0.1832 | 0.855 | -0.045810227 | count | 1 |

| | | | | | | | |
|---|---|---|---|---|---|---|---|
| SP2 | -0.04117 | 0.2490023 | -0.1653 | 0.869 | -0.045791771 | count | 1 |
| BCORL1 | -0.1430604 | 0.432885 | -0.3305 | 0.741 | -0.045754832 | count | 1 |
| TOR3A | -0.0343591 | 0.1450399 | -0.2369 | 0.813 | -0.0457198 | count | 1 |
| ZNF667-AS1 | -0.261095 | 0.4792857 | -0.5448 | 0.586 | -0.045630378 | count | 1 |
| FAHD2A | -0.0443428 | 0.2563865 | -0.173 | 0.863 | -0.045621071 | count | 1 |
| SLC25A40 | -0.0513149 | 0.2872902 | -0.1786 | 0.858 | -0.045618781 | count | 1 |
| RAB8A | -0.0324791 | 0.0854941 | -0.3799 | 0.704 | -0.045603816 | count | 1 |
| FAM162A | -0.0324018 | 0.0885595 | -0.3659 | 0.714 | -0.04555155 | count | 1 |
| TAOK1 | -0.0370947 | 0.1590836 | -0.2332 | 0.816 | -0.04554521 | count | 1 |
| TNRC6B | -0.032491 | 0.0741126 | -0.4384 | 0.661 | -0.045484099 | count | 1 |
| PRDX5 | -0.0319805 | 0.0679684 | -0.4705 | 0.638 | -0.045479908 | count | 1 |
| CXorf57 | -0.0511067 | 0.2952882 | -0.1731 | 0.863 | -0.045434658 | count | 1 |
| ACIN1 | -0.0330723 | 0.0976754 | -0.3386 | 0.735 | -0.04540297 | count | 1 |
| TMEM30A | -0.0340533 | 0.1444729 | -0.2357 | 0.814 | -0.045356128 | count | 1 |
| PIGF | -0.0346876 | 0.1742716 | -0.199 | 0.842 | -0.045355949 | count | 1 |
| ASXL2 | -0.0348888 | 0.1563393 | -0.2232 | 0.823 | -0.045344756 | count | 1 |
| KLHDC9 | -0.2593361 | 0.7429554 | -0.3491 | 0.727 | -0.045342878 | count | 1 |
| TMEM9B | -0.0323371 | 0.0961238 | -0.3364 | 0.737 | -0.045165155 | count | 1 |
| CRACR2A | -0.050744 | 0.2859912 | -0.1774 | 0.859 | -0.045113884 | count | 1 |
| SRP19 | -0.0321962 | 0.0862822 | -0.3731 | 0.709 | -0.045065492 | count | 1 |
| TMEM62 | -0.0437985 | 0.3536013 | -0.1239 | 0.901 | -0.045062948 | count | 1 |
| ESF1 | -0.0331253 | 0.1154642 | -0.2869 | 0.774 | -0.044951708 | count | 1 |
| PDCD10 | -0.0317886 | 0.0735589 | -0.4322 | 0.666 | -0.044944057 | count | 1 |
| REXO1 | -0.0345676 | 0.1571422 | -0.22 | 0.826 | -0.044927673 | count | 1 |
| SRSF8 | -0.0323604 | 0.093618 | -0.3457 | 0.73 | -0.044855147 | count | 1 |
| FAM213A | -0.0847235 | 0.4265155 | -0.1986 | 0.843 | -0.044805246 | count | 1 |
| HTATIP2 | -0.0325364 | 0.1318878 | -0.2467 | 0.805 | -0.044789736 | count | 1 |
| ECHDC2 | -0.0333479 | 0.1468891 | -0.227 | 0.82 | -0.044757067 | count | 1 |
| GDI1 | -0.0369045 | 0.1640022 | -0.225 | 0.822 | -0.044756522 | count | 1 |
| MED31 | -0.0328453 | 0.130713 | -0.2513 | 0.802 | -0.044666665 | count | 1 |
| PIGZ | -0.1395104 | 0.7020425 | -0.1987 | 0.842 | -0.044653269 | count | 1 |
| POP5 | -0.0322854 | 0.0995964 | -0.3242 | 0.746 | -0.044627806 | count | 1 |
| AC048382.6 | -0.2549195 | 1.012569 | -0.2518 | 0.801 | -0.04461979 | count | 1 |
| SUPT20H | -0.0336874 | 0.1335752 | -0.2522 | 0.801 | -0.044499747 | count | 1 |
| KCNQ1OT1 | -0.0458758 | 0.2093057 | -0.2192 | 0.827 | -0.044459056 | count | 1 |
| SLC37A1 | -0.0660645 | 0.4051736 | -0.1631 | 0.87 | -0.044406686 | count | 1 |
| NKILA | -0.1020246 | 0.5412235 | -0.1885 | 0.85 | -0.044404975 | count | 1 |
| CRY2 | -0.0660312 | 0.3045444 | -0.2168 | 0.828 | -0.044384515 | count | 1 |
| DCBLD1 | -0.1019514 | 0.3669539 | -0.2778 | 0.781 | -0.044373731 | count | 1 |
| SLC11A2 | -0.0731115 | 0.3404421 | -0.2148 | 0.83 | -0.044352665 | count | 1 |
| DMAC1 | -0.0322301 | 0.1302422 | -0.2475 | 0.805 | -0.044339231 | count | 1 |
| RAB6A | -0.0322227 | 0.117754 | -0.2736 | 0.784 | -0.044329055 | count | 1 |
| LRIG2 | -0.0482224 | 0.2840485 | -0.1698 | 0.865 | -0.044299122 | count | 1 |
| PLCXD2 | -0.0516303 | 0.4426322 | -0.1166 | 0.907 | -0.044201277 | count | 1 |
| CCDC51 | -0.0410863 | 0.3112244 | -0.132 | 0.895 | -0.044181091 | count | 1 |

| | | | | | | | |
|---|---|---|---|---|---|---|---|
| WDR86 | -0.1377133 | 0.5284099 | -0.2606 | 0.794 | -0.044094963 | count | 1 |
| VRK3 | -0.0346753 | 0.1740615 | -0.1992 | 0.842 | -0.044020387 | count | 1 |
| CD3D | -0.0305881 | 0.0325624 | -0.9394 | 0.348 | -0.044009061 | count | 1 |
| TOR4A | -0.0603132 | 0.5362295 | -0.1125 | 0.91 | -0.043922658 | count | 1 |
| TCTE3 | -0.0434408 | 0.2938036 | -0.1479 | 0.882 | -0.043909033 | count | 1 |
| ERI1 | -0.0463458 | 0.2500216 | -0.1854 | 0.853 | -0.04380651 | count | 1 |
| ARHGEF37 | -0.0828068 | 0.5606492 | -0.1477 | 0.883 | -0.043805929 | count | 1 |
| TMEM243 | -0.0311417 | 0.0744474 | -0.4183 | 0.676 | -0.04380154 | count | 1 |
| ZER1 | -0.0462233 | 0.3269207 | -0.1414 | 0.888 | -0.043691212 | count | 1 |
| NUTM2A-AS1 | -0.0351666 | 0.1769943 | -0.1987 | 0.843 | -0.043645292 | count | 1 |
| PNO1 | -0.0328085 | 0.1576437 | -0.2081 | 0.835 | -0.043527915 | count | 1 |
| POLR2K | -0.0306915 | 0.0713702 | -0.43 | 0.667 | -0.043460393 | count | 1 |
| TMX4 | -0.0308619 | 0.0861878 | -0.3581 | 0.72 | -0.04345527 | count | 1 |
| B3GAT3 | -0.0317253 | 0.1088269 | -0.2915 | 0.771 | -0.043421062 | count | 1 |
| GNAO1 | -0.0507029 | 0.4011357 | -0.1264 | 0.899 | -0.043411674 | count | 1 |
| ERLIN1 | -0.0558687 | 0.3299927 | -0.1693 | 0.866 | -0.043394297 | count | 1 |
| RTCB | -0.0320596 | 0.1407192 | -0.2278 | 0.82 | -0.043381235 | count | 1 |
| LINC02447 | -0.0993383 | 0.7453657 | -0.1333 | 0.894 | -0.043257748 | count | 1 |
| SPATA6 | -0.0992114 | 0.5861924 | -0.1692 | 0.866 | -0.043203524 | count | 1 |
| ZNF264 | -0.0352629 | 0.1973152 | -0.1787 | 0.858 | -0.042953554 | count | 1 |
| SYF2 | -0.030008 | 0.0589957 | -0.5086 | 0.611 | -0.042785447 | count | 1 |
| C20orf204 | -0.0550757 | 0.2698121 | -0.2041 | 0.838 | -0.042782531 | count | 1 |
| SOCS3 | -0.0303126 | 0.0763811 | -0.3969 | 0.691 | -0.042766966 | count | 1 |
| NSA2 | -0.0299458 | 0.0520854 | -0.5749 | 0.5654 | -0.042731585 | count | 1 |
| BCL2L15 | -0.2432657 | 0.6973621 | -0.3488 | 0.727 | -0.042703541 | count | 1 |
| BMP4 | -0.2432657 | 0.7449462 | -0.3266 | 0.744 | -0.042703541 | count | 1 |
| ELOVL6 | -0.0703015 | 0.3689555 | -0.1905 | 0.849 | -0.042666605 | count | 1 |
| SF3B5 | -0.0300114 | 0.0639435 | -0.4693 | 0.639 | -0.042634139 | count | 1 |
| BAG2 | -0.0351377 | 0.1921424 | -0.1829 | 0.855 | -0.042616981 | count | 1 |
| MCRIP2 | -0.0318415 | 0.1674188 | -0.1902 | 0.849 | -0.04248974 | count | 1 |
| SNRPA1 | -0.0303276 | 0.0892693 | -0.3397 | 0.734 | -0.042439121 | count | 1 |
| WRAP53 | -0.037091 | 0.327033 | -0.1134 | 0.91 | -0.042384226 | count | 1 |
| ARMC5 | -0.0545292 | 0.3559356 | -0.1532 | 0.878 | -0.042360855 | count | 1 |
| SDK1 | -0.241172 | 1.230101 | -0.1961 | 0.845 | -0.042357999 | count | 1 |
| AC004839.1 | -0.241172 | 1.230101 | -0.1961 | 0.845 | -0.042357999 | count | 1 |
| CX3CL1 | -0.241172 | 1.230101 | -0.1961 | 0.845 | -0.042357999 | count | 1 |
| WTIP | -0.241172 | 1.230101 | -0.1961 | 0.845 | -0.042357999 | count | 1 |
| UPB1 | -0.241172 | 1.230101 | -0.1961 | 0.845 | -0.042357999 | count | 1 |
| AC005037.1 | -0.241172 | 1.238935 | -0.1947 | 0.846 | -0.042357999 | count | 1 |
| AC009686.2 | -0.241172 | 1.238935 | -0.1947 | 0.846 | -0.042357999 | count | 1 |
| STARD6 | -0.241172 | 1.238935 | -0.1947 | 0.846 | -0.042357999 | count | 1 |
| AC068633.1 | -0.241172 | 1.280149 | -0.1884 | 0.851 | -0.042357999 | count | 1 |
| PTPN20 | -0.241172 | 1.280149 | -0.1884 | 0.851 | -0.042357999 | count | 1 |
| ZNF738 | -0.0460939 | 0.3750109 | -0.1229 | 0.902 | -0.042352485 | count | 1 |
| GEMIN5 | -0.0545091 | 0.3905475 | -0.1396 | 0.889 | -0.042345345 | count | 1 |

| | | | | | | | |
|---|---|---|---|---|---|---|---|
| BCL7C | -0.0297611 | 0.073185 | -0.4067 | 0.684 | -0.042278606 | count | 1 |
| SDCCAG8 | -0.0328653 | 0.1560447 | -0.2106 | 0.833 | -0.042268416 | count | 1 |
| ARFIP1 | -0.0327207 | 0.1710558 | -0.1913 | 0.848 | -0.042240703 | count | 1 |
| NOM1 | -0.0375267 | 0.1791425 | -0.2095 | 0.834 | -0.042150644 | count | 1 |
| S100A4 | -0.0292332 | 0.0319006 | -0.9164 | 0.36 | -0.042147483 | count | 1 |
| MEGF6 | -0.1310079 | 0.6123706 | -0.2139 | 0.831 | -0.042007846 | count | 1 |
| ARHGDIB | -0.0291515 | 0.0253732 | -1.1489 | 0.251 | -0.04197623 | count | 1 |
| LINC00667 | -0.0304034 | 0.1431506 | -0.2124 | 0.832 | -0.041770144 | count | 1 |
| SKP1 | -0.0290245 | 0.0348562 | -0.8327 | 0.405 | -0.041693992 | count | 1 |
| KIDINS220 | -0.0311737 | 0.1338433 | -0.2329 | 0.816 | -0.041671892 | count | 1 |
| LINC00167 | -0.0954677 | 0.7662105 | -0.1246 | 0.901 | -0.04160262 | count | 1 |
| AC100778.3 | -0.0954677 | 1.003728 | -0.0951 | 0.924 | -0.04160262 | count | 1 |
| SIK3 | -0.0319642 | 0.1371749 | -0.233 | 0.816 | -0.041546854 | count | 1 |
| BNIP3 | -0.0303484 | 0.1225504 | -0.2476 | 0.804 | -0.041334501 | count | 1 |
| CISH | -0.0296546 | 0.1318709 | -0.2249 | 0.822 | -0.041255145 | count | 1 |
| PDCD6IP | -0.0302676 | 0.1264692 | -0.2393 | 0.811 | -0.041224501 | count | 1 |
| FAM208B | -0.0319853 | 0.1427525 | -0.2241 | 0.823 | -0.04121614 | count | 1 |
| CLEC11A | -0.046218 | 0.5780226 | -0.08 | 0.936 | -0.041109013 | count | 1 |
| RBM3 | -0.0287151 | 0.047153 | -0.609 | 0.5426 | -0.041004642 | count | 1 |
| NVL | -0.032807 | 0.2093875 | -0.1567 | 0.876 | -0.040980484 | count | 1 |
| TLK1 | -0.0289607 | 0.0717034 | -0.4039 | 0.686 | -0.040910694 | count | 1 |
| AC017083.1 | -0.042177 | 0.4112357 | -0.1026 | 0.918 | -0.040887642 | count | 1 |
| MAPK7 | -0.0371188 | 0.3611674 | -0.1028 | 0.918 | -0.040870781 | count | 1 |
| KIF27 | -0.0673076 | 0.4158663 | -0.1618 | 0.871 | -0.04086853 | count | 1 |
| CYP51A1 | -0.0607234 | 0.3021664 | -0.201 | 0.841 | -0.040847645 | count | 1 |
| CUL9 | -0.0432016 | 0.405591 | -0.1065 | 0.915 | -0.040846322 | count | 1 |
| MRPS18C | -0.0292 | 0.1022812 | -0.2855 | 0.775 | -0.040752515 | count | 1 |
| CAPNS1 | -0.0288948 | 0.0800411 | -0.361 | 0.718 | -0.04073621 | count | 1 |
| AC116407.2 | -0.037857 | 0.3708415 | -0.1021 | 0.919 | -0.040717407 | count | 1 |
| KIAA0319L | -0.0359219 | 0.2171576 | -0.1654 | 0.869 | -0.040712391 | count | 1 |
| SPOCK2 | -0.0284499 | 0.0527761 | -0.5391 | 0.5899 | -0.04062597 | count | 1 |
| TIMM10 | -0.0303382 | 0.1186262 | -0.2557 | 0.798 | -0.040623459 | count | 1 |
| AL583839.1 | -0.1265696 | 0.9781586 | -0.1294 | 0.897 | -0.040622928 | count | 1 |
| PITPNC1 | -0.029195 | 0.0968663 | -0.3014 | 0.763 | -0.040532774 | count | 1 |
| CSE1L | -0.0371973 | 0.2457337 | -0.1514 | 0.88 | -0.04050035 | count | 1 |
| RNF185 | -0.2294176 | 0.4019169 | -0.5708 | 0.568 | -0.040410865 | count | 1 |
| PPP2R2B | -0.0291535 | 0.125721 | -0.2319 | 0.817 | -0.040392177 | count | 1 |
| TMTC3 | -0.1258043 | 0.3643087 | -0.3453 | 0.73 | -0.040383859 | count | 1 |
| ITPKB | -0.0365192 | 0.1703085 | -0.2144 | 0.83 | -0.040212082 | count | 1 |
| TOB1 | -0.0281306 | 0.0613901 | -0.4582 | 0.647 | -0.040133643 | count | 1 |
| UBE2A | -0.028265 | 0.0831998 | -0.3397 | 0.734 | -0.0399459 | count | 1 |
| LSP1 | -0.0278899 | 0.0504212 | -0.5531 | 0.58 | -0.039932138 | count | 1 |
| FOXD1 | -0.1243202 | 0.6387829 | -0.1946 | 0.846 | -0.039919991 | count | 1 |
| DDIT4-AS1 | -0.124133 | 0.6923667 | -0.1793 | 0.8577 | -0.03986147 | count | 1 |
| SMPD1 | -0.0308846 | 0.1875521 | -0.1647 | 0.869 | -0.039799016 | count | 1 |

| | | | | | | | |
|---|---|---|---|---|---|---|---|
| NOLC1 | -0.0295107 | 0.1231297 | -0.2397 | 0.811 | -0.039698094 | count | 1 |
| HSPA5 | -0.0276348 | 0.0483786 | -0.5712 | 0.568 | -0.039563521 | count | 1 |
| DYRK2 | -0.030281 | 0.136787 | -0.2214 | 0.825 | -0.039541467 | count | 1 |
| C11orf58 | -0.0276603 | 0.0479056 | -0.5774 | 0.5637 | -0.039522052 | count | 1 |
| AL121944.1 | -0.0390774 | 0.2553105 | -0.1531 | 0.878 | -0.039512262 | count | 1 |
| SREK1IP1 | -0.0282012 | 0.1009317 | -0.2794 | 0.78 | -0.039490541 | count | 1 |
| STK11IP | -0.0903638 | 0.5759141 | -0.1569 | 0.875 | -0.039416278 | count | 1 |
| DGKZ | -0.0281642 | 0.0892264 | -0.3156 | 0.752 | -0.039401575 | count | 1 |
| LPGAT1 | -0.0286958 | 0.1192688 | -0.2406 | 0.81 | -0.039382616 | count | 1 |
| BNIP2 | -0.028483 | 0.0942812 | -0.3021 | 0.763 | -0.039373437 | count | 1 |
| VAV3 | -0.0297361 | 0.1627532 | -0.1827 | 0.855 | -0.039327892 | count | 1 |
| SMC6 | -0.0298232 | 0.1892392 | -0.1576 | 0.875 | -0.039209798 | count | 1 |
| PPARG | -0.1214648 | 0.4774248 | -0.2544 | 0.799 | -0.039026682 | count | 1 |
| CREBL2 | -0.0327374 | 0.1973792 | -0.1659 | 0.868 | -0.038934844 | count | 1 |
| CEP131 | -0.2204568 | 0.496756 | -0.4438 | 0.657 | -0.038918297 | count | 1 |
| BLOC1S5 | -0.0293192 | 0.1869143 | -0.1569 | 0.875 | -0.038901444 | count | 1 |
| SEC11C | -0.0273071 | 0.0740897 | -0.3686 | 0.712 | -0.038646227 | count | 1 |
| FBXO9 | -0.0284928 | 0.1316878 | -0.2164 | 0.829 | -0.03846072 | count | 1 |
| TRBV20-1 | -0.1195964 | 0.5365818 | -0.2229 | 0.824 | -0.038441536 | count | 1 |
| MAGOH | -0.027094 | 0.0675896 | -0.4009 | 0.689 | -0.038421136 | count | 1 |
| HAX1 | -0.027166 | 0.0742607 | -0.3658 | 0.715 | -0.038409367 | count | 1 |
| THAP1 | -0.0284414 | 0.1491237 | -0.1907 | 0.849 | -0.038260388 | count | 1 |
| RPL13 | -0.0265251 | 0.0137331 | -1.9315 | 0.0535 | -0.038254132 | count | 1 |
| GART | -0.0294555 | 0.1689613 | -0.1743 | 0.862 | -0.038162642 | count | 1 |
| GBGT1 | -0.0566128 | 0.4934067 | -0.1147 | 0.909 | -0.038104756 | count | 1 |
| QTRT1 | -0.0293886 | 0.1886818 | -0.1558 | 0.876 | -0.038010352 | count | 1 |
| KBTBD8 | -0.0391185 | 0.3288394 | -0.119 | 0.905 | -0.037932704 | count | 1 |
| ZNF483 | -0.0563325 | 0.3936319 | -0.1431 | 0.886 | -0.0379176 | count | 1 |
| MCMBP | -0.0297192 | 0.1751034 | -0.1697 | 0.865 | -0.037909358 | count | 1 |
| WASHC5 | -0.0328548 | 0.2431052 | -0.1351 | 0.893 | -0.03784339 | count | 1 |
| EGR3 | -0.0486315 | 0.3339518 | -0.1456 | 0.884 | -0.037806558 | count | 1 |
| AC009061.2 | -0.0518098 | 0.4128347 | -0.1255 | 0.9 | -0.037772405 | count | 1 |
| ICE1 | -0.027887 | 0.1158936 | -0.2406 | 0.81 | -0.037691403 | count | 1 |
| AL391069.2 | -0.0619833 | 0.5905116 | -0.105 | 0.916 | -0.037666628 | count | 1 |
| CDC42EP4 | -0.0515905 | 0.3536784 | -0.1459 | 0.884 | -0.037613604 | count | 1 |
| GNAI1 | -0.2123625 | 0.7452603 | -0.285 | 0.776 | -0.037563977 | count | 1 |
| HOXB7 | -0.0370828 | 0.4860318 | -0.0763 | 0.939 | -0.037501376 | count | 1 |
| RSL1D1 | -0.0262947 | 0.0525676 | -0.5002 | 0.617 | -0.03747483 | count | 1 |
| CEP68 | -0.0360428 | 0.2056229 | -0.1753 | 0.861 | -0.037105164 | count | 1 |
| APEX1 | -0.0262476 | 0.0736655 | -0.3563 | 0.722 | -0.037095183 | count | 1 |
| TRPC4AP | -0.0298765 | 0.1904206 | -0.1569 | 0.875 | -0.037086972 | count | 1 |
| LINC01644 | -0.1151279 | 0.8411351 | -0.1369 | 0.891 | -0.037040126 | count | 1 |
| TNPO3 | -0.0336101 | 0.2386217 | -0.1409 | 0.888 | -0.037015534 | count | 1 |
| ATP6V0A1 | -0.2089631 | 0.4668135 | -0.4476 | 0.654 | -0.036993474 | count | 1 |
| C1orf131 | -0.0272279 | 0.1339512 | -0.2033 | 0.839 | -0.036889726 | count | 1 |

| | | | | | | | |
|---|---|---|---|---|---|---|---|
| NEDD8 | -0.0256867 | 0.0534103 | -0.4809 | 0.631 | -0.036736738 | count | 1 |
| POLM | -0.0303069 | 0.2205621 | -0.1374 | 0.891 | -0.036598963 | count | 1 |
| CCDC77 | -0.0328653 | 0.261259 | -0.1258 | 0.9 | -0.036573149 | count | 1 |
| LAPTM4B | -0.0397352 | 0.2694529 | -0.1475 | 0.883 | -0.036532229 | count | 1 |
| SLC39A7 | -0.0285914 | 0.1764407 | -0.162 | 0.871 | -0.036472007 | count | 1 |
| ITFG1 | -0.0278095 | 0.1663291 | -0.1672 | 0.867 | -0.036419462 | count | 1 |
| MZT1 | -0.0263728 | 0.1151786 | -0.229 | 0.819 | -0.036396387 | count | 1 |
| AP001160.3 | -0.0374637 | 0.4231186 | -0.0885 | 0.929 | -0.036333269 | count | 1 |
| MRGBP | -0.0268339 | 0.1489676 | -0.1801 | 0.857 | -0.036245806 | count | 1 |
| RAB38 | -0.0422768 | 0.5259231 | -0.0804 | 0.936 | -0.036230137 | count | 1 |
| PPM1K | -0.0259337 | 0.0950756 | -0.2728 | 0.785 | -0.036223488 | count | 1 |
| PIH1D1 | -0.0262696 | 0.1073978 | -0.2446 | 0.807 | -0.036141953 | count | 1 |
| UFSP2 | -0.0278251 | 0.1610325 | -0.1728 | 0.863 | -0.036112202 | count | 1 |
| UBR3 | -0.049461 | 0.2531129 | -0.1954 | 0.845 | -0.0360711 | count | 1 |
| SLC25A46 | -0.0318172 | 0.1984874 | -0.1603 | 0.873 | -0.036068662 | count | 1 |
| USP18 | -0.0437485 | 0.3238618 | -0.1351 | 0.893 | -0.035867144 | count | 1 |
| RAB11A | -0.0253966 | 0.0852712 | -0.2978 | 0.766 | -0.035790959 | count | 1 |
| IMMP1L | -0.0278023 | 0.1792828 | -0.1551 | 0.877 | -0.035762011 | count | 1 |
| TBL1X | -0.0400164 | 0.2353587 | -0.17 | 0.865 | -0.035615334 | count | 1 |
| ATP6V1G1 | -0.0246698 | 0.0396536 | -0.6221 | 0.534 | -0.035382402 | count | 1 |
| INF2 | -0.0666078 | 0.3095544 | -0.2152 | 0.83 | -0.035333284 | count | 1 |
| ZUP1 | -0.027012 | 0.18761 | -0.144 | 0.886 | -0.03522345 | count | 1 |
| CERS5 | -0.0275379 | 0.1654889 | -0.1664 | 0.868 | -0.035206162 | count | 1 |
| UBAC2 | -0.0251668 | 0.0881931 | -0.2854 | 0.775 | -0.035147098 | count | 1 |
| ARL10 | -0.1090866 | 0.5344306 | -0.2041 | 0.838 | -0.035141099 | count | 1 |
| ARL4D | -0.0259502 | 0.1878837 | -0.1381 | 0.89 | -0.035074798 | count | 1 |
| MTX2 | -0.0266781 | 0.1602006 | -0.1665 | 0.868 | -0.034938733 | count | 1 |
| GOLGA8B | -0.0266773 | 0.1718584 | -0.1552 | 0.877 | -0.034937686 | count | 1 |
| SFXN2 | -0.1966053 | 0.5698781 | -0.345 | 0.73 | -0.034910964 | count | 1 |
| TTYH3 | -0.0656581 | 0.5104837 | -0.1286 | 0.898 | -0.034835071 | count | 1 |
| DIABLO | -0.196149 | 0.5209982 | -0.3765 | 0.707 | -0.034833818 | count | 1 |
| AHCYL1 | -0.0337351 | 0.2145537 | -0.1572 | 0.875 | -0.034735493 | count | 1 |
| LINC01569 | -0.1077925 | 0.771685 | -0.1397 | 0.889 | -0.034733643 | count | 1 |
| CA2 | -0.1077925 | 0.8018943 | -0.1344 | 0.893 | -0.034733643 | count | 1 |
| OSTM1 | -0.0262128 | 0.1353719 | -0.1936 | 0.846 | -0.034670776 | count | 1 |
| ITPA | -0.0250029 | 0.1049635 | -0.2382 | 0.812 | -0.034600198 | count | 1 |
| HMMR | -0.107248 | 0.5801019 | -0.1849 | 0.853 | -0.034562142 | count | 1 |
| PPIL2 | -0.0271614 | 0.1802263 | -0.1507 | 0.88 | -0.034489661 | count | 1 |
| TFIP11 | -0.0272024 | 0.2136232 | -0.1273 | 0.899 | -0.034457406 | count | 1 |
| DCAKD | -0.0386261 | 0.3221978 | -0.1199 | 0.905 | -0.034382772 | count | 1 |
| MRPL47 | -0.0248775 | 0.1066311 | -0.2333 | 0.816 | -0.034372086 | count | 1 |
| GCNT2 | -0.0564457 | 0.6942802 | -0.0813 | 0.935 | -0.034330719 | count | 1 |
| VILL | -0.0315146 | 0.2914575 | -0.1081 | 0.914 | -0.034325642 | count | 1 |
| RPS29 | -0.0238196 | 0.0165023 | -1.4434 | 0.149 | -0.034313581 | count | 1 |
| ZNF441 | -0.0438627 | 0.347321 | -0.1263 | 0.9 | -0.034119094 | count | 1 |

| | | | | | | | |
|---|---|---|---|---|---|---|---|
| WDR74 | -0.0259999 | 0.1134988 | -0.2291 | 0.819 | -0.034097331 | count | 1 |
| GOSR2 | -0.0279596 | 0.1873726 | -0.1492 | 0.881 | -0.034067639 | count | 1 |
| RAN | -0.0237319 | 0.0377086 | -0.6293 | 0.529 | -0.03405392 | count | 1 |
| PPIP5K1 | -0.0641486 | 0.5809282 | -0.1104 | 0.912 | -0.03404285 | count | 1 |
| SNX14 | -0.0289396 | 0.198764 | -0.1456 | 0.884 | -0.034026054 | count | 1 |
| FAM53C | -0.0255208 | 0.1474384 | -0.1731 | 0.863 | -0.033997419 | count | 1 |
| CARS2 | -0.0272722 | 0.174213 | -0.1565 | 0.876 | -0.033967562 | count | 1 |
| TSPAN18 | -0.0315072 | 0.3332032 | -0.0946 | 0.925 | -0.03390226 | count | 1 |
| COX6C | -0.0236554 | 0.0425459 | -0.556 | 0.578 | -0.033877883 | count | 1 |
| SNAI3-AS1 | -0.1042546 | 0.6428577 | -0.1622 | 0.871 | -0.033618567 | count | 1 |
| SEM1 | -0.0235586 | 0.0642839 | -0.3665 | 0.714 | -0.033558841 | count | 1 |
| TMEM203 | -0.0247101 | 0.1361541 | -0.1815 | 0.856 | -0.033517955 | count | 1 |
| ARL17B | -0.0457615 | 0.348353 | -0.1314 | 0.895 | -0.033389269 | count | 1 |
| ALG3 | -0.0248156 | 0.1653086 | -0.1501 | 0.881 | -0.03338498 | count | 1 |
| ERO1A | -0.024224 | 0.1145117 | -0.2115 | 0.832 | -0.033259419 | count | 1 |
| RGP1 | -0.0269569 | 0.2183423 | -0.1235 | 0.902 | -0.033234564 | count | 1 |
| NORAD | -0.0239633 | 0.1043533 | -0.2296 | 0.818 | -0.033186827 | count | 1 |
| ELF4 | -0.0341818 | 0.3392822 | -0.1007 | 0.92 | -0.033159791 | count | 1 |
| SNAP47 | -0.0261566 | 0.1666875 | -0.1569 | 0.875 | -0.033133796 | count | 1 |
| LRRC7 | -0.1026863 | 0.6582683 | -0.156 | 0.876 | -0.033123719 | count | 1 |
| TRAV8-6 | -0.1026863 | 0.6698264 | -0.1533 | 0.878 | -0.033123719 | count | 1 |
| BNC2 | -0.1026863 | 0.8169013 | -0.1257 | 0.9 | -0.033123719 | count | 1 |
| VBP1 | -0.0241971 | 0.1078218 | -0.2244 | 0.822 | -0.033093559 | count | 1 |
| MRM3 | -0.0254429 | 0.1820568 | -0.1398 | 0.889 | -0.032910629 | count | 1 |
| CEP135 | -0.0255693 | 0.1727947 | -0.148 | 0.882 | -0.032827131 | count | 1 |
| NECAB3 | -0.036821 | 0.3663046 | -0.1005 | 0.92 | -0.032781946 | count | 1 |
| UCK2 | -0.1015218 | 0.3938957 | -0.2577 | 0.797 | -0.032756071 | count | 1 |
| CXorf38 | -0.024188 | 0.1289892 | -0.1875 | 0.851 | -0.032714166 | count | 1 |
| ZNF428 | -0.0232184 | 0.0863546 | -0.2689 | 0.788 | -0.032708271 | count | 1 |
| TMEM14B | -0.0229467 | 0.0673056 | -0.3409 | 0.733 | -0.03261654 | count | 1 |
| AGFG2 | -0.0261766 | 0.2095447 | -0.1249 | 0.901 | -0.032604272 | count | 1 |
| CLINT1 | -0.0233825 | 0.0885437 | -0.2641 | 0.792 | -0.032602532 | count | 1 |
| USF2 | -0.0231806 | 0.0811186 | -0.2858 | 0.775 | -0.032580398 | count | 1 |
| NUCB2 | -0.0230516 | 0.0749716 | -0.3075 | 0.759 | -0.032489268 | count | 1 |
| RRN3 | -0.0271459 | 0.1855459 | -0.1463 | 0.884 | -0.032465745 | count | 1 |
| TRAF5 | -0.0235758 | 0.1049097 | -0.2247 | 0.822 | -0.032458041 | count | 1 |
| CCDC66 | -0.0231745 | 0.0921808 | -0.2514 | 0.802 | -0.032431215 | count | 1 |
| TANC2 | -0.0610559 | 0.3745457 | -0.163 | 0.871 | -0.032418439 | count | 1 |
| RPS25 | -0.0224456 | 0.0159603 | -1.4063 | 0.16 | -0.032353173 | count | 1 |
| ZNF598 | -0.0325505 | 0.2312515 | -0.1408 | 0.888 | -0.032286576 | count | 1 |
| NIF3L1 | -0.0278103 | 0.2154625 | -0.1291 | 0.897 | -0.032273639 | count | 1 |
| GLRX5 | -0.0229938 | 0.096171 | -0.2391 | 0.811 | -0.03224774 | count | 1 |
| FOXP3 | -0.0414354 | 0.3985685 | -0.104 | 0.917 | -0.032240508 | count | 1 |
| C19orf66 | -0.0230331 | 0.0959813 | -0.24 | 0.81 | -0.032187413 | count | 1 |
| FBXW11 | -0.0254986 | 0.1535535 | -0.1661 | 0.868 | -0.032134216 | count | 1 |

| | | | | | | | |
|---|---|---|---|---|---|---|---|
| GNE | -0.0298575 | 0.2900075 | -0.103 | 0.918 | -0.032130696 | count | 1 |
| WRAP73 | -0.0240408 | 0.1705884 | -0.1409 | 0.888 | -0.032085313 | count | 1 |
| GTF3C4 | -0.0291199 | 0.2513067 | -0.1159 | 0.908 | -0.032079339 | count | 1 |
| USP42 | -0.0258118 | 0.2060523 | -0.1253 | 0.9 | -0.03204606 | count | 1 |
| SF3A2 | -0.0245759 | 0.1905834 | -0.129 | 0.897 | -0.031898029 | count | 1 |
| U2SURP | -0.0224784 | 0.0666079 | -0.3375 | 0.736 | -0.031866681 | count | 1 |
| PPM1M | -0.0248307 | 0.1655867 | -0.15 | 0.881 | -0.03167837 | count | 1 |
| C15orf40 | -0.022855 | 0.1310544 | -0.1744 | 0.862 | -0.031620451 | count | 1 |
| C10orf143 | -0.0243478 | 0.2126859 | -0.1145 | 0.909 | -0.031602158 | count | 1 |
| MARS2 | -0.072153 | 0.3851641 | -0.1873 | 0.851 | -0.031580029 | count | 1 |
| FTCDNL1 | -0.1769003 | 0.8623614 | -0.2051 | 0.837 | -0.031562583 | count | 1 |
| MAP3K4 | -0.0237462 | 0.1747816 | -0.1359 | 0.892 | -0.03151075 | count | 1 |
| MAP4K3 | -0.0975405 | 0.3441932 | -0.2834 | 0.777 | -0.031497699 | count | 1 |
| SULT1A1 | -0.0383047 | 0.4140531 | -0.0925 | 0.926 | -0.031423525 | count | 1 |
| ORC6 | -0.0383047 | 0.4276466 | -0.0896 | 0.929 | -0.031423525 | count | 1 |
| HOMEZ | -0.0351925 | 0.4524326 | -0.0778 | 0.938 | -0.031337225 | count | 1 |
| LRRC8A | -0.0330991 | 0.2627367 | -0.126 | 0.9 | -0.031323333 | count | 1 |
| UBA52 | -0.0217395 | 0.0201959 | -1.0764 | 0.282 | -0.03130635 | count | 1 |
| RASSF5 | -0.022993 | 0.0934279 | -0.2461 | 0.806 | -0.031304503 | count | 1 |
| UROD | -0.0231603 | 0.1658781 | -0.1396 | 0.889 | -0.031224131 | count | 1 |
| MBNL2 | -0.024032 | 0.1580698 | -0.152 | 0.879 | -0.031140456 | count | 1 |
| LINC01534 | -0.0508007 | 0.4671716 | -0.1087 | 0.913 | -0.030924119 | count | 1 |
| LOH12CR2 | -0.0957212 | 0.53618 | -0.1785 | 0.858 | -0.030921948 | count | 1 |
| IL10RB-DT | -0.0705194 | 0.4381919 | -0.1609 | 0.872 | -0.030874398 | count | 1 |
| GSPT2 | -0.0359952 | 0.2905659 | -0.1239 | 0.901 | -0.030867704 | count | 1 |
| PIGU | -0.031807 | 0.3661562 | -0.0869 | 0.931 | -0.030862306 | count | 1 |
| SGO2 | -0.0375604 | 0.374943 | -0.1002 | 0.92 | -0.03081554 | count | 1 |
| C19orf54 | -0.0392543 | 0.7233104 | -0.0543 | 0.957 | -0.030551501 | count | 1 |
| CARD6 | -0.0392543 | 0.828139 | -0.0474 | 0.962 | -0.030551501 | count | 1 |
| HSD17B7 | -0.0240241 | 0.1864576 | -0.1288 | 0.897 | -0.030508911 | count | 1 |
| PIEZO1 | -0.0331385 | 0.2666599 | -0.1243 | 0.901 | -0.030486473 | count | 1 |
| UBP1 | -0.0286878 | 0.2296713 | -0.1249 | 0.901 | -0.030467023 | count | 1 |
| SEC31B | -0.0940216 | 0.5164161 | -0.1821 | 0.856 | -0.030383666 | count | 1 |
| PRICKLE3 | -0.0353684 | 0.593248 | -0.0596 | 0.952 | -0.03033222 | count | 1 |
| ANKRD16 | -0.030568 | 0.4352718 | -0.0702 | 0.944 | -0.030325084 | count | 1 |
| GBF1 | -0.0285093 | 0.224399 | -0.127 | 0.899 | -0.030277826 | count | 1 |
| SNRPB | -0.0211743 | 0.0471063 | -0.4495 | 0.6531 | -0.030233832 | count | 1 |
| RNF214 | -0.0222474 | 0.1396125 | -0.1594 | 0.873 | -0.030195299 | count | 1 |
| PRKCH | -0.0221389 | 0.1065657 | -0.2077 | 0.835 | -0.030096261 | count | 1 |
| ACTR1A | -0.0226059 | 0.1365089 | -0.1656 | 0.868 | -0.030087667 | count | 1 |
| RAD23A | -0.0212155 | 0.0721985 | -0.2938 | 0.769 | -0.030076494 | count | 1 |
| AL358933.1 | -0.1679662 | 1.081149 | -0.1554 | 0.877 | -0.030033244 | count | 1 |
| AC010999.2 | -0.1679662 | 1.083053 | -0.1551 | 0.877 | -0.030033244 | count | 1 |
| TSHR | -0.1679662 | 1.323429 | -0.1269 | 0.899 | -0.030033244 | count | 1 |
| CAMK2G | -0.0243333 | 0.1756246 | -0.1386 | 0.89 | -0.029891584 | count | 1 |

| | | | | | | | |
|---|---|---|---|---|---|---|---|
| DIS3 | -0.0228822 | 0.1377038 | -0.1662 | 0.868 | -0.029795901 | count | 1 |
| ZFAND1 | -0.0214052 | 0.1146725 | -0.1867 | 0.852 | -0.029739071 | count | 1 |
| VASP | -0.0210917 | 0.0775958 | -0.2718 | 0.786 | -0.029720094 | count | 1 |
| AC022182.2 | -0.043998 | 0.520563 | -0.0845 | 0.933 | -0.029666802 | count | 1 |
| ABCC1 | -0.0305482 | 0.2256358 | -0.1354 | 0.892 | -0.0296441 | count | 1 |
| MMRN1 | -0.0557563 | 0.5771905 | -0.0966 | 0.923 | -0.029630848 | count | 1 |
| GEMIN4 | -0.0331967 | 0.4318445 | -0.0769 | 0.939 | -0.029565996 | count | 1 |
| ZYX | -0.0209567 | 0.0940907 | -0.2227 | 0.824 | -0.029437012 | count | 1 |
| ZDHHC21 | -0.0236277 | 0.2525128 | -0.0936 | 0.925 | -0.029432174 | count | 1 |
| FGD3 | -0.022321 | 0.1280688 | -0.1743 | 0.862 | -0.029423825 | count | 1 |
| CERS2 | -0.0215677 | 0.1170524 | -0.1843 | 0.854 | -0.029393121 | count | 1 |
| CCDC138 | -0.0272515 | 0.3676356 | -0.0741 | 0.941 | -0.029331381 | count | 1 |
| HSD11B1L | -0.0356911 | 0.4881634 | -0.0731 | 0.942 | -0.029288126 | count | 1 |
| RFXAP | -0.0301429 | 0.2385558 | -0.1264 | 0.899 | -0.029251813 | count | 1 |
| AC007114.2 | -0.0340971 | 0.4123072 | -0.0827 | 0.934 | -0.029245905 | count | 1 |
| ZNF783 | -0.090311 | 0.5080235 | -0.1778 | 0.859 | -0.029207113 | count | 1 |
| HDAC8 | -0.0257456 | 0.2396439 | -0.1074 | 0.914 | -0.029195728 | count | 1 |
| CYB561D2 | -0.023661 | 0.2537216 | -0.0933 | 0.926 | -0.029174785 | count | 1 |
| SCAMP2 | -0.02064 | 0.090761 | -0.2274 | 0.82 | -0.029074049 | count | 1 |
| CNEP1R1 | -0.0250417 | 0.2171702 | -0.1153 | 0.908 | -0.029064824 | count | 1 |
| MAPRE1 | -0.0209056 | 0.0980014 | -0.2133 | 0.831 | -0.029026592 | count | 1 |
| ENO3 | -0.0545778 | 0.6766158 | -0.0807 | 0.936 | -0.029010268 | count | 1 |
| CNPY3 | -0.020838 | 0.0956464 | -0.2179 | 0.828 | -0.028945116 | count | 1 |
| RBM4B | -0.0254251 | 0.2847091 | -0.0893 | 0.929 | -0.028832796 | count | 1 |
| RPL38 | -0.0200666 | 0.0240895 | -0.833 | 0.405 | -0.028827706 | count | 1 |
| SH2B1 | -0.0289902 | 0.25479 | -0.1138 | 0.909 | -0.02876354 | count | 1 |
| AK6 | -0.0210435 | 0.1236681 | -0.1702 | 0.865 | -0.028678942 | count | 1 |
| VPS28 | -0.0200674 | 0.0523393 | -0.3834 | 0.701 | -0.028676684 | count | 1 |
| DAAM1 | -0.0233669 | 0.2035402 | -0.1148 | 0.909 | -0.028593781 | count | 1 |
| FNDC10 | -0.0347435 | 0.3995686 | -0.087 | 0.931 | -0.028513586 | count | 1 |
| SLC44A1 | -0.0281268 | 0.3271048 | -0.086 | 0.931 | -0.028464307 | count | 1 |
| XPO6 | -0.0227839 | 0.1937445 | -0.1176 | 0.906 | -0.028381938 | count | 1 |
| ANKEF1 | -0.0647083 | 0.5835799 | -0.1109 | 0.912 | -0.028360719 | count | 1 |
| ACTR8 | -0.0247543 | 0.217348 | -0.1139 | 0.909 | -0.028306037 | count | 1 |
| FAM185A | -0.0874699 | 0.4477312 | -0.1954 | 0.845 | -0.028304987 | count | 1 |
| USP21 | -0.0531682 | 0.4326167 | -0.1229 | 0.902 | -0.028267665 | count | 1 |
| SEMA4A | -0.0256172 | 0.3777831 | -0.0678 | 0.946 | -0.028226786 | count | 1 |
| WDFY2 | -0.0233142 | 0.2258507 | -0.1032 | 0.918 | -0.028162908 | count | 1 |
| ARHGAP15 | -0.0200287 | 0.0739408 | -0.2709 | 0.787 | -0.028159362 | count | 1 |
| TCEANC2 | -0.0865508 | 0.3831414 | -0.2259 | 0.821 | -0.028012912 | count | 1 |
| MAFA | -0.0864948 | 0.4528734 | -0.191 | 0.849 | -0.027995113 | count | 1 |
| GOLGA1 | -0.0226961 | 0.2188683 | -0.1037 | 0.917 | -0.027986058 | count | 1 |
| FBXL18 | -0.1560121 | 0.6557476 | -0.2379 | 0.812 | -0.027976035 | count | 1 |
| SEC24A | -0.0251078 | 0.2870553 | -0.0875 | 0.93 | -0.027953455 | count | 1 |
| DUSP11 | -0.0202848 | 0.1150528 | -0.1763 | 0.86 | -0.02793717 | count | 1 |

| | | | | | | | |
|---|---|---|---|---|---|---|---|
| CLEC2D | -0.0196101 | 0.0564757 | -0.3472 | 0.728 | -0.027900536 | count | 1 |
| LRSAM1 | -0.0635781 | 0.3724964 | -0.1707 | 0.864 | -0.027871189 | count | 1 |
| ZNF202 | -0.0635181 | 0.6391826 | -0.0994 | 0.921 | -0.027845197 | count | 1 |
| C16orf45 | -0.0280477 | 0.5104907 | -0.0549 | 0.956 | -0.027830555 | count | 1 |
| AGFG1 | -0.020711 | 0.1371981 | -0.151 | 0.88 | -0.027781736 | count | 1 |
| OTUD1 | -0.0226133 | 0.1661816 | -0.1361 | 0.892 | -0.027780552 | count | 1 |
| PCTP | -0.0355538 | 0.428074 | -0.0831 | 0.934 | -0.027683815 | count | 1 |
| EPM2AIP1 | -0.0212873 | 0.1586764 | -0.1342 | 0.893 | -0.027676765 | count | 1 |
| ATRAID | -0.0194466 | 0.0753192 | -0.2582 | 0.796 | -0.027570244 | count | 1 |
| MUS81 | -0.0228782 | 0.19077 | -0.1199 | 0.905 | -0.02750514 | count | 1 |
| DDX19B | -0.0216625 | 0.1791994 | -0.1209 | 0.904 | -0.027303295 | count | 1 |
| CHUK | -0.0296223 | 0.3162629 | -0.0937 | 0.925 | -0.027260777 | count | 1 |
| CYP4F22 | -0.0512254 | 0.7976402 | -0.0642 | 0.949 | -0.027243577 | count | 1 |
| TGFB1I1 | -0.0511331 | 0.4779346 | -0.107 | 0.915 | -0.027194907 | count | 1 |
| ZFP91 | -0.0234097 | 0.2206011 | -0.1061 | 0.915 | -0.027172899 | count | 1 |
| FTH1 | -0.0188301 | 0.0289868 | -0.6496 | 0.516 | -0.027152696 | count | 1 |
| ITM2B | -0.0187974 | 0.0252934 | -0.7432 | 0.4574 | -0.027053678 | count | 1 |
| KDM5C | -0.0370255 | 0.2938679 | -0.126 | 0.9 | -0.027045902 | count | 1 |
| GPATCH11 | -0.0196515 | 0.1366134 | -0.1438 | 0.886 | -0.026992609 | count | 1 |
| QSOX2 | -0.1500948 | 0.3955458 | -0.3795 | 0.704 | -0.026953099 | count | 1 |
| ZNF865 | -0.0261586 | 0.4163165 | -0.0628 | 0.95 | -0.026949645 | count | 1 |
| GGA3 | -0.0260749 | 0.2617312 | -0.0996 | 0.921 | -0.026863582 | count | 1 |
| MT-ND4 | -0.0185982 | 0.0244171 | -0.7617 | 0.446 | -0.026793329 | count | 1 |
| ATP8B3 | -0.1487975 | 0.5371817 | -0.277 | 0.782 | -0.026728426 | count | 1 |
| ZNF407 | -0.0224428 | 0.2042266 | -0.1099 | 0.913 | -0.026704052 | count | 1 |
| ANAPC16 | -0.0185697 | 0.0480004 | -0.3869 | 0.699 | -0.026570051 | count | 1 |
| LKAAEAR1 | -0.0392663 | 0.6783213 | -0.0579 | 0.954 | -0.026493898 | count | 1 |
| RNF139 | -0.0195583 | 0.1101696 | -0.1775 | 0.859 | -0.026454518 | count | 1 |
| ODF2L | -0.0188562 | 0.0821256 | -0.2296 | 0.818 | -0.026445745 | count | 1 |
| GMDS | -0.0244258 | 0.2199924 | -0.111 | 0.912 | -0.026294955 | count | 1 |
| AC011825.2 | -0.0599289 | 0.8045764 | -0.0745 | 0.941 | -0.026289159 | count | 1 |
| SHOC2 | -0.0192289 | 0.1032696 | -0.1862 | 0.852 | -0.026255038 | count | 1 |
| CLPTM1 | -0.0221801 | 0.1841855 | -0.1204 | 0.904 | -0.026243444 | count | 1 |
| AC020765.2 | -0.0596803 | 0.4335313 | -0.1377 | 0.891 | -0.026181304 | count | 1 |
| C3orf62 | -0.0263547 | 0.3784486 | -0.0696 | 0.944 | -0.02615428 | count | 1 |
| ARID3B | -0.0242395 | 0.2560902 | -0.0947 | 0.925 | -0.026094722 | count | 1 |
| ZNF707 | -0.0268712 | 0.3734057 | -0.072 | 0.943 | -0.026084147 | count | 1 |
| TBC1D14 | -0.0262369 | 0.2415016 | -0.1086 | 0.913 | -0.026037627 | count | 1 |
| UBR2 | -0.0188779 | 0.1089527 | -0.1733 | 0.862 | -0.025991654 | count | 1 |
| WDTC1 | -0.0266016 | 0.2788556 | -0.0954 | 0.924 | -0.025823039 | count | 1 |
| COA6 | -0.0188347 | 0.1374759 | -0.137 | 0.891 | -0.025803342 | count | 1 |
| EEA1 | -0.0188204 | 0.1183463 | -0.159 | 0.874 | -0.025741908 | count | 1 |
| MRPL13 | -0.0186095 | 0.1282955 | -0.1451 | 0.885 | -0.025734437 | count | 1 |
| HPSE | -0.0313236 | 0.3845814 | -0.0814 | 0.935 | -0.025716856 | count | 1 |
| ZNF273 | -0.023554 | 0.2556665 | -0.0921 | 0.927 | -0.025668082 | count | 1 |

| | | | | | | | |
|---|---|---|---|---|---|---|---|
| EFNA3 | -0.0583802 | 0.3812206 | -0.1531 | 0.878 | -0.025617093 | count | 1 |
| DAD1 | -0.0178938 | 0.0529732 | -0.3378 | 0.736 | -0.025588724 | count | 1 |
| ABCC5 | -0.0350067 | 0.324038 | -0.108 | 0.914 | -0.025577926 | count | 1 |
| CSK | -0.0179501 | 0.0750485 | -0.2392 | 0.811 | -0.025430988 | count | 1 |
| SNRPD1 | -0.0178481 | 0.0734159 | -0.2431 | 0.808 | -0.025246564 | count | 1 |
| SYS1 | -0.0183254 | 0.1049429 | -0.1746 | 0.861 | -0.025206288 | count | 1 |
| MMACHC | -0.1395104 | 0.93244 | -0.1496 | 0.881 | -0.025115757 | count | 1 |
| ACTRT3 | -0.1395104 | 1.057703 | -0.1319 | 0.895 | -0.025115757 | count | 1 |
| AGMAT | -0.0371516 | 0.4570126 | -0.0813 | 0.935 | -0.025074473 | count | 1 |
| ERLIN2 | -0.0305379 | 0.3104336 | -0.0984 | 0.922 | -0.025074016 | count | 1 |
| AC009309.1 | -0.0570381 | 0.6780747 | -0.0841 | 0.933 | -0.025034366 | count | 1 |
| IER2 | -0.0172726 | 0.0441466 | -0.3913 | 0.696 | -0.024850278 | count | 1 |
| IL27RA | -0.0179976 | 0.1059011 | -0.1699 | 0.865 | -0.024795609 | count | 1 |
| CTSK | -0.0464884 | 0.730735 | -0.0636 | 0.949 | -0.024743768 | count | 1 |
| PSME3 | -0.0199815 | 0.2062151 | -0.0969 | 0.923 | -0.024728862 | count | 1 |
| RBL1 | -0.020746 | 0.2275725 | -0.0912 | 0.927 | -0.024548273 | count | 1 |
| NAGS | -0.0363523 | 0.5002361 | -0.0727 | 0.942 | -0.024537746 | count | 1 |
| GMPR2 | -0.0177999 | 0.128454 | -0.1386 | 0.89 | -0.024475366 | count | 1 |
| TACC3 | -0.0208075 | 0.1525078 | -0.1364 | 0.891 | -0.024474278 | count | 1 |
| GCNA | -0.1356651 | 0.4984673 | -0.2722 | 0.786 | -0.024445843 | count | 1 |
| AC145124.1 | -0.0294395 | 0.4987063 | -0.059 | 0.953 | -0.024175137 | count | 1 |
| SNRPE | -0.0171718 | 0.0853515 | -0.2012 | 0.841 | -0.024165939 | count | 1 |
| CASP4 | -0.0170051 | 0.068736 | -0.2474 | 0.805 | -0.024148289 | count | 1 |
| BATF3 | -0.0270232 | 0.4374672 | -0.0618 | 0.951 | -0.024082582 | count | 1 |
| SNHG8 | -0.0168916 | 0.0516132 | -0.3273 | 0.743 | -0.024026005 | count | 1 |
| TRBC1 | -0.0166769 | 0.0510394 | -0.3267 | 0.744 | -0.023994624 | count | 1 |
| ARL2BP | -0.0170572 | 0.0928978 | -0.1836 | 0.854 | -0.023934031 | count | 1 |
| SLC16A7 | -0.0178505 | 0.1257895 | -0.1419 | 0.887 | -0.023888119 | count | 1 |
| MARK4 | -0.0196833 | 0.2441919 | -0.0806 | 0.936 | -0.023667384 | count | 1 |
| C1QBP | -0.0166277 | 0.067681 | -0.2457 | 0.806 | -0.023621722 | count | 1 |
| VPS36 | -0.0170482 | 0.1013135 | -0.1683 | 0.866 | -0.023594106 | count | 1 |
| MTHFSD | -0.0321514 | 0.4691398 | -0.0685 | 0.945 | -0.023500355 | count | 1 |
| SAP30BP | -0.0170554 | 0.107035 | -0.1593 | 0.873 | -0.023410599 | count | 1 |
| USP48 | -0.0175499 | 0.1279552 | -0.1372 | 0.891 | -0.023404315 | count | 1 |
| DOLPP1 | -0.0214621 | 0.3522005 | -0.0609 | 0.951 | -0.023391548 | count | 1 |
| BTBD7 | -0.0186542 | 0.1635524 | -0.1141 | 0.909 | -0.023382753 | count | 1 |
| TJAP1 | -0.023528 | 0.323864 | -0.0726 | 0.942 | -0.023354458 | count | 1 |
| TBC1D25 | -0.0202377 | 0.2437557 | -0.083 | 0.934 | -0.023326067 | count | 1 |
| PINX1 | -0.0192185 | 0.2148145 | -0.0895 | 0.929 | -0.023324771 | count | 1 |
| INSL3 | -0.0714784 | 0.6198983 | -0.1153 | 0.908 | -0.023206772 | count | 1 |
| TCEA1 | -0.0162863 | 0.0606536 | -0.2685 | 0.788 | -0.023183896 | count | 1 |
| POLA2 | -0.0269877 | 0.4095686 | -0.0659 | 0.947 | -0.02316548 | count | 1 |
| LRR1 | -0.018829 | 0.2081066 | -0.0905 | 0.928 | -0.023134911 | count | 1 |
| CASP8 | -0.0163488 | 0.0729207 | -0.2242 | 0.823 | -0.022962794 | count | 1 |
| CCNF | -0.0703064 | 0.6339583 | -0.1109 | 0.912 | -0.022831766 | count | 1 |

| | | | | | | | |
|---|---|---|---|---|---|---|---|
| CCDC117 | -0.0198972 | 0.2609924 | -0.0762 | 0.939 | -0.022758024 | count | 1 |
| AP002884.1 | -0.0228165 | 0.3320418 | -0.0687 | 0.945 | -0.022649517 | count | 1 |
| COIL | -0.0170084 | 0.1598557 | -0.1064 | 0.915 | -0.022475715 | count | 1 |
| GTSE1 | -0.0508083 | 0.9008721 | -0.0564 | 0.955 | -0.022325593 | count | 1 |
| MRPL16 | -0.0160392 | 0.1116199 | -0.1437 | 0.886 | -0.022168819 | count | 1 |
| LINC01006 | -0.032802 | 0.5751305 | -0.057 | 0.955 | -0.022152261 | count | 1 |
| AC090204.1 | -0.067624 | 0.5220655 | -0.1295 | 0.897 | -0.021972775 | count | 1 |
| COQ3 | -0.0201543 | 0.3409715 | -0.0591 | 0.953 | -0.021968007 | count | 1 |
| SLC25A38 | -0.0159531 | 0.1112666 | -0.1434 | 0.886 | -0.021802408 | count | 1 |
| MIR4458HG | -0.0230001 | 0.5276016 | -0.0436 | 0.965 | -0.021785934 | count | 1 |
| TOP3B | -0.0199558 | 0.3967315 | -0.0503 | 0.96 | -0.021751919 | count | 1 |
| STXBP2 | -0.0155849 | 0.1232529 | -0.1264 | 0.899 | -0.021667254 | count | 1 |
| ANXA7 | -0.015337 | 0.0780232 | -0.1966 | 0.844 | -0.021643425 | count | 1 |
| C21orf91 | -0.0154936 | 0.1068139 | -0.1451 | 0.885 | -0.021632817 | count | 1 |
| GTF3A | -0.0150429 | 0.052467 | -0.2867 | 0.774 | -0.021541496 | count | 1 |
| ZNFX1 | -0.0168537 | 0.1616237 | -0.1043 | 0.917 | -0.021355696 | count | 1 |
| DDIT3 | -0.0150421 | 0.0766028 | -0.1964 | 0.844 | -0.021327178 | count | 1 |
| HK2 | -0.1170136 | 0.6706607 | -0.1745 | 0.862 | -0.02117826 | count | 1 |
| CGRRF1 | -0.0159658 | 0.1379072 | -0.1158 | 0.908 | -0.021122117 | count | 1 |
| PGAM1 | -0.0147891 | 0.0602354 | -0.2455 | 0.806 | -0.021102181 | count | 1 |
| PHF1 | -0.015867 | 0.1017428 | -0.156 | 0.876 | -0.021036801 | count | 1 |
| PLGRKT | -0.015151 | 0.1117934 | -0.1355 | 0.892 | -0.021023899 | count | 1 |
| AC006064.2 | -0.1160069 | 0.6723183 | -0.1725 | 0.863 | -0.02100104 | count | 1 |
| PCGF3 | -0.0227516 | 0.3242984 | -0.0702 | 0.944 | -0.020951427 | count | 1 |
| ZCCHC3 | -0.0342626 | 0.1938542 | -0.1767 | 0.86 | -0.020909295 | count | 1 |
| LRRC3 | -0.0392218 | 0.871998 | -0.045 | 0.964 | -0.020901235 | count | 1 |
| GATA3 | -0.0148206 | 0.0815856 | -0.1817 | 0.856 | -0.020895997 | count | 1 |
| AP003068.2 | -0.0391382 | 0.5894095 | -0.0664 | 0.947 | -0.020856973 | count | 1 |
| ICMT | -0.0174144 | 0.253961 | -0.0686 | 0.945 | -0.020836187 | count | 1 |
| HCST | -0.0144358 | 0.033736 | -0.4279 | 0.669 | -0.020767177 | count | 1 |
| CEP85L | -0.0154054 | 0.1084636 | -0.142 | 0.887 | -0.020545305 | count | 1 |
| STARD9 | -0.0464695 | 0.5230197 | -0.0888 | 0.929 | -0.020435306 | count | 1 |
| PNPLA6 | -0.0221742 | 0.3098001 | -0.0716 | 0.943 | -0.020420823 | count | 1 |
| CRADD | -0.0181383 | 0.2765213 | -0.0656 | 0.948 | -0.020396167 | count | 1 |
| RNF220 | -0.0156571 | 0.1832092 | -0.0855 | 0.932 | -0.02025795 | count | 1 |
| TNK2-AS1 | -0.0619833 | 0.8996045 | -0.0689 | 0.945 | -0.020163269 | count | 1 |
| POP4 | -0.0145045 | 0.1244586 | -0.1165 | 0.907 | -0.020063923 | count | 1 |
| GOLGA8A | -0.0153165 | 0.1710896 | -0.0895 | 0.929 | -0.019978463 | count | 1 |
| ARPC1B | -0.0139426 | 0.0551259 | -0.2529 | 0.8 | -0.019953188 | count | 1 |
| FAM102B | -0.0157518 | 0.2016336 | -0.0781 | 0.938 | -0.019857342 | count | 1 |
| LINC01480 | -0.1084069 | 0.6608705 | -0.164 | 0.87 | -0.019660305 | count | 1 |
| CMTM6 | -0.0140499 | 0.0793187 | -0.1771 | 0.859 | -0.019646849 | count | 1 |
| NRDC | -0.0140139 | 0.0943875 | -0.1485 | 0.882 | -0.01956704 | count | 1 |
| NRARP | -0.0366969 | 0.3475898 | -0.1056 | 0.916 | -0.019563879 | count | 1 |
| ZFP30 | -0.0365137 | 0.3754986 | -0.0972 | 0.923 | -0.0194668 | count | 1 |

| | | | | | | | |
|---|---|---|---|---|---|---|---|
| AL121983.2 | -0.0442004 | 0.799673 | -0.0553 | 0.956 | -0.019445508 | count | 1 |
| GALR2 | -0.0442004 | 0.8894708 | -0.0497 | 0.96 | -0.019445508 | count | 1 |
| C8orf37 | -0.0442004 | 0.9948164 | -0.0444 | 0.965 | -0.019445508 | count | 1 |
| TMSB10 | -0.0134813 | 0.0212576 | -0.6342 | 0.526 | -0.019435007 | count | 1 |
| PFKM | -0.028693 | 0.5561128 | -0.0516 | 0.959 | -0.019388389 | count | 1 |
| NXPE3 | -0.0173761 | 0.211492 | -0.0822 | 0.935 | -0.019354364 | count | 1 |
| VPS41 | -0.0163423 | 0.1867678 | -0.0875 | 0.93 | -0.019341459 | count | 1 |
| CDK6 | -0.0149819 | 0.1481079 | -0.1012 | 0.919 | -0.019278159 | count | 1 |
| TADA2B | -0.0178838 | 0.213324 | -0.0838 | 0.933 | -0.019260666 | count | 1 |
| DTWD2 | -0.0284754 | 0.4169011 | -0.0683 | 0.946 | -0.019241933 | count | 1 |
| TLR2 | -0.0586169 | 0.8591141 | -0.0682 | 0.946 | -0.019081307 | count | 1 |
| AL121845.1 | -0.0586169 | 0.8617815 | -0.068 | 0.946 | -0.019081307 | count | 1 |
| RRM2 | -0.0586169 | 0.9404896 | -0.0623 | 0.95 | -0.019081307 | count | 1 |
| DOCK9-DT | -0.1050903 | 0.6995907 | -0.1502 | 0.881 | -0.019073655 | count | 1 |
| TGFBR2 | -0.0136869 | 0.0930553 | -0.1471 | 0.883 | -0.018966236 | count | 1 |
| RCN2 | -0.0135522 | 0.1032301 | -0.1313 | 0.896 | -0.018849132 | count | 1 |
| CTSD | -0.0131634 | 0.0726165 | -0.1813 | 0.856 | -0.018766663 | count | 1 |
| FARSA | -0.0141819 | 0.1773235 | -0.08 | 0.936 | -0.01876281 | count | 1 |
| PLEKHM3 | -0.102984 | 0.3993415 | -0.2579 | 0.797 | -0.018700588 | count | 1 |
| NKTR | -0.0132765 | 0.0722198 | -0.1838 | 0.854 | -0.018450823 | count | 1 |
| ERMP1 | -0.0235034 | 0.5273305 | -0.0446 | 0.964 | -0.018327364 | count | 1 |
| DUT | -0.0128525 | 0.0663808 | -0.1936 | 0.846 | -0.018320352 | count | 1 |
| IL12RB1 | -0.0141294 | 0.1558643 | -0.0907 | 0.928 | -0.018282082 | count | 1 |
| RAD23B | -0.0133205 | 0.124845 | -0.1067 | 0.915 | -0.017996663 | count | 1 |
| CYC1 | -0.0127268 | 0.0954419 | -0.1333 | 0.894 | -0.017883377 | count | 1 |
| MMS19 | -0.0207523 | 0.3711746 | -0.0559 | 0.955 | -0.017824913 | count | 1 |
| DDX10 | -0.0143287 | 0.2132288 | -0.0672 | 0.946 | -0.017608513 | count | 1 |
| SIRT7 | -0.0127894 | 0.0988909 | -0.1293 | 0.897 | -0.017549492 | count | 1 |
| DROSHA | -0.0167411 | 0.3670989 | -0.0456 | 0.964 | -0.017537509 | count | 1 |
| MEA1 | -0.0125341 | 0.105504 | -0.1188 | 0.905 | -0.017522346 | count | 1 |
| DCPS | -0.0127937 | 0.1432976 | -0.0893 | 0.929 | -0.017376207 | count | 1 |
| DSTN | -0.0121427 | 0.0685502 | -0.1771 | 0.859 | -0.017277633 | count | 1 |
| AC108047.1 | -0.021016 | 0.6324249 | -0.0332 | 0.973 | -0.017274258 | count | 1 |
| ZZEF1 | -0.0146737 | 0.1859257 | -0.0789 | 0.937 | -0.017264639 | count | 1 |
| Z98884.1 | -0.0526718 | 0.8388391 | -0.0628 | 0.95 | -0.01716683 | count | 1 |
| GPR137C | -0.0526718 | 0.8986422 | -0.0586 | 0.953 | -0.01716683 | count | 1 |
| SLX4IP | -0.0171799 | 0.3034751 | -0.0566 | 0.955 | -0.017061969 | count | 1 |
| SLC5A6 | -0.0198402 | 0.4074004 | -0.0487 | 0.961 | -0.017043112 | count | 1 |
| CDKN2A | -0.0136955 | 0.1809388 | -0.0757 | 0.94 | -0.016830784 | count | 1 |
| ZFP64 | -0.017718 | 0.3662469 | -0.0484 | 0.961 | -0.016790593 | count | 1 |
| FAM167B | -0.0214987 | 0.7183553 | -0.0299 | 0.976 | -0.016768168 | count | 1 |
| DOLK | -0.031184 | 0.6050013 | -0.0515 | 0.959 | -0.016639944 | count | 1 |
| CD40 | -0.091352 | 0.4433808 | -0.206 | 0.837 | -0.016633517 | count | 1 |
| GHITM | -0.0117018 | 0.0655041 | -0.1786 | 0.858 | -0.016581516 | count | 1 |
| ZFR | -0.0118555 | 0.0892997 | -0.1328 | 0.894 | -0.016576183 | count | 1 |

| | | | | | | | |
|---|---|---|---|---|---|---|---|
| NDUFAF5 | -0.015575 | 0.2640404 | -0.059 | 0.953 | -0.016555881 | count | 1 |
| FAH | -0.014643 | 0.335629 | -0.0436 | 0.965 | -0.016469065 | count | 1 |
| LGALS8-AS1 | -0.0243046 | 0.7024253 | -0.0346 | 0.972 | -0.016433052 | count | 1 |
| AMDHD1 | -0.0243046 | 0.8344783 | -0.0291 | 0.977 | -0.016433052 | count | 1 |
| PPP4R1 | -0.0135331 | 0.2145253 | -0.0631 | 0.95 | -0.016354377 | count | 1 |
| AL132780.1 | -0.0500531 | 0.9215949 | -0.0543 | 0.957 | -0.016322039 | count | 1 |
| CDC42SE2 | -0.0114486 | 0.0521109 | -0.2197 | 0.826 | -0.016300979 | count | 1 |
| ACACA | -0.0176344 | 0.4657027 | -0.0379 | 0.97 | -0.01624693 | count | 1 |
| AC099791.2 | -0.0163436 | 0.780276 | -0.0209 | 0.983 | -0.016232507 | count | 1 |
| FAM168B | -0.0135607 | 0.2114219 | -0.0641 | 0.949 | -0.016228036 | count | 1 |
| EHMT1 | -0.0117315 | 0.1177753 | -0.0996 | 0.921 | -0.016115564 | count | 1 |
| GGA1 | -0.0123203 | 0.1373819 | -0.0897 | 0.929 | -0.016071491 | count | 1 |
| MED8 | -0.0121618 | 0.1652172 | -0.0736 | 0.941 | -0.015996153 | count | 1 |
| USP40 | -0.0299308 | 0.4425609 | -0.0676 | 0.946 | -0.01597452 | count | 1 |
| ZFPL1 | -0.0124771 | 0.2018547 | -0.0618 | 0.951 | -0.015958624 | count | 1 |
| CD2AP | -0.011793 | 0.1402811 | -0.0841 | 0.933 | -0.015809259 | count | 1 |
| RSAD1 | -0.0128571 | 0.2172521 | -0.0592 | 0.953 | -0.015675434 | count | 1 |
| AC245014.1 | -0.0355442 | 0.509621 | -0.0697 | 0.944 | -0.015661953 | count | 1 |
| BCL2L12 | -0.0134829 | 0.2017152 | -0.0668 | 0.947 | -0.015658223 | count | 1 |
| LLPH | -0.0113814 | 0.1280594 | -0.0889 | 0.929 | -0.015656244 | count | 1 |
| CYB5R4 | -0.0121378 | 0.1526705 | -0.0795 | 0.937 | -0.015557483 | count | 1 |
| MYO1E | -0.0291089 | 0.6958827 | -0.0418 | 0.967 | -0.015537957 | count | 1 |
| CAPN5 | -0.0291089 | 0.79922 | -0.0364 | 0.971 | -0.015537957 | count | 1 |
| MAP2K7 | -0.0135565 | 0.1893025 | -0.0716 | 0.943 | -0.015510947 | count | 1 |
| AC023509.4 | -0.0197907 | 0.3321231 | -0.0596 | 0.952 | -0.015439139 | count | 1 |
| SECISBP2L | -0.0112049 | 0.1333008 | -0.0841 | 0.933 | -0.015272656 | count | 1 |
| KIFAP3 | -0.0119492 | 0.1490468 | -0.0802 | 0.936 | -0.015250393 | count | 1 |
| P2RY8 | -0.0116343 | 0.1586752 | -0.0733 | 0.942 | -0.015176881 | count | 1 |
| DNM1L | -0.0113664 | 0.1269384 | -0.0895 | 0.929 | -0.015131706 | count | 1 |
| ABCB1 | -0.0137959 | 0.2351791 | -0.0587 | 0.953 | -0.015043477 | count | 1 |
| ZDHHC2 | -0.0112807 | 0.1547175 | -0.0729 | 0.942 | -0.0150318 | count | 1 |
| SMAD7 | -0.0118439 | 0.1850046 | -0.064 | 0.949 | -0.014972124 | count | 1 |
| TMEM79 | -0.0279846 | 0.6459895 | -0.0433 | 0.965 | -0.014940576 | count | 1 |
| NAE1 | -0.0108454 | 0.1160336 | -0.0935 | 0.926 | -0.01493369 | count | 1 |
| RPL36AL | -0.0103721 | 0.0230686 | -0.4496 | 0.653 | -0.014928011 | count | 1 |
| MFSD13A | -0.0203163 | 0.4345993 | -0.0467 | 0.963 | -0.014872357 | count | 1 |
| SLC25A3 | -0.0103403 | 0.0443234 | -0.2333 | 0.816 | -0.014815742 | count | 1 |
| CCP110 | -0.0116038 | 0.2243731 | -0.0517 | 0.959 | -0.014630194 | count | 1 |
| BLCAP | -0.0116392 | 0.1763879 | -0.066 | 0.947 | -0.014504702 | count | 1 |
| ITK | -0.0104054 | 0.0867291 | -0.12 | 0.905 | -0.014183073 | count | 1 |
| PIGS | -0.0125886 | 0.212778 | -0.0592 | 0.953 | -0.014160131 | count | 1 |
| NBPF14 | -0.0158622 | 0.3515662 | -0.0451 | 0.964 | -0.014151795 | count | 1 |
| RAB11B | -0.0103062 | 0.1113641 | -0.0925 | 0.926 | -0.01409809 | count | 1 |
| ACTG1 | -0.0097433 | 0.0255911 | -0.3807 | 0.703 | -0.014043065 | count | 1 |
| APEX2 | -0.0131015 | 0.3084196 | -0.0425 | 0.966 | -0.013928954 | count | 1 |

| | | | | | | | |
|---|---|---|---|---|---|---|---|
| LSM14B | -0.0126042 | 0.209082 | -0.0603 | 0.952 | -0.013899273 | count | 1 |
| PRKAA1 | -0.0101508 | 0.1183953 | -0.0857 | 0.932 | -0.013815843 | count | 1 |
| RPL31 | -0.0095288 | 0.0275562 | -0.3458 | 0.73 | -0.013693359 | count | 1 |
| RAB3GAP1 | -0.0109706 | 0.1694291 | -0.0648 | 0.948 | -0.013627631 | count | 1 |
| ATAD1 | -0.0100633 | 0.1262835 | -0.0797 | 0.936 | -0.013522116 | count | 1 |
| CD3EAP | -0.0412668 | 0.4274114 | -0.0966 | 0.923 | -0.013480882 | count | 1 |
| FITM2 | -0.0730062 | 0.711487 | -0.1026 | 0.918 | -0.013349786 | count | 1 |
| AL138781.1 | -0.0730062 | 1.0708052 | -0.0682 | 0.9456 | -0.013349786 | count | 1 |
| MLST8 | -0.0109364 | 0.2169491 | -0.0504 | 0.96 | -0.013277623 | count | 1 |
| ARGLU1 | -0.0091462 | 0.0517484 | -0.1767 | 0.8597 | -0.013061486 | count | 1 |
| PHF19 | -0.0096603 | 0.1517599 | -0.0637 | 0.949 | -0.012907767 | count | 1 |
| CENPL | -0.0118116 | 0.3346892 | -0.0353 | 0.972 | -0.01288135 | count | 1 |
| PLSCR2 | -0.0392218 | 0.9181612 | -0.0427 | 0.966 | -0.012818126 | count | 1 |
| TRIM31 | -0.0392218 | 1.0833183 | -0.0362 | 0.971 | -0.012818126 | count | 1 |
| BICDL2 | -0.0392218 | 1.0833183 | -0.0362 | 0.971 | -0.012818126 | count | 1 |
| OSCAR | -0.0392218 | 1.0781211 | -0.0364 | 0.971 | -0.012818126 | count | 1 |
| GUCY1B1 | -0.0392218 | 1.340908 | -0.0293 | 0.977 | -0.012818126 | count | 1 |
| ZNF670 | -0.0189043 | 0.6111037 | -0.0309 | 0.975 | -0.012791279 | count | 1 |
| CMAS | -0.0095893 | 0.1453083 | -0.066 | 0.947 | -0.012741462 | count | 1 |
| CAP1 | -0.008883 | 0.0505763 | -0.1756 | 0.861 | -0.012704957 | count | 1 |
| TAF7 | -0.0089251 | 0.0655513 | -0.1362 | 0.892 | -0.012623886 | count | 1 |
| AC005363.2 | -0.068848 | 0.7607898 | -0.0905 | 0.928 | -0.012601514 | count | 1 |
| ZNF503 | -0.068848 | 0.7946129 | -0.0866 | 0.931 | -0.012601514 | count | 1 |
| GNAI2 | -0.0088146 | 0.0487935 | -0.1807 | 0.8567 | -0.01259205 | count | 1 |
| CHST12 | -0.0089464 | 0.0859438 | -0.1041 | 0.917 | -0.012576641 | count | 1 |
| ROCK2 | -0.0095211 | 0.1570601 | -0.0606 | 0.952 | -0.012491225 | count | 1 |
| NUMB | -0.0113629 | 0.2056998 | -0.0552 | 0.956 | -0.012392363 | count | 1 |
| AL035587.1 | -0.067624 | 0.7445821 | -0.0908 | 0.928 | -0.012380974 | count | 1 |
| ELMO2 | -0.0113994 | 0.2730188 | -0.0418 | 0.967 | -0.012282263 | count | 1 |
| AL031777.3 | -0.0178328 | 0.4509641 | -0.0395 | 0.968 | -0.012068046 | count | 1 |
| TMEM258 | -0.0084847 | 0.0631888 | -0.1343 | 0.893 | -0.012027527 | count | 1 |
| ZNF286B | -0.0656581 | 0.9673153 | -0.0679 | 0.946 | -0.012026491 | count | 1 |
| LAPTM4A | -0.0084793 | 0.071584 | -0.1185 | 0.906 | -0.012002689 | count | 1 |
| TSPAN33 | -0.0223493 | 0.436921 | -0.0512 | 0.959 | -0.011942985 | count | 1 |
| ASF1A | -0.008415 | 0.0845023 | -0.0996 | 0.921 | -0.011847354 | count | 1 |
| CC2D1B | -0.0220467 | 0.3178489 | -0.0694 | 0.945 | -0.011781864 | count | 1 |
| TENT5C | -0.0082715 | 0.0692489 | -0.1194 | 0.905 | -0.011774047 | count | 1 |
| TBC1D17 | -0.0105295 | 0.2885241 | -0.0365 | 0.971 | -0.011733021 | count | 1 |
| HOXB2 | -0.0083855 | 0.1342062 | -0.0625 | 0.95 | -0.011603331 | count | 1 |
| NCOA7 | -0.0084252 | 0.1006645 | -0.0837 | 0.933 | -0.01159011 | count | 1 |
| AC009113.1 | -0.0632088 | 0.4356837 | -0.1451 | 0.885 | -0.011584384 | count | 1 |
| LRRC8B | -0.0158001 | 0.3464211 | -0.0456 | 0.964 | -0.011572987 | count | 1 |
| RPS15A | -0.008015 | 0.0137725 | -0.582 | 0.561 | -0.011556931 | count | 1 |
| GHRLOS | -0.0187798 | 0.744859 | -0.0252 | 0.98 | -0.011487343 | count | 1 |
| ANKFY1 | -0.0099238 | 0.2574535 | -0.0385 | 0.969 | -0.011444367 | count | 1 |

| | | | | | | | |
|---|---|---|---|---|---|---|---|
| IQCG | -0.0112454 | 0.2911269 | -0.0386 | 0.969 | -0.011395248 | count | 1 |
| AL357055.3 | -0.0619833 | 0.9338819 | -0.0664 | 0.947 | -0.011362985 | count | 1 |
| CDC42BPA | -0.0619833 | 0.9318405 | -0.0665 | 0.947 | -0.011362985 | count | 1 |
| ZNF763 | -0.0619833 | 1.045992 | -0.0593 | 0.953 | -0.011362985 | count | 1 |
| CEP170B | -0.0619833 | 1.148961 | -0.0539 | 0.957 | -0.011362985 | count | 1 |
| RNPS1 | -0.0080189 | 0.0710473 | -0.1129 | 0.91 | -0.011338184 | count | 1 |
| TMEM141 | -0.0083561 | 0.123904 | -0.0674 | 0.946 | -0.01129043 | count | 1 |
| CPNE5 | -0.0344801 | 0.6647435 | -0.0519 | 0.959 | -0.011279277 | count | 1 |
| GON4L | -0.0082463 | 0.1037668 | -0.0795 | 0.937 | -0.011240459 | count | 1 |
| GSTO2 | -0.0341105 | 0.625979 | -0.0545 | 0.957 | -0.0111592 | count | 1 |
| CMPK1 | -0.0079608 | 0.0765451 | -0.104 | 0.917 | -0.011157815 | count | 1 |
| PHKG1 | -0.0091136 | 0.2451121 | -0.0372 | 0.97 | -0.011065431 | count | 1 |
| SPATA7 | -0.0336671 | 0.538899 | -0.0625 | 0.95 | -0.01101513 | count | 1 |
| GINS4 | -0.0336657 | 0.3961727 | -0.085 | 0.932 | -0.011014674 | count | 1 |
| HOTAIRM1 | -0.0081835 | 0.1652685 | -0.0495 | 0.961 | -0.010934824 | count | 1 |
| NFX1 | -0.0087221 | 0.1918573 | -0.0455 | 0.964 | -0.010835429 | count | 1 |
| UBE2L6 | -0.007624 | 0.0765436 | -0.0996 | 0.921 | -0.01080242 | count | 1 |
| ANKDD1A | -0.0175118 | 0.3682826 | -0.0475 | 0.962 | -0.010713752 | count | 1 |
| NECAP2 | -0.0075586 | 0.0919904 | -0.0822 | 0.935 | -0.010545819 | count | 1 |
| GPKOW | -0.0081249 | 0.1855521 | -0.0438 | 0.965 | -0.0105326 | count | 1 |
| RNF149 | -0.0073782 | 0.0706543 | -0.1044 | 0.917 | -0.010438378 | count | 1 |
| MAN1C1 | -0.0101128 | 0.2918017 | -0.0347 | 0.972 | -0.010431025 | count | 1 |
| PNRC2 | -0.00726 | 0.065755 | -0.1104 | 0.912 | -0.010276247 | count | 1 |
| WBP2 | -0.0074209 | 0.1131204 | -0.0656 | 0.948 | -0.010268682 | count | 1 |
| LINC01970 | -0.0230792 | 0.5235447 | -0.0441 | 0.965 | -0.010192396 | count | 1 |
| ARL5B | -0.0082067 | 0.1809562 | -0.0454 | 0.964 | -0.010125025 | count | 1 |
| ENDOV | -0.0088356 | 0.2448851 | -0.0361 | 0.971 | -0.010111985 | count | 1 |
| FAM149B1 | -0.0550664 | 0.516451 | -0.1066 | 0.915 | -0.010110992 | count | 1 |
| MTHFD1 | -0.0116958 | 0.2508919 | -0.0466 | 0.963 | -0.010055491 | count | 1 |
| TRAF7 | -0.0084524 | 0.2154305 | -0.0392 | 0.969 | -0.010007263 | count | 1 |
| MDFIC | -0.0071109 | 0.0760769 | -0.0935 | 0.926 | -0.009989333 | count | 1 |
| NHLRC4 | -0.018622 | 0.7184517 | -0.0259 | 0.979 | -0.00995725 | count | 1 |
| MFSD8 | -0.0079575 | 0.1857766 | -0.0428 | 0.966 | -0.009917873 | count | 1 |
| NNT | -0.0078412 | 0.2211059 | -0.0355 | 0.972 | -0.009741398 | count | 1 |
| ERAL1 | -0.0085344 | 0.2570782 | -0.0332 | 0.974 | -0.009687332 | count | 1 |
| ZNF550 | -0.0097415 | 0.4231843 | -0.023 | 0.982 | -0.009680431 | count | 1 |
| CHRNA10 | -0.0526718 | 0.8388391 | -0.0628 | 0.95 | -0.009676613 | count | 1 |
| AC103691.1 | -0.0526718 | 0.8422179 | -0.0625 | 0.95 | -0.009676613 | count | 1 |
| NOCT | -0.0525863 | 0.4800186 | -0.1096 | 0.913 | -0.009661094 | count | 1 |
| GPR150 | -0.0293426 | 0.4477289 | -0.0655 | 0.948 | -0.009608603 | count | 1 |
| COL6A3 | -0.0293347 | 0.6207325 | -0.0473 | 0.962 | -0.009606031 | count | 1 |
| AC009118.2 | -0.0293347 | 0.6961832 | -0.0421 | 0.966 | -0.009606031 | count | 1 |
| FOPNL | -0.0073965 | 0.1591657 | -0.0465 | 0.963 | -0.009604502 | count | 1 |
| C17orf75 | -0.0071877 | 0.1728858 | -0.0416 | 0.967 | -0.009595984 | count | 1 |
| FOXN2 | -0.0069202 | 0.0955422 | -0.0724 | 0.942 | -0.009544061 | count | 1 |

| | | | | | | | |
|---|---|---|---|---|---|---|---|
| MIPEP | -0.0140817 | 0.4758149 | -0.0296 | 0.976 | -0.009534463 | count | 1 |
| ABHD12 | -0.0083255 | 0.2091832 | -0.0398 | 0.968 | -0.009528455 | count | 1 |
| SLC12A9 | -0.0081302 | 0.2162696 | -0.0376 | 0.97 | -0.009444468 | count | 1 |
| TOLLIP | -0.0070799 | 0.1655046 | -0.0428 | 0.966 | -0.009378432 | count | 1 |
| RPL41 | -0.0064831 | 0.0116086 | -0.5585 | 0.577 | -0.009349719 | count | 1 |
| RRP12 | -0.0507955 | 0.3790946 | -0.134 | 0.893 | -0.009335914 | count | 1 |
| ITGB3BP | -0.0071861 | 0.1899701 | -0.0378 | 0.97 | -0.009283361 | count | 1 |
| CHD1L | -0.0089957 | 0.2530469 | -0.0355 | 0.972 | -0.009279535 | count | 1 |
| FBXL17 | -0.0088161 | 0.2199891 | -0.0401 | 0.968 | -0.009240685 | count | 1 |
| DCAF13 | -0.0068132 | 0.1358172 | -0.0502 | 0.96 | -0.009155464 | count | 1 |
| APLP2 | -0.0069684 | 0.1618886 | -0.043 | 0.966 | -0.008968688 | count | 1 |
| HSCB | -0.0072897 | 0.20282 | -0.0359 | 0.971 | -0.008960713 | count | 1 |
| ARHGAP1 | -0.0076604 | 0.2108984 | -0.0363 | 0.971 | -0.008898933 | count | 1 |
| ATP6V1H | -0.0068339 | 0.1551077 | -0.0441 | 0.965 | -0.008812291 | count | 1 |
| PURB | -0.0073846 | 0.1843996 | -0.04 | 0.968 | -0.008792735 | count | 1 |
| SLC4A2 | -0.016361 | 0.3211996 | -0.0509 | 0.959 | -0.008751507 | count | 1 |
| EIF5A | -0.0060267 | 0.0541106 | -0.1114 | 0.911 | -0.008630338 | count | 1 |
| GIT1 | -0.0070185 | 0.2003593 | -0.035 | 0.972 | -0.008593975 | count | 1 |
| TRAPPC9 | -0.0077091 | 0.307941 | -0.025 | 0.98 | -0.008591672 | count | 1 |
| GIMAP7 | -0.0057671 | 0.0444713 | -0.1297 | 0.897 | -0.008296367 | count | 1 |
| AC018653.3 | -0.0447244 | 0.6004458 | -0.0745 | 0.941 | -0.008231482 | count | 1 |
| POC1A | -0.0447244 | 0.6213141 | -0.072 | 0.943 | -0.008231482 | count | 1 |
| SPPL2A | -0.0059211 | 0.1113514 | -0.0532 | 0.958 | -0.00821331 | count | 1 |
| ATXN7 | -0.0444014 | 0.317206 | -0.14 | 0.889 | -0.008172636 | count | 1 |
| ACVR2A | -0.0111141 | 0.3090708 | -0.036 | 0.971 | -0.008145519 | count | 1 |
| CENPBD1 | -0.0184157 | 0.7615259 | -0.0242 | 0.981 | -0.008139689 | count | 1 |
| PNISR | -0.0056762 | 0.0628746 | -0.0903 | 0.928 | -0.008067388 | count | 1 |
| RPL23A | -0.0055984 | 0.0173355 | -0.3229 | 0.747 | -0.008066246 | count | 1 |
| SLC25A17 | -0.0072292 | 0.2495391 | -0.029 | 0.977 | -0.008057057 | count | 1 |
| CCDC59 | -0.0055951 | 0.0888547 | -0.063 | 0.95 | -0.007874583 | count | 1 |
| TOR1B | -0.0061695 | 0.2433089 | -0.0254 | 0.98 | -0.007779946 | count | 1 |
| LRIF1 | -0.0056126 | 0.1390195 | -0.0404 | 0.968 | -0.007687488 | count | 1 |
| MCCC1 | -0.0072754 | 0.30094 | -0.0242 | 0.981 | -0.007626612 | count | 1 |
| PDIA6 | -0.0053124 | 0.0670263 | -0.0793 | 0.937 | -0.007548309 | count | 1 |
| STAT6 | -0.0067326 | 0.2144588 | -0.0314 | 0.975 | -0.007503807 | count | 1 |
| P2RY10 | -0.0055107 | 0.1012533 | -0.0544 | 0.957 | -0.007500898 | count | 1 |
| LRRC28 | -0.006506 | 0.2527352 | -0.0257 | 0.979 | -0.007446776 | count | 1 |
| MT1G | -0.0108212 | 1.0630992 | -0.0102 | 0.992 | -0.007330111 | count | 1 |
| REX1BD | -0.0051336 | 0.0575918 | -0.0891 | 0.929 | -0.007328225 | count | 1 |
| STOM | -0.0052027 | 0.0753068 | -0.0691 | 0.945 | -0.007321669 | count | 1 |
| ACSF2 | -0.0222113 | 0.4435011 | -0.0501 | 0.96 | -0.00728378 | count | 1 |
| ZNF75D | -0.0106741 | 0.3009228 | -0.0355 | 0.972 | -0.007230613 | count | 1 |
| DLGAP4 | -0.0056364 | 0.1642098 | -0.0343 | 0.973 | -0.007210585 | count | 1 |
| DHX32 | -0.0219671 | 0.4824373 | -0.0455 | 0.964 | -0.007204051 | count | 1 |
| RPL36 | -0.0049854 | 0.021906 | -0.2276 | 0.82 | -0.007174873 | count | 1 |

| | | | | | | | |
|---|---|---|---|---|---|---|---|
| NDUFAF7 | -0.0061233 | 0.2279884 | -0.0269 | 0.979 | -0.007161953 | count | 1 |
| ANKS6 | -0.0132936 | 0.4726729 | -0.0281 | 0.978 | -0.007114299 | count | 1 |
| DISC1 | -0.0215089 | 0.5230752 | -0.0411 | 0.967 | -0.007054432 | count | 1 |
| UMPS | -0.0062154 | 0.2462049 | -0.0252 | 0.98 | -0.006927573 | count | 1 |
| CAAP1 | -0.0050631 | 0.1499105 | -0.0338 | 0.973 | -0.006721244 | count | 1 |
| ZNF282 | -0.0060073 | 0.3471564 | -0.0173 | 0.986 | -0.00669571 | count | 1 |
| WDR4 | -0.0150472 | 0.4943326 | -0.0304 | 0.976 | -0.006654838 | count | 1 |
| ETFA | -0.0047152 | 0.105227 | -0.0448 | 0.964 | -0.006594046 | count | 1 |
| ZNF821 | -0.0051626 | 0.1928284 | -0.0268 | 0.979 | -0.006510424 | count | 1 |
| RASGRF2 | -0.00957 | 0.3009435 | -0.0318 | 0.975 | -0.006483677 | count | 1 |
| CELF2 | -0.0046081 | 0.0703324 | -0.0655 | 0.948 | -0.006472165 | count | 1 |
| YWHAG | -0.0048143 | 0.1284065 | -0.0375 | 0.97 | -0.006444224 | count | 1 |
| ZNF524 | -0.0046737 | 0.1197267 | -0.039 | 0.969 | -0.006404101 | count | 1 |
| ZC3H7A | -0.0047003 | 0.1182917 | -0.0397 | 0.968 | -0.006366761 | count | 1 |
| SDHAF3 | -0.0046834 | 0.1532997 | -0.0306 | 0.976 | -0.006351193 | count | 1 |
| ZNF443 | -0.014132 | 0.4447331 | -0.0318 | 0.975 | -0.006251101 | count | 1 |
| FLOT2 | -0.0049426 | 0.1738317 | -0.0284 | 0.977 | -0.006120284 | count | 1 |
| CCNT2 | -0.0047028 | 0.1721144 | -0.0273 | 0.978 | -0.006016405 | count | 1 |
| MT-ATP8 | -0.0047599 | 0.1338002 | -0.0356 | 0.972 | -0.006002667 | count | 1 |
| YDJC | -0.0042649 | 0.1096423 | -0.0389 | 0.969 | -0.005923869 | count | 1 |
| CHURC1 | -0.0041441 | 0.0757316 | -0.0547 | 0.956 | -0.005867451 | count | 1 |
| TNS3 | -0.0108212 | 1.0962291 | -0.0099 | 0.992 | -0.005793474 | count | 1 |
| MGST1 | -0.0108212 | 1.0962291 | -0.0099 | 0.992 | -0.005793474 | count | 1 |
| IGSF10 | -0.0108212 | 1.2343583 | -0.0088 | 0.993 | -0.005793474 | count | 1 |
| HAT1 | -0.0042191 | 0.1187483 | -0.0355 | 0.972 | -0.005766981 | count | 1 |
| EPHX2 | -0.005597 | 0.2242106 | -0.025 | 0.98 | -0.005674038 | count | 1 |
| HOOK2 | -0.0045361 | 0.2061512 | -0.022 | 0.982 | -0.0056718 | count | 1 |
| AC108134.2 | -0.0171005 | 0.5486883 | -0.0312 | 0.975 | -0.005613518 | count | 1 |
| ANKRD49 | -0.0041643 | 0.1669965 | -0.0249 | 0.98 | -0.005574228 | count | 1 |
| DDT | -0.0039265 | 0.0686482 | -0.0572 | 0.954 | -0.005556388 | count | 1 |
| NUP62 | -0.0042691 | 0.1706885 | -0.025 | 0.98 | -0.005552901 | count | 1 |
| WAS | -0.0039516 | 0.085113 | -0.0464 | 0.963 | -0.005532321 | count | 1 |
| BFAR | -0.0039336 | 0.0963342 | -0.0408 | 0.967 | -0.005485597 | count | 1 |
| SARS | -0.0039256 | 0.0925085 | -0.0424 | 0.966 | -0.005449067 | count | 1 |
| XRCC6 | -0.0038149 | 0.0628965 | -0.0607 | 0.952 | -0.005407943 | count | 1 |
| LIMD1 | -0.0100433 | 0.4502654 | -0.0223 | 0.982 | -0.005377679 | count | 1 |
| CFL2 | -0.0045082 | 0.2452143 | -0.0184 | 0.985 | -0.005338478 | count | 1 |
| AL445686.2 | -0.0044867 | 0.2282792 | -0.0197 | 0.984 | -0.005248166 | count | 1 |
| KAT5 | -0.0041627 | 0.1981043 | -0.021 | 0.983 | -0.005220443 | count | 1 |
| PRPF31 | -0.0037633 | 0.1050651 | -0.0358 | 0.971 | -0.005216652 | count | 1 |
| CPNE8 | -0.004544 | 0.2194008 | -0.0207 | 0.983 | -0.005158989 | count | 1 |
| PRKRIP1 | -0.003841 | 0.139259 | -0.0276 | 0.978 | -0.005132782 | count | 1 |
| LENG8 | -0.0042924 | 0.202503 | -0.0212 | 0.983 | -0.004987237 | count | 1 |
| FBRSL1 | -0.0050375 | 0.2354273 | -0.0214 | 0.983 | -0.004899001 | count | 1 |
| VSIG10L | -0.007834 | 0.8630766 | -0.0091 | 0.993 | -0.004799751 | count | 1 |

| | | | | | | | |
|---|---|---|---|---|---|---|---|
| AKR1A1 | -0.0034236 | 0.1036839 | -0.033 | 0.974 | -0.004780654 | count | 1 |
| CLP1 | -0.0037001 | 0.1589396 | -0.0233 | 0.981 | -0.004771675 | count | 1 |
| ARF4 | -0.003362 | 0.0698932 | -0.0481 | 0.962 | -0.004745417 | count | 1 |
| SIGIRR | -0.0032954 | 0.0592517 | -0.0556 | 0.956 | -0.004693654 | count | 1 |
| TMEM134 | -0.0033321 | 0.1023688 | -0.0326 | 0.974 | -0.004668664 | count | 1 |
| CCNJ | -0.010544 | 0.7550524 | -0.014 | 0.989 | -0.004666989 | count | 1 |
| IER5L | -0.0033687 | 0.1319173 | -0.0255 | 0.98 | -0.004614189 | count | 1 |
| PACS2 | -0.003991 | 0.2775811 | -0.0144 | 0.989 | -0.004491418 | count | 1 |
| FPR1 | -0.0060949 | 0.6677665 | -0.0091 | 0.993 | -0.004469789 | count | 1 |
| POLR1D | -0.0031238 | 0.0636815 | -0.0491 | 0.961 | -0.004429456 | count | 1 |
| TOE1 | -0.0037856 | 0.2463308 | -0.0154 | 0.988 | -0.004428229 | count | 1 |
| PSME1 | -0.0030212 | 0.0340618 | -0.0887 | 0.929 | -0.004345368 | count | 1 |
| TMSB15B | -0.0228613 | 0.420104 | -0.0544 | 0.957 | -0.00422851 | count | 1 |
| POLR3C | -0.0036306 | 0.2605473 | -0.0139 | 0.989 | -0.004188257 | count | 1 |
| TMEM68 | -0.0036121 | 0.2576209 | -0.014 | 0.989 | -0.004166919 | count | 1 |
| TTC30B | -0.0224864 | 0.7034701 | -0.032 | 0.975 | -0.004159519 | count | 1 |
| DAZAP1 | -0.0029791 | 0.0833698 | -0.0357 | 0.971 | -0.004143899 | count | 1 |
| PAAF1 | -0.0034107 | 0.2528077 | -0.0135 | 0.989 | -0.004123482 | count | 1 |
| COPS9 | -0.0028991 | 0.0669218 | -0.0433 | 0.965 | -0.004111023 | count | 1 |
| LITAF | -0.002801 | 0.0582544 | -0.0481 | 0.962 | -0.004003935 | count | 1 |
| EML5 | -0.0121278 | 0.7503379 | -0.0162 | 0.987 | -0.003985093 | count | 1 |
| MFSD4A | -0.0121278 | 0.847594 | -0.0143 | 0.989 | -0.003985093 | count | 1 |
| WSB1 | -0.0027241 | 0.0797055 | -0.0342 | 0.973 | -0.003772515 | count | 1 |
| TFCP2 | -0.0035933 | 0.2881572 | -0.0125 | 0.99 | -0.003767737 | count | 1 |
| PPIP5K2 | -0.0028053 | 0.1383621 | -0.0203 | 0.984 | -0.003748835 | count | 1 |
| DIMT1 | -0.0027896 | 0.1326719 | -0.021 | 0.983 | -0.003674443 | count | 1 |
| PMF1-BGLAP | -0.0108212 | 1.2343583 | -0.0088 | 0.993 | -0.003556679 | count | 1 |
| AL356272.1 | -0.0108212 | 1.2343583 | -0.0088 | 0.993 | -0.003556679 | count | 1 |
| AC012485.3 | -0.0108212 | 1.2343583 | -0.0088 | 0.993 | -0.003556679 | count | 1 |
| AC124944.1 | -0.0108212 | 1.2343583 | -0.0088 | 0.993 | -0.003556679 | count | 1 |
| AC010273.1 | -0.0108212 | 1.2343583 | -0.0088 | 0.993 | -0.003556679 | count | 1 |
| LUCAT1 | -0.0108212 | 1.2343583 | -0.0088 | 0.993 | -0.003556679 | count | 1 |
| TRBV30 | -0.0108212 | 1.2343583 | -0.0088 | 0.993 | -0.003556679 | count | 1 |
| CSF2RA | -0.0108212 | 1.2343583 | -0.0088 | 0.993 | -0.003556679 | count | 1 |
| TMEM136 | -0.0108212 | 1.2343583 | -0.0088 | 0.993 | -0.003556679 | count | 1 |
| LRP6 | -0.0108212 | 1.2343583 | -0.0088 | 0.993 | -0.003556679 | count | 1 |
| AC087241.3 | -0.0108212 | 1.2343583 | -0.0088 | 0.993 | -0.003556679 | count | 1 |
| AL358333.2 | -0.0108212 | 1.2343583 | -0.0088 | 0.993 | -0.003556679 | count | 1 |
| AC091153.3 | -0.0108212 | 1.2343583 | -0.0088 | 0.993 | -0.003556679 | count | 1 |
| TTC25 | -0.0108212 | 1.2343583 | -0.0088 | 0.993 | -0.003556679 | count | 1 |
| RUNDC3A-AS1 | -0.0108212 | 1.2343583 | -0.0088 | 0.993 | -0.003556679 | count | 1 |
| PTPRS | -0.0108212 | 1.2343583 | -0.0088 | 0.993 | -0.003556679 | count | 1 |
| AC020907.3 | -0.0108212 | 1.2343583 | -0.0088 | 0.993 | -0.003556679 | count | 1 |
| TRAK2 | -0.0029891 | 0.2131239 | -0.014 | 0.989 | -0.003539852 | count | 1 |
| RAD51B | -0.0034257 | 0.3617764 | -0.0095 | 0.992 | -0.003473428 | count | 1 |

| | | | | | | | |
|---|---|---|---|---|---|---|---|
| AAMP | -0.0025562 | 0.1418553 | -0.018 | 0.986 | -0.003445068 | count | 1 |
| MDH1 | -0.002426 | 0.067369 | -0.036 | 0.971 | -0.003441633 | count | 1 |
| TIGIT | -0.0024363 | 0.1008321 | -0.0242 | 0.981 | -0.003405156 | count | 1 |
| NFXL1 | -0.0045543 | 0.2835737 | -0.0161 | 0.987 | -0.003340617 | count | 1 |
| TSPOAP1-AS1 | -0.0026828 | 0.2231651 | -0.012 | 0.99 | -0.00325822 | count | 1 |
| CBWD5 | -0.0026839 | 0.1813023 | -0.0148 | 0.988 | -0.003213347 | count | 1 |
| THOC1 | -0.0025208 | 0.1587875 | -0.0159 | 0.987 | -0.003195599 | count | 1 |
| PSPC1 | -0.0024322 | 0.1438324 | -0.0169 | 0.987 | -0.003153416 | count | 1 |
| PKN2 | -0.0022424 | 0.0953386 | -0.0235 | 0.981 | -0.003129779 | count | 1 |
| BRMS1 | -0.0021697 | 0.1083894 | -0.02 | 0.984 | -0.003000932 | count | 1 |
| DGKD | -0.0023844 | 0.2111421 | -0.0113 | 0.991 | -0.002931521 | count | 1 |
| NUDT4 | -0.0022369 | 0.1378776 | -0.0162 | 0.987 | -0.002931245 | count | 1 |
| MSANTD4 | -0.0023046 | 0.2790242 | -0.0083 | 0.993 | -0.002786353 | count | 1 |
| LMOD1 | -0.008355 | 0.6986981 | -0.012 | 0.99 | -0.002747442 | count | 1 |
| HIPK2 | -0.0026413 | 0.2451015 | -0.0108 | 0.991 | -0.002725908 | count | 1 |
| FAM83D | -0.0036565 | 0.3643511 | -0.01 | 0.992 | -0.002682377 | count | 1 |
| MFF | -0.0018679 | 0.1154263 | -0.0162 | 0.987 | -0.002580012 | count | 1 |
| RPL22L1 | -0.0017866 | 0.0565269 | -0.0316 | 0.975 | -0.002547451 | count | 1 |
| AC136475.5 | -0.0132981 | 0.5430653 | -0.0245 | 0.98 | -0.002464964 | count | 1 |
| RDH5 | -0.0023613 | 0.3842784 | -0.0061 | 0.995 | -0.002436988 | count | 1 |
| MEF2D | -0.0019671 | 0.1914249 | -0.0103 | 0.992 | -0.002378335 | count | 1 |
| AP003774.4 | -0.0017835 | 0.2311497 | -0.0077 | 0.994 | -0.002300134 | count | 1 |
| GPATCH2L | -0.0016743 | 0.1506459 | -0.0111 | 0.991 | -0.0021671 | count | 1 |
| CEP70 | -0.0026714 | 0.3243414 | -0.0082 | 0.993 | -0.002088243 | count | 1 |
| SAP25 | -0.0046594 | 0.2861579 | -0.0163 | 0.987 | -0.002064509 | count | 1 |
| CCNL1 | -0.0014365 | 0.0529774 | -0.0271 | 0.978 | -0.002038884 | count | 1 |
| MCCC2 | -0.0016087 | 0.216273 | -0.0074 | 0.994 | -0.002005457 | count | 1 |
| TRAM1 | -0.001343 | 0.0627669 | -0.0214 | 0.983 | -0.001906621 | count | 1 |
| PRKCQ | -0.0012443 | 0.142294 | -0.0087 | 0.993 | -0.001607705 | count | 1 |
| TNKS2-AS1 | -0.0047522 | 0.7743725 | -0.0061 | 0.995 | -0.001563821 | count | 1 |
| CHML | -0.0017726 | 0.3296047 | -0.0054 | 0.996 | -0.001525569 | count | 1 |
| PUS10 | -0.0016416 | 0.2960427 | -0.0055 | 0.996 | -0.001514693 | count | 1 |
| POLR2A | -0.0010936 | 0.13076 | -0.0084 | 0.993 | -0.001448826 | count | 1 |
| CDC37 | -0.0010047 | 0.0629493 | -0.016 | 0.987 | -0.00142433 | count | 1 |
| NME7 | -0.0011606 | 0.2338279 | -0.005 | 0.996 | -0.001396627 | count | 1 |
| PKNOX1 | -0.0011216 | 0.2108314 | -0.0053 | 0.996 | -0.00138414 | count | 1 |
| THEM4 | -0.0008831 | 0.1321136 | -0.0067 | 0.995 | -0.001183137 | count | 1 |
| ZNF79 | -0.0024586 | 0.4546657 | -0.0054 | 0.996 | -0.001089794 | count | 1 |
| STX18 | -0.000878 | 0.182678 | -0.0048 | 0.996 | -0.001070973 | count | 1 |
| ZC3HC1 | -0.0007873 | 0.2828013 | -0.0028 | 0.998 | -0.000964262 | count | 1 |
| AC004982.2 | -0.004453 | 0.6047861 | -0.0074 | 0.994 | -0.000827054 | count | 1 |
| FLT3LG | -0.0005546 | 0.0874136 | -0.0063 | 0.995 | -0.00078145 | count | 1 |
| SGSM3 | -0.0020883 | 0.6192173 | -0.0034 | 0.997 | -0.000687566 | count | 1 |
| DENND6A | -0.0006759 | 0.2296921 | -0.0029 | 0.998 | -0.000657557 | count | 1 |
| AC026304.1 | -0.0028456 | 0.7358316 | -0.0039 | 0.997 | -0.000528702 | count | 1 |

| | | | | | | | |
|---|---|---|---|---|---|---|---|
| GPATCH8 | -0.0003366 | 0.1040487 | -0.0032 | 0.997 | -0.000463689 | count | 1 |
| OS9 | -0.0003165 | 0.1138959 | -0.0028 | 0.998 | -0.00043062 | count | 1 |
| MPPE1 | -0.0003089 | 0.1704749 | -0.0018 | 0.999 | -0.00039063 | count | 1 |
| C1orf56 | -0.0001267 | 0.0848367 | -0.0015 | 0.999 | -0.000178069 | count | 1 |
| CASP1 | -8.16E-05 | 0.0864741 | -9.00E-04 | 0.999 | -0.000115518 | count | 1 |
| TUBD1 | -3.98E-05 | 0.1774161 | -2.00E-04 | 1 | -4.99E-05 | count | 1 |
| IGHG2 | -1.597141 | 0.3761618 | -4.2459 | 2.24E-05 | -2.34E-07 | count | 0.5376448 |
| CCL19 | -1.4508893 | 0.3867946 | -3.7511 | 2.00E-04 | -2.14E-07 | count | 1 |
| ZNF80 | -0.8833512 | 0.5268271 | -1.6767 | 0.0937 | -1.53E-07 | count | 1 |
| ANGPT2 | -0.8634433 | 0.4148518 | -2.0813 | 0.0375 | -1.50E-07 | count | 1 |
| MAP1A | -0.7590089 | 0.4121591 | -1.8415 | 0.0656 | -1.36E-07 | count | 1 |
| AACS | -0.6003766 | 0.3439772 | -1.7454 | 0.081 | -1.12E-07 | count | 1 |
| AL158163.1 | -2.580035 | 1.3163089 | -1.9601 | 0.0501 | -1.01E-07 | count | 1 |
| ZNF319 | -2.569656 | 1.1297473 | -2.2745 | 0.023 | -1.01E-07 | count | 1 |
| PRMT5 | -0.5235175 | 0.2811527 | -1.862 | 0.0627 | -1.00E-07 | count | 1 |
| PPP2R3A | -2.391153 | 1.0411366 | -2.2967 | 0.0217 | -9.88E-08 | count | 1 |
| RCN3 | -2.3543935 | 1.2080528 | -1.9489 | 0.0514 | -9.82E-08 | count | 1 |
| COL14A1 | -2.018065 | 0.8885959 | -2.2711 | 0.0232 | -9.27E-08 | count | 1 |
| CALCRL | -1.7516803 | 0.7581551 | -2.3105 | 0.0209 | -8.66E-08 | count | 1 |
| WDR19 | -1.6962703 | 0.6616774 | -2.5636 | 0.0104 | -8.51E-08 | count | 1 |
| LTBP2 | -1.6384063 | 0.9716427 | -1.6862 | 0.0918 | -8.35E-08 | count | 1 |
| MPZ | -1.5697097 | 0.9514774 | -1.6498 | 0.0991 | -8.16E-08 | count | 1 |
| EXPH5 | -1.5697097 | 0.836282 | -1.877 | 0.0606 | -8.16E-08 | count | 1 |
| ZNF703 | -0.4140558 | 0.370271 | -1.1183 | 0.264 | -8.15E-08 | count | 1 |
| DNASE1L3 | -1.5638983 | 0.6635379 | -2.3569 | 0.0185 | -8.15E-08 | count | 1 |
| AL731577.1 | -0.412176 | 0.3309268 | -1.2455 | 0.213 | -8.12E-08 | count | 1 |
| KIF16B | -0.4104396 | 0.38656 | -1.0618 | 0.288 | -8.09E-08 | count | 1 |
| FANCC | -1.5352561 | 0.9777773 | -1.5701 | 0.116 | -8.06E-08 | count | 1 |
| CDYL2 | -1.520957 | 0.5762012 | -2.6396 | 0.0083 | -8.05E-08 | count | 1 |
| TMEM8B | -1.5173332 | 0.6203931 | -2.4458 | 0.0145 | -8.02E-08 | count | 1 |
| ITGA8 | -1.4922048 | 1.0053363 | -1.4843 | 0.138 | -7.93E-08 | count | 1 |
| MIR34AHG | -1.4491891 | 1.4392264 | -1.0069 | 0.314 | -7.79E-08 | count | 1 |
| BORA | -0.3909881 | 0.327489 | -1.1939 | 0.233 | -7.76E-08 | count | 1 |
| PLAT | -1.3963755 | 0.4744177 | -2.9433 | 0.0033 | -7.68E-08 | count | 1 |
| DEPP1 | -1.398298 | 0.7990229 | -1.75 | 0.0802 | -7.64E-08 | count | 1 |
| DENND2C | -1.3723402 | 0.7269468 | -1.8878 | 0.0591 | -7.55E-08 | count | 1 |
| KIF23 | -1.339843 | 0.6585828 | -2.0344 | 0.042 | -7.44E-08 | count | 1 |
| RNASEH2A | -1.3265241 | 0.6658266 | -1.9923 | 0.0464 | -7.40E-08 | count | 1 |
| SAMD4A | -1.3022951 | 0.6476264 | -2.0109 | 0.0444 | -7.32E-08 | count | 1 |
| JAG1 | -1.2787308 | 0.6465079 | -1.9779 | 0.048 | -7.23E-08 | count | 1 |
| U2AF1 | -1.2787308 | 0.6649667 | -1.923 | 0.0546 | -7.23E-08 | count | 1 |
| EPOP | -1.2783234 | 0.6553315 | -1.9507 | 0.0512 | -7.23E-08 | count | 1 |
| ZNF154 | -1.2113975 | 0.8236377 | -1.4708 | 0.141 | -6.98E-08 | count | 1 |
| ZNF404 | -1.2009103 | 0.7692672 | -1.5611 | 0.119 | -6.95E-08 | count | 1 |
| RGS12 | -1.175838 | 0.5884292 | -1.9983 | 0.0458 | -6.87E-08 | count | 1 |

| | | | | | | | |
|---|---|---|---|---|---|---|---|
| AC010542.2 | -1.1768387 | 0.7856919 | -1.4978 | 0.134 | -6.85E-08 | count | 1 |
| AC246785.3 | -1.175838 | 0.6438476 | -1.8263 | 0.0679 | -6.85E-08 | count | 1 |
| IL22 | -1.1748185 | 1.5221288 | -0.7718 | 0.44 | -6.84E-08 | count | 1 |
| TCEAL9 | -1.1388049 | 0.7246026 | -1.5716 | 0.116 | -6.70E-08 | count | 1 |
| PLN | -1.0923737 | 0.527901 | -2.0693 | 0.0386 | -6.52E-08 | count | 1 |
| TMEM201 | -1.0916881 | 0.5716944 | -1.9096 | 0.0563 | -6.52E-08 | count | 1 |
| ZNF436 | -1.0597504 | 0.4979054 | -2.1284 | 0.0334 | -6.39E-08 | count | 1 |
| JRK | -1.0231228 | 0.5851986 | -1.7483 | 0.0805 | -6.24E-08 | count | 1 |
| JDP2 | -1.0088522 | 0.5810057 | -1.7364 | 0.0826 | -6.18E-08 | count | 1 |
| SLC4A8 | -1.0048483 | 0.9369981 | -1.0724 | 0.284 | -6.15E-08 | count | 1 |
| AC006942.1 | -0.9954115 | 0.5901797 | -1.6866 | 0.0918 | -6.11E-08 | count | 1 |
| AC138207.7 | -0.9799282 | 0.7599077 | -1.2895 | 0.197 | -6.04E-08 | count | 1 |
| CPEB3 | -0.9799282 | 0.821898 | -1.1923 | 0.233 | -6.04E-08 | count | 1 |
| AP000487.1 | -0.9799282 | 1.0213714 | -0.9594 | 0.337 | -6.04E-08 | count | 1 |
| MANSC1 | -0.9678703 | 0.5831343 | -1.6598 | 0.0971 | -5.99E-08 | count | 1 |
| AC118553.2 | -0.9386625 | 0.8184963 | -1.1468 | 0.252 | -5.85E-08 | count | 1 |
| ZNF653 | -0.935139 | 0.5239405 | -1.7848 | 0.0744 | -5.85E-08 | count | 1 |
| CYP1B1 | -0.905705 | 0.9664568 | -0.9371 | 0.349 | -5.70E-08 | count | 1 |
| MAPKBP1 | -0.9038857 | 0.508803 | -1.7765 | 0.0757 | -5.70E-08 | count | 1 |
| LINC02076 | -0.8965072 | 0.6901863 | -1.2989 | 0.194 | -5.66E-08 | count | 1 |
| MED20 | -0.8819952 | 0.5180468 | -1.7025 | 0.0887 | -5.59E-08 | count | 1 |
| SYNPO2 | -0.8556292 | 0.6348622 | -1.3477 | 0.178 | -5.47E-08 | count | 1 |
| CAMKK1 | -0.8338498 | 0.7567362 | -1.1019 | 0.271 | -5.36E-08 | count | 1 |
| ZIK1 | -0.8198301 | 0.4979398 | -1.6464 | 0.0998 | -5.30E-08 | count | 1 |
| FGF7 | -0.8030314 | 0.4532409 | -1.7718 | 0.0765 | -5.22E-08 | count | 1 |
| SPRED2 | -0.8019011 | 0.62749 | -1.278 | 0.201 | -5.20E-08 | count | 1 |
| ADM2 | -0.7999193 | 0.8305732 | -0.9631 | 0.336 | -5.19E-08 | count | 1 |
| CUBN | -0.7999193 | 0.7353107 | -1.0879 | 0.277 | -5.19E-08 | count | 1 |
| RIMS3 | -0.7854013 | 0.6761556 | -1.1616 | 0.245 | -5.12E-08 | count | 1 |
| GCAT | -0.7788053 | 0.6709497 | -1.1608 | 0.246 | -5.09E-08 | count | 1 |
| UACA | -0.7541259 | 0.6810958 | -1.1072 | 0.268 | -4.96E-08 | count | 1 |
| AC005224.3 | -0.2400129 | 0.370443 | -0.6479 | 0.517 | -4.95E-08 | count | 1 |
| KIF3C | -0.7500531 | 1.1913253 | -0.6296 | 0.529 | -4.94E-08 | count | 1 |
| WDR78 | -0.7291838 | 0.6489966 | -1.1236 | 0.261 | -4.83E-08 | count | 1 |
| LINC01521 | -0.7180187 | 0.7480443 | -0.9599 | 0.337 | -4.77E-08 | count | 1 |
| CCL23 | -0.7163264 | 0.7287367 | -0.983 | 0.326 | -4.76E-08 | count | 1 |
| HLF | -0.70958 | 0.6611921 | -1.0732 | 0.283 | -4.73E-08 | count | 1 |
| PGBD1 | -0.7046625 | 0.6764497 | -1.0417 | 0.298 | -4.70E-08 | count | 1 |
| AFG1L | -0.690364 | 0.6777725 | -1.0186 | 0.308 | -4.62E-08 | count | 1 |
| HIST1H4J | -0.6744027 | 0.6426123 | -1.0495 | 0.294 | -4.54E-08 | count | 1 |
| CATSPER2 | -0.6666389 | 0.5271056 | -1.2647 | 0.206 | -4.50E-08 | count | 1 |
| AC011498.1 | -0.6666389 | 0.6806889 | -0.9794 | 0.327 | -4.49E-08 | count | 1 |
| PLXDC2 | -0.6558272 | 0.6087819 | -1.0773 | 0.281 | -4.44E-08 | count | 1 |
| FOXC1 | -0.6508119 | 0.7862605 | -0.8277 | 0.408 | -4.41E-08 | count | 1 |
| PAN3-AS1 | -0.6508119 | 0.787283 | -0.8267 | 0.408 | -4.41E-08 | count | 1 |

| | | | | | | | |
|---|---|---|---|---|---|---|---|
| SPIN3 | -0.6442042 | 0.6350062 | -1.0145 | 0.31 | -4.37E-08 | count | 1 |
| COL6A1 | -0.6335418 | 0.7441232 | -0.8514 | 0.395 | -4.31E-08 | count | 1 |
| AL353708.1 | -0.6280121 | 0.6926116 | -0.9067 | 0.365 | -4.28E-08 | count | 1 |
| ZDHHC9 | -0.6246271 | 0.5012727 | -1.2461 | 0.213 | -4.26E-08 | count | 1 |
| AC022364.1 | -0.6023298 | 0.5260221 | -1.1451 | 0.252 | -4.14E-08 | count | 1 |
| SPACA9 | -0.5999251 | 0.5802585 | -1.0339 | 0.301 | -4.12E-08 | count | 1 |
| HLA-DQA2 | -0.1961554 | 0.1035078 | -1.8951 | 0.0582 | -4.10E-08 | count | 1 |
| ERMN | -0.5930478 | 0.6181372 | -0.9594 | 0.337 | -4.08E-08 | count | 1 |
| TRAV6 | -0.5930478 | 0.5693098 | -1.0417 | 0.298 | -4.08E-08 | count | 1 |
| RBP7 | -0.5702521 | 0.6478448 | -0.8802 | 0.379 | -3.95E-08 | count | 1 |
| PTGIR | -0.5696068 | 0.6867041 | -0.8295 | 0.407 | -3.95E-08 | count | 1 |
| AC018557.1 | -0.5696068 | 0.8313681 | -0.6851 | 0.493 | -3.95E-08 | count | 1 |
| KRT1 | -0.5690096 | 0.5173923 | -1.0998 | 0.272 | -3.95E-08 | count | 1 |
| TNK1 | -0.5611077 | 0.578941 | -0.9692 | 0.333 | -3.90E-08 | count | 1 |
| ZFP57 | -0.5559286 | 0.509143 | -1.0919 | 0.275 | -3.87E-08 | count | 1 |
| AL360270.1 | -0.5274083 | 0.6306759 | -0.8363 | 0.403 | -3.70E-08 | count | 1 |
| AC132192.1 | -0.5182529 | 0.4546867 | -1.1398 | 0.254 | -3.64E-08 | count | 1 |
| HHAT | -0.5167695 | 0.4991466 | -1.0353 | 0.301 | -3.64E-08 | count | 1 |
| RARRES1 | -2.2713325 | 1.3245193 | -1.7148 | 0.0865 | -3.56E-08 | count | 1 |
| U73166.1 | -2.1864583 | 1.2902379 | -1.6946 | 0.0902 | -3.51E-08 | count | 1 |
| UST | -0.4965274 | 0.466762 | -1.0638 | 0.288 | -3.51E-08 | count | 1 |
| TMTC4 | -0.4952118 | 0.450116 | -1.1002 | 0.271 | -3.50E-08 | count | 1 |
| AGAP9 | -0.4922593 | 0.4599297 | -1.0703 | 0.285 | -3.49E-08 | count | 1 |
| TRAV8-3 | -0.4922593 | 0.670197 | -0.7345 | 0.463 | -3.49E-08 | count | 1 |
| ZNF460-AS1 | -2.1415922 | 1.2349304 | -1.7342 | 0.083 | -3.48E-08 | count | 1 |
| NAV1 | -2.1297287 | 1.1460158 | -1.8584 | 0.0632 | -3.48E-08 | count | 1 |
| DNAAF1 | -2.1069668 | 1.4695537 | -1.4337 | 0.152 | -3.46E-08 | count | 1 |
| AL662797.1 | -2.1069668 | 1.1115202 | -1.8956 | 0.0581 | -3.46E-08 | count | 1 |
| PEX5 | -0.4876748 | 0.4511612 | -1.0809 | 0.28 | -3.46E-08 | count | 1 |
| GPR34 | -2.0949711 | 0.9829174 | -2.1314 | 0.0331 | -3.45E-08 | count | 1 |
| AC084036.1 | -0.4858312 | 0.6825383 | -0.7118 | 0.477 | -3.45E-08 | count | 1 |
| PAK3 | -0.4838716 | 0.5786786 | -0.8362 | 0.403 | -3.43E-08 | count | 1 |
| CYP3A5 | -0.4838716 | 0.752314 | -0.6432 | 0.52 | -3.43E-08 | count | 1 |
| GRIP1 | -2.0584728 | 1.1535966 | -1.7844 | 0.0744 | -3.43E-08 | count | 1 |
| NDUFA4L2 | -0.4716437 | 0.5775749 | -0.8166 | 0.414 | -3.36E-08 | count | 1 |
| SOX8 | -1.9434615 | 1.2574842 | -1.5455 | 0.122 | -3.34E-08 | count | 1 |
| Z82206.1 | -0.4645277 | 0.6755752 | -0.6876 | 0.492 | -3.32E-08 | count | 1 |
| GPX3 | -1.9002209 | 1.1620186 | -1.6353 | 0.102 | -3.31E-08 | count | 1 |
| KRTCAP3 | -1.9002209 | 1.1839545 | -1.605 | 0.109 | -3.31E-08 | count | 1 |
| TUSC1 | -1.9002209 | 1.3205534 | -1.439 | 0.15 | -3.31E-08 | count | 1 |
| NBPF26 | -0.4590912 | 0.4402826 | -1.0427 | 0.297 | -3.28E-08 | count | 1 |
| TCN2 | -1.8541842 | 1.0942448 | -1.6945 | 0.0903 | -3.27E-08 | count | 1 |
| AC015987.1 | -1.8427521 | 1.1027767 | -1.671 | 0.0948 | -3.26E-08 | count | 1 |
| SH3D19 | -1.8427521 | 1.256069 | -1.4671 | 0.142 | -3.26E-08 | count | 1 |
| AC005670.2 | -1.8048961 | 1.3567494 | -1.3303 | 0.184 | -3.23E-08 | count | 1 |

| | | | | | | | |
|---|---|---|---|---|---|---|---|
| CNKSR1 | -1.8048961 | 1.4421428 | -1.2515 | 0.211 | -3.23E-08 | count | 1 |
| ACOXL | -1.8048961 | 1.4421428 | -1.2515 | 0.211 | -3.23E-08 | count | 1 |
| SSPN | -1.8048961 | 1.5615013 | -1.1559 | 0.248 | -3.23E-08 | count | 1 |
| MAFG-DT | -1.8048961 | 1.6723626 | -1.0792 | 0.281 | -3.23E-08 | count | 1 |
| TNKS1BP1 | -1.7936481 | 0.9906517 | -1.8106 | 0.0703 | -3.22E-08 | count | 1 |
| PABPC3 | -1.7936481 | 1.040309 | -1.7241 | 0.0848 | -3.22E-08 | count | 1 |
| HOMER2 | -1.7936481 | 1.0760835 | -1.6668 | 0.0956 | -3.22E-08 | count | 1 |
| AL592148.3 | -1.7936481 | 1.0760835 | -1.6668 | 0.0956 | -3.22E-08 | count | 1 |
| MCM10 | -1.7936481 | 1.2139433 | -1.4775 | 0.14 | -3.22E-08 | count | 1 |
| OR2A25 | -1.7826871 | 0.8926365 | -1.9971 | 0.0459 | -3.21E-08 | count | 1 |
| COBLL1 | -1.7826871 | 1.2766213 | -1.3964 | 0.163 | -3.20E-08 | count | 1 |
| SPRED3 | -1.7408204 | 1.0265152 | -1.6959 | 0.09 | -3.17E-08 | count | 1 |
| MEX3B | -1.7408204 | 1.1226949 | -1.5506 | 0.121 | -3.17E-08 | count | 1 |
| PPP1R2B | -1.7408204 | 1.0949483 | -1.5899 | 0.112 | -3.17E-08 | count | 1 |
| LINC01315 | -1.7408204 | 1.1591264 | -1.5018 | 0.133 | -3.17E-08 | count | 1 |
| ZNF781 | -0.4347774 | 0.5710282 | -0.7614 | 0.446 | -3.13E-08 | count | 1 |
| MSH5 | -1.6734209 | 1.0749275 | -1.5568 | 0.12 | -3.11E-08 | count | 1 |
| USP49 | -1.6734209 | 1.0749275 | -1.5568 | 0.12 | -3.11E-08 | count | 1 |
| LRP11 | -1.6734209 | 1.1377615 | -1.4708 | 0.141 | -3.11E-08 | count | 1 |
| AL606760.1 | -1.6734209 | 1.234124 | -1.356 | 0.175 | -3.11E-08 | count | 1 |
| LINC01635 | -1.6734209 | 1.3928956 | -1.2014 | 0.23 | -3.11E-08 | count | 1 |
| SLC2A14 | -0.1458845 | 0.3398385 | -0.4293 | 0.668 | -3.09E-08 | count | 1 |
| SORT1 | -1.6114706 | 1.0257012 | -1.5711 | 0.116 | -3.05E-08 | count | 1 |
| ATAD3C | -1.6114706 | 1.0974715 | -1.4683 | 0.142 | -3.05E-08 | count | 1 |
| ZFHX3 | -1.6114706 | 0.8460678 | -1.9047 | 0.0569 | -3.05E-08 | count | 1 |
| PHLDB1 | -1.6024799 | 1.0184294 | -1.5735 | 0.116 | -3.04E-08 | count | 1 |
| AL121985.1 | -1.6024799 | 1.2802099 | -1.2517 | 0.211 | -3.03E-08 | count | 1 |
| AC004771.4 | -1.6024799 | 1.3971492 | -1.147 | 0.251 | -3.03E-08 | count | 1 |
| AC112220.4 | -1.6024799 | 1.3971492 | -1.147 | 0.251 | -3.03E-08 | count | 1 |
| GHRL | -1.5938634 | 0.8606246 | -1.852 | 0.0641 | -3.02E-08 | count | 1 |
| LIMS2 | -0.4160996 | 0.4637923 | -0.8972 | 0.37 | -3.01E-08 | count | 1 |
| LINC01431 | -0.4154014 | 0.5389508 | -0.7708 | 0.441 | -3.01E-08 | count | 1 |
| PAWR | -1.5352561 | 0.9777773 | -1.5701 | 0.116 | -2.97E-08 | count | 1 |
| LBHD1 | -1.5352561 | 1.0835056 | -1.4169 | 0.157 | -2.97E-08 | count | 1 |
| KLHL32 | -1.5352561 | 1.0722717 | -1.4318 | 0.152 | -2.97E-08 | count | 1 |
| AL139412.1 | -1.5352561 | 1.1779608 | -1.3033 | 0.193 | -2.97E-08 | count | 1 |
| AC138028.1 | -1.5352561 | 1.2575014 | -1.2209 | 0.222 | -2.97E-08 | count | 1 |
| C1QTNF1 | -1.5352561 | 1.3248166 | -1.1588 | 0.247 | -2.97E-08 | count | 1 |
| C9orf24 | -1.5352561 | 1.3248166 | -1.1588 | 0.247 | -2.97E-08 | count | 1 |
| KYNU | -1.5277937 | 1.117734 | -1.3669 | 0.172 | -2.96E-08 | count | 1 |
| AL031714.1 | -0.4054965 | 0.4841505 | -0.8375 | 0.402 | -2.94E-08 | count | 1 |
| SFRP2 | -0.400404 | 0.5361439 | -0.7468 | 0.455 | -2.91E-08 | count | 1 |
| TPD52L1 | -0.3973017 | 0.7128829 | -0.5573 | 0.577 | -2.89E-08 | count | 1 |
| PDE1A | -1.4546259 | 1.0218115 | -1.4236 | 0.155 | -2.88E-08 | count | 1 |
| PDE6G | -1.4546259 | 1.0568275 | -1.3764 | 0.169 | -2.88E-08 | count | 1 |

| | | | | | | | |
|---|---|---|---|---|---|---|---|
| CD80 | -1.4546259 | 1.2058102 | -1.2063 | 0.228 | -2.88E-08 | count | 1 |
| CHDH | -1.4546259 | 1.1242485 | -1.2939 | 0.196 | -2.88E-08 | count | 1 |
| SPEG | -1.4546259 | 0.7895886 | -1.8423 | 0.0655 | -2.88E-08 | count | 1 |
| PTPRO | -1.4491891 | 1.3823972 | -1.0483 | 0.295 | -2.87E-08 | count | 1 |
| Z97192.1 | -1.4491891 | 0.8837883 | -1.6397 | 0.101 | -2.87E-08 | count | 1 |
| KCNK7 | -1.4491891 | 1.4079378 | -1.0293 | 0.303 | -2.87E-08 | count | 1 |
| PMCH | -0.3909203 | 0.6574549 | -0.5946 | 0.552 | -2.85E-08 | count | 1 |
| AL121658.1 | -0.3909203 | 0.4883491 | -0.8005 | 0.423 | -2.85E-08 | count | 1 |
| AMER1 | -1.417065 | 0.9614984 | -1.4738 | 0.141 | -2.83E-08 | count | 1 |
| PGF | -1.417065 | 1.0303577 | -1.3753 | 0.169 | -2.83E-08 | count | 1 |
| CD302 | -1.4147837 | 0.8142687 | -1.7375 | 0.0824 | -2.83E-08 | count | 1 |
| AC005944.1 | -1.4147837 | 0.8828195 | -1.6026 | 0.109 | -2.83E-08 | count | 1 |
| STAC3 | -1.3723402 | 1.2395141 | -1.1072 | 0.268 | -2.78E-08 | count | 1 |
| AC090198.1 | -1.3723402 | 1.2395141 | -1.1072 | 0.268 | -2.78E-08 | count | 1 |
| CDC42BPB | -1.3723402 | 1.2395141 | -1.1072 | 0.268 | -2.78E-08 | count | 1 |
| SYNPO | -1.3723402 | 1.0774461 | -1.2737 | 0.203 | -2.78E-08 | count | 1 |
| LAMA4 | -1.3723402 | 1.0774461 | -1.2737 | 0.203 | -2.78E-08 | count | 1 |
| REG4 | -1.3723402 | 1.3208301 | -1.039 | 0.299 | -2.78E-08 | count | 1 |
| ARPIN | -1.3723402 | 1.3208301 | -1.039 | 0.299 | -2.78E-08 | count | 1 |
| NPIPA5 | -1.3723402 | 1.3974224 | -0.9821 | 0.326 | -2.78E-08 | count | 1 |
| TMEM236 | -1.3723402 | 1.2558998 | -1.0927 | 0.275 | -2.78E-08 | count | 1 |
| AC104984.3 | -1.3708122 | 0.9075581 | -1.5104 | 0.131 | -2.78E-08 | count | 1 |
| C6orf52 | -1.3693392 | 1.13964 | -1.2016 | 0.23 | -2.77E-08 | count | 1 |
| NCR3LG1 | -1.3693392 | 1.13964 | -1.2016 | 0.23 | -2.77E-08 | count | 1 |
| AL160408.2 | -1.3693392 | 1.3712088 | -0.9986 | 0.318 | -2.77E-08 | count | 1 |
| CRHBP | -1.3693392 | 1.3712088 | -0.9986 | 0.318 | -2.77E-08 | count | 1 |
| AL096711.2 | -1.3693392 | 1.2336708 | -1.11 | 0.267 | -2.77E-08 | count | 1 |
| GUCY1A1 | -1.3693392 | 0.8088272 | -1.693 | 0.0905 | -2.77E-08 | count | 1 |
| RBMS2 | -0.378249 | 0.4474993 | -0.8453 | 0.398 | -2.77E-08 | count | 1 |
| GFPT2 | -0.3744364 | 0.578761 | -0.647 | 0.518 | -2.74E-08 | count | 1 |
| PNPLA7 | -0.371113 | 0.698122 | -0.5316 | 0.595 | -2.72E-08 | count | 1 |
| TCEAL2 | -0.371113 | 0.5792005 | -0.6407 | 0.522 | -2.72E-08 | count | 1 |
| ZNF155 | -0.3701844 | 0.3972293 | -0.9319 | 0.351 | -2.71E-08 | count | 1 |
| GVQW3 | -0.3683558 | 0.4002251 | -0.9204 | 0.357 | -2.70E-08 | count | 1 |
| AC037487.2 | -1.2785588 | 1.1461497 | -1.1155 | 0.265 | -2.66E-08 | count | 1 |
| TIMD4 | -1.2785588 | 1.1461497 | -1.1155 | 0.265 | -2.66E-08 | count | 1 |
| CPLANE2 | -1.2785588 | 1.1461497 | -1.1155 | 0.265 | -2.66E-08 | count | 1 |
| TMEM139 | -1.2785588 | 1.1461497 | -1.1155 | 0.265 | -2.66E-08 | count | 1 |
| VIL1 | -1.2785588 | 1.118245 | -1.1434 | 0.253 | -2.66E-08 | count | 1 |
| PAM16 | -1.2785588 | 1.118245 | -1.1434 | 0.253 | -2.66E-08 | count | 1 |
| AC022929.2 | -1.2785588 | 1.118245 | -1.1434 | 0.253 | -2.66E-08 | count | 1 |
| AC129492.1 | -1.2785588 | 1.2447376 | -1.0272 | 0.304 | -2.66E-08 | count | 1 |
| F13A1 | -1.2785588 | 0.6943115 | -1.8415 | 0.0656 | -2.66E-08 | count | 1 |
| NREP | -1.2792244 | 1.3680487 | -0.9351 | 0.35 | -2.66E-08 | count | 1 |
| SERINC2 | -1.278899 | 0.8759292 | -1.46 | 0.144 | -2.66E-08 | count | 1 |

| | | | | | | | |
|---|---|---|---|---|---|---|---|
| AC093525.4 | -1.278899 | 1.0969767 | -1.1658 | 0.244 | -2.66E-08 | count | 1 |
| HRH1 | -1.2792244 | 1.0097362 | -1.2669 | 0.205 | -2.66E-08 | count | 1 |
| PAGR1 | -1.278899 | 0.9427896 | -1.3565 | 0.175 | -2.66E-08 | count | 1 |
| KCNAB1 | -1.2792244 | 1.4941558 | -0.8562 | 0.392 | -2.66E-08 | count | 1 |
| CCDC96 | -0.3593633 | 0.6492649 | -0.5535 | 0.58 | -2.64E-08 | count | 1 |
| IGHA2 | -1.252763 | 0.6095037 | -2.0554 | 0.0399 | -2.63E-08 | count | 1 |
| FBN1 | -1.2294329 | 0.9116416 | -1.3486 | 0.178 | -2.59E-08 | count | 1 |
| SMOC2 | -1.2278726 | 0.9149321 | -1.342 | 0.18 | -2.59E-08 | count | 1 |
| CDH11 | -1.2278726 | 1.0419011 | -1.1785 | 0.239 | -2.59E-08 | count | 1 |
| TRIM66 | -0.3430978 | 0.5028976 | -0.6822 | 0.495 | -2.53E-08 | count | 1 |
| ANKRD23 | -1.1787855 | 1.0806482 | -1.0908 | 0.275 | -2.52E-08 | count | 1 |
| A4GALT | -1.1787855 | 1.1522421 | -1.023 | 0.306 | -2.52E-08 | count | 1 |
| AXL | -1.1787855 | 1.1370546 | -1.0367 | 0.3 | -2.52E-08 | count | 1 |
| ERVMER61-1 | -1.1787855 | 1.1370546 | -1.0367 | 0.3 | -2.52E-08 | count | 1 |
| AC008429.1 | -1.1787855 | 1.1370546 | -1.0367 | 0.3 | -2.52E-08 | count | 1 |
| DMD | -1.1787855 | 1.2835049 | -0.9184 | 0.358 | -2.52E-08 | count | 1 |
| C2orf92 | -1.1787855 | 1.2053027 | -0.978 | 0.328 | -2.52E-08 | count | 1 |
| TXNDC5 | -1.1787855 | 1.2053027 | -0.978 | 0.328 | -2.52E-08 | count | 1 |
| DND1 | -1.1787855 | 0.8688512 | -1.3567 | 0.175 | -2.52E-08 | count | 1 |
| IL33 | -1.1787855 | 0.8798326 | -1.3398 | 0.18 | -2.52E-08 | count | 1 |
| AL445248.1 | -1.1787855 | 0.8798326 | -1.3398 | 0.18 | -2.52E-08 | count | 1 |
| PDE9A | -1.175838 | 0.8199329 | -1.4341 | 0.152 | -2.52E-08 | count | 1 |
| CIT | -1.175838 | 1.0740507 | -1.0948 | 0.274 | -2.52E-08 | count | 1 |
| SPTBN4 | -1.175838 | 0.9111813 | -1.2905 | 0.197 | -2.52E-08 | count | 1 |
| USP44 | -0.3409017 | 0.3885688 | -0.8773 | 0.38 | -2.52E-08 | count | 1 |
| LINC01001 | -0.335764 | 0.4779892 | -0.7025 | 0.482 | -2.48E-08 | count | 1 |
| LINC01772 | -1.1308851 | 0.7378773 | -1.5326 | 0.125 | -2.45E-08 | count | 1 |
| AC015912.3 | -1.12207 | 0.8323985 | -1.348 | 0.178 | -2.44E-08 | count | 1 |
| LOX | -1.1172413 | 0.8777075 | -1.2729 | 0.203 | -2.43E-08 | count | 1 |
| ARMCX1 | -1.1172413 | 1.0804516 | -1.0341 | 0.301 | -2.43E-08 | count | 1 |
| YPEL4 | -0.3278176 | 0.6244196 | -0.525 | 0.6 | -2.43E-08 | count | 1 |
| CCDC180 | -0.3193462 | 0.6659724 | -0.4795 | 0.632 | -2.37E-08 | count | 1 |
| OR3A3 | -1.0729103 | 1.2943128 | -0.8289 | 0.407 | -2.37E-08 | count | 1 |
| FERMT1 | -1.0729103 | 1.3852484 | -0.7745 | 0.439 | -2.37E-08 | count | 1 |
| NTN4 | -1.0665546 | 0.9301722 | -1.1466 | 0.252 | -2.36E-08 | count | 1 |
| TMEM56-RWDD3 | -1.0597504 | 1.1093134 | -0.9553 | 0.339 | -2.35E-08 | count | 1 |
| AC021739.2 | -1.0597504 | 1.0203391 | -1.0386 | 0.299 | -2.35E-08 | count | 1 |
| EDAR | -1.0597504 | 1.0203391 | -1.0386 | 0.299 | -2.35E-08 | count | 1 |
| FRMD6 | -1.0597504 | 1.0203391 | -1.0386 | 0.299 | -2.35E-08 | count | 1 |
| PIK3R6 | -1.0597504 | 1.0203391 | -1.0386 | 0.299 | -2.35E-08 | count | 1 |
| TRIM58 | -1.0597504 | 1.2452872 | -0.851 | 0.395 | -2.35E-08 | count | 1 |
| AC027682.6 | -1.0597504 | 1.2452872 | -0.851 | 0.395 | -2.35E-08 | count | 1 |
| STXBP6 | -1.0597504 | 1.2452872 | -0.851 | 0.395 | -2.35E-08 | count | 1 |
| MPEG1 | -1.0597504 | 1.2452872 | -0.851 | 0.395 | -2.35E-08 | count | 1 |
| AP000704.1 | -1.0597504 | 1.1667287 | -0.9083 | 0.364 | -2.35E-08 | count | 1 |

| | | | | | | | |
|---|---|---|---|---|---|---|---|
| ZNF571-AS1 | -1.0597504 | 1.1667287 | -0.9083 | 0.364 | -2.35E-08 | count | 1 |
| KCNC3 | -1.0597504 | 1.1667287 | -0.9083 | 0.364 | -2.35E-08 | count | 1 |
| AC084809.1 | -1.0597504 | 1.1667287 | -0.9083 | 0.364 | -2.35E-08 | count | 1 |
| GPRC5C | -1.0597504 | 1.1667287 | -0.9083 | 0.364 | -2.35E-08 | count | 1 |
| WEE2-AS1 | -1.0597504 | 1.1667287 | -0.9083 | 0.364 | -2.35E-08 | count | 1 |
| SSC5D | -1.0597504 | 1.1667287 | -0.9083 | 0.364 | -2.35E-08 | count | 1 |
| LINC00654 | -1.0597504 | 1.4802257 | -0.7159 | 0.474 | -2.35E-08 | count | 1 |
| MCF2L | -0.3118 | 0.6331028 | -0.4925 | 0.622 | -2.32E-08 | count | 1 |
| ADAM23 | -0.3115044 | 0.6904627 | -0.4512 | 0.652 | -2.32E-08 | count | 1 |
| AC006480.2 | -0.3048861 | 0.5522745 | -0.5521 | 0.581 | -2.27E-08 | count | 1 |
| AC027020.2 | -0.3042331 | 0.5224238 | -0.5823 | 0.56 | -2.27E-08 | count | 1 |
| SEPT7-AS1 | -1.0092984 | 1.148745 | -0.8786 | 0.38 | -2.27E-08 | count | 1 |
| AC083798.2 | -0.2945976 | 0.4133375 | -0.7127 | 0.476 | -2.20E-08 | count | 1 |
| PAN2 | -0.2933123 | 0.4814849 | -0.6092 | 0.542 | -2.20E-08 | count | 1 |
| ASMT | -0.9386625 | 1.0740546 | -0.8739 | 0.382 | -2.15E-08 | count | 1 |
| SOWAHC | -0.9386625 | 0.9867437 | -0.9513 | 0.342 | -2.15E-08 | count | 1 |
| DPYSL3 | -0.9386625 | 1.1922279 | -0.7873 | 0.431 | -2.15E-08 | count | 1 |
| COL5A2 | -0.9386625 | 1.1922279 | -0.7873 | 0.431 | -2.15E-08 | count | 1 |
| SPATA6L | -0.9386625 | 1.2135348 | -0.7735 | 0.439 | -2.15E-08 | count | 1 |
| KRT7 | -0.9386625 | 1.2135348 | -0.7735 | 0.439 | -2.15E-08 | count | 1 |
| EGFL8 | -0.9386625 | 1.2135348 | -0.7735 | 0.439 | -2.15E-08 | count | 1 |
| AL603832.1 | -0.9386625 | 1.2135348 | -0.7735 | 0.439 | -2.15E-08 | count | 1 |
| IFT74-AS1 | -0.9386625 | 1.2135348 | -0.7735 | 0.439 | -2.15E-08 | count | 1 |
| MYO15B | -0.9386625 | 1.2135348 | -0.7735 | 0.439 | -2.15E-08 | count | 1 |
| ATP2A1-AS1 | -0.9386625 | 0.8501319 | -1.1041 | 0.27 | -2.15E-08 | count | 1 |
| RPL34-AS1 | -0.9386625 | 0.9255437 | -1.0142 | 0.311 | -2.15E-08 | count | 1 |
| PSMD6-AS2 | -0.2862832 | 0.4953249 | -0.578 | 0.563 | -2.15E-08 | count | 1 |
| ACE | -0.2862832 | 0.5989268 | -0.478 | 0.633 | -2.15E-08 | count | 1 |
| ARHGAP29 | -0.9259219 | 0.9163544 | -1.0104 | 0.312 | -2.13E-08 | count | 1 |
| AC007773.1 | -0.9259219 | 0.962711 | -0.9618 | 0.336 | -2.13E-08 | count | 1 |
| ZGPAT | -0.9259219 | 0.7872929 | -1.1761 | 0.24 | -2.13E-08 | count | 1 |
| IGLV1-51 | -0.9259219 | 0.962711 | -0.9618 | 0.336 | -2.13E-08 | count | 1 |
| EFCAB10 | -0.9259219 | 0.962711 | -0.9618 | 0.336 | -2.13E-08 | count | 1 |
| HIST1H3F | -0.9259219 | 0.9241585 | -1.0019 | 0.316 | -2.13E-08 | count | 1 |
| ZC3H12C | -0.9259219 | 1.2453616 | -0.7435 | 0.457 | -2.13E-08 | count | 1 |
| GSTM1 | -0.9214058 | 0.6717771 | -1.3716 | 0.17 | -2.12E-08 | count | 1 |
| AC008378.1 | -0.9214058 | 0.7466806 | -1.234 | 0.217 | -2.12E-08 | count | 1 |
| ITPK1-AS1 | -0.2796823 | 0.6498924 | -0.4304 | 0.667 | -2.10E-08 | count | 1 |
| ATXN7 | -0.2778908 | 0.5311499 | -0.5232 | 0.601 | -2.09E-08 | count | 1 |
| SRGAP1 | -0.8897186 | 0.7908872 | -1.125 | 0.261 | -2.07E-08 | count | 1 |
| SLC17A9 | -0.8751018 | 1.0759086 | -0.8134 | 0.416 | -2.04E-08 | count | 1 |
| AC103736.1 | -0.2706738 | 0.4977824 | -0.5438 | 0.587 | -2.04E-08 | count | 1 |
| CACFD1 | -0.8590005 | 0.9748788 | -0.8811 | 0.378 | -2.02E-08 | count | 1 |
| AC018362.2 | -0.8590005 | 0.9564815 | -0.8981 | 0.369 | -2.02E-08 | count | 1 |
| NTN5 | -0.8590005 | 0.9564815 | -0.8981 | 0.369 | -2.02E-08 | count | 1 |

| | | | | | | | |
|---|---|---|---|---|---|---|---|
| LOXL2 | -0.8590005 | 0.8135993 | -1.0558 | 0.291 | -2.02E-08 | count | 1 |
| POLE2 | -0.8590005 | 1.0625992 | -0.8084 | 0.419 | -2.02E-08 | count | 1 |
| MORN1 | -0.8590005 | 1.1053373 | -0.7771 | 0.437 | -2.02E-08 | count | 1 |
| ZNF704 | -0.8534822 | 0.6600339 | -1.2931 | 0.196 | -2.01E-08 | count | 1 |
| NCKIPSD | -0.2654782 | 0.5131328 | -0.5174 | 0.605 | -2.00E-08 | count | 1 |
| PPARGC1B | -0.8299564 | 0.6691693 | -1.2403 | 0.215 | -1.97E-08 | count | 1 |
| UBAC2-AS1 | -0.8299564 | 0.6691693 | -1.2403 | 0.215 | -1.97E-08 | count | 1 |
| ZNF619 | -0.2553177 | 0.5249026 | -0.4864 | 0.627 | -1.93E-08 | count | 1 |
| IL17RD | -0.8096158 | 1.2733752 | -0.6358 | 0.525 | -1.93E-08 | count | 1 |
| DACT1 | -0.8096158 | 0.7450617 | -1.0866 | 0.277 | -1.93E-08 | count | 1 |
| GREB1L | -0.8096158 | 1.0436413 | -0.7758 | 0.438 | -1.93E-08 | count | 1 |
| TRAV40 | -0.8096158 | 1.4099424 | -0.5742 | 0.566 | -1.93E-08 | count | 1 |
| FXYD6 | -0.8096158 | 1.4099424 | -0.5742 | 0.566 | -1.93E-08 | count | 1 |
| DNAH6 | -0.8096158 | 0.8766346 | -0.9235 | 0.356 | -1.93E-08 | count | 1 |
| AC022167.3 | -0.8096158 | 1.2733752 | -0.6358 | 0.525 | -1.93E-08 | count | 1 |
| TMEM98 | -0.7896694 | 1.0115071 | -0.7807 | 0.435 | -1.89E-08 | count | 1 |
| TSC22D1-AS1 | -0.7896694 | 1.0115071 | -0.7807 | 0.435 | -1.89E-08 | count | 1 |
| FAM83H | -0.7896694 | 1.0846144 | -0.7281 | 0.467 | -1.89E-08 | count | 1 |
| ADGRA2 | -0.7896694 | 0.8996682 | -0.8777 | 0.38 | -1.89E-08 | count | 1 |
| CCDC3 | -0.7824991 | 0.7764117 | -1.0078 | 0.314 | -1.88E-08 | count | 1 |
| LINC00544 | -0.7824991 | 0.7060667 | -1.1083 | 0.268 | -1.88E-08 | count | 1 |
| DLX2 | -0.7672552 | 0.6131833 | -1.2513 | 0.211 | -1.85E-08 | count | 1 |
| PSD | -0.2438661 | 0.4900653 | -0.4976 | 0.619 | -1.85E-08 | count | 1 |
| AL137003.2 | -0.2398913 | 0.4889101 | -0.4907 | 0.624 | -1.82E-08 | count | 1 |
| LINC02019 | -0.2309286 | 0.5004883 | -0.4614 | 0.645 | -1.76E-08 | count | 1 |
| AC009948.4 | -0.7180187 | 0.9201794 | -0.7803 | 0.435 | -1.76E-08 | count | 1 |
| TBC1D16 | -0.7180187 | 0.9872451 | -0.7273 | 0.467 | -1.75E-08 | count | 1 |
| DST | -0.2270861 | 0.5030092 | -0.4515 | 0.652 | -1.73E-08 | count | 1 |
| SV2A | -0.2235411 | 0.4749957 | -0.4706 | 0.638 | -1.70E-08 | count | 1 |
| TCIM | -0.690364 | 0.9709942 | -0.711 | 0.477 | -1.70E-08 | count | 1 |
| RND2 | -0.690364 | 1.0885667 | -0.6342 | 0.526 | -1.70E-08 | count | 1 |
| CSF1R | -0.690364 | 0.9591023 | -0.7198 | 0.472 | -1.70E-08 | count | 1 |
| IGHV1-2 | -0.690364 | 0.9591023 | -0.7198 | 0.472 | -1.70E-08 | count | 1 |
| CLDN5 | -0.6842452 | 0.7642759 | -0.8953 | 0.371 | -1.69E-08 | count | 1 |
| PRELP | -0.6842452 | 0.6998778 | -0.9777 | 0.328 | -1.69E-08 | count | 1 |
| GOLGA6L4 | -0.6442042 | 1.195036 | -0.5391 | 0.59 | -1.61E-08 | count | 1 |
| VWDE | -0.6442042 | 0.8457645 | -0.7617 | 0.446 | -1.61E-08 | count | 1 |
| AC090192.2 | -0.6442042 | 1.2682828 | -0.5079 | 0.612 | -1.61E-08 | count | 1 |
| IGHD | -17.5660755 | 1461.702181 | -0.012 | 0.99 | -1.57E-08 | count | 1 |
| AC021087.1 | -17.5660755 | 1461.702181 | -0.012 | 0.99 | -1.57E-08 | count | 1 |
| ANGPT1 | -17.5660755 | 1461.702181 | -0.012 | 0.99 | -1.57E-08 | count | 1 |
| GLT8D2 | -17.5660755 | 1461.702181 | -0.012 | 0.99 | -1.57E-08 | count | 1 |
| AC005899.8 | -17.5660755 | 1461.702181 | -0.012 | 0.99 | -1.57E-08 | count | 1 |
| TUBB4A | -17.5660755 | 1461.702181 | -0.012 | 0.99 | -1.57E-08 | count | 1 |
| CXorf65 | -17.5660755 | 1461.702181 | -0.012 | 0.99 | -1.57E-08 | count | 1 |

| | | | | | | | |
|---|---|---|---|---|---|---|---|
| NT5DC2 | -17.757037 | 1278.262095 | -0.0139 | 0.989 | -1.57E-08 | count | 1 |
| FAM53A | -17.4886483 | 1372.90403 | -0.0127 | 0.99 | -1.57E-08 | count | 1 |
| AC009812.4 | -17.4886483 | 1372.90403 | -0.0127 | 0.99 | -1.57E-08 | count | 1 |
| AL121917.1 | -17.5661961 | 1596.080928 | -0.011 | 0.991 | -1.57E-08 | count | 1 |
| BHLHE41 | -17.5661961 | 1596.080928 | -0.011 | 0.991 | -1.57E-08 | count | 1 |
| SHROOM4 | -17.4887811 | 1545.937659 | -0.0113 | 0.991 | -1.57E-08 | count | 1 |
| SDK2 | -17.4887811 | 1545.937659 | -0.0113 | 0.991 | -1.57E-08 | count | 1 |
| UGGT2 | -17.4887811 | 1545.937659 | -0.0113 | 0.991 | -1.57E-08 | count | 1 |
| TLR4 | -17.874386 | 1287.242952 | -0.0139 | 0.989 | -1.57E-08 | count | 1 |
| HCAR3 | -17.7574447 | 1512.695118 | -0.0117 | 0.991 | -1.57E-08 | count | 1 |
| KCNMA1 | -17.7574447 | 1512.695118 | -0.0117 | 0.991 | -1.57E-08 | count | 1 |
| EEF1AKMT3 | -17.4888037 | 1730.083932 | -0.0101 | 0.992 | -1.57E-08 | count | 1 |
| AC012645.1 | -17.8745616 | 1410.254442 | -0.0127 | 0.99 | -1.57E-08 | count | 1 |
| TEAD1 | -17.7013734 | 1632.500418 | -0.0108 | 0.991 | -1.57E-08 | count | 1 |
| AGT | -17.4889564 | 1870.439345 | -0.0094 | 0.993 | -1.56E-08 | count | 1 |
| PEX11G | -17.8745418 | 1578.687584 | -0.0113 | 0.991 | -1.56E-08 | count | 1 |
| AL162377.3 | -17.7014436 | 1755.172903 | -0.0101 | 0.992 | -1.56E-08 | count | 1 |
| FHL5 | -17.6183502 | 1369.231212 | -0.0129 | 0.99 | -1.56E-08 | count | 1 |
| KHDRBS3 | -17.8745418 | 1578.687584 | -0.0113 | 0.991 | -1.56E-08 | count | 1 |
| ADAMTSL4 | -17.9173513 | 1544.731639 | -0.0116 | 0.991 | -1.56E-08 | count | 1 |
| AC016027.1 | -18.0541969 | 1410.560974 | -0.0128 | 0.99 | -1.56E-08 | count | 1 |
| TRIO | -18.0543343 | 1410.657905 | -0.0128 | 0.99 | -1.56E-08 | count | 1 |
| BBS1 | -17.6186022 | 1486.678731 | -0.0119 | 0.991 | -1.56E-08 | count | 1 |
| AL078581.1 | -17.6186022 | 1486.678731 | -0.0119 | 0.991 | -1.56E-08 | count | 1 |
| PAGE5 | -17.7016168 | 1955.195198 | -0.0091 | 0.993 | -1.56E-08 | count | 1 |
| ZNF433 | -17.8748959 | 1821.003603 | -0.0098 | 0.992 | -1.56E-08 | count | 1 |
| AIRN | -17.8747905 | 1776.538832 | -0.0101 | 0.992 | -1.56E-08 | count | 1 |
| LINC02175 | -17.8115278 | 1579.873424 | -0.0113 | 0.991 | -1.56E-08 | count | 1 |
| REPS2 | -17.3740264 | 1517.117829 | -0.0115 | 0.991 | -1.56E-08 | count | 1 |
| NOTCH3 | -17.3734693 | 1567.025808 | -0.0111 | 0.991 | -1.56E-08 | count | 1 |
| AC019069.1 | -17.3734693 | 1567.025808 | -0.0111 | 0.991 | -1.56E-08 | count | 1 |
| AC087289.5 | -17.6189118 | 1697.350688 | -0.0104 | 0.992 | -1.56E-08 | count | 1 |
| P2RY13 | -17.6189118 | 1697.350688 | -0.0104 | 0.992 | -1.56E-08 | count | 1 |
| PLSCR3 | -17.811562 | 1706.303867 | -0.0104 | 0.992 | -1.56E-08 | count | 1 |
| AL441883.1 | -17.6186503 | 1820.203515 | -0.0097 | 0.992 | -1.56E-08 | count | 1 |
| ZNF185 | -18.1825128 | 1460.844384 | -0.0124 | 0.99 | -1.56E-08 | count | 1 |
| IQCC | -17.8117013 | 1780.2857 | -0.01 | 0.992 | -1.56E-08 | count | 1 |
| ADPRHL1 | -17.3740088 | 1765.990439 | -0.0098 | 0.992 | -1.56E-08 | count | 1 |
| LRRK1 | -17.3740088 | 1765.990439 | -0.0098 | 0.992 | -1.56E-08 | count | 1 |
| AC239803.2 | -17.3740088 | 1765.990439 | -0.0098 | 0.992 | -1.56E-08 | count | 1 |
| AC006504.1 | -17.3740088 | 1765.990439 | -0.0098 | 0.992 | -1.56E-08 | count | 1 |
| ARHGEF10L | -17.3740088 | 1765.990439 | -0.0098 | 0.992 | -1.56E-08 | count | 1 |
| MALL | -17.3740088 | 1765.990439 | -0.0098 | 0.992 | -1.56E-08 | count | 1 |
| BICC1 | -17.3740088 | 1765.990439 | -0.0098 | 0.992 | -1.56E-08 | count | 1 |
| TGM2 | -18.0548516 | 1497.608728 | -0.0121 | 0.99 | -1.56E-08 | count | 1 |

| | | | | | | | |
|---|---|---|---|---|---|---|---|
| NUDT13 | -17.508066 | 1239.135154 | -0.0141 | 0.989 | -1.56E-08 | count | 1 |
| CRISPLD1 | -17.508066 | 1239.135154 | -0.0141 | 0.989 | -1.56E-08 | count | 1 |
| RASAL2 | -18.0549974 | 1681.546792 | -0.0107 | 0.991 | -1.56E-08 | count | 1 |
| AC007262.2 | -17.7296212 | 1535.852177 | -0.0115 | 0.991 | -1.56E-08 | count | 1 |
| AL031590.1 | -18.0489965 | 1449.432786 | -0.0125 | 0.99 | -1.56E-08 | count | 1 |
| MIPOL1 | -17.5084796 | 1334.571487 | -0.0131 | 0.99 | -1.56E-08 | count | 1 |
| PRCD | -17.9184049 | 2318.4178 | -0.0077 | 0.994 | -1.56E-08 | count | 1 |
| CDS1 | -17.5086538 | 1423.525161 | -0.0123 | 0.99 | -1.56E-08 | count | 1 |
| EDIL3 | -18.0674377 | 1212.482682 | -0.0149 | 0.988 | -1.56E-08 | count | 1 |
| TFPI2 | -17.5079782 | 1530.200335 | -0.0114 | 0.991 | -1.56E-08 | count | 1 |
| PKN3 | -18.0551071 | 1847.272215 | -0.0098 | 0.992 | -1.56E-08 | count | 1 |
| PCDH9 | -17.7301306 | 1832.435825 | -0.0097 | 0.992 | -1.56E-08 | count | 1 |
| SOCS6 | -17.7301306 | 1832.435825 | -0.0097 | 0.992 | -1.56E-08 | count | 1 |
| AC092745.1 | -17.9913456 | 1654.35057 | -0.0109 | 0.991 | -1.56E-08 | count | 1 |
| AL157392.4 | -18.0498549 | 1907.069941 | -0.0095 | 0.992 | -1.56E-08 | count | 1 |
| TTC34 | -17.9162237 | 2012.048955 | -0.0089 | 0.993 | -1.56E-08 | count | 1 |
| TIAF1 | -17.9162237 | 2012.048955 | -0.0089 | 0.993 | -1.56E-08 | count | 1 |
| CDA | -18.2733703 | 1995.461856 | -0.0092 | 0.993 | -1.56E-08 | count | 1 |
| LRMDA | -17.9916368 | 1826.922085 | -0.0098 | 0.992 | -1.56E-08 | count | 1 |
| ANTXR1 | -18.0679148 | 1541.567791 | -0.0117 | 0.991 | -1.56E-08 | count | 1 |
| ENTPD2 | -18.0501066 | 1956.952061 | -0.0092 | 0.993 | -1.56E-08 | count | 1 |
| AC005224.4 | -17.6249601 | 1289.240301 | -0.0137 | 0.989 | -1.56E-08 | count | 1 |
| CILP2 | -17.5092348 | 1889.532201 | -0.0093 | 0.993 | -1.56E-08 | count | 1 |
| IGLV4-69 | -17.9168541 | 2316.607034 | -0.0077 | 0.994 | -1.56E-08 | count | 1 |
| AC003956.1 | -17.9168541 | 2316.607034 | -0.0077 | 0.994 | -1.56E-08 | count | 1 |
| SORBS2 | -17.9147783 | 1575.267806 | -0.0114 | 0.991 | -1.56E-08 | count | 1 |
| OLFML2B | -18.3740169 | 1742.120715 | -0.0105 | 0.992 | -1.56E-08 | count | 1 |
| RERG | -17.6254391 | 1687.545895 | -0.0104 | 0.992 | -1.56E-08 | count | 1 |
| AC005288.1 | -17.2109707 | 1627.764444 | -0.0106 | 0.992 | -1.56E-08 | count | 1 |
| AL021707.7 | -17.2109707 | 1627.764444 | -0.0106 | 0.992 | -1.56E-08 | count | 1 |
| DACT3 | -17.7288968 | 1734.736535 | -0.0102 | 0.992 | -1.56E-08 | count | 1 |
| BOLA2 | -17.3590018 | 1526.921481 | -0.0114 | 0.991 | -1.56E-08 | count | 1 |
| AGAP4 | -0.2014524 | 0.6592883 | -0.3056 | 0.76 | -1.54E-08 | count | 1 |
| CCL21 | -0.6131045 | 0.4940271 | -1.241 | 0.215 | -1.54E-08 | count | 1 |
| SH3PXD2B | -0.6105498 | 1.0279203 | -0.594 | 0.553 | -1.54E-08 | count | 1 |
| DPH1 | -0.6105498 | 0.7800235 | -0.7827 | 0.434 | -1.54E-08 | count | 1 |
| TMEM47 | -0.6105498 | 1.0249691 | -0.5957 | 0.551 | -1.54E-08 | count | 1 |
| LAMB2 | -0.6105498 | 1.0249691 | -0.5957 | 0.551 | -1.54E-08 | count | 1 |
| KIF5A | -0.6105498 | 1.1014154 | -0.5543 | 0.579 | -1.54E-08 | count | 1 |
| RAD54B | -0.6105498 | 0.6885963 | -0.8867 | 0.375 | -1.54E-08 | count | 1 |
| AC025263.1 | -0.6105498 | 0.9019965 | -0.6769 | 0.499 | -1.54E-08 | count | 1 |
| ALDH1A1 | -0.6105498 | 0.9782421 | -0.6241 | 0.533 | -1.54E-08 | count | 1 |
| CRABP2 | -0.5916148 | 0.6491026 | -0.9114 | 0.362 | -1.50E-08 | count | 1 |
| IL21R-AS1 | -0.5818768 | 0.8485737 | -0.6857 | 0.493 | -1.48E-08 | count | 1 |
| AC003681.1 | -0.0681386 | 0.3687477 | -0.1848 | 0.853 | -1.47E-08 | count | 1 |

| | | | | | | | |
|---|---|---|---|---|---|---|---|
| SPACA6 | -0.1911138 | 0.5703247 | -0.3351 | 0.738 | -1.47E-08 | count | 1 |
| AC025682.1 | -0.5684554 | 0.791544 | -0.7182 | 0.473 | -1.45E-08 | count | 1 |
| SHPK | -0.5684554 | 0.7990494 | -0.7114 | 0.477 | -1.45E-08 | count | 1 |
| RN7SL832P | -0.1881934 | 0.5773306 | -0.326 | 0.744 | -1.45E-08 | count | 1 |
| USP30 | -0.1875071 | 0.5486669 | -0.3418 | 0.733 | -1.44E-08 | count | 1 |
| TOGARAM2 | -0.1868371 | 0.4520098 | -0.4133 | 0.679 | -1.44E-08 | count | 1 |
| ZNF710-AS1 | -0.5558139 | 0.8552281 | -0.6499 | 0.516 | -1.42E-08 | count | 1 |
| SMARCA1 | -0.5558139 | 0.9037511 | -0.615 | 0.539 | -1.42E-08 | count | 1 |
| ZBTB26 | -0.5486891 | 0.6833637 | -0.8029 | 0.422 | -1.41E-08 | count | 1 |
| AC240274.1 | -0.5293389 | 0.781872 | -0.677 | 0.498 | -1.36E-08 | count | 1 |
| LRRC17 | -0.5280193 | 1.0563526 | -0.4999 | 0.617 | -1.36E-08 | count | 1 |
| LINC01252 | -0.5280193 | 0.8660452 | -0.6097 | 0.542 | -1.36E-08 | count | 1 |
| CPXM2 | -0.5280193 | 1.1567192 | -0.4565 | 0.648 | -1.36E-08 | count | 1 |
| PCAT19 | -0.5280193 | 1.1567192 | -0.4565 | 0.648 | -1.36E-08 | count | 1 |
| PTPRB | -0.5280193 | 0.9046135 | -0.5837 | 0.559 | -1.36E-08 | count | 1 |
| PPL | -0.5280193 | 0.9640461 | -0.5477 | 0.584 | -1.36E-08 | count | 1 |
| HMSD | -0.5280193 | 0.9859781 | -0.5355 | 0.592 | -1.36E-08 | count | 1 |
| AL590822.2 | -0.5280193 | 1.1015635 | -0.4793 | 0.632 | -1.36E-08 | count | 1 |
| AC005726.1 | -0.5265461 | 0.8996379 | -0.5853 | 0.558 | -1.36E-08 | count | 1 |
| GDPD3 | -0.5265461 | 0.7369616 | -0.7145 | 0.475 | -1.36E-08 | count | 1 |
| AL606807.1 | -0.5049809 | 0.71435 | -0.7069 | 0.48 | -1.31E-08 | count | 1 |
| DERL3 | -2.1535495 | 0.9082222 | -2.3712 | 0.0178 | -1.29E-08 | count | 1 |
| OR52N4 | -0.4760342 | 0.8965105 | -0.531 | 0.595 | -1.25E-08 | count | 1 |
| PLEK2 | -0.4666956 | 0.7406514 | -0.6301 | 0.529 | -1.22E-08 | count | 1 |
| AC011450.1 | -0.4666956 | 0.8925867 | -0.5229 | 0.601 | -1.22E-08 | count | 1 |
| AP001062.1 | -0.4666956 | 0.8642904 | -0.54 | 0.589 | -1.22E-08 | count | 1 |
| AC074386.1 | -0.4666956 | 0.7698923 | -0.6062 | 0.544 | -1.22E-08 | count | 1 |
| KCTD12 | -0.4605339 | 0.7018419 | -0.6562 | 0.512 | -1.21E-08 | count | 1 |
| BUB1B | -1.8658674 | 0.8541472 | -2.1845 | 0.029 | -1.21E-08 | count | 1 |
| TRBV11-2 | -1.8658674 | 0.911481 | -2.0471 | 0.0407 | -1.21E-08 | count | 1 |
| NPIPB15 | -1.8658674 | 0.911481 | -2.0471 | 0.0407 | -1.21E-08 | count | 1 |
| MT1A | -1.8658674 | 0.9654159 | -1.9327 | 0.0534 | -1.21E-08 | count | 1 |
| MBOAT2 | -0.1535763 | 0.6714176 | -0.2287 | 0.819 | -1.19E-08 | count | 1 |
| IQCH-AS1 | -0.1532102 | 0.515288 | -0.2973 | 0.766 | -1.19E-08 | count | 1 |
| DTX1 | -0.4430939 | 1.1005442 | -0.4026 | 0.687 | -1.17E-08 | count | 1 |
| RPS6KL1 | -0.4430939 | 0.8270857 | -0.5357 | 0.592 | -1.17E-08 | count | 1 |
| AC002553.1 | -0.4430939 | 0.8307843 | -0.5333 | 0.594 | -1.17E-08 | count | 1 |
| LINC00271 | -0.4430939 | 1.2112071 | -0.3658 | 0.715 | -1.17E-08 | count | 1 |
| RBMS3 | -0.4430939 | 0.9539334 | -0.4645 | 0.642 | -1.17E-08 | count | 1 |
| MOB3B | -0.4430939 | 1.0962905 | -0.4042 | 0.686 | -1.17E-08 | count | 1 |
| CLBA1 | -1.6835459 | 0.878402 | -1.9166 | 0.0554 | -1.15E-08 | count | 1 |
| CTNND1 | -1.6835459 | 0.9468564 | -1.778 | 0.0755 | -1.15E-08 | count | 1 |
| SLCO4A1 | -1.6835459 | 0.9468564 | -1.778 | 0.0755 | -1.15E-08 | count | 1 |
| KRT85 | -1.6835459 | 1.0707152 | -1.5724 | 0.116 | -1.15E-08 | count | 1 |
| AL022322.2 | -0.4324822 | 0.6844149 | -0.6319 | 0.527 | -1.15E-08 | count | 1 |

| | | | | | | | |
|---|---|---|---|---|---|---|---|
| DENND5A | -0.1454798 | 0.4991826 | -0.2914 | 0.771 | -1.13E-08 | count | 1 |
| CDK20 | -0.142862 | 0.5532263 | -0.2582 | 0.796 | -1.11E-08 | count | 1 |
| PHGDH | -0.4154014 | 0.7313009 | -0.568 | 0.57 | -1.11E-08 | count | 1 |
| TRAV9-2 | -0.4105802 | 0.5697299 | -0.7207 | 0.471 | -1.09E-08 | count | 1 |
| FAM239B | -0.4054965 | 0.8206903 | -0.4941 | 0.621 | -1.08E-08 | count | 1 |
| BDNF-AS | -0.4054965 | 0.8206903 | -0.4941 | 0.621 | -1.08E-08 | count | 1 |
| AL034417.4 | -0.4054965 | 0.7270009 | -0.5578 | 0.577 | -1.08E-08 | count | 1 |
| AL162457.1 | -0.4054965 | 0.7836078 | -0.5175 | 0.605 | -1.08E-08 | count | 1 |
| COL15A1 | -0.4054965 | 0.785976 | -0.5159 | 0.606 | -1.08E-08 | count | 1 |
| IGKV1-5 | -1.4604023 | 0.9146836 | -1.5966 | 0.11 | -1.06E-08 | count | 1 |
| FAM218A | -1.4604023 | 0.9996687 | -1.4609 | 0.144 | -1.06E-08 | count | 1 |
| RHOXF1 | -1.4604023 | 1.0779744 | -1.3548 | 0.176 | -1.06E-08 | count | 1 |
| ZNF471 | -1.4604023 | 1.0779744 | -1.3548 | 0.176 | -1.06E-08 | count | 1 |
| HAVCR1 | -1.4604023 | 1.2845633 | -1.1369 | 0.256 | -1.06E-08 | count | 1 |
| AC007342.5 | -1.4604023 | 1.2845633 | -1.1369 | 0.256 | -1.06E-08 | count | 1 |
| ADAMTS17 | -0.1346965 | 0.6061455 | -0.2222 | 0.824 | -1.05E-08 | count | 1 |
| CCNA2 | -0.1346285 | 0.563855 | -0.2388 | 0.811 | -1.05E-08 | count | 1 |
| POU5F2 | -0.1346285 | 0.5108636 | -0.2635 | 0.792 | -1.05E-08 | count | 1 |
| KCNMB4 | -0.3853594 | 0.675849 | -0.5702 | 0.569 | -1.03E-08 | count | 1 |
| C20orf96 | -0.3845236 | 0.9683409 | -0.3971 | 0.691 | -1.03E-08 | count | 1 |
| AC009133.2 | -0.3845236 | 1.061761 | -0.3622 | 0.717 | -1.03E-08 | count | 1 |
| RNF208 | -0.3845236 | 0.8118505 | -0.4736 | 0.636 | -1.03E-08 | count | 1 |
| CLIC2 | -0.3845236 | 1.061761 | -0.3622 | 0.717 | -1.03E-08 | count | 1 |
| GPBAR1 | -0.3845236 | 1.061761 | -0.3622 | 0.717 | -1.03E-08 | count | 1 |
| U62317.2 | -0.3845236 | 1.061761 | -0.3622 | 0.717 | -1.03E-08 | count | 1 |
| GLIDR | -0.3845236 | 0.9212782 | -0.4174 | 0.676 | -1.03E-08 | count | 1 |
| NBPF10 | -0.3845236 | 0.9457106 | -0.4066 | 0.684 | -1.03E-08 | count | 1 |
| JMJD1C-AS1 | -0.3845236 | 0.9457106 | -0.4066 | 0.684 | -1.03E-08 | count | 1 |
| ANGPTL4 | -0.3845236 | 0.9457106 | -0.4066 | 0.684 | -1.03E-08 | count | 1 |
| FZD2 | -0.3845236 | 0.9457106 | -0.4066 | 0.684 | -1.03E-08 | count | 1 |
| AL117381.1 | -0.3845236 | 0.9457106 | -0.4066 | 0.684 | -1.03E-08 | count | 1 |
| AC073508.3 | -0.3845236 | 0.9212782 | -0.4174 | 0.676 | -1.03E-08 | count | 1 |
| SLC6A8 | -0.3845236 | 0.9212782 | -0.4174 | 0.676 | -1.03E-08 | count | 1 |
| NLRP6 | -0.3845236 | 0.7665882 | -0.5016 | 0.616 | -1.03E-08 | count | 1 |
| AL139011.1 | -0.3845236 | 0.7354024 | -0.5229 | 0.601 | -1.03E-08 | count | 1 |
| AF213884.3 | -0.3845236 | 1.1285712 | -0.3407 | 0.733 | -1.03E-08 | count | 1 |
| KIR2DL4 | -0.3845236 | 0.7816887 | -0.4919 | 0.623 | -1.03E-08 | count | 1 |
| TTLL7 | -1.3665476 | 0.7406994 | -1.8449 | 0.0651 | -1.02E-08 | count | 1 |
| RB1-DT | -0.36179 | 0.6051808 | -0.5978 | 0.55 | -9.77E-09 | count | 1 |
| ZNF473 | -0.1233983 | 0.7174198 | -0.172 | 0.863 | -9.66E-09 | count | 1 |
| ABCG2 | -0.1233983 | 0.7958635 | -0.155 | 0.877 | -9.66E-09 | count | 1 |
| POMT2 | -0.1233983 | 0.7662619 | -0.161 | 0.872 | -9.66E-09 | count | 1 |
| TEAD3 | -0.3562146 | 0.8048636 | -0.4426 | 0.658 | -9.64E-09 | count | 1 |
| PCSK4 | -0.3562146 | 0.8470799 | -0.4205 | 0.674 | -9.64E-09 | count | 1 |
| AC011446.2 | -0.3562146 | 0.8665053 | -0.4111 | 0.681 | -9.64E-09 | count | 1 |

| | | | | | | | |
|---|---|---|---|---|---|---|---|
| TNNC1 | -0.3562146 | 0.8665053 | -0.4111 | 0.681 | -9.64E-09 | count | 1 |
| SOGA3 | -0.3562146 | 0.9257548 | -0.3848 | 0.7 | -9.64E-09 | count | 1 |
| AC092794.1 | -0.3562146 | 0.9435619 | -0.3775 | 0.706 | -9.64E-09 | count | 1 |
| AC079807.1 | -0.3430978 | 0.7893273 | -0.4347 | 0.664 | -9.31E-09 | count | 1 |
| KCNRG | -0.3430978 | 0.8852625 | -0.3876 | 0.698 | -9.31E-09 | count | 1 |
| AL031056.1 | -1.184226 | 1.0347325 | -1.1445 | 0.253 | -9.30E-09 | count | 1 |
| AL354707.1 | -1.1727203 | 0.8481023 | -1.3828 | 0.167 | -9.26E-09 | count | 1 |
| AC012511.1 | -1.1727203 | 0.9750184 | -1.2028 | 0.229 | -9.26E-09 | count | 1 |
| HIST1H2AB | -1.1727203 | 0.9750184 | -1.2028 | 0.229 | -9.26E-09 | count | 1 |
| DOCK6 | -1.1727203 | 0.9750184 | -1.2028 | 0.229 | -9.26E-09 | count | 1 |
| GJA4 | -1.1727203 | 0.9750184 | -1.2028 | 0.229 | -9.26E-09 | count | 1 |
| PARS2 | -1.1727203 | 0.9750184 | -1.2028 | 0.229 | -9.26E-09 | count | 1 |
| SCGB3A1 | -1.1727203 | 0.9750184 | -1.2028 | 0.229 | -9.26E-09 | count | 1 |
| AC022730.4 | -1.1727203 | 1.1888766 | -0.9864 | 0.324 | -9.25E-09 | count | 1 |
| AL592211.1 | -1.1727203 | 1.1888766 | -0.9864 | 0.324 | -9.25E-09 | count | 1 |
| VASH1-AS1 | -1.1727203 | 0.8481023 | -1.3828 | 0.167 | -9.25E-09 | count | 1 |
| AC048341.1 | -0.1174273 | 0.5384676 | -0.2181 | 0.827 | -9.21E-09 | count | 1 |
| AC009093.2 | -0.116604 | 0.5982572 | -0.1949 | 0.845 | -9.14E-09 | count | 1 |
| KCNC1 | -0.116604 | 0.7104547 | -0.1641 | 0.87 | -9.14E-09 | count | 1 |
| TENM1 | -0.335764 | 0.7817687 | -0.4295 | 0.668 | -9.13E-09 | count | 1 |
| STIL | -0.335764 | 0.6250151 | -0.5372 | 0.591 | -9.13E-09 | count | 1 |
| MAPK8IP1 | -0.335764 | 0.6708384 | -0.5005 | 0.617 | -9.13E-09 | count | 1 |
| CYB5RL | -0.3233434 | 0.6518468 | -0.496 | 0.62 | -8.83E-09 | count | 1 |
| STAM-AS1 | -0.1072018 | 0.476494 | -0.225 | 0.822 | -8.43E-09 | count | 1 |
| GSTM2 | -0.1057833 | 0.4951983 | -0.2136 | 0.831 | -8.32E-09 | count | 1 |
| REXO5 | -0.9903987 | 0.9299334 | -1.065 | 0.287 | -8.24E-09 | count | 1 |
| AL022328.3 | -0.2934426 | 0.7426639 | -0.3951 | 0.693 | -8.07E-09 | count | 1 |
| GHET1 | -0.2934426 | 1.0796877 | -0.2718 | 0.786 | -8.07E-09 | count | 1 |
| TBC1D9 | -0.2862832 | 0.96291 | -0.2973 | 0.766 | -7.89E-09 | count | 1 |
| BEND5 | -0.2862832 | 0.8183918 | -0.3498 | 0.727 | -7.89E-09 | count | 1 |
| FAM171A1 | -0.2862832 | 0.8202878 | -0.349 | 0.727 | -7.89E-09 | count | 1 |
| FP565260.6 | -0.2862832 | 1.1118651 | -0.2575 | 0.797 | -7.89E-09 | count | 1 |
| UNC45B | -0.2862832 | 0.8269323 | -0.3462 | 0.729 | -7.89E-09 | count | 1 |
| ICAM4 | -0.2862832 | 0.9685766 | -0.2956 | 0.768 | -7.89E-09 | count | 1 |
| CLDN12 | -0.2862832 | 0.9685766 | -0.2956 | 0.768 | -7.89E-09 | count | 1 |
| LHX4 | -0.2862832 | 1.3126315 | -0.2181 | 0.827 | -7.89E-09 | count | 1 |
| HM13-AS1 | -0.2862832 | 1.3126315 | -0.2181 | 0.827 | -7.89E-09 | count | 1 |
| WDR63 | -0.2862832 | 0.6844328 | -0.4183 | 0.676 | -7.89E-09 | count | 1 |
| IL1RAP | -0.0921399 | 0.3732688 | -0.2468 | 0.805 | -7.27E-09 | count | 1 |
| IGLV3-21 | -0.7672552 | 0.8438093 | -0.9093 | 0.363 | -6.80E-09 | count | 1 |
| ABCC2 | -0.7672552 | 0.8997938 | -0.8527 | 0.394 | -6.80E-09 | count | 1 |
| THY1 | -0.7672552 | 0.8997938 | -0.8527 | 0.394 | -6.80E-09 | count | 1 |
| MICALL2 | -0.7672552 | 0.9524935 | -0.8055 | 0.421 | -6.80E-09 | count | 1 |
| SCUBE1 | -0.7672552 | 0.7741974 | -0.991 | 0.322 | -6.80E-09 | count | 1 |
| TSKS | -0.7672552 | 0.7741974 | -0.991 | 0.322 | -6.80E-09 | count | 1 |

| | | | | | | | |
|---|---|---|---|---|---|---|---|
| PCDHGC3 | -0.7672552 | 1.0954854 | -0.7004 | 0.484 | -6.80E-09 | count | 1 |
| AL136320.1 | -0.7672552 | 1.0954854 | -0.7004 | 0.484 | -6.80E-09 | count | 1 |
| AC087164.1 | -0.2438661 | 0.7072622 | -0.3448 | 0.73 | -6.80E-09 | count | 1 |
| AC106791.1 | -0.2438661 | 0.6391642 | -0.3815 | 0.703 | -6.80E-09 | count | 1 |
| SPRY4 | -0.2438661 | 0.7755242 | -0.3145 | 0.753 | -6.80E-09 | count | 1 |
| AC090948.1 | -0.0838774 | 0.4378947 | -0.1915 | 0.848 | -6.63E-09 | count | 1 |
| VPS33B-DT | -0.2361746 | 0.753023 | -0.3136 | 0.754 | -6.60E-09 | count | 1 |
| AL358781.1 | -0.2294273 | 0.8322896 | -0.2757 | 0.783 | -6.42E-09 | count | 1 |
| LINC02580 | -0.2282587 | 0.3704483 | -0.6162 | 0.538 | -6.39E-09 | count | 1 |
| AC147651.1 | -0.0781241 | 0.7833575 | -0.0997 | 0.921 | -6.19E-09 | count | 1 |
| ANKRD33B | -0.0781241 | 0.7440675 | -0.105 | 0.916 | -6.19E-09 | count | 1 |
| LZTS3 | -0.0774944 | 0.5731358 | -0.1352 | 0.892 | -6.14E-09 | count | 1 |
| HSF4 | -0.0774944 | 0.6612614 | -0.1172 | 0.907 | -6.14E-09 | count | 1 |
| MDK | -0.0770355 | 0.6486699 | -0.1188 | 0.905 | -6.10E-09 | count | 1 |
| AC145207.5 | -0.2126234 | 0.7024305 | -0.3027 | 0.762 | -5.98E-09 | count | 1 |
| DMXL2 | -0.2126234 | 0.6492242 | -0.3275 | 0.743 | -5.98E-09 | count | 1 |
| AC021086.1 | -18.0072308 | 1902.257827 | -0.0095 | 0.992 | -5.77E-09 | count | 1 |
| CDCA8 | -18.0072308 | 1902.257827 | -0.0095 | 0.992 | -5.77E-09 | count | 1 |
| RAVER2 | -18.0072308 | 1902.257827 | -0.0095 | 0.992 | -5.77E-09 | count | 1 |
| AL162274.2 | -18.0072308 | 1902.257827 | -0.0095 | 0.992 | -5.77E-09 | count | 1 |
| PLAU | -18.0072308 | 1902.257827 | -0.0095 | 0.992 | -5.77E-09 | count | 1 |
| AC138028.2 | -17.5186638 | 1898.440621 | -0.0092 | 0.993 | -5.77E-09 | count | 1 |
| LILRB5 | -17.5186638 | 1898.440621 | -0.0092 | 0.993 | -5.77E-09 | count | 1 |
| AC104109.3 | -17.5186638 | 1898.440621 | -0.0092 | 0.993 | -5.77E-09 | count | 1 |
| AL590708.1 | -17.5186638 | 1898.440621 | -0.0092 | 0.993 | -5.77E-09 | count | 1 |
| DIO2 | -17.5186638 | 1898.440621 | -0.0092 | 0.993 | -5.77E-09 | count | 1 |
| AC005264.1 | -17.5186638 | 1898.440621 | -0.0092 | 0.993 | -5.77E-09 | count | 1 |
| AC096586.1 | -17.5186638 | 1898.440621 | -0.0092 | 0.993 | -5.77E-09 | count | 1 |
| AC012358.1 | -17.5186638 | 1898.440621 | -0.0092 | 0.993 | -5.77E-09 | count | 1 |
| AC008771.1 | -17.5186638 | 1898.440621 | -0.0092 | 0.993 | -5.77E-09 | count | 1 |
| PAPLN | -17.5186638 | 1898.440621 | -0.0092 | 0.993 | -5.77E-09 | count | 1 |
| ABCB5 | -17.5186638 | 1898.440621 | -0.0092 | 0.993 | -5.77E-09 | count | 1 |
| AL158207.2 | -17.5186638 | 1898.440621 | -0.0092 | 0.993 | -5.77E-09 | count | 1 |
| AL357556.4 | -17.5186638 | 1898.440621 | -0.0092 | 0.993 | -5.77E-09 | count | 1 |
| TDRP | -17.5186638 | 1898.440621 | -0.0092 | 0.993 | -5.77E-09 | count | 1 |
| JCAD | -17.5186638 | 1898.440621 | -0.0092 | 0.993 | -5.77E-09 | count | 1 |
| AC007608.1 | -17.5186638 | 1898.440621 | -0.0092 | 0.993 | -5.77E-09 | count | 1 |
| C13orf46 | -17.5186638 | 1898.440621 | -0.0092 | 0.993 | -5.77E-09 | count | 1 |
| AL137145.2 | -17.5186638 | 1898.440621 | -0.0092 | 0.993 | -5.77E-09 | count | 1 |
| BMPER | -17.5186638 | 1898.440621 | -0.0092 | 0.993 | -5.77E-09 | count | 1 |
| CXorf58 | -17.5186638 | 1898.440621 | -0.0092 | 0.993 | -5.77E-09 | count | 1 |
| AC068282.1 | -17.5186638 | 1898.440621 | -0.0092 | 0.993 | -5.77E-09 | count | 1 |
| NID2 | -17.5186638 | 1898.440621 | -0.0092 | 0.993 | -5.77E-09 | count | 1 |
| AC113382.1 | -17.5186638 | 1898.440621 | -0.0092 | 0.993 | -5.77E-09 | count | 1 |
| AC021752.1 | -17.5186638 | 1898.440621 | -0.0092 | 0.993 | -5.77E-09 | count | 1 |

| | | | | | | | |
|---|---|---|---|---|---|---|---|
| OMG | -17.5186638 | 1898.440621 | -0.0092 | 0.993 | -5.77E-09 | count | 1 |
| AC009123.1 | -17.5186638 | 1898.440621 | -0.0092 | 0.993 | -5.77E-09 | count | 1 |
| PBX1 | -17.5186638 | 1898.440621 | -0.0092 | 0.993 | -5.77E-09 | count | 1 |
| AC068025.2 | -17.5186638 | 1898.440621 | -0.0092 | 0.993 | -5.77E-09 | count | 1 |
| LAMA3 | -17.5186638 | 1898.440621 | -0.0092 | 0.993 | -5.77E-09 | count | 1 |
| EML1 | -17.5186638 | 1898.440621 | -0.0092 | 0.993 | -5.77E-09 | count | 1 |
| KRT23 | -17.5186638 | 1898.440621 | -0.0092 | 0.993 | -5.77E-09 | count | 1 |
| AC123768.4 | -17.5186638 | 1898.440621 | -0.0092 | 0.993 | -5.77E-09 | count | 1 |
| LINC00982 | -17.5186638 | 1898.440621 | -0.0092 | 0.993 | -5.77E-09 | count | 1 |
| STARD13 | -17.5186638 | 1898.440621 | -0.0092 | 0.993 | -5.77E-09 | count | 1 |
| AC027682.4 | -17.5186638 | 1898.440621 | -0.0092 | 0.993 | -5.77E-09 | count | 1 |
| AC011815.1 | -17.5186638 | 1898.440621 | -0.0092 | 0.993 | -5.77E-09 | count | 1 |
| SLC16A14 | -17.5186638 | 1898.440621 | -0.0092 | 0.993 | -5.77E-09 | count | 1 |
| AC015727.1 | -18.0074419 | 2423.98417 | -0.0074 | 0.994 | -5.77E-09 | count | 1 |
| AC022113.1 | -18.0074419 | 2423.98417 | -0.0074 | 0.994 | -5.77E-09 | count | 1 |
| KSR2 | -18.0074419 | 2423.98417 | -0.0074 | 0.994 | -5.77E-09 | count | 1 |
| CATSPERE | -18.0074419 | 2423.98417 | -0.0074 | 0.994 | -5.77E-09 | count | 1 |
| TRPM3 | -18.0074419 | 2423.98417 | -0.0074 | 0.994 | -5.77E-09 | count | 1 |
| MIOX | -18.0074419 | 2423.98417 | -0.0074 | 0.994 | -5.77E-09 | count | 1 |
| LCA5 | -18.0074419 | 2423.98417 | -0.0074 | 0.994 | -5.77E-09 | count | 1 |
| RARB | -18.0074419 | 2423.98417 | -0.0074 | 0.994 | -5.77E-09 | count | 1 |
| AL022328.1 | -18.0074419 | 2423.98417 | -0.0074 | 0.994 | -5.77E-09 | count | 1 |
| LYG2 | -18.0074419 | 2423.98417 | -0.0074 | 0.994 | -5.77E-09 | count | 1 |
| PLCD3 | -18.0074419 | 2423.98417 | -0.0074 | 0.994 | -5.77E-09 | count | 1 |
| AL049712.1 | -18.0074419 | 2423.98417 | -0.0074 | 0.994 | -5.77E-09 | count | 1 |
| ANKRD29 | -18.0074419 | 2423.98417 | -0.0074 | 0.994 | -5.77E-09 | count | 1 |
| OTUD6A | -18.0074419 | 2423.98417 | -0.0074 | 0.994 | -5.77E-09 | count | 1 |
| TNFAIP8L3 | -18.0074419 | 2423.98417 | -0.0074 | 0.994 | -5.77E-09 | count | 1 |
| VWA1 | -18.0635519 | 1437.653979 | -0.0126 | 0.99 | -5.77E-09 | count | 1 |
| HIST1H2BL | -18.0635519 | 1437.653979 | -0.0126 | 0.99 | -5.77E-09 | count | 1 |
| CR936218.1 | -18.0074419 | 2423.98417 | -0.0074 | 0.994 | -5.77E-09 | count | 1 |
| TEX29 | -18.0074419 | 2423.98417 | -0.0074 | 0.994 | -5.77E-09 | count | 1 |
| TMEM184A | -18.0074419 | 2423.98417 | -0.0074 | 0.994 | -5.77E-09 | count | 1 |
| AL133551.1 | -18.0074419 | 2423.98417 | -0.0074 | 0.994 | -5.77E-09 | count | 1 |
| ADRA2B | -18.0074419 | 2423.98417 | -0.0074 | 0.994 | -5.77E-09 | count | 1 |
| MYRF | -18.0074419 | 2423.98417 | -0.0074 | 0.994 | -5.77E-09 | count | 1 |
| ACKR4 | -18.0074419 | 2423.98417 | -0.0074 | 0.994 | -5.77E-09 | count | 1 |
| AC096734.1 | -18.0074419 | 2423.98417 | -0.0074 | 0.994 | -5.77E-09 | count | 1 |
| GGT5 | -18.0074419 | 2423.98417 | -0.0074 | 0.994 | -5.77E-09 | count | 1 |
| AC009812.3 | -18.0074419 | 2423.98417 | -0.0074 | 0.994 | -5.77E-09 | count | 1 |
| HCAR2 | -18.0074419 | 2423.98417 | -0.0074 | 0.994 | -5.77E-09 | count | 1 |
| FMOD | -18.0074419 | 2423.98417 | -0.0074 | 0.994 | -5.77E-09 | count | 1 |
| KDELR3 | -18.3269205 | 2257.089008 | -0.0081 | 0.994 | -5.77E-09 | count | 1 |
| LINC01220 | -18.3269205 | 2257.089008 | -0.0081 | 0.994 | -5.77E-09 | count | 1 |
| AC087393.2 | -18.3269205 | 2257.089008 | -0.0081 | 0.994 | -5.77E-09 | count | 1 |

| | | | | | | | |
|---|---|---|---|---|---|---|---|
| SNORC | -18.3269205 | 2257.089008 | -0.0081 | 0.994 | -5.77E-09 | count | 1 |
| ZNF618 | -18.3269205 | 2257.089008 | -0.0081 | 0.994 | -5.77E-09 | count | 1 |
| LINC00545 | -18.064095 | 1857.771311 | -0.0097 | 0.992 | -5.77E-09 | count | 1 |
| CCNB3 | -18.064095 | 1857.771311 | -0.0097 | 0.992 | -5.77E-09 | count | 1 |
| AL159169.3 | -18.064095 | 1857.771311 | -0.0097 | 0.992 | -5.77E-09 | count | 1 |
| IGHV2-5 | -18.064095 | 1857.771311 | -0.0097 | 0.992 | -5.77E-09 | count | 1 |
| TWIST2 | -18.064095 | 1857.771311 | -0.0097 | 0.992 | -5.77E-09 | count | 1 |
| CFI | -18.064095 | 1857.771311 | -0.0097 | 0.992 | -5.77E-09 | count | 1 |
| GPR85 | -18.064095 | 1857.771311 | -0.0097 | 0.992 | -5.77E-09 | count | 1 |
| FAM133A | -18.064095 | 1857.771311 | -0.0097 | 0.992 | -5.77E-09 | count | 1 |
| IL13RA2 | -18.064095 | 1857.771311 | -0.0097 | 0.992 | -5.77E-09 | count | 1 |
| RBP5 | -18.064095 | 1857.771311 | -0.0097 | 0.992 | -5.77E-09 | count | 1 |
| AC025034.1 | -18.064095 | 1857.771311 | -0.0097 | 0.992 | -5.77E-09 | count | 1 |
| CCSER1 | -18.064095 | 1857.771311 | -0.0097 | 0.992 | -5.77E-09 | count | 1 |
| RTN1 | -18.064095 | 1857.771311 | -0.0097 | 0.992 | -5.77E-09 | count | 1 |
| AC135068.9 | -18.064095 | 1857.771311 | -0.0097 | 0.992 | -5.77E-09 | count | 1 |
| CPT1C | -18.064095 | 1857.771311 | -0.0097 | 0.992 | -5.77E-09 | count | 1 |
| DNM3OS | -18.064095 | 1857.771311 | -0.0097 | 0.992 | -5.77E-09 | count | 1 |
| CXCL9 | -18.064095 | 1857.771311 | -0.0097 | 0.992 | -5.77E-09 | count | 1 |
| ZG16B | -18.3520432 | 1437.419299 | -0.0128 | 0.99 | -5.77E-09 | count | 1 |
| SPC24 | -18.5657928 | 2165.726883 | -0.0086 | 0.993 | -5.76E-09 | count | 1 |
| AC012510.1 | -18.2124093 | 1897.669873 | -0.0096 | 0.992 | -5.76E-09 | count | 1 |
| C1orf115 | -18.2124093 | 1897.669873 | -0.0096 | 0.992 | -5.76E-09 | count | 1 |
| ARHGAP22 | -18.2124093 | 1897.669873 | -0.0096 | 0.992 | -5.76E-09 | count | 1 |
| FCRL5 | -18.5657928 | 2165.726883 | -0.0086 | 0.993 | -5.76E-09 | count | 1 |
| AL512625.2 | -18.3272904 | 2844.344462 | -0.0064 | 0.995 | -5.76E-09 | count | 1 |
| MERTK | -18.3272904 | 2844.344462 | -0.0064 | 0.995 | -5.76E-09 | count | 1 |
| GBP6 | -18.3272904 | 2844.344462 | -0.0064 | 0.995 | -5.76E-09 | count | 1 |
| MIR99AHG | -18.3272904 | 2844.344462 | -0.0064 | 0.995 | -5.76E-09 | count | 1 |
| FGF13 | -18.3272904 | 2844.344462 | -0.0064 | 0.995 | -5.76E-09 | count | 1 |
| AC010327.4 | -18.3272904 | 2844.344462 | -0.0064 | 0.995 | -5.76E-09 | count | 1 |
| AF131216.4 | -18.3272904 | 2844.344462 | -0.0064 | 0.995 | -5.76E-09 | count | 1 |
| KCTD16 | -18.3272904 | 2844.344462 | -0.0064 | 0.995 | -5.76E-09 | count | 1 |
| IGKV3-11 | -18.0648541 | 2494.538168 | -0.0072 | 0.994 | -5.76E-09 | count | 1 |
| IGKV3-15 | -18.0648541 | 2494.538168 | -0.0072 | 0.994 | -5.76E-09 | count | 1 |
| FOCAD-AS1 | -18.0648541 | 2494.538168 | -0.0072 | 0.994 | -5.76E-09 | count | 1 |
| ZNF521 | -18.0648541 | 2494.538168 | -0.0072 | 0.994 | -5.76E-09 | count | 1 |
| AC010198.1 | -18.0648541 | 2494.538168 | -0.0072 | 0.994 | -5.76E-09 | count | 1 |
| AL590133.1 | -18.0648541 | 2494.538168 | -0.0072 | 0.994 | -5.76E-09 | count | 1 |
| OSR1 | -18.0648541 | 2494.538168 | -0.0072 | 0.994 | -5.76E-09 | count | 1 |
| AC018742.1 | -18.0648541 | 2494.538168 | -0.0072 | 0.994 | -5.76E-09 | count | 1 |
| ANKHD1-EIF4EBP3 | -18.0648541 | 2494.538168 | -0.0072 | 0.994 | -5.76E-09 | count | 1 |
| DLGAP5 | -18.0648541 | 2494.538168 | -0.0072 | 0.994 | -5.76E-09 | count | 1 |
| SIRPD | -18.0648541 | 2494.538168 | -0.0072 | 0.994 | -5.76E-09 | count | 1 |
| AC079305.3 | -18.0648541 | 2494.538168 | -0.0072 | 0.994 | -5.76E-09 | count | 1 |

| | | | | | | | |
|---|---|---|---|---|---|---|---|
| SCG2 | -18.0648541 | 2494.538168 | -0.0072 | 0.994 | -5.76E-09 | count | 1 |
| NPNT | -18.0648541 | 2494.538168 | -0.0072 | 0.994 | -5.76E-09 | count | 1 |
| AL157832.3 | -18.0648541 | 2494.538168 | -0.0072 | 0.994 | -5.76E-09 | count | 1 |
| AL355810.1 | -18.0648541 | 2494.538168 | -0.0072 | 0.994 | -5.76E-09 | count | 1 |
| IGHV1-45 | -18.0648541 | 2494.538168 | -0.0072 | 0.994 | -5.76E-09 | count | 1 |
| PLA2G10 | -18.0648541 | 2494.538168 | -0.0072 | 0.994 | -5.76E-09 | count | 1 |
| AC135012.1 | -18.0648541 | 2494.538168 | -0.0072 | 0.994 | -5.76E-09 | count | 1 |
| ZNF396 | -18.0648541 | 2494.538168 | -0.0072 | 0.994 | -5.76E-09 | count | 1 |
| LILRA2 | -18.0648541 | 2494.538168 | -0.0072 | 0.994 | -5.76E-09 | count | 1 |
| LINC01116 | -18.0648541 | 2494.538168 | -0.0072 | 0.994 | -5.76E-09 | count | 1 |
| AIF1L | -18.0648541 | 2494.538168 | -0.0072 | 0.994 | -5.76E-09 | count | 1 |
| SMTNL1 | -18.0648541 | 2494.538168 | -0.0072 | 0.994 | -5.76E-09 | count | 1 |
| AP001893.1 | -18.0648541 | 2494.538168 | -0.0072 | 0.994 | -5.76E-09 | count | 1 |
| AL132639.3 | -18.0648541 | 2494.538168 | -0.0072 | 0.994 | -5.76E-09 | count | 1 |
| GLIS1 | -18.0648541 | 2494.538168 | -0.0072 | 0.994 | -5.76E-09 | count | 1 |
| TMEM233 | -18.0648541 | 2494.538168 | -0.0072 | 0.994 | -5.76E-09 | count | 1 |
| STK24-AS1 | -18.0648541 | 2494.538168 | -0.0072 | 0.994 | -5.76E-09 | count | 1 |
| NDRG4 | -18.0648541 | 2494.538168 | -0.0072 | 0.994 | -5.76E-09 | count | 1 |
| FAM20A | -18.0648541 | 2494.538168 | -0.0072 | 0.994 | -5.76E-09 | count | 1 |
| TNFAIP6 | -18.0648541 | 2494.538168 | -0.0072 | 0.994 | -5.76E-09 | count | 1 |
| FOXS1 | -18.0648541 | 2494.538168 | -0.0072 | 0.994 | -5.76E-09 | count | 1 |
| AL357033.1 | -18.0648541 | 2494.538168 | -0.0072 | 0.994 | -5.76E-09 | count | 1 |
| LINC00240 | -18.3525469 | 1762.248898 | -0.0104 | 0.992 | -5.76E-09 | count | 1 |
| CD24 | -18.3525469 | 1762.248898 | -0.0104 | 0.992 | -5.76E-09 | count | 1 |
| CMTM1 | -18.2126942 | 2228.742416 | -0.0082 | 0.993 | -5.76E-09 | count | 1 |
| S1PR3 | -18.2124495 | 2418.258721 | -0.0075 | 0.994 | -5.76E-09 | count | 1 |
| MRGPRF | -18.2124495 | 2418.258721 | -0.0075 | 0.994 | -5.76E-09 | count | 1 |
| FAM198B | -18.2124495 | 2418.258721 | -0.0075 | 0.994 | -5.76E-09 | count | 1 |
| LIMCH1 | -18.2124495 | 2418.258721 | -0.0075 | 0.994 | -5.76E-09 | count | 1 |
| EBF1 | -18.2124495 | 2418.258721 | -0.0075 | 0.994 | -5.76E-09 | count | 1 |
| SPATA41 | -18.2124495 | 2418.258721 | -0.0075 | 0.994 | -5.76E-09 | count | 1 |
| AD000671.3 | -18.2124495 | 2418.258721 | -0.0075 | 0.994 | -5.76E-09 | count | 1 |
| SDC3 | -18.2124495 | 2418.258721 | -0.0075 | 0.994 | -5.76E-09 | count | 1 |
| AL080317.3 | -18.3530367 | 2036.137805 | -0.009 | 0.993 | -5.76E-09 | count | 1 |
| IGLV6-57 | -18.3530367 | 2036.137805 | -0.009 | 0.993 | -5.76E-09 | count | 1 |
| CYP2E1 | -18.3530367 | 2036.137805 | -0.009 | 0.993 | -5.76E-09 | count | 1 |
| TM4SF18 | -18.3530367 | 2036.137805 | -0.009 | 0.993 | -5.76E-09 | count | 1 |
| AC005537.1 | -18.3530367 | 2036.137805 | -0.009 | 0.993 | -5.76E-09 | count | 1 |
| CADPS2 | -18.3530367 | 2036.137805 | -0.009 | 0.993 | -5.76E-09 | count | 1 |
| SLX1A | -18.3530367 | 2036.137805 | -0.009 | 0.993 | -5.76E-09 | count | 1 |
| AC114488.3 | -18.3530367 | 2036.137805 | -0.009 | 0.993 | -5.76E-09 | count | 1 |
| FNDC11 | -18.5763248 | 1702.159815 | -0.0109 | 0.991 | -5.76E-09 | count | 1 |
| AGBL5-AS1 | -18.2127478 | 2686.058324 | -0.0068 | 0.995 | -5.76E-09 | count | 1 |
| AP001469.3 | -18.2127478 | 2686.058324 | -0.0068 | 0.995 | -5.76E-09 | count | 1 |
| ICAM5 | -18.2127478 | 2686.058324 | -0.0068 | 0.995 | -5.76E-09 | count | 1 |

| | | | | | | | |
|---|---|---|---|---|---|---|---|
| AL157895.1 | -18.2127478 | 2686.058324 | -0.0068 | 0.995 | -5.76E-09 | count | 1 |
| FKBP9 | -18.2127478 | 2686.058324 | -0.0068 | 0.995 | -5.76E-09 | count | 1 |
| SMIM10 | -18.2127478 | 2686.058324 | -0.0068 | 0.995 | -5.76E-09 | count | 1 |
| AL354793.1 | -18.2127478 | 2686.058324 | -0.0068 | 0.995 | -5.76E-09 | count | 1 |
| ATOH8 | -18.3532496 | 2277.229745 | -0.0081 | 0.994 | -5.76E-09 | count | 1 |
| ASIP | -18.3532496 | 2277.229745 | -0.0081 | 0.994 | -5.76E-09 | count | 1 |
| TTTY14 | -18.2127478 | 2686.058324 | -0.0068 | 0.995 | -5.76E-09 | count | 1 |
| FILIP1 | -17.8131883 | 1873.361942 | -0.0095 | 0.992 | -5.76E-09 | count | 1 |
| PVR | -17.8131883 | 1873.361942 | -0.0095 | 0.992 | -5.76E-09 | count | 1 |
| AC020978.5 | -17.8131883 | 1873.361942 | -0.0095 | 0.992 | -5.76E-09 | count | 1 |
| ARHGAP24 | -18.5767769 | 1931.37411 | -0.0096 | 0.992 | -5.76E-09 | count | 1 |
| L1CAM | -18.5767769 | 1931.37411 | -0.0096 | 0.992 | -5.76E-09 | count | 1 |
| MMP15 | -18.5767769 | 1931.37411 | -0.0096 | 0.992 | -5.76E-09 | count | 1 |
| RYR2 | -17.8137536 | 2200.288997 | -0.0081 | 0.994 | -5.76E-09 | count | 1 |
| SERPINF2 | -17.8137536 | 2200.288997 | -0.0081 | 0.994 | -5.76E-09 | count | 1 |
| NR6A1 | -17.8137536 | 2200.288997 | -0.0081 | 0.994 | -5.76E-09 | count | 1 |
| SLC7A7 | -17.8137536 | 2200.288997 | -0.0081 | 0.994 | -5.76E-09 | count | 1 |
| ALDH1A2 | -17.8137536 | 2200.288997 | -0.0081 | 0.994 | -5.76E-09 | count | 1 |
| HEATR4 | -17.8137536 | 2200.288997 | -0.0081 | 0.994 | -5.76E-09 | count | 1 |
| TP53I3 | -17.8137536 | 2200.288997 | -0.0081 | 0.994 | -5.76E-09 | count | 1 |
| CD300LF | -17.8137536 | 2200.288997 | -0.0081 | 0.994 | -5.76E-09 | count | 1 |
| COL27A1 | -17.8137536 | 2200.288997 | -0.0081 | 0.994 | -5.76E-09 | count | 1 |
| LONRF2 | -17.8137536 | 2200.288997 | -0.0081 | 0.994 | -5.76E-09 | count | 1 |
| AC104365.3 | -17.8137536 | 2200.288997 | -0.0081 | 0.994 | -5.76E-09 | count | 1 |
| MFAP3L | -17.8137536 | 2200.288997 | -0.0081 | 0.994 | -5.76E-09 | count | 1 |
| PCA3 | -17.8137536 | 2200.288997 | -0.0081 | 0.994 | -5.76E-09 | count | 1 |
| MGARP | -17.8137536 | 2200.288997 | -0.0081 | 0.994 | -5.76E-09 | count | 1 |
| CNKSR3 | -17.8137536 | 2200.288997 | -0.0081 | 0.994 | -5.76E-09 | count | 1 |
| AC074387.1 | -18.373109 | 1885.381631 | -0.0097 | 0.992 | -5.76E-09 | count | 1 |
| FOXB1 | -18.5769731 | 2136.035754 | -0.0087 | 0.993 | -5.76E-09 | count | 1 |
| SRPX | -18.5769731 | 2136.035754 | -0.0087 | 0.993 | -5.76E-09 | count | 1 |
| NEGR1 | -18.3540567 | 2882.63542 | -0.0064 | 0.995 | -5.76E-09 | count | 1 |
| AL589765.5 | -18.3540567 | 2882.63542 | -0.0064 | 0.995 | -5.76E-09 | count | 1 |
| ST3GAL6-AS1 | -18.3540567 | 2882.63542 | -0.0064 | 0.995 | -5.76E-09 | count | 1 |
| AP006216.1 | -18.3540567 | 2882.63542 | -0.0064 | 0.995 | -5.76E-09 | count | 1 |
| CD177 | -18.3540567 | 2882.63542 | -0.0064 | 0.995 | -5.76E-09 | count | 1 |
| RUNX1T1 | -18.5774056 | 2323.0783 | -0.008 | 0.994 | -5.76E-09 | count | 1 |
| SP5 | -18.5774056 | 2323.0783 | -0.008 | 0.994 | -5.76E-09 | count | 1 |
| FGD2 | -18.5774056 | 2323.0783 | -0.008 | 0.994 | -5.76E-09 | count | 1 |
| AC245128.3 | -18.5774056 | 2323.0783 | -0.008 | 0.994 | -5.76E-09 | count | 1 |
| OSR2 | -18.5774056 | 2323.0783 | -0.008 | 0.994 | -5.76E-09 | count | 1 |
| UHRF1 | -18.3730589 | 2402.816989 | -0.0076 | 0.994 | -5.76E-09 | count | 1 |
| AC007228.2 | -18.9141417 | 1803.672574 | -0.0105 | 0.992 | -5.75E-09 | count | 1 |
| MIR9-3HG | -18.9145078 | 1961.921733 | -0.0096 | 0.992 | -5.75E-09 | count | 1 |
| ASPN | -18.759992 | 2036.499989 | -0.0092 | 0.993 | -5.75E-09 | count | 1 |

| | | | | | | | |
|---|---|---|---|---|---|---|---|
| FZD1 | -18.759992 | 2036.499989 | -0.0092 | 0.993 | -5.75E-09 | count | 1 |
| GYPE | -18.759992 | 2036.499989 | -0.0092 | 0.993 | -5.75E-09 | count | 1 |
| TTC24 | -18.760166 | 2200.397782 | -0.0085 | 0.993 | -5.75E-09 | count | 1 |
| LCN6 | -18.760166 | 2200.397782 | -0.0085 | 0.993 | -5.75E-09 | count | 1 |
| MAGI1 | -18.760166 | 2200.397782 | -0.0085 | 0.993 | -5.75E-09 | count | 1 |
| FGF17 | -18.5777157 | 2657.635064 | -0.007 | 0.994 | -5.75E-09 | count | 1 |
| SLCO2B1 | -19.0483165 | 1903.883074 | -0.01 | 0.992 | -5.75E-09 | count | 1 |
| IGHGP | -19.0483165 | 1903.883074 | -0.01 | 0.992 | -5.75E-09 | count | 1 |
| CD276 | -19.0483165 | 1903.883074 | -0.01 | 0.992 | -5.75E-09 | count | 1 |
| APBB2 | -18.7607141 | 2496.509206 | -0.0075 | 0.994 | -5.75E-09 | count | 1 |
| AC015726.1 | -18.0321796 | 1851.019927 | -0.0097 | 0.992 | -5.75E-09 | count | 1 |
| FAM114A1 | -18.0321796 | 1851.019927 | -0.0097 | 0.992 | -5.75E-09 | count | 1 |
| ACAN | -18.7608222 | 2631.992379 | -0.0071 | 0.994 | -5.75E-09 | count | 1 |
| AL009178.2 | -18.5785034 | 3224.984872 | -0.0058 | 0.995 | -5.75E-09 | count | 1 |
| AC121338.1 | -18.9152611 | 2496.532102 | -0.0076 | 0.994 | -5.75E-09 | count | 1 |
| SNRK-AS1 | -18.032813 | 2072.349009 | -0.0087 | 0.993 | -5.75E-09 | count | 1 |
| RIPOR3 | -18.032813 | 2072.349009 | -0.0087 | 0.993 | -5.75E-09 | count | 1 |
| AC007292.2 | -18.032813 | 2072.349009 | -0.0087 | 0.993 | -5.75E-09 | count | 1 |
| MILR1 | -18.032813 | 2072.349009 | -0.0087 | 0.993 | -5.75E-09 | count | 1 |
| SPRY3 | -18.7611667 | 3002.156598 | -0.0062 | 0.995 | -5.75E-09 | count | 1 |
| AC022113.2 | -18.0336783 | 2456.057215 | -0.0073 | 0.994 | -5.75E-09 | count | 1 |
| KIF18B | -18.0336783 | 2456.057215 | -0.0073 | 0.994 | -5.75E-09 | count | 1 |
| UNC5B | -18.0336783 | 2456.057215 | -0.0073 | 0.994 | -5.75E-09 | count | 1 |
| SASH1 | -18.0336783 | 2456.057215 | -0.0073 | 0.994 | -5.75E-09 | count | 1 |
| NARF-AS1 | -18.0336783 | 2456.057215 | -0.0073 | 0.994 | -5.75E-09 | count | 1 |
| FBLN2 | -18.2091053 | 2000.152124 | -0.0091 | 0.993 | -5.74E-09 | count | 1 |
| ZBTB47 | -18.2097215 | 2154.330998 | -0.0085 | 0.993 | -5.74E-09 | count | 1 |
| YAP1 | -18.2097215 | 2154.330998 | -0.0085 | 0.993 | -5.74E-09 | count | 1 |
| KIZ-AS1 | -18.2099861 | 2297.957171 | -0.0079 | 0.994 | -5.74E-09 | count | 1 |
| CCDC74B | -18.2099861 | 2297.957171 | -0.0079 | 0.994 | -5.74E-09 | count | 1 |
| AL355353.1 | -18.2099861 | 2297.957171 | -0.0079 | 0.994 | -5.74E-09 | count | 1 |
| ABI3BP | -17.9016609 | 1213.712808 | -0.0147 | 0.988 | -5.73E-09 | count | 1 |
| TNFRSF21 | -17.9016609 | 1213.712808 | -0.0147 | 0.988 | -5.73E-09 | count | 1 |
| AC084824.5 | -17.9028429 | 1524.22103 | -0.0117 | 0.991 | -5.72E-09 | count | 1 |
| AC232271.1 | -17.9028429 | 1524.22103 | -0.0117 | 0.991 | -5.72E-09 | count | 1 |
| ATP8B1 | -17.9028429 | 1524.22103 | -0.0117 | 0.991 | -5.72E-09 | count | 1 |
| AL590666.3 | -17.6801449 | 2058.162211 | -0.0086 | 0.993 | -5.72E-09 | count | 1 |
| Z95114.2 | -17.6801449 | 2058.162211 | -0.0086 | 0.993 | -5.72E-09 | count | 1 |
| INHBA | -18.0864121 | 1453.205354 | -0.0124 | 0.99 | -5.72E-09 | count | 1 |
| PEAR1 | -17.9041992 | 1897.562515 | -0.0094 | 0.992 | -5.72E-09 | count | 1 |
| SLC3A1 | -17.9041992 | 1897.562515 | -0.0094 | 0.992 | -5.72E-09 | count | 1 |
| PLA2G4B | -17.9041992 | 1897.562515 | -0.0094 | 0.992 | -5.72E-09 | count | 1 |
| LRRC6 | -18.3754311 | 1452.81491 | -0.0126 | 0.99 | -5.72E-09 | count | 1 |
| ADARB2 | -18.4946658 | 1644.174543 | -0.0112 | 0.991 | -5.72E-09 | count | 1 |
| AC009950.1 | -18.4946658 | 1644.174543 | -0.0112 | 0.991 | -5.72E-09 | count | 1 |

| | | | | | | | |
|---|---|---|---|---|---|---|---|
| AC005697.2 | -18.0898779 | 2526.108386 | -0.0072 | 0.994 | -5.72E-09 | count | 1 |
| AC120349.1 | -18.0898779 | 2526.108386 | -0.0072 | 0.994 | -5.72E-09 | count | 1 |
| SLC4A11 | -0.6105498 | 1.1014154 | -0.5543 | 0.579 | -5.66E-09 | count | 1 |
| AL161756.1 | -0.6105498 | 1.1060342 | -0.552 | 0.581 | -5.66E-09 | count | 1 |
| AL080250.1 | -0.6105498 | 1.1014154 | -0.5543 | 0.579 | -5.66E-09 | count | 1 |
| ERG | -0.6105498 | 1.1014154 | -0.5543 | 0.579 | -5.66E-09 | count | 1 |
| PRR19 | -0.6105498 | 1.1014154 | -0.5543 | 0.579 | -5.66E-09 | count | 1 |
| F3 | -0.6105498 | 1.3598878 | -0.449 | 0.653 | -5.66E-09 | count | 1 |
| PLA2R1 | -0.6105498 | 1.3598878 | -0.449 | 0.653 | -5.66E-09 | count | 1 |
| AC135050.1 | -0.6105498 | 1.3598878 | -0.449 | 0.653 | -5.66E-09 | count | 1 |
| AL590723.1 | -0.6105498 | 1.3598878 | -0.449 | 0.653 | -5.66E-09 | count | 1 |
| FMNL2 | -0.6105498 | 1.3598878 | -0.449 | 0.653 | -5.66E-09 | count | 1 |
| AC025287.3 | -0.6105498 | 1.3598878 | -0.449 | 0.653 | -5.66E-09 | count | 1 |
| LYNX1 | -0.6105498 | 1.3598878 | -0.449 | 0.653 | -5.66E-09 | count | 1 |
| PCDHB2 | -0.6105498 | 1.3598878 | -0.449 | 0.653 | -5.66E-09 | count | 1 |
| AL021707.1 | -0.6105498 | 1.3598878 | -0.449 | 0.653 | -5.66E-09 | count | 1 |
| EXOC3L1 | -0.6105498 | 1.3598878 | -0.449 | 0.653 | -5.66E-09 | count | 1 |
| AL590006.1 | -0.6105498 | 1.3598878 | -0.449 | 0.653 | -5.66E-09 | count | 1 |
| ADCY2 | -0.6105498 | 1.3598878 | -0.449 | 0.653 | -5.66E-09 | count | 1 |
| AC006213.1 | -0.6105498 | 1.3598878 | -0.449 | 0.653 | -5.66E-09 | count | 1 |
| TTC28 | -0.6105498 | 1.3598878 | -0.449 | 0.653 | -5.66E-09 | count | 1 |
| AC130650.2 | -0.6105498 | 1.3598878 | -0.449 | 0.653 | -5.66E-09 | count | 1 |
| TRAV24 | -0.6105498 | 1.3598878 | -0.449 | 0.653 | -5.66E-09 | count | 1 |
| SLC8A1 | -0.6105498 | 1.3598878 | -0.449 | 0.653 | -5.66E-09 | count | 1 |
| IGLV3-1 | -0.5849336 | 0.8675813 | -0.6742 | 0.5 | -5.46E-09 | count | 1 |
| AP002026.1 | -0.5849336 | 0.6309888 | -0.927 | 0.354 | -5.46E-09 | count | 1 |
| HLA-DQB2 | -0.5849336 | 0.6309888 | -0.927 | 0.354 | -5.46E-09 | count | 1 |
| ZNF233 | -0.5849336 | 0.78031 | -0.7496 | 0.454 | -5.46E-09 | count | 1 |
| ARAP3 | -0.1896173 | 0.8302114 | -0.2284 | 0.819 | -5.37E-09 | count | 1 |
| IFNLR1 | -0.1896173 | 0.7550076 | -0.2511 | 0.802 | -5.37E-09 | count | 1 |
| PFKFB2 | -0.185544 | 1.0995875 | -0.1687 | 0.866 | -5.26E-09 | count | 1 |
| AL137786.1 | -0.185544 | 1.1604428 | -0.1599 | 0.873 | -5.26E-09 | count | 1 |
| SH3RF3 | -0.185544 | 1.1604428 | -0.1599 | 0.873 | -5.26E-09 | count | 1 |
| AC124068.2 | -0.185544 | 0.8237639 | -0.2252 | 0.822 | -5.26E-09 | count | 1 |
| CDCA5 | -0.185544 | 1.1102593 | -0.1671 | 0.867 | -5.26E-09 | count | 1 |
| DRC3 | -0.185544 | 0.8408775 | -0.2207 | 0.825 | -5.26E-09 | count | 1 |
| CBX2 | -0.185544 | 1.2734587 | -0.1457 | 0.884 | -5.26E-09 | count | 1 |
| SHISA8 | -0.185544 | 0.9190265 | -0.2019 | 0.84 | -5.26E-09 | count | 1 |
| TTC21A | -0.185544 | 0.734891 | -0.2525 | 0.801 | -5.26E-09 | count | 1 |
| FAM69B | -0.185544 | 1.2734587 | -0.1457 | 0.884 | -5.26E-09 | count | 1 |
| C7orf25 | -0.185544 | 0.9033946 | -0.2054 | 0.837 | -5.26E-09 | count | 1 |
| AC108673.2 | -0.185544 | 1.0330059 | -0.1796 | 0.857 | -5.26E-09 | count | 1 |
| C9orf64 | -0.0612581 | 0.4061425 | -0.1508 | 0.88 | -4.87E-09 | count | 1 |
| TONSL | -0.1682569 | 0.6533957 | -0.2575 | 0.797 | -4.79E-09 | count | 1 |
| TP53TG3D | -0.4910789 | 0.5852325 | -0.8391 | 0.401 | -4.71E-09 | count | 1 |

| | | | | | | | |
|---|---|---|---|---|---|---|---|
| P4HA3 | -0.4910789 | 0.5197569 | -0.9448 | 0.345 | -4.71E-09 | count | 1 |
| AC004069.1 | -0.4795731 | 0.8263844 | -0.5803 | 0.562 | -4.61E-09 | count | 1 |
| SDC1 | -0.4795731 | 0.9096845 | -0.5272 | 0.598 | -4.61E-09 | count | 1 |
| CLEC18A | -0.4795731 | 0.9096845 | -0.5272 | 0.598 | -4.61E-09 | count | 1 |
| AL360270.3 | -0.4795731 | 0.7706605 | -0.6223 | 0.534 | -4.61E-09 | count | 1 |
| AC008736.1 | -0.4795731 | 0.7706605 | -0.6223 | 0.534 | -4.61E-09 | count | 1 |
| GATA2 | -0.4795731 | 0.7706605 | -0.6223 | 0.534 | -4.61E-09 | count | 1 |
| POF1B | -0.4795731 | 0.7706605 | -0.6223 | 0.534 | -4.61E-09 | count | 1 |
| CHGB | -0.4795731 | 0.7706605 | -0.6223 | 0.534 | -4.61E-09 | count | 1 |
| AC006547.3 | -0.4795731 | 0.9397589 | -0.5103 | 0.61 | -4.61E-09 | count | 1 |
| B9D1 | -0.1525153 | 0.8552998 | -0.1783 | 0.858 | -4.36E-09 | count | 1 |
| SLC5A5 | -0.1525153 | 0.7861664 | -0.194 | 0.846 | -4.36E-09 | count | 1 |
| MST1 | -0.4430939 | 1.1754776 | -0.3769 | 0.706 | -4.31E-09 | count | 1 |
| LINC02539 | -0.4430939 | 1.1754776 | -0.3769 | 0.706 | -4.31E-09 | count | 1 |
| AC011491.3 | -0.4430939 | 1.0506422 | -0.4217 | 0.673 | -4.31E-09 | count | 1 |
| ALDH4A1 | -0.4430939 | 1.0506422 | -0.4217 | 0.673 | -4.31E-09 | count | 1 |
| AC079610.2 | -0.4430939 | 1.0506422 | -0.4217 | 0.673 | -4.31E-09 | count | 1 |
| LINC01715 | -0.4430939 | 1.1754776 | -0.3769 | 0.706 | -4.31E-09 | count | 1 |
| GPX8 | -0.4430939 | 1.0506422 | -0.4217 | 0.673 | -4.31E-09 | count | 1 |
| AP006623.1 | -0.4430939 | 1.1754776 | -0.3769 | 0.706 | -4.31E-09 | count | 1 |
| AC138969.1 | -0.4430939 | 1.0506422 | -0.4217 | 0.673 | -4.31E-09 | count | 1 |
| AL512306.3 | -0.4430939 | 1.1754776 | -0.3769 | 0.706 | -4.31E-09 | count | 1 |
| AC018638.7 | -0.4430939 | 1.1754776 | -0.3769 | 0.706 | -4.31E-09 | count | 1 |
| SMTN | -0.4430939 | 1.0506422 | -0.4217 | 0.673 | -4.31E-09 | count | 1 |
| AC025175.1 | -0.4430939 | 1.0506422 | -0.4217 | 0.673 | -4.31E-09 | count | 1 |
| ADM5 | -0.4430939 | 1.0506422 | -0.4217 | 0.673 | -4.31E-09 | count | 1 |
| AL160269.1 | -0.4430939 | 1.0506422 | -0.4217 | 0.673 | -4.31E-09 | count | 1 |
| PDGFRA | -0.4430939 | 1.1754776 | -0.3769 | 0.706 | -4.31E-09 | count | 1 |
| AC008264.2 | -0.4430939 | 1.1754776 | -0.3769 | 0.706 | -4.31E-09 | count | 1 |
| AC036108.3 | -0.4430939 | 1.0506422 | -0.4217 | 0.673 | -4.31E-09 | count | 1 |
| CSF3 | -0.4430939 | 1.3125729 | -0.3376 | 0.736 | -4.31E-09 | count | 1 |
| GTF2H4 | -0.4430939 | 1.3125729 | -0.3376 | 0.736 | -4.31E-09 | count | 1 |
| AC091057.6 | -0.4430939 | 1.3125729 | -0.3376 | 0.736 | -4.31E-09 | count | 1 |
| AL031963.1 | -0.4430939 | 1.3125729 | -0.3376 | 0.736 | -4.31E-09 | count | 1 |
| PCDH1 | -0.4430939 | 1.3125729 | -0.3376 | 0.736 | -4.31E-09 | count | 1 |
| LINC01730 | -0.4430939 | 1.3125729 | -0.3376 | 0.736 | -4.31E-09 | count | 1 |
| CACNB2 | -0.4430939 | 1.3125729 | -0.3376 | 0.736 | -4.31E-09 | count | 1 |
| AL021707.5 | -0.4430939 | 1.3125729 | -0.3376 | 0.736 | -4.31E-09 | count | 1 |
| WWC2-AS2 | -0.4430939 | 1.3125729 | -0.3376 | 0.736 | -4.31E-09 | count | 1 |
| PKN2-AS1 | -0.4430939 | 1.3125729 | -0.3376 | 0.736 | -4.31E-09 | count | 1 |
| AC087473.1 | -0.4430939 | 1.3125729 | -0.3376 | 0.736 | -4.31E-09 | count | 1 |
| LINC00266-1 | -0.4430939 | 1.3125729 | -0.3376 | 0.736 | -4.31E-09 | count | 1 |
| CPLX1 | -0.0502757 | 0.5575578 | -0.0902 | 0.928 | -4.01E-09 | count | 1 |
| KCTD21-AS1 | -0.0502757 | 0.4689364 | -0.1072 | 0.915 | -4.01E-09 | count | 1 |
| AL358072.1 | -0.1346285 | 0.8323561 | -0.1617 | 0.872 | -3.87E-09 | count | 1 |

| | | | | | | | |
|---|---|---|---|---|---|---|---|
| TRBV6-2 | -0.1346285 | 0.6643892 | -0.2026 | 0.839 | -3.87E-09 | count | 1 |
| TXNRD3 | -0.36179 | 0.7064761 | -0.5121 | 0.609 | -3.59E-09 | count | 1 |
| FAM171B | -0.36179 | 0.6245729 | -0.5793 | 0.562 | -3.59E-09 | count | 1 |
| C11orf95 | -0.36179 | 0.6826521 | -0.53 | 0.596 | -3.59E-09 | count | 1 |
| SLC17A3 | -0.36179 | 0.806817 | -0.4484 | 0.654 | -3.59E-09 | count | 1 |
| AC108463.3 | -1.184226 | 0.6225446 | -1.9022 | 0.0572 | -3.42E-09 | count | 1 |
| ZBTB20-AS4 | -1.184226 | 0.734414 | -1.6125 | 0.107 | -3.42E-09 | count | 1 |
| AL513550.1 | -0.0390453 | 0.8162874 | -0.0478 | 0.962 | -3.13E-09 | count | 1 |
| IGFL2 | -0.2972515 | 0.7245871 | -0.4102 | 0.682 | -3.01E-09 | count | 1 |
| ADCY4 | -0.9610825 | 0.6510332 | -1.4762 | 0.14 | -2.96E-09 | count | 1 |
| RAB3IL1 | -0.9610825 | 0.7229618 | -1.3294 | 0.184 | -2.96E-09 | count | 1 |
| ZNF492 | -0.9610825 | 0.9581188 | -1.0031 | 0.316 | -2.96E-09 | count | 1 |
| AP001267.2 | -0.2679353 | 0.5674473 | -0.4722 | 0.637 | -2.73E-09 | count | 1 |
| EDC4 | -0.0331477 | 0.6803382 | -0.0487 | 0.961 | -2.66E-09 | count | 1 |
| AC084116.3 | -0.7672552 | 0.8997938 | -0.8527 | 0.394 | -2.50E-09 | count | 1 |
| AC001226.2 | -0.7672552 | 0.8997938 | -0.8527 | 0.394 | -2.50E-09 | count | 1 |
| CYP7B1 | -0.7672552 | 0.8997938 | -0.8527 | 0.394 | -2.50E-09 | count | 1 |
| AC011773.4 | -0.7672552 | 0.8997938 | -0.8527 | 0.394 | -2.50E-09 | count | 1 |
| ATP8B4 | -0.7672552 | 0.8997938 | -0.8527 | 0.394 | -2.50E-09 | count | 1 |
| AC138627.1 | -0.7672552 | 0.8997938 | -0.8527 | 0.394 | -2.50E-09 | count | 1 |
| MATN2 | -0.7672552 | 0.8997938 | -0.8527 | 0.394 | -2.50E-09 | count | 1 |
| AC009318.1 | -0.7672552 | 0.8997938 | -0.8527 | 0.394 | -2.50E-09 | count | 1 |
| GJC1 | -0.7672552 | 0.8997938 | -0.8527 | 0.394 | -2.50E-09 | count | 1 |
| AC004846.1 | -0.7672552 | 0.8997938 | -0.8527 | 0.394 | -2.50E-09 | count | 1 |
| AC138150.2 | -0.7672552 | 1.0954854 | -0.7004 | 0.484 | -2.50E-09 | count | 1 |
| AL354872.2 | -0.7672552 | 1.0954854 | -0.7004 | 0.484 | -2.50E-09 | count | 1 |
| FBXL13 | -0.7672552 | 1.0954854 | -0.7004 | 0.484 | -2.50E-09 | count | 1 |
| IL3RA | -0.7672552 | 1.0954854 | -0.7004 | 0.484 | -2.50E-09 | count | 1 |
| AL928970.1 | -0.7672552 | 1.0954854 | -0.7004 | 0.484 | -2.50E-09 | count | 1 |
| TMEM86A | -0.7672552 | 1.0954854 | -0.7004 | 0.484 | -2.50E-09 | count | 1 |
| MTRNR2L1 | -0.7672552 | 1.0954854 | -0.7004 | 0.484 | -2.50E-09 | count | 1 |
| PRKCZ-AS1 | -0.7672552 | 1.0954854 | -0.7004 | 0.484 | -2.50E-09 | count | 1 |
| LINC01144 | -0.7672552 | 1.0954854 | -0.7004 | 0.484 | -2.50E-09 | count | 1 |
| AC093277.1 | -0.7672552 | 1.0954854 | -0.7004 | 0.484 | -2.50E-09 | count | 1 |
| AC018754.1 | -0.7672552 | 1.0954854 | -0.7004 | 0.484 | -2.50E-09 | count | 1 |
| KCNQ3 | -0.7672552 | 1.0954854 | -0.7004 | 0.484 | -2.50E-09 | count | 1 |
| AC130324.1 | -0.7672552 | 1.0954854 | -0.7004 | 0.484 | -2.50E-09 | count | 1 |
| FOXP4-AS1 | -0.7672552 | 1.0954854 | -0.7004 | 0.484 | -2.50E-09 | count | 1 |
| CLEC2L | -0.7672552 | 1.0954854 | -0.7004 | 0.484 | -2.50E-09 | count | 1 |
| AC096711.1 | -0.7672552 | 1.0954854 | -0.7004 | 0.484 | -2.50E-09 | count | 1 |
| PCDHGB6 | -0.7672552 | 1.0954854 | -0.7004 | 0.484 | -2.50E-09 | count | 1 |
| TSPAN6 | -0.7672552 | 1.0954854 | -0.7004 | 0.484 | -2.50E-09 | count | 1 |
| SLC7A2 | -0.7672552 | 1.0954854 | -0.7004 | 0.484 | -2.50E-09 | count | 1 |
| AC138466.1 | -0.7672552 | 1.0954854 | -0.7004 | 0.484 | -2.50E-09 | count | 1 |
| FST | -0.7672552 | 1.0954854 | -0.7004 | 0.484 | -2.50E-09 | count | 1 |

| | | | | | | | |
|---|---|---|---|---|---|---|---|
| RBFOX2 | -0.0831921 | 0.8310214 | -0.1001 | 0.92 | -2.42E-09 | count | 1 |
| ZNF343 | -0.0831921 | 0.8310214 | -0.1001 | 0.92 | -2.42E-09 | count | 1 |
| LARGE2 | -0.0831921 | 0.8740429 | -0.0952 | 0.924 | -2.42E-09 | count | 1 |
| LRRC39 | -0.0831921 | 1.0051464 | -0.0828 | 0.934 | -2.42E-09 | count | 1 |
| L1TD1 | -0.0831921 | 0.8740429 | -0.0952 | 0.924 | -2.42E-09 | count | 1 |
| GREM2 | -0.0294059 | 0.5110511 | -0.0575 | 0.954 | -2.36E-09 | count | 1 |
| AC011481.2 | -0.0805019 | 1.1059694 | -0.0728 | 0.942 | -2.34E-09 | count | 1 |
| SCNN1A | -0.0805019 | 0.8702319 | -0.0925 | 0.926 | -2.34E-09 | count | 1 |
| SAG | -0.0805019 | 0.8666092 | -0.0929 | 0.926 | -2.34E-09 | count | 1 |
| EDRF1-AS1 | -0.0805019 | 1.1718699 | -0.0687 | 0.945 | -2.34E-09 | count | 1 |
| AL049840.2 | -0.0805019 | 0.7426832 | -0.1084 | 0.914 | -2.34E-09 | count | 1 |
| AC007014.1 | -0.0805019 | 0.8188497 | -0.0983 | 0.922 | -2.34E-09 | count | 1 |
| FAM153A | -0.028251 | 0.6168378 | -0.0458 | 0.963 | -2.27E-09 | count | 1 |
| ALDH8A1 | -0.6734004 | 0.8767825 | -0.768 | 0.443 | -2.26E-09 | count | 1 |
| AL671710.1 | -0.6734004 | 0.8767825 | -0.768 | 0.443 | -2.26E-09 | count | 1 |
| BAIAP3 | -0.074108 | 0.653089 | -0.1135 | 0.91 | -2.16E-09 | count | 1 |
| CRISPLD2 | -0.074108 | 0.6229607 | -0.119 | 0.905 | -2.16E-09 | count | 1 |
| AMPD1 | -17.9629662 | 2370.639356 | -0.0076 | 0.994 | -2.13E-09 | count | 1 |
| FIGN | -17.9629662 | 2370.639356 | -0.0076 | 0.994 | -2.13E-09 | count | 1 |
| AC026785.2 | -17.9629662 | 2370.639356 | -0.0076 | 0.994 | -2.13E-09 | count | 1 |
| ZAN | -17.9629662 | 2370.639356 | -0.0076 | 0.994 | -2.13E-09 | count | 1 |
| FJX1 | -17.9629662 | 2370.639356 | -0.0076 | 0.994 | -2.13E-09 | count | 1 |
| TMPRSS4 | -17.9629662 | 2370.639356 | -0.0076 | 0.994 | -2.13E-09 | count | 1 |
| AL731571.1 | -17.9629662 | 2370.639356 | -0.0076 | 0.994 | -2.13E-09 | count | 1 |
| GLDN | -17.9629662 | 2370.639356 | -0.0076 | 0.994 | -2.13E-09 | count | 1 |
| HSF5 | -17.9629662 | 2370.639356 | -0.0076 | 0.994 | -2.13E-09 | count | 1 |
| NPB | -17.9629662 | 2370.639356 | -0.0076 | 0.994 | -2.13E-09 | count | 1 |
| ASAP3 | -17.9629662 | 2370.639356 | -0.0076 | 0.994 | -2.13E-09 | count | 1 |
| OPRD1 | -17.9629662 | 2370.639356 | -0.0076 | 0.994 | -2.13E-09 | count | 1 |
| ADGRB2 | -17.9629662 | 2370.639356 | -0.0076 | 0.994 | -2.13E-09 | count | 1 |
| PPM1J | -17.9629662 | 2370.639356 | -0.0076 | 0.994 | -2.13E-09 | count | 1 |
| AL137856.1 | -17.9629662 | 2370.639356 | -0.0076 | 0.994 | -2.13E-09 | count | 1 |
| HIST2H3D | -17.9629662 | 2370.639356 | -0.0076 | 0.994 | -2.13E-09 | count | 1 |
| AC097468.3 | -17.9629662 | 2370.639356 | -0.0076 | 0.994 | -2.13E-09 | count | 1 |
| AC073052.2 | -17.9629662 | 2370.639356 | -0.0076 | 0.994 | -2.13E-09 | count | 1 |
| LINC01967 | -17.9629662 | 2370.639356 | -0.0076 | 0.994 | -2.13E-09 | count | 1 |
| CADPS | -17.9629662 | 2370.639356 | -0.0076 | 0.994 | -2.13E-09 | count | 1 |
| HAPLN1 | -17.9629662 | 2370.639356 | -0.0076 | 0.994 | -2.13E-09 | count | 1 |
| LINC00992 | -17.9629662 | 2370.639356 | -0.0076 | 0.994 | -2.13E-09 | count | 1 |
| KIF20A | -17.9629662 | 2370.639356 | -0.0076 | 0.994 | -2.13E-09 | count | 1 |
| LINC01556 | -17.9629662 | 2370.639356 | -0.0076 | 0.994 | -2.13E-09 | count | 1 |
| Z98200.1 | -17.9629662 | 2370.639356 | -0.0076 | 0.994 | -2.13E-09 | count | 1 |
| NYAP1 | -17.9629662 | 2370.639356 | -0.0076 | 0.994 | -2.13E-09 | count | 1 |
| FAM66D | -17.9629662 | 2370.639356 | -0.0076 | 0.994 | -2.13E-09 | count | 1 |
| AL162412.1 | -17.9629662 | 2370.639356 | -0.0076 | 0.994 | -2.13E-09 | count | 1 |

| | | | | | | | |
|---|---|---|---|---|---|---|---|
| SHANK2-AS1 | -17.9629662 | 2370.639356 | -0.0076 | 0.994 | -2.13E-09 | count | 1 |
| CMA1 | -17.9629662 | 2370.639356 | -0.0076 | 0.994 | -2.13E-09 | count | 1 |
| IGHV1-46 | -17.9629662 | 2370.639356 | -0.0076 | 0.994 | -2.13E-09 | count | 1 |
| CLDN9 | -17.9629662 | 2370.639356 | -0.0076 | 0.994 | -2.13E-09 | count | 1 |
| AC026401.1 | -17.9629662 | 2370.639356 | -0.0076 | 0.994 | -2.13E-09 | count | 1 |
| AC092119.3 | -17.9629662 | 2370.639356 | -0.0076 | 0.994 | -2.13E-09 | count | 1 |
| SPIRE2 | -17.9629662 | 2370.639356 | -0.0076 | 0.994 | -2.13E-09 | count | 1 |
| CCDC40 | -17.9629662 | 2370.639356 | -0.0076 | 0.994 | -2.13E-09 | count | 1 |
| ADCYAP1 | -17.9629662 | 2370.639356 | -0.0076 | 0.994 | -2.13E-09 | count | 1 |
| AL022238.4 | -17.9629662 | 2370.639356 | -0.0076 | 0.994 | -2.13E-09 | count | 1 |
| AL109917.1 | -17.9629662 | 2370.639356 | -0.0076 | 0.994 | -2.13E-09 | count | 1 |
| TNFRSF8 | -17.9629662 | 2370.639356 | -0.0076 | 0.994 | -2.13E-09 | count | 1 |
| SLC25A34-AS1 | -17.9629662 | 2370.639356 | -0.0076 | 0.994 | -2.13E-09 | count | 1 |
| MATN1 | -17.9629662 | 2370.639356 | -0.0076 | 0.994 | -2.13E-09 | count | 1 |
| LINC01389 | -17.9629662 | 2370.639356 | -0.0076 | 0.994 | -2.13E-09 | count | 1 |
| LINC01781 | -17.9629662 | 2370.639356 | -0.0076 | 0.994 | -2.13E-09 | count | 1 |
| LINC01675 | -17.9629662 | 2370.639356 | -0.0076 | 0.994 | -2.13E-09 | count | 1 |
| AL357793.2 | -17.9629662 | 2370.639356 | -0.0076 | 0.994 | -2.13E-09 | count | 1 |
| AC092809.4 | -17.9629662 | 2370.639356 | -0.0076 | 0.994 | -2.13E-09 | count | 1 |
| AL732292.2 | -17.9629662 | 2370.639356 | -0.0076 | 0.994 | -2.13E-09 | count | 1 |
| AC010729.1 | -17.9629662 | 2370.639356 | -0.0076 | 0.994 | -2.13E-09 | count | 1 |
| CDKL4 | -17.9629662 | 2370.639356 | -0.0076 | 0.994 | -2.13E-09 | count | 1 |
| AC016722.3 | -17.9629662 | 2370.639356 | -0.0076 | 0.994 | -2.13E-09 | count | 1 |
| AC096558.1 | -17.9629662 | 2370.639356 | -0.0076 | 0.994 | -2.13E-09 | count | 1 |
| AC053503.5 | -17.9629662 | 2370.639356 | -0.0076 | 0.994 | -2.13E-09 | count | 1 |
| COL4A4 | -17.9629662 | 2370.639356 | -0.0076 | 0.994 | -2.13E-09 | count | 1 |
| AC078795.2 | -17.9629662 | 2370.639356 | -0.0076 | 0.994 | -2.13E-09 | count | 1 |
| KCNMB2-AS1 | -17.9629662 | 2370.639356 | -0.0076 | 0.994 | -2.13E-09 | count | 1 |
| CNGA1 | -17.9629662 | 2370.639356 | -0.0076 | 0.994 | -2.13E-09 | count | 1 |
| AC083829.2 | -17.9629662 | 2370.639356 | -0.0076 | 0.994 | -2.13E-09 | count | 1 |
| TRIML2 | -17.9629662 | 2370.639356 | -0.0076 | 0.994 | -2.13E-09 | count | 1 |
| AC008957.1 | -17.9629662 | 2370.639356 | -0.0076 | 0.994 | -2.13E-09 | count | 1 |
| OSMR | -17.9629662 | 2370.639356 | -0.0076 | 0.994 | -2.13E-09 | count | 1 |
| GHR | -17.9629662 | 2370.639356 | -0.0076 | 0.994 | -2.13E-09 | count | 1 |
| ABLIM3 | -17.9629662 | 2370.639356 | -0.0076 | 0.994 | -2.13E-09 | count | 1 |
| AFAP1L1 | -17.9629662 | 2370.639356 | -0.0076 | 0.994 | -2.13E-09 | count | 1 |
| ZBED9 | -17.9629662 | 2370.639356 | -0.0076 | 0.994 | -2.13E-09 | count | 1 |
| DNAH8 | -17.9629662 | 2370.639356 | -0.0076 | 0.994 | -2.13E-09 | count | 1 |
| AL355297.2 | -17.9629662 | 2370.639356 | -0.0076 | 0.994 | -2.13E-09 | count | 1 |
| PKD1L1 | -17.9629662 | 2370.639356 | -0.0076 | 0.994 | -2.13E-09 | count | 1 |
| AC092634.3 | -17.9629662 | 2370.639356 | -0.0076 | 0.994 | -2.13E-09 | count | 1 |
| CCL26 | -17.9629662 | 2370.639356 | -0.0076 | 0.994 | -2.13E-09 | count | 1 |
| TSPAN12 | -17.9629662 | 2370.639356 | -0.0076 | 0.994 | -2.13E-09 | count | 1 |
| ATP6V1FNB | -17.9629662 | 2370.639356 | -0.0076 | 0.994 | -2.13E-09 | count | 1 |
| PIR | -17.9629662 | 2370.639356 | -0.0076 | 0.994 | -2.13E-09 | count | 1 |

| | | | | | | | |
|---|---|---|---|---|---|---|---|
| SVEP1 | -17.9629662 | 2370.639356 | -0.0076 | 0.994 | -2.13E-09 | count | 1 |
| STKLD1 | -17.9629662 | 2370.639356 | -0.0076 | 0.994 | -2.13E-09 | count | 1 |
| CEND1 | -17.9629662 | 2370.639356 | -0.0076 | 0.994 | -2.13E-09 | count | 1 |
| TRIM6 | -17.9629662 | 2370.639356 | -0.0076 | 0.994 | -2.13E-09 | count | 1 |
| ST5 | -17.9629662 | 2370.639356 | -0.0076 | 0.994 | -2.13E-09 | count | 1 |
| AC010768.1 | -17.9629662 | 2370.639356 | -0.0076 | 0.994 | -2.13E-09 | count | 1 |
| AC024475.1 | -17.9629662 | 2370.639356 | -0.0076 | 0.994 | -2.13E-09 | count | 1 |
| TCN1 | -17.9629662 | 2370.639356 | -0.0076 | 0.994 | -2.13E-09 | count | 1 |
| AP003733.3 | -17.9629662 | 2370.639356 | -0.0076 | 0.994 | -2.13E-09 | count | 1 |
| BATF2 | -17.9629662 | 2370.639356 | -0.0076 | 0.994 | -2.13E-09 | count | 1 |
| SNX15 | -17.9629662 | 2370.639356 | -0.0076 | 0.994 | -2.13E-09 | count | 1 |
| OVOL1 | -17.9629662 | 2370.639356 | -0.0076 | 0.994 | -2.13E-09 | count | 1 |
| AP000763.3 | -17.9629662 | 2370.639356 | -0.0076 | 0.994 | -2.13E-09 | count | 1 |
| APOC3 | -17.9629662 | 2370.639356 | -0.0076 | 0.994 | -2.13E-09 | count | 1 |
| AKR1E2 | -17.9629662 | 2370.639356 | -0.0076 | 0.994 | -2.13E-09 | count | 1 |
| AKR1C2 | -17.9629662 | 2370.639356 | -0.0076 | 0.994 | -2.13E-09 | count | 1 |
| AC005840.4 | -17.9629662 | 2370.639356 | -0.0076 | 0.994 | -2.13E-09 | count | 1 |
| NANOGNB | -17.9629662 | 2370.639356 | -0.0076 | 0.994 | -2.13E-09 | count | 1 |
| A2ML1 | -17.9629662 | 2370.639356 | -0.0076 | 0.994 | -2.13E-09 | count | 1 |
| LINC02397 | -17.9629662 | 2370.639356 | -0.0076 | 0.994 | -2.13E-09 | count | 1 |
| LINC02249 | -17.9629662 | 2370.639356 | -0.0076 | 0.994 | -2.13E-09 | count | 1 |
| AC023034.1 | -17.9629662 | 2370.639356 | -0.0076 | 0.994 | -2.13E-09 | count | 1 |
| AC003965.1 | -17.9629662 | 2370.639356 | -0.0076 | 0.994 | -2.13E-09 | count | 1 |
| AC106782.1 | -17.9629662 | 2370.639356 | -0.0076 | 0.994 | -2.13E-09 | count | 1 |
| AC010547.1 | -17.9629662 | 2370.639356 | -0.0076 | 0.994 | -2.13E-09 | count | 1 |
| AC092718.6 | -17.9629662 | 2370.639356 | -0.0076 | 0.994 | -2.13E-09 | count | 1 |
| AC100788.1 | -17.9629662 | 2370.639356 | -0.0076 | 0.994 | -2.13E-09 | count | 1 |
| LINC01909 | -17.9629662 | 2370.639356 | -0.0076 | 0.994 | -2.13E-09 | count | 1 |
| AC018413.1 | -17.9629662 | 2370.639356 | -0.0076 | 0.994 | -2.13E-09 | count | 1 |
| MYH7B | -17.9629662 | 2370.639356 | -0.0076 | 0.994 | -2.13E-09 | count | 1 |
| CSNK1G2-AS1 | -17.9629662 | 2370.639356 | -0.0076 | 0.994 | -2.13E-09 | count | 1 |
| AC020917.3 | -17.9629662 | 2370.639356 | -0.0076 | 0.994 | -2.13E-09 | count | 1 |
| TEAD2 | -17.9629662 | 2370.639356 | -0.0076 | 0.994 | -2.13E-09 | count | 1 |
| RPS4Y2 | -17.9629662 | 2370.639356 | -0.0076 | 0.994 | -2.13E-09 | count | 1 |
| AC007326.4 | -17.9629662 | 2370.639356 | -0.0076 | 0.994 | -2.13E-09 | count | 1 |
| AL035681.1 | -17.9629662 | 2370.639356 | -0.0076 | 0.994 | -2.13E-09 | count | 1 |
| HES5 | -17.9629662 | 2370.639356 | -0.0076 | 0.994 | -2.13E-09 | count | 1 |
| DNALI1 | -17.9629662 | 2370.639356 | -0.0076 | 0.994 | -2.13E-09 | count | 1 |
| MTMR11 | -17.9629662 | 2370.639356 | -0.0076 | 0.994 | -2.13E-09 | count | 1 |
| AL513329.1 | -17.9629662 | 2370.639356 | -0.0076 | 0.994 | -2.13E-09 | count | 1 |
| KIAA1614-AS1 | -17.9629662 | 2370.639356 | -0.0076 | 0.994 | -2.13E-09 | count | 1 |
| NEK2 | -17.9629662 | 2370.639356 | -0.0076 | 0.994 | -2.13E-09 | count | 1 |
| KLHL29 | -17.9629662 | 2370.639356 | -0.0076 | 0.994 | -2.13E-09 | count | 1 |
| IGKV1-16 | -17.9629662 | 2370.639356 | -0.0076 | 0.994 | -2.13E-09 | count | 1 |
| AC011893.1 | -17.9629662 | 2370.639356 | -0.0076 | 0.994 | -2.13E-09 | count | 1 |

| | | | | | | | |
|---|---|---|---|---|---|---|---|
| AC019186.1 | -17.9629662 | 2370.639356 | -0.0076 | 0.994 | -2.13E-09 | count | 1 |
| DLX1 | -17.9629662 | 2370.639356 | -0.0076 | 0.994 | -2.13E-09 | count | 1 |
| RUFY4 | -17.9629662 | 2370.639356 | -0.0076 | 0.994 | -2.13E-09 | count | 1 |
| LINC02033 | -17.9629662 | 2370.639356 | -0.0076 | 0.994 | -2.13E-09 | count | 1 |
| HHLA2 | -17.9629662 | 2370.639356 | -0.0076 | 0.994 | -2.13E-09 | count | 1 |
| AC112503.1 | -17.9629662 | 2370.639356 | -0.0076 | 0.994 | -2.13E-09 | count | 1 |
| NME9 | -17.9629662 | 2370.639356 | -0.0076 | 0.994 | -2.13E-09 | count | 1 |
| AC114947.2 | -17.9629662 | 2370.639356 | -0.0076 | 0.994 | -2.13E-09 | count | 1 |
| AL121936.1 | -17.9629662 | 2370.639356 | -0.0076 | 0.994 | -2.13E-09 | count | 1 |
| PSORS1C1 | -17.9629662 | 2370.639356 | -0.0076 | 0.994 | -2.13E-09 | count | 1 |
| HOXA5 | -17.9629662 | 2370.639356 | -0.0076 | 0.994 | -2.13E-09 | count | 1 |
| JAZF1-AS1 | -17.9629662 | 2370.639356 | -0.0076 | 0.994 | -2.13E-09 | count | 1 |
| AC018647.1 | -17.9629662 | 2370.639356 | -0.0076 | 0.994 | -2.13E-09 | count | 1 |
| AC003991.1 | -17.9629662 | 2370.639356 | -0.0076 | 0.994 | -2.13E-09 | count | 1 |
| TRBV7-1 | -17.9629662 | 2370.639356 | -0.0076 | 0.994 | -2.13E-09 | count | 1 |
| PRSS2 | -17.9629662 | 2370.639356 | -0.0076 | 0.994 | -2.13E-09 | count | 1 |
| SYTL4 | -17.9629662 | 2370.639356 | -0.0076 | 0.994 | -2.13E-09 | count | 1 |
| GRIA3 | -17.9629662 | 2370.639356 | -0.0076 | 0.994 | -2.13E-09 | count | 1 |
| PNOC | -17.9629662 | 2370.639356 | -0.0076 | 0.994 | -2.13E-09 | count | 1 |
| AP000424.1 | -17.9629662 | 2370.639356 | -0.0076 | 0.994 | -2.13E-09 | count | 1 |
| AL583785.1 | -17.9629662 | 2370.639356 | -0.0076 | 0.994 | -2.13E-09 | count | 1 |
| AL158071.1 | -17.9629662 | 2370.639356 | -0.0076 | 0.994 | -2.13E-09 | count | 1 |
| AL589843.1 | -17.9629662 | 2370.639356 | -0.0076 | 0.994 | -2.13E-09 | count | 1 |
| PTGS1 | -17.9629662 | 2370.639356 | -0.0076 | 0.994 | -2.13E-09 | count | 1 |
| AP001528.2 | -17.9629662 | 2370.639356 | -0.0076 | 0.994 | -2.13E-09 | count | 1 |
| AP000880.1 | -17.9629662 | 2370.639356 | -0.0076 | 0.994 | -2.13E-09 | count | 1 |
| CYP2C8 | -17.9629662 | 2370.639356 | -0.0076 | 0.994 | -2.13E-09 | count | 1 |
| PTHLH | -17.9629662 | 2370.639356 | -0.0076 | 0.994 | -2.13E-09 | count | 1 |
| BCDIN3D-AS1 | -17.9629662 | 2370.639356 | -0.0076 | 0.994 | -2.13E-09 | count | 1 |
| CFAP73 | -17.9629662 | 2370.639356 | -0.0076 | 0.994 | -2.13E-09 | count | 1 |
| LINC01198 | -17.9629662 | 2370.639356 | -0.0076 | 0.994 | -2.13E-09 | count | 1 |
| TRAV26-2 | -17.9629662 | 2370.639356 | -0.0076 | 0.994 | -2.13E-09 | count | 1 |
| IGHV1-17 | -17.9629662 | 2370.639356 | -0.0076 | 0.994 | -2.13E-09 | count | 1 |
| AC025580.3 | -17.9629662 | 2370.639356 | -0.0076 | 0.994 | -2.13E-09 | count | 1 |
| AC087477.2 | -17.9629662 | 2370.639356 | -0.0076 | 0.994 | -2.13E-09 | count | 1 |
| CACNA1H | -17.9629662 | 2370.639356 | -0.0076 | 0.994 | -2.13E-09 | count | 1 |
| AC106820.2 | -17.9629662 | 2370.639356 | -0.0076 | 0.994 | -2.13E-09 | count | 1 |
| NPIPA2 | -17.9629662 | 2370.639356 | -0.0076 | 0.994 | -2.13E-09 | count | 1 |
| PDF | -17.9629662 | 2370.639356 | -0.0076 | 0.994 | -2.13E-09 | count | 1 |
| HES7 | -17.9629662 | 2370.639356 | -0.0076 | 0.994 | -2.13E-09 | count | 1 |
| DNAH17 | -17.9629662 | 2370.639356 | -0.0076 | 0.994 | -2.13E-09 | count | 1 |
| DSC2 | -17.9629662 | 2370.639356 | -0.0076 | 0.994 | -2.13E-09 | count | 1 |
| HSPA12B | -17.9629662 | 2370.639356 | -0.0076 | 0.994 | -2.13E-09 | count | 1 |
| SNAP25 | -17.9629662 | 2370.639356 | -0.0076 | 0.994 | -2.13E-09 | count | 1 |
| AL390198.1 | -17.9629662 | 2370.639356 | -0.0076 | 0.994 | -2.13E-09 | count | 1 |

| | | | | | | | |
|---|---|---|---|---|---|---|---|
| AC093227.1 | -17.9629662 | 2370.639356 | -0.0076 | 0.994 | -2.13E-09 | count | 1 |
| RIMBP3C | -17.9629662 | 2370.639356 | -0.0076 | 0.994 | -2.13E-09 | count | 1 |
| Z83844.2 | -17.9629662 | 2370.639356 | -0.0076 | 0.994 | -2.13E-09 | count | 1 |
| AGRN | -17.9629662 | 2370.639356 | -0.0076 | 0.994 | -2.13E-09 | count | 1 |
| PLA2G2D | -17.9629662 | 2370.639356 | -0.0076 | 0.994 | -2.13E-09 | count | 1 |
| DCDC2B | -17.9629662 | 2370.639356 | -0.0076 | 0.994 | -2.13E-09 | count | 1 |
| TMEM125 | -17.9629662 | 2370.639356 | -0.0076 | 0.994 | -2.13E-09 | count | 1 |
| GPR61 | -17.9629662 | 2370.639356 | -0.0076 | 0.994 | -2.13E-09 | count | 1 |
| LINC01691 | -17.9629662 | 2370.639356 | -0.0076 | 0.994 | -2.13E-09 | count | 1 |
| AL355388.2 | -17.9629662 | 2370.639356 | -0.0076 | 0.994 | -2.13E-09 | count | 1 |
| FCRLA | -17.9629662 | 2370.639356 | -0.0076 | 0.994 | -2.13E-09 | count | 1 |
| LINC00582 | -17.9629662 | 2370.639356 | -0.0076 | 0.994 | -2.13E-09 | count | 1 |
| ARHGEF33 | -17.9629662 | 2370.639356 | -0.0076 | 0.994 | -2.13E-09 | count | 1 |
| IGKV1D-43 | -17.9629662 | 2370.639356 | -0.0076 | 0.994 | -2.13E-09 | count | 1 |
| AC092587.1 | -17.9629662 | 2370.639356 | -0.0076 | 0.994 | -2.13E-09 | count | 1 |
| AC091488.1 | -17.9629662 | 2370.639356 | -0.0076 | 0.994 | -2.13E-09 | count | 1 |
| OBSL1 | -17.9629662 | 2370.639356 | -0.0076 | 0.994 | -2.13E-09 | count | 1 |
| LINC01238 | -17.9629662 | 2370.639356 | -0.0076 | 0.994 | -2.13E-09 | count | 1 |
| LINC00691 | -17.9629662 | 2370.639356 | -0.0076 | 0.994 | -2.13E-09 | count | 1 |
| VIPR1-AS1 | -17.9629662 | 2370.639356 | -0.0076 | 0.994 | -2.13E-09 | count | 1 |
| CDC25A | -17.9629662 | 2370.639356 | -0.0076 | 0.994 | -2.13E-09 | count | 1 |
| AC012467.2 | -17.9629662 | 2370.639356 | -0.0076 | 0.994 | -2.13E-09 | count | 1 |
| PRICKLE2 | -17.9629662 | 2370.639356 | -0.0076 | 0.994 | -2.13E-09 | count | 1 |
| BOC | -17.9629662 | 2370.639356 | -0.0076 | 0.994 | -2.13E-09 | count | 1 |
| LINC01972 | -17.9629662 | 2370.639356 | -0.0076 | 0.994 | -2.13E-09 | count | 1 |
| ADGRA3 | -17.9629662 | 2370.639356 | -0.0076 | 0.994 | -2.13E-09 | count | 1 |
| BANK1 | -17.9629662 | 2370.639356 | -0.0076 | 0.994 | -2.13E-09 | count | 1 |
| PCDHGA3 | -17.9629662 | 2370.639356 | -0.0076 | 0.994 | -2.13E-09 | count | 1 |
| PCDHGA7 | -17.9629662 | 2370.639356 | -0.0076 | 0.994 | -2.13E-09 | count | 1 |
| AC136604.3 | -17.9629662 | 2370.639356 | -0.0076 | 0.994 | -2.13E-09 | count | 1 |
| FOXQ1 | -17.9629662 | 2370.639356 | -0.0076 | 0.994 | -2.13E-09 | count | 1 |
| AL158198.1 | -17.9629662 | 2370.639356 | -0.0076 | 0.994 | -2.13E-09 | count | 1 |
| C2 | -17.9629662 | 2370.639356 | -0.0076 | 0.994 | -2.13E-09 | count | 1 |
| COL11A2 | -17.9629662 | 2370.639356 | -0.0076 | 0.994 | -2.13E-09 | count | 1 |
| AL157823.2 | -17.9629662 | 2370.639356 | -0.0076 | 0.994 | -2.13E-09 | count | 1 |
| IL17A | -17.9629662 | 2370.639356 | -0.0076 | 0.994 | -2.13E-09 | count | 1 |
| C6orf163 | -17.9629662 | 2370.639356 | -0.0076 | 0.994 | -2.13E-09 | count | 1 |
| SLC22A3 | -17.9629662 | 2370.639356 | -0.0076 | 0.994 | -2.13E-09 | count | 1 |
| LINC01372 | -17.9629662 | 2370.639356 | -0.0076 | 0.994 | -2.13E-09 | count | 1 |
| AC093726.2 | -17.9629662 | 2370.639356 | -0.0076 | 0.994 | -2.13E-09 | count | 1 |
| RHOXF1-AS1 | -17.9629662 | 2370.639356 | -0.0076 | 0.994 | -2.13E-09 | count | 1 |
| AC120193.1 | -17.9629662 | 2370.639356 | -0.0076 | 0.994 | -2.13E-09 | count | 1 |
| AC067817.2 | -17.9629662 | 2370.639356 | -0.0076 | 0.994 | -2.13E-09 | count | 1 |
| AC022182.1 | -17.9629662 | 2370.639356 | -0.0076 | 0.994 | -2.13E-09 | count | 1 |
| PREX2 | -17.9629662 | 2370.639356 | -0.0076 | 0.994 | -2.13E-09 | count | 1 |

| | | | | | | | |
|---|---|---|---|---|---|---|---|
| AC022973.3 | -17.9629662 | 2370.639356 | -0.0076 | 0.994 | -2.13E-09 | count | 1 |
| MAMDC2 | -17.9629662 | 2370.639356 | -0.0076 | 0.994 | -2.13E-09 | count | 1 |
| DAPK1 | -17.9629662 | 2370.639356 | -0.0076 | 0.994 | -2.13E-09 | count | 1 |
| ECM2 | -17.9629662 | 2370.639356 | -0.0076 | 0.994 | -2.13E-09 | count | 1 |
| AL158152.2 | -17.9629662 | 2370.639356 | -0.0076 | 0.994 | -2.13E-09 | count | 1 |
| TEX48 | -17.9629662 | 2370.639356 | -0.0076 | 0.994 | -2.13E-09 | count | 1 |
| PLPP7 | -17.9629662 | 2370.639356 | -0.0076 | 0.994 | -2.13E-09 | count | 1 |
| LYVE1 | -17.9629662 | 2370.639356 | -0.0076 | 0.994 | -2.13E-09 | count | 1 |
| CREB3L1 | -17.9629662 | 2370.639356 | -0.0076 | 0.994 | -2.13E-09 | count | 1 |
| LINC00619 | -17.9629662 | 2370.639356 | -0.0076 | 0.994 | -2.13E-09 | count | 1 |
| RAPGEF3 | -17.9629662 | 2370.639356 | -0.0076 | 0.994 | -2.13E-09 | count | 1 |
| TROAP | -17.9629662 | 2370.639356 | -0.0076 | 0.994 | -2.13E-09 | count | 1 |
| AC069234.2 | -17.9629662 | 2370.639356 | -0.0076 | 0.994 | -2.13E-09 | count | 1 |
| HPD | -17.9629662 | 2370.639356 | -0.0076 | 0.994 | -2.13E-09 | count | 1 |
| AL512506.3 | -17.9629662 | 2370.639356 | -0.0076 | 0.994 | -2.13E-09 | count | 1 |
| RUBCNL | -17.9629662 | 2370.639356 | -0.0076 | 0.994 | -2.13E-09 | count | 1 |
| FARP1-AS1 | -17.9629662 | 2370.639356 | -0.0076 | 0.994 | -2.13E-09 | count | 1 |
| SMOC1 | -17.9629662 | 2370.639356 | -0.0076 | 0.994 | -2.13E-09 | count | 1 |
| OTUB2 | -17.9629662 | 2370.639356 | -0.0076 | 0.994 | -2.13E-09 | count | 1 |
| IGHV3-23 | -17.9629662 | 2370.639356 | -0.0076 | 0.994 | -2.13E-09 | count | 1 |
| STRC | -17.9629662 | 2370.639356 | -0.0076 | 0.994 | -2.13E-09 | count | 1 |
| CCNB2 | -17.9629662 | 2370.639356 | -0.0076 | 0.994 | -2.13E-09 | count | 1 |
| PAQR5 | -17.9629662 | 2370.639356 | -0.0076 | 0.994 | -2.13E-09 | count | 1 |
| DNM1P35 | -17.9629662 | 2370.639356 | -0.0076 | 0.994 | -2.13E-09 | count | 1 |
| AC090181.1 | -17.9629662 | 2370.639356 | -0.0076 | 0.994 | -2.13E-09 | count | 1 |
| CRABP1 | -17.9629662 | 2370.639356 | -0.0076 | 0.994 | -2.13E-09 | count | 1 |
| TICRR | -17.9629662 | 2370.639356 | -0.0076 | 0.994 | -2.13E-09 | count | 1 |
| Z97986.1 | -17.9629662 | 2370.639356 | -0.0076 | 0.994 | -2.13E-09 | count | 1 |
| PMFBP1 | -17.9629662 | 2370.639356 | -0.0076 | 0.994 | -2.13E-09 | count | 1 |
| LINC01229 | -17.9629662 | 2370.639356 | -0.0076 | 0.994 | -2.13E-09 | count | 1 |
| AC138028.4 | -17.9629662 | 2370.639356 | -0.0076 | 0.994 | -2.13E-09 | count | 1 |
| NXN | -17.9629662 | 2370.639356 | -0.0076 | 0.994 | -2.13E-09 | count | 1 |
| AC004706.1 | -17.9629662 | 2370.639356 | -0.0076 | 0.994 | -2.13E-09 | count | 1 |
| MIR497HG | -17.9629662 | 2370.639356 | -0.0076 | 0.994 | -2.13E-09 | count | 1 |
| BCL6B | -17.9629662 | 2370.639356 | -0.0076 | 0.994 | -2.13E-09 | count | 1 |
| AC007952.7 | -17.9629662 | 2370.639356 | -0.0076 | 0.994 | -2.13E-09 | count | 1 |
| SLC4A1 | -17.9629662 | 2370.639356 | -0.0076 | 0.994 | -2.13E-09 | count | 1 |
| AC015909.2 | -17.9629662 | 2370.639356 | -0.0076 | 0.994 | -2.13E-09 | count | 1 |
| ABCC3 | -17.9629662 | 2370.639356 | -0.0076 | 0.994 | -2.13E-09 | count | 1 |
| MPO | -17.9629662 | 2370.639356 | -0.0076 | 0.994 | -2.13E-09 | count | 1 |
| SCN4A | -17.9629662 | 2370.639356 | -0.0076 | 0.994 | -2.13E-09 | count | 1 |
| AC087741.2 | -17.9629662 | 2370.639356 | -0.0076 | 0.994 | -2.13E-09 | count | 1 |
| DSG2 | -17.9629662 | 2370.639356 | -0.0076 | 0.994 | -2.13E-09 | count | 1 |
| AC090229.1 | -17.9629662 | 2370.639356 | -0.0076 | 0.994 | -2.13E-09 | count | 1 |
| AC118757.1 | -17.9629662 | 2370.639356 | -0.0076 | 0.994 | -2.13E-09 | count | 1 |

| | | | | | | | |
|---|---|---|---|---|---|---|---|
| FAM182B | -17.9629662 | 2370.639356 | -0.0076 | 0.994 | -2.13E-09 | count | 1 |
| ITCH-AS1 | -17.9629662 | 2370.639356 | -0.0076 | 0.994 | -2.13E-09 | count | 1 |
| AL035420.3 | -17.9629662 | 2370.639356 | -0.0076 | 0.994 | -2.13E-09 | count | 1 |
| SOGA1 | -17.9629662 | 2370.639356 | -0.0076 | 0.994 | -2.13E-09 | count | 1 |
| SMIM24 | -17.9629662 | 2370.639356 | -0.0076 | 0.994 | -2.13E-09 | count | 1 |
| RAVER1 | -17.9629662 | 2370.639356 | -0.0076 | 0.994 | -2.13E-09 | count | 1 |
| C19orf67 | -17.9629662 | 2370.639356 | -0.0076 | 0.994 | -2.13E-09 | count | 1 |
| UPK1A | -17.9629662 | 2370.639356 | -0.0076 | 0.994 | -2.13E-09 | count | 1 |
| AC074138.1 | -17.9629662 | 2370.639356 | -0.0076 | 0.994 | -2.13E-09 | count | 1 |
| IL11 | -17.9629662 | 2370.639356 | -0.0076 | 0.994 | -2.13E-09 | count | 1 |
| HDHD5-AS1 | -17.9629662 | 2370.639356 | -0.0076 | 0.994 | -2.13E-09 | count | 1 |
| IGLV3-27 | -17.9629662 | 2370.639356 | -0.0076 | 0.994 | -2.13E-09 | count | 1 |
| IGLV2-11 | -17.9629662 | 2370.639356 | -0.0076 | 0.994 | -2.13E-09 | count | 1 |
| RASL10A | -17.9629662 | 2370.639356 | -0.0076 | 0.994 | -2.13E-09 | count | 1 |
| RIPK4 | -17.9629662 | 2370.639356 | -0.0076 | 0.994 | -2.13E-09 | count | 1 |
| CORIN | -17.9629662 | 2370.639356 | -0.0076 | 0.994 | -2.13E-09 | count | 1 |
| CLTRN | -17.9629662 | 2370.639356 | -0.0076 | 0.994 | -2.13E-09 | count | 1 |
| SLX1B | -17.9629662 | 2370.639356 | -0.0076 | 0.994 | -2.13E-09 | count | 1 |
| CADM4 | -17.9629662 | 2370.639356 | -0.0076 | 0.994 | -2.13E-09 | count | 1 |
| SLC29A4 | -18.6571555 | 2370.531453 | -0.0079 | 0.994 | -2.12E-09 | count | 1 |
| SLC52A1 | -18.6571555 | 2370.531453 | -0.0079 | 0.994 | -2.12E-09 | count | 1 |
| HTRA3 | -18.6571555 | 2370.531453 | -0.0079 | 0.994 | -2.12E-09 | count | 1 |
| PPP1R26 | -18.6571555 | 2370.531453 | -0.0079 | 0.994 | -2.12E-09 | count | 1 |
| AC026740.1 | -18.6571555 | 2370.531453 | -0.0079 | 0.994 | -2.12E-09 | count | 1 |
| AC007383.3 | -18.6571555 | 2370.531453 | -0.0079 | 0.994 | -2.12E-09 | count | 1 |
| TG | -18.6571555 | 2370.531453 | -0.0079 | 0.994 | -2.12E-09 | count | 1 |
| CLEC4E | -18.6571555 | 2370.531453 | -0.0079 | 0.994 | -2.12E-09 | count | 1 |
| AL136368.1 | -18.6571555 | 2370.531453 | -0.0079 | 0.994 | -2.12E-09 | count | 1 |
| CCL13 | -18.6571555 | 2370.531453 | -0.0079 | 0.994 | -2.12E-09 | count | 1 |
| CLC | -18.6571555 | 2370.531453 | -0.0079 | 0.994 | -2.12E-09 | count | 1 |
| AC135178.2 | -18.6571555 | 2370.531453 | -0.0079 | 0.994 | -2.12E-09 | count | 1 |
| PTGER3 | -18.6571555 | 2370.531453 | -0.0079 | 0.994 | -2.12E-09 | count | 1 |
| ST7-AS1 | -18.6571555 | 2370.531453 | -0.0079 | 0.994 | -2.12E-09 | count | 1 |
| CD33 | -18.6571555 | 2370.531453 | -0.0079 | 0.994 | -2.12E-09 | count | 1 |
| BX004987.1 | -18.6571555 | 2370.531453 | -0.0079 | 0.994 | -2.12E-09 | count | 1 |
| U62317.5 | -18.6571555 | 2370.531453 | -0.0079 | 0.994 | -2.12E-09 | count | 1 |
| AL133383.1 | -18.6577407 | 3355.31924 | -0.0056 | 0.996 | -2.12E-09 | count | 1 |
| MEIS1 | -18.6577407 | 3355.31924 | -0.0056 | 0.996 | -2.12E-09 | count | 1 |
| RSPO3 | -18.6577407 | 3355.31924 | -0.0056 | 0.996 | -2.12E-09 | count | 1 |
| ILDR2 | -18.6577407 | 3355.31924 | -0.0056 | 0.996 | -2.12E-09 | count | 1 |
| AL031731.1 | -18.6577407 | 3355.31924 | -0.0056 | 0.996 | -2.12E-09 | count | 1 |
| LGI2 | -18.6577407 | 3355.31924 | -0.0056 | 0.996 | -2.12E-09 | count | 1 |
| PARD3 | -18.6577407 | 3355.31924 | -0.0056 | 0.996 | -2.12E-09 | count | 1 |
| AC034102.8 | -18.6577407 | 3355.31924 | -0.0056 | 0.996 | -2.12E-09 | count | 1 |
| REM1 | -18.6577407 | 3355.31924 | -0.0056 | 0.996 | -2.12E-09 | count | 1 |

| | | | | | | | |
|---|---|---|---|---|---|---|---|
| ERICH3 | -18.6577407 | 3355.31924 | -0.0056 | 0.996 | -2.12E-09 | count | 1 |
| AL160272.1 | -18.6577407 | 3355.31924 | -0.0056 | 0.996 | -2.12E-09 | count | 1 |
| LINC02312 | -18.6577407 | 3355.31924 | -0.0056 | 0.996 | -2.12E-09 | count | 1 |
| AL583722.2 | -18.6577407 | 3355.31924 | -0.0056 | 0.996 | -2.12E-09 | count | 1 |
| AC092338.2 | -18.6577407 | 3355.31924 | -0.0056 | 0.996 | -2.12E-09 | count | 1 |
| VSTM1 | -18.6577407 | 3355.31924 | -0.0056 | 0.996 | -2.12E-09 | count | 1 |
| PRODH | -18.6577407 | 3355.31924 | -0.0056 | 0.996 | -2.12E-09 | count | 1 |
| SUSD2 | -18.6577407 | 3355.31924 | -0.0056 | 0.996 | -2.12E-09 | count | 1 |
| LINC01119 | -18.6577407 | 3355.31924 | -0.0056 | 0.996 | -2.12E-09 | count | 1 |
| CSRNP3 | -18.6577407 | 3355.31924 | -0.0056 | 0.996 | -2.12E-09 | count | 1 |
| AC093895.1 | -18.6577407 | 3355.31924 | -0.0056 | 0.996 | -2.12E-09 | count | 1 |
| LINC02362 | -18.6577407 | 3355.31924 | -0.0056 | 0.996 | -2.12E-09 | count | 1 |
| ASB11 | -18.6577407 | 3355.31924 | -0.0056 | 0.996 | -2.12E-09 | count | 1 |
| ZFPM2 | -18.6577407 | 3355.31924 | -0.0056 | 0.996 | -2.12E-09 | count | 1 |
| TRDV3 | -18.6577407 | 3355.31924 | -0.0056 | 0.996 | -2.12E-09 | count | 1 |
| AC025287.2 | -18.6577407 | 3355.31924 | -0.0056 | 0.996 | -2.12E-09 | count | 1 |
| AC090617.3 | -18.6577407 | 3355.31924 | -0.0056 | 0.996 | -2.12E-09 | count | 1 |
| IGLV7-46 | -18.6577407 | 3355.31924 | -0.0056 | 0.996 | -2.12E-09 | count | 1 |
| SAMD11 | -18.6577407 | 3355.31924 | -0.0056 | 0.996 | -2.12E-09 | count | 1 |
| IGSF21 | -18.6577407 | 3355.31924 | -0.0056 | 0.996 | -2.12E-09 | count | 1 |
| AL020998.1 | -18.6577407 | 3355.31924 | -0.0056 | 0.996 | -2.12E-09 | count | 1 |
| SFN | -18.6577407 | 3355.31924 | -0.0056 | 0.996 | -2.12E-09 | count | 1 |
| AC009226.1 | -18.6577407 | 3355.31924 | -0.0056 | 0.996 | -2.12E-09 | count | 1 |
| MYH15 | -18.6577407 | 3355.31924 | -0.0056 | 0.996 | -2.12E-09 | count | 1 |
| AC068631.1 | -18.6577407 | 3355.31924 | -0.0056 | 0.996 | -2.12E-09 | count | 1 |
| BEND4 | -18.6577407 | 3355.31924 | -0.0056 | 0.996 | -2.12E-09 | count | 1 |
| HAND2-AS1 | -18.6577407 | 3355.31924 | -0.0056 | 0.996 | -2.12E-09 | count | 1 |
| ADAM29 | -18.6577407 | 3355.31924 | -0.0056 | 0.996 | -2.12E-09 | count | 1 |
| STOX2 | -18.6577407 | 3355.31924 | -0.0056 | 0.996 | -2.12E-09 | count | 1 |
| AC035140.1 | -18.6577407 | 3355.31924 | -0.0056 | 0.996 | -2.12E-09 | count | 1 |
| AC123595.1 | -18.6577407 | 3355.31924 | -0.0056 | 0.996 | -2.12E-09 | count | 1 |
| AC008467.1 | -18.6577407 | 3355.31924 | -0.0056 | 0.996 | -2.12E-09 | count | 1 |
| COL21A1 | -18.6577407 | 3355.31924 | -0.0056 | 0.996 | -2.12E-09 | count | 1 |
| AL589666.1 | -18.6577407 | 3355.31924 | -0.0056 | 0.996 | -2.12E-09 | count | 1 |
| RAET1G | -18.6577407 | 3355.31924 | -0.0056 | 0.996 | -2.12E-09 | count | 1 |
| TRGV1 | -18.6577407 | 3355.31924 | -0.0056 | 0.996 | -2.12E-09 | count | 1 |
| AC004835.1 | -18.6577407 | 3355.31924 | -0.0056 | 0.996 | -2.12E-09 | count | 1 |
| GLUD2 | -18.6577407 | 3355.31924 | -0.0056 | 0.996 | -2.12E-09 | count | 1 |
| AC019257.1 | -18.6577407 | 3355.31924 | -0.0056 | 0.996 | -2.12E-09 | count | 1 |
| AC084082.1 | -18.6577407 | 3355.31924 | -0.0056 | 0.996 | -2.12E-09 | count | 1 |
| BX649632.1 | -18.6577407 | 3355.31924 | -0.0056 | 0.996 | -2.12E-09 | count | 1 |
| AP001001.1 | -18.6577407 | 3355.31924 | -0.0056 | 0.996 | -2.12E-09 | count | 1 |
| TTC36 | -18.6577407 | 3355.31924 | -0.0056 | 0.996 | -2.12E-09 | count | 1 |
| SKIDA1 | -18.6577407 | 3355.31924 | -0.0056 | 0.996 | -2.12E-09 | count | 1 |
| LINC00866 | -18.6577407 | 3355.31924 | -0.0056 | 0.996 | -2.12E-09 | count | 1 |

| | | | | | | | |
|---|---|---|---|---|---|---|---|
| AC007406.3 | -18.6577407 | 3355.31924 | -0.0056 | 0.996 | -2.12E-09 | count | 1 |
| TSPAN11 | -18.6577407 | 3355.31924 | -0.0056 | 0.996 | -2.12E-09 | count | 1 |
| MAB21L1 | -18.6577407 | 3355.31924 | -0.0056 | 0.996 | -2.12E-09 | count | 1 |
| LINC00598 | -18.6577407 | 3355.31924 | -0.0056 | 0.996 | -2.12E-09 | count | 1 |
| TRIM9 | -18.6577407 | 3355.31924 | -0.0056 | 0.996 | -2.12E-09 | count | 1 |
| SYNJ2BP-COX16 | -18.6577407 | 3355.31924 | -0.0056 | 0.996 | -2.12E-09 | count | 1 |
| ACOT1 | -18.6577407 | 3355.31924 | -0.0056 | 0.996 | -2.12E-09 | count | 1 |
| IGHV3-41 | -18.6577407 | 3355.31924 | -0.0056 | 0.996 | -2.12E-09 | count | 1 |
| HDC | -18.6577407 | 3355.31924 | -0.0056 | 0.996 | -2.12E-09 | count | 1 |
| AC066613.1 | -18.6577407 | 3355.31924 | -0.0056 | 0.996 | -2.12E-09 | count | 1 |
| CGNL1 | -18.6577407 | 3355.31924 | -0.0056 | 0.996 | -2.12E-09 | count | 1 |
| NOX5 | -18.6577407 | 3355.31924 | -0.0056 | 0.996 | -2.12E-09 | count | 1 |
| AC004233.4 | -18.6577407 | 3355.31924 | -0.0056 | 0.996 | -2.12E-09 | count | 1 |
| INCA1 | -18.6577407 | 3355.31924 | -0.0056 | 0.996 | -2.12E-09 | count | 1 |
| AC002091.1 | -18.6577407 | 3355.31924 | -0.0056 | 0.996 | -2.12E-09 | count | 1 |
| C17orf99 | -18.6577407 | 3355.31924 | -0.0056 | 0.996 | -2.12E-09 | count | 1 |
| AP000919.3 | -18.6577407 | 3355.31924 | -0.0056 | 0.996 | -2.12E-09 | count | 1 |
| LAMP5 | -18.6577407 | 3355.31924 | -0.0056 | 0.996 | -2.12E-09 | count | 1 |
| DOK5 | -18.6577407 | 3355.31924 | -0.0056 | 0.996 | -2.12E-09 | count | 1 |
| AMH | -18.6577407 | 3355.31924 | -0.0056 | 0.996 | -2.12E-09 | count | 1 |
| ZNF878 | -18.6577407 | 3355.31924 | -0.0056 | 0.996 | -2.12E-09 | count | 1 |
| KLF1 | -18.6577407 | 3355.31924 | -0.0056 | 0.996 | -2.12E-09 | count | 1 |
| COMP | -18.6577407 | 3355.31924 | -0.0056 | 0.996 | -2.12E-09 | count | 1 |
| GALR3 | -18.6577407 | 3355.31924 | -0.0056 | 0.996 | -2.12E-09 | count | 1 |
| FCN3 | -18.6577407 | 3355.31924 | -0.0056 | 0.996 | -2.12E-09 | count | 1 |
| AC005014.2 | -18.6577407 | 3355.31924 | -0.0056 | 0.996 | -2.12E-09 | count | 1 |
| C9orf170 | -18.6577407 | 3355.31924 | -0.0056 | 0.996 | -2.12E-09 | count | 1 |
| AL359091.4 | -18.6577407 | 3355.31924 | -0.0056 | 0.996 | -2.12E-09 | count | 1 |
| ACVRL1 | -18.6577407 | 3355.31924 | -0.0056 | 0.996 | -2.12E-09 | count | 1 |
| LINC02280 | -18.6577407 | 3355.31924 | -0.0056 | 0.996 | -2.12E-09 | count | 1 |
| AC013553.4 | -18.6577407 | 3355.31924 | -0.0056 | 0.996 | -2.12E-09 | count | 1 |
| P2RX1 | -18.6577407 | 3355.31924 | -0.0056 | 0.996 | -2.12E-09 | count | 1 |
| NOTUM | -18.6577407 | 3355.31924 | -0.0056 | 0.996 | -2.12E-09 | count | 1 |
| TIE1 | -18.6577407 | 3355.31924 | -0.0056 | 0.996 | -2.12E-09 | count | 1 |
| AL162430.2 | -18.6577407 | 3355.31924 | -0.0056 | 0.996 | -2.12E-09 | count | 1 |
| TCF7L1 | -18.6577407 | 3355.31924 | -0.0056 | 0.996 | -2.12E-09 | count | 1 |
| ASTL | -18.6577407 | 3355.31924 | -0.0056 | 0.996 | -2.12E-09 | count | 1 |
| AC096649.1 | -18.6577407 | 3355.31924 | -0.0056 | 0.996 | -2.12E-09 | count | 1 |
| FBXL2 | -18.6577407 | 3355.31924 | -0.0056 | 0.996 | -2.12E-09 | count | 1 |
| TGM4 | -18.6577407 | 3355.31924 | -0.0056 | 0.996 | -2.12E-09 | count | 1 |
| LRRC66 | -18.6577407 | 3355.31924 | -0.0056 | 0.996 | -2.12E-09 | count | 1 |
| HAND2 | -18.6577407 | 3355.31924 | -0.0056 | 0.996 | -2.12E-09 | count | 1 |
| LINC00461 | -18.6577407 | 3355.31924 | -0.0056 | 0.996 | -2.12E-09 | count | 1 |
| FBLL1 | -18.6577407 | 3355.31924 | -0.0056 | 0.996 | -2.12E-09 | count | 1 |
| AL356234.3 | -18.6577407 | 3355.31924 | -0.0056 | 0.996 | -2.12E-09 | count | 1 |

| | | | | | | | |
|---|---|---|---|---|---|---|---|
| OCM | -18.6577407 | 3355.31924 | -0.0056 | 0.996 | -2.12E-09 | count | 1 |
| PGAM2 | -18.6577407 | 3355.31924 | -0.0056 | 0.996 | -2.12E-09 | count | 1 |
| ZNF727 | -18.6577407 | 3355.31924 | -0.0056 | 0.996 | -2.12E-09 | count | 1 |
| IQUB | -18.6577407 | 3355.31924 | -0.0056 | 0.996 | -2.12E-09 | count | 1 |
| MID1IP1-AS1 | -18.6577407 | 3355.31924 | -0.0056 | 0.996 | -2.12E-09 | count | 1 |
| HEPH | -18.6577407 | 3355.31924 | -0.0056 | 0.996 | -2.12E-09 | count | 1 |
| TCEAL7 | -18.6577407 | 3355.31924 | -0.0056 | 0.996 | -2.12E-09 | count | 1 |
| AF131215.3 | -18.6577407 | 3355.31924 | -0.0056 | 0.996 | -2.12E-09 | count | 1 |
| MBOAT4 | -18.6577407 | 3355.31924 | -0.0056 | 0.996 | -2.12E-09 | count | 1 |
| AC027702.1 | -18.6577407 | 3355.31924 | -0.0056 | 0.996 | -2.12E-09 | count | 1 |
| FOXH1 | -18.6577407 | 3355.31924 | -0.0056 | 0.996 | -2.12E-09 | count | 1 |
| C9orf84 | -18.6577407 | 3355.31924 | -0.0056 | 0.996 | -2.12E-09 | count | 1 |
| AL606469.1 | -18.6577407 | 3355.31924 | -0.0056 | 0.996 | -2.12E-09 | count | 1 |
| COL17A1 | -18.6577407 | 3355.31924 | -0.0056 | 0.996 | -2.12E-09 | count | 1 |
| CLEC9A | -18.6577407 | 3355.31924 | -0.0056 | 0.996 | -2.12E-09 | count | 1 |
| PTPRR | -18.6577407 | 3355.31924 | -0.0056 | 0.996 | -2.12E-09 | count | 1 |
| GAS2L3 | -18.6577407 | 3355.31924 | -0.0056 | 0.996 | -2.12E-09 | count | 1 |
| AC090617.5 | -18.6577407 | 3355.31924 | -0.0056 | 0.996 | -2.12E-09 | count | 1 |
| AC093484.3 | -18.6577407 | 3355.31924 | -0.0056 | 0.996 | -2.12E-09 | count | 1 |
| AC015911.6 | -18.6577407 | 3355.31924 | -0.0056 | 0.996 | -2.12E-09 | count | 1 |
| ZNF334 | -18.6577407 | 3355.31924 | -0.0056 | 0.996 | -2.12E-09 | count | 1 |
| AC005775.1 | -18.6577407 | 3355.31924 | -0.0056 | 0.996 | -2.12E-09 | count | 1 |
| AC008764.3 | -18.6577407 | 3355.31924 | -0.0056 | 0.996 | -2.12E-09 | count | 1 |
| APLP1 | -18.6577407 | 3355.31924 | -0.0056 | 0.996 | -2.12E-09 | count | 1 |
| AC005393.1 | -18.6577407 | 3355.31924 | -0.0056 | 0.996 | -2.12E-09 | count | 1 |
| AC020909.3 | -18.6577407 | 3355.31924 | -0.0056 | 0.996 | -2.12E-09 | count | 1 |
| OGFRP1 | -18.6577407 | 3355.31924 | -0.0056 | 0.996 | -2.12E-09 | count | 1 |
| NEXN-AS1 | -18.6577407 | 3355.31924 | -0.0056 | 0.996 | -2.12E-09 | count | 1 |
| OLFML3 | -18.6577407 | 3355.31924 | -0.0056 | 0.996 | -2.12E-09 | count | 1 |
| KCNT2 | -18.6577407 | 3355.31924 | -0.0056 | 0.996 | -2.12E-09 | count | 1 |
| AL592402.1 | -18.6577407 | 3355.31924 | -0.0056 | 0.996 | -2.12E-09 | count | 1 |
| PROX1 | -18.6577407 | 3355.31924 | -0.0056 | 0.996 | -2.12E-09 | count | 1 |
| LYPLAL1-DT | -18.6577407 | 3355.31924 | -0.0056 | 0.996 | -2.12E-09 | count | 1 |
| TMEM178A | -18.6577407 | 3355.31924 | -0.0056 | 0.996 | -2.12E-09 | count | 1 |
| LINC01888 | -18.6577407 | 3355.31924 | -0.0056 | 0.996 | -2.12E-09 | count | 1 |
| AC107027.1 | -18.6577407 | 3355.31924 | -0.0056 | 0.996 | -2.12E-09 | count | 1 |
| NKX6-1 | -18.6577407 | 3355.31924 | -0.0056 | 0.996 | -2.12E-09 | count | 1 |
| LRRTM2 | -18.6577407 | 3355.31924 | -0.0056 | 0.996 | -2.12E-09 | count | 1 |
| MEOX2 | -18.6577407 | 3355.31924 | -0.0056 | 0.996 | -2.12E-09 | count | 1 |
| FAM167A | -18.6577407 | 3355.31924 | -0.0056 | 0.996 | -2.12E-09 | count | 1 |
| MYO1A | -18.6577407 | 3355.31924 | -0.0056 | 0.996 | -2.12E-09 | count | 1 |
| EDNRB | -18.6577407 | 3355.31924 | -0.0056 | 0.996 | -2.12E-09 | count | 1 |
| AL358333.3 | -18.6577407 | 3355.31924 | -0.0056 | 0.996 | -2.12E-09 | count | 1 |
| AC010809.2 | -18.6577407 | 3355.31924 | -0.0056 | 0.996 | -2.12E-09 | count | 1 |
| AC240565.2 | -18.6577407 | 3355.31924 | -0.0056 | 0.996 | -2.12E-09 | count | 1 |

| | | | | | | | |
|---|---|---|---|---|---|---|---|
| CEP295NL | -18.6577407 | 3355.31924 | -0.0056 | 0.996 | -2.12E-09 | count | 1 |
| AC090377.1 | -18.6577407 | 3355.31924 | -0.0056 | 0.996 | -2.12E-09 | count | 1 |
| AL023803.2 | -18.6577407 | 3355.31924 | -0.0056 | 0.996 | -2.12E-09 | count | 1 |
| ZNF491 | -18.6577407 | 3355.31924 | -0.0056 | 0.996 | -2.12E-09 | count | 1 |
| FAM3D | -18.6577407 | 3355.31924 | -0.0056 | 0.996 | -2.12E-09 | count | 1 |
| HMHB1 | -18.6577407 | 3355.31924 | -0.0056 | 0.996 | -2.12E-09 | count | 1 |
| AC007029.1 | -18.6577407 | 3355.31924 | -0.0056 | 0.996 | -2.12E-09 | count | 1 |
| MXRA5 | -18.6577407 | 3355.31924 | -0.0056 | 0.996 | -2.12E-09 | count | 1 |
| CPA6 | -18.6577407 | 3355.31924 | -0.0056 | 0.996 | -2.12E-09 | count | 1 |
| CA3-AS1 | -18.6577407 | 3355.31924 | -0.0056 | 0.996 | -2.12E-09 | count | 1 |
| CLEC4D | -18.6577407 | 3355.31924 | -0.0056 | 0.996 | -2.12E-09 | count | 1 |
| AC131238.1 | -18.6577407 | 3355.31924 | -0.0056 | 0.996 | -2.12E-09 | count | 1 |
| TRPC4 | -18.6577407 | 3355.31924 | -0.0056 | 0.996 | -2.12E-09 | count | 1 |
| CLMN | -18.6577407 | 3355.31924 | -0.0056 | 0.996 | -2.12E-09 | count | 1 |
| ITGAD | -18.6577407 | 3355.31924 | -0.0056 | 0.996 | -2.12E-09 | count | 1 |
| AC005358.2 | -18.6577407 | 3355.31924 | -0.0056 | 0.996 | -2.12E-09 | count | 1 |
| ARHGEF18 | -18.6577407 | 3355.31924 | -0.0056 | 0.996 | -2.12E-09 | count | 1 |
| HPN | -18.6577407 | 3355.31924 | -0.0056 | 0.996 | -2.12E-09 | count | 1 |
| AL109811.1 | -18.6577407 | 3355.31924 | -0.0056 | 0.996 | -2.12E-09 | count | 1 |
| SMPDL3B | -18.6577407 | 3355.31924 | -0.0056 | 0.996 | -2.12E-09 | count | 1 |
| AC007681.1 | -18.6577407 | 3355.31924 | -0.0056 | 0.996 | -2.12E-09 | count | 1 |
| HAGLROS | -18.6577407 | 3355.31924 | -0.0056 | 0.996 | -2.12E-09 | count | 1 |
| OOEP | -18.6577407 | 3355.31924 | -0.0056 | 0.996 | -2.12E-09 | count | 1 |
| ZFP92 | -18.6577407 | 3355.31924 | -0.0056 | 0.996 | -2.12E-09 | count | 1 |
| AC023509.2 | -18.6577407 | 3355.31924 | -0.0056 | 0.996 | -2.12E-09 | count | 1 |
| AL138820.1 | -18.6577407 | 3355.31924 | -0.0056 | 0.996 | -2.12E-09 | count | 1 |
| AC012173.1 | -18.6577407 | 3355.31924 | -0.0056 | 0.996 | -2.12E-09 | count | 1 |
| LINC01660 | -18.6577407 | 3355.31924 | -0.0056 | 0.996 | -2.12E-09 | count | 1 |
| HSF2BP | -18.6577407 | 3355.31924 | -0.0056 | 0.996 | -2.12E-09 | count | 1 |
| TAS1R3 | -18.6577407 | 3355.31924 | -0.0056 | 0.996 | -2.12E-09 | count | 1 |
| CYP2J2 | -18.6577407 | 3355.31924 | -0.0056 | 0.996 | -2.12E-09 | count | 1 |
| LINC01106 | -18.6577407 | 3355.31924 | -0.0056 | 0.996 | -2.12E-09 | count | 1 |
| AC010680.5 | -18.6577407 | 3355.31924 | -0.0056 | 0.996 | -2.12E-09 | count | 1 |
| AC022001.3 | -18.6577407 | 3355.31924 | -0.0056 | 0.996 | -2.12E-09 | count | 1 |
| CDHR4 | -18.6577407 | 3355.31924 | -0.0056 | 0.996 | -2.12E-09 | count | 1 |
| AC109779.1 | -18.6577407 | 3355.31924 | -0.0056 | 0.996 | -2.12E-09 | count | 1 |
| AC107214.2 | -18.6577407 | 3355.31924 | -0.0056 | 0.996 | -2.12E-09 | count | 1 |
| AC091965.4 | -18.6577407 | 3355.31924 | -0.0056 | 0.996 | -2.12E-09 | count | 1 |
| AL136307.1 | -18.6577407 | 3355.31924 | -0.0056 | 0.996 | -2.12E-09 | count | 1 |
| AL008729.2 | -18.6577407 | 3355.31924 | -0.0056 | 0.996 | -2.12E-09 | count | 1 |
| AC004540.1 | -18.6577407 | 3355.31924 | -0.0056 | 0.996 | -2.12E-09 | count | 1 |
| KCND2 | -18.6577407 | 3355.31924 | -0.0056 | 0.996 | -2.12E-09 | count | 1 |
| TRBV23-1 | -18.6577407 | 3355.31924 | -0.0056 | 0.996 | -2.12E-09 | count | 1 |
| IDO1 | -18.6577407 | 3355.31924 | -0.0056 | 0.996 | -2.12E-09 | count | 1 |
| AP000424.2 | -18.6577407 | 3355.31924 | -0.0056 | 0.996 | -2.12E-09 | count | 1 |

| | | | | | | | |
|---|---|---|---|---|---|---|---|
| AC090587.1 | -18.6577407 | 3355.31924 | -0.0056 | 0.996 | -2.12E-09 | count | 1 |
| MYBPC3 | -18.6577407 | 3355.31924 | -0.0056 | 0.996 | -2.12E-09 | count | 1 |
| PROSER2-AS1 | -18.6577407 | 3355.31924 | -0.0056 | 0.996 | -2.12E-09 | count | 1 |
| AL161935.3 | -18.6577407 | 3355.31924 | -0.0056 | 0.996 | -2.12E-09 | count | 1 |
| AL132657.1 | -18.6577407 | 3355.31924 | -0.0056 | 0.996 | -2.12E-09 | count | 1 |
| ELOVL3 | -18.6577407 | 3355.31924 | -0.0056 | 0.996 | -2.12E-09 | count | 1 |
| AC078777.1 | -18.6577407 | 3355.31924 | -0.0056 | 0.996 | -2.12E-09 | count | 1 |
| AC124947.1 | -18.6577407 | 3355.31924 | -0.0056 | 0.996 | -2.12E-09 | count | 1 |
| GAS6-DT | -18.6577407 | 3355.31924 | -0.0056 | 0.996 | -2.12E-09 | count | 1 |
| AC016705.1 | -18.6577407 | 3355.31924 | -0.0056 | 0.996 | -2.12E-09 | count | 1 |
| TNFRSF17 | -18.6577407 | 3355.31924 | -0.0056 | 0.996 | -2.12E-09 | count | 1 |
| AC133555.3 | -18.6577407 | 3355.31924 | -0.0056 | 0.996 | -2.12E-09 | count | 1 |
| GPT2 | -18.6577407 | 3355.31924 | -0.0056 | 0.996 | -2.12E-09 | count | 1 |
| CCL17 | -18.6577407 | 3355.31924 | -0.0056 | 0.996 | -2.12E-09 | count | 1 |
| KRT14 | -18.6577407 | 3355.31924 | -0.0056 | 0.996 | -2.12E-09 | count | 1 |
| AL035541.1 | -18.6577407 | 3355.31924 | -0.0056 | 0.996 | -2.12E-09 | count | 1 |
| SLC25A41 | -18.6577407 | 3355.31924 | -0.0056 | 0.996 | -2.12E-09 | count | 1 |
| AC011477.4 | -18.6577407 | 3355.31924 | -0.0056 | 0.996 | -2.12E-09 | count | 1 |
| LINC00960 | -18.6577407 | 3355.31924 | -0.0056 | 0.996 | -2.12E-09 | count | 1 |
| AL133264.2 | -18.6577407 | 3355.31924 | -0.0056 | 0.996 | -2.12E-09 | count | 1 |
| CDK14 | -18.6577407 | 3355.31924 | -0.0056 | 0.996 | -2.12E-09 | count | 1 |
| CXorf36 | -18.6577407 | 3355.31924 | -0.0056 | 0.996 | -2.12E-09 | count | 1 |
| DLG3-AS1 | -18.6577407 | 3355.31924 | -0.0056 | 0.996 | -2.12E-09 | count | 1 |
| AL139384.2 | -18.6577407 | 3355.31924 | -0.0056 | 0.996 | -2.12E-09 | count | 1 |
| OR11G2 | -18.6577407 | 3355.31924 | -0.0056 | 0.996 | -2.12E-09 | count | 1 |
| AF127577.3 | -18.6577407 | 3355.31924 | -0.0056 | 0.996 | -2.12E-09 | count | 1 |
| HIST2H2AA3 | -18.6577407 | 3355.31924 | -0.0056 | 0.996 | -2.12E-09 | count | 1 |
| AC108062.1 | -18.6577407 | 3355.31924 | -0.0056 | 0.996 | -2.12E-09 | count | 1 |
| HCG14 | -18.6577407 | 3355.31924 | -0.0056 | 0.996 | -2.12E-09 | count | 1 |
| AC002310.2 | -18.6577407 | 3355.31924 | -0.0056 | 0.996 | -2.12E-09 | count | 1 |
| CCDC153 | -18.6577407 | 3355.31924 | -0.0056 | 0.996 | -2.12E-09 | count | 1 |
| FKBP10 | -18.6577407 | 3355.31924 | -0.0056 | 0.996 | -2.12E-09 | count | 1 |
| KIF14 | -18.6577407 | 3355.31924 | -0.0056 | 0.996 | -2.12E-09 | count | 1 |
| CTAGE6 | -17.980269 | 1689.39115 | -0.0106 | 0.992 | -2.11E-09 | count | 1 |
| AC073657.1 | -17.980269 | 1689.39115 | -0.0106 | 0.992 | -2.11E-09 | count | 1 |
| AL139424.3 | -17.980269 | 1689.39115 | -0.0106 | 0.992 | -2.11E-09 | count | 1 |
| ALS2CL | -17.980269 | 1689.39115 | -0.0106 | 0.992 | -2.11E-09 | count | 1 |
| SEZ6 | -17.980269 | 1689.39115 | -0.0106 | 0.992 | -2.11E-09 | count | 1 |
| AC005899.5 | -17.980269 | 1689.39115 | -0.0106 | 0.992 | -2.11E-09 | count | 1 |
| AC061992.1 | -17.980269 | 1689.39115 | -0.0106 | 0.992 | -2.11E-09 | count | 1 |
| AC011455.1 | -17.980269 | 1689.39115 | -0.0106 | 0.992 | -2.11E-09 | count | 1 |
| AL117350.1 | -17.980269 | 1689.39115 | -0.0106 | 0.992 | -2.11E-09 | count | 1 |
| KCNJ3 | -17.980269 | 1689.39115 | -0.0106 | 0.992 | -2.11E-09 | count | 1 |
| AC026979.3 | -17.980269 | 1689.39115 | -0.0106 | 0.992 | -2.11E-09 | count | 1 |
| PLBD1 | -17.980269 | 1689.39115 | -0.0106 | 0.992 | -2.11E-09 | count | 1 |

| | | | | | | | |
|---|---|---|---|---|---|---|---|
| JUP | -17.980269 | 1689.39115 | -0.0106 | 0.992 | -2.11E-09 | count | 1 |
| AC011471.2 | -17.980269 | 1689.39115 | -0.0106 | 0.992 | -2.11E-09 | count | 1 |
| KLB | -17.980269 | 1689.39115 | -0.0106 | 0.992 | -2.11E-09 | count | 1 |
| PDE3A | -17.980269 | 1689.39115 | -0.0106 | 0.992 | -2.11E-09 | count | 1 |
| AC089984.1 | -17.980269 | 1689.39115 | -0.0106 | 0.992 | -2.11E-09 | count | 1 |
| AL356019.2 | -17.980269 | 1689.39115 | -0.0106 | 0.992 | -2.11E-09 | count | 1 |
| PITPNM3 | -17.980269 | 1689.39115 | -0.0106 | 0.992 | -2.11E-09 | count | 1 |
| AP001059.2 | -17.980269 | 1689.39115 | -0.0106 | 0.992 | -2.11E-09 | count | 1 |
| AL031728.1 | -17.980269 | 1689.39115 | -0.0106 | 0.992 | -2.11E-09 | count | 1 |
| AL391335.1 | -17.980269 | 1689.39115 | -0.0106 | 0.992 | -2.11E-09 | count | 1 |
| MYOZ2 | -17.980269 | 1689.39115 | -0.0106 | 0.992 | -2.11E-09 | count | 1 |
| RNF180 | -17.980269 | 1689.39115 | -0.0106 | 0.992 | -2.11E-09 | count | 1 |
| AC027575.2 | -17.980269 | 1689.39115 | -0.0106 | 0.992 | -2.11E-09 | count | 1 |
| AL034417.3 | -17.980269 | 1689.39115 | -0.0106 | 0.992 | -2.11E-09 | count | 1 |
| AL355816.1 | -17.980269 | 1689.39115 | -0.0106 | 0.992 | -2.11E-09 | count | 1 |
| LINC01031 | -17.980269 | 1689.39115 | -0.0106 | 0.992 | -2.11E-09 | count | 1 |
| C2orf27B | -17.980269 | 1689.39115 | -0.0106 | 0.992 | -2.11E-09 | count | 1 |
| LINC01088 | -17.980269 | 1689.39115 | -0.0106 | 0.992 | -2.11E-09 | count | 1 |
| C1QTNF2 | -17.980269 | 1689.39115 | -0.0106 | 0.992 | -2.11E-09 | count | 1 |
| AL355312.2 | -17.980269 | 1689.39115 | -0.0106 | 0.992 | -2.11E-09 | count | 1 |
| MEDAG | -17.980269 | 1689.39115 | -0.0106 | 0.992 | -2.11E-09 | count | 1 |
| DLEU7-AS1 | -17.980269 | 1689.39115 | -0.0106 | 0.992 | -2.11E-09 | count | 1 |
| AP000317.1 | -17.980269 | 1689.39115 | -0.0106 | 0.992 | -2.11E-09 | count | 1 |
| DLGAP2 | -17.980269 | 1689.39115 | -0.0106 | 0.992 | -2.11E-09 | count | 1 |
| SHE | -17.9813226 | 2392.651737 | -0.0075 | 0.994 | -2.11E-09 | count | 1 |
| AL391422.3 | -17.9813226 | 2392.651737 | -0.0075 | 0.994 | -2.11E-09 | count | 1 |
| EMX2OS | -17.9813226 | 2392.651737 | -0.0075 | 0.994 | -2.11E-09 | count | 1 |
| LINC01133 | -17.9813226 | 2392.651737 | -0.0075 | 0.994 | -2.11E-09 | count | 1 |
| AC087623.4 | -17.9813226 | 2392.651737 | -0.0075 | 0.994 | -2.11E-09 | count | 1 |
| LINC01505 | -17.9813226 | 2392.651737 | -0.0075 | 0.994 | -2.11E-09 | count | 1 |
| NOX4 | -17.9813226 | 2392.651737 | -0.0075 | 0.994 | -2.11E-09 | count | 1 |
| BCL2L2 | -17.9813226 | 2392.651737 | -0.0075 | 0.994 | -2.11E-09 | count | 1 |
| AL035461.2 | -17.9813226 | 2392.651737 | -0.0075 | 0.994 | -2.11E-09 | count | 1 |
| CYP4X1 | -17.9813226 | 2392.651737 | -0.0075 | 0.994 | -2.11E-09 | count | 1 |
| AL139147.1 | -17.9813226 | 2392.651737 | -0.0075 | 0.994 | -2.11E-09 | count | 1 |
| AC243772.2 | -17.9813226 | 2392.651737 | -0.0075 | 0.994 | -2.11E-09 | count | 1 |
| AC104463.2 | -17.9813226 | 2392.651737 | -0.0075 | 0.994 | -2.11E-09 | count | 1 |
| ST6GAL2 | -17.9813226 | 2392.651737 | -0.0075 | 0.994 | -2.11E-09 | count | 1 |
| AC009948.3 | -17.9813226 | 2392.651737 | -0.0075 | 0.994 | -2.11E-09 | count | 1 |
| AC078785.2 | -17.9813226 | 2392.651737 | -0.0075 | 0.994 | -2.11E-09 | count | 1 |
| AC080013.6 | -17.9813226 | 2392.651737 | -0.0075 | 0.994 | -2.11E-09 | count | 1 |
| SOX2 | -17.9813226 | 2392.651737 | -0.0075 | 0.994 | -2.11E-09 | count | 1 |
| ADGRL3 | -17.9813226 | 2392.651737 | -0.0075 | 0.994 | -2.11E-09 | count | 1 |
| AP001330.5 | -17.9813226 | 2392.651737 | -0.0075 | 0.994 | -2.11E-09 | count | 1 |
| AC233992.2 | -17.9813226 | 2392.651737 | -0.0075 | 0.994 | -2.11E-09 | count | 1 |

| | | | | | | | |
|---|---|---|---|---|---|---|---|
| DMRT2 | -17.9813226 | 2392.651737 | -0.0075 | 0.994 | -2.11E-09 | count | 1 |
| FRMD3 | -17.9813226 | 2392.651737 | -0.0075 | 0.994 | -2.11E-09 | count | 1 |
| STPG3-AS1 | -17.9813226 | 2392.651737 | -0.0075 | 0.994 | -2.11E-09 | count | 1 |
| CCDC73 | -17.9813226 | 2392.651737 | -0.0075 | 0.994 | -2.11E-09 | count | 1 |
| GPC6 | -17.9813226 | 2392.651737 | -0.0075 | 0.994 | -2.11E-09 | count | 1 |
| SLC25A21 | -17.9813226 | 2392.651737 | -0.0075 | 0.994 | -2.11E-09 | count | 1 |
| MNS1 | -17.9813226 | 2392.651737 | -0.0075 | 0.994 | -2.11E-09 | count | 1 |
| LINC01273 | -17.9813226 | 2392.651737 | -0.0075 | 0.994 | -2.11E-09 | count | 1 |
| TULP2 | -17.9813226 | 2392.651737 | -0.0075 | 0.994 | -2.11E-09 | count | 1 |
| AC006946.2 | -17.9813226 | 2392.651737 | -0.0075 | 0.994 | -2.11E-09 | count | 1 |
| AP000345.2 | -17.9813226 | 2392.651737 | -0.0075 | 0.994 | -2.11E-09 | count | 1 |
| JAM2 | -17.9813226 | 2392.651737 | -0.0075 | 0.994 | -2.11E-09 | count | 1 |
| SLC2A5 | -17.9813226 | 2392.651737 | -0.0075 | 0.994 | -2.11E-09 | count | 1 |
| AKR7A3 | -17.9813226 | 2392.651737 | -0.0075 | 0.994 | -2.11E-09 | count | 1 |
| FLG-AS1 | -17.9813226 | 2392.651737 | -0.0075 | 0.994 | -2.11E-09 | count | 1 |
| OSBPL10-AS1 | -17.9813226 | 2392.651737 | -0.0075 | 0.994 | -2.11E-09 | count | 1 |
| LRRC70 | -17.9813226 | 2392.651737 | -0.0075 | 0.994 | -2.11E-09 | count | 1 |
| VIP | -17.9813226 | 2392.651737 | -0.0075 | 0.994 | -2.11E-09 | count | 1 |
| SEMA3D | -17.9813226 | 2392.651737 | -0.0075 | 0.994 | -2.11E-09 | count | 1 |
| PIWIL2 | -17.9813226 | 2392.651737 | -0.0075 | 0.994 | -2.11E-09 | count | 1 |
| AL162586.1 | -17.9813226 | 2392.651737 | -0.0075 | 0.994 | -2.11E-09 | count | 1 |
| TMEM254-AS1 | -17.9813226 | 2392.651737 | -0.0075 | 0.994 | -2.11E-09 | count | 1 |
| LINC00857 | -17.9813226 | 2392.651737 | -0.0075 | 0.994 | -2.11E-09 | count | 1 |
| LMO3 | -17.9813226 | 2392.651737 | -0.0075 | 0.994 | -2.11E-09 | count | 1 |
| AC025031.4 | -17.9813226 | 2392.651737 | -0.0075 | 0.994 | -2.11E-09 | count | 1 |
| AC012435.2 | -17.9813226 | 2392.651737 | -0.0075 | 0.994 | -2.11E-09 | count | 1 |
| LINC01197 | -17.9813226 | 2392.651737 | -0.0075 | 0.994 | -2.11E-09 | count | 1 |
| AOC3 | -17.9813226 | 2392.651737 | -0.0075 | 0.994 | -2.11E-09 | count | 1 |
| AC015802.4 | -17.9813226 | 2392.651737 | -0.0075 | 0.994 | -2.11E-09 | count | 1 |
| AP005899.1 | -17.9813226 | 2392.651737 | -0.0075 | 0.994 | -2.11E-09 | count | 1 |
| AC008752.2 | -17.9813226 | 2392.651737 | -0.0075 | 0.994 | -2.11E-09 | count | 1 |
| FUT2 | -17.9813226 | 2392.651737 | -0.0075 | 0.994 | -2.11E-09 | count | 1 |
| LINC01725 | -17.9813226 | 2392.651737 | -0.0075 | 0.994 | -2.11E-09 | count | 1 |
| POTEE | -17.9813226 | 2392.651737 | -0.0075 | 0.994 | -2.11E-09 | count | 1 |
| LINC00607 | -17.9813226 | 2392.651737 | -0.0075 | 0.994 | -2.11E-09 | count | 1 |
| DENND6A-AS1 | -17.9813226 | 2392.651737 | -0.0075 | 0.994 | -2.11E-09 | count | 1 |
| ADCY1 | -17.9813226 | 2392.651737 | -0.0075 | 0.994 | -2.11E-09 | count | 1 |
| KCND1 | -17.9813226 | 2392.651737 | -0.0075 | 0.994 | -2.11E-09 | count | 1 |
| PCAT7 | -17.9813226 | 2392.651737 | -0.0075 | 0.994 | -2.11E-09 | count | 1 |
| VAV2 | -17.9813226 | 2392.651737 | -0.0075 | 0.994 | -2.11E-09 | count | 1 |
| CD44-AS1 | -17.9813226 | 2392.651737 | -0.0075 | 0.994 | -2.11E-09 | count | 1 |
| LDB3 | -17.9813226 | 2392.651737 | -0.0075 | 0.994 | -2.11E-09 | count | 1 |
| SLC16A12 | -17.9813226 | 2392.651737 | -0.0075 | 0.994 | -2.11E-09 | count | 1 |
| LINC02455 | -17.9813226 | 2392.651737 | -0.0075 | 0.994 | -2.11E-09 | count | 1 |
| AC127164.1 | -17.9813226 | 2392.651737 | -0.0075 | 0.994 | -2.11E-09 | count | 1 |

| | | | | | | | |
|---|---|---|---|---|---|---|---|
| AC117503.1 | -17.9813226 | 2392.651737 | -0.0075 | 0.994 | -2.11E-09 | count | 1 |
| AC004817.4 | -17.9813226 | 2392.651737 | -0.0075 | 0.994 | -2.11E-09 | count | 1 |
| LINC02185 | -17.9813226 | 2392.651737 | -0.0075 | 0.994 | -2.11E-09 | count | 1 |
| IL34 | -17.9813226 | 2392.651737 | -0.0075 | 0.994 | -2.11E-09 | count | 1 |
| HIGD1B | -17.9813226 | 2392.651737 | -0.0075 | 0.994 | -2.11E-09 | count | 1 |
| AC145207.3 | -17.9813226 | 2392.651737 | -0.0075 | 0.994 | -2.11E-09 | count | 1 |
| AP001496.1 | -17.9813226 | 2392.651737 | -0.0075 | 0.994 | -2.11E-09 | count | 1 |
| AC012615.2 | -17.9813226 | 2392.651737 | -0.0075 | 0.994 | -2.11E-09 | count | 1 |
| C2orf50 | -17.9813226 | 2392.651737 | -0.0075 | 0.994 | -2.11E-09 | count | 1 |
| C4orf36 | -17.9813226 | 2392.651737 | -0.0075 | 0.994 | -2.11E-09 | count | 1 |
| LIFR | -17.9813226 | 2392.651737 | -0.0075 | 0.994 | -2.11E-09 | count | 1 |
| AC007349.3 | -17.9813226 | 2392.651737 | -0.0075 | 0.994 | -2.11E-09 | count | 1 |
| NRG1 | -17.9813226 | 2392.651737 | -0.0075 | 0.994 | -2.11E-09 | count | 1 |
| AC006581.1 | -17.9813226 | 2392.651737 | -0.0075 | 0.994 | -2.11E-09 | count | 1 |
| LINC00943 | -17.9813226 | 2392.651737 | -0.0075 | 0.994 | -2.11E-09 | count | 1 |
| EPHA3 | -17.9813226 | 2392.651737 | -0.0075 | 0.994 | -2.11E-09 | count | 1 |
| AL023583.1 | -17.9813226 | 2392.651737 | -0.0075 | 0.994 | -2.11E-09 | count | 1 |
| FZD4 | -17.9813226 | 2392.651737 | -0.0075 | 0.994 | -2.11E-09 | count | 1 |
| SMIM35 | -17.9813226 | 2392.651737 | -0.0075 | 0.994 | -2.11E-09 | count | 1 |
| TMEM52B | -17.9813226 | 2392.651737 | -0.0075 | 0.994 | -2.11E-09 | count | 1 |
| SLC10A1 | -17.9813226 | 2392.651737 | -0.0075 | 0.994 | -2.11E-09 | count | 1 |
| CPAMD8 | -17.9813226 | 2392.651737 | -0.0075 | 0.994 | -2.11E-09 | count | 1 |
| FOXRED2 | -17.9813226 | 2392.651737 | -0.0075 | 0.994 | -2.11E-09 | count | 1 |
| LAMC1 | -17.9813226 | 2392.651737 | -0.0075 | 0.994 | -2.11E-09 | count | 1 |
| LCT | -17.9813226 | 2392.651737 | -0.0075 | 0.994 | -2.11E-09 | count | 1 |
| WNT5B | -17.9813226 | 2392.651737 | -0.0075 | 0.994 | -2.11E-09 | count | 1 |
| AC078778.1 | -17.9813226 | 2392.651737 | -0.0075 | 0.994 | -2.11E-09 | count | 1 |
| KCTD1 | -17.9813226 | 2392.651737 | -0.0075 | 0.994 | -2.11E-09 | count | 1 |
| RBP4 | -17.9813226 | 2392.651737 | -0.0075 | 0.994 | -2.11E-09 | count | 1 |
| ZNF713 | -17.9813226 | 2392.651737 | -0.0075 | 0.994 | -2.11E-09 | count | 1 |
| SRC | -18.3873838 | 1689.204272 | -0.0109 | 0.991 | -2.11E-09 | count | 1 |
| C1QTNF7 | -18.3873838 | 1689.204272 | -0.0109 | 0.991 | -2.11E-09 | count | 1 |
| LINC00562 | -18.3873838 | 1689.204272 | -0.0109 | 0.991 | -2.11E-09 | count | 1 |
| CACHD1 | -18.3873838 | 1689.204272 | -0.0109 | 0.991 | -2.11E-09 | count | 1 |
| LINC02021 | -18.3873838 | 1689.204272 | -0.0109 | 0.991 | -2.11E-09 | count | 1 |
| B4GALT6 | -18.3873838 | 1689.204272 | -0.0109 | 0.991 | -2.11E-09 | count | 1 |
| ARHGEF10 | -18.3883671 | 2184.275888 | -0.0084 | 0.993 | -2.11E-09 | count | 1 |
| AC008676.1 | -18.3883671 | 2184.275888 | -0.0084 | 0.993 | -2.11E-09 | count | 1 |
| TLR7 | -18.3883671 | 2184.275888 | -0.0084 | 0.993 | -2.11E-09 | count | 1 |
| LINC00102 | -18.3883671 | 2184.275888 | -0.0084 | 0.993 | -2.11E-09 | count | 1 |
| MFAP2 | -18.3883671 | 2184.275888 | -0.0084 | 0.993 | -2.11E-09 | count | 1 |
| PRUNE2 | -18.3883671 | 2184.275888 | -0.0084 | 0.993 | -2.11E-09 | count | 1 |
| LINC00884 | -18.3883671 | 2184.275888 | -0.0084 | 0.993 | -2.11E-09 | count | 1 |
| AP001350.1 | -18.3883671 | 2184.275888 | -0.0084 | 0.993 | -2.11E-09 | count | 1 |
| AC018845.3 | -18.3883671 | 2184.275888 | -0.0084 | 0.993 | -2.11E-09 | count | 1 |

| | | | | | | | |
|---|---|---|---|---|---|---|---|
| KDM4A-AS1 | -18.3883671 | 2184.275888 | -0.0084 | 0.993 | -2.11E-09 | count | 1 |
| EXTL3-AS1 | -18.3883671 | 2184.275888 | -0.0084 | 0.993 | -2.11E-09 | count | 1 |
| TEX12 | -18.3883671 | 2184.275888 | -0.0084 | 0.993 | -2.11E-09 | count | 1 |
| PCDHGA6 | -18.3883671 | 2184.275888 | -0.0084 | 0.993 | -2.11E-09 | count | 1 |
| AC097359.3 | -18.3883671 | 2184.275888 | -0.0084 | 0.993 | -2.11E-09 | count | 1 |
| SLIT3 | -18.6764713 | 1688.808225 | -0.0111 | 0.991 | -2.11E-09 | count | 1 |
| WISP2 | -18.6773866 | 2071.892659 | -0.009 | 0.993 | -2.11E-09 | count | 1 |
| GJA5 | -18.6773866 | 2071.892659 | -0.009 | 0.993 | -2.11E-09 | count | 1 |
| PARD6G-AS1 | -18.3897451 | 2934.724354 | -0.0063 | 0.995 | -2.11E-09 | count | 1 |
| AL158211.4 | -18.3897451 | 2934.724354 | -0.0063 | 0.995 | -2.11E-09 | count | 1 |
| AC079174.2 | -18.3897451 | 2934.724354 | -0.0063 | 0.995 | -2.11E-09 | count | 1 |
| SIX1 | -18.3897451 | 2934.724354 | -0.0063 | 0.995 | -2.11E-09 | count | 1 |
| CDH19 | -18.3897451 | 2934.724354 | -0.0063 | 0.995 | -2.11E-09 | count | 1 |
| SLC22A15 | -18.3897451 | 2934.724354 | -0.0063 | 0.995 | -2.11E-09 | count | 1 |
| AC079089.1 | -18.3897451 | 2934.724354 | -0.0063 | 0.995 | -2.11E-09 | count | 1 |
| AC004264.1 | -18.3897451 | 2934.724354 | -0.0063 | 0.995 | -2.11E-09 | count | 1 |
| RANBP17 | -18.3897451 | 2934.724354 | -0.0063 | 0.995 | -2.11E-09 | count | 1 |
| CREB5 | -18.3897451 | 2934.724354 | -0.0063 | 0.995 | -2.11E-09 | count | 1 |
| UBE2C | -18.3897451 | 2934.724354 | -0.0063 | 0.995 | -2.11E-09 | count | 1 |
| AC021092.1 | -18.3897451 | 2934.724354 | -0.0063 | 0.995 | -2.11E-09 | count | 1 |
| SLC26A4 | -18.3897451 | 2934.724354 | -0.0063 | 0.995 | -2.11E-09 | count | 1 |
| LINC02388 | -18.3897451 | 2934.724354 | -0.0063 | 0.995 | -2.11E-09 | count | 1 |
| EME1 | -18.3897451 | 2934.724354 | -0.0063 | 0.995 | -2.11E-09 | count | 1 |
| ZNRF3-AS1 | -18.3897451 | 2934.724354 | -0.0063 | 0.995 | -2.11E-09 | count | 1 |
| HJURP | -18.3897451 | 2934.724354 | -0.0063 | 0.995 | -2.11E-09 | count | 1 |
| DEPTOR | -18.3897451 | 2934.724354 | -0.0063 | 0.995 | -2.11E-09 | count | 1 |
| AL161725.1 | -18.3897451 | 2934.724354 | -0.0063 | 0.995 | -2.11E-09 | count | 1 |
| AC015674.1 | -18.3897451 | 2934.724354 | -0.0063 | 0.995 | -2.11E-09 | count | 1 |
| AL136172.1 | -18.3897451 | 2934.724354 | -0.0063 | 0.995 | -2.11E-09 | count | 1 |
| LURAP1L | -18.3897451 | 2934.724354 | -0.0063 | 0.995 | -2.11E-09 | count | 1 |
| SFTPD | -18.3897451 | 2934.724354 | -0.0063 | 0.995 | -2.11E-09 | count | 1 |
| FGFR2 | -18.3897451 | 2934.724354 | -0.0063 | 0.995 | -2.11E-09 | count | 1 |
| AC006449.3 | -18.3897451 | 2934.724354 | -0.0063 | 0.995 | -2.11E-09 | count | 1 |
| ADRA2C | -18.3897451 | 2934.724354 | -0.0063 | 0.995 | -2.11E-09 | count | 1 |
| LRAT | -18.3897451 | 2934.724354 | -0.0063 | 0.995 | -2.11E-09 | count | 1 |
| AC026725.1 | -18.3897451 | 2934.724354 | -0.0063 | 0.995 | -2.11E-09 | count | 1 |
| PCOLCE-AS1 | -18.3897451 | 2934.724354 | -0.0063 | 0.995 | -2.11E-09 | count | 1 |
| AC079329.1 | -18.3897451 | 2934.724354 | -0.0063 | 0.995 | -2.11E-09 | count | 1 |
| AL683807.2 | -18.3897451 | 2934.724354 | -0.0063 | 0.995 | -2.11E-09 | count | 1 |
| AC133550.1 | -18.3897451 | 2934.724354 | -0.0063 | 0.995 | -2.11E-09 | count | 1 |
| MAOB | -18.3897451 | 2934.724354 | -0.0063 | 0.995 | -2.11E-09 | count | 1 |
| TEK | -18.3897451 | 2934.724354 | -0.0063 | 0.995 | -2.11E-09 | count | 1 |
| AC245052.4 | -18.3897451 | 2934.724354 | -0.0063 | 0.995 | -2.11E-09 | count | 1 |
| GPX2 | -18.3897451 | 2934.724354 | -0.0063 | 0.995 | -2.11E-09 | count | 1 |
| FAM177B | -18.3897451 | 2934.724354 | -0.0063 | 0.995 | -2.11E-09 | count | 1 |

| | | | | | | | |
|---|---|---|---|---|---|---|---|
| ASB15 | -18.6786664 | 2679.184142 | -0.007 | 0.994 | -2.11E-09 | count | 1 |
| CNN1 | -18.6786664 | 2679.184142 | -0.007 | 0.994 | -2.11E-09 | count | 1 |
| AL158212.2 | -18.6786664 | 2679.184142 | -0.007 | 0.994 | -2.11E-09 | count | 1 |
| UBE2L5 | -18.6786664 | 2679.184142 | -0.007 | 0.994 | -2.11E-09 | count | 1 |
| TIGD2 | -0.0240226 | 0.5739428 | -0.0419 | 0.967 | -1.93E-09 | count | 1 |
| SRSF12 | -0.2679353 | 0.6252208 | -0.4285 | 0.668 | -1.00E-09 | count | 1 |
| BMP8B | -0.2679353 | 0.6252208 | -0.4285 | 0.668 | -1.00E-09 | count | 1 |
| AC116096.1 | -0.2679353 | 0.6252208 | -0.4285 | 0.668 | -1.00E-09 | count | 1 |
| ITIH4 | -0.2679353 | 0.6252208 | -0.4285 | 0.668 | -1.00E-09 | count | 1 |
| AC008079.2 | -0.2679353 | 0.4413479 | -0.6071 | 0.544 | -1.00E-09 | count | 1 |
| SKA1 | -0.2679353 | 0.7925224 | -0.3381 | 0.735 | -1.00E-09 | count | 1 |
| BTBD3 | -0.2679353 | 0.7925224 | -0.3381 | 0.735 | -1.00E-09 | count | 1 |
| FAM227A | -0.2679353 | 0.7925224 | -0.3381 | 0.735 | -1.00E-09 | count | 1 |
| AL365255.1 | -0.2679353 | 0.7925224 | -0.3381 | 0.735 | -1.00E-09 | count | 1 |
| STK33 | -0.2679353 | 0.7925224 | -0.3381 | 0.735 | -1.00E-09 | count | 1 |
| SLC49A3 | -0.074108 | 0.7742925 | -0.0957 | 0.924 | -7.95E-10 | count | 1 |
| DDIT4L | -0.074108 | 0.7742925 | -0.0957 | 0.924 | -7.95E-10 | count | 1 |
| AC008014.1 | -0.074108 | 0.7742925 | -0.0957 | 0.924 | -7.95E-10 | count | 1 |
| CLNK | -0.074108 | 0.9054214 | -0.0818 | 0.935 | -7.95E-10 | count | 1 |
| IL21-AS1 | -0.074108 | 0.9054214 | -0.0818 | 0.935 | -7.95E-10 | count | 1 |
| SH2B2 | -0.074108 | 0.9054214 | -0.0818 | 0.935 | -7.95E-10 | count | 1 |
| IGHV1-69-2 | -0.074108 | 0.9054214 | -0.0818 | 0.935 | -7.95E-10 | count | 1 |
| KCNJ14 | -0.074108 | 0.9054214 | -0.0818 | 0.935 | -7.95E-10 | count | 1 |
| IRS1 | -0.074108 | 0.6358452 | -0.1166 | 0.907 | -7.95E-10 | count | 1 |
| LINC02166 | -0.074108 | 0.8942955 | -0.0829 | 0.934 | -7.95E-10 | count | 1 |
| ADD2 | -0.074108 | 1.0392758 | -0.0713 | 0.943 | -7.95E-10 | count | 1 |
| AC109587.1 | -0.074108 | 1.0392758 | -0.0713 | 0.943 | -7.95E-10 | count | 1 |
| SLC26A5 | -0.074108 | 1.0392758 | -0.0713 | 0.943 | -7.95E-10 | count | 1 |
| AC092681.3 | -0.074108 | 1.0392758 | -0.0713 | 0.943 | -7.95E-10 | count | 1 |
| MCMDC2 | -0.074108 | 1.0392758 | -0.0713 | 0.943 | -7.95E-10 | count | 1 |
| AL133415.1 | -0.074108 | 1.0392758 | -0.0713 | 0.943 | -7.95E-10 | count | 1 |
| NTF3 | -0.074108 | 1.0392758 | -0.0713 | 0.943 | -7.95E-10 | count | 1 |
| CLYBL-AS1 | -0.074108 | 1.0392758 | -0.0713 | 0.943 | -7.95E-10 | count | 1 |
| AC007220.1 | -0.074108 | 1.0392758 | -0.0713 | 0.943 | -7.95E-10 | count | 1 |
| AC135782.3 | -0.074108 | 1.0392758 | -0.0713 | 0.943 | -7.95E-10 | count | 1 |
| AC005696.1 | -0.074108 | 1.0392758 | -0.0713 | 0.943 | -7.95E-10 | count | 1 |
| FBXO15 | -0.074108 | 1.0392758 | -0.0713 | 0.943 | -7.95E-10 | count | 1 |
| AC011451.1 | -0.074108 | 1.0392758 | -0.0713 | 0.943 | -7.95E-10 | count | 1 |
| SYT5 | -0.074108 | 1.0392758 | -0.0713 | 0.943 | -7.95E-10 | count | 1 |
| AP001347.1 | -0.074108 | 1.0392758 | -0.0713 | 0.943 | -7.95E-10 | count | 1 |
| CHODL | -0.074108 | 1.0392758 | -0.0713 | 0.943 | -7.95E-10 | count | 1 |
| AC036214.3 | -0.074108 | 1.0392758 | -0.0713 | 0.943 | -7.95E-10 | count | 1 |
| AC092718.1 | -0.074108 | 1.0392758 | -0.0713 | 0.943 | -7.95E-10 | count | 1 |
| PRTFDC1 | -0.074108 | 0.7344781 | -0.1009 | 0.92 | -7.95E-10 | count | 1 |
| KNDC1 | -0.074108 | 0.7344781 | -0.1009 | 0.92 | -7.95E-10 | count | 1 |

| | | | | | | | |
|---|---|---|---|---|---|---|---|
| ADAMTSL3 | -0.074108 | 0.7344781 | -0.1009 | 0.92 | -7.95E-10 | count | 1 |
| SERHL2 | -0.074108 | 0.7344781 | -0.1009 | 0.92 | -7.95E-10 | count | 1 |
| LINC01801 | -0.074108 | 0.7310624 | -0.1014 | 0.919 | -7.95E-10 | count | 1 |
| AL359921.2 | -18.2852655 | 2785.350449 | -0.0066 | 0.995 | -7.76E-10 | count | 1 |
| AC093788.1 | -18.2852655 | 2785.350449 | -0.0066 | 0.995 | -7.76E-10 | count | 1 |
| LRRC4 | -18.2852655 | 2785.350449 | -0.0066 | 0.995 | -7.76E-10 | count | 1 |
| ADGRG2 | -18.2852655 | 2785.350449 | -0.0066 | 0.995 | -7.76E-10 | count | 1 |
| AL157935.2 | -18.2852655 | 2785.350449 | -0.0066 | 0.995 | -7.76E-10 | count | 1 |
| AL590226.1 | -18.2852655 | 2785.350449 | -0.0066 | 0.995 | -7.76E-10 | count | 1 |
| VWCE | -18.2852655 | 2785.350449 | -0.0066 | 0.995 | -7.76E-10 | count | 1 |
| AC078962.3 | -18.2852655 | 2785.350449 | -0.0066 | 0.995 | -7.76E-10 | count | 1 |
| AC079315.1 | -18.2852655 | 2785.350449 | -0.0066 | 0.995 | -7.76E-10 | count | 1 |
| TAL1 | -18.2852655 | 2785.350449 | -0.0066 | 0.995 | -7.76E-10 | count | 1 |
| AL356356.1 | -18.2852655 | 2785.350449 | -0.0066 | 0.995 | -7.76E-10 | count | 1 |
| AC013726.1 | -18.2852655 | 2785.350449 | -0.0066 | 0.995 | -7.76E-10 | count | 1 |
| SAMMSON | -18.2852655 | 2785.350449 | -0.0066 | 0.995 | -7.76E-10 | count | 1 |
| EDA2R | -18.2852655 | 2785.350449 | -0.0066 | 0.995 | -7.76E-10 | count | 1 |
| LRRC19 | -18.2852655 | 2785.350449 | -0.0066 | 0.995 | -7.76E-10 | count | 1 |
| KIF24 | -18.2852655 | 2785.350449 | -0.0066 | 0.995 | -7.76E-10 | count | 1 |
| LPAR1 | -18.2852655 | 2785.350449 | -0.0066 | 0.995 | -7.76E-10 | count | 1 |
| PMEL | -18.2852655 | 2785.350449 | -0.0066 | 0.995 | -7.76E-10 | count | 1 |
| ATP11A-AS1 | -18.2852655 | 2785.350449 | -0.0066 | 0.995 | -7.76E-10 | count | 1 |
| FAM71D | -18.2852655 | 2785.350449 | -0.0066 | 0.995 | -7.76E-10 | count | 1 |
| NPIPA8 | -18.2852655 | 2785.350449 | -0.0066 | 0.995 | -7.76E-10 | count | 1 |
| LGALS9B | -18.2852655 | 2785.350449 | -0.0066 | 0.995 | -7.76E-10 | count | 1 |
| HSPB9 | -18.2852655 | 2785.350449 | -0.0066 | 0.995 | -7.76E-10 | count | 1 |
| SP6 | -18.2852655 | 2785.350449 | -0.0066 | 0.995 | -7.76E-10 | count | 1 |
| LINC01638 | -18.2852655 | 2785.350449 | -0.0066 | 0.995 | -7.76E-10 | count | 1 |
| RIBC2 | -18.2852655 | 2785.350449 | -0.0066 | 0.995 | -7.76E-10 | count | 1 |
| AL031283.2 | -18.2852655 | 2785.350449 | -0.0066 | 0.995 | -7.76E-10 | count | 1 |
| FNDC5 | -18.2852655 | 2785.350449 | -0.0066 | 0.995 | -7.76E-10 | count | 1 |
| AL606760.2 | -18.2852655 | 2785.350449 | -0.0066 | 0.995 | -7.76E-10 | count | 1 |
| HIST2H4A | -18.2852655 | 2785.350449 | -0.0066 | 0.995 | -7.76E-10 | count | 1 |
| AL450992.1 | -18.2852655 | 2785.350449 | -0.0066 | 0.995 | -7.76E-10 | count | 1 |
| FCRL2 | -18.2852655 | 2785.350449 | -0.0066 | 0.995 | -7.76E-10 | count | 1 |
| AL138899.1 | -18.2852655 | 2785.350449 | -0.0066 | 0.995 | -7.76E-10 | count | 1 |
| VANGL2 | -18.2852655 | 2785.350449 | -0.0066 | 0.995 | -7.76E-10 | count | 1 |
| ELF3 | -18.2852655 | 2785.350449 | -0.0066 | 0.995 | -7.76E-10 | count | 1 |
| C1orf229 | -18.2852655 | 2785.350449 | -0.0066 | 0.995 | -7.76E-10 | count | 1 |
| CYS1 | -18.2852655 | 2785.350449 | -0.0066 | 0.995 | -7.76E-10 | count | 1 |
| AC013403.2 | -18.2852655 | 2785.350449 | -0.0066 | 0.995 | -7.76E-10 | count | 1 |
| AC010680.2 | -18.2852655 | 2785.350449 | -0.0066 | 0.995 | -7.76E-10 | count | 1 |
| LINC01857 | -18.2852655 | 2785.350449 | -0.0066 | 0.995 | -7.76E-10 | count | 1 |
| CCR3 | -18.2852655 | 2785.350449 | -0.0066 | 0.995 | -7.76E-10 | count | 1 |
| TMIE | -18.2852655 | 2785.350449 | -0.0066 | 0.995 | -7.76E-10 | count | 1 |

| | | | | | | | |
|---|---|---|---|---|---|---|---|
| AC117394.2 | -18.2852655 | 2785.350449 | -0.0066 | 0.995 | -7.76E-10 | count | 1 |
| NLGN1 | -18.2852655 | 2785.350449 | -0.0066 | 0.995 | -7.76E-10 | count | 1 |
| KLHL6-AS1 | -18.2852655 | 2785.350449 | -0.0066 | 0.995 | -7.76E-10 | count | 1 |
| CC2D2A | -18.2852655 | 2785.350449 | -0.0066 | 0.995 | -7.76E-10 | count | 1 |
| LRIT3 | -18.2852655 | 2785.350449 | -0.0066 | 0.995 | -7.76E-10 | count | 1 |
| SLC45A2 | -18.2852655 | 2785.350449 | -0.0066 | 0.995 | -7.76E-10 | count | 1 |
| PART1 | -18.2852655 | 2785.350449 | -0.0066 | 0.995 | -7.76E-10 | count | 1 |
| AC008522.1 | -18.2852655 | 2785.350449 | -0.0066 | 0.995 | -7.76E-10 | count | 1 |
| C5orf66 | -18.2852655 | 2785.350449 | -0.0066 | 0.995 | -7.76E-10 | count | 1 |
| PCDHB4 | -18.2852655 | 2785.350449 | -0.0066 | 0.995 | -7.76E-10 | count | 1 |
| SLC25A2 | -18.2852655 | 2785.350449 | -0.0066 | 0.995 | -7.76E-10 | count | 1 |
| FGFR4 | -18.2852655 | 2785.350449 | -0.0066 | 0.995 | -7.76E-10 | count | 1 |
| AC008443.4 | -18.2852655 | 2785.350449 | -0.0066 | 0.995 | -7.76E-10 | count | 1 |
| HIST1H3E | -18.2852655 | 2785.350449 | -0.0066 | 0.995 | -7.76E-10 | count | 1 |
| AL136304.1 | -18.2852655 | 2785.350449 | -0.0066 | 0.995 | -7.76E-10 | count | 1 |
| AL137784.2 | -18.2852655 | 2785.350449 | -0.0066 | 0.995 | -7.76E-10 | count | 1 |
| SOBP | -18.2852655 | 2785.350449 | -0.0066 | 0.995 | -7.76E-10 | count | 1 |
| ULBP1 | -18.2852655 | 2785.350449 | -0.0066 | 0.995 | -7.76E-10 | count | 1 |
| LINC00574 | -18.2852655 | 2785.350449 | -0.0066 | 0.995 | -7.76E-10 | count | 1 |
| RSPH10B | -18.2852655 | 2785.350449 | -0.0066 | 0.995 | -7.76E-10 | count | 1 |
| HOXA2 | -18.2852655 | 2785.350449 | -0.0066 | 0.995 | -7.76E-10 | count | 1 |
| GLI3 | -18.2852655 | 2785.350449 | -0.0066 | 0.995 | -7.76E-10 | count | 1 |
| FAM71F2 | -18.2852655 | 2785.350449 | -0.0066 | 0.995 | -7.76E-10 | count | 1 |
| ARHGAP6 | -18.2852655 | 2785.350449 | -0.0066 | 0.995 | -7.76E-10 | count | 1 |
| PCDH11X | -18.2852655 | 2785.350449 | -0.0066 | 0.995 | -7.76E-10 | count | 1 |
| TAF7L | -18.2852655 | 2785.350449 | -0.0066 | 0.995 | -7.76E-10 | count | 1 |
| LINC01285 | -18.2852655 | 2785.350449 | -0.0066 | 0.995 | -7.76E-10 | count | 1 |
| PNMA6A | -18.2852655 | 2785.350449 | -0.0066 | 0.995 | -7.76E-10 | count | 1 |
| AF106564.1 | -18.2852655 | 2785.350449 | -0.0066 | 0.995 | -7.76E-10 | count | 1 |
| AC087627.1 | -18.2852655 | 2785.350449 | -0.0066 | 0.995 | -7.76E-10 | count | 1 |
| CHMP4C | -18.2852655 | 2785.350449 | -0.0066 | 0.995 | -7.76E-10 | count | 1 |
| AC084116.1 | -18.2852655 | 2785.350449 | -0.0066 | 0.995 | -7.76E-10 | count | 1 |
| FAM225B | -18.2852655 | 2785.350449 | -0.0066 | 0.995 | -7.76E-10 | count | 1 |
| MS4A2 | -18.2852655 | 2785.350449 | -0.0066 | 0.995 | -7.76E-10 | count | 1 |
| KCNJ5 | -18.2852655 | 2785.350449 | -0.0066 | 0.995 | -7.76E-10 | count | 1 |
| FZD8 | -18.2852655 | 2785.350449 | -0.0066 | 0.995 | -7.76E-10 | count | 1 |
| PPP1R3C | -18.2852655 | 2785.350449 | -0.0066 | 0.995 | -7.76E-10 | count | 1 |
| SFRP5 | -18.2852655 | 2785.350449 | -0.0066 | 0.995 | -7.76E-10 | count | 1 |
| DOCK1 | -18.2852655 | 2785.350449 | -0.0066 | 0.995 | -7.76E-10 | count | 1 |
| AL354950.1 | -18.2852655 | 2785.350449 | -0.0066 | 0.995 | -7.76E-10 | count | 1 |
| NRIP2 | -18.2852655 | 2785.350449 | -0.0066 | 0.995 | -7.76E-10 | count | 1 |
| AC006207.1 | -18.2852655 | 2785.350449 | -0.0066 | 0.995 | -7.76E-10 | count | 1 |
| FGF23 | -18.2852655 | 2785.350449 | -0.0066 | 0.995 | -7.76E-10 | count | 1 |
| AC083806.2 | -18.2852655 | 2785.350449 | -0.0066 | 0.995 | -7.76E-10 | count | 1 |
| PLA2G1B | -18.2852655 | 2785.350449 | -0.0066 | 0.995 | -7.76E-10 | count | 1 |

| | | | | | | | |
|---|---|---|---|---|---|---|---|
| LMO7-AS1 | -18.2852655 | 2785.350449 | -0.0066 | 0.995 | -7.76E-10 | count | 1 |
| DZIP1 | -18.2852655 | 2785.350449 | -0.0066 | 0.995 | -7.76E-10 | count | 1 |
| ARHGEF7-AS2 | -18.2852655 | 2785.350449 | -0.0066 | 0.995 | -7.76E-10 | count | 1 |
| CYP46A1 | -18.2852655 | 2785.350449 | -0.0066 | 0.995 | -7.76E-10 | count | 1 |
| MKRN3 | -18.2852655 | 2785.350449 | -0.0066 | 0.995 | -7.76E-10 | count | 1 |
| AC091057.3 | -18.2852655 | 2785.350449 | -0.0066 | 0.995 | -7.76E-10 | count | 1 |
| DLL4 | -18.2852655 | 2785.350449 | -0.0066 | 0.995 | -7.76E-10 | count | 1 |
| AC021739.5 | -18.2852655 | 2785.350449 | -0.0066 | 0.995 | -7.76E-10 | count | 1 |
| CD19 | -18.2852655 | 2785.350449 | -0.0066 | 0.995 | -7.76E-10 | count | 1 |
| ZNF629 | -18.2852655 | 2785.350449 | -0.0066 | 0.995 | -7.76E-10 | count | 1 |
| LINC01227 | -18.2852655 | 2785.350449 | -0.0066 | 0.995 | -7.76E-10 | count | 1 |
| FBXO39 | -18.2852655 | 2785.350449 | -0.0066 | 0.995 | -7.76E-10 | count | 1 |
| NTN1 | -18.2852655 | 2785.350449 | -0.0066 | 0.995 | -7.76E-10 | count | 1 |
| AC015813.2 | -18.2852655 | 2785.350449 | -0.0066 | 0.995 | -7.76E-10 | count | 1 |
| AC145343.1 | -18.2852655 | 2785.350449 | -0.0066 | 0.995 | -7.76E-10 | count | 1 |
| ZACN | -18.2852655 | 2785.350449 | -0.0066 | 0.995 | -7.76E-10 | count | 1 |
| C1QTNF1-AS1 | -18.2852655 | 2785.350449 | -0.0066 | 0.995 | -7.76E-10 | count | 1 |
| AC007998.4 | -18.2852655 | 2785.350449 | -0.0066 | 0.995 | -7.76E-10 | count | 1 |
| AL109935.1 | -18.2852655 | 2785.350449 | -0.0066 | 0.995 | -7.76E-10 | count | 1 |
| AL133230.1 | -18.2852655 | 2785.350449 | -0.0066 | 0.995 | -7.76E-10 | count | 1 |
| AC011498.4 | -18.2852655 | 2785.350449 | -0.0066 | 0.995 | -7.76E-10 | count | 1 |
| NANOS3 | -18.2852655 | 2785.350449 | -0.0066 | 0.995 | -7.76E-10 | count | 1 |
| AC022148.1 | -18.2852655 | 2785.350449 | -0.0066 | 0.995 | -7.76E-10 | count | 1 |
| GRIK5 | -18.2852655 | 2785.350449 | -0.0066 | 0.995 | -7.76E-10 | count | 1 |
| ZNF112 | -18.2852655 | 2785.350449 | -0.0066 | 0.995 | -7.76E-10 | count | 1 |
| CEACAM19 | -18.2852655 | 2785.350449 | -0.0066 | 0.995 | -7.76E-10 | count | 1 |
| AC020909.2 | -18.2852655 | 2785.350449 | -0.0066 | 0.995 | -7.76E-10 | count | 1 |
| TMEM240 | -18.2852655 | 2785.350449 | -0.0066 | 0.995 | -7.76E-10 | count | 1 |
| PRDM16 | -18.2852655 | 2785.350449 | -0.0066 | 0.995 | -7.76E-10 | count | 1 |
| AL109936.2 | -18.2852655 | 2785.350449 | -0.0066 | 0.995 | -7.76E-10 | count | 1 |
| AC099062.1 | -18.2852655 | 2785.350449 | -0.0066 | 0.995 | -7.76E-10 | count | 1 |
| TMEM56 | -18.2852655 | 2785.350449 | -0.0066 | 0.995 | -7.76E-10 | count | 1 |
| FP700111.1 | -18.2852655 | 2785.350449 | -0.0066 | 0.995 | -7.76E-10 | count | 1 |
| OR2L2 | -18.2852655 | 2785.350449 | -0.0066 | 0.995 | -7.76E-10 | count | 1 |
| AC013472.3 | -18.2852655 | 2785.350449 | -0.0066 | 0.995 | -7.76E-10 | count | 1 |
| AC108058.1 | -18.2852655 | 2785.350449 | -0.0066 | 0.995 | -7.76E-10 | count | 1 |
| AC016717.2 | -18.2852655 | 2785.350449 | -0.0066 | 0.995 | -7.76E-10 | count | 1 |
| ACVR2B-AS1 | -18.2852655 | 2785.350449 | -0.0066 | 0.995 | -7.76E-10 | count | 1 |
| COL7A1 | -18.2852655 | 2785.350449 | -0.0066 | 0.995 | -7.76E-10 | count | 1 |
| IGSF11 | -18.2852655 | 2785.350449 | -0.0066 | 0.995 | -7.76E-10 | count | 1 |
| ALDH1L1 | -18.2852655 | 2785.350449 | -0.0066 | 0.995 | -7.76E-10 | count | 1 |
| PTX3 | -18.2852655 | 2785.350449 | -0.0066 | 0.995 | -7.76E-10 | count | 1 |
| AC078795.3 | -18.2852655 | 2785.350449 | -0.0066 | 0.995 | -7.76E-10 | count | 1 |
| LIPH | -18.2852655 | 2785.350449 | -0.0066 | 0.995 | -7.76E-10 | count | 1 |
| LINC02513 | -18.2852655 | 2785.350449 | -0.0066 | 0.995 | -7.76E-10 | count | 1 |

| | | | | | | | |
|---|---|---|---|---|---|---|---|
| AC091891.2 | -18.2852655 | 2785.350449 | -0.0066 | 0.995 | -7.76E-10 | count | 1 |
| NPR3 | -18.2852655 | 2785.350449 | -0.0066 | 0.995 | -7.76E-10 | count | 1 |
| AC114956.1 | -18.2852655 | 2785.350449 | -0.0066 | 0.995 | -7.76E-10 | count | 1 |
| MEIKIN | -18.2852655 | 2785.350449 | -0.0066 | 0.995 | -7.76E-10 | count | 1 |
| AL133351.1 | -18.2852655 | 2785.350449 | -0.0066 | 0.995 | -7.76E-10 | count | 1 |
| AL353597.1 | -18.2852655 | 2785.350449 | -0.0066 | 0.995 | -7.76E-10 | count | 1 |
| CALHM5 | -18.2852655 | 2785.350449 | -0.0066 | 0.995 | -7.76E-10 | count | 1 |
| MACC1 | -18.2852655 | 2785.350449 | -0.0066 | 0.995 | -7.76E-10 | count | 1 |
| AMPH | -18.2852655 | 2785.350449 | -0.0066 | 0.995 | -7.76E-10 | count | 1 |
| NUPR2 | -18.2852655 | 2785.350449 | -0.0066 | 0.995 | -7.76E-10 | count | 1 |
| AC004990.1 | -18.2852655 | 2785.350449 | -0.0066 | 0.995 | -7.76E-10 | count | 1 |
| TRBV4-1 | -18.2852655 | 2785.350449 | -0.0066 | 0.995 | -7.76E-10 | count | 1 |
| KLHL34 | -18.2852655 | 2785.350449 | -0.0066 | 0.995 | -7.76E-10 | count | 1 |
| SMIM10L2B | -18.2852655 | 2785.350449 | -0.0066 | 0.995 | -7.76E-10 | count | 1 |
| AF131216.5 | -18.2852655 | 2785.350449 | -0.0066 | 0.995 | -7.76E-10 | count | 1 |
| AC087362.2 | -18.2852655 | 2785.350449 | -0.0066 | 0.995 | -7.76E-10 | count | 1 |
| AC090739.1 | -18.2852655 | 2785.350449 | -0.0066 | 0.995 | -7.76E-10 | count | 1 |
| AC103843.1 | -18.2852655 | 2785.350449 | -0.0066 | 0.995 | -7.76E-10 | count | 1 |
| AC107952.2 | -18.2852655 | 2785.350449 | -0.0066 | 0.995 | -7.76E-10 | count | 1 |
| AC016877.1 | -18.2852655 | 2785.350449 | -0.0066 | 0.995 | -7.76E-10 | count | 1 |
| POU5F1B | -18.2852655 | 2785.350449 | -0.0066 | 0.995 | -7.76E-10 | count | 1 |
| DMRTA1 | -18.2852655 | 2785.350449 | -0.0066 | 0.995 | -7.76E-10 | count | 1 |
| LINGO2 | -18.2852655 | 2785.350449 | -0.0066 | 0.995 | -7.76E-10 | count | 1 |
| C9orf106 | -18.2852655 | 2785.350449 | -0.0066 | 0.995 | -7.76E-10 | count | 1 |
| AP006333.1 | -18.2852655 | 2785.350449 | -0.0066 | 0.995 | -7.76E-10 | count | 1 |
| AP003119.2 | -18.2852655 | 2785.350449 | -0.0066 | 0.995 | -7.76E-10 | count | 1 |
| SLN | -18.2852655 | 2785.350449 | -0.0066 | 0.995 | -7.76E-10 | count | 1 |
| BARX2 | -18.2852655 | 2785.350449 | -0.0066 | 0.995 | -7.76E-10 | count | 1 |
| AL137145.1 | -18.2852655 | 2785.350449 | -0.0066 | 0.995 | -7.76E-10 | count | 1 |
| AL731563.3 | -18.2852655 | 2785.350449 | -0.0066 | 0.995 | -7.76E-10 | count | 1 |
| LINC02367 | -18.2852655 | 2785.350449 | -0.0066 | 0.995 | -7.76E-10 | count | 1 |
| AC008115.1 | -18.2852655 | 2785.350449 | -0.0066 | 0.995 | -7.76E-10 | count | 1 |
| ART4 | -18.2852655 | 2785.350449 | -0.0066 | 0.995 | -7.76E-10 | count | 1 |
| AC046130.1 | -18.2852655 | 2785.350449 | -0.0066 | 0.995 | -7.76E-10 | count | 1 |
| AC025165.2 | -18.2852655 | 2785.350449 | -0.0066 | 0.995 | -7.76E-10 | count | 1 |
| AC078962.4 | -18.2852655 | 2785.350449 | -0.0066 | 0.995 | -7.76E-10 | count | 1 |
| AC079907.1 | -18.2852655 | 2785.350449 | -0.0066 | 0.995 | -7.76E-10 | count | 1 |
| CCDC62 | -18.2852655 | 2785.350449 | -0.0066 | 0.995 | -7.76E-10 | count | 1 |
| RFLNA | -18.2852655 | 2785.350449 | -0.0066 | 0.995 | -7.76E-10 | count | 1 |
| GRTP1 | -18.2852655 | 2785.350449 | -0.0066 | 0.995 | -7.76E-10 | count | 1 |
| NFATC4 | -18.2852655 | 2785.350449 | -0.0066 | 0.995 | -7.76E-10 | count | 1 |
| AF107885.2 | -18.2852655 | 2785.350449 | -0.0066 | 0.995 | -7.76E-10 | count | 1 |
| IGHV3-29 | -18.2852655 | 2785.350449 | -0.0066 | 0.995 | -7.76E-10 | count | 1 |
| GREM1 | -18.2852655 | 2785.350449 | -0.0066 | 0.995 | -7.76E-10 | count | 1 |
| LIPC | -18.2852655 | 2785.350449 | -0.0066 | 0.995 | -7.76E-10 | count | 1 |

| | | | | | | | |
|---|---|---|---|---|---|---|---|
| AC118658.1 | -18.2852655 | 2785.350449 | -0.0066 | 0.995 | -7.76E-10 | count | 1 |
| AC009034.1 | -18.2852655 | 2785.350449 | -0.0066 | 0.995 | -7.76E-10 | count | 1 |
| SULT1A2 | -18.2852655 | 2785.350449 | -0.0066 | 0.995 | -7.76E-10 | count | 1 |
| AC009097.4 | -18.2852655 | 2785.350449 | -0.0066 | 0.995 | -7.76E-10 | count | 1 |
| ZMYND15 | -18.2852655 | 2785.350449 | -0.0066 | 0.995 | -7.76E-10 | count | 1 |
| ASGR2 | -18.2852655 | 2785.350449 | -0.0066 | 0.995 | -7.76E-10 | count | 1 |
| RNF222 | -18.2852655 | 2785.350449 | -0.0066 | 0.995 | -7.76E-10 | count | 1 |
| TMEM220-AS1 | -18.2852655 | 2785.350449 | -0.0066 | 0.995 | -7.76E-10 | count | 1 |
| CCDC144NL-AS1 | -18.2852655 | 2785.350449 | -0.0066 | 0.995 | -7.76E-10 | count | 1 |
| AC084809.2 | -18.2852655 | 2785.350449 | -0.0066 | 0.995 | -7.76E-10 | count | 1 |
| AC243830.2 | -18.2852655 | 2785.350449 | -0.0066 | 0.995 | -7.76E-10 | count | 1 |
| LINC00511 | -18.2852655 | 2785.350449 | -0.0066 | 0.995 | -7.76E-10 | count | 1 |
| AC021683.3 | -18.2852655 | 2785.350449 | -0.0066 | 0.995 | -7.76E-10 | count | 1 |
| AC021683.2 | -18.2852655 | 2785.350449 | -0.0066 | 0.995 | -7.76E-10 | count | 1 |
| AC145207.6 | -18.2852655 | 2785.350449 | -0.0066 | 0.995 | -7.76E-10 | count | 1 |
| LINC00683 | -18.2852655 | 2785.350449 | -0.0066 | 0.995 | -7.76E-10 | count | 1 |
| SIGLEC1 | -18.2852655 | 2785.350449 | -0.0066 | 0.995 | -7.76E-10 | count | 1 |
| LAMA5 | -18.2852655 | 2785.350449 | -0.0066 | 0.995 | -7.76E-10 | count | 1 |
| AC011477.3 | -18.2852655 | 2785.350449 | -0.0066 | 0.995 | -7.76E-10 | count | 1 |
| RHPN2 | -18.2852655 | 2785.350449 | -0.0066 | 0.995 | -7.76E-10 | count | 1 |
| WDR62 | -18.2852655 | 2785.350449 | -0.0066 | 0.995 | -7.76E-10 | count | 1 |
| AC092301.1 | -18.2852655 | 2785.350449 | -0.0066 | 0.995 | -7.76E-10 | count | 1 |
| AC006115.1 | -18.2852655 | 2785.350449 | -0.0066 | 0.995 | -7.76E-10 | count | 1 |
| PLA2G5 | -18.2852655 | 2785.350449 | -0.0066 | 0.995 | -7.76E-10 | count | 1 |
| CSMD2 | -18.2852655 | 2785.350449 | -0.0066 | 0.995 | -7.76E-10 | count | 1 |
| COL8A2 | -18.2852655 | 2785.350449 | -0.0066 | 0.995 | -7.76E-10 | count | 1 |
| TTC39A-AS1 | -18.2852655 | 2785.350449 | -0.0066 | 0.995 | -7.76E-10 | count | 1 |
| NPR1 | -18.2852655 | 2785.350449 | -0.0066 | 0.995 | -7.76E-10 | count | 1 |
| DCST1 | -18.2852655 | 2785.350449 | -0.0066 | 0.995 | -7.76E-10 | count | 1 |
| CD1D | -18.2852655 | 2785.350449 | -0.0066 | 0.995 | -7.76E-10 | count | 1 |
| ANGPTL1 | -18.2852655 | 2785.350449 | -0.0066 | 0.995 | -7.76E-10 | count | 1 |
| PLA2G4A | -18.2852655 | 2785.350449 | -0.0066 | 0.995 | -7.76E-10 | count | 1 |
| RAB7B | -18.2852655 | 2785.350449 | -0.0066 | 0.995 | -7.76E-10 | count | 1 |
| GDF7 | -18.2852655 | 2785.350449 | -0.0066 | 0.995 | -7.76E-10 | count | 1 |
| BCL11A | -18.2852655 | 2785.350449 | -0.0066 | 0.995 | -7.76E-10 | count | 1 |
| AC007040.2 | -18.2852655 | 2785.350449 | -0.0066 | 0.995 | -7.76E-10 | count | 1 |
| AC114730.2 | -18.2852655 | 2785.350449 | -0.0066 | 0.995 | -7.76E-10 | count | 1 |
| MITF | -18.2852655 | 2785.350449 | -0.0066 | 0.995 | -7.76E-10 | count | 1 |
| COL6A6 | -18.2852655 | 2785.350449 | -0.0066 | 0.995 | -7.76E-10 | count | 1 |
| AC072039.2 | -18.2852655 | 2785.350449 | -0.0066 | 0.995 | -7.76E-10 | count | 1 |
| MELTF-AS1 | -18.2852655 | 2785.350449 | -0.0066 | 0.995 | -7.76E-10 | count | 1 |
| SPATA18 | -18.2852655 | 2785.350449 | -0.0066 | 0.995 | -7.76E-10 | count | 1 |
| RASL11B | -18.2852655 | 2785.350449 | -0.0066 | 0.995 | -7.76E-10 | count | 1 |
| AC020741.1 | -18.2852655 | 2785.350449 | -0.0066 | 0.995 | -7.76E-10 | count | 1 |
| HHIP | -18.2852655 | 2785.350449 | -0.0066 | 0.995 | -7.76E-10 | count | 1 |

| | | | | | | | |
|---|---|---|---|---|---|---|---|
| SH3RF1 | -18.2852655 | 2785.350449 | -0.0066 | 0.995 | -7.76E-10 | count | 1 |
| C6 | -18.2852655 | 2785.350449 | -0.0066 | 0.995 | -7.76E-10 | count | 1 |
| AC008875.1 | -18.2852655 | 2785.350449 | -0.0066 | 0.995 | -7.76E-10 | count | 1 |
| MAST4-AS1 | -18.2852655 | 2785.350449 | -0.0066 | 0.995 | -7.76E-10 | count | 1 |
| AC010501.1 | -18.2852655 | 2785.350449 | -0.0066 | 0.995 | -7.76E-10 | count | 1 |
| ZBED3-AS1 | -18.2852655 | 2785.350449 | -0.0066 | 0.995 | -7.76E-10 | count | 1 |
| FAM151B | -18.2852655 | 2785.350449 | -0.0066 | 0.995 | -7.76E-10 | count | 1 |
| AC116366.2 | -18.2852655 | 2785.350449 | -0.0066 | 0.995 | -7.76E-10 | count | 1 |
| AC135457.1 | -18.2852655 | 2785.350449 | -0.0066 | 0.995 | -7.76E-10 | count | 1 |
| PCDHB11 | -18.2852655 | 2785.350449 | -0.0066 | 0.995 | -7.76E-10 | count | 1 |
| PCDHGA9 | -18.2852655 | 2785.350449 | -0.0066 | 0.995 | -7.76E-10 | count | 1 |
| SPINK5 | -18.2852655 | 2785.350449 | -0.0066 | 0.995 | -7.76E-10 | count | 1 |
| SGCD | -18.2852655 | 2785.350449 | -0.0066 | 0.995 | -7.76E-10 | count | 1 |
| CCNJL | -18.2852655 | 2785.350449 | -0.0066 | 0.995 | -7.76E-10 | count | 1 |
| AC008443.6 | -18.2852655 | 2785.350449 | -0.0066 | 0.995 | -7.76E-10 | count | 1 |
| C6orf201 | -18.2852655 | 2785.350449 | -0.0066 | 0.995 | -7.76E-10 | count | 1 |
| DCDC2 | -18.2852655 | 2785.350449 | -0.0066 | 0.995 | -7.76E-10 | count | 1 |
| HCG15 | -18.2852655 | 2785.350449 | -0.0066 | 0.995 | -7.76E-10 | count | 1 |
| COL12A1 | -18.2852655 | 2785.350449 | -0.0066 | 0.995 | -7.76E-10 | count | 1 |
| GVQW2 | -18.2852655 | 2785.350449 | -0.0066 | 0.995 | -7.76E-10 | count | 1 |
| AL031056.2 | -18.2852655 | 2785.350449 | -0.0066 | 0.995 | -7.76E-10 | count | 1 |
| LRRD1 | -18.2852655 | 2785.350449 | -0.0066 | 0.995 | -7.76E-10 | count | 1 |
| GPR22 | -18.2852655 | 2785.350449 | -0.0066 | 0.995 | -7.76E-10 | count | 1 |
| DOCK4 | -18.2852655 | 2785.350449 | -0.0066 | 0.995 | -7.76E-10 | count | 1 |
| AC073320.1 | -18.2852655 | 2785.350449 | -0.0066 | 0.995 | -7.76E-10 | count | 1 |
| AKR1B10 | -18.2852655 | 2785.350449 | -0.0066 | 0.995 | -7.76E-10 | count | 1 |
| ARSD-AS1 | -18.2852655 | 2785.350449 | -0.0066 | 0.995 | -7.76E-10 | count | 1 |
| SRPX2 | -18.2852655 | 2785.350449 | -0.0066 | 0.995 | -7.76E-10 | count | 1 |
| FAM86B2 | -18.2852655 | 2785.350449 | -0.0066 | 0.995 | -7.76E-10 | count | 1 |
| AC013643.2 | -18.2852655 | 2785.350449 | -0.0066 | 0.995 | -7.76E-10 | count | 1 |
| AC064807.4 | -18.2852655 | 2785.350449 | -0.0066 | 0.995 | -7.76E-10 | count | 1 |
| AC104958.1 | -18.2852655 | 2785.350449 | -0.0066 | 0.995 | -7.76E-10 | count | 1 |
| CAVIN4 | -18.2852655 | 2785.350449 | -0.0066 | 0.995 | -7.76E-10 | count | 1 |
| AC136475.8 | -18.2852655 | 2785.350449 | -0.0066 | 0.995 | -7.76E-10 | count | 1 |
| PRR33 | -18.2852655 | 2785.350449 | -0.0066 | 0.995 | -7.76E-10 | count | 1 |
| BDNF | -18.2852655 | 2785.350449 | -0.0066 | 0.995 | -7.76E-10 | count | 1 |
| PAX6 | -18.2852655 | 2785.350449 | -0.0066 | 0.995 | -7.76E-10 | count | 1 |
| AL138921.1 | -18.2852655 | 2785.350449 | -0.0066 | 0.995 | -7.76E-10 | count | 1 |
| AL121929.2 | -18.2852655 | 2785.350449 | -0.0066 | 0.995 | -7.76E-10 | count | 1 |
| SPX | -18.2852655 | 2785.350449 | -0.0066 | 0.995 | -7.76E-10 | count | 1 |
| AC009318.2 | -18.2852655 | 2785.350449 | -0.0066 | 0.995 | -7.76E-10 | count | 1 |
| RDH16 | -18.2852655 | 2785.350449 | -0.0066 | 0.995 | -7.76E-10 | count | 1 |
| IGF1 | -18.2852655 | 2785.350449 | -0.0066 | 0.995 | -7.76E-10 | count | 1 |
| SVOP | -18.2852655 | 2785.350449 | -0.0066 | 0.995 | -7.76E-10 | count | 1 |
| ZFHX2 | -18.2852655 | 2785.350449 | -0.0066 | 0.995 | -7.76E-10 | count | 1 |

| | | | | | | | |
|---|---|---|---|---|---|---|---|
| TGM1 | -18.2852655 | 2785.350449 | -0.0066 | 0.995 | -7.76E-10 | count | 1 |
| AL359317.2 | -18.2852655 | 2785.350449 | -0.0066 | 0.995 | -7.76E-10 | count | 1 |
| PTPN21 | -18.2852655 | 2785.350449 | -0.0066 | 0.995 | -7.76E-10 | count | 1 |
| LINC00637 | -18.2852655 | 2785.350449 | -0.0066 | 0.995 | -7.76E-10 | count | 1 |
| GPR176 | -18.2852655 | 2785.350449 | -0.0066 | 0.995 | -7.76E-10 | count | 1 |
| SEMA6D | -18.2852655 | 2785.350449 | -0.0066 | 0.995 | -7.76E-10 | count | 1 |
| RASL12 | -18.2852655 | 2785.350449 | -0.0066 | 0.995 | -7.76E-10 | count | 1 |
| IGDCC3 | -18.2852655 | 2785.350449 | -0.0066 | 0.995 | -7.76E-10 | count | 1 |
| AC009690.2 | -18.2852655 | 2785.350449 | -0.0066 | 0.995 | -7.76E-10 | count | 1 |
| AC027020.1 | -18.2852655 | 2785.350449 | -0.0066 | 0.995 | -7.76E-10 | count | 1 |
| ALDH1A3 | -18.2852655 | 2785.350449 | -0.0066 | 0.995 | -7.76E-10 | count | 1 |
| AC023024.1 | -18.2852655 | 2785.350449 | -0.0066 | 0.995 | -7.76E-10 | count | 1 |
| AJ003147.2 | -18.2852655 | 2785.350449 | -0.0066 | 0.995 | -7.76E-10 | count | 1 |
| TBX6 | -18.2852655 | 2785.350449 | -0.0066 | 0.995 | -7.76E-10 | count | 1 |
| AC027682.5 | -18.2852655 | 2785.350449 | -0.0066 | 0.995 | -7.76E-10 | count | 1 |
| CDH3 | -18.2852655 | 2785.350449 | -0.0066 | 0.995 | -7.76E-10 | count | 1 |
| AC009148.1 | -18.2852655 | 2785.350449 | -0.0066 | 0.995 | -7.76E-10 | count | 1 |
| LINC00304 | -18.2852655 | 2785.350449 | -0.0066 | 0.995 | -7.76E-10 | count | 1 |
| RAPGEFL1 | -18.2852655 | 2785.350449 | -0.0066 | 0.995 | -7.76E-10 | count | 1 |
| CNTNAP1 | -18.2852655 | 2785.350449 | -0.0066 | 0.995 | -7.76E-10 | count | 1 |
| NPTX1 | -18.2852655 | 2785.350449 | -0.0066 | 0.995 | -7.76E-10 | count | 1 |
| AC132938.1 | -18.2852655 | 2785.350449 | -0.0066 | 0.995 | -7.76E-10 | count | 1 |
| AP005329.2 | -18.2852655 | 2785.350449 | -0.0066 | 0.995 | -7.76E-10 | count | 1 |
| AL031666.2 | -18.2852655 | 2785.350449 | -0.0066 | 0.995 | -7.76E-10 | count | 1 |
| CCDC151 | -18.2852655 | 2785.350449 | -0.0066 | 0.995 | -7.76E-10 | count | 1 |
| AC002398.1 | -18.2852655 | 2785.350449 | -0.0066 | 0.995 | -7.76E-10 | count | 1 |
| KLC3 | -18.2852655 | 2785.350449 | -0.0066 | 0.995 | -7.76E-10 | count | 1 |
| NOVA2 | -18.2852655 | 2785.350449 | -0.0066 | 0.995 | -7.76E-10 | count | 1 |
| PNMA8B | -18.2852655 | 2785.350449 | -0.0066 | 0.995 | -7.76E-10 | count | 1 |
| CRX | -18.2852655 | 2785.350449 | -0.0066 | 0.995 | -7.76E-10 | count | 1 |
| SPIB | -18.2852655 | 2785.350449 | -0.0066 | 0.995 | -7.76E-10 | count | 1 |
| AC008750.2 | -18.2852655 | 2785.350449 | -0.0066 | 0.995 | -7.76E-10 | count | 1 |
| LILRA4 | -18.2852655 | 2785.350449 | -0.0066 | 0.995 | -7.76E-10 | count | 1 |
| COX6B2 | -18.2852655 | 2785.350449 | -0.0066 | 0.995 | -7.76E-10 | count | 1 |
| SHANK3 | -18.2852655 | 2785.350449 | -0.0066 | 0.995 | -7.76E-10 | count | 1 |
| AF127577.2 | -18.2852655 | 2785.350449 | -0.0066 | 0.995 | -7.76E-10 | count | 1 |
| CTRC | -18.2852655 | 2785.350449 | -0.0066 | 0.995 | -7.76E-10 | count | 1 |
| CYP4B1 | -18.2852655 | 2785.350449 | -0.0066 | 0.995 | -7.76E-10 | count | 1 |
| PODN | -18.2852655 | 2785.350449 | -0.0066 | 0.995 | -7.76E-10 | count | 1 |
| KIAA1614 | -18.2852655 | 2785.350449 | -0.0066 | 0.995 | -7.76E-10 | count | 1 |
| VASH2 | -18.2852655 | 2785.350449 | -0.0066 | 0.995 | -7.76E-10 | count | 1 |
| MYT1L | -18.2852655 | 2785.350449 | -0.0066 | 0.995 | -7.76E-10 | count | 1 |
| AC006030.1 | -18.2852655 | 2785.350449 | -0.0066 | 0.995 | -7.76E-10 | count | 1 |
| TRPM8 | -18.2852655 | 2785.350449 | -0.0066 | 0.995 | -7.76E-10 | count | 1 |
| AC022001.2 | -18.2852655 | 2785.350449 | -0.0066 | 0.995 | -7.76E-10 | count | 1 |

| | | | | | | | |
|---|---|---|---|---|---|---|---|
| PDZRN3 | -18.2852655 | 2785.350449 | -0.0066 | 0.995 | -7.76E-10 | count | 1 |
| AC110491.3 | -18.2852655 | 2785.350449 | -0.0066 | 0.995 | -7.76E-10 | count | 1 |
| OR5H14 | -18.2852655 | 2785.350449 | -0.0066 | 0.995 | -7.76E-10 | count | 1 |
| LINC01618 | -18.2852655 | 2785.350449 | -0.0066 | 0.995 | -7.76E-10 | count | 1 |
| SGMS2 | -18.2852655 | 2785.350449 | -0.0066 | 0.995 | -7.76E-10 | count | 1 |
| LINC02060 | -18.2852655 | 2785.350449 | -0.0066 | 0.995 | -7.76E-10 | count | 1 |
| PPT2-EGFL8 | -18.2852655 | 2785.350449 | -0.0066 | 0.995 | -7.76E-10 | count | 1 |
| SCUBE3 | -18.2852655 | 2785.350449 | -0.0066 | 0.995 | -7.76E-10 | count | 1 |
| AC093627.6 | -18.2852655 | 2785.350449 | -0.0066 | 0.995 | -7.76E-10 | count | 1 |
| DNAH11 | -18.2852655 | 2785.350449 | -0.0066 | 0.995 | -7.76E-10 | count | 1 |
| AC074183.1 | -18.2852655 | 2785.350449 | -0.0066 | 0.995 | -7.76E-10 | count | 1 |
| DLX5 | -18.2852655 | 2785.350449 | -0.0066 | 0.995 | -7.76E-10 | count | 1 |
| PLXNA4 | -18.2852655 | 2785.350449 | -0.0066 | 0.995 | -7.76E-10 | count | 1 |
| FAM9C | -18.2852655 | 2785.350449 | -0.0066 | 0.995 | -7.76E-10 | count | 1 |
| AC011008.1 | -18.2852655 | 2785.350449 | -0.0066 | 0.995 | -7.76E-10 | count | 1 |
| PURG | -18.2852655 | 2785.350449 | -0.0066 | 0.995 | -7.76E-10 | count | 1 |
| HEY1 | -18.2852655 | 2785.350449 | -0.0066 | 0.995 | -7.76E-10 | count | 1 |
| LY6K | -18.2852655 | 2785.350449 | -0.0066 | 0.995 | -7.76E-10 | count | 1 |
| NALT1 | -18.2852655 | 2785.350449 | -0.0066 | 0.995 | -7.76E-10 | count | 1 |
| CFAP300 | -18.2852655 | 2785.350449 | -0.0066 | 0.995 | -7.76E-10 | count | 1 |
| VENTX | -18.2852655 | 2785.350449 | -0.0066 | 0.995 | -7.76E-10 | count | 1 |
| U47924.3 | -18.2852655 | 2785.350449 | -0.0066 | 0.995 | -7.76E-10 | count | 1 |
| CLEC4C | -18.2852655 | 2785.350449 | -0.0066 | 0.995 | -7.76E-10 | count | 1 |
| ENDOU | -18.2852655 | 2785.350449 | -0.0066 | 0.995 | -7.76E-10 | count | 1 |
| RNASE2 | -18.2852655 | 2785.350449 | -0.0066 | 0.995 | -7.76E-10 | count | 1 |
| AL355922.2 | -18.2852655 | 2785.350449 | -0.0066 | 0.995 | -7.76E-10 | count | 1 |
| TRAV35 | -18.2852655 | 2785.350449 | -0.0066 | 0.995 | -7.76E-10 | count | 1 |
| AKAP6 | -18.2852655 | 2785.350449 | -0.0066 | 0.995 | -7.76E-10 | count | 1 |
| AC005480.1 | -18.2852655 | 2785.350449 | -0.0066 | 0.995 | -7.76E-10 | count | 1 |
| SLC24A4 | -18.2852655 | 2785.350449 | -0.0066 | 0.995 | -7.76E-10 | count | 1 |
| MYZAP | -18.2852655 | 2785.350449 | -0.0066 | 0.995 | -7.76E-10 | count | 1 |
| AC084855.2 | -18.2852655 | 2785.350449 | -0.0066 | 0.995 | -7.76E-10 | count | 1 |
| RHBDF1 | -18.2852655 | 2785.350449 | -0.0066 | 0.995 | -7.76E-10 | count | 1 |
| AC009065.8 | -18.2852655 | 2785.350449 | -0.0066 | 0.995 | -7.76E-10 | count | 1 |
| AC092140.2 | -18.2852655 | 2785.350449 | -0.0066 | 0.995 | -7.76E-10 | count | 1 |
| RTN4RL1 | -18.2852655 | 2785.350449 | -0.0066 | 0.995 | -7.76E-10 | count | 1 |
| AC024267.6 | -18.2852655 | 2785.350449 | -0.0066 | 0.995 | -7.76E-10 | count | 1 |
| AC015795.1 | -18.2852655 | 2785.350449 | -0.0066 | 0.995 | -7.76E-10 | count | 1 |
| AP000919.4 | -18.2852655 | 2785.350449 | -0.0066 | 0.995 | -7.76E-10 | count | 1 |
| AL031673.1 | -18.2852655 | 2785.350449 | -0.0066 | 0.995 | -7.76E-10 | count | 1 |
| AC008555.2 | -18.2852655 | 2785.350449 | -0.0066 | 0.995 | -7.76E-10 | count | 1 |
| ZNF135 | -18.2852655 | 2785.350449 | -0.0066 | 0.995 | -7.76E-10 | count | 1 |
| AL022328.2 | -18.2852655 | 2785.350449 | -0.0066 | 0.995 | -7.76E-10 | count | 1 |
| AC106900.1 | -18.2852655 | 2785.350449 | -0.0066 | 0.995 | -7.76E-10 | count | 1 |
| UNC5C | -18.2852655 | 2785.350449 | -0.0066 | 0.995 | -7.76E-10 | count | 1 |

| | | | | | | | |
|---|---|---|---|---|---|---|---|
| MYOCD | -18.2852655 | 2785.350449 | -0.0066 | 0.995 | -7.76E-10 | count | 1 |
| EMID1 | -18.2852655 | 2785.350449 | -0.0066 | 0.995 | -7.76E-10 | count | 1 |
| CACNA1C | -0.0230123 | 0.8241214 | -0.0279 | 0.978 | -6.80E-10 | count | 1 |
| AL035563.1 | -0.0079055 | 0.5755606 | -0.0137 | 0.989 | -6.38E-10 | count | 1 |
| RAPH1 | -0.0036738 | 0.6207583 | -0.0059 | 0.995 | -2.97E-10 | count | 1 |
| BX293535.1 | -0.074108 | 1.0392758 | -0.0713 | 0.943 | -2.93E-10 | count | 1 |
| PROC | -0.074108 | 1.0392758 | -0.0713 | 0.943 | -2.93E-10 | count | 1 |
| CLEC3B | -0.074108 | 1.0392758 | -0.0713 | 0.943 | -2.93E-10 | count | 1 |
| AC104078.1 | -0.074108 | 1.0392758 | -0.0713 | 0.943 | -2.93E-10 | count | 1 |
| AL138831.2 | -0.074108 | 1.0392758 | -0.0713 | 0.943 | -2.93E-10 | count | 1 |
| AL109914.1 | -0.074108 | 1.0392758 | -0.0713 | 0.943 | -2.93E-10 | count | 1 |
| ZNF391 | -0.074108 | 1.0392758 | -0.0713 | 0.943 | -2.93E-10 | count | 1 |
| AC007938.2 | -0.074108 | 1.0392758 | -0.0713 | 0.943 | -2.93E-10 | count | 1 |
| EFHC2 | -0.074108 | 1.0392758 | -0.0713 | 0.943 | -2.93E-10 | count | 1 |
| RUSC2 | -0.074108 | 1.0392758 | -0.0713 | 0.943 | -2.93E-10 | count | 1 |
| USP2 | -0.074108 | 1.0392758 | -0.0713 | 0.943 | -2.93E-10 | count | 1 |
| GLB1L3 | -0.074108 | 1.0392758 | -0.0713 | 0.943 | -2.93E-10 | count | 1 |
| INA | -0.074108 | 1.0392758 | -0.0713 | 0.943 | -2.93E-10 | count | 1 |
| GPR84 | -0.074108 | 1.0392758 | -0.0713 | 0.943 | -2.93E-10 | count | 1 |
| LINC02341 | -0.074108 | 1.0392758 | -0.0713 | 0.943 | -2.93E-10 | count | 1 |
| TRAV2 | -0.074108 | 1.0392758 | -0.0713 | 0.943 | -2.93E-10 | count | 1 |
| AC068831.1 | -0.074108 | 1.0392758 | -0.0713 | 0.943 | -2.93E-10 | count | 1 |
| LDHD | -0.074108 | 1.0392758 | -0.0713 | 0.943 | -2.93E-10 | count | 1 |
| FOXL1 | -0.074108 | 1.0392758 | -0.0713 | 0.943 | -2.93E-10 | count | 1 |
| AOC2 | -0.074108 | 1.0392758 | -0.0713 | 0.943 | -2.93E-10 | count | 1 |
| LINC01841 | -0.074108 | 1.0392758 | -0.0713 | 0.943 | -2.93E-10 | count | 1 |
| ZNF418 | -0.074108 | 1.0392758 | -0.0713 | 0.943 | -2.93E-10 | count | 1 |
| TCP10L | -0.074108 | 1.0392758 | -0.0713 | 0.943 | -2.93E-10 | count | 1 |
| PIK3CD-AS2 | -0.074108 | 1.0392758 | -0.0713 | 0.943 | -2.93E-10 | count | 1 |
| GIPC2 | -0.074108 | 1.0392758 | -0.0713 | 0.943 | -2.93E-10 | count | 1 |
| KCNS3 | -0.074108 | 1.0392758 | -0.0713 | 0.943 | -2.93E-10 | count | 1 |
| AC104184.1 | -0.074108 | 1.0392758 | -0.0713 | 0.943 | -2.93E-10 | count | 1 |
| FKBP6 | -0.074108 | 1.0392758 | -0.0713 | 0.943 | -2.93E-10 | count | 1 |
| STRIP2 | -0.074108 | 1.0392758 | -0.0713 | 0.943 | -2.93E-10 | count | 1 |
| AC115618.1 | -0.074108 | 1.0392758 | -0.0713 | 0.943 | -2.93E-10 | count | 1 |
| KBTBD11-OT1 | -0.074108 | 1.0392758 | -0.0713 | 0.943 | -2.93E-10 | count | 1 |
| DLC1 | -0.074108 | 1.0392758 | -0.0713 | 0.943 | -2.93E-10 | count | 1 |
| HSPA12A | -0.074108 | 1.0392758 | -0.0713 | 0.943 | -2.93E-10 | count | 1 |
| FAM30A | -0.074108 | 1.0392758 | -0.0713 | 0.943 | -2.93E-10 | count | 1 |
| GLIS2 | -0.074108 | 1.0392758 | -0.0713 | 0.943 | -2.93E-10 | count | 1 |
| AC024267.4 | -0.074108 | 1.0392758 | -0.0713 | 0.943 | -2.93E-10 | count | 1 |
| MEIOC | -0.074108 | 1.0392758 | -0.0713 | 0.943 | -2.93E-10 | count | 1 |
| LINC02073 | -0.074108 | 1.0392758 | -0.0713 | 0.943 | -2.93E-10 | count | 1 |
| RNFT1-DT | -0.074108 | 1.0392758 | -0.0713 | 0.943 | -2.93E-10 | count | 1 |
| AC144831.1 | -0.074108 | 1.0392758 | -0.0713 | 0.943 | -2.93E-10 | count | 1 |

| | | | | | | | |
|---|---|---|---|---|---|---|---|
| AL031670.1 | -0.074108 | 1.0392758 | -0.0713 | 0.943 | -2.93E-10 | count | 1 |
| TINCR | -0.074108 | 1.0392758 | -0.0713 | 0.943 | -2.93E-10 | count | 1 |
| LGI4 | -0.074108 | 1.0392758 | -0.0713 | 0.943 | -2.93E-10 | count | 1 |
| SPACA6P-AS | -0.074108 | 1.0392758 | -0.0713 | 0.943 | -2.93E-10 | count | 1 |
| TNC | -0.0079055 | 0.9894921 | -0.008 | 0.994 | -2.35E-10 | count | 1 |
| ARF4-AS1 | 0.0023313 | 0.4357649 | 0.0053 | 0.996 | 1.89E-10 | count | 1 |
| AC107375.1 | 0.0081604 | 0.8266071 | 0.0099 | 0.992 | 2.43E-10 | count | 1 |
| CATSPERB | 0.0081604 | 0.9106566 | 0.009 | 0.993 | 2.43E-10 | count | 1 |
| PRKN | 0.0081604 | 0.7337915 | 0.0111 | 0.991 | 2.43E-10 | count | 1 |
| FBXL19-AS1 | 0.0081604 | 0.9106566 | 0.009 | 0.993 | 2.43E-10 | count | 1 |
| AC092490.1 | 0.0081604 | 0.9863762 | 0.0083 | 0.993 | 2.43E-10 | count | 1 |
| NR5A2 | 0.024767 | 0.8389414 | 0.0295 | 0.976 | 7.41E-10 | count | 1 |
| SRCAP | 0.0133323 | 0.4743075 | 0.0281 | 0.978 | 1.08E-09 | count | 1 |
| LLGL1 | 0.0163586 | 0.4935472 | 0.0331 | 0.974 | 1.33E-09 | count | 1 |
| AL162431.2 | 0.0497532 | 1.080724 | 0.046 | 0.963 | 1.50E-09 | count | 1 |
| AASS | 0.0497532 | 1.080724 | 0.046 | 0.963 | 1.50E-09 | count | 1 |
| SCARF2 | 0.0497532 | 0.7661095 | 0.0649 | 0.948 | 1.50E-09 | count | 1 |
| LRRC24 | 0.0497532 | 0.9010418 | 0.0552 | 0.956 | 1.50E-09 | count | 1 |
| ANKRD22 | 0.0497532 | 1.080724 | 0.046 | 0.963 | 1.50E-09 | count | 1 |
| TYRO3 | 0.0497532 | 1.080724 | 0.046 | 0.963 | 1.50E-09 | count | 1 |
| AC104590.1 | 0.0497532 | 1.080724 | 0.046 | 0.963 | 1.50E-09 | count | 1 |
| AL357874.1 | 0.0497532 | 0.9010418 | 0.0552 | 0.956 | 1.50E-09 | count | 1 |
| FAM78B | 0.0497532 | 0.9010418 | 0.0552 | 0.956 | 1.50E-09 | count | 1 |
| FAM209B | 0.0497532 | 0.8988109 | 0.0554 | 0.956 | 1.50E-09 | count | 1 |
| MAK | 0.0497532 | 0.9010418 | 0.0552 | 0.956 | 1.50E-09 | count | 1 |
| AC100803.3 | 0.0497532 | 1.0872505 | 0.0458 | 0.964 | 1.50E-09 | count | 1 |
| AC106886.5 | 0.0497532 | 0.875198 | 0.0568 | 0.955 | 1.50E-09 | count | 1 |
| GNA11 | 0.0497532 | 1.0872505 | 0.0458 | 0.964 | 1.50E-09 | count | 1 |
| AL445228.2 | 0.1375298 | 0.5743567 | 0.2395 | 0.811 | 1.56E-09 | count | 1 |
| CFLAR-AS1 | 0.4252119 | 0.6030449 | 0.7051 | 0.481 | 1.90E-09 | count | 1 |
| HIF3A | 0.4252119 | 0.6030449 | 0.7051 | 0.481 | 1.90E-09 | count | 1 |
| TTC39A | 0.4252119 | 0.7223505 | 0.5887 | 0.556 | 1.90E-09 | count | 1 |
| SELENBP1 | 0.4252119 | 0.7223505 | 0.5887 | 0.556 | 1.90E-09 | count | 1 |
| ACHE | 0.4252119 | 0.7223505 | 0.5887 | 0.556 | 1.90E-09 | count | 1 |
| HCAR1 | 0.4252119 | 0.7223505 | 0.5887 | 0.556 | 1.90E-09 | count | 1 |
| LINC00330 | 0.4252119 | 0.7223505 | 0.5887 | 0.556 | 1.90E-09 | count | 1 |
| MYH3 | 0.4252119 | 0.7223505 | 0.5887 | 0.556 | 1.90E-09 | count | 1 |
| AC005339.1 | 0.4252119 | 0.7223505 | 0.5887 | 0.556 | 1.90E-09 | count | 1 |
| AL021707.4 | 0.4252119 | 0.7223505 | 0.5887 | 0.556 | 1.90E-09 | count | 1 |
| SLFNL1-AS1 | 0.4252119 | 0.7223505 | 0.5887 | 0.556 | 1.90E-09 | count | 1 |
| PARD3B | 0.4252119 | 0.7223505 | 0.5887 | 0.556 | 1.90E-09 | count | 1 |
| AC079834.2 | 0.4252119 | 0.7223505 | 0.5887 | 0.556 | 1.90E-09 | count | 1 |
| AL159163.1 | 0.4252119 | 0.7223505 | 0.5887 | 0.556 | 1.90E-09 | count | 1 |
| TMPRSS13 | 0.4252119 | 0.7223505 | 0.5887 | 0.556 | 1.90E-09 | count | 1 |
| VSTM4 | 0.4252119 | 0.7223505 | 0.5887 | 0.556 | 1.90E-09 | count | 1 |

| | | | | | | | |
|---|---|---|---|---|---|---|---|
| AC012377.1 | 0.4252119 | 0.7223505 | 0.5887 | 0.556 | 1.90E-09 | count | 1 |
| AC009133.4 | 0.4252119 | 0.7223505 | 0.5887 | 0.556 | 1.90E-09 | count | 1 |
| AC015917.2 | 0.4252119 | 0.7223505 | 0.5887 | 0.556 | 1.90E-09 | count | 1 |
| AC103810.2 | 0.4252119 | 0.7223505 | 0.5887 | 0.556 | 1.90E-09 | count | 1 |
| LINC01483 | 0.4252119 | 0.7223505 | 0.5887 | 0.556 | 1.90E-09 | count | 1 |
| AC010319.4 | 0.4252119 | 0.7223505 | 0.5887 | 0.556 | 1.90E-09 | count | 1 |
| LINC00624 | 0.4252119 | 0.7223505 | 0.5887 | 0.556 | 1.90E-09 | count | 1 |
| IL12A | 0.4252119 | 0.7223505 | 0.5887 | 0.556 | 1.90E-09 | count | 1 |
| AC137810.1 | 0.4252119 | 0.7223505 | 0.5887 | 0.556 | 1.90E-09 | count | 1 |
| AL356417.3 | 0.4252119 | 0.7223505 | 0.5887 | 0.556 | 1.90E-09 | count | 1 |
| AL732314.4 | 0.4252119 | 0.7223505 | 0.5887 | 0.556 | 1.90E-09 | count | 1 |
| AC079209.2 | 0.4252119 | 0.7223505 | 0.5887 | 0.556 | 1.90E-09 | count | 1 |
| TNNT3 | 0.4252119 | 0.7223505 | 0.5887 | 0.556 | 1.90E-09 | count | 1 |
| SORCS3 | 0.4252119 | 0.7223505 | 0.5887 | 0.556 | 1.90E-09 | count | 1 |
| KRT72 | 0.4252119 | 0.7223505 | 0.5887 | 0.556 | 1.90E-09 | count | 1 |
| AC073592.1 | 0.4252119 | 0.7223505 | 0.5887 | 0.556 | 1.90E-09 | count | 1 |
| FITM1 | 0.4252119 | 0.7223505 | 0.5887 | 0.556 | 1.90E-09 | count | 1 |
| AL118558.4 | 0.4252119 | 0.7223505 | 0.5887 | 0.556 | 1.90E-09 | count | 1 |
| AC068446.2 | 0.4252119 | 0.7223505 | 0.5887 | 0.556 | 1.90E-09 | count | 1 |
| AC079322.1 | 0.4252119 | 0.7223505 | 0.5887 | 0.556 | 1.90E-09 | count | 1 |
| AC002558.2 | 0.4252119 | 0.7223505 | 0.5887 | 0.556 | 1.90E-09 | count | 1 |
| AC010754.1 | 0.4252119 | 0.7223505 | 0.5887 | 0.556 | 1.90E-09 | count | 1 |
| AP000253.1 | 0.4252119 | 0.6468643 | 0.6573 | 0.511 | 1.90E-09 | count | 1 |
| AC104389.4 | 0.0648997 | 0.817639 | 0.0794 | 0.937 | 1.96E-09 | count | 1 |
| PACSIN3 | 0.1755519 | 1.2583036 | 0.1395 | 0.889 | 2.01E-09 | count | 1 |
| VPREB3 | 0.1755519 | 1.2983581 | 0.1352 | 0.892 | 2.01E-09 | count | 1 |
| ZNF385C | 0.1755519 | 1.2583036 | 0.1395 | 0.889 | 2.01E-09 | count | 1 |
| AC021351.1 | 0.1755519 | 1.2983581 | 0.1352 | 0.892 | 2.01E-09 | count | 1 |
| AC073957.3 | 0.1755519 | 1.2583036 | 0.1395 | 0.889 | 2.01E-09 | count | 1 |
| PXDN | 0.1755519 | 1.2583036 | 0.1395 | 0.889 | 2.01E-09 | count | 1 |
| WLS | 0.1755519 | 1.2583036 | 0.1395 | 0.889 | 2.01E-09 | count | 1 |
| AL121574.1 | 0.1755519 | 1.2583036 | 0.1395 | 0.889 | 2.01E-09 | count | 1 |
| AC008758.4 | 0.1755519 | 1.2983581 | 0.1352 | 0.892 | 2.01E-09 | count | 1 |
| ADAMTS12 | 0.1755519 | 1.2583036 | 0.1395 | 0.889 | 2.01E-09 | count | 1 |
| BOK | 0.1755519 | 1.2583036 | 0.1395 | 0.889 | 2.01E-09 | count | 1 |
| CARD14 | 0.1755519 | 1.2583036 | 0.1395 | 0.889 | 2.01E-09 | count | 1 |
| REEP2 | 0.1755519 | 1.2583036 | 0.1395 | 0.889 | 2.01E-09 | count | 1 |
| THNSL2 | 0.1755519 | 1.2583036 | 0.1395 | 0.889 | 2.01E-09 | count | 1 |
| LINC02012 | 0.1755519 | 1.2583036 | 0.1395 | 0.889 | 2.01E-09 | count | 1 |
| PTTG2 | 0.1755519 | 1.2983581 | 0.1352 | 0.892 | 2.01E-09 | count | 1 |
| PRDM6 | 0.1755519 | 1.2583036 | 0.1395 | 0.889 | 2.01E-09 | count | 1 |
| AC092620.3 | 0.1755519 | 1.2983581 | 0.1352 | 0.892 | 2.01E-09 | count | 1 |
| KCNK3 | 0.1755519 | 1.2983581 | 0.1352 | 0.892 | 2.01E-09 | count | 1 |
| AF230666.1 | 0.1755519 | 1.2583036 | 0.1395 | 0.889 | 2.01E-09 | count | 1 |
| SLC5A10 | 0.1755519 | 1.2583036 | 0.1395 | 0.889 | 2.01E-09 | count | 1 |

| | | | | | | | |
|---|---|---|---|---|---|---|---|
| AL021368.1 | 0.1755519 | 1.2983581 | 0.1352 | 0.892 | 2.01E-09 | count | 1 |
| ELFN1-AS1 | 0.1755519 | 1.2583036 | 0.1395 | 0.889 | 2.01E-09 | count | 1 |
| LINC01513 | 0.1755519 | 1.2983581 | 0.1352 | 0.892 | 2.01E-09 | count | 1 |
| GP5 | 0.1755519 | 1.2583036 | 0.1395 | 0.889 | 2.01E-09 | count | 1 |
| GAS6-AS1 | 0.1755519 | 1.2583036 | 0.1395 | 0.889 | 2.01E-09 | count | 1 |
| AC010904.2 | 0.1755519 | 1.2583036 | 0.1395 | 0.889 | 2.01E-09 | count | 1 |
| DRD4 | 0.1755519 | 1.2583036 | 0.1395 | 0.889 | 2.01E-09 | count | 1 |
| LINC00900 | 0.1755519 | 1.2983581 | 0.1352 | 0.892 | 2.01E-09 | count | 1 |
| AC009501.1 | 0.1755519 | 1.2583036 | 0.1395 | 0.889 | 2.01E-09 | count | 1 |
| AC130324.3 | 0.1755519 | 1.2583036 | 0.1395 | 0.889 | 2.01E-09 | count | 1 |
| LINC01503 | 0.1755519 | 1.2983581 | 0.1352 | 0.892 | 2.01E-09 | count | 1 |
| LINC00551 | 0.1755519 | 1.2583036 | 0.1395 | 0.889 | 2.01E-09 | count | 1 |
| AC034231.1 | 0.1755519 | 1.2583036 | 0.1395 | 0.889 | 2.01E-09 | count | 1 |
| AL133406.3 | 0.1755519 | 1.2583036 | 0.1395 | 0.889 | 2.01E-09 | count | 1 |
| MAS1 | 0.1755519 | 1.2583036 | 0.1395 | 0.889 | 2.01E-09 | count | 1 |
| TTYH1 | 0.1755519 | 1.2583036 | 0.1395 | 0.889 | 2.01E-09 | count | 1 |
| PRC1-AS1 | 0.1755519 | 1.2983581 | 0.1352 | 0.892 | 2.01E-09 | count | 1 |
| AC032044.1 | 0.1755519 | 1.2583036 | 0.1395 | 0.889 | 2.01E-09 | count | 1 |
| SLC35G3 | 0.1755519 | 1.2583036 | 0.1395 | 0.889 | 2.01E-09 | count | 1 |
| ERICD | 0.1755519 | 1.2983581 | 0.1352 | 0.892 | 2.01E-09 | count | 1 |
| AC069213.1 | 0.1755519 | 1.2983581 | 0.1352 | 0.892 | 2.01E-09 | count | 1 |
| RBFOX3 | 0.1755519 | 1.2583036 | 0.1395 | 0.889 | 2.01E-09 | count | 1 |
| AC106886.3 | 0.1755519 | 1.2983581 | 0.1352 | 0.892 | 2.01E-09 | count | 1 |
| WDR97 | 0.1755519 | 1.2583036 | 0.1395 | 0.889 | 2.01E-09 | count | 1 |
| AC009021.1 | 0.1755519 | 1.2583036 | 0.1395 | 0.889 | 2.01E-09 | count | 1 |
| CCBE1 | 0.1755519 | 1.2583036 | 0.1395 | 0.889 | 2.01E-09 | count | 1 |
| F7 | 0.1755519 | 1.2583036 | 0.1395 | 0.889 | 2.01E-09 | count | 1 |
| ELMO3 | 0.1755519 | 1.2983581 | 0.1352 | 0.892 | 2.01E-09 | count | 1 |
| LINC01764 | 0.1755519 | 1.2983581 | 0.1352 | 0.892 | 2.01E-09 | count | 1 |
| GRIN2D | 0.1755519 | 1.2583036 | 0.1395 | 0.889 | 2.01E-09 | count | 1 |
| AC022167.1 | 0.1755519 | 1.2983581 | 0.1352 | 0.892 | 2.01E-09 | count | 1 |
| SLC46A1 | 0.1755519 | 1.2583036 | 0.1395 | 0.889 | 2.01E-09 | count | 1 |
| ZNF676 | 0.1755519 | 1.2983581 | 0.1352 | 0.892 | 2.01E-09 | count | 1 |
| AL592494.1 | 0.1755519 | 1.2583036 | 0.1395 | 0.889 | 2.01E-09 | count | 1 |
| FPGT-TNNI3K | 0.1755519 | 1.2583036 | 0.1395 | 0.889 | 2.01E-09 | count | 1 |
| AC055811.3 | 0.1755519 | 1.2583036 | 0.1395 | 0.889 | 2.01E-09 | count | 1 |
| LGALS9C | 0.1755519 | 1.2983581 | 0.1352 | 0.892 | 2.01E-09 | count | 1 |
| SMAD6 | 0.1755519 | 1.2583036 | 0.1395 | 0.889 | 2.01E-09 | count | 1 |
| ANKDD1B | 0.1755519 | 1.2983581 | 0.1352 | 0.892 | 2.01E-09 | count | 1 |
| LNP1 | 0.1755519 | 1.2583036 | 0.1395 | 0.889 | 2.01E-09 | count | 1 |
| ZNF788P | 0.1755519 | 1.2583036 | 0.1395 | 0.889 | 2.01E-09 | count | 1 |
| HRCT1 | 0.1755519 | 1.2983581 | 0.1352 | 0.892 | 2.01E-09 | count | 1 |
| AL158070.1 | 0.1755519 | 1.2983581 | 0.1352 | 0.892 | 2.01E-09 | count | 1 |
| KRT73-AS1 | 0.2020683 | 0.3412847 | 0.5921 | 0.554 | 2.32E-09 | count | 1 |
| XXYLT1-AS2 | 0.2135741 | 0.6911543 | 0.309 | 0.757 | 2.46E-09 | count | 1 |

| | | | | | | | |
|---|---|---|---|---|---|---|---|
| MEX3A | 0.2135741 | 0.9518277 | 0.2244 | 0.822 | 2.46E-09 | count | 1 |
| ZBTB12 | 0.2135741 | 0.7050969 | 0.3029 | 0.762 | 2.46E-09 | count | 1 |
| VSIG2 | 0.0915635 | 0.8459696 | 0.1082 | 0.914 | 2.79E-09 | count | 1 |
| TMEM52 | 0.0915635 | 0.948209 | 0.0966 | 0.923 | 2.79E-09 | count | 1 |
| ICOSLG | 0.0915635 | 0.948209 | 0.0966 | 0.923 | 2.79E-09 | count | 1 |
| ZNF70 | 0.0915635 | 0.952797 | 0.0961 | 0.923 | 2.79E-09 | count | 1 |
| DSC1 | 0.0915635 | 0.856044 | 0.107 | 0.915 | 2.79E-09 | count | 1 |
| Z69706.1 | 0.0915635 | 1.0567691 | 0.0866 | 0.931 | 2.79E-09 | count | 1 |
| C1QTNF4 | 0.6190392 | 1.1091094 | 0.5581 | 0.577 | 2.88E-09 | count | 1 |
| AL139339.1 | 0.6190392 | 1.1091094 | 0.5581 | 0.577 | 2.88E-09 | count | 1 |
| AL355488.1 | 0.6190392 | 1.1091094 | 0.5581 | 0.577 | 2.88E-09 | count | 1 |
| SELE | 0.6190392 | 1.1091094 | 0.5581 | 0.577 | 2.88E-09 | count | 1 |
| TMEM17 | 0.6190392 | 1.1091094 | 0.5581 | 0.577 | 2.88E-09 | count | 1 |
| CCDC150 | 0.6190392 | 1.1091094 | 0.5581 | 0.577 | 2.88E-09 | count | 1 |
| AC009570.1 | 0.6190392 | 1.1091094 | 0.5581 | 0.577 | 2.88E-09 | count | 1 |
| DNM1 | 0.6190392 | 1.1091094 | 0.5581 | 0.577 | 2.88E-09 | count | 1 |
| ASAH2 | 0.6190392 | 1.1091094 | 0.5581 | 0.577 | 2.88E-09 | count | 1 |
| GJB6 | 0.6190392 | 1.1091094 | 0.5581 | 0.577 | 2.88E-09 | count | 1 |
| DLGAP4-AS1 | 0.6190392 | 1.1091094 | 0.5581 | 0.577 | 2.88E-09 | count | 1 |
| AC011484.1 | 0.6190392 | 1.1091094 | 0.5581 | 0.577 | 2.88E-09 | count | 1 |
| AC012313.1 | 0.6190392 | 1.1091094 | 0.5581 | 0.577 | 2.88E-09 | count | 1 |
| CLCN1 | 0.6190392 | 1.1091094 | 0.5581 | 0.577 | 2.88E-09 | count | 1 |
| AL157832.1 | 0.6190392 | 1.1091094 | 0.5581 | 0.577 | 2.88E-09 | count | 1 |
| SCN8A | 0.6190392 | 1.1091094 | 0.5581 | 0.577 | 2.88E-09 | count | 1 |
| AL137230.1 | 0.6190392 | 1.1091094 | 0.5581 | 0.577 | 2.88E-09 | count | 1 |
| AC008731.1 | 0.6190392 | 1.1091094 | 0.5581 | 0.577 | 2.88E-09 | count | 1 |
| GGT6 | 0.6190392 | 1.1091094 | 0.5581 | 0.577 | 2.88E-09 | count | 1 |
| EVPL | 0.6190392 | 1.1091094 | 0.5581 | 0.577 | 2.88E-09 | count | 1 |
| SLC7A4 | 0.6190392 | 1.1091094 | 0.5581 | 0.577 | 2.88E-09 | count | 1 |
| NPW | 0.6190392 | 0.8997938 | 0.688 | 0.492 | 2.88E-09 | count | 1 |
| TRPV3 | 0.6190392 | 0.8997938 | 0.688 | 0.492 | 2.88E-09 | count | 1 |
| FGF2 | 0.6190392 | 0.8997938 | 0.688 | 0.492 | 2.88E-09 | count | 1 |
| SPATA9 | 0.6190392 | 0.8997938 | 0.688 | 0.492 | 2.88E-09 | count | 1 |
| TLDC2 | 0.6190392 | 0.8997938 | 0.688 | 0.492 | 2.88E-09 | count | 1 |
| DHDH | 0.6190392 | 0.8997938 | 0.688 | 0.492 | 2.88E-09 | count | 1 |
| SELP | 0.6190392 | 0.8997938 | 0.688 | 0.492 | 2.88E-09 | count | 1 |
| POLN | 0.6190392 | 0.8997938 | 0.688 | 0.492 | 2.88E-09 | count | 1 |
| SPATA17 | 18.4790936 | 3429.526272 | 0.0054 | 0.996 | 2.98E-09 | count | 1 |
| AC006059.1 | 18.4790936 | 3429.526272 | 0.0054 | 0.996 | 2.98E-09 | count | 1 |
| ARHGEF16 | 18.4790935 | 3429.526432 | 0.0054 | 0.996 | 2.98E-09 | count | 1 |
| AC092807.2 | 18.4790935 | 3429.526352 | 0.0054 | 0.996 | 2.98E-09 | count | 1 |
| AC092903.2 | 18.4790935 | 3429.526224 | 0.0054 | 0.996 | 2.98E-09 | count | 1 |
| NAALADL2 | 18.4790934 | 3429.526176 | 0.0054 | 0.996 | 2.98E-09 | count | 1 |
| LINC00887 | 18.4790935 | 3429.525952 | 0.0054 | 0.996 | 2.98E-09 | count | 1 |
| CFAP99 | 18.4790935 | 3429.526176 | 0.0054 | 0.996 | 2.98E-09 | count | 1 |

| | | | | | | | |
|---|---|---|---|---|---|---|---|
| SEMA6A-AS1 | 18.4790936 | 3429.526464 | 0.0054 | 0.996 | 2.98E-09 | count | 1 |
| AL365275.1 | 18.4790934 | 3429.526448 | 0.0054 | 0.996 | 2.98E-09 | count | 1 |
| ECT2L | 18.4790936 | 3429.526464 | 0.0054 | 0.996 | 2.98E-09 | count | 1 |
| STC1 | 18.4790936 | 3429.526464 | 0.0054 | 0.996 | 2.98E-09 | count | 1 |
| ARHGAP39 | 18.4790936 | 3429.52624 | 0.0054 | 0.996 | 2.98E-09 | count | 1 |
| ARMS2 | 18.4790935 | 3429.526352 | 0.0054 | 0.996 | 2.98E-09 | count | 1 |
| AC004466.1 | 18.4790935 | 3429.526432 | 0.0054 | 0.996 | 2.98E-09 | count | 1 |
| USP12-AS2 | 18.4790935 | 3429.526304 | 0.0054 | 0.996 | 2.98E-09 | count | 1 |
| UNC79 | 18.4790935 | 3429.526416 | 0.0054 | 0.996 | 2.98E-09 | count | 1 |
| CTXND1 | 18.4790935 | 3429.526432 | 0.0054 | 0.996 | 2.98E-09 | count | 1 |
| CPEB1-AS1 | 18.4790935 | 3429.526416 | 0.0054 | 0.996 | 2.98E-09 | count | 1 |
| AC015574.1 | 18.4790936 | 3429.52608 | 0.0054 | 0.996 | 2.98E-09 | count | 1 |
| AL023881.1 | 18.4790936 | 3429.526464 | 0.0054 | 0.996 | 2.98E-09 | count | 1 |
| AC009093.6 | 18.4790936 | 3429.52608 | 0.0054 | 0.996 | 2.98E-09 | count | 1 |
| AC009145.3 | 18.4790935 | 3429.526432 | 0.0054 | 0.996 | 2.98E-09 | count | 1 |
| ALOX12B | 18.4790935 | 3429.526304 | 0.0054 | 0.996 | 2.98E-09 | count | 1 |
| PPP1R27 | 18.4790936 | 3429.52624 | 0.0054 | 0.996 | 2.98E-09 | count | 1 |
| MIR1-1HG-AS1 | 18.4790934 | 3429.526176 | 0.0054 | 0.996 | 2.98E-09 | count | 1 |
| COL9A3 | 18.4790936 | 3429.526464 | 0.0054 | 0.996 | 2.98E-09 | count | 1 |
| AC022098.3 | 18.4790935 | 3429.526416 | 0.0054 | 0.996 | 2.98E-09 | count | 1 |
| AP001432.1 | 18.4790934 | 3429.526448 | 0.0054 | 0.996 | 2.98E-09 | count | 1 |
| KRTAP12-3 | 18.4790935 | 3429.526384 | 0.0054 | 0.996 | 2.98E-09 | count | 1 |
| DAB1 | 18.4790934 | 3429.526208 | 0.0054 | 0.996 | 2.98E-09 | count | 1 |
| AC097381.1 | 18.4790934 | 3429.52632 | 0.0054 | 0.996 | 2.98E-09 | count | 1 |
| VSTM2L | 18.4790935 | 3429.526192 | 0.0054 | 0.996 | 2.98E-09 | count | 1 |
| AC011472.1 | 18.4790935 | 3429.526192 | 0.0054 | 0.996 | 2.98E-09 | count | 1 |
| LINC01623 | 18.4790935 | 3429.526208 | 0.0054 | 0.996 | 2.98E-09 | count | 1 |
| AC118758.3 | 18.4790934 | 3429.526128 | 0.0054 | 0.996 | 2.98E-09 | count | 1 |
| PPP1R26-AS1 | 18.4790933 | 3429.526192 | 0.0054 | 0.996 | 2.98E-09 | count | 1 |
| FGF8 | 18.4790934 | 3429.526208 | 0.0054 | 0.996 | 2.98E-09 | count | 1 |
| BEGAIN | 18.4790933 | 3429.5264 | 0.0054 | 0.996 | 2.98E-09 | count | 1 |
| AC093525.6 | 18.4790935 | 3429.526208 | 0.0054 | 0.996 | 2.98E-09 | count | 1 |
| AC141424.1 | 18.4790935 | 3429.526208 | 0.0054 | 0.996 | 2.98E-09 | count | 1 |
| TMEM200B | 18.4790934 | 3429.526304 | 0.0054 | 0.996 | 2.98E-09 | count | 1 |
| KIT | 18.4790934 | 3429.526256 | 0.0054 | 0.996 | 2.98E-09 | count | 1 |
| AL021807.1 | 18.4790934 | 3429.526416 | 0.0054 | 0.996 | 2.98E-09 | count | 1 |
| AC092647.5 | 18.4790933 | 3429.526304 | 0.0054 | 0.996 | 2.98E-09 | count | 1 |
| CASC8 | 18.4790934 | 3429.526416 | 0.0054 | 0.996 | 2.98E-09 | count | 1 |
| LINC00941 | 18.4790936 | 3429.526368 | 0.0054 | 0.996 | 2.98E-09 | count | 1 |
| AC126755.3 | 18.4790936 | 3429.526368 | 0.0054 | 0.996 | 2.98E-09 | count | 1 |
| SLC2A4 | 18.4790935 | 3429.526096 | 0.0054 | 0.996 | 2.98E-09 | count | 1 |
| AC015908.2 | 18.4790935 | 3429.5264 | 0.0054 | 0.996 | 2.98E-09 | count | 1 |
| MOCOS | 18.4790934 | 3429.526256 | 0.0054 | 0.996 | 2.98E-09 | count | 1 |
| EPB41L1 | 18.4790934 | 3429.526256 | 0.0054 | 0.996 | 2.98E-09 | count | 1 |
| INHBB | 18.4790933 | 3429.526144 | 0.0054 | 0.996 | 2.98E-09 | count | 1 |

| | | | | | | | |
|---|---|---|---|---|---|---|---|
| PCDHA11 | 18.4790934 | 3429.526224 | 0.0054 | 0.996 | 2.98E-09 | count | 1 |
| SCGB3A2 | 18.4790934 | 3429.526192 | 0.0054 | 0.996 | 2.98E-09 | count | 1 |
| AC004922.1 | 18.4790934 | 3429.526192 | 0.0054 | 0.996 | 2.98E-09 | count | 1 |
| TRBV6-8 | 18.4790936 | 3429.52632 | 0.0054 | 0.996 | 2.98E-09 | count | 1 |
| PNMA2 | 18.4790934 | 3429.526224 | 0.0054 | 0.996 | 2.98E-09 | count | 1 |
| PGM5 | 18.4790934 | 3429.526416 | 0.0054 | 0.996 | 2.98E-09 | count | 1 |
| FAM189A2 | 18.4790934 | 3429.52624 | 0.0054 | 0.996 | 2.98E-09 | count | 1 |
| TRPM6 | 18.4790934 | 3429.526192 | 0.0054 | 0.996 | 2.98E-09 | count | 1 |
| GFI1B | 18.4790933 | 3429.52616 | 0.0054 | 0.996 | 2.98E-09 | count | 1 |
| ADAMTS13 | 18.4790935 | 3429.526288 | 0.0054 | 0.996 | 2.98E-09 | count | 1 |
| MS4A3 | 18.4790934 | 3429.526192 | 0.0054 | 0.996 | 2.98E-09 | count | 1 |
| LGALS12 | 18.4790934 | 3429.526192 | 0.0054 | 0.996 | 2.98E-09 | count | 1 |
| AP001107.1 | 18.4790934 | 3429.526192 | 0.0054 | 0.996 | 2.98E-09 | count | 1 |
| AC090510.1 | 18.4790933 | 3429.526176 | 0.0054 | 0.996 | 2.98E-09 | count | 1 |
| HYDIN | 18.4790932 | 3429.526112 | 0.0054 | 0.996 | 2.98E-09 | count | 1 |
| ADAD2 | 18.4790932 | 3429.526272 | 0.0054 | 0.996 | 2.98E-09 | count | 1 |
| CDR2L | 18.4790932 | 3429.526272 | 0.0054 | 0.996 | 2.98E-09 | count | 1 |
| AL121583.1 | 18.4790933 | 3429.52616 | 0.0054 | 0.996 | 2.98E-09 | count | 1 |
| MTRNR2L3 | 18.4790934 | 3429.526224 | 0.0054 | 0.996 | 2.98E-09 | count | 1 |
| AC011444.1 | 18.4790933 | 3429.526144 | 0.0054 | 0.996 | 2.98E-09 | count | 1 |
| RASGRP4 | 18.4790934 | 3429.526192 | 0.0054 | 0.996 | 2.98E-09 | count | 1 |
| C5AR2 | 18.4790934 | 3429.526192 | 0.0054 | 0.996 | 2.98E-09 | count | 1 |
| CACNG6 | 18.4790934 | 3429.526192 | 0.0054 | 0.996 | 2.98E-09 | count | 1 |
| NFAM1 | 18.4790933 | 3429.526016 | 0.0054 | 0.996 | 2.98E-09 | count | 1 |
| AC008063.1 | 18.4790934 | 3429.526192 | 0.0054 | 0.996 | 2.98E-09 | count | 1 |
| TF | 18.4790936 | 3429.526304 | 0.0054 | 0.996 | 2.98E-09 | count | 1 |
| AC025178.1 | 18.4790934 | 3429.526272 | 0.0054 | 0.996 | 2.98E-09 | count | 1 |
| AC092634.5 | 18.4790934 | 3429.526208 | 0.0054 | 0.996 | 2.98E-09 | count | 1 |
| AL391056.1 | 18.4790934 | 3429.526304 | 0.0054 | 0.996 | 2.98E-09 | count | 1 |
| AC091564.4 | 18.4790933 | 3429.526368 | 0.0054 | 0.996 | 2.98E-09 | count | 1 |
| SAA2 | 18.4790935 | 3429.526272 | 0.0054 | 0.996 | 2.98E-09 | count | 1 |
| DUOX2 | 18.4790933 | 3429.526176 | 0.0054 | 0.996 | 2.98E-09 | count | 1 |
| AC015818.2 | 18.4790935 | 3429.526176 | 0.0054 | 0.996 | 2.98E-09 | count | 1 |
| SP2-AS1 | 18.4790935 | 3429.526176 | 0.0054 | 0.996 | 2.98E-09 | count | 1 |
| LINC01993 | 18.4790935 | 3429.526192 | 0.0054 | 0.996 | 2.98E-09 | count | 1 |
| AP001469.2 | 18.4790934 | 3429.526288 | 0.0054 | 0.996 | 2.98E-09 | count | 1 |
| AL512408.1 | 18.4790935 | 3429.52632 | 0.0054 | 0.996 | 2.98E-09 | count | 1 |
| AL157402.2 | 18.4790933 | 3429.526192 | 0.0054 | 0.996 | 2.98E-09 | count | 1 |
| PPARGC1A | 18.4790934 | 3429.526128 | 0.0054 | 0.996 | 2.98E-09 | count | 1 |
| FABP2 | 18.4790932 | 3429.526032 | 0.0054 | 0.996 | 2.98E-09 | count | 1 |
| NIM1K | 18.4790933 | 3429.526128 | 0.0054 | 0.996 | 2.98E-09 | count | 1 |
| AC244517.11 | 18.4790935 | 3429.526336 | 0.0054 | 0.996 | 2.98E-09 | count | 1 |
| AL390208.1 | 18.4790935 | 3429.526336 | 0.0054 | 0.996 | 2.98E-09 | count | 1 |
| GFRA2 | 18.4790935 | 3429.526288 | 0.0054 | 0.996 | 2.98E-09 | count | 1 |
| LINC01030 | 18.4790935 | 3429.526336 | 0.0054 | 0.996 | 2.98E-09 | count | 1 |

| | | | | | | | |
|---|---|---|---|---|---|---|---|
| PDCD1LG2 | 18.4790932 | 3429.526144 | 0.0054 | 0.996 | 2.98E-09 | count | 1 |
| PIH1D2 | 18.4790934 | 3429.526048 | 0.0054 | 0.996 | 2.98E-09 | count | 1 |
| AC022400.6 | 18.4790933 | 3429.526128 | 0.0054 | 0.996 | 2.98E-09 | count | 1 |
| MFAP5 | 18.4790934 | 3429.52616 | 0.0054 | 0.996 | 2.98E-09 | count | 1 |
| AC022509.2 | 18.4790935 | 3429.526336 | 0.0054 | 0.996 | 2.98E-09 | count | 1 |
| HSD17B6 | 18.4790934 | 3429.526416 | 0.0054 | 0.996 | 2.98E-09 | count | 1 |
| AC040934.1 | 18.4790931 | 3429.526224 | 0.0054 | 0.996 | 2.98E-09 | count | 1 |
| SPATA25 | 18.4790932 | 3429.526384 | 0.0054 | 0.996 | 2.98E-09 | count | 1 |
| SHC2 | 18.4790935 | 3429.52632 | 0.0054 | 0.996 | 2.98E-09 | count | 1 |
| AC007292.1 | 18.4790933 | 3429.526048 | 0.0054 | 0.996 | 2.98E-09 | count | 1 |
| SLC17A7 | 18.4790935 | 3429.526336 | 0.0054 | 0.996 | 2.98E-09 | count | 1 |
| AL645608.9 | 18.4790931 | 3429.526352 | 0.0054 | 0.996 | 2.98E-09 | count | 1 |
| LINC01778 | 18.4790932 | 3429.526224 | 0.0054 | 0.996 | 2.98E-09 | count | 1 |
| LINC01362 | 18.4790933 | 3429.526224 | 0.0054 | 0.996 | 2.98E-09 | count | 1 |
| AL590560.1 | 18.4790933 | 3429.526112 | 0.0054 | 0.996 | 2.98E-09 | count | 1 |
| AL357793.1 | 18.479093 | 3429.52608 | 0.0054 | 0.996 | 2.98E-09 | count | 1 |
| AC096637.2 | 18.4790932 | 3429.526224 | 0.0054 | 0.996 | 2.98E-09 | count | 1 |
| PGBD5 | 18.4790931 | 3429.526208 | 0.0054 | 0.996 | 2.98E-09 | count | 1 |
| ID2-AS1 | 18.4790933 | 3429.526352 | 0.0054 | 0.996 | 2.98E-09 | count | 1 |
| AC080162.1 | 18.4790931 | 3429.52624 | 0.0054 | 0.996 | 2.98E-09 | count | 1 |
| AC083949.1 | 18.4790931 | 3429.526224 | 0.0054 | 0.996 | 2.98E-09 | count | 1 |
| DCTN1-AS1 | 18.4790931 | 3429.526208 | 0.0054 | 0.996 | 2.98E-09 | count | 1 |
| MYO7B | 18.4790933 | 3429.526016 | 0.0054 | 0.996 | 2.98E-09 | count | 1 |
| CERKL | 18.4790933 | 3429.526288 | 0.0054 | 0.996 | 2.98E-09 | count | 1 |
| IDH1-AS1 | 18.4790933 | 3429.526096 | 0.0054 | 0.996 | 2.98E-09 | count | 1 |
| AC114730.1 | 18.4790931 | 3429.526416 | 0.0054 | 0.996 | 2.98E-09 | count | 1 |
| AC097359.2 | 18.4790933 | 3429.52632 | 0.0054 | 0.996 | 2.98E-09 | count | 1 |
| AC024560.3 | 18.4790932 | 3429.526224 | 0.0054 | 0.996 | 2.98E-09 | count | 1 |
| AC109347.2 | 18.4790933 | 3429.526112 | 0.0054 | 0.996 | 2.98E-09 | count | 1 |
| LINC02365 | 18.4790932 | 3429.52624 | 0.0054 | 0.996 | 2.98E-09 | count | 1 |
| AC106897.1 | 18.4790934 | 3429.526112 | 0.0054 | 0.996 | 2.98E-09 | count | 1 |
| SH3RF2 | 18.4790933 | 3429.526208 | 0.0054 | 0.996 | 2.98E-09 | count | 1 |
| KIAA0319 | 18.479093 | 3429.52616 | 0.0054 | 0.996 | 2.98E-09 | count | 1 |
| AC092849.2 | 18.4790935 | 3429.526256 | 0.0054 | 0.996 | 2.98E-09 | count | 1 |
| AC005064.1 | 18.4790933 | 3429.526096 | 0.0054 | 0.996 | 2.98E-09 | count | 1 |
| AC023632.2 | 18.4790932 | 3429.52624 | 0.0054 | 0.996 | 2.98E-09 | count | 1 |
| FBXO43 | 18.4790933 | 3429.526256 | 0.0054 | 0.996 | 2.98E-09 | count | 1 |
| AF178030.1 | 18.479093 | 3429.52616 | 0.0054 | 0.996 | 2.98E-09 | count | 1 |
| AKR1C1 | 18.4790931 | 3429.526032 | 0.0054 | 0.996 | 2.98E-09 | count | 1 |
| HTR7 | 18.4790934 | 3429.526112 | 0.0054 | 0.996 | 2.98E-09 | count | 1 |
| AL133352.1 | 18.4790934 | 3429.526224 | 0.0054 | 0.996 | 2.98E-09 | count | 1 |
| AL390763.2 | 18.4790931 | 3429.526032 | 0.0054 | 0.996 | 2.98E-09 | count | 1 |
| LINC02449 | 18.4790932 | 3429.525984 | 0.0054 | 0.996 | 2.98E-09 | count | 1 |
| AL139383.1 | 18.4790933 | 3429.52624 | 0.0054 | 0.996 | 2.98E-09 | count | 1 |
| SALL2 | 18.4790931 | 3429.526208 | 0.0054 | 0.996 | 2.98E-09 | count | 1 |

| | | | | | | | |
|---|---|---|---|---|---|---|---|
| AC013652.1 | 18.4790931 | 3429.526208 | 0.0054 | 0.996 | 2.98E-09 | count | 1 |
| AC009097.2 | 18.4790931 | 3429.526208 | 0.0054 | 0.996 | 2.98E-09 | count | 1 |
| OR3A1 | 18.4790932 | 3429.526288 | 0.0054 | 0.996 | 2.98E-09 | count | 1 |
| CNTD1 | 18.4790931 | 3429.52624 | 0.0054 | 0.996 | 2.98E-09 | count | 1 |
| AL390037.1 | 18.4790933 | 3429.526176 | 0.0054 | 0.996 | 2.98E-09 | count | 1 |
| ZBTB46 | 18.4790932 | 3429.526288 | 0.0054 | 0.996 | 2.98E-09 | count | 1 |
| AC007193.1 | 18.4790933 | 3429.526176 | 0.0054 | 0.996 | 2.98E-09 | count | 1 |
| LINC01869 | 18.4790931 | 3429.526256 | 0.0054 | 0.996 | 2.98E-09 | count | 1 |
| AP000350.6 | 18.4790934 | 3429.526144 | 0.0054 | 0.996 | 2.98E-09 | count | 1 |
| MYO18B | 18.4790934 | 3429.526224 | 0.0054 | 0.996 | 2.98E-09 | count | 1 |
| SEC14L3 | 18.4790931 | 3429.526112 | 0.0054 | 0.996 | 2.98E-09 | count | 1 |
| AL008635.1 | 18.4790933 | 3429.526096 | 0.0054 | 0.996 | 2.98E-09 | count | 1 |
| AC254562.2 | 18.4790931 | 3429.526352 | 0.0054 | 0.996 | 2.98E-09 | count | 1 |
| LINC01191 | 18.4790932 | 3429.526368 | 0.0054 | 0.996 | 2.98E-09 | count | 1 |
| IHH | 18.4790932 | 3429.526368 | 0.0054 | 0.996 | 2.98E-09 | count | 1 |
| AC005237.1 | 18.4790934 | 3429.526304 | 0.0054 | 0.996 | 2.98E-09 | count | 1 |
| TREX1 | 18.4790933 | 3429.526048 | 0.0054 | 0.996 | 2.98E-09 | count | 1 |
| ART3 | 18.4790933 | 3429.526272 | 0.0054 | 0.996 | 2.98E-09 | count | 1 |
| AL096678.1 | 18.4790933 | 3429.526304 | 0.0054 | 0.996 | 2.98E-09 | count | 1 |
| SLC22A1 | 18.4790934 | 3429.526352 | 0.0054 | 0.996 | 2.98E-09 | count | 1 |
| OPN1SW | 18.4790931 | 3429.526112 | 0.0054 | 0.996 | 2.98E-09 | count | 1 |
| TRBV10-1 | 18.4790931 | 3429.526064 | 0.0054 | 0.996 | 2.98E-09 | count | 1 |
| TRBV5-5 | 18.4790932 | 3429.526096 | 0.0054 | 0.996 | 2.98E-09 | count | 1 |
| MRLN | 18.4790931 | 3429.526 | 0.0054 | 0.996 | 2.98E-09 | count | 1 |
| MYOF | 18.4790934 | 3429.526256 | 0.0054 | 0.996 | 2.98E-09 | count | 1 |
| HOXC6 | 18.4790933 | 3429.526224 | 0.0054 | 0.996 | 2.98E-09 | count | 1 |
| AF111167.1 | 18.4790934 | 3429.526192 | 0.0054 | 0.996 | 2.98E-09 | count | 1 |
| AC068722.2 | 18.4790929 | 3429.52616 | 0.0054 | 0.996 | 2.98E-09 | count | 1 |
| AC010531.6 | 18.4790933 | 3429.526144 | 0.0054 | 0.996 | 2.98E-09 | count | 1 |
| AC012301.1 | 18.4790933 | 3429.526176 | 0.0054 | 0.996 | 2.98E-09 | count | 1 |
| LYPD5 | 18.4790933 | 3429.526336 | 0.0054 | 0.996 | 2.98E-09 | count | 1 |
| AL162741.1 | 18.4790932 | 3429.526368 | 0.0054 | 0.996 | 2.98E-09 | count | 1 |
| IQGAP3 | 18.4790932 | 3429.526368 | 0.0054 | 0.996 | 2.98E-09 | count | 1 |
| DYSF | 18.4790931 | 3429.526048 | 0.0054 | 0.996 | 2.98E-09 | count | 1 |
| AC021851.1 | 18.4790933 | 3429.526464 | 0.0054 | 0.996 | 2.98E-09 | count | 1 |
| STEAP1 | 18.4790931 | 3429.526064 | 0.0054 | 0.996 | 2.98E-09 | count | 1 |
| KPNA7 | 18.4790932 | 3429.526448 | 0.0054 | 0.996 | 2.98E-09 | count | 1 |
| SOX7 | 18.4790931 | 3429.526048 | 0.0054 | 0.996 | 2.98E-09 | count | 1 |
| AC100812.1 | 18.4790932 | 3429.526368 | 0.0054 | 0.996 | 2.98E-09 | count | 1 |
| LINC01251 | 18.4790933 | 3429.526256 | 0.0054 | 0.996 | 2.98E-09 | count | 1 |
| AP000977.1 | 18.4790933 | 3429.526288 | 0.0054 | 0.996 | 2.98E-09 | count | 1 |
| AC018653.1 | 18.4790932 | 3429.526448 | 0.0054 | 0.996 | 2.98E-09 | count | 1 |
| LINC02295 | 18.4790932 | 3429.52632 | 0.0054 | 0.996 | 2.98E-09 | count | 1 |
| AC074050.2 | 18.4790931 | 3429.526048 | 0.0054 | 0.996 | 2.98E-09 | count | 1 |
| AC026461.3 | 18.4790932 | 3429.526128 | 0.0054 | 0.996 | 2.98E-09 | count | 1 |

| | | | | | | | |
|---|---|---|---|---|---|---|---|
| AC087498.1 | 18.4790933 | 3429.526288 | 0.0054 | 0.996 | 2.98E-09 | count | 1 |
| C17orf64 | 18.4790932 | 3429.52632 | 0.0054 | 0.996 | 2.98E-09 | count | 1 |
| AC093330.1 | 18.4790932 | 3429.526448 | 0.0054 | 0.996 | 2.98E-09 | count | 1 |
| C20orf202 | 18.4790931 | 3429.526352 | 0.0054 | 0.996 | 2.98E-09 | count | 1 |
| AL354956.1 | 18.4790931 | 3429.525984 | 0.0054 | 0.996 | 2.98E-09 | count | 1 |
| ZBTB8B | 18.4790932 | 3429.526176 | 0.0054 | 0.996 | 2.98E-09 | count | 1 |
| RAB3B | 18.4790934 | 3429.526144 | 0.0054 | 0.996 | 2.98E-09 | count | 1 |
| AC239804.1 | 18.4790932 | 3429.52608 | 0.0054 | 0.996 | 2.98E-09 | count | 1 |
| HCN3 | 18.479093 | 3429.52624 | 0.0054 | 0.996 | 2.98E-09 | count | 1 |
| BCAN | 18.4790933 | 3429.526064 | 0.0054 | 0.996 | 2.98E-09 | count | 1 |
| AC244034.2 | 18.4790931 | 3429.526368 | 0.0054 | 0.996 | 2.98E-09 | count | 1 |
| TLX2 | 18.4790931 | 3429.526224 | 0.0054 | 0.996 | 2.98E-09 | count | 1 |
| STEAP3-AS1 | 18.4790933 | 3429.526416 | 0.0054 | 0.996 | 2.98E-09 | count | 1 |
| LINC02042 | 18.4790933 | 3429.526416 | 0.0054 | 0.996 | 2.98E-09 | count | 1 |
| AC108693.2 | 18.4790931 | 3429.526368 | 0.0054 | 0.996 | 2.98E-09 | count | 1 |
| ZBTB20-AS5 | 18.4790933 | 3429.526416 | 0.0054 | 0.996 | 2.98E-09 | count | 1 |
| PLCH1 | 18.479093 | 3429.526032 | 0.0054 | 0.996 | 2.98E-09 | count | 1 |
| P3H2 | 18.4790931 | 3429.526048 | 0.0054 | 0.996 | 2.98E-09 | count | 1 |
| UBXN7-AS1 | 18.479093 | 3429.526256 | 0.0054 | 0.996 | 2.98E-09 | count | 1 |
| ODAPH | 18.4790931 | 3429.526 | 0.0054 | 0.996 | 2.98E-09 | count | 1 |
| AC010280.2 | 18.479093 | 3429.526 | 0.0054 | 0.996 | 2.98E-09 | count | 1 |
| AC022217.3 | 18.4790933 | 3429.526416 | 0.0054 | 0.996 | 2.98E-09 | count | 1 |
| FKBP1C | 18.4790932 | 3429.526192 | 0.0054 | 0.996 | 2.98E-09 | count | 1 |
| AL158850.1 | 18.4790931 | 3429.52608 | 0.0054 | 0.996 | 2.98E-09 | count | 1 |
| SOD2 | 18.4790932 | 3429.526176 | 0.0054 | 0.996 | 2.98E-09 | count | 1 |
| EVX1 | 18.4790933 | 3429.526416 | 0.0054 | 0.996 | 2.98E-09 | count | 1 |
| TFEC | 18.479093 | 3429.526096 | 0.0054 | 0.996 | 2.98E-09 | count | 1 |
| AC006372.3 | 18.4790931 | 3429.526176 | 0.0054 | 0.996 | 2.98E-09 | count | 1 |
| ASB12 | 18.4790929 | 3429.526112 | 0.0054 | 0.996 | 2.98E-09 | count | 1 |
| MAFA-AS1 | 18.4790933 | 3429.526416 | 0.0054 | 0.996 | 2.98E-09 | count | 1 |
| AL158071.5 | 18.4790931 | 3429.526128 | 0.0054 | 0.996 | 2.98E-09 | count | 1 |
| AL158151.3 | 18.4790931 | 3429.526352 | 0.0054 | 0.996 | 2.98E-09 | count | 1 |
| LINC02552 | 18.4790931 | 3429.526352 | 0.0054 | 0.996 | 2.98E-09 | count | 1 |
| AL513303.1 | 18.4790931 | 3429.526368 | 0.0054 | 0.996 | 2.98E-09 | count | 1 |
| ZMIZ1-AS1 | 18.4790931 | 3429.525984 | 0.0054 | 0.996 | 2.98E-09 | count | 1 |
| PRH2 | 18.4790931 | 3429.526048 | 0.0054 | 0.996 | 2.98E-09 | count | 1 |
| TAS2R43 | 18.4790931 | 3429.526128 | 0.0054 | 0.996 | 2.98E-09 | count | 1 |
| PRB3 | 18.4790931 | 3429.526192 | 0.0054 | 0.996 | 2.98E-09 | count | 1 |
| GLI1 | 18.4790931 | 3429.525984 | 0.0054 | 0.996 | 2.98E-09 | count | 1 |
| ATXN2-AS | 18.4790933 | 3429.526416 | 0.0054 | 0.996 | 2.98E-09 | count | 1 |
| AL355001.1 | 18.479093 | 3429.52616 | 0.0054 | 0.996 | 2.98E-09 | count | 1 |
| AL135744.1 | 18.4790933 | 3429.526416 | 0.0054 | 0.996 | 2.98E-09 | count | 1 |
| AC140725.1 | 18.4790931 | 3429.52608 | 0.0054 | 0.996 | 2.98E-09 | count | 1 |
| Z92544.1 | 18.4790933 | 3429.526336 | 0.0054 | 0.996 | 2.98E-09 | count | 1 |
| AC007611.1 | 18.4790931 | 3429.526144 | 0.0054 | 0.996 | 2.98E-09 | count | 1 |

| | | | | | | | |
|---|---|---|---|---|---|---|---|
| MAFTRR | 18.4790931 | 3429.526352 | 0.0054 | 0.996 | 2.98E-09 | count | 1 |
| AC068418.1 | 18.4790933 | 3429.526416 | 0.0054 | 0.996 | 2.98E-09 | count | 1 |
| LPO | 18.4790933 | 3429.526096 | 0.0054 | 0.996 | 2.98E-09 | count | 1 |
| OR4F17 | 18.4790932 | 3429.526288 | 0.0054 | 0.996 | 2.98E-09 | count | 1 |
| AC004449.1 | 18.4790931 | 3429.526368 | 0.0054 | 0.996 | 2.98E-09 | count | 1 |
| MUC16 | 18.4790929 | 3429.52616 | 0.0054 | 0.996 | 2.98E-09 | count | 1 |
| ZNF625-ZNF20 | 18.4790933 | 3429.526416 | 0.0054 | 0.996 | 2.98E-09 | count | 1 |
| ZNF98 | 18.479093 | 3429.526096 | 0.0054 | 0.996 | 2.98E-09 | count | 1 |
| AC010271.1 | 18.4790931 | 3429.526032 | 0.0054 | 0.996 | 2.98E-09 | count | 1 |
| CACNG8 | 18.4790931 | 3429.52608 | 0.0054 | 0.996 | 2.98E-09 | count | 1 |
| C19orf18 | 18.4790932 | 3429.526288 | 0.0054 | 0.996 | 2.98E-09 | count | 1 |
| LINC01311 | 18.4790932 | 3429.526288 | 0.0054 | 0.996 | 2.98E-09 | count | 1 |
| AL022314.1 | 18.4790934 | 3429.526144 | 0.0054 | 0.996 | 2.98E-09 | count | 1 |
| LINC00158 | 18.479093 | 3429.526032 | 0.0054 | 0.996 | 2.98E-09 | count | 1 |
| STPG2 | 18.479093 | 3429.52616 | 0.0054 | 0.996 | 2.98E-09 | count | 1 |
| AC073343.2 | 18.4790932 | 3429.526064 | 0.0054 | 0.996 | 2.98E-09 | count | 1 |
| PTCHD1 | 18.4790932 | 3429.526128 | 0.0054 | 0.996 | 2.98E-09 | count | 1 |
| NUDT10 | 18.4790931 | 3429.526096 | 0.0054 | 0.996 | 2.98E-09 | count | 1 |
| FOXD4 | 18.4790931 | 3429.526352 | 0.0054 | 0.996 | 2.98E-09 | count | 1 |
| SPAAR | 18.4790931 | 3429.526096 | 0.0054 | 0.996 | 2.98E-09 | count | 1 |
| FAM74A1 | 18.4790932 | 3429.526256 | 0.0054 | 0.996 | 2.98E-09 | count | 1 |
| AP003717.1 | 18.479093 | 3429.526192 | 0.0054 | 0.996 | 2.98E-09 | count | 1 |
| TSKU | 18.479093 | 3429.526192 | 0.0054 | 0.996 | 2.98E-09 | count | 1 |
| AC007569.1 | 18.4790931 | 3429.526176 | 0.0054 | 0.996 | 2.98E-09 | count | 1 |
| LINC02285 | 18.4790932 | 3429.526128 | 0.0054 | 0.996 | 2.98E-09 | count | 1 |
| LINC00638 | 18.479093 | 3429.526192 | 0.0054 | 0.996 | 2.98E-09 | count | 1 |
| AP3B2 | 18.4790932 | 3429.526256 | 0.0054 | 0.996 | 2.98E-09 | count | 1 |
| MTRNR2L4 | 18.4790932 | 3429.526208 | 0.0054 | 0.996 | 2.98E-09 | count | 1 |
| C17orf53 | 18.4790931 | 3429.526176 | 0.0054 | 0.996 | 2.98E-09 | count | 1 |
| VSX1 | 18.479093 | 3429.52608 | 0.0054 | 0.996 | 2.98E-09 | count | 1 |
| AC000068.1 | 18.479093 | 3429.526192 | 0.0054 | 0.996 | 2.98E-09 | count | 1 |
| SCNN1D | 18.4790931 | 3429.52608 | 0.0054 | 0.996 | 2.98E-09 | count | 1 |
| C1orf189 | 18.4790931 | 3429.526064 | 0.0054 | 0.996 | 2.98E-09 | count | 1 |
| GPR161 | 18.4790931 | 3429.526208 | 0.0054 | 0.996 | 2.98E-09 | count | 1 |
| LAD1 | 18.479093 | 3429.52608 | 0.0054 | 0.996 | 2.98E-09 | count | 1 |
| AC016737.1 | 18.4790929 | 3429.526224 | 0.0054 | 0.996 | 2.98E-09 | count | 1 |
| ABCB6 | 18.4790932 | 3429.526288 | 0.0054 | 0.996 | 2.98E-09 | count | 1 |
| AC084035.1 | 18.4790929 | 3429.526144 | 0.0054 | 0.996 | 2.98E-09 | count | 1 |
| AC007823.1 | 18.479093 | 3429.526192 | 0.0054 | 0.996 | 2.98E-09 | count | 1 |
| AC069431.1 | 18.479093 | 3429.52608 | 0.0054 | 0.996 | 2.98E-09 | count | 1 |
| AC096711.2 | 18.4790931 | 3429.526208 | 0.0054 | 0.996 | 2.98E-09 | count | 1 |
| LINC02108 | 18.4790929 | 3429.526224 | 0.0054 | 0.996 | 2.98E-09 | count | 1 |
| CKMT2 | 18.4790932 | 3429.526288 | 0.0054 | 0.996 | 2.98E-09 | count | 1 |
| AC006004.1 | 18.4790928 | 3429.526128 | 0.0054 | 0.996 | 2.98E-09 | count | 1 |
| MAOA | 18.4790929 | 3429.526224 | 0.0054 | 0.996 | 2.98E-09 | count | 1 |

| | | | | | | | |
|---|---|---|---|---|---|---|---|
| H2AFB1 | 18.4790929 | 3429.52608 | 0.0054 | 0.996 | 2.98E-09 | count | 1 |
| BX571846.1 | 18.4790931 | 3429.526128 | 0.0054 | 0.996 | 2.98E-09 | count | 1 |
| FBXO16 | 18.479093 | 3429.52608 | 0.0054 | 0.996 | 2.98E-09 | count | 1 |
| ANKRD20A2 | 18.479093 | 3429.526256 | 0.0054 | 0.996 | 2.98E-09 | count | 1 |
| CTSV | 18.4790931 | 3429.52608 | 0.0054 | 0.996 | 2.98E-09 | count | 1 |
| PALM2 | 18.4790932 | 3429.526288 | 0.0054 | 0.996 | 2.98E-09 | count | 1 |
| TRIM34 | 18.4790929 | 3429.526224 | 0.0054 | 0.996 | 2.98E-09 | count | 1 |
| MIR202HG | 18.4790932 | 3429.526112 | 0.0054 | 0.996 | 2.98E-09 | count | 1 |
| TAS2R10 | 18.4790929 | 3429.525984 | 0.0054 | 0.996 | 2.98E-09 | count | 1 |
| AC023794.3 | 18.4790932 | 3429.526224 | 0.0054 | 0.996 | 2.98E-09 | count | 1 |
| AC026367.1 | 18.479093 | 3429.526256 | 0.0054 | 0.996 | 2.98E-09 | count | 1 |
| AL590096.1 | 18.479093 | 3429.526176 | 0.0054 | 0.996 | 2.98E-09 | count | 1 |
| PPP1R36 | 18.4790929 | 3429.526208 | 0.0054 | 0.996 | 2.98E-09 | count | 1 |
| AC005520.5 | 18.4790929 | 3429.526224 | 0.0054 | 0.996 | 2.98E-09 | count | 1 |
| C14orf178 | 18.4790932 | 3429.52624 | 0.0054 | 0.996 | 2.98E-09 | count | 1 |
| IGHV4-34 | 18.4790932 | 3429.52624 | 0.0054 | 0.996 | 2.98E-09 | count | 1 |
| CACNA1I | 18.4790929 | 3429.526224 | 0.0054 | 0.996 | 2.98E-09 | count | 1 |
| AL049795.1 | 18.4790931 | 3429.526064 | 0.0054 | 0.996 | 2.98E-09 | count | 1 |
| C1orf116 | 18.479093 | 3429.526384 | 0.0054 | 0.996 | 2.98E-09 | count | 1 |
| CHRM3 | 18.479093 | 3429.526112 | 0.0054 | 0.996 | 2.98E-09 | count | 1 |
| RAD21-AS1 | 18.479093 | 3429.52608 | 0.0054 | 0.996 | 2.98E-09 | count | 1 |
| AC073911.1 | 18.4790929 | 3429.526224 | 0.0054 | 0.996 | 2.98E-09 | count | 1 |
| THAP10 | 18.4790931 | 3429.526192 | 0.0054 | 0.996 | 2.98E-09 | count | 1 |
| MPP2 | 18.479093 | 3429.52608 | 0.0054 | 0.996 | 2.98E-09 | count | 1 |
| AL121772.3 | 18.4790929 | 3429.526176 | 0.0054 | 0.996 | 2.98E-09 | count | 1 |
| LCA5L | 18.479093 | 3429.526112 | 0.0054 | 0.996 | 2.98E-09 | count | 1 |
| GUCA1B | 18.4790931 | 3429.526192 | 0.0054 | 0.996 | 2.98E-09 | count | 1 |
| KANK4 | 18.4790928 | 3429.526016 | 0.0054 | 0.996 | 2.98E-09 | count | 1 |
| ANKUB1 | 18.4790929 | 3429.526096 | 0.0054 | 0.996 | 2.98E-09 | count | 1 |
| AL590617.2 | 18.4790928 | 3429.526016 | 0.0054 | 0.996 | 2.98E-09 | count | 1 |
| TRBV5-6 | 18.4790931 | 3429.526128 | 0.0054 | 0.996 | 2.98E-09 | count | 1 |
| AC011611.3 | 18.4790928 | 3429.526016 | 0.0054 | 0.996 | 2.98E-09 | count | 1 |
| TRAJ37 | 18.4790931 | 3429.526128 | 0.0054 | 0.996 | 2.98E-09 | count | 1 |
| AL133367.1 | 18.4790931 | 3429.526128 | 0.0054 | 0.996 | 2.98E-09 | count | 1 |
| COL25A1 | 18.4790929 | 3429.526048 | 0.0054 | 0.996 | 2.98E-09 | count | 1 |
| AC087286.3 | 18.4790927 | 3429.526128 | 0.0054 | 0.996 | 2.98E-09 | count | 1 |
| AL627171.2 | 0.036701 | 0.4849003 | 0.0757 | 0.94 | 2.99E-09 | count | 1 |
| AL121999.1 | 18.8788949 | 1979.562493 | 0.0095 | 0.992 | 3.01E-09 | count | 1 |
| AC103563.7 | 18.8788948 | 1979.562552 | 0.0095 | 0.992 | 3.01E-09 | count | 1 |
| AC226118.1 | 18.8788948 | 1979.562552 | 0.0095 | 0.992 | 3.01E-09 | count | 1 |
| NR2E3 | 18.8788948 | 1979.562552 | 0.0095 | 0.992 | 3.01E-09 | count | 1 |
| AC053527.1 | 18.8788949 | 1979.562568 | 0.0095 | 0.992 | 3.01E-09 | count | 1 |
| PTGFRN | 18.8788947 | 1979.56256 | 0.0095 | 0.992 | 3.01E-09 | count | 1 |
| AL121972.1 | 18.8788949 | 1979.562527 | 0.0095 | 0.992 | 3.01E-09 | count | 1 |
| SH3BGRL2 | 18.8788949 | 1979.562527 | 0.0095 | 0.992 | 3.01E-09 | count | 1 |

| | | | | | | | |
|---|---|---|---|---|---|---|---|
| ABCB4 | 18.8788947 | 1979.56256 | 0.0095 | 0.992 | 3.01E-09 | count | 1 |
| AL157400.4 | 18.8788947 | 1979.56256 | 0.0095 | 0.992 | 3.01E-09 | count | 1 |
| FAAH | 18.8788947 | 1979.562393 | 0.0095 | 0.992 | 3.01E-09 | count | 1 |
| EXO1 | 18.8788947 | 1979.562535 | 0.0095 | 0.992 | 3.01E-09 | count | 1 |
| AC017071.1 | 18.8788947 | 1979.562543 | 0.0095 | 0.992 | 3.01E-09 | count | 1 |
| AC026355.1 | 18.8788947 | 1979.562485 | 0.0095 | 0.992 | 3.01E-09 | count | 1 |
| FAM153B | 18.8788946 | 1979.562552 | 0.0095 | 0.992 | 3.01E-09 | count | 1 |
| AC003991.2 | 18.8788947 | 1979.562543 | 0.0095 | 0.992 | 3.01E-09 | count | 1 |
| PTPRCAP | 18.8788947 | 1979.562644 | 0.0095 | 0.992 | 3.01E-09 | count | 1 |
| AC126175.1 | 18.8788947 | 1979.562493 | 0.0095 | 0.992 | 3.01E-09 | count | 1 |
| AC012170.2 | 18.8788949 | 1979.562644 | 0.0095 | 0.992 | 3.01E-09 | count | 1 |
| AC100830.3 | 18.8788948 | 1979.562543 | 0.0095 | 0.992 | 3.01E-09 | count | 1 |
| AC087392.5 | 18.8788947 | 1979.562644 | 0.0095 | 0.992 | 3.01E-09 | count | 1 |
| AC129492.4 | 18.8788948 | 1979.562577 | 0.0095 | 0.992 | 3.01E-09 | count | 1 |
| AC010325.1 | 18.8788947 | 1979.562476 | 0.0095 | 0.992 | 3.01E-09 | count | 1 |
| NPAS2 | 18.8788948 | 1979.562435 | 0.0095 | 0.992 | 3.01E-09 | count | 1 |
| CATSPER3 | 18.8788946 | 1979.562543 | 0.0095 | 0.992 | 3.01E-09 | count | 1 |
| AL031118.1 | 18.8788946 | 1979.562543 | 0.0095 | 0.992 | 3.01E-09 | count | 1 |
| AL158055.1 | 18.8788946 | 1979.562485 | 0.0095 | 0.992 | 3.01E-09 | count | 1 |
| AP006287.2 | 18.8788947 | 1979.562535 | 0.0095 | 0.992 | 3.01E-09 | count | 1 |
| SH3PXD2A-AS1 | 18.8788949 | 1979.562602 | 0.0095 | 0.992 | 3.01E-09 | count | 1 |
| AC010536.1 | 18.8788946 | 1979.562543 | 0.0095 | 0.992 | 3.01E-09 | count | 1 |
| ZYG11A | 18.8788948 | 1979.562518 | 0.0095 | 0.992 | 3.01E-09 | count | 1 |
| PDLIM4 | 18.8788948 | 1979.562518 | 0.0095 | 0.992 | 3.01E-09 | count | 1 |
| TWIST1 | 18.8788948 | 1979.562518 | 0.0095 | 0.992 | 3.01E-09 | count | 1 |
| WIPF3 | 18.8788947 | 1979.562493 | 0.0095 | 0.992 | 3.01E-09 | count | 1 |
| AC100821.2 | 18.8788946 | 1979.562552 | 0.0095 | 0.992 | 3.01E-09 | count | 1 |
| AC087672.2 | 18.8788945 | 1979.562468 | 0.0095 | 0.992 | 3.01E-09 | count | 1 |
| PGM5P3-AS1 | 18.8788948 | 1979.562401 | 0.0095 | 0.992 | 3.01E-09 | count | 1 |
| AC005899.7 | 18.8788947 | 1979.562493 | 0.0095 | 0.992 | 3.01E-09 | count | 1 |
| LINC01140 | 18.8788946 | 1979.562568 | 0.0095 | 0.992 | 3.01E-09 | count | 1 |
| RBM15-AS1 | 18.8788945 | 1979.562502 | 0.0095 | 0.992 | 3.01E-09 | count | 1 |
| AQP10 | 18.8788945 | 1979.562476 | 0.0095 | 0.992 | 3.01E-09 | count | 1 |
| AC010680.4 | 18.8788948 | 1979.562568 | 0.0095 | 0.992 | 3.01E-09 | count | 1 |
| PTPRG | 18.8788946 | 1979.562568 | 0.0095 | 0.992 | 3.01E-09 | count | 1 |
| GXYLT2 | 18.8788947 | 1979.562426 | 0.0095 | 0.992 | 3.01E-09 | count | 1 |
| KLF15 | 18.8788946 | 1979.562602 | 0.0095 | 0.992 | 3.01E-09 | count | 1 |
| REELD1 | 18.8788948 | 1979.562418 | 0.0095 | 0.992 | 3.01E-09 | count | 1 |
| AC008781.2 | 18.8788946 | 1979.562493 | 0.0095 | 0.992 | 3.01E-09 | count | 1 |
| AL049697.1 | 18.8788946 | 1979.562568 | 0.0095 | 0.992 | 3.01E-09 | count | 1 |
| CA8 | 18.8788949 | 1979.562577 | 0.0095 | 0.992 | 3.01E-09 | count | 1 |
| AL158151.1 | 18.8788945 | 1979.562476 | 0.0095 | 0.992 | 3.01E-09 | count | 1 |
| AL731533.2 | 18.8788946 | 1979.56251 | 0.0095 | 0.992 | 3.01E-09 | count | 1 |
| AL158834.1 | 18.8788945 | 1979.562552 | 0.0095 | 0.992 | 3.01E-09 | count | 1 |
| CUZD1 | 18.8788947 | 1979.562426 | 0.0095 | 0.992 | 3.01E-09 | count | 1 |

| | | | | | | | |
|---|---|---|---|---|---|---|---|
| AC140847.2 | 18.8788946 | 1979.562568 | 0.0095 | 0.992 | 3.01E-09 | count | 1 |
| SUOX | 18.8788948 | 1979.562568 | 0.0095 | 0.992 | 3.01E-09 | count | 1 |
| SLITRK5 | 18.8788949 | 1979.562577 | 0.0095 | 0.992 | 3.01E-09 | count | 1 |
| IGHV4-28 | 18.8788947 | 1979.562577 | 0.0095 | 0.992 | 3.01E-09 | count | 1 |
| AC138904.3 | 18.8788946 | 1979.562568 | 0.0095 | 0.992 | 3.01E-09 | count | 1 |
| DPEP3 | 18.8788945 | 1979.56246 | 0.0095 | 0.992 | 3.01E-09 | count | 1 |
| AC026464.4 | 18.8788947 | 1979.562426 | 0.0095 | 0.992 | 3.01E-09 | count | 1 |
| DNAI2 | 18.8788948 | 1979.562602 | 0.0095 | 0.992 | 3.01E-09 | count | 1 |
| SLC32A1 | 18.8788948 | 1979.562602 | 0.0095 | 0.992 | 3.01E-09 | count | 1 |
| Z73429.1 | 18.8788945 | 1979.562476 | 0.0095 | 0.992 | 3.01E-09 | count | 1 |
| Z95114.4 | 18.8788948 | 1979.562602 | 0.0095 | 0.992 | 3.01E-09 | count | 1 |
| PRR34 | 18.8788948 | 1979.562602 | 0.0095 | 0.992 | 3.01E-09 | count | 1 |
| CHD5 | 18.8788947 | 1979.56261 | 0.0095 | 0.992 | 3.01E-09 | count | 1 |
| GSTM5 | 18.8788948 | 1979.562619 | 0.0095 | 0.992 | 3.01E-09 | count | 1 |
| AL390066.1 | 18.8788944 | 1979.56246 | 0.0095 | 0.992 | 3.01E-09 | count | 1 |
| PDZK1 | 18.8788945 | 1979.562435 | 0.0095 | 0.992 | 3.01E-09 | count | 1 |
| LENEP | 18.8788947 | 1979.562627 | 0.0095 | 0.992 | 3.01E-09 | count | 1 |
| DNM3 | 18.8788947 | 1979.562627 | 0.0095 | 0.992 | 3.01E-09 | count | 1 |
| AC093388.1 | 18.8788944 | 1979.56246 | 0.0095 | 0.992 | 3.01E-09 | count | 1 |
| AC079610.1 | 18.8788946 | 1979.562518 | 0.0095 | 0.992 | 3.01E-09 | count | 1 |
| LINC01238 | 18.8788944 | 1979.56246 | 0.0095 | 0.992 | 3.01E-09 | count | 1 |
| HESX1 | 18.8788946 | 1979.562552 | 0.0095 | 0.992 | 3.01E-09 | count | 1 |
| ANK2 | 18.8788947 | 1979.562627 | 0.0095 | 0.992 | 3.01E-09 | count | 1 |
| STK31 | 18.8788946 | 1979.562577 | 0.0095 | 0.992 | 3.01E-09 | count | 1 |
| SPDYE3 | 18.8788947 | 1979.562627 | 0.0095 | 0.992 | 3.01E-09 | count | 1 |
| ANKRD7 | 18.8788946 | 1979.562518 | 0.0095 | 0.992 | 3.01E-09 | count | 1 |
| LMOD2 | 18.8788947 | 1979.562527 | 0.0095 | 0.992 | 3.01E-09 | count | 1 |
| HYAL4 | 18.8788946 | 1979.562518 | 0.0095 | 0.992 | 3.01E-09 | count | 1 |
| ZNF157 | 18.8788947 | 1979.562627 | 0.0095 | 0.992 | 3.01E-09 | count | 1 |
| AC100810.3 | 18.8788947 | 1979.562627 | 0.0095 | 0.992 | 3.01E-09 | count | 1 |
| RFX3-AS1 | 18.8788947 | 1979.562627 | 0.0095 | 0.992 | 3.01E-09 | count | 1 |
| KRTAP5-2 | 18.8788946 | 1979.562493 | 0.0095 | 0.992 | 3.01E-09 | count | 1 |
| C12orf40 | 18.8788947 | 1979.562627 | 0.0095 | 0.992 | 3.01E-09 | count | 1 |
| AC069234.4 | 18.8788947 | 1979.56261 | 0.0095 | 0.992 | 3.01E-09 | count | 1 |
| AC055876.5 | 18.8788947 | 1979.562627 | 0.0095 | 0.992 | 3.01E-09 | count | 1 |
| CEMP1 | 18.8788947 | 1979.562627 | 0.0095 | 0.992 | 3.01E-09 | count | 1 |
| IRX5 | 18.8788947 | 1979.562527 | 0.0095 | 0.992 | 3.01E-09 | count | 1 |
| AC004223.2 | 18.8788945 | 1979.562485 | 0.0095 | 0.992 | 3.01E-09 | count | 1 |
| AC091132.1 | 18.8788947 | 1979.562627 | 0.0095 | 0.992 | 3.01E-09 | count | 1 |
| NXPH3 | 18.8788947 | 1979.562418 | 0.0095 | 0.992 | 3.01E-09 | count | 1 |
| GRIN2C | 18.8788947 | 1979.562627 | 0.0095 | 0.992 | 3.01E-09 | count | 1 |
| LINC02080 | 18.8788946 | 1979.562493 | 0.0095 | 0.992 | 3.01E-09 | count | 1 |
| AC010336.4 | 18.8788947 | 1979.562627 | 0.0095 | 0.992 | 3.01E-09 | count | 1 |
| AC010323.1 | 18.8788947 | 1979.562527 | 0.0095 | 0.992 | 3.01E-09 | count | 1 |
| AC024575.1 | 18.8788947 | 1979.562627 | 0.0095 | 0.992 | 3.01E-09 | count | 1 |

| | | | | | | | |
|---|---|---|---|---|---|---|---|
| CYP2A6 | 18.8788947 | 1979.562627 | 0.0095 | 0.992 | 3.01E-09 | count | 1 |
| CAMTA1-DT | 18.8788946 | 1979.562577 | 0.0095 | 0.992 | 3.01E-09 | count | 1 |
| AL023755.1 | 18.8788945 | 1979.562418 | 0.0095 | 0.992 | 3.01E-09 | count | 1 |
| SLC4A3 | 18.8788945 | 1979.562619 | 0.0095 | 0.992 | 3.01E-09 | count | 1 |
| LINC00971 | 18.8788946 | 1979.562543 | 0.0095 | 0.992 | 3.01E-09 | count | 1 |
| SOWAHA | 18.8788945 | 1979.562568 | 0.0095 | 0.992 | 3.01E-09 | count | 1 |
| MYOZ3 | 18.8788945 | 1979.562568 | 0.0095 | 0.992 | 3.01E-09 | count | 1 |
| AGBL2 | 18.8788945 | 1979.562619 | 0.0095 | 0.992 | 3.01E-09 | count | 1 |
| ME3 | 18.8788947 | 1979.562418 | 0.0095 | 0.992 | 3.01E-09 | count | 1 |
| TLL2 | 18.8788945 | 1979.562568 | 0.0095 | 0.992 | 3.01E-09 | count | 1 |
| CASC1 | 18.8788945 | 1979.562568 | 0.0095 | 0.992 | 3.01E-09 | count | 1 |
| LINC00384 | 18.8788946 | 1979.562577 | 0.0095 | 0.992 | 3.01E-09 | count | 1 |
| TRAV10 | 18.8788945 | 1979.562619 | 0.0095 | 0.992 | 3.01E-09 | count | 1 |
| AC093249.2 | 18.8788945 | 1979.562451 | 0.0095 | 0.992 | 3.01E-09 | count | 1 |
| AC006441.4 | 18.8788945 | 1979.562619 | 0.0095 | 0.992 | 3.01E-09 | count | 1 |
| HOXB-AS3 | 18.8788947 | 1979.562418 | 0.0095 | 0.992 | 3.01E-09 | count | 1 |
| ADAM33 | 18.8788945 | 1979.562619 | 0.0095 | 0.992 | 3.01E-09 | count | 1 |
| PRR22 | 18.8788947 | 1979.562485 | 0.0095 | 0.992 | 3.01E-09 | count | 1 |
| AC024592.1 | 18.8788945 | 1979.562451 | 0.0095 | 0.992 | 3.01E-09 | count | 1 |
| CCER2 | 18.8788945 | 1979.562493 | 0.0095 | 0.992 | 3.01E-09 | count | 1 |
| MAPK12 | 18.8788946 | 1979.562577 | 0.0095 | 0.992 | 3.01E-09 | count | 1 |
| SLC44A3 | 18.8788947 | 1979.562585 | 0.0095 | 0.992 | 3.01E-09 | count | 1 |
| BX470102.1 | 18.8788946 | 1979.562527 | 0.0095 | 0.992 | 3.01E-09 | count | 1 |
| AL590714.1 | 18.8788947 | 1979.562527 | 0.0095 | 0.992 | 3.01E-09 | count | 1 |
| AC104135.1 | 18.8788947 | 1979.562502 | 0.0095 | 0.992 | 3.01E-09 | count | 1 |
| AC073869.3 | 18.8788947 | 1979.562635 | 0.0095 | 0.992 | 3.01E-09 | count | 1 |
| TERT | 18.8788945 | 1979.56251 | 0.0095 | 0.992 | 3.01E-09 | count | 1 |
| AC018645.2 | 18.8788947 | 1979.562585 | 0.0095 | 0.992 | 3.01E-09 | count | 1 |
| AL390294.1 | 18.8788946 | 1979.56251 | 0.0095 | 0.992 | 3.01E-09 | count | 1 |
| AL137025.1 | 18.8788947 | 1979.56251 | 0.0095 | 0.992 | 3.01E-09 | count | 1 |
| AC008115.4 | 18.8788946 | 1979.562602 | 0.0095 | 0.992 | 3.01E-09 | count | 1 |
| GALNT4 | 18.8788946 | 1979.562527 | 0.0095 | 0.992 | 3.01E-09 | count | 1 |
| SPTB | 18.8788947 | 1979.562527 | 0.0095 | 0.992 | 3.01E-09 | count | 1 |
| AC005954.2 | 18.8788947 | 1979.562502 | 0.0095 | 0.992 | 3.01E-09 | count | 1 |
| AC010422.3 | 18.8788946 | 1979.56251 | 0.0095 | 0.992 | 3.01E-09 | count | 1 |
| LINC00334 | 18.8788946 | 1979.56256 | 0.0095 | 0.992 | 3.01E-09 | count | 1 |
| AL034417.2 | 18.8788945 | 1979.562451 | 0.0095 | 0.992 | 3.01E-09 | count | 1 |
| AL050343.1 | 18.8788946 | 1979.562518 | 0.0095 | 0.992 | 3.01E-09 | count | 1 |
| TFCP2L1 | 18.8788945 | 1979.562418 | 0.0095 | 0.992 | 3.01E-09 | count | 1 |
| AC010680.1 | 18.8788945 | 1979.56251 | 0.0095 | 0.992 | 3.01E-09 | count | 1 |
| GCK | 18.8788945 | 1979.56261 | 0.0095 | 0.992 | 3.01E-09 | count | 1 |
| FSCN3 | 18.8788944 | 1979.562451 | 0.0095 | 0.992 | 3.01E-09 | count | 1 |
| AL157938.2 | 18.8788944 | 1979.56246 | 0.0095 | 0.992 | 3.01E-09 | count | 1 |
| ENTPD7 | 18.8788946 | 1979.562485 | 0.0095 | 0.992 | 3.01E-09 | count | 1 |
| TMEM272 | 18.8788944 | 1979.562451 | 0.0095 | 0.992 | 3.01E-09 | count | 1 |

| | | | | | | | |
|---|---|---|---|---|---|---|---|
| AC105020.5 | 18.8788946 | 1979.562518 | 0.0095 | 0.992 | 3.01E-09 | count | 1 |
| AC007336.1 | 18.8788944 | 1979.562493 | 0.0095 | 0.992 | 3.01E-09 | count | 1 |
| AC145207.8 | 18.8788945 | 1979.562527 | 0.0095 | 0.992 | 3.01E-09 | count | 1 |
| CIDEC | 18.8788945 | 1979.562443 | 0.0095 | 0.992 | 3.01E-09 | count | 1 |
| AC010609.1 | 18.8788946 | 1979.562468 | 0.0095 | 0.992 | 3.01E-09 | count | 1 |
| SMO | 18.8788946 | 1979.562443 | 0.0095 | 0.992 | 3.01E-09 | count | 1 |
| LINC00865 | 18.8788945 | 1979.56251 | 0.0095 | 0.992 | 3.01E-09 | count | 1 |
| AC108134.1 | 18.8788946 | 1979.562468 | 0.0095 | 0.992 | 3.01E-09 | count | 1 |
| CA5A | 18.8788946 | 1979.562468 | 0.0095 | 0.992 | 3.01E-09 | count | 1 |
| AC015911.2 | 18.8788944 | 1979.562451 | 0.0095 | 0.992 | 3.01E-09 | count | 1 |
| C17orf113 | 18.8788945 | 1979.562543 | 0.0095 | 0.992 | 3.01E-09 | count | 1 |
| MMP24 | 18.8788944 | 1979.562451 | 0.0095 | 0.992 | 3.01E-09 | count | 1 |
| CYP3A43 | 18.8788944 | 1979.562485 | 0.0095 | 0.992 | 3.01E-09 | count | 1 |
| TRBV25-1 | 18.8788944 | 1979.562568 | 0.0095 | 0.992 | 3.01E-09 | count | 1 |
| SLC16A2 | 18.8788944 | 1979.562552 | 0.0095 | 0.992 | 3.01E-09 | count | 1 |
| GRIK4 | 18.8788944 | 1979.562485 | 0.0095 | 0.992 | 3.01E-09 | count | 1 |
| FAM90A1 | 18.8788944 | 1979.562568 | 0.0095 | 0.992 | 3.01E-09 | count | 1 |
| SPATA13-AS1 | 18.8788944 | 1979.562393 | 0.0095 | 0.992 | 3.01E-09 | count | 1 |
| AANAT | 18.8788945 | 1979.562493 | 0.0095 | 0.992 | 3.01E-09 | count | 1 |
| SERPINB2 | 18.8788944 | 1979.562485 | 0.0095 | 0.992 | 3.01E-09 | count | 1 |
| LEFTY1 | 18.8788944 | 1979.562401 | 0.0095 | 0.992 | 3.01E-09 | count | 1 |
| CLDN1 | 18.8788945 | 1979.562543 | 0.0095 | 0.992 | 3.01E-09 | count | 1 |
| AC010275.1 | 18.8788945 | 1979.562543 | 0.0095 | 0.992 | 3.01E-09 | count | 1 |
| AMZ1 | 18.8788944 | 1979.562568 | 0.0095 | 0.992 | 3.01E-09 | count | 1 |
| TRBV12-4 | 18.8788944 | 1979.562468 | 0.0095 | 0.992 | 3.01E-09 | count | 1 |
| AC084024.1 | 18.8788944 | 1979.562568 | 0.0095 | 0.992 | 3.01E-09 | count | 1 |
| AL133410.2 | 18.8788944 | 1979.562401 | 0.0095 | 0.992 | 3.01E-09 | count | 1 |
| AC125807.2 | 18.8788945 | 1979.562543 | 0.0095 | 0.992 | 3.01E-09 | count | 1 |
| AC079035.1 | 18.8788944 | 1979.562401 | 0.0095 | 0.992 | 3.01E-09 | count | 1 |
| PIWIL1 | 18.8788944 | 1979.562568 | 0.0095 | 0.992 | 3.01E-09 | count | 1 |
| AC005520.1 | 18.8788944 | 1979.562401 | 0.0095 | 0.992 | 3.01E-09 | count | 1 |
| CRYBA4 | 18.8788945 | 1979.562418 | 0.0095 | 0.992 | 3.01E-09 | count | 1 |
| MDH1B | 18.8788944 | 1979.562435 | 0.0095 | 0.992 | 3.01E-09 | count | 1 |
| AL365226.1 | 18.8788944 | 1979.562527 | 0.0095 | 0.992 | 3.01E-09 | count | 1 |
| HOXA7 | 18.8788944 | 1979.562527 | 0.0095 | 0.992 | 3.01E-09 | count | 1 |
| HOXA9 | 18.8788944 | 1979.562527 | 0.0095 | 0.992 | 3.01E-09 | count | 1 |
| AL359076.1 | 18.8788944 | 1979.562527 | 0.0095 | 0.992 | 3.01E-09 | count | 1 |
| AL355512.1 | 18.8788944 | 1979.562518 | 0.0095 | 0.992 | 3.01E-09 | count | 1 |
| C12orf56 | 18.8788943 | 1979.562401 | 0.0095 | 0.992 | 3.01E-09 | count | 1 |
| AC134669.1 | 18.8788943 | 1979.562476 | 0.0095 | 0.992 | 3.01E-09 | count | 1 |
| REEP1 | 18.8788943 | 1979.562468 | 0.0095 | 0.992 | 3.01E-09 | count | 1 |
| ARPC4-TTLL3 | 18.8788944 | 1979.562518 | 0.0095 | 0.992 | 3.01E-09 | count | 1 |
| GAL3ST4 | 18.8788944 | 1979.562585 | 0.0095 | 0.992 | 3.01E-09 | count | 1 |
| HBA2 | 18.8788944 | 1979.562518 | 0.0095 | 0.992 | 3.01E-09 | count | 1 |
| AC098483.1 | 18.8788944 | 1979.562435 | 0.0095 | 0.992 | 3.01E-09 | count | 1 |

| | | | | | | | |
|---|---|---|---|---|---|---|---|
| EML6 | 18.8788944 | 1979.562435 | 0.0095 | 0.992 | 3.01E-09 | count | 1 |
| AC007620.3 | 18.8788941 | 1979.562393 | 0.0095 | 0.992 | 3.01E-09 | count | 1 |
| DLG1-AS1 | 18.8788944 | 1979.562602 | 0.0095 | 0.992 | 3.01E-09 | count | 1 |
| C6orf132 | 18.8788944 | 1979.562435 | 0.0095 | 0.992 | 3.01E-09 | count | 1 |
| UPK3BL1 | 18.8788944 | 1979.562435 | 0.0095 | 0.992 | 3.01E-09 | count | 1 |
| ANTXRL | 18.8788943 | 1979.562493 | 0.0095 | 0.992 | 3.01E-09 | count | 1 |
| SPERT | 18.8788944 | 1979.562435 | 0.0095 | 0.992 | 3.01E-09 | count | 1 |
| MMP14 | 18.8788944 | 1979.562435 | 0.0095 | 0.992 | 3.01E-09 | count | 1 |
| AL138995.1 | 18.8788944 | 1979.562435 | 0.0095 | 0.992 | 3.01E-09 | count | 1 |
| PLEKHD1 | 18.8788944 | 1979.562435 | 0.0095 | 0.992 | 3.01E-09 | count | 1 |
| PLA2G4D | 18.8788944 | 1979.562435 | 0.0095 | 0.992 | 3.01E-09 | count | 1 |
| ANKS4B | 18.8788944 | 1979.562435 | 0.0095 | 0.992 | 3.01E-09 | count | 1 |
| AC090912.2 | 18.8788941 | 1979.562393 | 0.0095 | 0.992 | 3.01E-09 | count | 1 |
| CYP24A1 | 18.8788944 | 1979.562435 | 0.0095 | 0.992 | 3.01E-09 | count | 1 |
| AP000350.7 | 18.8788944 | 1979.562435 | 0.0095 | 0.992 | 3.01E-09 | count | 1 |
| AC100793.3 | 18.8788941 | 1979.562401 | 0.0095 | 0.992 | 3.01E-09 | count | 1 |
| TSSK3 | 18.8788943 | 1979.562476 | 0.0095 | 0.992 | 3.01E-09 | count | 1 |
| AL513548.3 | 18.8788943 | 1979.562476 | 0.0095 | 0.992 | 3.01E-09 | count | 1 |
| AP002336.2 | 18.8788941 | 1979.562334 | 0.0095 | 0.992 | 3.01E-09 | count | 1 |
| PRPH | 18.8788943 | 1979.562476 | 0.0095 | 0.992 | 3.01E-09 | count | 1 |
| SLC28A2 | 18.8788943 | 1979.562476 | 0.0095 | 0.992 | 3.01E-09 | count | 1 |
| AC130456.5 | 18.8788943 | 1979.562476 | 0.0095 | 0.992 | 3.01E-09 | count | 1 |
| PLIN4 | 18.8788943 | 1979.562476 | 0.0095 | 0.992 | 3.01E-09 | count | 1 |
| MPZL2 | 0.1082136 | 0.7601638 | 0.1424 | 0.887 | 3.31E-09 | count | 1 |
| AC010616.1 | 0.1125283 | 0.7759424 | 0.145 | 0.885 | 3.44E-09 | count | 1 |
| COLEC12 | 0.1125283 | 0.7911835 | 0.1422 | 0.887 | 3.44E-09 | count | 1 |
| ZCCHC24 | 0.1125283 | 0.6377054 | 0.1765 | 0.86 | 3.44E-09 | count | 1 |
| PJVK | 0.1125283 | 0.9158991 | 0.1229 | 0.902 | 3.44E-09 | count | 1 |
| AC068888.1 | 0.125121 | 0.6735749 | 0.1858 | 0.853 | 3.84E-09 | count | 1 |
| ZNF711 | 0.3313571 | 0.7778444 | 0.426 | 0.67 | 3.93E-09 | count | 1 |
| MED12L | 0.3313571 | 1.064632 | 0.3112 | 0.756 | 3.93E-09 | count | 1 |
| PSD3 | 0.3313571 | 0.9066221 | 0.3655 | 0.715 | 3.93E-09 | count | 1 |
| LRG1 | 0.3313571 | 0.816226 | 0.406 | 0.685 | 3.93E-09 | count | 1 |
| AP001065.1 | 0.3313571 | 0.816226 | 0.406 | 0.685 | 3.93E-09 | count | 1 |
| RGS18 | 0.048431 | 0.4584068 | 0.1057 | 0.916 | 3.96E-09 | count | 1 |
| OLFML1 | 0.830677 | 0.7216028 | 1.1512 | 0.25 | 4.02E-09 | count | 1 |
| MYO3B | 0.830677 | 0.5235427 | 1.5866 | 0.113 | 4.02E-09 | count | 1 |
| AL359636.1 | 0.830677 | 0.5235427 | 1.5866 | 0.113 | 4.02E-09 | count | 1 |
| KIFC1 | 0.830677 | 0.637079 | 1.3039 | 0.192 | 4.02E-09 | count | 1 |
| HIST3H2BB | 0.0497532 | 0.6566862 | 0.0758 | 0.94 | 4.07E-09 | count | 1 |
| BBS5 | 0.0497532 | 0.6258366 | 0.0795 | 0.937 | 4.07E-09 | count | 1 |
| LAMB1 | 0.0497532 | 0.6542877 | 0.076 | 0.939 | 4.07E-09 | count | 1 |
| ADAM12 | 0.1335207 | 0.5208206 | 0.2564 | 0.798 | 4.11E-09 | count | 1 |
| RNF175 | 0.1513917 | 0.7660959 | 0.1976 | 0.843 | 4.68E-09 | count | 1 |
| SOAT2 | 0.4367177 | 0.859336 | 0.5082 | 0.611 | 5.31E-09 | count | 1 |

| | | | | | | | |
|---|---|---|---|---|---|---|---|
| AL356417.1 | 0.4367177 | 0.8626435 | 0.5063 | 0.613 | 5.31E-09 | count | 1 |
| AL928654.3 | 0.4367177 | 0.8626435 | 0.5063 | 0.613 | 5.31E-09 | count | 1 |
| WASF1 | 0.1755519 | 1.1529744 | 0.1523 | 0.879 | 5.45E-09 | count | 1 |
| PTK6 | 0.1755519 | 1.1391237 | 0.1541 | 0.878 | 5.45E-09 | count | 1 |
| AC020928.2 | 0.1755519 | 1.0401876 | 0.1688 | 0.866 | 5.45E-09 | count | 1 |
| AC106795.2 | 0.1755519 | 1.1391237 | 0.1541 | 0.878 | 5.45E-09 | count | 1 |
| AC007786.1 | 0.1755519 | 1.1391237 | 0.1541 | 0.878 | 5.45E-09 | count | 1 |
| AL513320.1 | 0.1755519 | 1.1391237 | 0.1541 | 0.878 | 5.45E-09 | count | 1 |
| FLJ45513 | 0.1755519 | 1.1391237 | 0.1541 | 0.878 | 5.45E-09 | count | 1 |
| TTYH2 | 0.1755519 | 0.6363544 | 0.2759 | 0.783 | 5.45E-09 | count | 1 |
| AC012557.2 | 0.1755519 | 1.0401876 | 0.1688 | 0.866 | 5.45E-09 | count | 1 |
| MIR193BHG | 0.1755519 | 1.1391237 | 0.1541 | 0.878 | 5.45E-09 | count | 1 |
| KANK2 | 0.1755519 | 1.1391237 | 0.1541 | 0.878 | 5.45E-09 | count | 1 |
| FOXC2 | 0.1755519 | 1.1391237 | 0.1541 | 0.878 | 5.45E-09 | count | 1 |
| AP000911.1 | 0.1755519 | 0.7742097 | 0.2267 | 0.821 | 5.45E-09 | count | 1 |
| AC240565.1 | 0.1755519 | 1.2583036 | 0.1395 | 0.889 | 5.45E-09 | count | 1 |
| AP002884.4 | 0.1755519 | 1.2983581 | 0.1352 | 0.892 | 5.45E-09 | count | 1 |
| THBS3 | 0.1755519 | 0.733728 | 0.2393 | 0.811 | 5.45E-09 | count | 1 |
| TCERG1L | 0.1755519 | 1.2983581 | 0.1352 | 0.892 | 5.45E-09 | count | 1 |
| EPHX1 | 0.1755519 | 0.6713143 | 0.2615 | 0.794 | 5.45E-09 | count | 1 |
| FAM85B | 0.1755519 | 0.9036964 | 0.1943 | 0.846 | 5.45E-09 | count | 1 |
| ZNF843 | 0.1755519 | 1.2983581 | 0.1352 | 0.892 | 5.45E-09 | count | 1 |
| C19orf38 | 0.1755519 | 1.2983581 | 0.1352 | 0.892 | 5.45E-09 | count | 1 |
| AC011468.5 | 0.1755519 | 0.7652564 | 0.2294 | 0.819 | 5.45E-09 | count | 1 |
| CDKN2B-AS1 | 0.1755519 | 0.9036964 | 0.1943 | 0.846 | 5.45E-09 | count | 1 |
| PLEKHH3 | 0.1755519 | 1.2983581 | 0.1352 | 0.892 | 5.45E-09 | count | 1 |
| DLX4 | 0.1755519 | 1.2983581 | 0.1352 | 0.892 | 5.45E-09 | count | 1 |
| AC011611.4 | 0.1755519 | 0.9036964 | 0.1943 | 0.846 | 5.45E-09 | count | 1 |
| TAF1A-AS1 | 0.1755519 | 0.733728 | 0.2393 | 0.811 | 5.45E-09 | count | 1 |
| LGALSL | 0.1755519 | 0.8894188 | 0.1974 | 0.844 | 5.45E-09 | count | 1 |
| BEX1 | 0.1755519 | 1.2583036 | 0.1395 | 0.889 | 5.45E-09 | count | 1 |
| MEG8 | 0.1755519 | 0.9036964 | 0.1943 | 0.846 | 5.45E-09 | count | 1 |
| CCL27 | 0.1755519 | 1.2583036 | 0.1395 | 0.889 | 5.45E-09 | count | 1 |
| LINC01891 | 0.1755519 | 0.6484597 | 0.2707 | 0.787 | 5.45E-09 | count | 1 |
| AL391988.1 | 0.1755519 | 1.2983581 | 0.1352 | 0.892 | 5.45E-09 | count | 1 |
| AC021097.1 | 0.1755519 | 0.917752 | 0.1913 | 0.848 | 5.45E-09 | count | 1 |
| AC137630.2 | 0.1755519 | 0.8156873 | 0.2152 | 0.83 | 5.45E-09 | count | 1 |
| AC006252.1 | 0.1755519 | 0.6828001 | 0.2571 | 0.797 | 5.45E-09 | count | 1 |
| GNG8 | 0.1755519 | 1.2025024 | 0.146 | 0.884 | 5.45E-09 | count | 1 |
| LINC01840 | 0.1755519 | 1.2025024 | 0.146 | 0.884 | 5.46E-09 | count | 1 |
| AL355490.2 | 0.1755519 | 0.9528616 | 0.1842 | 0.854 | 5.46E-09 | count | 1 |
| ZDHHC11B | 0.1755519 | 1.1688049 | 0.1502 | 0.881 | 5.46E-09 | count | 1 |
| LMTK3 | 0.0676494 | 0.6263994 | 0.108 | 0.914 | 5.56E-09 | count | 1 |
| AC008892.1 | 1.1183591 | 0.4832668 | 2.3142 | 0.0207 | 5.67E-09 | count | 1 |
| AC090922.1 | 1.1183591 | 0.7388751 | 1.5136 | 0.13 | 5.68E-09 | count | 1 |

| | | | | | | | |
|---|---|---|---|---|---|---|---|
| FAM181B | 1.1183591 | 0.7388751 | 1.5136 | 0.13 | 5.68E-09 | count | 1 |
| AC108102.1 | 1.1183591 | 0.7388751 | 1.5136 | 0.13 | 5.68E-09 | count | 1 |
| CNNM3-DT | 1.1183591 | 0.7388751 | 1.5136 | 0.13 | 5.68E-09 | count | 1 |
| AC011405.1 | 1.1183591 | 0.7388751 | 1.5136 | 0.13 | 5.68E-09 | count | 1 |
| AC004494.1 | 1.1183591 | 0.7388751 | 1.5136 | 0.13 | 5.68E-09 | count | 1 |
| ZC3H11B | 1.1183591 | 0.7388751 | 1.5136 | 0.13 | 5.68E-09 | count | 1 |
| AL109797.1 | 1.1183591 | 0.7388751 | 1.5136 | 0.13 | 5.68E-09 | count | 1 |
| CDCA2 | 1.1183591 | 0.7388751 | 1.5136 | 0.13 | 5.68E-09 | count | 1 |
| SCML2 | 1.1183591 | 0.7388751 | 1.5136 | 0.13 | 5.68E-09 | count | 1 |
| AC022382.1 | 1.1183591 | 0.6252208 | 1.7887 | 0.0737 | 5.68E-09 | count | 1 |
| ZNF674 | 0.0748178 | 0.5713721 | 0.1309 | 0.896 | 6.16E-09 | count | 1 |
| SNX21 | 0.1997122 | 0.6975203 | 0.2863 | 0.775 | 6.24E-09 | count | 1 |
| TRBV7-2 | 0.2050957 | 0.9536775 | 0.2151 | 0.83 | 6.42E-09 | count | 1 |
| MEG3 | 0.2050957 | 0.8933257 | 0.2296 | 0.818 | 6.42E-09 | count | 1 |
| LINC02062 | 0.2050957 | 0.7381171 | 0.2779 | 0.781 | 6.42E-09 | count | 1 |
| AC004520.1 | 0.0810944 | 0.6001057 | 0.1351 | 0.893 | 6.69E-09 | count | 1 |
| PEAK3 | 1.3415026 | 0.4588419 | 2.9237 | 0.0035 | 7.01E-09 | count | 1 |
| AL137077.2 | 0.2259829 | 0.6691709 | 0.3377 | 0.736 | 7.11E-09 | count | 1 |
| AL031595.2 | 0.2259829 | 0.7676009 | 0.2944 | 0.768 | 7.11E-09 | count | 1 |
| BRIP1 | 0.2385756 | 0.8294022 | 0.2876 | 0.774 | 7.53E-09 | count | 1 |
| AL596094.1 | 0.2385756 | 0.8548314 | 0.2791 | 0.78 | 7.53E-09 | count | 1 |
| RND3 | 0.24572 | 0.6220715 | 0.395 | 0.693 | 7.76E-09 | count | 1 |
| ZNF417 | 0.24572 | 0.6785155 | 0.3621 | 0.717 | 7.76E-09 | count | 1 |
| CDHR1 | 0.6190392 | 0.7042205 | 0.879 | 0.379 | 7.82E-09 | count | 1 |
| LINC01842 | 0.6190392 | 0.848247 | 0.7298 | 0.466 | 7.82E-09 | count | 1 |
| AC020922.4 | 0.6190392 | 0.8997938 | 0.688 | 0.492 | 7.82E-09 | count | 1 |
| AC010834.2 | 0.6190392 | 0.8997938 | 0.688 | 0.492 | 7.82E-09 | count | 1 |
| DSCR9 | 0.6190392 | 0.8393481 | 0.7375 | 0.461 | 7.82E-09 | count | 1 |
| AC009630.2 | 0.6190392 | 0.8393481 | 0.7375 | 0.461 | 7.82E-09 | count | 1 |
| SMARCA5-AS1 | 0.6190392 | 0.8393481 | 0.7375 | 0.461 | 7.82E-09 | count | 1 |
| AC136475.2 | 0.6190392 | 0.8393481 | 0.7375 | 0.461 | 7.82E-09 | count | 1 |
| TRGV5 | 0.6190392 | 0.7741974 | 0.7996 | 0.424 | 7.82E-09 | count | 1 |
| MATN3 | 18.5800652 | 3606.96877 | 0.0052 | 0.996 | 8.08E-09 | count | 1 |
| AC018690.1 | 18.5800652 | 3606.96877 | 0.0052 | 0.996 | 8.08E-09 | count | 1 |
| TMEM212 | 18.5800652 | 3606.96877 | 0.0052 | 0.996 | 8.08E-09 | count | 1 |
| AC067838.1 | 18.5800651 | 3606.968485 | 0.0052 | 0.996 | 8.08E-09 | count | 1 |
| LINC02452 | 18.5800652 | 3606.968733 | 0.0052 | 0.996 | 8.08E-09 | count | 1 |
| ABCA6 | 18.5800652 | 3606.968733 | 0.0052 | 0.996 | 8.08E-09 | count | 1 |
| NOSTRIN | 18.5800652 | 3606.968733 | 0.0052 | 0.996 | 8.08E-09 | count | 1 |
| CEP83-DT | 18.5800651 | 3606.968311 | 0.0052 | 0.996 | 8.08E-09 | count | 1 |
| A1CF | 18.5800646 | 3606.968435 | 0.0052 | 0.996 | 8.08E-09 | count | 1 |
| AC113382.2 | 18.5800647 | 3606.968286 | 0.0052 | 0.996 | 8.08E-09 | count | 1 |
| AC136475.4 | 18.5800647 | 3606.968286 | 0.0052 | 0.996 | 8.08E-09 | count | 1 |
| OR51E1 | 18.5800647 | 3606.968286 | 0.0052 | 0.996 | 8.08E-09 | count | 1 |
| AC008747.1 | 18.5800647 | 3606.968286 | 0.0052 | 0.996 | 8.08E-09 | count | 1 |

| | | | | | | | |
|---|---|---|---|---|---|---|---|
| AL603832.2 | 18.5800649 | 3606.968708 | 0.0052 | 0.996 | 8.08E-09 | count | 1 |
| AL590068.2 | 18.5800647 | 3606.968497 | 0.0052 | 0.996 | 8.08E-09 | count | 1 |
| AC138932.5 | 18.5800648 | 3606.968584 | 0.0052 | 0.996 | 8.08E-09 | count | 1 |
| AL035252.2 | 18.5800648 | 3606.968584 | 0.0052 | 0.996 | 8.08E-09 | count | 1 |
| BEND7 | 18.5800646 | 3606.968584 | 0.0052 | 0.996 | 8.08E-09 | count | 1 |
| DMC1 | 18.5800647 | 3606.968497 | 0.0052 | 0.996 | 8.08E-09 | count | 1 |
| AL121890.5 | 18.5800646 | 3606.968509 | 0.0052 | 0.996 | 8.08E-09 | count | 1 |
| PRSS51 | 18.5800648 | 3606.968361 | 0.0052 | 0.996 | 8.08E-09 | count | 1 |
| AC008569.1 | 18.5800648 | 3606.968361 | 0.0052 | 0.996 | 8.08E-09 | count | 1 |
| AC093827.5 | 18.5800644 | 3606.968485 | 0.0052 | 0.996 | 8.08E-09 | count | 1 |
| AC103923.1 | 18.5795359 | 2686.633299 | 0.0069 | 0.994 | 8.08E-09 | count | 1 |
| AC112220.2 | 18.5795357 | 2686.633058 | 0.0069 | 0.994 | 8.08E-09 | count | 1 |
| FHDC1 | 18.5795355 | 2686.633215 | 0.0069 | 0.994 | 8.08E-09 | count | 1 |
| PLGLB2 | 18.5795354 | 2686.633151 | 0.0069 | 0.994 | 8.08E-09 | count | 1 |
| AC009237.14 | 18.5795355 | 2686.633197 | 0.0069 | 0.994 | 8.08E-09 | count | 1 |
| CRMP1 | 18.5795353 | 2686.633188 | 0.0069 | 0.994 | 8.08E-09 | count | 1 |
| LINC02288 | 18.5795357 | 2686.633197 | 0.0069 | 0.994 | 8.08E-09 | count | 1 |
| RAB26 | 18.5795354 | 2686.633114 | 0.0069 | 0.994 | 8.08E-09 | count | 1 |
| ZNF660 | 18.5795352 | 2686.632892 | 0.0069 | 0.994 | 8.08E-09 | count | 1 |
| ARTN | 18.5795353 | 2686.633179 | 0.0069 | 0.994 | 8.08E-09 | count | 1 |
| PITX1 | 18.5795352 | 2686.632902 | 0.0069 | 0.994 | 8.08E-09 | count | 1 |
| KLHL31 | 18.5795355 | 2686.633243 | 0.0069 | 0.994 | 8.08E-09 | count | 1 |
| AC092849.1 | 18.579535 | 2686.633234 | 0.0069 | 0.994 | 8.08E-09 | count | 1 |
| AC080013.5 | 18.5795353 | 2686.632938 | 0.0069 | 0.994 | 8.08E-09 | count | 1 |
| AC097460.1 | 18.5795351 | 2686.632975 | 0.0069 | 0.994 | 8.08E-09 | count | 1 |
| RPS6KA6 | 18.5795351 | 2686.633114 | 0.0069 | 0.994 | 8.08E-09 | count | 1 |
| AC121338.2 | 18.5795351 | 2686.63292 | 0.0069 | 0.994 | 8.08E-09 | count | 1 |
| AL136115.1 | 18.5795353 | 2686.633068 | 0.0069 | 0.994 | 8.08E-09 | count | 1 |
| NKAPL | 18.5795348 | 2686.632966 | 0.0069 | 0.994 | 8.08E-09 | count | 1 |
| C16orf71 | 18.5795348 | 2686.632975 | 0.0069 | 0.994 | 8.08E-09 | count | 1 |
| LINC01694 | 18.5795349 | 2686.63316 | 0.0069 | 0.994 | 8.08E-09 | count | 1 |
| MIA-RAB4B | 18.5795348 | 2686.632966 | 0.0069 | 0.994 | 8.08E-09 | count | 1 |
| AC009119.2 | 18.579535 | 2686.633012 | 0.0069 | 0.994 | 8.08E-09 | count | 1 |
| P2RX5-TAX1BP3 | 18.5791616 | 2079.353677 | 0.0089 | 0.993 | 8.09E-09 | count | 1 |
| BX539320.1 | 18.5791616 | 2079.353849 | 0.0089 | 0.993 | 8.09E-09 | count | 1 |
| AC126614.1 | 18.5791613 | 2079.353685 | 0.0089 | 0.993 | 8.09E-09 | count | 1 |
| AL359834.1 | 18.5791612 | 2079.353913 | 0.0089 | 0.993 | 8.09E-09 | count | 1 |
| AC010538.1 | 18.1734474 | 2943.402224 | 0.0062 | 0.995 | 8.09E-09 | count | 1 |
| SCN5A | 18.1734473 | 2943.402143 | 0.0062 | 0.995 | 8.09E-09 | count | 1 |
| PRRT4 | 18.1734473 | 2943.402062 | 0.0062 | 0.995 | 8.09E-09 | count | 1 |
| C10orf105 | 18.1734473 | 2943.402062 | 0.0062 | 0.995 | 8.09E-09 | count | 1 |
| AC008763.1 | 18.1734472 | 2943.402203 | 0.0062 | 0.995 | 8.09E-09 | count | 1 |
| LINC02269 | 18.1734471 | 2943.402143 | 0.0062 | 0.995 | 8.09E-09 | count | 1 |
| NEUROG3 | 18.1734472 | 2943.402133 | 0.0062 | 0.995 | 8.09E-09 | count | 1 |
| RAD9B | 18.1734472 | 2943.402173 | 0.0062 | 0.995 | 8.09E-09 | count | 1 |

| | | | | | | | |
|---|---|---|---|---|---|---|---|
| C20orf203 | 18.1734471 | 2943.402153 | 0.0062 | 0.995 | 8.09E-09 | count | 1 |
| AL445490.1 | 18.1734471 | 2943.402102 | 0.0062 | 0.995 | 8.09E-09 | count | 1 |
| AL449106.1 | 18.1734472 | 2943.402214 | 0.0062 | 0.995 | 8.09E-09 | count | 1 |
| AC138356.1 | 18.1734471 | 2943.402102 | 0.0062 | 0.995 | 8.09E-09 | count | 1 |
| LINC00958 | 18.1734471 | 2943.402355 | 0.0062 | 0.995 | 8.09E-09 | count | 1 |
| KCNIP2 | 18.1734472 | 2943.402143 | 0.0062 | 0.995 | 8.09E-09 | count | 1 |
| TRAV1-1 | 18.173447 | 2943.402173 | 0.0062 | 0.995 | 8.09E-09 | count | 1 |
| CCDC85A | 18.1734472 | 2943.402183 | 0.0062 | 0.995 | 8.09E-09 | count | 1 |
| AC073046.1 | 18.1734471 | 2943.402163 | 0.0062 | 0.995 | 8.09E-09 | count | 1 |
| AP001999.1 | 18.1734471 | 2943.402032 | 0.0062 | 0.995 | 8.09E-09 | count | 1 |
| KIAA1217 | 18.1734471 | 2943.402082 | 0.0062 | 0.995 | 8.09E-09 | count | 1 |
| IQSEC3 | 18.1734471 | 2943.402193 | 0.0062 | 0.995 | 8.09E-09 | count | 1 |
| ATP2C2 | 18.1734471 | 2943.402072 | 0.0062 | 0.995 | 8.09E-09 | count | 1 |
| ZNF559-ZNF177 | 18.1734472 | 2943.402274 | 0.0062 | 0.995 | 8.09E-09 | count | 1 |
| AC010643.1 | 18.1734471 | 2943.402173 | 0.0062 | 0.995 | 8.09E-09 | count | 1 |
| TMEM150C | 18.1734471 | 2943.401961 | 0.0062 | 0.995 | 8.09E-09 | count | 1 |
| CRIP3 | 18.1734471 | 2943.401981 | 0.0062 | 0.995 | 8.09E-09 | count | 1 |
| TAC1 | 18.1734471 | 2943.402062 | 0.0062 | 0.995 | 8.09E-09 | count | 1 |
| AC244197.3 | 18.173447 | 2943.402284 | 0.0062 | 0.995 | 8.09E-09 | count | 1 |
| AP003472.2 | 18.1734471 | 2943.402052 | 0.0062 | 0.995 | 8.09E-09 | count | 1 |
| KC877392.1 | 18.1734471 | 2943.401981 | 0.0062 | 0.995 | 8.09E-09 | count | 1 |
| MYRIP | 18.173447 | 2943.402052 | 0.0062 | 0.995 | 8.09E-09 | count | 1 |
| TRGV11 | 18.1734468 | 2943.402193 | 0.0062 | 0.995 | 8.09E-09 | count | 1 |
| MIR503HG | 18.173447 | 2943.402052 | 0.0062 | 0.995 | 8.09E-09 | count | 1 |
| PKP2 | 18.1734469 | 2943.402234 | 0.0062 | 0.995 | 8.09E-09 | count | 1 |
| WNT1 | 18.173447 | 2943.402001 | 0.0062 | 0.995 | 8.09E-09 | count | 1 |
| LINC02404 | 18.1734471 | 2943.401991 | 0.0062 | 0.995 | 8.09E-09 | count | 1 |
| AC068831.6 | 18.173447 | 2943.402092 | 0.0062 | 0.995 | 8.09E-09 | count | 1 |
| AC126763.1 | 18.1734468 | 2943.402193 | 0.0062 | 0.995 | 8.09E-09 | count | 1 |
| AC010319.3 | 18.1734468 | 2943.402193 | 0.0062 | 0.995 | 8.09E-09 | count | 1 |
| AC011043.1 | 18.173447 | 2943.402001 | 0.0062 | 0.995 | 8.09E-09 | count | 1 |
| PIK3CD-AS1 | 18.173447 | 2943.402214 | 0.0062 | 0.995 | 8.09E-09 | count | 1 |
| AL451070.1 | 18.173447 | 2943.402305 | 0.0062 | 0.995 | 8.09E-09 | count | 1 |
| AC126283.1 | 18.1734471 | 2943.402042 | 0.0062 | 0.995 | 8.09E-09 | count | 1 |
| TCEAL6 | 18.1734469 | 2943.402214 | 0.0062 | 0.995 | 8.09E-09 | count | 1 |
| TUB | 18.173447 | 2943.402193 | 0.0062 | 0.995 | 8.09E-09 | count | 1 |
| AC008149.1 | 18.173447 | 2943.402203 | 0.0062 | 0.995 | 8.09E-09 | count | 1 |
| TRAV34 | 18.173447 | 2943.402082 | 0.0062 | 0.995 | 8.09E-09 | count | 1 |
| AC005329.1 | 18.1734471 | 2943.401981 | 0.0062 | 0.995 | 8.09E-09 | count | 1 |
| AL022313.2 | 18.173447 | 2943.402153 | 0.0062 | 0.995 | 8.09E-09 | count | 1 |
| AL157402.1 | 18.1734469 | 2943.402123 | 0.0062 | 0.995 | 8.09E-09 | count | 1 |
| CPO | 18.1734471 | 2943.402153 | 0.0062 | 0.995 | 8.09E-09 | count | 1 |
| SUSD5 | 18.1734469 | 2943.402315 | 0.0062 | 0.995 | 8.09E-09 | count | 1 |
| GRM2 | 18.173447 | 2943.402345 | 0.0062 | 0.995 | 8.09E-09 | count | 1 |
| CDC20B | 18.173447 | 2943.402345 | 0.0062 | 0.995 | 8.09E-09 | count | 1 |

| | | | | | | | |
|---|---|---|---|---|---|---|---|
| AL138720.1 | 18.1734469 | 2943.402123 | 0.0062 | 0.995 | 8.09E-09 | count | 1 |
| DSCAML1 | 18.1734469 | 2943.402224 | 0.0062 | 0.995 | 8.09E-09 | count | 1 |
| NEBL | 18.1734469 | 2943.402315 | 0.0062 | 0.995 | 8.09E-09 | count | 1 |
| B3GNT4 | 18.173447 | 2943.402345 | 0.0062 | 0.995 | 8.09E-09 | count | 1 |
| ASPHD1 | 18.173447 | 2943.402315 | 0.0062 | 0.995 | 8.09E-09 | count | 1 |
| AC092145.1 | 18.173447 | 2943.402193 | 0.0062 | 0.995 | 8.09E-09 | count | 1 |
| CASKIN2 | 18.1734471 | 2943.402153 | 0.0062 | 0.995 | 8.09E-09 | count | 1 |
| AC020908.3 | 18.1734469 | 2943.402224 | 0.0062 | 0.995 | 8.09E-09 | count | 1 |
| TRGV6 | 18.173447 | 2943.402102 | 0.0062 | 0.995 | 8.09E-09 | count | 1 |
| CELF4 | 18.173447 | 2943.402163 | 0.0062 | 0.995 | 8.09E-09 | count | 1 |
| NUP210L | 18.1734469 | 2943.402214 | 0.0062 | 0.995 | 8.09E-09 | count | 1 |
| AC007250.1 | 18.1734468 | 2943.402082 | 0.0062 | 0.995 | 8.09E-09 | count | 1 |
| AL662890.1 | 18.1734469 | 2943.402214 | 0.0062 | 0.995 | 8.09E-09 | count | 1 |
| AC007349.2 | 18.1734469 | 2943.401971 | 0.0062 | 0.995 | 8.09E-09 | count | 1 |
| AC073335.2 | 18.1734468 | 2943.402183 | 0.0062 | 0.995 | 8.09E-09 | count | 1 |
| AL136141.1 | 18.1734467 | 2943.402032 | 0.0062 | 0.995 | 8.09E-09 | count | 1 |
| POC1B-GALNT4 | 18.1734468 | 2943.40193 | 0.0062 | 0.995 | 8.09E-09 | count | 1 |
| C15orf62 | 18.173447 | 2943.402102 | 0.0062 | 0.995 | 8.09E-09 | count | 1 |
| AC093525.7 | 18.173447 | 2943.402274 | 0.0062 | 0.995 | 8.09E-09 | count | 1 |
| AP001010.1 | 18.173447 | 2943.402163 | 0.0062 | 0.995 | 8.09E-09 | count | 1 |
| CBARP | 18.1734469 | 2943.402214 | 0.0062 | 0.995 | 8.09E-09 | count | 1 |
| AF127577.1 | 18.1734469 | 2943.402163 | 0.0062 | 0.995 | 8.09E-09 | count | 1 |
| C21orf91-OT1 | 18.1734468 | 2943.402001 | 0.0062 | 0.995 | 8.09E-09 | count | 1 |
| TRPM2-AS | 18.173447 | 2943.402173 | 0.0062 | 0.995 | 8.09E-09 | count | 1 |
| KCTD3 | 18.1734469 | 2943.402193 | 0.0062 | 0.995 | 8.09E-09 | count | 1 |
| AC068051.1 | 18.1734467 | 2943.40188 | 0.0062 | 0.995 | 8.09E-09 | count | 1 |
| AC131025.1 | 18.1734467 | 2943.402254 | 0.0062 | 0.995 | 8.09E-09 | count | 1 |
| AF131216.1 | 18.1734469 | 2943.402092 | 0.0062 | 0.995 | 8.09E-09 | count | 1 |
| PAEP | 18.1734468 | 2943.402062 | 0.0062 | 0.995 | 8.09E-09 | count | 1 |
| RBM20 | 18.173447 | 2943.402274 | 0.0062 | 0.995 | 8.09E-09 | count | 1 |
| TMEM63C | 18.1734469 | 2943.402193 | 0.0062 | 0.995 | 8.09E-09 | count | 1 |
| AC093525.3 | 18.1734468 | 2943.402123 | 0.0062 | 0.995 | 8.09E-09 | count | 1 |
| C19orf81 | 18.1734467 | 2943.40188 | 0.0062 | 0.995 | 8.09E-09 | count | 1 |
| AL023802.1 | 18.1734469 | 2943.402112 | 0.0062 | 0.995 | 8.09E-09 | count | 1 |
| SCO2 | 18.1734468 | 2943.402143 | 0.0062 | 0.995 | 8.09E-09 | count | 1 |
| TEX43 | 18.1734468 | 2943.402143 | 0.0062 | 0.995 | 8.09E-09 | count | 1 |
| HIST1H3G | 18.1734468 | 2943.401991 | 0.0062 | 0.995 | 8.09E-09 | count | 1 |
| KLHL33 | 18.1734468 | 2943.402032 | 0.0062 | 0.995 | 8.09E-09 | count | 1 |
| AL162311.3 | 18.1734468 | 2943.401991 | 0.0062 | 0.995 | 8.09E-09 | count | 1 |
| AC015922.4 | 18.1734469 | 2943.402001 | 0.0062 | 0.995 | 8.09E-09 | count | 1 |
| AC015849.3 | 18.1734467 | 2943.402032 | 0.0062 | 0.995 | 8.09E-09 | count | 1 |
| SRCIN1 | 18.1734469 | 2943.402042 | 0.0062 | 0.995 | 8.09E-09 | count | 1 |
| GPR52 | 18.1734468 | 2943.402092 | 0.0062 | 0.995 | 8.09E-09 | count | 1 |
| LMNB1-DT | 18.1734467 | 2943.402052 | 0.0062 | 0.995 | 8.09E-09 | count | 1 |
| DDX39B-AS1 | 18.1734469 | 2943.401961 | 0.0062 | 0.995 | 8.09E-09 | count | 1 |

| | | | | | | | |
|---|---|---|---|---|---|---|---|
| AC092171.2 | 18.1734467 | 2943.402052 | 0.0062 | 0.995 | 8.09E-09 | count | 1 |
| TBX20 | 18.1734469 | 2943.401961 | 0.0062 | 0.995 | 8.09E-09 | count | 1 |
| AL391834.1 | 18.1734468 | 2943.402032 | 0.0062 | 0.995 | 8.09E-09 | count | 1 |
| TJP2 | 18.1734468 | 2943.401941 | 0.0062 | 0.995 | 8.09E-09 | count | 1 |
| ST8SIA6-AS1 | 18.1734468 | 2943.402092 | 0.0062 | 0.995 | 8.09E-09 | count | 1 |
| BX248123.1 | 18.1734468 | 2943.402032 | 0.0062 | 0.995 | 8.09E-09 | count | 1 |
| AKAP3 | 18.1734467 | 2943.402052 | 0.0062 | 0.995 | 8.09E-09 | count | 1 |
| LINC02399 | 18.1734467 | 2943.402052 | 0.0062 | 0.995 | 8.09E-09 | count | 1 |
| TRAV8-5 | 18.1734467 | 2943.402052 | 0.0062 | 0.995 | 8.09E-09 | count | 1 |
| TRAJ28 | 18.1734467 | 2943.402052 | 0.0062 | 0.995 | 8.09E-09 | count | 1 |
| SEC23A-AS1 | 18.1734467 | 2943.401951 | 0.0062 | 0.995 | 8.09E-09 | count | 1 |
| NOXRED1 | 18.1734468 | 2943.402133 | 0.0062 | 0.995 | 8.09E-09 | count | 1 |
| CCDC9B | 18.1734468 | 2943.402173 | 0.0062 | 0.995 | 8.09E-09 | count | 1 |
| NKX2-2 | 18.1734467 | 2943.402032 | 0.0062 | 0.995 | 8.09E-09 | count | 1 |
| AC005625.1 | 18.1734467 | 2943.402224 | 0.0062 | 0.995 | 8.09E-09 | count | 1 |
| AL929472.2 | 18.1734467 | 2943.40193 | 0.0062 | 0.995 | 8.09E-09 | count | 1 |
| AC098934.4 | 18.1734467 | 2943.401961 | 0.0062 | 0.995 | 8.09E-09 | count | 1 |
| C2orf81 | 18.1734468 | 2943.402092 | 0.0062 | 0.995 | 8.09E-09 | count | 1 |
| ADGRV1 | 18.1734468 | 2943.402082 | 0.0062 | 0.995 | 8.09E-09 | count | 1 |
| AC091948.1 | 18.1734467 | 2943.402193 | 0.0062 | 0.995 | 8.09E-09 | count | 1 |
| AP003680.1 | 18.1734467 | 2943.402214 | 0.0062 | 0.995 | 8.09E-09 | count | 1 |
| FLRT2 | 18.1734467 | 2943.402193 | 0.0062 | 0.995 | 8.09E-09 | count | 1 |
| AC004233.3 | 18.1734467 | 2943.401971 | 0.0062 | 0.995 | 8.09E-09 | count | 1 |
| Z98752.4 | 18.1734467 | 2943.40192 | 0.0062 | 0.995 | 8.09E-09 | count | 1 |
| TENT5B | 18.1734467 | 2943.402123 | 0.0062 | 0.995 | 8.09E-09 | count | 1 |
| LRFN2 | 18.1734468 | 2943.402234 | 0.0062 | 0.995 | 8.09E-09 | count | 1 |
| SOD2-OT1 | 18.1734467 | 2943.402032 | 0.0062 | 0.995 | 8.09E-09 | count | 1 |
| AC012213.4 | 18.1734467 | 2943.402021 | 0.0062 | 0.995 | 8.09E-09 | count | 1 |
| LGR4 | 18.1734468 | 2943.402214 | 0.0062 | 0.995 | 8.09E-09 | count | 1 |
| FAM180B | 18.1734468 | 2943.402214 | 0.0062 | 0.995 | 8.09E-09 | count | 1 |
| AL360181.2 | 18.1734467 | 2943.402021 | 0.0062 | 0.995 | 8.09E-09 | count | 1 |
| ATCAY | 18.1734467 | 2943.401941 | 0.0062 | 0.995 | 8.09E-09 | count | 1 |
| NECTIN3-AS1 | 18.1734467 | 2943.402112 | 0.0062 | 0.995 | 8.09E-09 | count | 1 |
| AC073073.2 | 18.1734466 | 2943.402092 | 0.0062 | 0.995 | 8.09E-09 | count | 1 |
| AC022167.4 | 18.1734466 | 2943.402062 | 0.0062 | 0.995 | 8.09E-09 | count | 1 |
| AP005131.1 | 18.1734466 | 2943.402153 | 0.0062 | 0.995 | 8.09E-09 | count | 1 |
| NRIR | 18.1734466 | 2943.402153 | 0.0062 | 0.995 | 8.09E-09 | count | 1 |
| AC093915.1 | 18.1734467 | 2943.402153 | 0.0062 | 0.995 | 8.09E-09 | count | 1 |
| RNF152 | 18.1734466 | 2943.401941 | 0.0062 | 0.995 | 8.09E-09 | count | 1 |
| AL445231.1 | 18.1734467 | 2943.402173 | 0.0062 | 0.995 | 8.09E-09 | count | 1 |
| AC093274.1 | 18.1734467 | 2943.402112 | 0.0062 | 0.995 | 8.09E-09 | count | 1 |
| AC106038.1 | 18.1734467 | 2943.402112 | 0.0062 | 0.995 | 8.09E-09 | count | 1 |
| CFAP157 | 18.1734466 | 2943.402052 | 0.0062 | 0.995 | 8.09E-09 | count | 1 |
| TMEM262 | 18.1734466 | 2943.402052 | 0.0062 | 0.995 | 8.09E-09 | count | 1 |
| AL589986.2 | 18.1734466 | 2943.402102 | 0.0062 | 0.995 | 8.09E-09 | count | 1 |

| | | | | | | | |
|---|---|---|---|---|---|---|---|
| AC113361.1 | 18.1734467 | 2943.402062 | 0.0062 | 0.995 | 8.09E-09 | count | 1 |
| RAB39A | 18.1734467 | 2943.402153 | 0.0062 | 0.995 | 8.09E-09 | count | 1 |
| RASSF8-AS1 | 18.1734466 | 2943.401991 | 0.0062 | 0.995 | 8.09E-09 | count | 1 |
| AC084855.1 | 18.1734467 | 2943.402153 | 0.0062 | 0.995 | 8.09E-09 | count | 1 |
| IGKV1D-12 | 18.1734467 | 2943.402072 | 0.0062 | 0.995 | 8.09E-09 | count | 1 |
| CSPG5 | 18.1734467 | 2943.402072 | 0.0062 | 0.995 | 8.09E-09 | count | 1 |
| C4orf47 | 18.1734466 | 2943.401961 | 0.0062 | 0.995 | 8.09E-09 | count | 1 |
| AMOTL1 | 18.1734466 | 2943.401961 | 0.0062 | 0.995 | 8.09E-09 | count | 1 |
| AP001781.2 | 18.1734466 | 2943.402062 | 0.0062 | 0.995 | 8.09E-09 | count | 1 |
| AL136418.1 | 18.1734466 | 2943.402062 | 0.0062 | 0.995 | 8.09E-09 | count | 1 |
| OR1A1 | 18.1734466 | 2943.402001 | 0.0062 | 0.995 | 8.09E-09 | count | 1 |
| GTSE1-DT | 18.1734465 | 2943.401941 | 0.0062 | 0.995 | 8.09E-09 | count | 1 |
| CEL | 18.1734464 | 2943.402042 | 0.0062 | 0.995 | 8.09E-09 | count | 1 |
| DLGAP1 | 18.1730554 | 2079.797662 | 0.0087 | 0.993 | 8.09E-09 | count | 1 |
| AC073352.2 | 18.1730553 | 2079.797605 | 0.0087 | 0.993 | 8.09E-09 | count | 1 |
| LINC01535 | 18.1730553 | 2079.797741 | 0.0087 | 0.993 | 8.09E-09 | count | 1 |
| ATP6V1G3 | 18.1730553 | 2079.797598 | 0.0087 | 0.993 | 8.09E-09 | count | 1 |
| TBC1D3D | 18.1730552 | 2079.797677 | 0.0087 | 0.993 | 8.09E-09 | count | 1 |
| IGKV1-6 | 18.1730551 | 2079.797562 | 0.0087 | 0.993 | 8.09E-09 | count | 1 |
| KCNK15 | 18.1730552 | 2079.797734 | 0.0087 | 0.993 | 8.09E-09 | count | 1 |
| PRRG3 | 18.173055 | 2079.797634 | 0.0087 | 0.993 | 8.09E-09 | count | 1 |
| AL136982.7 | 18.1730551 | 2079.79762 | 0.0087 | 0.993 | 8.09E-09 | count | 1 |
| AL121983.1 | 18.173055 | 2079.797598 | 0.0087 | 0.993 | 8.09E-09 | count | 1 |
| CPNE9 | 18.1730551 | 2079.797584 | 0.0087 | 0.993 | 8.09E-09 | count | 1 |
| RPS6KB2-AS1 | 18.1730552 | 2079.797662 | 0.0087 | 0.993 | 8.09E-09 | count | 1 |
| TSNAXIP1 | 18.1730552 | 2079.797491 | 0.0087 | 0.993 | 8.09E-09 | count | 1 |
| LINC02055 | 18.1730552 | 2079.797684 | 0.0087 | 0.993 | 8.09E-09 | count | 1 |
| AL354710.2 | 18.1730551 | 2079.797627 | 0.0087 | 0.993 | 8.09E-09 | count | 1 |
| C10orf25 | 18.1730549 | 2079.797512 | 0.0087 | 0.993 | 8.09E-09 | count | 1 |
| PYCR1 | 18.1730552 | 2079.797655 | 0.0087 | 0.993 | 8.09E-09 | count | 1 |
| FAM209A | 18.1730549 | 2079.797698 | 0.0087 | 0.993 | 8.09E-09 | count | 1 |
| CCDC78 | 18.1730548 | 2079.797591 | 0.0087 | 0.993 | 8.09E-09 | count | 1 |
| ARL11 | 18.1730549 | 2079.797548 | 0.0087 | 0.993 | 8.09E-09 | count | 1 |
| SEMA3B | 18.1730547 | 2079.797627 | 0.0087 | 0.993 | 8.09E-09 | count | 1 |
| AC091959.3 | 0.0979023 | 0.5014958 | 0.1952 | 0.845 | 8.11E-09 | count | 1 |
| AC127070.2 | 1.5238242 | 0.7891092 | 1.9311 | 0.0536 | 8.12E-09 | count | 1 |
| AIPL1 | 19.2745975 | 2412.653984 | 0.008 | 0.994 | 8.12E-09 | count | 1 |
| AC069287.2 | 19.2745976 | 2412.653818 | 0.008 | 0.994 | 8.12E-09 | count | 1 |
| AC131011.1 | 19.2745971 | 2412.653833 | 0.008 | 0.994 | 8.12E-09 | count | 1 |
| NRG4 | 1.5238242 | 0.6625313 | 2.3 | 0.0215 | 8.12E-09 | count | 1 |
| DSG3 | 1.5238242 | 0.6625313 | 2.3 | 0.0215 | 8.12E-09 | count | 1 |
| AC013468.1 | 1.5238242 | 0.6625313 | 2.3 | 0.0215 | 8.12E-09 | count | 1 |
| LRRN4CL | 1.5238242 | 0.5891307 | 2.5866 | 0.0097 | 8.13E-09 | count | 1 |
| CFAP97D2 | 19.2715705 | 1471.69177 | 0.0131 | 0.99 | 8.14E-09 | count | 1 |
| CDON | 18.9840485 | 2086.426243 | 0.0091 | 0.993 | 8.14E-09 | count | 1 |

| | | | | | | | |
|---|---|---|---|---|---|---|---|
| AC024075.3 | 18.9840484 | 2086.426158 | 0.0091 | 0.993 | 8.14E-09 | count | 1 |
| LBX2-AS1 | 18.9840482 | 2086.426021 | 0.0091 | 0.993 | 8.14E-09 | count | 1 |
| AC096564.2 | 18.9840478 | 2086.426165 | 0.0091 | 0.993 | 8.14E-09 | count | 1 |
| ADAMTSL1 | 18.9840478 | 2086.426165 | 0.0091 | 0.993 | 8.14E-09 | count | 1 |
| TAS2R19 | 18.9840478 | 2086.426165 | 0.0091 | 0.993 | 8.14E-09 | count | 1 |
| XG | 18.9840483 | 2086.426158 | 0.0091 | 0.993 | 8.14E-09 | count | 1 |
| LINC02391 | 18.984048 | 2086.426113 | 0.0091 | 0.993 | 8.14E-09 | count | 1 |
| SEMA3C | 18.982507 | 1552.155452 | 0.0122 | 0.99 | 8.15E-09 | count | 1 |
| CHRDL1 | 18.9825067 | 1552.155394 | 0.0122 | 0.99 | 8.15E-09 | count | 1 |
| AC024896.1 | 18.9825067 | 1552.155336 | 0.0122 | 0.99 | 8.15E-09 | count | 1 |
| AL356020.1 | 18.9814015 | 1199.563582 | 0.0158 | 0.987 | 8.15E-09 | count | 1 |
| KRT73 | 18.9814013 | 1199.563545 | 0.0158 | 0.987 | 8.15E-09 | count | 1 |
| C8G | 18.9814014 | 1199.563575 | 0.0158 | 0.987 | 8.15E-09 | count | 1 |
| ATP1A4 | 18.9814014 | 1199.563556 | 0.0158 | 0.987 | 8.15E-09 | count | 1 |
| AC064834.1 | 18.5753108 | 1700.774696 | 0.0109 | 0.991 | 8.16E-09 | count | 1 |
| AC005534.1 | 18.5753108 | 1700.774696 | 0.0109 | 0.991 | 8.16E-09 | count | 1 |
| AC008105.1 | 18.5753109 | 1700.774781 | 0.0109 | 0.991 | 8.16E-09 | count | 1 |
| AC092436.4 | 18.5753108 | 1700.774722 | 0.0109 | 0.991 | 8.16E-09 | count | 1 |
| ATP5MF-PTCD1 | 18.5753108 | 1700.77477 | 0.0109 | 0.991 | 8.16E-09 | count | 1 |
| CCDC183-AS1 | 18.5753107 | 1700.774738 | 0.0109 | 0.991 | 8.16E-09 | count | 1 |
| DCT | 18.5753108 | 1700.77477 | 0.0109 | 0.991 | 8.16E-09 | count | 1 |
| AC087500.2 | 18.5753108 | 1700.774722 | 0.0109 | 0.991 | 8.16E-09 | count | 1 |
| AC104982.1 | 18.5753108 | 1700.774722 | 0.0109 | 0.991 | 8.16E-09 | count | 1 |
| AC024084.1 | 18.5753106 | 1700.774728 | 0.0109 | 0.991 | 8.16E-09 | count | 1 |
| FXYD4 | 18.5753108 | 1700.774754 | 0.0109 | 0.991 | 8.16E-09 | count | 1 |
| AC012615.5 | 18.5753107 | 1700.77468 | 0.0109 | 0.991 | 8.16E-09 | count | 1 |
| AC012368.2 | 18.5753106 | 1700.774643 | 0.0109 | 0.991 | 8.16E-09 | count | 1 |
| SLC22A14 | 18.5753106 | 1700.774643 | 0.0109 | 0.991 | 8.16E-09 | count | 1 |
| LINC02043 | 18.5753107 | 1700.774685 | 0.0109 | 0.991 | 8.16E-09 | count | 1 |
| AC092535.4 | 18.5753106 | 1700.774722 | 0.0109 | 0.991 | 8.16E-09 | count | 1 |
| AC243829.2 | 18.5753107 | 1700.774712 | 0.0109 | 0.991 | 8.16E-09 | count | 1 |
| OR10H5 | 18.5753106 | 1700.774643 | 0.0109 | 0.991 | 8.16E-09 | count | 1 |
| LINC01381 | 18.5753107 | 1700.774701 | 0.0109 | 0.991 | 8.16E-09 | count | 1 |
| AC093677.2 | 18.5753107 | 1700.774743 | 0.0109 | 0.991 | 8.16E-09 | count | 1 |
| C9 | 18.5753107 | 1700.774701 | 0.0109 | 0.991 | 8.16E-09 | count | 1 |
| AR | 18.5753106 | 1700.774728 | 0.0109 | 0.991 | 8.16E-09 | count | 1 |
| AC005225.2 | 18.5753107 | 1700.774759 | 0.0109 | 0.991 | 8.16E-09 | count | 1 |
| SSBP3-AS1 | 18.5753106 | 1700.774706 | 0.0109 | 0.991 | 8.16E-09 | count | 1 |
| MARVELD2 | 18.5753107 | 1700.774754 | 0.0109 | 0.991 | 8.16E-09 | count | 1 |
| AC138035.1 | 18.5753106 | 1700.774706 | 0.0109 | 0.991 | 8.16E-09 | count | 1 |
| AC005046.1 | 18.5753106 | 1700.774839 | 0.0109 | 0.991 | 8.16E-09 | count | 1 |
| APOF | 18.5753107 | 1700.774733 | 0.0109 | 0.991 | 8.16E-09 | count | 1 |
| WNT5A | 18.5753105 | 1700.774685 | 0.0109 | 0.991 | 8.16E-09 | count | 1 |
| LINC02145 | 18.5753106 | 1700.774669 | 0.0109 | 0.991 | 8.16E-09 | count | 1 |
| GJB7 | 18.5753106 | 1700.774669 | 0.0109 | 0.991 | 8.16E-09 | count | 1 |

| | | | | | | | |
|---|---|---|---|---|---|---|---|
| AP001107.7 | 18.5753106 | 1700.774669 | 0.0109 | 0.991 | 8.16E-09 | count | 1 |
| AL136038.5 | 18.5753106 | 1700.774728 | 0.0109 | 0.991 | 8.16E-09 | count | 1 |
| AC004477.3 | 18.5753105 | 1700.774717 | 0.0109 | 0.991 | 8.16E-09 | count | 1 |
| FAM224B | 18.5753105 | 1700.774643 | 0.0109 | 0.991 | 8.16E-09 | count | 1 |
| STAR | 18.5753104 | 1700.774754 | 0.0109 | 0.991 | 8.16E-09 | count | 1 |
| AC120498.9 | 18.5753104 | 1700.774754 | 0.0109 | 0.991 | 8.16E-09 | count | 1 |
| AL009181.1 | 18.5753105 | 1700.774696 | 0.0109 | 0.991 | 8.16E-09 | count | 1 |
| AC022613.2 | 18.5753104 | 1700.77468 | 0.0109 | 0.991 | 8.16E-09 | count | 1 |
| AC106820.3 | 18.5741126 | 1200.19079 | 0.0155 | 0.988 | 8.16E-09 | count | 1 |
| PDE6A | 18.5741124 | 1200.190768 | 0.0155 | 0.988 | 8.16E-09 | count | 1 |
| AL451050.2 | 18.5741124 | 1200.190749 | 0.0155 | 0.988 | 8.16E-09 | count | 1 |
| GPR12 | 18.5741124 | 1200.190715 | 0.0155 | 0.988 | 8.16E-09 | count | 1 |
| TAS2R30 | 18.5741123 | 1200.190813 | 0.0155 | 0.988 | 8.16E-09 | count | 1 |
| GALNT18 | 18.5741122 | 1200.190738 | 0.0155 | 0.988 | 8.16E-09 | count | 1 |
| DEPDC4 | 18.5741122 | 1200.190749 | 0.0155 | 0.988 | 8.16E-09 | count | 1 |
| TSACC | 18.5741123 | 1200.190771 | 0.0155 | 0.988 | 8.16E-09 | count | 1 |
| CTDSPL | 18.5741123 | 1200.190771 | 0.0155 | 0.988 | 8.16E-09 | count | 1 |
| CCDC154 | 18.5741122 | 1200.190734 | 0.0155 | 0.988 | 8.16E-09 | count | 1 |
| ALOXE3 | 18.5741122 | 1200.190753 | 0.0155 | 0.988 | 8.16E-09 | count | 1 |
| PADI6 | 18.5741123 | 1200.190779 | 0.0155 | 0.988 | 8.16E-09 | count | 1 |
| AC097662.1 | 18.5741123 | 1200.190734 | 0.0155 | 0.988 | 8.16E-09 | count | 1 |
| CRLF2 | 18.5741121 | 1200.190734 | 0.0155 | 0.988 | 8.16E-09 | count | 1 |
| ZP1 | 18.5741121 | 1200.190738 | 0.0155 | 0.988 | 8.16E-09 | count | 1 |
| AP001107.2 | 18.5741123 | 1200.190786 | 0.0155 | 0.988 | 8.16E-09 | count | 1 |
| AP003392.5 | 18.5741121 | 1200.19076 | 0.0155 | 0.988 | 8.16E-09 | count | 1 |
| OSBP2 | 18.5741121 | 1200.190786 | 0.0155 | 0.988 | 8.16E-09 | count | 1 |
| UCKL1-AS1 | 18.5341637 | 2534.006124 | 0.0073 | 0.994 | 8.21E-09 | count | 1 |
| AC009802.1 | 18.5341464 | 2533.994071 | 0.0073 | 0.994 | 8.21E-09 | count | 1 |
| AL109811.3 | 18.5341431 | 2533.977596 | 0.0073 | 0.994 | 8.21E-09 | count | 1 |
| SAMD14 | 18.5341309 | 2533.994411 | 0.0073 | 0.994 | 8.21E-09 | count | 1 |
| STOX1 | 18.5341399 | 2534.006482 | 0.0073 | 0.994 | 8.21E-09 | count | 1 |
| LINC00310 | 18.5341335 | 2534.002616 | 0.0073 | 0.994 | 8.21E-09 | count | 1 |
| SLC18A2 | 18.5341486 | 2533.998146 | 0.0073 | 0.994 | 8.21E-09 | count | 1 |
| FAM124B | 18.5341218 | 2533.990318 | 0.0073 | 0.994 | 8.21E-09 | count | 1 |
| CYSRT1 | 18.534136 | 2534.006643 | 0.0073 | 0.994 | 8.21E-09 | count | 1 |
| ITGB8 | 18.5341254 | 2534.006765 | 0.0073 | 0.994 | 8.21E-09 | count | 1 |
| AC107068.1 | 18.534137 | 2534.002446 | 0.0073 | 0.994 | 8.21E-09 | count | 1 |
| C5 | 18.5341144 | 2533.98233 | 0.0073 | 0.994 | 8.21E-09 | count | 1 |
| OPLAH | 18.534111 | 2534.002899 | 0.0073 | 0.994 | 8.21E-09 | count | 1 |
| RFPL2 | 18.534129 | 2533.977841 | 0.0073 | 0.994 | 8.21E-09 | count | 1 |
| AL590867.1 | 18.534123 | 2534.002738 | 0.0073 | 0.994 | 8.21E-09 | count | 1 |
| AP003392.1 | 18.5341018 | 2533.986678 | 0.0073 | 0.994 | 8.21E-09 | count | 1 |
| AC016745.2 | 18.5340807 | 2533.99939 | 0.0073 | 0.994 | 8.21E-09 | count | 1 |
| KCTD15 | 18.5340889 | 2533.991073 | 0.0073 | 0.994 | 8.21E-09 | count | 1 |
| NLGN3 | 18.534069 | 2533.966638 | 0.0073 | 0.994 | 8.21E-09 | count | 1 |

| | | | | | | | |
|---|---|---|---|---|---|---|---|
| CUEDC1 | 18.5340868 | 2533.978671 | 0.0073 | 0.994 | 8.21E-09 | count | 1 |
| AC136475.9 | 18.5340701 | 2533.974908 | 0.0073 | 0.994 | 8.21E-09 | count | 1 |
| DES | 18.5340777 | 2533.982896 | 0.0073 | 0.994 | 8.21E-09 | count | 1 |
| GTF3C2-AS1 | 18.5340736 | 2533.978916 | 0.0073 | 0.994 | 8.21E-09 | count | 1 |
| CDC42EP1 | 0.2595404 | 0.9664752 | 0.2685 | 0.788 | 8.23E-09 | count | 1 |
| ZBTB34 | 0.2595404 | 0.7396339 | 0.3509 | 0.726 | 8.23E-09 | count | 1 |
| AC093297.2 | 0.2595404 | 0.8036029 | 0.323 | 0.747 | 8.23E-09 | count | 1 |
| LINC00996 | 0.2595404 | 1.0027573 | 0.2588 | 0.796 | 8.23E-09 | count | 1 |
| AC009118.3 | 0.2614139 | 0.9084061 | 0.2878 | 0.774 | 8.29E-09 | count | 1 |
| ZCCHC12 | 0.2614139 | 0.8272908 | 0.316 | 0.752 | 8.29E-09 | count | 1 |
| LINC01393 | 0.2614139 | 0.7119136 | 0.3672 | 0.713 | 8.29E-09 | count | 1 |
| CCDC121 | 0.2614139 | 0.6665864 | 0.3922 | 0.695 | 8.29E-09 | count | 1 |
| BRDT | 0.2614139 | 0.8179999 | 0.3196 | 0.749 | 8.29E-09 | count | 1 |
| CYBRD1 | 0.2762861 | 0.7634404 | 0.3619 | 0.717 | 8.80E-09 | count | 1 |
| ZNF772 | 0.2862042 | 0.926979 | 0.3087 | 0.758 | 9.13E-09 | count | 1 |
| AL121601.1 | 0.2862042 | 0.8370355 | 0.3419 | 0.732 | 9.13E-09 | count | 1 |
| AC233976.1 | 0.2862042 | 0.7612937 | 0.3759 | 0.707 | 9.13E-09 | count | 1 |
| LRP2BP | 0.1125283 | 0.4816659 | 0.2336 | 0.815 | 9.36E-09 | count | 1 |
| ZNF792 | 0.1125283 | 0.6556293 | 0.1716 | 0.864 | 9.36E-09 | count | 1 |
| AL392046.1 | 0.3013507 | 1.0213149 | 0.2951 | 0.768 | 9.65E-09 | count | 1 |
| TLN2 | 0.3013507 | 0.8673919 | 0.3474 | 0.728 | 9.65E-09 | count | 1 |
| ZNF705E | 0.3013507 | 0.9457715 | 0.3186 | 0.75 | 9.65E-09 | count | 1 |
| C16orf95 | 0.3013507 | 0.9457715 | 0.3186 | 0.75 | 9.65E-09 | count | 1 |
| FAM57A | 0.1162191 | 0.6602551 | 0.176 | 0.86 | 9.67E-09 | count | 1 |
| AC119428.2 | 0.1185119 | 0.6398074 | 0.1852 | 0.853 | 9.87E-09 | count | 1 |
| RAC3 | 0.1195231 | 0.4771118 | 0.2505 | 0.802 | 9.96E-09 | count | 1 |
| AL162231.2 | 0.3178137 | 0.8233064 | 0.386 | 0.7 | 1.02E-08 | count | 1 |
| EPB41L5 | 0.0461788 | 0.3795262 | 0.1217 | 0.903 | 1.03E-08 | count | 1 |
| LDHC | 0.7941978 | 1.3400863 | 0.5926 | 0.553 | 1.04E-08 | count | 1 |
| IL17D | 0.7941978 | 1.3400863 | 0.5926 | 0.553 | 1.04E-08 | count | 1 |
| ZNF221 | 0.7941978 | 1.3400863 | 0.5926 | 0.553 | 1.04E-08 | count | 1 |
| SPTBN5 | 0.7941978 | 1.3400863 | 0.5926 | 0.553 | 1.04E-08 | count | 1 |
| NFASC | 0.7941978 | 1.3400863 | 0.5926 | 0.553 | 1.04E-08 | count | 1 |
| RBFADN | 0.7941978 | 1.3400863 | 0.5926 | 0.553 | 1.04E-08 | count | 1 |
| FOXE1 | 0.7941978 | 1.3400863 | 0.5926 | 0.553 | 1.04E-08 | count | 1 |
| CNTN4 | 0.7941978 | 1.3400863 | 0.5926 | 0.553 | 1.04E-08 | count | 1 |
| PTK7 | 0.7941978 | 1.3400863 | 0.5926 | 0.553 | 1.04E-08 | count | 1 |
| LRRC74B | 0.7941978 | 1.3400863 | 0.5926 | 0.553 | 1.04E-08 | count | 1 |
| RIBC1 | 0.7941978 | 1.3400863 | 0.5926 | 0.553 | 1.04E-08 | count | 1 |
| FAM47E | 0.7941978 | 1.3400863 | 0.5926 | 0.553 | 1.04E-08 | count | 1 |
| FAM72B | 0.7941978 | 1.3400863 | 0.5926 | 0.553 | 1.04E-08 | count | 1 |
| TACR2 | 0.7941978 | 1.3400863 | 0.5926 | 0.553 | 1.04E-08 | count | 1 |
| AC005523.1 | 0.7941978 | 1.3400863 | 0.5926 | 0.553 | 1.04E-08 | count | 1 |
| PEBP4 | 0.7941978 | 1.3400863 | 0.5926 | 0.553 | 1.04E-08 | count | 1 |
| EIF3CL | 0.7941978 | 1.3400863 | 0.5926 | 0.553 | 1.04E-08 | count | 1 |

| | | | | | | | |
|---|---|---|---|---|---|---|---|
| LRP5 | 0.7941978 | 1.1115989 | 0.7145 | 0.475 | 1.04E-08 | count | 1 |
| AC234582.1 | 0.7941978 | 1.0913867 | 0.7277 | 0.467 | 1.04E-08 | count | 1 |
| CENPS-CORT | 0.7941978 | 1.0913867 | 0.7277 | 0.467 | 1.04E-08 | count | 1 |
| SERF1B | 0.7941978 | 1.1115989 | 0.7145 | 0.475 | 1.04E-08 | count | 1 |
| LCTL | 0.7941978 | 1.1115989 | 0.7145 | 0.475 | 1.04E-08 | count | 1 |
| AC023055.1 | 0.7941978 | 1.1115989 | 0.7145 | 0.475 | 1.04E-08 | count | 1 |
| RBM5-AS1 | 0.7941978 | 1.1115989 | 0.7145 | 0.475 | 1.04E-08 | count | 1 |
| LINC02210-CRHR1 | 0.7941978 | 1.1115989 | 0.7145 | 0.475 | 1.04E-08 | count | 1 |
| EFCAB12 | 0.7941978 | 1.0913867 | 0.7277 | 0.467 | 1.04E-08 | count | 1 |
| AC100861.2 | 0.7941978 | 1.1115989 | 0.7145 | 0.475 | 1.04E-08 | count | 1 |
| AC012603.1 | 0.7941978 | 1.1115989 | 0.7145 | 0.475 | 1.04E-08 | count | 1 |
| GOLGA6L10 | 0.7941978 | 1.1115989 | 0.7145 | 0.475 | 1.04E-08 | count | 1 |
| GNMT | 0.7941978 | 1.1115989 | 0.7145 | 0.475 | 1.04E-08 | count | 1 |
| AC016394.2 | 0.7941978 | 1.0913867 | 0.7277 | 0.467 | 1.04E-08 | count | 1 |
| RASGRF2-AS1 | 0.7941978 | 1.0913867 | 0.7277 | 0.467 | 1.04E-08 | count | 1 |
| PTPRF | 0.7941978 | 1.1115989 | 0.7145 | 0.475 | 1.04E-08 | count | 1 |
| AC122129.1 | 0.7941978 | 1.0913867 | 0.7277 | 0.467 | 1.04E-08 | count | 1 |
| AC073610.3 | 0.7941978 | 1.0913867 | 0.7277 | 0.467 | 1.04E-08 | count | 1 |
| TNNI2 | 0.7941978 | 1.1115989 | 0.7145 | 0.475 | 1.04E-08 | count | 1 |
| FLT1 | 0.7941978 | 1.1115989 | 0.7145 | 0.475 | 1.04E-08 | count | 1 |
| AL031665.2 | 0.7941978 | 1.0913867 | 0.7277 | 0.467 | 1.04E-08 | count | 1 |
| TTC23 | 0.7941978 | 1.0913867 | 0.7277 | 0.467 | 1.04E-08 | count | 1 |
| AC211476.2 | 0.7941978 | 1.1115989 | 0.7145 | 0.475 | 1.04E-08 | count | 1 |
| CAPN3 | 0.7941978 | 1.0913867 | 0.7277 | 0.467 | 1.04E-08 | count | 1 |
| AP001020.1 | 0.7941978 | 1.0913867 | 0.7277 | 0.467 | 1.04E-08 | count | 1 |
| AURKB | 0.7941978 | 1.0913867 | 0.7277 | 0.467 | 1.04E-08 | count | 1 |
| UAP1L1 | 0.1260837 | 0.4031923 | 0.3127 | 0.755 | 1.05E-08 | count | 1 |
| ATP1B2 | 0.3313571 | 0.6406 | 0.5173 | 0.605 | 1.07E-08 | count | 1 |
| AL513314.2 | 0.3429435 | 0.7947877 | 0.4315 | 0.666 | 1.11E-08 | count | 1 |
| ATP6AP1L | 0.8421828 | 0.9208455 | 0.9146 | 0.36 | 1.11E-08 | count | 1 |
| TRIP13 | 0.8421828 | 0.8462868 | 0.9952 | 0.32 | 1.11E-08 | count | 1 |
| ACTG2 | 2.0346498 | 0.780595 | 2.6065 | 0.0092 | 1.12E-08 | count | 1 |
| COL5A3 | 2.0346498 | 0.6410019 | 3.1742 | 0.0015 | 1.12E-08 | count | 1 |
| TEF | 0.1349924 | 0.5198592 | 0.2597 | 0.795 | 1.13E-08 | count | 1 |
| AC005076.1 | 0.3590094 | 0.76515 | 0.4692 | 0.639 | 1.17E-08 | count | 1 |
| GPR135 | 0.3590094 | 0.6901059 | 0.5202 | 0.603 | 1.17E-08 | count | 1 |
| MELTF | 0.3590094 | 0.7930513 | 0.4527 | 0.651 | 1.17E-08 | count | 1 |
| ALG10B | 0.1395224 | 0.600834 | 0.2322 | 0.816 | 1.17E-08 | count | 1 |
| ADAMTSL5 | 0.1395224 | 0.6239036 | 0.2236 | 0.823 | 1.17E-08 | count | 1 |
| CCDC173 | 0.1405109 | 0.4729873 | 0.2971 | 0.766 | 1.18E-08 | count | 1 |
| SH3BP5-AS1 | 0.3741162 | 0.8197107 | 0.4564 | 0.648 | 1.22E-08 | count | 1 |
| PM20D1 | 0.3741162 | 0.8310396 | 0.4502 | 0.653 | 1.22E-08 | count | 1 |
| DNAJB5 | 0.3741162 | 0.8584398 | 0.4358 | 0.663 | 1.22E-08 | count | 1 |
| CCDC183 | 0.3741162 | 0.7155815 | 0.5228 | 0.601 | 1.22E-08 | count | 1 |
| GLIS3 | 0.3760159 | 0.6121283 | 0.6143 | 0.539 | 1.22E-08 | count | 1 |

| | | | | | | | |
|---|---|---|---|---|---|---|---|
| C9orf135 | 0.9360375 | 0.3058647 | 3.0603 | 0.0022 | 1.25E-08 | count | 1 |
| RAB19 | 0.1538454 | 0.7601067 | 0.2024 | 0.84 | 1.29E-08 | count | 1 |
| CCDC87 | 0.9616537 | 1.4154585 | 0.6794 | 0.497 | 1.30E-08 | count | 1 |
| PPP1R9A | 0.9616537 | 1.4154585 | 0.6794 | 0.497 | 1.30E-08 | count | 1 |
| AC104113.1 | 0.9616537 | 1.4154585 | 0.6794 | 0.497 | 1.30E-08 | count | 1 |
| PRX | 0.9616537 | 1.4154585 | 0.6794 | 0.497 | 1.30E-08 | count | 1 |
| FAXDC2 | 0.9616537 | 1.0121267 | 0.9501 | 0.342 | 1.30E-08 | count | 1 |
| AC012313.8 | 0.9616537 | 1.0121267 | 0.9501 | 0.342 | 1.30E-08 | count | 1 |
| TPST1 | 0.9616537 | 1.0121267 | 0.9501 | 0.342 | 1.30E-08 | count | 1 |
| AC105760.2 | 0.9616537 | 1.0121267 | 0.9501 | 0.342 | 1.30E-08 | count | 1 |
| CACNB4 | 0.9616537 | 1.0121267 | 0.9501 | 0.342 | 1.30E-08 | count | 1 |
| SLC16A5 | 0.1614978 | 0.6790576 | 0.2378 | 0.812 | 1.36E-08 | count | 1 |
| ALG1L2 | 1.0245043 | 0.8105443 | 1.264 | 0.206 | 1.39E-08 | count | 1 |
| HTD2 | 1.0245043 | 1.2111249 | 0.8459 | 0.398 | 1.39E-08 | count | 1 |
| AL021368.3 | 1.0245043 | 1.2111249 | 0.8459 | 0.398 | 1.39E-08 | count | 1 |
| MET | 1.0245043 | 1.2111249 | 0.8459 | 0.398 | 1.39E-08 | count | 1 |
| AGAP2-AS1 | 1.0245043 | 1.2111249 | 0.8459 | 0.398 | 1.39E-08 | count | 1 |
| CITED1 | 1.0245043 | 0.9841034 | 1.0411 | 0.298 | 1.39E-08 | count | 1 |
| HCFC1-AS1 | 1.0245043 | 0.9841034 | 1.0411 | 0.298 | 1.39E-08 | count | 1 |
| ORC1 | 1.0245043 | 0.9841034 | 1.0411 | 0.298 | 1.39E-08 | count | 1 |
| LINC00880 | 1.0245043 | 0.9841034 | 1.0411 | 0.298 | 1.39E-08 | count | 1 |
| INTU | 1.0245043 | 0.9841034 | 1.0411 | 0.298 | 1.39E-08 | count | 1 |
| IGLV2-14 | 1.0245043 | 0.9841034 | 1.0411 | 0.298 | 1.39E-08 | count | 1 |
| AC010245.1 | 1.0245043 | 0.9841034 | 1.0411 | 0.298 | 1.39E-08 | count | 1 |
| AL136531.1 | 1.0245043 | 0.9841034 | 1.0411 | 0.298 | 1.39E-08 | count | 1 |
| PRRT2 | 0.1673109 | 0.3957261 | 0.4228 | 0.672 | 1.41E-08 | count | 1 |
| AL117336.3 | 0.429228 | 0.5719598 | 0.7505 | 0.453 | 1.42E-08 | count | 1 |
| MTRNR2L10 | 0.4301452 | 0.7447731 | 0.5776 | 0.564 | 1.42E-08 | count | 1 |
| HEATR5A | 0.4316058 | 0.6933158 | 0.6225 | 0.534 | 1.42E-08 | count | 1 |
| AC104825.1 | 0.4316058 | 0.8514948 | 0.5069 | 0.612 | 1.42E-08 | count | 1 |
| SH3D21 | 0.4316058 | 0.7694052 | 0.561 | 0.575 | 1.42E-08 | count | 1 |
| AL022157.1 | 0.4316058 | 1.0911523 | 0.3956 | 0.692 | 1.42E-08 | count | 1 |
| SKAP1-AS1 | 0.434296 | 1.1234895 | 0.3866 | 0.699 | 1.43E-08 | count | 1 |
| LINGO3 | 0.434296 | 0.9404022 | 0.4618 | 0.644 | 1.43E-08 | count | 1 |
| SIK1B | 0.434296 | 0.9404022 | 0.4618 | 0.644 | 1.43E-08 | count | 1 |
| KIAA1522 | 0.434296 | 0.917351 | 0.4734 | 0.636 | 1.43E-08 | count | 1 |
| AC004943.2 | 0.434296 | 0.9404022 | 0.4618 | 0.644 | 1.43E-08 | count | 1 |
| GNAL | 0.434296 | 0.9404022 | 0.4618 | 0.644 | 1.43E-08 | count | 1 |
| AC034102.5 | 0.434296 | 1.0645312 | 0.408 | 0.683 | 1.43E-08 | count | 1 |
| AL022323.4 | 0.434296 | 1.0645312 | 0.408 | 0.683 | 1.43E-08 | count | 1 |
| CELSR1 | 0.434296 | 1.0645312 | 0.408 | 0.683 | 1.43E-08 | count | 1 |
| ZNF132 | 0.434296 | 1.0645312 | 0.408 | 0.683 | 1.43E-08 | count | 1 |
| TMEM81 | 0.434296 | 0.9717461 | 0.4469 | 0.655 | 1.43E-08 | count | 1 |
| AC104596.1 | 0.1755519 | 0.7857422 | 0.2234 | 0.823 | 1.48E-08 | count | 1 |
| CMTM2 | 0.1755519 | 0.9561319 | 0.1836 | 0.854 | 1.48E-08 | count | 1 |

| | | | | | | | |
|---|---|---|---|---|---|---|---|
| KDM8 | 0.1755519 | 0.4375564 | 0.4012 | 0.688 | 1.48E-08 | count | 1 |
| AL512625.1 | 0.1881981 | 0.8038795 | 0.2341 | 0.815 | 1.59E-08 | count | 1 |
| IL21 | 0.4855078 | 0.6229881 | 0.7793 | 0.436 | 1.62E-08 | count | 1 |
| ZNF594 | 0.4855078 | 0.6385766 | 0.7603 | 0.447 | 1.62E-08 | count | 1 |
| ITPKB-AS1 | 0.1911759 | 0.5600277 | 0.3414 | 0.733 | 1.62E-08 | count | 1 |
| CLIP2 | 0.4856118 | 0.6918196 | 0.7019 | 0.483 | 1.62E-08 | count | 1 |
| GPR55 | 0.4857324 | 0.8013467 | 0.6061 | 0.544 | 1.62E-08 | count | 1 |
| AC092953.2 | 1.178655 | 0.9067098 | 1.2999 | 0.194 | 1.64E-08 | count | 1 |
| IDI2-AS1 | 1.178655 | 0.8778573 | 1.3426 | 0.179 | 1.64E-08 | count | 1 |
| AF129075.2 | 1.178655 | 0.8171017 | 1.4425 | 0.149 | 1.64E-08 | count | 1 |
| AC002306.1 | 0.1935697 | 0.4378942 | 0.442 | 0.658 | 1.64E-08 | count | 1 |
| ACCS | 0.1959894 | 0.5956283 | 0.329 | 0.742 | 1.66E-08 | count | 1 |
| BBS10 | 0.5017921 | 0.8364449 | 0.5999 | 0.549 | 1.68E-08 | count | 1 |
| AC010894.2 | 0.5017921 | 0.7952665 | 0.631 | 0.528 | 1.68E-08 | count | 1 |
| SIRPA | 0.5036192 | 1.088265 | 0.4628 | 0.644 | 1.69E-08 | count | 1 |
| AC005899.6 | 0.5036192 | 0.8388043 | 0.6004 | 0.548 | 1.69E-08 | count | 1 |
| ACER2 | 0.1997122 | 0.9155056 | 0.2181 | 0.827 | 1.70E-08 | count | 1 |
| YJEFN3 | 0.2007754 | 0.4694155 | 0.4277 | 0.669 | 1.71E-08 | count | 1 |
| TRAV12-3 | 0.5136787 | 0.5073967 | 1.0124 | 0.311 | 1.72E-08 | count | 1 |
| PWAR6 | 0.2070794 | 0.6586887 | 0.3144 | 0.753 | 1.76E-08 | count | 1 |
| DPYSL4 | 0.2070794 | 0.5371604 | 0.3855 | 0.7 | 1.76E-08 | count | 1 |
| CNKSR2 | 0.2070794 | 0.5448251 | 0.3801 | 0.704 | 1.76E-08 | count | 1 |
| OBSCN-AS1 | 0.533107 | 0.6815969 | 0.7821 | 0.434 | 1.80E-08 | count | 1 |
| MYO16 | 0.5366479 | 1.1743661 | 0.457 | 0.648 | 1.81E-08 | count | 1 |
| BNIPL | 0.5366479 | 1.2893067 | 0.4162 | 0.677 | 1.81E-08 | count | 1 |
| AP003486.1 | 0.5366479 | 1.0838197 | 0.4951 | 0.621 | 1.81E-08 | count | 1 |
| RNF157-AS1 | 0.5366479 | 1.13394 | 0.4733 | 0.636 | 1.81E-08 | count | 1 |
| RGS11 | 0.5366479 | 1.13394 | 0.4733 | 0.636 | 1.81E-08 | count | 1 |
| FAM129C | 0.5366479 | 0.8047769 | 0.6668 | 0.505 | 1.81E-08 | count | 1 |
| INKA2 | 1.3121864 | 1.3134654 | 0.999 | 0.318 | 1.86E-08 | count | 1 |
| EPHB1 | 1.3121864 | 1.3134654 | 0.999 | 0.318 | 1.86E-08 | count | 1 |
| AL137779.2 | 1.3121864 | 1.0952502 | 1.1981 | 0.231 | 1.86E-08 | count | 1 |
| LINC01063 | 1.3121864 | 0.9214992 | 1.424 | 0.155 | 1.86E-08 | count | 1 |
| AL137186.2 | 1.3121864 | 0.9214992 | 1.424 | 0.155 | 1.86E-08 | count | 1 |
| KIAA0895 | 0.566569 | 0.8143383 | 0.6957 | 0.487 | 1.92E-08 | count | 1 |
| ADAMTS10 | 0.574702 | 0.7893636 | 0.7281 | 0.467 | 1.96E-08 | count | 1 |
| GPRASP2 | 0.574702 | 0.8659924 | 0.6636 | 0.507 | 1.96E-08 | count | 1 |
| FRS3 | 0.2315808 | 0.6832055 | 0.339 | 0.735 | 1.98E-08 | count | 1 |
| AC114271.1 | 0.5872785 | 0.7934177 | 0.7402 | 0.459 | 2.00E-08 | count | 1 |
| AC099522.2 | 0.5872785 | 0.6760173 | 0.8687 | 0.385 | 2.00E-08 | count | 1 |
| SLC9A7 | 0.2348848 | 0.6082321 | 0.3862 | 0.699 | 2.01E-08 | count | 1 |
| LINC01952 | 1.4108543 | 0.9208972 | 1.532 | 0.126 | 2.02E-08 | count | 1 |
| SAPCD1-AS1 | 1.4108543 | 0.9208972 | 1.532 | 0.126 | 2.02E-08 | count | 1 |
| LMLN | 0.2398482 | 0.4750745 | 0.5049 | 0.614 | 2.06E-08 | count | 1 |
| LINC00663 | 0.24572 | 0.6995057 | 0.3513 | 0.725 | 2.11E-08 | count | 1 |

| | | | | | | | |
|---|---|---|---|---|---|---|---|
| LINC00278 | 0.24572 | 0.7152091 | 0.3436 | 0.731 | 2.11E-08 | count | 1 |
| LINC02325 | 0.2498434 | 0.6118043 | 0.4084 | 0.683 | 2.15E-08 | count | 1 |
| HES1 | 0.6276772 | 0.6912643 | 0.908 | 0.364 | 2.16E-08 | count | 1 |
| SIAE | 0.6307862 | 0.8822271 | 0.715 | 0.475 | 2.17E-08 | count | 1 |
| NFIB | 0.6307862 | 0.9725351 | 0.6486 | 0.517 | 2.17E-08 | count | 1 |
| DPY19L2 | 0.6307862 | 0.8744059 | 0.7214 | 0.471 | 2.17E-08 | count | 1 |
| AL359541.1 | 0.6307862 | 0.9890559 | 0.6378 | 0.524 | 2.17E-08 | count | 1 |
| AC078785.1 | 18.565146 | 3164.085052 | 0.0059 | 0.995 | 2.18E-08 | count | 1 |
| SAA1 | 18.4313895 | 3348.669492 | 0.0055 | 0.996 | 2.18E-08 | count | 1 |
| AIRE | 18.6826543 | 2494.162924 | 0.0075 | 0.994 | 2.18E-08 | count | 1 |
| FPR2 | 18.4307971 | 2908.522519 | 0.0063 | 0.995 | 2.18E-08 | count | 1 |
| FBXO24 | 18.27636 | 3098.886334 | 0.0059 | 0.995 | 2.19E-08 | count | 1 |
| AC092112.1 | 18.2763599 | 3098.886224 | 0.0059 | 0.995 | 2.19E-08 | count | 1 |
| PRAM1 | 18.2763601 | 3098.886114 | 0.0059 | 0.995 | 2.19E-08 | count | 1 |
| GNG4 | 18.2763597 | 3098.886287 | 0.0059 | 0.995 | 2.19E-08 | count | 1 |
| WNT9A | 18.27636 | 3098.886279 | 0.0059 | 0.995 | 2.19E-08 | count | 1 |
| C3orf67 | 18.5635882 | 1894.701564 | 0.0098 | 0.992 | 2.19E-08 | count | 1 |
| AP003419.2 | 18.5631911 | 1669.742967 | 0.0111 | 0.991 | 2.19E-08 | count | 1 |
| GALNT14 | 18.093031 | 2827.458472 | 0.0064 | 0.995 | 2.19E-08 | count | 1 |
| LIN7A | 18.093031 | 2827.458472 | 0.0064 | 0.995 | 2.19E-08 | count | 1 |
| C20orf144 | 18.093031 | 2827.458472 | 0.0064 | 0.995 | 2.19E-08 | count | 1 |
| MYO10 | 18.0930308 | 2827.458422 | 0.0064 | 0.995 | 2.19E-08 | count | 1 |
| LINC01973 | 18.0930306 | 2827.458429 | 0.0064 | 0.995 | 2.19E-08 | count | 1 |
| AL359962.2 | 18.274939 | 1929.526654 | 0.0095 | 0.992 | 2.19E-08 | count | 1 |
| AC092287.1 | 18.2744898 | 1629.497944 | 0.0112 | 0.991 | 2.19E-08 | count | 1 |
| MAMDC4 | 18.2744897 | 1629.497845 | 0.0112 | 0.991 | 2.19E-08 | count | 1 |
| AC022098.2 | 18.2744894 | 1629.497807 | 0.0112 | 0.991 | 2.19E-08 | count | 1 |
| LINC02413 | 18.0921798 | 2037.052491 | 0.0089 | 0.993 | 2.19E-08 | count | 1 |
| TRBV6-6 | 18.0921797 | 2037.052475 | 0.0089 | 0.993 | 2.19E-08 | count | 1 |
| AL645941.3 | 17.8688086 | 2527.592164 | 0.0071 | 0.994 | 2.19E-08 | count | 1 |
| RASIP1 | 17.8688086 | 2527.592125 | 0.0071 | 0.994 | 2.19E-08 | count | 1 |
| AC046136.1 | 17.8688084 | 2527.592138 | 0.0071 | 0.994 | 2.19E-08 | count | 1 |
| PRR16 | 17.8688086 | 2527.5921 | 0.0071 | 0.994 | 2.19E-08 | count | 1 |
| AL645939.4 | 17.8688084 | 2527.592106 | 0.0071 | 0.994 | 2.19E-08 | count | 1 |
| SCAMP5 | 17.8688084 | 2527.592106 | 0.0071 | 0.994 | 2.19E-08 | count | 1 |
| PLCD4 | 17.8688084 | 2527.592093 | 0.0071 | 0.994 | 2.19E-08 | count | 1 |
| AC107214.1 | 18.0918537 | 1873.165484 | 0.0097 | 0.992 | 2.19E-08 | count | 1 |
| AC128688.2 | 18.0917066 | 1693.766869 | 0.0107 | 0.991 | 2.19E-08 | count | 1 |
| XK | 18.0917066 | 1693.766873 | 0.0107 | 0.991 | 2.19E-08 | count | 1 |
| LINC02416 | 18.0917066 | 1693.766899 | 0.0107 | 0.991 | 2.19E-08 | count | 1 |
| AC023906.5 | 18.0917066 | 1693.766899 | 0.0107 | 0.991 | 2.19E-08 | count | 1 |
| AP000936.1 | 18.0913725 | 1492.888 | 0.0121 | 0.99 | 2.19E-08 | count | 1 |
| KCNH2 | 18.0913726 | 1492.888072 | 0.0121 | 0.99 | 2.19E-08 | count | 1 |
| SLC9A5 | 17.8682152 | 1997.014029 | 0.0089 | 0.993 | 2.19E-08 | count | 1 |
| HAS3 | 17.8682151 | 1997.014054 | 0.0089 | 0.993 | 2.19E-08 | count | 1 |

| | | | | | | | |
|---|---|---|---|---|---|---|---|
| CBFA2T3 | 17.8682151 | 1997.014024 | 0.0089 | 0.993 | 2.19E-08 | count | 1 |
| FAM66B | 17.8680616 | 1785.670524 | 0.01 | 0.992 | 2.19E-08 | count | 1 |
| CRTC3-AS1 | 17.8680616 | 1785.670573 | 0.01 | 0.992 | 2.19E-08 | count | 1 |
| AC092620.1 | 17.8680614 | 1785.670564 | 0.01 | 0.992 | 2.19E-08 | count | 1 |
| AC008403.2 | 17.8680613 | 1785.67051 | 0.01 | 0.992 | 2.19E-08 | count | 1 |
| AL158163.2 | 17.8680615 | 1785.670591 | 0.01 | 0.992 | 2.19E-08 | count | 1 |
| AP000223.1 | 17.8680614 | 1785.670528 | 0.01 | 0.992 | 2.19E-08 | count | 1 |
| AC013731.1 | 17.8680614 | 1785.670524 | 0.01 | 0.992 | 2.19E-08 | count | 1 |
| LINC02321 | 17.8680615 | 1785.670551 | 0.01 | 0.992 | 2.19E-08 | count | 1 |
| IGLC6 | 17.8680613 | 1785.670555 | 0.01 | 0.992 | 2.19E-08 | count | 1 |
| IL5 | 19.3756212 | 1741.047605 | 0.0111 | 0.991 | 2.19E-08 | count | 1 |
| AC073263.2 | 17.8677103 | 1545.621888 | 0.0116 | 0.991 | 2.20E-08 | count | 1 |
| NUDT12 | 17.8677103 | 1545.621907 | 0.0116 | 0.991 | 2.20E-08 | count | 1 |
| AL356234.2 | 17.8677102 | 1545.6219 | 0.0116 | 0.991 | 2.20E-08 | count | 1 |
| LINC01572 | 17.8677101 | 1545.621915 | 0.0116 | 0.991 | 2.20E-08 | count | 1 |
| RGPD4-AS1 | 17.8677102 | 1545.621876 | 0.0116 | 0.991 | 2.20E-08 | count | 1 |
| C9orf163 | 17.8677101 | 1545.621911 | 0.0116 | 0.991 | 2.20E-08 | count | 1 |
| AC007216.4 | 17.8677101 | 1545.621892 | 0.0116 | 0.991 | 2.20E-08 | count | 1 |
| CDCA3 | 17.8677102 | 1545.621876 | 0.0116 | 0.991 | 2.20E-08 | count | 1 |
| LINC00449 | 17.8673542 | 1260.883909 | 0.0142 | 0.989 | 2.20E-08 | count | 1 |
| CCL14 | 0.6373871 | 1.3449518 | 0.4739 | 0.636 | 2.20E-08 | count | 1 |
| PFKFB4 | 0.6373871 | 1.3449518 | 0.4739 | 0.636 | 2.20E-08 | count | 1 |
| ARHGEF39 | 0.6373871 | 0.8454448 | 0.7539 | 0.451 | 2.20E-08 | count | 1 |
| AC145285.2 | 0.6373871 | 1.0753014 | 0.5928 | 0.553 | 2.20E-08 | count | 1 |
| PINK1-AS | 0.6373871 | 1.0753014 | 0.5928 | 0.553 | 2.20E-08 | count | 1 |
| POLR2J3 | 0.6373871 | 0.7770755 | 0.8202 | 0.412 | 2.20E-08 | count | 1 |
| ZBTB3 | 0.6373871 | 0.7770755 | 0.8202 | 0.412 | 2.20E-08 | count | 1 |
| L3MBTL4 | 0.6373871 | 0.8451853 | 0.7541 | 0.451 | 2.20E-08 | count | 1 |
| ZNF223 | 0.6373871 | 0.8451853 | 0.7541 | 0.451 | 2.20E-08 | count | 1 |
| GJC2 | 0.6373871 | 0.8451853 | 0.7541 | 0.451 | 2.20E-08 | count | 1 |
| CACNA1D | 0.6373871 | 1.2642156 | 0.5042 | 0.614 | 2.20E-08 | count | 1 |
| CACNA1C-AS1 | 0.6373871 | 0.8961452 | 0.7113 | 0.477 | 2.20E-08 | count | 1 |
| TLR8 | 18.6842718 | 1796.004457 | 0.0104 | 0.992 | 2.20E-08 | count | 1 |
| CFAP58-DT | 18.4710724 | 2281.378195 | 0.0081 | 0.994 | 2.21E-08 | count | 1 |
| AC116914.2 | 18.4710595 | 2281.378404 | 0.0081 | 0.994 | 2.21E-08 | count | 1 |
| RHOV | 18.4708369 | 2121.598891 | 0.0087 | 0.993 | 2.21E-08 | count | 1 |
| ALKAL2 | 18.4706134 | 1759.285535 | 0.0105 | 0.992 | 2.21E-08 | count | 1 |
| KIAA1257 | 18.3114233 | 2773.568143 | 0.0066 | 0.995 | 2.21E-08 | count | 1 |
| PRRG4 | 18.5207569 | 1998.411484 | 0.0093 | 0.993 | 2.21E-08 | count | 1 |
| GLT1D1 | 18.6809762 | 2211.2914 | 0.0084 | 0.993 | 2.22E-08 | count | 1 |
| AL355297.4 | 18.8117762 | 1810.501745 | 0.0104 | 0.992 | 2.22E-08 | count | 1 |
| TLE6 | 18.1184203 | 2437.734719 | 0.0074 | 0.994 | 2.22E-08 | count | 1 |
| ARHGEF28 | 18.118415 | 2437.718806 | 0.0074 | 0.994 | 2.22E-08 | count | 1 |
| SLC35G5 | 18.118401 | 2437.735804 | 0.0074 | 0.994 | 2.22E-08 | count | 1 |
| LIF | 18.6810992 | 1792.467299 | 0.0104 | 0.992 | 2.22E-08 | count | 1 |

| | | | | | | | |
|---|---|---|---|---|---|---|---|
| AC010680.3 | 18.4824859 | 2297.051183 | 0.008 | 0.994 | 2.22E-08 | count | 1 |
| TNNC2 | 18.6346213 | 1984.855507 | 0.0094 | 0.993 | 2.22E-08 | count | 1 |
| MYOM1 | 18.1182532 | 1893.930005 | 0.0096 | 0.992 | 2.22E-08 | count | 1 |
| SLC44A4 | 18.1182564 | 1893.929628 | 0.0096 | 0.992 | 2.22E-08 | count | 1 |
| AC140125.2 | 18.1182563 | 1893.929634 | 0.0096 | 0.992 | 2.22E-08 | count | 1 |
| FRG1-DT | 18.1182515 | 1893.930217 | 0.0096 | 0.992 | 2.22E-08 | count | 1 |
| AL096855.1 | 18.1182373 | 1893.930476 | 0.0096 | 0.992 | 2.22E-08 | count | 1 |
| SGMS1-AS1 | 18.4148413 | 2051.205253 | 0.009 | 0.993 | 2.22E-08 | count | 1 |
| AC084346.2 | 18.2896587 | 1856.26676 | 0.0099 | 0.992 | 2.22E-08 | count | 1 |
| ATP1B3-AS1 | 18.1181477 | 1552.221689 | 0.0117 | 0.991 | 2.22E-08 | count | 1 |
| AL353622.2 | 18.1181253 | 1552.212045 | 0.0117 | 0.991 | 2.22E-08 | count | 1 |
| LINC02081 | 18.2885307 | 1623.830313 | 0.0113 | 0.991 | 2.22E-08 | count | 1 |
| AC005034.3 | 18.4817041 | 1654.164324 | 0.0112 | 0.991 | 2.22E-08 | count | 1 |
| PRG4 | 18.2885207 | 1623.832411 | 0.0113 | 0.991 | 2.22E-08 | count | 1 |
| AC025259.3 | 18.2285737 | 2174.98178 | 0.0084 | 0.993 | 2.22E-08 | count | 1 |
| PHYHD1 | 18.2285669 | 2174.976687 | 0.0084 | 0.993 | 2.22E-08 | count | 1 |
| AC107871.1 | 18.2285574 | 2174.976818 | 0.0084 | 0.993 | 2.22E-08 | count | 1 |
| ZNF667 | 1.5353299 | 1.0248502 | 1.4981 | 0.134 | 2.22E-08 | count | 1 |
| LINC02478 | 1.5353299 | 0.9569533 | 1.6044 | 0.109 | 2.22E-08 | count | 1 |
| LINC01816 | 1.5353299 | 0.9569533 | 1.6044 | 0.109 | 2.22E-08 | count | 1 |
| CELA1 | 18.2877099 | 1494.156234 | 0.0122 | 0.99 | 2.22E-08 | count | 1 |
| BEST4 | 17.8738127 | 2041.672793 | 0.0088 | 0.993 | 2.22E-08 | count | 1 |
| SSTR2 | 17.873813 | 2041.676307 | 0.0088 | 0.993 | 2.23E-08 | count | 1 |
| CNTN2 | 17.8738131 | 2041.672776 | 0.0088 | 0.993 | 2.23E-08 | count | 1 |
| AL136162.1 | 18.2286378 | 1732.029219 | 0.0105 | 0.992 | 2.23E-08 | count | 1 |
| AC127496.5 | 17.873813 | 2041.665791 | 0.0088 | 0.993 | 2.23E-08 | count | 1 |
| BAAT | 18.2286141 | 1732.033692 | 0.0105 | 0.992 | 2.23E-08 | count | 1 |
| AC109460.2 | 17.8737849 | 2041.673605 | 0.0088 | 0.993 | 2.23E-08 | count | 1 |
| AC009549.1 | 17.8737826 | 2041.666783 | 0.0088 | 0.993 | 2.23E-08 | count | 1 |
| AC068025.1 | 17.8737753 | 2041.673896 | 0.0088 | 0.993 | 2.23E-08 | count | 1 |
| MB | 17.873782 | 2041.673836 | 0.0088 | 0.993 | 2.23E-08 | count | 1 |
| KIAA0408 | 17.873762 | 2041.670972 | 0.0088 | 0.993 | 2.23E-08 | count | 1 |
| AC244021.1 | 17.8737718 | 2041.663665 | 0.0088 | 0.993 | 2.23E-08 | count | 1 |
| BACH1-IT2 | 17.8737723 | 2041.663623 | 0.0088 | 0.993 | 2.23E-08 | count | 1 |
| LRRC75B | 17.8737735 | 2041.674015 | 0.0088 | 0.993 | 2.23E-08 | count | 1 |
| AL662884.4 | 17.8737616 | 2041.660482 | 0.0088 | 0.993 | 2.23E-08 | count | 1 |
| PCDHGB5 | 17.8737607 | 2041.660562 | 0.0088 | 0.993 | 2.23E-08 | count | 1 |
| AC112721.2 | 17.8737641 | 2041.663785 | 0.0088 | 0.993 | 2.23E-08 | count | 1 |
| AP001160.2 | 17.8737604 | 1551.176961 | 0.0115 | 0.991 | 2.23E-08 | count | 1 |
| AC100814.1 | 17.8737517 | 1551.190414 | 0.0115 | 0.991 | 2.23E-08 | count | 1 |
| VXN | 17.8737397 | 1551.17483 | 0.0115 | 0.991 | 2.23E-08 | count | 1 |
| RFTN2 | 17.873711 | 1551.178145 | 0.0115 | 0.991 | 2.23E-08 | count | 1 |
| NEK10 | 18.2281005 | 1536.398357 | 0.0119 | 0.991 | 2.23E-08 | count | 1 |
| KLRC4-KLRK1 | 18.228107 | 1536.403785 | 0.0119 | 0.991 | 2.23E-08 | count | 1 |
| POT1-AS1 | 17.8737126 | 1551.183369 | 0.0115 | 0.991 | 2.23E-08 | count | 1 |

| | | | | | | | |
|---|---|---|---|---|---|---|---|
| AC004803.1 | 17.9854386 | 1703.459447 | 0.0106 | 0.992 | 2.23E-08 | count | 1 |
| AC021321.1 | 17.9854441 | 1703.462594 | 0.0106 | 0.992 | 2.23E-08 | count | 1 |
| FSBP | 17.9854431 | 1703.463394 | 0.0106 | 0.992 | 2.23E-08 | count | 1 |
| SNX32 | 17.9854265 | 1703.45872 | 0.0106 | 0.992 | 2.23E-08 | count | 1 |
| CEBPE | 17.9846444 | 1510.972486 | 0.0119 | 0.991 | 2.23E-08 | count | 1 |
| PDE7B | 17.9846461 | 1510.968951 | 0.0119 | 0.991 | 2.23E-08 | count | 1 |
| DLL3 | 17.9846481 | 1510.97599 | 0.0119 | 0.991 | 2.23E-08 | count | 1 |
| AC009404.1 | 0.2606603 | 0.5774352 | 0.4514 | 0.652 | 2.25E-08 | count | 1 |
| AL683813.1 | 0.269281 | 0.5174713 | 0.5204 | 0.603 | 2.33E-08 | count | 1 |
| DMGDH | 0.2722384 | 0.4931846 | 0.552 | 0.581 | 2.35E-08 | count | 1 |
| AC008735.2 | 0.6780174 | 0.8027312 | 0.8446 | 0.398 | 2.36E-08 | count | 1 |
| AP003472.1 | 0.6780174 | 0.7002263 | 0.9683 | 0.333 | 2.36E-08 | count | 1 |
| LINC01004 | 0.2738071 | 0.6002089 | 0.4562 | 0.648 | 2.37E-08 | count | 1 |
| CCDC17 | 0.6942017 | 0.9549807 | 0.7269 | 0.467 | 2.42E-08 | count | 1 |
| AC127002.1 | 0.7073185 | 0.9469833 | 0.7469 | 0.455 | 2.47E-08 | count | 1 |
| FOXO6 | 0.7073185 | 0.834073 | 0.848 | 0.396 | 2.47E-08 | count | 1 |
| AC112236.2 | 0.7073185 | 0.8792858 | 0.8044 | 0.421 | 2.47E-08 | count | 1 |
| TRBV7-9 | 0.2862042 | 0.7225971 | 0.3961 | 0.692 | 2.48E-08 | count | 1 |
| AC145207.2 | 0.2862042 | 0.6777199 | 0.4223 | 0.673 | 2.48E-08 | count | 1 |
| CCNI2 | 0.2862042 | 0.5434739 | 0.5266 | 0.598 | 2.48E-08 | count | 1 |
| KIR3DX1 | 0.7356275 | 0.9880674 | 0.7445 | 0.457 | 2.59E-08 | count | 1 |
| SLC4A5 | 0.7356275 | 0.896781 | 0.8203 | 0.412 | 2.59E-08 | count | 1 |
| SLC22A23 | 0.7356275 | 0.7561556 | 0.9729 | 0.331 | 2.59E-08 | count | 1 |
| EXTL1 | 0.7364633 | 0.8801867 | 0.8367 | 0.403 | 2.59E-08 | count | 1 |
| SLC35F3 | 0.7364633 | 0.7335385 | 1.004 | 0.315 | 2.59E-08 | count | 1 |
| TSPAN7 | 0.304924 | 0.6656839 | 0.4581 | 0.647 | 2.66E-08 | count | 1 |
| LINC02035 | 0.7566004 | 0.7468395 | 1.0131 | 0.311 | 2.67E-08 | count | 1 |
| GPC2 | 0.7665053 | 0.6168389 | 1.2426 | 0.214 | 2.71E-08 | count | 1 |
| CYP4F12 | 0.3126974 | 0.6948219 | 0.45 | 0.653 | 2.73E-08 | count | 1 |
| AP000866.1 | 0.7731899 | 0.6731276 | 1.1487 | 0.251 | 2.74E-08 | count | 1 |
| SEMA6B | 0.7731899 | 0.6731276 | 1.1487 | 0.251 | 2.74E-08 | count | 1 |
| SERP2 | 1.8718022 | 1.0357889 | 1.8071 | 0.0708 | 2.77E-08 | count | 1 |
| TRAV8-1 | 1.8718022 | 0.9432848 | 1.9843 | 0.0473 | 2.77E-08 | count | 1 |
| CFAP54 | 1.8718022 | 0.8934484 | 2.095 | 0.0362 | 2.77E-08 | count | 1 |
| ASAH2B | 0.3178137 | 0.5840279 | 0.5442 | 0.586 | 2.78E-08 | count | 1 |
| IPO4 | 0.7941978 | 0.8184452 | 0.9704 | 0.332 | 2.82E-08 | count | 1 |
| LIN37 | 0.322609 | 0.5444839 | 0.5925 | 0.554 | 2.82E-08 | count | 1 |
| TIGD6 | 0.7941978 | 0.8301138 | 0.9567 | 0.339 | 2.82E-08 | count | 1 |
| ST3GAL5-AS1 | 0.7941978 | 1.1115988 | 0.7145 | 0.475 | 2.82E-08 | count | 1 |
| PAK6 | 0.7941978 | 0.8701722 | 0.9127 | 0.361 | 2.82E-08 | count | 1 |
| C11orf74 | 0.7941978 | 0.771292 | 1.0297 | 0.303 | 2.82E-08 | count | 1 |
| CIART | 0.7941978 | 0.7784749 | 1.0202 | 0.308 | 2.82E-08 | count | 1 |
| ADNP-AS1 | 0.7941978 | 1.0501593 | 0.7563 | 0.45 | 2.82E-08 | count | 1 |
| AC027682.1 | 0.7941978 | 0.9755296 | 0.8141 | 0.416 | 2.82E-08 | count | 1 |
| SLC39A14 | 0.3248961 | 0.5199673 | 0.6248 | 0.532 | 2.84E-08 | count | 1 |

| | | | | | | | |
|---|---|---|---|---|---|---|---|
| LINC01473 | 0.3279762 | 0.7115646 | 0.4609 | 0.645 | 2.87E-08 | count | 1 |
| CDNF | 0.8177995 | 0.758516 | 1.0782 | 0.281 | 2.92E-08 | count | 1 |
| ZDHHC11 | 0.8271381 | 0.857732 | 0.9643 | 0.335 | 2.96E-08 | count | 1 |
| AC006213.2 | 0.8271381 | 0.7838846 | 1.0552 | 0.291 | 2.96E-08 | count | 1 |
| AL359513.1 | 0.3369405 | 0.387891 | 0.8686 | 0.385 | 2.96E-08 | count | 1 |
| LINC01465 | 0.3459882 | 0.6034259 | 0.5734 | 0.566 | 3.04E-08 | count | 1 |
| AC004771.3 | 0.8510615 | 0.9025577 | 0.9429 | 0.346 | 3.06E-08 | count | 1 |
| FCER1A | 0.8791232 | 1.0648239 | 0.8256 | 0.409 | 3.17E-08 | count | 1 |
| TNXB | 0.8791232 | 0.9728518 | 0.9037 | 0.366 | 3.17E-08 | count | 1 |
| PIP5KL1 | 0.8791232 | 0.9728518 | 0.9037 | 0.366 | 3.17E-08 | count | 1 |
| KRT2 | 0.8791232 | 0.7883703 | 1.1151 | 0.265 | 3.17E-08 | count | 1 |
| AC007216.2 | 0.8791232 | 0.8657676 | 1.0154 | 0.31 | 3.17E-08 | count | 1 |
| ZKSCAN2 | 0.8804428 | 0.9181645 | 0.9589 | 0.338 | 3.18E-08 | count | 1 |
| ZNF793-AS1 | 0.8804428 | 0.7442463 | 1.183 | 0.237 | 3.18E-08 | count | 1 |
| KCNH3 | 0.9195593 | 0.9963526 | 0.9229 | 0.356 | 3.34E-08 | count | 1 |
| CAND2 | 0.9195593 | 0.9642253 | 0.9537 | 0.34 | 3.34E-08 | count | 1 |
| FUK | 0.3870159 | 0.4767668 | 0.8118 | 0.417 | 3.44E-08 | count | 1 |
| URB1 | 0.3880284 | 0.3957044 | 0.9806 | 0.327 | 3.45E-08 | count | 1 |
| RMRP | 0.3901776 | 0.7036631 | 0.5545 | 0.579 | 3.47E-08 | count | 1 |
| SENP8 | 0.9564443 | 0.727834 | 1.3141 | 0.189 | 3.50E-08 | count | 1 |
| PHEX | 0.9564443 | 0.6070438 | 1.5756 | 0.115 | 3.50E-08 | count | 1 |
| PIK3IP1-AS1 | 0.3953575 | 0.6723908 | 0.588 | 0.557 | 3.52E-08 | count | 1 |
| ISLR | 0.9616537 | 0.7897781 | 1.2176 | 0.223 | 3.52E-08 | count | 1 |
| AC024361.2 | 0.9616537 | 1.4154585 | 0.6794 | 0.497 | 3.52E-08 | count | 1 |
| AL121845.4 | 0.9616537 | 0.9383958 | 1.0248 | 0.306 | 3.52E-08 | count | 1 |
| AC124242.1 | 0.9616537 | 1.0121267 | 0.9501 | 0.342 | 3.52E-08 | count | 1 |
| TRBV6-1 | 0.9616537 | 1.0121267 | 0.9501 | 0.342 | 3.52E-08 | count | 1 |
| DBNDD1 | 0.9616537 | 0.9154123 | 1.0505 | 0.294 | 3.52E-08 | count | 1 |
| PCBP3 | 0.9616537 | 0.7152132 | 1.3446 | 0.179 | 3.52E-08 | count | 1 |
| AC104035.1 | 2.371122 | 0.7296513 | 3.2497 | 0.0012 | 3.54E-08 | count | 1 |
| TRBV21-1 | 0.9673459 | 0.4729666 | 2.0453 | 0.0409 | 3.54E-08 | count | 1 |
| AC040977.1 | 0.4021756 | 0.5547969 | 0.7249 | 0.469 | 3.58E-08 | count | 1 |
| MRVI1 | 0.9846457 | 1.1262 | 0.8743 | 0.382 | 3.61E-08 | count | 1 |
| AC007993.3 | 0.9846457 | 0.8398505 | 1.1724 | 0.241 | 3.62E-08 | count | 1 |
| CDO1 | 0.4066228 | 0.6186265 | 0.6573 | 0.511 | 3.63E-08 | count | 1 |
| LAMA2 | 0.9953081 | 1.1451574 | 0.8691 | 0.385 | 3.66E-08 | count | 1 |
| TRPV1 | 0.9953081 | 0.8846778 | 1.1251 | 0.261 | 3.66E-08 | count | 1 |
| AC092053.3 | 0.9953081 | 0.8846778 | 1.1251 | 0.261 | 3.66E-08 | count | 1 |
| AP003392.6 | 0.9953081 | 0.8878298 | 1.1211 | 0.262 | 3.66E-08 | count | 1 |
| AMY2B | 0.9953081 | 1.0976168 | 0.9068 | 0.365 | 3.66E-08 | count | 1 |
| AC090948.2 | 0.9953081 | 1.0479216 | 0.9498 | 0.342 | 3.66E-08 | count | 1 |
| SYK | 1.01518 | 0.7782715 | 1.3044 | 0.192 | 3.75E-08 | count | 1 |
| TMC3-AS1 | 1.0245043 | 0.7800833 | 1.3133 | 0.189 | 3.78E-08 | count | 1 |
| PLEKHH2 | 0.4271103 | 0.4639426 | 0.9206 | 0.357 | 3.83E-08 | count | 1 |
| PXT1 | 0.4307813 | 0.6202224 | 0.6946 | 0.487 | 3.86E-08 | count | 1 |

| | | | | | | | |
|---|---|---|---|---|---|---|---|
| AL137003.1 | 0.4331785 | 0.6633703 | 0.653 | 0.514 | 3.89E-08 | count | 1 |
| NKD1 | 0.434296 | 0.548287 | 0.7921 | 0.428 | 3.90E-08 | count | 1 |
| LINC01679 | 0.4357785 | 0.6514764 | 0.6689 | 0.504 | 3.91E-08 | count | 1 |
| AC096992.2 | 1.0537404 | 0.6792589 | 1.5513 | 0.121 | 3.91E-08 | count | 1 |
| GRAMD1C | 1.0674303 | 0.9762793 | 1.0934 | 0.274 | 3.97E-08 | count | 1 |
| PZP | 1.0674303 | 0.6162989 | 1.732 | 0.0834 | 3.97E-08 | count | 1 |
| UCN | 1.0691226 | 0.8800905 | 1.2148 | 0.225 | 3.98E-08 | count | 1 |
| NT5E | 1.0691226 | 1.0607096 | 1.0079 | 0.314 | 3.98E-08 | count | 1 |
| MCOLN3 | 1.0691226 | 0.7543212 | 1.4173 | 0.156 | 3.98E-08 | count | 1 |
| AL031663.3 | 1.0934287 | 0.8355869 | 1.3086 | 0.191 | 4.09E-08 | count | 1 |
| AC012358.3 | 1.0934287 | 0.9168668 | 1.1926 | 0.233 | 4.09E-08 | count | 1 |
| C2CD2 | 0.4556929 | 0.5134166 | 0.8876 | 0.375 | 4.11E-08 | count | 1 |
| EVI5 | 0.4667918 | 0.6079771 | 0.7678 | 0.443 | 4.22E-08 | count | 1 |
| AL024507.2 | 1.1298648 | 0.636394 | 1.7754 | 0.0759 | 4.24E-08 | count | 1 |
| DOK6 | 0.4714661 | 0.4384991 | 1.0752 | 0.282 | 4.27E-08 | count | 1 |
| ZNF610 | 1.1407733 | 0.907411 | 1.2572 | 0.209 | 4.30E-08 | count | 1 |
| LINC00504 | 1.1407733 | 0.8547658 | 1.3346 | 0.182 | 4.30E-08 | count | 1 |
| AC018362.1 | 0.4750965 | 0.7256949 | 0.6547 | 0.513 | 4.30E-08 | count | 1 |
| YY2 | 1.1607197 | 1.0168764 | 1.1415 | 0.254 | 4.38E-08 | count | 1 |
| ZNF556 | 1.1607197 | 1.4550887 | 0.7977 | 0.425 | 4.38E-08 | count | 1 |
| GP1BA | 1.1607197 | 1.4550887 | 0.7977 | 0.425 | 4.38E-08 | count | 1 |
| Z95115.1 | 1.1607197 | 1.171489 | 0.9908 | 0.322 | 4.38E-08 | count | 1 |
| IL20RB | 1.1607197 | 1.171489 | 0.9908 | 0.322 | 4.38E-08 | count | 1 |
| AC139795.2 | 1.1607197 | 1.171489 | 0.9908 | 0.322 | 4.38E-08 | count | 1 |
| TMEM232 | 1.1607197 | 1.2114579 | 0.9581 | 0.338 | 4.38E-08 | count | 1 |
| SHB | 1.1607197 | 1.2114579 | 0.9581 | 0.338 | 4.38E-08 | count | 1 |
| AC016588.2 | 1.1607197 | 1.2114579 | 0.9581 | 0.338 | 4.38E-08 | count | 1 |
| AL356215.1 | 1.1607197 | 1.171489 | 0.9908 | 0.322 | 4.38E-08 | count | 1 |
| AC010186.1 | 1.1607197 | 1.2114579 | 0.9581 | 0.338 | 4.38E-08 | count | 1 |
| AC107241.1 | 1.1607197 | 1.2114579 | 0.9581 | 0.338 | 4.38E-08 | count | 1 |
| SYCE2 | 1.1607197 | 1.2114579 | 0.9581 | 0.338 | 4.38E-08 | count | 1 |
| ACY3 | 1.1607197 | 1.2114579 | 0.9581 | 0.338 | 4.38E-08 | count | 1 |
| TRIM7 | 1.1607197 | 1.2114579 | 0.9581 | 0.338 | 4.38E-08 | count | 1 |
| OR4D9 | 1.1607197 | 1.2114579 | 0.9581 | 0.338 | 4.38E-08 | count | 1 |
| AC008878.3 | 1.1607197 | 1.2114579 | 0.9581 | 0.338 | 4.38E-08 | count | 1 |
| AL022341.1 | 1.1607197 | 1.2114579 | 0.9581 | 0.338 | 4.38E-08 | count | 1 |
| TBC1D8 | 1.1607197 | 1.2114579 | 0.9581 | 0.338 | 4.38E-08 | count | 1 |
| TBC1D12 | 1.1607197 | 1.0235038 | 1.1341 | 0.257 | 4.39E-08 | count | 1 |
| AC006042.4 | 1.1607197 | 1.06902 | 1.0858 | 0.278 | 4.39E-08 | count | 1 |
| ZSCAN5A | 0.4900253 | 0.4205951 | 1.1651 | 0.244 | 4.45E-08 | count | 1 |
| FASN | 0.4933125 | 0.3895169 | 1.2665 | 0.205 | 4.49E-08 | count | 1 |
| XPNPEP2 | 1.2101044 | 1.3888675 | 0.8713 | 0.384 | 4.60E-08 | count | 1 |
| RECQL4 | 1.2262057 | 0.833395 | 1.4713 | 0.141 | 4.67E-08 | count | 1 |
| CTTN | 0.5122019 | 0.6633135 | 0.7722 | 0.44 | 4.68E-08 | count | 1 |
| AVIL | 0.5172431 | 0.7237994 | 0.7146 | 0.475 | 4.73E-08 | count | 1 |

| | | | | | | | |
|---|---|---|---|---|---|---|---|
| AL596202.1 | 0.5172431 | 0.5969246 | 0.8665 | 0.386 | 4.73E-08 | count | 1 |
| AL139099.1 | 1.2408225 | 0.9146827 | 1.3566 | 0.175 | 4.73E-08 | count | 1 |
| PNMA5 | 1.2408225 | 0.6823729 | 1.8184 | 0.0691 | 4.73E-08 | count | 1 |
| AC008914.1 | 1.2476111 | 0.6457894 | 1.9319 | 0.0535 | 4.76E-08 | count | 1 |
| EGFL6 | 0.533107 | 0.7531178 | 0.7079 | 0.479 | 4.89E-08 | count | 1 |
| EPB41L3 | 0.533107 | 0.583726 | 0.9133 | 0.361 | 4.89E-08 | count | 1 |
| PRR29-AS1 | 1.2770258 | 1.0186153 | 1.2537 | 0.21 | 4.90E-08 | count | 1 |
| AP000442.2 | 1.2897664 | 0.8511664 | 1.5153 | 0.13 | 4.95E-08 | count | 1 |
| ZNF501 | 1.2897664 | 0.8215539 | 1.5699 | 0.117 | 4.95E-08 | count | 1 |
| AC009283.1 | 1.2897664 | 1.2224008 | 1.0551 | 0.291 | 4.96E-08 | count | 1 |
| AC092375.2 | 1.2897664 | 1.1861074 | 1.0874 | 0.277 | 4.96E-08 | count | 1 |
| DENND2A | 1.2897664 | 1.1861074 | 1.0874 | 0.277 | 4.96E-08 | count | 1 |
| RORB | 1.2897664 | 1.1861074 | 1.0874 | 0.277 | 4.96E-08 | count | 1 |
| AC067747.1 | 1.2897664 | 1.1861074 | 1.0874 | 0.277 | 4.96E-08 | count | 1 |
| AL512353.1 | 1.2897664 | 0.9654361 | 1.3359 | 0.182 | 4.96E-08 | count | 1 |
| AL160006.1 | 1.2897664 | 0.9654361 | 1.3359 | 0.182 | 4.96E-08 | count | 1 |
| SEMA5A | 1.2897664 | 1.138477 | 1.1329 | 0.257 | 4.96E-08 | count | 1 |
| PARD6G | 0.5413041 | 0.4602863 | 1.176 | 0.24 | 4.97E-08 | count | 1 |
| RASSF8 | 1.3513274 | 0.9737954 | 1.3877 | 0.165 | 5.23E-08 | count | 1 |
| AC139887.4 | 1.3513274 | 0.797519 | 1.6944 | 0.0903 | 5.23E-08 | count | 1 |
| TCAP | 1.3604023 | 0.8538466 | 1.5933 | 0.111 | 5.27E-08 | count | 1 |
| CTPS2 | 0.5704541 | 0.6155326 | 0.9268 | 0.354 | 5.27E-08 | count | 1 |
| FSTL3 | 0.5805312 | 0.8141797 | 0.713 | 0.476 | 5.37E-08 | count | 1 |
| AP000904.1 | 1.4108543 | 1.569771 | 0.8988 | 0.369 | 5.50E-08 | count | 1 |
| CMYA5 | 1.4108543 | 1.569771 | 0.8988 | 0.369 | 5.50E-08 | count | 1 |
| RGPD8 | 1.4108543 | 0.9875098 | 1.4287 | 0.153 | 5.50E-08 | count | 1 |
| AC022098.4 | 1.4108543 | 0.9875098 | 1.4287 | 0.153 | 5.50E-08 | count | 1 |
| CELSR2 | 1.4108543 | 0.9875098 | 1.4287 | 0.153 | 5.50E-08 | count | 1 |
| SHISA4 | 0.5939002 | 0.6297879 | 0.943 | 0.346 | 5.51E-08 | count | 1 |
| AC124016.2 | 1.4176585 | 0.7974839 | 1.7777 | 0.0755 | 5.53E-08 | count | 1 |
| OSBPL1A | 1.4240142 | 0.7979748 | 1.7845 | 0.0744 | 5.55E-08 | count | 1 |
| AC097724.1 | 1.4240142 | 1.5703163 | 0.9068 | 0.365 | 5.55E-08 | count | 1 |
| SYCP3 | 1.4240142 | 1.2636411 | 1.1269 | 0.26 | 5.56E-08 | count | 1 |
| ALPK3 | 1.4240142 | 1.2636411 | 1.1269 | 0.26 | 5.56E-08 | count | 1 |
| Z97989.1 | 1.4240142 | 1.2656776 | 1.1251 | 0.261 | 5.56E-08 | count | 1 |
| AL121987.2 | 1.4240142 | 1.2656776 | 1.1251 | 0.261 | 5.56E-08 | count | 1 |
| KCNN1 | 1.4240142 | 1.2656776 | 1.1251 | 0.261 | 5.56E-08 | count | 1 |
| AC008906.1 | 1.4240142 | 1.1772644 | 1.2096 | 0.227 | 5.56E-08 | count | 1 |
| Z83847.1 | 1.4240142 | 1.1772644 | 1.2096 | 0.227 | 5.56E-08 | count | 1 |
| ADPGK-AS1 | 1.4240142 | 1.1772644 | 1.2096 | 0.227 | 5.56E-08 | count | 1 |
| AC006160.1 | 1.4240142 | 1.1772644 | 1.2096 | 0.227 | 5.56E-08 | count | 1 |
| USP54 | 1.481989 | 0.8514595 | 1.7405 | 0.0819 | 5.80E-08 | count | 1 |
| AC108866.1 | 1.481989 | 0.9281724 | 1.5967 | 0.11 | 5.80E-08 | count | 1 |
| POU2AF1 | 0.6276772 | 0.8621982 | 0.728 | 0.467 | 5.87E-08 | count | 1 |
| STK36 | 0.6276772 | 0.8469675 | 0.7411 | 0.459 | 5.87E-08 | count | 1 |

| | | | | | | | |
|---|---|---|---|---|---|---|---|
| CYP4F3 | 17.9720884 | 1881.030208 | 0.0096 | 0.992 | 5.92E-08 | count | 1 |
| HIST1H3I | 18.1245047 | 1042.549167 | 0.0174 | 0.986 | 5.93E-08 | count | 1 |
| TERC | 17.9702876 | 1127.235911 | 0.0159 | 0.987 | 5.93E-08 | count | 1 |
| CXCL14 | 18.1700581 | 1841.699079 | 0.0099 | 0.992 | 5.95E-08 | count | 1 |
| LINC00886 | 17.8335201 | 1571.495427 | 0.0113 | 0.991 | 5.98E-08 | count | 1 |
| LRRK2 | 18.4858162 | 1898.006846 | 0.0097 | 0.992 | 5.98E-08 | count | 1 |
| AL109767.1 | 17.9284343 | 1136.200589 | 0.0158 | 0.987 | 5.98E-08 | count | 1 |
| AC010336.1 | 17.8331807 | 1291.984178 | 0.0138 | 0.989 | 5.98E-08 | count | 1 |
| ABALON | 17.7267201 | 1192.807012 | 0.0149 | 0.988 | 5.99E-08 | count | 1 |
| AL358472.2 | 18.3652789 | 1483.300495 | 0.0124 | 0.99 | 5.99E-08 | count | 1 |
| AL451060.1 | 17.6980701 | 1185.272405 | 0.0149 | 0.988 | 6.02E-08 | count | 1 |
| CCDC114 | 17.9594146 | 1202.806306 | 0.0149 | 0.988 | 6.02E-08 | count | 1 |
| C1QTNF3 | 1.5326811 | 1.1244963 | 1.363 | 0.173 | 6.03E-08 | count | 1 |
| SYT15 | 1.5298894 | 1.3271914 | 1.1527 | 0.249 | 6.04E-08 | count | 1 |
| AC034102.4 | 1.5298894 | 1.3271914 | 1.1527 | 0.249 | 6.04E-08 | count | 1 |
| ASB9 | 1.5298894 | 1.0929907 | 1.3997 | 0.162 | 6.04E-08 | count | 1 |
| FFAR3 | 1.5298894 | 1.0929907 | 1.3997 | 0.162 | 6.04E-08 | count | 1 |
| AL390957.1 | 1.5298894 | 1.0929907 | 1.3997 | 0.162 | 6.04E-08 | count | 1 |
| AC010503.4 | 1.5298894 | 1.0929907 | 1.3997 | 0.162 | 6.04E-08 | count | 1 |
| WFS1 | 1.5298894 | 1.0929907 | 1.3997 | 0.162 | 6.04E-08 | count | 1 |
| AL122035.2 | 1.5298894 | 1.0929907 | 1.3997 | 0.162 | 6.04E-08 | count | 1 |
| CLIP3 | 1.5298894 | 1.1802593 | 1.2962 | 0.195 | 6.04E-08 | count | 1 |
| AC012640.2 | 0.6473755 | 0.5830644 | 1.1103 | 0.267 | 6.08E-08 | count | 1 |
| AC023355.1 | 0.6629039 | 0.6644683 | 0.9976 | 0.319 | 6.24E-08 | count | 1 |
| AC105277.1 | 0.6633154 | 0.8694395 | 0.7629 | 0.446 | 6.24E-08 | count | 1 |
| AL451074.2 | 1.5820218 | 1.0033554 | 1.5767 | 0.115 | 6.26E-08 | count | 1 |
| BACE1-AS | 1.5820218 | 0.8876072 | 1.7823 | 0.0748 | 6.26E-08 | count | 1 |
| WDFY3 | 0.6721052 | 0.7973751 | 0.8429 | 0.399 | 6.34E-08 | count | 1 |
| ADORA2A-AS1 | 0.682179 | 0.8848143 | 0.771 | 0.441 | 6.45E-08 | count | 1 |
| CHRNA1 | 1.6306401 | 0.788424 | 2.0682 | 0.0387 | 6.46E-08 | count | 1 |
| LINC01551 | 1.6303283 | 1.3658462 | 1.1936 | 0.233 | 6.48E-08 | count | 1 |
| FSD1 | 1.6303283 | 1.0992148 | 1.4832 | 0.138 | 6.48E-08 | count | 1 |
| AF129408.1 | 1.6303283 | 1.0992148 | 1.4832 | 0.138 | 6.48E-08 | count | 1 |
| RAB9B | 1.6303283 | 1.0992148 | 1.4832 | 0.138 | 6.48E-08 | count | 1 |
| AP001412.1 | 1.6303283 | 1.023866 | 1.5923 | 0.111 | 6.48E-08 | count | 1 |
| NUDT11 | 1.6303283 | 1.023866 | 1.5923 | 0.111 | 6.48E-08 | count | 1 |
| FAM95C | 1.6296627 | 1.1610155 | 1.4037 | 0.161 | 6.49E-08 | count | 1 |
| MYO5C | 1.6303283 | 1.1070904 | 1.4726 | 0.141 | 6.49E-08 | count | 1 |
| TRBV7-3 | 1.6296627 | 0.964408 | 1.6898 | 0.0912 | 6.49E-08 | count | 1 |
| GPA33 | 1.6296627 | 0.9039514 | 1.8028 | 0.0715 | 6.49E-08 | count | 1 |
| DNAH1 | 1.675996 | 0.8163048 | 2.0531 | 0.0401 | 6.68E-08 | count | 1 |
| CIPC | 0.7073185 | 0.5258087 | 1.3452 | 0.179 | 6.72E-08 | count | 1 |
| CDK3 | 0.7104672 | 0.6693171 | 1.0615 | 0.289 | 6.75E-08 | count | 1 |
| IL1RL1 | 1.7219161 | 1.0199307 | 1.6883 | 0.0915 | 6.88E-08 | count | 1 |
| TTC7B | 1.7234441 | 1.0061791 | 1.7129 | 0.0868 | 6.90E-08 | count | 1 |

| | | | | | | | |
|---|---|---|---|---|---|---|---|
| PID1 | 1.7234441 | 1.6469779 | 1.0464 | 0.295 | 6.90E-08 | count | 1 |
| AC133919.1 | 1.7234441 | 0.9203605 | 1.8726 | 0.0612 | 6.90E-08 | count | 1 |
| ZNF81 | 0.729193 | 0.5047491 | 1.4447 | 0.149 | 6.96E-08 | count | 1 |
| RERGL | 0.7356275 | 0.6788167 | 1.0837 | 0.279 | 7.02E-08 | count | 1 |
| BACE1 | 1.7636814 | 0.8355979 | 2.1107 | 0.0349 | 7.08E-08 | count | 1 |
| HOMER1 | 0.7428895 | 0.436428 | 1.7022 | 0.0888 | 7.11E-08 | count | 1 |
| MCTP1 | 0.7447586 | 0.6282049 | 1.1855 | 0.236 | 7.13E-08 | count | 1 |
| ACRBP | 1.800293 | 1.4529054 | 1.2391 | 0.215 | 7.23E-08 | count | 1 |
| DNAJC25-GNG10 | 1.800293 | 1.4328971 | 1.2564 | 0.209 | 7.23E-08 | count | 1 |
| DNMBP-AS1 | 1.800293 | 1.1631322 | 1.5478 | 0.122 | 7.23E-08 | count | 1 |
| TTC26 | 1.800293 | 1.3418302 | 1.3417 | 0.18 | 7.23E-08 | count | 1 |
| AC004130.1 | 1.800293 | 1.1922581 | 1.51 | 0.131 | 7.24E-08 | count | 1 |
| UBAP1L | 1.8057298 | 1.0843389 | 1.6653 | 0.0959 | 7.27E-08 | count | 1 |
| HEATR9 | 1.8057298 | 1.0351002 | 1.7445 | 0.0812 | 7.28E-08 | count | 1 |
| HIST1H2BD | 0.7654299 | 0.5331087 | 1.4358 | 0.151 | 7.35E-08 | count | 1 |
| AC008440.1 | 1.8718022 | 0.8269399 | 2.2635 | 0.0237 | 7.52E-08 | count | 1 |
| PLS1 | 0.7813026 | 0.5513467 | 1.4171 | 0.157 | 7.53E-08 | count | 1 |
| SMIM33 | 1.8788976 | 0.9690206 | 1.939 | 0.0526 | 7.60E-08 | count | 1 |
| PTGDR2 | 1.8902352 | 1.215917 | 1.5546 | 0.12 | 7.62E-08 | count | 1 |
| U91328.1 | 1.9449673 | 1.3374289 | 1.4543 | 0.146 | 7.86E-08 | count | 1 |
| ZCWPW2 | 1.9449673 | 1.3374289 | 1.4543 | 0.146 | 7.86E-08 | count | 1 |
| HEMGN | 0.8144499 | 0.6303479 | 1.2921 | 0.196 | 7.89E-08 | count | 1 |
| AC083973.1 | 0.8144499 | 0.5565651 | 1.4634 | 0.143 | 7.90E-08 | count | 1 |
| E2F2 | 1.9535838 | 1.3194334 | 1.4806 | 0.139 | 7.92E-08 | count | 1 |
| DUOX1 | 1.9580315 | 0.8465281 | 2.313 | 0.0208 | 7.92E-08 | count | 1 |
| IL17RB | 1.9625745 | 1.7107378 | 1.1472 | 0.251 | 7.96E-08 | count | 1 |
| AC106047.1 | 0.8227476 | 0.6724776 | 1.2235 | 0.221 | 7.99E-08 | count | 1 |
| AL357033.4 | 0.8369351 | 0.8770092 | 0.9543 | 0.34 | 8.14E-08 | count | 1 |
| SGCE | 0.8369351 | 0.703792 | 1.1892 | 0.234 | 8.14E-08 | count | 1 |
| SLC5A2 | 0.8369351 | 0.7166944 | 1.1678 | 0.243 | 8.15E-08 | count | 1 |
| PRR36 | 2.0147554 | 1.2043737 | 1.6729 | 0.0944 | 8.17E-08 | count | 1 |
| TCTEX1D4 | 2.0147554 | 1.1311592 | 1.7811 | 0.075 | 8.17E-08 | count | 1 |
| LINC01422 | 2.0812979 | 1.5376305 | 1.3536 | 0.176 | 8.45E-08 | count | 1 |
| ST6GALNAC3 | 2.0812979 | 1.0573702 | 1.9684 | 0.0491 | 8.46E-08 | count | 1 |
| S100A12 | 2.0919243 | 1.0289169 | 2.0331 | 0.0421 | 8.50E-08 | count | 1 |
| LRRC37A3 | 2.0919243 | 1.0388755 | 2.0136 | 0.0441 | 8.51E-08 | count | 1 |
| ESR2 | 2.133791 | 1.0963501 | 1.9463 | 0.0517 | 8.68E-08 | count | 1 |
| AL359711.2 | 0.8963757 | 0.568425 | 1.5769 | 0.115 | 8.82E-08 | count | 1 |
| DLGAP1-AS2 | 0.9001814 | 0.5445756 | 1.653 | 0.0984 | 8.87E-08 | count | 1 |
| ATP6V0E2-AS1 | 2.193856 | 1.128667 | 1.9438 | 0.052 | 8.93E-08 | count | 1 |
| GPR27 | 2.2052881 | 1.0038763 | 2.1968 | 0.0281 | 8.98E-08 | count | 1 |
| EPGN | 2.2052881 | 0.9758261 | 2.2599 | 0.0239 | 8.99E-08 | count | 1 |
| NLGN2 | 0.9275408 | 0.6038951 | 1.5359 | 0.125 | 9.17E-08 | count | 1 |
| ZNF768 | 0.9584132 | 0.6208951 | 1.5436 | 0.123 | 9.53E-08 | count | 1 |
| KLRC3 | 0.9584132 | 0.6142896 | 1.5602 | 0.119 | 9.53E-08 | count | 1 |

| | | | | | | | |
|---|---|---|---|---|---|---|---|
| BAIAP2L1 | 2.3471256 | 1.2375304 | 1.8966 | 0.058 | 9.54E-08 | count | 1 |
| GASAL1 | 0.9718402 | 0.5727906 | 1.6967 | 0.0898 | 9.68E-08 | count | 1 |
| LINC02029 | 0.974132 | 0.8508799 | 1.1449 | 0.252 | 9.70E-08 | count | 1 |
| AC074117.1 | 0.979116 | 0.564277 | 1.7352 | 0.0828 | 9.77E-08 | count | 1 |
| AP002986.1 | 2.446075 | 1.3531209 | 1.8077 | 0.0707 | 9.92E-08 | count | 1 |
| SNPH | 2.6169699 | 1.1237061 | 2.3289 | 0.0199 | 1.05E-07 | count | 1 |
| EPPK1 | 1.0557664 | 0.6693144 | 1.5774 | 0.115 | 1.07E-07 | count | 1 |
| SNX33 | 1.1160501 | 0.7072072 | 1.5781 | 0.115 | 1.14E-07 | count | 1 |
| VASN | 1.1607197 | 0.7092809 | 1.6365 | 0.102 | 1.19E-07 | count | 1 |
| AC022021.1 | 1.1607197 | 0.7240474 | 1.6031 | 0.109 | 1.19E-07 | count | 1 |
| AC106739.1 | 1.1607197 | 0.7924313 | 1.4648 | 0.143 | 1.19E-07 | count | 1 |
| VDR | 1.1607197 | 0.7664931 | 1.5143 | 0.13 | 1.19E-07 | count | 1 |
| BX323046.1 | 1.1728771 | 0.8483935 | 1.3825 | 0.167 | 1.20E-07 | count | 1 |
| LINC01132 | 1.2152053 | 0.7143482 | 1.7011 | 0.089 | 1.26E-07 | count | 1 |
| ZKSCAN7 | 1.2183574 | 0.8157481 | 1.4935 | 0.135 | 1.26E-07 | count | 1 |
| AC025171.5 | 1.3051157 | 0.8792019 | 1.4844 | 0.138 | 1.36E-07 | count | 1 |
| CCDC168 | 1.3377207 | 0.9226358 | 1.4499 | 0.147 | 1.40E-07 | count | 1 |
| AC022075.1 | 1.3848753 | 0.5890265 | 2.3511 | 0.0188 | 1.46E-07 | count | 1 |
| AL353759.1 | 1.4000952 | 0.6119573 | 2.2879 | 0.0222 | 1.48E-07 | count | 1 |
| AKAP12 | 1.4108543 | 0.8639773 | 1.633 | 0.103 | 1.50E-07 | count | 1 |
| PAXBP1-AS1 | 1.4599812 | 0.7738868 | 1.8866 | 0.0593 | 1.55E-07 | count | 1 |
| AC103724.3 | 1.505772 | 0.6306321 | 2.3877 | 0.017 | 1.61E-07 | count | 1 |
| TRAV5 | 1.6753874 | 0.9717438 | 1.7241 | 0.0848 | 1.81E-07 | count | 1 |
| PCP2 | 1.675996 | 0.9292652 | 1.8036 | 0.0714 | 1.81E-07 | count | 1 |
| CR1 | 1.6766291 | 1.2838927 | 1.3059 | 0.192 | 1.82E-07 | count | 1 |
| NCKAP1 | 1.69156 | 0.7918536 | 2.1362 | 0.0327 | 1.83E-07 | count | 1 |
| MME | 1.7190224 | 1.2201284 | 1.4089 | 0.159 | 1.87E-07 | count | 1 |
| PLB1 | 2.1080331 | 0.5606645 | 3.7599 | 2.00E-04 | 2.34E-07 | count | 1 |
| FBLN1 | 2.3785743 | 0.7398109 | 3.2151 | 0.0013 | 2.63E-07 | count | 1 |
| AC139720.1 | 0.9718236 | 0.3541637 | 2.744 | 0.0061 | 2.65E-07 | count | 1 |
| HPGDS | 2.6369353 | 1.1951985 | 2.2063 | 0.0274 | 2.89E-07 | count | 1 |
| SPDYA | 2.6450996 | 1.2803916 | 2.0659 | 0.0389 | 2.89E-07 | count | 1 |
| CXCL13 | 1.0916436 | 0.561368 | 1.9446 | 0.0519 | 3.20E-07 | count | 1 |
| IL13 | 3.5729609 | 2.0342394 | 1.7564 | 0.0791 | 1.10E-06 | count | 1 |
| LINC00909 | 1.46E-05 | 0.1796232 | 1.00E-04 | 1 | 1.91E-05 | count | 1 |
| AC090114.2 | 6.46E-05 | 0.3477364 | 2.00E-04 | 1 | 2.13E-05 | count | 1 |
| PIM1 | 5.44E-05 | 0.08147 | 7.00E-04 | 0.999 | 7.70E-05 | count | 1 |
| PARL | 7.49E-05 | 0.1285425 | 6.00E-04 | 1 | 0.000101448 | count | 1 |
| POR | 0.0001321 | 0.168436 | 8.00E-04 | 0.999 | 0.000158973 | count | 1 |
| TBC1D15 | 0.000211 | 0.1221942 | 0.0017 | 0.999 | 0.000275329 | count | 1 |
| AL139393.2 | 0.0013057 | 0.4611296 | 0.0028 | 0.998 | 0.000700417 | count | 1 |
| THAP11 | 0.0007003 | 0.093755 | 0.0075 | 0.994 | 0.000974143 | count | 1 |
| ATP2A3 | 0.0007595 | 0.1461706 | 0.0052 | 0.996 | 0.001010367 | count | 1 |
| URI1 | 0.0007215 | 0.0760032 | 0.0095 | 0.992 | 0.00101797 | count | 1 |
| HIPK3 | 0.0007894 | 0.1385579 | 0.0057 | 0.995 | 0.00104911 | count | 1 |

| | | | | | | | |
|---|---|---|---|---|---|---|---|
| HMGN4 | 0.0008763 | 0.2101342 | 0.0042 | 0.997 | 0.001081501 | count | 1 |
| NT5C2 | 0.0013347 | 0.2864733 | 0.0047 | 0.996 | 0.001298695 | count | 1 |
| NUTF2 | 0.0009311 | 0.1055771 | 0.0088 | 0.993 | 0.001301793 | count | 1 |
| NSDHL | 0.0012071 | 0.3147112 | 0.0038 | 0.997 | 0.001466236 | count | 1 |
| GSTZ1 | 0.0015077 | 0.3270522 | 0.0046 | 0.996 | 0.001467049 | count | 1 |
| GAPLINC | 0.0034656 | 0.8331217 | 0.0042 | 0.997 | 0.001537768 | count | 1 |
| DHX35 | 0.0082786 | 0.3981326 | 0.0208 | 0.983 | 0.001541947 | count | 1 |
| KLF8 | 0.0083926 | 0.5356038 | 0.0157 | 0.987 | 0.00156322 | count | 1 |
| MCM2 | 0.0048946 | 0.6850807 | 0.0071 | 0.994 | 0.001613752 | count | 1 |
| SLC6A16 | 0.0088106 | 0.777153 | 0.0113 | 0.991 | 0.00164123 | count | 1 |
| AC021028.1 | 0.0088106 | 1.067117 | 0.0083 | 0.993 | 0.00164123 | count | 1 |
| CARD16 | 0.001192 | 0.0814292 | 0.0146 | 0.988 | 0.001690178 | count | 1 |
| BMPR1A | 0.003272 | 0.452962 | 0.0072 | 0.994 | 0.001755754 | count | 1 |
| AC098818.2 | 0.002589 | 0.4839793 | 0.0053 | 0.996 | 0.00175695 | count | 1 |
| MAML1 | 0.0016653 | 0.2503482 | 0.0067 | 0.995 | 0.001772243 | count | 1 |
| GNB5 | 0.0014501 | 0.1645985 | 0.0088 | 0.993 | 0.001818912 | count | 1 |
| ZNF460 | 0.0034461 | 0.327474 | 0.0105 | 0.992 | 0.001849228 | count | 1 |
| TIPRL | 0.0013733 | 0.0941811 | 0.0146 | 0.988 | 0.001918324 | count | 1 |
| OLMALINC | 0.003245 | 0.5777191 | 0.0056 | 0.996 | 0.001991402 | count | 1 |
| ZNF354A | 0.0019606 | 0.2004616 | 0.0098 | 0.992 | 0.002024081 | count | 1 |
| ATG14 | 0.00161 | 0.1888445 | 0.0085 | 0.993 | 0.002055619 | count | 1 |
| BBS9 | 0.0026874 | 0.3417509 | 0.0079 | 0.994 | 0.00210207 | count | 1 |
| PHLPP2 | 0.0116097 | 0.4787793 | 0.0242 | 0.981 | 0.002163986 | count | 1 |
| ECH1 | 0.0016161 | 0.0648188 | 0.0249 | 0.98 | 0.002301574 | count | 1 |
| ZNF543 | 0.0053235 | 0.6854077 | 0.0078 | 0.994 | 0.002362938 | count | 1 |
| ARL6IP1 | 0.0017707 | 0.0848542 | 0.0209 | 0.983 | 0.002482284 | count | 1 |
| CAPN2 | 0.0017884 | 0.0897113 | 0.0199 | 0.984 | 0.002499686 | count | 1 |
| SRP72 | 0.0017812 | 0.082988 | 0.0215 | 0.983 | 0.002514564 | count | 1 |
| MINDY3 | 0.0023416 | 0.2495985 | 0.0094 | 0.993 | 0.002525206 | count | 1 |
| GNB1L | 0.002093 | 0.2274386 | 0.0092 | 0.993 | 0.002553313 | count | 1 |
| SPECC1L | 0.0032043 | 0.33358 | 0.0096 | 0.992 | 0.002640867 | count | 1 |
| MIER2 | 0.0025656 | 0.3437431 | 0.0075 | 0.994 | 0.002691306 | count | 1 |
| CENPC | 0.001935 | 0.0762345 | 0.0254 | 0.98 | 0.002727405 | count | 1 |
| UTP23 | 0.0020562 | 0.1204286 | 0.0171 | 0.986 | 0.002828011 | count | 1 |
| LCP2 | 0.0020589 | 0.0981478 | 0.021 | 0.983 | 0.002866248 | count | 1 |
| CD99 | 0.0020056 | 0.0342867 | 0.0585 | 0.953 | 0.002885247 | count | 1 |
| THSD1 | 0.0088106 | 0.6868916 | 0.0128 | 0.99 | 0.002907097 | count | 1 |
| CHCHD2 | 0.0020538 | 0.0336273 | 0.0611 | 0.951 | 0.002949937 | count | 1 |
| TM9SF1 | 0.0033298 | 0.3754667 | 0.0089 | 0.993 | 0.002976343 | count | 1 |
| CNOT10 | 0.0024376 | 0.1811062 | 0.0135 | 0.989 | 0.00309811 | count | 1 |
| CXXC1 | 0.0025998 | 0.1842423 | 0.0141 | 0.989 | 0.003196949 | count | 1 |
| TRIT1 | 0.0061454 | 0.3748185 | 0.0164 | 0.987 | 0.003299139 | count | 1 |
| TMED9 | 0.0023821 | 0.0799802 | 0.0298 | 0.976 | 0.003341784 | count | 1 |
| DCAF6 | 0.0033577 | 0.2133882 | 0.0157 | 0.987 | 0.003406237 | count | 1 |
| ZNF106 | 0.00262 | 0.1248898 | 0.021 | 0.983 | 0.003573529 | count | 1 |

| | | | | | | | |
|---|---|---|---|---|---|---|---|
| RPL24 | 0.002573 | 0.0179401 | 0.1434 | 0.886 | 0.003707806 | count | 1 |
| FAM216A | 0.0040745 | 0.3631933 | 0.0112 | 0.991 | 0.003761492 | count | 1 |
| ZXDA | 0.0070169 | 0.4289425 | 0.0164 | 0.987 | 0.003767526 | count | 1 |
| PTPRM | 0.0085667 | 0.5077828 | 0.0169 | 0.987 | 0.003804671 | count | 1 |
| TMEM242 | 0.0028502 | 0.1480082 | 0.0193 | 0.985 | 0.003827824 | count | 1 |
| ZDHHC12 | 0.0027739 | 0.1267726 | 0.0219 | 0.983 | 0.003830527 | count | 1 |
| SPON1 | 0.0086605 | 0.4886845 | 0.0177 | 0.986 | 0.003846393 | count | 1 |
| TTC37 | 0.0030178 | 0.1379089 | 0.0219 | 0.983 | 0.003869708 | count | 1 |
| MEAF6 | 0.0027358 | 0.0674751 | 0.0405 | 0.968 | 0.003890407 | count | 1 |
| SRRM2 | 0.0027644 | 0.0483546 | 0.0572 | 0.954 | 0.00394985 | count | 1 |
| MRPS18B | 0.002945 | 0.1303208 | 0.0226 | 0.982 | 0.003984842 | count | 1 |
| PHB2 | 0.0028167 | 0.0673985 | 0.0418 | 0.967 | 0.003990194 | count | 1 |
| CDC34 | 0.0029272 | 0.1055091 | 0.0277 | 0.978 | 0.004003208 | count | 1 |
| GORAB | 0.0034004 | 0.2574654 | 0.0132 | 0.989 | 0.004028114 | count | 1 |
| AL691432.2 | 0.0090892 | 0.5706543 | 0.0159 | 0.987 | 0.004037097 | count | 1 |
| LINC00893 | 0.021861 | 0.3993702 | 0.0547 | 0.956 | 0.004084018 | count | 1 |
| ZCCHC14 | 0.0049615 | 0.3705931 | 0.0134 | 0.989 | 0.004089873 | count | 1 |
| PTBP3 | 0.0029564 | 0.0971195 | 0.0304 | 0.976 | 0.004103102 | count | 1 |
| FAM210B | 0.0031923 | 0.2073139 | 0.0154 | 0.988 | 0.004153066 | count | 1 |
| SMDT1 | 0.0029757 | 0.0582725 | 0.0511 | 0.9593 | 0.004242041 | count | 1 |
| ZNF451 | 0.0032465 | 0.1342592 | 0.0242 | 0.981 | 0.004248882 | count | 1 |
| STRN3 | 0.0032736 | 0.1625384 | 0.0201 | 0.984 | 0.004251996 | count | 1 |
| PPOX | 0.0035966 | 0.2582612 | 0.0139 | 0.989 | 0.004260571 | count | 1 |
| PDE8A | 0.0050831 | 0.2926191 | 0.0174 | 0.986 | 0.004377806 | count | 1 |
| MAN1B1-DT | 0.0044372 | 0.3543054 | 0.0125 | 0.99 | 0.004501709 | count | 1 |
| HSD17B11 | 0.0032709 | 0.0850604 | 0.0385 | 0.969 | 0.004596206 | count | 1 |
| ATP5F1D | 0.003269 | 0.0439812 | 0.0743 | 0.941 | 0.004685651 | count | 1 |
| UBXN6 | 0.0039145 | 0.2206997 | 0.0177 | 0.986 | 0.004711591 | count | 1 |
| CYSLTR1 | 0.0035965 | 0.1476541 | 0.0244 | 0.981 | 0.004713517 | count | 1 |
| QKI | 0.0034746 | 0.1175097 | 0.0296 | 0.976 | 0.004720303 | count | 1 |
| DHRS4-AS1 | 0.0037609 | 0.2303381 | 0.0163 | 0.987 | 0.004851065 | count | 1 |
| C8orf33 | 0.0039883 | 0.1775013 | 0.0225 | 0.982 | 0.004865809 | count | 1 |
| SHMT1 | 0.0051487 | 0.2661158 | 0.0193 | 0.985 | 0.004889042 | count | 1 |
| TGS1 | 0.0037219 | 0.0998664 | 0.0373 | 0.97 | 0.005129017 | count | 1 |
| CUTA | 0.0036372 | 0.0498949 | 0.0729 | 0.9419 | 0.005200039 | count | 1 |
| NDUFA12 | 0.0036946 | 0.0637779 | 0.0579 | 0.954 | 0.00526482 | count | 1 |
| RBM48 | 0.0042127 | 0.1865404 | 0.0226 | 0.982 | 0.005284627 | count | 1 |
| LINC01353 | 0.0086367 | 0.4516165 | 0.0191 | 0.985 | 0.005304384 | count | 1 |
| EXD3 | 0.004644 | 0.2796367 | 0.0166 | 0.987 | 0.005359585 | count | 1 |
| RPS2 | 0.0037963 | 0.0146092 | 0.2599 | 0.795 | 0.00547371 | count | 1 |
| CCDC124 | 0.0039685 | 0.1150742 | 0.0345 | 0.972 | 0.00551024 | count | 1 |
| ZNF506 | 0.0042671 | 0.1554455 | 0.0275 | 0.978 | 0.00556016 | count | 1 |
| ADPGK | 0.0043552 | 0.159656 | 0.0273 | 0.978 | 0.005584861 | count | 1 |
| TPRKB | 0.0041175 | 0.1213073 | 0.0339 | 0.973 | 0.005599633 | count | 1 |
| NUAK1 | 0.0068475 | 0.7976007 | 0.0086 | 0.993 | 0.005645706 | count | 1 |

| | | | | | | | |
|---|---|---|---|---|---|---|---|
| SLC20A1 | 0.0040924 | 0.1022091 | 0.04 | 0.968 | 0.005683508 | count | 1 |
| SNURF | 0.0176459 | 0.6522512 | 0.0271 | 0.978 | 0.005832431 | count | 1 |
| ZNF468 | 0.0068091 | 0.3872957 | 0.0176 | 0.986 | 0.005865358 | count | 1 |
| TNFSF8 | 0.0044649 | 0.1067219 | 0.0418 | 0.967 | 0.005928289 | count | 1 |
| TAF10 | 0.0042295 | 0.0610843 | 0.0692 | 0.945 | 0.006024099 | count | 1 |
| POLR1C | 0.0050897 | 0.1810537 | 0.0281 | 0.978 | 0.006063611 | count | 1 |
| SLC25A1 | 0.0045343 | 0.1563597 | 0.029 | 0.977 | 0.006103208 | count | 1 |
| SH3BGRL | 0.0043263 | 0.0569742 | 0.0759 | 0.939 | 0.006163736 | count | 1 |
| STT3A | 0.0060112 | 0.3754853 | 0.016 | 0.987 | 0.006207657 | count | 1 |
| TSEN15 | 0.0046185 | 0.152089 | 0.0304 | 0.976 | 0.006296581 | count | 1 |
| SNHG25 | 0.0093824 | 0.2390829 | 0.0392 | 0.969 | 0.006372935 | count | 1 |
| ARL6IP5 | 0.0044733 | 0.0372471 | 0.1201 | 0.904 | 0.006429368 | count | 1 |
| MT-CO3 | 0.0045831 | 0.0205989 | 0.2225 | 0.824 | 0.00660689 | count | 1 |
| MMADHC | 0.0048925 | 0.1180089 | 0.0415 | 0.967 | 0.006673298 | count | 1 |
| SLC16A10 | 0.0356598 | 0.4459196 | 0.08 | 0.936 | 0.006682093 | count | 1 |
| ZNF692 | 0.0060999 | 0.285526 | 0.0214 | 0.983 | 0.006803708 | count | 1 |
| LY75 | 0.0067111 | 0.3323591 | 0.0202 | 0.984 | 0.006809837 | count | 1 |
| ABHD5 | 0.0053284 | 0.1563535 | 0.0341 | 0.973 | 0.006846859 | count | 1 |
| DAB2IP | 0.0367079 | 0.7402329 | 0.0496 | 0.96 | 0.006880068 | count | 1 |
| ATP6AP1 | 0.0050994 | 0.1241217 | 0.0411 | 0.967 | 0.006938628 | count | 1 |
| ADARB1 | 0.005725 | 0.1897964 | 0.0302 | 0.976 | 0.006955288 | count | 1 |
| YTHDF1 | 0.0056803 | 0.1710263 | 0.0332 | 0.974 | 0.0069588 | count | 1 |
| ZNF747 | 0.008893 | 0.3657263 | 0.0243 | 0.981 | 0.006961092 | count | 1 |
| MTAP | 0.0055003 | 0.1727939 | 0.0318 | 0.975 | 0.007237318 | count | 1 |
| LIMK1 | 0.0098734 | 0.290371 | 0.034 | 0.973 | 0.00725534 | count | 1 |
| STAMBP | 0.0053446 | 0.1469393 | 0.0364 | 0.971 | 0.007315543 | count | 1 |
| AC009779.2 | 0.0094674 | 0.5616144 | 0.0169 | 0.987 | 0.007411204 | count | 1 |
| SDAD1 | 0.0053199 | 0.1056296 | 0.0504 | 0.96 | 0.007414302 | count | 1 |
| TNFAIP3 | 0.0052968 | 0.0536567 | 0.0987 | 0.921 | 0.007563117 | count | 1 |
| ACER3 | 0.0141119 | 0.3474456 | 0.0406 | 0.968 | 0.007585567 | count | 1 |
| PRPS1 | 0.0055799 | 0.0951532 | 0.0586 | 0.953 | 0.007694342 | count | 1 |
| GABPA | 0.0062908 | 0.1850054 | 0.034 | 0.973 | 0.00776545 | count | 1 |
| RAB33B | 0.0066573 | 0.1747173 | 0.0381 | 0.97 | 0.007791346 | count | 1 |
| NFYC-AS1 | 0.0418618 | 0.4963319 | 0.0843 | 0.933 | 0.007854887 | count | 1 |
| FAM239A | 0.0177289 | 0.5655496 | 0.0313 | 0.975 | 0.007886501 | count | 1 |
| MAPK1 | 0.0056825 | 0.1085837 | 0.0523 | 0.958 | 0.007939219 | count | 1 |
| RIOK1 | 0.0059092 | 0.1199174 | 0.0493 | 0.961 | 0.007954023 | count | 1 |
| POPDC2 | 0.0425589 | 0.6694789 | 0.0636 | 0.949 | 0.007986903 | count | 1 |
| NUP93 | 0.0071252 | 0.261955 | 0.0272 | 0.978 | 0.008023611 | count | 1 |
| OGA | 0.0059326 | 0.1036659 | 0.0572 | 0.954 | 0.008142565 | count | 1 |
| TXN2 | 0.0058933 | 0.0971654 | 0.0607 | 0.952 | 0.008229927 | count | 1 |
| ITGA4 | 0.0058079 | 0.0701822 | 0.0828 | 0.934 | 0.008260146 | count | 1 |
| CCDC184 | 0.0249555 | 0.3245246 | 0.0769 | 0.939 | 0.008260209 | count | 1 |
| PKIA | 0.0112915 | 0.3279734 | 0.0344 | 0.973 | 0.008298878 | count | 1 |
| IFNAR2 | 0.0064972 | 0.1840912 | 0.0353 | 0.972 | 0.008365335 | count | 1 |

| | | | | | | | |
|---|---|---|---|---|---|---|---|
| TBX2 | 0.0446124 | 0.931107 | 0.0479 | 0.962 | 0.008376024 | count | 1 |
| CDADC1 | 0.0067776 | 0.1967146 | 0.0345 | 0.973 | 0.008502893 | count | 1 |
| ZNF345 | 0.0125215 | 0.3964541 | 0.0316 | 0.975 | 0.008508734 | count | 1 |
| CTNNB1 | 0.0063583 | 0.1233556 | 0.0515 | 0.959 | 0.008608987 | count | 1 |
| LPCAT3 | 0.0071766 | 0.2134629 | 0.0336 | 0.973 | 0.008639125 | count | 1 |
| SNHG15 | 0.0061487 | 0.0740569 | 0.083 | 0.934 | 0.008713451 | count | 1 |
| IMP3 | 0.0061538 | 0.072051 | 0.0854 | 0.932 | 0.008737313 | count | 1 |
| AC025423.1 | 0.0264034 | 0.9553625 | 0.0276 | 0.978 | 0.008741918 | count | 1 |
| THRIL | 0.0264034 | 0.9553625 | 0.0276 | 0.978 | 0.008741918 | count | 1 |
| CPNE1 | 0.0063947 | 0.0873872 | 0.0732 | 0.942 | 0.008798406 | count | 1 |
| CDK12 | 0.0065078 | 0.1001548 | 0.065 | 0.948 | 0.008911457 | count | 1 |
| PTPRC | 0.0063343 | 0.0240356 | 0.2635 | 0.792 | 0.009123211 | count | 1 |
| 2-Mar | 0.0068896 | 0.1235338 | 0.0558 | 0.956 | 0.009414545 | count | 1 |
| HSPA14 | 0.0071975 | 0.1595689 | 0.0451 | 0.964 | 0.009420663 | count | 1 |
| HDAC7 | 0.008186 | 0.2031787 | 0.0403 | 0.968 | 0.009449067 | count | 1 |
| RUNDC1 | 0.0088977 | 0.2996412 | 0.0297 | 0.976 | 0.009473672 | count | 1 |
| HSPA1A | 0.0066207 | 0.1026984 | 0.0645 | 0.9486 | 0.009484206 | count | 1 |
| SLC25A6 | 0.0066704 | 0.0315448 | 0.2115 | 0.833 | 0.009589751 | count | 1 |
| RABL2A | 0.0156283 | 0.3659287 | 0.0427 | 0.966 | 0.009608184 | count | 1 |
| NEK1 | 0.0072635 | 0.1808238 | 0.0402 | 0.968 | 0.009663925 | count | 1 |
| LAMP3 | 0.0159693 | 0.8354635 | 0.0191 | 0.985 | 0.009818315 | count | 1 |
| AL442128.2 | 0.0159693 | 0.8389678 | 0.019 | 0.985 | 0.009818315 | count | 1 |
| RAD54L2 | 0.0126325 | 0.2678475 | 0.0472 | 0.962 | 0.00989252 | count | 1 |
| CCDC102B | 0.0107617 | 0.4595902 | 0.0234 | 0.981 | 0.009941057 | count | 1 |
| ZNF880 | 0.0076615 | 0.2057497 | 0.0372 | 0.97 | 0.009952435 | count | 1 |
| RPS15 | 0.0069744 | 0.0147437 | 0.473 | 0.636 | 0.010054486 | count | 1 |
| CCDC25 | 0.0072875 | 0.1090948 | 0.0668 | 0.947 | 0.010114533 | count | 1 |
| HIGD2A | 0.0070932 | 0.0436535 | 0.1625 | 0.871 | 0.010171436 | count | 1 |
| MIB1 | 0.0098836 | 0.251129 | 0.0394 | 0.969 | 0.010209468 | count | 1 |
| MT-CYB | 0.0070839 | 0.0219741 | 0.3224 | 0.747 | 0.010214507 | count | 1 |
| IKZF2 | 0.013922 | 0.2597804 | 0.0536 | 0.957 | 0.010235556 | count | 1 |
| CDK11B | 0.0075943 | 0.1356593 | 0.056 | 0.955 | 0.010288782 | count | 1 |
| POFUT1 | 0.0310985 | 0.4480488 | 0.0694 | 0.945 | 0.010305809 | count | 1 |
| RSU1 | 0.0075247 | 0.1201564 | 0.0626 | 0.95 | 0.01030821 | count | 1 |
| PDE6D | 0.0078094 | 0.1677443 | 0.0466 | 0.963 | 0.010325234 | count | 1 |
| ZNF76 | 0.0087463 | 0.2387766 | 0.0366 | 0.971 | 0.010363388 | count | 1 |
| 3-Mar | 0.0112231 | 0.4426353 | 0.0254 | 0.98 | 0.010367709 | count | 1 |
| ADIPOR1 | 0.0076752 | 0.1346218 | 0.057 | 0.955 | 0.010474104 | count | 1 |
| CDC73 | 0.0076094 | 0.1035336 | 0.0735 | 0.941 | 0.010486705 | count | 1 |
| UNC45A | 0.0088139 | 0.1822158 | 0.0484 | 0.961 | 0.010502165 | count | 1 |
| DISP1 | 0.0559069 | 0.5912271 | 0.0946 | 0.925 | 0.010522346 | count | 1 |
| SNAPC1 | 0.0078226 | 0.1132718 | 0.0691 | 0.945 | 0.010616253 | count | 1 |
| PSMC4 | 0.0075825 | 0.0841058 | 0.0902 | 0.928 | 0.010632663 | count | 1 |
| PPP3CA | 0.0075775 | 0.0942715 | 0.0804 | 0.936 | 0.010665504 | count | 1 |
| ZNF611 | 0.0096681 | 0.297701 | 0.0325 | 0.974 | 0.010675807 | count | 1 |

| | | | | | | | |
|---|---|---|---|---|---|---|---|
| APEH | 0.0083724 | 0.1691907 | 0.0495 | 0.961 | 0.010715058 | count | 1 |
| NDUFA5 | 0.0076242 | 0.0763715 | 0.0998 | 0.92 | 0.010727306 | count | 1 |
| BBOF1 | 0.0175673 | 0.5200749 | 0.0338 | 0.973 | 0.010803311 | count | 1 |
| ARL3 | 0.0083797 | 0.1720546 | 0.0487 | 0.961 | 0.011012725 | count | 1 |
| CFDP1 | 0.0079849 | 0.1022136 | 0.0781 | 0.938 | 0.011132643 | count | 1 |
| RPLP2 | 0.0077605 | 0.0162021 | 0.479 | 0.632 | 0.011185673 | count | 1 |
| STK39 | 0.0086171 | 0.1530695 | 0.0563 | 0.955 | 0.011229543 | count | 1 |
| SUCLG1 | 0.0080804 | 0.0997541 | 0.081 | 0.935 | 0.011286151 | count | 1 |
| BTRC | 0.0184526 | 0.2671922 | 0.0691 | 0.945 | 0.011349199 | count | 1 |
| LDHB | 0.0079118 | 0.0381858 | 0.2072 | 0.836 | 0.011352151 | count | 1 |
| HIST1H2AG | 0.0098418 | 0.2697551 | 0.0365 | 0.971 | 0.011361305 | count | 1 |
| ZNF124 | 0.0102181 | 0.2523356 | 0.0405 | 0.968 | 0.011399771 | count | 1 |
| PCYT1A | 0.0097855 | 0.1925632 | 0.0508 | 0.959 | 0.01145414 | count | 1 |
| PIGA | 0.0116447 | 0.3369255 | 0.0346 | 0.972 | 0.011591373 | count | 1 |
| UBXN10-AS1 | 0.0119664 | 0.3379713 | 0.0354 | 0.972 | 0.011653844 | count | 1 |
| ADCK5 | 0.0119687 | 0.3443628 | 0.0348 | 0.972 | 0.011656086 | count | 1 |
| RNPEPL1 | 0.0083098 | 0.0835098 | 0.0995 | 0.921 | 0.011660757 | count | 1 |
| FERMT3 | 0.0082994 | 0.0808671 | 0.1026 | 0.918 | 0.011679523 | count | 1 |
| PLEKHA3 | 0.0084812 | 0.1030538 | 0.0823 | 0.934 | 0.011760749 | count | 1 |
| RUSC1 | 0.0095997 | 0.2160662 | 0.0444 | 0.965 | 0.01176213 | count | 1 |
| DIP2B | 0.0193661 | 0.3288408 | 0.0589 | 0.953 | 0.01191262 | count | 1 |
| UBTD2 | 0.0267906 | 0.4147699 | 0.0646 | 0.949 | 0.011936364 | count | 1 |
| RGS14 | 0.008633 | 0.0914779 | 0.0944 | 0.925 | 0.012000055 | count | 1 |
| DENR | 0.0088178 | 0.105763 | 0.0834 | 0.934 | 0.012028004 | count | 1 |
| NUP35 | 0.0100089 | 0.2087665 | 0.0479 | 0.962 | 0.012050056 | count | 1 |
| ICA1L | 0.0178426 | 0.5098516 | 0.035 | 0.972 | 0.012133222 | count | 1 |
| ZFC3H1 | 0.0088937 | 0.0996169 | 0.0893 | 0.929 | 0.012282285 | count | 1 |
| CACUL1 | 0.0094973 | 0.1692759 | 0.0561 | 0.955 | 0.012296253 | count | 1 |
| TMEM253 | 0.0653512 | 0.9291994 | 0.0703 | 0.944 | 0.012324976 | count | 1 |
| RNF38 | 0.0100407 | 0.2017987 | 0.0498 | 0.96 | 0.012439849 | count | 1 |
| APPL2 | 0.0107083 | 0.2661909 | 0.0402 | 0.968 | 0.012534851 | count | 1 |
| MON1B | 0.0113587 | 0.2356509 | 0.0482 | 0.962 | 0.012543879 | count | 1 |
| ATG4D | 0.0098946 | 0.1932919 | 0.0512 | 0.959 | 0.012607928 | count | 1 |
| FKBP11 | 0.0089946 | 0.0830419 | 0.1083 | 0.914 | 0.012701887 | count | 1 |
| VPS54 | 0.0127754 | 0.3121681 | 0.0409 | 0.967 | 0.012718024 | count | 1 |
| PEX19 | 0.0134675 | 0.2807047 | 0.048 | 0.962 | 0.012797543 | count | 1 |
| MAVS | 0.0126491 | 0.2765442 | 0.0457 | 0.964 | 0.012840937 | count | 1 |
| P3H4 | 0.0685447 | 0.5088162 | 0.1347 | 0.893 | 0.012936141 | count | 1 |
| ATP5PB | 0.0092223 | 0.0683999 | 0.1348 | 0.893 | 0.013097134 | count | 1 |
| GLUD1 | 0.0098138 | 0.1168396 | 0.084 | 0.933 | 0.013210649 | count | 1 |
| RNF144A | 0.0135973 | 0.3792033 | 0.0359 | 0.971 | 0.013243923 | count | 1 |
| NINL | 0.0708889 | 0.7067802 | 0.1003 | 0.92 | 0.01338529 | count | 1 |
| ARHGEF7 | 0.0162345 | 0.2297298 | 0.0707 | 0.944 | 0.013398864 | count | 1 |
| COLGALT1 | 0.0126073 | 0.2445753 | 0.0515 | 0.959 | 0.01342671 | count | 1 |
| TBC1D10C | 0.0096245 | 0.0577282 | 0.1667 | 0.8676 | 0.013720004 | count | 1 |

| | | | | | | | |
|---|---|---|---|---|---|---|---|
| MAP3K9 | 0.0415267 | 0.6318237 | 0.0657 | 0.948 | 0.013789379 | count | 1 |
| MTOR | 0.0309429 | 0.2834689 | 0.1092 | 0.913 | 0.013796344 | count | 1 |
| AC023908.3 | 0.0204008 | 0.7036155 | 0.029 | 0.977 | 0.013877571 | count | 1 |
| RPS6 | 0.0096446 | 0.0189104 | 0.51 | 0.61 | 0.013896678 | count | 1 |
| SERINC1 | 0.0101263 | 0.0941942 | 0.1075 | 0.914 | 0.013919084 | count | 1 |
| VPS18 | 0.0179819 | 0.3695428 | 0.0487 | 0.961 | 0.01409035 | count | 1 |
| MXD1 | 0.0152589 | 0.2149351 | 0.071 | 0.943 | 0.014101092 | count | 1 |
| SRSF9 | 0.0099098 | 0.0537383 | 0.1844 | 0.854 | 0.014152664 | count | 1 |
| FBXO5 | 0.0118987 | 0.264081 | 0.0451 | 0.964 | 0.014179775 | count | 1 |
| ACP6 | 0.012264 | 0.2941106 | 0.0417 | 0.967 | 0.014260942 | count | 1 |
| EOMES | 0.0108087 | 0.160916 | 0.0672 | 0.946 | 0.014275353 | count | 1 |
| GPR146 | 0.0194043 | 0.4565024 | 0.0425 | 0.966 | 0.014275878 | count | 1 |
| ZNF585A | 0.0194043 | 0.4567517 | 0.0425 | 0.966 | 0.014275878 | count | 1 |
| SF1 | 0.0100861 | 0.0471611 | 0.2139 | 0.831 | 0.014363476 | count | 1 |
| AC245140.2 | 0.0267382 | 0.5037321 | 0.0531 | 0.958 | 0.014401347 | count | 1 |
| FCF1 | 0.0123245 | 0.2396318 | 0.0514 | 0.959 | 0.014519101 | count | 1 |
| FBXO48 | 0.0190102 | 0.2962114 | 0.0642 | 0.949 | 0.014897877 | count | 1 |
| TLE2 | 0.0336992 | 0.5515343 | 0.0611 | 0.951 | 0.015032462 | count | 1 |
| HIF1AN | 0.0154667 | 0.2709472 | 0.0571 | 0.954 | 0.015067052 | count | 1 |
| SMARCA4 | 0.0114712 | 0.1427324 | 0.0804 | 0.936 | 0.015076672 | count | 1 |
| TAF9 | 0.0109104 | 0.1116848 | 0.0977 | 0.922 | 0.015172161 | count | 1 |
| UXT | 0.0106941 | 0.0493015 | 0.2169 | 0.8283 | 0.015251956 | count | 1 |
| TMEM11 | 0.0123381 | 0.1762282 | 0.07 | 0.944 | 0.015339004 | count | 1 |
| NOP53 | 0.0107584 | 0.0383528 | 0.2805 | 0.779 | 0.01539009 | count | 1 |
| COQ10B | 0.0110096 | 0.0738091 | 0.1492 | 0.881 | 0.015458169 | count | 1 |
| ZFAS1 | 0.0109171 | 0.0469417 | 0.2326 | 0.816 | 0.015535004 | count | 1 |
| PRKRA | 0.0114362 | 0.1319487 | 0.0867 | 0.931 | 0.015562378 | count | 1 |
| 10-Sep | 0.0199408 | 0.6439374 | 0.031 | 0.975 | 0.015628843 | count | 1 |
| PPP1R11 | 0.0112687 | 0.0988625 | 0.114 | 0.909 | 0.015654325 | count | 1 |
| NRTN | 0.0834806 | 0.9772827 | 0.0854 | 0.932 | 0.015805369 | count | 1 |
| ZNF184 | 0.0125704 | 0.167231 | 0.0752 | 0.94 | 0.015818101 | count | 1 |
| P2RY14 | 0.0836145 | 0.4977619 | 0.168 | 0.867 | 0.015831172 | count | 1 |
| AC245297.2 | 0.0188091 | 0.5670794 | 0.0332 | 0.974 | 0.016221996 | count | 1 |
| ZSCAN16-AS1 | 0.0133512 | 0.1826704 | 0.0731 | 0.942 | 0.016225288 | count | 1 |
| OGFOD2 | 0.0861141 | 0.3607077 | 0.2387 | 0.811 | 0.016313115 | count | 1 |
| AC139530.1 | 0.0135639 | 0.2875336 | 0.0472 | 0.962 | 0.016332472 | count | 1 |
| DNAJB11 | 0.0122829 | 0.1355124 | 0.0906 | 0.928 | 0.016359432 | count | 1 |
| AF165147.1 | 0.0494598 | 0.6015641 | 0.0822 | 0.934 | 0.01644868 | count | 1 |
| H1FX | 0.0115036 | 0.0627224 | 0.1834 | 0.854 | 0.016492297 | count | 1 |
| AP1B1 | 0.0224918 | 0.3004028 | 0.0749 | 0.94 | 0.016553683 | count | 1 |
| NAF1 | 0.01396 | 0.1637071 | 0.0853 | 0.932 | 0.016725889 | count | 1 |
| MRPL19 | 0.0125962 | 0.1467783 | 0.0858 | 0.932 | 0.016837668 | count | 1 |
| BRIX1 | 0.0122355 | 0.1144777 | 0.1069 | 0.915 | 0.016929999 | count | 1 |
| PPP2R5E | 0.0126443 | 0.119627 | 0.1057 | 0.916 | 0.017100964 | count | 1 |
| LINC02315 | 0.0318118 | 0.8155343 | 0.039 | 0.969 | 0.017147697 | count | 1 |

| | | | | | | | |
|---|---|---|---|---|---|---|---|
| HBQ1 | 0.0318118 | 0.8155343 | 0.039 | 0.969 | 0.017147697 | count | 1 |
| TOMM40L | 0.0318819 | 0.5752258 | 0.0554 | 0.956 | 0.017185673 | count | 1 |
| PTCD2 | 0.0181429 | 0.5156253 | 0.0352 | 0.972 | 0.017247307 | count | 1 |
| BCS1L | 0.0174211 | 0.3066403 | 0.0568 | 0.955 | 0.017349168 | count | 1 |
| CTH | 0.0521664 | 0.412582 | 0.1264 | 0.899 | 0.017357797 | count | 1 |
| JAM3 | 0.0323637 | 0.567941 | 0.057 | 0.955 | 0.017446702 | count | 1 |
| ZNF790 | 0.0172281 | 0.3231109 | 0.0533 | 0.957 | 0.017495377 | count | 1 |
| AC106707.1 | 0.0189424 | 0.3114791 | 0.0608 | 0.952 | 0.017510945 | count | 1 |
| MICALL1 | 0.0531816 | 0.4436523 | 0.1199 | 0.905 | 0.017699026 | count | 1 |
| SZT2 | 0.0164058 | 0.3282068 | 0.05 | 0.96 | 0.017708118 | count | 1 |
| AL353194.1 | 0.0198181 | 0.3682334 | 0.0538 | 0.957 | 0.017742578 | count | 1 |
| ZNF682 | 0.0533987 | 0.3456815 | 0.1545 | 0.877 | 0.017772015 | count | 1 |
| FBXO41 | 0.0405693 | 0.5852548 | 0.0693 | 0.945 | 0.018118555 | count | 1 |
| ANXA11 | 0.0127716 | 0.0641248 | 0.1992 | 0.842 | 0.018138623 | count | 1 |
| LPCAT2 | 0.0248177 | 0.7636845 | 0.0325 | 0.974 | 0.018270748 | count | 1 |
| GANC | 0.0183623 | 0.3282741 | 0.0559 | 0.955 | 0.018287825 | count | 1 |
| EIF4G3 | 0.0135734 | 0.143697 | 0.0945 | 0.925 | 0.018310593 | count | 1 |
| FAM78A | 0.014522 | 0.1909626 | 0.076 | 0.939 | 0.018419094 | count | 1 |
| KAT6A | 0.0139398 | 0.1163524 | 0.1198 | 0.905 | 0.018493074 | count | 1 |
| PRRC1 | 0.0141824 | 0.1659022 | 0.0855 | 0.932 | 0.018710491 | count | 1 |
| ACAP3 | 0.0210411 | 0.2977042 | 0.0707 | 0.944 | 0.018839695 | count | 1 |
| METAP1 | 0.016066 | 0.1755861 | 0.0915 | 0.927 | 0.018930123 | count | 1 |
| AC062029.1 | 0.030833 | 0.5610558 | 0.055 | 0.956 | 0.018997615 | count | 1 |
| AL512329.2 | 0.0281197 | 0.7128097 | 0.0394 | 0.969 | 0.019147953 | count | 1 |
| RHPN1 | 0.0576339 | 0.3524819 | 0.1635 | 0.87 | 0.019197068 | count | 1 |
| HPS5 | 0.0166244 | 0.2354202 | 0.0706 | 0.944 | 0.019335471 | count | 1 |
| UTP11 | 0.014727 | 0.163371 | 0.0901 | 0.928 | 0.019405817 | count | 1 |
| EHD1 | 0.0149433 | 0.1249266 | 0.1196 | 0.905 | 0.019446376 | count | 1 |
| C6orf62 | 0.0142671 | 0.099959 | 0.1427 | 0.887 | 0.019522627 | count | 1 |
| N6AMT1 | 0.0175414 | 0.346918 | 0.0506 | 0.96 | 0.019578179 | count | 1 |
| MKKS | 0.0171271 | 0.3257592 | 0.0526 | 0.958 | 0.019628037 | count | 1 |
| PDK2 | 0.015368 | 0.2090703 | 0.0735 | 0.941 | 0.019791525 | count | 1 |
| HSD17B8 | 0.0156452 | 0.1804234 | 0.0867 | 0.931 | 0.019844393 | count | 1 |
| DPY19L3 | 0.1045058 | 0.7840724 | 0.1333 | 0.894 | 0.019874387 | count | 1 |
| IGFBP6 | 0.0170701 | 0.3053173 | 0.0559 | 0.955 | 0.019987859 | count | 1 |
| RAB3GAP2 | 0.0155848 | 0.1898625 | 0.0821 | 0.935 | 0.020180985 | count | 1 |
| SSR3 | 0.0142987 | 0.0743183 | 0.1924 | 0.847 | 0.020220886 | count | 1 |
| NDE1 | 0.0152651 | 0.1333974 | 0.1144 | 0.909 | 0.020230406 | count | 1 |
| ULBP2 | 0.0166233 | 0.2456201 | 0.0677 | 0.946 | 0.020290785 | count | 1 |
| ITPKA | 0.0612208 | 0.8181695 | 0.0748 | 0.94 | 0.020405733 | count | 1 |
| ACSM1 | 0.0612208 | 0.8216216 | 0.0745 | 0.941 | 0.020405733 | count | 1 |
| ZNF425 | 0.0612208 | 0.880164 | 0.0696 | 0.945 | 0.020405733 | count | 1 |
| CNOT4 | 0.0147699 | 0.0990587 | 0.1491 | 0.881 | 0.020523138 | count | 1 |
| SRM | 0.0146794 | 0.0929321 | 0.158 | 0.874 | 0.020592713 | count | 1 |
| ACSM3 | 0.0336474 | 0.4884471 | 0.0689 | 0.945 | 0.020740062 | count | 1 |

| | | | | | | | |
|---|---|---|---|---|---|---|---|
| CDK5R1 | 0.1094443 | 0.3828894 | 0.2858 | 0.775 | 0.020835186 | count | 1 |
| NUAK2 | 0.0283124 | 0.4304526 | 0.0658 | 0.948 | 0.020852492 | count | 1 |
| EEF1E1 | 0.01523 | 0.1296528 | 0.1175 | 0.906 | 0.020997124 | count | 1 |
| CFAP298 | 0.0152006 | 0.1190017 | 0.1277 | 0.898 | 0.021011079 | count | 1 |
| SF3B1 | 0.0149017 | 0.0525387 | 0.2836 | 0.777 | 0.021123268 | count | 1 |
| HGH1 | 0.0229404 | 0.3384552 | 0.0678 | 0.946 | 0.021214481 | count | 1 |
| TTLL11 | 0.0396786 | 0.5529215 | 0.0718 | 0.943 | 0.021414538 | count | 1 |
| GNL3L | 0.0231803 | 0.2581675 | 0.0898 | 0.928 | 0.021436795 | count | 1 |
| HES6 | 0.0192375 | 0.2854276 | 0.0674 | 0.946 | 0.021473292 | count | 1 |
| SERPINB9 | 0.0154869 | 0.097572 | 0.1587 | 0.874 | 0.021486776 | count | 1 |
| LBR | 0.0152618 | 0.0861322 | 0.1772 | 0.859 | 0.021499225 | count | 1 |
| SECTM1 | 0.1128895 | 0.5160156 | 0.2188 | 0.827 | 0.021506592 | count | 1 |
| LEKR1 | 0.1130986 | 0.5954741 | 0.1899 | 0.849 | 0.02154737 | count | 1 |
| IMP4 | 0.0156386 | 0.1141421 | 0.137 | 0.891 | 0.021610689 | count | 1 |
| BIN1 | 0.0152655 | 0.0774413 | 0.1971 | 0.844 | 0.02168075 | count | 1 |
| PCAT6 | 0.1138824 | 0.7138535 | 0.1595 | 0.873 | 0.02170026 | count | 1 |
| ZBED2 | 0.1138824 | 0.7185707 | 0.1585 | 0.874 | 0.02170026 | count | 1 |
| ZNF426-DT | 0.1138824 | 0.9052866 | 0.1258 | 0.9 | 0.02170026 | count | 1 |
| RIMS2 | 0.1138824 | 1.051427 | 0.1083 | 0.914 | 0.02170026 | count | 1 |
| LY6H | 0.1138824 | 1.051427 | 0.1083 | 0.914 | 0.02170026 | count | 1 |
| CNN3 | 0.0652132 | 0.6967226 | 0.0936 | 0.925 | 0.02175294 | count | 1 |
| LSM12 | 0.0155293 | 0.0937176 | 0.1657 | 0.868 | 0.021825463 | count | 1 |
| SUN1 | 0.0184338 | 0.2149363 | 0.0858 | 0.932 | 0.02185154 | count | 1 |
| GOLGA5 | 0.017111 | 0.2047439 | 0.0836 | 0.933 | 0.021856873 | count | 1 |
| RARS2 | 0.0178157 | 0.1926873 | 0.0925 | 0.926 | 0.02192025 | count | 1 |
| FN3K | 0.0355527 | 0.7893048 | 0.045 | 0.964 | 0.021920454 | count | 1 |
| STK38 | 0.0161594 | 0.1248085 | 0.1295 | 0.897 | 0.021956928 | count | 1 |
| IL21R | 0.0182551 | 0.2051406 | 0.089 | 0.929 | 0.021985506 | count | 1 |
| RWDD4 | 0.016072 | 0.1332821 | 0.1206 | 0.904 | 0.0220109 | count | 1 |
| ST20-AS1 | 0.0299252 | 0.5186999 | 0.0577 | 0.954 | 0.0220447 | count | 1 |
| THRAP3 | 0.0155172 | 0.0615605 | 0.2521 | 0.801 | 0.022078161 | count | 1 |
| TRAK1 | 0.0281501 | 0.4605714 | 0.0611 | 0.951 | 0.022083765 | count | 1 |
| TUFM | 0.0156227 | 0.0681678 | 0.2292 | 0.819 | 0.022159141 | count | 1 |
| DIS3L | 0.0208224 | 0.3165575 | 0.0658 | 0.948 | 0.02218777 | count | 1 |
| LATS1 | 0.0178665 | 0.2120504 | 0.0843 | 0.933 | 0.022216315 | count | 1 |
| STX12 | 0.0168886 | 0.1351804 | 0.1249 | 0.901 | 0.022255214 | count | 1 |
| MPHOSPH8 | 0.0156367 | 0.0551262 | 0.2837 | 0.7767 | 0.022354482 | count | 1 |
| 8-Sep | 0.0250176 | 0.2833378 | 0.0883 | 0.93 | 0.022408645 | count | 1 |
| TANGO6 | 0.1175345 | 0.3218882 | 0.3651 | 0.715 | 0.022413283 | count | 1 |
| ZKSCAN3 | 0.0674554 | 0.6707595 | 0.1006 | 0.92 | 0.022510415 | count | 1 |
| LDLRAP1 | 0.0172452 | 0.1548271 | 0.1114 | 0.911 | 0.022512106 | count | 1 |
| TRAC | 0.0156742 | 0.0343515 | 0.4563 | 0.648 | 0.022566079 | count | 1 |
| RPL17-C18orf32 | 0.0420021 | 1.0582817 | 0.0397 | 0.968 | 0.022676746 | count | 1 |
| PTGES2 | 0.017259 | 0.1400112 | 0.1233 | 0.902 | 0.02279758 | count | 1 |
| ARMC7 | 0.018343 | 0.2699854 | 0.0679 | 0.946 | 0.022809202 | count | 1 |

| | | | | | | | |
|---|---|---|---|---|---|---|---|
| LSMEM1 | 0.0685518 | 0.6697274 | 0.1024 | 0.918 | 0.022881047 | count | 1 |
| ZNF557 | 0.0201538 | 0.2642565 | 0.0763 | 0.939 | 0.022911971 | count | 1 |
| WDYHV1 | 0.0216414 | 0.3226205 | 0.0671 | 0.947 | 0.023061713 | count | 1 |
| MPND | 0.0205124 | 0.2256667 | 0.0909 | 0.928 | 0.023115926 | count | 1 |
| ZNF614 | 0.0517309 | 0.4091078 | 0.1264 | 0.899 | 0.023147722 | count | 1 |
| SCAI | 0.0228215 | 0.3061204 | 0.0746 | 0.941 | 0.023185205 | count | 1 |
| ACOT13 | 0.0177832 | 0.1706219 | 0.1042 | 0.917 | 0.023214717 | count | 1 |
| RMDN3 | 0.0183643 | 0.1867964 | 0.0983 | 0.922 | 0.023236779 | count | 1 |
| C1orf123 | 0.016958 | 0.1192765 | 0.1422 | 0.887 | 0.023269596 | count | 1 |
| TRAV13-2 | 0.0222132 | 0.4349853 | 0.0511 | 0.959 | 0.023333167 | count | 1 |
| ACO2 | 0.0183855 | 0.1473031 | 0.1248 | 0.901 | 0.023536608 | count | 1 |
| REV1 | 0.0181614 | 0.1313137 | 0.1383 | 0.89 | 0.023598232 | count | 1 |
| ZNF512B | 0.0264795 | 0.376083 | 0.0704 | 0.944 | 0.023721385 | count | 1 |
| UBA3 | 0.0176525 | 0.1389408 | 0.1271 | 0.899 | 0.023731235 | count | 1 |
| TBL3 | 0.0207532 | 0.2697023 | 0.0769 | 0.939 | 0.023788087 | count | 1 |
| C1orf74 | 0.0713321 | 0.9465691 | 0.0754 | 0.94 | 0.023821556 | count | 1 |
| SHCBP1 | 0.0713321 | 1.0322752 | 0.0691 | 0.945 | 0.023821556 | count | 1 |
| LINC02170 | 0.0713321 | 1.2375139 | 0.0576 | 0.954 | 0.023821556 | count | 1 |
| NOL11 | 0.0185712 | 0.1540502 | 0.1206 | 0.904 | 0.023872248 | count | 1 |
| ZNF770 | 0.0183301 | 0.1566255 | 0.117 | 0.907 | 0.023964042 | count | 1 |
| MOB3A | 0.0182632 | 0.1818302 | 0.1004 | 0.92 | 0.023975768 | count | 1 |
| PPARA | 0.0217353 | 0.2888199 | 0.0753 | 0.94 | 0.024017902 | count | 1 |
| MALINC1 | 0.0352772 | 0.4485235 | 0.0787 | 0.937 | 0.024044524 | count | 1 |
| GSTCD | 0.0291789 | 0.4577198 | 0.0637 | 0.949 | 0.024115831 | count | 1 |
| GTF2IRD2B | 0.0291789 | 0.4981353 | 0.0586 | 0.953 | 0.024115831 | count | 1 |
| TAGAP | 0.0168565 | 0.0490689 | 0.3435 | 0.7312 | 0.024123621 | count | 1 |
| STARD3NL | 0.0180206 | 0.1507121 | 0.1196 | 0.905 | 0.024151421 | count | 1 |
| EBP | 0.0174829 | 0.0969464 | 0.1803 | 0.857 | 0.024401376 | count | 1 |
| TMEM183A | 0.0179277 | 0.1130819 | 0.1585 | 0.874 | 0.024410517 | count | 1 |
| EIF3K | 0.0170059 | 0.0324939 | 0.5234 | 0.601 | 0.024443778 | count | 1 |
| TMEM170A | 0.017795 | 0.1062364 | 0.1675 | 0.867 | 0.024487231 | count | 1 |
| EGR2 | 0.0210567 | 0.2187946 | 0.0962 | 0.923 | 0.024495869 | count | 1 |
| TTC8 | 0.0737281 | 0.6588202 | 0.1119 | 0.911 | 0.024632845 | count | 1 |
| GDPGP1 | 0.0737281 | 0.6586176 | 0.1119 | 0.911 | 0.024632845 | count | 1 |
| FOXP4 | 0.0275011 | 0.3354575 | 0.082 | 0.935 | 0.024638963 | count | 1 |
| PPP1R13L | 0.1296417 | 0.895485 | 0.1448 | 0.885 | 0.02478446 | count | 1 |
| HNRNPA2B1 | 0.0173253 | 0.0311309 | 0.5565 | 0.578 | 0.024886922 | count | 1 |
| KDM2B | 0.0288463 | 0.2322503 | 0.1242 | 0.901 | 0.024903839 | count | 1 |
| HPCAL4 | 0.0461214 | 0.7866349 | 0.0586 | 0.953 | 0.024916697 | count | 1 |
| ZNF414 | 0.0200726 | 0.2072356 | 0.0969 | 0.923 | 0.024961427 | count | 1 |
| PPME1 | 0.0234963 | 0.2575919 | 0.0912 | 0.927 | 0.025041394 | count | 1 |
| AL035071.1 | 0.0750568 | 0.3283904 | 0.2286 | 0.819 | 0.025083048 | count | 1 |
| MAP4K5 | 0.0185791 | 0.157362 | 0.1181 | 0.906 | 0.025218235 | count | 1 |
| TOMM70 | 0.0198589 | 0.1504188 | 0.132 | 0.895 | 0.025312088 | count | 1 |
| ZNF574 | 0.0237739 | 0.2780045 | 0.0855 | 0.932 | 0.025337709 | count | 1 |

| | | | | | | | |
|---|---|---|---|---|---|---|---|
| PSMG1 | 0.0192814 | 0.1637934 | 0.1177 | 0.906 | 0.025440267 | count | 1 |
| RBM42 | 0.0184473 | 0.1180487 | 0.1563 | 0.876 | 0.025485675 | count | 1 |
| TRAF3IP2-AS1 | 0.0346764 | 0.3421444 | 0.1014 | 0.919 | 0.025559561 | count | 1 |
| FHIT | 0.0212459 | 0.2126795 | 0.0999 | 0.92 | 0.025590646 | count | 1 |
| RNF207 | 0.0473815 | 0.5503066 | 0.0861 | 0.931 | 0.025602462 | count | 1 |
| TBC1D32 | 0.0286401 | 0.3889162 | 0.0736 | 0.941 | 0.025662194 | count | 1 |
| SH3YL1 | 0.018784 | 0.1126186 | 0.1668 | 0.868 | 0.025746315 | count | 1 |
| SPEF2 | 0.0213082 | 0.2342613 | 0.091 | 0.928 | 0.025787397 | count | 1 |
| C4orf3 | 0.0181179 | 0.0553681 | 0.3272 | 0.744 | 0.025814525 | count | 1 |
| C16orf86 | 0.0313069 | 0.5399497 | 0.058 | 0.954 | 0.025880462 | count | 1 |
| NSMCE4A | 0.0189315 | 0.1217813 | 0.1555 | 0.876 | 0.025927983 | count | 1 |
| EIF1 | 0.018013 | 0.017824 | 1.0106 | 0.312 | 0.025962607 | count | 1 |
| NCBP3 | 0.0193122 | 0.1222569 | 0.158 | 0.874 | 0.026000893 | count | 1 |
| RPLP0 | 0.018102 | 0.0211552 | 0.8557 | 0.3922 | 0.026085547 | count | 1 |
| COPA | 0.0201634 | 0.1288576 | 0.1565 | 0.876 | 0.026157569 | count | 1 |
| ZNF514 | 0.0319002 | 0.5145996 | 0.062 | 0.951 | 0.026372593 | count | 1 |
| PCYOX1L | 0.0245477 | 0.3216882 | 0.0763 | 0.939 | 0.026509978 | count | 1 |
| TIMP3 | 0.0430459 | 0.6329351 | 0.068 | 0.946 | 0.026568846 | count | 1 |
| UBR5-AS1 | 0.1389885 | 0.6837928 | 0.2033 | 0.839 | 0.026622769 | count | 1 |
| HIST1H2AI | 0.1389885 | 0.7344188 | 0.1892 | 0.85 | 0.026622769 | count | 1 |
| CCDC144A | 0.0244003 | 0.3252216 | 0.075 | 0.94 | 0.02666989 | count | 1 |
| KRBA2 | 0.1393023 | 0.5366506 | 0.2596 | 0.795 | 0.026684604 | count | 1 |
| SEH1L | 0.0248404 | 0.2553015 | 0.0973 | 0.922 | 0.02682657 | count | 1 |
| MTMR6 | 0.0209194 | 0.1625932 | 0.1287 | 0.898 | 0.026838312 | count | 1 |
| RPS24 | 0.0186365 | 0.0137582 | 1.3546 | 0.176 | 0.026872305 | count | 1 |
| PLPP5 | 0.0210456 | 0.1744065 | 0.1207 | 0.904 | 0.027000313 | count | 1 |
| KLF11 | 0.0267001 | 0.2905178 | 0.0919 | 0.927 | 0.027133411 | count | 1 |
| P2RX4 | 0.0233348 | 0.2266016 | 0.103 | 0.918 | 0.02714905 | count | 1 |
| UROS | 0.0198501 | 0.1277252 | 0.1554 | 0.877 | 0.027152825 | count | 1 |
| NBPF12 | 0.0606214 | 0.3781201 | 0.1603 | 0.873 | 0.027167036 | count | 1 |
| MICU3 | 0.0285555 | 0.3044817 | 0.0938 | 0.925 | 0.027170091 | count | 1 |
| AL049597.2 | 0.0607881 | 0.6231205 | 0.0976 | 0.922 | 0.027242514 | count | 1 |
| VIPAS39 | 0.0316975 | 0.3344969 | 0.0948 | 0.925 | 0.027373187 | count | 1 |
| UBA5 | 0.0208002 | 0.1510232 | 0.1377 | 0.89 | 0.027598171 | count | 1 |
| TRAV12-2 | 0.0447492 | 0.7076604 | 0.0632 | 0.95 | 0.027626842 | count | 1 |
| TSEN54 | 0.0197457 | 0.1005169 | 0.1964 | 0.844 | 0.027638157 | count | 1 |
| PIGC | 0.0200699 | 0.1366964 | 0.1468 | 0.883 | 0.027696121 | count | 1 |
| LINC01146 | 0.0828328 | 0.8166151 | 0.1014 | 0.919 | 0.027722154 | count | 1 |
| Z94721.1 | 0.0828328 | 0.9320398 | 0.0889 | 0.929 | 0.027722154 | count | 1 |
| PRMT3 | 0.0269147 | 0.3123438 | 0.0862 | 0.931 | 0.027835927 | count | 1 |
| NEAT1 | 0.0195188 | 0.0558365 | 0.3496 | 0.727 | 0.027877685 | count | 1 |
| ANGEL1 | 0.0834806 | 0.6358944 | 0.1313 | 0.896 | 0.027942347 | count | 1 |
| PLAGL1 | 0.0835868 | 0.5100456 | 0.1639 | 0.87 | 0.027978459 | count | 1 |
| AIMP2 | 0.0215846 | 0.2235763 | 0.0965 | 0.923 | 0.0280486 | count | 1 |
| CBX5 | 0.0205385 | 0.1240461 | 0.1656 | 0.869 | 0.028118056 | count | 1 |

| | | | | | | | |
|---|---|---|---|---|---|---|---|
| AP5B1 | 0.0522795 | 0.3561886 | 0.1468 | 0.883 | 0.028270517 | count | 1 |
| PDCD4-AS1 | 0.022784 | 0.2393181 | 0.0952 | 0.924 | 0.028335865 | count | 1 |
| PREB | 0.0224809 | 0.1999425 | 0.1124 | 0.91 | 0.028375062 | count | 1 |
| SURF1 | 0.020582 | 0.1018275 | 0.2021 | 0.84 | 0.028419438 | count | 1 |
| MPDU1 | 0.0257234 | 0.2409225 | 0.1068 | 0.915 | 0.028431469 | count | 1 |
| CYFIP1 | 0.1484139 | 0.4572464 | 0.3246 | 0.746 | 0.028483331 | count | 1 |
| AP001157.1 | 0.0310426 | 0.3618766 | 0.0858 | 0.932 | 0.028728003 | count | 1 |
| RNF130 | 0.0225449 | 0.1983663 | 0.1137 | 0.91 | 0.028802408 | count | 1 |
| HLA-B | 0.0199842 | 0.0159218 | 1.2551 | 0.21 | 0.028821356 | count | 1 |
| TMEM119 | 0.1505766 | 0.7037059 | 0.214 | 0.831 | 0.028911203 | count | 1 |
| TRMO | 0.0225597 | 0.1614098 | 0.1398 | 0.889 | 0.029002403 | count | 1 |
| DECR1 | 0.0205768 | 0.0808197 | 0.2546 | 0.799 | 0.029019987 | count | 1 |
| EXOSC2 | 0.0304963 | 0.2763327 | 0.1104 | 0.912 | 0.029021516 | count | 1 |
| EVI2A | 0.0210696 | 0.0735949 | 0.2863 | 0.775 | 0.029445131 | count | 1 |
| GPX7 | 0.0221207 | 0.1722484 | 0.1284 | 0.898 | 0.029522375 | count | 1 |
| LSM5 | 0.0211728 | 0.0875333 | 0.2419 | 0.809 | 0.029602559 | count | 1 |
| RPTOR | 0.0332758 | 0.3632797 | 0.0916 | 0.927 | 0.029828955 | count | 1 |
| SHF | 0.0666004 | 0.5705391 | 0.1167 | 0.907 | 0.029876751 | count | 1 |
| SCN1B | 0.0284871 | 0.4103048 | 0.0694 | 0.945 | 0.029936159 | count | 1 |
| RNH1 | 0.0212435 | 0.0710361 | 0.2991 | 0.765 | 0.030001349 | count | 1 |
| FDFT1 | 0.0218775 | 0.1116653 | 0.1959 | 0.845 | 0.030043946 | count | 1 |
| LTV1 | 0.0224389 | 0.1539667 | 0.1457 | 0.884 | 0.030075245 | count | 1 |
| ERAP1 | 0.0242694 | 0.1939469 | 0.1251 | 0.9 | 0.030083577 | count | 1 |
| RPL26L1 | 0.0219848 | 0.1606474 | 0.1369 | 0.891 | 0.030098657 | count | 1 |
| TBL1XR1 | 0.0217117 | 0.0919571 | 0.2361 | 0.813 | 0.030177149 | count | 1 |
| GTPBP4 | 0.0221782 | 0.1212881 | 0.1829 | 0.855 | 0.030184712 | count | 1 |
| TOP3A | 0.0291854 | 0.3428298 | 0.0851 | 0.932 | 0.030189192 | count | 1 |
| SNAPC5 | 0.0222544 | 0.1478527 | 0.1505 | 0.88 | 0.030257305 | count | 1 |
| FIZ1 | 0.0903386 | 0.3322783 | 0.2719 | 0.786 | 0.030276619 | count | 1 |
| AL355075.4 | 0.0676018 | 0.5495055 | 0.123 | 0.902 | 0.030331111 | count | 1 |
| NAALADL1 | 0.0240337 | 0.2082926 | 0.1154 | 0.908 | 0.030336458 | count | 1 |
| CAMSAP1 | 0.0312418 | 0.2616138 | 0.1194 | 0.905 | 0.030473627 | count | 1 |
| TRAPPC8 | 0.0278899 | 0.2493151 | 0.1119 | 0.911 | 0.030490517 | count | 1 |
| STK16 | 0.0229104 | 0.1628251 | 0.1407 | 0.888 | 0.030604056 | count | 1 |
| AC090152.1 | 0.0223653 | 0.152169 | 0.147 | 0.883 | 0.03069147 | count | 1 |
| RNF187 | 0.0224985 | 0.103994 | 0.2163 | 0.829 | 0.030709188 | count | 1 |
| ERCC2 | 0.0308066 | 0.3917503 | 0.0786 | 0.937 | 0.03071126 | count | 1 |
| LZTS2 | 0.0332052 | 0.3465581 | 0.0958 | 0.924 | 0.030735293 | count | 1 |
| POM121C | 0.0250415 | 0.1636116 | 0.1531 | 0.878 | 0.030932629 | count | 1 |
| INPP5K | 0.0237843 | 0.1626649 | 0.1462 | 0.884 | 0.031006408 | count | 1 |
| SLC37A4 | 0.0503463 | 0.3669662 | 0.1372 | 0.891 | 0.03110697 | count | 1 |
| SUSD4 | 0.1619311 | 0.7056136 | 0.2295 | 0.819 | 0.031163431 | count | 1 |
| ZNF708 | 0.0235966 | 0.1299776 | 0.1815 | 0.856 | 0.031173155 | count | 1 |
| NAA80 | 0.0240477 | 0.2287387 | 0.1051 | 0.916 | 0.031349989 | count | 1 |
| MLLT3 | 0.0232753 | 0.1239112 | 0.1878 | 0.851 | 0.03152245 | count | 1 |

| | | | | | | | |
|---|---|---|---|---|---|---|---|
| PSMC3IP | 0.0940296 | 0.5192781 | 0.1811 | 0.856 | 0.031535312 | count | 1 |
| CDC42EP3 | 0.0222542 | 0.0742961 | 0.2995 | 0.765 | 0.031550824 | count | 1 |
| R3HCC1 | 0.0245355 | 0.1777391 | 0.138 | 0.89 | 0.031665099 | count | 1 |
| PARG | 0.0279215 | 0.1913419 | 0.1459 | 0.884 | 0.031755803 | count | 1 |
| ZNF561 | 0.0294115 | 0.333864 | 0.0881 | 0.93 | 0.031772278 | count | 1 |
| ERC1 | 0.0256125 | 0.1992283 | 0.1286 | 0.898 | 0.031856703 | count | 1 |
| ATP5MC2 | 0.0221586 | 0.0314257 | 0.7051 | 0.481 | 0.031874901 | count | 1 |
| NMNAT3 | 0.0710208 | 0.5746399 | 0.1236 | 0.902 | 0.031883523 | count | 1 |
| ITGA6 | 0.0255209 | 0.1604871 | 0.159 | 0.874 | 0.032037132 | count | 1 |
| SLC2A4RG | 0.0227496 | 0.0788728 | 0.2884 | 0.773 | 0.032151699 | count | 1 |
| PYGM | 0.1674509 | 0.68292 | 0.2452 | 0.806 | 0.032261838 | count | 1 |
| CANX | 0.023137 | 0.0855972 | 0.2703 | 0.787 | 0.032267552 | count | 1 |
| MT-CO2 | 0.0224343 | 0.018705 | 1.1994 | 0.23 | 0.032344593 | count | 1 |
| MIRLET7BHG | 0.1684148 | 0.643001 | 0.2619 | 0.793 | 0.032453884 | count | 1 |
| PBX4 | 0.0262004 | 0.1425257 | 0.1838 | 0.854 | 0.032479388 | count | 1 |
| MYL12A | 0.0225929 | 0.0241275 | 0.9364 | 0.349 | 0.032557007 | count | 1 |
| SRSF1 | 0.0249209 | 0.1323806 | 0.1883 | 0.851 | 0.032585633 | count | 1 |
| ST13 | 0.0228827 | 0.0585052 | 0.3911 | 0.696 | 0.032593898 | count | 1 |
| DHX57 | 0.0477895 | 0.4016008 | 0.119 | 0.905 | 0.032626164 | count | 1 |
| YAF2 | 0.0243002 | 0.1256434 | 0.1934 | 0.847 | 0.032647395 | count | 1 |
| GRB10 | 0.0974497 | 0.6492711 | 0.1501 | 0.881 | 0.032703094 | count | 1 |
| SMG8 | 0.0975632 | 0.762315 | 0.128 | 0.898 | 0.032741873 | count | 1 |
| HEXA-AS1 | 0.1699913 | 0.6403277 | 0.2655 | 0.791 | 0.032768134 | count | 1 |
| RABEPK | 0.0254929 | 0.2100457 | 0.1214 | 0.903 | 0.032839715 | count | 1 |
| SLAIN2 | 0.0238928 | 0.1095778 | 0.218 | 0.827 | 0.032982641 | count | 1 |
| CUEDC2 | 0.023741 | 0.1204067 | 0.1972 | 0.844 | 0.032984503 | count | 1 |
| ACTR10 | 0.0237078 | 0.1005944 | 0.2357 | 0.814 | 0.03299805 | count | 1 |
| DDRGK1 | 0.0237871 | 0.1049024 | 0.2268 | 0.821 | 0.033282167 | count | 1 |
| COX7A2L | 0.0236007 | 0.0661833 | 0.3566 | 0.721 | 0.03351741 | count | 1 |
| ITGB2-AS1 | 0.0257919 | 0.1465229 | 0.176 | 0.86 | 0.033773127 | count | 1 |
| SLC25A28 | 0.0251616 | 0.1417626 | 0.1775 | 0.859 | 0.033855236 | count | 1 |
| LMNB2 | 0.0378333 | 0.3136525 | 0.1206 | 0.904 | 0.033928916 | count | 1 |
| SH3PXD2A | 0.0497816 | 0.4198465 | 0.1186 | 0.906 | 0.033994991 | count | 1 |
| LAMTOR4 | 0.0238071 | 0.0563736 | 0.4223 | 0.673 | 0.034014658 | count | 1 |
| SFT2D1 | 0.0246438 | 0.1173344 | 0.21 | 0.834 | 0.034029416 | count | 1 |
| TFRC | 0.0262066 | 0.1331175 | 0.1969 | 0.844 | 0.034113231 | count | 1 |
| MRPL45 | 0.0266766 | 0.1935672 | 0.1378 | 0.89 | 0.034158621 | count | 1 |
| COX17 | 0.0242137 | 0.0780582 | 0.3102 | 0.756 | 0.034165265 | count | 1 |
| AC007038.1 | 0.0760632 | 0.9463131 | 0.0804 | 0.936 | 0.034176212 | count | 1 |
| MTG1 | 0.0370297 | 0.3800012 | 0.0974 | 0.922 | 0.03428699 | count | 1 |
| EIF4E3 | 0.0261271 | 0.1644502 | 0.1589 | 0.874 | 0.034305501 | count | 1 |
| LACTB2-AS1 | 0.028528 | 0.3253673 | 0.0877 | 0.93 | 0.03437224 | count | 1 |
| COX6B1 | 0.0240049 | 0.042887 | 0.5597 | 0.576 | 0.034434504 | count | 1 |
| SRGAP2C | 0.036183 | 0.2482197 | 0.1458 | 0.884 | 0.034449791 | count | 1 |
| FNTA | 0.0250819 | 0.110007 | 0.228 | 0.82 | 0.034482382 | count | 1 |

| | | | | | | | |
|---|---|---|---|---|---|---|---|
| CD3G | 0.0239808 | 0.0353738 | 0.6779 | 0.498 | 0.034487366 | count | 1 |
| AC009961.1 | 0.0384653 | 0.5417854 | 0.071 | 0.943 | 0.034497741 | count | 1 |
| ZNF316 | 0.0416878 | 0.3158491 | 0.132 | 0.895 | 0.034500015 | count | 1 |
| NUP210 | 0.028213 | 0.1697833 | 0.1662 | 0.868 | 0.034592594 | count | 1 |
| GET4 | 0.1030457 | 0.9869835 | 0.1044 | 0.917 | 0.034616911 | count | 1 |
| CCNDBP1 | 0.0244278 | 0.072928 | 0.335 | 0.738 | 0.034624827 | count | 1 |
| CCDC142 | 0.1795439 | 0.5102113 | 0.3519 | 0.725 | 0.034676279 | count | 1 |
| PRPF39 | 0.0278257 | 0.189431 | 0.1469 | 0.883 | 0.034830267 | count | 1 |
| ARAP1 | 0.0421507 | 0.3191622 | 0.1321 | 0.895 | 0.034884807 | count | 1 |
| NDUFA9 | 0.0253074 | 0.1063529 | 0.238 | 0.812 | 0.03497539 | count | 1 |
| TOR1AIP2 | 0.0262762 | 0.1157156 | 0.2271 | 0.82 | 0.035039063 | count | 1 |
| SOX4 | 0.0475282 | 0.5519252 | 0.0861 | 0.931 | 0.035087116 | count | 1 |
| ZNF841 | 0.1816618 | 0.4875586 | 0.3726 | 0.709 | 0.035100256 | count | 1 |
| PRKCB | 0.0258593 | 0.1121352 | 0.2306 | 0.818 | 0.035160385 | count | 1 |
| B4GALT4 | 0.0290662 | 0.1597709 | 0.1819 | 0.856 | 0.035187186 | count | 1 |
| GRK3 | 0.1822219 | 0.5792726 | 0.3146 | 0.753 | 0.035212437 | count | 1 |
| DCXR | 0.0250117 | 0.0780177 | 0.3206 | 0.749 | 0.035220778 | count | 1 |
| SLAMF8 | 0.0515805 | 0.6022635 | 0.0856 | 0.932 | 0.03523166 | count | 1 |
| AL136038.3 | 0.0515805 | 0.6049464 | 0.0853 | 0.932 | 0.03523166 | count | 1 |
| TMEM18 | 0.0254252 | 0.1298412 | 0.1958 | 0.845 | 0.035254342 | count | 1 |
| ACAD9 | 0.0361373 | 0.3165574 | 0.1142 | 0.909 | 0.035262653 | count | 1 |
| LRRC29 | 0.0785791 | 0.8443368 | 0.0931 | 0.926 | 0.035321551 | count | 1 |
| PHF12 | 0.0283708 | 0.1603779 | 0.1769 | 0.86 | 0.035513223 | count | 1 |
| INSIG2 | 0.0275688 | 0.1968096 | 0.1401 | 0.889 | 0.035515838 | count | 1 |
| POU2F2 | 0.0277147 | 0.1757945 | 0.1577 | 0.875 | 0.035562946 | count | 1 |
| PNRC1 | 0.0247403 | 0.0239731 | 1.032 | 0.3021 | 0.035629118 | count | 1 |
| PFDN1 | 0.0256424 | 0.1060307 | 0.2418 | 0.809 | 0.035691358 | count | 1 |
| THEM6 | 0.0293692 | 0.2120644 | 0.1385 | 0.89 | 0.03571402 | count | 1 |
| IL15RA | 0.0297895 | 0.216368 | 0.1377 | 0.891 | 0.035715656 | count | 1 |
| RPN2 | 0.0254039 | 0.0807316 | 0.3147 | 0.753 | 0.035753994 | count | 1 |
| MED16 | 0.0302059 | 0.1844898 | 0.1637 | 0.87 | 0.035825043 | count | 1 |
| DUS1L | 0.0261999 | 0.1005925 | 0.2605 | 0.795 | 0.035885856 | count | 1 |
| DRG1 | 0.0277544 | 0.1646668 | 0.1685 | 0.866 | 0.035887578 | count | 1 |
| IL7 | 0.0662394 | 0.5082247 | 0.1303 | 0.896 | 0.035896378 | count | 1 |
| INTS7 | 0.0664584 | 0.4047259 | 0.1642 | 0.87 | 0.036016265 | count | 1 |
| NME1 | 0.0263041 | 0.1399135 | 0.188 | 0.851 | 0.036028626 | count | 1 |
| PABPC1 | 0.0251155 | 0.0346726 | 0.7244 | 0.469 | 0.036040795 | count | 1 |
| QRICH1 | 0.0363158 | 0.2075619 | 0.175 | 0.861 | 0.036218727 | count | 1 |
| HIST1H1C | 0.0264928 | 0.1293886 | 0.2048 | 0.838 | 0.036459703 | count | 1 |
| GPR82 | 0.0810774 | 0.3881977 | 0.2089 | 0.835 | 0.036459799 | count | 1 |
| RHOBTB3 | 0.0394273 | 0.4711348 | 0.0837 | 0.933 | 0.036514776 | count | 1 |
| EIF2B1 | 0.0273827 | 0.1324436 | 0.2067 | 0.836 | 0.036548655 | count | 1 |
| NUDCD1 | 0.0318946 | 0.2486996 | 0.1282 | 0.898 | 0.036579658 | count | 1 |
| NOMO3 | 0.0535818 | 0.4751021 | 0.1128 | 0.91 | 0.036608131 | count | 1 |
| LINC02273 | 0.0272539 | 0.1787705 | 0.1525 | 0.879 | 0.036671739 | count | 1 |

| | | | | | | | |
|---|---|---|---|---|---|---|---|
| AL161772.1 | 0.1897582 | 0.4966959 | 0.382 | 0.702 | 0.036724129 | count | 1 |
| KLHDC3 | 0.0310472 | 0.2033747 | 0.1527 | 0.879 | 0.036824218 | count | 1 |
| ELAC1 | 0.0305878 | 0.2583327 | 0.1184 | 0.906 | 0.036857123 | count | 1 |
| STXBP4 | 0.1096909 | 0.4105618 | 0.2672 | 0.789 | 0.036894481 | count | 1 |
| RILP | 0.038756 | 0.4016833 | 0.0965 | 0.923 | 0.036907545 | count | 1 |
| CRY1 | 0.0427006 | 0.2591414 | 0.1648 | 0.869 | 0.036915637 | count | 1 |
| CYP4V2 | 0.0357701 | 0.2815265 | 0.1271 | 0.899 | 0.037017507 | count | 1 |
| NIFK-AS1 | 0.0299067 | 0.2382292 | 0.1255 | 0.9 | 0.037078726 | count | 1 |
| SMC1A | 0.0274914 | 0.1167143 | 0.2355 | 0.814 | 0.037118959 | count | 1 |
| NUF2 | 0.0503395 | 0.4772281 | 0.1055 | 0.916 | 0.037175104 | count | 1 |
| ARMC8 | 0.0281579 | 0.1412349 | 0.1994 | 0.842 | 0.037286316 | count | 1 |
| POLR2C | 0.0268509 | 0.1059084 | 0.2535 | 0.8 | 0.037290375 | count | 1 |
| DUSP18 | 0.039429 | 0.293736 | 0.1342 | 0.893 | 0.037550574 | count | 1 |
| INTS6 | 0.0274685 | 0.0873197 | 0.3146 | 0.753 | 0.037561755 | count | 1 |
| ZNF528 | 0.0405693 | 0.313523 | 0.1294 | 0.897 | 0.037576217 | count | 1 |
| HYKK | 0.1941709 | 0.9121242 | 0.2129 | 0.831 | 0.037611217 | count | 1 |
| PTGER4 | 0.0265931 | 0.0570429 | 0.4662 | 0.641 | 0.037681881 | count | 1 |
| CCNT1 | 0.0293766 | 0.1587335 | 0.1851 | 0.853 | 0.037697185 | count | 1 |
| NADSYN1 | 0.0291543 | 0.2030827 | 0.1436 | 0.886 | 0.037699068 | count | 1 |
| SLC35A2 | 0.0354185 | 0.2340006 | 0.1514 | 0.88 | 0.037776874 | count | 1 |
| KLF3 | 0.0269082 | 0.0774628 | 0.3474 | 0.728 | 0.037956431 | count | 1 |
| SAMM50 | 0.0294518 | 0.1618644 | 0.182 | 0.856 | 0.038084053 | count | 1 |
| RPUSD1 | 0.0301716 | 0.1986983 | 0.1518 | 0.879 | 0.038091283 | count | 1 |
| AC093512.1 | 0.197408 | 0.5701389 | 0.3462 | 0.729 | 0.038262874 | count | 1 |
| SLC22A17 | 0.197408 | 0.5719379 | 0.3452 | 0.73 | 0.038262874 | count | 1 |
| HLA-F | 0.0268073 | 0.045832 | 0.5849 | 0.559 | 0.038394242 | count | 1 |
| SPR | 0.0520361 | 0.7092651 | 0.0734 | 0.942 | 0.038435857 | count | 1 |
| ASNS | 0.0360575 | 0.2773904 | 0.13 | 0.897 | 0.03846001 | count | 1 |
| RACK1 | 0.0267419 | 0.0181614 | 1.4725 | 0.141 | 0.038542298 | count | 1 |
| DCAF10 | 0.0328989 | 0.2342689 | 0.1404 | 0.888 | 0.038550923 | count | 1 |
| ABI1 | 0.0280395 | 0.1047735 | 0.2676 | 0.789 | 0.038588974 | count | 1 |
| RRP9 | 0.0367684 | 0.2534691 | 0.1451 | 0.885 | 0.038660301 | count | 1 |
| MRPS21 | 0.0272594 | 0.0693866 | 0.3929 | 0.694 | 0.038712572 | count | 1 |
| ARHGAP10 | 0.0301808 | 0.1835051 | 0.1645 | 0.869 | 0.038730021 | count | 1 |
| ZNF816 | 0.0568093 | 0.4554787 | 0.1247 | 0.901 | 0.038829433 | count | 1 |
| LINC02195 | 0.049445 | 0.6966329 | 0.071 | 0.943 | 0.038883208 | count | 1 |
| CSGALNACT2 | 0.0289878 | 0.1272693 | 0.2278 | 0.82 | 0.038888307 | count | 1 |
| AC034102.6 | 0.2005751 | 1.040908 | 0.1927 | 0.847 | 0.038901212 | count | 1 |
| FCHO2 | 0.0496544 | 0.3437391 | 0.1445 | 0.885 | 0.039048796 | count | 1 |
| C21orf58 | 0.0392355 | 0.4573997 | 0.0858 | 0.932 | 0.039139357 | count | 1 |
| FAU | 0.0271561 | 0.0148348 | 1.8306 | 0.0673 | 0.039153014 | count | 1 |
| TFR2 | 0.1163594 | 0.6640113 | 0.1752 | 0.861 | 0.039185374 | count | 1 |
| RPS6KA5 | 0.0316072 | 0.1594134 | 0.1983 | 0.843 | 0.039189356 | count | 1 |
| GMIP | 0.0324502 | 0.1878258 | 0.1728 | 0.863 | 0.039289127 | count | 1 |
| TM7SF3 | 0.0312083 | 0.1939386 | 0.1609 | 0.872 | 0.039294694 | count | 1 |

| | | | | | | | |
|---|---|---|---|---|---|---|---|
| ARL8A | 0.0284977 | 0.1226053 | 0.2324 | 0.816 | 0.039374884 | count | 1 |
| USPL1 | 0.0353229 | 0.254041 | 0.139 | 0.889 | 0.039463929 | count | 1 |
| ABTB1 | 0.0290624 | 0.1265346 | 0.2297 | 0.818 | 0.039496554 | count | 1 |
| RPSA | 0.027445 | 0.0196776 | 1.3947 | 0.163 | 0.039543497 | count | 1 |
| SGCB | 0.0579706 | 0.2573225 | 0.2253 | 0.822 | 0.039629141 | count | 1 |
| ZNF35 | 0.0731575 | 0.6631947 | 0.1103 | 0.912 | 0.039687263 | count | 1 |
| ASB8 | 0.0288125 | 0.1178095 | 0.2446 | 0.807 | 0.039873998 | count | 1 |
| PFDN6 | 0.0293027 | 0.1270356 | 0.2307 | 0.818 | 0.039981803 | count | 1 |
| RNPEP | 0.0324162 | 0.2003981 | 0.1618 | 0.872 | 0.040052822 | count | 1 |
| HIST1H1D | 0.028588 | 0.0941895 | 0.3035 | 0.762 | 0.040261181 | count | 1 |
| GMFB | 0.0318359 | 0.2002269 | 0.159 | 0.874 | 0.040400415 | count | 1 |
| AC034111.1 | 0.1199309 | 0.8238954 | 0.1456 | 0.884 | 0.040414503 | count | 1 |
| LRRC69 | 0.1199309 | 0.8295472 | 0.1446 | 0.885 | 0.040414503 | count | 1 |
| LATS2-AS1 | 0.1199309 | 0.9028147 | 0.1328 | 0.894 | 0.040414503 | count | 1 |
| BANF1 | 0.0289156 | 0.0848364 | 0.3408 | 0.733 | 0.04063008 | count | 1 |
| EEF1AKMT2 | 0.0316613 | 0.1611816 | 0.1964 | 0.844 | 0.040631547 | count | 1 |
| ADAP1 | 0.0338117 | 0.2453273 | 0.1378 | 0.89 | 0.040747167 | count | 1 |
| HSPBAP1 | 0.0350881 | 0.2578999 | 0.1361 | 0.892 | 0.040846619 | count | 1 |
| AP5Z1 | 0.0331943 | 0.1918849 | 0.173 | 0.863 | 0.040864963 | count | 1 |
| KLF4 | 0.0519508 | 0.3186533 | 0.163 | 0.871 | 0.040865209 | count | 1 |
| PPP6R3 | 0.0316014 | 0.1374598 | 0.2299 | 0.818 | 0.04093787 | count | 1 |
| ARMCX6 | 0.0310337 | 0.1423301 | 0.218 | 0.827 | 0.041004602 | count | 1 |
| NARS | 0.0309759 | 0.1245823 | 0.2486 | 0.804 | 0.041107883 | count | 1 |
| LRRC47 | 0.034976 | 0.2005501 | 0.1744 | 0.862 | 0.041246882 | count | 1 |
| FBXO30 | 0.0603528 | 0.4028925 | 0.1498 | 0.881 | 0.041270293 | count | 1 |
| PLXNA3 | 0.0369553 | 0.2628608 | 0.1406 | 0.888 | 0.041291464 | count | 1 |
| FAM219A | 0.0917524 | 0.5122059 | 0.1791 | 0.858 | 0.041333746 | count | 1 |
| ZNF324 | 0.0371228 | 0.2052988 | 0.1808 | 0.857 | 0.041479006 | count | 1 |
| ARMH3 | 0.0371309 | 0.243577 | 0.1524 | 0.879 | 0.041488074 | count | 1 |
| TSPAN31 | 0.0314559 | 0.1528193 | 0.2058 | 0.837 | 0.041514303 | count | 1 |
| KDM4D | 0.2136957 | 0.8872431 | 0.2409 | 0.81 | 0.041553469 | count | 1 |
| OXCT2 | 0.2136957 | 1.065637 | 0.2005 | 0.841 | 0.041553469 | count | 1 |
| VEGFC | 0.2136957 | 1.065637 | 0.2005 | 0.841 | 0.041553469 | count | 1 |
| SIAH3 | 0.2136957 | 1.065637 | 0.2005 | 0.841 | 0.041553469 | count | 1 |
| KNTC1 | 0.0529471 | 0.4532805 | 0.1168 | 0.907 | 0.04165355 | count | 1 |
| ZBTB7B | 0.0348766 | 0.2673588 | 0.1304 | 0.896 | 0.041823668 | count | 1 |
| SPAG7 | 0.0304873 | 0.1041039 | 0.2929 | 0.77 | 0.041958899 | count | 1 |
| CHKB-DT | 0.056785 | 0.4469572 | 0.127 | 0.899 | 0.041967452 | count | 1 |
| SQSTM1 | 0.0294948 | 0.0614153 | 0.4803 | 0.6311 | 0.042033541 | count | 1 |
| RAB7A | 0.0297675 | 0.0763916 | 0.3897 | 0.697 | 0.042044163 | count | 1 |
| TSHZ1 | 0.0421399 | 0.2224533 | 0.1894 | 0.85 | 0.042045945 | count | 1 |
| RPS6KB1 | 0.0335095 | 0.1608425 | 0.2083 | 0.835 | 0.042076297 | count | 1 |
| B4GALT1 | 0.0303386 | 0.0998392 | 0.3039 | 0.761 | 0.042144478 | count | 1 |
| AC010761.1 | 0.216646 | 0.6507847 | 0.3329 | 0.739 | 0.042151596 | count | 1 |
| ERP29 | 0.0294656 | 0.0426962 | 0.6901 | 0.49 | 0.042172196 | count | 1 |

| | | | | | | | |
|---|---|---|---|---|---|---|---|
| DNAJC16 | 0.0456488 | 0.3193405 | 0.1429 | 0.886 | 0.042299943 | count | 1 |
| USP11 | 0.0332735 | 0.1795875 | 0.1853 | 0.853 | 0.042328449 | count | 1 |
| SCPEP1 | 0.0369082 | 0.2306233 | 0.16 | 0.873 | 0.042340494 | count | 1 |
| SHQ1 | 0.0434356 | 0.2977439 | 0.1459 | 0.884 | 0.042409059 | count | 1 |
| IDUA | 0.0459384 | 0.3301364 | 0.1391 | 0.889 | 0.042569383 | count | 1 |
| RNASEH1 | 0.0343015 | 0.1867295 | 0.1837 | 0.854 | 0.042676731 | count | 1 |
| TAPBP | 0.0299862 | 0.062971 | 0.4762 | 0.634 | 0.042683584 | count | 1 |
| EEF2KMT | 0.0382661 | 0.2713488 | 0.141 | 0.888 | 0.042759192 | count | 1 |
| CHD8 | 0.0319818 | 0.1391128 | 0.2299 | 0.818 | 0.042973287 | count | 1 |
| SH2D3C | 0.0374977 | 0.2549311 | 0.1471 | 0.883 | 0.043018044 | count | 1 |
| GRAMD1A | 0.0320223 | 0.1204565 | 0.2658 | 0.79 | 0.043211178 | count | 1 |
| GRAP2 | 0.031437 | 0.1233875 | 0.2548 | 0.799 | 0.043237362 | count | 1 |
| GLCE | 0.0340605 | 0.2149864 | 0.1584 | 0.874 | 0.043330618 | count | 1 |
| SMCO4 | 0.0434614 | 0.2964093 | 0.1466 | 0.883 | 0.043368856 | count | 1 |
| AXIN2 | 0.0963067 | 0.4341247 | 0.2218 | 0.824 | 0.043418188 | count | 1 |
| MAN2A2 | 0.0524057 | 0.3206512 | 0.1634 | 0.87 | 0.043418794 | count | 1 |
| CHD2 | 0.0314602 | 0.0947542 | 0.332 | 0.74 | 0.04349335 | count | 1 |
| EIF4B | 0.0308068 | 0.0547076 | 0.5631 | 0.573 | 0.04349751 | count | 1 |
| CDHR3 | 0.0804104 | 0.5944752 | 0.1353 | 0.892 | 0.043669881 | count | 1 |
| TSC22D4 | 0.0312458 | 0.0851177 | 0.3671 | 0.714 | 0.04372666 | count | 1 |
| P3H3 | 0.1304283 | 0.8162314 | 0.1598 | 0.873 | 0.044036001 | count | 1 |
| GSTK1 | 0.0307443 | 0.0432111 | 0.7115 | 0.477 | 0.044071483 | count | 1 |
| LINC00243 | 0.2261341 | 0.3068304 | 0.737 | 0.461 | 0.044079371 | count | 1 |
| TATDN1 | 0.0327466 | 0.1300552 | 0.2518 | 0.801 | 0.044129005 | count | 1 |
| MCFD2 | 0.0360736 | 0.1803984 | 0.2 | 0.842 | 0.044243591 | count | 1 |
| SH2D4A | 0.2271226 | 0.5441229 | 0.4174 | 0.676 | 0.044280585 | count | 1 |
| MTREX | 0.0371286 | 0.1674509 | 0.2217 | 0.825 | 0.044294905 | count | 1 |
| ATG7 | 0.0428009 | 0.3440497 | 0.1244 | 0.901 | 0.044315242 | count | 1 |
| HLA-C | 0.0307843 | 0.0199172 | 1.5456 | 0.122 | 0.044369711 | count | 1 |
| TMEM154 | 0.0357107 | 0.1744828 | 0.2047 | 0.838 | 0.044432139 | count | 1 |
| MRPS7 | 0.0320342 | 0.1225114 | 0.2615 | 0.794 | 0.044519365 | count | 1 |
| RELT | 0.0375681 | 0.2463603 | 0.1525 | 0.879 | 0.044571271 | count | 1 |
| MRPL2 | 0.0336756 | 0.170679 | 0.1973 | 0.844 | 0.044597791 | count | 1 |
| ICAM1 | 0.0359663 | 0.2141658 | 0.1679 | 0.867 | 0.04460091 | count | 1 |
| CNNM3 | 0.0651842 | 0.3429836 | 0.1901 | 0.849 | 0.044601775 | count | 1 |
| TMCO1 | 0.0316435 | 0.0709073 | 0.4463 | 0.655 | 0.044734471 | count | 1 |
| UNG | 0.0420308 | 0.1938625 | 0.2168 | 0.828 | 0.044848552 | count | 1 |
| KDELC1 | 0.2305502 | 0.7141616 | 0.3228 | 0.747 | 0.04497884 | count | 1 |
| PPP1R37 | 0.041154 | 0.3029589 | 0.1358 | 0.892 | 0.045027258 | count | 1 |
| CPLANE1 | 0.0502087 | 0.3713628 | 0.1352 | 0.892 | 0.045079198 | count | 1 |
| AC064807.1 | 0.0417088 | 0.3820335 | 0.1092 | 0.913 | 0.045091115 | count | 1 |
| LAMTOR1 | 0.0318175 | 0.0793365 | 0.401 | 0.688 | 0.045115071 | count | 1 |
| ZDHHC18 | 0.0346898 | 0.1704721 | 0.2035 | 0.839 | 0.045165129 | count | 1 |
| DNAAF2 | 0.0332302 | 0.1276456 | 0.2603 | 0.795 | 0.045210603 | count | 1 |
| HNRNPDL | 0.0314902 | 0.0337462 | 0.9331 | 0.351 | 0.045235781 | count | 1 |

| | | | | | | | |
|---|---|---|---|---|---|---|---|
| NR1H2 | 0.0326466 | 0.0956206 | 0.3414 | 0.733 | 0.045259971 | count | 1 |
| SPSB3 | 0.0322538 | 0.0881917 | 0.3657 | 0.715 | 0.045316933 | count | 1 |
| FAHD2B | 0.0392035 | 0.3367687 | 0.1164 | 0.907 | 0.04532252 | count | 1 |
| TMEM161A | 0.0356846 | 0.1968911 | 0.1812 | 0.856 | 0.045398898 | count | 1 |
| NUP188 | 0.0506275 | 0.3152816 | 0.1606 | 0.872 | 0.045456976 | count | 1 |
| NABP2 | 0.034236 | 0.1685133 | 0.2032 | 0.839 | 0.045782288 | count | 1 |
| AP000692.2 | 0.0480687 | 0.4970837 | 0.0967 | 0.923 | 0.045811806 | count | 1 |
| CNOT2 | 0.0328577 | 0.0894874 | 0.3672 | 0.714 | 0.046007925 | count | 1 |
| LSS | 0.0515423 | 0.4555454 | 0.1131 | 0.91 | 0.046282271 | count | 1 |
| PYCR3 | 0.0559408 | 0.4613361 | 0.1213 | 0.903 | 0.046364764 | count | 1 |
| MSL2 | 0.0358829 | 0.1421777 | 0.2524 | 0.801 | 0.046489341 | count | 1 |
| PPP2R3C | 0.034022 | 0.1165222 | 0.292 | 0.77 | 0.046546974 | count | 1 |
| ZNF490 | 0.0488938 | 0.4244257 | 0.1152 | 0.908 | 0.046601371 | count | 1 |
| PFDN5 | 0.0324175 | 0.0256727 | 1.2627 | 0.2068 | 0.046655264 | count | 1 |
| RHOH | 0.0326609 | 0.049559 | 0.659 | 0.51 | 0.046678919 | count | 1 |
| ZFP14 | 0.035823 | 0.1840806 | 0.1946 | 0.846 | 0.046714104 | count | 1 |
| PTBP1 | 0.0341379 | 0.0922739 | 0.37 | 0.711 | 0.046744258 | count | 1 |
| MT-ND2 | 0.0324995 | 0.0302496 | 1.0744 | 0.283 | 0.046805962 | count | 1 |
| CASP2 | 0.0397832 | 0.2040606 | 0.195 | 0.845 | 0.046926136 | count | 1 |
| SSR1 | 0.033802 | 0.1045446 | 0.3233 | 0.746 | 0.046926571 | count | 1 |
| ATXN7L3B | 0.0342981 | 0.1148788 | 0.2986 | 0.765 | 0.046944446 | count | 1 |
| TTLL1 | 0.0420178 | 0.3382771 | 0.1242 | 0.901 | 0.046961196 | count | 1 |
| LRWD1 | 0.0430072 | 0.2605995 | 0.165 | 0.869 | 0.047060062 | count | 1 |
| MICA | 0.0407431 | 0.215151 | 0.1894 | 0.85 | 0.047105976 | count | 1 |
| BHLHE40-AS1 | 0.2415484 | 0.4883469 | 0.4946 | 0.621 | 0.047225005 | count | 1 |
| PMPCB | 0.0350417 | 0.1076374 | 0.3256 | 0.745 | 0.047287865 | count | 1 |
| NBPF3 | 0.0570671 | 0.4451539 | 0.1282 | 0.898 | 0.047303808 | count | 1 |
| PXYLP1 | 0.2423757 | 0.4853957 | 0.4993 | 0.618 | 0.047394312 | count | 1 |
| ZBTB38 | 0.0339898 | 0.0810682 | 0.4193 | 0.675 | 0.047624517 | count | 1 |
| UQCRC2 | 0.0339773 | 0.081865 | 0.415 | 0.678 | 0.047676236 | count | 1 |
| PCGF5 | 0.0346663 | 0.0996389 | 0.3479 | 0.728 | 0.047712621 | count | 1 |
| NDUFA1 | 0.0334846 | 0.0524561 | 0.6383 | 0.5233 | 0.047778685 | count | 1 |
| PEX11B | 0.0394999 | 0.2779693 | 0.1421 | 0.887 | 0.047837951 | count | 1 |
| TMEM126B | 0.0353064 | 0.1182181 | 0.2987 | 0.765 | 0.047934724 | count | 1 |
| ANKMY1 | 0.0608866 | 0.3385959 | 0.1798 | 0.857 | 0.047941894 | count | 1 |
| TMEM184C | 0.0480587 | 0.3233044 | 0.1486 | 0.882 | 0.047973079 | count | 1 |
| AL023806.1 | 0.0882729 | 1.01184 | 0.0872 | 0.93 | 0.047996735 | count | 1 |
| AL354696.2 | 0.0882729 | 1.01184 | 0.0872 | 0.93 | 0.047996735 | count | 1 |
| ARHGAP45 | 0.0349111 | 0.0979792 | 0.3563 | 0.722 | 0.048065472 | count | 1 |
| RCOR3 | 0.037551 | 0.1620112 | 0.2318 | 0.817 | 0.048097536 | count | 1 |
| ANKRD6 | 0.142367 | 0.5736728 | 0.2482 | 0.804 | 0.048170582 | count | 1 |
| MYC | 0.03723 | 0.1438447 | 0.2588 | 0.796 | 0.048318348 | count | 1 |
| POLD1 | 0.2485025 | 0.6018471 | 0.4129 | 0.68 | 0.048649674 | count | 1 |
| INPP5A | 0.0562668 | 0.4494954 | 0.1252 | 0.9 | 0.048708919 | count | 1 |
| AL645933.2 | 0.0658441 | 0.4403935 | 0.1495 | 0.881 | 0.048715179 | count | 1 |

| | | | | | | | |
|---|---|---|---|---|---|---|---|
| SATB1 | 0.0389106 | 0.1349347 | 0.2884 | 0.773 | 0.048723251 | count | 1 |
| NKIRAS1 | 0.0587795 | 0.316804 | 0.1855 | 0.853 | 0.048731914 | count | 1 |
| AC012360.3 | 0.0659299 | 0.3346285 | 0.197 | 0.844 | 0.04877916 | count | 1 |
| SON | 0.0340672 | 0.0384346 | 0.8864 | 0.375 | 0.048813711 | count | 1 |
| CEP295 | 0.037379 | 0.1708852 | 0.2187 | 0.827 | 0.048818257 | count | 1 |
| TRAV29DV5 | 0.1081342 | 0.7083758 | 0.1527 | 0.879 | 0.048845482 | count | 1 |
| TRAV13-1 | 0.1081342 | 0.740895 | 0.146 | 0.884 | 0.048845482 | count | 1 |
| LBH | 0.0343161 | 0.0597003 | 0.5748 | 0.5655 | 0.048896286 | count | 1 |
| TP53I11 | 0.0398781 | 0.2836579 | 0.1406 | 0.888 | 0.048916613 | count | 1 |
| 8-Mar | 0.0545045 | 0.2377037 | 0.2293 | 0.819 | 0.048955575 | count | 1 |
| ERH | 0.0345519 | 0.0626866 | 0.5512 | 0.582 | 0.049198822 | count | 1 |
| DBR1 | 0.0457715 | 0.2424556 | 0.1888 | 0.85 | 0.049495663 | count | 1 |
| PRPF4B | 0.034885 | 0.0605227 | 0.5764 | 0.564 | 0.049526776 | count | 1 |
| SND1-IT1 | 0.1463563 | 0.4837478 | 0.3025 | 0.762 | 0.049555867 | count | 1 |
| ANP32A | 0.0351853 | 0.0812313 | 0.4331 | 0.665 | 0.049562758 | count | 1 |
| RPS5 | 0.034409 | 0.0194262 | 1.7713 | 0.0766 | 0.049575338 | count | 1 |
| ZFP36L2 | 0.0344467 | 0.0295882 | 1.1642 | 0.244 | 0.049616874 | count | 1 |
| SIPA1L2 | 0.1098575 | 0.6034306 | 0.1821 | 0.856 | 0.049637935 | count | 1 |
| HMGXB3 | 0.0377069 | 0.1446578 | 0.2607 | 0.794 | 0.049649976 | count | 1 |
| SRPK2 | 0.0352979 | 0.0892608 | 0.3954 | 0.693 | 0.049654261 | count | 1 |
| ZNF134 | 0.0465292 | 0.2821365 | 0.1649 | 0.869 | 0.04966282 | count | 1 |
| F5 | 0.1467323 | 0.7413946 | 0.1979 | 0.843 | 0.04968653 | count | 1 |
| HMGCL | 0.0423896 | 0.2130834 | 0.1989 | 0.842 | 0.049693966 | count | 1 |
| FCHSD2 | 0.0396977 | 0.1815349 | 0.2187 | 0.827 | 0.049710118 | count | 1 |
| SNX27 | 0.0407231 | 0.2036617 | 0.2 | 0.842 | 0.049753366 | count | 1 |
| ANXA9 | 0.091553 | 0.543391 | 0.1685 | 0.866 | 0.049804725 | count | 1 |
| FAF2 | 0.0391941 | 0.1829153 | 0.2143 | 0.83 | 0.049868873 | count | 1 |
| FAAH2 | 0.0729034 | 0.3119972 | 0.2337 | 0.815 | 0.049932815 | count | 1 |
| SDHD | 0.0355408 | 0.0903324 | 0.3934 | 0.694 | 0.050010145 | count | 1 |
| MSH2 | 0.0415109 | 0.2265876 | 0.1832 | 0.855 | 0.050041273 | count | 1 |
| KIAA0930 | 0.0420066 | 0.2806565 | 0.1497 | 0.881 | 0.050124842 | count | 1 |
| TBC1D23 | 0.0387263 | 0.1737876 | 0.2228 | 0.824 | 0.050176791 | count | 1 |
| TBPL1 | 0.0363917 | 0.1071189 | 0.3397 | 0.734 | 0.050366797 | count | 1 |
| NRBP2 | 0.0472274 | 0.3577589 | 0.132 | 0.895 | 0.050410289 | count | 1 |
| RARG | 0.0495945 | 0.3439697 | 0.1442 | 0.885 | 0.050483681 | count | 1 |
| KLF10 | 0.0365151 | 0.1154697 | 0.3162 | 0.752 | 0.050484318 | count | 1 |
| KLHL2 | 0.081388 | 0.3143237 | 0.2589 | 0.796 | 0.050504555 | count | 1 |
| BUD23 | 0.0365228 | 0.0939088 | 0.3889 | 0.697 | 0.050574019 | count | 1 |
| RPL7L1 | 0.0362734 | 0.0921291 | 0.3937 | 0.694 | 0.05064366 | count | 1 |
| AC005726.5 | 0.1121397 | 0.5689786 | 0.1971 | 0.844 | 0.050688047 | count | 1 |
| MDM2 | 0.037586 | 0.1259694 | 0.2984 | 0.765 | 0.050689289 | count | 1 |
| KLHDC1 | 0.0643794 | 0.3647076 | 0.1765 | 0.86 | 0.05071171 | count | 1 |
| SPTLC2 | 0.0391526 | 0.1809846 | 0.2163 | 0.829 | 0.050729681 | count | 1 |
| VTI1A | 0.0389624 | 0.1885248 | 0.2067 | 0.836 | 0.050733143 | count | 1 |
| USP34 | 0.0362835 | 0.0879008 | 0.4128 | 0.68 | 0.050963876 | count | 1 |

| | | | | | | | |
|---|---|---|---|---|---|---|---|
| MCM3 | 0.0408604 | 0.1801003 | 0.2269 | 0.821 | 0.051011415 | count | 1 |
| SLC35G1 | 0.26013 | 0.5410756 | 0.4808 | 0.631 | 0.05103937 | count | 1 |
| FUNDC2 | 0.0364638 | 0.0930357 | 0.3919 | 0.695 | 0.051078711 | count | 1 |
| SRSF10 | 0.0360604 | 0.0707528 | 0.5097 | 0.61 | 0.051089055 | count | 1 |
| MAP3K6 | 0.0478796 | 0.3139991 | 0.1525 | 0.879 | 0.051108569 | count | 1 |
| M6PR | 0.0361656 | 0.0750902 | 0.4816 | 0.63 | 0.051128519 | count | 1 |
| POLR2L | 0.0360006 | 0.0624292 | 0.5767 | 0.564 | 0.051233125 | count | 1 |
| MFNG | 0.0367067 | 0.1039152 | 0.3532 | 0.724 | 0.051273111 | count | 1 |
| NDUFAF3 | 0.0362709 | 0.0772762 | 0.4694 | 0.639 | 0.051430662 | count | 1 |
| PEX3 | 0.0386897 | 0.2086541 | 0.1854 | 0.853 | 0.051456842 | count | 1 |
| VPS37C | 0.0594359 | 0.6452103 | 0.0921 | 0.927 | 0.051468255 | count | 1 |
| HNRNPK | 0.0360341 | 0.0460327 | 0.7828 | 0.4338 | 0.05154353 | count | 1 |
| ZNF862 | 0.0752639 | 0.4516002 | 0.1667 | 0.868 | 0.051565049 | count | 1 |
| CYTH3 | 0.0830932 | 0.4014477 | 0.207 | 0.836 | 0.051574795 | count | 1 |
| KDM4C | 0.0400538 | 0.1481834 | 0.2703 | 0.787 | 0.051713678 | count | 1 |
| COMMD10 | 0.0398477 | 0.1584789 | 0.2514 | 0.801 | 0.051719033 | count | 1 |
| SERGEF | 0.0417451 | 0.1718466 | 0.2429 | 0.808 | 0.0517774 | count | 1 |
| ATL3 | 0.0389731 | 0.1628251 | 0.2394 | 0.811 | 0.051934214 | count | 1 |
| ADAM19 | 0.0389168 | 0.1465282 | 0.2656 | 0.791 | 0.051954703 | count | 1 |
| B4GALNT4 | 0.1533879 | 0.7530019 | 0.2037 | 0.839 | 0.052002099 | count | 1 |
| CRELD1 | 0.0441854 | 0.2673784 | 0.1653 | 0.869 | 0.052129021 | count | 1 |
| FAM193B | 0.0629501 | 0.4073184 | 0.1545 | 0.877 | 0.052212171 | count | 1 |
| NUDT18 | 0.0416662 | 0.2444127 | 0.1705 | 0.865 | 0.052331914 | count | 1 |
| C17orf107 | 0.1157158 | 0.5866033 | 0.1973 | 0.844 | 0.052335019 | count | 1 |
| SNRPA | 0.0384326 | 0.1282899 | 0.2996 | 0.765 | 0.052345132 | count | 1 |
| ZNF292 | 0.0384079 | 0.102051 | 0.3764 | 0.707 | 0.052387255 | count | 1 |
| SLC25A13 | 0.0474759 | 0.2813733 | 0.1687 | 0.866 | 0.052539965 | count | 1 |
| DARS-AS1 | 0.2677525 | 0.5201868 | 0.5147 | 0.607 | 0.052611071 | count | 1 |
| C2orf74 | 0.0393762 | 0.2011778 | 0.1957 | 0.845 | 0.052660922 | count | 1 |
| TRIM44 | 0.0388321 | 0.1139959 | 0.3406 | 0.733 | 0.052752947 | count | 1 |
| AC044839.1 | 0.1555668 | 0.5868424 | 0.2651 | 0.791 | 0.052761285 | count | 1 |
| CDK9 | 0.0389189 | 0.1335701 | 0.2914 | 0.771 | 0.052870929 | count | 1 |
| RASA3 | 0.0410295 | 0.1469101 | 0.2793 | 0.78 | 0.052875555 | count | 1 |
| EIF4H | 0.038262 | 0.0944825 | 0.405 | 0.686 | 0.053009528 | count | 1 |
| CWC25 | 0.0381604 | 0.0939456 | 0.4062 | 0.685 | 0.053013828 | count | 1 |
| TAF4B | 0.117245 | 0.2770706 | 0.4232 | 0.672 | 0.053039833 | count | 1 |
| SLC18B1 | 0.0556771 | 0.3854178 | 0.1445 | 0.885 | 0.053096517 | count | 1 |
| MRPS31 | 0.0386944 | 0.1275672 | 0.3033 | 0.762 | 0.053148912 | count | 1 |
| AC118549.1 | 0.0422665 | 0.1593327 | 0.2653 | 0.791 | 0.053236651 | count | 1 |
| SERP1 | 0.0371844 | 0.0442905 | 0.8396 | 0.401 | 0.053244635 | count | 1 |
| CCDC159 | 0.0454819 | 0.2195167 | 0.2072 | 0.836 | 0.053326698 | count | 1 |
| NF2 | 0.056 | 0.2526358 | 0.2217 | 0.825 | 0.053405877 | count | 1 |
| UBOX5 | 0.1575607 | 0.5608513 | 0.2809 | 0.779 | 0.053456509 | count | 1 |
| FBXO42 | 0.0489392 | 0.2723359 | 0.1797 | 0.857 | 0.053569893 | count | 1 |
| ZCCHC7 | 0.0393773 | 0.1186561 | 0.3319 | 0.74 | 0.053735243 | count | 1 |

| | | | | | | | |
|---|---|---|---|---|---|---|---|
| ING5 | 0.0400088 | 0.1415449 | 0.2827 | 0.777 | 0.0537663 | count | 1 |
| HYLS1 | 0.050365 | 0.258354 | 0.1949 | 0.845 | 0.053770087 | count | 1 |
| MANEA | 0.052856 | 0.2842129 | 0.186 | 0.852 | 0.053816289 | count | 1 |
| SAMHD1 | 0.0383127 | 0.0773757 | 0.4952 | 0.621 | 0.053826213 | count | 1 |
| NDUFAF6 | 0.0466293 | 0.2124879 | 0.2194 | 0.826 | 0.053926897 | count | 1 |
| MVB12A | 0.0416273 | 0.1804799 | 0.2306 | 0.818 | 0.054031176 | count | 1 |
| WDR45 | 0.0401075 | 0.1404068 | 0.2857 | 0.775 | 0.054054944 | count | 1 |
| C1GALT1 | 0.0395214 | 0.1069132 | 0.3697 | 0.712 | 0.054183957 | count | 1 |
| SNRPD2 | 0.0377681 | 0.0381883 | 0.989 | 0.323 | 0.05421611 | count | 1 |
| ZNF287 | 0.0509552 | 0.2785635 | 0.1829 | 0.855 | 0.054402231 | count | 1 |
| COX7C | 0.0378817 | 0.0296556 | 1.2774 | 0.202 | 0.054431513 | count | 1 |
| AIG1 | 0.0474543 | 0.2134361 | 0.2223 | 0.824 | 0.054467715 | count | 1 |
| CDK13 | 0.0394129 | 0.0939251 | 0.4196 | 0.675 | 0.054492318 | count | 1 |
| KCTD5 | 0.0479002 | 0.2259405 | 0.212 | 0.832 | 0.054534894 | count | 1 |
| ISYNA1 | 0.0450898 | 0.2527853 | 0.1784 | 0.858 | 0.054619857 | count | 1 |
| NUDCD2 | 0.0391292 | 0.1040952 | 0.3759 | 0.707 | 0.054623263 | count | 1 |
| AC004812.2 | 0.0519536 | 0.3495673 | 0.1486 | 0.882 | 0.054682125 | count | 1 |
| AC116366.3 | 0.0660793 | 0.3579684 | 0.1846 | 0.854 | 0.05482531 | count | 1 |
| RCHY1 | 0.0444447 | 0.1665069 | 0.2669 | 0.79 | 0.054938349 | count | 1 |
| LINC02453 | 0.2798831 | 0.731212 | 0.3828 | 0.702 | 0.055120589 | count | 1 |
| GCDH | 0.0451476 | 0.2219621 | 0.2034 | 0.839 | 0.055168159 | count | 1 |
| TRAF6 | 0.0488977 | 0.2297533 | 0.2128 | 0.831 | 0.055188689 | count | 1 |
| PSMC3 | 0.0396574 | 0.0980229 | 0.4046 | 0.686 | 0.05530546 | count | 1 |
| WDR75 | 0.0435799 | 0.1850363 | 0.2355 | 0.814 | 0.055323046 | count | 1 |
| IAH1 | 0.0392353 | 0.0859927 | 0.4563 | 0.648 | 0.055344483 | count | 1 |
| RAB39B | 0.0451532 | 0.1678793 | 0.269 | 0.788 | 0.05539807 | count | 1 |
| FTO | 0.0703619 | 0.2887451 | 0.2437 | 0.807 | 0.055460667 | count | 1 |
| PPP1R9B | 0.056777 | 0.2718763 | 0.2088 | 0.835 | 0.055493704 | count | 1 |
| ZDBF2 | 0.0750301 | 0.3727577 | 0.2013 | 0.84 | 0.055571748 | count | 1 |
| AL135925.1 | 0.0453299 | 0.1848673 | 0.2452 | 0.806 | 0.055615222 | count | 1 |
| PRR3 | 0.0515497 | 0.2751109 | 0.1874 | 0.851 | 0.055763742 | count | 1 |
| NUDCD3 | 0.0438499 | 0.1750255 | 0.2505 | 0.802 | 0.055800238 | count | 1 |
| KLHL9 | 0.0708153 | 0.3083616 | 0.2297 | 0.818 | 0.055820825 | count | 1 |
| C22orf46 | 0.0504583 | 0.2596384 | 0.1943 | 0.846 | 0.055849972 | count | 1 |
| FAM204A | 0.0397448 | 0.0840041 | 0.4731 | 0.636 | 0.055861829 | count | 1 |
| THUMPD3 | 0.0409889 | 0.1438173 | 0.285 | 0.776 | 0.055909597 | count | 1 |
| MALAT1 | 0.0387552 | 0.0132386 | 2.9274 | 0.00344 | 0.055910532 | count | 1 |
| SETD6 | 0.0674222 | 0.2808512 | 0.2401 | 0.81 | 0.055947239 | count | 1 |
| TTLL10 | 0.1236811 | 0.9406943 | 0.1315 | 0.895 | 0.056009963 | count | 1 |
| PITPNA-AS1 | 0.0417329 | 0.148244 | 0.2815 | 0.778 | 0.056084877 | count | 1 |
| FBXL5 | 0.0418151 | 0.1342964 | 0.3114 | 0.756 | 0.056237282 | count | 1 |
| C3orf58 | 0.0475733 | 0.1747498 | 0.2722 | 0.785 | 0.056466193 | count | 1 |
| HYOU1 | 0.0460235 | 0.240917 | 0.191 | 0.849 | 0.056467633 | count | 1 |
| CFAP97 | 0.0400806 | 0.0838799 | 0.4778 | 0.633 | 0.056506431 | count | 1 |
| ASNA1 | 0.0409797 | 0.1123463 | 0.3648 | 0.715 | 0.056612652 | count | 1 |

| | | | | | | | |
|---|---|---|---|---|---|---|---|
| DBH-AS1 | 0.2871786 | 0.9528899 | 0.3014 | 0.763 | 0.056634697 | count | 1 |
| RGS19 | 0.0400196 | 0.0738261 | 0.5421 | 0.588 | 0.056718409 | count | 1 |
| HEXA | 0.0413475 | 0.1419253 | 0.2913 | 0.771 | 0.056753366 | count | 1 |
| MSRA | 0.0473632 | 0.1847962 | 0.2563 | 0.798 | 0.056826887 | count | 1 |
| KAT8 | 0.0431217 | 0.1608177 | 0.2681 | 0.789 | 0.056856249 | count | 1 |
| AC005070.3 | 0.0687126 | 0.3898078 | 0.1763 | 0.86 | 0.057025592 | count | 1 |
| SNX4 | 0.0445978 | 0.1610506 | 0.2769 | 0.782 | 0.057134576 | count | 1 |
| CCPG1 | 0.0411813 | 0.0938832 | 0.4386 | 0.661 | 0.057175003 | count | 1 |
| FUCA1 | 0.0511663 | 0.2582945 | 0.1981 | 0.843 | 0.057214887 | count | 1 |
| OSGEP | 0.042756 | 0.1441084 | 0.2967 | 0.767 | 0.057372542 | count | 1 |
| NAA60 | 0.0442908 | 0.1826808 | 0.2424 | 0.808 | 0.057492094 | count | 1 |
| DDOST | 0.0410393 | 0.0922093 | 0.4451 | 0.656 | 0.057519241 | count | 1 |
| SENP3 | 0.2921417 | 0.4361172 | 0.6699 | 0.503 | 0.057666809 | count | 1 |
| CCDC82 | 0.0413107 | 0.0952587 | 0.4337 | 0.665 | 0.057723997 | count | 1 |
| ZBED6 | 0.2929616 | 0.6276886 | 0.4667 | 0.641 | 0.057837473 | count | 1 |
| CIR1 | 0.0408143 | 0.0778193 | 0.5245 | 0.6 | 0.057930193 | count | 1 |
| ZNF844 | 0.0781886 | 0.5184921 | 0.1508 | 0.88 | 0.057932594 | count | 1 |
| SPCS3 | 0.0408485 | 0.0695937 | 0.587 | 0.557 | 0.057945355 | count | 1 |
| ATF1 | 0.0425951 | 0.1150182 | 0.3703 | 0.711 | 0.058155463 | count | 1 |
| PDIA3 | 0.0406656 | 0.0450991 | 0.9017 | 0.367 | 0.058267753 | count | 1 |
| CYHR1 | 0.0429274 | 0.1389863 | 0.3089 | 0.757 | 0.058288015 | count | 1 |
| CFAP20 | 0.0421576 | 0.1073041 | 0.3929 | 0.694 | 0.058478271 | count | 1 |
| POC1B-AS1 | 0.0675035 | 0.3742666 | 0.1804 | 0.857 | 0.05850015 | count | 1 |
| NMI | 0.0436352 | 0.1259167 | 0.3465 | 0.729 | 0.058506727 | count | 1 |
| MAN2B1 | 0.0675367 | 0.3260862 | 0.2071 | 0.836 | 0.058529111 | count | 1 |
| ZNF827 | 0.0708355 | 0.3243312 | 0.2184 | 0.827 | 0.05880024 | count | 1 |
| CHFR | 0.0467024 | 0.2047247 | 0.2281 | 0.82 | 0.058831983 | count | 1 |
| TAPBPL | 0.0447237 | 0.1433238 | 0.312 | 0.755 | 0.058970487 | count | 1 |
| MRPL43 | 0.0417458 | 0.0813824 | 0.513 | 0.608 | 0.058985589 | count | 1 |
| STK19 | 0.0479628 | 0.2070848 | 0.2316 | 0.817 | 0.059077642 | count | 1 |
| CYSLTR2 | 0.0749776 | 0.4576213 | 0.1638 | 0.87 | 0.059128758 | count | 1 |
| LYAR | 0.0415575 | 0.072766 | 0.5711 | 0.568 | 0.059140698 | count | 1 |
| ZNF22 | 0.0426481 | 0.1236307 | 0.345 | 0.73 | 0.059185905 | count | 1 |
| GARS | 0.0471661 | 0.1732067 | 0.2723 | 0.785 | 0.059249999 | count | 1 |
| CRLF3 | 0.0440639 | 0.1246 | 0.3536 | 0.724 | 0.059349462 | count | 1 |
| DNAJC4 | 0.0431315 | 0.1168049 | 0.3693 | 0.712 | 0.059445616 | count | 1 |
| DDX19A | 0.0583994 | 0.2590038 | 0.2255 | 0.822 | 0.059484022 | count | 1 |
| TST | 0.0717277 | 0.645354 | 0.1111 | 0.912 | 0.059546302 | count | 1 |
| CPSF1 | 0.0687727 | 0.3711087 | 0.1853 | 0.853 | 0.059607378 | count | 1 |
| COX11 | 0.0464296 | 0.1440667 | 0.3223 | 0.747 | 0.059727453 | count | 1 |
| EXOSC1 | 0.0433431 | 0.1182572 | 0.3665 | 0.714 | 0.05973739 | count | 1 |
| FBXL8 | 0.0576913 | 0.3208004 | 0.1798 | 0.857 | 0.059793877 | count | 1 |
| KALRN | 0.3033552 | 0.4227535 | 0.7176 | 0.473 | 0.060004842 | count | 1 |
| SCAF4 | 0.0472682 | 0.1904648 | 0.2482 | 0.804 | 0.060011701 | count | 1 |
| ASXL1 | 0.0433589 | 0.0943056 | 0.4598 | 0.646 | 0.060013703 | count | 1 |

| | | | | | | | |
|---|---|---|---|---|---|---|---|
| E2F5 | 0.1766794 | 0.5846137 | 0.3022 | 0.763 | 0.060145776 | count | 1 |
| PRDM11 | 0.1769215 | 0.6554041 | 0.2699 | 0.787 | 0.060230747 | count | 1 |
| SNTB1 | 0.0557255 | 0.2664962 | 0.2091 | 0.834 | 0.060296243 | count | 1 |
| CENPJ | 0.0564776 | 0.3655612 | 0.1545 | 0.877 | 0.060319301 | count | 1 |
| ISCA2 | 0.0441527 | 0.1210735 | 0.3647 | 0.715 | 0.060337222 | count | 1 |
| PIGN | 0.0617324 | 0.3776722 | 0.1635 | 0.87 | 0.060360533 | count | 1 |
| DIP2A | 0.0558134 | 0.1829707 | 0.305 | 0.76 | 0.060391675 | count | 1 |
| VMA21 | 0.0447076 | 0.1279523 | 0.3494 | 0.727 | 0.060419685 | count | 1 |
| PITRM1 | 0.0633367 | 0.3184932 | 0.1989 | 0.842 | 0.060439223 | count | 1 |
| BRCA1 | 0.1111665 | 0.4736019 | 0.2347 | 0.814 | 0.060650953 | count | 1 |
| PRR13 | 0.0424642 | 0.0516374 | 0.8224 | 0.411 | 0.060710422 | count | 1 |
| FUT4 | 0.0770464 | 0.4179523 | 0.1843 | 0.854 | 0.060773988 | count | 1 |
| SMARCB1 | 0.0441234 | 0.1176833 | 0.3749 | 0.708 | 0.06079423 | count | 1 |
| KRT10 | 0.0427252 | 0.0592526 | 0.7211 | 0.471 | 0.060928273 | count | 1 |
| CCR1 | 0.0980228 | 0.3971857 | 0.2468 | 0.805 | 0.060965543 | count | 1 |
| ZNF671 | 0.0773414 | 0.4110349 | 0.1882 | 0.851 | 0.061008647 | count | 1 |
| ALG13 | 0.0445004 | 0.1016052 | 0.438 | 0.661 | 0.061060503 | count | 1 |
| L3MBTL1 | 0.0982295 | 0.6485828 | 0.1515 | 0.88 | 0.061095813 | count | 1 |
| OPRL1 | 0.1793993 | 0.8619024 | 0.2081 | 0.835 | 0.061100784 | count | 1 |
| CABYR | 0.1793993 | 1.068844 | 0.1678 | 0.867 | 0.061100784 | count | 1 |
| RC3H2 | 0.048187 | 0.1731143 | 0.2784 | 0.781 | 0.061179855 | count | 1 |
| ALKBH2 | 0.0518672 | 0.2691144 | 0.1927 | 0.847 | 0.061212773 | count | 1 |
| AQR | 0.0481714 | 0.1792964 | 0.2687 | 0.788 | 0.061307102 | count | 1 |
| AC008764.8 | 0.0614613 | 0.381505 | 0.1611 | 0.872 | 0.061413552 | count | 1 |
| MAK16 | 0.0614679 | 0.2965823 | 0.2073 | 0.836 | 0.061420177 | count | 1 |
| LINC01560 | 0.1125778 | 0.5642442 | 0.1995 | 0.842 | 0.06143371 | count | 1 |
| EIF3H | 0.0429092 | 0.0428609 | 1.0011 | 0.317 | 0.061449779 | count | 1 |
| PSRC1 | 0.1126883 | 0.9540398 | 0.1181 | 0.906 | 0.061495008 | count | 1 |
| AL358852.1 | 0.1810596 | 1.0135806 | 0.1786 | 0.858 | 0.061684167 | count | 1 |
| C10orf67 | 0.1810596 | 1.0135806 | 0.1786 | 0.858 | 0.061684167 | count | 1 |
| AC024940.1 | 0.1810596 | 1.0135806 | 0.1786 | 0.858 | 0.061684167 | count | 1 |
| PYGL | 0.1810596 | 1.1400299 | 0.1588 | 0.874 | 0.061684167 | count | 1 |
| MYCBPAP | 0.1360062 | 0.9396406 | 0.1447 | 0.885 | 0.061714008 | count | 1 |
| AC073611.1 | 0.1360062 | 0.979273 | 0.1389 | 0.89 | 0.061714008 | count | 1 |
| IFT46 | 0.1363263 | 0.5064829 | 0.2692 | 0.788 | 0.061862431 | count | 1 |
| KATNB1 | 0.0588141 | 0.2835496 | 0.2074 | 0.836 | 0.061930877 | count | 1 |
| POLRMT | 0.0508944 | 0.221279 | 0.23 | 0.818 | 0.061940871 | count | 1 |
| NCAPD2 | 0.0747489 | 0.3277537 | 0.2281 | 0.82 | 0.062073615 | count | 1 |
| MIR181A1HG | 0.0554985 | 0.372391 | 0.149 | 0.882 | 0.062073934 | count | 1 |
| SEC31A | 0.0456272 | 0.1153192 | 0.3957 | 0.692 | 0.062119314 | count | 1 |
| NDUFB9 | 0.0436749 | 0.0662666 | 0.6591 | 0.51 | 0.062259468 | count | 1 |
| ZNF213 | 0.0793673 | 0.6070474 | 0.1307 | 0.896 | 0.062620548 | count | 1 |
| ZNF613 | 0.1385049 | 0.5789947 | 0.2392 | 0.811 | 0.062872992 | count | 1 |
| NCK1 | 0.0454391 | 0.1018019 | 0.4463 | 0.655 | 0.062877982 | count | 1 |
| NFKBIB | 0.0478687 | 0.1406787 | 0.3403 | 0.734 | 0.062882945 | count | 1 |

| | | | | | | | |
|---|---|---|---|---|---|---|---|
| PIM2 | 0.0452295 | 0.0775837 | 0.583 | 0.56 | 0.06295304 | count | 1 |
| SUCLG2 | 0.0472137 | 0.1178349 | 0.4007 | 0.689 | 0.063040777 | count | 1 |
| PI4K2B | 0.0619346 | 0.3764613 | 0.1645 | 0.869 | 0.06310078 | count | 1 |
| CNRIP1 | 0.1852369 | 1.0017281 | 0.1849 | 0.853 | 0.06315333 | count | 1 |
| AL023584.2 | 0.1852369 | 1.0017281 | 0.1849 | 0.853 | 0.06315333 | count | 1 |
| ATP12A | 0.1852369 | 1.0017281 | 0.1849 | 0.853 | 0.06315333 | count | 1 |
| CEACAM4 | 0.1852369 | 1.0017281 | 0.1849 | 0.853 | 0.06315333 | count | 1 |
| BCAM | 0.1852369 | 1.0017281 | 0.1849 | 0.853 | 0.06315333 | count | 1 |
| AC073115.1 | 0.0760632 | 0.5311069 | 0.1432 | 0.886 | 0.06317353 | count | 1 |
| DCAF4 | 0.1015362 | 0.3813025 | 0.2663 | 0.79 | 0.06318077 | count | 1 |
| LZTR1 | 0.1017717 | 0.2990673 | 0.3403 | 0.734 | 0.063329327 | count | 1 |
| CXADR | 0.3193537 | 0.4653639 | 0.6862 | 0.493 | 0.063355065 | count | 1 |
| PIK3CD | 0.0525538 | 0.1794997 | 0.2928 | 0.77 | 0.063381555 | count | 1 |
| LINC00239 | 0.0731101 | 0.2260087 | 0.3235 | 0.746 | 0.063393216 | count | 1 |
| PRKAG2 | 0.0480403 | 0.1254573 | 0.3829 | 0.702 | 0.063423747 | count | 1 |
| COASY | 0.0557227 | 0.2241716 | 0.2486 | 0.804 | 0.063466383 | count | 1 |
| ZAP70 | 0.0457555 | 0.0906908 | 0.5045 | 0.614 | 0.063812927 | count | 1 |
| CDK5RAP2 | 0.0479538 | 0.147793 | 0.3245 | 0.746 | 0.063912298 | count | 1 |
| GLE1 | 0.0669621 | 0.2898891 | 0.231 | 0.817 | 0.063917742 | count | 1 |
| ATP6V1A | 0.0627572 | 0.237985 | 0.2637 | 0.792 | 0.06394261 | count | 1 |
| SPATA24 | 0.0931349 | 0.4541992 | 0.2051 | 0.838 | 0.063952827 | count | 1 |
| HMBS | 0.0770687 | 0.274192 | 0.2811 | 0.779 | 0.064015206 | count | 1 |
| TMSB15B | 0.3226955 | 0.5571186 | 0.5792 | 0.562 | 0.064056993 | count | 1 |
| B4GALT1-AS1 | 0.3228475 | 0.8731714 | 0.3697 | 0.712 | 0.064088942 | count | 1 |
| TSHZ2 | 0.0515169 | 0.1854252 | 0.2778 | 0.781 | 0.064132724 | count | 1 |
| ZNF280C | 0.1413124 | 0.46307 | 0.3052 | 0.76 | 0.064176245 | count | 1 |
| GIMAP2 | 0.0465588 | 0.1127517 | 0.4129 | 0.68 | 0.064428125 | count | 1 |
| NGDN | 0.0470588 | 0.1126461 | 0.4178 | 0.676 | 0.064498468 | count | 1 |
| SIRPB1 | 0.0696066 | 0.5256399 | 0.1324 | 0.895 | 0.064634779 | count | 1 |
| CXorf56 | 0.0778273 | 0.3052262 | 0.255 | 0.799 | 0.064650317 | count | 1 |
| TMEM175 | 0.0518272 | 0.2372618 | 0.2184 | 0.827 | 0.064726047 | count | 1 |
| MBTPS1 | 0.0484095 | 0.1250558 | 0.3871 | 0.699 | 0.064752305 | count | 1 |
| TXLNB | 0.3262096 | 0.5098792 | 0.6398 | 0.522 | 0.064795902 | count | 1 |
| EIF3E | 0.0453595 | 0.0430841 | 1.0528 | 0.293 | 0.064825343 | count | 1 |
| AL109955.1 | 0.1430177 | 0.4181664 | 0.342 | 0.732 | 0.064968389 | count | 1 |
| DUSP2 | 0.045129 | 0.0450151 | 1.0025 | 0.316 | 0.065010969 | count | 1 |
| FDPS | 0.0464262 | 0.0927584 | 0.5005 | 0.617 | 0.065047077 | count | 1 |
| MAP3K20 | 0.190812 | 0.400435 | 0.4765 | 0.634 | 0.065117195 | count | 1 |
| RICTOR | 0.0508413 | 0.1291854 | 0.3936 | 0.694 | 0.065144274 | count | 1 |
| KDM1A | 0.0516075 | 0.1907755 | 0.2705 | 0.787 | 0.065196634 | count | 1 |
| TAF13 | 0.0546321 | 0.2093704 | 0.2609 | 0.794 | 0.065225083 | count | 1 |
| TBX19 | 0.1435942 | 0.8271909 | 0.1736 | 0.862 | 0.065236275 | count | 1 |
| ALG1 | 0.0611383 | 0.3435067 | 0.178 | 0.859 | 0.065316194 | count | 1 |
| VPS16 | 0.0492284 | 0.1996274 | 0.2466 | 0.805 | 0.065354064 | count | 1 |
| ZNF684 | 0.058144 | 0.2535523 | 0.2293 | 0.819 | 0.065656707 | count | 1 |

| | | | | | | | |
|---|---|---|---|---|---|---|---|
| SENP6 | 0.0471736 | 0.0907628 | 0.5197 | 0.603 | 0.065802672 | count | 1 |
| PRR7 | 0.0485158 | 0.12785 | 0.3795 | 0.704 | 0.065844887 | count | 1 |
| C8orf59 | 0.0463817 | 0.0706239 | 0.6567 | 0.511 | 0.065849238 | count | 1 |
| PPIL1 | 0.0554667 | 0.2642362 | 0.2099 | 0.834 | 0.065857529 | count | 1 |
| KAT2A | 0.1058424 | 0.4298589 | 0.2462 | 0.806 | 0.065898593 | count | 1 |
| NAT1 | 0.3314934 | 0.5686284 | 0.583 | 0.56 | 0.065908442 | count | 1 |
| TBCK | 0.0602425 | 0.2929664 | 0.2056 | 0.837 | 0.065986455 | count | 1 |
| MSH3 | 0.0537917 | 0.1714407 | 0.3138 | 0.754 | 0.066017345 | count | 1 |
| AC245595.1 | 0.0836519 | 0.577401 | 0.1449 | 0.885 | 0.066031821 | count | 1 |
| CCDC9 | 0.0560009 | 0.2277175 | 0.2459 | 0.806 | 0.066103369 | count | 1 |
| TRBV3-1 | 0.1941709 | 0.7983751 | 0.2432 | 0.808 | 0.066302032 | count | 1 |
| ARHGEF6 | 0.0509697 | 0.1394593 | 0.3655 | 0.715 | 0.066386875 | count | 1 |
| ZNF251 | 0.0664916 | 0.3824533 | 0.1739 | 0.862 | 0.066464783 | count | 1 |
| BCL2L14 | 0.3342678 | 0.6594309 | 0.5069 | 0.612 | 0.066493333 | count | 1 |
| GAR1 | 0.0491521 | 0.1544328 | 0.3183 | 0.75 | 0.066513939 | count | 1 |
| SETDB2 | 0.0561141 | 0.1950848 | 0.2876 | 0.774 | 0.06662806 | count | 1 |
| CHST11 | 0.0485531 | 0.1099956 | 0.4414 | 0.659 | 0.066673929 | count | 1 |
| VDAC3 | 0.0474759 | 0.0893434 | 0.5314 | 0.595 | 0.06670367 | count | 1 |
| NHLRC3 | 0.0558738 | 0.1904897 | 0.2933 | 0.769 | 0.066711008 | count | 1 |
| MOSPD1 | 0.0558933 | 0.2460551 | 0.2272 | 0.82 | 0.066734345 | count | 1 |
| PHF5A | 0.0480715 | 0.0998147 | 0.4816 | 0.63 | 0.066830557 | count | 1 |
| ZNF600 | 0.0578257 | 0.2698396 | 0.2143 | 0.83 | 0.066911701 | count | 1 |
| C15orf65 | 0.0772328 | 0.8661992 | 0.0892 | 0.929 | 0.066994465 | count | 1 |
| FNIP2 | 0.0702235 | 0.4439553 | 0.1582 | 0.874 | 0.067048705 | count | 1 |
| HCLS1 | 0.0472665 | 0.0589426 | 0.8019 | 0.423 | 0.067113345 | count | 1 |
| RRM2B | 0.0606099 | 0.2840551 | 0.2134 | 0.831 | 0.067124817 | count | 1 |
| RAI2 | 0.3375276 | 0.7396117 | 0.4564 | 0.648 | 0.067181193 | count | 1 |
| MEST | 0.3375276 | 0.8174659 | 0.4129 | 0.68 | 0.067181193 | count | 1 |
| AC009133.3 | 0.3375276 | 0.8833647 | 0.3821 | 0.702 | 0.067181193 | count | 1 |
| ZSWIM7 | 0.0492794 | 0.1261673 | 0.3906 | 0.696 | 0.067225402 | count | 1 |
| TNFRSF1A | 0.0493029 | 0.1429697 | 0.3448 | 0.73 | 0.067349896 | count | 1 |
| EAF1 | 0.0522559 | 0.1805087 | 0.2895 | 0.772 | 0.067362925 | count | 1 |
| PAIP1 | 0.0490402 | 0.1309549 | 0.3745 | 0.708 | 0.067391641 | count | 1 |
| CHTF8 | 0.0523443 | 0.1820776 | 0.2875 | 0.774 | 0.067477035 | count | 1 |
| UFC1 | 0.0473156 | 0.0604682 | 0.7825 | 0.434 | 0.06752725 | count | 1 |
| PIK3R5 | 0.0624205 | 0.2487692 | 0.2509 | 0.802 | 0.067567771 | count | 1 |
| IKBKE | 0.0663145 | 0.2175619 | 0.3048 | 0.761 | 0.067584157 | count | 1 |
| SMU1 | 0.0490343 | 0.1028289 | 0.4769 | 0.633 | 0.067606387 | count | 1 |
| MMAA | 0.0916245 | 0.3660887 | 0.2503 | 0.802 | 0.067994053 | count | 1 |
| PRMT7 | 0.0542767 | 0.2494704 | 0.2176 | 0.828 | 0.067998097 | count | 1 |
| AKIRIN2 | 0.0489162 | 0.0854389 | 0.5725 | 0.567 | 0.068060078 | count | 1 |
| RHOA | 0.0473909 | 0.03821 | 1.2403 | 0.215 | 0.068066861 | count | 1 |
| HPS4 | 0.057666 | 0.2014828 | 0.2862 | 0.775 | 0.068073841 | count | 1 |
| MLF1 | 0.0547156 | 0.1908108 | 0.2868 | 0.774 | 0.068121995 | count | 1 |
| SNIP1 | 0.0588835 | 0.1811317 | 0.3251 | 0.745 | 0.068139164 | count | 1 |

| | | | | | | | |
|---|---|---|---|---|---|---|---|
| VAMP5 | 0.0484908 | 0.0931696 | 0.5205 | 0.603 | 0.068237664 | count | 1 |
| HIRA | 0.0919664 | 0.3957103 | 0.2324 | 0.816 | 0.068250475 | count | 1 |
| SPCS2 | 0.0476688 | 0.0477733 | 0.9978 | 0.318 | 0.068304046 | count | 1 |
| ZSCAN30 | 0.2002963 | 0.3293808 | 0.6081 | 0.543 | 0.068465991 | count | 1 |
| APBA2 | 0.0789662 | 0.2131207 | 0.3705 | 0.711 | 0.068509435 | count | 1 |
| ARMH1 | 0.0633008 | 0.2080404 | 0.3043 | 0.761 | 0.068524298 | count | 1 |
| TBC1D7 | 0.0589054 | 0.2824339 | 0.2086 | 0.835 | 0.06864976 | count | 1 |
| EXOSC5 | 0.0539411 | 0.2151271 | 0.2507 | 0.802 | 0.068661465 | count | 1 |
| TMEM230 | 0.0486133 | 0.0743173 | 0.6541 | 0.513 | 0.068678079 | count | 1 |
| SRPRA | 0.0488928 | 0.0726397 | 0.6731 | 0.501 | 0.068809894 | count | 1 |
| FAM213B | 0.0675399 | 0.3089527 | 0.2186 | 0.827 | 0.068838987 | count | 1 |
| AL121935.1 | 0.3453717 | 1.022695 | 0.3377 | 0.736 | 0.068839189 | count | 1 |
| C14orf132 | 0.3453717 | 1.022695 | 0.3377 | 0.736 | 0.068839189 | count | 1 |
| AC026471.3 | 0.3453717 | 1.022695 | 0.3377 | 0.736 | 0.068839189 | count | 1 |
| NUP62CL | 0.3453717 | 1.039067 | 0.3324 | 0.74 | 0.068839189 | count | 1 |
| TRAIP | 0.3453717 | 1.335208 | 0.2587 | 0.796 | 0.068839189 | count | 1 |
| ARHGAP42 | 0.3453717 | 1.335208 | 0.2587 | 0.796 | 0.068839189 | count | 1 |
| PIGH | 0.0518595 | 0.1868711 | 0.2775 | 0.781 | 0.068921008 | count | 1 |
| EID1 | 0.0482546 | 0.0507173 | 0.9514 | 0.3414 | 0.068941499 | count | 1 |
| CCDC122 | 0.0623002 | 0.4297957 | 0.145 | 0.885 | 0.069003352 | count | 1 |
| ZC3H12D | 0.057494 | 0.1932378 | 0.2975 | 0.766 | 0.069010524 | count | 1 |
| KLHL6 | 0.0665874 | 0.239624 | 0.2779 | 0.781 | 0.069056014 | count | 1 |
| ZNF66 | 0.0796473 | 0.3537616 | 0.2251 | 0.822 | 0.069104839 | count | 1 |
| AC069224.1 | 0.1263881 | 0.8205518 | 0.154 | 0.878 | 0.069109542 | count | 1 |
| AL445472.1 | 0.0657305 | 0.3050294 | 0.2155 | 0.829 | 0.069245063 | count | 1 |
| C11orf54 | 0.0540602 | 0.223985 | 0.2414 | 0.809 | 0.069274785 | count | 1 |
| FMN1 | 0.0877322 | 0.561588 | 0.1562 | 0.876 | 0.069283254 | count | 1 |
| RASGEF1B | 0.0501526 | 0.1255414 | 0.3995 | 0.69 | 0.069291405 | count | 1 |
| PNKD | 0.0495215 | 0.1036867 | 0.4776 | 0.633 | 0.069376437 | count | 1 |
| SLC35B2 | 0.0543989 | 0.2269808 | 0.2397 | 0.811 | 0.069405251 | count | 1 |
| COPS8 | 0.0511431 | 0.124644 | 0.4103 | 0.682 | 0.069413243 | count | 1 |
| SNX11 | 0.0748284 | 0.2982792 | 0.2509 | 0.802 | 0.069514702 | count | 1 |
| URGCP | 0.0611623 | 0.2829632 | 0.2161 | 0.829 | 0.069681224 | count | 1 |
| ZSCAN26 | 0.058386 | 0.2335317 | 0.25 | 0.803 | 0.069717779 | count | 1 |
| COMMD6 | 0.0485957 | 0.0344397 | 1.411 | 0.158 | 0.069780352 | count | 1 |
| TPRG1L | 0.0525812 | 0.1529078 | 0.3439 | 0.731 | 0.069809668 | count | 1 |
| SACM1L | 0.0526289 | 0.1340493 | 0.3926 | 0.695 | 0.069873062 | count | 1 |
| GLB1L2 | 0.0752143 | 0.8593096 | 0.0875 | 0.93 | 0.069875503 | count | 1 |
| NCAPH | 0.204527 | 0.4296356 | 0.476 | 0.634 | 0.069963033 | count | 1 |
| VPS13C | 0.0502741 | 0.0758132 | 0.6631 | 0.507 | 0.069963901 | count | 1 |
| THAP5 | 0.051537 | 0.1328182 | 0.388 | 0.698 | 0.070025061 | count | 1 |
| AC020911.2 | 0.0733399 | 0.3557537 | 0.2062 | 0.837 | 0.070041958 | count | 1 |
| IFNGR2 | 0.0701012 | 0.2843323 | 0.2465 | 0.805 | 0.070091637 | count | 1 |
| ARF1 | 0.0490107 | 0.0463418 | 1.0576 | 0.2903 | 0.070117772 | count | 1 |
| CSNK2B | 0.0493384 | 0.0658749 | 0.749 | 0.454 | 0.0701894 | count | 1 |

| | | | | | | | |
|---|---|---|---|---|---|---|---|
| UBXN11 | 0.0560458 | 0.2015802 | 0.278 | 0.781 | 0.070218392 | count | 1 |
| RGS2 | 0.050332 | 0.0975262 | 0.5161 | 0.606 | 0.070245353 | count | 1 |
| MBLAC2 | 0.0718969 | 0.3785868 | 0.1899 | 0.849 | 0.070354708 | count | 1 |
| PCDHGA10 | 0.2057726 | 0.7945023 | 0.259 | 0.796 | 0.070404145 | count | 1 |
| CAHM | 0.2057726 | 0.8293432 | 0.2481 | 0.804 | 0.070404145 | count | 1 |
| AL603756.1 | 0.2057726 | 0.8421903 | 0.2443 | 0.807 | 0.070404145 | count | 1 |
| SMAD1 | 0.1132238 | 0.7739991 | 0.1463 | 0.884 | 0.070564242 | count | 1 |
| AC010247.2 | 0.1132238 | 0.7739991 | 0.1463 | 0.884 | 0.070564242 | count | 1 |
| UQCRC1 | 0.0513899 | 0.1297607 | 0.396 | 0.692 | 0.070571811 | count | 1 |
| CTSC | 0.0492057 | 0.0588615 | 0.836 | 0.403 | 0.070598565 | count | 1 |
| C18orf25 | 0.0577521 | 0.1930855 | 0.2991 | 0.765 | 0.070603321 | count | 1 |
| C12orf75 | 0.0495193 | 0.067356 | 0.7352 | 0.462 | 0.070754573 | count | 1 |
| RNF138 | 0.053028 | 0.1213792 | 0.4369 | 0.662 | 0.070811607 | count | 1 |
| BTN3A2 | 0.0501791 | 0.081888 | 0.6128 | 0.54 | 0.070817225 | count | 1 |
| VEZT | 0.0552887 | 0.1470476 | 0.376 | 0.707 | 0.070851387 | count | 1 |
| PPP3CB | 0.0543649 | 0.1808171 | 0.3007 | 0.764 | 0.070924044 | count | 1 |
| COP1 | 0.0601042 | 0.2168624 | 0.2772 | 0.782 | 0.070959699 | count | 1 |
| RNF2 | 0.0554884 | 0.1638589 | 0.3386 | 0.735 | 0.071107682 | count | 1 |
| AL355816.2 | 0.0900483 | 0.6518235 | 0.1381 | 0.89 | 0.071130078 | count | 1 |
| NAA15 | 0.0536548 | 0.1319603 | 0.4066 | 0.684 | 0.071380577 | count | 1 |
| ATMIN | 0.0558616 | 0.1768351 | 0.3159 | 0.752 | 0.071433172 | count | 1 |
| XPR1 | 0.1038925 | 0.3312978 | 0.3136 | 0.754 | 0.071435306 | count | 1 |
| MAD2L1BP | 0.0539865 | 0.1599774 | 0.3375 | 0.736 | 0.071446478 | count | 1 |
| ACAA2 | 0.0509226 | 0.1047086 | 0.4863 | 0.627 | 0.071532728 | count | 1 |
| MRPS27 | 0.0633453 | 0.2109147 | 0.3003 | 0.764 | 0.07154959 | count | 1 |
| SMN2 | 0.2090486 | 0.3659365 | 0.5713 | 0.568 | 0.071565183 | count | 1 |
| RP2 | 0.114843 | 0.2895621 | 0.3966 | 0.692 | 0.071588866 | count | 1 |
| PAXX | 0.0505822 | 0.0662699 | 0.7633 | 0.445 | 0.071971693 | count | 1 |
| HIST1H2BJ | 0.0799909 | 0.4969844 | 0.161 | 0.872 | 0.072014333 | count | 1 |
| DUSP10 | 0.0589581 | 0.1561056 | 0.3777 | 0.706 | 0.07208089 | count | 1 |
| CASP9 | 0.072161 | 0.300883 | 0.2398 | 0.81 | 0.072162114 | count | 1 |
| UFM1 | 0.0518409 | 0.0934763 | 0.5546 | 0.579 | 0.07239884 | count | 1 |
| SEC61G | 0.0510592 | 0.0650988 | 0.7843 | 0.433 | 0.072410756 | count | 1 |
| HENMT1 | 0.0538466 | 0.1800423 | 0.2991 | 0.765 | 0.072485542 | count | 1 |
| NOXA1 | 0.1592364 | 0.6133014 | 0.2596 | 0.795 | 0.07252225 | count | 1 |
| AC078846.1 | 0.212327 | 0.4880175 | 0.4351 | 0.664 | 0.072728232 | count | 1 |
| YTHDF3-AS1 | 0.1329406 | 0.6113192 | 0.2175 | 0.828 | 0.07276163 | count | 1 |
| ZNF335 | 0.0634655 | 0.2616102 | 0.2426 | 0.808 | 0.072903076 | count | 1 |
| NOL4L | 0.0634724 | 0.20074 | 0.3162 | 0.752 | 0.072911028 | count | 1 |
| ZC3H11A | 0.3654618 | 0.7013407 | 0.5211 | 0.602 | 0.073103393 | count | 1 |
| SLC51A | 0.3654618 | 0.7227846 | 0.5056 | 0.613 | 0.073103393 | count | 1 |
| AL137779.1 | 0.3654618 | 0.9736011 | 0.3754 | 0.7074 | 0.073103393 | count | 1 |
| TRGV8 | 0.2136066 | 0.5255821 | 0.4064 | 0.684 | 0.073182487 | count | 1 |
| OTOF | 0.2136171 | 0.446206 | 0.4787 | 0.632 | 0.073186215 | count | 1 |
| ATP6V1C2 | 0.2136957 | 1.065637 | 0.2005 | 0.841 | 0.073214128 | count | 1 |

| | | | | | | | |
|---|---|---|---|---|---|---|---|
| AC093827.4 | 0.2143671 | 0.4927387 | 0.4351 | 0.664 | 0.073452578 | count | 1 |
| ZSCAN29 | 0.1068792 | 0.5529463 | 0.1933 | 0.847 | 0.073516076 | count | 1 |
| AC239800.3 | 0.0617605 | 0.2220767 | 0.2781 | 0.781 | 0.073757564 | count | 1 |
| SLC35C1 | 0.0594911 | 0.2665335 | 0.2232 | 0.823 | 0.07383281 | count | 1 |
| TMEM44 | 0.0888222 | 0.5759535 | 0.1542 | 0.877 | 0.073865939 | count | 1 |
| TTC27 | 0.0821557 | 0.2760433 | 0.2976 | 0.766 | 0.073977647 | count | 1 |
| ABHD18 | 0.0756189 | 0.2723961 | 0.2776 | 0.781 | 0.074018118 | count | 1 |
| C17orf97 | 0.2160729 | 0.6608779 | 0.3269 | 0.744 | 0.074058577 | count | 1 |
| ATP6V0A2 | 0.062019 | 0.2111002 | 0.2938 | 0.769 | 0.074067072 | count | 1 |
| SNRPB2 | 0.0521369 | 0.0676054 | 0.7712 | 0.441 | 0.074073482 | count | 1 |
| ACSL5 | 0.0594993 | 0.2157438 | 0.2758 | 0.783 | 0.074089529 | count | 1 |
| CNST | 0.0580542 | 0.1412398 | 0.411 | 0.681 | 0.074241433 | count | 1 |
| MAGEE1 | 0.0999636 | 0.617534 | 0.1619 | 0.871 | 0.074253803 | count | 1 |
| DAPL1 | 0.0893351 | 0.4619954 | 0.1934 | 0.847 | 0.074296316 | count | 1 |
| HRASLS2 | 0.1080703 | 0.6487478 | 0.1666 | 0.868 | 0.074346294 | count | 1 |
| XRCC3 | 0.1080703 | 0.7898334 | 0.1368 | 0.891 | 0.074346294 | count | 1 |
| TOMM22 | 0.0528356 | 0.0754034 | 0.7007 | 0.484 | 0.074420175 | count | 1 |
| ACYP1 | 0.0563699 | 0.1636526 | 0.3444 | 0.731 | 0.074519901 | count | 1 |
| ZNF263 | 0.0683353 | 0.2444412 | 0.2796 | 0.78 | 0.07488602 | count | 1 |
| AC103706.1 | 0.1645968 | 0.4334505 | 0.3797 | 0.704 | 0.075026754 | count | 1 |
| TMEM167B | 0.056833 | 0.1510894 | 0.3762 | 0.707 | 0.075132813 | count | 1 |
| PLEKHF2 | 0.0582907 | 0.1674254 | 0.3482 | 0.728 | 0.075154041 | count | 1 |
| SMAD4 | 0.060612 | 0.177066 | 0.3423 | 0.732 | 0.075226769 | count | 1 |
| AL117339.5 | 0.1651233 | 0.3615978 | 0.4566 | 0.648 | 0.075272956 | count | 1 |
| ZNF773 | 0.0952567 | 0.4182481 | 0.2278 | 0.82 | 0.075286385 | count | 1 |
| HARS | 0.0557631 | 0.1418095 | 0.3932 | 0.694 | 0.075324831 | count | 1 |
| CAMKMT | 0.0728612 | 0.3338731 | 0.2182 | 0.827 | 0.075594417 | count | 1 |
| STARD3 | 0.0561802 | 0.1384665 | 0.4057 | 0.685 | 0.075629769 | count | 1 |
| MAF1 | 0.0540582 | 0.0789823 | 0.6844 | 0.494 | 0.075674246 | count | 1 |
| CUL2 | 0.0589939 | 0.1505094 | 0.392 | 0.695 | 0.075763739 | count | 1 |
| CXorf40A | 0.0911222 | 0.2969688 | 0.3068 | 0.759 | 0.075796208 | count | 1 |
| RASSF1 | 0.0539807 | 0.0937617 | 0.5757 | 0.565 | 0.075797216 | count | 1 |
| TMEM41B | 0.061144 | 0.1713755 | 0.3568 | 0.721 | 0.075888404 | count | 1 |
| KIAA1191 | 0.0570697 | 0.1591452 | 0.3586 | 0.72 | 0.075928524 | count | 1 |
| NSUN6 | 0.0610622 | 0.1831579 | 0.3334 | 0.739 | 0.076039598 | count | 1 |
| MRPL48 | 0.0572121 | 0.1549201 | 0.3693 | 0.712 | 0.07604234 | count | 1 |
| FLNA | 0.0539755 | 0.0896721 | 0.6019 | 0.547 | 0.076095551 | count | 1 |
| ATAD3A | 0.0694873 | 0.2830364 | 0.2455 | 0.806 | 0.076153513 | count | 1 |
| NPTN | 0.0567504 | 0.1388331 | 0.4088 | 0.683 | 0.076505788 | count | 1 |
| TMEM237 | 0.0850136 | 0.4587061 | 0.1853 | 0.853 | 0.076570663 | count | 1 |
| BLOC1S3 | 0.0698699 | 0.3178459 | 0.2198 | 0.826 | 0.076574506 | count | 1 |
| PSTK | 0.068557 | 0.2368193 | 0.2895 | 0.772 | 0.076733879 | count | 1 |
| C1orf216 | 0.092252 | 0.3157526 | 0.2922 | 0.77 | 0.0767447 | count | 1 |
| EPHA1 | 0.0768599 | 0.2469249 | 0.3113 | 0.756 | 0.076887622 | count | 1 |
| CHMP3 | 0.0581458 | 0.1437312 | 0.4045 | 0.686 | 0.076957341 | count | 1 |

| | | | | | | | |
|---|---|---|---|---|---|---|---|
| BPHL | 0.0755743 | 0.3773466 | 0.2003 | 0.841 | 0.0770715 | count | 1 |
| TMEM39A | 0.0662868 | 0.2238759 | 0.2961 | 0.767 | 0.077278612 | count | 1 |
| UBB | 0.0537372 | 0.0295668 | 1.8175 | 0.0692 | 0.077336739 | count | 1 |
| STXBP1 | 0.141288 | 0.6570808 | 0.215 | 0.83 | 0.077423491 | count | 1 |
| FOXD2-AS1 | 0.141288 | 0.7107542 | 0.1988 | 0.842 | 0.077423491 | count | 1 |
| COG2 | 0.0645354 | 0.1898945 | 0.3398 | 0.734 | 0.077484456 | count | 1 |
| MAPKAPK3 | 0.0587033 | 0.1508025 | 0.3893 | 0.697 | 0.077518225 | count | 1 |
| RCC1 | 0.0893934 | 0.309016 | 0.2893 | 0.772 | 0.077632752 | count | 1 |
| SIPA1L3 | 0.0861858 | 0.4989221 | 0.1727 | 0.863 | 0.077634582 | count | 1 |
| LINC01871 | 0.0575205 | 0.1295841 | 0.4439 | 0.657 | 0.077649857 | count | 1 |
| PTPRA | 0.0551836 | 0.0883959 | 0.6243 | 0.532 | 0.077659058 | count | 1 |
| WDR12 | 0.0717409 | 0.2608288 | 0.275 | 0.783 | 0.077700185 | count | 1 |
| TUBE1 | 0.0624165 | 0.2162324 | 0.2887 | 0.773 | 0.077729546 | count | 1 |
| BTG2 | 0.0541165 | 0.0467522 | 1.1575 | 0.247 | 0.077754666 | count | 1 |
| DENND1C | 0.0580777 | 0.1600763 | 0.3628 | 0.717 | 0.077827731 | count | 1 |
| RGMB | 0.22669 | 0.4514404 | 0.5021 | 0.616 | 0.077837474 | count | 1 |
| EXTL3 | 0.22669 | 0.4901722 | 0.4625 | 0.644 | 0.077837474 | count | 1 |
| ISOC1 | 0.0611558 | 0.1557593 | 0.3926 | 0.695 | 0.077860985 | count | 1 |
| NFIA | 0.0690369 | 0.2872258 | 0.2404 | 0.81 | 0.078001497 | count | 1 |
| ENTPD4 | 0.0765177 | 0.2284973 | 0.3349 | 0.738 | 0.078038745 | count | 1 |
| AAED1 | 0.0561452 | 0.1024059 | 0.5483 | 0.584 | 0.078076341 | count | 1 |
| NOD2 | 0.1134278 | 0.6549651 | 0.1732 | 0.863 | 0.078083412 | count | 1 |
| SLC8B1 | 0.0939147 | 0.2872379 | 0.327 | 0.744 | 0.078140944 | count | 1 |
| XRCC4 | 0.0867456 | 0.2450227 | 0.354 | 0.723 | 0.078142748 | count | 1 |
| SHMT2 | 0.0604294 | 0.1980239 | 0.3052 | 0.76 | 0.078202314 | count | 1 |
| UCKL1 | 0.0573 | 0.1497116 | 0.3827 | 0.702 | 0.078282322 | count | 1 |
| MAPKAPK5 | 0.0614404 | 0.1756318 | 0.3498 | 0.726 | 0.078404598 | count | 1 |
| KCTD20 | 0.0610501 | 0.1831816 | 0.3333 | 0.739 | 0.078408704 | count | 1 |
| CD2BP2 | 0.0570368 | 0.1173365 | 0.4861 | 0.627 | 0.078572682 | count | 1 |
| CENPV | 0.0702321 | 0.236318 | 0.2972 | 0.766 | 0.078615844 | count | 1 |
| UTY | 0.0604545 | 0.1511158 | 0.4001 | 0.689 | 0.078633119 | count | 1 |
| GPSM3 | 0.0549427 | 0.0419953 | 1.3083 | 0.191 | 0.078690198 | count | 1 |
| AC021016.3 | 0.1726515 | 0.5450027 | 0.3168 | 0.751 | 0.078797385 | count | 1 |
| AL365205.1 | 0.3921107 | 0.532501 | 0.7364 | 0.462 | 0.078798351 | count | 1 |
| C12orf73 | 0.0805174 | 0.30594 | 0.2632 | 0.792 | 0.078842567 | count | 1 |
| GTF2H1 | 0.064959 | 0.1876497 | 0.3462 | 0.729 | 0.079100141 | count | 1 |
| PPP4C | 0.055758 | 0.0808481 | 0.6897 | 0.49 | 0.079103261 | count | 1 |
| LAMP2 | 0.0575551 | 0.1254052 | 0.459 | 0.646 | 0.079182792 | count | 1 |
| AC093495.1 | 0.1735187 | 0.574678 | 0.3019 | 0.763 | 0.079203863 | count | 1 |
| RARS | 0.0615655 | 0.200937 | 0.3064 | 0.759 | 0.079229828 | count | 1 |
| LINC002481 | 0.0695528 | 0.3169913 | 0.2194 | 0.826 | 0.079273993 | count | 1 |
| ING4 | 0.0636789 | 0.208933 | 0.3048 | 0.761 | 0.07930495 | count | 1 |
| PDAP1 | 0.0562796 | 0.0800935 | 0.7027 | 0.482 | 0.079319288 | count | 1 |
| Z68871.1 | 0.3950297 | 0.7555917 | 0.5228 | 0.601 | 0.079424769 | count | 1 |
| NIP7 | 0.0578774 | 0.1151873 | 0.5025 | 0.615 | 0.079457693 | count | 1 |

| | | | | | | | |
|---|---|---|---|---|---|---|---|
| PPDPF | 0.0554812 | 0.0454072 | 1.2219 | 0.222 | 0.079568352 | count | 1 |
| POLG2 | 0.0663512 | 0.2305643 | 0.2878 | 0.774 | 0.079670421 | count | 1 |
| CLNS1A | 0.0564746 | 0.0770774 | 0.7327 | 0.464 | 0.079687138 | count | 1 |
| RPL10A | 0.0553509 | 0.0185432 | 2.985 | 0.00286 | 0.079756461 | count | 1 |
| DUS3L | 0.0736625 | 0.2600366 | 0.2833 | 0.777 | 0.079790546 | count | 1 |
| AC116913.1 | 0.1457926 | 0.6252669 | 0.2332 | 0.816 | 0.079943557 | count | 1 |
| ETV3 | 0.0610413 | 0.1390693 | 0.4389 | 0.661 | 0.079993844 | count | 1 |
| CMIP | 0.0623748 | 0.1719411 | 0.3628 | 0.717 | 0.08011286 | count | 1 |
| TYSND1 | 0.0675602 | 0.1922075 | 0.3515 | 0.725 | 0.080257912 | count | 1 |
| SLC30A5 | 0.0750908 | 0.2224948 | 0.3375 | 0.736 | 0.080291899 | count | 1 |
| VPS11 | 0.1285798 | 0.2830303 | 0.4543 | 0.65 | 0.08029828 | count | 1 |
| C3orf38 | 0.0594207 | 0.1454119 | 0.4086 | 0.683 | 0.080372326 | count | 1 |
| UBE2L3 | 0.0565227 | 0.0661047 | 0.855 | 0.393 | 0.080409446 | count | 1 |
| MECP2 | 0.0581465 | 0.1007715 | 0.577 | 0.564 | 0.080451407 | count | 1 |
| ELOF1 | 0.0596041 | 0.1261212 | 0.4726 | 0.637 | 0.080465208 | count | 1 |
| POLE | 0.0927601 | 0.5911278 | 0.1569 | 0.875 | 0.080582126 | count | 1 |
| KAT6B | 0.0603349 | 0.1177381 | 0.5124 | 0.608 | 0.080720598 | count | 1 |
| PHYH | 0.0635655 | 0.1666144 | 0.3815 | 0.703 | 0.080740887 | count | 1 |
| MBD5 | 0.0606735 | 0.1465058 | 0.4141 | 0.679 | 0.080807176 | count | 1 |
| YTHDC2 | 0.0602241 | 0.1545742 | 0.3896 | 0.697 | 0.080836169 | count | 1 |
| SRPRB | 0.0631377 | 0.1572801 | 0.4014 | 0.688 | 0.080926816 | count | 1 |
| KDM6A | 0.0730584 | 0.2471679 | 0.2956 | 0.768 | 0.080967604 | count | 1 |
| DHRS11 | 0.1024108 | 0.4722478 | 0.2169 | 0.828 | 0.081002536 | count | 1 |
| ZNF236 | 0.0649598 | 0.2543553 | 0.2554 | 0.798 | 0.081161625 | count | 1 |
| MRPS30 | 0.0585479 | 0.1121654 | 0.522 | 0.602 | 0.081169277 | count | 1 |
| RLN2 | 0.0873207 | 0.4008382 | 0.2178 | 0.828 | 0.081205985 | count | 1 |
| SF3A3 | 0.0586566 | 0.1157284 | 0.5068 | 0.612 | 0.081240773 | count | 1 |
| MAPK1IP1L | 0.0580466 | 0.0839249 | 0.6916 | 0.489 | 0.081324397 | count | 1 |
| MOB3C | 0.0874693 | 0.2715593 | 0.3221 | 0.747 | 0.081345199 | count | 1 |
| IL16 | 0.0578906 | 0.095422 | 0.6067 | 0.544 | 0.081399689 | count | 1 |
| AC091729.3 | 0.2367448 | 0.7401979 | 0.3198 | 0.749 | 0.081427399 | count | 1 |
| B3GNT7 | 0.2367448 | 0.8145557 | 0.2906 | 0.771 | 0.081427399 | count | 1 |
| CEBPB | 0.0575375 | 0.0682242 | 0.8434 | 0.399 | 0.0814921 | count | 1 |
| PBX3 | 0.068235 | 0.2569841 | 0.2655 | 0.791 | 0.081511554 | count | 1 |
| TNFAIP8L2 | 0.0636432 | 0.2385909 | 0.2667 | 0.79 | 0.081575845 | count | 1 |
| SPATA20 | 0.0854437 | 0.3464505 | 0.2466 | 0.805 | 0.081681163 | count | 1 |
| CD37 | 0.0568851 | 0.0344201 | 1.6527 | 0.0985 | 0.081701394 | count | 1 |
| CLPTM1L | 0.0612507 | 0.1429083 | 0.4286 | 0.668 | 0.08180395 | count | 1 |
| UBFD1 | 0.0835433 | 0.3150073 | 0.2652 | 0.791 | 0.081824451 | count | 1 |
| JPX | 0.0608542 | 0.1448173 | 0.4202 | 0.674 | 0.081928069 | count | 1 |
| GGT1 | 0.4074748 | 0.643716 | 0.633 | 0.527 | 0.082101205 | count | 1 |
| ZNF814 | 0.0883257 | 0.3156068 | 0.2799 | 0.78 | 0.082147569 | count | 1 |
| AC062017.1 | 0.1194535 | 0.4215321 | 0.2834 | 0.777 | 0.082292155 | count | 1 |
| DVL1 | 0.0806933 | 0.3163437 | 0.2551 | 0.799 | 0.082321354 | count | 1 |
| TBX1 | 0.1318303 | 0.894372 | 0.1474 | 0.883 | 0.082363509 | count | 1 |

| | | | | | | | |
|---|---|---|---|---|---|---|---|
| MAGI2 | 0.2394504 | 0.6218048 | 0.3851 | 0.7 | 0.082395246 | count | 1 |
| ENSA | 0.057876 | 0.0611644 | 0.9462 | 0.344 | 0.08240143 | count | 1 |
| TMEM229B | 0.0841609 | 0.4188845 | 0.2009 | 0.841 | 0.082433225 | count | 1 |
| MAN2A1 | 0.0631567 | 0.1763266 | 0.3582 | 0.72 | 0.082533661 | count | 1 |
| ARHGEF3 | 0.0601633 | 0.1071217 | 0.5616 | 0.574 | 0.082567388 | count | 1 |
| LINC01011 | 0.0917098 | 0.5003393 | 0.1833 | 0.855 | 0.082651161 | count | 1 |
| CPEB2 | 0.2402076 | 0.326294 | 0.7362 | 0.462 | 0.082666272 | count | 1 |
| TMEM167A | 0.0596125 | 0.1090267 | 0.5468 | 0.585 | 0.082684917 | count | 1 |
| PLCG2 | 0.0591712 | 0.0922856 | 0.6412 | 0.521 | 0.082716874 | count | 1 |
| LONRF1 | 0.0826878 | 0.2725993 | 0.3033 | 0.762 | 0.082752834 | count | 1 |
| BST2 | 0.0581702 | 0.0633851 | 0.9177 | 0.359 | 0.082900826 | count | 1 |
| TDP1 | 0.0679207 | 0.1909869 | 0.3556 | 0.722 | 0.08306564 | count | 1 |
| ZNF655 | 0.0613923 | 0.1178365 | 0.521 | 0.602 | 0.083092918 | count | 1 |
| PRPS2 | 0.0654271 | 0.1712604 | 0.382 | 0.702 | 0.083109925 | count | 1 |
| ZNF510 | 0.0814701 | 0.3662784 | 0.2224 | 0.824 | 0.083118322 | count | 1 |
| RAB1B | 0.0609053 | 0.1318049 | 0.4621 | 0.644 | 0.083211647 | count | 1 |
| AC005838.2 | 0.1517698 | 0.4682221 | 0.3241 | 0.746 | 0.083292108 | count | 1 |
| ASPHD2 | 0.0871744 | 0.3302734 | 0.2639 | 0.792 | 0.083347196 | count | 1 |
| RBL2 | 0.0595973 | 0.0902724 | 0.6602 | 0.509 | 0.08345404 | count | 1 |
| SAMD10 | 0.079193 | 0.2671485 | 0.2964 | 0.767 | 0.083499909 | count | 1 |
| CSNK1G1 | 0.1211938 | 0.2409353 | 0.503 | 0.615 | 0.083508803 | count | 1 |
| C5orf51 | 0.0753776 | 0.2717038 | 0.2774 | 0.781 | 0.083548594 | count | 1 |
| UIMC1 | 0.062798 | 0.1585 | 0.3962 | 0.692 | 0.083558704 | count | 1 |
| DAB2 | 0.0874839 | 0.5508418 | 0.1588 | 0.874 | 0.083645176 | count | 1 |
| TRIM59 | 0.0693187 | 0.2636171 | 0.263 | 0.793 | 0.083655909 | count | 1 |
| TMEM14A | 0.0619087 | 0.1529245 | 0.4048 | 0.686 | 0.083843322 | count | 1 |
| HS1BP3 | 0.0808987 | 0.3381497 | 0.2392 | 0.811 | 0.083978615 | count | 1 |
| TRMT5 | 0.1010655 | 0.4038124 | 0.2503 | 0.802 | 0.084150814 | count | 1 |
| RNPC3 | 0.064003 | 0.1544226 | 0.4145 | 0.679 | 0.084215893 | count | 1 |
| GPN1 | 0.0658342 | 0.1638571 | 0.4018 | 0.688 | 0.084389139 | count | 1 |
| WDR27 | 0.4180874 | 0.4897216 | 0.8537 | 0.393 | 0.08439075 | count | 1 |
| FUBP3 | 0.0829433 | 0.2315737 | 0.3582 | 0.72 | 0.084629998 | count | 1 |
| TAF1C | 0.1138827 | 0.362801 | 0.3139 | 0.754 | 0.084727449 | count | 1 |
| BCL2 | 0.0602433 | 0.076226 | 0.7903 | 0.429 | 0.084835552 | count | 1 |
| ASPH | 0.1019394 | 0.2775445 | 0.3673 | 0.713 | 0.084885837 | count | 1 |
| TMEM115 | 0.0675329 | 0.1824152 | 0.3702 | 0.711 | 0.084888618 | count | 1 |
| POLR2F | 0.0605021 | 0.0908095 | 0.6663 | 0.505 | 0.084900494 | count | 1 |
| PTPA | 0.0697475 | 0.2620774 | 0.2661 | 0.79 | 0.084946244 | count | 1 |
| TLE4 | 0.0606371 | 0.0895185 | 0.6774 | 0.498 | 0.084955264 | count | 1 |
| KLHL28 | 0.0628725 | 0.1217063 | 0.5166 | 0.605 | 0.085045622 | count | 1 |
| SLC35F5 | 0.079581 | 0.3013989 | 0.264 | 0.792 | 0.085116665 | count | 1 |
| WDR54 | 0.0622 | 0.1240815 | 0.5013 | 0.616 | 0.085128577 | count | 1 |
| LARP1B | 0.0720912 | 0.2200429 | 0.3276 | 0.743 | 0.085156126 | count | 1 |
| APOL2 | 0.066238 | 0.1767606 | 0.3747 | 0.708 | 0.085253264 | count | 1 |
| RNASEH2C | 0.0615658 | 0.0960744 | 0.6408 | 0.522 | 0.085395765 | count | 1 |

| | | | | | | | |
|---|---|---|---|---|---|---|---|
| JAK3 | 0.0626097 | 0.1152314 | 0.5433 | 0.587 | 0.085503748 | count | 1 |
| ZNF180 | 0.0949412 | 0.375122 | 0.2531 | 0.8 | 0.085587899 | count | 1 |
| LENG8-AS1 | 0.0895187 | 0.423564 | 0.2113 | 0.833 | 0.085604599 | count | 1 |
| SRD5A1 | 0.1028918 | 0.4413272 | 0.2331 | 0.816 | 0.085687023 | count | 1 |
| RASGRP3 | 0.1028918 | 0.5128799 | 0.2006 | 0.841 | 0.085687023 | count | 1 |
| CNOT9 | 0.0638557 | 0.1314265 | 0.4859 | 0.627 | 0.085715902 | count | 1 |
| MBNL1 | 0.0604708 | 0.055537 | 1.0888 | 0.276 | 0.085787246 | count | 1 |
| RTKN2 | 0.0727706 | 0.2563883 | 0.2838 | 0.777 | 0.085961177 | count | 1 |
| WDR20 | 0.0642138 | 0.1482501 | 0.4331 | 0.665 | 0.085988469 | count | 1 |
| TERF2 | 0.0688217 | 0.1741521 | 0.3952 | 0.693 | 0.085997375 | count | 1 |
| INTS11 | 0.0620565 | 0.1049038 | 0.5916 | 0.554 | 0.086076788 | count | 1 |
| SLC35A4 | 0.0805128 | 0.2937224 | 0.2741 | 0.784 | 0.086118211 | count | 1 |
| IQSEC1 | 0.0710291 | 0.2016534 | 0.3522 | 0.725 | 0.086127949 | count | 1 |
| AC119396.1 | 0.2499718 | 0.5433112 | 0.4601 | 0.645 | 0.086166315 | count | 1 |
| TWISTNB | 0.0624516 | 0.1238397 | 0.5043 | 0.614 | 0.08617007 | count | 1 |
| RITA1 | 0.0713994 | 0.2409551 | 0.2963 | 0.767 | 0.086173958 | count | 1 |
| ZNF519 | 0.108912 | 0.3840368 | 0.2836 | 0.777 | 0.086204157 | count | 1 |
| MKL2 | 0.1379144 | 0.3267695 | 0.4221 | 0.673 | 0.086233507 | count | 1 |
| PITHD1 | 0.0621193 | 0.0847824 | 0.7327 | 0.464 | 0.086261191 | count | 1 |
| SUGP2 | 0.0663172 | 0.1786476 | 0.3712 | 0.71 | 0.086270656 | count | 1 |
| COA1 | 0.0656761 | 0.1369098 | 0.4797 | 0.631 | 0.086308835 | count | 1 |
| HS3ST3B1 | 0.0740671 | 0.228321 | 0.3244 | 0.746 | 0.086379871 | count | 1 |
| VPS37A | 0.0667455 | 0.1808982 | 0.369 | 0.712 | 0.086389509 | count | 1 |
| TARDBP | 0.0832386 | 0.1667237 | 0.4993 | 0.618 | 0.08642106 | count | 1 |
| KLHL12 | 0.0847353 | 0.356377 | 0.2378 | 0.812 | 0.086469198 | count | 1 |
| RSRP1 | 0.0605451 | 0.0462442 | 1.3092 | 0.191 | 0.086533048 | count | 1 |
| EDRF1 | 0.0789746 | 0.1849011 | 0.4271 | 0.669 | 0.086598059 | count | 1 |
| C1orf174 | 0.0635732 | 0.1330528 | 0.4778 | 0.633 | 0.086740799 | count | 1 |
| UPRT | 0.1389502 | 0.3180757 | 0.4368 | 0.662 | 0.086892934 | count | 1 |
| ABHD17C | 0.0837441 | 0.3818388 | 0.2193 | 0.826 | 0.086948809 | count | 1 |
| PDCL | 0.0650449 | 0.1347029 | 0.4829 | 0.629 | 0.086953997 | count | 1 |
| CPSF6 | 0.0690618 | 0.1568093 | 0.4404 | 0.66 | 0.087058037 | count | 1 |
| TEX261 | 0.1263911 | 0.3243644 | 0.3897 | 0.697 | 0.087145019 | count | 1 |
| FBXL12 | 0.0688007 | 0.1764968 | 0.3898 | 0.697 | 0.087186794 | count | 1 |
| C18orf32 | 0.0735884 | 0.2078129 | 0.3541 | 0.723 | 0.087441307 | count | 1 |
| TCF7L2 | 0.1400105 | 0.4893374 | 0.2861 | 0.775 | 0.087568129 | count | 1 |
| AKAP10 | 0.0775547 | 0.2365199 | 0.3279 | 0.743 | 0.087663957 | count | 1 |
| VAMP3 | 0.065719 | 0.1540285 | 0.4267 | 0.67 | 0.087778602 | count | 1 |
| ALMS1 | 0.1918317 | 0.3686544 | 0.5204 | 0.603 | 0.087811003 | count | 1 |
| EPS15L1 | 0.0821062 | 0.2379644 | 0.345 | 0.73 | 0.087831133 | count | 1 |
| ITPR1-DT | 0.1919139 | 0.8169072 | 0.2349 | 0.814 | 0.087849738 | count | 1 |
| HLA-E | 0.0610201 | 0.0218539 | 2.7922 | 0.00526 | 0.087901926 | count | 1 |
| ANKHD1 | 0.0759291 | 0.2202368 | 0.3448 | 0.73 | 0.087935098 | count | 1 |
| NEB | 0.2550036 | 0.9654219 | 0.2641 | 0.792 | 0.087973916 | count | 1 |
| WDCP | 0.1111664 | 0.4644894 | 0.2393 | 0.811 | 0.088009477 | count | 1 |

| | | | | | | | |
|---|---|---|---|---|---|---|---|
| MAZ | 0.0622209 | 0.0751507 | 0.8279 | 0.408 | 0.088027017 | count | 1 |
| TMEM179B | 0.0628624 | 0.093436 | 0.6728 | 0.501 | 0.088366633 | count | 1 |
| RPS6KA1 | 0.0695815 | 0.1627534 | 0.4275 | 0.669 | 0.088397607 | count | 1 |
| VAMP7 | 0.072578 | 0.1782636 | 0.4071 | 0.684 | 0.088402835 | count | 1 |
| ATF5 | 0.070131 | 0.1930452 | 0.3633 | 0.716 | 0.088408696 | count | 1 |
| ZBTB21 | 0.0776814 | 0.1566116 | 0.496 | 0.62 | 0.088574724 | count | 1 |
| NRM | 0.071143 | 0.2543329 | 0.2797 | 0.78 | 0.088622269 | count | 1 |
| POLR2D | 0.0678591 | 0.1775382 | 0.3822 | 0.702 | 0.088688184 | count | 1 |
| CERS6 | 0.0983527 | 0.289348 | 0.3399 | 0.734 | 0.08869004 | count | 1 |
| GMPPA | 0.0719592 | 0.2148804 | 0.3349 | 0.738 | 0.088709781 | count | 1 |
| SIRT2 | 0.0666232 | 0.1408021 | 0.4732 | 0.636 | 0.088740913 | count | 1 |
| CHTF18 | 0.2571473 | 0.6891592 | 0.3731 | 0.709 | 0.088744813 | count | 1 |
| RAB40C | 0.0953749 | 0.3080259 | 0.3096 | 0.757 | 0.08875619 | count | 1 |
| ENDOD1 | 0.0747318 | 0.2146501 | 0.3482 | 0.728 | 0.088804211 | count | 1 |
| IARS2 | 0.0747786 | 0.1661007 | 0.4502 | 0.653 | 0.088859998 | count | 1 |
| AL121603.2 | 0.0767718 | 0.2343555 | 0.3276 | 0.743 | 0.088914559 | count | 1 |
| DSE | 0.1125609 | 0.3312055 | 0.3399 | 0.734 | 0.089126599 | count | 1 |
| ZNF28 | 0.1025269 | 0.501504 | 0.2044 | 0.838 | 0.089148265 | count | 1 |
| HDAC3 | 0.0646588 | 0.1215579 | 0.5319 | 0.595 | 0.089163626 | count | 1 |
| FSIP1 | 0.112631 | 0.5539813 | 0.2033 | 0.839 | 0.08918276 | count | 1 |
| RNLS | 0.1623659 | 0.6701526 | 0.2423 | 0.809 | 0.089241196 | count | 1 |
| MBOAT7 | 0.0760726 | 0.2247645 | 0.3385 | 0.735 | 0.089316786 | count | 1 |
| VPS26B | 0.0677469 | 0.1690791 | 0.4007 | 0.689 | 0.089370394 | count | 1 |
| HCG11 | 0.1626875 | 0.4185413 | 0.3887 | 0.698 | 0.089422014 | count | 1 |
| RBM10 | 0.0791724 | 0.2239495 | 0.3535 | 0.724 | 0.089499966 | count | 1 |
| AHCYL2 | 0.1075094 | 0.4156534 | 0.2587 | 0.796 | 0.089573499 | count | 1 |
| ZNF444 | 0.0817125 | 0.2738299 | 0.2984 | 0.765 | 0.089614214 | count | 1 |
| RAB30-AS1 | 0.0714988 | 0.1857815 | 0.3849 | 0.7 | 0.08962273 | count | 1 |
| ZNF276 | 0.0671182 | 0.1425341 | 0.4709 | 0.638 | 0.089649686 | count | 1 |
| ZNF593 | 0.066144 | 0.1578503 | 0.419 | 0.675 | 0.089792165 | count | 1 |
| LMAN1 | 0.0636836 | 0.0817766 | 0.7788 | 0.436 | 0.089815125 | count | 1 |
| ZFP3 | 0.443123 | 0.6513578 | 0.6803 | 0.496 | 0.089817607 | count | 1 |
| AP4M1 | 0.0782596 | 0.2502043 | 0.3128 | 0.754 | 0.089961848 | count | 1 |
| AC004825.2 | 0.4439376 | 0.4856246 | 0.9142 | 0.361 | 0.089994762 | count | 1 |
| SPG7 | 0.0686792 | 0.1416766 | 0.4848 | 0.628 | 0.09001828 | count | 1 |
| BRCA2 | 0.0998298 | 0.3339375 | 0.2989 | 0.765 | 0.090033742 | count | 1 |
| FAM49B | 0.0632868 | 0.0615875 | 1.0276 | 0.304 | 0.090080124 | count | 1 |
| PKN1 | 0.0647094 | 0.1018119 | 0.6356 | 0.525 | 0.090148824 | count | 1 |
| CCNB1 | 0.1968509 | 0.4220611 | 0.4664 | 0.641 | 0.090177741 | count | 1 |
| APOPT1 | 0.0659668 | 0.1326645 | 0.4972 | 0.619 | 0.090211607 | count | 1 |
| IVNS1ABP | 0.0659493 | 0.0921806 | 0.7154 | 0.474 | 0.090264122 | count | 1 |
| TGOLN2 | 0.0643029 | 0.0769402 | 0.8358 | 0.403 | 0.090326504 | count | 1 |
| SYNGR3 | 0.0921845 | 0.4296603 | 0.2146 | 0.83 | 0.090347139 | count | 1 |
| SLC39A10 | 0.0659724 | 0.1147984 | 0.5747 | 0.566 | 0.090645366 | count | 1 |
| ZNF17 | 0.4479253 | 0.3598263 | 1.2448 | 0.213 | 0.090862608 | count | 1 |

| | | | | | | | |
|---|---|---|---|---|---|---|---|
| KIAA1328 | 0.0850555 | 0.2614415 | 0.3253 | 0.745 | 0.0910025 | count | 1 |
| ZNF358 | 0.090928 | 0.3804002 | 0.239 | 0.811 | 0.091053866 | count | 1 |
| RGS9 | 0.0685214 | 0.1622655 | 0.4223 | 0.673 | 0.091093287 | count | 1 |
| CLMP | 0.1320614 | 0.9215849 | 0.1433 | 0.886 | 0.091117073 | count | 1 |
| SLC25A43 | 0.1456711 | 0.368882 | 0.3949 | 0.693 | 0.091175722 | count | 1 |
| SPATA1 | 0.4494583 | 0.7216683 | 0.6228 | 0.533 | 0.091196478 | count | 1 |
| SLC39A13 | 0.0777366 | 0.2241557 | 0.3468 | 0.729 | 0.091277198 | count | 1 |
| ZNF529 | 0.0865686 | 0.2733093 | 0.3167 | 0.751 | 0.091319574 | count | 1 |
| MIAT | 0.0699884 | 0.190195 | 0.368 | 0.713 | 0.091339078 | count | 1 |
| GTF2F1 | 0.066526 | 0.1051563 | 0.6326 | 0.527 | 0.091406581 | count | 1 |
| TCIRG1 | 0.0708047 | 0.1407733 | 0.503 | 0.615 | 0.091487569 | count | 1 |
| SLC35D2 | 0.0676008 | 0.1253803 | 0.5392 | 0.59 | 0.091504359 | count | 1 |
| MCL1 | 0.0639902 | 0.0463952 | 1.3792 | 0.168 | 0.091556837 | count | 1 |
| COQ4 | 0.067668 | 0.1381511 | 0.4898 | 0.624 | 0.091811403 | count | 1 |
| ARRDC5 | 0.2005751 | 0.6266622 | 0.3201 | 0.749 | 0.091935959 | count | 1 |
| MFSD12 | 0.0754951 | 0.1780791 | 0.4239 | 0.672 | 0.091965905 | count | 1 |
| SKAP2 | 0.0674956 | 0.1508898 | 0.4473 | 0.655 | 0.092053904 | count | 1 |
| PIGO | 0.090207 | 0.3126208 | 0.2886 | 0.773 | 0.092087697 | count | 1 |
| MRPL32 | 0.0669326 | 0.1137113 | 0.5886 | 0.556 | 0.092214782 | count | 1 |
| PCMTD1 | 0.0663936 | 0.0948771 | 0.6998 | 0.484 | 0.09222024 | count | 1 |
| TIA1 | 0.0693714 | 0.1538892 | 0.4508 | 0.652 | 0.092224754 | count | 1 |
| NSL1 | 0.0667026 | 0.1170324 | 0.57 | 0.569 | 0.092248373 | count | 1 |
| AC009041.2 | 0.2013346 | 0.428263 | 0.4701 | 0.638 | 0.092294748 | count | 1 |
| AL080276.2 | 0.1678024 | 0.5520719 | 0.304 | 0.761 | 0.092299835 | count | 1 |
| PSPH | 0.2671099 | 0.5516744 | 0.4842 | 0.628 | 0.092333739 | count | 1 |
| NAT10 | 0.1239756 | 0.2786983 | 0.4448 | 0.656 | 0.092341464 | count | 1 |
| VIPR2 | 0.4554238 | 0.4390221 | 1.0374 | 0.3 | 0.092496899 | count | 1 |
| PIK3C2B | 0.0825893 | 0.2997074 | 0.2756 | 0.783 | 0.092509034 | count | 1 |
| MAP2K5 | 0.0967809 | 0.2999229 | 0.3227 | 0.747 | 0.092602678 | count | 1 |
| KCNA2 | 0.2020399 | 0.9344912 | 0.2162 | 0.829 | 0.092627999 | count | 1 |
| U62317.3 | 0.2020399 | 1.0391026 | 0.1944 | 0.846 | 0.092627999 | count | 1 |
| ATP5F1A | 0.0654209 | 0.0631517 | 1.0359 | 0.3 | 0.09268866 | count | 1 |
| RPS14 | 0.06457 | 0.013832 | 4.6682 | 3.16E-06 | 0.093118207 | count | 0.07606436 |
| PNPO | 0.095018 | 0.3595035 | 0.2643 | 0.792 | 0.09314408 | count | 1 |
| UBE2G1 | 0.067953 | 0.1129188 | 0.6018 | 0.547 | 0.093159284 | count | 1 |
| MOB1A | 0.0657484 | 0.0709195 | 0.9271 | 0.354 | 0.093218997 | count | 1 |
| INTS4 | 0.0913218 | 0.2341941 | 0.3899 | 0.697 | 0.093232898 | count | 1 |
| RORC | 0.1252752 | 0.6071145 | 0.2063 | 0.837 | 0.093323041 | count | 1 |
| IRAK3 | 0.1252752 | 0.6682561 | 0.1875 | 0.851 | 0.093323041 | count | 1 |
| ARC | 0.2698571 | 0.6642689 | 0.4062 | 0.685 | 0.093325157 | count | 1 |
| PHF2 | 0.0975517 | 0.2346236 | 0.4158 | 0.678 | 0.093345889 | count | 1 |
| RPL21 | 0.0648632 | 0.0186407 | 3.4797 | 0.000508 | 0.093476834 | count | 1 |
| HEXIM1 | 0.0722671 | 0.1203037 | 0.6007 | 0.548 | 0.093548833 | count | 1 |
| NIPBL | 0.0674967 | 0.0938282 | 0.7194 | 0.472 | 0.093561111 | count | 1 |
| FGF14-AS2 | 0.4604262 | 0.925911 | 0.4973 | 0.619 | 0.093588883 | count | 1 |

| | | | | | | | |
|---|---|---|---|---|---|---|---|
| OSTF1 | 0.065803 | 0.0617376 | 1.0658 | 0.287 | 0.093952693 | count | 1 |
| B3GALT4 | 0.0696565 | 0.1641989 | 0.4242 | 0.671 | 0.093987636 | count | 1 |
| RPL12 | 0.065196 | 0.0173493 | 3.7578 | 0.000174 | 0.094003609 | count | 1 |
| RCOR1 | 0.0726899 | 0.1698818 | 0.4279 | 0.669 | 0.094097112 | count | 1 |
| SLC38A1 | 0.0676218 | 0.0802785 | 0.8423 | 0.4 | 0.094137793 | count | 1 |
| AC005332.3 | 0.2723455 | 0.8091529 | 0.3366 | 0.736 | 0.094223871 | count | 1 |
| SDHC | 0.0673521 | 0.087372 | 0.7709 | 0.441 | 0.094267005 | count | 1 |
| OSBPL10 | 0.2056288 | 0.5585995 | 0.3681 | 0.713 | 0.09432472 | count | 1 |
| EIF2A | 0.0678706 | 0.083167 | 0.8161 | 0.415 | 0.09435281 | count | 1 |
| WDR44 | 0.0804709 | 0.2044871 | 0.3935 | 0.694 | 0.094499162 | count | 1 |
| TAF4 | 0.0964083 | 0.3222222 | 0.2992 | 0.765 | 0.094516854 | count | 1 |
| ZNF687 | 0.1271004 | 0.4848072 | 0.2622 | 0.793 | 0.094702062 | count | 1 |
| AC084018.2 | 0.1049695 | 0.3783861 | 0.2774 | 0.781 | 0.094711832 | count | 1 |
| UBE2V1 | 0.0734854 | 0.1944827 | 0.3779 | 0.706 | 0.094781092 | count | 1 |
| IGHV1-3 | 0.4659759 | 0.5815976 | 0.8012 | 0.423 | 0.094801931 | count | 1 |
| ATG12 | 0.0685286 | 0.0937628 | 0.7309 | 0.465 | 0.094898868 | count | 1 |
| ELOVL5 | 0.0688846 | 0.1079381 | 0.6382 | 0.523 | 0.094936182 | count | 1 |
| TMEM109 | 0.0671952 | 0.0768165 | 0.8747 | 0.382 | 0.094962921 | count | 1 |
| FAM184B | 0.1139385 | 0.4375241 | 0.2604 | 0.795 | 0.094990206 | count | 1 |
| CD8B2 | 0.1376026 | 0.5641133 | 0.2439 | 0.807 | 0.095003572 | count | 1 |
| SH3BP1 | 0.0688725 | 0.120758 | 0.5703 | 0.568 | 0.095064061 | count | 1 |
| ARSD | 0.0949425 | 0.4720225 | 0.2011 | 0.841 | 0.09510139 | count | 1 |
| RBM19 | 0.0850323 | 0.2296385 | 0.3703 | 0.711 | 0.095257749 | count | 1 |
| ACTL6A | 0.0727729 | 0.1646761 | 0.4419 | 0.659 | 0.095258459 | count | 1 |
| PPP1R18 | 0.0668421 | 0.0659604 | 1.0134 | 0.311 | 0.095307139 | count | 1 |
| ARAF | 0.0756411 | 0.1969575 | 0.384 | 0.701 | 0.095370602 | count | 1 |
| HSPA13 | 0.0783036 | 0.1410894 | 0.555 | 0.579 | 0.095397015 | count | 1 |
| NDOR1 | 0.2080207 | 0.7598515 | 0.2738 | 0.784 | 0.095456456 | count | 1 |
| ITGB4 | 0.2760046 | 0.6169093 | 0.4474 | 0.655 | 0.095546566 | count | 1 |
| PLA2G15 | 0.2760969 | 0.5414341 | 0.5099 | 0.61 | 0.095579944 | count | 1 |
| RPL18A | 0.0663095 | 0.016145 | 4.1071 | 4.10E-05 | 0.095604395 | count | 0.982934 |
| ITFG2-AS1 | 0.4697727 | 0.5915249 | 0.7942 | 0.427 | 0.095632786 | count | 1 |
| DMTF1 | 0.0732093 | 0.1529523 | 0.4786 | 0.632 | 0.095692059 | count | 1 |
| BLOC1S4 | 0.0684274 | 0.0980745 | 0.6977 | 0.485 | 0.09573249 | count | 1 |
| FAM120C | 0.0884157 | 0.3104149 | 0.2848 | 0.776 | 0.095854532 | count | 1 |
| AL645568.1 | 0.0864525 | 0.2362036 | 0.366 | 0.714 | 0.095882271 | count | 1 |
| GNAI3 | 0.0680338 | 0.0762593 | 0.8921 | 0.372 | 0.095922527 | count | 1 |
| CTC1 | 0.088593 | 0.2781286 | 0.3185 | 0.75 | 0.096047747 | count | 1 |
| RPL7A | 0.0666256 | 0.0140741 | 4.7339 | 2.29E-06 | 0.096072291 | count | 0.05514778 |
| AUP1 | 0.069572 | 0.0984992 | 0.7063 | 0.48 | 0.096086044 | count | 1 |
| PRMT2 | 0.0678724 | 0.067491 | 1.0057 | 0.315 | 0.096314492 | count | 1 |
| DCUN1D2 | 0.2099164 | 0.5723854 | 0.3667 | 0.714 | 0.09635394 | count | 1 |
| JAKMIP2 | 0.0764361 | 0.2092511 | 0.3653 | 0.715 | 0.09637525 | count | 1 |
| NAA35 | 0.0807037 | 0.1887479 | 0.4276 | 0.669 | 0.096455423 | count | 1 |
| SUMO3 | 0.0689483 | 0.0911402 | 0.7565 | 0.449 | 0.09646161 | count | 1 |

| | | | | | | | |
|---|---|---|---|---|---|---|---|
| LY96 | 0.0788843 | 0.1666471 | 0.4734 | 0.636 | 0.09651218 | count | 1 |
| CHP1 | 0.0727679 | 0.1589318 | 0.4579 | 0.647 | 0.096546999 | count | 1 |
| MAGEH1 | 0.0833702 | 0.2041029 | 0.4085 | 0.683 | 0.096586346 | count | 1 |
| PRPF18 | 0.0723383 | 0.1547619 | 0.4674 | 0.64 | 0.09663106 | count | 1 |
| SNX18 | 0.078379 | 0.2483562 | 0.3156 | 0.752 | 0.096645383 | count | 1 |
| PRR7-AS1 | 0.4749195 | 0.6696605 | 0.7092 | 0.478 | 0.096760304 | count | 1 |
| EIF3F | 0.0674691 | 0.0390673 | 1.727 | 0.0843 | 0.096778812 | count | 1 |
| ZNF248 | 0.1113024 | 0.3098427 | 0.3592 | 0.719 | 0.096857519 | count | 1 |
| MTCH2 | 0.0727882 | 0.1435029 | 0.5072 | 0.612 | 0.096963488 | count | 1 |
| PRMT9 | 0.0784651 | 0.177143 | 0.4429 | 0.658 | 0.097104572 | count | 1 |
| ZNF219 | 0.0969569 | 0.3279383 | 0.2957 | 0.768 | 0.097133192 | count | 1 |
| AKR7A2 | 0.070499 | 0.121489 | 0.5803 | 0.562 | 0.097310729 | count | 1 |
| POLR3F | 0.0814701 | 0.2732531 | 0.2981 | 0.766 | 0.097374432 | count | 1 |
| MIR29B2CHG | 0.2124017 | 0.3778793 | 0.5621 | 0.574 | 0.097531252 | count | 1 |
| ZNF37A | 0.0825811 | 0.2100814 | 0.3931 | 0.694 | 0.097591048 | count | 1 |
| SDF4 | 0.0699377 | 0.0929529 | 0.7524 | 0.452 | 0.097606711 | count | 1 |
| ZNF440 | 0.212756 | 0.4937979 | 0.4309 | 0.667 | 0.097699153 | count | 1 |
| C2CD4D | 0.1051074 | 0.423702 | 0.2481 | 0.804 | 0.097892492 | count | 1 |
| CIRBP | 0.0683163 | 0.0411358 | 1.6607 | 0.0969 | 0.097894921 | count | 1 |
| SOCS1 | 0.0684479 | 0.0513443 | 1.3331 | 0.183 | 0.097903226 | count | 1 |
| STAM2 | 0.08286 | 0.2708263 | 0.306 | 0.76 | 0.097921807 | count | 1 |
| AC139887.2 | 0.2134519 | 0.3554709 | 0.6005 | 0.548 | 0.09802898 | count | 1 |
| DNAJC6 | 0.2136957 | 0.8544277 | 0.2501 | 0.803 | 0.098144548 | count | 1 |
| RPL18 | 0.0680852 | 0.015714 | 4.3328 | 1.52E-05 | 0.098169103 | count | 0.3650736 |
| PARP12 | 0.089516 | 0.2263715 | 0.3954 | 0.693 | 0.09821567 | count | 1 |
| BAIAP2-DT | 0.2138848 | 0.873147 | 0.245 | 0.807 | 0.098234191 | count | 1 |
| AL357078.1 | 0.2138848 | 1.0988698 | 0.1946 | 0.846 | 0.098234191 | count | 1 |
| ZMYND10 | 0.2138848 | 1.2092927 | 0.1769 | 0.86 | 0.098234191 | count | 1 |
| NPY1R | 0.2138848 | 1.2092927 | 0.1769 | 0.86 | 0.098234191 | count | 1 |
| ZFP2 | 0.2138848 | 1.2092927 | 0.1769 | 0.86 | 0.098234191 | count | 1 |
| CASP5 | 0.2138848 | 1.2092927 | 0.1769 | 0.86 | 0.098234191 | count | 1 |
| RNFT2 | 0.2138848 | 1.2092927 | 0.1769 | 0.86 | 0.098234191 | count | 1 |
| AC010542.4 | 0.2138848 | 1.2092927 | 0.1769 | 0.86 | 0.098234191 | count | 1 |
| ALPK2 | 0.2138848 | 1.2092927 | 0.1769 | 0.86 | 0.098234191 | count | 1 |
| ZNF780B | 0.1239528 | 0.3161036 | 0.3921 | 0.695 | 0.098264012 | count | 1 |
| NUDT19 | 0.0833242 | 0.2316289 | 0.3597 | 0.719 | 0.098472335 | count | 1 |
| AC040162.1 | 0.102916 | 0.3234202 | 0.3182 | 0.75 | 0.098520565 | count | 1 |
| TRAPPC1 | 0.0691237 | 0.0679901 | 1.0167 | 0.309 | 0.098567088 | count | 1 |
| BTN3A3 | 0.0743721 | 0.1683768 | 0.4417 | 0.659 | 0.098572874 | count | 1 |
| CYTH1 | 0.0710765 | 0.0912723 | 0.7787 | 0.436 | 0.098641515 | count | 1 |
| KIAA1147 | 0.1244548 | 0.2399521 | 0.5187 | 0.604 | 0.098667132 | count | 1 |
| SNRPD3 | 0.0695656 | 0.0623499 | 1.1157 | 0.265 | 0.098721925 | count | 1 |
| SLC19A2 | 0.1324484 | 0.4242951 | 0.3122 | 0.755 | 0.098745719 | count | 1 |
| C11orf1 | 0.0770986 | 0.1531939 | 0.5033 | 0.615 | 0.098857828 | count | 1 |
| ST8SIA4 | 0.071314 | 0.1075173 | 0.6633 | 0.507 | 0.098926004 | count | 1 |

| | | | | | | | |
|---|---|---|---|---|---|---|---|
| GPAA1 | 0.0726134 | 0.1385069 | 0.5243 | 0.6 | 0.098992993 | count | 1 |
| FAM200A | 0.0902556 | 0.3177635 | 0.284 | 0.776 | 0.09903127 | count | 1 |
| GID8 | 0.0719517 | 0.1131414 | 0.6359 | 0.525 | 0.099103206 | count | 1 |
| CHRNB1 | 0.0764306 | 0.1818762 | 0.4202 | 0.674 | 0.099120883 | count | 1 |
| SRSF7 | 0.0692291 | 0.038924 | 1.7786 | 0.0754 | 0.09914197 | count | 1 |
| ACSS2 | 0.1435942 | 0.5955148 | 0.2411 | 0.809 | 0.099211366 | count | 1 |
| MAP3K12 | 0.0752926 | 0.1609996 | 0.4677 | 0.64 | 0.09921862 | count | 1 |
| BTN3A1 | 0.0730861 | 0.116325 | 0.6283 | 0.53 | 0.099225902 | count | 1 |
| AC012306.2 | 0.0808145 | 0.2989162 | 0.2704 | 0.787 | 0.099277383 | count | 1 |
| ANXA4 | 0.0730219 | 0.1451894 | 0.5029 | 0.615 | 0.099299743 | count | 1 |
| IRF5 | 0.2868159 | 0.5907218 | 0.4855 | 0.627 | 0.099462491 | count | 1 |
| PIP4K2C | 0.0973904 | 0.3419416 | 0.2848 | 0.776 | 0.099469898 | count | 1 |
| DBNL | 0.0717744 | 0.0997821 | 0.7193 | 0.472 | 0.099633122 | count | 1 |
| AL161729.1 | 0.1810596 | 0.7163003 | 0.2528 | 0.8 | 0.099776349 | count | 1 |
| HIST1H2BB | 0.1810596 | 0.7203555 | 0.2513 | 0.802 | 0.099776349 | count | 1 |
| MELK | 0.1810596 | 0.9190042 | 0.197 | 0.844 | 0.099776349 | count | 1 |
| HIST1H2BN | 0.1258373 | 0.2528042 | 0.4978 | 0.619 | 0.099777522 | count | 1 |
| ZNF551 | 0.1594979 | 0.4473247 | 0.3566 | 0.721 | 0.100008336 | count | 1 |
| FBXO4 | 0.0805956 | 0.2238658 | 0.36 | 0.719 | 0.100095285 | count | 1 |
| RGN | 0.2885986 | 0.8353476 | 0.3455 | 0.73 | 0.100109298 | count | 1 |
| BIRC5 | 0.2885986 | 1.0178124 | 0.2835 | 0.777 | 0.100109298 | count | 1 |
| GTPBP10 | 0.0812083 | 0.20094 | 0.4041 | 0.686 | 0.100143777 | count | 1 |
| SLC17A5 | 0.1075104 | 0.308806 | 0.3481 | 0.728 | 0.100150425 | count | 1 |
| COA3 | 0.0734476 | 0.1390981 | 0.528 | 0.598 | 0.100179274 | count | 1 |
| TMEM187 | 0.0801474 | 0.2559022 | 0.3132 | 0.754 | 0.100490612 | count | 1 |
| SELENOW | 0.0703548 | 0.0530825 | 1.3254 | 0.185 | 0.100577571 | count | 1 |
| SDHAF2 | 0.0721404 | 0.1068611 | 0.6751 | 0.5 | 0.100648902 | count | 1 |
| LUC7L | 0.0745905 | 0.1237763 | 0.6026 | 0.547 | 0.100652709 | count | 1 |
| UNC50 | 0.0734304 | 0.1235172 | 0.5945 | 0.552 | 0.100864756 | count | 1 |
| BTD | 0.0832267 | 0.2017836 | 0.4125 | 0.68 | 0.100964916 | count | 1 |
| PCED1B | 0.0734522 | 0.1532086 | 0.4794 | 0.632 | 0.101037024 | count | 1 |
| ZNF486 | 0.105654 | 0.3844531 | 0.2748 | 0.783 | 0.10116336 | count | 1 |
| DHFR | 0.1356631 | 0.334766 | 0.4052 | 0.685 | 0.101178541 | count | 1 |
| ACACB | 0.1467323 | 0.3674084 | 0.3994 | 0.69 | 0.101417417 | count | 1 |
| SPTSSA | 0.076972 | 0.1535887 | 0.5012 | 0.616 | 0.101435174 | count | 1 |
| SLC16A1 | 0.0794936 | 0.1650325 | 0.4817 | 0.63 | 0.101492713 | count | 1 |
| CALCOCO2 | 0.0731373 | 0.0899979 | 0.8127 | 0.416 | 0.10152626 | count | 1 |
| PSKH1 | 0.1217315 | 0.4430431 | 0.2748 | 0.784 | 0.101564629 | count | 1 |
| TMOD2 | 0.128199 | 0.3099863 | 0.4136 | 0.679 | 0.101675067 | count | 1 |
| IL32 | 0.0706084 | 0.0249938 | 2.825 | 0.00476 | 0.101750561 | count | 1 |
| KYAT1 | 0.1847897 | 0.4658457 | 0.3967 | 0.692 | 0.101884492 | count | 1 |
| AZIN2 | 0.1847897 | 0.4947155 | 0.3735 | 0.709 | 0.101884492 | count | 1 |
| TBRG4 | 0.0837069 | 0.1693938 | 0.4942 | 0.621 | 0.102000057 | count | 1 |
| MRAP2 | 0.1852369 | 0.7145813 | 0.2592 | 0.795 | 0.102137371 | count | 1 |
| PC | 0.1852369 | 0.7145813 | 0.2592 | 0.795 | 0.102137371 | count | 1 |

| | | | | | | | |
|---|---|---|---|---|---|---|---|
| FAM86C1 | 0.2222414 | 0.6066897 | 0.3663 | 0.714 | 0.102200147 | count | 1 |
| HS2ST1 | 0.1853734 | 0.3279606 | 0.5652 | 0.572 | 0.102214563 | count | 1 |
| PIGG | 0.1479082 | 0.2886746 | 0.5124 | 0.608 | 0.102244457 | count | 1 |
| TTC5 | 0.0779701 | 0.1922168 | 0.4056 | 0.685 | 0.102355985 | count | 1 |
| HUWE1 | 0.0840708 | 0.1349356 | 0.623 | 0.533 | 0.102444845 | count | 1 |
| AMMECR1 | 0.0872455 | 0.2652154 | 0.329 | 0.742 | 0.102485176 | count | 1 |
| CDKAL1 | 0.1022805 | 0.2568568 | 0.3982 | 0.691 | 0.102505438 | count | 1 |
| ABCA5 | 0.0834699 | 0.2130286 | 0.3918 | 0.695 | 0.10254896 | count | 1 |
| TNFRSF25 | 0.0821132 | 0.1308648 | 0.6275 | 0.53 | 0.102649241 | count | 1 |
| CA5B | 0.0759641 | 0.1273664 | 0.5964 | 0.551 | 0.102774147 | count | 1 |
| AL683807.1 | 0.1487373 | 0.5471147 | 0.2719 | 0.786 | 0.102827704 | count | 1 |
| C12orf43 | 0.0789285 | 0.1969201 | 0.4008 | 0.689 | 0.102869263 | count | 1 |
| IGFBP4 | 0.0937447 | 0.2699634 | 0.3472 | 0.728 | 0.102879771 | count | 1 |
| CARM1 | 0.1107136 | 0.3326172 | 0.3329 | 0.739 | 0.103161554 | count | 1 |
| RPS27 | 0.0718143 | 0.0142293 | 5.0469 | 4.73E-07 | 0.103524331 | count | 0.011408287 |
| ZNF582 | 0.1305728 | 0.5967344 | 0.2188 | 0.827 | 0.103583206 | count | 1 |
| AUH | 0.0877908 | 0.2019033 | 0.4348 | 0.664 | 0.103770659 | count | 1 |
| PDCD11 | 0.0983128 | 0.2962355 | 0.3319 | 0.74 | 0.103785153 | count | 1 |
| DHCR24 | 0.5069926 | 0.7327803 | 0.6919 | 0.489 | 0.10381785 | count | 1 |
| TIMM29 | 0.0813454 | 0.1845859 | 0.4407 | 0.659 | 0.103862193 | count | 1 |
| EMC4 | 0.0740521 | 0.0863576 | 0.8575 | 0.391 | 0.104113731 | count | 1 |
| SNX29 | 0.0871046 | 0.2327932 | 0.3742 | 0.708 | 0.104132557 | count | 1 |
| ZNF142 | 0.2997758 | 0.3901262 | 0.7684 | 0.442 | 0.104172077 | count | 1 |
| KIAA0825 | 0.0973218 | 0.2699333 | 0.3605 | 0.718 | 0.104203947 | count | 1 |
| YARS2 | 0.0876663 | 0.2185511 | 0.4011 | 0.688 | 0.104230419 | count | 1 |
| PHF8 | 0.1197037 | 0.2676912 | 0.4472 | 0.655 | 0.104249019 | count | 1 |
| TPD52 | 0.0775819 | 0.1354889 | 0.5726 | 0.567 | 0.10432369 | count | 1 |
| YY1AP1 | 0.0838003 | 0.1949929 | 0.4298 | 0.667 | 0.104432016 | count | 1 |
| CAPZB | 0.0728757 | 0.0437302 | 1.6665 | 0.0957 | 0.104439064 | count | 1 |
| SIDT1-AS1 | 0.1157104 | 0.3344046 | 0.346 | 0.729 | 0.104500812 | count | 1 |
| TRAFD1 | 0.0832129 | 0.2081117 | 0.3998 | 0.689 | 0.104648301 | count | 1 |
| FAAP24 | 0.1897673 | 0.5650926 | 0.3358 | 0.737 | 0.104700758 | count | 1 |
| STAT2 | 0.0832672 | 0.1794926 | 0.4639 | 0.643 | 0.104716759 | count | 1 |
| CCT7 | 0.0742933 | 0.0726723 | 1.0223 | 0.307 | 0.104808269 | count | 1 |
| AC072061.1 | 0.5117457 | 0.5852864 | 0.8744 | 0.382 | 0.104868225 | count | 1 |
| SEC11A | 0.0736327 | 0.064521 | 1.1412 | 0.254 | 0.104869782 | count | 1 |
| ARMCX4 | 0.1126883 | 0.5739656 | 0.1963 | 0.844 | 0.105018583 | count | 1 |
| CMTM8 | 0.0790128 | 0.1212491 | 0.6517 | 0.515 | 0.105061172 | count | 1 |
| SMIM30 | 0.0866523 | 0.1792564 | 0.4834 | 0.629 | 0.105134085 | count | 1 |
| GABPB1-AS1 | 0.0754739 | 0.091322 | 0.8265 | 0.409 | 0.105135055 | count | 1 |
| NOA1 | 0.0818444 | 0.1443608 | 0.5669 | 0.571 | 0.105381862 | count | 1 |
| TRIM32 | 0.1524539 | 0.4551544 | 0.3349 | 0.738 | 0.105443543 | count | 1 |
| TAGLN2 | 0.0733463 | 0.0361421 | 2.0294 | 0.0425 | 0.105498981 | count | 1 |
| NECAP1 | 0.0785857 | 0.1313421 | 0.5983 | 0.55 | 0.105514157 | count | 1 |
| REPIN1 | 0.0911376 | 0.2131791 | 0.4275 | 0.669 | 0.105623008 | count | 1 |

| | | | | | | | |
|---|---|---|---|---|---|---|---|
| ARL4A | 0.0746733 | 0.0772908 | 0.9661 | 0.334 | 0.105720302 | count | 1 |
| ZSWIM5 | 0.2297141 | 0.7670092 | 0.2995 | 0.765 | 0.105754073 | count | 1 |
| LMAN2 | 0.0747723 | 0.0746175 | 1.0021 | 0.316 | 0.10588842 | count | 1 |
| CLPX | 0.0862636 | 0.1726369 | 0.4997 | 0.617 | 0.105991549 | count | 1 |
| ZNF211 | 0.1058118 | 0.3165638 | 0.3343 | 0.738 | 0.106071117 | count | 1 |
| KIF1BP | 0.0857511 | 0.2165025 | 0.3961 | 0.692 | 0.10614725 | count | 1 |
| ESM1 | 0.1272091 | 0.5722801 | 0.2223 | 0.824 | 0.106191279 | count | 1 |
| ANXA2R | 0.0759576 | 0.1034795 | 0.734 | 0.463 | 0.106211841 | count | 1 |
| COMMD9 | 0.0810563 | 0.1777816 | 0.4559 | 0.648 | 0.106269472 | count | 1 |
| ARFRP1 | 0.0786012 | 0.1249155 | 0.6292 | 0.529 | 0.106279318 | count | 1 |
| TRAV14DV4 | 0.1341085 | 0.6656161 | 0.2015 | 0.84 | 0.10642693 | count | 1 |
| AC079305.1 | 0.3062216 | 0.602065 | 0.5086 | 0.611 | 0.106520611 | count | 1 |
| CHRM3-AS2 | 0.1087962 | 0.2190927 | 0.4966 | 0.62 | 0.106760477 | count | 1 |
| CD164 | 0.0754346 | 0.0701254 | 1.0757 | 0.282 | 0.10676953 | count | 1 |
| KARS | 0.0820722 | 0.1473917 | 0.5568 | 0.578 | 0.106805133 | count | 1 |
| CLU | 0.0802016 | 0.1372097 | 0.5845 | 0.559 | 0.106954463 | count | 1 |
| CSF2 | 0.5216783 | 0.7998895 | 0.6522 | 0.5143 | 0.107066824 | count | 1 |
| GKAP1 | 0.0931541 | 0.1581056 | 0.5892 | 0.556 | 0.107159783 | count | 1 |
| CHORDC1 | 0.0772776 | 0.0894807 | 0.8636 | 0.388 | 0.107482852 | count | 1 |
| PCGF6 | 0.0921034 | 0.2915145 | 0.3159 | 0.752 | 0.107501977 | count | 1 |
| ABRAXAS2 | 0.0816734 | 0.177107 | 0.4612 | 0.645 | 0.10750636 | count | 1 |
| BROX | 0.07867 | 0.1139559 | 0.6904 | 0.49 | 0.1075066 | count | 1 |
| ERG28 | 0.0781971 | 0.1168992 | 0.6689 | 0.504 | 0.107641991 | count | 1 |
| IKZF5 | 0.0936358 | 0.2172511 | 0.431 | 0.666 | 0.107716364 | count | 1 |
| NAP1L2 | 0.1124425 | 0.3324632 | 0.3382 | 0.735 | 0.107720391 | count | 1 |
| NPM3 | 0.1192934 | 0.3144713 | 0.3793 | 0.704 | 0.107770062 | count | 1 |
| CD48 | 0.0750693 | 0.0304171 | 2.468 | 0.0136 | 0.107900047 | count | 1 |
| MTMR1 | 0.0963349 | 0.2672999 | 0.3604 | 0.719 | 0.107983436 | count | 1 |
| BRPF3 | 0.1560723 | 0.385015 | 0.4054 | 0.685 | 0.10799229 | count | 1 |
| SETMAR | 0.2346914 | 0.4657614 | 0.5039 | 0.614 | 0.108125094 | count | 1 |
| SLC25A42 | 0.0853303 | 0.2143601 | 0.3981 | 0.691 | 0.108185058 | count | 1 |
| MRPL27 | 0.078323 | 0.1273194 | 0.6152 | 0.538 | 0.108272664 | count | 1 |
| PIK3R4 | 0.1964573 | 0.3783901 | 0.5192 | 0.604 | 0.108491355 | count | 1 |
| AL391244.3 | 0.5281581 | 1.206944 | 0.4376 | 0.662 | 0.108503754 | count | 1 |
| LPAR3 | 0.5281581 | 1.206944 | 0.4376 | 0.662 | 0.108503754 | count | 1 |
| AC007495.1 | 0.5281581 | 1.206944 | 0.4376 | 0.662 | 0.108503754 | count | 1 |
| AC080112.1 | 0.5281581 | 1.206944 | 0.4376 | 0.662 | 0.108503754 | count | 1 |
| KCNG1 | 0.5281581 | 1.206944 | 0.4376 | 0.662 | 0.108503754 | count | 1 |
| AC027307.1 | 0.5281581 | 1.206944 | 0.4376 | 0.662 | 0.108503754 | count | 1 |
| FBXO27 | 0.5281581 | 1.206944 | 0.4376 | 0.662 | 0.108503754 | count | 1 |
| SLC44A5 | 0.5281581 | 1.381269 | 0.3824 | 0.702 | 0.108503754 | count | 1 |
| ADAMTS9 | 0.5281581 | 1.381269 | 0.3824 | 0.702 | 0.108503754 | count | 1 |
| TIGD4 | 0.5281581 | 1.381269 | 0.3824 | 0.702 | 0.108503754 | count | 1 |
| SLC26A7 | 0.5281581 | 1.381269 | 0.3824 | 0.702 | 0.108503754 | count | 1 |
| FAM13C | 0.5281581 | 1.381269 | 0.3824 | 0.702 | 0.108503754 | count | 1 |

| | | | | | | | |
|---|---|---|---|---|---|---|---|
| RASSF9 | 0.5281581 | 1.381269 | 0.3824 | 0.702 | 0.108503754 | count | 1 |
| FGF14 | 0.5281581 | 1.381269 | 0.3824 | 0.702 | 0.108503754 | count | 1 |
| AC018904.1 | 0.5281581 | 1.381269 | 0.3824 | 0.702 | 0.108503754 | count | 1 |
| PRSS27 | 0.5281581 | 1.381269 | 0.3824 | 0.702 | 0.108503754 | count | 1 |
| CDH5 | 0.5281581 | 1.381269 | 0.3824 | 0.702 | 0.108503754 | count | 1 |
| LINC00896 | 0.5281581 | 1.381269 | 0.3824 | 0.702 | 0.108503754 | count | 1 |
| UBR4 | 0.0943936 | 0.2071059 | 0.4558 | 0.649 | 0.108592012 | count | 1 |
| C1RL | 0.124844 | 0.3115494 | 0.4007 | 0.689 | 0.108776713 | count | 1 |
| FDX1 | 0.0775155 | 0.0901284 | 0.8601 | 0.39 | 0.108941015 | count | 1 |
| SARS2 | 0.3133128 | 0.425475 | 0.7364 | 0.462 | 0.109109044 | count | 1 |
| EIF2B5 | 0.0838715 | 0.171838 | 0.4881 | 0.626 | 0.109151178 | count | 1 |
| OR4D1 | 0.1737872 | 0.4969779 | 0.3497 | 0.727 | 0.109166615 | count | 1 |
| MIF-AS1 | 0.3138908 | 0.59645 | 0.5263 | 0.599 | 0.109320241 | count | 1 |
| TRAF1 | 0.0834715 | 0.1422419 | 0.5868 | 0.557 | 0.109441599 | count | 1 |
| ERCC6 | 0.0884157 | 0.2249049 | 0.3931 | 0.694 | 0.109455331 | count | 1 |
| TMEM209 | 0.1010159 | 0.2640457 | 0.3826 | 0.702 | 0.109595046 | count | 1 |
| IDS | 0.0771112 | 0.0625592 | 1.2326 | 0.218 | 0.109595744 | count | 1 |
| MTPN | 0.0771199 | 0.0646849 | 1.1922 | 0.233 | 0.109608113 | count | 1 |
| RAB18 | 0.0797476 | 0.1132696 | 0.7041 | 0.481 | 0.109628173 | count | 1 |
| PODXL2 | 0.1313841 | 0.5031601 | 0.2611 | 0.794 | 0.109720754 | count | 1 |
| PRDX6 | 0.0772556 | 0.062945 | 1.2274 | 0.22 | 0.109793585 | count | 1 |
| RBM6 | 0.0811889 | 0.1048665 | 0.7742 | 0.439 | 0.109851339 | count | 1 |
| DNASE1L1 | 0.2385368 | 0.3639085 | 0.6555 | 0.512 | 0.109959021 | count | 1 |
| PACRGL | 0.2391501 | 0.4672467 | 0.5118 | 0.609 | 0.110251685 | count | 1 |
| PCSK1N | 0.0932598 | 0.3141352 | 0.2969 | 0.767 | 0.110260614 | count | 1 |
| SLC36A4 | 0.0944813 | 0.2359201 | 0.4005 | 0.689 | 0.110289121 | count | 1 |
| RHCE | 0.3166253 | 0.9224232 | 0.3433 | 0.731 | 0.110319802 | count | 1 |
| TTC30A | 0.3166253 | 0.9224232 | 0.3433 | 0.731 | 0.110319802 | count | 1 |
| NOMO2 | 0.3173528 | 0.680307 | 0.4665 | 0.641 | 0.110585856 | count | 1 |
| FXYD5 | 0.0769363 | 0.0317482 | 2.4233 | 0.0154 | 0.110684243 | count | 1 |
| ERI3 | 0.0876194 | 0.1824002 | 0.4804 | 0.631 | 0.110808889 | count | 1 |
| RAB9A | 0.0792679 | 0.0866771 | 0.9145 | 0.361 | 0.110826921 | count | 1 |
| TRIM37 | 0.0877412 | 0.2114686 | 0.4149 | 0.678 | 0.110963314 | count | 1 |
| LINC01089 | 0.0921792 | 0.2591051 | 0.3558 | 0.722 | 0.111342664 | count | 1 |
| KIAA0556 | 0.1162067 | 0.4081264 | 0.2847 | 0.776 | 0.111359031 | count | 1 |
| SIKE1 | 0.083517 | 0.1313566 | 0.6358 | 0.525 | 0.111382334 | count | 1 |
| SYCE1L | 0.5417467 | 0.4209516 | 1.287 | 0.198 | 0.11152366 | count | 1 |
| GORASP1 | 0.2018851 | 0.4411809 | 0.4576 | 0.647 | 0.111571374 | count | 1 |
| FAN1 | 0.1016637 | 0.385194 | 0.2639 | 0.792 | 0.111619722 | count | 1 |
| ZNF589 | 0.1612852 | 0.3760812 | 0.4289 | 0.668 | 0.111667677 | count | 1 |
| ARFGAP1 | 0.140824 | 0.2631823 | 0.5351 | 0.593 | 0.111833414 | count | 1 |
| LASP1 | 0.0915971 | 0.1482509 | 0.6179 | 0.537 | 0.112116642 | count | 1 |
| SELENOT | 0.0789997 | 0.0645036 | 1.2247 | 0.221 | 0.112145567 | count | 1 |
| HDAC2 | 0.0832142 | 0.1353806 | 0.6147 | 0.539 | 0.112150283 | count | 1 |
| RBM26-AS1 | 0.2029293 | 0.5264772 | 0.3854 | 0.7 | 0.112164372 | count | 1 |

| | | | | | | | |
|---|---|---|---|---|---|---|---|
| IFNL1 | 0.1503484 | 0.693068 | 0.2169 | 0.828 | 0.112312334 | count | 1 |
| KCNC4 | 0.5454778 | 0.5645006 | 0.9663 | 0.334 | 0.112354382 | count | 1 |
| GOT2 | 0.1002551 | 0.2501412 | 0.4008 | 0.689 | 0.11240052 | count | 1 |
| MT-ND4L | 0.0792657 | 0.0578295 | 1.3707 | 0.171 | 0.112463808 | count | 1 |
| FEN1 | 0.0957648 | 0.2759292 | 0.3471 | 0.729 | 0.112534195 | count | 1 |
| EFEMP2 | 0.3228475 | 0.612239 | 0.5273 | 0.598 | 0.112596993 | count | 1 |
| MUC1 | 0.3234583 | 0.659913 | 0.4902 | 0.624 | 0.112820734 | count | 1 |
| USP35 | 0.1423314 | 0.5656995 | 0.2516 | 0.801 | 0.113047906 | count | 1 |
| ATP1A3 | 0.2045901 | 0.5657545 | 0.3616 | 0.718 | 0.113107851 | count | 1 |
| INTS5 | 0.163399 | 0.3992662 | 0.4092 | 0.682 | 0.113159192 | count | 1 |
| IKZF4 | 0.1073001 | 0.4223355 | 0.2541 | 0.799 | 0.113336159 | count | 1 |
| MRPS18A | 0.0867734 | 0.1651692 | 0.5254 | 0.599 | 0.113619529 | count | 1 |
| UBE2W | 0.0867366 | 0.150113 | 0.5778 | 0.563 | 0.113730479 | count | 1 |
| KDELR1 | 0.0807215 | 0.0846053 | 0.9541 | 0.34 | 0.113754318 | count | 1 |
| CROT | 0.1048894 | 0.2558614 | 0.4099 | 0.682 | 0.113822877 | count | 1 |
| FICD | 0.1050302 | 0.2867586 | 0.3663 | 0.714 | 0.113976589 | count | 1 |
| ALOX5 | 0.1646355 | 0.8533928 | 0.1929 | 0.847 | 0.114031994 | count | 1 |
| RELA | 0.0838932 | 0.1356828 | 0.6183 | 0.536 | 0.114099957 | count | 1 |
| PPIA | 0.0793844 | 0.0285161 | 2.7838 | 0.0054 | 0.114163775 | count | 1 |
| IKBKG | 0.087351 | 0.1653822 | 0.5282 | 0.597 | 0.114212379 | count | 1 |
| TP53TG1 | 0.0828447 | 0.130487 | 0.6349 | 0.526 | 0.114263391 | count | 1 |
| FARP2 | 0.2067992 | 0.4879982 | 0.4238 | 0.672 | 0.114363398 | count | 1 |
| SH3TC1 | 0.1068175 | 0.3397455 | 0.3144 | 0.753 | 0.11443619 | count | 1 |
| TTI1 | 0.3280039 | 0.3426789 | 0.9572 | 0.339 | 0.114486966 | count | 1 |
| MTX1 | 0.0865849 | 0.159839 | 0.5417 | 0.588 | 0.114532065 | count | 1 |
| SWSAP1 | 0.1195595 | 0.241441 | 0.4952 | 0.62 | 0.114601656 | count | 1 |
| FANK1 | 0.1823101 | 0.5150386 | 0.354 | 0.723 | 0.114643484 | count | 1 |
| DCLRE1C | 0.0933346 | 0.1916252 | 0.4871 | 0.626 | 0.114707734 | count | 1 |
| ANKS1A | 0.1269917 | 0.3133178 | 0.4053 | 0.685 | 0.11480059 | count | 1 |
| ZNF304 | 0.2076816 | 0.6162537 | 0.337 | 0.736 | 0.114865105 | count | 1 |
| SRGAP2 | 0.0965847 | 0.2014943 | 0.4793 | 0.632 | 0.114875944 | count | 1 |
| PPIB | 0.0799507 | 0.0373229 | 2.1421 | 0.0323 | 0.114898813 | count | 1 |
| ZNF829 | 0.1827284 | 0.4886082 | 0.374 | 0.708 | 0.114912562 | count | 1 |
| IER3-AS1 | 0.1659291 | 0.655736 | 0.253 | 0.8 | 0.114945339 | count | 1 |
| DUSP12 | 0.0883152 | 0.1712836 | 0.5156 | 0.606 | 0.114945947 | count | 1 |
| RARRES3 | 0.0801192 | 0.0404843 | 1.979 | 0.0479 | 0.115050895 | count | 1 |
| CSAD | 0.0907656 | 0.1760117 | 0.5157 | 0.606 | 0.115093853 | count | 1 |
| FER | 0.1147902 | 0.3321328 | 0.3456 | 0.73 | 0.115144433 | count | 1 |
| ACSL6 | 0.1126855 | 0.2613433 | 0.4312 | 0.666 | 0.115210937 | count | 1 |
| ZNF30 | 0.330238 | 0.645758 | 0.5114 | 0.609 | 0.115306564 | count | 1 |
| ERLEC1 | 0.0830274 | 0.0952219 | 0.8719 | 0.383 | 0.115316292 | count | 1 |
| TMX3 | 0.087825 | 0.1613373 | 0.5444 | 0.586 | 0.115317084 | count | 1 |
| ABHD10 | 0.0882417 | 0.2191186 | 0.4027 | 0.687 | 0.115379226 | count | 1 |
| GPI | 0.0832995 | 0.1088023 | 0.7656 | 0.444 | 0.115483137 | count | 1 |
| SLC4A4 | 0.138202 | 0.3523098 | 0.3923 | 0.695 | 0.115490144 | count | 1 |

| | | | | | | | |
|---|---|---|---|---|---|---|---|
| CDCA7L | 0.2093718 | 0.5060457 | 0.4137 | 0.679 | 0.115826392 | count | 1 |
| COG7 | 0.1328681 | 0.3247847 | 0.4091 | 0.682 | 0.115852378 | count | 1 |
| TSC1 | 0.1180289 | 0.270642 | 0.4361 | 0.663 | 0.115899366 | count | 1 |
| LRRC40 | 0.0936482 | 0.2207638 | 0.4242 | 0.671 | 0.115953043 | count | 1 |
| LRP10 | 0.0836865 | 0.1012404 | 0.8266 | 0.409 | 0.115992113 | count | 1 |
| AC106739.2 | 0.5620768 | 0.3804852 | 1.4773 | 0.14 | 0.116057952 | count | 1 |
| NUP160 | 0.1001119 | 0.2005823 | 0.4991 | 0.618 | 0.116071365 | count | 1 |
| COA5 | 0.0860712 | 0.1492666 | 0.5766 | 0.564 | 0.11608623 | count | 1 |
| APOL6 | 0.0841204 | 0.095066 | 0.8849 | 0.376 | 0.116162447 | count | 1 |
| NACA | 0.0806198 | 0.019586 | 4.1162 | 3.94E-05 | 0.116190522 | count | 0.9446938 |
| FPGS | 0.0929555 | 0.2092854 | 0.4442 | 0.657 | 0.116242468 | count | 1 |
| NEURL1 | 0.1677727 | 0.5288223 | 0.3173 | 0.751 | 0.116247451 | count | 1 |
| AC100793.2 | 0.1677727 | 0.6185394 | 0.2712 | 0.786 | 0.116247451 | count | 1 |
| TFDP1 | 0.0880068 | 0.1740163 | 0.5057 | 0.613 | 0.116281924 | count | 1 |
| NEDD1 | 0.0882263 | 0.1498752 | 0.5887 | 0.556 | 0.116434452 | count | 1 |
| EIF3L | 0.0825198 | 0.0659351 | 1.2515 | 0.211 | 0.116536171 | count | 1 |
| IGBP1 | 0.0829287 | 0.0784943 | 1.0565 | 0.291 | 0.116564585 | count | 1 |
| BTF3 | 0.0810038 | 0.0250973 | 3.2276 | 0.00126 | 0.116601391 | count | 1 |
| ANKRD44 | 0.0848917 | 0.0932716 | 0.9102 | 0.363 | 0.11662314 | count | 1 |
| TIAM1 | 0.1089119 | 0.2257251 | 0.4825 | 0.629 | 0.116694507 | count | 1 |
| GRK6 | 0.0835692 | 0.0914485 | 0.9138 | 0.361 | 0.116700289 | count | 1 |
| CCL5 | 0.0810422 | 0.0392054 | 2.0671 | 0.0388 | 0.116851883 | count | 1 |
| AC024257.3 | 0.5659243 | 0.481013 | 1.1765 | 0.239 | 0.116918192 | count | 1 |
| REEP6 | 0.1858501 | 0.6164126 | 0.3015 | 0.763 | 0.116921426 | count | 1 |
| HERPUD2 | 0.0838704 | 0.0918438 | 0.9132 | 0.361 | 0.116964961 | count | 1 |
| DHTKD1 | 0.1295416 | 0.4647445 | 0.2787 | 0.78 | 0.117131199 | count | 1 |
| P4HB | 0.0830252 | 0.0737746 | 1.1254 | 0.261 | 0.117266231 | count | 1 |
| ZNF680 | 0.0950526 | 0.2572031 | 0.3696 | 0.712 | 0.117271275 | count | 1 |
| RPL35A | 0.0814217 | 0.0162488 | 5.0109 | 5.69E-07 | 0.117370199 | count | 0.013722004 |
| FCMR | 0.0858284 | 0.1060045 | 0.8097 | 0.418 | 0.117401549 | count | 1 |
| LCOR | 0.0895489 | 0.1339178 | 0.6687 | 0.504 | 0.117425032 | count | 1 |
| PIGV | 0.1081896 | 0.2947236 | 0.3671 | 0.714 | 0.117426351 | count | 1 |
| GTF2E1 | 0.1132173 | 0.2839428 | 0.3987 | 0.69 | 0.117776452 | count | 1 |
| IRGM | 0.255164 | 0.8244314 | 0.3095 | 0.757 | 0.117909669 | count | 1 |
| SSR2 | 0.0821139 | 0.0357561 | 2.2965 | 0.0217 | 0.117933055 | count | 1 |
| NR4A2 | 0.0828995 | 0.0603629 | 1.3734 | 0.17 | 0.118062219 | count | 1 |
| MED13 | 0.0870134 | 0.1251501 | 0.6953 | 0.487 | 0.118091074 | count | 1 |
| DDX6 | 0.0834699 | 0.0649326 | 1.2855 | 0.199 | 0.118140334 | count | 1 |
| ACADVL | 0.0855501 | 0.118589 | 0.7214 | 0.471 | 0.118205896 | count | 1 |
| SSH3 | 0.1414109 | 0.4626113 | 0.3057 | 0.76 | 0.118207956 | count | 1 |
| IRF3 | 0.0842858 | 0.0907692 | 0.9286 | 0.353 | 0.11821002 | count | 1 |
| MAP2K6 | 0.0970148 | 0.1917604 | 0.5059 | 0.613 | 0.11827329 | count | 1 |
| WDR3 | 0.1006771 | 0.1943243 | 0.5181 | 0.604 | 0.118331707 | count | 1 |
| TMEM163 | 0.2137978 | 0.8217391 | 0.2602 | 0.795 | 0.118345493 | count | 1 |
| KIFC2 | 0.1357068 | 0.3608421 | 0.3761 | 0.707 | 0.118357816 | count | 1 |

| | | | | | | | |
|---|---|---|---|---|---|---|---|
| CMTR2 | 0.1038284 | 0.2089434 | 0.4969 | 0.619 | 0.118540162 | count | 1 |
| NDUFA3 | 0.0835026 | 0.0730984 | 1.1423 | 0.253 | 0.118776978 | count | 1 |
| TMEM245 | 0.1109037 | 0.2287201 | 0.4849 | 0.628 | 0.118842677 | count | 1 |
| CD200 | 0.3401078 | 0.473342 | 0.7185 | 0.472 | 0.118933194 | count | 1 |
| CASTOR2 | 0.2577437 | 0.7072186 | 0.3644 | 0.716 | 0.119146212 | count | 1 |
| AC121761.1 | 0.2577437 | 0.7719912 | 0.3339 | 0.738 | 0.119146212 | count | 1 |
| TMBIM6 | 0.0831342 | 0.038707 | 2.1478 | 0.0318 | 0.119342484 | count | 1 |
| XXYLT1 | 0.1151657 | 0.3736482 | 0.3082 | 0.758 | 0.119818285 | count | 1 |
| ZNF138 | 0.0944927 | 0.1676786 | 0.5635 | 0.573 | 0.119832511 | count | 1 |
| LSG1 | 0.0904916 | 0.1475628 | 0.6132 | 0.54 | 0.1199745 | count | 1 |
| DPH5 | 0.0948533 | 0.1708239 | 0.5553 | 0.579 | 0.119982254 | count | 1 |
| SZRD1 | 0.0882469 | 0.126808 | 0.6959 | 0.487 | 0.12002824 | count | 1 |
| EPG5 | 0.1051535 | 0.2849399 | 0.369 | 0.712 | 0.120060685 | count | 1 |
| MPRIP | 0.0925445 | 0.1464427 | 0.632 | 0.527 | 0.120064328 | count | 1 |
| MR1 | 0.0929467 | 0.193797 | 0.4796 | 0.632 | 0.120162616 | count | 1 |
| ANKRD54 | 0.0991339 | 0.2069691 | 0.479 | 0.632 | 0.120333426 | count | 1 |
| CTBP1-DT | 0.1178047 | 0.2416845 | 0.4874 | 0.626 | 0.120486164 | count | 1 |
| ZNF662 | 0.5822012 | 0.920383 | 0.6326 | 0.527 | 0.120564756 | count | 1 |
| SCOC | 0.0899778 | 0.13637 | 0.6598 | 0.509 | 0.120737326 | count | 1 |
| HAR1B | 0.1335716 | 0.3994856 | 0.3344 | 0.738 | 0.120816525 | count | 1 |
| NFS1 | 0.1067369 | 0.2654078 | 0.4022 | 0.688 | 0.120828177 | count | 1 |
| RLN1 | 0.3458026 | 0.5200477 | 0.6649 | 0.506 | 0.121029841 | count | 1 |
| SEC61A2 | 0.1924418 | 0.4135407 | 0.4654 | 0.642 | 0.121167937 | count | 1 |
| TGFB1 | 0.0848465 | 0.0523407 | 1.621 | 0.105 | 0.121397825 | count | 1 |
| DNAJC3 | 0.0871632 | 0.0989804 | 0.8806 | 0.379 | 0.121474073 | count | 1 |
| LY9 | 0.0874417 | 0.1024051 | 0.8539 | 0.393 | 0.121504279 | count | 1 |
| PIK3CG | 0.099241 | 0.2472365 | 0.4014 | 0.688 | 0.121505602 | count | 1 |
| ZRANB3 | 0.1455148 | 0.445168 | 0.3269 | 0.744 | 0.121686034 | count | 1 |
| SAV1 | 0.2197336 | 0.2137122 | 1.0282 | 0.304 | 0.121728096 | count | 1 |
| TTC4 | 0.1627385 | 0.4548374 | 0.3578 | 0.721 | 0.121731541 | count | 1 |
| MDC1 | 0.1531941 | 0.3439925 | 0.4453 | 0.656 | 0.121810112 | count | 1 |
| RFX5 | 0.1270275 | 0.2426842 | 0.5234 | 0.601 | 0.121829841 | count | 1 |
| LINC00649 | 0.0952231 | 0.1697449 | 0.561 | 0.575 | 0.121895319 | count | 1 |
| SLC35F6 | 0.103102 | 0.2349232 | 0.4389 | 0.661 | 0.121947223 | count | 1 |
| DNTTIP2 | 0.0861051 | 0.0696791 | 1.2357 | 0.217 | 0.122045907 | count | 1 |
| PEX2 | 0.0900145 | 0.1418197 | 0.6347 | 0.526 | 0.122099544 | count | 1 |
| TTPAL | 0.1633327 | 0.2532774 | 0.6449 | 0.519 | 0.122183852 | count | 1 |
| TP53INP2 | 0.2641366 | 0.4150931 | 0.6363 | 0.525 | 0.122214015 | count | 1 |
| NMNAT1 | 0.1941709 | 0.4167585 | 0.4659 | 0.641 | 0.122282895 | count | 1 |
| NTAN1 | 0.088995 | 0.1225187 | 0.7264 | 0.468 | 0.122479084 | count | 1 |
| CLCN3 | 0.1057406 | 0.2218753 | 0.4766 | 0.634 | 0.122628683 | count | 1 |
| FAAP20 | 0.087533 | 0.09441 | 0.9272 | 0.354 | 0.122721808 | count | 1 |
| NPDC1 | 0.0959249 | 0.2400062 | 0.3997 | 0.689 | 0.122795944 | count | 1 |
| ZNF845 | 0.1408022 | 0.4469985 | 0.315 | 0.753 | 0.122857983 | count | 1 |
| DNASE2 | 0.0911726 | 0.1578393 | 0.5776 | 0.564 | 0.122891055 | count | 1 |

| | | | | | | | |
|---|---|---|---|---|---|---|---|
| PRKCZ | 0.1281453 | 0.2609289 | 0.4911 | 0.623 | 0.122912405 | count | 1 |
| CHMP6 | 0.0971675 | 0.1863578 | 0.5214 | 0.602 | 0.122917661 | count | 1 |
| SSBP4 | 0.0866872 | 0.0728353 | 1.1902 | 0.234 | 0.122976274 | count | 1 |
| ASCC1 | 0.1000539 | 0.2163788 | 0.4624 | 0.644 | 0.122994092 | count | 1 |
| CADM1 | 0.1645585 | 0.5717947 | 0.2878 | 0.774 | 0.123117086 | count | 1 |
| HAUS6 | 0.0918254 | 0.1773573 | 0.5177 | 0.605 | 0.123121364 | count | 1 |
| B3GNT8 | 0.2222607 | 0.5071908 | 0.4382 | 0.661 | 0.123169643 | count | 1 |
| TUBGCP4 | 0.1646415 | 0.3227929 | 0.5101 | 0.61 | 0.123180295 | count | 1 |
| NRDE2 | 0.1055339 | 0.2075276 | 0.5085 | 0.611 | 0.123251151 | count | 1 |
| HNRNPUL2 | 0.113524 | 0.2661521 | 0.4265 | 0.67 | 0.123253639 | count | 1 |
| RCE1 | 0.0935558 | 0.168741 | 0.5544 | 0.579 | 0.123330961 | count | 1 |
| DCTN2 | 0.0884923 | 0.1034861 | 0.8551 | 0.393 | 0.123349839 | count | 1 |
| TTC17 | 0.1071716 | 0.1529133 | 0.7009 | 0.483 | 0.123365964 | count | 1 |
| CDC42 | 0.0859465 | 0.0363477 | 2.3646 | 0.0181 | 0.123466624 | count | 1 |
| CLECL1 | 0.1138214 | 0.3574208 | 0.3185 | 0.75 | 0.123578616 | count | 1 |
| TRGC2 | 0.0872961 | 0.1108592 | 0.7875 | 0.431 | 0.123686871 | count | 1 |
| MICAL1 | 0.1039803 | 0.2256271 | 0.4609 | 0.645 | 0.12370928 | count | 1 |
| OGG1 | 0.0969289 | 0.2085848 | 0.4647 | 0.642 | 0.123810958 | count | 1 |
| C6orf106 | 0.0965603 | 0.1740278 | 0.5549 | 0.579 | 0.123874703 | count | 1 |
| BBS4 | 0.1117459 | 0.2357549 | 0.474 | 0.636 | 0.124102946 | count | 1 |
| CLOCK | 0.1174512 | 0.2114971 | 0.5553 | 0.579 | 0.124135906 | count | 1 |
| FKRP | 0.3543872 | 0.4116337 | 0.8609 | 0.389 | 0.124196085 | count | 1 |
| TM6SF1 | 0.1971631 | 0.9399059 | 0.2098 | 0.834 | 0.124213335 | count | 1 |
| COL9A2 | 0.2684666 | 0.5747615 | 0.4671 | 0.64 | 0.124294645 | count | 1 |
| ANO8 | 0.3547405 | 0.6953792 | 0.5101 | 0.61 | 0.124326505 | count | 1 |
| SORD | 0.1296984 | 0.4208037 | 0.3082 | 0.758 | 0.124416831 | count | 1 |
| ARL16 | 0.0995454 | 0.1286765 | 0.7736 | 0.439 | 0.124508569 | count | 1 |
| GAB3 | 0.1090842 | 0.2143851 | 0.5088 | 0.611 | 0.124572144 | count | 1 |
| RPRD2 | 0.0959444 | 0.2076268 | 0.4621 | 0.644 | 0.124897312 | count | 1 |
| LINC02018 | 0.2699505 | 0.7951122 | 0.3395 | 0.734 | 0.125008199 | count | 1 |
| FMO4 | 0.6021704 | 0.7321248 | 0.8225 | 0.411 | 0.125054122 | count | 1 |
| WIZ | 0.1339607 | 0.3654789 | 0.3665 | 0.714 | 0.125058658 | count | 1 |
| CGGBP1 | 0.0889979 | 0.0829799 | 1.0725 | 0.284 | 0.125061153 | count | 1 |
| AARS | 0.167492 | 0.3147501 | 0.5321 | 0.595 | 0.125351366 | count | 1 |
| NFKBIE | 0.0994256 | 0.1790334 | 0.5553 | 0.579 | 0.125446565 | count | 1 |
| AGTRAP | 0.0895328 | 0.1017353 | 0.8801 | 0.379 | 0.125447892 | count | 1 |
| NPIPB4 | 0.1684366 | 0.4206591 | 0.4004 | 0.689 | 0.126071076 | count | 1 |
| AP3S2 | 0.1255967 | 0.3640957 | 0.345 | 0.73 | 0.126079271 | count | 1 |
| ELP1 | 0.1393254 | 0.3537652 | 0.3938 | 0.694 | 0.126082248 | count | 1 |
| AC114939.1 | 0.6070142 | 1.095044 | 0.5543 | 0.579 | 0.126145591 | count | 1 |
| ARHGDIA | 0.0888107 | 0.0619035 | 1.4347 | 0.151 | 0.126181074 | count | 1 |
| MSANTD2 | 0.1148437 | 0.286171 | 0.4013 | 0.688 | 0.126181976 | count | 1 |
| HNRNPA0 | 0.0888111 | 0.0545859 | 1.627 | 0.104 | 0.126186098 | count | 1 |
| RHEBL1 | 0.1212765 | 0.3700652 | 0.3277 | 0.743 | 0.126225182 | count | 1 |
| NDUFB5 | 0.0897589 | 0.0841826 | 1.0662 | 0.286 | 0.126387382 | count | 1 |

| | | | | | | | |
|---|---|---|---|---|---|---|---|
| RNF144B | 0.3608143 | 0.6812061 | 0.5297 | 0.596 | 0.126570997 | count | 1 |
| BORCS8 | 0.097749 | 0.1584633 | 0.6169 | 0.537 | 0.126612232 | count | 1 |
| DAG1 | 0.1319827 | 0.3893365 | 0.339 | 0.735 | 0.126630137 | count | 1 |
| CAD | 0.1400111 | 0.4295866 | 0.3259 | 0.745 | 0.126710094 | count | 1 |
| PIP4P2 | 0.1048602 | 0.1886508 | 0.5558 | 0.578 | 0.126720474 | count | 1 |
| TSN | 0.0962446 | 0.1404735 | 0.6851 | 0.493 | 0.126882309 | count | 1 |
| IL24 | 0.6104835 | 0.7282688 | 0.8383 | 0.402 | 0.126927925 | count | 1 |
| AP004609.3 | 0.3620016 | 0.4245639 | 0.8526 | 0.394 | 0.127010082 | count | 1 |
| MAL | 0.0917231 | 0.111309 | 0.824 | 0.41 | 0.127078143 | count | 1 |
| CLIP4 | 0.0981582 | 0.1797001 | 0.5462 | 0.585 | 0.127143487 | count | 1 |
| C3orf18 | 0.1519785 | 0.4393379 | 0.3459 | 0.729 | 0.12716908 | count | 1 |
| MBD6 | 0.1075031 | 0.2076862 | 0.5176 | 0.605 | 0.12717598 | count | 1 |
| MTMR12 | 0.1096559 | 0.2291269 | 0.4786 | 0.632 | 0.127191765 | count | 1 |
| TXNDC12 | 0.0906279 | 0.0910836 | 0.995 | 0.32 | 0.127405421 | count | 1 |
| LINC00921 | 0.2752731 | 0.4107242 | 0.6702 | 0.503 | 0.127569762 | count | 1 |
| SLC25A25 | 0.1366384 | 0.2328806 | 0.5867 | 0.557 | 0.127585744 | count | 1 |
| ZNF438 | 0.275433 | 0.368227 | 0.748 | 0.455 | 0.127646768 | count | 1 |
| EXOSC4 | 0.097919 | 0.2031304 | 0.482 | 0.63 | 0.127869804 | count | 1 |
| DPYD | 0.0989357 | 0.1814704 | 0.5452 | 0.586 | 0.127923676 | count | 1 |
| PDCD7 | 0.0929708 | 0.0915745 | 1.0152 | 0.31 | 0.127998878 | count | 1 |
| TUBGCP6 | 0.1304283 | 0.3355118 | 0.3887 | 0.697 | 0.128190856 | count | 1 |
| RPS23 | 0.0889508 | 0.0160974 | 5.5258 | 3.53E-08 | 0.128261418 | count | 0.000852919 |
| GTF2A2 | 0.0907514 | 0.0757247 | 1.1984 | 0.231 | 0.128300181 | count | 1 |
| RPS4Y1 | 0.0891642 | 0.0291142 | 3.0626 | 0.00221 | 0.128310568 | count | 1 |
| VPS51 | 0.0947637 | 0.1010963 | 0.9374 | 0.349 | 0.128399659 | count | 1 |
| TNFRSF14-AS1 | 0.1850041 | 0.3368175 | 0.5493 | 0.583 | 0.128442129 | count | 1 |
| FBXO33 | 0.0981195 | 0.1452239 | 0.6756 | 0.499 | 0.128507147 | count | 1 |
| PUSL1 | 0.1024529 | 0.2085071 | 0.4914 | 0.623 | 0.128544999 | count | 1 |
| RAB2B | 0.1069403 | 0.1944991 | 0.5498 | 0.582 | 0.128611843 | count | 1 |
| ARHGAP12 | 0.1184931 | 0.2058459 | 0.5756 | 0.565 | 0.128684827 | count | 1 |
| LINC00944 | 0.1128214 | 0.2693584 | 0.4189 | 0.675 | 0.12886298 | count | 1 |
| PREP | 0.118712 | 0.2093345 | 0.5671 | 0.571 | 0.128924147 | count | 1 |
| CPTP | 0.1044774 | 0.2139896 | 0.4882 | 0.625 | 0.128939767 | count | 1 |
| KLHL23 | 0.6199463 | 0.66607 | 0.9308 | 0.352 | 0.129064269 | count | 1 |
| RBM22 | 0.0926744 | 0.0918483 | 1.009 | 0.313 | 0.129089219 | count | 1 |
| RPS12 | 0.0895373 | 0.0155611 | 5.7539 | 9.49E-09 | 0.129140184 | count | 0.000229478 |
| DKK3 | 0.1384705 | 0.3017689 | 0.4589 | 0.646 | 0.129315364 | count | 1 |
| FALEC | 0.1349645 | 0.7387988 | 0.1827 | 0.855 | 0.129520325 | count | 1 |
| PARPBP | 0.3689509 | 0.4873251 | 0.7571 | 0.449 | 0.129582918 | count | 1 |
| FLT4 | 0.279516 | 0.9614742 | 0.2907 | 0.771 | 0.129614098 | count | 1 |
| C9orf43 | 0.279516 | 1.0809647 | 0.2586 | 0.796 | 0.129614098 | count | 1 |
| AC109460.1 | 0.279516 | 1.1271202 | 0.248 | 0.804 | 0.129614098 | count | 1 |
| MARCKSL1 | 0.0922505 | 0.0919761 | 1.003 | 0.316 | 0.129700832 | count | 1 |
| YEATS4 | 0.0965874 | 0.1424277 | 0.6782 | 0.498 | 0.129722048 | count | 1 |
| CBX7 | 0.112671 | 0.2273939 | 0.4955 | 0.62 | 0.129729445 | count | 1 |

| | | | | | | | |
|---|---|---|---|---|---|---|---|
| NGRN | 0.2058564 | 0.3066927 | 0.6712 | 0.502 | 0.129829109 | count | 1 |
| FAM199X | 0.1008408 | 0.177705 | 0.5675 | 0.57 | 0.129902992 | count | 1 |
| CHID1 | 0.0984321 | 0.1774295 | 0.5548 | 0.579 | 0.130083127 | count | 1 |
| METTL1 | 0.1296817 | 0.3504919 | 0.37 | 0.711 | 0.13021674 | count | 1 |
| WDR35 | 0.13575 | 0.4433359 | 0.3062 | 0.759 | 0.130281893 | count | 1 |
| RTTN | 0.3709126 | 0.4337666 | 0.8551 | 0.393 | 0.130309965 | count | 1 |
| EML2 | 0.0985042 | 0.1625967 | 0.6058 | 0.545 | 0.130328495 | count | 1 |
| TMEM106B | 0.0980689 | 0.1302517 | 0.7529 | 0.452 | 0.130573767 | count | 1 |
| SF3B4 | 0.0946796 | 0.108857 | 0.8698 | 0.384 | 0.130641948 | count | 1 |
| DDI2 | 0.1167342 | 0.3311909 | 0.3525 | 0.725 | 0.130986565 | count | 1 |
| 5-Mar | 0.0997721 | 0.1535884 | 0.6496 | 0.516 | 0.131036581 | count | 1 |
| ARMC6 | 0.108457 | 0.2564859 | 0.4229 | 0.672 | 0.131084675 | count | 1 |
| GATAD1 | 0.1080107 | 0.1705785 | 0.6332 | 0.527 | 0.13115101 | count | 1 |
| ESRRA | 0.1004838 | 0.1631991 | 0.6157 | 0.538 | 0.13122646 | count | 1 |
| SHLD1 | 0.102911 | 0.2014038 | 0.511 | 0.609 | 0.13176294 | count | 1 |
| COX4I1 | 0.0915542 | 0.0257265 | 3.5588 | 0.000378 | 0.131769624 | count | 1 |
| SNX17 | 0.0941643 | 0.0936649 | 1.0053 | 0.315 | 0.132040884 | count | 1 |
| P3H1 | 0.1249647 | 0.2872158 | 0.4351 | 0.664 | 0.132137542 | count | 1 |
| LSM6 | 0.0940676 | 0.0872281 | 1.0784 | 0.281 | 0.132614794 | count | 1 |
| SNAP23 | 0.0968153 | 0.1179746 | 0.8206 | 0.412 | 0.132720117 | count | 1 |
| ABCD1 | 0.3774681 | 0.5508169 | 0.6853 | 0.493 | 0.132742057 | count | 1 |
| COG1 | 0.1350241 | 0.2596935 | 0.5199 | 0.603 | 0.132751844 | count | 1 |
| TIMM44 | 0.104254 | 0.2183972 | 0.4774 | 0.633 | 0.132889185 | count | 1 |
| FLNB | 0.2106766 | 0.5127946 | 0.4108 | 0.681 | 0.132947502 | count | 1 |
| CAPN12 | 0.104816 | 0.2040272 | 0.5137 | 0.607 | 0.132962448 | count | 1 |
| RAPGEF1 | 0.1041309 | 0.1706383 | 0.6102 | 0.542 | 0.133035628 | count | 1 |
| EDA | 0.2866499 | 0.4181383 | 0.6855 | 0.493 | 0.133056104 | count | 1 |
| PPP3CC | 0.0993147 | 0.1276913 | 0.7778 | 0.437 | 0.133070343 | count | 1 |
| PTOV1 | 0.0988778 | 0.1582466 | 0.6248 | 0.532 | 0.133102622 | count | 1 |
| S100A5 | 0.2871786 | 0.6245755 | 0.4598 | 0.646 | 0.133311423 | count | 1 |
| RUNX2 | 0.1088488 | 0.1910818 | 0.5696 | 0.569 | 0.133313464 | count | 1 |
| ZNF146 | 0.1041449 | 0.1524722 | 0.683 | 0.495 | 0.133347024 | count | 1 |
| PDRG1 | 0.1022642 | 0.1942199 | 0.5265 | 0.599 | 0.133556748 | count | 1 |
| SLA2 | 0.106132 | 0.164735 | 0.6443 | 0.519 | 0.133562223 | count | 1 |
| CNTLN | 0.2878158 | 0.8364215 | 0.3441 | 0.731 | 0.133619192 | count | 1 |
| ARHGAP32 | 0.2878158 | 1.048003 | 0.2746 | 0.784 | 0.133619192 | count | 1 |
| AC083862.2 | 0.3798449 | 0.7177803 | 0.5292 | 0.597 | 0.133624746 | count | 1 |
| PRNP | 0.0964433 | 0.0901001 | 1.0704 | 0.285 | 0.133656442 | count | 1 |
| MVB12B | 0.1393676 | 0.3627351 | 0.3842 | 0.701 | 0.133790343 | count | 1 |
| NEURL4 | 0.2408574 | 0.5343253 | 0.4508 | 0.652 | 0.133804062 | count | 1 |
| ANKRD36B | 0.1074861 | 0.1710952 | 0.6282 | 0.53 | 0.13404934 | count | 1 |
| NDUFAF1 | 0.143518 | 0.2916585 | 0.4921 | 0.623 | 0.134082918 | count | 1 |
| SCD5 | 0.1535192 | 0.7706627 | 0.1992 | 0.842 | 0.134105774 | count | 1 |
| ANKRD36 | 0.1006658 | 0.132258 | 0.7611 | 0.447 | 0.134166822 | count | 1 |
| ZNF398 | 0.1791172 | 0.339332 | 0.5279 | 0.598 | 0.134218071 | count | 1 |

| | | | | | | | |
|---|---|---|---|---|---|---|---|
| FBRS | 0.104018 | 0.1605723 | 0.6478 | 0.517 | 0.134262892 | count | 1 |
| PQLC3 | 0.0966731 | 0.1149457 | 0.841 | 0.4 | 0.134342298 | count | 1 |
| AASDHPPT | 0.0981769 | 0.123801 | 0.793 | 0.428 | 0.134425578 | count | 1 |
| ARHGAP33 | 0.3820122 | 0.5388973 | 0.7089 | 0.478 | 0.134430133 | count | 1 |
| RMC1 | 0.1031086 | 0.1746889 | 0.5902 | 0.555 | 0.134456573 | count | 1 |
| VTI1B | 0.096767 | 0.1105476 | 0.8753 | 0.381 | 0.134794323 | count | 1 |
| ZMYND11 | 0.1046942 | 0.1516627 | 0.6903 | 0.49 | 0.134879717 | count | 1 |
| FN3KRP | 0.1041168 | 0.1958197 | 0.5317 | 0.595 | 0.134880489 | count | 1 |
| HIBADH | 0.1133319 | 0.2267752 | 0.4998 | 0.617 | 0.134885913 | count | 1 |
| CCDC22 | 0.1086474 | 0.1805913 | 0.6016 | 0.547 | 0.135056148 | count | 1 |
| MRPL50 | 0.097891 | 0.128852 | 0.7597 | 0.447 | 0.135077494 | count | 1 |
| RPS3 | 0.0936754 | 0.0134956 | 6.9412 | 4.64E-12 | 0.135088821 | count | 1.12E-07 |
| TRIM21 | 0.1071484 | 0.2137052 | 0.5014 | 0.616 | 0.135220612 | count | 1 |
| PLA2G16 | 0.0973107 | 0.1059442 | 0.9185 | 0.358 | 0.135422655 | count | 1 |
| RPS7 | 0.0939417 | 0.0143701 | 6.5373 | 7.20E-11 | 0.135452646 | count | 1.74E-06 |
| AL669831.5 | 0.3848372 | 0.4878922 | 0.7888 | 0.43 | 0.135480493 | count | 1 |
| LCLAT1 | 0.1949763 | 0.2481274 | 0.7858 | 0.432 | 0.135519309 | count | 1 |
| TET2 | 0.1196517 | 0.1874482 | 0.6383 | 0.523 | 0.135534169 | count | 1 |
| PROSER3 | 0.2146776 | 0.3800223 | 0.5649 | 0.572 | 0.135538389 | count | 1 |
| CTDSPL2 | 0.1033367 | 0.1251747 | 0.8255 | 0.409 | 0.13572814 | count | 1 |
| C9orf66 | 0.3858318 | 0.7642741 | 0.5048 | 0.614 | 0.135850426 | count | 1 |
| TTC21B | 0.1267226 | 0.2181399 | 0.5809 | 0.561 | 0.135920097 | count | 1 |
| SMG5 | 0.1957442 | 0.3318225 | 0.5899 | 0.555 | 0.13606485 | count | 1 |
| TRAF2 | 0.1143324 | 0.2456496 | 0.4654 | 0.642 | 0.136082129 | count | 1 |
| LRRC56 | 0.1957816 | 0.5689421 | 0.3441 | 0.731 | 0.13609142 | count | 1 |
| YTHDF3 | 0.122506 | 0.2037722 | 0.6012 | 0.548 | 0.136129819 | count | 1 |
| ZNF576 | 0.1158218 | 0.2620371 | 0.442 | 0.659 | 0.136219811 | count | 1 |
| EIF4E2 | 0.0992461 | 0.1199328 | 0.8275 | 0.408 | 0.136315106 | count | 1 |
| SLC1A4 | 0.2934272 | 0.3839328 | 0.7643 | 0.445 | 0.136331489 | count | 1 |
| SLC12A2 | 0.138715 | 0.2481598 | 0.559 | 0.576 | 0.136416793 | count | 1 |
| AKT1S1 | 0.127295 | 0.3042427 | 0.4184 | 0.676 | 0.136538585 | count | 1 |
| RPL19 | 0.0947197 | 0.0141781 | 6.6807 | 2.77E-11 | 0.136601988 | count | 6.71E-07 |
| COG8 | 0.653295 | 0.5903196 | 1.1067 | 0.269 | 0.136620835 | count | 1 |
| FAM45A | 0.1008919 | 0.1447775 | 0.6969 | 0.486 | 0.136632593 | count | 1 |
| TMCC3 | 0.1716152 | 0.4362559 | 0.3934 | 0.694 | 0.136709166 | count | 1 |
| NCALD | 0.1275099 | 0.236605 | 0.5389 | 0.59 | 0.136770796 | count | 1 |
| TMED10 | 0.0970718 | 0.0700287 | 1.3862 | 0.166 | 0.137289838 | count | 1 |
| TFDP2 | 0.1036957 | 0.1523082 | 0.6808 | 0.496 | 0.1375145 | count | 1 |
| TARBP2 | 0.1162548 | 0.2574638 | 0.4515 | 0.652 | 0.13757874 | count | 1 |
| SRP9 | 0.0966585 | 0.0648389 | 1.4907 | 0.136 | 0.137609365 | count | 1 |
| PRR34-AS1 | 0.1369875 | 0.2333055 | 0.5872 | 0.557 | 0.137621719 | count | 1 |
| PLXDC1 | 0.1400061 | 0.3041589 | 0.4603 | 0.645 | 0.137699235 | count | 1 |
| CCDC102A | 0.1128898 | 0.267791 | 0.4216 | 0.673 | 0.137704684 | count | 1 |
| SIMC1 | 0.1254596 | 0.2825653 | 0.444 | 0.657 | 0.137925382 | count | 1 |
| GCC1 | 0.1436523 | 0.2910909 | 0.4935 | 0.622 | 0.13794803 | count | 1 |

| | | | | | | | |
|---|---|---|---|---|---|---|---|
| AC092123.1 | 0.1984921 | 0.6564335 | 0.3024 | 0.762 | 0.138017821 | count | 1 |
| ZNF771 | 0.1374109 | 0.3122733 | 0.44 | 0.66 | 0.138051077 | count | 1 |
| ZNF420 | 0.1403831 | 0.2925819 | 0.4798 | 0.631 | 0.138073748 | count | 1 |
| RDH11 | 0.104425 | 0.129994 | 0.8033 | 0.422 | 0.13833252 | count | 1 |
| PPRC1 | 0.130838 | 0.307005 | 0.4262 | 0.67 | 0.138397108 | count | 1 |
| AC027031.2 | 0.6613968 | 0.9181696 | 0.7203 | 0.471 | 0.138462854 | count | 1 |
| BCO2 | 0.661568 | 0.8582763 | 0.7708 | 0.441 | 0.138501802 | count | 1 |
| AL449266.1 | 0.661568 | 0.9349003 | 0.7076 | 0.479 | 0.138501802 | count | 1 |
| CSGALNACT1 | 0.1145916 | 0.2147013 | 0.5337 | 0.594 | 0.13853063 | count | 1 |
| RNF169 | 0.1010959 | 0.1279704 | 0.79 | 0.43 | 0.138594999 | count | 1 |
| AL512791.2 | 0.662421 | 0.5691619 | 1.1639 | 0.245 | 0.138695915 | count | 1 |
| TMA16 | 0.1040936 | 0.1470574 | 0.7078 | 0.479 | 0.138743679 | count | 1 |
| FAM217B | 0.1071131 | 0.1817097 | 0.5895 | 0.556 | 0.138771823 | count | 1 |
| RAB5C | 0.0983368 | 0.0814249 | 1.2077 | 0.227 | 0.13877545 | count | 1 |
| DGCR8 | 0.1588322 | 0.3605279 | 0.4406 | 0.66 | 0.138811785 | count | 1 |
| AP4E1 | 0.1535233 | 0.3298295 | 0.4655 | 0.642 | 0.139095741 | count | 1 |
| ELK1 | 0.1198824 | 0.2515121 | 0.4766 | 0.634 | 0.139117237 | count | 1 |
| NR2C2 | 0.1450937 | 0.2468876 | 0.5877 | 0.557 | 0.139347251 | count | 1 |
| RPL6 | 0.0966823 | 0.0157767 | 6.1282 | 9.91E-10 | 0.139396075 | count | 2.40E-05 |
| RPL8 | 0.0967558 | 0.0169032 | 5.7241 | 1.13E-08 | 0.139487542 | count | 0.000273211 |
| CCDC28B | 0.1084774 | 0.1863388 | 0.5822 | 0.561 | 0.13949046 | count | 1 |
| SCAPER | 0.1083385 | 0.1375068 | 0.7879 | 0.431 | 0.139587189 | count | 1 |
| DTD1 | 0.1026954 | 0.1157304 | 0.8874 | 0.375 | 0.139633109 | count | 1 |
| ZNF669 | 0.119607 | 0.2197126 | 0.5444 | 0.586 | 0.139772427 | count | 1 |
| ZMYM5 | 0.101186 | 0.1099761 | 0.9201 | 0.358 | 0.139792066 | count | 1 |
| RHBDD2 | 0.1011613 | 0.1000861 | 1.0107 | 0.312 | 0.139797416 | count | 1 |
| AL359504.2 | 0.2513206 | 0.8133414 | 0.309 | 0.757 | 0.139807349 | count | 1 |
| BCAP31 | 0.0983255 | 0.0693663 | 1.4175 | 0.156 | 0.139842723 | count | 1 |
| ATP10D | 0.115191 | 0.2115184 | 0.5446 | 0.586 | 0.139905921 | count | 1 |
| PFKL | 0.1057604 | 0.1506344 | 0.7021 | 0.483 | 0.139948301 | count | 1 |
| TMA7 | 0.097355 | 0.0317255 | 3.0687 | 0.00217 | 0.139972517 | count | 1 |
| R3HDM2 | 0.0995643 | 0.0813035 | 1.2246 | 0.221 | 0.140031475 | count | 1 |
| TMEM234 | 0.1236766 | 0.3156837 | 0.3918 | 0.695 | 0.140120819 | count | 1 |
| PRDM1 | 0.0985645 | 0.0561105 | 1.7566 | 0.0791 | 0.140147508 | count | 1 |
| AC024060.1 | 0.3015355 | 0.5715245 | 0.5276 | 0.598 | 0.140257021 | count | 1 |
| POFUT2 | 0.1604923 | 0.2537385 | 0.6325 | 0.527 | 0.140283042 | count | 1 |
| GRWD1 | 0.1136672 | 0.1913685 | 0.594 | 0.553 | 0.140323867 | count | 1 |
| AGER | 0.6702163 | 0.529604 | 1.2655 | 0.206 | 0.140470682 | count | 1 |
| CWF19L1 | 0.1134898 | 0.2537284 | 0.4473 | 0.655 | 0.140611252 | count | 1 |
| CCDC69 | 0.1032821 | 0.1277581 | 0.8084 | 0.419 | 0.140650612 | count | 1 |
| FANCA | 0.1877547 | 0.3663193 | 0.5125 | 0.608 | 0.140818751 | count | 1 |
| ZBTB22 | 0.1170586 | 0.2606527 | 0.4491 | 0.653 | 0.140834718 | count | 1 |
| MRPS28 | 0.1107513 | 0.1865498 | 0.5937 | 0.553 | 0.140861157 | count | 1 |
| NKIRAS2 | 0.1155442 | 0.2123492 | 0.5441 | 0.586 | 0.140955715 | count | 1 |
| MREG | 0.2231491 | 0.4263188 | 0.5234 | 0.601 | 0.141031508 | count | 1 |

| | | | | | | | |
|---|---|---|---|---|---|---|---|
| AMN | 0.3033175 | 0.5456794 | 0.5559 | 0.578 | 0.141120755 | count | 1 |
| SAMD4B | 0.1135765 | 0.2283077 | 0.4975 | 0.619 | 0.141205313 | count | 1 |
| ZMIZ2 | 0.1378802 | 0.3331389 | 0.4139 | 0.679 | 0.141205532 | count | 1 |
| EEF1B2 | 0.0981169 | 0.0239203 | 4.1018 | 4.20E-05 | 0.141293473 | count | 1 |
| BAZ2B | 0.1080318 | 0.1680488 | 0.6429 | 0.52 | 0.141518918 | count | 1 |
| CASP8AP2 | 0.1071469 | 0.1337912 | 0.8009 | 0.423 | 0.14162393 | count | 1 |
| DET1 | 0.2244199 | 0.489547 | 0.4584 | 0.647 | 0.141856376 | count | 1 |
| PLA2G12A | 0.1693198 | 0.3271842 | 0.5175 | 0.605 | 0.141909374 | count | 1 |
| PGGHG | 0.1242519 | 0.1910662 | 0.6503 | 0.516 | 0.141995573 | count | 1 |
| DEPDC1B | 0.6782952 | 0.9059895 | 0.7487 | 0.454 | 0.142312257 | count | 1 |
| SPRY1 | 0.1522889 | 0.1991405 | 0.7647 | 0.444 | 0.142375635 | count | 1 |
| TEFM | 0.1268297 | 0.2611692 | 0.4856 | 0.627 | 0.142387103 | count | 1 |
| ANKRA2 | 0.1131677 | 0.1973643 | 0.5734 | 0.566 | 0.142445522 | count | 1 |
| REEP4 | 0.1390818 | 0.2847024 | 0.4885 | 0.625 | 0.142447264 | count | 1 |
| MTG2 | 0.1368418 | 0.2114367 | 0.6472 | 0.518 | 0.142565387 | count | 1 |
| ARL14EP | 0.1054718 | 0.1294802 | 0.8146 | 0.415 | 0.142844084 | count | 1 |
| ATP11C | 0.1371538 | 0.2610132 | 0.5255 | 0.599 | 0.142893219 | count | 1 |
| NOSIP | 0.1009125 | 0.0732007 | 1.3786 | 0.168 | 0.142900313 | count | 1 |
| ZNF100 | 0.1300032 | 0.2528969 | 0.5141 | 0.607 | 0.142955345 | count | 1 |
| LEPROT | 0.104202 | 0.1139941 | 0.9141 | 0.361 | 0.14296933 | count | 1 |
| KBTBD11 | 0.4050299 | 0.8449974 | 0.4793 | 0.632 | 0.143008309 | count | 1 |
| ABHD1 | 0.4050299 | 0.9199343 | 0.4403 | 0.66 | 0.143008309 | count | 1 |
| SAP30L-AS1 | 0.4050299 | 0.9199343 | 0.4403 | 0.66 | 0.143008309 | count | 1 |
| AL138966.2 | 0.4050299 | 0.9310541 | 0.435 | 0.664 | 0.143008309 | count | 1 |
| LINC01358 | 0.4050299 | 1.0308758 | 0.3929 | 0.694 | 0.143008309 | count | 1 |
| EGOT | 0.4050299 | 1.0308758 | 0.3929 | 0.694 | 0.143008309 | count | 1 |
| CGN | 0.4050299 | 1.0408108 | 0.3891 | 0.697 | 0.143008309 | count | 1 |
| RNF217 | 0.4050299 | 1.0408108 | 0.3891 | 0.697 | 0.143008309 | count | 1 |
| ZDHHC15 | 0.4050299 | 1.0408108 | 0.3891 | 0.697 | 0.143008309 | count | 1 |
| AL136115.2 | 0.4050299 | 1.0828362 | 0.374 | 0.708 | 0.143008309 | count | 1 |
| DENND6A-DT | 0.4050299 | 1.092299 | 0.3708 | 0.711 | 0.143008309 | count | 1 |
| SCIN | 0.4050299 | 1.231316 | 0.3289 | 0.742 | 0.143008309 | count | 1 |
| AC009812.1 | 0.6815844 | 0.5148114 | 1.3239 | 0.186 | 0.143062672 | count | 1 |
| CEBPA | 0.1579456 | 0.3411806 | 0.4629 | 0.643 | 0.143154849 | count | 1 |
| AC010654.1 | 0.3075117 | 0.5142109 | 0.598 | 0.55 | 0.143155076 | count | 1 |
| ZNF513 | 0.1533236 | 0.3498253 | 0.4383 | 0.661 | 0.143354607 | count | 1 |
| HUS1 | 0.1077481 | 0.1546154 | 0.6969 | 0.486 | 0.143485645 | count | 1 |
| TADA3 | 0.1033632 | 0.1064632 | 0.9709 | 0.332 | 0.143766591 | count | 1 |
| DHCR7 | 0.1201321 | 0.2463471 | 0.4877 | 0.626 | 0.143803494 | count | 1 |
| PXMP2 | 0.1324308 | 0.3459767 | 0.3828 | 0.702 | 0.143933577 | count | 1 |
| MIF4GD | 0.1034981 | 0.1241915 | 0.8334 | 0.405 | 0.143954381 | count | 1 |
| CCDC61 | 0.1260523 | 0.2730254 | 0.4617 | 0.644 | 0.144065256 | count | 1 |
| AGAP5 | 0.6863094 | 1.073132 | 0.6395 | 0.523 | 0.144141266 | count | 1 |
| CNIH1 | 0.103291 | 0.0930915 | 1.1096 | 0.267 | 0.144284739 | count | 1 |
| DPY19L4 | 0.1721791 | 0.3240022 | 0.5314 | 0.595 | 0.144343945 | count | 1 |

| | | | | | | | |
|---|---|---|---|---|---|---|---|
| DDX50 | 0.1048134 | 0.120125 | 0.8725 | 0.383 | 0.144368605 | count | 1 |
| TBL2 | 0.1545043 | 0.3432974 | 0.4501 | 0.653 | 0.144471896 | count | 1 |
| IFITM2 | 0.1003871 | 0.0369362 | 2.7179 | 0.0066 | 0.144486059 | count | 1 |
| MANBA | 0.1724174 | 0.2282539 | 0.7554 | 0.45 | 0.144546897 | count | 1 |
| DCAF5 | 0.1093495 | 0.1404506 | 0.7786 | 0.436 | 0.144869345 | count | 1 |
| CYBA | 0.1006754 | 0.0301028 | 3.3444 | 0.000834 | 0.144960463 | count | 1 |
| EHD3 | 0.4103462 | 0.8826844 | 0.4649 | 0.642 | 0.144995962 | count | 1 |
| AL354920.1 | 0.4103462 | 0.8949543 | 0.4585 | 0.647 | 0.144995962 | count | 1 |
| CORO6 | 0.4103462 | 0.8971523 | 0.4574 | 0.647 | 0.144995962 | count | 1 |
| 10-Mar | 0.4103462 | 1.19831 | 0.3424 | 0.732 | 0.144995962 | count | 1 |
| KAT2B | 0.1258576 | 0.2063929 | 0.6098 | 0.542 | 0.14500011 | count | 1 |
| CAPZA1 | 0.1019266 | 0.0596248 | 1.7095 | 0.0875 | 0.145127043 | count | 1 |
| ALDH7A1 | 0.6912001 | 0.6924599 | 0.9982 | 0.318 | 0.14525845 | count | 1 |
| ANKMY2 | 0.1372822 | 0.2710783 | 0.5064 | 0.613 | 0.14526979 | count | 1 |
| CCDC137 | 0.113082 | 0.1669098 | 0.6775 | 0.498 | 0.145428048 | count | 1 |
| RPA2 | 0.1042893 | 0.0979306 | 1.0649 | 0.287 | 0.145633821 | count | 1 |
| SOCS7 | 0.6928612 | 0.4484403 | 1.545 | 0.122 | 0.145638069 | count | 1 |
| SART1 | 0.1249057 | 0.2197111 | 0.5685 | 0.57 | 0.145997716 | count | 1 |
| SLC25A14 | 0.1452649 | 0.392304 | 0.3703 | 0.711 | 0.146019664 | count | 1 |
| GNPDA2 | 0.1234684 | 0.2369514 | 0.5211 | 0.602 | 0.146158405 | count | 1 |
| ZNF623 | 0.1948079 | 0.4207771 | 0.463 | 0.643 | 0.146216664 | count | 1 |
| TMEM71 | 0.109219 | 0.152352 | 0.7169 | 0.473 | 0.146242233 | count | 1 |
| ACER1 | 0.3139401 | 0.9391453 | 0.3343 | 0.738 | 0.146276866 | count | 1 |
| UBE2D4 | 0.1291043 | 0.2244862 | 0.5751 | 0.565 | 0.146308663 | count | 1 |
| NBR1 | 0.1232285 | 0.1714386 | 0.7188 | 0.472 | 0.146722321 | count | 1 |
| ABCF2 | 0.6976275 | 0.6570726 | 1.0617 | 0.288 | 0.146727841 | count | 1 |
| POLR3B | 0.1956397 | 0.3888188 | 0.5032 | 0.615 | 0.146853739 | count | 1 |
| ZBTB37 | 0.2321297 | 0.3069914 | 0.7561 | 0.45 | 0.146865474 | count | 1 |
| BRWD1 | 0.1119485 | 0.1447188 | 0.7736 | 0.439 | 0.146866244 | count | 1 |
| ZNF197 | 0.2636527 | 0.4163596 | 0.6332 | 0.527 | 0.146901003 | count | 1 |
| CTU2 | 0.1370625 | 0.3033688 | 0.4518 | 0.651 | 0.147098295 | count | 1 |
| WDR92 | 0.1574246 | 0.3827067 | 0.4113 | 0.681 | 0.147236158 | count | 1 |
| MBNL3 | 0.127789 | 0.282498 | 0.4524 | 0.651 | 0.14723818 | count | 1 |
| SMPD2 | 0.1391748 | 0.303049 | 0.4592 | 0.646 | 0.147289153 | count | 1 |
| AC074366.1 | 0.4168769 | 0.8956922 | 0.4654 | 0.642 | 0.147440879 | count | 1 |
| PLEKHG5 | 0.4168769 | 0.9047384 | 0.4608 | 0.645 | 0.147440879 | count | 1 |
| CPQ | 0.1110689 | 0.1440202 | 0.7712 | 0.441 | 0.14746887 | count | 1 |
| KATNBL1 | 0.1071711 | 0.1239913 | 0.8643 | 0.387 | 0.147472479 | count | 1 |
| IFT52 | 0.1122635 | 0.1954742 | 0.5743 | 0.566 | 0.147479967 | count | 1 |
| PLPP1 | 0.1415382 | 0.2570989 | 0.5505 | 0.582 | 0.147501336 | count | 1 |
| PARD6B | 0.1271546 | 0.296178 | 0.4293 | 0.668 | 0.147603684 | count | 1 |
| SLFN12L | 0.1112467 | 0.1346829 | 0.826 | 0.409 | 0.147858424 | count | 1 |
| SRSF5 | 0.1039519 | 0.0483022 | 2.1521 | 0.0315 | 0.148181823 | count | 1 |
| CTSS | 0.1048012 | 0.0769028 | 1.3628 | 0.173 | 0.148190955 | count | 1 |
| CLCN2 | 0.4195746 | 0.7062309 | 0.5941 | 0.552 | 0.148451857 | count | 1 |

| | | | | | | | |
|---|---|---|---|---|---|---|---|
| THAP9 | 0.1637759 | 0.4764832 | 0.3437 | 0.731 | 0.148510423 | count | 1 |
| CNPPD1 | 0.1121033 | 0.1383236 | 0.8104 | 0.418 | 0.148845051 | count | 1 |
| HIPK1 | 0.1087423 | 0.1124521 | 0.967 | 0.334 | 0.148848213 | count | 1 |
| GSC | 0.2138848 | 0.9103078 | 0.235 | 0.814 | 0.148977395 | count | 1 |
| AC060780.1 | 0.1432024 | 0.3204988 | 0.4468 | 0.655 | 0.14925105 | count | 1 |
| RABEP2 | 0.1357503 | 0.2477501 | 0.5479 | 0.584 | 0.149320852 | count | 1 |
| AP005019.1 | 0.3202457 | 0.6178769 | 0.5183 | 0.604 | 0.149343457 | count | 1 |
| TMEM104 | 0.164848 | 0.4178696 | 0.3945 | 0.693 | 0.149495733 | count | 1 |
| AC022916.1 | 0.2364408 | 0.39498 | 0.5986 | 0.549 | 0.149669935 | count | 1 |
| ZNF652 | 0.1087228 | 0.1104351 | 0.9845 | 0.325 | 0.149710324 | count | 1 |
| ICAM3 | 0.1050398 | 0.0504674 | 2.0813 | 0.0375 | 0.149849337 | count | 1 |
| SUV39H2 | 0.3214402 | 0.51548 | 0.6236 | 0.533 | 0.149924877 | count | 1 |
| AL359232.1 | 0.7116032 | 0.7988835 | 0.8907 | 0.373 | 0.149927342 | count | 1 |
| WDR6 | 0.1523467 | 0.2019899 | 0.7542 | 0.451 | 0.149967928 | count | 1 |
| C10orf88 | 0.1998409 | 0.3472982 | 0.5754 | 0.565 | 0.15007282 | count | 1 |
| PTOV1-AS1 | 0.4239597 | 0.6010903 | 0.7053 | 0.481 | 0.150096506 | count | 1 |
| CTDNEP1 | 0.1065073 | 0.0816018 | 1.3052 | 0.192 | 0.150244304 | count | 1 |
| ARID2 | 0.1157682 | 0.169192 | 0.6842 | 0.494 | 0.150274402 | count | 1 |
| ALG5 | 0.1101266 | 0.1227644 | 0.8971 | 0.37 | 0.15027723 | count | 1 |
| EMC10 | 0.1114763 | 0.1135534 | 0.9817 | 0.326 | 0.150304196 | count | 1 |
| LINC01184 | 0.1144152 | 0.1863391 | 0.614 | 0.539 | 0.150313196 | count | 1 |
| FADD | 0.1142735 | 0.1707404 | 0.6693 | 0.503 | 0.150324175 | count | 1 |
| LENG9 | 0.133915 | 0.3241113 | 0.4132 | 0.68 | 0.150394646 | count | 1 |
| DALRD3 | 0.1129536 | 0.1342279 | 0.8415 | 0.4 | 0.150576042 | count | 1 |
| CATSPERG | 0.152972 | 0.4346538 | 0.3519 | 0.725 | 0.150590099 | count | 1 |
| TMEM268 | 0.3228475 | 0.5320496 | 0.6068 | 0.544 | 0.150610058 | count | 1 |
| ENTPD5 | 0.2702994 | 0.4717274 | 0.573 | 0.567 | 0.150732286 | count | 1 |
| ELP4 | 0.123525 | 0.2136082 | 0.5783 | 0.563 | 0.150733717 | count | 1 |
| CYB5D1 | 0.3231974 | 0.5648259 | 0.5722 | 0.567 | 0.150780454 | count | 1 |
| AKT2 | 0.1191275 | 0.1714691 | 0.6947 | 0.487 | 0.150790231 | count | 1 |
| ACTR3B | 0.4258136 | 0.468934 | 0.908 | 0.364 | 0.150792266 | count | 1 |
| TMEM63B | 0.4259144 | 0.5528485 | 0.7704 | 0.441 | 0.150830097 | count | 1 |
| ARHGEF40 | 0.4259786 | 0.5041963 | 0.8449 | 0.398 | 0.15085419 | count | 1 |
| DCAF8 | 0.1500915 | 0.2133126 | 0.7036 | 0.482 | 0.150920516 | count | 1 |
| TRIM4 | 0.1183625 | 0.1424184 | 0.8311 | 0.406 | 0.150929201 | count | 1 |
| NFATC3 | 0.1111293 | 0.1369968 | 0.8112 | 0.417 | 0.15103615 | count | 1 |
| BHLHE40 | 0.1075147 | 0.0836625 | 1.2851 | 0.199 | 0.151307056 | count | 1 |
| THUMPD1 | 0.1071237 | 0.0657422 | 1.6295 | 0.103 | 0.151540078 | count | 1 |
| TM2D1 | 0.1119139 | 0.1220361 | 0.9171 | 0.359 | 0.151582428 | count | 1 |
| PLPPR2 | 0.324964 | 0.9914616 | 0.3278 | 0.743 | 0.151640994 | count | 1 |
| AC118553.1 | 0.324964 | 1.0879987 | 0.2987 | 0.765 | 0.151640994 | count | 1 |
| AC027607.1 | 0.324964 | 1.0879987 | 0.2987 | 0.765 | 0.151640994 | count | 1 |
| AC145422.1 | 0.324964 | 1.0879987 | 0.2987 | 0.765 | 0.151640994 | count | 1 |
| RANBP10 | 0.1809956 | 0.3797079 | 0.4767 | 0.634 | 0.151858042 | count | 1 |
| CCNYL1 | 0.1436463 | 0.2661149 | 0.5398 | 0.589 | 0.152061795 | count | 1 |

| | | | | | | | |
|---|---|---|---|---|---|---|---|
| CYBC1 | 0.1088013 | 0.0901158 | 1.2073 | 0.227 | 0.152260087 | count | 1 |
| MED12 | 0.273263 | 0.4241645 | 0.6442 | 0.519 | 0.15244235 | count | 1 |
| BICRA | 0.3270313 | 0.3374355 | 0.9692 | 0.333 | 0.152648357 | count | 1 |
| TBCE | 0.1425231 | 0.2287639 | 0.623 | 0.533 | 0.15300651 | count | 1 |
| PDPR | 0.1469006 | 0.2850426 | 0.5154 | 0.606 | 0.153140452 | count | 1 |
| C1GALT1C1 | 0.1181809 | 0.1634593 | 0.723 | 0.47 | 0.153150059 | count | 1 |
| NHLRC2 | 0.159435 | 0.3513272 | 0.4538 | 0.65 | 0.153283772 | count | 1 |
| AP001437.1 | 0.7262735 | 0.9050303 | 0.8025 | 0.422 | 0.153292186 | count | 1 |
| TNS2 | 0.7262735 | 1.052556 | 0.69 | 0.49 | 0.153292186 | count | 1 |
| TMEM222 | 0.1169228 | 0.161827 | 0.7225 | 0.47 | 0.153615339 | count | 1 |
| B2M | 0.1065258 | 0.0109877 | 9.695 | 6.08E-22 | 0.15366743 | count | 1.48E-17 |
| PIGT | 0.1125661 | 0.1350489 | 0.8335 | 0.405 | 0.15368242 | count | 1 |
| PDSS1 | 0.1598564 | 0.4013588 | 0.3983 | 0.69 | 0.153693682 | count | 1 |
| APBA3 | 0.1283818 | 0.2296076 | 0.5591 | 0.576 | 0.153727381 | count | 1 |
| SLC25A23 | 0.2205627 | 0.4000905 | 0.5513 | 0.581 | 0.153742343 | count | 1 |
| NOTCH4 | 0.1599776 | 0.4735795 | 0.3378 | 0.736 | 0.153811582 | count | 1 |
| CBWD2 | 0.2048347 | 0.2541369 | 0.806 | 0.42 | 0.153902455 | count | 1 |
| MT-CO1 | 0.1067285 | 0.0160277 | 6.659 | 3.20E-11 | 0.153945281 | count | 7.75E-07 |
| C19orf12 | 0.1139464 | 0.1323337 | 0.8611 | 0.389 | 0.154051406 | count | 1 |
| ZDHHC16 | 0.1435729 | 0.2743865 | 0.5233 | 0.601 | 0.154142746 | count | 1 |
| KIAA1324L | 0.1837089 | 0.4610416 | 0.3985 | 0.69 | 0.154172704 | count | 1 |
| MED11 | 0.1124744 | 0.1430763 | 0.7861 | 0.432 | 0.154274206 | count | 1 |
| SPATA5 | 0.128213 | 0.2515555 | 0.5097 | 0.61 | 0.154319164 | count | 1 |
| CSNK1G3 | 0.1185579 | 0.1283968 | 0.9234 | 0.356 | 0.154412529 | count | 1 |
| MOB2 | 0.1122402 | 0.1071064 | 1.0479 | 0.295 | 0.154559361 | count | 1 |
| SYT11 | 0.1204734 | 0.1622614 | 0.7425 | 0.458 | 0.154645462 | count | 1 |
| SNAPIN | 0.1132982 | 0.1329153 | 0.8524 | 0.394 | 0.154683303 | count | 1 |
| ZFPM1 | 0.1166203 | 0.1579183 | 0.7385 | 0.46 | 0.154690929 | count | 1 |
| EEF1D | 0.1074184 | 0.0225836 | 4.7565 | 2.05E-06 | 0.154726702 | count | 0.04937425 |
| DNAJC17 | 0.1169846 | 0.1762387 | 0.6638 | 0.507 | 0.154833435 | count | 1 |
| UGGT1 | 0.138333 | 0.2184266 | 0.6333 | 0.527 | 0.155390315 | count | 1 |
| IMMP2L | 0.1412829 | 0.2430185 | 0.5814 | 0.561 | 0.155452135 | count | 1 |
| RPL14 | 0.1078483 | 0.0152713 | 7.0622 | 1.98E-12 | 0.155496902 | count | 4.80E-08 |
| SF3B3 | 0.1330587 | 0.1930387 | 0.6893 | 0.491 | 0.155581491 | count | 1 |
| GTPBP8 | 0.1137666 | 0.1416621 | 0.8031 | 0.422 | 0.155601973 | count | 1 |
| AL096865.1 | 0.2458054 | 0.5389017 | 0.4561 | 0.648 | 0.155770365 | count | 1 |
| UBE2O | 0.1471326 | 0.2374503 | 0.6196 | 0.536 | 0.155784481 | count | 1 |
| FPGT | 0.1664674 | 0.4374158 | 0.3806 | 0.704 | 0.155802962 | count | 1 |
| NDUFV3 | 0.1149911 | 0.1332688 | 0.8629 | 0.388 | 0.156120488 | count | 1 |
| HIST1H4A | 0.7392177 | 0.5227735 | 1.414 | 0.157 | 0.15626623 | count | 1 |
| SMAD3 | 0.136672 | 0.2246518 | 0.6084 | 0.543 | 0.156279824 | count | 1 |
| PRPF19 | 0.1281352 | 0.1703049 | 0.7524 | 0.452 | 0.156384359 | count | 1 |
| CYCS | 0.1093723 | 0.0498536 | 2.1939 | 0.0283 | 0.156403056 | count | 1 |
| DNMT3A | 0.1438285 | 0.2067093 | 0.6958 | 0.487 | 0.156419482 | count | 1 |
| FBF1 | 0.2083245 | 0.3458694 | 0.6023 | 0.547 | 0.156580771 | count | 1 |

| | | | | | | | |
|---|---|---|---|---|---|---|---|
| DPH3 | 0.1133772 | 0.1162162 | 0.9756 | 0.329 | 0.15660829 | count | 1 |
| LIMD2 | 0.1093427 | 0.0449757 | 2.4311 | 0.0151 | 0.156730492 | count | 1 |
| RRAGA | 0.1130606 | 0.1154596 | 0.9792 | 0.328 | 0.156746944 | count | 1 |
| ADAM15 | 0.1631529 | 0.3192048 | 0.5111 | 0.609 | 0.156901092 | count | 1 |
| PELI2 | 0.7427735 | 0.5585645 | 1.3298 | 0.184 | 0.157084017 | count | 1 |
| C1orf50 | 0.1337193 | 0.2947563 | 0.4537 | 0.65 | 0.157386505 | count | 1 |
| ASH1L-AS1 | 0.1314399 | 0.2945482 | 0.4462 | 0.655 | 0.157407543 | count | 1 |
| OXT | 0.7446072 | 0.7312674 | 1.0182 | 0.309 | 0.15750588 | count | 1 |
| RDH14 | 0.1158468 | 0.1158586 | 0.9999 | 0.317 | 0.15762484 | count | 1 |
| SLC30A1 | 0.1339653 | 0.2202122 | 0.6083 | 0.543 | 0.157677641 | count | 1 |
| NAAA | 0.1146529 | 0.1414746 | 0.8104 | 0.418 | 0.157726244 | count | 1 |
| ABCA2 | 0.141869 | 0.3225876 | 0.4398 | 0.66 | 0.157803574 | count | 1 |
| PELI1 | 0.1326048 | 0.1794536 | 0.7389 | 0.46 | 0.157944277 | count | 1 |
| NSMF | 0.1603627 | 0.2984407 | 0.5373 | 0.591 | 0.157947603 | count | 1 |
| WDR45B | 0.1216808 | 0.1509075 | 0.8063 | 0.42 | 0.157970422 | count | 1 |
| SLC25A29 | 0.1327246 | 0.2396452 | 0.5538 | 0.58 | 0.158087707 | count | 1 |
| SLC25A51 | 0.1382531 | 0.2813658 | 0.4914 | 0.623 | 0.158099315 | count | 1 |
| AC007038.2 | 0.2831241 | 0.7290428 | 0.3884 | 0.698 | 0.158140247 | count | 1 |
| HEATR3 | 0.4454921 | 0.3497659 | 1.2737 | 0.203 | 0.158194499 | count | 1 |
| RPS8 | 0.1097246 | 0.017992 | 6.0985 | 1.19E-09 | 0.158230176 | count | 2.88E-05 |
| NQO1 | 0.1543586 | 0.3877544 | 0.3981 | 0.691 | 0.158249578 | count | 1 |
| AC023590.1 | 0.747854 | 0.6998337 | 1.0686 | 0.285 | 0.158253069 | count | 1 |
| ATP23 | 0.1253627 | 0.2194669 | 0.5712 | 0.568 | 0.158288583 | count | 1 |
| ROGDI | 0.1363098 | 0.2418587 | 0.5636 | 0.573 | 0.158294609 | count | 1 |
| HACD4 | 0.1157146 | 0.1395129 | 0.8294 | 0.407 | 0.158404794 | count | 1 |
| DPM2 | 0.1169378 | 0.1325027 | 0.8825 | 0.378 | 0.158492993 | count | 1 |
| AL022328.4 | 0.7497617 | 0.5531973 | 1.3553 | 0.175 | 0.158692185 | count | 1 |
| AL158152.1 | 0.4468276 | 0.4496389 | 0.9937 | 0.32 | 0.158697966 | count | 1 |
| WDR60 | 0.1176669 | 0.1769931 | 0.6648 | 0.506 | 0.15877472 | count | 1 |
| AC010883.1 | 0.1991871 | 0.6350961 | 0.3136 | 0.754 | 0.159100629 | count | 1 |
| SYNJ1 | 0.1583812 | 0.3381418 | 0.4684 | 0.64 | 0.159344456 | count | 1 |
| PLXND1 | 0.1702087 | 0.3397729 | 0.5009 | 0.616 | 0.159350478 | count | 1 |
| PPP4R3B | 0.1166819 | 0.1010168 | 1.1551 | 0.248 | 0.159525154 | count | 1 |
| ERAP2 | 0.1248105 | 0.1319419 | 0.946 | 0.344 | 0.159540923 | count | 1 |
| APC | 0.1384037 | 0.2090459 | 0.6621 | 0.508 | 0.159544653 | count | 1 |
| GPR180 | 0.1451086 | 0.2810636 | 0.5163 | 0.606 | 0.15969372 | count | 1 |
| AC008741.2 | 0.2518656 | 0.3645527 | 0.6909 | 0.49 | 0.159724386 | count | 1 |
| AC104187.1 | 0.2521934 | 0.8079894 | 0.3121 | 0.755 | 0.1599384 | count | 1 |
| PTPMT1 | 0.1232102 | 0.1957281 | 0.6295 | 0.529 | 0.159961441 | count | 1 |
| DMTN | 0.1590985 | 0.2778106 | 0.5727 | 0.567 | 0.160073769 | count | 1 |
| SDR39U1 | 0.1242853 | 0.1655362 | 0.7508 | 0.453 | 0.160195286 | count | 1 |
| TENT2 | 0.1255759 | 0.1579598 | 0.795 | 0.427 | 0.160522443 | count | 1 |
| HAGHL | 0.1291372 | 0.2557379 | 0.505 | 0.614 | 0.160629107 | count | 1 |
| SPIN4 | 0.2875894 | 0.6279091 | 0.458 | 0.647 | 0.160724288 | count | 1 |
| ANKRD40 | 0.1335478 | 0.1795692 | 0.7437 | 0.457 | 0.160771977 | count | 1 |

| | | | | | | | |
|---|---|---|---|---|---|---|---|
| AC078883.3 | 0.2013464 | 0.5831325 | 0.3453 | 0.73 | 0.160858719 | count | 1 |
| LINC02470 | 0.2141334 | 0.5834687 | 0.367 | 0.714 | 0.161042649 | count | 1 |
| KCNQ1 | 0.2019205 | 0.2614635 | 0.7723 | 0.44 | 0.16132626 | count | 1 |
| NAT9 | 0.1333738 | 0.2177927 | 0.6124 | 0.54 | 0.161346974 | count | 1 |
| TRIM39 | 0.2145731 | 0.309642 | 0.693 | 0.488 | 0.161380589 | count | 1 |
| USP53 | 0.138063 | 0.2154092 | 0.6409 | 0.522 | 0.161466996 | count | 1 |
| LRRC27 | 0.3453717 | 0.6835456 | 0.5053 | 0.613 | 0.161605732 | count | 1 |
| AAGAB | 0.128057 | 0.1888641 | 0.678 | 0.498 | 0.161702695 | count | 1 |
| POLR2G | 0.1155477 | 0.0917833 | 1.2589 | 0.208 | 0.161775878 | count | 1 |
| ABCC4 | 0.2151929 | 0.4398327 | 0.4893 | 0.625 | 0.161856967 | count | 1 |
| LSM3 | 0.115369 | 0.088376 | 1.3054 | 0.192 | 0.161967755 | count | 1 |
| SESN3 | 0.1333044 | 0.1322417 | 1.008 | 0.314 | 0.162009924 | count | 1 |
| TSNAX | 0.1196232 | 0.109319 | 1.0943 | 0.274 | 0.162138604 | count | 1 |
| JOSD1 | 0.1189931 | 0.109637 | 1.0853 | 0.278 | 0.162316968 | count | 1 |
| RHOC | 0.1139703 | 0.072143 | 1.5798 | 0.114 | 0.162445804 | count | 1 |
| NAPA-AS1 | 0.2036081 | 0.4188758 | 0.4861 | 0.627 | 0.162700861 | count | 1 |
| GTF3C5 | 0.1402219 | 0.2240419 | 0.6259 | 0.531 | 0.162865305 | count | 1 |
| AC040970.1 | 0.768337 | 0.688757 | 1.1155 | 0.265 | 0.162972931 | count | 1 |
| ARHGAP26 | 0.12695 | 0.1504392 | 0.8439 | 0.399 | 0.162985222 | count | 1 |
| RCSD1 | 0.1166294 | 0.0807612 | 1.4441 | 0.149 | 0.163106256 | count | 1 |
| SCLY | 0.3486137 | 0.5082149 | 0.686 | 0.493 | 0.163192805 | count | 1 |
| AC005261.1 | 0.2918731 | 0.27203 | 1.0729 | 0.283 | 0.163205511 | count | 1 |
| HHLA3 | 0.1797878 | 0.3103026 | 0.5794 | 0.562 | 0.163242154 | count | 1 |
| MRPL40 | 0.1181896 | 0.1211683 | 0.9754 | 0.329 | 0.163263196 | count | 1 |
| AC004817.3 | 0.292001 | 0.4931085 | 0.5922 | 0.554 | 0.163279628 | count | 1 |
| NKAPD1 | 0.1257227 | 0.166013 | 0.7573 | 0.449 | 0.163505173 | count | 1 |
| TMEM33 | 0.1232679 | 0.1583837 | 0.7783 | 0.436 | 0.163528301 | count | 1 |
| IMPAD1 | 0.1335057 | 0.1757461 | 0.7597 | 0.448 | 0.163649448 | count | 1 |
| MRPL46 | 0.1228249 | 0.1714968 | 0.7162 | 0.474 | 0.163762887 | count | 1 |
| KAT14 | 0.2178585 | 0.4632859 | 0.4702 | 0.638 | 0.163906387 | count | 1 |
| FGD6 | 0.3504776 | 0.6950589 | 0.5042 | 0.614 | 0.164105751 | count | 1 |
| TESMIN | 0.2937647 | 0.5607653 | 0.5239 | 0.6 | 0.164301882 | count | 1 |
| FRYL | 0.1235178 | 0.1194101 | 1.0344 | 0.301 | 0.164369176 | count | 1 |
| EXOSC6 | 0.1203806 | 0.1055562 | 1.1404 | 0.254 | 0.164516179 | count | 1 |
| SNX19 | 0.1710325 | 0.2893298 | 0.5911 | 0.554 | 0.164573377 | count | 1 |
| CDC7 | 0.1636369 | 0.4724111 | 0.3464 | 0.729 | 0.164689613 | count | 1 |
| ATP5S | 0.121685 | 0.1330054 | 0.9149 | 0.36 | 0.164738565 | count | 1 |
| ELOVL1 | 0.1194876 | 0.1300193 | 0.919 | 0.358 | 0.164813363 | count | 1 |
| CAPN10-DT | 0.2191679 | 0.5809471 | 0.3773 | 0.706 | 0.16491346 | count | 1 |
| AP001462.1 | 0.2948446 | 0.5104712 | 0.5776 | 0.564 | 0.164927983 | count | 1 |
| JAZF1 | 0.1253684 | 0.1882383 | 0.666 | 0.505 | 0.164955512 | count | 1 |
| AP2S1 | 0.1170005 | 0.08889 | 1.3162 | 0.188 | 0.164991045 | count | 1 |
| IPO11 | 0.2363978 | 0.3711549 | 0.6369 | 0.524 | 0.165065704 | count | 1 |
| AC008083.1 | 0.777818 | 0.9436094 | 0.8243 | 0.41 | 0.1651611 | count | 1 |
| SPATA32 | 0.777818 | 0.9436094 | 0.8243 | 0.41 | 0.1651611 | count | 1 |

| | | | | | | | |
|---|---|---|---|---|---|---|---|
| AC007663.3 | 0.777818 | 0.9436094 | 0.8243 | 0.41 | 0.1651611 | count | 1 |
| DDR2 | 0.777818 | 1.0158164 | 0.7657 | 0.444 | 0.1651611 | count | 1 |
| AC016700.3 | 0.777818 | 1.0158164 | 0.7657 | 0.444 | 0.1651611 | count | 1 |
| LINC00881 | 0.777818 | 1.0158164 | 0.7657 | 0.444 | 0.1651611 | count | 1 |
| CCDC38 | 0.777818 | 1.0158164 | 0.7657 | 0.444 | 0.1651611 | count | 1 |
| LMBRD1 | 0.125072 | 0.1216396 | 1.0282 | 0.304 | 0.165178583 | count | 1 |
| ITGA10 | 0.7781568 | 0.7551389 | 1.0305 | 0.303 | 0.165239325 | count | 1 |
| TRAV20 | 0.7781568 | 0.769185 | 1.0117 | 0.312 | 0.165239325 | count | 1 |
| HNRNPL | 0.11865 | 0.0940173 | 1.262 | 0.207 | 0.165274305 | count | 1 |
| SLC7A1 | 0.2071308 | 0.3400852 | 0.6091 | 0.543 | 0.165571488 | count | 1 |
| KLHL8 | 0.1541849 | 0.2690783 | 0.573 | 0.567 | 0.165635384 | count | 1 |
| NEU1 | 0.1214458 | 0.1128462 | 1.0762 | 0.282 | 0.16574611 | count | 1 |
| YWHAQ | 0.1161614 | 0.0584341 | 1.9879 | 0.0469 | 0.165992443 | count | 1 |
| KIF13B | 0.129613 | 0.1600724 | 0.8097 | 0.418 | 0.166063632 | count | 1 |
| TEN1 | 0.1315451 | 0.1894432 | 0.6944 | 0.487 | 0.166123371 | count | 1 |
| MKNK1 | 0.1316982 | 0.1814212 | 0.7259 | 0.468 | 0.166317422 | count | 1 |
| WASHC1 | 0.1252599 | 0.174799 | 0.7166 | 0.474 | 0.166352896 | count | 1 |
| PMM2 | 0.1382656 | 0.1991427 | 0.6943 | 0.488 | 0.166480414 | count | 1 |
| RPL10 | 0.1154221 | 0.0128743 | 8.9653 | 5.00E-19 | 0.16648686 | count | 1.21E-14 |
| THAP3 | 0.1389941 | 0.2372388 | 0.5859 | 0.558 | 0.166501713 | count | 1 |
| SVIL | 0.1377116 | 0.2101447 | 0.6553 | 0.512 | 0.166620501 | count | 1 |
| PAFAH1B3 | 0.1213308 | 0.1400193 | 0.8665 | 0.386 | 0.166627317 | count | 1 |
| AMACR | 0.3556455 | 1.0118467 | 0.3515 | 0.725 | 0.166638896 | count | 1 |
| AP4B1 | 0.1497685 | 0.1962805 | 0.763 | 0.445 | 0.166657016 | count | 1 |
| LARS2 | 0.2084768 | 0.4346534 | 0.4796 | 0.632 | 0.166668775 | count | 1 |
| PYHIN1 | 0.11819 | 0.0800015 | 1.4773 | 0.14 | 0.166734232 | count | 1 |
| UHRF2 | 0.1317719 | 0.1600759 | 0.8232 | 0.41 | 0.166853266 | count | 1 |
| TMEM50B | 0.1229584 | 0.1084415 | 1.1339 | 0.257 | 0.167138859 | count | 1 |
| UBXN2A | 0.1242652 | 0.1605112 | 0.7742 | 0.439 | 0.167219374 | count | 1 |
| RARA-AS1 | 0.7872546 | 0.8866383 | 0.8879 | 0.375 | 0.167341067 | count | 1 |
| AC079447.1 | 0.7872546 | 1.392439 | 0.5654 | 0.572 | 0.167341067 | count | 1 |
| TSR1 | 0.147582 | 0.2213008 | 0.6669 | 0.505 | 0.16739623 | count | 1 |
| AC092279.1 | 0.3573562 | 0.7636702 | 0.4679 | 0.64 | 0.16747803 | count | 1 |
| TRIM14 | 0.1289984 | 0.1800121 | 0.7166 | 0.474 | 0.167497824 | count | 1 |
| ASPM | 0.2399313 | 0.6777935 | 0.354 | 0.723 | 0.167597053 | count | 1 |
| ANKRD28 | 0.1203701 | 0.0914563 | 1.3161 | 0.188 | 0.167609082 | count | 1 |
| PIBF1 | 0.124861 | 0.1228446 | 1.0164 | 0.31 | 0.167638341 | count | 1 |
| ZNF786 | 0.166843 | 0.3456087 | 0.4828 | 0.629 | 0.167951927 | count | 1 |
| EIF2S3 | 0.121466 | 0.0814708 | 1.4909 | 0.136 | 0.167982114 | count | 1 |
| ZNF337-AS1 | 0.7909702 | 0.7434177 | 1.064 | 0.287 | 0.168199958 | count | 1 |
| AP006621.3 | 0.1673616 | 0.3664398 | 0.4567 | 0.648 | 0.168479732 | count | 1 |
| DLST | 0.1407976 | 0.1885849 | 0.7466 | 0.455 | 0.168673562 | count | 1 |
| ZDHHC24 | 0.1220856 | 0.1296252 | 0.9418 | 0.346 | 0.168793682 | count | 1 |
| NFIL3 | 0.1264553 | 0.1303168 | 0.9704 | 0.332 | 0.16892582 | count | 1 |
| MATK | 0.1204518 | 0.0927544 | 1.2986 | 0.194 | 0.168950928 | count | 1 |

| | | | | | | | |
|---|---|---|---|---|---|---|---|
| NEPRO | 0.1277491 | 0.1443734 | 0.8849 | 0.376 | 0.169114303 | count | 1 |
| ZFP41 | 0.4743877 | 0.8102466 | 0.5855 | 0.558 | 0.169118145 | count | 1 |
| NGLY1 | 0.1236722 | 0.1168305 | 1.0586 | 0.29 | 0.169169682 | count | 1 |
| SEL1L3 | 0.1340226 | 0.1709913 | 0.7838 | 0.433 | 0.169263729 | count | 1 |
| SLC25A35 | 0.4748132 | 0.5747883 | 0.8261 | 0.409 | 0.169279518 | count | 1 |
| MED14 | 0.1353752 | 0.2567483 | 0.5273 | 0.598 | 0.169505627 | count | 1 |
| RNF14 | 0.138977 | 0.2390099 | 0.5815 | 0.561 | 0.169679413 | count | 1 |
| ARMC10 | 0.1316998 | 0.1549519 | 0.8499 | 0.395 | 0.169781982 | count | 1 |
| WRB | 0.1385024 | 0.2363689 | 0.586 | 0.558 | 0.169802636 | count | 1 |
| PROSER2 | 0.3038141 | 0.6584074 | 0.4614 | 0.645 | 0.170133665 | count | 1 |
| HMGA1P4 | 0.3038141 | 0.952921 | 0.3188 | 0.75 | 0.170133665 | count | 1 |
| CD84 | 0.1271968 | 0.1244143 | 1.0224 | 0.307 | 0.170505598 | count | 1 |
| STK26 | 0.1277707 | 0.1278598 | 0.9993 | 0.318 | 0.170686705 | count | 1 |
| RPL29 | 0.1184079 | 0.0172771 | 6.8534 | 8.53E-12 | 0.1707057 | count | 2.07E-07 |
| RPL32 | 0.1185931 | 0.017692 | 6.7032 | 2.38E-11 | 0.171009782 | count | 5.77E-07 |
| AC011374.2 | 0.3053643 | 0.9390978 | 0.3252 | 0.745 | 0.171034328 | count | 1 |
| MPI | 0.1522911 | 0.2469277 | 0.6167 | 0.537 | 0.171186415 | count | 1 |
| RPL34 | 0.1187418 | 0.0169153 | 7.0198 | 2.67E-12 | 0.171210947 | count | 6.47E-08 |
| ACADM | 0.1254957 | 0.1278321 | 0.9817 | 0.326 | 0.171281669 | count | 1 |
| EIF2AK1 | 0.1266003 | 0.119681 | 1.0578 | 0.29 | 0.171509039 | count | 1 |
| ACOT7 | 0.1360311 | 0.2362067 | 0.5759 | 0.565 | 0.17180989 | count | 1 |
| SCFD2 | 0.1743293 | 0.4063246 | 0.429 | 0.668 | 0.171870048 | count | 1 |
| FAM53B | 0.1376053 | 0.2117605 | 0.6498 | 0.516 | 0.17230921 | count | 1 |
| GPAT3 | 0.1897285 | 0.5420019 | 0.3501 | 0.726 | 0.172405152 | count | 1 |
| PPM1N | 0.1749607 | 0.3691848 | 0.4739 | 0.636 | 0.172500015 | count | 1 |
| TTLL3 | 0.1902238 | 0.364277 | 0.5222 | 0.602 | 0.172862038 | count | 1 |
| RPL30 | 0.1199422 | 0.0147125 | 8.1524 | 4.97E-16 | 0.172971363 | count | 1.21E-11 |
| FAM20B | 0.2297554 | 0.3546898 | 0.6478 | 0.517 | 0.173064952 | count | 1 |
| EWSR1 | 0.1240878 | 0.0888388 | 1.3968 | 0.163 | 0.173074578 | count | 1 |
| RALGAPB | 0.1417524 | 0.2634723 | 0.538 | 0.591 | 0.173084259 | count | 1 |
| RHOT2 | 0.1309204 | 0.1474776 | 0.8877 | 0.375 | 0.173124525 | count | 1 |
| TCTN1 | 0.8124437 | 0.51865 | 1.5665 | 0.117 | 0.173169387 | count | 1 |
| KIAA1586 | 0.13388 | 0.1533106 | 0.8733 | 0.383 | 0.173248053 | count | 1 |
| LRFN3 | 0.2480848 | 0.4603123 | 0.5389 | 0.59 | 0.173444427 | count | 1 |
| RAB4A | 0.1263356 | 0.118377 | 1.0672 | 0.286 | 0.173509869 | count | 1 |
| DHX29 | 0.1248891 | 0.1141607 | 1.094 | 0.274 | 0.173556576 | count | 1 |
| DDX52 | 0.1312649 | 0.1393934 | 0.9417 | 0.346 | 0.173581186 | count | 1 |
| DNAJC19 | 0.1235296 | 0.0905141 | 1.3648 | 0.172 | 0.173638858 | count | 1 |
| KYAT3 | 0.1358217 | 0.1639231 | 0.8286 | 0.407 | 0.173664627 | count | 1 |
| ETV7 | 0.1505734 | 0.3242073 | 0.4644 | 0.642 | 0.173667009 | count | 1 |
| OCLN | 0.2171095 | 0.6962402 | 0.3118 | 0.755 | 0.173712176 | count | 1 |
| MCRS1 | 0.1290938 | 0.1486402 | 0.8685 | 0.385 | 0.173729608 | count | 1 |
| UAP1 | 0.1348691 | 0.1610513 | 0.8374 | 0.402 | 0.173880711 | count | 1 |
| SUCLG2-AS1 | 0.2736488 | 0.7058575 | 0.3877 | 0.698 | 0.173976313 | count | 1 |
| ZNF615 | 0.1916143 | 0.6527585 | 0.2935 | 0.769 | 0.17414487 | count | 1 |

| | | | | | | | |
|---|---|---|---|---|---|---|---|
| RETREG2 | 0.1290518 | 0.1509092 | 0.8552 | 0.393 | 0.174165653 | count | 1 |
| CNDP2 | 0.1326219 | 0.1562866 | 0.8486 | 0.396 | 0.174295668 | count | 1 |
| RNF165 | 0.4881017 | 0.745711 | 0.6545 | 0.513 | 0.174324296 | count | 1 |
| KIAA0753 | 0.2073394 | 0.4513299 | 0.4594 | 0.646 | 0.174373969 | count | 1 |
| URB1-AS1 | 0.1919659 | 0.3810471 | 0.5038 | 0.614 | 0.174469281 | count | 1 |
| RNF125 | 0.125434 | 0.0750989 | 1.6703 | 0.095 | 0.17453072 | count | 1 |
| AL450384.2 | 0.3717765 | 0.6050777 | 0.6144 | 0.539 | 0.17456339 | count | 1 |
| RNF135 | 0.1418487 | 0.2743645 | 0.517 | 0.605 | 0.17461412 | count | 1 |
| POLR2E | 0.1245541 | 0.0843252 | 1.4771 | 0.14 | 0.174688268 | count | 1 |
| RAMP1 | 0.2498603 | 0.61539 | 0.406 | 0.685 | 0.174718916 | count | 1 |
| HSD17B4 | 0.1432529 | 0.2080125 | 0.6887 | 0.491 | 0.174925303 | count | 1 |
| ZNF573 | 0.2753654 | 0.3399069 | 0.8101 | 0.418 | 0.175101989 | count | 1 |
| ZBTB33 | 0.2324999 | 0.365925 | 0.6354 | 0.525 | 0.175180445 | count | 1 |
| ACADSB | 0.1870376 | 0.2509473 | 0.7453 | 0.456 | 0.175330077 | count | 1 |
| SULT1B1 | 0.8218018 | 0.4724541 | 1.7394 | 0.082 | 0.175337884 | count | 1 |
| PPIH | 0.1261299 | 0.1125215 | 1.1209 | 0.262 | 0.175393117 | count | 1 |
| KDM4A | 0.1680508 | 0.2994173 | 0.5613 | 0.575 | 0.175414697 | count | 1 |
| NRAS | 0.1415571 | 0.2059076 | 0.6875 | 0.492 | 0.175541314 | count | 1 |
| RETN | 0.491776 | 0.9062851 | 0.5426 | 0.587 | 0.175721434 | count | 1 |
| AC022144.1 | 0.491776 | 0.9062851 | 0.5426 | 0.587 | 0.175721434 | count | 1 |
| AL162426.1 | 0.491776 | 1.183575 | 0.4155 | 0.678 | 0.175721434 | count | 1 |
| AGBL3 | 0.2200883 | 0.6143462 | 0.3582 | 0.72 | 0.17614489 | count | 1 |
| CHADL | 0.8259193 | 0.8465957 | 0.9756 | 0.329 | 0.176292521 | count | 1 |
| AL359258.2 | 0.8259193 | 0.8913078 | 0.9266 | 0.354 | 0.176292521 | count | 1 |
| DNAJB14 | 0.1255447 | 0.0816478 | 1.5376 | 0.124 | 0.176303701 | count | 1 |
| PMPCA | 0.1353315 | 0.1727628 | 0.7833 | 0.433 | 0.176322911 | count | 1 |
| CASS4 | 0.1884354 | 0.2854942 | 0.66 | 0.509 | 0.176658957 | count | 1 |
| ZFP37 | 0.8277481 | 0.6822886 | 1.2132 | 0.225 | 0.176716614 | count | 1 |
| ERCC8 | 0.1604786 | 0.3173375 | 0.5057 | 0.613 | 0.176750012 | count | 1 |
| NPM2 | 0.1944613 | 0.347907 | 0.5589 | 0.576 | 0.176772197 | count | 1 |
| MRPL53 | 0.2348063 | 0.4568866 | 0.5139 | 0.607 | 0.176958987 | count | 1 |
| MED27 | 0.1342138 | 0.1522937 | 0.8813 | 0.378 | 0.177068762 | count | 1 |
| TRA2A | 0.1263513 | 0.0786548 | 1.6064 | 0.108 | 0.177100762 | count | 1 |
| U2AF2 | 0.1339556 | 0.1442406 | 0.9287 | 0.353 | 0.177148096 | count | 1 |
| ABCE1 | 0.1329374 | 0.1176097 | 1.1303 | 0.258 | 0.17727613 | count | 1 |
| SYNE3 | 0.1592821 | 0.2571658 | 0.6194 | 0.536 | 0.177327897 | count | 1 |
| TRIAP1 | 0.12989 | 0.1482029 | 0.8764 | 0.381 | 0.177370955 | count | 1 |
| AC010978.1 | 0.3166253 | 0.7533292 | 0.4203 | 0.674 | 0.177585419 | count | 1 |
| VRK2 | 0.1500088 | 0.1956831 | 0.7666 | 0.443 | 0.177763984 | count | 1 |
| MTRF1L | 0.1475907 | 0.192785 | 0.7656 | 0.444 | 0.177768834 | count | 1 |
| TMEM30B | 0.3784322 | 0.6105356 | 0.6198 | 0.535 | 0.177840686 | count | 1 |
| ACLY | 0.1469418 | 0.2047488 | 0.7177 | 0.473 | 0.177846713 | count | 1 |
| PRSS36 | 0.497471 | 0.6741231 | 0.738 | 0.461 | 0.177888846 | count | 1 |
| TRAV22 | 0.497471 | 0.749485 | 0.6638 | 0.507 | 0.177888846 | count | 1 |
| PANK1 | 0.497471 | 0.7642886 | 0.6509 | 0.515 | 0.177888846 | count | 1 |

| | | | | | | | |
|---|---|---|---|---|---|---|---|
| NUDT16 | 0.1416242 | 0.1995044 | 0.7099 | 0.478 | 0.177895776 | count | 1 |
| SIDT1 | 0.1531153 | 0.2131674 | 0.7183 | 0.473 | 0.177939156 | count | 1 |
| MATN1-AS1 | 0.2224028 | 0.3020429 | 0.7363 | 0.462 | 0.178035905 | count | 1 |
| ZDHHC4 | 0.1345069 | 0.1910836 | 0.7039 | 0.482 | 0.178082259 | count | 1 |
| HIST4H4 | 0.180579 | 0.2769957 | 0.6519 | 0.514 | 0.178107714 | count | 1 |
| ECSIT | 0.1371472 | 0.1654511 | 0.8289 | 0.407 | 0.178110758 | count | 1 |
| TMEM206 | 0.1706154 | 0.2510499 | 0.6796 | 0.497 | 0.178119068 | count | 1 |
| DTX3 | 0.17696 | 0.3016349 | 0.5867 | 0.557 | 0.178254342 | count | 1 |
| ZNF570 | 0.1618584 | 0.2292203 | 0.7061 | 0.48 | 0.178282387 | count | 1 |
| BMT2 | 0.1851058 | 0.293327 | 0.6311 | 0.528 | 0.178295829 | count | 1 |
| DUSP28 | 0.1388444 | 0.2019598 | 0.6875 | 0.492 | 0.178307638 | count | 1 |
| MED24 | 0.4987659 | 0.3817665 | 1.3065 | 0.191 | 0.178382018 | count | 1 |
| NDST2 | 0.1852229 | 0.4714902 | 0.3928 | 0.694 | 0.178410111 | count | 1 |
| MSI2 | 0.1371609 | 0.1595074 | 0.8599 | 0.39 | 0.178425406 | count | 1 |
| NFE2L1 | 0.1516868 | 0.2510966 | 0.6041 | 0.546 | 0.178664541 | count | 1 |
| CHCHD3 | 0.1303344 | 0.1314656 | 0.9914 | 0.322 | 0.178737184 | count | 1 |
| OTUB1 | 0.1285909 | 0.0995612 | 1.2916 | 0.197 | 0.178818848 | count | 1 |
| CDC42SE1 | 0.1258003 | 0.0634356 | 1.9831 | 0.0474 | 0.178956013 | count | 1 |
| EVA1C | 0.1577484 | 0.2333089 | 0.6761 | 0.499 | 0.179012793 | count | 1 |
| STX2 | 0.1493908 | 0.1846513 | 0.809 | 0.419 | 0.179025478 | count | 1 |
| PGM1 | 0.1479187 | 0.2147468 | 0.6888 | 0.491 | 0.179035254 | count | 1 |
| DDHD1 | 0.1352624 | 0.121231 | 1.1157 | 0.265 | 0.179084973 | count | 1 |
| RPS4X | 0.1242913 | 0.0167548 | 7.4183 | 1.49E-13 | 0.179206545 | count | 3.61E-09 |
| ARSK | 0.1581733 | 0.2180622 | 0.7254 | 0.468 | 0.179498517 | count | 1 |
| MFSD10 | 0.1273835 | 0.084491 | 1.5077 | 0.132 | 0.179656371 | count | 1 |
| FKBP8 | 0.1270564 | 0.0696804 | 1.8234 | 0.0683 | 0.179678053 | count | 1 |
| SERTAD2 | 0.1783658 | 0.2042869 | 0.8731 | 0.383 | 0.179686862 | count | 1 |
| C12orf10 | 0.130491 | 0.1184815 | 1.1014 | 0.271 | 0.179724774 | count | 1 |
| XBP1 | 0.1265166 | 0.0638924 | 1.9802 | 0.0478 | 0.180038908 | count | 1 |
| CDC25B | 0.1393976 | 0.1628612 | 0.8559 | 0.392 | 0.180079857 | count | 1 |
| EFCAB7 | 0.2574083 | 0.3279747 | 0.7848 | 0.433 | 0.18014156 | count | 1 |
| GTF3C3 | 0.1550768 | 0.2529419 | 0.6131 | 0.54 | 0.180233682 | count | 1 |
| AC008035.1 | 0.2833305 | 0.8222662 | 0.3446 | 0.73 | 0.180330015 | count | 1 |
| IFT172 | 0.8434727 | 0.6445422 | 1.3086 | 0.191 | 0.180365493 | count | 1 |
| RPRD1B | 0.2060511 | 0.2665842 | 0.7729 | 0.44 | 0.180806484 | count | 1 |
| CRTC2 | 0.1486931 | 0.2100318 | 0.708 | 0.479 | 0.180809086 | count | 1 |
| SLAMF7 | 0.1367639 | 0.1712609 | 0.7986 | 0.425 | 0.181077863 | count | 1 |
| WDSUB1 | 0.1482794 | 0.2634795 | 0.5628 | 0.574 | 0.181093818 | count | 1 |
| SFXN1 | 0.1294739 | 0.0841664 | 1.5383 | 0.124 | 0.181339254 | count | 1 |
| RPS27A | 0.1258078 | 0.014235 | 8.8379 | 1.54E-18 | 0.181436379 | count | 3.74E-14 |
| PDE6B | 0.1599123 | 0.3362506 | 0.4756 | 0.634 | 0.181486635 | count | 1 |
| GNG2 | 0.1287066 | 0.0776033 | 1.6585 | 0.0973 | 0.181551857 | count | 1 |
| DCTN5 | 0.1541932 | 0.2182216 | 0.7066 | 0.48 | 0.18163496 | count | 1 |
| RIOX1 | 0.1494009 | 0.225949 | 0.6612 | 0.509 | 0.181674183 | count | 1 |
| SMIM15 | 0.1341065 | 0.1359466 | 0.9865 | 0.324 | 0.181804808 | count | 1 |

| | | | | | | | |
|---|---|---|---|---|---|---|---|
| FLYWCH1 | 0.2270706 | 0.2416421 | 0.9397 | 0.347 | 0.181851777 | count | 1 |
| EFCAB14 | 0.1368582 | 0.1215376 | 1.1261 | 0.26 | 0.181980579 | count | 1 |
| ZPR1 | 0.1366356 | 0.1318843 | 1.036 | 0.3 | 0.182044826 | count | 1 |
| GATA6 | 0.8519332 | 0.8025141 | 1.0616 | 0.289 | 0.182330386 | count | 1 |
| MSTO1 | 0.227817 | 0.3482292 | 0.6542 | 0.513 | 0.182462224 | count | 1 |
| GPR171 | 0.1356835 | 0.0978873 | 1.3861 | 0.166 | 0.182615645 | count | 1 |
| RANBP6 | 0.1468007 | 0.2067286 | 0.7101 | 0.478 | 0.182698286 | count | 1 |
| KRCC1 | 0.1318749 | 0.1149947 | 1.1468 | 0.252 | 0.182726958 | count | 1 |
| SNCA | 0.5104821 | 0.7068074 | 0.7222 | 0.47 | 0.182849064 | count | 1 |
| SYNGAP1-AS1 | 0.5104821 | 0.8147702 | 0.6265 | 0.531 | 0.182849064 | count | 1 |
| EPC1 | 0.1288533 | 0.0592257 | 2.1756 | 0.0297 | 0.182883788 | count | 1 |
| PGRMC2 | 0.139353 | 0.1295273 | 1.0759 | 0.282 | 0.182919661 | count | 1 |
| TRAV12-1 | 0.2427336 | 0.7025623 | 0.3455 | 0.73 | 0.183077424 | count | 1 |
| RPS26 | 0.1270842 | 0.0159072 | 7.9891 | 1.84E-15 | 0.183191691 | count | 4.47E-11 |
| FAM120AOS | 0.1349252 | 0.1242846 | 1.0856 | 0.278 | 0.183232387 | count | 1 |
| RPS13 | 0.1272522 | 0.0194803 | 6.5323 | 7.44E-11 | 0.183467721 | count | 1.80E-06 |
| CAT | 0.1360362 | 0.1288469 | 1.0558 | 0.291 | 0.183484143 | count | 1 |
| GDAP2 | 0.1709058 | 0.2057803 | 0.8305 | 0.406 | 0.18376879 | count | 1 |
| SIN3B | 0.1440764 | 0.1940796 | 0.7424 | 0.458 | 0.183839909 | count | 1 |
| PIGQ | 0.2094673 | 0.3437077 | 0.6094 | 0.542 | 0.183856208 | count | 1 |
| GABPB2 | 0.1668945 | 0.2102516 | 0.7938 | 0.427 | 0.183877014 | count | 1 |
| MYEF2 | 0.2022444 | 0.3527279 | 0.5734 | 0.566 | 0.183960037 | count | 1 |
| SERPINI1 | 0.1910821 | 0.2948925 | 0.648 | 0.517 | 0.184130544 | count | 1 |
| EEF1G | 0.8611175 | 0.6683589 | 1.2884 | 0.198 | 0.184464581 | count | 1 |
| SLC2A11 | 0.1914449 | 0.2852641 | 0.6711 | 0.502 | 0.184484888 | count | 1 |
| ANAPC15 | 0.1341788 | 0.1201307 | 1.1169 | 0.264 | 0.184496634 | count | 1 |
| TRAPPC12 | 0.1468841 | 0.185854 | 0.7903 | 0.429 | 0.184530321 | count | 1 |
| NXT2 | 0.1870274 | 0.2449658 | 0.7635 | 0.445 | 0.184548628 | count | 1 |
| VMP1 | 0.1315252 | 0.0798327 | 1.6475 | 0.0995 | 0.184688144 | count | 1 |
| PPCS | 0.1319804 | 0.0981925 | 1.3441 | 0.179 | 0.184700316 | count | 1 |
| SLC9A3 | 0.1569406 | 0.3127604 | 0.5018 | 0.616 | 0.184891613 | count | 1 |
| ATXN2L | 0.1495998 | 0.1592704 | 0.9393 | 0.348 | 0.184896101 | count | 1 |
| FAM122A | 0.1435239 | 0.1830061 | 0.7843 | 0.433 | 0.18507643 | count | 1 |
| URM1 | 0.1368508 | 0.1334908 | 1.0252 | 0.305 | 0.185308544 | count | 1 |
| CBX6 | 0.1359017 | 0.1246458 | 1.0903 | 0.276 | 0.185330614 | count | 1 |
| AC008040.5 | 0.2315276 | 0.5066419 | 0.457 | 0.648 | 0.185497988 | count | 1 |
| OARD1 | 0.1363363 | 0.1252963 | 1.0881 | 0.277 | 0.18564711 | count | 1 |
| ZNF252P-AS1 | 0.394665 | 0.7380409 | 0.5347 | 0.593 | 0.185852031 | count | 1 |
| LINC01410 | 0.394665 | 0.8144227 | 0.4846 | 0.628 | 0.185852031 | count | 1 |
| NEU3 | 0.8680614 | 0.718142 | 1.2088 | 0.227 | 0.186078928 | count | 1 |
| ZNF493 | 0.1481194 | 0.1813719 | 0.8167 | 0.414 | 0.186088716 | count | 1 |
| TMX1 | 0.1336726 | 0.1079793 | 1.2379 | 0.216 | 0.186186559 | count | 1 |
| ARL1 | 0.1382717 | 0.1299946 | 1.0637 | 0.288 | 0.186241936 | count | 1 |
| RPS28 | 0.1293818 | 0.0162758 | 7.9493 | 2.53E-15 | 0.186498913 | count | 6.14E-11 |
| RAC2 | 0.1297057 | 0.0335988 | 3.8604 | 0.000115 | 0.18656228 | count | 1 |

| | | | | | | | |
|---|---|---|---|---|---|---|---|
| RCAN2 | 0.2663635 | 0.3301609 | 0.8068 | 0.42 | 0.186584694 | count | 1 |
| SLC39A6 | 0.150007 | 0.1750631 | 0.8569 | 0.392 | 0.18670659 | count | 1 |
| SYVN1 | 0.1818541 | 0.2460887 | 0.739 | 0.46 | 0.186760496 | count | 1 |
| TMEM91 | 0.1819208 | 0.2843031 | 0.6399 | 0.522 | 0.186829766 | count | 1 |
| TMEM143 | 0.2935325 | 0.4756559 | 0.6171 | 0.537 | 0.187037725 | count | 1 |
| ACYP2 | 0.1450861 | 0.1650632 | 0.879 | 0.379 | 0.187097703 | count | 1 |
| ACTR1B | 0.1392167 | 0.145476 | 0.957 | 0.339 | 0.187241238 | count | 1 |
| SEC24D | 0.1555038 | 0.2577137 | 0.6034 | 0.546 | 0.187353351 | count | 1 |
| SAR1B | 0.1360084 | 0.1160143 | 1.1723 | 0.241 | 0.187455433 | count | 1 |
| ATG4C | 0.1702168 | 0.2705338 | 0.6292 | 0.529 | 0.187569172 | count | 1 |
| FAM83G | 0.294364 | 0.6044079 | 0.487 | 0.626 | 0.187584993 | count | 1 |
| RNF8 | 0.1402915 | 0.1657424 | 0.8464 | 0.397 | 0.187618255 | count | 1 |
| CPPED1 | 0.1652802 | 0.2895332 | 0.5709 | 0.568 | 0.187625299 | count | 1 |
| BICRAL | 0.1626903 | 0.2349235 | 0.6925 | 0.489 | 0.187741607 | count | 1 |
| TTTY15 | 0.1406365 | 0.1265275 | 1.1115 | 0.266 | 0.187742236 | count | 1 |
| XKR8 | 0.1653841 | 0.244942 | 0.6752 | 0.5 | 0.187744147 | count | 1 |
| TRBV24-1 | 0.3342449 | 0.8419909 | 0.397 | 0.691 | 0.187864856 | count | 1 |
| C8orf58 | 0.234451 | 0.3593955 | 0.6523 | 0.514 | 0.187890984 | count | 1 |
| FAM120B | 0.1747679 | 0.2845894 | 0.6141 | 0.539 | 0.187961385 | count | 1 |
| AC005253.1 | 0.5238747 | 0.5392612 | 0.9715 | 0.331 | 0.187966769 | count | 1 |
| PHC2 | 0.1460934 | 0.1798989 | 0.8121 | 0.417 | 0.188031621 | count | 1 |
| XAB2 | 0.174996 | 0.2214589 | 0.7902 | 0.429 | 0.188209052 | count | 1 |
| VSIG1 | 0.1952781 | 0.3352499 | 0.5825 | 0.56 | 0.18822973 | count | 1 |
| FBXW4 | 0.1503279 | 0.2049566 | 0.7335 | 0.463 | 0.188309782 | count | 1 |
| WIPF2 | 0.1588671 | 0.209007 | 0.7601 | 0.447 | 0.188326161 | count | 1 |
| LETMD1 | 0.1390419 | 0.122804 | 1.1322 | 0.258 | 0.188833003 | count | 1 |
| OPN3 | 0.2502583 | 0.4660468 | 0.537 | 0.591 | 0.188892708 | count | 1 |
| AC244090.1 | 0.2013464 | 0.4245221 | 0.4743 | 0.635 | 0.188944914 | count | 1 |
| NARFL | 0.16378 | 0.267511 | 0.6122 | 0.54 | 0.189008017 | count | 1 |
| FGF9 | 0.4011815 | 0.5273118 | 0.7608 | 0.447 | 0.18907524 | count | 1 |
| AC246817.2 | 0.269846 | 0.8057659 | 0.3349 | 0.738 | 0.189093009 | count | 1 |
| RYK | 0.1541648 | 0.1628095 | 0.9469 | 0.344 | 0.189102004 | count | 1 |
| ALYREF | 0.1343512 | 0.0977036 | 1.3751 | 0.169 | 0.189102177 | count | 1 |
| RANBP3 | 0.1500856 | 0.1846314 | 0.8129 | 0.416 | 0.189111898 | count | 1 |
| TNFRSF14 | 0.134132 | 0.0706247 | 1.8992 | 0.0576 | 0.189165626 | count | 1 |
| GCSH | 0.1843001 | 0.2903929 | 0.6347 | 0.526 | 0.189301036 | count | 1 |
| SOS2 | 0.147773 | 0.1619125 | 0.9127 | 0.361 | 0.189415397 | count | 1 |
| ASCL5 | 0.5281581 | 0.9167937 | 0.5761 | 0.565 | 0.189606011 | count | 1 |
| LINC02574 | 0.5281581 | 0.9911305 | 0.5329 | 0.594 | 0.189606011 | count | 1 |
| ARSJ | 0.5281581 | 0.9911305 | 0.5329 | 0.594 | 0.189606011 | count | 1 |
| TRBV6-5 | 0.5281581 | 0.9911305 | 0.5329 | 0.594 | 0.189606011 | count | 1 |
| MTRNR2L6 | 0.5281581 | 1.0504569 | 0.5028 | 0.615 | 0.189606011 | count | 1 |
| FAM131C | 0.5281581 | 1.206944 | 0.4376 | 0.662 | 0.189606011 | count | 1 |
| RHD | 0.5281581 | 1.206944 | 0.4376 | 0.662 | 0.189606011 | count | 1 |
| TM4SF19-AS1 | 0.5281581 | 1.206944 | 0.4376 | 0.662 | 0.189606011 | count | 1 |

| | | | | | | | |
|---|---|---|---|---|---|---|---|
| FRMPD3 | 0.5281581 | 1.206944 | 0.4376 | 0.662 | 0.189606011 | count | 1 |
| LINC01366 | 0.5281581 | 1.381269 | 0.3824 | 0.702 | 0.189606011 | count | 1 |
| AC138230.1 | 0.5281581 | 1.381269 | 0.3824 | 0.702 | 0.189606011 | count | 1 |
| AC135048.3 | 0.5281581 | 1.381269 | 0.3824 | 0.702 | 0.189606011 | count | 1 |
| RBM44 | 0.8843223 | 0.9735012 | 0.9084 | 0.364 | 0.189861723 | count | 1 |
| SPTSSB | 0.8843223 | 1.058115 | 0.8358 | 0.403 | 0.189861723 | count | 1 |
| HEG1 | 0.2254924 | 0.2609317 | 0.8642 | 0.388 | 0.189943067 | count | 1 |
| IMPACT | 0.1725664 | 0.2782025 | 0.6203 | 0.535 | 0.190181015 | count | 1 |
| ANGEL2 | 0.1436686 | 0.1545251 | 0.9297 | 0.353 | 0.190661097 | count | 1 |
| MYL6B | 0.1610876 | 0.270082 | 0.5964 | 0.551 | 0.190974788 | count | 1 |
| RNF212 | 0.5317941 | 0.8405253 | 0.6327 | 0.527 | 0.190998486 | count | 1 |
| PDHB | 0.1376979 | 0.1076468 | 1.2792 | 0.201 | 0.191035181 | count | 1 |
| POLR2M | 0.1621345 | 0.2237718 | 0.7246 | 0.469 | 0.191049977 | count | 1 |
| ITPRIP | 0.1622393 | 0.1982598 | 0.8183 | 0.413 | 0.191174259 | count | 1 |
| METTL8 | 0.1586683 | 0.1844392 | 0.8603 | 0.39 | 0.191187626 | count | 1 |
| C6orf99 | 0.8902493 | 0.7011236 | 1.2697 | 0.204 | 0.191241288 | count | 1 |
| CALHM2 | 0.1439889 | 0.1639115 | 0.8785 | 0.38 | 0.191288883 | count | 1 |
| ZNF710 | 0.2533772 | 0.6543892 | 0.3872 | 0.699 | 0.19130521 | count | 1 |
| AL138963.3 | 0.5329464 | 0.9969329 | 0.5346 | 0.593 | 0.191439961 | count | 1 |
| DDX31 | 0.2180133 | 0.4243198 | 0.5138 | 0.607 | 0.191491965 | count | 1 |
| ERI2 | 0.2732285 | 0.5901753 | 0.463 | 0.643 | 0.191530861 | count | 1 |
| TMEM128 | 0.1758622 | 0.2350374 | 0.7482 | 0.454 | 0.191586224 | count | 1 |
| LINC01336 | 0.534281 | 0.640373 | 0.8343 | 0.404 | 0.19195138 | count | 1 |
| PAQR7 | 0.534281 | 0.6837085 | 0.7814 | 0.435 | 0.19195138 | count | 1 |
| HPS3 | 0.1490108 | 0.1965181 | 0.7583 | 0.448 | 0.192176303 | count | 1 |
| VSIR | 0.1365339 | 0.0742065 | 1.8399 | 0.0659 | 0.192176617 | count | 1 |
| TTC12 | 0.2546764 | 0.4120992 | 0.618 | 0.537 | 0.192310519 | count | 1 |
| AC016831.1 | 0.3019628 | 0.1765496 | 1.7104 | 0.0873 | 0.192590123 | count | 1 |
| TYW5 | 0.19512 | 0.2843509 | 0.6862 | 0.493 | 0.192638807 | count | 1 |
| PCBP2 | 0.1357571 | 0.0560215 | 2.4233 | 0.0154 | 0.192864677 | count | 1 |
| PALB2 | 0.1732085 | 0.3226973 | 0.5368 | 0.591 | 0.19296462 | count | 1 |
| ANKRD36C | 0.1448465 | 0.1587663 | 0.9123 | 0.362 | 0.193011056 | count | 1 |
| ZNF689 | 0.2057666 | 0.3264471 | 0.6303 | 0.529 | 0.193155852 | count | 1 |
| FAM117A | 0.1431945 | 0.1309279 | 1.0937 | 0.274 | 0.193158884 | count | 1 |
| MZF1 | 0.175345 | 0.2346506 | 0.7473 | 0.455 | 0.193270448 | count | 1 |
| FKTN | 0.2757252 | 0.4295912 | 0.6418 | 0.521 | 0.193331174 | count | 1 |
| MFSD6 | 0.1884759 | 0.2523624 | 0.7468 | 0.455 | 0.19363978 | count | 1 |
| OSTC | 0.1365254 | 0.0721725 | 1.8917 | 0.0586 | 0.193664012 | count | 1 |
| PTRH1 | 0.1508278 | 0.1926809 | 0.7828 | 0.434 | 0.193752755 | count | 1 |
| MIEN1 | 0.1376402 | 0.1018756 | 1.3511 | 0.177 | 0.193857819 | count | 1 |
| FCRL3 | 0.1782006 | 0.2444296 | 0.729 | 0.466 | 0.19415751 | count | 1 |
| CCNB1IP1 | 0.1419402 | 0.1230575 | 1.1534 | 0.249 | 0.194281693 | count | 1 |
| ZNF816-ZNF321P | 0.9037639 | 0.6627785 | 1.3636 | 0.173 | 0.194388289 | count | 1 |
| GZMM | 0.1357026 | 0.0528371 | 2.5683 | 0.0103 | 0.194529129 | count | 1 |
| VPS25 | 0.1500596 | 0.177435 | 0.8457 | 0.398 | 0.194600772 | count | 1 |

| | | | | | | | |
|---|---|---|---|---|---|---|---|
| ARHGAP9 | 0.1398345 | 0.093476 | 1.4959 | 0.135 | 0.194666343 | count | 1 |
| FBXO8 | 0.1515467 | 0.2138615 | 0.7086 | 0.479 | 0.194679579 | count | 1 |
| ZBTB1 | 0.1422469 | 0.0846744 | 1.6799 | 0.0931 | 0.194783973 | count | 1 |
| ZDHHC5 | 0.15081 | 0.1625448 | 0.9278 | 0.354 | 0.19487365 | count | 1 |
| L3MBTL2 | 0.1534735 | 0.1970976 | 0.7787 | 0.436 | 0.194940118 | count | 1 |
| RPL5 | 0.1352521 | 0.0181557 | 7.4495 | 1.18E-13 | 0.194981325 | count | 2.86E-09 |
| ZBTB24 | 0.1412502 | 0.1261983 | 1.1193 | 0.263 | 0.195104431 | count | 1 |
| ANKRD42 | 0.3059534 | 0.3160766 | 0.968 | 0.333 | 0.1952214 | count | 1 |
| ADPRHL2 | 0.145391 | 0.1346463 | 1.0798 | 0.28 | 0.195414758 | count | 1 |
| SLC29A3 | 0.2146864 | 0.3603234 | 0.5958 | 0.551 | 0.195466308 | count | 1 |
| SOAT1 | 0.1562211 | 0.1790419 | 0.8725 | 0.383 | 0.195724922 | count | 1 |
| FASTKD1 | 0.2227711 | 0.3372328 | 0.6606 | 0.509 | 0.195747008 | count | 1 |
| MMP25-AS1 | 0.1984284 | 0.2277815 | 0.8711 | 0.384 | 0.195948426 | count | 1 |
| CYB5R3 | 0.1404219 | 0.1100323 | 1.2762 | 0.202 | 0.196141339 | count | 1 |
| CDKN2B | 0.2446007 | 0.4072596 | 0.6006 | 0.548 | 0.196207746 | count | 1 |
| STX3 | 0.1781515 | 0.2324955 | 0.7663 | 0.444 | 0.196391681 | count | 1 |
| DCUN1D3 | 0.185512 | 0.3535544 | 0.5247 | 0.6 | 0.196855821 | count | 1 |
| C5orf22 | 0.1767167 | 0.2674355 | 0.6608 | 0.509 | 0.196906656 | count | 1 |
| LRPPRC | 0.1599612 | 0.1629524 | 0.9816 | 0.326 | 0.197024372 | count | 1 |
| CXorf40B | 0.1661739 | 0.2208001 | 0.7526 | 0.452 | 0.197043269 | count | 1 |
| RAP2C | 0.1461839 | 0.1426359 | 1.0249 | 0.305 | 0.197334617 | count | 1 |
| TRAV25 | 0.9168213 | 0.7959271 | 1.1519 | 0.2494 | 0.197430381 | count | 1 |
| C15orf39 | 0.1999102 | 0.3079777 | 0.6491 | 0.516 | 0.197431189 | count | 1 |
| EFNA5 | 0.2818272 | 0.6449902 | 0.4369 | 0.662 | 0.197734455 | count | 1 |
| PABPN1 | 0.143164 | 0.1093673 | 1.309 | 0.191 | 0.197808293 | count | 1 |
| ZFAND3 | 0.1689868 | 0.1732003 | 0.9757 | 0.329 | 0.197885182 | count | 1 |
| LINC00891 | 0.225195 | 0.4786105 | 0.4705 | 0.638 | 0.197915867 | count | 1 |
| SLC26A1 | 0.3103075 | 0.788406 | 0.3936 | 0.694 | 0.198094502 | count | 1 |
| MPV17 | 0.1488856 | 0.1564112 | 0.9519 | 0.341 | 0.198212664 | count | 1 |
| AC093323.1 | 0.1564398 | 0.1610026 | 0.9717 | 0.331 | 0.198219222 | count | 1 |
| RIC8A | 0.1440536 | 0.1221511 | 1.1793 | 0.238 | 0.198232099 | count | 1 |
| AL390728.6 | 0.2112599 | 0.2335205 | 0.9047 | 0.366 | 0.198392399 | count | 1 |
| USF1 | 0.1646242 | 0.289689 | 0.5683 | 0.57 | 0.19840619 | count | 1 |
| RNF167 | 0.1408864 | 0.0927085 | 1.5197 | 0.129 | 0.19843323 | count | 1 |
| S100B | 0.2627833 | 0.5392043 | 0.4874 | 0.626 | 0.198588423 | count | 1 |
| METTL2B | 0.1665283 | 0.2219428 | 0.7503 | 0.453 | 0.1986067 | count | 1 |
| PFAS | 0.9223456 | 0.4872357 | 1.893 | 0.0584 | 0.198717829 | count | 1 |
| SORL1 | 0.1513812 | 0.1293621 | 1.1702 | 0.242 | 0.198755253 | count | 1 |
| ASH2L | 0.1936121 | 0.2854452 | 0.6783 | 0.498 | 0.198979063 | count | 1 |
| PSMF1 | 0.1410788 | 0.0880455 | 1.6023 | 0.109 | 0.19906148 | count | 1 |
| HM13 | 0.1424921 | 0.0922802 | 1.5441 | 0.123 | 0.199280735 | count | 1 |
| AP005482.1 | 0.1663002 | 0.3162036 | 0.5259 | 0.599 | 0.199412813 | count | 1 |
| ZNF347 | 0.2638586 | 0.562311 | 0.4692 | 0.639 | 0.19942174 | count | 1 |
| HSP90B1 | 0.1392813 | 0.0474839 | 2.9332 | 0.00338 | 0.199492305 | count | 1 |
| SRSF6 | 0.1464078 | 0.1016834 | 1.4398 | 0.15 | 0.199682958 | count | 1 |

| | | | | | | | |
|---|---|---|---|---|---|---|---|
| TRIP11 | 0.1462184 | 0.1207705 | 1.2107 | 0.226 | 0.199974976 | count | 1 |
| AAAS | 0.1858357 | 0.2309232 | 0.8048 | 0.421 | 0.199984929 | count | 1 |
| LINC00937 | 0.2647532 | 0.6892898 | 0.3841 | 0.701 | 0.200115142 | count | 1 |
| GABARAP | 0.2374946 | 0.3661553 | 0.6486 | 0.517 | 0.200260135 | count | 1 |
| CAMK2N1 | 0.1525257 | 0.1636032 | 0.9323 | 0.351 | 0.200262381 | count | 1 |
| EIF4G1 | 0.1555672 | 0.1336879 | 1.1637 | 0.245 | 0.200269407 | count | 1 |
| AC239800.2 | 0.9291266 | 0.9920857 | 0.9365 | 0.349 | 0.200298414 | count | 1 |
| LINC02265 | 0.9291266 | 0.9920857 | 0.9365 | 0.349 | 0.200298414 | count | 1 |
| ZNF835 | 0.9291266 | 1.063562 | 0.8736 | 0.382 | 0.200298414 | count | 1 |
| HNRNPA1 | 0.1396554 | 0.0327288 | 4.267 | 2.03E-05 | 0.200363996 | count | 0.4874233 |
| C19orf73 | 0.3137447 | 0.4813166 | 0.6518 | 0.515 | 0.200364166 | count | 1 |
| TCEA2 | 0.153295 | 0.1831892 | 0.8368 | 0.403 | 0.200417608 | count | 1 |
| MTMR14 | 0.1513466 | 0.1737083 | 0.8713 | 0.384 | 0.200438028 | count | 1 |
| AL135999.1 | 0.5565366 | 0.5528512 | 1.0067 | 0.314 | 0.200496336 | count | 1 |
| CDKL1 | 0.1889278 | 0.3168563 | 0.5963 | 0.551 | 0.20051893 | count | 1 |
| XCL2 | 0.3149668 | 0.1580185 | 1.9932 | 0.0463 | 0.201171479 | count | 1 |
| LRTOMT | 0.3150039 | 0.7250109 | 0.4345 | 0.664 | 0.201195988 | count | 1 |
| FGF22 | 0.5583958 | 0.8382559 | 0.6661 | 0.505 | 0.201211491 | count | 1 |
| ZHX2 | 0.2386722 | 0.272506 | 0.8758 | 0.381 | 0.201273371 | count | 1 |
| ELP5 | 0.1533569 | 0.1921787 | 0.798 | 0.425 | 0.201356976 | count | 1 |
| GSS | 0.1551332 | 0.1704172 | 0.9103 | 0.363 | 0.201547665 | count | 1 |
| WDR1 | 0.1442903 | 0.0917344 | 1.5729 | 0.116 | 0.201582152 | count | 1 |
| DCK | 0.1496444 | 0.1230976 | 1.2157 | 0.224 | 0.201736913 | count | 1 |
| HACD2 | 0.1596729 | 0.2214519 | 0.721 | 0.471 | 0.20179932 | count | 1 |
| ASAH1 | 0.147111 | 0.1178871 | 1.2479 | 0.212 | 0.201859051 | count | 1 |
| ST3GAL2 | 0.1931628 | 0.2406936 | 0.8025 | 0.422 | 0.201926613 | count | 1 |
| PPP5C | 0.1779385 | 0.2087987 | 0.8522 | 0.394 | 0.202111798 | count | 1 |
| RNF6 | 0.1619636 | 0.1669406 | 0.9702 | 0.332 | 0.202319594 | count | 1 |
| SESN1 | 0.1568776 | 0.171908 | 0.9126 | 0.362 | 0.202358489 | count | 1 |
| CEBPZOS | 0.1520523 | 0.1293827 | 1.1752 | 0.24 | 0.202439229 | count | 1 |
| CD59 | 0.1601879 | 0.1908931 | 0.8391 | 0.401 | 0.202452974 | count | 1 |
| CYB5R1 | 0.1489932 | 0.1456024 | 1.0233 | 0.306 | 0.202485334 | count | 1 |
| GATB | 0.2522767 | 0.3823176 | 0.6599 | 0.509 | 0.202506224 | count | 1 |
| WNT7A | 0.5617898 | 0.5363467 | 1.0474 | 0.295 | 0.202517706 | count | 1 |
| IRF8 | 0.3170112 | 0.3604638 | 0.8795 | 0.379 | 0.202522391 | count | 1 |
| COQ5 | 0.1783339 | 0.229598 | 0.7767 | 0.437 | 0.202564541 | count | 1 |
| POMGNT2 | 0.2680817 | 0.4374261 | 0.6129 | 0.54 | 0.202695912 | count | 1 |
| TBCC | 0.1440824 | 0.0802029 | 1.7965 | 0.0725 | 0.20271359 | count | 1 |
| IL5RA | 0.9402914 | 0.8019268 | 1.1725 | 0.241 | 0.202901451 | count | 1 |
| AL356488.2 | 0.9402914 | 0.8352531 | 1.1258 | 0.26 | 0.202901451 | count | 1 |
| GLYCTK | 0.1864064 | 0.2599823 | 0.717 | 0.473 | 0.203184898 | count | 1 |
| STRN4 | 0.1889706 | 0.2982737 | 0.6335 | 0.526 | 0.203392833 | count | 1 |
| PTS | 0.1512818 | 0.1444043 | 1.0476 | 0.295 | 0.203504498 | count | 1 |
| WIPI1 | 0.1764678 | 0.2586838 | 0.6822 | 0.495 | 0.203761043 | count | 1 |
| AFF3 | 0.4308256 | 0.578875 | 0.7442 | 0.457 | 0.203787735 | count | 1 |

| | | | | | | | |
|---|---|---|---|---|---|---|---|
| CERNA1 | 0.2320669 | 0.4944169 | 0.4694 | 0.639 | 0.204068669 | count | 1 |
| ELMOD3 | 0.1814248 | 0.2660962 | 0.6818 | 0.495 | 0.20421773 | count | 1 |
| MIS12 | 0.1852004 | 0.1897449 | 0.976 | 0.329 | 0.204234484 | count | 1 |
| RNF26 | 0.1898149 | 0.3486925 | 0.5444 | 0.586 | 0.20431083 | count | 1 |
| ATPAF1 | 0.1578101 | 0.1801131 | 0.8762 | 0.381 | 0.20432461 | count | 1 |
| MROH8 | 0.946407 | 0.6909578 | 1.3697 | 0.171 | 0.204327546 | count | 1 |
| SRGN | 0.1418976 | 0.0280287 | 5.0626 | 4.36E-07 | 0.204379036 | count | 0.010517192 |
| FRRS1 | 0.4321037 | 0.8691682 | 0.4971 | 0.619 | 0.204423849 | count | 1 |
| WAC-AS1 | 0.1614578 | 0.148513 | 1.0872 | 0.277 | 0.204604249 | count | 1 |
| ZNF449 | 0.4325401 | 0.5901004 | 0.733 | 0.464 | 0.204641092 | count | 1 |
| GPATCH3 | 0.2246489 | 0.3264992 | 0.6881 | 0.491 | 0.204693333 | count | 1 |
| ANAPC10 | 0.1508638 | 0.1629318 | 0.9259 | 0.355 | 0.2049192 | count | 1 |
| TMEM216 | 0.1537651 | 0.188619 | 0.8152 | 0.415 | 0.204925506 | count | 1 |
| ZNF416 | 0.2552681 | 0.5636727 | 0.4529 | 0.651 | 0.204962779 | count | 1 |
| ZBTB39 | 0.4331902 | 0.5122762 | 0.8456 | 0.398 | 0.204964725 | count | 1 |
| ZNF616 | 0.5681446 | 0.4351189 | 1.3057 | 0.192 | 0.204965141 | count | 1 |
| CDK2AP1 | 0.4332103 | 0.8064057 | 0.5372 | 0.591 | 0.204974729 | count | 1 |
| AC074050.4 | 0.4332103 | 0.8869287 | 0.4884 | 0.625 | 0.204974729 | count | 1 |
| METAP1D | 0.3635918 | 0.3395886 | 1.0707 | 0.284 | 0.205062494 | count | 1 |
| PNPLA2 | 0.1516479 | 0.117322 | 1.2926 | 0.196 | 0.205129458 | count | 1 |
| ARHGAP5-AS1 | 0.4337557 | 0.6166566 | 0.7034 | 0.482 | 0.20524628 | count | 1 |
| CARS | 0.1649124 | 0.1683937 | 0.9793 | 0.327 | 0.205349301 | count | 1 |
| RWDD2B | 0.1934384 | 0.3263088 | 0.5928 | 0.553 | 0.20535798 | count | 1 |
| ZNF480 | 0.175479 | 0.1814512 | 0.9671 | 0.334 | 0.205541284 | count | 1 |
| EZH1 | 0.1683024 | 0.1751304 | 0.961 | 0.337 | 0.205684365 | count | 1 |
| GAS7 | 0.3648005 | 0.4611149 | 0.7911 | 0.429 | 0.205772793 | count | 1 |
| TRIM61 | 0.4349489 | 0.7118691 | 0.611 | 0.541 | 0.205840439 | count | 1 |
| SMYD3 | 0.1670909 | 0.1924353 | 0.8683 | 0.385 | 0.205852102 | count | 1 |
| ARRB2 | 0.1463037 | 0.0904159 | 1.6181 | 0.106 | 0.205861027 | count | 1 |
| VHL | 0.1590474 | 0.1684156 | 0.9444 | 0.345 | 0.205932221 | count | 1 |
| TMEM116 | 0.1575183 | 0.1689589 | 0.9323 | 0.351 | 0.205956719 | count | 1 |
| C19orf44 | 0.5708098 | 0.6929323 | 0.8238 | 0.41 | 0.205992248 | count | 1 |
| HOXB3 | 0.5708098 | 0.8079724 | 0.7065 | 0.48 | 0.205992248 | count | 1 |
| POMK | 0.4353673 | 0.8579288 | 0.5075 | 0.612 | 0.206048811 | count | 1 |
| AC040169.1 | 0.4353673 | 0.910783 | 0.478 | 0.633 | 0.206048811 | count | 1 |
| LAMB3 | 0.4353673 | 1.0678309 | 0.4077 | 0.684 | 0.206048811 | count | 1 |
| NACC2 | 0.1890201 | 0.3795546 | 0.498 | 0.619 | 0.2060617 | count | 1 |
| HEATR1 | 0.1673411 | 0.2039937 | 0.8203 | 0.412 | 0.206161951 | count | 1 |
| PLRG1 | 0.1532821 | 0.1333995 | 1.149 | 0.251 | 0.206201317 | count | 1 |
| MAFF | 0.1566021 | 0.1296424 | 1.208 | 0.227 | 0.206437101 | count | 1 |
| DCAF4L1 | 0.4368643 | 1.093531 | 0.3995 | 0.69 | 0.20679449 | count | 1 |
| CENPO | 0.4372769 | 0.7231654 | 0.6047 | 0.545 | 0.207000048 | count | 1 |
| AC067735.1 | 0.4372769 | 0.7231654 | 0.6047 | 0.545 | 0.207000048 | count | 1 |
| AC005730.2 | 0.4372769 | 0.7231654 | 0.6047 | 0.545 | 0.207000048 | count | 1 |
| ARVCF | 0.4372769 | 0.7231654 | 0.6047 | 0.545 | 0.207000048 | count | 1 |

| | | | | | | | |
|---|---|---|---|---|---|---|---|
| MACF1 | 0.1484707 | 0.0805257 | 1.8438 | 0.0653 | 0.207047533 | count | 1 |
| PXK | 0.170255 | 0.1879322 | 0.9059 | 0.365 | 0.207179471 | count | 1 |
| OXSM | 0.1758304 | 0.2784845 | 0.6314 | 0.528 | 0.207299816 | count | 1 |
| TMEM127 | 0.1689617 | 0.2117345 | 0.798 | 0.425 | 0.207351034 | count | 1 |
| FNIP1 | 0.1524273 | 0.1239874 | 1.2294 | 0.219 | 0.207492204 | count | 1 |
| PXMP4 | 0.1771528 | 0.3327826 | 0.5323 | 0.595 | 0.207515727 | count | 1 |
| FBXO36 | 0.5750336 | 0.5344084 | 1.076 | 0.282 | 0.207621008 | count | 1 |
| DUSP6 | 0.1523217 | 0.130758 | 1.1649 | 0.244 | 0.207662166 | count | 1 |
| NAP1L5 | 0.2058125 | 0.3302337 | 0.6232 | 0.533 | 0.207700869 | count | 1 |
| NRF1 | 0.3248664 | 0.2671616 | 1.216 | 0.224 | 0.207717494 | count | 1 |
| ZBTB17 | 0.1723044 | 0.2541915 | 0.6779 | 0.498 | 0.207718577 | count | 1 |
| VIPR1 | 0.5754285 | 0.5891482 | 0.9767 | 0.329 | 0.207773256 | count | 1 |
| RPL39 | 0.1442886 | 0.0165523 | 8.7171 | 4.39E-18 | 0.207999487 | count | 1.07E-13 |
| AL603839.3 | 0.5762863 | 0.8917911 | 0.6462 | 0.518 | 0.208104171 | count | 1 |
| LRRC32 | 0.5762863 | 0.9079731 | 0.6347 | 0.526 | 0.208104171 | count | 1 |
| CAMTA2 | 0.1909146 | 0.2358979 | 0.8093 | 0.418 | 0.208147334 | count | 1 |
| FAM136A | 0.1546568 | 0.1258639 | 1.2288 | 0.219 | 0.208209271 | count | 1 |
| MYCBP | 0.1632627 | 0.2000457 | 0.8161 | 0.414 | 0.20842128 | count | 1 |
| MT3 | 0.9650375 | 0.8986799 | 1.0738 | 0.283 | 0.208672812 | count | 1 |
| MED15 | 0.1607496 | 0.1520636 | 1.0571 | 0.291 | 0.209215919 | count | 1 |
| CNIH2 | 0.2766272 | 0.5925595 | 0.4668 | 0.641 | 0.209327976 | count | 1 |
| TMED1 | 0.170034 | 0.1813623 | 0.9375 | 0.349 | 0.209497153 | count | 1 |
| NGFR | 0.2982115 | 0.7527498 | 0.3962 | 0.692 | 0.209580098 | count | 1 |
| RPL37 | 0.1456434 | 0.01777 | 8.196 | 3.48E-16 | 0.209855724 | count | 8.45E-12 |
| ARHGAP27 | 0.1791431 | 0.2514122 | 0.7125 | 0.476 | 0.209863817 | count | 1 |
| TAP2 | 0.1643999 | 0.1437156 | 1.1439 | 0.253 | 0.209878908 | count | 1 |
| COMT | 0.1502886 | 0.1099111 | 1.3674 | 0.172 | 0.209903764 | count | 1 |
| TSPYL4 | 0.1781508 | 0.2521586 | 0.7065 | 0.48 | 0.21005443 | count | 1 |
| PUS7L | 0.1545621 | 0.1390308 | 1.1117 | 0.266 | 0.210068224 | count | 1 |
| RPL11 | 0.1457662 | 0.0160272 | 9.0949 | 1.57E-19 | 0.210214826 | count | 3.81E-15 |
| PPP1CB | 0.1479958 | 0.0549359 | 2.694 | 0.0071 | 0.210290318 | count | 1 |
| TNPO1 | 0.1795152 | 0.161605 | 1.1108 | 0.267 | 0.210302844 | count | 1 |
| DLD | 0.1606537 | 0.1486196 | 1.081 | 0.28 | 0.210377298 | count | 1 |
| DAP | 0.1664344 | 0.1516777 | 1.0973 | 0.273 | 0.210382476 | count | 1 |
| PROB1 | 0.5822012 | 0.6714928 | 0.867 | 0.386 | 0.21038708 | count | 1 |
| H1FX-AS1 | 0.5822012 | 0.6748901 | 0.8627 | 0.388 | 0.21038708 | count | 1 |
| TNFRSF13C | 0.9735131 | 0.4524613 | 2.1516 | 0.0315 | 0.210649907 | count | 1 |
| IGFLR1 | 0.2014201 | 0.2150961 | 0.9364 | 0.349 | 0.210659149 | count | 1 |
| KRTCAP2 | 0.148767 | 0.0741121 | 2.0073 | 0.0448 | 0.210854918 | count | 1 |
| ZNF84 | 0.1718941 | 0.2217597 | 0.7751 | 0.438 | 0.210969382 | count | 1 |
| EEF1A1 | 0.1463578 | 0.0140347 | 10.4283 | 4.39E-25 | 0.21109621 | count | 1.07E-20 |
| PSEN2 | 0.2630281 | 0.4769887 | 0.5514 | 0.581 | 0.211340515 | count | 1 |
| NT5C | 0.150939 | 0.1031714 | 1.463 | 0.144 | 0.211374921 | count | 1 |
| TRABD2A | 0.1645476 | 0.133148 | 1.2358 | 0.217 | 0.211445514 | count | 1 |
| NDC1 | 0.2404347 | 0.3987297 | 0.603 | 0.547 | 0.211568647 | count | 1 |

| | | | | | | | |
|---|---|---|---|---|---|---|---|
| MVD | 0.1693681 | 0.1949467 | 0.8688 | 0.385 | 0.211613979 | count | 1 |
| BEX3 | 0.1629633 | 0.2682441 | 0.6075 | 0.544 | 0.211755847 | count | 1 |
| AC005332.5 | 0.190017 | 0.2887842 | 0.658 | 0.511 | 0.211862389 | count | 1 |
| CIAPIN1 | 0.1690446 | 0.1627374 | 1.0388 | 0.299 | 0.211867738 | count | 1 |
| TIMM13 | 0.1586651 | 0.1263144 | 1.2561 | 0.209 | 0.211871135 | count | 1 |
| BTBD19 | 0.4470887 | 0.4838978 | 0.9239 | 0.356 | 0.211892623 | count | 1 |
| SLF2 | 0.1583321 | 0.1554527 | 1.0185 | 0.309 | 0.211988709 | count | 1 |
| APLF | 0.586357 | 0.5554759 | 1.0556 | 0.291 | 0.211992266 | count | 1 |
| AC124283.1 | 0.586571 | 0.4042995 | 1.4508 | 0.147 | 0.212074931 | count | 1 |
| MRPS14 | 0.1596556 | 0.1649277 | 0.968 | 0.333 | 0.212158827 | count | 1 |
| TRIM16 | 0.3023224 | 0.3696384 | 0.8179 | 0.413 | 0.212557295 | count | 1 |
| MEPCE | 0.1772006 | 0.1738137 | 1.0195 | 0.308 | 0.212566977 | count | 1 |
| DPAGT1 | 0.1764585 | 0.227861 | 0.7744 | 0.439 | 0.21275734 | count | 1 |
| NAPA | 0.1523353 | 0.1054009 | 1.4453 | 0.148 | 0.212799415 | count | 1 |
| KLHL3 | 0.4493411 | 0.5123164 | 0.8771 | 0.381 | 0.213016931 | count | 1 |
| DPCD | 0.5898954 | 0.804009 | 0.7337 | 0.463 | 0.213359651 | count | 1 |
| ATP13A4 | 0.5898954 | 0.8623316 | 0.6841 | 0.494 | 0.213359651 | count | 1 |
| LINC00299 | 0.5898954 | 1.130866 | 0.5216 | 0.602 | 0.213359651 | count | 1 |
| SYTL1 | 0.1520196 | 0.0978148 | 1.5542 | 0.12 | 0.213419735 | count | 1 |
| YME1L1 | 0.1530163 | 0.0829337 | 1.845 | 0.0651 | 0.213468041 | count | 1 |
| KCNA3 | 0.1640944 | 0.1539839 | 1.0657 | 0.287 | 0.213584125 | count | 1 |
| SLC16A6 | 0.1960189 | 0.3531345 | 0.5551 | 0.579 | 0.213768343 | count | 1 |
| S100A9 | 0.1696067 | 0.3042253 | 0.5575 | 0.577 | 0.213822523 | count | 1 |
| USP38 | 0.2014751 | 0.2193115 | 0.9187 | 0.358 | 0.213985113 | count | 1 |
| CTDSP1 | 0.1535674 | 0.0931748 | 1.6482 | 0.0994 | 0.214008602 | count | 1 |
| GRK4 | 0.2538186 | 0.5795117 | 0.438 | 0.661 | 0.214321044 | count | 1 |
| HKR1 | 0.1922127 | 0.3252879 | 0.5909 | 0.555 | 0.214332979 | count | 1 |
| ITGAL | 0.155822 | 0.1221202 | 1.276 | 0.202 | 0.214451703 | count | 1 |
| CD8B | 0.1504229 | 0.0774125 | 1.9431 | 0.0521 | 0.214541212 | count | 1 |
| DAGLB | 0.3352744 | 0.4241224 | 0.7905 | 0.429 | 0.214611757 | count | 1 |
| ZNF665 | 0.4530685 | 0.5516951 | 0.8212 | 0.412 | 0.214878453 | count | 1 |
| IRAK2 | 0.2226268 | 0.6436305 | 0.3459 | 0.729 | 0.214998553 | count | 1 |
| ZNF562 | 0.2227352 | 0.3013171 | 0.7392 | 0.46 | 0.215104824 | count | 1 |
| MGAT4A | 0.1528067 | 0.079919 | 1.912 | 0.056 | 0.215197519 | count | 1 |
| DDX3Y | 0.1560866 | 0.0887168 | 1.7594 | 0.0786 | 0.215371174 | count | 1 |
| NPIPA1 | 0.5959017 | 0.6385255 | 0.9332 | 0.351 | 0.215682384 | count | 1 |
| PPM1A | 0.1716778 | 0.1455647 | 1.1794 | 0.238 | 0.215827172 | count | 1 |
| EXT2 | 0.2453811 | 0.2877004 | 0.8529 | 0.394 | 0.216006048 | count | 1 |
| TNFRSF4 | 0.1596603 | 0.1770221 | 0.9019 | 0.367 | 0.216126743 | count | 1 |
| ORC5 | 0.173564 | 0.259188 | 0.6696 | 0.503 | 0.216176776 | count | 1 |
| TOMM40 | 0.1653192 | 0.142108 | 1.1633 | 0.245 | 0.216190256 | count | 1 |
| TBC1D13 | 0.2857214 | 0.3940302 | 0.7251 | 0.468 | 0.216395679 | count | 1 |
| NRSN2-AS1 | 0.3829912 | 0.7364565 | 0.52 | 0.603 | 0.216480947 | count | 1 |
| SELENOF | 0.1533887 | 0.063752 | 2.406 | 0.0162 | 0.21658571 | count | 1 |
| HSD11B1 | 0.3832281 | 0.9813934 | 0.3905 | 0.696 | 0.216620617 | count | 1 |

| | | | | | | | |
|---|---|---|---|---|---|---|---|
| CST7 | 0.1505671 | 0.045634 | 3.2995 | 0.000979 | 0.216698173 | count | 1 |
| TMEM42 | 0.1773111 | 0.2045033 | 0.867 | 0.386 | 0.216757412 | count | 1 |
| CCR4 | 0.1892961 | 0.1956133 | 0.9677 | 0.333 | 0.216963601 | count | 1 |
| PRELID2 | 1.000842 | 0.5809863 | 1.7227 | 0.085 | 0.217025047 | count | 1 |
| DPYSL2 | 0.1717613 | 0.2341138 | 0.7337 | 0.463 | 0.21714643 | count | 1 |
| PFN1 | 0.1506661 | 0.0244927 | 6.1515 | 8.57E-10 | 0.217168933 | count | 2.07E-05 |
| ZNF737 | 0.1911687 | 0.289851 | 0.6595 | 0.51 | 0.217268375 | count | 1 |
| MINPP1 | 0.2382369 | 0.371331 | 0.6416 | 0.521 | 0.21729748 | count | 1 |
| ATM | 0.1578884 | 0.0855299 | 1.846 | 0.065 | 0.217373595 | count | 1 |
| OTUD6B-AS1 | 0.1564077 | 0.1041237 | 1.5021 | 0.133 | 0.217592755 | count | 1 |
| PRR5L | 0.1683469 | 0.2326778 | 0.7235 | 0.469 | 0.217619812 | count | 1 |
| GADD45B | 0.151592 | 0.0764491 | 1.9829 | 0.0475 | 0.217834588 | count | 1 |
| SYCP2 | 0.2317383 | 0.4744518 | 0.4884 | 0.625 | 0.217945302 | count | 1 |
| SNX5 | 0.1575266 | 0.1066657 | 1.4768 | 0.14 | 0.217981727 | count | 1 |
| MGAT2 | 0.1603776 | 0.1255465 | 1.2774 | 0.202 | 0.218105823 | count | 1 |
| IMPDH1 | 0.1649884 | 0.1589199 | 1.0382 | 0.299 | 0.218309139 | count | 1 |
| SYNM | 0.248078 | 0.2548728 | 0.9733 | 0.33 | 0.218426659 | count | 1 |
| LINC00630 | 0.3106464 | 0.6149125 | 0.5052 | 0.613 | 0.218591803 | count | 1 |
| ATP8A1 | 0.1981051 | 0.1811477 | 1.0936 | 0.274 | 0.218605057 | count | 1 |
| HIST1H4D | 1.008297 | 0.7984629 | 1.2628 | 0.207 | 0.218763908 | count | 1 |
| LRRC37A2 | 0.3110017 | 0.3262602 | 0.9532 | 0.341 | 0.218849555 | count | 1 |
| CFAP45 | 0.6041095 | 0.6829297 | 0.8846 | 0.376 | 0.218859593 | count | 1 |
| PICK1 | 0.2092617 | 0.3442775 | 0.6078 | 0.543 | 0.218958701 | count | 1 |
| PCIF1 | 0.1620512 | 0.1454614 | 1.114 | 0.265 | 0.219091527 | count | 1 |
| TOMM34 | 0.1785563 | 0.2194692 | 0.8136 | 0.416 | 0.219192197 | count | 1 |
| MAN1A1 | 0.1898106 | 0.2228459 | 0.8518 | 0.394 | 0.219290757 | count | 1 |
| PECR | 0.1947219 | 0.2530122 | 0.7696 | 0.442 | 0.219320226 | count | 1 |
| MYADM | 0.1539068 | 0.060484 | 2.5446 | 0.011 | 0.219327172 | count | 1 |
| TRAPPC2 | 0.1695078 | 0.1576996 | 1.0749 | 0.283 | 0.219526382 | count | 1 |
| RTCA | 0.1617213 | 0.1205582 | 1.3414 | 0.18 | 0.219573678 | count | 1 |
| DCTN1 | 0.1729045 | 0.2054392 | 0.8416 | 0.4 | 0.219730241 | count | 1 |
| PRKAR2A-AS1 | 1.0130763 | 0.9578876 | 1.0576 | 0.29 | 0.219878597 | count | 1 |
| FADS1 | 0.4635815 | 0.6888496 | 0.673 | 0.501 | 0.220135076 | count | 1 |
| LINC01003 | 0.1671723 | 0.2127678 | 0.7857 | 0.432 | 0.220135247 | count | 1 |
| RPS3A | 0.1526986 | 0.0189377 | 8.0632 | 1.02E-15 | 0.220168979 | count | 2.48E-11 |
| MYO9A | 0.1786784 | 0.2216028 | 0.8063 | 0.42 | 0.22020668 | count | 1 |
| UTS2 | 0.3133145 | 0.4505149 | 0.6955 | 0.487 | 0.220527759 | count | 1 |
| ABHD14B | 0.1580841 | 0.0903165 | 1.7503 | 0.0802 | 0.220545854 | count | 1 |
| PTPN9 | 0.1895257 | 0.2369416 | 0.7999 | 0.424 | 0.22058574 | count | 1 |
| TDRD7 | 0.2283467 | 0.3039954 | 0.7512 | 0.453 | 0.220608096 | count | 1 |
| FCHSD1 | 0.2418373 | 0.290055 | 0.8338 | 0.404 | 0.22064085 | count | 1 |
| DPF3 | 0.6090415 | 0.6057699 | 1.0054 | 0.315 | 0.220770476 | count | 1 |
| UNK | 0.1912707 | 0.2194606 | 0.8715 | 0.384 | 0.220991088 | count | 1 |
| ZNF136 | 0.1914449 | 0.2148905 | 0.8909 | 0.373 | 0.221193964 | count | 1 |
| APH1A | 0.1591821 | 0.0919189 | 1.7318 | 0.0834 | 0.221366371 | count | 1 |

| | | | | | | | |
|---|---|---|---|---|---|---|---|
| HDHD2 | 0.2922175 | 0.2510455 | 1.164 | 0.245 | 0.221450293 | count | 1 |
| TRAPPC11 | 0.1889822 | 0.3083462 | 0.6129 | 0.54 | 0.221476425 | count | 1 |
| GNPAT | 0.1761686 | 0.1681313 | 1.0478 | 0.295 | 0.221499898 | count | 1 |
| BCL2L13 | 0.1932648 | 0.2110045 | 0.9159 | 0.36 | 0.221550206 | count | 1 |
| CRYBG2 | 0.1950274 | 0.3675389 | 0.5306 | 0.596 | 0.221691853 | count | 1 |
| 6-Mar | 0.1658252 | 0.1165719 | 1.4225 | 0.155 | 0.221854606 | count | 1 |
| CHMP1A | 0.1705508 | 0.1511014 | 1.1287 | 0.259 | 0.2220175 | count | 1 |
| ZNF212 | 1.022541 | 0.499378 | 2.0476 | 0.0407 | 0.222085915 | count | 1 |
| AC116366.1 | 0.3155884 | 0.4597021 | 0.6865 | 0.492 | 0.222178338 | count | 1 |
| AP002360.1 | 0.2199648 | 0.3229973 | 0.681 | 0.496 | 0.22217859 | count | 1 |
| LRRC1 | 0.4679489 | 0.6155716 | 0.7602 | 0.447 | 0.222321526 | count | 1 |
| CYB5B | 0.1629942 | 0.1273405 | 1.28 | 0.201 | 0.222762639 | count | 1 |
| AC103724.4 | 0.2020686 | 0.3677208 | 0.5495 | 0.583 | 0.223021952 | count | 1 |
| AFMID | 0.6155743 | 0.5792928 | 1.0626 | 0.288 | 0.223303572 | count | 1 |
| MAU2 | 0.2047378 | 0.2254868 | 0.908 | 0.364 | 0.223375721 | count | 1 |
| CYREN | 0.1933694 | 0.2087176 | 0.9265 | 0.354 | 0.223435404 | count | 1 |
| TSPAN14 | 0.1626331 | 0.121165 | 1.3422 | 0.18 | 0.223685851 | count | 1 |
| MAN1A2 | 0.1631226 | 0.1190359 | 1.3704 | 0.171 | 0.223692561 | count | 1 |
| AC034236.2 | 0.2539831 | 0.5609446 | 0.4528 | 0.651 | 0.223729803 | count | 1 |
| AL390728.5 | 0.3177755 | 0.7424223 | 0.428 | 0.669 | 0.223766464 | count | 1 |
| ZNF226 | 0.1746262 | 0.1822104 | 0.9584 | 0.338 | 0.223979273 | count | 1 |
| GOLGA7 | 0.159485 | 0.0882193 | 1.8078 | 0.0707 | 0.224154642 | count | 1 |
| MRPS17 | 0.2456553 | 0.3744017 | 0.6561 | 0.512 | 0.224187931 | count | 1 |
| TIMM23B | 0.2657194 | 0.2795647 | 0.9505 | 0.342 | 0.224592354 | count | 1 |
| DOHH | 0.1714751 | 0.1986574 | 0.8632 | 0.388 | 0.22459567 | count | 1 |
| MIF | 0.1581236 | 0.0579825 | 2.7271 | 0.00642 | 0.224788718 | count | 1 |
| ZNF649 | 0.2463186 | 0.2791948 | 0.8822 | 0.378 | 0.224804342 | count | 1 |
| HVCN1 | 0.2148226 | 0.3465209 | 0.6199 | 0.535 | 0.22484822 | count | 1 |
| TMEM131L | 0.1799073 | 0.1707216 | 1.0538 | 0.292 | 0.22484908 | count | 1 |
| PTBP2 | 0.1978285 | 0.2566469 | 0.7708 | 0.441 | 0.224903754 | count | 1 |
| MEIS3 | 0.2795933 | 0.5232689 | 0.5343 | 0.593 | 0.224979506 | count | 1 |
| ZNF141 | 0.1840098 | 0.1897864 | 0.9696 | 0.332 | 0.224994772 | count | 1 |
| ASB6 | 0.1998161 | 0.2204771 | 0.9063 | 0.365 | 0.225110326 | count | 1 |
| DOCK11 | 0.1682236 | 0.1256708 | 1.3386 | 0.181 | 0.225456852 | count | 1 |
| KANTR | 0.4748605 | 0.4708423 | 1.0085 | 0.313 | 0.22578482 | count | 1 |
| AP000894.4 | 0.4749546 | 0.6651888 | 0.714 | 0.475 | 0.225831993 | count | 1 |
| ADAM22 | 0.4749546 | 0.7259314 | 0.6543 | 0.513 | 0.225831993 | count | 1 |
| MTA3 | 0.2341351 | 0.2793758 | 0.8381 | 0.402 | 0.226288566 | count | 1 |
| AC093462.1 | 0.3528987 | 0.4832567 | 0.7303 | 0.465 | 0.226313623 | count | 1 |
| GNPTG | 0.1645654 | 0.126165 | 1.3044 | 0.192 | 0.22650431 | count | 1 |
| ORC3 | 0.171458 | 0.169478 | 1.0117 | 0.312 | 0.226631268 | count | 1 |
| LIPE-AS1 | 0.2409187 | 0.500908 | 0.481 | 0.631 | 0.226726626 | count | 1 |
| PHTF1 | 0.1900259 | 0.1839135 | 1.0332 | 0.302 | 0.226825784 | count | 1 |
| SULT2B1 | 0.4006357 | 0.7564427 | 0.5296 | 0.596 | 0.226899562 | count | 1 |
| C15orf41 | 0.4007488 | 0.425253 | 0.9424 | 0.346 | 0.226966442 | count | 1 |

| | | | | | | | |
|---|---|---|---|---|---|---|---|
| HELQ | 0.1751114 | 0.1709858 | 1.0241 | 0.306 | 0.227211265 | count | 1 |
| NCAPH2 | 0.2297153 | 0.2356547 | 0.9748 | 0.33 | 0.227308441 | count | 1 |
| LAT2 | 0.1968163 | 0.3296095 | 0.5971 | 0.55 | 0.227450739 | count | 1 |
| APOO | 0.1941328 | 0.2045195 | 0.9492 | 0.343 | 0.22755854 | count | 1 |
| BCL2A1 | 0.1639075 | 0.1220991 | 1.3424 | 0.18 | 0.227703211 | count | 1 |
| RAB22A | 0.1645545 | 0.1114131 | 1.477 | 0.14 | 0.227720664 | count | 1 |
| PPP2R1A | 0.1653117 | 0.1108537 | 1.4913 | 0.136 | 0.227760695 | count | 1 |
| ACOT4 | 0.2302288 | 0.3912317 | 0.5885 | 0.556 | 0.227824045 | count | 1 |
| VSIG8 | 0.3554044 | 0.7430064 | 0.4783 | 0.632 | 0.227980062 | count | 1 |
| RORA-AS1 | 0.3554044 | 0.7878242 | 0.4511 | 0.652 | 0.227980062 | count | 1 |
| PMS1 | 0.1712718 | 0.1808348 | 0.9471 | 0.344 | 0.228099719 | count | 1 |
| AL358472.4 | 0.4797589 | 0.6475588 | 0.7409 | 0.459 | 0.228241646 | count | 1 |
| CCDC157 | 0.6283611 | 0.4873229 | 1.2894 | 0.197 | 0.228267702 | count | 1 |
| ACAD11 | 1.0496453 | 1.026644 | 1.0224 | 0.307 | 0.228404244 | count | 1 |
| UHRF1BP1 | 0.2837965 | 0.4640883 | 0.6115 | 0.541 | 0.228445415 | count | 1 |
| TMCC1 | 0.2049984 | 0.2997815 | 0.6838 | 0.494 | 0.228728224 | count | 1 |
| DZANK1 | 0.2842279 | 0.4164779 | 0.6825 | 0.495 | 0.22880126 | count | 1 |
| C16orf70 | 0.2367701 | 0.3422971 | 0.6917 | 0.489 | 0.228875666 | count | 1 |
| CREB3L2 | 0.1850038 | 0.2147386 | 0.8615 | 0.389 | 0.228900538 | count | 1 |
| GPLD1 | 0.324964 | 0.8651271 | 0.3756 | 0.707 | 0.228990163 | count | 1 |
| FASTKD5 | 0.2268242 | 0.2489038 | 0.9113 | 0.362 | 0.229203496 | count | 1 |
| ENTPD6 | 0.2055642 | 0.2754279 | 0.7463 | 0.456 | 0.229365597 | count | 1 |
| SLC39A9 | 0.271293 | 0.3838555 | 0.7068 | 0.48 | 0.229408573 | count | 1 |
| ACTL10 | 0.357656 | 0.5197958 | 0.6881 | 0.491 | 0.229478081 | count | 1 |
| SCAANT1 | 0.4050299 | 0.7360811 | 0.5503 | 0.582 | 0.229498977 | count | 1 |
| TMEM150A | 0.2082282 | 0.3404631 | 0.6116 | 0.541 | 0.229889075 | count | 1 |
| CD53 | 0.1602469 | 0.0449943 | 3.5615 | 0.000374 | 0.229920884 | count | 1 |
| RPL22 | 0.1597183 | 0.0242 | 6.5999 | 4.75E-11 | 0.230039417 | count | 1.15E-06 |
| ZCCHC17 | 0.1655305 | 0.1110249 | 1.4909 | 0.136 | 0.230060537 | count | 1 |
| PAPOLG | 0.1976933 | 0.1942896 | 1.0175 | 0.309 | 0.230167542 | count | 1 |
| DPF2 | 0.176518 | 0.1532874 | 1.1515 | 0.25 | 0.230181735 | count | 1 |
| HIST1H3B | 0.6345952 | 0.9132613 | 0.6949 | 0.487 | 0.230690741 | count | 1 |
| AC009065.4 | 0.4847135 | 0.8253815 | 0.5873 | 0.557 | 0.230728592 | count | 1 |
| F8A1 | 0.168361 | 0.1252891 | 1.3438 | 0.179 | 0.230888716 | count | 1 |
| PPFIBP2 | 0.6355534 | 0.6683034 | 0.951 | 0.342 | 0.231063346 | count | 1 |
| ASB1 | 0.1874875 | 0.2254111 | 0.8318 | 0.406 | 0.231125355 | count | 1 |
| GIGYF1 | 0.1798375 | 0.1664695 | 1.0803 | 0.28 | 0.231174786 | count | 1 |
| CPT1A | 0.1792625 | 0.2077562 | 0.8628 | 0.388 | 0.231349332 | count | 1 |
| FZR1 | 0.2211279 | 0.2169454 | 1.0193 | 0.308 | 0.231529939 | count | 1 |
| AEBP2 | 0.1800017 | 0.130583 | 1.3784 | 0.168 | 0.231854523 | count | 1 |
| MRPL44 | 0.1808748 | 0.1876689 | 0.9638 | 0.335 | 0.232027632 | count | 1 |
| PPP1R15A | 0.1619897 | 0.0497539 | 3.2558 | 0.00114 | 0.232036638 | count | 1 |
| FAIM | 0.2079404 | 0.2767322 | 0.7514 | 0.452 | 0.232042685 | count | 1 |
| DNASE1L2 | 1.0671373 | 1.0350485 | 1.031 | 0.303 | 0.232479053 | count | 1 |
| AP000845.1 | 1.0671373 | 1.154511 | 0.9243 | 0.355 | 0.232479053 | count | 1 |

| | | | | | | | |
|---|---|---|---|---|---|---|---|
| EIF4EBP3 | 1.0671373 | 1.292699 | 0.8255 | 0.409 | 0.232479053 | count | 1 |
| HCG25 | 1.0671373 | 1.292699 | 0.8255 | 0.409 | 0.232479053 | count | 1 |
| MTURN | 0.1947808 | 0.2411326 | 0.8078 | 0.419 | 0.232541209 | count | 1 |
| SLC25A12 | 0.2107031 | 0.2274294 | 0.9265 | 0.354 | 0.232649239 | count | 1 |
| SPN | 0.1665939 | 0.100702 | 1.6543 | 0.0982 | 0.232702661 | count | 1 |
| AC008440.2 | 1.0685188 | 0.986138 | 1.0835 | 0.279 | 0.232800788 | count | 1 |
| P2RX7 | 0.2409442 | 0.4025827 | 0.5985 | 0.55 | 0.232975462 | count | 1 |
| C20orf27 | 0.1730226 | 0.1637387 | 1.0567 | 0.291 | 0.232995216 | count | 1 |
| ABLIM2 | 0.6410546 | 0.9563531 | 0.6703 | 0.503 | 0.233203367 | count | 1 |
| AL627309.1 | 0.6410546 | 1.065743 | 0.6015 | 0.548 | 0.233203367 | count | 1 |
| KIF9 | 0.2163648 | 0.3747097 | 0.5774 | 0.564 | 0.233214518 | count | 1 |
| AF117829.1 | 0.3633597 | 0.5218156 | 0.6963 | 0.486 | 0.233275246 | count | 1 |
| TTLL12 | 0.641413 | 0.5420857 | 1.1832 | 0.237 | 0.233342823 | count | 1 |
| TMEM185B | 0.2308649 | 0.3293907 | 0.7009 | 0.483 | 0.233344021 | count | 1 |
| CDAN1 | 0.2647404 | 0.4624963 | 0.5724 | 0.567 | 0.233400991 | count | 1 |
| MTR | 0.2232186 | 0.1973814 | 1.1309 | 0.258 | 0.233746336 | count | 1 |
| INO80C | 0.1877031 | 0.1777054 | 1.0563 | 0.291 | 0.233882071 | count | 1 |
| PI4KA | 0.3643158 | 0.3015617 | 1.2081 | 0.227 | 0.233912093 | count | 1 |
| USE1 | 0.1777394 | 0.1352479 | 1.3142 | 0.189 | 0.234095629 | count | 1 |
| FOXRED1 | 0.2655634 | 0.3243551 | 0.8187 | 0.413 | 0.234141447 | count | 1 |
| AC021054.1 | 0.2656194 | 0.3998324 | 0.6643 | 0.507 | 0.234191832 | count | 1 |
| PTPDC1 | 0.4922575 | 0.5851279 | 0.8413 | 0.4 | 0.234518938 | count | 1 |
| CNIH3 | 0.4137691 | 0.5927866 | 0.698 | 0.485 | 0.234674207 | count | 1 |
| AARSD1 | 0.2776661 | 0.2731615 | 1.0165 | 0.309 | 0.234920081 | count | 1 |
| TMEM241 | 0.6459038 | 0.8841578 | 0.7305 | 0.465 | 0.235090904 | count | 1 |
| PLEKHG3 | 0.20878 | 0.2767574 | 0.7544 | 0.451 | 0.235304334 | count | 1 |
| PABPC1L | 0.3339768 | 0.5536195 | 0.6033 | 0.546 | 0.235547693 | count | 1 |
| TMEM106C | 0.1770199 | 0.1542246 | 1.1478 | 0.251 | 0.235776905 | count | 1 |
| HACD1 | 0.3106007 | 0.4622497 | 0.6719 | 0.502 | 0.235781019 | count | 1 |
| FBXL14 | 0.4951706 | 0.5188702 | 0.9543 | 0.34 | 0.235983747 | count | 1 |
| TLNRD1 | 0.1812464 | 0.173316 | 1.0458 | 0.296 | 0.23599245 | count | 1 |
| AC093010.2 | 0.2137022 | 0.2392581 | 0.8932 | 0.372 | 0.235994779 | count | 1 |
| GSTM3 | 0.495379 | 0.2099172 | 2.3599 | 0.0183 | 0.236088574 | count | 1 |
| ARRDC2 | 0.1830586 | 0.1507001 | 1.2147 | 0.225 | 0.236268194 | count | 1 |
| PRRG2 | 0.2294961 | 0.2850755 | 0.805 | 0.421 | 0.236361236 | count | 1 |
| PGPEP1 | 0.209744 | 0.2214372 | 0.9472 | 0.344 | 0.236401042 | count | 1 |
| SPIN2B | 0.2259373 | 0.2852027 | 0.7922 | 0.428 | 0.236629145 | count | 1 |
| MRM2 | 0.1926815 | 0.2247302 | 0.8574 | 0.391 | 0.236636092 | count | 1 |
| RABL6 | 0.1767748 | 0.1310382 | 1.349 | 0.177 | 0.236747775 | count | 1 |
| ECE1 | 0.1994587 | 0.2177797 | 0.9159 | 0.36 | 0.236805994 | count | 1 |
| THAP2 | 0.1895868 | 0.1941523 | 0.9765 | 0.329 | 0.23701058 | count | 1 |
| DDX41 | 0.1946321 | 0.2088043 | 0.9321 | 0.351 | 0.237033019 | count | 1 |
| IDE | 0.2022462 | 0.2509196 | 0.806 | 0.42 | 0.237143558 | count | 1 |
| DCAF1 | 0.2397452 | 0.2775984 | 0.8636 | 0.388 | 0.237384613 | count | 1 |
| GLIPR1 | 0.1675257 | 0.0646327 | 2.592 | 0.00958 | 0.237401489 | count | 1 |

| | | | | | | | |
|---|---|---|---|---|---|---|---|
| PUF60 | 0.1686212 | 0.0796384 | 2.1173 | 0.0343 | 0.237402243 | count | 1 |
| POLG | 0.2522903 | 0.2576297 | 0.9793 | 0.328 | 0.237617073 | count | 1 |
| AL137802.2 | 0.4190782 | 0.5427642 | 0.7721 | 0.44 | 0.237821707 | count | 1 |
| ARRB1 | 0.2696769 | 0.3230591 | 0.8348 | 0.404 | 0.237843532 | count | 1 |
| MPZL3 | 0.1833602 | 0.1688073 | 1.0862 | 0.277 | 0.2379556 | count | 1 |
| MRPS35 | 0.1724253 | 0.1276627 | 1.3506 | 0.177 | 0.238023241 | count | 1 |
| SELENOI | 0.2608712 | 0.2395517 | 1.089 | 0.276 | 0.238340738 | count | 1 |
| ZNF830 | 0.1820385 | 0.1413219 | 1.2881 | 0.198 | 0.238480097 | count | 1 |
| CHAC1 | 1.0936224 | 0.9280015 | 1.1785 | 0.239 | 0.238643104 | count | 1 |
| RFPL1S | 0.5006674 | 0.9486386 | 0.5278 | 0.598 | 0.238749489 | count | 1 |
| MMP11 | 0.5006674 | 0.9530332 | 0.5253 | 0.599 | 0.238749489 | count | 1 |
| YIPF4 | 0.186526 | 0.1373614 | 1.3579 | 0.175 | 0.238790059 | count | 1 |
| PIP4P1 | 0.1739502 | 0.1121776 | 1.5507 | 0.121 | 0.238933255 | count | 1 |
| TTC32 | 0.1765291 | 0.1572188 | 1.1228 | 0.262 | 0.23901394 | count | 1 |
| MAST2 | 0.421118 | 0.6224239 | 0.6766 | 0.499 | 0.239031698 | count | 1 |
| PCCB | 0.2039148 | 0.2624847 | 0.7769 | 0.437 | 0.239115449 | count | 1 |
| IGSF9B | 0.6566277 | 1.076327 | 0.6101 | 0.542 | 0.239268911 | count | 1 |
| AL136961.1 | 0.6566277 | 1.076327 | 0.6101 | 0.542 | 0.239268911 | count | 1 |
| BRSK1 | 0.6566277 | 1.076327 | 0.6101 | 0.542 | 0.239268911 | count | 1 |
| EPHB4 | 0.6566277 | 1.2255571 | 0.5358 | 0.592 | 0.239268911 | count | 1 |
| HPS6 | 0.214476 | 0.2877615 | 0.7453 | 0.456 | 0.239408475 | count | 1 |
| RPL17 | 0.1698152 | 0.0704761 | 2.4095 | 0.016 | 0.2394352 | count | 1 |
| DYM | 0.1944706 | 0.2022753 | 0.9614 | 0.336 | 0.239784331 | count | 1 |
| RAP1B | 0.1673909 | 0.0447086 | 3.744 | 0.000184 | 0.239838766 | count | 1 |
| AC022087.1 | 0.6581179 | 0.7439199 | 0.8847 | 0.376 | 0.239849888 | count | 1 |
| ZNF256 | 0.2258023 | 0.3913231 | 0.577 | 0.564 | 0.240139231 | count | 1 |
| AC133550.2 | 0.3161908 | 0.5053679 | 0.6257 | 0.532 | 0.240146494 | count | 1 |
| ANKRD26 | 0.2079175 | 0.2079395 | 0.9999 | 0.317 | 0.240389311 | count | 1 |
| TSPYL2 | 0.1726651 | 0.0733286 | 2.3547 | 0.0186 | 0.240429426 | count | 1 |
| GABARAPL1 | 0.1727573 | 0.0870281 | 1.9851 | 0.0472 | 0.240650044 | count | 1 |
| SC5D | 0.1963069 | 0.1121418 | 1.7505 | 0.0801 | 0.241115397 | count | 1 |
| MYO1G | 0.1755293 | 0.1099092 | 1.597 | 0.11 | 0.241194932 | count | 1 |
| CES2 | 0.2269759 | 0.3368161 | 0.6739 | 0.5 | 0.24140244 | count | 1 |
| YPEL5 | 0.1688789 | 0.0513835 | 3.2866 | 0.001 | 0.241412085 | count | 1 |
| TAF6L | 0.2105383 | 0.315314 | 0.6677 | 0.504 | 0.24152863 | count | 1 |
| TRAF3IP1 | 0.2344776 | 0.2916064 | 0.8041 | 0.421 | 0.241561325 | count | 1 |
| RGL2 | 0.2093387 | 0.2108116 | 0.993 | 0.321 | 0.242046462 | count | 1 |
| ACVR1B | 0.3765988 | 0.5731087 | 0.6571 | 0.511 | 0.242102142 | count | 1 |
| MIR4422HG | 0.3430909 | 0.7632363 | 0.4495 | 0.653 | 0.242188087 | count | 1 |
| C14orf93 | 0.2351563 | 0.4028654 | 0.5837 | 0.559 | 0.24227 | count | 1 |
| HAUS4 | 0.2572308 | 0.4104178 | 0.6268 | 0.531 | 0.24235299 | count | 1 |
| C18orf21 | 0.1831809 | 0.1621364 | 1.1298 | 0.259 | 0.242457746 | count | 1 |
| BPNT1 | 0.2155875 | 0.2579359 | 0.8358 | 0.403 | 0.243050707 | count | 1 |
| NIPAL2 | 0.6668695 | 0.6241691 | 1.0684 | 0.285 | 0.243263854 | count | 1 |
| HSDL1 | 0.2228867 | 0.3481591 | 0.6402 | 0.522 | 0.24339717 | count | 1 |

| | | | | | | | |
|---|---|---|---|---|---|---|---|
| KLF2 | 0.1690459 | 0.0437749 | 3.8617 | 0.000115 | 0.243462867 | count | 1 |
| AL133338.1 | 0.6674347 | 0.5657726 | 1.1797 | 0.238 | 0.243484458 | count | 1 |
| RAB29 | 0.1765646 | 0.1186648 | 1.4879 | 0.137 | 0.243672944 | count | 1 |
| CYB5D2 | 0.2233117 | 0.2544677 | 0.8776 | 0.38 | 0.243866387 | count | 1 |
| SUN2 | 0.1739304 | 0.0848308 | 2.0503 | 0.0404 | 0.244097958 | count | 1 |
| SNRNP40 | 0.1743126 | 0.1038858 | 1.6779 | 0.0935 | 0.244142667 | count | 1 |
| METTL17 | 0.18963 | 0.1657033 | 1.1444 | 0.253 | 0.24430884 | count | 1 |
| ZNF333 | 0.3460685 | 0.3790603 | 0.913 | 0.361 | 0.244359474 | count | 1 |
| PHB | 0.1741159 | 0.0881125 | 1.9761 | 0.0482 | 0.244443488 | count | 1 |
| TRAV8-4 | 1.118681 | 1.004871 | 1.1133 | 0.266 | 0.24446708 | count | 1 |
| CDPF1 | 0.2373241 | 0.2903236 | 0.8174 | 0.414 | 0.244533853 | count | 1 |
| RABGGTA | 0.1919796 | 0.1937806 | 0.9907 | 0.322 | 0.244679594 | count | 1 |
| RFT1 | 0.2241613 | 0.2703732 | 0.8291 | 0.407 | 0.244804423 | count | 1 |
| OPRM1 | 0.6711602 | 0.6639491 | 1.0109 | 0.312 | 0.244938699 | count | 1 |
| PPIE | 0.1797478 | 0.1247441 | 1.4409 | 0.15 | 0.245009826 | count | 1 |
| PIP4K2A | 0.1725109 | 0.0630261 | 2.7371 | 0.00623 | 0.245203052 | count | 1 |
| RABGGTB | 0.1807944 | 0.1176672 | 1.5365 | 0.125 | 0.245245574 | count | 1 |
| ZNF227 | 0.3472854 | 0.267404 | 1.2987 | 0.194 | 0.245247239 | count | 1 |
| ABR | 0.2091053 | 0.2405334 | 0.8693 | 0.385 | 0.245250774 | count | 1 |
| JMJD8 | 0.1894178 | 0.1551599 | 1.2208 | 0.222 | 0.245415902 | count | 1 |
| ARHGAP5 | 0.2015385 | 0.1215587 | 1.658 | 0.0974 | 0.245498412 | count | 1 |
| CASP10 | 0.2249058 | 0.2949582 | 0.7625 | 0.446 | 0.245626484 | count | 1 |
| TGFBR1 | 0.1910874 | 0.2215623 | 0.8625 | 0.388 | 0.245698708 | count | 1 |
| ENO2 | 0.1904199 | 0.1651704 | 1.1529 | 0.249 | 0.245808736 | count | 1 |
| TXK | 0.2036885 | 0.1610269 | 1.2649 | 0.206 | 0.245817295 | count | 1 |
| TM2D2 | 0.1869492 | 0.1770649 | 1.0558 | 0.291 | 0.245947661 | count | 1 |
| CTNNBIP1 | 0.1891822 | 0.1849657 | 1.0228 | 0.306 | 0.245959146 | count | 1 |
| ZKSCAN4 | 0.6741067 | 0.4909954 | 1.3729 | 0.17 | 0.246089404 | count | 1 |
| ZNF721 | 0.1842368 | 0.1292078 | 1.4259 | 0.154 | 0.246116355 | count | 1 |
| PRH1 | 0.1896614 | 0.1716998 | 1.1046 | 0.269 | 0.246165283 | count | 1 |
| AC022211.2 | 0.3239886 | 0.6729109 | 0.4815 | 0.63 | 0.246241876 | count | 1 |
| METTL13 | 0.254737 | 0.2827632 | 0.9009 | 0.368 | 0.246536159 | count | 1 |
| NEMF | 0.1777525 | 0.1011449 | 1.7574 | 0.0789 | 0.24662795 | count | 1 |
| CYB561D2 | 0.1781245 | 0.112922 | 1.5774 | 0.115 | 0.24672119 | count | 1 |
| YPEL3 | 0.1730075 | 0.061896 | 2.7951 | 0.00522 | 0.24676104 | count | 1 |
| WDR7 | 0.4345833 | 0.3797913 | 1.1443 | 0.253 | 0.247028809 | count | 1 |
| GPR108 | 0.1789752 | 0.1150318 | 1.5559 | 0.12 | 0.247225492 | count | 1 |
| NPRL2 | 0.1840238 | 0.1548486 | 1.1884 | 0.235 | 0.247287331 | count | 1 |
| ATAD3B | 0.2709153 | 0.332575 | 0.8146 | 0.415 | 0.247697309 | count | 1 |
| GINS3 | 0.3507412 | 0.4295772 | 0.8165 | 0.414 | 0.24776892 | count | 1 |
| CDT1 | 0.5193386 | 0.5637906 | 0.9212 | 0.357 | 0.248160612 | count | 1 |
| SNX8 | 0.216557 | 0.3417624 | 0.6336 | 0.526 | 0.248495644 | count | 1 |
| HDAC10 | 0.2304103 | 0.248669 | 0.9266 | 0.354 | 0.248532792 | count | 1 |
| ENOSF1 | 0.1965342 | 0.1701515 | 1.1551 | 0.248 | 0.248623797 | count | 1 |
| AC044802.2 | 0.3522262 | 0.7195787 | 0.4895 | 0.625 | 0.248852942 | count | 1 |

| | | | | | | | |
|---|---|---|---|---|---|---|---|
| AC127024.5 | 0.5207712 | 0.7348604 | 0.7087 | 0.479 | 0.248883752 | count | 1 |
| C2CD2L | 0.2941698 | 0.3172534 | 0.9272 | 0.354 | 0.249214155 | count | 1 |
| AAMDC | 0.1885269 | 0.2082655 | 0.9052 | 0.365 | 0.249267024 | count | 1 |
| TPRA1 | 0.1897738 | 0.2056336 | 0.9229 | 0.356 | 0.249343895 | count | 1 |
| ZNF395 | 0.4385352 | 0.4657294 | 0.9416 | 0.346 | 0.249378983 | count | 1 |
| PHKG2 | 0.1898481 | 0.1498721 | 1.2667 | 0.205 | 0.249441858 | count | 1 |
| ZNF518B | 0.2265329 | 0.226139 | 1.0017 | 0.317 | 0.250316779 | count | 1 |
| ADO | 0.2037534 | 0.2209078 | 0.9223 | 0.356 | 0.250318468 | count | 1 |
| HUS1B | 0.3104762 | 0.3899372 | 0.7962 | 0.426 | 0.250492594 | count | 1 |
| CEACAM1 | 0.6856421 | 0.4626929 | 1.4819 | 0.138 | 0.250597411 | count | 1 |
| JMJD4 | 0.218575 | 0.2501224 | 0.8739 | 0.382 | 0.250832261 | count | 1 |
| VPS53 | 0.2050466 | 0.1965165 | 1.0434 | 0.297 | 0.250883286 | count | 1 |
| MTFR1 | 0.2662282 | 0.3440975 | 0.7737 | 0.439 | 0.250984619 | count | 1 |
| CD226 | 0.1838873 | 0.1085371 | 1.6942 | 0.0903 | 0.251028369 | count | 1 |
| ZNF236-DT | 0.3899848 | 0.6837518 | 0.5704 | 0.568 | 0.251045182 | count | 1 |
| MFSD1 | 0.1899696 | 0.1746943 | 1.0874 | 0.277 | 0.251180777 | count | 1 |
| MSC | 0.1997736 | 0.2908038 | 0.687 | 0.492 | 0.251336969 | count | 1 |
| AC004918.1 | 0.2537751 | 0.3511011 | 0.7228 | 0.47 | 0.251497075 | count | 1 |
| CCDC84 | 0.2064847 | 0.1738596 | 1.1877 | 0.235 | 0.251563084 | count | 1 |
| ERCC5 | 0.2277868 | 0.3071316 | 0.7417 | 0.458 | 0.251717205 | count | 1 |
| LAX1 | 0.19342 | 0.1648059 | 1.1736 | 0.241 | 0.252307153 | count | 1 |
| IL2RG | 0.1760629 | 0.04553 | 3.867 | 0.000112 | 0.25246812 | count | 1 |
| SESTD1 | 0.3573562 | 0.5077082 | 0.7039 | 0.482 | 0.252599536 | count | 1 |
| ZNF442 | 0.5281581 | 1.206944 | 0.4376 | 0.662 | 0.252614712 | count | 1 |
| AC008074.3 | 0.4443876 | 0.6300033 | 0.7054 | 0.481 | 0.252861893 | count | 1 |
| ACVR2B | 1.1551136 | 0.5292918 | 2.1824 | 0.0292 | 0.252916914 | count | 1 |
| LGR6 | 0.268532 | 0.4280607 | 0.6273 | 0.53 | 0.253196163 | count | 1 |
| CRYZL1 | 0.1924178 | 0.1816887 | 1.0591 | 0.29 | 0.253495237 | count | 1 |
| RNF121 | 0.2383241 | 0.2794054 | 0.853 | 0.394 | 0.253623874 | count | 1 |
| LINC02328 | 0.2463353 | 0.3398491 | 0.7248 | 0.469 | 0.253949343 | count | 1 |
| DMWD | 0.2116043 | 0.2388181 | 0.886 | 0.376 | 0.25414166 | count | 1 |
| ADK | 0.1918682 | 0.155015 | 1.2377 | 0.216 | 0.254280848 | count | 1 |
| SNN | 0.2088356 | 0.1970178 | 1.06 | 0.289 | 0.254446153 | count | 1 |
| FES | 0.269846 | 0.6271645 | 0.4303 | 0.667 | 0.254457818 | count | 1 |
| FBXO7 | 0.1831027 | 0.0964385 | 1.8986 | 0.0577 | 0.254462564 | count | 1 |
| FAM32A | 0.1820276 | 0.1038034 | 1.7536 | 0.0796 | 0.254485507 | count | 1 |
| AC025171.3 | 0.2391312 | 0.2594496 | 0.9217 | 0.357 | 0.254493542 | count | 1 |
| DEGS1 | 0.1830358 | 0.0936374 | 1.9547 | 0.0507 | 0.254584321 | count | 1 |
| NEFL | 0.3006164 | 0.5208499 | 0.5772 | 0.564 | 0.254805892 | count | 1 |
| TRRAP | 0.2634222 | 0.2528768 | 1.0417 | 0.298 | 0.255085588 | count | 1 |
| ZNF500 | 0.3163341 | 0.3816846 | 0.8288 | 0.407 | 0.255344035 | count | 1 |
| PNPLA4 | 0.2791442 | 0.379171 | 0.7362 | 0.462 | 0.255371152 | count | 1 |
| NPHP4 | 0.4486493 | 0.7586774 | 0.5914 | 0.554 | 0.255400035 | count | 1 |
| EBLN3P | 0.1885006 | 0.1138655 | 1.6555 | 0.0979 | 0.255418532 | count | 1 |
| CIAO1 | 0.1927598 | 0.1335671 | 1.4432 | 0.149 | 0.25546628 | count | 1 |

| | | | | | | | |
|---|---|---|---|---|---|---|---|
| FUCA2 | 0.2311944 | 0.2817779 | 0.8205 | 0.412 | 0.255523703 | count | 1 |
| WDR5 | 0.2525367 | 0.3142041 | 0.8037 | 0.422 | 0.255580042 | count | 1 |
| GANAB | 0.2050678 | 0.2065718 | 0.9927 | 0.321 | 0.255643246 | count | 1 |
| AC007613.1 | 0.2894427 | 0.4326105 | 0.6691 | 0.504 | 0.255658861 | count | 1 |
| CEP63 | 0.1898182 | 0.1602012 | 1.1849 | 0.236 | 0.255856345 | count | 1 |
| LCORL | 0.216733 | 0.223985 | 0.9676 | 0.333 | 0.255918204 | count | 1 |
| RIPK3 | 0.2155084 | 0.283538 | 0.7601 | 0.447 | 0.25600933 | count | 1 |
| PIP5K1A | 0.2534104 | 0.2622441 | 0.9663 | 0.334 | 0.256477487 | count | 1 |
| AC026471.1 | 0.290358 | 0.405304 | 0.7164 | 0.474 | 0.256484879 | count | 1 |
| AC055822.1 | 0.5358966 | 0.7069358 | 0.7581 | 0.448 | 0.256527315 | count | 1 |
| MAPK8 | 0.2446976 | 0.2422225 | 1.0102 | 0.312 | 0.25654169 | count | 1 |
| CLCN5 | 0.700839 | 1.05296 | 0.6656 | 0.506 | 0.256543988 | count | 1 |
| AP001505.1 | 1.1712812 | 0.9345901 | 1.2533 | 0.21 | 0.256658739 | count | 1 |
| TRGV4 | 0.22989 | 0.4502634 | 0.5106 | 0.61 | 0.256795263 | count | 1 |
| NDRG3 | 0.2107939 | 0.2012034 | 1.0477 | 0.295 | 0.256848027 | count | 1 |
| DDX28 | 0.2450643 | 0.3411443 | 0.7184 | 0.473 | 0.256931243 | count | 1 |
| CCDC174 | 0.1857808 | 0.1092707 | 1.7002 | 0.0892 | 0.257072618 | count | 1 |
| RASL11A | 0.2539977 | 0.3509981 | 0.7236 | 0.469 | 0.257080789 | count | 1 |
| AVEN | 0.2222481 | 0.2282283 | 0.9738 | 0.33 | 0.257106458 | count | 1 |
| MARK2 | 0.1954079 | 0.1643207 | 1.1892 | 0.234 | 0.257115201 | count | 1 |
| C7orf43 | 0.2260897 | 0.2496643 | 0.9056 | 0.365 | 0.257347092 | count | 1 |
| ARSG | 0.2596095 | 0.3128609 | 0.8298 | 0.407 | 0.257371799 | count | 1 |
| ARIH1 | 0.2051936 | 0.1365737 | 1.5024 | 0.133 | 0.257427507 | count | 1 |
| PIGB | 0.2455549 | 0.3686562 | 0.6661 | 0.505 | 0.257452457 | count | 1 |
| AFG3L2 | 0.2107383 | 0.1628476 | 1.2941 | 0.196 | 0.257892655 | count | 1 |
| DCTD | 0.1911202 | 0.1536715 | 1.2437 | 0.214 | 0.25797633 | count | 1 |
| TMEM107 | 0.1931801 | 0.1316278 | 1.4676 | 0.142 | 0.258098616 | count | 1 |
| RELB | 0.1930711 | 0.121222 | 1.5927 | 0.111 | 0.258412757 | count | 1 |
| SCIMP | 0.453721 | 0.8889189 | 0.5104 | 0.61 | 0.258422624 | count | 1 |
| PIGX | 0.2023665 | 0.1975705 | 1.0243 | 0.306 | 0.258583339 | count | 1 |
| GSTP1 | 0.1803912 | 0.0559496 | 3.2242 | 0.00128 | 0.258587842 | count | 1 |
| LINC01126 | 0.7066026 | 0.3305297 | 2.1378 | 0.0326 | 0.258801601 | count | 1 |
| LRRC8C-DT | 0.4017789 | 0.425257 | 0.9448 | 0.345 | 0.258939413 | count | 1 |
| TSFM | 0.1901504 | 0.136314 | 1.3949 | 0.163 | 0.258958395 | count | 1 |
| PRR14 | 0.195083 | 0.1647539 | 1.1841 | 0.236 | 0.259117091 | count | 1 |
| ZBTB49 | 0.2401567 | 0.2636339 | 0.9109 | 0.362 | 0.259173175 | count | 1 |
| RAB24 | 0.4022613 | 0.4874872 | 0.8252 | 0.409 | 0.25926258 | count | 1 |
| RASSF7 | 0.1909407 | 0.1387518 | 1.3761 | 0.169 | 0.25962546 | count | 1 |
| LINC02256 | 0.2753724 | 0.3828444 | 0.7193 | 0.472 | 0.259765934 | count | 1 |
| AC008966.1 | 0.3217904 | 0.6694465 | 0.4807 | 0.631 | 0.259866224 | count | 1 |
| NRG2 | 0.5425451 | 0.4613115 | 1.1761 | 0.24 | 0.259892029 | count | 1 |
| RPS6KA4 | 0.2326992 | 0.2627307 | 0.8857 | 0.376 | 0.259966225 | count | 1 |
| HSH2D | 0.1960383 | 0.1768046 | 1.1088 | 0.268 | 0.260111247 | count | 1 |
| GOLGA8R | 0.2685651 | 0.6069508 | 0.4425 | 0.658 | 0.260151786 | count | 1 |
| AMIGO1 | 0.4579309 | 0.5511985 | 0.8308 | 0.406 | 0.260933276 | count | 1 |

| | | | | | | | |
|---|---|---|---|---|---|---|---|
| NAP1L3 | 0.4048276 | 0.5141736 | 0.7873 | 0.431 | 0.260982203 | count | 1 |
| CD274 | 0.4048276 | 0.5208403 | 0.7773 | 0.437 | 0.260982203 | count | 1 |
| MYO19 | 0.2337135 | 0.3168468 | 0.7376 | 0.461 | 0.261111306 | count | 1 |
| MSRB3 | 1.1910189 | 0.8327822 | 1.4302 | 0.153 | 0.261219317 | count | 1 |
| C6orf226 | 0.1968523 | 0.1728884 | 1.1386 | 0.255 | 0.261474641 | count | 1 |
| AC026801.2 | 0.7137402 | 0.9486698 | 0.7524 | 0.452 | 0.261598818 | count | 1 |
| GXYLT1 | 0.2098523 | 0.1921233 | 1.0923 | 0.275 | 0.261642252 | count | 1 |
| PRKCD | 0.2774304 | 0.3046282 | 0.9107 | 0.363 | 0.261743472 | count | 1 |
| ILF3-DT | 0.189167 | 0.1201836 | 1.574 | 0.116 | 0.262106514 | count | 1 |
| NR3C1 | 0.1859424 | 0.0670414 | 2.7735 | 0.00558 | 0.262108147 | count | 1 |
| GDPD5 | 0.4607479 | 0.3446708 | 1.3368 | 0.181 | 0.26261409 | count | 1 |
| OSER1-DT | 0.233125 | 0.3020294 | 0.7719 | 0.44 | 0.263025073 | count | 1 |
| ZNF71 | 0.2598232 | 0.3650089 | 0.7118 | 0.477 | 0.26306687 | count | 1 |
| NEK8 | 0.4085878 | 0.4836629 | 0.8448 | 0.398 | 0.263502965 | count | 1 |
| CPT2 | 0.2723806 | 0.3790704 | 0.7185 | 0.472 | 0.263912142 | count | 1 |
| RALGDS | 0.2080174 | 0.156011 | 1.3334 | 0.183 | 0.263916109 | count | 1 |
| NOB1 | 0.2001507 | 0.1390387 | 1.4395 | 0.15 | 0.264050608 | count | 1 |
| CRAT | 0.2725529 | 0.3581078 | 0.7611 | 0.447 | 0.264081987 | count | 1 |
| ZNF607 | 0.7211893 | 0.6236788 | 1.1563 | 0.248 | 0.264520126 | count | 1 |
| HDAC6 | 0.3119173 | 0.3096166 | 1.0074 | 0.314 | 0.264619126 | count | 1 |
| PRRT1 | 0.7216462 | 0.675872 | 1.0677 | 0.286 | 0.264699257 | count | 1 |
| IL10RA | 0.1883163 | 0.0663249 | 2.8393 | 0.00455 | 0.265228934 | count | 1 |
| PHETA2 | 1.2086648 | 0.8877965 | 1.3614 | 0.173 | 0.265288873 | count | 1 |
| MORN4 | 1.2086648 | 1.000707 | 1.2078 | 0.227 | 0.265288873 | count | 1 |
| HACE1 | 0.4653799 | 0.3591175 | 1.2959 | 0.195 | 0.265379276 | count | 1 |
| CLASRP | 0.2221493 | 0.226507 | 0.9808 | 0.327 | 0.265470254 | count | 1 |
| BAHD1 | 0.4124924 | 0.3390988 | 1.2164 | 0.224 | 0.26612195 | count | 1 |
| SLC2A6 | 0.3293344 | 0.4987278 | 0.6603 | 0.509 | 0.26612396 | count | 1 |
| AC099778.1 | 0.4125294 | 0.3743406 | 1.102 | 0.271 | 0.266146764 | count | 1 |
| AC005523.2 | 0.2581173 | 0.3385538 | 0.7624 | 0.446 | 0.266271898 | count | 1 |
| CREB3L4 | 0.2473883 | 0.3849352 | 0.6427 | 0.52 | 0.267073654 | count | 1 |
| ANO6 | 0.220196 | 0.2172033 | 1.0138 | 0.311 | 0.267164655 | count | 1 |
| POLL | 0.2309978 | 0.2417606 | 0.9555 | 0.339 | 0.267321194 | count | 1 |
| SMIM4 | 0.2082918 | 0.1984294 | 1.0497 | 0.294 | 0.267364908 | count | 1 |
| SEPSECS | 0.3513019 | 0.4176291 | 0.8412 | 0.4 | 0.267644153 | count | 1 |
| THOC5 | 0.2371893 | 0.2527124 | 0.9386 | 0.348 | 0.267657762 | count | 1 |
| ZNF512 | 0.3155223 | 0.4283682 | 0.7366 | 0.461 | 0.267752429 | count | 1 |
| PIP5K1B | 0.5580796 | 0.9070773 | 0.6153 | 0.538 | 0.267765132 | count | 1 |
| PPP1R10 | 0.1885191 | 0.0624171 | 3.0203 | 0.00254 | 0.267857364 | count | 1 |
| SIK1 | 0.469611 | 0.4163395 | 1.128 | 0.259 | 0.267906694 | count | 1 |
| AL356488.3 | 0.4698787 | 0.5948055 | 0.79 | 0.43 | 0.268066648 | count | 1 |
| MTERF3 | 0.2150811 | 0.2111408 | 1.0187 | 0.308 | 0.26819986 | count | 1 |
| JUNB | 0.1863197 | 0.0321577 | 5.7939 | 7.50E-09 | 0.268496389 | count | 0.000181373 |
| RETSAT | 0.3792704 | 0.4862186 | 0.78 | 0.435 | 0.268634768 | count | 1 |
| UNKL | 0.2772178 | 0.2914635 | 0.9511 | 0.342 | 0.268681553 | count | 1 |

| | | | | | | | |
|---|---|---|---|---|---|---|---|
| GTF2IRD2 | 0.4713556 | 0.4175195 | 1.1289 | 0.259 | 0.26894924 | count | 1 |
| PRDM5 | 1.2262026 | 1.232262 | 0.9951 | 0.32 | 0.269325712 | count | 1 |
| BX322234.1 | 1.2262026 | 1.232262 | 0.9951 | 0.32 | 0.269325712 | count | 1 |
| AC022306.2 | 1.2262026 | 1.232262 | 0.9951 | 0.32 | 0.269325712 | count | 1 |
| BIN2 | 0.1891362 | 0.0610605 | 3.0975 | 0.00197 | 0.269399575 | count | 1 |
| RNF123 | 0.2660121 | 0.3763995 | 0.7067 | 0.48 | 0.269430012 | count | 1 |
| MED23 | 0.2147458 | 0.1748395 | 1.2282 | 0.219 | 0.269479089 | count | 1 |
| ATG16L2 | 0.2126175 | 0.1652993 | 1.2863 | 0.198 | 0.269782522 | count | 1 |
| SPHK1 | 0.5621109 | 0.6034413 | 0.9315 | 0.352 | 0.26981075 | count | 1 |
| MTFMT | 0.2195733 | 0.2206958 | 0.9949 | 0.32 | 0.269881794 | count | 1 |
| LETM2 | 0.239522 | 0.2380224 | 1.0063 | 0.314 | 0.270317293 | count | 1 |
| ZBTB40 | 0.250433 | 0.2486874 | 1.007 | 0.314 | 0.270401365 | count | 1 |
| TMEM182 | 0.5635461 | 0.5001844 | 1.1267 | 0.26 | 0.270539274 | count | 1 |
| INCENP | 0.2794521 | 0.5093325 | 0.5487 | 0.583 | 0.270885339 | count | 1 |
| CLASP2 | 0.2626257 | 0.250443 | 1.0486 | 0.294 | 0.270990663 | count | 1 |
| AL590705.1 | 0.4748192 | 0.6393996 | 0.7426 | 0.458 | 0.271019766 | count | 1 |
| GSTA4 | 0.4748192 | 0.6746041 | 0.7038 | 0.482 | 0.271019766 | count | 1 |
| ANAPC13 | 0.2116015 | 0.1484916 | 1.425 | 0.154 | 0.271047738 | count | 1 |
| AC108673.3 | 0.2960889 | 0.3733005 | 0.7932 | 0.428 | 0.271195582 | count | 1 |
| COQ8B | 0.2208245 | 0.2502329 | 0.8825 | 0.378 | 0.271429726 | count | 1 |
| POMC | 0.2088854 | 0.2063301 | 1.0124 | 0.311 | 0.271683326 | count | 1 |
| AARS2 | 0.356643 | 0.5678746 | 0.628 | 0.53 | 0.271838518 | count | 1 |
| DGAT1 | 0.2145111 | 0.1999586 | 1.0728 | 0.283 | 0.272197715 | count | 1 |
| TGDS | 0.2074345 | 0.2057006 | 1.0084 | 0.313 | 0.272263136 | count | 1 |
| NAA40 | 0.3078989 | 0.389754 | 0.79 | 0.43 | 0.272332014 | count | 1 |
| TRIM16L | 0.4223261 | 0.6924864 | 0.6099 | 0.542 | 0.2727241 | count | 1 |
| AC006449.6 | 0.2144005 | 0.257557 | 0.8324 | 0.405 | 0.272740799 | count | 1 |
| LINC02132 | 0.3849797 | 0.5556496 | 0.6928 | 0.488 | 0.272820349 | count | 1 |
| MYPOP | 0.2346806 | 0.303168 | 0.7741 | 0.439 | 0.273624949 | count | 1 |
| RUFY2 | 0.2180829 | 0.206281 | 1.0572 | 0.29 | 0.273690552 | count | 1 |
| FBXO38 | 0.3097327 | 0.2701875 | 1.1464 | 0.252 | 0.273990602 | count | 1 |
| ARNT | 0.265593 | 0.2952186 | 0.8996 | 0.368 | 0.27409746 | count | 1 |
| CSF1 | 0.2238955 | 0.231764 | 0.966 | 0.334 | 0.274103699 | count | 1 |
| ZNF77 | 0.245221 | 0.3979029 | 0.6163 | 0.538 | 0.274108522 | count | 1 |
| CCDC127 | 0.2249151 | 0.2198525 | 1.023 | 0.306 | 0.27417514 | count | 1 |
| EML3 | 0.2574448 | 0.3205635 | 0.8031 | 0.422 | 0.274243018 | count | 1 |
| HINT3 | 0.2271306 | 0.2867503 | 0.7921 | 0.428 | 0.274319605 | count | 1 |
| AC099850.1 | 0.3870321 | 0.7343609 | 0.527 | 0.598 | 0.274325773 | count | 1 |
| SCCPDH | 0.2261232 | 0.2295322 | 0.9851 | 0.325 | 0.274407748 | count | 1 |
| TMEM25 | 0.2995379 | 0.3592458 | 0.8338 | 0.404 | 0.27442021 | count | 1 |
| NISCH | 0.2509902 | 0.2640124 | 0.9507 | 0.342 | 0.274459372 | count | 1 |
| ZNF624 | 0.3233936 | 0.4548229 | 0.711 | 0.477 | 0.27459854 | count | 1 |
| ZNF567 | 0.211181 | 0.164078 | 1.2871 | 0.198 | 0.27468154 | count | 1 |
| ILVBL | 0.2373601 | 0.2553045 | 0.9297 | 0.353 | 0.274752463 | count | 1 |
| REXO4 | 0.2083979 | 0.1648739 | 1.264 | 0.206 | 0.274969987 | count | 1 |

| | | | | | | | |
|---|---|---|---|---|---|---|---|
| FAM98B | 0.2376008 | 0.2339946 | 1.0154 | 0.31 | 0.27503366 | count | 1 |
| AC011445.2 | 0.4818941 | 0.7085824 | 0.6801 | 0.496 | 0.275252117 | count | 1 |
| MAP3K7CL | 0.2583891 | 0.4440964 | 0.5818 | 0.561 | 0.275262182 | count | 1 |
| DAZAP2 | 0.1935704 | 0.0610104 | 3.1727 | 0.00152 | 0.275332974 | count | 1 |
| TGFA | 0.5731258 | 0.7274264 | 0.7879 | 0.431 | 0.2754052 | count | 1 |
| EDDM13 | 0.3887179 | 0.6164534 | 0.6306 | 0.528 | 0.275562621 | count | 1 |
| LRP5L | 0.2842483 | 0.5306871 | 0.5356 | 0.592 | 0.275617706 | count | 1 |
| DTD2 | 0.4269253 | 0.4832829 | 0.8834 | 0.377 | 0.275814965 | count | 1 |
| ZNF776 | 0.5739995 | 0.5638135 | 1.0181 | 0.309 | 0.275849277 | count | 1 |
| FGD5-AS1 | 0.2047291 | 0.1244973 | 1.6444 | 0.1 | 0.276394921 | count | 1 |
| TATDN2 | 0.259465 | 0.3071627 | 0.8447 | 0.398 | 0.276423476 | count | 1 |
| AK2 | 0.2074509 | 0.157199 | 1.3197 | 0.187 | 0.27644074 | count | 1 |
| CEP120 | 0.3020708 | 0.2317433 | 1.3035 | 0.193 | 0.276789109 | count | 1 |
| AL592295.4 | 0.3021215 | 0.7908756 | 0.382 | 0.702 | 0.276836535 | count | 1 |
| FXYD7 | 0.2734216 | 0.2524929 | 1.0829 | 0.279 | 0.277052971 | count | 1 |
| FBLN5 | 0.2360746 | 0.2250158 | 1.0491 | 0.294 | 0.277161914 | count | 1 |
| NOG | 0.7545937 | 0.62094 | 1.2152 | 0.224 | 0.277640579 | count | 1 |
| AC092683.1 | 0.2570621 | 0.1848178 | 1.3909 | 0.164 | 0.277649475 | count | 1 |
| ABCF2 | 0.2690416 | 0.3156586 | 0.8523 | 0.394 | 0.277709213 | count | 1 |
| NEMP1 | 0.7551415 | 0.4424979 | 1.7065 | 0.088 | 0.277856079 | count | 1 |
| GUSB | 0.2080394 | 0.1427537 | 1.4573 | 0.145 | 0.278013231 | count | 1 |
| CCNT2-AS1 | 0.5788952 | 0.7749329 | 0.747 | 0.455 | 0.278338378 | count | 1 |
| PILRB | 0.2218554 | 0.1610515 | 1.3775 | 0.168 | 0.278452227 | count | 1 |
| SLC46A3 | 0.2518116 | 0.2727301 | 0.9233 | 0.356 | 0.278575676 | count | 1 |
| SYNE1 | 0.2007514 | 0.0973752 | 2.0616 | 0.0393 | 0.278780473 | count | 1 |
| ASB16-AS1 | 0.2469841 | 0.2255583 | 1.095 | 0.274 | 0.278827835 | count | 1 |
| ZNF232 | 0.4314913 | 0.4057353 | 1.0635 | 0.288 | 0.27888538 | count | 1 |
| ZNF578 | 0.3657376 | 0.5543216 | 0.6598 | 0.509 | 0.278987177 | count | 1 |
| TRBV18 | 0.7581936 | 0.7212029 | 1.0513 | 0.293 | 0.279056407 | count | 1 |
| UMAD1 | 0.2160688 | 0.1854083 | 1.1654 | 0.244 | 0.279070877 | count | 1 |
| E2F4 | 0.2021174 | 0.111063 | 1.8198 | 0.0689 | 0.279329238 | count | 1 |
| AC084033.3 | 0.232425 | 0.2257155 | 1.0297 | 0.303 | 0.2793425 | count | 1 |
| SLC9A3R1 | 0.1981391 | 0.0862905 | 2.2962 | 0.0217 | 0.279452707 | count | 1 |
| ABHD14A-ACY1 | 1.2709321 | 0.9037755 | 1.4062 | 0.16 | 0.279582459 | count | 1 |
| SNX16 | 0.2556323 | 0.3314748 | 0.7712 | 0.441 | 0.279596817 | count | 1 |
| CCSAP | 0.2624816 | 0.2300114 | 1.1412 | 0.254 | 0.279680048 | count | 1 |
| ESYT2 | 0.2125169 | 0.1430597 | 1.4855 | 0.138 | 0.279712016 | count | 1 |
| AL139353.1 | 0.7598776 | 0.7655127 | 0.9926 | 0.321 | 0.279718835 | count | 1 |
| AC138894.1 | 0.7598776 | 0.8461043 | 0.8981 | 0.369 | 0.279718835 | count | 1 |
| AC092835.1 | 0.7598776 | 0.9322418 | 0.8151 | 0.415 | 0.279718835 | count | 1 |
| YARS | 0.2087885 | 0.1525837 | 1.3684 | 0.171 | 0.279753588 | count | 1 |
| COPG1 | 0.2437816 | 0.2759601 | 0.8834 | 0.377 | 0.280045848 | count | 1 |
| AL359265.3 | 1.2746484 | 0.9968313 | 1.2787 | 0.201 | 0.280431888 | count | 1 |
| TMEM259 | 0.2051698 | 0.0916396 | 2.2389 | 0.0252 | 0.280656114 | count | 1 |
| IL17RA | 0.2275694 | 0.1876911 | 1.2125 | 0.225 | 0.280867989 | count | 1 |

| | | | | | | | |
|---|---|---|---|---|---|---|---|
| TRAV26-1 | 0.4913274 | 0.6467262 | 0.7597 | 0.447 | 0.280901424 | count | 1 |
| MFSD9 | 0.2973969 | 0.3415202 | 0.8708 | 0.384 | 0.280951843 | count | 1 |
| SLC38A9 | 0.3175822 | 0.3393162 | 0.9359 | 0.349 | 0.281094027 | count | 1 |
| LTN1 | 0.2105934 | 0.1280926 | 1.6441 | 0.1 | 0.281177983 | count | 1 |
| UEVLD | 0.3067733 | 0.3607553 | 0.8504 | 0.395 | 0.281188847 | count | 1 |
| BRI3BP | 0.2205882 | 0.2044108 | 1.0791 | 0.281 | 0.28133106 | count | 1 |
| AC239868.3 | 0.4922838 | 0.5718242 | 0.8609 | 0.389 | 0.281474565 | count | 1 |
| SCP2 | 0.1986135 | 0.072131 | 2.7535 | 0.00593 | 0.28162217 | count | 1 |
| GALT | 0.2111308 | 0.1471771 | 1.4345 | 0.152 | 0.282157293 | count | 1 |
| C10orf91 | 0.3979484 | 0.5983187 | 0.6651 | 0.506 | 0.282339691 | count | 1 |
| EEPD1 | 0.2525839 | 0.2978245 | 0.8481 | 0.396 | 0.282430304 | count | 1 |
| ZNF362 | 0.2280452 | 0.2647965 | 0.8612 | 0.389 | 0.282505228 | count | 1 |
| PLPP6 | 0.58725 | 0.5207027 | 1.1278 | 0.259 | 0.282589424 | count | 1 |
| VAC14 | 0.3491781 | 0.4042053 | 0.8639 | 0.388 | 0.282612627 | count | 1 |
| VKORC1L1 | 0.2737452 | 0.2642447 | 1.036 | 0.3 | 0.282637119 | count | 1 |
| FAM173A | 0.2039607 | 0.1203237 | 1.6951 | 0.0901 | 0.282708539 | count | 1 |
| CPSF3 | 0.231103 | 0.2128336 | 1.0858 | 0.278 | 0.282988645 | count | 1 |
| SYNJ2BP | 0.2187137 | 0.1803702 | 1.2126 | 0.225 | 0.283031515 | count | 1 |
| SLC44A2 | 0.2191777 | 0.1790231 | 1.2243 | 0.221 | 0.283104635 | count | 1 |
| TMEM138 | 0.2204977 | 0.1733201 | 1.2722 | 0.203 | 0.283108701 | count | 1 |
| TRUB1 | 0.3337027 | 0.3027705 | 1.1022 | 0.27 | 0.283574607 | count | 1 |
| TLE1 | 0.2925214 | 0.3987765 | 0.7335 | 0.463 | 0.283786017 | count | 1 |
| DBN1 | 0.2861122 | 0.4371009 | 0.6546 | 0.513 | 0.284100341 | count | 1 |
| IGSF8 | 0.2077872 | 0.1379955 | 1.5058 | 0.132 | 0.284118461 | count | 1 |
| AC091271.1 | 0.2320489 | 0.2393448 | 0.9695 | 0.332 | 0.284154922 | count | 1 |
| SMAGP | 0.2330857 | 0.2577721 | 0.9042 | 0.366 | 0.284206447 | count | 1 |
| WWC2 | 0.5906343 | 0.5450041 | 1.0837 | 0.279 | 0.284312531 | count | 1 |
| AGPS | 0.2601222 | 0.2143859 | 1.2133 | 0.225 | 0.284567549 | count | 1 |
| ZBTB42 | 0.5918261 | 0.4393612 | 1.347 | 0.178 | 0.284919473 | count | 1 |
| METTL4 | 0.2210822 | 0.1715867 | 1.2885 | 0.198 | 0.285023768 | count | 1 |
| ZNF43 | 0.2264809 | 0.2157762 | 1.0496 | 0.294 | 0.285133158 | count | 1 |
| ITFG2 | 0.2414735 | 0.1982167 | 1.2182 | 0.223 | 0.28538611 | count | 1 |
| AMOT | 0.3525153 | 0.5838573 | 0.6038 | 0.546 | 0.285389523 | count | 1 |
| TRIM23 | 0.2464823 | 0.2222773 | 1.1089 | 0.268 | 0.285412621 | count | 1 |
| THAP9-AS1 | 0.2097024 | 0.1238512 | 1.6932 | 0.0905 | 0.285501158 | count | 1 |
| ERMARD | 0.2681327 | 0.3341063 | 0.8025 | 0.422 | 0.285782847 | count | 1 |
| GPR107 | 0.3231445 | 0.3595145 | 0.8988 | 0.369 | 0.286131414 | count | 1 |
| POLDIP3 | 0.2337154 | 0.1969785 | 1.1865 | 0.236 | 0.286209824 | count | 1 |
| SLC35E3 | 0.2238793 | 0.2137196 | 1.0475 | 0.295 | 0.286213284 | count | 1 |
| AC007448.3 | 0.2726249 | 0.3549286 | 0.7681 | 0.442 | 0.286245857 | count | 1 |
| MEN1 | 0.3033656 | 0.3543421 | 0.8561 | 0.392 | 0.286701655 | count | 1 |
| ZNF764 | 0.337295 | 0.2909481 | 1.1593 | 0.246 | 0.286704923 | count | 1 |
| DNAJC9-AS1 | 0.777818 | 0.6927936 | 1.1227 | 0.262 | 0.286780196 | count | 1 |
| FRY-AS1 | 0.777818 | 0.8419439 | 0.9238 | 0.356 | 0.286780196 | count | 1 |
| KATNAL2 | 0.777818 | 0.8419439 | 0.9238 | 0.356 | 0.286780196 | count | 1 |

| | | | | | | | |
|---|---|---|---|---|---|---|---|
| GALNT15 | 0.777818 | 0.9436094 | 0.8243 | 0.41 | 0.286780196 | count | 1 |
| EPB41L4A-DT | 0.777818 | 1.0158164 | 0.7657 | 0.444 | 0.286780196 | count | 1 |
| AC009093.5 | 0.777818 | 1.0158164 | 0.7657 | 0.444 | 0.286780196 | count | 1 |
| AC091153.2 | 0.777818 | 1.0158164 | 0.7657 | 0.444 | 0.286780196 | count | 1 |
| MFN2 | 0.3036828 | 0.3684954 | 0.8241 | 0.41 | 0.287007322 | count | 1 |
| HCN2 | 0.7785647 | 0.51357 | 1.516 | 0.13 | 0.28707438 | count | 1 |
| TRBV5-1 | 0.4050299 | 0.6558284 | 0.6176 | 0.537 | 0.287544432 | count | 1 |
| LILRB2 | 0.37675 | 0.8470632 | 0.4448 | 0.657 | 0.287654269 | count | 1 |
| PRR12 | 0.4446818 | 0.4423305 | 1.0053 | 0.315 | 0.287765716 | count | 1 |
| PMS2 | 0.2450342 | 0.2479936 | 0.9881 | 0.323 | 0.287774966 | count | 1 |
| CHCHD4 | 0.2408671 | 0.2905016 | 0.8291 | 0.407 | 0.288021042 | count | 1 |
| MCPH1 | 0.2281553 | 0.1526583 | 1.4945 | 0.135 | 0.288067861 | count | 1 |
| VPS26C | 0.2288918 | 0.1720264 | 1.3306 | 0.183 | 0.288185867 | count | 1 |
| CD3E | 0.2004443 | 0.0323369 | 6.1986 | 6.38E-10 | 0.28819275 | count | 1.54E-05 |
| CSTF1 | 0.2310462 | 0.1862458 | 1.2405 | 0.215 | 0.288231799 | count | 1 |
| NSMCE3 | 0.2041122 | 0.0687624 | 2.9684 | 0.00301 | 0.288271681 | count | 1 |
| LIPT1 | 0.2411356 | 0.2353348 | 1.0246 | 0.306 | 0.288344695 | count | 1 |
| PAK1IP1 | 0.2161912 | 0.1734778 | 1.2462 | 0.213 | 0.28840425 | count | 1 |
| AC007342.4 | 0.5041673 | 0.7869651 | 0.6406 | 0.522 | 0.288601757 | count | 1 |
| DNAJC28 | 0.3566157 | 0.604188 | 0.5902 | 0.555 | 0.288803001 | count | 1 |
| FAM160A2 | 0.2582445 | 0.2366078 | 1.0914 | 0.275 | 0.28883106 | count | 1 |
| WDR5B | 0.4071795 | 0.5092126 | 0.7996 | 0.424 | 0.289125251 | count | 1 |
| AL161457.2 | 0.7844472 | 0.9494832 | 0.8262 | 0.409 | 0.289391313 | count | 1 |
| RBM12B | 0.327441 | 0.3035824 | 1.0786 | 0.281 | 0.290024555 | count | 1 |
| SERINC3 | 0.212961 | 0.1041573 | 2.0446 | 0.041 | 0.290531999 | count | 1 |
| PATZ1 | 0.2925178 | 0.2986129 | 0.9796 | 0.327 | 0.29057069 | count | 1 |
| RNF219 | 0.2208423 | 0.1584328 | 1.3939 | 0.163 | 0.290711962 | count | 1 |
| LMF2 | 0.21717 | 0.1461798 | 1.4856 | 0.137 | 0.291500679 | count | 1 |
| LEF1 | 0.2119394 | 0.1037159 | 2.0435 | 0.0411 | 0.29173955 | count | 1 |
| HOOK1 | 0.382212 | 0.2799296 | 1.3654 | 0.172 | 0.291957431 | count | 1 |
| STX17-AS1 | 0.791351 | 0.5252405 | 1.5066 | 0.132 | 0.292111667 | count | 1 |
| PPP2R5C | 0.2045723 | 0.0500327 | 4.0888 | 4.44E-05 | 0.292162264 | count | 1 |
| C2orf42 | 0.3608143 | 0.3392328 | 1.0636 | 0.288 | 0.292299955 | count | 1 |
| EFL1 | 0.2642135 | 0.246105 | 1.0736 | 0.283 | 0.292459251 | count | 1 |
| LST1 | 0.227326 | 0.2063729 | 1.1015 | 0.271 | 0.292525564 | count | 1 |
| PTPN18 | 0.2182685 | 0.1417853 | 1.5394 | 0.124 | 0.29273997 | count | 1 |
| ITCH | 0.2239251 | 0.1681238 | 1.3319 | 0.183 | 0.293157561 | count | 1 |
| ATP2A2 | 0.2597122 | 0.2151188 | 1.2073 | 0.227 | 0.293354407 | count | 1 |
| ALDOA | 0.2337261 | 0.2002104 | 1.1674 | 0.243 | 0.293440506 | count | 1 |
| TVP23B | 0.2303516 | 0.2039708 | 1.1293 | 0.259 | 0.293848624 | count | 1 |
| TIMM8A | 0.2537154 | 0.2590413 | 0.9794 | 0.327 | 0.293869527 | count | 1 |
| FANCF | 0.2409725 | 0.2085935 | 1.1552 | 0.248 | 0.293893222 | count | 1 |
| ZNF525 | 0.2844918 | 0.3706426 | 0.7676 | 0.443 | 0.293903747 | count | 1 |
| ALKBH1 | 0.275665 | 0.3340519 | 0.8252 | 0.409 | 0.293921534 | count | 1 |
| TAF8 | 0.234968 | 0.1969954 | 1.1928 | 0.233 | 0.294101024 | count | 1 |

| | | | | | | | |
|---|---|---|---|---|---|---|---|
| DMAC2 | 0.2562779 | 0.2079432 | 1.2324 | 0.218 | 0.294546667 | count | 1 |
| CCR2 | 0.245 | 0.2953044 | 0.8297 | 0.407 | 0.294576867 | count | 1 |
| NUP155 | 0.296574 | 0.2839502 | 1.0445 | 0.296 | 0.294669873 | count | 1 |
| SELPLG | 0.211182 | 0.1011406 | 2.088 | 0.0369 | 0.294779413 | count | 1 |
| AC019129.2 | 1.338457 | 1.205188 | 1.1106 | 0.267 | 0.29494258 | count | 1 |
| TMEM267 | 0.2389798 | 0.235513 | 1.0147 | 0.31 | 0.295046257 | count | 1 |
| SPATA5L1 | 0.2971319 | 0.4104378 | 0.7239 | 0.469 | 0.295233802 | count | 1 |
| APMAP | 0.2115908 | 0.0991922 | 2.1331 | 0.033 | 0.295641629 | count | 1 |
| NR4A3 | 0.2559156 | 0.1849737 | 1.3835 | 0.167 | 0.296442736 | count | 1 |
| ARHGEF11 | 0.6145623 | 1.0552126 | 0.5824 | 0.56 | 0.296513214 | count | 1 |
| LAMTOR3 | 0.2226872 | 0.1651007 | 1.3488 | 0.177 | 0.296519502 | count | 1 |
| SH3BP5 | 0.2128216 | 0.1013668 | 2.0995 | 0.0358 | 0.296652614 | count | 1 |
| LIAS | 0.2543549 | 0.2401369 | 1.0592 | 0.29 | 0.296782492 | count | 1 |
| LINC02482 | 0.3493826 | 0.3084481 | 1.1327 | 0.257 | 0.297247373 | count | 1 |
| UGDH-AS1 | 0.6160104 | 0.8543205 | 0.7211 | 0.471 | 0.297252546 | count | 1 |
| ALG10 | 0.4587597 | 0.4608165 | 0.9955 | 0.32 | 0.297259909 | count | 1 |
| PET100 | 0.2136376 | 0.0936431 | 2.2814 | 0.0226 | 0.297274756 | count | 1 |
| ZNF169 | 0.3356802 | 0.404936 | 0.829 | 0.407 | 0.297495345 | count | 1 |
| CYB561A3 | 0.2588422 | 0.241689 | 1.071 | 0.284 | 0.29752373 | count | 1 |
| GLUL | 0.214539 | 0.1004834 | 2.1351 | 0.0328 | 0.297957761 | count | 1 |
| MAP3K21 | 0.6177185 | 0.6290835 | 0.9819 | 0.326 | 0.298124752 | count | 1 |
| PEX1 | 0.226831 | 0.1491765 | 1.5206 | 0.128 | 0.298231086 | count | 1 |
| HSPA1B | 0.2098973 | 0.0985581 | 2.1297 | 0.0333 | 0.298465765 | count | 1 |
| TIMM22 | 0.239203 | 0.1669501 | 1.4328 | 0.152 | 0.298471835 | count | 1 |
| ACTB | 0.2069465 | 0.0235166 | 8.8 | 2.14E-18 | 0.298472651 | count | 5.20E-14 |
| LACTB2 | 0.2400179 | 0.2472568 | 0.9707 | 0.332 | 0.298487576 | count | 1 |
| BTN2A1 | 0.2371759 | 0.1507136 | 1.5737 | 0.116 | 0.298677582 | count | 1 |
| SPRYD3 | 0.2485294 | 0.2319556 | 1.0715 | 0.284 | 0.298854487 | count | 1 |
| SNRNP70 | 0.2152891 | 0.0959456 | 2.2439 | 0.0249 | 0.298865128 | count | 1 |
| CYP2U1 | 0.3257071 | 0.4844601 | 0.6723 | 0.501 | 0.298925762 | count | 1 |
| MCTP2 | 0.2581513 | 0.2519263 | 1.0247 | 0.306 | 0.299057822 | count | 1 |
| OGT | 0.2160735 | 0.1021544 | 2.1152 | 0.0345 | 0.299451651 | count | 1 |
| RIPOR2 | 0.2119566 | 0.0754572 | 2.809 | 0.005 | 0.299503862 | count | 1 |
| ZNF91 | 0.232346 | 0.1411012 | 1.6467 | 0.0997 | 0.299615857 | count | 1 |
| FRY | 0.4214301 | 0.3865355 | 1.0903 | 0.276 | 0.299615891 | count | 1 |
| NEK3 | 0.2708438 | 0.3507486 | 0.7722 | 0.44 | 0.299886766 | count | 1 |
| ETFRF1 | 0.2221309 | 0.1369207 | 1.6223 | 0.105 | 0.299954804 | count | 1 |
| GCSAM | 0.2611535 | 0.1962386 | 1.3308 | 0.183 | 0.30020748 | count | 1 |
| MTRF1 | 0.2659158 | 0.3103041 | 0.857 | 0.392 | 0.3004391 | count | 1 |
| MMP24OS | 0.2144446 | 0.0903571 | 2.3733 | 0.0177 | 0.300733641 | count | 1 |
| PLD6 | 0.5245404 | 0.4585942 | 1.1438 | 0.253 | 0.300844578 | count | 1 |
| ACOT8 | 0.2358594 | 0.1936386 | 1.218 | 0.223 | 0.300912137 | count | 1 |
| DFFA | 0.255131 | 0.2014304 | 1.2666 | 0.205 | 0.301671515 | count | 1 |
| BFSP1 | 0.6250439 | 0.8532058 | 0.7326 | 0.464 | 0.301867025 | count | 1 |
| ZBED3 | 1.3692789 | 0.7421871 | 1.8449 | 0.0651 | 0.301896851 | count | 1 |

| | | | | | | | |
|---|---|---|---|---|---|---|---|
| KCP | 0.8162476 | 0.7864346 | 1.0379 | 0.299 | 0.301928623 | count | 1 |
| WDR64 | 1.3703991 | 0.8232552 | 1.6646 | 0.0961 | 0.302148875 | count | 1 |
| LINC01376 | 1.3703991 | 0.8232552 | 1.6646 | 0.0961 | 0.302148875 | count | 1 |
| MMP28 | 1.3703991 | 0.8232552 | 1.6646 | 0.0961 | 0.302148875 | count | 1 |
| AL353135.1 | 1.3703991 | 0.983237 | 1.3938 | 0.163 | 0.302148875 | count | 1 |
| HOXB4 | 0.2728675 | 0.2732947 | 0.9984 | 0.318 | 0.302154487 | count | 1 |
| AP003352.1 | 0.8169223 | 0.5990815 | 1.3636 | 0.173 | 0.302194819 | count | 1 |
| GPR68 | 0.2541478 | 0.2618944 | 0.9704 | 0.332 | 0.302315619 | count | 1 |
| F11R | 0.3293222 | 0.3358323 | 0.9806 | 0.327 | 0.302316275 | count | 1 |
| TMBIM1 | 0.2169731 | 0.1201593 | 1.8057 | 0.0711 | 0.302335522 | count | 1 |
| USP28 | 0.241563 | 0.1979776 | 1.2202 | 0.222 | 0.302407118 | count | 1 |
| IRAK4 | 0.2202072 | 0.1103497 | 1.9955 | 0.0461 | 0.302493829 | count | 1 |
| SIPA1L1 | 0.2798649 | 0.2076072 | 1.348 | 0.178 | 0.302610259 | count | 1 |
| GTPBP3 | 0.2348581 | 0.1984026 | 1.1837 | 0.237 | 0.302871009 | count | 1 |
| AC016065.1 | 0.2635478 | 0.3855051 | 0.6836 | 0.494 | 0.302988011 | count | 1 |
| RABL2B | 0.3048216 | 0.2700505 | 1.1288 | 0.259 | 0.303009524 | count | 1 |
| AC087071.1 | 0.5281581 | 0.8496571 | 0.6216 | 0.534 | 0.303021615 | count | 1 |
| IPO9-AS1 | 0.5281581 | 1.0963969 | 0.4817 | 0.63 | 0.303021615 | count | 1 |
| OTUD5 | 0.2406506 | 0.1387743 | 1.7341 | 0.083 | 0.303079294 | count | 1 |
| GALNT12 | 0.2986924 | 0.3904095 | 0.7651 | 0.444 | 0.303090262 | count | 1 |
| NUDT8 | 0.2685674 | 0.3389698 | 0.7923 | 0.428 | 0.303468189 | count | 1 |
| PLEKHA1 | 0.224052 | 0.1197529 | 1.871 | 0.0614 | 0.303722007 | count | 1 |
| ADHFE1 | 0.3751952 | 0.4431363 | 0.8467 | 0.397 | 0.304290405 | count | 1 |
| ZNF518A | 0.2455448 | 0.1774263 | 1.3839 | 0.166 | 0.304330743 | count | 1 |
| AP3M1 | 0.2543991 | 0.2193476 | 1.1598 | 0.246 | 0.304338514 | count | 1 |
| GMPPB | 0.248438 | 0.2094059 | 1.1864 | 0.236 | 0.304370888 | count | 1 |
| AIDA | 0.230319 | 0.1582326 | 1.4556 | 0.146 | 0.304736381 | count | 1 |
| ACADS | 0.245076 | 0.2490043 | 0.9842 | 0.325 | 0.304819056 | count | 1 |
| AL512625.3 | 0.8244697 | 0.9614937 | 0.8575 | 0.391 | 0.305172907 | count | 1 |
| AC146944.4 | 0.8244697 | 0.9937279 | 0.8297 | 0.407 | 0.305172907 | count | 1 |
| ZC3H12B | 0.8244697 | 1.102356 | 0.7479 | 0.455 | 0.305172907 | count | 1 |
| TRPM2 | 0.3990612 | 0.7873607 | 0.5068 | 0.612 | 0.30524944 | count | 1 |
| MRPS10 | 0.2250615 | 0.1302287 | 1.7282 | 0.084 | 0.305631369 | count | 1 |
| CENPP | 0.8259193 | 0.5745132 | 1.4376 | 0.151 | 0.305744973 | count | 1 |
| TRBV9 | 0.5327019 | 0.738792 | 0.721 | 0.471 | 0.305757199 | count | 1 |
| AK3 | 0.2375825 | 0.2085979 | 1.1389 | 0.255 | 0.305789844 | count | 1 |
| TRAV16 | 0.8269898 | 0.7066721 | 1.1703 | 0.242 | 0.306167465 | count | 1 |
| TRBV4-2 | 1.3885612 | 1.195834 | 1.1612 | 0.246 | 0.306227435 | count | 1 |
| TDRD1 | 1.3885612 | 1.400048 | 0.9918 | 0.321 | 0.306227435 | count | 1 |
| LOXL1 | 0.3597339 | 0.4651341 | 0.7734 | 0.439 | 0.306286696 | count | 1 |
| TBP | 0.2624735 | 0.2392154 | 1.0972 | 0.273 | 0.306346417 | count | 1 |
| RUVBL2 | 0.2439834 | 0.2081779 | 1.172 | 0.241 | 0.306397621 | count | 1 |
| TRIM69 | 0.2308263 | 0.1527576 | 1.5111 | 0.131 | 0.306439467 | count | 1 |
| ZNF439 | 0.2798927 | 0.3080001 | 0.9087 | 0.364 | 0.306475074 | count | 1 |
| GPATCH1 | 0.2874619 | 0.2304321 | 1.2475 | 0.212 | 0.306677816 | count | 1 |

| | | | | | | | |
|---|---|---|---|---|---|---|---|
| ASTE1 | 0.2316832 | 0.186357 | 1.2432 | 0.214 | 0.307245478 | count | 1 |
| ZNF445 | 0.3248779 | 0.3245323 | 1.0011 | 0.317 | 0.307453427 | count | 1 |
| RTN4IP1 | 0.3095613 | 0.4693026 | 0.6596 | 0.51 | 0.30780492 | count | 1 |
| MINCR | 0.2979581 | 0.3926158 | 0.7589 | 0.448 | 0.308036149 | count | 1 |
| UTP15 | 0.2848899 | 0.2924513 | 0.9741 | 0.33 | 0.308116613 | count | 1 |
| CCND3 | 0.2157594 | 0.0562736 | 3.8341 | 0.000128 | 0.308160703 | count | 1 |
| AC093484.2 | 0.4334291 | 0.5585214 | 0.776 | 0.438 | 0.308462965 | count | 1 |
| TMEM170B | 0.5372678 | 0.6910643 | 0.7774 | 0.437 | 0.308507496 | count | 1 |
| ELMOD2 | 0.2565344 | 0.2225078 | 1.1529 | 0.249 | 0.308559417 | count | 1 |
| COPG2 | 0.4032702 | 0.3659182 | 1.1021 | 0.271 | 0.308573926 | count | 1 |
| TINF2 | 0.2219468 | 0.1071899 | 2.0706 | 0.0385 | 0.308599063 | count | 1 |
| C11orf68 | 0.278871 | 0.2871975 | 0.971 | 0.332 | 0.308883777 | count | 1 |
| TMPRSS3 | 0.4341074 | 0.7798506 | 0.5567 | 0.578 | 0.308963471 | count | 1 |
| TMEM135 | 0.3483482 | 0.352149 | 0.9892 | 0.323 | 0.308994587 | count | 1 |
| DHX33 | 0.3110122 | 0.4185664 | 0.743 | 0.458 | 0.309273268 | count | 1 |
| TRPC1 | 1.4037219 | 0.6962679 | 2.0161 | 0.0439 | 0.309620964 | count | 1 |
| ZNF606 | 0.4353673 | 0.6861558 | 0.6345 | 0.526 | 0.309893205 | count | 1 |
| CCDC71 | 0.2866112 | 0.35559 | 0.806 | 0.42 | 0.310003271 | count | 1 |
| TSPAN3 | 0.2676098 | 0.2249078 | 1.1899 | 0.234 | 0.310125272 | count | 1 |
| CDK8 | 0.3644657 | 0.3257448 | 1.1189 | 0.263 | 0.310422133 | count | 1 |
| TRGV7 | 0.4363779 | 0.5128317 | 0.8509 | 0.395 | 0.310639082 | count | 1 |
| SASH3 | 0.2298619 | 0.142002 | 1.6187 | 0.106 | 0.311039076 | count | 1 |
| BSG | 0.2184145 | 0.0675426 | 3.2337 | 0.00123 | 0.311110025 | count | 1 |
| MBNL1-AS1 | 0.31296 | 0.3495901 | 0.8952 | 0.371 | 0.311244777 | count | 1 |
| EIF2B3 | 0.2471566 | 0.2217381 | 1.1146 | 0.265 | 0.311322666 | count | 1 |
| MFN1 | 0.2781607 | 0.2416552 | 1.1511 | 0.25 | 0.311371312 | count | 1 |
| DLG5 | 1.412272 | 0.6545159 | 2.1577 | 0.031 | 0.311530185 | count | 1 |
| ZMYM1 | 0.2634089 | 0.2223069 | 1.1849 | 0.236 | 0.311548262 | count | 1 |
| STX7 | 0.2383139 | 0.1922035 | 1.2399 | 0.215 | 0.311612212 | count | 1 |
| KATNAL1 | 0.2814528 | 0.2747698 | 1.0243 | 0.306 | 0.311778554 | count | 1 |
| UTP6 | 0.2356379 | 0.1445756 | 1.6299 | 0.103 | 0.311800528 | count | 1 |
| AC025043.1 | 0.842291 | 1.003302 | 0.8395 | 0.401 | 0.312207822 | count | 1 |
| NPIPB6 | 0.8424313 | 1.24294 | 0.6778 | 0.498 | 0.312263223 | count | 1 |
| NR3C2 | 0.3847611 | 0.3386166 | 1.1363 | 0.256 | 0.312277 | count | 1 |
| STAG3 | 0.3078253 | 0.3394842 | 0.9067 | 0.365 | 0.312514293 | count | 1 |
| MAP1LC3A | 0.2718815 | 0.2125849 | 1.2789 | 0.201 | 0.312669237 | count | 1 |
| CDIPT | 0.2333951 | 0.1274775 | 1.8309 | 0.0672 | 0.312831529 | count | 1 |
| MOV10 | 0.3304729 | 0.3748436 | 0.8816 | 0.378 | 0.312857698 | count | 1 |
| ARL15 | 0.2932009 | 0.2924114 | 1.0027 | 0.316 | 0.31288771 | count | 1 |
| MRRF | 0.2799512 | 0.227295 | 1.2317 | 0.218 | 0.313399202 | count | 1 |
| CDC37L1-DT | 0.5459618 | 0.4596273 | 1.1878 | 0.235 | 0.313748119 | count | 1 |
| FBXO44 | 0.2493044 | 0.2136261 | 1.167 | 0.243 | 0.314044481 | count | 1 |
| CKAP4 | 0.3540424 | 0.3992494 | 0.8868 | 0.375 | 0.314168326 | count | 1 |
| AC010969.2 | 0.5470677 | 0.5502986 | 0.9941 | 0.32 | 0.31441508 | count | 1 |
| AL133410.1 | 0.5470677 | 0.6546808 | 0.8356 | 0.403 | 0.31441508 | count | 1 |

| | | | | | | | |
|---|---|---|---|---|---|---|---|
| ALG11 | 0.3690537 | 0.4040955 | 0.9133 | 0.361 | 0.31443387 | count | 1 |
| ZNF250 | 0.2615055 | 0.190386 | 1.3736 | 0.17 | 0.314588165 | count | 1 |
| GPC1 | 0.5475136 | 0.6394154 | 0.8563 | 0.392 | 0.31468403 | count | 1 |
| PEX13 | 0.2549637 | 0.1936971 | 1.3163 | 0.188 | 0.314919845 | count | 1 |
| MED29 | 0.237485 | 0.1475718 | 1.6093 | 0.108 | 0.31531183 | count | 1 |
| TMEM129 | 0.2596526 | 0.2021677 | 1.2843 | 0.199 | 0.315422317 | count | 1 |
| GPR19 | 0.6519148 | 0.662174 | 0.9845 | 0.325 | 0.315615922 | count | 1 |
| PRUNE1 | 0.3333577 | 0.3924494 | 0.8494 | 0.396 | 0.315645265 | count | 1 |
| SH3BP2 | 0.2744674 | 0.2136728 | 1.2845 | 0.199 | 0.315674261 | count | 1 |
| ZNF12 | 0.2883282 | 0.2467901 | 1.1683 | 0.243 | 0.315831901 | count | 1 |
| DRG2 | 0.233774 | 0.140167 | 1.6678 | 0.0954 | 0.315935235 | count | 1 |
| TMEM161B-AS1 | 0.265646 | 0.2588067 | 1.0264 | 0.305 | 0.316114501 | count | 1 |
| TNFSF11 | 0.4868566 | 0.6094142 | 0.7989 | 0.424 | 0.316256529 | count | 1 |
| PTGS2 | 0.5501544 | 0.681364 | 0.8074 | 0.419 | 0.31627709 | count | 1 |
| STMP1 | 0.2232855 | 0.0802522 | 2.7823 | 0.00543 | 0.316290653 | count | 1 |
| ARF5 | 0.225106 | 0.083497 | 2.696 | 0.00705 | 0.316359502 | count | 1 |
| ZSWIM1 | 0.8529755 | 0.7961756 | 1.0713 | 0.284 | 0.316427131 | count | 1 |
| HYAL3 | 0.3445284 | 0.4701749 | 0.7328 | 0.464 | 0.316591135 | count | 1 |
| CCRL2 | 0.5508659 | 0.5547394 | 0.993 | 0.321 | 0.316706375 | count | 1 |
| COG5 | 0.2510193 | 0.2510422 | 0.9999 | 0.317 | 0.317111031 | count | 1 |
| EDEM1 | 0.2419109 | 0.1427606 | 1.6945 | 0.0903 | 0.317263712 | count | 1 |
| C6orf136 | 0.2545614 | 0.2147557 | 1.1854 | 0.236 | 0.317762378 | count | 1 |
| DPP3 | 0.2593252 | 0.2272317 | 1.1412 | 0.254 | 0.317808874 | count | 1 |
| TSPYL1 | 0.2268528 | 0.0987504 | 2.2972 | 0.0217 | 0.317870448 | count | 1 |
| ACKR1 | 1.4409573 | 0.6046568 | 2.3831 | 0.0172 | 0.317910892 | count | 1 |
| MSX2 | 0.4896493 | 1.0838958 | 0.4517 | 0.651 | 0.318148036 | count | 1 |
| CNNM2 | 1.442793 | 0.4390286 | 3.2863 | 0.00103 | 0.318317809 | count | 1 |
| PM20D2 | 0.3587034 | 0.3165042 | 1.1333 | 0.257 | 0.318405522 | count | 1 |
| DENND1B | 0.2522706 | 0.1601132 | 1.5756 | 0.115 | 0.318701246 | count | 1 |
| ZNF527 | 0.5544383 | 0.3914398 | 1.4164 | 0.157 | 0.318862271 | count | 1 |
| C12orf4 | 0.2848837 | 0.2342637 | 1.2161 | 0.224 | 0.318986891 | count | 1 |
| AC025164.1 | 0.2332652 | 0.142261 | 1.6397 | 0.101 | 0.31917964 | count | 1 |
| CCDC65 | 0.3746242 | 0.3794199 | 0.9874 | 0.324 | 0.319307287 | count | 1 |
| AC005842.1 | 0.4913979 | 0.3283544 | 1.4965 | 0.135 | 0.319332672 | count | 1 |
| LGALS3BP | 0.2882248 | 0.2230503 | 1.2922 | 0.196 | 0.319373913 | count | 1 |
| TRBV14 | 0.6598729 | 0.7067738 | 0.9336 | 0.351 | 0.319693929 | count | 1 |
| SCRIB | 0.3378759 | 0.3957823 | 0.8537 | 0.393 | 0.320012677 | count | 1 |
| MAPK14 | 0.252637 | 0.1815493 | 1.3916 | 0.164 | 0.320031314 | count | 1 |
| AL355338.1 | 0.4179111 | 0.5736131 | 0.7286 | 0.466 | 0.320150325 | count | 1 |
| MT1M | 0.8624358 | 0.7882078 | 1.0942 | 0.274 | 0.320163815 | count | 1 |
| SMG9 | 0.2859492 | 0.299881 | 0.9535 | 0.34 | 0.320194164 | count | 1 |
| TMEM101 | 0.2435941 | 0.1680251 | 1.4497 | 0.147 | 0.320362865 | count | 1 |
| UTP4 | 0.2925165 | 0.2705523 | 1.0812 | 0.28 | 0.320479702 | count | 1 |
| CASTOR1 | 1.453686 | 0.5671647 | 2.5631 | 0.0104 | 0.32072939 | count | 1 |
| KLF6 | 0.2228548 | 0.0409562 | 5.4413 | 5.66E-08 | 0.320868287 | count | 0.00136723 |

| | | | | | | | |
|---|---|---|---|---|---|---|---|
| LRRC57 | 0.2729573 | 0.2391794 | 1.1412 | 0.254 | 0.320886535 | count | 1 |
| CHMP1B | 0.2323506 | 0.0873302 | 2.6606 | 0.00784 | 0.321461038 | count | 1 |
| PTRH2 | 0.2533125 | 0.2047612 | 1.2371 | 0.216 | 0.321726232 | count | 1 |
| PEPD | 0.2353257 | 0.120811 | 1.9479 | 0.0515 | 0.322417537 | count | 1 |
| HIPK1-AS1 | 0.868599 | 0.4377963 | 1.984 | 0.0473 | 0.322598682 | count | 1 |
| C12orf66 | 0.3406545 | 0.3656765 | 0.9316 | 0.352 | 0.322699447 | count | 1 |
| FBXO11 | 0.2519971 | 0.1552262 | 1.6234 | 0.105 | 0.323769827 | count | 1 |
| AKAP17A | 0.2362397 | 0.1012272 | 2.3338 | 0.0197 | 0.323937637 | count | 1 |
| TRUB2 | 0.2738061 | 0.224003 | 1.2223 | 0.222 | 0.323959924 | count | 1 |
| TRMT13 | 0.2669177 | 0.1996479 | 1.3369 | 0.181 | 0.324318161 | count | 1 |
| ARPC4 | 0.2277779 | 0.0609458 | 3.7374 | 0.000189 | 0.324821009 | count | 1 |
| TRAF3IP3 | 0.2281763 | 0.061556 | 3.7068 | 0.000213 | 0.325014333 | count | 1 |
| ZNF2 | 0.4558339 | 0.5710807 | 0.7982 | 0.425 | 0.325015023 | count | 1 |
| C17orf49 | 0.2805807 | 0.2728793 | 1.0282 | 0.304 | 0.32531263 | count | 1 |
| ZNF74 | 0.3350852 | 0.4857256 | 0.6899 | 0.49 | 0.325912109 | count | 1 |
| TIPARP-AS1 | 0.672303 | 0.4392741 | 1.5305 | 0.126 | 0.326068659 | count | 1 |
| NRN1 | 0.6728806 | 1.0704598 | 0.6286 | 0.53 | 0.32636503 | count | 1 |
| FKBP1B | 0.3677167 | 0.4306621 | 0.8538 | 0.393 | 0.326604828 | count | 1 |
| GATC | 0.3359528 | 0.2454852 | 1.3685 | 0.171 | 0.326772475 | count | 1 |
| TMEM254 | 0.3290693 | 0.2827577 | 1.1638 | 0.245 | 0.327562901 | count | 1 |
| ZNF626 | 0.2769941 | 0.2339736 | 1.1839 | 0.237 | 0.327766976 | count | 1 |
| ZNF26 | 0.2827094 | 0.2316839 | 1.2202 | 0.222 | 0.327806159 | count | 1 |
| COX10 | 0.4033991 | 0.2780654 | 1.4507 | 0.147 | 0.327861576 | count | 1 |
| AC104958.2 | 0.5694578 | 0.5567817 | 1.0228 | 0.306 | 0.32793483 | count | 1 |
| HECTD3 | 0.3371457 | 0.3978698 | 0.8474 | 0.397 | 0.327955529 | count | 1 |
| AC093157.1 | 0.3295307 | 0.3133647 | 1.0516 | 0.293 | 0.328030612 | count | 1 |
| FUT7 | 0.2875661 | 0.4424001 | 0.65 | 0.516 | 0.32813905 | count | 1 |
| STIMATE | 0.4037769 | 0.4110658 | 0.9823 | 0.326 | 0.328177785 | count | 1 |
| MRI1 | 0.2691517 | 0.2095674 | 1.2843 | 0.199 | 0.328533683 | count | 1 |
| ATP13A1 | 0.3852468 | 0.3511186 | 1.0972 | 0.273 | 0.328608282 | count | 1 |
| AC092803.2 | 0.8839219 | 0.7097153 | 1.2455 | 0.213 | 0.328652468 | count | 1 |
| CARD8-AS1 | 0.2525084 | 0.2104011 | 1.2001 | 0.23 | 0.328695528 | count | 1 |
| IFT88 | 0.2683009 | 0.2271037 | 1.1814 | 0.238 | 0.328892512 | count | 1 |
| FAM13A | 0.3579402 | 0.3657967 | 0.9785 | 0.328 | 0.329198883 | count | 1 |
| MOSMO | 0.2886731 | 0.2345285 | 1.2309 | 0.218 | 0.329416321 | count | 1 |
| CDK17 | 0.2570488 | 0.1428312 | 1.7997 | 0.072 | 0.329588653 | count | 1 |
| MECR | 0.3311101 | 0.40064 | 0.8265 | 0.409 | 0.329631732 | count | 1 |
| NEIL3 | 0.8874887 | 1.252055 | 0.7088 | 0.478 | 0.330061786 | count | 1 |
| BRAT1 | 0.2922538 | 0.205507 | 1.4221 | 0.155 | 0.330549566 | count | 1 |
| LAIR1 | 0.2465805 | 0.1644381 | 1.4995 | 0.134 | 0.330839193 | count | 1 |
| LINC00954 | 0.3597222 | 0.4041487 | 0.8901 | 0.373 | 0.330875234 | count | 1 |
| SPRYD4 | 0.4070347 | 0.4110404 | 0.9903 | 0.322 | 0.330905101 | count | 1 |
| MLH3 | 0.2954922 | 0.2114349 | 1.3976 | 0.162 | 0.331010573 | count | 1 |
| WDR89 | 0.2750475 | 0.2408557 | 1.142 | 0.254 | 0.331018911 | count | 1 |
| TBC1D10B | 0.2755023 | 0.2150466 | 1.2811 | 0.2 | 0.331570915 | count | 1 |

| | | | | | | | |
|---|---|---|---|---|---|---|---|
| PYCR2 | 0.2435401 | 0.121167 | 2.01 | 0.0445 | 0.331692579 | count | 1 |
| HSD17B12 | 0.2485797 | 0.1530448 | 1.6242 | 0.104 | 0.331767848 | count | 1 |
| PIGK | 0.2577062 | 0.1723248 | 1.4955 | 0.135 | 0.33182823 | count | 1 |
| ZNF117 | 0.3737517 | 0.4411324 | 0.8473 | 0.397 | 0.3320988 | count | 1 |
| AL050341.2 | 0.8933964 | 0.5662648 | 1.5777 | 0.115 | 0.332396257 | count | 1 |
| ITGAV | 0.3342743 | 0.369461 | 0.9048 | 0.366 | 0.332840092 | count | 1 |
| AP001830.1 | 1.5089604 | 1.088893 | 1.3858 | 0.166 | 0.332873339 | count | 1 |
| AC090912.1 | 1.5089604 | 1.210249 | 1.2468 | 0.213 | 0.332873339 | count | 1 |
| SORBS1 | 1.5089604 | 1.392364 | 1.0837 | 0.279 | 0.332873339 | count | 1 |
| PIANP | 1.5089604 | 1.392364 | 1.0837 | 0.279 | 0.332873339 | count | 1 |
| RFK | 0.2542332 | 0.1499237 | 1.6958 | 0.09 | 0.333015842 | count | 1 |
| GLO1 | 0.2451305 | 0.1128246 | 2.1727 | 0.0299 | 0.333147662 | count | 1 |
| MIS18A | 0.300551 | 0.2710492 | 1.1088 | 0.268 | 0.333207548 | count | 1 |
| ZNF627 | 0.6865386 | 0.5416118 | 1.2676 | 0.205 | 0.333376558 | count | 1 |
| TRAV36DV7 | 0.3348317 | 0.4752004 | 0.7046 | 0.481 | 0.333405357 | count | 1 |
| FHAD1 | 0.5785683 | 0.7775592 | 0.7441 | 0.457 | 0.333444494 | count | 1 |
| FTSJ1 | 0.2721394 | 0.1776307 | 1.5321 | 0.126 | 0.333633828 | count | 1 |
| CLHC1 | 0.5789924 | 0.4306859 | 1.3443 | 0.179 | 0.333701087 | count | 1 |
| AC243829.4 | 0.5126532 | 0.5614709 | 0.9131 | 0.361 | 0.33375025 | count | 1 |
| TRAPPC5 | 0.6879494 | 0.4834887 | 1.4229 | 0.155 | 0.334101195 | count | 1 |
| DYNC2LI1 | 0.3179252 | 0.3731853 | 0.8519 | 0.394 | 0.334573345 | count | 1 |
| NUP50-DT | 0.6890873 | 0.4568712 | 1.5083 | 0.132 | 0.334685699 | count | 1 |
| EXTL2 | 0.3236286 | 0.3630234 | 0.8915 | 0.373 | 0.335018993 | count | 1 |
| SURF6 | 0.2757355 | 0.2348006 | 1.1743 | 0.24 | 0.335119278 | count | 1 |
| ATG10 | 0.2722534 | 0.2148515 | 1.2672 | 0.205 | 0.335134272 | count | 1 |
| KANSL2 | 0.2481748 | 0.1304308 | 1.9027 | 0.0572 | 0.33545561 | count | 1 |
| AC020910.4 | 0.5821214 | 0.4578703 | 1.2714 | 0.204 | 0.335594525 | count | 1 |
| SNAI1 | 0.5825019 | 0.5063204 | 1.1505 | 0.25 | 0.335824809 | count | 1 |
| CDCA7 | 0.3101974 | 0.3918643 | 0.7916 | 0.429 | 0.335878708 | count | 1 |
| NEFM | 0.9039884 | 0.5373372 | 1.6823 | 0.0926 | 0.33658156 | count | 1 |
| SLAMF6 | 0.2617672 | 0.15407 | 1.699 | 0.0894 | 0.337084886 | count | 1 |
| CTSF | 0.2868113 | 0.2945194 | 0.9738 | 0.33 | 0.337333759 | count | 1 |
| AKAP5 | 0.5863919 | 0.4836073 | 1.2125 | 0.225 | 0.338179571 | count | 1 |
| HSBP1L1 | 0.3019922 | 0.2016302 | 1.4978 | 0.134 | 0.338381666 | count | 1 |
| GTF2H2C | 0.3271126 | 0.3714821 | 0.8806 | 0.379 | 0.338685245 | count | 1 |
| RBM18 | 0.2570061 | 0.1401558 | 1.8337 | 0.0668 | 0.338956512 | count | 1 |
| NSG1 | 0.2487585 | 0.1908208 | 1.3036 | 0.192 | 0.338992406 | count | 1 |
| RBM23 | 0.267057 | 0.1322266 | 2.0197 | 0.0435 | 0.339287672 | count | 1 |
| ZFAT | 0.5208529 | 0.5996487 | 0.8686 | 0.385 | 0.339320569 | count | 1 |
| GDF11 | 0.2808112 | 0.2041434 | 1.3756 | 0.169 | 0.339719175 | count | 1 |
| HMGN3 | 0.2414781 | 0.0955129 | 2.5282 | 0.0115 | 0.339809981 | count | 1 |
| KLF9 | 0.2520279 | 0.0920479 | 2.738 | 0.00621 | 0.340218363 | count | 1 |
| PXN | 0.2826865 | 0.185205 | 1.5263 | 0.127 | 0.340292191 | count | 1 |
| AC095055.1 | 0.5903161 | 0.5869084 | 1.0058 | 0.315 | 0.340555884 | count | 1 |
| AES | 0.2371734 | 0.0383705 | 6.1811 | 7.12E-10 | 0.340768418 | count | 1.72E-05 |

| | | | | | | | |
|---|---|---|---|---|---|---|---|
| CDK7 | 0.2633586 | 0.2110437 | 1.2479 | 0.212 | 0.341106318 | count | 1 |
| SPSB1 | 0.2820384 | 0.2472216 | 1.1408 | 0.254 | 0.341216275 | count | 1 |
| CD55 | 0.2452742 | 0.0782789 | 3.1333 | 0.00174 | 0.341506813 | count | 1 |
| FAM157C | 0.7024266 | 0.7633172 | 0.9202 | 0.358 | 0.341541076 | count | 1 |
| TCTN3 | 0.3118682 | 0.2102698 | 1.4832 | 0.138 | 0.341971641 | count | 1 |
| SGSM1 | 0.4455502 | 0.641964 | 0.694 | 0.488 | 0.34205377 | count | 1 |
| RPUSD3 | 0.2611203 | 0.1772511 | 1.4732 | 0.141 | 0.342077828 | count | 1 |
| CIZ1 | 0.312085 | 0.2151909 | 1.4503 | 0.147 | 0.342212572 | count | 1 |
| MRPL10 | 0.2476266 | 0.1137053 | 2.1778 | 0.0295 | 0.342820984 | count | 1 |
| ZBED5-AS1 | 0.2785005 | 0.2185908 | 1.2741 | 0.203 | 0.342882441 | count | 1 |
| LYPD3 | 0.325785 | 0.2869284 | 1.1354 | 0.256 | 0.342975323 | count | 1 |
| GALNT3 | 0.2899566 | 0.2717546 | 1.067 | 0.286 | 0.343253017 | count | 1 |
| PLPBP | 0.2767523 | 0.1546345 | 1.7897 | 0.0736 | 0.343293852 | count | 1 |
| PDIA4 | 0.257464 | 0.1523885 | 1.6895 | 0.0912 | 0.343668334 | count | 1 |
| TRMT61A | 0.4025667 | 0.2904448 | 1.386 | 0.166 | 0.343794248 | count | 1 |
| IL9R | 0.3734557 | 0.5091379 | 0.7335 | 0.463 | 0.343803672 | count | 1 |
| THAP6 | 0.2628427 | 0.1754686 | 1.4979 | 0.134 | 0.343834876 | count | 1 |
| PANX1 | 0.4227975 | 0.3361079 | 1.2579 | 0.209 | 0.344113684 | count | 1 |
| PCSK5 | 0.3738758 | 0.5485272 | 0.6816 | 0.496 | 0.344199403 | count | 1 |
| AL035530.2 | 0.4483252 | 0.6647012 | 0.6745 | 0.5 | 0.344256309 | count | 1 |
| CDC23 | 0.4819705 | 0.3342797 | 1.4418 | 0.149 | 0.344374332 | count | 1 |
| ZNF621 | 0.3632084 | 0.3419443 | 1.0622 | 0.288 | 0.344532595 | count | 1 |
| CARF | 0.4035322 | 0.3066621 | 1.3159 | 0.188 | 0.344641537 | count | 1 |
| SUMF1 | 0.3462601 | 0.3148719 | 1.0997 | 0.272 | 0.345000627 | count | 1 |
| COL6A2 | 0.2998286 | 0.2467996 | 1.2149 | 0.225 | 0.345170177 | count | 1 |
| BCLAF1 | 0.2449065 | 0.0652637 | 3.7526 | 0.000178 | 0.345590368 | count | 1 |
| PSMB8-AS1 | 0.245015 | 0.0895483 | 2.7361 | 0.00625 | 0.345899207 | count | 1 |
| THNSL1 | 0.5992979 | 0.5223809 | 1.1472 | 0.251 | 0.345997861 | count | 1 |
| IFNG-AS1 | 0.3758813 | 0.3053586 | 1.231 | 0.218 | 0.346088733 | count | 1 |
| ZDHHC23 | 0.4847135 | 0.517998 | 0.9357 | 0.349 | 0.346409034 | count | 1 |
| ANXA6 | 0.245094 | 0.0730871 | 3.3535 | 0.000807 | 0.346442315 | count | 1 |
| TMSB15B-AS1 | 0.6000823 | 0.891441 | 0.6732 | 0.501 | 0.346473339 | count | 1 |
| PDE5A | 0.7124788 | 0.6733071 | 1.0582 | 0.29 | 0.346710875 | count | 1 |
| AC068473.5 | 1.5734642 | 0.8949719 | 1.7581 | 0.0788 | 0.346832484 | count | 1 |
| PAM | 0.3094848 | 0.278812 | 1.11 | 0.267 | 0.34688204 | count | 1 |
| DPH2 | 0.3900565 | 0.3548123 | 1.0993 | 0.272 | 0.346957498 | count | 1 |
| G2E3 | 0.265231 | 0.1593292 | 1.6647 | 0.0961 | 0.346973481 | count | 1 |
| AC242426.2 | 0.426377 | 0.4452529 | 0.9576 | 0.338 | 0.347115997 | count | 1 |
| LINC00484 | 1.5752023 | 1.064509 | 1.4797 | 0.139 | 0.347205271 | count | 1 |
| AC109826.1 | 0.3566952 | 0.3181478 | 1.1212 | 0.262 | 0.347360697 | count | 1 |
| SLC25A15 | 0.4267587 | 0.5537515 | 0.7707 | 0.441 | 0.347436213 | count | 1 |
| PYGO2 | 0.2918454 | 0.2512065 | 1.1618 | 0.245 | 0.347586791 | count | 1 |
| DAPK2 | 0.3778527 | 0.3795246 | 0.9956 | 0.32 | 0.347946255 | count | 1 |
| FCGBP | 0.7150834 | 0.7772265 | 0.92 | 0.358 | 0.348050891 | count | 1 |
| AC021188.1 | 0.4276525 | 0.4326799 | 0.9884 | 0.323 | 0.348186068 | count | 1 |

| | | | | | | | |
|---|---|---|---|---|---|---|---|
| PRCP | 0.2675362 | 0.1777738 | 1.5049 | 0.132 | 0.348353219 | count | 1 |
| TMEM97 | 0.4283212 | 0.3975957 | 1.0773 | 0.281 | 0.348747127 | count | 1 |
| NFKBIA | 0.2422753 | 0.0430069 | 5.6334 | 1.91E-08 | 0.348858966 | count | 0.000461685 |
| TRPT1 | 0.2732496 | 0.1785175 | 1.5307 | 0.126 | 0.348900009 | count | 1 |
| RBM7 | 0.2552976 | 0.1291073 | 1.9774 | 0.0481 | 0.349248767 | count | 1 |
| CCND1 | 0.3927987 | 0.2930656 | 1.3403 | 0.18 | 0.349458669 | count | 1 |
| PDLIM1 | 0.2751567 | 0.2146987 | 1.2816 | 0.2 | 0.349640661 | count | 1 |
| CCDC7 | 0.3151866 | 0.2368105 | 1.331 | 0.183 | 0.349647268 | count | 1 |
| NADK | 0.3439048 | 0.2956767 | 1.1631 | 0.245 | 0.349812546 | count | 1 |
| LTBP4 | 0.263196 | 0.1593561 | 1.6516 | 0.0987 | 0.34995395 | count | 1 |
| COPS5 | 0.2510019 | 0.0998591 | 2.5136 | 0.012 | 0.350085344 | count | 1 |
| PRKAG2-AS1 | 0.3689845 | 0.2754831 | 1.3394 | 0.181 | 0.350130949 | count | 1 |
| FBXO2 | 0.2779488 | 0.2181594 | 1.2741 | 0.203 | 0.350365487 | count | 1 |
| RMDN2 | 0.3806465 | 0.5563037 | 0.6842 | 0.494 | 0.350579224 | count | 1 |
| PTPN23 | 0.4104093 | 0.342678 | 1.1977 | 0.231 | 0.350678815 | count | 1 |
| GSTM4 | 0.3807957 | 0.2736738 | 1.3914 | 0.164 | 0.350719863 | count | 1 |
| RNF43 | 0.7203502 | 0.8476298 | 0.8498 | 0.395 | 0.35076116 | count | 1 |
| AASDH | 0.2653597 | 0.1813131 | 1.4635 | 0.143 | 0.350879423 | count | 1 |
| PTP4A3 | 0.3387219 | 0.2814838 | 1.2033 | 0.229 | 0.350908666 | count | 1 |
| VPS9D1 | 0.4908061 | 0.385258 | 1.274 | 0.203 | 0.350930322 | count | 1 |
| C1orf53 | 0.9412056 | 1.0841555 | 0.8681 | 0.385 | 0.351285017 | count | 1 |
| MAFIP | 0.6080669 | 0.8082893 | 0.7523 | 0.452 | 0.351314878 | count | 1 |
| AC091132.5 | 0.6080669 | 0.8230413 | 0.7388 | 0.46 | 0.351314878 | count | 1 |
| RIN3 | 0.3168579 | 0.2719622 | 1.1651 | 0.244 | 0.351525546 | count | 1 |
| NUCB1 | 0.2572989 | 0.0992147 | 2.5934 | 0.00955 | 0.351676922 | count | 1 |
| BTBD9 | 0.3821464 | 0.2937574 | 1.3009 | 0.193 | 0.351993044 | count | 1 |
| MYBL1 | 0.2494104 | 0.0871072 | 2.8633 | 0.00422 | 0.352005852 | count | 1 |
| PARP15 | 0.2763647 | 0.1858426 | 1.4871 | 0.137 | 0.35205865 | count | 1 |
| INTS12 | 0.2719372 | 0.2011797 | 1.3517 | 0.177 | 0.352274691 | count | 1 |
| MBP | 0.2471809 | 0.0567402 | 4.3564 | 1.36E-05 | 0.352352596 | count | 0.3267128 |
| TTLL4 | 0.7236251 | 0.7372874 | 0.9815 | 0.326 | 0.352446805 | count | 1 |
| RGPD2 | 0.9442137 | 0.549492 | 1.7183 | 0.0858 | 0.352473252 | count | 1 |
| TSGA10 | 0.2929046 | 0.2320736 | 1.2621 | 0.207 | 0.352701534 | count | 1 |
| POMZP3 | 0.3627356 | 0.3327831 | 1.09 | 0.276 | 0.353362818 | count | 1 |
| TP53INP1 | 0.3100957 | 0.1813317 | 1.7101 | 0.0873 | 0.354150283 | count | 1 |
| LZTS1 | 0.9486627 | 0.7173545 | 1.3224 | 0.186 | 0.354230123 | count | 1 |
| TPTEP2-CSNK1E | 0.9487933 | 0.71431 | 1.3283 | 0.184 | 0.35428172 | count | 1 |
| ITPKC | 0.4151609 | 0.5202617 | 0.798 | 0.425 | 0.354852386 | count | 1 |
| USB1 | 0.2660095 | 0.1462554 | 1.8188 | 0.069 | 0.355117186 | count | 1 |
| AC097103.2 | 0.9512656 | 0.692203 | 1.3743 | 0.169 | 0.355258012 | count | 1 |
| ZFY | 0.2749024 | 0.1604784 | 1.713 | 0.0868 | 0.355476653 | count | 1 |
| PTPN6 | 0.2535495 | 0.0937334 | 2.705 | 0.00686 | 0.355691815 | count | 1 |
| PXN-AS1 | 0.3435768 | 0.3549635 | 0.9679 | 0.333 | 0.356023448 | count | 1 |
| ICAM2 | 0.2576721 | 0.1025802 | 2.5119 | 0.0121 | 0.356266854 | count | 1 |
| QPCTL | 0.7310681 | 0.6666477 | 1.0966 | 0.273 | 0.356278899 | count | 1 |

| | | | | | | | |
|---|---|---|---|---|---|---|---|
| COPS7A | 0.2797379 | 0.1996417 | 1.4012 | 0.161 | 0.356381501 | count | 1 |
| LINC02084 | 0.2707537 | 0.2174148 | 1.2453 | 0.213 | 0.356710244 | count | 1 |
| C6orf47-AS1 | 1.6200514 | 1.06235 | 1.525 | 0.127 | 0.356760157 | count | 1 |
| XKR9 | 1.6200514 | 1.672472 | 0.9687 | 0.333 | 0.356760157 | count | 1 |
| PPP1R3F | 0.4380982 | 0.3114396 | 1.4067 | 0.16 | 0.356954292 | count | 1 |
| TMEM217 | 1.621236 | 1.083923 | 1.4957 | 0.135 | 0.357010895 | count | 1 |
| ZNF709 | 1.621236 | 1.095246 | 1.4802 | 0.139 | 0.357010895 | count | 1 |
| THBS1 | 0.6182691 | 0.5494237 | 1.1253 | 0.261 | 0.357505612 | count | 1 |
| SMIM14 | 0.2726414 | 0.1671922 | 1.6307 | 0.103 | 0.35775366 | count | 1 |
| SMIM3 | 0.2790856 | 0.1552994 | 1.7971 | 0.0724 | 0.358006925 | count | 1 |
| C19orf48 | 0.2885804 | 0.1897802 | 1.5206 | 0.128 | 0.358074415 | count | 1 |
| C11orf80 | 0.4658026 | 0.5788329 | 0.8047 | 0.421 | 0.358141849 | count | 1 |
| PIK3IP1 | 0.2557144 | 0.0606618 | 4.2154 | 2.56E-05 | 0.358368138 | count | 0.614272 |
| RASSF3 | 0.2699022 | 0.1456818 | 1.8527 | 0.064 | 0.358526449 | count | 1 |
| TMEM191B | 0.2924446 | 0.2430347 | 1.2033 | 0.229 | 0.35872782 | count | 1 |
| ZNF672 | 0.3048474 | 0.2633075 | 1.1578 | 0.247 | 0.358763685 | count | 1 |
| ATF6B | 0.268129 | 0.1381259 | 1.9412 | 0.0523 | 0.358936831 | count | 1 |
| NCAPG2 | 0.3313291 | 0.3321981 | 0.9974 | 0.319 | 0.359096898 | count | 1 |
| LINC01588 | 0.3056305 | 0.2362084 | 1.2939 | 0.196 | 0.359694586 | count | 1 |
| ISM1 | 0.6222662 | 0.5043361 | 1.2338 | 0.217 | 0.359932375 | count | 1 |
| AL118516.1 | 0.2653029 | 0.1449065 | 1.8309 | 0.0672 | 0.360024406 | count | 1 |
| MPST | 0.2743854 | 0.1782699 | 1.5392 | 0.124 | 0.360052505 | count | 1 |
| POMGNT1 | 0.2884087 | 0.2354328 | 1.225 | 0.221 | 0.360317455 | count | 1 |
| ERCC3 | 0.2863847 | 0.1853084 | 1.5454 | 0.122 | 0.361069411 | count | 1 |
| CENPM | 0.3433017 | 0.2676092 | 1.2828 | 0.2 | 0.361717179 | count | 1 |
| ATE1 | 0.282238 | 0.1641937 | 1.7189 | 0.0857 | 0.362073823 | count | 1 |
| CYB561 | 0.3026748 | 0.2139678 | 1.4146 | 0.157 | 0.362643013 | count | 1 |
| AC010618.3 | 0.5553886 | 0.5276698 | 1.0525 | 0.293 | 0.362829191 | count | 1 |
| AL355472.1 | 0.310395 | 0.2957908 | 1.0494 | 0.294 | 0.362887615 | count | 1 |
| GADD45G | 0.2898703 | 0.2023381 | 1.4326 | 0.152 | 0.363314538 | count | 1 |
| CSTF2T | 0.3242257 | 0.2153008 | 1.5059 | 0.132 | 0.36361675 | count | 1 |
| MYO1B | 0.4461474 | 0.4858691 | 0.9182 | 0.359 | 0.363716583 | count | 1 |
| CAPN15 | 0.3053303 | 0.2255388 | 1.3538 | 0.176 | 0.363801437 | count | 1 |
| MMAB | 0.3186755 | 0.2970185 | 1.0729 | 0.283 | 0.364064691 | count | 1 |
| GSE1 | 0.37391 | 0.339245 | 1.1022 | 0.27 | 0.364473956 | count | 1 |
| AL138724.1 | 0.319114 | 0.3256867 | 0.9798 | 0.327 | 0.364571524 | count | 1 |
| CD101 | 0.9749193 | 0.9818343 | 0.993 | 0.321 | 0.364595435 | count | 1 |
| AC078881.1 | 0.9749193 | 0.99724 | 0.9776 | 0.328 | 0.364595435 | count | 1 |
| EPS8 | 0.3143363 | 0.281275 | 1.1175 | 0.264 | 0.364887847 | count | 1 |
| CMC1 | 0.2585959 | 0.0863339 | 2.9953 | 0.00276 | 0.364955772 | count | 1 |
| SLC25A45 | 0.2811792 | 0.1460459 | 1.9253 | 0.0543 | 0.364962063 | count | 1 |
| MRPL9 | 0.2647441 | 0.1138443 | 2.3255 | 0.0201 | 0.36507506 | count | 1 |
| PIK3C2A | 0.3101858 | 0.1828806 | 1.6961 | 0.09 | 0.365110325 | count | 1 |
| SMN1 | 0.4478239 | 0.3059565 | 1.4637 | 0.143 | 0.365125668 | count | 1 |
| SGK1 | 0.2767958 | 0.1539606 | 1.7978 | 0.0723 | 0.365170946 | count | 1 |

| | | | | | | | |
|---|---|---|---|---|---|---|---|
| SLC35F2 | 0.3747208 | 0.3557877 | 1.0532 | 0.292 | 0.365280545 | count | 1 |
| PHLDB2 | 0.3847034 | 0.3416908 | 1.1259 | 0.26 | 0.365379621 | count | 1 |
| ACP2 | 0.3966129 | 0.365027 | 1.0865 | 0.277 | 0.365638471 | count | 1 |
| CITED2 | 0.2567034 | 0.0684393 | 3.7508 | 0.000179 | 0.366153283 | count | 1 |
| AL365226.2 | 0.7509519 | 0.8597934 | 0.8734 | 0.383 | 0.366522789 | count | 1 |
| MED7 | 0.2830032 | 0.2445192 | 1.1574 | 0.247 | 0.366685481 | count | 1 |
| ABI3 | 0.2666943 | 0.1222331 | 2.1819 | 0.0292 | 0.366773834 | count | 1 |
| TWNK | 0.3979393 | 0.5211427 | 0.7636 | 0.445 | 0.366890395 | count | 1 |
| NPIPB2 | 0.5618334 | 0.5759638 | 0.9755 | 0.329 | 0.367224133 | count | 1 |
| PRR5 | 0.267042 | 0.1290852 | 2.0687 | 0.0386 | 0.367253106 | count | 1 |
| AC111182.1 | 0.3122199 | 0.2525503 | 1.2363 | 0.216 | 0.367529028 | count | 1 |
| AC099811.4 | 1.6736628 | 0.9245639 | 1.8102 | 0.0704 | 0.368013192 | count | 1 |
| UCP2 | 0.2578395 | 0.0628499 | 4.1025 | 4.18E-05 | 0.368314264 | count | 1 |
| EID2B | 0.3150044 | 0.2920436 | 1.0786 | 0.281 | 0.368333619 | count | 1 |
| SLC10A3 | 0.3015458 | 0.1762124 | 1.7113 | 0.0871 | 0.36840979 | count | 1 |
| EIF5A2 | 0.2817011 | 0.1812128 | 1.5545 | 0.12 | 0.368623211 | count | 1 |
| GNPNAT1 | 0.2971236 | 0.2409941 | 1.2329 | 0.218 | 0.368754329 | count | 1 |
| AL049780.2 | 0.5644425 | 0.8924354 | 0.6325 | 0.527 | 0.369004044 | count | 1 |
| AC018797.2 | 0.4524873 | 0.5245673 | 0.8626 | 0.388 | 0.369046277 | count | 1 |
| CSTF2 | 0.3035798 | 0.2728787 | 1.1125 | 0.266 | 0.369254259 | count | 1 |
| CDK16 | 0.3561343 | 0.2632502 | 1.3528 | 0.176 | 0.369261265 | count | 1 |
| AL121748.1 | 0.7568446 | 0.5418702 | 1.3967 | 0.163 | 0.369560299 | count | 1 |
| C15orf53 | 0.3332514 | 0.2586881 | 1.2882 | 0.198 | 0.369959293 | count | 1 |
| UBA7 | 0.3894938 | 0.2700018 | 1.4426 | 0.149 | 0.37003052 | count | 1 |
| ITGB7 | 0.2658653 | 0.0983761 | 2.7025 | 0.00692 | 0.370079112 | count | 1 |
| PGS1 | 0.2952358 | 0.1997155 | 1.4783 | 0.139 | 0.370086349 | count | 1 |
| CMKLR1 | 0.7579288 | 0.4838975 | 1.5663 | 0.117 | 0.370119232 | count | 1 |
| RALGPS2 | 0.4333045 | 0.3671277 | 1.1803 | 0.238 | 0.370805019 | count | 1 |
| SCAP | 0.4333424 | 0.3011503 | 1.439 | 0.15 | 0.370838368 | count | 1 |
| RAB11FIP5 | 0.6402183 | 0.4535009 | 1.4117 | 0.158 | 0.370840398 | count | 1 |
| ZNF75A | 0.2891297 | 0.1611344 | 1.7943 | 0.0728 | 0.370966142 | count | 1 |
| LETM1 | 0.2907565 | 0.1757741 | 1.6541 | 0.0982 | 0.371389607 | count | 1 |
| NOS3 | 1.6904358 | 0.7661395 | 2.2064 | 0.0274 | 0.371494514 | count | 1 |
| ZNF470 | 1.691067 | 0.8584707 | 1.9699 | 0.0489 | 0.37162514 | count | 1 |
| GALE | 0.3179412 | 0.2631326 | 1.2083 | 0.227 | 0.371804098 | count | 1 |
| TTN-AS1 | 0.4033317 | 0.3252384 | 1.2401 | 0.215 | 0.371981446 | count | 1 |
| ZNF302 | 0.2977254 | 0.2195287 | 1.3562 | 0.175 | 0.372040588 | count | 1 |
| DHRS7 | 0.2601744 | 0.0541019 | 4.809 | 1.58E-06 | 0.372053668 | count | 0.03806694 |
| MORC2-AS1 | 0.4561496 | 0.3382987 | 1.3484 | 0.178 | 0.372126359 | count | 1 |
| TMEM147-AS1 | 0.9958323 | 0.7607319 | 1.309 | 0.191 | 0.372844523 | count | 1 |
| TCEA3 | 0.34868 | 0.2888982 | 1.2069 | 0.228 | 0.373050643 | count | 1 |
| ZNF765 | 0.3601835 | 0.2572151 | 1.4003 | 0.162 | 0.373532278 | count | 1 |
| AC087500.1 | 0.4370063 | 0.657324 | 0.6648 | 0.506 | 0.374062856 | count | 1 |
| ADD3 | 0.2688455 | 0.0943726 | 2.8488 | 0.00442 | 0.374082732 | count | 1 |
| JKAMP | 0.2782888 | 0.1447292 | 1.9228 | 0.0546 | 0.374134313 | count | 1 |

| | | | | | | | |
|---|---|---|---|---|---|---|---|
| ACSS1 | 0.3411579 | 0.2275205 | 1.4995 | 0.134 | 0.374551601 | count | 1 |
| AL118506.1 | 1.0025796 | 0.632812 | 1.5843 | 0.113 | 0.375504389 | count | 1 |
| ZNF517 | 1.0025796 | 0.6742222 | 1.487 | 0.137 | 0.375504389 | count | 1 |
| VPS37D | 0.574216 | 0.6802225 | 0.8442 | 0.399 | 0.37567474 | count | 1 |
| RNF44 | 0.3018908 | 0.1510338 | 1.9988 | 0.0457 | 0.376026931 | count | 1 |
| NEIL1 | 0.3425896 | 0.2887938 | 1.1863 | 0.236 | 0.376145621 | count | 1 |
| SOX13 | 1.0044955 | 0.5572398 | 1.8026 | 0.0715 | 0.376259526 | count | 1 |
| AP000766.1 | 0.4885757 | 0.5892266 | 0.8292 | 0.407 | 0.376268091 | count | 1 |
| CC2D1A | 0.3321764 | 0.2801106 | 1.1859 | 0.236 | 0.376281671 | count | 1 |
| SRSF3 | 0.2634133 | 0.0446089 | 5.9049 | 3.88E-09 | 0.376445878 | count | 9.38E-05 |
| ABHD17A | 0.2631608 | 0.0545545 | 4.8238 | 1.47E-06 | 0.376641919 | count | 0.03542112 |
| KIAA1958 | 0.7707207 | 0.8564529 | 0.8999 | 0.368 | 0.376715564 | count | 1 |
| PTGER2 | 0.2675537 | 0.0889431 | 3.0081 | 0.00265 | 0.376830198 | count | 1 |
| COPZ1 | 0.2689225 | 0.0989814 | 2.7169 | 0.00662 | 0.376904608 | count | 1 |
| AP000254.1 | 1.719327 | 1.161241 | 1.4806 | 0.139 | 0.377445503 | count | 1 |
| AC005498.2 | 1.719327 | 1.278002 | 1.3453 | 0.179 | 0.377445503 | count | 1 |
| LINC01814 | 1.719327 | 1.330103 | 1.2926 | 0.196 | 0.377445503 | count | 1 |
| PLGLB1 | 1.719327 | 1.330103 | 1.2926 | 0.196 | 0.377445503 | count | 1 |
| USP27X-AS1 | 1.719327 | 1.52931 | 1.1243 | 0.261 | 0.377445503 | count | 1 |
| ATP6V1C1 | 0.3641167 | 0.2491921 | 1.4612 | 0.144 | 0.377682055 | count | 1 |
| PSMD6-AS1 | 1.0082871 | 0.8078463 | 1.2481 | 0.212 | 0.377753709 | count | 1 |
| AP002449.1 | 1.0082871 | 0.9280539 | 1.0865 | 0.277 | 0.377753709 | count | 1 |
| AC083843.2 | 1.0082871 | 0.9979013 | 1.0104 | 0.312 | 0.377753709 | count | 1 |
| AL596325.1 | 1.7214271 | 0.7959079 | 2.1628 | 0.0306 | 0.377875806 | count | 1 |
| LBX2 | 1.7225327 | 0.8209545 | 2.0982 | 0.036 | 0.378102212 | count | 1 |
| AC137630.4 | 1.7225327 | 0.8209545 | 2.0982 | 0.036 | 0.378102212 | count | 1 |
| G6PC3 | 0.3788511 | 0.4103196 | 0.9233 | 0.356 | 0.378123654 | count | 1 |
| HIST2H2BF | 0.4242955 | 0.4938057 | 0.8592 | 0.39 | 0.378229491 | count | 1 |
| OGDH | 0.3213496 | 0.1847399 | 1.7395 | 0.082 | 0.378387853 | count | 1 |
| ZNF746 | 0.4101339 | 0.3282144 | 1.2496 | 0.212 | 0.378406628 | count | 1 |
| CCDC163 | 0.4422949 | 0.4504464 | 0.9819 | 0.326 | 0.378718922 | count | 1 |
| MRPL30 | 0.3177965 | 0.2221699 | 1.4304 | 0.153 | 0.3788002 | count | 1 |
| LRFN1 | 0.4920648 | 0.4824142 | 1.02 | 0.308 | 0.379048398 | count | 1 |
| ZBTB10 | 0.3346698 | 0.181011 | 1.8489 | 0.0646 | 0.37914131 | count | 1 |
| DPP9 | 0.3065445 | 0.2207239 | 1.3888 | 0.165 | 0.379144428 | count | 1 |
| ZNF749 | 0.42555 | 0.4320539 | 0.9849 | 0.325 | 0.379376978 | count | 1 |
| RHBDD3 | 1.012587 | 0.4074584 | 2.4851 | 0.013 | 0.379448059 | count | 1 |
| AC025159.1 | 0.3068222 | 0.2141518 | 1.4327 | 0.152 | 0.379490552 | count | 1 |
| WDR83 | 0.3057751 | 0.2164602 | 1.4126 | 0.158 | 0.379573111 | count | 1 |
| SNHG10 | 0.5298194 | 0.3952472 | 1.3405 | 0.18 | 0.379941402 | count | 1 |
| MMP25 | 1.0143572 | 0.5753312 | 1.7631 | 0.078 | 0.380145377 | count | 1 |
| PPP2R5B | 0.5304355 | 0.36158 | 1.467 | 0.142 | 0.380400317 | count | 1 |
| C1orf159 | 0.5305521 | 0.390968 | 1.357 | 0.175 | 0.380487176 | count | 1 |
| FAM201A | 0.7780788 | 0.613428 | 1.2684 | 0.205 | 0.380511137 | count | 1 |
| KEAP1 | 0.2877023 | 0.1774731 | 1.6211 | 0.105 | 0.380551538 | count | 1 |

| | | | | | | | |
|---|---|---|---|---|---|---|---|
| C2orf68 | 0.3304332 | 0.2291215 | 1.4422 | 0.149 | 0.380819408 | count | 1 |
| AL450998.2 | 0.3200142 | 0.2145426 | 1.4916 | 0.136 | 0.381469317 | count | 1 |
| LINC01136 | 1.018442 | 0.5366604 | 1.8977 | 0.0578 | 0.381754099 | count | 1 |
| NME4 | 0.2969474 | 0.2007279 | 1.4794 | 0.139 | 0.381866564 | count | 1 |
| SMIM8 | 0.2918124 | 0.1866937 | 1.5631 | 0.118 | 0.38191893 | count | 1 |
| ZNF329 | 0.4463442 | 0.40105 | 1.1129 | 0.266 | 0.382285281 | count | 1 |
| ZNF429 | 0.3490904 | 0.2722002 | 1.2825 | 0.2 | 0.383385141 | count | 1 |
| HDAC9 | 0.4696028 | 0.4751582 | 0.9883 | 0.323 | 0.383449032 | count | 1 |
| HIST1H3H | 0.3930201 | 0.3324329 | 1.1823 | 0.237 | 0.383497729 | count | 1 |
| LINC02193 | 1.749765 | 0.6487559 | 2.6971 | 0.00703 | 0.383651362 | count | 1 |
| TMSB4Y | 1.749765 | 0.7315831 | 2.3918 | 0.0168 | 0.383651362 | count | 1 |
| APOBR | 0.3932133 | 0.2926147 | 1.3438 | 0.179 | 0.383690195 | count | 1 |
| AXIN1 | 0.308054 | 0.2067232 | 1.4902 | 0.136 | 0.38376074 | count | 1 |
| JUND | 0.2663273 | 0.0258093 | 10.319 | 1.33E-24 | 0.383835823 | count | 3.23E-20 |
| PBX2 | 0.3260638 | 0.2274494 | 1.4336 | 0.152 | 0.383996725 | count | 1 |
| UHMK1 | 0.281286 | 0.1130644 | 2.4878 | 0.0129 | 0.384551059 | count | 1 |
| RHOB | 0.3067728 | 0.1913006 | 1.6036 | 0.109 | 0.38465153 | count | 1 |
| RRP15 | 0.286455 | 0.1289934 | 2.2207 | 0.0264 | 0.384847218 | count | 1 |
| CMC4 | 0.4997376 | 0.3946754 | 1.2662 | 0.206 | 0.385165324 | count | 1 |
| RAB37 | 0.285507 | 0.1920523 | 1.4866 | 0.137 | 0.385296724 | count | 1 |
| MKLN1 | 0.3182388 | 0.1817474 | 1.751 | 0.08 | 0.38541478 | count | 1 |
| PTPN13 | 0.588954 | 0.3539716 | 1.6638 | 0.0962 | 0.38574332 | count | 1 |
| ZNF808 | 0.3476426 | 0.2622776 | 1.3255 | 0.185 | 0.386156057 | count | 1 |
| FMO5 | 1.0308056 | 0.8790853 | 1.1726 | 0.241 | 0.386621518 | count | 1 |
| AC009275.1 | 1.0308056 | 0.9527714 | 1.0819 | 0.279 | 0.386621518 | count | 1 |
| GCNT4 | 0.3563906 | 0.2281147 | 1.5623 | 0.118 | 0.38667336 | count | 1 |
| TUT1 | 0.3667621 | 0.2909776 | 1.2604 | 0.208 | 0.386852978 | count | 1 |
| PAFAH2 | 0.3667621 | 0.3279202 | 1.1184 | 0.263 | 0.386852978 | count | 1 |
| ABHD6 | 0.451667 | 0.3535232 | 1.2776 | 0.201 | 0.386975029 | count | 1 |
| BCAT2 | 0.3087713 | 0.2624793 | 1.1764 | 0.24 | 0.387175175 | count | 1 |
| ATPAF2 | 0.4073218 | 0.3023464 | 1.3472 | 0.178 | 0.387354363 | count | 1 |
| ZNF529-AS1 | 0.5025558 | 0.5372138 | 0.9355 | 0.35 | 0.387413012 | count | 1 |
| C5orf15 | 0.2970724 | 0.1707862 | 1.7394 | 0.082 | 0.387639222 | count | 1 |
| HSPA2 | 0.3624535 | 0.2630282 | 1.378 | 0.168 | 0.388021209 | count | 1 |
| FAM118B | 0.3532954 | 0.315615 | 1.1194 | 0.263 | 0.388069423 | count | 1 |
| IL17RC | 0.6686961 | 0.9295162 | 0.7194 | 0.472 | 0.388170437 | count | 1 |
| AL159169.2 | 1.0349371 | 1.136108 | 0.9109 | 0.362 | 0.388247236 | count | 1 |
| ZNF879 | 0.503661 | 0.7086059 | 0.7108 | 0.477 | 0.388294622 | count | 1 |
| WDR77 | 0.3205996 | 0.2153257 | 1.4889 | 0.137 | 0.388299524 | count | 1 |
| AC092295.2 | 0.5036891 | 0.8056734 | 0.6252 | 0.532 | 0.388317017 | count | 1 |
| AC006033.2 | 0.5036891 | 0.9102092 | 0.5534 | 0.58 | 0.388317017 | count | 1 |
| VPS52 | 0.34972 | 0.4809903 | 0.7271 | 0.467 | 0.388495184 | count | 1 |
| DRAM2 | 0.2843394 | 0.1290516 | 2.2033 | 0.0276 | 0.38873654 | count | 1 |
| STX5 | 0.2992557 | 0.143669 | 2.083 | 0.0373 | 0.389221576 | count | 1 |
| CSRNP1 | 0.2775335 | 0.0762971 | 3.6375 | 0.000279 | 0.38984094 | count | 1 |

| | | | | | | | |
|---|---|---|---|---|---|---|---|
| GAREM2 | 0.5951573 | 0.664365 | 0.8958 | 0.37 | 0.389984476 | count | 1 |
| UBE2F | 0.2870993 | 0.1381156 | 2.0787 | 0.0377 | 0.39014196 | count | 1 |
| TWSG1 | 0.437475 | 0.3915956 | 1.1172 | 0.264 | 0.39029035 | count | 1 |
| FUNDC1 | 0.3029045 | 0.1990367 | 1.5219 | 0.128 | 0.390370569 | count | 1 |
| OSBPL7 | 0.544092 | 0.3286759 | 1.6554 | 0.0979 | 0.390578506 | count | 1 |
| PLCXD1 | 0.7985363 | 0.592602 | 1.3475 | 0.178 | 0.391067705 | count | 1 |
| HDGFL3 | 0.3708325 | 0.243089 | 1.5255 | 0.127 | 0.391217891 | count | 1 |
| CDK10 | 0.3035598 | 0.1730661 | 1.754 | 0.0795 | 0.391219903 | count | 1 |
| PTCH2 | 0.6737566 | 0.4931166 | 1.3663 | 0.172 | 0.391253015 | count | 1 |
| PRAG1 | 0.4015413 | 0.276837 | 1.4505 | 0.147 | 0.39198894 | count | 1 |
| SRR | 0.4799944 | 0.5686739 | 0.8441 | 0.399 | 0.392203445 | count | 1 |
| SDF2 | 0.2807711 | 0.0999584 | 2.8089 | 0.005 | 0.392211217 | count | 1 |
| IL18RAP | 0.3493931 | 0.196688 | 1.7764 | 0.0758 | 0.39222064 | count | 1 |
| CORO2A | 0.3929925 | 0.2717038 | 1.4464 | 0.148 | 0.392520186 | count | 1 |
| AC015802.6 | 0.5990715 | 0.3808939 | 1.5728 | 0.116 | 0.392661517 | count | 1 |
| AC132192.2 | 0.6765093 | 0.3408025 | 1.985 | 0.0472 | 0.392930167 | count | 1 |
| SRD5A3 | 0.3110377 | 0.2253226 | 1.3804 | 0.168 | 0.393465438 | count | 1 |
| NPC1 | 0.4815831 | 0.2583583 | 1.864 | 0.0624 | 0.393542485 | count | 1 |
| FAM41C | 0.441094 | 0.522449 | 0.8443 | 0.399 | 0.393604311 | count | 1 |
| ZNF18 | 0.3862577 | 0.3171448 | 1.2179 | 0.223 | 0.393724199 | count | 1 |
| EEFSEC | 0.3322414 | 0.1946349 | 1.707 | 0.0879 | 0.393837811 | count | 1 |
| LINC00847 | 0.3050506 | 0.2294123 | 1.3297 | 0.184 | 0.393929735 | count | 1 |
| NPHP3 | 0.3630199 | 0.2399459 | 1.5129 | 0.13 | 0.393974851 | count | 1 |
| ZNF461 | 0.8051827 | 0.6106602 | 1.3185 | 0.187 | 0.394498413 | count | 1 |
| MKL1 | 0.3450521 | 0.3108549 | 1.11 | 0.267 | 0.394572565 | count | 1 |
| SNX12 | 0.4150437 | 0.3351377 | 1.2384 | 0.216 | 0.394864968 | count | 1 |
| B3GLCT | 0.6023325 | 0.4499705 | 1.3386 | 0.181 | 0.39489238 | count | 1 |
| ZSCAN21 | 0.511996 | 0.4321289 | 1.1848 | 0.236 | 0.394945769 | count | 1 |
| ZNF717 | 1.0538117 | 0.570895 | 1.8459 | 0.065 | 0.39566874 | count | 1 |
| B4GALT7 | 0.3096827 | 0.1973596 | 1.5691 | 0.117 | 0.395716147 | count | 1 |
| ZNF8 | 0.8080128 | 0.4242453 | 1.9046 | 0.0569 | 0.395959336 | count | 1 |
| BCAR3 | 1.811195 | 0.9270322 | 1.9538 | 0.0508 | 0.39596986 | count | 1 |
| AC002467.1 | 0.4059278 | 0.4704766 | 0.8628 | 0.388 | 0.396361981 | count | 1 |
| NCS1 | 0.5142575 | 0.6030313 | 0.8528 | 0.394 | 0.39675114 | count | 1 |
| HIST2H2AB | 0.5142575 | 0.6657918 | 0.7724 | 0.44 | 0.39675114 | count | 1 |
| ANGPTL6 | 0.8098247 | 0.7906047 | 1.0243 | 0.306 | 0.396894718 | count | 1 |
| LPCAT1 | 0.3107535 | 0.2361353 | 1.316 | 0.188 | 0.397092892 | count | 1 |
| NUGGC | 0.514813 | 0.3578974 | 1.4384 | 0.15 | 0.397194687 | count | 1 |
| PGBD4 | 0.683687 | 0.6622669 | 1.0323 | 0.302 | 0.397304457 | count | 1 |
| BLK | 0.4868449 | 0.6364622 | 0.7649 | 0.444 | 0.397978546 | count | 1 |
| AL162377.1 | 0.3545679 | 0.2996575 | 1.1832 | 0.237 | 0.398106853 | count | 1 |
| TMEM19 | 0.3364434 | 0.2785586 | 1.2078 | 0.227 | 0.398869947 | count | 1 |
| DOK2 | 0.2791861 | 0.0606535 | 4.603 | 4.32E-06 | 0.399047174 | count | 0.10395216 |
| MYLPF | 0.5555694 | 0.5670354 | 0.9798 | 0.327 | 0.399140783 | count | 1 |
| TMEM214 | 0.3915617 | 0.2950021 | 1.3273 | 0.184 | 0.399232305 | count | 1 |

| | | | | | | | |
|---|---|---|---|---|---|---|---|
| HSPA14 | 0.3731476 | 0.2380602 | 1.5675 | 0.117 | 0.399653345 | count | 1 |
| LINC01918 | 1.0671373 | 0.9598901 | 1.1117 | 0.266 | 0.40090239 | count | 1 |
| CTXN1 | 1.0671373 | 1.0350485 | 1.031 | 0.303 | 0.40090239 | count | 1 |
| AP001267.1 | 1.0671373 | 1.08764 | 0.9811 | 0.327 | 0.40090239 | count | 1 |
| AC007383.2 | 0.5580796 | 0.5846393 | 0.9546 | 0.34 | 0.401014368 | count | 1 |
| METTL7A | 1.0678675 | 0.5749661 | 1.8573 | 0.0634 | 0.401189029 | count | 1 |
| C22orf15 | 1.0681966 | 0.7407817 | 1.442 | 0.149 | 0.401318215 | count | 1 |
| EGLN2 | 1.0681966 | 0.8801521 | 1.2137 | 0.225 | 0.401318215 | count | 1 |
| AL157938.3 | 0.3697399 | 0.3911067 | 0.9454 | 0.345 | 0.401379068 | count | 1 |
| ANAPC2 | 0.8198356 | 0.4239715 | 1.9337 | 0.0532 | 0.402063107 | count | 1 |
| NLRC5 | 0.3052337 | 0.1582853 | 1.9284 | 0.0539 | 0.402351601 | count | 1 |
| ZNF726 | 0.6927462 | 0.6927935 | 0.9999 | 0.317 | 0.402827597 | count | 1 |
| DUBR | 0.6927462 | 0.7796334 | 0.8886 | 0.374 | 0.402827597 | count | 1 |
| CPSF4 | 0.3295049 | 0.2218655 | 1.4852 | 0.138 | 0.402869159 | count | 1 |
| QSER1 | 0.3523101 | 0.3078985 | 1.1442 | 0.253 | 0.402974515 | count | 1 |
| COPS7B | 0.3423698 | 0.2361246 | 1.45 | 0.147 | 0.403406595 | count | 1 |
| PVALB | 1.073696 | 0.3943897 | 2.7224 | 0.00651 | 0.403476306 | count | 1 |
| FBXO46 | 0.3767327 | 0.3201656 | 1.1767 | 0.239 | 0.403554538 | count | 1 |
| C2CD3 | 0.4524297 | 0.3691116 | 1.2257 | 0.22 | 0.403990329 | count | 1 |
| LINC00861 | 0.298078 | 0.1264928 | 2.3565 | 0.0185 | 0.404642235 | count | 1 |
| RRP8 | 0.3228514 | 0.1954061 | 1.6522 | 0.0986 | 0.404959821 | count | 1 |
| PHOSPHO2 | 0.4953357 | 0.4110379 | 1.2051 | 0.228 | 0.405140596 | count | 1 |
| AC011476.3 | 0.4956005 | 0.5995385 | 0.8266 | 0.409 | 0.405364033 | count | 1 |
| ATL1 | 0.826568 | 0.5766625 | 1.4334 | 0.152 | 0.405539209 | count | 1 |
| ALPK1 | 0.4400008 | 0.3830945 | 1.1485 | 0.251 | 0.406657164 | count | 1 |
| TRERF1 | 0.4273856 | 0.2857218 | 1.4958 | 0.135 | 0.406877638 | count | 1 |
| POC5 | 0.3101896 | 0.1876513 | 1.653 | 0.0984 | 0.407269332 | count | 1 |
| PUDP | 0.3995154 | 0.3742049 | 1.0676 | 0.286 | 0.407495533 | count | 1 |
| DNLZ | 0.566963 | 0.313707 | 1.8073 | 0.0708 | 0.407647483 | count | 1 |
| VARS2 | 0.7013975 | 0.460103 | 1.5244 | 0.127 | 0.408104184 | count | 1 |
| CHPF2 | 0.3810761 | 0.2503018 | 1.5225 | 0.128 | 0.408281951 | count | 1 |
| PGBD2 | 0.4293722 | 0.2662439 | 1.6127 | 0.107 | 0.408812187 | count | 1 |
| HAR1A | 0.418516 | 0.3509439 | 1.1925 | 0.233 | 0.408918737 | count | 1 |
| LDAH | 0.4094419 | 0.3219042 | 1.2719 | 0.203 | 0.409283531 | count | 1 |
| AZIN1-AS1 | 0.7034362 | 0.6894239 | 1.0203 | 0.308 | 0.409347929 | count | 1 |
| CYTH4 | 0.3119471 | 0.1474626 | 2.1154 | 0.0345 | 0.410154923 | count | 1 |
| TNFSF4 | 0.4784487 | 0.4154481 | 1.1516 | 0.25 | 0.410600486 | count | 1 |
| NEK4 | 0.4787307 | 0.3519017 | 1.3604 | 0.174 | 0.4108495 | count | 1 |
| RNF166 | 0.2923833 | 0.0851792 | 3.4326 | 0.000605 | 0.410953423 | count | 1 |
| RAB25 | 1.8882125 | 1.184123 | 1.5946 | 0.111 | 0.411007768 | count | 1 |
| EHHADH | 0.6269631 | 0.6769564 | 0.9261 | 0.354 | 0.411757141 | count | 1 |
| RINT1 | 0.3797881 | 0.2672562 | 1.4211 | 0.155 | 0.412455522 | count | 1 |
| VARS | 0.4332499 | 0.3250617 | 1.3328 | 0.183 | 0.412589036 | count | 1 |
| ARL6IP6 | 0.3018561 | 0.1132248 | 2.666 | 0.00771 | 0.412751136 | count | 1 |
| LINC01597 | 0.6287225 | 0.7287443 | 0.8627 | 0.388 | 0.412962765 | count | 1 |

| | | | | | | | |
|---|---|---|---|---|---|---|---|
| TMEM202-AS1 | 0.7102795 | 0.4338998 | 1.637 | 0.102 | 0.413523486 | count | 1 |
| PEAK1 | 0.4820159 | 0.4305773 | 1.1195 | 0.263 | 0.413750763 | count | 1 |
| PSTPIP2 | 0.3468371 | 0.1810336 | 1.9159 | 0.0555 | 0.41377219 | count | 1 |
| FOXA3 | 0.8426205 | 0.9116083 | 0.9243 | 0.355 | 0.413828023 | count | 1 |
| DDX55 | 0.3187414 | 0.1671901 | 1.9065 | 0.0567 | 0.414703328 | count | 1 |
| GIN1 | 0.3870678 | 0.3282909 | 1.179 | 0.238 | 0.414805301 | count | 1 |
| KAZN | 1.1027351 | 0.8862149 | 1.2443 | 0.213 | 0.414855781 | count | 1 |
| TMUB2 | 0.3065492 | 0.1454082 | 2.1082 | 0.0351 | 0.415168966 | count | 1 |
| AC020659.1 | 0.6320951 | 0.5199705 | 1.2156 | 0.224 | 0.415274168 | count | 1 |
| AC005921.2 | 0.3288367 | 0.1793975 | 1.833 | 0.0669 | 0.416137978 | count | 1 |
| MCM4 | 0.4849559 | 0.4909651 | 0.9878 | 0.323 | 0.416347741 | count | 1 |
| CRIP1 | 0.2926285 | 0.0640839 | 4.5663 | 5.14E-06 | 0.416607437 | count | 0.1236427 |
| MYO1F | 0.2991617 | 0.105859 | 2.826 | 0.00474 | 0.416996688 | count | 1 |
| RNF146 | 0.3248217 | 0.1734106 | 1.8731 | 0.0611 | 0.417047781 | count | 1 |
| MSANTD3 | 0.349597 | 0.2104367 | 1.6613 | 0.0967 | 0.417097973 | count | 1 |
| RAP1GAP2 | 1.108778 | 0.3534113 | 3.1374 | 0.00172 | 0.417220043 | count | 1 |
| ZNF831 | 0.3044556 | 0.0999975 | 3.0446 | 0.00235 | 0.418377624 | count | 1 |
| CCL3L1 | 0.3118435 | 0.2045864 | 1.5243 | 0.128 | 0.418746812 | count | 1 |
| AC018647.2 | 0.5114606 | 0.5545213 | 0.9223 | 0.356 | 0.418754124 | count | 1 |
| GATD1 | 0.3766128 | 0.2924007 | 1.288 | 0.198 | 0.418799433 | count | 1 |
| STARD4 | 0.4528422 | 0.3767462 | 1.202 | 0.229 | 0.418821993 | count | 1 |
| TBKBP1 | 0.5117803 | 0.507956 | 1.0075 | 0.314 | 0.41902417 | count | 1 |
| KLF7 | 0.5422409 | 0.3788913 | 1.4311 | 0.152 | 0.419114943 | count | 1 |
| FOXK1 | 0.3337775 | 0.2125773 | 1.5701 | 0.116 | 0.420038256 | count | 1 |
| SLFN13 | 0.397793 | 0.3442484 | 1.1555 | 0.248 | 0.420155949 | count | 1 |
| THRA | 0.3216618 | 0.1824353 | 1.7632 | 0.078 | 0.420551479 | count | 1 |
| CD69 | 0.292028 | 0.0394851 | 7.3959 | 1.76E-13 | 0.420787596 | count | 4.27E-09 |
| MHENCR | 0.3156844 | 0.1558952 | 2.025 | 0.0429 | 0.420899687 | count | 1 |
| SFXN4 | 0.3831555 | 0.2763061 | 1.3867 | 0.166 | 0.421364091 | count | 1 |
| LHPP | 0.3990057 | 0.255948 | 1.5589 | 0.119 | 0.421458656 | count | 1 |
| UBXN8 | 0.4215614 | 0.3312433 | 1.2727 | 0.203 | 0.421645538 | count | 1 |
| CDIP1 | 0.3376719 | 0.2039137 | 1.656 | 0.0978 | 0.422347707 | count | 1 |
| MMP2 | 0.5464421 | 0.7417053 | 0.7367 | 0.461 | 0.422476163 | count | 1 |
| ALDH18A1 | 0.4579376 | 0.354132 | 1.2931 | 0.196 | 0.423651796 | count | 1 |
| FAM122B | 0.4014456 | 0.254346 | 1.5783 | 0.115 | 0.424079896 | count | 1 |
| GTF2H2 | 0.4450815 | 0.3309758 | 1.3448 | 0.179 | 0.424118813 | count | 1 |
| PTCD1 | 0.5898954 | 0.5291805 | 1.1147 | 0.265 | 0.42478866 | count | 1 |
| TMEM45B | 0.8639401 | 0.6989426 | 1.2361 | 0.217 | 0.424836479 | count | 1 |
| MRPS30-DT | 0.8639401 | 0.8978142 | 0.9623 | 0.336 | 0.424836479 | count | 1 |
| BORCS5 | 0.3149952 | 0.1385062 | 2.2742 | 0.023 | 0.424932572 | count | 1 |
| CHST7 | 0.3399739 | 0.2067133 | 1.6447 | 0.1 | 0.42524875 | count | 1 |
| ZNF181 | 0.3824879 | 0.3294995 | 1.1608 | 0.246 | 0.425425358 | count | 1 |
| NSUN2 | 0.3869019 | 0.2313133 | 1.6726 | 0.0945 | 0.425545088 | count | 1 |
| ARRDC4 | 1.130424 | 0.6300298 | 1.7942 | 0.0729 | 0.425677057 | count | 1 |
| AC120049.1 | 1.130424 | 0.6300298 | 1.7942 | 0.0729 | 0.425677057 | count | 1 |

| | | | | | | | |
|---|---|---|---|---|---|---|---|
| RHOQ | 0.3382377 | 0.2120939 | 1.5948 | 0.111 | 0.425691812 | count | 1 |
| ANKRD53 | 1.96659 | 1.257035 | 1.5645 | 0.118 | 0.425825734 | count | 1 |
| RBM43 | 0.3297721 | 0.1883328 | 1.751 | 0.08 | 0.426043389 | count | 1 |
| FCGR3B | 0.6484004 | 0.4431922 | 1.463 | 0.144 | 0.426454857 | count | 1 |
| PLD1 | 1.1329868 | 0.7695102 | 1.4723 | 0.141 | 0.426677256 | count | 1 |
| S100P | 1.1329868 | 0.9959766 | 1.1376 | 0.255 | 0.426677256 | count | 1 |
| RNF20 | 0.3285287 | 0.1962172 | 1.6743 | 0.0942 | 0.426773537 | count | 1 |
| AC005332.1 | 0.3699228 | 0.3138592 | 1.1786 | 0.239 | 0.426898428 | count | 1 |
| DDIT4 | 0.2978707 | 0.0395808 | 7.5256 | 6.68E-14 | 0.428286601 | count | 1.62E-09 |
| RFESD | 0.4380085 | 0.4013071 | 1.0915 | 0.275 | 0.42838267 | count | 1 |
| RGS3 | 0.3503487 | 0.243564 | 1.4384 | 0.15 | 0.428582413 | count | 1 |
| GFOD1 | 0.38559 | 0.3193418 | 1.2075 | 0.227 | 0.42892466 | count | 1 |
| TMEM231 | 0.8718592 | 0.6057804 | 1.4392 | 0.15 | 0.428925104 | count | 1 |
| AC012360.1 | 1.1392166 | 0.8259033 | 1.3794 | 0.168 | 0.429107028 | count | 1 |
| AC004241.1 | 0.5554774 | 0.5559465 | 0.9992 | 0.318 | 0.429708043 | count | 1 |
| RNF11 | 0.3167214 | 0.1304075 | 2.4287 | 0.0152 | 0.429779247 | count | 1 |
| ITPK1 | 0.3563261 | 0.2434401 | 1.4637 | 0.143 | 0.429845594 | count | 1 |
| G6PD | 0.3365397 | 0.1829528 | 1.8395 | 0.0659 | 0.430259163 | count | 1 |
| ZNF664 | 0.3959452 | 0.253044 | 1.5647 | 0.118 | 0.430278476 | count | 1 |
| AL031848.2 | 0.5009476 | 0.4542204 | 1.1029 | 0.27 | 0.430482568 | count | 1 |
| SPATC1L | 0.3360613 | 0.2805923 | 1.1977 | 0.231 | 0.430623106 | count | 1 |
| DCLRE1A | 0.4653726 | 0.3998781 | 1.1638 | 0.245 | 0.430702098 | count | 1 |
| TEPSIN | 0.4520813 | 0.291409 | 1.5514 | 0.121 | 0.430944097 | count | 1 |
| ZNF469 | 0.7396908 | 0.8489115 | 0.8713 | 0.384 | 0.431480853 | count | 1 |
| AC019131.2 | 0.5270778 | 0.6917894 | 0.7619 | 0.446 | 0.431953156 | count | 1 |
| TKTL1 | 0.5995209 | 0.5848614 | 1.0251 | 0.305 | 0.431990486 | count | 1 |
| AC068620.1 | 1.1485522 | 1.111318 | 1.0335 | 0.301 | 0.432745014 | count | 1 |
| SENCR | 0.6007353 | 0.4985525 | 1.205 | 0.228 | 0.432899376 | count | 1 |
| AC020916.1 | 0.3080277 | 0.1043598 | 2.9516 | 0.00318 | 0.433460167 | count | 1 |
| UBL7 | 0.3292119 | 0.1815152 | 1.8137 | 0.0698 | 0.4335478 | count | 1 |
| PDIA5 | 1.153101 | 0.8040185 | 1.4342 | 0.152 | 0.434516224 | count | 1 |
| C16orf54 | 0.3068108 | 0.0833262 | 3.682 | 0.000235 | 0.434814283 | count | 1 |
| DDX20 | 0.3515666 | 0.2294565 | 1.5322 | 0.126 | 0.43530418 | count | 1 |
| GPR160 | 0.4567396 | 0.3596398 | 1.27 | 0.204 | 0.435487867 | count | 1 |
| DPH7 | 0.3550443 | 0.1951747 | 1.8191 | 0.069 | 0.436216607 | count | 1 |
| MYO6 | 0.40207 | 0.3241867 | 1.2402 | 0.215 | 0.437038622 | count | 1 |
| AC110769.2 | 0.7490696 | 0.3836512 | 1.9525 | 0.051 | 0.437210421 | count | 1 |
| TRBV2 | 2.0299301 | 1.123452 | 1.8069 | 0.0709 | 0.437429556 | count | 1 |
| BLZF1 | 0.3372885 | 0.1756873 | 1.9198 | 0.055 | 0.437440642 | count | 1 |
| MACROD1 | 0.459075 | 0.4176074 | 1.0993 | 0.272 | 0.437766329 | count | 1 |
| ATAD5 | 0.3524975 | 0.2327225 | 1.5147 | 0.13 | 0.438056326 | count | 1 |
| AC007384.1 | 0.3863076 | 0.2430889 | 1.5892 | 0.112 | 0.438444638 | count | 1 |
| TELO2 | 0.4153621 | 0.2789251 | 1.4892 | 0.137 | 0.439037335 | count | 1 |
| SH2B3 | 0.4487512 | 0.3197998 | 1.4032 | 0.161 | 0.439119481 | count | 1 |
| NECAB1 | 2.0404035 | 1.204331 | 1.6942 | 0.0903 | 0.439315656 | count | 1 |

| | | | | | | | |
|---|---|---|---|---|---|---|---|
| AC087190.1 | 0.3563479 | 0.2378 | 1.4985 | 0.134 | 0.439592373 | count | 1 |
| MXRA7 | 0.3216105 | 0.1518556 | 2.1179 | 0.0343 | 0.439840131 | count | 1 |
| GLMP | 0.4168426 | 0.2719473 | 1.5328 | 0.125 | 0.440629225 | count | 1 |
| AGO4 | 0.3924939 | 0.2555325 | 1.536 | 0.125 | 0.441293059 | count | 1 |
| TRNT1 | 0.3285976 | 0.1775317 | 1.8509 | 0.0643 | 0.441330842 | count | 1 |
| WDR11 | 0.3925762 | 0.2157755 | 1.8194 | 0.0689 | 0.44138686 | count | 1 |
| AGTPBP1 | 0.3436954 | 0.178738 | 1.9229 | 0.0546 | 0.441433412 | count | 1 |
| PRCC | 0.3427094 | 0.1517357 | 2.2586 | 0.024 | 0.441988072 | count | 1 |
| KLHDC4 | 0.3352058 | 0.1646941 | 2.0353 | 0.0419 | 0.442053975 | count | 1 |
| HIST1H2AM | 0.6131551 | 0.8165072 | 0.7509 | 0.453 | 0.442198165 | count | 1 |
| AC092142.1 | 0.7572888 | 0.9382392 | 0.8071 | 0.42 | 0.442232581 | count | 1 |
| AL034397.3 | 0.7572888 | 0.9401629 | 0.8055 | 0.421 | 0.442232581 | count | 1 |
| TMEM53 | 0.4023029 | 0.4306227 | 0.9342 | 0.35 | 0.44274076 | count | 1 |
| ALKBH4 | 0.3808688 | 0.3053916 | 1.2471 | 0.212 | 0.443084238 | count | 1 |
| ATR | 0.3737172 | 0.2038397 | 1.8334 | 0.0668 | 0.443545673 | count | 1 |
| HIST1H1A | 0.376201 | 0.4564681 | 0.8242 | 0.41 | 0.443720584 | count | 1 |
| TIGD1 | 0.5730815 | 0.4544691 | 1.261 | 0.207 | 0.443809691 | count | 1 |
| BAIAP2 | 0.4203751 | 0.513837 | 0.8181 | 0.413 | 0.444427973 | count | 1 |
| GBA2 | 0.4544771 | 0.362756 | 1.2528 | 0.21 | 0.444844964 | count | 1 |
| BCDIN3D | 0.4045926 | 0.3013305 | 1.3427 | 0.179 | 0.44529836 | count | 1 |
| C11orf71 | 0.3827894 | 0.2666015 | 1.4358 | 0.151 | 0.445344913 | count | 1 |
| ZNF213-AS1 | 0.4284851 | 0.3327728 | 1.2876 | 0.198 | 0.445738073 | count | 1 |
| CHCHD6 | 0.4979131 | 0.3300327 | 1.5087 | 0.131 | 0.445744126 | count | 1 |
| SLC23A2 | 0.5187638 | 0.3523475 | 1.4723 | 0.141 | 0.446246932 | count | 1 |
| AC105020.6 | 0.6779285 | 0.4369729 | 1.5514 | 0.121 | 0.446724377 | count | 1 |
| AC009831.1 | 0.5768493 | 0.4883941 | 1.1811 | 0.238 | 0.446829704 | count | 1 |
| NCF2 | 0.7657749 | 0.6017436 | 1.2726 | 0.203 | 0.447418652 | count | 1 |
| C3orf33 | 0.6789992 | 0.4867378 | 1.395 | 0.163 | 0.44745984 | count | 1 |
| ZNF883 | 0.7662769 | 0.7778828 | 0.9851 | 0.325 | 0.447725478 | count | 1 |
| BCAR1 | 2.0877644 | 1.7931967 | 1.1643 | 0.2444 | 0.447727315 | count | 1 |
| ARIH2OS | 0.3880684 | 0.2627225 | 1.4771 | 0.14 | 0.448099167 | count | 1 |
| PTPN12 | 0.344277 | 0.185831 | 1.8526 | 0.064 | 0.448114483 | count | 1 |
| AC005920.1 | 0.9096016 | 0.625492 | 1.4542 | 0.146 | 0.448404042 | count | 1 |
| SAP130 | 0.5212037 | 0.3464398 | 1.5045 | 0.133 | 0.448407144 | count | 1 |
| LINC02298 | 0.6218353 | 0.6200044 | 1.003 | 0.316 | 0.448700574 | count | 1 |
| TMED7 | 0.3570253 | 0.1782651 | 2.0028 | 0.0453 | 0.449514192 | count | 1 |
| AC020656.2 | 2.0987239 | 1.167968 | 1.7969 | 0.0724 | 0.449646282 | count | 1 |
| AL356481.1 | 2.0987239 | 1.300182 | 1.6142 | 0.107 | 0.449646282 | count | 1 |
| XPNPEP3 | 0.4712778 | 0.2773027 | 1.6995 | 0.0893 | 0.449676636 | count | 1 |
| ITGB2 | 0.3140947 | 0.0479831 | 6.5459 | 6.80E-11 | 0.449935255 | count | 1.65E-06 |
| SMAD2 | 0.3291369 | 0.1254775 | 2.6231 | 0.00875 | 0.450162746 | count | 1 |
| POLA1 | 0.771042 | 0.6151229 | 1.2535 | 0.21 | 0.450637868 | count | 1 |
| CPEB4 | 0.3630033 | 0.2013864 | 1.8025 | 0.0716 | 0.451218713 | count | 1 |
| SLC27A3 | 0.3643178 | 0.2588334 | 1.4075 | 0.159 | 0.451224912 | count | 1 |
| KBTBD4 | 0.3941906 | 0.2185339 | 1.8038 | 0.0714 | 0.451510981 | count | 1 |

| | | | | | | | |
|---|---|---|---|---|---|---|---|
| SPA17 | 0.9163544 | 0.6364525 | 1.4398 | 0.15 | 0.451887302 | count | 1 |
| NPRL3 | 0.3573222 | 0.2201867 | 1.6228 | 0.105 | 0.452447398 | count | 1 |
| AC078883.1 | 0.7740342 | 0.4907121 | 1.5774 | 0.115 | 0.452466813 | count | 1 |
| FAM27C | 0.5261843 | 0.4158966 | 1.2652 | 0.206 | 0.452817709 | count | 1 |
| AC006299.1 | 0.6873206 | 0.7688176 | 0.894 | 0.371 | 0.453176591 | count | 1 |
| MED18 | 0.3924336 | 0.345686 | 1.1352 | 0.256 | 0.453201691 | count | 1 |
| SRP14-AS1 | 0.4526005 | 0.3260828 | 1.388 | 0.165 | 0.453345732 | count | 1 |
| GPAT2 | 0.5272566 | 0.6695683 | 0.7875 | 0.431 | 0.453767459 | count | 1 |
| B4GALT5 | 0.3762622 | 0.2101651 | 1.7903 | 0.0735 | 0.454134212 | count | 1 |
| C11orf98 | 0.453629 | 0.3579138 | 1.2674 | 0.205 | 0.454397072 | count | 1 |
| ENO1-AS1 | 0.9215564 | 0.535083 | 1.7223 | 0.0851 | 0.454570106 | count | 1 |
| LAMP1 | 0.3225343 | 0.0808894 | 3.9873 | 6.82E-05 | 0.454608665 | count | 1 |
| NR4A1 | 0.3268866 | 0.1162594 | 2.8117 | 0.00496 | 0.45491274 | count | 1 |
| CAPS | 0.4456253 | 0.3738419 | 1.192 | 0.233 | 0.455475901 | count | 1 |
| HIST1H2BI | 0.9244859 | 1.025184 | 0.9018 | 0.367 | 0.456080714 | count | 1 |
| DARS2 | 0.5891005 | 0.4383214 | 1.344 | 0.179 | 0.456653566 | count | 1 |
| SNHG22 | 0.9256867 | 0.4825995 | 1.9181 | 0.0552 | 0.456699859 | count | 1 |
| AC087623.3 | 0.4029749 | 0.3148269 | 1.28 | 0.201 | 0.457616315 | count | 1 |
| ZNF280B | 0.6948436 | 0.4380805 | 1.5861 | 0.113 | 0.458346255 | count | 1 |
| PTPN4 | 0.3265669 | 0.0878383 | 3.7178 | 0.000204 | 0.458610409 | count | 1 |
| LPP-AS2 | 0.7844472 | 0.9209816 | 0.8518 | 0.394 | 0.458832008 | count | 1 |
| AL021068.1 | 2.1523547 | 1.332971 | 1.6147 | 0.106 | 0.458886115 | count | 1 |
| LINC00235 | 2.1523547 | 1.384089 | 1.5551 | 0.12 | 0.458886115 | count | 1 |
| BOLA3-AS1 | 0.5136645 | 0.529378 | 0.9703 | 0.332 | 0.460230992 | count | 1 |
| CHST14 | 0.4704611 | 0.4008526 | 1.1737 | 0.241 | 0.460837312 | count | 1 |
| PLAG1 | 1.2212073 | 0.5739705 | 2.1276 | 0.0334 | 0.460913585 | count | 1 |
| IL4 | 0.6993481 | 0.4976267 | 1.4054 | 0.16 | 0.461442228 | count | 1 |
| COX16 | 0.4067381 | 0.3073084 | 1.3235 | 0.186 | 0.461946811 | count | 1 |
| PLD3 | 0.3437848 | 0.1552761 | 2.214 | 0.0269 | 0.462177961 | count | 1 |
| WDPCP | 0.4848939 | 0.4557961 | 1.0638 | 0.287 | 0.462975886 | count | 1 |
| HIST1H4H | 0.4729286 | 0.415255 | 1.1389 | 0.255 | 0.463307301 | count | 1 |
| MAIP1 | 0.41747 | 0.305097 | 1.3683 | 0.171 | 0.46491575 | count | 1 |
| TMEM69 | 0.3915503 | 0.2444981 | 1.6014 | 0.109 | 0.464942646 | count | 1 |
| FBXL19 | 0.540276 | 0.5511175 | 0.9803 | 0.327 | 0.465303184 | count | 1 |
| BTLA | 0.4469837 | 0.2176948 | 2.0533 | 0.0401 | 0.465341131 | count | 1 |
| TAZ | 0.3467239 | 0.1381914 | 2.509 | 0.0122 | 0.465390063 | count | 1 |
| AL357060.1 | 0.3697418 | 0.1894601 | 1.9516 | 0.0511 | 0.465645534 | count | 1 |
| DNA2 | 0.7066145 | 0.6013202 | 1.1751 | 0.24 | 0.466437337 | count | 1 |
| AL135791.1 | 0.7975185 | 0.4299633 | 1.8549 | 0.0637 | 0.466822871 | count | 1 |
| SERPINB6 | 0.3432266 | 0.1248306 | 2.7495 | 0.006 | 0.467183081 | count | 1 |
| LINC00471 | 2.2020837 | 1.02778 | 2.1426 | 0.0322 | 0.467228434 | count | 1 |
| NDUFV2-AS1 | 0.3761914 | 0.1819404 | 2.0677 | 0.0387 | 0.467747397 | count | 1 |
| CCDC86 | 0.4580682 | 0.3151532 | 1.4535 | 0.146 | 0.468443784 | count | 1 |
| TXNL4B | 0.4502002 | 0.2540876 | 1.7718 | 0.0765 | 0.468751461 | count | 1 |
| MPRIP-AS1 | 0.8011832 | 0.567568 | 1.4116 | 0.158 | 0.469063305 | count | 1 |

| | | | | | | | |
|---|---|---|---|---|---|---|---|
| AL157756.1 | 2.2138075 | 1.159166 | 1.9098 | 0.0562 | 0.469163367 | count | 1 |
| AL157955.1 | 1.2432033 | 1.346111 | 0.9236 | 0.356 | 0.469384934 | count | 1 |
| XKR6 | 0.6051994 | 0.3572809 | 1.6939 | 0.0904 | 0.469571864 | count | 1 |
| AL353708.3 | 0.6497639 | 0.4695514 | 1.3838 | 0.167 | 0.469639344 | count | 1 |
| SLC2A1 | 0.3664851 | 0.1976611 | 1.8541 | 0.0638 | 0.469866928 | count | 1 |
| CD7 | 0.3286156 | 0.0595046 | 5.5225 | 3.59E-08 | 0.469945931 | count | 0.00086738 |
| DLEC1 | 2.2257838 | 1.285506 | 1.7314 | 0.0835 | 0.471127446 | count | 1 |
| AC005498.3 | 0.8052457 | 0.8402485 | 0.9583 | 0.338 | 0.471546916 | count | 1 |
| CENPT | 0.4151265 | 0.1979331 | 2.0973 | 0.036 | 0.47160208 | count | 1 |
| LINC00659 | 0.9568394 | 0.7620505 | 1.2556 | 0.209 | 0.472752247 | count | 1 |
| TULP3 | 0.6539998 | 0.4259509 | 1.5354 | 0.125 | 0.472817161 | count | 1 |
| SKIV2L | 0.471657 | 0.2955956 | 1.5956 | 0.111 | 0.472834177 | count | 1 |
| KLRC4 | 0.3879288 | 0.2509788 | 1.5457 | 0.122 | 0.472886406 | count | 1 |
| KLRK1 | 0.471934 | 0.4883152 | 0.9665 | 0.334 | 0.473117586 | count | 1 |
| RFC2 | 0.3640721 | 0.2109968 | 1.7255 | 0.0845 | 0.473218754 | count | 1 |
| C1orf61 | 0.8098622 | 0.5085943 | 1.5924 | 0.111 | 0.474369226 | count | 1 |
| ZNF599 | 0.5506907 | 0.5049882 | 1.0905 | 0.276 | 0.474536604 | count | 1 |
| AL136295.5 | 0.810617 | 0.3544368 | 2.2871 | 0.0223 | 0.474830669 | count | 1 |
| ETNK2 | 0.6117614 | 0.5546428 | 1.103 | 0.27 | 0.474840065 | count | 1 |
| FASTKD2 | 0.3783748 | 0.1879912 | 2.0127 | 0.0442 | 0.475165963 | count | 1 |
| RASGRP2 | 0.3427001 | 0.1165747 | 2.9397 | 0.00331 | 0.475360865 | count | 1 |
| RUNX1 | 0.3685423 | 0.1517003 | 2.4294 | 0.0152 | 0.475513855 | count | 1 |
| AF196972.1 | 0.8122326 | 1.0009789 | 0.8114 | 0.417 | 0.475818371 | count | 1 |
| CHRNE | 0.6134447 | 0.5397284 | 1.1366 | 0.256 | 0.476191743 | count | 1 |
| FDXACB1 | 0.4376173 | 0.3873709 | 1.1297 | 0.259 | 0.476311928 | count | 1 |
| TOGARAM1 | 0.415658 | 0.215657 | 1.9274 | 0.054 | 0.476423301 | count | 1 |
| AL645728.1 | 0.4861516 | 0.3286631 | 1.4792 | 0.139 | 0.476548862 | count | 1 |
| UCP3 | 2.2603104 | 1.502625 | 1.5042 | 0.133 | 0.476718503 | count | 1 |
| TAF9B | 0.4237915 | 0.2671281 | 1.5865 | 0.113 | 0.476987191 | count | 1 |
| WDHD1 | 0.5535119 | 0.4558363 | 1.2143 | 0.225 | 0.477038624 | count | 1 |
| LRRC2 | 0.6597748 | 0.5752361 | 1.147 | 0.251 | 0.477150365 | count | 1 |
| SLC39A4 | 0.3837683 | 0.2027587 | 1.8927 | 0.0585 | 0.477246309 | count | 1 |
| AC009041.1 | 0.66013 | 0.4164352 | 1.5852 | 0.113 | 0.477416933 | count | 1 |
| PWWP2B | 0.4132761 | 0.2659041 | 1.5542 | 0.12 | 0.477576566 | count | 1 |
| AC009163.7 | 2.2723332 | 1.237758 | 1.8358 | 0.0665 | 0.478640591 | count | 1 |
| ZSCAN31 | 2.2723332 | 1.274874 | 1.7824 | 0.0748 | 0.478640591 | count | 1 |
| NAIF1 | 1.2678525 | 0.6545485 | 1.937 | 0.0528 | 0.478843042 | count | 1 |
| AL162258.2 | 1.2678525 | 0.9218738 | 1.3753 | 0.169 | 0.478843042 | count | 1 |
| PANK4 | 0.5011481 | 0.3704251 | 1.3529 | 0.176 | 0.478864101 | count | 1 |
| TM7SF2 | 0.4182081 | 0.2438082 | 1.7153 | 0.0864 | 0.479383984 | count | 1 |
| PDZD4 | 0.7254592 | 0.40943 | 1.7719 | 0.0765 | 0.479395842 | count | 1 |
| DHX34 | 0.5562873 | 0.4825182 | 1.1529 | 0.249 | 0.479500358 | count | 1 |
| SLC24A1 | 0.8189933 | 0.7874485 | 1.0401 | 0.298 | 0.479951407 | count | 1 |
| INPP5E | 0.7263675 | 0.3293795 | 2.2053 | 0.0275 | 0.480020545 | count | 1 |
| RPH3AL | 0.9714522 | 0.6264805 | 1.5507 | 0.121 | 0.480273863 | count | 1 |

| | | | | | | | |
|---|---|---|---|---|---|---|---|
| SFXN5 | 0.584145 | 0.389152 | 1.5011 | 0.133 | 0.480285702 | count | 1 |
| DNAL4 | 0.406855 | 0.2694408 | 1.51 | 0.131 | 0.480293641 | count | 1 |
| ERGIC1 | 0.3944331 | 0.1760217 | 2.2408 | 0.0251 | 0.480889875 | count | 1 |
| MCEE | 0.4419268 | 0.307691 | 1.4363 | 0.151 | 0.481077321 | count | 1 |
| ZNF724 | 0.6660554 | 0.7475698 | 0.891 | 0.373 | 0.481863916 | count | 1 |
| NUDT17 | 0.6208662 | 0.5132437 | 1.2097 | 0.226 | 0.482152184 | count | 1 |
| ATRN | 0.5205713 | 0.4049954 | 1.2854 | 0.199 | 0.483139157 | count | 1 |
| DEGS2 | 0.5875357 | 0.6826557 | 0.8607 | 0.389 | 0.483161736 | count | 1 |
| AC007032.1 | 0.668561 | 0.59155 | 1.1302 | 0.258 | 0.483744608 | count | 1 |
| GUF1 | 0.4651848 | 0.276369 | 1.6832 | 0.0924 | 0.484645675 | count | 1 |
| NRL | 0.669846 | 0.4876431 | 1.3736 | 0.17 | 0.484709206 | count | 1 |
| TDRD12 | 0.6704844 | 0.6895268 | 0.9724 | 0.331 | 0.485188407 | count | 1 |
| CUL4B | 0.4269515 | 0.237217 | 1.7998 | 0.072 | 0.485218279 | count | 1 |
| GEMIN6 | 0.3733886 | 0.2082092 | 1.7933 | 0.073 | 0.486226977 | count | 1 |
| MAST3 | 0.4074752 | 0.2500727 | 1.6294 | 0.103 | 0.486921575 | count | 1 |
| AL035413.1 | 0.6728805 | 0.6585582 | 1.0217 | 0.307 | 0.486987166 | count | 1 |
| KCTD2 | 0.4969289 | 0.376149 | 1.3211 | 0.187 | 0.487347471 | count | 1 |
| AC099568.2 | 2.3281351 | 1.291272 | 1.803 | 0.0715 | 0.487393209 | count | 1 |
| FEM1A | 0.5104671 | 0.313591 | 1.6278 | 0.104 | 0.487978893 | count | 1 |
| IVD | 0.3952661 | 0.2004021 | 1.9724 | 0.0486 | 0.488034882 | count | 1 |
| NR2C1 | 0.4191251 | 0.2033592 | 2.061 | 0.0394 | 0.488145523 | count | 1 |
| POU6F1 | 0.6294696 | 0.6245696 | 1.0078 | 0.314 | 0.489063962 | count | 1 |
| CDKN2AIPNL | 0.3865999 | 0.1962439 | 1.97 | 0.0489 | 0.489794847 | count | 1 |
| ST6GAL1 | 0.4062587 | 0.1769987 | 2.2953 | 0.0218 | 0.490709849 | count | 1 |
| NEK5 | 1.300116 | 0.854854 | 1.5209 | 0.128 | 0.491163009 | count | 1 |
| C3 | 1.300116 | 0.9884464 | 1.3153 | 0.188 | 0.491163009 | count | 1 |
| LINC01684 | 1.3008376 | 0.76889 | 1.6918 | 0.0908 | 0.491437707 | count | 1 |
| MCU | 0.6794547 | 0.5202972 | 1.3059 | 0.192 | 0.491923011 | count | 1 |
| NSUN4 | 0.4810859 | 0.3287337 | 1.4635 | 0.143 | 0.492452607 | count | 1 |
| TINAGL1 | 0.6801655 | 0.591892 | 1.1491 | 0.251 | 0.492456715 | count | 1 |
| RPE | 0.4468988 | 0.2697559 | 1.6567 | 0.0977 | 0.492600573 | count | 1 |
| COQ7 | 0.3977575 | 0.2047629 | 1.9425 | 0.0522 | 0.493004697 | count | 1 |
| SLC2A8 | 0.4915131 | 0.3747127 | 1.3117 | 0.19 | 0.493159158 | count | 1 |
| ARHGEF19 | 0.4180841 | 0.3119167 | 1.3404 | 0.18 | 0.493700626 | count | 1 |
| SLC9A1 | 0.5338273 | 0.369903 | 1.4432 | 0.149 | 0.495753718 | count | 1 |
| HNRNPH1 | 0.347412 | 0.0526007 | 6.6047 | 4.61E-11 | 0.496010504 | count | 1.12E-06 |
| AC108488.1 | 1.313008 | 1.050812 | 1.2495 | 0.212 | 0.49606577 | count | 1 |
| ALAD | 0.4371702 | 0.2677351 | 1.6328 | 0.103 | 0.496989624 | count | 1 |
| HOXA4 | 0.6394392 | 0.9876623 | 0.6474 | 0.517 | 0.497076006 | count | 1 |
| CCDC43 | 0.3759145 | 0.1815794 | 2.0702 | 0.0385 | 0.497245008 | count | 1 |
| ZNF350 | 0.4116277 | 0.2409066 | 1.7087 | 0.0876 | 0.497259962 | count | 1 |
| AGPAT1 | 0.4080998 | 0.2178008 | 1.8737 | 0.0611 | 0.497711497 | count | 1 |
| ARV1 | 0.3928929 | 0.2267969 | 1.7324 | 0.0833 | 0.497825783 | count | 1 |
| AC009053.2 | 0.8521535 | 0.5721731 | 1.4893 | 0.136 | 0.500219518 | count | 1 |
| IQCK | 2.420021 | 1.065781 | 2.2707 | 0.0232 | 0.501202202 | count | 1 |

| | | | | | | | |
|---|---|---|---|---|---|---|---|
| PHLDA1 | 0.3591445 | 0.1054846 | 3.4047 | 0.00067 | 0.501397679 | count | 1 |
| ZNF646 | 0.7576468 | 0.5426469 | 1.3962 | 0.163 | 0.50153933 | count | 1 |
| THUMPD2 | 0.4411218 | 0.2444541 | 1.8045 | 0.0712 | 0.501542773 | count | 1 |
| ETV1 | 0.8543475 | 0.6947783 | 1.2297 | 0.219 | 0.501560143 | count | 1 |
| AC091814.1 | 1.0130763 | 0.6058861 | 1.6721 | 0.0946 | 0.50166296 | count | 1 |
| TRGV2 | 0.4064706 | 0.3283768 | 1.2378 | 0.216 | 0.501991197 | count | 1 |
| IGF1R | 0.7584756 | 0.4285198 | 1.77 | 0.0768 | 0.50210961 | count | 1 |
| MUC12 | 0.5405977 | 0.5452698 | 0.9914 | 0.322 | 0.502199304 | count | 1 |
| SUV39H1 | 0.8561437 | 0.5166577 | 1.6571 | 0.0976 | 0.502657667 | count | 1 |
| MAGED1 | 0.4357439 | 0.2567227 | 1.6973 | 0.0897 | 0.503872662 | count | 1 |
| ZEB2 | 0.3634832 | 0.1158032 | 3.1388 | 0.00171 | 0.504003175 | count | 1 |
| FAM3A | 0.3816331 | 0.1640531 | 2.3263 | 0.0201 | 0.504230718 | count | 1 |
| CTSO | 0.4438412 | 0.3553623 | 1.249 | 0.212 | 0.504676491 | count | 1 |
| AL355001.2 | 0.5625306 | 0.3593324 | 1.5655 | 0.118 | 0.505247189 | count | 1 |
| COQ8A | 0.4167226 | 0.246621 | 1.6897 | 0.0912 | 0.505964126 | count | 1 |
| ADAM8 | 0.3754192 | 0.1317329 | 2.8499 | 0.0044 | 0.506403466 | count | 1 |
| TMEM94 | 0.6991206 | 0.5264712 | 1.3279 | 0.184 | 0.506692766 | count | 1 |
| AC245060.6 | 1.3414229 | 0.8125415 | 1.6509 | 0.0989 | 0.506829173 | count | 1 |
| MTO1 | 0.4500677 | 0.2075923 | 2.168 | 0.0302 | 0.506988337 | count | 1 |
| TMEM220 | 1.0236787 | 0.6769287 | 1.5122 | 0.131 | 0.507101285 | count | 1 |
| GATD3A | 0.865672 | 1.0931948 | 0.7919 | 0.428 | 0.508478957 | count | 1 |
| LLGL2 | 0.3854623 | 0.1555755 | 2.4777 | 0.0133 | 0.509314948 | count | 1 |
| PECAM1 | 0.461887 | 0.356196 | 1.2967 | 0.195 | 0.509377881 | count | 1 |
| ZNF595 | 0.703376 | 0.5210199 | 1.35 | 0.177 | 0.509889549 | count | 1 |
| ZNF322 | 0.4294579 | 0.2684408 | 1.5998 | 0.11 | 0.510468408 | count | 1 |
| BOLA1 | 0.445647 | 0.2823262 | 1.5785 | 0.115 | 0.511257494 | count | 1 |
| PLEKHA4 | 0.8708547 | 0.6303451 | 1.3816 | 0.167 | 0.511644741 | count | 1 |
| RBM14-RBM4 | 1.3550087 | 0.9167969 | 1.478 | 0.14 | 0.51195377 | count | 1 |
| AL356512.1 | 0.7738902 | 0.4037379 | 1.9168 | 0.0553 | 0.512716322 | count | 1 |
| ZNF628 | 0.4920385 | 0.3220432 | 1.5279 | 0.127 | 0.513154685 | count | 1 |
| IL1R2 | 0.7081794 | 0.6876089 | 1.0299 | 0.303 | 0.513498237 | count | 1 |
| AC087741.1 | 0.7752397 | 0.4395127 | 1.7639 | 0.0778 | 0.513644936 | count | 1 |
| EPS8L2 | 0.3980768 | 0.1741293 | 2.2861 | 0.0223 | 0.513866056 | count | 1 |
| LINC00987 | 0.493316 | 0.3516448 | 1.4029 | 0.161 | 0.514511676 | count | 1 |
| ZNF532 | 0.7793304 | 0.4616602 | 1.6881 | 0.0915 | 0.51645975 | count | 1 |
| ATF3 | 0.3781017 | 0.1393954 | 2.7124 | 0.00671 | 0.516626582 | count | 1 |
| OR2A1-AS1 | 1.3695306 | 0.7563403 | 1.8107 | 0.0703 | 0.517415521 | count | 1 |
| GNS | 0.4749742 | 0.3856521 | 1.2316 | 0.218 | 0.517647423 | count | 1 |
| TMEM130 | 1.3703991 | 0.6784266 | 2.02 | 0.0435 | 0.517741599 | count | 1 |
| TMEM80 | 0.4096753 | 0.1974232 | 2.0751 | 0.0381 | 0.519248401 | count | 1 |
| FBXL4 | 0.4829251 | 0.2528278 | 1.9101 | 0.0562 | 0.519426528 | count | 1 |
| PGGT1B | 0.4124418 | 0.1822781 | 2.2627 | 0.0237 | 0.519849373 | count | 1 |
| RAP2A | 0.4125398 | 0.2036638 | 2.0256 | 0.0429 | 0.519973832 | count | 1 |
| AGK | 0.4434634 | 0.2231172 | 1.9876 | 0.0469 | 0.520539593 | count | 1 |
| THOC3 | 0.4011072 | 0.1659663 | 2.4168 | 0.0157 | 0.520733455 | count | 1 |

| | | | | | | | |
|---|---|---|---|---|---|---|---|
| ZNF888 | 1.0503834 | 0.8234221 | 1.2756 | 0.202 | 0.52077837 | count | 1 |
| AP002433.1 | 1.3798612 | 1.0019912 | 1.3771 | 0.169 | 0.521290535 | count | 1 |
| FAM86B1 | 1.3798612 | 1.018418 | 1.3549 | 0.176 | 0.521290535 | count | 1 |
| SLC12A5 | 1.3798612 | 1.173737 | 1.1756 | 0.24 | 0.521290535 | count | 1 |
| MBD1 | 0.4181979 | 0.208854 | 2.0023 | 0.0453 | 0.522223776 | count | 1 |
| DNAJC24 | 0.4042776 | 0.2101612 | 1.9237 | 0.0545 | 0.522937909 | count | 1 |
| SEMA4B | 0.7888187 | 0.670502 | 1.1765 | 0.239 | 0.522988632 | count | 1 |
| ZNF415 | 0.8924333 | 0.8960484 | 0.996 | 0.319 | 0.524820515 | count | 1 |
| LINC00115 | 0.8926216 | 0.5109948 | 1.7468 | 0.0808 | 0.524935449 | count | 1 |
| ARHGEF9 | 0.3956694 | 0.1564025 | 2.5298 | 0.0115 | 0.525318651 | count | 1 |
| AC092117.1 | 2.5986224 | 1.566724 | 1.6586 | 0.0973 | 0.525912508 | count | 1 |
| CCDC58 | 0.4139767 | 0.2362661 | 1.7522 | 0.0798 | 0.526126706 | count | 1 |
| AC044849.1 | 0.3976339 | 0.1203061 | 3.3052 | 0.000959 | 0.526740691 | count | 1 |
| KIF19 | 0.6100535 | 0.415165 | 1.4694 | 0.142 | 0.527245336 | count | 1 |
| TMEM67 | 0.5867922 | 0.3580838 | 1.6387 | 0.101 | 0.527631295 | count | 1 |
| YPEL1 | 0.4413545 | 0.2082943 | 2.1189 | 0.0342 | 0.527853638 | count | 1 |
| LIM2 | 0.8975181 | 0.6762778 | 1.3271 | 0.185 | 0.52792385 | count | 1 |
| CD8A | 0.3692426 | 0.0687272 | 5.3726 | 8.28E-08 | 0.528521658 | count | 0.001999289 |
| SSX2IP | 0.5068903 | 0.3028245 | 1.6739 | 0.0942 | 0.528934445 | count | 1 |
| CROCC | 0.5080637 | 0.2990284 | 1.699 | 0.0894 | 0.530181515 | count | 1 |
| WWOX | 0.4930077 | 0.285677 | 1.7258 | 0.0845 | 0.530454639 | count | 1 |
| RWDD3 | 0.6436022 | 0.4969134 | 1.2952 | 0.195 | 0.530770351 | count | 1 |
| MRPL23 | 0.3828216 | 0.1198244 | 3.1949 | 0.00141 | 0.530878284 | count | 1 |
| AC093249.6 | 0.903034 | 0.6452327 | 1.3995 | 0.162 | 0.531289609 | count | 1 |
| PPP1R16A | 0.732356 | 0.459277 | 1.5946 | 0.111 | 0.531665295 | count | 1 |
| AC008555.5 | 0.4451449 | 0.2818961 | 1.5791 | 0.114 | 0.532435477 | count | 1 |
| UBA6-AS1 | 0.5304481 | 0.3923256 | 1.3521 | 0.176 | 0.533061905 | count | 1 |
| LINC00476 | 0.4269586 | 0.2399445 | 1.7794 | 0.0753 | 0.53325405 | count | 1 |
| MTMR9 | 0.4835469 | 0.1942903 | 2.4888 | 0.0129 | 0.533638635 | count | 1 |
| ARMC2 | 0.5431769 | 0.4308978 | 1.2606 | 0.208 | 0.533742171 | count | 1 |
| AL358472.5 | 1.0766043 | 0.6074417 | 1.7724 | 0.0764 | 0.53417572 | count | 1 |
| SIK2 | 0.5587165 | 0.3081408 | 1.8132 | 0.0699 | 0.535227459 | count | 1 |
| CD200R1 | 0.4360041 | 0.214187 | 2.0356 | 0.0419 | 0.536662515 | count | 1 |
| AL034549.1 | 0.5970819 | 0.4991376 | 1.1962 | 0.232 | 0.537130185 | count | 1 |
| DGKQ | 0.577602 | 0.2849695 | 2.0269 | 0.0428 | 0.537457696 | count | 1 |
| GNPTAB | 0.3854423 | 0.1146337 | 3.3624 | 0.000781 | 0.537512385 | count | 1 |
| AC025171.2 | 0.65157 | 0.4635333 | 1.4057 | 0.16 | 0.537542586 | count | 1 |
| THG1L | 0.4777753 | 0.2443936 | 1.9549 | 0.0507 | 0.538652702 | count | 1 |
| AC012615.1 | 0.403006 | 0.1751961 | 2.3003 | 0.0215 | 0.539397658 | count | 1 |
| CCR8 | 1.4300087 | 0.7181145 | 1.9913 | 0.0465 | 0.539973461 | count | 1 |
| MEOX1 | 1.0886912 | 0.6498327 | 1.6753 | 0.094 | 0.540339683 | count | 1 |
| PTAFR | 0.9183623 | 0.8328526 | 1.1027 | 0.27 | 0.54063876 | count | 1 |
| ZNF548 | 0.5809425 | 0.2874716 | 2.0209 | 0.0434 | 0.540642742 | count | 1 |
| AL512770.1 | 1.4322591 | 0.7847701 | 1.8251 | 0.0681 | 0.540806699 | count | 1 |
| TLR3 | 0.9187043 | 0.5966136 | 1.5399 | 0.124 | 0.540847298 | count | 1 |

| | | | | | | | |
|---|---|---|---|---|---|---|---|
| DDN-AS1 | 1.4337271 | 0.5672093 | 2.5277 | 0.0115 | 0.541350022 | count | 1 |
| KLRG1 | 0.3835612 | 0.0879958 | 4.3589 | 1.35E-05 | 0.543396077 | count | 0.324324 |
| PPP1R32 | 1.095538 | 0.8448438 | 1.2967 | 0.195 | 0.543828035 | count | 1 |
| ALG14 | 0.5536947 | 0.3244413 | 1.7066 | 0.088 | 0.544304122 | count | 1 |
| FOXJ2 | 0.5214477 | 0.3065645 | 1.7009 | 0.089 | 0.544409082 | count | 1 |
| CACNA1A | 1.4438212 | 0.9344489 | 1.5451 | 0.122 | 0.545080705 | count | 1 |
| LINC00852 | 1.4438212 | 0.9727038 | 1.4843 | 0.138 | 0.545080705 | count | 1 |
| EPHX4 | 1.4438212 | 1.091268 | 1.3231 | 0.186 | 0.545080705 | count | 1 |
| AL035661.1 | 1.0992465 | 0.8570673 | 1.2826 | 0.2 | 0.545716077 | count | 1 |
| GOLIM4 | 0.5551441 | 0.3220942 | 1.7235 | 0.0849 | 0.54575987 | count | 1 |
| SGPP1 | 0.4894649 | 0.3001551 | 1.6307 | 0.103 | 0.546360728 | count | 1 |
| EIF2D | 0.43913 | 0.1934775 | 2.2697 | 0.0233 | 0.546705881 | count | 1 |
| TRAV19 | 1.4499583 | 0.9875069 | 1.4683 | 0.142 | 0.547344356 | count | 1 |
| GPR15 | 1.4499583 | 1.018064 | 1.4242 | 0.154 | 0.547344356 | count | 1 |
| FO393401.1 | 0.4235941 | 0.1994925 | 2.1234 | 0.0338 | 0.548081823 | count | 1 |
| SPAG8 | 1.104111 | 0.7694465 | 1.4349 | 0.151 | 0.548191731 | count | 1 |
| C2CD4B | 1.104111 | 0.9412034 | 1.1731 | 0.241 | 0.548191731 | count | 1 |
| IPP | 0.4822355 | 0.3101424 | 1.5549 | 0.12 | 0.548949115 | count | 1 |
| GTF2H3 | 0.4208705 | 0.1892093 | 2.2244 | 0.0262 | 0.549333612 | count | 1 |
| TMEM251 | 0.4239491 | 0.19501 | 2.174 | 0.0298 | 0.549573226 | count | 1 |
| FAM49A | 0.4517093 | 0.3204112 | 1.4098 | 0.159 | 0.551428132 | count | 1 |
| AC005614.1 | 0.8307931 | 0.8948264 | 0.9284 | 0.353 | 0.551863851 | count | 1 |
| NRROS | 0.5207396 | 0.4448939 | 1.1705 | 0.242 | 0.552600572 | count | 1 |
| ZNF678 | 0.5132455 | 0.3080835 | 1.6659 | 0.0958 | 0.552600906 | count | 1 |
| SIRT4 | 0.7088866 | 0.6178646 | 1.1473 | 0.251 | 0.552943733 | count | 1 |
| ZNF254 | 0.4494395 | 0.2471549 | 1.8185 | 0.0691 | 0.553351464 | count | 1 |
| TMIGD2 | 0.4198902 | 0.2304785 | 1.8218 | 0.0686 | 0.553636023 | count | 1 |
| FAM19A1 | 0.9404973 | 0.5531097 | 1.7004 | 0.0892 | 0.554127445 | count | 1 |
| EFCAB13 | 0.9404973 | 0.6918887 | 1.3593 | 0.174 | 0.554127445 | count | 1 |
| SLC45A1 | 0.9412056 | 0.9025284 | 1.0429 | 0.297 | 0.55455877 | count | 1 |
| GAS8 | 0.8347459 | 0.6311591 | 1.3226 | 0.186 | 0.554582005 | count | 1 |
| AL391121.1 | 0.6414211 | 0.3746089 | 1.7122 | 0.0869 | 0.555138057 | count | 1 |
| MLLT11 | 0.7127137 | 0.2897017 | 2.4602 | 0.0139 | 0.556024329 | count | 1 |
| ZNF852 | 0.5655837 | 0.4228067 | 1.3377 | 0.181 | 0.556247022 | count | 1 |
| BDH1 | 0.5973867 | 0.3129081 | 1.9091 | 0.0563 | 0.556326119 | count | 1 |
| CCL28 | 0.4677858 | 0.2583988 | 1.8103 | 0.0703 | 0.556549295 | count | 1 |
| ERICH6-AS1 | 0.5533573 | 0.336105 | 1.6464 | 0.0998 | 0.556565577 | count | 1 |
| MAP3K10 | 0.4771477 | 0.3540834 | 1.3476 | 0.178 | 0.556596617 | count | 1 |
| SMC5-AS1 | 1.1218306 | 0.9695658 | 1.157 | 0.247 | 0.557197957 | count | 1 |
| PGAP3 | 0.4993063 | 0.3155033 | 1.5826 | 0.114 | 0.557508501 | count | 1 |
| SLC39A11 | 0.5051678 | 0.30043 | 1.6815 | 0.0928 | 0.557871811 | count | 1 |
| VPS72 | 0.4169772 | 0.1577359 | 2.6435 | 0.00824 | 0.558177949 | count | 1 |
| ZNF14 | 0.478525 | 0.1849976 | 2.5867 | 0.00973 | 0.558222777 | count | 1 |
| ZNF487 | 0.4419001 | 0.2765915 | 1.5977 | 0.11 | 0.55886942 | count | 1 |
| IL10RB | 1.4820835 | 0.9377436 | 1.5805 | 0.114 | 0.559136304 | count | 1 |

| | | | | | | | |
|---|---|---|---|---|---|---|---|
| SH2D6 | 1.482328 | 1.624621 | 0.9124 | 0.362 | 0.559225608 | count | 1 |
| AC120053.1 | 1.482328 | 1.624621 | 0.9124 | 0.362 | 0.559225608 | count | 1 |
| FAM95B1 | 1.482328 | 1.624621 | 0.9124 | 0.362 | 0.559225608 | count | 1 |
| LIN9 | 0.6776293 | 0.481076 | 1.4086 | 0.159 | 0.559699205 | count | 1 |
| GSAP | 0.4605749 | 0.2718573 | 1.6942 | 0.0903 | 0.559754453 | count | 1 |
| JARID2-AS1 | 0.9503985 | 0.7209207 | 1.3183 | 0.187 | 0.560155788 | count | 1 |
| AC048341.2 | 1.1280447 | 0.5749019 | 1.9622 | 0.0498 | 0.560351814 | count | 1 |
| NOL12 | 0.4807385 | 0.2826751 | 1.7007 | 0.0891 | 0.560836341 | count | 1 |
| CDKL3 | 0.8443779 | 0.4969983 | 1.699 | 0.0894 | 0.561204464 | count | 1 |
| ADGRL1 | 1.1300114 | 0.7070831 | 1.5981 | 0.11 | 0.561349472 | count | 1 |
| KIAA1468 | 0.5214162 | 0.2436378 | 2.1401 | 0.0324 | 0.561545816 | count | 1 |
| LAIR2 | 0.4363963 | 0.2727301 | 1.6001 | 0.11 | 0.562525771 | count | 1 |
| TSC22D3 | 0.3908177 | 0.0290055 | 13.4739 | 2.38E-40 | 0.562624739 | count | 5.79E-36 |
| PYGB | 0.5605278 | 0.3702055 | 1.5141 | 0.13 | 0.563925331 | count | 1 |
| CD247 | 0.3938597 | 0.0580436 | 6.7856 | 1.36E-11 | 0.564259889 | count | 3.30E-07 |
| ZNF701 | 0.4461518 | 0.2379133 | 1.8753 | 0.0608 | 0.564287107 | count | 1 |
| FANCB | 0.6523467 | 0.3838277 | 1.6996 | 0.0893 | 0.564857865 | count | 1 |
| MED9 | 0.6070236 | 0.2792768 | 2.1736 | 0.0298 | 0.565520197 | count | 1 |
| H1F0 | 1.1391558 | 0.7464028 | 1.5262 | 0.127 | 0.565985026 | count | 1 |
| TLR1 | 1.502043 | 0.4721388 | 3.1814 | 0.00148 | 0.566413263 | count | 1 |
| B4GAT1 | 0.6545419 | 0.412918 | 1.5852 | 0.113 | 0.566811015 | count | 1 |
| TPCN2 | 2.9515644 | 1.221458 | 2.4164 | 0.0157 | 0.566824319 | count | 1 |
| KPNA5 | 0.4495495 | 0.1955227 | 2.2992 | 0.0216 | 0.566994499 | count | 1 |
| FUZ | 0.7265784 | 0.4041419 | 1.7978 | 0.0723 | 0.567185022 | count | 1 |
| CXCR5 | 0.9620638 | 0.4481022 | 2.147 | 0.0319 | 0.567253628 | count | 1 |
| AL031432.3 | 1.504984 | 0.9890888 | 1.5216 | 0.128 | 0.567481635 | count | 1 |
| FCRLB | 0.6093823 | 0.452068 | 1.348 | 0.178 | 0.56777083 | count | 1 |
| TNF | 0.3958498 | 0.0857849 | 4.6144 | 4.09E-06 | 0.568785947 | count | 0.09842585 |
| RRAS2 | 0.4584267 | 0.1974097 | 2.3222 | 0.0203 | 0.568893216 | count | 1 |
| AC107081.2 | 1.5089604 | 0.9849919 | 1.532 | 0.126 | 0.568925459 | count | 1 |
| ZC2HC1C | 1.5089604 | 1.012901 | 1.4897 | 0.136 | 0.568925459 | count | 1 |
| AC078802.1 | 1.5089604 | 1.043415 | 1.4462 | 0.148 | 0.568925459 | count | 1 |
| ANKAR | 0.4524192 | 0.2205527 | 2.0513 | 0.0403 | 0.568941916 | count | 1 |
| AK5 | 1.5091534 | 0.4522048 | 3.3373 | 0.000855 | 0.568995477 | count | 1 |
| PSMB10 | 0.4043136 | 0.1005428 | 4.0213 | 5.91E-05 | 0.568996995 | count | 1 |
| MORC2 | 0.6109931 | 0.3071163 | 1.9895 | 0.0467 | 0.569307892 | count | 1 |
| AREG | 0.402663 | 0.1170645 | 3.4397 | 0.000589 | 0.569499814 | count | 1 |
| AC013394.1 | 0.4507061 | 0.1588637 | 2.8371 | 0.00458 | 0.570090813 | count | 1 |
| TAF2 | 0.446187 | 0.1870911 | 2.3849 | 0.0171 | 0.57151175 | count | 1 |
| AC007881.3 | 1.5187357 | 0.8247539 | 1.8414 | 0.0656 | 0.572468033 | count | 1 |
| ZHX3 | 0.8631623 | 0.5612955 | 1.5378 | 0.124 | 0.574114488 | count | 1 |
| MAPT | 0.7892102 | 0.6629063 | 1.1905 | 0.234 | 0.574392661 | count | 1 |
| IFNG | 0.4031282 | 0.1340676 | 3.0069 | 0.0027 | 0.576469394 | count | 1 |
| AC008393.1 | 1.1599236 | 0.7857829 | 1.4761 | 0.14 | 0.576492365 | count | 1 |
| FXN | 0.4377728 | 0.2019928 | 2.1673 | 0.0303 | 0.576577167 | count | 1 |

| | | | | | | | |
|---|---|---|---|---|---|---|---|
| IL23A | 0.4635282 | 0.258138 | 1.7957 | 0.0726 | 0.57734317 | count | 1 |
| HAVCR2 | 0.4804299 | 0.2737095 | 1.7553 | 0.0793 | 0.5782754 | count | 1 |
| AL353622.1 | 1.163595 | 0.63369 | 1.8362 | 0.0664 | 0.578346995 | count | 1 |
| KIF13A | 0.5748681 | 0.6520367 | 0.8816 | 0.378 | 0.578648097 | count | 1 |
| HIST1H2AK | 0.6034662 | 0.3262169 | 1.8499 | 0.0644 | 0.579114949 | count | 1 |
| NLRP2 | 0.6037397 | 0.5025077 | 1.2015 | 0.23 | 0.579383333 | count | 1 |
| BET1 | 0.4509281 | 0.2028713 | 2.2227 | 0.0263 | 0.580172291 | count | 1 |
| ST7L | 0.5009723 | 0.242814 | 2.0632 | 0.0392 | 0.580315195 | count | 1 |
| ZMAT5 | 0.4340759 | 0.1642091 | 2.6434 | 0.00824 | 0.581682519 | count | 1 |
| SLC9B1 | 0.8742863 | 0.6719013 | 1.3012 | 0.193 | 0.581755933 | count | 1 |
| TTC13 | 0.5333805 | 0.260165 | 2.0502 | 0.0404 | 0.582375702 | count | 1 |
| FOSB | 0.4080168 | 0.0619889 | 6.5821 | 5.35E-11 | 0.582500927 | count | 1.30E-06 |
| BOP1 | 0.5672568 | 0.2523689 | 2.2477 | 0.0247 | 0.582517081 | count | 1 |
| ENPP4 | 0.455193 | 0.1958923 | 2.3237 | 0.0202 | 0.583126466 | count | 1 |
| MED22 | 0.626248 | 0.3930823 | 1.5932 | 0.111 | 0.583866894 | count | 1 |
| CBWD6 | 1.1748546 | 1.0336846 | 1.1366 | 0.256 | 0.584028206 | count | 1 |
| PAN3 | 0.4307328 | 0.1259196 | 3.4207 | 0.000632 | 0.584393475 | count | 1 |
| MDS2 | 0.674824 | 0.387674 | 1.7407 | 0.0818 | 0.584859614 | count | 1 |
| OTUD7B | 0.8823914 | 0.3494787 | 2.5249 | 0.0116 | 0.587321532 | count | 1 |
| EXT1 | 0.9965324 | 0.4754659 | 2.0959 | 0.0362 | 0.588193494 | count | 1 |
| ZBTB16 | 0.8078608 | 0.3326547 | 2.4285 | 0.0152 | 0.588404326 | count | 1 |
| TSNARE1 | 0.9969226 | 0.5688971 | 1.7524 | 0.0798 | 0.588430228 | count | 1 |
| MINDY4 | 1.56347 | 0.6306626 | 2.4791 | 0.0132 | 0.588554602 | count | 1 |
| CH25H | 0.6793721 | 0.2606625 | 2.6063 | 0.00919 | 0.58890747 | count | 1 |
| XYLB | 1.5676726 | 0.9293952 | 1.6868 | 0.0917 | 0.590055055 | count | 1 |
| WNT6 | 0.9997618 | 1.018872 | 0.9812 | 0.327 | 0.590152601 | count | 1 |
| EGR1 | 0.4552866 | 0.1611029 | 2.8261 | 0.00474 | 0.59151884 | count | 1 |
| ZNF805 | 0.7155157 | 0.3274287 | 2.1853 | 0.0289 | 0.591924053 | count | 1 |
| AC008267.5 | 0.5300422 | 0.3505457 | 1.512 | 0.131 | 0.592341876 | count | 1 |
| AL627171.1 | 0.6173142 | 0.1995392 | 3.0937 | 0.00199 | 0.592705208 | count | 1 |
| NCSTN | 0.4925012 | 0.2719743 | 1.8108 | 0.0703 | 0.59295716 | count | 1 |
| HIST1H4B | 1.5760385 | 0.8265688 | 1.9067 | 0.0566 | 0.593036269 | count | 1 |
| AOAH | 0.4333103 | 0.1308253 | 3.3121 | 0.000936 | 0.593120824 | count | 1 |
| AC104109.2 | 0.7591954 | 0.6929901 | 1.0955 | 0.273 | 0.593441043 | count | 1 |
| TMPPE | 0.7591954 | 0.7516717 | 1.01 | 0.313 | 0.593441043 | count | 1 |
| GEMIN7 | 0.4715484 | 0.246557 | 1.9125 | 0.0559 | 0.594958508 | count | 1 |
| RAD52 | 0.7629515 | 0.4656332 | 1.6385 | 0.101 | 0.596464323 | count | 1 |
| XCL1 | 0.4178052 | 0.1591992 | 2.6244 | 0.00872 | 0.596647542 | count | 1 |
| MTFR1L | 0.4691774 | 0.2371932 | 1.978 | 0.048 | 0.598321937 | count | 1 |
| RIPOR1 | 0.8217729 | 0.4321797 | 1.9015 | 0.0573 | 0.598852692 | count | 1 |
| ARSB | 0.9016737 | 0.5296077 | 1.7025 | 0.0887 | 0.600554509 | count | 1 |
| AL451165.2 | 0.55725 | 0.3027316 | 1.8407 | 0.0657 | 0.600796051 | count | 1 |
| CD38 | 0.494782 | 0.2919448 | 1.6948 | 0.0902 | 0.601748724 | count | 1 |
| EDEM2 | 0.4852955 | 0.1940726 | 2.5006 | 0.0124 | 0.602531186 | count | 1 |
| ZWINT | 0.9058064 | 0.7653254 | 1.1836 | 0.237 | 0.603389133 | count | 1 |

| | | | | | | | |
|---|---|---|---|---|---|---|---|
| TSSK6 | 1.608308 | 0.7533659 | 2.1348 | 0.0328 | 0.60446426 | count | 1 |
| AC105285.1 | 1.608308 | 1.0740553 | 1.4974 | 0.134 | 0.60446426 | count | 1 |
| AL080317.1 | 1.608308 | 1.1288581 | 1.4247 | 0.154 | 0.60446426 | count | 1 |
| AC090186.1 | 1.608308 | 1.1288581 | 1.4247 | 0.154 | 0.60446426 | count | 1 |
| AC106782.2 | 1.608308 | 1.1288581 | 1.4247 | 0.154 | 0.60446426 | count | 1 |
| FBXO6 | 0.4608586 | 0.1874387 | 2.4587 | 0.014 | 0.604608177 | count | 1 |
| PIK3AP1 | 0.561234 | 0.4179453 | 1.3428 | 0.179 | 0.605161718 | count | 1 |
| AP005329.3 | 0.6713269 | 0.3889363 | 1.7261 | 0.0844 | 0.605734792 | count | 1 |
| ADRB2 | 0.4323574 | 0.1197964 | 3.6091 | 0.000312 | 0.605760665 | count | 1 |
| ERVK3-1 | 0.5063623 | 0.2462941 | 2.0559 | 0.0399 | 0.606489296 | count | 1 |
| KDM4B | 0.5570102 | 0.2435157 | 2.2874 | 0.0222 | 0.608589609 | count | 1 |
| CCDC110 | 1.620658 | 1.150351 | 1.4088 | 0.159 | 0.608806918 | count | 1 |
| AC136475.3 | 0.5303195 | 0.1906032 | 2.7823 | 0.00543 | 0.609771864 | count | 1 |
| AP002748.3 | 0.838028 | 0.3827979 | 2.1892 | 0.0286 | 0.611055975 | count | 1 |
| GK5 | 0.5541689 | 0.289297 | 1.9156 | 0.0555 | 0.612841193 | count | 1 |
| PELI3 | 0.9208264 | 0.4950395 | 1.8601 | 0.063 | 0.613686172 | count | 1 |
| AC007388.1 | 0.6804531 | 0.4552645 | 1.4946 | 0.135 | 0.614172413 | count | 1 |
| TSPAN17 | 0.5267603 | 0.2105998 | 2.5012 | 0.0124 | 0.615203731 | count | 1 |
| TFEB | 0.5901657 | 0.2298818 | 2.5673 | 0.0103 | 0.617536639 | count | 1 |
| SETBP1 | 0.7123091 | 0.5074659 | 1.4037 | 0.161 | 0.618224327 | count | 1 |
| RMND5B | 0.5184898 | 0.258362 | 2.0068 | 0.0448 | 0.621169859 | count | 1 |
| BABAM2 | 0.4685298 | 0.1471445 | 3.1841 | 0.00146 | 0.623221334 | count | 1 |
| PLCH2 | 0.6677722 | 0.3469022 | 1.925 | 0.0543 | 0.623513004 | count | 1 |
| AC005229.4 | 0.6064359 | 0.4239956 | 1.4303 | 0.153 | 0.623532085 | count | 1 |
| ZNF286A | 0.8551624 | 0.5471301 | 1.563 | 0.118 | 0.623912609 | count | 1 |
| AC083880.1 | 1.25574 | 0.3758329 | 3.3412 | 0.000843 | 0.624554018 | count | 1 |
| MZF1-AS1 | 0.8016329 | 0.2409929 | 3.3264 | 0.000889 | 0.627589639 | count | 1 |
| PSMD9 | 0.4729446 | 0.1650785 | 2.865 | 0.0042 | 0.629121034 | count | 1 |
| ACBD4 | 0.6381078 | 0.3539578 | 1.8028 | 0.0715 | 0.629167565 | count | 1 |
| AL357054.4 | 0.8042197 | 0.4304652 | 1.8683 | 0.0618 | 0.629670203 | count | 1 |
| OSM | 0.479315 | 0.1652392 | 2.9007 | 0.00375 | 0.629858043 | count | 1 |
| RHBDL1 | 1.267037 | 0.8043316 | 1.5753 | 0.115 | 0.630170266 | count | 1 |
| AC092069.1 | 0.5764816 | 0.2486568 | 2.3184 | 0.0205 | 0.630198706 | count | 1 |
| BCKDK | 0.5529348 | 0.2723291 | 2.0304 | 0.0424 | 0.630582753 | count | 1 |
| LPAR6 | 0.4590503 | 0.1214752 | 3.779 | 0.00016 | 0.630797942 | count | 1 |
| AC092053.2 | 1.2701536 | 0.6327763 | 2.0073 | 0.0448 | 0.63171765 | count | 1 |
| CAB39L | 0.6767629 | 0.3886829 | 1.7412 | 0.0817 | 0.632098844 | count | 1 |
| KLC2 | 0.6774542 | 0.303796 | 2.23 | 0.0258 | 0.632759029 | count | 1 |
| HILPDA | 0.5421166 | 0.3539242 | 1.5317 | 0.126 | 0.633354582 | count | 1 |
| TMEM273 | 0.4895509 | 0.1938857 | 2.5249 | 0.0116 | 0.635168974 | count | 1 |
| GPR89B | 0.5741977 | 0.4005746 | 1.4334 | 0.152 | 0.635323964 | count | 1 |
| C11orf49 | 0.5035743 | 0.2425247 | 2.0764 | 0.0379 | 0.635683995 | count | 1 |
| FAM189B | 1.2788165 | 0.538256 | 2.3759 | 0.0176 | 0.636014089 | count | 1 |
| ZNF658 | 0.7344199 | 0.6264665 | 1.1723 | 0.241 | 0.637904485 | count | 1 |
| CABLES2 | 1.285502 | 0.9042696 | 1.4216 | 0.155 | 0.639325032 | count | 1 |

| | | | | | | | |
|---|---|---|---|---|---|---|---|
| AL356599.1 | 0.6350228 | 0.4786967 | 1.3266 | 0.185 | 0.640449688 | count | 1 |
| LIPE | 0.9600935 | 0.4768808 | 2.0133 | 0.0442 | 0.640562534 | count | 1 |
| KCTD7 | 0.5482918 | 0.3305243 | 1.6589 | 0.0972 | 0.640654641 | count | 1 |
| SLC15A4 | 0.4960498 | 0.1920513 | 2.5829 | 0.00984 | 0.641222393 | count | 1 |
| CTIF | 1.718016 | 1.318999 | 1.3025 | 0.193 | 0.642421418 | count | 1 |
| BEND3 | 1.719327 | 1.030679 | 1.6681 | 0.0954 | 0.642866441 | count | 1 |
| RCBTB2 | 0.5026996 | 0.1808739 | 2.7793 | 0.00548 | 0.644416523 | count | 1 |
| FBXL6 | 0.4944825 | 0.1993875 | 2.48 | 0.0132 | 0.646005468 | count | 1 |
| SPPL2B | 0.6716191 | 0.2604062 | 2.5791 | 0.00995 | 0.646022506 | count | 1 |
| ALDH5A1 | 0.7437772 | 0.526262 | 1.4133 | 0.158 | 0.646232147 | count | 1 |
| AC004832.6 | 1.3033972 | 0.7333284 | 1.7774 | 0.0756 | 0.648166624 | count | 1 |
| ANKS3 | 0.5750732 | 0.2823972 | 2.0364 | 0.0418 | 0.650015594 | count | 1 |
| FOS | 0.4519963 | 0.0567616 | 7.9631 | 2.27E-15 | 0.651428848 | count | 5.51E-11 |
| VNN2 | 0.6225269 | 0.3296946 | 1.8882 | 0.0591 | 0.652004795 | count | 1 |
| AC108718.1 | 1.1027351 | 0.6363878 | 1.7328 | 0.0832 | 0.652311706 | count | 1 |
| KLHL17 | 0.7528487 | 0.3586171 | 2.0993 | 0.0359 | 0.654304571 | count | 1 |
| IL18BP | 0.5849058 | 0.3292768 | 1.7763 | 0.0758 | 0.654569341 | count | 1 |
| CERS4 | 0.5607732 | 0.2469474 | 2.2708 | 0.0232 | 0.655411295 | count | 1 |
| AC026979.2 | 0.5236196 | 0.1597554 | 3.2776 | 0.00106 | 0.657193021 | count | 1 |
| LPAR5 | 0.6285272 | 0.4077903 | 1.5413 | 0.123 | 0.658396891 | count | 1 |
| FGL2 | 0.4879664 | 0.1697729 | 2.8742 | 0.00408 | 0.658398023 | count | 1 |
| AL513548.1 | 1.7658885 | 0.9669098 | 1.8263 | 0.0679 | 0.658527589 | count | 1 |
| GHDC | 0.582646 | 0.2777704 | 2.0976 | 0.036 | 0.658690499 | count | 1 |
| CD320 | 0.4714833 | 0.1034917 | 4.5558 | 5.40E-06 | 0.658891683 | count | 0.1298808 |
| MTSS1 | 0.4952522 | 0.2030215 | 2.4394 | 0.0148 | 0.660960859 | count | 1 |
| CCR10 | 0.6869159 | 0.4798283 | 1.4316 | 0.152 | 0.661043875 | count | 1 |
| ZFP82 | 0.6553325 | 0.3949715 | 1.6592 | 0.0972 | 0.661324375 | count | 1 |
| IL6R-AS1 | 1.3318468 | 0.9875656 | 1.3486 | 0.178 | 0.662157918 | count | 1 |
| TMEM117 | 1.1198738 | 0.609485 | 1.8374 | 0.0662 | 0.662588924 | count | 1 |
| AC007541.1 | 0.7986984 | 0.4739293 | 1.6853 | 0.092 | 0.662659303 | count | 1 |
| UTP25 | 0.5702447 | 0.2562249 | 2.2256 | 0.0261 | 0.666610677 | count | 1 |
| LIG4 | 0.6178794 | 0.3273462 | 1.8875 | 0.0592 | 0.66726062 | count | 1 |
| GDPD1 | 0.7379853 | 0.4820831 | 1.5308 | 0.126 | 0.66736298 | count | 1 |
| IGF2R | 0.4945526 | 0.1461279 | 3.3844 | 0.000721 | 0.668727828 | count | 1 |
| AC116614.1 | 1.130424 | 0.6300298 | 1.7942 | 0.0729 | 0.668904236 | count | 1 |
| PPP1R14C | 1.130424 | 0.6300298 | 1.7942 | 0.0729 | 0.668904236 | count | 1 |
| ABCA10 | 1.130424 | 0.6300298 | 1.7942 | 0.0729 | 0.668904236 | count | 1 |
| BCKDHA | 1.130424 | 0.6300298 | 1.7942 | 0.0729 | 0.668904236 | count | 1 |
| CU638689.4 | 1.130424 | 0.6300298 | 1.7942 | 0.0729 | 0.668904236 | count | 1 |
| RASD1 | 0.564586 | 0.2618371 | 2.1562 | 0.0311 | 0.668954533 | count | 1 |
| MED14OS | 0.7717198 | 0.5823004 | 1.3253 | 0.185 | 0.671093706 | count | 1 |
| DDX47 | 1.8078451 | 0.8666181 | 2.0861 | 0.037 | 0.672403355 | count | 1 |
| AL021707.6 | 1.8108722 | 1.107288 | 1.6354 | 0.102 | 0.673395614 | count | 1 |
| FNDC3B | 0.7208511 | 0.3154505 | 2.2851 | 0.0224 | 0.674200953 | count | 1 |
| BMP1 | 0.8619762 | 0.5622813 | 1.533 | 0.125 | 0.676082766 | count | 1 |

| | | | | | | | |
|---|---|---|---|---|---|---|---|
| FAM229A | 1.143367 | 0.8142831 | 1.4041 | 0.16 | 0.676639366 | count | 1 |
| AC239868.2 | 1.3633418 | 0.7063807 | 1.93 | 0.0537 | 0.677549324 | count | 1 |
| PPP1R8 | 0.575618 | 0.2146565 | 2.6816 | 0.00736 | 0.677704942 | count | 1 |
| CCL4 | 0.4702137 | 0.0695072 | 6.765 | 1.56E-11 | 0.678152981 | count | 3.78E-07 |
| TAF1A | 0.6473128 | 0.362651 | 1.7849 | 0.0744 | 0.67841037 | count | 1 |
| GOLM1 | 0.726037 | 0.5482794 | 1.3242 | 0.186 | 0.679152486 | count | 1 |
| LIN52 | 1.019459 | 0.4494456 | 2.2683 | 0.0234 | 0.681046935 | count | 1 |
| L3HYPDH | 0.6032192 | 0.3845501 | 1.5686 | 0.117 | 0.682260675 | count | 1 |
| FAM169A | 0.6763804 | 0.3452244 | 1.9592 | 0.0502 | 0.682958838 | count | 1 |
| AC015468.3 | 17.9548809 | 1276.789545 | 0.0141 | 0.989 | 0.683526307 | count | 1 |
| RTKN | 17.9560931 | 1099.855615 | 0.0163 | 0.987 | 0.683526307 | count | 1 |
| AC092718.5 | 18.2203373 | 1411.957629 | 0.0129 | 0.99 | 0.68352631 | count | 1 |
| ZNF630 | 18.2203525 | 1922.863424 | 0.0095 | 0.992 | 0.68352631 | count | 1 |
| KAZALD1 | 18.2203568 | 1922.863118 | 0.0095 | 0.992 | 0.68352631 | count | 1 |
| AC009560.1 | 18.2203623 | 1922.859187 | 0.0095 | 0.992 | 0.68352631 | count | 1 |
| C9orf41-AS1 | 18.2203804 | 1922.864589 | 0.0095 | 0.992 | 0.68352631 | count | 1 |
| AL450306.1 | 18.2804554 | 1511.231146 | 0.0121 | 0.99 | 0.683526311 | count | 1 |
| AP006565.1 | 18.3956755 | 1669.174186 | 0.011 | 0.9912 | 0.683526312 | count | 1 |
| AC093512.2 | 18.4089377 | 1783.429831 | 0.0103 | 0.992 | 0.683526312 | count | 1 |
| ZNF497 | 18.4090546 | 2343.368392 | 0.0079 | 0.994 | 0.683526312 | count | 1 |
| AC084026.2 | 18.5631991 | 1945.596126 | 0.0095 | 0.992 | 0.683526313 | count | 1 |
| ME1 | 18.6942192 | 1656.740099 | 0.0113 | 0.991 | 0.683526314 | count | 1 |
| MTFR2 | 18.6946607 | 2500.134209 | 0.0075 | 0.994 | 0.683526314 | count | 1 |
| AC098936.1 | 18.9715187 | 2281.412776 | 0.0083 | 0.9934 | 0.683526316 | count | 1 |
| VLDLR | 18.9715189 | 2281.41276 | 0.0083 | 0.9934 | 0.683526316 | count | 1 |
| NIPSNAP3B | 18.971541 | 2281.388845 | 0.0083 | 0.9934 | 0.683526316 | count | 1 |
| AL353593.1 | 19.0862953 | 1530.17427 | 0.0125 | 0.99 | 0.683526317 | count | 1 |
| CARMIL3 | 19.0862964 | 1530.169597 | 0.0125 | 0.99 | 0.683526317 | count | 1 |
| AL033528.2 | 19.3865617 | 1539.898263 | 0.0126 | 0.99 | 0.683526318 | count | 1 |
| RUSC1-AS1 | 19.3865685 | 1539.897967 | 0.0126 | 0.99 | 0.683526318 | count | 1 |
| AC011921.1 | 19.3872577 | 1999.108908 | 0.0097 | 0.992 | 0.683526318 | count | 1 |
| FCRL1 | 19.5377703 | 1324.021366 | 0.0148 | 0.988 | 0.683526318 | count | 1 |
| AL121655.1 | 19.5377703 | 1324.021345 | 0.0148 | 0.988 | 0.683526318 | count | 1 |
| HTR2B | 19.5377703 | 1324.021361 | 0.0148 | 0.988 | 0.683526318 | count | 1 |
| FAT1 | 19.5377703 | 1324.021345 | 0.0148 | 0.988 | 0.683526318 | count | 1 |
| AC139491.7 | 19.5377703 | 1324.021323 | 0.0148 | 0.988 | 0.683526318 | count | 1 |
| AL157373.2 | 19.5377703 | 1324.021345 | 0.0148 | 0.988 | 0.683526318 | count | 1 |
| MOCS1 | 19.5377703 | 1324.021323 | 0.0148 | 0.988 | 0.683526318 | count | 1 |
| SSC4D | 19.5377703 | 1324.021355 | 0.0148 | 0.988 | 0.683526318 | count | 1 |
| DLX6-AS1 | 19.5377703 | 1324.021361 | 0.0148 | 0.988 | 0.683526318 | count | 1 |
| OCRL | 19.5377703 | 1324.021323 | 0.0148 | 0.988 | 0.683526318 | count | 1 |
| RAG1 | 19.5377703 | 1324.021355 | 0.0148 | 0.988 | 0.683526318 | count | 1 |
| NRXN2 | 19.5377703 | 1324.021387 | 0.0148 | 0.988 | 0.683526318 | count | 1 |
| STT3A-AS1 | 19.5377703 | 1324.021361 | 0.0148 | 0.988 | 0.683526318 | count | 1 |
| TEX22 | 19.5377703 | 1324.021361 | 0.0148 | 0.988 | 0.683526318 | count | 1 |

| | | | | | | | |
|---|---|---|---|---|---|---|---|
| PCSK6 | 19.5377703 | 1324.021387 | 0.0148 | 0.988 | 0.683526318 | count | 1 |
| TMEM256-PLSCR3 | 19.5377703 | 1324.021345 | 0.0148 | 0.988 | 0.683526318 | count | 1 |
| AC016876.3 | 19.5377703 | 1324.021387 | 0.0148 | 0.988 | 0.683526318 | count | 1 |
| AC060766.6 | 19.5377703 | 1324.021361 | 0.0148 | 0.988 | 0.683526318 | count | 1 |
| AC015849.2 | 19.5377703 | 1324.021355 | 0.0148 | 0.988 | 0.683526318 | count | 1 |
| AC068234.2 | 19.5377703 | 1324.021323 | 0.0148 | 0.988 | 0.683526318 | count | 1 |
| AC005920.4 | 19.5377702 | 1324.021361 | 0.0148 | 0.988 | 0.683526318 | count | 1 |
| AC245052.1 | 19.5377703 | 1324.021355 | 0.0148 | 0.988 | 0.683526318 | count | 1 |
| HORMAD2 | 19.5377703 | 1324.021371 | 0.0148 | 0.988 | 0.683526318 | count | 1 |
| LARGE1 | 19.5377702 | 1324.021345 | 0.0148 | 0.988 | 0.683526318 | count | 1 |
| APOBEC3B | 19.5377703 | 1324.021382 | 0.0148 | 0.988 | 0.683526318 | count | 1 |
| AL844908.1 | 19.5377703 | 1324.021355 | 0.0148 | 0.988 | 0.683526318 | count | 1 |
| OSCP1 | 19.5377705 | 1324.02135 | 0.0148 | 0.988 | 0.683526318 | count | 1 |
| RTCA-AS1 | 19.5377705 | 1324.021355 | 0.0148 | 0.988 | 0.683526318 | count | 1 |
| IRF6 | 19.5377705 | 1324.021377 | 0.0148 | 0.988 | 0.683526318 | count | 1 |
| MYCN | 19.5377704 | 1324.021382 | 0.0148 | 0.988 | 0.683526318 | count | 1 |
| AL133245.1 | 19.5377705 | 1324.021403 | 0.0148 | 0.988 | 0.683526318 | count | 1 |
| HSD17B13 | 19.5377704 | 1324.021377 | 0.0148 | 0.988 | 0.683526318 | count | 1 |
| IRX2 | 19.5377705 | 1324.021318 | 0.0148 | 0.988 | 0.683526318 | count | 1 |
| ZC3HAV1L | 19.5377705 | 1324.02135 | 0.0148 | 0.988 | 0.683526318 | count | 1 |
| LINC01402 | 19.5377704 | 1324.021361 | 0.0148 | 0.988 | 0.683526318 | count | 1 |
| MCPH1-AS1 | 19.5377705 | 1324.021377 | 0.0148 | 0.988 | 0.683526318 | count | 1 |
| TSPYL5 | 19.5377704 | 1324.021361 | 0.0148 | 0.988 | 0.683526318 | count | 1 |
| AC018630.2 | 19.5377704 | 1324.021377 | 0.0148 | 0.988 | 0.683526318 | count | 1 |
| CDH1 | 19.5377704 | 1324.021329 | 0.0148 | 0.988 | 0.683526318 | count | 1 |
| CA4 | 19.5377705 | 1324.021377 | 0.0148 | 0.988 | 0.683526318 | count | 1 |
| VAV3-AS1 | 19.5377706 | 1324.021371 | 0.0148 | 0.988 | 0.683526318 | count | 1 |
| SEMA6C | 19.5377706 | 1324.021371 | 0.0148 | 0.988 | 0.683526318 | count | 1 |
| ZNF462 | 19.5377706 | 1324.021371 | 0.0148 | 0.988 | 0.683526318 | count | 1 |
| CCDC113 | 19.5377706 | 1324.021371 | 0.0148 | 0.988 | 0.683526318 | count | 1 |
| ARHGEF4 | 19.6095698 | 1539.205047 | 0.0127 | 0.99 | 0.683526318 | count | 1 |
| LINC01547 | 17.7279278 | 1238.288129 | 0.0143 | 0.989 | 0.683526358 | count | 1 |
| MSS51 | 18.1011172 | 1126.364442 | 0.0161 | 0.9872 | 0.683526363 | count | 1 |
| GATA3-AS1 | 18.1043852 | 968.9228083 | 0.0187 | 0.9851 | 0.683526363 | count | 1 |
| TBXA2R | 18.2203362 | 1411.952693 | 0.0129 | 0.99 | 0.683526365 | count | 1 |
| FAM66C | 18.6352955 | 1665.573535 | 0.0112 | 0.9911 | 0.683526368 | count | 1 |
| AL158211.2 | 18.7887228 | 931.5653934 | 0.0202 | 0.984 | 0.683526369 | count | 1 |
| WDR11-AS1 | 18.9715186 | 2281.412663 | 0.0083 | 0.9934 | 0.68352637 | count | 1 |
| AL354977.2 | 18.9715411 | 2281.388901 | 0.0083 | 0.9934 | 0.68352637 | count | 1 |
| ITGB1-DT | 19.0863025 | 1530.174238 | 0.0125 | 0.99 | 0.683526371 | count | 1 |
| SEMA3G | 19.3872783 | 1999.115881 | 0.0097 | 0.992 | 0.683526372 | count | 1 |
| LINC01776 | 19.5377703 | 1324.021329 | 0.0148 | 0.988 | 0.683526373 | count | 1 |
| AC100830.1 | 19.5377703 | 1324.021329 | 0.0148 | 0.988 | 0.683526373 | count | 1 |
| AC136944.2 | 19.5377703 | 1324.021329 | 0.0148 | 0.988 | 0.683526373 | count | 1 |
| ATP2B2 | 19.5377704 | 1324.021334 | 0.0148 | 0.988 | 0.683526373 | count | 1 |

| | | | | | | | |
|---|---|---|---|---|---|---|---|
| DOCK3 | 19.5377705 | 1324.021393 | 0.0148 | 0.988 | 0.683526373 | count | 1 |
| AFDN-DT | 19.5377704 | 1324.021371 | 0.0148 | 0.988 | 0.683526373 | count | 1 |
| CYP51A1-AS1 | 19.5377705 | 1324.021393 | 0.0148 | 0.988 | 0.683526373 | count | 1 |
| RNASEH2B-AS1 | 19.5377704 | 1324.021371 | 0.0148 | 0.988 | 0.683526373 | count | 1 |
| TRARG1 | 19.5377704 | 1324.021323 | 0.0148 | 0.988 | 0.683526373 | count | 1 |
| C20orf197 | 19.5377705 | 1324.021366 | 0.0148 | 0.988 | 0.683526373 | count | 1 |
| AL391987.4 | 19.5377706 | 1324.021366 | 0.0148 | 0.988 | 0.683526373 | count | 1 |
| HDX | 1.1551736 | 0.5256884 | 2.1974 | 0.0281 | 0.683683557 | count | 1 |
| FAM174A | 0.5324794 | 0.2037266 | 2.6137 | 0.009 | 0.6843689 | count | 1 |
| AP005329.1 | 0.9361939 | 0.7085244 | 1.3213 | 0.186 | 0.684578495 | count | 1 |
| UBXN10 | 0.9361939 | 0.7286038 | 1.2849 | 0.199 | 0.684578495 | count | 1 |
| SLC2A1-AS1 | 1.3828405 | 0.829687 | 1.6667 | 0.0957 | 0.687024505 | count | 1 |
| HIST1H2AE | 0.8275798 | 0.5763557 | 1.4359 | 0.151 | 0.68719099 | count | 1 |
| ALDH16A1 | 0.5365466 | 0.1804548 | 2.9733 | 0.00297 | 0.688101547 | count | 1 |
| ZNF761 | 0.6828172 | 0.356433 | 1.9157 | 0.0555 | 0.689574872 | count | 1 |
| XPO4 | 0.5909444 | 0.2570364 | 2.2991 | 0.0216 | 0.691089625 | count | 1 |
| TPST2 | 0.4914084 | 0.1003916 | 4.8949 | 1.03E-06 | 0.692774164 | count | 0.02483021 |
| TXNRD2 | 0.6146213 | 0.2810707 | 2.1867 | 0.0288 | 0.695325044 | count | 1 |
| AL136962.1 | 0.8391436 | 0.5674533 | 1.4788 | 0.139 | 0.697006542 | count | 1 |
| CBLN3 | 1.884293 | 0.9650517 | 1.9525 | 0.051 | 0.697089652 | count | 1 |
| NBPF1 | 0.8020037 | 0.5098027 | 1.5732 | 0.116 | 0.698022228 | count | 1 |
| ZDHHC8 | 0.7066859 | 0.3119163 | 2.2656 | 0.0235 | 0.698163543 | count | 1 |
| MAGEF1 | 0.5543844 | 0.2204738 | 2.5145 | 0.012 | 0.698261743 | count | 1 |
| CAGE1 | 1.8902189 | 0.9197915 | 2.0551 | 0.04 | 0.698970318 | count | 1 |
| AC009126.1 | 0.749032 | 0.5171893 | 1.4483 | 0.148 | 0.701104264 | count | 1 |
| C11orf65 | 1.898656 | 1.1473531 | 1.6548 | 0.0981 | 0.701639344 | count | 1 |
| GOLGA8Q | 1.898656 | 1.187723 | 1.5986 | 0.11 | 0.701639344 | count | 1 |
| SELL | 0.5026404 | 0.0967806 | 5.1936 | 2.18E-07 | 0.702627981 | count | 0.005261212 |
| FSIP2 | 1.907589 | 0.9279474 | 2.0557 | 0.0399 | 0.704455516 | count | 1 |
| CARMN | 1.907589 | 0.9279474 | 2.0557 | 0.0399 | 0.704455516 | count | 1 |
| GLS2 | 1.907589 | 0.9279474 | 2.0557 | 0.0399 | 0.704455516 | count | 1 |
| THAP7-AS1 | 0.8098247 | 0.4455183 | 1.8177 | 0.0692 | 0.704973049 | count | 1 |
| AC027449.1 | 1.42 | 0.8525772 | 1.666 | 0.0958 | 0.70502371 | count | 1 |
| PLK1 | 1.425922 | 0.9630888 | 1.4806 | 0.139 | 0.707806002 | count | 1 |
| SSBP3 | 0.549764 | 0.1838474 | 2.9903 | 0.00281 | 0.708236934 | count | 1 |
| ZNF555 | 0.782673 | 0.4643033 | 1.6857 | 0.0919 | 0.708654425 | count | 1 |
| AL391834.2 | 0.9711677 | 0.5851362 | 1.6597 | 0.0971 | 0.710669012 | count | 1 |
| WDFY1 | 0.6427553 | 0.2343459 | 2.7428 | 0.00612 | 0.712314314 | count | 1 |
| AC104794.2 | 0.9774433 | 0.5421874 | 1.8028 | 0.0715 | 0.715343271 | count | 1 |
| ATRIP | 0.6635472 | 0.3719708 | 1.7839 | 0.0745 | 0.717342391 | count | 1 |
| AC108471.2 | 0.980253 | 0.8359167 | 1.1727 | 0.241 | 0.71743523 | count | 1 |
| ANKRD20A4 | 1.212558 | 1.1672807 | 1.0388 | 0.299 | 0.717743729 | count | 1 |
| SEC22A | 0.610356 | 0.2440094 | 2.5014 | 0.0124 | 0.719060763 | count | 1 |
| RDH13 | 0.6420696 | 0.2904071 | 2.2109 | 0.0271 | 0.719440811 | count | 1 |
| SH3BGR | 1.21694 | 0.8367672 | 1.4543 | 0.146 | 0.720332137 | count | 1 |

| | | | | | | | |
|---|---|---|---|---|---|---|---|
| NME2 | 1.0779568 | 0.4812582 | 2.2399 | 0.0252 | 0.720718604 | count | 1 |
| C1orf162 | 0.5165503 | 0.1056092 | 4.8911 | 1.05E-06 | 0.721751628 | count | 0.02531025 |
| NUDT9 | 0.5627305 | 0.2082005 | 2.7028 | 0.00691 | 0.721902595 | count | 1 |
| TTC16 | 0.6379911 | 0.2576328 | 2.4764 | 0.0133 | 0.722103115 | count | 1 |
| KLHDC8B | 1.96659 | 0.8974662 | 2.1913 | 0.0285 | 0.722774143 | count | 1 |
| CCL4L2 | 0.502125 | 0.1154515 | 4.3492 | 1.41E-05 | 0.723253259 | count | 0.338682 |
| ZNF674-AS1 | 0.7157164 | 0.4105304 | 1.7434 | 0.0814 | 0.723385839 | count | 1 |
| WDR37 | 0.6104803 | 0.2175691 | 2.8059 | 0.00505 | 0.723929279 | count | 1 |
| ATP2B1-AS1 | 0.5174738 | 0.1329125 | 3.8933 | 0.000101 | 0.724721519 | count | 1 |
| HSPA1L | 0.7755923 | 0.5181555 | 1.4968 | 0.135 | 0.726448389 | count | 1 |
| AC015819.2 | 1.9804773 | 1.102874 | 1.7957 | 0.0726 | 0.727015024 | count | 1 |
| ATXN7L2 | 1.0890194 | 0.7752955 | 1.4047 | 0.16 | 0.728192109 | count | 1 |
| SETD1A | 0.7780602 | 0.3687451 | 2.11 | 0.0349 | 0.728802507 | count | 1 |
| NSUN3 | 0.6190223 | 0.2224637 | 2.7826 | 0.00542 | 0.729378784 | count | 1 |
| JRKL | 0.6386009 | 0.3071253 | 2.0793 | 0.0377 | 0.729599025 | count | 1 |
| PARN | 0.5676502 | 0.1972627 | 2.8776 | 0.00403 | 0.729866283 | count | 1 |
| SMKR1 | 0.6334969 | 0.3206459 | 1.9757 | 0.0483 | 0.729996653 | count | 1 |
| NTHL1 | 0.612935 | 0.2494942 | 2.4567 | 0.0141 | 0.731339203 | count | 1 |
| PRMT5-AS1 | 1.477012 | 0.7144055 | 2.0675 | 0.0388 | 0.732161825 | count | 1 |
| LOXL1-AS1 | 0.7107921 | 0.3812559 | 1.8643 | 0.0624 | 0.732836648 | count | 1 |
| AAR2 | 0.7605229 | 0.3026513 | 2.5129 | 0.012 | 0.733301152 | count | 1 |
| HSPBP1 | 0.570944 | 0.191764 | 2.9773 | 0.00293 | 0.735692115 | count | 1 |
| AC016355.1 | 1.1001524 | 0.7481735 | 1.4705 | 0.142 | 0.735703053 | count | 1 |
| GPAM | 0.9379011 | 0.5687948 | 1.6489 | 0.0993 | 0.736906674 | count | 1 |
| GZMH | 0.5123866 | 0.0631143 | 8.1184 | 6.54E-16 | 0.737213043 | count | 1.59E-11 |
| AP000769.2 | 2.0299302 | 1.014378 | 2.0012 | 0.0455 | 0.74189416 | count | 1 |
| C2orf15 | 0.8924334 | 0.7041471 | 1.2674 | 0.205 | 0.742175368 | count | 1 |
| TBX21 | 0.5302992 | 0.1113868 | 4.7609 | 2.01E-06 | 0.743432984 | count | 0.04841487 |
| PRKAB1 | 0.6885511 | 0.3298868 | 2.0872 | 0.0369 | 0.7447609 | count | 1 |
| ZNF385A | 2.0464443 | 1.1116947 | 1.8408 | 0.0657 | 0.746784798 | count | 1 |
| AL451085.1 | 0.8585526 | 0.4162548 | 2.0626 | 0.0392 | 0.748234796 | count | 1 |
| TRBV5-4 | 1.5114746 | 0.7420128 | 2.037 | 0.0417 | 0.748403151 | count | 1 |
| LINC00092 | 1.5128302 | 0.6996795 | 2.1622 | 0.0307 | 0.749038824 | count | 1 |
| ADAMTS1 | 0.8307719 | 0.6582754 | 1.262 | 0.207 | 0.753044023 | count | 1 |
| APOM | 0.7621085 | 0.404333 | 1.8849 | 0.0595 | 0.753896262 | count | 1 |
| PTDSS2 | 0.7824603 | 0.3643044 | 2.1478 | 0.0318 | 0.754818306 | count | 1 |
| AL590399.1 | 1.03081 | 0.9095681 | 1.1333 | 0.257 | 0.754988313 | count | 1 |
| AC137767.1 | 0.8076422 | 0.3323261 | 2.4303 | 0.0151 | 0.757007697 | count | 1 |
| SHLD3 | 0.8351972 | 0.4038977 | 2.0678 | 0.0387 | 0.757124255 | count | 1 |
| PI16 | 0.9636176 | 0.5413735 | 1.7799 | 0.0752 | 0.757440749 | count | 1 |
| AL139274.2 | 0.8102906 | 0.384869 | 2.1054 | 0.0353 | 0.759531542 | count | 1 |
| AL662844.4 | 0.7364607 | 0.211617 | 3.4802 | 0.000507 | 0.759712087 | count | 1 |
| ZNF683 | 0.5535997 | 0.1683886 | 3.2876 | 0.00102 | 0.75991585 | count | 1 |
| AC144652.1 | 0.7263391 | 0.3239297 | 2.2423 | 0.025 | 0.76259023 | count | 1 |
| RCAN1 | 0.9167534 | 0.3828171 | 2.3948 | 0.0167 | 0.762746332 | count | 1 |

| | | | | | | | |
|---|---|---|---|---|---|---|---|
| AC010491.1 | 1.5429506 | 0.8274283 | 1.8648 | 0.0623 | 0.763098712 | count | 1 |
| SPECC1 | 0.6324471 | 0.2773798 | 2.2801 | 0.0227 | 0.763316726 | count | 1 |
| MTFP1 | 0.7560162 | 0.3267075 | 2.314 | 0.0207 | 0.764782699 | count | 1 |
| AC013400.1 | 2.117469 | 1.288499 | 1.6434 | 0.1 | 0.767368698 | count | 1 |
| AL590764.1 | 0.7638357 | 0.4488252 | 1.7019 | 0.0889 | 0.772811239 | count | 1 |
| MANEAL | 1.056974 | 0.6108937 | 1.7302 | 0.0837 | 0.774348977 | count | 1 |
| TCHH | 2.1466815 | 1.211086 | 1.7725 | 0.0764 | 0.775620587 | count | 1 |
| PLEKHG4 | 1.313008 | 0.7215804 | 1.8196 | 0.0689 | 0.776561697 | count | 1 |
| METTL3 | 0.6603884 | 0.2964505 | 2.2277 | 0.026 | 0.778627386 | count | 1 |
| EFHD2 | 0.5441008 | 0.0701796 | 7.753 | 1.18E-14 | 0.778720792 | count | 2.86E-10 |
| NOMO1 | 1.3218019 | 0.8529354 | 1.5497 | 0.121 | 0.781656422 | count | 1 |
| NBPF11 | 0.9003162 | 0.4459607 | 2.0188 | 0.0436 | 0.78523544 | count | 1 |
| NAGLU | 0.662112 | 0.2550031 | 2.5965 | 0.00946 | 0.785780349 | count | 1 |
| AC009495.3 | 2.184332 | 1.487015 | 1.4689 | 0.142 | 0.786071482 | count | 1 |
| APOD | 1.0754412 | 0.8095679 | 1.3284 | 0.184 | 0.787981048 | count | 1 |
| LINC01727 | 1.5981379 | 0.9304907 | 1.7175 | 0.086 | 0.788531315 | count | 1 |
| BX284668.5 | 0.697181 | 0.4175859 | 1.6696 | 0.0951 | 0.789916822 | count | 1 |
| VAMP4 | 0.5945385 | 0.1615234 | 3.6808 | 0.000236 | 0.794841682 | count | 1 |
| TBXAS1 | 0.7169629 | 0.2688167 | 2.6671 | 0.00769 | 0.795643407 | count | 1 |
| EFCAB2 | 0.7290885 | 0.3605839 | 2.022 | 0.0433 | 0.799624652 | count | 1 |
| CCDC120 | 1.1955946 | 0.7378208 | 1.6204 | 0.105 | 0.79962899 | count | 1 |
| PLA2G2A | 2.2348986 | 1.505857 | 1.4841 | 0.138 | 0.79977796 | count | 1 |
| CEACAM21 | 0.6832692 | 0.2719274 | 2.5127 | 0.012 | 0.800280125 | count | 1 |
| CDC14B | 0.7033602 | 0.3293782 | 2.1354 | 0.0328 | 0.804449656 | count | 1 |
| TGFBR3 | 0.659737 | 0.2028825 | 3.2518 | 0.00116 | 0.804461381 | count | 1 |
| AL355581.1 | 2.2555466 | 0.875944 | 2.575 | 0.0101 | 0.805265944 | count | 1 |
| HIST1H2BE | 0.9729138 | 0.3214414 | 3.0267 | 0.00249 | 0.810119362 | count | 1 |
| CHI3L2 | 0.6752693 | 0.2953806 | 2.2861 | 0.0223 | 0.811073492 | count | 1 |
| KCTD11 | 1.6548301 | 0.9217524 | 1.7953 | 0.0727 | 0.814194389 | count | 1 |
| CRAMP1 | 1.0364873 | 0.5196719 | 1.9945 | 0.0462 | 0.815383542 | count | 1 |
| AP001020.3 | 2.294376 | 1.430417 | 1.604 | 0.109 | 0.815415137 | count | 1 |
| ZEB2-AS1 | 2.3005619 | 0.9807237 | 2.3458 | 0.019 | 0.817011501 | count | 1 |
| SLCO4C1 | 1.673328 | 0.8034606 | 2.0827 | 0.0374 | 0.822462397 | count | 1 |
| CERCAM | 1.2327486 | 0.7046837 | 1.7494 | 0.0803 | 0.824260661 | count | 1 |
| GBA | 0.7845666 | 0.360096 | 2.1788 | 0.0294 | 0.824552338 | count | 1 |
| ADCY9 | 1.1252607 | 0.5296463 | 2.1246 | 0.0337 | 0.824603975 | count | 1 |
| LINC01176 | 1.23918 | 0.8287676 | 1.4952 | 0.135 | 0.828508268 | count | 1 |
| FCGR1B | 1.405059 | 0.6979242 | 2.0132 | 0.0442 | 0.829405538 | count | 1 |
| TGFB3 | 1.405059 | 0.6979242 | 2.0132 | 0.0442 | 0.829405538 | count | 1 |
| FAM229B | 1.0574011 | 0.4536898 | 2.3307 | 0.0198 | 0.831936741 | count | 1 |
| MTERF1 | 0.7075349 | 0.2888247 | 2.4497 | 0.0143 | 0.834741987 | count | 1 |
| EPB41L4A | 1.1405994 | 0.73054 | 1.5613 | 0.119 | 0.835831367 | count | 1 |
| B3GAT1 | 0.8663484 | 0.5084249 | 1.704 | 0.0885 | 0.836953522 | count | 1 |
| AC112907.3 | 1.4195621 | 0.8327086 | 1.7048 | 0.0883 | 0.83762886 | count | 1 |
| LRRN3 | 2.387608 | 0.7048116 | 3.3876 | 0.000713 | 0.838872333 | count | 1 |

| | | | | | | | |
|---|---|---|---|---|---|---|---|
| PTGDR | 0.6010772 | 0.1411472 | 4.2585 | 2.11E-05 | 0.843036346 | count | 0.5065688 |
| ABCA3 | 1.1520167 | 0.5760062 | 2 | 0.0456 | 0.844172706 | count | 1 |
| AL353719.1 | 1.2676137 | 0.6863438 | 1.8469 | 0.0648 | 0.847229108 | count | 1 |
| BRD3OS | 0.782949 | 0.3427219 | 2.2845 | 0.0224 | 0.848189496 | count | 1 |
| BTBD8 | 0.8071244 | 0.4028781 | 2.0034 | 0.0452 | 0.848530946 | count | 1 |
| PPT2 | 1.0219264 | 0.5960962 | 1.7144 | 0.0866 | 0.851284468 | count | 1 |
| CFD | 0.7317883 | 0.2220472 | 3.2956 | 0.000992 | 0.851287575 | count | 1 |
| MKS1 | 0.9775638 | 0.594741 | 1.6437 | 0.1 | 0.853413592 | count | 1 |
| LINC00910 | 0.8837291 | 0.5869323 | 1.5057 | 0.132 | 0.853932678 | count | 1 |
| SLC25A20 | 0.6772853 | 0.252457 | 2.6828 | 0.00734 | 0.854217108 | count | 1 |
| AC026471.4 | 2.4557923 | 0.7593948 | 3.2339 | 0.00123 | 0.855215859 | count | 1 |
| AL132639.2 | 2.4674093 | 1.101661 | 2.2397 | 0.0252 | 0.857932506 | count | 1 |
| AC007952.4 | 0.7378746 | 0.2174058 | 3.394 | 0.000697 | 0.858428362 | count | 1 |
| SPDL1 | 0.8053416 | 0.4250715 | 1.8946 | 0.0582 | 0.860226968 | count | 1 |
| AC002091.2 | 1.459947 | 0.7943835 | 1.8378 | 0.0662 | 0.860368711 | count | 1 |
| PALLD | 0.6994103 | 0.3362816 | 2.0798 | 0.0376 | 0.860769977 | count | 1 |
| SMPD3 | 0.9177288 | 0.3532622 | 2.5979 | 0.00942 | 0.861665528 | count | 1 |
| FRMD6-AS1 | 1.7646809 | 0.8845795 | 1.9949 | 0.0461 | 0.862499341 | count | 1 |
| DCHS1 | 2.4921636 | 1.194089 | 2.0871 | 0.037 | 0.863655611 | count | 1 |
| HOPX | 0.6054716 | 0.0769617 | 7.8672 | 4.84E-15 | 0.867720142 | count | 1.17E-10 |
| ZNF436-AS1 | 2.5144761 | 1.2122367 | 2.0742 | 0.0381 | 0.868737906 | count | 1 |
| ZNF554 | 0.9975882 | 0.5173665 | 1.9282 | 0.0539 | 0.871019797 | count | 1 |
| SYNGR1 | 0.672931 | 0.2271599 | 2.9624 | 0.00307 | 0.874617158 | count | 1 |
| AP000787.1 | 1.4866428 | 0.4476149 | 3.3213 | 0.000906 | 0.875268581 | count | 1 |
| FHL3 | 0.6868967 | 0.2642991 | 2.5989 | 0.00939 | 0.87810755 | count | 1 |
| AC243965.1 | 1.2013045 | 0.4864778 | 2.4694 | 0.0136 | 0.880018352 | count | 1 |
| VLDLR-AS1 | 1.823571 | 0.6083924 | 2.9974 | 0.00274 | 0.887580889 | count | 1 |
| RECQL5 | 1.3299674 | 0.7414746 | 1.7937 | 0.073 | 0.887924026 | count | 1 |
| PROK2 | 0.8769172 | 0.4095604 | 2.1411 | 0.0323 | 0.888684784 | count | 1 |
| Z93930.2 | 0.8466709 | 0.2489421 | 3.4011 | 0.000679 | 0.890521338 | count | 1 |
| CARNS1 | 0.7939399 | 0.3882671 | 2.0448 | 0.0409 | 0.891660276 | count | 1 |
| JUN | 0.6203484 | 0.0646634 | 9.5935 | 1.59E-21 | 0.892694725 | count | 3.86E-17 |
| COPZ2 | 0.7456336 | 0.4019637 | 1.855 | 0.0637 | 0.896262319 | count | 1 |
| AL360012.1 | 2.6589202 | 0.6496531 | 4.0928 | 4.36E-05 | 0.899912136 | count | 1 |
| C1orf21 | 0.6612071 | 0.1610458 | 4.1057 | 4.13E-05 | 0.901354186 | count | 0.9900849 |
| TRGV3 | 0.8727847 | 0.4597111 | 1.8986 | 0.0577 | 0.902134958 | count | 1 |
| RAB27B | 0.7835999 | 0.2425324 | 3.2309 | 0.00125 | 0.904820801 | count | 1 |
| FZD3 | 1.1502246 | 0.5125158 | 2.2443 | 0.0249 | 0.904908172 | count | 1 |
| AKR1C3 | 0.7919902 | 0.3138097 | 2.5238 | 0.0117 | 0.906777588 | count | 1 |
| PCGF1 | 0.7433506 | 0.2865709 | 2.594 | 0.00953 | 0.907179156 | count | 1 |
| AC016405.3 | 2.701043 | 1.1533763 | 2.3419 | 0.0192 | 0.908449562 | count | 1 |
| ATN1 | 1.0910971 | 0.375483 | 2.9058 | 0.00369 | 0.909033854 | count | 1 |
| BX284668.6 | 1.0425996 | 0.3653016 | 2.8541 | 0.00434 | 0.910476013 | count | 1 |
| VCL | 0.7374586 | 0.2376531 | 3.1031 | 0.00193 | 0.911589874 | count | 1 |
| AQP11 | 1.0946318 | 0.6409272 | 1.7079 | 0.0877 | 0.911972637 | count | 1 |

| | | | | | | | |
|---|---|---|---|---|---|---|---|
| TK1 | 1.159442 | 0.6763411 | 1.7143 | 0.0866 | 0.912104862 | count | 1 |
| SBK1 | 0.774755 | 0.3347313 | 2.3146 | 0.0207 | 0.914681384 | count | 1 |
| DUSP1 | 0.638523 | 0.0442153 | 14.4412 | 6.36E-46 | 0.918777675 | count | 1.55E-41 |
| ADAMTS4 | 1.2552598 | 0.9341249 | 1.3438 | 0.179 | 0.918928142 | count | 1 |
| CEP78 | 0.7098078 | 0.1974875 | 3.5942 | 0.00033 | 0.919336925 | count | 1 |
| NNT-AS1 | 0.730683 | 0.2270024 | 3.2188 | 0.0013 | 0.927203272 | count | 1 |
| AC007365.1 | 1.267037 | 0.5357406 | 2.365 | 0.0181 | 0.927371735 | count | 1 |
| CACNA2D2 | 2.817025 | 1.205637 | 2.3365 | 0.0195 | 0.930704122 | count | 1 |
| KLHL26 | 1.0264933 | 0.4207599 | 2.4396 | 0.0148 | 0.932477557 | count | 1 |
| MCM3AP-AS1 | 1.2761964 | 0.7176126 | 1.7784 | 0.0754 | 0.933925829 | count | 1 |
| SGK494 | 1.072486 | 0.681669 | 1.5733 | 0.116 | 0.936573216 | count | 1 |
| ACKR3 | 1.406389 | 0.8769256 | 1.6038 | 0.109 | 0.937063797 | count | 1 |
| AL161421.1 | 1.1261274 | 0.423704 | 2.6578 | 0.0079 | 0.938100875 | count | 1 |
| HS6ST1 | 0.9704511 | 0.4683565 | 2.072 | 0.0383 | 0.938380575 | count | 1 |
| AC107464.3 | 0.9477273 | 0.5473306 | 1.7315 | 0.0834 | 0.939779393 | count | 1 |
| AL021453.1 | 0.8497698 | 0.2922303 | 2.9079 | 0.00366 | 0.944479448 | count | 1 |
| TRAV21 | 1.13866 | 0.7341971 | 1.5509 | 0.121 | 0.948467651 | count | 1 |
| KMT2E-AS1 | 0.7763464 | 0.1854576 | 4.1861 | 2.91E-05 | 0.951958441 | count | 0.698109 |
| TRAV4 | 1.4310611 | 0.4872285 | 2.9371 | 0.00333 | 0.952740883 | count | 1 |
| AF001548.2 | 1.3045555 | 0.6792163 | 1.9207 | 0.0549 | 0.954145114 | count | 1 |
| KLKB1 | 1.996777 | 1.834623 | 1.0884 | 0.277 | 0.95784963 | count | 1 |
| VSTM2B | 1.996777 | 1.834623 | 1.0884 | 0.277 | 0.95784963 | count | 1 |
| FCHO1 | 0.8544247 | 0.2635631 | 3.2418 | 0.0012 | 0.960072901 | count | 1 |
| SDR42E2 | 0.8251002 | 0.3264965 | 2.5271 | 0.0115 | 0.960654622 | count | 1 |
| KIR2DL3 | 0.8575593 | 0.5279475 | 1.6243 | 0.104 | 0.963614065 | count | 1 |
| PYCARD-AS1 | 2.0128638 | 0.8616855 | 2.336 | 0.0196 | 0.964101829 | count | 1 |
| TGFBR3L | 0.849732 | 0.3530309 | 2.407 | 0.0161 | 0.964370285 | count | 1 |
| AC092329.3 | 1.320149 | 0.8960336 | 1.4733 | 0.141 | 0.965214107 | count | 1 |
| RBKS | 1.169002 | 0.3653506 | 3.1997 | 0.00139 | 0.973491837 | count | 1 |
| AC007728.2 | 1.6860523 | 0.9048359 | 1.8634 | 0.0625 | 0.982907415 | count | 1 |
| FCER1G | 0.7014567 | 0.2034218 | 3.4483 | 0.000571 | 0.985108335 | count | 1 |
| USP46 | 0.8062646 | 0.2578896 | 3.1264 | 0.00178 | 0.993037706 | count | 1 |
| AL078644.2 | 2.1022351 | 0.9104909 | 2.3089 | 0.021 | 0.997962701 | count | 1 |
| EPDR1 | 1.718016 | 1.220424 | 1.4077 | 0.159 | 0.99951218 | count | 1 |
| CLIC3 | 0.71543 | 0.1558689 | 4.5899 | 4.59E-06 | 1.003363284 | count | 0.11043081 |
| NOL6 | 1.2791232 | 0.5695669 | 2.2458 | 0.0248 | 1.004597607 | count | 1 |
| GNGT2 | 0.8518197 | 0.3070856 | 2.7739 | 0.00557 | 1.00616367 | count | 1 |
| CTSW | 0.7012079 | 0.0546923 | 12.821 | 8.80E-37 | 1.00855308 | count | 2.14E-32 |
| FAM234B | 2.1414103 | 0.8796797 | 2.4343 | 0.015 | 1.012334355 | count | 1 |
| C22orf34 | 1.744818 | 0.7061808 | 2.4708 | 0.0135 | 1.013288923 | count | 1 |
| BSPRY | 1.533505 | 0.9443182 | 1.6239 | 0.104 | 1.016769477 | count | 1 |
| TRIM17 | 1.535889 | 0.609118 | 2.5215 | 0.0117 | 1.018238429 | count | 1 |
| DHX37 | 1.0293045 | 0.3672198 | 2.803 | 0.00509 | 1.02079595 | count | 1 |
| CXCR1 | 1.768448 | 0.8956727 | 1.9744 | 0.0484 | 1.025321244 | count | 1 |
| FADS2 | 1.768448 | 1.178864 | 1.5001 | 0.134 | 1.025321244 | count | 1 |

| | | | | | | | |
|---|---|---|---|---|---|---|---|
| USP46-AS1 | 1.7711038 | 0.6763363 | 2.6187 | 0.00887 | 1.026666681 | count | 1 |
| TSPAN2 | 0.7536974 | 0.182881 | 4.1212 | 3.86E-05 | 1.028244016 | count | 0.9255894 |
| Z93241.1 | 1.0640742 | 0.3564868 | 2.9849 | 0.00286 | 1.028899352 | count | 1 |
| PLAC8 | 0.7363116 | 0.0964506 | 7.6341 | 2.94E-14 | 1.038565843 | count | 7.13E-10 |
| SUSD1 | 0.9404649 | 0.3554004 | 2.6462 | 0.00818 | 1.045681295 | count | 1 |
| PEX11A | 1.201884 | 0.5038557 | 2.3854 | 0.0171 | 1.048456211 | count | 1 |
| ITGAM | 0.9986639 | 0.4027083 | 2.4799 | 0.0132 | 1.051101285 | count | 1 |
| RASSF4 | 0.8508056 | 0.3419073 | 2.4884 | 0.0129 | 1.060223459 | count | 1 |
| MORF4L2-AS1 | 1.8401438 | 0.7610887 | 2.4178 | 0.0157 | 1.061162053 | count | 1 |
| AC087239.1 | 1.0092723 | 0.2466577 | 4.0918 | 4.38E-05 | 1.062247386 | count | 1 |
| AC011511.1 | 1.4593164 | 0.9503372 | 1.5356 | 0.125 | 1.062335918 | count | 1 |
| AC005837.1 | 1.0290575 | 0.4436062 | 2.3198 | 0.0204 | 1.064159794 | count | 1 |
| ISPD | 1.6125049 | 1.1140729 | 1.4474 | 0.148 | 1.064882812 | count | 1 |
| KLRC1 | 0.7938399 | 0.2294572 | 3.4596 | 0.000548 | 1.066888598 | count | 1 |
| C9orf139 | 1.6169843 | 0.6152944 | 2.628 | 0.00863 | 1.06757596 | count | 1 |
| SPIN2A | 1.859113 | 1.128521 | 1.6474 | 0.0996 | 1.070473608 | count | 1 |
| KLRC2 | 0.9639172 | 0.3932086 | 2.4514 | 0.0143 | 1.071768155 | count | 1 |
| C7orf31 | 1.3741033 | 0.5121386 | 2.6831 | 0.00733 | 1.07655989 | count | 1 |
| CD300A | 0.7803334 | 0.147962 | 5.2739 | 1.42E-07 | 1.085341113 | count | 0.003427596 |
| PPFIA3 | 2.361462 | 1.3433095 | 1.7579 | 0.0788 | 1.087657862 | count | 1 |
| CHIC1 | 0.9495341 | 0.3093898 | 3.0691 | 0.00216 | 1.087932161 | count | 1 |
| FUT8-AS1 | 2.372188 | 1.251478 | 1.8955 | 0.0581 | 1.091095046 | count | 1 |
| EARS2 | 1.903064 | 1.46468 | 1.2993 | 0.194 | 1.091767957 | count | 1 |
| CTBP1-AS | 1.907589 | 0.6555455 | 2.9099 | 0.00364 | 1.093937621 | count | 1 |
| ZFP36 | 0.761096 | 0.0422829 | 18.0001 | 2.92E-69 | 1.095251308 | count | 7.10E-65 |
| NKG7 | 0.7607415 | 0.0573494 | 13.265 | 3.43E-39 | 1.096427136 | count | 8.34E-35 |
| IL2 | 1.2586467 | 0.4746307 | 2.6518 | 0.00804 | 1.096870691 | count | 1 |
| CD244 | 1.0279433 | 0.3138392 | 3.2754 | 0.00107 | 1.099045683 | count | 1 |
| SLC12A7 | 1.272542 | 0.8803523 | 1.4455 | 0.148 | 1.108652264 | count | 1 |
| AC145285.6 | 1.3363505 | 0.6366942 | 2.0989 | 0.0359 | 1.109322627 | count | 1 |
| AC093635.1 | 1.5310383 | 0.5992222 | 2.555 | 0.0107 | 1.111098095 | count | 1 |
| LILRB1 | 1.155343 | 0.4024191 | 2.871 | 0.00412 | 1.116299049 | count | 1 |
| CSF3R | 1.9590605 | 0.7483861 | 2.6177 | 0.00889 | 1.118319779 | count | 1 |
| GPR153 | 1.719566 | 0.8992171 | 1.9123 | 0.0559 | 1.128171669 | count | 1 |
| DOCK5 | 1.0273928 | 0.4966912 | 2.0685 | 0.0387 | 1.128754812 | count | 1 |
| PYROXD2 | 0.9740851 | 0.3682595 | 2.6451 | 0.0082 | 1.134496554 | count | 1 |
| AC048382.5 | 2.5161298 | 2.1905699 | 1.1486 | 0.2508 | 1.135116348 | count | 1 |
| AL158071.4 | 1.0479141 | 0.4308933 | 2.432 | 0.0151 | 1.13637567 | count | 1 |
| AC008537.4 | 17.9294129 | 1061.238892 | 0.0169 | 0.9865 | 1.145430406 | count | 1 |
| FZD5 | 18.0413412 | 1263.050821 | 0.0143 | 0.9886 | 1.145430409 | count | 1 |
| HOXA1 | 18.1042927 | 1306.36339 | 0.0139 | 0.989 | 1.14543041 | count | 1 |
| TFAP2E | 18.2205188 | 1516.873656 | 0.012 | 0.9904 | 1.145430413 | count | 1 |
| ZBTB8A | 18.4139134 | 1699.778685 | 0.0108 | 0.991 | 1.145430417 | count | 1 |
| MSX1 | 18.4215274 | 1462.818904 | 0.0126 | 0.99 | 1.145430417 | count | 1 |
| AL031281.2 | 18.4997027 | 1450.922601 | 0.0128 | 0.9898 | 1.145430418 | count | 1 |

| | | | | | | | |
|---|---|---|---|---|---|---|---|
| AC135279.3 | 18.4997026 | 1450.922578 | 0.0128 | 0.9898 | 1.145430418 | count | 1 |
| AC017100.1 | 18.6682395 | 1960.410411 | 0.0095 | 0.9924 | 1.145430421 | count | 1 |
| AC087386.1 | 18.6682396 | 1960.410416 | 0.0095 | 0.9924 | 1.145430421 | count | 1 |
| TRBV22-1 | 18.6682563 | 1960.418884 | 0.0095 | 0.9924 | 1.145430421 | count | 1 |
| AC135050.3 | 18.6682965 | 1484.133222 | 0.0126 | 0.99 | 1.145430421 | count | 1 |
| AC010746.1 | 18.7840983 | 1315.569464 | 0.0143 | 0.989 | 1.145430422 | count | 1 |
| RBPMS2 | 18.7901456 | 1717.880593 | 0.0109 | 0.9913 | 1.145430422 | count | 1 |
| AC010271.2 | 18.8024727 | 2370.967249 | 0.0079 | 0.994 | 1.145430422 | count | 1 |
| AL133215.2 | 18.802512 | 2370.972538 | 0.0079 | 0.994 | 1.145430422 | count | 1 |
| CXXC4 | 18.8025544 | 1838.057249 | 0.0102 | 0.9918 | 1.145430422 | count | 1 |
| TRAV41 | 18.9161427 | 1773.059048 | 0.0107 | 0.9915 | 1.145430424 | count | 1 |
| AC018410.1 | 18.9161674 | 2202.756216 | 0.0086 | 0.993 | 1.145430424 | count | 1 |
| AL512598.1 | 19.576161 | 1737.677734 | 0.0113 | 0.991 | 1.145430429 | count | 1 |
| AC098614.4 | 19.576191 | 1737.690864 | 0.0113 | 0.991 | 1.145430429 | count | 1 |
| INTS6L-AS1 | 19.5761911 | 1737.690893 | 0.0113 | 0.991 | 1.145430429 | count | 1 |
| AL590226.2 | 19.5761909 | 1737.690829 | 0.0113 | 0.991 | 1.145430429 | count | 1 |
| TMEM88 | 19.576191 | 1737.690893 | 0.0113 | 0.991 | 1.145430429 | count | 1 |
| AP000350.5 | 19.576191 | 1737.690811 | 0.0113 | 0.991 | 1.145430429 | count | 1 |
| TM4SF19 | 20.2349033 | 1322.407182 | 0.0153 | 0.988 | 1.145430431 | count | 1 |
| AARD | 20.2349035 | 1322.407097 | 0.0153 | 0.988 | 1.145430431 | count | 1 |
| CD300C | 20.2349031 | 1322.407118 | 0.0153 | 0.988 | 1.145430431 | count | 1 |
| AL121992.1 | 20.2370456 | 1877.903377 | 0.0108 | 0.991 | 1.145430431 | count | 1 |
| KIF2C | 20.2370454 | 1877.903301 | 0.0108 | 0.991 | 1.145430431 | count | 1 |
| AC243547.2 | 20.2370458 | 1877.903667 | 0.0108 | 0.991 | 1.145430431 | count | 1 |
| AC020594.1 | 20.2370455 | 1877.903545 | 0.0108 | 0.991 | 1.145430431 | count | 1 |
| SCN2A | 20.2370454 | 1877.903545 | 0.0108 | 0.991 | 1.145430431 | count | 1 |
| CPS1 | 20.2370457 | 1877.90356 | 0.0108 | 0.991 | 1.145430431 | count | 1 |
| COL4A3 | 20.2370458 | 1877.903667 | 0.0108 | 0.991 | 1.145430431 | count | 1 |
| AC073352.1 | 20.2370452 | 1877.903545 | 0.0108 | 0.991 | 1.145430431 | count | 1 |
| RAB23 | 20.2370453 | 1877.903438 | 0.0108 | 0.991 | 1.145430431 | count | 1 |
| AC073342.1 | 20.2370458 | 1877.903743 | 0.0108 | 0.991 | 1.145430431 | count | 1 |
| OPHN1 | 20.2370456 | 1877.903728 | 0.0108 | 0.991 | 1.145430431 | count | 1 |
| SMC2-AS1 | 20.237046 | 1877.903606 | 0.0108 | 0.991 | 1.145430431 | count | 1 |
| ABTB2 | 20.2370451 | 1877.903499 | 0.0108 | 0.991 | 1.145430431 | count | 1 |
| SYT12 | 20.2370454 | 1877.903286 | 0.0108 | 0.991 | 1.145430431 | count | 1 |
| MEIG1 | 20.2370458 | 1877.903484 | 0.0108 | 0.991 | 1.145430431 | count | 1 |
| PDE6H | 20.2370455 | 1877.903575 | 0.0108 | 0.991 | 1.145430431 | count | 1 |
| INSM2 | 20.2370461 | 1877.903438 | 0.0108 | 0.991 | 1.145430431 | count | 1 |
| PAX9 | 20.2370456 | 1877.903575 | 0.0108 | 0.991 | 1.145430431 | count | 1 |
| PLD4 | 20.2370461 | 1877.903438 | 0.0108 | 0.991 | 1.145430431 | count | 1 |
| AC026150.3 | 20.2370457 | 1877.903423 | 0.0108 | 0.991 | 1.145430431 | count | 1 |
| AC090971.2 | 20.2370456 | 1877.903575 | 0.0108 | 0.991 | 1.145430431 | count | 1 |
| SRRM2-AS1 | 20.2370458 | 1877.903636 | 0.0108 | 0.991 | 1.145430431 | count | 1 |
| ZNF205 | 20.2370458 | 1877.903484 | 0.0108 | 0.991 | 1.145430431 | count | 1 |
| AC109597.2 | 20.2370457 | 1877.903484 | 0.0108 | 0.991 | 1.145430431 | count | 1 |

| | | | | | | | |
|---|---|---|---|---|---|---|---|
| IL17C | 20.237046 | 1877.903621 | 0.0108 | 0.991 | 1.145430431 | count | 1 |
| AC087501.1 | 20.2370456 | 1877.903606 | 0.0108 | 0.991 | 1.145430431 | count | 1 |
| SLC47A1 | 20.2370458 | 1877.903743 | 0.0108 | 0.991 | 1.145430431 | count | 1 |
| FBXO47 | 20.2370453 | 1877.903438 | 0.0108 | 0.991 | 1.145430431 | count | 1 |
| AC087289.2 | 20.2370458 | 1877.903667 | 0.0108 | 0.991 | 1.145430431 | count | 1 |
| LINC00237 | 20.2370456 | 1877.903423 | 0.0108 | 0.991 | 1.145430431 | count | 1 |
| CCM2L | 20.237046 | 1877.903575 | 0.0108 | 0.991 | 1.145430431 | count | 1 |
| SLC7A9 | 20.2370458 | 1877.903469 | 0.0108 | 0.991 | 1.145430431 | count | 1 |
| CDC45 | 20.2370453 | 1877.903438 | 0.0108 | 0.991 | 1.145430431 | count | 1 |
| AC253536.6 | 20.2370459 | 1877.903514 | 0.0108 | 0.991 | 1.145430431 | count | 1 |
| TAPT1-AS1 | 18.4997026 | 1450.92258 | 0.0128 | 0.9898 | 1.145430497 | count | 1 |
| GJC3 | 18.4997026 | 1450.922567 | 0.0128 | 0.9898 | 1.145430497 | count | 1 |
| AP000802.1 | 18.4997027 | 1450.922594 | 0.0128 | 0.9898 | 1.145430497 | count | 1 |
| ERFE | 20.2370456 | 1877.90353 | 0.0108 | 0.991 | 1.14543051 | count | 1 |
| PCDHGB7 | 20.2370456 | 1877.90353 | 0.0108 | 0.991 | 1.14543051 | count | 1 |
| AC006027.1 | 20.2370453 | 1877.903362 | 0.0108 | 0.991 | 1.14543051 | count | 1 |
| KRT6B | 20.2370452 | 1877.903484 | 0.0108 | 0.991 | 1.14543051 | count | 1 |
| TP73 | 20.2370461 | 1877.903697 | 0.0108 | 0.991 | 1.14543051 | count | 1 |
| AC084756.1 | 20.2370462 | 1877.90359 | 0.0108 | 0.991 | 1.14543051 | count | 1 |
| AC009095.1 | 20.2370462 | 1877.90359 | 0.0108 | 0.991 | 1.14543051 | count | 1 |
| PRSS22 | 1.587588 | 0.7200633 | 2.2048 | 0.0275 | 1.148863279 | count | 1 |
| AC015982.1 | 1.2695081 | 0.4427765 | 2.8672 | 0.00417 | 1.150322493 | count | 1 |
| PPM1L | 1.194455 | 0.5593221 | 2.1355 | 0.0328 | 1.15345075 | count | 1 |
| PLCD1 | 1.280228 | 0.423524 | 3.0228 | 0.00252 | 1.159754106 | count | 1 |
| SPAG4 | 1.7752285 | 0.600997 | 2.9538 | 0.00316 | 1.16015298 | count | 1 |
| CCL3 | 0.8097656 | 0.1774859 | 4.5624 | 5.24E-06 | 1.160251372 | count | 0.12604296 |
| ITGAX | 0.995316 | 0.4811159 | 2.0688 | 0.0386 | 1.167711802 | count | 1 |
| PTK2 | 1.4101707 | 0.5920712 | 2.3818 | 0.0173 | 1.167870695 | count | 1 |
| AC090517.4 | 2.656304 | 1.2964251 | 2.0489 | 0.0405 | 1.174296057 | count | 1 |
| IFITM1 | 0.8255514 | 0.0619753 | 13.3206 | 1.69E-39 | 1.17757487 | count | 4.11E-35 |
| LINC00920 | 1.1748629 | 0.3758774 | 3.1257 | 0.00179 | 1.189783218 | count | 1 |
| GZMB | 0.8330532 | 0.1035231 | 8.047 | 1.16E-15 | 1.194545346 | count | 2.82E-11 |
| AC078845.1 | 2.735355 | 1.431481 | 1.9109 | 0.0561 | 1.194830524 | count | 1 |
| KIR3DL2 | 1.0533418 | 0.3165277 | 3.3278 | 0.000885 | 1.195426705 | count | 1 |
| ADGRG5 | 1.182056 | 0.4207536 | 2.8094 | 0.00499 | 1.196939463 | count | 1 |
| GOLGA8N | 1.3310331 | 0.6726711 | 1.9787 | 0.0479 | 1.204210352 | count | 1 |
| LEXM | 2.1513953 | 0.9319773 | 2.3084 | 0.021 | 1.20443551 | count | 1 |
| AC008443.5 | 1.55346 | 0.7144468 | 2.1744 | 0.0297 | 1.208248972 | count | 1 |
| RTL6 | 1.4112075 | 0.5289691 | 2.6678 | 0.00767 | 1.224547385 | count | 1 |
| TSPAN32 | 0.9419461 | 0.2085837 | 4.5159 | 6.52E-06 | 1.229336913 | count | 0.15679948 |
| PLEK | 0.8643554 | 0.0996129 | 8.6771 | 6.19E-18 | 1.231183793 | count | 1.50E-13 |
| GPR141 | 1.172785 | 0.6326562 | 1.8537 | 0.0639 | 1.232664166 | count | 1 |
| TRGC1 | 0.8946401 | 0.1797229 | 4.9779 | 6.75E-07 | 1.235133264 | count | 0.016276275 |
| PATL2 | 0.9422839 | 0.2012073 | 4.6831 | 2.94E-06 | 1.235634075 | count | 0.07077756 |
| GSDMA | 1.7441814 | 0.7823881 | 2.2293 | 0.0259 | 1.250025821 | count | 1 |

| | | | | | | | |
|---|---|---|---|---|---|---|---|
| CATSPER1 | 1.612368 | 1.0290194 | 1.5669 | 0.117 | 1.250143752 | count | 1 |
| SH3BP5L | 1.3442643 | 0.463815 | 2.8983 | 0.00378 | 1.257002411 | count | 1 |
| AL035701.1 | 1.3084754 | 0.5500696 | 2.3787 | 0.0174 | 1.260549479 | count | 1 |
| RASSF1-AS1 | 1.4950947 | 0.5000694 | 2.9898 | 0.00281 | 1.293010977 | count | 1 |
| TYROBP | 0.9091843 | 0.1686979 | 5.3894 | 7.55E-08 | 1.293504583 | count | 0.001823325 |
| NUMBL | 1.6800071 | 0.7823956 | 2.1473 | 0.0318 | 1.297338442 | count | 1 |
| MLC1 | 2.0306818 | 1.487798 | 1.3649 | 0.172 | 1.298165724 | count | 1 |
| S1PR5 | 0.9576724 | 0.1967714 | 4.8669 | 1.19E-06 | 1.303073259 | count | 0.028679 |
| NCR1 | 1.8311465 | 0.5984353 | 3.0599 | 0.00223 | 1.303880382 | count | 1 |
| LINC02446 | 1.0119234 | 0.2406818 | 4.2044 | 2.69E-05 | 1.320430383 | count | 0.6454117 |
| DNAJC27-AS1 | 1.7188288 | 0.6546188 | 2.6257 | 0.00869 | 1.323968478 | count | 1 |
| GPRIN1 | 2.091889 | 0.7898468 | 2.6485 | 0.00812 | 1.328990583 | count | 1 |
| LINC01504 | 2.097854 | 1.408787 | 1.4891 | 0.137 | 1.331947341 | count | 1 |
| TLR6 | 1.482328 | 0.5665788 | 2.6163 | 0.00893 | 1.333972614 | count | 1 |
| PIF1 | 1.165909 | 0.488939 | 2.3846 | 0.0172 | 1.345308163 | count | 1 |
| ROPN1L | 1.466261 | 0.5840085 | 2.5107 | 0.0121 | 1.365280371 | count | 1 |
| CXXC5 | 1.1227471 | 0.346615 | 3.2392 | 0.00121 | 1.376025564 | count | 1 |
| HOXA10 | 1.9574624 | 1.165822 | 1.679 | 0.0932 | 1.378932277 | count | 1 |
| PROCR | 1.699466 | 0.6610064 | 2.571 | 0.0102 | 1.387225306 | count | 1 |
| HIST1H3A | 1.62292 | 0.4281619 | 3.7904 | 0.000153 | 1.394610293 | count | 1 |
| NCR3 | 1.0124447 | 0.1612776 | 6.2777 | 3.87E-10 | 1.415549144 | count | 9.37E-06 |
| PDGFD | 1.1505172 | 0.2910503 | 3.953 | 7.88E-05 | 1.426886489 | count | 1 |
| GTF3C1 | 1.05566 | 0.1722765 | 6.1277 | 9.94E-10 | 1.458012625 | count | 2.41E-05 |
| WDR81 | 1.4326193 | 0.4293614 | 3.3366 | 0.000857 | 1.470161257 | count | 1 |
| CNR2 | 2.9634089 | 1.4707126 | 2.0149 | 0.044 | 1.479728394 | count | 1 |
| AP001269.4 | 2.9736131 | 1.0183552 | 2.92 | 0.00352 | 1.482327964 | count | 1 |
| NCAM1 | 1.740026 | 0.8814681 | 1.974 | 0.0485 | 1.484503338 | count | 1 |
| HBA1 | 18.175971 | 1299.421528 | 0.014 | 0.9888 | 1.494764645 | count | 1 |
| HOXC4 | 18.3254835 | 1095.639854 | 0.0167 | 0.9867 | 1.494764649 | count | 1 |
| IDI2 | 19.6451196 | 1038.698885 | 0.0189 | 0.985 | 1.49476467 | count | 1 |
| SLC25A30-AS1 | 19.6451196 | 1038.698907 | 0.0189 | 0.985 | 1.49476467 | count | 1 |
| SYT6 | 19.6478274 | 1398.864737 | 0.014 | 0.989 | 1.49476467 | count | 1 |
| DGKK | 18.9683319 | 1911.221144 | 0.0099 | 0.9921 | 1.494764756 | count | 1 |
| HOXC5 | 19.6451199 | 1038.698928 | 0.0189 | 0.985 | 1.494764763 | count | 1 |
| EBF4 | 18.83633 | 1337.066522 | 0.0141 | 0.989 | 1.494764847 | count | 1 |
| AC007405.3 | 18.837984 | 1402.741004 | 0.0134 | 0.989 | 1.494764847 | count | 1 |
| CYTL1 | 18.837984 | 1402.74098 | 0.0134 | 0.989 | 1.494764847 | count | 1 |
| TTC38 | 1.0970822 | 0.1981405 | 5.5369 | 3.31E-08 | 1.499234376 | count | 0.000799828 |
| ST3GAL4 | 1.179373 | 0.2917726 | 4.0421 | 5.42E-05 | 1.506803006 | count | 1 |
| OSBPL5 | 1.3358657 | 0.4099702 | 3.2584 | 0.00113 | 1.509585741 | count | 1 |
| ERBB2 | 1.883419 | 0.7876974 | 2.391 | 0.0169 | 1.516886702 | count | 1 |
| AL031283.1 | 2.232107 | 1.47104 | 1.5174 | 0.129 | 1.528356714 | count | 1 |
| KLRD1 | 1.0733473 | 0.0967948 | 11.0889 | 4.29E-28 | 1.533855066 | count | 1.04E-23 |
| FCRL6 | 1.1421269 | 0.1707241 | 6.6899 | 2.60E-11 | 1.541152197 | count | 6.30E-07 |
| PRF1 | 1.1001882 | 0.0894405 | 12.3008 | 4.71E-34 | 1.576294711 | count | 1.14E-29 |

| | | | | | | | |
|---|---|---|---|---|---|---|---|
| LYN | 1.3106391 | 0.351139 | 3.7325 | 0.000193 | 1.57933328 | count | 1 |
| KIR3DL1 | 1.503443 | 0.6422698 | 2.3408 | 0.0193 | 1.591314278 | count | 1 |
| PLVAP | 1.609165 | 0.9016146 | 1.7848 | 0.0744 | 1.607036408 | count | 1 |
| SLC1A7 | 1.8539873 | 0.6734949 | 2.7528 | 0.00594 | 1.632204181 | count | 1 |
| AL158071.3 | 1.638847 | 0.580865 | 2.8214 | 0.00481 | 1.634224318 | count | 1 |
| MMP23B | 1.3692081 | 0.3416258 | 4.0079 | 6.26E-05 | 1.639086278 | count | 1 |
| CHST2 | 1.462328 | 0.5286858 | 2.766 | 0.00571 | 1.67651355 | count | 1 |
| FEZ1 | 1.382487 | 0.3693032 | 3.7435 | 0.000184 | 1.708590843 | count | 1 |
| LINC02384 | 1.3447797 | 0.3069024 | 4.3818 | 1.21E-05 | 1.718611851 | count | 0.2907146 |
| SIGLEC7 | 1.977722 | 0.5319454 | 3.7179 | 0.000204 | 1.723855703 | count | 1 |
| AC103591.3 | 1.6347643 | 0.3230112 | 5.061 | 4.39E-07 | 1.74573162 | count | 0.010589119 |
| TRBV13 | 19.196157 | 1403.873085 | 0.0137 | 0.989 | 1.775783921 | count | 1 |
| MIR181A2HG | 18.4497844 | 1205.685575 | 0.0153 | 0.9878 | 1.775784822 | count | 1 |
| SIGLEC9 | 18.5597617 | 1053.002042 | 0.0176 | 0.9859 | 1.775784826 | count | 1 |
| PRDM12 | 19.1979105 | 1955.020676 | 0.0098 | 0.9922 | 1.77578484 | count | 1 |
| KIR2DL1 | 2.8997968 | 2.5535101 | 1.1356 | 0.2562 | 1.810307148 | count | 1 |
| GNLY | 1.267818 | 0.1139948 | 11.1217 | 3.01E-28 | 1.827522932 | count | 7.32E-24 |
| TRDC | 1.3026982 | 0.2348066 | 5.548 | 3.11E-08 | 1.82789735 | count | 0.000751532 |
| LCNL1 | 3.0637126 | 2.7193279 | 1.1266 | 0.26 | 1.862764456 | count | 1 |
| C11orf21 | 1.5179689 | 0.2806105 | 5.4095 | 6.76E-08 | 1.902567484 | count | 0.001632743 |
| AC245014.3 | 1.6595195 | 0.266443 | 6.2284 | 5.29E-10 | 1.932861057 | count | 1.28E-05 |
| ZNF625 | 2.432335 | 0.7745199 | 3.1404 | 0.0017 | 1.941612526 | count | 1 |
| PRSS23 | 1.4543552 | 0.2331641 | 6.2375 | 4.99E-10 | 1.975182919 | count | 1.21E-05 |
| NMUR1 | 1.6618225 | 0.4720128 | 3.5207 | 0.000436 | 1.983198348 | count | 1 |
| ENPP5 | 2.784221 | 0.8218658 | 3.3877 | 0.000713 | 2.018369046 | count | 1 |
| S100A8 | 1.7233916 | 0.6347863 | 2.7149 | 0.00666 | 2.062763157 | count | 1 |
| CD160 | 1.542867 | 0.2667865 | 5.7832 | 7.99E-09 | 2.084201433 | count | 0.000193214 |
| ASCL2 | 1.60183 | 0.253876 | 6.3095 | 3.16E-10 | 2.152857546 | count | 7.65E-06 |
| CX3CR1 | 1.609111 | 0.2619409 | 6.143 | 9.03E-10 | 2.200019821 | count | 2.19E-05 |
| FGR | 1.8498691 | 0.3273945 | 5.6503 | 1.73E-08 | 2.213703141 | count | 0.00041821 |
| SPON2 | 1.562978 | 0.1589106 | 9.8356 | 1.58E-22 | 2.218499595 | count | 3.84E-18 |
| GOLGA8M | 2.335426 | 0.5494292 | 4.2506 | 2.19E-05 | 2.253268356 | count | 0.5256876 |
| CES1 | 2.02294 | 0.4295727 | 4.7092 | 2.59E-06 | 2.40050531 | count | 0.06235943 |
| ADGRG1 | 1.8017191 | 0.2225562 | 8.0956 | 7.86E-16 | 2.402900946 | count | 1.91E-11 |
| CXCR2 | 2.440979 | 0.5780867 | 4.2225 | 2.48E-05 | 2.417078954 | count | 0.5951008 |
| SH2D1B | 3.121451 | 0.8803115 | 3.5458 | 0.000397 | 2.447540982 | count | 1 |
| FCGR3A | 1.7422479 | 0.1691575 | 10.2996 | 1.62E-24 | 2.488157672 | count | 3.94E-20 |
| PRSS57 | 19.751448 | 991.3638884 | 0.0199 | 0.984 | 2.548062818 | count | 1 |
| B3GALNT1 | 19.755532 | 1465.78083 | 0.0135 | 0.989 | 2.548062818 | count | 1 |
| KLRF1 | 1.8581154 | 0.2757154 | 6.7393 | 1.86E-11 | 2.549812538 | count | 4.51E-07 |
| FGFBP2 | 1.8104716 | 0.1477384 | 12.2546 | 8.14E-34 | 2.597979266 | count | 1.98E-29 |
| NME8 | 3.312978 | 1.1902349 | 2.7835 | 0.00541 | 2.821213448 | count | 1 |
| MYOM2 | 2.375792 | 0.7430392 | 3.1974 | 0.0014 | 2.838474552 | count | 1 |
| PTGDS | 2.132601 | 0.3990947 | 5.3436 | 9.71E-08 | 3.016428631 | count | 0.002344382 |
