# Supplementary material for: Decoding the transcriptome of calcified atherosclerotic plaque at single-cell resolution
Source: Commun Biol. 2022 Oct 12;5:1084. doi: 10.1038/s42003-022-04056-7 (PMC9556750; doi:10.1038/s42003-022-04056-7)
Supplement: Supplementary file 5 — Supplementary Data 3 [file 42003_2022_4056_MOESM5_ESM.pdf]

Full differential gene expression results for B-cells.

gene_short_name	estimate	std_err	test_val	p_value	normalized_effect	model_component	q_value
FTCDNL1	-17.8333156	1605.870665	-0.0111	0.9911	-5.988683106	count	1
DHX58	-18.2761568	1690.986029	-0.0108	0.991	-5.26678585	count	1
P2RX7	-18.0277693	2443.263452	-0.0074	0.994	-5.266785673	count	1
NAT1	-17.9025282	2325.090025	-0.0077	0.994	-5.266785566	count	1
EGFL7	-17.9025282	2325.090025	-0.0077	0.994	-5.266785566	count	1
AL022328.3	-17.9025282	2325.090025	-0.0077	0.994	-5.266785566	count	1
SAMD11	-17.386259	1284.202387	-0.0135	0.989	-5.266784953	count	1
TGFB2	-17.386259	1284.202387	-0.0135	0.989	-5.266784953	count	1
FBXO36	-17.386259	1284.202387	-0.0135	0.989	-5.266784953	count	1
AC233280.1	-17.386259	1284.202387	-0.0135	0.989	-5.266784953	count	1
SGMS2	-17.386259	1284.202387	-0.0135	0.989	-5.266784953	count	1
P2RY6	-17.386259	1284.202387	-0.0135	0.989	-5.266784953	count	1
HIST2H2BF	-17.2924151	1668.857595	-0.0104	0.992	-5.266784803	count	1
PRELP	-18.262667	2757.882578	-0.0066	0.995	-4.700439797	count	1
PRICKLE3	-18.262667	2757.882578	-0.0066	0.995	-4.700439797	count	1
LINC02156	-18.262667	2757.882578	-0.0066	0.995	-4.700439797	count	1
LRRC45	-18.262667	2757.882578	-0.0066	0.995	-4.700439797	count	1
VPS11	-18.058693	1926.344679	-0.0094	0.993	-4.700439701	count	1
NPL	-18.001623	1746.859997	-0.0103	0.992	-4.700439671	count	1
DISC1	-18.001623	1746.859997	-0.0103	0.992	-4.700439671	count	1
SH3BP5-AS1	-18.001623	1746.859997	-0.0103	0.992	-4.700439671	count	1
APBB2	-18.001623	1746.859997	-0.0103	0.992	-4.700439671	count	1
CPVL	-18.001623	1746.859997	-0.0103	0.992	-4.700439671	count	1
OR2A1-AS1	-18.001623	1746.859997	-0.0103	0.992	-4.700439671	count	1
LRRC69	-18.001623	1746.859997	-0.0103	0.992	-4.700439671	count	1
ZFP41	-18.001623	1746.859997	-0.0103	0.992	-4.700439671	count	1
CLIC3	-18.001623	1746.859997	-0.0103	0.992	-4.700439671	count	1
KIF11	-18.001623	1746.859997	-0.0103	0.992	-4.700439671	count	1
AL138762.1	-18.001623	1746.859997	-0.0103	0.992	-4.700439671	count	1
NEURL1	-18.001623	1746.859997	-0.0103	0.992	-4.700439671	count	1
VWF	-18.001623	1746.859997	-0.0103	0.992	-4.700439671	count	1
NRG4	-18.001623	1746.859997	-0.0103	0.992	-4.700439671	count	1
CLEC10A	-18.001623	1746.859997	-0.0103	0.992	-4.700439671	count	1
AC005224.3	-18.001623	1746.859997	-0.0103	0.992	-4.700439671	count	1
HOXB6	-18.001623	1746.859997	-0.0103	0.992	-4.700439671	count	1
SERPINB2	-18.001623	1746.859997	-0.0103	0.992	-4.700439671	count	1
AC011445.2	-18.001623	1746.859997	-0.0103	0.992	-4.700439671	count	1
CASTOR1	-18.001623	1746.859997	-0.0103	0.992	-4.700439671	count	1
S100B	-18.001623	1746.859997	-0.0103	0.992	-4.700439671	count	1
LANCL1	-17.894585	1682.864199	-0.0106	0.992	-4.700439609	count	1
COL5A2	-17.63961	2049.801115	-0.0086	0.993	-4.700439431	count	1
PI4K2B	-17.63961	2049.801115	-0.0086	0.993	-4.700439431	count	1
RNF32	-17.63961	2049.801115	-0.0086	0.993	-4.700439431	count	1
GDPD3	-17.63961	2049.801115	-0.0086	0.993	-4.700439431	count	1

AC087741.1	-17.63961	2049.801115	-0.0086	0.993	-4.700439431	count	1
CMPK2	-18.5590123	2043.080761	-0.0091	0.9928	-4.70043935	count	1
TBC1D31	-18.2833331	2860.729905	-0.0064	0.9949	-4.70043925	count	1
TMOD1	-18.2814003	2148.755715	-0.0085	0.9932	-4.70043925	count	1
FN1	-18.1640251	1358.447403	-0.0134	0.9893	-4.700439198	count	1
DHFR2	-18.1418687	1461.240711	-0.0124	0.9901	-4.700439188	count	1
APOE	-18.1104181	1396.465871	-0.013	0.9897	-4.700439172	count	1
LYZ	-18.10338	1184.363123	-0.0153	0.9878	-4.700439169	count	1
HTRA1	-18.0431481	1494.405832	-0.0121	0.9904	-4.700439138	count	1
CCL18	-17.5878119	1969.741963	-0.0089	0.9929	-4.700438835	count	1
CD33	-17.4473243	2154.888831	-0.0081	0.9935	-4.70043871	count	1
RNF139-AS1	-17.3948502	1521.79768	-0.0114	0.9909	-4.700438658	count	1
PRRT3	-18.2738842	2144.591331	-0.0085	0.9932	-3.754887349	count	1
GPR146	-18.2186743	2750.472106	-0.0066	0.9947	-3.754887337	count	1
AC090825.1	-17.336925	1917.29881	-0.009	0.993	-3.754886357	count	1
MTTP	-17.318963	1241.710551	-0.0139	0.989	-3.754886347	count	1
RUNDC1	-2.8734139	1.0951173	-2.6238	0.009	-3.426000137	count	1
HMBOX1	-2.6871283	1.2202826	-2.2021	0.0282	-3.262398923	count	1
KIAA0355	-2.6212999	1.2652826	-2.0717	0.0389	-3.201776322	count	1
ANXA1	-2.1139002	0.4985101	-4.2404	2.72E-05	-2.68897783	count	0.660688
IFITM3	-2.0860466	0.781582	-2.669	0.0079	-2.658674146	count	1
U62317.5	-1.8671619	0.6296049	-2.9656	0.0032	-2.572361288	count	1
IFI6	-1.9876938	0.6708817	-2.9628	0.0032	-2.550049686	count	1
MGP	-1.8353281	0.3318812	-5.5301	5.47E-08	-2.503384726	count	0.00132921
GATAD2A	-1.9375648	0.7290879	-2.6575	0.0082	-2.493744039	count	1
TYROBP	-2.0052707	0.9120928	-2.1985	0.0284	-2.323925129	count	1
TMEM260	-1.7729541	1.0649275	-1.6649	0.0966	-2.304637757	count	1
GAA	-1.6906673	0.8690875	-1.9453	0.0524	-2.207824791	count	1
SOD3	-1.6613286	1.290804	-1.287	0.1987	-2.172960076	count	1
PPP1R14A	-1.747019	1.2616922	-1.3847	0.1669	-2.078941002	count	1
FGGY	-1.5612406	1.1450496	-1.3635	0.1734	-2.052710859	count	1
C16orf86	-1.4700702	0.8700208	-1.6897	0.0918	-1.941499071	count	1
MXD4	-1.4633942	0.4263345	-3.4325	7.00E-04	-1.933295577	count	1
ABCA1	-1.4577589	0.7807716	-1.8671	0.0625	-1.926364684	count	1
PSMD10	-1.3802838	0.6282796	-2.1969	0.0285	-1.880745391	count	1
CHST2	-1.400069	0.6071773	-2.3059	0.0216	-1.855089893	count	1
CERK	-1.3836018	0.6799389	-2.0349	0.0425	-1.834639549	count	1
SLK	-1.3113442	0.5820755	-2.2529	0.0248	-1.81474403	count	1
LGALS3BP	-1.4700702	1.024786	-1.4345	0.1521	-1.793517807	count	1
MAK16	-1.3468887	0.6920505	-1.9462	0.0523	-1.788882127	count	1
DDX58	-1.3365088	0.7100214	-1.8824	0.0604	-1.775904696	count	1
RAB12	-1.33312	1.0705198	-1.2453	0.2137	-1.771664062	count	1
TMEM223	-1.2871767	0.4889512	-2.6325	0.0088	-1.758846103	count	1
PRMT3	-1.2734357	1.3735814	-0.9271	0.3544	-1.740770073	count	1
TUBB6	-1.2968792	0.8513496	-1.5233	0.1284	-1.726197644	count	1

RALGAPA2	-1.2968792	0.9612849	-1.3491	0.178	-1.726197644	count	1
TRIP4	-1.2968792	1.0903614	-1.1894	0.2349	-1.726197644	count	1
MT1F	-1.3758621	0.7501703	-1.8341	0.0673	-1.691532028	count	1
CLCC1	-1.2683004	0.8473197	-1.4968	0.1351	-1.690196925	count	1
CEMIP2	-1.2140287	0.3217554	-3.7731	2.00E-04	-1.683696503	count	1
TMEM14A	-1.3681753	0.4822989	-2.8368	0.0048	-1.683107679	count	1
GUCY2C	-1.2264171	0.7483275	-1.6389	0.1019	-1.678758126	count	1
PRMT9	-1.2574231	0.99796	-1.26	0.2083	-1.676461442	count	1
ATP1B3	-1.1788568	0.347549	-3.3919	8.00E-04	-1.648615419	count	1
SLC31A2	-1.2272942	1.1923837	-1.0293	0.3039	-1.63832138	count	1
DMD	-1.2272942	1.3747601	-0.8927	0.3725	-1.63832138	count	1
GALNT12	-1.2272942	1.3747601	-0.8927	0.3725	-1.63832138	count	1
IPP	-1.3267663	0.7682194	-1.7271	0.0849	-1.637465131	count	1
CSAD	-1.2964341	0.5519505	-2.3488	0.0193	-1.603757201	count	1
SERGEF	-1.1932867	0.7880955	-1.5141	0.131	-1.595108164	count	1
HMGCL	-1.1732981	0.7647859	-1.5342	0.126	-1.569629706	count	1
IRF9	-1.170955	0.3514647	-3.3316	9.00E-04	-1.566639304	count	1
ZSCAN16-AS1	-1.2597277	0.5162497	-2.4402	0.0151	-1.562661435	count	1
ENTPD3-AS1	-1.1446724	0.6103432	-1.8755	0.0614	-1.533042298	count	1
LYPD6B	-1.1442318	1.1486435	-0.9962	0.3197	-1.532478244	count	1
CLDND2	-1.1442318	1.3484994	-0.8485	0.3966	-1.532478244	count	1
GMPR2	-1.2308211	0.4374803	-2.8134	0.0051	-1.530067982	count	1
GLIPR2	-1.141315	0.5610347	-2.0343	0.0425	-1.528743513	count	1
SCLT1	-1.1357351	0.5120376	-2.2181	0.0271	-1.521595574	count	1
PI4KB	-1.1312421	0.802989	-1.4088	0.1596	-1.515836817	count	1
SPP1	-1.0553338	0.6224512	-1.6954	0.0907	-1.50231505	count	1
TIMP1	-1.058647	0.3198014	-3.3103	0.001	-1.499530434	count	1
TBC1D10B	-1.1005777	0.7723885	-1.4249	0.1549	-1.476459429	count	1
TULP4	-1.0438751	0.4149138	-2.5159	0.0122	-1.469997708	count	1
PDIA4	-1.1737375	0.4494655	-2.6114	0.0093	-1.465122264	count	1
IMPA2	-1.0882405	0.8152435	-1.3349	0.1826	-1.46058064	count	1
ZNF765	-1.0882405	0.8152435	-1.3349	0.1826	-1.46058064	count	1
C1orf162	-1.0789337	0.4977246	-2.1677	0.0307	-1.448588665	count	1
SLC35A5	-1.1584589	0.665482	-1.7408	0.0824	-1.447611039	count	1
PIN4	-1.0767396	0.468657	-2.2975	0.0221	-1.445759851	count	1
SLC16A7	-1.0443267	0.512145	-2.0391	0.042	-1.436423388	count	1
ANKMY2	-1.068467	0.85637	-1.2477	0.2128	-1.435088381	count	1
RASGRP1	-1.068467	0.8902779	-1.2001	0.2307	-1.435088381	count	1
COQ7	-1.0648218	0.5953871	-1.7885	0.0744	-1.430383292	count	1
USP7	-1.0395625	0.3397615	-3.0597	0.0024	-1.430039527	count	1
SDHAF3	-1.0636014	0.4643372	-2.2906	0.0225	-1.428807648	count	1
CCL4	-1.0539055	0.382155	-2.7578	0.0061	-1.416282496	count	1
PEAK1	-1.0482853	0.5800942	-1.8071	0.0714	-1.409016711	count	1
HERC4	-1.0386928	0.4185286	-2.4818	0.0134	-1.396606085	count	1
ZNF331	-0.9767252	0.2201851	-4.4359	1.16E-05	-1.38457405	count	0.2817988

UROS	-1.0235216	0.3459482	-2.9586	0.0033	-1.376953709	count	1
AC114760.2	-1.0969208	0.5361747	-2.0458	0.0414	-1.376547045	count	1
ZFX	-0.980962	0.3881509	-2.5273	0.0118	-1.376143362	count	1
YAF2	-0.990942	0.4276429	-2.3172	0.0209	-1.364771703	count	1
SAMD9L	-0.9752646	0.4077598	-2.3918	0.0172	-1.358933681	count	1
FEZ2	-0.9629413	0.4350968	-2.2132	0.0274	-1.351217178	count	1
DSP	-1.0027785	1.5144913	-0.6621	0.5082	-1.350036394	count	1
APLP2	-0.9614299	0.5378782	-1.7874	0.0745	-1.32505257	count	1
HERC5	-0.9411734	0.4588068	-2.0514	0.0408	-1.321084301	count	1
TRAK2	-1.0443156	0.7025883	-1.4864	0.138	-1.315139522	count	1
DNAJC30	-0.9735666	0.7842338	-1.2414	0.2151	-1.312038642	count	1
UNC50	-0.9622151	0.3655408	-2.6323	0.0088	-1.297244849	count	1
IGFBP7	-0.9551333	0.3142575	-3.0393	0.0025	-1.288007638	count	1
PEG10	-1.0177801	0.6190761	-1.644	0.101	-1.283940335	count	1
TOR1A	-0.9161925	0.5415043	-1.6919	0.0914	-1.277955881	count	1
KAT5	-0.9227658	0.5137385	-1.7962	0.0731	-1.272903238	count	1
NIPSNAP3A	-0.9418564	0.5335842	-1.7652	0.0782	-1.270673621	count	1
PHF12	-0.940365	0.4989298	-1.8848	0.0601	-1.26872517	count	1
MCRIP2	-1.0016762	0.419862	-2.3857	0.0175	-1.264934553	count	1
FZD3	-0.9362825	0.8917013	-1.05	0.2943	-1.263390224	count	1
TAPBPL	-0.9123246	0.459596	-1.9851	0.0478	-1.258798926	count	1
CRKL	-0.9305717	0.6975879	-1.334	0.1829	-1.25592413	count	1
PRDX4	-0.9266153	0.3860873	-2.4	0.0168	-1.250749433	count	1
GPR18	-0.9219378	0.3562457	-2.5879	0.01	-1.244629229	count	1
GAPT	-0.9138846	0.2635755	-3.4673	6.00E-04	-1.234086164	count	1
TCHP	-0.9123173	0.5241904	-1.7404	0.0825	-1.232033414	count	1
ISG15	-0.8640059	0.3561801	-2.4258	0.0157	-1.228254498	count	1
EXOC6	-0.8748012	0.3798089	-2.3033	0.0217	-1.221081904	count	1
LY6E	-0.8673927	0.2422283	-3.5809	4.00E-04	-1.210890991	count	1
SLC10A3	-0.9518688	0.5629188	-1.691	0.0915	-1.205817026	count	1
SAP25	-0.9509791	0.8925559	-1.0655	0.2872	-1.204756485	count	1
AGPS	-0.8895664	0.8808154	-1.0099	0.3131	-1.202203993	count	1
ZFYVE21	-0.8895664	0.9753899	-0.912	0.3623	-1.202203993	count	1
SPIN2B	-0.8883147	0.6539069	-1.3585	0.175	-1.200561135	count	1
ZNF286A	-0.8659553	0.8514606	-1.017	0.3097	-1.196054784	count	1
PRAF2	-0.9418564	0.6717868	-1.402	0.1616	-1.193873024	count	1
DHX16	-0.859958	0.5079692	-1.6929	0.0912	-1.187927091	count	1
STARD10	-0.8486817	0.4048849	-2.0961	0.0366	-1.185137842	count	1
LGALS9	-0.8514753	0.3757552	-2.266	0.0239	-1.176426317	count	1
C16orf58	-0.8633367	0.7643696	-1.1295	0.259	-1.16774067	count	1
UACA	-0.8337136	1.1474627	-0.7266	0.4679	-1.152327111	count	1
ZBED5-AS1	-0.8486817	0.498661	-1.7019	0.0895	-1.148452108	count	1
TXNIP	-0.795638	0.1079407	-7.3711	8.34E-13	-1.140849098	count	2.03E-08
SSX2IP	-0.8243082	1.2502851	-0.6593	0.51	-1.139556016	count	1
GRK3	-0.8223891	0.3682385	-2.2333	0.026	-1.136949348	count	1

CD72	-0.8935173	0.3844197	-2.3243	0.0206	-1.135932553	count	1
DLGAP4	-0.8110208	0.3753352	-2.1608	0.0312	-1.133239547	count	1
DIDO1	-0.8276379	0.4953473	-1.6708	0.0955	-1.120713755	count	1
C1RL-AS1	-0.8058895	0.7547263	-1.0678	0.2862	-1.114526898	count	1
STARD7	-0.8704689	0.4186064	-2.0794	0.0381	-1.108148394	count	1
CCNT1	-0.7899156	0.5003912	-1.5786	0.1151	-1.104119691	count	1
BANP	-0.815045	0.5739346	-1.4201	0.156	-1.104091958	count	1
HCK	-0.8105259	0.5173355	-1.5667	0.118	-1.098122924	count	1
C6orf226	-0.7845024	0.5791598	-1.3546	0.176	-1.085432395	count	1
TIMM21	-0.7935007	0.7851851	-1.0106	0.313	-1.075615964	count	1
AZI2	-0.7751068	0.5043602	-1.5368	0.1251	-1.072640262	count	1
PRKD2	-0.8394244	0.4927842	-1.7034	0.0892	-1.070568119	count	1
IGHG3	-0.7603083	0.2638667	-2.8814	0.0042	-1.069786079	count	1
ZNF667-AS1	-0.7884936	0.7116052	-1.108	0.2684	-1.068990913	count	1
SH3GL1	-0.8313624	0.5512827	-1.5081	0.132	-1.060779787	count	1
PEF1	-0.829792	0.4670289	-1.7767	0.0763	-1.058871726	count	1
PCSK7	-0.7558298	0.3263233	-2.3162	0.021	-1.057036875	count	1
LIPT1	-0.7527002	1.0484552	-0.7179	0.4732	-1.052710729	count	1
CKS2	-0.7493348	0.2224667	-3.3683	8.00E-04	-1.048058035	count	1
NAGPA	-0.7698156	0.6348839	-1.2125	0.226	-1.044254638	count	1
TTC37	-0.7689959	0.3372855	-2.28	0.0231	-1.043168251	count	1
ZCCHC9	-0.7667144	0.4958756	-1.5462	0.123	-1.040144098	count	1
DHRS4L2	-0.7425241	0.3957323	-1.8763	0.0613	-1.038640298	count	1
VPS37B	-0.8076465	0.3239389	-2.4932	0.013	-1.0319172	count	1
HES1	-0.8056597	0.6106701	-1.3193	0.188	-1.029494643	count	1
BCL2L13	-0.7967059	0.6410566	-1.2428	0.215	-1.018568318	count	1
IRF2BPL	-0.7498729	0.447007	-1.6775	0.0941	-1.017804211	count	1
C16orf91	-0.7208914	0.3486529	-2.0676	0.0393	-1.014815597	count	1
USP44	-0.7452599	1.121158	-0.6647	0.5066	-1.011680193	count	1
TOMM34	-0.7294301	0.6995111	-1.0428	0.298	-1.010361481	count	1
KCTD20	-0.7210542	0.5122761	-1.4075	0.16	-1.008935708	count	1
NCKAP1L	-0.7417861	0.3622281	-2.0478	0.0412	-1.00706713	count	1
ABHD18	-0.7414453	0.7369423	-1.0061	0.3149	-1.006614494	count	1
FXYD1	-0.7414453	0.7369423	-1.0061	0.3149	-1.006614494	count	1
GCDH	-0.7262159	0.6293675	-1.1539	0.2492	-1.005973507	count	1
RPS6KA4	-0.7224804	0.7762377	-0.9307	0.3525	-1.000872967	count	1
MRPL36	-0.7366663	0.3656573	-2.0146	0.0445	-1.000266055	count	1
TRIP10	-0.7199095	0.8210446	-0.8768	0.3811	-0.997362043	count	1
MIEN1	-0.7192529	0.3692944	-1.9476	0.0521	-0.99646529	count	1
TRMU	-0.7184013	0.7249891	-0.9909	0.3223	-0.995302173	count	1
FMR1	-0.7729349	0.3578489	-2.1599	0.0313	-0.98949181	count	1
NDUFA8	-0.7028202	0.3389598	-2.0735	0.0387	-0.983688963	count	1
PPIF	-0.6983978	0.5154339	-1.355	0.176	-0.983416078	count	1
FBXO6	-0.7623362	0.6649934	-1.1464	0.2523	-0.976495713	count	1
TXN2	-0.7124905	0.3216584	-2.2151	0.0273	-0.968116416	count	1

SLC25A29	-0.7101599	0.6619581	-1.0728	0.2839	-0.965014109	count	1
HERPUD1	-0.6704852	0.1189209	-5.6381	3.06E-08	-0.963966485	count	0.000743611
ANKRD49	-0.7092855	0.4032414	-1.759	0.0793	-0.963850046	count	1
LAMP2	-0.7061914	0.422252	-1.6724	0.0951	-0.959730356	count	1
DDX60	-0.7483984	0.6861333	-1.0907	0.276	-0.959375711	count	1
ROM1	-0.6911991	1.2312941	-0.5614	0.5748	-0.958123444	count	1
ECHDC2	-0.689519	0.4295098	-1.6054	0.1091	-0.955825529	count	1
RAB31	-0.7009049	0.2785297	-2.5164	0.0122	-0.952689439	count	1
C12orf65	-0.671016	0.2806167	-2.3912	0.0172	-0.951593811	count	1
CKLF	-0.6848914	0.2539101	-2.6974	0.0072	-0.949495273	count	1
CST3	-0.7375709	0.558969	-1.3195	0.188	-0.946053189	count	1
HMOX1	-0.6741455	0.4983789	-1.3527	0.1768	-0.934790114	count	1
PHKG2	-0.6741455	0.5748853	-1.1727	0.2416	-0.934790114	count	1
SFSWAP	-0.6863885	0.3583489	-1.9154	0.0561	-0.93334179	count	1
BMPR2	-0.7269214	0.6062616	-1.199	0.2312	-0.932930297	count	1
NFX1	-0.7232764	0.4064008	-1.7797	0.0758	-0.928434338	count	1
DPEP2	-0.7219987	0.651501	-1.1082	0.2684	-0.926857821	count	1
COX11	-0.654546	0.4640395	-1.4105	0.1591	-0.916766042	count	1
TXNL1	-0.6721107	0.2363901	-2.8432	0.0047	-0.91429272	count	1
CSTF1	-0.7105977	0.7092914	-1.0018	0.317	-0.912778397	count	1
G6PD	-0.6564632	0.6703541	-0.9793	0.328	-0.910576436	count	1
DICER1	-0.6685948	0.4423605	-1.5114	0.131	-0.909598977	count	1
SLC35E1	-0.7057559	0.4896866	-1.4412	0.1502	-0.906792568	count	1
SRM	-0.6518074	0.2295114	-2.84	0.0047	-0.904197539	count	1
NCBP2	-0.6488262	0.347767	-1.8657	0.0627	-0.900112273	count	1
LEPROTL1	-0.6607055	0.2999404	-2.2028	0.0281	-0.899062537	count	1
CTSC	-0.6471514	0.3068741	-2.1088	0.0355	-0.897816974	count	1
RAB5IF	-0.6330696	0.2959063	-2.1394	0.0329	-0.895569627	count	1
NMRAL1	-0.6448693	0.4900682	-1.3159	0.189	-0.894689089	count	1
IER5L	-0.6571273	0.6739819	-0.975	0.33	-0.894281826	count	1
TMEM189	-0.6567074	0.8074575	-0.8133	0.4165	-0.893720733	count	1
SLC2A3	-0.62283	0.1554781	-4.0059	7.24E-05	-0.891952322	count	1
RAP2B	-0.652698	0.3905216	-1.6713	0.0954	-0.888362361	count	1
MESD	-0.6519122	0.3008984	-2.1666	0.0308	-0.887312006	count	1
FAM98C	-0.6512453	0.430782	-1.5118	0.1313	-0.886420534	count	1
CISD3	-0.6711796	0.5530121	-1.2137	0.2255	-0.863934796	count	1
MYSM1	-0.6340782	0.3386029	-1.8726	0.0618	-0.863458674	count	1
FAM30A	-0.6042366	0.197291	-3.0627	0.0023	-0.861619266	count	1
EIF3D	-0.6317003	0.1702006	-3.7115	2.00E-04	-0.860276013	count	1
KMT2B	-0.6189202	0.314684	-1.9668	0.0498	-0.859099622	count	1
C14orf28	-0.6099282	0.6492855	-0.9394	0.348	-0.854809235	count	1
C4orf48	-0.6025018	0.2723824	-2.212	0.0275	-0.849318785	count	1
CXXC5	-0.6051177	0.2388127	-2.5339	0.0116	-0.848123609	count	1
MRPL28	-0.598641	0.4000048	-1.4966	0.1352	-0.843912401	count	1
THOC6	-0.6185776	0.4551469	-1.3591	0.1748	-0.842703039	count	1

BIN2	-0.6514012	0.3570916	-1.8242	0.0688	-0.83933241	count	1
SNRNP200	-0.651034	0.3008404	-2.1641	0.031	-0.838875058	count	1
BCL6	-0.6148585	0.529805	-1.1605	0.246	-0.837719905	count	1
TRAF3IP2-AS1	-0.61421	0.7526219	-0.8161	0.4149	-0.836850868	count	1
EAF1	-0.6015553	0.8133034	-0.7396	0.4599	-0.835260041	count	1
CCR6	-0.6112645	0.2895117	-2.1114	0.0353	-0.832903226	count	1
ZNF83	-0.5986711	0.500524	-1.1961	0.232	-0.831298643	count	1
MIR22HG	-0.6089266	0.5755873	-1.0579	0.2907	-0.82976936	count	1
HSD17B10	-0.5885376	0.3112891	-1.8906	0.0593	-0.829761662	count	1
MARF1	-0.6086578	0.3701559	-1.6443	0.1008	-0.829409014	count	1
GLRX	-0.6431245	0.3071763	-2.0937	0.0369	-0.829018566	count	1
MAPKAP1	-0.6049705	0.3768965	-1.6051	0.109	-0.824465267	count	1
RFK	-0.6039898	0.3706037	-1.6297	0.104	-0.823150192	count	1
ZNF721	-0.5915427	0.3350354	-1.7656	0.0781	-0.821505745	count	1
ACAA2	-0.5773689	0.4669443	-1.2365	0.2169	-0.817195473	count	1
CSRP1	-0.6291916	0.4853518	-1.2964	0.1955	-0.811632088	count	1
BHLHE40	-0.6291916	0.5724593	-1.0991	0.272	-0.811632088	count	1
GLOD4	-0.5827276	0.2942971	-1.9801	0.0483	-0.809391433	count	1
USP20	-0.5936311	1.2446974	-0.4769	0.6337	-0.809254521	count	1
IGFBP6	-0.5936311	1.2446974	-0.4769	0.6337	-0.809254521	count	1
EIF3A	-0.5920129	0.1747905	-3.387	8.00E-04	-0.807082945	count	1
CDK4	-0.5790761	0.3585541	-1.615	0.107	-0.804371923	count	1
IGLL5	-0.5732339	0.5275599	-1.0866	0.278	-0.803784234	count	1
FIGNL1	-0.5700496	0.5582175	-1.0212	0.3077	-0.799353398	count	1
ZFAND2B	-0.5656662	0.3621712	-1.5619	0.119	-0.797714035	count	1
VPS72	-0.5815589	0.4435943	-1.311	0.191	-0.793048556	count	1
MAPK6	-0.5700849	0.4545249	-1.2542	0.2104	-0.792008844	count	1
SNX29	-0.5639337	0.3421213	-1.6483	0.1	-0.790842053	count	1
PSMC6	-0.5607367	0.2676797	-2.0948	0.0368	-0.790804237	count	1
AC118549.1	-0.60991	0.5327725	-1.1448	0.2529	-0.787521402	count	1
SLC30A4	-0.5728111	1.0434252	-0.549	0.5833	-0.781297522	count	1
ATG2B	-0.5728111	1.1598762	-0.4939	0.6217	-0.781297522	count	1
C1orf123	-0.5507947	0.37877	-1.4542	0.1466	-0.779769434	count	1
ASAP1	-0.5709266	0.4183308	-1.3648	0.173	-0.778765196	count	1
MX2	-0.560303	0.3830487	-1.4627	0.144	-0.778553103	count	1
ANKS1A	-0.5502246	0.9321096	-0.5903	0.555	-0.776066213	count	1
POLR3GL	-0.5499522	0.2326986	-2.3634	0.0185	-0.771378018	count	1
TNK2	-0.5625605	0.6953045	-0.8091	0.419	-0.767519487	count	1
DBF4	-0.5931834	0.3207874	-1.8491	0.0651	-0.766559505	count	1
TMEM9	-0.561454	0.5182861	-1.0833	0.2793	-0.766031689	count	1
FAM122B	-0.5506789	0.7579359	-0.7266	0.4679	-0.765308949	count	1
MYL6B	-0.5603736	0.4599194	-1.2184	0.2237	-0.764578887	count	1
LYRM7	-0.5603736	0.4908296	-1.1417	0.2542	-0.764578887	count	1
N4BP2	-0.5599007	0.6672892	-0.8391	0.4019	-0.763942952	count	1
UBQLN2	-0.5587726	0.4259713	-1.3118	0.1903	-0.762425854	count	1

PRPS2	-0.557263	0.4553233	-1.2239	0.2216	-0.760395542	count	1
GPN3	-0.5866532	0.3800734	-1.5435	0.1234	-0.758364321	count	1
CHAMP1	-0.58631	0.7414571	-0.7908	0.4295	-0.757933441	count	1
TRAPPC4	-0.5525614	0.2552508	-2.1648	0.0309	-0.754070983	count	1
OSGEPL1	-0.5790761	0.7221602	-0.8019	0.4231	-0.748847294	count	1
TMED3	-0.548461	0.3656544	-1.4999	0.1343	-0.748553649	count	1
FH	-0.5483135	0.4073984	-1.3459	0.179	-0.748355155	count	1
CCND3	-0.5345595	0.2162078	-2.4724	0.0138	-0.743114299	count	1
TMEM33	-0.5335668	0.4989382	-1.0694	0.2855	-0.741746971	count	1
PAWR	-0.5428755	0.3217927	-1.687	0.0923	-0.741035782	count	1
CDK12	-0.5424875	0.2496634	-2.1729	0.0303	-0.740513453	count	1
LONP2	-0.5321352	0.3682494	-1.445	0.149	-0.739775013	count	1
H2AFJ	-0.5718368	0.3853349	-1.484	0.1385	-0.739746557	count	1
PPIB	-0.5158329	0.1276792	-4.0401	6.30E-05	-0.739297746	count	1
ANKRD10	-0.5395125	0.3427729	-1.574	0.1162	-0.73650807	count	1
SNHG9	-0.5390304	0.4467214	-1.2066	0.2282	-0.735858926	count	1
HVCN1	-0.5288596	0.1934043	-2.7345	0.0065	-0.735262594	count	1
EFR3A	-0.5198765	0.3117926	-1.6674	0.0961	-0.733495722	count	1
WDR45	-0.5145348	0.4015024	-1.2815	0.201	-0.730586239	count	1
USP15	-0.5103621	0.1969307	-2.5916	0.0099	-0.727232151	count	1
LINC01857	-0.5097465	0.2486407	-2.0501	0.0409	-0.723813131	count	1
SMS	-0.5127654	0.3044807	-1.6841	0.0929	-0.723515991	count	1
AHI1	-0.511666	0.4025631	-1.271	0.2044	-0.721972934	count	1
CASP4	-0.5099546	0.2023433	-2.5202	0.0121	-0.719570824	count	1
DERA	-0.5264673	0.4399424	-1.1967	0.2321	-0.718936133	count	1
DECR1	-0.5119864	0.236532	-2.1646	0.031	-0.718481175	count	1
AC004687.1	-0.5162719	0.2750144	-1.8773	0.0611	-0.717916306	count	1
WAPL	-0.5538494	0.285892	-1.9373	0.0533	-0.717100531	count	1
SIPA1	-0.5531916	0.2738151	-2.0203	0.044	-0.716271466	count	1
PCED1B	-0.5240647	0.4316146	-1.2142	0.2253	-0.715698308	count	1
GIGYF1	-0.5225216	0.3863114	-1.3526	0.1769	-0.713618528	count	1
TAF12	-0.5043365	0.3314402	-1.5217	0.129	-0.707815195	count	1
GYPC	-0.4963204	0.1886559	-2.6308	0.0088	-0.707280981	count	1
FGD6	-0.508157	0.6846008	-0.7423	0.4583	-0.706728972	count	1
SYMPK	-0.508157	0.6912789	-0.7351	0.4627	-0.706728972	count	1
PIK3C3	-0.5074375	0.5881718	-0.8627	0.3887	-0.705736879	count	1
BAG1	-0.5165467	0.26846	-1.9241	0.055	-0.705563779	count	1
KIZ	-0.5071479	0.483323	-1.0493	0.295	-0.705337554	count	1
NKRF	-0.5423757	0.5565614	-0.9745	0.3303	-0.702630501	count	1
LY96	-0.5403801	0.2873055	-1.8809	0.0606	-0.7001118	count	1
SUGT1	-0.4942741	0.2675709	-1.8473	0.0654	-0.700097285	count	1
NPRL2	-0.5111672	0.5836032	-0.8759	0.3816	-0.698309228	count	1
RNF130	-0.536885	0.4169221	-1.2877	0.1985	-0.695699166	count	1
NELFE	-0.5085892	0.4310279	-1.1799	0.2387	-0.694831833	count	1
ETHE1	-0.4988538	0.2906456	-1.7164	0.0868	-0.693898921	count	1

F8A1	-0.5049917	0.3956377	-1.2764	0.2025	-0.689978375	count	1
GSTO1	-0.4894942	0.2096985	-2.3343	0.02	-0.687114027	count	1
AC104024.1	-0.4935146	0.4106348	-1.2018	0.23	-0.686533468	count	1
DDAH2	-0.4929733	0.1777145	-2.774	0.0058	-0.685786655	count	1
ABCE1	-0.4999632	0.3129128	-1.5978	0.1108	-0.683192599	count	1
CNR2	-0.4994867	0.5363015	-0.9314	0.352	-0.682549476	count	1
LPP	-0.525344	0.5734913	-0.916	0.36	-0.681115965	count	1
CHD8	-0.524528	0.4086549	-1.2835	0.2	-0.680084146	count	1
IGLC3	-0.4717888	0.2793661	-1.6888	0.092	-0.679286403	count	1
CRK	-0.523488	0.4485807	-1.167	0.2438	-0.678768945	count	1
TAF9	-0.4803736	0.3028786	-1.586	0.1134	-0.678035209	count	1
IFT80	-0.4951225	0.7237611	-0.6841	0.4943	-0.676658363	count	1
KBTBD2	-0.4939091	0.3565233	-1.3853	0.1666	-0.675020159	count	1
NQO2	-0.484556	0.5095664	-0.9509	0.3422	-0.674171528	count	1
OSTF1	-0.4787944	0.1857016	-2.5783	0.0102	-0.672184842	count	1
ZNF277	-0.5161125	0.3523044	-1.465	0.1436	-0.669437373	count	1
SLC25A38	-0.4867313	0.2998986	-1.623	0.105	-0.665327099	count	1
TBC1D10C	-0.4852724	0.2062183	-2.3532	0.019	-0.663356473	count	1
MHENCR	-0.4750097	0.5180564	-0.9169	0.3597	-0.660993822	count	1
ULK3	-0.4833257	0.6923199	-0.6981	0.485	-0.66072669	count	1
TMEM167A	-0.4740858	0.3467634	-1.3672	0.172	-0.659718212	count	1
DHX29	-0.4739632	0.3878727	-1.222	0.222	-0.659548935	count	1
NHP2	-0.4735402	0.2277496	-2.0792	0.0382	-0.65896489	count	1
HHEX	-0.4691061	0.3369046	-1.3924	0.1645	-0.658662888	count	1
INTS11	-0.4815335	0.3664194	-1.3142	0.1895	-0.658305361	count	1
NUDT22	-0.5067878	0.3749282	-1.3517	0.177	-0.657628656	count	1
NDUFV1	-0.5061891	0.366049	-1.3828	0.1674	-0.656870045	count	1
ATP10D	-0.4703371	0.4993168	-0.942	0.3467	-0.65454198	count	1
CDK13	-0.4635684	0.2383858	-1.9446	0.0525	-0.654425326	count	1
CNPY2	-0.4699755	0.2777403	-1.6921	0.0913	-0.65404264	count	1
PUDP	-0.4779654	0.9057349	-0.5277	0.598	-0.653483973	count	1
PABPC1L	-0.4779654	1.2721145	-0.3757	0.7073	-0.653483973	count	1
KIF5B	-0.4688565	0.2221783	-2.1103	0.0354	-0.65249735	count	1
ERCC1	-0.4688345	0.2454962	-1.9097	0.0568	-0.652466968	count	1
FAM120A	-0.5027102	0.4055701	-1.2395	0.2158	-0.65246098	count	1
POLL	-0.5019188	0.5499901	-0.9126	0.362	-0.65145774	count	1
TUBA1C	-0.4666774	0.2453732	-1.9019	0.0578	-0.649487913	count	1
RIPK1	-0.4742527	0.4424852	-1.0718	0.2844	-0.648466151	count	1
RNF169	-0.4590583	0.4131621	-1.1111	0.2671	-0.648087423	count	1
ASPH	-0.4588164	0.6593196	-0.6959	0.487	-0.647747469	count	1
FAM120AOS	-0.4540695	0.3241715	-1.4007	0.162	-0.643368227	count	1
PPP1CA	-0.4580448	0.1912557	-2.3949	0.017	-0.643219937	count	1
CREBZF	-0.4577538	0.2823013	-1.6215	0.106	-0.642813598	count	1
GUSB	-0.4698435	0.5232442	-0.8979	0.3697	-0.642505605	count	1
IKBKB	-0.4698435	0.5724438	-0.8208	0.412	-0.642505605	count	1

ZDHHC4	-0.4696697	0.5469887	-0.8586	0.391	-0.642270624	count	1
PSMB4	-0.4939636	0.2133327	-2.3155	0.021	-0.6413683	count	1
P4HTM	-0.4687766	0.4021099	-1.1658	0.2443	-0.641063102	count	1
UBQLN1	-0.4605556	0.3478638	-1.324	0.186	-0.641032079	count	1
NDUFA1	-0.4485423	0.1646192	-2.7247	0.0067	-0.640132773	count	1
UQCR11	-0.4492484	0.1628034	-2.7595	0.006	-0.634299568	count	1
DALRD3	-0.4871006	0.36505	-1.3343	0.1828	-0.632657027	count	1
S100A11	-0.439496	0.1815796	-2.4204	0.0159	-0.630035884	count	1
CYB5R2	-0.4846641	0.6811635	-0.7115	0.4771	-0.629562789	count	1
SELENOW	-0.4472568	0.2025399	-2.2082	0.0277	-0.628153768	count	1
CLIP1	-0.4456736	0.4033972	-1.1048	0.2698	-0.625942328	count	1
OSTC	-0.4809694	0.2435877	-1.9745	0.0489	-0.624869139	count	1
TRAF3IP3	-0.4391665	0.2287713	-1.9197	0.0555	-0.62391146	count	1
ARL2	-0.455832	0.2392964	-1.9049	0.0574	-0.62355437	count	1
ZNF337	-0.4779654	1.1247592	-0.4249	0.6711	-0.621051557	count	1
ADM	-0.4779654	1.2721145	-0.3757	0.7073	-0.621051557	count	1
ZBTB38	-0.4434927	0.3015474	-1.4707	0.142	-0.617453224	count	1
GRAP	-0.4738354	0.406658	-1.1652	0.245	-0.615801009	count	1
HM13	-0.4735188	0.418608	-1.1312	0.2586	-0.61539841	count	1
VDAC3	-0.4497726	0.3014699	-1.4919	0.1364	-0.615354115	count	1
PWWP2B	-0.4397446	0.5223658	-0.8418	0.4003	-0.612271765	count	1
RAB5A	-0.4461284	0.3157964	-1.4127	0.1584	-0.61042104	count	1
BTN3A2	-0.446095	0.3151676	-1.4154	0.1576	-0.610375825	count	1
GPR137	-0.4460752	0.5275042	-0.8456	0.3982	-0.610349019	count	1
AGA	-0.4460752	0.5790807	-0.7703	0.442	-0.610349019	count	1
SPICE1	-0.4460752	0.6083477	-0.7333	0.464	-0.610349019	count	1
EIF1AY	-0.4268973	0.1515108	-2.8176	0.005	-0.609899368	count	1
PICALM	-0.4369652	0.5177857	-0.8439	0.3992	-0.608428988	count	1
ORAI3	-0.4368031	0.4560637	-0.9578	0.339	-0.608204857	count	1
SF3B5	-0.4293968	0.1982984	-2.1654	0.0309	-0.606388512	count	1
BRI3	-0.4354536	0.1984811	-2.1939	0.0288	-0.606338893	count	1
ABRACL	-0.434656	0.3243004	-1.3403	0.181	-0.605236001	count	1
FABP5	-0.4250907	0.2268432	-1.8739	0.0616	-0.605120942	count	1
PLEKHA2	-0.4635119	0.3766445	-1.2306	0.2191	-0.602666393	count	1
VPS35	-0.4263959	0.1950537	-2.186	0.0293	-0.602168191	count	1
STOML2	-0.4400211	0.2645124	-1.6635	0.0969	-0.602151492	count	1
TMSB4X	-0.4154141	0.0520792	-7.9766	1.29E-14	-0.598766433	count	3.14E-10
STMN1	-0.4366037	0.2810579	-1.5534	0.121	-0.597522969	count	1
NBEAL1	-0.4360444	0.2939195	-1.4836	0.1386	-0.596765371	count	1
EVI5	-0.4283836	0.467862	-0.9156	0.36	-0.596561616	count	1
ZFP36L2	-0.4238341	0.1260691	-3.3619	8.00E-04	-0.595426402	count	1
TMEM8A	-0.4578013	0.7141427	-0.6411	0.5218	-0.595394594	count	1
UMAD1	-0.42744	0.5906231	-0.7237	0.4696	-0.595256493	count	1
CHKA	-0.4574028	0.5966769	-0.7666	0.4437	-0.594886987	count	1
ADCY3	-0.4338478	0.83875	-0.5173	0.6052	-0.593789745	count	1

SETD7	-0.4338478	0.8839814	-0.4908	0.6238	-0.593789745	count	1
PARM1	-0.4338478	0.9617049	-0.4511	0.6521	-0.593789745	count	1
TRAPPC8	-0.4559	0.5406494	-0.8432	0.3995	-0.59297253	count	1
NCR3	-0.4211717	0.227546	-1.8509	0.0648	-0.591704996	count	1
SIDT1	-0.4320739	0.3486998	-1.2391	0.216	-0.591386471	count	1
VAMP2	-0.4243903	0.1494813	-2.8391	0.0047	-0.591038048	count	1
CALR	-0.4243063	0.1491767	-2.8443	0.0047	-0.590921849	count	1
DDIT4	-0.4304703	0.1597519	-2.6946	0.0073	-0.58921372	count	1
CCNG1	-0.4288592	0.4101572	-1.0456	0.2963	-0.587030613	count	1
IFI30	-0.4208404	0.2938105	-1.4324	0.153	-0.586127111	count	1
UNC93B1	-0.4270048	0.3665178	-1.165	0.245	-0.584517589	count	1
DDHD1	-0.4142855	0.3842129	-1.0783	0.281	-0.582078434	count	1
TBK1	-0.4473306	0.4060558	-1.1016	0.2712	-0.582049987	count	1
NOP16	-0.4136795	0.4144863	-0.9981	0.3188	-0.58123119	count	1
TUBA4A	-0.4135206	0.1573056	-2.6288	0.0089	-0.58100903	count	1
AP2S1	-0.4088209	0.2573036	-1.5889	0.1128	-0.580922836	count	1
DNAJB9	-0.4463967	0.2976578	-1.4997	0.1344	-0.580859045	count	1
ZNF581	-0.4127059	0.2454861	-1.6812	0.0934	-0.579869974	count	1
TERF1	-0.4445535	0.3680334	-1.2079	0.228	-0.578508198	count	1
HSPE1	-0.406024	0.1294443	-3.1367	0.0018	-0.578046035	count	1
PYCARD	-0.4138306	0.2194479	-1.8858	0.06	-0.576427863	count	1
VPS36	-0.4137923	0.259634	-1.5938	0.112	-0.57637486	count	1
TFDP2	-0.412857	0.4413896	-0.9354	0.3501	-0.575080524	count	1
DDX6	-0.4077193	0.1744427	-2.3373	0.0199	-0.572897508	count	1
CAPN2	-0.4182297	0.3313446	-1.2622	0.2075	-0.572622419	count	1
NDUFB8	-0.3979992	0.1768663	-2.2503	0.0249	-0.569525813	count	1
ZNF274	-0.4374855	0.4420943	-0.9896	0.3229	-0.569489379	count	1
ABHD15	-0.4339262	0.4971815	-0.8728	0.3833	-0.564945186	count	1
ITM2C	-0.41158	0.3229198	-1.2746	0.2031	-0.563604606	count	1
TUSC2	-0.4008246	0.3115399	-1.2866	0.199	-0.563255487	count	1
ECHDC1	-0.3984933	0.2708737	-1.4711	0.142	-0.562913746	count	1
METRNL	-0.4108675	0.2504926	-1.6402	0.102	-0.562638178	count	1
RALGAPA1	-0.4104753	0.3971127	-1.0336	0.302	-0.562106183	count	1
FAM120C	-0.410462	0.9618606	-0.4267	0.6698	-0.562088143	count	1
IDH3B	-0.4316325	0.3654231	-1.1812	0.238	-0.562015918	count	1
ASL	-0.4316176	0.8028032	-0.5376	0.5911	-0.561996888	count	1
NEMF	-0.4021758	0.274813	-1.4635	0.144	-0.560295981	count	1
RUBCNL	-0.4088778	0.3467406	-1.1792	0.239	-0.55993917	count	1
PSMB9	-0.3890768	0.122043	-3.188	0.00153	-0.559122583	count	1
ALDH9A1	-0.4078167	0.287495	-1.4185	0.1567	-0.558499683	count	1
NSD1	-0.4078098	0.2609715	-1.5627	0.119	-0.558490323	count	1
ANKS3	-0.4001388	0.7018042	-0.5702	0.5689	-0.557475789	count	1
MCRS1	-0.4065446	0.4398422	-0.9243	0.3558	-0.556773846	count	1
EIF2AK2	-0.3996318	0.2645819	-1.5104	0.132	-0.556773824	count	1
ORMDL2	-0.4269175	0.3176712	-1.3439	0.1797	-0.555992254	count	1

NDUFAB1	-0.4046586	0.2084627	-1.9412	0.0529	-0.554214928	count	1
DPM3	-0.4045003	0.4129263	-0.9796	0.328	-0.554000135	count	1
AKAP17A	-0.3969782	0.3151212	-1.2598	0.208	-0.553099581	count	1
SHKBP1	-0.4035039	0.2732377	-1.4768	0.1405	-0.552648106	count	1
L3MBTL2	-0.4032526	0.5621298	-0.7174	0.4735	-0.552307104	count	1
HNRNPH3	-0.3904208	0.2119667	-1.8419	0.0662	-0.551552535	count	1
THAP11	-0.4021016	0.3577617	-1.1239	0.2616	-0.550745187	count	1
AC025164.1	-0.3947217	0.2733577	-1.444	0.149	-0.5499749	count	1
JADE1	-0.4008246	0.4404437	-0.91	0.3633	-0.549012177	count	1
TUBE1	-0.4008246	0.5373481	-0.7459	0.456	-0.549012177	count	1
TM9SF2	-0.4209736	0.2812867	-1.4966	0.1352	-0.548394467	count	1
NDUFS2	-0.3928561	0.3867633	-1.0158	0.31	-0.547391324	count	1
NRM	-0.3922711	0.6119168	-0.6411	0.5218	-0.546581153	count	1
HUS1	-0.419389	0.4283923	-0.979	0.3281	-0.546368179	count	1
FCRL2	-0.3920469	0.2942113	-1.3325	0.183	-0.546270653	count	1
RBM33	-0.398175	0.3020353	-1.3183	0.188	-0.545416045	count	1
PSMC3	-0.418489	0.3126443	-1.3385	0.1814	-0.545217167	count	1
NFYC	-0.3972448	0.3904428	-1.0174	0.3095	-0.544153424	count	1
TMEM259	-0.3968459	0.2405034	-1.6501	0.0996	-0.543611953	count	1
RRAGA	-0.3892526	0.3074535	-1.2661	0.206	-0.542400533	count	1
ZBTB6	-0.3959448	0.5901261	-0.6709	0.503	-0.542388748	count	1
IGLC2	-0.3756561	0.1973376	-1.9036	0.0576	-0.541625182	count	1
SNHG15	-0.3803511	0.2736432	-1.39	0.1652	-0.540572601	count	1
NEDD8	-0.381145	0.160813	-2.3701	0.0182	-0.540358911	count	1
METTL23	-0.3876077	0.3242705	-1.1953	0.233	-0.54012216	count	1
PLEKHO1	-0.3840775	0.2025944	-1.8958	0.0586	-0.539827663	count	1
AGFG1	-0.4142629	0.3352079	-1.2358	0.2172	-0.539811004	count	1
ARHGAP9	-0.3935111	0.2946301	-1.3356	0.182	-0.539084811	count	1
AIM2	-0.3813548	0.2026306	-1.882	0.0605	-0.538790701	count	1
FCGRT	-0.3921606	0.3611062	-1.086	0.2781	-0.537251221	count	1
PTPN2	-0.3788353	0.2448407	-1.5473	0.123	-0.537094083	count	1
ATP6V0B	-0.3821138	0.146979	-2.5998	0.0096	-0.537079915	count	1
ARRB2	-0.3900313	0.542801	-0.7186	0.4728	-0.534359981	count	1
UBA6	-0.3829537	0.4137116	-0.9257	0.355	-0.533675122	count	1
IL16	-0.4084318	0.2130132	-1.9174	0.0558	-0.532347882	count	1
MAP1LC3B	-0.3750931	0.1614065	-2.3239	0.0206	-0.531804088	count	1
R3HDM1	-0.407859	0.3474835	-1.1738	0.241	-0.531614533	count	1
MRPS2	-0.3810684	0.5101691	-0.7469	0.4555	-0.531063174	count	1
RPL27A	-0.3696939	0.052047	-7.1031	4.88E-12	-0.530928381	count	1.19E-07
DENR	-0.3737271	0.3126047	-1.1955	0.2325	-0.529873014	count	1
FAM76A	-0.3746097	0.4153676	-0.9019	0.3676	-0.529294287	count	1
MDFIC	-0.3796167	0.3126068	-1.2144	0.225	-0.529051829	count	1
ATG4A	-0.3860749	0.6163307	-0.6264	0.5314	-0.528986987	count	1
SCAMP1	-0.3795673	0.5391693	-0.704	0.4818	-0.528983383	count	1
KIF3B	-0.4056683	0.6467605	-0.6272	0.531	-0.528809405	count	1

AGL	-0.4050647	0.4456877	-0.9089	0.3639	-0.528036402	count	1
PSMG3	-0.3851474	0.4436505	-0.8681	0.3858	-0.527727233	count	1
LAMTOR4	-0.3680534	0.1525615	-2.4125	0.0162	-0.526380206	count	1
NDUFAF2	-0.4035187	0.3902496	-1.034	0.302	-0.526056306	count	1
AKR1A1	-0.3761641	0.2406742	-1.563	0.119	-0.524267806	count	1
BABAM1	-0.4005914	0.2644984	-1.5145	0.1306	-0.522306218	count	1
TMPO	-0.3709549	0.2211063	-1.6777	0.0941	-0.521462862	count	1
ZDHHC21	-0.3800775	0.3820423	-0.9949	0.3203	-0.520840098	count	1
RNF7	-0.3643175	0.1910107	-1.9073	0.0571	-0.520634751	count	1
FYN	-0.398873	0.5739693	-0.6949	0.487	-0.520104311	count	1
PIGK	-0.3789089	0.6198574	-0.6113	0.5413	-0.519252372	count	1
GNPNAT1	-0.3789089	0.7251621	-0.5225	0.602	-0.519252372	count	1
SIRT1	-0.3980342	0.4291166	-0.9276	0.3541	-0.519029363	count	1
HMGA1	-0.3691269	0.2232734	-1.6533	0.099	-0.518904107	count	1
PPP1R2	-0.3671994	0.1586399	-2.3147	0.0211	-0.518859762	count	1
MRPL42	-0.3784419	0.2948424	-1.2835	0.2	-0.518617854	count	1
DMAP1	-0.37705	0.3560111	-1.0591	0.29	-0.516726572	count	1
CLPTM1L	-0.3766869	0.4862739	-0.7746	0.439	-0.516233176	count	1
SLC25A4	-0.3745455	0.3790971	-0.988	0.324	-0.51332317	count	1
DHPS	-0.3742149	0.2202132	-1.6993	0.09	-0.512873879	count	1
TFRC	-0.3931652	0.3234251	-1.2156	0.225	-0.512787809	count	1
MFAP1	-0.3675418	0.2932576	-1.2533	0.211	-0.512317971	count	1
BIN1	-0.3737763	0.2043281	-1.8293	0.068	-0.512277805	count	1
LARP1B	-0.3734736	0.6122037	-0.61	0.5421	-0.511866415	count	1
PQLC1	-0.372785	0.3713728	-1.0038	0.316	-0.510930537	count	1
TRMT1	-0.372785	0.3766999	-0.9896	0.323	-0.510930537	count	1
ALDOA	-0.3727258	0.5044442	-0.7389	0.4604	-0.510850077	count	1
TNFSF12	-0.3632926	0.2262245	-1.6059	0.109	-0.510736686	count	1
GOLGA4	-0.3903272	0.2375272	-1.6433	0.101	-0.509148401	count	1
PLSCR1	-0.3710545	0.3351859	-1.107	0.269	-0.508578467	count	1
ORMDL3	-0.3885303	0.4650923	-0.8354	0.404	-0.506843557	count	1
MAF1	-0.3598325	0.2145963	-1.6768	0.0943	-0.50589231	count	1
S100A6	-0.3522236	0.111269	-3.1655	0.0016	-0.504105995	count	1
NIPSNAP3B	-0.3860749	0.7338041	-0.5261	0.5991	-0.503693416	count	1
KLHL22	-0.3860749	0.8493135	-0.4546	0.6496	-0.503693416	count	1
GPATCH11	-0.3605622	0.3426314	-1.0523	0.293	-0.502642167	count	1
EI24	-0.3847987	0.5969158	-0.6446	0.5195	-0.50205582	count	1
PRELID1	-0.3518298	0.1449	-2.4281	0.0156	-0.500131567	count	1
C20orf27	-0.3832671	0.3708302	-1.0335	0.3019	-0.500090229	count	1
SRSF8	-0.358547	0.3095132	-1.1584	0.247	-0.499848067	count	1
CDKN1B	-0.3533186	0.1652901	-2.1376	0.0331	-0.499309659	count	1
PTGER4	-0.3550708	0.5745959	-0.6179	0.5369	-0.499224893	count	1
NR1D2	-0.3629951	0.375342	-0.9671	0.334	-0.497621507	count	1
ECE1	-0.3629474	0.5392991	-0.673	0.501	-0.497556644	count	1
DTNBP1	-0.3620966	0.3309651	-1.0941	0.2745	-0.496399695	count	1

PPP1R14B	-0.3802043	0.1933819	-1.9661	0.0499	-0.49615868	count	1
METTL9	-0.3800558	0.235241	-1.6156	0.1069	-0.495968028	count	1
PET100	-0.3608259	0.2343739	-1.5395	0.1244	-0.494671656	count	1
PSAP	-0.3603557	0.2670776	-1.3493	0.178	-0.494032198	count	1
ATP5F1A	-0.3491837	0.1698635	-2.0557	0.0404	-0.493484855	count	1
ABT1	-0.3778702	0.2651515	-1.4251	0.1548	-0.493161733	count	1
TMEM131L	-0.3533308	0.3287923	-1.0746	0.283	-0.492614839	count	1
RIOK1	-0.3455172	0.3305805	-1.0452	0.2965	-0.492078798	count	1
S1PR4	-0.3437029	0.3055429	-1.1249	0.261	-0.490758485	count	1
IDNK	-0.3578538	0.5363741	-0.6672	0.505	-0.490629433	count	1
ARL6IP1	-0.3576128	0.2413704	-1.4816	0.139	-0.490301632	count	1
RAC1	-0.3428673	0.1494918	-2.2936	0.0223	-0.490025216	count	1
IRF7	-0.3442215	0.2965583	-1.1607	0.2464	-0.489340636	count	1
FIBP	-0.3451151	0.3141847	-1.0984	0.2726	-0.487752957	count	1
ANP32B	-0.3401688	0.1320706	-2.5757	0.0103	-0.486873874	count	1
CLK3	-0.3729139	0.3146059	-1.1853	0.2365	-0.486795669	count	1
KLF9	-0.3533348	0.3030085	-1.1661	0.244	-0.484482175	count	1
LCOR	-0.3705343	0.4089208	-0.9061	0.365	-0.483738134	count	1
LAMTOR2	-0.3527626	0.2980272	-1.1837	0.237	-0.483703703	count	1
SYPL1	-0.3374008	0.1315387	-2.565	0.0106	-0.482916832	count	1
PHB2	-0.3383058	0.1651108	-2.049	0.0411	-0.481828324	count	1
CHMP4B	-0.3451865	0.337832	-1.0218	0.3074	-0.481318683	count	1
COMMD7	-0.3451865	0.3701074	-0.9327	0.351	-0.481318683	count	1
SHISAL2A	-0.3422565	0.2755703	-1.242	0.215	-0.481277997	count	1
GABPB1-AS1	-0.3681487	0.2455467	-1.4993	0.135	-0.480672183	count	1
BRD7	-0.3432388	0.244548	-1.4036	0.161	-0.478616757	count	1
EPSTI1	-0.3381227	0.3302949	-1.0237	0.3065	-0.477900849	count	1
ACBD3	-0.3425237	0.2648285	-1.2934	0.197	-0.477624698	count	1
KIAA1551	-0.3645112	0.1346874	-2.7063	0.0071	-0.475995945	count	1
SERPINB1	-0.3405878	0.1861644	-1.8295	0.068	-0.474938901	count	1
LDHA	-0.334313	0.1630644	-2.0502	0.0409	-0.474134476	count	1
CEP350	-0.3456219	0.2879207	-1.2004	0.2306	-0.473986988	count	1
FGFR1OP2	-0.3363104	0.198994	-1.6901	0.0917	-0.472948264	count	1
LRRC41	-0.361996	0.6118061	-0.5917	0.5544	-0.472761543	count	1
OGT	-0.3356481	0.3006944	-1.1162	0.265	-0.472020387	count	1
DCTN3	-0.3332207	0.2266196	-1.4704	0.142	-0.470993217	count	1
MCAT	-0.3377254	0.7777077	-0.4343	0.6643	-0.470967389	count	1
MEPCE	-0.3345937	0.3923149	-0.8529	0.394	-0.470543149	count	1
FAM110A	-0.3365171	0.6910949	-0.4869	0.6266	-0.469290788	count	1
NDUFB9	-0.3592386	0.1906564	-1.8842	0.0602	-0.469214786	count	1
NAGK	-0.3313351	0.2424217	-1.3668	0.1724	-0.468335947	count	1
RNPEPL1	-0.3357278	0.2851687	-1.1773	0.24	-0.46819554	count	1
NUDT21	-0.3394582	0.396358	-0.8564	0.392	-0.465596945	count	1
CSDE1	-0.3558376	0.1825812	-1.9489	0.0519	-0.464838904	count	1
MRPS18C	-0.3287966	0.3135091	-1.0488	0.295	-0.464758422	count	1

AIDA	-0.3378389	0.2771245	-1.2191	0.2235	-0.463392324	count	1
MRPL11	-0.3545599	0.2747076	-1.2907	0.197	-0.463194588	count	1
DBNL	-0.3264407	0.23763	-1.3737	0.1702	-0.462997096	count	1
H1FX	-0.3254285	0.4879792	-0.6669	0.5052	-0.462681124	count	1
BLK	-0.3264072	0.1739838	-1.8761	0.0613	-0.459072342	count	1
BST2	-0.3242992	0.1869234	-1.7349	0.0834	-0.458419753	count	1
HSPA8	-0.3187809	0.0901817	-3.5349	5.00E-04	-0.458159547	count	1
POLR2F	-0.3283252	0.3184044	-1.0312	0.303	-0.457922157	count	1
LY9	-0.3231406	0.2505173	-1.2899	0.198	-0.45678672	count	1
EXOC1	-0.3495294	0.4519074	-0.7735	0.4397	-0.456718739	count	1
RASGRP3	-0.349014	0.389774	-0.8954	0.371	-0.456055079	count	1
DCAF5	-0.3484521	0.4557507	-0.7646	0.4449	-0.45533151	count	1
GDI2	-0.3483896	0.2160427	-1.6126	0.108	-0.455251024	count	1
ISG20	-0.3172507	0.122404	-2.5918	0.0099	-0.455235108	count	1
GPSM3	-0.3183603	0.1141931	-2.7879	0.0055	-0.454112113	count	1
UBL7	-0.320122	0.4112271	-0.7785	0.4367	-0.454056605	count	1
UQCRH	-0.3148263	0.0976367	-3.2245	0.00136	-0.452650256	count	1
SFT2D2	-0.3238107	0.4010201	-0.8075	0.42	-0.451655656	count	1
CSNK1A1	-0.3176123	0.1583702	-2.0055	0.0455	-0.451591009	count	1
CAMLG	-0.3187891	0.2383015	-1.3378	0.182	-0.450652994	count	1
SCAND1	-0.3445683	0.1952305	-1.7649	0.0783	-0.450329207	count	1
RBBP7	-0.3436809	0.2460116	-1.397	0.1631	-0.449185988	count	1
NIT2	-0.3434374	0.3937189	-0.8723	0.3835	-0.448872276	count	1
RCC2	-0.3270983	0.4340746	-0.7536	0.4515	-0.448764917	count	1
TSPAN31	-0.3269279	0.3655235	-0.8944	0.3716	-0.448532791	count	1
PGLS	-0.3156546	0.1693427	-1.864	0.063	-0.447734993	count	1
SHISA5	-0.3420077	0.360896	-0.9477	0.344	-0.44703018	count	1
ZNF32	-0.3253225	0.4012451	-0.8108	0.4179	-0.44634575	count	1
LSM6	-0.3250796	0.2266097	-1.4345	0.1521	-0.44601483	count	1
COA1	-0.3240298	0.3223797	-1.0051	0.315	-0.444584574	count	1
GRN	-0.322569	0.2428753	-1.3281	0.185	-0.442594249	count	1
CYTOR	-0.3384013	0.2811925	-1.2035	0.2294	-0.442382416	count	1
IL18BP	-0.3207769	0.5406437	-0.5933	0.553	-0.440152334	count	1
ARHGAP15	-0.3151773	0.2268761	-1.3892	0.165	-0.439669177	count	1
TACC3	-0.3195027	0.6651108	-0.4804	0.631	-0.438415982	count	1
MAP3K13	-0.3097353	0.2993032	-1.0349	0.3013	-0.437889351	count	1
GSPT1	-0.3344608	0.1948812	-1.7162	0.0868	-0.43730229	count	1
UBE2G2	-0.3338072	0.3394074	-0.9835	0.3259	-0.436459482	count	1
APPL1	-0.3173401	0.1689351	-1.8785	0.061	-0.435468762	count	1
EMD	-0.3172483	0.2372723	-1.3371	0.182	-0.43534365	count	1
REEP5	-0.3045589	0.1885074	-1.6156	0.1069	-0.434943448	count	1
ZC3H18	-0.3161935	0.4263193	-0.7417	0.459	-0.433906035	count	1
GLB1	-0.3160301	0.5441593	-0.5808	0.5617	-0.433683327	count	1
ZNF428	-0.3156437	0.29056	-1.0863	0.2779	-0.43315667	count	1
SC5D	-0.3311519	0.4942374	-0.67	0.503	-0.433034985	count	1

DRAM2	-0.3103519	0.1938524	-1.601	0.11	-0.432968186	count	1
ATF6B	-0.3152859	0.4001128	-0.788	0.4311	-0.432668985	count	1
ICAM3	-0.3049819	0.1604545	-1.9007	0.058	-0.432630638	count	1
BIRC6	-0.305927	0.3198817	-0.9564	0.339	-0.432519894	count	1
GTF2I	-0.3145034	0.2786543	-1.1287	0.26	-0.431602402	count	1
IGHA1	-0.3000451	0.1316383	-2.2793	0.0231	-0.43096359	count	1
TAF8	-0.3294823	0.5137491	-0.6413	0.5216	-0.4308813	count	1
DNAJC10	-0.3127217	0.2229525	-1.4026	0.161	-0.429173712	count	1
MTERF4	-0.3126981	0.2383516	-1.3119	0.19	-0.429141543	count	1
SERINC1	-0.3124321	0.3105591	-1.006	0.315	-0.428778932	count	1
URI1	-0.3123383	0.2478446	-1.2602	0.208	-0.428651062	count	1
PRRC2B	-0.3069233	0.4373679	-0.7018	0.483	-0.428206278	count	1
PDE4B	-0.3028131	0.1844006	-1.6421	0.101	-0.428129194	count	1
DGKZ	-0.3270983	0.580984	-0.563	0.5737	-0.427805502	count	1
SND1	-0.3014755	0.2976735	-1.0128	0.3117	-0.427667683	count	1
TAZ	-0.3058359	0.3935513	-0.7771	0.4375	-0.426695902	count	1
RAB35	-0.3103431	0.6475915	-0.4792	0.632	-0.425931049	count	1
WASF2	-0.300192	0.1732949	-1.7323	0.0839	-0.425850946	count	1
YY1	-0.2993607	0.1456672	-2.0551	0.0405	-0.425689646	count	1
TMEM120B	-0.3252197	0.3591276	-0.9056	0.3656	-0.425381287	count	1
MPHOSPH8	-0.2975232	0.1577127	-1.8865	0.0599	-0.42443369	count	1
CDC40	-0.324266	0.1659999	-1.9534	0.0514	-0.424150433	count	1
MRPS34	-0.2997373	0.2150059	-1.3941	0.164	-0.423791952	count	1
ATP5MC2	-0.2940643	0.0899737	-3.2683	0.0012	-0.422526836	count	1
TFEB	-0.296914	0.2872296	-1.0337	0.3018	-0.422216955	count	1
MINDY2	-0.3070106	0.5039853	-0.6092	0.543	-0.42138734	count	1
STK11	-0.3019424	0.4930473	-0.6124	0.541	-0.421287471	count	1
GRB2	-0.2935892	0.1256744	-2.3361	0.0199	-0.421042251	count	1
AL392172.1	-0.3066304	0.4299187	-0.7132	0.4761	-0.420868909	count	1
C19orf38	-0.305883	0.6839195	-0.4473	0.6549	-0.419849746	count	1
FAM136A	-0.3058135	0.3956732	-0.7729	0.44	-0.419754975	count	1
CSNK2A2	-0.3055104	0.3173756	-0.9626	0.3363	-0.419341651	count	1
NDUFAF8	-0.2977172	0.2356521	-1.2634	0.207	-0.418853823	count	1
SCAF4	-0.2974853	0.4600364	-0.6467	0.5182	-0.418528623	count	1
STAT3	-0.3046064	0.2226258	-1.3682	0.172	-0.418108878	count	1
UTRN	-0.2970758	0.2754057	-1.0787	0.281	-0.417954364	count	1
SHPRH	-0.3044552	0.3499385	-0.87	0.385	-0.417902682	count	1
TSPAN3	-0.2981108	0.1882035	-1.584	0.114	-0.41596436	count	1
ARMC10	-0.3179125	0.4845755	-0.6561	0.512	-0.415947816	count	1
NUDT1	-0.3028872	0.2764778	-1.0955	0.274	-0.415764278	count	1
RASGRP2	-0.2977819	0.2569288	-1.159	0.247	-0.4155074	count	1
MSN	-0.3162574	0.1705751	-1.8541	0.0644	-0.413810233	count	1
DUSP23	-0.2955737	0.4082947	-0.7239	0.4695	-0.412439293	count	1
SOD1	-0.2875786	0.1159084	-2.4811	0.0135	-0.411905569	count	1
TYMP	-0.2907294	0.2014902	-1.4429	0.15	-0.411088244	count	1

CCDC138	-0.294281	0.6573608	-0.4477	0.6546	-0.410643095	count	1
PLCL2	-0.3132338	0.4162178	-0.7526	0.452	-0.409904376	count	1
NAA16	-0.2935436	0.5530918	-0.5307	0.5959	-0.40961845	count	1
PNKD	-0.2934571	0.306666	-0.9569	0.339	-0.409498253	count	1
HP1BP3	-0.2878449	0.1689581	-1.7036	0.0891	-0.408372233	count	1
TENT2	-0.3117728	0.4461749	-0.6988	0.4851	-0.408016687	count	1
MIDN	-0.2971294	0.1914881	-1.5517	0.121	-0.407910544	count	1
CD48	-0.2831524	0.0856283	-3.3068	0.00102	-0.406855912	count	1
FBP1	-0.2888167	0.8064295	-0.3581	0.7204	-0.406371076	count	1
RARS	-0.2956769	0.5769944	-0.5124	0.609	-0.405928972	count	1
FTL	-0.281604	0.0495139	-5.6874	2.34E-08	-0.40585417	count	0.00056869
PMEPA1	-0.2947769	0.399813	-0.7373	0.461	-0.404701078	count	1
EIF4A1	-0.2876136	0.1985995	-1.4482	0.148	-0.40468355	count	1
SAT1	-0.2814461	0.0984224	-2.8596	0.0044	-0.403998572	count	1
ACSL4	-0.3081277	0.4588172	-0.6716	0.5022	-0.403305934	count	1
CCDC25	-0.3076658	0.3085648	-0.9971	0.3193	-0.402708884	count	1
OSER1	-0.2856746	0.1807716	-1.5803	0.115	-0.401963712	count	1
PDCD2	-0.2876167	0.2552155	-1.127	0.26	-0.401381916	count	1
CAPN12	-0.291742	0.4984059	-0.5854	0.559	-0.400560103	count	1
NDUFA4	-0.2813325	0.113471	-2.4793	0.0135	-0.400098765	count	1
RAB5C	-0.2911576	0.2611868	-1.1147	0.2656	-0.39976265	count	1
XRN1	-0.2814898	0.2864545	-0.9827	0.3263	-0.399374506	count	1
PSMA3-AS1	-0.3046263	0.2938573	-1.0366	0.3005	-0.398779428	count	1
RPS27L	-0.2789848	0.1716532	-1.6253	0.1048	-0.398465744	count	1
PLEKHA3	-0.2852972	0.2840603	-1.0044	0.316	-0.398158114	count	1
PSMB8	-0.2780994	0.1641512	-1.6942	0.0909	-0.397202687	count	1
DDX39B	-0.2819048	0.3080597	-0.9151	0.361	-0.396675446	count	1
BTBD9	-0.2888167	0.8879508	-0.3253	0.7451	-0.396568115	count	1
VPS35L	-0.2888167	0.9332235	-0.3095	0.7571	-0.396568115	count	1
AC010226.1	-0.28037	0.4888254	-0.5736	0.5666	-0.396475787	count	1
PNPLA8	-0.280256	0.2056101	-1.363	0.174	-0.396314968	count	1
STX16	-0.2885654	0.584845	-0.4934	0.622	-0.396225155	count	1
PPP1R15B	-0.288423	0.3764617	-0.7661	0.444	-0.396030813	count	1
CPNE1	-0.2832399	0.2853746	-0.9925	0.321	-0.39529854	count	1
CARS2	-0.3016233	0.4082053	-0.7389	0.4604	-0.394896097	count	1
CALM1	-0.2740412	0.0742169	-3.6924	0.00025	-0.394388447	count	1
NDUFB10	-0.2758191	0.1920678	-1.4361	0.1517	-0.394307595	count	1
LBR	-0.282415	0.2849975	-0.9909	0.322	-0.394151904	count	1
WAC	-0.2787185	0.2128389	-1.3095	0.191	-0.394145993	count	1
TTC3	-0.2861332	0.2659394	-1.0759	0.283	-0.392905614	count	1
CASP8AP2	-0.2858207	0.4346362	-0.6576	0.511	-0.392479077	count	1
SLC2A11	-0.2852258	0.4683561	-0.609	0.5428	-0.39166707	count	1
SMCHD1	-0.275124	0.1376399	-1.9989	0.0462	-0.391284366	count	1
SPRYD7	-0.2977372	0.4639961	-0.6417	0.5214	-0.389869228	count	1
NHLRC2	-0.2829371	0.6021445	-0.4699	0.6387	-0.388542907	count	1

POLR2H	-0.282896	0.3829465	-0.7387	0.4605	-0.3884868	count	1
ODC1	-0.2735247	0.1571402	-1.7406	0.0824	-0.388095996	count	1
ADRM1	-0.2729674	0.1889237	-1.4449	0.1492	-0.387306811	count	1
BCL11A	-0.2738406	0.1846223	-1.4832	0.139	-0.387264242	count	1
SAFB	-0.2819953	0.2597534	-1.0856	0.2782	-0.387257217	count	1
ATP5PO	-0.2699657	0.1394209	-1.9363	0.0535	-0.387186942	count	1
KBTBD3	-0.2814294	0.5140985	-0.5474	0.584	-0.386484656	count	1
IMP4	-0.2945421	0.288671	-1.0203	0.3081	-0.385734895	count	1
LTB	-0.267245	0.0938646	-2.8471	0.00462	-0.384948645	count	1
CXCR4	-0.2685359	0.0875127	-3.0685	0.0023	-0.384442302	count	1
ZBTB21	-0.2794368	0.4070857	-0.6864	0.493	-0.38376422	count	1
SOD2	-0.2706521	0.3149889	-0.8592	0.3907	-0.382765553	count	1
SMARCD1	-0.2781665	0.2909894	-0.9559	0.3396	-0.382029785	count	1
MT-ATP6	-0.2646141	0.0438657	-6.0324	3.41E-09	-0.381119908	count	8.29E-05
JTB	-0.2678962	0.1659805	-1.614	0.107	-0.380125226	count	1
PPP4R3A	-0.2716913	0.2562331	-1.0603	0.29	-0.379242941	count	1
CDV3	-0.2656913	0.1414869	-1.8779	0.0611	-0.379082221	count	1
ATP6V0D1	-0.2756328	0.2334319	-1.1808	0.2383	-0.378570034	count	1
BORCS8	-0.2748521	0.443571	-0.6196	0.5358	-0.377503911	count	1
AC010642.2	-0.2748521	0.4798953	-0.5727	0.567	-0.377503911	count	1
ILF3	-0.270172	0.2321529	-1.1638	0.245	-0.377130282	count	1
DUSP4	-0.2729839	0.5639001	-0.4841	0.629	-0.374952543	count	1
PDIA3	-0.2635208	0.1407436	-1.8723	0.0618	-0.374808936	count	1
RBM17	-0.2683983	0.2269756	-1.1825	0.238	-0.374663738	count	1
CD79B	-0.2613614	0.1542792	-1.6941	0.091	-0.374180051	count	1
MRPL57	-0.2679979	0.2626064	-1.0205	0.308	-0.374106916	count	1
HLA-DRB5	-0.2590212	0.0657006	-3.9425	9.37E-05	-0.37270657	count	1
GTF2E2	-0.2627749	0.290439	-0.9048	0.3661	-0.371650361	count	1
AES	-0.2592619	0.1458807	-1.7772	0.0762	-0.371379624	count	1
RPL13A	-0.2576239	0.0366917	-7.0213	8.29E-12	-0.371297431	count	2.02E-07
HMGN1	-0.2624349	0.1020958	-2.5705	0.0105	-0.371170565	count	1
PSMD8	-0.261505	0.1561785	-1.6744	0.0948	-0.371073502	count	1
BRK1	-0.2613049	0.1626592	-1.6065	0.109	-0.369575922	count	1
MRPS5	-0.2646684	0.326039	-0.8118	0.417	-0.369476423	count	1
NOC3L	-0.2689422	0.3979998	-0.6757	0.5	-0.369432118	count	1
USE1	-0.2644539	0.4635681	-0.5705	0.569	-0.369178091	count	1
XPA	-0.2643692	0.3145694	-0.8404	0.401	-0.369060287	count	1
MKNK1	-0.2637625	0.6605418	-0.3993	0.6899	-0.368216462	count	1
GMFG	-0.2806301	0.1676193	-1.6742	0.0948	-0.367719607	count	1
ALOX5AP	-0.2599834	0.1729803	-1.503	0.134	-0.367710994	count	1
CD52	-0.2543381	0.0675444	-3.7655	0.000189	-0.366228577	count	1
UBE2L6	-0.2622583	0.2671634	-0.9816	0.327	-0.366124284	count	1
ZNHIT3	-0.2622539	0.2354852	-1.1137	0.266	-0.366118164	count	1
RRBP1	-0.2664154	0.5590161	-0.4766	0.6339	-0.365980324	count	1
FUT8	-0.2621166	0.3249397	-0.8067	0.42	-0.36592719	count	1

PSMA7	-0.2555087	0.1201669	-2.1263	0.034	-0.365808073	count	1
TIGD3	-0.2661078	0.5746135	-0.4631	0.6435	-0.365560092	count	1
MZF1-AS1	-0.2661078	0.7758315	-0.343	0.7318	-0.365560092	count	1
C12orf10	-0.2617449	0.3552462	-0.7368	0.462	-0.365410178	count	1
TTC9	-0.2657922	0.4539231	-0.5855	0.5585	-0.365128925	count	1
HSP90AA1	-0.2547046	0.0965061	-2.6393	0.0086	-0.364657824	count	1
RAB14	-0.2549891	0.2121684	-1.2018	0.2301	-0.364559104	count	1
TP53TG1	-0.2653028	0.3668395	-0.7232	0.47	-0.364460304	count	1
GSDMD	-0.256911	0.2605403	-0.9861	0.325	-0.363374982	count	1
CYB561A3	-0.2600171	0.2483914	-1.0468	0.296	-0.36300684	count	1
PLEKHJ1	-0.2636829	0.266004	-0.9913	0.3221	-0.36224708	count	1
STAMBP	-0.2552101	0.4023762	-0.6343	0.5262	-0.362157275	count	1
HSPD1	-0.2572518	0.1474008	-1.7453	0.0816	-0.362080614	count	1
TSPAN13	-0.25502	0.324914	-0.7849	0.4329	-0.361887999	count	1
MAP2K2	-0.2570024	0.1739115	-1.4778	0.14	-0.361730535	count	1
PEX16	-0.2588096	0.3604053	-0.7181	0.4731	-0.361327153	count	1
SYNCRIP	-0.2561083	0.2125445	-1.205	0.229	-0.360475487	count	1
PHPT1	-0.254638	0.2885335	-0.8825	0.378	-0.358411571	count	1
CCDC186	-0.254638	0.2889372	-0.8813	0.379	-0.358411571	count	1
TOMM40	-0.2566484	0.3884921	-0.6606	0.509	-0.358320671	count	1
TSPYL2	-0.2605718	0.2965577	-0.8787	0.38	-0.357996012	count	1
SF3B3	-0.2729839	0.616752	-0.4426	0.6583	-0.35780878	count	1
LINC00996	-0.2729839	0.7963383	-0.3428	0.732	-0.35780878	count	1
HSPH1	-0.2556358	0.2719849	-0.9399	0.348	-0.356911957	count	1
TMEM160	-0.2518249	0.1915773	-1.3145	0.189	-0.356196532	count	1
VPS29	-0.2716135	0.2329587	-1.1659	0.244	-0.356031796	count	1
NORAD	-0.2708864	0.2771204	-0.9775	0.329	-0.35508889	count	1
SIT1	-0.2707271	0.2190878	-1.2357	0.2172	-0.354882299	count	1
NFKB2	-0.2706443	0.2673381	-1.0124	0.312	-0.354774919	count	1
PSMD13	-0.2580452	0.2436502	-1.0591	0.29	-0.354543175	count	1
RECQL	-0.2575167	0.2634191	-0.9776	0.3288	-0.353820879	count	1
RSRP1	-0.2486933	0.1253749	-1.9836	0.0479	-0.353751542	count	1
TXNDC17	-0.2480392	0.2800712	-0.8856	0.3763	-0.35282252	count	1
FYTTD1	-0.268755	0.1893212	-1.4196	0.156	-0.352324532	count	1
ACTG1	-0.2442191	0.0772676	-3.1607	0.00168	-0.351708857	count	1
LYL1	-0.2486394	0.2284381	-1.0884	0.277	-0.351700215	count	1
HECTD1	-0.2660849	0.295266	-0.9012	0.368	-0.348860774	count	1
PML	-0.2537412	0.5455293	-0.4651	0.6421	-0.348660443	count	1
EPB41L4A-AS1	-0.2463452	0.2495412	-0.9872	0.324	-0.348461799	count	1
MYCBP2	-0.2493462	0.1473928	-1.6917	0.0914	-0.348160982	count	1
PCMTD2	-0.2488651	0.7313772	-0.3403	0.7338	-0.347491538	count	1
ATM	-0.2527636	0.2210736	-1.1433	0.254	-0.347324094	count	1
DHX36	-0.2454448	0.195309	-1.2567	0.21	-0.347190787	count	1
C4orf3	-0.2431605	0.1452756	-1.6738	0.0949	-0.346972905	count	1
NIPA2	-0.2484043	0.2352353	-1.056	0.292	-0.346850333	count	1

BCL3	-0.2522914	0.3400316	-0.742	0.459	-0.346678591	count	1
PTPN11	-0.2640106	0.3198608	-0.8254	0.4096	-0.346169359	count	1
IGHM	-0.2402874	0.155911	-1.5412	0.124	-0.345757758	count	1
RBX1	-0.2422989	0.1645885	-1.4721	0.142	-0.345744864	count	1
SMIM15	-0.2636434	0.3440522	-0.7663	0.4439	-0.345692867	count	1
H2AFZ	-0.2412466	0.1147063	-2.1032	0.036	-0.345405405	count	1
CCNH	-0.2456742	0.1944599	-1.2634	0.207	-0.343051204	count	1
MAPRE1	-0.2410762	0.275491	-0.8751	0.382	-0.342932434	count	1
SRP9	-0.2409377	0.1931571	-1.2474	0.2129	-0.342735703	count	1
CAPNS1	-0.2448673	0.2961983	-0.8267	0.409	-0.341928285	count	1
EBAG9	-0.2470831	0.3372431	-0.7327	0.464	-0.339557868	count	1
BHLHE41	-0.2585317	0.3058785	-0.8452	0.398	-0.33905814	count	1
PTPRJ	-0.2582645	0.9039047	-0.2857	0.7752	-0.338711248	count	1
MFF	-0.2392323	0.2588454	-0.9242	0.3559	-0.338420597	count	1
DUSP2	-0.235584	0.1523002	-1.5468	0.123	-0.337304184	count	1
ID3	-0.2435772	0.2955853	-0.8241	0.41	-0.334763709	count	1
GINM1	-0.2395646	0.2674234	-0.8958	0.371	-0.334548119	count	1
FCRL3	-0.2548332	0.349788	-0.7285	0.4667	-0.334255858	count	1
TPR	-0.2354025	0.1552174	-1.5166	0.13	-0.334095689	count	1
CCDC50	-0.235305	0.1451674	-1.6209	0.106	-0.333957538	count	1
IFITM2	-0.2365558	0.1559393	-1.517	0.13	-0.333023176	count	1
CD2AP	-0.2419209	0.3902746	-0.6199	0.536	-0.332498534	count	1
SNRPF	-0.2379774	0.1716166	-1.3867	0.166	-0.332338864	count	1
TMEM256	-0.2416056	0.2383606	-1.0136	0.3113	-0.332067307	count	1
PHACTR1	-0.2307358	0.2241346	-1.0295	0.3038	-0.330700765	count	1
TTC9C	-0.2365397	0.382891	-0.6178	0.537	-0.33033761	count	1
OAS1	-0.236443	0.4261947	-0.5548	0.579	-0.330203002	count	1
ATG4C	-0.2363738	0.4418031	-0.535	0.5929	-0.330106675	count	1
MED30	-0.2340044	0.2349891	-0.9958	0.32	-0.329440027	count	1
GTPBP4	-0.2511248	0.2954673	-0.8499	0.3958	-0.329439195	count	1
ATF2	-0.2358091	0.4598805	-0.5128	0.6084	-0.329320595	count	1
KLHDC4	-0.2505651	0.5984707	-0.4187	0.6757	-0.328712095	count	1
KCNAB2	-0.2505651	0.7098346	-0.353	0.724	-0.328712095	count	1
UBE2D2	-0.2300651	0.1332102	-1.7271	0.0848	-0.328663961	count	1
IFNAR1	-0.2502694	0.2850697	-0.8779	0.3805	-0.328327941	count	1
TMEM205	-0.2332036	0.3414501	-0.683	0.495	-0.328315352	count	1
SERBP1	-0.2325192	0.1364673	-1.7038	0.0891	-0.327354139	count	1
C19orf24	-0.2378915	0.2573137	-0.9245	0.356	-0.326987182	count	1
TMEM156	-0.2338427	0.2640654	-0.8855	0.376	-0.3265832	count	1
STK26	-0.2369234	0.3779243	-0.6269	0.531	-0.325662884	count	1
PPP1CC	-0.2367595	0.2124877	-1.1142	0.266	-0.325438672	count	1
COMMD4	-0.236443	0.5066629	-0.4667	0.641	-0.325005705	count	1
RPL26	-0.2249926	0.0409796	-5.4904	6.76E-08	-0.324351407	count	0.001642612
CD200	-0.2349297	0.6416029	-0.3662	0.7144	-0.32293545	count	1
ABCB4	-0.2342235	0.7174967	-0.3264	0.7442	-0.321969293	count	1

CEP164	-0.2441295	0.4767678	-0.5121	0.6089	-0.320349193	count	1
NUFIP2	-0.2330007	0.2219536	-1.0498	0.2944	-0.3202963	count	1
CHCHD5	-0.2329017	0.3764272	-0.6187	0.5364	-0.320160847	count	1
TRNAU1AP	-0.2325284	0.406868	-0.5715	0.568	-0.319650091	count	1
MYL12A	-0.221787	0.0690895	-3.2101	0.00142	-0.318957207	count	1
STAT1	-0.2319325	0.3368445	-0.6885	0.491	-0.318834752	count	1
HTATSF1	-0.2279131	0.3552076	-0.6416	0.5214	-0.318327721	count	1
LUC7L3	-0.2310129	0.2051193	-1.1262	0.261	-0.317576468	count	1
PRRC2C	-0.2309793	0.1261386	-1.8312	0.0677	-0.317530493	count	1
MIER1	-0.2218843	0.2030704	-1.0926	0.2751	-0.317269379	count	1
TMSB10	-0.2198875	0.0738314	-2.9782	0.00306	-0.316930577	count	1
NKAP	-0.2305017	0.2384845	-0.9665	0.334	-0.316876975	count	1
LINC00324	-0.2266278	0.3495225	-0.6484	0.517	-0.316538072	count	1
FAM162A	-0.2265519	0.2532755	-0.8945	0.372	-0.316432387	count	1
CCT4	-0.2300479	0.2045666	-1.1246	0.261	-0.31625601	count	1
SNRPD1	-0.2252567	0.1922252	-1.1718	0.242	-0.31462888	count	1
ANXA2	-0.2393618	0.2070484	-1.1561	0.248	-0.314150745	count	1
TMEM219	-0.2206943	0.2212309	-0.9976	0.319	-0.313252962	count	1
NDUFA2	-0.2240138	0.2103442	-1.065	0.287	-0.312898133	count	1
MDH1	-0.2204237	0.1972092	-1.1177	0.264	-0.312869456	count	1
C6orf48	-0.2269764	0.1980594	-1.146	0.2524	-0.312052747	count	1
FAM89B	-0.2215775	0.2045848	-1.0831	0.279	-0.311984918	count	1
RAB8A	-0.2229006	0.2315658	-0.9626	0.336	-0.31134794	count	1
NELFB	-0.2371838	0.486053	-0.488	0.626	-0.311318319	count	1
IGBP1	-0.2225723	0.2068884	-1.0758	0.283	-0.310890754	count	1
CFL1	-0.2160411	0.0724608	-2.9815	0.00303	-0.310460802	count	1
PSMB1	-0.2166799	0.145968	-1.4844	0.1384	-0.310256605	count	1
ALG2	-0.2220091	0.3838473	-0.5784	0.563	-0.31010644	count	1
DYNLL1	-0.2164126	0.1287063	-1.6814	0.0934	-0.309680454	count	1
PYURF	-0.2216818	0.2054619	-1.0789	0.281	-0.309650634	count	1
ABCF1	-0.2194393	0.2060491	-1.065	0.287	-0.308981062	count	1
UBE2Q2	-0.2193256	0.4041553	-0.5427	0.5876	-0.308821326	count	1
SNX1	-0.2244306	0.3216802	-0.6977	0.4857	-0.308568462	count	1
NDUFA11	-0.2173622	0.1736766	-1.2515	0.211	-0.308530461	count	1
CPT1A	-0.2342235	0.8368477	-0.2799	0.7797	-0.307467699	count	1
OAZ1	-0.2135012	0.0635998	-3.3569	0.000856	-0.307458086	count	1
IGHE	-0.2200995	0.4901392	-0.4491	0.654	-0.307447027	count	1
TGIF1	-0.215259	0.1881731	-1.1439	0.253	-0.305549527	count	1
ARL16	-0.2218619	0.3734315	-0.5941	0.553	-0.305052444	count	1
ZNF506	-0.2181942	0.3668663	-0.5948	0.552	-0.304793453	count	1
NSMCE3	-0.2140556	0.2116254	-1.0115	0.312	-0.304544443	count	1
TLK1	-0.2212056	0.1927022	-1.1479	0.252	-0.304154041	count	1
CNIH1	-0.2142068	0.3031671	-0.7066	0.48	-0.304058175	count	1
MITD1	-0.2176289	0.3347927	-0.65	0.516	-0.304006112	count	1
NUP35	-0.2175175	0.5331413	-0.408	0.6835	-0.303850955	count	1

MGST3	-0.2139239	0.1999231	-1.07	0.285	-0.303657198	count	1
MARCKSL1	-0.2151938	0.175996	-1.2227	0.222	-0.303016337	count	1
DNTTIP2	-0.2200326	0.19196	-1.1462	0.252	-0.30254827	count	1
HK1	-0.2165705	0.4556815	-0.4753	0.6348	-0.302531955	count	1
AC007388.1	-0.219753	1.2913333	-0.1702	0.8649	-0.302165501	count	1
TMEM220	-0.219753	1.3485594	-0.163	0.8706	-0.302165501	count	1
HMGB1	-0.2102196	0.0772418	-2.7216	0.0068	-0.302048808	count	1
NOP53	-0.210174	0.0894996	-2.3483	0.0193	-0.301660334	count	1
MRPL21	-0.2106347	0.2751647	-0.7655	0.4444	-0.300201834	count	1
C17orf100	-0.2183155	0.727494	-0.3001	0.7642	-0.30019751	count	1
EIF6	-0.2119106	0.2080061	-1.0188	0.309	-0.299838773	count	1
SLIRP	-0.2178484	0.3421943	-0.6366	0.525	-0.299558006	count	1
RRP36	-0.2279377	0.3693953	-0.6171	0.5375	-0.299288266	count	1
CSTB	-0.2089722	0.3178906	-0.6574	0.511	-0.298821095	count	1
SDHAF2	-0.2273811	0.290838	-0.7818	0.4347	-0.298563781	count	1
PGAM1	-0.2119344	0.1889459	-1.1217	0.263	-0.298436655	count	1
KIAA2026	-0.2163362	0.3041532	-0.7113	0.477	-0.297487573	count	1
IFNGR1	-0.2081974	0.1930669	-1.0784	0.281	-0.296731853	count	1
DDX17	-0.2121427	0.1414184	-1.5001	0.134	-0.296364346	count	1
NUDT5	-0.2255625	0.3509145	-0.6428	0.5207	-0.296196406	count	1
SCPEP1	-0.2153268	0.234478	-0.9183	0.359	-0.296105474	count	1
TOMM20	-0.2080856	0.1124033	-1.8512	0.0648	-0.296061097	count	1
NOP10	-0.2057568	0.1664189	-1.2364	0.217	-0.294443119	count	1
IRF2	-0.2078758	0.2126386	-0.9776	0.329	-0.294139504	count	1
CLTB	-0.2088673	0.2590603	-0.8062	0.4205	-0.294126866	count	1
RGS10	-0.2077281	0.2523653	-0.8231	0.411	-0.293930866	count	1
IFI35	-0.2136544	0.3638705	-0.5872	0.5574	-0.293815446	count	1
BNIP2	-0.2074906	0.2170991	-0.9557	0.34	-0.293595377	count	1
SUCLA2	-0.2235583	0.4527534	-0.4938	0.6217	-0.293587017	count	1
RAD9A	-0.2132611	0.4761673	-0.4479	0.6545	-0.293276873	count	1
PLD4	-0.208078	0.3764603	-0.5527	0.5807	-0.29301772	count	1
PSME2	-0.2050311	0.1762926	-1.163	0.245	-0.292616625	count	1
VAMP8	-0.2069517	0.1615197	-1.2813	0.201	-0.29143498	count	1
ZNF226	-0.2117523	0.5256006	-0.4029	0.687	-0.291210686	count	1
BLOC1S5	-0.2117523	0.5426741	-0.3902	0.697	-0.291210686	count	1
DNAJA2	-0.2052844	0.1892845	-1.0845	0.279	-0.290478865	count	1
UTP6	-0.2076893	0.341831	-0.6076	0.544	-0.290160274	count	1
SP140	-0.2207893	0.1788757	-1.2343	0.218	-0.289981171	count	1
SKAP2	-0.210362	0.1848921	-1.1378	0.2558	-0.289306657	count	1
CD63	-0.2031966	0.2031472	-1.0002	0.318	-0.289113368	count	1
MMADHC	-0.2035866	0.29905	-0.6808	0.4964	-0.289004163	count	1
ARHGAP25	-0.2041333	0.2423942	-0.8422	0.4	-0.288852755	count	1
TLR10	-0.2029715	0.2886052	-0.7033	0.4822	-0.288132193	count	1
IDH3A	-0.2192134	0.4031083	-0.5438	0.5868	-0.287928638	count	1
ATP1A1	-0.2085794	0.286678	-0.7276	0.4673	-0.286865204	count	1

BTG1	-0.1989207	0.055126	-3.6085	0.000343	-0.286754416	count	1
SRRM2	-0.1998945	0.1081524	-1.8483	0.0652	-0.285292436	count	1
PSMA1	-0.2023919	0.2073228	-0.9762	0.329	-0.285026891	count	1
GLCCI1	-0.2039358	0.4424878	-0.4609	0.6451	-0.284930616	count	1
GBA	-0.2165705	0.8352308	-0.2593	0.7955	-0.284485786	count	1
SEC31A	-0.2035302	0.2977904	-0.6835	0.495	-0.284365469	count	1
NOSIP	-0.2003507	0.2609299	-0.7678	0.443	-0.283509004	count	1
MEAF6	-0.198988	0.1971661	-1.0092	0.313	-0.283132207	count	1
ORMDL1	-0.1994061	0.2017383	-0.9884	0.323	-0.283077705	count	1
ATP6V1G1	-0.1969512	0.0985167	-1.9992	0.0462	-0.282990901	count	1
SECISBP2L	-0.2056367	0.2841601	-0.7237	0.4697	-0.282834467	count	1
ZNF326	-0.2021309	0.315412	-0.6408	0.522	-0.282415691	count	1
ITSN2	-0.2021147	0.188363	-1.073	0.284	-0.282393117	count	1
NSL1	-0.2143947	0.2586298	-0.829	0.4076	-0.281650859	count	1
EIF4A2	-0.1981222	0.1353831	-1.4634	0.144	-0.28125752	count	1
ARRDC2	-0.2040542	0.3601466	-0.5666	0.5713	-0.280666642	count	1
MRPS16	-0.2036037	0.3340892	-0.6094	0.5426	-0.280049486	count	1
ANKRD11	-0.1965617	0.2096944	-0.9374	0.349	-0.279683864	count	1
1-Mar	-0.2127117	0.1875489	-1.1342	0.2573	-0.279457668	count	1
NDUFS6	-0.2000066	0.2084226	-0.9596	0.338	-0.279455551	count	1
BAX	-0.1981862	0.1998525	-0.9917	0.322	-0.279115869	count	1
RTF2	-0.2026528	0.2164916	-0.9361	0.35	-0.278746779	count	1
RBCK1	-0.2019467	0.2756384	-0.7327	0.4642	-0.277779406	count	1
ATP5F1D	-0.1937879	0.125104	-1.549	0.122	-0.277326666	count	1
FBXO21	-0.198469	0.300778	-0.6599	0.51	-0.277312844	count	1
HSH2D	-0.1948355	0.2515446	-0.7746	0.439	-0.277230464	count	1
ATG101	-0.1957028	0.1833767	-1.0672	0.286	-0.276942343	count	1
PUF60	-0.1981447	0.2063933	-0.96	0.3376	-0.276860906	count	1
DCK	-0.2009786	0.2508812	-0.8011	0.4235	-0.27645304	count	1
GTF2F1	-0.2004594	0.2847609	-0.704	0.482	-0.275741678	count	1
JUNB	-0.1916407	0.0960311	-1.9956	0.0466	-0.275666465	count	1
OPA1	-0.2002436	0.4260661	-0.47	0.639	-0.275446001	count	1
SNRPE	-0.1937607	0.2157609	-0.898	0.37	-0.275073966	count	1
ZNF829	-0.1998523	0.9559114	-0.2091	0.8345	-0.274909861	count	1
AATF	-0.1997288	0.3091154	-0.6461	0.519	-0.274740644	count	1
GTF3A	-0.1947963	0.1390577	-1.4008	0.162	-0.274351043	count	1
RETREG2	-0.2085296	0.4438426	-0.4698	0.6387	-0.27400649	count	1
NDUFS4	-0.1983451	0.3324043	-0.5967	0.551	-0.272844683	count	1
HTATIP2	-0.1983451	0.3950545	-0.5021	0.616	-0.272844683	count	1
ATP5MF	-0.198103	0.2012245	-0.9845	0.325	-0.272512943	count	1
PRDX2	-0.1913822	0.2615734	-0.7317	0.4648	-0.271701662	count	1
RPS11	-0.1883674	0.051921	-3.628	0.000319	-0.271196175	count	1
GNAI2	-0.2061611	0.1521721	-1.3548	0.1762	-0.27091843	count	1
BPTF	-0.1913914	0.1680557	-1.1389	0.255	-0.270850622	count	1
ECHS1	-0.193533	0.2786227	-0.6946	0.488	-0.270433692	count	1

PPP2R5E	-0.1932364	0.3831521	-0.5043	0.614	-0.270020299	count	1
EDF1	-0.1890442	0.1245864	-1.5174	0.13	-0.269819967	count	1
PDCD4-AS1	-0.2049029	0.606171	-0.338	0.7355	-0.269277744	count	1
DCTN4	-0.2049029	0.6836478	-0.2997	0.7645	-0.269277744	count	1
7-Sep	-0.1872927	0.0974838	-1.9213	0.0553	-0.269237985	count	1
PEBP1	-0.1873252	0.1259755	-1.487	0.1377	-0.268803074	count	1
TAF10	-0.1892092	0.1808247	-1.0464	0.296	-0.26862062	count	1
ESF1	-0.2043249	0.2810314	-0.7271	0.468	-0.26852398	count	1
PSMA4	-0.1950008	0.1785084	-1.0924	0.2753	-0.268261823	count	1
EIF3G	-0.1881055	0.1399899	-1.3437	0.18	-0.268123471	count	1
TRADD	-0.1918047	0.3196959	-0.6	0.549	-0.268024784	count	1
NUMB	-0.1947903	0.4317054	-0.4512	0.652	-0.267973343	count	1
OST4	-0.1865456	0.1130727	-1.6498	0.0997	-0.267275259	count	1
ITGB2-AS1	-0.1908647	0.5683906	-0.3358	0.7372	-0.26671456	count	1
VPS50	-0.2023916	0.4959293	-0.4081	0.6834	-0.26600252	count	1
SP100	-0.1903499	0.1212985	-1.5693	0.1173	-0.265996989	count	1
QTRT1	-0.1932863	0.4422878	-0.437	0.6623	-0.265912109	count	1
NUFIP1	-0.1899138	0.4618013	-0.4112	0.681	-0.265389107	count	1
EIF4G2	-0.1874248	0.1664227	-1.1262	0.261	-0.265245677	count	1
PRPF6	-0.2017043	0.2527644	-0.798	0.4253	-0.265106033	count	1
FKBP1A	-0.2017043	0.266248	-0.7576	0.4491	-0.265106033	count	1
HSPB11	-0.2009716	0.2644367	-0.76	0.4477	-0.26415027	count	1
CMAS	-0.1875295	0.4963845	-0.3778	0.7058	-0.26413569	count	1
DNAJC19	-0.1919833	0.2491887	-0.7704	0.441	-0.264126239	count	1
ARF6	-0.1871588	0.1467427	-1.2754	0.203	-0.263614533	count	1
ADAR	-0.2005393	0.2925921	-0.6854	0.493	-0.263586335	count	1
ASNA1	-0.2003615	0.3526039	-0.5682	0.5702	-0.263354389	count	1
USP24	-0.1878817	0.3554132	-0.5286	0.597	-0.262556458	count	1
KHDRBS1	-0.1862762	0.1440079	-1.2935	0.197	-0.262373693	count	1
NSA2	-0.1831447	0.1280587	-1.4302	0.153	-0.262266452	count	1
RIPOR2	-0.183682	0.1639837	-1.1201	0.263	-0.262172897	count	1
ESD	-0.1874818	0.2196254	-0.8536	0.394	-0.261998997	count	1
BAZ1A	-0.1851213	0.2142892	-0.8639	0.388	-0.261990575	count	1
EMC6	-0.1841342	0.2227701	-0.8266	0.409	-0.261424528	count	1
ITM2B	-0.1845242	0.1146721	-1.6091	0.1083	-0.261146785	count	1
UBAC2	-0.1845525	0.3930501	-0.4695	0.6389	-0.259950291	count	1
BORCS6	-0.184547	0.3880519	-0.4756	0.6346	-0.259942558	count	1
TIMM17B	-0.1835002	0.218787	-0.8387	0.402	-0.259699702	count	1
ATG12	-0.1857926	0.2332969	-0.7964	0.426	-0.259644183	count	1
STUB1	-0.1857484	0.2022466	-0.9184	0.359	-0.259582564	count	1
MZT1	-0.182768	0.2565534	-0.7124	0.4766	-0.259487235	count	1
ANAPC5	-0.1879823	0.3021891	-0.6221	0.534	-0.258641928	count	1
TAF7	-0.1826301	0.1534941	-1.1898	0.235	-0.258470086	count	1
TUBGCP2	-0.1876895	0.3036609	-0.6181	0.537	-0.25824054	count	1
HLA-DQB1	-0.1789312	0.0743371	-2.407	0.0165	-0.257576722	count	1

PHF11	-0.180976	0.3463384	-0.5225	0.6016	-0.25753043	count	1
ENO1	-0.1796088	0.1510561	-1.189	0.235	-0.256860687	count	1
ARF3	-0.1862052	0.5532004	-0.3366	0.7366	-0.256205699	count	1
CCDC102A	-0.1808241	0.5654713	-0.3198	0.7493	-0.255917807	count	1
PTMA	-0.17731	0.0394811	-4.491	9.05E-06	-0.2556699	count	0.2198607
GNL3	-0.1784313	0.2644885	-0.6746	0.5003	-0.254684454	count	1
DAD1	-0.1788474	0.1360233	-1.3148	0.189	-0.254504517	count	1
TCF25	-0.1820331	0.1622339	-1.122	0.262	-0.25440288	count	1
POP7	-0.1836822	0.3880055	-0.4734	0.6362	-0.252746604	count	1
MRPS15	-0.1774977	0.3423557	-0.5185	0.6044	-0.251216659	count	1
GPS2	-0.1909154	0.277924	-0.6869	0.492	-0.251026882	count	1
XRCC5	-0.1754615	0.1357094	-1.2929	0.197	-0.250116761	count	1
VRK1	-0.1814723	0.399741	-0.454	0.65	-0.249716479	count	1
STX8	-0.1812425	0.386924	-0.4684	0.64	-0.24940137	count	1
HBP1	-0.1782052	0.2016449	-0.8838	0.377	-0.249065647	count	1
AMZ2	-0.1893728	0.359735	-0.5264	0.5989	-0.249012846	count	1
SMAD3	-0.1806104	0.5514816	-0.3275	0.743	-0.248534601	count	1
SWAP70	-0.180501	0.1468606	-1.2291	0.22	-0.248384583	count	1
SETX	-0.1888508	0.263145	-0.7177	0.4733	-0.248331259	count	1
DIAPH1	-0.1802945	0.3993574	-0.4515	0.6519	-0.248101413	count	1
HDAC2	-0.1800522	0.3144007	-0.5727	0.5671	-0.247769148	count	1
NUCKS1	-0.1772673	0.1449723	-1.2228	0.222	-0.247757847	count	1
PIP4P1	-0.188255	0.2420824	-0.7776	0.437	-0.247553277	count	1
ACAP3	-0.1881126	1.2354579	-0.1523	0.879	-0.247367328	count	1
UGCG	-0.1746206	0.2386958	-0.7316	0.465	-0.247150284	count	1
SDHB	-0.1795273	0.3012276	-0.596	0.5515	-0.247049343	count	1
SMAP1	-0.1767225	0.2735945	-0.6459	0.519	-0.246998167	count	1
RNGTT	-0.1785009	0.3207078	-0.5566	0.578	-0.245641777	count	1
C16orf72	-0.1750592	0.421671	-0.4152	0.678	-0.244678756	count	1
DOK7	-0.1859608	0.6951898	-0.2675	0.7892	-0.244557218	count	1
RPS4Y1	-0.1694504	0.0744232	-2.2768	0.0233	-0.243793943	count	1
MAP3K8	-0.1730448	0.1737173	-0.9961	0.32	-0.243768981	count	1
CYBA	-0.1691913	0.0578991	-2.9222	0.00365	-0.24360239	count	1
APRT	-0.1696082	0.1375883	-1.2327	0.2183	-0.243227747	count	1
PPP1R10	-0.1724604	0.165888	-1.0396	0.299	-0.242947134	count	1
CCT7	-0.1764934	0.1883788	-0.9369	0.3493	-0.242888594	count	1
ARPP19	-0.1720008	0.234178	-0.7345	0.463	-0.242300787	count	1
YBX1	-0.1678889	0.0785217	-2.1381	0.0331	-0.241202449	count	1
ABHD17A	-0.1723564	0.255897	-0.6735	0.501	-0.24090957	count	1
CAPN1	-0.1697903	0.3851799	-0.4408	0.6596	-0.240322889	count	1
GOT1	-0.1826203	0.4586383	-0.3982	0.6907	-0.240193791	count	1
NR2C2AP	-0.1822862	0.5049597	-0.361	0.718	-0.23975732	count	1
DPH7	-0.1822862	0.5523238	-0.33	0.7415	-0.23975732	count	1
UBAC1	-0.1714337	0.5012971	-0.342	0.7325	-0.239622755	count	1
MAGED2	-0.1735744	0.2754509	-0.6301	0.529	-0.238884928	count	1

NOB1	-0.1705735	0.3203889	-0.5324	0.595	-0.238423074	count	1
TMEM159	-0.1809134	0.5210823	-0.3472	0.729	-0.237963759	count	1
CWC15	-0.1724277	0.2819242	-0.6116	0.541	-0.237311996	count	1
RNF213	-0.1673066	0.2300286	-0.7273	0.467	-0.236812082	count	1
XRCC6	-0.1662953	0.1576965	-1.0545	0.292	-0.236659468	count	1
RNF114	-0.1712918	0.2502774	-0.6844	0.4941	-0.235753805	count	1
PNN	-0.166514	0.1397105	-1.1918	0.234	-0.235691681	count	1
EP300	-0.170741	0.4193737	-0.4071	0.684	-0.23499821	count	1
RNF126	-0.1659665	0.2524374	-0.6575	0.511	-0.234917738	count	1
TNF	-0.16658	0.1740738	-0.9569	0.339	-0.234676911	count	1
PDCD4	-0.1674563	0.1710729	-0.9789	0.328	-0.234075419	count	1
CGGBP1	-0.1661306	0.2358113	-0.7045	0.481	-0.234044831	count	1
DDX27	-0.164576	0.2398607	-0.6861	0.493	-0.232952103	count	1
RPS20	-0.1616395	0.0524442	-3.0821	0.00218	-0.23275809	count	1
SMDT1	-0.1628716	0.1304349	-1.2487	0.212	-0.232737223	count	1
CLTA	-0.163647	0.1795711	-0.9113	0.363	-0.23236941	count	1
METTL5	-0.1639319	0.2362518	-0.6939	0.488	-0.232041577	count	1
HIKESHI	-0.1642594	0.3041737	-0.54	0.589	-0.231412927	count	1
SRP14	-0.1611787	0.0812469	-1.9838	0.0479	-0.231297577	count	1
LINC01184	-0.1652085	0.4085314	-0.4044	0.6861	-0.230940109	count	1
RRNAD1	-0.1677434	0.5633102	-0.2978	0.766	-0.230885758	count	1
GKAP1	-0.1672269	0.4136817	-0.4042	0.686	-0.230177112	count	1
HMGN2	-0.1607752	0.1261177	-1.2748	0.203	-0.230108222	count	1
SDHC	-0.1632016	0.2630768	-0.6204	0.535	-0.229925052	count	1
RFC1	-0.1746703	0.2473274	-0.7062	0.4804	-0.229804711	count	1
POLR2G	-0.1745642	0.2811049	-0.621	0.5349	-0.229666016	count	1
ENTPD1	-0.1745185	0.2716408	-0.6425	0.5209	-0.229606275	count	1
TRIM52	-0.1668005	0.5495683	-0.3035	0.762	-0.229592074	count	1
GPX4	-0.1597701	0.1172927	-1.3621	0.174	-0.228810107	count	1
EIF5	-0.1623761	0.1347906	-1.2047	0.229	-0.228763901	count	1
CWC22	-0.1661242	0.3341244	-0.4972	0.619	-0.228664143	count	1
XBP1	-0.1658711	0.3263731	-0.5082	0.612	-0.228316866	count	1
CAMK1D	-0.1619708	0.2533616	-0.6393	0.523	-0.228193797	count	1
IDI1	-0.163236	0.1806142	-0.9038	0.367	-0.228188638	count	1
TUT4	-0.1616282	0.1417601	-1.1402	0.255	-0.227711885	count	1
ETFB	-0.1627497	0.1994439	-0.816	0.415	-0.227510267	count	1
SERINC3	-0.1648387	0.2917063	-0.5651	0.5723	-0.226900276	count	1
DNAJB14	-0.1596236	0.2528392	-0.6313	0.528	-0.225950933	count	1
TAF1D	-0.1584888	0.1244772	-1.2732	0.204	-0.225941582	count	1
ENY2	-0.1584012	0.1822908	-0.8689	0.3853	-0.225816797	count	1
SELENOF	-0.1589905	0.171977	-0.9245	0.356	-0.224001496	count	1
ANKRD12	-0.1576026	0.1312778	-1.2005	0.231	-0.223795586	count	1
CMC2	-0.1625238	0.2343447	-0.6935	0.4883	-0.223723709	count	1
TIMM10	-0.1597813	0.2552972	-0.6259	0.532	-0.223369261	count	1
RPL23	-0.1551701	0.0653128	-2.3758	0.0179	-0.223300165	count	1

MTPN	-0.156549	0.1244537	-1.2579	0.209	-0.223178339	count	1
ALCAM	-0.1619512	0.4646811	-0.3485	0.728	-0.222937926	count	1
SINHCAF	-0.157999	0.1714244	-0.9217	0.357	-0.222606725	count	1
TRAF1	-0.1690255	0.4920959	-0.3435	0.7314	-0.222424132	count	1
FCMR	-0.1569277	0.1657976	-0.9465	0.344	-0.222139516	count	1
CAST	-0.1561302	0.1332605	-1.1716	0.242	-0.221706915	count	1
COTL1	-0.1545531	0.128059	-1.2069	0.228	-0.221457941	count	1
PSMB5	-0.1582864	0.3771572	-0.4197	0.675	-0.221283702	count	1
ELOA	-0.1561983	0.2537539	-0.6156	0.539	-0.221108274	count	1
TNFRSF14	-0.1553728	0.2134958	-0.7278	0.467	-0.219941147	count	1
CCNI	-0.1536944	0.085609	-1.7953	0.0733	-0.219820848	count	1
NCOR1	-0.1555717	0.1712366	-0.9085	0.364	-0.219192051	count	1
BZW2	-0.1591254	0.4758891	-0.3344	0.738	-0.219059793	count	1
TATDN3	-0.1590076	0.5072865	-0.3134	0.7541	-0.218898114	count	1
MAPKAPK2	-0.1563589	0.3853977	-0.4057	0.685	-0.218594491	count	1
C12orf57	-0.1537552	0.1519202	-1.0121	0.312	-0.218337772	count	1
C9orf16	-0.1533747	0.1740181	-0.8814	0.379	-0.217797989	count	1
LRIG2	-0.1579127	0.7856736	-0.201	0.8408	-0.217395345	count	1
AKIRIN2	-0.1645558	0.2505343	-0.6568	0.5116	-0.216577691	count	1
LAMTOR1	-0.1528862	0.1898217	-0.8054	0.421	-0.216425392	count	1
TRAPPC2B	-0.1533256	0.2771334	-0.5533	0.58	-0.216032132	count	1
H2AFV	-0.1516921	0.1972881	-0.7689	0.4424	-0.215895004	count	1
HDDC3	-0.1541617	0.3901609	-0.3951	0.6929	-0.215528827	count	1
HLA-DMA	-0.1497345	0.109691	-1.3651	0.173	-0.215286275	count	1
LCP1	-0.1635294	0.1335093	-1.2249	0.2213	-0.215234861	count	1
TIMM13	-0.1558875	0.3493252	-0.4463	0.656	-0.214615548	count	1
MS4A7	-0.1503186	0.3482414	-0.4317	0.6662	-0.213462461	count	1
DDX24	-0.1484409	0.1185612	-1.252	0.211	-0.212311041	count	1
BOD1L1	-0.1499198	0.1396411	-1.0736	0.284	-0.212231068	count	1
EVI2B	-0.1477373	0.1418056	-1.0418	0.298	-0.211456909	count	1
LITAF	-0.149875	0.2111961	-0.7096	0.478	-0.211177382	count	1
VEGFB	-0.1603772	0.1981617	-0.8093	0.4188	-0.211110178	count	1
C11orf58	-0.1471619	0.1209202	-1.217	0.224	-0.210966812	count	1
SPCS1	-0.1481973	0.1381024	-1.0731	0.284	-0.210452977	count	1
RPS16	-0.1459203	0.0414994	-3.5162	0.000483	-0.210257894	count	1
CCT3	-0.1487946	0.2097116	-0.7095	0.478	-0.209657265	count	1
FAM118A	-0.1497975	0.3112498	-0.4813	0.631	-0.209439089	count	1
SLC38A1	-0.1467967	0.2313674	-0.6345	0.526	-0.207814957	count	1
YTHDF2	-0.1456599	0.2416956	-0.6027	0.547	-0.207316691	count	1
PDAP1	-0.156856	0.2356931	-0.6655	0.5061	-0.206501473	count	1
SSR4	-0.1439312	0.1127771	-1.2762	0.203	-0.206244067	count	1
TNFSF9	-0.1438115	0.2049392	-0.7017	0.483	-0.205516516	count	1
MED11	-0.1560594	0.3823252	-0.4082	0.6833	-0.205458674	count	1
ARGLU1	-0.1438133	0.12259	-1.1731	0.241	-0.205303635	count	1
RTRAF	-0.1438054	0.1782101	-0.8069	0.4201	-0.205292364	count	1

CCNL2	-0.1448568	0.3000048	-0.4828	0.6294	-0.205071794	count	1
IST1	-0.1451528	0.2964864	-0.4896	0.625	-0.204533023	count	1
MRPS36	-0.1462542	0.2277491	-0.6422	0.521	-0.204494292	count	1
NT5C3A	-0.1435118	0.2865779	-0.5008	0.6168	-0.204261758	count	1
TNFRSF10B	-0.1447258	0.5221706	-0.2772	0.7818	-0.203932182	count	1
RANBP1	-0.1442732	0.211421	-0.6824	0.495	-0.203295313	count	1
COX5B	-0.1417353	0.1268314	-1.1175	0.264	-0.202870204	count	1
UBA52	-0.140711	0.0494109	-2.8478	0.00461	-0.202675929	count	1
LRIF1	-0.1536881	0.2949712	-0.521	0.603	-0.202354117	count	1
HIBCH	-0.1534984	0.510525	-0.3007	0.764	-0.202105733	count	1
SH3BGRL3	-0.1403556	0.0827135	-1.6969	0.0904	-0.201997165	count	1
COPS5	-0.1464903	0.3310716	-0.4425	0.6584	-0.20171395	count	1
ZNF791	-0.1463577	0.2059871	-0.7105	0.478	-0.201531866	count	1
SOS1	-0.1528614	0.3966979	-0.3853	0.7002	-0.20127165	count	1
LSM10	-0.14269	0.2469225	-0.5779	0.564	-0.201067492	count	1
LAPTM5	-0.1394923	0.0855512	-1.6305	0.1037	-0.200241728	count	1
YBX3	-0.1401765	0.3164661	-0.4429	0.658	-0.200115	count	1
IFI16	-0.1407405	0.1588496	-0.886	0.376	-0.199873331	count	1
ZNF692	-0.1448047	0.8264778	-0.1752	0.861	-0.199399244	count	1
METTL26	-0.1514073	0.2773895	-0.5458	0.5855	-0.19936751	count	1
UTP3	-0.1446455	0.3550485	-0.4074	0.684	-0.199180619	count	1
WSB1	-0.139799	0.1667482	-0.8384	0.402	-0.198981396	count	1
UQCC2	-0.1443361	0.3816088	-0.3782	0.7054	-0.198755724	count	1
REV1	-0.141005	0.4208942	-0.335	0.7378	-0.198696343	count	1
RNF181	-0.1508858	0.2641532	-0.5712	0.5681	-0.198684556	count	1
STAP1	-0.1420824	0.2869152	-0.4952	0.621	-0.198671795	count	1
HNRNPA3	-0.1416654	0.1141485	-1.2411	0.2152	-0.19808976	count	1
ARPC5L	-0.1386481	0.1562357	-0.8874	0.375	-0.196291644	count	1
RPS10	-0.1363532	0.0721459	-1.89	0.0594	-0.196133456	count	1
CAT	-0.1423213	0.2847199	-0.4999	0.617	-0.195988696	count	1
NDUFB2	-0.1378015	0.1566361	-0.8798	0.379	-0.195703206	count	1
HIST1H1E	-0.1379378	0.3203825	-0.4305	0.667	-0.195287103	count	1
HSP90AB1	-0.1355036	0.0627183	-2.1605	0.0313	-0.195246249	count	1
CTDSPL2	-0.1394482	0.3706181	-0.3763	0.707	-0.194994957	count	1
REL	-0.1347914	0.0893837	-1.508	0.1323	-0.193781083	count	1
TPI1	-0.1355528	0.1301263	-1.0417	0.2981	-0.193720502	count	1
LBH	-0.1374629	0.181344	-0.758	0.449	-0.193711599	count	1
PTPN7	-0.1466902	0.2738029	-0.5358	0.5924	-0.193189026	count	1
ARPC2	-0.134132	0.08043	-1.6677	0.0961	-0.192748838	count	1
SS18	-0.139359	0.3411026	-0.4086	0.6831	-0.191920018	count	1
NIF3L1	-0.139241	0.4230538	-0.3291	0.742	-0.191757936	count	1
MT-ND1	-0.133232	0.0578675	-2.3024	0.0218	-0.191677935	count	1
POLE3	-0.13885	0.2642998	-0.5254	0.5996	-0.191220865	count	1
LZIC	-0.1361143	0.5363307	-0.2538	0.7998	-0.190341101	count	1
MBNL1	-0.1380169	0.1892664	-0.7292	0.466	-0.190076505	count	1

TRIM38	-0.137521	0.1904307	-0.7222	0.471	-0.18939531	count	1
ZFC3H1	-0.1374787	0.2870268	-0.479	0.632	-0.189337203	count	1
UBTF	-0.1336425	0.2663301	-0.5018	0.616	-0.189212233	count	1
NDUFB4	-0.1371876	0.1603271	-0.8557	0.3926	-0.188937326	count	1
CCT5	-0.1348475	0.193094	-0.6984	0.485	-0.188572641	count	1
RTN4	-0.1322724	0.2280183	-0.5801	0.562	-0.187857555	count	1
CYTH4	-0.1426205	0.4914636	-0.2902	0.7718	-0.187856728	count	1
RPA2	-0.1361802	0.2183489	-0.6237	0.5332	-0.187553448	count	1
LY86-AS1	-0.1358914	1.253897	-0.1084	0.9137	-0.18715671	count	1
SH3GLB1	-0.133423	0.2169184	-0.6151	0.539	-0.18658396	count	1
EXOSC6	-0.1322921	0.2880195	-0.4593	0.646	-0.186434168	count	1
RPS9	-0.1293085	0.0433591	-2.9823	0.00302	-0.186349711	count	1
DARS	-0.1348146	0.2254427	-0.598	0.55	-0.185677419	count	1
NOL7	-0.1297995	0.1528021	-0.8495	0.396	-0.185309153	count	1
IMP3	-0.1406331	0.204983	-0.6861	0.493	-0.185252153	count	1
RPS19	-0.1280309	0.0499269	-2.5644	0.0107	-0.18458002	count	1
PLK3	-0.1315459	0.4259957	-0.3088	0.7576	-0.183963308	count	1
LARP4	-0.1315013	0.3795579	-0.3465	0.729	-0.18390104	count	1
SH3BGRL	-0.1286804	0.1207649	-1.0655	0.287	-0.183712344	count	1
PLRG1	-0.1333638	0.3362348	-0.3966	0.692	-0.183684233	count	1
GADD45B	-0.1281177	0.2246528	-0.5703	0.569	-0.183592429	count	1
MZT2B	-0.1278645	0.1291608	-0.99	0.3227	-0.183554354	count	1
CTDNEP1	-0.1392351	0.2417994	-0.5758	0.565	-0.183419778	count	1
OTUD6B-AS1	-0.1330835	0.290435	-0.4582	0.647	-0.18329913	count	1
ALKBH7	-0.1274871	0.1649054	-0.7731	0.44	-0.181773028	count	1
DNAJC2	-0.1296179	0.3399842	-0.3812	0.703	-0.181271459	count	1
SKP1	-0.1260859	0.092384	-1.3648	0.173	-0.180762808	count	1
CWC25	-0.1371541	0.256648	-0.5344	0.593	-0.180691828	count	1
IER2	-0.1255777	0.1522309	-0.8249	0.41	-0.18031709	count	1
NOL9	-0.1306813	0.541786	-0.2412	0.81	-0.179998574	count	1
ANXA11	-0.1285444	0.1906864	-0.6741	0.501	-0.179772592	count	1
RPL27	-0.1248885	0.0561303	-2.225	0.0266	-0.179644885	count	1
RPL15	-0.1245164	0.0438012	-2.8428	0.00468	-0.179452648	count	1
OXLD1	-0.1361675	0.4497805	-0.3027	0.7622	-0.17939836	count	1
LGALS8	-0.1361675	0.5132496	-0.2653	0.791	-0.17939836	count	1
HEXB	-0.1301778	0.3541301	-0.3676	0.7133	-0.17930674	count	1
NUTM2B-AS1	-0.129377	0.2983204	-0.4337	0.6647	-0.178206372	count	1
PSPC1	-0.1349371	0.325425	-0.4146	0.6786	-0.177785127	count	1
NUMA1	-0.1348807	0.3867524	-0.3488	0.7274	-0.177711175	count	1
PDCD6IP	-0.1289892	0.2657673	-0.4853	0.628	-0.17767349	count	1
PXK	-0.125015	0.3158172	-0.3958	0.6924	-0.17700903	count	1
PTEN	-0.1258968	0.2816213	-0.447	0.655	-0.176075715	count	1
VTI1B	-0.127769	0.249158	-0.5128	0.608	-0.175996739	count	1
ERP44	-0.1238465	0.2627427	-0.4714	0.6376	-0.175900293	count	1
PNISR	-0.1236243	0.1275161	-0.9695	0.333	-0.175584948	count	1

C8orf76	-0.1330398	0.3986099	-0.3338	0.7387	-0.1752972	count	1
NDUFC1	-0.1251628	0.2631913	-0.4756	0.635	-0.175050777	count	1
SRP72	-0.1239972	0.2186886	-0.567	0.571	-0.174758271	count	1
RPL13	-0.1209032	0.0284916	-4.2435	2.68E-05	-0.174387041	count	0.6509988
ATP5MG	-0.1212912	0.0907393	-1.3367	0.182	-0.17372377	count	1
NCOA3	-0.1314837	0.2156645	-0.6097	0.5424	-0.173256424	count	1
H2AFY	-0.1257166	0.247802	-0.5073	0.612	-0.173176232	count	1
KHDRBS2	-0.1254453	0.6585791	-0.1905	0.849	-0.172803381	count	1
PRDX3	-0.1251543	0.3360941	-0.3724	0.7098	-0.172403453	count	1
FAM204A	-0.1307864	0.2143554	-0.6101	0.542	-0.17234186	count	1
IDH3G	-0.1298265	0.271696	-0.4778	0.633	-0.171082797	count	1
LMBRD1	-0.119725	0.2332979	-0.5132	0.6081	-0.170712064	count	1
C3orf58	-0.1238749	0.4286022	-0.289	0.773	-0.170645087	count	1
SH3GLB2	-0.1238547	0.4162833	-0.2975	0.7662	-0.170617324	count	1
SAMD4B	-0.1238272	0.497115	-0.2491	0.803	-0.170579528	count	1
SSR2	-0.1190961	0.1109216	-1.0737	0.284	-0.170357603	count	1
PMF1	-0.1229926	0.2651495	-0.4639	0.643	-0.169432432	count	1
UQCR10	-0.1200201	0.1732384	-0.6928	0.489	-0.169159422	count	1
RRP1B	-0.1280659	0.3583204	-0.3574	0.721	-0.168773254	count	1
TRAPPC3	-0.1206411	0.3385549	-0.3563	0.722	-0.168736354	count	1
MRPL4	-0.1278663	0.2749795	-0.465	0.6422	-0.168511401	count	1
CIB1	-0.1173971	0.1379127	-0.8512	0.3951	-0.168485351	count	1
HSPA1B	-0.1272727	0.2997355	-0.4246	0.671	-0.167732641	count	1
RCN2	-0.1187296	0.212166	-0.5596	0.576	-0.167342597	count	1
CTSH	-0.1171163	0.134261	-0.8723	0.384	-0.167210948	count	1
C19orf25	-0.1213195	0.3651113	-0.3323	0.7398	-0.167132766	count	1
TSTD1	-0.1179086	0.168027	-0.7017	0.483	-0.166956084	count	1
TMA7	-0.1161474	0.0972346	-1.1945	0.233	-0.166444494	count	1
NAA10	-0.1177873	0.2511708	-0.469	0.639	-0.166015955	count	1
MRPL22	-0.1199454	0.33476	-0.3583	0.72	-0.165243963	count	1
POMC	-0.1245459	0.4578257	-0.272	0.7857	-0.164154834	count	1
NEAT1	-0.1145252	0.1714002	-0.6682	0.504	-0.163301886	count	1
SERF2	-0.1135788	0.0708093	-1.604	0.1094	-0.163268209	count	1
MIF4GD	-0.1184298	0.3435388	-0.3447	0.7305	-0.163160542	count	1
AQR	-0.1237105	0.4643679	-0.2664	0.79	-0.163058568	count	1
PNOC	-0.1235211	0.2596859	-0.4757	0.6346	-0.162810016	count	1
MBD6	-0.1162955	0.6423158	-0.1811	0.8564	-0.162667142	count	1
PSMB3	-0.1144215	0.1596379	-0.7168	0.474	-0.1625236	count	1
TNFAIP8	-0.1136888	0.1499526	-0.7582	0.449	-0.162488014	count	1
HLA-DPB1	-0.1122061	0.0508286	-2.2075	0.0278	-0.161734208	count	1
PARP14	-0.1217203	0.192418	-0.6326	0.527	-0.160446628	count	1
ELAVL1	-0.1163919	0.2982957	-0.3902	0.697	-0.160358947	count	1
TMEM14C	-0.1144462	0.2590675	-0.4418	0.659	-0.160084138	count	1
NDUFV2	-0.1119378	0.1706701	-0.6559	0.5122	-0.159820933	count	1
CXorf38	-0.1209091	0.3626015	-0.3334	0.739	-0.159381897	count	1

PLEKHF1	-0.1155334	0.4879075	-0.2368	0.8129	-0.159178662	count	1
MTDH	-0.1109881	0.1389199	-0.7989	0.4248	-0.159053225	count	1
CDC26	-0.1125669	0.2376289	-0.4737	0.636	-0.158665829	count	1
COA3	-0.120329	0.4652468	-0.2586	0.796	-0.158620455	count	1
IGHG1	-0.1115963	0.2690674	-0.4148	0.679	-0.158513531	count	1
CISD2	-0.120084	0.3332865	-0.3603	0.7188	-0.158298858	count	1
PIGT	-0.1114253	0.2924693	-0.381	0.7034	-0.15827081	count	1
TWF2	-0.1118965	0.2934934	-0.3813	0.703	-0.157721876	count	1
SAT2	-0.1104338	0.2616326	-0.4221	0.6732	-0.157471006	count	1
TEX264	-0.1194439	0.2405831	-0.4965	0.6198	-0.157458606	count	1
STX5	-0.1103631	0.2947332	-0.3745	0.7082	-0.157370246	count	1
ARMCX3	-0.1115101	0.2569377	-0.434	0.665	-0.157177802	count	1
SEL1L3	-0.1114531	0.2498968	-0.446	0.656	-0.157097543	count	1
ARPC1B	-0.1094421	0.1591712	-0.6876	0.4921	-0.156908143	count	1
GNPTG	-0.1120761	0.3800536	-0.2949	0.7682	-0.156773528	count	1
VPS13C	-0.1103183	0.1706024	-0.6466	0.518	-0.156699498	count	1
UQCRB	-0.1089959	0.0843355	-1.2924	0.1969	-0.15638317	count	1
COPS6	-0.1108535	0.2158555	-0.5136	0.608	-0.156253262	count	1
PDCD10	-0.1184039	0.2411807	-0.4909	0.6237	-0.156093327	count	1
HCCS	-0.1131624	0.3140592	-0.3603	0.7188	-0.155918755	count	1
SRSF7	-0.1105448	0.1177602	-0.9387	0.3484	-0.155818588	count	1
RNMT	-0.1084889	0.1476105	-0.735	0.4627	-0.155472766	count	1
KDM1A	-0.1123009	0.4658631	-0.2411	0.8096	-0.154734198	count	1
NOL11	-0.1123009	0.4797935	-0.2341	0.815	-0.154734198	count	1
CSNK1G2	-0.1095943	0.273141	-0.4012	0.688	-0.154480183	count	1
CDC123	-0.1164017	0.2204484	-0.528	0.5977	-0.153464606	count	1
TUBA1B	-0.107549	0.1125442	-0.9556	0.34	-0.153359577	count	1
BLOC1S2	-0.1075306	0.2563314	-0.4195	0.6751	-0.153333352	count	1
IKZF1	-0.1078747	0.255911	-0.4215	0.6736	-0.15323089	count	1
ERGIC2	-0.1104589	0.2511259	-0.4399	0.66	-0.152201329	count	1
U2AF1L4	-0.110356	0.3150097	-0.3503	0.726	-0.152059829	count	1
SEMA7A	-0.1072864	0.3746368	-0.2864	0.7747	-0.151927456	count	1
AGTRAP	-0.115151	0.5412012	-0.2128	0.8316	-0.151822345	count	1
HLA-C	-0.1050764	0.048949	-2.1466	0.0324	-0.151360338	count	1
HPS3	-0.1096086	0.5317837	-0.2061	0.8368	-0.151032051	count	1
NUBP1	-0.1092622	0.3223916	-0.3389	0.7348	-0.150555693	count	1
PGK1	-0.1052942	0.1314369	-0.8011	0.424	-0.15049496	count	1
MINOS1	-0.1074635	0.2226087	-0.4827	0.63	-0.150329956	count	1
RPSA	-0.103811	0.0463445	-2.24	0.0256	-0.149649658	count	1
EIF3L	-0.1051141	0.1503113	-0.6993	0.485	-0.14964139	count	1
NR4A2	-0.1048019	0.1484604	-0.7059	0.481	-0.149637043	count	1
SEC62	-0.1043078	0.1337786	-0.7797	0.436	-0.149211751	count	1
RAB22A	-0.1052641	0.3190495	-0.3299	0.7416	-0.149065957	count	1
RPL35	-0.1032578	0.0513347	-2.0115	0.0449	-0.14871595	count	1
FKBP8	-0.1040016	0.2063095	-0.5041	0.6144	-0.148058512	count	1

TPM3	-0.1034192	0.0936328	-1.1045	0.27	-0.147473563	count	1
RAB2A	-0.1053093	0.2013374	-0.523	0.601	-0.147320383	count	1
COQ10B	-0.1112923	0.2003582	-0.5555	0.5789	-0.146754641	count	1
CBX3	-0.1048132	0.1826131	-0.574	0.566	-0.146627272	count	1
SLC25A36	-0.1033216	0.2701233	-0.3825	0.702	-0.145646948	count	1
SLC39A10	-0.1038104	0.3547425	-0.2926	0.77	-0.145226213	count	1
NPM1	-0.1008692	0.0650591	-1.5504	0.122	-0.145038208	count	1
DEK	-0.1051453	0.1498678	-0.7016	0.483	-0.144893786	count	1
AC245297.3	-0.1049957	0.2867945	-0.3661	0.7145	-0.144688027	count	1
ARAP2	-0.1049957	0.3052344	-0.344	0.731	-0.144688027	count	1
THG1L	-0.1033476	0.4831944	-0.2139	0.8307	-0.144579601	count	1
LINC01781	-0.1030922	0.2117413	-0.4869	0.627	-0.144222759	count	1
TMEM147	-0.1030458	0.270962	-0.3803	0.704	-0.14415793		1
FAM96A	-0.1009359	0.2372679	-0.4254	0.6707	-0.143696496	count	1
MRPL51	-0.1011631	0.2333073	-0.4336	0.665	-0.143262875	count	1
CAPRIN2	-0.1081355	0.5383627	-0.2009	0.8409	-0.142607691	count	1
GRK6	-0.1034067	0.2738921	-0.3775	0.7059	-0.142502451	count	1
SLC25A5	-0.0999126	0.1118453	-0.8933	0.372	-0.14247558	count	1
CD81	-0.0991408	0.0922809	-1.0743	0.2833	-0.142423863	count	1
GTF2A2	-0.1027789	0.209541	-0.4905	0.624	-0.141638912	count	1
RPL24	-0.0977775	0.0429189	-2.2782	0.0232	-0.140927173	count	1
PTPRA	-0.1022308	0.2984221	-0.3426	0.7321	-0.140884984	count	1
MNDA	-0.099131	0.2932852	-0.338	0.736	-0.140818593	count	1
ARHGAP24	-0.0989956	0.154935	-0.6389	0.523	-0.140626372	count	1
HDAC3	-0.106278	0.2780883	-0.3822	0.703	-0.140167129	count	1
RPL23A	-0.0971635	0.0403134	-2.4102	0.0163	-0.140021367	count	1
NGLY1	-0.0983508	0.2294925	-0.4286	0.668	-0.139710975	count	1
CDK17	-0.0985965	0.4808478	-0.205	0.8376	-0.139630843	count	1
NIPSNAP2	-0.0996855	0.3535773	-0.2819	0.778	-0.139462742	count	1
MRPS7	-0.0988069	0.2594559	-0.3808	0.704	-0.139288667	count	1
BCAS4	-0.0968105	0.2036991	-0.4753	0.6348	-0.138490468	count	1
LINC01504	-0.1049531	0.8713027	-0.1205	0.9042	-0.138426146	count	1
COX6A1	-0.0967701	0.1117869	-0.8657	0.387	-0.138316081	count	1
M6PR	-0.0972339	0.2219695	-0.4381	0.662	-0.138125339	count	1
FBL	-0.0955905	0.1942902	-0.492	0.623	-0.135792202	count	1
INSIG2	-0.0984719	0.4449421	-0.2213	0.8249	-0.135714083	count	1
UGP2	-0.0966346	0.2650108	-0.3646	0.716	-0.135199513	count	1
PPP1R12A	-0.0958955	0.1796998	-0.5336	0.594	-0.1351881	count	1
SSU72	-0.0953209	0.1678097	-0.568	0.57	-0.134995285	count	1
CUL3	-0.0979314	0.2155802	-0.4543	0.65	-0.134970488	count	1
RPL9	-0.0933934	0.0415472	-2.2479	0.0251	-0.134628851	count	1
RPL3	-0.0933203	0.0393813	-2.3697	0.0182	-0.134515317	count	1
ERICH1	-0.0942454	0.2436613	-0.3868	0.6991	-0.134176434	count	1
WASHC4	-0.0951715	0.288219	-0.3302	0.741	-0.133154907	count	1
PTPN22	-0.0964911	0.3691843	-0.2614	0.794	-0.132988916	count	1

OGFOD3	-0.0943244	0.4257372	-0.2216	0.8248	-0.132975185	count	1
SLA	-0.1006487	0.4516557	-0.2228	0.824	-0.132768802	count	1
SNX22	-0.095998	0.3746206	-0.2563	0.798	-0.132310482	count	1
SDAD1	-0.1000551	0.3198258	-0.3128	0.7545	-0.131988485	count	1
ARPC3	-0.0916444	0.0643507	-1.4241	0.155	-0.131973395	count	1
PPM1K	-0.0941262	0.1848558	-0.5092	0.611	-0.131694108	count	1
SIVA1	-0.0996063	0.1851493	-0.538	0.5909	-0.131398493	count	1
TGFB1	-0.0920425	0.1764195	-0.5217	0.602	-0.130754932	count	1
ASAH1	-0.0923964	0.2395643	-0.3857	0.7	-0.130259481	count	1
GGNBP2	-0.0911694	0.1657482	-0.55	0.583	-0.130179861	count	1
HSP90B1	-0.0906762	0.1295947	-0.6997	0.4845	-0.130178865	count	1
TMEM242	-0.0984288	0.3586871	-0.2744	0.7839	-0.129850463	count	1
TXLNB	-0.0937722	0.4081924	-0.2297	0.818	-0.129247947	count	1
SPCS2	-0.0900094	0.1175623	-0.7656	0.4443	-0.128996687	count	1
GNA13	-0.0920534	0.3409528	-0.27	0.787	-0.128797271	count	1
MZT2A	-0.0975414	0.1646438	-0.5924	0.5539	-0.128683732	count	1
CHST11	-0.093322	0.3040785	-0.3069	0.7591	-0.128628474	count	1
PHC3	-0.0931915	0.3672449	-0.2538	0.8	-0.128448905	count	1
RPL4	-0.0892432	0.0713749	-1.2503	0.2118	-0.128252435	count	1
EIF4A3	-0.0901894	0.2117829	-0.4259	0.67	-0.128123903	count	1
CCDC9	-0.0970022	0.5207803	-0.1863	0.8523	-0.12797477	count	1
MT-ND3	-0.0888146	0.04796	-1.8518	0.0647	-0.127855179	count	1
ZNHIT1	-0.090166	0.2531854	-0.3561	0.722	-0.127117704	count	1
HMGB2	-0.0901523	0.191832	-0.47	0.639	-0.127098405	count	1
CANX	-0.0907562	0.2928041	-0.31	0.757	-0.126984295	count	1
TRIM44	-0.0919752	0.2785872	-0.3301	0.741	-0.126775222	count	1
PPCS	-0.0905563	0.2940777	-0.3079	0.758	-0.126704908	count	1
LAMTOR5	-0.0886556	0.1593803	-0.5563	0.578	-0.126591753	count	1
ATP6V1F	-0.0890346	0.1782035	-0.4996	0.618	-0.126484288	count	1
HSPA4	-0.0900571	0.2140083	-0.4208	0.674	-0.126007203	count	1
FBXW5	-0.0953943	0.37768	-0.2526	0.8007	-0.125860474	count	1
SRGAP2	-0.0953943	0.4332247	-0.2202	0.8258	-0.125860474	count	1
PIN1	-0.0911938	0.3066919	-0.2973	0.7663	-0.125699941	count	1
SNHG7	-0.0952385	0.1587386	-0.6	0.549	-0.125655593	count	1
MEF2C	-0.0879407	0.1146355	-0.7671	0.443	-0.125410652	count	1
CAP1	-0.0884562	0.1615407	-0.5476	0.584	-0.125279749	count	1
RPL18A	-0.0868517	0.042223	-2.057	0.0403	-0.12526175	count	1
EBPL	-0.0894998	0.3105328	-0.2882	0.773	-0.125228284	count	1
RNASEH2B	-0.0875223	0.1819912	-0.4809	0.631	-0.124609271	count	1
SNHG8	-0.0944058	0.1184874	-0.7968	0.426	-0.124560533	count	1
CEBPB	-0.0943739	0.2799332	-0.3371	0.7362	-0.124518579	count	1
G3BP1	-0.0941949	0.2532644	-0.3719	0.7101	-0.124283172	count	1
RPS6	-0.0859942	0.0377669	-2.277	0.0233	-0.123987445	count	1
NDUFS8	-0.0876829	0.2540989	-0.3451	0.73	-0.123619811	count	1
ETS1	-0.088332	0.21964	-0.4022	0.688	-0.123596055	count	1

RRAS2	-0.0881439	0.2844516	-0.3099	0.757	-0.123333144	count	1
MFNG	-0.0894324	0.3746252	-0.2387	0.8114	-0.12327597	count	1
PRDX5	-0.089211	0.1776289	-0.5022	0.6158	-0.122971277	count	1
KMT2A	-0.0861864	0.2019879	-0.4267	0.67	-0.122708163	count	1
TMC6	-0.0863659	0.2790425	-0.3095	0.7571	-0.122695106	count	1
NDUFC2	-0.0857757	0.2040554	-0.4204	0.6744	-0.122123694	count	1
RRAGC	-0.0881291	0.3407881	-0.2586	0.796	-0.121482316	count	1
HELZ	-0.0865853	0.3970141	-0.2181	0.8275	-0.12115461	count	1
PRKCB	-0.0859038	0.2216419	-0.3876	0.699	-0.121113527	count	1
RPL36	-0.0838696	0.0499122	-1.6803	0.0936	-0.120702717	count	1
DCAF7	-0.0913569	0.4017632	-0.2274	0.8202	-0.120550449	count	1
ABHD14B	-0.0873809	0.2404976	-0.3633	0.7165	-0.120452575	count	1
ITGB1	-0.0846678	0.2011641	-0.4209	0.674	-0.120283973	count	1
USP3	-0.0869083	0.2104323	-0.413	0.68	-0.119802124	count	1
ITGA4	-0.084396	0.2444122	-0.3453	0.73	-0.119532909	count	1
FAM168B	-0.0860775	0.5217328	-0.165	0.869	-0.118658646	count	1
CHD2	-0.0860367	0.2508511	-0.343	0.7318	-0.11860249	count	1
PKM	-0.0898282	0.1887233	-0.476	0.634	-0.118539488	count	1
NRROS	-0.0896162	0.5039953	-0.1778	0.859	-0.118260591	count	1
CLNS1A	-0.0827971	0.231122	-0.3582	0.7203	-0.117884753	count	1
RNASEH2C	-0.0831466	0.250046	-0.3325	0.7396	-0.117764426	count	1
ZNF280D	-0.0841163	0.5303416	-0.1586	0.8741	-0.117703389	count	1
HLA-DPA1	-0.0815487	0.0508717	-1.603	0.11	-0.117567113	count	1
MRPL34	-0.0852416	0.2409821	-0.3537	0.724	-0.117508113	count	1
MT-ND4	-0.081499	0.0450354	-1.8097	0.071	-0.117467909	count	1
CHCHD1	-0.0831998	0.3669095	-0.2268	0.821	-0.116422232	count	1
RAB1A	-0.0881077	0.3518803	-0.2504	0.8024	-0.116275958	count	1
FAU	-0.0804681	0.0369175	-2.1797	0.0298	-0.116039093	count	1
PARP1	-0.0812037	0.1494992	-0.5432	0.587	-0.115954722	count	1
MYL6	-0.0805489	0.0754641	-1.0674	0.286	-0.115916398	count	1
SUB1	-0.0805419	0.1056878	-0.7621	0.4464	-0.115749269	count	1
AC026250.1	-0.0874398	0.786352	-0.1112	0.912	-0.115397179	count	1
COL4A3BP	-0.0809026	0.3357532	-0.241	0.8097	-0.114937577	count	1
NCL	-0.0799109	0.0828333	-0.9647	0.335	-0.114918946	count	1
KIAA1109	-0.0830276	0.4301803	-0.193	0.847	-0.114460591	count	1
FIS1	-0.0805066	0.2122254	-0.3793	0.7046	-0.114375262	count	1
RPS15	-0.0787117	0.0366733	-2.1463	0.0324	-0.113492331	count	1
SUMO3	-0.0804582	0.259067	-0.3106	0.756	-0.113441581	count	1
SLC25A28	-0.0820544	0.3368858	-0.2436	0.8077	-0.113120924	count	1
EIF3F	-0.0786835	0.0979561	-0.8033	0.4223	-0.113078831	count	1
EIF2S2	-0.0804605	0.1710117	-0.4705	0.638	-0.112592841	count	1
MBD4	-0.0815312	0.175835	-0.4637	0.643	-0.112400688	count	1
FAM27C	-0.0797368	1.2348312	-0.0646	0.9485	-0.111581105	count	1
RAB21	-0.0809224	0.2361999	-0.3426	0.7321	-0.111562598	count	1
RBM39	-0.0768481	0.09551	-0.8046	0.4215	-0.109180104	count	1

PLEK	-0.0761435	0.192863	-0.3948	0.6932	-0.108842306	count	1
H3F3A	-0.0751573	0.0636952	-1.18	0.239	-0.108279224	count	1
YPEL3	-0.0767836	0.1574095	-0.4878	0.626	-0.108264234	count	1
LSP1	-0.07572	0.1355605	-0.5586	0.577	-0.108237109	count	1
RPS25	-0.0750379	0.0338651	-2.2158	0.0272	-0.108183595	count	1
FCGR2B	-0.0766415	0.2092455	-0.3663	0.714	-0.108064014	count	1
CXXC1	-0.0782994	0.4413054	-0.1774	0.8593	-0.107951492	count	1
DCP2	-0.0816561	0.2606651	-0.3133	0.754	-0.107785616	count	1
ATXN10	-0.0769163	0.2614981	-0.2941	0.769	-0.107637856	count	1
ARL6IP4	-0.0759002	0.1269059	-0.5981	0.55	-0.107506734	count	1
FAM177B	-0.0814169	0.4727949	-0.1722	0.863	-0.107470753	count	1
FCRL1	-0.081383	0.2740346	-0.297	0.7666	-0.107426129	count	1
DNAJC8	-0.0778429	0.1819674	-0.4278	0.669	-0.107322989	count	1
CAPZB	-0.076502	0.1321305	-0.579	0.563	-0.107058613	count	1
GID8	-0.0754059	0.365392	-0.2064	0.8366	-0.106806983	count	1
TRIP11	-0.0774397	0.3566863	-0.2171	0.828	-0.10676786	count	1
THAP1	-0.0804435	0.5281169	-0.1523	0.879	-0.106189394	count	1
RPL21	-0.0734558	0.0388379	-1.8913	0.0592	-0.105911414	count	1
AURKAIP1	-0.0738812	0.1997989	-0.3698	0.7117	-0.105501742	count	1
EEF1D	-0.0727168	0.0643435	-1.1301	0.259	-0.104771484	count	1
IRF8	-0.0759253	0.1458456	-0.5206	0.6029	-0.104682748	count	1
NCF2	-0.0741342	0.6216694	-0.1193	0.9051	-0.104531125	count	1
PRPF40A	-0.0730958	0.1953043	-0.3743	0.708	-0.104077569	count	1
ADH5	-0.0742519	0.2642492	-0.281	0.779	-0.103912591	count	1
PTP4A3	-0.0749899	0.5447798	-0.1377	0.8906	-0.103394779	count	1
TRAPPC10	-0.0728982	0.2531214	-0.288	0.773	-0.103256902	count	1
ANP32A	-0.0726076	0.2193973	-0.3309	0.741	-0.102380001	count	1
BORCS5	-0.0727484	0.4463622	-0.163	0.8706	-0.101810345	count	1
EXOC5	-0.0737545	0.3968718	-0.1858	0.8527	-0.101693665	count	1
FNBP1	-0.076916	0.1712276	-0.4492	0.654	-0.101545149	count	1
ACTR3	-0.0708562	0.118122	-0.5999	0.549	-0.101286422	count	1
RTCB	-0.0734534	0.2839283	-0.2587	0.796	-0.101279047	count	1
C19orf66	-0.0733542	0.2611593	-0.2809	0.7789	-0.101142446	count	1
BCL2	-0.0764	0.1814802	-0.421	0.674	-0.100865695	count	1
PTGES3	-0.07036	0.1231955	-0.5711	0.568	-0.100731237	count	1
ATP5F1E	-0.0700468	0.0715108	-0.9795	0.3279	-0.10066807	count	1
TOB2	-0.072718	0.299825	-0.2425	0.8085	-0.100266372	count	1
PRPF38A	-0.0757914	0.3392769	-0.2234	0.8233	-0.100064277	count	1
RPS24	-0.0693824	0.0397832	-1.744	0.0818	-0.100036363	count	1
CD27	-0.0709309	0.1870469	-0.3792	0.705	-0.100017302	count	1
YTHDC1	-0.0752917	0.2106832	-0.3574	0.721	-0.099406235	count	1
U2SURP	-0.0715856	0.2064908	-0.3467	0.729	-0.098706961	count	1
TMEM258	-0.0691802	0.1967546	-0.3516	0.725	-0.098290722	count	1
HLA-A	-0.0681915	0.0483619	-1.41	0.159	-0.09828077	count	1
RPS2	-0.0681206	0.0348562	-1.9543	0.0513	-0.098240628	count	1

AIMP1	-0.0743387	0.2942012	-0.2527	0.801	-0.098151191	count	1
NDUFS7	-0.0742514	0.1996011	-0.372	0.7101	-0.098036218	count	1
PPIA	-0.0681707	0.0761717	-0.895	0.3713	-0.097704289	count	1
NAP1L1	-0.067994	0.1295636	-0.5248	0.6	-0.097344649	count	1
RPL28	-0.0674425	0.0316878	-2.1283	0.0339	-0.097266737	count	1
PRCP	-0.0694177	0.4538802	-0.1529	0.8785	-0.097152964	count	1
EIF1AX	-0.0731052	0.1908953	-0.383	0.7019	-0.096526621	count	1
GSTP1	-0.0670326	0.1465847	-0.4573	0.6477	-0.096152235	count	1
S100A4	-0.0668575	0.1496936	-0.4466	0.6554	-0.096051533	count	1
ECI1	-0.0672993	0.3164024	-0.2127	0.8317	-0.095330212	count	1
SSSCA1	-0.0669957	0.3113375	-0.2152	0.8297	-0.095188266	count	1
ASH1L	-0.0674236	0.2377819	-0.2836	0.777	-0.095074797	count	1
RACK1	-0.0658123	0.0448583	-1.4671	0.143	-0.094884476	count	1
SKI	-0.0716219	0.6039835	-0.1186	0.9057	-0.094572867	count	1
ATP5PF	-0.0660459	0.1564667	-0.4221	0.673	-0.094411938	count	1
HNRNPF	-0.0656689	0.1229283	-0.5342	0.5935	-0.094196425	count	1
RPL38	-0.0656333	0.0542363	-1.2101	0.2269	-0.094068239	count	1
TPT1	-0.0647438	0.0354214	-1.8278	0.0682	-0.093365277	count	1
PMPCB	-0.0703291	0.248062	-0.2835	0.7769	-0.092869868	count	1
MTRF1L	-0.0672841	0.4924291	-0.1366	0.8914	-0.09278284	count	1
TMEM43	-0.0672841	0.5160411	-0.1304	0.8963	-0.09278284	count	1
SLC9B2	-0.0672841	0.5771359	-0.1166	0.9072	-0.09278284	count	1
PRRC1	-0.0672841	0.5967896	-0.1127	0.91	-0.09278284	count	1
MLF2	-0.0699738	0.2656646	-0.2634	0.7924	-0.092401807	count	1
RGS19	-0.0649587	0.1590514	-0.4084	0.683	-0.092295214	count	1
TMEM80	-0.0665531	0.5132061	-0.1297	0.8969	-0.091775998	count	1
LONRF1	-0.0694382	0.3194664	-0.2174	0.828	-0.091696201	count	1
TXNL4A	-0.0652541	0.1945858	-0.3353	0.7375	-0.091330385	count	1
ANP32E	-0.0651236	0.1572176	-0.4142	0.679	-0.091147878	count	1
RBM8A	-0.0637264	0.1121879	-0.568	0.57	-0.090741379	count	1
RAB4A	-0.0652573	0.3929074	-0.1661	0.868	-0.089991164	count	1
SPCS3	-0.0650662	0.2305508	-0.2822	0.7779	-0.089727936	count	1
PSMA5	-0.0649857	0.2877939	-0.2258	0.8215	-0.089617052	count	1
TMBIM4	-0.0624443	0.1505336	-0.4148	0.6785	-0.089601888	count	1
CHMP5	-0.063972	0.2486463	-0.2573	0.797	-0.089537315	count	1
SNRPD3	-0.0649034	0.152692	-0.4251	0.671	-0.089503688	count	1
HNRNPC	-0.0635232	0.1407693	-0.4513	0.652	-0.088909636	count	1
TMEM50A	-0.0629776	0.166823	-0.3775	0.706	-0.088809008	count	1
SYNRG	-0.0643022	0.2370285	-0.2713	0.7863	-0.088675557	count	1
P3H1	-0.0669645	1.2545481	-0.0534	0.9575	-0.08843698	count	1
MRPL41	-0.0624763	0.2126937	-0.2937	0.769	-0.087445445	count	1
RBM25	-0.0614048	0.1332742	-0.4607	0.645	-0.087436659	count	1
RBBP6	-0.0633027	0.2741362	-0.2309	0.817	-0.087298742	count	1
LSM8	-0.0660788	0.1581348	-0.4179	0.6762	-0.087269891	count	1
GNB2	-0.0620786	0.1910112	-0.325	0.745	-0.086889213	count	1

GLRX5	-0.0629839	0.3295313	-0.1911	0.8485	-0.086859583	count	1
ARPC5	-0.0611235	0.1306826	-0.4677	0.64	-0.086848069	count	1
NT5C	-0.0617676	0.2042191	-0.3025	0.762	-0.086454238	count	1
POLR2K	-0.0616747	0.2212457	-0.2788	0.781	-0.086324304	count	1
SAP18	-0.0607821	0.1161498	-0.5233	0.601	-0.086102596	count	1
CLEC2D	-0.0614074	0.309971	-0.1981	0.843	-0.085950446	count	1
SELENOT	-0.0613521	0.1464433	-0.4189	0.675	-0.085873101	count	1
LSM2	-0.0599275	0.2194041	-0.2731	0.785	-0.083880544	count	1
NCK2	-0.0634935	0.3439592	-0.1846	0.8536	-0.083862822	count	1
ERP29	-0.0586327	0.1002847	-0.5847	0.559	-0.083732208	count	1
LSM1	-0.0628065	0.3272601	-0.1919	0.8479	-0.082957349	count	1
SZRD1	-0.0588056	0.308977	-0.1903	0.849	-0.082311319	count	1
EIF3E	-0.0572448	0.1112433	-0.5146	0.607	-0.082083056	count	1
AP1S2	-0.0569103	0.2680146	-0.2123	0.8319	-0.081273049	count	1
ARL4C	-0.0613778	0.40981	-0.1498	0.881	-0.081074168	count	1
SSPN	-0.0611529	0.2861626	-0.2137	0.831	-0.080777709	count	1
NDUFA3	-0.0565723	0.191854	-0.2949	0.7682	-0.08038365	count	1
GCC2	-0.0579052	0.179601	-0.3224	0.747	-0.079862804	count	1
LINC02132	-0.0577974	0.7198907	-0.0803	0.936	-0.079714277	count	1
ANAPC11	-0.06025	0.2397466	-0.2513	0.8017	-0.079587476	count	1
PTPN18	-0.0566759	0.3991317	-0.142	0.887	-0.079332344	count	1
ZFAS1	-0.0551916	0.0947265	-0.5826	0.56	-0.079139377	count	1
WASHC3	-0.0573527	0.2910577	-0.197	0.8439	-0.079101564	count	1
COX7A2	-0.0553861	0.1211204	-0.4573	0.648	-0.078996938	count	1
ZNF787	-0.0562736	0.3030959	-0.1857	0.853	-0.078769599	count	1
MED13L	-0.0570393	0.3191406	-0.1787	0.8582	-0.078669751	count	1
SRA1	-0.0552217	0.3618137	-0.1526	0.8788	-0.078465219	count	1
HNRNPA0	-0.0535385	0.1315892	-0.4069	0.684	-0.076362353	count	1
EVL	-0.0536312	0.1964432	-0.273	0.785	-0.075976706	count	1
DDOST	-0.0549052	0.3153546	-0.1741	0.862	-0.07572919	count	1
6-Sep	-0.0530756	0.1493052	-0.3555	0.722	-0.075702274	count	1
SYK	-0.0540616	0.3032297	-0.1783	0.859	-0.075675312	count	1
LETMD1	-0.0540181	0.3981538	-0.1357	0.8921	-0.07561446	count	1
KTN1	-0.0531058	0.1565834	-0.3392	0.735	-0.07545967	count	1
LEMD2	-0.053854	0.5653606	-0.0953	0.9242	-0.0753849	count	1
NOP56	-0.0528196	0.2739794	-0.1928	0.8472	-0.074827389	count	1
RPL12	-0.0518	0.0411439	-1.259	0.209	-0.074708102	count	1
TRIM22	-0.0521139	0.2014036	-0.2588	0.7959	-0.074561773	count	1
PPDPF	-0.0521018	0.1438462	-0.3622	0.717	-0.074482776	count	1
UBE2A	-0.0520643	0.2104299	-0.2474	0.8047	-0.073980233	count	1
BCAS2	-0.0516813	0.2070165	-0.2496	0.803	-0.073942937	count	1
RB1	-0.0523277	0.2753428	-0.19	0.849	-0.073797914	count	1
RBM38	-0.0556568	0.1776573	-0.3133	0.7542	-0.073531416	count	1
CDK5R1	-0.0524912	1.007981	-0.0521	0.9585	-0.073478435	count	1
PFDN5	-0.0509412	0.0549189	-0.9276	0.354	-0.073390512	count	1

CORO1A	-0.0505338	0.0868546	-0.5818	0.561	-0.072682437	count	1
RASSF1	-0.0508211	0.2429899	-0.2091	0.8344	-0.072214259	count	1
RPS18	-0.0498797	0.0370977	-1.3445	0.179	-0.071937273	count	1
VPS28	-0.050056	0.1551973	-0.3225	0.747	-0.071396362	count	1
FAM96B	-0.0503795	0.1833735	-0.2747	0.784	-0.071371848	count	1
PAFAH1B2	-0.0501911	0.3033162	-0.1655	0.8686	-0.07131933	count	1
EEF2	-0.0497213	0.0930078	-0.5346	0.593	-0.071296445	count	1
SEC11A	-0.0499758	0.1983581	-0.2519	0.8012	-0.071166674	count	1
TBCA	-0.0515614	0.1493271	-0.3453	0.73	-0.07112134	count	1
LAT2	-0.0496272	0.2336299	-0.2124	0.8319	-0.070874203	count	1
DMTF1	-0.0534584	0.4003449	-0.1335	0.8938	-0.070632187	count	1
GSTK1	-0.0500142	0.1709502	-0.2926	0.77	-0.070536635	count	1
PARVG	-0.0503616	0.3311482	-0.1521	0.879	-0.070499145	count	1
POP5	-0.0509514	0.2968434	-0.1716	0.864	-0.070280684	count	1
RPS15A	-0.0487086	0.0336037	-1.4495	0.148	-0.070245244	count	1
ATP6V1E1	-0.0531383	0.2952175	-0.18	0.8572	-0.070210005	count	1
GPR183	-0.0487018	0.1263012	-0.3856	0.7	-0.070032388	count	1
GOSR2	-0.0503184	0.4480465	-0.1123	0.9106	-0.069408311	count	1
SELENOH	-0.0480577	0.1417469	-0.339	0.7347	-0.068937435	count	1
SNX3	-0.0479598	0.1188997	-0.4034	0.687	-0.068770885	count	1
HLA-DRA	-0.0475249	0.0411313	-1.1554	0.249	-0.068545771	count	1
MRPL40	-0.0483357	0.3009703	-0.1606	0.8725	-0.068477431	count	1
POLR3K	-0.0492473	0.2848358	-0.1729	0.8628	-0.067932124	count	1
ZBTB8OS	-0.049153	0.3215867	-0.1528	0.879	-0.067802157	count	1
GNG5	-0.0478486	0.1526874	-0.3134	0.754	-0.067787591	count	1
UBE2V2	-0.0479778	0.2883305	-0.1664	0.8679	-0.067665863	count	1
ZNF330	-0.0479778	0.3197763	-0.15	0.8808	-0.067665863	count	1
UFC1	-0.0472821	0.1685904	-0.2805	0.779	-0.067525725	count	1
RPL36AL	-0.0467626	0.0600874	-0.7782	0.4368	-0.067184227	count	1
UBA3	-0.048398	0.3691041	-0.1311	0.8957	-0.066761581	count	1
TADA3	-0.0471079	0.3744061	-0.1258	0.8999	-0.066738587	count	1
CYB5R4	-0.04726	0.2854876	-0.1655	0.869	-0.066159762	count	1
MAML2	-0.0464416	0.422471	-0.1099	0.9125	-0.065500167	count	1
UBE2D3	-0.0456366	0.1317161	-0.3465	0.729	-0.065176141	count	1
SLC25A6	-0.0451399	0.0727953	-0.6201	0.536	-0.06492485	count	1
RPL36A	-0.045497	0.1199947	-0.3792	0.705	-0.064895057	count	1
EIF4B	-0.0454599	0.1461779	-0.311	0.756	-0.064598333	count	1
UBLCP1	-0.0463392	0.5197248	-0.0892	0.929	-0.063923904	count	1
POLK	-0.0483178	0.2919303	-0.1655	0.8686	-0.063851109	count	1
RPL7	-0.0438947	0.0572818	-0.7663	0.444	-0.063221413	count	1
SLC25A26	-0.0450488	0.3831564	-0.1176	0.9065	-0.063065921	count	1
HELQ	-0.0441091	0.4026389	-0.1096	0.9128	-0.062679357	count	1
PSMG2	-0.0438181	0.2225356	-0.1969	0.844	-0.062265952	count	1
HSPB1	-0.0470428	0.2458008	-0.1914	0.8483	-0.062168861	count	1
SHOC2	-0.0435808	0.1850441	-0.2355	0.814	-0.061928834	count	1

PPIG	-0.0435784	0.1483546	-0.2937	0.769	-0.061925424	count	1
EIF3H	-0.0431366	0.1115037	-0.3869	0.699	-0.061828697	count	1
CARD16	-0.043179	0.344735	-0.1253	0.9004	-0.061174162	count	1
RPL37A	-0.0420274	0.0516874	-0.8131	0.417	-0.060524137	count	1
GNL2	-0.0456353	0.348947	-0.1308	0.896	-0.060311623	count	1
CSK	-0.0425966	0.2166411	-0.1966	0.844	-0.060079319	count	1
ITGB2	-0.0423996	0.2940122	-0.1442	0.885	-0.059801569	count	1
SMARCA5	-0.0421902	0.1812245	-0.2328	0.816	-0.059773694	count	1
RALGPS2	-0.041343	0.1044514	-0.3958	0.692	-0.059153439	count	1
COX4I1	-0.0404379	0.0650508	-0.6216	0.535	-0.058246119	count	1
CYBB	-0.0409929	0.1654952	-0.2477	0.804	-0.057818241	count	1
SNN	-0.043608	0.2844926	-0.1533	0.8782	-0.057636228	count	1
DGUOK	-0.0403875	0.1843605	-0.2191	0.8267	-0.057392208	count	1
HLA-DQA1	-0.0397334	0.0705777	-0.563	0.574	-0.057252453	count	1
PSIP1	-0.0411429	0.171165	-0.2404	0.81	-0.056760842	count	1
PTTG1	-0.0409317	0.4767477	-0.0859	0.932	-0.056469676	count	1
HLA-DRB1	-0.0391113	0.052116	-0.7505	0.453	-0.056404638	count	1
DDX5	-0.0393466	0.0682694	-0.5763	0.5647	-0.05633605	count	1
CMSS1	-0.0397364	0.4027471	-0.0987	0.9214	-0.05604664	count	1
MRPL20	-0.0396026	0.1902298	-0.2082	0.835	-0.055857986	count	1
CPQ	-0.0397652	0.5893626	-0.0675	0.9462	-0.054861472	count	1
POU2F2	-0.0381026	0.1685574	-0.2261	0.821	-0.053743005	count	1
NOP58	-0.0388809	0.2166949	-0.1794	0.8577	-0.053642281	count	1
ATP5IF1	-0.0375358	0.1475131	-0.2545	0.799	-0.053181252	count	1
ZBTB24	-0.038423	0.2436693	-0.1577	0.875	-0.053010955	count	1
CORO1B	-0.0381333	0.2712065	-0.1406	0.888	-0.052611529	count	1
SNU13	-0.0365655	0.1111584	-0.3289	0.742	-0.052354535	count	1
PPP2CA	-0.0375346	0.2086021	-0.1799	0.857	-0.051786054	count	1
DAZAP1	-0.0372967	0.2440535	-0.1528	0.8786	-0.051458037	count	1
KDM5A	-0.0363029	0.2724352	-0.1333	0.894	-0.051205375	count	1
CD164	-0.036918	0.2089276	-0.1767	0.8598	-0.05093588	count	1
G3BP2	-0.036284	0.1561881	-0.2323	0.816	-0.050800884	count	1
CSNK2B	-0.036816	0.1903851	-0.1934	0.8468	-0.05079524	count	1
PSMD2	-0.0359337	0.2826738	-0.1271	0.899	-0.050310638	count	1
SSBP4	-0.0353853	0.2597936	-0.1362	0.892	-0.050135158	count	1
RSL24D1	-0.0347676	0.1478464	-0.2352	0.8142	-0.049809477	count	1
PPM1G	-0.0355322	0.2124522	-0.1672	0.867	-0.049748732	count	1
NUPL2	-0.0358348	0.4421786	-0.081	0.935	-0.04944231	count	1
EIF5A	-0.0342647	0.1460766	-0.2346	0.815	-0.049197322	count	1
RPL41	-0.0335488	0.0281415	-1.1921	0.234	-0.048389344	count	1
ACADM	-0.0347603	0.3170817	-0.1096	0.9128	-0.047960679	count	1
VIM	-0.0328347	0.1186761	-0.2767	0.7822	-0.047226943	count	1
TELO2	-0.0341963	0.6275731	-0.0545	0.9566	-0.047182956	count	1
TCTA	-0.0341044	0.8211267	-0.0415	0.9669	-0.04705623	count	1
NOC2L	-0.0334763	0.4724875	-0.0709	0.9435	-0.046871384	count	1

APBB1IP	-0.0326362	0.1489817	-0.2191	0.827	-0.046379439	count	1
FOS	-0.0322413	0.2971206	-0.1085	0.914	-0.04630335	count	1
PPP1R8	-0.0342257	0.7615988	-0.0449	0.9642	-0.045249795	count	1
YJU2	-0.0335294	0.4358712	-0.0769	0.9387	-0.044330234	count	1
NUDCD2	-0.0333069	0.297022	-0.1121	0.9108	-0.044036383	count	1
HCG18	-0.0314	0.2835407	-0.1107	0.912	-0.043965343	count	1
SRRM1	-0.0305674	0.1080346	-0.2829	0.777	-0.043737276	count	1
RHOH	-0.0304574	0.1345617	-0.2263	0.821	-0.043692023	count	1
BDP1	-0.0306217	0.2024346	-0.1513	0.88	-0.043680441	count	1
SUPT16H	-0.0316424	0.253249	-0.1249	0.901	-0.043661085	count	1
CREB1	-0.0309995	0.2942414	-0.1054	0.916	-0.043404777	count	1
HTRA2	-0.0322427	0.3195736	-0.1009	0.9197	-0.042630857	count	1
ZCCHC10	-0.0317207	0.2409042	-0.1317	0.895	-0.041941396	count	1
MTRNR2L12	-0.0285993	0.0958025	-0.2985	0.765	-0.040973214	count	1
DNPH1	-0.0308968	0.2487125	-0.1242	0.9012	-0.040853136	count	1
SARS	-0.0295238	0.2933245	-0.1007	0.9199	-0.040739262	count	1
PSME4	-0.0294406	0.5087092	-0.0579	0.9539	-0.040624514	count	1
TFG	-0.0287439	0.2965944	-0.0969	0.923	-0.039663626	count	1
DCTN6	-0.0283011	0.3355672	-0.0843	0.933	-0.039627782	count	1
CLK1	-0.0277687	0.1967403	-0.1411	0.888	-0.039547739	count	1
PHB	-0.0278552	0.2283083	-0.122	0.903	-0.039003625	count	1
EZR	-0.0268351	0.0883648	-0.3037	0.7615	-0.038574531	count	1
PRKD3	-0.0279492	0.2830277	-0.0988	0.921	-0.038567547	count	1
PAX5	-0.0268395	0.4233755	-0.0634	0.9495	-0.038029457	count	1
CD79A	-0.0263894	0.0635398	-0.4153	0.678	-0.037996743	count	1
GUK1	-0.0263907	0.1509847	-0.1748	0.8613	-0.037871157	count	1
CYCS	-0.0265018	0.1357499	-0.1952	0.845	-0.037851554	count	1
SPAG7	-0.027416	0.2729085	-0.1005	0.92	-0.037832121	count	1
TRIR	-0.0262732	0.1075716	-0.2442	0.8072	-0.037702551	count	1
PLPP5	-0.0269897	0.3047518	-0.0886	0.929	-0.037244129	count	1
PAK2	-0.0260315	0.1492005	-0.1745	0.862	-0.037179911	count	1
DIS3	-0.0257095	0.325695	-0.0789	0.937	-0.036674213	count	1
TCF4	-0.0260635	0.1636567	-0.1593	0.874	-0.0359666	count	1
SEM1	-0.0251196	0.1884126	-0.1333	0.894	-0.035832823	count	1
CD53	-0.0245596	0.1032689	-0.2378	0.8121	-0.035271758	count	1
TCEA1	-0.0246772	0.1281979	-0.1925	0.847	-0.035245798	count	1
RERE	-0.0265268	0.2949256	-0.0899	0.928	-0.03507996	count	1
QARS	-0.0246782	0.3969114	-0.0622	0.95	-0.034967596	count	1
MT-CO2	-0.0242234	0.0376691	-0.6431	0.521	-0.034927746	count	1
HAUS3	-0.0252314	0.7327442	-0.0344	0.9725	-0.034818831	count	1
CEP57	-0.0246675	0.2724057	-0.0906	0.928	-0.034541394	count	1
HNRNPU	-0.0238101	0.1206536	-0.1973	0.8436	-0.033910641	count	1
MAZ	-0.0237166	0.275929	-0.086	0.9315	-0.033705528	count	1
COBLL1	-0.0242877	0.2310228	-0.1051	0.916	-0.033517084	count	1
PRKCD	-0.0230454	0.4269363	-0.054	0.957	-0.031803381	count	1

ATG3	-0.0224024	0.2745183	-0.0816	0.935	-0.031743424	count	1
STK4	-0.0219794	0.0939094	-0.234	0.8151	-0.031236974	count	1
MTSS1	-0.0218893	0.231533	-0.0945	0.925	-0.031016491	count	1
OTULIN	-0.0219031	0.3488587	-0.0628	0.95	-0.03067144	count	1
RTF1	-0.0214974	0.2352567	-0.0914	0.927	-0.030461264	count	1
CHMP4A	-0.0227602	0.2268661	-0.1003	0.9201	-0.03010259	count	1
PDCD6	-0.0216565	0.1818847	-0.1191	0.9053	-0.029887363	count	1
GPR174	-0.0210922	0.4655799	-0.0453	0.9639	-0.029887191	count	1
ESRRA	-0.0211245	0.5683528	-0.0372	0.9704	-0.029581413	count	1
NDUFA9	-0.022039	0.3107817	-0.0709	0.9435	-0.029149419	count	1
POMP	-0.0208861	0.1555403	-0.1343	0.8932	-0.02882454	count	1
SRI	-0.0201013	0.1899982	-0.1058	0.916	-0.028483304	count	1
MOGS	-0.0214809	0.5323633	-0.0404	0.9678	-0.028411778	count	1
SLC35E2B	-0.0205667	0.6565447	-0.0313	0.975	-0.028383896	count	1
MNAT1	-0.0201981	0.2815316	-0.0717	0.9428	-0.02787537	count	1
NDUFA12	-0.0198355	0.2070603	-0.0958	0.924	-0.027776792	count	1
LDHB	-0.0194035	0.1438827	-0.1349	0.893	-0.027714084	count	1
LSM7	-0.018359	0.1252754	-0.1465	0.884	-0.026248502	count	1
PSMC5	-0.0194826	0.2225291	-0.0876	0.9303	-0.025770401	count	1
ACSF3	-0.0186514	0.4425621	-0.0421	0.9664	-0.025741449	count	1
RPL14	-0.0175638	0.0374976	-0.4684	0.64	-0.025325707	count	1
RPLP2	-0.0172963	0.0358958	-0.4818	0.63	-0.024933469	count	1
FDPS	-0.0175948	0.3119633	-0.0564	0.955	-0.02493206	count	1
TSEN34	-0.018634	0.3593694	-0.0519	0.9587	-0.024648606	count	1
RPS21	-0.0170709	0.0449815	-0.3795	0.704	-0.024590341	count	1
RPF2	-0.0184817	0.3300124	-0.056	0.9554	-0.024447268	count	1
RPL19	-0.016883	0.0289519	-0.5831	0.56	-0.024350212	count	1
SLC25A45	-0.0173485	0.3691384	-0.047	0.9625	-0.023943801	count	1
ENTR1	-0.0173203	0.3583451	-0.0483	0.961	-0.023904892	count	1
ATP5S	-0.017298	0.3487944	-0.0496	0.96	-0.023874124	count	1
SPAG9	-0.0170011	0.2522888	-0.0674	0.946	-0.023808394	count	1
C7orf50	-0.0171754	0.1688404	-0.1017	0.919	-0.023704965	count	1
RPLP0	-0.0158919	0.0527582	-0.3012	0.763	-0.022915239	count	1
ARPC4	-0.0156603	0.163254	-0.0959	0.924	-0.022257133	count	1
SAV1	-0.0157932	0.3195081	-0.0494	0.9606	-0.021797806	count	1
HLA-DMB	-0.015066	0.1320674	-0.1141	0.909	-0.021572893	count	1
RHOB	-0.0153165	0.3627803	-0.0422	0.966	-0.02144969	count	1
DDX3X	-0.0149438	0.1403717	-0.1065	0.915	-0.021284093	count	1
KPNA1	-0.0151277	0.4301452	-0.0352	0.972	-0.020879517	count	1
HLA-F	-0.0145879	0.1410723	-0.1034	0.918	-0.020429507	count	1
JAK1	-0.014311	0.1147169	-0.1248	0.901	-0.020339606	count	1
EIF3K	-0.0138358	0.0950534	-0.1456	0.8843	-0.019848587	count	1
C1orf52	-0.0147902	0.2564554	-0.0577	0.954	-0.019566567	count	1
SMARCA2	-0.0141741	0.2903443	-0.0488	0.961	-0.018751878	count	1
FBXO9	-0.0141462	0.3142266	-0.045	0.9641	-0.018714984	count	1

TMEM154	-0.0131644	0.2013874	-0.0654	0.948	-0.018710118	count	1
ZFAND6	-0.0127849	0.1970423	-0.0649	0.9483	-0.018294283	count	1
RPS12	-0.0126227	0.0379825	-0.3323	0.74	-0.018207339	count	1
ZNF22	-0.0126358	0.2956311	-0.0427	0.966	-0.0176961	count	1
DUSP22	-0.0119179	0.2698156	-0.0442	0.9648	-0.016888486	count	1
PSMD4	-0.0118353	0.1996435	-0.0593	0.953	-0.016697301	count	1
DTD1	-0.0118793	0.3628315	-0.0327	0.9739	-0.01639692	count	1
SLC7A6OS	-0.0114485	0.4718779	-0.0243	0.9807	-0.016033536	count	1
AC016831.5	-0.0114913	0.3537833	-0.0325	0.9741	-0.01586147	count	1
POU2AF1	-0.0114381	0.278037	-0.0411	0.967	-0.015788052	count	1
RNF219	-0.0113302	0.4852319	-0.0234	0.9814	-0.015639146	count	1
FAM3C	-0.0112859	0.2581118	-0.0437	0.9651	-0.01557801	count	1
ZNF706	-0.0107071	0.1301151	-0.0823	0.934	-0.015331612	count	1
MED29	-0.0107242	0.4482711	-0.0239	0.9809	-0.01524216	count	1
FTH1	-0.0104767	0.0595331	-0.176	0.86	-0.015108023	count	1
UBL5	-0.0103909	0.1344332	-0.0773	0.938	-0.014841879	count	1
IK	-0.0099415	0.1662962	-0.0598	0.952	-0.014159791	count	1
CAPG	-0.0096189	0.1656267	-0.0581	0.9537	-0.013781371	count	1
UBXN1	-0.0095055	0.1179515	-0.0806	0.936	-0.013577261	count	1
ATP23	-0.0096176	0.5985035	-0.0161	0.987	-0.013275618	count	1
PLGRKT	-0.0099568	0.3626064	-0.0275	0.9781	-0.013174327	count	1
SSR1	-0.0099313	0.290281	-0.0342	0.973	-0.013140597	count	1
MT-CO3	-0.0090715	0.0405369	-0.2238	0.823	-0.013078658	count	1
PCBP2	-0.0090294	0.1408413	-0.0641	0.9489	-0.01295355	count	1
WDR82	-0.0089168	0.3385726	-0.0263	0.979	-0.012308417	count	1
HNRNPR	-0.0091091	0.1688814	-0.0539	0.957	-0.012053024	count	1
RAB8B	-0.0084622	0.1873278	-0.0452	0.964	-0.01185165	count	1
C15orf40	-0.0082158	0.3392717	-0.0242	0.981	-0.011340916	count	1
TIFA	-0.0085572	0.2970374	-0.0288	0.977	-0.01132296	count	1
TMEM216	-0.0079412	0.402735	-0.0197	0.9843	-0.011253538	count	1
EIF5B	-0.0078872	0.1766744	-0.0446	0.964	-0.011251851	count	1
RPS17	-0.008465	0.2657202	-0.0319	0.9746	-0.011200994	count	1
RCSD1	-0.0077884	0.1828453	-0.0426	0.966	-0.011037016	count	1
ELF1	-0.007286	0.1020892	-0.0714	0.943	-0.010417453	count	1
STK24	-0.0073388	0.2673272	-0.0275	0.978	-0.010278415	count	1
SETD5	-0.0072297	0.3637163	-0.0199	0.984	-0.010125627	count	1
RPS5	-0.0066338	0.0404387	-0.164	0.87	-0.009565088	count	1
ZFP14	-0.0065486	0.4365667	-0.015	0.988	-0.009039803	count	1
NBDY	-0.0058604	0.2615676	-0.0224	0.9821	-0.007755207	count	1
MARS	-0.0056965	0.4797957	-0.0119	0.9905	-0.007538354	count	1
TMEM123	-0.0052861	0.2286016	-0.0231	0.982	-0.007513299	count	1
TRIM8	-0.0050058	0.3727241	-0.0134	0.9893	-0.006910274	count	1
COX6C	-0.0047184	0.1203718	-0.0392	0.969	-0.006746383	count	1
C1QBP	-0.0046955	0.1787315	-0.0263	0.9791	-0.0062139	count	1
PSENEN	-0.0041118	0.2471164	-0.0166	0.987	-0.005759026	count	1

MRFAP1	-0.0038563	0.2140489	-0.018	0.986	-0.005401186	count	1
BTF3L4	-0.0037364	0.2488096	-0.015	0.988	-0.005233259	count	1
LY86	-0.0034905	0.138021	-0.0253	0.98	-0.004961208	count	1
SUMO2	-0.0030815	0.082851	-0.0372	0.97	-0.004430311	count	1
CHD1	-0.0030213	0.1748923	-0.0173	0.9862	-0.003998524	count	1
HSPA5	-0.0027379	0.1780314	-0.0154	0.988	-0.003899762	count	1
LIMD2	-0.0026716	0.1110619	-0.0241	0.981	-0.003819889	count	1
SIGIRR	-0.0025789	0.231942	-0.0111	0.9911	-0.003560197	count	1
DDX46	-0.0023842	0.1721458	-0.0139	0.989	-0.003411733	count	1
IFI27L2	-0.0019788	0.2600593	-0.0076	0.9939	-0.002804289	count	1
YWHAZ	-0.0018976	0.0827796	-0.0229	0.982	-0.002728556	count	1
ISCU	-0.0018465	0.1307976	-0.0141	0.989	-0.002630097	count	1
ATP5MD	-0.0017786	0.1911515	-0.0093	0.993	-0.002520576	count	1
PRDX1	-0.001746	0.1256871	-0.0139	0.989	-0.002490904	count	1
LGALS1	-0.0015968	0.180103	-0.0089	0.993	-0.002283134	count	1
SH3BP5	-0.0016858	0.1916632	-0.0088	0.993	-0.002231159	count	1
C19orf53	-0.0013429	0.1304185	-0.0103	0.9918	-0.001918203	count	1
ARL1	-0.0014107	0.3202179	-0.0044	0.9965	-0.00186708	count	1
ACTR2	-0.0007995	0.1394707	-0.0057	0.995	-0.00110375	count	1
SMAP2	-0.0007407	0.0987381	-0.0075	0.994	-0.001061255	count	1
CHMP2A	-0.0004671	0.2407966	-0.0019	0.9985	-0.000654252	count	1
KDELR1	-0.0002536	0.2343866	-0.0011	0.9991	-0.000335655	count	1
SS18L2	-0.0001772	0.1890763	-9.00E-04	0.999	-0.00025002	count	1
IGLC7	-3.9015907	2.7017927	-1.4441	0.149	-2.19E-05	count	1
RGS1	-1.1864544	0.5677661	-2.0897	0.0372	-9.77E-06	count	1
CCL4L2	-1.1408519	0.4132528	-2.7607	0.006	-9.29E-06	count	1
UBA2	-0.9651771	0.24759	-3.8983	1.00E-04	-8.26E-06	count	1
TIPARP	-0.8795963	0.3927611	-2.2395	0.0256	-7.81E-06	count	1
TTC14	-0.8755713	0.3436539	-2.5478	0.0112	-7.74E-06	count	1
KCMF1	-0.8677961	0.3212851	-2.701	0.0072	-7.68E-06	count	1
OSGEP	-0.808155	0.2986184	-2.7063	0.0071	-7.30E-06	count	1
MX1	-0.7881084	0.3648332	-2.1602	0.0313	-7.23E-06	count	1
ST6GAL1	-0.7881084	0.2811738	-2.8029	0.0053	-7.13E-06	count	1
SLAMF1	-0.784021	0.375533	-2.0878	0.0374	-7.11E-06	count	1
EMC3	-0.7732002	0.3485848	-2.2181	0.0271	-7.07E-06	count	1
DUSP5	-0.7608574	0.4270477	-1.7817	0.0755	-7.06E-06	count	1
HAUS1	-0.7760155	0.3562444	-2.1783	0.0299	-7.02E-06	count	1
FBXL5	-0.7653053	0.4202944	-1.8209	0.0693	-6.99E-06	count	1
TMOD3	-0.7031996	0.3084241	-2.28	0.0231	-6.57E-06	count	1
TAP1	-0.6953742	0.2633329	-2.6407	0.0086	-6.47E-06	count	1
RIF1	-0.6738102	0.4006582	-1.6818	0.0933	-6.37E-06	count	1
KANSL1-AS1	-0.6659696	0.3336409	-1.9961	0.0465	-6.35E-06	count	1
COMMD3	-0.6732801	0.2770579	-2.4301	0.0155	-6.34E-06	count	1
MICU2	-0.6697055	0.2619615	-2.5565	0.0109	-6.32E-06	count	1
THAP12	-0.6644369	0.3314477	-2.0047	0.0456	-6.30E-06	count	1

ZNF107	-0.6480859	0.3387953	-1.9129	0.0564	-6.16E-06	count	1
MED28	-0.6353055	0.2434079	-2.61	0.0094	-6.04E-06	count	1
PSMA2	-0.6130995	0.2814944	-2.178	0.0299	-5.88E-06	count	1
C9orf72	-0.6068453	0.33939	-1.788	0.0745	-5.88E-06	count	1
CHML	-0.5969044	0.4299922	-1.3882	0.166	-5.81E-06	count	1
APOBEC3G	-0.5948966	0.2466946	-2.4115	0.0163	-5.79E-06	count	1
ZNF830	-0.5894947	0.3374046	-1.7471	0.0813	-5.76E-06	count	1
SMIM29	-0.5924262	0.3613896	-1.6393	0.102	-5.76E-06	count	1
KIAA0040	-0.5906367	0.308319	-1.9157	0.056	-5.72E-06	count	1
MXI1	-0.583348	0.3498985	-1.6672	0.0962	-5.65E-06	count	1
ZBTB32	-0.5773298	0.34115	-1.6923	0.0913	-5.62E-06	count	1
N4BP2L1	-0.5645652	0.372138	-1.5171	0.13	-5.57E-06	count	1
NSMCE2	-0.5714685	0.3351146	-1.7053	0.0888	-5.57E-06	count	1
ZMAT2	-0.5684005	0.2764366	-2.0562	0.0404	-5.53E-06	count	1
ECI2	-0.5547951	0.3358357	-1.652	0.0992	-5.42E-06	count	1
DNAJC9	-0.5502385	0.3147352	-1.7483	0.0811	-5.40E-06	count	1
RNASE1	-3.3374109	1.1146064	-2.9943	0.0029	-5.38E-06	count	1
FKBP3	-0.5337826	0.3150067	-1.6945	0.0909	-5.27E-06	count	1
FUS	-0.1713159	0.113442	-1.5102	0.132	-5.23E-06	count	1
STX12	-0.5206292	0.2672669	-1.948	0.052	-5.13E-06	count	1
RASA2	-0.5110876	0.3781092	-1.3517	0.177	-5.12E-06	count	1
MCRIP1	-0.5049702	0.3102747	-1.6275	0.104	-5.05E-06	count	1
UBE2K	-0.4978091	0.2921446	-1.704	0.0891	-5.01E-06	count	1
RB1CC1	-0.4976192	0.3797846	-1.3103	0.191	-4.96E-06	count	1
PHRF1	-2.7145349	1.0702034	-2.5365	0.0115	-4.95E-06	count	1
IRF4	-0.490631	0.3745901	-1.3098	0.191	-4.93E-06	count	1
PPP2R2D	-0.4874365	0.3083876	-1.5806	0.115	-4.90E-06	count	1
BLOC1S1	-0.4908919	0.174222	-2.8176	0.005	-4.90E-06	count	1
CYFIP2	-0.4901733	0.3343079	-1.4662	0.143	-4.89E-06	count	1
LINC01480	-0.4875483	0.3547762	-1.3742	0.17	-4.88E-06	count	1
FUBP1	-0.4856473	0.2729478	-1.7793	0.0759	-4.86E-06	count	1
DNAJC7	-0.4836719	0.1983262	-2.4388	0.0151	-4.85E-06	count	1
NUCB1	-0.4768131	0.3107811	-1.5342	0.126	-4.80E-06	count	1
ZNF184	-2.3035191	0.845404	-2.7248	0.0067	-4.80E-06	count	1
CUEDC2	-0.4698848	0.3538371	-1.328	0.185	-4.74E-06	count	1
RXRB	-2.1081173	0.7396135	-2.8503	0.0046	-4.73E-06	count	1
MLX	-0.46392	0.3453465	-1.3433	0.18	-4.70E-06	count	1
ZBTB16	-2.3048331	1.6301213	-1.4139	0.158	-4.68E-06	count	1
QRSL1	-0.4617529	0.2993957	-1.5423	0.124	-4.67E-06	count	1
COX19	-0.4517631	0.3159226	-1.43	0.153	-4.58E-06	count	1
CEP295	-0.4517743	0.4420082	-1.0221	0.307	-4.58E-06	count	1
WDR47	-2.0695307	0.8901244	-2.325	0.0205	-4.57E-06	count	1
SNRNP27	-0.4422868	0.3594301	-1.2305	0.219	-4.51E-06	count	1
TPM1	-1.8346876	0.6583971	-2.7866	0.0056	-4.51E-06	count	1
RGS2	-0.4400202	0.2627715	-1.6745	0.0947	-4.48E-06	count	1

CKAP2	-0.4364574	0.2862591	-1.5247	0.128	-4.43E-06	count	1
GOLGA7	-0.4293924	0.3227857	-1.3303	0.184	-4.41E-06	count	1
RICTOR	-0.4260655	0.3253668	-1.3095	0.191	-4.39E-06	count	1
IFI44L	-1.7642464	0.5276206	-3.3438	9.00E-04	-4.37E-06	count	1
HOPX	-0.4257115	0.2765814	-1.5392	0.124	-4.35E-06	count	1
CALD1	-1.7783973	0.7192998	-2.4724	0.0138	-4.28E-06	count	1
FKBP15	-1.8059448	0.9147417	-1.9743	0.049	-4.26E-06	count	1
NCBP3	-0.408601	0.3640159	-1.1225	0.262	-4.24E-06	count	1
METTL7A	-1.6211943	0.5453078	-2.973	0.0031	-4.20E-06	count	1
TRIM56	-1.7125559	0.6812979	-2.5137	0.0123	-4.19E-06	count	1
ADD1	-0.4054651	0.2680947	-1.5124	0.131	-4.18E-06	count	1
UBN1	-0.4029578	0.3447415	-1.1689	0.243	-4.18E-06	count	1
FAM13B	-0.4021899	0.4096128	-0.9819	0.327	-4.17E-06	count	1
IL7R	-1.6659173	0.9850659	-1.6912	0.0915	-4.16E-06	count	1
OXSR1	-1.6106261	0.5856718	-2.75	0.0062	-4.13E-06	count	1
ZNF207	-0.3991432	0.2266854	-1.7608	0.079	-4.12E-06	count	1
TNFSF10	-1.6127004	0.6944121	-2.3224	0.0207	-4.12E-06	count	1
IDH1	-1.5816097	0.6651165	-2.3779	0.0178	-4.11E-06	count	1
XAF1	-1.5619012	0.4168409	-3.747	2.00E-04	-4.11E-06	count	1
MILR1	-1.5744022	0.8842835	-1.7804	0.0757	-4.07E-06	count	1
KNOP1	-1.5997746	0.6580244	-2.4312	0.0154	-4.07E-06	count	1
CEBPD	-1.4430231	0.9817131	-1.4699	0.142	-4.07E-06	count	1
PEPD	-0.392613	0.3301568	-1.1892	0.235	-4.06E-06	count	1
FCER1G	-1.513172	0.7950491	-1.9032	0.0577	-4.05E-06	count	1
RBM4	-0.3903545	0.3639792	-1.0725	0.284	-4.05E-06	count	1
ACTA2	-1.5128824	0.6153182	-2.4587	0.0143	-4.04E-06	count	1
SERTAD1	-0.387717	0.2788674	-1.3903	0.165	-4.03E-06	count	1
CCDC57	-1.5347307	0.5192196	-2.9558	0.0033	-4.03E-06	count	1
SFXN1	-0.3829266	0.3846565	-0.9955	0.32	-3.99E-06	count	1
METTL15	-1.5223395	0.649221	-2.3449	0.0195	-3.98E-06	count	1
MT2A	-1.4246655	0.4397271	-3.2399	0.0013	-3.94E-06	count	1
NIN	-0.3773798	0.2752752	-1.3709	0.171	-3.93E-06	count	1
NME3	-0.3761563	0.2337596	-1.6092	0.108	-3.92E-06	count	1
TCP1	-0.3757928	0.2085655	-1.8018	0.0723	-3.91E-06	count	1
PPP2R2A	-0.3744161	0.2951084	-1.2687	0.205	-3.89E-06	count	1
RPS19BP1	-0.374227	0.1801396	-2.0774	0.0383	-3.89E-06	count	1
CCDC88A	-0.373131	0.4725231	-0.7897	0.43	-3.89E-06	count	1
TUBD1	-1.4482429	0.7209325	-2.0088	0.0452	-3.87E-06	count	1
USP45	-1.4482429	0.7067156	-2.0493	0.041	-3.87E-06	count	1
MTRNR2L8	-0.3678359	0.3135101	-1.1733	0.241	-3.85E-06	count	1
TNFRSF17	-1.4482429	0.8074039	-1.7937	0.0735	-3.85E-06	count	1
DMXL1	-0.3624787	0.290658	-1.2471	0.213	-3.82E-06	count	1
MCTS1	-0.3657162	0.2921536	-1.2518	0.211	-3.82E-06	count	1
ABCG1	-1.4130019	0.6517899	-2.1679	0.0307	-3.81E-06	count	1
GLTP	-1.378467	0.4522168	-3.0482	0.0024	-3.81E-06	count	1

TRAPPC1	-0.3629722	0.1817288	-1.9973	0.0464	-3.79E-06	count	1
IQSEC1	-1.3530439	0.480833	-2.814	0.0051	-3.79E-06	count	1
CHRAC1	-0.3597708	0.2894472	-1.243	0.215	-3.78E-06	count	1
TXLNG	-1.3413179	0.5627102	-2.3837	0.0176	-3.76E-06	count	1
DCAF6	-1.3800309	0.6201567	-2.2253	0.0266	-3.76E-06	count	1
MIA3	-0.3581392	0.2712081	-1.3205	0.187	-3.76E-06	count	1
TPM2	-1.377236	0.728865	-1.8896	0.0595	-3.75E-06	count	1
SART3	-1.35054	0.5152491	-2.6211	0.0091	-3.75E-06	count	1
CTCF	-0.3571988	0.2594184	-1.3769	0.169	-3.74E-06	count	1
SSH2	-0.3532292	0.2759277	-1.2802	0.201	-3.72E-06	count	1
ARHGAP30	-0.3540423	0.2729369	-1.2972	0.195	-3.72E-06	count	1
ANAPC15	-0.3536681	0.3493205	-1.0124	0.312	-3.72E-06	count	1
JPT1	-0.3536886	0.2206765	-1.6027	0.11	-3.72E-06	count	1
GLRX3	-0.3530211	0.3029032	-1.1655	0.244	-3.71E-06	count	1
GSS	-1.3174401	0.4827301	-2.7291	0.0066	-3.70E-06	count	1
TMEM134	-0.3521039	0.2078074	-1.6944	0.0909	-3.69E-06	count	1
TMEM165	-0.3509025	0.2105182	-1.6669	0.0962	-3.68E-06	count	1
STAG3	-0.3492039	0.3211905	-1.0872	0.278	-3.68E-06	count	1
AHCY	-1.2884946	0.4829832	-2.6678	0.0079	-3.67E-06	count	1
PRKRA	-0.3481657	0.3539439	-0.9837	0.326	-3.67E-06	count	1
PDHB	-0.3482323	0.2856266	-1.2192	0.223	-3.67E-06	count	1
UBE4A	-1.3255323	0.7002125	-1.893	0.059	-3.66E-06	count	1
MED17	-1.3031398	0.5818255	-2.2397	0.0256	-3.66E-06	count	1
TMEM41A	-1.3031398	0.6221987	-2.0944	0.0368	-3.65E-06	count	1
MTHFD1	-1.3043814	0.6174204	-2.1126	0.0352	-3.65E-06	count	1
GTPBP3	-1.3043814	0.6140708	-2.1242	0.0342	-3.65E-06	count	1
FAM200B	-1.2580634	0.5027896	-2.5022	0.0127	-3.64E-06	count	1
POLR3E	-1.3074321	0.7258053	-1.8014	0.0723	-3.63E-06	count	1
ATF1	-0.3425596	0.3113659	-1.1002	0.272	-3.62E-06	count	1
CLUAP1	-1.2880203	0.7480071	-1.7219	0.0858	-3.59E-06	count	1
KIAA2013	-1.2632913	0.5383653	-2.3465	0.0194	-3.58E-06	count	1
PRKX	-1.2689042	0.7535936	-1.6838	0.0929	-3.58E-06	count	1
CPLANE1	-1.2697711	0.7980008	-1.5912	0.112	-3.57E-06	count	1
PRKAR2A	-0.3380743	0.3888843	-0.8693	0.385	-3.57E-06	count	1
ITFG1	-1.200475	0.5152597	-2.3298	0.0203	-3.52E-06	count	1
MICAL1	-1.2259454	0.5359648	-2.2874	0.0226	-3.51E-06	count	1
KYAT3	-1.1866522	0.4674493	-2.5386	0.0115	-3.51E-06	count	1
CHMP1B	-0.3301446	0.2684831	-1.2297	0.219	-3.51E-06	count	1
PSMC2	-0.3287702	0.3024312	-1.0871	0.278	-3.50E-06	count	1
OXA1L	-0.3298704	0.2551094	-1.2931	0.197	-3.50E-06	count	1
PFDN6	-0.328956	0.3548715	-0.927	0.354	-3.49E-06	count	1
DBP	-1.1911503	0.5022497	-2.3716	0.0181	-3.49E-06	count	1
TFEC	-1.1731235	0.3800444	-3.0868	0.0022	-3.48E-06	count	1
APP	-0.3273797	0.3468096	-0.944	0.346	-3.47E-06	count	1
INTS6	-0.3262702	0.2288404	-1.4258	0.155	-3.47E-06	count	1

CLNK	-1.1731235	0.6016306	-1.9499	0.0518	-3.46E-06	count	1
PXDC1	-0.3253624	0.3494002	-0.9312	0.352	-3.46E-06	count	1
EAPP	-0.3248877	0.2156787	-1.5064	0.133	-3.45E-06	count	1
TRAC	-0.3255585	0.2058224	-1.5817	0.114	-3.44E-06	count	1
RHBDF2	-0.320701	0.3441446	-0.9319	0.352	-3.42E-06	count	1
PTPA	-1.1886856	0.6980566	-1.7028	0.0893	-3.42E-06	count	1
DUS3L	-1.1886856	0.7009565	-1.6958	0.0906	-3.41E-06	count	1
ITGB1BP1	-0.3210365	0.2984504	-1.0757	0.283	-3.41E-06	count	1
LNPEP	-0.317522	0.3410189	-0.9311	0.352	-3.40E-06	count	1
AAAS	-1.1670905	0.7408262	-1.5754	0.116	-3.39E-06	count	1
RORA	-0.3179686	0.3320995	-0.9574	0.339	-3.39E-06	count	1
SEMA4B	-1.1586298	0.6428817	-1.8022	0.0722	-3.39E-06	count	1
ZFAND3	-1.1529804	0.5532451	-2.084	0.0377	-3.38E-06	count	1
NASP	-0.3179177	0.1793029	-1.7731	0.0769	-3.38E-06	count	1
NDUFA10	-0.3167715	0.277893	-1.1399	0.255	-3.37E-06	count	1
SLBP	-0.3171499	0.2291466	-1.384	0.167	-3.37E-06	count	1
FAM173A	-0.315811	0.2718173	-1.1619	0.246	-3.36E-06	count	1
ATP5MC1	-0.3149987	0.1946193	-1.6185	0.106	-3.35E-06	count	1
TTC19	-1.1075643	0.4961779	-2.2322	0.0261	-3.34E-06	count	1
CHERP	-1.1246367	0.5088023	-2.2104	0.0276	-3.34E-06	count	1
AFF4	-0.3128604	0.2497261	-1.2528	0.211	-3.34E-06	count	1
SMARCC1	-0.3138421	0.2753665	-1.1397	0.255	-3.34E-06	count	1
ZNF106	-0.3119918	0.310466	-1.0049	0.315	-3.33E-06	count	1
ATF7IP2	-0.3119918	0.2802685	-1.1132	0.266	-3.32E-06	count	1
TBC1D5	-1.1025485	0.328912	-3.3521	9.00E-04	-3.31E-06	count	1
PPP3R1	-1.1115493	0.5350923	-2.0773	0.0383	-3.31E-06	count	1
PTPRN2	-1.1246367	0.7533244	-1.4929	0.136	-3.31E-06	count	1
XPO7	-1.128275	0.885791	-1.2737	0.203	-3.28E-06	count	1
PPP2R5A	-1.089662	0.4891418	-2.2277	0.0264	-3.27E-06	count	1
ZNF708	-0.3051052	0.3322357	-0.9183	0.359	-3.26E-06	count	1
FAM217B	-1.0477036	0.5187302	-2.0197	0.044	-3.26E-06	count	1
LNPK	-1.1015208	0.7573302	-1.4545	0.147	-3.26E-06	count	1
MATR3	-1.0611527	0.4569483	-2.3223	0.0207	-3.25E-06	count	1
CKAP5	-1.0872651	0.6162306	-1.7644	0.0784	-3.24E-06	count	1
MKNK2	-0.3034013	0.2041944	-1.4858	0.138	-3.24E-06	count	1
FUBP3	-1.0677768	0.7426386	-1.4378	0.151	-3.23E-06	count	1
SMARCAD1	-1.0374345	0.4301028	-2.4121	0.0163	-3.22E-06	count	1
TET2	-1.045703	0.5542712	-1.8866	0.0599	-3.21E-06	count	1
SEC16A	-1.0675941	0.832339	-1.2826	0.2	-3.20E-06	count	1
NUP107	-1.0407703	0.4149977	-2.5079	0.0125	-3.20E-06	count	1
ARMH3	-1.0442982	0.6050495	-1.726	0.085	-3.19E-06	count	1
CTBS	-1.0377065	0.4166608	-2.4905	0.0131	-3.18E-06	count	1
AL390728.6	-1.0527789	0.698223	-1.5078	0.132	-3.18E-06	count	1
OTUD5	-0.2960167	0.3215256	-0.9207	0.358	-3.18E-06	count	1
TMEM251	-1.0301623	0.5084211	-2.0262	0.0433	-3.17E-06	count	1

CAMTA1	-0.2935115	0.2574447	-1.1401	0.255	-3.16E-06	count	1
MCM3AP	-1.0320028	0.7011681	-1.4718	0.142	-3.15E-06	count	1
PPP2CB	-1.0064793	0.4503966	-2.2347	0.0259	-3.14E-06	count	1
PARL	-0.2908628	0.3431498	-0.8476	0.397	-3.14E-06	count	1
INKA1	-1.008533	0.4688975	-2.1509	0.032	-3.14E-06	count	1
ZNF580	-0.2918954	0.3176581	-0.9189	0.359	-3.14E-06	count	1
C12orf42	-1.0113504	0.742642	-1.3618	0.174	-3.12E-06	count	1
CISD1	-1.0157759	0.5839032	-1.7396	0.0826	-3.12E-06	count	1
MSL1	-0.992895	0.361934	-2.7433	0.0063	-3.12E-06	count	1
DDX42	-0.2897902	0.3255008	-0.8903	0.374	-3.11E-06	count	1
CFDP1	-0.2877795	0.2909642	-0.9891	0.323	-3.11E-06	count	1
IQCB1	-0.2883183	0.3350007	-0.8606	0.39	-3.11E-06	count	1
TES	-0.2877504	0.3145786	-0.9147	0.361	-3.10E-06	count	1
CBL	-0.9977737	0.4316879	-2.3113	0.0213	-3.09E-06	count	1
SMC2	-1.0113504	0.6123284	-1.6516	0.0993	-3.09E-06	count	1
MUM1	-0.2872969	0.4131566	-0.6954	0.487	-3.09E-06	count	1
AUTS2	-0.9903855	0.477064	-2.076	0.0385	-3.08E-06	count	1
ACADSB	-1.0075088	0.6684015	-1.5073	0.132	-3.08E-06	count	1
SPARCL1	-1.0011702	0.6397811	-1.5649	0.118	-3.08E-06	count	1
SPINK2	-0.9900421	0.6951916	-1.4241	0.155	-3.07E-06	count	1
TMEM70	-0.283642	0.3039557	-0.9332	0.351	-3.06E-06	count	1
PIGF	-0.9850885	0.5390072	-1.8276	0.0683	-3.05E-06	count	1
ZC3H6	-0.2809745	0.2934678	-0.9574	0.339	-3.04E-06	count	1
ZBTB11	-0.2809521	0.3038539	-0.9246	0.356	-3.03E-06	count	1
GRK2	-0.2807608	0.2620813	-1.0713	0.285	-3.03E-06	count	1
SREK1IP1	-0.2798736	0.276426	-1.0125	0.312	-3.02E-06	count	1
RBM12	-0.948702	0.5205072	-1.8226	0.069	-3.01E-06	count	1
P4HA1	-0.2780718	0.3582709	-0.7761	0.438	-3.01E-06	count	1
CCSAP	-0.9680274	0.6731139	-1.4381	0.151	-3.01E-06	count	1
KIAA1147	-0.9680274	0.5800376	-1.6689	0.0958	-3.01E-06	count	1
TMCO1	-0.2778167	0.2411492	-1.1521	0.25	-3.01E-06	count	1
PRAG1	-0.9592735	0.5991516	-1.6011	0.11	-2.98E-06	count	1
SPATS2L	-0.9529613	0.7858477	-1.2127	0.226	-2.97E-06	count	1
EMILIN2	-0.9275679	0.7225152	-1.2838	0.2	-2.97E-06	count	1
BICD2	-0.9452491	0.6250214	-1.5123	0.131	-2.97E-06	count	1
SEH1L	-0.938592	0.5995914	-1.5654	0.118	-2.95E-06	count	1
PTDSS1	-0.9275679	0.5421456	-1.7109	0.0878	-2.94E-06	count	1
TOP2B	-0.2720509	0.3201434	-0.8498	0.396	-2.94E-06	count	1
SCYL1	-0.9188152	0.4984478	-1.8434	0.0659	-2.93E-06	count	1
AC007384.1	-0.9182468	0.4960181	-1.8512	0.0648	-2.93E-06	count	1
SUSD3	-0.9166974	0.3722465	-2.4626	0.0142	-2.92E-06	count	1
ARCN1	-0.2700904	0.3160454	-0.8546	0.393	-2.92E-06	count	1
TOR1AIP2	-0.9144686	0.4574076	-1.9992	0.0462	-2.92E-06	count	1
CBR1	-0.930423	0.6275512	-1.4826	0.139	-2.92E-06	count	1
SLC35E3	-0.9161434	0.7695777	-1.1904	0.235	-2.91E-06	count	1

CEPT1	-0.8909393	0.571024	-1.5602	0.119	-2.89E-06	count	1
CLPX	-0.9081385	0.5086451	-1.7854	0.0749	-2.89E-06	count	1
UBXN7	-0.8997873	0.5127281	-1.7549	0.08	-2.89E-06	count	1
RIOK3	-0.2657481	0.3097061	-0.8581	0.391	-2.89E-06	count	1
SDE2	-0.2659842	0.2722149	-0.9771	0.329	-2.88E-06	count	1
UBE3C	-0.9078194	0.6243851	-1.4539	0.147	-2.88E-06	count	1
CREBBP	-0.8997873	0.5207645	-1.7278	0.0847	-2.88E-06	count	1
ITPK1	-0.9058025	0.7190132	-1.2598	0.208	-2.87E-06	count	1
FANCF	-0.9058025	0.7325873	-1.2364	0.217	-2.87E-06	count	1
HCST	-0.2637081	0.1850303	-1.4252	0.155	-2.87E-06	count	1
ERCC6	-0.8908796	0.5507935	-1.6174	0.106	-2.84E-06	count	1
SLC7A6	-0.8874543	0.6085713	-1.4583	0.145	-2.84E-06	count	1
TLE1	-0.2616572	0.2969508	-0.8811	0.379	-2.84E-06	count	1
TAF15	-0.2607492	0.2937734	-0.8876	0.375	-2.84E-06	count	1
ARHGAP12	-0.8874543	0.5767476	-1.5387	0.125	-2.83E-06	count	1
WDR74	-0.2604145	0.2951442	-0.8823	0.378	-2.83E-06	count	1
PPM1D	-0.8698814	0.4250688	-2.0464	0.0413	-2.82E-06	count	1
LIG1	-0.882717	0.5852443	-1.5083	0.132	-2.82E-06	count	1
DSN1	-0.882717	0.715951	-1.2329	0.218	-2.82E-06	count	1
PARP9	-0.8561949	0.4581312	-1.8689	0.0623	-2.81E-06	count	1
ATP13A1	-0.8885897	0.983295	-0.9037	0.367	-2.81E-06	count	1
TBL1X	-0.8719251	0.5377811	-1.6213	0.106	-2.81E-06	count	1
ZC3H4	-0.8628209	0.6595605	-1.3082	0.191	-2.81E-06	count	1
CPSF7	-0.8757375	0.7525218	-1.1637	0.245	-2.80E-06	count	1
TAF13	-0.8628209	0.564441	-1.5286	0.127	-2.79E-06	count	1
WAC-AS1	-0.8574939	0.4190214	-2.0464	0.0413	-2.79E-06	count	1
NIPAL3	-0.8628209	0.5362129	-1.6091	0.108	-2.79E-06	count	1
SMC5	-0.2559441	0.3012412	-0.8496	0.396	-2.78E-06	count	1
TRAPPC11	-0.8574939	0.6858604	-1.2502	0.212	-2.78E-06	count	1
WDR34	-0.85926	0.5083551	-1.6903	0.0917	-2.78E-06	count	1
ORAI2	-0.2555378	0.248466	-1.0285	0.304	-2.78E-06	count	1
SRRD	-0.8324289	0.4824601	-1.7254	0.0852	-2.78E-06	count	1
COPRS	-0.8589437	0.6768628	-1.269	0.205	-2.77E-06	count	1
PTPN12	-0.8407255	0.4147628	-2.027	0.0433	-2.77E-06	count	1
TEX9	-0.2538416	0.2453893	-1.0344	0.301	-2.77E-06	count	1
EBLN3P	-0.2534171	0.2297582	-1.103	0.271	-2.76E-06	count	1
SIDT2	-0.8527536	0.5083445	-1.6775	0.0941	-2.76E-06	count	1
ARIH2	-0.8445436	0.3828854	-2.2057	0.0279	-2.76E-06	count	1
SURF6	-0.8574939	0.7200636	-1.1909	0.234	-2.76E-06	count	1
ZFYVE16	-0.8517848	0.5498087	-1.5492	0.122	-2.76E-06	count	1
PCNA	-0.8393269	0.3796833	-2.2106	0.0276	-2.75E-06	count	1
SLC25A46	-0.8371088	0.5144291	-1.6273	0.104	-2.74E-06	count	1
FAM208A	-0.251256	0.3321958	-0.7563	0.45	-2.74E-06	count	1
GOLGA8B	-0.856742	0.5947826	-1.4404	0.15	-2.74E-06	count	1
TMEM99	-0.8442428	0.8359717	-1.0099	0.313	-2.74E-06	count	1

WDR12	-0.8348893	0.5778354	-1.4449	0.149	-2.74E-06	count	1
RAB2B	-0.8333987	0.6438576	-1.2944	0.196	-2.73E-06	count	1
NMRK1	-0.8333987	0.6729719	-1.2384	0.216	-2.73E-06	count	1
C6orf106	-0.2493541	0.3508127	-0.7108	0.478	-2.72E-06	count	1
PABPN1	-0.2493409	0.2745299	-0.9082	0.364	-2.72E-06	count	1
RNF139	-0.8254931	0.4226292	-1.9532	0.0514	-2.72E-06	count	1
ERVK3-1	-0.8254931	0.5556293	-1.4857	0.138	-2.71E-06	count	1
FBXO11	-0.247519	0.3155853	-0.7843	0.433	-2.70E-06	count	1
PSMD14	-0.2475228	0.305085	-0.8113	0.418	-2.70E-06	count	1
THOP1	-0.8312425	0.5770046	-1.4406	0.15	-2.70E-06	count	1
KIF2A	-0.2473298	0.2763343	-0.895	0.371	-2.70E-06	count	1
FIG4	-0.8312425	0.6913411	-1.2024	0.23	-2.70E-06	count	1
MOSPD3	-0.8312425	0.6323676	-1.3145	0.189	-2.70E-06	count	1
ZNF720	-0.8312425	0.6323676	-1.3145	0.189	-2.70E-06	count	1
OXR1	-0.2461969	0.2906918	-0.8469	0.397	-2.69E-06	count	1
GM2A	-0.8083875	0.4314921	-1.8735	0.0617	-2.68E-06	count	1
WDR77	-0.8181048	0.5982533	-1.3675	0.172	-2.67E-06	count	1
RBMS1	-0.8111503	0.5076593	-1.5978	0.111	-2.67E-06	count	1
RP9	-0.2436657	0.3339658	-0.7296	0.466	-2.67E-06	count	1
CCDC93	-0.8026047	0.3989234	-2.0119	0.0448	-2.67E-06	count	1
FCRL5	-0.2435378	0.3387289	-0.719	0.473	-2.67E-06	count	1
ARPC1A	-0.8009318	0.3639005	-2.201	0.0283	-2.66E-06	count	1
RPRD2	-0.8000292	0.4975233	-1.608	0.109	-2.66E-06	count	1
FAM207A	-0.8078797	0.4670273	-1.7298	0.0844	-2.66E-06	count	1
ARL5B	-0.8071426	0.5484063	-1.4718	0.142	-2.66E-06	count	1
SETD4	-0.8059785	0.5889849	-1.3684	0.172	-2.65E-06	count	1
BRCC3	-0.8071432	0.4970684	-1.6238	0.105	-2.65E-06	count	1
MRPL49	-0.8059785	0.5308208	-1.5184	0.13	-2.65E-06	count	1
HIST2H2AC	-0.7995485	0.4280161	-1.868	0.0624	-2.64E-06	count	1
SIMC1	-0.784647	0.6126826	-1.2807	0.201	-2.64E-06	count	1
TAGLN	-0.7939433	0.3907472	-2.0319	0.0428	-2.64E-06	count	1
LRCH4	-0.7916127	0.5782054	-1.3691	0.172	-2.64E-06	count	1
SLC25A42	-0.7939433	0.5965646	-1.3309	0.184	-2.62E-06	count	1
ATG2A	-0.7939433	0.6075108	-1.3069	0.192	-2.62E-06	count	1
PARP8	-0.7762581	0.5056103	-1.5353	0.125	-2.62E-06	count	1
LINC00662	-0.7851624	0.4209531	-1.8652	0.0628	-2.62E-06	count	1
MPLKIP	-0.2383094	0.2724135	-0.8748	0.382	-2.62E-06	count	1
TRAF7	-0.7911569	0.6240555	-1.2678	0.206	-2.61E-06	count	1
SAFB2	-0.2382046	0.2890566	-0.8241	0.41	-2.61E-06	count	1
PHYKPL	-0.2381286	0.2919633	-0.8156	0.415	-2.61E-06	count	1
STOM	-0.7955081	0.889892	-0.8939	0.372	-2.61E-06	count	1
SYNC	-0.7712313	0.4540804	-1.6984	0.0901	-2.58E-06	count	1
SPINT2	-0.2350177	0.2341133	-1.0039	0.316	-2.57E-06	count	1
AAMDC	-0.7641865	0.7418371	-1.0301	0.304	-2.57E-06	count	1
FAM210A	-0.7634658	0.4037396	-1.891	0.0593	-2.57E-06	count	1

LCK	-0.7641865	0.4460331	-1.7133	0.0874	-2.57E-06	count	1
SH2B2	-0.7641865	0.4397708	-1.7377	0.083	-2.56E-06	count	1
DCAF11	-0.7684567	0.6074988	-1.265	0.207	-2.55E-06	count	1
WDFY2	-0.745934	0.4924957	-1.5146	0.131	-2.55E-06	count	1
ATG5	-0.2319797	0.2471208	-0.9387	0.348	-2.55E-06	count	1
JMJD6	-0.2314971	0.3094199	-0.7482	0.455	-2.55E-06	count	1
SPATA2L	-0.2310344	0.3287056	-0.7029	0.483	-2.54E-06	count	1
EXOSC4	-0.7564495	0.5336153	-1.4176	0.157	-2.54E-06	count	1
ZMAT1	-0.7503314	0.5723854	-1.3109	0.191	-2.54E-06	count	1
PTCD3	-0.745934	0.4421694	-1.687	0.0923	-2.54E-06	count	1
RNASE6	-0.2307347	0.3485166	-0.662	0.508	-2.53E-06	count	1
EP400	-0.7564495	0.5975433	-1.2659	0.206	-2.53E-06	count	1
ZNF146	-0.7486631	0.4269091	-1.7537	0.0802	-2.53E-06	count	1
GGPS1	-0.7474338	0.4114737	-1.8165	0.07	-2.53E-06	count	1
C12orf43	-0.7527252	0.4920863	-1.5297	0.127	-2.53E-06	count	1
RDX	-0.7500994	0.4759882	-1.5759	0.116	-2.53E-06	count	1
EPN1	-0.7487622	0.3712259	-2.017	0.0443	-2.52E-06	count	1
CCDC88C	-0.7430967	0.3582029	-2.0745	0.0386	-2.52E-06	count	1
ING2	-0.2286452	0.2585297	-0.8844	0.377	-2.52E-06	count	1
NEK8	-0.7527252	0.524335	-1.4356	0.152	-2.51E-06	count	1
DEGS1	-0.2281363	0.225942	-1.0097	0.313	-2.51E-06	count	1
DDX39A	-0.2279519	0.2729897	-0.835	0.404	-2.51E-06	count	1
CBLL1	-0.2281363	0.3510205	-0.6499	0.516	-2.51E-06	count	1
CRYL1	-0.7361389	0.5440645	-1.353	0.177	-2.50E-06	count	1
KPNA4	-0.227202	0.2505276	-0.9069	0.365	-2.50E-06	count	1
SPG21	-0.7357452	0.3988842	-1.8445	0.0658	-2.50E-06	count	1
FNIP1	-0.2263736	0.2985742	-0.7582	0.449	-2.49E-06	count	1
ZNF12	-0.7475819	0.6536736	-1.1437	0.253	-2.49E-06	count	1
FAM114A2	-0.7361389	0.5550634	-1.3262	0.185	-2.48E-06	count	1
ENDOD1	-0.7211112	0.4798801	-1.5027	0.134	-2.48E-06	count	1
CDKN2AIPNL	-0.7361389	0.5362188	-1.3728	0.17	-2.48E-06	count	1
FNBP4	-0.2250838	0.1968746	-1.1433	0.254	-2.48E-06	count	1
AC012368.1	-0.7361389	0.5558765	-1.3243	0.186	-2.48E-06	count	1
NSUN2	-0.7352401	0.5241682	-1.4027	0.161	-2.47E-06	count	1
CBWD5	-0.2246687	0.3074161	-0.7308	0.465	-2.47E-06	count	1
LINC01003	-0.7352401	0.5570992	-1.3198	0.188	-2.47E-06	count	1
CDC27	-0.7272117	0.4052428	-1.7945	0.0734	-2.47E-06	count	1
TTPAL	-0.725436	0.5739631	-1.2639	0.207	-2.47E-06	count	1
ITCH	-0.7299419	0.4671289	-1.5626	0.119	-2.46E-06	count	1
HSF2	-0.7336337	0.6700384	-1.0949	0.274	-2.46E-06	count	1
PARP10	-0.7361223	0.7051169	-1.044	0.297	-2.46E-06	count	1
LINC01678	-0.7336337	0.7695522	-0.9533	0.341	-2.46E-06	count	1
GFPT1	-0.7299419	0.5431476	-1.3439	0.18	-2.46E-06	count	1
ZNF880	-0.7191006	0.5473643	-1.3138	0.19	-2.46E-06	count	1
HEATR5B	-0.7299419	0.6658278	-1.0963	0.274	-2.46E-06	count	1

PI4K2A	-0.7299419	0.6082579	-1.2001	0.231	-2.46E-06	count	1
DESI1	-0.7184007	0.4918008	-1.4608	0.145	-2.45E-06	count	1
SGF29	-0.7243608	0.4420673	-1.6386	0.102	-2.45E-06	count	1
SUPT6H	-0.7173735	0.4437848	-1.6165	0.107	-2.45E-06	count	1
AKNA	-0.2215828	0.3254307	-0.6809	0.496	-2.44E-06	count	1
AP2B1	-0.7132883	0.4223956	-1.6887	0.092	-2.44E-06	count	1
GOLIM4	-0.725436	0.7847191	-0.9245	0.356	-2.44E-06	count	1
CHRNB1	-0.7227894	0.7028389	-1.0284	0.304	-2.44E-06	count	1
HACL1	-0.7092272	0.4352815	-1.6294	0.104	-2.43E-06	count	1
DDX23	-0.7083646	0.4701988	-1.5065	0.133	-2.42E-06	count	1
SLC26A2	-0.7131975	0.5867354	-1.2155	0.225	-2.42E-06	count	1
TMC8	-0.7052155	0.4764589	-1.4801	0.14	-2.42E-06	count	1
CTNNBL1	-0.2185012	0.2911899	-0.7504	0.453	-2.42E-06	count	1
ZNF395	-0.7045515	0.6590925	-1.069	0.286	-2.41E-06	count	1
ARHGEF7	-0.7132883	0.606585	-1.1759	0.24	-2.41E-06	count	1
VAMP7	-0.7045515	0.6026764	-1.169	0.243	-2.40E-06	count	1
SYNJ2	-0.7045515	0.6424994	-1.0966	0.273	-2.40E-06	count	1
CS	-0.7045515	0.6424994	-1.0966	0.273	-2.40E-06	count	1
KIAA1328	-0.7027876	0.7743843	-0.9075	0.365	-2.40E-06	count	1
EXOC4	-0.6991114	0.4827181	-1.4483	0.148	-2.40E-06	count	1
FAM49A	-0.2165957	0.3141637	-0.6894	0.491	-2.39E-06	count	1
SH2D3C	-0.7027876	0.727955	-0.9654	0.335	-2.39E-06	count	1
AC242426.2	-0.7027876	0.728419	-0.9648	0.335	-2.38E-06	count	1
CCDC124	-0.2147351	0.3481706	-0.6168	0.538	-2.38E-06	count	1
ODF3B	-0.6935592	0.5156561	-1.345	0.179	-2.38E-06	count	1
CCL3	-0.6911951	0.4684627	-1.4755	0.141	-2.37E-06	count	1
SLC25A11	-0.6835028	0.3891833	-1.7562	0.0797	-2.37E-06	count	1
AP1G1	-0.685751	0.4016963	-1.7071	0.0885	-2.36E-06	count	1
HCFC1R1	-0.6804583	0.3442308	-1.9768	0.0487	-2.35E-06	count	1
CHD9	-0.2125847	0.2113188	-1.006	0.315	-2.35E-06	count	1
RASSF2	-0.2121641	0.3025181	-0.7013	0.483	-2.35E-06	count	1
SF3A1	-0.2120293	0.3261802	-0.65	0.516	-2.34E-06	count	1
HIVEP3	-0.6873639	0.9653889	-0.712	0.477	-2.34E-06	count	1
OGFR	-0.2108952	0.2511487	-0.8397	0.402	-2.34E-06	count	1
ZDBF2	-0.6873639	0.8388471	-0.8194	0.413	-2.34E-06	count	1
LPGAT1	-0.2103729	0.2347837	-0.896	0.371	-2.33E-06	count	1
OAS2	-0.6780969	0.5855492	-1.1581	0.247	-2.33E-06	count	1
TM9SF4	-0.6728657	0.4722554	-1.4248	0.155	-2.33E-06	count	1
TRAPPC6A	-0.2106186	0.2461424	-0.8557	0.393	-2.33E-06	count	1
ACSL3	-0.6671931	0.4144196	-1.6099	0.108	-2.33E-06	count	1
FGD2	-0.2105407	0.3567816	-0.5901	0.555	-2.33E-06	count	1
C12orf45	-0.2104696	0.3612902	-0.5826	0.56	-2.33E-06	count	1
IL32	-0.6780969	0.6645963	-1.0203	0.308	-2.33E-06	count	1
LINC02397	-0.2094986	0.2409123	-0.8696	0.385	-2.32E-06	count	1
GALK2	-0.6732801	0.5581616	-1.2062	0.228	-2.32E-06	count	1

GRAMD1C	-0.6598158	0.7216645	-0.9143	0.361	-2.32E-06	count	1
THUMPD2	-0.6515166	0.5348505	-1.2181	0.224	-2.32E-06	count	1
TMX3	-0.2079214	0.3881233	-0.5357	0.592	-2.31E-06	count	1
ORC4	-0.667444	0.508421	-1.3128	0.19	-2.31E-06	count	1
POU2F1	-0.667444	0.602413	-1.108	0.268	-2.30E-06	count	1
GALM	-0.6703285	0.8272847	-0.8103	0.418	-2.30E-06	count	1
TLE4	-0.2062715	0.28423	-0.7257	0.468	-2.29E-06	count	1
CRYBG1	-0.2061294	0.258841	-0.7964	0.426	-2.29E-06	count	1
DHX40	-0.6554289	0.4348762	-1.5072	0.132	-2.29E-06	count	1
ZCRB1	-0.2065684	0.2998726	-0.6889	0.491	-2.29E-06	count	1
ZNF264	-0.6594996	0.5197122	-1.269	0.205	-2.28E-06	count	1
DYM	-0.6603036	0.5488548	-1.2031	0.23	-2.28E-06	count	1
B3GNT2	-0.2054555	0.2693653	-0.7627	0.446	-2.28E-06	count	1
RANBP3	-0.6603036	0.6262691	-1.0543	0.292	-2.28E-06	count	1
MCCC1	-0.6611548	0.7681539	-0.8607	0.39	-2.28E-06	count	1
GRHPR	-0.6541352	0.3529628	-1.8533	0.0645	-2.28E-06	count	1
MAGEF1	-0.6504684	0.4115363	-1.5806	0.115	-2.27E-06	count	1
FAM208B	-0.651143	0.3666788	-1.7758	0.0765	-2.27E-06	count	1
CAND1	-0.20461	0.2458328	-0.8323	0.406	-2.27E-06	count	1
TSC22D2	-0.2039186	0.3484928	-0.5851	0.559	-2.27E-06	count	1
ETFDH	-0.6504684	0.6127774	-1.0615	0.289	-2.27E-06	count	1
ERAP2	-0.6447909	0.4107665	-1.5697	0.117	-2.26E-06	count	1
MSANTD4	-0.6504684	0.6109994	-1.0646	0.288	-2.25E-06	count	1
BTBD1	-0.6504684	0.6109994	-1.0646	0.288	-2.25E-06	count	1
SHLD2	-0.6447763	0.4581973	-1.4072	0.16	-2.25E-06	count	1
SLC23A2	-0.6504684	0.7065164	-0.9207	0.358	-2.25E-06	count	1
PHF2	-0.6504684	0.6302252	-1.0321	0.303	-2.25E-06	count	1
TENT4B	-0.6447763	0.4947122	-1.3033	0.193	-2.24E-06	count	1
NUDT3	-0.6417025	0.5206089	-1.2326	0.218	-2.24E-06	count	1
PIK3C2B	-0.6409765	0.5513796	-1.1625	0.246	-2.24E-06	count	1
MRPL48	-0.6428223	0.4319378	-1.4882	0.137	-2.24E-06	count	1
CSGALNACT2	-0.6361747	0.4512289	-1.4099	0.159	-2.24E-06	count	1
HNRNPL	-0.2010538	0.2455325	-0.8188	0.413	-2.24E-06	count	1
PSMD3	-0.641327	0.3762119	-1.7047	0.089	-2.24E-06	count	1
TMCO4	-0.6417025	0.6357484	-1.0094	0.313	-2.23E-06	count	1
RPIA	-0.6313547	0.3563129	-1.7719	0.0771	-2.22E-06	count	1
TRIM28	-0.1995511	0.3360561	-0.5938	0.553	-2.22E-06	count	1
APOL3	-0.6319198	0.4757663	-1.3282	0.185	-2.22E-06	count	1
TIMM8B	-0.6362661	0.6198305	-1.0265	0.305	-2.22E-06	count	1
CD2BP2	-0.6299211	0.2967528	-2.1227	0.0343	-2.22E-06	count	1
ADK	-0.1986165	0.2694517	-0.7371	0.461	-2.22E-06	count	1
SERTAD2	-0.198149	0.380969	-0.5201	0.603	-2.21E-06	count	1
ARV1	-0.6319198	0.5393671	-1.1716	0.242	-2.21E-06	count	1
TSC22D4	-0.198671	0.2947383	-0.6741	0.501	-2.21E-06	count	1
ARHGEF3	-0.636774	0.9462748	-0.6729	0.501	-2.21E-06	count	1

GPN1	-0.6256091	0.5524526	-1.1324	0.258	-2.20E-06	count	1
TBRG4	-0.6305757	0.5931149	-1.0632	0.288	-2.20E-06	count	1
IQCG	-0.6279629	0.7886012	-0.7963	0.426	-2.20E-06	count	1
PPP1R21	-0.6208804	0.5466195	-1.1359	0.257	-2.19E-06	count	1
IMPACT	-0.6256091	0.6373716	-0.9815	0.327	-2.19E-06	count	1
ABCF2	-0.6279629	0.7367505	-0.8523	0.394	-2.19E-06	count	1
CCDC14	-0.6256091	0.5106428	-1.2251	0.221	-2.19E-06	count	1
ARID3B	-0.6279629	0.7163187	-0.8767	0.381	-2.18E-06	count	1
PPP1R7	-0.1964335	0.2715711	-0.7233	0.47	-2.18E-06	count	1
MAPK1	-0.1961923	0.3520018	-0.5574	0.578	-2.18E-06	count	1
STIM1	-0.6252019	0.5450707	-1.147	0.252	-2.18E-06	count	1
STAT2	-0.6152827	0.4645473	-1.3245	0.186	-2.17E-06	count	1
TMEM183A	-0.6182583	0.3122026	-1.9803	0.0483	-2.17E-06	count	1
MRPL46	-0.614332	0.4790463	-1.2824	0.2	-2.17E-06	count	1
ALKBH5	-0.6164988	0.4289779	-1.4371	0.151	-2.17E-06	count	1
ZPR1	-0.614332	0.3415925	-1.7984	0.0728	-2.17E-06	count	1
GPAA1	-0.6130638	0.3831789	-1.5999	0.11	-2.16E-06	count	1
MYD88	-0.617319	0.6337154	-0.9741	0.331	-2.16E-06	count	1
RBM19	-0.608461	0.4665348	-1.3042	0.193	-2.16E-06	count	1
ALG8	-0.6112539	0.5624213	-1.0868	0.278	-2.16E-06	count	1
KCNQ1OT1	-0.6112539	0.4773566	-1.2805	0.201	-2.16E-06	count	1
ZDHHC17	-0.6112539	0.559589	-1.0923	0.275	-2.15E-06	count	1
MAP3K11	-0.6075624	0.382437	-1.5887	0.113	-2.15E-06	count	1
NDUFS1	-0.608461	0.520853	-1.1682	0.243	-2.15E-06	count	1
TBPL1	-0.1923647	0.3144876	-0.6117	0.541	-2.15E-06	count	1
RPUSD1	-0.6112539	0.5919278	-1.0326	0.302	-2.15E-06	count	1
HDGF	-0.1927937	0.346843	-0.5559	0.579	-2.15E-06	count	1
ATL3	-0.6071594	0.4162191	-1.4587	0.145	-2.14E-06	count	1
NEDD1	-0.6048203	0.4033004	-1.4997	0.134	-2.14E-06	count	1
NADK	-0.6112539	0.6361191	-0.9609	0.337	-2.14E-06	count	1
TMEM222	-0.6035858	0.540448	-1.1168	0.265	-2.13E-06	count	1
TRMT61B	-0.6035858	0.4857683	-1.2425	0.215	-2.13E-06	count	1
NIPSNAP1	-0.6035858	0.5700034	-1.0589	0.29	-2.13E-06	count	1
DGAT1	-0.6035858	0.6094632	-0.9904	0.323	-2.13E-06	count	1
RAD51C	-0.5994733	0.4864996	-1.2322	0.219	-2.13E-06	count	1
SCRN2	-0.5976493	0.5708277	-1.047	0.296	-2.12E-06	count	1
GPX7	-0.5959578	0.4524293	-1.3172	0.188	-2.12E-06	count	1
PTK2B	-0.5953486	0.4261725	-1.397	0.163	-2.11E-06	count	1
BRD8	-0.5884515	0.539587	-1.0906	0.276	-2.11E-06	count	1
TBC1D23	-0.5884515	0.4125551	-1.4264	0.154	-2.11E-06	count	1
ICK	-0.5919704	0.504561	-1.1732	0.241	-2.10E-06	count	1
ZNF566	-0.5953486	0.8418956	-0.7072	0.48	-2.10E-06	count	1
ZNF576	-0.5953486	0.7883651	-0.7552	0.451	-2.09E-06	count	1
TMEM63A	-0.5953486	0.7681798	-0.775	0.439	-2.09E-06	count	1
AC092140.1	-0.592918	0.8193302	-0.7237	0.47	-2.09E-06	count	1

DNAL4	-0.592918	0.7229342	-0.8202	0.413	-2.09E-06	count	1
MDH2	-0.1866189	0.1752289	-1.065	0.287	-2.09E-06	count	1
MRPL44	-0.5807817	0.3756876	-1.5459	0.123	-2.08E-06	count	1
ARHGAP18	-0.5828016	0.4085305	-1.4266	0.154	-2.08E-06	count	1
KMO	-0.5858517	0.5771395	-1.0151	0.311	-2.08E-06	count	1
RBSN	-0.5867393	0.7127857	-0.8232	0.411	-2.07E-06	count	1
TAB2	-0.5828016	0.4375438	-1.332	0.184	-2.07E-06	count	1
KIAA0930	-0.5807408	0.6125799	-0.948	0.344	-2.07E-06	count	1
WNT10A	-0.5807408	0.5972188	-0.9724	0.331	-2.07E-06	count	1
PGM2L1	-0.5807408	0.5582588	-1.0403	0.299	-2.07E-06	count	1
TP53RK	-0.5807408	0.5992526	-0.9691	0.333	-2.07E-06	count	1
INPP5K	-0.5773298	0.5296392	-1.09	0.276	-2.06E-06	count	1
MRPL45	-0.5807408	0.5573617	-1.0419	0.298	-2.06E-06	count	1
TMED9	-0.1841907	0.2426285	-0.7591	0.448	-2.06E-06	count	1
ZMIZ2	-0.5773298	0.5599324	-1.0311	0.303	-2.05E-06	count	1
THOC2	-0.1836024	0.2602774	-0.7054	0.481	-2.05E-06	count	1
RAB3GAP2	-0.5666143	0.5208403	-1.0879	0.277	-2.05E-06	count	1
CINP	-0.5741588	0.5331435	-1.0769	0.282	-2.05E-06	count	1
RBM41	-0.5767582	0.6132635	-0.9405	0.347	-2.05E-06	count	1
CUL1	-0.5696862	0.4140041	-1.376	0.17	-2.05E-06	count	1
ZC3H7A	-0.1831147	0.3156789	-0.5801	0.562	-2.05E-06	count	1
SNX4	-0.5718333	0.489612	-1.1679	0.243	-2.05E-06	count	1
HAUS8	-0.5689871	0.3960522	-1.4366	0.152	-2.04E-06	count	1
NEK7	-0.5546479	0.5190631	-1.0686	0.286	-2.02E-06	count	1
VPS26C	-0.5565199	0.4313927	-1.2901	0.198	-2.01E-06	count	1
IARS2	-0.5546479	0.4306715	-1.2879	0.198	-2.00E-06	count	1
STIP1	-0.1783617	0.3308165	-0.5392	0.59	-2.00E-06	count	1
GNL3L	-0.5563084	0.5212491	-1.0673	0.286	-2.00E-06	count	1
POLR3D	-0.5546479	0.435418	-1.2738	0.203	-2.00E-06	count	1
PSTPIP1	-0.5563084	0.5429006	-1.0247	0.306	-2.00E-06	count	1
SPOUT1	-0.554955	0.6729146	-0.8247	0.41	-1.99E-06	count	1
C11orf96	-0.554955	0.583903	-0.9504	0.342	-1.99E-06	count	1
ZBTB14	-0.5557626	0.8141712	-0.6826	0.495	-1.99E-06	count	1
PATL2	-0.5543862	0.6359665	-0.8717	0.384	-1.99E-06	count	1
TBP	-0.5539639	0.6400595	-0.8655	0.387	-1.99E-06	count	1
NKIRAS2	-0.553638	0.5275578	-1.0494	0.295	-1.99E-06	count	1
SLC35A1	-0.5557626	0.7595177	-0.7317	0.465	-1.99E-06	count	1
TOGARAM1	-0.5533788	0.4890477	-1.1315	0.258	-1.99E-06	count	1
DUSP18	-0.5557626	0.7656594	-0.7259	0.468	-1.99E-06	count	1
RNF145	-0.1769675	0.3412575	-0.5186	0.604	-1.98E-06	count	1
CBX4	-0.5463594	0.3509385	-1.5569	0.12	-1.98E-06	count	1
SEC22C	-0.5511077	0.4819461	-1.1435	0.253	-1.98E-06	count	1
NBR1	-0.5511077	0.8543151	-0.6451	0.519	-1.97E-06	count	1
JOSD1	-0.5439425	0.3620482	-1.5024	0.134	-1.97E-06	count	1
DHX15	-0.5435526	0.3373132	-1.6114	0.108	-1.97E-06	count	1

ARRDC1	-0.542227	0.4187836	-1.2948	0.196	-1.97E-06	count	1
CHI3L2	-0.5426298	0.3965069	-1.3685	0.172	-1.96E-06	count	1
ALDH2	-0.5428554	0.4015101	-1.352	0.177	-1.96E-06	count	1
SGCB	-0.5362965	0.5591766	-0.9591	0.338	-1.95E-06	count	1
LTBP3	-0.5398888	0.4607919	-1.1717	0.242	-1.94E-06	count	1
AK9	-0.5377251	0.4865585	-1.1052	0.27	-1.94E-06	count	1
TMA16	-0.5361065	0.364778	-1.4697	0.142	-1.94E-06	count	1
PPP3CC	-0.1726606	0.3065516	-0.5632	0.574	-1.94E-06	count	1
UHRF2	-0.1713775	0.4540498	-0.3774	0.706	-1.94E-06	count	1
ZNF407	-0.5343603	0.5814198	-0.9191	0.359	-1.94E-06	count	1
DKC1	-0.5334555	0.3893438	-1.3701	0.171	-1.93E-06	count	1
TMEM141	-0.1719949	0.3577055	-0.4808	0.631	-1.93E-06	count	1
CCDC28B	-0.5324151	0.4983239	-1.0684	0.286	-1.93E-06	count	1
JDP2	-0.5325199	0.5109699	-1.0422	0.298	-1.93E-06	count	1
MRM2	-0.5299582	0.3725008	-1.4227	0.156	-1.93E-06	count	1
SDHAF1	-0.5310976	0.3844102	-1.3816	0.168	-1.93E-06	count	1
SNAPC5	-0.5325199	0.5437104	-0.9794	0.328	-1.92E-06	count	1
RHBDD1	-0.5296652	0.5316298	-0.9963	0.32	-1.92E-06	count	1
IKZF3	-0.1701477	0.2402962	-0.7081	0.479	-1.91E-06	count	1
ELL	-0.5267571	0.6245804	-0.8434	0.399	-1.91E-06	count	1
PHACTR2	-0.5267571	0.5729163	-0.9194	0.358	-1.91E-06	count	1
TMEM126B	-0.5240235	0.3754127	-1.3959	0.163	-1.91E-06	count	1
HMGN5	-0.5224126	0.8709181	-0.5998	0.549	-1.90E-06	count	1
SMIM30	-0.524763	0.3949544	-1.3287	0.185	-1.90E-06	count	1
C9orf40	-0.5224126	0.4344548	-1.2025	0.23	-1.90E-06	count	1
SNAI3	-0.5224126	0.7538327	-0.693	0.489	-1.90E-06	count	1
ZNF780A	-0.5224126	0.6820765	-0.7659	0.444	-1.90E-06	count	1
NFAT5	-0.5210872	0.430936	-1.2092	0.227	-1.90E-06	count	1
TRAFD1	-0.5224126	0.7197143	-0.7259	0.468	-1.90E-06	count	1
ARHGAP1	-0.5224126	0.6345433	-0.8233	0.411	-1.89E-06	count	1
CIC	-0.5224126	0.609388	-0.8573	0.392	-1.89E-06	count	1
MYLIP	-0.5182158	0.4952807	-1.0463	0.296	-1.89E-06	count	1
ZNF486	-0.5224126	0.6267556	-0.8335	0.405	-1.89E-06	count	1
TGFBRAP1	-0.5198184	0.7425292	-0.7001	0.484	-1.88E-06	count	1
ATG14	-0.5198184	0.6134239	-0.8474	0.397	-1.88E-06	count	1
RFTN1	-0.5115039	0.4426796	-1.1555	0.249	-1.87E-06	count	1
ANKAR	-0.5104321	0.577031	-0.8846	0.377	-1.87E-06	count	1
DOCK10	-0.1660994	0.2872153	-0.5783	0.563	-1.87E-06	count	1
GAMT	-0.5133703	0.7814678	-0.6569	0.512	-1.87E-06	count	1
TUBGCP4	-0.5133703	0.6592281	-0.7787	0.437	-1.87E-06	count	1
UTP14A	-0.5133703	0.6827886	-0.7519	0.453	-1.86E-06	count	1
BAZ2A	-0.1655204	0.3531055	-0.4688	0.639	-1.86E-06	count	1
ZNF250	-0.5104321	0.6109994	-0.8354	0.404	-1.86E-06	count	1
PDCL	-0.5078733	0.3851227	-1.3187	0.188	-1.86E-06	count	1
ZCCHC2	-0.5042595	0.449253	-1.1224	0.262	-1.86E-06	count	1

ATP8A1	-0.5044937	0.5192003	-0.9717	0.332	-1.85E-06	count	1
GTF2H5	-0.1644974	0.279131	-0.5893	0.556	-1.85E-06	count	1
EPS8	-2.7463786	1.3817638	-1.9876	0.0475	-1.85E-06	count	1
LAIR1	-0.5022652	0.6235452	-0.8055	0.421	-1.85E-06	count	1
RINL	-0.5020659	0.3819296	-1.3146	0.189	-1.85E-06	count	1
XPO4	-0.5054578	0.4907087	-1.0301	0.304	-1.85E-06	count	1
STK25	-0.4978091	0.4721454	-1.0544	0.292	-1.84E-06	count	1
AL118516.1	-0.5049702	0.623092	-0.8104	0.418	-1.84E-06	count	1
RBM15	-0.4999655	0.5459004	-0.9159	0.36	-1.84E-06	count	1
SP1	-0.5035725	0.6090523	-0.8268	0.409	-1.83E-06	count	1
ZC3HAV1	-0.1622752	0.3177739	-0.5107	0.61	-1.83E-06	count	1
PMM2	-0.4999655	0.5629336	-0.8881	0.375	-1.83E-06	count	1
PTBP3	-0.1614038	0.261022	-0.6184	0.537	-1.83E-06	count	1
DNAJB12	-0.4948995	0.3690246	-1.3411	0.181	-1.82E-06	count	1
CNOT6L	-0.1616864	0.2316701	-0.6979	0.486	-1.82E-06	count	1
XRRA1	-0.4978091	0.6115374	-0.814	0.416	-1.82E-06	count	1
CRTC3	-0.4936186	0.558283	-0.8842	0.377	-1.82E-06	count	1
AVL9	-0.4978091	0.6427547	-0.7745	0.439	-1.82E-06	count	1
PI4KA	-0.4978091	0.6427547	-0.7745	0.439	-1.82E-06	count	1
UQCC3	-0.4978091	0.5438958	-0.9153	0.361	-1.82E-06	count	1
ATG16L2	-0.4912371	0.3934007	-1.2487	0.212	-1.82E-06	count	1
SUPT5H	-0.4908336	0.4038793	-1.2153	0.225	-1.81E-06	count	1
FBXO28	-0.4936186	0.4992684	-0.9887	0.323	-1.81E-06	count	1
VGLL4	-0.4916527	0.3593538	-1.3682	0.172	-1.81E-06	count	1
KIF3A	-0.4873258	0.4402342	-1.107	0.269	-1.80E-06	count	1
ITGAE	-0.1595026	0.3521499	-0.4529	0.651	-1.80E-06	count	1
SEC63	-0.1595028	0.3326586	-0.4795	0.632	-1.80E-06	count	1
KMT2C	-0.1595027	0.2772349	-0.5753	0.565	-1.80E-06	count	1
IGHG2	-0.1595023	0.2932622	-0.5439	0.587	-1.80E-06	count	1
AL354733.3	-2.5669356	1.4331875	-1.7911	0.074	-1.80E-06	count	1
TWF1	-0.4874365	0.4620022	-1.0551	0.292	-1.80E-06	count	1
SWSAP1	-0.4874365	0.569694	-0.8556	0.393	-1.79E-06	count	1
RING1	-0.4876234	0.5618352	-0.8679	0.386	-1.79E-06	count	1
PTPRK	-0.4868932	0.5761995	-0.845	0.399	-1.79E-06	count	1
ACTR6	-0.1580919	0.3390294	-0.4663	0.641	-1.79E-06	count	1
IL6ST	-0.4868932	0.6445695	-0.7554	0.45	-1.79E-06	count	1
NPIPB5	-0.4868932	0.5520731	-0.8819	0.378	-1.79E-06	count	1
MAT2A	-0.4837786	0.3337616	-1.4495	0.148	-1.78E-06	count	1
DERL1	-0.1576124	0.2865906	-0.55	0.583	-1.78E-06	count	1
SPART	-0.482411	0.4006544	-1.2041	0.229	-1.78E-06	count	1
MAD2L2	-0.4811501	0.4819995	-0.9982	0.319	-1.77E-06	count	1
LINC02001	-0.4811501	0.5092493	-0.9448	0.345	-1.77E-06	count	1
ABLIM1	-0.48021	0.3785353	-1.2686	0.205	-1.77E-06	count	1
CLPTM1	-0.4811501	0.4988685	-0.9645	0.335	-1.77E-06	count	1
ACTR10	-0.1566391	0.3243351	-0.483	0.629	-1.77E-06	count	1

MED16	-0.4783827	0.5526696	-0.8656	0.387	-1.77E-06	count	1
PACSIN2	-0.4789018	0.6040484	-0.7928	0.428	-1.77E-06	count	1
FKBPL	-0.4789018	0.5620863	-0.852	0.395	-1.77E-06	count	1
MRPL39	-0.1562276	0.3370493	-0.4635	0.643	-1.76E-06	count	1
SPDL1	-2.4927089	1.1123691	-2.2409	0.0255	-1.76E-06	count	1
ATXN1L	-0.4783827	0.5199195	-0.9201	0.358	-1.76E-06	count	1
CSKMT	-0.4734809	0.521184	-0.9085	0.364	-1.76E-06	count	1
SQOR	-0.4733921	0.4149887	-1.1407	0.255	-1.76E-06	count	1
DHRS1	-0.4735715	0.4105303	-1.1536	0.249	-1.75E-06	count	1
WDR11	-0.4733921	0.4397549	-1.0765	0.282	-1.75E-06	count	1
KIF16B	-0.4732195	0.8037837	-0.5887	0.556	-1.75E-06	count	1
RMDN1	-0.4735715	0.4144603	-1.1426	0.254	-1.75E-06	count	1
CCS	-0.1542484	0.2449055	-0.6298	0.529	-1.75E-06	count	1
IPO9	-0.4732195	0.7375614	-0.6416	0.521	-1.74E-06	count	1
FAM227B	-0.4732195	0.7375614	-0.6416	0.521	-1.74E-06	count	1
PDCD5	-0.1543796	0.2354543	-0.6557	0.512	-1.74E-06	count	1
MPZL1	-0.4734809	0.5932869	-0.7981	0.425	-1.74E-06	count	1
ARHGAP27	-0.4732195	0.7596978	-0.6229	0.534	-1.74E-06	count	1
AP4B1	-2.433855	1.2193227	-1.9961	0.0465	-1.74E-06	count	1
CHCHD7	-0.1537069	0.26089	-0.5892	0.556	-1.74E-06	count	1
ZMAT5	-0.4698848	0.4422429	-1.0625	0.289	-1.74E-06	count	1
TENT5C	-0.1534196	0.294184	-0.5215	0.602	-1.74E-06	count	1
CASP7	-0.4672654	0.6108352	-0.765	0.445	-1.73E-06	count	1
PUS10	-0.4672654	0.6528222	-0.7158	0.475	-1.73E-06	count	1
ZNF814	-0.4672654	0.7253234	-0.6442	0.52	-1.72E-06	count	1
RPL39L	-0.4639136	0.4986298	-0.9304	0.353	-1.72E-06	count	1
IGF2R	-0.4639136	0.59239	-0.7831	0.434	-1.72E-06	count	1
TMEM199	-0.4639136	0.5432708	-0.8539	0.394	-1.72E-06	count	1
KPNB1	-0.1518374	0.2666323	-0.5695	0.569	-1.72E-06	count	1
MAST4	-0.4602254	0.465601	-0.9885	0.323	-1.72E-06	count	1
CHD7	-0.151399	0.2443917	-0.6195	0.536	-1.71E-06	count	1
ZNF101	-0.4617277	0.4299534	-1.0739	0.283	-1.71E-06	count	1
PSMB7	-0.1507031	0.2564107	-0.5877	0.557	-1.71E-06	count	1
RSAD1	-2.3266145	1.1549992	-2.0144	0.0446	-1.71E-06	count	1
CFH	-2.3035191	1.2538248	-1.8372	0.0668	-1.70E-06	count	1
FOXO1	-0.1504134	0.3503716	-0.4293	0.668	-1.70E-06	count	1
NSF	-0.456565	0.3299498	-1.3837	0.167	-1.70E-06	count	1
CPEB2	-2.3035191	1.1544874	-1.9953	0.0466	-1.70E-06	count	1
IFI44	-2.3035191	1.1544874	-1.9953	0.0466	-1.70E-06	count	1
LSM14A	-0.1497191	0.2043999	-0.7325	0.464	-1.70E-06	count	1
NCOA4	-0.4519508	0.3384204	-1.3355	0.182	-1.69E-06	count	1
LRBA	-0.4518041	0.4282112	-1.0551	0.292	-1.69E-06	count	1
PHF21A	-0.4518041	0.4979789	-0.9073	0.365	-1.69E-06	count	1
NUP214	-0.452425	0.5132563	-0.8815	0.379	-1.69E-06	count	1
PHF20L1	-0.1486369	0.3286987	-0.4522	0.651	-1.68E-06	count	1

STYX	-0.4518041	0.4965158	-0.9099	0.363	-1.68E-06	count	1
TNFRSF10A	-0.4518506	0.4472455	-1.0103	0.313	-1.68E-06	count	1
VAMP4	-0.4521224	0.6102431	-0.7409	0.459	-1.68E-06	count	1
PRPF19	-0.4525707	0.5561902	-0.8137	0.416	-1.68E-06	count	1
TUT7	-0.1481935	0.2867412	-0.5168	0.606	-1.68E-06	count	1
RGS16	-0.1476245	0.3794458	-0.3891	0.697	-1.68E-06	count	1
DCTD	-0.4433129	0.4123302	-1.0751	0.283	-1.67E-06	count	1
TMUB1	-0.147276	0.280493	-0.5251	0.6	-1.67E-06	count	1
TMED1	-0.4449252	0.4118839	-1.0802	0.281	-1.67E-06	count	1
UBR2	-0.1465046	0.330934	-0.4427	0.658	-1.66E-06	count	1
AEBP1	-2.1844188	1.2309293	-1.7746	0.0766	-1.66E-06	count	1
SPARC	-2.1808899	1.1235091	-1.9411	0.0529	-1.66E-06	count	1
GCC1	-2.1808899	1.1235091	-1.9411	0.0529	-1.66E-06	count	1
DNAJB2	-0.4438104	0.476275	-0.9318	0.352	-1.66E-06	count	1
PITPNB	-0.1458805	0.2956384	-0.4934	0.622	-1.65E-06	count	1
ICE2	-0.4405604	0.408174	-1.0793	0.281	-1.65E-06	count	1
CD2	-2.1537203	1.1844753	-1.8183	0.0697	-1.65E-06	count	1
VRK3	-0.441651	0.4611891	-0.9576	0.339	-1.65E-06	count	1
ATAD5	-0.4400794	0.6200311	-0.7098	0.478	-1.65E-06	count	1
ATAD1	-0.4396554	0.3646083	-1.2058	0.229	-1.65E-06	count	1
UBAP2	-0.4400794	0.6108946	-0.7204	0.472	-1.65E-06	count	1
DNASE1L1	-2.1537203	1.0577689	-2.0361	0.0423	-1.65E-06	count	1
VAV1	-0.4380479	0.4083457	-1.0727	0.284	-1.65E-06	count	1
COQ10A	-0.4380479	0.4609047	-0.9504	0.342	-1.64E-06	count	1
NAPRT	-0.4377724	0.6454823	-0.6782	0.498	-1.64E-06	count	1
ADNP	-0.1441825	0.3502461	-0.4117	0.681	-1.64E-06	count	1
ELP3	-0.4365942	0.649276	-0.6724	0.502	-1.64E-06	count	1
DEF8	-0.4377724	0.5713781	-0.7662	0.444	-1.64E-06	count	1
PYHIN1	-0.4361775	0.3750639	-1.1629	0.245	-1.64E-06	count	1
PAFAH1B3	-0.4372291	0.3421713	-1.2778	0.202	-1.64E-06	count	1
TMEM214	-0.4365942	0.6073089	-0.7189	0.473	-1.63E-06	count	1
ZNF141	-0.4359643	0.4755276	-0.9168	0.36	-1.63E-06	count	1
FARS2	-0.4359643	0.4577783	-0.9523	0.341	-1.63E-06	count	1
TRIM26	-0.4344462	0.4049287	-1.0729	0.284	-1.63E-06	count	1
SEC23A	-0.4331562	0.4902729	-0.8835	0.377	-1.63E-06	count	1
CYP20A1	-0.143462	0.3258645	-0.4403	0.66	-1.63E-06	count	1
CHMP7	-0.4357478	0.3820962	-1.1404	0.255	-1.63E-06	count	1
FAM91A1	-0.4339671	0.5573425	-0.7786	0.437	-1.63E-06	count	1
ACBD6	-0.1429867	0.2763287	-0.5175	0.605	-1.62E-06	count	1
C21orf2	-0.4302699	0.4901249	-0.8779	0.38	-1.62E-06	count	1
PARP6	-2.0422416	1.1813896	-1.7287	0.0846	-1.62E-06	count	1
RNF103	-0.4302699	0.5825658	-0.7386	0.461	-1.61E-06	count	1
ZNF608	-2.0105986	1.3574783	-1.4811	0.139	-1.61E-06	count	1
SLC44A1	-0.4302699	0.8584103	-0.5012	0.616	-1.61E-06	count	1
PLTP	-0.4302699	0.7776123	-0.5533	0.58	-1.61E-06	count	1

RNF44	-0.426837	0.4439374	-0.9615	0.337	-1.61E-06	count	1
DHX32	-0.4302699	0.8054939	-0.5342	0.593	-1.61E-06	count	1
CUX1	-0.4261887	0.5857542	-0.7276	0.467	-1.61E-06	count	1
ENOX2	-2.0105986	1.0513695	-1.9124	0.0565	-1.61E-06	count	1
PFKP	-0.4261887	0.4983542	-0.8552	0.393	-1.60E-06	count	1
GRPEL1	-0.1412178	0.2440668	-0.5786	0.563	-1.60E-06	count	1
REPS1	-1.9776586	1.3604372	-1.4537	0.147	-1.60E-06	count	1
NDUFA5	-0.1407968	0.2078211	-0.6775	0.498	-1.60E-06	count	1
C6orf62	-0.1406094	0.2629307	-0.5348	0.593	-1.60E-06	count	1
ZNF655	-0.1406588	0.3363796	-0.4182	0.676	-1.60E-06	count	1
RPP14	-1.9776586	1.22546	-1.6138	0.107	-1.60E-06	count	1
ELOVL1	-0.4238807	0.3918385	-1.0818	0.28	-1.60E-06	count	1
AC239800.3	-0.4246814	0.6082693	-0.6982	0.485	-1.60E-06	count	1
PRIM1	-0.4246814	0.5911858	-0.7184	0.473	-1.59E-06	count	1
SGSM3	-1.9776586	1.1520562	-1.7166	0.0867	-1.59E-06	count	1
CTBP1-DT	-1.9776586	1.1520562	-1.7166	0.0867	-1.59E-06	count	1
CEP162	-0.4221239	0.5628491	-0.75	0.454	-1.59E-06	count	1
MTM1	-1.9776586	1.0736456	-1.842	0.0661	-1.59E-06	count	1
ANKRD26	-0.4177407	0.5387802	-0.7753	0.439	-1.59E-06	count	1
KIDINS220	-0.4188528	0.3884973	-1.0781	0.282	-1.58E-06	count	1
CCDC112	-0.4206576	0.644266	-0.6529	0.514	-1.58E-06	count	1
SMC4	-0.4183051	0.3916021	-1.0682	0.286	-1.58E-06	count	1
EIF2AK3	-0.4199831	0.643653	-0.6525	0.514	-1.58E-06	count	1
AP1AR	-0.4152503	0.491125	-0.8455	0.398	-1.57E-06	count	1
KIAA0586	-0.4162789	0.4656395	-0.894	0.372	-1.57E-06	count	1
MUC20-OT1	-0.4162789	0.4063329	-1.0245	0.306	-1.57E-06	count	1
GBP4	-0.4104786	0.4858416	-0.8449	0.399	-1.57E-06	count	1
CAV1	-1.8832202	1.4685861	-1.2823	0.2	-1.57E-06	count	1
DHRS4-AS1	-0.4152503	0.5792517	-0.7169	0.474	-1.57E-06	count	1
NSD2	-0.4152503	0.6957584	-0.5968	0.551	-1.57E-06	count	1
DTWD1	-0.4152503	0.6494213	-0.6394	0.523	-1.57E-06	count	1
SPAG16	-0.4152503	0.6862267	-0.6051	0.545	-1.56E-06	count	1
TLR6	-0.4152503	0.6687412	-0.6209	0.535	-1.56E-06	count	1
VPS45	-1.8832202	1.3159123	-1.4311	0.153	-1.56E-06	count	1
LAP3	-0.1370438	0.2966272	-0.462	0.644	-1.56E-06	count	1
NNMT	-1.8832202	1.190458	-1.5819	0.114	-1.56E-06	count	1
NAGA	-1.8832202	1.2325037	-1.528	0.127	-1.56E-06	count	1
COG4	-1.8832202	1.2325037	-1.528	0.127	-1.56E-06	count	1
TRIM41	-1.8832202	1.1669824	-1.6138	0.107	-1.56E-06	count	1
ZNF37A	-0.409372	0.5522204	-0.7413	0.459	-1.55E-06	count	1
CCDC91	-0.136092	0.2339384	-0.5817	0.561	-1.55E-06	count	1
LSG1	-0.1361897	0.4261739	-0.3196	0.749	-1.55E-06	count	1
TBRG1	-0.4075972	0.3363157	-1.2119	0.226	-1.55E-06	count	1
SERPINB9P1	-1.8455256	1.27686	-1.4454	0.149	-1.55E-06	count	1
RBM45	-1.8455256	1.27686	-1.4454	0.149	-1.55E-06	count	1

LMAN2L	-1.8455256	1.220219	-1.5125	0.131	-1.55E-06	count	1
AC008105.3	-0.4093826	0.5373927	-0.7618	0.447	-1.55E-06	count	1
ZNF654	-1.8455256	1.1891401	-1.552	0.121	-1.55E-06	count	1
TPGS1	-0.404287	0.3546091	-1.1401	0.255	-1.54E-06	count	1
MS4A6A	-1.7425177	1.1228663	-1.5518	0.121	-1.54E-06	count	1
UBE2D4	-1.7425177	0.8742126	-1.9932	0.0468	-1.54E-06	count	1
CCDC126	-1.7425177	0.8742126	-1.9932	0.0468	-1.54E-06	count	1
AGPAT5	-0.4030898	0.4661341	-0.8648	0.388	-1.54E-06	count	1
ALG11	-1.8059448	1.1921029	-1.5149	0.131	-1.53E-06	count	1
AC026401.3	-1.8059448	1.1921029	-1.5149	0.131	-1.53E-06	count	1
BGN	-1.8059448	1.1921029	-1.5149	0.131	-1.53E-06	count	1
ACLY	-0.404287	0.6164442	-0.6558	0.512	-1.53E-06	count	1
MTURN	-0.404287	0.5939937	-0.6806	0.496	-1.53E-06	count	1
PMM1	-0.404287	0.6276773	-0.6441	0.52	-1.53E-06	count	1
AC100810.1	-0.404287	0.5704056	-0.7088	0.479	-1.53E-06	count	1
YTHDF1	-0.404287	0.5704056	-0.7088	0.479	-1.53E-06	count	1
LIPA	-0.4012298	0.3963569	-1.0123	0.312	-1.53E-06	count	1
ITPR3	-0.404287	0.7381705	-0.5477	0.584	-1.53E-06	count	1
BRAP	-0.404287	0.604438	-0.6689	0.504	-1.53E-06	count	1
PUS3	-0.404287	0.6375701	-0.6341	0.526	-1.53E-06	count	1
BATF	-0.4024401	0.4158057	-0.9679	0.334	-1.53E-06	count	1
EPHA1-AS1	-1.7642464	1.5978188	-1.1042	0.27	-1.53E-06	count	1
ARHGAP4	-0.4012618	0.3599971	-1.1146	0.266	-1.53E-06	count	1
RETREG1	-0.4012618	0.5472392	-0.7332	0.464	-1.52E-06	count	1
MBD5	-0.4012896	0.4393095	-0.9135	0.361	-1.52E-06	count	1
RGS5	-1.7642464	1.3700258	-1.2877	0.199	-1.52E-06	count	1
TMEM273	-0.4012896	0.4507659	-0.8902	0.374	-1.52E-06	count	1
PUS1	-1.7642464	1.2851535	-1.3728	0.171	-1.52E-06	count	1
PWWP2A	-0.3978671	0.4016535	-0.9906	0.322	-1.52E-06	count	1
LTC4S	-1.7642464	1.2784807	-1.38	0.168	-1.52E-06	count	1
SPTY2D1	-0.3998447	0.439291	-0.9102	0.363	-1.52E-06	count	1
NUDT7	-1.7642464	1.1942646	-1.4773	0.14	-1.52E-06	count	1
ABAT	-1.7642464	1.1942646	-1.4773	0.14	-1.52E-06	count	1
NAPA-AS1	-1.7642464	1.1942646	-1.4773	0.14	-1.52E-06	count	1
IGKV3-15	-1.7642464	1.1942646	-1.4773	0.14	-1.52E-06	count	1
CREB3L4	-1.7642464	1.1942646	-1.4773	0.14	-1.52E-06	count	1
CDKN1C	-1.7642464	1.1942646	-1.4773	0.14	-1.52E-06	count	1
IGHV1-3	-1.7642464	1.1942646	-1.4773	0.14	-1.52E-06	count	1
COG7	-1.7642464	1.1942646	-1.4773	0.14	-1.52E-06	count	1
MOSPD1	-1.7642464	1.0958634	-1.6099	0.108	-1.51E-06	count	1
ADAM10	-0.132759	0.3536067	-0.3754	0.708	-1.51E-06	count	1
IP6K2	-0.1324783	0.2996892	-0.4421	0.659	-1.51E-06	count	1
CR1	-0.3986482	0.5795755	-0.6878	0.492	-1.51E-06	count	1
FILIP1L	-0.3959304	0.6361497	-0.6224	0.534	-1.51E-06	count	1
AKT1S1	-1.6378974	1.1501963	-1.424	0.155	-1.51E-06	count	1

PDE8A	-1.741409	1.2187262	-1.4289	0.154	-1.50E-06	count	1
RBM15B	-0.3959304	0.3940675	-1.0047	0.316	-1.50E-06	count	1
MSH3	-0.3959304	0.4560152	-0.8682	0.386	-1.50E-06	count	1
TESK2	-0.3959304	0.5270253	-0.7513	0.453	-1.50E-06	count	1
NCOA6	-1.6622761	0.9556917	-1.7393	0.0827	-1.50E-06	count	1
BRCA2	-0.3947754	0.3852792	-1.0246	0.306	-1.50E-06	count	1
CKS1B	-0.3924549	0.3789651	-1.0356	0.301	-1.50E-06	count	1
CFD	-1.6378974	1.029497	-1.591	0.112	-1.50E-06	count	1
P2RY14	-0.3840298	0.7479126	-0.5135	0.608	-1.50E-06	count	1
ATP6V1A	-0.3932077	0.5153946	-0.7629	0.446	-1.50E-06	count	1
WDR48	-0.3932077	0.4434803	-0.8866	0.376	-1.50E-06	count	1
AC027097.1	-0.3924549	0.4474246	-0.8771	0.381	-1.49E-06	count	1
SESTD1	-1.6515729	0.9225319	-1.7903	0.0741	-1.49E-06	count	1
MPHOSPH6	-0.3913815	0.4554415	-0.8593	0.391	-1.49E-06	count	1
ARFGAP3	-0.3893485	0.4135511	-0.9415	0.347	-1.49E-06	count	1
NVL	-0.3893485	0.5483977	-0.71	0.478	-1.49E-06	count	1
EPB41	-0.1301898	0.2062768	-0.6311	0.528	-1.48E-06	count	1
ADAT1	-1.6378974	0.8214978	-1.9938	0.0468	-1.48E-06	count	1
NR2C2	-0.3893485	0.5707381	-0.6822	0.495	-1.48E-06	count	1
CASP10	-0.3891008	0.6616792	-0.5881	0.557	-1.48E-06	count	1
PTBP2	-0.3891008	0.6855033	-0.5676	0.571	-1.48E-06	count	1
PCDH9	-0.3874544	0.5344704	-0.7249	0.469	-1.48E-06	count	1
SGK494	-0.3891008	0.7296852	-0.5332	0.594	-1.48E-06	count	1
UGT8	-0.3891008	0.7003193	-0.5556	0.579	-1.48E-06	count	1
VPS4B	-0.1293561	0.2369455	-0.5459	0.585	-1.48E-06	count	1
IFRD2	-0.3857992	0.4266331	-0.9043	0.366	-1.48E-06	count	1
UBE2J1	-0.129139	0.2213481	-0.5834	0.56	-1.47E-06	count	1
LSM5	-0.1288156	0.2066288	-0.6234	0.533	-1.47E-06	count	1
NOL8	-0.3857992	0.4276241	-0.9022	0.367	-1.47E-06	count	1
CLASP1	-1.6127004	1.0389603	-1.5522	0.121	-1.47E-06	count	1
LMBRD2	-1.6515729	1.3104019	-1.2604	0.208	-1.47E-06	count	1
RARA-AS1	-1.6515729	1.3104019	-1.2604	0.208	-1.47E-06	count	1
RAD51D	-1.6515729	1.2894047	-1.2809	0.201	-1.47E-06	count	1
FAM160B2	-0.384969	0.7681523	-0.5012	0.617	-1.46E-06	count	1
IL23A	-0.3821833	0.4937103	-0.7741	0.439	-1.46E-06	count	1
CCDC107	-0.1278689	0.2204426	-0.5801	0.562	-1.46E-06	count	1
AC124312.1	-1.6024741	1.2216077	-1.3118	0.19	-1.46E-06	count	1
RAD18	-0.3796422	0.5722569	-0.6634	0.507	-1.46E-06	count	1
MGAT4B	-0.3796422	0.4983059	-0.7619	0.447	-1.45E-06	count	1
PIGH	-0.3759608	0.5779383	-0.6505	0.516	-1.45E-06	count	1
MRM1	-1.6024741	1.3354454	-1.2	0.231	-1.45E-06	count	1
CDC23	-1.6024741	0.9101411	-1.7607	0.079	-1.45E-06	count	1
BACE2	-0.3784124	0.4493245	-0.8422	0.4	-1.45E-06	count	1
MEF2C-AS1	-0.3759608	0.8673231	-0.4335	0.665	-1.45E-06	count	1
BBS2	-1.6024741	1.1252136	-1.4242	0.155	-1.45E-06	count	1

POC1B	-0.3759608	0.6648045	-0.5655	0.572	-1.44E-06	count	1
SLC25A19	-0.3759608	0.4744728	-0.7924	0.429	-1.44E-06	count	1
FAM221A	-0.3759608	0.66895	-0.562	0.574	-1.44E-06	count	1
GCFC2	-0.3759608	0.6349642	-0.5921	0.554	-1.44E-06	count	1
UTP11	-0.3759608	0.5128979	-0.733	0.464	-1.44E-06	count	1
NEIL2	-0.3759608	0.677987	-0.5545	0.579	-1.44E-06	count	1
NFE2L1	-0.3759608	0.677987	-0.5545	0.579	-1.44E-06	count	1
SLC25A24	-0.3728276	0.5019599	-0.7427	0.458	-1.44E-06	count	1
NR2C1	-0.3728276	0.4637137	-0.804	0.422	-1.43E-06	count	1
IFNAR2	-0.3745564	0.7126658	-0.5256	0.599	-1.43E-06	count	1
PYM1	-0.3738188	0.3730654	-1.002	0.317	-1.43E-06	count	1
LIN52	-1.5223395	0.9056556	-1.6809	0.0935	-1.43E-06	count	1
BTK	-0.1249184	0.263107	-0.4748	0.635	-1.43E-06	count	1
PIK3CD	-0.3701286	0.7697801	-0.4808	0.631	-1.43E-06	count	1
LINC00494	-1.5223395	0.8398175	-1.8127	0.0706	-1.43E-06	count	1
MED14	-1.5223395	0.8398175	-1.8127	0.0706	-1.43E-06	count	1
ZCCHC8	-0.3677795	0.4736293	-0.7765	0.438	-1.43E-06	count	1
DPH5	-1.4935963	0.6712152	-2.2252	0.0266	-1.43E-06	count	1
PCNT	-1.5223395	1.083799	-1.4046	0.161	-1.43E-06	count	1
ARHGEF12	-1.5223395	1.083799	-1.4046	0.161	-1.43E-06	count	1
MYO15B	-1.5500099	1.2542239	-1.2358	0.217	-1.43E-06	count	1
ZBED5	-0.3718844	0.3659096	-1.0163	0.31	-1.43E-06	count	1
TCAIM	-1.5223395	1.0294181	-1.4788	0.14	-1.43E-06	count	1
RABL2B	-1.5500099	1.1264855	-1.376	0.17	-1.43E-06	count	1
EGR3	-0.1244903	0.2949096	-0.4221	0.673	-1.42E-06	count	1
NDUFAF7	-0.3701286	0.5667961	-0.653	0.514	-1.42E-06	count	1
NARFL	-0.3698265	0.5372762	-0.6883	0.492	-1.42E-06	count	1
EIF2AK4	-0.3698265	0.4275497	-0.865	0.388	-1.42E-06	count	1
YIPF2	-0.3701286	0.6023585	-0.6145	0.539	-1.42E-06	count	1
MTMR14	-0.124157	0.3150085	-0.3941	0.694	-1.42E-06	count	1
PIAS4	-0.3701286	0.5718312	-0.6473	0.518	-1.42E-06	count	1
CNNM4	-1.4935963	0.9310672	-1.6042	0.109	-1.42E-06	count	1
KMT2E-AS1	-0.3701286	0.6678263	-0.5542	0.58	-1.42E-06	count	1
TMPO-AS1	-1.4935963	1.1034589	-1.3536	0.177	-1.42E-06	count	1
ATF3	-0.3701286	0.5829694	-0.6349	0.526	-1.42E-06	count	1
RUNX1	-0.3696083	0.6171191	-0.5989	0.55	-1.42E-06	count	1
HNRNPUL2	-1.4534249	0.750317	-1.9371	0.0534	-1.42E-06	count	1
TRMT5	-0.3701286	0.9143565	-0.4048	0.686	-1.42E-06	count	1
NCAPD3	-0.3660829	0.5635113	-0.6496	0.516	-1.41E-06	count	1
BLCAP	-0.3660829	0.5275797	-0.6939	0.488	-1.41E-06	count	1
ZNF736	-1.4534249	0.7007606	-2.0741	0.0386	-1.41E-06	count	1
YPEL2	-1.4534249	0.7007606	-2.0741	0.0386	-1.41E-06	count	1
SAMD10	-1.4636784	1.0563776	-1.3856	0.167	-1.41E-06	count	1
COQ8A	-1.4534249	0.8251224	-1.7615	0.0788	-1.41E-06	count	1
TSTD2	-0.3657162	0.6005199	-0.609	0.543	-1.41E-06	count	1

GART	-0.3659138	0.4092318	-0.8941	0.372	-1.41E-06	count	1
NFATC3	-0.3630459	0.6255252	-0.5804	0.562	-1.41E-06	count	1
HSPBAP1	-0.3657162	0.6005199	-0.609	0.543	-1.41E-06	count	1
C2orf69	-0.3660829	0.5778783	-0.6335	0.527	-1.41E-06	count	1
DXO	-0.3657162	0.6265007	-0.5837	0.56	-1.41E-06	count	1
RASA4	-1.4636784	0.9353719	-1.5648	0.118	-1.41E-06	count	1
EXD2	-1.4636784	1.1412553	-1.2825	0.2	-1.40E-06	count	1
KATNB1	-1.4935963	1.4902924	-1.0022	0.317	-1.40E-06	count	1
GGT5	-1.4935963	1.4902924	-1.0022	0.317	-1.40E-06	count	1
LINC02447	-1.4935963	1.4902924	-1.0022	0.317	-1.40E-06	count	1
DDX60L	-1.4534249	0.7803313	-1.8626	0.0632	-1.40E-06	count	1
TOR1AIP1	-0.3644747	0.3042103	-1.1981	0.232	-1.40E-06	count	1
GZMA	-1.4636784	0.8913939	-1.642	0.101	-1.40E-06	count	1
TET3	-1.4636784	0.8913939	-1.642	0.101	-1.40E-06	count	1
RBKS	-1.4636784	0.8913939	-1.642	0.101	-1.40E-06	count	1
OTULINL	-1.4935963	1.2612977	-1.1842	0.237	-1.40E-06	count	1
TBC1D14	-1.4935963	1.2612977	-1.1842	0.237	-1.40E-06	count	1
ABCC4	-1.4935963	1.2612977	-1.1842	0.237	-1.40E-06	count	1
AQP1	-1.4935963	1.2612977	-1.1842	0.237	-1.40E-06	count	1
A2M	-1.4935963	1.2612977	-1.1842	0.237	-1.40E-06	count	1
EFCAB11	-1.4935963	1.2612977	-1.1842	0.237	-1.40E-06	count	1
EARS2	-1.4935963	1.2036897	-1.2408	0.215	-1.40E-06	count	1
AL731577.1	-1.4636784	1.068545	-1.3698	0.171	-1.40E-06	count	1
SOS2	-0.3630459	0.5103569	-0.7114	0.477	-1.40E-06	count	1
GBF1	-0.3630459	0.5190407	-0.6995	0.485	-1.40E-06	count	1
FMC1	-0.3608002	0.394866	-0.9137	0.361	-1.40E-06	count	1
USP39	-0.3622611	0.5313656	-0.6818	0.496	-1.40E-06	count	1
ZNF253	-0.3622611	0.5768742	-0.628	0.53	-1.39E-06	count	1
C19orf12	-0.3622611	0.4122759	-0.8787	0.38	-1.39E-06	count	1
NUDT14	-0.3622611	0.5594878	-0.6475	0.518	-1.39E-06	count	1
NEDD9	-0.3605325	0.5261294	-0.6853	0.494	-1.39E-06	count	1
NEK3	-0.3594823	0.5678022	-0.6331	0.527	-1.39E-06	count	1
TNS3	-1.4255081	0.850185	-1.6767	0.0943	-1.39E-06	count	1
FAM192A	-0.3588943	0.3746739	-0.9579	0.339	-1.38E-06	count	1
FCF1	-0.3594823	0.5228878	-0.6875	0.492	-1.38E-06	count	1
PRR14L	-0.3571988	0.5521375	-0.6469	0.518	-1.38E-06	count	1
HACD4	-0.358418	0.4955804	-0.7232	0.47	-1.38E-06	count	1
POGZ	-0.357844	0.3492314	-1.0247	0.306	-1.38E-06	count	1
FAF1	-0.3571988	0.5303777	-0.6735	0.501	-1.38E-06	count	1
HPS4	-0.3566143	0.4650026	-0.7669	0.444	-1.38E-06	count	1
TCEAL3	-0.1198454	0.3755526	-0.3191	0.75	-1.37E-06	count	1
PITPNA	-0.355289	0.4475872	-0.7938	0.428	-1.37E-06	count	1
INPP5B	-0.3550575	0.4735667	-0.7498	0.454	-1.37E-06	count	1
VEZF1	-0.3505033	0.4352042	-0.8054	0.421	-1.37E-06	count	1
GRK5	-0.3536681	0.4394953	-0.8047	0.421	-1.37E-06	count	1

C2orf68	-1.4189334	1.3451278	-1.0549	0.292	-1.36E-06	count	1
SEC14L1	-0.3525063	0.4408345	-0.7996	0.424	-1.36E-06	count	1
MAGOHB	-0.3500045	0.4259423	-0.8217	0.412	-1.36E-06	count	1
MOSPD2	-0.3500045	0.4287109	-0.8164	0.415	-1.36E-06	count	1
CPEB4	-0.3510653	0.4868093	-0.7212	0.471	-1.35E-06	count	1
IWS1	-0.1179532	0.2836035	-0.4159	0.678	-1.35E-06	count	1
MFSD4B	-1.3387906	0.7739302	-1.7299	0.0844	-1.35E-06	count	1
SPINT1-AS1	-1.3387906	0.7465962	-1.7932	0.0736	-1.35E-06	count	1
FBXL20	-1.3387906	0.7465962	-1.7932	0.0736	-1.35E-06	count	1
ST3GAL1	-0.3461763	0.4239776	-0.8165	0.415	-1.35E-06	count	1
SMIM27	-0.3481938	0.3585136	-0.9712	0.332	-1.35E-06	count	1
AL445472.1	-0.3474845	0.4553972	-0.763	0.446	-1.35E-06	count	1
BARD1	-0.3451372	0.3603666	-0.9577	0.339	-1.34E-06	count	1
GCNT2	-1.3604102	1.0321813	-1.318	0.188	-1.34E-06	count	1
CDC42BPB	-1.3604102	1.0126417	-1.3434	0.18	-1.34E-06	count	1
STK39	-1.3387906	0.7954981	-1.683	0.0931	-1.34E-06	count	1
CFL2	-0.3420025	0.5033078	-0.6795	0.497	-1.33E-06	count	1
ABL2	-1.3555697	0.9075123	-1.4937	0.136	-1.33E-06	count	1
PMVK	-0.1157551	0.3391304	-0.3413	0.733	-1.33E-06	count	1
BNIP3	-0.3427562	0.7838558	-0.4373	0.662	-1.33E-06	count	1
ENC1	-1.3255323	1.132548	-1.1704	0.242	-1.33E-06	count	1
DEXI	-1.3255323	0.904168	-1.466	0.143	-1.33E-06	count	1
SEC61A2	-1.3255323	0.8697904	-1.524	0.128	-1.33E-06	count	1
MARK4	-1.3255323	0.8697904	-1.524	0.128	-1.33E-06	count	1
ST20	-1.3255323	0.8697904	-1.524	0.128	-1.33E-06	count	1
ZNF432	-1.3255323	0.8697904	-1.524	0.128	-1.33E-06	count	1
SREBF2	-1.3255323	0.8697904	-1.524	0.128	-1.33E-06	count	1
PPA2	-0.3399416	0.3948124	-0.861	0.39	-1.32E-06	count	1
CGRRF1	-0.3399416	0.4416796	-0.7697	0.442	-1.32E-06	count	1
AMDHD2	-0.3371959	0.484956	-0.6953	0.487	-1.32E-06	count	1
TMEM62	-0.3403524	0.6417085	-0.5304	0.596	-1.32E-06	count	1
NTHL1	-0.3403524	0.617413	-0.5513	0.582	-1.32E-06	count	1
CELF1	-0.3395796	0.368996	-0.9203	0.358	-1.32E-06	count	1
PDXK	-0.3384796	0.4896039	-0.6913	0.49	-1.32E-06	count	1
RDH14	-0.1145932	0.294464	-0.3892	0.697	-1.32E-06	count	1
UBE2G1	-0.1145932	0.279075	-0.4106	0.682	-1.31E-06	count	1
FADS3	-0.114425	0.2760444	-0.4145	0.679	-1.31E-06	count	1
TIMM50	-0.3384796	0.550956	-0.6143	0.539	-1.31E-06	count	1
ZBTB25	-0.337445	0.4953172	-0.6813	0.496	-1.31E-06	count	1
NR4A3	-0.3384796	0.5372558	-0.63	0.529	-1.31E-06	count	1
AFF1	-0.337445	0.4483201	-0.7527	0.452	-1.31E-06	count	1
UPF3B	-0.1141704	0.3307027	-0.3452	0.73	-1.31E-06	count	1
DSE	-0.3380743	0.5670989	-0.5961	0.551	-1.31E-06	count	1
MTHFD2L	-1.2888551	1.0325339	-1.2482	0.213	-1.31E-06	count	1
NARF	-0.3367887	0.4409861	-0.7637	0.445	-1.31E-06	count	1

CAMK2N1	-1.2888551	1.2770723	-1.0092	0.313	-1.31E-06	count	1
STAG2	-0.1140753	0.2513524	-0.4538	0.65	-1.31E-06	count	1
NUBP2	-0.3369791	0.4228299	-0.797	0.426	-1.31E-06	count	1
BTN2A1	-0.3367887	0.4638001	-0.7262	0.468	-1.31E-06	count	1
TTC39C	-0.3363354	0.43044	-0.7814	0.435	-1.31E-06	count	1
STMP1	-0.1135413	0.2584874	-0.4393	0.661	-1.30E-06	count	1
TMEM268	-1.2864161	0.9306188	-1.3823	0.168	-1.30E-06	count	1
SLC43A2	-1.2888551	1.0626402	-1.2129	0.226	-1.30E-06	count	1
NHSL2	-1.2888551	1.0626402	-1.2129	0.226	-1.30E-06	count	1
CYP51A1	-1.2888551	0.8153274	-1.5808	0.115	-1.30E-06	count	1
PGM1	-1.2627989	0.6997002	-1.8048	0.0718	-1.30E-06	count	1
GTF3C3	-1.2864161	0.9384614	-1.3708	0.171	-1.29E-06	count	1
TAB1	-1.2864161	1.3766876	-0.9344	0.351	-1.29E-06	count	1
ICA1L	-1.2864161	1.2667164	-1.0156	0.31	-1.29E-06	count	1
AC091132.5	-1.2864161	1.2667164	-1.0156	0.31	-1.29E-06	count	1
DNASE1	-1.2864161	1.2667164	-1.0156	0.31	-1.29E-06	count	1
HINT2	-0.1125272	0.2967021	-0.3793	0.705	-1.29E-06	count	1
TNIP1	-0.3302767	0.4075531	-0.8104	0.418	-1.29E-06	count	1
CEP290	-0.1118953	0.4317727	-0.2592	0.796	-1.29E-06	count	1
SDHAF4	-1.2366398	0.8691514	-1.4228	0.155	-1.28E-06	count	1
GLA	-1.2366398	0.7361571	-1.6799	0.0937	-1.28E-06	count	1
NCKAP5L	-1.2492586	0.9306651	-1.3423	0.18	-1.28E-06	count	1
MTX2	-0.3260194	0.4703867	-0.6931	0.489	-1.27E-06	count	1
USP33	-0.110634	0.3310083	-0.3342	0.738	-1.27E-06	count	1
HRAS	-0.3232589	0.3869095	-0.8355	0.404	-1.27E-06	count	1
TLNRD1	-0.3206406	0.5027061	-0.6378	0.524	-1.27E-06	count	1
STARD3NL	-0.3232295	0.4847222	-0.6668	0.505	-1.27E-06	count	1
FBXO3	-0.3248158	0.5223292	-0.6219	0.534	-1.26E-06	count	1
DOPEY2	-1.2100985	0.9937581	-1.2177	0.224	-1.26E-06	count	1
C1orf174	-0.3220937	0.4036087	-0.798	0.425	-1.26E-06	count	1
ARFGAP2	-0.3189249	0.367705	-0.8673	0.386	-1.25E-06	count	1
SRPK2	-0.1089447	0.2288551	-0.476	0.634	-1.25E-06	count	1
TSTA3	-0.320701	0.4932712	-0.6502	0.516	-1.25E-06	count	1
FAM126A	-0.3208092	0.5878209	-0.5458	0.586	-1.25E-06	count	1
LINC00342	-1.2100985	1.4071935	-0.8599	0.39	-1.25E-06	count	1
FAM131A	-1.2100985	1.362438	-0.8882	0.375	-1.25E-06	count	1
YTHDF3	-0.3195679	0.5262146	-0.6073	0.544	-1.25E-06	count	1
FAM219B	-0.3197706	0.4434309	-0.7211	0.471	-1.25E-06	count	1
ASNS	-1.2100985	1.1751336	-1.0298	0.304	-1.25E-06	count	1
ENG	-1.2100985	1.1751336	-1.0298	0.304	-1.25E-06	count	1
LDOC1	-1.2100985	1.1751336	-1.0298	0.304	-1.25E-06	count	1
CDK10	-1.1827492	0.7352634	-1.6086	0.108	-1.25E-06	count	1
ZNF136	-0.3176349	0.4384184	-0.7245	0.469	-1.24E-06	count	1
IP6K1	-0.3176349	0.4850554	-0.6548	0.513	-1.24E-06	count	1
MPRIP	-0.3176349	0.4799883	-0.6618	0.508	-1.24E-06	count	1

ZNF318	-0.3170988	0.8632031	-0.3674	0.714	-1.24E-06	count	1
PBX2	-0.3163044	0.4737663	-0.6676	0.505	-1.24E-06	count	1
TRMT13	-0.3163044	0.5283732	-0.5986	0.55	-1.24E-06	count	1
GOLGA8A	-0.3165951	0.5233728	-0.6049	0.546	-1.24E-06	count	1
PPM1B	-0.3146733	0.414647	-0.7589	0.448	-1.24E-06	count	1
KCNQ1	-0.3147362	0.4717739	-0.6671	0.505	-1.24E-06	count	1
SMIM19	-0.1069843	0.3216238	-0.3326	0.74	-1.23E-06	count	1
CCDC69	-0.3147362	0.3469811	-0.9071	0.365	-1.23E-06	count	1
PBRM1	-0.1070164	0.3307266	-0.3236	0.746	-1.23E-06	count	1
ZNF121	-0.3147362	0.5481613	-0.5742	0.566	-1.23E-06	count	1
PAXIP1-AS2	-0.3147362	0.4998615	-0.6296	0.529	-1.23E-06	count	1
MAP2K4	-0.3145916	0.5445801	-0.5777	0.564	-1.23E-06	count	1
COPG1	-1.1686678	0.9688939	-1.2062	0.228	-1.23E-06	count	1
C16orf87	-0.3134049	0.3930674	-0.7973	0.426	-1.23E-06	count	1
SIKE1	-0.3138421	0.3541993	-0.8861	0.376	-1.23E-06	count	1
ALOX5	-0.313125	0.3584187	-0.8736	0.383	-1.23E-06	count	1
ATL2	-0.3128604	0.5074251	-0.6166	0.538	-1.23E-06	count	1
YWHAG	-0.1064894	0.3465622	-0.3073	0.759	-1.23E-06	count	1
TM7SF3	-0.3128604	0.6123011	-0.511	0.61	-1.23E-06	count	1
GATAD2B	-0.3125192	0.4747037	-0.6583	0.511	-1.22E-06	count	1
FLOT2	-0.3105767	0.3817714	-0.8135	0.416	-1.22E-06	count	1
ZRANB1	-0.3105767	0.5676155	-0.5472	0.585	-1.22E-06	count	1
FUOM	-0.1056105	0.3750145	-0.2816	0.778	-1.22E-06	count	1
PLEKHB2	-0.3098208	0.5034377	-0.6154	0.539	-1.22E-06	count	1
ALDH16A1	-0.309238	0.4315079	-0.7166	0.474	-1.22E-06	count	1
TMEM38B	-0.3087518	0.3987594	-0.7743	0.439	-1.21E-06	count	1
ABL1	-1.1246367	0.8813571	-1.276	0.203	-1.21E-06	count	1
NPRL3	-0.307736	0.5588933	-0.5506	0.582	-1.21E-06	count	1
CTSO	-1.1246367	0.8820583	-1.275	0.203	-1.21E-06	count	1
SEC24D	-0.307736	0.7032958	-0.4376	0.662	-1.21E-06	count	1
OTUD4	-0.307736	0.6681212	-0.4606	0.645	-1.21E-06	count	1
METTL16	-0.3073542	0.3774017	-0.8144	0.416	-1.21E-06	count	1
APOPT1	-0.3057919	0.4242969	-0.7207	0.471	-1.21E-06	count	1
DPP9	-1.1246367	0.8719666	-1.2898	0.198	-1.20E-06	count	1
SLC30A7	-1.1246367	1.1590983	-0.9703	0.332	-1.20E-06	count	1
LINC00476	-1.1246367	0.9082708	-1.2382	0.216	-1.20E-06	count	1
AP002360.1	-1.1347909	1.2853641	-0.8829	0.378	-1.20E-06	count	1
PKN1	-0.1042111	0.3131254	-0.3328	0.739	-1.20E-06	count	1
AC087482.1	-1.1246367	1.1028982	-1.0197	0.308	-1.20E-06	count	1
HS2ST1	-1.1246367	1.1028982	-1.0197	0.308	-1.20E-06	count	1
KPNA5	-0.3040338	0.3936394	-0.7724	0.44	-1.20E-06	count	1
UTS2B	-1.1246367	1.2878042	-0.8733	0.383	-1.20E-06	count	1
METTL3	-1.1246367	1.2878042	-0.8733	0.383	-1.20E-06	count	1
AC008741.2	-1.1246367	1.3845431	-0.8123	0.417	-1.20E-06	count	1
AC016588.2	-1.1246367	1.3845431	-0.8123	0.417	-1.20E-06	count	1

EXT2	-1.1246367	1.3845431	-0.8123	0.417	-1.20E-06	count	1
AZIN1-AS1	-1.1246367	1.3845431	-0.8123	0.417	-1.20E-06	count	1
EZH2	-1.1246367	1.3845431	-0.8123	0.417	-1.20E-06	count	1
CRY2	-1.1246367	1.3845431	-0.8123	0.417	-1.20E-06	count	1
TLCD1	-1.1246367	1.3845431	-0.8123	0.417	-1.20E-06	count	1
DARS-AS1	-1.1246367	1.3845431	-0.8123	0.417	-1.20E-06	count	1
CRY1	-1.1246367	1.3845431	-0.8123	0.417	-1.20E-06	count	1
WDR17	-1.1246367	1.3845431	-0.8123	0.417	-1.20E-06	count	1
GZMH	-1.1246367	1.3845431	-0.8123	0.417	-1.20E-06	count	1
AGAP9	-1.1246367	1.3845431	-0.8123	0.417	-1.20E-06	count	1
RCBTB1	-1.1246367	1.3845431	-0.8123	0.417	-1.20E-06	count	1
CDC42EP2	-1.1246367	1.3845431	-0.8123	0.417	-1.20E-06	count	1
NCK1	-0.1037997	0.3754686	-0.2765	0.782	-1.20E-06	count	1
AC025171.3	-1.1246367	1.0723534	-1.0488	0.295	-1.20E-06	count	1
C9orf64	-1.1246367	1.0723534	-1.0488	0.295	-1.20E-06	count	1
RTL10	-1.1246367	1.0723534	-1.0488	0.295	-1.20E-06	count	1
AC124016.1	-1.1246367	1.1867864	-0.9476	0.344	-1.20E-06	count	1
FDXACB1	-1.1246367	1.1867864	-0.9476	0.344	-1.20E-06	count	1
KCNMB4	-1.1246367	1.1867864	-0.9476	0.344	-1.20E-06	count	1
ILVBL	-1.1246367	1.1867864	-0.9476	0.344	-1.20E-06	count	1
ZNF17	-1.1246367	1.1867864	-0.9476	0.344	-1.20E-06	count	1
MLLT11	-1.1246367	1.1867864	-0.9476	0.344	-1.20E-06	count	1
LINC01006	-1.1246367	1.1867864	-0.9476	0.344	-1.20E-06	count	1
TPTEP2-CSNK1E	-1.1246367	1.1867864	-0.9476	0.344	-1.20E-06	count	1
UHRF1BP1	-1.1246367	1.1867864	-0.9476	0.344	-1.20E-06	count	1
AC022167.3	-1.1246367	1.1867864	-0.9476	0.344	-1.20E-06	count	1
LINC01004	-1.1246367	1.1867864	-0.9476	0.344	-1.20E-06	count	1
CHAC2	-1.1246367	1.1867864	-0.9476	0.344	-1.20E-06	count	1
SNX13	-0.3041065	0.5799799	-0.5243	0.6	-1.20E-06	count	1
RIMKLB	-0.3041065	0.6834897	-0.4449	0.657	-1.20E-06	count	1
ZFYVE27	-0.3041065	0.6596189	-0.461	0.645	-1.20E-06	count	1
ZNF460	-0.3043126	0.7523186	-0.4045	0.686	-1.19E-06	count	1
BCL9L	-1.093638	0.8353893	-1.3091	0.191	-1.19E-06	count	1
DDX59	-0.3027118	0.3592503	-0.8426	0.4	-1.19E-06	count	1
MAPK9	-1.093638	0.7912573	-1.3822	0.168	-1.19E-06	count	1
IFIT3	-1.093638	0.7628087	-1.4337	0.152	-1.19E-06	count	1
ILK	-0.3013867	0.3376206	-0.8927	0.373	-1.19E-06	count	1
UBP1	-1.093638	0.8689137	-1.2586	0.209	-1.19E-06	count	1
SCO1	-0.3020409	0.4962391	-0.6087	0.543	-1.19E-06	count	1
PDLIM2	-0.1027875	0.3093036	-0.3323	0.74	-1.18E-06	count	1
GNG11	-1.093638	0.826511	-1.3232	0.186	-1.18E-06	count	1
ASMTL	-0.2997625	0.4530022	-0.6617	0.508	-1.18E-06	count	1
USP28	-1.0775915	0.6178179	-1.7442	0.0818	-1.18E-06	count	1
TCEAL4	-0.1024601	0.2951157	-0.3472	0.729	-1.18E-06	count	1
HERC2	-0.2993071	0.4017179	-0.7451	0.457	-1.18E-06	count	1

EFL1	-0.2993071	0.7235268	-0.4137	0.679	-1.18E-06	count	1
MASTL	-0.2993071	0.6738938	-0.4441	0.657	-1.18E-06	count	1
MFHAS1	-0.2993071	0.6768406	-0.4422	0.659	-1.18E-06	count	1
BCS1L	-0.2993071	0.7330373	-0.4083	0.683	-1.18E-06	count	1
AMIGO2	-0.2993071	0.7330373	-0.4083	0.683	-1.18E-06	count	1
TTL	-0.2993071	0.7330373	-0.4083	0.683	-1.18E-06	count	1
HIPK3	-0.2982541	0.3858192	-0.773	0.44	-1.18E-06	count	1
AC090204.1	-1.0775915	1.0515254	-1.0248	0.306	-1.17E-06	count	1
ZNF281	-0.2962767	0.4987303	-0.5941	0.553	-1.17E-06	count	1
GNPDA2	-0.2971329	0.456343	-0.6511	0.515	-1.17E-06	count	1
REXO4	-0.2971329	0.456343	-0.6511	0.515	-1.17E-06	count	1
GPR82	-0.2979349	0.956435	-0.3115	0.756	-1.17E-06	count	1
CASC3	-0.2962767	0.4755093	-0.6231	0.534	-1.17E-06	count	1
ARMC5	-1.0775915	0.9170865	-1.175	0.241	-1.17E-06	count	1
FPGT	-1.0775915	0.9170865	-1.175	0.241	-1.17E-06	count	1
ACBD4	-1.0775915	0.9170865	-1.175	0.241	-1.17E-06	count	1
PIP5K1C	-1.0775915	0.9170865	-1.175	0.241	-1.17E-06	count	1
PCGF6	-1.0775915	0.9170865	-1.175	0.241	-1.17E-06	count	1
NUSAP1	-1.0775915	0.9170865	-1.175	0.241	-1.17E-06	count	1
EID3	-1.0775915	0.9170865	-1.175	0.241	-1.17E-06	count	1
Z84485.1	-1.0775915	0.9177752	-1.1741	0.241	-1.17E-06	count	1
KCNG1	-1.0775915	1.1089531	-0.9717	0.332	-1.17E-06	count	1
ALDH5A1	-1.0775915	1.1089531	-0.9717	0.332	-1.17E-06	count	1
C17orf80	-1.0775915	1.1089531	-0.9717	0.332	-1.17E-06	count	1
S100Z	-1.0775915	0.8418543	-1.28	0.201	-1.17E-06	count	1
ATG7	-1.0775915	0.8418543	-1.28	0.201	-1.17E-06	count	1
OXSM	-1.0775915	0.8426045	-1.2789	0.202	-1.17E-06	count	1
KMT5B	-0.2958535	0.3778776	-0.7829	0.434	-1.17E-06	count	1
PTGES2	-0.2956599	0.3272704	-0.9034	0.367	-1.17E-06	count	1
FEM1B	-1.0775915	1.0475915	-1.0286	0.304	-1.17E-06	count	1
GNB4	-1.0611527	0.7687678	-1.3803	0.168	-1.17E-06	count	1
ASB3	-1.0611527	0.90726	-1.1696	0.243	-1.17E-06	count	1
ZBTB20-AS2	-1.0611527	1.108007	-0.9577	0.339	-1.17E-06	count	1
CLEC16A	-1.0611527	0.873754	-1.2145	0.225	-1.17E-06	count	1
FANCA	-1.0611527	0.873754	-1.2145	0.225	-1.17E-06	count	1
GGA2	-0.100846	0.2762273	-0.3651	0.715	-1.16E-06	count	1
CARNMT1	-1.0611527	0.8389108	-1.2649	0.207	-1.16E-06	count	1
ALKBH3	-1.0527789	0.6463888	-1.6287	0.104	-1.16E-06	count	1
SPATC1L	-0.2940638	0.5381214	-0.5465	0.585	-1.16E-06	count	1
EDEM2	-0.2940638	0.4707729	-0.6246	0.533	-1.16E-06	count	1
SSR3	-0.1001502	0.2688191	-0.3726	0.71	-1.16E-06	count	1
ING1	-0.100396	0.22037	-0.4556	0.649	-1.16E-06	count	1
IMMT	-0.2915208	0.363434	-0.8021	0.423	-1.15E-06	count	1
DCUN1D1	-0.2911527	0.3790756	-0.7681	0.443	-1.15E-06	count	1
ZNF493	-0.2904353	0.4995147	-0.5814	0.561	-1.15E-06	count	1

ZADH2	-0.2904353	0.4862073	-0.5973	0.551	-1.15E-06	count	1
PCF11-AS1	-1.0494289	1.2988684	-0.808	0.42	-1.15E-06	count	1
CACNA1A	-1.0494289	1.2801341	-0.8198	0.413	-1.15E-06	count	1
AC067852.2	-1.0494289	1.2801341	-0.8198	0.413	-1.15E-06	count	1
ZNF90	-1.0494289	1.241818	-0.8451	0.399	-1.15E-06	count	1
IGFLR1	-0.2897419	0.3762122	-0.7702	0.442	-1.15E-06	count	1
IRGQ	-1.0389827	1.1877257	-0.8748	0.382	-1.14E-06	count	1
RNF141	-0.2883612	0.3619948	-0.7966	0.426	-1.14E-06	count	1
STAU2	-0.2882864	0.5454834	-0.5285	0.597	-1.14E-06	count	1
TSPAN33	-0.0986134	0.2417795	-0.4079	0.684	-1.14E-06	count	1
NDE1	-0.2866045	0.3169305	-0.9043	0.366	-1.14E-06	count	1
RLIM	-0.2860791	0.491941	-0.5815	0.561	-1.13E-06	count	1
PITPNA-AS1	-0.2851266	0.4635597	-0.6151	0.539	-1.13E-06	count	1
EPRS	-0.097655	0.254178	-0.3842	0.701	-1.13E-06	count	1
ARL14EP	-0.0971659	0.2420954	-0.4014	0.688	-1.12E-06	count	1
ATXN2	-0.2828697	0.4529641	-0.6245	0.533	-1.12E-06	count	1
OPA3	-0.2828697	0.4984011	-0.5676	0.571	-1.12E-06	count	1
THOC7	-0.0967238	0.2535497	-0.3815	0.703	-1.12E-06	count	1
ERO1B	-0.2807522	0.4190624	-0.67	0.503	-1.12E-06	count	1
DZIP3	-0.2807522	0.5328013	-0.5269	0.599	-1.11E-06	count	1
RANGAP1	-0.9887348	0.7860896	-1.2578	0.209	-1.11E-06	count	1
DNAAF5	-0.9887348	0.9614722	-1.0284	0.304	-1.11E-06	count	1
PPP1R12B	-0.9887348	1.1215566	-0.8816	0.378	-1.11E-06	count	1
PRKAR1A	-0.0954551	0.2602241	-0.3668	0.714	-1.10E-06	count	1
TBCB	-0.0952594	0.1935535	-0.4922	0.623	-1.10E-06	count	1
DCPS	-0.2776174	0.4371348	-0.6351	0.526	-1.10E-06	count	1
MPP5	-0.2759973	0.686924	-0.4018	0.688	-1.10E-06	count	1
2-Sep	-0.0946339	0.2483866	-0.381	0.703	-1.09E-06	count	1
CHAF1A	-0.2759973	0.700744	-0.3939	0.694	-1.09E-06	count	1
THEMIS2	-0.0946295	0.2491941	-0.3797	0.704	-1.09E-06	count	1
RNF10	-0.2751912	0.4016729	-0.6851	0.494	-1.09E-06	count	1
NPIPB4	-0.9653779	0.8717983	-1.1073	0.269	-1.09E-06	count	1
RAPGEF6	-0.9653779	0.8360557	-1.1547	0.249	-1.09E-06	count	1
NAF1	-0.2750095	0.4831049	-0.5693	0.569	-1.09E-06	count	1
CSNK1E	-0.2740897	0.5380734	-0.5094	0.611	-1.09E-06	count	1
KDM5B	-0.2740897	0.5971233	-0.459	0.646	-1.09E-06	count	1
CNKSR2	-0.2740897	0.6636805	-0.413	0.68	-1.09E-06	count	1
MCTP2	-0.2740897	0.6245582	-0.4389	0.661	-1.09E-06	count	1
EIF4EBP1	-0.2740897	0.4034124	-0.6794	0.497	-1.09E-06	count	1
PAXIP1-AS1	-0.2709387	0.5133139	-0.5278	0.598	-1.08E-06	count	1
CCDC92	-0.2709387	0.541429	-0.5004	0.617	-1.08E-06	count	1
AC104532.2	-0.9533746	1.3713316	-0.6952	0.487	-1.08E-06	count	1
VPS37C	-0.9533746	1.3004227	-0.7331	0.464	-1.08E-06	count	1
CDK16	-0.9533746	1.2509191	-0.7621	0.446	-1.08E-06	count	1
ERGIC1	-0.2700904	0.4046918	-0.6674	0.505	-1.08E-06	count	1

C9orf85	-0.2709387	0.6812409	-0.3977	0.691	-1.08E-06	count	1
CNST	-0.2697098	0.3735353	-0.722	0.471	-1.08E-06	count	1
CDK5RAP3	-0.2689179	0.3912975	-0.6872	0.492	-1.07E-06	count	1
SNX14	-0.2689179	0.3970137	-0.6774	0.499	-1.07E-06	count	1
ARNTL	-0.9238992	0.7686117	-1.202	0.23	-1.07E-06	count	1
DCAF1	-0.9275679	0.7982603	-1.162	0.246	-1.07E-06	count	1
AC005726.5	-0.9345794	0.9759357	-0.9576	0.339	-1.07E-06	count	1
AEN	-0.2669947	0.4485871	-0.5952	0.552	-1.07E-06	count	1
ZNF678	-0.9345794	1.1421917	-0.8182	0.414	-1.07E-06	count	1
SUGP1	-0.9345794	1.1421917	-0.8182	0.414	-1.07E-06	count	1
EXOC3	-0.9275679	0.8649358	-1.0724	0.284	-1.06E-06	count	1
NPLOC4	-0.9275679	0.821922	-1.1285	0.26	-1.06E-06	count	1
GSKIP	-0.2655182	0.5530846	-0.4801	0.631	-1.06E-06	count	1
PHF13	-0.26525	0.3820124	-0.6943	0.488	-1.06E-06	count	1
TIA1	-0.2655182	0.4630349	-0.5734	0.567	-1.06E-06	count	1
ATP9B	-0.26525	0.4072131	-0.6514	0.515	-1.06E-06	count	1
CTDSP2	-0.2655182	0.478837	-0.5545	0.58	-1.06E-06	count	1
TBC1D9B	-0.2655182	0.479045	-0.5543	0.58	-1.06E-06	count	1
PHACTR4	-0.2657948	0.4520509	-0.588	0.557	-1.06E-06	count	1
GDE1	-0.2655182	0.621081	-0.4275	0.669	-1.06E-06	count	1
RRAS	-0.2650757	0.5048804	-0.525	0.6	-1.06E-06	count	1
ING4	-0.2650757	0.5215868	-0.5082	0.612	-1.06E-06	count	1
TXNRD1	-0.2620881	0.4303623	-0.609	0.543	-1.05E-06	count	1
GATAD1	-0.0901019	0.4129429	-0.2182	0.827	-1.05E-06	count	1
MCPH1	-0.0900094	0.4020538	-0.2239	0.823	-1.05E-06	count	1
VEZT	-0.0901019	0.3383788	-0.2663	0.79	-1.04E-06	count	1
ROCK2	-0.0900094	0.3710447	-0.2426	0.808	-1.04E-06	count	1
PDS5B	-0.2607767	0.4395731	-0.5932	0.553	-1.04E-06	count	1
AKT1	-0.2602408	0.462806	-0.5623	0.574	-1.04E-06	count	1
FLNA	-0.0896483	0.3120265	-0.2873	0.774	-1.04E-06	count	1
MPND	-0.8874543	0.8617271	-1.0299	0.304	-1.04E-06	count	1
AC109446.3	-0.2580079	0.6118817	-0.4217	0.673	-1.03E-06	count	1
INTS6-AS1	-0.8874543	0.9139672	-0.971	0.332	-1.03E-06	count	1
SKA2	-0.8874543	0.7593736	-1.1687	0.243	-1.03E-06	count	1
MAPK8IP3	-0.8874543	0.7593736	-1.1687	0.243	-1.03E-06	count	1
TSEN54	-0.2580079	0.5351759	-0.4821	0.63	-1.03E-06	count	1
TSC22D1	-0.2586011	0.5382666	-0.4804	0.631	-1.03E-06	count	1
ZNF490	-0.8874543	0.8717187	-1.0181	0.309	-1.03E-06	count	1
WDPCP	-0.8874543	0.8717187	-1.0181	0.309	-1.03E-06	count	1
ZNF154	-0.8874543	0.8717187	-1.0181	0.309	-1.03E-06	count	1
NDUFA7	-0.8874543	0.8273155	-1.0727	0.284	-1.03E-06	count	1
ARL17A	-0.8874543	0.8273155	-1.0727	0.284	-1.03E-06	count	1
CEP70	-0.8874543	1.0613124	-0.8362	0.404	-1.03E-06	count	1
KIAA1671	-0.8912662	1.6185399	-0.5507	0.582	-1.03E-06	count	1
SUSD6	-0.2571502	0.4267343	-0.6026	0.547	-1.03E-06	count	1

CES2	-0.8757375	0.9260392	-0.9457	0.345	-1.02E-06	count	1
ZNF25	-0.8757375	1.0018397	-0.8741	0.383	-1.02E-06	count	1
RPUSD2	-0.8757375	0.9657454	-0.9068	0.365	-1.02E-06	count	1
CCDC146	-0.8757375	0.9085184	-0.9639	0.336	-1.02E-06	count	1
POLR3C	-0.8757375	0.8685546	-1.0083	0.314	-1.02E-06	count	1
RNF215	-0.8757375	1.1195472	-0.7822	0.434	-1.02E-06	count	1
LASP1	-0.2540818	0.4816953	-0.5275	0.598	-1.02E-06	count	1
SPECC1L	-0.8757375	1.0873672	-0.8054	0.421	-1.02E-06	count	1
HDHD2	-0.2546988	0.7900828	-0.3224	0.747	-1.02E-06	count	1
RNF38	-0.2540818	0.6883772	-0.3691	0.712	-1.02E-06	count	1
SAC3D1	-0.2540818	0.7309213	-0.3476	0.728	-1.02E-06	count	1
ADIPOR2	-0.2540818	0.5275465	-0.4816	0.63	-1.02E-06	count	1
TLR7	-0.2540818	0.7414493	-0.3427	0.732	-1.02E-06	count	1
GABPB2	-0.2540818	0.7811081	-0.3253	0.745	-1.02E-06	count	1
UFD1	-0.0871303	0.3156584	-0.276	0.783	-1.01E-06	count	1
SESN3	-0.0868568	0.2339216	-0.3713	0.711	-1.01E-06	count	1
EXOC7	-0.251387	0.4112543	-0.6113	0.541	-1.01E-06	count	1
PGGHG	-0.251256	0.4819073	-0.5214	0.602	-1.01E-06	count	1
UBE2W	-0.2493217	0.3955216	-0.6304	0.529	-1.00E-06	count	1
BCLAF3	-0.2494231	0.6180315	-0.4036	0.687	-1.00E-06	count	1
SHLD1	-0.2493217	0.5339316	-0.467	0.641	-1.00E-06	count	1
KRI1	-0.2493217	0.5973947	-0.4173	0.677	-1.00E-06	count	1
MBD3	-0.2495385	0.6130012	-0.4071	0.684	-1.00E-06	count	1
AC009403.1	-0.2493217	0.6259563	-0.3983	0.691	-9.99E-07	count	1
WDHD1	-0.8430639	1.3394883	-0.6294	0.529	-9.93E-07	count	1
RPAP1	-0.8430639	1.2711388	-0.6632	0.508	-9.93E-07	count	1
ATMIN	-0.2468007	0.5203963	-0.4743	0.636	-9.91E-07	count	1
RUBCN	-0.2452317	0.4727448	-0.5187	0.604	-9.87E-07	count	1
AGO3	-0.2441874	0.3978163	-0.6138	0.54	-9.82E-07	count	1
PLEC	-0.2441874	0.4387595	-0.5565	0.578	-9.82E-07	count	1
SPATA13	-0.8225679	0.8148868	-1.0094	0.313	-9.81E-07	count	1
AKIRIN1	-0.0843944	0.1900229	-0.4441	0.657	-9.80E-07	count	1
EIF3J-DT	-0.8225679	0.7110341	-1.1569	0.248	-9.79E-07	count	1
VNN2	-0.8225679	0.8386733	-0.9808	0.327	-9.78E-07	count	1
ITPKB	-0.8111503	0.6837486	-1.1863	0.236	-9.74E-07	count	1
C1orf220	-0.8111503	0.8667017	-0.9359	0.35	-9.71E-07	count	1
BAMBI	-0.8111503	1.1604401	-0.699	0.485	-9.71E-07	count	1
CEACAM21	-0.8111503	1.0633089	-0.7629	0.446	-9.70E-07	count	1
FBXL4	-0.8111503	0.9831004	-0.8251	0.41	-9.69E-07	count	1
AC002467.1	-0.8111503	0.9831004	-0.8251	0.41	-9.69E-07	count	1
TTC8	-0.8111503	0.9831004	-0.8251	0.41	-9.69E-07	count	1
DLEU2	-0.8111503	0.9831004	-0.8251	0.41	-9.69E-07	count	1
CRTC1	-0.8111503	0.9831004	-0.8251	0.41	-9.69E-07	count	1
NEMP1	-0.8111503	0.9831004	-0.8251	0.41	-9.69E-07	count	1
ZNF671	-0.8111503	0.9831004	-0.8251	0.41	-9.69E-07	count	1

PACRGL	-0.8111503	0.9831004	-0.8251	0.41	-9.69E-07	count	1
LMNB2	-0.8111503	0.9831004	-0.8251	0.41	-9.69E-07	count	1
HS1BP3	-0.8111503	0.9698239	-0.8364	0.403	-9.69E-07	count	1
TFAP4	-0.8111503	0.9698239	-0.8364	0.403	-9.69E-07	count	1
ACACB	-0.8111503	1.1807761	-0.687	0.492	-9.68E-07	count	1
POT1	-0.8111503	1.1807761	-0.687	0.492	-9.68E-07	count	1
KCNA3	-0.8111503	1.1807761	-0.687	0.492	-9.68E-07	count	1
PIGR	-0.8111503	1.1807761	-0.687	0.492	-9.68E-07	count	1
POLR3F	-0.8111503	0.8811463	-0.9206	0.358	-9.68E-07	count	1
AMN	-0.8111503	0.8811463	-0.9206	0.358	-9.68E-07	count	1
ORC5	-0.8111503	0.8811463	-0.9206	0.358	-9.68E-07	count	1
ANKRD27	-0.8111503	0.8811463	-0.9206	0.358	-9.68E-07	count	1
FMNL2	-0.8111503	1.0973444	-0.7392	0.46	-9.67E-07	count	1
MREG	-0.8111503	1.0973444	-0.7392	0.46	-9.67E-07	count	1
DDX50	-0.0832411	0.3072417	-0.2709	0.787	-9.67E-07	count	1
CYHR1	-0.2382046	0.4276657	-0.557	0.578	-9.62E-07	count	1
CRTC2	-0.2383894	0.6280014	-0.3796	0.704	-9.61E-07	count	1
TEN1	-0.2383894	0.4408299	-0.5408	0.589	-9.61E-07	count	1
MTMR4	-0.2382608	0.4748799	-0.5017	0.616	-9.61E-07	count	1
CHST12	-0.2383894	0.4503616	-0.5293	0.597	-9.61E-07	count	1
TTF2	-0.2380611	0.5798186	-0.4106	0.682	-9.60E-07	count	1
AL592183.1	-0.2383894	0.5838173	-0.4083	0.683	-9.60E-07	count	1
KHSRP	-0.2383894	0.5838173	-0.4083	0.683	-9.60E-07	count	1
CMTR2	-0.2380611	0.5065507	-0.47	0.639	-9.60E-07	count	1
LMNB1	-0.2383894	0.6599782	-0.3612	0.718	-9.60E-07	count	1
KIF22	-0.2383894	0.6122264	-0.3894	0.697	-9.60E-07	count	1
FARP2	-0.2380611	0.591541	-0.4024	0.688	-9.59E-07	count	1
ZNF770	-0.0824842	0.3847181	-0.2144	0.83	-9.58E-07	count	1
LMF2	-0.2378529	0.4795248	-0.496	0.62	-9.58E-07	count	1
MRPL30	-0.7762581	0.7718808	-1.0057	0.315	-9.43E-07	count	1
TKFC	-0.7762581	0.7718808	-1.0057	0.315	-9.43E-07	count	1
MAN2B2	-0.7762581	0.7718808	-1.0057	0.315	-9.43E-07	count	1
IL13RA1	-0.2334143	0.3806036	-0.6133	0.54	-9.43E-07	count	1
ASB1	-0.7778938	0.7835249	-0.9928	0.321	-9.42E-07	count	1
FAHD1	-0.7762581	0.7051489	-1.1008	0.272	-9.41E-07	count	1
FAM213B	-0.7762581	0.8721992	-0.89	0.374	-9.40E-07	count	1
SLFN5	-0.7762581	0.8721992	-0.89	0.374	-9.40E-07	count	1
VAMP3	-0.2317901	0.4334845	-0.5347	0.593	-9.37E-07	count	1
SPRTN	-0.2317901	0.4649855	-0.4985	0.618	-9.36E-07	count	1
ARHGAP5	-0.2301015	0.3502015	-0.6571	0.511	-9.31E-07	count	1
FBXW4	-0.2301015	0.4618175	-0.4983	0.619	-9.30E-07	count	1
GPR160	-0.2301015	0.505804	-0.4549	0.649	-9.30E-07	count	1
MAP2K3	-0.2301015	0.3618085	-0.636	0.525	-9.29E-07	count	1
MRPS10	-0.2289205	0.4077177	-0.5615	0.575	-9.29E-07	count	1
NKX3-1	-0.758855	0.9389362	-0.8082	0.419	-9.24E-07	count	1

INPP5F	-0.2279519	0.4244248	-0.5371	0.591	-9.23E-07	count	1
MRPS30	-0.2279519	0.3782727	-0.6026	0.547	-9.22E-07	count	1
ING5	-0.2273777	0.3849694	-0.5906	0.555	-9.21E-07	count	1
MICU1	-0.2279519	0.4150763	-0.5492	0.583	-9.21E-07	count	1
MTHFR	-0.2273777	0.5856889	-0.3882	0.698	-9.20E-07	count	1
IL10RA	-0.0789963	0.2282626	-0.3461	0.729	-9.20E-07	count	1
IARS	-0.2273777	0.6345856	-0.3583	0.72	-9.19E-07	count	1
DUSP12	-0.2260199	0.4572031	-0.4944	0.621	-9.16E-07	count	1
AKAP8	-0.2254771	0.3562713	-0.6329	0.527	-9.13E-07	count	1
EFCAB13	-0.2242112	0.8302651	-0.27	0.787	-9.12E-07	count	1
EXOSC1	-0.2242112	0.3711991	-0.604	0.546	-9.12E-07	count	1
KDM3A	-0.224482	0.5393235	-0.4162	0.677	-9.09E-07	count	1
MRPS28	-0.2242112	0.5655996	-0.3964	0.692	-9.08E-07	count	1
CXorf40A	-0.2242112	0.6955865	-0.3223	0.747	-9.08E-07	count	1
CCL28	-0.2242112	0.6955865	-0.3223	0.747	-9.08E-07	count	1
MAP7D3	-0.2242112	0.5102506	-0.4394	0.661	-9.08E-07	count	1
AC119396.1	-0.2242112	0.6671688	-0.3361	0.737	-9.08E-07	count	1
DNAJC13	-0.2242112	0.568227	-0.3946	0.693	-9.08E-07	count	1
TGDS	-0.2242112	0.6693976	-0.3349	0.738	-9.08E-07	count	1
QSER1	-0.2242112	0.6063117	-0.3698	0.712	-9.08E-07	count	1
ZFP90	-0.2242112	0.5358493	-0.4184	0.676	-9.07E-07	count	1
XRCC1	-0.2242112	0.6112442	-0.3668	0.714	-9.07E-07	count	1
TRAPPC9	-0.2242112	0.7918466	-0.2831	0.777	-9.07E-07	count	1
SRRT	-0.0777137	0.331806	-0.2342	0.815	-9.06E-07	count	1
IPO7	-0.221884	0.4006338	-0.5538	0.58	-9.04E-07	count	1
AC012306.2	-0.7299419	0.8477159	-0.8611	0.39	-9.01E-07	count	1
DGCR8	-0.7299419	0.8929099	-0.8175	0.414	-8.99E-07	count	1
INTS6L	-0.7299419	0.9338817	-0.7816	0.435	-8.99E-07	count	1
GPAT4	-0.7299419	0.9338817	-0.7816	0.435	-8.99E-07	count	1
FGR	-0.7299419	0.9061949	-0.8055	0.421	-8.99E-07	count	1
NF2	-0.7299419	0.9061949	-0.8055	0.421	-8.99E-07	count	1
CD9	-0.2199383	0.6500626	-0.3383	0.735	-8.98E-07	count	1
RREB1	-0.7299419	0.851924	-0.8568	0.392	-8.98E-07	count	1
FBXO25	-0.725436	0.6675667	-1.0867	0.278	-8.97E-07	count	1
VCPIP1	-0.2204222	0.5435917	-0.4055	0.685	-8.93E-07	count	1
ABHD3	-0.2204222	0.5598708	-0.3937	0.694	-8.93E-07	count	1
HLA-DQA2	-0.0276816	0.0821501	-0.337	0.736	-8.93E-07	count	1
PDS5A	-0.076389	0.3491785	-0.2188	0.827	-8.90E-07	count	1
CLN8	-0.7126147	0.7977456	-0.8933	0.372	-8.89E-07	count	1
1-Sep	-0.0761904	0.1940913	-0.3925	0.695	-8.88E-07	count	1
RABGAP1L	-0.0759496	0.2991625	-0.2539	0.8	-8.85E-07	count	1
REXO2	-0.0758851	0.3762324	-0.2017	0.84	-8.84E-07	count	1
NMI	-0.2176928	0.4100723	-0.5309	0.596	-8.84E-07	count	1
HENMT1	-0.7126147	1.0004111	-0.7123	0.477	-8.82E-07	count	1
PEX26	-0.7126147	0.885233	-0.805	0.421	-8.81E-07	count	1

GALNT6	-0.7126147	0.885233	-0.805	0.421	-8.81E-07	count	1
CRYAB	-0.7126147	1.1741924	-0.6069	0.544	-8.81E-07	count	1
ZNF517	-0.7126147	1.3137274	-0.5424	0.588	-8.81E-07	count	1
ABCA2	-0.7126147	1.3137274	-0.5424	0.588	-8.81E-07	count	1
ARIH1	-0.2163334	0.3665252	-0.5902	0.555	-8.79E-07	count	1
UTP4	-0.7027876	0.5957024	-1.1798	0.239	-8.78E-07	count	1
GMPPB	-0.7027876	0.6521423	-1.0777	0.282	-8.77E-07	count	1
NUP133	-0.7027876	0.6521423	-1.0777	0.282	-8.77E-07	count	1
DCAF10	-0.7027876	0.6521423	-1.0777	0.282	-8.77E-07	count	1
MTO1	-0.7027876	0.5656722	-1.2424	0.215	-8.77E-07	count	1
TMEM238	-0.0750043	0.3212802	-0.2335	0.816	-8.74E-07	count	1
NAB2	-0.2140242	0.5195102	-0.412	0.681	-8.72E-07	count	1
DFFA	-0.2140242	0.573115	-0.3734	0.709	-8.71E-07	count	1
GEN1	-0.2140242	0.5089256	-0.4205	0.674	-8.70E-07	count	1
CENPX	-0.6756049	0.3034319	-2.2265	0.0265	-8.65E-07	count	1
RAB33A	-0.6873639	0.7981906	-0.8612	0.39	-8.63E-07	count	1
AC005523.2	-0.6873639	0.8425356	-0.8158	0.415	-8.63E-07	count	1
XPOT	-0.6873639	0.804414	-0.8545	0.393	-8.61E-07	count	1
NOA1	-0.2102884	0.4693393	-0.4481	0.654	-8.58E-07	count	1
PGP	-0.2104696	0.3434819	-0.6128	0.54	-8.58E-07	count	1
RNF2	-0.2102884	0.5336801	-0.394	0.694	-8.58E-07	count	1
PRPF4	-0.2100363	0.4554869	-0.4611	0.645	-8.56E-07	count	1
ZNF528	-0.6780969	0.8646015	-0.7843	0.433	-8.52E-07	count	1
DGCR6L	-0.2088326	0.4794771	-0.4355	0.663	-8.52E-07	count	1
PORCN	-0.6780969	0.9715642	-0.6979	0.486	-8.52E-07	count	1
ABHD17B	-0.6780969	0.8025636	-0.8449	0.399	-8.52E-07	count	1
SIK2	-0.6780969	0.8025636	-0.8449	0.399	-8.52E-07	count	1
YPEL1	-0.6780969	0.9181442	-0.7386	0.461	-8.51E-07	count	1
RECK	-0.6780969	0.9181442	-0.7386	0.461	-8.51E-07	count	1
PMS2	-0.6780969	0.7369941	-0.9201	0.358	-8.51E-07	count	1
BISPR	-0.6780969	0.9194933	-0.7375	0.461	-8.51E-07	count	1
C19orf73	-0.6780969	0.9194933	-0.7375	0.461	-8.51E-07	count	1
CXorf40B	-0.6780969	0.7386741	-0.918	0.359	-8.51E-07	count	1
MZB1	-0.0727942	0.3288005	-0.2214	0.825	-8.50E-07	count	1
LINC01089	-0.6780969	0.8614178	-0.7872	0.432	-8.50E-07	count	1
SLC25A23	-0.6780969	0.8614178	-0.7872	0.432	-8.50E-07	count	1
HDHD5	-0.2087559	0.5279619	-0.3954	0.693	-8.50E-07	count	1
CSGALNACT1	-0.2087559	0.6493038	-0.3215	0.748	-8.50E-07	count	1
RBM6	-0.0724255	0.2442098	-0.2966	0.767	-8.48E-07	count	1
SLC38A11	-0.670331	1.3542971	-0.495	0.621	-8.42E-07	count	1
TRIM5	-0.6630165	0.8074342	-0.8211	0.412	-8.41E-07	count	1
ZSWIM6	-0.6630165	1.0215063	-0.6491	0.517	-8.40E-07	count	1
FAM214A	-0.0720008	0.3434589	-0.2096	0.834	-8.40E-07	count	1
ZNF567	-0.2050909	0.4915753	-0.4172	0.677	-8.39E-07	count	1
GRIPAP1	-0.2050909	0.3588674	-0.5715	0.568	-8.38E-07	count	1

ZBTB41	-0.2050909	0.7876427	-0.2604	0.795	-8.36E-07	count	1
CENPK	-0.2050909	0.8257843	-0.2484	0.804	-8.36E-07	count	1
S100A13	-0.2050909	0.7948534	-0.258	0.797	-8.36E-07	count	1
RLF	-0.0712611	0.3224434	-0.221	0.825	-8.32E-07	count	1
PURA	-0.0711883	0.2961707	-0.2404	0.81	-8.31E-07	count	1
ZCWPW1	-0.2031023	0.5927783	-0.3426	0.732	-8.31E-07	count	1
AASDH	-0.2031023	0.5527644	-0.3674	0.713	-8.30E-07	count	1
CLCN6	-0.646876	0.7137081	-0.9064	0.365	-8.25E-07	count	1
CNNM3	-0.646876	0.8256511	-0.7835	0.434	-8.24E-07	count	1
SMN1	-0.646876	0.8010524	-0.8075	0.42	-8.23E-07	count	1
NABP2	-0.646876	0.7627865	-0.848	0.397	-8.22E-07	count	1
ID1	-0.2009269	0.8050901	-0.2496	0.803	-8.20E-07	count	1
STX18	-0.2009269	0.5065589	-0.3967	0.692	-8.20E-07	count	1
PLBD2	-0.636774	1.103213	-0.5772	0.564	-8.11E-07	count	1
TIMP2	-0.636774	1.031812	-0.6171	0.537	-8.11E-07	count	1
ZNF485	-0.636774	1.2025741	-0.5295	0.597	-8.10E-07	count	1
ZNF549	-0.636774	1.2025741	-0.5295	0.597	-8.10E-07	count	1
AC009779.2	-0.636774	1.2025741	-0.5295	0.597	-8.10E-07	count	1
TAF1B	-0.1968639	0.6296661	-0.3126	0.755	-8.06E-07	count	1
C11orf1	-0.1968639	0.4473857	-0.44	0.66	-8.05E-07	count	1
DGKA	-0.1955081	0.4011884	-0.4873	0.626	-8.03E-07	count	1
FRA10AC1	-0.1955081	0.4621537	-0.423	0.672	-8.00E-07	count	1
UPF1	-0.1944229	0.5167524	-0.3762	0.707	-7.96E-07	count	1
TFB1M	-0.1944229	0.4469727	-0.435	0.664	-7.96E-07	count	1
DDX19B	-0.1944229	0.5395718	-0.3603	0.719	-7.96E-07	count	1
VPS41	-0.1927937	0.5150298	-0.3743	0.708	-7.90E-07	count	1
OAZ2	-0.1927937	0.4846133	-0.3978	0.691	-7.90E-07	count	1
SHARPIN	-0.0674714	0.3851486	-0.1752	0.861	-7.88E-07	count	1
ABHD10	-0.1916289	0.45058	-0.4253	0.671	-7.86E-07	count	1
WDR60	-0.1916289	0.5369021	-0.3569	0.721	-7.85E-07	count	1
TARDBP	-0.191432	0.3592566	-0.5329	0.594	-7.85E-07	count	1
CBX6	-0.191432	0.3808918	-0.5026	0.616	-7.85E-07	count	1
ZNF740	-0.6094495	1.0150952	-0.6004	0.549	-7.84E-07	count	1
ABR	-0.6094495	0.7864722	-0.7749	0.439	-7.84E-07	count	1
AC006449.6	-0.6094495	0.9137844	-0.667	0.505	-7.84E-07	count	1
RGL2	-0.1911405	0.618234	-0.3092	0.757	-7.83E-07	count	1
DAG1	-0.6035858	0.9488608	-0.6361	0.525	-7.81E-07	count	1
IRS2	-0.6035858	0.7435758	-0.8117	0.417	-7.81E-07	count	1
CC2D1A	-0.6035858	0.7435758	-0.8117	0.417	-7.81E-07	count	1
B3GAT2	-0.6035858	0.812152	-0.7432	0.458	-7.80E-07	count	1
ZNF687	-0.6035858	0.694064	-0.8696	0.385	-7.80E-07	count	1
ZBP1	-0.6035858	0.694064	-0.8696	0.385	-7.80E-07	count	1
MIB2	-0.6035858	0.7724707	-0.7814	0.435	-7.79E-07	count	1
TEP1	-0.6035858	0.8436215	-0.7155	0.475	-7.79E-07	count	1
TNNI2	-0.6035858	0.9092213	-0.6638	0.507	-7.79E-07	count	1

TEX10	-0.1892055	0.5121382	-0.3694	0.712	-7.78E-07	count	1
SLC25A32	-0.1877929	0.415597	-0.4519	0.652	-7.71E-07	count	1
SLC35B3	-0.592918	0.637976	-0.9294	0.353	-7.69E-07	count	1
EIF3B	-0.1863124	0.4318835	-0.4314	0.666	-7.65E-07	count	1
NCOR2	-0.1861122	0.4603128	-0.4043	0.686	-7.64E-07	count	1
DIAPH2	-0.1859543	0.4108226	-0.4526	0.651	-7.64E-07	count	1
CCDC6	-0.0650483	0.2799478	-0.2324	0.816	-7.62E-07	count	1
SP3	-0.0650743	0.2898887	-0.2245	0.822	-7.61E-07	count	1
COCH	-0.0648953	0.3339202	-0.1943	0.846	-7.59E-07	count	1
NUB1	-0.0647772	0.2333577	-0.2776	0.781	-7.59E-07	count	1
EIF4E	-0.0633918	0.2395954	-0.2646	0.791	-7.42E-07	count	1
YME1L1	-0.0631726	0.2360171	-0.2677	0.789	-7.41E-07	count	1
TNFRSF1B	-0.1789435	0.4040426	-0.4429	0.658	-7.38E-07	count	1
CCNQ	-0.0628754	0.2881457	-0.2182	0.827	-7.36E-07	count	1
CABIN1	-0.1779028	0.6747641	-0.2637	0.792	-7.36E-07	count	1
TTN	-0.1784668	0.4115238	-0.4337	0.665	-7.35E-07	count	1
TPST2	-0.1779028	0.4600908	-0.3867	0.699	-7.34E-07	count	1
BRD3	-0.1779028	0.5277536	-0.3371	0.736	-7.34E-07	count	1
MSL2	-0.1779028	0.3962974	-0.4489	0.654	-7.34E-07	count	1
MAEA	-0.1779028	0.5314413	-0.3348	0.738	-7.33E-07	count	1
AP1S1	-0.1779028	0.5519815	-0.3223	0.747	-7.33E-07	count	1
POR	-0.1779028	0.6130506	-0.2902	0.772	-7.33E-07	count	1
KAT2A	-0.1779028	0.5753986	-0.3092	0.757	-7.33E-07	count	1
NCLN	-0.5557626	0.6605299	-0.8414	0.401	-7.32E-07	count	1
APPBP2	-0.5557626	0.6271331	-0.8862	0.376	-7.31E-07	count	1
PRKAB1	-0.5557626	0.6774186	-0.8204	0.412	-7.31E-07	count	1
SNAP47	-0.5557626	0.5838877	-0.9518	0.342	-7.31E-07	count	1
SFXN4	-0.5557626	0.5838877	-0.9518	0.342	-7.31E-07	count	1
OXCT1	-0.1769675	0.4543833	-0.3895	0.697	-7.30E-07	count	1
FRMD8	-0.1769675	0.5483214	-0.3227	0.747	-7.29E-07	count	1
ETAA1	-0.1763949	0.6473972	-0.2725	0.785	-7.28E-07	count	1
FASTKD5	-0.1763949	0.624066	-0.2827	0.778	-7.28E-07	count	1
TK2	-0.5511077	0.7793951	-0.7071	0.48	-7.26E-07	count	1
HIST3H2A	-0.5511077	0.8243953	-0.6685	0.504	-7.26E-07	count	1
POGLUT1	-0.5511077	0.7392735	-0.7455	0.456	-7.26E-07	count	1
FHL1	-0.5511077	0.8061008	-0.6837	0.495	-7.26E-07	count	1
AL645728.1	-0.5511077	0.8477793	-0.6501	0.516	-7.25E-07	count	1
MARK3	-0.1753551	0.4570544	-0.3837	0.701	-7.25E-07	count	1
HMGCR	-0.5511077	0.8158077	-0.6755	0.5	-7.25E-07	count	1
PMS1	-0.1753551	0.4345858	-0.4035	0.687	-7.25E-07	count	1
TMEM245	-0.5511077	1.0600497	-0.5199	0.603	-7.24E-07	count	1
STARD4	-0.5511077	1.0600497	-0.5199	0.603	-7.24E-07	count	1
IGLV6-57	-0.5511077	0.9397541	-0.5864	0.558	-7.24E-07	count	1
TSC1	-0.5511077	1.1711178	-0.4706	0.638	-7.24E-07	count	1
ST3GAL4	-0.5511077	1.3017441	-0.4234	0.672	-7.24E-07	count	1

ABHD16A	-0.5511077	0.9606201	-0.5737	0.566	-7.24E-07	count	1
MYO1D	-0.5511077	0.9606201	-0.5737	0.566	-7.24E-07	count	1
GNS	-0.5511077	0.9173598	-0.6008	0.548	-7.24E-07	count	1
CDR2	-0.5511077	0.9173598	-0.6008	0.548	-7.24E-07	count	1
CNPY4	-0.5511077	1.1884365	-0.4637	0.643	-7.24E-07	count	1
UBA6-AS1	-0.5511077	1.1537501	-0.4777	0.633	-7.24E-07	count	1
RBFA	-0.1753551	0.4870656	-0.36	0.719	-7.23E-07	count	1
APH1B	-0.1751148	0.4208573	-0.4161	0.678	-7.22E-07	count	1
RBBP9	-0.544262	0.7309597	-0.7446	0.457	-7.18E-07	count	1
CHPF2	-0.1740147	0.6303926	-0.276	0.783	-7.18E-07	count	1
FN3KRP	-0.1740147	0.7504711	-0.2319	0.817	-7.18E-07	count	1
WDR41	-0.1740147	0.6752232	-0.2577	0.797	-7.18E-07	count	1
CREB3L2	-0.1740147	0.748363	-0.2325	0.816	-7.18E-07	count	1
CD59	-0.1740147	0.4833781	-0.36	0.719	-7.18E-07	count	1
MIR29B2CHG	-0.0603725	0.3208352	-0.1882	0.851	-7.08E-07	count	1
DIP2C	-0.5332447	1.4070888	-0.379	0.705	-7.05E-07	count	1
DENND6B	-0.169954	0.4233303	-0.4015	0.688	-7.03E-07	count	1
GOSR1	-0.0595898	0.2862956	-0.2081	0.835	-6.99E-07	count	1
URM1	-0.1682503	0.4587272	-0.3668	0.714	-6.96E-07	count	1
MLLT10	-0.1682503	0.4452819	-0.3779	0.706	-6.96E-07	count	1
RUFY1	-0.1675131	0.3946343	-0.4245	0.671	-6.94E-07	count	1
CBR4	-0.1673614	0.7732658	-0.2164	0.829	-6.93E-07	count	1
MRRF	-0.1660994	0.4522223	-0.3673	0.714	-6.88E-07	count	1
SYNE1	-0.1660994	0.4994375	-0.3326	0.74	-6.87E-07	count	1
EHMT2	-0.1655204	0.415768	-0.3981	0.691	-6.86E-07	count	1
HOOK2	-0.1650848	0.3312974	-0.4983	0.619	-6.83E-07	count	1
SEPHS1	-0.1637716	0.5367572	-0.3051	0.76	-6.79E-07	count	1
JAM3	-0.1637716	0.5035735	-0.3252	0.745	-6.79E-07	count	1
FAM228B	-0.1637716	0.5393782	-0.3036	0.762	-6.78E-07	count	1
CDYL	-0.1635214	0.3820393	-0.428	0.669	-6.78E-07	count	1
UTY	-0.1632988	0.3462373	-0.4716	0.637	-6.77E-07	count	1
ARID1B	-0.0576822	0.2400051	-0.2403	0.81	-6.77E-07	count	1
ASPHD2	-0.5035725	0.7080169	-0.7112	0.477	-6.75E-07	count	1
MTFR1L	-0.5035725	0.8004614	-0.6291	0.53	-6.75E-07	count	1
C9orf3	-0.5035725	0.8419797	-0.5981	0.55	-6.75E-07	count	1
AC147651.4	-0.5035725	0.7902703	-0.6372	0.524	-6.75E-07	count	1
DRAM1	-0.5035725	0.8808522	-0.5717	0.568	-6.74E-07	count	1
CCDC71L	-0.161835	0.378829	-0.4272	0.669	-6.72E-07	count	1
SEC22B	-0.0572089	0.3214643	-0.178	0.859	-6.71E-07	count	1
MLEC	-0.1615556	0.3521826	-0.4587	0.647	-6.70E-07	count	1
NUDCD3	-0.1595023	0.4352952	-0.3664	0.714	-6.62E-07	count	1
MRPL19	-0.1595023	0.4202774	-0.3795	0.704	-6.62E-07	count	1
SLC6A6	-0.1595025	0.5969075	-0.2672	0.789	-6.62E-07	count	1
SMAD4	-0.1595027	0.4325433	-0.3688	0.712	-6.62E-07	count	1
TCEAL1	-0.1595028	0.589759	-0.2705	0.787	-6.62E-07	count	1

GTF2H1	-0.1595023	0.4554651	-0.3502	0.726	-6.62E-07	count	1
ZNF668	-0.4868932	0.8301697	-0.5865	0.558	-6.57E-07	count	1
BRAT1	-0.1563773	0.4244328	-0.3684	0.713	-6.50E-07	count	1
MRPL58	-0.1540686	0.5765703	-0.2672	0.789	-6.41E-07	count	1
KIF13B	-0.1540686	0.6018598	-0.256	0.798	-6.41E-07	count	1
TFE3	-0.1530412	0.7331945	-0.2087	0.835	-6.38E-07	count	1
IL2RA	-0.1530412	0.5500946	-0.2782	0.781	-6.37E-07	count	1
TOPBP1	-0.1526065	0.4105847	-0.3717	0.71	-6.36E-07	count	1
NAA38	-0.0540141	0.2876018	-0.1878	0.851	-6.35E-07	count	1
SUN1	-0.1523664	0.5139619	-0.2965	0.767	-6.34E-07	count	1
PPP6R3	-0.0539567	0.3361832	-0.1605	0.873	-6.34E-07	count	1
GPI	-0.152109	0.3958345	-0.3843	0.701	-6.33E-07	count	1
LINC00513	-0.1496472	0.5497696	-0.2722	0.786	-6.23E-07	count	1
EBF1	-0.1485752	0.4136171	-0.3592	0.72	-6.19E-07	count	1
SORL1	-0.4529874	0.6435583	-0.7039	0.482	-6.19E-07	count	1
LUM	-0.4537384	1.2213379	-0.3715	0.71	-6.19E-07	count	1
SLC16A4	-0.4537384	0.9563367	-0.4745	0.635	-6.18E-07	count	1
ZNF711	-0.4521224	0.6490036	-0.6966	0.486	-6.18E-07	count	1
PRKAG2	-0.1477479	0.3534675	-0.418	0.676	-6.18E-07	count	1
IFI27L1	-0.4521224	0.7968457	-0.5674	0.571	-6.18E-07	count	1
CENPN	-0.4521224	0.8301146	-0.5447	0.586	-6.18E-07	count	1
COA7	-0.4521224	0.8031215	-0.563	0.574	-6.17E-07	count	1
SFI1	-0.4521224	0.8031215	-0.563	0.574	-6.17E-07	count	1
MSANTD2	-0.4521224	0.8031215	-0.563	0.574	-6.17E-07	count	1
ZNF418	-0.4521224	1.081438	-0.4181	0.676	-6.17E-07	count	1
LIMS2	-0.4521224	1.081438	-0.4181	0.676	-6.17E-07	count	1
BRD3OS	-0.4521224	1.081438	-0.4181	0.676	-6.17E-07	count	1
LIMK2	-0.4521224	0.7521886	-0.6011	0.548	-6.17E-07	count	1
COX18	-0.4521224	0.7521886	-0.6011	0.548	-6.17E-07	count	1
EPM2A	-0.4521224	1.0998509	-0.4111	0.681	-6.17E-07	count	1
SEC23B	-0.4521224	1.0998509	-0.4111	0.681	-6.17E-07	count	1
ZNF627	-0.4521224	0.9233865	-0.4896	0.625	-6.17E-07	count	1
TNS1	-0.4521224	1.3067792	-0.346	0.73	-6.17E-07	count	1
SLC43A3	-0.4521224	1.3067792	-0.346	0.73	-6.17E-07	count	1
NETO2	-0.4521224	1.3067792	-0.346	0.73	-6.17E-07	count	1
AL359711.2	-0.4521224	1.3067792	-0.346	0.73	-6.17E-07	count	1
AC104506.1	-0.4521224	1.3067792	-0.346	0.73	-6.17E-07	count	1
SSFA2	-0.4521224	0.9448844	-0.4785	0.633	-6.17E-07	count	1
PLCD1	-0.4521224	0.9448844	-0.4785	0.633	-6.17E-07	count	1
NR2F6	-0.4521224	0.9448844	-0.4785	0.633	-6.17E-07	count	1
CAPN10	-0.4521224	0.9448844	-0.4785	0.633	-6.17E-07	count	1
ERCC5	-0.4521224	0.9448844	-0.4785	0.633	-6.17E-07	count	1
ZWILCH	-0.4521224	0.9448844	-0.4785	0.633	-6.17E-07	count	1
EIF5AL1	-0.4521224	0.9448844	-0.4785	0.633	-6.17E-07	count	1
ZNF845	-0.4521224	0.9448844	-0.4785	0.633	-6.17E-07	count	1

GASAL1	-0.4521224	1.1793246	-0.3834	0.702	-6.17E-07	count	1
KNTC1	-0.4521224	1.1793246	-0.3834	0.702	-6.17E-07	count	1
INPP4A	-0.4521224	1.1793246	-0.3834	0.702	-6.17E-07	count	1
NQO1	-0.4521224	1.1793246	-0.3834	0.702	-6.17E-07	count	1
AC004982.2	-0.4521224	1.1793246	-0.3834	0.702	-6.17E-07	count	1
RNF146	-0.1476245	0.4394821	-0.3359	0.737	-6.15E-07	count	1
LMBR1L	-0.1470323	0.5192517	-0.2832	0.777	-6.13E-07	count	1
STAM	-0.1470323	0.5798941	-0.2536	0.8	-6.13E-07	count	1
SLC12A9	-0.1470323	0.5798941	-0.2536	0.8	-6.13E-07	count	1
MTX3	-2.0386037	0.8391392	-2.4294	0.0155	-6.12E-07	count	1
AKIP1	-2.0386037	0.8624609	-2.3637	0.0185	-6.10E-07	count	1
HOOK1	-0.1456529	0.7291591	-0.1998	0.842	-6.09E-07	count	1
CCDC159	-0.1453024	0.5573361	-0.2607	0.794	-6.07E-07	count	1
COPS7A	-0.1453024	0.5002328	-0.2905	0.772	-6.06E-07	count	1
UFL1	-0.0512887	0.3306708	-0.1551	0.877	-6.03E-07	count	1
NAIP	-1.9432935	1.0012155	-1.9409	0.0529	-6.02E-07	count	1
AL121944.1	-0.0504258	0.2946862	-0.1711	0.864	-5.94E-07	count	1
FAM50B	-0.4302699	0.7323789	-0.5875	0.557	-5.93E-07	count	1
TNFRSF14-AS1	-0.4302699	0.6959087	-0.6183	0.537	-5.93E-07	count	1
NAA30	-0.4302699	0.5970247	-0.7207	0.471	-5.93E-07	count	1
SNX18	-0.4302699	0.7541374	-0.5705	0.569	-5.92E-07	count	1
HIBADH	-0.4302699	0.7724833	-0.557	0.578	-5.92E-07	count	1
MBD2	-0.0499833	0.1719151	-0.2907	0.771	-5.88E-07	count	1
WAS	-0.0497577	0.2367675	-0.2102	0.834	-5.85E-07	count	1
FARSB	-0.1395946	0.6200765	-0.2251	0.822	-5.84E-07	count	1
SAMM50	-0.0495028	0.300849	-0.1645	0.869	-5.83E-07	count	1
RAI1	-0.4199831	0.8677493	-0.484	0.629	-5.81E-07	count	1
USP38	-0.4199831	0.7706873	-0.5449	0.586	-5.81E-07	count	1
JMJD4	-0.4199831	0.8338933	-0.5036	0.615	-5.80E-07	count	1
MANBA	-0.1376831	0.3996317	-0.3445	0.731	-5.77E-07	count	1
TRPV3	-0.4152503	1.1282304	-0.3681	0.713	-5.75E-07	count	1
APOL2	-0.4152503	1.1282304	-0.3681	0.713	-5.75E-07	count	1
SLC2A14	-0.4152503	1.0577155	-0.3926	0.695	-5.75E-07	count	1
C4orf46	-0.4152503	0.8686886	-0.478	0.633	-5.75E-07	count	1
WWOX	-0.4152503	0.8686886	-0.478	0.633	-5.75E-07	count	1
MAP2K6	-0.4152503	0.864834	-0.4802	0.631	-5.75E-07	count	1
ZNF266	-0.4152503	0.864834	-0.4802	0.631	-5.75E-07	count	1
LRRCC1	-0.4152503	0.988931	-0.4199	0.675	-5.75E-07	count	1
SLC33A1	-0.4152503	0.988931	-0.4199	0.675	-5.75E-07	count	1
PPP5D1	-0.4152503	0.988931	-0.4199	0.675	-5.75E-07	count	1
TRIB2	-0.4152503	0.988931	-0.4199	0.675	-5.75E-07	count	1
ZNF677	-0.4152503	0.988931	-0.4199	0.675	-5.75E-07	count	1
TRAM2	-0.4152503	0.988931	-0.4199	0.675	-5.75E-07	count	1
OGFOD2	-0.4152503	0.9855468	-0.4213	0.674	-5.75E-07	count	1
KDELC2	-0.4152503	0.9855468	-0.4213	0.674	-5.75E-07	count	1

SLC39A8	-0.4152503	0.7792048	-0.5329	0.594	-5.75E-07	count	1
TXNDC16	-0.4152503	0.9113313	-0.4557	0.649	-5.74E-07	count	1
PRICKLE1	-0.4152503	0.9113313	-0.4557	0.649	-5.74E-07	count	1
TRIM39	-0.4152503	0.9113313	-0.4557	0.649	-5.74E-07	count	1
ALG3	-0.1361897	0.6248135	-0.218	0.828	-5.70E-07	count	1
ZNF276	-0.1361897	0.4638393	-0.2936	0.769	-5.70E-07	count	1
NPEPPS	-0.1361897	0.415455	-0.3278	0.743	-5.70E-07	count	1
AL450998.2	-0.1361897	0.5089946	-0.2676	0.789	-5.70E-07	count	1
ANAPC10	-0.1344757	0.4219067	-0.3187	0.75	-5.63E-07	count	1
TM4SF1	-1.7201499	1.1249374	-1.5291	0.127	-5.63E-07	count	1
RSU1	-0.133733	0.3889578	-0.3438	0.731	-5.62E-07	count	1
ZNF211	-0.3959304	0.8452986	-0.4684	0.64	-5.54E-07	count	1
JADE3	-0.3959304	0.7945972	-0.4983	0.619	-5.53E-07	count	1
TPCN1	-0.3959304	0.9169817	-0.4318	0.666	-5.53E-07	count	1
AL357060.1	-0.3959304	0.7945972	-0.4983	0.619	-5.53E-07	count	1
ZNF358	-0.3959304	0.8693857	-0.4554	0.649	-5.53E-07	count	1
AC110769.2	-0.3959304	0.8190284	-0.4834	0.629	-5.52E-07	count	1
ZNF641	-0.3959304	0.8190284	-0.4834	0.629	-5.52E-07	count	1
CASP6	-0.3959304	0.8190284	-0.4834	0.629	-5.52E-07	count	1
CACFD1	-0.3959304	1.0042324	-0.3943	0.694	-5.52E-07	count	1
AL391121.1	-0.3959304	0.8201747	-0.4827	0.63	-5.52E-07	count	1
ZNF846	-0.3959304	0.8928231	-0.4435	0.658	-5.52E-07	count	1
SEC24C	-0.3959304	0.8928231	-0.4435	0.658	-5.52E-07	count	1
SERPING1	-0.3959304	0.8928231	-0.4435	0.658	-5.52E-07	count	1
AC040162.1	-0.3959304	0.765365	-0.5173	0.605	-5.52E-07	count	1
ZNF439	-0.1312473	0.3897673	-0.3367	0.736	-5.52E-07	count	1
ZNF224	-0.0465064	0.3893942	-0.1194	0.905	-5.48E-07	count	1
ZNF639	-0.0464672	0.3332633	-0.1394	0.889	-5.48E-07	count	1
NME1	-0.1303348	0.3502243	-0.3721	0.71	-5.47E-07	count	1
MYO1C	-0.1298507	0.46278	-0.2806	0.779	-5.45E-07	count	1
NUDCD1	-0.1298507	0.522143	-0.2487	0.804	-5.45E-07	count	1
ZNF891	-0.1298507	0.5157657	-0.2518	0.801	-5.45E-07	count	1
ZMYND8	-0.1296267	0.3818056	-0.3395	0.734	-5.44E-07	count	1
DLG1	-0.1282732	0.4410571	-0.2908	0.771	-5.41E-07	count	1
PYCARD-AS1	-0.3840298	0.8192753	-0.4687	0.639	-5.40E-07	count	1
ZNF764	-0.3840298	0.7313083	-0.5251	0.6	-5.40E-07	count	1
CYREN	-0.3840298	0.6924944	-0.5546	0.579	-5.39E-07	count	1
TMEM116	-0.3840298	0.7877682	-0.4875	0.626	-5.39E-07	count	1
CEP152	-0.3840298	0.7109305	-0.5402	0.589	-5.38E-07	count	1
RBM43	-0.3840298	0.7109305	-0.5402	0.589	-5.38E-07	count	1
HIC2	-0.3840298	0.8502702	-0.4517	0.652	-5.38E-07	count	1
MAPKAPK5-AS1	-0.1273542	0.6526582	-0.1951	0.845	-5.38E-07	count	1
MBD1	-0.3840298	0.7141793	-0.5377	0.591	-5.38E-07	count	1
SEC23IP	-0.3840298	0.7658715	-0.5014	0.616	-5.38E-07	count	1
SLC46A3	-0.3840298	0.7658715	-0.5014	0.616	-5.38E-07	count	1

CBFB	-0.1278493	0.4449811	-0.2873	0.774	-5.37E-07	count	1
PARP15	-0.0454675	0.3212119	-0.1415	0.887	-5.37E-07	count	1
ANKRD36C	-0.1273542	0.5505054	-0.2313	0.817	-5.35E-07	count	1
SPINDOC	-0.1273542	0.6045097	-0.2107	0.833	-5.35E-07	count	1
UAP1	-0.1266686	0.4990298	-0.2538	0.8	-5.33E-07	count	1
ZZEF1	-0.3759608	0.5888061	-0.6385	0.523	-5.29E-07	count	1
TCTN3	-0.0446379	0.388371	-0.1149	0.909	-5.27E-07	count	1
DAXX	-0.0445319	0.2675816	-0.1664	0.868	-5.25E-07	count	1
ZNF236	-1.4324679	0.9858864	-1.453	0.147	-5.21E-07	count	1
BORCS7	-0.0440331	0.3566958	-0.1234	0.902	-5.20E-07	count	1
PIGBOS1	-1.4324679	0.6042476	-2.3707	0.0182	-5.19E-07	count	1
ARHGEF35	-0.3682543	1.1829932	-0.3113	0.756	-5.19E-07	count	1
ZNF573	-0.3682543	1.4358662	-0.2565	0.798	-5.19E-07	count	1
CERS6	-0.3682543	1.4358662	-0.2565	0.798	-5.19E-07	count	1
ZNF618	-0.3682543	1.4358662	-0.2565	0.798	-5.19E-07	count	1
BCORL1	-0.3682543	1.4358662	-0.2565	0.798	-5.19E-07	count	1
STX18-AS1	-0.3682543	1.4358662	-0.2565	0.798	-5.19E-07	count	1
PSMC3IP	-0.3682543	1.4358662	-0.2565	0.798	-5.19E-07	count	1
SAAL1	-1.4324679	0.5870218	-2.4402	0.0151	-5.19E-07	count	1
TPP1	-0.1221433	0.3783086	-0.3229	0.747	-5.16E-07	count	1
PHLDB3	-0.1223286	0.5829401	-0.2098	0.834	-5.15E-07	count	1
BNIP1	-0.1223286	0.416151	-0.294	0.769	-5.15E-07	count	1
PPP1R37	-0.1223286	0.7148629	-0.1711	0.864	-5.15E-07	count	1
TCTN1	-0.1223286	0.5608651	-0.2181	0.827	-5.15E-07	count	1
HMGXB3	-0.121912	0.3989822	-0.3056	0.76	-5.14E-07	count	1
LPXN	-0.0435342	0.2884576	-0.1509	0.88	-5.14E-07	count	1
FRAT1	-1.4324679	0.7554348	-1.8962	0.0586	-5.11E-07	count	1
TOP1	-0.0431989	0.2028092	-0.213	0.831	-5.10E-07	count	1
CDKN2A	-1.363475	0.492908	-2.7662	0.0059	-5.04E-07	count	1
MED21	-0.1192923	0.3843639	-0.3104	0.756	-5.03E-07	count	1
MIGA2	-0.353877	0.9872521	-0.3584	0.72	-5.01E-07	count	1
SLC36A4	-0.1182361	0.4955395	-0.2386	0.812	-5.00E-07	count	1
PNO1	-0.1176195	0.3781241	-0.3111	0.756	-4.97E-07	count	1
BACH2	-0.1175888	0.4649825	-0.2529	0.8	-4.96E-07	count	1
NFYA	-0.3479872	0.8195542	-0.4246	0.671	-4.94E-07	count	1
TTI1	-0.3479872	0.9570905	-0.3636	0.716	-4.94E-07	count	1
SIK3	-0.1168556	0.4020358	-0.2907	0.771	-4.93E-07	count	1
ZNF217	-0.041731	0.3271493	-0.1276	0.899	-4.93E-07	count	1
SPNS3	-1.3146848	0.7784277	-1.6889	0.0919	-4.92E-07	count	1
IFI27	-1.3146848	1.1171523	-1.1768	0.24	-4.92E-07	count	1
AL157938.3	-1.3146848	0.8738105	-1.5045	0.133	-4.91E-07	count	1
GOLGA2	-0.116276	0.4092731	-0.2841	0.776	-4.91E-07	count	1
IZUMO4	-0.3447772	0.8617297	-0.4001	0.689	-4.90E-07	count	1
TRBC1	-1.3146848	0.8239524	-1.5956	0.111	-4.89E-07	count	1
CD96	-0.114989	0.5090255	-0.2259	0.821	-4.87E-07	count	1

AL161781.2	-1.2501463	0.5361226	-2.3318	0.0202	-4.86E-07	count	1
TRIOBP	-0.1146084	0.6675965	-0.1717	0.864	-4.84E-07	count	1
MINDY3	-0.1146084	0.6279722	-0.1825	0.855	-4.84E-07	count	1
CNDP2	-0.0408137	0.2828648	-0.1443	0.885	-4.82E-07	count	1
FKBP4	-0.1137403	0.4178757	-0.2722	0.786	-4.82E-07	count	1
NENF	-0.0406261	0.2663443	-0.1525	0.879	-4.80E-07	count	1
LRRC8D	-1.2501463	0.8203424	-1.5239	0.128	-4.76E-07	count	1
ERCC4	-1.2501463	0.6638765	-1.8831	0.0603	-4.76E-07	count	1
NUDT16	-1.2501463	0.6638765	-1.8831	0.0603	-4.76E-07	count	1
SETDB2	-0.1125272	0.4295591	-0.262	0.793	-4.75E-07	count	1
AL158850.1	-1.2093243	0.6396672	-1.8906	0.0593	-4.69E-07	count	1
ME2	-0.1105969	0.3970379	-0.2786	0.781	-4.68E-07	count	1
UBR5	-0.1090428	0.3973165	-0.2744	0.784	-4.61E-07	count	1
KARS	-0.1086035	0.36807	-0.2951	0.768	-4.60E-07	count	1
ANKRD37	-0.0387761	0.2895169	-0.1339	0.894	-4.58E-07	count	1
GANAB	-0.107744	0.5138534	-0.2097	0.834	-4.56E-07	count	1
TUBGCP3	-1.1447858	0.6308576	-1.8147	0.0703	-4.53E-07	count	1
NFYB	-0.1069843	0.4371987	-0.2447	0.807	-4.53E-07	count	1
EXTL2	-1.1447858	0.8021917	-1.4271	0.154	-4.53E-07	count	1
OGFOD1	-1.1447858	0.7007893	-1.6336	0.103	-4.52E-07	count	1
TPMT	-1.1447858	0.7447075	-1.5372	0.125	-4.50E-07	count	1
PPP2R3B	-0.1057019	0.6561103	-0.1611	0.872	-4.48E-07	count	1
USP25	-0.1057019	0.4808646	-0.2198	0.826	-4.48E-07	count	1
TRIO	-0.1057019	0.6915866	-0.1528	0.879	-4.48E-07	count	1
FZR1	-0.1057019	0.6219523	-0.17	0.865	-4.48E-07	count	1
ZNF844	-0.1057019	0.859815	-0.1229	0.902	-4.48E-07	count	1
PSMD9	-0.1057019	0.622467	-0.1698	0.865	-4.48E-07	count	1
FAM53B	-0.1057019	0.7286282	-0.1451	0.885	-4.48E-07	count	1
SLC16A1-AS1	-0.1057019	0.6832287	-0.1547	0.877	-4.48E-07	count	1
LIN54	-0.1057019	0.6832287	-0.1547	0.877	-4.48E-07	count	1
GDAP2	-0.1057019	0.6597549	-0.1602	0.873	-4.48E-07	count	1
PNMA1	-0.1057019	0.6131677	-0.1724	0.863	-4.48E-07	count	1
TAMM41	-0.1057019	0.5868984	-0.1801	0.857	-4.48E-07	count	1
CD320	-0.1057019	0.5868984	-0.1801	0.857	-4.48E-07	count	1
CSNK2A1	-0.0378511	0.3555576	-0.1065	0.915	-4.48E-07	count	1
GPS1	-1.1070455	0.5499394	-2.013	0.0447	-4.47E-07	count	1
ZNF302	-1.0959956	0.5559306	-1.9715	0.0493	-4.46E-07	count	1
LRPPRC	-0.0376167	0.3382486	-0.1112	0.911	-4.45E-07	count	1
EWSR1	-0.0374887	0.244571	-0.1533	0.878	-4.43E-07	count	1
ING3	-0.1042111	0.4498117	-0.2317	0.817	-4.42E-07	count	1
PIH1D1	-0.1036234	0.3215716	-0.3222	0.747	-4.39E-07	count	1
KBTBD8	-0.3030839	0.2934539	-1.0328	0.302	-4.39E-07	count	1
GTF2F2	-0.037026	0.2798006	-0.1323	0.895	-4.38E-07	count	1
CCSER1	-0.103075	0.5526267	-0.1865	0.852	-4.37E-07	count	1
P2RY8	-0.103075	0.6819623	-0.1511	0.88	-4.37E-07	count	1

SMAD2	-0.1027875	0.4541729	-0.2263	0.821	-4.36E-07	count	1
VRK2	-0.101881	0.4429401	-0.23	0.818	-4.32E-07	count	1
ESYT1	-0.1014579	0.5256985	-0.193	0.847	-4.31E-07	count	1
WDR37	-1.0270028	0.6362722	-1.6141	0.107	-4.26E-07	count	1
ARFIP2	-1.0270028	0.5618636	-1.8279	0.0682	-4.25E-07	count	1
PLEKHO2	-0.2926647	0.6799004	-0.4305	0.667	-4.25E-07	count	1
XIAP	-0.291296	0.3263859	-0.8925	0.373	-4.24E-07	count	1
TDRKH	-1.0270028	1.2505254	-0.8213	0.412	-4.22E-07	count	1
POLD4	-1.0270028	0.6253377	-1.6423	0.101	-4.22E-07	count	1
UCK1	-1.0270028	0.6253377	-1.6423	0.101	-4.22E-07	count	1
CRBN	-0.0355779	0.340073	-0.1046	0.917	-4.21E-07	count	1
CCL2	-1.0270028	0.7868213	-1.3053	0.192	-4.21E-07	count	1
CARMIL2	-1.0270028	0.846046	-1.2139	0.225	-4.21E-07	count	1
NAT9	-0.0990029	0.5177557	-0.1912	0.848	-4.20E-07	count	1
CETN3	-1.0270028	0.6731456	-1.5257	0.128	-4.20E-07	count	1
ACYP1	-0.2873356	0.3798579	-0.7564	0.45	-4.18E-07	count	1
CTSD	-0.0976692	0.3859678	-0.2531	0.8	-4.15E-07	count	1
NCBP2-AS2	-0.0971659	0.3820997	-0.2543	0.799	-4.14E-07	count	1
CCDC191	-0.0969234	0.4102824	-0.2362	0.813	-4.12E-07	count	1
ALDH3A2	-0.2828697	0.8132781	-0.3478	0.728	-4.12E-07	count	1
INTS10	-0.0969234	0.3890252	-0.2491	0.803	-4.12E-07	count	1
KLF4	-0.2828697	0.6726569	-0.4205	0.674	-4.12E-07	count	1
SLC9A9	-0.2828697	0.7472958	-0.3785	0.705	-4.12E-07	count	1
NAXD	-0.2828697	0.7472958	-0.3785	0.705	-4.12E-07	count	1
S1PR2	-0.2828697	0.7951265	-0.3558	0.722	-4.12E-07	count	1
RHOF	-0.0346006	0.2102315	-0.1646	0.869	-4.10E-07	count	1
HNRNPAB	-0.0342154	0.1611059	-0.2124	0.832	-4.05E-07	count	1
DPYSL2	-0.9528948	0.5044701	-1.8889	0.0596	-4.04E-07	count	1
ZDHHC3	-0.0946667	0.561827	-0.1685	0.866	-4.03E-07	count	1
LIMK1	-0.9399914	0.6598432	-1.4246	0.155	-4.02E-07	count	1
MTR	-0.093582	0.5080972	-0.1842	0.854	-3.99E-07	count	1
PMPCA	-0.0937475	0.4617325	-0.203	0.839	-3.99E-07	count	1
MAP3K4	-0.0933437	0.4394061	-0.2124	0.832	-3.98E-07	count	1
UNC45A	-0.0933437	0.5629329	-0.1658	0.868	-3.98E-07	count	1
SMAD5	-0.0933437	0.6330045	-0.1475	0.883	-3.97E-07	count	1
LRRC28	-0.0933437	0.5832104	-0.1601	0.873	-3.97E-07	count	1
NFKBIL1	-0.0933437	0.6545927	-0.1426	0.887	-3.97E-07	count	1
KDM5D	-0.0929713	0.5245904	-0.1772	0.859	-3.96E-07	count	1
DLGAP1-AS1	-0.0929713	0.4551888	-0.2042	0.838	-3.96E-07	count	1
TTC33	-0.0929713	0.7460646	-0.1246	0.901	-3.96E-07	count	1
COPS4	-0.0929713	0.3706505	-0.2508	0.802	-3.96E-07	count	1
TRIAP1	-0.0929713	0.5799701	-0.1603	0.873	-3.96E-07	count	1
PSD4	-0.0929713	0.6107972	-0.1522	0.879	-3.96E-07	count	1
TCF3	-0.0929713	0.3606601	-0.2578	0.797	-3.96E-07	count	1
VMAC	-0.9216422	0.6571882	-1.4024	0.161	-3.94E-07	count	1

DUS4L	-0.9216422	0.685962	-1.3436	0.18	-3.93E-07	count	1
YARS	-0.9216422	0.6171086	-1.4935	0.136	-3.93E-07	count	1
AIF1	-0.9216422	0.7572157	-1.2171	0.224	-3.91E-07	count	1
ZNF507	-0.2669947	1.0456135	-0.2553	0.799	-3.91E-07	count	1
GINS3	-0.2669947	0.8585602	-0.311	0.756	-3.91E-07	count	1
AC003102.1	-0.2669947	0.9503154	-0.281	0.779	-3.91E-07	count	1
RER1	-0.0325246	0.2618848	-0.1242	0.901	-3.85E-07	count	1
TMEM106A	-0.8934714	0.7098155	-1.2587	0.209	-3.85E-07	count	1
E4F1	-0.8934714	0.7779133	-1.1485	0.251	-3.84E-07	count	1
MALT1	-0.0323899	0.2218088	-0.146	0.884	-3.84E-07	count	1
GSPT2	-0.8934714	0.7226545	-1.2364	0.217	-3.83E-07	count	1
AP2A2	-0.8934714	0.7226545	-1.2364	0.217	-3.83E-07	count	1
AUH	-0.8934714	0.6933756	-1.2886	0.198	-3.83E-07	count	1
SYNE3	-0.8934714	0.6933756	-1.2886	0.198	-3.83E-07	count	1
USP42	-0.8934714	0.7629422	-1.1711	0.242	-3.82E-07	count	1
GALK1	-0.8728521	0.501708	-1.7398	0.0826	-3.79E-07	count	1
CASP3	-0.0883569	0.3979213	-0.222	0.824	-3.77E-07	count	1
FMNL3	-0.0878118	0.4995411	-0.1758	0.861	-3.75E-07	count	1
ARHGEF2	-0.0315614	0.3200489	-0.0986	0.921	-3.74E-07	count	1
FAM45A	-0.2540818	0.6165987	-0.4121	0.68	-3.74E-07	count	1
ABI3	-0.8446812	0.4287235	-1.9702	0.0494	-3.73E-07	count	1
PIAS2	-0.8446812	0.5756486	-1.4674	0.143	-3.71E-07	count	1
SRD5A3	-0.8446812	0.515583	-1.6383	0.102	-3.71E-07	count	1
HIP1R	-0.0866506	0.5256595	-0.1648	0.869	-3.70E-07	count	1
HMG20A	-0.0866506	0.5247387	-0.1651	0.869	-3.70E-07	count	1
ACSL1	-0.8446812	0.650433	-1.2986	0.195	-3.70E-07	count	1
RAB5B	-0.8446812	0.5979277	-1.4127	0.158	-3.69E-07	count	1
APOL6	-0.8446812	0.5403443	-1.5632	0.119	-3.69E-07	count	1
CCNC	-0.0865202	0.3284007	-0.2635	0.792	-3.69E-07	count	1
SNTB2	-0.8446812	0.6194059	-1.3637	0.173	-3.69E-07	count	1
STK19	-0.0851798	0.4916435	-0.1733	0.863	-3.64E-07	count	1
DUS2	-0.0850154	0.5134609	-0.1656	0.869	-3.63E-07	count	1
RBMX2	-0.0850154	0.3885744	-0.2188	0.827	-3.63E-07	count	1
CPSF3	-0.0850154	0.4971722	-0.171	0.864	-3.63E-07	count	1
BTN3A1	-0.0839331	0.4057982	-0.2068	0.836	-3.59E-07	count	1
DENND5B	-0.0839493	0.4488194	-0.187	0.852	-3.59E-07	count	1
RNF216	-0.0301471	0.3089951	-0.0976	0.922	-3.58E-07	count	1
NTPCR	-0.8038592	0.6310295	-1.2739	0.203	-3.58E-07	count	1
ZBTB11-AS1	-0.8038592	0.636573	-1.2628	0.207	-3.56E-07	count	1
GIT1	-0.8038592	0.636573	-1.2628	0.207	-3.56E-07	count	1
AL133467.1	-0.8038592	0.665029	-1.2088	0.227	-3.55E-07	count	1
UBN2	-0.7858407	0.6004413	-1.3088	0.191	-3.53E-07	count	1
CWF19L1	-0.2380611	0.6765229	-0.3519	0.725	-3.53E-07	count	1
MUL1	-0.2380611	0.6765229	-0.3519	0.725	-3.53E-07	count	1
FLI1	-0.0824842	0.3809878	-0.2165	0.829	-3.53E-07	count	1

IQGAP2	-0.2380611	0.620072	-0.3839	0.701	-3.53E-07	count	1
NMT2	-0.2380611	0.7652884	-0.3111	0.756	-3.53E-07	count	1
MICB	-0.2380611	0.7386303	-0.3223	0.747	-3.53E-07	count	1
IQCN	-0.2380611	0.7186057	-0.3313	0.741	-3.52E-07	count	1
CENPV	-0.7858407	0.615856	-1.276	0.203	-3.52E-07	count	1
SSRP1	-0.7646385	0.5148662	-1.4851	0.138	-3.52E-07	count	1
CCDC117	-0.0823519	0.6528327	-0.1261	0.9	-3.52E-07	count	1
MUS81	-0.2369232	0.833357	-0.2843	0.776	-3.51E-07	count	1
ZNF646	-0.2369232	1.2434149	-0.1905	0.849	-3.51E-07	count	1
C2CD2L	-0.2369232	1.2434149	-0.1905	0.849	-3.51E-07	count	1
EPB41L5	-0.2369232	1.2434149	-0.1905	0.849	-3.51E-07	count	1
LINC00449	-0.2369232	1.2905256	-0.1836	0.854	-3.51E-07	count	1
NOTCH4	-0.2369232	1.2905256	-0.1836	0.854	-3.51E-07	count	1
IGSF10	-0.2369232	1.0378041	-0.2283	0.82	-3.51E-07	count	1
ORC2	-0.7756883	0.4599635	-1.6864	0.0924	-3.50E-07	count	1
MCUR1	-0.0289396	0.3346112	-0.0865	0.931	-3.43E-07	count	1
SLC25A51	-0.7393207	0.9340422	-0.7915	0.429	-3.38E-07	count	1
RFX7	-0.7393207	0.650539	-1.1365	0.256	-3.36E-07	count	1
GTSF1	-0.7393207	0.5665209	-1.305	0.193	-3.36E-07	count	1
ADGRG5	-0.7393207	0.632772	-1.1684	0.243	-3.36E-07	count	1
HMGN4	-0.7393207	0.632772	-1.1684	0.243	-3.36E-07	count	1
AC009061.2	-0.7393207	0.787894	-0.9384	0.349	-3.35E-07	count	1
AC133644.2	-0.7393207	0.7042106	-1.0499	0.294	-3.35E-07	count	1
POMZP3	-0.7393207	0.7546361	-0.9797	0.328	-3.35E-07	count	1
LY75	-0.7393207	0.7546361	-0.9797	0.328	-3.35E-07	count	1
KLHDC3	-0.7393207	0.6344725	-1.1653	0.245	-3.35E-07	count	1
AC005332.5	-0.7393207	0.8227557	-0.8986	0.369	-3.34E-07	count	1
PXN-AS1	-0.7393207	0.856199	-0.8635	0.388	-3.34E-07	count	1
CALU	-0.0778023	0.491457	-0.1583	0.874	-3.33E-07	count	1
HELB	-0.0778023	0.491457	-0.1583	0.874	-3.33E-07	count	1
GMNN	-0.0775912	0.5272169	-0.1472	0.883	-3.32E-07	count	1
TFDP1	-0.0775912	0.4595842	-0.1688	0.866	-3.32E-07	count	1
CLTC	-0.0774799	0.4084281	-0.1897	0.85	-3.32E-07	count	1
DIS3L2	-0.0771202	0.622466	-0.1239	0.901	-3.31E-07	count	1
EPB41L2	-0.0755605	0.4531566	-0.1667	0.868	-3.24E-07	count	1
MINK1	-0.7126147	1.2574139	-0.5667	0.571	-3.24E-07	count	1
CDK8	-0.7126147	1.2574139	-0.5667	0.571	-3.24E-07	count	1
AC090152.1	-0.7015804	0.5829697	-1.2035	0.229	-3.23E-07	count	1
C5orf63	-0.6905305	0.6486639	-1.0645	0.288	-3.22E-07	count	1
IFT88	-0.0750043	0.4877114	-0.1538	0.878	-3.22E-07	count	1
TCF7	-0.6821623	0.4958475	-1.3758	0.17	-3.21E-07	count	1
TTC39B	-0.2140242	0.8404009	-0.2547	0.799	-3.20E-07	count	1
PCBP4	-0.2140242	0.775258	-0.2761	0.783	-3.20E-07	count	1
SMYD4	-0.2140242	0.889317	-0.2407	0.81	-3.20E-07	count	1
AC027097.2	-0.2140242	0.889317	-0.2407	0.81	-3.20E-07	count	1

C8orf82	-0.6905305	0.4914717	-1.405	0.161	-3.20E-07	count	1
MAGEH1	-0.2140242	0.7338881	-0.2916	0.771	-3.20E-07	count	1
SPNS1	-0.2140242	0.6988036	-0.3063	0.76	-3.20E-07	count	1
PRKCI	-0.2140242	0.6988036	-0.3063	0.76	-3.20E-07	count	1
TDRD7	-0.2140242	0.7876997	-0.2717	0.786	-3.20E-07	count	1
ZNF805	-0.2140242	0.938197	-0.2281	0.82	-3.20E-07	count	1
AC064807.1	-0.2140242	0.7924145	-0.2701	0.787	-3.20E-07	count	1
QDPR	-0.2140242	0.7924145	-0.2701	0.787	-3.20E-07	count	1
ZNF609	-0.2140242	0.7924145	-0.2701	0.787	-3.20E-07	count	1
ARSA	-0.6905305	0.5907636	-1.1689	0.243	-3.20E-07	count	1
C1orf35	-0.0269257	0.313165	-0.086	0.932	-3.20E-07	count	1
TMEM131	-0.026795	0.2719427	-0.0985	0.922	-3.18E-07	count	1
SMARCA4	-0.0742245	0.4457936	-0.1665	0.868	-3.18E-07	count	1
TNKS2	-0.0267238	0.3469417	-0.077	0.939	-3.17E-07	count	1
PLEKHF2	-0.0266919	0.2826117	-0.0944	0.925	-3.17E-07	count	1
SPRY1	-0.2090461	0.8421531	-0.2482	0.804	-3.13E-07	count	1
CAMK4	-0.6703278	0.713915	-0.9389	0.348	-3.12E-07	count	1
MTA1	-0.6703278	0.6054542	-1.1071	0.269	-3.12E-07	count	1
PQLC2	-0.6703278	0.574797	-1.1662	0.244	-3.11E-07	count	1
SCX	-0.6703278	0.7127706	-0.9405	0.347	-3.11E-07	count	1
SYBU	-0.6703278	0.6333445	-1.0584	0.29	-3.11E-07	count	1
RBM11	-0.6703278	0.6333445	-1.0584	0.29	-3.11E-07	count	1
C12orf66	-0.6703278	0.6333445	-1.0584	0.29	-3.11E-07	count	1
CEP104	-0.0723856	0.6675074	-0.1084	0.914	-3.11E-07	count	1
RNF121	-0.0723856	0.6087046	-0.1189	0.905	-3.11E-07	count	1
CWC27	-0.0718476	0.4410035	-0.1629	0.871	-3.09E-07	count	1
TIMM8A	-0.2050909	0.6618384	-0.3099	0.757	-3.08E-07	count	1
HCFC2	-0.2050909	0.7051977	-0.2908	0.771	-3.08E-07	count	1
SAE1	-0.2050909	0.7068017	-0.2902	0.772	-3.08E-07	count	1
DMAC2	-0.2050909	0.7132424	-0.2875	0.774	-3.08E-07	count	1
AKAP5	-0.2050909	0.7211974	-0.2844	0.776	-3.08E-07	count	1
BPHL	-0.2050909	0.7211974	-0.2844	0.776	-3.08E-07	count	1
MGRN1	-0.6523093	0.5031989	-1.2963	0.196	-3.07E-07	count	1
TADA2A	-0.6523093	0.5301403	-1.2304	0.219	-3.06E-07	count	1
ARID1A	-0.0256981	0.3499774	-0.0734	0.941	-3.05E-07	count	1
NAE1	-0.07087	0.3720406	-0.1905	0.849	-3.04E-07	count	1
ERG28	-0.0706994	0.3322841	-0.2128	0.832	-3.04E-07	count	1
FCHO1	-0.2009269	0.6501991	-0.309	0.757	-3.02E-07	count	1
MAD1L1	-0.6361364	0.3723691	-1.7083	0.0883	-3.02E-07	count	1
DHRSX	-0.0701826	0.588232	-0.1193	0.905	-3.01E-07	count	1
XPO6	-0.0689089	0.5274655	-0.1306	0.896	-2.96E-07	count	1
ASPSCR1	-0.6215376	0.4827839	-1.2874	0.199	-2.96E-07	count	1
GYG1	-0.6215376	0.5970607	-1.041	0.298	-2.95E-07	count	1
FOXK2	-0.6215376	0.6557858	-0.9478	0.344	-2.94E-07	count	1
PHYH	-0.5962198	0.462707	-1.2885	0.198	-2.86E-07	count	1

BLVRB	-0.5916847	0.3942652	-1.5007	0.134	-2.84E-07	count	1
ANTXR2	-0.0652001	0.4073184	-0.1601	0.873	-2.81E-07	count	1
NT5E	-0.0653652	0.4507927	-0.145	0.885	-2.81E-07	count	1
RHOC	-0.0653652	0.4435447	-0.1474	0.883	-2.81E-07	count	1
NCOA1	-0.0652001	0.3812706	-0.171	0.864	-2.81E-07	count	1
MYO9A	-0.58517	0.5233695	-1.1181	0.264	-2.81E-07	count	1
MGME1	-0.0652001	0.4094081	-0.1593	0.874	-2.81E-07	count	1
GALNT3	-0.58517	0.6972482	-0.8393	0.402	-2.80E-07	count	1
LINC02482	-0.064144	0.8102609	-0.0792	0.937	-2.76E-07	count	1
RCAN3	-0.064144	0.4615897	-0.139	0.89	-2.76E-07	count	1
DRG1	-0.064144	0.5479046	-0.1171	0.907	-2.76E-07	count	1
CARD8	-0.064144	0.3887946	-0.165	0.869	-2.76E-07	count	1
TMEM42	-0.064144	0.6072317	-0.1056	0.916	-2.76E-07	count	1
PRCD	-0.064144	0.5025001	-0.1276	0.898	-2.76E-07	count	1
KCNH8	-0.064144	0.7653478	-0.0838	0.933	-2.76E-07	count	1
HADHB	-0.0228862	0.2839933	-0.0806	0.936	-2.72E-07	count	1
SLC39A3	-0.0228614	0.2897351	-0.0789	0.937	-2.72E-07	count	1
EXOSC5	-0.5569991	0.5190189	-1.0732	0.284	-2.72E-07	count	1
KIF21B	-0.5569991	0.5185658	-1.0741	0.283	-2.71E-07	count	1
DLAT	-0.5569991	0.8781699	-0.6343	0.526	-2.70E-07	count	1
AC008074.3	-0.1779028	0.8057415	-0.2208	0.825	-2.70E-07	count	1
RUVBL2	-0.1779028	0.6956526	-0.2557	0.798	-2.70E-07	count	1
ADPRM	-0.1779028	0.8063372	-0.2206	0.825	-2.70E-07	count	1
USP5	-0.1779028	0.8063372	-0.2206	0.825	-2.70E-07	count	1
SESN2	-0.1779028	0.9510424	-0.1871	0.852	-2.70E-07	count	1
ADAL	-0.1779028	0.8597644	-0.2069	0.836	-2.70E-07	count	1
PIP4P2	-0.5569991	0.5144697	-1.0827	0.28	-2.70E-07	count	1
NIPA1	-0.1779028	0.7575671	-0.2348	0.814	-2.70E-07	count	1
RXYLT1	-0.1779028	0.7575671	-0.2348	0.814	-2.70E-07	count	1
ABHD12	-0.1779028	0.7575671	-0.2348	0.814	-2.70E-07	count	1
ZNF780B	-0.1779028	0.6392347	-0.2783	0.781	-2.70E-07	count	1
NAA40	-0.1779028	0.8147903	-0.2183	0.827	-2.70E-07	count	1
PPOX	-0.1779028	0.8147903	-0.2183	0.827	-2.70E-07	count	1
TAX1BP3	-0.1779028	0.7061136	-0.2519	0.801	-2.70E-07	count	1
GMDS-DT	-0.1779028	0.7061136	-0.2519	0.801	-2.70E-07	count	1
ZNF316	-0.1779028	0.7061136	-0.2519	0.801	-2.70E-07	count	1
LRWD1	-0.1779028	0.7061136	-0.2519	0.801	-2.70E-07	count	1
PACS2	-0.5511077	1.5898897	-0.3466	0.729	-2.66E-07	count	1
ZNF676	-0.5511077	1.5898897	-0.3466	0.729	-2.66E-07	count	1
AQP11	-0.5511077	1.5898897	-0.3466	0.729	-2.66E-07	count	1
DARS2	-0.5511077	1.1828456	-0.4659	0.642	-2.66E-07	count	1
SLC11A2	-0.5511077	1.1828456	-0.4659	0.642	-2.66E-07	count	1
ADORA2A-AS1	-0.5511077	1.1828456	-0.4659	0.642	-2.66E-07	count	1
ALKBH6	-0.5511077	1.1828456	-0.4659	0.642	-2.66E-07	count	1
AC008083.2	-0.5511077	1.1828456	-0.4659	0.642	-2.66E-07	count	1

AC007114.1	-0.5511077	1.1828456	-0.4659	0.642	-2.66E-07	count	1
UVSSA	-0.5511077	1.1828456	-0.4659	0.642	-2.66E-07	count	1
LRRC27	-0.5511077	1.1828456	-0.4659	0.642	-2.66E-07	count	1
C3orf62	-0.5511077	1.3017441	-0.4234	0.672	-2.66E-07	count	1
CTGF	-0.5511077	1.3017441	-0.4234	0.672	-2.66E-07	count	1
AL162377.1	-0.5511077	1.3017441	-0.4234	0.672	-2.66E-07	count	1
DPYD	-0.5511077	1.3017441	-0.4234	0.672	-2.66E-07	count	1
ADAM15	-0.5511077	1.3017441	-0.4234	0.672	-2.66E-07	count	1
TRPS1	-0.5511077	1.3017441	-0.4234	0.672	-2.66E-07	count	1
SGSM2	-0.5511077	1.3017441	-0.4234	0.672	-2.66E-07	count	1
FLVCR1	-0.5511077	1.3017441	-0.4234	0.672	-2.66E-07	count	1
AC108718.1	-0.5511077	1.3017441	-0.4234	0.672	-2.66E-07	count	1
CELSR1	-0.5511077	1.3017441	-0.4234	0.672	-2.66E-07	count	1
CDCA7	-0.5511077	1.3017441	-0.4234	0.672	-2.66E-07	count	1
AC004951.1	-0.5511077	1.3017441	-0.4234	0.672	-2.66E-07	count	1
ACACA	-0.5511077	1.3017441	-0.4234	0.672	-2.66E-07	count	1
ZNF483	-0.5511077	1.3017441	-0.4234	0.672	-2.66E-07	count	1
PLA1A	-0.5511077	1.3017441	-0.4234	0.672	-2.66E-07	count	1
CAHM	-0.5511077	1.3017441	-0.4234	0.672	-2.66E-07	count	1
ZBTB5	-0.5511077	1.3017441	-0.4234	0.672	-2.66E-07	count	1
ZNF621	-0.5511077	1.3017441	-0.4234	0.672	-2.66E-07	count	1
WDR27	-0.5511077	1.3017441	-0.4234	0.672	-2.66E-07	count	1
NBEA	-0.5511077	1.3017441	-0.4234	0.672	-2.66E-07	count	1
SNX16	-0.5511077	1.3017441	-0.4234	0.672	-2.66E-07	count	1
RBAK	-0.5511077	1.3017441	-0.4234	0.672	-2.66E-07	count	1
TYW1B	-0.5511077	1.3017441	-0.4234	0.672	-2.66E-07	count	1
CNNM2	-0.5511077	1.3017441	-0.4234	0.672	-2.66E-07	count	1
GDPGP1	-0.5511077	1.3017441	-0.4234	0.672	-2.66E-07	count	1
CRYBG3	-0.5511077	1.3017441	-0.4234	0.672	-2.66E-07	count	1
BICRA	-0.5511077	1.3017441	-0.4234	0.672	-2.66E-07	count	1
PDE6B	-0.5511077	1.3017441	-0.4234	0.672	-2.66E-07	count	1
ZNF726	-0.5511077	1.3017441	-0.4234	0.672	-2.66E-07	count	1
PWAR6	-0.5511077	1.3017441	-0.4234	0.672	-2.66E-07	count	1
CDC42EP1	-0.5511077	1.3017441	-0.4234	0.672	-2.66E-07	count	1
STK3	-0.5511077	1.3017441	-0.4234	0.672	-2.66E-07	count	1
TARBP2	-0.1740147	0.9243293	-0.1883	0.851	-2.64E-07	count	1
NUAK2	-0.1740147	0.7911827	-0.2199	0.826	-2.64E-07	count	1
HMBS	-0.1740147	0.9672818	-0.1799	0.857	-2.64E-07	count	1
RGS5	-0.1740147	1.0912469	-0.1595	0.873	-2.64E-07	count	1
CCHCR1	-0.1740147	1.0599795	-0.1642	0.87	-2.64E-07	count	1
PLCB2	-0.1740147	1.0599795	-0.1642	0.87	-2.64E-07	count	1
ATG13	-0.1740147	0.8492382	-0.2049	0.838	-2.64E-07	count	1
CHD3	-0.1740147	0.9881251	-0.1761	0.86	-2.64E-07	count	1
BPGM	-0.1740147	0.8086669	-0.2152	0.83	-2.64E-07	count	1
RAP1GDS1	-0.0610311	0.3786264	-0.1612	0.872	-2.63E-07	count	1

CITED2	-0.0218584	0.3359106	-0.0651	0.948	-2.60E-07	count	1
NUTM2A-AS1	-0.059847	0.4523066	-0.1323	0.895	-2.58E-07	count	1
FUT11	-0.1696997	1.053115	-0.1611	0.872	-2.58E-07	count	1
DYRK2	-0.0588378	0.5360584	-0.1098	0.913	-2.54E-07	count	1
ARHGEF9	-0.0588378	0.520317	-0.1131	0.91	-2.54E-07	count	1
TAF1C	-0.5161771	0.7256353	-0.7113	0.477	-2.54E-07	count	1
B3GLCT	-0.5161771	0.693831	-0.744	0.457	-2.54E-07	count	1
ELK1	-0.5161771	0.6005086	-0.8596	0.39	-2.54E-07	count	1
ZBTB7B	-0.5161771	0.660497	-0.7815	0.435	-2.53E-07	count	1
DDX28	-0.5161771	0.7154732	-0.7214	0.471	-2.53E-07	count	1
URB1	-0.5161771	0.7154732	-0.7214	0.471	-2.53E-07	count	1
BEND4	-0.5161771	0.7154732	-0.7214	0.471	-2.53E-07	count	1
ZNF597	-0.5161771	0.9271621	-0.5567	0.578	-2.53E-07	count	1
RC3H1	-0.058238	0.3992573	-0.1459	0.884	-2.52E-07	count	1
EFNA4	-0.5057058	0.4178449	-1.2103	0.227	-2.50E-07	count	1
UBL7-AS1	-0.5009097	0.4841322	-1.0347	0.301	-2.49E-07	count	1
B3GALT4	-0.4963745	0.4005835	-1.2391	0.216	-2.47E-07	count	1
CDK19	-0.0572089	0.6668487	-0.0858	0.932	-2.47E-07	count	1
PHF10	-0.0572089	0.4537296	-0.1261	0.9	-2.47E-07	count	1
DPH6	-0.0572089	0.651686	-0.0878	0.93	-2.47E-07	count	1
PEX13	-0.0572089	0.574901	-0.0995	0.921	-2.47E-07	count	1
ZC3H7B	-0.159505	0.9159853	-0.1741	0.862	-2.43E-07	count	1
LINC01023	-0.159505	1.3988336	-0.114	0.909	-2.43E-07	count	1
MED12	-0.159505	1.3988336	-0.114	0.909	-2.43E-07	count	1
CCDC42	-0.159505	1.3988336	-0.114	0.909	-2.43E-07	count	1
TRAPPC13	-0.4880063	0.6289428	-0.7759	0.438	-2.43E-07	count	1
CEP192	-0.4880063	0.6192353	-0.7881	0.431	-2.42E-07	count	1
EYA3	-0.4880063	0.6608073	-0.7385	0.461	-2.42E-07	count	1
CRYZ	-0.4880063	0.5922727	-0.824	0.41	-2.42E-07	count	1
NFS1	-0.4880063	0.5922727	-0.824	0.41	-2.42E-07	count	1
MAP4	-0.0203322	0.2712616	-0.075	0.94	-2.42E-07	count	1
CCDC22	-0.0559514	0.4632096	-0.1208	0.904	-2.42E-07	count	1
FTSJ3	-0.0559514	0.4610229	-0.1214	0.903	-2.42E-07	count	1
GPBP1L1	-0.0554243	0.4134212	-0.1341	0.893	-2.40E-07	count	1
POLDIP2	-0.4769564	0.4216148	-1.1313	0.259	-2.38E-07	count	1
RASL11A	-0.467387	0.8439297	-0.5538	0.58	-2.35E-07	count	1
AC106707.1	-0.0541366	0.9349131	-0.0579	0.954	-2.35E-07	count	1
C6orf136	-0.467387	0.6371205	-0.7336	0.464	-2.34E-07	count	1
XPNPEP1	-0.467387	0.6174042	-0.757	0.449	-2.34E-07	count	1
USB1	-0.0541366	0.7780324	-0.0696	0.945	-2.34E-07	count	1
PILRB	-0.0541366	0.4858215	-0.1114	0.911	-2.34E-07	count	1
GON7	-0.0541366	0.4839697	-0.1119	0.911	-2.34E-07	count	1
ZNF681	-0.467387	0.6174042	-0.757	0.449	-2.34E-07	count	1
DPM1	-0.0541366	0.4282594	-0.1264	0.899	-2.34E-07	count	1
MRS2	-0.0541366	0.6377787	-0.0849	0.932	-2.34E-07	count	1

TSGA10	-0.0541366	0.7108584	-0.0762	0.939	-2.34E-07	count	1
RBL1	-0.0541366	0.7090399	-0.0764	0.939	-2.34E-07	count	1
QSOX2	-0.0541366	0.7750696	-0.0698	0.944	-2.34E-07	count	1
SAP130	-2.4132971	1.1334145	-2.1292	0.0338	-2.34E-07	count	1
CYFIP1	-0.0541366	0.6750995	-0.0802	0.936	-2.34E-07	count	1
FAM98B	-0.467387	0.5970372	-0.7828	0.434	-2.34E-07	count	1
NME7	-0.467387	0.6292259	-0.7428	0.458	-2.34E-07	count	1
EFCAB7	-2.4132971	1.0866201	-2.2209	0.0269	-2.34E-07	count	1
TENT5A	-2.4132971	1.0866201	-2.2209	0.0269	-2.34E-07	count	1
PTER	-0.467387	0.744186	-0.6281	0.53	-2.33E-07	count	1
ARRDC3	-0.151534	0.4292058	-0.3531	0.724	-2.33E-07	count	1
CDK9	-0.019513	0.3011926	-0.0648	0.948	-2.32E-07	count	1
RFX3	-2.2797657	1.5224012	-1.4975	0.135	-2.32E-07	count	1
MAN1B1	-0.0535316	0.5850898	-0.0915	0.927	-2.32E-07	count	1
SGPP1	-0.0535316	0.6497281	-0.0824	0.934	-2.31E-07	count	1
C14orf93	-2.2797657	1.1631089	-1.9601	0.0506	-2.29E-07	count	1
MRGBP	-0.0528901	0.3443548	-0.1536	0.878	-2.29E-07	count	1
C11orf49	-2.2797657	0.9954853	-2.2901	0.0225	-2.28E-07	count	1
AP2M1	-0.019086	0.2767883	-0.069	0.945	-2.27E-07	count	1
TADA2B	-0.4516386	0.574495	-0.7861	0.432	-2.27E-07	count	1
ZNF43	-0.0524026	0.4598311	-0.114	0.909	-2.27E-07	count	1
JOSD2	-0.0524026	0.4666074	-0.1123	0.911	-2.27E-07	count	1
IGSF6	-2.125615	1.5654714	-1.3578	0.175	-2.25E-07	count	1
ELMOD2	-0.4392161	0.5291524	-0.83	0.407	-2.23E-07	count	1
MED22	-2.125615	1.2604915	-1.6863	0.0924	-2.23E-07	count	1
COQ9	-0.4392161	0.5730576	-0.7664	0.444	-2.23E-07	count	1
SAMHD1	-2.125615	1.2021627	-1.7682	0.0777	-2.23E-07	count	1
STK33	-2.125615	1.1408556	-1.8632	0.0631	-2.22E-07	count	1
GNLY	-2.125615	1.1408556	-1.8632	0.0631	-2.22E-07	count	1
OGG1	-0.4392161	0.5451641	-0.8057	0.421	-2.22E-07	count	1
DENND3	-0.0512887	0.4014477	-0.1278	0.898	-2.22E-07	count	1
EMG1	-0.0512891	0.3822073	-0.1342	0.893	-2.22E-07	count	1
TSEN15	-0.0512887	0.541289	-0.0948	0.925	-2.22E-07	count	1
GCLC	-0.0512887	0.5951359	-0.0862	0.931	-2.22E-07	count	1
AL645568.1	-2.125615	1.0760613	-1.9754	0.0488	-2.22E-07	count	1
USP9X	-0.0512891	0.3922916	-0.1307	0.896	-2.22E-07	count	1
RANBP6	-0.0512887	0.5776673	-0.0888	0.929	-2.22E-07	count	1
ESCO1	-0.0183934	0.295042	-0.0623	0.95	-2.19E-07	count	1
CRLS1	-0.4291658	0.4937096	-0.8693	0.385	-2.19E-07	count	1
RNASEH1-AS1	-0.1421294	0.7326642	-0.194	0.846	-2.18E-07	count	1
INO80E	-0.1421294	0.6690189	-0.2124	0.832	-2.18E-07	count	1
DHDDS	-0.4291658	0.5208035	-0.824	0.41	-2.17E-07	count	1
AL662796.1	-0.0499413	0.4966947	-0.1005	0.92	-2.16E-07	count	1
NTNG1	-0.420867	0.5291825	-0.7953	0.427	-2.15E-07	count	1
ACER3	-1.9432935	1.3248151	-1.4668	0.143	-2.15E-07	count	1

NPM3	-1.9432935	1.2561201	-1.5471	0.123	-2.14E-07	count	1
STK32C	-1.9432935	1.2561201	-1.5471	0.123	-2.14E-07	count	1
WDR81	-1.9432935	1.1834444	-1.6421	0.101	-2.14E-07	count	1
KRBA2	-1.9432935	1.1834444	-1.6421	0.101	-2.14E-07	count	1
TSTD3	-1.9432935	1.1834444	-1.6421	0.101	-2.14E-07	count	1
IL18	-1.9432935	1.1834444	-1.6421	0.101	-2.14E-07	count	1
ACO1	-1.9432935	1.1834444	-1.6421	0.101	-2.14E-07	count	1
C11orf68	-1.9432935	1.1060033	-1.757	0.0796	-2.14E-07	count	1
FBXO48	-1.9432935	1.1060033	-1.757	0.0796	-2.14E-07	count	1
BEX3	-1.9432935	1.1060033	-1.757	0.0796	-2.14E-07	count	1
ANO10	-1.9432935	1.1060033	-1.757	0.0796	-2.14E-07	count	1
DHX57	-1.9432935	1.022715	-1.9001	0.0581	-2.14E-07	count	1
PES1	-1.9432935	1.022715	-1.9001	0.0581	-2.14E-07	count	1
TMED2	-0.017672	0.247601	-0.0714	0.943	-2.11E-07	count	1
SLC39A13	-0.1361897	0.8040682	-0.1694	0.866	-2.10E-07	count	1
MIB1	-0.1361897	0.8040682	-0.1694	0.866	-2.10E-07	count	1
ATP2B4	-0.1361897	0.8040682	-0.1694	0.866	-2.10E-07	count	1
SLC18B1	-0.1361897	0.8040682	-0.1694	0.866	-2.10E-07	count	1
AGGF1	-0.1361897	0.803301	-0.1695	0.865	-2.10E-07	count	1
C8orf58	-0.1361897	0.803301	-0.1695	0.865	-2.10E-07	count	1
DIP2A	-0.1361897	0.9754305	-0.1396	0.889	-2.10E-07	count	1
TTC13	-0.1361897	0.8993187	-0.1514	0.88	-2.10E-07	count	1
C11orf71	-0.1361897	0.8890722	-0.1532	0.878	-2.10E-07	count	1
TTLL3	-0.1361897	0.8890722	-0.1532	0.878	-2.10E-07	count	1
LMTK2	-0.1361897	0.8890722	-0.1532	0.878	-2.10E-07	count	1
AC092069.1	-0.1361897	0.8890722	-0.1532	0.878	-2.10E-07	count	1
PTMS	-0.1361897	0.8890722	-0.1532	0.878	-2.10E-07	count	1
ZNF852	-0.1361897	0.8890722	-0.1532	0.878	-2.10E-07	count	1
DEPDC5	-0.1361897	0.8890722	-0.1532	0.878	-2.10E-07	count	1
PPARA	-0.1361897	0.8883784	-0.1533	0.878	-2.10E-07	count	1
SLC17A5	-0.1361897	0.8883784	-0.1533	0.878	-2.10E-07	count	1
KLHL42	-0.1361897	0.7208977	-0.1889	0.85	-2.10E-07	count	1
MAIP1	-0.1361897	0.7208977	-0.1889	0.85	-2.10E-07	count	1
PIGQ	-0.1361897	0.7217526	-0.1887	0.85	-2.10E-07	count	1
RMDN3	-0.1361897	0.8153836	-0.167	0.867	-2.10E-07	count	1
RTL8C	-0.1361897	0.8153836	-0.167	0.867	-2.10E-07	count	1
FHIT	-0.1361897	0.8153836	-0.167	0.867	-2.10E-07	count	1
SIPA1L3	-0.1361897	0.8153836	-0.167	0.867	-2.10E-07	count	1
TSHZ1	-0.1361897	1.0384107	-0.1312	0.896	-2.10E-07	count	1
ERV3-1	-0.4079635	0.5320607	-0.7668	0.444	-2.09E-07	count	1
CDADC1	-1.7201499	0.8439637	-2.0382	0.0421	-2.07E-07	count	1
MAP3K20	-1.7201499	0.8090493	-2.1261	0.034	-2.06E-07	count	1
ANGPTL6	-1.7201499	0.7725586	-2.2266	0.0265	-2.06E-07	count	1
ZNF524	-0.3983941	0.4572859	-0.8712	0.384	-2.04E-07	count	1
TMEM106B	-0.0468883	0.4952119	-0.0947	0.925	-2.03E-07	count	1

MCM8	-1.7201499	1.3360052	-1.2875	0.199	-2.03E-07	count	1
FNDC11	-1.7201499	1.3360052	-1.2875	0.199	-2.03E-07	count	1
AC009118.3	-1.7201499	1.1503035	-1.4954	0.136	-2.03E-07	count	1
LINC02413	-1.7201499	1.1503035	-1.4954	0.136	-2.03E-07	count	1
BTN3A3	-1.7201499	1.1503035	-1.4954	0.136	-2.03E-07	count	1
TAOK2	-1.7201499	1.1503035	-1.4954	0.136	-2.03E-07	count	1
ADAP2	-1.7201499	1.1503035	-1.4954	0.136	-2.03E-07	count	1
SNX30	-1.7201499	1.1503035	-1.4954	0.136	-2.03E-07	count	1
MFSD2A	-1.7201499	1.1503035	-1.4954	0.136	-2.03E-07	count	1
ZNF169	-1.7201499	1.1503035	-1.4954	0.136	-2.03E-07	count	1
DNAJA4	-1.7201499	1.1503035	-1.4954	0.136	-2.03E-07	count	1
ZNF174	-1.7201499	1.1503035	-1.4954	0.136	-2.03E-07	count	1
TNFAIP1	-1.7201499	1.1503035	-1.4954	0.136	-2.03E-07	count	1
SMAD7	-1.7201499	1.1503035	-1.4954	0.136	-2.03E-07	count	1
PCNX4	-0.0169743	0.436455	-0.0389	0.969	-2.03E-07	count	1
ETFBKMT	-1.7201499	1.0451518	-1.6458	0.101	-2.02E-07	count	1
DCUN1D3	-1.7201499	1.0451518	-1.6458	0.101	-2.02E-07	count	1
BMPR1A	-1.7201499	1.0451518	-1.6458	0.101	-2.02E-07	count	1
FUT7	-1.7201499	1.0451518	-1.6458	0.101	-2.02E-07	count	1
GPR180	-1.5866185	0.9813484	-1.6168	0.107	-1.99E-07	count	1
MAP3K10	-1.5866185	0.9095922	-1.7443	0.0818	-1.98E-07	count	1
ZNF75D	-1.5866185	0.8715014	-1.8206	0.0693	-1.98E-07	count	1
SPATA6	-1.5866185	1.0765322	-1.4738	0.141	-1.98E-07	count	1
MUTYH	-1.5866185	0.8316678	-1.9078	0.0571	-1.98E-07	count	1
TCF20	-1.5866185	1.0115508	-1.5685	0.117	-1.97E-07	count	1
FLCN	-1.5866185	1.0115508	-1.5685	0.117	-1.97E-07	count	1
NUP43	-1.5866185	0.7898278	-2.0088	0.0452	-1.97E-07	count	1
TLK2	-0.0164918	0.3234866	-0.051	0.959	-1.97E-07	count	1
AC012645.3	-1.5866185	0.9420978	-1.6841	0.0929	-1.97E-07	count	1
GBP1	-0.043656	0.492884	-0.0886	0.929	-1.89E-07	count	1
GABARAP	-1.4324679	0.9079231	-1.5777	0.115	-1.88E-07	count	1
KCTD7	-1.4324679	0.8615348	-1.6627	0.0971	-1.87E-07	count	1
PIK3CD-AS2	-1.4324679	0.8615348	-1.6627	0.0971	-1.87E-07	count	1
PLPP1	-1.4324679	0.8615348	-1.6627	0.0971	-1.87E-07	count	1
POU3F1	-1.4324679	1.0421545	-1.3745	0.17	-1.87E-07	count	1
RAB39A	-1.4324679	1.0421545	-1.3745	0.17	-1.87E-07	count	1
AL139246.3	-1.4324679	0.8125024	-1.763	0.0786	-1.87E-07	count	1
SNAPC4	-1.4324679	0.8125024	-1.763	0.0786	-1.87E-07	count	1
CNEP1R1	-1.4324679	0.7603145	-1.884	0.0602	-1.87E-07	count	1
FAM229B	-0.1198491	0.7888448	-0.1519	0.879	-1.86E-07	count	1
AC025423.1	-1.4324679	1.4673626	-0.9762	0.329	-1.86E-07	count	1
HECW2	-1.4324679	1.4673626	-0.9762	0.329	-1.86E-07	count	1
PDE2A	-1.4324679	1.4673626	-0.9762	0.329	-1.86E-07	count	1
C17orf58	-1.4324679	1.4673626	-0.9762	0.329	-1.86E-07	count	1
TTC30A	-1.4324679	1.4673626	-0.9762	0.329	-1.86E-07	count	1

MPZL3	-1.4324679	1.4673626	-0.9762	0.329	-1.86E-07	count	1
DCAF13	-0.0155725	0.2836467	-0.0549	0.956	-1.86E-07	count	1
AL118506.1	-1.4324679	1.2233137	-1.171	0.242	-1.85E-07	count	1
IGLV3-1	-1.4324679	1.2233137	-1.171	0.242	-1.85E-07	count	1
NBPF15	-1.4324679	1.2233137	-1.171	0.242	-1.85E-07	count	1
ST5	-1.4324679	1.2233137	-1.171	0.242	-1.85E-07	count	1
AACS	-1.4324679	1.2233137	-1.171	0.242	-1.85E-07	count	1
RFX2	-1.4324679	1.2233137	-1.171	0.242	-1.85E-07	count	1
AC098483.1	-1.4324679	1.2233137	-1.171	0.242	-1.85E-07	count	1
IL12A	-1.4324679	1.2233137	-1.171	0.242	-1.85E-07	count	1
AC091959.3	-1.4324679	1.2233137	-1.171	0.242	-1.85E-07	count	1
HACE1	-1.4324679	1.2233137	-1.171	0.242	-1.85E-07	count	1
ATG9B	-1.4324679	1.2233137	-1.171	0.242	-1.85E-07	count	1
ZNF79	-1.4324679	1.2233137	-1.171	0.242	-1.85E-07	count	1
SUFU	-1.4324679	1.2233137	-1.171	0.242	-1.85E-07	count	1
AC048341.1	-1.4324679	1.2233137	-1.171	0.242	-1.85E-07	count	1
AC004812.2	-1.4324679	1.2233137	-1.171	0.242	-1.85E-07	count	1
TEPSIN	-1.4324679	1.2233137	-1.171	0.242	-1.85E-07	count	1
AL590705.1	-1.4324679	1.2233137	-1.171	0.242	-1.85E-07	count	1
PIF1	-1.4324679	1.2233137	-1.171	0.242	-1.85E-07	count	1
HIST1H2AE	-1.4324679	1.2233137	-1.171	0.242	-1.85E-07	count	1
ARHGAP20	-1.4324679	1.2233137	-1.171	0.242	-1.85E-07	count	1
AC116913.1	-1.4324679	1.2233137	-1.171	0.242	-1.85E-07	count	1
ZNF710	-1.4324679	1.2233137	-1.171	0.242	-1.85E-07	count	1
GEMIN4	-1.4324679	1.2233137	-1.171	0.242	-1.85E-07	count	1
CCDC127	-1.4324679	1.2233137	-1.171	0.242	-1.85E-07	count	1
AC008393.1	-1.4324679	1.2233137	-1.171	0.242	-1.85E-07	count	1
FANCE	-1.4324679	1.2233137	-1.171	0.242	-1.85E-07	count	1
PET117	-1.4324679	1.2233137	-1.171	0.242	-1.85E-07	count	1
PRUNE1	-1.4324679	1.2233137	-1.171	0.242	-1.85E-07	count	1
HYAL3	-1.4324679	1.2233137	-1.171	0.242	-1.85E-07	count	1
AL365205.1	-1.4324679	1.2233137	-1.171	0.242	-1.85E-07	count	1
ZNF543	-1.4324679	1.2233137	-1.171	0.242	-1.85E-07	count	1
TMEM68	-1.4324679	1.0808183	-1.3254	0.186	-1.85E-07	count	1
MRVI1-AS1	-1.4324679	1.0808183	-1.3254	0.186	-1.85E-07	count	1
CLUH	-1.4324679	1.0808183	-1.3254	0.186	-1.85E-07	count	1
ZNF440	-1.4324679	1.0808183	-1.3254	0.186	-1.85E-07	count	1
MTHFD1L	-1.4324679	1.0808183	-1.3254	0.186	-1.85E-07	count	1
ZNF16	-1.4324679	1.0808183	-1.3254	0.186	-1.85E-07	count	1
FOXN3-AS1	-1.4324679	1.0808183	-1.3254	0.186	-1.85E-07	count	1
SORBS3	-1.4324679	1.0808183	-1.3254	0.186	-1.85E-07	count	1
ZNF200	-1.4324679	1.0808183	-1.3254	0.186	-1.85E-07	count	1
SLC1A4	-1.4324679	1.0808183	-1.3254	0.186	-1.85E-07	count	1
ZNF500	-1.4324679	1.0808183	-1.3254	0.186	-1.85E-07	count	1
RRP8	-0.041786	0.5342214	-0.0782	0.938	-1.81E-07	count	1

REXO1	-0.0417436	0.383345	-0.1089	0.913	-1.81E-07	count	1
TMEM203	-0.0417436	0.3328117	-0.1254	0.9	-1.81E-07	count	1
RAP2A	-1.3146848	0.6920831	-1.8996	0.0581	-1.80E-07	count	1
RABL6	-0.0408388	0.4854238	-0.0841	0.933	-1.78E-07	count	1
API5	-0.0408388	0.4322029	-0.0945	0.925	-1.78E-07	count	1
FKBP5	-0.0407857	0.4376738	-0.0932	0.926	-1.77E-07	count	1
SLFN12	-0.3338556	0.6135535	-0.5441	0.587	-1.76E-07	count	1
SPSB2	-0.3338556	0.7227325	-0.4619	0.644	-1.76E-07	count	1
LRR1	-0.3338556	0.6067274	-0.5503	0.582	-1.76E-07	count	1
CKAP4	-0.3338556	0.4996384	-0.6682	0.504	-1.76E-07	count	1
MACC1	-0.3338556	0.7515416	-0.4442	0.657	-1.76E-07	count	1
BEX5	-0.3338556	0.4607329	-0.7246	0.469	-1.76E-07	count	1
SLC25A13	-0.3338556	0.5391487	-0.6192	0.536	-1.76E-07	count	1
P2RY13	-0.3338556	0.6373693	-0.5238	0.601	-1.76E-07	count	1
QPCT	-0.3338556	0.6806197	-0.4905	0.624	-1.76E-07	count	1
ZFP91	-0.3338556	0.4255833	-0.7845	0.433	-1.76E-07	count	1
CASP1	-0.0147287	0.3403011	-0.0433	0.965	-1.76E-07	count	1
DGAT2	-0.3338556	0.7777306	-0.4293	0.668	-1.76E-07	count	1
ZNF737	-0.3338556	0.6522464	-0.5119	0.609	-1.76E-07	count	1
RAB11FIP4	-0.3338556	0.6522464	-0.5119	0.609	-1.76E-07	count	1
RNASEH1	-0.3338556	0.4494602	-0.7428	0.458	-1.76E-07	count	1
FBXO44	-0.3338556	0.5682576	-0.5875	0.557	-1.76E-07	count	1
GNPDA1	-0.3338556	0.5682576	-0.5875	0.557	-1.76E-07	count	1
FANCL	-0.3338556	0.5430146	-0.6148	0.539	-1.76E-07	count	1
AMPD2	-0.3338556	0.7387596	-0.4519	0.652	-1.76E-07	count	1
SLC35D1	-0.3338556	0.7387596	-0.4519	0.652	-1.76E-07	count	1
SMG9	-0.3338556	0.5622657	-0.5938	0.553	-1.76E-07	count	1
EXOC2	-0.3338556	0.5622657	-0.5938	0.553	-1.76E-07	count	1
AC093462.1	-0.3338556	0.8290883	-0.4027	0.687	-1.76E-07	count	1
TMCC1	-0.3338556	0.6947897	-0.4805	0.631	-1.76E-07	count	1
TBC1D7	-0.3338556	0.6947897	-0.4805	0.631	-1.76E-07	count	1
STAT5B	-0.3338556	0.6199874	-0.5385	0.591	-1.75E-07	count	1
DHFR	-0.3338556	0.6199874	-0.5385	0.591	-1.75E-07	count	1
C5orf30	-0.3338556	0.6199874	-0.5385	0.591	-1.75E-07	count	1
ANKDD1A	-0.3338556	0.8774451	-0.3805	0.704	-1.75E-07	count	1
GEMIN8	-0.3338556	0.5348234	-0.6242	0.533	-1.75E-07	count	1
C11orf54	-0.3338556	0.697615	-0.4786	0.632	-1.75E-07	count	1
IQCH-AS1	-0.3338556	0.697615	-0.4786	0.632	-1.75E-07	count	1
BCAS3	-0.3338556	0.697615	-0.4786	0.632	-1.75E-07	count	1
DNAJC5	-0.3338556	0.697615	-0.4786	0.632	-1.75E-07	count	1
TLR1	-0.3338556	0.5150877	-0.6482	0.517	-1.75E-07	count	1
STT3B	-0.0403426	0.3758211	-0.1073	0.915	-1.75E-07	count	1
ZNF559	-0.3338556	0.6035742	-0.5531	0.58	-1.75E-07	count	1
SIK1	-0.3338556	0.6327316	-0.5276	0.598	-1.75E-07	count	1
GATM	-0.3338556	0.7544463	-0.4425	0.658	-1.75E-07	count	1

WHRN	-0.3338556	0.8072866	-0.4136	0.679	-1.75E-07	count	1
HERC6	-0.3338556	0.6329697	-0.5274	0.598	-1.75E-07	count	1
PPP1R12C	-0.3338556	0.7458594	-0.4476	0.655	-1.75E-07	count	1
PCCA	-0.3338556	0.7142059	-0.4675	0.64	-1.75E-07	count	1
FERMT3	-0.0146934	0.3126396	-0.047	0.963	-1.75E-07	count	1
KEAP1	-0.3338556	0.7178198	-0.4651	0.642	-1.75E-07	count	1
PARP3	-0.3338556	0.7698134	-0.4337	0.665	-1.75E-07	count	1
TNFAIP8L1	-0.3338556	0.7698134	-0.4337	0.665	-1.75E-07	count	1
SPTY2D1OS	-1.2501463	1.4155666	-0.8831	0.378	-1.75E-07	count	1
TRAM2-AS1	-1.2501463	1.0110649	-1.2365	0.217	-1.74E-07	count	1
SPINT1	-1.2501463	1.0110649	-1.2365	0.217	-1.74E-07	count	1
UBE2E3	-0.0145785	0.315157	-0.0463	0.963	-1.74E-07	count	1
FBXL19-AS1	-1.2501463	1.1761328	-1.0629	0.288	-1.74E-07	count	1
ERICH6-AS1	-1.2501463	0.9585556	-1.3042	0.193	-1.74E-07	count	1
ENTPD1-AS1	-1.2501463	0.9585556	-1.3042	0.193	-1.74E-07	count	1
RENBP	-1.2501463	0.902998	-1.3844	0.167	-1.74E-07	count	1
FBXO30	-1.2501463	0.902998	-1.3844	0.167	-1.74E-07	count	1
PLBD1	-1.2501463	1.1313111	-1.105	0.27	-1.73E-07	count	1
SWT1	-1.2501463	1.0846388	-1.1526	0.25	-1.73E-07	count	1
MCU	-1.2501463	1.0846388	-1.1526	0.25	-1.73E-07	count	1
PTAFR	-1.2501463	0.8437902	-1.4816	0.139	-1.73E-07	count	1
CAMTA2	-1.2501463	0.8437902	-1.4816	0.139	-1.73E-07	count	1
ADCY7	-1.2501463	0.8437902	-1.4816	0.139	-1.73E-07	count	1
MRPL52	-0.0145187	0.2165252	-0.0671	0.947	-1.73E-07	count	1
INTS4	-1.2501463	1.0358658	-1.2069	0.228	-1.73E-07	count	1
MT1X	-1.2501463	0.7801016	-1.6025	0.11	-1.73E-07	count	1
CEP250	-1.2501463	0.9846799	-1.2696	0.205	-1.73E-07	count	1
SPTLC1	-0.0395459	0.3594376	-0.11	0.912	-1.72E-07	count	1
TMEM179B	-0.0143992	0.2708148	-0.0532	0.958	-1.72E-07	count	1
LMO2	-0.0394696	0.5217992	-0.0756	0.94	-1.72E-07	count	1
SPHK2	-1.1811534	0.7493537	-1.5762	0.116	-1.69E-07	count	1
MIATNB	-1.1811534	0.821591	-1.4376	0.151	-1.68E-07	count	1
EMC4	-0.0140756	0.2236907	-0.0629	0.95	-1.68E-07	count	1
REEP3	-0.0374899	0.41139	-0.0911	0.927	-1.63E-07	count	1
PSMD1	-0.0374899	0.3785065	-0.099	0.921	-1.63E-07	count	1
HERC1	-0.0371554	0.6165544	-0.0603	0.952	-1.62E-07	count	1
ADIRF	-0.0371554	0.6158607	-0.0603	0.952	-1.62E-07	count	1
TP53	-0.0356065	0.5860165	-0.0608	0.952	-1.55E-07	count	1
ARHGAP6	-1.0270028	0.9222801	-1.1135	0.266	-1.55E-07	count	1
PIGU	-1.0270028	1.2185226	-0.8428	0.4	-1.54E-07	count	1
ARL17B	-1.0270028	0.8529229	-1.2041	0.229	-1.54E-07	count	1
TJAP1	-1.0270028	0.8160367	-1.2585	0.209	-1.54E-07	count	1
PCBD2	-1.0270028	1.0340487	-0.9932	0.321	-1.54E-07	count	1
ZFP64	-1.0270028	1.0340487	-0.9932	0.321	-1.54E-07	count	1
METTL2A	-1.0270028	0.7774023	-1.3211	0.187	-1.54E-07	count	1

PHLDA2	-1.0270028	1.2066818	-0.8511	0.395	-1.54E-07	count	1
KLRB1	-1.0270028	1.2066818	-0.8511	0.395	-1.54E-07	count	1
ZNF761	-1.0270028	1.2066818	-0.8511	0.395	-1.54E-07	count	1
FKTN	-1.0270028	1.2066818	-0.8511	0.395	-1.54E-07	count	1
GAS7	-1.0270028	1.2066818	-0.8511	0.395	-1.54E-07	count	1
CXCL3	-1.0270028	1.1478535	-0.8947	0.371	-1.54E-07	count	1
THAP7-AS1	-1.0270028	0.8900729	-1.1538	0.249	-1.54E-07	count	1
UBFD1	-1.0270028	0.8900729	-1.1538	0.249	-1.54E-07	count	1
NADK2	-1.0270028	0.8900729	-1.1538	0.249	-1.54E-07	count	1
STMN3	-1.0270028	0.8900729	-1.1538	0.249	-1.54E-07	count	1
HSDL1	-1.0270028	0.8900729	-1.1538	0.249	-1.54E-07	count	1
ZNF562	-1.0270028	0.8900729	-1.1538	0.249	-1.54E-07	count	1
TMEM135	-1.0270028	0.8900729	-1.1538	0.249	-1.54E-07	count	1
COL9A2	-1.0270028	0.8900729	-1.1538	0.249	-1.54E-07	count	1
NUP98	-0.0352745	0.4054573	-0.087	0.931	-1.54E-07	count	1
NSMF	-1.0270028	1.0858425	-0.9458	0.345	-1.53E-07	count	1
MUT	-1.0270028	1.0858425	-0.9458	0.345	-1.53E-07	count	1
CITED4	-1.0270028	1.0858425	-0.9458	0.345	-1.53E-07	count	1
ARSG	-1.0270028	1.0858425	-0.9458	0.345	-1.53E-07	count	1
ACAD8	-1.0270028	1.0858425	-0.9458	0.345	-1.53E-07	count	1
IFT122	-1.0270028	1.0858425	-0.9458	0.345	-1.53E-07	count	1
STX3	-1.0270028	1.0858425	-0.9458	0.345	-1.53E-07	count	1
SVIL	-1.0270028	0.8085272	-1.2702	0.205	-1.53E-07	count	1
GDPD5	-1.0270028	0.8085272	-1.2702	0.205	-1.53E-07	count	1
ZNF367	-1.0270028	0.8085272	-1.2702	0.205	-1.53E-07	count	1
ZNF182	-1.0270028	0.8085272	-1.2702	0.205	-1.53E-07	count	1
RMI2	-1.0270028	0.8085272	-1.2702	0.205	-1.53E-07	count	1
FBF1	-1.0270028	0.8085272	-1.2702	0.205	-1.53E-07	count	1
MORC2	-1.0270028	0.8085272	-1.2702	0.205	-1.53E-07	count	1
CDK6	-1.0270028	1.0200689	-1.0068	0.315	-1.53E-07	count	1
ZBTB42	-1.0270028	1.0200689	-1.0068	0.315	-1.53E-07	count	1
NLGN4Y	-1.0270028	1.0200689	-1.0068	0.315	-1.53E-07	count	1
NCAPG2	-1.0270028	1.0200689	-1.0068	0.315	-1.53E-07	count	1
AL365361.1	-1.0270028	1.3680722	-0.7507	0.453	-1.53E-07	count	1
LY6G5C	-1.0270028	1.3680722	-0.7507	0.453	-1.53E-07	count	1
ZBTB20-AS5	-1.0270028	1.3680722	-0.7507	0.453	-1.53E-07	count	1
ZNF595	-1.0270028	1.3680722	-0.7507	0.453	-1.53E-07	count	1
XRCC3	-1.0270028	1.3680722	-0.7507	0.453	-1.53E-07	count	1
LDAH	-1.0270028	1.3680722	-0.7507	0.453	-1.53E-07	count	1
EPHA4	-1.0270028	1.3680722	-0.7507	0.453	-1.53E-07	count	1
SLC39A14	-1.0270028	1.3680722	-0.7507	0.453	-1.53E-07	count	1
TNFRSF10D	-1.0270028	1.3680722	-0.7507	0.453	-1.53E-07	count	1
KYAT1	-1.0270028	1.3680722	-0.7507	0.453	-1.53E-07	count	1
SPACA9	-1.0270028	1.3680722	-0.7507	0.453	-1.53E-07	count	1
LRP5	-1.0270028	1.3680722	-0.7507	0.453	-1.53E-07	count	1

LINC02416	-1.0270028	1.3680722	-0.7507	0.453	-1.53E-07	count	1
KLF5	-1.0270028	1.3680722	-0.7507	0.453	-1.53E-07	count	1
AC027307.2	-1.0270028	1.3680722	-0.7507	0.453	-1.53E-07	count	1
PPP1R13L	-1.0270028	1.3680722	-0.7507	0.453	-1.53E-07	count	1
MAMSTR	-1.0270028	1.3680722	-0.7507	0.453	-1.53E-07	count	1
ZNF579	-1.0270028	1.3680722	-0.7507	0.453	-1.53E-07	count	1
ADAMTS1	-1.0270028	1.3680722	-0.7507	0.453	-1.53E-07	count	1
IL3RA	-1.0270028	1.3680722	-0.7507	0.453	-1.53E-07	count	1
PAN2	-1.0270028	1.3680722	-0.7507	0.453	-1.53E-07	count	1
AC007541.1	-1.0270028	1.3680722	-0.7507	0.453	-1.53E-07	count	1
AC004825.2	-1.0270028	1.3680722	-0.7507	0.453	-1.53E-07	count	1
IGHV1-69D	-1.0270028	1.3680722	-0.7507	0.453	-1.53E-07	count	1
LINC02352	-1.0270028	1.3680722	-0.7507	0.453	-1.53E-07	count	1
CD7	-1.0270028	1.3680722	-0.7507	0.453	-1.53E-07	count	1
NFYC-AS1	-1.0270028	1.3680722	-0.7507	0.453	-1.53E-07	count	1
TRIM61	-1.0270028	1.3680722	-0.7507	0.453	-1.53E-07	count	1
AC025171.2	-1.0270028	1.3680722	-0.7507	0.453	-1.53E-07	count	1
ZNF300	-1.0270028	1.3680722	-0.7507	0.453	-1.53E-07	count	1
PDE7B	-1.0270028	1.3680722	-0.7507	0.453	-1.53E-07	count	1
SLC25A40	-1.0270028	1.3680722	-0.7507	0.453	-1.53E-07	count	1
LAMB1	-1.0270028	1.3680722	-0.7507	0.453	-1.53E-07	count	1
PHKA2	-1.0270028	1.3680722	-0.7507	0.453	-1.53E-07	count	1
RND1	-1.0270028	1.3680722	-0.7507	0.453	-1.53E-07	count	1
AC005476.2	-1.0270028	1.3680722	-0.7507	0.453	-1.53E-07	count	1
CCDC151	-1.0270028	1.3680722	-0.7507	0.453	-1.53E-07	count	1
ZNF155	-1.0270028	1.3680722	-0.7507	0.453	-1.53E-07	count	1
ZNF114	-1.0270028	1.3680722	-0.7507	0.453	-1.53E-07	count	1
AC006157.1	-1.0270028	1.3680722	-0.7507	0.453	-1.53E-07	count	1
HSPA6	-1.0270028	1.3680722	-0.7507	0.453	-1.53E-07	count	1
CAVIN2	-1.0270028	1.3680722	-0.7507	0.453	-1.53E-07	count	1
HDAC4	-1.0270028	1.3680722	-0.7507	0.453	-1.53E-07	count	1
HYAL2	-1.0270028	1.3680722	-0.7507	0.453	-1.53E-07	count	1
NUP62CL	-1.0270028	1.3680722	-0.7507	0.453	-1.53E-07	count	1
MROH1	-1.0270028	1.3680722	-0.7507	0.453	-1.53E-07	count	1
MTMR2	-1.0270028	1.3680722	-0.7507	0.453	-1.53E-07	count	1
SRGAP1	-1.0270028	1.3680722	-0.7507	0.453	-1.53E-07	count	1
SYCP3	-1.0270028	1.3680722	-0.7507	0.453	-1.53E-07	count	1
SLC8B1	-1.0270028	1.3680722	-0.7507	0.453	-1.53E-07	count	1
LINC01754	-1.0270028	1.3680722	-0.7507	0.453	-1.53E-07	count	1
MIER2	-1.0270028	1.3680722	-0.7507	0.453	-1.53E-07	count	1
NIPAL4	-1.0270028	1.3680722	-0.7507	0.453	-1.53E-07	count	1
INVS	-1.0270028	1.3680722	-0.7507	0.453	-1.53E-07	count	1
ERLIN1	-1.0270028	1.3680722	-0.7507	0.453	-1.53E-07	count	1
C16orf46	-1.0270028	1.3680722	-0.7507	0.453	-1.53E-07	count	1
VPS26B	-0.0946667	1.2277209	-0.0771	0.939	-1.48E-07	count	1

PECAM1	-0.0946667	0.7085025	-0.1336	0.894	-1.48E-07	count	1
SOX4	-0.0946667	1.2588833	-0.0752	0.94	-1.48E-07	count	1
DENND1A	-0.0946667	1.2588833	-0.0752	0.94	-1.48E-07	count	1
SNHG25	-0.0946667	1.2588833	-0.0752	0.94	-1.48E-07	count	1
SGMS1-AS1	-0.0946667	0.9763929	-0.097	0.923	-1.48E-07	count	1
ATAD3A	-0.0946667	0.9763929	-0.097	0.923	-1.48E-07	count	1
ZNF587B	-0.0946667	0.9763929	-0.097	0.923	-1.48E-07	count	1
NOL4L	-0.0946667	1.298398	-0.0729	0.942	-1.48E-07	count	1
ALKBH1	-0.0946667	0.8866506	-0.1068	0.915	-1.48E-07	count	1
TSPAN14	-0.0946667	0.8088359	-0.117	0.907	-1.48E-07	count	1
NDRG1	-0.0946667	0.9878401	-0.0958	0.924	-1.48E-07	count	1
OSM	-0.0946667	0.9878401	-0.0958	0.924	-1.48E-07	count	1
FCHO2	-0.0946667	0.9878401	-0.0958	0.924	-1.48E-07	count	1
DHRS11	-0.0946667	0.9878401	-0.0958	0.924	-1.48E-07	count	1
BRCA1	-0.0946667	0.9615811	-0.0984	0.922	-1.48E-07	count	1
WDR54	-0.0334537	0.4365449	-0.0766	0.939	-1.46E-07	count	1
HPF1	-0.2693171	0.4104414	-0.6562	0.512	-1.46E-07	count	1
SERPINI1	-0.0331322	0.5947978	-0.0557	0.956	-1.45E-07	count	1
FBXO34	-0.0331322	0.3836158	-0.0864	0.931	-1.44E-07	count	1
EPM2AIP1	-0.0331322	0.370758	-0.0894	0.929	-1.44E-07	count	1
HGSNAT	-0.0324032	0.6263633	-0.0517	0.959	-1.41E-07	count	1
SGTB	-0.0324032	0.6246793	-0.0519	0.959	-1.41E-07	count	1
EPOR	-0.2597476	0.5522394	-0.4704	0.638	-1.41E-07	count	1
FAM104B	-0.2538129	0.5083798	-0.4993	0.618	-1.38E-07	count	1
UBIAD1	-0.2538129	0.4649054	-0.5459	0.585	-1.37E-07	count	1
ZUP1	-0.0313118	0.3447399	-0.0908	0.928	-1.37E-07	count	1
KIF5C	-0.8446812	0.980327	-0.8616	0.389	-1.36E-07	count	1
KLHL20	-0.8446812	0.8359778	-1.0104	0.313	-1.35E-07	count	1
PTCH2	-0.8446812	0.9392775	-0.8993	0.369	-1.35E-07	count	1
GATC	-0.8446812	0.8512606	-0.9923	0.322	-1.35E-07	count	1
CPTP	-0.8446812	0.6800459	-1.2421	0.215	-1.35E-07	count	1
HNRNPA1P48	-0.8446812	0.6800459	-1.2421	0.215	-1.35E-07	count	1
TIRAP	-0.8446812	1.0447064	-0.8085	0.419	-1.35E-07	count	1
ARL3	-0.030852	0.4300561	-0.0717	0.943	-1.35E-07	count	1
BTAF1	-0.030852	0.4129875	-0.0747	0.94	-1.35E-07	count	1
QTRT2	-0.030852	0.4716341	-0.0654	0.948	-1.35E-07	count	1
POLD3	-0.2468442	0.5134424	-0.4808	0.631	-1.34E-07	count	1
TIMMDC1	-0.0306251	0.3838689	-0.0798	0.936	-1.34E-07	count	1
FAR1	-0.2385454	0.4732467	-0.5041	0.614	-1.30E-07	count	1
WWP1	-0.2284951	0.5391615	-0.4238	0.672	-1.25E-07	count	1
LMCD1	-0.7393207	0.7164676	-1.0319	0.303	-1.23E-07	count	1
WSB2	-0.7393207	0.6408769	-1.1536	0.249	-1.23E-07	count	1
ABHD6	-0.7393207	0.6408769	-1.1536	0.249	-1.23E-07	count	1
NAGLU	-0.7393207	0.7198433	-1.0271	0.305	-1.23E-07	count	1
STARD3	-0.7393207	0.7198433	-1.0271	0.305	-1.23E-07	count	1

SCAI	-0.7393207	0.7198433	-1.0271	0.305	-1.23E-07	count	1
BTD	-0.7393207	0.7198433	-1.0271	0.305	-1.23E-07	count	1
PANK4	-0.7393207	1.1592428	-0.6378	0.524	-1.23E-07	count	1
USP6	-0.7393207	1.1592428	-0.6378	0.524	-1.23E-07	count	1
AL592295.4	-0.7393207	1.331784	-0.5551	0.579	-1.23E-07	count	1
ALKAL2	-0.7393207	1.331784	-0.5551	0.579	-1.23E-07	count	1
PIGM	-0.7393207	1.331784	-0.5551	0.579	-1.23E-07	count	1
TMEM176A	-0.7393207	1.331784	-0.5551	0.579	-1.23E-07	count	1
MIEF2	-0.7393207	1.331784	-0.5551	0.579	-1.23E-07	count	1
HOXB4	-0.7393207	1.331784	-0.5551	0.579	-1.23E-07	count	1
AC084018.2	-0.7393207	1.331784	-0.5551	0.579	-1.23E-07	count	1
SPRY2	-0.7393207	0.9661436	-0.7652	0.445	-1.23E-07	count	1
KATNAL1	-0.7393207	0.9661436	-0.7652	0.445	-1.23E-07	count	1
C17orf53	-0.7393207	0.9661436	-0.7652	0.445	-1.23E-07	count	1
U2AF1L5	-0.7393207	0.9661436	-0.7652	0.445	-1.23E-07	count	1
MOXD1	-0.7393207	0.9661436	-0.7652	0.445	-1.23E-07	count	1
AL159169.2	-0.7393207	0.9661436	-0.7652	0.445	-1.23E-07	count	1
SLC22A18	-0.7393207	0.9661436	-0.7652	0.445	-1.23E-07	count	1
GPR89A	-0.7393207	0.9661436	-0.7652	0.445	-1.23E-07	count	1
TMEM56	-0.7393207	0.9661436	-0.7652	0.445	-1.23E-07	count	1
NAP1L2	-0.7393207	0.9661436	-0.7652	0.445	-1.23E-07	count	1
HIRA	-0.7393207	0.9661436	-0.7652	0.445	-1.23E-07	count	1
TCEAL9	-0.7393207	0.9661436	-0.7652	0.445	-1.23E-07	count	1
TPRN	-0.7393207	0.9661436	-0.7652	0.445	-1.23E-07	count	1
FUT4	-0.7393207	0.9661436	-0.7652	0.445	-1.23E-07	count	1
ATAD3B	-0.7393207	0.9661436	-0.7652	0.445	-1.23E-07	count	1
MBTPS2	-0.7393207	1.1675779	-0.6332	0.527	-1.23E-07	count	1
MT1M	-0.7393207	1.1675779	-0.6332	0.527	-1.23E-07	count	1
MED24	-0.7393207	1.1675779	-0.6332	0.527	-1.23E-07	count	1
AL356512.1	-0.7393207	1.1675779	-0.6332	0.527	-1.23E-07	count	1
LYG2	-0.7393207	1.1675779	-0.6332	0.527	-1.23E-07	count	1
NEMP2	-0.7393207	1.1675779	-0.6332	0.527	-1.23E-07	count	1
WWTR1	-0.7393207	1.1675779	-0.6332	0.527	-1.23E-07	count	1
HNRNPA1L2	-0.7393207	1.1675779	-0.6332	0.527	-1.23E-07	count	1
TNC	-0.7393207	1.1675779	-0.6332	0.527	-1.23E-07	count	1
MYO19	-0.7393207	1.1675779	-0.6332	0.527	-1.23E-07	count	1
AC011481.2	-0.7393207	1.1675779	-0.6332	0.527	-1.23E-07	count	1
AC010618.3	-0.7393207	1.1675779	-0.6332	0.527	-1.23E-07	count	1
HIST1H2AL	-0.7393207	0.8533629	-0.8664	0.387	-1.23E-07	count	1
TK1	-0.7393207	0.8533629	-0.8664	0.387	-1.23E-07	count	1
CHCHD6	-0.7393207	0.8533629	-0.8664	0.387	-1.23E-07	count	1
OSER1-DT	-0.7393207	0.8533629	-0.8664	0.387	-1.23E-07	count	1
BEND3	-0.7393207	0.8533629	-0.8664	0.387	-1.23E-07	count	1
ALG1	-0.7393207	0.8533629	-0.8664	0.387	-1.23E-07	count	1
ZSWIM9	-0.7393207	0.8533629	-0.8664	0.387	-1.23E-07	count	1

HAUS4	-0.7393207	0.8533629	-0.8664	0.387	-1.23E-07	count	1
GZMK	-0.7393207	0.8533629	-0.8664	0.387	-1.23E-07	count	1
GTPBP2	-0.7393207	0.8533629	-0.8664	0.387	-1.23E-07	count	1
FAM160B1	-0.7393207	1.0761194	-0.687	0.492	-1.22E-07	count	1
PKD2	-0.7393207	1.0761194	-0.687	0.492	-1.22E-07	count	1
SPIRE1	-0.7393207	1.0761194	-0.687	0.492	-1.22E-07	count	1
HEATR5A	-0.7393207	1.0761194	-0.687	0.492	-1.22E-07	count	1
BATF3	-0.7393207	1.0761194	-0.687	0.492	-1.22E-07	count	1
WDR25	-0.7393207	1.0761194	-0.687	0.492	-1.22E-07	count	1
USP30	-0.7393207	1.0761194	-0.687	0.492	-1.22E-07	count	1
AC008764.8	-0.7393207	1.0761194	-0.687	0.492	-1.22E-07	count	1
SPATA5	-0.0771202	0.7041357	-0.1095	0.913	-1.22E-07	count	1
RNF40	-0.0771202	0.7674617	-0.1005	0.92	-1.22E-07	count	1
GCH1	-0.0274817	0.60068	-0.0458	0.964	-1.20E-07	count	1
IFIT2	-0.2160725	0.6246203	-0.3459	0.73	-1.19E-07	count	1
RALB	-0.2160725	0.370883	-0.5826	0.56	-1.19E-07	count	1
IL1RAP	-0.2160725	0.5886803	-0.367	0.714	-1.19E-07	count	1
FAM69A	-0.2160725	0.5503984	-0.3926	0.695	-1.19E-07	count	1
SH3BP2	-0.0751448	0.7654036	-0.0982	0.922	-1.18E-07	count	1
TRMT12	-0.0269257	0.715959	-0.0376	0.97	-1.18E-07	count	1
CIB2	-0.0269257	0.5506207	-0.0489	0.961	-1.18E-07	count	1
FAS	-0.0269257	0.6887089	-0.0391	0.969	-1.18E-07	count	1
PKNOX1	-0.0269257	0.615403	-0.0438	0.965	-1.18E-07	count	1
GALNT11	-0.0269257	0.6880159	-0.0391	0.969	-1.18E-07	count	1
MEX3C	-0.0266481	0.4453961	-0.0598	0.952	-1.16E-07	count	1
EXOC8	-0.0264817	0.4780988	-0.0554	0.956	-1.16E-07	count	1
RNF11	-0.0264817	0.5040669	-0.0525	0.958	-1.16E-07	count	1
CHCHD4	-0.2003242	0.5593252	-0.3582	0.72	-1.11E-07	count	1
AC044849.1	-0.2003242	0.6179972	-0.3242	0.746	-1.11E-07	count	1
MRI1	-0.2003242	0.5649022	-0.3546	0.723	-1.11E-07	count	1
MTAP	-0.2003242	0.4064906	-0.4928	0.622	-1.11E-07	count	1
ZSCAN26	-0.2003242	0.6453359	-0.3104	0.756	-1.11E-07	count	1
ASH2L	-0.2003242	0.5393018	-0.3715	0.71	-1.11E-07	count	1
FYB1	-0.2003242	0.7057434	-0.2838	0.777	-1.11E-07	count	1
ITGAL	-0.2003242	0.5704247	-0.3512	0.726	-1.11E-07	count	1
UBALD1	-0.2003242	0.6051381	-0.331	0.741	-1.11E-07	count	1
AC073352.2	-0.6215376	1.0740244	-0.5787	0.563	-1.08E-07	count	1
Z93241.1	-0.6215376	0.9112246	-0.6821	0.496	-1.08E-07	count	1
SNHG19	-0.6215376	0.8500574	-0.7312	0.465	-1.08E-07	count	1
USP22	-0.0090172	0.3091937	-0.0292	0.977	-1.08E-07	count	1
ZNF251	-0.6215376	0.7841332	-0.7926	0.428	-1.08E-07	count	1
IL1B	-0.6215376	1.1966859	-0.5194	0.604	-1.08E-07	count	1
IRAK1BP1	-0.6215376	0.9030174	-0.6883	0.492	-1.08E-07	count	1
NDUFAF1	-0.6215376	0.9030174	-0.6883	0.492	-1.08E-07	count	1
EOGT	-0.6215376	0.9030174	-0.6883	0.492	-1.08E-07	count	1

IDUA	-0.6215376	0.9030174	-0.6883	0.492	-1.08E-07	count	1
GMEB2	-0.6215376	0.9030174	-0.6883	0.492	-1.08E-07	count	1
TNR	-0.6215376	0.9030174	-0.6883	0.492	-1.08E-07	count	1
FEM1A	-0.6215376	0.7121321	-0.8728	0.383	-1.08E-07	count	1
GPR137B	-0.6215376	0.7121321	-0.8728	0.383	-1.08E-07	count	1
NKG7	-0.6215376	0.7121321	-0.8728	0.383	-1.08E-07	count	1
C11orf80	-0.6215376	0.7121321	-0.8728	0.383	-1.08E-07	count	1
XAB2	-0.6215376	0.8412536	-0.7388	0.46	-1.08E-07	count	1
MRPS11	-0.1907547	0.4286407	-0.445	0.657	-1.06E-07	count	1
WASHC2C	-0.0239794	0.5590993	-0.0429	0.966	-1.05E-07	count	1
ICE1	-0.0238254	0.3350238	-0.0711	0.943	-1.04E-07	count	1
C1QA	-19.0712598	1219.810268	-0.0156	0.988	-1.04E-07	count	1
MGAT2	-0.0232985	0.4617087	-0.0505	0.96	-1.02E-07	count	1
ANKRD52	-0.064144	1.3144978	-0.0488	0.961	-1.02E-07	count	1
NDC1	-0.064144	0.9805658	-0.0654	0.948	-1.02E-07	count	1
MTFR1	-0.064144	0.9805658	-0.0654	0.948	-1.02E-07	count	1
AC087239.1	-0.064144	0.9822502	-0.0653	0.948	-1.02E-07	count	1
SRGAP2B	-0.064144	0.9822502	-0.0653	0.948	-1.02E-07	count	1
GAB1	-0.064144	0.9822502	-0.0653	0.948	-1.02E-07	count	1
AC016957.2	-0.064144	0.9822502	-0.0653	0.948	-1.02E-07	count	1
CCDC171	-0.064144	0.9822502	-0.0653	0.948	-1.02E-07	count	1
HMCES	-0.064144	0.6969487	-0.092	0.927	-1.02E-07	count	1
TPCN2	-0.064144	1.3282795	-0.0483	0.962	-1.02E-07	count	1
MADD	-0.064144	1.1081575	-0.0579	0.954	-1.02E-07	count	1
SEMA4F	-0.064144	1.3282795	-0.0483	0.962	-1.02E-07	count	1
LACC1	-0.064144	0.9906429	-0.0647	0.948	-1.02E-07	count	1
SOCS5	-0.064144	0.9906429	-0.0647	0.948	-1.02E-07	count	1
TTC21A	-0.064144	0.6939727	-0.0924	0.926	-1.02E-07	count	1
TOR1B	-0.064144	0.6939727	-0.0924	0.926	-1.02E-07	count	1
NCEH1	-0.064144	0.6473883	-0.0991	0.921	-1.02E-07	count	1
KDM1B	-0.064144	0.8435423	-0.076	0.939	-1.02E-07	count	1
YOD1	-0.064144	0.8435423	-0.076	0.939	-1.02E-07	count	1
SZT2	-0.064144	1.1096482	-0.0578	0.954	-1.02E-07	count	1
C1RL	-0.064144	0.8454996	-0.0759	0.94	-1.02E-07	count	1
SLC5A6	-0.064144	0.8454996	-0.0759	0.94	-1.02E-07	count	1
POLI	-0.064144	0.8454996	-0.0759	0.94	-1.02E-07	count	1
NGRN	-0.064144	0.8454996	-0.0759	0.94	-1.02E-07	count	1
CCL3L1	-18.6966334	1592.818742	-0.0117	0.991	-1.02E-07	count	1
SLC9A3R1	-0.1797049	0.5840606	-0.3077	0.758	-1.00E-07	count	1
C17orf67	-0.1797049	0.6000269	-0.2995	0.765	-1.00E-07	count	1
TMEM185A	-0.1797049	0.6575338	-0.2733	0.785	-1.00E-07	count	1
SGO2	-0.1797049	0.6575338	-0.2733	0.785	-1.00E-07	count	1
VPS13B	-0.1797049	0.6575338	-0.2733	0.785	-1.00E-07	count	1
SPIN1	-0.1797049	0.4256598	-0.4222	0.673	-1.00E-07	count	1
KLC1	-0.1797049	0.5904483	-0.3044	0.761	-1.00E-07	count	1

PLD3	-0.1797049	0.7183816	-0.2502	0.803	-1.00E-07	count	1
TMEM161B-AS1	-0.1797049	0.6913484	-0.2599	0.795	-1.00E-07	count	1
FBXO45	-0.1797049	0.5967677	-0.3011	0.763	-1.00E-07	count	1
FAM111B	-18.4390161	1819.192517	-0.0101	0.992	-1.00E-07	count	1
CCL19	-18.6221084	1257.221219	-0.0148	0.988	-9.98E-08	count	1
SELENOP	-18.5226797	1422.15283	-0.013	0.99	-9.94E-08	count	1
NAA80	-0.5569991	0.7672344	-0.726	0.468	-9.93E-08	count	1
IGKV3-20	-0.5569991	0.7226079	-0.7708	0.441	-9.92E-08	count	1
MACROD2	-0.5569991	0.8394617	-0.6635	0.507	-9.92E-08	count	1
AC012236.1	-0.5569991	0.7988801	-0.6972	0.486	-9.91E-08	count	1
MANEA	-0.5569991	0.7988801	-0.6972	0.486	-9.91E-08	count	1
PRR12	-0.5569991	0.7988801	-0.6972	0.486	-9.91E-08	count	1
MFSD5	-0.5569991	0.6750375	-0.8251	0.41	-9.91E-08	count	1
MZF1	-0.5569991	0.8684796	-0.6413	0.522	-9.90E-08	count	1
NRP2	-18.4419584	1388.351397	-0.0133	0.989	-9.90E-08	count	1
SLC37A4	-0.5569991	0.7561236	-0.7367	0.462	-9.90E-08	count	1
UTP20	-0.5569991	0.7561236	-0.7367	0.462	-9.90E-08	count	1
LINC01268	-0.5569991	0.8293192	-0.6716	0.502	-9.89E-08	count	1
IL12RB1	-0.5569991	0.8293192	-0.6716	0.502	-9.89E-08	count	1
SOX12	-0.5569991	0.7107999	-0.7836	0.434	-9.89E-08	count	1
ZNF707	-0.5569991	0.8965587	-0.6213	0.535	-9.89E-08	count	1
XRCC4	-0.0224113	0.6918363	-0.0324	0.974	-9.82E-08	count	1
NUBPL	-18.3713352	1363.782107	-0.0135	0.989	-9.82E-08	count	1
ARL11	-0.0224113	0.6701192	-0.0334	0.973	-9.82E-08	count	1
WDSUB1	-0.0224113	0.6256165	-0.0358	0.971	-9.81E-08	count	1
HDAC8	-0.0224113	0.549034	-0.0408	0.967	-9.81E-08	count	1
CLN5	-0.0224113	0.6009883	-0.0373	0.97	-9.81E-08	count	1
SMUG1	-18.3715707	1278.805236	-0.0144	0.989	-9.81E-08	count	1
AP002495.2	-0.0224113	0.6908793	-0.0324	0.974	-9.81E-08	count	1
EMC8	-0.0224113	0.6009883	-0.0373	0.97	-9.81E-08	count	1
FAM193A	-0.0224113	0.6009883	-0.0373	0.97	-9.81E-08	count	1
AP002748.3	-0.0224113	0.5752736	-0.039	0.969	-9.80E-08	count	1
TPK1	-0.0224113	0.7147145	-0.0314	0.975	-9.80E-08	count	1
ACTR5	-18.1866915	1600.595175	-0.0114	0.991	-9.79E-08	count	1
ZBED3	-18.1262419	1713.81758	-0.0106	0.992	-9.75E-08	count	1
TCEANC2	-18.159571	1489.373367	-0.0122	0.99	-9.73E-08	count	1
CD14	-18.12663	1535.830793	-0.0118	0.991	-9.73E-08	count	1
MTMR1	-18.0421044	1552.524373	-0.0116	0.991	-9.71E-08	count	1
C1QC	-18.1498074	1333.102399	-0.0136	0.989	-9.71E-08	count	1
COL14A1	-17.9763889	1798.766599	-0.01	0.992	-9.70E-08	count	1
C1QB	-18.0483218	1382.151558	-0.0131	0.99	-9.68E-08	count	1
COL1A2	-18.0021894	1489.393334	-0.0121	0.99	-9.67E-08	count	1
AL353194.1	-18.0010116	1453.454658	-0.0124	0.99	-9.65E-08	count	1
OSBPL10-AS1	-18.0021915	1370.971473	-0.0131	0.99	-9.65E-08	count	1
VCAN	-17.8286159	1909.014936	-0.0093	0.993	-9.64E-08	count	1

CHRNA5	-17.8275494	1750.433499	-0.0102	0.992	-9.63E-08	count	1
YLPM1	-17.978034	1243.83132	-0.0145	0.988	-9.61E-08	count	1
ICOSLG	-17.8284799	1421.057507	-0.0125	0.99	-9.59E-08	count	1
AC093157.1	-17.8376596	1427.857093	-0.0125	0.99	-9.59E-08	count	1
MID1IP1	-17.8009697	1560.43826	-0.0114	0.991	-9.59E-08	count	1
HEG1	-17.8011041	1424.284309	-0.0125	0.99	-9.58E-08	count	1
LINC00467	-17.8504306	1223.471396	-0.0146	0.988	-9.57E-08	count	1
ZNF714	-17.8261648	1358.037784	-0.0131	0.99	-9.57E-08	count	1
KLHL2	-17.6293339	1509.505642	-0.0117	0.991	-9.54E-08	count	1
MT1E	-17.6288553	1459.880869	-0.0121	0.99	-9.53E-08	count	1
SLC7A1	-17.6383263	1357.468276	-0.013	0.99	-9.53E-08	count	1
MYPOP	-17.4168595	1451.564688	-0.012	0.99	-9.50E-08	count	1
MAFB	-17.3647686	1330.513786	-0.0131	0.99	-9.48E-08	count	1
GLMN	-0.1668015	0.5535959	-0.3013	0.763	-9.34E-08	count	1
TPGS2	-0.0204385	0.4885276	-0.0418	0.967	-8.96E-08	count	1
MFAP4	-0.159505	1.2801846	-0.1246	0.901	-8.96E-08	count	1
PKD2L2	-0.159505	1.320919	-0.1208	0.904	-8.96E-08	count	1
PSME3	-0.0204385	0.4295761	-0.0476	0.962	-8.95E-08	count	1
EMC9	-0.151534	0.5328663	-0.2844	0.776	-8.54E-08	count	1
SCRIB	-0.151534	0.8492798	-0.1784	0.858	-8.54E-08	count	1
ELMOD3	-0.151534	0.8856152	-0.1711	0.864	-8.54E-08	count	1
ZNF408	-0.151534	0.7415456	-0.2043	0.838	-8.54E-08	count	1
VPS39	-0.151534	0.7415456	-0.2043	0.838	-8.54E-08	count	1
ZNF227	-0.151534	0.7103376	-0.2133	0.831	-8.54E-08	count	1
ANKFY1	-0.151534	0.8647935	-0.1752	0.861	-8.54E-08	count	1
ERAL1	-0.151534	0.7040708	-0.2152	0.83	-8.53E-08	count	1
NARS2	-0.151534	0.6644859	-0.228	0.82	-8.53E-08	count	1
DDX52	-0.0068636	0.4213964	-0.0163	0.987	-8.24E-08	count	1
UBR3	-0.0186246	0.7478099	-0.0249	0.98	-8.18E-08	count	1
FAM129A	-0.0186246	0.5858636	-0.0318	0.975	-8.16E-08	count	1
ENTPD4	-0.0186246	0.6062048	-0.0307	0.976	-8.16E-08	count	1
PTK2	-0.0186246	0.6062048	-0.0307	0.976	-8.16E-08	count	1
ESS2	-0.0186246	0.5856699	-0.0318	0.975	-8.16E-08	count	1
TRAF6	-0.0186246	0.6054593	-0.0308	0.975	-8.16E-08	count	1
PHC2	-0.0186246	0.5643884	-0.033	0.974	-8.16E-08	count	1
RAB33B	-0.0186246	0.5643884	-0.033	0.974	-8.16E-08	count	1
CCDC152	-0.0186246	0.6633295	-0.0281	0.978	-8.16E-08	count	1
STK10	-0.0183934	0.3598784	-0.0511	0.959	-8.06E-08	count	1
CCDC141	-0.0182234	0.5582758	-0.0326	0.974	-8.00E-08	count	1
MOB3B	-0.0182234	0.5035251	-0.0362	0.971	-7.99E-08	count	1
ZNHIT6	-0.0179901	0.4365033	-0.0412	0.967	-7.89E-08	count	1
CNTRL	-0.0064882	0.2211964	-0.0293	0.977	-7.76E-08	count	1
SNX12	-0.0478494	0.7302372	-0.0655	0.948	-7.63E-08	count	1
TMEM218	-0.1331849	0.4882688	-0.2728	0.785	-7.57E-08	count	1
ANKRD36B	-0.1331849	0.573066	-0.2324	0.816	-7.56E-08	count	1

LRCH1	-0.1331849	0.5096208	-0.2613	0.794	-7.56E-08	count	1
SLC38A10	-0.0171087	0.8022158	-0.0213	0.983	-7.53E-08	count	1
PAG1	-0.016951	0.5118852	-0.0331	0.974	-7.44E-08	count	1
AC103591.3	-0.0461735	0.4925445	-0.0937	0.925	-7.36E-08	count	1
RBM28	-0.0160845	0.5586017	-0.0288	0.977	-7.07E-08	count	1
RNF167	-0.005513	0.3190835	-0.0173	0.986	-6.60E-08	count	1
GBP5	-0.3338556	0.8429624	-0.3961	0.692	-6.46E-08	count	1
CABYR	-0.3338556	1.0361339	-0.3222	0.747	-6.46E-08	count	1
CATSPER2	-0.3338556	0.9162571	-0.3644	0.716	-6.46E-08	count	1
MICA	-0.3338556	0.9162571	-0.3644	0.716	-6.46E-08	count	1
PKIA	-0.3338556	0.6357233	-0.5252	0.6	-6.46E-08	count	1
TMEM44-AS1	-0.3338556	1.1414039	-0.2925	0.77	-6.46E-08	count	1
ZGRF1	-0.3338556	0.7251902	-0.4604	0.645	-6.45E-08	count	1
HIST1H2BN	-0.3338556	0.9841079	-0.3392	0.735	-6.45E-08	count	1
RAP2C-AS1	-0.3338556	0.9841079	-0.3392	0.735	-6.45E-08	count	1
TMEM44	-0.3338556	0.9360161	-0.3567	0.722	-6.45E-08	count	1
SPATA5L1	-0.3338556	0.8632759	-0.3867	0.699	-6.45E-08	count	1
ADAT2	-0.3338556	0.8632759	-0.3867	0.699	-6.45E-08	count	1
IDE	-0.3338556	0.8632759	-0.3867	0.699	-6.45E-08	count	1
IKBKG	-0.3338556	0.8092233	-0.4126	0.68	-6.45E-08	count	1
ARHGEF19	-0.3338556	0.8092233	-0.4126	0.68	-6.45E-08	count	1
NACA2	-0.3338556	0.8092233	-0.4126	0.68	-6.45E-08	count	1
EFCAB2	-0.3338556	0.8092233	-0.4126	0.68	-6.45E-08	count	1
FXR2	-0.3338556	0.8092233	-0.4126	0.68	-6.45E-08	count	1
ZNF808	-0.3338556	0.8092233	-0.4126	0.68	-6.45E-08	count	1
ARSB	-0.3338556	0.8092233	-0.4126	0.68	-6.45E-08	count	1
JAML	-0.3338556	1.3271252	-0.2516	0.801	-6.45E-08	count	1
SH3RF1	-0.3338556	1.3271252	-0.2516	0.801	-6.45E-08	count	1
ABCD3	-0.3338556	0.6538865	-0.5106	0.61	-6.45E-08	count	1
TOM1L2	-0.3338556	1.1160177	-0.2991	0.765	-6.45E-08	count	1
FNDC10	-0.3338556	1.1160177	-0.2991	0.765	-6.45E-08	count	1
CYB561D2	-0.3338556	1.1160177	-0.2991	0.765	-6.45E-08	count	1
NT5C2	-0.3338556	1.1160177	-0.2991	0.765	-6.45E-08	count	1
STPG1	-0.3338556	1.1160177	-0.2991	0.765	-6.45E-08	count	1
IGKV1-5	-0.3338556	1.1160177	-0.2991	0.765	-6.45E-08	count	1
TRAIP	-0.3338556	1.1160177	-0.2991	0.765	-6.45E-08	count	1
GAS6-AS1	-0.3338556	1.1160177	-0.2991	0.765	-6.45E-08	count	1
AC010883.1	-0.3338556	1.1160177	-0.2991	0.765	-6.45E-08	count	1
DDX43	-0.3338556	1.1160177	-0.2991	0.765	-6.45E-08	count	1
ZFP30	-0.3338556	1.1160177	-0.2991	0.765	-6.45E-08	count	1
LENG9	-0.3338556	1.1160177	-0.2991	0.765	-6.45E-08	count	1
KCNC1	-0.3338556	1.1160177	-0.2991	0.765	-6.45E-08	count	1
MKL2	-0.3338556	1.1160177	-0.2991	0.765	-6.45E-08	count	1
HS6ST1	-0.3338556	1.1160177	-0.2991	0.765	-6.45E-08	count	1
CD200R1	-0.3338556	0.9871547	-0.3382	0.735	-6.45E-08	count	1

UBE3D	-0.3338556	0.9871547	-0.3382	0.735	-6.45E-08	count	1
AC016876.1	-0.3338556	0.9871547	-0.3382	0.735	-6.45E-08	count	1
CRACR2A	-0.3338556	0.9871547	-0.3382	0.735	-6.45E-08	count	1
DNAJC28	-0.3338556	0.9871547	-0.3382	0.735	-6.45E-08	count	1
LINC01679	-0.3338556	0.9871547	-0.3382	0.735	-6.45E-08	count	1
CHRNA1	-0.3338556	0.9871547	-0.3382	0.735	-6.45E-08	count	1
AC016065.1	-0.3338556	0.9871547	-0.3382	0.735	-6.45E-08	count	1
ITGAV	-0.3338556	0.9871547	-0.3382	0.735	-6.45E-08	count	1
NBEAL2	-0.3338556	0.9871547	-0.3382	0.735	-6.45E-08	count	1
ALG9	-0.3338556	0.9871547	-0.3382	0.735	-6.45E-08	count	1
NUDT13	-0.3338556	0.9871547	-0.3382	0.735	-6.45E-08	count	1
FBXO31	-0.3338556	0.9871547	-0.3382	0.735	-6.45E-08	count	1
TBX21	-0.3338556	0.9871547	-0.3382	0.735	-6.45E-08	count	1
AC027307.3	-0.3338556	0.9871547	-0.3382	0.735	-6.45E-08	count	1
C2orf88	-0.3338556	0.9871547	-0.3382	0.735	-6.45E-08	count	1
FUT8-AS1	-0.3338556	0.9871547	-0.3382	0.735	-6.45E-08	count	1
C2orf15	-0.3338556	0.9871547	-0.3382	0.735	-6.45E-08	count	1
ZNF382	-0.3338556	0.8853156	-0.3771	0.706	-6.45E-08	count	1
PRADC1	-0.3338556	0.8853156	-0.3771	0.706	-6.45E-08	count	1
FAM57A	-0.3338556	0.8853156	-0.3771	0.706	-6.45E-08	count	1
PLAGL2	-0.3338556	0.7622867	-0.438	0.662	-6.45E-08	count	1
SYCE1L	-0.3338556	0.7622867	-0.438	0.662	-6.45E-08	count	1
ARMCX2	-0.3338556	0.7622867	-0.438	0.662	-6.45E-08	count	1
NSUN3	-0.3338556	0.7622867	-0.438	0.662	-6.45E-08	count	1
RGL4	-0.3338556	0.7622867	-0.438	0.662	-6.45E-08	count	1
ANO9	-0.3338556	0.75	-0.4451	0.656	-6.45E-08	count	1
RILP	-0.3338556	0.9553665	-0.3495	0.727	-6.45E-08	count	1
IGLV2-14	-0.3338556	0.9553665	-0.3495	0.727	-6.45E-08	count	1
PPCDC	-0.3338556	1.1263964	-0.2964	0.767	-6.45E-08	count	1
AC073332.1	-0.3338556	1.1263964	-0.2964	0.767	-6.45E-08	count	1
NOXA1	-0.3338556	1.3271252	-0.2516	0.801	-6.45E-08	count	1
IGLV1-44	-0.3338556	1.3271252	-0.2516	0.801	-6.45E-08	count	1
AC018797.2	-0.3338556	1.3271252	-0.2516	0.801	-6.45E-08	count	1
IGLV2-8	-0.3338556	1.3271252	-0.2516	0.801	-6.45E-08	count	1
TMSB15B-AS1	-0.3338556	1.3271252	-0.2516	0.801	-6.45E-08	count	1
GSTM2	-0.3338556	1.3271252	-0.2516	0.801	-6.45E-08	count	1
AC007381.1	-0.3338556	1.3271252	-0.2516	0.801	-6.45E-08	count	1
DCBLD2	-0.3338556	1.3271252	-0.2516	0.801	-6.45E-08	count	1
HIST1H2BF	-0.3338556	1.3271252	-0.2516	0.801	-6.45E-08	count	1
NEO1	-0.3338556	1.3271252	-0.2516	0.801	-6.45E-08	count	1
EVA1C	-0.3338556	1.3271252	-0.2516	0.801	-6.45E-08	count	1
AC012510.1	-0.3338556	1.3271252	-0.2516	0.801	-6.45E-08	count	1
HIST1H4A	-0.3338556	1.3271252	-0.2516	0.801	-6.45E-08	count	1
PRDM11	-0.3338556	1.3271252	-0.2516	0.801	-6.45E-08	count	1
AL359198.1	-0.3338556	1.3271252	-0.2516	0.801	-6.45E-08	count	1

APOLD1	-0.3338556	1.3271252	-0.2516	0.801	-6.45E-08	count	1
AC127002.1	-0.3338556	1.3271252	-0.2516	0.801	-6.45E-08	count	1
TSPOAP1	-0.3338556	1.3271252	-0.2516	0.801	-6.45E-08	count	1
ZSWIM4	-0.3338556	1.3271252	-0.2516	0.801	-6.45E-08	count	1
LBHD1	-0.3338556	1.3271252	-0.2516	0.801	-6.45E-08	count	1
FGFBP3	-0.3338556	1.3271252	-0.2516	0.801	-6.45E-08	count	1
AL356019.2	-0.3338556	1.3271252	-0.2516	0.801	-6.45E-08	count	1
RPP25	-0.3338556	1.3271252	-0.2516	0.801	-6.45E-08	count	1
SFXN3	-0.3338556	0.9001724	-0.3709	0.711	-6.45E-08	count	1
KCTD2	-0.3338556	0.9001724	-0.3709	0.711	-6.45E-08	count	1
AC137767.1	-0.3338556	0.9001724	-0.3709	0.711	-6.45E-08	count	1
CSNK1G1	-0.3338556	0.9001724	-0.3709	0.711	-6.45E-08	count	1
FAM102B	-0.3338556	0.9001724	-0.3709	0.711	-6.45E-08	count	1
ADAMDEC1	-0.3338556	0.9001724	-0.3709	0.711	-6.45E-08	count	1
FICD	-0.3338556	0.9001724	-0.3709	0.711	-6.45E-08	count	1
CDPF1	-0.3338556	0.9001724	-0.3709	0.711	-6.45E-08	count	1
AARS	-0.3338556	1.1970518	-0.2789	0.78	-6.45E-08	count	1
PPME1	-0.3338556	0.9360161	-0.3567	0.722	-6.45E-08	count	1
C5orf22	-0.3338556	0.9360161	-0.3567	0.722	-6.45E-08	count	1
SNHG21	-0.3338556	0.9360161	-0.3567	0.722	-6.45E-08	count	1
MAST3	-0.3338556	0.9360161	-0.3567	0.722	-6.45E-08	count	1
ZNF419	-0.3338556	0.9360161	-0.3567	0.722	-6.45E-08	count	1
SMIM1	-0.3338556	0.9360161	-0.3567	0.722	-6.45E-08	count	1
SLAMF7	-0.3338556	0.9360161	-0.3567	0.722	-6.45E-08	count	1
AC021054.1	-0.3338556	0.9360161	-0.3567	0.722	-6.45E-08	count	1
BLMH	-0.3338556	0.9360161	-0.3567	0.722	-6.45E-08	count	1
RPTOR	-0.3338556	0.9360161	-0.3567	0.722	-6.45E-08	count	1
LINC01534	-0.3338556	0.9360161	-0.3567	0.722	-6.45E-08	count	1
SLC22A15	-0.3338556	0.9360161	-0.3567	0.722	-6.45E-08	count	1
TNFAIP8L2	-0.3338556	0.9360161	-0.3567	0.722	-6.45E-08	count	1
PRSS16	-0.3338556	0.9360161	-0.3567	0.722	-6.45E-08	count	1
FECH	-0.3338556	0.9360161	-0.3567	0.722	-6.45E-08	count	1
LINC01353	-0.3338556	0.9360161	-0.3567	0.722	-6.45E-08	count	1
EXOC3-AS1	-0.3338556	0.9360161	-0.3567	0.722	-6.45E-08	count	1
C11orf98	-0.3338556	0.9360161	-0.3567	0.722	-6.45E-08	count	1
CRADD	-0.3338556	0.9360161	-0.3567	0.722	-6.45E-08	count	1
ALDOC	-0.3338556	0.9360161	-0.3567	0.722	-6.45E-08	count	1
HCN2	-0.3338556	0.9360161	-0.3567	0.722	-6.45E-08	count	1
SELENON	-0.3338556	1.1797847	-0.283	0.777	-6.45E-08	count	1
CD3E	-0.3338556	1.1797847	-0.283	0.777	-6.45E-08	count	1
ADGRL4	-0.3338556	1.1797847	-0.283	0.777	-6.45E-08	count	1
ZNF519	-0.3338556	1.1797847	-0.283	0.777	-6.45E-08	count	1
A4GALT	-0.3338556	1.1797847	-0.283	0.777	-6.45E-08	count	1
SHMT1	-0.3338556	1.1797847	-0.283	0.777	-6.45E-08	count	1
APOL1	-0.3338556	1.1797847	-0.283	0.777	-6.45E-08	count	1

LINC01136	-0.3338556	1.1797847	-0.283	0.777	-6.45E-08	count	1
TFPI	-0.3338556	1.1797847	-0.283	0.777	-6.45E-08	count	1
GPRASP1	-0.3338556	1.1797847	-0.283	0.777	-6.45E-08	count	1
LINC01001	-0.3338556	1.1797847	-0.283	0.777	-6.45E-08	count	1
ZBED2	-0.3338556	1.1797847	-0.283	0.777	-6.45E-08	count	1
PELI3	-0.3338556	1.1797847	-0.283	0.777	-6.45E-08	count	1
AL355001.2	-0.3338556	1.1797847	-0.283	0.777	-6.45E-08	count	1
AC015802.6	-0.3338556	1.1797847	-0.283	0.777	-6.45E-08	count	1
DPY19L3	-0.3338556	1.1797847	-0.283	0.777	-6.45E-08	count	1
PLXND1	-0.3338556	1.1797847	-0.283	0.777	-6.45E-08	count	1
PIGA	-0.3338556	1.1797847	-0.283	0.777	-6.45E-08	count	1
ZNF582-AS1	-0.3338556	1.1797847	-0.283	0.777	-6.45E-08	count	1
CEP41	-0.3338556	1.1797847	-0.283	0.777	-6.45E-08	count	1
ARHGAP5-AS1	-0.3338556	1.1797847	-0.283	0.777	-6.45E-08	count	1
AL117332.1	-0.3338556	1.1797847	-0.283	0.777	-6.45E-08	count	1
ZNF304	-0.3338556	1.1797847	-0.283	0.777	-6.45E-08	count	1
MIRLET7BHG	-0.3338556	1.1797847	-0.283	0.777	-6.45E-08	count	1
ARHGAP31	-0.110712	0.5854659	-0.1891	0.85	-6.34E-08	count	1
DYRK4	-0.110712	0.4705278	-0.2353	0.814	-6.34E-08	count	1
CXorf56	-0.110712	0.5272635	-0.21	0.834	-6.34E-08	count	1
AC116366.1	-0.110712	0.8608606	-0.1286	0.898	-6.34E-08	count	1
GALT	-0.110712	0.5717151	-0.1936	0.847	-6.34E-08	count	1
MFN2	-0.110712	0.9185035	-0.1205	0.904	-6.34E-08	count	1
MCM6	-0.110712	0.7073695	-0.1565	0.876	-6.33E-08	count	1
DDX41	-0.110712	0.5071782	-0.2183	0.827	-6.33E-08	count	1
FRYL	-0.005151	0.3470234	-0.0148	0.988	-6.17E-08	count	1
KHDC4	-0.013687	0.5222717	-0.0262	0.979	-6.02E-08	count	1
GMEB1	-0.013687	0.521932	-0.0262	0.979	-6.01E-08	count	1
ITGB3BP	-0.013687	0.489895	-0.0279	0.978	-6.01E-08	count	1
ZNF821	-0.013687	0.4176775	-0.0328	0.974	-6.01E-08	count	1
TBL2	-1.0270028	1.1478535	-0.8947	0.371	-5.63E-08	count	1
MAP2	-1.0270028	1.1478535	-0.8947	0.371	-5.63E-08	count	1
F11R	-1.0270028	1.1478535	-0.8947	0.371	-5.63E-08	count	1
NIFK-AS1	-1.0270028	1.1478535	-0.8947	0.371	-5.63E-08	count	1
TRIT1	-1.0270028	1.1478535	-0.8947	0.371	-5.63E-08	count	1
C1orf50	-1.0270028	1.1478535	-0.8947	0.371	-5.63E-08	count	1
MDC1	-1.0270028	1.1478535	-0.8947	0.371	-5.63E-08	count	1
AC026471.1	-1.0270028	1.1478535	-0.8947	0.371	-5.63E-08	count	1
KNSTRN	-1.0270028	1.1478535	-0.8947	0.371	-5.63E-08	count	1
AC015726.1	-1.0270028	1.1478535	-0.8947	0.371	-5.63E-08	count	1
ZNF175	-1.0270028	1.1478535	-0.8947	0.371	-5.63E-08	count	1
AC025181.2	-1.0270028	1.1478535	-0.8947	0.371	-5.63E-08	count	1
UBTD2	-1.0270028	1.1478535	-0.8947	0.371	-5.63E-08	count	1
RRAGD	-1.0270028	1.1478535	-0.8947	0.371	-5.63E-08	count	1
TFCP2	-1.0270028	1.1478535	-0.8947	0.371	-5.63E-08	count	1

LAT	-1.0270028	1.1478535	-0.8947	0.371	-5.63E-08	count	1
UQCC1	-1.0270028	1.1478535	-0.8947	0.371	-5.63E-08	count	1
AL355472.1	-1.0270028	1.1478535	-0.8947	0.371	-5.63E-08	count	1
CKMT2-AS1	-1.0270028	1.1478535	-0.8947	0.371	-5.63E-08	count	1
FLT3LG	-1.0270028	1.1478535	-0.8947	0.371	-5.63E-08	count	1
GPR89B	-1.0270028	1.1478535	-0.8947	0.371	-5.63E-08	count	1
ERCC8	-1.0270028	1.1478535	-0.8947	0.371	-5.63E-08	count	1
KLRG1	-1.0270028	1.1478535	-0.8947	0.371	-5.63E-08	count	1
UTP14C	-1.0270028	1.1478535	-0.8947	0.371	-5.63E-08	count	1
ZNF48	-1.0270028	1.1478535	-0.8947	0.371	-5.63E-08	count	1
TLDC1	-1.0270028	1.1478535	-0.8947	0.371	-5.63E-08	count	1
TTLL4	-1.0270028	1.1478535	-0.8947	0.371	-5.63E-08	count	1
HDX	-1.0270028	1.1478535	-0.8947	0.371	-5.63E-08	count	1
LINC01410	-1.0270028	1.1478535	-0.8947	0.371	-5.63E-08	count	1
SH3PXD2A	-1.0270028	1.1478535	-0.8947	0.371	-5.63E-08	count	1
ST3GAL2	-1.0270028	1.1478535	-0.8947	0.371	-5.63E-08	count	1
AC008691.1	-1.0270028	1.1478535	-0.8947	0.371	-5.63E-08	count	1
MMS19	-1.0270028	1.1478535	-0.8947	0.371	-5.63E-08	count	1
TIMELESS	-1.0270028	1.1478535	-0.8947	0.371	-5.63E-08	count	1
LMOD3	-1.0270028	1.1478535	-0.8947	0.371	-5.63E-08	count	1
AC093827.4	-1.0270028	1.1478535	-0.8947	0.371	-5.63E-08	count	1
ZNF879	-1.0270028	1.1478535	-0.8947	0.371	-5.63E-08	count	1
SPAG8	-1.0270028	1.1478535	-0.8947	0.371	-5.63E-08	count	1
AAK1	-1.0270028	1.1478535	-0.8947	0.371	-5.63E-08	count	1
SLC2A8	-1.0270028	1.1478535	-0.8947	0.371	-5.63E-08	count	1
SFRP2	-1.0270028	1.1478535	-0.8947	0.371	-5.63E-08	count	1
INPP5E	-1.0270028	1.1478535	-0.8947	0.371	-5.63E-08	count	1
C16orf45	-1.0270028	1.1478535	-0.8947	0.371	-5.63E-08	count	1
PHLPP2	-1.0270028	1.1478535	-0.8947	0.371	-5.63E-08	count	1
SFRP4	-1.0270028	1.1478535	-0.8947	0.371	-5.63E-08	count	1
LPAR6	-1.0270028	1.1478535	-0.8947	0.371	-5.63E-08	count	1
FGL2	-0.0926935	0.6221793	-0.149	0.882	-5.34E-08	count	1
ATG10	-0.0825411	0.5504268	-0.15	0.881	-4.78E-08	count	1
HABP4	-0.0825411	0.5971384	-0.1382	0.89	-4.77E-08	count	1
TTC21B	-0.0825411	0.6440429	-0.1282	0.898	-4.77E-08	count	1
MYBL2	-0.0291577	0.963202	-0.0303	0.976	-4.68E-08	count	1
SMPD1	-0.0291577	1.0105173	-0.0289	0.977	-4.68E-08	count	1
ARIH2OS	-0.0291577	0.9578517	-0.0304	0.976	-4.68E-08	count	1
PGPEP1	-0.0094839	0.73473	-0.0129	0.99	-4.17E-08	count	1
SPATA2	-0.0714913	0.5934671	-0.1205	0.904	-4.15E-08	count	1
ZNF91	-0.0033457	0.377559	-0.0089	0.993	-4.01E-08	count	1
TOPORS	-0.0031373	0.2874076	-0.0109	0.991	-3.76E-08	count	1
IFNG	-19.2229848	2148.526333	-0.0089	0.993	-3.59E-08	count	1
THBD	-19.2229848	2148.526333	-0.0089	0.993	-3.59E-08	count	1
ANGEL1	-19.0895157	2832.846038	-0.0067	0.995	-3.59E-08	count	1

ARHGAP19	-18.93635	3416.314542	-0.0055	0.996	-3.57E-08	count	1
HSD17B14	-19.0907492	2388.743609	-0.008	0.994	-3.57E-08	count	1
BOLA1	-19.0910481	2220.651881	-0.0086	0.993	-3.57E-08	count	1
AC026904.2	-19.0916968	2038.990789	-0.0094	0.993	-3.56E-08	count	1
MTFP1	-19.0916968	2038.990789	-0.0094	0.993	-3.56E-08	count	1
PARD6B	-18.9377349	2839.127272	-0.0067	0.995	-3.55E-08	count	1
CFI	-18.7557596	3677.229281	-0.0051	0.996	-3.55E-08	count	1
FCN1	-18.7557596	3677.229281	-0.0051	0.996	-3.55E-08	count	1
PLCXD1	-18.9382195	2500.060617	-0.0076	0.994	-3.55E-08	count	1
MPPED2	-18.9382195	2500.060617	-0.0076	0.994	-3.55E-08	count	1
CXCL12	-18.9385007	2311.856919	-0.0082	0.993	-3.54E-08	count	1
FABP4	-18.9391053	2107.127707	-0.009	0.993	-3.54E-08	count	1
TSPAN4	-18.9391053	2107.127707	-0.009	0.993	-3.54E-08	count	1
IFIT1	-18.9391053	2107.127707	-0.009	0.993	-3.54E-08	count	1
ZNF738	-18.9391053	2107.127707	-0.009	0.993	-3.54E-08	count	1
FXYD7	-18.9391053	2107.127707	-0.009	0.993	-3.54E-08	count	1
LRRIQ3	-18.9391053	2107.127707	-0.009	0.993	-3.54E-08	count	1
IGLV3-21	-18.9391053	2107.127707	-0.009	0.993	-3.54E-08	count	1
LCAT	-18.7575069	2646.479884	-0.0071	0.994	-3.53E-08	count	1
NHLRC4	-18.7575069	2646.479884	-0.0071	0.994	-3.53E-08	count	1
PEX11B	-18.9401854	1620.792354	-0.0117	0.991	-3.52E-08	count	1
OGN	-18.7580719	2432.467184	-0.0077	0.994	-3.52E-08	count	1
OSBPL7	-18.7580719	2432.467184	-0.0077	0.994	-3.52E-08	count	1
AC116366.3	-18.7580719	2432.467184	-0.0077	0.994	-3.52E-08	count	1
GPR155	-18.7583071	2197.069503	-0.0085	0.993	-3.52E-08	count	1
AL445524.1	-18.7583071	2197.069503	-0.0085	0.993	-3.52E-08	count	1
APOC1	-18.7583071	2197.069503	-0.0085	0.993	-3.52E-08	count	1
BAIAP3	-18.7583071	2197.069503	-0.0085	0.993	-3.52E-08	count	1
HIST1H2BK	-18.7583071	2197.069503	-0.0085	0.993	-3.52E-08	count	1
AC116407.2	-18.7583071	2197.069503	-0.0085	0.993	-3.52E-08	count	1
PRDM15	-18.801352	2099.954919	-0.009	0.993	-3.52E-08	count	1
CXCL2	-18.758801	1933.335453	-0.0097	0.992	-3.51E-08	count	1
FYCO1	-18.758801	1933.335453	-0.0097	0.992	-3.51E-08	count	1
MADCAM1	-18.758801	1933.335453	-0.0097	0.992	-3.51E-08	count	1
TONSL	-18.758801	1933.335453	-0.0097	0.992	-3.51E-08	count	1
ZNF577	-18.758801	1933.335453	-0.0097	0.992	-3.51E-08	count	1
CIDEB	-18.758801	1933.335453	-0.0097	0.992	-3.51E-08	count	1
TNFSF13B	-18.5856177	2732.867622	-0.0068	0.995	-3.51E-08	count	1
SPIN3	-18.5856177	2732.867622	-0.0068	0.995	-3.51E-08	count	1
EXOC6B	-18.5856177	2732.867622	-0.0068	0.995	-3.51E-08	count	1
NBPF9	-18.5856177	2732.867622	-0.0068	0.995	-3.51E-08	count	1
COX7A1	-18.5857117	2467.12271	-0.0075	0.994	-3.50E-08	count	1
SLCO4A1	-18.5857117	2467.12271	-0.0075	0.994	-3.50E-08	count	1
STAT4	-18.617168	2160.441749	-0.0086	0.993	-3.50E-08	count	1
AC016737.1	-18.5364072	2600.328179	-0.0071	0.994	-3.50E-08	count	1

AP5S1	-18.5364072	2600.328179	-0.0071	0.994	-3.50E-08	count	1
HSBP1L1	-18.5364072	2600.328179	-0.0071	0.994	-3.50E-08	count	1
LINC00562	-18.5364072	2600.328179	-0.0071	0.994	-3.50E-08	count	1
ID2	-18.5366043	2323.167165	-0.008	0.994	-3.50E-08	count	1
SLC38A5	-18.5366043	2323.167165	-0.008	0.994	-3.50E-08	count	1
PCSK4	-18.5366043	2323.167165	-0.008	0.994	-3.50E-08	count	1
LTB4R	-18.5366043	2323.167165	-0.008	0.994	-3.50E-08	count	1
ANKRD9	-18.5366043	2323.167165	-0.008	0.994	-3.50E-08	count	1
ZXDA	-18.5366043	2323.167165	-0.008	0.994	-3.50E-08	count	1
DHRS7B	-18.5366043	2323.167165	-0.008	0.994	-3.50E-08	count	1
MRPS24	-18.5366043	2323.167165	-0.008	0.994	-3.50E-08	count	1
CPE	-18.5366043	2323.167165	-0.008	0.994	-3.50E-08	count	1
ZNF256	-18.5858588	2169.058134	-0.0086	0.993	-3.50E-08	count	1
C1orf115	-18.5858588	2169.058134	-0.0086	0.993	-3.50E-08	count	1
KANK1	-18.382617	2944.494793	-0.0062	0.995	-3.49E-08	count	1
MFGE8	-18.3470853	3066.05779	-0.006	0.995	-3.49E-08	count	1
FOXO4	-18.5370057	2008.180727	-0.0092	0.993	-3.49E-08	count	1
IGHGP	-18.5370057	2008.180727	-0.0092	0.993	-3.49E-08	count	1
DSEL	-18.5370057	2008.180727	-0.0092	0.993	-3.49E-08	count	1
VPS54	-18.5370057	2008.180727	-0.0092	0.993	-3.49E-08	count	1
PTGDS	-18.5370057	2008.180727	-0.0092	0.993	-3.49E-08	count	1
ESR2	-18.5370057	2008.180727	-0.0092	0.993	-3.49E-08	count	1
ZNF347	-18.5370057	2008.180727	-0.0092	0.993	-3.49E-08	count	1
RAB13	-18.5370057	2008.180727	-0.0092	0.993	-3.49E-08	count	1
ATL1	-18.5370057	2008.180727	-0.0092	0.993	-3.49E-08	count	1
LRRC61	-18.5370057	2008.180727	-0.0092	0.993	-3.49E-08	count	1
GLMP	-18.5370057	2008.180727	-0.0092	0.993	-3.49E-08	count	1
DDR1	-18.5859119	1822.69384	-0.0102	0.992	-3.49E-08	count	1
HLA-G	-18.5859119	1822.69384	-0.0102	0.992	-3.49E-08	count	1
STRN4	-18.3655919	2490.580441	-0.0074	0.994	-3.49E-08	count	1
B4GALT5	-18.3655919	2490.580441	-0.0074	0.994	-3.49E-08	count	1
GPNMB	-18.3097036	2977.498836	-0.0061	0.995	-3.49E-08	count	1
DNAJC5B	-18.537345	1633.160152	-0.0114	0.991	-3.49E-08	count	1
CATSPERG	-18.537345	1633.160152	-0.0114	0.991	-3.49E-08	count	1
SGCA	-18.3455426	2395.966132	-0.0077	0.994	-3.48E-08	count	1
PLEKHA8	-18.3486604	2263.4208	-0.0081	0.994	-3.48E-08	count	1
OASL	-18.2500664	2855.688498	-0.0064	0.995	-3.48E-08	count	1
PREX2	-18.2500664	2855.688498	-0.0064	0.995	-3.48E-08	count	1
SERPINA1	-18.2500664	2855.688498	-0.0064	0.995	-3.48E-08	count	1
LILRB2	-18.2500664	2855.688498	-0.0064	0.995	-3.48E-08	count	1
AIFM2	-18.2500664	2855.688498	-0.0064	0.995	-3.48E-08	count	1
DKK3	-18.2500664	2855.688498	-0.0064	0.995	-3.48E-08	count	1
IL1R1	-18.2500664	2855.688498	-0.0064	0.995	-3.48E-08	count	1
AL360091.3	-18.2500664	2855.688498	-0.0064	0.995	-3.48E-08	count	1
LINC00240	-18.2500664	2855.688498	-0.0064	0.995	-3.48E-08	count	1

IGHV4-39	-18.2500664	2855.688498	-0.0064	0.995	-3.48E-08	count	1
TMEM225B	-18.2500664	2855.688498	-0.0064	0.995	-3.48E-08	count	1
AC097468.3	-18.2500664	2855.688498	-0.0064	0.995	-3.48E-08	count	1
FCAR	-18.2500664	2855.688498	-0.0064	0.995	-3.48E-08	count	1
TNNT3	-18.2500664	2855.688498	-0.0064	0.995	-3.48E-08	count	1
SLC12A9-AS1	-18.3097913	2276.054326	-0.008	0.994	-3.48E-08	count	1
EFEMP1	-18.3097913	2276.054326	-0.008	0.994	-3.48E-08	count	1
TMEM97	-18.3097913	2276.054326	-0.008	0.994	-3.48E-08	count	1
TMEM181	-18.3097913	2276.054326	-0.008	0.994	-3.48E-08	count	1
MAP1LC3B2	-18.3097913	2276.054326	-0.008	0.994	-3.48E-08	count	1
RCAN2	-18.3097913	2276.054326	-0.008	0.994	-3.48E-08	count	1
DDN-AS1	-18.3097913	2276.054326	-0.008	0.994	-3.48E-08	count	1
ATRIP	-18.3097913	2276.054326	-0.008	0.994	-3.48E-08	count	1
PPARD	-18.3097913	2276.054326	-0.008	0.994	-3.48E-08	count	1
AC009961.1	-18.3097913	2276.054326	-0.008	0.994	-3.48E-08	count	1
FKBP14	-18.3097913	2276.054326	-0.008	0.994	-3.48E-08	count	1
PCYOX1	-18.3442484	1978.08088	-0.0093	0.993	-3.48E-08	count	1
CCDC80	-18.3442484	1978.08088	-0.0093	0.993	-3.48E-08	count	1
NSDHL	-18.3097177	1826.814301	-0.01	0.992	-3.48E-08	count	1
NRL	-18.3097177	1826.814301	-0.01	0.992	-3.48E-08	count	1
FARP1	-18.3097177	1826.814301	-0.01	0.992	-3.48E-08	count	1
SPIN2A	-18.3097177	1826.814301	-0.01	0.992	-3.48E-08	count	1
FCRLB	-18.2504884	2123.722878	-0.0086	0.993	-3.48E-08	count	1
AP006621.3	-18.2504884	2123.722878	-0.0086	0.993	-3.48E-08	count	1
PEX12	-18.2504884	2123.722878	-0.0086	0.993	-3.48E-08	count	1
FDX2	-18.2504884	2123.722878	-0.0086	0.993	-3.48E-08	count	1
AC231981.1	-18.2504884	2123.722878	-0.0086	0.993	-3.48E-08	count	1
EEF1AKMT4	-18.2504884	2123.722878	-0.0086	0.993	-3.48E-08	count	1
GRPEL2	-18.2504884	2123.722878	-0.0086	0.993	-3.48E-08	count	1
TCF7L2	-18.2504884	2123.722878	-0.0086	0.993	-3.48E-08	count	1
SCML2	-18.2504884	2123.722878	-0.0086	0.993	-3.48E-08	count	1
C6orf163	-18.2504884	2123.722878	-0.0086	0.993	-3.48E-08	count	1
AC005332.1	-18.2504884	2123.722878	-0.0086	0.993	-3.48E-08	count	1
KHK	-18.2504884	2123.722878	-0.0086	0.993	-3.48E-08	count	1
ATP6AP1L	-18.2504884	2123.722878	-0.0086	0.993	-3.48E-08	count	1
GPRC5D-AS1	-18.2504884	2123.722878	-0.0086	0.993	-3.48E-08	count	1
LINC00663	-18.2504884	2123.722878	-0.0086	0.993	-3.48E-08	count	1
GZMM	-18.2504884	2123.722878	-0.0086	0.993	-3.48E-08	count	1
ZNF799	-18.2504884	2123.722878	-0.0086	0.993	-3.48E-08	count	1
AC109826.1	-18.2504884	2123.722878	-0.0086	0.993	-3.48E-08	count	1
PPP2R3A	-18.2504884	2123.722878	-0.0086	0.993	-3.48E-08	count	1
CHST7	-18.2504884	2123.722878	-0.0086	0.993	-3.48E-08	count	1
ZNF496	-18.2504884	2123.722878	-0.0086	0.993	-3.48E-08	count	1
SLC8A1	-18.2504884	2123.722878	-0.0086	0.993	-3.48E-08	count	1
CCNT2-AS1	-18.2504884	2123.722878	-0.0086	0.993	-3.48E-08	count	1

MFAP5	-18.2504884	2123.722878	-0.0086	0.993	-3.48E-08	count	1
ITPK1-AS1	-18.2504884	2123.722878	-0.0086	0.993	-3.48E-08	count	1
SDR42E1	-18.2504884	2123.722878	-0.0086	0.993	-3.48E-08	count	1
FAM198B	-18.2504884	2123.722878	-0.0086	0.993	-3.48E-08	count	1
ZBTB37	-18.2504884	2123.722878	-0.0086	0.993	-3.48E-08	count	1
AC013264.1	-18.2504884	2123.722878	-0.0086	0.993	-3.48E-08	count	1
QRICH2	-18.2504884	2123.722878	-0.0086	0.993	-3.48E-08	count	1
LINC01473	-18.2504884	2123.722878	-0.0086	0.993	-3.48E-08	count	1
MAML3	-18.2504884	2123.722878	-0.0086	0.993	-3.48E-08	count	1
OLFML2A	-18.2504884	2123.722878	-0.0086	0.993	-3.48E-08	count	1
DLEC1	-18.2504884	2123.722878	-0.0086	0.993	-3.48E-08	count	1
KSR1	-18.2504884	2123.722878	-0.0086	0.993	-3.48E-08	count	1
PAGR1	-18.2504884	2123.722878	-0.0086	0.993	-3.48E-08	count	1
AC008752.2	-18.2504884	2123.722878	-0.0086	0.993	-3.48E-08	count	1
AC104596.1	-18.2504884	2123.722878	-0.0086	0.993	-3.48E-08	count	1
AC004241.1	-18.2504884	2123.722878	-0.0086	0.993	-3.48E-08	count	1
IL10	-18.2504884	2123.722878	-0.0086	0.993	-3.48E-08	count	1
AC018682.1	-18.2504884	2123.722878	-0.0086	0.993	-3.48E-08	count	1
SRGAP3	-18.2504884	2123.722878	-0.0086	0.993	-3.48E-08	count	1
AL355075.4	-18.2504884	2123.722878	-0.0086	0.993	-3.48E-08	count	1
ZNF619	-18.2504884	2123.722878	-0.0086	0.993	-3.48E-08	count	1
AC055822.1	-18.2504884	2123.722878	-0.0086	0.993	-3.48E-08	count	1
CLCN5	-18.2504884	2123.722878	-0.0086	0.993	-3.48E-08	count	1
LAPTM4B	-18.2504884	2123.722878	-0.0086	0.993	-3.48E-08	count	1
SRCAP	-18.2506906	1639.097896	-0.0111	0.991	-3.47E-08	count	1
TMEM176B	-18.2506906	1639.097896	-0.0111	0.991	-3.47E-08	count	1
ZNF324B	-18.2506906	1639.097896	-0.0111	0.991	-3.47E-08	count	1
ATXN7	-18.2506906	1639.097896	-0.0111	0.991	-3.47E-08	count	1
AC040970.1	-18.2506906	1639.097896	-0.0111	0.991	-3.47E-08	count	1
ADSL	-18.2506906	1639.097896	-0.0111	0.991	-3.47E-08	count	1
ZNF345	-18.2506906	1639.097896	-0.0111	0.991	-3.47E-08	count	1
AMMECR1	-18.2506906	1639.097896	-0.0111	0.991	-3.47E-08	count	1
LIPE-AS1	-18.2506906	1639.097896	-0.0111	0.991	-3.47E-08	count	1
FES	-18.2506906	1639.097896	-0.0111	0.991	-3.47E-08	count	1
FAM184B	-18.2506906	1639.097896	-0.0111	0.991	-3.47E-08	count	1
AC008014.1	-18.2506906	1639.097896	-0.0111	0.991	-3.47E-08	count	1
GTF2H2	-18.2506906	1639.097896	-0.0111	0.991	-3.47E-08	count	1
MAP3K21	-18.2506906	1639.097896	-0.0111	0.991	-3.47E-08	count	1
ZFYVE28	-18.2506906	1639.097896	-0.0111	0.991	-3.47E-08	count	1
RBM47	-18.1343947	1860.400131	-0.0097	0.992	-3.47E-08	count	1
DNASE1L3	-18.0468284	2557.459386	-0.0071	0.994	-3.47E-08	count	1
AP001059.2	-18.0468284	2557.459386	-0.0071	0.994	-3.47E-08	count	1
PLN	-18.0468284	2557.459386	-0.0071	0.994	-3.47E-08	count	1
GIMAP4	-18.0091417	2647.00011	-0.0068	0.995	-3.47E-08	count	1
AC019131.2	-17.976247	2571.453362	-0.007	0.994	-3.47E-08	count	1

POLE	-17.976247	2571.453362	-0.007	0.994	-3.47E-08	count	1
LMOD1	-17.976247	2571.453362	-0.007	0.994	-3.47E-08	count	1
ZNF785	-18.0054582	2088.308662	-0.0086	0.993	-3.47E-08	count	1
ZNF607	-18.0054582	2088.308662	-0.0086	0.993	-3.47E-08	count	1
IGLC5	-17.9796232	2450.394825	-0.0073	0.994	-3.47E-08	count	1
C3AR1	-17.9796232	2450.394825	-0.0073	0.994	-3.47E-08	count	1
AC010969.2	-17.9796232	2450.394825	-0.0073	0.994	-3.47E-08	count	1
AL390728.5	-17.9255695	2470.641666	-0.0073	0.994	-3.47E-08	count	1
HEXIM2	-17.9255695	2470.641666	-0.0073	0.994	-3.47E-08	count	1
AC019197.1	-17.9255695	2470.641666	-0.0073	0.994	-3.47E-08	count	1
ITGB5	-17.9255695	2470.641666	-0.0073	0.994	-3.47E-08	count	1
SLC35F6	-17.9255695	2470.641666	-0.0073	0.994	-3.47E-08	count	1
HSF5	-17.9255695	2470.641666	-0.0073	0.994	-3.47E-08	count	1
LMBR1	-17.9255695	2470.641666	-0.0073	0.994	-3.47E-08	count	1
GSTO2	-17.9255695	2470.641666	-0.0073	0.994	-3.47E-08	count	1
C12orf60	-17.9255695	2470.641666	-0.0073	0.994	-3.47E-08	count	1
APBB1	-17.9255695	2470.641666	-0.0073	0.994	-3.47E-08	count	1
RAB25	-17.9255695	2470.641666	-0.0073	0.994	-3.47E-08	count	1
CTBP2	-17.9255695	2470.641666	-0.0073	0.994	-3.47E-08	count	1
STK11IP	-17.9255695	2470.641666	-0.0073	0.994	-3.47E-08	count	1
PTGIR	-17.9255695	2470.641666	-0.0073	0.994	-3.47E-08	count	1
HES6	-17.9255695	2470.641666	-0.0073	0.994	-3.47E-08	count	1
CCR1	-17.9255695	2470.641666	-0.0073	0.994	-3.47E-08	count	1
ACOX3	-17.9255695	2470.641666	-0.0073	0.994	-3.47E-08	count	1
AC015967.1	-17.9255695	2470.641666	-0.0073	0.994	-3.47E-08	count	1
LYPLAL1-DT	-17.9255695	2470.641666	-0.0073	0.994	-3.47E-08	count	1
ZNF202	-17.9747142	1973.65758	-0.0091	0.993	-3.47E-08	count	1
ZNF445	-17.9787287	1811.424068	-0.0099	0.992	-3.47E-08	count	1
ZNF257	-17.9787287	1811.424068	-0.0099	0.992	-3.47E-08	count	1
AC099778.1	-17.9253874	1829.125288	-0.0098	0.992	-3.46E-08	count	1
SPATA7	-17.9253874	1829.125288	-0.0098	0.992	-3.46E-08	count	1
IGSF22	-17.9253874	1829.125288	-0.0098	0.992	-3.46E-08	count	1
AC107884.1	-17.9253874	1829.125288	-0.0098	0.992	-3.46E-08	count	1
AC025569.1	-17.9253874	1829.125288	-0.0098	0.992	-3.46E-08	count	1
ZNF433	-17.9253874	1829.125288	-0.0098	0.992	-3.46E-08	count	1
FOCAD	-17.9253874	1829.125288	-0.0098	0.992	-3.46E-08	count	1
SPON1	-17.9253874	1829.125288	-0.0098	0.992	-3.46E-08	count	1
SIRPA	-17.9253874	1829.125288	-0.0098	0.992	-3.46E-08	count	1
TNFRSF18	-17.9253874	1829.125288	-0.0098	0.992	-3.46E-08	count	1
POFUT2	-17.9253874	1829.125288	-0.0098	0.992	-3.46E-08	count	1
GIMAP1	-17.9253874	1829.125288	-0.0098	0.992	-3.46E-08	count	1
MAP1B	-17.9253874	1829.125288	-0.0098	0.992	-3.46E-08	count	1
TMEM234	-17.9253874	1829.125288	-0.0098	0.992	-3.46E-08	count	1
ZNF585B	-17.9253874	1829.125288	-0.0098	0.992	-3.46E-08	count	1
HMMR	-17.9253874	1829.125288	-0.0098	0.992	-3.46E-08	count	1

ZSCAN12	-17.5528127	2019.790605	-0.0087	0.993	-3.46E-08	count	1
CACNA2D4	-17.4928174	2133.463129	-0.0082	0.993	-3.46E-08	count	1
PTGIS	-17.5163872	1950.92206	-0.009	0.993	-3.46E-08	count	1
ENAH	-17.5163872	1950.92206	-0.009	0.993	-3.46E-08	count	1
ZPBP2	-17.5163872	1950.92206	-0.009	0.993	-3.46E-08	count	1
TTLL1	-17.4488954	2058.214364	-0.0085	0.993	-3.46E-08	count	1
NBL1	-17.4488954	2058.214364	-0.0085	0.993	-3.46E-08	count	1
ZBTB3	-17.4488954	2058.214364	-0.0085	0.993	-3.46E-08	count	1
AL163051.1	-17.4488954	2058.214364	-0.0085	0.993	-3.46E-08	count	1
PTPRO	-17.4488954	2058.214364	-0.0085	0.993	-3.46E-08	count	1
DLGAP3	-17.4488954	2058.214364	-0.0085	0.993	-3.46E-08	count	1
HSD11B1L	-17.4488954	2058.214364	-0.0085	0.993	-3.46E-08	count	1
ACOT7	-17.4488954	2058.214364	-0.0085	0.993	-3.46E-08	count	1
C6orf99	-17.4488954	2058.214364	-0.0085	0.993	-3.46E-08	count	1
FOXJ2	-17.4488954	2058.214364	-0.0085	0.993	-3.46E-08	count	1
AGAP5	-17.3816025	1955.140307	-0.0089	0.993	-3.46E-08	count	1
AC006946.2	-17.3816025	1955.140307	-0.0089	0.993	-3.46E-08	count	1
PTCH1	-17.3816025	1955.140307	-0.0089	0.993	-3.46E-08	count	1
ERRFI1	-17.3816025	1955.140307	-0.0089	0.993	-3.46E-08	count	1
RARRES1	-17.3816025	1955.140307	-0.0089	0.993	-3.46E-08	count	1
TBX6	-17.3816025	1955.140307	-0.0089	0.993	-3.46E-08	count	1
CDC42EP4	-17.3851414	1794.728549	-0.0097	0.992	-3.46E-08	count	1
RTTN	-17.3851414	1794.728549	-0.0097	0.992	-3.46E-08	count	1
PLCG1	-17.4608552	1869.343674	-0.0093	0.993	-3.46E-08	count	1
RNLS	-17.466271	1764.90359	-0.0099	0.992	-3.46E-08	count	1
PCNX1	-0.0075254	0.5034153	-0.0149	0.988	-3.31E-08	count	1
SYS1	-0.0024575	0.2541851	-0.0097	0.992	-2.95E-08	count	1
PIGS	-0.0066279	0.7109258	-0.0093	0.993	-2.92E-08	count	1
PHF8	-0.0066279	0.610162	-0.0109	0.991	-2.92E-08	count	1
CEP95	-0.0063745	0.379181	-0.0168	0.987	-2.81E-08	count	1
GLE1	-0.0171087	0.8548275	-0.02	0.984	-2.76E-08	count	1
SLCO5A1	-0.0171087	0.9116525	-0.0188	0.985	-2.76E-08	count	1
GATD1	-0.0461735	0.8035451	-0.0575	0.954	-2.72E-08	count	1
LLGL2	-0.0461735	0.6279398	-0.0735	0.941	-2.71E-08	count	1
IKZF5	-0.0461735	0.5432509	-0.085	0.932	-2.71E-08	count	1
PISD	-0.0461735	0.5693712	-0.0811	0.935	-2.71E-08	count	1
ZNF584	-0.0461735	0.6306365	-0.0732	0.942	-2.71E-08	count	1
ZNF414	-0.0461735	0.567879	-0.0813	0.935	-2.71E-08	count	1
TM2D2	-0.0461735	0.6000787	-0.0769	0.939	-2.71E-08	count	1
C10orf143	-0.0461735	0.6000787	-0.0769	0.939	-2.71E-08	count	1
GALNT7	-0.0461735	0.5115586	-0.0903	0.928	-2.71E-08	count	1
REX1BD	-0.0020779	0.1766966	-0.0118	0.991	-2.49E-08	count	1
ZFP28	-0.3338556	1.3271252	-0.2516	0.801	-2.37E-08	count	1
MFSD13A	-0.3338556	1.3271252	-0.2516	0.801	-2.37E-08	count	1
MINDY4	-0.3338556	1.3271252	-0.2516	0.801	-2.37E-08	count	1

POP1	-0.3338556	1.3271252	-0.2516	0.801	-2.37E-08	count	1
ZNF653	-0.3338556	1.3271252	-0.2516	0.801	-2.37E-08	count	1
PRRG2	-0.3338556	1.3271252	-0.2516	0.801	-2.37E-08	count	1
COL3A1	-0.3338556	1.3271252	-0.2516	0.801	-2.37E-08	count	1
ZNF862	-0.3338556	1.3271252	-0.2516	0.801	-2.37E-08	count	1
TMEM8B	-0.3338556	1.3271252	-0.2516	0.801	-2.37E-08	count	1
TOLLIP-AS1	-0.3338556	1.3271252	-0.2516	0.801	-2.37E-08	count	1
MS4A14	-0.3338556	1.3271252	-0.2516	0.801	-2.37E-08	count	1
SERPINA9	-0.3338556	1.3271252	-0.2516	0.801	-2.37E-08	count	1
PLEKHG4	-0.3338556	1.3271252	-0.2516	0.801	-2.37E-08	count	1
ZNF181	-0.3338556	1.3271252	-0.2516	0.801	-2.37E-08	count	1
IL10RB	-0.3338556	1.3271252	-0.2516	0.801	-2.37E-08	count	1
LDLRAP1	-0.3338556	1.3271252	-0.2516	0.801	-2.37E-08	count	1
PLPP3	-0.3338556	1.3271252	-0.2516	0.801	-2.37E-08	count	1
LGALSL	-0.3338556	1.3271252	-0.2516	0.801	-2.37E-08	count	1
LINC01800	-0.3338556	1.3271252	-0.2516	0.801	-2.37E-08	count	1
ARL6	-0.3338556	1.3271252	-0.2516	0.801	-2.37E-08	count	1
GFM2	-0.3338556	1.3271252	-0.2516	0.801	-2.37E-08	count	1
NUDT12	-0.3338556	1.3271252	-0.2516	0.801	-2.37E-08	count	1
PILRA	-0.3338556	1.3271252	-0.2516	0.801	-2.37E-08	count	1
AC004918.1	-0.3338556	1.3271252	-0.2516	0.801	-2.37E-08	count	1
XKR9	-0.3338556	1.3271252	-0.2516	0.801	-2.37E-08	count	1
EFEMP2	-0.3338556	1.3271252	-0.2516	0.801	-2.37E-08	count	1
NLRX1	-0.3338556	1.3271252	-0.2516	0.801	-2.37E-08	count	1
ARHGEF40	-0.3338556	1.3271252	-0.2516	0.801	-2.37E-08	count	1
CCDC144A	-0.3338556	1.3271252	-0.2516	0.801	-2.37E-08	count	1
ZNF286B	-0.3338556	1.3271252	-0.2516	0.801	-2.37E-08	count	1
FAAP24	-0.3338556	1.3271252	-0.2516	0.801	-2.37E-08	count	1
ZNF28	-0.3338556	1.3271252	-0.2516	0.801	-2.37E-08	count	1
LILRB4	-0.3338556	1.3271252	-0.2516	0.801	-2.37E-08	count	1
LSS	-0.3338556	1.3271252	-0.2516	0.801	-2.37E-08	count	1
AL592494.3	-0.3338556	1.3271252	-0.2516	0.801	-2.37E-08	count	1
CLEC20A	-0.3338556	1.3271252	-0.2516	0.801	-2.37E-08	count	1
CD8B	-0.3338556	1.3271252	-0.2516	0.801	-2.37E-08	count	1
IGKV2D-29	-0.3338556	1.3271252	-0.2516	0.801	-2.37E-08	count	1
IL18R1	-0.3338556	1.3271252	-0.2516	0.801	-2.37E-08	count	1
VIPR1	-0.3338556	1.3271252	-0.2516	0.801	-2.37E-08	count	1
AC097534.2	-0.3338556	1.3271252	-0.2516	0.801	-2.37E-08	count	1
ICA1	-0.3338556	1.3271252	-0.2516	0.801	-2.37E-08	count	1
NFE2L3	-0.3338556	1.3271252	-0.2516	0.801	-2.37E-08	count	1
THEM6	-0.3338556	1.3271252	-0.2516	0.801	-2.37E-08	count	1
CNTLN	-0.3338556	1.3271252	-0.2516	0.801	-2.37E-08	count	1
ST6GALNAC6	-0.3338556	1.3271252	-0.2516	0.801	-2.37E-08	count	1
RXRA	-0.3338556	1.3271252	-0.2516	0.801	-2.37E-08	count	1
HARBI1	-0.3338556	1.3271252	-0.2516	0.801	-2.37E-08	count	1

TNKS2-AS1	-0.3338556	1.3271252	-0.2516	0.801	-2.37E-08	count	1
KCNA5	-0.3338556	1.3271252	-0.2516	0.801	-2.37E-08	count	1
LINC02422	-0.3338556	1.3271252	-0.2516	0.801	-2.37E-08	count	1
STAC3	-0.3338556	1.3271252	-0.2516	0.801	-2.37E-08	count	1
IGHV4-34	-0.3338556	1.3271252	-0.2516	0.801	-2.37E-08	count	1
CHRFAM7A	-0.3338556	1.3271252	-0.2516	0.801	-2.37E-08	count	1
AC068338.2	-0.3338556	1.3271252	-0.2516	0.801	-2.37E-08	count	1
BRICD5	-0.3338556	1.3271252	-0.2516	0.801	-2.37E-08	count	1
NOMO3	-0.3338556	1.3271252	-0.2516	0.801	-2.37E-08	count	1
AC105105.1	-0.3338556	1.3271252	-0.2516	0.801	-2.37E-08	count	1
NECTIN2	-0.3338556	1.3271252	-0.2516	0.801	-2.37E-08	count	1
DCLRE1B	-0.3338556	1.3271252	-0.2516	0.801	-2.37E-08	count	1
RGS13	-0.3338556	1.3271252	-0.2516	0.801	-2.37E-08	count	1
EMILIN1	-0.3338556	1.3271252	-0.2516	0.801	-2.37E-08	count	1
CMTM8	-0.3338556	1.3271252	-0.2516	0.801	-2.37E-08	count	1
DGKQ	-0.3338556	1.3271252	-0.2516	0.801	-2.37E-08	count	1
EREG	-0.3338556	1.3271252	-0.2516	0.801	-2.37E-08	count	1
PPARGC1B	-0.3338556	1.3271252	-0.2516	0.801	-2.37E-08	count	1
HSPA1L	-0.3338556	1.3271252	-0.2516	0.801	-2.37E-08	count	1
VPS52	-0.3338556	1.3271252	-0.2516	0.801	-2.37E-08	count	1
STEAP1	-0.3338556	1.3271252	-0.2516	0.801	-2.37E-08	count	1
VSIG1	-0.3338556	1.3271252	-0.2516	0.801	-2.37E-08	count	1
AL512625.2	-0.3338556	1.3271252	-0.2516	0.801	-2.37E-08	count	1
AP000442.2	-0.3338556	1.3271252	-0.2516	0.801	-2.37E-08	count	1
AP5B1	-0.3338556	1.3271252	-0.2516	0.801	-2.37E-08	count	1
FAM86C1	-0.3338556	1.3271252	-0.2516	0.801	-2.37E-08	count	1
MPP7	-0.3338556	1.3271252	-0.2516	0.801	-2.37E-08	count	1
AVPI1	-0.3338556	1.3271252	-0.2516	0.801	-2.37E-08	count	1
TAS2R14	-0.3338556	1.3271252	-0.2516	0.801	-2.37E-08	count	1
TCTN2	-0.3338556	1.3271252	-0.2516	0.801	-2.37E-08	count	1
SLC25A30	-0.3338556	1.3271252	-0.2516	0.801	-2.37E-08	count	1
CDKL1	-0.3338556	1.3271252	-0.2516	0.801	-2.37E-08	count	1
GALC	-0.3338556	1.3271252	-0.2516	0.801	-2.37E-08	count	1
IGHV2-5	-0.3338556	1.3271252	-0.2516	0.801	-2.37E-08	count	1
POLG	-0.3338556	1.3271252	-0.2516	0.801	-2.37E-08	count	1
ATP6V0C	-0.3338556	1.3271252	-0.2516	0.801	-2.37E-08	count	1
AC106739.1	-0.3338556	1.3271252	-0.2516	0.801	-2.37E-08	count	1
AC009093.2	-0.3338556	1.3271252	-0.2516	0.801	-2.37E-08	count	1
PSKH1	-0.3338556	1.3271252	-0.2516	0.801	-2.37E-08	count	1
AC009113.1	-0.3338556	1.3271252	-0.2516	0.801	-2.37E-08	count	1
KCTD11	-0.3338556	1.3271252	-0.2516	0.801	-2.37E-08	count	1
LINC02210-CRHR1	-0.3338556	1.3271252	-0.2516	0.801	-2.37E-08	count	1
SMG8	-0.3338556	1.3271252	-0.2516	0.801	-2.37E-08	count	1
AC127496.5	-0.3338556	1.3271252	-0.2516	0.801	-2.37E-08	count	1
KAT14	-0.3338556	1.3271252	-0.2516	0.801	-2.37E-08	count	1

ZNF599	-0.3338556	1.3271252	-0.2516	0.801	-2.37E-08	count	1
SERHL2	-0.3338556	1.3271252	-0.2516	0.801	-2.37E-08	count	1
KLHDC9	-0.3338556	1.3271252	-0.2516	0.801	-2.37E-08	count	1
RNASEL	-0.3338556	1.3271252	-0.2516	0.801	-2.37E-08	count	1
ZNF512	-0.3338556	1.3271252	-0.2516	0.801	-2.37E-08	count	1
CYBRD1	-0.3338556	1.3271252	-0.2516	0.801	-2.37E-08	count	1
NAP1L5	-0.3338556	1.3271252	-0.2516	0.801	-2.37E-08	count	1
C6orf47-AS1	-0.3338556	1.3271252	-0.2516	0.801	-2.37E-08	count	1
HLA-DQB2	-0.3338556	1.3271252	-0.2516	0.801	-2.37E-08	count	1
AC087672.2	-0.3338556	1.3271252	-0.2516	0.801	-2.37E-08	count	1
ALDH1B1	-0.3338556	1.3271252	-0.2516	0.801	-2.37E-08	count	1
OLFM1	-0.3338556	1.3271252	-0.2516	0.801	-2.37E-08	count	1
DHCR7	-0.3338556	1.3271252	-0.2516	0.801	-2.37E-08	count	1
AL132656.2	-0.3338556	1.3271252	-0.2516	0.801	-2.37E-08	count	1
RAD52	-0.3338556	1.3271252	-0.2516	0.801	-2.37E-08	count	1
H3F3C	-0.3338556	1.3271252	-0.2516	0.801	-2.37E-08	count	1
ENTPD5	-0.3338556	1.3271252	-0.2516	0.801	-2.37E-08	count	1
ZKSCAN2	-0.3338556	1.3271252	-0.2516	0.801	-2.37E-08	count	1
C17orf97	-0.3338556	1.3271252	-0.2516	0.801	-2.37E-08	count	1
AC002091.2	-0.3338556	1.3271252	-0.2516	0.801	-2.37E-08	count	1
PLVAP	-0.3338556	1.3271252	-0.2516	0.801	-2.37E-08	count	1
ZNF429	-0.3338556	1.3271252	-0.2516	0.801	-2.37E-08	count	1
ATP5MGL	-0.3338556	1.3271252	-0.2516	0.801	-2.37E-08	count	1
RTL6	-0.3338556	1.3271252	-0.2516	0.801	-2.37E-08	count	1
AP001059.3	-0.3338556	1.3271252	-0.2516	0.801	-2.37E-08	count	1
PEX14	-0.3338556	1.3271252	-0.2516	0.801	-2.37E-08	count	1
CPT2	-0.3338556	1.3271252	-0.2516	0.801	-2.37E-08	count	1
ZEB2-AS1	-0.3338556	1.3271252	-0.2516	0.801	-2.37E-08	count	1
CYP27A1	-0.3338556	1.3271252	-0.2516	0.801	-2.37E-08	count	1
AC062017.1	-0.3338556	1.3271252	-0.2516	0.801	-2.37E-08	count	1
SETMAR	-0.3338556	1.3271252	-0.2516	0.801	-2.37E-08	count	1
AMACR	-0.3338556	1.3271252	-0.2516	0.801	-2.37E-08	count	1
AGER	-0.3338556	1.3271252	-0.2516	0.801	-2.37E-08	count	1
AC004466.1	-0.3338556	1.3271252	-0.2516	0.801	-2.37E-08	count	1
PARP2	-0.3338556	1.3271252	-0.2516	0.801	-2.37E-08	count	1
HYPK	-0.3338556	1.3271252	-0.2516	0.801	-2.37E-08	count	1
AC093512.1	-0.3338556	1.3271252	-0.2516	0.801	-2.37E-08	count	1
AC007728.2	-0.3338556	1.3271252	-0.2516	0.801	-2.37E-08	count	1
LZTS3	-0.3338556	1.3271252	-0.2516	0.801	-2.37E-08	count	1
MOCS3	-0.3338556	1.3271252	-0.2516	0.801	-2.37E-08	count	1
LARGE1	-0.3338556	1.3271252	-0.2516	0.801	-2.37E-08	count	1
FP565260.1	-0.3338556	1.3271252	-0.2516	0.801	-2.37E-08	count	1
MCM3AP-AS1	-0.3338556	1.3271252	-0.2516	0.801	-2.37E-08	count	1
TTC22	-0.3338556	1.3271252	-0.2516	0.801	-2.37E-08	count	1
ARF4-AS1	-0.3338556	1.3271252	-0.2516	0.801	-2.37E-08	count	1

AL355338.1	-0.3338556	1.3271252	-0.2516	0.801	-2.37E-08	count	1
NDRG2	-0.3338556	1.3271252	-0.2516	0.801	-2.37E-08	count	1
USP40	-0.3338556	1.3271252	-0.2516	0.801	-2.37E-08	count	1
EFCAB12	-0.3338556	1.3271252	-0.2516	0.801	-2.37E-08	count	1
RGS12	-0.3338556	1.3271252	-0.2516	0.801	-2.37E-08	count	1
NEXMIF	-0.3338556	1.3271252	-0.2516	0.801	-2.37E-08	count	1
ARMCX5	-0.3338556	1.3271252	-0.2516	0.801	-2.37E-08	count	1
ENPP2	-0.3338556	1.3271252	-0.2516	0.801	-2.37E-08	count	1
REM2	-0.3338556	1.3271252	-0.2516	0.801	-2.37E-08	count	1
AC068446.2	-0.3338556	1.3271252	-0.2516	0.801	-2.37E-08	count	1
ZNF283	-0.3338556	1.3271252	-0.2516	0.801	-2.37E-08	count	1
CHKB	-0.3338556	1.3271252	-0.2516	0.801	-2.37E-08	count	1
MARK1	-0.3338556	1.3271252	-0.2516	0.801	-2.37E-08	count	1
AC012360.3	-0.3338556	1.3271252	-0.2516	0.801	-2.37E-08	count	1
NEB	-0.3338556	1.3271252	-0.2516	0.801	-2.37E-08	count	1
FBLN2	-0.3338556	1.3271252	-0.2516	0.801	-2.37E-08	count	1
POLR3G	-0.3338556	1.3271252	-0.2516	0.801	-2.37E-08	count	1
RAD54B	-0.3338556	1.3271252	-0.2516	0.801	-2.37E-08	count	1
CCL21	-0.3338556	1.3271252	-0.2516	0.801	-2.37E-08	count	1
AP000880.1	-0.3338556	1.3271252	-0.2516	0.801	-2.37E-08	count	1
CCT6B	-0.3338556	1.3271252	-0.2516	0.801	-2.37E-08	count	1
AL021707.6	-0.3338556	1.3271252	-0.2516	0.801	-2.37E-08	count	1
SUSD1	-0.3338556	1.3271252	-0.2516	0.801	-2.37E-08	count	1
ZBTB26	-0.3338556	1.3271252	-0.2516	0.801	-2.37E-08	count	1
MIR210HG	-0.3338556	1.3271252	-0.2516	0.801	-2.37E-08	count	1
HSD17B1	-0.3338556	1.3271252	-0.2516	0.801	-2.37E-08	count	1
PHLDB2	-0.3338556	1.3271252	-0.2516	0.801	-2.37E-08	count	1
CDCA7L	-0.0053713	0.4031569	-0.0133	0.989	-2.37E-08	count	1
BMT2	-0.110712	0.8051355	-0.1375	0.891	-2.33E-08	count	1
CPSF4	-0.110712	0.6872389	-0.1611	0.872	-2.33E-08	count	1
RIOX1	-0.110712	0.7517547	-0.1473	0.883	-2.33E-08	count	1
USP51	-0.110712	0.7517547	-0.1473	0.883	-2.33E-08	count	1
GDAP1	-0.110712	0.7517547	-0.1473	0.883	-2.33E-08	count	1
ZNF611	-0.110712	0.7581984	-0.146	0.884	-2.33E-08	count	1
HSPA12A	-0.110712	0.7581984	-0.146	0.884	-2.33E-08	count	1
INPPL1	-0.110712	0.8111553	-0.1365	0.891	-2.33E-08	count	1
SGCE	-0.110712	0.8111553	-0.1365	0.891	-2.33E-08	count	1
PPP5C	-0.110712	0.6942817	-0.1595	0.873	-2.33E-08	count	1
HEMK1	-0.110712	0.6942817	-0.1595	0.873	-2.33E-08	count	1
ECSIT	-0.0042114	0.4445962	-0.0095	0.992	-1.86E-08	count	1
BTLA	-0.0042114	0.4318882	-0.0098	0.992	-1.86E-08	count	1
SLTM	-0.0012926	0.1773915	-0.0073	0.994	-1.55E-08	count	1
AP4S1	-0.0094839	0.8182871	-0.0116	0.991	-1.53E-08	count	1
UPRT	-0.0094839	0.8402896	-0.0113	0.991	-1.53E-08	count	1
ZDHHC7	-0.0094839	0.8826508	-0.0107	0.991	-1.53E-08	count	1

REC8	-0.0094839	0.8818122	-0.0108	0.991	-1.53E-08	count	1
KLHL32	-18.8458926	3846.740106	-0.0049	0.996	-1.27E-08	count	1
LANCL2	-18.8458926	3846.740106	-0.0049	0.996	-1.27E-08	count	1
AC022540.1	-18.8458926	3846.740106	-0.0049	0.996	-1.27E-08	count	1
ACSM3	-18.8458926	3846.740106	-0.0049	0.996	-1.27E-08	count	1
CDH11	-18.8458926	3846.740106	-0.0049	0.996	-1.27E-08	count	1
LILRB3	-18.8458926	3846.740106	-0.0049	0.996	-1.27E-08	count	1
AC233755.2	-18.8458926	3846.740106	-0.0049	0.996	-1.27E-08	count	1
TRBV7-2	-18.8458926	3846.740106	-0.0049	0.996	-1.27E-08	count	1
AC024060.1	-18.8458926	3846.740106	-0.0049	0.996	-1.27E-08	count	1
AC092849.1	-18.8458926	3846.740106	-0.0049	0.996	-1.27E-08	count	1
HMGA1P4	-18.8458926	3846.740106	-0.0049	0.996	-1.27E-08	count	1
AL137779.1	-18.8458926	3846.740106	-0.0049	0.996	-1.27E-08	count	1
FAM110D	-18.8458926	3846.740106	-0.0049	0.996	-1.27E-08	count	1
FAM8A1	-18.8458926	3846.740106	-0.0049	0.996	-1.27E-08	count	1
STEAP1B	-18.8458926	3846.740106	-0.0049	0.996	-1.27E-08	count	1
AC145207.2	-18.8458926	3846.740106	-0.0049	0.996	-1.27E-08	count	1
IGLV3-19	-18.8458926	3846.740106	-0.0049	0.996	-1.27E-08	count	1
ZNF70	-18.8458926	3846.740106	-0.0049	0.996	-1.27E-08	count	1
ZKSCAN3	-18.8458926	3846.740106	-0.0049	0.996	-1.27E-08	count	1
AC083862.2	-18.8458926	3846.740106	-0.0049	0.996	-1.27E-08	count	1
ADAMTS17	-18.8458926	3846.740106	-0.0049	0.996	-1.27E-08	count	1
ERI2	-18.8458926	3846.740106	-0.0049	0.996	-1.27E-08	count	1
GINS1	-18.8458926	3846.740106	-0.0049	0.996	-1.27E-08	count	1
ZNF30	-18.8458926	3846.740106	-0.0049	0.996	-1.27E-08	count	1
AC007228.2	-18.8458926	3846.740106	-0.0049	0.996	-1.27E-08	count	1
ANTXR1	-18.8458926	3846.740106	-0.0049	0.996	-1.27E-08	count	1
AC004834.1	-18.8458926	3846.740106	-0.0049	0.996	-1.27E-08	count	1
TCEAL2	-18.8458926	3846.740106	-0.0049	0.996	-1.27E-08	count	1
ADCK5	-18.8458926	3846.740106	-0.0049	0.996	-1.27E-08	count	1
ZNF404	-18.8458926	3846.740106	-0.0049	0.996	-1.27E-08	count	1
AL589666.1	-18.8458926	3846.740106	-0.0049	0.996	-1.27E-08	count	1
SLC43A1	-18.8458926	3846.740106	-0.0049	0.996	-1.27E-08	count	1
RASSF4	-18.8458926	3846.740106	-0.0049	0.996	-1.27E-08	count	1
CASTOR2	-18.8458926	3846.740106	-0.0049	0.996	-1.27E-08	count	1
ITGBL1	-18.8458926	3846.740106	-0.0049	0.996	-1.27E-08	count	1
AC017083.1	-18.8458926	3846.740106	-0.0049	0.996	-1.27E-08	count	1
IDH1-AS1	-18.8458926	3846.740106	-0.0049	0.996	-1.27E-08	count	1
CACNA2D3	-18.8458926	3846.740106	-0.0049	0.996	-1.27E-08	count	1
FZD6	-18.8458926	3846.740106	-0.0049	0.996	-1.27E-08	count	1
AP003392.3	-18.8458926	3846.740106	-0.0049	0.996	-1.27E-08	count	1
AL136295.5	-18.8458926	3846.740106	-0.0049	0.996	-1.27E-08	count	1
IGLV3-25	-18.8458926	3846.740106	-0.0049	0.996	-1.27E-08	count	1
C21orf58	-18.8458926	3846.740106	-0.0049	0.996	-1.27E-08	count	1
KCND3	-18.8458926	3846.740106	-0.0049	0.996	-1.27E-08	count	1

XCL1	-18.8458926	3846.740106	-0.0049	0.996	-1.27E-08	count	1
IGKV2D-28	-18.8458926	3846.740106	-0.0049	0.996	-1.27E-08	count	1
FHL3	-18.8458926	3846.740106	-0.0049	0.996	-1.27E-08	count	1
CHPF	-18.8458926	3846.740106	-0.0049	0.996	-1.27E-08	count	1
ABTB2	-18.8458926	3846.740106	-0.0049	0.996	-1.27E-08	count	1
TANC2	-18.8458926	3846.740106	-0.0049	0.996	-1.27E-08	count	1
LINC01415	-18.8458926	3846.740106	-0.0049	0.996	-1.27E-08	count	1
SLC35G2	-18.8458926	3846.740106	-0.0049	0.996	-1.27E-08	count	1
AC090061.1	-18.8458926	3846.740106	-0.0049	0.996	-1.27E-08	count	1
MAPKBP1	-18.8458926	3846.740106	-0.0049	0.996	-1.27E-08	count	1
MT1G	-18.8458926	3846.740106	-0.0049	0.996	-1.27E-08	count	1
AL353807.2	-18.8458926	3846.740106	-0.0049	0.996	-1.27E-08	count	1
PRRT2	-18.8458926	3846.740106	-0.0049	0.996	-1.27E-08	count	1
GPR52	-18.8458926	3846.740106	-0.0049	0.996	-1.27E-08	count	1
STON1	-18.8458926	3846.740106	-0.0049	0.996	-1.27E-08	count	1
ROBO1	-18.8458926	3846.740106	-0.0049	0.996	-1.27E-08	count	1
HIST1H3A	-18.8458926	3846.740106	-0.0049	0.996	-1.27E-08	count	1
SMIM10L2B	-18.8458926	3846.740106	-0.0049	0.996	-1.27E-08	count	1
NOL6	-18.8458926	3846.740106	-0.0049	0.996	-1.27E-08	count	1
DOLPP1	-18.8458926	3846.740106	-0.0049	0.996	-1.27E-08	count	1
AC011603.2	-18.8458926	3846.740106	-0.0049	0.996	-1.27E-08	count	1
AC011611.4	-18.8458926	3846.740106	-0.0049	0.996	-1.27E-08	count	1
FNTB	-18.8458926	3846.740106	-0.0049	0.996	-1.27E-08	count	1
C2CD4B	-18.8458926	3846.740106	-0.0049	0.996	-1.27E-08	count	1
TP53INP2	-18.8458926	3846.740106	-0.0049	0.996	-1.27E-08	count	1
LINC01094	-18.8458926	3846.740106	-0.0049	0.996	-1.27E-08	count	1
STK24-AS1	-18.8458926	3846.740106	-0.0049	0.996	-1.27E-08	count	1
TCAP	-18.8458926	3846.740106	-0.0049	0.996	-1.27E-08	count	1
NNAT	-18.8458926	3846.740106	-0.0049	0.996	-1.27E-08	count	1
INKA2	-18.8458926	3846.740106	-0.0049	0.996	-1.27E-08	count	1
KIFC3	-18.8458926	3846.740106	-0.0049	0.996	-1.27E-08	count	1
RBP7	-18.8458926	3846.740106	-0.0049	0.996	-1.27E-08	count	1
NFIA	-18.8458926	3846.740106	-0.0049	0.996	-1.27E-08	count	1
SHROOM3	-18.8458926	3846.740106	-0.0049	0.996	-1.27E-08	count	1
PRDM6	-18.8458926	3846.740106	-0.0049	0.996	-1.27E-08	count	1
ULBP3	-18.8458926	3846.740106	-0.0049	0.996	-1.27E-08	count	1
GPC4	-18.8458926	3846.740106	-0.0049	0.996	-1.27E-08	count	1
TNFRSF11B	-18.8458926	3846.740106	-0.0049	0.996	-1.27E-08	count	1
CRAT	-18.8458926	3846.740106	-0.0049	0.996	-1.27E-08	count	1
DNAJC9-AS1	-18.8458926	3846.740106	-0.0049	0.996	-1.27E-08	count	1
TNFRSF1A	-18.8458926	3846.740106	-0.0049	0.996	-1.27E-08	count	1
AC009053.2	-18.8458926	3846.740106	-0.0049	0.996	-1.27E-08	count	1
AC004771.1	-18.8458926	3846.740106	-0.0049	0.996	-1.27E-08	count	1
DBNDD2	-18.8458926	3846.740106	-0.0049	0.996	-1.27E-08	count	1
ZBED6	-18.8458926	3846.740106	-0.0049	0.996	-1.27E-08	count	1

TMEM177	-18.8458926	3846.740106	-0.0049	0.996	-1.27E-08	count	1
AC108471.2	-18.8458926	3846.740106	-0.0049	0.996	-1.27E-08	count	1
LRIG3	-18.8458926	3846.740106	-0.0049	0.996	-1.27E-08	count	1
AL121809.1	-18.8458926	3846.740106	-0.0049	0.996	-1.27E-08	count	1
AL118558.3	-18.8458926	3846.740106	-0.0049	0.996	-1.27E-08	count	1
GOLGA8H	-18.8458926	3846.740106	-0.0049	0.996	-1.27E-08	count	1
AC015819.2	-18.8458926	3846.740106	-0.0049	0.996	-1.27E-08	count	1
AL451085.1	-18.8458926	3846.740106	-0.0049	0.996	-1.27E-08	count	1
TDRD15	-18.8458926	3846.740106	-0.0049	0.996	-1.27E-08	count	1
AC009501.1	-18.8458926	3846.740106	-0.0049	0.996	-1.27E-08	count	1
SEPSECS-AS1	-18.8458926	3846.740106	-0.0049	0.996	-1.27E-08	count	1
HPGD	-18.8458926	3846.740106	-0.0049	0.996	-1.27E-08	count	1
PPIC	-18.8458926	3846.740106	-0.0049	0.996	-1.27E-08	count	1
BSPRY	-18.8458926	3846.740106	-0.0049	0.996	-1.27E-08	count	1
DLG2	-18.8458926	3846.740106	-0.0049	0.996	-1.27E-08	count	1
TGFB3	-18.8458926	3846.740106	-0.0049	0.996	-1.27E-08	count	1
KLK2	-18.8458926	3846.740106	-0.0049	0.996	-1.27E-08	count	1
MUC1	-18.8458926	3846.740106	-0.0049	0.996	-1.27E-08	count	1
ITPR1-DT	-18.8458926	3846.740106	-0.0049	0.996	-1.27E-08	count	1
AC008522.1	-18.8458926	3846.740106	-0.0049	0.996	-1.27E-08	count	1
TFR2	-18.8458926	3846.740106	-0.0049	0.996	-1.27E-08	count	1
LINC02547	-18.8458926	3846.740106	-0.0049	0.996	-1.27E-08	count	1
UBAP1L	-18.8458926	3846.740106	-0.0049	0.996	-1.27E-08	count	1
EDC4	-18.8458926	3846.740106	-0.0049	0.996	-1.27E-08	count	1
MYRIP	-18.8458926	3846.740106	-0.0049	0.996	-1.27E-08	count	1
USP46-AS1	-18.8458926	3846.740106	-0.0049	0.996	-1.27E-08	count	1
SND1-IT1	-18.8458926	3846.740106	-0.0049	0.996	-1.27E-08	count	1
CDK1	-18.8458926	3846.740106	-0.0049	0.996	-1.27E-08	count	1
TPSAB1	-18.8458926	3846.740106	-0.0049	0.996	-1.27E-08	count	1
AC025283.2	-18.8458926	3846.740106	-0.0049	0.996	-1.27E-08	count	1
C9orf43	-18.8458926	3846.740106	-0.0049	0.996	-1.27E-08	count	1
MICAL2	-18.8458926	3846.740106	-0.0049	0.996	-1.27E-08	count	1
TMEM94	-18.8458926	3846.740106	-0.0049	0.996	-1.27E-08	count	1
CP	-18.8458926	3846.740106	-0.0049	0.996	-1.27E-08	count	1
HIST1H4H	-18.8458926	3846.740106	-0.0049	0.996	-1.27E-08	count	1
DOCK5	-18.8458926	3846.740106	-0.0049	0.996	-1.27E-08	count	1
AL358472.5	-18.8458926	3846.740106	-0.0049	0.996	-1.27E-08	count	1
TMSB15B	-18.8458926	3846.740106	-0.0049	0.996	-1.27E-08	count	1
SUOX	-18.8458926	3846.740106	-0.0049	0.996	-1.27E-08	count	1
FSIP1	-18.8458926	3846.740106	-0.0049	0.996	-1.27E-08	count	1
ZNF888	-18.8458926	3846.740106	-0.0049	0.996	-1.27E-08	count	1
AC103736.1	-18.8458926	3846.740106	-0.0049	0.996	-1.27E-08	count	1
AP002360.3	-18.8458926	3846.740106	-0.0049	0.996	-1.27E-08	count	1
STAM-AS1	-18.8458926	3846.740106	-0.0049	0.996	-1.27E-08	count	1
GNA15	-18.8458926	3846.740106	-0.0049	0.996	-1.27E-08	count	1

AC090948.1	-18.8458926	3846.740106	-0.0049	0.996	-1.27E-08	count	1
LAMB2	-18.8458926	3846.740106	-0.0049	0.996	-1.27E-08	count	1
TREM1	-18.8458926	3846.740106	-0.0049	0.996	-1.27E-08	count	1
RPS6KA6	-18.8458926	3846.740106	-0.0049	0.996	-1.27E-08	count	1
AC022021.1	-18.8458926	3846.740106	-0.0049	0.996	-1.27E-08	count	1
WDR62	-18.8458926	3846.740106	-0.0049	0.996	-1.27E-08	count	1
AL121574.1	-18.8458926	3846.740106	-0.0049	0.996	-1.27E-08	count	1
SRRM3	-18.8458926	3846.740106	-0.0049	0.996	-1.27E-08	count	1
AC246817.2	-18.8458926	3846.740106	-0.0049	0.996	-1.27E-08	count	1
GATA3	-18.8458926	3846.740106	-0.0049	0.996	-1.27E-08	count	1
TIE1	-18.8458926	3846.740106	-0.0049	0.996	-1.27E-08	count	1
LIX1L-AS1	-18.8458926	3846.740106	-0.0049	0.996	-1.27E-08	count	1
PCAT6	-18.8458926	3846.740106	-0.0049	0.996	-1.27E-08	count	1
ATOH8	-18.8458926	3846.740106	-0.0049	0.996	-1.27E-08	count	1
ITGA9	-18.8458926	3846.740106	-0.0049	0.996	-1.27E-08	count	1
LINC02014	-18.8458926	3846.740106	-0.0049	0.996	-1.27E-08	count	1
LMLN	-18.8458926	3846.740106	-0.0049	0.996	-1.27E-08	count	1
ANKRD50	-18.8458926	3846.740106	-0.0049	0.996	-1.27E-08	count	1
SMAD1	-18.8458926	3846.740106	-0.0049	0.996	-1.27E-08	count	1
ANKRD31	-18.8458926	3846.740106	-0.0049	0.996	-1.27E-08	count	1
ARMC2	-18.8458926	3846.740106	-0.0049	0.996	-1.27E-08	count	1
LINC01615	-18.8458926	3846.740106	-0.0049	0.996	-1.27E-08	count	1
MTBP	-18.8458926	3846.740106	-0.0049	0.996	-1.27E-08	count	1
B3GNT10	-18.8458926	3846.740106	-0.0049	0.996	-1.27E-08	count	1
CHRNA10	-18.8458926	3846.740106	-0.0049	0.996	-1.27E-08	count	1
AP003086.1	-18.8458926	3846.740106	-0.0049	0.996	-1.27E-08	count	1
AL512770.1	-18.8458926	3846.740106	-0.0049	0.996	-1.27E-08	count	1
AC074032.1	-18.8458926	3846.740106	-0.0049	0.996	-1.27E-08	count	1
IGF1	-18.8458926	3846.740106	-0.0049	0.996	-1.27E-08	count	1
BCL2L10	-18.8458926	3846.740106	-0.0049	0.996	-1.27E-08	count	1
AC100830.1	-18.8458926	3846.740106	-0.0049	0.996	-1.27E-08	count	1
STOML1	-18.8458926	3846.740106	-0.0049	0.996	-1.27E-08	count	1
SPNS2	-18.8458926	3846.740106	-0.0049	0.996	-1.27E-08	count	1
DOCK6	-18.8458926	3846.740106	-0.0049	0.996	-1.27E-08	count	1
UPK1A	-18.8458926	3846.740106	-0.0049	0.996	-1.27E-08	count	1
LINC01424	-18.8458926	3846.740106	-0.0049	0.996	-1.27E-08	count	1
IGHEP1	-18.8458926	3846.740106	-0.0049	0.996	-1.27E-08	count	1
AC087164.1	-18.8458926	3846.740106	-0.0049	0.996	-1.27E-08	count	1
ABHD8	-18.8458926	3846.740106	-0.0049	0.996	-1.27E-08	count	1
AC006547.3	-18.8458926	3846.740106	-0.0049	0.996	-1.27E-08	count	1
S100A16	-18.8458926	3846.740106	-0.0049	0.996	-1.27E-08	count	1
CCL20	-18.8458926	3846.740106	-0.0049	0.996	-1.27E-08	count	1
EFNB2	-18.8458926	3846.740106	-0.0049	0.996	-1.27E-08	count	1
IQCK	-18.8458926	3846.740106	-0.0049	0.996	-1.27E-08	count	1
CNIH3	-18.8459109	2711.724213	-0.0069	0.994	-1.27E-08	count	1

ULK1	-18.8459109	2711.724213	-0.0069	0.994	-1.27E-08	count	1
RNF125	-18.8459109	2711.724213	-0.0069	0.994	-1.27E-08	count	1
NAGS	-18.8459109	2711.724213	-0.0069	0.994	-1.27E-08	count	1
BX255925.3	-18.8459109	2711.724213	-0.0069	0.994	-1.27E-08	count	1
ACSM1	-18.8459109	2711.724213	-0.0069	0.994	-1.27E-08	count	1
SLC14A1	-18.8459109	2711.724213	-0.0069	0.994	-1.27E-08	count	1
NDST1	-18.8459109	2711.724213	-0.0069	0.994	-1.27E-08	count	1
AL158835.1	-18.8459109	2711.724213	-0.0069	0.994	-1.27E-08	count	1
VEGFA	-18.8459109	2711.724213	-0.0069	0.994	-1.27E-08	count	1
FBLIM1	-18.8459109	2711.724213	-0.0069	0.994	-1.27E-08	count	1
LINGO3	-18.8459109	2711.724213	-0.0069	0.994	-1.27E-08	count	1
AC007686.3	-18.8459109	2711.724213	-0.0069	0.994	-1.27E-08	count	1
BRIP1	-18.8459109	2711.724213	-0.0069	0.994	-1.27E-08	count	1
AL356599.1	-18.8459109	2711.724213	-0.0069	0.994	-1.27E-08	count	1
RBPMS	-18.8459109	2711.724213	-0.0069	0.994	-1.27E-08	count	1
AC127070.1	-18.8459109	2711.724213	-0.0069	0.994	-1.27E-08	count	1
IPO13	-18.8459109	2711.724213	-0.0069	0.994	-1.27E-08	count	1
CTBP1-AS	-18.8459109	2711.724213	-0.0069	0.994	-1.27E-08	count	1
TNFAIP2	-18.8459109	2711.724213	-0.0069	0.994	-1.27E-08	count	1
MED18	-18.8459109	2711.724213	-0.0069	0.994	-1.27E-08	count	1
F13A1	-18.8459109	2711.724213	-0.0069	0.994	-1.27E-08	count	1
MIR193BHG	-18.8459109	2711.724213	-0.0069	0.994	-1.27E-08	count	1
PCTP	-18.8459109	2711.724213	-0.0069	0.994	-1.27E-08	count	1
GPRC5C	-18.8459109	2711.724213	-0.0069	0.994	-1.27E-08	count	1
ZNF749	-18.8459109	2711.724213	-0.0069	0.994	-1.27E-08	count	1
CLIP4	-18.8459109	2711.724213	-0.0069	0.994	-1.27E-08	count	1
ZNF589	-18.8459109	2711.724213	-0.0069	0.994	-1.27E-08	count	1
C11orf65	-18.8459109	2711.724213	-0.0069	0.994	-1.27E-08	count	1
BCL2L14	-18.8459109	2711.724213	-0.0069	0.994	-1.27E-08	count	1
LINC00944	-18.8459109	2711.724213	-0.0069	0.994	-1.27E-08	count	1
CEP128	-18.8459109	2711.724213	-0.0069	0.994	-1.27E-08	count	1
H1F0	-18.8459109	2711.724213	-0.0069	0.994	-1.27E-08	count	1
TNFRSF25	-18.8459109	2711.724213	-0.0069	0.994	-1.27E-08	count	1
MRC1	-18.8459109	2711.724213	-0.0069	0.994	-1.27E-08	count	1
NR2F2	-18.8459109	2711.724213	-0.0069	0.994	-1.27E-08	count	1
VSIG10L	-18.8459109	2711.724213	-0.0069	0.994	-1.27E-08	count	1
HIST1H1B	-18.8459109	2711.724213	-0.0069	0.994	-1.27E-08	count	1
KISS1R	-18.8459109	2711.724213	-0.0069	0.994	-1.27E-08	count	1
NUMBL	-18.8459109	2711.724213	-0.0069	0.994	-1.27E-08	count	1
NR1H3	-18.8459109	2711.724213	-0.0069	0.994	-1.27E-08	count	1
RSAD2	-18.8459109	2711.724213	-0.0069	0.994	-1.27E-08	count	1
DFFB	-18.8459109	2711.724213	-0.0069	0.994	-1.27E-08	count	1
NLE1	-18.8459109	2711.724213	-0.0069	0.994	-1.27E-08	count	1
CHIT1	-18.8459109	2711.724213	-0.0069	0.994	-1.27E-08	count	1
RPH3AL	-18.8459109	2711.724213	-0.0069	0.994	-1.27E-08	count	1

COL6A3	-18.8459109	2711.724213	-0.0069	0.994	-1.27E-08	count	1
RTP4	-18.8459109	2711.724213	-0.0069	0.994	-1.27E-08	count	1
AL157392.5	-18.8459109	2711.724213	-0.0069	0.994	-1.27E-08	count	1
AL450311.2	-18.8459109	2711.724213	-0.0069	0.994	-1.27E-08	count	1
IGHV3-74	-18.8459109	2711.724213	-0.0069	0.994	-1.27E-08	count	1
HIC1	-18.8459109	2711.724213	-0.0069	0.994	-1.27E-08	count	1
ZNF317	-18.8459109	2711.724213	-0.0069	0.994	-1.27E-08	count	1
NAV2	-18.8459109	2711.724213	-0.0069	0.994	-1.27E-08	count	1
PEX5	-18.8459109	2711.724213	-0.0069	0.994	-1.27E-08	count	1
BCL11B	-18.8459109	2711.724213	-0.0069	0.994	-1.27E-08	count	1
AL049597.2	-18.8459109	2711.724213	-0.0069	0.994	-1.27E-08	count	1
CDKN2C	-18.8459109	2711.724213	-0.0069	0.994	-1.27E-08	count	1
ARHGAP29	-18.8459109	2711.724213	-0.0069	0.994	-1.27E-08	count	1
SH3BP5L	-18.8459109	2711.724213	-0.0069	0.994	-1.27E-08	count	1
AL139274.2	-18.8459109	2711.724213	-0.0069	0.994	-1.27E-08	count	1
PANO1	-18.8459109	2711.724213	-0.0069	0.994	-1.27E-08	count	1
RARG	-18.8459109	2711.724213	-0.0069	0.994	-1.27E-08	count	1
LINC02324	-18.8459109	2711.724213	-0.0069	0.994	-1.27E-08	count	1
SPN	-18.8459109	2711.724213	-0.0069	0.994	-1.27E-08	count	1
DPH1	-18.8459109	2711.724213	-0.0069	0.994	-1.27E-08	count	1
KREMEN2	-18.8459109	2711.724213	-0.0069	0.994	-1.27E-08	count	1
ADNP-AS1	-18.8459109	2711.724213	-0.0069	0.994	-1.27E-08	count	1
CCDC120	-18.8459109	2711.724213	-0.0069	0.994	-1.27E-08	count	1
CTSK	-18.8459109	2711.724213	-0.0069	0.994	-1.27E-08	count	1
AP004609.3	-18.8459109	2711.724213	-0.0069	0.994	-1.27E-08	count	1
NPIPB2	-18.8459109	2711.724213	-0.0069	0.994	-1.27E-08	count	1
P2RX5-TAX1BP3	-18.8459109	2711.724213	-0.0069	0.994	-1.27E-08	count	1
C1QTNF2	-18.8459109	2711.724213	-0.0069	0.994	-1.27E-08	count	1
BBS5	-18.8459109	2711.724213	-0.0069	0.994	-1.27E-08	count	1
C18orf54	-18.8459109	2711.724213	-0.0069	0.994	-1.27E-08	count	1
CTSF	-18.8459109	2711.724213	-0.0069	0.994	-1.27E-08	count	1
PRKG1	-18.8459109	2711.724213	-0.0069	0.994	-1.27E-08	count	1
AL596325.1	-18.8459109	2711.724213	-0.0069	0.994	-1.27E-08	count	1
PLAC9	-18.8459109	2711.724213	-0.0069	0.994	-1.27E-08	count	1
ZNF781	-18.8459109	2711.724213	-0.0069	0.994	-1.27E-08	count	1
GPD1L	-18.8459109	2711.724213	-0.0069	0.994	-1.27E-08	count	1
AC005670.2	-18.8459109	2711.724213	-0.0069	0.994	-1.27E-08	count	1
SRD5A1	-18.8459109	2711.724213	-0.0069	0.994	-1.27E-08	count	1
AP002026.1	-18.8459109	2711.724213	-0.0069	0.994	-1.27E-08	count	1
USP18	-18.8459109	2711.724213	-0.0069	0.994	-1.27E-08	count	1
APLNR	-18.8459109	2711.724213	-0.0069	0.994	-1.27E-08	count	1
MYH15	-18.8459109	2711.724213	-0.0069	0.994	-1.27E-08	count	1
ZNF554	-18.8459109	2711.724213	-0.0069	0.994	-1.27E-08	count	1
AL359644.1	-18.8459109	2711.724213	-0.0069	0.994	-1.27E-08	count	1
FAM19A2	-18.8459109	2711.724213	-0.0069	0.994	-1.27E-08	count	1

ERN1	-18.8459109	2711.724213	-0.0069	0.994	-1.27E-08	count	1
FAM167B	-18.1529225	2720.296955	-0.0067	0.995	-1.27E-08	count	1
PIK3R3	-18.1529225	2720.296955	-0.0067	0.995	-1.27E-08	count	1
AL589765.4	-18.1529225	2720.296955	-0.0067	0.995	-1.27E-08	count	1
EFNA1	-18.1529225	2720.296955	-0.0067	0.995	-1.27E-08	count	1
PFKFB2	-18.1529225	2720.296955	-0.0067	0.995	-1.27E-08	count	1
KRTCAP3	-18.1529225	2720.296955	-0.0067	0.995	-1.27E-08	count	1
AC083949.1	-18.1529225	2720.296955	-0.0067	0.995	-1.27E-08	count	1
RND3	-18.1529225	2720.296955	-0.0067	0.995	-1.27E-08	count	1
AC009495.3	-18.1529225	2720.296955	-0.0067	0.995	-1.27E-08	count	1
AC009950.1	-18.1529225	2720.296955	-0.0067	0.995	-1.27E-08	count	1
PDCD1	-18.1529225	2720.296955	-0.0067	0.995	-1.27E-08	count	1
ALG1L	-18.1529225	2720.296955	-0.0067	0.995	-1.27E-08	count	1
ATP1B3-AS1	-18.1529225	2720.296955	-0.0067	0.995	-1.27E-08	count	1
SPON2	-18.1529225	2720.296955	-0.0067	0.995	-1.27E-08	count	1
SORBS2	-18.1529225	2720.296955	-0.0067	0.995	-1.27E-08	count	1
DROSHA	-18.1529225	2720.296955	-0.0067	0.995	-1.27E-08	count	1
LSM11	-18.1529225	2720.296955	-0.0067	0.995	-1.27E-08	count	1
FAM193B	-18.1529225	2720.296955	-0.0067	0.995	-1.27E-08	count	1
RPS6KA2	-18.1529225	2720.296955	-0.0067	0.995	-1.27E-08	count	1
SPDYE3	-18.1529225	2720.296955	-0.0067	0.995	-1.27E-08	count	1
AC034111.1	-18.1529225	2720.296955	-0.0067	0.995	-1.27E-08	count	1
UBXN8	-18.1529225	2720.296955	-0.0067	0.995	-1.27E-08	count	1
CTHRC1	-18.1529225	2720.296955	-0.0067	0.995	-1.27E-08	count	1
AC083843.2	-18.1529225	2720.296955	-0.0067	0.995	-1.27E-08	count	1
STX17-AS1	-18.1529225	2720.296955	-0.0067	0.995	-1.27E-08	count	1
CSNK2A3	-18.1529225	2720.296955	-0.0067	0.995	-1.27E-08	count	1
SNX15	-18.1529225	2720.296955	-0.0067	0.995	-1.27E-08	count	1
SERPINH1	-18.1529225	2720.296955	-0.0067	0.995	-1.27E-08	count	1
TEX12	-18.1529225	2720.296955	-0.0067	0.995	-1.27E-08	count	1
PBLD	-18.1529225	2720.296955	-0.0067	0.995	-1.27E-08	count	1
VAMP1	-18.1529225	2720.296955	-0.0067	0.995	-1.27E-08	count	1
AC053513.1	-18.1529225	2720.296955	-0.0067	0.995	-1.27E-08	count	1
RACGAP1	-18.1529225	2720.296955	-0.0067	0.995	-1.27E-08	count	1
LINC02381	-18.1529225	2720.296955	-0.0067	0.995	-1.27E-08	count	1
HSPB8	-18.1529225	2720.296955	-0.0067	0.995	-1.27E-08	count	1
AL136962.1	-18.1529225	2720.296955	-0.0067	0.995	-1.27E-08	count	1
AL139384.1	-18.1529225	2720.296955	-0.0067	0.995	-1.27E-08	count	1
AL139099.1	-18.1529225	2720.296955	-0.0067	0.995	-1.27E-08	count	1
LINC01569	-18.1529225	2720.296955	-0.0067	0.995	-1.27E-08	count	1
TP53TG3D	-18.1529225	2720.296955	-0.0067	0.995	-1.27E-08	count	1
VMO1	-18.1529225	2720.296955	-0.0067	0.995	-1.27E-08	count	1
AC005304.3	-18.1529225	2720.296955	-0.0067	0.995	-1.27E-08	count	1
OMG	-18.1529225	2720.296955	-0.0067	0.995	-1.27E-08	count	1
RUNDC3A	-18.1529225	2720.296955	-0.0067	0.995	-1.27E-08	count	1

SRC	-18.1529225	2720.296955	-0.0067	0.995	-1.27E-08	count	1
SLPI	-18.1529225	2720.296955	-0.0067	0.995	-1.27E-08	count	1
ZSWIM3	-18.1529225	2720.296955	-0.0067	0.995	-1.27E-08	count	1
AL121829.2	-18.1529225	2720.296955	-0.0067	0.995	-1.27E-08	count	1
ZNF442	-18.1529225	2720.296955	-0.0067	0.995	-1.27E-08	count	1
TMEM91	-18.1529225	2720.296955	-0.0067	0.995	-1.27E-08	count	1
CLEC11A	-18.1529225	2720.296955	-0.0067	0.995	-1.27E-08	count	1
BRSK1	-18.1529225	2720.296955	-0.0067	0.995	-1.27E-08	count	1
FOXD2	-18.1529225	2720.296955	-0.0067	0.995	-1.27E-08	count	1
AL353719.1	-18.1529225	2720.296955	-0.0067	0.995	-1.27E-08	count	1
OSGEPL1-AS1	-18.1529225	2720.296955	-0.0067	0.995	-1.27E-08	count	1
ADAM23	-18.1529225	2720.296955	-0.0067	0.995	-1.27E-08	count	1
SH3BP4	-18.1529225	2720.296955	-0.0067	0.995	-1.27E-08	count	1
CD5	-18.1529225	2720.296955	-0.0067	0.995	-1.27E-08	count	1
KIF26A	-18.1529225	2720.296955	-0.0067	0.995	-1.27E-08	count	1
IGHV3-73	-18.1529225	2720.296955	-0.0067	0.995	-1.27E-08	count	1
AL031705.1	-18.1529225	2720.296955	-0.0067	0.995	-1.27E-08	count	1
SGIP1	-18.1529225	2720.296955	-0.0067	0.995	-1.27E-08	count	1
F3	-18.1529225	2720.296955	-0.0067	0.995	-1.27E-08	count	1
DPT	-18.1529225	2720.296955	-0.0067	0.995	-1.27E-08	count	1
TMCC2	-18.1529225	2720.296955	-0.0067	0.995	-1.27E-08	count	1
TAF1A-AS1	-18.1529225	2720.296955	-0.0067	0.995	-1.27E-08	count	1
CAPN14	-18.1529225	2720.296955	-0.0067	0.995	-1.27E-08	count	1
AC008676.1	-18.1529225	2720.296955	-0.0067	0.995	-1.27E-08	count	1
SMARCA1	-18.1529225	2720.296955	-0.0067	0.995	-1.27E-08	count	1
C11orf74	-18.1529225	2720.296955	-0.0067	0.995	-1.27E-08	count	1
SPX	-18.1529225	2720.296955	-0.0067	0.995	-1.27E-08	count	1
MMP19	-18.1529225	2720.296955	-0.0067	0.995	-1.27E-08	count	1
AGAP2-AS1	-18.1529225	2720.296955	-0.0067	0.995	-1.27E-08	count	1
FGF7	-18.1529225	2720.296955	-0.0067	0.995	-1.27E-08	count	1
AC015813.1	-18.1529225	2720.296955	-0.0067	0.995	-1.27E-08	count	1
NAV1	-18.1529225	2720.296955	-0.0067	0.995	-1.27E-08	count	1
CXCL11	-18.1529225	2720.296955	-0.0067	0.995	-1.27E-08	count	1
PALLD	-18.1529225	2720.296955	-0.0067	0.995	-1.27E-08	count	1
DST	-18.1529225	2720.296955	-0.0067	0.995	-1.27E-08	count	1
SNTB1	-18.1529225	2720.296955	-0.0067	0.995	-1.27E-08	count	1
NRP1	-18.1529225	2720.296955	-0.0067	0.995	-1.27E-08	count	1
ZNF503	-18.1529225	2720.296955	-0.0067	0.995	-1.27E-08	count	1
KCNMA1	-18.1529225	2720.296955	-0.0067	0.995	-1.27E-08	count	1
ZNRF1	-18.1529225	2720.296955	-0.0067	0.995	-1.27E-08	count	1
CCL13	-18.1529225	2720.296955	-0.0067	0.995	-1.27E-08	count	1
PRELID3A	-18.1529225	2720.296955	-0.0067	0.995	-1.27E-08	count	1
SDCBP2	-18.1529225	2720.296955	-0.0067	0.995	-1.27E-08	count	1
NBPF1	-18.1529225	2720.296955	-0.0067	0.995	-1.27E-08	count	1
AL358472.4	-18.1529225	2720.296955	-0.0067	0.995	-1.27E-08	count	1

AL121987.2	-18.1529225	2720.296955	-0.0067	0.995	-1.27E-08	count	1
BX323046.1	-18.1529225	2720.296955	-0.0067	0.995	-1.27E-08	count	1
AC007681.1	-18.1529225	2720.296955	-0.0067	0.995	-1.27E-08	count	1
C2orf40	-18.1529225	2720.296955	-0.0067	0.995	-1.27E-08	count	1
LINC01934	-18.1529225	2720.296955	-0.0067	0.995	-1.27E-08	count	1
LRRN1	-18.1529225	2720.296955	-0.0067	0.995	-1.27E-08	count	1
THRB	-18.1529225	2720.296955	-0.0067	0.995	-1.27E-08	count	1
EOMES	-18.1529225	2720.296955	-0.0067	0.995	-1.27E-08	count	1
AC006058.1	-18.1529225	2720.296955	-0.0067	0.995	-1.27E-08	count	1
ATXN7	-18.1529225	2720.296955	-0.0067	0.995	-1.27E-08	count	1
UGDH-AS1	-18.1529225	2720.296955	-0.0067	0.995	-1.27E-08	count	1
HPSE	-18.1529225	2720.296955	-0.0067	0.995	-1.27E-08	count	1
GPRIN3	-18.1529225	2720.296955	-0.0067	0.995	-1.27E-08	count	1
SKP2	-18.1529225	2720.296955	-0.0067	0.995	-1.27E-08	count	1
SNHG4	-18.1529225	2720.296955	-0.0067	0.995	-1.27E-08	count	1
AC008429.1	-18.1529225	2720.296955	-0.0067	0.995	-1.27E-08	count	1
ATAT1	-18.1529225	2720.296955	-0.0067	0.995	-1.27E-08	count	1
TNFRSF21	-18.1529225	2720.296955	-0.0067	0.995	-1.27E-08	count	1
ZNF853	-18.1529225	2720.296955	-0.0067	0.995	-1.27E-08	count	1
CTTNBP2	-18.1529225	2720.296955	-0.0067	0.995	-1.27E-08	count	1
PODXL	-18.1529225	2720.296955	-0.0067	0.995	-1.27E-08	count	1
NDUFB2-AS1	-18.1529225	2720.296955	-0.0067	0.995	-1.27E-08	count	1
ARMCX1	-18.1529225	2720.296955	-0.0067	0.995	-1.27E-08	count	1
YTHDF3-AS1	-18.1529225	2720.296955	-0.0067	0.995	-1.27E-08	count	1
PHLDB1	-18.1529225	2720.296955	-0.0067	0.995	-1.27E-08	count	1
PLXDC2	-18.1529225	2720.296955	-0.0067	0.995	-1.27E-08	count	1
PLAU	-18.1529225	2720.296955	-0.0067	0.995	-1.27E-08	count	1
CD163	-18.1529225	2720.296955	-0.0067	0.995	-1.27E-08	count	1
AC027288.3	-18.1529225	2720.296955	-0.0067	0.995	-1.27E-08	count	1
SOCS2	-18.1529225	2720.296955	-0.0067	0.995	-1.27E-08	count	1
RCBTB2	-18.1529225	2720.296955	-0.0067	0.995	-1.27E-08	count	1
PLEKHH1	-18.1529225	2720.296955	-0.0067	0.995	-1.27E-08	count	1
MAP3K9	-18.1529225	2720.296955	-0.0067	0.995	-1.27E-08	count	1
LINC02285	-18.1529225	2720.296955	-0.0067	0.995	-1.27E-08	count	1
AC091057.6	-18.1529225	2720.296955	-0.0067	0.995	-1.27E-08	count	1
FBN1	-18.1529225	2720.296955	-0.0067	0.995	-1.27E-08	count	1
AC023906.5	-18.1529225	2720.296955	-0.0067	0.995	-1.27E-08	count	1
LCTL	-18.1529225	2720.296955	-0.0067	0.995	-1.27E-08	count	1
TMC3-AS1	-18.1529225	2720.296955	-0.0067	0.995	-1.27E-08	count	1
AL031600.3	-18.1529225	2720.296955	-0.0067	0.995	-1.27E-08	count	1
TEDC2	-18.1529225	2720.296955	-0.0067	0.995	-1.27E-08	count	1
AC012645.1	-18.1529225	2720.296955	-0.0067	0.995	-1.27E-08	count	1
CES4A	-18.1529225	2720.296955	-0.0067	0.995	-1.27E-08	count	1
AC087500.2	-18.1529225	2720.296955	-0.0067	0.995	-1.27E-08	count	1
CLDN7	-18.1529225	2720.296955	-0.0067	0.995	-1.27E-08	count	1

KRT17	-18.1529225	2720.296955	-0.0067	0.995	-1.27E-08	count	1
COPZ2	-18.1529225	2720.296955	-0.0067	0.995	-1.27E-08	count	1
AC011825.2	-18.1529225	2720.296955	-0.0067	0.995	-1.27E-08	count	1
POFUT1	-18.1529225	2720.296955	-0.0067	0.995	-1.27E-08	count	1
TRERNA1	-18.1529225	2720.296955	-0.0067	0.995	-1.27E-08	count	1
CRB3	-18.1529225	2720.296955	-0.0067	0.995	-1.27E-08	count	1
TTTY14	-18.1529225	2720.296955	-0.0067	0.995	-1.27E-08	count	1
IGLV2-11	-18.1529225	2720.296955	-0.0067	0.995	-1.27E-08	count	1
IGLJ2	-18.1529225	2720.296955	-0.0067	0.995	-1.27E-08	count	1
BX537318.1	-18.1529225	2720.296955	-0.0067	0.995	-1.27E-08	count	1
RGS7	-18.1529225	2720.296955	-0.0067	0.995	-1.27E-08	count	1
ZAP70	-18.1529225	2720.296955	-0.0067	0.995	-1.27E-08	count	1
SLC6A20	-18.1529225	2720.296955	-0.0067	0.995	-1.27E-08	count	1
PSMD6-AS2	-18.1529225	2720.296955	-0.0067	0.995	-1.27E-08	count	1
NEK11	-18.1529225	2720.296955	-0.0067	0.995	-1.27E-08	count	1
TIGD2	-18.1529225	2720.296955	-0.0067	0.995	-1.27E-08	count	1
CARD6	-18.1529225	2720.296955	-0.0067	0.995	-1.27E-08	count	1
WWC1	-18.1529225	2720.296955	-0.0067	0.995	-1.27E-08	count	1
ATP6V1G2	-18.1529225	2720.296955	-0.0067	0.995	-1.27E-08	count	1
SH3BGRL2	-18.1529225	2720.296955	-0.0067	0.995	-1.27E-08	count	1
AC015987.1	-18.1529225	2720.296955	-0.0067	0.995	-1.27E-08	count	1
TMEM164	-18.1529225	2720.296955	-0.0067	0.995	-1.27E-08	count	1
CRISPLD1	-18.1529225	2720.296955	-0.0067	0.995	-1.27E-08	count	1
AL353608.2	-18.1529225	2720.296955	-0.0067	0.995	-1.27E-08	count	1
NALT1	-18.1529225	2720.296955	-0.0067	0.995	-1.27E-08	count	1
AL133523.1	-18.1529225	2720.296955	-0.0067	0.995	-1.27E-08	count	1
BEGAIN	-18.1529225	2720.296955	-0.0067	0.995	-1.27E-08	count	1
IGHV1-18	-18.1529225	2720.296955	-0.0067	0.995	-1.27E-08	count	1
STX1B	-18.1529225	2720.296955	-0.0067	0.995	-1.27E-08	count	1
AC107926.1	-18.1529225	2720.296955	-0.0067	0.995	-1.27E-08	count	1
LINC02081	-18.1529225	2720.296955	-0.0067	0.995	-1.27E-08	count	1
AL117335.1	-18.1529225	2720.296955	-0.0067	0.995	-1.27E-08	count	1
COL16A1	-18.1529225	2720.296955	-0.0067	0.995	-1.27E-08	count	1
ZNF449	-18.1529225	2720.296955	-0.0067	0.995	-1.27E-08	count	1
KPTN	-18.1529225	2720.296955	-0.0067	0.995	-1.27E-08	count	1
RPS4Y2	-18.1529225	2720.296955	-0.0067	0.995	-1.27E-08	count	1
CRYBB2	-18.1529225	2720.296955	-0.0067	0.995	-1.27E-08	count	1
AC079922.2	-18.1529225	2720.296955	-0.0067	0.995	-1.27E-08	count	1
MAST4-AS1	-18.1529225	2720.296955	-0.0067	0.995	-1.27E-08	count	1
TM6SF1	-18.1529225	2720.296955	-0.0067	0.995	-1.27E-08	count	1
BOLA2-SMG1P6	-18.1529225	2720.296955	-0.0067	0.995	-1.27E-08	count	1
AC133919.1	-18.1529225	2720.296955	-0.0067	0.995	-1.27E-08	count	1
ZNF816-ZNF321P	-18.1529225	2720.296955	-0.0067	0.995	-1.27E-08	count	1
AL021707.7	-18.1529225	2720.296955	-0.0067	0.995	-1.27E-08	count	1
TMEM201	-18.1529225	2720.296955	-0.0067	0.995	-1.27E-08	count	1

TINAGL1	-18.1529225	2720.296955	-0.0067	0.995	-1.27E-08	count	1
BEST4	-18.1529225	2720.296955	-0.0067	0.995	-1.27E-08	count	1
CYP2J2	-18.1529225	2720.296955	-0.0067	0.995	-1.27E-08	count	1
GBP7	-18.1529225	2720.296955	-0.0067	0.995	-1.27E-08	count	1
AL355816.2	-18.1529225	2720.296955	-0.0067	0.995	-1.27E-08	count	1
S100A3	-18.1529225	2720.296955	-0.0067	0.995	-1.27E-08	count	1
DCST1-AS1	-18.1529225	2720.296955	-0.0067	0.995	-1.27E-08	count	1
VASH2	-18.1529225	2720.296955	-0.0067	0.995	-1.27E-08	count	1
HLX-AS1	-18.1529225	2720.296955	-0.0067	0.995	-1.27E-08	count	1
TLR5	-18.1529225	2720.296955	-0.0067	0.995	-1.27E-08	count	1
AC093635.1	-18.1529225	2720.296955	-0.0067	0.995	-1.27E-08	count	1
ST3GAL5-AS1	-18.1529225	2720.296955	-0.0067	0.995	-1.27E-08	count	1
KLHL23	-18.1529225	2720.296955	-0.0067	0.995	-1.27E-08	count	1
TMEM237	-18.1529225	2720.296955	-0.0067	0.995	-1.27E-08	count	1
CD28	-18.1529225	2720.296955	-0.0067	0.995	-1.27E-08	count	1
GPR35	-18.1529225	2720.296955	-0.0067	0.995	-1.27E-08	count	1
CPNE9	-18.1529225	2720.296955	-0.0067	0.995	-1.27E-08	count	1
FAM198A	-18.1529225	2720.296955	-0.0067	0.995	-1.27E-08	count	1
CSTA	-18.1529225	2720.296955	-0.0067	0.995	-1.27E-08	count	1
WDR5B	-18.1529225	2720.296955	-0.0067	0.995	-1.27E-08	count	1
MSANTD1	-18.1529225	2720.296955	-0.0067	0.995	-1.27E-08	count	1
AC105345.1	-18.1529225	2720.296955	-0.0067	0.995	-1.27E-08	count	1
AC111006.1	-18.1529225	2720.296955	-0.0067	0.995	-1.27E-08	count	1
WDFY3	-18.1529225	2720.296955	-0.0067	0.995	-1.27E-08	count	1
POU5F2	-18.1529225	2720.296955	-0.0067	0.995	-1.27E-08	count	1
AC008549.2	-18.1529225	2720.296955	-0.0067	0.995	-1.27E-08	count	1
HRH2	-18.1529225	2720.296955	-0.0067	0.995	-1.27E-08	count	1
AL358933.1	-18.1529225	2720.296955	-0.0067	0.995	-1.27E-08	count	1
LY6G5B	-18.1529225	2720.296955	-0.0067	0.995	-1.27E-08	count	1
XPO5	-18.1529225	2720.296955	-0.0067	0.995	-1.27E-08	count	1
HMGN3-AS1	-18.1529225	2720.296955	-0.0067	0.995	-1.27E-08	count	1
THEMIS	-18.1529225	2720.296955	-0.0067	0.995	-1.27E-08	count	1
AL159163.1	-18.1529225	2720.296955	-0.0067	0.995	-1.27E-08	count	1
DLL1	-18.1529225	2720.296955	-0.0067	0.995	-1.27E-08	count	1
AC073957.3	-18.1529225	2720.296955	-0.0067	0.995	-1.27E-08	count	1
HOXA1	-18.1529225	2720.296955	-0.0067	0.995	-1.27E-08	count	1
AC007036.1	-18.1529225	2720.296955	-0.0067	0.995	-1.27E-08	count	1
CD36	-18.1529225	2720.296955	-0.0067	0.995	-1.27E-08	count	1
HGF	-18.1529225	2720.296955	-0.0067	0.995	-1.27E-08	count	1
PDK4	-18.1529225	2720.296955	-0.0067	0.995	-1.27E-08	count	1
TLR8	-18.1529225	2720.296955	-0.0067	0.995	-1.27E-08	count	1
KCND1	-18.1529225	2720.296955	-0.0067	0.995	-1.27E-08	count	1
SH2D1A	-18.1529225	2720.296955	-0.0067	0.995	-1.27E-08	count	1
OCRL	-18.1529225	2720.296955	-0.0067	0.995	-1.27E-08	count	1
ELF4	-18.1529225	2720.296955	-0.0067	0.995	-1.27E-08	count	1

F8	-18.1529225	2720.296955	-0.0067	0.995	-1.27E-08	count	1
CHRNA6	-18.1529225	2720.296955	-0.0067	0.995	-1.27E-08	count	1
POMK	-18.1529225	2720.296955	-0.0067	0.995	-1.27E-08	count	1
C8orf37	-18.1529225	2720.296955	-0.0067	0.995	-1.27E-08	count	1
EXT1	-18.1529225	2720.296955	-0.0067	0.995	-1.27E-08	count	1
C9orf66	-18.1529225	2720.296955	-0.0067	0.995	-1.27E-08	count	1
NTRK2	-18.1529225	2720.296955	-0.0067	0.995	-1.27E-08	count	1
GOLM1	-18.1529225	2720.296955	-0.0067	0.995	-1.27E-08	count	1
PTPDC1	-18.1529225	2720.296955	-0.0067	0.995	-1.27E-08	count	1
AL359091.4	-18.1529225	2720.296955	-0.0067	0.995	-1.27E-08	count	1
AL158151.1	-18.1529225	2720.296955	-0.0067	0.995	-1.27E-08	count	1
AL358781.1	-18.1529225	2720.296955	-0.0067	0.995	-1.27E-08	count	1
AP003721.4	-18.1529225	2720.296955	-0.0067	0.995	-1.27E-08	count	1
AP000759.1	-18.1529225	2720.296955	-0.0067	0.995	-1.27E-08	count	1
KCNE3	-18.1529225	2720.296955	-0.0067	0.995	-1.27E-08	count	1
KLHL35	-18.1529225	2720.296955	-0.0067	0.995	-1.27E-08	count	1
NAALAD2	-18.1529225	2720.296955	-0.0067	0.995	-1.27E-08	count	1
TMPRSS13	-18.1529225	2720.296955	-0.0067	0.995	-1.27E-08	count	1
CD3D	-18.1529225	2720.296955	-0.0067	0.995	-1.27E-08	count	1
OAF	-18.1529225	2720.296955	-0.0067	0.995	-1.27E-08	count	1
DCLRE1A	-18.1529225	2720.296955	-0.0067	0.995	-1.27E-08	count	1
TRUB1	-18.1529225	2720.296955	-0.0067	0.995	-1.27E-08	count	1
ENO4	-18.1529225	2720.296955	-0.0067	0.995	-1.27E-08	count	1
CACNA1C-AS2	-18.1529225	2720.296955	-0.0067	0.995	-1.27E-08	count	1
AC107032.2	-18.1529225	2720.296955	-0.0067	0.995	-1.27E-08	count	1
ACSS3	-18.1529225	2720.296955	-0.0067	0.995	-1.27E-08	count	1
AC007569.1	-18.1529225	2720.296955	-0.0067	0.995	-1.27E-08	count	1
RNFT2	-18.1529225	2720.296955	-0.0067	0.995	-1.27E-08	count	1
NRAV	-18.1529225	2720.296955	-0.0067	0.995	-1.27E-08	count	1
THRIL	-18.1529225	2720.296955	-0.0067	0.995	-1.27E-08	count	1
PRKCH	-18.1529225	2720.296955	-0.0067	0.995	-1.27E-08	count	1
AC005520.2	-18.1529225	2720.296955	-0.0067	0.995	-1.27E-08	count	1
ZNF410	-18.1529225	2720.296955	-0.0067	0.995	-1.27E-08	count	1
DICER1-AS1	-18.1529225	2720.296955	-0.0067	0.995	-1.27E-08	count	1
TECPR2	-18.1529225	2720.296955	-0.0067	0.995	-1.27E-08	count	1
ARHGAP11B	-18.1529225	2720.296955	-0.0067	0.995	-1.27E-08	count	1
HEXA-AS1	-18.1529225	2720.296955	-0.0067	0.995	-1.27E-08	count	1
ZNF319	-18.1529225	2720.296955	-0.0067	0.995	-1.27E-08	count	1
RRAD	-18.1529225	2720.296955	-0.0067	0.995	-1.27E-08	count	1
TAT-AS1	-18.1529225	2720.296955	-0.0067	0.995	-1.27E-08	count	1
CENPBD1	-18.1529225	2720.296955	-0.0067	0.995	-1.27E-08	count	1
AC015727.1	-18.1529225	2720.296955	-0.0067	0.995	-1.27E-08	count	1
ASGR2	-18.1529225	2720.296955	-0.0067	0.995	-1.27E-08	count	1
MYH10	-18.1529225	2720.296955	-0.0067	0.995	-1.27E-08	count	1
TVP23C	-18.1529225	2720.296955	-0.0067	0.995	-1.27E-08	count	1

AC005726.1	-18.1529225	2720.296955	-0.0067	0.995	-1.27E-08	count	1
AC005899.7	-18.1529225	2720.296955	-0.0067	0.995	-1.27E-08	count	1
AC138150.2	-18.1529225	2720.296955	-0.0067	0.995	-1.27E-08	count	1
AC011825.4	-18.1529225	2720.296955	-0.0067	0.995	-1.27E-08	count	1
AC018529.2	-18.1529225	2720.296955	-0.0067	0.995	-1.27E-08	count	1
PLCB1	-18.1529225	2720.296955	-0.0067	0.995	-1.27E-08	count	1
MCEMP1	-18.1529225	2720.296955	-0.0067	0.995	-1.27E-08	count	1
TRAPPC5	-18.1529225	2720.296955	-0.0067	0.995	-1.27E-08	count	1
NANOS3	-18.1529225	2720.296955	-0.0067	0.995	-1.27E-08	count	1
AC010331.1	-18.1529225	2720.296955	-0.0067	0.995	-1.27E-08	count	1
Z99774.1	-18.1529225	2720.296955	-0.0067	0.995	-1.27E-08	count	1
PLA2G6	-18.1529225	2720.296955	-0.0067	0.995	-1.27E-08	count	1
APOBEC3A	-18.1529225	2720.296955	-0.0067	0.995	-1.27E-08	count	1
TTC34	-18.1529225	2720.296955	-0.0067	0.995	-1.27E-08	count	1
CRYBG2	-18.1529225	2720.296955	-0.0067	0.995	-1.27E-08	count	1
S100A5	-18.1529225	2720.296955	-0.0067	0.995	-1.27E-08	count	1
AL358472.2	-18.1529225	2720.296955	-0.0067	0.995	-1.27E-08	count	1
AL591846.2	-18.1529225	2720.296955	-0.0067	0.995	-1.27E-08	count	1
AC092164.1	-18.1529225	2720.296955	-0.0067	0.995	-1.27E-08	count	1
HNRNPLL	-18.1529225	2720.296955	-0.0067	0.995	-1.27E-08	count	1
AC012447.1	-18.1529225	2720.296955	-0.0067	0.995	-1.27E-08	count	1
KCNE4	-18.1529225	2720.296955	-0.0067	0.995	-1.27E-08	count	1
GATA2	-18.1529225	2720.296955	-0.0067	0.995	-1.27E-08	count	1
TMCO6	-18.1529225	2720.296955	-0.0067	0.995	-1.27E-08	count	1
TRERF1	-18.1529225	2720.296955	-0.0067	0.995	-1.27E-08	count	1
AL590764.1	-18.1529225	2720.296955	-0.0067	0.995	-1.27E-08	count	1
AC104986.2	-18.1529225	2720.296955	-0.0067	0.995	-1.27E-08	count	1
TRIM6	-18.1529225	2720.296955	-0.0067	0.995	-1.27E-08	count	1
DENND5A	-18.1529225	2720.296955	-0.0067	0.995	-1.27E-08	count	1
C11orf95	-18.1529225	2720.296955	-0.0067	0.995	-1.27E-08	count	1
CDNF	-18.1529225	2720.296955	-0.0067	0.995	-1.27E-08	count	1
LINC00843	-18.1529225	2720.296955	-0.0067	0.995	-1.27E-08	count	1
CRTAC1	-18.1529225	2720.296955	-0.0067	0.995	-1.27E-08	count	1
WDR11-AS1	-18.1529225	2720.296955	-0.0067	0.995	-1.27E-08	count	1
AL162274.2	-18.1529225	2720.296955	-0.0067	0.995	-1.27E-08	count	1
TSPAN9	-18.1529225	2720.296955	-0.0067	0.995	-1.27E-08	count	1
LAG3	-18.1529225	2720.296955	-0.0067	0.995	-1.27E-08	count	1
VSIG10	-18.1529225	2720.296955	-0.0067	0.995	-1.27E-08	count	1
AL139353.1	-18.1529225	2720.296955	-0.0067	0.995	-1.27E-08	count	1
PTGDR	-18.1529225	2720.296955	-0.0067	0.995	-1.27E-08	count	1
POMT2	-18.1529225	2720.296955	-0.0067	0.995	-1.27E-08	count	1
CCNB2	-18.1529225	2720.296955	-0.0067	0.995	-1.27E-08	count	1
SLC24A1	-18.1529225	2720.296955	-0.0067	0.995	-1.27E-08	count	1
ZNF213-AS1	-18.1529225	2720.296955	-0.0067	0.995	-1.27E-08	count	1
AC106779.1	-18.1529225	2720.296955	-0.0067	0.995	-1.27E-08	count	1

GAPLINC	-18.1529225	2720.296955	-0.0067	0.995	-1.27E-08	count	1
TWSG1	-18.1529225	2720.296955	-0.0067	0.995	-1.27E-08	count	1
AC011815.2	-18.1529225	2720.296955	-0.0067	0.995	-1.27E-08	count	1
LKAAEAR1	-18.1529225	2720.296955	-0.0067	0.995	-1.27E-08	count	1
AC012615.5	-18.1529225	2720.296955	-0.0067	0.995	-1.27E-08	count	1
DLL3	-18.1529225	2720.296955	-0.0067	0.995	-1.27E-08	count	1
AC008079.2	-18.1529225	2720.296955	-0.0067	0.995	-1.27E-08	count	1
SCO2	-18.1529225	2720.296955	-0.0067	0.995	-1.27E-08	count	1
LINC00115	-18.1529225	2720.296955	-0.0067	0.995	-1.27E-08	count	1
AC013403.2	-18.1529225	2720.296955	-0.0067	0.995	-1.27E-08	count	1
AC012358.3	-18.1529225	2720.296955	-0.0067	0.995	-1.27E-08	count	1
AC016745.2	-18.1529225	2720.296955	-0.0067	0.995	-1.27E-08	count	1
HCG22	-18.1529225	2720.296955	-0.0067	0.995	-1.27E-08	count	1
AC011978.2	-18.1529225	2720.296955	-0.0067	0.995	-1.27E-08	count	1
AC010834.2	-18.1529225	2720.296955	-0.0067	0.995	-1.27E-08	count	1
GNAQ	-18.1529225	2720.296955	-0.0067	0.995	-1.27E-08	count	1
FZD4	-18.1529225	2720.296955	-0.0067	0.995	-1.27E-08	count	1
KLRF1	-18.1529225	2720.296955	-0.0067	0.995	-1.27E-08	count	1
AC008035.1	-18.1529225	2720.296955	-0.0067	0.995	-1.27E-08	count	1
MYL2	-18.1529225	2720.296955	-0.0067	0.995	-1.27E-08	count	1
GCN1	-18.1529225	2720.296955	-0.0067	0.995	-1.27E-08	count	1
BUB1B	-18.1529225	2720.296955	-0.0067	0.995	-1.27E-08	count	1
ZGLP1	-18.1529225	2720.296955	-0.0067	0.995	-1.27E-08	count	1
NAT14	-18.1529225	2720.296955	-0.0067	0.995	-1.27E-08	count	1
U62317.2	-18.1529225	2720.296955	-0.0067	0.995	-1.27E-08	count	1
BCL2L15	-18.1529225	2720.296955	-0.0067	0.995	-1.27E-08	count	1
AC008438.1	-18.1529225	2720.296955	-0.0067	0.995	-1.27E-08	count	1
TRDC	-18.1529225	2720.296955	-0.0067	0.995	-1.27E-08	count	1
AL136038.3	-18.1529225	2720.296955	-0.0067	0.995	-1.27E-08	count	1
AC020915.1	-18.1529225	2720.296955	-0.0067	0.995	-1.27E-08	count	1
AL031432.3	-18.1529225	2720.296955	-0.0067	0.995	-1.27E-08	count	1
FAM85B	-18.1529225	2720.296955	-0.0067	0.995	-1.27E-08	count	1
AL591368.1	-18.1529225	2720.296955	-0.0067	0.995	-1.27E-08	count	1
FRMD3	-18.1529225	2720.296955	-0.0067	0.995	-1.27E-08	count	1
NPDC1	-18.1529225	2720.296955	-0.0067	0.995	-1.27E-08	count	1
C2CD4D	-18.1529225	2720.296955	-0.0067	0.995	-1.27E-08	count	1
FCER1A	-18.1529225	2720.296955	-0.0067	0.995	-1.27E-08	count	1
LINC01871	-18.1529225	2720.296955	-0.0067	0.995	-1.27E-08	count	1
TP53I3	-18.1529225	2720.296955	-0.0067	0.995	-1.27E-08	count	1
AC107081.2	-18.1529225	2720.296955	-0.0067	0.995	-1.27E-08	count	1
AC078883.1	-18.1529225	2720.296955	-0.0067	0.995	-1.27E-08	count	1
CALCRL	-18.1529225	2720.296955	-0.0067	0.995	-1.27E-08	count	1
NREP	-18.1529225	2720.296955	-0.0067	0.995	-1.27E-08	count	1
SLC45A4	-18.1529225	2720.296955	-0.0067	0.995	-1.27E-08	count	1
ARHGEF39	-18.1529225	2720.296955	-0.0067	0.995	-1.27E-08	count	1

VWCE	-18.1529225	2720.296955	-0.0067	0.995	-1.27E-08	count	1
AP001453.1	-18.1529225	2720.296955	-0.0067	0.995	-1.27E-08	count	1
CPXM2	-18.1529225	2720.296955	-0.0067	0.995	-1.27E-08	count	1
LIN7A	-18.1529225	2720.296955	-0.0067	0.995	-1.27E-08	count	1
AC027290.1	-18.1529225	2720.296955	-0.0067	0.995	-1.27E-08	count	1
SCARB1	-18.1529225	2720.296955	-0.0067	0.995	-1.27E-08	count	1
AF111167.2	-18.1529225	2720.296955	-0.0067	0.995	-1.27E-08	count	1
KIAA0513	-18.1529225	2720.296955	-0.0067	0.995	-1.27E-08	count	1
CCDC144NL-AS1	-18.1529225	2720.296955	-0.0067	0.995	-1.27E-08	count	1
AC130324.1	-18.1529225	2720.296955	-0.0067	0.995	-1.27E-08	count	1
CELF4	-18.1529225	2720.296955	-0.0067	0.995	-1.27E-08	count	1
CD93	-18.1529225	2720.296955	-0.0067	0.995	-1.27E-08	count	1
AL357033.1	-18.1529225	2720.296955	-0.0067	0.995	-1.27E-08	count	1
ICAM4	-18.1529225	2720.296955	-0.0067	0.995	-1.27E-08	count	1
F2RL3	-18.1529225	2720.296955	-0.0067	0.995	-1.27E-08	count	1
PNMA8B	-18.1529225	2720.296955	-0.0067	0.995	-1.27E-08	count	1
ZNF837	-18.1529225	2720.296955	-0.0067	0.995	-1.27E-08	count	1
VWA1	-18.1529225	2720.296955	-0.0067	0.995	-1.27E-08	count	1
HIST2H2AB	-18.1529225	2720.296955	-0.0067	0.995	-1.27E-08	count	1
LINC01126	-18.1529225	2720.296955	-0.0067	0.995	-1.27E-08	count	1
TUBB2B	-18.1529225	2720.296955	-0.0067	0.995	-1.27E-08	count	1
GNMT	-18.1529225	2720.296955	-0.0067	0.995	-1.27E-08	count	1
AL080317.1	-18.1529225	2720.296955	-0.0067	0.995	-1.27E-08	count	1
PRSS1	-18.1529225	2720.296955	-0.0067	0.995	-1.27E-08	count	1
AC109322.1	-18.1529225	2720.296955	-0.0067	0.995	-1.27E-08	count	1
SMC2-AS1	-18.1529225	2720.296955	-0.0067	0.995	-1.27E-08	count	1
AMER2	-18.1529225	2720.296955	-0.0067	0.995	-1.27E-08	count	1
POSTN	-18.1529225	2720.296955	-0.0067	0.995	-1.27E-08	count	1
CTSG	-18.1529225	2720.296955	-0.0067	0.995	-1.27E-08	count	1
PPP1R36	-18.1529225	2720.296955	-0.0067	0.995	-1.27E-08	count	1
IGHV2-70D	-18.1529225	2720.296955	-0.0067	0.995	-1.27E-08	count	1
B4GALT6	-18.1529225	2720.296955	-0.0067	0.995	-1.27E-08	count	1
BTBD8	-18.1529225	2720.296955	-0.0067	0.995	-1.27E-08	count	1
PALMD	-18.1529225	2720.296955	-0.0067	0.995	-1.27E-08	count	1
SELE	-18.1529225	2720.296955	-0.0067	0.995	-1.27E-08	count	1
FAM72A	-18.1529225	2720.296955	-0.0067	0.995	-1.27E-08	count	1
LINC01918	-18.1529225	2720.296955	-0.0067	0.995	-1.27E-08	count	1
AC092053.3	-18.1529225	2720.296955	-0.0067	0.995	-1.27E-08	count	1
LRRC34	-18.1529225	2720.296955	-0.0067	0.995	-1.27E-08	count	1
AC104825.1	-18.1529225	2720.296955	-0.0067	0.995	-1.27E-08	count	1
STPG2	-18.1529225	2720.296955	-0.0067	0.995	-1.27E-08	count	1
AC095050.1	-18.1529225	2720.296955	-0.0067	0.995	-1.27E-08	count	1
KCNQ5	-18.1529225	2720.296955	-0.0067	0.995	-1.27E-08	count	1
AL356417.1	-18.1529225	2720.296955	-0.0067	0.995	-1.27E-08	count	1
EEF1G	-18.1529225	2720.296955	-0.0067	0.995	-1.27E-08	count	1

AP001825.1	-18.1529225	2720.296955	-0.0067	0.995	-1.27E-08	count	1
AP002433.1	-18.1529225	2720.296955	-0.0067	0.995	-1.27E-08	count	1
COL4A2	-18.1529225	2720.296955	-0.0067	0.995	-1.27E-08	count	1
RNASE4	-18.1529225	2720.296955	-0.0067	0.995	-1.27E-08	count	1
NDN	-18.1529225	2720.296955	-0.0067	0.995	-1.27E-08	count	1
CCNF	-18.1529225	2720.296955	-0.0067	0.995	-1.27E-08	count	1
TPPP3	-18.1529225	2720.296955	-0.0067	0.995	-1.27E-08	count	1
MC1R	-18.1529225	2720.296955	-0.0067	0.995	-1.27E-08	count	1
SENP3	-18.1529225	2720.296955	-0.0067	0.995	-1.27E-08	count	1
EFCAB5	-18.1529225	2720.296955	-0.0067	0.995	-1.27E-08	count	1
MMP28	-18.1529225	2720.296955	-0.0067	0.995	-1.27E-08	count	1
PROSER3	-18.1529225	2720.296955	-0.0067	0.995	-1.27E-08	count	1
RYR1	-18.1529225	2720.296955	-0.0067	0.995	-1.27E-08	count	1
ZNF470	-18.1529225	2720.296955	-0.0067	0.995	-1.27E-08	count	1
AC245060.6	-18.1529225	2720.296955	-0.0067	0.995	-1.27E-08	count	1
AL050343.1	-18.1529225	2720.296955	-0.0067	0.995	-1.27E-08	count	1
XCL2	-18.1529225	2720.296955	-0.0067	0.995	-1.27E-08	count	1
NR5A2	-18.1529225	2720.296955	-0.0067	0.995	-1.27E-08	count	1
VIL1	-18.1529225	2720.296955	-0.0067	0.995	-1.27E-08	count	1
SLC6A1	-18.1529225	2720.296955	-0.0067	0.995	-1.27E-08	count	1
FNDC3B	-18.1529225	2720.296955	-0.0067	0.995	-1.27E-08	count	1
MED20	-18.1529225	2720.296955	-0.0067	0.995	-1.27E-08	count	1
AC005091.1	-18.1529225	2720.296955	-0.0067	0.995	-1.27E-08	count	1
ANKRD7	-18.1529225	2720.296955	-0.0067	0.995	-1.27E-08	count	1
SPIN4	-18.1529225	2720.296955	-0.0067	0.995	-1.27E-08	count	1
ZCCHC18	-18.1529225	2720.296955	-0.0067	0.995	-1.27E-08	count	1
PCAT1	-18.1529225	2720.296955	-0.0067	0.995	-1.27E-08	count	1
MKI67	-18.1529225	2720.296955	-0.0067	0.995	-1.27E-08	count	1
AL360181.3	-18.1529225	2720.296955	-0.0067	0.995	-1.27E-08	count	1
AL138966.2	-18.1529225	2720.296955	-0.0067	0.995	-1.27E-08	count	1
THBS1	-18.1529225	2720.296955	-0.0067	0.995	-1.27E-08	count	1
SENP8	-18.1529225	2720.296955	-0.0067	0.995	-1.27E-08	count	1
LINC01572	-18.1529225	2720.296955	-0.0067	0.995	-1.27E-08	count	1
TAS1R3	-18.1529225	2720.296955	-0.0067	0.995	-1.27E-08	count	1
TNFRSF9	-18.1529225	2720.296955	-0.0067	0.995	-1.27E-08	count	1
HIST3H2BB	-18.1529225	2720.296955	-0.0067	0.995	-1.27E-08	count	1
GRHL1	-18.1529225	2720.296955	-0.0067	0.995	-1.27E-08	count	1
SNED1	-18.1529225	2720.296955	-0.0067	0.995	-1.27E-08	count	1
TAPT1-AS1	-18.1529225	2720.296955	-0.0067	0.995	-1.27E-08	count	1
ADCY2	-18.1529225	2720.296955	-0.0067	0.995	-1.27E-08	count	1
GCNT4	-18.1529225	2720.296955	-0.0067	0.995	-1.27E-08	count	1
PDE8B	-18.1529225	2720.296955	-0.0067	0.995	-1.27E-08	count	1
PCDHGB3	-18.1529225	2720.296955	-0.0067	0.995	-1.27E-08	count	1
AL365275.1	-18.1529225	2720.296955	-0.0067	0.995	-1.27E-08	count	1
ULBP2	-18.1529225	2720.296955	-0.0067	0.995	-1.27E-08	count	1

CLEC5A	-18.1529225	2720.296955	-0.0067	0.995	-1.27E-08	count	1
AC083973.1	-18.1529225	2720.296955	-0.0067	0.995	-1.27E-08	count	1
MROH6	-18.1529225	2720.296955	-0.0067	0.995	-1.27E-08	count	1
GAS1RR	-18.1529225	2720.296955	-0.0067	0.995	-1.27E-08	count	1
GADD45G	-18.1529225	2720.296955	-0.0067	0.995	-1.27E-08	count	1
ANKS6	-18.1529225	2720.296955	-0.0067	0.995	-1.27E-08	count	1
RIN1	-18.1529225	2720.296955	-0.0067	0.995	-1.27E-08	count	1
KCTD21-AS1	-18.1529225	2720.296955	-0.0067	0.995	-1.27E-08	count	1
MINPP1	-18.1529225	2720.296955	-0.0067	0.995	-1.27E-08	count	1
UBTD1	-18.1529225	2720.296955	-0.0067	0.995	-1.27E-08	count	1
LINC01481	-18.1529225	2720.296955	-0.0067	0.995	-1.27E-08	count	1
AC073912.2	-18.1529225	2720.296955	-0.0067	0.995	-1.27E-08	count	1
DOCK9	-18.1529225	2720.296955	-0.0067	0.995	-1.27E-08	count	1
FITM1	-18.1529225	2720.296955	-0.0067	0.995	-1.27E-08	count	1
AL136295.2	-18.1529225	2720.296955	-0.0067	0.995	-1.27E-08	count	1
SHCBP1	-18.1529225	2720.296955	-0.0067	0.995	-1.27E-08	count	1
CETP	-18.1529225	2720.296955	-0.0067	0.995	-1.27E-08	count	1
ENO3	-18.1529225	2720.296955	-0.0067	0.995	-1.27E-08	count	1
JUP	-18.1529225	2720.296955	-0.0067	0.995	-1.27E-08	count	1
KLHL11	-18.1529225	2720.296955	-0.0067	0.995	-1.27E-08	count	1
ACE	-18.1529225	2720.296955	-0.0067	0.995	-1.27E-08	count	1
CYGB	-18.1529225	2720.296955	-0.0067	0.995	-1.27E-08	count	1
AC145207.5	-18.1529225	2720.296955	-0.0067	0.995	-1.27E-08	count	1
SKA1	-18.1529225	2720.296955	-0.0067	0.995	-1.27E-08	count	1
UBOX5	-18.1529225	2720.296955	-0.0067	0.995	-1.27E-08	count	1
MKKS	-18.1529225	2720.296955	-0.0067	0.995	-1.27E-08	count	1
LINC00237	-18.1529225	2720.296955	-0.0067	0.995	-1.27E-08	count	1
AC092279.1	-18.1529225	2720.296955	-0.0067	0.995	-1.27E-08	count	1
SGSM1	-18.1529225	2720.296955	-0.0067	0.995	-1.27E-08	count	1
MGAT3	-18.1529225	2720.296955	-0.0067	0.995	-1.27E-08	count	1
FAM83F	-18.1529225	2720.296955	-0.0067	0.995	-1.27E-08	count	1
HSF2BP	-18.1529225	2720.296955	-0.0067	0.995	-1.27E-08	count	1
ITIH4	-18.1529225	2720.296955	-0.0067	0.995	-1.27E-08	count	1
ADAM29	-18.1529225	2720.296955	-0.0067	0.995	-1.27E-08	count	1
AC018638.7	-18.1529225	2720.296955	-0.0067	0.995	-1.27E-08	count	1
CCDC136	-18.1529225	2720.296955	-0.0067	0.995	-1.27E-08	count	1
KLF14	-18.1529225	2720.296955	-0.0067	0.995	-1.27E-08	count	1
AP001636.3	-18.1529225	2720.296955	-0.0067	0.995	-1.27E-08	count	1
CD3G	-18.1529225	2720.296955	-0.0067	0.995	-1.27E-08	count	1
ESPL1	-18.1529225	2720.296955	-0.0067	0.995	-1.27E-08	count	1
AC107958.2	-18.1529225	2720.296955	-0.0067	0.995	-1.27E-08	count	1
AC022167.2	-18.1529225	2720.296955	-0.0067	0.995	-1.27E-08	count	1
ZNF234	-18.1529225	2720.296955	-0.0067	0.995	-1.27E-08	count	1
SERINC2	-18.1529225	2720.296955	-0.0067	0.995	-1.27E-08	count	1
SCN11A	-18.1529225	2720.296955	-0.0067	0.995	-1.27E-08	count	1

MYLK	-18.1529225	2720.296955	-0.0067	0.995	-1.27E-08	count	1
AC008914.1	-18.1529225	2720.296955	-0.0067	0.995	-1.27E-08	count	1
MTMR8	-18.1529225	2720.296955	-0.0067	0.995	-1.27E-08	count	1
TSPAN6	-18.1529225	2720.296955	-0.0067	0.995	-1.27E-08	count	1
VSTM4	-18.1529225	2720.296955	-0.0067	0.995	-1.27E-08	count	1
SEC31B	-18.1529225	2720.296955	-0.0067	0.995	-1.27E-08	count	1
AC006064.4	-18.1529225	2720.296955	-0.0067	0.995	-1.27E-08	count	1
TOP2A	-18.1529225	2720.296955	-0.0067	0.995	-1.27E-08	count	1
AC037487.2	-18.1529225	2720.296955	-0.0067	0.995	-1.27E-08	count	1
RAB27B	-18.1529225	2720.296955	-0.0067	0.995	-1.27E-08	count	1
SLC27A1	-18.1529225	2720.296955	-0.0067	0.995	-1.27E-08	count	1
AHDC1	-18.1529225	2720.296955	-0.0067	0.995	-1.27E-08	count	1
TMEM54	-18.1529225	2720.296955	-0.0067	0.995	-1.27E-08	count	1
FASLG	-18.1529225	2720.296955	-0.0067	0.995	-1.27E-08	count	1
AC108488.1	-18.1529225	2720.296955	-0.0067	0.995	-1.27E-08	count	1
CYP1B1-AS1	-18.1529225	2720.296955	-0.0067	0.995	-1.27E-08	count	1
NECTIN3	-18.1529225	2720.296955	-0.0067	0.995	-1.27E-08	count	1
KLB	-18.1529225	2720.296955	-0.0067	0.995	-1.27E-08	count	1
HPGDS	-18.1529225	2720.296955	-0.0067	0.995	-1.27E-08	count	1
PRR7-AS1	-18.1529225	2720.296955	-0.0067	0.995	-1.27E-08	count	1
PERP	-18.1529225	2720.296955	-0.0067	0.995	-1.27E-08	count	1
SLC22A3	-18.1529225	2720.296955	-0.0067	0.995	-1.27E-08	count	1
AC067838.1	-18.1529225	2720.296955	-0.0067	0.995	-1.27E-08	count	1
GLIS3	-18.1529225	2720.296955	-0.0067	0.995	-1.27E-08	count	1
CHEK1	-18.1529225	2720.296955	-0.0067	0.995	-1.27E-08	count	1
FZD8	-18.1529225	2720.296955	-0.0067	0.995	-1.27E-08	count	1
AC091057.2	-18.1529225	2720.296955	-0.0067	0.995	-1.27E-08	count	1
AC020659.1	-18.1529225	2720.296955	-0.0067	0.995	-1.27E-08	count	1
MAP3K14-AS1	-18.1529225	2720.296955	-0.0067	0.995	-1.27E-08	count	1
AL139260.1	-18.1529225	2720.296955	-0.0067	0.995	-1.27E-08	count	1
AL513493.1	-18.1529225	2720.296955	-0.0067	0.995	-1.27E-08	count	1
NES	-18.1529225	2720.296955	-0.0067	0.995	-1.27E-08	count	1
BUB1	-18.1529225	2720.296955	-0.0067	0.995	-1.27E-08	count	1
TANC1	-18.1529225	2720.296955	-0.0067	0.995	-1.27E-08	count	1
CHST13	-18.1529225	2720.296955	-0.0067	0.995	-1.27E-08	count	1
LAMP3	-18.1529225	2720.296955	-0.0067	0.995	-1.27E-08	count	1
KIAA1211	-18.1529225	2720.296955	-0.0067	0.995	-1.27E-08	count	1
PCDHGC3	-18.1529225	2720.296955	-0.0067	0.995	-1.27E-08	count	1
FGFR4	-18.1529225	2720.296955	-0.0067	0.995	-1.27E-08	count	1
AC145098.1	-18.1529225	2720.296955	-0.0067	0.995	-1.27E-08	count	1
LINC01446	-18.1529225	2720.296955	-0.0067	0.995	-1.27E-08	count	1
MIR3150BHG	-18.1529225	2720.296955	-0.0067	0.995	-1.27E-08	count	1
DCAF12	-18.1529225	2720.296955	-0.0067	0.995	-1.27E-08	count	1
NDOR1	-18.1529225	2720.296955	-0.0067	0.995	-1.27E-08	count	1
TRIM34	-18.1529225	2720.296955	-0.0067	0.995	-1.27E-08	count	1

PDE3B	-18.1529225	2720.296955	-0.0067	0.995	-1.27E-08	count	1
AP003108.2	-18.1529225	2720.296955	-0.0067	0.995	-1.27E-08	count	1
TESMIN	-18.1529225	2720.296955	-0.0067	0.995	-1.27E-08	count	1
SIAE	-18.1529225	2720.296955	-0.0067	0.995	-1.27E-08	count	1
TMTC2	-18.1529225	2720.296955	-0.0067	0.995	-1.27E-08	count	1
AL122035.1	-18.1529225	2720.296955	-0.0067	0.995	-1.27E-08	count	1
AL359232.1	-18.1529225	2720.296955	-0.0067	0.995	-1.27E-08	count	1
CCDC9B	-18.1529225	2720.296955	-0.0067	0.995	-1.27E-08	count	1
WNT9B	-18.1529225	2720.296955	-0.0067	0.995	-1.27E-08	count	1
CIRBP-AS1	-18.1529225	2720.296955	-0.0067	0.995	-1.27E-08	count	1
PLXNB2	-18.1529225	2720.296955	-0.0067	0.995	-1.27E-08	count	1
AL449106.1	-18.1529225	2720.296955	-0.0067	0.995	-1.27E-08	count	1
PLEKHA6	-18.1529225	2720.296955	-0.0067	0.995	-1.27E-08	count	1
HESX1	-18.1529225	2720.296955	-0.0067	0.995	-1.27E-08	count	1
AC107464.3	-18.1529225	2720.296955	-0.0067	0.995	-1.27E-08	count	1
CSRP2	-18.1529225	2720.296955	-0.0067	0.995	-1.27E-08	count	1
AL121658.1	-18.1529225	2720.296955	-0.0067	0.995	-1.27E-08	count	1
HNMT	-18.1529225	2720.296955	-0.0067	0.995	-1.27E-08	count	1
PLCL1	-18.1529225	2720.296955	-0.0067	0.995	-1.27E-08	count	1
AC096711.1	-18.1529225	2720.296955	-0.0067	0.995	-1.27E-08	count	1
IGFBP2	-18.1529225	2720.296955	-0.0067	0.995	-1.27E-08	count	1
IGFBP5	-18.1529225	2720.296955	-0.0067	0.995	-1.27E-08	count	1
TRIM68	-18.1529225	2720.296955	-0.0067	0.995	-1.27E-08	count	1
DND1	-18.1529225	2720.296955	-0.0067	0.995	-1.27E-08	count	1
DDX39B-AS1	-18.1529225	2720.296955	-0.0067	0.995	-1.27E-08	count	1
MSR1	-18.1529225	2720.296955	-0.0067	0.995	-1.27E-08	count	1
JMJD1C-AS1	-18.1529225	2720.296955	-0.0067	0.995	-1.27E-08	count	1
AC008083.3	-18.1529225	2720.296955	-0.0067	0.995	-1.27E-08	count	1
FAM209B	-18.1529225	2720.296955	-0.0067	0.995	-1.27E-08	count	1
CU638689.4	-18.1529225	2720.296955	-0.0067	0.995	-1.27E-08	count	1
PRRX1	-18.2893503	3007.240567	-0.0061	0.995	-1.27E-08	count	1
LINC01816	-18.2893503	3007.240567	-0.0061	0.995	-1.27E-08	count	1
TBX19	-18.2893503	3007.240567	-0.0061	0.995	-1.27E-08	count	1
USP19	-18.2893503	3007.240567	-0.0061	0.995	-1.27E-08	count	1
KIAA1522	-18.2893503	3007.240567	-0.0061	0.995	-1.27E-08	count	1
SLC37A2	-18.2893503	3007.240567	-0.0061	0.995	-1.27E-08	count	1
MMP17	-18.2893503	3007.240567	-0.0061	0.995	-1.27E-08	count	1
OPRL1	-18.2893503	3007.240567	-0.0061	0.995	-1.27E-08	count	1
AC120498.9	-18.2893503	3007.240567	-0.0061	0.995	-1.27E-08	count	1
LRRC29	-18.2893503	3007.240567	-0.0061	0.995	-1.27E-08	count	1
CAPS2	-18.2893503	3007.240567	-0.0061	0.995	-1.27E-08	count	1
ZCCHC24	-18.2893503	3007.240567	-0.0061	0.995	-1.27E-08	count	1
AGAP6	-18.2893503	3007.240567	-0.0061	0.995	-1.27E-08	count	1
FOSL2	-18.2893503	3007.240567	-0.0061	0.995	-1.27E-08	count	1
IGKV3-11	-18.2893503	3007.240567	-0.0061	0.995	-1.27E-08	count	1

UGGT2	-18.2893503	3007.240567	-0.0061	0.995	-1.27E-08	count	1
AC005498.3	-18.2893503	3007.240567	-0.0061	0.995	-1.27E-08	count	1
CASZ1	-18.2893503	3007.240567	-0.0061	0.995	-1.27E-08	count	1
GHRL	-18.2893503	3007.240567	-0.0061	0.995	-1.27E-08	count	1
ANAPC2	-18.2893503	3007.240567	-0.0061	0.995	-1.27E-08	count	1
P3H4	-18.2893503	3007.240567	-0.0061	0.995	-1.27E-08	count	1
CLDN15	-18.2893503	3007.240567	-0.0061	0.995	-1.27E-08	count	1
STAB1	-18.2893503	3007.240567	-0.0061	0.995	-1.27E-08	count	1
UCN	-18.2893503	3007.240567	-0.0061	0.995	-1.27E-08	count	1
ZFYVE26	-18.2893503	3007.240567	-0.0061	0.995	-1.27E-08	count	1
GCNT1	-18.2893503	3007.240567	-0.0061	0.995	-1.27E-08	count	1
ZNF674-AS1	-18.2893503	3007.240567	-0.0061	0.995	-1.27E-08	count	1
IL9R	-18.2893503	3007.240567	-0.0061	0.995	-1.27E-08	count	1
DLC1	-18.2893503	3007.240567	-0.0061	0.995	-1.27E-08	count	1
URB2	-18.2893503	3007.240567	-0.0061	0.995	-1.27E-08	count	1
SLC6A16	-18.2893503	3007.240567	-0.0061	0.995	-1.27E-08	count	1
ETS2	-18.2893503	3007.240567	-0.0061	0.995	-1.27E-08	count	1
IGLC6	-17.5024295	2411.088862	-0.0073	0.994	-1.27E-08	count	1
VAV3-AS1	-17.5761534	1744.847773	-0.0101	0.992	-1.27E-08	count	1
ITPKB-AS1	-17.2221755	1643.412824	-0.0105	0.992	-1.27E-08	count	1
KDF1	-17.2046244	2077.52212	-0.0083	0.993	-1.27E-08	count	1
RFTN2	-17.2046244	2077.52212	-0.0083	0.993	-1.27E-08	count	1
CTLA4	-17.2046244	2077.52212	-0.0083	0.993	-1.27E-08	count	1
AC018816.1	-17.2046244	2077.52212	-0.0083	0.993	-1.27E-08	count	1
CMYA5	-17.2046244	2077.52212	-0.0083	0.993	-1.27E-08	count	1
CMC1	-0.0010327	0.3570181	-0.0029	0.998	-1.24E-08	count	1
POM121C	-0.0027967	0.4451644	-0.0063	0.995	-1.23E-08	count	1
NLRP1	-0.0027967	0.4623071	-0.006	0.995	-1.23E-08	count	1
GUF1	-0.0461735	1.0433095	-0.0443	0.965	-9.97E-09	count	1
RAB40C	-0.0461735	1.0433095	-0.0443	0.965	-9.97E-09	count	1
E2F7	-0.0461735	1.246341	-0.037	0.97	-9.97E-09	count	1
Z93930.2	-0.0461735	0.9789929	-0.0472	0.962	-9.97E-09	count	1
TADA1	-0.0461735	0.8067067	-0.0572	0.954	-9.97E-09	count	1
CNR1	-0.0461735	0.8067067	-0.0572	0.954	-9.97E-09	count	1
ATP6V1E2	-0.0461735	0.8067067	-0.0572	0.954	-9.97E-09	count	1
POLR1B	-0.0461735	0.8109031	-0.0569	0.955	-9.97E-09	count	1
THAP9	-0.0461735	0.8109031	-0.0569	0.955	-9.97E-09	count	1
LRRC14	-0.0461735	0.8109031	-0.0569	0.955	-9.97E-09	count	1
CYP2U1	-0.0461735	0.8109031	-0.0569	0.955	-9.97E-09	count	1
WDYHV1	-0.0461735	0.8109031	-0.0569	0.955	-9.97E-09	count	1
GGH	-0.0461735	0.8109031	-0.0569	0.955	-9.97E-09	count	1
TACO1	-0.0461735	0.8109031	-0.0569	0.955	-9.97E-09	count	1
DHX33	-0.0461735	0.8109031	-0.0569	0.955	-9.97E-09	count	1
VKORC1L1	-0.0461735	0.8951019	-0.0516	0.959	-9.97E-09	count	1
PITPNM2	-0.0461735	0.8951019	-0.0516	0.959	-9.97E-09	count	1

PSMA6	-0.0461735	0.8951019	-0.0516	0.959	-9.97E-09	count	1
AC005037.1	-0.0461735	0.8951019	-0.0516	0.959	-9.97E-09	count	1
SLC9A3	-0.0461735	0.8951019	-0.0516	0.959	-9.97E-09	count	1
TCTE3	-0.0461735	0.8951019	-0.0516	0.959	-9.97E-09	count	1
PSTK	-0.0461735	0.8951019	-0.0516	0.959	-9.97E-09	count	1
FUCA1	-0.0461735	0.8951019	-0.0516	0.959	-9.97E-09	count	1
PHLDA1	-0.0461735	0.8951019	-0.0516	0.959	-9.97E-09	count	1
PCDHGA10	-0.0461735	0.9755199	-0.0473	0.962	-9.96E-09	count	1
ZYG11A	-0.0461735	0.9755199	-0.0473	0.962	-9.96E-09	count	1
THNSL1	-0.0461735	0.9755199	-0.0473	0.962	-9.96E-09	count	1
AC123023.1	-0.0461735	0.9755199	-0.0473	0.962	-9.96E-09	count	1
CYR61	-0.0461735	0.9755199	-0.0473	0.962	-9.96E-09	count	1
TMEM25	-0.0461735	0.9755199	-0.0473	0.962	-9.96E-09	count	1
ITFG2-AS1	-0.0461735	0.9755199	-0.0473	0.962	-9.96E-09	count	1
CST7	-0.0461735	0.9755199	-0.0473	0.962	-9.96E-09	count	1
PHF7	-0.0461735	0.9755199	-0.0473	0.962	-9.96E-09	count	1
RAB24	-0.0461735	0.9755199	-0.0473	0.962	-9.96E-09	count	1
COQ5	-0.0461735	0.7121321	-0.0648	0.948	-9.96E-09	count	1
COPS7B	-0.0021398	0.5457963	-0.0039	0.997	-9.46E-09	count	1
TRAF3IP2	-0.0021398	0.5403419	-0.004	0.997	-9.44E-09	count	1
GANC	-0.0021398	0.5403014	-0.004	0.997	-9.44E-09	count	1
NINJ1	-0.0021398	0.5070917	-0.0042	0.997	-9.44E-09	count	1
DUSP3	-0.0021398	0.5459284	-0.0039	0.997	-9.44E-09	count	1
TMEM175	-0.0154018	0.5861194	-0.0263	0.979	-9.16E-09	count	1
TVP23B	-0.0154018	0.5502113	-0.028	0.978	-9.14E-09	count	1
DENND1C	-0.0154018	0.471226	-0.0327	0.974	-9.14E-09	count	1
CFLAR	-0.0006915	0.1998272	-0.0035	0.997	-8.29E-09	count	1
LINC00847	-0.0042114	0.7293595	-0.0058	0.995	-6.83E-09	count	1
GATB	-0.0042114	0.7293595	-0.0058	0.995	-6.83E-09	count	1
EDC3	-0.0042114	0.7941668	-0.0053	0.996	-6.83E-09	count	1
PBX3	-0.0042114	0.8544443	-0.0049	0.996	-6.83E-09	count	1
RABL2A	-0.0042114	0.881155	-0.0048	0.996	-6.83E-09	count	1
ITM2A	-0.0036139	0.3240578	-0.0112	0.991	-5.86E-09	count	1
TMED7	-0.0012108	0.3810125	-0.0032	0.997	-5.34E-09	count	1
PDGFRA	-17.9563581	2230.487198	-0.0081	0.994	-4.66E-09	count	1
ZBED3-AS1	-17.9563581	2230.487198	-0.0081	0.994	-4.66E-09	count	1
POLR2J2	-17.9563581	2230.487198	-0.0081	0.994	-4.66E-09	count	1
CATSPERE	-17.9493107	3014.756518	-0.006	0.995	-4.66E-09	count	1
AC007249.2	-17.9493107	3014.756518	-0.006	0.995	-4.66E-09	count	1
FST	-17.9493107	3014.756518	-0.006	0.995	-4.66E-09	count	1
GTF2H4	-17.9493107	3014.756518	-0.006	0.995	-4.66E-09	count	1
ETV7	-17.9493107	3014.756518	-0.006	0.995	-4.66E-09	count	1
IGHV3-53	-17.9493107	3014.756518	-0.006	0.995	-4.66E-09	count	1
MKS1	-17.9493107	3014.756518	-0.006	0.995	-4.66E-09	count	1
ICOS	-17.9493107	3014.756518	-0.006	0.995	-4.66E-09	count	1

THBS2	-17.9493107	3014.756518	-0.006	0.995	-4.66E-09	count	1
ITGA10	-17.9493107	3014.756518	-0.006	0.995	-4.66E-09	count	1
DGKI	-17.9493107	3014.756518	-0.006	0.995	-4.66E-09	count	1
ZNF20	-17.9493107	3014.756518	-0.006	0.995	-4.66E-09	count	1
CHST10	-17.9493107	3014.756518	-0.006	0.995	-4.66E-09	count	1
AC073611.1	-17.9493107	3014.756518	-0.006	0.995	-4.66E-09	count	1
ZNF492	-17.9493107	3014.756518	-0.006	0.995	-4.66E-09	count	1
AC092329.3	-17.9493107	3014.756518	-0.006	0.995	-4.66E-09	count	1
AP001432.1	-17.9493107	3014.756518	-0.006	0.995	-4.66E-09	count	1
FGF18	-17.9493107	3014.756518	-0.006	0.995	-4.66E-09	count	1
BICC1	-17.9493107	3014.756518	-0.006	0.995	-4.66E-09	count	1
LINC02298	-17.9493107	3014.756518	-0.006	0.995	-4.66E-09	count	1
MANSC1	-17.5606354	1731.361908	-0.0101	0.992	-4.66E-09	count	1
Z97192.1	-17.5606354	1731.361908	-0.0101	0.992	-4.66E-09	count	1
ZC3H12B	-17.5606354	1731.361908	-0.0101	0.992	-4.66E-09	count	1
EMG1	-17.5606354	1731.361908	-0.0101	0.992	-4.66E-09	count	1
MORF4L2-AS1	-17.5606354	1731.361908	-0.0101	0.992	-4.66E-09	count	1
PLS3	-17.5606354	1731.361908	-0.0101	0.992	-4.66E-09	count	1
DAB2IP	-17.5606354	1731.361908	-0.0101	0.992	-4.66E-09	count	1
CACNB2	-17.5606354	1731.361908	-0.0101	0.992	-4.66E-09	count	1
C1R	-17.5606354	1731.361908	-0.0101	0.992	-4.66E-09	count	1
TREML2	-17.5606354	1731.361908	-0.0101	0.992	-4.66E-09	count	1
SLC4A8	-17.5606354	1731.361908	-0.0101	0.992	-4.66E-09	count	1
AC079305.1	-17.5606354	1731.361908	-0.0101	0.992	-4.66E-09	count	1
BCL2L2	-17.5606354	1731.361908	-0.0101	0.992	-4.66E-09	count	1
RAVER2	-17.5603336	2481.9152	-0.0071	0.994	-4.66E-09	count	1
AC007365.1	-17.5603336	2481.9152	-0.0071	0.994	-4.66E-09	count	1
P4HA2	-17.5603336	2481.9152	-0.0071	0.994	-4.66E-09	count	1
AC005696.1	-17.5603336	2481.9152	-0.0071	0.994	-4.66E-09	count	1
TEX14	-17.5603336	2481.9152	-0.0071	0.994	-4.66E-09	count	1
ZNF497	-17.5603336	2481.9152	-0.0071	0.994	-4.66E-09	count	1
AC064836.3	-17.5603336	2481.9152	-0.0071	0.994	-4.66E-09	count	1
ADGRF3	-17.5603336	2481.9152	-0.0071	0.994	-4.66E-09	count	1
EEPD1	-17.5603336	2481.9152	-0.0071	0.994	-4.66E-09	count	1
AC145124.1	-17.5603336	2481.9152	-0.0071	0.994	-4.66E-09	count	1
AC021678.2	-17.5603336	2481.9152	-0.0071	0.994	-4.66E-09	count	1
AC100812.1	-17.5603336	2481.9152	-0.0071	0.994	-4.66E-09	count	1
FAM149B1	-17.5603336	2481.9152	-0.0071	0.994	-4.66E-09	count	1
RGS11	-17.5603336	2481.9152	-0.0071	0.994	-4.66E-09	count	1
UPK3A	-17.5603336	2481.9152	-0.0071	0.994	-4.66E-09	count	1
NACC2	-17.5603336	2481.9152	-0.0071	0.994	-4.66E-09	count	1
CPEB1-AS1	-17.5603336	2481.9152	-0.0071	0.994	-4.66E-09	count	1
ELMO3	-17.5603336	2481.9152	-0.0071	0.994	-4.66E-09	count	1
PTPRM	-17.5603336	2481.9152	-0.0071	0.994	-4.66E-09	count	1
KANK2	-17.5603336	2481.9152	-0.0071	0.994	-4.66E-09	count	1

AC096642.1	-17.5603336	2481.9152	-0.0071	0.994	-4.66E-09	count	1
AL450344.2	-17.5603336	2481.9152	-0.0071	0.994	-4.66E-09	count	1
FAM84B	-17.5603336	2481.9152	-0.0071	0.994	-4.66E-09	count	1
FERMT2	-17.5603336	2481.9152	-0.0071	0.994	-4.66E-09	count	1
UCKL1-AS1	-17.5603336	2481.9152	-0.0071	0.994	-4.66E-09	count	1
AXL	-17.5603336	2481.9152	-0.0071	0.994	-4.66E-09	count	1
COIL	-0.0003437	0.3292537	-0.001	0.999	-4.12E-09	count	1
HK2	-17.8696159	2896.988219	-0.0062	0.995	-1.71E-09	count	1
KCNJ3	-17.8696159	2896.988219	-0.0062	0.995	-1.71E-09	count	1
AC013468.1	-17.8696159	2896.988219	-0.0062	0.995	-1.71E-09	count	1
LINC00702	-17.8696159	2896.988219	-0.0062	0.995	-1.71E-09	count	1
AL450326.1	-17.8696159	2896.988219	-0.0062	0.995	-1.71E-09	count	1
TMEM229B	-17.8696159	2896.988219	-0.0062	0.995	-1.71E-09	count	1
AC005253.1	-17.8696159	2896.988219	-0.0062	0.995	-1.71E-09	count	1
IRS1	-17.8696159	2896.988219	-0.0062	0.995	-1.71E-09	count	1
ABI3BP	-17.8696159	2896.988219	-0.0062	0.995	-1.71E-09	count	1
MYO10	-17.8696159	2896.988219	-0.0062	0.995	-1.71E-09	count	1
DAB2	-17.8696159	2896.988219	-0.0062	0.995	-1.71E-09	count	1
BHMT2	-17.8696159	2896.988219	-0.0062	0.995	-1.71E-09	count	1
DBN1	-17.8696159	2896.988219	-0.0062	0.995	-1.71E-09	count	1
ZFP2	-17.8696159	2896.988219	-0.0062	0.995	-1.71E-09	count	1
TREML4	-17.8696159	2896.988219	-0.0062	0.995	-1.71E-09	count	1
AC091729.3	-17.8696159	2896.988219	-0.0062	0.995	-1.71E-09	count	1
CDHR3	-17.8696159	2896.988219	-0.0062	0.995	-1.71E-09	count	1
HTRA4	-17.8696159	2896.988219	-0.0062	0.995	-1.71E-09	count	1
AC027702.1	-17.8696159	2896.988219	-0.0062	0.995	-1.71E-09	count	1
RORB	-17.8696159	2896.988219	-0.0062	0.995	-1.71E-09	count	1
ASPN	-17.8696159	2896.988219	-0.0062	0.995	-1.71E-09	count	1
MEIG1	-17.8696159	2896.988219	-0.0062	0.995	-1.71E-09	count	1
ZNF365	-17.8696159	2896.988219	-0.0062	0.995	-1.71E-09	count	1
RASSF8	-17.8696159	2896.988219	-0.0062	0.995	-1.71E-09	count	1
AC009318.3	-17.8696159	2896.988219	-0.0062	0.995	-1.71E-09	count	1
AC007014.1	-17.8696159	2896.988219	-0.0062	0.995	-1.71E-09	count	1
B3GNT9	-17.8696159	2896.988219	-0.0062	0.995	-1.71E-09	count	1
AC137932.3	-17.8696159	2896.988219	-0.0062	0.995	-1.71E-09	count	1
AC026954.3	-17.8696159	2896.988219	-0.0062	0.995	-1.71E-09	count	1
TBC1D3L	-17.8696159	2896.988219	-0.0062	0.995	-1.71E-09	count	1
PCGF2	-17.8696159	2896.988219	-0.0062	0.995	-1.71E-09	count	1
AC090844.2	-17.8696159	2896.988219	-0.0062	0.995	-1.71E-09	count	1
CSF3	-17.8696159	2896.988219	-0.0062	0.995	-1.71E-09	count	1
AC090912.1	-17.8696159	2896.988219	-0.0062	0.995	-1.71E-09	count	1
CCBE1	-17.8696159	2896.988219	-0.0062	0.995	-1.71E-09	count	1
SDCBP2-AS1	-17.8696159	2896.988219	-0.0062	0.995	-1.71E-09	count	1
SPAG4	-17.8696159	2896.988219	-0.0062	0.995	-1.71E-09	count	1
NTN5	-17.8696159	2896.988219	-0.0062	0.995	-1.71E-09	count	1

CHAF1B	-17.8696159	2896.988219	-0.0062	0.995	-1.71E-09	count	1
FRZB	-17.8696159	2896.988219	-0.0062	0.995	-1.71E-09	count	1
RAB43	-17.8696159	2896.988219	-0.0062	0.995	-1.71E-09	count	1
CD163L1	-17.8696159	2896.988219	-0.0062	0.995	-1.71E-09	count	1
HDGFL3	-17.8696159	2896.988219	-0.0062	0.995	-1.71E-09	count	1
CHTF18	-17.8696159	2896.988219	-0.0062	0.995	-1.71E-09	count	1
SLC25A34	-17.8696159	2896.988219	-0.0062	0.995	-1.71E-09	count	1
ITLN1	-17.8696159	2896.988219	-0.0062	0.995	-1.71E-09	count	1
STPG4	-17.8696159	2896.988219	-0.0062	0.995	-1.71E-09	count	1
ARHGAP10	-17.8696159	2896.988219	-0.0062	0.995	-1.71E-09	count	1
AL009179.1	-17.8696159	2896.988219	-0.0062	0.995	-1.71E-09	count	1
DTX4	-17.8696159	2896.988219	-0.0062	0.995	-1.71E-09	count	1
OR4D9	-17.8696159	2896.988219	-0.0062	0.995	-1.71E-09	count	1
RERGL	-17.8696159	2896.988219	-0.0062	0.995	-1.71E-09	count	1
TBC1D24	-17.8696159	2896.988219	-0.0062	0.995	-1.71E-09	count	1
ELMO2	-17.8696159	2896.988219	-0.0062	0.995	-1.71E-09	count	1
GNG8	-17.8696159	2896.988219	-0.0062	0.995	-1.71E-09	count	1
GULP1	-17.8696159	2896.988219	-0.0062	0.995	-1.71E-09	count	1
TRMT9B	-17.8696159	2896.988219	-0.0062	0.995	-1.71E-09	count	1
TRIM66	-17.8696159	2896.988219	-0.0062	0.995	-1.71E-09	count	1
AP003392.1	-17.8696159	2896.988219	-0.0062	0.995	-1.71E-09	count	1
ESAM	-17.8696159	2896.988219	-0.0062	0.995	-1.71E-09	count	1
DOCK1	-17.8696159	2896.988219	-0.0062	0.995	-1.71E-09	count	1
AL138820.1	-17.8696159	2896.988219	-0.0062	0.995	-1.71E-09	count	1
AC135782.1	-17.8696159	2896.988219	-0.0062	0.995	-1.71E-09	count	1
PGAP1	-17.8696159	2896.988219	-0.0062	0.995	-1.71E-09	count	1
AL121972.1	-17.8696159	2896.988219	-0.0062	0.995	-1.71E-09	count	1
LINC00957	-17.8696159	2896.988219	-0.0062	0.995	-1.71E-09	count	1
LINC01285	-17.8696159	2896.988219	-0.0062	0.995	-1.71E-09	count	1
GALR2	-17.8696159	2896.988219	-0.0062	0.995	-1.71E-09	count	1
ARL9	-17.8696159	2896.988219	-0.0062	0.995	-1.71E-09	count	1
GSG1	-17.8696159	2896.988219	-0.0062	0.995	-1.71E-09	count	1
MYT1	-17.8696159	2896.988219	-0.0062	0.995	-1.71E-09	count	1
Z95115.1	-17.8696159	2896.988219	-0.0062	0.995	-1.71E-09	count	1
AP4B1-AS1	-17.8696159	2896.988219	-0.0062	0.995	-1.71E-09	count	1
CRIM1	-17.8696159	2896.988219	-0.0062	0.995	-1.71E-09	count	1
ADH1B	-17.8696159	2896.988219	-0.0062	0.995	-1.71E-09	count	1
LHFPL2	-17.8696159	2896.988219	-0.0062	0.995	-1.71E-09	count	1
EIF4EBP3	-17.8696159	2896.988219	-0.0062	0.995	-1.71E-09	count	1
CASC15	-17.8696159	2896.988219	-0.0062	0.995	-1.71E-09	count	1
AC084824.5	-17.8696159	2896.988219	-0.0062	0.995	-1.71E-09	count	1
DIAPH3	-17.8696159	2896.988219	-0.0062	0.995	-1.71E-09	count	1
PGBD1	-17.8696159	2896.988219	-0.0062	0.995	-1.71E-09	count	1
MYO6	-17.8696159	2896.988219	-0.0062	0.995	-1.71E-09	count	1
HEY2	-17.8696159	2896.988219	-0.0062	0.995	-1.71E-09	count	1

AC004846.2	-17.8696159	2896.988219	-0.0062	0.995	-1.71E-09	count	1
AC005523.1	-17.8696159	2896.988219	-0.0062	0.995	-1.71E-09	count	1
LINC00954	-17.8696159	2896.988219	-0.0062	0.995	-1.71E-09	count	1
NCKAP5	-17.8696159	2896.988219	-0.0062	0.995	-1.71E-09	count	1
PTH2R	-17.8696159	2896.988219	-0.0062	0.995	-1.71E-09	count	1
MST1	-17.8696159	2896.988219	-0.0062	0.995	-1.71E-09	count	1
AC008040.1	-17.8696159	2896.988219	-0.0062	0.995	-1.71E-09	count	1
AC008957.1	-17.8696159	2896.988219	-0.0062	0.995	-1.71E-09	count	1
SPATA24	-17.8696159	2896.988219	-0.0062	0.995	-1.71E-09	count	1
FOXC1	-17.8696159	2896.988219	-0.0062	0.995	-1.71E-09	count	1
HCG21	-17.8696159	2896.988219	-0.0062	0.995	-1.71E-09	count	1
IGFBP3	-17.8696159	2896.988219	-0.0062	0.995	-1.71E-09	count	1
PPP1R9A	-17.8696159	2896.988219	-0.0062	0.995	-1.71E-09	count	1
AC244090.1	-17.8696159	2896.988219	-0.0062	0.995	-1.71E-09	count	1
SULF1	-17.8696159	2896.988219	-0.0062	0.995	-1.71E-09	count	1
ANGPT1	-17.8696159	2896.988219	-0.0062	0.995	-1.71E-09	count	1
ENHO	-17.8696159	2896.988219	-0.0062	0.995	-1.71E-09	count	1
IL11RA	-17.8696159	2896.988219	-0.0062	0.995	-1.71E-09	count	1
PIP5KL1	-17.8696159	2896.988219	-0.0062	0.995	-1.71E-09	count	1
OR13A1	-17.8696159	2896.988219	-0.0062	0.995	-1.71E-09	count	1
KRT8	-17.8696159	2896.988219	-0.0062	0.995	-1.71E-09	count	1
USP12-AS2	-17.8696159	2896.988219	-0.0062	0.995	-1.71E-09	count	1
SMAD9	-17.8696159	2896.988219	-0.0062	0.995	-1.71E-09	count	1
AC025580.3	-17.8696159	2896.988219	-0.0062	0.995	-1.71E-09	count	1
CPEB1	-17.8696159	2896.988219	-0.0062	0.995	-1.71E-09	count	1
NOMO1	-17.8696159	2896.988219	-0.0062	0.995	-1.71E-09	count	1
AC004943.2	-17.8696159	2896.988219	-0.0062	0.995	-1.71E-09	count	1
JAG1	-17.8696159	2896.988219	-0.0062	0.995	-1.71E-09	count	1
SLA2	-17.8696159	2896.988219	-0.0062	0.995	-1.71E-09	count	1
TGM2	-17.8696159	2896.988219	-0.0062	0.995	-1.71E-09	count	1
FITM2	-17.8696159	2896.988219	-0.0062	0.995	-1.71E-09	count	1
LILRA4	-17.8696159	2896.988219	-0.0062	0.995	-1.71E-09	count	1
GTSE1	-17.8696159	2896.988219	-0.0062	0.995	-1.71E-09	count	1
COL6A2	-17.8696159	2896.988219	-0.0062	0.995	-1.71E-09	count	1
AL391834.2	-17.8696159	2896.988219	-0.0062	0.995	-1.71E-09	count	1
LAYN	-17.8696159	2896.988219	-0.0062	0.995	-1.71E-09	count	1
NET1	-17.8696159	2896.988219	-0.0062	0.995	-1.71E-09	count	1
ANO8	-17.8696159	2896.988219	-0.0062	0.995	-1.71E-09	count	1
AC010247.2	-17.8696159	2896.988219	-0.0062	0.995	-1.71E-09	count	1
LINC00528	-17.8696159	2896.988219	-0.0062	0.995	-1.71E-09	count	1
SEC24B-AS1	-17.8696159	2896.988219	-0.0062	0.995	-1.71E-09	count	1
HIP1	-17.8696159	2896.988219	-0.0062	0.995	-1.71E-09	count	1
AC025419.1	-17.8696159	2896.988219	-0.0062	0.995	-1.71E-09	count	1
AL135999.1	-17.8696159	2896.988219	-0.0062	0.995	-1.71E-09	count	1
AL132639.2	-17.8696159	2896.988219	-0.0062	0.995	-1.71E-09	count	1

AC010401.1	-17.8696159	2896.988219	-0.0062	0.995	-1.71E-09	count	1
C1QTNF1	-17.8696159	2896.988219	-0.0062	0.995	-1.71E-09	count	1
OXT	-17.8696159	2896.988219	-0.0062	0.995	-1.71E-09	count	1
PRR19	-17.8696159	2896.988219	-0.0062	0.995	-1.71E-09	count	1
MXRA8	-17.8696159	2896.988219	-0.0062	0.995	-1.71E-09	count	1
RAB23	-17.8696159	2896.988219	-0.0062	0.995	-1.71E-09	count	1
AC093627.4	-17.8696159	2896.988219	-0.0062	0.995	-1.71E-09	count	1
SATL1	-17.8696159	2896.988219	-0.0062	0.995	-1.71E-09	count	1
FAM69B	-17.8696159	2896.988219	-0.0062	0.995	-1.71E-09	count	1
RAMP2-AS1	-17.8696159	2896.988219	-0.0062	0.995	-1.71E-09	count	1
SKAP1-AS1	-17.8696159	2896.988219	-0.0062	0.995	-1.71E-09	count	1
PNPLA6	-17.8696159	2896.988219	-0.0062	0.995	-1.71E-09	count	1
URB1-AS1	-17.8696159	2896.988219	-0.0062	0.995	-1.71E-09	count	1
AL354822.1	-17.8696159	2896.988219	-0.0062	0.995	-1.71E-09	count	1
SAMD13	-17.8696159	2896.988219	-0.0062	0.995	-1.71E-09	count	1
TGFBI	-17.8696159	2896.988219	-0.0062	0.995	-1.71E-09	count	1
AL109914.1	-17.8696159	2896.988219	-0.0062	0.995	-1.71E-09	count	1
MSRB3	-17.8696159	2896.988219	-0.0062	0.995	-1.71E-09	count	1
PKD1L3	-17.8696159	2896.988219	-0.0062	0.995	-1.71E-09	count	1
ZNF850	-17.8696159	2896.988219	-0.0062	0.995	-1.71E-09	count	1
LINC01311	-17.8696159	2896.988219	-0.0062	0.995	-1.71E-09	count	1
CFHR1	-17.8696159	2896.988219	-0.0062	0.995	-1.71E-09	count	1
CSF1R	-17.8696159	2896.988219	-0.0062	0.995	-1.71E-09	count	1
AC005076.1	-17.8696159	2896.988219	-0.0062	0.995	-1.71E-09	count	1
AC067817.2	-17.8696159	2896.988219	-0.0062	0.995	-1.71E-09	count	1
MATN2	-17.8696159	2896.988219	-0.0062	0.995	-1.71E-09	count	1
MS4A2	-17.8696159	2896.988219	-0.0062	0.995	-1.71E-09	count	1
EGLN3	-17.8696159	2896.988219	-0.0062	0.995	-1.71E-09	count	1
PFAS	-17.8696159	2896.988219	-0.0062	0.995	-1.71E-09	count	1
EFHD1	-17.8696159	2896.988219	-0.0062	0.995	-1.71E-09	count	1
MAFA	-17.8696159	2896.988219	-0.0062	0.995	-1.71E-09	count	1
ZNF883	-17.8696159	2896.988219	-0.0062	0.995	-1.71E-09	count	1
AP001107.1	-17.8696159	2896.988219	-0.0062	0.995	-1.71E-09	count	1
GPR68	-17.8696159	2896.988219	-0.0062	0.995	-1.71E-09	count	1
PLCD4	-17.8696159	2896.988219	-0.0062	0.995	-1.71E-09	count	1
RASSF1-AS1	-17.8696159	2896.988219	-0.0062	0.995	-1.71E-09	count	1
WFS1	-17.8696159	2896.988219	-0.0062	0.995	-1.71E-09	count	1
GSDME	-17.8696159	2896.988219	-0.0062	0.995	-1.71E-09	count	1
AL157935.2	-17.8696159	2896.988219	-0.0062	0.995	-1.71E-09	count	1
SLCO2B1	-17.8696159	2896.988219	-0.0062	0.995	-1.71E-09	count	1
RNF31	-17.8696159	2896.988219	-0.0062	0.995	-1.71E-09	count	1
ATXN7L2	-17.8696159	2896.988219	-0.0062	0.995	-1.71E-09	count	1
AC092953.2	-17.8696159	2896.988219	-0.0062	0.995	-1.71E-09	count	1
FNIP2	-17.8696159	2896.988219	-0.0062	0.995	-1.71E-09	count	1
HMGB3	-17.8696159	2896.988219	-0.0062	0.995	-1.71E-09	count	1

AP003696.1	-17.8696159	2896.988219	-0.0062	0.995	-1.71E-09	count	1
ZNF239	-17.8696159	2896.988219	-0.0062	0.995	-1.71E-09	count	1
ABHD17C	-17.8696159	2896.988219	-0.0062	0.995	-1.71E-09	count	1
NEURL4	-17.8696159	2896.988219	-0.0062	0.995	-1.71E-09	count	1
MYO5B	-17.8696159	2896.988219	-0.0062	0.995	-1.71E-09	count	1
ZNF625-ZNF20	-17.8696159	2896.988219	-0.0062	0.995	-1.71E-09	count	1
F12	-17.8696159	2896.988219	-0.0062	0.995	-1.71E-09	count	1
AC107375.1	-17.8696159	2896.988219	-0.0062	0.995	-1.71E-09	count	1
PAX8	-17.8696159	2896.988219	-0.0062	0.995	-1.71E-09	count	1
EPHA3	-17.8696159	2896.988219	-0.0062	0.995	-1.71E-09	count	1
AC107067.1	-17.8696159	2896.988219	-0.0062	0.995	-1.71E-09	count	1
LINC01596	-17.8696159	2896.988219	-0.0062	0.995	-1.71E-09	count	1
IRGM	-17.8696159	2896.988219	-0.0062	0.995	-1.71E-09	count	1
KCNMB1	-17.8696159	2896.988219	-0.0062	0.995	-1.71E-09	count	1
AL513550.1	-17.8696159	2896.988219	-0.0062	0.995	-1.71E-09	count	1
ZKSCAN5	-17.8696159	2896.988219	-0.0062	0.995	-1.71E-09	count	1
BNC2	-17.8696159	2896.988219	-0.0062	0.995	-1.71E-09	count	1
CH25H	-17.8696159	2896.988219	-0.0062	0.995	-1.71E-09	count	1
ZNF385A	-17.8696159	2896.988219	-0.0062	0.995	-1.71E-09	count	1
TBX3	-17.8696159	2896.988219	-0.0062	0.995	-1.71E-09	count	1
MEG8	-17.8696159	2896.988219	-0.0062	0.995	-1.71E-09	count	1
AC233723.1	-17.8696159	2896.988219	-0.0062	0.995	-1.71E-09	count	1
AC009283.1	-17.8696159	2896.988219	-0.0062	0.995	-1.71E-09	count	1
MMP23B	-17.8696159	2896.988219	-0.0062	0.995	-1.71E-09	count	1
GLDC	-17.8696159	2896.988219	-0.0062	0.995	-1.71E-09	count	1
KIAA0895L	-17.8696159	2896.988219	-0.0062	0.995	-1.71E-09	count	1
AC002094.2	-17.8696159	2896.988219	-0.0062	0.995	-1.71E-09	count	1
U2AF1	-17.8696159	2896.988219	-0.0062	0.995	-1.71E-09	count	1
ABHD14A	-0.0003437	0.3892789	-9.00E-04	0.999	-1.52E-09	count	1
PGBD2	-0.0003437	0.5501138	-6.00E-04	1	-1.52E-09	count	1
GLT8D1	-0.0003437	0.6094083	-6.00E-04	1	-1.52E-09	count	1
PUM2	-0.0003437	0.4665219	-7.00E-04	0.999	-1.52E-09	count	1
HIVEP2	-0.0003437	0.5179354	-7.00E-04	0.999	-1.52E-09	count	1
CLU	-0.0003437	0.8869831	-4.00E-04	1	-1.52E-09	count	1
EEFSEC	-0.0003437	0.4836263	-7.00E-04	0.999	-1.52E-09	count	1
CLOCK	-0.0003437	0.5370061	-6.00E-04	0.999	-1.52E-09	count	1
ERCC6L2	-0.0003437	0.7619334	-5.00E-04	1	-5.58E-10	count	1
ALOX12-AS1	-0.0003437	0.7619334	-5.00E-04	1	-5.58E-10	count	1
RBM14	-0.0003437	0.7153981	-5.00E-04	1	-5.58E-10	count	1
PASK	-0.0003437	0.7153981	-5.00E-04	1	-5.58E-10	count	1
CEP68	-0.0003437	0.7619037	-5.00E-04	1	-5.58E-10	count	1
TAF6L	-0.0003437	0.6656189	-5.00E-04	1	-5.58E-10	count	1
HAUS5	-0.0003437	0.6656174	-5.00E-04	1	-5.58E-10	count	1
CORO1C	-0.0003437	0.7619179	-5.00E-04	1	-5.58E-10	count	1
DPH2	-0.0003437	0.7619179	-5.00E-04	1	-5.58E-10	count	1

PPP1R13B	-0.0003437	0.7153816	-5.00E-04	1	-5.58E-10	count	1
BCKDK	-0.0003437	0.7153816	-5.00E-04	1	-5.58E-10	count	1
SLC25A14	-0.0003437	0.7153816	-5.00E-04	1	-5.58E-10	count	1
ERAP1	-0.0003437	0.8057575	-4.00E-04	1	-5.58E-10	count	1
HLX	0.0026167	0.7731427	0.0034	0.997	1.56E-09	count	1
COG1	0.0026167	0.6677671	0.0039	0.997	1.56E-09	count	1
MRPL35	0.0026167	0.7045827	0.0037	0.997	1.56E-09	count	1
ZRSR2	0.0026167	0.3991441	0.0066	0.995	1.56E-09	count	1
MTOR	0.002617	0.6286943	0.0042	0.997	1.57E-09	count	1
RPS6KC1	0.002617	0.739568	0.0035	0.997	1.57E-09	count	1
TYW5	0.002617	0.6286943	0.0042	0.997	1.57E-09	count	1
NUDT8	0.002617	0.739747	0.0035	0.997	1.57E-09	count	1
THOC5	0.002617	0.739568	0.0035	0.997	1.57E-09	count	1
TIMM22	0.0007267	0.4013256	0.0018	0.999	3.21E-09	count	1
SUCO	0.0007267	0.4341512	0.0017	0.999	3.21E-09	count	1
TRANK1	0.0007267	0.4637218	0.0016	0.999	3.21E-09	count	1
SWI5	0.0012286	0.5364339	0.0023	0.998	5.43E-09	count	1
KIF21A	0.0012286	0.5083898	0.0024	0.998	5.43E-09	count	1
ZNF831	0.1718503	0.5252627	0.3272	0.744	5.46E-09	count	1
MOAP1	0.0016893	0.4963152	0.0034	0.997	7.47E-09	count	1
EMC2	0.0017101	0.4199224	0.0041	0.997	7.55E-09	count	1
TRAPPC2	0.0144511	0.460358	0.0314	0.975	8.68E-09	count	1
CYBC1	0.0010952	0.2271092	0.0048	0.996	1.32E-08	count	1
EHBP1	0.0228194	0.5304672	0.043	0.966	1.38E-08	count	1
GPALPP1	0.0228194	0.5304672	0.043	0.966	1.38E-08	count	1
SLC38A6	0.0228194	0.597602	0.0382	0.97	1.38E-08	count	1
SP2	0.0228194	0.531012	0.043	0.966	1.38E-08	count	1
AL078459.1	0.0228194	0.6182068	0.0369	0.971	1.38E-08	count	1
WRN	0.0228194	0.6376934	0.0358	0.971	1.38E-08	count	1
CNTNAP2	0.0228194	0.7297181	0.0313	0.975	1.38E-08	count	1
ADAM17	0.0012281	0.3967662	0.0031	0.998	1.47E-08	count	1
BLOC1S3	0.0716095	0.8533629	0.0839	0.933	1.62E-08	count	1
STK35	0.0716095	0.8533629	0.0839	0.933	1.62E-08	count	1
CDKL3	0.0716095	0.8533629	0.0839	0.933	1.62E-08	count	1
TTBK2	0.0716095	0.8533629	0.0839	0.933	1.62E-08	count	1
MCEE	0.0716095	0.8533629	0.0839	0.933	1.62E-08	count	1
EPG5	0.0716095	0.8533629	0.0839	0.933	1.62E-08	count	1
TRIM16	0.0716095	1.1500315	0.0623	0.95	1.62E-08	count	1
C8orf88	0.0716095	1.1500315	0.0623	0.95	1.62E-08	count	1
RFESD	0.0716095	1.1500315	0.0623	0.95	1.62E-08	count	1
ZNF461	0.0716095	1.1500315	0.0623	0.95	1.62E-08	count	1
POLH	0.0716095	1.1500315	0.0623	0.95	1.62E-08	count	1
LINC01361	0.0716095	1.1500315	0.0623	0.95	1.62E-08	count	1
AL034397.3	0.0716095	1.1500315	0.0623	0.95	1.62E-08	count	1
PYGM	0.0716095	1.1500315	0.0623	0.95	1.62E-08	count	1

SMURF1	0.0716095	1.1500315	0.0623	0.95	1.62E-08	count	1
MSC	0.0716095	1.1500315	0.0623	0.95	1.62E-08	count	1
SLC35G1	0.0716095	1.1500315	0.0623	0.95	1.62E-08	count	1
INSR	0.0716095	1.1500315	0.0623	0.95	1.62E-08	count	1
CAMSAP2	0.0716095	1.1500315	0.0623	0.95	1.62E-08	count	1
MYLK-AS1	0.0716095	1.1500315	0.0623	0.95	1.62E-08	count	1
CPEB3	0.0716095	1.1500315	0.0623	0.95	1.62E-08	count	1
ZNF527	0.0716095	1.1500315	0.0623	0.95	1.62E-08	count	1
IFNLR1	0.0716095	1.1500315	0.0623	0.95	1.62E-08	count	1
SOX18	0.0716095	1.1500315	0.0623	0.95	1.62E-08	count	1
SLC38A7	0.0716095	1.0073553	0.0711	0.943	1.62E-08	count	1
APBA3	0.0716095	1.0073553	0.0711	0.943	1.62E-08	count	1
FBXW8	0.0716095	1.0073553	0.0711	0.943	1.62E-08	count	1
FAN1	0.0716095	1.0073553	0.0711	0.943	1.62E-08	count	1
ZNF865	0.0716095	1.0073553	0.0711	0.943	1.62E-08	count	1
TOP3B	0.0716095	1.0073553	0.0711	0.943	1.62E-08	count	1
ANKRD6	0.0716095	1.0073553	0.0711	0.943	1.62E-08	count	1
VWA8	0.0716095	1.0073553	0.0711	0.943	1.62E-08	count	1
CXorf57	0.0716095	1.0073553	0.0711	0.943	1.62E-08	count	1
SGPL1	0.0716095	1.0073553	0.0711	0.943	1.62E-08	count	1
SLC29A2	0.0716095	1.0178585	0.0704	0.944	1.62E-08	count	1
AC002310.1	0.0716095	1.0178585	0.0704	0.944	1.62E-08	count	1
PTCD2	0.0716095	1.0178585	0.0704	0.944	1.62E-08	count	1
MDP1	0.0716095	1.0178585	0.0704	0.944	1.62E-08	count	1
SLC11A1	0.0716095	1.3768245	0.052	0.959	1.62E-08	count	1
SERPINF1	0.0716095	1.3768245	0.052	0.959	1.62E-08	count	1
WNT2B	0.0716095	1.3768245	0.052	0.959	1.62E-08	count	1
ZNF354C	0.0716095	1.3768245	0.052	0.959	1.62E-08	count	1
ZNF778	0.0716095	1.3768245	0.052	0.959	1.62E-08	count	1
AC079807.1	0.0716095	1.3768245	0.052	0.959	1.62E-08	count	1
ZNF718	0.0716095	1.3768245	0.052	0.959	1.62E-08	count	1
CAVIN3	0.0716095	1.3768245	0.052	0.959	1.62E-08	count	1
SLC23A3	0.0716095	1.3768245	0.052	0.959	1.62E-08	count	1
TNFSF8	0.0716095	0.6565486	0.1091	0.913	1.62E-08	count	1
ZMYND19	0.0716095	0.6565486	0.1091	0.913	1.62E-08	count	1
ABHD11	0.0716095	0.6565486	0.1091	0.913	1.62E-08	count	1
TMEM206	0.0716095	0.6565486	0.1091	0.913	1.62E-08	count	1
AC022182.2	0.0716095	0.6565486	0.1091	0.913	1.62E-08	count	1
AC136475.3	0.0716095	1.2600944	0.0568	0.955	1.62E-08	count	1
SLC25A25-AS1	0.0716095	1.2600944	0.0568	0.955	1.62E-08	count	1
TACC1	0.001512	0.3237658	0.0047	0.996	1.81E-08	count	1
ZNF44	0.0338692	0.4661691	0.0727	0.942	2.05E-08	count	1
AC107068.1	21.0922268	1328.869578	0.0159	0.987	2.07E-08	count	1
AGAP3	21.0922268	1328.869578	0.0159	0.987	2.07E-08	count	1
JPH1	21.0922268	1328.869578	0.0159	0.987	2.07E-08	count	1

LINC02361	21.0922268	1328.869578	0.0159	0.987	2.07E-08	count	1
SH3BGR	21.0922268	1328.869578	0.0159	0.987	2.07E-08	count	1
AL121935.1	21.0922267	1328.869826	0.0159	0.987	2.07E-08	count	1
AL121928.1	21.0922267	1328.869826	0.0159	0.987	2.07E-08	count	1
AC105020.6	21.0922267	1328.869826	0.0159	0.987	2.07E-08	count	1
UGDH	21.0922263	1328.869644	0.0159	0.987	2.07E-08	count	1
FOXD1	21.0922263	1328.869644	0.0159	0.987	2.07E-08	count	1
ADAM9	21.0922263	1328.869644	0.0159	0.987	2.07E-08	count	1
LY6E-DT	21.0922263	1328.869644	0.0159	0.987	2.07E-08	count	1
AP003486.1	21.0922263	1328.869644	0.0159	0.987	2.07E-08	count	1
ABCC9	21.0922263	1328.869644	0.0159	0.987	2.07E-08	count	1
LINC01134	21.0922258	1328.869562	0.0159	0.987	2.07E-08	count	1
AC239868.2	21.0922258	1328.869562	0.0159	0.987	2.07E-08	count	1
SEMA4A	21.0922258	1328.869562	0.0159	0.987	2.07E-08	count	1
FAM218A	21.0922258	1328.869562	0.0159	0.987	2.07E-08	count	1
RHOD	21.0922258	1328.869562	0.0159	0.987	2.07E-08	count	1
AC079907.2	21.0922258	1328.869562	0.0159	0.987	2.07E-08	count	1
LINC02175	21.0922258	1328.869562	0.0159	0.987	2.07E-08	count	1
LINC00672	21.0922258	1328.869562	0.0159	0.987	2.07E-08	count	1
HOXB-AS1	21.0922258	1328.869562	0.0159	0.987	2.07E-08	count	1
LRRC37A3	21.0922258	1328.869562	0.0159	0.987	2.07E-08	count	1
CEACAM3	21.0922258	1328.869562	0.0159	0.987	2.07E-08	count	1
ZBTB8A	21.0922258	1328.869429	0.0159	0.987	2.07E-08	count	1
FMO5	21.0922258	1328.869429	0.0159	0.987	2.07E-08	count	1
ERMP1	21.0922258	1328.869429	0.0159	0.987	2.07E-08	count	1
FOSL1	21.0922258	1328.869429	0.0159	0.987	2.07E-08	count	1
CTNNA3	21.0922258	1328.869429	0.0159	0.987	2.07E-08	count	1
PRC1	21.0922258	1328.869429	0.0159	0.987	2.07E-08	count	1
ZNF763	21.0922258	1328.869429	0.0159	0.987	2.07E-08	count	1
ELF3-AS1	21.0922259	1328.869446	0.0159	0.987	2.07E-08	count	1
MAP10	21.0922259	1328.869446	0.0159	0.987	2.07E-08	count	1
AC008608.2	21.0922259	1328.869446	0.0159	0.987	2.07E-08	count	1
PCDHGA7	21.0922259	1328.869446	0.0159	0.987	2.07E-08	count	1
AC006027.1	21.0922259	1328.869446	0.0159	0.987	2.07E-08	count	1
AC016405.3	21.0922259	1328.869446	0.0159	0.987	2.07E-08	count	1
AL359636.1	21.0922259	1328.869446	0.0159	0.987	2.07E-08	count	1
GPRC5D	21.0922259	1328.869446	0.0159	0.987	2.07E-08	count	1
PCLAF	21.0922259	1328.869446	0.0159	0.987	2.07E-08	count	1
FBXL8	21.0922259	1328.869446	0.0159	0.987	2.07E-08	count	1
ITGA3	21.0922259	1328.869446	0.0159	0.987	2.07E-08	count	1
CHAD	21.0922259	1328.869446	0.0159	0.987	2.07E-08	count	1
C19orf57	21.0922259	1328.869446	0.0159	0.987	2.07E-08	count	1
TST	21.0922259	1328.869446	0.0159	0.987	2.07E-08	count	1
DYNC1H1	0.0020596	0.3289079	0.0063	0.995	2.47E-08	count	1
ANG	0.0408379	0.5269731	0.0775	0.938	2.48E-08	count	1

WDR44	0.0408379	0.5070456	0.0805	0.936	2.48E-08	count	1
TBCE	1.5581446	0.4451946	3.4999	5.00E-04	3.03E-08	count	1
LINC00294	1.5581446	0.4451946	3.4999	5.00E-04	3.03E-08	count	1
SULT1A3	1.5581446	0.4451946	3.4999	5.00E-04	3.03E-08	count	1
ZNF773	1.5581446	0.4451946	3.4999	5.00E-04	3.03E-08	count	1
LRP8	1.5581446	0.4451946	3.4999	5.00E-04	3.03E-08	count	1
FUK	1.5581446	0.4451946	3.4999	5.00E-04	3.03E-08	count	1
DIRAS1	1.5581446	0.4451946	3.4999	5.00E-04	3.03E-08	count	1
MAFG	0.0025679	0.2609767	0.0098	0.992	3.09E-08	count	1
CAAP1	0.0072078	0.357168	0.0202	0.984	3.19E-08	count	1
SYVN1	0.0073695	0.458943	0.0161	0.987	3.26E-08	count	1
SH2D3A	0.0206752	0.6798493	0.0304	0.976	3.38E-08	count	1
GSE1	0.0206752	0.8633114	0.0239	0.981	3.39E-08	count	1
INTS3	0.0217405	0.6046843	0.036	0.971	3.56E-08	count	1
KDM5C	0.0217405	0.6460421	0.0337	0.973	3.56E-08	count	1
CUL2	0.0080965	0.4636325	0.0175	0.986	3.59E-08	count	1
UPF3A	0.0031041	0.230868	0.0134	0.989	3.73E-08	count	1
NUP58	0.0087658	0.4096761	0.0214	0.983	3.88E-08	count	1
SMG6	0.0087658	0.3938295	0.0223	0.982	3.88E-08	count	1
ACBD5	0.0090582	0.4964005	0.0182	0.985	4.01E-08	count	1
ARHGAP42	0.17697	1.0375531	0.1706	0.865	4.17E-08	count	1
INTS8	0.17697	0.7705802	0.2297	0.818	4.17E-08	count	1
WDR5	0.17697	0.8389421	0.2109	0.833	4.17E-08	count	1
CROCC	0.17697	0.8389421	0.2109	0.833	4.17E-08	count	1
MLLT1	0.17697	0.8389421	0.2109	0.833	4.17E-08	count	1
FNBP1L	0.17697	0.8389421	0.2109	0.833	4.17E-08	count	1
LXN	0.17697	0.6800459	0.2602	0.795	4.17E-08	count	1
MOB3C	0.17697	0.9021385	0.1962	0.845	4.17E-08	count	1
EIF5A2	0.17697	0.9021385	0.1962	0.845	4.17E-08	count	1
ZRANB3	0.17697	0.7566323	0.2339	0.815	4.17E-08	count	1
SLC25A22	0.17697	0.7566323	0.2339	0.815	4.17E-08	count	1
TMEM67	0.17697	0.7566323	0.2339	0.815	4.17E-08	count	1
RNF185	0.17697	0.8261492	0.2142	0.83	4.17E-08	count	1
INTS5	0.17697	0.8261492	0.2142	0.83	4.17E-08	count	1
WDR59	0.17697	0.8261492	0.2142	0.83	4.17E-08	count	1
HRK	0.17697	1.1198098	0.158	0.875	4.17E-08	count	1
NINJ2	0.17697	1.1198098	0.158	0.875	4.17E-08	count	1
MPST	0.17697	0.9500435	0.1863	0.852	4.17E-08	count	1
EPB41L4A	0.17697	0.9500435	0.1863	0.852	4.17E-08	count	1
MAGED1	0.0716095	0.7127468	0.1005	0.92	4.40E-08	count	1
ZNF35	0.0716095	0.7127468	0.1005	0.92	4.40E-08	count	1
METTL25	0.0716095	0.7127468	0.1005	0.92	4.40E-08	count	1
ATP1B1	0.0716095	0.6070939	0.118	0.906	4.40E-08	count	1
COG3	0.0716095	0.8103049	0.0884	0.93	4.40E-08	count	1
PIP5K1B	0.0716095	0.5500225	0.1302	0.896	4.40E-08	count	1

ERMAP	0.0716095	0.9363836	0.0765	0.939	4.40E-08	count	1
ARHGAP35	0.0716095	0.632772	0.1132	0.91	4.40E-08	count	1
PRKCZ	0.0716095	0.7613423	0.0941	0.925	4.40E-08	count	1
SOX5	0.0716095	0.7613423	0.0941	0.925	4.40E-08	count	1
CDKAL1	0.0716095	0.7613423	0.0941	0.925	4.40E-08	count	1
CUL9	0.0716095	0.7648267	0.0936	0.925	4.40E-08	count	1
SARNP	0.0716095	0.5782405	0.1238	0.901	4.40E-08	count	1
SLC7A5	0.0716095	0.8070169	0.0887	0.929	4.40E-08	count	1
USP37	0.0716095	0.7924576	0.0904	0.928	4.40E-08	count	1
TRMT2A	0.0716095	0.6051442	0.1183	0.906	4.40E-08	count	1
WDR70	0.0716095	0.5504883	0.1301	0.897	4.40E-08	count	1
TBCK	0.0716095	0.7090065	0.101	0.92	4.40E-08	count	1
TRIM24	0.0716095	0.7090065	0.101	0.92	4.40E-08	count	1
ABCA5	0.0716095	0.7090065	0.101	0.92	4.40E-08	count	1
CASTOR3	0.0716095	0.9711491	0.0737	0.941	4.40E-08	count	1
RUFY3	0.0716095	0.5478698	0.1307	0.896	4.40E-08	count	1
ARHGAP26	0.0716095	0.7042106	0.1017	0.919	4.40E-08	count	1
KHNYN	0.0716095	0.6290258	0.1138	0.909	4.41E-08	count	1
RNF144B	0.0716095	0.4585248	0.1562	0.876	4.41E-08	count	1
CXCL8	0.0716095	1.2600944	0.0568	0.955	4.41E-08	count	1
IFT74	0.0099569	0.4805824	0.0207	0.983	4.43E-08	count	1
SLC15A4	0.0104891	0.3996159	0.0262	0.979	4.65E-08	count	1
TAOK3	0.0041563	0.2583667	0.0161	0.987	4.99E-08	count	1
NMD3	0.011924	0.5298566	0.0225	0.982	5.29E-08	count	1
MAP3K7CL	0.011924	0.4824076	0.0247	0.98	5.29E-08	count	1
SPSB3	0.0044536	0.2986519	0.0149	0.988	5.35E-08	count	1
HHIP	21.0647983	1310.769664	0.0161	0.987	5.58E-08	count	1
AL356417.3	21.0647983	1310.769664	0.0161	0.987	5.58E-08	count	1
BCO2	21.0647983	1310.769664	0.0161	0.987	5.58E-08	count	1
IGHV3-23	21.0647983	1310.769664	0.0161	0.987	5.58E-08	count	1
CGNL1	21.0647983	1310.769664	0.0161	0.987	5.58E-08	count	1
MYZAP	21.0647983	1310.769664	0.0161	0.987	5.58E-08	count	1
LINC00921	21.0647983	1310.769664	0.0161	0.987	5.58E-08	count	1
DBF4B	21.0647983	1310.769664	0.0161	0.987	5.58E-08	count	1
C2CD2	21.0647983	1310.769664	0.0161	0.987	5.58E-08	count	1
MBOAT2	20.7516591	1120.801881	0.0185	0.985	5.61E-08	count	1
IL1RN	20.7516591	1120.801881	0.0185	0.985	5.61E-08	count	1
CHL1	20.7516591	1120.801881	0.0185	0.985	5.61E-08	count	1
LINC01506	20.7516591	1120.801881	0.0185	0.985	5.61E-08	count	1
AL158801.2	20.7516591	1120.801881	0.0185	0.985	5.61E-08	count	1
FBXL22	20.7516591	1120.801881	0.0185	0.985	5.61E-08	count	1
CHRNE	20.7516591	1120.801881	0.0185	0.985	5.61E-08	count	1
AL133342.1	0.0126629	0.4811379	0.0263	0.979	5.62E-08	count	1
CERS2	0.0126629	0.4940239	0.0256	0.98	5.62E-08	count	1
NFATC2IP	0.0126629	0.4808007	0.0263	0.979	5.62E-08	count	1

TARS	0.0126629	0.5648335	0.0224	0.982	5.63E-08	count	1
DPP8	0.0133228	0.4611447	0.0289	0.977	5.91E-08	count	1
LYSMD3	0.0969273	0.4198238	0.2309	0.818	6.02E-08	count	1
IRAK3	1.2704625	0.3835976	3.312	0.001	6.10E-08	count	1
DOK1	0.0139156	0.4548168	0.0306	0.976	6.17E-08	count	1
USPL1	0.0139156	0.4438299	0.0314	0.975	6.18E-08	count	1
ARL13B	0.0139156	0.5053772	0.0275	0.978	6.18E-08	count	1
COL9A3	0.0151387	0.5114829	0.0296	0.976	6.77E-08	count	1
C8orf44	2.656757	0.4173418	6.3659	4.85E-10	6.79E-08	count	1.18E-05
SART1	0.042959	0.6125053	0.0701	0.944	7.10E-08	count	1
MRNIP	0.0162019	0.3611573	0.0449	0.964	7.20E-08	count	1
UHMK1	0.0060358	0.2783921	0.0217	0.983	7.26E-08	count	1
BRI3BP	0.0165072	0.4207349	0.0392	0.969	7.33E-08	count	1
WRAP73	0.0165072	0.3661072	0.0451	0.964	7.33E-08	count	1
AP003774.4	0.1181295	0.6015676	0.1964	0.844	7.39E-08	count	1
HIRIP3	0.1181295	0.5590555	0.2113	0.833	7.39E-08	count	1
NXPE3	0.1181295	0.5759478	0.2051	0.838	7.40E-08	count	1
ZMYM4	0.1256768	0.4331706	0.2901	0.772	7.89E-08	count	1
ELOF1	0.0067111	0.2966349	0.0226	0.982	8.07E-08	count	1
BCCIP	0.0067111	0.3132721	0.0214	0.983	8.07E-08	count	1
HBEGF	0.1287169	1.3804934	0.0932	0.926	8.08E-08	count	1
SLC25A16	0.1287169	1.3804934	0.0932	0.926	8.08E-08	count	1
PRKCA	0.1287169	1.3804934	0.0932	0.926	8.08E-08	count	1
ZNF616	0.1287169	1.3804934	0.0932	0.926	8.08E-08	count	1
TMPPE	0.1287169	1.3804934	0.0932	0.926	8.08E-08	count	1
ACY1	0.1287169	1.3804934	0.0932	0.926	8.08E-08	count	1
INAFM2	0.1287169	1.3804934	0.0932	0.926	8.08E-08	count	1
CYB5D1	0.1287169	1.3086167	0.0984	0.922	8.08E-08	count	1
AL353622.1	0.1287169	1.3086167	0.0984	0.922	8.08E-08	count	1
PCBP3	0.1287169	1.3086167	0.0984	0.922	8.08E-08	count	1
CLEC4A	0.1287169	1.3086167	0.0984	0.922	8.08E-08	count	1
C2CD3	0.1287169	1.3086167	0.0984	0.922	8.08E-08	count	1
CCDC180	0.1287169	1.3086167	0.0984	0.922	8.08E-08	count	1
LINC00685	0.1287169	1.3824563	0.0931	0.926	8.08E-08	count	1
TTYH3	0.1287169	1.3824563	0.0931	0.926	8.08E-08	count	1
ZNF561	0.1287169	1.3824563	0.0931	0.926	8.08E-08	count	1
E2F3	0.1361481	0.7986545	0.1705	0.865	8.57E-08	count	1
BOP1	0.1361481	0.6767578	0.2012	0.841	8.57E-08	count	1
IGKV4-1	0.1361481	0.7680046	0.1773	0.859	8.58E-08	count	1
GGT7	0.1361481	0.7070411	0.1926	0.847	8.58E-08	count	1
SCAP	0.1361481	0.7640151	0.1782	0.859	8.58E-08	count	1
CHEK2	0.1361481	0.6722269	0.2025	0.84	8.58E-08	count	1
SIRT3	0.1361481	0.7027055	0.1937	0.846	8.58E-08	count	1
ACOX1	0.1361481	0.7027055	0.1937	0.846	8.58E-08	count	1
GMIP	0.1361481	0.4197912	0.3243	0.746	8.60E-08	count	1

AIMP2	0.1361481	0.7559728	0.1801	0.857	8.61E-08	count	1
SOCS7	0.3592916	1.1478534	0.313	0.754	9.08E-08	count	1
ZFAND4	0.3592916	1.1478534	0.313	0.754	9.08E-08	count	1
CABP4	0.3592916	1.1478534	0.313	0.754	9.08E-08	count	1
PXMP4	0.3592916	1.1478534	0.313	0.754	9.08E-08	count	1
AL645933.2	0.3592916	1.1478534	0.313	0.754	9.08E-08	count	1
KRBA1	0.3592916	1.1478534	0.313	0.754	9.08E-08	count	1
HAUS7	0.3592916	1.1478534	0.313	0.754	9.08E-08	count	1
PPP1R3B	0.3592916	1.1478534	0.313	0.754	9.08E-08	count	1
RAD51AP1	0.3592916	1.1478534	0.313	0.754	9.08E-08	count	1
ABCB9	0.3592916	1.1478534	0.313	0.754	9.08E-08	count	1
WDR7	0.3592916	1.1478534	0.313	0.754	9.08E-08	count	1
NOTCH2NL	0.3592916	1.1478534	0.313	0.754	9.08E-08	count	1
MMS22L	0.3592916	1.1478534	0.313	0.754	9.08E-08	count	1
CD99L2	0.3592916	1.1478534	0.313	0.754	9.08E-08	count	1
AC091814.1	0.3592916	1.1478534	0.313	0.754	9.08E-08	count	1
AC079630.1	0.3592916	1.1478534	0.313	0.754	9.08E-08	count	1
PALB2	0.3592916	1.1478534	0.313	0.754	9.08E-08	count	1
MTCL1	0.3592916	1.1478534	0.313	0.754	9.08E-08	count	1
RWDD3	0.3592916	1.1478534	0.313	0.754	9.08E-08	count	1
MFSD6	0.3592916	1.1478534	0.313	0.754	9.08E-08	count	1
OXNAD1	0.3592916	1.1478534	0.313	0.754	9.08E-08	count	1
FLNB	0.3592916	1.1478534	0.313	0.754	9.08E-08	count	1
GRAMD2B	0.3592916	1.1478534	0.313	0.754	9.08E-08	count	1
CKB	0.3592916	1.1478534	0.313	0.754	9.08E-08	count	1
ZSCAN32	0.3592916	1.1478534	0.313	0.754	9.08E-08	count	1
CTU2	0.3592916	1.1478534	0.313	0.754	9.08E-08	count	1
SLC16A6	0.3592916	1.1478534	0.313	0.754	9.08E-08	count	1
SIGLEC6	0.3592916	1.1478534	0.313	0.754	9.08E-08	count	1
WDR4	0.3592916	1.1478534	0.313	0.754	9.08E-08	count	1
LARS2	0.3592916	1.1478534	0.313	0.754	9.08E-08	count	1
CCNE2	0.3592916	1.1478534	0.313	0.754	9.08E-08	count	1
KNL1	0.3592916	1.1478534	0.313	0.754	9.08E-08	count	1
MMP25-AS1	0.3592916	1.1478534	0.313	0.754	9.08E-08	count	1
CNTROB	0.3592916	1.1478534	0.313	0.754	9.08E-08	count	1
EPHX1	0.3592916	1.1478534	0.313	0.754	9.08E-08	count	1
NFXL1	0.3592916	1.1478534	0.313	0.754	9.08E-08	count	1
NLN	0.3592916	1.1478534	0.313	0.754	9.08E-08	count	1
AC106791.1	0.3592916	1.1478534	0.313	0.754	9.08E-08	count	1
ZEB1-AS1	0.3592916	1.1478534	0.313	0.754	9.08E-08	count	1
SLC48A1	0.3592916	1.1478534	0.313	0.754	9.08E-08	count	1
ABHD4	0.3592916	1.1478534	0.313	0.754	9.08E-08	count	1
IL4I1	0.3592916	1.1478534	0.313	0.754	9.08E-08	count	1
EXO5	0.3592916	1.1478534	0.313	0.754	9.08E-08	count	1
FLVCR1-DT	0.3592916	1.1478534	0.313	0.754	9.08E-08	count	1

DLG3	0.3592916	1.1478534	0.313	0.754	9.08E-08	count	1
SENCR	0.3592916	1.1478534	0.313	0.754	9.08E-08	count	1
CCNJ	0.3592916	1.1478534	0.313	0.754	9.08E-08	count	1
ZNF555	0.3592916	1.1478534	0.313	0.754	9.08E-08	count	1
ZMYM3	0.3592916	1.1478534	0.313	0.754	9.08E-08	count	1
SULT1A1	0.3592916	1.1478534	0.313	0.754	9.08E-08	count	1
C20orf194	0.3592916	1.1478534	0.313	0.754	9.08E-08	count	1
AC011416.3	0.3592916	1.4461038	0.2485	0.804	9.08E-08	count	1
CEP85	0.3592916	1.4461038	0.2485	0.804	9.08E-08	count	1
AL683813.1	0.3592916	1.4461038	0.2485	0.804	9.08E-08	count	1
KCNIP2	0.3592916	1.4461038	0.2485	0.804	9.08E-08	count	1
UNC119B	0.3592916	1.4461038	0.2485	0.804	9.08E-08	count	1
DTD2	0.3592916	1.4461038	0.2485	0.804	9.08E-08	count	1
LIN7B	0.3592916	1.4461038	0.2485	0.804	9.08E-08	count	1
ARNT	0.3592916	1.4461038	0.2485	0.804	9.08E-08	count	1
MSH5	0.3592916	1.4461038	0.2485	0.804	9.08E-08	count	1
MAOA	0.3592916	1.4461038	0.2485	0.804	9.08E-08	count	1
POC1B-GALNT4	0.3592916	1.4461038	0.2485	0.804	9.08E-08	count	1
SGSH	0.3592916	1.4461038	0.2485	0.804	9.08E-08	count	1
LINC00158	0.3592916	1.4461038	0.2485	0.804	9.08E-08	count	1
IGKV1-39	0.3592916	1.4461038	0.2485	0.804	9.08E-08	count	1
DNAH1	0.3592916	1.4461038	0.2485	0.804	9.08E-08	count	1
AL365203.2	0.3592916	1.4461038	0.2485	0.804	9.08E-08	count	1
ZBTB39	0.3592916	1.4461038	0.2485	0.804	9.08E-08	count	1
VASH1-AS1	0.3592916	1.4461038	0.2485	0.804	9.08E-08	count	1
BMF	0.3592916	1.4461038	0.2485	0.804	9.08E-08	count	1
FAM171A2	0.3592916	1.4461038	0.2485	0.804	9.08E-08	count	1
LAMA5	0.3592916	1.4461038	0.2485	0.804	9.08E-08	count	1
POLR1A	0.3592916	1.4461038	0.2485	0.804	9.08E-08	count	1
COL8A1	0.3592916	1.4461038	0.2485	0.804	9.08E-08	count	1
CXCL13	0.3592916	1.4461038	0.2485	0.804	9.08E-08	count	1
PDE5A	0.3592916	1.4461038	0.2485	0.804	9.08E-08	count	1
ADAP1	0.3592916	1.4461038	0.2485	0.804	9.08E-08	count	1
ELFN1-AS1	0.3592916	1.4461038	0.2485	0.804	9.08E-08	count	1
FANCG	0.3592916	1.4461038	0.2485	0.804	9.08E-08	count	1
CTSL	0.3592916	1.4461038	0.2485	0.804	9.08E-08	count	1
KIAA1958	0.3592916	1.4461038	0.2485	0.804	9.08E-08	count	1
NTNG2	0.3592916	1.4461038	0.2485	0.804	9.08E-08	count	1
PAN3-AS1	0.3592916	1.4461038	0.2485	0.804	9.08E-08	count	1
AL161421.1	0.3592916	1.4461038	0.2485	0.804	9.08E-08	count	1
GZMB	0.3592916	1.4461038	0.2485	0.804	9.08E-08	count	1
CIPC	0.3592916	1.4461038	0.2485	0.804	9.08E-08	count	1
CCDC78	0.3592916	1.4461038	0.2485	0.804	9.08E-08	count	1
RAB3D	0.3592916	1.4461038	0.2485	0.804	9.08E-08	count	1
NACC1	0.3592916	1.4461038	0.2485	0.804	9.08E-08	count	1

LINC02576	0.3592916	1.4461038	0.2485	0.804	9.08E-08	count	1
DAPL1	0.3592916	1.4461038	0.2485	0.804	9.08E-08	count	1
POMGNT2	0.3592916	1.4461038	0.2485	0.804	9.08E-08	count	1
RNF123	0.3592916	1.4461038	0.2485	0.804	9.08E-08	count	1
GYPE	0.3592916	1.4461038	0.2485	0.804	9.08E-08	count	1
SEPT7-AS1	0.3592916	1.4461038	0.2485	0.804	9.08E-08	count	1
RLN2	0.3592916	1.4461038	0.2485	0.804	9.08E-08	count	1
NAIF1	0.3592916	1.4461038	0.2485	0.804	9.08E-08	count	1
PNPLA7	0.3592916	1.4461038	0.2485	0.804	9.08E-08	count	1
CLSTN3	0.3592916	1.4461038	0.2485	0.804	9.08E-08	count	1
ALG10B	0.3592916	1.4461038	0.2485	0.804	9.08E-08	count	1
CAB39L	0.3592916	1.4461038	0.2485	0.804	9.08E-08	count	1
PAQR4	0.3592916	1.4461038	0.2485	0.804	9.08E-08	count	1
LINC00910	0.3592916	1.4461038	0.2485	0.804	9.08E-08	count	1
MAL	0.3592916	1.4461038	0.2485	0.804	9.08E-08	count	1
STK36	0.3592916	1.4461038	0.2485	0.804	9.08E-08	count	1
AGPAT4	0.3592916	1.4461038	0.2485	0.804	9.08E-08	count	1
AC022182.1	0.3592916	1.4461038	0.2485	0.804	9.08E-08	count	1
SMC5-AS1	0.3592916	1.4461038	0.2485	0.804	9.08E-08	count	1
SFXN2	0.3592916	1.4461038	0.2485	0.804	9.08E-08	count	1
GRAPL	0.3592916	1.4461038	0.2485	0.804	9.08E-08	count	1
FCRL4	0.3592916	1.4461038	0.2485	0.804	9.08E-08	count	1
AC091982.3	0.3592916	1.4461038	0.2485	0.804	9.08E-08	count	1
TWNK	0.3592916	1.4461038	0.2485	0.804	9.08E-08	count	1
RIC8B	0.3592916	1.4461038	0.2485	0.804	9.08E-08	count	1
MRPL53	0.3592916	0.9647503	0.3724	0.71	9.08E-08	count	1
DBR1	0.3592916	0.9647503	0.3724	0.71	9.08E-08	count	1
AP003068.2	0.3592916	0.9647503	0.3724	0.71	9.08E-08	count	1
LINC02158	0.3592916	0.9647503	0.3724	0.71	9.08E-08	count	1
LINC01358	0.3592916	1.0602619	0.3389	0.735	9.08E-08	count	1
MAP3K6	0.3592916	1.0602619	0.3389	0.735	9.08E-08	count	1
MBNL1-AS1	0.3592916	1.0602619	0.3389	0.735	9.08E-08	count	1
RCC1	0.3592916	1.0602619	0.3389	0.735	9.08E-08	count	1
ZNF551	0.3592916	1.0602619	0.3389	0.735	9.08E-08	count	1
NUP205	0.3592916	1.0602619	0.3389	0.735	9.08E-08	count	1
AL627422.2	0.3592916	1.0602619	0.3389	0.735	9.08E-08	count	1
ARMC7	0.3592916	1.0602619	0.3389	0.735	9.08E-08	count	1
LINC00539	0.3592916	1.0602619	0.3389	0.735	9.08E-08	count	1
TBC1D19	0.3592916	1.0602619	0.3389	0.735	9.08E-08	count	1
PINK1	0.3592916	0.8085272	0.4444	0.657	9.08E-08	count	1
ZNF208	0.3592916	0.8085272	0.4444	0.657	9.08E-08	count	1
TBC1D8	0.3592916	0.8085272	0.4444	0.657	9.08E-08	count	1
TBC1D17	0.3592916	0.8085272	0.4444	0.657	9.08E-08	count	1
TMEM182	0.3592916	0.8085272	0.4444	0.657	9.08E-08	count	1
PIGN	0.3592916	0.8085272	0.4444	0.657	9.08E-08	count	1

SLC9A8	0.3592916	0.745356	0.482	0.63	9.08E-08	count	1
SLC16A13	0.3592916	1.1478534	0.313	0.754	9.08E-08	count	1
EID2B	0.3592916	1.1478534	0.313	0.754	9.08E-08	count	1
DOLK	0.3592916	1.1478534	0.313	0.754	9.08E-08	count	1
ZNF548	0.3592916	1.1478534	0.313	0.754	9.08E-08	count	1
SETD1A	0.3592916	1.1478534	0.313	0.754	9.08E-08	count	1
MTRR	0.3592916	0.9203958	0.3904	0.696	9.08E-08	count	1
ATP8B2	0.3592916	0.9203958	0.3904	0.696	9.08E-08	count	1
PCNX2	0.3592916	0.9203958	0.3904	0.696	9.08E-08	count	1
MORC2-AS1	0.3592916	0.9203958	0.3904	0.696	9.08E-08	count	1
FASTKD3	0.3592916	0.9203958	0.3904	0.696	9.08E-08	count	1
NDC80	0.3592916	0.9203958	0.3904	0.696	9.08E-08	count	1
PEX10	0.3592916	0.9203958	0.3904	0.696	9.08E-08	count	1
TAF1A	0.3592916	0.9203958	0.3904	0.696	9.08E-08	count	1
DHX35	0.3592916	0.9203958	0.3904	0.696	9.08E-08	count	1
ZNF550	0.3592916	0.9203958	0.3904	0.696	9.08E-08	count	1
NUDT18	0.3592916	0.9203958	0.3904	0.696	9.08E-08	count	1
ARRDC3-AS1	0.3592916	0.9203958	0.3904	0.696	9.08E-08	count	1
VPS9D1	0.3592916	0.9203958	0.3904	0.696	9.08E-08	count	1
MYBBP1A	0.3592916	0.9203958	0.3904	0.696	9.08E-08	count	1
CAMSAP1	0.3592916	0.9203958	0.3904	0.696	9.08E-08	count	1
NRF1	0.3592916	0.6575959	0.5464	0.585	9.08E-08	count	1
NOCT	0.3592916	0.8529229	0.4212	0.674	9.09E-08	count	1
KANSL3	0.3592916	1.0200689	0.3522	0.725	9.09E-08	count	1
APBB3	0.3592916	1.0200689	0.3522	0.725	9.09E-08	count	1
YAE1D1	0.3592916	1.0200689	0.3522	0.725	9.09E-08	count	1
ZNF180	0.3592916	1.0200689	0.3522	0.725	9.09E-08	count	1
ZNF280B	0.3592916	1.0200689	0.3522	0.725	9.09E-08	count	1
ZFYVE1	0.3592916	1.0200689	0.3522	0.725	9.09E-08	count	1
GIN1	0.3592916	1.0200689	0.3522	0.725	9.09E-08	count	1
SLC16A1	0.3592916	0.7199954	0.499	0.618	9.09E-08	count	1
GNB1L	0.3592916	0.7774023	0.4622	0.644	9.10E-08	count	1
PIK3R6	0.3592916	0.7774023	0.4622	0.644	9.10E-08	count	1
NUS1	0.3592916	0.8308522	0.4324	0.666	9.10E-08	count	1
ANKRD46	0.3592916	0.8308522	0.4324	0.666	9.11E-08	count	1
XPR1	0.3592916	0.8308522	0.4324	0.666	9.11E-08	count	1
CREB5	0.3592916	1.3469184	0.2668	0.79	9.11E-08	count	1
CCDC68	0.3592916	1.3469184	0.2668	0.79	9.11E-08	count	1
BAZ1B	0.0077406	0.3202434	0.0242	0.981	9.33E-08	count	1
NRBP1	0.0078738	0.2911474	0.027	0.978	9.48E-08	count	1
SNX25	0.0215264	0.5822676	0.037	0.971	9.58E-08	count	1
PAPSS1	0.1516522	0.4856869	0.3122	0.755	9.62E-08	count	1
C4orf33	0.0217405	0.7122557	0.0305	0.976	9.68E-08	count	1
KCTD6	0.0217405	0.6898915	0.0315	0.975	9.68E-08	count	1
RIC1	0.0217405	0.7378163	0.0295	0.977	9.69E-08	count	1

APPL2	0.0220986	0.7339581	0.0301	0.976	9.84E-08	count	1
MAP3K7	0.0220986	0.6421646	0.0344	0.973	9.84E-08	count	1
MEMO1	0.0220986	0.7285898	0.0303	0.976	9.84E-08	count	1
ELP5	0.0220986	0.4243954	0.0521	0.958	9.84E-08	count	1
ACTN4	0.0220986	0.3758592	0.0588	0.953	9.84E-08	count	1
TAF6	0.0220986	0.7195972	0.0307	0.976	9.84E-08	count	1
ARAP1	0.0220986	0.680173	0.0325	0.974	9.85E-08	count	1
RPS6KA1	0.0220986	0.5775287	0.0383	0.969	9.88E-08	count	1
RPS6KB1	0.0222498	0.487412	0.0456	0.964	9.91E-08	count	1
CCDC58	0.0222498	0.4970712	0.0448	0.964	9.91E-08	count	1
AMPD3	0.0223861	0.5973552	0.0375	0.97	9.97E-08	count	1
FBXL12	0.0223861	0.4617268	0.0485	0.961	9.97E-08	count	1
FBXO38	0.0223861	0.660415	0.0339	0.973	9.97E-08	count	1
TRAF3IP1	0.0223861	0.660415	0.0339	0.973	9.97E-08	count	1
MIER3	0.0223861	0.6293224	0.0356	0.972	9.97E-08	count	1
CREB3	0.0223861	0.4695744	0.0477	0.962	9.97E-08	count	1
ZDHHC14	0.0223861	0.5244531	0.0427	0.966	9.97E-08	count	1
KIF27	0.0223861	0.6597425	0.0339	0.973	9.97E-08	count	1
NUP85	0.0223861	0.6610867	0.0339	0.973	9.97E-08	count	1
GOLGA5	0.0223861	0.7104256	0.0315	0.975	9.97E-08	count	1
CEP83	0.0223861	0.6590694	0.034	0.973	9.98E-08	count	1
CRLF3	0.0224698	0.3394195	0.0662	0.947	1.00E-07	count	1
SPPL2B	0.0226222	0.4552643	0.0497	0.96	1.01E-07	count	1
RNF187	0.0226222	0.4286125	0.0528	0.958	1.01E-07	count	1
POC5	0.0226222	0.5879299	0.0385	0.969	1.01E-07	count	1
ADA2	0.0226222	0.6632415	0.0341	0.973	1.01E-07	count	1
AKAP1	0.0226222	0.6627076	0.0341	0.973	1.01E-07	count	1
SLC25A43	0.0226222	0.5877073	0.0385	0.969	1.01E-07	count	1
UXS1	0.0226222	0.6136766	0.0369	0.971	1.01E-07	count	1
CDC14A	0.0226222	0.6637749	0.0341	0.973	1.01E-07	count	1
2-Mar	0.1586209	0.4487619	0.3535	0.724	1.01E-07	count	1
PREPL	0.1586209	0.5182038	0.3061	0.76	1.01E-07	count	1
YWHAE	0.0085132	0.1998655	0.0426	0.966	1.02E-07	count	1
CCNG2	0.0092724	0.3347418	0.0277	0.978	1.12E-07	count	1
RANBP9	0.17697	0.5355061	0.3305	0.741	1.13E-07	count	1
AC093010.2	0.17697	0.5039186	0.3512	0.726	1.13E-07	count	1
ZNF680	0.17697	0.5846211	0.3027	0.762	1.13E-07	count	1
STARD5	0.17697	0.5846211	0.3027	0.762	1.13E-07	count	1
CLYBL	0.17697	0.6299181	0.2809	0.779	1.13E-07	count	1
DAPK3	0.17697	0.5002858	0.3537	0.724	1.14E-07	count	1
DHX30	0.17697	0.4240753	0.4173	0.677	1.14E-07	count	1
REV3L	0.0100223	0.3404155	0.0294	0.977	1.21E-07	count	1
CDK14	0.0102278	0.2760214	0.0371	0.97	1.23E-07	count	1
KATNBL1	0.0103131	0.2857703	0.0361	0.971	1.24E-07	count	1
NECAP2	0.0103131	0.2851477	0.0362	0.971	1.24E-07	count	1

CASK	0.0278674	0.3722247	0.0749	0.94	1.24E-07	count	1
ILF3-DT	0.0107038	0.3344518	0.032	0.974	1.29E-07	count	1
SLF1	0.0291995	0.4222845	0.0691	0.945	1.30E-07	count	1
PURB	0.0295347	0.4220354	0.07	0.944	1.32E-07	count	1
SCYL3	0.0791277	0.8272283	0.0957	0.924	1.32E-07	count	1
SORD	0.0791277	0.8269867	0.0957	0.924	1.32E-07	count	1
CERNA1	0.0791277	0.8269867	0.0957	0.924	1.32E-07	count	1
TRMT2B	0.0791277	0.8269867	0.0957	0.924	1.32E-07	count	1
KLHL9	0.0791277	0.8272283	0.0957	0.924	1.32E-07	count	1
IFNG-AS1	0.0791277	0.8269867	0.0957	0.924	1.32E-07	count	1
IFFO1	0.0791277	0.8269867	0.0957	0.924	1.32E-07	count	1
C7orf26	0.0791277	0.6845332	0.1156	0.908	1.32E-07	count	1
PDK3	0.0791277	0.6842413	0.1156	0.908	1.32E-07	count	1
TATDN2	0.0791277	1.0060582	0.0787	0.937	1.32E-07	count	1
ACSF2	0.0791277	0.8874133	0.0892	0.929	1.33E-07	count	1
LPCAT3	0.0791277	0.8874133	0.0892	0.929	1.33E-07	count	1
YIPF1	0.0791277	0.8874133	0.0892	0.929	1.33E-07	count	1
KDM4A	0.0791277	0.7561623	0.1046	0.917	1.33E-07	count	1
ZNF260	0.0791277	0.7561623	0.1046	0.917	1.33E-07	count	1
NMB	0.0791277	0.953183	0.083	0.934	1.33E-07	count	1
ZBTB49	0.0791277	0.8825939	0.0897	0.929	1.33E-07	count	1
AL133453.1	0.0297232	0.4197305	0.0708	0.944	1.33E-07	count	1
ADTRP	0.2051409	0.7687315	0.2669	0.79	1.33E-07	count	1
SLC35F5	0.2051409	0.5957623	0.3443	0.731	1.33E-07	count	1
SNIP1	0.0303968	0.4943846	0.0615	0.951	1.36E-07	count	1
WDR13	0.0303968	0.481039	0.0632	0.95	1.36E-07	count	1
LIPE	0.5134423	0.773434	0.6638	0.507	1.38E-07	count	1
MLYCD	0.5134423	0.773434	0.6638	0.507	1.38E-07	count	1
LINC00665	0.5134423	0.6409118	0.8011	0.423	1.38E-07	count	1
TRIM21	0.5134423	0.6934985	0.7404	0.459	1.38E-07	count	1
PELO	0.5134423	0.6934985	0.7404	0.459	1.38E-07	count	1
AC004865.2	0.5134423	0.7423694	0.6916	0.49	1.38E-07	count	1
POMGNT1	0.5134423	0.7423694	0.6916	0.49	1.38E-07	count	1
TICAM1	0.0312954	0.4801758	0.0652	0.948	1.40E-07	count	1
SPATC1	18.2510171	3328.981499	0.0055	0.996	1.44E-07	count	1
MMAA	18.2510171	3328.98145	0.0055	0.996	1.44E-07	count	1
CENPI	18.2510171	3328.981547	0.0055	0.996	1.44E-07	count	1
USP31	18.2510171	3328.98145	0.0055	0.996	1.44E-07	count	1
C19orf47	18.2510171	3328.98145	0.0055	0.996	1.44E-07	count	1
PLD2	18.251017	3328.981466	0.0055	0.996	1.44E-07	count	1
MFSD4A	18.2510169	3328.98145	0.0055	0.996	1.44E-07	count	1
TTC26	18.2510169	3328.981515	0.0055	0.996	1.44E-07	count	1
SPATA1	18.2510168	3328.98137	0.0055	0.996	1.44E-07	count	1
KIAA0895	18.251017	3328.98145	0.0055	0.996	1.44E-07	count	1
MAGI2	18.2510169	3328.981354	0.0055	0.996	1.44E-07	count	1

MORN3	18.2510169	3328.981434	0.0055	0.996	1.44E-07	count	1
DHRS13	18.2510167	3328.981466	0.0055	0.996	1.44E-07	count	1
WNT16	18.2510169	3328.981418	0.0055	0.996	1.44E-07	count	1
TRIQK	18.2510168	3328.981306	0.0055	0.996	1.44E-07	count	1
DSCC1	18.2510168	3328.981306	0.0055	0.996	1.44E-07	count	1
ARHGAP44	18.2510169	3328.981418	0.0055	0.996	1.44E-07	count	1
ZNF391	18.2510168	3328.981258	0.0055	0.996	1.44E-07	count	1
AC034236.2	18.2510167	3328.981434	0.0055	0.996	1.44E-07	count	1
KLHL26	18.2510169	3328.981466	0.0055	0.996	1.44E-07	count	1
NPIPB15	18.2510168	3328.981226	0.0055	0.996	1.44E-07	count	1
ZNF547	18.2510169	3328.981322	0.0055	0.996	1.44E-07	count	1
SNHG10	18.2510167	3328.98145	0.0055	0.996	1.44E-07	count	1
TMEM38A	18.2510168	3328.981483	0.0055	0.996	1.44E-07	count	1
NCKIPSD	18.2510166	3328.981499	0.0055	0.996	1.44E-07	count	1
AL109811.3	18.2510167	3328.981226	0.0055	0.996	1.44E-07	count	1
GSTM1	18.2510167	3328.981226	0.0055	0.996	1.44E-07	count	1
CEP131	18.2510165	3328.981434	0.0055	0.996	1.44E-07	count	1
KCTD17	18.2510169	3328.981643	0.0055	0.996	1.44E-07	count	1
PDIA5	18.2510165	3328.981434	0.0055	0.996	1.44E-07	count	1
DUSP19	18.2510167	3328.981306	0.0055	0.996	1.44E-07	count	1
PAM	18.2510167	3328.981402	0.0055	0.996	1.44E-07	count	1
LRRC24	18.2510166	3328.981418	0.0055	0.996	1.44E-07	count	1
AC142472.1	18.2510166	3328.981258	0.0055	0.996	1.44E-07	count	1
FAM214B	18.2510166	3328.981402	0.0055	0.996	1.44E-07	count	1
ITPRIPL2	18.2510165	3328.981466	0.0055	0.996	1.44E-07	count	1
CAMK2N2	18.2510166	3328.981226	0.0055	0.996	1.44E-07	count	1
SPRED1	18.2510167	3328.981354	0.0055	0.996	1.44E-07	count	1
MCOLN1	18.2510167	3328.981258	0.0055	0.996	1.44E-07	count	1
RIN2	18.2510165	3328.981402	0.0055	0.996	1.44E-07	count	1
FAM13A-AS1	18.2510164	3328.981418	0.0055	0.996	1.44E-07	count	1
ZNF582	18.2510164	3328.981547	0.0055	0.996	1.44E-07	count	1
AL358072.1	18.2510167	3328.981274	0.0055	0.996	1.44E-07	count	1
ANKRD65	18.2510165	3328.981322	0.0055	0.996	1.44E-07	count	1
LYSMD4	18.2510166	3328.981483	0.0055	0.996	1.44E-07	count	1
AL669831.5	18.2510166	3328.981338	0.0055	0.996	1.44E-07	count	1
ABCB10	18.2510165	3328.981338	0.0055	0.996	1.44E-07	count	1
KCNRG	18.2510166	3328.981354	0.0055	0.996	1.44E-07	count	1
C21orf62-AS1	18.2510167	3328.981402	0.0055	0.996	1.44E-07	count	1
ELP1	18.2510164	3328.981226	0.0055	0.996	1.44E-07	count	1
TLR4	18.2510166	3328.981483	0.0055	0.996	1.44E-07	count	1
HLCS	18.2510166	3328.981322	0.0055	0.996	1.44E-07	count	1
BOLA2B	18.2510163	3328.981162	0.0055	0.996	1.44E-07	count	1
ZMAT3	18.2510166	3328.981386	0.0055	0.996	1.44E-07	count	1
GOLGA8N	18.2510166	3328.981531	0.0055	0.996	1.44E-07	count	1
P2RY11	18.2510165	3328.981226	0.0055	0.996	1.44E-07	count	1

GPR135	18.2510163	3328.981402	0.0055	0.996	1.44E-07	count	1
IGKV2-24	18.2510164	3328.981563	0.0055	0.996	1.44E-07	count	1
LINC01521	18.2510165	3328.98145	0.0055	0.996	1.44E-07	count	1
SEC61A1	18.2510163	3328.98129	0.0055	0.996	1.44E-07	count	1
ZNF282	18.2510163	3328.981338	0.0055	0.996	1.44E-07	count	1
TRIP6	18.2510164	3328.981531	0.0055	0.996	1.44E-07	count	1
VARS2	18.2510164	3328.981386	0.0055	0.996	1.44E-07	count	1
AC011773.4	18.2510164	3328.981402	0.0055	0.996	1.44E-07	count	1
ZNF438	18.2510164	3328.981386	0.0055	0.996	1.44E-07	count	1
CCDC51	18.2510165	3328.981322	0.0055	0.996	1.44E-07	count	1
PANK1	18.2510162	3328.98137	0.0055	0.996	1.44E-07	count	1
LINC02202	18.2510166	3328.981499	0.0055	0.996	1.44E-07	count	1
BIK	18.2510163	3328.981418	0.0055	0.996	1.44E-07	count	1
ELOA-AS1	18.2510163	3328.981338	0.0055	0.996	1.44E-07	count	1
OBSCN	18.2510164	3328.981306	0.0055	0.996	1.44E-07	count	1
HGH1	18.2510163	3328.981338	0.0055	0.996	1.44E-07	count	1
CCDC157	18.2510163	3328.981338	0.0055	0.996	1.44E-07	count	1
AL080276.2	18.2510162	3328.98145	0.0055	0.996	1.44E-07	count	1
AC021016.3	18.2510163	3328.981402	0.0055	0.996	1.44E-07	count	1
RAP1GAP2	18.2510163	3328.981402	0.0055	0.996	1.44E-07	count	1
LLGL1	18.2510163	3328.981402	0.0055	0.996	1.44E-07	count	1
HMSD	18.2510163	3328.981402	0.0055	0.996	1.44E-07	count	1
ANKMY1	18.251016	3328.981306	0.0055	0.996	1.44E-07	count	1
AC068338.3	18.2510163	3328.981226	0.0055	0.996	1.44E-07	count	1
HIST1H2BH	18.2510163	3328.981434	0.0055	0.996	1.44E-07	count	1
ADHFE1	18.2510161	3328.981194	0.0055	0.996	1.44E-07	count	1
MACROD1	18.2510162	3328.981466	0.0055	0.996	1.44E-07	count	1
IL2RB	18.2510162	3328.981242	0.0055	0.996	1.44E-07	count	1
AC023906.3	18.2510162	3328.981402	0.0055	0.996	1.44E-07	count	1
CD247	18.2510159	3328.981306	0.0055	0.996	1.44E-07	count	1
C1orf198	18.2510161	3328.981515	0.0055	0.996	1.44E-07	count	1
GLYCTK	18.2510161	3328.981515	0.0055	0.996	1.44E-07	count	1
TCEANC	18.2510161	3328.981434	0.0055	0.996	1.44E-07	count	1
TDG	0.011924	0.3059061	0.039	0.969	1.44E-07	count	1
TTC23	17.5574105	3335.552586	0.0053	0.996	1.44E-07	count	1
AC024592.3	17.5574105	3335.552586	0.0053	0.996	1.44E-07	count	1
STRIP2	17.5574103	3335.552586	0.0053	0.996	1.44E-07	count	1
CDC42BPG	17.5574103	3335.552586	0.0053	0.996	1.44E-07	count	1
LIPT2	17.5574103	3335.552586	0.0053	0.996	1.44E-07	count	1
AC025034.1	17.5574103	3335.552586	0.0053	0.996	1.44E-07	count	1
PTK6	17.5574103	3335.552586	0.0053	0.996	1.44E-07	count	1
ZNF436	17.5574104	3335.552626	0.0053	0.996	1.44E-07	count	1
FAM229A	17.5574104	3335.552538	0.0053	0.996	1.44E-07	count	1
EPB41L4A-DT	17.5574104	3335.552538	0.0053	0.996	1.44E-07	count	1
AC006333.2	17.5574104	3335.552538	0.0053	0.996	1.44E-07	count	1

RMRP	17.5574104	3335.552626	0.0053	0.996	1.44E-07	count	1
TMEM9B-AS1	17.5574104	3335.552538	0.0053	0.996	1.44E-07	count	1
AC069544.1	17.5574104	3335.552538	0.0053	0.996	1.44E-07	count	1
C14orf132	17.5574104	3335.552626	0.0053	0.996	1.44E-07	count	1
AC021739.2	17.5574104	3335.552538	0.0053	0.996	1.44E-07	count	1
CCL14	17.5574104	3335.552538	0.0053	0.996	1.44E-07	count	1
AL035413.1	17.5574104	3335.552513	0.0053	0.996	1.44E-07	count	1
AL353708.3	17.5574104	3335.552513	0.0053	0.996	1.44E-07	count	1
AC137630.3	17.5574104	3335.552513	0.0053	0.996	1.44E-07	count	1
GPER1	17.5574104	3335.552513	0.0053	0.996	1.44E-07	count	1
TRGV5	17.5574104	3335.552513	0.0053	0.996	1.44E-07	count	1
MAGEE1	17.5574104	3335.552513	0.0053	0.996	1.44E-07	count	1
NLRP6	17.5574103	3335.55257	0.0053	0.996	1.44E-07	count	1
OVOL1	17.5574103	3335.55257	0.0053	0.996	1.44E-07	count	1
AL117336.3	17.5574104	3335.552513	0.0053	0.996	1.44E-07	count	1
CCDC189	17.5574104	3335.552513	0.0053	0.996	1.44E-07	count	1
AC092723.5	17.5574104	3335.552513	0.0053	0.996	1.44E-07	count	1
4-Sep	17.5574104	3335.552513	0.0053	0.996	1.44E-07	count	1
AC123912.1	17.5574104	3335.552513	0.0053	0.996	1.44E-07	count	1
AC020910.4	17.5574104	3335.552513	0.0053	0.996	1.44E-07	count	1
SIK1B	17.5574104	3335.552513	0.0053	0.996	1.44E-07	count	1
DHCR24	17.5574103	3335.552522	0.0053	0.996	1.44E-07	count	1
GPAT2	17.5574104	3335.552586	0.0053	0.996	1.44E-07	count	1
RARB	17.5574103	3335.552473	0.0053	0.996	1.44E-07	count	1
EIF4E3	17.5574103	3335.552522	0.0053	0.996	1.44E-07	count	1
AC097382.2	17.5574103	3335.552538	0.0053	0.996	1.44E-07	count	1
GALNTL6	17.5574103	3335.552473	0.0053	0.996	1.44E-07	count	1
CLDN12	17.5574103	3335.552473	0.0053	0.996	1.44E-07	count	1
TRIM3	17.5574104	3335.552586	0.0053	0.996	1.44E-07	count	1
CSRNP2	17.5574103	3335.552538	0.0053	0.996	1.44E-07	count	1
IGHV4-28	17.5574104	3335.552586	0.0053	0.996	1.44E-07	count	1
AC027601.1	17.5574104	3335.552586	0.0053	0.996	1.44E-07	count	1
AC012123.1	17.5574103	3335.552538	0.0053	0.996	1.44E-07	count	1
HM13-AS1	17.5574103	3335.552522	0.0053	0.996	1.44E-07	count	1
AC007876.1	17.5574103	3335.552538	0.0053	0.996	1.44E-07	count	1
LINC02562	17.5574103	3335.552505	0.0053	0.996	1.44E-07	count	1
AMER1	17.5574103	3335.55257	0.0053	0.996	1.44E-07	count	1
ANK1	17.5574103	3335.55257	0.0053	0.996	1.44E-07	count	1
AC079209.1	17.5574103	3335.552505	0.0053	0.996	1.44E-07	count	1
LRRC8A	17.5574103	3335.552505	0.0053	0.996	1.44E-07	count	1
DDX47	17.5574104	3335.552626	0.0053	0.996	1.44E-07	count	1
ASPHD1	17.5574103	3335.552505	0.0053	0.996	1.44E-07	count	1
ZNF469	17.5574104	3335.552626	0.0053	0.996	1.44E-07	count	1
RFLNB	17.5574104	3335.552626	0.0053	0.996	1.44E-07	count	1
AC084809.1	17.5574103	3335.552505	0.0053	0.996	1.44E-07	count	1

CD1A	17.5574104	3335.55253	0.0053	0.996	1.44E-07	count	1
IGKV1-9	17.5574103	3335.552546	0.0053	0.996	1.44E-07	count	1
CFAP69	17.5574103	3335.552546	0.0053	0.996	1.44E-07	count	1
AC008264.2	17.5574104	3335.55253	0.0053	0.996	1.44E-07	count	1
WDCP	17.5574104	3335.552497	0.0053	0.996	1.44E-07	count	1
MYLK4	17.5574104	3335.552497	0.0053	0.996	1.44E-07	count	1
AC104958.2	17.5574104	3335.552497	0.0053	0.996	1.44E-07	count	1
AC090192.2	17.5574104	3335.552497	0.0053	0.996	1.44E-07	count	1
KBTBD7	17.5574104	3335.552497	0.0053	0.996	1.44E-07	count	1
COLEC12	17.5574104	3335.552497	0.0053	0.996	1.44E-07	count	1
OSBPL1A	17.5574104	3335.552497	0.0053	0.996	1.44E-07	count	1
LINC02055	17.5574104	3335.552578	0.0053	0.996	1.44E-07	count	1
C1S	17.5574104	3335.552578	0.0053	0.996	1.44E-07	count	1
LINC00937	17.5574104	3335.552578	0.0053	0.996	1.44E-07	count	1
RAMP2	17.5574103	3335.552562	0.0053	0.996	1.44E-07	count	1
ZSCAN5A	17.5574103	3335.552562	0.0053	0.996	1.44E-07	count	1
AC104123.1	17.5574103	3335.552497	0.0053	0.996	1.44E-07	count	1
LINC01551	17.5574103	3335.552497	0.0053	0.996	1.44E-07	count	1
AC004771.3	17.5574103	3335.552497	0.0053	0.996	1.44E-07	count	1
AL031772.1	17.5574103	3335.552634	0.0053	0.996	1.44E-07	count	1
AC131011.1	17.5574103	3335.552634	0.0053	0.996	1.44E-07	count	1
AC124242.1	17.5574103	3335.552634	0.0053	0.996	1.44E-07	count	1
AP000424.1	17.5574103	3335.552562	0.0053	0.996	1.44E-07	count	1
TMEM255B	17.5574103	3335.552562	0.0053	0.996	1.44E-07	count	1
GPAT3	17.5574104	3335.552513	0.0053	0.996	1.44E-07	count	1
C6orf52	17.5574104	3335.552513	0.0053	0.996	1.44E-07	count	1
DEPTOR	17.5574104	3335.552513	0.0053	0.996	1.44E-07	count	1
CUL7	17.5574103	3335.552618	0.0053	0.996	1.44E-07	count	1
LRP11	17.5574103	3335.552618	0.0053	0.996	1.44E-07	count	1
PROC	17.5574104	3335.55261	0.0053	0.996	1.44E-07	count	1
CCRL2	17.5574104	3335.55261	0.0053	0.996	1.44E-07	count	1
MON1A	17.5574103	3335.552602	0.0053	0.996	1.44E-07	count	1
LPP-AS1	17.5574103	3335.552618	0.0053	0.996	1.44E-07	count	1
CENPE	17.5574103	3335.552602	0.0053	0.996	1.44E-07	count	1
DMGDH	17.5574103	3335.552602	0.0053	0.996	1.44E-07	count	1
LCP2	17.5574103	3335.552602	0.0053	0.996	1.44E-07	count	1
ZNF713	17.5574104	3335.55261	0.0053	0.996	1.44E-07	count	1
AP003352.1	17.5574103	3335.552618	0.0053	0.996	1.44E-07	count	1
AC023908.3	17.5574103	3335.552602	0.0053	0.996	1.44E-07	count	1
AC010542.2	17.5574103	3335.552618	0.0053	0.996	1.44E-07	count	1
ZNF19	17.5574103	3335.552602	0.0053	0.996	1.44E-07	count	1
SLFN12L	17.5574104	3335.55261	0.0053	0.996	1.44E-07	count	1
YES1	17.5574103	3335.552618	0.0053	0.996	1.44E-07	count	1
AC009005.1	17.5574103	3335.552602	0.0053	0.996	1.44E-07	count	1
DYRK3	17.5574104	3335.552618	0.0053	0.996	1.44E-07	count	1

AC021321.1	17.5574104	3335.552618	0.0053	0.996	1.44E-07	count	1
RSPO2	17.5574104	3335.552618	0.0053	0.996	1.44E-07	count	1
TBCD	17.5574104	3335.552618	0.0053	0.996	1.44E-07	count	1
BCAM	17.5574102	3335.552497	0.0053	0.996	1.44E-07	count	1
MICALL1	17.5574104	3335.552618	0.0053	0.996	1.44E-07	count	1
LINC01063	17.5574103	3335.552505	0.0053	0.996	1.44E-07	count	1
HCG25	17.5574103	3335.552602	0.0053	0.996	1.44E-07	count	1
ADGRF5	17.5574103	3335.552505	0.0053	0.996	1.44E-07	count	1
RWDD2A	17.5574103	3335.552481	0.0053	0.996	1.44E-07	count	1
SPDYE16	17.5574103	3335.552481	0.0053	0.996	1.44E-07	count	1
ZNF623	17.5574103	3335.552602	0.0053	0.996	1.44E-07	count	1
AL161725.1	17.5574103	3335.552505	0.0053	0.996	1.44E-07	count	1
ARHGEF17	17.5574103	3335.552481	0.0053	0.996	1.44E-07	count	1
FAM241B	17.5574103	3335.552578	0.0053	0.996	1.44E-07	count	1
TMEM254	17.5574103	3335.552505	0.0053	0.996	1.44E-07	count	1
ITGA5	17.5574103	3335.552578	0.0053	0.996	1.44E-07	count	1
NEK5	17.5574103	3335.552602	0.0053	0.996	1.44E-07	count	1
NOL3	17.5574103	3335.552578	0.0053	0.996	1.44E-07	count	1
MYO18A	17.5574103	3335.552602	0.0053	0.996	1.44E-07	count	1
RND2	17.5574103	3335.552602	0.0053	0.996	1.44E-07	count	1
PLEKHA4	17.5574103	3335.552578	0.0053	0.996	1.44E-07	count	1
LEPR	17.5574103	3335.552586	0.0053	0.996	1.44E-07	count	1
LYPD1	17.5574103	3335.552554	0.0053	0.996	1.44E-07	count	1
AP003392.5	17.5574103	3335.552554	0.0053	0.996	1.44E-07	count	1
SLC25A15	17.5574103	3335.552554	0.0053	0.996	1.44E-07	count	1
AL928654.1	17.5574103	3335.552586	0.0053	0.996	1.44E-07	count	1
GPR153	17.5574103	3335.552562	0.0053	0.996	1.44E-07	count	1
PARS2	17.5574103	3335.552562	0.0053	0.996	1.44E-07	count	1
VANGL1	17.5574103	3335.552562	0.0053	0.996	1.44E-07	count	1
C2orf42	17.5574103	3335.552562	0.0053	0.996	1.44E-07	count	1
AC104076.1	17.5574103	3335.552562	0.0053	0.996	1.44E-07	count	1
ODAPH	17.5574103	3335.552562	0.0053	0.996	1.44E-07	count	1
HIST1H2AH	17.5574103	3335.552562	0.0053	0.996	1.44E-07	count	1
WEE2-AS1	17.5574103	3335.552562	0.0053	0.996	1.44E-07	count	1
TSPYL5	17.5574103	3335.55265	0.0053	0.996	1.44E-07	count	1
TMEM132A	17.5574103	3335.552562	0.0053	0.996	1.44E-07	count	1
AP001160.3	17.5574103	3335.552562	0.0053	0.996	1.44E-07	count	1
TET1	17.5574103	3335.552562	0.0053	0.996	1.44E-07	count	1
MEG3	17.5574103	3335.552562	0.0053	0.996	1.44E-07	count	1
NPW	17.5574103	3335.55265	0.0053	0.996	1.44E-07	count	1
OVCA2	17.5574103	3335.552562	0.0053	0.996	1.44E-07	count	1
PSTPIP2	17.5574103	3335.552562	0.0053	0.996	1.44E-07	count	1
KCNJ14	17.5574103	3335.552562	0.0053	0.996	1.44E-07	count	1
AL022322.2	17.5574103	3335.552562	0.0053	0.996	1.44E-07	count	1
AL356488.3	17.5574103	3335.552465	0.0053	0.996	1.44E-07	count	1

LINC01118	17.5574103	3335.552465	0.0053	0.996	1.44E-07	count	1
IL17RC	17.5574103	3335.552578	0.0053	0.996	1.44E-07	count	1
ABLIM2	17.5574103	3335.552465	0.0053	0.996	1.44E-07	count	1
CAGE1	17.5574103	3335.552465	0.0053	0.996	1.44E-07	count	1
INE2	17.5574103	3335.552578	0.0053	0.996	1.44E-07	count	1
PLAT	17.5574103	3335.552465	0.0053	0.996	1.44E-07	count	1
AP000866.2	17.5574103	3335.552578	0.0053	0.996	1.44E-07	count	1
AL442128.2	17.5574103	3335.552465	0.0053	0.996	1.44E-07	count	1
THAP10	17.5574103	3335.552465	0.0053	0.996	1.44E-07	count	1
CRNDE	17.5574103	3335.552578	0.0053	0.996	1.44E-07	count	1
CPNE2	17.5574103	3335.552578	0.0053	0.996	1.44E-07	count	1
ZNF624	17.5574103	3335.552465	0.0053	0.996	1.44E-07	count	1
AC003681.1	17.5574103	3335.552578	0.0053	0.996	1.44E-07	count	1
KCTD3	17.5574104	3335.55261	0.0053	0.996	1.44E-07	count	1
LINC00471	17.5574103	3335.552546	0.0053	0.996	1.44E-07	count	1
C3orf18	17.5574103	3335.552546	0.0053	0.996	1.44E-07	count	1
LINC00635	17.5574103	3335.552546	0.0053	0.996	1.44E-07	count	1
SMIM3	17.5574103	3335.552546	0.0053	0.996	1.44E-07	count	1
DOCK4	17.5574103	3335.552546	0.0053	0.996	1.44E-07	count	1
FAM219A	17.5574103	3335.552546	0.0053	0.996	1.44E-07	count	1
CARD9	17.5574103	3335.552546	0.0053	0.996	1.44E-07	count	1
AC010864.1	17.5574103	3335.552546	0.0053	0.996	1.44E-07	count	1
AL137077.2	17.5574103	3335.552546	0.0053	0.996	1.44E-07	count	1
ZFP69	17.5574103	3335.552554	0.0053	0.996	1.44E-07	count	1
HIST2H2BE	17.5574103	3335.552578	0.0053	0.996	1.44E-07	count	1
AC007881.3	17.5574103	3335.552554	0.0053	0.996	1.44E-07	count	1
VGLL3	17.5574103	3335.552554	0.0053	0.996	1.44E-07	count	1
8-Sep	17.5574103	3335.552554	0.0053	0.996	1.44E-07	count	1
AL157373.2	17.5574103	3335.552578	0.0053	0.996	1.44E-07	count	1
AL137003.2	17.5574103	3335.552578	0.0053	0.996	1.44E-07	count	1
TRG-AS1	17.5574103	3335.552578	0.0053	0.996	1.44E-07	count	1
SCARA3	17.5574103	3335.552554	0.0053	0.996	1.44E-07	count	1
AC073508.3	17.5574103	3335.552578	0.0053	0.996	1.44E-07	count	1
AC011498.1	17.5574103	3335.552554	0.0053	0.996	1.44E-07	count	1
KLK15	17.5574103	3335.552578	0.0053	0.996	1.44E-07	count	1
ZNF613	17.5574103	3335.552578	0.0053	0.996	1.44E-07	count	1
BACH1-AS1	17.5574103	3335.552578	0.0053	0.996	1.44E-07	count	1
ABHD14A-ACY1	17.5574103	3335.552602	0.0053	0.996	1.44E-07	count	1
AC068888.1	17.5574103	3335.552602	0.0053	0.996	1.44E-07	count	1
SPACA6	17.5574103	3335.552602	0.0053	0.996	1.44E-07	count	1
LINC01376	17.5574102	3335.552578	0.0053	0.996	1.44E-07	count	1
CAPN10-DT	17.5574103	3335.552578	0.0053	0.996	1.44E-07	count	1
POPDC2	17.5574102	3335.552578	0.0053	0.996	1.44E-07	count	1
KCNMB3	17.5574102	3335.552578	0.0053	0.996	1.44E-07	count	1
AC106882.1	17.5574102	3335.55253	0.0053	0.996	1.44E-07	count	1

AL137784.2	17.5574103	3335.552489	0.0053	0.996	1.44E-07	count	1
STEAP4	17.5574103	3335.552449	0.0053	0.996	1.44E-07	count	1
UFSP1	17.5574103	3335.552489	0.0053	0.996	1.44E-07	count	1
ZNF425	17.5574102	3335.552578	0.0053	0.996	1.44E-07	count	1
KDM4D	17.5574102	3335.55253	0.0053	0.996	1.44E-07	count	1
USP2	17.5574103	3335.552578	0.0053	0.996	1.44E-07	count	1
EIF2S3B	17.5574102	3335.552578	0.0053	0.996	1.44E-07	count	1
HCAR3	17.5574103	3335.552489	0.0053	0.996	1.44E-07	count	1
CCDC154	17.5574103	3335.552449	0.0053	0.996	1.44E-07	count	1
MMP9	17.5574102	3335.552578	0.0053	0.996	1.44E-07	count	1
EYA2	17.5574103	3335.552489	0.0053	0.996	1.44E-07	count	1
SSC5D	17.5574102	3335.55253	0.0053	0.996	1.44E-07	count	1
KIRREL1	17.5574102	3335.55261	0.0053	0.996	1.44E-07	count	1
DNM3OS	17.5574103	3335.552634	0.0053	0.996	1.44E-07	count	1
C1orf74	17.5574102	3335.55261	0.0053	0.996	1.44E-07	count	1
MMRN1	17.5574102	3335.55261	0.0053	0.996	1.44E-07	count	1
SMPDL3A	17.5574102	3335.552449	0.0053	0.996	1.44E-07	count	1
XK	17.5574102	3335.55261	0.0053	0.996	1.44E-07	count	1
KANTR	17.5574103	3335.552634	0.0053	0.996	1.44E-07	count	1
ZNF696	17.5574102	3335.552497	0.0053	0.996	1.44E-07	count	1
TAL2	17.5574102	3335.552449	0.0053	0.996	1.44E-07	count	1
MEN1	17.5574102	3335.552449	0.0053	0.996	1.44E-07	count	1
AP000487.1	17.5574102	3335.55261	0.0053	0.996	1.44E-07	count	1
PABPC3	17.5574102	3335.552449	0.0053	0.996	1.44E-07	count	1
SYNJ2BP-COX16	17.5574102	3335.552497	0.0053	0.996	1.44E-07	count	1
FBLN5	17.5574102	3335.552449	0.0053	0.996	1.44E-07	count	1
AC107241.1	17.5574102	3335.552449	0.0053	0.996	1.44E-07	count	1
AC068831.1	17.5574103	3335.552634	0.0053	0.996	1.44E-07	count	1
AC027682.3	17.5574102	3335.552449	0.0053	0.996	1.44E-07	count	1
AL022332.1	17.5574103	3335.552634	0.0053	0.996	1.44E-07	count	1
TTC24	17.5574103	3335.552473	0.0053	0.996	1.44E-07	count	1
SNTG2	17.5574102	3335.552602	0.0053	0.996	1.44E-07	count	1
PLEKHM3	17.5574103	3335.55261	0.0053	0.996	1.44E-07	count	1
IKZF2	17.5574102	3335.552602	0.0053	0.996	1.44E-07	count	1
ESPNL	17.5574103	3335.55261	0.0053	0.996	1.44E-07	count	1
PODXL2	17.5574102	3335.552602	0.0053	0.996	1.44E-07	count	1
RNF212	17.5574102	3335.55253	0.0053	0.996	1.44E-07	count	1
MGST2	17.5574103	3335.552473	0.0053	0.996	1.44E-07	count	1
FHDC1	17.5574102	3335.55253	0.0053	0.996	1.44E-07	count	1
KHDC1	17.5574102	3335.55253	0.0053	0.996	1.44E-07	count	1
AC073073.2	17.5574103	3335.55261	0.0053	0.996	1.44E-07	count	1
CACNA1F	17.5574102	3335.552602	0.0053	0.996	1.44E-07	count	1
LRRC4C	17.5574102	3335.55253	0.0053	0.996	1.44E-07	count	1
CLLU1OS	17.5574103	3335.552473	0.0053	0.996	1.44E-07	count	1
LINC01550	17.5574103	3335.552473	0.0053	0.996	1.44E-07	count	1

AC018512.1	17.5574103	3335.55261	0.0053	0.996	1.44E-07	count	1
WNT3	17.5574103	3335.55261	0.0053	0.996	1.44E-07	count	1
AC120024.1	17.5574103	3335.55261	0.0053	0.996	1.44E-07	count	1
ZNF565	17.5574103	3335.552473	0.0053	0.996	1.44E-07	count	1
AC010487.2	17.5574102	3335.55253	0.0053	0.996	1.44E-07	count	1
ZNF776	17.5574103	3335.55261	0.0053	0.996	1.44E-07	count	1
AL136115.2	17.5574102	3335.552497	0.0053	0.996	1.44E-07	count	1
AC245297.2	17.5574103	3335.55261	0.0053	0.996	1.44E-07	count	1
AC007405.3	17.5574102	3335.552546	0.0053	0.996	1.44E-07	count	1
AC026202.2	17.5574102	3335.552546	0.0053	0.996	1.44E-07	count	1
AL035701.1	17.5574103	3335.55261	0.0053	0.996	1.44E-07	count	1
LRRC1	17.5574102	3335.552546	0.0053	0.996	1.44E-07	count	1
AC105446.1	17.5574102	3335.552497	0.0053	0.996	1.44E-07	count	1
CTPS2	17.5574103	3335.55261	0.0053	0.996	1.44E-07	count	1
TBC1D8B	17.5574102	3335.552497	0.0053	0.996	1.44E-07	count	1
FANCC	17.5574102	3335.552546	0.0053	0.996	1.44E-07	count	1
AP005019.1	17.5574103	3335.55261	0.0053	0.996	1.44E-07	count	1
AC008537.4	17.5574103	3335.55261	0.0053	0.996	1.44E-07	count	1
ADGRB2	17.5574102	3335.552538	0.0053	0.996	1.44E-07	count	1
AC123595.1	17.5574102	3335.552505	0.0053	0.996	1.44E-07	count	1
KLHL3	17.5574102	3335.552538	0.0053	0.996	1.44E-07	count	1
TIGD6	17.5574102	3335.552505	0.0053	0.996	1.44E-07	count	1
AASS	17.5574102	3335.552505	0.0053	0.996	1.44E-07	count	1
L1CAM	17.5574102	3335.552538	0.0053	0.996	1.44E-07	count	1
SLC35G5	17.5574103	3335.552489	0.0053	0.996	1.44E-07	count	1
FAM181B	17.5574102	3335.552578	0.0053	0.996	1.44E-07	count	1
PRKD1	17.5574103	3335.552489	0.0053	0.996	1.44E-07	count	1
IGHV3-15	17.5574102	3335.552505	0.0053	0.996	1.44E-07	count	1
XYLT1	17.5574102	3335.552505	0.0053	0.996	1.44E-07	count	1
AC111182.1	17.5574102	3335.552505	0.0053	0.996	1.44E-07	count	1
AL034417.4	17.5574102	3335.552538	0.0053	0.996	1.44E-07	count	1
ZSCAN20	17.5574102	3335.55257	0.0053	0.996	1.44E-07	count	1
ZMYND12	17.5574102	3335.55257	0.0053	0.996	1.44E-07	count	1
USP21	17.5574103	3335.552586	0.0053	0.996	1.44E-07	count	1
AC006058.3	17.5574103	3335.552586	0.0053	0.996	1.44E-07	count	1
PLSCR4	17.5574103	3335.552586	0.0053	0.996	1.44E-07	count	1
SPINK9	17.5574102	3335.55257	0.0053	0.996	1.44E-07	count	1
AL139393.2	17.5574103	3335.552586	0.0053	0.996	1.44E-07	count	1
LINC02099	17.5574103	3335.55257	0.0053	0.996	1.44E-07	count	1
MYBL1	17.5574102	3335.55257	0.0053	0.996	1.44E-07	count	1
SVEP1	17.5574103	3335.552497	0.0053	0.996	1.44E-07	count	1
AC090587.1	17.5574102	3335.552538	0.0053	0.996	1.44E-07	count	1
RPS6KB2-AS1	17.5574102	3335.55257	0.0053	0.996	1.44E-07	count	1
VWA5A	17.5574103	3335.552586	0.0053	0.996	1.44E-07	count	1
AL391988.1	17.5574102	3335.552538	0.0053	0.996	1.44E-07	count	1

C12orf40	17.5574102	3335.552538	0.0053	0.996	1.44E-07	count	1
USP3-AS1	17.5574102	3335.55257	0.0053	0.996	1.44E-07	count	1
AC092295.2	17.5574103	3335.552586	0.0053	0.996	1.44E-07	count	1
AC114490.2	17.5574101	3335.552522	0.0053	0.996	1.44E-07	count	1
NBPF26	17.5574102	3335.552546	0.0053	0.996	1.44E-07	count	1
GPR55	17.5574102	3335.552546	0.0053	0.996	1.44E-07	count	1
XYLB	17.5574102	3335.552546	0.0053	0.996	1.44E-07	count	1
PTPN23	17.5574101	3335.552522	0.0053	0.996	1.44E-07	count	1
POLA1	17.5574102	3335.552546	0.0053	0.996	1.44E-07	count	1
OVCH1-AS1	17.5574102	3335.552546	0.0053	0.996	1.44E-07	count	1
PLOD1	17.5574103	3335.552522	0.0053	0.996	1.44E-07	count	1
PHLDA3	17.5574103	3335.552554	0.0053	0.996	1.44E-07	count	1
C5orf38	17.5574103	3335.552522	0.0053	0.996	1.44E-07	count	1
CXCL14	17.5574103	3335.552554	0.0053	0.996	1.44E-07	count	1
AL096865.1	17.5574103	3335.552554	0.0053	0.996	1.44E-07	count	1
SLC39A9	17.5574103	3335.552522	0.0053	0.996	1.44E-07	count	1
AC137723.1	17.5574103	3335.552522	0.0053	0.996	1.44E-07	count	1
ELAC1	17.5574103	3335.552554	0.0053	0.996	1.44E-07	count	1
ZSWIM1	17.5574103	3335.552554	0.0053	0.996	1.44E-07	count	1
CLHC1	17.5574102	3335.552578	0.0053	0.996	1.44E-07	count	1
FHL2	17.5574102	3335.552578	0.0053	0.996	1.44E-07	count	1
CYTL1	17.5574102	3335.552578	0.0053	0.996	1.44E-07	count	1
AC010768.1	17.5574103	3335.552465	0.0053	0.996	1.44E-07	count	1
CCDC15	17.5574103	3335.552465	0.0053	0.996	1.44E-07	count	1
NTRK3	17.5574103	3335.552465	0.0053	0.996	1.44E-07	count	1
DHX34	17.5574103	3335.552465	0.0053	0.996	1.44E-07	count	1
REEP6	17.5574102	3335.552489	0.0053	0.996	1.44E-07	count	1
CYB561D1	17.5574103	3335.552554	0.0053	0.996	1.44E-07	count	1
AC062029.1	17.5574103	3335.552554	0.0053	0.996	1.44E-07	count	1
SOWAHC	17.5574103	3335.552481	0.0053	0.996	1.44E-07	count	1
AC114811.2	17.5574103	3335.552481	0.0053	0.996	1.44E-07	count	1
AC091965.4	17.5574103	3335.552554	0.0053	0.996	1.44E-07	count	1
JAZF1-AS1	17.5574103	3335.552554	0.0053	0.996	1.44E-07	count	1
PSPH	17.5574103	3335.552481	0.0053	0.996	1.44E-07	count	1
MAGIX	17.5574103	3335.552481	0.0053	0.996	1.44E-07	count	1
AMOT	17.5574103	3335.552481	0.0053	0.996	1.44E-07	count	1
TMEM71	17.5574103	3335.552481	0.0053	0.996	1.44E-07	count	1
AL158163.1	17.5574103	3335.552554	0.0053	0.996	1.44E-07	count	1
U47924.2	17.5574103	3335.552554	0.0053	0.996	1.44E-07	count	1
AC020656.1	17.5574103	3335.552481	0.0053	0.996	1.44E-07	count	1
LINC02453	17.5574103	3335.552481	0.0053	0.996	1.44E-07	count	1
RNASEH2B-AS1	17.5574103	3335.552554	0.0053	0.996	1.44E-07	count	1
AC008915.2	17.5574103	3335.552554	0.0053	0.996	1.44E-07	count	1
AC027682.6	17.5574103	3335.552481	0.0053	0.996	1.44E-07	count	1
EXO1	17.5574103	3335.552666	0.0053	0.996	1.44E-07	count	1

IFT172	17.5574103	3335.552666	0.0053	0.996	1.44E-07	count	1
GALNT14	17.5574103	3335.552666	0.0053	0.996	1.44E-07	count	1
SIX3	17.5574103	3335.552666	0.0053	0.996	1.44E-07	count	1
EFHB	17.5574103	3335.552666	0.0053	0.996	1.44E-07	count	1
AP000442.1	17.5574103	3335.552666	0.0053	0.996	1.44E-07	count	1
Z69706.1	17.5574103	3335.552666	0.0053	0.996	1.44E-07	count	1
IRF6	17.5574102	3335.55257	0.0053	0.996	1.44E-07	count	1
MCM2	17.5574102	3335.55257	0.0053	0.996	1.44E-07	count	1
AC112907.3	17.5574102	3335.552586	0.0053	0.996	1.44E-07	count	1
KIF4A	17.5574102	3335.552586	0.0053	0.996	1.44E-07	count	1
FAM133A	17.5574102	3335.55257	0.0053	0.996	1.44E-07	count	1
SYDE2	17.5574101	3335.552473	0.0053	0.996	1.44E-07	count	1
KIF9-AS1	17.5574102	3335.552489	0.0053	0.996	1.44E-07	count	1
ZCCHC12	17.5574101	3335.552473	0.0053	0.996	1.44E-07	count	1
NECTIN1	17.5574102	3335.552489	0.0053	0.996	1.44E-07	count	1
WASIR2	17.5574102	3335.552489	0.0053	0.996	1.44E-07	count	1
OLFML3	17.5574102	3335.552497	0.0053	0.996	1.44E-07	count	1
FAM72B	17.5574102	3335.552497	0.0053	0.996	1.44E-07	count	1
DNAH14	17.5574102	3335.552497	0.0053	0.996	1.44E-07	count	1
AC096992.2	17.5574101	3335.552497	0.0053	0.996	1.44E-07	count	1
AC008892.1	17.5574102	3335.55265	0.0053	0.996	1.44E-07	count	1
TMEM184A	17.5574101	3335.552497	0.0053	0.996	1.44E-07	count	1
SLC9A6	17.5574102	3335.552497	0.0053	0.996	1.44E-07	count	1
SLC12A3	17.5574102	3335.55265	0.0053	0.996	1.44E-07	count	1
SLC12A4	17.5574102	3335.552497	0.0053	0.996	1.44E-07	count	1
SNAP25	17.5574101	3335.552497	0.0053	0.996	1.44E-07	count	1
AC008686.1	17.5574101	3335.552497	0.0053	0.996	1.44E-07	count	1
AC006213.2	17.5574102	3335.55265	0.0053	0.996	1.44E-07	count	1
GATD3B	17.5574101	3335.552497	0.0053	0.996	1.44E-07	count	1
TMEM240	17.5574102	3335.552481	0.0053	0.996	1.44E-07	count	1
AL359541.1	17.5574102	3335.552562	0.0053	0.996	1.44E-07	count	1
IGKV5-2	17.5574102	3335.552562	0.0053	0.996	1.44E-07	count	1
AC109587.1	17.5574102	3335.552505	0.0053	0.996	1.44E-07	count	1
OMD	17.5574102	3335.552481	0.0053	0.996	1.44E-07	count	1
CARNS1	17.5574102	3335.552481	0.0053	0.996	1.44E-07	count	1
AC073896.3	17.5574102	3335.552505	0.0053	0.996	1.44E-07	count	1
THSD1	17.5574102	3335.552562	0.0053	0.996	1.44E-07	count	1
AC005695.2	17.5574102	3335.552481	0.0053	0.996	1.44E-07	count	1
SP2-AS1	17.5574102	3335.552481	0.0053	0.996	1.44E-07	count	1
C1orf159	17.5574102	3335.55257	0.0053	0.996	1.44E-07	count	1
AL009181.1	17.5574102	3335.55257	0.0053	0.996	1.44E-07	count	1
BCL9	17.5574102	3335.55257	0.0053	0.996	1.44E-07	count	1
TEX35	17.5574102	3335.552578	0.0053	0.996	1.44E-07	count	1
C1orf53	17.5574102	3335.55257	0.0053	0.996	1.44E-07	count	1
TRABD2A	17.5574102	3335.55257	0.0053	0.996	1.44E-07	count	1

SATB2	17.5574102	3335.552578	0.0053	0.996	1.44E-07	count	1
PRKAR2A-AS1	17.5574102	3335.55257	0.0053	0.996	1.44E-07	count	1
AF213884.3	17.5574102	3335.55257	0.0053	0.996	1.44E-07	count	1
AC106897.1	17.5574102	3335.55257	0.0053	0.996	1.44E-07	count	1
AL391422.3	17.5574102	3335.55257	0.0053	0.996	1.44E-07	count	1
PNMA5	17.5574102	3335.552578	0.0053	0.996	1.44E-07	count	1
JRK	17.5574102	3335.552578	0.0053	0.996	1.44E-07	count	1
CDC37L1-DT	17.5574102	3335.55257	0.0053	0.996	1.44E-07	count	1
SPA17	17.5574102	3335.55257	0.0053	0.996	1.44E-07	count	1
BBS10	17.5574102	3335.55257	0.0053	0.996	1.44E-07	count	1
AL139089.1	17.5574102	3335.55257	0.0053	0.996	1.44E-07	count	1
AC084782.1	17.5574102	3335.55257	0.0053	0.996	1.44E-07	count	1
AC009560.1	17.5574102	3335.552578	0.0053	0.996	1.44E-07	count	1
VPS33B	17.5574102	3335.55257	0.0053	0.996	1.44E-07	count	1
ZNF23	17.5574102	3335.55257	0.0053	0.996	1.44E-07	count	1
AC015912.3	17.5574102	3335.55257	0.0053	0.996	1.44E-07	count	1
TNNC2	17.5574102	3335.55257	0.0053	0.996	1.44E-07	count	1
ZNF45	17.5574102	3335.55257	0.0053	0.996	1.44E-07	count	1
NPAS1	17.5574102	3335.552578	0.0053	0.996	1.44E-07	count	1
AP001065.1	17.5574102	3335.552578	0.0053	0.996	1.44E-07	count	1
DENND6A-AS1	17.5574101	3335.552497	0.0053	0.996	1.44E-07	count	1
OLAH	17.5574101	3335.552497	0.0053	0.996	1.44E-07	count	1
C19orf44	17.5574101	3335.552497	0.0053	0.996	1.44E-07	count	1
ZNF99	17.5574101	3335.552497	0.0053	0.996	1.44E-07	count	1
TIMP3	17.5574101	3335.552497	0.0053	0.996	1.44E-07	count	1
PLA2G2A	17.5574102	3335.552602	0.0053	0.996	1.44E-07	count	1
AL449266.1	17.5574102	3335.552602	0.0053	0.996	1.44E-07	count	1
ASPM	17.5574102	3335.552546	0.0053	0.996	1.44E-07	count	1
LINC00862	17.5574102	3335.552618	0.0053	0.996	1.44E-07	count	1
CLSTN2	17.5574102	3335.552602	0.0053	0.996	1.44E-07	count	1
LINC01991	17.5574102	3335.552602	0.0053	0.996	1.44E-07	count	1
SERF1B	17.5574102	3335.552602	0.0053	0.996	1.44E-07	count	1
LINC01301	17.5574102	3335.552602	0.0053	0.996	1.44E-07	count	1
MAPK8IP1	17.5574102	3335.552602	0.0053	0.996	1.44E-07	count	1
SAMD15	17.5574102	3335.552602	0.0053	0.996	1.44E-07	count	1
FRMD5	17.5574102	3335.552618	0.0053	0.996	1.44E-07	count	1
ZNF625	17.5574102	3335.552546	0.0053	0.996	1.44E-07	count	1
LINC01801	17.5574102	3335.552546	0.0053	0.996	1.44E-07	count	1
TMEM145	17.5574102	3335.552618	0.0053	0.996	1.44E-07	count	1
ZNF417	17.5574102	3335.552546	0.0053	0.996	1.44E-07	count	1
AC005540.1	17.5574102	3335.552522	0.0053	0.996	1.44E-07	count	1
AC074044.1	17.5574102	3335.552522	0.0053	0.996	1.44E-07	count	1
MRPL38	17.5574102	3335.552522	0.0053	0.996	1.44E-07	count	1
BOLA3-AS1	17.5574101	3335.552513	0.0053	0.996	1.44E-07	count	1
GET4	17.5574101	3335.552513	0.0053	0.996	1.44E-07	count	1

NR6A1	17.5574101	3335.552513	0.0053	0.996	1.44E-07	count	1
NCS1	17.5574101	3335.552513	0.0053	0.996	1.44E-07	count	1
ITIH5	17.5574101	3335.552513	0.0053	0.996	1.44E-07	count	1
ENTPD7	17.5574101	3335.552513	0.0053	0.996	1.44E-07	count	1
AC008736.1	17.5574101	3335.552513	0.0053	0.996	1.44E-07	count	1
FRRS1	17.5574101	3335.552522	0.0053	0.996	1.44E-07	count	1
FRMD6-AS1	17.5574101	3335.552522	0.0053	0.996	1.44E-07	count	1
AC007998.4	17.5574101	3335.552522	0.0053	0.996	1.44E-07	count	1
NID1	17.5574102	3335.55265	0.0053	0.996	1.44E-07	count	1
IGKV3D-11	17.5574102	3335.552538	0.0053	0.996	1.44E-07	count	1
LINC01238	17.5574102	3335.55265	0.0053	0.996	1.44E-07	count	1
NMNAT3	17.5574102	3335.55265	0.0053	0.996	1.44E-07	count	1
AC002480.4	17.5574102	3335.55265	0.0053	0.996	1.44E-07	count	1
PON2	17.5574101	3335.552473	0.0053	0.996	1.44E-07	count	1
AC007032.1	17.5574101	3335.552473	0.0053	0.996	1.44E-07	count	1
CYP17A1-AS1	17.5574101	3335.552473	0.0053	0.996	1.44E-07	count	1
AL513190.1	17.5574102	3335.55265	0.0053	0.996	1.44E-07	count	1
PRPF40B	17.5574102	3335.552538	0.0053	0.996	1.44E-07	count	1
CDAN1	17.5574101	3335.552473	0.0053	0.996	1.44E-07	count	1
MEX3B	17.5574102	3335.55265	0.0053	0.996	1.44E-07	count	1
ATRN	17.5574102	3335.55265	0.0053	0.996	1.44E-07	count	1
ENTPD6	17.5574101	3335.552473	0.0053	0.996	1.44E-07	count	1
ZNF564	17.5574101	3335.552473	0.0053	0.996	1.44E-07	count	1
IGLV5-45	17.5574102	3335.552538	0.0053	0.996	1.44E-07	count	1
PLK4	17.5574101	3335.552513	0.0053	0.996	1.44E-07	count	1
IGF2BP3	17.5574101	3335.552513	0.0053	0.996	1.44E-07	count	1
KCTD21	17.5574101	3335.552513	0.0053	0.996	1.44E-07	count	1
LTBP1	17.5574102	3335.55253	0.0053	0.996	1.44E-07	count	1
INPP1	17.5574101	3335.552554	0.0053	0.996	1.44E-07	count	1
GRK4	17.5574101	3335.552554	0.0053	0.996	1.44E-07	count	1
LNX1	17.5574102	3335.55253	0.0053	0.996	1.44E-07	count	1
AL357054.2	17.5574102	3335.55253	0.0053	0.996	1.44E-07	count	1
HCG15	17.5574101	3335.552554	0.0053	0.996	1.44E-07	count	1
AL355297.4	17.5574101	3335.552489	0.0053	0.996	1.44E-07	count	1
CYTH3	17.5574102	3335.55253	0.0053	0.996	1.44E-07	count	1
STEAP2	17.5574102	3335.55253	0.0053	0.996	1.44E-07	count	1
XRCC2	17.5574101	3335.552554	0.0053	0.996	1.44E-07	count	1
PTCHD1	17.5574102	3335.55253	0.0053	0.996	1.44E-07	count	1
AC087354.1	17.5574101	3335.552554	0.0053	0.996	1.44E-07	count	1
FJX1	17.5574101	3335.552554	0.0053	0.996	1.44E-07	count	1
AC073389.1	17.5574102	3335.55253	0.0053	0.996	1.44E-07	count	1
AL355073.2	17.5574101	3335.552489	0.0053	0.996	1.44E-07	count	1
AL139022.2	17.5574102	3335.55253	0.0053	0.996	1.44E-07	count	1
PTGR2	17.5574101	3335.552554	0.0053	0.996	1.44E-07	count	1
IL21R	17.5574101	3335.552554	0.0053	0.996	1.44E-07	count	1

AC009133.1	17.5574102	3335.55253	0.0053	0.996	1.44E-07	count	1
RAPGEFL1	17.5574101	3335.552489	0.0053	0.996	1.44E-07	count	1
ZHX3	17.5574101	3335.552554	0.0053	0.996	1.44E-07	count	1
ARHGAP33	17.5574101	3335.552554	0.0053	0.996	1.44E-07	count	1
LYPD3	17.5574101	3335.552554	0.0053	0.996	1.44E-07	count	1
CDKL5	17.5574101	3335.552489	0.0053	0.996	1.44E-07	count	1
ATAD3C	17.55741	3335.552481	0.0053	0.996	1.44E-07	count	1
ENO1-AS1	17.55741	3335.552481	0.0053	0.996	1.44E-07	count	1
MIR4422HG	17.55741	3335.552481	0.0053	0.996	1.44E-07	count	1
AC092053.2	17.55741	3335.552481	0.0053	0.996	1.44E-07	count	1
LINC00882	17.55741	3335.552481	0.0053	0.996	1.44E-07	count	1
AC016575.1	17.55741	3335.552481	0.0053	0.996	1.44E-07	count	1
FAM169A	17.55741	3335.552481	0.0053	0.996	1.44E-07	count	1
TNXB	17.55741	3335.552481	0.0053	0.996	1.44E-07	count	1
PPT2	17.55741	3335.552481	0.0053	0.996	1.44E-07	count	1
AC002480.2	17.55741	3335.552481	0.0053	0.996	1.44E-07	count	1
LINC01393	17.55741	3335.552481	0.0053	0.996	1.44E-07	count	1
KCP	17.55741	3335.552481	0.0053	0.996	1.44E-07	count	1
KCNH2	17.55741	3335.552481	0.0053	0.996	1.44E-07	count	1
ABCF2	17.55741	3335.552481	0.0053	0.996	1.44E-07	count	1
VSIG4	17.55741	3335.552481	0.0053	0.996	1.44E-07	count	1
ANKRD20A2	17.55741	3335.552481	0.0053	0.996	1.44E-07	count	1
CCDC183	17.55741	3335.552481	0.0053	0.996	1.44E-07	count	1
PIDD1	17.55741	3335.552481	0.0053	0.996	1.44E-07	count	1
DNHD1	17.55741	3335.552481	0.0053	0.996	1.44E-07	count	1
DIXDC1	17.55741	3335.552481	0.0053	0.996	1.44E-07	count	1
AP001267.3	17.55741	3335.552481	0.0053	0.996	1.44E-07	count	1
AC034102.6	17.55741	3335.552481	0.0053	0.996	1.44E-07	count	1
AL355076.2	17.55741	3335.552481	0.0053	0.996	1.44E-07	count	1
AC010809.3	17.55741	3335.552481	0.0053	0.996	1.44E-07	count	1
AC078909.1	17.55741	3335.552481	0.0053	0.996	1.44E-07	count	1
C15orf65	17.55741	3335.552481	0.0053	0.996	1.44E-07	count	1
ZFHX3	17.55741	3335.552481	0.0053	0.996	1.44E-07	count	1
CDYL2	17.55741	3335.552481	0.0053	0.996	1.44E-07	count	1
FAM157C	17.55741	3335.552481	0.0053	0.996	1.44E-07	count	1
C20orf96	17.55741	3335.552481	0.0053	0.996	1.44E-07	count	1
ZNF343	17.55741	3335.552481	0.0053	0.996	1.44E-07	count	1
NPEPL1	17.55741	3335.552481	0.0053	0.996	1.44E-07	count	1
CABLES2	17.55741	3335.552481	0.0053	0.996	1.44E-07	count	1
ADM5	17.55741	3335.552481	0.0053	0.996	1.44E-07	count	1
IGLV9-49	17.55741	3335.552481	0.0053	0.996	1.44E-07	count	1
IGLV7-43	17.55741	3335.552481	0.0053	0.996	1.44E-07	count	1
AP000692.2	17.55741	3335.552481	0.0053	0.996	1.44E-07	count	1
AC007038.2	17.5574101	3335.552562	0.0053	0.996	1.44E-07	count	1
SLC27A4	17.5574101	3335.552562	0.0053	0.996	1.44E-07	count	1

ACTL10	17.5574101	3335.552562	0.0053	0.996	1.44E-07	count	1
AC020922.4	17.5574101	3335.552562	0.0053	0.996	1.44E-07	count	1
AC105935.1	17.5574101	3335.55261	0.0053	0.996	1.44E-07	count	1
DUSP7	17.5574101	3335.55261	0.0053	0.996	1.44E-07	count	1
ACTR3B	17.5574101	3335.55261	0.0053	0.996	1.44E-07	count	1
AL354710.2	17.5574101	3335.55261	0.0053	0.996	1.44E-07	count	1
ZNF385C	17.5574101	3335.55261	0.0053	0.996	1.44E-07	count	1
MUC16	17.5574101	3335.55261	0.0053	0.996	1.44E-07	count	1
GNAZ	17.5574101	3335.55261	0.0053	0.996	1.44E-07	count	1
RFC5	0.2257602	0.7355271	0.3069	0.759	1.47E-07	count	1
C5orf51	0.2257602	0.6407444	0.3523	0.725	1.47E-07	count	1
TXNL4B	0.2257602	0.6407444	0.3523	0.725	1.47E-07	count	1
ASB2	0.2257602	0.8540135	0.2644	0.792	1.47E-07	count	1
INTS1	0.2257602	0.6291985	0.3588	0.72	1.47E-07	count	1
SUPT3H	0.2257602	0.6291985	0.3588	0.72	1.47E-07	count	1
AL512631.1	0.2257602	0.7643491	0.2954	0.768	1.47E-07	count	1
MED12L	0.2257602	0.7643491	0.2954	0.768	1.47E-07	count	1
SCCPDH	0.2257602	0.4595842	0.4912	0.624	1.47E-07	count	1
AGPAT1	0.2257602	0.801325	0.2817	0.778	1.47E-07	count	1
PUSL1	0.2257602	0.6736341	0.3351	0.738	1.47E-07	count	1
TBCE	0.2257602	0.6736341	0.3351	0.738	1.47E-07	count	1
TOX	0.2257602	0.5167912	0.4368	0.662	1.49E-07	count	1
PPM1F	0.0894925	1.0597092	0.0845	0.933	1.50E-07	count	1
YEATS2	0.0894925	1.0597092	0.0845	0.933	1.50E-07	count	1
TAF9B	0.0894925	1.0597092	0.0845	0.933	1.50E-07	count	1
RBM26-AS1	0.0894925	1.0597092	0.0845	0.933	1.50E-07	count	1
CAMP	0.0894925	1.0597092	0.0845	0.933	1.50E-07	count	1
VILL	0.0894925	1.0597092	0.0845	0.933	1.50E-07	count	1
GPR107	0.0894925	1.0597092	0.0845	0.933	1.50E-07	count	1
SLC10A7	0.0894925	1.0597092	0.0845	0.933	1.50E-07	count	1
TANGO2	0.0894925	1.0597092	0.0845	0.933	1.50E-07	count	1
DNMBP	0.0894925	1.0597092	0.0845	0.933	1.50E-07	count	1
SOGA1	0.0894925	1.0597092	0.0845	0.933	1.50E-07	count	1
DNAJC25-GNG10	0.0894925	1.0597092	0.0845	0.933	1.50E-07	count	1
GBGT1	0.0894925	0.7492516	0.1194	0.905	1.50E-07	count	1
ZFAT	0.0894925	0.857456	0.1044	0.917	1.50E-07	count	1
CD300A	0.0894925	0.9480685	0.0944	0.925	1.50E-07	count	1
ZIK1	0.0894925	1.0692288	0.0837	0.933	1.50E-07	count	1
RCN1	0.0894925	1.0692288	0.0837	0.933	1.50E-07	count	1
AL359513.1	0.0894925	1.0692288	0.0837	0.933	1.50E-07	count	1
TRPM2	0.0894925	1.0692288	0.0837	0.933	1.50E-07	count	1
SCMH1	0.0894925	0.8514582	0.1051	0.916	1.50E-07	count	1
USP9Y	0.0894925	0.7458628	0.12	0.905	1.50E-07	count	1
IPPK	0.0894925	1.3331902	0.0671	0.947	1.50E-07	count	1
BCR	0.0894925	1.3331902	0.0671	0.947	1.50E-07	count	1

HIST1H4E	0.0894925	1.3331902	0.0671	0.947	1.50E-07	count	1
AC040977.1	0.0894925	1.3331902	0.0671	0.947	1.50E-07	count	1
AC083798.2	0.0894925	1.3331902	0.0671	0.947	1.50E-07	count	1
CRHBP	0.0894925	1.3331902	0.0671	0.947	1.50E-07	count	1
FAM213A	0.0894925	1.3331902	0.0671	0.947	1.50E-07	count	1
KIAA0825	0.0894925	1.3331902	0.0671	0.947	1.50E-07	count	1
ZNF41	0.0894925	1.3331902	0.0671	0.947	1.50E-07	count	1
FAM161B	0.0894925	1.3331902	0.0671	0.947	1.50E-07	count	1
AL161457.2	0.0894925	1.3331902	0.0671	0.947	1.50E-07	count	1
CCDC34	0.0894925	1.3331902	0.0671	0.947	1.50E-07	count	1
BBS12	0.0894925	1.3331902	0.0671	0.947	1.50E-07	count	1
MTHFSD	0.0894925	1.3331902	0.0671	0.947	1.50E-07	count	1
ALPL	0.0894925	1.3331902	0.0671	0.947	1.50E-07	count	1
AC104653.1	0.0894925	1.3331902	0.0671	0.947	1.50E-07	count	1
AC108673.3	0.0894925	0.8604054	0.104	0.917	1.50E-07	count	1
SLC2A13	0.0894925	0.8834371	0.1013	0.919	1.50E-07	count	1
EME2	0.0894925	1.3634252	0.0656	0.948	1.51E-07	count	1
LINC02328	0.0894925	1.3634252	0.0656	0.948	1.51E-07	count	1
CR2	0.0894925	1.1072669	0.0808	0.936	1.51E-07	count	1
D2HGDH	0.0894925	0.8484777	0.1055	0.916	1.51E-07	count	1
UQCRHL	0.0894925	0.8484777	0.1055	0.916	1.51E-07	count	1
MVD	0.0894925	0.6718958	0.1332	0.894	1.51E-07	count	1
C15orf39	0.0894925	1.1232718	0.0797	0.937	1.51E-07	count	1
IFIT5	0.0894925	0.8633447	0.1037	0.917	1.51E-07	count	1
B3GALNT2	0.0894925	0.9399561	0.0952	0.924	1.51E-07	count	1
ZNF546	20.2034602	2863.06839	0.0071	0.994	1.51E-07	count	1
HS3ST1	19.4057862	3088.634149	0.0063	0.995	1.52E-07	count	1
AC018413.1	19.4057842	3088.630898	0.0063	0.995	1.52E-07	count	1
C11orf21	19.405784	3088.634433	0.0063	0.995	1.52E-07	count	1
DBNDD1	19.405784	3088.632258	0.0063	0.995	1.52E-07	count	1
STKLD1	19.4057834	3088.634162	0.0063	0.995	1.52E-07	count	1
TPST1	19.405783	3088.632802	0.0063	0.995	1.52E-07	count	1
ARHGEF5	19.4057825	3088.633346	0.0063	0.995	1.52E-07	count	1
UAP1L1	19.4057823	3088.632814	0.0063	0.995	1.52E-07	count	1
S1PR1	19.9457748	2953.219157	0.0068	0.995	1.52E-07	count	1
AGPAT2	0.0126629	0.3464637	0.0365	0.971	1.53E-07	count	1
SLFN11	0.0344685	0.559046	0.0617	0.951	1.54E-07	count	1
UBXN6	0.0351547	0.5559279	0.0632	0.95	1.57E-07	count	1
MSRB2	0.0351547	0.5361321	0.0656	0.948	1.57E-07	count	1
GABPA	0.0351547	0.4949221	0.071	0.943	1.57E-07	count	1
ZNF322	0.2415086	0.4660107	0.5182	0.605	1.59E-07	count	1
PDCD11	0.5824352	0.9311912	0.6255	0.532	1.60E-07	count	1
ZSCAN29	0.5824352	1.0057492	0.5791	0.563	1.60E-07	count	1
TBC1D25	0.5824352	1.0057492	0.5791	0.563	1.60E-07	count	1
INAFM1	0.5824352	1.0057492	0.5791	0.563	1.60E-07	count	1

CLK2	0.5824352	1.0057492	0.5791	0.563	1.60E-07	count	1
BX284668.5	0.5824352	1.0057492	0.5791	0.563	1.60E-07	count	1
UBR5-AS1	0.5824352	1.0057492	0.5791	0.563	1.60E-07	count	1
FANCM	0.5824352	0.7801016	0.7466	0.456	1.61E-07	count	1
ALG6	0.5824352	0.7801016	0.7466	0.456	1.61E-07	count	1
ITPRIPL1	0.5824352	1.0751493	0.5417	0.588	1.61E-07	count	1
FBXO10	0.5824352	1.0751493	0.5417	0.588	1.61E-07	count	1
FAM135A	0.5824352	1.0751493	0.5417	0.588	1.61E-07	count	1
ZNF71	0.5824352	1.0751493	0.5417	0.588	1.61E-07	count	1
DECR2	0.5824352	1.0751493	0.5417	0.588	1.61E-07	count	1
PGAP3	0.5824352	1.0751493	0.5417	0.588	1.61E-07	count	1
HHAT	0.5824352	1.1403334	0.5108	0.61	1.61E-07	count	1
TOM1	0.5824352	0.8677401	0.6712	0.502	1.61E-07	count	1
LINC00309	0.5824352	0.8677401	0.6712	0.502	1.61E-07	count	1
ZNF513	0.5824352	0.8677401	0.6712	0.502	1.61E-07	count	1
RMDN2	0.5824352	0.8677401	0.6712	0.502	1.61E-07	count	1
ZNF700	0.5824352	0.8677401	0.6712	0.502	1.61E-07	count	1
FAM173B	0.5824352	0.8677401	0.6712	0.502	1.61E-07	count	1
PRIMPOL	0.5824352	0.8677401	0.6712	0.502	1.61E-07	count	1
SDF2L1	0.0133228	0.3293566	0.0405	0.968	1.61E-07	count	1
AC015982.1	0.5824352	0.9473053	0.6148	0.539	1.61E-07	count	1
TOX2	0.5824352	0.9473053	0.6148	0.539	1.61E-07	count	1
AL359764.1	0.5824352	0.9473053	0.6148	0.539	1.61E-07	count	1
FAM234A	0.5824352	0.9473053	0.6148	0.539	1.61E-07	count	1
MYO1B	0.5824352	1.0206869	0.5706	0.569	1.61E-07	count	1
NCBP1	0.5824352	1.0891355	0.5348	0.593	1.61E-07	count	1
CPOX	0.5824352	1.0891355	0.5348	0.593	1.61E-07	count	1
ZBED1	0.0359482	0.5435388	0.0661	0.947	1.61E-07	count	1
RNPC3	0.0363933	0.4165711	0.0874	0.93	1.63E-07	count	1
DLST	0.0368761	0.584656	0.0631	0.95	1.65E-07	count	1
RPRD1A	0.0368761	0.5633106	0.0655	0.948	1.65E-07	count	1
AGAP2	0.0368761	0.7280068	0.0507	0.96	1.65E-07	count	1
ORAOV1	0.0988773	0.816085	0.1212	0.904	1.67E-07	count	1
NAA60	0.0988773	0.8148707	0.1213	0.903	1.67E-07	count	1
RABGGTA	0.2539311	0.5892278	0.431	0.667	1.68E-07	count	1
MAML1	0.2539311	0.6187465	0.4104	0.682	1.68E-07	count	1
SLC35A3	0.2539311	0.6187465	0.4104	0.682	1.68E-07	count	1
NIPAL2	0.2539311	0.6187465	0.4104	0.682	1.68E-07	count	1
DHRS12	0.2539311	0.5066074	0.5012	0.616	1.68E-07	count	1
MCM9	0.2539311	0.6739162	0.3768	0.707	1.68E-07	count	1
WDFY4	0.038474	0.3728817	0.1032	0.918	1.72E-07	count	1
COMT	0.0388928	0.3460383	0.1124	0.911	1.74E-07	count	1
MTA2	0.0392991	0.8313602	0.0473	0.962	1.77E-07	count	1
ZNF526	0.1046468	0.833133	0.1256	0.9	1.77E-07	count	1
RCCD1	0.1046468	0.9106998	0.1149	0.909	1.77E-07	count	1

USP30-AS1	0.1046468	0.8246434	0.1269	0.899	1.77E-07	count	1
ZNF383	0.2722802	0.5460306	0.4987	0.618	1.81E-07	count	1
AC023157.3	0.2722802	0.6530545	0.4169	0.677	1.81E-07	count	1
TMEM41B	0.2722802	0.4291325	0.6345	0.526	1.81E-07	count	1
PGRMC1	0.2722802	0.5390642	0.5051	0.614	1.81E-07	count	1
RPS6KA3	0.0408642	0.3889857	0.1051	0.916	1.84E-07	count	1
AKAP10	0.0409222	0.8108703	0.0505	0.96	1.85E-07	count	1
RBBP8	0.0416994	0.4558416	0.0915	0.927	1.87E-07	count	1
CNOT1	0.0156479	0.2973301	0.0526	0.958	1.89E-07	count	1
TMEM208	0.0421663	0.3401753	0.124	0.901	1.89E-07	count	1
NAPG	0.0157006	0.2999185	0.0523	0.958	1.90E-07	count	1
AP3M1	0.2851836	0.5322984	0.5358	0.592	1.91E-07	count	1
GXYLT1	0.2851836	0.5431627	0.525	0.6	1.91E-07	count	1
DDB2	0.0426599	0.4746902	0.0899	0.928	1.91E-07	count	1
AIFM1	0.0426599	0.4745943	0.0899	0.928	1.91E-07	count	1
CEP44	0.0426599	0.4880324	0.0874	0.93	1.91E-07	count	1
RASGRF1	0.0426599	0.5011103	0.0851	0.932	1.92E-07	count	1
NT5C3B	0.2851836	0.5743729	0.4965	0.62	1.92E-07	count	1
ZNF669	0.0426599	0.4782876	0.0892	0.929	1.92E-07	count	1
DGKG	0.0426599	0.5504052	0.0775	0.938	1.92E-07	count	1
SERTAD3	0.016129	0.1838025	0.0878	0.93	1.95E-07	count	1
SMIM8	0.0437373	0.5810916	0.0753	0.94	1.96E-07	count	1
UBXN11	0.2947531	0.5870484	0.5021	0.616	1.99E-07	count	1
VCL	0.2947531	0.6303559	0.4676	0.64	1.99E-07	count	1
STK38L	0.0449542	0.4870064	0.0923	0.926	2.02E-07	count	1
HECTD4	0.0449542	0.4853414	0.0926	0.926	2.02E-07	count	1
ZNF100	0.0449542	0.5025948	0.0894	0.929	2.02E-07	count	1
TMUB2	0.0449542	0.4034011	0.1114	0.911	2.02E-07	count	1
UBL3	0.0451493	0.428451	0.1054	0.916	2.03E-07	count	1
C6orf47	0.0456237	0.3980784	0.1146	0.909	2.05E-07	count	1
SUPV3L1	0.0463396	0.5722834	0.081	0.935	2.08E-07	count	1
ODR4	0.0463396	0.6548631	0.0708	0.944	2.09E-07	count	1
MRPL32	0.0172386	0.3773017	0.0457	0.964	2.09E-07	count	1
SEL1L	0.0173807	0.3856073	0.0451	0.964	2.10E-07	count	1
GRSF1	0.0471066	0.3879131	0.1214	0.903	2.12E-07	count	1
CLINT1	0.0180633	0.2375359	0.076	0.939	2.18E-07	count	1
PAK1IP1	0.3200709	0.3788794	0.8448	0.399	2.19E-07	count	1
CCP110	0.0487319	0.4567797	0.1067	0.915	2.19E-07	count	1
ZNFX1	0.0487319	0.4675357	0.1042	0.917	2.19E-07	count	1
CAPS	0.1287169	1.0677123	0.1206	0.904	2.20E-07	count	1
PPP1R3D	0.1287169	1.0677123	0.1206	0.904	2.20E-07	count	1
GPATCH1	0.1287169	0.9975201	0.129	0.897	2.20E-07	count	1
ZNF782	0.1287169	1.3269509	0.097	0.923	2.20E-07	count	1
PTPN4	0.0499812	0.5070222	0.0986	0.922	2.25E-07	count	1
NUDT2	0.0500806	0.3917706	0.1278	0.898	2.25E-07	count	1

METTL2B	0.0500806	0.5044091	0.0993	0.921	2.26E-07	count	1
POLR3B	0.7647567	1.0808183	0.7076	0.48	2.26E-07	count	1
CCNB1	0.7647567	1.0808183	0.7076	0.48	2.26E-07	count	1
ZNF66	0.7647567	1.0808183	0.7076	0.48	2.26E-07	count	1
CENPS	0.7647567	1.0808183	0.7076	0.48	2.26E-07	count	1
ANKRD34A	0.7647567	1.0808183	0.7076	0.48	2.26E-07	count	1
TSPAN17	0.7647567	1.0808183	0.7076	0.48	2.26E-07	count	1
TEX261	0.7647567	1.0808183	0.7076	0.48	2.26E-07	count	1
AP3S2	0.7647567	1.0808183	0.7076	0.48	2.26E-07	count	1
IGFBP4	0.7647567	1.0808183	0.7076	0.48	2.26E-07	count	1
LNX2	0.7647567	1.0808183	0.7076	0.48	2.26E-07	count	1
TMEM150A	0.7647567	1.0808183	0.7076	0.48	2.26E-07	count	1
WRAP53	0.7647567	1.0808183	0.7076	0.48	2.26E-07	count	1
ZNF18	0.7647567	1.0808183	0.7076	0.48	2.26E-07	count	1
ZDHHC8	0.7647567	1.0808183	0.7076	0.48	2.26E-07	count	1
PHYHD1	0.7647567	1.0808183	0.7076	0.48	2.26E-07	count	1
AP001160.1	0.7647567	1.0808183	0.7076	0.48	2.26E-07	count	1
LINC01465	0.7647567	1.0808183	0.7076	0.48	2.26E-07	count	1
GAB3	0.7647567	1.0808183	0.7076	0.48	2.26E-07	count	1
SLC26A4-AS1	0.7647567	1.2753927	0.5996	0.549	2.26E-07	count	1
PTDSS2	0.7647567	1.2753927	0.5996	0.549	2.26E-07	count	1
ZNF333	0.7647567	1.2753927	0.5996	0.549	2.26E-07	count	1
AL691432.2	0.7647567	1.2753927	0.5996	0.549	2.26E-07	count	1
ATP13A2	0.7647567	1.2753927	0.5996	0.549	2.26E-07	count	1
AC119428.2	0.7647567	1.2753927	0.5996	0.549	2.26E-07	count	1
BTN1A1	0.7647567	1.2753927	0.5996	0.549	2.26E-07	count	1
GSTM3	0.7647567	1.2753927	0.5996	0.549	2.26E-07	count	1
AC245014.1	0.7647567	1.2753927	0.5996	0.549	2.26E-07	count	1
UBE2QL1	0.7647567	1.2753927	0.5996	0.549	2.26E-07	count	1
SLC4A2	0.7647567	1.2753927	0.5996	0.549	2.26E-07	count	1
COLCA1	0.7647567	1.2753927	0.5996	0.549	2.26E-07	count	1
MATK	0.7647567	1.2753927	0.5996	0.549	2.26E-07	count	1
ZNF682	0.7647567	1.2753927	0.5996	0.549	2.26E-07	count	1
L1TD1	0.7647567	1.2753927	0.5996	0.549	2.26E-07	count	1
UCK2	0.7647567	1.2753927	0.5996	0.549	2.26E-07	count	1
SNCG	0.7647567	1.2753927	0.5996	0.549	2.26E-07	count	1
LIMA1	0.7647567	1.2753927	0.5996	0.549	2.26E-07	count	1
RGS9	0.7647567	1.2753927	0.5996	0.549	2.26E-07	count	1
ITPKC	0.7647567	1.2753927	0.5996	0.549	2.26E-07	count	1
AC124016.2	0.7647567	1.2753927	0.5996	0.549	2.26E-07	count	1
AMZ1	0.7647567	1.2753927	0.5996	0.549	2.26E-07	count	1
MRPS17	0.7647567	1.2753927	0.5996	0.549	2.26E-07	count	1
AL133551.1	0.7647567	1.2753927	0.5996	0.549	2.26E-07	count	1
ABCD2	0.7647567	1.2753927	0.5996	0.549	2.26E-07	count	1
MYO5C	0.7647567	1.2753927	0.5996	0.549	2.26E-07	count	1

MYLPF	0.7647567	1.2753927	0.5996	0.549	2.26E-07	count	1
PYGB	0.7647567	1.2753927	0.5996	0.549	2.26E-07	count	1
ZNF558	0.7647567	1.2753927	0.5996	0.549	2.26E-07	count	1
IL10RB-DT	0.7647567	1.2753927	0.5996	0.549	2.26E-07	count	1
LINC00649	0.7647567	1.2753927	0.5996	0.549	2.26E-07	count	1
EFNA3	0.7647567	1.2753927	0.5996	0.549	2.26E-07	count	1
ZSCAN21	0.7647567	1.2753927	0.5996	0.549	2.26E-07	count	1
SDR16C5	0.7647567	1.2753927	0.5996	0.549	2.26E-07	count	1
C12orf74	0.7647567	1.2753927	0.5996	0.549	2.26E-07	count	1
GGACT	0.7647567	1.2753927	0.5996	0.549	2.26E-07	count	1
FBXL19	0.7647567	1.2753927	0.5996	0.549	2.26E-07	count	1
AP001347.1	0.7647567	1.2753927	0.5996	0.549	2.26E-07	count	1
ASH1L-AS1	0.7647567	1.2753927	0.5996	0.549	2.26E-07	count	1
PIK3R4	0.7647567	1.2753927	0.5996	0.549	2.26E-07	count	1
ERCC2	0.7647567	1.2753927	0.5996	0.549	2.26E-07	count	1
JAM2	0.7647567	1.2753927	0.5996	0.549	2.26E-07	count	1
HOMER1	0.7647567	1.2753927	0.5996	0.549	2.26E-07	count	1
IFT81	0.7647567	1.2753927	0.5996	0.549	2.26E-07	count	1
OAS3	0.7647567	1.2753927	0.5996	0.549	2.26E-07	count	1
ADAT3	0.7647567	1.2753927	0.5996	0.549	2.26E-07	count	1
CBR3	0.7647567	1.2753927	0.5996	0.549	2.26E-07	count	1
PDIK1L	0.7647567	1.594849	0.4795	0.632	2.26E-07	count	1
FAM66C	0.7647567	1.594849	0.4795	0.632	2.26E-07	count	1
TIGD1	0.7647567	1.594849	0.4795	0.632	2.26E-07	count	1
AC083843.3	0.7647567	1.594849	0.4795	0.632	2.26E-07	count	1
INTS7	0.7647567	1.594849	0.4795	0.632	2.26E-07	count	1
C15orf48	0.7647567	1.594849	0.4795	0.632	2.26E-07	count	1
LRG1	0.7647567	1.594849	0.4795	0.632	2.26E-07	count	1
C19orf18	0.7647567	1.594849	0.4795	0.632	2.26E-07	count	1
AP000911.1	0.7647567	1.594849	0.4795	0.632	2.26E-07	count	1
CRAMP1	0.7647567	1.594849	0.4795	0.632	2.26E-07	count	1
APOBR	0.7647567	1.594849	0.4795	0.632	2.26E-07	count	1
LRRC37A	0.7647567	1.594849	0.4795	0.632	2.26E-07	count	1
MORN1	0.7647567	1.594849	0.4795	0.632	2.26E-07	count	1
CDA	0.7647567	1.594849	0.4795	0.632	2.26E-07	count	1
ASF1B	0.7647567	1.594849	0.4795	0.632	2.26E-07	count	1
RAB34	0.7647567	0.9079231	0.8423	0.4	2.26E-07	count	1
ZNF670	0.7647567	0.9689886	0.7892	0.43	2.26E-07	count	1
PFKFB3	0.7647567	0.9689886	0.7892	0.43	2.26E-07	count	1
TMLHE	0.7647567	0.9689886	0.7892	0.43	2.26E-07	count	1
AC104699.1	0.7647567	0.7603144	1.0058	0.315	2.26E-07	count	1
LINC02541	0.7647567	1.0264276	0.7451	0.457	2.26E-07	count	1
GALNS	0.7647567	1.0264276	0.7451	0.457	2.26E-07	count	1
DTWD2	0.7647567	1.0264276	0.7451	0.457	2.26E-07	count	1
SLC38A9	0.7647567	1.0264276	0.7451	0.457	2.26E-07	count	1

ZP3	0.7647567	0.8322816	0.9189	0.359	2.26E-07	count	1
GOT2	0.7647567	0.8322816	0.9189	0.359	2.26E-07	count	1
EXOSC2	0.7647567	0.8322816	0.9189	0.359	2.26E-07	count	1
LINC01684	0.7647567	1.1326	0.6752	0.5	2.26E-07	count	1
PRKAB2	0.7647567	0.8985028	0.8511	0.395	2.27E-07	count	1
TRDMT1	0.7647567	0.8985028	0.8511	0.395	2.27E-07	count	1
FAAH2	0.7647567	0.8985028	0.8511	0.395	2.27E-07	count	1
ZNF514	0.7647567	0.9601676	0.7965	0.426	2.27E-07	count	1
FKRP	0.7647567	1.0181044	0.7512	0.453	2.27E-07	count	1
RNF115	0.0188072	0.3181646	0.0591	0.953	2.27E-07	count	1
ABCC5	0.7647567	1.1748959	0.6509	0.515	2.28E-07	count	1
DAPP1	0.0189629	0.2170995	0.0873	0.93	2.29E-07	count	1
BCAP31	0.0191712	0.2006261	0.0956	0.924	2.32E-07	count	1
ACD	0.0527261	0.3759574	0.1402	0.889	2.38E-07	count	1
SETD2	0.0197918	0.3104528	0.0638	0.949	2.39E-07	count	1
ZBTB20	0.0197884	0.2145978	0.0922	0.927	2.39E-07	count	1
RNF220	0.0534119	0.4113653	0.1298	0.897	2.41E-07	count	1
POLD2	0.0537102	0.4062837	0.1322	0.895	2.42E-07	count	1
RPS6KB2	0.0547269	0.4036206	0.1356	0.892	2.47E-07	count	1
DNAJC11	0.3592916	1.0703483	0.3357	0.737	2.47E-07	count	1
LETM2	0.3592916	0.6123724	0.5867	0.558	2.47E-07	count	1
PHTF1	0.3592916	0.6710218	0.5354	0.593	2.47E-07	count	1
NEK9	0.3592916	0.6628728	0.542	0.588	2.47E-07	count	1
TAF5L	0.3592916	0.6936975	0.5179	0.605	2.47E-07	count	1
WDR18	0.3592916	0.660978	0.5436	0.587	2.47E-07	count	1
S100A12	0.3592916	1.1478534	0.313	0.754	2.47E-07	count	1
ZNF134	0.3592916	0.6983934	0.5145	0.607	2.47E-07	count	1
AL031663.3	0.3592916	0.6983934	0.5145	0.607	2.47E-07	count	1
PIK3CG	0.05466	0.5909705	0.0925	0.926	2.47E-07	count	1
AC145285.6	0.3592916	0.9019082	0.3984	0.691	2.47E-07	count	1
C6orf203	0.3592916	0.6065985	0.5923	0.554	2.47E-07	count	1
AC093673.1	0.3592916	0.6083943	0.5906	0.555	2.47E-07	count	1
IFT52	0.3592916	0.6083943	0.5906	0.555	2.47E-07	count	1
UBE2O	0.3592916	0.6013498	0.5975	0.55	2.47E-07	count	1
LRRC40	0.3592916	0.5806728	0.6188	0.536	2.47E-07	count	1
GOLPH3L	0.3592916	0.7321929	0.4907	0.624	2.47E-07	count	1
TMEM161B	0.3592916	0.5216626	0.6887	0.491	2.47E-07	count	1
TRIM59	0.3592916	0.7644995	0.47	0.639	2.47E-07	count	1
POLG2	0.3592916	0.6469131	0.5554	0.579	2.47E-07	count	1
MLKL	0.3592916	0.6469131	0.5554	0.579	2.47E-07	count	1
THAP4	0.3592916	0.6469131	0.5554	0.579	2.47E-07	count	1
RFT1	0.3592916	0.6469131	0.5554	0.579	2.47E-07	count	1
AGO1	0.3592916	0.683264	0.5258	0.599	2.48E-07	count	1
PRKAG2-AS1	0.3592916	0.683264	0.5258	0.599	2.48E-07	count	1
AP4M1	0.3592916	0.6337599	0.5669	0.571	2.48E-07	count	1

TMEM64	0.3592916	0.6741361	0.533	0.594	2.48E-07	count	1
SCNM1	0.3592916	0.4833044	0.7434	0.458	2.48E-07	count	1
NUP188	0.3592916	0.7185759	0.5	0.617	2.48E-07	count	1
CDIPT	0.3592916	0.4484761	0.8011	0.423	2.49E-07	count	1
PELP1	0.3592916	0.7092059	0.5066	0.613	2.49E-07	count	1
ZNF75A	0.0557302	0.4117422	0.1354	0.892	2.52E-07	count	1
SLC30A9	0.0559028	0.4444986	0.1258	0.9	2.52E-07	count	1
GCLM	0.0559028	0.4091764	0.1366	0.891	2.52E-07	count	1
PER1	0.0561688	0.446799	0.1257	0.9	2.53E-07	count	1
PGAP2	0.0566317	0.5743204	0.0986	0.921	2.56E-07	count	1
SLAMF6	0.1493594	0.6429665	0.2323	0.816	2.57E-07	count	1
SLC25A12	0.1493594	0.68445	0.2182	0.827	2.57E-07	count	1
SLC39A7	0.1493594	0.6360309	0.2348	0.814	2.57E-07	count	1
MTA3	0.1493594	0.6779389	0.2203	0.826	2.57E-07	count	1
PLPBP	0.0577937	0.3909464	0.1478	0.883	2.61E-07	count	1
IDH2	0.0582813	0.3357311	0.1736	0.862	2.63E-07	count	1
COMMD10	0.0589323	0.4063745	0.145	0.885	2.66E-07	count	1
LTV1	0.0589323	0.3971608	0.1484	0.882	2.66E-07	count	1
ZDHHC6	0.0589323	0.4054268	0.1454	0.884	2.67E-07	count	1
PIK3R1	0.0223861	0.4114888	0.0544	0.957	2.71E-07	count	1
ACTR1A	0.0601522	0.386881	0.1555	0.877	2.72E-07	count	1
WDR45B	0.0603361	0.3346648	0.1803	0.857	2.73E-07	count	1
HMGXB4	0.0614624	0.4456384	0.1379	0.89	2.78E-07	count	1
SENP5	0.0614644	0.4432389	0.1387	0.89	2.78E-07	count	1
DYNLL2	0.061461	0.4237869	0.145	0.885	2.79E-07	count	1
ENOSF1	0.1660166	0.7504038	0.2212	0.825	2.88E-07	count	1
CSE1L	0.1660166	0.6282209	0.2643	0.792	2.88E-07	count	1
BDH1	0.1660166	0.7594527	0.2186	0.827	2.88E-07	count	1
GCSH	0.1660166	0.7970487	0.2083	0.835	2.88E-07	count	1
DNMT3A	0.1660166	0.7970487	0.2083	0.835	2.88E-07	count	1
ACSL5	0.1660166	0.6857956	0.2421	0.809	2.88E-07	count	1
RNF4	0.0636035	0.3503769	0.1815	0.856	2.88E-07	count	1
DHODH	0.9189074	0.9424271	0.975	0.33	2.88E-07	count	1
DHTKD1	0.1660166	0.7388977	0.2247	0.822	2.88E-07	count	1
LONP1	0.9189074	0.9914138	0.9269	0.354	2.88E-07	count	1
BICRAL	0.9189074	0.8066703	1.1391	0.255	2.88E-07	count	1
HCG11	0.9189074	0.8066703	1.1391	0.255	2.88E-07	count	1
PITPNM1	0.9189074	1.0827586	0.8487	0.397	2.89E-07	count	1
GLCE	0.9189074	1.0827586	0.8487	0.397	2.89E-07	count	1
GBE1	0.9189074	0.8633942	1.0643	0.288	2.89E-07	count	1
DNM3	0.9189074	0.8633942	1.0643	0.288	2.89E-07	count	1
CMTR1	0.9189074	0.8633942	1.0643	0.288	2.89E-07	count	1
SIRT5	0.9189074	0.8633942	1.0643	0.288	2.89E-07	count	1
KANSL1L	0.9189074	0.9166144	1.0025	0.317	2.89E-07	count	1
ZMIZ1	0.9189074	0.9669098	0.9504	0.342	2.89E-07	count	1

TBC1D10A	0.4133588	0.5093995	0.8115	0.418	2.91E-07	count	1
UNK	0.4199162	0.4625466	0.9078	0.364	2.97E-07	count	1
GSAP	0.0660125	0.3761146	0.1755	0.861	2.99E-07	count	1
ZNF117	0.1754379	0.6908741	0.2539	0.8	3.06E-07	count	1
CCDC66	0.0256321	0.2858433	0.0897	0.929	3.11E-07	count	1
BMI1	0.4393343	0.5684668	0.7728	0.44	3.12E-07	count	1
AC027644.3	0.4393343	0.6015077	0.7304	0.466	3.13E-07	count	1
IRF2BP1	0.4393343	0.5590094	0.7859	0.432	3.13E-07	count	1
TYK2	0.4393343	0.5925778	0.7414	0.459	3.14E-07	count	1
KMT5A	0.0263506	0.3431336	0.0768	0.939	3.19E-07	count	1
GUCD1	0.0266437	0.3958497	0.0673	0.946	3.24E-07	count	1
TRIM37	0.4546018	0.5491548	0.8278	0.408	3.26E-07	count	1
C11orf24	0.4546018	0.6317535	0.7196	0.472	3.26E-07	count	1
ZNF10	0.4546018	0.6735524	0.6749	0.5	3.26E-07	count	1
DUSP14	0.4546018	0.5967674	0.7618	0.447	3.27E-07	count	1
PPRC1	0.1875943	0.6958059	0.2696	0.788	3.28E-07	count	1
PDZD8	0.1875943	0.8974772	0.209	0.835	3.28E-07	count	1
TRIM14	0.1875943	0.7490959	0.2504	0.802	3.28E-07	count	1
TOR4A	0.1875943	0.8400101	0.2233	0.823	3.28E-07	count	1
PCM1	0.0272682	0.1886946	0.1445	0.885	3.31E-07	count	1
PUM1	0.1893926	0.3672531	0.5157	0.606	3.32E-07	count	1
B4GALT3	0.0735376	0.4219312	0.1743	0.862	3.35E-07	count	1
SNUPN	0.0743539	0.5613092	0.1325	0.895	3.38E-07	count	1
WASHC2A	0.0743539	0.5239547	0.1419	0.887	3.38E-07	count	1
RCOR3	0.0743539	0.4826718	0.154	0.878	3.38E-07	count	1
TMEM106C	0.0750907	0.5688811	0.132	0.895	3.41E-07	count	1
C12orf4	0.0750907	0.5880231	0.1277	0.898	3.41E-07	count	1
AC084033.3	0.0750907	0.5284543	0.1421	0.887	3.41E-07	count	1
DTYMK	0.0750907	0.5268768	0.1425	0.887	3.41E-07	count	1
RAB3A	0.4770746	0.6458633	0.7387	0.461	3.44E-07	count	1
HHLA3	0.4770746	0.6458633	0.7387	0.461	3.44E-07	count	1
THTPA	0.4770746	0.7797415	0.6118	0.541	3.44E-07	count	1
PLEKHG7	0.4770746	0.5919862	0.8059	0.421	3.44E-07	count	1
ZNF605	0.4770746	0.7070478	0.6747	0.5	3.44E-07	count	1
ZFP1	0.4770746	0.6259683	0.7621	0.446	3.44E-07	count	1
PAXIP1	0.1959796	0.7728433	0.2536	0.8	3.45E-07	count	1
ZNF570	0.4770746	0.7633438	0.625	0.532	3.45E-07	count	1
ACY3	0.4770746	0.7633438	0.625	0.532	3.45E-07	count	1
JRKL	0.4770746	0.6581982	0.7248	0.469	3.45E-07	count	1
ACCS	1.0524388	1.0451518	1.007	0.314	3.45E-07	count	1
NLK	1.0524388	1.0451518	1.007	0.314	3.45E-07	count	1
MAK	1.0524388	1.0451518	1.007	0.314	3.45E-07	count	1
DVL2	1.0524388	1.0451518	1.007	0.314	3.45E-07	count	1
MARS2	1.0524388	1.0451518	1.007	0.314	3.45E-07	count	1
ZCCHC4	1.0524388	1.0451518	1.007	0.314	3.45E-07	count	1

FAM220A	1.0524388	1.0451518	1.007	0.314	3.45E-07	count	1
EEF2K	1.0524388	1.0451518	1.007	0.314	3.45E-07	count	1
TTC30B	1.0524388	1.1894099	0.8848	0.377	3.45E-07	count	1
AL683807.1	1.0524388	1.1894099	0.8848	0.377	3.45E-07	count	1
RITA1	1.0524388	1.1894099	0.8848	0.377	3.45E-07	count	1
PARPBP	1.0524388	1.1894099	0.8848	0.377	3.45E-07	count	1
NRARP	1.0524388	1.1894099	0.8848	0.377	3.45E-07	count	1
UEVLD	1.0524388	1.1894099	0.8848	0.377	3.45E-07	count	1
AGPAT3	1.0524388	1.1894099	0.8848	0.377	3.45E-07	count	1
TIMM23B	1.0524388	1.1894099	0.8848	0.377	3.45E-07	count	1
L3HYPDH	1.0524388	1.1894099	0.8848	0.377	3.45E-07	count	1
PRELID2	1.0524388	1.1894099	0.8848	0.377	3.45E-07	count	1
CCDC7	1.0524388	1.1894099	0.8848	0.377	3.45E-07	count	1
ATP11A	1.0524388	1.1894099	0.8848	0.377	3.45E-07	count	1
C1orf216	1.0524388	1.1894099	0.8848	0.377	3.45E-07	count	1
PDK1	1.0524388	1.1894099	0.8848	0.377	3.45E-07	count	1
HIST1H4I	1.0524388	1.1894099	0.8848	0.377	3.45E-07	count	1
CSTF2	1.0524388	1.1894099	0.8848	0.377	3.45E-07	count	1
NECAB3	1.0524388	1.1894099	0.8848	0.377	3.45E-07	count	1
ZNF578	1.0524388	1.1894099	0.8848	0.377	3.45E-07	count	1
SLC35E4	1.0524388	1.1894099	0.8848	0.377	3.45E-07	count	1
BYSL	1.0524388	1.1894099	0.8848	0.377	3.45E-07	count	1
FGF9	1.0524388	1.1894099	0.8848	0.377	3.45E-07	count	1
KDM8	1.0524388	1.1894099	0.8848	0.377	3.45E-07	count	1
PLEKHG2	1.0524388	1.1894099	0.8848	0.377	3.45E-07	count	1
IGIP	1.0524388	1.1894099	0.8848	0.377	3.45E-07	count	1
HOXC4	1.0524388	1.1894099	0.8848	0.377	3.45E-07	count	1
NR1D1	1.0524388	1.1894099	0.8848	0.377	3.45E-07	count	1
SLC17A9	1.0524388	1.1894099	0.8848	0.377	3.45E-07	count	1
GPR34	1.0524388	1.3179717	0.7985	0.425	3.46E-07	count	1
AC009126.1	1.0524388	1.3179717	0.7985	0.425	3.46E-07	count	1
KCNK12	1.0524388	1.3179717	0.7985	0.425	3.46E-07	count	1
AL035563.1	1.0524388	1.3179717	0.7985	0.425	3.46E-07	count	1
GRAMD1B	1.0524388	1.3179717	0.7985	0.425	3.46E-07	count	1
SELPLG	1.0524388	1.3179717	0.7985	0.425	3.46E-07	count	1
TBC1D4	1.0524388	1.3179717	0.7985	0.425	3.46E-07	count	1
SLC39A11	1.0524388	1.3179717	0.7985	0.425	3.46E-07	count	1
PRDM10	1.0524388	1.3179717	0.7985	0.425	3.46E-07	count	1
CBY1	1.0524388	1.3179717	0.7985	0.425	3.46E-07	count	1
TSPAN2	1.0524388	1.3179717	0.7985	0.425	3.46E-07	count	1
COL1A1	1.0524388	1.435062	0.7334	0.464	3.46E-07	count	1
EGR4	1.0524388	1.435062	0.7334	0.464	3.46E-07	count	1
SS18L1	1.0524388	1.435062	0.7334	0.464	3.46E-07	count	1
CDC14B	1.0524388	1.435062	0.7334	0.464	3.46E-07	count	1
RILPL1	1.0524388	1.435062	0.7334	0.464	3.46E-07	count	1

CYP4V2	1.0524388	1.435062	0.7334	0.464	3.46E-07	count	1
SNAI1	1.0524388	1.435062	0.7334	0.464	3.46E-07	count	1
WASF1	1.0524388	1.435062	0.7334	0.464	3.46E-07	count	1
HOXB7	1.0524388	1.435062	0.7334	0.464	3.46E-07	count	1
COQ2	0.0763684	0.6789938	0.1125	0.91	3.47E-07	count	1
ZNF684	0.0763684	0.6140384	0.1244	0.901	3.47E-07	count	1
FIZ1	0.0763684	0.6460922	0.1182	0.906	3.47E-07	count	1
FAM41C	0.0763684	0.7360818	0.1037	0.917	3.47E-07	count	1
UBA7	0.0763684	0.7075762	0.1079	0.914	3.47E-07	count	1
WDTC1	0.0763684	0.5774475	0.1323	0.895	3.47E-07	count	1
PAK1	0.0763684	0.6766292	0.1129	0.91	3.47E-07	count	1
THAP2	0.0763684	0.5774475	0.1323	0.895	3.47E-07	count	1
THAP3	0.0763684	0.6504504	0.1174	0.907	3.47E-07	count	1
DBT	0.0763684	0.7360818	0.1037	0.917	3.47E-07	count	1
ISYNA1	0.0763684	0.7126258	0.1072	0.915	3.47E-07	count	1
TCEAL8	0.0763684	0.5002575	0.1527	0.879	3.47E-07	count	1
ATP2C1	1.0524388	0.7872238	1.3369	0.182	3.48E-07	count	1
ATXN1	0.4770746	0.5190255	0.9192	0.359	3.48E-07	count	1
TMEM128	1.0524388	0.8836844	1.191	0.234	3.48E-07	count	1
P2RX4	1.0524388	0.8836844	1.191	0.234	3.49E-07	count	1
FBXL6	1.0524388	0.9281629	1.1339	0.257	3.49E-07	count	1
BCDIN3D	1.0524388	0.9281629	1.1339	0.257	3.49E-07	count	1
GLUD1	0.0770318	0.3759349	0.2049	0.838	3.50E-07	count	1
PQLC3	0.0770318	0.4137451	0.1862	0.852	3.51E-07	count	1
CAPZA2	0.0290065	0.2254909	0.1286	0.898	3.52E-07	count	1
HACD3	0.0774381	0.4892052	0.1583	0.874	3.52E-07	count	1
RASSF7	0.0774381	0.506928	0.1528	0.879	3.52E-07	count	1
SMIM37	0.0774381	0.3610583	0.2145	0.83	3.52E-07	count	1
CDK5RAP1	0.0779101	0.4185143	0.1862	0.852	3.54E-07	count	1
EIF2D	0.0779101	0.4565028	0.1707	0.865	3.54E-07	count	1
PDRG1	0.0779101	0.4445333	0.1753	0.861	3.55E-07	count	1
PPAN	0.487125	0.4530365	1.0752	0.283	3.56E-07	count	1
CEP120	0.0791277	0.5823965	0.1359	0.892	3.60E-07	count	1
OSBPL2	0.0791277	0.6024549	0.1313	0.896	3.60E-07	count	1
PDP1	0.0791277	0.4272202	0.1852	0.853	3.61E-07	count	1
KIAA0232	0.0791277	0.4419764	0.179	0.858	3.61E-07	count	1
XRN2	0.0297628	0.1965449	0.1514	0.88	3.61E-07	count	1
LAGE3	0.0299282	0.3138142	0.0954	0.924	3.64E-07	count	1
PPTC7	0.0300375	0.3442899	0.0872	0.931	3.65E-07	count	1
MED27	0.0804021	0.4857562	0.1655	0.869	3.66E-07	count	1
RDH11	0.0804021	0.4489633	0.1791	0.858	3.66E-07	count	1
PPIL3	0.2084424	0.9152806	0.2277	0.82	3.67E-07	count	1
PTRH1	0.2084424	0.9152806	0.2277	0.82	3.67E-07	count	1
FSCN1	0.2084424	1.0060798	0.2072	0.836	3.67E-07	count	1
MXRA7	0.2084424	1.0060798	0.2072	0.836	3.67E-07	count	1

AC008267.5	0.2084424	0.7937583	0.2626	0.793	3.67E-07	count	1
TBCCD1	0.2084424	0.9192074	0.2268	0.821	3.67E-07	count	1
RDH5	0.2084424	0.7892076	0.2641	0.792	3.67E-07	count	1
NKIRAS1	0.2084424	0.7892076	0.2641	0.792	3.67E-07	count	1
CCDC102B	0.2084424	0.8969449	0.2324	0.816	3.67E-07	count	1
AL021368.2	0.2084424	1.0893367	0.1913	0.848	3.67E-07	count	1
ARL15	0.2084424	0.8929203	0.2334	0.816	3.67E-07	count	1
ZER1	0.2084424	0.8929203	0.2334	0.816	3.67E-07	count	1
DNAJC25	0.2084424	0.8929203	0.2334	0.816	3.67E-07	count	1
MECR	0.2084424	0.8929203	0.2334	0.816	3.67E-07	count	1
E2F6	0.2084424	0.8929203	0.2334	0.816	3.67E-07	count	1
C7orf31	0.2084424	0.9857812	0.2114	0.833	3.67E-07	count	1
MBLAC1	0.2084424	0.9857812	0.2114	0.833	3.67E-07	count	1
DNLZ	0.2084424	0.9857812	0.2114	0.833	3.67E-07	count	1
TBC1D2B	0.2084424	0.9857812	0.2114	0.833	3.67E-07	count	1
IFT46	0.2084424	1.0706178	0.1947	0.846	3.67E-07	count	1
MTG1	0.2084424	1.2921286	0.1613	0.872	3.68E-07	count	1
FASTKD1	0.2084424	1.2921286	0.1613	0.872	3.68E-07	count	1
PLEKHG1	0.0809286	0.5297238	0.1528	0.879	3.69E-07	count	1
CAB39	0.0809286	0.5284922	0.1531	0.878	3.69E-07	count	1
ZNF649	0.0821964	0.6874959	0.1196	0.905	3.75E-07	count	1
STYXL1	0.0821964	0.7273699	0.113	0.91	3.75E-07	count	1
ERI3	0.0821964	0.4765683	0.1725	0.863	3.75E-07	count	1
MRPL10	0.0821964	0.5324558	0.1544	0.877	3.75E-07	count	1
LPCAT4	0.0821964	0.6998854	0.1174	0.907	3.75E-07	count	1
TNFRSF12A	0.0821964	0.6998854	0.1174	0.907	3.75E-07	count	1
PPFIA1	0.0821964	0.4934097	0.1666	0.868	3.75E-07	count	1
DNAL1	0.5134423	0.8992868	0.5709	0.568	3.76E-07	count	1
METRN	0.5134423	0.5472739	0.9382	0.349	3.77E-07	count	1
DDX49	0.5134423	0.5630735	0.9119	0.362	3.78E-07	count	1
CEP85L	0.0838633	0.4556757	0.184	0.854	3.82E-07	count	1
KCTD13	0.0838633	0.6078839	0.138	0.89	3.82E-07	count	1
FAM78A	0.0838633	0.6846898	0.1225	0.903	3.82E-07	count	1
HSPBP1	0.0838633	0.5761567	0.1456	0.884	3.83E-07	count	1
KIF1B	0.2164728	0.7193337	0.3009	0.764	3.83E-07	count	1
SMPD2	0.0849102	0.5397621	0.1573	0.875	3.87E-07	count	1
HDAC5	0.0849102	0.5770276	0.1472	0.883	3.88E-07	count	1
LPIN3	17.9453936	4049.643109	0.0044	0.996	3.88E-07	count	1
MNS1	17.9453936	4049.643253	0.0044	0.996	3.88E-07	count	1
MSTO1	17.9453936	4049.643209	0.0044	0.996	3.88E-07	count	1
CD8A	17.9453936	4049.643209	0.0044	0.996	3.88E-07	count	1
ARHGEF26	17.9453936	4049.643209	0.0044	0.996	3.88E-07	count	1
TRIM32	17.9453936	4049.643209	0.0044	0.996	3.88E-07	count	1
GARNL3	17.9453936	4049.643209	0.0044	0.996	3.88E-07	count	1
TSPAN18	17.9453936	4049.643209	0.0044	0.996	3.88E-07	count	1

AL359397.2	17.9453936	4049.643209	0.0044	0.996	3.88E-07	count	1
GNAO1	17.9453936	4049.643209	0.0044	0.996	3.88E-07	count	1
TLR2	17.6573425	3506.45471	0.005	0.996	3.89E-07	count	1
DIABLO	17.6573426	3506.454697	0.005	0.996	3.89E-07	count	1
REEP4	17.6573425	3506.454797	0.005	0.996	3.89E-07	count	1
FCGR3A	17.6573426	3506.454753	0.005	0.996	3.89E-07	count	1
1-Mar	17.6573426	3506.454753	0.005	0.996	3.89E-07	count	1
OR2T10	17.6573427	3506.454753	0.005	0.996	3.89E-07	count	1
IL18RAP	17.6573426	3506.454753	0.005	0.996	3.89E-07	count	1
S100P	17.6573426	3506.454753	0.005	0.996	3.89E-07	count	1
HAVCR2	17.6573427	3506.454753	0.005	0.996	3.89E-07	count	1
CYP3A5	17.6573427	3506.454753	0.005	0.996	3.89E-07	count	1
MGAM	17.6573426	3506.454753	0.005	0.996	3.89E-07	count	1
AC107959.4	17.6573426	3506.454753	0.005	0.996	3.89E-07	count	1
OLMALINC	17.6573426	3506.454753	0.005	0.996	3.89E-07	count	1
PI3	17.6573426	3506.454753	0.005	0.996	3.89E-07	count	1
AC022144.1	17.6573427	3506.454753	0.005	0.996	3.89E-07	count	1
EGLN2	17.6573426	3506.454753	0.005	0.996	3.89E-07	count	1
FPR1	17.6573426	3506.454753	0.005	0.996	3.89E-07	count	1
PGAM2	17.6573426	3506.454722	0.005	0.996	3.89E-07	count	1
C5orf34	17.6573426	3506.454735	0.005	0.996	3.89E-07	count	1
AQP9	17.6573426	3506.454846	0.005	0.996	3.89E-07	count	1
HIST1H4B	17.6573426	3506.454822	0.005	0.996	3.89E-07	count	1
AFF2	17.6573426	3506.454822	0.005	0.996	3.89E-07	count	1
GLT1D1	17.6573426	3506.454822	0.005	0.996	3.89E-07	count	1
NOP9	17.6573426	3506.454822	0.005	0.996	3.89E-07	count	1
ANPEP	17.6573426	3506.454822	0.005	0.996	3.89E-07	count	1
PRAM1	17.6573426	3506.454822	0.005	0.996	3.89E-07	count	1
CYP4F3	17.6573426	3506.454822	0.005	0.996	3.89E-07	count	1
FPR2	17.6573426	3506.454822	0.005	0.996	3.89E-07	count	1
AC074386.1	17.6573425	3506.454753	0.005	0.996	3.89E-07	count	1
RAD21-AS1	17.6573425	3506.454753	0.005	0.996	3.89E-07	count	1
ENKUR	17.6573425	3506.454753	0.005	0.996	3.89E-07	count	1
TSC22D1-AS1	17.6573425	3506.454753	0.005	0.996	3.89E-07	count	1
IGLV10-54	17.6573426	3506.454735	0.005	0.996	3.89E-07	count	1
VEGFC	17.6573425	3506.45484	0.005	0.996	3.89E-07	count	1
HOMER2	17.6573426	3506.454809	0.005	0.996	3.89E-07	count	1
AL451007.3	17.6573425	3506.454759	0.005	0.996	3.89E-07	count	1
ARAP3	17.6573425	3506.454759	0.005	0.996	3.89E-07	count	1
AL022157.1	17.6573425	3506.454759	0.005	0.996	3.89E-07	count	1
USP35	17.6573425	3506.454759	0.005	0.996	3.89E-07	count	1
EMP1	17.6573425	3506.454759	0.005	0.996	3.89E-07	count	1
TGM1	17.6573425	3506.454759	0.005	0.996	3.89E-07	count	1
AC091045.1	17.9450896	3196.822682	0.0056	0.996	3.89E-07	count	1
PTPN9	17.9450895	3196.822727	0.0056	0.996	3.89E-07	count	1

CTSW	17.9450896	3196.822739	0.0056	0.996	3.89E-07	count	1
AP001107.9	17.9450894	3196.822659	0.0056	0.996	3.89E-07	count	1
ZNF606	17.9450894	3196.822659	0.0056	0.996	3.89E-07	count	1
HIST1H2BI	17.9450895	3196.822795	0.0056	0.996	3.89E-07	count	1
AC103702.2	18.168463	3729.592872	0.0049	0.996	3.89E-07	count	1
AL035530.2	17.9450061	2856.689818	0.0063	0.995	3.89E-07	count	1
NEXN	17.9450059	2856.689787	0.0063	0.995	3.89E-07	count	1
LIN9	17.9450058	2856.689808	0.0063	0.995	3.89E-07	count	1
DNAAF4	17.9450059	2856.689919	0.0063	0.995	3.89E-07	count	1
FAH	17.9450059	2856.689868	0.0063	0.995	3.89E-07	count	1
ZKSCAN4	17.945006	2856.689909	0.0063	0.995	3.89E-07	count	1
AC008040.5	17.9450058	2856.689737	0.0063	0.995	3.89E-07	count	1
MIPEP	17.9450058	2856.689838	0.0063	0.995	3.89E-07	count	1
AC010654.1	17.9450059	2856.689787	0.0063	0.995	3.89E-07	count	1
ZNF540	17.9450058	2856.689696	0.0063	0.995	3.89E-07	count	1
OR2G6	17.9450059	2856.689767	0.0063	0.995	3.89E-07	count	1
B3GALNT1	17.9450059	2856.689818	0.0063	0.995	3.89E-07	count	1
MPZ	17.945006	2856.689848	0.0063	0.995	3.89E-07	count	1
ERICH3	17.9450059	2856.689676	0.0063	0.995	3.89E-07	count	1
TUT1	17.9450059	2856.689787	0.0063	0.995	3.89E-07	count	1
IGLV7-46	17.9450058	2856.689848	0.0063	0.995	3.89E-07	count	1
ZNF594	18.1683598	3258.02154	0.0056	0.996	3.89E-07	count	1
CD302	18.1683595	3258.02154	0.0056	0.996	3.89E-07	count	1
ENKD1	18.1683594	3258.02154	0.0056	0.996	3.89E-07	count	1
KIF1C	18.1683593	3258.02139	0.0056	0.996	3.89E-07	count	1
TTC12	18.1683596	3258.021528	0.0056	0.996	3.89E-07	count	1
AC023509.4	18.1683596	3258.021367	0.0056	0.996	3.89E-07	count	1
XXYLT1	18.1683593	3258.02154	0.0056	0.996	3.89E-07	count	1
POC1A	18.1683592	3258.021424	0.0056	0.996	3.89E-07	count	1
IGKV2-29	18.1683594	3258.021563	0.0056	0.996	3.89E-07	count	1
MAP7	18.1683593	3258.021517	0.0056	0.996	3.89E-07	count	1
TXNRD2	18.1682096	2994.411188	0.0061	0.995	3.89E-07	count	1
ZNF717	18.1682097	2994.411315	0.0061	0.995	3.89E-07	count	1
H6PD	18.1682096	2994.411326	0.0061	0.995	3.89E-07	count	1
FAM160A2	18.1682093	2994.411283	0.0061	0.995	3.89E-07	count	1
RNF24	18.1682095	2994.411156	0.0061	0.995	3.89E-07	count	1
SARM1	18.1681395	2705.433671	0.0067	0.995	3.89E-07	count	1
AC020911.2	18.1681396	2705.43346	0.0067	0.995	3.89E-07	count	1
PXMP2	18.1681395	2705.433623	0.0067	0.995	3.89E-07	count	1
TSNARE1	18.1681396	2705.433691	0.0067	0.995	3.89E-07	count	1
CPED1	18.1681394	2705.433643	0.0067	0.995	3.89E-07	count	1
SIGLEC14	18.1681395	2705.433604	0.0067	0.995	3.89E-07	count	1
AL591895.1	18.1681394	2705.433671	0.0067	0.995	3.89E-07	count	1
P2RY12	18.1681394	2705.433566	0.0067	0.995	3.89E-07	count	1
SMYD5	18.1681394	2705.433614	0.0067	0.995	3.89E-07	count	1

NBPF19	18.1681393	2705.43347	0.0067	0.995	3.89E-07	count	1
HIST1H2BC	18.1681392	2705.43348	0.0067	0.995	3.89E-07	count	1
AL391069.3	17.9448083	2470.086067	0.0073	0.994	3.89E-07	count	1
BFSP2	17.9448083	2470.086102	0.0073	0.994	3.89E-07	count	1
CLCN2	17.9448082	2470.086032	0.0073	0.994	3.89E-07	count	1
LINC01011	17.9448082	2470.086067	0.0073	0.994	3.89E-07	count	1
FO704657.1	17.944808	2470.086084	0.0073	0.994	3.89E-07	count	1
CELSR3	17.9448082	2470.085997	0.0073	0.994	3.89E-07	count	1
GPHN	17.9448082	2470.086111	0.0073	0.994	3.89E-07	count	1
DYNC2H1	17.9448081	2470.086058	0.0073	0.994	3.89E-07	count	1
ZNF436-AS1	17.9448081	2470.086006	0.0073	0.994	3.89E-07	count	1
ANGPT2	17.944808	2470.085979	0.0073	0.994	3.89E-07	count	1
DCP1B	17.944808	2470.086049	0.0073	0.994	3.89E-07	count	1
PIK3R5	17.9448081	2470.086058	0.0073	0.994	3.89E-07	count	1
QPCTL	17.9448081	2470.086111	0.0073	0.994	3.89E-07	count	1
TCFL5	17.9448079	2470.086032	0.0073	0.994	3.89E-07	count	1
AC009812.1	17.944808	2470.086049	0.0073	0.994	3.89E-07	count	1
ZNF529-AS1	17.9448081	2470.086111	0.0073	0.994	3.89E-07	count	1
SYNJ1	17.9448081	2470.086084	0.0073	0.994	3.89E-07	count	1
CTH	17.9448081	2470.086067	0.0073	0.994	3.89E-07	count	1
PDSS1	17.9448079	2470.085988	0.0073	0.994	3.89E-07	count	1
MAGI3	17.944808	2470.085944	0.0073	0.994	3.89E-07	count	1
CTNND1	17.9448081	2470.086084	0.0073	0.994	3.89E-07	count	1
CCDC184	17.944808	2470.086023	0.0073	0.994	3.89E-07	count	1
AL031728.1	17.9448081	2470.086102	0.0073	0.994	3.89E-07	count	1
AC006369.1	17.9448081	2470.086084	0.0073	0.994	3.89E-07	count	1
DDTL	17.9448079	2470.086014	0.0073	0.994	3.89E-07	count	1
RAPGEF5	17.9448079	2470.086093	0.0073	0.994	3.89E-07	count	1
TIGD7	17.944808	2470.086102	0.0073	0.994	3.89E-07	count	1
AHCYL2	17.9448079	2470.086076	0.0073	0.994	3.89E-07	count	1
AF001548.2	17.9448078	2470.086119	0.0073	0.994	3.89E-07	count	1
AL360270.1	17.9448078	2470.086041	0.0073	0.994	3.89E-07	count	1
AC100793.2	18.1679732	2381.581106	0.0076	0.994	3.89E-07	count	1
TM7SF2	18.1679733	2381.581089	0.0076	0.994	3.89E-07	count	1
NBPF10	18.1679732	2381.580996	0.0076	0.994	3.89E-07	count	1
ST7	18.1679731	2381.58103	0.0076	0.994	3.89E-07	count	1
CAD	18.1679732	2381.580954	0.0076	0.994	3.89E-07	count	1
TCAF2	18.1679732	2381.581097	0.0076	0.994	3.89E-07	count	1
ZNF747	18.1679728	2381.581038	0.0076	0.994	3.89E-07	count	1
DNAJC14	18.167973	2381.580895	0.0076	0.994	3.89E-07	count	1
FAM234B	18.1679729	2381.580954	0.0076	0.994	3.89E-07	count	1
RELL1	18.167973	2381.581097	0.0076	0.994	3.89E-07	count	1
MEGF9	18.1679729	2381.581021	0.0076	0.994	3.89E-07	count	1
AL357054.4	18.1679729	2381.581089	0.0076	0.994	3.89E-07	count	1
MCTP1	17.6570072	2608.530197	0.0068	0.995	3.89E-07	count	1

LINC00893	17.6570072	2608.530248	0.0068	0.995	3.89E-07	count	1
PARP16	17.6570072	2608.530183	0.0068	0.995	3.89E-07	count	1
NRN1	17.6570071	2608.530151	0.0068	0.995	3.89E-07	count	1
PSMG3-AS1	17.6570071	2608.530142	0.0068	0.995	3.89E-07	count	1
ZNF771	17.6570072	2608.530146	0.0068	0.995	3.89E-07	count	1
AC099850.1	17.6570071	2608.530234	0.0068	0.995	3.89E-07	count	1
AL049840.2	17.6570072	2608.530179	0.0068	0.995	3.89E-07	count	1
LINC01431	17.6570071	2608.53016	0.0068	0.995	3.89E-07	count	1
STIMATE	17.657007	2608.530179	0.0068	0.995	3.89E-07	count	1
DNMBP-AS1	17.6570071	2608.530202	0.0068	0.995	3.89E-07	count	1
CBX8	17.6570072	2608.53022	0.0068	0.995	3.89E-07	count	1
AC007773.1	17.657007	2608.530206	0.0068	0.995	3.89E-07	count	1
TMEM121	17.6570071	2608.530188	0.0068	0.995	3.89E-07	count	1
AP002449.1	17.6570072	2608.530225	0.0068	0.995	3.89E-07	count	1
AP001505.1	17.6570071	2608.530119	0.0068	0.995	3.89E-07	count	1
HOXB2	17.6570071	2608.530202	0.0068	0.995	3.89E-07	count	1
TC2N	17.657007	2608.530109	0.0068	0.995	3.89E-07	count	1
AC087386.1	17.6570071	2608.530211	0.0068	0.995	3.89E-07	count	1
GIMAP7	17.6570072	2608.530202	0.0068	0.995	3.89E-07	count	1
ECT2	17.6570071	2608.530174	0.0068	0.995	3.89E-07	count	1
SCAF1	17.6570071	2608.530137	0.0068	0.995	3.89E-07	count	1
DGCR2	17.6570071	2608.530188	0.0068	0.995	3.89E-07	count	1
PRRT3-AS1	17.657007	2608.530174	0.0068	0.995	3.89E-07	count	1
GORASP1	17.6570071	2608.530197	0.0068	0.995	3.89E-07	count	1
AP000766.1	17.6570071	2608.530188	0.0068	0.995	3.89E-07	count	1
PIGW	17.6570072	2608.530202	0.0068	0.995	3.89E-07	count	1
ABALON	17.6570071	2608.530146	0.0068	0.995	3.89E-07	count	1
AC025171.4	17.6570072	2608.530119	0.0068	0.995	3.89E-07	count	1
AFG1L	17.6570071	2608.530174	0.0068	0.995	3.89E-07	count	1
GTF2IRD2B	17.6570071	2608.530179	0.0068	0.995	3.89E-07	count	1
SBF2	17.657007	2608.530114	0.0068	0.995	3.89E-07	count	1
PPP2R5D	17.657007	2608.530151	0.0068	0.995	3.89E-07	count	1
PANX1	17.6570071	2608.530179	0.0068	0.995	3.89E-07	count	1
UBQLN4	17.6570071	2608.530206	0.0068	0.995	3.89E-07	count	1
TBC1D13	17.657007	2608.530211	0.0068	0.995	3.89E-07	count	1
LINC00221	17.6570072	2608.530132	0.0068	0.995	3.89E-07	count	1
ZNF77	17.6570071	2608.530225	0.0068	0.995	3.89E-07	count	1
IGKV1-27	17.6570072	2608.53016	0.0068	0.995	3.89E-07	count	1
F2RL1	17.6570071	2608.530197	0.0068	0.995	3.89E-07	count	1
FAM185A	17.6570071	2608.530179	0.0068	0.995	3.89E-07	count	1
AL353597.1	17.6570071	2608.530128	0.0068	0.995	3.89E-07	count	1
DPY19L4	17.6570069	2608.530193	0.0068	0.995	3.89E-07	count	1
AC139887.2	17.6570071	2608.530225	0.0068	0.995	3.89E-07	count	1
PDLIM3	17.6570072	2608.530193	0.0068	0.995	3.89E-07	count	1
IGHV5-51	17.657007	2608.530193	0.0068	0.995	3.89E-07	count	1

FBLN1	17.6570072	2608.530202	0.0068	0.995	3.89E-07	count	1
LINC00265	17.657007	2608.530229	0.0068	0.995	3.89E-07	count	1
C15orf41	17.6570071	2608.530183	0.0068	0.995	3.89E-07	count	1
TMEM147-AS1	17.657007	2608.530229	0.0068	0.995	3.89E-07	count	1
AREL1	17.657007	2608.530225	0.0068	0.995	3.89E-07	count	1
LRIG1	17.657007	2608.530243	0.0068	0.995	3.89E-07	count	1
PTPN20	17.6570071	2608.530132	0.0068	0.995	3.89E-07	count	1
AC009041.2	17.657007	2608.530169	0.0068	0.995	3.89E-07	count	1
RHOBTB3	17.657007	2608.530188	0.0068	0.995	3.89E-07	count	1
MBOAT1	17.6570071	2608.530188	0.0068	0.995	3.89E-07	count	1
TNKS1BP1	17.6570071	2608.530169	0.0068	0.995	3.89E-07	count	1
TBCEL	17.657007	2608.530132	0.0068	0.995	3.89E-07	count	1
CBWD6	17.6570071	2608.530197	0.0068	0.995	3.89E-07	count	1
AC007114.2	17.657007	2608.530174	0.0068	0.995	3.89E-07	count	1
PLCH2	17.657007	2608.5301	0.0068	0.995	3.89E-07	count	1
LINC01943	17.657007	2608.530202	0.0068	0.995	3.89E-07	count	1
TMEM241	17.6570069	2608.530132	0.0068	0.995	3.89E-07	count	1
AC010894.2	17.657007	2608.53016	0.0068	0.995	3.89E-07	count	1
CA13	17.657007	2608.530179	0.0068	0.995	3.89E-07	count	1
AC073072.1	17.6570071	2608.530202	0.0068	0.995	3.89E-07	count	1
SCOC-AS1	17.6570071	2608.530179	0.0068	0.995	3.89E-07	count	1
PAQR7	17.6570069	2608.53016	0.0068	0.995	3.89E-07	count	1
PRMT6	17.657007	2608.530188	0.0068	0.995	3.89E-07	count	1
SLC29A1	17.657007	2608.530174	0.0068	0.995	3.89E-07	count	1
CPNE8	17.657007	2608.530142	0.0068	0.995	3.89E-07	count	1
AC018647.2	17.6570071	2608.53022	0.0068	0.995	3.89E-07	count	1
TMEM184B	17.657007	2608.530151	0.0068	0.995	3.89E-07	count	1
AC078883.3	17.6570069	2608.530169	0.0068	0.995	3.89E-07	count	1
SLC19A2	17.657007	2608.530114	0.0068	0.995	3.89E-07	count	1
ACAD11	17.657007	2608.530174	0.0068	0.995	3.89E-07	count	1
CNIH2	17.6570069	2608.530105	0.0068	0.995	3.89E-07	count	1
POLRMT	17.657007	2608.530137	0.0068	0.995	3.89E-07	count	1
RN7SL832P	17.657007	2608.530156	0.0068	0.995	3.89E-07	count	1
U91328.1	17.657007	2608.530119	0.0068	0.995	3.89E-07	count	1
MTCP1	17.6570069	2608.530137	0.0068	0.995	3.89E-07	count	1
ZNF823	17.657007	2608.530188	0.0068	0.995	3.89E-07	count	1
ARHGAP22	17.6570071	2608.530174	0.0068	0.995	3.89E-07	count	1
IL1A	17.657007	2608.530105	0.0068	0.995	3.89E-07	count	1
ZDHHC9	17.657007	2608.530132	0.0068	0.995	3.89E-07	count	1
LINC01963	17.6570068	2608.530132	0.0068	0.995	3.89E-07	count	1
IPCEF1	17.9445976	2010.655842	0.0089	0.993	3.90E-07	count	1
SYT17	17.9445975	2010.655842	0.0089	0.993	3.90E-07	count	1
PLOD3	17.9445974	2010.655878	0.0089	0.993	3.90E-07	count	1
CHST14	18.3505596	3081.827964	0.006	0.995	3.90E-07	count	1
KBTBD6	18.3505595	3081.827931	0.006	0.995	3.90E-07	count	1

AL139020.1	18.3505597	3081.828062	0.006	0.995	3.90E-07	count	1
TBC1D22B	18.3505596	3081.828117	0.006	0.995	3.90E-07	count	1
AC124283.1	18.3505596	3081.828259	0.006	0.995	3.90E-07	count	1
PHOSPHO2	18.3505056	2850.439567	0.0064	0.995	3.90E-07	count	1
ZNF793	18.3505056	2850.439789	0.0064	0.995	3.90E-07	count	1
MTERF2	18.3506452	3499.021024	0.0052	0.996	3.90E-07	count	1
AL451165.2	18.3504344	2850.338755	0.0064	0.995	3.90E-07	count	1
SMIM25	18.3507222	3499.155101	0.0052	0.996	3.90E-07	count	1
FAM118B	18.3503763	2598.458833	0.0071	0.994	3.90E-07	count	1
PLPP6	18.3503764	2598.458833	0.0071	0.994	3.90E-07	count	1
ROGDI	18.3503761	2598.458897	0.0071	0.994	3.90E-07	count	1
KLHL15	18.3503759	2598.458833	0.0071	0.994	3.90E-07	count	1
COQ3	18.3503759	2598.458861	0.0071	0.994	3.90E-07	count	1
ZNF329	18.3503756	2598.458815	0.0071	0.994	3.90E-07	count	1
BAHD1	18.3503755	2598.458759	0.0071	0.994	3.90E-07	count	1
MOK	18.3503758	2598.458787	0.0071	0.994	3.90E-07	count	1
SRR	18.3507523	3690.114012	0.005	0.996	3.90E-07	count	1
PPM1N	18.3502357	2319.325003	0.0079	0.994	3.90E-07	count	1
RTN4IP1	18.3502356	2319.324913	0.0079	0.994	3.90E-07	count	1
DCN	18.3502357	2319.324904	0.0079	0.994	3.90E-07	count	1
C10orf88	17.6567578	2014.964507	0.0088	0.993	3.90E-07	count	1
MLLT3	17.2513052	2862.186587	0.006	0.995	3.90E-07	count	1
LINC01374	17.2513052	2862.186587	0.006	0.995	3.90E-07	count	1
DDX11-AS1	17.2513052	2862.186587	0.006	0.995	3.90E-07	count	1
BTNL9	17.2513052	2862.186623	0.006	0.995	3.90E-07	count	1
USP54	17.2513052	2862.186623	0.006	0.995	3.90E-07	count	1
JSRP1	17.2513052	2862.186623	0.006	0.995	3.90E-07	count	1
ZNF225	17.2513052	2862.186623	0.006	0.995	3.90E-07	count	1
RSPH14	17.2513052	2862.186623	0.006	0.995	3.90E-07	count	1
AL359915.2	17.2513051	2862.186587	0.006	0.995	3.90E-07	count	1
AC009948.3	17.2513051	2862.186587	0.006	0.995	3.90E-07	count	1
GAS1	17.2513051	2862.186587	0.006	0.995	3.90E-07	count	1
KIAA0556	17.2513051	2862.186587	0.006	0.995	3.90E-07	count	1
AC106739.2	17.6567578	2014.964504	0.0088	0.993	3.90E-07	count	1
SLC3A1	17.2513051	2862.186567	0.006	0.995	3.90E-07	count	1
FZD7	17.2513051	2862.186567	0.006	0.995	3.90E-07	count	1
PTPRG	17.2513051	2862.186567	0.006	0.995	3.90E-07	count	1
SUGCT	17.2513051	2862.186567	0.006	0.995	3.90E-07	count	1
AC115618.1	17.2513051	2862.186633	0.006	0.995	3.90E-07	count	1
ULK2	17.2513051	2862.186567	0.006	0.995	3.90E-07	count	1
C19orf33	17.2513051	2862.186567	0.006	0.995	3.90E-07	count	1
FRMD4A	17.2513051	2862.186602	0.006	0.995	3.90E-07	count	1
Z83851.1	17.2513051	2862.186602	0.006	0.995	3.90E-07	count	1
ST6GALNAC3	17.6567578	2014.964507	0.0088	0.993	3.90E-07	count	1
AC073046.1	17.2513051	2862.186521	0.006	0.995	3.90E-07	count	1

AL121601.1	17.2513051	2862.186521	0.006	0.995	3.90E-07	count	1
NATD1	17.2513051	2862.186521	0.006	0.995	3.90E-07	count	1
ZNF512B	17.2513051	2862.186521	0.006	0.995	3.90E-07	count	1
RNASEH2A	17.2513051	2862.186521	0.006	0.995	3.90E-07	count	1
AC245595.1	17.2513051	2862.186618	0.006	0.995	3.90E-07	count	1
AC017071.1	17.2513051	2862.186618	0.006	0.995	3.90E-07	count	1
PEBP4	17.2513051	2862.186618	0.006	0.995	3.90E-07	count	1
AC091057.3	17.2513051	2862.186618	0.006	0.995	3.90E-07	count	1
RTCA-AS1	17.2513051	2862.186597	0.006	0.995	3.90E-07	count	1
TDRKH-AS1	17.2513051	2862.186537	0.006	0.995	3.90E-07	count	1
EVC2	17.2513051	2862.186537	0.006	0.995	3.90E-07	count	1
AC113361.1	17.2513051	2862.186537	0.006	0.995	3.90E-07	count	1
HIST1H2BJ	17.2513051	2862.186557	0.006	0.995	3.90E-07	count	1
HIST1H2AI	17.2513051	2862.186597	0.006	0.995	3.90E-07	count	1
AC246817.1	17.251305	2862.186582	0.006	0.995	3.90E-07	count	1
EXTL3-AS1	17.2513051	2862.186557	0.006	0.995	3.90E-07	count	1
LHX2	17.251305	2862.186582	0.006	0.995	3.90E-07	count	1
TDRD1	17.2513051	2862.186597	0.006	0.995	3.90E-07	count	1
TMEM88	17.2513051	2862.186557	0.006	0.995	3.90E-07	count	1
BMP2	17.2513051	2862.186557	0.006	0.995	3.90E-07	count	1
AC012615.1	17.2513051	2862.186537	0.006	0.995	3.90E-07	count	1
LPP-AS2	17.2513051	2862.186633	0.006	0.995	3.90E-07	count	1
ALDH7A1	17.2513051	2862.186633	0.006	0.995	3.90E-07	count	1
AC034243.1	17.2513051	2862.186521	0.006	0.995	3.90E-07	count	1
CHMP4C	17.2513051	2862.186633	0.006	0.995	3.90E-07	count	1
RNF157	17.2513051	2862.186521	0.006	0.995	3.90E-07	count	1
SLC26A11	17.2513051	2862.186633	0.006	0.995	3.90E-07	count	1
FBXW9	17.2513051	2862.186633	0.006	0.995	3.90E-07	count	1
SH2B1	17.6567577	2014.964514	0.0088	0.993	3.90E-07	count	1
DUBR	17.2513051	2862.186552	0.006	0.995	3.90E-07	count	1
RAB3C	17.251305	2862.186547	0.006	0.995	3.90E-07	count	1
TMEM47	17.2513051	2862.186552	0.006	0.995	3.90E-07	count	1
COL15A1	17.2513051	2862.186552	0.006	0.995	3.90E-07	count	1
LINC00167	17.2513051	2862.186552	0.006	0.995	3.90E-07	count	1
GLS2	17.2513051	2862.186592	0.006	0.995	3.90E-07	count	1
NDUFA4L2	17.2513051	2862.186552	0.006	0.995	3.90E-07	count	1
KTN1-AS1	17.2513051	2862.186552	0.006	0.995	3.90E-07	count	1
AL031055.1	17.2513051	2862.186552	0.006	0.995	3.90E-07	count	1
NUDT4B	17.2513051	2862.186592	0.006	0.995	3.90E-07	count	1
FKBP1B	17.251305	2862.186633	0.006	0.995	3.90E-07	count	1
ALS2CR12	17.251305	2862.186577	0.006	0.995	3.90E-07	count	1
ZNF197	17.251305	2862.186577	0.006	0.995	3.90E-07	count	1
LNP1	17.251305	2862.186633	0.006	0.995	3.90E-07	count	1
TRAT1	17.251305	2862.186633	0.006	0.995	3.90E-07	count	1
CCDC96	17.251305	2862.186633	0.006	0.995	3.90E-07	count	1

HIST1H2BE	17.2513051	2862.186592	0.006	0.995	3.90E-07	count	1
AC004130.1	17.251305	2862.186633	0.006	0.995	3.90E-07	count	1
ADAM22	17.251305	2862.186633	0.006	0.995	3.90E-07	count	1
TRBV28	17.2513051	2862.186592	0.006	0.995	3.90E-07	count	1
CXorf36	17.2513051	2862.186592	0.006	0.995	3.90E-07	count	1
ZNF81	17.251305	2862.186633	0.006	0.995	3.90E-07	count	1
FAM167A	17.251305	2862.186577	0.006	0.995	3.90E-07	count	1
CDKN2B	17.2513051	2862.186633	0.006	0.995	3.90E-07	count	1
GNG10	17.2513051	2862.186633	0.006	0.995	3.90E-07	count	1
PPP2R5B	17.251305	2862.186633	0.006	0.995	3.90E-07	count	1
CADM1	17.2513051	2862.186633	0.006	0.995	3.90E-07	count	1
AGAP4	17.2513051	2862.186633	0.006	0.995	3.90E-07	count	1
AL450306.1	17.251305	2862.186633	0.006	0.995	3.90E-07	count	1
CLEC7A	17.2513051	2862.186633	0.006	0.995	3.90E-07	count	1
DUSP16	17.2513051	2862.186592	0.006	0.995	3.90E-07	count	1
AC125603.4	17.251305	2862.186633	0.006	0.995	3.90E-07	count	1
AL354696.2	17.251305	2862.186633	0.006	0.995	3.90E-07	count	1
DGKH	17.2513051	2862.186592	0.006	0.995	3.90E-07	count	1
AC243965.1	17.2513051	2862.186592	0.006	0.995	3.90E-07	count	1
AC005280.2	17.2513051	2862.186633	0.006	0.995	3.90E-07	count	1
FLRT2	17.2513051	2862.186592	0.006	0.995	3.90E-07	count	1
MAP1A	17.251305	2862.186633	0.006	0.995	3.90E-07	count	1
TMEM104	17.251305	2862.186633	0.006	0.995	3.90E-07	count	1
AC100788.1	17.2513051	2862.186592	0.006	0.995	3.90E-07	count	1
TBC1D16	17.2513051	2862.186633	0.006	0.995	3.90E-07	count	1
AC015819.1	17.251305	2862.186633	0.006	0.995	3.90E-07	count	1
ONECUT3	17.2513051	2862.186592	0.006	0.995	3.90E-07	count	1
ZNF57	17.2513051	2862.186592	0.006	0.995	3.90E-07	count	1
YJEFN3	17.2513051	2862.186633	0.006	0.995	3.90E-07	count	1
ZNF223	17.2513051	2862.186592	0.006	0.995	3.90E-07	count	1
ZNF233	17.251305	2862.186633	0.006	0.995	3.90E-07	count	1
AL031846.2	17.251305	2862.186633	0.006	0.995	3.90E-07	count	1
LRRC7	17.2513051	2862.186618	0.006	0.995	3.90E-07	count	1
NCAPH	17.2513051	2862.186572	0.006	0.995	3.90E-07	count	1
ADAMTS9	17.2513051	2862.186618	0.006	0.995	3.90E-07	count	1
AC078795.2	17.2513051	2862.186547	0.006	0.995	3.90E-07	count	1
AC008771.1	17.2513051	2862.186618	0.006	0.995	3.90E-07	count	1
AC007285.1	17.2513051	2862.186547	0.006	0.995	3.90E-07	count	1
SLC7A8	17.2513051	2862.186572	0.006	0.995	3.90E-07	count	1
ATP8B4	17.2513051	2862.186572	0.006	0.995	3.90E-07	count	1
TSPAN10	17.2513051	2862.186547	0.006	0.995	3.90E-07	count	1
ZFY-AS1	17.2513051	2862.186618	0.006	0.995	3.90E-07	count	1
PIP4K2C	17.6567578	2014.964529	0.0088	0.993	3.90E-07	count	1
GPR75	17.2513051	2862.186623	0.006	0.995	3.90E-07	count	1
CLIP2	17.2513051	2862.186623	0.006	0.995	3.90E-07	count	1

CCR2	17.251305	2862.186531	0.006	0.995	3.90E-07	count	1
CCR5	17.251305	2862.186531	0.006	0.995	3.90E-07	count	1
AC008467.1	17.2513051	2862.186607	0.006	0.995	3.90E-07	count	1
HOXA10	17.251305	2862.186531	0.006	0.995	3.90E-07	count	1
AC073335.2	17.251305	2862.186531	0.006	0.995	3.90E-07	count	1
PDE1B	17.251305	2862.186531	0.006	0.995	3.90E-07	count	1
AC106028.4	17.2513051	2862.186607	0.006	0.995	3.90E-07	count	1
CLN3	17.251305	2862.186531	0.006	0.995	3.90E-07	count	1
MYOM1	17.251305	2862.186531	0.006	0.995	3.90E-07	count	1
RSPO3	17.251305	2862.186567	0.006	0.995	3.90E-07	count	1
FAM71D	17.251305	2862.186567	0.006	0.995	3.90E-07	count	1
AL354872.2	17.251305	2862.186562	0.006	0.995	3.90E-07	count	1
FREM3	17.251305	2862.186562	0.006	0.995	3.90E-07	count	1
NUGGC	17.251305	2862.186562	0.006	0.995	3.90E-07	count	1
IDI2-AS1	17.251305	2862.186562	0.006	0.995	3.90E-07	count	1
DDIT4-AS1	17.251305	2862.186562	0.006	0.995	3.90E-07	count	1
PEX11A	17.251305	2862.186582	0.006	0.995	3.90E-07	count	1
MT1A	17.251305	2862.186562	0.006	0.995	3.90E-07	count	1
AC024940.1	17.2513051	2862.186552	0.006	0.995	3.90E-07	count	1
CLN6	17.2513051	2862.186552	0.006	0.995	3.90E-07	count	1
AC117382.2	17.251305	2862.186582	0.006	0.995	3.90E-07	count	1
RBP5	17.2513051	2862.186552	0.006	0.995	3.90E-07	count	1
ZNF341	17.2513051	2862.186547	0.006	0.995	3.90E-07	count	1
GDF5	17.251305	2862.186582	0.006	0.995	3.90E-07	count	1
RAB11B-AS1	17.2513051	2862.186547	0.006	0.995	3.90E-07	count	1
SNX7	17.251305	2862.186592	0.006	0.995	3.90E-07	count	1
CFAP45	17.251305	2862.186592	0.006	0.995	3.90E-07	count	1
PBX1	17.251305	2862.186592	0.006	0.995	3.90E-07	count	1
TNFSF4	17.251305	2862.186537	0.006	0.995	3.90E-07	count	1
WBP1	17.251305	2862.186537	0.006	0.995	3.90E-07	count	1
NEK10	17.251305	2862.186592	0.006	0.995	3.90E-07	count	1
LSAMP	17.251305	2862.186633	0.006	0.995	3.90E-07	count	1
ILDR1	17.251305	2862.186633	0.006	0.995	3.90E-07	count	1
MAPK10	17.251305	2862.186577	0.006	0.995	3.90E-07	count	1
AC010491.1	17.251305	2862.186537	0.006	0.995	3.90E-07	count	1
AL603910.1	17.251305	2862.186537	0.006	0.995	3.90E-07	count	1
TRGC2	17.251305	2862.186592	0.006	0.995	3.90E-07	count	1
CLIC2	17.251305	2862.186542	0.006	0.995	3.90E-07	count	1
KIF24	17.251305	2862.186592	0.006	0.995	3.90E-07	count	1
GPD1	17.251305	2862.186537	0.006	0.995	3.90E-07	count	1
AC018362.2	17.251305	2862.186537	0.006	0.995	3.90E-07	count	1
AC084782.3	17.251305	2862.186577	0.006	0.995	3.90E-07	count	1
OSGIN1	17.251305	2862.186577	0.006	0.995	3.90E-07	count	1
AL023803.2	17.251305	2862.186537	0.006	0.995	3.90E-07	count	1
ZNF473	17.251305	2862.186592	0.006	0.995	3.90E-07	count	1

CADM2	17.251305	2862.186567	0.006	0.995	3.90E-07	count	1
SH3D19	17.2513049	2862.186552	0.006	0.995	3.90E-07	count	1
GPX8	17.2513049	2862.186552	0.006	0.995	3.90E-07	count	1
SERF1A	17.251305	2862.186552	0.006	0.995	3.90E-07	count	1
PCDHB1	17.251305	2862.186552	0.006	0.995	3.90E-07	count	1
GCK	17.251305	2862.186567	0.006	0.995	3.90E-07	count	1
TSPAN7	17.2513049	2862.186552	0.006	0.995	3.90E-07	count	1
TUSC3	17.2513049	2862.186552	0.006	0.995	3.90E-07	count	1
LINC01030	17.251305	2862.186567	0.006	0.995	3.90E-07	count	1
AC069234.5	17.251305	2862.186567	0.006	0.995	3.90E-07	count	1
L2HGDH	17.251305	2862.186567	0.006	0.995	3.90E-07	count	1
PGBD4	17.2513049	2862.186552	0.006	0.995	3.90E-07	count	1
AC018362.1	17.251305	2862.186552	0.006	0.995	3.90E-07	count	1
AC106886.5	17.2513049	2862.186552	0.006	0.995	3.90E-07	count	1
AC027682.2	17.251305	2862.186602	0.006	0.995	3.90E-07	count	1
AC007292.2	17.2513049	2862.186552	0.006	0.995	3.90E-07	count	1
AL365330.1	17.251305	2862.186552	0.006	0.995	3.90E-07	count	1
AC009948.1	17.251305	2862.186572	0.006	0.995	3.90E-07	count	1
CXXC5-AS1	17.251305	2862.186552	0.006	0.995	3.90E-07	count	1
AC008443.6	17.251305	2862.186572	0.006	0.995	3.90E-07	count	1
AC068389.4	17.251305	2862.186552	0.006	0.995	3.90E-07	count	1
AC008764.2	17.251305	2862.186552	0.006	0.995	3.90E-07	count	1
SLC44A5	17.251305	2862.186537	0.006	0.995	3.90E-07	count	1
PCYOX1L	17.251305	2862.186577	0.006	0.995	3.90E-07	count	1
GPR63	17.251305	2862.186577	0.006	0.995	3.90E-07	count	1
ZC3H10	17.251305	2862.186577	0.006	0.995	3.90E-07	count	1
RPS6KL1	17.251305	2862.186537	0.006	0.995	3.90E-07	count	1
ATP2A1	17.251305	2862.186537	0.006	0.995	3.90E-07	count	1
AC109460.1	17.251305	2862.186537	0.006	0.995	3.90E-07	count	1
TTLL9	17.251305	2862.186577	0.006	0.995	3.90E-07	count	1
AC005498.2	17.251305	2862.186537	0.006	0.995	3.90E-07	count	1
AL008719.1	17.251305	2862.186537	0.006	0.995	3.90E-07	count	1
AC007262.2	17.251305	2862.186552	0.006	0.995	3.90E-07	count	1
AC008440.1	17.251305	2862.186607	0.006	0.995	3.90E-07	count	1
AC008946.1	17.2513051	2862.186567	0.006	0.995	3.90E-07	count	1
ITGA6	17.251305	2862.186582	0.006	0.995	3.90E-07	count	1
F2R	17.251305	2862.186582	0.006	0.995	3.90E-07	count	1
SCUBE3	17.251305	2862.186582	0.006	0.995	3.90E-07	count	1
GPRASP2	17.2513049	2862.186531	0.006	0.995	3.90E-07	count	1
CNTNAP3	17.251305	2862.186597	0.006	0.995	3.90E-07	count	1
KLLN	17.251305	2862.186597	0.006	0.995	3.90E-07	count	1
AP003900.1	17.251305	2862.186597	0.006	0.995	3.90E-07	count	1
KLRD1	17.251305	2862.186623	0.006	0.995	3.90E-07	count	1
TMEM86B	17.6567577	2014.964557	0.0088	0.993	3.90E-07	count	1
IGKV2D-26	17.6567577	2014.964514	0.0088	0.993	3.90E-07	count	1

AC007952.7	17.6567578	2014.964564	0.0088	0.993	3.90E-07	count	1
CLEC3B	17.251305	2862.186547	0.006	0.995	3.90E-07	count	1
AC093274.1	17.251305	2862.186547	0.006	0.995	3.90E-07	count	1
DSG2	17.251305	2862.186547	0.006	0.995	3.90E-07	count	1
AC010335.1	17.251305	2862.186547	0.006	0.995	3.90E-07	count	1
AC007785.3	17.251305	2862.186547	0.006	0.995	3.90E-07	count	1
AC025259.3	17.251305	2862.186582	0.006	0.995	3.90E-07	count	1
IKZF4	17.251305	2862.186582	0.006	0.995	3.90E-07	count	1
BORA	17.251305	2862.186582	0.006	0.995	3.90E-07	count	1
KLHL25	17.251305	2862.186582	0.006	0.995	3.90E-07	count	1
CSF1	17.251305	2862.186587	0.006	0.995	3.90E-07	count	1
PIFO	17.251305	2862.186572	0.006	0.995	3.90E-07	count	1
BRPF1	17.251305	2862.186592	0.006	0.995	3.90E-07	count	1
SLC22A5	17.251305	2862.186587	0.006	0.995	3.90E-07	count	1
KIAA0319	17.251305	2862.186572	0.006	0.995	3.90E-07	count	1
PI16	17.251305	2862.186572	0.006	0.995	3.90E-07	count	1
FRK	17.251305	2862.186572	0.006	0.995	3.90E-07	count	1
NRCAM	17.251305	2862.186587	0.006	0.995	3.90E-07	count	1
RBM14-RBM4	17.251305	2862.186572	0.006	0.995	3.90E-07	count	1
PDGFD	17.251305	2862.186572	0.006	0.995	3.90E-07	count	1
CCDC65	17.251305	2862.186587	0.006	0.995	3.90E-07	count	1
RHPN2	17.251305	2862.186572	0.006	0.995	3.90E-07	count	1
AC016027.1	17.251305	2862.186572	0.006	0.995	3.90E-07	count	1
TRIM46	17.251305	2862.186613	0.006	0.995	3.90E-07	count	1
RAB11FIP5	17.2513049	2862.186572	0.006	0.995	3.90E-07	count	1
HDAC11	17.251305	2862.186613	0.006	0.995	3.90E-07	count	1
DCDC2	17.2513049	2862.186572	0.006	0.995	3.90E-07	count	1
AC044839.1	17.2513049	2862.186572	0.006	0.995	3.90E-07	count	1
FEZ1	17.251305	2862.186613	0.006	0.995	3.90E-07	count	1
AC011731.1	17.251305	2862.186613	0.006	0.995	3.90E-07	count	1
ZNF530	17.251305	2862.186613	0.006	0.995	3.90E-07	count	1
LRRC8C-DT	17.6567578	2014.964571	0.0088	0.993	3.90E-07	count	1
AC011944.1	17.6567577	2014.964507	0.0088	0.993	3.90E-07	count	1
AMBRA1	17.2513049	2862.186552	0.006	0.995	3.90E-07	count	1
ARNTL2	17.2513049	2862.186552	0.006	0.995	3.90E-07	count	1
IGHV1-24	17.2513049	2862.186552	0.006	0.995	3.90E-07	count	1
AC002553.1	17.2513049	2862.186552	0.006	0.995	3.90E-07	count	1
MESTIT1	17.2513049	2862.186547	0.006	0.995	3.90E-07	count	1
CDK3	17.2513049	2862.186547	0.006	0.995	3.90E-07	count	1
AC022098.2	17.2513049	2862.186547	0.006	0.995	3.90E-07	count	1
MFSD3	17.6567578	2014.964582	0.0088	0.993	3.90E-07	count	1
COL18A1	17.6567576	2014.964457	0.0088	0.993	3.90E-07	count	1
FAM83G	17.251305	2862.186562	0.006	0.995	3.90E-07	count	1
AC022098.4	17.251305	2862.186562	0.006	0.995	3.90E-07	count	1
AC008966.1	17.6567576	2014.964522	0.0088	0.993	3.90E-07	count	1

CCDC24	17.251305	2862.186537	0.006	0.995	3.90E-07	count	1
PLOD2	17.251305	2862.186537	0.006	0.995	3.90E-07	count	1
AARS2	17.251305	2862.186537	0.006	0.995	3.90E-07	count	1
AC068189.1	17.251305	2862.186537	0.006	0.995	3.90E-07	count	1
UCP3	17.251305	2862.186537	0.006	0.995	3.90E-07	count	1
GPAM	17.251305	2862.186537	0.006	0.995	3.90E-07	count	1
GALNT4	17.251305	2862.186537	0.006	0.995	3.90E-07	count	1
AC123768.3	17.251305	2862.186537	0.006	0.995	3.90E-07	count	1
PRSS27	17.251305	2862.186537	0.006	0.995	3.90E-07	count	1
AC008105.1	17.251305	2862.186537	0.006	0.995	3.90E-07	count	1
ABCA10	17.251305	2862.186537	0.006	0.995	3.90E-07	count	1
CEBPA	17.251305	2862.186537	0.006	0.995	3.90E-07	count	1
LINC01535	17.251305	2862.186537	0.006	0.995	3.90E-07	count	1
ZSCAN22	17.251305	2862.186537	0.006	0.995	3.90E-07	count	1
C1QTNF6	17.251305	2862.186537	0.006	0.995	3.90E-07	count	1
AC104971.3	17.6567576	2014.964479	0.0088	0.993	3.90E-07	count	1
LEKR1	17.6567577	2014.964464	0.0088	0.993	3.90E-07	count	1
GYS1	17.6567576	2014.964543	0.0088	0.993	3.90E-07	count	1
HAAO	17.6567577	2014.964472	0.0088	0.993	3.90E-07	count	1
ZC4H2	17.6567576	2014.964482	0.0088	0.993	3.90E-07	count	1
CSTF3	0.0856288	0.521683	0.1641	0.87	3.91E-07	count	1
CMC4	18.5042674	2272.600708	0.0081	0.994	3.91E-07	count	1
AAR2	18.5042667	2272.600547	0.0081	0.994	3.91E-07	count	1
ZSCAN9	18.5043852	2518.159543	0.0073	0.994	3.91E-07	count	1
TSEN2	18.5043848	2518.159499	0.0073	0.994	3.91E-07	count	1
AL158152.1	18.5044928	2741.85014	0.0067	0.995	3.91E-07	count	1
FBXO32	18.504493	2741.850053	0.0067	0.995	3.91E-07	count	1
CYB5R1	0.0856288	0.4804612	0.1782	0.859	3.91E-07	count	1
RPP40	18.6376356	2235.838429	0.0083	0.993	3.92E-07	count	1
UBE2V1	0.5311419	0.5129732	1.0354	0.301	3.93E-07	count	1
AKR1B1	0.0324825	0.2899179	0.112	0.911	3.97E-07	count	1
RTL8A	18.9928354	2384.467803	0.008	0.994	4.03E-07	count	1
LZTR1	18.9928333	2384.465019	0.008	0.994	4.03E-07	count	1
CASP9	18.9928314	2384.465649	0.008	0.994	4.03E-07	count	1
GALNT2	18.9926474	2017.550619	0.0094	0.992	4.04E-07	count	1
AC083809.1	18.8828639	2446.525302	0.0077	0.994	4.04E-07	count	1
HECTD2	18.8828629	2446.525844	0.0077	0.994	4.04E-07	count	1
AF131216.5	18.7557374	3406.284706	0.0055	0.996	4.04E-07	count	1
RAB11FIP3	18.7555427	2544.513498	0.0074	0.994	4.05E-07	count	1
CFLAR-AS1	18.7555423	2544.514198	0.0074	0.994	4.05E-07	count	1
SLC30A1	18.7555422	2544.514214	0.0074	0.994	4.05E-07	count	1
WIZ	18.7555392	2544.514256	0.0074	0.994	4.05E-07	count	1
SLC27A3	18.7555384	2544.514029	0.0074	0.994	4.05E-07	count	1
NDUFAF5	18.7553679	1977.602438	0.0095	0.992	4.06E-07	count	1
SLC29A3	18.7553669	1977.602078	0.0095	0.992	4.06E-07	count	1

HARS2	18.7553647	1977.601554	0.0095	0.992	4.06E-07	count	1
RABGEF1	0.2293444	0.6411981	0.3577	0.721	4.07E-07	count	1
AK8	19.2681138	2705.276662	0.0071	0.994	4.07E-07	count	1
NDST2	18.6026059	2728.22755	0.0068	0.995	4.08E-07	count	1
STAP2	18.6026056	2728.227338	0.0068	0.995	4.08E-07	count	1
GDPD1	18.6026055	2728.226257	0.0068	0.995	4.08E-07	count	1
SLC35E2A	18.602603	2728.225215	0.0068	0.995	4.08E-07	count	1
DONSON	18.6026025	2728.226296	0.0068	0.995	4.08E-07	count	1
ZNF552	19.2771738	2334.961325	0.0083	0.993	4.09E-07	count	1
FLYWCH1	18.6024029	1939.339945	0.0096	0.992	4.09E-07	count	1
AC132192.1	18.6024026	1939.339182	0.0096	0.992	4.09E-07	count	1
CACTIN	18.6024003	1939.340117	0.0096	0.992	4.09E-07	count	1
CSTF2T	0.0894925	0.6355536	0.1408	0.888	4.09E-07	count	1
DIS3L	0.0894925	0.7747428	0.1155	0.908	4.09E-07	count	1
ECD	0.0894925	0.4566362	0.196	0.845	4.09E-07	count	1
CBFA2T2	0.0894925	0.6360835	0.1407	0.888	4.09E-07	count	1
KLHL18	0.0894925	0.4854351	0.1844	0.854	4.09E-07	count	1
SCML1	0.0894925	0.529246	0.1691	0.866	4.09E-07	count	1
HIF1AN	0.0894925	0.8413097	0.1064	0.915	4.09E-07	count	1
SRP68	0.0894925	0.6670206	0.1342	0.893	4.09E-07	count	1
PAFAH2	19.0675487	2608.071902	0.0073	0.994	4.09E-07	count	1
IGHV3-7	19.0675483	2608.072073	0.0073	0.994	4.09E-07	count	1
TMEM186	0.0894925	0.7700757	0.1162	0.908	4.10E-07	count	1
TIMM44	0.0894925	0.5397261	0.1658	0.868	4.10E-07	count	1
ZNF841	19.4776666	2241.05446	0.0087	0.993	4.10E-07	count	1
AGBL5	19.0648811	1865.093199	0.0102	0.992	4.10E-07	count	1
POLDIP3	0.5534476	0.5655028	0.9787	0.328	4.10E-07	count	1
FBRSL1	19.4373789	2330.814666	0.0083	0.993	4.11E-07	count	1
GOLPH3	0.0908055	0.3421781	0.2654	0.791	4.16E-07	count	1
DDX56	0.5599623	0.5545718	1.0097	0.313	4.17E-07	count	1
STRBP	0.0348049	0.2204338	0.1579	0.875	4.23E-07	count	1
METAP2	0.0349152	0.2416881	0.1445	0.885	4.25E-07	count	1
POLB	0.0938725	0.6688935	0.1403	0.888	4.33E-07	count	1
YIF1A	0.0356703	0.3384857	0.1054	0.916	4.34E-07	count	1
CENPJ	0.5824352	0.7515899	0.7749	0.439	4.37E-07	count	1
TEFM	0.5824352	0.7752361	0.7513	0.453	4.37E-07	count	1
TMEM163	0.5824352	0.6600061	0.8825	0.378	4.38E-07	count	1
ATP6V0A1	0.0954652	0.4390452	0.2174	0.828	4.38E-07	count	1
EPS8L2	0.5824352	0.6090718	0.9563	0.339	4.39E-07	count	1
CENPT	0.5824352	0.7375086	0.7897	0.43	4.39E-07	count	1
CEP63	0.5824352	0.5125014	1.1365	0.256	4.40E-07	count	1
TBC1D12	0.5824352	0.6923	0.8413	0.401	4.40E-07	count	1
GEMIN2	0.5824352	0.5517739	1.0556	0.292	4.41E-07	count	1
MYO1E	0.0962889	0.6844844	0.1407	0.888	4.42E-07	count	1
SCYL2	0.0962889	0.5958393	0.1616	0.872	4.42E-07	count	1

PARP4	0.0973654	0.3609402	0.2698	0.787	4.47E-07	count	1
SMCR8	1.2755823	1.022715	1.2473	0.213	4.53E-07	count	1
AL035071.1	1.2755823	1.022715	1.2473	0.213	4.53E-07	count	1
ZNF93	1.2755823	1.1373273	1.1216	0.263	4.53E-07	count	1
ZNF280C	1.2755823	1.1373273	1.1216	0.263	4.53E-07	count	1
FKBP7	1.2755823	1.1373273	1.1216	0.263	4.53E-07	count	1
TMEM184C	1.2755823	1.1373273	1.1216	0.263	4.53E-07	count	1
INTS2	1.2755823	1.1373273	1.1216	0.263	4.53E-07	count	1
SLC27A5	1.2755823	1.1373273	1.1216	0.263	4.53E-07	count	1
RGMB	1.2755823	1.1373273	1.1216	0.263	4.53E-07	count	1
TULP3	1.2755823	1.1373273	1.1216	0.263	4.53E-07	count	1
FAM43A	1.2755823	1.2414028	1.0275	0.305	4.53E-07	count	1
ARHGAP11A	1.2755823	1.2414028	1.0275	0.305	4.53E-07	count	1
GSTA4	1.2755823	1.2414028	1.0275	0.305	4.53E-07	count	1
CENPF	1.2755823	1.2414028	1.0275	0.305	4.53E-07	count	1
ZFP3	1.2755823	1.2414028	1.0275	0.305	4.53E-07	count	1
AC015917.2	1.2755823	1.2414028	1.0275	0.305	4.53E-07	count	1
CAMKMT	1.2755823	1.2414028	1.0275	0.305	4.53E-07	count	1
ZNF816	1.2755823	1.2414028	1.0275	0.305	4.53E-07	count	1
FDXR	1.2755823	1.2414028	1.0275	0.305	4.53E-07	count	1
CENPP	1.2755823	1.2414028	1.0275	0.305	4.53E-07	count	1
CEP57L1	1.2755823	1.2414028	1.0275	0.305	4.53E-07	count	1
HSPA2	1.2755823	1.3374037	0.9538	0.341	4.54E-07	count	1
TMEM63B	1.2755823	1.3374037	0.9538	0.341	4.54E-07	count	1
ZDHHC19	1.2755823	1.4269604	0.8939	0.372	4.54E-07	count	1
AC005261.3	1.2755823	1.4269604	0.8939	0.372	4.54E-07	count	1
DDX31	1.2755823	1.4269604	0.8939	0.372	4.54E-07	count	1
SLC16A11	1.2755823	1.4269604	0.8939	0.372	4.54E-07	count	1
EDRF1	0.0989907	0.513674	0.1927	0.847	4.54E-07	count	1
GDF7	0.2543021	0.9490314	0.268	0.789	4.56E-07	count	1
CNOT6	0.2543021	0.7320403	0.3474	0.728	4.56E-07	count	1
DSTYK	0.2543021	0.7846517	0.3241	0.746	4.56E-07	count	1
SMIM13	0.2543021	0.6575459	0.3867	0.699	4.56E-07	count	1
NFATC2	0.2543021	0.6575459	0.3867	0.699	4.56E-07	count	1
PIGV	0.2543021	0.8335817	0.3051	0.76	4.56E-07	count	1
ABCF3	0.2543021	0.8804937	0.2888	0.773	4.56E-07	count	1
DNAJC27	0.2543021	0.8801442	0.2889	0.773	4.56E-07	count	1
CHUK	0.2543021	0.715655	0.3553	0.723	4.56E-07	count	1
SBF2-AS1	0.2543021	0.715655	0.3553	0.723	4.56E-07	count	1
GSTZ1	0.2543021	0.715655	0.3553	0.723	4.56E-07	count	1
DCUN1D4	0.2543021	0.7693877	0.3305	0.741	4.57E-07	count	1
CLCN7	0.2543021	0.7693877	0.3305	0.741	4.57E-07	count	1
FEN1	0.2543021	0.7693877	0.3305	0.741	4.57E-07	count	1
RBM12B	0.2552531	0.9500667	0.2687	0.788	4.58E-07	count	1
ZNF275	0.2552531	0.9212806	0.2771	0.782	4.58E-07	count	1

ZNF346	0.2552531	0.8729063	0.2924	0.77	4.58E-07	count	1
SIGMAR1	0.2552531	1.1006276	0.2319	0.817	4.58E-07	count	1
EBLN2	0.2552531	1.1006276	0.2319	0.817	4.58E-07	count	1
NBPF12	0.2552531	1.0809666	0.2361	0.813	4.58E-07	count	1
FBXL18	0.2552531	1.0400463	0.2454	0.806	4.58E-07	count	1
PNPO	0.2552531	1.0400463	0.2454	0.806	4.58E-07	count	1
NAPEPLD	0.2552531	1.0400463	0.2454	0.806	4.58E-07	count	1
ATN1	0.2552531	1.0400463	0.2454	0.806	4.58E-07	count	1
ZNF583	0.2552531	1.0400463	0.2454	0.806	4.58E-07	count	1
RAB4B	0.2552531	1.0400463	0.2454	0.806	4.58E-07	count	1
POLD1	0.2552531	1.0400463	0.2454	0.806	4.58E-07	count	1
NXF1	0.2552531	0.8249166	0.3094	0.757	4.59E-07	count	1
AC005070.3	0.2566426	0.8467493	0.3031	0.762	4.60E-07	count	1
PHLPP1	0.2566426	1.0507166	0.2443	0.807	4.62E-07	count	1
DNAJC18	0.2566426	1.0092362	0.2543	0.799	4.62E-07	count	1
MPP1	0.6121445	1.4355437	0.4264	0.67	4.63E-07	count	1
MCF2L-AS1	0.6121445	1.4355437	0.4264	0.67	4.63E-07	count	1
AL627171.2	0.6121445	1.4355437	0.4264	0.67	4.63E-07	count	1
GNGT2	0.6121445	1.4355437	0.4264	0.67	4.63E-07	count	1
AC006942.1	0.6121445	1.4355437	0.4264	0.67	4.63E-07	count	1
CCDC30	0.6121445	1.4355437	0.4264	0.67	4.63E-07	count	1
LINC01825	0.6121445	1.4355437	0.4264	0.67	4.63E-07	count	1
AC026979.2	0.6121445	1.4355437	0.4264	0.67	4.63E-07	count	1
BAALC	0.6121445	1.4355437	0.4264	0.67	4.63E-07	count	1
TMEM185B	0.6121445	1.4355437	0.4264	0.67	4.63E-07	count	1
AC136475.1	0.6121445	1.4355437	0.4264	0.67	4.63E-07	count	1
ECHDC3	0.6121445	1.4355437	0.4264	0.67	4.63E-07	count	1
AL356488.2	0.6121445	1.4355437	0.4264	0.67	4.63E-07	count	1
FAM153A	0.6121445	1.4355437	0.4264	0.67	4.63E-07	count	1
AC002456.1	0.6121445	1.4355437	0.4264	0.67	4.63E-07	count	1
SLC41A2	0.6121445	1.4355437	0.4264	0.67	4.63E-07	count	1
GAS6	0.6121445	1.4355437	0.4264	0.67	4.63E-07	count	1
MYLK3	0.6121445	1.4355437	0.4264	0.67	4.63E-07	count	1
SH2D2A	0.6121445	1.4355437	0.4264	0.67	4.63E-07	count	1
NFIB	0.6121445	1.4355437	0.4264	0.67	4.63E-07	count	1
FAM168A	0.6121445	1.4355437	0.4264	0.67	4.63E-07	count	1
LOXL3	0.6121445	1.4355437	0.4264	0.67	4.63E-07	count	1
PCOLCE	0.6121445	1.4355437	0.4264	0.67	4.63E-07	count	1
BDNF-AS	0.6121445	1.4355437	0.4264	0.67	4.63E-07	count	1
ACP6	0.6121445	1.4355437	0.4264	0.67	4.63E-07	count	1
CEP72	0.6121445	1.4355437	0.4264	0.67	4.63E-07	count	1
AC245452.1	0.6121445	1.4355437	0.4264	0.67	4.63E-07	count	1
AURKA	0.6121445	1.4355437	0.4264	0.67	4.63E-07	count	1
NMNAT1	0.6121445	1.4355437	0.4264	0.67	4.63E-07	count	1
AC109347.2	0.6121445	1.4355437	0.4264	0.67	4.63E-07	count	1

DDIAS	0.6121445	1.4355437	0.4264	0.67	4.63E-07	count	1
MIR762HG	0.6121445	1.4355437	0.4264	0.67	4.63E-07	count	1
RECQL4	0.6121445	1.4355437	0.4264	0.67	4.63E-07	count	1
SUV39H2	0.6121445	1.4355437	0.4264	0.67	4.63E-07	count	1
WBP2NL	0.6121445	1.4355437	0.4264	0.67	4.63E-07	count	1
AL022328.4	0.6121445	1.4355437	0.4264	0.67	4.63E-07	count	1
AGRN	0.6121445	1.4355437	0.4264	0.67	4.63E-07	count	1
ENGASE	0.6121445	1.4355437	0.4264	0.67	4.63E-07	count	1
AC074387.1	0.6121445	1.4355437	0.4264	0.67	4.63E-07	count	1
REPS2	0.6121445	1.4355437	0.4264	0.67	4.63E-07	count	1
SLC25A44	0.6121445	1.4355437	0.4264	0.67	4.63E-07	count	1
DOCK9-DT	0.6121445	1.4355437	0.4264	0.67	4.63E-07	count	1
LMNTD2	0.6121445	1.0123256	0.6047	0.546	4.63E-07	count	1
MPV17L	0.6121445	1.0123256	0.6047	0.546	4.63E-07	count	1
UST	0.6121445	1.1688396	0.5237	0.601	4.63E-07	count	1
AP002884.1	0.6121445	1.1688396	0.5237	0.601	4.63E-07	count	1
SCFD2	0.6121445	1.8510314	0.3307	0.741	4.64E-07	count	1
RHBDD3	0.6121445	1.8510314	0.3307	0.741	4.64E-07	count	1
AC009686.2	0.6121445	1.8510314	0.3307	0.741	4.64E-07	count	1
CDCA8	0.6121445	1.8510314	0.3307	0.741	4.64E-07	count	1
BTBD19	0.6121445	1.8510314	0.3307	0.741	4.64E-07	count	1
DMKN	0.6121445	1.8510314	0.3307	0.741	4.64E-07	count	1
AC011447.3	0.6121445	1.8510314	0.3307	0.741	4.64E-07	count	1
ZNF786	0.6121445	1.8510314	0.3307	0.741	4.64E-07	count	1
AC139887.4	0.6121445	1.8510314	0.3307	0.741	4.64E-07	count	1
TMED5	0.0386802	0.2715426	0.1424	0.887	4.72E-07	count	1
RAF1	0.1035265	0.43216	0.2396	0.811	4.76E-07	count	1
DDA1	0.1035265	0.43216	0.2396	0.811	4.76E-07	count	1
B4GALT7	0.1035265	0.4502211	0.2299	0.818	4.76E-07	count	1
KAT7	0.1038418	0.3842607	0.2702	0.787	4.78E-07	count	1
GTPBP10	0.6216559	0.6546882	0.9495	0.343	4.79E-07	count	1
AHR	0.6216559	0.7338146	0.8472	0.397	4.81E-07	count	1
OSBPL8	0.0393193	0.304558	0.1291	0.897	4.81E-07	count	1
C12orf75	0.0395162	0.2610409	0.1514	0.88	4.82E-07	count	1
GEMIN6	0.1054469	0.5883311	0.1792	0.858	4.87E-07	count	1
TMEM120A	0.6469737	0.8899542	0.727	0.468	4.97E-07	count	1
TMEM53	0.6469737	0.9225599	0.7013	0.483	4.97E-07	count	1
EDEM3	0.6469737	0.7908167	0.8181	0.414	4.98E-07	count	1
CAMKK2	0.6469737	1.0707976	0.6042	0.546	4.99E-07	count	1
RETREG3	0.6469737	0.8273386	0.782	0.435	4.99E-07	count	1
N6AMT1	0.6469737	0.7560597	0.8557	0.393	5.00E-07	count	1
PIP5K1A	0.6469737	0.7941816	0.8146	0.416	5.00E-07	count	1
FRAT2	0.1088702	0.4520201	0.2409	0.81	5.02E-07	count	1
TIGIT	0.6469737	1.0219645	0.6331	0.527	5.04E-07	count	1
SNRNP48	0.6469737	0.5005862	1.2924	0.197	5.04E-07	count	1

WDR26	0.1093908	0.4530797	0.2414	0.809	5.04E-07	count	1
RAB37	0.109862	0.4831153	0.2274	0.82	5.06E-07	count	1
IPO8	0.109862	0.4831153	0.2274	0.82	5.06E-07	count	1
USP34	0.0415923	0.1945666	0.2138	0.831	5.08E-07	count	1
ACIN1	0.0418558	0.2670436	0.1567	0.876	5.11E-07	count	1
LIN7C	0.1114339	0.4525002	0.2463	0.806	5.14E-07	count	1
VSIR	0.1114459	0.3800136	0.2933	0.769	5.14E-07	count	1
TIGAR	0.1114339	0.4142273	0.269	0.788	5.14E-07	count	1
MYL9	0.1113651	0.6352105	0.1753	0.861	5.19E-07	count	1
CD80	0.6646733	0.6094825	1.0906	0.276	5.22E-07	count	1
TMEM192	0.6646733	0.49588	1.3404	0.181	5.23E-07	count	1
CARS	0.1132973	0.5143154	0.2203	0.826	5.23E-07	count	1
MCM3	0.1132973	0.5740664	0.1974	0.844	5.23E-07	count	1
ARRDC1-AS1	0.2879977	0.8188995	0.3517	0.725	5.23E-07	count	1
FKBP11	0.0426599	0.4358083	0.0979	0.922	5.24E-07	count	1
ZDHHC13	0.2879977	0.8785305	0.3278	0.743	5.25E-07	count	1
HDDC2	0.0431827	0.3750215	0.1151	0.908	5.28E-07	count	1
ZNF14	0.6777453	0.620615	1.0921	0.275	5.29E-07	count	1
TESC	0.6777453	0.6458576	1.0494	0.295	5.29E-07	count	1
CBWD3	0.11478	0.4961711	0.2313	0.817	5.31E-07	count	1
AC147067.1	0.2916482	0.790758	0.3688	0.712	5.31E-07	count	1
MAPK11	0.6777453	0.693592	0.9772	0.329	5.31E-07	count	1
HIST1H2AG	0.6777453	0.693592	0.9772	0.329	5.31E-07	count	1
POF1B	0.6777453	0.693592	0.9772	0.329	5.31E-07	count	1
GPM6B	0.2916482	0.7827258	0.3726	0.71	5.31E-07	count	1
DNAJB4	0.2916482	0.7827258	0.3726	0.71	5.31E-07	count	1
ARL5A	0.11478	0.5614855	0.2044	0.838	5.31E-07	count	1
HDAC6	0.2916482	0.8542326	0.3414	0.733	5.31E-07	count	1
BTRC	0.2916482	0.9505457	0.3068	0.759	5.32E-07	count	1
RFWD3	0.2916482	0.9817435	0.2971	0.767	5.32E-07	count	1
TSNAX	0.114955	0.3775007	0.3045	0.761	5.32E-07	count	1
TMCO3	0.6777453	0.7169491	0.9453	0.345	5.35E-07	count	1
BCL2L12	0.1154904	0.3572922	0.3232	0.747	5.35E-07	count	1
MFSD14C	0.1160032	0.5288246	0.2194	0.826	5.36E-07	count	1
LSR	0.116868	0.3473125	0.3365	0.737	5.40E-07	count	1
UFM1	0.0445245	0.2370409	0.1878	0.851	5.44E-07	count	1
CD1D	0.6957638	0.6395676	1.0879	0.277	5.45E-07	count	1
MRPS31	0.044844	0.3949603	0.1135	0.91	5.48E-07	count	1
TRIP12	0.044875	0.3296976	0.1361	0.892	5.48E-07	count	1
DTNB	1.4579039	1.0071068	1.4476	0.148	5.50E-07	count	1
BRF2	1.4579039	1.1021977	1.3227	0.187	5.51E-07	count	1
LCLAT1	1.4579039	1.1021977	1.3227	0.187	5.51E-07	count	1
DCAKD	1.4579039	1.1021977	1.3227	0.187	5.51E-07	count	1
EMC1	1.4579039	1.1021977	1.3227	0.187	5.51E-07	count	1
TRIM47	1.4579039	1.1021977	1.3227	0.187	5.51E-07	count	1

ZNF790	1.4579039	1.1021977	1.3227	0.187	5.51E-07	count	1
SLFN13	1.4579039	1.1021977	1.3227	0.187	5.51E-07	count	1
RFC4	1.4579039	1.1021977	1.3227	0.187	5.51E-07	count	1
ZNF626	0.6957638	0.5372458	1.2951	0.196	5.51E-07	count	1
KLHDC7B	1.4579039	1.1897123	1.2254	0.221	5.51E-07	count	1
TUBG1	1.4579039	1.1897123	1.2254	0.221	5.51E-07	count	1
AGO4	1.4579039	1.1897123	1.2254	0.221	5.51E-07	count	1
NOD1	1.4579039	1.1897123	1.2254	0.221	5.51E-07	count	1
ZNF510	1.4579039	1.1897123	1.2254	0.221	5.51E-07	count	1
TMEM231	1.4579039	1.1897123	1.2254	0.221	5.51E-07	count	1
NPHP1	1.4579039	1.2712163	1.1469	0.252	5.52E-07	count	1
MAPK14	0.1193323	0.6045714	0.1974	0.844	5.52E-07	count	1
FAM189B	1.4579039	1.3478006	1.0817	0.28	5.53E-07	count	1
PER3	1.4579039	1.3478006	1.0817	0.28	5.53E-07	count	1
RRP12	1.4579039	1.3478006	1.0817	0.28	5.53E-07	count	1
ARMC6	1.4579039	1.3478006	1.0817	0.28	5.53E-07	count	1
PPP1R16B	0.1193323	0.3669954	0.3252	0.745	5.53E-07	count	1
ANXA2R	0.6957638	0.5904492	1.1784	0.239	5.54E-07	count	1
CCDC110	1.4579039	1.5550862	0.9375	0.349	5.54E-07	count	1
TCEA2	0.1198369	0.3566089	0.336	0.737	5.55E-07	count	1
CCNY	0.12029	0.3727865	0.3227	0.747	5.56E-07	count	1
MRPS14	0.12029	0.6035527	0.1993	0.842	5.57E-07	count	1
COG2	0.1210706	0.4831811	0.2506	0.802	5.61E-07	count	1
AC016831.1	0.1218564	0.4728693	0.2577	0.797	5.64E-07	count	1
JADE2	0.1222851	0.4361968	0.2803	0.779	5.66E-07	count	1
SMG5	0.3091216	0.7175509	0.4308	0.667	5.66E-07	count	1
RIDA	0.3091216	0.7584849	0.4076	0.684	5.66E-07	count	1
POLR2M	0.3091216	0.7138331	0.433	0.665	5.66E-07	count	1
ZNF532	0.3091216	0.6577033	0.47	0.639	5.67E-07	count	1
NSUN4	0.3091216	0.7549686	0.4094	0.682	5.67E-07	count	1
CAPN15	0.3091216	0.7549686	0.4094	0.682	5.67E-07	count	1
FMNL1	0.0467163	0.2828203	0.1652	0.869	5.71E-07	count	1
AP5M1	0.7159665	0.4888878	1.4645	0.144	5.75E-07	count	1
EML6	0.3149313	0.929477	0.3388	0.735	5.78E-07	count	1
IGSF8	0.1257505	0.3391478	0.3708	0.711	5.83E-07	count	1
CNFN	0.1258878	0.6412649	0.1963	0.844	5.83E-07	count	1
CPPED1	0.1258878	0.5725341	0.2199	0.826	5.83E-07	count	1
UBR7	0.1258878	0.5442591	0.2313	0.817	5.84E-07	count	1
CDK7	0.1258878	0.569407	0.2211	0.825	5.84E-07	count	1
TIGD5	0.1258878	0.569407	0.2211	0.825	5.84E-07	count	1
B4GALT4	0.1258878	0.5171239	0.2434	0.808	5.84E-07	count	1
MAPK13	0.1258878	0.5923196	0.2125	0.832	5.84E-07	count	1
DOK2	0.1258878	0.6762507	0.1862	0.852	5.85E-07	count	1
RAD51B	0.1258878	0.5892975	0.2136	0.831	5.85E-07	count	1
BUD13	0.1262327	0.5674364	0.2225	0.824	5.85E-07	count	1

SURF4	0.1262327	0.5427854	0.2326	0.816	5.85E-07	count	1
EPN2	0.1262327	0.5626839	0.2243	0.823	5.85E-07	count	1
HEXIM1	0.1262327	0.3822208	0.3303	0.741	5.85E-07	count	1
RABEPK	0.1262327	0.6204965	0.2034	0.839	5.86E-07	count	1
ATF6	0.1262327	0.4816241	0.2621	0.793	5.86E-07	count	1
LCMT1	0.1260715	0.4330645	0.2911	0.771	5.86E-07	count	1
HOMER3	0.1262327	0.6256813	0.2018	0.84	5.86E-07	count	1
C16orf74	0.1265027	0.5869414	0.2155	0.829	5.86E-07	count	1
ACOT8	0.1267198	0.5525262	0.2293	0.819	5.88E-07	count	1
ZFAND2A	0.1300662	0.4227897	0.3076	0.759	6.04E-07	count	1
KDM7A	0.1300662	0.4350678	0.299	0.765	6.05E-07	count	1
GLRX2	0.1302617	0.4698555	0.2772	0.782	6.07E-07	count	1
BCL2L1	0.3302351	1.0247182	0.3223	0.747	6.10E-07	count	1
SCN3A	0.3302351	1.0610175	0.3112	0.756	6.10E-07	count	1
RSPH3	0.3302351	1.0610175	0.3112	0.756	6.10E-07	count	1
LRRC37A2	0.3302351	0.9506952	0.3474	0.728	6.10E-07	count	1
CFAP44	0.3302351	1.3902397	0.2375	0.812	6.10E-07	count	1
PTCD1	0.3302351	1.3902397	0.2375	0.812	6.10E-07	count	1
FAM222A	0.3302351	1.3059993	0.2529	0.8	6.10E-07	count	1
RANGRF	0.3311207	0.3315595	0.9987	0.318	6.13E-07	count	1
TMEM161A	0.7647567	0.7698726	0.9934	0.321	6.17E-07	count	1
COX10	0.7647567	0.6807815	1.1234	0.262	6.17E-07	count	1
DHX38	0.1321386	0.4575381	0.2888	0.773	6.18E-07	count	1
FEM1C	0.7647567	0.6339573	1.2063	0.228	6.19E-07	count	1
AVEN	0.7647567	0.7308904	1.0463	0.296	6.19E-07	count	1
LINC02384	0.7647567	0.7412317	1.0317	0.303	6.19E-07	count	1
CTNNBIP1	0.7647567	0.8924478	0.8569	0.392	6.20E-07	count	1
ENDOG	0.7647567	0.5833869	1.3109	0.191	6.20E-07	count	1
MAP7D1	0.1330535	0.4888184	0.2722	0.786	6.21E-07	count	1
CERS5	0.1335572	0.5110648	0.2613	0.794	6.21E-07	count	1
ZNF143	0.7647567	0.4923031	1.5534	0.121	6.21E-07	count	1
MCM7	0.1335572	0.5619206	0.2377	0.812	6.21E-07	count	1
TGFBR1	0.7647567	0.8288669	0.9227	0.357	6.21E-07	count	1
KLF12	0.1341935	0.6570497	0.2042	0.838	6.24E-07	count	1
MFN1	0.1341935	0.4357063	0.308	0.758	6.26E-07	count	1
S100PBP	0.1341935	0.5644379	0.2377	0.812	6.26E-07	count	1
SRBD1	0.1350226	0.5698529	0.2369	0.813	6.28E-07	count	1
GMPPA	0.1350226	0.6323207	0.2135	0.831	6.29E-07	count	1
KPNA3	0.1350226	0.3748244	0.3602	0.719	6.29E-07	count	1
ALAS1	0.1350226	0.5981254	0.2257	0.822	6.29E-07	count	1
HYLS1	0.3376584	0.6975715	0.484	0.629	6.29E-07	count	1
ALPK1	0.1350226	0.6012456	0.2246	0.822	6.29E-07	count	1
MAGT1	0.1353578	0.3471123	0.39	0.697	6.30E-07	count	1
USP53	0.1355391	0.5259373	0.2577	0.797	6.31E-07	count	1
FBH1	0.1350226	0.790993	0.1707	0.865	6.32E-07	count	1

IBA57	0.3420533	0.7743569	0.4417	0.659	6.34E-07	count	1
ZNF135	0.3420533	0.9441389	0.3623	0.717	6.35E-07	count	1
ARMH1	0.3420533	0.8698424	0.3932	0.694	6.36E-07	count	1
MINCR	0.3420533	1.1267099	0.3036	0.762	6.36E-07	count	1
LATS1	0.3420533	1.1267099	0.3036	0.762	6.36E-07	count	1
PIGP	0.3398735	0.3200931	1.0618	0.289	6.37E-07	count	1
ZFYVE19	1.6120546	1.0767563	1.4971	0.135	6.41E-07	count	1
EHD3	1.6120546	1.0767563	1.4971	0.135	6.41E-07	count	1
ATPAF2	1.6120546	1.0767563	1.4971	0.135	6.41E-07	count	1
HDAC10	1.6120546	1.0767563	1.4971	0.135	6.41E-07	count	1
SLC35B4	1.6120546	1.1523096	1.399	0.163	6.42E-07	count	1
NOTCH1	1.6120546	1.1523096	1.399	0.163	6.42E-07	count	1
STT3A	1.6120546	1.1523096	1.399	0.163	6.42E-07	count	1
FHOD1	1.6120546	1.1523096	1.399	0.163	6.42E-07	count	1
ST3GAL3	1.6120546	1.2232051	1.3179	0.188	6.43E-07	count	1
WDR35	1.6120546	1.2232051	1.3179	0.188	6.43E-07	count	1
RPGRIP1L	1.6120546	1.4147334	1.1395	0.255	6.45E-07	count	1
RBM27	0.347029	0.7577929	0.4579	0.647	6.45E-07	count	1
PDCD2L	0.347029	0.9274412	0.3742	0.708	6.45E-07	count	1
TMEM117	0.347029	0.7664049	0.4528	0.651	6.46E-07	count	1
PPP2R5C	0.0529177	0.220481	0.24	0.81	6.50E-07	count	1
LRP10	0.0535774	0.2789816	0.192	0.848	6.57E-07	count	1
MAN2A1	0.1412135	0.4708208	0.2999	0.764	6.59E-07	count	1
SAP30	0.1412135	0.4932218	0.2863	0.775	6.59E-07	count	1
VAMP5	0.1416304	0.5280535	0.2682	0.789	6.61E-07	count	1
RNF20	0.1416304	0.5413762	0.2616	0.794	6.63E-07	count	1
SLC39A6	0.1423917	0.4872879	0.2922	0.77	6.64E-07	count	1
NUDT9	0.1423917	0.5434914	0.262	0.793	6.66E-07	count	1
SH3BP1	0.1423917	0.5326842	0.2673	0.789	6.66E-07	count	1
SPOP	0.0547422	0.2662063	0.2056	0.837	6.71E-07	count	1
C2orf49	0.8011244	0.4791739	1.6719	0.0953	6.72E-07	count	1
MOSMO	0.1434703	0.6140423	0.2336	0.815	6.72E-07	count	1
AAMP	0.1442689	0.306062	0.4714	0.638	6.74E-07	count	1
CHTOP	0.3592916	0.3414429	1.0523	0.293	6.75E-07	count	1
LMO7	0.1459234	0.6152515	0.2372	0.813	6.82E-07	count	1
ZNF76	0.1459234	0.6485559	0.225	0.822	6.82E-07	count	1
KIFAP3	0.1459234	0.4855322	0.3005	0.764	6.84E-07	count	1
PSMB2	0.0558377	0.2413908	0.2313	0.817	6.85E-07	count	1
STAMBPL1	0.1466477	0.3688998	0.3975	0.691	6.87E-07	count	1
RRP1	0.1474848	0.5048285	0.2921	0.77	6.90E-07	count	1
PRDM1	0.1474848	0.4992568	0.2954	0.768	6.90E-07	count	1
MAP4K2	0.1480705	0.4357738	0.3398	0.734	6.93E-07	count	1
SGTA	0.8292952	0.6141508	1.3503	0.178	6.96E-07	count	1
FAM172A	0.1488822	0.3743922	0.3977	0.691	6.96E-07	count	1
NSMCE4A	0.0567287	0.2876035	0.1972	0.844	6.97E-07	count	1

KCTD5	0.1493594	0.7290245	0.2049	0.838	6.99E-07	count	1
CC2D1B	0.1493594	0.7664685	0.1949	0.846	6.99E-07	count	1
SASS6	0.1493594	0.6293247	0.2373	0.813	6.99E-07	count	1
DHX37	0.1493594	0.7658606	0.195	0.845	6.99E-07	count	1
ST7L	0.1493594	0.723559	0.2064	0.837	6.99E-07	count	1
WDR76	0.1493594	0.723559	0.2064	0.837	6.99E-07	count	1
AL451085.2	0.1493594	0.7606599	0.1964	0.844	6.99E-07	count	1
SLC37A1	0.1493594	0.4172373	0.358	0.721	7.00E-07	count	1
EXOSC7	0.1493594	0.446706	0.3344	0.738	7.00E-07	count	1
MRPL2	0.1493594	0.504391	0.2961	0.767	7.00E-07	count	1
MIS18A	0.1493594	0.8572808	0.1742	0.862	7.01E-07	count	1
SYT1	0.1508331	0.4822773	0.3128	0.755	7.07E-07	count	1
CAMK2G	0.8447994	0.5733546	1.4734	0.141	7.08E-07	count	1
WDR55	0.1513345	0.4149177	0.3647	0.715	7.09E-07	count	1
ZYG11B	0.8447994	0.6376191	1.3249	0.186	7.13E-07	count	1
SULF2	0.8447994	0.6376191	1.3249	0.186	7.13E-07	count	1
ATXN7L3	0.1521983	0.4461437	0.3411	0.733	7.13E-07	count	1
SOWAHD	0.1521983	0.4888231	0.3114	0.756	7.14E-07	count	1
FOXO3	0.1521983	0.5357903	0.2841	0.776	7.16E-07	count	1
RMND1	1.745586	1.0573703	1.6509	0.0995	7.24E-07	count	1
AC020928.1	1.745586	1.0573703	1.6509	0.0995	7.24E-07	count	1
CAMK2D	0.1544043	0.667679	0.2313	0.817	7.24E-07	count	1
C17orf51	1.745586	1.1866551	1.471	0.142	7.26E-07	count	1
PIP4K2B	1.745586	1.1866551	1.471	0.142	7.26E-07	count	1
AC098818.2	1.745586	1.3031763	1.3395	0.181	7.28E-07	count	1
CCL22	1.745586	1.4101017	1.2379	0.216	7.30E-07	count	1
AL031714.1	1.745586	1.4101017	1.2379	0.216	7.31E-07	count	1
DPF3	0.8701172	0.7820976	1.1125	0.267	7.32E-07	count	1
SELENOO	0.8701172	0.7820976	1.1125	0.267	7.32E-07	count	1
TSPOAP1-AS1	0.8701172	0.8373028	1.0392	0.299	7.34E-07	count	1
ZNF544	0.8701172	0.7413971	1.1736	0.241	7.35E-07	count	1
EIF4ENIF1	0.8701172	0.8890869	0.9787	0.328	7.35E-07	count	1
RNF122	0.8701172	0.7413971	1.1736	0.241	7.36E-07	count	1
ATP5F1B	0.0600729	0.1432344	0.4194	0.675	7.38E-07	count	1
NFU1	0.3882791	0.3247977	1.1954	0.233	7.38E-07	count	1
TUBGCP5	0.1571807	0.5108942	0.3077	0.758	7.38E-07	count	1
PARN	0.1571807	0.5135164	0.3061	0.76	7.38E-07	count	1
COMMD2	0.1571807	0.3387327	0.464	0.643	7.39E-07	count	1
GFOD1	0.8701172	0.7994179	1.0884	0.277	7.39E-07	count	1
SRP54	0.0601522	0.2897465	0.2076	0.836	7.39E-07	count	1
DDX20	0.1571807	0.7659578	0.2052	0.838	7.45E-07	count	1
GPR132	0.1587774	0.3866784	0.4106	0.682	7.47E-07	count	1
ABCB8	0.159135	0.7486234	0.2126	0.832	7.47E-07	count	1
SRGAP2C	0.159135	0.6682008	0.2382	0.812	7.49E-07	count	1
GIPC1	0.395142	0.757117	0.5219	0.602	7.51E-07	count	1

FAAP100	0.395142	0.757117	0.5219	0.602	7.51E-07	count	1
ERI1	0.395142	0.8834423	0.4473	0.655	7.51E-07	count	1
TMEM209	0.1605654	0.5883692	0.2729	0.785	7.55E-07	count	1
HPS6	0.1605654	0.5523903	0.2907	0.771	7.55E-07	count	1
MBNL3	0.1605654	0.5523903	0.2907	0.771	7.55E-07	count	1
CRIPT	0.06146	0.4087799	0.1503	0.881	7.55E-07	count	1
UBE2F	0.1605654	0.5106895	0.3144	0.753	7.56E-07	count	1
RIOX2	0.1605654	0.5288491	0.3036	0.762	7.56E-07	count	1
SERPINB8	0.3970319	0.4558814	0.8709	0.384	7.59E-07	count	1
YKT6	0.4018913	0.7090001	0.5668	0.571	7.63E-07	count	1
SARS2	0.4018913	0.7528569	0.5338	0.594	7.64E-07	count	1
NUDT19	0.4018913	0.7944284	0.5059	0.613	7.64E-07	count	1
SLC35A2	0.4018913	0.7944284	0.5059	0.613	7.64E-07	count	1
STN1	0.4018913	0.6861952	0.5857	0.558	7.64E-07	count	1
SETD1B	0.4018913	0.6861952	0.5857	0.558	7.64E-07	count	1
CENPW	0.4018913	0.6861952	0.5857	0.558	7.64E-07	count	1
UBE2T	0.4018913	0.6860417	0.5858	0.558	7.64E-07	count	1
RELL2	0.4018913	0.6860417	0.5858	0.558	7.64E-07	count	1
TMEM102	0.8899199	0.5770916	1.5421	0.124	7.65E-07	count	1
CROT	0.4018913	0.7315646	0.5494	0.583	7.65E-07	count	1
NNT	0.4018913	0.8716435	0.4611	0.645	7.65E-07	count	1
PCBD1	0.8899199	0.5429494	1.639	0.102	7.67E-07	count	1
MRPL55	0.0625014	0.3521027	0.1775	0.859	7.69E-07	count	1
CLASRP	0.1634588	0.4680291	0.3492	0.727	7.71E-07	count	1
BCAR3	0.8899199	0.5847299	1.5219	0.129	7.72E-07	count	1
NRAS	0.1653004	0.4605081	0.359	0.72	7.79E-07	count	1
RHNO1	0.1653004	0.5015531	0.3296	0.742	7.79E-07	count	1
LINC01588	0.1654954	0.4088515	0.4048	0.686	7.80E-07	count	1
KIF1BP	0.1653004	0.4788405	0.3452	0.73	7.80E-07	count	1
SMYD2	0.1653004	0.4670703	0.3539	0.724	7.82E-07	count	1
ACADVL	0.1660166	0.4197551	0.3955	0.693	7.82E-07	count	1
CACUL1	0.0636035	0.3978904	0.1599	0.873	7.83E-07	count	1
TRIM4	0.1660166	0.4329186	0.3835	0.702	7.84E-07	count	1
NHLRC3	0.1665918	0.4014321	0.415	0.678	7.86E-07	count	1
PCNX3	0.4122878	0.926616	0.4449	0.657	7.86E-07	count	1
IFFO2	0.4122878	0.8117368	0.5079	0.612	7.87E-07	count	1
AHSA1	0.0640509	0.2958772	0.2165	0.829	7.88E-07	count	1
PWP1	0.0642154	0.2364521	0.2716	0.786	7.90E-07	count	1
FTX	0.1685446	0.4062236	0.4149	0.678	7.94E-07	count	1
ZNF33B	0.1683291	0.6317905	0.2664	0.79	7.96E-07	count	1
UBAP2L	0.169065	0.4559866	0.3708	0.711	7.97E-07	count	1
GAK	0.169065	0.5323667	0.3176	0.751	7.98E-07	count	1
ETV3	0.169065	0.4872499	0.347	0.729	7.99E-07	count	1
SCRN3	0.4183666	0.9466707	0.4419	0.659	7.99E-07	count	1
NAALADL1	0.4183666	0.9466707	0.4419	0.659	7.99E-07	count	1

MAP2K5	0.4183666	0.9411817	0.4445	0.657	8.00E-07	count	1
AL109955.1	0.4183666	0.9411817	0.4445	0.657	8.00E-07	count	1
MAP3K14	0.4183666	0.9411817	0.4445	0.657	8.00E-07	count	1
KIF13A	0.4183666	0.7522869	0.5561	0.578	8.00E-07	count	1
KCNK6	0.4183666	1.0063741	0.4157	0.678	8.00E-07	count	1
SSBP3	0.4183666	1.0724351	0.3901	0.697	8.00E-07	count	1
MYNN	0.169065	0.4872499	0.347	0.729	8.00E-07	count	1
ADD2	0.4183666	1.0675929	0.3919	0.695	8.00E-07	count	1
CARM1	0.4183666	1.0675929	0.3919	0.695	8.00E-07	count	1
UPP1	0.4183666	0.832407	0.5026	0.615	8.00E-07	count	1
AC090114.2	0.4183666	0.832407	0.5026	0.615	8.00E-07	count	1
PIEZO1	0.4183666	0.832407	0.5026	0.615	8.00E-07	count	1
OTUD3	0.4183666	0.9111693	0.4592	0.646	8.00E-07	count	1
SFMBT1	0.169065	0.5109377	0.3309	0.741	8.01E-07	count	1
DOCK7	0.4183666	0.9054651	0.462	0.644	8.01E-07	count	1
AC108863.1	0.4183666	0.9054651	0.462	0.644	8.01E-07	count	1
AL441992.1	0.4183666	0.9054651	0.462	0.644	8.01E-07	count	1
ERMARD	0.4183666	0.9054651	0.462	0.644	8.01E-07	count	1
ZMYND11	0.169065	0.4824577	0.3504	0.726	8.01E-07	count	1
SLC31A1	0.4183666	1.0362426	0.4037	0.687	8.02E-07	count	1
PNPLA4	1.863369	1.1014343	1.6918	0.0914	8.03E-07	count	1
NDRG3	1.863369	1.1014343	1.6918	0.0914	8.03E-07	count	1
CCZ1	0.169736	0.3737858	0.4541	0.65	8.03E-07	count	1
RBM48	0.9189074	0.467701	1.9647	0.0501	8.04E-07	count	1
RPGR	0.1693601	0.4815779	0.3517	0.725	8.04E-07	count	1
ZSCAN30	1.863369	1.1578027	1.6094	0.108	8.04E-07	count	1
COPG2	1.863369	1.2115513	1.538	0.125	8.05E-07	count	1
INO80B	1.863369	1.2115513	1.538	0.125	8.05E-07	count	1
DNAJC24	0.1707858	0.5184182	0.3294	0.742	8.06E-07	count	1
ULK4	1.863369	1.2115513	1.538	0.125	8.06E-07	count	1
DCLRE1C	0.1707858	0.602717	0.2834	0.777	8.06E-07	count	1
11-Sep	0.1726614	0.4629181	0.373	0.709	8.19E-07	count	1
RPP30	0.173664	0.4139192	0.4196	0.675	8.22E-07	count	1
BANF1	0.0667695	0.1933859	0.3453	0.73	8.22E-07	count	1
KAT8	0.1742881	0.3699695	0.4711	0.638	8.25E-07	count	1
RGP1	0.1746121	0.5571841	0.3134	0.754	8.26E-07	count	1
ZNF124	0.4308807	0.8183077	0.5266	0.599	8.28E-07	count	1
RPUSD3	0.432506	0.5877849	0.7358	0.462	8.33E-07	count	1
HSPA14	0.432506	0.6288007	0.6878	0.492	8.34E-07	count	1
SACM1L	0.1763319	0.4762489	0.3703	0.711	8.34E-07	count	1
EML2	0.9470783	0.5691632	1.664	0.0968	8.34E-07	count	1
ZFPL1	0.176069	0.6114928	0.2879	0.774	8.35E-07	count	1
HSF1	0.176567	0.394053	0.4481	0.654	8.36E-07	count	1
RABEP1	0.068262	0.3230532	0.2113	0.833	8.42E-07	count	1
KLC2	0.9654274	0.7390332	1.3063	0.192	8.42E-07	count	1

TSPAN5	0.9654274	0.7910356	1.2205	0.223	8.45E-07	count	1
BBIP1	0.179313	0.3804145	0.4714	0.638	8.50E-07	count	1
FCRLA	0.0692138	0.1835187	0.3771	0.706	8.53E-07	count	1
PPID	0.1798802	0.4668173	0.3853	0.7	8.55E-07	count	1
SLC35B1	0.1808097	0.5715901	0.3163	0.752	8.57E-07	count	1
ANAPC13	0.1814671	0.3440797	0.5274	0.598	8.62E-07	count	1
C17orf75	0.1826112	0.6540727	0.2792	0.78	8.69E-07	count	1
IER3	0.1826112	0.7416814	0.2462	0.806	8.69E-07	count	1
AL139246.5	0.1826112	0.4505099	0.4053	0.685	8.71E-07	count	1
SLC7A7	1.9687295	1.0294974	1.9123	0.0565	8.74E-07	count	1
RAD54L2	1.9687295	1.0832051	1.8175	0.0698	8.75E-07	count	1
AGK	1.9687295	1.0832051	1.8175	0.0698	8.75E-07	count	1
BAG3	1.9687295	1.0832051	1.8175	0.0698	8.75E-07	count	1
SMN2	1.9687295	1.1343728	1.7355	0.0833	8.77E-07	count	1
CTC1	1.9687295	1.1343728	1.7355	0.0833	8.77E-07	count	1
AC025159.1	0.9879003	0.7171955	1.3774	0.169	8.77E-07	count	1
PPP4R2	0.0711495	0.221137	0.3217	0.748	8.78E-07	count	1
MAD2L1	1.9687295	1.1833301	1.6637	0.0969	8.79E-07	count	1
CCDC61	1.9687295	1.1833301	1.6637	0.0969	8.79E-07	count	1
TRAK1	0.9879003	0.6751932	1.4631	0.144	8.81E-07	count	1
AP002387.2	0.9654274	0.6079278	1.5881	0.113	8.82E-07	count	1
LACTB2-AS1	1.9687295	1.3193466	1.4922	0.136	8.82E-07	count	1
CENPQ	0.9879003	0.6911314	1.4294	0.154	8.82E-07	count	1
ZSCAN25	0.4554843	0.990959	0.4596	0.646	8.83E-07	count	1
ZNF839	0.4554843	0.990959	0.4596	0.646	8.83E-07	count	1
C16orf70	0.4554843	0.990959	0.4596	0.646	8.83E-07	count	1
NRSN2-AS1	0.4554843	0.990959	0.4596	0.646	8.83E-07	count	1
NEK4	0.4554843	0.9583672	0.4753	0.635	8.83E-07	count	1
GPATCH3	0.4554843	0.9583672	0.4753	0.635	8.83E-07	count	1
KMT2D	0.4554843	0.9583672	0.4753	0.635	8.83E-07	count	1
ZNF398	0.4554843	0.9583672	0.4753	0.635	8.83E-07	count	1
AC132872.1	0.4554843	1.1577716	0.3934	0.694	8.83E-07	count	1
ZFP69B	0.4554843	1.1577716	0.3934	0.694	8.83E-07	count	1
AL035587.1	0.4554843	1.2438769	0.3662	0.714	8.83E-07	count	1
SYP	0.4554843	1.2438769	0.3662	0.714	8.83E-07	count	1
PEX7	0.4554843	1.1300014	0.4031	0.687	8.83E-07	count	1
ERLIN2	0.4554843	1.1300014	0.4031	0.687	8.83E-07	count	1
ST14	0.4554843	1.1300014	0.4031	0.687	8.83E-07	count	1
AP001462.1	0.4554843	1.1300014	0.4031	0.687	8.83E-07	count	1
AL354707.1	0.4554843	1.1300014	0.4031	0.687	8.83E-07	count	1
PLK2	0.4554843	1.1300014	0.4031	0.687	8.83E-07	count	1
PPIP5K1	0.4554843	1.1300014	0.4031	0.687	8.83E-07	count	1
SLX4	0.4554843	1.1300014	0.4031	0.687	8.83E-07	count	1
ZBED4	0.4554843	1.1300014	0.4031	0.687	8.83E-07	count	1
LEAP2	0.4554843	1.1300014	0.4031	0.687	8.83E-07	count	1

MAN2A2	0.4554843	0.8889658	0.5124	0.609	8.84E-07	count	1
MAN1B1-DT	0.4554843	0.8889658	0.5124	0.609	8.84E-07	count	1
DOT1L	0.4554843	0.8275073	0.5504	0.582	8.84E-07	count	1
ZNF571	0.4554843	0.8275073	0.5504	0.582	8.84E-07	count	1
NCK1-DT	0.4554843	0.9380133	0.4856	0.628	8.84E-07	count	1
ZBTB33	0.4554843	0.6736854	0.6761	0.499	8.84E-07	count	1
METTL13	0.4554843	0.879987	0.5176	0.605	8.84E-07	count	1
PRR5	0.4554843	0.805576	0.5654	0.572	8.84E-07	count	1
KRT18	0.4554843	0.859396	0.53	0.596	8.85E-07	count	1
MRE11	0.1862297	0.5435934	0.3426	0.732	8.85E-07	count	1
IMMP2L	0.1862297	0.5142251	0.3622	0.717	8.86E-07	count	1
WDR20	0.1866194	0.440963	0.4232	0.672	8.88E-07	count	1
MTMR10	0.4571741	1.0350718	0.4417	0.659	8.89E-07	count	1
CREBL2	0.4571741	0.7888956	0.5795	0.563	8.89E-07	count	1
VKORC1	0.1862297	0.4138474	0.45	0.653	8.90E-07	count	1
NECAP1	0.0718002	0.392014	0.1832	0.855	8.90E-07	count	1
DOPEY1	0.9879003	0.6960672	1.4193	0.157	8.94E-07	count	1
ADNP2	0.4565519	0.6466595	0.706	0.481	8.94E-07	count	1
NEK1	0.1892432	0.4412812	0.4288	0.668	9.01E-07	count	1
LYAR	1.0011455	0.608871	1.6443	0.101	9.09E-07	count	1
PER2	0.1912809	0.7095765	0.2696	0.788	9.10E-07	count	1
B3GNT7	0.1912809	0.6232293	0.3069	0.759	9.10E-07	count	1
ZBTB43	0.1912809	0.6232293	0.3069	0.759	9.10E-07	count	1
CREG1	0.1912809	0.6232293	0.3069	0.759	9.10E-07	count	1
LIG3	0.1912809	0.6500687	0.2942	0.769	9.11E-07	count	1
AC087500.1	0.1912809	0.5890978	0.3247	0.746	9.11E-07	count	1
PCGF1	0.1912809	0.70067	0.273	0.785	9.11E-07	count	1
OTUD6B	0.1912809	0.6174227	0.3098	0.757	9.11E-07	count	1
CCDC88B	0.1917014	0.4599983	0.4167	0.677	9.13E-07	count	1
CHID1	0.1912809	0.4692774	0.4076	0.684	9.13E-07	count	1
VAV2	0.1912809	0.4816665	0.3971	0.691	9.14E-07	count	1
B3GAT3	0.0740912	0.3324928	0.2228	0.824	9.16E-07	count	1
PPP2R1A	0.0740912	0.3324928	0.2228	0.824	9.16E-07	count	1
TMEM109	0.0748047	0.2216346	0.3375	0.736	9.24E-07	count	1
ARMC1	0.1948363	0.3734456	0.5217	0.602	9.30E-07	count	1
EIF2S3	0.0753246	0.1813317	0.4154	0.678	9.31E-07	count	1
MAPK3	0.1964318	0.6425418	0.3057	0.76	9.36E-07	count	1
TPD52L2	0.1964318	0.6172244	0.3183	0.75	9.37E-07	count	1
PDGFA	0.1964318	0.722812	0.2718	0.786	9.38E-07	count	1
TRMT61A	0.1966469	0.7038664	0.2794	0.78	9.38E-07	count	1
SOAT1	0.1964318	0.6443635	0.3048	0.761	9.38E-07	count	1
SIN3A	0.0759255	0.2842812	0.2671	0.79	9.38E-07	count	1
APAF1	0.1966469	0.5902273	0.3332	0.739	9.39E-07	count	1
PLAGL1	0.1964318	0.7161611	0.2743	0.784	9.39E-07	count	1
PEX6	0.4808271	0.6871072	0.6998	0.484	9.42E-07	count	1

TAF5	0.1966469	0.6646505	0.2959	0.767	9.43E-07	count	1
NAT10	0.4808271	0.7685308	0.6256	0.532	9.44E-07	count	1
CNN3	0.4808271	0.7315536	0.6573	0.511	9.44E-07	count	1
RPRD1B	0.4808271	0.688157	0.6987	0.485	9.45E-07	count	1
MRPS12	0.0763684	0.3192542	0.2392	0.811	9.46E-07	count	1
TMEM191C	2.0640397	1.114908	1.8513	0.0648	9.46E-07	count	1
EMC7	0.0766785	0.2644487	0.29	0.772	9.48E-07	count	1
TSHZ2	1.0524388	1.1550357	0.9112	0.363	9.49E-07	count	1
SIDT1-AS1	1.0524388	0.9706054	1.0843	0.279	9.50E-07	count	1
ICMT	1.0524388	1.0503578	1.002	0.317	9.52E-07	count	1
DUSP6	0.2000064	0.6045101	0.3309	0.741	9.55E-07	count	1
GTF3C4	1.0524388	0.7977579	1.3192	0.188	9.55E-07	count	1
RRM1	1.0524388	0.6798389	1.5481	0.122	9.56E-07	count	1
RAB1B	0.2006398	0.4161462	0.4821	0.63	9.58E-07	count	1
USP11	0.2003985	0.3984839	0.5029	0.615	9.60E-07	count	1
ZBTB17	0.2011856	0.4427388	0.4544	0.65	9.61E-07	count	1
AL360012.1	1.0524388	0.7547398	1.3944	0.164	9.62E-07	count	1
ROMO1	0.0777124	0.245069	0.3171	0.751	9.62E-07	count	1
AMN1	0.2011856	0.4454224	0.4517	0.652	9.63E-07	count	1
CBFA2T3	0.0783465	0.381819	0.2052	0.838	9.70E-07	count	1
SPG11	0.2031539	0.4835156	0.4202	0.675	9.72E-07	count	1
HSPA14	1.0524388	0.5814063	1.8102	0.0709	9.78E-07	count	1
MED4	0.0791277	0.2520344	0.314	0.754	9.80E-07	count	1
C1GALT1	0.0791277	0.2747668	0.288	0.773	9.80E-07	count	1
RRS1	0.4991823	0.8373267	0.5962	0.551	9.84E-07	count	1
SDK2	0.4991823	0.8373267	0.5962	0.551	9.84E-07	count	1
DEDD	0.4991823	0.8063821	0.619	0.536	9.85E-07	count	1
CHIC2	0.2056358	0.351803	0.5845	0.559	9.85E-07	count	1
AKTIP	0.4991823	0.9252231	0.5395	0.59	9.85E-07	count	1
CD68	0.2056358	0.4023609	0.5111	0.61	9.86E-07	count	1
LIMD1	0.4991823	1.0304482	0.4844	0.628	9.86E-07	count	1
AC103691.1	0.4991823	1.0304482	0.4844	0.628	9.86E-07	count	1
ALKBH8	0.4991823	1.1258815	0.4434	0.658	9.87E-07	count	1
MRPS27	0.2060016	0.4873	0.4227	0.673	9.87E-07	count	1
GEM	1.0524388	1.0855976	0.9695	0.333	9.87E-07	count	1
HOOK3	0.0798066	0.3211683	0.2485	0.804	9.89E-07	count	1
C6orf120	0.2060016	0.5504533	0.3742	0.708	9.90E-07	count	1
VTA1	0.2070728	0.4049793	0.5113	0.609	9.91E-07	count	1
CCL8	1.0955721	1.6035614	0.6832	0.495	9.91E-07	count	1
AL022238.2	1.0955721	1.6035614	0.6832	0.495	9.91E-07	count	1
INCENP	1.0955721	1.6035614	0.6832	0.495	9.91E-07	count	1
KLC4	1.0955721	1.6035614	0.6832	0.495	9.91E-07	count	1
AC069185.1	1.0955721	1.6035614	0.6832	0.495	9.91E-07	count	1
LINC01685	1.0955721	1.6035614	0.6832	0.495	9.91E-07	count	1
PIK3R2	1.0955721	1.6035614	0.6832	0.495	9.91E-07	count	1

TUBGCP6	1.0955721	1.6035614	0.6832	0.495	9.91E-07	count	1
RARRES2	1.0955721	1.6035614	0.6832	0.495	9.91E-07	count	1
HAPLN3	1.0955721	1.6035614	0.6832	0.495	9.91E-07	count	1
ZNF724	1.0955721	1.6035614	0.6832	0.495	9.91E-07	count	1
TMEM170B	1.0955721	1.6035614	0.6832	0.495	9.91E-07	count	1
MEST	1.0955721	1.6035614	0.6832	0.495	9.91E-07	count	1
ZNF703	1.0955721	1.6035614	0.6832	0.495	9.91E-07	count	1
MSMP	1.0955721	1.6035614	0.6832	0.495	9.91E-07	count	1
CAVIN1	1.0955721	1.6035614	0.6832	0.495	9.91E-07	count	1
AL157838.1	1.0955721	1.6035614	0.6832	0.495	9.91E-07	count	1
AC008443.5	1.0955721	1.6035614	0.6832	0.495	9.91E-07	count	1
GNE	1.0955721	1.6035614	0.6832	0.495	9.91E-07	count	1
LINC00526	1.0955721	1.6035614	0.6832	0.495	9.91E-07	count	1
B3GNT5	1.0955721	1.6035614	0.6832	0.495	9.92E-07	count	1
CD40LG	1.0955721	1.6035614	0.6832	0.495	9.92E-07	count	1
C8orf89	1.0955721	1.6035614	0.6832	0.495	9.92E-07	count	1
TM9SF1	1.0955721	1.1860088	0.9237	0.356	9.92E-07	count	1
PLA2G12A	1.0955721	1.1860088	0.9237	0.356	9.92E-07	count	1
WDR92	1.0955721	1.1860088	0.9237	0.356	9.92E-07	count	1
MED14OS	1.0955721	1.1860088	0.9237	0.356	9.92E-07	count	1
LINC02021	1.0955721	1.1860088	0.9237	0.356	9.92E-07	count	1
AC093249.6	1.0955721	1.1860088	0.9237	0.356	9.92E-07	count	1
IFRD1	0.0799319	0.2790039	0.2865	0.775	9.92E-07	count	1
CFAP36	0.2070728	0.3915778	0.5288	0.597	9.92E-07	count	1
TSPYL4	1.0955721	1.5482052	0.7076	0.48	9.92E-07	count	1
NKILA	1.0955721	1.5482052	0.7076	0.48	9.92E-07	count	1
SAMD4A	1.0955721	1.5482052	0.7076	0.48	9.92E-07	count	1
ALKBH4	1.0955721	1.5482052	0.7076	0.48	9.92E-07	count	1
SCLY	1.0955721	1.5482052	0.7076	0.48	9.92E-07	count	1
ZBTB45	1.0955721	1.5482052	0.7076	0.48	9.92E-07	count	1
MVK	1.0955721	1.5482052	0.7076	0.48	9.92E-07	count	1
MANEAL	1.0955721	1.5482052	0.7076	0.48	9.92E-07	count	1
FMO4	1.0955721	1.5482052	0.7076	0.48	9.92E-07	count	1
LIX1-AS1	1.0955721	1.5482052	0.7076	0.48	9.92E-07	count	1
TPH1	1.0955721	1.5482052	0.7076	0.48	9.92E-07	count	1
NADSYN1	0.2060016	0.5046817	0.4082	0.683	9.92E-07	count	1
SURF2	0.2068381	0.4065777	0.5087	0.611	9.93E-07	count	1
TRAPPC6B	0.2084424	0.5812483	0.3586	0.72	9.98E-07	count	1
APEX2	0.2084424	0.668818	0.3117	0.755	9.99E-07	count	1
TMEM168	0.2084424	0.5923168	0.3519	0.725	9.99E-07	count	1
LACTB2	0.2084424	0.5326365	0.3913	0.696	9.99E-07	count	1
TOR2A	0.2084424	0.6273716	0.3322	0.74	1.00E-06	count	1
IMPAD1	0.2084424	0.6273716	0.3322	0.74	1.00E-06	count	1
ZNF292	0.0807511	0.2791282	0.2893	0.772	1.00E-06	count	1
PTS	0.2084424	0.4880052	0.4271	0.669	1.00E-06	count	1

MTFMT	0.2084424	0.6612182	0.3152	0.753	1.00E-06	count	1
INPP5A	0.2084424	0.6612182	0.3152	0.753	1.00E-06	count	1
GDF11	0.2084424	0.7536929	0.2766	0.782	1.00E-06	count	1
DIMT1	0.0812162	0.3569143	0.2276	0.82	1.01E-06	count	1
GPN2	2.1510511	1.0984308	1.9583	0.0508	1.01E-06	count	1
FNDC3A	0.2113762	0.3935464	0.5371	0.591	1.01E-06	count	1
CDC34	0.2119123	0.4340515	0.4882	0.626	1.02E-06	count	1
PTOV1	0.2119123	0.3492225	0.6068	0.544	1.02E-06	count	1
PRKAG1	0.2127659	0.539483	0.3944	0.693	1.02E-06	count	1
DDI2	0.2127659	0.6772533	0.3142	0.754	1.02E-06	count	1
UBE2J2	0.2127135	0.3782167	0.5624	0.574	1.02E-06	count	1
OSBPL11	0.2127659	0.4761832	0.4468	0.655	1.02E-06	count	1
EFCAB14	0.0821964	0.3620269	0.227	0.82	1.02E-06	count	1
LRRC59	0.2139857	0.3741115	0.572	0.568	1.03E-06	count	1
EZH1	0.2145554	0.5168239	0.4151	0.678	1.03E-06	count	1
TAOK1	0.2146882	0.4675967	0.4591	0.646	1.04E-06	count	1
KTI12	0.216236	0.4865826	0.4444	0.657	1.04E-06	count	1
LINS1	1.1130634	0.6250877	1.7807	0.0757	1.04E-06	count	1
NME2	0.5239353	0.9542262	0.5491	0.583	1.04E-06	count	1
ZNF263	0.5239353	0.817991	0.6405	0.522	1.04E-06	count	1
RUFY2	0.216236	0.5469807	0.3953	0.693	1.04E-06	count	1
SLC35C1	0.5239353	0.790433	0.6628	0.508	1.04E-06	count	1
MOB1B	0.5239353	0.9699158	0.5402	0.589	1.05E-06	count	1
PSEN1	0.2183573	0.4842292	0.4509	0.652	1.05E-06	count	1
TRIM33	0.0846074	0.3410797	0.2481	0.804	1.05E-06	count	1
NAA35	1.1130634	0.6092433	1.827	0.0684	1.05E-06	count	1
RABL3	0.2191914	0.6069155	0.3612	0.718	1.05E-06	count	1
GALNT10	0.2191914	0.6459374	0.3393	0.735	1.05E-06	count	1
HAUS6	1.1130634	0.6092433	1.827	0.0684	1.05E-06	count	1
KLHDC2	0.2186988	0.3610319	0.6058	0.545	1.05E-06	count	1
TMEM191B	0.2191914	0.6751045	0.3247	0.746	1.06E-06	count	1
KDM6A	0.2191914	0.5946057	0.3686	0.713	1.06E-06	count	1
RFFL	0.5293711	0.7642399	0.6927	0.489	1.06E-06	count	1
TFPI2	17.3508432	2554.353296	0.0068	0.995	1.06E-06	count	1
RNPEP	0.5293711	0.7025756	0.7535	0.452	1.06E-06	count	1
WWP2	0.5293711	0.7397135	0.7156	0.475	1.06E-06	count	1
WRB	1.1324815	0.6678384	1.6957	0.0906	1.06E-06	count	1
C17orf49	1.1324815	0.6678384	1.6957	0.0906	1.06E-06	count	1
PRR3	0.5293711	0.7747555	0.6833	0.495	1.06E-06	count	1
FBXL13	17.5046305	1905.605687	0.0092	0.993	1.06E-06	count	1
KCTD12	17.5046303	1905.605687	0.0092	0.993	1.06E-06	count	1
GABBR1	1.1324815	0.8029271	1.4104	0.159	1.06E-06	count	1
EHBP1L1	0.2205837	0.4480194	0.4924	0.623	1.06E-06	count	1
DZANK1	17.637823	1611.291806	0.0109	0.991	1.07E-06	count	1
HOMEZ	17.6378228	1611.291781	0.0109	0.991	1.07E-06	count	1

CCDC71	17.6379048	1724.805405	0.0102	0.992	1.07E-06	count	1
ANKRD44	0.0859945	0.1934135	0.4446	0.657	1.07E-06	count	1
AL135791.1	1.1324815	0.8424954	1.3442	0.18	1.07E-06	count	1
SBF1	17.7551237	1205.654101	0.0147	0.988	1.07E-06	count	1
GTPBP1	0.2218928	0.5246899	0.4229	0.673	1.07E-06	count	1
DENND4C	17.755215	1337.909579	0.0133	0.989	1.07E-06	count	1
ASXL2	1.1324815	0.7373384	1.5359	0.125	1.07E-06	count	1
CALHM2	17.755298	1458.234083	0.0122	0.99	1.07E-06	count	1
CARD11	0.2218928	0.5448704	0.4072	0.684	1.07E-06	count	1
BBS9	17.7555225	1863.440182	0.0095	0.992	1.07E-06	count	1
RBBP4	0.086319	0.249806	0.3455	0.73	1.07E-06	count	1
SKIV2L	17.8605315	1630.206896	0.011	0.991	1.08E-06	count	1
TRIM65	17.955516	1505.013755	0.0119	0.99	1.08E-06	count	1
TNFSF11	17.9555599	1593.801915	0.0113	0.991	1.08E-06	count	1
FCGR3B	17.7556831	2740.450917	0.0065	0.995	1.09E-06	count	1
ATP7A	0.5398267	0.8072558	0.6687	0.504	1.09E-06	count	1
WDR19	0.5398267	0.8072558	0.6687	0.504	1.09E-06	count	1
LRRC47	0.2242883	0.564799	0.3971	0.691	1.09E-06	count	1
CLSTN1	0.2242883	0.5565857	0.403	0.687	1.09E-06	count	1
NAAA	0.5369728	0.3772567	1.4234	0.155	1.09E-06	count	1
SLAIN1	0.5398267	0.8415948	0.6414	0.522	1.09E-06	count	1
PDK2	17.883211	2417.043677	0.0074	0.994	1.09E-06	count	1
MKL1	17.9929828	1638.890383	0.011	0.991	1.10E-06	count	1
SFR1	18.1220018	1535.02368	0.0118	0.991	1.10E-06	count	1
CENPL	18.1777488	1682.503654	0.0108	0.991	1.10E-06	count	1
CDCA4	0.2276406	0.5354442	0.4251	0.671	1.10E-06	count	1
LRRC23	1.1702218	1.0157118	1.1521	0.25	1.10E-06	count	1
EIF2B5	0.2270347	0.4346785	0.5223	0.602	1.10E-06	count	1
NFIC	0.2276406	0.5000438	0.4552	0.649	1.10E-06	count	1
ZNF699	1.1702218	0.8578476	1.3641	0.173	1.10E-06	count	1
ZBTB48	1.1702218	0.8578476	1.3641	0.173	1.10E-06	count	1
IFT22	1.1702218	0.8578476	1.3641	0.173	1.10E-06	count	1
EXD3	1.1702218	0.8977443	1.3035	0.193	1.10E-06	count	1
ENPP5	1.1702218	0.8977443	1.3035	0.193	1.10E-06	count	1
MTRF1	1.1702218	0.8977443	1.3035	0.193	1.10E-06	count	1
C12orf49	0.228227	0.413432	0.552	0.581	1.10E-06	count	1
BBS7	1.1702218	0.9359418	1.2503	0.212	1.11E-06	count	1
GBP3	0.2293444	0.8315	0.2758	0.783	1.11E-06	count	1
A1BG-AS1	0.2293444	0.7288488	0.3147	0.753	1.11E-06	count	1
PGD	0.2293444	0.70319	0.3261	0.744	1.11E-06	count	1
PAQR3	18.1958377	1647.557151	0.011	0.991	1.11E-06	count	1
PJA1	0.2293444	0.7713015	0.2973	0.766	1.11E-06	count	1
MXD1	0.5508807	0.7260119	0.7588	0.448	1.11E-06	count	1
TNRC6A	0.0891548	0.3061711	0.2912	0.771	1.11E-06	count	1
INPP5D	0.0894925	0.2547709	0.3513	0.726	1.11E-06	count	1

RAD17	0.2299249	0.4161214	0.5525	0.581	1.12E-06	count	1
BEND5	0.2309048	0.4313764	0.5353	0.593	1.12E-06	count	1
C5orf24	0.2315961	0.4816182	0.4809	0.631	1.12E-06	count	1
SPPL3	0.2309048	0.4106622	0.5623	0.574	1.12E-06	count	1
VBP1	0.0903242	0.2692891	0.3354	0.737	1.12E-06	count	1
CA2	18.2645126	1738.836592	0.0105	0.992	1.12E-06	count	1
ACTN1	18.0422718	2781.024864	0.0065	0.995	1.12E-06	count	1
SNX5	0.0910513	0.2431246	0.3745	0.708	1.13E-06	count	1
METTL14	0.2344856	0.4255725	0.551	0.582	1.13E-06	count	1
QKI	0.091585	0.3092995	0.2961	0.767	1.14E-06	count	1
IMPA1	0.2344856	0.4294977	0.546	0.585	1.14E-06	count	1
MAP3K3	0.2355068	0.6381024	0.3691	0.712	1.14E-06	count	1
TOE1	0.2355068	0.7017891	0.3356	0.737	1.14E-06	count	1
FBRS	0.0919448	0.3558549	0.2584	0.796	1.14E-06	count	1
ATP11B	0.2355068	0.670113	0.3514	0.725	1.14E-06	count	1
ZC3H12D	0.2355068	0.754939	0.312	0.755	1.14E-06	count	1
NOTCH2	0.2373478	0.6042163	0.3928	0.695	1.15E-06	count	1
BET1L	0.2391262	0.4857155	0.4923	0.623	1.16E-06	count	1
SLC2A1	0.238961	0.5125735	0.4662	0.641	1.16E-06	count	1
SMARCD2	0.2404156	0.6092882	0.3946	0.693	1.17E-06	count	1
AF117829.1	1.2065895	0.7882776	1.5307	0.127	1.17E-06	count	1
RAB30-AS1	0.0939663	0.358532	0.2621	0.793	1.17E-06	count	1
YARS2	0.2415745	0.5250046	0.4601	0.646	1.17E-06	count	1
ARMT1	0.2404156	0.5846662	0.4112	0.681	1.17E-06	count	1
AHCYL1	0.2415745	0.4978911	0.4852	0.628	1.18E-06	count	1
RFXANK	0.0946511	0.3953585	0.2394	0.811	1.18E-06	count	1
SMURF2	0.2415745	0.53999	0.4474	0.655	1.19E-06	count	1
TMED8	0.2441871	0.4733683	0.5159	0.606	1.19E-06	count	1
MMGT1	0.2439673	0.4739771	0.5147	0.607	1.19E-06	count	1
LINC00909	0.2441871	0.4918304	0.4965	0.62	1.19E-06	count	1
ZSCAN16	0.2446815	0.4866287	0.5028	0.615	1.19E-06	count	1
ANK3	0.2433707	0.549798	0.4427	0.658	1.19E-06	count	1
SUPT20H	0.2450878	0.4633134	0.529	0.597	1.19E-06	count	1
C12orf29	0.2453446	0.4636116	0.5292	0.597	1.19E-06	count	1
C22orf39	0.2450878	0.4381255	0.5594	0.576	1.19E-06	count	1
PGGT1B	0.2450878	0.4274814	0.5733	0.567	1.20E-06	count	1
JMY	0.2462354	0.4258694	0.5782	0.563	1.20E-06	count	1
ZNF335	0.5889782	0.8962515	0.6572	0.511	1.20E-06	count	1
NISCH	0.5889782	0.790937	0.7447	0.457	1.20E-06	count	1
FAHD2A	0.5889782	0.790937	0.7447	0.457	1.20E-06	count	1
NEDD4L	0.5889782	0.790937	0.7447	0.457	1.20E-06	count	1
AC005261.1	0.5889782	0.790937	0.7447	0.457	1.20E-06	count	1
AC132192.2	0.5889782	0.8509864	0.6921	0.489	1.21E-06	count	1
SPATA20	0.5889782	0.8509864	0.6921	0.489	1.21E-06	count	1
ACKR1	0.5889782	1.1385297	0.5173	0.605	1.21E-06	count	1

AP001816.1	0.5889782	1.1032502	0.5339	0.594	1.21E-06	count	1
LENG8-AS1	0.5931657	0.8298681	0.7148	0.475	1.21E-06	count	1
ZNF451	0.247993	0.3649664	0.6795	0.497	1.21E-06	count	1
MTERF1	0.5931657	0.7905442	0.7503	0.453	1.22E-06	count	1
ATR	0.5931657	0.6505418	0.9118	0.362	1.22E-06	count	1
MAN1A1	0.5931657	0.9260573	0.6405	0.522	1.22E-06	count	1
DVL3	0.5931657	0.7642121	0.7762	0.438	1.22E-06	count	1
IL17RA	0.5931657	0.8766831	0.6766	0.499	1.22E-06	count	1
CNOT3	0.5931657	0.787319	0.7534	0.452	1.22E-06	count	1
TOX4	0.0977676	0.2054268	0.4759	0.634	1.22E-06	count	1
IL15RA	0.5931657	0.7991397	0.7423	0.458	1.22E-06	count	1
NFATC1	0.0976367	0.4160639	0.2347	0.815	1.23E-06	count	1
RAB28	0.2540832	0.5198364	0.4888	0.625	1.24E-06	count	1
SSBP2	0.2543021	0.41073	0.6191	0.536	1.24E-06	count	1
TXNDC11	0.2543021	0.5260851	0.4834	0.629	1.24E-06	count	1
DTX2	0.254644	0.6330864	0.4022	0.688	1.24E-06	count	1
ZNF397	0.254644	0.7191622	0.3541	0.723	1.24E-06	count	1
CLASP2	0.2540832	0.5037078	0.5044	0.614	1.24E-06	count	1
AFG3L2	0.2540268	0.4051142	0.627	0.531	1.24E-06	count	1
WASL	0.2543021	0.56663	0.4488	0.654	1.24E-06	count	1
WDR3	0.2543021	0.5448561	0.4667	0.641	1.24E-06	count	1
TRPT1	0.2545106	0.4787388	0.5316	0.595	1.24E-06	count	1
TMEM267	0.254644	0.6943317	0.3667	0.714	1.24E-06	count	1
SENP2	0.2541469	0.4260612	0.5965	0.551	1.24E-06	count	1
ARID3A	0.2543021	0.5801828	0.4383	0.661	1.24E-06	count	1
PATJ	0.2543021	0.5034435	0.5051	0.614	1.24E-06	count	1
MAFF	0.2552531	0.5640786	0.4525	0.651	1.24E-06	count	1
FCHSD1	1.2755823	0.9602927	1.3283	0.185	1.25E-06	count	1
ATP6V1H	0.254644	0.4757519	0.5352	0.593	1.25E-06	count	1
NEIL1	0.2552531	0.6164337	0.4141	0.679	1.25E-06	count	1
IFIH1	0.2545611	0.3704674	0.6871	0.492	1.25E-06	count	1
LRRC58	0.254644	0.4691806	0.5427	0.588	1.25E-06	count	1
LARS	0.099906	0.2581232	0.387	0.699	1.25E-06	count	1
ARG2	1.2755823	0.836983	1.524	0.128	1.25E-06	count	1
HIST4H4	1.2755823	0.836983	1.524	0.128	1.25E-06	count	1
CDC37L1	0.2552531	0.5836666	0.4373	0.662	1.26E-06	count	1
AC073896.2	0.6121445	1.0827033	0.5654	0.572	1.26E-06	count	1
ZNF337-AS1	0.6121445	1.0827033	0.5654	0.572	1.26E-06	count	1
SCAMP1-AS1	0.6121445	1.1365026	0.5386	0.59	1.26E-06	count	1
ZNF74	0.6121445	1.4355437	0.4264	0.67	1.26E-06	count	1
P2RX1	0.6121445	1.3832363	0.4425	0.658	1.26E-06	count	1
MTRNR2L1	0.6121445	1.3832363	0.4425	0.658	1.26E-06	count	1
PBX4	1.2755823	0.8935202	1.4276	0.154	1.26E-06	count	1
ALS2	0.6121445	1.8510314	0.3307	0.741	1.26E-06	count	1
FRMD4B	0.6121445	1.8510314	0.3307	0.741	1.26E-06	count	1

GUCY1A1	0.6121445	1.8510314	0.3307	0.741	1.26E-06	count	1
RHEBL1	0.6121445	1.0817497	0.5659	0.572	1.26E-06	count	1
NCAPD2	0.6121445	1.1273744	0.543	0.587	1.26E-06	count	1
CCDC85C	0.6121445	1.1273744	0.543	0.587	1.26E-06	count	1
USP13	0.6121445	0.7373416	0.8302	0.407	1.26E-06	count	1
GLUL	0.2552531	1.2548249	0.2034	0.839	1.26E-06	count	1
SERINC5	0.6121445	0.8751043	0.6995	0.485	1.27E-06	count	1
CCDC174	0.1016306	0.2895778	0.351	0.726	1.27E-06	count	1
SRFBP1	0.102345	0.3025624	0.3383	0.735	1.28E-06	count	1
PNKP	0.2617712	0.3956806	0.6616	0.509	1.28E-06	count	1
SEMA4D	0.2626711	0.4563742	0.5756	0.565	1.29E-06	count	1
AEBP2	0.2638734	0.3919668	0.6732	0.501	1.30E-06	count	1
NUP153	0.2639427	0.4315247	0.6117	0.541	1.30E-06	count	1
DAAM1	0.104413	0.3123846	0.3342	0.738	1.31E-06	count	1
RIN3	0.2674037	0.5275186	0.5069	0.612	1.31E-06	count	1
TRIM25	1.2755823	1.6561922	0.7702	0.442	1.31E-06	count	1
SP4	0.2681261	0.5604607	0.4784	0.633	1.32E-06	count	1
WDR75	0.26827	0.3721615	0.7208	0.471	1.32E-06	count	1
MSH2	0.2678605	0.5600311	0.4783	0.633	1.32E-06	count	1
NTAN1	0.26827	0.3536702	0.7585	0.449	1.32E-06	count	1
APTX	0.2676209	0.5306909	0.5043	0.614	1.32E-06	count	1
H2AFX	0.2697057	0.488355	0.5523	0.581	1.32E-06	count	1
RNF5	0.1060968	0.2714476	0.3909	0.696	1.33E-06	count	1
RASA3	0.2676209	0.5172353	0.5174	0.605	1.33E-06	count	1
AP1M1	0.2711091	0.5367914	0.5051	0.614	1.33E-06	count	1
ZBTB4	0.2716552	0.5674937	0.4787	0.632	1.33E-06	count	1
C2CD5	0.2716552	0.6589526	0.4123	0.68	1.34E-06	count	1
NBPF14	0.2716552	0.5738863	0.4734	0.636	1.34E-06	count	1
PLEKHB1	0.6415501	0.6453866	0.9941	0.321	1.34E-06	count	1
CTU1	0.6415501	0.6453866	0.9941	0.321	1.34E-06	count	1
MAP3K12	0.2719568	0.4344086	0.626	0.532	1.34E-06	count	1
INF2	0.2716552	0.6462354	0.4204	0.674	1.35E-06	count	1
CARD8-AS1	0.2745624	0.4222036	0.6503	0.516	1.35E-06	count	1
AKAP11	0.1078463	0.2712988	0.3975	0.691	1.35E-06	count	1
OCEL1	0.2750403	0.4656037	0.5907	0.555	1.35E-06	count	1
PLIN2	0.2734851	0.3605976	0.7584	0.449	1.36E-06	count	1
ANKHD1	0.2761797	0.7415635	0.3724	0.71	1.36E-06	count	1
N4BP1	0.2761452	0.3866606	0.7142	0.475	1.36E-06	count	1
OSBPL9	0.2761452	0.4068546	0.6787	0.498	1.36E-06	count	1
TCTEX1D2	0.2780001	0.5170388	0.5377	0.591	1.37E-06	count	1
SPTBN1	0.2780001	0.439455	0.6326	0.527	1.37E-06	count	1
NF1	0.2773395	0.6611596	0.4195	0.675	1.37E-06	count	1
DTX3L	0.2761452	0.3987185	0.6926	0.489	1.37E-06	count	1
NDUFB6	0.1092728	0.2813943	0.3883	0.698	1.37E-06	count	1
PDPR	0.278725	0.6606932	0.4219	0.673	1.37E-06	count	1

GOLGA1	0.278725	0.6606932	0.4219	0.673	1.37E-06	count	1
SAMD12	0.278725	0.7668349	0.3635	0.716	1.37E-06	count	1
CDC25B	0.278725	0.6943589	0.4014	0.688	1.37E-06	count	1
SCARB2	0.278725	0.6943589	0.4014	0.688	1.37E-06	count	1
MAPK8	0.278725	0.6943589	0.4014	0.688	1.37E-06	count	1
EIF4EBP2	0.278725	0.52642	0.5295	0.597	1.37E-06	count	1
AC138150.1	0.278725	0.7252079	0.3843	0.701	1.37E-06	count	1
CNP	0.278725	0.7252079	0.3843	0.701	1.37E-06	count	1
WARS2	0.278725	0.6495011	0.4291	0.668	1.37E-06	count	1
FAM120B	0.278725	0.6495011	0.4291	0.668	1.37E-06	count	1
PIGL	0.278725	0.6480934	0.4301	0.667	1.37E-06	count	1
ALKBH2	0.278725	0.6837181	0.4077	0.684	1.37E-06	count	1
CDK5RAP2	0.278725	0.4213396	0.6615	0.509	1.37E-06	count	1
CXCL16	0.278725	0.7163025	0.3891	0.697	1.38E-06	count	1
CHIC1	0.278725	0.7150263	0.3898	0.697	1.38E-06	count	1
SLC2A4RG	0.109862	0.3672973	0.2991	0.765	1.38E-06	count	1
ZNF235	1.3837941	1.0915681	1.2677	0.206	1.38E-06	count	1
PFKM	1.3837941	1.0915681	1.2677	0.206	1.38E-06	count	1
USP27X	1.3837941	1.0915681	1.2677	0.206	1.38E-06	count	1
ABCA7	1.3837941	1.0915681	1.2677	0.206	1.38E-06	count	1
APTR	1.3837941	1.0915681	1.2677	0.206	1.38E-06	count	1
TMEM143	1.3837941	1.4001102	0.9883	0.324	1.38E-06	count	1
TCP11L1	1.3837941	1.4001102	0.9883	0.324	1.38E-06	count	1
SHLD3	1.3837941	1.4001102	0.9883	0.324	1.38E-06	count	1
GEMIN5	1.3837941	1.4001102	0.9883	0.324	1.38E-06	count	1
PIM2	0.1105735	0.2286878	0.4835	0.629	1.38E-06	count	1
KCTD18	0.661451	0.8260209	0.8008	0.424	1.39E-06	count	1
ZNF354A	1.3708925	0.9724444	1.4097	0.159	1.39E-06	count	1
9-Mar	0.1108352	0.1832529	0.6048	0.546	1.39E-06	count	1
STRN3	0.2810722	0.3985383	0.7053	0.481	1.39E-06	count	1
ZNF431	0.2812883	0.4307107	0.6531	0.514	1.39E-06	count	1
ISOC2	0.2838907	0.5535153	0.5129	0.608	1.40E-06	count	1
PPP3CB	0.2826679	0.4626912	0.6109	0.542	1.40E-06	count	1
RBBP5	1.3708925	0.9453163	1.4502	0.148	1.40E-06	count	1
NPHP3	0.661451	0.8148537	0.8117	0.417	1.40E-06	count	1
ODF2	0.2838907	0.4963412	0.572	0.568	1.40E-06	count	1
ACAT2	0.2845175	0.3665906	0.7761	0.438	1.41E-06	count	1
ZMYM2	0.111854	0.2732429	0.4094	0.682	1.41E-06	count	1
CEBPZOS	0.2845175	0.4418921	0.6439	0.52	1.41E-06	count	1
BLOC1S6	0.111854	0.2861461	0.3909	0.696	1.41E-06	count	1
PYROXD1	0.2871116	0.4786661	0.5998	0.549	1.42E-06	count	1
BTBD6	0.2871116	0.4364269	0.6579	0.511	1.42E-06	count	1
CA11	0.6714097	0.8328372	0.8062	0.421	1.42E-06	count	1
SACS	0.2871116	0.5951315	0.4824	0.63	1.43E-06	count	1
BOLA3	0.2890278	0.4211041	0.6864	0.493	1.43E-06	count	1

RABEP2	0.2881213	0.3740511	0.7703	0.442	1.43E-06	count	1
SLC52A2	0.2890278	0.3937069	0.7341	0.463	1.43E-06	count	1
SP140L	0.1141285	0.2529499	0.4512	0.652	1.43E-06	count	1
CHMP3	0.2873376	0.41148	0.6983	0.485	1.44E-06	count	1
ZMYM5	0.11478	0.2961398	0.3876	0.699	1.44E-06	count	1
MGAT1	0.2881539	0.5151439	0.5594	0.576	1.44E-06	count	1
ZNF160	0.2916482	0.6187372	0.4714	0.638	1.45E-06	count	1
DNM2	0.2927372	0.4763848	0.6145	0.539	1.45E-06	count	1
PNP	0.2925127	0.4079635	0.717	0.474	1.45E-06	count	1
CHD1L	0.2916482	0.6975493	0.4181	0.676	1.45E-06	count	1
ZNF628	0.6864523	0.9633314	0.7126	0.476	1.45E-06	count	1
CLCN3	0.2925127	0.5379592	0.5437	0.587	1.45E-06	count	1
MYEF2	0.2939141	0.6939993	0.4235	0.672	1.45E-06	count	1
STX2	0.6864523	0.8845084	0.7761	0.438	1.46E-06	count	1
ZNF525	0.6864523	0.8845084	0.7761	0.438	1.46E-06	count	1
SNX27	0.2939141	0.5797988	0.5069	0.612	1.46E-06	count	1
CYP2R1	0.6864523	0.8108878	0.8465	0.398	1.46E-06	count	1
UNG	0.6864523	0.8973078	0.765	0.445	1.46E-06	count	1
SLC25A25	0.2939141	0.6286256	0.4676	0.64	1.46E-06	count	1
ZC3H3	0.6864523	0.9761064	0.7033	0.482	1.46E-06	count	1
ZDHHC5	0.2939141	0.458057	0.6417	0.521	1.46E-06	count	1
YIF1B	0.2939141	0.673924	0.4361	0.663	1.46E-06	count	1
HINFP	0.2939141	0.673924	0.4361	0.663	1.46E-06	count	1
GDI1	0.2927372	0.4370981	0.6697	0.503	1.46E-06	count	1
CEP126	0.2931192	0.5393717	0.5434	0.587	1.46E-06	count	1
SOCS4	0.294412	0.3817332	0.7713	0.441	1.46E-06	count	1
CSNK1D	0.1162252	0.2587573	0.4492	0.654	1.46E-06	count	1
MDS2	0.2939141	0.6954677	0.4226	0.673	1.46E-06	count	1
C19orf48	0.2939141	0.3671448	0.8005	0.424	1.46E-06	count	1
FOXRED1	0.6864523	1.0823214	0.6342	0.526	1.46E-06	count	1
ACAA1	0.2959591	0.4321738	0.6848	0.494	1.47E-06	count	1
CXCL10	1.3243725	1.0620328	1.247	0.213	1.47E-06	count	1
PRKCSH	0.2951754	0.3604053	0.819	0.413	1.47E-06	count	1
ABHD13	0.2959591	0.4216685	0.7019	0.483	1.47E-06	count	1
ARFGEF1	0.1176429	0.347084	0.3389	0.735	1.48E-06	count	1
SOCS3	0.2982628	0.501653	0.5946	0.552	1.48E-06	count	1
PUM3	0.1180791	0.2210805	0.5341	0.594	1.48E-06	count	1
INTS12	1.4007455	0.7532604	1.8596	0.0636	1.49E-06	count	1
ABCD4	0.2986898	0.5451125	0.5479	0.584	1.49E-06	count	1
MYL5	0.7003537	0.8793689	0.7964	0.426	1.49E-06	count	1
STX6	0.2995431	0.4395658	0.6815	0.496	1.50E-06	count	1
CNOT11	0.2982628	0.4534132	0.6578	0.511	1.50E-06	count	1
CCDC84	0.3003302	0.5191231	0.5785	0.563	1.50E-06	count	1
MYO5A	0.3016859	0.6215494	0.4854	0.628	1.50E-06	count	1
SH2B3	0.2973865	0.5522051	0.5385	0.59	1.50E-06	count	1

TRAPPC12	0.3016859	0.5361623	0.5627	0.574	1.51E-06	count	1
TRIM69	0.3019385	0.3741457	0.807	0.42	1.51E-06	count	1
HACD2	0.3042786	0.7299073	0.4169	0.677	1.51E-06	count	1
CARHSP1	0.12029	0.3987301	0.3017	0.763	1.51E-06	count	1
AC091271.1	0.3045744	0.7671838	0.397	0.692	1.51E-06	count	1
GCSAM	0.3042786	0.584334	0.5207	0.603	1.51E-06	count	1
ANKRD36	0.3042786	0.5411413	0.5623	0.574	1.51E-06	count	1
SNRPB2	0.1205175	0.1812867	0.6648	0.507	1.52E-06	count	1
SCML4	0.3042786	0.5780198	0.5264	0.599	1.52E-06	count	1
PDE4DIP	0.3042786	0.5313791	0.5726	0.567	1.52E-06	count	1
ZNF789	0.3042786	0.5716359	0.5323	0.595	1.52E-06	count	1
STXBP5	0.7118861	1.1686987	0.6091	0.543	1.52E-06	count	1
AC016727.1	0.7118861	1.1686987	0.6091	0.543	1.52E-06	count	1
ZNF784	0.7118861	1.1686987	0.6091	0.543	1.52E-06	count	1
DPP3	0.7118861	0.9386978	0.7584	0.449	1.52E-06	count	1
DENND6A	0.7118861	0.9004806	0.7906	0.43	1.52E-06	count	1
WBP1L	0.7118861	0.9004806	0.7906	0.43	1.52E-06	count	1
AQP3	0.7118861	1.0578308	0.673	0.501	1.52E-06	count	1
AC144652.1	0.7118861	1.0578308	0.673	0.501	1.52E-06	count	1
ARHGAP21	0.7118861	1.0240692	0.6952	0.487	1.52E-06	count	1
MAP3K5	0.7118861	1.0240692	0.6952	0.487	1.52E-06	count	1
MORC4	0.7118861	1.0240692	0.6952	0.487	1.52E-06	count	1
RALGPS1	0.7118861	1.0240692	0.6952	0.487	1.52E-06	count	1
NBPF11	0.7118861	1.0240692	0.6952	0.487	1.52E-06	count	1
PITPNC1	0.7118861	1.0240692	0.6952	0.487	1.52E-06	count	1
KCTD10	0.7118861	1.0240692	0.6952	0.487	1.52E-06	count	1
AC036176.1	0.7118861	1.0240692	0.6952	0.487	1.52E-06	count	1
MON1B	0.7118861	1.0240692	0.6952	0.487	1.52E-06	count	1
KLHDC10	0.7118861	1.0240692	0.6952	0.487	1.52E-06	count	1
VPS18	0.7118861	1.0240692	0.6952	0.487	1.52E-06	count	1
NOL10	0.3042786	0.5867481	0.5186	0.604	1.52E-06	count	1
UBE2E1	0.1212378	0.3193079	0.3797	0.704	1.53E-06	count	1
LRRC57	0.7118861	0.8107666	0.878	0.38	1.53E-06	count	1
RABIF	0.3065365	0.364781	0.8403	0.401	1.53E-06	count	1
WDR1	0.1218564	0.3071417	0.3967	0.692	1.53E-06	count	1
CCAR2	1.4579039	0.8652484	1.685	0.0927	1.54E-06	count	1
SGK3	0.7118861	0.7603011	0.9363	0.35	1.54E-06	count	1
NUTF2	0.1220776	0.321886	0.3793	0.705	1.54E-06	count	1
FAM20B	0.3091216	0.8311543	0.3719	0.71	1.54E-06	count	1
KDSR	0.3091216	0.5246578	0.5892	0.556	1.54E-06	count	1
TRMT10A	0.3091216	0.9009203	0.3431	0.732	1.54E-06	count	1
EID2	0.3091216	0.5199592	0.5945	0.552	1.55E-06	count	1
EBI3	1.4579039	1.0244599	1.4231	0.155	1.55E-06	count	1
YEATS4	0.1231646	0.3647473	0.3377	0.736	1.55E-06	count	1
DERL3	0.1234039	0.3345371	0.3689	0.712	1.55E-06	count	1

ECPAS	0.311023	0.5970713	0.5209	0.603	1.56E-06	count	1
RINT1	0.311804	0.542702	0.5745	0.566	1.56E-06	count	1
SCAMP4	0.311804	0.542702	0.5745	0.566	1.56E-06	count	1
SLC4A1AP	0.3104236	0.396584	0.7827	0.434	1.56E-06	count	1
JAK3	0.314853	0.5209814	0.6043	0.546	1.57E-06	count	1
ARFGEF2	0.3148669	0.5934018	0.5306	0.596	1.57E-06	count	1
KAT2B	0.3148669	0.5824019	0.5406	0.589	1.57E-06	count	1
PTGS2	1.4579039	1.3290683	1.0969	0.273	1.58E-06	count	1
MCCC2	0.314853	0.524446	0.6004	0.549	1.58E-06	count	1
MKLN1	0.3148469	0.4893366	0.6434	0.52	1.58E-06	count	1
TAF4B	0.3148669	0.6577271	0.4787	0.632	1.58E-06	count	1
WASHC1	0.7326579	0.8154432	0.8985	0.369	1.58E-06	count	1
MARK2	0.314853	0.5197519	0.6058	0.545	1.58E-06	count	1
ATG16L1	0.7326579	0.8688835	0.8432	0.4	1.58E-06	count	1
NUP155	0.7326579	0.8688835	0.8432	0.4	1.58E-06	count	1
AGMAT	0.7326579	0.8688835	0.8432	0.4	1.58E-06	count	1
DYNC1LI2	0.3164825	0.383097	0.8261	0.409	1.58E-06	count	1
WDR83	0.7326579	0.7060735	1.0377	0.3	1.58E-06	count	1
CDK5	0.7326579	0.7671705	0.955	0.34	1.58E-06	count	1
FBXO5	0.7326579	0.7606053	0.9633	0.336	1.59E-06	count	1
ZNF563	0.7326579	0.7606053	0.9633	0.336	1.59E-06	count	1
MIEF1	0.7326579	0.8114807	0.9029	0.367	1.59E-06	count	1
STRIP1	0.7326579	0.8114807	0.9029	0.367	1.59E-06	count	1
SEC24A	0.7326579	0.9102156	0.8049	0.421	1.59E-06	count	1
AKAP2	0.7326579	0.8593495	0.8526	0.394	1.59E-06	count	1
TDRD3	0.3175171	0.48164	0.6592	0.51	1.59E-06	count	1
HMOX2	0.1262327	0.3083054	0.4094	0.682	1.59E-06	count	1
LGMN	0.3175171	0.4680486	0.6784	0.498	1.59E-06	count	1
ANAPC7	0.7326579	0.904689	0.8098	0.418	1.59E-06	count	1
ZBTB44	0.3186806	0.4215134	0.756	0.45	1.59E-06	count	1
ELP2	0.3182161	0.4040134	0.7876	0.431	1.60E-06	count	1
VPS13A	0.1266168	0.4134149	0.3063	0.76	1.60E-06	count	1
UCKL1	0.3182161	0.4324401	0.7359	0.462	1.60E-06	count	1
SHF	0.3194069	0.7322376	0.4362	0.663	1.60E-06	count	1
BAG6	0.3194069	0.465536	0.6861	0.493	1.60E-06	count	1
AC008124.1	0.3194069	0.64415	0.4959	0.62	1.60E-06	count	1
ZNF768	0.3194069	0.7227099	0.442	0.659	1.60E-06	count	1
SNX19	0.3194069	0.6593734	0.4844	0.628	1.60E-06	count	1
LDB1	0.3194069	0.5086056	0.628	0.53	1.60E-06	count	1
RIPOR1	0.3194069	0.4959887	0.644	0.52	1.60E-06	count	1
OSBPL10	0.127338	0.2676539	0.4758	0.634	1.61E-06	count	1
AL445686.2	0.3194069	0.774025	0.4127	0.68	1.61E-06	count	1
TIPRL	0.1277079	0.2815009	0.4537	0.65	1.61E-06	count	1
ANKRD13D	0.3211099	0.4054122	0.7921	0.429	1.62E-06	count	1
RNF41	0.3219566	0.3868383	0.8323	0.406	1.62E-06	count	1

FBXO22	0.3231128	0.4644398	0.6957	0.487	1.62E-06	count	1
FAM76B	0.1281812	0.32424	0.3953	0.693	1.62E-06	count	1
RFC2	0.3220024	0.4857902	0.6628	0.508	1.62E-06	count	1
AKAP7	0.3220024	0.5140864	0.6264	0.531	1.63E-06	count	1
CNOT9	0.3236225	0.3974203	0.8143	0.416	1.63E-06	count	1
RCOR1	0.3231128	0.4598796	0.7026	0.483	1.64E-06	count	1
CASD1	0.3256175	0.5546775	0.587	0.557	1.64E-06	count	1
RCC1L	0.3261877	0.5699667	0.5723	0.567	1.64E-06	count	1
PAOX	0.3261877	0.5933073	0.5498	0.583	1.64E-06	count	1
C22orf46	0.3261877	0.6112069	0.5337	0.594	1.64E-06	count	1
HIST1H2AC	0.3261877	0.6060086	0.5383	0.591	1.64E-06	count	1
CEP97	0.3269895	0.6493493	0.5036	0.615	1.65E-06	count	1
NFIL3	0.3285934	0.4323275	0.7601	0.448	1.65E-06	count	1
ANKRD40	0.3287803	0.4157997	0.7907	0.43	1.65E-06	count	1
FBXO8	1.5379466	0.9420053	1.6326	0.103	1.66E-06	count	1
ELP6	0.3287803	0.429115	0.7662	0.444	1.66E-06	count	1
P2RX5	0.3306052	0.3937695	0.8396	0.402	1.66E-06	count	1
POM121	0.330996	0.5543543	0.5971	0.551	1.67E-06	count	1
EGLN1	0.330996	0.5914721	0.5596	0.576	1.67E-06	count	1
WHAMM	0.1317936	0.2947826	0.4471	0.655	1.67E-06	count	1
RAE1	0.330996	0.5314963	0.6228	0.534	1.67E-06	count	1
CCND1	0.3308633	0.5692929	0.5812	0.561	1.68E-06	count	1
XPNPEP3	0.7671149	0.8765119	0.8752	0.382	1.68E-06	count	1
SLC2A5	0.7671149	0.8815181	0.8702	0.385	1.68E-06	count	1
STRADA	0.3339061	0.4646676	0.7186	0.473	1.68E-06	count	1
AC104794.2	0.7671149	0.9090023	0.8439	0.399	1.68E-06	count	1
TBC1D1	0.1330687	0.3028012	0.4395	0.661	1.68E-06	count	1
MPDU1	0.3339061	0.4894052	0.6823	0.495	1.68E-06	count	1
SH3YL1	0.3329114	0.3675385	0.9058	0.366	1.69E-06	count	1
IVD	0.3356041	0.4554521	0.7369	0.462	1.70E-06	count	1
TMOD2	0.3345837	0.5628698	0.5944	0.553	1.70E-06	count	1
AL662844.4	0.3363598	0.5379902	0.6252	0.532	1.70E-06	count	1
AC005921.2	0.3363598	0.4634247	0.7258	0.468	1.70E-06	count	1
HAGHL	0.3373635	0.498553	0.6767	0.499	1.71E-06	count	1
PCGF5	0.1350226	0.2888574	0.4674	0.64	1.71E-06	count	1
CSPP1	0.3373635	0.4726333	0.7138	0.476	1.71E-06	count	1
SMNDC1	0.135162	0.2464203	0.5485	0.584	1.71E-06	count	1
USP1	0.1352707	0.237765	0.5689	0.57	1.71E-06	count	1
ARID5A	0.1350226	0.337941	0.3995	0.69	1.71E-06	count	1
TIMM17A	0.1355391	0.3247157	0.4174	0.677	1.72E-06	count	1
PHF1	0.1369143	0.2732365	0.5011	0.617	1.73E-06	count	1
TBC1D15	0.3428961	0.4205243	0.8154	0.415	1.74E-06	count	1
BICD1	0.3427515	0.4059153	0.8444	0.399	1.74E-06	count	1
RAB7A	0.1375943	0.2043965	0.6732	0.501	1.74E-06	count	1
ARFRP1	0.3427515	0.4025346	0.8515	0.395	1.75E-06	count	1

RTN3	0.3441678	0.4480802	0.7681	0.443	1.75E-06	count	1
IREB2	0.3400736	0.4622715	0.7357	0.462	1.75E-06	count	1
DOCK11	0.138307	0.359217	0.385	0.7	1.76E-06	count	1
RSPRY1	1.5632644	0.661869	2.3619	0.0186	1.76E-06	count	1
APOO	0.7955109	0.7578548	1.0497	0.294	1.76E-06	count	1
RAP1B	0.1391132	0.1408048	0.988	0.324	1.77E-06	count	1
MPC1	0.1387274	0.3773221	0.3677	0.713	1.77E-06	count	1
HINT3	0.7955109	0.8806831	0.9033	0.367	1.77E-06	count	1
SLU7	0.1395821	0.2716727	0.5138	0.608	1.77E-06	count	1
PIGG	0.3497741	0.6797151	0.5146	0.607	1.77E-06	count	1
AP5Z1	0.3497741	0.6611928	0.529	0.597	1.77E-06	count	1
UGGT1	0.3497741	0.6611928	0.529	0.597	1.77E-06	count	1
AC096733.2	0.7955109	0.880588	0.9034	0.367	1.77E-06	count	1
ASB7	0.7955109	0.880588	0.9034	0.367	1.77E-06	count	1
CDS2	0.3490068	0.4034293	0.8651	0.387	1.78E-06	count	1
KAT6A	0.3516192	0.3836509	0.9165	0.36	1.78E-06	count	1
METTL18	0.3515144	0.6547163	0.5369	0.592	1.78E-06	count	1
TNFAIP6	1.6120546	2.1278322	0.7576	0.449	1.78E-06	count	1
CHSY1	0.3515144	0.6422505	0.5473	0.584	1.79E-06	count	1
CD180	0.3521659	0.462494	0.7614	0.447	1.79E-06	count	1
RPE	0.347029	0.6596335	0.5261	0.599	1.79E-06	count	1
GRAMD1A	0.3527165	0.6145955	0.5739	0.566	1.79E-06	count	1
CETN2	0.3527165	0.6188327	0.57	0.569	1.79E-06	count	1
VARS	0.3527165	0.5762843	0.6121	0.541	1.80E-06	count	1
MSANTD3	1.6120546	0.834096	1.9327	0.0539	1.80E-06	count	1
AKT3	0.3531882	0.424251	0.8325	0.406	1.80E-06	count	1
MRPL50	0.141776	0.3387321	0.4185	0.676	1.80E-06	count	1
JPT2	0.3535967	0.5959831	0.5933	0.553	1.80E-06	count	1
DCAF16	0.354269	0.5475383	0.647	0.518	1.80E-06	count	1
ORC6	0.8120012	0.8758116	0.9271	0.354	1.80E-06	count	1
PLCE1	0.8120012	1.0060471	0.8071	0.42	1.80E-06	count	1
LENG8	0.3527165	0.6451886	0.5467	0.585	1.80E-06	count	1
COLGALT1	0.8120012	0.9754368	0.8324	0.406	1.80E-06	count	1
FAM122A	0.354269	0.5434211	0.6519	0.515	1.80E-06	count	1
HSCB	0.3527165	0.4208027	0.8382	0.402	1.80E-06	count	1
HSD17B7	0.3535967	0.6196983	0.5706	0.569	1.81E-06	count	1
FAM122C	0.8120012	0.867031	0.9365	0.35	1.81E-06	count	1
CCDC86	0.8120012	1.1144112	0.7286	0.467	1.81E-06	count	1
MPHOSPH10	0.1426599	0.3078056	0.4635	0.643	1.81E-06	count	1
SLC35D2	0.3558798	0.4151063	0.8573	0.392	1.81E-06	count	1
PPP6C	0.1430061	0.3179925	0.4497	0.653	1.81E-06	count	1
NAA50	0.1430932	0.2379147	0.6014	0.548	1.82E-06	count	1
NAP1L4	0.1432332	0.28455	0.5034	0.615	1.82E-06	count	1
GAPVD1	0.3561331	0.3823001	0.9316	0.352	1.82E-06	count	1
GNB1	0.1429533	0.2811402	0.5085	0.611	1.82E-06	count	1

ZNF420	1.6120546	0.9519625	1.6934	0.0911	1.82E-06	count	1
TMEM129	0.816245	0.6942109	1.1758	0.24	1.84E-06	count	1
CLCF1	0.3671885	0.4739194	0.7748	0.439	1.88E-06	count	1
RBMX	0.1477909	0.1677271	0.8811	0.379	1.88E-06	count	1
RAB18	0.3661392	0.351283	1.0423	0.298	1.88E-06	count	1
CD58	0.3690811	0.3902431	0.9458	0.345	1.88E-06	count	1
MANBAL	0.8385374	0.8365882	1.0023	0.317	1.89E-06	count	1
MIR4435-2HG	0.8385374	1.0058855	0.8336	0.405	1.89E-06	count	1
PLEKHM1	0.8385374	0.8041957	1.0427	0.298	1.89E-06	count	1
SNRPN	0.148955	0.2278554	0.6537	0.514	1.89E-06	count	1
NUDT6	0.8385374	0.9932103	0.8443	0.399	1.90E-06	count	1
TCOF1	0.3702242	0.488653	0.7576	0.449	1.90E-06	count	1
NDUFS5	0.1500226	0.175953	0.8526	0.394	1.91E-06	count	1
GINS4	0.3741416	0.7252035	0.5159	0.606	1.91E-06	count	1
PLEKHA1	0.3748686	0.5640488	0.6646	0.507	1.92E-06	count	1
RMND5A	0.3752139	0.4760977	0.7881	0.431	1.92E-06	count	1
AUP1	0.1510186	0.2529919	0.5969	0.551	1.93E-06	count	1
ANKRA2	0.37707	0.534625	0.7053	0.481	1.93E-06	count	1
SPOPL	0.37707	0.493526	0.764	0.445	1.93E-06	count	1
HOTAIRM1	0.3752139	0.4677704	0.8021	0.423	1.93E-06	count	1
LTBP4	0.37707	0.5366388	0.7027	0.483	1.94E-06	count	1
NLRC5	0.3774369	0.5473276	0.6896	0.491	1.94E-06	count	1
ATG4D	0.8567517	0.8019927	1.0683	0.286	1.94E-06	count	1
MTG2	0.8567517	0.8019927	1.0683	0.286	1.94E-06	count	1
ZNF354B	1.6810474	0.8675613	1.9377	0.0533	1.94E-06	count	1
DMWD	0.3774369	0.6415728	0.5883	0.557	1.94E-06	count	1
APOM	0.8567517	0.8426929	1.0167	0.31	1.94E-06	count	1
BTBD10	0.3794157	0.5187777	0.7314	0.465	1.95E-06	count	1
CTDP1	0.8567517	0.9139499	0.9374	0.349	1.95E-06	count	1
NR3C2	0.8567517	0.9139499	0.9374	0.349	1.95E-06	count	1
ZNF140	0.8567517	0.9139499	0.9374	0.349	1.95E-06	count	1
ITGAM	0.8567517	0.787017	1.0886	0.277	1.95E-06	count	1
ATP6AP2	0.1529533	0.1909842	0.8009	0.424	1.95E-06	count	1
PLXNC1	0.3794157	0.5872832	0.6461	0.519	1.95E-06	count	1
PPP6R2	0.3794157	0.3863656	0.982	0.327	1.96E-06	count	1
CCZ1B	0.8567517	0.9372549	0.9141	0.361	1.96E-06	count	1
SEPSECS	0.3810172	0.6729525	0.5662	0.572	1.96E-06	count	1
GABPB1	0.1544759	0.2925364	0.5281	0.598	1.97E-06	count	1
LRRFIP2	0.1544883	0.4188391	0.3688	0.712	1.97E-06	count	1
RAD23B	0.1544611	0.3229603	0.4783	0.633	1.97E-06	count	1
GTF2IRD2	0.3824744	0.6298701	0.6072	0.544	1.97E-06	count	1
MFSD14B	0.3824744	0.5393171	0.7092	0.479	1.97E-06	count	1
PARVB	0.3843809	0.4143221	0.9277	0.354	1.98E-06	count	1
MAD2L1BP	0.3843809	0.4305931	0.8927	0.373	1.98E-06	count	1
PRRC2A	0.3839572	0.4855383	0.7908	0.429	1.99E-06	count	1

ANKRD42	0.3866288	0.6867999	0.5629	0.574	1.99E-06	count	1
MPEG1	0.3866288	0.5893638	0.656	0.512	1.99E-06	count	1
BRMS1L	0.3866288	0.610924	0.6329	0.527	1.99E-06	count	1
TMEM87B	0.3839572	0.4615066	0.832	0.406	1.99E-06	count	1
ST8SIA4	0.1563894	0.2345933	0.6666	0.505	1.99E-06	count	1
PTAR1	0.3863524	0.5568682	0.6938	0.488	2.00E-06	count	1
SNX11	0.3865122	0.4645398	0.832	0.406	2.00E-06	count	1
BCOR	0.8822358	0.9178247	0.9612	0.337	2.01E-06	count	1
ABCB1	1.7753967	1.075989	1.65	0.0996	2.01E-06	count	1
ZNF667	1.7753967	1.075989	1.65	0.0996	2.01E-06	count	1
BST1	1.7753967	2.4245418	0.7323	0.464	2.01E-06	count	1
RAB32	1.7753967	2.4245418	0.7323	0.464	2.01E-06	count	1
XXYLT1-AS2	1.7753967	2.4245418	0.7323	0.464	2.01E-06	count	1
ACOT9	0.389319	0.5718089	0.6809	0.496	2.01E-06	count	1
CD38	0.8822358	1.170754	0.7536	0.452	2.02E-06	count	1
SUPT7L	0.392596	0.7257438	0.541	0.589	2.02E-06	count	1
TECPR1	0.392596	0.7652339	0.513	0.608	2.02E-06	count	1
SNAPC3	0.392596	0.5931645	0.6619	0.508	2.02E-06	count	1
DCTN1	0.392596	0.6995954	0.5612	0.575	2.02E-06	count	1
DOHH	0.392596	0.7267673	0.5402	0.589	2.03E-06	count	1
CNOT8	0.1588609	0.3554389	0.4469	0.655	2.03E-06	count	1
COQ6	0.392596	0.8027838	0.489	0.625	2.03E-06	count	1
CSF3R	0.392596	1.1652251	0.3369	0.736	2.03E-06	count	1
SDF2	0.1597403	0.2786903	0.5732	0.567	2.04E-06	count	1
IGHMBP2	0.8940539	1.2459816	0.7175	0.473	2.05E-06	count	1
C17orf107	0.8940539	1.2459816	0.7175	0.473	2.05E-06	count	1
SERAC1	0.8940539	1.2004016	0.7448	0.457	2.05E-06	count	1
ACVR1	0.8940539	1.1811811	0.7569	0.45	2.05E-06	count	1
TTC4	0.8940539	1.1811811	0.7569	0.45	2.05E-06	count	1
IL5RA	0.8940539	1.1811811	0.7569	0.45	2.05E-06	count	1
KIFC2	0.8940539	1.1811811	0.7569	0.45	2.05E-06	count	1
MAPRE3	0.8940539	1.1234486	0.7958	0.427	2.05E-06	count	1
MAN2C1	0.8940539	1.1234486	0.7958	0.427	2.05E-06	count	1
ASRGL1	0.8940539	1.4746698	0.6063	0.545	2.05E-06	count	1
RSRC1	0.1608325	0.291698	0.5514	0.582	2.06E-06	count	1
AP1S3	0.3962548	0.503176	0.7875	0.431	2.06E-06	count	1
CCDC32	0.1610312	0.3018963	0.5334	0.594	2.06E-06	count	1
DGKD	0.1613396	0.3851508	0.4189	0.675	2.06E-06	count	1
PPAT	0.3987692	0.6190879	0.6441	0.52	2.07E-06	count	1
TTC32	0.398266	0.4695958	0.8481	0.397	2.07E-06	count	1
PDPK1	0.3962548	0.4510386	0.8785	0.38	2.07E-06	count	1
GADD45A	0.3987692	0.6363041	0.6267	0.531	2.07E-06	count	1
TSSC4	0.1624442	0.3124331	0.5199	0.603	2.08E-06	count	1
DENND4B	0.9027151	0.8416987	1.0725	0.284	2.08E-06	count	1
ALG10	0.4018913	0.9077915	0.4427	0.658	2.08E-06	count	1

MAFK	0.3987692	0.5434014	0.7338	0.463	2.08E-06	count	1
ZHX2	0.9027151	0.7721245	1.1691	0.243	2.08E-06	count	1
UROD	0.4018913	0.5198271	0.7731	0.44	2.08E-06	count	1
BEX4	0.162947	0.3186374	0.5114	0.609	2.08E-06	count	1
MRPS26	0.1623831	0.2860689	0.5676	0.571	2.08E-06	count	1
C18orf25	0.4019989	0.5138388	0.7823	0.434	2.09E-06	count	1
BCKDHB	0.4018913	0.4390068	0.9155	0.36	2.09E-06	count	1
PITHD1	0.1636269	0.2549608	0.6418	0.521	2.09E-06	count	1
HKR1	0.9093577	1.1918574	0.763	0.446	2.09E-06	count	1
ZSCAN2	0.9093577	1.1918574	0.763	0.446	2.09E-06	count	1
DET1	0.9093577	1.1918574	0.763	0.446	2.09E-06	count	1
GAB2	0.9093577	0.9011623	1.0091	0.313	2.09E-06	count	1
EML3	0.9093577	0.9933745	0.9154	0.36	2.10E-06	count	1
AL117339.5	0.9093577	0.9933745	0.9154	0.36	2.10E-06	count	1
PHC1	0.9093577	0.8625302	1.0543	0.292	2.10E-06	count	1
PLAG1	0.9093577	0.8625302	1.0543	0.292	2.10E-06	count	1
GBP2	0.3976222	0.5848434	0.6799	0.497	2.10E-06	count	1
KLF8	0.9093577	1.0777256	0.8438	0.399	2.10E-06	count	1
TBL3	0.9093577	1.0777256	0.8438	0.399	2.10E-06	count	1
AC022706.1	0.9093577	1.0777256	0.8438	0.399	2.10E-06	count	1
RUSC1	0.9093577	0.9584664	0.9488	0.343	2.10E-06	count	1
FAM200A	0.9093577	0.9584664	0.9488	0.343	2.10E-06	count	1
ZNF746	0.9093577	0.9584664	0.9488	0.343	2.10E-06	count	1
SLC25A53	0.9093577	0.9584664	0.9488	0.343	2.10E-06	count	1
LINC02256	0.9093577	0.9584664	0.9488	0.343	2.10E-06	count	1
AMMECR1L	0.9093577	0.9584664	0.9488	0.343	2.10E-06	count	1
LZTS2	0.9093577	0.9584664	0.9488	0.343	2.10E-06	count	1
PROSER1	0.9093577	1.0456373	0.8697	0.385	2.10E-06	count	1
RIPK3	0.9093577	1.0456373	0.8697	0.385	2.10E-06	count	1
TESPA1	0.9093577	1.0456373	0.8697	0.385	2.10E-06	count	1
GPSM2	0.9093577	1.0456373	0.8697	0.385	2.10E-06	count	1
USP16	0.1641551	0.1959352	0.8378	0.403	2.10E-06	count	1
ENPP4	0.4032958	0.3917687	1.0294	0.304	2.10E-06	count	1
NCOA5	0.9093577	1.1260803	0.8075	0.42	2.10E-06	count	1
ZNF689	0.9093577	1.1260803	0.8075	0.42	2.10E-06	count	1
COG5	0.4018913	0.5714796	0.7032	0.482	2.10E-06	count	1
CLMN	0.4057275	0.6104061	0.6647	0.507	2.10E-06	count	1
ANKLE2	0.1642295	0.2331246	0.7045	0.482	2.10E-06	count	1
DISP1	0.9093577	1.2011479	0.7571	0.449	2.10E-06	count	1
GSK3A	0.4062591	0.4055692	1.0017	0.317	2.11E-06	count	1
BRD1	0.4044089	0.3498113	1.1561	0.248	2.11E-06	count	1
ZNF384	0.9093577	0.6398727	1.4212	0.156	2.12E-06	count	1
STX4	0.1655578	0.2668362	0.6204	0.535	2.12E-06	count	1
C6orf89	0.4062591	0.5342991	0.7604	0.447	2.13E-06	count	1
TRIM73	0.4087965	0.6117591	0.6682	0.504	2.13E-06	count	1

BAD	0.1653004	0.341855	0.4835	0.629	2.13E-06	count	1
SUDS3	0.1665234	0.2530845	0.658	0.511	2.13E-06	count	1
RELA	0.4093036	0.4452822	0.9192	0.358	2.14E-06	count	1
TNRC6C	0.9234281	0.6108467	1.5117	0.131	2.15E-06	count	1
LRCH3	0.4104546	0.4036385	1.0169	0.31	2.16E-06	count	1
U2AF2	0.4156442	0.3416941	1.2164	0.224	2.17E-06	count	1
SYNJ2BP	0.4170715	0.4690856	0.8891	0.374	2.17E-06	count	1
OGFRL1	0.4170715	0.5231279	0.7973	0.426	2.17E-06	count	1
TESK1	0.4096822	0.5404785	0.758	0.449	2.17E-06	count	1
ZBTB18	0.4183666	0.6023664	0.6945	0.488	2.18E-06	count	1
SLC41A3	0.4183666	0.6002122	0.697	0.486	2.18E-06	count	1
HDAC7	0.4183666	0.7420802	0.5638	0.573	2.18E-06	count	1
MYDGF	0.169736	0.261972	0.6479	0.517	2.18E-06	count	1
ZNF701	0.4183666	0.7836874	0.5338	0.594	2.18E-06	count	1
TIMM23	0.4183666	0.6240236	0.6704	0.503	2.18E-06	count	1
FANCD2	0.4183666	0.6489583	0.6447	0.519	2.18E-06	count	1
QRICH1	0.4183666	0.5841019	0.7163	0.474	2.18E-06	count	1
PUS7L	0.4147911	0.377098	1.1	0.272	2.18E-06	count	1
HEATR6	0.4183666	0.6371749	0.6566	0.512	2.19E-06	count	1
ESYT2	0.4183666	0.6106689	0.6851	0.494	2.19E-06	count	1
RBM26	0.1708478	0.314564	0.5431	0.587	2.19E-06	count	1
ZNF394	0.1703987	0.23068	0.7387	0.46	2.19E-06	count	1
PPP6R1	0.4183666	0.4317406	0.969	0.333	2.19E-06	count	1
ERC1	0.4183666	0.4878937	0.8575	0.392	2.20E-06	count	1
STAM2	0.4183666	0.536368	0.78	0.436	2.20E-06	count	1
TOMM5	0.1713728	0.2873068	0.5965	0.551	2.21E-06	count	1
ASB8	0.1725685	0.3595966	0.4799	0.632	2.21E-06	count	1
UBE2E2	0.1726614	0.3701165	0.4665	0.641	2.22E-06	count	1
MAN2B1	0.4257331	0.544529	0.7818	0.435	2.22E-06	count	1
GOLGA3	0.4264408	0.419224	1.0172	0.31	2.22E-06	count	1
COQ4	0.4233142	0.4420129	0.9577	0.339	2.23E-06	count	1
DDB1	0.4246829	0.4426066	0.9595	0.338	2.23E-06	count	1
VTI1A	0.1739498	0.3837548	0.4533	0.651	2.23E-06	count	1
GMCL1	0.4231539	0.3992666	1.0598	0.29	2.23E-06	count	1
DNAJA3	0.4270986	0.5469744	0.7808	0.435	2.23E-06	count	1
NUP37	0.4270986	0.5160144	0.8277	0.408	2.24E-06	count	1
CUL4B	0.4270986	0.6070399	0.7036	0.482	2.25E-06	count	1
ZFAND5	0.1742639	0.2327688	0.7487	0.454	2.25E-06	count	1
KAT6B	0.4277117	0.385317	1.11	0.268	2.25E-06	count	1
FLII	0.4270986	0.5512041	0.7748	0.439	2.25E-06	count	1
DCAF15	0.4291632	0.4745412	0.9044	0.366	2.25E-06	count	1
EAF2	0.175657	0.2396751	0.7329	0.464	2.26E-06	count	1
DRG2	0.4298292	0.4622367	0.9299	0.353	2.26E-06	count	1
ADCK2	0.4291632	0.4611146	0.9307	0.353	2.26E-06	count	1
CD84	0.4291632	0.4859134	0.8832	0.378	2.26E-06	count	1

PEX2	0.4298292	0.4341166	0.9901	0.323	2.27E-06	count	1
PITRM1	0.432506	0.697474	0.6201	0.536	2.27E-06	count	1
SGK1	0.176567	0.4692462	0.3763	0.707	2.27E-06	count	1
FCGR2A	0.432506	0.7036966	0.6146	0.539	2.27E-06	count	1
TP53BP1	0.432506	0.4932563	0.8768	0.381	2.27E-06	count	1
EEF1E1	0.1767105	0.3130534	0.5645	0.573	2.28E-06	count	1
ENOPH1	0.1766297	0.3475559	0.5082	0.612	2.28E-06	count	1
ITPRIP	0.9676465	0.9706289	0.9969	0.319	2.28E-06	count	1
MED26	0.9676465	0.7914404	1.2226	0.222	2.28E-06	count	1
APOBEC3F	0.9690359	0.8723991	1.1108	0.267	2.28E-06	count	1
PCID2	0.432506	0.3880462	1.1146	0.266	2.28E-06	count	1
CD6	0.9676465	0.7498714	1.2904	0.198	2.29E-06	count	1
RPUSD4	0.432506	0.4661827	0.9278	0.354	2.29E-06	count	1
ALG12	0.9666808	0.767985	1.2587	0.209	2.29E-06	count	1
C1orf131	0.4331881	0.4904098	0.8833	0.378	2.29E-06	count	1
RGPD5	0.9676465	1.0107324	0.9574	0.339	2.29E-06	count	1
LRRC56	0.9666808	0.8685538	1.113	0.266	2.29E-06	count	1
FURIN	0.9676465	1.0923484	0.8858	0.376	2.29E-06	count	1
PPP2R1B	0.9666808	0.8250522	1.1717	0.242	2.29E-06	count	1
ZW10	1.9369038	1.3842026	1.3993	0.162	2.29E-06	count	1
ARL10	1.9369038	1.8820467	1.0291	0.304	2.30E-06	count	1
AC074117.1	1.9369038	1.8820467	1.0291	0.304	2.30E-06	count	1
ZNF416	0.9676465	1.1682765	0.8283	0.408	2.30E-06	count	1
GPANK1	0.432506	0.365296	1.184	0.237	2.30E-06	count	1
ABI2	0.9676465	1.261815	0.7669	0.444	2.30E-06	count	1
SBNO2	0.965971	0.8301036	1.1637	0.245	2.30E-06	count	1
MFSD11	0.9682726	0.6772353	1.4297	0.153	2.31E-06	count	1
ABCC1	0.9666808	1.0654867	0.9073	0.365	2.31E-06	count	1
SREK1	0.1794849	0.3103087	0.5784	0.563	2.31E-06	count	1
MR1	0.4388462	0.6275194	0.6993	0.485	2.31E-06	count	1
MTHFD2	0.4390997	0.4657421	0.9428	0.346	2.32E-06	count	1
LEO1	0.4390997	0.4833704	0.9084	0.364	2.33E-06	count	1
YDJC	0.1808092	0.2782068	0.6499	0.516	2.33E-06	count	1
SLC35B2	0.4424445	0.5108032	0.8662	0.387	2.33E-06	count	1
SNX6	0.1811346	0.1973291	0.9179	0.359	2.34E-06	count	1
AGO2	0.4447633	0.4516879	0.9847	0.325	2.35E-06	count	1
TRIM11	0.4447946	0.508762	0.8743	0.382	2.35E-06	count	1
OGA	0.1819444	0.2365013	0.7693	0.442	2.35E-06	count	1
GFM1	0.4447946	0.4717359	0.9429	0.346	2.35E-06	count	1
VPS33A	0.4447633	0.5150335	0.8636	0.388	2.35E-06	count	1
SAMD1	0.4426194	0.5169603	0.8562	0.392	2.36E-06	count	1
UBR4	0.4447287	0.5209802	0.8536	0.394	2.36E-06	count	1
R3HCC1L	0.4447946	0.4852749	0.9166	0.36	2.36E-06	count	1
SRPRB	0.4447633	0.5120323	0.8686	0.386	2.36E-06	count	1
ZFY	0.4463408	0.4520402	0.9874	0.324	2.38E-06	count	1

MDN1	0.9971409	0.8045026	1.2395	0.216	2.38E-06	count	1
YRDC	0.1845426	0.3578051	0.5158	0.606	2.38E-06	count	1
ZBTB1	0.1843171	0.2340447	0.7875	0.431	2.38E-06	count	1
CCNB1IP1	0.1844657	0.2764333	0.6673	0.505	2.38E-06	count	1
NDUFAF4	0.1841527	0.2573124	0.7157	0.475	2.39E-06	count	1
URGCP	1.0018015	0.9196061	1.0894	0.277	2.39E-06	count	1
PRKACA	1.0018015	0.8308905	1.2057	0.229	2.40E-06	count	1
AL031708.1	1.9687295	1.3193466	1.4922	0.136	2.40E-06	count	1
TYW1	0.4554843	0.6337267	0.7187	0.473	2.41E-06	count	1
HYOU1	1.0018015	0.7820866	1.2809	0.201	2.41E-06	count	1
DGLUCY	0.4554843	0.6131747	0.7428	0.458	2.41E-06	count	1
RBM34	0.4554843	0.6302607	0.7227	0.47	2.41E-06	count	1
KLHL36	0.4551343	0.4472951	1.0175	0.309	2.41E-06	count	1
PVT1	0.455023	0.4901958	0.9282	0.354	2.41E-06	count	1
CNOT10	0.4548077	0.5321426	0.8547	0.393	2.41E-06	count	1
ZSWIM7	0.186457	0.3454555	0.5397	0.59	2.41E-06	count	1
FUZ	1.9174362	0.8018937	2.3911	0.0172	2.41E-06	count	1
EIF4G1	0.4493379	0.3985051	1.1276	0.26	2.41E-06	count	1
ZNF7	0.4549357	0.5105134	0.8911	0.373	2.41E-06	count	1
PREB	0.4554843	0.6347019	0.7176	0.473	2.41E-06	count	1
SCRN1	0.4548077	0.4053158	1.1221	0.262	2.42E-06	count	1
SIL1	0.4548077	0.5773812	0.7877	0.431	2.42E-06	count	1
BTBD7	0.4549357	0.5035879	0.9034	0.367	2.42E-06	count	1
GFOD2	0.9971409	0.7696525	1.2956	0.196	2.43E-06	count	1
MYH11	0.4565519	0.849299	0.5376	0.591	2.45E-06	count	1
ZNF138	0.4640997	0.4296582	1.0802	0.281	2.47E-06	count	1
PIAS1	0.1906512	0.3347008	0.5696	0.569	2.47E-06	count	1
POLM	0.4629748	0.4194695	1.1037	0.27	2.48E-06	count	1
STX17	0.4677989	0.5096633	0.9179	0.359	2.49E-06	count	1
LARP4B	0.4677989	0.5589084	0.837	0.403	2.49E-06	count	1
G0S2	1.863369	1.3548891	1.3753	0.17	2.51E-06	count	1
STK16	0.4692801	0.3956557	1.1861	0.236	2.51E-06	count	1
FAIM	0.4711667	0.5764296	0.8174	0.414	2.51E-06	count	1
RTCA	0.4701239	0.4104677	1.1453	0.253	2.51E-06	count	1
RMND5B	0.4711667	0.596044	0.7905	0.43	2.51E-06	count	1
OSTN-AS1	0.4733281	0.6145781	0.7702	0.442	2.51E-06	count	1
PGS1	0.4711667	0.6114933	0.7705	0.441	2.52E-06	count	1
KLF11	0.4711667	0.5642959	0.835	0.404	2.52E-06	count	1
TRIM27	0.4692801	0.3979436	1.1793	0.239	2.52E-06	count	1
SMG7	0.4692532	0.5452302	0.8607	0.39	2.52E-06	count	1
LIG4	0.4733281	0.5926523	0.7987	0.425	2.52E-06	count	1
RABGAP1	0.464936	0.4656724	0.9984	0.319	2.52E-06	count	1
KLHL14	0.4758731	0.6410413	0.7423	0.458	2.53E-06	count	1
SMPD4	0.4733281	0.6763466	0.6998	0.484	2.53E-06	count	1
C8orf33	0.472205	0.4248375	1.1115	0.267	2.54E-06	count	1

PDE6D	0.4749761	0.5073659	0.9362	0.35	2.54E-06	count	1
MFSD12	0.4749761	0.4880199	0.9733	0.331	2.54E-06	count	1
MSRA	0.472205	0.4469063	1.0566	0.291	2.54E-06	count	1
TRIM13	0.47045	0.3145428	1.4957	0.135	2.55E-06	count	1
TRPC4AP	0.4808271	0.8714248	0.5518	0.581	2.56E-06	count	1
PSMF1	0.1966842	0.2573181	0.7644	0.445	2.56E-06	count	1
MAX	0.1975022	0.2578883	0.7658	0.444	2.56E-06	count	1
HERC3	0.478849	0.5194637	0.9218	0.357	2.56E-06	count	1
IPMK	0.478849	0.5303202	0.9029	0.367	2.56E-06	count	1
HSD17B4	0.4808271	0.5078455	0.9468	0.344	2.57E-06	count	1
MTMR9	0.478849	0.5781829	0.8282	0.408	2.58E-06	count	1
ZNF268	2.0640397	1.2031712	1.7155	0.087	2.58E-06	count	1
C7orf43	0.4846451	0.656646	0.7381	0.461	2.59E-06	count	1
PHF19	2.0640397	1.2449584	1.6579	0.098	2.59E-06	count	1
ZNF26	0.4808271	1.1789249	0.4079	0.684	2.59E-06	count	1
RBM7	0.4816406	0.407572	1.1817	0.238	2.60E-06	count	1
GNB5	0.1997951	0.3020453	0.6615	0.509	2.60E-06	count	1
ANGEL2	0.4794842	0.5095278	0.941	0.347	2.60E-06	count	1
DMAC1	0.1997012	0.3646721	0.5476	0.584	2.61E-06	count	1
DUSP28	0.4808271	0.6942897	0.6925	0.489	2.61E-06	count	1
LINC01215	0.4832276	0.4861324	0.994	0.321	2.61E-06	count	1
OAT	0.4832276	0.4359547	1.1084	0.268	2.61E-06	count	1
MYO9B	0.2007226	0.3402585	0.5899	0.556	2.62E-06	count	1
FAM206A	1.0700776	1.197083	0.8939	0.372	2.62E-06	count	1
BDH2	1.0700776	0.8356275	1.2806	0.201	2.62E-06	count	1
SLC5A3	0.4692801	0.54104	0.8674	0.386	2.62E-06	count	1
WDFY1	0.4883557	0.5106072	0.9564	0.339	2.62E-06	count	1
MGLL	1.0700776	1.0172307	1.052	0.293	2.62E-06	count	1
FTO	1.0700776	0.9137098	1.1711	0.242	2.62E-06	count	1
DIRC2	1.0700776	0.9137098	1.1711	0.242	2.62E-06	count	1
EEF2KMT	1.0700776	0.9137098	1.1711	0.242	2.62E-06	count	1
CDK2	1.0700776	0.9856256	1.0857	0.278	2.63E-06	count	1
PGM3	1.0700776	0.9856256	1.0857	0.278	2.63E-06	count	1
CTTNBP2NL	1.0700776	0.9856256	1.0857	0.278	2.63E-06	count	1
SFMBT2	1.0700776	0.9856256	1.0857	0.278	2.63E-06	count	1
EIPR1	1.0635154	0.7388273	1.4395	0.151	2.63E-06	count	1
VPS16	1.0635154	0.7388273	1.4395	0.151	2.63E-06	count	1
WDR43	0.2021932	0.2693202	0.7508	0.453	2.63E-06	count	1
WWC3	0.4862451	0.4969527	0.9785	0.328	2.63E-06	count	1
NELFCD	0.2015303	0.315019	0.6397	0.523	2.63E-06	count	1
PPM1M	1.0635154	0.7719496	1.3777	0.169	2.63E-06	count	1
RSF1	0.2024983	0.2006543	1.0092	0.313	2.63E-06	count	1
RNF113A	0.2018225	0.3015332	0.6693	0.504	2.64E-06	count	1
COX7A2L	0.20298	0.2049294	0.9905	0.322	2.64E-06	count	1
MMAB	0.4846451	0.7338183	0.6604	0.509	2.64E-06	count	1

MOCS2	0.4883557	0.5115559	0.9546	0.34	2.64E-06	count	1
RFX1	0.4860554	0.4419347	1.0998	0.272	2.64E-06	count	1
MAPRE2	0.4917037	0.3697377	1.3299	0.184	2.65E-06	count	1
MICU3	0.4942738	0.6910035	0.7153	0.475	2.65E-06	count	1
TSC2	0.4942738	0.6499575	0.7605	0.447	2.65E-06	count	1
PRKCE	1.0774911	0.7725354	1.3947	0.164	2.66E-06	count	1
TRIM23	0.4942738	0.675293	0.7319	0.465	2.66E-06	count	1
PTPRE	0.4932715	0.5657619	0.8719	0.384	2.66E-06	count	1
TNRC18	0.4942738	0.6359916	0.7772	0.437	2.66E-06	count	1
TAP2	0.4890374	0.4178161	1.1705	0.242	2.66E-06	count	1
CDK11B	0.4890374	0.3965901	1.2331	0.218	2.66E-06	count	1
ACAD10	1.0774911	0.9470218	1.1378	0.256	2.67E-06	count	1
PA2G4	0.2047032	0.1994286	1.0264	0.305	2.67E-06	count	1
AL136454.1	0.4991823	0.6693059	0.7458	0.456	2.68E-06	count	1
CYB561D2	0.2050515	0.3469435	0.591	0.555	2.68E-06	count	1
MYO1G	0.4942738	0.4018977	1.2298	0.219	2.70E-06	count	1
QSOX1	1.0955721	0.9719739	1.1272	0.26	2.70E-06	count	1
AC005332.7	1.0955721	1.1117245	0.9855	0.325	2.70E-06	count	1
SPRYD4	1.0955721	1.1117245	0.9855	0.325	2.70E-06	count	1
ZNF446	1.0955721	0.9078661	1.2068	0.228	2.70E-06	count	1
VIPAS39	1.0955721	0.9690592	1.1306	0.259	2.70E-06	count	1
TRIB1	1.0955721	1.3484537	0.8125	0.417	2.70E-06	count	1
RNF19B	1.0955721	0.9941583	1.102	0.271	2.70E-06	count	1
WIPI1	1.0955721	0.9941583	1.102	0.271	2.70E-06	count	1
C10orf95	1.0955721	0.9941583	1.102	0.271	2.70E-06	count	1
IGF1R	1.0955721	0.9941583	1.102	0.271	2.70E-06	count	1
CCNT2	0.4974453	0.4693161	1.0599	0.29	2.71E-06	count	1
KIAA1586	0.5019532	0.4829597	1.0393	0.299	2.71E-06	count	1
LPAR5	0.5019532	0.5180999	0.9688	0.333	2.72E-06	count	1
ILKAP	0.5013578	0.4184417	1.1982	0.231	2.73E-06	count	1
HCFC1	1.0955721	0.7626807	1.4365	0.152	2.73E-06	count	1
GPD2	1.0955721	1.0713423	1.0226	0.307	2.74E-06	count	1
OSTM1	0.5042488	0.4133202	1.22	0.223	2.74E-06	count	1
KYNU	0.2100976	0.3019812	0.6957	0.487	2.74E-06	count	1
ZKSCAN1	0.5076518	0.6534716	0.7769	0.438	2.74E-06	count	1
SRPK1	0.2100976	0.2842909	0.739	0.46	2.74E-06	count	1
RRP9	0.5076518	0.5703281	0.8901	0.374	2.74E-06	count	1
PM20D2	0.5076518	0.7286359	0.6967	0.486	2.75E-06	count	1
CRACR2B	0.5076518	0.6534716	0.7769	0.438	2.75E-06	count	1
SLC12A2	0.5076518	0.5941922	0.8544	0.393	2.75E-06	count	1
COPZ1	0.2099649	0.2918357	0.7195	0.472	2.75E-06	count	1
SNTA1	0.5052069	0.5258756	0.9607	0.337	2.75E-06	count	1
RBM4B	2.1510511	1.1400327	1.8868	0.0598	2.76E-06	count	1
TRPV2	2.1510511	1.1400327	1.8868	0.0598	2.76E-06	count	1
PICK1	0.5076518	0.7317714	0.6937	0.488	2.76E-06	count	1

TPRG1L	0.505239	0.4400938	1.148	0.252	2.76E-06	count	1
RNF26	1.0955721	0.8100125	1.3525	0.177	2.77E-06	count	1
CFAP20	0.5107949	0.4103006	1.2449	0.214	2.78E-06	count	1
IPO11	2.1510511	1.2931248	1.6635	0.0969	2.78E-06	count	1
USF1	0.5098088	0.4949285	1.0301	0.304	2.78E-06	count	1
VCPKMT	0.2141012	0.239261	0.8948	0.371	2.80E-06	count	1
POLR2I	0.2145554	0.3337401	0.6429	0.521	2.80E-06	count	1
USP4	0.5166354	0.4979727	1.0375	0.3	2.81E-06	count	1
SCOC	0.5153251	0.3751991	1.3735	0.17	2.81E-06	count	1
ZNF688	0.5158111	0.4200981	1.2278	0.22	2.82E-06	count	1
PIGX	0.5183392	0.6868465	0.7547	0.451	2.83E-06	count	1
DELE1	0.5183392	0.6428795	0.8063	0.421	2.83E-06	count	1
ATE1	0.5075092	0.4778784	1.062	0.289	2.83E-06	count	1
AL049840.1	0.5193804	0.5209199	0.997	0.319	2.84E-06	count	1
CALCOCO1	0.5239353	0.5870327	0.8925	0.373	2.84E-06	count	1
SMKR1	2.1084915	0.7724622	2.7296	0.0066	2.85E-06	count	1
HEATR1	0.5203615	0.4369238	1.191	0.234	2.85E-06	count	1
ELAC2	0.5220939	0.5410748	0.9649	0.335	2.85E-06	count	1
MLH3	0.5220939	0.5549929	0.9407	0.347	2.85E-06	count	1
EIF2B4	0.5220939	0.5818311	0.8973	0.37	2.86E-06	count	1
USP10	0.2163522	0.4002697	0.5405	0.589	2.86E-06	count	1
AL135925.1	0.5220939	0.5641117	0.9255	0.355	2.86E-06	count	1
SDHA	0.5245072	0.4148964	1.2642	0.207	2.86E-06	count	1
ZNF131	0.218572	0.3073777	0.7111	0.477	2.86E-06	count	1
TMEM140	0.5293711	0.9346502	0.5664	0.571	2.88E-06	count	1
RNF111	0.5246555	0.4009909	1.3084	0.191	2.88E-06	count	1
ZNF600	0.5293711	0.8316751	0.6365	0.525	2.88E-06	count	1
CHFR	0.5270651	0.6200101	0.8501	0.396	2.89E-06	count	1
MAPK7	0.5293711	0.862991	0.6134	0.54	2.89E-06	count	1
ISG20L2	0.2196676	0.3378974	0.6501	0.516	2.89E-06	count	1
DEGS2	0.5293711	0.9232412	0.5734	0.567	2.89E-06	count	1
DOCK2	0.2205837	0.3126277	0.7056	0.481	2.89E-06	count	1
SPATS2	0.5246555	0.5263854	0.9967	0.319	2.89E-06	count	1
CLK4	0.2205837	0.3080298	0.7161	0.474	2.90E-06	count	1
MAU2	0.5303747	0.6010642	0.8824	0.378	2.90E-06	count	1
GMPS	0.5293711	0.4402635	1.2024	0.23	2.90E-06	count	1
RNF8	0.5279076	0.486557	1.085	0.279	2.91E-06	count	1
PGRMC2	0.5344566	0.4405078	1.2133	0.226	2.93E-06	count	1
PEMT	2.2310938	1.1229757	1.9868	0.0476	2.93E-06	count	1
PSMG1	0.5355737	0.5100439	1.0501	0.294	2.93E-06	count	1
MSI2	0.5344566	0.443411	1.2053	0.229	2.94E-06	count	1
IAH1	0.2233176	0.2823979	0.7908	0.429	2.94E-06	count	1
CUL4A	0.5398267	0.5599111	0.9641	0.336	2.95E-06	count	1
BIN3	0.5409208	0.6709055	0.8063	0.421	2.95E-06	count	1
WDR61	0.5344566	0.4245961	1.2587	0.209	2.95E-06	count	1

MPV17L2	0.5409208	0.6144322	0.8804	0.379	2.96E-06	count	1
ZCCHC3	0.5368234	0.4294527	1.25	0.212	2.96E-06	count	1
LILRB1	0.5409208	0.6427062	0.8416	0.4	2.96E-06	count	1
FGFR1OP	0.5409208	0.7814178	0.6922	0.489	2.96E-06	count	1
COX15	0.5409208	0.7825868	0.6912	0.49	2.97E-06	count	1
POLR3A	0.5409208	0.7054032	0.7668	0.444	2.97E-06	count	1
RHOQ	0.2275521	0.2532961	0.8984	0.369	2.99E-06	count	1
THADA	0.540443	0.6029764	0.8963	0.371	2.99E-06	count	1
CHORDC1	0.2278589	0.2512476	0.9069	0.365	2.99E-06	count	1
IMMP1L	0.5455764	0.4825284	1.1307	0.259	3.00E-06	count	1
TRA2A	0.2276406	0.2567789	0.8865	0.376	3.00E-06	count	1
KLHL6	0.5413019	0.4632253	1.1686	0.243	3.01E-06	count	1
AL138724.1	1.1751767	0.7914577	1.4848	0.138	3.01E-06	count	1
MGAT5	0.229112	0.3153213	0.7266	0.468	3.01E-06	count	1
USP36	0.5488489	0.6298535	0.8714	0.384	3.02E-06	count	1
AP1G2	0.5455764	0.4929368	1.1068	0.269	3.02E-06	count	1
UBXN2A	0.545493	0.4648679	1.1734	0.241	3.02E-06	count	1
SLF2	0.5488489	0.6421439	0.8547	0.393	3.02E-06	count	1
RPF1	0.5441686	0.3463132	1.5713	0.117	3.02E-06	count	1
OSGIN2	0.5508807	0.8680385	0.6346	0.526	3.03E-06	count	1
ZNF766	0.2297855	0.3704835	0.6202	0.535	3.03E-06	count	1
ARL14	1.1751767	0.948937	1.2384	0.216	3.06E-06	count	1
LYRM9	0.5547516	0.5337933	1.0393	0.299	3.06E-06	count	1
INO80	0.2322544	0.3166692	0.7334	0.464	3.06E-06	count	1
KPNA2	0.2315272	0.2500507	0.9259	0.355	3.06E-06	count	1
SNX9	0.2327563	0.2686288	0.8665	0.387	3.07E-06	count	1
TMEM138	0.5578623	0.5527479	1.0093	0.313	3.08E-06	count	1
HIGD1A	0.551943	0.4224731	1.3065	0.192	3.08E-06	count	1
KIAA1143	0.5540545	0.4053401	1.3669	0.172	3.08E-06	count	1
COMMD1	0.2341084	0.2814152	0.8319	0.406	3.09E-06	count	1
ZNF664	0.5565102	0.699222	0.7959	0.427	3.09E-06	count	1
INTS9	2.3489257	2.8807484	0.8154	0.415	3.09E-06	count	1
GTF2H2C	0.5565102	0.7883719	0.7059	0.481	3.09E-06	count	1
ZNF587	1.2036138	0.9572579	1.2574	0.209	3.10E-06	count	1
DPF2	0.5596555	0.4955522	1.1294	0.259	3.10E-06	count	1
DHX8	0.5620106	0.6058576	0.9276	0.354	3.10E-06	count	1
LSM12	0.2355937	0.2368413	0.9947	0.32	3.11E-06	count	1
ZNF324	0.5620106	0.6330943	0.8877	0.375	3.11E-06	count	1
IRF5	1.2057067	0.8812293	1.3682	0.172	3.11E-06	count	1
TOP3A	1.2057067	0.9423199	1.2795	0.201	3.11E-06	count	1
AL162231.1	1.2057067	0.9423199	1.2795	0.201	3.11E-06	count	1
AARSD1	1.2057067	0.9423199	1.2795	0.201	3.11E-06	count	1
THRA	1.2036138	0.8744207	1.3765	0.169	3.11E-06	count	1
PIM1	0.5634877	0.5086109	1.1079	0.269	3.12E-06	count	1
PUS7	1.2057067	0.9996843	1.2061	0.228	3.12E-06	count	1

ZMYM6	1.2057067	0.9996843	1.2061	0.228	3.12E-06	count	1
NLRC3	1.2057067	0.9996843	1.2061	0.228	3.12E-06	count	1
PARG	0.5616651	0.5297826	1.0602	0.29	3.12E-06	count	1
BICDL1	1.2057067	1.0539309	1.144	0.253	3.12E-06	count	1
ATXN7L1	1.2057067	1.0539309	1.144	0.253	3.12E-06	count	1
LRRC8C	1.2057067	1.0539309	1.144	0.253	3.12E-06	count	1
ZNF598	0.5665257	0.5613128	1.0093	0.313	3.12E-06	count	1
FAM241A	1.2057067	1.1548047	1.0441	0.297	3.13E-06	count	1
RAB11FIP2	0.5637929	0.5465111	1.0316	0.303	3.14E-06	count	1
C3orf38	0.5633752	0.3141577	1.7933	0.0736	3.14E-06	count	1
PRDM4	0.5649741	0.4830762	1.1695	0.243	3.14E-06	count	1
KDM6B	0.2401063	0.1925874	1.2467	0.213	3.17E-06	count	1
ARF5	0.2387336	0.2659855	0.8975	0.37	3.17E-06	count	1
NBAS	0.5730546	0.4645332	1.2336	0.218	3.17E-06	count	1
TAF1	0.5678787	0.4769344	1.1907	0.234	3.17E-06	count	1
HSPA13	0.5704486	0.4387753	1.3001	0.194	3.18E-06	count	1
IRAK1	0.5682781	0.4114479	1.3812	0.168	3.19E-06	count	1
BOD1	0.2415402	0.3764011	0.6417	0.521	3.19E-06	count	1
ARMC8	0.5705586	0.3603527	1.5833	0.114	3.20E-06	count	1
SLC44A2	0.5607294	0.4691904	1.1951	0.233	3.20E-06	count	1
STAG1	0.5747903	0.4349072	1.3216	0.187	3.20E-06	count	1
COL4A4	0.5649741	0.6051076	0.9337	0.351	3.21E-06	count	1
RBL2	0.5704948	0.4602469	1.2395	0.216	3.21E-06	count	1
PPP4R3B	0.2433098	0.2862777	0.8499	0.396	3.21E-06	count	1
KIN	0.2432803	0.3562983	0.6828	0.495	3.22E-06	count	1
ATAD2B	0.2429357	0.3378512	0.7191	0.472	3.22E-06	count	1
MIIP	0.5721642	0.3812127	1.5009	0.134	3.23E-06	count	1
ISCA1	0.2451702	0.2626162	0.9336	0.351	3.24E-06	count	1
ZEB1	0.5747903	0.4344565	1.323	0.187	3.24E-06	count	1
NAPB	2.4343875	1.2873546	1.891	0.0593	3.25E-06	count	1
KDM2B	0.5659661	0.4171079	1.3569	0.176	3.25E-06	count	1
ZXDB	2.4343875	2.1734036	1.1201	0.263	3.25E-06	count	1
LARGE2	2.3741946	1.1280391	2.1047	0.0359	3.26E-06	count	1
NKAPD1	0.5861095	0.7360072	0.7963	0.426	3.26E-06	count	1
LYRM2	0.2468007	0.3248363	0.7598	0.448	3.26E-06	count	1
AOAH	1.2534467	1.5664287	0.8002	0.424	3.27E-06	count	1
LRRC8B	1.2534467	0.9208848	1.3611	0.174	3.27E-06	count	1
SLX4IP	0.5861095	0.6599495	0.8881	0.375	3.27E-06	count	1
CPSF1	1.2534467	1.0277493	1.2196	0.223	3.27E-06	count	1
AC016773.1	1.2534467	1.0277493	1.2196	0.223	3.27E-06	count	1
CDC16	0.5849895	0.5447231	1.0739	0.283	3.28E-06	count	1
CRELD1	1.2534467	0.9166428	1.3674	0.172	3.28E-06	count	1
RPS6KA5	0.247993	0.3989031	0.6217	0.534	3.28E-06	count	1
USP46	0.5861095	0.7239887	0.8096	0.419	3.28E-06	count	1
PPP1R3E	1.2534467	1.0239501	1.2241	0.222	3.28E-06	count	1

GTF2E1	1.2534467	1.0239501	1.2241	0.222	3.28E-06	count	1
AC006480.2	1.2534467	1.1210325	1.1181	0.264	3.29E-06	count	1
AIG1	0.5843928	0.4496384	1.2997	0.194	3.29E-06	count	1
MBTPS1	0.5867385	0.3991517	1.47	0.142	3.29E-06	count	1
HAUS2	0.5889782	0.5526434	1.0657	0.287	3.29E-06	count	1
GOLT1B	0.5861095	0.4279192	1.3697	0.171	3.29E-06	count	1
SNAPIN	0.5849895	0.3732704	1.5672	0.118	3.29E-06	count	1
LUC7L2	0.2487082	0.3603029	0.6903	0.49	3.30E-06	count	1
FAM89A	0.5889782	0.7508359	0.7844	0.433	3.30E-06	count	1
METAP1	0.5912793	0.5765347	1.0256	0.306	3.30E-06	count	1
POLR1C	0.5889782	0.5135439	1.1469	0.252	3.30E-06	count	1
SPIDR	0.247993	0.373764	0.6635	0.507	3.31E-06	count	1
TTC7A	0.5889782	0.4921323	1.1968	0.232	3.31E-06	count	1
ETFRF1	0.2495437	0.3296734	0.7569	0.449	3.31E-06	count	1
ATP6AP1	0.5861095	0.4296675	1.3641	0.173	3.31E-06	count	1
PDHX	0.5889782	0.6391703	0.9215	0.357	3.31E-06	count	1
ELMO1	0.2492952	0.3346451	0.745	0.457	3.31E-06	count	1
TRAF2	0.5912793	0.6286491	0.9406	0.347	3.31E-06	count	1
ANKRD16	0.5960735	0.7978282	0.7471	0.455	3.32E-06	count	1
TSR1	0.5912793	0.5630439	1.0501	0.294	3.32E-06	count	1
ELOVL5	0.2508626	0.304425	0.8241	0.41	3.33E-06	count	1
SPSB1	0.5931657	1.1274835	0.5261	0.599	3.33E-06	count	1
HECA	0.2500191	0.316917	0.7889	0.431	3.33E-06	count	1
RAB11B	0.5846517	0.3694461	1.5825	0.114	3.33E-06	count	1
TAF2	0.5982097	0.7550386	0.7923	0.429	3.33E-06	count	1
ICAM2	0.5942449	0.4154652	1.4303	0.153	3.33E-06	count	1
HNRNPUL1	0.2509773	0.2757664	0.9101	0.363	3.33E-06	count	1
HGS	0.5960735	0.7048131	0.8457	0.398	3.33E-06	count	1
STRN	0.5942449	0.4421866	1.3439	0.18	3.33E-06	count	1
AP001157.1	0.5960735	0.8605411	0.6927	0.489	3.33E-06	count	1
PCYT1A	1.26258	0.8868272	1.4237	0.155	3.34E-06	count	1
UBR1	0.5931657	0.5833564	1.0168	0.31	3.34E-06	count	1
FAM104A	0.5901887	0.4218909	1.3989	0.163	3.35E-06	count	1
MIS12	0.5861095	0.5151991	1.1376	0.256	3.35E-06	count	1
RHOBTB2	1.2721384	0.7972691	1.5956	0.111	3.36E-06	count	1
THOC3	0.6030492	0.58635	1.0285	0.304	3.37E-06	count	1
ZNF296	0.2546943	0.3170533	0.8033	0.422	3.38E-06	count	1
LETM1	0.5982097	0.474695	1.2602	0.208	3.39E-06	count	1
IMPDH2	0.2545106	0.2798564	0.9094	0.364	3.39E-06	count	1
AP2A1	0.5982097	0.7760868	0.7708	0.441	3.40E-06	count	1
GRWD1	0.5889782	0.4732194	1.2446	0.214	3.40E-06	count	1
AC097376.2	0.6011375	0.7298305	0.8237	0.411	3.40E-06	count	1
IGHG4	2.2310938	1.2575853	1.7741	0.0767	3.42E-06	count	1
EDEM1	0.2583476	0.2949921	0.8758	0.382	3.43E-06	count	1
PLIN3	0.602006	0.3761089	1.6006	0.11	3.44E-06	count	1

CRTAP	0.6052706	0.4204533	1.4396	0.151	3.44E-06	count	1
CEP170	0.258209	0.3053069	0.8457	0.398	3.44E-06	count	1
LYST	0.2588042	0.3196696	0.8096	0.419	3.45E-06	count	1
AXIN1	1.2994338	0.8351884	1.5559	0.12	3.45E-06	count	1
PRKAA1	0.2603683	0.3643926	0.7145	0.475	3.46E-06	count	1
THEM4	0.6107199	0.504268	1.2111	0.227	3.46E-06	count	1
ZNF350	0.6107199	0.4912769	1.2431	0.214	3.46E-06	count	1
SPEN	0.2597347	0.2967673	0.8752	0.382	3.47E-06	count	1
BIRC2	0.2590895	0.3323279	0.7796	0.436	3.47E-06	count	1
CTSA	0.2612855	0.2676486	0.9762	0.329	3.48E-06	count	1
FBXW2	0.6107199	0.5308944	1.1504	0.251	3.48E-06	count	1
RELT	0.6109037	0.4772344	1.2801	0.201	3.49E-06	count	1
EIF2B1	0.6187221	0.5573221	1.1102	0.268	3.49E-06	count	1
MAP2K1	0.6109788	0.5094416	1.1993	0.231	3.50E-06	count	1
COMTD1	0.6199211	0.579661	1.0695	0.285	3.50E-06	count	1
NAMPT	0.2580581	0.2786894	0.926	0.355	3.50E-06	count	1
AK6	0.2626757	0.2735511	0.9602	0.337	3.50E-06	count	1
SLC20A1	0.6192715	0.4985462	1.2422	0.215	3.51E-06	count	1
THAP8	1.3189557	1.1926318	1.1059	0.269	3.51E-06	count	1
PHKB	0.2632674	0.2968897	0.8868	0.376	3.52E-06	count	1
ANKRD33B	0.6192715	0.5102997	1.2135	0.226	3.52E-06	count	1
PCK2	0.6207013	0.5885034	1.0547	0.292	3.52E-06	count	1
ANKRD17	0.6207013	0.5885034	1.0547	0.292	3.52E-06	count	1
LYPLA2	0.6207013	0.40277	1.5411	0.124	3.52E-06	count	1
ELP4	0.6108181	0.5170685	1.1813	0.238	3.53E-06	count	1
CAPN7	0.6195821	0.3897739	1.5896	0.113	3.53E-06	count	1
CANT1	1.3189557	0.7651039	1.7239	0.0854	3.53E-06	count	1
ELK4	0.2647878	0.346509	0.7642	0.445	3.54E-06	count	1
ZNF248	0.6207013	0.6490277	0.9564	0.339	3.55E-06	count	1
DYRK1A	0.265723	0.3550574	0.7484	0.455	3.55E-06	count	1
SLC35F2	0.6207013	0.6632965	0.9358	0.35	3.55E-06	count	1
FAHD2B	1.323122	0.8863829	1.4927	0.136	3.55E-06	count	1
MRPL27	0.265723	0.2931688	0.9064	0.365	3.55E-06	count	1
PCED1A	1.323122	0.9863987	1.3414	0.18	3.57E-06	count	1
PIGO	1.323122	0.9096553	1.4545	0.147	3.57E-06	count	1
ARMCX6	0.2671584	0.2985548	0.8948	0.371	3.57E-06	count	1
RAD50	1.323122	0.9597534	1.3786	0.169	3.57E-06	count	1
SYT11	1.323122	1.0771677	1.2283	0.22	3.58E-06	count	1
VIM-AS1	1.3214178	0.6996585	1.8887	0.0596	3.58E-06	count	1
CD82	0.2691084	0.1814734	1.4829	0.139	3.60E-06	count	1
RAPGEF1	0.2690266	0.314155	0.8563	0.392	3.61E-06	count	1
ATP6V1C1	0.6322048	0.5805459	1.089	0.277	3.61E-06	count	1
MAP2K7	0.6287025	0.5704239	1.1022	0.271	3.62E-06	count	1
PREP	0.6322048	0.6773058	0.9334	0.351	3.62E-06	count	1
PAAF1	0.6375941	0.6758618	0.9434	0.346	3.63E-06	count	1

AMFR	0.2712833	0.351726	0.7713	0.441	3.63E-06	count	1
GRINA	0.6316139	0.3754146	1.6824	0.0932	3.63E-06	count	1
SLC25A39	0.2703085	0.3572861	0.7566	0.45	3.63E-06	count	1
ZNF189	0.6345793	0.7494094	0.8468	0.398	3.63E-06	count	1
HSD17B12	0.6316139	0.3716093	1.6997	0.0899	3.64E-06	count	1
CBWD2	0.6415501	0.8500867	0.7547	0.451	3.64E-06	count	1
EHD4	0.6256121	0.4950652	1.2637	0.207	3.64E-06	count	1
TRMO	0.6359914	0.490411	1.2969	0.195	3.64E-06	count	1
NELFA	0.6375941	0.485093	1.3144	0.189	3.64E-06	count	1
AP3M2	0.6375941	0.6787738	0.9393	0.348	3.64E-06	count	1
DDX19A	1.3441381	0.9377525	1.4334	0.152	3.65E-06	count	1
PCYT2	0.6415501	0.7711905	0.8319	0.406	3.65E-06	count	1
LMF1	0.6415501	0.8002729	0.8017	0.423	3.66E-06	count	1
CENPB	0.6415501	0.7126904	0.9002	0.369	3.66E-06	count	1
CASC4	0.2737536	0.3869992	0.7074	0.48	3.66E-06	count	1
PPP1R3F	0.6415501	0.7732918	0.8296	0.407	3.67E-06	count	1
ZFP62	1.3417097	0.7487829	1.7919	0.0738	3.67E-06	count	1
PIGB	1.3417097	0.7487829	1.7919	0.0738	3.67E-06	count	1
BBS4	0.6415501	0.774165	0.8287	0.408	3.67E-06	count	1
TTC5	0.6394287	0.4858069	1.3162	0.189	3.67E-06	count	1
SPAG1	0.6344199	0.453911	1.3977	0.163	3.68E-06	count	1
ZNF529	1.3552062	0.8508644	1.5927	0.112	3.68E-06	count	1
COP1	0.6440314	0.5464186	1.1786	0.239	3.68E-06	count	1
VPS53	0.6440314	0.5464186	1.1786	0.239	3.68E-06	count	1
SLC9A7	0.6490642	0.592269	1.0959	0.274	3.69E-06	count	1
UNC13D	1.3417097	0.7535443	1.7805	0.0757	3.71E-06	count	1
MDM2	0.2767403	0.3361617	0.8232	0.411	3.71E-06	count	1
IER3IP1	0.6491924	0.3745644	1.7332	0.0838	3.74E-06	count	1
DENND1B	0.6511335	0.4870143	1.337	0.182	3.75E-06	count	1
AP4E1	1.3837941	1.6120864	0.8584	0.391	3.76E-06	count	1
SLC9A1	1.3837941	1.3527879	1.0229	0.307	3.76E-06	count	1
FZD5	1.3837941	1.3527879	1.0229	0.307	3.76E-06	count	1
CBLN3	1.3837941	1.3527879	1.0229	0.307	3.76E-06	count	1
PTGS1	1.3837941	1.3527879	1.0229	0.307	3.76E-06	count	1
ZNF614	1.3837941	1.3527879	1.0229	0.307	3.76E-06	count	1
LRSAM1	1.3837941	1.3527879	1.0229	0.307	3.76E-06	count	1
RAB40B	1.3837941	1.3527879	1.0229	0.307	3.76E-06	count	1
CAMKK1	1.3837941	1.3527879	1.0229	0.307	3.76E-06	count	1
PAK4	1.3837941	1.3527879	1.0229	0.307	3.76E-06	count	1
HUS1B	1.3837941	1.3527879	1.0229	0.307	3.76E-06	count	1
CTNS	1.3837941	1.3527879	1.0229	0.307	3.76E-06	count	1
NOP2	1.3837941	1.7626865	0.785	0.433	3.76E-06	count	1
AL133338.1	1.3837941	1.7626865	0.785	0.433	3.76E-06	count	1
SFT2D3	1.3837941	1.7626865	0.785	0.433	3.76E-06	count	1
AC023590.1	1.3837941	1.7626865	0.785	0.433	3.76E-06	count	1

FGFRL1	1.3837941	1.7626865	0.785	0.433	3.76E-06	count	1
GSK3B	0.6511335	0.5472195	1.1899	0.235	3.77E-06	count	1
AFDN	0.6508621	0.4050156	1.607	0.109	3.77E-06	count	1
ZNF672	0.6347342	0.3956343	1.6043	0.109	3.78E-06	count	1
ARFGAP1	1.3837941	1.0488673	1.3193	0.188	3.78E-06	count	1
SQLE	0.2826679	0.3227985	0.8757	0.382	3.79E-06	count	1
ARSD	1.3837941	0.9841146	1.4061	0.16	3.79E-06	count	1
SCAMP3	0.6569252	0.4407791	1.4904	0.137	3.79E-06	count	1
SLC30A5	0.6545644	0.5441856	1.2028	0.23	3.79E-06	count	1
LTA	0.2823037	0.3626488	0.7784	0.437	3.79E-06	count	1
ACAD9	1.3837941	0.8650417	1.5997	0.11	3.79E-06	count	1
ATRNL1	1.3837941	1.015821	1.3622	0.174	3.80E-06	count	1
PRMT7	1.3837941	1.0332252	1.3393	0.181	3.80E-06	count	1
TFPT	0.6636447	0.6187352	1.0726	0.284	3.81E-06	count	1
ATG4B	0.6636447	0.6855277	0.9681	0.334	3.81E-06	count	1
TDP1	0.6636447	0.5830251	1.1383	0.256	3.82E-06	count	1
PEX1	0.6508621	0.4411333	1.4754	0.141	3.83E-06	count	1
THUMPD3	0.6636447	0.6845482	0.9695	0.333	3.83E-06	count	1
PDE4D	0.6610258	0.4565008	1.448	0.148	3.84E-06	count	1
CCDC97	0.6636447	0.6088177	1.0901	0.276	3.85E-06	count	1
MED19	0.2851832	0.3186504	0.895	0.371	3.85E-06	count	1
ZNF652	0.2851833	0.2834582	1.0061	0.315	3.85E-06	count	1
BROX	0.6599047	0.3845452	1.7161	0.0868	3.86E-06	count	1
DDHD2	0.6698362	0.7210634	0.929	0.353	3.86E-06	count	1
ATP6V0E2	0.6698362	0.6448675	1.0387	0.3	3.86E-06	count	1
BMP2K	0.2862366	0.3196469	0.8955	0.371	3.87E-06	count	1
SPRYD3	1.3837941	0.727743	1.9015	0.0579	3.87E-06	count	1
HDHD3	0.6698362	0.6611447	1.0131	0.312	3.87E-06	count	1
NARS	0.2871116	0.3633564	0.7902	0.43	3.88E-06	count	1
ZNF230	1.3837941	0.7749554	1.7856	0.0748	3.88E-06	count	1
RP2	0.6596814	0.4641012	1.4214	0.156	3.88E-06	count	1
SMIM7	0.2891252	0.2522095	1.1464	0.252	3.89E-06	count	1
TBL1XR1	0.2899095	0.2690555	1.0775	0.282	3.92E-06	count	1
ARHGEF6	0.6663371	0.5588282	1.1924	0.234	3.93E-06	count	1
SETBP1	0.6676251	0.3870945	1.7247	0.0853	3.94E-06	count	1
FAF2	0.6698362	0.4714367	1.4208	0.156	3.94E-06	count	1
HPS1	0.2928997	0.3668334	0.7985	0.425	3.95E-06	count	1
APEH	0.6808017	0.5443308	1.2507	0.212	3.96E-06	count	1
PTTG1IP	0.6864523	0.5345931	1.2841	0.2	3.98E-06	count	1
COASY	1.4267144	0.7878608	1.8109	0.0708	3.98E-06	count	1
GGCT	0.6864523	0.6254543	1.0975	0.273	3.98E-06	count	1
ZNF444	1.4267144	0.884206	1.6136	0.107	4.00E-06	count	1
ZNF33A	0.2961834	0.3663952	0.8084	0.419	4.00E-06	count	1
DCAF17	1.4267144	0.9286377	1.5364	0.125	4.01E-06	count	1
DAPK2	1.4267144	0.9286377	1.5364	0.125	4.01E-06	count	1

KRBOX4	1.4267144	0.9710385	1.4693	0.142	4.01E-06	count	1
GIT2	0.2961291	0.34313	0.863	0.389	4.01E-06	count	1
WDR89	0.6875362	0.6474288	1.0619	0.289	4.02E-06	count	1
ZNF254	0.6864523	0.6730175	1.02	0.308	4.02E-06	count	1
MED25	0.6875362	0.6557479	1.0485	0.295	4.02E-06	count	1
ERBIN	0.2968554	0.3111399	0.9541	0.341	4.03E-06	count	1
EPHX2	0.6972901	0.7505005	0.9291	0.353	4.04E-06	count	1
CD151	0.6972901	0.7305317	0.9545	0.34	4.05E-06	count	1
SF3A2	0.6972901	0.6284225	1.1096	0.268	4.05E-06	count	1
CYB5D2	0.6972901	0.7614479	0.9157	0.36	4.05E-06	count	1
HDGFL2	0.6822702	0.5106631	1.336	0.182	4.06E-06	count	1
NME6	0.6875362	0.6068227	1.133	0.258	4.06E-06	count	1
NUP54	0.6834588	0.4526542	1.5099	0.132	4.08E-06	count	1
PTP4A1	0.3011369	0.312885	0.9625	0.336	4.08E-06	count	1
RANBP10	1.4612122	1.3492087	1.083	0.279	4.08E-06	count	1
LUZP1	0.6914993	0.5100002	1.3559	0.176	4.08E-06	count	1
RGS14	0.3006556	0.309388	0.9718	0.332	4.08E-06	count	1
AC133550.2	1.4612122	0.7977398	1.8317	0.0677	4.08E-06	count	1
ZNF85	0.6972901	0.6865143	1.0157	0.31	4.09E-06	count	1
KPNA6	0.6905221	0.4806682	1.4366	0.152	4.10E-06	count	1
ELF2	0.3018619	0.3056585	0.9876	0.324	4.10E-06	count	1
VCP	0.3034956	0.2286923	1.3271	0.185	4.11E-06	count	1
ABCB7	0.6929664	0.579464	1.1959	0.232	4.12E-06	count	1
LRRK2	0.698804	0.4498812	1.5533	0.121	4.13E-06	count	1
TIAL1	0.3047283	0.2246826	1.3563	0.176	4.13E-06	count	1
CYSTM1	0.3054163	0.407697	0.7491	0.454	4.14E-06	count	1
ICAM1	0.6745654	0.4678707	1.4418	0.15	4.15E-06	count	1
GTF2A1	0.6914993	0.4765353	1.4511	0.147	4.16E-06	count	1
MORC3	0.3054163	0.2751233	1.1101	0.268	4.16E-06	count	1
MTF1	1.4597748	0.7974521	1.8305	0.0678	4.17E-06	count	1
NRBF2	0.305765	0.2159623	1.4158	0.158	4.17E-06	count	1
PAICS	0.7054782	0.4189414	1.684	0.0929	4.18E-06	count	1
SENP1	0.7118861	0.6016047	1.1833	0.237	4.18E-06	count	1
TP53BP2	0.7118861	0.4406565	1.6155	0.107	4.20E-06	count	1
DAP	0.7005528	0.4387124	1.5968	0.111	4.21E-06	count	1
ZSCAN18	0.7094955	0.4892793	1.4501	0.148	4.21E-06	count	1
RARA	0.3120517	0.302972	1.03	0.304	4.23E-06	count	1
RPAP2	0.311242	0.3748253	0.8304	0.407	4.23E-06	count	1
AAGAB	0.7118861	0.5409802	1.3159	0.189	4.24E-06	count	1
ADGRE5	0.3128111	0.2291448	1.3651	0.173	4.26E-06	count	1
ZNRF2	0.3138142	0.3068361	1.0227	0.307	4.26E-06	count	1
PRPF18	0.7143183	0.4445398	1.6069	0.109	4.26E-06	count	1
AP3D1	0.7248173	0.6171965	1.1744	0.241	4.27E-06	count	1
NSMAF	0.7118861	0.4749334	1.4989	0.135	4.27E-06	count	1
CRYZL1	0.7212744	0.5985533	1.205	0.229	4.28E-06	count	1

ASB16-AS1	0.7248173	0.6060333	1.196	0.232	4.28E-06	count	1
PCMT1	0.3153249	0.211113	1.4936	0.136	4.28E-06	count	1
GK5	1.4951383	0.970835	1.5401	0.124	4.29E-06	count	1
NUCB2	0.7118861	0.58387	1.2193	0.223	4.29E-06	count	1
ZNF836	0.7294304	0.7113977	1.0253	0.306	4.30E-06	count	1
CHD6	0.3158453	0.3490386	0.9049	0.366	4.30E-06	count	1
ARID2	0.7278253	0.5101515	1.4267	0.154	4.33E-06	count	1
PRPSAP1	0.7204992	0.4827284	1.4926	0.136	4.33E-06	count	1
FLOT1	0.7247644	0.4003024	1.8105	0.0709	4.34E-06	count	1
EFTUD2	0.7326579	0.5873766	1.2473	0.213	4.35E-06	count	1
SNHG12	0.3191718	0.2815345	1.1337	0.258	4.36E-06	count	1
TMEM115	0.7278253	0.5513644	1.32	0.188	4.36E-06	count	1
LINC00426	1.5194566	1.0191151	1.491	0.137	4.37E-06	count	1
PIBF1	0.319887	0.3654282	0.8754	0.382	4.37E-06	count	1
GMDS	0.7294304	0.6421133	1.136	0.257	4.39E-06	count	1
CCNYL1	0.7417019	0.6307616	1.1759	0.24	4.39E-06	count	1
GALE	1.5194566	0.8220608	1.8484	0.0652	4.39E-06	count	1
TBXAS1	0.7417019	0.6615102	1.1212	0.263	4.40E-06	count	1
SLC20A2	0.7417019	0.7190736	1.0315	0.303	4.41E-06	count	1
MTMR3	1.5194566	0.9037756	1.6812	0.0934	4.41E-06	count	1
C18orf32	1.5194566	0.9037756	1.6812	0.0934	4.41E-06	count	1
TCAF1	0.7417019	0.7446566	0.996	0.32	4.42E-06	count	1
MBTD1	0.7417019	0.8714552	0.8511	0.395	4.42E-06	count	1
RYK	0.7379223	0.5073198	1.4546	0.147	4.43E-06	count	1
STK40	0.7453795	0.7083099	1.0523	0.293	4.43E-06	count	1
PLEKHM2	0.7408405	0.4088366	1.8121	0.0707	4.44E-06	count	1
GBA2	0.7493126	0.6614759	1.1328	0.258	4.44E-06	count	1
EIF2B3	0.7467678	0.6319948	1.1816	0.238	4.45E-06	count	1
CNBD2	0.7326579	0.7451858	0.9832	0.326	4.45E-06	count	1
LPIN2	0.7379223	0.6177769	1.1945	0.233	4.46E-06	count	1
RHOT2	0.738188	0.4607751	1.6021	0.11	4.46E-06	count	1
GGA1	0.3234112	0.3086921	1.0477	0.295	4.48E-06	count	1
PPWD1	0.7355255	0.4365769	1.6848	0.0927	4.48E-06	count	1
RASAL3	0.328269	0.3031434	1.0829	0.279	4.49E-06	count	1
ZNF34	0.7500078	0.6352112	1.1807	0.238	4.50E-06	count	1
ATXN7L3B	0.3283807	0.2595942	1.265	0.207	4.50E-06	count	1
PLXNA3	0.7493126	0.6177787	1.2129	0.226	4.51E-06	count	1
TASP1	1.528967	0.8383705	1.8237	0.0689	4.51E-06	count	1
EFHC1	0.7522586	0.5424976	1.3867	0.166	4.51E-06	count	1
GNAI3	0.3310615	0.2259825	1.465	0.144	4.52E-06	count	1
PRKRIP1	0.3308633	0.3899947	0.8484	0.397	4.54E-06	count	1
ARL2BP	0.3310384	0.2909028	1.138	0.256	4.54E-06	count	1
SNRNP70	0.3278228	0.2819355	1.1628	0.246	4.54E-06	count	1
TRMT11	0.7391808	0.4139806	1.7855	0.0749	4.54E-06	count	1
SPI1	0.3320814	0.2991718	1.11	0.268	4.54E-06	count	1

PRR14	0.7436324	0.5250084	1.4164	0.157	4.55E-06	count	1
SAR1B	0.7551531	0.43684	1.7287	0.0846	4.55E-06	count	1
CALHM6	0.7671149	0.6651495	1.1533	0.249	4.56E-06	count	1
STK38	0.7660416	0.5586364	1.3713	0.171	4.56E-06	count	1
MRPL17	0.7562277	0.4506773	1.678	0.0941	4.56E-06	count	1
MSRB1	0.7660416	0.639213	1.1984	0.231	4.57E-06	count	1
DNPEP	0.7570859	0.3999003	1.8932	0.059	4.57E-06	count	1
PATL1	0.7519166	0.6200647	1.2126	0.226	4.60E-06	count	1
C1orf112	1.5925433	1.4649334	1.0871	0.278	4.63E-06	count	1
AC087623.3	1.5925433	1.4649334	1.0871	0.278	4.63E-06	count	1
ABCC10	1.5925433	1.4649334	1.0871	0.278	4.63E-06	count	1
FAM24B	1.5925433	1.4649334	1.0871	0.278	4.63E-06	count	1
TRIM62	1.5925433	1.3115726	1.2142	0.225	4.63E-06	count	1
TPRA1	1.5925433	1.3115726	1.2142	0.225	4.63E-06	count	1
FAM222B	1.5925433	1.3115726	1.2142	0.225	4.63E-06	count	1
PAM16	1.5925433	1.0423034	1.5279	0.127	4.63E-06	count	1
SLC41A1	1.5925433	1.0423034	1.5279	0.127	4.63E-06	count	1
GAR1	0.7655202	0.4763658	1.607	0.109	4.63E-06	count	1
BRPF3	1.5925433	1.2297168	1.295	0.196	4.63E-06	count	1
SLC25A20	1.5925433	1.2297168	1.295	0.196	4.63E-06	count	1
GSDMB	1.5925433	1.2297168	1.295	0.196	4.63E-06	count	1
MFSD9	1.5925433	1.2297168	1.295	0.196	4.63E-06	count	1
SNAI3-AS1	1.5925433	1.2297168	1.295	0.196	4.63E-06	count	1
WDR24	1.5925433	1.3921245	1.144	0.253	4.64E-06	count	1
AL034417.3	1.5925433	1.3921245	1.144	0.253	4.64E-06	count	1
AL008729.2	1.5925433	1.5374712	1.0358	0.301	4.64E-06	count	1
AC092803.2	1.5925433	1.5374712	1.0358	0.301	4.64E-06	count	1
TSPYL1	0.3380462	0.2714539	1.2453	0.214	4.64E-06	count	1
FBXL3	0.3375017	0.3727217	0.9055	0.366	4.65E-06	count	1
UMPS	1.5781661	0.5575622	2.8305	0.0049	4.67E-06	count	1
MKRN1	0.340929	0.2555577	1.3341	0.183	4.68E-06	count	1
VPS13D	0.7656396	0.5862385	1.306	0.192	4.71E-06	count	1
OSBP	0.3398545	0.3031571	1.1211	0.263	4.72E-06	count	1
HILPDA	0.7867023	0.7083905	1.1105	0.267	4.72E-06	count	1
LPAR2	0.7797473	0.6118373	1.2744	0.203	4.72E-06	count	1
MED23	0.7775577	0.5765775	1.3486	0.178	4.72E-06	count	1
LUC7L	0.7717998	0.3722051	2.0736	0.0387	4.72E-06	count	1
FSD1L	0.7828393	0.810857	0.9654	0.335	4.72E-06	count	1
EIF1AD	0.7561832	0.5377648	1.4062	0.16	4.73E-06	count	1
COPS8	0.7759259	0.5010518	1.5486	0.122	4.74E-06	count	1
PELI1	0.344986	0.261764	1.3179	0.188	4.76E-06	count	1
WIPI2	0.3454451	0.3275879	1.0545	0.292	4.80E-06	count	1
DNM1L	0.7797473	0.4642222	1.6797	0.0937	4.80E-06	count	1
BET1	0.7797473	0.6095198	1.2793	0.201	4.80E-06	count	1
LGALS3	0.7775577	0.5923262	1.3127	0.19	4.80E-06	count	1

UBE3B	1.6034536	0.9700705	1.6529	0.0991	4.81E-06	count	1
COX20	0.348902	0.263295	1.3251	0.186	4.82E-06	count	1
ETV5	1.6034536	0.9517435	1.6848	0.0927	4.83E-06	count	1
MTPAP	1.6034536	0.9840849	1.6294	0.104	4.83E-06	count	1
SENP6	0.3519612	0.2641925	1.3322	0.183	4.85E-06	count	1
RC3H2	0.7909304	0.5217834	1.5158	0.13	4.85E-06	count	1
RCL1	1.6034536	1.0457715	1.5333	0.126	4.86E-06	count	1
MED1	0.7875348	0.5288377	1.4892	0.137	4.86E-06	count	1
RAB3IP	0.7846468	0.4226989	1.8563	0.0641	4.88E-06	count	1
BAG5	0.3532568	0.2745442	1.2867	0.199	4.89E-06	count	1
WDR36	0.7875348	0.5697774	1.3822	0.168	4.91E-06	count	1
MRPS18A	0.7963569	0.446503	1.7835	0.0752	4.92E-06	count	1
PHF6	0.3553777	0.2277065	1.5607	0.119	4.92E-06	count	1
NAA15	0.3574787	0.3204705	1.1155	0.265	4.93E-06	count	1
AC060780.1	0.8042747	0.6381981	1.2602	0.208	4.94E-06	count	1
SRP14-AS1	0.816245	0.8085879	1.0095	0.313	4.96E-06	count	1
SYTL3	0.8086506	0.6126186	1.32	0.188	4.96E-06	count	1
JMJD8	0.8021301	0.5008082	1.6017	0.11	4.97E-06	count	1
EMSY	0.8020233	0.6090606	1.3168	0.189	4.98E-06	count	1
TCP11L2	0.7940473	0.4321444	1.8375	0.0668	4.98E-06	count	1
RFC3	0.816245	0.7304645	1.1174	0.264	4.99E-06	count	1
CCDC82	0.3577426	0.284085	1.2593	0.209	5.00E-06	count	1
TEC	0.816245	0.5484389	1.4883	0.137	5.00E-06	count	1
RASA1	0.8222157	0.720748	1.1408	0.255	5.00E-06	count	1
5-Mar	0.7963569	0.4879672	1.632	0.103	5.01E-06	count	1
KLF7	0.8179238	0.6208701	1.3174	0.188	5.02E-06	count	1
YTHDC2	0.3629277	0.4235816	0.8568	0.392	5.02E-06	count	1
RIPK2	0.3629495	0.3113064	1.1659	0.244	5.03E-06	count	1
CCDC142	1.6780274	1.231146	1.363	0.174	5.03E-06	count	1
TOMM70	0.8086506	0.6556836	1.2333	0.218	5.04E-06	count	1
TRUB2	0.8179238	0.5930407	1.3792	0.169	5.04E-06	count	1
POGK	0.816245	0.5296233	1.5412	0.124	5.05E-06	count	1
PNPT1	0.7955109	0.6345981	1.2536	0.211	5.05E-06	count	1
MAN1A2	0.8219899	0.4339778	1.8941	0.0589	5.06E-06	count	1
NT5DC1	0.3618312	0.2952607	1.2255	0.221	5.06E-06	count	1
AK2	0.3629277	0.3472688	1.0451	0.297	5.06E-06	count	1
METTL4	0.3565413	0.4936157	0.7223	0.47	5.07E-06	count	1
DCAF4	1.6780274	1.135746	1.4775	0.14	5.07E-06	count	1
POLR2E	0.3655542	0.2505337	1.4591	0.145	5.07E-06	count	1
RAB20	0.8321552	0.7303588	1.1394	0.255	5.11E-06	count	1
ISOC1	0.8220193	0.4645027	1.7697	0.0775	5.12E-06	count	1
MAT2B	0.368411	0.2849151	1.2931	0.197	5.12E-06	count	1
CTNNA1	0.8246862	0.5650211	1.4596	0.145	5.13E-06	count	1
ZNF3	0.8321552	0.6984938	1.1914	0.234	5.14E-06	count	1
CIZ1	1.6802498	0.833676	2.0155	0.0445	5.16E-06	count	1

USP32	1.6792676	0.9852415	1.7044	0.089	5.17E-06	count	1
UTP23	0.3720101	0.3386098	1.0986	0.273	5.18E-06	count	1
COL19A1	0.8350193	0.4829401	1.729	0.0845	5.19E-06	count	1
RRN3	0.8385374	0.5974729	1.4035	0.161	5.20E-06	count	1
WARS	0.8246862	0.5090976	1.6199	0.106	5.21E-06	count	1
ERF	0.8205992	0.37117	2.2108	0.0276	5.23E-06	count	1
ZNF165	0.8219899	0.5022613	1.6366	0.102	5.23E-06	count	1
TTN-AS1	0.8385374	0.7460434	1.124	0.262	5.23E-06	count	1
RNF166	0.3760758	0.4307333	0.8731	0.383	5.24E-06	count	1
ZC3H14	0.8292435	0.4916742	1.6866	0.0924	5.25E-06	count	1
ZNF426	1.7076041	0.9098919	1.8767	0.0612	5.29E-06	count	1
CXCR5	0.3760414	0.2930838	1.2831	0.2	5.30E-06	count	1
SEC22A	0.8588049	0.6906923	1.2434	0.214	5.31E-06	count	1
ENDOV	1.7076041	0.8008102	2.1323	0.0335	5.31E-06	count	1
PPIL2	0.8415804	0.4824667	1.7443	0.0818	5.35E-06	count	1
NUP50	0.8415013	0.4936737	1.7046	0.089	5.36E-06	count	1
ATF7IP	0.3841111	0.2032156	1.8902	0.0594	5.36E-06	count	1
NEK6	0.3813565	0.3257625	1.1707	0.242	5.37E-06	count	1
MAP4K1	0.8467797	0.4601265	1.8403	0.0664	5.37E-06	count	1
CLPB	1.7575337	1.0129654	1.735	0.0834	5.38E-06	count	1
FER	1.7575337	1.1578832	1.5179	0.13	5.38E-06	count	1
NOL12	1.7575337	1.1578832	1.5179	0.13	5.38E-06	count	1
TMEM39A	1.7575337	1.1578832	1.5179	0.13	5.38E-06	count	1
TAB3	1.7575337	1.1578832	1.5179	0.13	5.38E-06	count	1
CHST15	1.7575337	1.1578832	1.5179	0.13	5.38E-06	count	1
GAN	1.7575337	1.1578832	1.5179	0.13	5.38E-06	count	1
GABARAPL1	1.7575337	1.28658	1.3661	0.173	5.39E-06	count	1
KIAA1324L	1.7575337	1.28658	1.3661	0.173	5.39E-06	count	1
RDH13	1.7575337	1.28658	1.3661	0.173	5.39E-06	count	1
MATN1	1.7575337	1.28658	1.3661	0.173	5.39E-06	count	1
SELENOI	1.7575337	1.28658	1.3661	0.173	5.39E-06	count	1
ZNF142	1.7575337	1.28658	1.3661	0.173	5.39E-06	count	1
GIMAP2	1.7575337	1.28658	1.3661	0.173	5.39E-06	count	1
TTLL5	1.7575337	1.28658	1.3661	0.173	5.39E-06	count	1
LINC02245	1.7575337	1.4035251	1.2522	0.211	5.39E-06	count	1
SGPP2	1.7575337	1.4035251	1.2522	0.211	5.39E-06	count	1
HSD17B6	1.7575337	1.858745	0.9455	0.345	5.39E-06	count	1
BMS1	0.849065	0.3875265	2.191	0.029	5.43E-06	count	1
TRIB3	0.8689694	0.7134197	1.218	0.224	5.43E-06	count	1
MTREX	0.8434363	0.4172657	2.0213	0.0438	5.43E-06	count	1
RBM10	0.8689694	0.6755424	1.2863	0.199	5.43E-06	count	1
PPIP5K2	0.3853241	0.3554246	1.0841	0.279	5.44E-06	count	1
SUMF1	1.7654963	1.2214744	1.4454	0.149	5.45E-06	count	1
TNPO2	1.7654963	1.0629335	1.661	0.0974	5.45E-06	count	1
AFMID	0.8689694	0.7033769	1.2354	0.217	5.46E-06	count	1

PGM2	0.8640526	0.5092249	1.6968	0.0904	5.46E-06	count	1
ELL2	0.3848756	0.3860022	0.9971	0.319	5.47E-06	count	1
AC020915.3	1.7654963	0.9554853	1.8477	0.0653	5.47E-06	count	1
PKD1	0.8689694	0.6911544	1.2573	0.209	5.50E-06	count	1
NPTN	0.849065	0.4134897	2.0534	0.0406	5.50E-06	count	1
ABHD5	0.3922746	0.3316345	1.1829	0.238	5.52E-06	count	1
AC092683.1	0.8579239	0.5952955	1.4412	0.15	5.52E-06	count	1
NDUFV3	0.8810994	0.5463988	1.6126	0.108	5.53E-06	count	1
NPAT	0.391905	0.2986453	1.3123	0.19	5.54E-06	count	1
SREBF1	1.7575337	0.8601307	2.0433	0.0416	5.55E-06	count	1
PRR34-AS1	0.8772392	0.6425484	1.3653	0.173	5.56E-06	count	1
RRP15	0.3952088	0.3529821	1.1196	0.263	5.56E-06	count	1
PDLIM5	0.8772392	0.6724744	1.3045	0.193	5.56E-06	count	1
DCUN1D5	0.3971906	0.3868795	1.0267	0.305	5.57E-06	count	1
6-Mar	0.3963667	0.2640483	1.5011	0.134	5.58E-06	count	1
OSBPL3	0.8841113	0.6212649	1.4231	0.155	5.59E-06	count	1
HSD17B8	0.8841113	0.6577326	1.3442	0.18	5.60E-06	count	1
TIMM10B	0.8733052	0.4339851	2.0123	0.0448	5.61E-06	count	1
MED15	0.8940539	0.7016475	1.2742	0.203	5.61E-06	count	1
MRPL3	0.3999551	0.2424851	1.6494	0.0998	5.64E-06	count	1
SDR42E2	1.7575337	1.1913615	1.4752	0.141	5.64E-06	count	1
ABRAXAS1	0.9000948	0.6293647	1.4302	0.153	5.64E-06	count	1
ZNF518B	0.8907528	0.5383418	1.6546	0.0987	5.65E-06	count	1
FAM102A	0.4018913	0.3524704	1.1402	0.255	5.67E-06	count	1
ACSS1	0.8978179	0.6062094	1.481	0.139	5.68E-06	count	1
VIRMA	0.4018913	0.3685933	1.0903	0.276	5.71E-06	count	1
SERPINB9	0.4058441	0.2021672	2.0075	0.0453	5.71E-06	count	1
LAX1	0.9093577	0.7313711	1.2434	0.214	5.73E-06	count	1
PDXDC1	0.9051149	0.6768777	1.3372	0.182	5.73E-06	count	1
TOLLIP	0.9041796	0.4891186	1.8486	0.0652	5.78E-06	count	1
CORO7	0.9051149	0.6045092	1.4973	0.135	5.78E-06	count	1
GSR	0.9093577	0.6108224	1.4887	0.137	5.80E-06	count	1
GTF3C1	0.9093577	0.7642883	1.1898	0.235	5.81E-06	count	1
ANKH	0.9146242	0.7118241	1.2849	0.199	5.81E-06	count	1
USP12	0.4092661	0.2553702	1.6026	0.11	5.82E-06	count	1
ZNF484	1.84353	1.0510663	1.754	0.0801	5.83E-06	count	1
POC1B-AS1	1.84353	1.1590426	1.5906	0.112	5.84E-06	count	1
CENPU	1.8231984	0.868518	2.0992	0.0364	5.84E-06	count	1
BRWD3	0.9146242	0.6812435	1.3426	0.18	5.85E-06	count	1
GTF3C5	0.9051149	0.609002	1.4862	0.138	5.85E-06	count	1
TUBB2A	0.4122171	0.317303	1.2991	0.195	5.85E-06	count	1
TIPIN	0.9146242	0.8170832	1.1194	0.264	5.85E-06	count	1
NOLC1	0.411623	0.3216517	1.2797	0.201	5.86E-06	count	1
CARD19	0.9093577	0.6366088	1.4284	0.154	5.87E-06	count	1
FBXL17	0.9146242	0.7470548	1.2243	0.221	5.88E-06	count	1

CLCN4	1.861063	0.9627601	1.933	0.0539	5.89E-06	count	1
ADRB2	0.9168704	0.5046882	1.8167	0.0699	5.90E-06	count	1
NCAPH2	0.9093577	0.5967941	1.5237	0.128	5.90E-06	count	1
SAMD8	0.9041796	0.4956216	1.8243	0.0688	5.92E-06	count	1
CEP135	0.9116034	0.5383006	1.6935	0.0911	5.93E-06	count	1
IFITM1	0.9161464	0.572065	1.6015	0.11	5.93E-06	count	1
PAF1	0.4183666	0.3459915	1.2092	0.227	5.95E-06	count	1
SPG7	0.4170715	0.3798468	1.098	0.273	5.96E-06	count	1
TMX4	0.4203299	0.2349239	1.7892	0.0743	5.98E-06	count	1
KLHL7	0.9431771	0.6347236	1.486	0.138	6.01E-06	count	1
ZFP82	1.8946201	1.2086754	1.5675	0.118	6.03E-06	count	1
ADPRH	1.8946201	1.2086754	1.5675	0.118	6.03E-06	count	1
PSEN2	1.8946201	1.2086754	1.5675	0.118	6.03E-06	count	1
NANP	1.8946201	1.3070274	1.4496	0.148	6.04E-06	count	1
ZNF222	1.8946201	1.2179944	1.5555	0.121	6.05E-06	count	1
ZNF592	1.8946201	1.2179944	1.5555	0.121	6.05E-06	count	1
TMEM187	1.8946201	1.2179944	1.5555	0.121	6.05E-06	count	1
SYNGAP1	1.8946201	1.3156499	1.4401	0.151	6.06E-06	count	1
NLRP3	1.8946201	1.3156499	1.4401	0.151	6.06E-06	count	1
METTL22	1.8946201	1.3156499	1.4401	0.151	6.06E-06	count	1
B9D2	0.9323241	0.5288027	1.7631	0.0786	6.06E-06	count	1
MCM4	1.8946201	1.5726434	1.2047	0.229	6.07E-06	count	1
MIA2	0.4251523	0.2801258	1.5177	0.13	6.08E-06	count	1
POLR2D	0.9333391	0.5513698	1.6928	0.0912	6.08E-06	count	1
LHPP	0.4211501	0.3910016	1.0771	0.282	6.08E-06	count	1
RASSF6	0.4267812	0.4652411	0.9173	0.359	6.10E-06	count	1
ASF1A	0.9258715	0.4336891	2.1349	0.0333	6.10E-06	count	1
AC016831.7	0.4307093	0.2647688	1.6267	0.105	6.13E-06	count	1
PPFIBP2	0.9459796	0.6461823	1.464	0.144	6.15E-06	count	1
IFT27	1.8778592	0.777214	2.4161	0.0161	6.18E-06	count	1
RFNG	0.9548145	0.5127098	1.8623	0.0632	6.20E-06	count	1
ACTR8	1.8946201	0.8519741	2.2238	0.0267	6.20E-06	count	1
ADA	1.8946201	0.9622059	1.969	0.0496	6.21E-06	count	1
FAM161A	0.9379045	0.6872249	1.3648	0.173	6.21E-06	count	1
PSMD6	0.4350956	0.2784453	1.5626	0.119	6.21E-06	count	1
MAP3K2	0.4309536	0.2484629	1.7345	0.0835	6.21E-06	count	1
NRIP1	0.9343386	0.4880499	1.9144	0.0562	6.23E-06	count	1
CERS4	0.4350324	0.3249579	1.3387	0.181	6.25E-06	count	1
JPX	0.4345045	0.3784926	1.148	0.252	6.25E-06	count	1
SPECC1	0.9682726	0.7619952	1.2707	0.204	6.26E-06	count	1
VMP1	0.436394	0.3419214	1.2763	0.203	6.28E-06	count	1
GSTCD	1.9141174	0.9443192	2.027	0.0433	6.28E-06	count	1
APMAP	0.9666808	0.599775	1.6117	0.108	6.30E-06	count	1
FIP1L1	0.4391481	0.2651278	1.6564	0.0984	6.31E-06	count	1
PPP4R1	0.9524116	0.4627243	2.0583	0.0401	6.34E-06	count	1

ZHX1	1.8946201	0.9576438	1.9784	0.0485	6.35E-06	count	1
CCDC137	0.9663007	0.6392604	1.5116	0.131	6.38E-06	count	1
MNT	0.9919779	0.8040224	1.2338	0.218	6.41E-06	count	1
PFDN1	0.448822	0.2967465	1.5125	0.131	6.44E-06	count	1
MOB4	0.4474964	0.2925604	1.5296	0.127	6.50E-06	count	1
NSUN5	0.9904073	0.7214236	1.3729	0.17	6.50E-06	count	1
LZTFL1	0.9904073	0.7407633	1.337	0.182	6.51E-06	count	1
ZBTB40	0.9934295	0.6962178	1.4269	0.154	6.51E-06	count	1
RCHY1	0.9971409	0.7718725	1.2918	0.197	6.55E-06	count	1
POLR2J3	0.4522889	0.319179	1.417	0.157	6.55E-06	count	1
AC013394.1	0.965971	0.5413939	1.7842	0.0751	6.57E-06	count	1
SCAPER	0.450829	0.382484	1.1787	0.239	6.57E-06	count	1
PAXBP1	0.4517475	0.3142669	1.4375	0.151	6.59E-06	count	1
SLC35A4	0.9934295	0.7885149	1.2599	0.208	6.59E-06	count	1
RABGGTB	0.4549683	0.2807022	1.6208	0.106	6.62E-06	count	1
KIF18A	2.0122699	1.2589797	1.5983	0.111	6.63E-06	count	1
KLHL21	2.0122699	1.3371435	1.5049	0.133	6.64E-06	count	1
TNK1	2.0122699	1.4109839	1.4261	0.155	6.64E-06	count	1
IQCE	2.0051576	0.9599694	2.0888	0.0373	6.65E-06	count	1
DGKE	0.9951854	0.5568382	1.7872	0.0746	6.66E-06	count	1
ZNF468	2.0122699	1.2543097	1.6043	0.109	6.66E-06	count	1
RAPGEF2	2.0122699	1.3327474	1.5099	0.132	6.67E-06	count	1
THAP6	0.9802063	0.5904839	1.66	0.0976	6.68E-06	count	1
DENND2C	2.0122699	1.4068186	1.4304	0.153	6.68E-06	count	1
SLMAP	1.0113514	0.5948946	1.7001	0.0898	6.70E-06	count	1
RALGDS	1.0040591	0.5700313	1.7614	0.0789	6.70E-06	count	1
ST3GAL5	0.4608667	0.374572	1.2304	0.219	6.71E-06	count	1
TRMT6	0.9847763	0.5260987	1.8718	0.0619	6.71E-06	count	1
ACTR1B	1.0113514	0.6918896	1.4617	0.145	6.72E-06	count	1
NUDT4	0.4607007	0.2960438	1.5562	0.12	6.74E-06	count	1
FBXW7	0.4637074	0.3299722	1.4053	0.161	6.76E-06	count	1
KIF9	1.0074241	0.6310903	1.5963	0.111	6.77E-06	count	1
UTP18	0.4652586	0.3221386	1.4443	0.149	6.79E-06	count	1
MAP1LC3A	1.0032453	0.4748659	2.1127	0.0352	6.79E-06	count	1
ODF2L	0.4688726	0.309365	1.5156	0.13	6.80E-06	count	1
SLC16A3	0.4682476	0.3347488	1.3988	0.163	6.82E-06	count	1
RIT1	0.47045	0.3311315	1.4207	0.156	6.84E-06	count	1
FPGS	2.0122699	0.822677	2.446	0.0148	6.87E-06	count	1
CORO2A	2.067353	1.807387	1.1438	0.253	6.89E-06	count	1
ZNF775	2.067353	1.2296793	1.6812	0.0934	6.89E-06	count	1
DVL1	2.067353	1.2296793	1.6812	0.0934	6.89E-06	count	1
TMX1	0.4735835	0.2971001	1.594	0.112	6.89E-06	count	1
EXOSC10	0.9985728	0.5141364	1.9422	0.0527	6.89E-06	count	1
PARP12	2.0265014	1.0138705	1.9988	0.0462	6.91E-06	count	1
DR1	0.4756845	0.3703096	1.2846	0.2	6.94E-06	count	1

DUS1L	0.4768027	0.3095146	1.5405	0.124	6.95E-06	count	1
MRPL9	0.4742992	0.3447619	1.3757	0.17	6.96E-06	count	1
RNF170	1.0434746	0.6230687	1.6747	0.0947	6.96E-06	count	1
EEA1	0.4751597	0.3721001	1.277	0.202	6.96E-06	count	1
HLTF	0.473539	0.4460522	1.0616	0.289	6.96E-06	count	1
DNAJC16	2.067353	1.0358005	1.9959	0.0466	6.97E-06	count	1
ASNSD1	1.012781	0.427661	2.3682	0.0183	7.00E-06	count	1
UNC119	0.4818169	0.3897808	1.2361	0.217	7.01E-06	count	1
CIAO1	1.0261151	0.4764122	2.1538	0.0318	7.01E-06	count	1
SIGLEC10	0.4824177	0.3785536	1.2744	0.203	7.01E-06	count	1
ZDHHC2	0.4804416	0.3740667	1.2844	0.2	7.04E-06	count	1
RWDD4	0.4798106	0.4399182	1.0907	0.276	7.06E-06	count	1
CSNK1G3	0.4869865	0.2431752	2.0026	0.0458	7.11E-06	count	1
XPC	0.4837563	0.3839284	1.26	0.208	7.13E-06	count	1
PRKACB	0.4862451	0.3390631	1.4341	0.152	7.13E-06	count	1
SHC1	1.0635154	0.8893203	1.1959	0.232	7.14E-06	count	1
C1GALT1C1	1.0605109	0.6855256	1.547	0.123	7.15E-06	count	1
TENT4A	1.0635154	0.9439128	1.1267	0.26	7.17E-06	count	1
ZNF430	0.4905578	0.3149688	1.5575	0.12	7.17E-06	count	1
MIAT	1.0605109	0.7220977	1.4687	0.143	7.17E-06	count	1
PDSS2	1.0605109	0.7220977	1.4687	0.143	7.17E-06	count	1
SNX20	2.1155552	1.221752	1.7316	0.084	7.17E-06	count	1
IL7	1.0635154	0.8952183	1.188	0.235	7.19E-06	count	1
VAT1	1.0635154	0.9198415	1.1562	0.248	7.22E-06	count	1
LSM14B	1.0700776	0.653111	1.6384	0.102	7.22E-06	count	1
DNMT1	0.4936418	0.3804653	1.2975	0.195	7.27E-06	count	1
RMI1	1.0733389	0.6666074	1.6102	0.108	7.27E-06	count	1
PEX3	1.0660472	0.6115312	1.7432	0.082	7.27E-06	count	1
ZNF800	0.4939236	0.3387841	1.4579	0.146	7.28E-06	count	1
AC139530.1	1.0744138	0.6795246	1.5811	0.115	7.29E-06	count	1
PATZ1	1.0815671	0.7679752	1.4083	0.16	7.29E-06	count	1
KLHL24	0.4943971	0.2974972	1.6619	0.0972	7.31E-06	count	1
ABHD2	1.0841443	0.7358727	1.4733	0.141	7.33E-06	count	1
ATXN3	0.4883557	0.4250822	1.1489	0.251	7.36E-06	count	1
GNA12	1.0731418	0.6027355	1.7805	0.0757	7.41E-06	count	1
WDR6	1.0635154	0.6006686	1.7706	0.0773	7.43E-06	count	1
CRNKL1	1.0421854	0.5060234	2.0596	0.04	7.47E-06	count	1
MTCH1	0.5066426	0.3480485	1.4557	0.146	7.48E-06	count	1
FAM215B	1.1067041	0.6776886	1.6331	0.103	7.56E-06	count	1
PPIL1	2.1776636	1.1594092	1.8783	0.061	7.57E-06	count	1
NETO1	1.1196422	0.7923212	1.4131	0.158	7.57E-06	count	1
PEX19	2.1452046	0.9656878	2.2214	0.0268	7.58E-06	count	1
ACYP2	1.0635154	0.5170611	2.0568	0.0403	7.60E-06	count	1
PFDN4	0.5132155	0.3750802	1.3683	0.172	7.65E-06	count	1
SLC15A3	1.0774911	0.7532044	1.4305	0.153	7.70E-06	count	1

MDM1	1.1105342	0.6223676	1.7844	0.075	7.77E-06	count	1
SAP30L	1.1105342	0.6223676	1.7844	0.075	7.77E-06	count	1
TTC17	0.5183392	0.3557569	1.457	0.146	7.80E-06	count	1
VPS51	0.5242889	0.3072698	1.7063	0.0887	7.82E-06	count	1
INSIG1	0.5217646	0.4410109	1.1831	0.237	7.86E-06	count	1
UBXN2B	1.139148	0.7756672	1.4686	0.143	7.87E-06	count	1
PPP1R9B	1.1405223	0.711252	1.6035	0.11	7.89E-06	count	1
TRMT10B	1.1405223	0.7325183	1.557	0.12	7.91E-06	count	1
ITPR2	0.5242889	0.3490665	1.502	0.134	7.92E-06	count	1
METTL17	1.1113493	0.488983	2.2728	0.0235	7.96E-06	count	1
PRELID3B	0.5330315	0.2846457	1.8726	0.0618	7.97E-06	count	1
AC008555.5	2.2737179	1.0896261	2.0867	0.0375	7.98E-06	count	1
RBM18	1.139148	0.7018033	1.6232	0.105	8.00E-06	count	1
AC006504.5	1.1516553	0.8669859	1.3283	0.185	8.01E-06	count	1
C12orf73	1.1516553	0.8921678	1.2909	0.197	8.02E-06	count	1
FAM174A	1.1516553	0.784256	1.4685	0.143	8.02E-06	count	1
FXN	1.1516553	0.7531466	1.5291	0.127	8.02E-06	count	1
RNF25	1.1405223	0.7909893	1.4419	0.15	8.03E-06	count	1
UBAP1	1.1468777	0.6187196	1.8536	0.0645	8.06E-06	count	1
EGR1	0.5304265	0.4167862	1.2727	0.204	8.07E-06	count	1
CARF	1.1516553	0.8367354	1.3764	0.169	8.08E-06	count	1
DCTPP1	0.5397911	0.2657819	2.031	0.0429	8.09E-06	count	1
KIAA0753	1.1468777	0.755005	1.519	0.129	8.11E-06	count	1
TMEM126A	0.5386922	0.3626145	1.4856	0.138	8.12E-06	count	1
CA5B	1.1156363	0.4755239	2.3461	0.0194	8.13E-06	count	1
RPL7L1	0.5425426	0.2886987	1.8793	0.0609	8.16E-06	count	1
BAP1	1.18133	0.996329	1.1857	0.236	8.17E-06	count	1
WIPF2	1.151875	0.5407132	2.1303	0.0337	8.17E-06	count	1
TEX30	1.1277726	0.6172903	1.827	0.0684	8.19E-06	count	1
LRRC75A	1.1192486	0.4888598	2.2895	0.0225	8.21E-06	count	1
SNX24	2.2911488	1.1505507	1.9913	0.0471	8.22E-06	count	1
ALDH6A1	2.2911488	1.2058441	1.9	0.0581	8.24E-06	count	1
RBMXL1	1.1786999	0.5813816	2.0274	0.0432	8.27E-06	count	1
AC245140.2	2.3102042	0.848136	2.7239	0.0067	8.36E-06	count	1
REPIN1	1.1488708	0.5255286	2.1861	0.0293	8.37E-06	count	1
MPI	1.1867075	0.6712906	1.7678	0.0778	8.43E-06	count	1
WDR53	2.3590799	1.0518997	2.2427	0.0254	8.46E-06	count	1
TP53INP1	0.5582717	0.2540319	2.1976	0.0285	8.46E-06	count	1
BX890604.1	2.3590799	1.157865	2.0374	0.0422	8.47E-06	count	1
KIAA1324	2.3590799	1.6746608	1.4087	0.16	8.47E-06	count	1
FBXO46	2.3590799	1.3449787	1.754	0.0801	8.49E-06	count	1
PYROXD2	2.3542825	1.6650445	1.4139	0.158	8.49E-06	count	1
WRNIP1	1.1442134	0.5481888	2.0873	0.0374	8.56E-06	count	1
COG6	1.1879625	0.7282317	1.6313	0.104	8.59E-06	count	1
ZNF804A	2.3673322	1.289876	1.8353	0.0671	8.64E-06	count	1

TIAM2	1.2050144	0.7500162	1.6067	0.109	8.66E-06	count	1
XPO1	0.5671475	0.2576036	2.2016	0.0282	8.66E-06	count	1
AC099524.1	2.3673322	1.1808882	2.0047	0.0456	8.70E-06	count	1
ZC2HC1A	0.5660875	0.3586621	1.5783	0.115	8.73E-06	count	1
ZFR	0.5778888	0.3055582	1.8913	0.0592	8.74E-06	count	1
GMFB	1.2244396	0.7630601	1.6046	0.109	8.76E-06	count	1
FAM126B	1.2057067	0.6296331	1.9149	0.0561	8.81E-06	count	1
BLZF1	1.2320593	0.8245156	1.4943	0.136	8.82E-06	count	1
IRAK2	1.2050144	0.6616333	1.8213	0.0692	8.84E-06	count	1
ADAM8	1.2062694	0.6268101	1.9245	0.0549	8.84E-06	count	1
MIR222HG	1.2277419	0.7123628	1.7235	0.0855	8.86E-06	count	1
RPP21	1.2320593	0.7638492	1.613	0.107	8.89E-06	count	1
PYGO2	1.2320593	0.8151757	1.5114	0.131	8.91E-06	count	1
SATB1	2.4360871	1.1154971	2.1839	0.0295	8.92E-06	count	1
ATP2A2	1.2320593	0.8141441	1.5133	0.131	8.93E-06	count	1
GPATCH4	0.5793514	0.3613392	1.6033	0.11	8.94E-06	count	1
NONO	0.5824781	0.2802627	2.0783	0.0383	8.95E-06	count	1
NOP14-AS1	2.4360871	1.4227248	1.7123	0.0875	8.95E-06	count	1
AL021453.1	1.2358678	0.6400817	1.9308	0.0541	8.96E-06	count	1
PAPOLG	1.2277419	0.7169176	1.7125	0.0875	8.96E-06	count	1
PLA2G16	1.2219547	0.6931982	1.7628	0.0786	9.02E-06	count	1
ADO	1.2439625	0.6007074	2.0708	0.039	9.09E-06	count	1
SKAP1	1.2634839	0.5042219	2.5058	0.0126	9.14E-06	count	1
PDLIM7	1.2534467	1.0146498	1.2353	0.217	9.16E-06	count	1
PTPMT1	1.2431066	0.6098035	2.0385	0.0421	9.17E-06	count	1
CRIP2	1.2439625	0.856688	1.4521	0.147	9.30E-06	count	1
RIOK2	1.1958627	0.5516229	2.1679	0.0307	9.31E-06	count	1
DNTTIP1	1.2187631	0.6404019	1.9031	0.0577	9.40E-06	count	1
S100A9	0.6006772	0.9172339	0.6549	0.513	9.50E-06	count	1
ZNF860	2.5026172	1.3106013	1.9095	0.0568	9.54E-06	count	1
ZNF586	2.508451	0.9350677	2.6826	0.0076	9.61E-06	count	1
PNPLA2	1.2407919	0.4095166	3.0299	0.0026	9.68E-06	count	1
OGDH	2.5710832	1.247939	2.0603	0.04	9.68E-06	count	1
SETD9	1.3059891	0.7240734	1.8037	0.072	9.68E-06	count	1
APOOL	1.2507496	0.7231011	1.7297	0.0844	9.72E-06	count	1
KCNN4	0.6267235	0.3365669	1.8621	0.0632	9.84E-06	count	1
MTMR12	1.2697669	0.5701545	2.2271	0.0264	9.90E-06	count	1
ABI1	0.6367561	0.3271331	1.9465	0.0522	9.94E-06	count	1
HSBP1	0.6352562	0.2710808	2.3434	0.0195	9.96E-06	count	1
PRNP	0.6369278	0.3105608	2.0509	0.0409	9.98E-06	count	1
UTP25	1.3417097	0.7807376	1.7185	0.0864	1.00E-05	count	1
RHEX	2.6432224	1.6723581	1.5805	0.115	1.00E-05	count	1
BAZ2B	1.31401	0.619272	2.1219	0.0344	1.00E-05	count	1
KLHL8	2.6311319	1.2094945	2.1754	0.0301	1.02E-05	count	1
PIK3AP1	0.6473854	0.3006446	2.1533	0.0318	1.03E-05	count	1

BRD9	0.6530671	0.4097726	1.5937	0.112	1.03E-05	count	1
IFT43	1.3307409	0.545027	2.4416	0.015	1.03E-05	count	1
KLF10	0.6649622	0.3133886	2.1218	0.0344	1.05E-05	count	1
NEPRO	1.3473066	0.5392601	2.4984	0.0128	1.05E-05	count	1
TCF12	1.3372883	0.528951	2.5282	0.0118	1.05E-05	count	1
GPKOW	1.3744211	0.7571789	1.8152	0.0702	1.05E-05	count	1
SYNGR3	1.3744211	0.7992546	1.7196	0.0862	1.06E-05	count	1
ADCK1	2.7742989	1.2398617	2.2376	0.0257	1.07E-05	count	1
AC245060.5	2.7742989	1.2398617	2.2376	0.0257	1.07E-05	count	1
DYNC1LI1	0.6778545	0.2485434	2.7273	0.0066	1.08E-05	count	1
NCOA2	1.3837941	0.661782	2.091	0.0371	1.08E-05	count	1
MPHOSPH9	1.4140375	0.8166451	1.7315	0.0841	1.09E-05	count	1
ZXDC	1.4140375	0.7048041	2.0063	0.0454	1.09E-05	count	1
MBOAT7	2.7397939	1.3826501	1.9816	0.0481	1.09E-05	count	1
THOC1	0.6817789	0.409552	1.6647	0.0967	1.10E-05	count	1
SGMS1	1.3973755	0.6212664	2.2492	0.025	1.10E-05	count	1
ZBTB10	0.6914993	0.3605645	1.9178	0.0558	1.10E-05	count	1
PBXIP1	0.6909434	0.3227216	2.141	0.0328	1.10E-05	count	1
SLC12A6	1.3837941	0.5719302	2.4195	0.0159	1.11E-05	count	1
LAS1L	1.3621476	0.6205509	2.1951	0.0287	1.11E-05	count	1
CHP1	1.4473935	0.6917854	2.0923	0.037	1.13E-05	count	1
KLHL28	0.6929664	0.328525	2.1093	0.0355	1.13E-05	count	1
PIK3IP1	0.699105	0.3366802	2.0765	0.0384	1.13E-05	count	1
TP53I13	1.386996	0.4598076	3.0165	0.0027	1.14E-05	count	1
ASXL1	0.7110667	0.2528493	2.8122	0.0051	1.15E-05	count	1
R3HDM4	0.7248173	0.2684966	2.6995	0.0072	1.18E-05	count	1
MOB2	1.3913634	0.5092083	2.7324	0.0065	1.18E-05	count	1
SRF	1.4806731	0.8193116	1.8072	0.0714	1.18E-05	count	1
ZYX	1.4411839	0.5005701	2.8791	0.0042	1.20E-05	count	1
ZNF84	1.4977258	0.7329908	2.0433	0.0416	1.21E-05	count	1
TMEM127	1.445552	0.6651077	2.1734	0.0303	1.21E-05	count	1
RPA1	1.4742943	0.771403	1.9112	0.0566	1.22E-05	count	1
AC020916.1	0.7495944	0.2967	2.5264	0.0119	1.23E-05	count	1
IL4R	0.7632099	0.4709339	1.6206	0.106	1.23E-05	count	1
SNRK	0.7544627	0.3805653	1.9825	0.048	1.24E-05	count	1
JAGN1	0.7562047	0.3159456	2.3935	0.0171	1.26E-05	count	1
SMAGP	0.7538439	0.4037108	1.8673	0.0625	1.26E-05	count	1
MATN1-AS1	1.5550164	0.6410559	2.4257	0.0157	1.27E-05	count	1
SHQ1	1.5424743	0.800432	1.9271	0.0546	1.28E-05	count	1
ZNF441	1.5925433	1.0043059	1.5857	0.114	1.29E-05	count	1
FBXO33	0.7713052	0.3900766	1.9773	0.0486	1.30E-05	count	1
HMGCS1	0.7920149	0.3426171	2.3117	0.0213	1.30E-05	count	1
PLAUR	1.5424743	1.1699257	1.3184	0.188	1.31E-05	count	1
CIITA	0.7817513	0.3503495	2.2313	0.0262	1.32E-05	count	1
RFXAP	3.2110634	1.7021341	1.8865	0.0599	1.32E-05	count	1

SMIM20	0.7907388	0.3261792	2.4242	0.0157	1.33E-05	count	1
MRPL24	1.6065366	0.7309164	2.198	0.0285	1.36E-05	count	1
AC245014.3	0.8258084	0.3878447	2.1292	0.0338	1.40E-05	count	1
SBNO1	0.8141109	0.3118736	2.6104	0.0094	1.40E-05	count	1
TRMT1L	1.6370832	0.7495768	2.184	0.0295	1.44E-05	count	1
ARHGEF1	0.8452277	0.2822591	2.9945	0.0029	1.45E-05	count	1
TARSL2	0.8499631	0.33085	2.569	0.0105	1.45E-05	count	1
SUN2	0.8369794	0.4060501	2.0613	0.0399	1.47E-05	count	1
PSMD5	1.7510125	0.9105251	1.9231	0.0551	1.52E-05	count	1
PGAM5	1.7510125	0.9381089	1.8665	0.0626	1.52E-05	count	1
GNPTAB	0.8724072	0.4032639	2.1634	0.031	1.54E-05	count	1
XKR6	1.7575337	0.7514792	2.3388	0.0198	1.55E-05	count	1
PRPF39	1.6949789	0.6878261	2.4643	0.0141	1.57E-05	count	1
PRDM8	1.7530499	0.7746888	2.2629	0.0241	1.60E-05	count	1
PIAS3	1.8166445	0.9310919	1.9511	0.0517	1.61E-05	count	1
HIPK2	1.6802498	0.8385473	2.0038	0.0457	1.63E-05	count	1
GPATCH2	0.916563	0.4002053	2.2902	0.0225	1.63E-05	count	1
BCL2L11	0.9400315	0.3633423	2.5872	0.01	1.69E-05	count	1
TMCC3	1.8946201	1.3070273	1.4496	0.148	1.70E-05	count	1
DCAF8	1.955503	1.1711836	1.6697	0.0957	1.77E-05	count	1
CCDC43	1.8831726	0.710844	2.6492	0.0084	1.80E-05	count	1
MED31	0.9548409	0.369379	2.585	0.0101	1.81E-05	count	1
MMD	1.9356969	0.7186377	2.6936	0.0073	1.85E-05	count	1
TEDC1	1.9106637	0.7050213	2.7101	0.007	1.85E-05	count	1
KIAA0319L	1.9914655	0.7747138	2.5706	0.0105	1.91E-05	count	1
LINC00667	1.9763066	0.5921117	3.3377	9.00E-04	1.95E-05	count	1
CCDC77	1.9748529	0.8684518	2.274	0.0234	1.96E-05	count	1
AC120193.1	2.2110563	0.8752798	2.5261	0.0119	2.21E-05	count	1
ZC3HC1	2.3241429	1.199234	1.938	0.0533	2.38E-05	count	1
SEC24B	2.2287011	0.8122374	2.7439	0.0063	2.44E-05	count	1
PINX1	2.3634065	0.9846454	2.4003	0.0168	2.48E-05	count	1
LRRC42	2.6943146	0.9048633	2.9776	0.0031	3.06E-05	count	1
LMNA	1.0026426	0.2942727	3.4072	7.00E-04	5.00E-05	count	1
RBM5	0.0008691	0.316416	0.0027	0.9978	0.00115035	count	1
TLE3	0.0009394	0.2555757	0.0037	0.997	0.001325456	count	1
AFF3	0.0015535	0.2516793	0.0062	0.9951	0.00214477	count	1
MGAT4A	0.0016241	0.3241461	0.005	0.996	0.002274878	count	1
UPF2	0.0016378	0.1407002	0.0116	0.9907	0.002346611	count	1
RHOA	0.0019275	0.1029065	0.0187	0.9851	0.002767694	count	1
PHAX	0.0020176	0.3646894	0.0055	0.9956	0.002846779	count	1
ZC3H15	0.0024619	0.1644977	0.015	0.988	0.003448417	count	1
MEA1	0.0032516	0.3032793	0.0107	0.9915	0.00430418	count	1
EXOG	0.0031244	0.4650604	0.0067	0.995	0.004313677	count	1
COPS2	0.0033972	0.2757981	0.0123	0.99	0.004758555	count	1
SNRPC	0.0038829	0.2316992	0.0168	0.9866	0.005139943	count	1

LRPAP1	0.0041208	0.2350513	0.0175	0.986	0.005879025	count	1
PHF3	0.0042394	0.183947	0.023	0.982	0.006008192	count	1
CLECL1	0.0047054	0.2428897	0.0194	0.985	0.006228882	count	1
EEF1AKMT2	0.0047206	0.3831049	0.0123	0.9902	0.00651763	count	1
NCF1	0.0046801	0.1548434	0.0302	0.976	0.006697261	count	1
SMIM26	0.0051677	0.1974889	0.0262	0.9791	0.006840963	count	1
LST1	0.0052002	0.5300165	0.0098	0.992	0.006883994	count	1
PAIP2	0.0049678	0.1689707	0.0294	0.977	0.00708744	count	1
RPL10A	0.0050423	0.0446391	0.113	0.91	0.007267406	count	1
TNIP2	0.0052002	0.2965273	0.0175	0.986	0.007284217	count	1
PIP4K2A	0.0053862	0.2479104	0.0217	0.9827	0.007436692	count	1
RALY	0.0057069	0.233637	0.0244	0.981	0.007554882	count	1
SLC25A3	0.0053012	0.1200511	0.0442	0.9648	0.007612032	count	1
UBE2H	0.0056994	0.2676821	0.0213	0.983	0.007869167	count	1
ATP5MPL	0.0060611	0.1964031	0.0309	0.975	0.008590061	count	1
MIS18BP1	0.0065962	0.2051681	0.0322	0.974	0.009307424	count	1
SMIM4	0.0070381	0.4132988	0.017	0.9864	0.009317542	count	1
LINC00926	0.0068922	0.1887149	0.0365	0.971	0.009516256	count	1
HINT1	0.0068022	0.0778591	0.0874	0.93	0.00978975	count	1
ANXA5	0.0071165	0.2044173	0.0348	0.972	0.00982599	count	1
VASP	0.0070243	0.1595192	0.044	0.965	0.009911521	count	1
UQCRC2	0.0072012	0.2260433	0.0319	0.975	0.010205941	count	1
RPS3A	0.0070975	0.0379281	0.1871	0.852	0.010236223	count	1
FAM177A1	0.00749	0.2230198	0.0336	0.973	0.010568681	count	1
SCIMP	0.0076093	0.2135898	0.0356	0.972	0.010784354	count	1
LSM3	0.0077156	0.2081283	0.0371	0.97	0.010807988	count	1
BAK1	0.0085631	0.4382883	0.0195	0.9844	0.011336998	count	1
FTSJ1	0.0082262	0.6626534	0.0124	0.9901	0.011358399	count	1
TARBP1	0.0082262	0.6913512	0.0119	0.9905	0.011358399	count	1
C2orf74	0.0082262	0.6912915	0.0119	0.9905	0.011358399	count	1
THAP7	0.0086096	0.3640216	0.0237	0.9811	0.011887858	count	1
MORF4L1	0.0085159	0.1079803	0.0789	0.937	0.012069316	count	1
RILPL2	0.0088169	0.1193918	0.0738	0.941	0.012559174	count	1
HSPA9	0.0093693	0.2144052	0.0437	0.965	0.013220657	count	1
METTL8	0.00961	0.2577524	0.0373	0.97	0.013461944	count	1
PRPF38B	0.0095961	0.159402	0.0602	0.952	0.013600349	count	1
KDM2A	0.0098026	0.2639949	0.0371	0.97	0.013731773	count	1
BANK1	0.0096841	0.0896494	0.108	0.914	0.013875502	count	1
E2F4	0.0106686	0.3015646	0.0354	0.9718	0.014125493	count	1
PPP1R35	0.0102672	0.3141362	0.0327	0.9739	0.014177009	count	1
ARL6IP5	0.0099323	0.1200501	0.0827	0.934	0.014244144	count	1
HPS5	0.0105136	0.4041483	0.026	0.9793	0.014517299	count	1
ATP6V0A2	0.0110312	0.6551437	0.0168	0.987	0.014605753	count	1
TMEM230	0.0116388	0.2274363	0.0512	0.9592	0.015410538	count	1
TROVE2	0.0113941	0.2613027	0.0436	0.965	0.015961479	count	1

NCF4	0.0114812	0.1965816	0.0584	0.953	0.016083509	count	1
PSME1	0.0114566	0.1063864	0.1077	0.914	0.016394835	count	1
IRF2BP2	0.0119277	0.2163937	0.0551	0.956	0.016709075	count	1
POLR3H	0.0127778	0.495694	0.0258	0.9794	0.016919262	count	1
NXT1	0.0119438	0.1488216	0.0803	0.936	0.017013527	count	1
KANSL1	0.012856	0.2504959	0.0513	0.9591	0.01702285	count	1
WDR83OS	0.0121661	0.1596152	0.0762	0.939	0.017293786	count	1
RAD21	0.0127066	0.2197741	0.0578	0.954	0.017800363	count	1
TRA2B	0.0128467	0.144809	0.0887	0.929	0.017996653	count	1
RPL7A	0.0124937	0.0349661	0.3573	0.721	0.018017459	count	1
SRSF11	0.0138976	0.1511629	0.0919	0.927	0.019755262	count	1
PTP4A2	0.0143868	0.1863244	0.0772	0.938	0.020154493	count	1
HEBP2	0.014755	0.1965593	0.0751	0.94	0.020375305	count	1
COA6	0.0152479	0.4438538	0.0344	0.9726	0.021056127	count	1
CDC42SE2	0.0150389	0.1694649	0.0887	0.929	0.021315104	count	1
PLCG2	0.0150656	0.1686354	0.0893	0.929	0.021494659	count	1
TRAP1	0.0165026	0.4481048	0.0368	0.971	0.021853905	count	1
7-Mar	0.0161777	0.2634765	0.0614	0.951	0.022340449	count	1
PPP1R18	0.0161805	0.245543	0.0659	0.947	0.022832995	count	1
PCF11	0.0178468	0.1959831	0.0911	0.9275	0.023634996	count	1
MT-ND5	0.0167297	0.0708633	0.2361	0.8135	0.023970974	count	1
COPE	0.0169707	0.1560453	0.1088	0.913	0.024174763	count	1
ATP6V0E1	0.0173893	0.1064707	0.1633	0.87	0.024901946	count	1
SNRPA1	0.0182081	0.2429875	0.0749	0.94	0.025508847	count	1
ATP5MC3	0.0182459	0.1448315	0.126	0.9	0.025936954	count	1
HDAC1	0.0181408	0.2106018	0.0861	0.9314	0.025939818	count	1
RPL11	0.0181072	0.0308405	0.5871	0.557	0.026116402	count	1
CCDC12	0.0188031	0.1823818	0.1031	0.918	0.026650903	count	1
CEBPG	0.0188117	0.2628366	0.0716	0.943	0.026741337	count	1
SMC6	0.0190094	0.2463301	0.0772	0.939	0.026943343	count	1
PPP3CA	0.0190702	0.191748	0.0995	0.921	0.027029531	count	1
TMEM243	0.0189349	0.1202238	0.1575	0.875	0.027144063	count	1
CCM2	0.0198955	0.3079814	0.0646	0.949	0.027873354	count	1
LSM4	0.0199806	0.2853381	0.07	0.944	0.027992605	count	1
PPP1R11	0.0204789	0.2223009	0.0921	0.9266	0.028282159	count	1
MPG	0.0198831	0.2170251	0.0916	0.927	0.028323878	count	1
DRAP1	0.0199048	0.2050318	0.0971	0.9227	0.028434395	count	1
YWHAB	0.0199278	0.1091796	0.1825	0.855	0.028579578	count	1
GNAS	0.0201306	0.0741383	0.2715	0.786	0.028961495	count	1
IL2RG	0.0205431	0.1649486	0.1245	0.9009	0.029449609	count	1
ELOB	0.0208636	0.1349904	0.1546	0.877	0.029857488	count	1
DDRGK1	0.0230146	0.3412909	0.0674	0.946	0.030483828	count	1
HEBP1	0.0233775	0.5335282	0.0438	0.965	0.030964859	count	1
COX8A	0.0222005	0.1585225	0.14	0.889	0.031625416	count	1
VAPA	0.0226555	0.16962	0.1336	0.894	0.031741055	count	1

RBPJ	0.0231649	0.3163594	0.0732	0.9417	0.031993044	count	1
ZDHHC24	0.0231796	0.3691107	0.0628	0.95	0.032013353	count	1
XKR8	0.0244297	0.7453194	0.0328	0.974	0.032359632	count	1
NCOA7	0.0231796	0.3295971	0.0703	0.944	0.032475525	count	1
TMEM167B	0.0237009	0.4052478	0.0585	0.9534	0.032733601	count	1
CDC5L	0.0233197	0.1998478	0.1167	0.907	0.033219937	count	1
LIAS	0.0244297	0.5711641	0.0428	0.9659	0.033740559	count	1
BPNT1	0.0244297	0.5927107	0.0412	0.9671	0.033740559	count	1
C3orf14	0.0245091	0.6191949	0.0396	0.968	0.033850265	count	1
USP8	0.0258413	0.2068381	0.1249	0.9006	0.034230962	count	1
UBE2L3	0.0249913	0.2277243	0.1097	0.9127	0.03451652	count	1
NR4A1	0.0242272	0.140573	0.1723	0.8632	0.034789207	count	1
ZNF638	0.0253781	0.3012198	0.0843	0.933	0.035050967	count	1
STK17A	0.025231	0.1268591	0.1989	0.842	0.035943	count	1
RPL22L1	0.0251215	0.1326214	0.1894	0.85	0.035951359	count	1
RPL32	0.0250743	0.0323694	0.7746	0.439	0.036164526	count	1
CNOT7	0.0273073	0.2013985	0.1356	0.8922	0.036174582	count	1
THRAP3	0.0255318	0.1531777	0.1667	0.868	0.03637156	count	1
METTL6	0.0266892	0.6945482	0.0384	0.9694	0.037394067	count	1
SRSF9	0.026593	0.1375841	0.1933	0.847	0.037529803	count	1
CDKN1A	0.0269811	0.2672613	0.101	0.92	0.037803169	count	1
DNAJA1	0.0264739	0.1027499	0.2577	0.7968	0.03781942	count	1
EXOSC9	0.0277917	0.3545956	0.0784	0.9376	0.038386029	count	1
CCL5	0.027203	0.2540184	0.1071	0.9148	0.038671625	count	1
IGHD	0.0273031	0.2693687	0.1014	0.919	0.038813948	count	1
BSG	0.0273413	0.2108252	0.1297	0.8969	0.039010898	count	1
EIF4E2	0.0280768	0.3272977	0.0858	0.9317	0.039624332	count	1
PPHLN1	0.0283455	0.2499408	0.1134	0.91	0.039715427	count	1
MTIF3	0.0302197	0.2473962	0.1222	0.903	0.040036362	count	1
LMO4	0.0284681	0.1999102	0.1424	0.887	0.040352388	count	1
AHCTF1	0.0296141	0.4383335	0.0676	0.946	0.040904356	count	1
TOMM7	0.0291656	0.0747438	0.3902	0.6966	0.04189067	count	1
DNAJB6	0.0302665	0.1582035	0.1913	0.848	0.042407877	count	1
KDM4B	0.0308287	0.4860602	0.0634	0.949	0.042582862	count	1
B2M	0.0295896	0.0304148	0.9729	0.331	0.042681452	count	1
TSR2	0.0326643	0.3221108	0.1014	0.9193	0.043278386	count	1
RECQL5	0.0320067	0.9915885	0.0323	0.9743	0.044210852	count	1
NPC1	0.0320067	0.9915885	0.0323	0.9743	0.044210852	count	1
TBC1D22A	0.0320744	0.325894	0.0984	0.9216	0.044304415	count	1
SESN1	0.0324613	0.3967088	0.0818	0.935	0.044839123	count	1
COX7C	0.0312075	0.0714312	0.4369	0.6624	0.044841659	count	1
RPL35A	0.0311885	0.0348735	0.8943	0.372	0.044967726	count	1
SLC25A17	0.0320067	0.8064679	0.0397	0.968	0.045172017	count	1
PPA1	0.0342332	0.2418469	0.1415	0.8875	0.045359317	count	1
SVIP	0.0319587	0.2081947	0.1535	0.8781	0.045599948	count	1

HLA-DOB	0.0345589	0.2095478	0.1649	0.8691	0.045791339	count	1
RPS13	0.0320178	0.0375452	0.8528	0.394	0.0461719	count	1
ZNF480	0.0335925	0.4156225	0.0808	0.9356	0.046402517	count	1
PSMB6	0.0343498	0.1730449	0.1985	0.8427	0.047449187	count	1
TMF1	0.0344841	0.1955408	0.1764	0.8601	0.047634807	count	1
RAB39B	0.0346305	0.5286833	0.0655	0.9478	0.047837151	count	1
ATP5PD	0.0365115	0.196664	0.1857	0.8528	0.048381527	count	1
STX7	0.0346201	0.2212312	0.1565	0.876	0.049074622	count	1
RPS23	0.0341016	0.0316883	1.0762	0.282	0.049185284	count	1
CYTH1	0.0374697	0.2454818	0.1526	0.8788	0.049652725	count	1
CLIC1	0.0347141	0.079068	0.439	0.661	0.049923526	count	1
WBP4	0.0363795	0.3160294	0.1151	0.908	0.050254578	count	1
CBX7	0.0353868	0.4277789	0.0827	0.934	0.050307971	count	1
C1orf43	0.036771	0.2555959	0.1439	0.8857	0.050795718	count	1
FAM50A	0.0359131	0.3215286	0.1117	0.9111	0.05116289	count	1
SYNGR1	0.0370925	0.1774299	0.2091	0.8345	0.051240108	count	1
PLAC8	0.0359521	0.2752362	0.1306	0.8961	0.051298751	count	1
RPP38	0.0367685	0.321078	0.1145	0.909	0.051521838	count	1
GPATCH8	0.0373339	0.2918078	0.1279	0.898	0.051573784	count	1
TMEM248	0.0361625	0.2334701	0.1549	0.877	0.051712492	count	1
ZBTB7A	0.036922	0.18353	0.2012	0.841	0.052490951	count	1
SNX17	0.0368604	0.2937815	0.1255	0.9	0.052594972	count	1
ZFAND1	0.0396981	0.3110205	0.1276	0.8985	0.052609326	count	1
TRMT112	0.0367762	0.114562	0.321	0.7483	0.052697151	count	1
RAP1A	0.0384604	0.203393	0.1891	0.85	0.053130924	count	1
MOB1A	0.0389857	0.1451275	0.2686	0.7883	0.053857056	count	1
THUMPD3-AS1	0.0408978	0.2330315	0.1755	0.8608	0.054201232	count	1
RASSF5	0.0387914	0.2901555	0.1337	0.894	0.054357632	count	1
BIRC3	0.0381566	0.1611276	0.2368	0.8129	0.054359632	count	1
FAM117B	0.0412006	0.5726894	0.0719	0.9427	0.054603043	count	1
ABRAXAS2	0.0388343	0.4934733	0.0787	0.937	0.055049874	count	1
TSPO	0.0389671	0.2525089	0.1543	0.877	0.055399042	count	1
SCAF11	0.0401245	0.1394139	0.2878	0.7736	0.055431282	count	1
RNF138	0.0401949	0.3537334	0.1136	0.9096	0.056325195	count	1
PDE7A	0.0432654	0.2805648	0.1542	0.8775	0.057343193	count	1
TRPM7	0.0434693	0.4148592	0.1048	0.9166	0.057613802	count	1
SIAH1	0.0434693	0.442255	0.0983	0.9217	0.057613802	count	1
ACP1	0.0411965	0.2390772	0.1723	0.863	0.057729372	count	1
TPM4	0.0404614	0.1609674	0.2514	0.802	0.057733999	count	1
RPS14	0.0406799	0.0343907	1.1829	0.237	0.058673921	count	1
PMAIP1	0.0415315	0.1699696	0.2443	0.807	0.058874367	count	1
RPS29	0.0410947	0.0369721	1.1115	0.267	0.059235674	count	1
P2RY10	0.0428863	0.2389497	0.1795	0.858	0.059249307	count	1
PIK3C2A	0.0429855	0.4734658	0.0908	0.9277	0.059386452	count	1
CACYBP	0.0425688	0.2589068	0.1644	0.869	0.059653295	count	1

MICAL3	0.0433422	0.4085137	0.1061	0.916	0.059879594	count	1
RABAC1	0.0426187	0.1814999	0.2348	0.814	0.060154345	count	1
NDUFB7	0.0423476	0.1992442	0.2125	0.8318	0.060331527	count	1
SNRNP25	0.0455239	0.3807773	0.1196	0.9049	0.060340792	count	1
BUD31	0.0424672	0.2203089	0.1928	0.847	0.060376264	count	1
NACA	0.0420757	0.0450578	0.9338	0.351	0.060660107	count	1
EIF3J	0.0429969	0.2498309	0.1721	0.863	0.060688346	count	1
MED6	0.0467384	0.338542	0.1381	0.8903	0.061952911	count	1
GPATCH2L	0.0477174	0.3929678	0.1214	0.9034	0.063252514	count	1
ARID4B	0.0459794	0.1626827	0.2826	0.778	0.063525732	count	1
MCOLN2	0.0456404	0.3922512	0.1164	0.907	0.063959791	count	1
RPS7	0.0445412	0.0339368	1.3125	0.19	0.06423539	count	1
RNASET2	0.0447269	0.1072503	0.417	0.677	0.06435982	count	1
MSL3	0.0486308	0.3397661	0.1431	0.886	0.064465105	count	1
RPL34	0.0448964	0.0333976	1.3443	0.18	0.064748719	count	1
JHY	0.0462838	0.5767464	0.0802	0.9361	0.064861897	count	1
RPL30	0.0455029	0.0289999	1.5691	0.117	0.06563064	count	1
YWHAQ	0.0475774	0.1835277	0.2592	0.7956	0.065735242	count	1
MYL12B	0.0458101	0.0981586	0.4667	0.6409	0.065812876	count	1
SELL	0.0471725	0.1748088	0.2699	0.787	0.066584323	count	1
PSMC1	0.0470232	0.2609602	0.1802	0.8571	0.066855283	count	1
PSMB8-AS1	0.0476214	0.2391983	0.1991	0.842	0.067705998	count	1
IGKC	0.0470454	0.1023789	0.4595	0.646	0.067839038	count	1
HLA-B	0.0470707	0.0398242	1.182	0.238	0.067873787	count	1
EMB	0.0492063	0.1928508	0.2552	0.7987	0.067987591	count	1
HNRNPA2B1	0.0477296	0.0779309	0.6125	0.5405	0.068522874	count	1
EPS15L1	0.0522502	0.456003	0.1146	0.9088	0.069270723	count	1
GEMIN7	0.0522502	0.5027157	0.1039	0.9173	0.069270723	count	1
TTC1	0.0488944	0.2545524	0.1921	0.848	0.069516381	count	1
RPS27A	0.0484948	0.0311653	1.5561	0.12	0.069948318	count	1
NDUFB1	0.0501807	0.1997889	0.2512	0.802	0.070325975	count	1
CPNE3	0.050182	0.2418652	0.2075	0.836	0.070327798	count	1
PDIA6	0.04974	0.180949	0.2749	0.784	0.070718956	count	1
MT-ND6	0.0501991	0.2518907	0.1993	0.842	0.071165493	count	1
CCDC85B	0.0504736	0.1595336	0.3164	0.752	0.071911202	count	1
PDE12	0.0521472	0.4224386	0.1234	0.9018	0.07205439	count	1
CIR1	0.0522744	0.1965376	0.266	0.79	0.072230296	count	1
CHMP1A	0.052171	0.4716989	0.1106	0.912	0.073116869	count	1
DOCK8	0.0515954	0.1877487	0.2748	0.784	0.07314565	count	1
RPS27	0.0509213	0.0305895	1.6647	0.0967	0.073420994	count	1
EIF2B2	0.0556001	0.5463881	0.1018	0.919	0.07371945	count	1
CENPH	0.0559548	0.5957869	0.0939	0.9252	0.07419055	count	1
POLR1D	0.0523888	0.1838137	0.285	0.776	0.074486032	count	1
TMEM59	0.052098	0.1607564	0.3241	0.746	0.074563549	count	1
PRMT1	0.053339	0.1680022	0.3175	0.751	0.07475475	count	1

TMEM250	0.0543536	0.7241907	0.0751	0.9402	0.075105738	count	1
RPL31	0.0529249	0.0710279	0.7451	0.4566	0.076019287	count	1
ZC3H13	0.0552207	0.2851848	0.1936	0.8466	0.076304955	count	1
TMEM87A	0.0555006	0.3533847	0.1571	0.875	0.076692069	count	1
HPRT1	0.0545244	0.3442215	0.1584	0.8742	0.076966221	count	1
SURF1	0.0549945	0.3179098	0.173	0.863	0.077076318	count	1
KLF13	0.0552662	0.1855637	0.2978	0.766	0.077457341	count	1
COPS9	0.0560939	0.2067832	0.2713	0.7863	0.077512641	count	1
NUP88	0.0589852	0.3484467	0.1693	0.8657	0.078215832	count	1
ROCK1	0.0556319	0.1842121	0.302	0.763	0.078870198	count	1
CDC37	0.0573616	0.1893335	0.303	0.7621	0.079266001	count	1
PLP2	0.0560904	0.1255708	0.4467	0.6553	0.079915737	count	1
DNAJC21	0.0563579	0.2385366	0.2363	0.8133	0.080421875	count	1
MALAT1	0.0560518	0.0334414	1.6761	0.0944	0.080863482	count	1
SERPINB6	0.0586756	0.2654427	0.221	0.8252	0.081083472	count	1
CMPK1	0.0573112	0.2073471	0.2764	0.782	0.081486866	count	1
SARAF	0.0576978	0.0981976	0.5876	0.5571	0.082206528	count	1
NIT1	0.0598228	0.8548882	0.07	0.9442	0.082670293	count	1
PABPC1	0.0574994	0.0788683	0.7291	0.466	0.082684497	count	1
CD40	0.0579005	0.1541577	0.3756	0.707	0.082803899	count	1
ERCC3	0.0625019	0.5683286	0.11	0.9125	0.082887979	count	1
GTF3C6	0.058076	0.2204928	0.2634	0.7924	0.082974493	count	1
RPL29	0.0577391	0.0379074	1.5232	0.128	0.083264159	count	1
ATP5F1C	0.0590242	0.2038925	0.2895	0.772	0.083321162	count	1
TFAM	0.0604303	0.2366848	0.2553	0.7986	0.083510617	count	1
GAPDH	0.0582631	0.0744338	0.7828	0.434	0.083856418	count	1
TNPO1	0.061145	0.3866527	0.1581	0.8744	0.084499246	count	1
SYAP1	0.0598957	0.2401172	0.2494	0.803	0.084552009	count	1
INIP	0.0598566	0.4702728	0.1273	0.8988	0.084861967	count	1
CD37	0.0595393	0.0592603	1.0047	0.316	0.085827145	count	1
DESI2	0.0649165	0.4119684	0.1576	0.8749	0.086096486	count	1
CHD4	0.0652729	0.2733145	0.2388	0.8114	0.086570108	count	1
PABPC4	0.0626646	0.2911309	0.2152	0.8297	0.086601349	count	1
CHCHD2	0.0606577	0.094518	0.6418	0.521	0.08706025	count	1
LIMS1	0.0657896	0.3493781	0.1883	0.8507	0.08725677	count	1
TAPBP	0.0622015	0.1568527	0.3966	0.692	0.087808647	count	1
PDHA1	0.0664055	0.3441232	0.193	0.8471	0.088075291	count	1
ADAM28	0.0625873	0.1894748	0.3303	0.741	0.08835355	count	1
PPIL4	0.0639871	0.1705863	0.3751	0.708	0.088430879	count	1
GIGYF2	0.062642	0.4252296	0.1473	0.883	0.088812596	count	1
APH1A	0.0633867	0.2366552	0.2678	0.789	0.088846254	count	1
UHRF1BP1L	0.0645403	0.6228518	0.1036	0.9175	0.08919619	count	1
CLEC17A	0.0645403	0.7041873	0.0917	0.927	0.08919619	count	1
HLA-E	0.0620714	0.0577235	1.0753	0.283	0.089401855	count	1
LENG1	0.0641039	0.3407172	0.1881	0.851	0.089852213	count	1

PRPS1	0.0679362	0.2766467	0.2456	0.8061	0.090109694	count	1
FGD5-AS1	0.0656905	0.3494055	0.188	0.851	0.09078745	count	1
NIFK	0.0646682	0.2507026	0.2579	0.797	0.091292665	count	1
CYB5B	0.0654649	0.3667784	0.1785	0.858	0.091761219	count	1
EIF3M	0.0646312	0.1637573	0.3947	0.693	0.092088072	count	1
SEC61B	0.064823	0.1335478	0.4854	0.628	0.092171157	count	1
RPL10	0.0639057	0.0337754	1.8921	0.0591	0.092181527	count	1
ASCC3	0.0697858	0.3941614	0.177	0.86	0.092568183	count	1
CNOT2	0.0670565	0.2618586	0.2561	0.798	0.092677334	count	1
CDC42SE1	0.0670731	0.1966142	0.3411	0.733	0.092700301	count	1
HIF1A	0.0652233	0.211558	0.3083	0.758	0.092740541	count	1
SUMO4	0.07008	0.5128463	0.1366	0.8914	0.092959258	count	1
MTF2	0.0673607	0.2487548	0.2708	0.7867	0.093098211	count	1
FAM32A	0.0674521	0.3325697	0.2028	0.839	0.093224669	count	1
SSBP1	0.0668515	0.1669882	0.4003	0.689	0.093706188	count	1
CCDC18	0.0665604	0.4369621	0.1523	0.879	0.09396534	count	1
AMD1	0.0659386	0.1803589	0.3656	0.715	0.094097091	count	1
CLIC4	0.0683965	0.4890688	0.1399	0.8888	0.094531324	count	1
SLC1A5	0.0683965	0.5013443	0.1364	0.8915	0.094531324	count	1
SNRPB	0.0661554	0.1289213	0.5131	0.6081	0.094979049	count	1
CNOT4	0.0688053	0.3099211	0.222	0.824	0.095096945	count	1
SRSF2	0.0688295	0.1515483	0.4542	0.65	0.095130429	count	1
PRPSAP2	0.0688908	0.4066969	0.1694	0.8656	0.095215245	count	1
PCNP	0.0675711	0.2112885	0.3198	0.749	0.095392955	count	1
DPP7	0.0674501	0.2088011	0.323	0.747	0.095632417	count	1
MT-CYB	0.0663354	0.0437397	1.5166	0.13	0.095654864	count	1
PHTF2	0.0721217	0.3803548	0.1896	0.8497	0.095673444	count	1
LYRM4	0.0723447	0.4173982	0.1733	0.862	0.095969915	count	1
RNF19A	0.0672325	0.1607775	0.4182	0.676	0.096059801	count	1
RPL6	0.0666893	0.0418415	1.5939	0.112	0.096162986	count	1
ARHGAP17	0.0686581	0.4157793	0.1651	0.869	0.096240372	count	1
ARHGDIB	0.0671021	0.0821653	0.8167	0.415	0.096494375	count	1
CNPY3	0.0675583	0.1612533	0.419	0.6754	0.096859251	count	1
NDUFB11	0.0676171	0.1148044	0.589	0.5562	0.097124957	count	1
KBTBD4	0.0704949	0.552276	0.1276	0.8985	0.097434771	count	1
ARF4	0.070508	0.1846959	0.3818	0.7028	0.097452897	count	1
GADD45GIP1	0.0708238	0.1891678	0.3744	0.7083	0.09788987	count	1
SMYD3	0.0708531	0.4490574	0.1578	0.8747	0.097930412	count	1
ITPA	0.0698657	0.2972367	0.2351	0.814	0.097934369	count	1
ATXN2L	0.0743352	0.4271089	0.174	0.8619	0.098616385	count	1
DSTN	0.0699168	0.2871318	0.2435	0.808	0.099131351	count	1
RPA3	0.0701447	0.2150436	0.3262	0.7444	0.100101087	count	1
BTN2A2	0.0717027	0.2364655	0.3032	0.762	0.100511357	count	1
MSH6	0.0757826	0.2518616	0.3009	0.7636	0.10054097	count	1
BUB3	0.0727724	0.1984198	0.3668	0.714	0.10058624	count	1

AIP	0.0709824	0.217111	0.3269	0.744	0.100642903	count	1
IQGAP1	0.071333	0.2203763	0.3237	0.746	0.100706843	count	1
FAM3A	0.0729972	0.4759643	0.1534	0.8782	0.100897316	count	1
STRAP	0.0721683	0.2423621	0.2978	0.766	0.101164528	count	1
CHTF8	0.0736997	0.6474255	0.1138	0.909	0.101869445	count	1
SF1	0.0717235	0.1130451	0.6345	0.526	0.102196725	count	1
FAM49B	0.0717392	0.1544326	0.4645	0.6425	0.102680406	count	1
LIX1L	0.0743747	0.4029727	0.1846	0.8537	0.102803539	count	1
KRIT1	0.0734144	0.3080181	0.2383	0.812	0.10291266	count	1
CD46	0.0725072	0.2268302	0.3197	0.749	0.103101536	count	1
CHCHD10	0.072418	0.1113751	0.6502	0.516	0.103720426	count	1
TMEM14B	0.0739955	0.1803621	0.4103	0.682	0.10372789	count	1
BLM	0.0753492	0.6055056	0.1244	0.901	0.104152127	count	1
ST13	0.0730592	0.1412999	0.5171	0.605	0.104261506	count	1
SUMO1	0.0737239	0.1697186	0.4344	0.664	0.105048011	count	1
OS9	0.0754241	0.3484309	0.2165	0.829	0.105732127	count	1
EVI2A	0.0799832	0.1822121	0.439	0.6609	0.106127365	count	1
RPLP1	0.0739547	0.0353133	2.0942	0.0368	0.106674221	count	1
ECH1	0.0748199	0.1687324	0.4434	0.658	0.106774933	count	1
PJA2	0.0775851	0.1810629	0.4285	0.6685	0.107246488	count	1
NIPBL	0.0767798	0.243807	0.3149	0.753	0.107634144	count	1
DYNLRB1	0.0759762	0.2109283	0.3602	0.719	0.107726873	count	1
EPC2	0.078228	0.3655705	0.214	0.8307	0.108136265	count	1
HADHA	0.0759319	0.1881474	0.4036	0.6867	0.108597141	count	1
SECISBP2	0.077825	0.2161823	0.36	0.719	0.109100574	count	1
PHF23	0.0778975	0.2129741	0.3658	0.715	0.109202293	count	1
ANXA4	0.0770343	0.2888529	0.2667	0.7898	0.109227901	count	1
SPTAN1	0.0824062	0.3163142	0.2605	0.7946	0.109350344	count	1
TMEM18	0.0792027	0.3059878	0.2588	0.7959	0.109485288	count	1
SNW1	0.0792724	0.1975743	0.4012	0.688	0.109581757	count	1
CBWD1	0.0801332	0.3926187	0.2041	0.8384	0.110773173	count	1
PSMD12	0.0776925	0.2756513	0.2819	0.7782	0.11087569	count	1
EID1	0.0839275	0.1829711	0.4587	0.6467	0.111374149	count	1
RPN2	0.0790014	0.240642	0.3283	0.743	0.112018494	count	1
REST	0.0802257	0.2246916	0.357	0.721	0.113269504	count	1
TAX1BP1	0.0809567	0.1580229	0.5123	0.609	0.113494576	count	1
SCFD1	0.0855811	0.3794884	0.2255	0.8217	0.113574157	count	1
SGO1	0.0813223	0.7121118	0.1142	0.909	0.114007558	count	1
GABARAPL2	0.0804254	0.1499374	0.5364	0.592	0.115025381	count	1
H3F3B	0.080525	0.0786496	1.0238	0.306	0.116066387	count	1
IPO5	0.0840019	0.4487964	0.1872	0.8516	0.116128147	count	1
CDKN2D	0.0813248	0.1502719	0.5412	0.589	0.116403584	count	1
STAT5A	0.0842639	0.7016889	0.1201	0.9045	0.116490825	count	1
TALDO1	0.0822958	0.2049535	0.4015	0.6882	0.117026587	count	1
SSNA1	0.0849778	0.230802	0.3682	0.7129	0.117479066	count	1

C19orf70	0.0838403	0.2019897	0.4151	0.678	0.117540726	count	1
DYNLT1	0.0838425	0.2293471	0.3656	0.715	0.117543812	count	1
WTAP	0.0829769	0.1765294	0.47	0.639	0.117658478	count	1
TRAM1	0.083792	0.1904169	0.44	0.66	0.118308079	count	1
SMC3	0.083356	0.220186	0.3786	0.705	0.118777918	count	1
COQ8B	0.0837709	0.6068502	0.138	0.89	0.118784948	count	1
HUWE1	0.0850514	0.3901816	0.218	0.828	0.119240162	count	1
ADI1	0.0845031	0.2873266	0.2941	0.769	0.119312775	count	1
CCDC130	0.0863814	0.3559684	0.2427	0.8084	0.119422111	count	1
CHPT1	0.090211	0.1808901	0.4987	0.6182	0.119735056	count	1
GNPAT	0.0856445	0.4887424	0.1752	0.861	0.120072426	count	1
RPL17	0.0858283	0.1745526	0.4917	0.623	0.120330344	count	1
VOPP1	0.087454	0.2259774	0.387	0.6989	0.120906998	count	1
SELENOS	0.0862645	0.2486352	0.347	0.729	0.120942447	count	1
RHOT1	0.0868087	0.636852	0.1363	0.8916	0.12170611	count	1
RSBN1	0.0880343	0.239925	0.3669	0.714	0.121710375	count	1
SRSF10	0.0855482	0.1577394	0.5423	0.588	0.121902914	count	1
SLC38A2	0.0859921	0.2533375	0.3394	0.734	0.121936284	count	1
CD47	0.0854977	0.1332049	0.6419	0.521	0.122526041	count	1
AKAP13	0.0888487	0.1542612	0.576	0.565	0.122837866	count	1
PHF14	0.0889376	0.2477859	0.3589	0.72	0.122960945	count	1
HPCAL1	0.0871081	0.2785817	0.3127	0.755	0.122993411	count	1
CD86	0.0881646	0.2438904	0.3615	0.718	0.12360885	count	1
NPC2	0.0872183	0.202286	0.4312	0.667	0.124283694	count	1
SYF2	0.0881073	0.1398672	0.6299	0.529	0.124405232	count	1
RAP2C	0.0937368	0.3615033	0.2593	0.7955	0.124427855	count	1
MRPL15	0.090034	0.3957188	0.2275	0.8201	0.124478897	count	1
ATP5PB	0.087677	0.2202248	0.3981	0.691	0.124682367	count	1
SUPT4H1	0.0890286	0.2160732	0.412	0.681	0.124821332	count	1
BRD4	0.0891086	0.2240638	0.3977	0.691	0.1249336	count	1
FASTK	0.0887737	0.4255233	0.2086	0.8348	0.125346835	count	1
PHIP	0.0895173	0.3673297	0.2437	0.8076	0.12639753	count	1
TGFBR2	0.0954411	0.231288	0.4127	0.6801	0.126696599	count	1
HSD17B11	0.0956365	0.2479598	0.3857	0.6999	0.126956727	count	1
KRR1	0.0958451	0.244968	0.3913	0.6958	0.127234431	count	1
SPPL2A	0.092078	0.3169944	0.2905	0.772	0.127308923	count	1
PTPRC	0.0886958	0.1050105	0.8446	0.3988	0.127345407	count	1
MRPS22	0.0914931	0.3049963	0.3	0.7643	0.128279968	count	1
WIPF1	0.0916	0.2105441	0.4351	0.664	0.128429994	count	1
PTPN6	0.0904481	0.1568763	0.5766	0.565	0.129085953	count	1
LYPLAL1	0.0921039	0.252436	0.3649	0.715	0.129137181	count	1
UXT	0.0904863	0.1211077	0.7472	0.455	0.129738338	count	1
UBB	0.0902752	0.0783279	1.1525	0.2497	0.129821461	count	1
LEPROT	0.0939562	0.3478775	0.2701	0.7872	0.129909541	count	1
NUDC	0.0980126	0.1918776	0.5108	0.6097	0.130120165	count	1

PSMD7	0.0917756	0.2105663	0.4359	0.663	0.130513688	count	1
CCDC47	0.0930516	0.269465	0.3453	0.73	0.131391603	count	1
FCHSD2	0.095063	0.2044293	0.465	0.642	0.131442121	count	1
APC	0.0991756	0.4674408	0.2122	0.8321	0.13166869	count	1
PRH1	0.0953826	0.4283047	0.2227	0.8239	0.131884679	count	1
MT-ND2	0.0915776	0.0626288	1.4622	0.144	0.131930563	count	1
CALM2	0.0923619	0.0897493	1.0291	0.304	0.132292121	count	1
HNRNPD	0.0931512	0.1578463	0.5901	0.555	0.13247089	count	1
JMJD1C	0.0945917	0.1801104	0.5252	0.6	0.132628733	count	1
MAVS	0.0959291	0.7693349	0.1247	0.9008	0.13264144	count	1
ATP6V1D	0.0939404	0.4138785	0.227	0.8206	0.132647546	count	1
LLPH	0.0932848	0.3546487	0.263	0.7926	0.132660978	count	1
CNBP	0.0936666	0.113573	0.8247	0.41	0.13282517	count	1
ATRX	0.0933753	0.1695713	0.5507	0.582	0.133060994	count	1
PCBP1	0.0932074	0.1169613	0.7969	0.426	0.133577297	count	1
C1D	0.0946146	0.259247	0.365	0.715	0.133600255	count	1
RPS3	0.0927646	0.0351951	2.6357	0.00869	0.133767867	count	1
BECN1	0.0954765	0.3922758	0.2434	0.808	0.134818215	count	1
BBC3	0.0976426	0.2578932	0.3786	0.705	0.135014275	count	1
DDX3Y	0.0980896	0.1911564	0.5131	0.6081	0.135633295	count	1
CUTC	0.0981198	0.3059384	0.3207	0.7486	0.135675117	count	1
RALBP1	0.0952631	0.2480032	0.3841	0.7011	0.1357523	count	1
FOXN3	0.0982208	0.317136	0.3097	0.757	0.135814987	count	1
WNK1	0.098298	0.3230961	0.3042	0.761	0.135921897	count	1
MRPL13	0.0972643	0.3401522	0.2859	0.7751	0.137344625	count	1
COX5A	0.0968246	0.1866331	0.5188	0.604	0.137697534	count	1
NABP1	0.1045854	0.2774805	0.3769	0.7064	0.138873136	count	1
HNRNPM	0.1046922	0.1576025	0.6643	0.5069	0.139015389	count	1
BRD2	0.0995383	0.1878273	0.5299	0.596	0.139571653	count	1
FAM133B	0.097603	0.1934464	0.5045	0.6141	0.139709896	count	1
CEBPZ	0.1021806	0.2632757	0.3881	0.698	0.141299025	count	1
HNRNPK	0.1009173	0.1130714	0.8925	0.3726	0.141507305	count	1
MS4A1	0.0983643	0.0825621	1.1914	0.234	0.141568745	count	1
MAP4K4	0.1001125	0.2339041	0.428	0.669	0.141971657	count	1
RPL8	0.0987262	0.0348852	2.83	0.00487	0.142374923	count	1
NSRP1	0.1031736	0.2614192	0.3947	0.693	0.142674358	count	1
LAPTM4A	0.1007098	0.1648663	0.6109	0.542	0.14373715	count	1
RNF13	0.1043437	0.2778433	0.3755	0.707	0.144295032	count	1
CCNK	0.1043551	0.3327525	0.3136	0.754	0.144310822	count	1
CTDSP1	0.1046729	0.2714862	0.3856	0.7	0.144751007	count	1
LMAN2	0.1022073	0.2289298	0.4465	0.6555	0.145356582	count	1
EIF4H	0.1036955	0.2404248	0.4313	0.666	0.145407122	count	1
ZNF148	0.1033057	0.2728956	0.3786	0.705	0.145882468	count	1
TERF2IP	0.1028618	0.1077532	0.9546	0.34	0.146809825	count	1
SNRPD2	0.1025688	0.1062576	0.9653	0.335	0.146820102	count	1

STAU1	0.1065534	0.2670187	0.399	0.69	0.147355779	count	1
PRMT2	0.1065534	0.3039998	0.3505	0.726	0.147355779	count	1
CBX5	0.1066913	0.2734954	0.3901	0.6966	0.147546796	count	1
NRDC	0.1046028	0.2284632	0.4579	0.647	0.14771566	count	1
NFE2L2	0.1042473	0.1692244	0.616	0.538	0.147839155	count	1
THAP5	0.1070339	0.3415488	0.3134	0.754	0.148021365	count	1
TMEM65	0.107285	0.5119334	0.2096	0.8341	0.148369192	count	1
CASP2	0.1120541	0.8137498	0.1377	0.891	0.148823112	count	1
CEP76	0.1120541	0.816909	0.1372	0.891	0.148823112	count	1
TP53I11	0.1080474	0.5235195	0.2064	0.8366	0.149425292	count	1
RAB3GAP1	0.108223	0.3014162	0.359	0.7197	0.149668542	count	1
PFN1	0.1041108	0.0694888	1.4982	0.135	0.14981891	count	1
RPL22	0.1041645	0.0483258	2.1555	0.0317	0.150143695	count	1
RPL37	0.1041711	0.0388577	2.6808	0.00762	0.150170087	count	1
DDX18	0.1065611	0.1664649	0.6401	0.522	0.150483393	count	1
GPBP1	0.1057446	0.1296525	0.8156	0.415	0.150695851	count	1
RRAGB	0.1137744	0.5277012	0.2156	0.8294	0.151115526	count	1
UBE2R2	0.1079037	0.2512335	0.4295	0.668	0.151314668	count	1
TM2D1	0.1082552	0.3189061	0.3395	0.734	0.151808133	count	1
DCP1A	0.1098598	0.3092345	0.3553	0.7226	0.151935979	count	1
RPS28	0.1055946	0.0352597	2.9948	0.0029	0.152263812	count	1
SPOCK2	0.1149679	0.2919313	0.3938	0.6939	0.152706074	count	1
HIST1H4C	0.1074971	0.1528746	0.7032	0.4823	0.153976813	count	1
UBE2Q1	0.1086645	0.3715742	0.2924	0.7701	0.154545084	count	1
SF3B1	0.1102557	0.1303401	0.8459	0.3981	0.154616663	count	1
NAPA	0.109858	0.3555584	0.309	0.7575	0.155143198	count	1
RPS8	0.1079635	0.0342467	3.1525	0.00173	0.155725023	count	1
SUZ12	0.1128567	0.2299488	0.4908	0.6238	0.156087824	count	1
BBX	0.1101356	0.1812188	0.6077	0.544	0.156195465	count	1
PPIH	0.11396	0.3448528	0.3305	0.7412	0.157616405	count	1
SMC1A	0.1125529	0.2884019	0.3903	0.697	0.157841868	count	1
EEF1B2	0.1097024	0.0537298	2.0417	0.0418	0.1581494	count	1
CDC42EP3	0.1146084	0.2640207	0.4341	0.664	0.158514761	count	1
PNRC2	0.1133446	0.1679296	0.675	0.5	0.158953427	count	1
RBM23	0.1153238	0.3008501	0.3833	0.702	0.159505966	count	1
PRPF3	0.1126729	0.3604486	0.3126	0.7547	0.159796424	count	1
PSMB10	0.1155368	0.3193234	0.3618	0.718	0.159801087	count	1
FAM210B	0.1139584	1.0047618	0.1134	0.9098	0.159815223	count	1
RPS26	0.1113861	0.0414946	2.6844	0.00754	0.160575695	count	1
OTUD1	0.1127207	0.2523616	0.4467	0.6553	0.160642475	count	1
BZW1	0.1125384	0.1282058	0.8778	0.381	0.160970282	count	1
NANS	0.1164248	0.3766617	0.3091	0.7574	0.161031469	count	1
EEF1A1	0.1117411	0.0295516	3.7812	0.000177	0.16118001	count	1
MIOS	0.1168755	0.2980321	0.3922	0.6951	0.161655955	count	1
DDX10	0.1168896	0.5429203	0.2153	0.8296	0.161675492	count	1

HMG20B	0.1218561	0.446537	0.2729	0.7851	0.161887861	count	1
SNAP29	0.1218561	0.514496	0.2368	0.8129	0.161887861	count	1
OARD1	0.1218563	0.3376537	0.3609	0.7184	0.161888128	count	1
IRF3	0.1170508	0.2688728	0.4353	0.664	0.161898852	count	1
MMP24OS	0.1147823	0.3055997	0.3756	0.707	0.162103578	count	1
RSBN1L	0.1163061	0.1672129	0.6956	0.487	0.163111571	count	1
PPP2R3C	0.1180471	0.3323309	0.3552	0.7226	0.163279353	count	1
UQCRC1	0.1185452	0.3575797	0.3315	0.7404	0.163969549	count	1
SCAF8	0.1170849	0.5092725	0.2299	0.8183	0.164205098	count	1
ZFPM1	0.1170849	0.5678837	0.2062	0.8367	0.164205098	count	1
DNAJC3	0.1166324	0.4084828	0.2855	0.7754	0.16471878	count	1
DCXR	0.1177004	0.214476	0.5488	0.583	0.166228481	count	1
SPTLC2	0.1205898	0.4197259	0.2873	0.774	0.166802769	count	1
RPL18	0.1158597	0.0344638	3.3618	0.000841	0.167090491	count	1
RPL39	0.1158767	0.0357097	3.245	0.00126	0.167104179	count	1
RPS4X	0.1160508	0.0390168	2.9744	0.0031	0.167348415	count	1
SELENOK	0.116895	0.1329921	0.879	0.38	0.167534136	count	1
CCNL1	0.1176674	0.1431572	0.8219	0.412	0.168149844	count	1
COMMD8	0.1265702	0.4034811	0.3137	0.754	0.168173646	count	1
RUVBL1	0.1199416	0.3628007	0.3306	0.741	0.168216383	count	1
EXOSC8	0.1206938	0.2863353	0.4215	0.674	0.169272635	count	1
SNRPG	0.1187465	0.1386378	0.8565	0.392	0.169692543	count	1
STK17B	0.1196835	0.1049643	1.1402	0.2548	0.169746495	count	1
BLOC1S4	0.1189422	0.2522455	0.4715	0.6375	0.169771359	count	1
SEPHS2	0.1207734	0.3589427	0.3365	0.7367	0.171293453	count	1
ATAD2	0.1242397	0.4129439	0.3009	0.7637	0.171860879	count	1
AGTPBP1	0.1227188	0.4966295	0.2471	0.8049	0.172116251	count	1
IL27RA	0.1229005	0.3225649	0.381	0.703	0.172371409	count	1
ALG13	0.1247119	0.1989473	0.6269	0.531	0.172515302	count	1
N4BP2L2	0.120911	0.1281598	0.9434	0.346	0.173372921	count	1
9-Sep	0.1223117	0.2208103	0.5539	0.58	0.173966791	count	1
PDCL3	0.1265132	0.3039219	0.4163	0.677	0.175011811	count	1
SEC13	0.1316993	0.3457646	0.3809	0.7035	0.175014637	count	1
TGS1	0.1228938	0.3064687	0.401	0.6886	0.175148486	count	1
ASCC2	0.1266371	0.3786812	0.3344	0.7382	0.175183534	count	1
ZNRD1	0.1271574	0.2207662	0.576	0.565	0.17590467	count	1
GLIPR1	0.1245674	0.1739399	0.7162	0.474	0.175936078	count	1
ZNF675	0.1274479	0.4155421	0.3067	0.7592	0.176307307	count	1
HIST1H1D	0.1240872	0.3314292	0.3744	0.7083	0.176493746	count	1
DAP3	0.1328462	0.2724196	0.4877	0.626	0.176544589	count	1
ACTB	0.1237376	0.0574258	2.1547	0.0317	0.178426519	count	1
TTC31	0.1290679	0.678532	0.1902	0.8492	0.178552712	count	1
KLHDC1	0.129136	0.7350237	0.1757	0.8606	0.178647104	count	1
CMTM3	0.129136	0.7350237	0.1757	0.8606	0.178647104	count	1
DBI	0.124737	0.161918	0.7704	0.441	0.178996892	count	1

BTF3	0.1245286	0.065249	1.9085	0.057	0.179126405	count	1
SRSF5	0.1255286	0.1511729	0.8304	0.407	0.179177007	count	1
C8orf59	0.12702	0.1699254	0.7475	0.455	0.179403453	count	1
SON	0.1249868	0.0858824	1.4553	0.1463	0.179583649	count	1
ZCCHC7	0.1268791	0.2478511	0.5119	0.609	0.181319394	count	1
TM9SF3	0.1311702	0.3115061	0.4211	0.6739	0.181466764	count	1
DYNC1I2	0.1370618	0.3278562	0.4181	0.676	0.182168972	count	1
MBIP	0.1319034	0.4765183	0.2768	0.782	0.182483112	count	1
ALYREF	0.1296025	0.2925993	0.4429	0.658	0.183825826	count	1
SHISA8	0.1329874	0.3055266	0.4353	0.664	0.183985771	count	1
GLS	0.1313517	0.1749733	0.7507	0.453	0.184240283	count	1
LRMP	0.1335994	0.3245602	0.4116	0.681	0.184834155	count	1
TMBIM6	0.1311618	0.1080568	1.2138	0.225	0.186039292	count	1
NAA25	0.1309075	0.4572622	0.2863	0.775	0.186201046	count	1
MAPKAPK5	0.140231	0.5315916	0.2638	0.7921	0.18639811	count	1
SUCLG2	0.1407082	0.3902008	0.3606	0.719	0.187034972	count	1
BCL2A1	0.1308115	0.1523653	0.8585	0.391	0.187485556	count	1
PSMC4	0.1341063	0.2691004	0.4984	0.618	0.188109261	count	1
SRGN	0.1319721	0.1006754	1.3109	0.1906	0.188924719	count	1
ATP5ME	0.1350429	0.175312	0.7703	0.442	0.18942481	count	1
RUNX3	0.1375157	0.2842599	0.4838	0.6288	0.190263472	count	1
SFPQ	0.1339022	0.1036263	1.2922	0.197	0.190846962	count	1
ANXA7	0.1352305	0.2093188	0.6461	0.519	0.191011984	count	1
UBE2B	0.134152	0.1489135	0.9009	0.368	0.19149226	count	1
UCHL3	0.1370419	0.3555623	0.3854	0.7001	0.192232686	count	1
PFDN2	0.1345115	0.1553432	0.8659	0.387	0.192412875	count	1
ELMSAN1	0.1373508	0.2695096	0.5096	0.611	0.192666588	count	1
AL627171.1	0.1385524	0.5933597	0.2335	0.8155	0.194354465	count	1
3-Mar	0.1462341	0.5467233	0.2675	0.7892	0.194410924	count	1
MT-CO1	0.1348453	0.0344212	3.9175	0.000104	0.194503833	count	1
BCAP29	0.1465428	0.3014913	0.4861	0.6272	0.19482304	count	1
THUMPD1	0.1472634	0.1927685	0.7639	0.4453	0.19578507	count	1
WBP2	0.1387702	0.3066163	0.4526	0.6511	0.196017061	count	1
SERP1	0.1364603	0.1082888	1.2602	0.2083	0.196032603	count	1
ARHGAP45	0.139757	0.3205633	0.436	0.663	0.196046594	count	1
USP47	0.1474785	0.3482586	0.4235	0.6722	0.196072245	count	1
ANAPC16	0.1373472	0.1240726	1.107	0.2689	0.197027491	count	1
SQSTM1	0.1396929	0.143046	0.9766	0.329	0.197321785	count	1
DNAJC15	0.137578	0.1679828	0.819	0.4132	0.197428575	count	1
CLDND1	0.1407883	0.2601844	0.5411	0.589	0.197495315	count	1
CREBRF	0.1417462	0.2195886	0.6455	0.519	0.198840953	count	1
HNRNPH2	0.1439665	0.2615792	0.5504	0.5823	0.199207762	count	1
RPAIN	0.1500524	0.2683434	0.5592	0.576	0.199508851	count	1
MRPL54	0.1446194	0.1884034	0.7676	0.4431	0.200113123	count	1
CUL5	0.1508823	0.2825755	0.534	0.5936	0.200617012	count	1

HNRNPDL	0.1397468	0.0823352	1.6973	0.0903	0.200660068	count	1
C21orf91	0.1432081	0.4707458	0.3042	0.7611	0.200894645	count	1
C12orf76	0.1521644	0.5515445	0.2759	0.783	0.20232909	count	1
SIRT2	0.1462267	0.5342825	0.2737	0.7845	0.202341998	count	1
SF3B2	0.1436846	0.1748702	0.8217	0.412	0.202966359	count	1
CAPRIN1	0.1468857	0.3309657	0.4438	0.6574	0.203255876	count	1
CCPG1	0.1472381	0.2807824	0.5244	0.6003	0.203744578	count	1
BRAF	0.1462304	0.4005758	0.3651	0.7152	0.205140584	count	1
FASTKD2	0.148273	0.4953937	0.2993	0.7648	0.205179788	count	1
FAM129C	0.1463171	0.3475204	0.421	0.674	0.20526239	count	1
SEC61G	0.1454169	0.2038792	0.7133	0.476	0.205416065	count	1
POLR2L	0.1443511	0.2041422	0.7071	0.4799	0.206058795	count	1
EIF1	0.1434327	0.04877	2.941	0.00344	0.206714662	count	1
AKAP9	0.1460592	0.2164082	0.6749	0.5	0.207768671	count	1
BTG2	0.1448486	0.1141206	1.2693	0.205	0.207791607	count	1
ZCCHC17	0.1502872	0.3201167	0.4695	0.639	0.207973218	count	1
TUBB	0.156736	0.145474	1.0774	0.2819	0.208434814	count	1
BTG3	0.1512129	0.2370631	0.6379	0.524	0.209257094	count	1
RAC2	0.1471559	0.1077121	1.3662	0.173	0.209749232	count	1
COX6B1	0.146994	0.1275134	1.1528	0.25	0.209833606	count	1
MBNL2	0.151668	0.3599781	0.4213	0.6737	0.209888295	count	1
UBALD2	0.1466336	0.1443867	1.0156	0.31	0.209922002	count	1
PCMTD1	0.1521847	0.2315884	0.6571	0.5114	0.210604942	count	1
POLE4	0.1489605	0.248524	0.5994	0.5492	0.211307807	count	1
NMT1	0.152945	0.3554285	0.4303	0.6672	0.211659473	count	1
TRBC2	0.1477587	0.1653699	0.8935	0.3721	0.211668851	count	1
UBC	0.1470834	0.0591928	2.4848	0.0133	0.211856053	count	1
CTSS	0.1481491	0.1181673	1.2537	0.211	0.212527749	count	1
SKIL	0.1484914	0.1550898	0.9575	0.339	0.212582741	count	1
CRIP3	0.1515924	1.2485439	0.1214	0.903	0.212674086	count	1
NAA20	0.1537225	0.2778956	0.5532	0.5804	0.212737884	count	1
ZDHHC16	0.1540153	0.8258129	0.1865	0.8521	0.21314401	count	1
DEF6	0.1511093	0.2967428	0.5092	0.6108	0.21346631	count	1
RNFT1	0.1544017	0.3970962	0.3888	0.698	0.213679969	count	1
TRAF5	0.1544031	0.2590814	0.596	0.552	0.21368191	count	1
CLEC2B	0.1526164	0.3958285	0.3856	0.7	0.214112871	count	1
DPM2	0.1610745	0.4038676	0.3988	0.6902	0.214230575	count	1
NFKBIZ	0.1535885	0.31173	0.4927	0.622	0.215478758	count	1
LEMD3	0.162139	0.5003999	0.324	0.7461	0.215652832	count	1
PARK7	0.1506709	0.1376182	1.0948	0.274	0.215704252	count	1
EIF3I	0.157341	0.1785346	0.8813	0.3786	0.217757129	count	1
KRT10	0.1528248	0.1379652	1.1077	0.269	0.217834772	count	1
FOXK1	0.1574449	0.5634725	0.2794	0.7801	0.217901256	count	1
TMEM30A	0.1639694	0.3876202	0.423	0.6725	0.218098579	count	1
ANKRD13A	0.1643208	0.2132666	0.7705	0.4414	0.21856814	count	1

EMP3	0.152208	0.1150548	1.3229	0.1865	0.218744481	count	1
2-Mar	0.15813	0.4237871	0.3731	0.7092	0.218851621	count	1
SLC25A1	0.158161	0.3515765	0.4499	0.653	0.218894625	count	1
LAMTOR3	0.1590478	0.4960728	0.3206	0.749	0.220124815	count	1
ADAM19	0.1656403	0.3498223	0.4735	0.6361	0.220331407	count	1
GLYR1	0.1561258	0.425784	0.3667	0.714	0.220561223	count	1
PCIF1	0.1577095	0.3428918	0.4599	0.6458	0.221269404	count	1
NBN	0.1664066	0.3837211	0.4337	0.6647	0.221355482	count	1
DDX21	0.1549027	0.1138328	1.3608	0.174	0.221765197	count	1
AL137796.1	0.1603797	0.8343821	0.1922	0.8477	0.221972512	count	1
RPL5	0.1542051	0.0398789	3.8668	0.000127	0.22237356	count	1
GHITM	0.1563852	0.1851638	0.8446	0.399	0.22246895	count	1
HSPA1A	0.1672557	0.3441025	0.4861	0.627	0.222490257	count	1
ATF7	0.1672826	0.6925323	0.2416	0.8092	0.222526207	count	1
SNX2	0.1565197	0.1148502	1.3628	0.174	0.222660436	count	1
NME4	0.1595831	0.5410903	0.2949	0.7682	0.223902249	count	1
CRCP	0.1687189	0.3240822	0.5206	0.6029	0.22444586	count	1
NDUFAF3	0.1571795	0.2047197	0.7678	0.443	0.224854042	count	1
SRSF4	0.1626399	0.2653943	0.6128	0.54	0.225108161	count	1
TECR	0.1626671	0.2825137	0.5758	0.5651	0.225145898	count	1
PAFAH1B1	0.159552	0.1836259	0.8689	0.385	0.225407236	count	1
SYTL1	0.1633561	0.3083702	0.5297	0.5966	0.226101809	count	1
DHX9	0.1609109	0.252264	0.6379	0.524	0.227329326	count	1
APLF	0.1710548	1.0719332	0.1596	0.8733	0.227568155	count	1
LARP7	0.1644843	0.242758	0.6776	0.498	0.227667099	count	1
STXBP2	0.1647635	0.3935908	0.4186	0.676	0.228054476	count	1
GRASP	0.1605095	0.1593735	1.0071	0.314	0.228340775	count	1
UVRAG	0.1722284	0.1975174	0.872	0.3837	0.229136999	count	1
HEXA	0.1634011	0.3858611	0.4235	0.6722	0.229267709	count	1
MPV17	0.1625454	0.4452503	0.3651	0.7152	0.229641285	count	1
MPC2	0.160482	0.2334814	0.6873	0.4922	0.229756282	count	1
SAR1A	0.1661584	0.2625997	0.6327	0.527	0.229989873	count	1
HIGD2A	0.1603048	0.1288326	1.2443	0.2141	0.230186984	count	1
ALG5	0.1669377	0.4324636	0.386	0.6997	0.231071168	count	1
EBNA1BP2	0.1647254	0.3090292	0.533	0.5943	0.231128844	count	1
TRAPPC2L	0.1644038	0.3299345	0.4983	0.619	0.232270009	count	1
MPPE1	0.1680401	0.5492175	0.306	0.7598	0.232600811	count	1
RSRC2	0.1634678	0.1360671	1.2014	0.23	0.233364452	count	1
TSR3	0.1664894	0.2942696	0.5658	0.572	0.233607991	count	1
RNH1	0.166231	0.210072	0.7913	0.429	0.234854664	count	1
BASP1	0.1642156	0.1446867	1.135	0.257	0.235666304	count	1
SIPA1L1	0.1775719	0.3112416	0.5705	0.5686	0.23628127	count	1
CIRBP	0.1667882	0.0984248	1.6946	0.0909	0.237280281	count	1
IFNGR2	0.1676032	0.2234173	0.7502	0.454	0.237779978	count	1
POLR1E	0.1722817	0.4453438	0.3869	0.699	0.238486693	count	1

RSL1D1	0.1663718	0.1362205	1.2213	0.223	0.238677974	count	1
DNAJB1	0.1705601	0.2578455	0.6615	0.509	0.239329306	count	1
PAXX	0.1752367	0.248167	0.7061	0.4805	0.242587613	count	1
NDFIP1	0.1825615	0.3039375	0.6007	0.5484	0.242954142	count	1
MYC	0.1701112	0.2125107	0.8005	0.424	0.243136759	count	1
AK3	0.1756394	0.4343621	0.4044	0.6861	0.243146501	count	1
MRPS21	0.171048	0.1706449	1.0024	0.317	0.243828975	count	1
BCL7C	0.1834512	0.2606012	0.704	0.4818	0.244144167	count	1
HELLS	0.1835772	0.4491321	0.4087	0.683	0.244312702	count	1
MRPS33	0.1744207	0.3159605	0.552	0.5812	0.244755716	count	1
COPB2	0.1841035	0.2714044	0.6783	0.4979	0.245016689	count	1
TUFM	0.1779212	0.2037138	0.8734	0.3829	0.246313415	count	1
CRELD2	0.1850786	0.4076728	0.454	0.6501	0.246321047	count	1
ADSS	0.174014	0.2927034	0.5945	0.5525	0.246884417	count	1
ZEB2	0.1860217	0.3015506	0.6169	0.5376	0.247582659	count	1
CDKN2AIP	0.1790386	0.3143206	0.5696	0.5692	0.247864325	count	1
PSMA3	0.1752481	0.2105284	0.8324	0.406	0.249326158	count	1
POP4	0.1777598	0.3658427	0.4859	0.6273	0.249449417	count	1
PANK2	0.1777598	0.3873326	0.4589	0.6465	0.249449417	count	1
BTBD2	0.1874625	0.4695532	0.3992	0.6899	0.249510177	count	1
ZNF273	0.1804015	0.6218177	0.2901	0.7719	0.249756043	count	1
LRRFIP1	0.1756345	0.1221935	1.4373	0.151	0.249876368	count	1
VPS26A	0.1880543	0.2676895	0.7025	0.483	0.250301933	count	1
FXYD5	0.1742042	0.1024083	1.7011	0.0896	0.250318181	count	1
NDUFA6	0.1758983	0.2151866	0.8174	0.4141	0.250748241	count	1
GTPBP6	0.1820026	0.3019058	0.6028	0.5469	0.251978473	count	1
CTSZ	0.1787335	0.1380135	1.295	0.196	0.253587326	count	1
SASH3	0.1838155	0.3900815	0.4712	0.6377	0.254495008	count	1
NUP160	0.1919261	0.4272246	0.4492	0.653	0.255482518	count	1
SDCCAG8	0.1920927	0.3423978	0.561	0.5751	0.255705457	count	1
ZBTB22	0.1847646	0.5268297	0.3507	0.726	0.255812527	count	1
SNRNP40	0.1923207	0.2701627	0.7119	0.4769	0.256010561	count	1
NUP62	0.1818679	0.3690304	0.4928	0.6224	0.256976394	count	1
SRSF6	0.1833648	0.3077087	0.5959	0.552	0.257328875	count	1
NDUFB5	0.1837873	0.2108718	0.8716	0.3839	0.257922854	count	1
LYSMD2	0.1825649	0.2469274	0.7393	0.46	0.257962559	count	1
NRDE2	0.1864356	0.4768448	0.391	0.696	0.25813225	count	1
ILF2	0.1844548	0.2031364	0.908	0.364	0.258861278	count	1
PBDC1	0.185029	0.3140707	0.5891	0.556	0.259668543	count	1
BNIP3L	0.1838622	0.2205963	0.8335	0.405	0.259798094	count	1
ZSWIM8	0.1956082	0.768004	0.2547	0.7991	0.260410213	count	1
VAV3	0.196052	0.4267883	0.4594	0.646	0.261004207	count	1
SF3B6	0.1847451	0.1835715	1.0064	0.315	0.261047317	count	1
LACTB	0.1861462	0.2565002	0.7257	0.468	0.261239233	count	1
ATOX1	0.1840913	0.357456	0.515	0.6068	0.261918799	count	1

CEP78	0.1893353	0.7597929	0.2492	0.8033	0.262157927	count	1
RBM22	0.1978521	0.2450692	0.8073	0.4199	0.263413636	count	1
CFAP97	0.1860896	0.2380366	0.7818	0.435	0.26403557	count	1
VDAC2	0.1853161	0.1391574	1.3317	0.184	0.264576274	count	1
RWDD1	0.18651	0.1985097	0.9396	0.348	0.265363182	count	1
C1orf122	0.1916782	0.3279157	0.5845	0.5592	0.265410816	count	1
VDAC1	0.1893732	0.2144701	0.883	0.378	0.2657763	count	1
CCT8	0.1862916	0.1912778	0.9739	0.3306	0.265969952	count	1
VHL	0.2010252	0.3709049	0.542	0.5881	0.267661348	count	1
SET	0.1868388	0.1025231	1.8224	0.0691	0.267678454	count	1
PSMG4	0.1909443	0.3565452	0.5355	0.5925	0.267985314	count	1
UQCRFS1	0.1914783	0.2116317	0.9048	0.366	0.268736148	count	1
TMED10	0.1904329	0.2091401	0.9106	0.363	0.26909539	count	1
ZMPSTE24	0.1908797	0.4682908	0.4076	0.684	0.270839637	count	1
DAZAP2	0.191287	0.1756352	1.0891	0.277	0.272166152	count	1
LINC00623	0.1956082	0.4565432	0.4285	0.6685	0.274543255	count	1
PDLIM1	0.1924345	0.1655098	1.1627	0.246	0.274746406	count	1
MDM4	0.198899	0.2224352	0.8942	0.372	0.275437442	count	1
EIF1B	0.1936129	0.1330343	1.4554	0.146	0.27766307	count	1
ATRAID	0.194001	0.2048342	0.9471	0.3441	0.277769388	count	1
TNRC6B	0.2086922	0.1702085	1.2261	0.2208	0.277927636	count	1
ARFIP1	0.2006948	0.4328126	0.4637	0.6431	0.277931336	count	1
COX7B	0.1973467	0.145803	1.3535	0.177	0.278879034	count	1
OCIAD2	0.1974498	0.2040888	0.9675	0.334	0.279024937	count	1
MRPS35	0.2096715	0.3898155	0.5379	0.5909	0.279239219	count	1
PPP4C	0.1960565	0.2261499	0.8669	0.3864	0.279508001	count	1
RBAK-RBAKDN	0.2019762	0.4272472	0.4727	0.637	0.279710933	count	1
CD24	0.2101703	0.1656592	1.2687	0.205	0.279907291	count	1
CHCHD3	0.2021687	0.2778862	0.7275	0.4673	0.279978281	count	1
FGD3	0.1982696	0.5279792	0.3755	0.7074	0.280185088	count	1
FOLR2	0.2112265	0.5620544	0.3758	0.707	0.281321974	count	1
NCSTN	0.2112265	0.5679301	0.3719	0.71	0.281321974	count	1
SLC2A6	0.2113134	0.3611508	0.5851	0.5588	0.281438372	count	1
HCLS1	0.1985876	0.1579256	1.2575	0.209	0.281789001	count	1
ETF1	0.2035387	0.3396072	0.5993	0.549	0.281881	count	1
RHEB	0.2120083	0.2401711	0.8827	0.3779	0.28236917	count	1
FUCA2	0.2125145	0.5748715	0.3697	0.7118	0.283047229	count	1
KMT2E	0.1976664	0.1111261	1.7788	0.076	0.28359094	count	1
DPY30	0.2051861	0.282557	0.7262	0.468	0.284169073	count	1
EED	0.2057687	0.3291735	0.6251	0.532	0.284978269	count	1
JCHAIN	0.2018065	0.2976566	0.678	0.498	0.285190522	count	1
AC243960.1	0.2010314	0.2825004	0.7116	0.477	0.285260698	count	1
SLC25A37	0.2002863	0.2466249	0.8121	0.4172	0.285543231	count	1
MBP	0.2008723	0.2032776	0.9882	0.324	0.286379372	count	1
LRRC37B	0.2068128	0.8673558	0.2384	0.8116	0.286428491	count	1

DLD	0.2045463	0.4351768	0.47	0.6386	0.287112668	count	1
ALMS1	0.2076937	0.7254342	0.2863	0.7748	0.287652063	count	1
USF2	0.2029207	0.2000858	1.0142	0.311	0.287944725	count	1
PRR13	0.201676	0.149408	1.3498	0.178	0.287950552	count	1
PRDX6	0.2039299	0.1788033	1.1405	0.255	0.289378463	count	1
ANXA6	0.2050817	0.2080312	0.9858	0.325	0.289825804	count	1
SUCLG1	0.2177588	0.2947894	0.7387	0.4605	0.290073007	count	1
EBP	0.2097685	0.3790768	0.5534	0.5803	0.290534073	count	1
CCDC59	0.2055581	0.2274937	0.9036	0.367	0.292491473	count	1
COMMD6	0.2047668	0.0931121	2.1991	0.0284	0.294124422	count	1
ACOT13	0.2125145	0.425651	0.4993	0.6178	0.294348649	count	1
FOXJ3	0.2107803	0.3834728	0.5497	0.583	0.295880523	count	1
NR3C1	0.2113059	0.2404096	0.8789	0.38	0.296619801	count	1
BLVRA	0.2144502	0.4804587	0.4463	0.656	0.297037765	count	1
SP110	0.2084961	0.1369538	1.5224	0.129	0.298036902	count	1
DEDD2	0.2154032	0.3429212	0.6281	0.5302	0.29836174	count	1
TM2D3	0.2103188	0.2983819	0.7049	0.4813	0.298455318	count	1
SYNGR2	0.2080494	0.1200337	1.7333	0.0837	0.298841006	count	1
CUTA	0.2084476	0.1558004	1.3379	0.1816	0.298946959	count	1
RRM2B	0.2162048	0.4139685	0.5223	0.602	0.299475406	count	1
BEX2	0.214171	0.3064239	0.6989	0.485	0.300649791	count	1
ZRANB2	0.2262076	0.2199311	1.0285	0.3043	0.301395602	count	1
SFT2D1	0.2264665	0.2740426	0.8264	0.409	0.301742639	count	1
PIK3CA	0.2269936	0.4132882	0.5492	0.5831	0.302449189	count	1
PTRHD1	0.2138616	0.2535603	0.8434	0.3994	0.303488914	count	1
CARMIL1	0.2192336	0.5019128	0.4368	0.662	0.303683528	count	1
MED10	0.2150618	0.1895433	1.1346	0.257	0.303951519	count	1
TCERG1	0.2196991	0.3183903	0.69	0.4905	0.304330308	count	1
HMGN3	0.214182	0.2581222	0.8298	0.4071	0.304775112	count	1
ZDHHC12	0.2172408	0.3870069	0.5613	0.5749	0.304967927	count	1
TATDN1	0.2218744	0.3254254	0.6818	0.4957	0.307352835	count	1
SETD3	0.2307885	0.5456773	0.4229	0.6725	0.307536582	count	1
ELOC	0.2220085	0.1928548	1.1512	0.2503	0.307539169	count	1
BFAR	0.2181052	0.279914	0.7792	0.436	0.308259472	count	1
ZNF518A	0.2184102	0.5410764	0.4037	0.6867	0.30869121	count	1
MVB12A	0.2321155	0.6722915	0.3453	0.7301	0.309315758	count	1
UQCRQ	0.223411	0.2114156	1.0567	0.2912	0.309488003	count	1
VPS37A	0.22351	0.5051655	0.4424	0.6584	0.30962557	count	1
POLR2A	0.2192482	0.3512516	0.6242	0.5328	0.309877439	count	1
HNRNPA1	0.2159853	0.0934307	2.3117	0.0213	0.309990698	count	1
CTBP1	0.2326425	0.4012299	0.5798	0.5623	0.310022365	count	1
RNF168	0.2243967	0.2522938	0.8894	0.374	0.310857716	count	1
DDX1	0.2250451	0.4524488	0.4974	0.6192	0.311758741	count	1
PNRC1	0.2165064	0.0572638	3.7809	0.000178	0.311934632	count	1
VPREB3	0.2176116	0.1436269	1.5151	0.13	0.311953948	count	1

CCT2	0.2210714	0.180428	1.2253	0.221	0.312458309	count	1
SCAMP2	0.2203322	0.2590429	0.8506	0.3955	0.312682788	count	1
GTPBP8	0.2261396	0.4794468	0.4717	0.6374	0.313279704	count	1
TIMM29	0.2265565	0.6218666	0.3643	0.7158	0.313859056	count	1
TRMT44	0.2265565	0.7997896	0.2833	0.7771	0.313859056	count	1
ACTL6A	0.2363927	0.4267056	0.554	0.5799	0.31505118	count	1
FAM111A	0.2274478	0.3353571	0.6782	0.498	0.315097688	count	1
COPA	0.2238701	0.3197294	0.7002	0.4842	0.316420183	count	1
PHF5A	0.2288305	0.2292455	0.9982	0.3187	0.317019267	count	1
TPD52	0.2242921	0.2260873	0.9921	0.322	0.318309587	count	1
SLC25A33	0.2300264	0.339857	0.6768	0.4989	0.318681298	count	1
RBM3	0.2229325	0.1240727	1.7968	0.073	0.318978973	count	1
C15orf61	0.2396963	0.4110687	0.5831	0.5601	0.319481867	count	1
TOMM22	0.224644	0.1874727	1.1983	0.231	0.319678111	count	1
HAX1	0.2279385	0.1946689	1.1709	0.242	0.320017599	count	1
RASSF3	0.2403729	0.3775797	0.6366	0.525	0.320389387	count	1
CD74	0.2223057	0.0333608	6.6637	7.93E-11	0.320670803	count	1.93E-06
CSRNP1	0.2258193	0.2670501	0.8456	0.3982	0.321978667	count	1
ORC3	0.2295904	0.5470676	0.4197	0.675	0.322341755	count	1
GTF2H3	0.229719	0.5067476	0.4533	0.6505	0.322522692	count	1
MRM3	0.229719	0.5637136	0.4075	0.6838	0.322522692	count	1
OLA1	0.2332034	0.2916865	0.7995	0.4244	0.323096849	count	1
TGOLN2	0.2428199	0.1899048	1.2786	0.2017	0.323671777	count	1
DHRS7	0.2278024	0.1983682	1.1484	0.2514	0.324808824	count	1
GCHFR	0.2446368	0.5758084	0.4249	0.671	0.326109199	count	1
ORAI1	0.2291605	0.171398	1.337	0.182	0.326747044	count	1
MYCBP	0.232775	0.4990558	0.4664	0.6411	0.326822537	count	1
SYNE2	0.2474929	0.4004371	0.6181	0.5369	0.329941153	count	1
KXD1	0.2486583	0.3473775	0.7158	0.4745	0.331504886	count	1
RHBDD2	0.2369525	0.4589269	0.5163	0.6059	0.332700703	count	1
EIF4G3	0.2497734	0.3474382	0.7189	0.473	0.333001207	count	1
FBXW11	0.2499741	0.2888667	0.8654	0.387	0.33327053	count	1
SMIM12	0.2409296	0.3675088	0.6556	0.5124	0.333836531	count	1
GOPC	0.2366685	0.3331468	0.7104	0.4778	0.334539538	count	1
RYBP	0.2429567	0.3868607	0.628	0.53	0.336654604	count	1
KDELR2	0.2430435	0.2741386	0.8866	0.3758	0.336775277	count	1
PKIG	0.2528965	0.1858238	1.3609	0.1742	0.337192415	count	1
TMED4	0.2444851	0.2451528	0.9973	0.319	0.338779476	count	1
FLYWCH2	0.2546384	0.4342545	0.5864	0.5579	0.339530311	count	1
IL15	0.2552477	0.6147368	0.4152	0.6782	0.340348127	count	1
TNKS	0.2552499	0.4611364	0.5535	0.58	0.34035108	count	1
AC087190.1	0.2425657	0.596568	0.4066	0.6845	0.340599679	count	1
UIMC1	0.2554579	0.4400576	0.5805	0.562	0.340630269	count	1
NUP93	0.2557786	0.7207842	0.3549	0.723	0.341060736	count	1
DDIT3	0.2560192	0.2009655	1.2739	0.2033	0.341383688	count	1

NKTR	0.2415216	0.2313439	1.044	0.297	0.341411077	count	1
CEP19	0.2566593	0.783807	0.3275	0.7435	0.342242902	count	1
CCDC125	0.2566593	1.0117967	0.2537	0.7999	0.342242902	count	1
R3HDM2	0.2442644	0.218608	1.1174	0.264	0.342990257	count	1
CDK2AP2	0.2436133	0.248431	0.9806	0.3273	0.345767159	count	1
TAF3	0.2446017	0.3368329	0.7262	0.4681	0.345772425	count	1
MRPL33	0.2463802	0.2487108	0.9906	0.322	0.345967911	count	1
MRPS25	0.2464862	0.3362215	0.7331	0.464	0.346117092	count	1
DUSP11	0.2497864	0.2916159	0.8566	0.3922	0.346150262	count	1
GPCPD1	0.2609409	0.2660537	0.9808	0.3272	0.347990792	count	1
PPM1A	0.2480694	0.4215051	0.5885	0.5565	0.348345268	count	1
LYN	0.2454547	0.1526461	1.608	0.109	0.348384268	count	1
COX14	0.2438135	0.1829754	1.3325	0.1834	0.349352097	count	1
PPIE	0.2627222	0.4057819	0.6474	0.5177	0.350382455	count	1
BSDC1	0.2627222	0.4068791	0.6457	0.5188	0.350382455	count	1
GPR65	0.2469347	0.2570331	0.9607	0.3372	0.350487768	count	1
ETNK1	0.2505464	0.2989988	0.838	0.4025	0.351831479	count	1
GNG7	0.2461467	0.1882666	1.3074	0.1917	0.352479048	count	1
SLAIN2	0.2548003	0.3652371	0.6976	0.486	0.353122316	count	1
NDEL1	0.2548936	0.3902202	0.6532	0.514	0.353252061	count	1
DUSP1	0.2457152	0.1870835	1.3134	0.1897	0.353329454	count	1
ZC3H12A	0.2552512	0.3013615	0.847	0.397	0.353749353	count	1
SENP7	0.2554344	0.3691364	0.692	0.4893	0.35400412	count	1
USO1	0.2654392	0.5401579	0.4914	0.623	0.354030806	count	1
NAXE	0.2504751	0.3290414	0.7612	0.4469	0.354089477	count	1
FAAP20	0.2479656	0.2480971	0.9995	0.3181	0.354100469	count	1
DDX54	0.2555226	0.4608703	0.5544	0.5796	0.354126774	count	1
TUBB4B	0.265886	0.2070796	1.284	0.2	0.354630807	count	1
GON4L	0.2513435	0.285969	0.8789	0.3799	0.355319228	count	1
RAB10	0.267113	0.3043181	0.8777	0.3806	0.356278585	count	1
SNF8	0.2548949	0.2497592	1.0206	0.308	0.357952037	count	1
TMEM39B	0.2583983	0.7802535	0.3312	0.7407	0.358126008	count	1
ERH	0.2506229	0.1669245	1.5014	0.134	0.358302962	count	1
RPAP3	0.2693491	0.2991332	0.9004	0.3684	0.359281756	count	1
UFSP2	0.2558761	0.4561189	0.561	0.5751	0.359333146	count	1
POLR2J	0.2527897	0.2081084	1.2147	0.2251	0.359777833	count	1
ZNF593	0.2703479	0.372942	0.7249	0.469	0.360623281	count	1
UBE2I	0.2527679	0.147878	1.7093	0.0881	0.360964273	count	1
CPNE5	0.2605058	0.3391365	0.7681	0.4428	0.361057081	count	1
SUGP2	0.2712366	0.5783043	0.469	0.6393	0.361816976	count	1
EPS15	0.2614295	0.2907221	0.8992	0.369	0.362341791	count	1
LRRK1	0.2717257	0.4649956	0.5844	0.5593	0.362473953	count	1
MFSD10	0.2554843	0.2944526	0.8677	0.3861	0.362639772	count	1
IGHA2	0.2561039	0.257675	0.9939	0.321	0.363520482	count	1
WBP11	0.2589507	0.2142444	1.2087	0.227	0.363661002	count	1

CDK11A	0.2732864	0.382409	0.7146	0.4752	0.364570439	count	1
TTC38	0.2634221	1.0041787	0.2623	0.7932	0.365113255	count	1
CALCOCO2	0.2639079	0.2787856	0.9466	0.344	0.365788964	count	1
RAB11A	0.2751298	0.2584644	1.0645	0.2877	0.36704686	count	1
ZNF622	0.2602664	0.3230526	0.8056	0.4209	0.367955804	count	1
TNFRSF13C	0.2581889	0.120743	2.1383	0.033	0.369461489	count	1
NAB1	0.2635243	0.8427727	0.3127	0.755	0.370099267	count	1
ZKSCAN8	0.2635243	1.0116135	0.2605	0.7946	0.370099267	count	1
SRPRA	0.2645147	0.2014786	1.3129	0.19	0.371493516	count	1
UBE3A	0.2789382	0.2887148	0.9661	0.3345	0.372163711	count	1
CD83	0.2593179	0.0814493	3.1838	0.00156	0.373405579	count	1
SCP2	0.2621478	0.2217783	1.182	0.2378	0.373830791	count	1
SAMD9	0.2623664	0.2779548	0.9439	0.3457	0.374142841	count	1
NSUN6	0.2704133	0.5422063	0.4987	0.6182	0.374838187	count	1
MRPL12	0.2651407	0.4338225	0.6112	0.5414	0.374859339	count	1
RNF34	0.2652175	0.4214719	0.6293	0.5295	0.374968115	count	1
MGMT	0.2651867	0.2399148	1.1053	0.27	0.376431529	count	1
STIM2	0.2715953	0.2637834	1.0296	0.304	0.376482536	count	1
TMEM107	0.266363	0.369893	0.7201	0.4718	0.376590561	count	1
MOB3A	0.2725494	0.3403742	0.8007	0.424	0.377809871	count	1
DDT	0.2672318	0.1576571	1.695	0.0908	0.377821114	count	1
SDF4	0.2691221	0.3769499	0.7139	0.4756	0.377979932	count	1
LFNG	0.2735002	0.4515528	0.6057	0.545	0.379132646	count	1
ERGIC3	0.2842511	0.3301472	0.861	0.3897	0.379303408	count	1
SBDS	0.2648005	0.1250153	2.1181	0.0347	0.379442053	count	1
MAPK1IP1L	0.2685375	0.1956609	1.3725	0.171	0.379670508	count	1
TMX2	0.2853544	0.4746783	0.6012	0.548	0.38078628	count	1
STAT6	0.2684656	0.4174212	0.6432	0.5205	0.381092707	count	1
PRPF4B	0.2756696	0.1626506	1.6949	0.0908	0.382150871	count	1
KRTCAP2	0.2872279	0.2219797	1.2939	0.1964	0.38330449	count	1
TBCC	0.2768223	0.2953516	0.9373	0.3491	0.383754652	count	1
LARP1	0.2881747	0.3125401	0.922	0.357	0.38457718	count	1
IFT57	0.2741247	0.1562366	1.7545	0.08	0.385023252	count	1
MRPL14	0.2699546	0.2926965	0.9223	0.357	0.385530418	count	1
HNRNPH1	0.2716309	0.1532684	1.7723	0.077	0.385592531	count	1
ADIPOR1	0.2750377	0.3261804	0.8432	0.4	0.386308753	count	1
CCR7	0.2707127	0.1262086	2.145	0.0325	0.386614081	count	1
CCDC106	0.2900463	0.5989224	0.4843	0.6284	0.387093147	count	1
COA5	0.2905595	0.4382363	0.663	0.5077	0.38778307	count	1
KDM3B	0.2797622	0.4471189	0.6257	0.532	0.387845202	count	1
NDUFS3	0.2797848	0.28889	0.9685	0.3333	0.387876649	count	1
MRPS6	0.276458	0.2406305	1.1489	0.251	0.388308566	count	1
TRRAP	0.2802746	0.5226166	0.5363	0.592	0.38855818	count	1
SMIM14	0.271525	0.1292949	2.1	0.0363	0.388559302	count	1
RHOG	0.2912599	0.1627598	1.7895	0.0742	0.388724684	count	1

AKAP8L	0.2770553	0.2940486	0.9422	0.347	0.389149592	count	1
TANK	0.2826315	0.2115298	1.3361	0.182	0.391837787	count	1
JKAMP	0.2798699	0.404955	0.6911	0.4899	0.393112777	count	1
RPN1	0.2767989	0.3285741	0.8424	0.4	0.393991462	count	1
CCT6A	0.2782456	0.2086548	1.3335	0.183	0.394996494	count	1
FXR1	0.2820705	0.2023239	1.3942	0.164	0.396211521	count	1
PDCD7	0.2971271	0.2853088	1.0414	0.2982	0.396613629	count	1
MYH9	0.297229	0.2541023	1.1697	0.2427	0.396750659	count	1
TKT	0.2806074	0.2055188	1.3654	0.173	0.396767665	count	1
CHM	0.2973275	0.3570927	0.8326	0.405	0.396883121	count	1
ACAT1	0.2974655	0.4578801	0.6497	0.5162	0.397068699	count	1
RAD23A	0.2773748	0.1770106	1.567	0.1178	0.397677641	count	1
ANKRD39	0.2870531	0.5073769	0.5658	0.5718	0.397990881	count	1
SRSF3	0.2806001	0.1438047	1.9513	0.0517	0.398343971	count	1
SSB	0.2799704	0.1816715	1.5411	0.124	0.398511405	count	1
ZC3H8	0.2985842	0.3687244	0.8098	0.419	0.398573134	count	1
FAM107B	0.2805788	0.1325214	2.1172	0.0348	0.40116596	count	1
MTCH2	0.2911372	0.4609871	0.6316	0.528	0.403674857	count	1
DNAJC4	0.3035722	0.2235049	1.3582	0.1751	0.405281918	count	1
METTL21A	0.3044538	0.2455832	1.2397	0.2157	0.406467807	count	1
TRABD	0.2894801	0.2494042	1.1607	0.2464	0.406646012	count	1
GORASP2	0.3046771	0.3712199	0.8207	0.4122	0.406768189	count	1
HAT1	0.293658	0.2893166	1.015	0.3107	0.4071834	count	1
DIP2B	0.3053793	0.7302347	0.4182	0.676	0.407712795	count	1
MORF4L2	0.2883485	0.3062493	0.9415	0.3469	0.407734289	count	1
HLA-DOA	0.2944681	0.354789	0.83	0.407	0.408310968	count	1
MPP6	0.287664	0.6135033	0.4689	0.6394	0.408387413	count	1
DNASE2	0.2870019	0.4908869	0.5847	0.5591	0.408532919	count	1
ETFA	0.307077	0.2803942	1.0952	0.274	0.40999668	count	1
IKBIP	0.3073253	0.2603266	1.1805	0.2384	0.410330729	count	1
ARHGDIA	0.2890534	0.1830224	1.5793	0.115	0.410362937	count	1
TANGO6	0.2923524	1.0025705	0.2916	0.7707	0.410691215	count	1
MFSD8	0.2962442	0.4983346	0.5945	0.5525	0.410783173	count	1
C9orf78	0.2865115	0.1883949	1.5208	0.129	0.410966975	count	1
YIPF3	0.3087716	0.3963908	0.779	0.4364	0.41227656	count	1
CCAR1	0.2975891	0.344542	0.8637	0.3882	0.412655243	count	1
FKBP2	0.2977701	0.2182186	1.3645	0.1731	0.412907196	count	1
FBXO7	0.2991045	0.2997526	0.9978	0.3189	0.414764713	count	1
PKN2	0.3109981	0.2641224	1.1775	0.2396	0.415272295	count	1
SMG1	0.3112921	0.2764441	1.1261	0.2607	0.415667892	count	1
MTHFS	0.3117297	0.5469733	0.5699	0.569	0.41625672	count	1
SOBP	0.2965964	0.8574205	0.3459	0.7296	0.416668573	count	1
CYB5R3	0.3120511	0.3026094	1.0312	0.303	0.416689196	count	1
MT-ATP8	0.3134791	0.2890002	1.0847	0.2786	0.418610793	count	1
GTF2B	0.2931658	0.1528352	1.9182	0.0557	0.418711774	count	1

SOCS1	0.3140151	0.377748	0.8313	0.406	0.419332094	count	1
CCDC90B	0.3031066	0.3129281	0.9686	0.3333	0.420336063	count	1
RALA	0.2967176	0.2939229	1.0095	0.3133	0.421260761	count	1
RAN	0.2934633	0.1091671	2.6882	0.0074	0.421371588	count	1
TTI2	0.3157066	0.5899957	0.5351	0.593	0.421608472	count	1
TMEM11	0.3001529	0.4458776	0.6732	0.5012	0.421677932	count	1
ARID4A	0.3041632	0.2387606	1.2739	0.203	0.421807046	count	1
MRPL16	0.3158842	0.3170327	0.9964	0.3196	0.421847491	count	1
TBC1D32	0.2965964	0.768038	0.3862	0.6996	0.422208176	count	1
TUBA1A	0.2942336	0.1567868	1.8766	0.0612	0.422211429	count	1
SMARCB1	0.2956965	0.1699969	1.7394	0.0827	0.422329783	count	1
LPIN1	0.3050208	0.3279241	0.9302	0.3528	0.423001009	count	1
KDM4C	0.2983975	0.4079853	0.7314	0.465	0.423649535	count	1
NDUFAF6	0.3174942	0.4418054	0.7186	0.4727	0.424014353	count	1
TSN	0.3179965	0.4622682	0.6879	0.4919	0.424690419	count	1
ARF1	0.2989753	0.1502552	1.9898	0.0472	0.425599031	count	1
PFKL	0.3188654	0.5495846	0.5802	0.5621	0.425859935	count	1
C14orf119	0.3195732	0.2643892	1.2087	0.2274	0.426812651	count	1
IER5	0.3083378	0.1725794	1.7866	0.0747	0.427619196	count	1
CHMP6	0.3089499	0.5763728	0.536	0.5922	0.428471446	count	1
RRP7A	0.3050524	0.3325372	0.9173	0.359	0.428579352	count	1
CD44	0.3021983	0.1308261	2.3099	0.0214	0.429054305	count	1
TSG101	0.3100835	0.2816742	1.1009	0.272	0.430049831	count	1
PAPOLA	0.3105527	0.1743391	1.7813	0.0755	0.430703139	count	1
FOXN2	0.3240924	0.2729694	1.1873	0.2358	0.432896249	count	1
JARID2	0.3084072	0.4462637	0.6911	0.4899	0.433305203	count	1
TXN	0.3046946	0.1771226	1.7202	0.0861	0.433751499	count	1
CGAS	0.3092805	0.3622756	0.8537	0.3937	0.434535445	count	1
STRADB	0.3253386	0.6507881	0.4999	0.6174	0.434574046	count	1
CCSER2	0.3133456	0.2914616	1.0751	0.2829	0.434592081	count	1
ADPGK	0.3133456	0.311893	1.0047	0.3156	0.434592081	count	1
CDC42	0.3027762	0.0969971	3.1215	0.0019	0.434621601	count	1
FOSB	0.3071526	0.2070381	1.4836	0.139	0.436099641	count	1
RNF135	0.3108848	0.5663438	0.5489	0.5833	0.436795507	count	1
ADAMTS6	0.3273797	0.4019881	0.8144	0.4159	0.437322225	count	1
PDZD11	0.327776	0.5512534	0.5946	0.5524	0.437855837	count	1
ARID5B	0.3160336	0.1349674	2.3416	0.0196	0.438335179	count	1
ASCC1	0.328188	0.4893806	0.6706	0.5028	0.4384106	count	1
COMMD9	0.328188	0.5047156	0.6502	0.5159	0.4384106	count	1
OFD1	0.310441	0.2313973	1.3416	0.18	0.439037502	count	1
CTSB	0.3084072	0.3514903	0.8774	0.3807	0.439043742	count	1
SIRT6	0.3126306	0.6359802	0.4916	0.6233	0.439254969	count	1
ACAP2	0.3095826	0.2061518	1.5017	0.134	0.439555373	count	1
MCUB	0.3291063	0.2112029	1.5582	0.1199	0.439647134	count	1
NFIX	0.3173878	0.7010628	0.4527	0.651	0.440221014	count	1

TNFRSF13B	0.3078793	0.1607241	1.9156	0.0561	0.440626151	count	1
UBE2N	0.3076609	0.1435261	2.1436	0.0326	0.441126909	count	1
SELENOM	0.318626	0.2171487	1.4673	0.143	0.441945357	count	1
RAB6A	0.3310839	0.3728757	0.8879	0.3751	0.442310228	count	1
COA4	0.3311092	0.3025416	1.0944	0.2744	0.442344297	count	1
BID	0.3190105	0.3935584	0.8106	0.418	0.44248083	count	1
HADH	0.3314017	0.4671141	0.7095	0.4784	0.442738207	count	1
MRPL23	0.3111784	0.3214236	0.9681	0.3335	0.442994131	count	1
MRPS23	0.331608	0.4678921	0.7087	0.4789	0.443016032	count	1
EML4	0.3106546	0.1624013	1.9129	0.0564	0.443085218	count	1
SIRT7	0.3154005	0.2888273	1.092	0.275	0.443157297	count	1
MAPKAPK3	0.3324014	0.834778	0.3982	0.6907	0.444084532	count	1
SYNPO	0.3324014	0.8428498	0.3944	0.693	0.444084532	count	1
CAPZA1	0.3112714	0.1553273	2.004	0.0457	0.444597543	count	1
GPR108	0.3335597	0.3725249	0.8954	0.3711	0.445644516	count	1
MAGOH	0.3213182	0.1900233	1.6909	0.0915	0.445694733	count	1
GLO1	0.3337697	0.2840803	1.1749	0.2407	0.445927346	count	1
NFKBID	0.3110616	0.1361342	2.285	0.0228	0.446005935	count	1
PRDM2	0.3136976	0.1393284	2.2515	0.0248	0.44743052	count	1
ARL8A	0.3362626	0.3094203	1.0868	0.2777	0.449285034	count	1
ERLEC1	0.324401	0.3384392	0.9585	0.338	0.449988353	count	1
THYN1	0.3280008	0.3679611	0.8914	0.3732	0.455002394	count	1
ATIC	0.3408981	0.3809501	0.8949	0.3713	0.455529496	count	1
FDFT1	0.3202638	0.2599992	1.2318	0.2187	0.455946082	count	1
UBXN4	0.3198487	0.1700967	1.8804	0.0607	0.458613158	count	1
SMU1	0.3437491	0.2361321	1.4557	0.1462	0.459370649	count	1
DENND2D	0.3324014	0.5672515	0.586	0.5582	0.46113236	count	1
GZF1	0.333343	0.3762568	0.8859	0.3761	0.462444068	count	1
TRMT10C	0.3468971	0.2556447	1.357	0.175	0.463612461	count	1
STXBP3	0.334843	0.3364687	0.9952	0.32	0.464533718	count	1
CD22	0.3309197	0.2655903	1.246	0.213	0.465024175	count	1
RARRES3	0.335238	0.2700407	1.2414	0.2151	0.465084002	count	1
GTDC1	0.3482245	0.5490844	0.6342	0.5263	0.465401242	count	1
UBE2S	0.3270287	0.2180884	1.4995	0.134	0.466467865	count	1
MLST8	0.3364817	0.5487191	0.6132	0.54	0.466816664	count	1
IBTK	0.3364817	0.5594617	0.6014	0.5479	0.466816664	count	1
ATPAF1	0.3496431	0.5219442	0.6699	0.503	0.46731303	count	1
ATF4	0.3304213	0.1884568	1.7533	0.0802	0.467354514	count	1
MACF1	0.3314186	0.3025351	1.0955	0.274	0.468768095	count	1
HDLBP	0.3507392	0.4914068	0.7137	0.4758	0.468790271	count	1
MRPL37	0.3337761	0.3792759	0.88	0.3793	0.469049432	count	1
CBX1	0.3386238	0.3290072	1.0292	0.3039	0.469801038	count	1
COX16	0.3361909	0.5457739	0.616	0.5382	0.472452511	count	1
CHURC1	0.3551507	0.2507942	1.4161	0.1574	0.474736412	count	1
SUMF2	0.3424319	0.3294423	1.0394	0.2992	0.475106811	count	1

PRKDC	0.3376225	0.2183104	1.5465	0.123	0.47756189	count	1
YBEY	0.3445194	0.3882449	0.8874	0.3754	0.478015476	count	1
USP14	0.336791	0.3316761	1.0154	0.3105	0.478253799	count	1
PANK3	0.3580388	0.6078663	0.589	0.5562	0.478629765	count	1
CHMP2B	0.3583533	0.2225459	1.6102	0.1081	0.47905376	count	1
KANSL2	0.3455697	0.4826427	0.716	0.474	0.479478982	count	1
TTTY15	0.358834	0.3407116	1.0532	0.2928	0.479701829	count	1
CYLD	0.339746	0.2121466	1.6015	0.11	0.480572004	count	1
AHNAK	0.3604616	0.1901293	1.8959	0.0586	0.481896209	count	1
TOB1	0.340255	0.4075045	0.835	0.4042	0.483181289	count	1
BUD23	0.3416687	0.2661573	1.2837	0.2	0.483297538	count	1
SHMT2	0.3484427	0.3969652	0.8778	0.3805	0.483482432	count	1
CMTM6	0.3441763	0.1207253	2.8509	0.0046	0.483706832	count	1
TLN1	0.3443073	0.1729994	1.9902	0.0472	0.483891468	count	1
MRPL18	0.3620199	0.2388209	1.5159	0.1303	0.483997284	count	1
SPIB	0.3389025	0.1350747	2.509	0.0125	0.486163461	count	1
CCND2	0.3437918	0.3483339	0.987	0.3242	0.488212485	count	1
TXNDC12	0.3453646	0.2864862	1.2055	0.2286	0.488536836	count	1
STX10	0.3656004	0.3234653	1.1303	0.259	0.488825407	count	1
SH3KBP1	0.3688749	0.2450305	1.5054	0.1329	0.493241495	count	1
GOLGB1	0.3488385	0.2044295	1.7064	0.0886	0.493461613	count	1
BRF1	0.3524591	0.6161971	0.572	0.5676	0.495381618	count	1
TMBIM1	0.3502961	0.3529025	0.9926	0.3214	0.495528034	count	1
SLC3A2	0.3477043	0.1798863	1.9329	0.0539	0.495996555	count	1
APEX1	0.3575388	0.2007374	1.7811	0.0756	0.496159161	count	1
MCFD2	0.3595221	0.5326876	0.6749	0.5001	0.498923486	count	1
MIGA1	0.3595221	0.5584038	0.6438	0.52	0.498923486	count	1
VPS8	0.3595542	0.6638516	0.5416	0.588	0.498968228	count	1
RPL26L1	0.3596555	0.7217866	0.4983	0.6185	0.499109423	count	1
INTS13	0.373623	0.6293227	0.5937	0.553	0.499645919	count	1
LYPLA1	0.3556059	0.2709865	1.3123	0.19	0.499817451	count	1
COX17	0.3567136	0.1790927	1.9918	0.047	0.501378946	count	1
PIGC	0.3625327	0.3836365	0.945	0.3452	0.503119875	count	1
BRIX1	0.3768742	0.4160656	0.9058	0.3655	0.504031939	count	1
SMCO4	0.3768864	0.8002689	0.4709	0.638	0.504048397	count	1
ZFP36L1	0.3508866	0.1092785	3.2109	0.0014	0.504114436	count	1
ZNF644	0.36339	0.2215457	1.6402	0.1017	0.504314888	count	1
SRSF1	0.3654321	0.3310117	1.104	0.2702	0.507161507	count	1
NSFL1C	0.3663836	0.3366524	1.0883	0.277	0.508487906	count	1
CENPC	0.3803096	0.2291629	1.6596	0.0977	0.508667043	count	1
USP48	0.3684615	0.2923848	1.2602	0.2083	0.511384603	count	1
SETDB1	0.3687195	0.6709955	0.5495	0.5829	0.511744276	count	1
COPB1	0.3614358	0.2443259	1.4793	0.1398	0.513313784	count	1
LMAN1	0.3655247	0.2524756	1.4478	0.148	0.513800562	count	1
NSD3	0.3596467	0.120417	2.9867	0.003	0.514333154	count	1

GSTM4	0.3712562	0.7582019	0.4897	0.6246	0.515280744	count	1
ZNF585A	0.3712562	0.7982409	0.4651	0.6421	0.515280744	count	1
SLC35C2	0.3673478	0.3828536	0.9595	0.3378	0.516370896	count	1
CCDC28A	0.3726219	0.3394928	1.0976	0.273	0.517184769	count	1
APIP	0.373623	0.503338	0.7423	0.458	0.518580511	count	1
TMEM19	0.3653801	0.3422286	1.0676	0.2863	0.518925657	count	1
FAM199X	0.3881136	0.598877	0.6481	0.517	0.51919859	count	1
YIPF4	0.374116	0.3536282	1.0579	0.2907	0.519267866	count	1
PQBP1	0.3741444	0.2520292	1.4845	0.1384	0.519307461	count	1
RNASEK	0.364141	0.2161292	1.6848	0.0927	0.519473909	count	1
HIVEP1	0.3743294	0.3110968	1.2033	0.2295	0.519565394	count	1
N4BP3	0.3885595	0.5585895	0.6956	0.487	0.519800429	count	1
CYB5A	0.3700105	0.3700372	0.9999	0.318	0.52012507	count	1
KLHL5	0.3900708	0.4673449	0.8347	0.4044	0.521840329	count	1
CMTM7	0.3770078	0.2904247	1.2981	0.1949	0.523299828	count	1
FRG1	0.3777181	0.294516	1.2825	0.2003	0.524290217	count	1
KRCC1	0.3730936	0.2860986	1.3041	0.193	0.524472137	count	1
HEXDC	0.3694321	0.3585357	1.0304	0.3034	0.524690949	count	1
CALM3	0.36796	0.2705736	1.3599	0.1745	0.524929101	count	1
MEI1	0.3784236	0.483287	0.783	0.434	0.525273927	count	1
BCL7B	0.3716616	0.2179714	1.7051	0.0889	0.525821181	count	1
MEF2D	0.3931293	0.4741286	0.8292	0.4075	0.52596894	count	1
INO80C	0.3813069	0.3433994	1.1104	0.2674	0.529294377	count	1
CD69	0.3686292	0.1388632	2.6546	0.0082	0.530821048	count	1
ATP13A3	0.3977447	0.5725974	0.6946	0.4876	0.532200068	count	1
ACP5	0.3849954	0.2504455	1.5372	0.1249	0.534437923	count	1
PRKAR1B	0.3851705	1.0538548	0.3655	0.7149	0.534682106	count	1
DAGLB	0.3851705	1.0538548	0.3655	0.7149	0.534682106	count	1
ARHGEF37	0.3851705	1.3347236	0.2886	0.773	0.534682106	count	1
TSFM	0.3803489	0.3344249	1.1373	0.256	0.534702568	count	1
AKR7A2	0.3997552	0.3423233	1.1678	0.2435	0.534914722	count	1
MRPL1	0.4000035	0.4302907	0.9296	0.3531	0.535250001	count	1
CWF19L2	0.3810756	0.2326365	1.6381	0.102	0.535727315	count	1
PRPF31	0.4016985	0.3185183	1.2611	0.2079	0.537538832	count	1
CLPP	0.3792618	0.3249061	1.1673	0.2437	0.538677689	count	1
TBC1D9	0.3891132	0.3204063	1.2144	0.225	0.540180553	count	1
KIAA1191	0.3845487	0.4744587	0.8105	0.4181	0.540624997	count	1
CTR9	0.4042672	0.394804	1.024	0.306	0.541007731	count	1
ITFG2	0.3849468	0.4701153	0.8188	0.4133	0.541186403	count	1
GGCX	0.3905662	0.5274269	0.7405	0.4594	0.542206996	count	1
GCA	0.3910105	0.3237982	1.2076	0.2279	0.542826656	count	1
MANF	0.3923818	0.334238	1.174	0.241	0.544739224	count	1
MT-ND4L	0.3851637	0.1138347	3.3835	8.00E-04	0.544968542	count	1
TBC1D20	0.3929624	0.5492386	0.7155	0.4747	0.545549008	count	1
RBM42	0.4076727	0.3064724	1.3302	0.1841	0.545607184	count	1

MRPL43	0.3864788	0.2376371	1.6263	0.105	0.546833624	count	1
FNTA	0.4091984	0.3566231	1.1474	0.2518	0.547667973	count	1
ATP2A3	0.3947814	0.4406214	0.896	0.371	0.548086094	count	1
POLR2B	0.394973	0.303106	1.3031	0.1932	0.548353336	count	1
ACAP1	0.3876331	0.1895099	2.0455	0.0414	0.54847068	count	1
MYO1F	0.3908416	0.559167	0.699	0.4849	0.549499657	count	1
VPS25	0.3964581	0.6235981	0.6358	0.5253	0.550424782	count	1
ATP6V1B2	0.3964581	0.6601874	0.6005	0.5485	0.550424782	count	1
DENND4A	0.4118363	0.271434	1.5173	0.1299	0.551231298	count	1
UCHL5	0.4132366	0.3016567	1.3699	0.1714	0.553122988	count	1
WDR33	0.3909185	0.2652982	1.4735	0.141	0.553130211	count	1
NOM1	0.4135043	0.4695666	0.8806	0.379	0.553484641	count	1
SPATA33	0.4000346	0.7292817	0.5485	0.5836	0.555413588	count	1
TAGAP	0.3910646	0.1311939	2.9808	0.003	0.555473405	count	1
FUNDC2	0.3902942	0.2366288	1.6494	0.0998	0.55820921	count	1
SDCBP	0.3930523	0.1541308	2.5501	0.0111	0.55830211	count	1
UBE2M	0.394973	0.2229106	1.7719	0.0771	0.558880726	count	1
ARL8B	0.4028034	0.368075	1.0944	0.2744	0.559275977	count	1
ZDHHC23	0.4036312	0.5048186	0.7996	0.4244	0.56043077	count	1
CELF2	0.3990016	0.2170053	1.8387	0.0666	0.561008494	count	1
ANO6	0.419387	0.5890923	0.7119	0.4769	0.561432797	count	1
PSMD11	0.394398	0.323396	1.2196	0.2233	0.561661248	count	1
PARD6A	0.4058964	0.4411536	0.9201	0.358	0.563590853	count	1
ATP2B1-AS1	0.3957836	0.2614988	1.5135	0.1309	0.563637613	count	1
CREM	0.398726	0.2522777	1.5805	0.115	0.564203823	count	1
MCM5	0.4218526	0.3268757	1.2906	0.1975	0.564764582	count	1
PRIM2	0.3993889	0.5764565	0.6928	0.4888	0.565144072	count	1
SLC39A1	0.4224016	0.3532581	1.1957	0.2324	0.56550649	count	1
DNAJB11	0.409054	0.3150534	1.2984	0.1948	0.567996106	count	1
MRPL47	0.4257783	0.2898087	1.4692	0.1425	0.570070013	count	1
DUT	0.3992662	0.1615932	2.4708	0.0139	0.571054967	count	1
SNAP23	0.4067749	0.2338124	1.7397	0.0826	0.571973041	count	1
CD55	0.405591	0.104739	3.8724	1.00E-04	0.573941344	count	1
ARAF	0.4292706	0.5136491	0.8357	0.4038	0.574790339	count	1
GK	0.4292706	0.7138182	0.6014	0.548	0.574790339	count	1
PTBP1	0.4147416	0.3451958	1.2015	0.2302	0.575931678	count	1
GLG1	0.4315907	0.3760264	1.1478	0.2517	0.577926593	count	1
SNX8	0.4170054	0.2925723	1.4253	0.1548	0.579090456	count	1
UNKL	0.4188688	0.8206458	0.5104	0.61	0.581690638	count	1
ZNF24	0.4346	0.2135889	2.0348	0.0425	0.581994865	count	1
SIN3B	0.4347115	0.5132689	0.8469	0.3975	0.582145612	count	1
CCDC115	0.4142034	0.3289324	1.2592	0.2086	0.582452232	count	1
TXLNA	0.4361124	0.7921496	0.5505	0.5822	0.584039643	count	1
FRY	0.4206331	1.2782253	0.3291	0.7423	0.584152618	count	1
AC005363.2	0.4206331	1.2782253	0.3291	0.7423	0.584152618	count	1

CYTIP	0.4075038	0.1015673	4.0122	7.06E-05	0.585365634	count	1
NIP7	0.4218808	0.3765657	1.1203	0.263	0.585893758	count	1
PHF20	0.4090972	0.1802633	2.2694	0.0237	0.586046651	count	1
TWISTNB	0.4232601	0.2508574	1.6873	0.0923	0.587818591	count	1
IVNS1ABP	0.4239302	0.2690892	1.5754	0.1159	0.588753743	count	1
DCTN2	0.415564	0.3353184	1.2393	0.2159	0.590341628	count	1
TINF2	0.4171925	0.2356952	1.7701	0.0774	0.59039869	count	1
CASP8	0.4137035	0.2826366	1.4637	0.144	0.591726758	count	1
YIPF5	0.4267642	0.4480457	0.9525	0.341	0.592708828	count	1
HARS	0.4427821	0.3841156	1.1527	0.2496	0.593058397	count	1
HTT	0.4460037	0.520176	0.8574	0.3917	0.597415375	count	1
EIF2A	0.4309389	0.2307925	1.8672	0.0625	0.598535339	count	1
P4HB	0.422718	0.2943371	1.4362	0.1517	0.600524612	count	1
AKT2	0.4490668	0.4744592	0.9465	0.3444	0.601558438	count	1
SRP19	0.4334397	0.266748	1.6249	0.105	0.602025845	count	1
CD99	0.4202139	0.1327577	3.1653	0.0017	0.602338378	count	1
ACO2	0.4511617	0.3966012	1.1376	0.2559	0.604392188	count	1
TTF1	0.4248107	0.3625103	1.1719	0.2419	0.60615048	count	1
ERO1A	0.4366649	0.4111158	1.0621	0.289	0.606527664	count	1
PIKFYVE	0.4534143	0.2794409	1.6226	0.1054	0.607439476	count	1
CNN2	0.4276748	0.2309635	1.8517	0.0647	0.607580423	count	1
USP6NL	0.438457	0.3846715	1.1398	0.255	0.609029235	count	1
PRPF8	0.4364488	0.2723139	1.6027	0.1097	0.613838947	count	1
RAB29	0.4426332	0.3411785	1.2974	0.195	0.614859051	count	1
BRMS1	0.4429167	0.3439747	1.2876	0.1985	0.615254821	count	1
DNAJC1	0.4318032	0.3874787	1.1144	0.2657	0.61614224	count	1
FUNDC1	0.4357499	0.4972705	0.8763	0.3814	0.616727017	count	1
TMTC3	0.444416	0.911238	0.4877	0.626	0.617347902	count	1
WDR46	0.4395184	0.4448535	0.988	0.3237	0.618170601	count	1
KRAS	0.4369207	0.1792224	2.4379	0.0152	0.618388238	count	1
BAG2	0.4397447	0.5067022	0.8679	0.3859	0.618489949	count	1
IDS	0.4464346	0.1672893	2.6686	0.0079	0.620166031	count	1
RNF149	0.4478303	0.3670432	1.2201	0.2231	0.6221146	count	1
RELB	0.4495142	0.2704934	1.6618	0.0973	0.624465593	count	1
ADGRE2	0.4686194	0.9361118	0.5006	0.6169	0.628014747	count	1
BLNK	0.4522234	0.2412292	1.8747	0.0615	0.628248212	count	1
HIPK1	0.4690863	0.5052483	0.9284	0.3537	0.628646708	count	1
OTUB1	0.4429886	0.3185077	1.3908	0.165	0.629380618	count	1
OCIAD1	0.4424798	0.1774318	2.4938	0.013	0.630253248	count	1
TYW3	0.4497832	0.3243498	1.3867	0.166	0.632656863	count	1
SEC11C	0.4728885	0.2010176	2.3525	0.0191	0.633793464	count	1
MCL1	0.4462624	0.1608656	2.7741	0.0058	0.634041396	count	1
AC058791.1	0.4568823	0.2210208	2.0671	0.0393	0.634753437	count	1
TMEM9B	0.4574801	0.2648139	1.7276	0.0848	0.635588185	count	1
RANBP2	0.4521583	0.3135166	1.4422	0.15	0.636008985	count	1

THAP9-AS1	0.459219	0.3943359	1.1645	0.245	0.63801637	count	1
VPS4A	0.4599878	0.3792889	1.2128	0.226	0.639089937	count	1
MALSU1	0.4556205	0.3887281	1.1721	0.2418	0.640895562	count	1
A1BG	0.4781416	0.3056798	1.5642	0.1185	0.640905238	count	1
SNAPC2	0.4561975	0.4356178	1.0472	0.2956	0.641709962	count	1
OPN3	0.4621914	0.4758938	0.9712	0.332	0.642167169	count	1
NTMT1	0.4634022	0.3202955	1.4468	0.1487	0.643858047	count	1
ANKRD13C	0.4581019	0.7309993	0.6267	0.5312	0.644397948	count	1
EXOSC3	0.482041	0.3952793	1.2195	0.2233	0.646185114	count	1
COMMD5	0.4651336	0.3903602	1.1915	0.234	0.646276001	count	1
SNRNP35	0.460391	0.3478073	1.3237	0.186	0.647629	count	1
HERPUD2	0.4666911	0.3283045	1.4215	0.1559	0.648451159	count	1
FGD4	0.4686194	0.6964323	0.6729	0.5014	0.651144244	count	1
OPTN	0.4697022	0.3954012	1.1879	0.236	0.652656531	count	1
MRPS18B	0.4612508	0.2928283	1.5752	0.1159	0.652913554	count	1
GALNT1	0.4705446	0.2938756	1.6012	0.1101	0.653833084	count	1
DDX11	0.4881342	0.5783902	0.844	0.3992	0.654436756	count	1
SDR39U1	0.4722316	0.5182656	0.9112	0.3627	0.656189312	count	1
TMEM50B	0.4728053	0.4103487	1.1522	0.25	0.656990613	count	1
SLC50A1	0.4626003	0.307203	1.5058	0.1328	0.657302606	count	1
FAM98A	0.4674121	0.5513028	0.8478	0.397	0.657539766	count	1
ST6GALNAC4	0.4628496	0.4408273	1.05	0.294	0.657657569	count	1
SMIM10L1	0.4915024	0.3523823	1.3948	0.1638	0.658998781	count	1
APOBEC3C	0.4669921	0.2415012	1.9337	0.0538	0.661061732	count	1
AREG	0.462673	0.350004	1.3219	0.187	0.661153263	count	1
MAP1S	0.4939068	0.5989176	0.8247	0.41	0.662255695	count	1
ABTB1	0.4941372	0.3961795	1.2473	0.213	0.662567798	count	1
PIM3	0.4647433	0.1810026	2.5676	0.0106	0.663215983	count	1
ENSA	0.4786715	0.180955	2.6453	0.0084	0.665184507	count	1
PRR7	0.4683282	0.3522204	1.3296	0.1843	0.665458367	count	1
BACH1	0.4671527	0.2070461	2.2563	0.0245	0.665458539	count	1
EHMT1	0.4753946	0.3394572	1.4005	0.162	0.668808564	count	1
COPS3	0.4768717	0.2641614	1.8052	0.0717	0.670893877	count	1
FOXP1	0.4733014	0.2205492	2.146	0.0324	0.672539791	count	1
INTS14	0.4843913	0.6252537	0.7747	0.4389	0.673174669	count	1
MECP2	0.5023277	0.2936216	1.7108	0.0878	0.673664248	count	1
RIC3	0.4789776	0.234852	2.0395	0.042	0.673866965	count	1
UCP2	0.4710148	0.1496916	3.1466	0.0018	0.674367646	count	1
SIAH2	0.4734806	0.2109201	2.2448	0.0253	0.674488499	count	1
SNX10	0.5035104	0.3238689	1.5547	0.121	0.675266794	count	1
NDUFB3	0.4754944	0.2931323	1.6221	0.1055	0.675662517	count	1
DCTN5	0.4877643	0.6286116	0.7759	0.4382	0.677886862	count	1
NDUFA13	0.482526	0.265831	1.8152	0.0702	0.678876716	count	1
MTX1	0.4795839	0.3978098	1.2056	0.2286	0.678933667	count	1
GNL1	0.4811734	0.3201015	1.5032	0.1335	0.681189827	count	1

MRTO4	0.4907528	0.4015786	1.2221	0.2223	0.68206211	count	1
PPP1CB	0.5111498	0.2253712	2.268	0.0238	0.685619504	count	1
CCNDBP1	0.4820487	0.2178126	2.2131	0.0274	0.686715751	count	1
BCLAF1	0.4862162	0.1602249	3.0346	0.0026	0.688347846	count	1
RAB9A	0.4850317	0.3092195	1.5686	0.1175	0.689243699	count	1
ZNF287	0.4959289	0.6650833	0.7457	0.4563	0.689294135	count	1
NUP210	0.5141492	0.4764542	1.0791	0.2811	0.68968486	count	1
ZNF568	0.5162244	0.6950194	0.7427	0.458	0.692497785	count	1
BRWD1	0.4983165	0.439086	1.1349	0.257	0.692630277	count	1
AZIN1	0.4985704	0.3533869	1.4108	0.159	0.692985055	count	1
BABAM2	0.5200699	0.4820091	1.079	0.2812	0.697710795	count	1
PPP1R15A	0.4871663	0.1456319	3.3452	9.00E-04	0.6979971	count	1
SMARCE1	0.5208751	0.442812	1.1763	0.2401	0.698802413	count	1
DERL2	0.4982413	0.3182134	1.5657	0.1181	0.701066417	count	1
PTPN1	0.4982829	0.1672181	2.9798	0.003	0.70112516	count	1
KLF3	0.4993	0.3425359	1.4577	0.146	0.702561415	count	1
DNAAF2	0.5057661	0.2743058	1.8438	0.0659	0.703040256	count	1
DYNLT3	0.5248722	0.3018186	1.739	0.0827	0.704221692	count	1
CD19	0.5006127	0.2061855	2.428	0.0156	0.704415112	count	1
ACADS	0.5069629	1.0988012	0.4614	0.6448	0.704712764	count	1
AP3S1	0.500978	0.2900311	1.7273	0.0848	0.704930965	count	1
YWHAH	0.5072058	0.178601	2.8399	0.0047	0.705052215	count	1
MEF2A	0.5264705	0.2538069	2.0743	0.0386	0.70638885	count	1
LTA4H	0.5277009	0.3520603	1.4989	0.1346	0.708057237	count	1
MKRN2	0.5115021	0.6407154	0.7983	0.4251	0.711056494	count	1
FRS2	0.5115021	0.6919751	0.7392	0.4602	0.711056494	count	1
NUDT15	0.5123202	0.5250007	0.9758	0.3297	0.712199871	count	1
B4GALT1	0.532354	0.2346777	2.2684	0.0238	0.714367246	count	1
HAGH	0.5326449	0.4281268	1.2441	0.214	0.714761764	count	1
FAM216A	0.5327136	0.7326656	0.7271	0.4676	0.714854932	count	1
NSMCE1	0.5045813	0.4349229	1.1602	0.2466	0.717085099	count	1
8-Mar	0.5137156	0.6362068	0.8075	0.4198	0.722919436	count	1
PREX1	0.5207448	0.3753416	1.3874	0.166	0.723974933	count	1
FAM53C	0.5231823	0.3681481	1.4211	0.156	0.727382107	count	1
YPEL5	0.5074856	0.1286904	3.9435	9.33E-05	0.728206427	count	1
NR1H2	0.5122413	0.2154541	2.3775	0.0179	0.729807133	count	1
KIAA0100	0.5449811	0.4871135	1.1188	0.2638	0.731494954	count	1
TDP2	0.5167522	0.3946148	1.3095	0.191	0.731698463	count	1
SNAPC1	0.5300761	0.3084511	1.7185	0.0864	0.737019032	count	1
EPC1	0.530126	0.2374753	2.2323	0.0261	0.737088793	count	1
TMEM170A	0.5305669	0.305954	1.7341	0.0836	0.737705165	count	1
MARCKS	0.5139985	0.1028647	4.9968	8.40E-07	0.738054935	count	0.02040948
NFKBIE	0.5203665	0.242723	2.1439	0.0326	0.73956797	count	1
MAP3K1	0.552177	0.2825603	1.9542	0.0513	0.741258388	count	1
ANKZF1	0.5274128	0.5694828	0.9261	0.3549	0.742265706	count	1

SMARCC2	0.5542457	0.3310622	1.6741	0.0948	0.744065576	count	1
SLC4A7	0.5359986	0.4037379	1.3276	0.185	0.745298953	count	1
TPRKB	0.5304241	0.3472314	1.5276	0.127	0.746519299	count	1
EGR2	0.5381215	0.5024607	1.071	0.285	0.748267038	count	1
UBE2Z	0.5407345	0.4296995	1.2584	0.209	0.751920469	count	1
ASTE1	0.5414743	0.5644674	0.9593	0.3379	0.752954864	count	1
VAC14	0.5619825	0.9023484	0.6228	0.5337	0.754565676	count	1
ATP2B1	0.5371097	0.1276002	4.2093	3.10E-05	0.755963453	count	0.752959
LYRM1	0.5445415	0.3862178	1.4099	0.1593	0.757243571	count	1
CNIH4	0.5458745	0.3242491	1.6835	0.093	0.759107492	count	1
TMEM69	0.5657727	0.6452804	0.8768	0.3811	0.759710405	count	1
PCCB	0.5657727	0.7645101	0.74	0.4597	0.759710405	count	1
RNPS1	0.5403	0.1912428	2.8252	0.0049	0.76047432	count	1
CXCR3	0.5473304	0.4836542	1.1317	0.258	0.761143306	count	1
CD70	0.5426867	0.1838972	2.951	0.0033	0.76384206	count	1
SF3A3	0.5433911	0.3427578	1.5854	0.1136	0.764837193	count	1
FDX1	0.5447358	0.2829095	1.9255	0.0548	0.766736922	count	1
PARP11	0.5724246	0.7200553	0.795	0.4271	0.768740815	count	1
SNRPA	0.5439369	0.3678327	1.4788	0.1399	0.770299945	count	1
ADARB1	0.5498085	0.6523998	0.8427	0.3998	0.773903615	count	1
MED13	0.5565272	0.3550385	1.5675	0.1177	0.774004345	count	1
GHDC	0.5566368	0.8490741	0.6556	0.5124	0.774157621	count	1
TNPO3	0.5774798	0.6285877	0.9187	0.359	0.775604665	count	1
EFHD2	0.5584069	0.2537662	2.2005	0.0283	0.77663317	count	1
ARL4D	0.5484197	0.6854739	0.8001	0.4241	0.776666132	count	1
VAPB	0.5587107	0.6285761	0.8889	0.3746	0.77705805	count	1
TGIF2	0.5526141	0.3042797	1.8161	0.07	0.777867506	count	1
FBXL15	0.5453286	0.337366	1.6164	0.1067	0.778409025	count	1
EIF2S1	0.5503616	0.3179286	1.7311	0.0841	0.779423959	count	1
IRAK4	0.5806325	0.3659087	1.5868	0.1133	0.779885798	count	1
PPT1	0.5547785	0.4327728	1.2819	0.2005	0.780925549	count	1
MVP	0.5518486	0.4432721	1.2449	0.2138	0.781535777	count	1
ITGAX	0.5619825	0.7092234	0.7924	0.4286	0.781633958	count	1
SAMSN1	0.5468955	0.1563434	3.498	5.00E-04	0.783136406	count	1
TYSND1	0.5521267	0.5893795	0.9368	0.3494	0.78481053	count	1
CDIP1	0.5843992	0.6009422	0.9725	0.3313	0.785001163	count	1
PRMT5	0.5854876	0.882766	0.6632	0.508	0.78647936	count	1
BAG4	0.5859332	0.4120807	1.4219	0.1558	0.787084557	count	1
PAIP1	0.5619945	0.4050222	1.3876	0.166	0.795945441	count	1
RAMMET	0.5956197	0.3336967	1.7849	0.075	0.80024211	count	1
AFTPH	0.5685339	0.265246	2.1434	0.0326	0.800361761	count	1
ARL4A	0.5760292	0.2200255	2.618	0.0092	0.801281869	count	1
NUDT16L1	0.5709608	0.387581	1.4731	0.1414	0.808680632	count	1
SAP30BP	0.5826902	0.3378284	1.7248	0.0853	0.810600284	count	1
CLP1	0.6053009	0.5117192	1.1829	0.2375	0.813395685	count	1

KLF2	0.5644803	0.119102	4.7395	2.89E-06	0.813480886	count	0.07021544
KLF16	0.6057058	0.3331287	1.8182	0.0697	0.813945884	count	1
AASDHPPT	0.6058113	0.5334244	1.1357	0.2567	0.81408924	count	1
S100A10	0.5718333	0.1539639	3.7141	2.00E-04	0.820285956	count	1
FARSA	0.5772797	0.4767996	1.2107	0.2266	0.820646943	count	1
MIF	0.5778077	0.1855116	3.1147	0.002	0.821399258	count	1
ZBTB2	0.6135746	0.550889	1.1138	0.266	0.824639434	count	1
SPAST	0.614154	0.6744085	0.9107	0.363	0.82542691	count	1
RALGAPB	0.6146646	0.6467195	0.9504	0.3424	0.826120889	count	1
C1orf56	0.5870461	0.2504689	2.3438	0.0195	0.826523111	count	1
UBE2D1	0.5836817	0.280101	2.0838	0.0377	0.829768897	count	1
CTNNB1	0.6186217	0.284641	2.1733	0.0303	0.831499431	count	1
METAP1D	0.6254353	0.6512982	0.9603	0.3374	0.840761764	count	1
AL121603.2	0.5899238	0.4784175	1.2331	0.218	0.842172806	count	1
PKHD1L1	0.6053819	0.7002146	0.8646	0.3877	0.842350953	count	1
CBLB	0.6062523	0.5907572	1.0262	0.3053	0.843569017	count	1
CCDC122	0.6074588	0.6760846	0.8985	0.3694	0.84525745	count	1
SF3B4	0.6012467	0.3299078	1.8225	0.0691	0.846594212	count	1
NGDN	0.6334126	0.3039256	2.0841	0.0377	0.851607909	count	1
TMEM60	0.6053295	0.4090864	1.4797	0.1397	0.852365285	count	1
JUND	0.5946124	0.0605309	9.8233	9.70E-21	0.857267929	count	2.36E-16
FAM111A-DT	0.6090247	1.09982	0.5537	0.58	0.857588653	count	1
PSD3	0.6100205	0.8236408	0.7406	0.459	0.858996297	count	1
DHRS4	0.6407269	0.8003744	0.8005	0.4238	0.861554384	count	1
MRPS9	0.643789	0.389105	1.6545	0.0987	0.865718932	count	1
ATP11C	0.6441986	0.5796917	1.1113	0.2671	0.866276019	count	1
SETD6	0.6233635	0.8163459	0.7636	0.4455	0.867517597	count	1
TXNDC15	0.6466097	0.3828396	1.689	0.0919	0.869555422	count	1
TRAF4	0.6178204	0.2575183	2.3991	0.0168	0.870022498	count	1
CYC1	0.625481	0.2622549	2.385	0.0175	0.870481564	count	1
USF3	0.6204413	0.3729964	1.6634	0.0969	0.873727647	count	1
EPHB6	0.6310504	0.3997797	1.5785	0.1152	0.87827768	count	1
YIPF6	0.6245939	0.4140898	1.5084	0.1322	0.879598312	count	1
UBE4B	0.6192193	0.6901988	0.8972	0.37	0.88041052	count	1
TAPT1	0.6333851	0.5434958	1.1654	0.2445	0.881545975	count	1
CD1C	0.6555639	0.5077743	1.2911	0.197	0.881735802	count	1
PRCC	0.6560259	0.4234099	1.5494	0.122	0.882364324	count	1
BCL10	0.6563787	0.1907055	3.4418	6.00E-04	0.882844291	count	1
RAD1	0.6274317	0.5059181	1.2402	0.2156	0.883610316	count	1
IRF1	0.6155662	0.1440502	4.2733	2.36E-05	0.883741177	count	0.5732912
ANKRD28	0.6351618	0.3106278	2.0448	0.0415	0.884033198	count	1
PYCR2	0.657372	0.4144889	1.586	0.1135	0.884195652	count	1
TMEM263	0.6366656	0.4089385	1.5569	0.1202	0.886138426	count	1
CNPPD1	0.632261	0.2846621	2.2211	0.0268	0.890438052	count	1
FCER2	0.642316	0.3422613	1.8767	0.0612	0.894048965	count	1

MLLT6	0.646807	0.4403246	1.4689	0.1426	0.900336714	count	1
RPP25L	0.6695122	0.6748632	0.9921	0.3217	0.900714407	count	1
SAYSD1	0.6695122	0.7266875	0.9213	0.3574	0.900714407	count	1
KLF6	0.6301474	0.1257272	5.012	7.79E-07	0.907360843	count	0.018928142
ZNF195	0.6407269	0.4757758	1.3467	0.1788	0.90779762	count	1
WDR73	0.6754849	0.5588324	1.2087	0.2274	0.908842828	count	1
NOP14	0.6781495	0.3989849	1.6997	0.0899	0.912469486	count	1
ZFP36	0.6378424	0.1409414	4.5256	7.74E-06	0.914135557	count	0.1880433
TOR3A	0.6438115	0.2429553	2.6499	0.0083	0.915459971	count	1
WEE1	0.6615096	0.2091625	3.1627	0.0017	0.931795582	count	1
MON2	0.6701146	0.531832	1.26	0.2083	0.932974223	count	1
OMA1	0.6737723	0.5214522	1.2921	0.197	0.93809683	count	1
ISCA2	0.6739546	0.3546103	1.9006	0.058	0.938352143	count	1
RAB30	0.7015365	0.3186865	2.2013	0.0282	0.944308846	count	1
NOC4L	0.7018917	0.8214971	0.8544	0.3933	0.944792536	count	1
GORAB	0.7023147	0.5852201	1.2001	0.2307	0.945368554	count	1
TMEM101	0.7023147	0.7634575	0.9199	0.358	0.945368554	count	1
STX11	0.7043201	0.6491675	1.085	0.279	0.948099458	count	1
TSC22D3	0.6608833	0.0968903	6.8209	2.97E-11	0.948527673	count	7.22E-07
ANKIB1	0.6817214	0.4415004	1.5441	0.1233	0.949230214	count	1
ANAPC1	0.6828559	0.560734	1.2178	0.224	0.950819254	count	1
ZNF487	0.6828559	0.5831057	1.1711	0.2422	0.950819254	count	1
RASGEF1B	0.663639	0.1891685	3.5082	5.00E-04	0.95210816	count	1
MLH1	0.6839428	0.5418335	1.2623	0.2075	0.952341638	count	1
MSMO1	0.7114728	0.5380483	1.3223	0.1867	0.957840692	count	1
AP3B1	0.713377	0.3710969	1.9223	0.0552	0.960434241	count	1
ZDHHC20	0.6901325	0.5192119	1.3292	0.1845	0.961011666	count	1
ZNF691	0.6822097	0.899264	0.7586	0.4485	0.961070893	count	1
FBXO4	0.6842899	0.6096804	1.1224	0.2623	0.964013072	count	1
PAN3	0.6941595	0.2951058	2.3522	0.0191	0.96665265	count	1
TARS2	0.7206312	0.7943562	0.9072	0.3648	0.970315407	count	1
HSDL2	0.7206312	0.7943562	0.9072	0.3648	0.970315407	count	1
LCORL	0.6904069	0.5561677	1.2414	0.2151	0.972665026	count	1
MFSD1	0.7001281	0.5049771	1.3865	0.1663	0.975013827	count	1
INO80D	0.6884674	0.3039506	2.2651	0.024	0.979114661	count	1
TXNDC9	0.6954339	0.3879243	1.7927	0.0737	0.97977554	count	1
TAGLN2	0.6825098	0.0896202	7.6156	1.60E-13	0.981813543	count	3.89E-09
RAB27A	0.7330815	0.693028	1.0578	0.291	0.987277392	count	1
ZNF511	0.7373228	0.3996274	1.845	0.0657	0.993056505	count	1
GGA3	0.7400411	0.5972971	1.239	0.216	0.996760634	count	1
RGCC	0.7027024	0.2281948	3.0794	0.0022	1.004749769	count	1
ISY1	0.7218184	0.4966989	1.4532	0.1469	1.005402962	count	1
NFKBIA	0.6982326	0.1066973	6.5441	1.65E-10	1.006186101	count	4.01E-06
LDLRAD4	0.7142828	0.2322978	3.0749	0.0022	1.00643872	count	1
MRFAP1L1	0.7472473	0.4396743	1.6995	0.0899	1.006581094	count	1

ZNF574	0.749421	0.8386437	0.8936	0.372	1.009543609	count	1
ARL6IP6	0.7184257	0.3741455	1.9202	0.0555	1.01229959	count	1
AC007952.4	0.7238072	0.3730507	1.9402	0.053	1.019912903	count	1
IMPDH1	0.7259285	0.5264176	1.379	0.1686	1.022914017	count	1
JUN	0.7127143	0.1919315	3.7134	2.00E-04	1.025502751	count	1
MED8	0.7383207	0.507727	1.4542	0.1466	1.028527538	count	1
KIF20B	0.7422821	0.4158012	1.7852	0.0749	1.034079125	count	1
FOXP4	0.7443342	0.7491412	0.9936	0.321	1.036955053	count	1
PEA15	0.74486	0.3153693	2.3619	0.0186	1.037691949	count	1
CMIP	0.7457985	0.5057461	1.4747	0.141	1.039007237	count	1
RAB11FIP1	0.7237959	0.128025	5.6536	2.82E-08	1.03925648	count	0.000685316
ZNF92	0.7461626	0.4039996	1.8469	0.0654	1.039517521	count	1
JAZF1	0.7378927	0.4547085	1.6228	0.1053	1.039841105	count	1
VMA21	0.7380887	0.3767467	1.9591	0.0507	1.040118419	count	1
TRAF3	0.7487965	0.3783624	1.979	0.0484	1.043208947	count	1
UBL4A	0.7494211	0.6548921	1.1443	0.2531	1.044084341	count	1
C2orf76	0.7762027	0.6264595	1.239	0.216	1.046052777	count	1
FBXO42	0.779443	0.7288822	1.0694	0.2855	1.050471047	count	1
DPAGT1	0.7795081	0.7232545	1.0778	0.2817	1.050559811	count	1
NNT-AS1	0.7795081	0.8760708	0.8898	0.374	1.050559811	count	1
MAP4K5	0.754116	0.4273715	1.7645	0.0783	1.05066451	count	1
GARS	0.7545163	0.5673626	1.3299	0.1842	1.051225567	count	1
YY1AP1	0.7463684	0.6207912	1.2023	0.2299	1.051833335	count	1
MGA	0.7593746	0.4706072	1.6136	0.1073	1.058035042	count	1
DPH3	0.7448053	0.3107231	2.397	0.0169	1.059437703	count	1
KCTD9	0.7458214	0.6186088	1.2056	0.229	1.060886545	count	1
ELK3	0.7871763	0.4394117	1.7914	0.0739	1.061016569	count	1
EIF2AK1	0.791765	0.3528374	2.244	0.0253	1.067274538	count	1
ADPRHL2	0.7666849	0.396226	1.935	0.0536	1.068281787	count	1
DNAJC17	0.7538064	0.5473378	1.3772	0.1691	1.072272407	count	1
ALG14	0.7705648	0.8412228	0.916	0.3602	1.07372043	count	1
PHKG1	0.799639	1.0180299	0.7855	0.4326	1.078013889	count	1
RIC8A	0.801684	0.4652199	1.7232	0.0855	1.080803258	count	1
GLI4	0.8030943	0.6002901	1.3378	0.1816	1.082726951	count	1
EIF3C	0.7783976	0.870988	0.8937	0.372	1.084700542	count	1
TRNT1	0.7794738	0.5193785	1.5008	0.1341	1.086209223	count	1
TCIRG1	0.7688152	0.434694	1.7686	0.0776	1.089872026	count	1
TIMM9	0.784178	0.4434161	1.7685	0.0777	1.092803994	count	1
HDAC9	0.766858	0.4209846	1.8216	0.0692	1.095253851	count	1
KLK1	0.7738847	0.7299942	1.0601	0.29	1.097080353	count	1
TERF2	0.8141006	0.4199151	1.9387	0.0532	1.097741171	count	1
ITGB7	0.7795942	0.3665632	2.1268	0.034	1.098849755	count	1
FOPNL	0.7891694	0.4935984	1.5988	0.1106	1.099801639	count	1
C5orf15	0.7913553	0.457028	1.7315	0.0841	1.102866223	count	1
ADD3	0.782836	0.3452016	2.2678	0.0238	1.103437525	count	1

MTMR6	0.8212542	0.3344309	2.4557	0.0144	1.107500933	count	1
ZNF557	0.7963503	0.5747828	1.3855	0.1666	1.109869281	count	1
DUSP10	0.7974268	0.4854086	1.6428	0.101	1.111378582	count	1
CPD	0.7889354	0.9471145	0.833	0.4053	1.112069542	count	1
HBS1L	0.8251128	0.4588928	1.7981	0.0728	1.112765661	count	1
AP005329.3	0.799639	0.8133513	0.9831	0.3261	1.114480217	count	1
E2F5	0.8277176	0.41017	2.018	0.0442	1.116319827	count	1
CCDC167	0.8287922	0.3240401	2.5577	0.0109	1.11778612	count	1
ZNHIT2	0.8044455	0.6384144	1.2601	0.2083	1.121219383	count	1
CPSF2	0.7969782	0.5419855	1.4705	0.1421	1.123452298	count	1
TNFAIP3	0.8334207	0.2998831	2.7792	0.0057	1.124101952	count	1
RNF214	0.8084268	0.5435081	1.4874	0.1376	1.126801713	count	1
CFAP298	0.8132235	0.3822454	2.1275	0.0339	1.13352754	count	1
PCGF3	0.8444408	0.80289	1.0518	0.2935	1.139140884	count	1
ZNF267	0.8173641	0.3104602	2.6328	0.0088	1.139333571	count	1
RNF14	0.8176179	0.7686412	1.0637	0.288	1.13968946	count	1
AAED1	0.8213269	0.2279092	3.6037	3.00E-04	1.144890434	count	1
BCAT2	0.8505329	0.6215716	1.3684	0.1719	1.147455516	count	1
C18orf21	0.8510671	0.5534604	1.5377	0.1248	1.148184629	count	1
CYSLTR1	0.8083986	0.2473203	3.2686	0.0012	1.15012336	count	1
AC073111.5	0.8138068	0.6358565	1.2799	0.201	1.15385032	count	1
NFKB1	0.8593876	0.3084816	2.7859	0.0056	1.159541633	count	1
LTN1	0.8185159	0.3711817	2.2052	0.028	1.164552411	count	1
RNF6	0.8307309	0.6441221	1.2897	0.1978	1.171226085	count	1
DOK3	0.8693763	0.6368141	1.3652	0.1729	1.173177011	count	1
TPP2	0.8693763	0.6947882	1.2513	0.2115	1.173177011	count	1
TMSB4Y	0.872793	1.3940752	0.6261	0.5316	1.177841415	count	1
MCMBP	0.8464799	0.4855418	1.7434	0.082	1.180164579	count	1
FAM117A	0.8388071	0.5357691	1.5656	0.1182	1.182658195	count	1
LAMP1	0.8869009	0.2967436	2.9888	0.003	1.197102926	count	1
CPSF6	0.8547024	0.4559503	1.8746	0.0615	1.205159528	count	1
TAF11	0.8551709	0.4372717	1.9557	0.0511	1.205822755	count	1
GFER	0.8667216	0.4368125	1.9842	0.0478	1.208554904	count	1
GTF3C2	0.8986356	0.757851	1.1858	0.2363	1.213126305	count	1
PYCR3	0.9013782	0.9630915	0.9359	0.3498	1.216871485	count	1
EEF1AKMT1	0.9013782	1.0472688	0.8607	0.3899	1.216871485	count	1
HIST1H2AK	0.872793	0.9991922	0.8735	0.383	1.217071024	count	1
DNAJC27-AS1	0.872793	1.3940752	0.6261	0.5316	1.217071032	count	1
ALDH1L2	0.872793	1.3940752	0.6261	0.5316	1.217071032	count	1
AL031666.1	0.872793	1.3940752	0.6261	0.5316	1.217071032	count	1
TCL1A	0.8550888	0.474867	1.8007	0.0724	1.225111714	count	1
WDR91	0.910365	0.749816	1.2141	0.2253	1.229144138	count	1
ZNF569	0.872793	0.8266589	1.0558	0.292	1.230770071	count	1
CYTH2	0.8839058	0.4490508	1.9684	0.0496	1.232659211	count	1
MORN2	0.8851023	0.9274929	0.9543	0.3404	1.23433762	count	1

EHD1	0.9143879	0.485278	1.8843	0.0602	1.234638234	count	1
G2E3	0.8838561	0.4066408	2.1736	0.0303	1.246432678	count	1
RARS2	0.9013782	0.6140912	1.4678	0.1429	1.257169766	count	1
EMC10	0.9022797	0.3706324	2.4344	0.0153	1.258434454	count	1
L3MBTL3	0.9350772	0.4726768	1.9783	0.0485	1.262896486	count	1
ZNF827	0.9359007	0.6973637	1.3421	0.1803	1.264021341	count	1
ENO2	0.8976107	0.6327234	1.4186	0.1567	1.265906507	count	1
HIST1H1C	0.904325	0.3982977	2.2705	0.0237	1.282594285	count	1
SVBP	0.9530314	0.3937907	2.4201	0.0159	1.287422425	count	1
DYRK1B	0.9533535	0.6793631	1.4033	0.161	1.287862446	count	1
COL4A3	0.9035223	0.6486475	1.3929	0.164	1.288715172	count	1
GNG2	0.956451	0.3041365	3.1448	0.0018	1.292094013	count	1
AP1B1	0.9234255	0.506415	1.8235	0.0689	1.302457207	count	1
LPCAT1	0.9252212	0.6438402	1.437	0.1514	1.304999792	count	1
MLXIP	0.9288167	0.7144324	1.3001	0.1942	1.310090799	count	1
CENPM	0.9274719	0.4648899	1.995	0.0466	1.315520422	count	1
CEP89	0.944107	0.6815976	1.3851	0.1667	1.317117556	count	1
NXT2	0.944107	0.7731465	1.2211	0.2227	1.317117556	count	1
HELZ2	0.9808631	1.0220202	0.9597	0.338	1.32544653	count	1
PECR	0.9495084	1.0570496	0.8983	0.3695	1.33938974	count	1
SDHD	0.9947936	0.311051	3.1982	0.0015	1.344480588	count	1
NEU1	0.9662678	0.3735282	2.5869	0.01	1.34821204	count	1
KATNA1	0.9667948	0.4918411	1.9657	0.05	1.348951513	count	1
RGPD2	0.9691398	1.012047	0.9576	0.3388	1.352241959	count	1
GPM6A	1.0087164	1.5149152	0.6659	0.506	1.36350519	count	1
POLR2C	0.9804144	0.3769113	2.6012	0.0096	1.368062467	count	1
C1orf109	0.9838727	0.7550966	1.303	0.1933	1.388051123	count	1
HYI	1.0292065	0.9474798	1.0863	0.278	1.391505117	count	1
MTIF2	1.0346118	0.6220474	1.6632	0.097	1.39889176	count	1
UBA1	1.002626	0.7302079	1.3731	0.1704	1.399230777	count	1
ANAPC4	1.0366032	0.8222952	1.2606	0.2081	1.401613136	count	1
CDC73	0.988614	0.3692028	2.6777	0.0077	1.40249967	count	1
C16orf54	1.0058535	0.3623603	2.7758	0.0057	1.419178064	count	1
RCE1	1.053724	0.4663312	2.2596	0.0243	1.425010182	count	1
R3HCC1	1.0545767	0.603289	1.748	0.0811	1.426175484	count	1
RFX5	1.0654804	0.443031	2.405	0.0166	1.441076574	count	1
ITPR1	1.0366303	0.4846445	2.1389	0.033	1.446948949	count	1
NFKBIB	1.0574831	0.4053955	2.6085	0.0094	1.476212173	count	1
CIAPIN1	1.0991285	0.5951532	1.8468	0.0654	1.487060352	count	1
MLF1	1.059806	0.9152882	1.1579	0.248	1.495581856	count	1
KLHL17	1.0598062	1.0493977	1.0099	0.3131	1.495582141	count	1
DDX55	1.077814	0.5973323	1.8044	0.0718	1.504743	count	1
MAP9	1.0777494	0.4466957	2.4127	0.0162	1.529308129	count	1
KLHL12	1.0963352	0.8912518	1.2301	0.2193	1.530733953	count	1
KIAA1468	1.1395801	1.0075588	1.131	0.2587	1.542338893	count	1

FANCB	1.148899	0.8472407	1.356	0.1758	1.55507259	count	1
PACS1	1.1508161	0.5577484	2.0633	0.0397	1.557692128	count	1
ALAD	1.1206266	1.027872	1.0902	0.2762	1.564821465	count	1
TFB2M	1.1652218	0.748866	1.556	0.1204	1.577375532	count	1
FBXO2	1.1687651	0.7164251	1.6314	0.1035	1.58221676	count	1
ARSK	1.1365612	1.1293225	1.0064	0.3148	1.58718135	count	1
AC016394.1	1.1365612	1.1293225	1.0064	0.3148	1.58718135	count	1
CXorf21	1.1081258	0.3727554	2.9728	0.00311	1.589063046	count	1
ANKRD54	1.1386072	0.7428357	1.5328	0.126	1.607172382	count	1
TTC27	1.1496897	0.7875228	1.4599	0.145	1.622865497	count	1
PTRH2	1.1555138	0.5095801	2.2676	0.0238	1.631112455	count	1
JAK2	1.2046067	0.6598004	1.8257	0.0686	1.631182088	count	1
SPTSSA	1.168268	0.5723276	2.0413	0.0418	1.631670806	count	1
SLC39A4	1.2060801	0.6808697	1.7714	0.0772	1.633194753	count	1
MYADM	1.1681557	0.280479	4.1649	3.74E-05	1.649013131	count	0.9083712
FLAD1	1.2196768	0.5875126	2.076	0.0385	1.651766885	count	1
NFRKB	1.2367482	0.9451413	1.3085	0.1914	1.675082486	count	1
MED9	1.2367482	0.9451413	1.3085	0.1914	1.675082486	count	1
ZDHHC18	1.2367482	1.0273269	1.2039	0.2293	1.675082496	count	1
DUSP8	1.2049534	0.4957373	2.4306	0.0155	1.683140623	count	1
ATF5	1.2051133	0.4739905	2.5425	0.0113	1.683364952	count	1
MED7	1.2059588	0.7965477	1.514	0.1307	1.684551108	count	1
PIK3CB	1.2059588	0.8457082	1.426	0.1546	1.684551108	count	1
MIR155HG	1.1968515	0.3157866	3.7901	2.00E-04	1.68964393	count	1
B3GALT6	1.2254794	0.7211157	1.6994	0.0899	1.711935541	count	1
ZMYM1	1.2355197	0.8388271	1.4729	0.1415	1.726019626	count	1
ASB6	1.2436714	0.8743678	1.4224	0.1556	1.737453964	count	1
AC093323.1	1.2436714	0.8955282	1.3888	0.1656	1.737453964	count	1
CRIP1	1.2374203	0.2076515	5.9591	5.17E-09	1.747080435	count	0.000125652
MACO1	1.2915554	0.5664602	2.28	0.0231	1.749912094	count	1
ETV6	1.2678776	0.3760579	3.3715	8.00E-04	1.790196144	count	1
BCL7A	1.3036419	1.1401346	1.1434	0.2535	1.821558367	count	1
MMP11	1.3249529	0.9937685	1.3333	0.1831	1.851437834	count	1
TFIP11	1.313389	0.7426918	1.7684	0.0777	1.854612647	count	1
G6PC3	1.3719018	0.8904268	1.5407	0.1241	1.859524025	count	1
UBA5	1.4244655	0.520768	2.7353	0.0065	1.931161996	count	1
IFT20	1.3759079	0.5450503	2.5244	0.0119	1.943077819	count	1
NDUFV2-AS1	1.3996025	0.5986657	2.3379	0.0198	1.956062501	count	1
SMARCAL1	1.4457495	0.7381428	1.9586	0.0508	2.020703889	count	1
UTP15	1.4659346	0.719507	2.0374	0.0422	2.048968784	count	1
SLC30A6	1.472388	0.7763369	1.8966	0.0585	2.05800402	count	1
EML5	1.4723884	1.2275679	1.1994	0.231	2.058004602	count	1
IL6	1.4582907	0.867783	1.6805	0.0936	2.059598772	count	1
IKBKE	1.5772424	1.4237646	1.1078	0.2685	2.138947611	count	1
SH3TC1	1.5832662	0.8789546	1.8013	0.0723	2.147124689	count	1

TOP1MT	1.5931965	0.8642202	1.8435	0.0659	2.160601772	count	1
DYNC2LI1	1.6049616	0.8732919	1.8378	0.0668	2.176564166	count	1
FADD	1.5603326	0.619069	2.5205	0.0121	2.181061923	count	1
FO393401.1	1.6839001	0.7269348	2.3164	0.021	2.283520574	count	1
HEATR3	1.7718242	1.0854651	1.6323	0.1033	2.402322254	count	1
CDKN3	1.7884971	1.5873644	1.1267	0.2605	2.42480698	count	1
ZNF620	1.818793	0.633557	2.8708	0.00429	2.465625304	count	1
MTERF3	1.788497	0.7248636	2.4674	0.014	2.539099986	count	1
METTL1	1.843278	0.8061572	2.2865	0.0227	2.602887291	count	1
PLAA	1.9382136	0.8041364	2.4103	0.0163	2.626003772	count	1
DDX51	1.9395039	0.9903465	1.9584	0.0508	2.627731742	count	1
CTPS1	1.9395039	1.0577737	1.8336	0.0674	2.627731742	count	1
S100A8	1.995365	1.3495731	1.4785	0.14	2.70243329	count	1
PAIP2B	2.038322	0.7071294	2.8825	0.00414	2.846454994	count	1
ZNF665	2.079442	0.1775712	11.7105	8.67E-28	2.903324593	count	2.11E-23
RMC1	2.2400475	0.8998097	2.4895	0.0132	3.02681852	count	1
DEAF1	2.2638244	1.5068445	1.5024	0.134	3.058062747	count	1
AC022098.1	2.3503881	1.2492605	1.8814	0.0606	3.171340595	count	1
RORA-AS1	2.4363995	1.066995	2.2834	0.0229	3.283111254	count	1
GRAMD4	18.5920837	2055.90075	0.009	0.9928	6.658210267	count	1
GID4	18.7653396	1619.642587	0.0116	0.9908	6.658210461	count	1
WASHC5	19.03719	1782.864668	0.0107	0.991	6.658210704	count	1
MBLAC2	19.1318349	2932.851783	0.0065	0.9948	6.658210774	count	1
ZNF232	19.647329	2718.495218	0.0072	0.994	6.65821106	count	1
TSPAN32	19.8062953	4001.432166	0.0049	0.9961	6.658211122	count	1
