# Supplementary material for: Decoding the transcriptome of calcified atherosclerotic plaque at single-cell resolution
Source: Commun Biol. 2022 Oct 12;5:1084. doi: 10.1038/s42003-022-04056-7 (PMC9556750; doi:10.1038/s42003-022-04056-7)
Supplement: Supplementary file 6 — Supplementary Data 4 [file 42003_2022_4056_MOESM6_ESM.pdf]

Full differential gene expression results for NKT cells.

gene_short_name	estimate	std_err	test_val	p_value	normalized_effect	model_component	q_value
ITLN1	-18.2092193	1940.675848	-0.0094	0.993	-5.302374675	count	1
FABP4	-18.0224398	1386.559223	-0.013	0.99	-4.752830404	count	1
LYZ	-18.1086113	1531.232534	-0.0118	0.991	-4.700439171	count	1
APOC1	-17.996985	1574.930343	-0.0114	0.991	-4.646073588	count	1
IGKC	-3.5589412	0.4281817	-8.3118	2.63E-15	-4.621374634	count	6.40E-11
CD68	-18.8235365	1973.402103	-0.0095	0.992	-4.405468543	count	1
APOE	-3.6237701	1.3378808	-2.7086	0.00712	-4.368028938	count	1
SPP1	-3.1104657	0.944079	-3.2947	0.0011	-4.184372664	count	1
C17orf80	-17.4391946	1988.920425	-0.0088	0.993	-3.854148574	count	1
SPIRE1	-17.839187	1709.968985	-0.0104	0.992	-3.854148208	count	1
CD14	-17.826345	1781.912414	-0.01	0.992	-3.854148204	count	1
POLR3F	-17.581876	1797.638639	-0.0098	0.992	-3.854148106	count	1
ZMIZ1	-18.1420647	2250.5915	-0.0081	0.994	-3.648288282	count	1
TMEM204	-17.599713	1492.719383	-0.0118	0.991	-3.648288123	count	1
UBE2E2	-17.32499	2839.976206	-0.0061	0.995	-3.53317901	count	1
PRR12	-17.32499	2839.976206	-0.0061	0.995	-3.53317901	count	1
LRRC45	-17.9664017	3311.351897	-0.0054	0.996	-3.408084528	count	1
URGCP	-17.2458277	2257.96026	-0.0076	0.9939	-3.408084298	count	1
AC034236.2	-17.839717	2110.407802	-0.0085	0.993	-3.408084237	count	1
SYNGAP1	-17.508759	3159.754246	-0.0055	0.996	-3.40808414	count	1
NAPEPLD	-18.27311	2630.783423	-0.0069	0.994	-3.271104107	count	1
FCGR2A	-18.25931	2944.371466	-0.0062	0.995	-3.271104105	count	1
ARMC2	-17.7023065	2644.792316	-0.0067	0.995	-3.271103608	count	1
MCAT	-17.4071471	1420.341086	-0.0123	0.99	-3.27110352	count	1
MZB1	-3.1649566	1.2698391	-2.4924	0.0132	-3.247344749	count	1
TMEM161B-AS1	-18.182468	2353.503978	-0.0077	0.994	-3.11973956	count	1
TNK1	-18.16949	2756.793651	-0.0066	0.995	-3.119739558	count	1
ZNF485	-18.16397	2995.299773	-0.0061	0.995	-3.119739557	count	1
AC019205.1	-18.148105	3407.870732	-0.0053	0.996	-3.119739555	count	1
LANCL2	-18.148105	3407.870732	-0.0053	0.996	-3.119739555	count	1
NXPH4	-18.148105	3407.870732	-0.0053	0.996	-3.119739555	count	1
RNASE1	-18.0527492	2747.493587	-0.0066	0.995	-3.11973903	count	1
ZNF420	-17.85706	1591.399702	-0.0112	0.991	-3.119738995	count	1
MPHOSPH9	-2.9381018	1.4143196	-2.0774	0.0386	-3.061847813	count	1
ABCA1	-18.4468488	2585.083844	-0.0071	0.994	-2.950611816	count	1
SPSB1	-18.0333761	1919.112093	-0.0094	0.993	-2.950611767	count	1
PHF7	-18.0316444	2015.21758	-0.0089	0.993	-2.950611767	count	1
MGLL	-18.0273064	2103.887245	-0.0086	0.993	-2.950611766	count	1
TM6SF1	-18.0251886	2190.658885	-0.0082	0.993	-2.950611766	count	1
SCAI	-17.8633744	2509.66105	-0.0071	0.994	-2.950611741	count	1
CCDC144A	-17.6114186	2024.521006	-0.0087	0.993	-2.950611692	count	1
ITFG2-AS1	-18.068353	2292.65868	-0.0079	0.994	-2.950611521	count	1
C7orf31	-18.059902	2622.226168	-0.0069	0.995	-2.95061152	count	1
GYPE	-18.041281	3413.087098	-0.0053	0.996	-2.950611517	count	1

AP001107.9	-18.041281	3413.087098	-0.0053	0.996	-2.950611517	count	1
CHTF18	-18.041281	3413.087098	-0.0053	0.996	-2.950611517	count	1
DDAH1	-18.032346	3844.881507	-0.0047	0.996	-2.950611516	count	1
ZNF112	-18.012425	4387.724358	-0.0041	0.997	-2.950611513	count	1
MAML3	-2.6604677	1.3463586	-1.976	0.049	-2.830248679	count	1
KIAA0355	-2.4643556	1.3896585	-1.7734	0.0771	-2.808919825	count	1
SMIM10	-18.280513	3202.803524	-0.0057	0.995	-2.758992333	count	1
ZC3HC1	-17.91699	2943.623308	-0.0061	0.995	-2.758992291	count	1
SOWAHD	-17.91699	2943.623308	-0.0061	0.995	-2.758992291	count	1
ENTPD5	-17.91699	2943.623308	-0.0061	0.995	-2.758992291	count	1
CPNE2	-17.91699	2943.623308	-0.0061	0.995	-2.758992291	count	1
AC004816.1	-17.915224	3103.151685	-0.0058	0.995	-2.758992291	count	1
PAK6	-17.915224	3103.151685	-0.0058	0.995	-2.758992291	count	1
AC009118.3	-17.915224	3103.151685	-0.0058	0.995	-2.758992291	count	1
AC007448.3	-17.915224	3103.151685	-0.0058	0.995	-2.758992291	count	1
COL9A3	-17.915224	3103.151685	-0.0058	0.995	-2.758992291	count	1
EEF1AKMT3	-17.909133	3534.539695	-0.0051	0.996	-2.75899229	count	1
MECOM	-17.893723	4134.88489	-0.0043	0.997	-2.758992288	count	1
PFAS	-17.893723	4134.88489	-0.0043	0.997	-2.758992288	count	1
COL1A1	-17.893723	4134.88489	-0.0043	0.997	-2.758992288	count	1
CD40	-17.893723	4134.88489	-0.0043	0.997	-2.758992288	count	1
AIF1	-17.855652	1943.61915	-0.0092	0.993	-2.758992283	count	1
IFNGR2	-17.84416	2363.039369	-0.0076	0.994	-2.758992281	count	1
AC136475.1	-17.607295	1900.098687	-0.0093	0.993	-2.758992241	count	1
GEN1	-17.589866	2476.160348	-0.0071	0.994	-2.758992238	count	1
NUBPL	-18.6613116	3113.289209	-0.006	0.995	-2.758991872	count	1
VWF	-18.5053871	3499.450077	-0.0053	0.996	-2.758991861	count	1
UGDH	-18.4955206	2088.859947	-0.0089	0.993	-2.75899186	count	1
CYR61	-18.4073537	2376.431264	-0.0077	0.994	-2.758991853	count	1
AC073896.2	-18.3998217	3079.902081	-0.006	0.995	-2.758991852	count	1
KIAA0319L	-18.2207192	1922.281537	-0.0095	0.992	-2.758991836	count	1
AL138724.1	-18.2207192	1922.281537	-0.0095	0.992	-2.758991836	count	1
CLN5	-18.2207192	1922.281537	-0.0095	0.992	-2.758991836	count	1
AC012306.2	-18.0062515	1936.214194	-0.0093	0.993	-2.758991811	count	1
VPS52	-17.9974565	2441.849293	-0.0074	0.994	-2.75899181	count	1
TPK1	-17.9714325	1980.215326	-0.0091	0.993	-2.758991807	count	1
LETM2	-17.9699045	2089.903444	-0.0086	0.993	-2.758991807	count	1
IFT46	-17.9626824	2384.070273	-0.0075	0.994	-2.758991806	count	1
LGMN	-17.955546	2236.097507	-0.008	0.994	-2.758991805	count	1
GORASP1	-17.9449103	3018.902436	-0.0059	0.995	-2.758991803	count	1
RAB33A	-2.1042928	0.945196	-2.2263	0.0267	-2.719858904	count	1
HIST1H2AC	-2.2173949	1.2825797	-1.7289	0.0848	-2.647602441	count	1
GLE1	-2.4695697	1.9468805	-1.2685	0.2055	-2.644608335	count	1
MS4A6A	-18.6453778	2795.681254	-0.0067	0.995	-2.53796495	count	1
SLC38A5	-18.5891892	3082.148622	-0.006	0.995	-2.537964947	count	1

C5AR1	-18.5724008	4242.782293	-0.0044	0.997	-2.537964946	count	1
AC027031.2	-18.4466117	2614.730464	-0.0071	0.994	-2.537964938	count	1
DMKN	-18.3591223	2161.410661	-0.0085	0.993	-2.537964932	count	1
NCF2	-18.1653462	3030.670042	-0.006	0.995	-2.537964916	count	1
KIF9-AS1	-18.1653462	3030.670042	-0.006	0.995	-2.537964916	count	1
AMZ1	-18.1653462	3030.670042	-0.006	0.995	-2.537964916	count	1
MED9	-18.1653462	3030.670042	-0.006	0.995	-2.537964916	count	1
RFC5	-18.1634089	2128.494571	-0.0085	0.993	-2.537964916	count	1
AL357054.4	-18.10746	2341.880007	-0.0077	0.994	-2.53796491	count	1
PPIAL4G	-17.8989704	2419.928873	-0.0074	0.994	-2.537964888	count	1
TYMS	-17.764284	2025.094063	-0.0088	0.993	-2.537964872	count	1
AL606807.1	-17.763955	2823.595649	-0.0063	0.995	-2.537964872	count	1
AC017100.1	-17.761097	2301.62549	-0.0077	0.994	-2.537964871	count	1
CYP4F22	-17.761097	2301.62549	-0.0077	0.994	-2.537964871	count	1
UBXN10	-17.755421	2769.991833	-0.0064	0.995	-2.53796487	count	1
KCNK12	-17.755421	2769.991833	-0.0064	0.995	-2.53796487	count	1
RETSAT	-17.755421	2769.991833	-0.0064	0.995	-2.53796487	count	1
CHST10	-17.755421	2769.991833	-0.0064	0.995	-2.53796487	count	1
SLC49A3	-17.755421	2769.991833	-0.0064	0.995	-2.53796487	count	1
AC098818.2	-17.755421	2769.991833	-0.0064	0.995	-2.53796487	count	1
AC008393.1	-17.755421	2769.991833	-0.0064	0.995	-2.53796487	count	1
HIST1H2BG	-17.755421	2769.991833	-0.0064	0.995	-2.53796487	count	1
C9orf66	-17.755421	2769.991833	-0.0064	0.995	-2.53796487	count	1
MMP7	-17.755421	2769.991833	-0.0064	0.995	-2.53796487	count	1
RNLS	-17.755421	2769.991833	-0.0064	0.995	-2.53796487	count	1
SLC14A1	-17.755421	2769.991833	-0.0064	0.995	-2.53796487	count	1
SIRPA	-17.755421	2769.991833	-0.0064	0.995	-2.53796487	count	1
ZNF133	-17.755421	2769.991833	-0.0064	0.995	-2.53796487	count	1
TBX1	-17.755421	2769.991833	-0.0064	0.995	-2.53796487	count	1
FAM166B	-17.752031	3169.242827	-0.0056	0.996	-2.53796487	count	1
ZBTB42	-17.752031	3169.242827	-0.0056	0.996	-2.53796487	count	1
AC106782.2	-17.752031	3169.242827	-0.0056	0.996	-2.53796487	count	1
AL669831.5	-17.741413	3831.684559	-0.0046	0.996	-2.537964869	count	1
IBA57-DT	-17.741413	3831.684559	-0.0046	0.996	-2.537964869	count	1
AC073263.2	-17.741413	3831.684559	-0.0046	0.996	-2.537964869	count	1
AC108047.1	-17.741413	3831.684559	-0.0046	0.996	-2.537964869	count	1
PTPN23	-17.741413	3831.684559	-0.0046	0.996	-2.537964869	count	1
FZD3	-17.741413	3831.684559	-0.0046	0.996	-2.537964869	count	1
PCAT7	-17.741413	3831.684559	-0.0046	0.996	-2.537964869	count	1
AL136295.5	-17.741413	3831.684559	-0.0046	0.996	-2.537964869	count	1
WDR24	-17.741413	3831.684559	-0.0046	0.996	-2.537964869	count	1
RPGRIP1L	-17.741413	3831.684559	-0.0046	0.996	-2.537964869	count	1
MT1H	-17.741413	3831.684559	-0.0046	0.996	-2.537964869	count	1
AC011447.3	-17.741413	3831.684559	-0.0046	0.996	-2.537964869	count	1
DLL3	-17.741413	3831.684559	-0.0046	0.996	-2.537964869	count	1

AC008079.2	-17.741413	3831.684559	-0.0046	0.996	-2.537964869	count	1
CNTLN	-17.516075	2009.064822	-0.0087	0.993	-2.537964834	count	1
AC011416.3	-17.512105	2254.830629	-0.0078	0.994	-2.537964833	count	1
YIPF5	-1.8834806	0.7220299	-2.6086	0.0095	-2.511282587	count	1
AP5M1	-2.101706	0.9825674	-2.139	0.0332	-2.394200706	count	1
PBX3	-2.1153037	1.0474038	-2.0196	0.0443	-2.366880564	count	1
C1QA	-1.9653751	0.865449	-2.2709	0.0238	-2.330635929	count	1
RASSF8	-2.3796706	1.236925	-1.9239	0.0552	-2.318947053	count	1
SPINK2	-1.8673944	1.0662122	-1.7514	0.08082	-2.314802068	count	1
OSBPL11	-2.8175542	1.4060394	-2.0039	0.0459	-2.311157926	count	1
CAMKK2	-2.3666283	1.3734852	-1.7231	0.0858	-2.310936852	count	1
LRRC41	-2.0440575	1.0793981	-1.8937	0.0592	-2.305746659	count	1
EXOC5	-1.9065855	0.8495022	-2.2444	0.0255	-2.297124516	count	1
PPP1R9A	-2.7431959	1.3154251	-2.0854	0.0378	-2.28185263	count	1
FAM30A	-18.8284263	2685.087575	-0.007	0.994	-2.276840212	count	1
DCAF4	-18.4906552	3730.935886	-0.005	0.996	-2.276840197	count	1
TMCC3	-18.4742814	3858.390578	-0.0048	0.996	-2.276840196	count	1
RILPL1	-18.4168481	3001.995938	-0.0061	0.995	-2.276840193	count	1
HERC6	-18.3015083	1961.910542	-0.0093	0.993	-2.276840186	count	1
ANO9	-18.3015083	1961.910542	-0.0093	0.993	-2.276840186	count	1
SETD9	-18.2982157	2038.662236	-0.009	0.993	-2.276840186	count	1
TOLLIP-AS1	-18.2955009	2857.14303	-0.0064	0.995	-2.276840186	count	1
TSPAN18	-18.2952585	3127.472681	-0.0058	0.995	-2.276840186	count	1
COLQ	-18.2928864	3821.752308	-0.0048	0.996	-2.276840186	count	1
AC007541.1	-18.2928864	3821.752308	-0.0048	0.996	-2.276840186	count	1
AC133919.1	-18.2928864	3821.752308	-0.0048	0.996	-2.276840186	count	1
SERPINE1	-18.1696836	2240.483114	-0.0081	0.994	-2.276840178	count	1
CCDC146	-18.0416744	2319.025726	-0.0078	0.994	-2.276840168	count	1
TRGV7	-18.0247012	2715.058593	-0.0066	0.995	-2.276840166	count	1
NCAPG2	-18.0247012	2715.058593	-0.0066	0.995	-2.276840166	count	1
RCN1	-17.8649386	1957.723591	-0.0091	0.993	-2.276840152	count	1
CDCA7	-17.8281178	1820.535821	-0.0098	0.992	-2.276840149	count	1
USP19	-17.8271419	1999.957553	-0.0089	0.993	-2.276840148	count	1
CYP4V2	-17.8271419	1999.957553	-0.0089	0.993	-2.276840148	count	1
AF117829.1	-17.8271419	1999.957553	-0.0089	0.993	-2.276840148	count	1
FAM167B	-17.8264696	2164.576583	-0.0082	0.993	-2.276840148	count	1
BAP1	-17.8264696	2164.576583	-0.0082	0.993	-2.276840148	count	1
CRKL	-17.8264696	2164.576583	-0.0082	0.993	-2.276840148	count	1
NRP2	-17.8224005	2592.822695	-0.0069	0.995	-2.276840148	count	1
CTNND1	-17.8224005	2592.822695	-0.0069	0.995	-2.276840148	count	1
CRAMP1	-17.8224005	2592.822695	-0.0069	0.995	-2.276840148	count	1
STXBP4	-17.8224005	2592.822695	-0.0069	0.995	-2.276840148	count	1
COL1A2	-17.5789379	1648.673998	-0.0107	0.991	-2.27684012	count	1
GM2A	-17.4776366	1755.46735	-0.01	0.992	-2.276840106	count	1
AC002467.1	-17.4074645	2446.332378	-0.0071	0.994	-2.276840096	count	1

TBC1D13	-17.4074645	2446.332378	-0.0071	0.994	-2.276840096	count	1
A4GALT	-17.4074645	2446.332378	-0.0071	0.994	-2.276840096	count	1
JCHAIN	-19.306995	1689.904168	-0.0114	0.991	-2.276839654	count	1
ANXA9	-17.550609	2115.584996	-0.0083	0.993	-2.276839544	count	1
AGMAT	-17.548459	2448.255539	-0.0072	0.994	-2.276839544	count	1
RSAD2	-17.548459	2448.255539	-0.0072	0.994	-2.276839544	count	1
CD28	-17.548459	2448.255539	-0.0072	0.994	-2.276839544	count	1
LINC00884	-17.548459	2448.255539	-0.0072	0.994	-2.276839544	count	1
SRD5A1	-17.548459	2448.255539	-0.0072	0.994	-2.276839544	count	1
ECSCR	-17.548459	2448.255539	-0.0072	0.994	-2.276839544	count	1
PRELID2	-17.548459	2448.255539	-0.0072	0.994	-2.276839544	count	1
TMSB15B-AS1	-17.548459	2448.255539	-0.0072	0.994	-2.276839544	count	1
NARS2	-17.548459	2448.255539	-0.0072	0.994	-2.276839544	count	1
LIG4	-17.548459	2448.255539	-0.0072	0.994	-2.276839544	count	1
AC051619.5	-17.548459	2448.255539	-0.0072	0.994	-2.276839544	count	1
C15orf48	-17.548459	2448.255539	-0.0072	0.994	-2.276839544	count	1
VPS33B	-17.548459	2448.255539	-0.0072	0.994	-2.276839544	count	1
S1PR5	-17.548459	2448.255539	-0.0072	0.994	-2.276839544	count	1
AL139260.1	-17.547294	2740.997978	-0.0064	0.995	-2.276839544	count	1
NT5DC2	-17.547294	2740.997978	-0.0064	0.995	-2.276839544	count	1
LRRC1	-17.547294	2740.997978	-0.0064	0.995	-2.276839544	count	1
EEPD1	-17.547294	2740.997978	-0.0064	0.995	-2.276839544	count	1
ZHX1-C8orf76	-17.547294	2740.997978	-0.0064	0.995	-2.276839544	count	1
NDOR1	-17.547294	2740.997978	-0.0064	0.995	-2.276839544	count	1
PCLAF	-17.547294	2740.997978	-0.0064	0.995	-2.276839544	count	1
KDELR3	-17.547294	2740.997978	-0.0064	0.995	-2.276839544	count	1
MMEL1	-17.541476	3467.161152	-0.0051	0.996	-2.276839543	count	1
AL139011.1	-17.541476	3467.161152	-0.0051	0.996	-2.276839543	count	1
FLVCR1	-17.541476	3467.161152	-0.0051	0.996	-2.276839543	count	1
MAL	-17.541476	3467.161152	-0.0051	0.996	-2.276839543	count	1
IL1R2	-17.541476	3467.161152	-0.0051	0.996	-2.276839543	count	1
TMEM163	-17.541476	3467.161152	-0.0051	0.996	-2.276839543	count	1
AC012510.1	-17.541476	3467.161152	-0.0051	0.996	-2.276839543	count	1
PLA1A	-17.541476	3467.161152	-0.0051	0.996	-2.276839543	count	1
KIAA1257	-17.541476	3467.161152	-0.0051	0.996	-2.276839543	count	1
RBM47	-17.541476	3467.161152	-0.0051	0.996	-2.276839543	count	1
ODAPH	-17.541476	3467.161152	-0.0051	0.996	-2.276839543	count	1
LY86	-17.541476	3467.161152	-0.0051	0.996	-2.276839543	count	1
TNFRSF21	-17.541476	3467.161152	-0.0051	0.996	-2.276839543	count	1
CPVL	-17.541476	3467.161152	-0.0051	0.996	-2.276839543	count	1
PILRA	-17.541476	3467.161152	-0.0051	0.996	-2.276839543	count	1
POLR2J3	-17.541476	3467.161152	-0.0051	0.996	-2.276839543	count	1
AC006333.2	-17.541476	3467.161152	-0.0051	0.996	-2.276839543	count	1
HEY1	-17.541476	3467.161152	-0.0051	0.996	-2.276839543	count	1
C8orf88	-17.541476	3467.161152	-0.0051	0.996	-2.276839543	count	1

NRIP3	-17.541476	3467.161152	-0.0051	0.996	-2.276839543	count	1
MSRB3	-17.541476	3467.161152	-0.0051	0.996	-2.276839543	count	1
GLT1D1	-17.541476	3467.161152	-0.0051	0.996	-2.276839543	count	1
RNASE2	-17.541476	3467.161152	-0.0051	0.996	-2.276839543	count	1
NUPR1	-17.541476	3467.161152	-0.0051	0.996	-2.276839543	count	1
AC010542.2	-17.541476	3467.161152	-0.0051	0.996	-2.276839543	count	1
ZNF19	-17.541476	3467.161152	-0.0051	0.996	-2.276839543	count	1
PMFBP1	-17.541476	3467.161152	-0.0051	0.996	-2.276839543	count	1
VMO1	-17.541476	3467.161152	-0.0051	0.996	-2.276839543	count	1
SERPINB2	-17.541476	3467.161152	-0.0051	0.996	-2.276839543	count	1
ZNF283	-17.541476	3467.161152	-0.0051	0.996	-2.276839543	count	1
AC008440.2	-17.541476	3467.161152	-0.0051	0.996	-2.276839543	count	1
MRPL30	-2.4633791	1.5718366	-1.5672	0.118	-2.272205718	count	1
UBXN11	-2.2860046	1.3025854	-1.755	0.0802	-2.260103894	count	1
TRIM73	-1.9907712	1.2667634	-1.5715	0.117	-2.258900336	count	1
IFI44L	-1.7836579	1.0670693	-1.6715	0.0956	-2.239290336	count	1
ELMO2	-2.2227	1.0677234	-2.0817	0.0382	-2.218594821	count	1
ZNF609	-2.345694	1.0588105	-2.2154	0.0274	-2.206470043	count	1
ZNF814	-1.7768205	0.9149445	-1.942	0.053	-2.201067918	count	1
ORMDL3	-1.8284998	0.9013972	-2.0285	0.0433	-2.195193793	count	1
TIGAR	-1.7867434	0.8862685	-2.016	0.0446	-2.193995414	count	1
PDE6B	-2.4608141	1.2955823	-1.8994	0.0584	-2.155651434	count	1
PIGG	-2.2568181	1.3439437	-1.6793	0.0941	-2.153704921	count	1
RBMX2	-1.7346977	0.8063134	-2.1514	0.0322	-2.138563176	count	1
MARCKS	-1.8201715	2.1008407	-0.8664	0.3869	-2.134069966	count	1
ORC2	-2.0115534	1.6082061	-1.2508	0.212	-2.13397057	count	1
SMIM8	-1.7374717	0.8345959	-2.0818	0.0381	-2.122809768	count	1
MYCBP	-1.9210084	0.7522513	-2.5537	0.0111	-2.113104252	count	1
QTRT1	-2.0686756	0.911639	-2.2692	0.0239	-2.111706288	count	1
TMEM127	-2.7004844	1.4432042	-1.8712	0.0622	-2.111649097	count	1
SCRN1	-2.692677	1.4627825	-1.8408	0.0666	-2.108772524	count	1
MRPL46	-1.792142	0.8445238	-2.1221	0.0346	-2.106791016	count	1
PLEKHA5	-2.6349912	1.3149566	-2.0039	0.0459	-2.087002085	count	1
DHRS4-AS1	-1.7004284	0.982076	-1.7315	0.0843	-2.08355599	count	1
PGRMC2	-1.5162246	0.5922086	-2.5603	0.0109	-2.055817606	count	1
DNPH1	-1.5969999	0.565591	-2.8236	0.00504	-2.051088424	count	1
CCNYL1	-2.5286371	1.3807804	-1.8313	0.068	-2.044417114	count	1
ASMTL	-1.8322736	0.9133405	-2.0061	0.0457	-2.036350935	count	1
RABEPK	-1.750203	1.0713693	-1.6336	0.103	-2.035827374	count	1
HVCN1	-1.776957	0.8149153	-2.1805	0.0299	-2.026954587	count	1
FADD	-1.6468697	0.7575802	-2.1739	0.0304	-2.026149491	count	1
CTSB	-1.593522	0.8018769	-1.9872	0.0477	-2.025301904	count	1
LDOC1	-1.593522	0.9539704	-1.6704	0.0958	-2.025301904	count	1
CBY1	-1.736774	1.1002012	-1.5786	0.115	-2.022834289	count	1
DAPK3	-2.2104497	1.384328	-1.5968	0.111	-2.022614129	count	1

ADCY3	-2.4487727	1.1578919	-2.1149	0.0352	-2.010280549	count	1
MGP	-1.4424291	0.3789014	-3.8069	2.00E-04	-2.008203637	count	1
PIK3R4	-1.9263874	1.3485401	-1.4285	0.154	-2.005542736	count	1
JMJD4	-1.9263874	1.5037913	-1.281	0.201	-2.005542736	count	1
PHLDA2	-1.881515	1.0281305	-1.83	0.0682	-1.970594758	count	1
IGLC2	-1.7134873	0.7274564	-2.3555	0.0191	-1.967330346	count	1
NAE1	-1.5089076	0.6087889	-2.4785	0.0137	-1.964327583	count	1
FBXO48	-1.8019997	1.216617	-1.4812	0.14	-1.962428145	count	1
MOSPD1	-1.8019997	1.3490289	-1.3358	0.183	-1.962428145	count	1
DIRC2	-18.7380547	3108.2316	-0.006	0.995	-1.957771746	count	1
FKTN	-18.7380547	3108.2316	-0.006	0.995	-1.957771746	count	1
C1R	-18.7380547	3108.2316	-0.006	0.995	-1.957771746	count	1
IZUMO4	-18.6354211	2554.488298	-0.0073	0.994	-1.957771742	count	1
ST6GALNAC6	-18.4735674	2421.521715	-0.0076	0.994	-1.957771736	count	1
ZNF410	-18.4465369	2508.817515	-0.0074	0.994	-1.957771735	count	1
HMOX1	-18.3281331	2486.073278	-0.0074	0.994	-1.95777173	count	1
CALHM6	-18.2946073	2814.463459	-0.0065	0.995	-1.957771729	count	1
CMC4	-18.2946073	2814.463459	-0.0065	0.995	-1.957771729	count	1
TBCE	-18.2909465	3589.135805	-0.0051	0.996	-1.957771728	count	1
SLC5A6	-18.2909465	3589.135805	-0.0051	0.996	-1.957771728	count	1
KIAA1211L	-18.2909465	3589.135805	-0.0051	0.996	-1.957771728	count	1
AMT	-18.2909465	3589.135805	-0.0051	0.996	-1.957771728	count	1
SLC39A8	-18.2909465	3589.135805	-0.0051	0.996	-1.957771728	count	1
SGMS2	-18.2909465	3589.135805	-0.0051	0.996	-1.957771728	count	1
IGHG4	-18.2909465	3589.135805	-0.0051	0.996	-1.957771728	count	1
DHX34	-18.2909465	3589.135805	-0.0051	0.996	-1.957771728	count	1
FUK	-18.1630976	3243.319423	-0.0056	0.996	-1.957771722	count	1
RASSF8-AS1	-18.1583734	3376.618256	-0.0054	0.996	-1.957771722	count	1
YJEFN3	-18.1583734	3376.618256	-0.0054	0.996	-1.957771722	count	1
IL1RN	-18.1573313	2661.995379	-0.0068	0.995	-1.957771722	count	1
ORAOV1	-18.1573313	2661.995379	-0.0068	0.995	-1.957771722	count	1
BCAS3	-18.1573313	2661.995379	-0.0068	0.995	-1.957771722	count	1
TIGD7	-18.0983244	2539.360022	-0.0071	0.994	-1.957771718	count	1
MRPS24	-18.0295171	2252.613041	-0.008	0.994	-1.957771714	count	1
KCNMB4	-18.0218995	2428.589325	-0.0074	0.994	-1.957771714	count	1
IL23A	-18.0183146	2022.325508	-0.0089	0.993	-1.957771714	count	1
MKL1	-17.8749756	1974.099997	-0.0091	0.993	-1.957771704	count	1
AFF3	-17.848519	2519.780525	-0.0071	0.994	-1.957771702	count	1
STXBP5	-17.848519	2519.780525	-0.0071	0.994	-1.957771702	count	1
AFAP1L2	-17.848519	2519.780525	-0.0071	0.994	-1.957771702	count	1
TRAK1	-17.8462389	3277.449289	-0.0054	0.996	-1.957771702	count	1
ACACB	-17.8462389	3277.449289	-0.0054	0.996	-1.957771702	count	1
MDP1	-17.8462389	3277.449289	-0.0054	0.996	-1.957771702	count	1
PNPO	-17.8462389	3277.449289	-0.0054	0.996	-1.957771702	count	1
FBF1	-17.8462389	3277.449289	-0.0054	0.996	-1.957771702	count	1

AC145207.5	-17.8462389	3277.449289	-0.0054	0.996	-1.957771702	count	1
ZNF90	-17.8462389	3277.449289	-0.0054	0.996	-1.957771702	count	1
LINC01954	-17.7375446	2321.214442	-0.0076	0.994	-1.957771693	count	1
EPAS1	-17.7375446	2321.214442	-0.0076	0.994	-1.957771693	count	1
C6orf52	-17.7375446	2321.214442	-0.0076	0.994	-1.957771693	count	1
PHACTR1	-17.7375446	2321.214442	-0.0076	0.994	-1.957771693	count	1
HIST1H2AL	-17.7375446	2321.214442	-0.0076	0.994	-1.957771693	count	1
TBC1D32	-17.7375446	2321.214442	-0.0076	0.994	-1.957771693	count	1
GPRASP1	-17.7375446	2321.214442	-0.0076	0.994	-1.957771693	count	1
INPP5A	-17.7375446	2321.214442	-0.0076	0.994	-1.957771693	count	1
PARPBP	-17.7375446	2321.214442	-0.0076	0.994	-1.957771693	count	1
SPHK1	-17.7375446	2321.214442	-0.0076	0.994	-1.957771693	count	1
ZNF57	-17.5972148	2323.778459	-0.0076	0.994	-1.957771681	count	1
SLC35B4	-18.645741	1732.906111	-0.0108	0.991	-1.957771314	count	1
MTA3	-18.528884	1778.620216	-0.0104	0.992	-1.95777131	count	1
CHAF1A	-18.51206	2608.775498	-0.0071	0.994	-1.957771309	count	1
MAP4K2	-18.355876	3415.934334	-0.0054	0.996	-1.957771303	count	1
LPAR5	-18.270601	2854.190206	-0.0064	0.995	-1.957771299	count	1
UQCRHL	-18.270415	3706.224659	-0.0049	0.996	-1.957771299	count	1
LINC00954	-18.270415	3706.224659	-0.0049	0.996	-1.957771299	count	1
MAP3K20	-18.270415	3706.224659	-0.0049	0.996	-1.957771299	count	1
EBLN2	-18.270415	3706.224659	-0.0049	0.996	-1.957771299	count	1
GPR15	-18.270415	3706.224659	-0.0049	0.996	-1.957771299	count	1
PFN2	-18.270415	3706.224659	-0.0049	0.996	-1.957771299	count	1
AC107464.3	-18.270415	3706.224659	-0.0049	0.996	-1.957771299	count	1
FAM13A-AS1	-18.270415	3706.224659	-0.0049	0.996	-1.957771299	count	1
AL356481.1	-18.270415	3706.224659	-0.0049	0.996	-1.957771299	count	1
IDE	-18.270415	3706.224659	-0.0049	0.996	-1.957771299	count	1
FGF7	-18.270415	3706.224659	-0.0049	0.996	-1.957771299	count	1
ANPEP	-18.270415	3706.224659	-0.0049	0.996	-1.957771299	count	1
PRC1	-18.270415	3706.224659	-0.0049	0.996	-1.957771299	count	1
CTF1	-18.270415	3706.224659	-0.0049	0.996	-1.957771299	count	1
PRAM1	-18.270415	3706.224659	-0.0049	0.996	-1.957771299	count	1
PINLYP	-18.270415	3706.224659	-0.0049	0.996	-1.957771299	count	1
ATAD3B	-18.268912	4988.095595	-0.0037	0.997	-1.957771299	count	1
ENO1-AS1	-18.268912	4988.095595	-0.0037	0.997	-1.957771299	count	1
AL035413.1	-18.268912	4988.095595	-0.0037	0.997	-1.957771299	count	1
TFAP2E	-18.268912	4988.095595	-0.0037	0.997	-1.957771299	count	1
RAD54L	-18.268912	4988.095595	-0.0037	0.997	-1.957771299	count	1
GNG12	-18.268912	4988.095595	-0.0037	0.997	-1.957771299	count	1
PTGER3	-18.268912	4988.095595	-0.0037	0.997	-1.957771299	count	1
FRRS1	-18.268912	4988.095595	-0.0037	0.997	-1.957771299	count	1
FCGR1A	-18.268912	4988.095595	-0.0037	0.997	-1.957771299	count	1
AL356356.1	-18.268912	4988.095595	-0.0037	0.997	-1.957771299	count	1
AXDND1	-18.268912	4988.095595	-0.0037	0.997	-1.957771299	count	1

TRAPPC12-AS1	-18.268912	4988.095595	-0.0037	0.997	-1.957771299	count	1
TTC27	-18.268912	4988.095595	-0.0037	0.997	-1.957771299	count	1
LBX2-AS1	-18.268912	4988.095595	-0.0037	0.997	-1.957771299	count	1
ANKRD23	-18.268912	4988.095595	-0.0037	0.997	-1.957771299	count	1
HNMT	-18.268912	4988.095595	-0.0037	0.997	-1.957771299	count	1
CYBRD1	-18.268912	4988.095595	-0.0037	0.997	-1.957771299	count	1
PLCL1	-18.268912	4988.095595	-0.0037	0.997	-1.957771299	count	1
IDH1-AS1	-18.268912	4988.095595	-0.0037	0.997	-1.957771299	count	1
MAP2	-18.268912	4988.095595	-0.0037	0.997	-1.957771299	count	1
AC021016.3	-18.268912	4988.095595	-0.0037	0.997	-1.957771299	count	1
TRPM8	-18.268912	4988.095595	-0.0037	0.997	-1.957771299	count	1
AC073352.2	-18.268912	4988.095595	-0.0037	0.997	-1.957771299	count	1
PODXL2	-18.268912	4988.095595	-0.0037	0.997	-1.957771299	count	1
CAMK2N2	-18.268912	4988.095595	-0.0037	0.997	-1.957771299	count	1
FAT4	-18.268912	4988.095595	-0.0037	0.997	-1.957771299	count	1
GATB	-18.268912	4988.095595	-0.0037	0.997	-1.957771299	count	1
SLC12A7	-18.268912	4988.095595	-0.0037	0.997	-1.957771299	count	1
LHFPL2	-18.268912	4988.095595	-0.0037	0.997	-1.957771299	count	1
LUCAT1	-18.268912	4988.095595	-0.0037	0.997	-1.957771299	count	1
RFESD	-18.268912	4988.095595	-0.0037	0.997	-1.957771299	count	1
DBN1	-18.268912	4988.095595	-0.0037	0.997	-1.957771299	count	1
CD109	-18.268912	4988.095595	-0.0037	0.997	-1.957771299	count	1
HMGN3-AS1	-18.268912	4988.095595	-0.0037	0.997	-1.957771299	count	1
CENPW	-18.268912	4988.095595	-0.0037	0.997	-1.957771299	count	1
PSMG3-AS1	-18.268912	4988.095595	-0.0037	0.997	-1.957771299	count	1
IGF2BP3	-18.268912	4988.095595	-0.0037	0.997	-1.957771299	count	1
HOXA4	-18.268912	4988.095595	-0.0037	0.997	-1.957771299	count	1
HOXA6	-18.268912	4988.095595	-0.0037	0.997	-1.957771299	count	1
KCTD7	-18.268912	4988.095595	-0.0037	0.997	-1.957771299	count	1
CADPS2	-18.268912	4988.095595	-0.0037	0.997	-1.957771299	count	1
AC016831.1	-18.268912	4988.095595	-0.0037	0.997	-1.957771299	count	1
AC074386.1	-18.268912	4988.095595	-0.0037	0.997	-1.957771299	count	1
KIF4A	-18.268912	4988.095595	-0.0037	0.997	-1.957771299	count	1
NHSL2	-18.268912	4988.095595	-0.0037	0.997	-1.957771299	count	1
MAGEE1	-18.268912	4988.095595	-0.0037	0.997	-1.957771299	count	1
NAT1	-18.268912	4988.095595	-0.0037	0.997	-1.957771299	count	1
EPHX2	-18.268912	4988.095595	-0.0037	0.997	-1.957771299	count	1
FUT10	-18.268912	4988.095595	-0.0037	0.997	-1.957771299	count	1
FAM83H	-18.268912	4988.095595	-0.0037	0.997	-1.957771299	count	1
C9orf47	-18.268912	4988.095595	-0.0037	0.997	-1.957771299	count	1
WDR34	-18.268912	4988.095595	-0.0037	0.997	-1.957771299	count	1
CARNS1	-18.268912	4988.095595	-0.0037	0.997	-1.957771299	count	1
AP005019.1	-18.268912	4988.095595	-0.0037	0.997	-1.957771299	count	1
SIAE	-18.268912	4988.095595	-0.0037	0.997	-1.957771299	count	1
HECTD2	-18.268912	4988.095595	-0.0037	0.997	-1.957771299	count	1

UBTD1	-18.268912	4988.095595	-0.0037	0.997	-1.957771299	count	1
PI4K2A	-18.268912	4988.095595	-0.0037	0.997	-1.957771299	count	1
SH3PXD2A	-18.268912	4988.095595	-0.0037	0.997	-1.957771299	count	1
ABCD2	-18.268912	4988.095595	-0.0037	0.997	-1.957771299	count	1
KRT85	-18.268912	4988.095595	-0.0037	0.997	-1.957771299	count	1
HIP1R	-18.268912	4988.095595	-0.0037	0.997	-1.957771299	count	1
KBTBD7	-18.268912	4988.095595	-0.0037	0.997	-1.957771299	count	1
CTSG	-18.268912	4988.095595	-0.0037	0.997	-1.957771299	count	1
EML5	-18.268912	4988.095595	-0.0037	0.997	-1.957771299	count	1
AC055876.5	-18.268912	4988.095595	-0.0037	0.997	-1.957771299	count	1
AC100830.3	-18.268912	4988.095595	-0.0037	0.997	-1.957771299	count	1
SLC24A1	-18.268912	4988.095595	-0.0037	0.997	-1.957771299	count	1
STOML1	-18.268912	4988.095595	-0.0037	0.997	-1.957771299	count	1
AC090826.2	-18.268912	4988.095595	-0.0037	0.997	-1.957771299	count	1
AC008731.1	-18.268912	4988.095595	-0.0037	0.997	-1.957771299	count	1
ZNF778	-18.268912	4988.095595	-0.0037	0.997	-1.957771299	count	1
AC092123.1	-18.268912	4988.095595	-0.0037	0.997	-1.957771299	count	1
KCNAB3	-18.268912	4988.095595	-0.0037	0.997	-1.957771299	count	1
RDM1	-18.268912	4988.095595	-0.0037	0.997	-1.957771299	count	1
TBKBP1	-18.268912	4988.095595	-0.0037	0.997	-1.957771299	count	1
BTBD17	-18.268912	4988.095595	-0.0037	0.997	-1.957771299	count	1
LINC01970	-18.268912	4988.095595	-0.0037	0.997	-1.957771299	count	1
SOX12	-18.268912	4988.095595	-0.0037	0.997	-1.957771299	count	1
SPTLC3	-18.268912	4988.095595	-0.0037	0.997	-1.957771299	count	1
CD93	-18.268912	4988.095595	-0.0037	0.997	-1.957771299	count	1
PEX11G	-18.268912	4988.095595	-0.0037	0.997	-1.957771299	count	1
FBXW9	-18.268912	4988.095595	-0.0037	0.997	-1.957771299	count	1
COX7A1	-18.268912	4988.095595	-0.0037	0.997	-1.957771299	count	1
ZNF793	-18.268912	4988.095595	-0.0037	0.997	-1.957771299	count	1
AC005614.1	-18.268912	4988.095595	-0.0037	0.997	-1.957771299	count	1
LIPE-AS1	-18.268912	4988.095595	-0.0037	0.997	-1.957771299	count	1
PPP5D1	-18.268912	4988.095595	-0.0037	0.997	-1.957771299	count	1
AC020922.3	-18.268912	4988.095595	-0.0037	0.997	-1.957771299	count	1
AC005498.2	-18.268912	4988.095595	-0.0037	0.997	-1.957771299	count	1
AIFM3	-18.268912	4988.095595	-0.0037	0.997	-1.957771299	count	1
MB	-18.268912	4988.095595	-0.0037	0.997	-1.957771299	count	1
FSD1L	-18.23898	2268.449126	-0.008	0.994	-1.957771297	count	1
SERP2	-18.23898	2268.449126	-0.008	0.994	-1.957771297	count	1
TM9SF1	-18.234327	1835.28681	-0.0099	0.992	-1.957771297	count	1
LRRC59	-1.6161612	0.6904573	-2.3407	0.0199	-1.953511661	count	1
PIK3CB	-1.5788604	1.0462181	-1.5091	0.132	-1.952174673	count	1
SNAPC5	-1.4612429	0.5840029	-2.5021	0.0128	-1.948246686	count	1
TNFSF13B	-1.7689936	1.2475987	-1.4179	0.157	-1.934044962	count	1
CRTC2	-1.522472	1.2017739	-1.2669	0.206	-1.931702072	count	1
CD27	-1.5024751	0.7249785	-2.0724	0.039	-1.919887301	count	1

RRS1	-1.6147351	0.7607158	-2.1227	0.0345	-1.902255523	count	1
EVA1C	-2.1971963	1.683263	-1.3053	0.193	-1.889988345	count	1
LARP1B	-1.625418	0.8745708	-1.8585	0.064	-1.882516622	count	1
KATNB1	-1.5834623	1.2652956	-1.2515	0.2117	-1.870648441	count	1
EMC1	-1.6893817	1.0582683	-1.5964	0.111	-1.864092463	count	1
HACL1	-1.4974236	0.5784854	-2.5885	0.0101	-1.862064588	count	1
ERLIN2	-2.1425119	1.1633872	-1.8416	0.0664	-1.861175103	count	1
VPS45	-1.945289	1.3750281	-1.4147	0.158	-1.858657519	count	1
MFHAS1	-2.4827267	1.2131178	-2.0466	0.0415	-1.848617522	count	1
MSRB2	-2.4827267	1.5377923	-1.6145	0.107	-1.848617522	count	1
ASL	-1.8109047	0.9537677	-1.8987	0.0585	-1.847480374	count	1
MYL6B	-1.7295106	1.2833638	-1.3476	0.179	-1.847050728	count	1
ZNF384	-1.9185736	1.2877962	-1.4898	0.137	-1.84079219	count	1
SNTB1	-1.9185736	1.4321529	-1.3396	0.181	-1.84079219	count	1
AC009403.1	-1.7925831	1.216459	-1.4736	0.142	-1.833413926	count	1
GTF3C1	-2.0823123	1.3697141	-1.5203	0.129	-1.828320903	count	1
RBMXL1	-1.5404693	0.9478416	-1.6252	0.105	-1.826737759	count	1
NADK	-1.6454457	0.866464	-1.899	0.0584	-1.824597142	count	1
DNAJC13	-1.6921124	1.3216209	-1.2803	0.201	-1.815450669	count	1
DNAJC14	-1.7587789	1.6628773	-1.0577	0.291	-1.807156231	count	1
MTPAP	-1.47887	0.8858479	-1.6694	0.096	-1.806769028	count	1
EPSTI1	-1.3000449	0.5544959	-2.3446	0.0197	-1.79117115	count	1
KLHL22	-1.45897	1.164602	-1.2528	0.211	-1.785092268	count	1
SELL	-1.3242722	0.6372275	-2.0782	0.0385	-1.778450129	count	1
EFL1	-1.8217329	1.3855688	-1.3148	0.19	-1.773953768	count	1
CCL2	-1.3731569	0.8253562	-1.6637	0.0971	-1.767163304	count	1
NUDT9	-1.9738518	1.3063823	-1.5109	0.132	-1.76608265	count	1
GLRX2	-1.3947145	0.6165015	-2.2623	0.0243	-1.759605181	count	1
KCTD18	-1.9479014	1.2024897	-1.6199	0.106	-1.750604686	count	1
RASSF4	-1.7887196	1.1187867	-1.5988	0.111	-1.750424163	count	1
MRPS16	-1.330474	0.6585057	-2.0204	0.0442	-1.746525726	count	1
C15orf40	-1.3197569	0.5654321	-2.3341	0.0202	-1.745177985	count	1
STAT1	-1.2365899	0.459983	-2.6883	0.0076	-1.738286392	count	1
PGM2L1	-1.5450661	0.6417154	-2.4077	0.0166	-1.732041624	count	1
ZNF324	-1.4642468	1.1214633	-1.3057	0.193	-1.721296754	count	1
YIF1A	-1.2916149	0.4722638	-2.7349	0.00658	-1.71992183	count	1
SETD4	-1.5788605	1.4547651	-1.0853	0.279	-1.716921736	count	1
GPR183	-1.2278774	0.3785113	-3.244	0.0013	-1.716388741	count	1
ATP9B	-1.5284148	0.8590451	-1.7792	0.0761	-1.716381581	count	1
CPOX	-1.8876176	1.2261005	-1.5395	0.125	-1.713767031	count	1
ATR	-1.4851892	0.7389713	-2.0098	0.0453	-1.711633495	count	1
UBE2Z	-1.3486756	0.6107677	-2.2082	0.0279	-1.706229313	count	1
FBLN5	-1.47553	0.9549717	-1.5451	0.123	-1.702066551	count	1
FAM102A	-1.2662778	0.5556043	-2.2791	0.0233	-1.700185251	count	1
AP001157.1	-1.6249973	1.3028213	-1.2473	0.213	-1.699403775	count	1

PXN	-1.7187322	1.4576903	-1.1791	0.239	-1.699294227	count	1
IP6K1	-1.2913006	0.5947052	-2.1713	0.0306	-1.697975339	count	1
FN1	-1.3394495	0.8977914	-1.4919	0.137	-1.695478722	count	1
AGA	-1.502408	0.8463348	-1.7752	0.0768	-1.691751394	count	1
ZNF213-AS1	-1.5499937	1.2257807	-1.2645	0.207	-1.691136784	count	1
CPNE7	-1.3014215	0.8041293	-1.6184	0.107	-1.689343667	count	1
ATF7	-1.705162	0.7514759	-2.2691	0.0239	-1.689184714	count	1
MIR4458HG	-2.730497	1.3098044	-2.0847	0.0379	-1.687314573	count	1
HNRNPLL	-1.607952	1.2952347	-1.2414	0.215	-1.685239719	count	1
TRPT1	-1.317008	0.6218276	-2.118	0.0349	-1.680433599	count	1
TMEM209	-1.6020961	1.2161352	-1.3174	0.189	-1.680351221	count	1
NOM1	-1.3168142	0.651016	-2.0227	0.0439	-1.680204311	count	1
MAPK9	-1.4514442	0.8061102	-1.8006	0.0727	-1.678091751	count	1
KLHL42	-1.4478863	0.9662518	-1.4985	0.135	-1.674535996	count	1
LIG1	-1.3943835	0.6660448	-2.0935	0.0371	-1.673710466	count	1
NPEPPS	-1.5275882	0.7631394	-2.0017	0.0461	-1.670938548	count	1
TRMU	-1.6802885	1.2900216	-1.3025	0.194	-1.670489912	count	1
HGH1	-1.6802885	1.4316149	-1.1737	0.241	-1.670489912	count	1
NUP133	-1.4409299	1.6946908	-0.8503	0.3958	-1.667573157	count	1
EXOC3	-1.5817507	1.093483	-1.4465	0.149	-1.663277911	count	1
DESI1	-1.3832762	0.940837	-1.4703	0.142	-1.661841966	count	1
SMUG1	-1.3832761	1.0978861	-1.2599	0.209	-1.661841862	count	1
GCDH	-1.3100927	0.742279	-1.765	0.0785	-1.661153106	count	1
THEM4	-1.4020903	0.686331	-2.0429	0.0419	-1.657140376	count	1
HIPK2	-1.4290542	0.683612	-2.0904	0.0374	-1.655654299	count	1
FAM217B	-1.3128541	0.6822821	-1.9242	0.0552	-1.652064731	count	1
AP3S2	-1.395864	1.2879546	-1.0838	0.279	-1.650655176	count	1
SRGAP3	-1.1796297	0.3557812	-3.3156	0.001	-1.64197653	count	1
UBR7	-1.493638	0.7266094	-2.0556	0.0406	-1.640027771	count	1
ASNS	-1.2787922	0.9819147	-1.3023	0.194	-1.635075734	count	1
ACOT7	-1.2854887	0.7821217	-1.6436	0.101	-1.632248415	count	1
SMARCC2	-1.2108069	0.4280479	-2.8287	0.00497	-1.632089504	count	1
AK9	-1.3506899	0.9130129	-1.4794	0.14	-1.626838613	count	1
PRAG1	-1.752678	1.222892	-1.4332	0.153	-1.626814553	count	1
SMG5	-1.7482809	1.3719259	-1.2743	0.203	-1.623876261	count	1
CC2D1A	-1.7482809	1.3719259	-1.2743	0.203	-1.623876261	count	1
GAA	-1.6175747	1.1601485	-1.3943	0.164	-1.622413453	count	1
TAP2	-1.4290328	0.5924825	-2.4119	0.0164	-1.621149518	count	1
ZFYVE16	-1.2967997	0.5405001	-2.3993	0.017	-1.619952869	count	1
CEP152	-1.528026	0.9711074	-1.5735	0.117	-1.617533855	count	1
FBXO3	-1.3400364	0.6858813	-1.9537	0.0516	-1.615336037	count	1
CRACR2A	-1.9563067	0.8847709	-2.2111	0.0277	-1.614887984	count	1
ABHD13	-1.304096	0.704883	-1.8501	0.0652	-1.613077741	count	1
SLFN12	-1.4177274	1.0269966	-1.3805	0.168	-1.610127833	count	1
AC006369.1	-1.459244	1.5033546	-0.9707	0.332	-1.608342387	count	1

NAB1	-1.936279	0.9150199	-2.1161	0.0351	-1.604314736	count	1
METTL1	-1.710639	1.3426761	-1.2741	0.204	-1.59845141	count	1
C1orf109	-1.290874	1.2129404	-1.0643	0.288	-1.598130641	count	1
ZNF595	-1.2885435	0.8530846	-1.5105	0.1319	-1.595491963	count	1
SNRNP25	-1.1575454	0.5300909	-2.1837	0.0297	-1.593816459	count	1
CD300A	-1.1597417	0.5536458	-2.0947	0.037	-1.593474707	count	1
THAP8	-2.37003	1.3216633	-1.7932	0.07387	-1.585025336	count	1
XPNPEP1	-1.3899918	0.8138922	-1.7078	0.0886	-1.582928409	count	1
MFSD5	-1.4863226	1.5464886	-0.9611	0.337	-1.581371717	count	1
SLC35A5	-2.3455858	1.5645857	-1.4992	0.135	-1.576999153	count	1
OSGEPL1	-1.881515	1.200352	-1.5675	0.118	-1.574741081	count	1
TAF1C	-1.881515	1.2821448	-1.4675	0.143	-1.574741081	count	1
YEATS4	-1.1529647	0.5545394	-2.0791	0.0384	-1.572680022	count	1
PIGBOS1	-1.218872	0.6969427	-1.7489	0.0813	-1.572468333	count	1
KANSL1L	-2.331795	1.6349042	-1.4263	0.155	-1.572405662	count	1
PAICS	-1.3791346	0.7254279	-1.9011	0.0582	-1.572219677	count	1
FAM160B1	-1.377316	0.9374241	-1.4693	0.143	-1.570422593	count	1
SEC22A	-1.8727329	1.0133761	-1.848	0.0655	-1.569907421	count	1
USP30-AS1	-1.2234106	0.683868	-1.789	0.0746	-1.568844609	count	1
HMGB3	-2.317554	1.2241892	-1.8931	0.0592	-1.567612325	count	1
DLG3	-1.2146221	0.9829638	-1.2357	0.217	-1.567313684	count	1
TMEM115	-2.3028277	1.1913701	-1.9329	0.0541	-1.562601545	count	1
SPRYD7	-1.2335001	0.6615802	-1.8645	0.0632	-1.559491768	count	1
HSBP1L1	-1.4003652	1.17568	-1.1911	0.234	-1.553247089	count	1
SLC39A11	-18.6344385	3015.40259	-0.0062	0.995	-1.547487791	count	1
FAM124B	-18.6321587	3295.112049	-0.0057	0.995	-1.54748779	count	1
TMEM44	-18.6321587	3295.112049	-0.0057	0.995	-1.54748779	count	1
CYTL1	-18.6321587	3295.112049	-0.0057	0.995	-1.54748779	count	1
PLA2G12A	-18.6321587	3295.112049	-0.0057	0.995	-1.54748779	count	1
KCNK5	-18.6321587	3295.112049	-0.0057	0.995	-1.54748779	count	1
ZC3H3	-18.6321587	3295.112049	-0.0057	0.995	-1.54748779	count	1
C11orf74	-18.6321587	3295.112049	-0.0057	0.995	-1.54748779	count	1
AKR1C3	-18.6321587	3295.112049	-0.0057	0.995	-1.54748779	count	1
PLAU	-18.6321587	3295.112049	-0.0057	0.995	-1.54748779	count	1
FAM234B	-18.6321587	3295.112049	-0.0057	0.995	-1.54748779	count	1
SLC30A4	-18.6321587	3295.112049	-0.0057	0.995	-1.54748779	count	1
BICDL2	-18.6321587	3295.112049	-0.0057	0.995	-1.54748779	count	1
AC005224.3	-18.6321587	3295.112049	-0.0057	0.995	-1.54748779	count	1
CCL8	-18.6321587	3295.112049	-0.0057	0.995	-1.54748779	count	1
EPB41L3	-18.6321587	3295.112049	-0.0057	0.995	-1.54748779	count	1
SLC23A2	-18.6321587	3295.112049	-0.0057	0.995	-1.54748779	count	1
ZNF681	-18.6321587	3295.112049	-0.0057	0.995	-1.54748779	count	1
PLEKHA4	-18.6321587	3295.112049	-0.0057	0.995	-1.54748779	count	1
ZSCAN22	-18.6321587	3295.112049	-0.0057	0.995	-1.54748779	count	1
SNX8	-18.6306614	2670.688587	-0.007	0.994	-1.54748779	count	1

AP000894.4	-18.6306614	2670.688587	-0.007	0.994	-1.54748779	count	1
CCL18	-18.4668501	2133.121065	-0.0087	0.993	-1.547487786	count	1
PLAC9	-18.2330226	2115.573401	-0.0086	0.993	-1.547487779	count	1
RNF123	-18.1883911	2132.141326	-0.0085	0.993	-1.547487778	count	1
CPT2	-18.1424078	3042.716765	-0.006	0.995	-1.547487776	count	1
MORN2	-18.1424078	3042.716765	-0.006	0.995	-1.547487776	count	1
FANCE	-18.1424078	3042.716765	-0.006	0.995	-1.547487776	count	1
KCNK17	-18.1424078	3042.716765	-0.006	0.995	-1.547487776	count	1
TUSC1	-18.1424078	3042.716765	-0.006	0.995	-1.547487776	count	1
SS18L1	-18.1424078	3042.716765	-0.006	0.995	-1.547487776	count	1
ZNF256	-18.1424078	3042.716765	-0.006	0.995	-1.547487776	count	1
CENPE	-18.1367444	2070.444394	-0.0088	0.993	-1.547487776	count	1
RNASEL	-18.0161185	2133.181508	-0.0084	0.993	-1.547487771	count	1
PRDM8	-18.0097075	2220.347307	-0.0081	0.994	-1.547487771	count	1
ZNF674	-18.0097075	2220.347307	-0.0081	0.994	-1.547487771	count	1
ACSM3	-18.0097075	2220.347307	-0.0081	0.994	-1.547487771	count	1
MLC1	-18.0097075	2220.347307	-0.0081	0.994	-1.547487771	count	1
ANKFY1	-17.8751865	1984.706628	-0.009	0.993	-1.547487765	count	1
NUAK2	-17.872533	2150.052248	-0.0083	0.993	-1.547487765	count	1
GRHL1	-17.872533	2150.052248	-0.0083	0.993	-1.547487765	count	1
KDELC1	-17.8710988	2028.98584	-0.0088	0.993	-1.547487765	count	1
FTO	-17.804693	1970.922728	-0.009	0.993	-1.547487762	count	1
PAQR4	-17.6646633	2050.767092	-0.0086	0.993	-1.547487754	count	1
SULT1A1	-17.6646633	2050.767092	-0.0086	0.993	-1.547487754	count	1
WWC3	-17.6617971	1673.381404	-0.0106	0.992	-1.547487754	count	1
MATN1	-17.622966	2733.953826	-0.0064	0.995	-1.547487751	count	1
AC139887.2	-17.622966	2733.953826	-0.0064	0.995	-1.547487751	count	1
DGKQ	-17.622966	2733.953826	-0.0064	0.995	-1.547487751	count	1
MFAP3	-17.622966	2733.953826	-0.0064	0.995	-1.547487751	count	1
TMEM170B	-17.622966	2733.953826	-0.0064	0.995	-1.547487751	count	1
TREM2	-17.622966	2733.953826	-0.0064	0.995	-1.547487751	count	1
TMEM176B	-17.622966	2733.953826	-0.0064	0.995	-1.547487751	count	1
SPIN3	-17.622966	2733.953826	-0.0064	0.995	-1.547487751	count	1
ANGPT2	-17.622966	2733.953826	-0.0064	0.995	-1.547487751	count	1
SIT1	-17.622966	2733.953826	-0.0064	0.995	-1.547487751	count	1
TRIM3	-17.622966	2733.953826	-0.0064	0.995	-1.547487751	count	1
PEAK1	-17.622966	2733.953826	-0.0064	0.995	-1.547487751	count	1
PALB2	-17.622966	2733.953826	-0.0064	0.995	-1.547487751	count	1
NAT14	-17.622966	2733.953826	-0.0064	0.995	-1.547487751	count	1
FP565260.6	-17.622966	2733.953826	-0.0064	0.995	-1.547487751	count	1
CU638689.4	-17.622966	2733.953826	-0.0064	0.995	-1.547487751	count	1
AP001462.1	-17.4803529	1993.785119	-0.0088	0.993	-1.547487742	count	1
MAPKBP1	-17.4803529	1993.785119	-0.0088	0.993	-1.547487742	count	1
PI4KA	-17.4803529	1993.785119	-0.0088	0.993	-1.547487742	count	1
C1QC	-19.159542	3191.552823	-0.006	0.995	-1.547487515	count	1

CTPS1	-18.942818	3160.108784	-0.006	0.995	-1.547487512	count	1
CD40LG	-18.942818	3160.108784	-0.006	0.995	-1.547487512	count	1
GCN1	-18.942818	3160.108784	-0.006	0.995	-1.547487512	count	1
ABCF2	-18.459772	1917.603771	-0.0096	0.992	-1.547487502	count	1
HNRNPA1P48	-18.429709	2090.968367	-0.0088	0.993	-1.547487501	count	1
TMEM44-AS1	-18.427185	2888.535923	-0.0064	0.995	-1.547487501	count	1
F13A1	-18.427185	2888.535923	-0.0064	0.995	-1.547487501	count	1
MSR1	-18.427185	2888.535923	-0.0064	0.995	-1.547487501	count	1
OTUD6B	-18.395697	2089.471975	-0.0088	0.993	-1.5474875	count	1
FZD6	-18.33416	1876.271632	-0.0098	0.992	-1.547487498	count	1
VPS37C	-18.279811	2273.189949	-0.008	0.994	-1.547487496	count	1
WDR47	-18.254484	2065.06617	-0.0088	0.993	-1.547487495	count	1
TAF9B	-18.254484	2065.06617	-0.0088	0.993	-1.547487495	count	1
CAPN10-DT	-18.238232	2731.655048	-0.0067	0.995	-1.547487495	count	1
RCC1	-18.212967	2902.773387	-0.0063	0.995	-1.547487494	count	1
AC004241.1	-18.212967	2902.773387	-0.0063	0.995	-1.547487494	count	1
CDC42BPB	-17.998179	2689.320456	-0.0067	0.995	-1.547487486	count	1
CEP76	-17.998179	2689.320456	-0.0067	0.995	-1.547487486	count	1
CFAP45	-17.990943	2846.199342	-0.0063	0.995	-1.547487486	count	1
TCEAL2	-17.990943	2846.199342	-0.0063	0.995	-1.547487486	count	1
OMD	-17.990943	2846.199342	-0.0063	0.995	-1.547487486	count	1
MS4A4A	-17.990943	2846.199342	-0.0063	0.995	-1.547487486	count	1
ZDHHC16	-17.990943	2846.199342	-0.0063	0.995	-1.547487486	count	1
SYCE1L	-17.990943	2846.199342	-0.0063	0.995	-1.547487486	count	1
PLEKHN1	-17.869595	4085.301308	-0.0044	0.997	-1.54748748	count	1
MORN1	-17.869595	4085.301308	-0.0044	0.997	-1.54748748	count	1
AL031848.2	-17.869595	4085.301308	-0.0044	0.997	-1.54748748	count	1
PIK3CD-AS1	-17.869595	4085.301308	-0.0044	0.997	-1.54748748	count	1
SH3D21	-17.869595	4085.301308	-0.0044	0.997	-1.54748748	count	1
FAAH	-17.869595	4085.301308	-0.0044	0.997	-1.54748748	count	1
LRP8	-17.869595	4085.301308	-0.0044	0.997	-1.54748748	count	1
ERICH3	-17.869595	4085.301308	-0.0044	0.997	-1.54748748	count	1
SORT1	-17.869595	4085.301308	-0.0044	0.997	-1.54748748	count	1
AL157904.1	-17.869595	4085.301308	-0.0044	0.997	-1.54748748	count	1
AC246785.3	-17.869595	4085.301308	-0.0044	0.997	-1.54748748	count	1
ITGA10	-17.869595	4085.301308	-0.0044	0.997	-1.54748748	count	1
NOTCH2NL	-17.869595	4085.301308	-0.0044	0.997	-1.54748748	count	1
OAZ3	-17.869595	4085.301308	-0.0044	0.997	-1.54748748	count	1
S100A5	-17.869595	4085.301308	-0.0044	0.997	-1.54748748	count	1
MEX3A	-17.869595	4085.301308	-0.0044	0.997	-1.54748748	count	1
AL021068.1	-17.869595	4085.301308	-0.0044	0.997	-1.54748748	count	1
C1orf53	-17.869595	4085.301308	-0.0044	0.997	-1.54748748	count	1
CHIT1	-17.869595	4085.301308	-0.0044	0.997	-1.54748748	count	1
MIR29B2CHG	-17.869595	4085.301308	-0.0044	0.997	-1.54748748	count	1
INTS7	-17.869595	4085.301308	-0.0044	0.997	-1.54748748	count	1

WDR64	-17.869595	4085.301308	-0.0044	0.997	-1.54748748	count	1
MBOAT2	-17.869595	4085.301308	-0.0044	0.997	-1.54748748	count	1
GTF3C2-AS1	-17.869595	4085.301308	-0.0044	0.997	-1.54748748	count	1
AC074117.1	-17.869595	4085.301308	-0.0044	0.997	-1.54748748	count	1
KRTCAP3	-17.869595	4085.301308	-0.0044	0.997	-1.54748748	count	1
SPDYA	-17.869595	4085.301308	-0.0044	0.997	-1.54748748	count	1
APLF	-17.869595	4085.301308	-0.0044	0.997	-1.54748748	count	1
SLC4A5	-17.869595	4085.301308	-0.0044	0.997	-1.54748748	count	1
MRPL53	-17.869595	4085.301308	-0.0044	0.997	-1.54748748	count	1
EVA1A	-17.869595	4085.301308	-0.0044	0.997	-1.54748748	count	1
IGKV3-20	-17.869595	4085.301308	-0.0044	0.997	-1.54748748	count	1
C2orf27B	-17.869595	4085.301308	-0.0044	0.997	-1.54748748	count	1
ZEB2-AS1	-17.869595	4085.301308	-0.0044	0.997	-1.54748748	count	1
LINC01806	-17.869595	4085.301308	-0.0044	0.997	-1.54748748	count	1
CHN1	-17.869595	4085.301308	-0.0044	0.997	-1.54748748	count	1
OSGEPL1-AS1	-17.869595	4085.301308	-0.0044	0.997	-1.54748748	count	1
AC064836.3	-17.869595	4085.301308	-0.0044	0.997	-1.54748748	count	1
AC007383.2	-17.869595	4085.301308	-0.0044	0.997	-1.54748748	count	1
AC007038.2	-17.869595	4085.301308	-0.0044	0.997	-1.54748748	count	1
AC079834.2	-17.869595	4085.301308	-0.0044	0.997	-1.54748748	count	1
FARP2	-17.869595	4085.301308	-0.0044	0.997	-1.54748748	count	1
AC112220.4	-17.869595	4085.301308	-0.0044	0.997	-1.54748748	count	1
DLEC1	-17.869595	4085.301308	-0.0044	0.997	-1.54748748	count	1
LRRC2	-17.869595	4085.301308	-0.0044	0.997	-1.54748748	count	1
ARF4-AS1	-17.869595	4085.301308	-0.0044	0.997	-1.54748748	count	1
SUCLG2-AS1	-17.869595	4085.301308	-0.0044	0.997	-1.54748748	count	1
GBE1	-17.869595	4085.301308	-0.0044	0.997	-1.54748748	count	1
PDIA5	-17.869595	4085.301308	-0.0044	0.997	-1.54748748	count	1
PLXNA1	-17.869595	4085.301308	-0.0044	0.997	-1.54748748	count	1
RAB43	-17.869595	4085.301308	-0.0044	0.997	-1.54748748	count	1
PPP2R3A	-17.869595	4085.301308	-0.0044	0.997	-1.54748748	count	1
AC097103.2	-17.869595	4085.301308	-0.0044	0.997	-1.54748748	count	1
AC007620.2	-17.869595	4085.301308	-0.0044	0.997	-1.54748748	count	1
AC016773.1	-17.869595	4085.301308	-0.0044	0.997	-1.54748748	count	1
HS3ST1	-17.869595	4085.301308	-0.0044	0.997	-1.54748748	count	1
AC025539.1	-17.869595	4085.301308	-0.0044	0.997	-1.54748748	count	1
SEPSECS	-17.869595	4085.301308	-0.0044	0.997	-1.54748748	count	1
TLR6	-17.869595	4085.301308	-0.0044	0.997	-1.54748748	count	1
WDR19	-17.869595	4085.301308	-0.0044	0.997	-1.54748748	count	1
EXOC1L	-17.869595	4085.301308	-0.0044	0.997	-1.54748748	count	1
TMPRSS11E	-17.869595	4085.301308	-0.0044	0.997	-1.54748748	count	1
IBSP	-17.869595	4085.301308	-0.0044	0.997	-1.54748748	count	1
AC004069.1	-17.869595	4085.301308	-0.0044	0.997	-1.54748748	count	1
SEC24B-AS1	-17.869595	4085.301308	-0.0044	0.997	-1.54748748	count	1
1-Mar	-17.869595	4085.301308	-0.0044	0.997	-1.54748748	count	1

WWC2	-17.869595	4085.301308	-0.0044	0.997	-1.54748748	count	1
GPX8	-17.869595	4085.301308	-0.0044	0.997	-1.54748748	count	1
FOXD1	-17.869595	4085.301308	-0.0044	0.997	-1.54748748	count	1
RGMB	-17.869595	4085.301308	-0.0044	0.997	-1.54748748	count	1
CDO1	-17.869595	4085.301308	-0.0044	0.997	-1.54748748	count	1
PCDHGA10	-17.869595	4085.301308	-0.0044	0.997	-1.54748748	count	1
ARHGEF37	-17.869595	4085.301308	-0.0044	0.997	-1.54748748	count	1
AC091982.3	-17.869595	4085.301308	-0.0044	0.997	-1.54748748	count	1
GEMIN5	-17.869595	4085.301308	-0.0044	0.997	-1.54748748	count	1
NRN1	-17.869595	4085.301308	-0.0044	0.997	-1.54748748	count	1
CAGE1	-17.869595	4085.301308	-0.0044	0.997	-1.54748748	count	1
AL353759.1	-17.869595	4085.301308	-0.0044	0.997	-1.54748748	count	1
ENPP5	-17.869595	4085.301308	-0.0044	0.997	-1.54748748	count	1
CRISP3	-17.869595	4085.301308	-0.0044	0.997	-1.54748748	count	1
TPBG	-17.869595	4085.301308	-0.0044	0.997	-1.54748748	count	1
AL024507.2	-17.869595	4085.301308	-0.0044	0.997	-1.54748748	count	1
AL356417.3	-17.869595	4085.301308	-0.0044	0.997	-1.54748748	count	1
PRKN	-17.869595	4085.301308	-0.0044	0.997	-1.54748748	count	1
HOXA-AS2	-17.869595	4085.301308	-0.0044	0.997	-1.54748748	count	1
TFR2	-17.869595	4085.301308	-0.0044	0.997	-1.54748748	count	1
RASA4	-17.869595	4085.301308	-0.0044	0.997	-1.54748748	count	1
POT1-AS1	-17.869595	4085.301308	-0.0044	0.997	-1.54748748	count	1
SVOPL	-17.869595	4085.301308	-0.0044	0.997	-1.54748748	count	1
AC083880.1	-17.869595	4085.301308	-0.0044	0.997	-1.54748748	count	1
EPHB6	-17.869595	4085.301308	-0.0044	0.997	-1.54748748	count	1
NOS3	-17.869595	4085.301308	-0.0044	0.997	-1.54748748	count	1
KLF8	-17.869595	4085.301308	-0.0044	0.997	-1.54748748	count	1
MTMR8	-17.869595	4085.301308	-0.0044	0.997	-1.54748748	count	1
CXorf57	-17.869595	4085.301308	-0.0044	0.997	-1.54748748	count	1
PAK3	-17.869595	4085.301308	-0.0044	0.997	-1.54748748	count	1
PLS3	-17.869595	4085.301308	-0.0044	0.997	-1.54748748	count	1
KLHL13	-17.869595	4085.301308	-0.0044	0.997	-1.54748748	count	1
AL121601.1	-17.869595	4085.301308	-0.0044	0.997	-1.54748748	count	1
CD99L2	-17.869595	4085.301308	-0.0044	0.997	-1.54748748	count	1
HCFC1-AS1	-17.869595	4085.301308	-0.0044	0.997	-1.54748748	count	1
AC145124.1	-17.869595	4085.301308	-0.0044	0.997	-1.54748748	count	1
MTUS1	-17.869595	4085.301308	-0.0044	0.997	-1.54748748	count	1
AC107959.1	-17.869595	4085.301308	-0.0044	0.997	-1.54748748	count	1
ESCO2	-17.869595	4085.301308	-0.0044	0.997	-1.54748748	count	1
PLAG1	-17.869595	4085.301308	-0.0044	0.997	-1.54748748	count	1
AC011978.2	-17.869595	4085.301308	-0.0044	0.997	-1.54748748	count	1
AC103706.1	-17.869595	4085.301308	-0.0044	0.997	-1.54748748	count	1
ZNF517	-17.869595	4085.301308	-0.0044	0.997	-1.54748748	count	1
AL391834.2	-17.869595	4085.301308	-0.0044	0.997	-1.54748748	count	1
ACO1	-17.869595	4085.301308	-0.0044	0.997	-1.54748748	count	1

AL162231.1	-17.869595	4085.301308	-0.0044	0.997	-1.54748748	count	1
FAM95B1	-17.869595	4085.301308	-0.0044	0.997	-1.54748748	count	1
AL512625.1	-17.869595	4085.301308	-0.0044	0.997	-1.54748748	count	1
PSAT1	-17.869595	4085.301308	-0.0044	0.997	-1.54748748	count	1
ASPN	-17.869595	4085.301308	-0.0044	0.997	-1.54748748	count	1
PTGR1	-17.869595	4085.301308	-0.0044	0.997	-1.54748748	count	1
MIR181A2HG	-17.869595	4085.301308	-0.0044	0.997	-1.54748748	count	1
SLC2A6	-17.869595	4085.301308	-0.0044	0.997	-1.54748748	count	1
BDNF-AS	-17.869595	4085.301308	-0.0044	0.997	-1.54748748	count	1
TNKS1BP1	-17.869595	4085.301308	-0.0044	0.997	-1.54748748	count	1
AP003108.2	-17.869595	4085.301308	-0.0044	0.997	-1.54748748	count	1
PPP1R32	-17.869595	4085.301308	-0.0044	0.997	-1.54748748	count	1
AP001160.3	-17.869595	4085.301308	-0.0044	0.997	-1.54748748	count	1
CADM1	-17.869595	4085.301308	-0.0044	0.997	-1.54748748	count	1
TIRAP	-17.869595	4085.301308	-0.0044	0.997	-1.54748748	count	1
HACD1	-17.869595	4085.301308	-0.0044	0.997	-1.54748748	count	1
ZEB1-AS1	-17.869595	4085.301308	-0.0044	0.997	-1.54748748	count	1
AGAP6	-17.869595	4085.301308	-0.0044	0.997	-1.54748748	count	1
PALD1	-17.869595	4085.301308	-0.0044	0.997	-1.54748748	count	1
UNC5B-AS1	-17.869595	4085.301308	-0.0044	0.997	-1.54748748	count	1
CFAP70	-17.869595	4085.301308	-0.0044	0.997	-1.54748748	count	1
TNKS2-AS1	-17.869595	4085.301308	-0.0044	0.997	-1.54748748	count	1
CCNJ	-17.869595	4085.301308	-0.0044	0.997	-1.54748748	count	1
AL158835.1	-17.869595	4085.301308	-0.0044	0.997	-1.54748748	count	1
RBP5	-17.869595	4085.301308	-0.0044	0.997	-1.54748748	count	1
LINC01252	-17.869595	4085.301308	-0.0044	0.997	-1.54748748	count	1
LINC00941	-17.869595	4085.301308	-0.0044	0.997	-1.54748748	count	1
ETFBKMT	-17.869595	4085.301308	-0.0044	0.997	-1.54748748	count	1
TMEM117	-17.869595	4085.301308	-0.0044	0.997	-1.54748748	count	1
ESPL1	-17.869595	4085.301308	-0.0044	0.997	-1.54748748	count	1
SPRYD4	-17.869595	4085.301308	-0.0044	0.997	-1.54748748	count	1
LINC02453	-17.869595	4085.301308	-0.0044	0.997	-1.54748748	count	1
PHETA1	-17.869595	4085.301308	-0.0044	0.997	-1.54748748	count	1
ALDH2	-17.869595	4085.301308	-0.0044	0.997	-1.54748748	count	1
OAS3	-17.869595	4085.301308	-0.0044	0.997	-1.54748748	count	1
AL138966.2	-17.869595	4085.301308	-0.0044	0.997	-1.54748748	count	1
LRRC63	-17.869595	4085.301308	-0.0044	0.997	-1.54748748	count	1
FARP1-AS1	-17.869595	4085.301308	-0.0044	0.997	-1.54748748	count	1
GAS6-AS1	-17.869595	4085.301308	-0.0044	0.997	-1.54748748	count	1
TRAV27	-17.869595	4085.301308	-0.0044	0.997	-1.54748748	count	1
HAUS4	-17.869595	4085.301308	-0.0044	0.997	-1.54748748	count	1
IPO4	-17.869595	4085.301308	-0.0044	0.997	-1.54748748	count	1
PTPN21	-17.869595	4085.301308	-0.0044	0.997	-1.54748748	count	1
DICER1-AS1	-17.869595	4085.301308	-0.0044	0.997	-1.54748748	count	1
INF2	-17.869595	4085.301308	-0.0044	0.997	-1.54748748	count	1

MKRN3	-17.869595	4085.301308	-0.0044	0.997	-1.54748748	count	1
ACTC1	-17.869595	4085.301308	-0.0044	0.997	-1.54748748	count	1
C15orf62	-17.869595	4085.301308	-0.0044	0.997	-1.54748748	count	1
OIP5	-17.869595	4085.301308	-0.0044	0.997	-1.54748748	count	1
NUSAP1	-17.869595	4085.301308	-0.0044	0.997	-1.54748748	count	1
C2CD4B	-17.869595	4085.301308	-0.0044	0.997	-1.54748748	count	1
TMC3-AS1	-17.869595	4085.301308	-0.0044	0.997	-1.54748748	count	1
ADAMTSL3	-17.869595	4085.301308	-0.0044	0.997	-1.54748748	count	1
EEF2KMT	-17.869595	4085.301308	-0.0044	0.997	-1.54748748	count	1
MYH11	-17.869595	4085.301308	-0.0044	0.997	-1.54748748	count	1
KIAA0895L	-17.869595	4085.301308	-0.0044	0.997	-1.54748748	count	1
LINC02166	-17.869595	4085.301308	-0.0044	0.997	-1.54748748	count	1
TLCD2	-17.869595	4085.301308	-0.0044	0.997	-1.54748748	count	1
CXCL16	-17.869595	4085.301308	-0.0044	0.997	-1.54748748	count	1
ZNF594	-17.869595	4085.301308	-0.0044	0.997	-1.54748748	count	1
SLC16A11	-17.869595	4085.301308	-0.0044	0.997	-1.54748748	count	1
NEURL4	-17.869595	4085.301308	-0.0044	0.997	-1.54748748	count	1
NLGN2	-17.869595	4085.301308	-0.0044	0.997	-1.54748748	count	1
AC093484.3	-17.869595	4085.301308	-0.0044	0.997	-1.54748748	count	1
MTRNR2L1	-17.869595	4085.301308	-0.0044	0.997	-1.54748748	count	1
KSR1	-17.869595	4085.301308	-0.0044	0.997	-1.54748748	count	1
PROCA1	-17.869595	4085.301308	-0.0044	0.997	-1.54748748	count	1
RAB34	-17.869595	4085.301308	-0.0044	0.997	-1.54748748	count	1
HCRT	-17.869595	4085.301308	-0.0044	0.997	-1.54748748	count	1
AC100793.2	-17.869595	4085.301308	-0.0044	0.997	-1.54748748	count	1
10-Mar	-17.869595	4085.301308	-0.0044	0.997	-1.54748748	count	1
CD300LF	-17.869595	4085.301308	-0.0044	0.997	-1.54748748	count	1
CDK3	-17.869595	4085.301308	-0.0044	0.997	-1.54748748	count	1
LIPG	-17.869595	4085.301308	-0.0044	0.997	-1.54748748	count	1
ACTL10	-17.869595	4085.301308	-0.0044	0.997	-1.54748748	count	1
LPIN3	-17.869595	4085.301308	-0.0044	0.997	-1.54748748	count	1
ZNF334	-17.869595	4085.301308	-0.0044	0.997	-1.54748748	count	1
ADNP-AS1	-17.869595	4085.301308	-0.0044	0.997	-1.54748748	count	1
BCAS1	-17.869595	4085.301308	-0.0044	0.997	-1.54748748	count	1
LINGO3	-17.869595	4085.301308	-0.0044	0.997	-1.54748748	count	1
GNG7	-17.869595	4085.301308	-0.0044	0.997	-1.54748748	count	1
C3	-17.869595	4085.301308	-0.0044	0.997	-1.54748748	count	1
ZNF69	-17.869595	4085.301308	-0.0044	0.997	-1.54748748	count	1
LYL1	-17.869595	4085.301308	-0.0044	0.997	-1.54748748	count	1
HOMER3	-17.869595	4085.301308	-0.0044	0.997	-1.54748748	count	1
TDRD12	-17.869595	4085.301308	-0.0044	0.997	-1.54748748	count	1
AC010616.1	-17.869595	4085.301308	-0.0044	0.997	-1.54748748	count	1
ZNF235	-17.869595	4085.301308	-0.0044	0.997	-1.54748748	count	1
PPM1N	-17.869595	4085.301308	-0.0044	0.997	-1.54748748	count	1
AC010331.1	-17.869595	4085.301308	-0.0044	0.997	-1.54748748	count	1

ZNF114	-17.869595	4085.301308	-0.0044	0.997	-1.54748748	count	1
AC006942.1	-17.869595	4085.301308	-0.0044	0.997	-1.54748748	count	1
LILRB2	-17.869595	4085.301308	-0.0044	0.997	-1.54748748	count	1
LILRB4	-17.869595	4085.301308	-0.0044	0.997	-1.54748748	count	1
ZSCAN5A	-17.869595	4085.301308	-0.0044	0.997	-1.54748748	count	1
AC005261.3	-17.869595	4085.301308	-0.0044	0.997	-1.54748748	count	1
ZNF749	-17.869595	4085.301308	-0.0044	0.997	-1.54748748	count	1
ZNF772	-17.869595	4085.301308	-0.0044	0.997	-1.54748748	count	1
ZIK1	-17.869595	4085.301308	-0.0044	0.997	-1.54748748	count	1
USP9Y	-17.869595	4085.301308	-0.0044	0.997	-1.54748748	count	1
AC007663.3	-17.869595	4085.301308	-0.0044	0.997	-1.54748748	count	1
AL022323.4	-17.869595	4085.301308	-0.0044	0.997	-1.54748748	count	1
RASD2	-17.869595	4085.301308	-0.0044	0.997	-1.54748748	count	1
RBFOX2	-17.869595	4085.301308	-0.0044	0.997	-1.54748748	count	1
AL022322.2	-17.869595	4085.301308	-0.0044	0.997	-1.54748748	count	1
Z83851.1	-17.869595	4085.301308	-0.0044	0.997	-1.54748748	count	1
SHISAL1	-17.869595	4085.301308	-0.0044	0.997	-1.54748748	count	1
C22orf34	-17.869595	4085.301308	-0.0044	0.997	-1.54748748	count	1
U62317.3	-17.869595	4085.301308	-0.0044	0.997	-1.54748748	count	1
CU633967.1	-17.869595	4085.301308	-0.0044	0.997	-1.54748748	count	1
MPIG6B	-17.868205	2872.690294	-0.0062	0.995	-1.54748748	count	1
TBC1D4	-17.868205	2872.690294	-0.0062	0.995	-1.54748748	count	1
ST6GALNAC1	-17.868205	2872.690294	-0.0062	0.995	-1.54748748	count	1
NDC80	-17.868205	2872.690294	-0.0062	0.995	-1.54748748	count	1
THBD	-17.868205	2872.690294	-0.0062	0.995	-1.54748748	count	1
ZNF701	-1.445849	0.9583754	-1.5086	0.132	-1.545737087	count	1
CUL3	-1.2033413	0.4241724	-2.8369	0.00484	-1.544701805	count	1
PHF2	-1.5148485	1.4378315	-1.0536	0.293	-1.540778621	count	1
ACP5	-1.1923343	0.6240006	-1.9108	0.0569	-1.540228694	count	1
RMDN1	-1.312973	0.6640741	-1.9771	0.0489	-1.537058279	count	1
ZNF382	-1.8123541	1.510493	-1.1998	0.231	-1.53599272	count	1
PAFAH1B3	-1.196045	0.6540439	-1.8287	0.0684	-1.535906267	count	1
CELF1	-1.3807887	0.7091265	-1.9472	0.0524	-1.534693401	count	1
LINC01089	-1.5940525	1.7019365	-0.9366	0.35	-1.516626518	count	1
HLA-DQA1	-1.2687841	0.6325778	-2.0057	0.0457	-1.516043112	count	1
NIPSNAP1	-1.2918451	0.813323	-1.5884	0.113	-1.515074358	count	1
HDAC3	-1.2053735	0.5588876	-2.1567	0.0318	-1.514194157	count	1
CHTF8	-1.185801	1.0653459	-1.1131	0.267	-1.513909582	count	1
GEM	-1.290503	0.8851085	-1.458	0.146	-1.513673927	count	1
QRSL1	-1.290503	0.9705305	-1.3297	0.185	-1.513673927	count	1
CTSL	-1.290503	1.0490195	-1.2302	0.22	-1.513673927	count	1
PRKCSH	-1.1464801	0.5995309	-1.9123	0.0567	-1.510984562	count	1
COG5	-1.3171848	0.8946253	-1.4723	0.142	-1.510466525	count	1
ZNF667	-1.3171848	1.1718067	-1.1241	0.262	-1.510466525	count	1
PLPP5	-1.212011	0.6361817	-1.9051	0.0576	-1.508167499	count	1

DOPEY2	-1.743949	1.2385015	-1.4081	0.16	-1.496116954	count	1
SQOR	-1.1384168	0.4925822	-2.3111	0.0215	-1.495031247	count	1
CERK	-1.247437	0.8971948	-1.3904	0.165	-1.493022073	count	1
SLC25A16	-1.3761407	1.0087013	-1.3643	0.173	-1.483137779	count	1
ORC3	-1.3249135	0.7887109	-1.6798	0.094	-1.481103539	count	1
SYCP2	-1.7076408	1.6446552	-1.0383	0.3	-1.474320104	count	1
GATC	-1.5353348	0.8588547	-1.7877	0.0748	-1.473661258	count	1
TMEM251	-1.278395	0.7738519	-1.652	0.0995	-1.471246109	count	1
AC064807.1	-1.168197	1.2446562	-0.9386	0.349	-1.470692646	count	1
NOP56	-1.0965436	0.4391993	-2.4967	0.013	-1.470084525	count	1
FRMD8	-1.3597897	1.0386996	-1.3091	0.191	-1.468232973	count	1
ZUP1	-1.2466294	0.6942678	-1.7956	0.0735	-1.467627598	count	1
PLGRKT	-1.132132	0.4548166	-2.4892	0.0133	-1.466638624	count	1
TFB1M	-1.0955305	0.5937092	-1.8452	0.0659	-1.460883934	count	1
INAFM1	-1.1836546	0.7006244	-1.6894	0.0921	-1.460433144	count	1
ATG2B	-1.1836546	0.7427427	-1.5936	0.112	-1.460433144	count	1
BICD1	-1.3003325	0.9515599	-1.3665	0.173	-1.457234757	count	1
ZNFX1	-1.2611531	0.6715651	-1.8779	0.0613	-1.453678579	count	1
SFXN3	-1.6715658	1.101129	-1.518	0.13	-1.452228164	count	1
RAMP1	-1.6680811	1.2758673	-1.3074	0.192	-1.450071169	count	1
ARAP1	-1.6680811	1.3245241	-1.2594	0.209	-1.450071169	count	1
BRCC3	-1.2042785	0.7733625	-1.5572	0.12	-1.446133491	count	1
PCGF3	-1.3353639	0.821415	-1.6257	0.105	-1.445812967	count	1
ERMP1	-1.9915389	1.3948185	-1.4278	0.154	-1.443213621	count	1
LINC01684	-1.6554612	1.0987387	-1.5067	0.133	-1.442225494	count	1
CAMK2N1	-1.3924211	0.856336	-1.626	0.105	-1.438893874	count	1
SWT1	-1.4839551	0.9956881	-1.4904	0.137	-1.435107913	count	1
CSTF2T	-1.1030296	0.7650455	-1.4418	0.15	-1.430846063	count	1
GRN	-1.2359025	0.7071908	-1.7476	0.0815	-1.427804081	count	1
CAT	-1.0777248	0.5286434	-2.0387	0.0423	-1.424160462	count	1
C6orf136	-1.4694619	1.1453587	-1.283	0.2	-1.424072025	count	1
RPE	-1.4694619	1.3873525	-1.0592	0.29	-1.424072025	count	1
PDIK1L	-1.4694619	1.544725	-0.9513	0.342	-1.424072025	count	1
DNAJC3-DT	-1.6245612	1.2560316	-1.2934	0.197	-1.422790332	count	1
ZNF81	-1.6245612	1.559039	-1.042	0.298	-1.422790332	count	1
LSG1	-1.1813013	0.8136256	-1.4519	0.147	-1.420985052	count	1
PDZD4	-1.3040959	1.1623974	-1.1219	0.263	-1.416844802	count	1
MTA2	-1.1577879	0.8286034	-1.3973	0.163	-1.414267418	count	1
PDK2	-1.1559227	0.9293048	-1.2439	0.214	-1.412169331	count	1
CHCHD6	-1.2541973	1.0479649	-1.1968	0.232	-1.411961843	count	1
MFSD6	-1.2190513	0.8721751	-1.3977	0.163	-1.41044032	count	1
IGHG1	-1.251484	0.6791505	-1.8427	0.0663	-1.40928022	count	1
TTLL10	-1.1518756	0.6963578	-1.6541	0.0991	-1.40761424	count	1
MADD	-1.3554799	1.1513729	-1.1773	0.24	-1.407190501	count	1
ACAD9	-1.596402	1.1254875	-1.4184	0.157	-1.404800369	count	1

SLC16A1-AS1	-1.290874	1.3987504	-0.9229	0.357	-1.404505818	count	1
PHLPP1	-1.290874	1.3987504	-0.9229	0.357	-1.404505818	count	1
PRPF40B	-1.290874	1.5626198	-0.8261	0.409	-1.404505818	count	1
FAM161B	-1.290874	1.6384199	-0.7879	0.431	-1.404505818	count	1
VSIG4	-1.2908735	1.9742378	-0.6539	0.5137	-1.404505266	count	1
TRIM4	-1.2101435	0.6399838	-1.8909	0.0595	-1.401230759	count	1
TLR1	-1.4362962	0.9617971	-1.4933	0.136	-1.398553149	count	1
RILP	-1.586022	1.0130172	-1.5656	0.118	-1.398101942	count	1
CCR1	-1.0691853	0.6888489	-1.5521	0.122	-1.395936953	count	1
TMEM254	-1.2022728	1.0163133	-1.183	0.238	-1.393075806	count	1
IFI44	-1.0269913	0.4686592	-2.1913	0.0291	-1.391737748	count	1
MTERF3	-1.0710542	0.8790105	-1.2185	0.224	-1.391361291	count	1
TRMT61A	-1.8728864	1.3005351	-1.4401	0.151	-1.390521812	count	1
PFKP	-1.1349734	0.6125975	-1.8527	0.0648	-1.388550804	count	1
LZIC	-1.1968009	0.7158349	-1.6719	0.0955	-1.38739656	count	1
CCDC102A	-1.1064512	0.6866169	-1.6115	0.108	-1.385672642	count	1
DUSP12	-1.0393761	0.5000377	-2.0786	0.0384	-1.384553035	count	1
SERPINI1	-1.078531	0.7858153	-1.3725	0.171	-1.384496066	count	1
AL133342.1	-1.1437621	0.8005264	-1.4288	0.154	-1.379627352	count	1
LST1	-1.263891	0.7286804	-1.7345	0.0838	-1.379160552	count	1
RMND1	-1.4104564	1.2478555	-1.1303	0.259	-1.378416779	count	1
RAB13	-1.4104564	1.3159676	-1.0718	0.285	-1.378416779	count	1
BRCA1	-1.4104564	1.3344344	-1.057	0.291	-1.378416779	count	1
SFXN5	-1.8423155	1.0621211	-1.7346	0.0838	-1.376268697	count	1
ZNF512	-1.8423155	1.2139292	-1.5176	0.13	-1.376268697	count	1
RCAN1	-1.8423155	1.5215859	-1.2108	0.227	-1.376268697	count	1
CLCN3	-1.3172252	0.9093777	-1.4485	0.148	-1.373899153	count	1
PEX6	-1.3168142	1.128621	-1.1667	0.244	-1.373538964	count	1
EDRF1	-1.4021484	0.9716639	-1.443	0.15	-1.371895388	count	1
SEC24B	-1.2131483	1.0368135	-1.1701	0.243	-1.371168693	count	1
VIM-AS1	-1.1789015	0.6560375	-1.797	0.0733	-1.368763665	count	1
ITGA1	-1.0161687	0.5674689	-1.7907	0.0743	-1.365751843	count	1
ARMT1	-1.079109	0.9259652	-1.1654	0.245	-1.365338231	count	1
UCHL5	-0.988693	0.4417124	-2.2383	0.0259	-1.363333841	count	1
MASTL	-1.1733804	1.000693	-1.1726	0.242	-1.362999313	count	1
CNOT3	-1.147732	0.9341604	-1.2286	0.2201	-1.362012886	count	1
SUPT7L	-1.041661	0.8012537	-1.3	0.195	-1.361570917	count	1
FANCG	-1.8093842	1.3585858	-1.3318	0.184	-1.360599343	count	1
MCRS1	-1.0510065	0.6359507	-1.6527	0.0994	-1.35912764	count	1
TMEM120A	-1.1237772	0.7030602	-1.5984	0.111	-1.357474736	count	1
PIK3C3	-1.380985	0.918802	-1.503	0.134	-1.355179956	count	1
ATP6V0A2	-1.0537873	1.0046623	-1.0489	0.295	-1.354357287	count	1
KIT	-2.701943	1.1814107	-2.287	0.0228	-1.351556506	count	1
TSPAN17	-1.232187	1.0870127	-1.1336	0.258	-1.349103177	count	1
FBXO22	-1.1907717	0.7377269	-1.6141	0.107	-1.348732402	count	1

ITPR3	-1.1596455	1.0146479	-1.1429	0.254	-1.348624742	count	1
SDHAF2	-0.9703277	0.4034695	-2.405	0.0167	-1.348502556	count	1
AC104532.2	-1.5085524	1.2655482	-1.192	0.234	-1.346969467	count	1
JRKL	-1.1578467	0.9884948	-1.1713	0.242	-1.346738552	count	1
AC008124.1	-1.2287346	0.8947828	-1.3732	0.171	-1.345812079	count	1
EIF2B5	-1.3654529	1.1101479	-1.23	0.22	-1.342818271	count	1
FAM78A	-1.0932999	0.6758764	-1.6176	0.107	-1.341279328	count	1
C1QB	-1.1518756	0.8026453	-1.4351	0.152	-1.340471293	count	1
LYRM4	-1.1275887	0.68964	-1.635	0.103	-1.340202196	count	1
OLMALINC	-1.498191	1.2897207	-1.1616	0.246	-1.33997821	count	1
RTL6	-1.498191	1.587852	-0.9435	0.346	-1.33997821	count	1
JAML	-1.498191	1.67273	-0.8957	0.371	-1.33997821	count	1
SMARCAD1	-1.0291144	0.6800218	-1.5134	0.131	-1.339327087	count	1
MAP4K1	-1.0773023	0.7279535	-1.4799	0.14	-1.338173155	count	1
UHRF2	-1.0274402	0.5421121	-1.8953	0.0589	-1.337244279	count	1
KXD1	-0.9750144	0.384303	-2.5371	0.0116	-1.335596279	count	1
ATP1A1	-1.146311	0.4785681	-2.3953	0.0172	-1.334622517	count	1
ZRANB1	-1.354076	0.8819988	-1.5352	0.126	-1.333713284	count	1
THBS1	-1.0284819	1.0440413	-0.9851	0.3253	-1.331357962	count	1
JPT2	-1.050425	1.0613552	-0.9897	0.323	-1.331096639	count	1
RRP1	-1.2653061	0.8694776	-1.4552	0.147	-1.32797675	count	1
TMEM150A	-1.039189	0.8500589	-1.2225	0.222	-1.327603411	count	1
PPM1B	-1.0808009	0.578223	-1.8692	0.0625	-1.327027859	count	1
LZTR1	-1.7374814	1.2432018	-1.3976	0.163	-1.325235566	count	1
EEF1AKMT1	-1.7374814	1.4325659	-1.2128	0.226	-1.325235566	count	1
FAM210B	-1.091938	1.0541556	-1.0358	0.301	-1.321992129	count	1
PAK1	-1.2569	0.7378818	-1.7034	0.0895	-1.320462179	count	1
PDGFA	-1.0595807	0.8817172	-1.2017	0.23	-1.317572209	count	1
MTMR10	-1.1985309	0.9778713	-1.2257	0.221	-1.31687062	count	1
TRIM21	-1.3300526	1.1789822	-1.1281	0.26	-1.314347951	count	1
OSER1-DT	-1.2443901	1.2317361	-1.0103	0.313	-1.309238957	count	1
GDI1	-0.9894054	0.6249121	-1.5833	0.114	-1.306886704	count	1
ARSG	-1.1879984	1.1079849	-1.0722	0.284	-1.306715772	count	1
EHBP1L1	-1.309766	0.626995	-2.089	0.0375	-1.297848255	count	1
APOL1	-2.419425	1.2595681	-1.9208	0.0556	-1.293649268	count	1
TPGS2	-0.9717378	0.4967542	-1.9562	0.0513	-1.293363473	count	1
AC015982.1	-1.038322	0.8803191	-1.1795	0.239	-1.292776364	count	1
HAGHL	-1.673048	0.7040321	-2.3764	0.0181	-1.292187039	count	1
HDAC2	-0.9645312	0.461551	-2.0898	0.0374	-1.291714068	count	1
DKC1	-0.9435554	0.4819638	-1.9577	0.0511	-1.29165734	count	1
NDRG3	-1.0818993	0.8234131	-1.3139	0.19	-1.290368051	count	1
CPTP	-1.0473899	0.7127652	-1.4695	0.143	-1.28876939	count	1
LINC01871	-1.032259	0.4242089	-2.4334	0.0155	-1.285688095	count	1
TLK2	-0.976693	0.5364882	-1.8205	0.0696	-1.285582997	count	1
ILVBL	-1.4191243	0.9075274	-1.5637	0.119	-1.285447407	count	1

NECAB3	-1.4191243	0.9328912	-1.5212	0.129	-1.285447407	count	1
SPTSSB	-0.9474418	0.6620396	-1.4311	0.153	-1.284190169	count	1
SLC25A43	-0.989728	0.986044	-1.0037	0.316	-1.283392259	count	1
OXSM	-1.2915397	1.0828419	-1.1927	0.234	-1.282910553	count	1
ZW10	-1.2915397	1.1358498	-1.1371	0.256	-1.282910553	count	1
BPGM	-0.9942849	0.9801369	-1.0144	0.311	-1.281462586	count	1
PPM1D	-0.9820777	0.7845038	-1.2518	0.212	-1.280649761	count	1
GIT1	-1.1232546	0.6350015	-1.7689	0.0779	-1.280202481	count	1
B3GNTL1	-1.027487	0.9677665	-1.0617	0.289	-1.280104149	count	1
MTRNR2L8	-1.054353	0.4737352	-2.2256	0.0267	-1.279812334	count	1
BRD8	-1.0936244	0.8267962	-1.3227	0.187	-1.278852968	count	1
ZFP90	-1.0244985	0.585046	-1.7511	0.0809	-1.276604813	count	1
TRUB2	-1.0132926	0.8384708	-1.2085	0.228	-1.275721127	count	1
METTL14	-1.205895	0.9228055	-1.3068	0.192	-1.274400979	count	1
LYAR	-1.0011852	0.6071639	-1.649	0.1	-1.271968007	count	1
RUFY1	-1.0646673	0.6780542	-1.5702	0.117	-1.271444422	count	1
ADK	-0.9995966	0.7550087	-1.324	0.186	-1.270053198	count	1
DYRK1A	-1.0449209	0.6201665	-1.6849	0.093	-1.269178043	count	1
LIMD1	-1.1492314	0.8630664	-1.3316	0.184	-1.269063861	count	1
BAG6	-1.2738704	0.8279647	-1.5386	0.125	-1.268326932	count	1
DYRK1B	-1.2738704	0.8843267	-1.4405	0.151	-1.268326932	count	1
INTS6-AS1	-1.0437836	0.8586331	-1.2156	0.225	-1.267894475	count	1
SLC4A2	-1.1987086	1.0079668	-1.1892	0.235	-1.267847128	count	1
SLC9A8	-1.625412	1.4262891	-1.1396	0.255	-1.266918255	count	1
IL7R	-0.9580044	0.3902595	-2.4548	0.0146	-1.26684997	count	1
PTPN11	-0.9961456	0.4678789	-2.1291	0.034	-1.265892032	count	1
DPY19L4	-1.1450806	1.8202051	-0.6291	0.53	-1.265007042	count	1
HLA-DRA	-0.9043749	0.3448942	-2.6222	0.0092	-1.262723686	count	1
NANP	-1.2668163	1.4849494	-0.8531	0.394	-1.2624767	count	1
NBEAL1	-0.9624152	0.4621702	-2.0824	0.0381	-1.262005759	count	1
TUBE1	-1.1419356	1.1425293	-0.9995	0.318	-1.261930012	count	1
ATG4C	-1.1406945	0.9787206	-1.1655	0.245	-1.260714962	count	1
PDE6D	-1.1393333	0.6682229	-1.705	0.0891	-1.259381833	count	1
HMGXB3	-1.0356558	0.8186542	-1.2651	0.207	-1.258713128	count	1
FXYD1	-1.3793182	1.025085	-1.3456	0.179	-1.257206046	count	1
PLCG1	-1.3793182	1.1102197	-1.2424	0.215	-1.257206046	count	1
HYOU1	-1.1866702	0.8385747	-1.4151	0.158	-1.256833451	count	1
SHLD2	-1.130415	0.8049813	-1.4043	0.161	-1.25063446	count	1
STT3B	-0.9321065	0.3783112	-2.4639	0.0143	-1.249529968	count	1
ZNF770	-0.9311367	0.5751962	-1.6188	0.106	-1.248266464	count	1
Z93930.2	-1.1759432	0.8909061	-1.3199	0.188	-1.246982757	count	1
TASP1	-1.0902013	1.0082528	-1.0813	0.28	-1.246207346	count	1
SLC27A2	-1.3627618	1.1513352	-1.1836	0.237	-1.245305028	count	1
RGS10	-0.8815519	0.3097531	-2.846	0.0047	-1.245190494	count	1
ZC3H6	-0.9179179	0.5383119	-1.7052	0.0891	-1.245119762	count	1

STARD9	-1.5796165	1.2471063	-1.2666	0.206	-1.241950426	count	1
HPS5	-1.0378235	0.8242008	-1.2592	0.209	-1.241828066	count	1
PRKAG1	-0.9664624	0.5716696	-1.6906	0.0919	-1.23907186	count	1
TMEM184C	-2.200989	1.28408	-1.7141	0.0875	-1.238739498	count	1
AGPAT5	-1.0038551	0.8513967	-1.1791	0.239	-1.238569993	count	1
AL450326.1	-1.0824584	1.0408716	-1.04	0.299	-1.238202297	count	1
EXOC2	-1.0157827	0.6535706	-1.5542	0.121	-1.236203818	count	1
TAF13	-0.9571465	0.8174043	-1.171	0.242	-1.235675708	count	1
PLIN2	-0.8850754	0.3307654	-2.6758	0.0078	-1.235424082	count	1
CXCL3	-1.163261	1.3255389	-0.8776	0.381	-1.235292029	count	1
AC005520.2	-1.163261	1.3255389	-0.8776	0.381	-1.235292029	count	1
ST6GAL1	-1.163261	1.380033	-0.8429	0.4	-1.235292029	count	1
SDHAF4	-1.163261	1.380033	-0.8429	0.4	-1.235292029	count	1
VPS4A	-0.9206828	0.5225722	-1.7618	0.079	-1.234640023	count	1
QPCT	-2.1831301	1.5339783	-1.4232	0.1556	-1.233815892	count	1
KLHL26	-1.5624047	1.1682112	-1.3374	0.182	-1.232394436	count	1
CACNB1	-1.0760046	1.1712213	-0.9187	0.359	-1.231518045	count	1
MPST	-0.8942049	0.3572091	-2.5033	0.0128	-1.231413067	count	1
HDHD5	-0.9675101	0.6281099	-1.5404	0.124	-1.231284032	count	1
CEP290	-1.229029	0.6123102	-2.0072	0.0456	-1.230867435	count	1
FBXO25	-1.1083666	0.7740554	-1.4319	0.153	-1.228913199	count	1
TIAM1	-1.2264214	0.8928664	-1.3736	0.171	-1.228669481	count	1
HMGA1	-0.9059867	0.3586081	-2.5264	0.012	-1.226883716	count	1
NMT1	-0.9191652	0.6950103	-1.3225	0.187	-1.225501908	count	1
EEFSEC	-0.9191652	0.6507679	-1.4124	0.159	-1.225501899	count	1
CALCOCO1	-1.070175	0.5247666	-2.0393	0.0422	-1.225470883	count	1
ALG10	-1.1518756	1.0920003	-1.0548	0.292	-1.22475586	count	1
ZSCAN30	-1.1518756	1.1982847	-0.9613	0.337	-1.22475586	count	1
N4BP2L1	-0.9129775	0.4658365	-1.9599	0.0509	-1.224588675	count	1
PINX1	-1.1475368	0.8715524	-1.3167	0.189	-1.220730517	count	1
INTS13	-1.0620094	0.8535777	-1.2442	0.214	-1.216986031	count	1
VIPR2	-1.014382	1.0269169	-0.9878	0.324	-1.215830628	count	1
CENPK	-0.9255837	0.777383	-1.1906	0.235	-1.215429533	count	1
UQCC1	-1.2099965	1.1548554	-1.0477	0.296	-1.214775079	count	1
ZMYND19	-1.5284148	1.2106369	-1.2625	0.208	-1.213246071	count	1
SIK1	-1.5284148	1.2106369	-1.2625	0.208	-1.213246071	count	1
NBPF11	-1.5284148	1.4337801	-1.066	0.287	-1.213246071	count	1
USP51	-1.5284148	1.4337801	-1.066	0.287	-1.213246071	count	1
RFC3	-1.5284148	1.4337801	-1.066	0.287	-1.213246071	count	1
PDP1	-1.208047	0.6785475	-1.7803	0.076	-1.213120248	count	1
ANKRD17	-0.9702875	0.7275776	-1.3336	0.183	-1.212828813	count	1
RAB32	-0.9049301	0.5814269	-1.5564	0.121	-1.210707152	count	1
IRF2BP1	-2.082852	1.2181599	-1.7098	0.0883	-1.204875334	count	1
TCP11L2	-1.024263	0.504013	-2.0322	0.0429	-1.204381426	count	1
NIPSNAP3A	-0.952996	0.7622354	-1.2503	0.212	-1.203691253	count	1

IGHA1	-2.070828	0.4858739	-4.2621	2.66E-05	-1.201254133	count	0.6467524
RBM28	-1.12627	0.4744516	-2.3738	0.0182	-1.200919868	count	1
TYMP	-0.8565794	0.4345296	-1.9713	0.0495	-1.200776801	count	1
PER2	-0.9842319	0.7490233	-1.314	0.19	-1.20029472	count	1
AK6	-0.9495194	0.6097354	-1.5573	0.12	-1.199518285	count	1
C12orf43	-0.9494017	0.6694191	-1.4182	0.157	-1.199376973	count	1
ICMT	-1.1227294	0.9813147	-1.1441	0.253	-1.19760861	count	1
ARHGAP5	-1.122145	0.5316664	-2.1106	0.0356	-1.197061699	count	1
MEF2A	-0.9387961	0.4912177	-1.9112	0.0569	-1.196440695	count	1
UPF3B	-0.9805734	0.5859852	-1.6734	0.0952	-1.196117303	count	1
SPATA2L	-0.9045553	0.5560079	-1.6269	0.105	-1.19379266	count	1
UACA	-1.2913006	1.2365958	-1.0442	0.297	-1.192900811	count	1
ERMARD	-1.2913006	1.2435488	-1.0384	0.3	-1.192900811	count	1
NUP205	-1.2913006	1.2435488	-1.0384	0.3	-1.192900811	count	1
SGSM3	-1.2913006	1.568748	-0.8231	0.411	-1.192900811	count	1
ATP2A2	-2.0426953	1.211915	-1.6855	0.0929	-1.192651454	count	1
CLIC5	-2.0426953	1.4632227	-1.396	0.164	-1.192651454	count	1
GNPAT	-1.0086866	0.647599	-1.5576	0.12	-1.187497925	count	1
CRTC3	-1.033072	0.6579108	-1.5702	0.117	-1.186779422	count	1
BRWD3	-1.2826996	1.0004087	-1.2822	0.201	-1.186480549	count	1
CD38	-0.9111215	0.7116706	-1.2803	0.201	-1.185400867	count	1
HIC1	-0.8634114	0.594843	-1.4515	0.1476	-1.183972968	count	1
MPRIP	-1.107929	0.6917877	-1.6015	0.11	-1.183727804	count	1
SUPT6H	-0.9360394	0.6126036	-1.528	0.127	-1.183318163	count	1
GPR132	-1.0605549	0.7236678	-1.4655	0.144	-1.181348859	count	1
CEP135	-0.9826601	0.7389105	-1.3299	0.184	-1.180453662	count	1
SDE2	-0.8939158	0.55151	-1.6209	0.106	-1.180197596	count	1
PPP6R2	-1.272464	0.8054181	-1.5799	0.115	-1.17880855	count	1
CXCL8	-1.056722	1.4106576	-0.7491	0.454	-1.177508782	count	1
ERAP2	-1.464946	0.9017991	-1.6245	0.105	-1.176501927	count	1
PAPSS1	-0.9789219	0.6842922	-1.4306	0.154	-1.176270067	count	1
IPO7	-1.0991942	0.7775735	-1.4136	0.158	-1.17550563	count	1
CDK19	-1.0991942	0.8801037	-1.2489	0.213	-1.17550563	count	1
RPA1	-0.9778221	0.7627146	-1.282	0.201	-1.175038638	count	1
FCGRT	-0.8892345	0.6797906	-1.3081	0.192	-1.174211353	count	1
MRPS28	-1.2653061	1.0718012	-1.1805	0.239	-1.173423344	count	1
NARFL	-1.2653061	1.091697	-1.159	0.247	-1.173423344	count	1
GFM2	-1.458776	0.9245169	-1.5779	0.116	-1.172861123	count	1
ERMN	-1.4518193	1.4688121	-0.9884	0.324	-1.168741249	count	1
ALG8	-1.256215	0.8720737	-1.4405	0.151	-1.166559479	count	1
ARL3	-0.9415271	0.8361481	-1.126	0.261	-1.166037816	count	1
SOS1	-0.8695412	0.5100848	-1.7047	0.0892	-1.164602889	count	1
STK38L	-1.251583	0.8005243	-1.5635	0.119	-1.163051958	count	1
PDRG1	-0.9184997	0.5511692	-1.6665	0.0966	-1.162191915	count	1
C6orf48	-0.9104255	0.4032212	-2.2579	0.0246	-1.161879226	count	1

CETN3	-0.8919857	0.8491844	-1.0504	0.294	-1.161409706	count	1
TAF1	-1.438536	0.8388264	-1.7149	0.0873	-1.160831611	count	1
SLC25A45	-0.909181	0.5531619	-1.6436	0.101	-1.160360133	count	1
VRK3	-0.8686187	0.7141754	-1.2163	0.225	-1.159952509	count	1
CISD1	-0.8553311	0.6083641	-1.406	0.161	-1.159779538	count	1
IFFO2	-0.8811667	0.5602628	-1.5728	0.117	-1.159019004	count	1
IMMT	-0.8561984	0.4408432	-1.9422	0.053	-1.158535844	count	1
ARID3B	-1.0808009	1.0025589	-1.078	0.282	-1.158119723	count	1
ENOX2	-0.9624891	0.888898	-1.0828	0.28	-1.157843144	count	1
MON2	-0.9996128	0.8982599	-1.1128	0.267	-1.151588786	count	1
SAMM50	-0.8880177	0.5752527	-1.5437	0.124	-1.149883509	count	1
CAMKMT	-1.9024405	1.2550459	-1.5158	0.131	-1.146975338	count	1
AC027097.1	-0.994454	1.1093935	-0.8964	0.371	-1.146138239	count	1
TM7SF2	-1.1291612	0.9055686	-1.2469	0.213	-1.145164337	count	1
RAB5IF	-0.8244193	0.3515794	-2.3449	0.0196	-1.14504423	count	1
ZNF266	-1.2276265	1.0274763	-1.1948	0.233	-1.144800325	count	1
TCF7	-0.8262314	0.5484954	-1.5064	0.133	-1.143744515	count	1
ZNF439	-1.0640118	1.0305066	-1.0325	0.303	-1.142165231	count	1
PDCD2L	-1.1253205	1.0585136	-1.0631	0.289	-1.14180669	count	1
FAM8A1	-1.1253205	1.061318	-1.0603	0.29	-1.14180669	count	1
LINC00528	-1.1253205	1.3053063	-0.8621	0.389	-1.14180669	count	1
APOL3	-1.0635364	1.0088049	-1.0543	0.293	-1.141712279	count	1
CARD11	-1.0631526	0.7647643	-1.3902	0.165	-1.141346555	count	1
DDX60L	-0.9658387	0.6378638	-1.5142	0.131	-1.140760667	count	1
GNLY	-0.790979	0.2502255	-3.1611	0.00172	-1.139355112	count	1
ZNF280D	-0.9644335	0.760528	-1.2681	0.206	-1.139220731	count	1
ACSF2	-1.2191519	1.0763201	-1.1327	0.258	-1.138299375	count	1
DGCR2	-1.2191519	1.0763201	-1.1327	0.258	-1.138299375	count	1
C14orf119	-0.8153728	0.3917407	-2.0814	0.0382	-1.135814665	count	1
THADA	-0.927237	1.0337567	-0.897	0.37	-1.134902866	count	1
TMTC3	-1.1153982	1.0960187	-1.0177	0.31	-1.133111494	count	1
ZFR	-0.8330798	0.365414	-2.2798	0.0233	-1.132399225	count	1
RAB3GAP1	-1.0113621	0.5559982	-1.819	0.0698	-1.131764792	count	1
MAD2L1	-1.3899918	1.3516673	-1.0284	0.305	-1.131443704	count	1
PORCN	-1.3899918	1.3516673	-1.0284	0.305	-1.131443704	count	1
PRRT3	-1.3899918	1.556503	-0.893	0.373	-1.131443704	count	1
EDC4	-1.3899918	1.556503	-0.893	0.373	-1.131443704	count	1
DGCR8	-1.3899918	1.556503	-0.893	0.373	-1.131443704	count	1
BLVRB	-0.8329196	0.4564863	-1.8246	0.069	-1.130015334	count	1
HIF1A	-0.8231429	0.3794041	-2.1696	0.0308	-1.129677385	count	1
CALD1	-0.9337287	0.8641195	-1.0806	0.2807	-1.125452314	count	1
VANGL1	-1.2013076	0.9846442	-1.22	0.223	-1.124535264	count	1
PPP2R2D	-0.8529186	0.7383932	-1.1551	0.249	-1.123010344	count	1
ZNF10	-1.103764	0.8896854	-1.2406	0.216	-1.122877859	count	1
DENR	-0.8282868	0.4217021	-1.9642	0.0504	-1.121563112	count	1

FAM76A	-1.041797	0.4730227	-2.2024	0.0283	-1.120931369	count	1
CCDC115	-0.8175175	0.4114765	-1.9868	0.0478	-1.120573299	count	1
USP36	-0.8909237	0.6317912	-1.4102	0.159	-1.11847556	count	1
TAP1	-0.8021148	0.3624429	-2.2131	0.0276	-1.118262787	count	1
FASTK	-0.8615172	0.5392731	-1.5976	0.111	-1.116807598	count	1
ZC3H7B	-0.9098328	0.8231472	-1.1053	0.27	-1.114803676	count	1
EP300	-0.8648103	0.5444811	-1.5883	0.113	-1.113893455	count	1
AP002360.1	-1.09288	0.9111717	-1.1994	0.231	-1.113267051	count	1
NR2C2	-1.09288	0.956527	-1.1426	0.254	-1.113267051	count	1
AC006504.5	-1.09288	1.0188948	-1.0726	0.284	-1.113267051	count	1
CALY	-1.358131	1.1276511	-1.2044	0.229	-1.111743469	count	1
NELL2	-1.8027355	1.349242	-1.3361	0.182	-1.111563108	count	1
ZNF827	-1.8027355	1.4445437	-1.248	0.213	-1.111563108	count	1
FILIP1L	-1.1836879	1.3251764	-0.8932	0.372	-1.110844162	count	1
FBXO11	-0.8277924	0.4638088	-1.7848	0.0752	-1.110040652	count	1
SUN1	-0.9602589	0.8563101	-1.1214	0.263	-1.109844271	count	1
TMEM222	-0.8271005	0.6123569	-1.3507	0.178	-1.109134866	count	1
ADO	-0.8914051	0.6052411	-1.4728	0.142	-1.10716645	count	1
PEMT	-0.8412506	0.7072046	-1.1895	0.235	-1.103082267	count	1
G2E3	-0.931339	0.7787826	-1.1959	0.233	-1.102823041	count	1
ASXL2	-0.9130061	0.671595	-1.3595	0.175	-1.102004983	count	1
ACTR1A	-0.8324855	0.6091684	-1.3666	0.173	-1.101428489	count	1
ZNF33A	-0.854392	0.5759102	-1.4836	0.139	-1.100974374	count	1
ZSCAN32	-0.9293189	1.015309	-0.9153	0.361	-1.100593316	count	1
AAR2	-1.1698096	0.8305699	-1.4084	0.16	-1.099990408	count	1
POLR2M	-1.1698096	0.87546	-1.3362	0.182	-1.099990408	count	1
USP7	-0.8847696	0.5416298	-1.6335	0.103	-1.099337465	count	1
TMEM129	-0.9793649	0.8412114	-1.1642	0.245	-1.099169981	count	1
CACUL1	-0.8304886	0.5383392	-1.5427	0.124	-1.098860308	count	1
BRAP	-0.845357	0.6003469	-1.4081	0.16	-1.096588354	count	1
ARAF	-0.8715989	0.7141042	-1.2205	0.223	-1.095330688	count	1
ZNF596	-1.0718302	1.133042	-0.946	0.345	-1.094578195	count	1
DHX32	-1.1623886	0.8737079	-1.3304	0.184	-1.09416152	count	1
HLA-DQB1	-0.8799769	0.5067402	-1.7365	0.0834	-1.093677713	count	1
UBE4A	-0.8915736	0.586634	-1.5198	0.13	-1.093652846	count	1
ISG20	-0.7661219	0.2539698	-3.0166	0.0028	-1.093484256	count	1
MPPE1	-0.8902319	0.7345499	-1.2119	0.226	-1.0920961	count	1
UBE2O	-0.9017211	0.7217308	-1.2494	0.212	-1.089198358	count	1
PLAA	-0.968581	0.8410074	-1.1517	0.25	-1.088124836	count	1
ADGRG5	-1.315793	1.2350137	-1.0654	0.287	-1.08506039	count	1
TMEM182	-1.004095	1.1079643	-0.9063	0.365	-1.084576538	count	1
R3HDM4	-0.8186698	0.5051287	-1.6207	0.106	-1.083650701	count	1
NAB2	-0.8714932	0.7210865	-1.2086	0.228	-1.083648914	count	1
KIR2DL4	-0.7835095	0.3681377	-2.1283	0.0341	-1.08229149	count	1
FAM161A	-1.7243603	1.0038578	-1.7177	0.0868	-1.081933773	count	1

BOD1	-1.057212	0.7005937	-1.509	0.132	-1.081521536	count	1
PPP1R3E	-1.1450019	0.9750354	-1.1743	0.241	-1.080436494	count	1
TRMT1	-0.9324256	0.7343382	-1.2697	0.205	-1.080093973	count	1
LRCH4	-0.9323084	0.9109552	-1.0234	0.307	-1.079968327	count	1
ATAD2	-0.7974003	0.5634281	-1.4153	0.158	-1.078236423	count	1
PPIE	-0.7703699	0.3177026	-2.4248	0.0159	-1.078045384	count	1
MCCC2	-0.890499	0.7539714	-1.1811	0.238	-1.07643699	count	1
STARD4	-1.0500345	0.9095257	-1.1545	0.249	-1.075087377	count	1
RHBDD2	-0.7792729	0.4807485	-1.621	0.106	-1.074241503	count	1
HACD3	-0.8879024	0.6595436	-1.3462	0.179	-1.073480544	count	1
VWA5A	-1.1358705	0.9959938	-1.1404	0.255	-1.073189862	count	1
C12orf4	-1.1349734	1.1491783	-0.9876	0.324	-1.072476519	count	1
SPG21	-0.8312715	0.7278642	-1.1421	0.254	-1.07224698	count	1
SLC25A44	-1.1325668	1.2804641	-0.8845	0.377	-1.070561592	count	1
NCEH1	-1.1325668	1.2804641	-0.8845	0.377	-1.070561592	count	1
KLF7	-1.1325668	1.3416153	-0.8442	0.399	-1.070561592	count	1
SLC43A3	-1.1325668	1.3416153	-0.8442	0.399	-1.070561592	count	1
CHKB	-1.1325668	1.3949058	-0.8119	0.417	-1.070561592	count	1
SFR1	-0.8418112	0.7897452	-1.0659	0.287	-1.069213429	count	1
MICAL3	-1.6903706	0.8955875	-1.8874	0.06	-1.068580436	count	1
RYK	-0.8182668	0.6510236	-1.2569	0.21	-1.068512352	count	1
MICAL2	-0.9874217	0.839039	-1.1768	0.24	-1.068373024	count	1
SART3	-0.9203594	0.7512153	-1.2252	0.221	-1.067139519	count	1
KIAA1147	-0.9468638	0.6511065	-1.4542	0.147	-1.065791155	count	1
MRPS27	-0.8809518	0.9693354	-0.9088	0.364	-1.065559905	count	1
PSMD6	-0.7774603	0.4080318	-1.9054	0.0576	-1.063455556	count	1
DENND1B	-0.7812292	0.5945916	-1.3139	0.19	-1.063196781	count	1
PTOV1	-0.7961787	0.5275981	-1.5091	0.132	-1.06219188	count	1
LLPH	-0.8177834	0.5223393	-1.5656	0.118	-1.062004553	count	1
RBBP5	-1.2782515	1.1675028	-1.0949	0.2744	-1.060918688	count	1
IGFBP2	-0.7399255	0.2497239	-2.963	0.00327	-1.060194991	count	1
ENG	-0.9129424	0.9585543	-0.9524	0.342	-1.059159517	count	1
CRELD1	-0.8410736	0.6916151	-1.2161	0.225	-1.058640268	count	1
ALG14	-1.665026	1.0068087	-1.6538	0.09914	-1.058421798	count	1
YTHDF3	-1.665026	1.0101288	-1.6483	0.1	-1.058421798	count	1
USP39	-0.8059949	0.8075512	-0.9981	0.319	-1.058214736	count	1
BRWD1	-0.7881224	0.5041965	-1.5631	0.119	-1.058028924	count	1
VGLL4	-0.8323527	0.6418252	-1.2969	0.196	-1.057679189	count	1
WASHC5	-1.2716466	0.8447641	-1.5053	0.1332	-1.056624515	count	1
PTPN22	-0.7587041	0.3137003	-2.4186	0.0161	-1.056307521	count	1
SH2B3	-0.9734428	1.0680309	-0.9114	0.363	-1.054729373	count	1
KLHL23	-1.6554612	1.2979899	-1.2754	0.203	-1.05454323	count	1
ZCCHC2	-0.780706	0.4761868	-1.6395	0.102	-1.053678878	count	1
PACSIN2	-0.8452333	0.9532381	-0.8867	0.376	-1.052524018	count	1
ARID2	-0.8211507	0.7153666	-1.1479	0.252	-1.052284957	count	1

C12orf45	-0.8146195	0.586215	-1.3896	0.166	-1.051508716	count	1
MED17	-1.106168	0.7036848	-1.572	0.117	-1.049436481	count	1
DTX3L	-0.7832973	0.6119431	-1.28	0.201	-1.048672084	count	1
HLA-DRB5	-0.7720359	0.4193807	-1.8409	0.0666	-1.046837238	count	1
TWISTNB	-0.7671241	0.3932862	-1.9505	0.052	-1.046171165	count	1
SNRPN	-0.741792	0.2843002	-2.6092	0.0095	-1.044266298	count	1
IFNL1	-1.0991942	1.0890046	-1.0094	0.314	-1.04381948	count	1
MFSD4B	-1.014382	1.2149497	-0.8349	0.404	-1.042902097	count	1
CBWD6	-1.014382	1.4270923	-0.7108	0.478	-1.042902097	count	1
C20orf96	-1.014382	1.4270923	-0.7108	0.478	-1.042902097	count	1
MARS	-0.9612838	0.9033767	-1.0641	0.288	-1.042818751	count	1
INPP5F	-0.9612838	0.9361136	-1.0269	0.305	-1.042818751	count	1
HUS1	-0.8977912	0.6974379	-1.2873	0.199	-1.042818366	count	1
RANGRF	-0.7658178	0.4450275	-1.7208	0.0862	-1.04258481	count	1
LAMP2	-0.8067488	0.6080806	-1.3267	0.186	-1.041692813	count	1
NAPRT	-0.7925859	0.5796294	-1.3674	0.172	-1.041109052	count	1
CNP	-0.8461008	0.7283	-1.1617	0.246	-1.040698959	count	1
BPHL	-0.8113763	0.732606	-1.1075	0.269	-1.040211436	count	1
PMS1	-0.7991572	0.5129994	-1.5578	0.12	-1.038583853	count	1
RAD54L2	-1.243507	0.8800561	-1.413	0.159	-1.038173222	count	1
FLII	-0.8434623	0.6770495	-1.2458	0.214	-1.037614346	count	1
JAKMIP1	-0.8724953	0.67696	-1.2888	0.198	-1.037504922	count	1
ZNF282	-1.2411454	1.2867413	-0.9646	0.335	-1.036613002	count	1
CCDC171	-1.2411454	1.6043919	-0.7736	0.44	-1.036613002	count	1
GATD1	-1.6104518	1.290376	-1.248	0.213	-1.035956632	count	1
ADPRM	-1.6104518	1.3178754	-1.222	0.223	-1.035956632	count	1
ZNF74	-1.6104518	1.3178754	-1.222	0.223	-1.035956632	count	1
DNTTIP1	-0.891066	0.7205427	-1.2367	0.217	-1.035547942	count	1
ZDHHC13	-1.2393147	1.0527827	-1.1772	0.24	-1.035402442	count	1
XRRA1	-1.2393146	1.1775956	-1.0524	0.293	-1.03540238	count	1
AC020928.1	-1.6031865	1.145417	-1.3997	0.1626	-1.03290447	count	1
KLHDC4	-0.7823442	0.4551731	-1.7188	0.0866	-1.032615327	count	1
R3HCC1	-0.7943289	0.610428	-1.3013	0.194	-1.032505071	count	1
PCK2	-1.600732	0.8258504	-1.9383	0.05346	-1.031869982	count	1
LEF1	-0.8104449	0.7932993	-1.0216	0.308	-1.030909158	count	1
MKLN1	-0.8502526	0.6669848	-1.2748	0.203	-1.030459336	count	1
AC009133.1	-0.8655498	0.9944192	-0.8704	0.385	-1.029745669	count	1
CBLB	-0.8357773	0.4335523	-1.9277	0.0548	-1.028622563	count	1
NBPF14	-0.9094991	0.8152868	-1.1156	0.265	-1.027087268	count	1
PPCDC	-0.8629102	0.676743	-1.2751	0.203	-1.026794101	count	1
NAF1	-0.8145845	0.5220043	-1.5605	0.12	-1.026674113	count	1
CCDC152	-0.7944461	0.6661345	-1.1926	0.234	-1.026332039	count	1
SP140	-0.7738345	0.5067892	-1.5269	0.128	-1.025805226	count	1
SLC25A42	-0.9955613	0.5861587	-1.6985	0.0904	-1.025761666	count	1
OSBPL9	-0.8044254	0.4289182	-1.8755	0.0616	-1.023540536	count	1

TMLHE	-1.0741208	1.1414793	-0.941	0.347	-1.023498811	count	1
TIMP2	-0.9058898	1.0145697	-0.8929	0.373	-1.023330191	count	1
TXNDC11	-0.7558589	0.6485253	-1.1655	0.245	-1.023117938	count	1
MKRN1	-0.7570567	0.3748771	-2.0195	0.0443	-1.022376071	count	1
HSPA4	-0.7737584	0.4857466	-1.5929	0.112	-1.021577594	count	1
PPFIBP1	-1.0711526	0.8451032	-1.2675	0.206	-1.021080329	count	1
CMTR2	-0.9036102	0.6682091	-1.3523	0.177	-1.020955601	count	1
TSPAN32	-0.7587381	0.4397928	-1.7252	0.0854	-1.019402242	count	1
QDPR	-0.7747158	0.5631269	-1.3757	0.17	-1.018278057	count	1
PDXK	-0.7490091	0.4584115	-1.6339	0.103	-1.018186434	count	1
DESI2	-0.815945	0.5059699	-1.6126	0.108	-1.017665353	count	1
GLYR1	-0.8542768	0.696583	-1.2264	0.221	-1.017130047	count	1
LRR1	-0.9347279	0.7599183	-1.23	0.22	-1.016666849	count	1
ACOT8	-0.9342163	0.5780067	-1.6163	0.107	-1.016161192	count	1
PRMT7	-0.8057037	0.7352382	-1.0958	0.274	-1.015931133	count	1
TMEM159	-0.7602993	0.6572524	-1.1568	0.248	-1.015405219	count	1
WDR54	-0.7763195	0.5231551	-1.4839	0.139	-1.015330304	count	1
MRPL19	-0.7478331	0.4908557	-1.5235	0.129	-1.014600576	count	1
BTN3A1	-0.8233437	0.4096691	-2.0098	0.0453	-1.014051504	count	1
CALU	-0.982297	0.5692135	-1.7257	0.0854	-1.013620109	count	1
CNST	-0.7954019	0.7192309	-1.1059	0.27	-1.012484133	count	1
TP53BP2	-0.8687857	0.5381087	-1.6145	0.107	-1.011387209	count	1
AFG3L2	-0.9282942	0.6561244	-1.4148	0.158	-1.010302686	count	1
C12orf10	-0.7584587	0.6084477	-1.2465	0.213	-1.009606577	count	1
TSPAN4	-0.7584587	0.8868448	-0.8552	0.3931	-1.009606577	count	1
HEXDC	-0.8195499	0.5671289	-1.4451	0.149	-1.009599838	count	1
PIK3CA	-0.9778221	1.0178233	-0.9607	0.337	-1.009512385	count	1
ANAPC13	-0.8668525	0.5700485	-1.5207	0.129	-1.009285538	count	1
CCT3	-0.7159248	0.273207	-2.6204	0.0092	-1.009040486	count	1
TM2D1	-0.7553674	0.411952	-1.8336	0.0676	-1.00896358	count	1
STK11	-0.8069624	0.8339884	-0.9676	0.334	-1.006944409	count	1
RABL6	-0.7588968	0.513153	-1.4789	0.14	-1.006482503	count	1
LSM1	-0.7188082	0.3413801	-2.1056	0.036	-1.004947299	count	1
TNFRSF14-AS1	-0.9221341	0.6960216	-1.3249	0.186	-1.004198897	count	1
ARMC8	-0.795891	0.5383434	-1.4784	0.14	-1.004045902	count	1
PIGP	-0.7762776	0.5095757	-1.5234	0.129	-1.003608771	count	1
DYNC1LI1	-0.7223361	0.3744269	-1.9292	0.0546	-1.003236021	count	1
CCNG2	-0.9704156	0.5947436	-1.6317	0.104	-1.002701206	count	1
ARFIP1	-0.7526356	0.4960764	-1.5172	0.13	-1.002031971	count	1
AL359504.2	-0.8850382	0.9354143	-0.9461	0.345	-1.001562222	count	1
GPRC5D-AS1	-0.8850382	0.9354143	-0.9461	0.345	-1.001562222	count	1
CKMT2-AS1	-0.8850382	0.9999142	-0.8851	0.377	-1.001562222	count	1
WDR53	-0.8850382	1.1723114	-0.755	0.451	-1.001562222	count	1
ACVR1	-0.8850382	1.540436	-0.5745	0.566	-1.001562222	count	1
CDC42EP1	-0.8850382	1.540436	-0.5745	0.566	-1.001562222	count	1

HDAC6	-1.0446102	1.2005549	-0.8701	0.385	-0.999332816	count	1
C1orf162	-0.716084	0.3469131	-2.0642	0.0398	-0.998845613	count	1
RPRD1A	-0.9652617	0.7827612	-1.2331	0.218	-0.99795232	count	1
PTBP2	-0.8370065	1.1465183	-0.73	0.466	-0.997750869	count	1
ABHD12	-0.9639501	0.8696984	-1.1084	0.269	-0.996742564	count	1
GK5	-0.9639501	0.9516911	-1.0129	0.312	-0.996742564	count	1
PRPS2	-0.8800258	0.6904201	-1.2746	0.203	-0.996313718	count	1
CDK10	-0.807574	0.5950807	-1.3571	0.176	-0.995529999	count	1
ILF2	-0.7034153	0.2516676	-2.795	0.0055	-0.995242268	count	1
FBXW7	-0.7435341	0.4638536	-1.6029	0.11	-0.993497809	count	1
ACOT9	-0.8491546	0.7134252	-1.1903	0.235	-0.990006058	count	1
CDK16	-0.9073101	0.9558876	-0.9492	0.343	-0.989469385	count	1
IDUA	-0.9073101	1.0973257	-0.8268	0.409	-0.989469385	count	1
B3GNT5	-0.9073101	1.1828398	-0.7671	0.444	-0.989469385	count	1
SPATS2	-1.502476	0.8103679	-1.8541	0.0646	-0.98908529	count	1
CLASRP	-0.8280468	0.6681806	-1.2393	0.216	-0.987672558	count	1
LGALS8	-0.7821784	0.522064	-1.4982	0.135	-0.987411058	count	1
ID1	-1.028552	0.636808	-1.6152	0.107	-0.986070487	count	1
MT2A	-0.6978777	0.2451229	-2.8471	0.0047	-0.985138073	count	1
FUCA2	-0.7551289	0.5467575	-1.3811	0.168	-0.983038396	count	1
IKBKB	-1.4879427	1.224483	-1.2152	0.2252	-0.982526879	count	1
SWAP70	-1.0241381	0.9153213	-1.1189	0.264	-0.982411402	count	1
TACC1	-0.7118165	0.3849393	-1.8492	0.0653	-0.982399602	count	1
IGLC3	-0.714273	0.3827941	-1.8659	0.063	-0.982298541	count	1
TCEAL8	-0.7234457	0.4151577	-1.7426	0.0824	-0.982068358	count	1
OGFOD1	-0.9479696	1.0812064	-0.8768	0.381	-0.98196383	count	1
SAE1	-0.7773705	0.5075226	-1.5317	0.127	-0.981571436	count	1
SARS2	-1.1577285	1.3289648	-0.8712	0.3843	-0.980368524	count	1
MINPP1	-1.1577283	1.0196022	-1.1355	0.257	-0.980368396	count	1
PLAUR	-0.8647612	1.1450993	-0.7552	0.451	-0.980292673	count	1
NCOA6	-1.481836	1.0263676	-1.4438	0.15	-0.979752972	count	1
SYNE1	-0.7245555	0.4602495	-1.5743	0.116	-0.97927492	count	1
ZNF714	-1.4807516	1.3009089	-1.1382	0.256	-0.979259536	count	1
ACAT2	-0.7234246	0.5294359	-1.3664	0.173	-0.977773516	count	1
MIGA1	-0.8368965	0.5412204	-1.5463	0.123	-0.976611485	count	1
MFN2	-1.1518756	1.3272722	-0.8679	0.386	-0.9763392	count	1
ACTN1	-1.1518756	1.3272722	-0.8679	0.386	-0.9763392	count	1
MAST2	-1.1518756	1.4439566	-0.7977	0.426	-0.9763392	count	1
TMEM147-AS1	-1.1518756	1.4439566	-0.7977	0.426	-0.9763392	count	1
METTL6	-1.1517449	1.0011688	-1.1504	0.251	-0.976249096	count	1
MMRN1	-0.8030745	0.8230053	-0.9758	0.33	-0.976153802	count	1
DPP8	-0.7542181	0.6804702	-1.1084	0.269	-0.975958117	count	1
FOXN3-AS1	-0.8024372	0.9306007	-0.8623	0.389	-0.975417279	count	1
EXOSC6	-0.7050139	0.4172094	-1.6898	0.092	-0.975127457	count	1
STK32C	-0.8021455	0.6395647	-1.2542	0.211	-0.975080137	count	1

CDK11A	-0.8595937	0.5557796	-1.5466	0.123	-0.974856399	count	1
R3HDM1	-0.7372965	0.5089977	-1.4485	0.148	-0.974608412	count	1
ELK1	-0.8350259	0.9050683	-0.9226	0.357	-0.974564519	count	1
LIN7C	-0.8350259	0.9050683	-0.9226	0.357	-0.974564519	count	1
GOLPH3L	-0.8350259	0.972214	-0.8589	0.391	-0.974564519	count	1
AARS	-0.8350259	1.3594119	-0.6143	0.539	-0.974564519	count	1
TRIM26	-0.937761	0.8002265	-1.1719	0.242	-0.97248507	count	1
ABCC5	-0.8143913	1.0631604	-0.766	0.444	-0.972280277	count	1
NF2	-0.8143913	1.0814931	-0.753	0.452	-0.972280277	count	1
URB2	-17.9460694	2338.232952	-0.0077	0.994	-0.971985601	count	1
FBLN7	-17.9460694	2338.232952	-0.0077	0.994	-0.971985601	count	1
C3orf33	-17.9460694	2338.232952	-0.0077	0.994	-0.971985601	count	1
LINC00504	-17.9460694	2338.232952	-0.0077	0.994	-0.971985601	count	1
ARL17A	-17.9460694	2338.232952	-0.0077	0.994	-0.971985601	count	1
SMCR5	-17.7702582	1599.589687	-0.0111	0.991	-0.971985597	count	1
ASH2L	-19.062968	2542.540249	-0.0075	0.994	-0.971985545	count	1
C12orf66	-18.834651	2488.842238	-0.0076	0.994	-0.971985543	count	1
ST7	-18.821666	2887.532543	-0.0065	0.995	-0.971985543	count	1
PGLYRP2	-18.821666	2887.532543	-0.0065	0.995	-0.971985543	count	1
DYRK3	-18.776763	3608.969544	-0.0052	0.996	-0.971985543	count	1
KLHL2	-18.776763	3608.969544	-0.0052	0.996	-0.971985543	count	1
CDCA7L	-18.776763	3608.969544	-0.0052	0.996	-0.971985543	count	1
CD4	-18.776763	3608.969544	-0.0052	0.996	-0.971985543	count	1
CLPB	-18.502376	2297.736204	-0.0081	0.994	-0.97198554	count	1
AREL1	-18.500018	1683.31026	-0.011	0.991	-0.97198554	count	1
AL359915.2	-18.498626	2646.033991	-0.007	0.994	-0.97198554	count	1
ZNF514	-18.498626	2646.033991	-0.007	0.994	-0.97198554	count	1
AC016596.1	-18.498626	2646.033991	-0.007	0.994	-0.97198554	count	1
GPR150	-18.498626	2646.033991	-0.007	0.994	-0.97198554	count	1
ALDH5A1	-18.498626	2646.033991	-0.007	0.994	-0.97198554	count	1
HIST1H2BK	-18.498626	2646.033991	-0.007	0.994	-0.97198554	count	1
OSBPL5	-18.498626	2646.033991	-0.007	0.994	-0.97198554	count	1
TIGD3	-18.498626	2646.033991	-0.007	0.994	-0.97198554	count	1
ERBB3	-18.498626	2646.033991	-0.007	0.994	-0.97198554	count	1
LATS2	-18.498626	2646.033991	-0.007	0.994	-0.97198554	count	1
L3HYPDH	-18.498626	2646.033991	-0.007	0.994	-0.97198554	count	1
CAPN15	-18.498626	2646.033991	-0.007	0.994	-0.97198554	count	1
RBM10	-18.430452	1909.193345	-0.0097	0.992	-0.971985539	count	1
HOXA7	-18.316107	3348.681508	-0.0055	0.996	-0.971985537	count	1
HRASLS2	-18.316107	3348.681508	-0.0055	0.996	-0.971985537	count	1
LIN52	-18.316107	3348.681508	-0.0055	0.996	-0.971985537	count	1
FBXL22	-18.316107	3348.681508	-0.0055	0.996	-0.971985537	count	1
PLTP	-18.235801	1700.211079	-0.0107	0.991	-0.971985536	count	1
CYP4X1	-18.173803	4756.440964	-0.0038	0.997	-0.971985535	count	1
AC105277.1	-18.173803	4756.440964	-0.0038	0.997	-0.971985535	count	1

RAVER2	-18.173803	4756.440964	-0.0038	0.997	-0.971985535	count	1
AK4	-18.173803	4756.440964	-0.0038	0.997	-0.971985535	count	1
ST6GALNAC3	-18.173803	4756.440964	-0.0038	0.997	-0.971985535	count	1
ADAM15	-18.173803	4756.440964	-0.0038	0.997	-0.971985535	count	1
FAM189B	-18.173803	4756.440964	-0.0038	0.997	-0.971985535	count	1
MSTO1	-18.173803	4756.440964	-0.0038	0.997	-0.971985535	count	1
KLHDC9	-18.173803	4756.440964	-0.0038	0.997	-0.971985535	count	1
AC017083.1	-18.173803	4756.440964	-0.0038	0.997	-0.971985535	count	1
AC009961.1	-18.173803	4756.440964	-0.0038	0.997	-0.971985535	count	1
ZNF142	-18.173803	4756.440964	-0.0038	0.997	-0.971985535	count	1
PDCD1	-18.173803	4756.440964	-0.0038	0.997	-0.971985535	count	1
DNAH1	-18.173803	4756.440964	-0.0038	0.997	-0.971985535	count	1
FAM53A	-18.173803	4756.440964	-0.0038	0.997	-0.971985535	count	1
AC019131.2	-18.173803	4756.440964	-0.0038	0.997	-0.971985535	count	1
STOX2	-18.173803	4756.440964	-0.0038	0.997	-0.971985535	count	1
LINC02363	-18.173803	4756.440964	-0.0038	0.997	-0.971985535	count	1
TPPP	-18.173803	4756.440964	-0.0038	0.997	-0.971985535	count	1
C1QTNF3	-18.173803	4756.440964	-0.0038	0.997	-0.971985535	count	1
CCNB1	-18.173803	4756.440964	-0.0038	0.997	-0.971985535	count	1
ZNF300	-18.173803	4756.440964	-0.0038	0.997	-0.971985535	count	1
ZNF311	-18.173803	4756.440964	-0.0038	0.997	-0.971985535	count	1
CCHCR1	-18.173803	4756.440964	-0.0038	0.997	-0.971985535	count	1
AL662796.1	-18.173803	4756.440964	-0.0038	0.997	-0.971985535	count	1
AARS2	-18.173803	4756.440964	-0.0038	0.997	-0.971985535	count	1
CENPQ	-18.173803	4756.440964	-0.0038	0.997	-0.971985535	count	1
SMPDL3A	-18.173803	4756.440964	-0.0038	0.997	-0.971985535	count	1
RADIL	-18.173803	4756.440964	-0.0038	0.997	-0.971985535	count	1
AC004895.1	-18.173803	4756.440964	-0.0038	0.997	-0.971985535	count	1
AC007029.1	-18.173803	4756.440964	-0.0038	0.997	-0.971985535	count	1
JAZF1-AS1	-18.173803	4756.440964	-0.0038	0.997	-0.971985535	count	1
AC004921.1	-18.173803	4756.440964	-0.0038	0.997	-0.971985535	count	1
ZBED6CL	-18.173803	4756.440964	-0.0038	0.997	-0.971985535	count	1
POLA1	-18.173803	4756.440964	-0.0038	0.997	-0.971985535	count	1
KCNE5	-18.173803	4756.440964	-0.0038	0.997	-0.971985535	count	1
TMEM164	-18.173803	4756.440964	-0.0038	0.997	-0.971985535	count	1
PRRG3	-18.173803	4756.440964	-0.0038	0.997	-0.971985535	count	1
MSC	-18.173803	4756.440964	-0.0038	0.997	-0.971985535	count	1
DSCC1	-18.173803	4756.440964	-0.0038	0.997	-0.971985535	count	1
AC040970.1	-18.173803	4756.440964	-0.0038	0.997	-0.971985535	count	1
ZBTB26	-18.173803	4756.440964	-0.0038	0.997	-0.971985535	count	1
CDC42EP2	-18.173803	4756.440964	-0.0038	0.997	-0.971985535	count	1
ACY3	-18.173803	4756.440964	-0.0038	0.997	-0.971985535	count	1
KCTD21	-18.173803	4756.440964	-0.0038	0.997	-0.971985535	count	1
FUT4	-18.173803	4756.440964	-0.0038	0.997	-0.971985535	count	1
MCAM	-18.173803	4756.440964	-0.0038	0.997	-0.971985535	count	1

IGSF9B	-18.173803	4756.440964	-0.0038	0.997	-0.971985535	count	1
AL392046.1	-18.173803	4756.440964	-0.0038	0.997	-0.971985535	count	1
ANKRD22	-18.173803	4756.440964	-0.0038	0.997	-0.971985535	count	1
AL157400.4	-18.173803	4756.440964	-0.0038	0.997	-0.971985535	count	1
CRTAC1	-18.173803	4756.440964	-0.0038	0.997	-0.971985535	count	1
AL353719.1	-18.173803	4756.440964	-0.0038	0.997	-0.971985535	count	1
AC008035.1	-18.173803	4756.440964	-0.0038	0.997	-0.971985535	count	1
TARBP2	-18.173803	4756.440964	-0.0038	0.997	-0.971985535	count	1
ZNF385A	-18.173803	4756.440964	-0.0038	0.997	-0.971985535	count	1
AC020656.2	-18.173803	4756.440964	-0.0038	0.997	-0.971985535	count	1
TMPO-AS1	-18.173803	4756.440964	-0.0038	0.997	-0.971985535	count	1
ALDH1L2	-18.173803	4756.440964	-0.0038	0.997	-0.971985535	count	1
HCAR2	-18.173803	4756.440964	-0.0038	0.997	-0.971985535	count	1
AC137767.1	-18.173803	4756.440964	-0.0038	0.997	-0.971985535	count	1
AC127070.2	-18.173803	4756.440964	-0.0038	0.997	-0.971985535	count	1
LINC02315	-18.173803	4756.440964	-0.0038	0.997	-0.971985535	count	1
TMEM260	-18.173803	4756.440964	-0.0038	0.997	-0.971985535	count	1
AC087386.1	-18.173803	4756.440964	-0.0038	0.997	-0.971985535	count	1
INAFM2	-18.173803	4756.440964	-0.0038	0.997	-0.971985535	count	1
PPIP5K1	-18.173803	4756.440964	-0.0038	0.997	-0.971985535	count	1
ALDH1A2	-18.173803	4756.440964	-0.0038	0.997	-0.971985535	count	1
NRG4	-18.173803	4756.440964	-0.0038	0.997	-0.971985535	count	1
SAXO2	-18.173803	4756.440964	-0.0038	0.997	-0.971985535	count	1
C17orf107	-18.173803	4756.440964	-0.0038	0.997	-0.971985535	count	1
FAM215A	-18.173803	4756.440964	-0.0038	0.997	-0.971985535	count	1
AC110285.6	-18.173803	4756.440964	-0.0038	0.997	-0.971985535	count	1
C18orf54	-18.173803	4756.440964	-0.0038	0.997	-0.971985535	count	1
AC036176.1	-18.173803	4756.440964	-0.0038	0.997	-0.971985535	count	1
MCM8	-18.173803	4756.440964	-0.0038	0.997	-0.971985535	count	1
SOGA1	-18.173803	4756.440964	-0.0038	0.997	-0.971985535	count	1
TSHZ2	-18.173803	4756.440964	-0.0038	0.997	-0.971985535	count	1
MIR646HG	-18.173803	4756.440964	-0.0038	0.997	-0.971985535	count	1
MIR1-1HG-AS1	-18.173803	4756.440964	-0.0038	0.997	-0.971985535	count	1
C20orf204	-18.173803	4756.440964	-0.0038	0.997	-0.971985535	count	1
STAP2	-18.173803	4756.440964	-0.0038	0.997	-0.971985535	count	1
PSPN	-18.173803	4756.440964	-0.0038	0.997	-0.971985535	count	1
MCOLN1	-18.173803	4756.440964	-0.0038	0.997	-0.971985535	count	1
NANOS3	-18.173803	4756.440964	-0.0038	0.997	-0.971985535	count	1
ASF1B	-18.173803	4756.440964	-0.0038	0.997	-0.971985535	count	1
ZNF30	-18.173803	4756.440964	-0.0038	0.997	-0.971985535	count	1
UPK1A	-18.173803	4756.440964	-0.0038	0.997	-0.971985535	count	1
PPP1R13L	-18.173803	4756.440964	-0.0038	0.997	-0.971985535	count	1
SPACA6	-18.173803	4756.440964	-0.0038	0.997	-0.971985535	count	1
ZNF530	-18.173803	4756.440964	-0.0038	0.997	-0.971985535	count	1
AC012313.6	-18.173803	4756.440964	-0.0038	0.997	-0.971985535	count	1

LINC01311	-18.173803	4756.440964	-0.0038	0.997	-0.971985535	count	1
PDXP	-18.173803	4756.440964	-0.0038	0.997	-0.971985535	count	1
DMC1	-18.173803	4756.440964	-0.0038	0.997	-0.971985535	count	1
AP000251.1	-18.173803	4756.440964	-0.0038	0.997	-0.971985535	count	1
N6AMT1	-18.037854	2290.605303	-0.0079	0.994	-0.971985532	count	1
AL359220.1	-17.760971	1563.245566	-0.0114	0.991	-0.971985526	count	1
CHEK1	-17.756888	1763.471006	-0.0101	0.992	-0.971985526	count	1
CACNA2D4	-17.756888	1763.471006	-0.0101	0.992	-0.971985526	count	1
UBR5	-0.7339228	0.5208688	-1.409	0.16	-0.970254904	count	1
USP25	-0.8879231	0.6897206	-1.2874	0.199	-0.97011923	count	1
TTC1	-0.692761	0.3199286	-2.1654	0.0311	-0.969838471	count	1
SF3A3	-0.7152302	0.45649	-1.5668	0.118	-0.969065022	count	1
MNAT1	-0.710528	0.3533375	-2.0109	0.0452	-0.968481743	count	1
IFI6	-0.7000515	0.4575943	-1.5299	0.127	-0.968340306	count	1
SURF1	-0.7018135	0.3935026	-1.7835	0.0754	-0.966562226	count	1
SVIP	-0.6916708	0.2978282	-2.3224	0.0208	-0.96509342	count	1
COIL	-0.7087998	0.515195	-1.3758	0.17	-0.962507642	count	1
PTRH2	-0.8476659	1.0768478	-0.7872	0.432	-0.962283867	count	1
PLSCR1	-0.6887911	0.4347314	-1.5844	0.1141	-0.962257016	count	1
TM9SF4	-0.7788943	0.7756248	-1.0042	0.316	-0.961730483	count	1
EFCAB14	-0.7146142	0.4954773	-1.4423	0.15	-0.961245155	count	1
JMJD1C	-0.6824316	0.2953025	-2.311	0.0215	-0.960922711	count	1
BAG2	-0.8043055	0.4371147	-1.84	0.0667	-0.960887198	count	1
BORCS6	-0.7333214	0.5873105	-1.2486	0.213	-0.960585788	count	1
KIAA1586	-0.713942	0.5853037	-1.2198	0.223	-0.960357766	count	1
FKBP5	-0.8219701	0.4041296	-2.0339	0.0428	-0.960256344	count	1
DLEU2	-0.9949668	0.7400045	-1.3445	0.18	-0.958079447	count	1
PAXIP1-AS1	-0.7350081	0.8693467	-0.8455	0.398	-0.957570486	count	1
SLC25A36	-0.6889497	0.3283942	-2.0979	0.0367	-0.956560053	count	1
TTC9C	-0.9925734	0.8458116	-1.1735	0.241	-0.956071678	count	1
TMEM161B	-0.799884	1.0073727	-0.794	0.428	-0.955886076	count	1
PLEC	-0.7369749	0.9252289	-0.7965	0.426	-0.954298843	count	1
CTBS	-0.6944428	0.4825087	-1.4392	0.151	-0.954139584	count	1
DFFA	-0.7625832	0.5116399	-1.4905	0.137	-0.953775479	count	1
HSPA9	-0.6771624	0.2718063	-2.4913	0.0132	-0.952907313	count	1
FHOD1	-1.4191243	1.0969403	-1.2937	0.197	-0.950655174	count	1
ZNF684	-1.4191243	1.2798928	-1.1088	0.268	-0.950655174	count	1
ZBED3	-1.4191243	1.2913349	-1.099	0.273	-0.950655174	count	1
NOXA1	-1.4191243	1.2913349	-1.099	0.273	-0.950655174	count	1
NME6	-0.7691685	0.587714	-1.3087	0.192	-0.950235232	count	1
SMARCC1	-0.7125011	0.394108	-1.8079	0.0716	-0.949729368	count	1
ACOX1	-0.8673766	0.8775255	-0.9884	0.324	-0.949505694	count	1
TNFRSF12A	-0.7167453	0.3869915	-1.8521	0.0649	-0.948069006	count	1
AHCY	-0.982585	0.9625152	-1.0209	0.308	-0.947674109	count	1
PER3	-0.982585	1.0778568	-0.9116	0.363	-0.947674109	count	1

LINC01126	-0.982585	1.2079461	-0.8134	0.417	-0.947674109	count	1
GDPD1	-0.982585	1.3017195	-0.7548	0.451	-0.947674109	count	1
PDK3	-0.9108349	0.8388471	-1.0858	0.278	-0.947343919	count	1
DSTYK	-1.4085244	1.0501124	-1.3413	0.181	-0.945625586	count	1
SSH1	-0.754747	0.7414452	-1.0179	0.309	-0.944353085	count	1
SATB1	-0.7050636	0.4369403	-1.6136	0.108	-0.943121248	count	1
EPB41L2	-0.8279525	0.8569032	-0.9662	0.335	-0.941431097	count	1
SRPK2	-0.675193	0.3395994	-1.9882	0.0476	-0.938390877	count	1
PSMA2	-0.6857914	0.3845563	-1.7833	0.0755	-0.938357757	count	1
TEC	-0.7829398	0.8098933	-0.9667	0.334	-0.936684149	count	1
TRAF4	-0.7473952	0.5856566	-1.2762	0.203	-0.935503996	count	1
TDP1	-0.7473616	0.5736813	-1.3027	0.194	-0.935463532	count	1
FOXK2	-1.0929722	1.0014519	-1.0914	0.2759	-0.935187252	count	1
PSMC1	-0.6770488	0.3170363	-2.1356	0.0335	-0.934953414	count	1
TRAK2	-0.8970899	0.8549217	-1.0493	0.295	-0.934432367	count	1
NR2C2AP	-0.9667786	0.927207	-1.0427	0.298	-0.934324103	count	1
ZNF786	-0.7666178	1.086104	-0.7058	0.481	-0.933897036	count	1
RDX	-0.780294	0.5678341	-1.3742	0.17	-0.933680607	count	1
NIFK	-0.6707035	0.3287034	-2.0405	0.0421	-0.93360238	count	1
REC8	-0.9656743	0.9929244	-0.9726	0.331	-0.933388631	count	1
CYB561	-0.9656743	1.1989848	-0.8054	0.421	-0.933388631	count	1
ITGAX	-1.382707	0.757237	-1.826	0.0688	-0.933239996	count	1
OSBPL2	-0.7374796	0.5351063	-1.3782	0.169	-0.932980343	count	1
MCM6	-1.377856	0.8724802	-1.5792	0.115	-0.930891435	count	1
TIMM23	-1.377856	0.8724802	-1.5792	0.115	-0.930891435	count	1
DDT	-0.6575617	0.2455141	-2.6783	0.0078	-0.930678652	count	1
PIK3AP1	-0.6993448	0.5370141	-1.3023	0.194	-0.929208085	count	1
IFT22	-0.7512285	0.6884033	-1.0913	0.276	-0.928987907	count	1
AL117339.5	-1.083314	1.0252442	-1.0566	0.291	-0.92833569	count	1
TRAF3IP1	-0.7413875	0.7629795	-0.9717	0.332	-0.928266209	count	1
ANKIB1	-0.7410203	0.4827214	-1.5351	0.126	-0.92782363	count	1
CDK4	-0.6980056	0.3657267	-1.9085	0.0572	-0.927466038	count	1
UBAP2	-0.8147679	0.9201287	-0.8855	0.377	-0.927433686	count	1
SMIM7	-0.6578181	0.3437856	-1.9135	0.0566	-0.926769795	count	1
PHF13	-1.368324	0.9913999	-1.3802	0.168	-0.926256351	count	1
EPHA1	-0.7252049	0.8136523	-0.8913	0.373	-0.926053251	count	1
EIPR1	-1.0798043	0.8849414	-1.2202	0.223	-0.925838829	count	1
CINP	-0.6992647	0.53613	-1.3043	0.193	-0.925458445	count	1
PDCD11	-0.8104449	1.1457154	-0.7074	0.48	-0.9228354	count	1
SLC10A3	-0.7872713	0.8175681	-0.9629	0.336	-0.922048853	count	1
CDC23	-0.7872713	0.9267683	-0.8495	0.396	-0.922048853	count	1
RHNO1	-0.7453175	0.6214085	-1.1994	0.231	-0.92197496	count	1
EI24	-0.6995131	0.5140269	-1.3608	0.175	-0.921783321	count	1
HPGD	-1.073919	0.6566855	-1.6354	0.103	-0.921643167	count	1
NPTN	-0.7215608	0.5905245	-1.2219	0.223	-0.921546644	count	1

IQSEC1	-1.0735307	1.2052085	-0.8907	0.374	-0.921365922	count	1
NOC4L	-0.7062212	0.6686385	-1.0562	0.292	-0.921044799	count	1
COX20	-0.6600871	0.3253078	-2.0291	0.0433	-0.919597614	count	1
PGGHG	-0.8374641	0.7318017	-1.1444	0.253	-0.919303265	count	1
CHCHD4	-0.8809518	1.0921406	-0.8066	0.42	-0.919206501	count	1
MED12	-0.8809518	1.1103439	-0.7934	0.428	-0.919206501	count	1
LHX4	-0.8809518	1.2043	-0.7315	0.465	-0.919206501	count	1
WDR44	-0.8801663	0.6750523	-1.3038	0.193	-0.918463596	count	1
PPP1R13B	-0.8801663	0.7822526	-1.1252	0.261	-0.918463596	count	1
SUMF2	-0.6788123	0.4017468	-1.6897	0.0921	-0.918458388	count	1
PCYT2	-0.7836132	1.0112214	-0.7749	0.439	-0.918005789	count	1
NOP14	-0.7080701	0.6378669	-1.1101	0.268	-0.917903463	count	1
MUM1	-0.7411074	0.5505627	-1.3461	0.179	-0.916976348	count	1
POLR3K	-0.6637524	0.3876841	-1.7121	0.0878	-0.916789382	count	1
AHI1	-0.6846442	0.4889618	-1.4002	0.162	-0.916323693	count	1
DEF8	-0.723243	0.8363448	-0.8648	0.3878	-0.915579291	count	1
ZBED4	-0.943855	0.7950545	-1.1872	0.236	-0.914830932	count	1
USP37	-0.7806766	0.6774753	-1.1523	0.25	-0.914758093	count	1
ATG14	-0.7046018	0.609476	-1.1561	0.249	-0.913529089	count	1
LPCAT4	-1.3410737	1.2189883	-1.1002	0.272	-0.912860608	count	1
TTC33	-0.7767124	0.840188	-0.9245	0.356	-0.910371046	count	1
SRFBP1	-0.6641557	0.4922934	-1.3491	0.178	-0.909136761	count	1
MIS12	-0.7112969	0.584165	-1.2176	0.224	-0.908843229	count	1
PDSS2	-0.7972732	0.6532477	-1.2205	0.223	-0.908798387	count	1
DDX39B	-0.744819	0.4111711	-1.8115	0.071	-0.908511982	count	1
ZCCHC17	-0.6498177	0.3906904	-1.6633	0.0972	-0.907165974	count	1
UBXN8	-1.0519169	1.073992	-0.9794	0.328	-0.905862002	count	1
FBXO2	-0.6828449	0.5368618	-1.2719	0.204	-0.904190024	count	1
PTGER2	-0.6587727	0.3733591	-1.7644	0.0786	-0.901861515	count	1
KRI1	-0.7686218	0.7238162	-1.0619	0.289	-0.901407162	count	1
TLE3	-0.8618684	0.7117825	-1.2109	0.227	-0.901110194	count	1
COL23A1	-1.0452544	0.8770177	-1.1918	0.234	-0.901053631	count	1
CD3G	-0.7504996	0.6232227	-1.2042	0.229	-0.899762304	count	1
ERO1B	-0.6893732	0.4386132	-1.5717	0.117	-0.899620197	count	1
PIGV	-0.9260269	0.9933707	-0.9322	0.352	-0.899563569	count	1
CAPRIN2	-0.6803626	0.6635266	-1.0254	0.306	-0.897107594	count	1
BRI3BP	-0.6946662	0.8418155	-0.8252	0.41	-0.894987331	count	1
C2orf76	-0.8536818	0.7622074	-1.12	0.264	-0.893316858	count	1
FUZ	-0.7448048	0.8571094	-0.869	0.386	-0.893259519	count	1
FBXO42	-1.3011332	0.9775119	-1.3311	0.1841	-0.892836289	count	1
GHDC	-0.6922202	0.7108835	-0.9737	0.331	-0.891923628	count	1
CSTF1	-0.7802936	0.7957513	-0.9806	0.328	-0.890644816	count	1
EMG1	-0.6964108	0.5245469	-1.3276	0.185	-0.890392497	count	1
TAF3	-0.7094374	0.4874967	-1.4553	0.147	-0.889676861	count	1
U2AF1L4	-0.7180221	0.5095826	-1.409	0.16	-0.889513766	count	1

ZNF226	-0.6471662	0.5692121	-1.137	0.256	-0.888747722	count	1
MRPS6	-0.6235242	0.2196812	-2.8383	0.0048	-0.888493829	count	1
VPS13D	-0.8069623	1.2647452	-0.638	0.524	-0.888275066	count	1
LINC01003	-0.7169291	0.6162004	-1.1635	0.245	-0.888211295	count	1
NUP93	-1.291799	1.0013868	-1.29	0.198	-0.88808933	count	1
PET117	-1.291799	1.0265543	-1.2584	0.209	-0.88808933	count	1
MPZL1	-1.291799	1.0511193	-1.229	0.22	-0.88808933	count	1
ERI3	-0.6724556	0.5297125	-1.2695	0.205	-0.886907506	count	1
BRD9	-0.6785216	0.4833933	-1.4037	0.161	-0.885802694	count	1
TKFC	-0.8455401	0.9406603	-0.8989	0.369	-0.885548399	count	1
FAN1	-0.7738345	1.1795981	-0.656	0.5123	-0.883722009	count	1
RPS6KB2	-0.6557282	0.4775375	-1.3731	0.171	-0.883354907	count	1
LIAS	-0.9063392	0.8362525	-1.0838	0.279	-0.882595614	count	1
UBXN2B	-0.9063392	0.8582105	-1.0561	0.292	-0.882595614	count	1
SENP1	-0.9063392	0.9284436	-0.9762	0.33	-0.882595614	count	1
JPT1	-0.6315545	0.2786646	-2.2664	0.0241	-0.882388538	count	1
CD3D	-0.6892869	0.8033716	-0.858	0.392	-0.88155172	count	1
CDK12	-0.6468361	0.3388269	-1.909	0.0571	-0.881247748	count	1
IKBKE	-1.0179335	0.9249467	-1.1005	0.272	-0.881192414	count	1
NRROS	-1.0179335	1.1607216	-0.877	0.3811	-0.881192414	count	1
NRIP1	-0.903831	0.6075533	-1.4877	0.138	-0.880425844	count	1
BASP1	-0.8388038	1.0359141	-0.8097	0.419	-0.879107523	count	1
ZNF213	-0.8388038	1.1208189	-0.7484	0.455	-0.879107523	count	1
ZNF117	-0.8388038	1.2320053	-0.6808	0.496	-0.879107523	count	1
NAGA	-0.8388038	1.2320053	-0.6808	0.496	-0.879107523	count	1
ARSA	-0.8388038	1.2320053	-0.6808	0.496	-0.879107523	count	1
RCOR3	-0.7005608	0.483409	-1.4492	0.148	-0.878927013	count	1
NCBP3	-0.6726968	0.3574641	-1.8819	0.0608	-0.878380092	count	1
ARID5B	-0.65781	0.5344589	-1.2308	0.219	-0.878195683	count	1
PTMS	-0.7314096	0.5805164	-1.2599	0.209	-0.877939125	count	1
DDX54	-0.7303379	0.5569701	-1.3113	0.191	-0.876711906	count	1
FBXO34	-0.6278301	0.3029013	-2.0727	0.039	-0.876702428	count	1
SHMT2	-0.6797629	0.5683063	-1.1961	0.233	-0.876308126	count	1
ANKRD10	-0.6672237	0.3587965	-1.8596	0.0638	-0.875998935	count	1
NSFL1C	-0.6437777	0.4823085	-1.3348	0.183	-0.875461207	count	1
MTMR6	-0.8977912	0.8528466	-1.0527	0.293	-0.875193425	count	1
AC008736.1	-0.8977912	1.2162501	-0.7382	0.461	-0.875193425	count	1
ZFP64	-1.2653061	1.3184107	-0.9597	0.338	-0.874477859	count	1
AL031316.1	-1.2653061	1.4280147	-0.8861	0.376	-0.874477859	count	1
CTSH	-1.2653061	1.4280147	-0.8861	0.376	-0.874477859	count	1
MAP2K7	-0.6768334	0.5931815	-1.141	0.255	-0.872632981	count	1
UBXN2A	-0.65474	0.3859114	-1.6966	0.0907	-0.871087187	count	1
EMC8	-0.7612642	0.7967023	-0.9555	0.34	-0.870222412	count	1
ZNF276	-0.6436174	0.5396239	-1.1927	0.234	-0.869499382	count	1
DNAJC8	-0.611901	0.2285367	-2.6775	0.0078	-0.869341085	count	1

SSH2	-0.6317532	0.3973934	-1.5897	0.113	-0.868972861	count	1
FAM129A	-0.6915981	0.5526701	-1.2514	0.212	-0.868060429	count	1
IFIH1	-0.6891285	0.6655872	-1.0354	0.301	-0.865064039	count	1
POLRMT	-1.246891	0.8084318	-1.5424	0.124	-0.864895362	count	1
SCLT1	-0.6543053	0.5024822	-1.3021	0.194	-0.863467839	count	1
CPQ	-0.6541268	0.5889014	-1.1108	0.267	-0.863237143	count	1
ARRDC2	-0.634652	0.4959268	-1.2797	0.202	-0.863218605	count	1
MRPL57	-0.6090817	0.2327337	-2.6171	0.0093	-0.861999561	count	1
ZNF638	-0.6247308	0.3636744	-1.7178	0.0868	-0.861521312	count	1
EIF4G3	-0.6450386	0.4419095	-1.4597	0.145	-0.861449468	count	1
GDAP2	-0.8811667	1.0598703	-0.8314	0.406	-0.860736945	count	1
AC004918.1	-0.8811667	1.1140363	-0.791	0.43	-0.860736945	count	1
ARFGEF2	-0.8811667	1.1368712	-0.7751	0.439	-0.860736945	count	1
ICA1	-0.8811667	1.2391637	-0.7111	0.478	-0.860736945	count	1
HOXB4	-0.7320456	0.8082524	-0.9057	0.366	-0.860712816	count	1
PLEKHA3	-0.637809	0.4107789	-1.5527	0.121	-0.859592692	count	1
TMEM191B	-0.8791748	1.1437183	-0.7687	0.443	-0.858999467	count	1
NNMT	-0.7767124	0.9035857	-0.8596	0.391	-0.857277531	count	1
PMM1	-0.6697304	0.5977781	-1.1204	0.263	-0.857245982	count	1
LRRC42	-0.7283315	0.8169471	-0.8915	0.373	-0.856565159	count	1
IFT74	-0.7283315	0.911141	-0.7994	0.425	-0.856565159	count	1
ANKRD26	-0.6639438	0.634587	-1.0463	0.296	-0.856449301	count	1
MYH9	-0.6086561	0.2912001	-2.0902	0.0374	-0.85613433	count	1
NDUFV3	-0.6217228	0.4661258	-1.3338	0.183	-0.855323319	count	1
CBWD5	-0.746612	0.709019	-1.053	0.293	-0.854442632	count	1
TMEM128	-0.8125926	0.8576383	-0.9475	0.344	-0.853931259	count	1
ZDHHC24	-0.6176636	0.4019368	-1.5367	0.125	-0.85374989	count	1
FAM228B	-0.7100749	0.5928519	-1.1977	0.232	-0.853467514	count	1
UCP2	-0.5988473	0.2247736	-2.6642	0.0081	-0.852800731	count	1
FAM215B	-0.9785837	0.7989606	-1.2248	0.222	-0.852183558	count	1
ZNF267	-0.6250896	0.4352748	-1.4361	0.152	-0.852000447	count	1
SLFN5	-0.6057119	0.3261735	-1.857	0.0642	-0.851698569	count	1
MGAT2	-0.6709585	0.554566	-1.2099	0.227	-0.851413839	count	1
SLC25A19	-0.7707736	0.8734718	-0.8824	0.378	-0.851165972	count	1
FCRL3	-0.7707736	1.0291847	-0.7489	0.454	-0.851165972	count	1
LENG1	-0.6275689	0.367847	-1.7061	0.089	-0.850124368	count	1
LEO1	-0.6469433	0.5409969	-1.1958	0.233	-0.84994515	count	1
PER1	-0.7691291	0.5401439	-1.4239	0.155	-0.849472153	count	1
PCNA	-0.663188	0.5630171	-1.1779	0.24	-0.849103112	count	1
POLR1C	-0.7058834	0.6442814	-1.0956	0.274	-0.848649645	count	1
TBL3	-0.9734428	1.3465819	-0.7229	0.47	-0.848358731	count	1
RNF185	-0.9734428	1.5702551	-0.6199	0.536	-0.848358731	count	1
HELLS	-0.7668314	0.7154129	-1.0719	0.285	-0.847104438	count	1
TARDBP	-0.8655216	0.570517	-1.5171	0.13	-0.847059748	count	1
LSM14A	-0.6167378	0.3430664	-1.7977	0.0732	-0.846234284	count	1

MPHOSPH6	-0.6662878	0.5799014	-1.149	0.251	-0.845662397	count	1
KIR3DL2	-0.6289096	0.5837205	-1.0774	0.282	-0.845474309	count	1
ATP7A	-0.7181967	0.6804836	-1.0554	0.292	-0.84523308	count	1
SMARCA2	-0.6132987	0.3145541	-1.9497	0.0521	-0.844910345	count	1
FDXACB1	-0.8030745	1.1497844	-0.6985	0.485	-0.844744224	count	1
ZNF180	-0.8030745	1.1531825	-0.6964	0.487	-0.844744224	count	1
KIF1C	-0.8030745	1.1761577	-0.6828	0.495	-0.844744224	count	1
RNF149	-0.6344156	0.4414197	-1.4372	0.152	-0.844538378	count	1
ANKHD1	-0.6651317	0.7430945	-0.8951	0.371	-0.844238308	count	1
HDLBP	-0.6719727	0.5321447	-1.2628	0.208	-0.844222945	count	1
AP4M1	-0.7172414	0.6914326	-1.0373	0.3	-0.844163861	count	1
OSTC	-0.5944278	0.2062288	-2.8824	0.0042	-0.843755918	count	1
SPHK2	-1.2065374	1.053955	-1.1448	0.253	-0.843548763	count	1
PRKAR2B	-0.6791582	0.7459083	-0.9105	0.363	-0.843080034	count	1
GALM	-0.8010116	0.7757132	-1.0326	0.303	-0.842749984	count	1
TIMP1	-0.5944843	0.2973848	-1.999	0.0464	-0.842104019	count	1
POC1B	-0.621458	0.6277996	-0.9899	0.323	-0.841961467	count	1
PDZD11	-0.6571371	0.6167439	-1.0655	0.287	-0.841566826	count	1
EFHC1	-0.698611	0.7388158	-0.9456	0.345	-0.840282689	count	1
ATP6V1E2	-0.698611	0.8920436	-0.7832	0.434	-0.840282689	count	1
EXOSC5	-0.6678708	0.4843752	-1.3788	0.169	-0.839233267	count	1
USP48	-0.6152992	0.4600671	-1.3374	0.182	-0.838822109	count	1
IRF7	-0.6070644	0.5185871	-1.1706	0.243	-0.838339246	count	1
THUMPD3	-0.6148321	0.488867	-1.2577	0.209	-0.838193202	count	1
COL6A1	-0.8553782	1.0089268	-0.8478	0.397	-0.838154956	count	1
TATDN1	-0.6286933	0.4806562	-1.308	0.192	-0.837056371	count	1
NCAPD3	-0.7569136	0.7396842	-1.0233	0.307	-0.836870008	count	1
HLA-DPA1	-0.5932704	0.2727119	-2.1754	0.0303	-0.836800502	count	1
TLK1	-0.6064983	0.3118119	-1.9451	0.0526	-0.836624349	count	1
RBBP7	-0.6062129	0.3755573	-1.6142	0.107	-0.836234478	count	1
MCPH1	-0.6477172	0.5014279	-1.2917	0.197	-0.836045417	count	1
ATXN10	-0.6024066	0.3905941	-1.5423	0.124	-0.836020779	count	1
TMEM241	-1.192168	1.0688858	-1.1153	0.266	-0.835832092	count	1
TRAPPC5	-1.192168	1.0688858	-1.1153	0.266	-0.835832092	count	1
ZMYND8	-0.6827878	0.6400916	-1.0667	0.287	-0.835808094	count	1
NT5C	-0.5917279	0.2791143	-2.12	0.0348	-0.835785734	count	1
NAA30	-0.755732	0.6550616	-1.1537	0.249	-0.835649126	count	1
LAPTM4B	-0.7290897	1.362379	-0.5352	0.593	-0.835509886	count	1
NUDT16	-0.6825139	0.6859336	-0.995	0.32	-0.835485563	count	1
URB1-AS1	-0.6427507	0.8458986	-0.7598	0.448	-0.835265999	count	1
TMEM14C	-0.5920227	0.3622192	-1.6344	0.1031	-0.834546121	count	1
TTLL3	-0.9547448	1.1204865	-0.8521	0.395	-0.834379788	count	1
DTNBP1	-0.621539	0.456276	-1.3622	0.174	-0.833260998	count	1
TMCC1	-0.7269542	0.7683413	-0.9461	0.345	-0.83319791	count	1
TGIF1	-0.6016709	0.3121097	-1.9278	0.0548	-0.831847792	count	1

PIN4	-0.608978	0.3728715	-1.6332	0.103	-0.831781476	count	1
TTC38	-0.6486636	0.4935828	-1.3142	0.19	-0.83100496	count	1
PEA15	-0.7042319	0.5269575	-1.3364	0.182	-0.829584694	count	1
TACC3	-0.9478618	0.9494413	-0.9983	0.319	-0.829207305	count	1
FAM13A	-0.9478618	1.0090167	-0.9394	0.348	-0.829207305	count	1
PDS5B	-0.6118046	0.446878	-1.3691	0.172	-0.829060636	count	1
PDHA1	-0.600526	0.4162412	-1.4427	0.15	-0.828464902	count	1
PDHX	-0.8443534	0.8895215	-0.9492	0.343	-0.828443348	count	1
SPATA20	-0.7225249	1.0476473	-0.6897	0.491	-0.828399432	count	1
PPM1G	-0.5855202	0.2360981	-2.48	0.0136	-0.82825097	count	1
TRIOBP	-0.6587461	0.7666838	-0.8592	0.391	-0.82812459	count	1
PRPF6	-0.5928713	0.3253677	-1.8222	0.0694	-0.827720805	count	1
P2RY11	-0.702567	0.7046868	-0.997	0.32	-0.827716464	count	1
ARMCX6	-0.6263947	0.5780611	-1.0836	0.279	-0.827355112	count	1
NUB1	-0.5899509	0.2794644	-2.111	0.0355	-0.826832773	count	1
FAM193B	-0.9442809	0.8911492	-1.0596	0.2901	-0.826510636	count	1
SOX4	-0.6447025	0.5419973	-1.1895	0.235	-0.826064327	count	1
PHF14	-0.5950174	0.3584827	-1.6598	0.0979	-0.825122961	count	1
ZFYVE19	-0.7819842	0.9733588	-0.8034	0.422	-0.824303624	count	1
ADD3	-0.6063083	0.5208087	-1.1642	0.245	-0.823491384	count	1
PIK3C2A	-0.7438703	0.7550807	-0.9852	0.325	-0.823374827	count	1
FDX1	-0.5964854	0.3488114	-1.7101	0.0882	-0.822943432	count	1
SMS	-0.5963361	0.3957057	-1.507	0.133	-0.822739395	count	1
CD83	-0.5970401	0.3930429	-1.519	0.1297	-0.822730887	count	1
RNF214	-0.6154882	0.5314634	-1.1581	0.248	-0.822643651	count	1
FAM102B	-0.6323117	0.6260439	-1.01	0.313	-0.822010871	count	1
TPRG1L	-0.6823178	0.7008903	-0.9735	0.331	-0.821501661	count	1
MRPL45	-0.619162	0.658166	-0.9407	0.348	-0.821436833	count	1
PPP2CB	-0.6356611	0.696611	-0.9125	0.362	-0.820864039	count	1
GARS	-0.635655	0.5295519	-1.2004	0.231	-0.820856351	count	1
RUSC1	-0.6523593	0.5551964	-1.175	0.241	-0.820341689	count	1
GGH	-0.8350259	1.2380923	-0.6744	0.501	-0.820200148	count	1
NR2F2	-0.8350259	1.3229663	-0.6312	0.528	-0.820200148	count	1
MTERF1	-0.8350259	1.3360296	-0.625	0.532	-0.820200148	count	1
RIC1	-0.8350259	1.3360296	-0.625	0.532	-0.820200148	count	1
HCFC2	-0.8350259	1.3360296	-0.625	0.532	-0.820200148	count	1
TTLL12	-0.8350259	1.3360296	-0.625	0.532	-0.820200148	count	1
SNCA	-0.8350259	1.4272624	-0.5851	0.559	-0.820200148	count	1
LINC02076	-0.8350259	1.4272624	-0.5851	0.559	-0.820200148	count	1
IFNG-AS1	-0.8350259	1.6713425	-0.4996	0.618	-0.820200148	count	1
ANKRD49	-0.6080748	0.5401996	-1.1256	0.261	-0.82010355	count	1
MINCR	-0.7141088	0.9083449	-0.7862	0.432	-0.819270213	count	1
CPSF6	-0.6796108	0.5357004	-1.2686	0.205	-0.818376601	count	1
MRPL40	-0.5853019	0.3065569	-1.9093	0.0571	-0.817721562	count	1
SURF6	-0.8319344	0.6398744	-1.3002	0.194	-0.817462643	count	1

TRABD2A	-0.7122912	0.9744187	-0.731	0.465	-0.817296602	count	1
SMDT1	-0.5729385	0.1893094	-3.0265	0.0027	-0.815510957	count	1
GOLGA7	-0.5856163	0.3264485	-1.7939	0.0738	-0.815475149	count	1
ACAP3	-0.9288323	0.7845146	-1.184	0.237	-0.814832539	count	1
PPIL3	-0.6140108	0.6417728	-0.9567	0.339	-0.814725197	count	1
RNF19A	-0.5732748	0.2008488	-2.8543	0.0046	-0.814189057	count	1
TEX30	-0.5991815	0.5063428	-1.1834	0.238	-0.813934713	count	1
KIAA0232	-0.6637624	0.580693	-1.1431	0.254	-0.813374899	count	1
AC084018.2	-0.8271545	1.0235295	-0.8081	0.42	-0.81322479	count	1
THAP5	-0.594082	0.473224	-1.2554	0.21	-0.813012739	count	1
ERBIN	-0.5959482	0.4021489	-1.4819	0.139	-0.812755005	count	1
RRAD	-0.586193	1.0615473	-0.5522	0.5812	-0.812239354	count	1
ARHGAP35	-0.6119846	0.6152108	-0.9948	0.321	-0.81208449	count	1
NUP98	-0.9250738	0.6552254	-1.4118	0.159	-0.811980538	count	1
CCDC28B	-0.6164557	0.504162	-1.2227	0.222	-0.810693226	count	1
VBP1	-0.589651	0.3607476	-1.6345	0.103	-0.810577306	count	1
NUDT18	-0.6519073	0.8192922	-0.7957	0.427	-0.810375159	count	1
TMEM242	-0.6077523	0.4716135	-1.2887	0.198	-0.809648992	count	1
TMPO	-0.6000097	0.2940118	-2.0408	0.0421	-0.809380024	count	1
ZNF721	-0.6083093	0.5125742	-1.1868	0.236	-0.807293519	count	1
JARID2	-0.6349832	0.6740334	-0.9421	0.3469	-0.80703457	count	1
MT-ND6	-0.6687273	0.4050288	-1.6511	0.0997	-0.805798817	count	1
FDXR	-0.6828449	0.8196032	-0.8331	0.405	-0.805544194	count	1
GEMIN8	-0.6828449	0.8646632	-0.7897	0.43	-0.805544194	count	1
QARS	-0.6280669	0.5090982	-1.2337	0.218	-0.805292309	count	1
PRAF2	-0.6026388	0.6329555	-0.9521	0.342	-0.802950216	count	1
HS2ST1	-2.209837	1.0189852	-2.1687	0.0308	-0.802819062	count	1
PPP1R35	-0.5773342	0.3319861	-1.739	0.083	-0.802816014	count	1
SNAPC1	-0.5913388	0.3689717	-1.6027	0.11	-0.801686564	count	1
ZNF441	-0.624365	0.9489833	-0.6579	0.511	-0.800665007	count	1
SEMA4D	-0.588028	0.368421	-1.5961	0.111	-0.80057523	count	1
CYB5D2	-0.6527661	0.8288428	-0.7876	0.432	-0.800380996	count	1
TMEM200A	-0.7571933	0.9139368	-0.8285	0.408	-0.800130254	count	1
TERF1	-0.6520752	0.5048156	-1.2917	0.197	-0.799563903	count	1
JMY	-0.6289412	0.5176405	-1.215	0.225	-0.799563385	count	1
LEMD3	-0.7207293	0.6012606	-1.1987	0.232	-0.799334049	count	1
ANKRD16	-2.187695	1.114439	-1.963	0.0505	-0.799330401	count	1
HSPH1	-0.5937063	0.4444868	-1.3357	0.183	-0.79884084	count	1
BCL2L11	-0.7195713	0.5623511	-1.2796	0.202	-0.79812776	count	1
USP24	-0.6762468	0.8245841	-0.8201	0.413	-0.798109342	count	1
IMMP2L	-0.6262481	0.8403076	-0.7453	0.457	-0.796231631	count	1
AC138150.1	-0.6603505	1.040497	-0.6346	0.526	-0.796103372	count	1
ZNF549	-0.6603505	1.040497	-0.6346	0.526	-0.796103372	count	1
EHBP1	-0.6921232	0.6303362	-1.098	0.273	-0.795350255	count	1
ZNF260	-0.6921232	1.0242816	-0.6757	0.4997	-0.795350255	count	1

SETD2	-0.5714561	0.3747706	-1.5248	0.1283	-0.795312388	count	1
ATRIP	-1.1172501	1.423455	-0.7849	0.4331	-0.794614021	count	1
BRPF3	-0.7161945	0.9534074	-0.7512	0.453	-0.794608359	count	1
MRPS9	-0.588547	0.4805967	-1.2246	0.222	-0.794129961	count	1
FH	-0.6381942	0.6021856	-1.0598	0.29	-0.793873121	count	1
MAGED1	-0.6098768	0.900314	-0.6774	0.499	-0.793480055	count	1
CEACAM21	-0.8985417	0.8537852	-1.0524	0.293	-0.791727503	count	1
GTF2IRD2	-0.8985417	0.9029431	-0.9951	0.32	-0.791727503	count	1
TNFRSF25	-0.8985417	0.9360397	-0.9599	0.338	-0.791727503	count	1
DOHH	-0.6445595	0.9034666	-0.7134	0.476	-0.790670378	count	1
ZDHHC17	-0.7475321	0.6739266	-1.1092	0.268	-0.790667437	count	1
OTUD4	-0.6871006	0.9955437	-0.6902	0.4906	-0.789871389	count	1
ZC3H4	-0.6198347	0.6084025	-1.0188	0.309	-0.788293344	count	1
AP1AR	-0.6022277	0.8612467	-0.6993	0.485	-0.788269022	count	1
ANKRD36C	-0.6260866	0.5955807	-1.0512	0.294	-0.788262624	count	1
BRD3	-0.5956171	0.5477142	-1.0875	0.278	-0.787439379	count	1
NLRC5	-0.8922144	0.8754402	-1.0192	0.309	-0.786866656	count	1
TRGC1	-0.5640628	0.3486599	-1.6178	0.1067	-0.78675801	count	1
NRARP	-2.1111524	1.9217427	-1.0986	0.2728	-0.78675569	count	1
ARCN1	-0.5826086	0.4341666	-1.3419	0.181	-0.786225349	count	1
RIOK2	-0.5772631	0.4337499	-1.3309	0.184	-0.786089689	count	1
ECHDC2	-0.6001596	0.6560889	-0.9148	0.361	-0.785616026	count	1
PFKFB3	-0.7071861	0.5111443	-1.3835	0.167	-0.78520676	count	1
KDM2B	-0.6647355	0.7026611	-0.946	0.345	-0.785117996	count	1
S1PR1	-1.0991942	1.4148668	-0.7769	0.438	-0.78443301	count	1
AC018816.1	-1.0991942	1.4679464	-0.7488	0.455	-0.78443301	count	1
ZNF48	-0.7063517	1.0317342	-0.6846	0.494	-0.784335	count	1
MRPL37	-0.5905169	0.4391244	-1.3448	0.18	-0.78408152	count	1
TMEM205	-0.5797599	0.507923	-1.1414	0.255	-0.782432453	count	1
MGAT5	-0.5892154	0.5226483	-1.1274	0.26	-0.782382384	count	1
CHD8	-0.5839538	0.5995686	-0.974	0.331	-0.781144755	count	1
IP6K2	-0.5701636	0.3977926	-1.4333	0.153	-0.780622849	count	1
COL21A1	-0.7027166	1.4350119	-0.4897	0.625	-0.78053529	count	1
AGPAT1	-0.5831666	0.7517237	-0.7758	0.438	-0.78010768	count	1
FAM214B	-0.6590804	0.9221465	-0.7147	0.475	-0.778726447	count	1
WRAP53	-0.8814049	0.9381283	-0.9395	0.348	-0.778535071	count	1
TRRAP	-0.8814049	1.2206139	-0.7221	0.471	-0.778535071	count	1
SMARCD3	-0.8814049	1.2206139	-0.7221	0.471	-0.778535071	count	1
SLC9A6	-0.8814049	1.2206139	-0.7221	0.471	-0.778535071	count	1
POLD1	-0.8814049	1.2206139	-0.7221	0.471	-0.778535071	count	1
MMADHC	-0.5694276	0.3621518	-1.5723	0.117	-0.776988252	count	1
VPS11	-0.8792581	1.0366183	-0.8482	0.397	-0.776876258	count	1
MFN1	-0.8782515	0.6629779	-1.3247	0.186	-0.776098032	count	1
COG4	-0.8782515	0.7500531	-1.1709	0.242	-0.776098032	count	1
EZH1	-0.6314788	0.6056336	-1.0427	0.298	-0.775169317	count	1

SCYL3	-0.7842849	0.8442482	-0.929	0.3536	-0.774933449	count	1
KCNQ1OT1	-1.0813319	1.3355243	-0.8097	0.4187	-0.774266807	count	1
UCK1	-1.0813319	1.4552987	-0.743	0.458	-0.774266807	count	1
C17orf75	-0.5844356	0.4778215	-1.2231	0.222	-0.772914326	count	1
PANK2	-0.8732257	0.5114241	-1.7074	0.0887	-0.772207894	count	1
TRANK1	-0.6125499	0.5826943	-1.0512	0.294	-0.771695044	count	1
DPM1	-1.076403	0.575093	-1.8717	0.0621	-0.771445169	count	1
CD6	-0.5747426	0.7294534	-0.7879	0.431	-0.771435976	count	1
ZBTB40	-1.0751829	0.8100789	-1.3273	0.185	-0.77074545	count	1
LRPPRC	-0.6520157	0.6266222	-1.0405	0.299	-0.770733127	count	1
UBA6	-0.6276225	0.5880688	-1.0673	0.287	-0.770594101	count	1
HPS1	-0.5618364	0.4198019	-1.3383	0.182	-0.770523739	count	1
ZNF678	-0.7767124	1.1818269	-0.6572	0.512	-0.7681172	count	1
PTS	-0.6095914	0.5405377	-1.1278	0.26	-0.768070635	count	1
SH3BGRL	-0.5436705	0.2204782	-2.4659	0.0142	-0.767984482	count	1
ARF3	-0.6893052	0.6371838	-1.0818	0.28	-0.76649058	count	1
AC006449.6	-0.6079746	0.8173866	-0.7438	0.458	-0.766089396	count	1
EDEM2	-0.6335581	0.591173	-1.0717	0.285	-0.765009346	count	1
METTL9	-0.5458565	0.2789391	-1.9569	0.0512	-0.763390391	count	1
SMIM4	-0.6321591	0.7476483	-0.8455	0.398	-0.76338226	count	1
SCO1	-0.6321591	0.782483	-0.8079	0.42	-0.76338226	count	1
BISPR	-0.6321591	0.8108955	-0.7796	0.436	-0.76338226	count	1
AC092069.1	-0.6321591	1.0804492	-0.5851	0.559	-0.76338226	count	1
FBXO32	-0.5826561	0.6224926	-0.936	0.35	-0.763143509	count	1
APPBP2	-0.6624954	0.5602599	-1.1825	0.238	-0.762954917	count	1
DIAPH2	-0.6310459	0.6694155	-0.9427	0.347	-0.762087318	count	1
PHF8	-1.059795	1.0513302	-1.0081	0.314	-0.761884656	count	1
NBPF3	-1.0597951	1.4141984	-0.7494	0.4542	-0.76188456	count	1
N4BP2	-0.7696044	0.6203172	-1.2407	0.216	-0.761704897	count	1
FAM120A	-0.6042883	0.4920213	-1.2282	0.22	-0.761570784	count	1
PTGES2	-0.5687981	0.4639871	-1.2259	0.221	-0.761168735	count	1
KRAS	-0.5376861	0.241916	-2.2226	0.0269	-0.761166433	count	1
RIPK3	-0.7686218	1.1119685	-0.6912	0.49	-0.760817392	count	1
C2orf68	-0.619162	0.9169163	-0.6753	0.5	-0.760547851	count	1
G3BP1	-0.5501082	0.4400019	-1.2502	0.2121	-0.760342432	count	1
NAALADL1	-0.5472061	0.4312529	-1.2689	0.205	-0.759321438	count	1
NEK4	-1.959276	1.215447	-1.612	0.108	-0.759311192	count	1
ZNF611	-0.7141906	0.8144975	-0.8768	0.381	-0.757831224	count	1
TP53BP1	-0.6082582	0.7583613	-0.8021	0.423	-0.757747883	count	1
USP14	-0.5611221	0.4044863	-1.3872	0.166	-0.757601513	count	1
PRMT3	-0.6571371	0.9019678	-0.7286	0.467	-0.757076697	count	1
MRS2	-0.7640223	0.7565661	-1.0099	0.313	-0.756659552	count	1
PPP3CB	-0.7632275	0.5139065	-1.4851	0.138	-0.755940488	count	1
DDX11	-0.6149073	0.643911	-0.955	0.34	-0.755491309	count	1
NUDT2	-0.5736495	0.5215036	-1.1	0.272	-0.755414519	count	1

HADHB	-0.5625638	0.3997689	-1.4072	0.16	-0.75531544	count	1
ALG12	-0.6370271	0.7390859	-0.8619	0.389	-0.753743001	count	1
IL9R	-0.7092586	0.9161543	-0.7742	0.439	-0.752950596	count	1
STRBP	-0.5458147	0.397694	-1.3724	0.171	-0.752753076	count	1
VAMP5	-0.5407415	0.3489354	-1.5497	0.122	-0.752282791	count	1
TPM2	-0.5907712	0.7606072	-0.7767	0.438	-0.752249796	count	1
DDX50	-0.5656313	0.4852072	-1.1658	0.245	-0.751565079	count	1
PSMB4	-0.534956	0.2757156	-1.9402	0.0532	-0.751188623	count	1
KLHL21	-0.7578963	1.0988815	-0.6897	0.491	-0.751112929	count	1
TAF10	-0.5279901	0.2405911	-2.1946	0.0289	-0.75065985	count	1
ANAPC5	-0.5568375	0.3760714	-1.4807	0.14	-0.749891201	count	1
RAB5C	-0.5298017	0.256983	-2.0616	0.04	-0.748848594	count	1
ARHGEF12	-0.7551356	0.9039804	-0.8353	0.404	-0.748610017	count	1
TAF6L	-1.903522	1.037463	-1.8348	0.0675	-0.748352698	count	1
GCSH	-0.7535113	0.8622845	-0.8739	0.383	-0.747136434	count	1
AC093673.1	-0.5639015	0.4569388	-1.2341	0.218	-0.746207926	count	1
KLF11	-0.7023969	0.8219995	-0.8545	0.393	-0.746150481	count	1
NFKB1	-0.5692895	0.3431992	-1.6588	0.0981	-0.745960071	count	1
DYNC1LI2	-0.6065657	0.6438863	-0.942	0.347	-0.745569187	count	1
PARP9	-0.5757389	0.5954588	-0.9669	0.334	-0.745143001	count	1
C16orf91	-0.5547063	0.5057844	-1.0967	0.274	-0.744908293	count	1
RIMKLB	-0.701066	1.1387133	-0.6157	0.5385	-0.744830192	count	1
LONP2	-0.5683864	0.5408231	-1.051	0.294	-0.744798401	count	1
HS3ST3B1	-0.5973561	0.9489624	-0.6295	0.529	-0.744558005	count	1
HSDL1	-0.5973561	1.1370347	-0.5254	0.6	-0.744558005	count	1
DLG1	-0.6286952	0.6334097	-0.9926	0.322	-0.744280215	count	1
CDKN1A	-0.6283649	0.4781022	-1.3143	0.19	-0.743904822	count	1
SLC35E1	-0.5445369	0.4140639	-1.3151	0.189	-0.743376984	count	1
DCTN3	-0.5233769	0.2450691	-2.1356	0.0335	-0.743312535	count	1
RUVBL1	-0.5741729	0.6246179	-0.9192	0.359	-0.743158344	count	1
TMEM18	-0.5548622	0.4478361	-1.239	0.216	-0.742782957	count	1
FXR2	-0.6988862	1.0139584	-0.6893	0.491	-0.742666827	count	1
AC023157.3	-1.0241381	1.194282	-0.8575	0.392	-0.741084818	count	1
DIP2A	-0.602564	0.5258127	-1.146	0.253	-0.740805308	count	1
MBD4	-0.5337298	0.3261877	-1.6363	0.103	-0.740754564	count	1
PPP1R12A	-0.5207355	0.1934307	-2.6921	0.0075	-0.740085731	count	1
MCM7	-0.7455367	0.4831313	-1.5431	0.124	-0.739891582	count	1
CIAPIN1	-0.5709271	0.5931587	-0.9625	0.337	-0.739043898	count	1
AC027644.3	-0.5709271	0.6904624	-0.8269	0.409	-0.739043898	count	1
PI4K2B	-0.6946662	1.090297	-0.6371	0.524	-0.73847539	count	1
NOA1	-0.5533953	0.615803	-0.8987	0.37	-0.738317582	count	1
DCUN1D5	-0.6097036	0.4730618	-1.2888	0.198	-0.737219109	count	1
MIS18BP1	-0.5301646	0.3238692	-1.637	0.103	-0.736475858	count	1
PSMC4	-0.5310729	0.3271522	-1.6233	0.105	-0.73642805	count	1
CD2BP2	-0.5726908	0.4507396	-1.2706	0.205	-0.735890403	count	1

TUSC2	-0.5775774	0.6144214	-0.94	0.348	-0.735850237	count	1
SNRPA1	-0.5334493	0.3760568	-1.4185	0.157	-0.735839462	count	1
ABCG1	-0.5395181	0.4903965	-1.1002	0.272	-0.735236269	count	1
RFX7	-0.6079746	0.7269847	-0.8363	0.404	-0.735201022	count	1
CHD1L	-0.6079746	0.9630379	-0.6313	0.528	-0.735201022	count	1
AC062017.1	-0.6079746	1.1244085	-0.5407	0.589	-0.735201022	count	1
SEPHS2	-0.5373547	0.4063578	-1.3224	0.187	-0.734940673	count	1
KLHDC10	-0.8252644	1.0446251	-0.79	0.43	-0.73471233	count	1
UQCRC1	-0.5468706	0.4773486	-1.1456	0.253	-0.734524982	count	1
CDC14A	-0.7394231	0.6368418	-1.1611	0.246	-0.734325901	count	1
AIG1	-0.5713788	0.6534297	-0.8744	0.383	-0.734241435	count	1
DMTF1	-0.5546247	0.4013934	-1.3817	0.168	-0.734129212	count	1
THAP9-AS1	-0.5660992	0.4579256	-1.2362	0.217	-0.732921672	count	1
CLPX	-0.5590211	0.6199249	-0.9018	0.368	-0.73274664	count	1
EMC7	-0.5298564	0.3237531	-1.6366	0.103	-0.732568224	count	1
FBP1	-0.5657684	0.5524139	-1.0242	0.307	-0.732502089	count	1
CYFIP2	-0.5384927	0.3546487	-1.5184	0.13	-0.732406585	count	1
ASCC1	-0.6341805	0.7967655	-0.7959	0.427	-0.73182638	count	1
LRRC58	-0.5516972	0.6001064	-0.9193	0.359	-0.730315794	count	1
ZNF217	-0.5281191	0.4009194	-1.3173	0.189	-0.730184645	count	1
CRNKL1	-0.5487069	0.4930816	-1.1128	0.267	-0.729418043	count	1
NOB1	-0.5384637	0.5476674	-0.9832	0.326	-0.729161111	count	1
SMARCD2	-0.6027254	0.6448943	-0.9346	0.351	-0.729071073	count	1
ARPC1A	-0.546197	0.5354677	-1.02	0.308	-0.728851538	count	1
REL	-0.5079495	0.1462043	-3.4742	0.000582	-0.727579457	count	1
RBM42	-0.5244656	0.3594286	-1.4592	0.145	-0.727330644	count	1
HDDC3	-0.6010426	0.5897261	-1.0192	0.309	-0.727104918	count	1
ZBTB37	-1.7965343	1.6469978	-1.0908	0.2762	-0.725907603	count	1
C2orf49	-0.5384191	0.515512	-1.0444	0.297	-0.72539695	count	1
TRIM5	-0.5892154	0.7801686	-0.7552	0.451	-0.724895883	count	1
CHMP7	-0.5637415	0.679118	-0.8301	0.407	-0.724638401	count	1
NDUFS6	-0.514279	0.2821417	-1.8228	0.0693	-0.723983513	count	1
SMARCE1	-0.5436462	0.431847	-1.2589	0.209	-0.722790586	count	1
AL445686.2	-0.586787	0.9524231	-0.6161	0.538	-0.721998593	count	1
TNFSF4	-0.9918337	1.0773217	-0.9206	0.358	-0.721919326	count	1
RBM41	-0.5504445	0.7471034	-0.7368	0.462	-0.721701756	count	1
RFNG	-0.5504319	0.5931899	-0.9279	0.354	-0.721685529	count	1
MVB12A	-0.5661469	0.5215499	-1.0855	0.279	-0.721624058	count	1
YEATS2	-0.8087072	0.7904747	-1.0231	0.307	-0.721613215	count	1
FAM126B	-0.624365	1.0342467	-0.6037	0.546	-0.720997765	count	1
ERCC6L2	-0.5607053	0.6574625	-0.8528	0.394	-0.720818756	count	1
WDR55	-0.5604572	0.6246374	-0.8973	0.37	-0.720506586	count	1
COX19	-0.5199211	0.3812399	-1.3638	0.174	-0.720393164	count	1
CYBC1	-0.5149362	0.3328213	-1.5472	0.123	-0.7203846	count	1
CDIPT	-0.5333953	0.4494582	-1.1868	0.236	-0.718711311	count	1

RPAP3	-0.5360275	0.5693931	-0.9414	0.347	-0.717907958	count	1
GZMB	-0.5105746	0.2496873	-2.0449	0.0417	-0.71770671	count	1
UBXN7	-0.6212707	0.6517588	-0.9532	0.341	-0.717580099	count	1
QKI	-0.5157863	0.3839844	-1.3432	0.18	-0.717222899	count	1
SLC30A6	-0.982585	1.2902886	-0.7615	0.447	-0.716376152	count	1
TPCN2	-0.982585	1.2902886	-0.7615	0.447	-0.716376152	count	1
AFMID	-0.982585	1.2902886	-0.7615	0.447	-0.716376152	count	1
DPH2	-0.982585	1.3017195	-0.7548	0.451	-0.716376152	count	1
SERPING1	-0.982585	1.3017195	-0.7548	0.451	-0.716376152	count	1
BSCL2	-0.982585	1.3017195	-0.7548	0.451	-0.716376152	count	1
MKL2	-0.982585	1.3017195	-0.7548	0.451	-0.716376152	count	1
EEF1AKMT2	-0.5232063	0.4418451	-1.1841	0.237	-0.715772123	count	1
CCNG1	-0.6190797	0.6471096	-0.9567	0.339	-0.715158995	count	1
TXNL1	-0.5061266	0.2910627	-1.7389	0.083	-0.714164031	count	1
SCAF4	-0.6691992	0.7450308	-0.8982	0.37	-0.713089506	count	1
RBM7	-0.5275618	0.4817956	-1.095	0.274	-0.712822413	count	1
CSNK2A1	-0.5879313	0.6308149	-0.932	0.352	-0.711769569	count	1
YTHDC1	-0.5104882	0.2438328	-2.0936	0.0371	-0.711506034	count	1
HADH	-0.5580204	0.6597931	-0.8458	0.398	-0.711499734	count	1
ASTE1	-0.5486617	0.5701427	-0.9623	0.337	-0.710786868	count	1
ITPK1	-0.5323535	0.4995488	-1.0657	0.287	-0.710634109	count	1
MAPK14	-0.548203	0.7037312	-0.779	0.437	-0.710204132	count	1
WWP2	-0.5481342	0.8111714	-0.6757	0.5	-0.710116728	count	1
TMEM65	-0.5380464	0.6169398	-0.8721	0.384	-0.709295011	count	1
OTUD5	-0.5743856	0.4903353	-1.1714	0.242	-0.707188274	count	1
SLAIN2	-0.5177201	0.4168287	-1.242	0.215	-0.707119994	count	1
SCCPDH	-0.5313738	0.4750163	-1.1186	0.264	-0.706709129	count	1
ZNF85	-0.6113605	0.8089783	-0.7557	0.45	-0.706621504	count	1
EFNA4	-0.6113605	0.9187651	-0.6654	0.506	-0.706621504	count	1
NSMCE1	-0.516402	0.3724088	-1.3867	0.167	-0.706549137	count	1
CMIP	-0.5537268	0.5450103	-1.016	0.31	-0.706147181	count	1
OGFOD3	-0.5727581	0.6643172	-0.8622	0.389	-0.705242866	count	1
NCSTN	-0.5727581	0.7653469	-0.7484	0.455	-0.705242866	count	1
JAKMIP2	-0.6312572	0.4848241	-1.302	0.194	-0.705239417	count	1
REEP5	-0.4939236	0.2014905	-2.4513	0.0148	-0.705148277	count	1
C2CD3	-0.7867003	1.0219014	-0.7698	0.442	-0.70408101	count	1
POLR2I	-0.511852	0.3906215	-1.3104	0.191	-0.703577266	count	1
HTATSF1	-0.5158888	0.3644826	-1.4154	0.158	-0.703352503	count	1
YOD1	-0.5924712	1.162291	-0.5097	0.611	-0.702990527	count	1
PEX11B	-0.9586092	1.1327992	-0.8462	0.398	-0.701890333	count	1
RECK	-0.9586092	1.1327992	-0.8462	0.398	-0.701890333	count	1
HILPDA	-0.9586092	1.3688137	-0.7003	0.4842	-0.701890333	count	1
POLG2	-0.6271565	0.7200273	-0.871	0.384	-0.70088462	count	1
CORO2A	-1.686474	1.1093	-1.5203	0.129	-0.700772798	count	1
UBR4	-0.7823347	0.6441303	-1.2146	0.225	-0.700586661	count	1

EXOC6	-0.5403151	0.592591	-0.9118	0.363	-0.700179571	count	1
AKR7A2	-0.5065241	0.4195362	-1.2073	0.228	-0.699782879	count	1
NDUFB10	-0.4930898	0.2177899	-2.2641	0.0242	-0.699050772	count	1
GKAP1	-0.5479795	0.5194774	-1.0549	0.292	-0.698978732	count	1
ZNF160	-0.6549488	0.6138395	-1.067	0.287	-0.698816923	count	1
KAT8	-0.5321031	0.5744068	-0.9264	0.355	-0.698056311	count	1
PCSK7	-0.5175764	0.3762267	-1.3757	0.17	-0.697646962	count	1
ELP2	-0.5345795	0.646024	-0.8275	0.409	-0.697304298	count	1
DCAF12	-0.6237523	0.7116038	-0.8765	0.381	-0.697266726	count	1
TCP11L1	-0.9502505	0.8125296	-1.1695	0.243	-0.696800802	count	1
UROD	-0.5516125	0.7206084	-0.7655	0.445	-0.696794691	count	1
SNRPF	-0.4944809	0.2988361	-1.6547	0.099	-0.695977019	count	1
RFXAP	-0.5450321	0.7842032	-0.695	0.488	-0.695300896	count	1
LIMS1	-0.4923042	0.3425997	-1.437	0.1517	-0.694732112	count	1
PIH1D1	-0.5067744	0.3718106	-1.363	0.174	-0.69461045	count	1
TPP2	-0.6001175	0.4264315	-1.4073	0.16	-0.694165722	count	1
NBPF19	-0.5634947	0.7657516	-0.7359	0.462	-0.694162208	count	1
CCDC9	-0.6953996	0.777294	-0.8946	0.372	-0.693956768	count	1
MCCC1	-0.6953996	0.824424	-0.8435	0.4	-0.693956768	count	1
ATXN2	-0.6953996	0.8392971	-0.8286	0.408	-0.693956768	count	1
KLHL18	-0.6953996	0.9873942	-0.7043	0.482	-0.693956768	count	1
MBD1	-0.6206246	0.7723223	-0.8036	0.422	-0.693940514	count	1
MED13L	-0.5127794	0.5277363	-0.9717	0.332	-0.693071787	count	1
TBC1D17	-0.9434204	0.6755355	-1.3966	0.164	-0.692626979	count	1
ECHS1	-0.5002784	0.3113635	-1.6067	0.109	-0.691966637	count	1
DOCK11	-0.5243478	0.6345627	-0.8263	0.409	-0.691516804	count	1
RNF227	-0.7707736	1.2593929	-0.612	0.541	-0.691306814	count	1
CBLL1	-0.5268387	0.5410762	-0.9737	0.331	-0.69126318	count	1
ATAD1	-0.512612	0.4363799	-1.1747	0.241	-0.691032476	count	1
THOC2	-0.5055662	0.3717608	-1.3599	0.175	-0.690672703	count	1
TIMM21	-1.6424179	1.6701261	-0.9834	0.3261	-0.690103076	count	1
RAMP2	-1.6424179	1.7082628	-0.9615	0.337	-0.690103076	count	1
SMCO4	-1.642418	1.038786	-1.5811	0.115	-0.690102863	count	1
NRBP1	-0.4966729	0.3186899	-1.5585	0.12	-0.68965666	count	1
MX1	-0.4928497	0.4641574	-1.0618	0.2891	-0.689252805	count	1
ARFGEF1	-0.5035694	0.3918793	-1.285	0.2	-0.689147373	count	1
AKR1B1	-0.4960689	0.3417989	-1.4513	0.148	-0.687565818	count	1
SPEN	-0.614435	0.4898239	-1.2544	0.211	-0.687351846	count	1
PHKG2	-0.5060671	0.4292267	-1.179	0.239	-0.687302366	count	1
HSD17B8	-0.5264391	0.688554	-0.7646	0.445	-0.686869531	count	1
AL450998.2	-0.6131922	0.6695397	-0.9158	0.36	-0.686028033	count	1
C21orf2	-0.5376019	0.5688354	-0.9451	0.345	-0.686024483	count	1
PSMG2	-0.491213	0.2760911	-1.7792	0.0761	-0.685716835	count	1
NRAS	-0.5650908	0.7098834	-0.796	0.427	-0.684986088	count	1
ZFAND5	-0.4906741	0.2880917	-1.7032	0.0895	-0.684516502	count	1

ZBTB4	-0.761596	0.5182341	-1.4696	0.143	-0.683913151	count	1
KAT6A	-0.5233876	0.4859385	-1.0771	0.282	-0.682956163	count	1
RRAS	-0.5403738	0.6699274	-0.8066	0.42	-0.682925451	count	1
NCLN	-0.9275932	0.9720756	-0.9542	0.341	-0.682903185	count	1
TLE1	-0.6386909	0.4654491	-1.3722	0.171	-0.682475311	count	1
CCDC6	-0.6828449	0.9628661	-0.7092	0.479	-0.682351662	count	1
PDE6G	-0.6828449	1.1048365	-0.6181	0.537	-0.682351662	count	1
APC	-0.5199193	0.6008031	-0.8654	0.387	-0.6823302	count	1
XIAP	-0.5105507	0.4222547	-1.2091	0.228	-0.681909284	count	1
ROMO1	-0.488527	0.3207661	-1.523	0.129	-0.681069648	count	1
DCUN1D1	-0.5291398	0.5339657	-0.991	0.322	-0.681042578	count	1
EIF3I	-0.4828144	0.2711231	-1.7808	0.0759	-0.680770848	count	1
HIST1H1B	-0.6079746	0.9383138	-0.6479	0.517	-0.680466398	count	1
GASAL1	-0.6079746	1.142242	-0.5323	0.595	-0.680466398	count	1
ZFYVE1	-0.6079746	1.1429054	-0.532	0.595	-0.680466398	count	1
ETV5	-0.6079746	1.5615897	-0.3893	0.6973	-0.680466398	count	1
WEE1	-0.5445066	0.7310297	-0.7448	0.457	-0.680370542	count	1
AL118516.1	-0.4998079	0.4584497	-1.0902	0.276	-0.680306072	count	1
DNAL1	-0.9231594	1.1239824	-0.8213	0.412	-0.680166147	count	1
USP18	-0.9231594	1.1239824	-0.8213	0.412	-0.680166147	count	1
WAPL	-0.4933256	0.451767	-1.092	0.276	-0.68008503	count	1
DCP2	-0.4931572	0.3255599	-1.5148	0.131	-0.679854605	count	1
ADPRHL2	-0.5108084	0.5394539	-0.9469	0.344	-0.679730662	count	1
ADAT1	-0.5603885	0.8223199	-0.6815	0.496	-0.67946135	count	1
TCHP	-0.5038411	0.5248165	-0.96	0.338	-0.679341751	count	1
GRB2	-0.4781175	0.2454626	-1.9478	0.0523	-0.67899892	count	1
CNOT11	-0.5230755	0.6813941	-0.7677	0.443	-0.678245786	count	1
RTF1	-0.4775814	0.2634769	-1.8126	0.0708	-0.677710242	count	1
CNOT6	-0.5487813	0.7365242	-0.7451	0.457	-0.676535416	count	1
MIR181A1HG	-0.632491	0.618558	-1.0225	0.307	-0.676227224	count	1
GTPBP8	-0.517888	0.3652954	-1.4177	0.157	-0.675900764	count	1
SLC1A4	-0.7513052	1.0242561	-0.7335	0.464	-0.675594811	count	1
IFIT2	-0.5477375	1.032124	-0.5307	0.596	-0.67528369	count	1
DCAKD	-0.7502056	0.8667059	-0.8656	0.387	-0.674704193	count	1
ZNF624	-0.6025173	0.8848504	-0.6809	0.496	-0.674643129	count	1
PPP1R14A	-0.6025173	1.0609615	-0.5679	0.57	-0.674643129	count	1
SMARCAL1	-1.5800295	1.2183296	-1.2969	0.1956	-0.674376023	count	1
FRS2	-0.7495736	0.960769	-0.7802	0.436	-0.674192151	count	1
BMPR2	-0.6019522	1.0460245	-0.5755	0.565	-0.67403978	count	1
COX10	-0.6019522	1.1083868	-0.5431	0.587	-0.67403978	count	1
ZBTB8OS	-0.4869359	0.3669743	-1.3269	0.185	-0.673637438	count	1
RAB5B	-0.6295078	0.9856665	-0.6387	0.523	-0.673217674	count	1
FAAP20	-0.4755596	0.3123382	-1.5226	0.1288	-0.67290426	count	1
KPNA2	-0.4891311	0.3487943	-1.4023	0.162	-0.672590624	count	1
DYNLT1	-0.4721234	0.2242868	-2.105	0.0361	-0.6722545	count	1

SP110	-0.4745712	0.237329	-1.9996	0.0464	-0.671659967	count	1
GEMIN6	-0.5178258	0.8466047	-0.6117	0.541	-0.671560075	count	1
MRRF	-0.579465	0.9054214	-0.64	0.523	-0.671221476	count	1
AC111182.1	-0.5643997	0.7966988	-0.7084	0.479	-0.670830474	count	1
DNAJC5	-0.5643997	1.0167467	-0.5551	0.5792	-0.670830474	count	1
CYB561D1	-0.6702334	1.0405721	-0.6441	0.52	-0.670653297	count	1
NASP	-0.4779457	0.247668	-1.9298	0.0545	-0.670590149	count	1
ANKRD39	-0.5246076	0.5299576	-0.9899	0.323	-0.669784901	count	1
CREBZF	-0.489859	0.3715483	-1.3184	0.188	-0.668188146	count	1
NLRP1	-0.50885	0.47497	-1.0713	0.285	-0.668029715	count	1
EGR3	-0.624074	1.0564063	-0.5908	0.555	-0.667730586	count	1
ZCCHC7	-0.4882254	0.3972696	-1.229	0.22	-0.667190493	count	1
PLCXD2	-1.550804	0.7391958	-2.098	0.0367	-0.666754446	count	1
LHFPL6	-0.9000457	1.2276632	-0.7331	0.464	-0.665806049	count	1
CPNE1	-0.4840624	0.3174757	-1.5247	0.128	-0.665674116	count	1
ATOX1	-0.5213201	0.4537285	-1.149	0.251	-0.665673053	count	1
IKZF2	-0.8993468	1.0888175	-0.826	0.4094	-0.665369436	count	1
ZNF764	-0.8993468	1.1060553	-0.8131	0.4168	-0.665369436	count	1
SCML1	-0.5737494	0.6743974	-0.8508	0.396	-0.664857177	count	1
CEP44	-0.5585359	0.6321184	-0.8836	0.378	-0.664095058	count	1
UQCR11	-0.4638425	0.2020253	-2.296	0.0223	-0.661911086	count	1
HELZ	-0.4771098	0.3685263	-1.2946	0.196	-0.661453966	count	1
PEX2	-0.478432	0.3578977	-1.3368	0.182	-0.661238379	count	1
ZNF706	-0.4661712	0.2188689	-2.1299	0.0339	-0.661110505	count	1
AK3	-0.4946041	0.5522654	-0.8956	0.371	-0.660874638	count	1
LGALS9	-0.4946041	0.5663242	-0.8734	0.383	-0.660874638	count	1
CAPN2	-0.4983691	0.4457335	-1.1181	0.264	-0.660709404	count	1
CENPF	-0.491043	0.7690808	-0.6385	0.524	-0.660404673	count	1
SCAMP2	-0.473889	0.3040603	-1.5585	0.12	-0.660301559	count	1
CBR1	-0.4772231	0.445159	-1.072	0.285	-0.66028917	count	1
ALG5	-0.4902585	0.4759691	-1.03	0.304	-0.659361407	count	1
MACF1	-0.4728117	0.2619317	-1.8051	0.072	-0.657809994	count	1
RGP1	-0.541491	0.6185003	-0.8755	0.382	-0.657222527	count	1
ZNF200	-0.5667857	0.8076734	-0.7018	0.483	-0.657094718	count	1
NKRF	-0.6135246	0.7333876	-0.8366	0.403	-0.657058363	count	1
MPND	-0.5252276	0.7047121	-0.7453	0.457	-0.656856257	count	1
AC073332.1	-0.5252276	0.7299722	-0.7195	0.472	-0.656856257	count	1
PRKAA1	-0.5096991	0.4514806	-1.129	0.26	-0.656486669	count	1
SNX12	-1.511263	1.155218	-1.3082	0.192	-0.65617998	count	1
C14orf28	-1.511263	1.155218	-1.3082	0.192	-0.65617998	count	1
CCNB1IP1	-0.4996609	0.416083	-1.2009	0.231	-0.656149058	count	1
DZIP3	-0.4816091	0.3778518	-1.2746	0.203	-0.655762095	count	1
UBAP2L	-0.5400731	0.6530339	-0.827	0.409	-0.655551579	count	1
MAPKAPK3	-0.4791427	0.4542726	-1.0547	0.292	-0.654883804	count	1
RSL24D1	-0.462571	0.2390255	-1.9352	0.0538	-0.654122729	count	1

MRPL4	-0.468067	0.3390087	-1.3807	0.168	-0.653982296	count	1
STK19	-0.5075665	0.7292394	-0.696	0.487	-0.653790287	count	1
SDHAF3	-0.4890956	0.5326867	-0.9182	0.359	-0.653603672	count	1
MTCH1	-0.4745591	0.3865815	-1.2276	0.22	-0.65357314	count	1
SYTL2	-0.5069808	0.4433325	-1.1436	0.254	-0.653049662	count	1
TC2N	-0.4754522	0.364499	-1.3044	0.193	-0.652999233	count	1
KDM3B	-0.8793473	1.1440842	-0.7686	0.4427	-0.652816052	count	1
MAP2K3	-0.4716903	0.3998946	-1.1795	0.239	-0.652683467	count	1
SH2D3A	-0.650897	0.6765006	-0.9622	0.337	-0.652638401	count	1
AC009831.1	-0.5288534	0.88405	-0.5982	0.55	-0.65260962	count	1
REXO4	-0.5104746	0.6331553	-0.8062	0.421	-0.65209866	count	1
TEP1	-1.489955	1.5334263	-0.9717	0.3319	-0.650353959	count	1
MYSM1	-0.47577	0.4395799	-1.0823	0.28	-0.65031268	count	1
PRR3	-0.5081301	0.7532357	-0.6746	0.5	-0.649162364	count	1
IER5L	-0.4823814	0.465104	-1.0371	0.3	-0.648883379	count	1
SLA	-0.4533869	0.164552	-2.7553	0.0062	-0.648210799	count	1
RPL27A	-0.4516531	0.1124254	-4.0174	7.32E-05	-0.647719844	count	1
XRN2	-0.4605769	0.2542104	-1.8118	0.0709	-0.647705022	count	1
COMMD3	-0.4679465	0.3363097	-1.3914	0.165	-0.64753621	count	1
PXMP2	-0.5328353	0.8567993	-0.6219	0.534	-0.647017214	count	1
AVEN	-0.5171045	0.5712373	-0.9052	0.366	-0.646933198	count	1
RAB18	-0.4985053	0.4250183	-1.1729	0.242	-0.646928675	count	1
RALA	-0.4600891	0.31379	-1.4662	0.1436	-0.646762792	count	1
FDFT1	-0.4680304	0.3599064	-1.3004	0.194	-0.646226186	count	1
TMED4	-0.4614171	0.2804848	-1.6451	0.1009	-0.64621432	count	1
ALKBH7	-0.4608569	0.263291	-1.7504	0.081	-0.646095891	count	1
ARHGAP12	-0.5053717	0.4619209	-1.0941	0.275	-0.645706853	count	1
AP2A2	-0.5751418	0.7586294	-0.7581	0.449	-0.645337981	count	1
NCR1	-0.4769583	0.4579004	-1.0416	0.298	-0.645146235	count	1
ZAP70	-0.4621545	0.3123502	-1.4796	0.14	-0.644933402	count	1
PSMG3	-0.530021	0.5660408	-0.9364	0.35	-0.643696527	count	1
CWC15	-0.4659993	0.3117104	-1.495	0.136	-0.643440469	count	1
AC147651.4	-0.5401638	0.9002289	-0.6	0.549	-0.642953413	count	1
HINT3	-0.5401638	0.9735099	-0.5549	0.579	-0.642953413	count	1
EIF5	-0.4498562	0.1542051	-2.9173	0.0038	-0.641747162	count	1
LINC00476	-0.5526346	0.759178	-0.7279	0.467	-0.641292311	count	1
MIER3	-0.5526346	0.9173473	-0.6024	0.547	-0.641292311	count	1
FAHD2B	-0.6386347	1.1335634	-0.5634	0.574	-0.641165323	count	1
POMGNT2	-0.6386347	1.1335634	-0.5634	0.574	-0.641165323	count	1
ZNF665	-0.6386347	1.1335634	-0.5634	0.574	-0.641165323	count	1
PSKH1	-0.6386347	1.2377364	-0.516	0.606	-0.641165323	count	1
ANO10	-1.456537	0.8439093	-1.7259	0.0853	-0.641035377	count	1
USF1	-0.5061837	0.900948	-0.5618	0.575	-0.640630784	count	1
ROCK2	-0.5381235	0.5379342	-1.0004	0.318	-0.640601955	count	1
TMEM60	-0.5174229	0.8542397	-0.6057	0.545	-0.638859403	count	1

UNC50	-0.4563598	0.3290479	-1.3869	0.166	-0.637703269	count	1
BTBD10	-0.4989611	0.6558504	-0.7608	0.447	-0.6376726	count	1
CLK4	-0.4823689	0.413335	-1.167	0.244	-0.636924739	count	1
RIPK1	-0.4732222	0.4575574	-1.0342	0.302	-0.63669406	count	1
GPKOW	-0.5670697	0.5806023	-0.9767	0.329	-0.636667393	count	1
BCLAF3	-0.4870065	0.768047	-0.6341	0.526	-0.636224862	count	1
PITPNM2	-0.8530539	1.3395575	-0.6368	0.5247	-0.636137742	count	1
AHDC1	-0.8530539	1.0367938	-0.8228	0.411	-0.636137712	count	1
DUS3L	-0.8530539	1.0426127	-0.8182	0.414	-0.636137712	count	1
MYPOP	-0.8530539	1.048068	-0.8139	0.416	-0.636137712	count	1
DUS2	-0.8530539	1.0516909	-0.8111	0.418	-0.636137712	count	1
PLEKHO2	-0.5665567	0.6939779	-0.8164	0.415	-0.636115912	count	1
LAPTM4A	-0.449461	0.2561907	-1.7544	0.0803	-0.635789757	count	1
DLD	-0.4814228	0.4543468	-1.0596	0.29	-0.635692482	count	1
PDCD5	-0.4527398	0.2780914	-1.628	0.104	-0.635072744	count	1
TP53	-0.5917163	0.8388774	-0.7054	0.481	-0.634915822	count	1
SURF2	-0.460604	0.3873911	-1.189	0.235	-0.634487425	count	1
HDHD2	-1.4333099	0.9892093	-1.4489	0.148	-0.634426421	count	1
LIN54	-0.5649568	0.9560364	-0.5909	0.555	-0.634395642	count	1
NAAA	-0.5061465	0.6996008	-0.7235	0.47	-0.633532748	count	1
ABHD14A	-0.4549025	0.363137	-1.2527	0.211	-0.633515382	count	1
ERCC3	-0.6994773	0.7678638	-0.9109	0.363	-0.633253236	count	1
HAPLN3	-1.4276404	0.9304807	-1.5343	0.1259	-0.63279661	count	1
PPA2	-0.4731883	0.5691383	-0.8314	0.406	-0.632592942	count	1
MSH2	-0.494614	0.5283862	-0.9361	0.35	-0.632221679	count	1
AC074044.1	-0.6971753	0.8708148	-0.8006	0.424	-0.631355461	count	1
DOCK10	-0.4565867	0.2956076	-1.5446	0.123	-0.631238516	count	1
PBRM1	-0.4653379	0.3587637	-1.2971	0.196	-0.631088131	count	1
PHF23	-0.477742	0.4207943	-1.1353	0.257	-0.630897592	count	1
ENSA	-0.4432298	0.1963915	-2.2569	0.0247	-0.630565949	count	1
GXYLT1	-0.477392	0.7345547	-0.6499	0.516	-0.630441592	count	1
PRELID3B	-0.4699091	0.4675998	-1.0049	0.316	-0.630356205	count	1
OTULINL	-0.4563193	0.375489	-1.2153	0.225	-0.630161294	count	1
SLC27A4	-1.416411	0.8855599	-1.5995	0.111	-0.629549052	count	1
HMGN3	-0.4432469	0.2347872	-1.8879	0.0599	-0.629402557	count	1
MIB1	-0.5178843	0.8807185	-0.588	0.557	-0.629361707	count	1
TTC3	-0.4484278	0.3432722	-1.3063	0.1924	-0.629343912	count	1
RPAIN	-0.4539931	0.3104341	-1.4624	0.145	-0.628985162	count	1
POLK	-0.4737429	0.529122	-0.8953	0.371	-0.628479213	count	1
TBXAS1	-0.5160097	1.005934	-0.513	0.608	-0.627145528	count	1
ZNF207	-0.4634344	0.3353007	-1.3821	0.168	-0.627028994	count	1
FAM193A	-0.5579833	0.7622663	-0.732	0.465	-0.626891423	count	1
ZC3H8	-0.4744784	0.4910815	-0.9662	0.335	-0.626645149	count	1
MARK3	-0.4590149	0.3890123	-1.1799	0.239	-0.62646553	count	1
WDR75	-0.5573756	0.741257	-0.7519	0.453	-0.626237012	count	1

SUPV3L1	-0.5573755	0.7524691	-0.7407	0.459	-0.626236907	count	1
KANSL1-AS1	-0.4564515	0.3687366	-1.2379	0.217	-0.626159688	count	1
WBP4	-0.4679639	0.4854899	-0.9639	0.336	-0.625687992	count	1
MSL2	-0.4891607	0.5493734	-0.8904	0.374	-0.625380504	count	1
SLMAP	-0.5143974	0.6834269	-0.7527	0.452	-0.625239008	count	1
WDR76	-0.6893052	0.7675764	-0.898	0.37	-0.62485649	count	1
SEC24C	-0.6893052	0.907106	-0.7599	0.448	-0.62485649	count	1
HLA-DMA	-0.4778741	0.497865	-0.9598	0.338	-0.624473132	count	1
TMEM131	-0.4746522	0.5291337	-0.897	0.37	-0.623773541	count	1
TMEM219	-0.441548	0.2850135	-1.5492	0.1223	-0.62277801	count	1
SPINT2	-1.393097	0.5998043	-2.3226	0.0208	-0.62272481	count	1
MPZL3	-0.4828708	0.7033041	-0.6866	0.493	-0.622528373	count	1
ZCCHC8	-0.5792386	0.6300774	-0.9193	0.359	-0.622198786	count	1
INTS11	-0.4443434	0.3909172	-1.1367	0.2565	-0.622070262	count	1
PTPN4	-0.4377487	0.2292065	-1.9098	0.057	-0.621706825	count	1
TTC13	-0.5350196	0.7631283	-0.7011	0.484	-0.621569712	count	1
SLC33A1	-0.5350196	0.8057873	-0.664	0.507	-0.621569712	count	1
HIST1H2AK	-0.6174351	0.9082907	-0.6798	0.497	-0.621241912	count	1
DNAJB2	-0.5212718	0.6389794	-0.8158	0.415	-0.621153247	count	1
TIPRL	-0.4441913	0.2967254	-1.497	0.135	-0.621151223	count	1
MSH6	-0.450427	0.446373	-1.0091	0.314	-0.620562581	count	1
SPAG1	-0.5205669	0.4627866	-1.1249	0.261	-0.620338667	count	1
TSEN2	-0.6827678	1.0444484	-0.6537	0.514	-0.619445257	count	1
GK	-0.6827678	1.0444484	-0.6537	0.514	-0.619445257	count	1
SLBP	-0.4483778	0.3503819	-1.2797	0.202	-0.619263652	count	1
RCHY1	-0.4797063	0.5784872	-0.8292	0.408	-0.618517541	count	1
MRNIP	-0.5186133	0.8753247	-0.5925	0.554	-0.618080673	count	1
CDK7	-0.4877751	0.8067289	-0.6046	0.546	-0.61779641	count	1
UIMC1	-0.4789711	0.5438192	-0.8808	0.379	-0.617585553	count	1
PPIL4	-0.4408113	0.2880925	-1.5301	0.127	-0.617478498	count	1
PITPNA-AS1	-0.4826066	0.6386014	-0.7557	0.45	-0.617153722	count	1
LRCH3	-0.8232185	0.8215073	-1.0021	0.317	-0.616974171	count	1
MT1X	-0.4475549	0.4163998	-1.0748	0.283	-0.616631837	count	1
DDX18	-0.438893	0.2823223	-1.5546	0.121	-0.616014816	count	1
PCIF1	-0.5300624	0.4751206	-1.1156	0.265	-0.616009098	count	1
STAMBP	-0.4621723	0.503863	-0.9173	0.36	-0.615782924	count	1
NRL	-1.3683244	1.6207993	-0.8442	0.3992	-0.61535074	count	1
SEH1L	-1.368324	1.162731	-1.1768	0.24	-0.615350718	count	1
KDM5C	-0.5162097	0.9895098	-0.5217	0.602	-0.615301672	count	1
LRIF1	-0.4768698	0.4892515	-0.9747	0.33	-0.614921486	count	1
MAPKAP1	-0.4906943	0.5120596	-0.9583	0.339	-0.614608804	count	1
DOCK8	-0.4394834	0.2490468	-1.7647	0.0786	-0.614596898	count	1
NDUFAF7	-0.4848344	0.8124406	-0.5968	0.551	-0.614144772	count	1
IFITM3	-0.4456319	0.2902765	-1.5352	0.126	-0.613189375	count	1
PAPOLA	-0.4327051	0.2364584	-1.8299	0.0682	-0.612954417	count	1

OAS2	-0.4478126	0.5154796	-0.8687	0.386	-0.612395395	count	1
BMT2	-0.6736959	1.0748386	-0.6268	0.531	-0.611917022	count	1
RAB14	-0.4353599	0.26752	-1.6274	0.105	-0.611618105	count	1
SKIV2L	-0.4827289	0.623963	-0.7737	0.44	-0.611529583	count	1
SIK3	-0.4942319	0.502746	-0.9831	0.326	-0.610903633	count	1
AC114760.2	-0.4775651	0.4730888	-1.0095	0.314	-0.61082214	count	1
ATP6V1D	-0.4581267	0.372765	-1.229	0.22	-0.610454768	count	1
GPR18	-0.4463336	0.4582645	-0.974	0.331	-0.609294967	count	1
AOAH	-0.4314557	0.2963374	-1.456	0.1464	-0.608774075	count	1
GPR137	-0.5660992	0.7835055	-0.7225	0.47	-0.608769994	count	1
LRRC37B	-0.5411759	0.7500206	-0.7215	0.471	-0.608764658	count	1
EXOSC7	-0.4429253	0.4292732	-1.0318	0.303	-0.60863877	count	1
CTSA	-0.4337096	0.3178721	-1.3644	0.1734	-0.608483591	count	1
PRKCB	-0.4756534	0.5110912	-0.9307	0.353	-0.608420471	count	1
ZMYM4	-0.4756534	0.5847896	-0.8134	0.417	-0.608420471	count	1
TRPS1	-0.5406569	0.8192569	-0.6599	0.51	-0.608204018	count	1
KLHL8	-0.5406569	0.8622982	-0.627	0.531	-0.608204018	count	1
BTN2A2	-0.5406569	0.8747489	-0.6181	0.537	-0.608204018	count	1
CAB39	-0.4715669	0.448327	-1.0518	0.294	-0.608196204	count	1
TPRN	-1.3431062	1.2123803	-1.1078	0.2688	-0.607712945	count	1
C1orf35	-0.4399416	0.3585419	-1.227	0.221	-0.607683871	count	1
B3GALNT2	-0.5400731	0.8521618	-0.6338	0.527	-0.607573313	count	1
NTPCR	-0.4848702	0.5163724	-0.939	0.348	-0.607467889	count	1
PPIF	-0.4790638	0.5743915	-0.834	0.405	-0.60697594	count	1
JTB	-0.427822	0.2105048	-2.0324	0.0429	-0.60674751	count	1
KDM6B	-0.4421614	0.403711	-1.0952	0.2742	-0.606696183	count	1
FOXO3	-0.4703496	0.5175731	-0.9088	0.364	-0.606651957	count	1
CACYBP	-0.4315525	0.2849575	-1.5144	0.1309	-0.606024526	count	1
HDAC7	-0.4734931	0.5212341	-0.9084	0.364	-0.605705977	count	1
IFT27	-0.4563636	0.5638112	-0.8094	0.419	-0.605701791	count	1
APOL6	-0.4379163	0.3562593	-1.2292	0.22	-0.605580898	count	1
PRR14	-0.507769	0.5761272	-0.8813	0.379	-0.605535069	count	1
LINC00467	-0.4778893	0.8383161	-0.5701	0.569	-0.605516352	count	1
NLE1	-0.6657116	0.9430185	-0.7059	0.481	-0.605273011	count	1
SMURF2	-0.4625435	0.4735402	-0.9768	0.329	-0.604726764	count	1
LZTS2	-0.5199377	0.9454088	-0.55	0.583	-0.604638068	count	1
TMEM187	-0.5199377	0.9942639	-0.5229	0.601	-0.604638068	count	1
ZNF736	-0.5199377	1.030973	-0.5043	0.614	-0.604638068	count	1
ALDH16A1	-0.4888509	0.6703426	-0.7293	0.466	-0.604406045	count	1
TRIM27	-0.438041	0.5140583	-0.8521	0.395	-0.604360871	count	1
WDR12	-0.4963944	0.7811113	-0.6355	0.526	-0.603923482	count	1
PYCARD	-0.4266851	0.2965035	-1.4391	0.1511	-0.603798642	count	1
CLN8	-0.6637279	0.8076619	-0.8218	0.412	-0.603619666	count	1
VPS25	-0.4420583	0.4950981	-0.8929	0.373	-0.603504041	count	1
MCM5	-0.4637564	0.5864644	-0.7908	0.43	-0.602527433	count	1

UBE2G2	-0.4430319	0.3809304	-1.163	0.246	-0.602427697	count	1
TRG-AS1	-0.4265809	0.29044	-1.4687	0.1429	-0.602269278	count	1
IDH3A	-0.4744784	0.5603299	-0.8468	0.398	-0.601276554	count	1
FKBP3	-0.4356033	0.4037909	-1.0788	0.281	-0.60027053	count	1
EXOSC3	-0.4542363	0.4197871	-1.0821	0.28	-0.60024841	count	1
XAB2	-0.473517	0.6035921	-0.7845	0.433	-0.600081264	count	1
TSPAN3	-0.7958682	0.6652891	-1.1963	0.232	-0.599185587	count	1
EEA1	-0.4312825	0.3934531	-1.0961	0.274	-0.597093781	count	1
CAMLG	-0.428941	0.3254635	-1.3179	0.188	-0.597079748	count	1
MAPKAPK2	-0.4459527	0.4982489	-0.895	0.371	-0.596572722	count	1
SLC39A6	-0.5127524	0.773021	-0.6633	0.508	-0.596557042	count	1
HINFP	-1.3055691	1.1311801	-1.1542	0.2493	-0.596096905	count	1
TBC1D22A	-0.4380042	0.5756473	-0.7609	0.447	-0.595647137	count	1
SLC25A46	-0.4893471	0.6276013	-0.7797	0.436	-0.595565973	count	1
ANGEL2	-0.4990912	0.5575	-0.8952	0.371	-0.595481817	count	1
POU2F2	-0.5282964	0.8770156	-0.6024	0.547	-0.594836058	count	1
DUSP23	-0.4273827	0.3616265	-1.1818	0.238	-0.594446803	count	1
B3GAT3	-0.4252084	0.3716893	-1.144	0.253	-0.594363013	count	1
THAP6	-0.4740669	0.8015568	-0.5914	0.555	-0.59421014	count	1
PEX1	-0.4280913	0.4472576	-0.9571	0.339	-0.593867973	count	1
SLC35F6	-0.5273223	0.7477505	-0.7052	0.481	-0.593781282	count	1
PCBP4	-0.4795731	0.8333333	-0.5755	0.565	-0.59319345	count	1
SEC63	-0.4448481	0.3433682	-1.2955	0.196	-0.592957158	count	1
LMBR1	-0.7860112	0.8534093	-0.921	0.3577	-0.59272304	count	1
TNFRSF4	-0.4229762	0.3194478	-1.3241	0.1864	-0.59259529	count	1
TMEM181	-1.292911	0.8519915	-1.5175	0.13	-0.592112516	count	1
TRIM32	-1.291799	1.121591	-1.1518	0.25	-0.591760872	count	1
MVB12B	-1.291799	1.121591	-1.1518	0.25	-0.591760872	count	1
TDRKH	-1.2917987	1.5152105	-0.8526	0.3945	-0.591760828	count	1
UBXN6	-0.5493689	0.7811094	-0.7033	0.482	-0.591616369	count	1
MBD3	-0.5493409	0.663012	-0.8286	0.408	-0.591587624	count	1
PHKB	-0.4379713	0.4944823	-0.8857	0.376	-0.591365251	count	1
ENOPH1	-0.4583057	0.4313867	-1.0624	0.289	-0.591364528	count	1
MOGS	-0.4580106	0.5491295	-0.8341	0.405	-0.590989754	count	1
RASGRP2	-0.4493642	0.6362281	-0.7063	0.481	-0.590976062	count	1
FAM222B	-0.5487327	0.9887834	-0.555	0.579	-0.590962872	count	1
YJU2	-0.5246909	0.589259	-0.8904	0.374	-0.590931014	count	1
WIPF3	-0.585289	0.7788961	-0.7514	0.453	-0.590820849	count	1
NDFIP2	-0.4318092	0.3974945	-1.0863	0.278	-0.590670496	count	1
SLC25A37	-0.46558	0.4558084	-1.0214	0.308	-0.590208986	count	1
TMED3	-0.4339727	0.4169587	-1.0408	0.299	-0.59020896	count	1
UXT	-0.4139083	0.1747369	-2.3688	0.0184	-0.590063022	count	1
TST	-0.523786	0.7707776	-0.6796	0.497	-0.589950535	count	1
AL645568.1	-0.523786	0.8782313	-0.5964	0.551	-0.589950535	count	1
CWC27	-0.427428	0.4223714	-1.012	0.312	-0.589806916	count	1

CLDND1	-0.4112659	0.1853693	-2.2186	0.0272	-0.589729579	count	1
ZFX	-0.4376013	0.4699337	-0.9312	0.352	-0.589230207	count	1
GRAMD2B	-1.282625	0.8108801	-1.5818	0.115	-0.588849748	count	1
TRIM33	-0.4560436	0.4946071	-0.922	0.357	-0.588491469	count	1
BCL2	-0.4219155	0.2944673	-1.4328	0.153	-0.587795213	count	1
ARV1	-0.4686312	0.5997101	-0.7814	0.435	-0.587533694	count	1
ETFB	-0.4158528	0.2997285	-1.3874	0.1663	-0.587491351	count	1
5-Mar	-0.474677	0.4431483	-1.0711	0.285	-0.587271451	count	1
SIL1	-0.4684155	0.587007	-0.798	0.425	-0.587268681	count	1
FBXO4	-0.4684155	0.6290018	-0.7447	0.457	-0.587268681	count	1
DYNC2LI1	-0.7767124	1.4359754	-0.5409	0.589	-0.586601527	count	1
AC009948.1	-0.7767124	1.4359754	-0.5409	0.589	-0.586601527	count	1
POLR3G	-0.7767124	1.4359754	-0.5409	0.589	-0.586601527	count	1
HMGA1P4	-0.7767124	1.4359754	-0.5409	0.589	-0.586601527	count	1
RAB11B-AS1	-0.7767124	1.4359754	-0.5409	0.589	-0.586601527	count	1
JPX	-0.4263994	0.3517845	-1.2121	0.226	-0.586079241	count	1
RIPOR2	-0.4413602	0.4170155	-1.0584	0.291	-0.586017568	count	1
MRPS30	-0.4206121	0.3492105	-1.2045	0.229	-0.584588731	count	1
ZNF287	-0.5787267	0.8995074	-0.6434	0.52	-0.584579993	count	1
GOT2	-0.5014738	1.0675816	-0.4697	0.639	-0.583853781	count	1
SNTB2	-0.4274935	0.3580589	-1.1939	0.233	-0.58376791	count	1
UBE2R2	-0.4196482	0.3327278	-1.2612	0.208	-0.583741237	count	1
ZBTB11-AS1	-0.4884047	0.7568054	-0.6454	0.519	-0.583084516	count	1
AHSA1	-0.4327505	0.3177604	-1.3619	0.174	-0.582759593	count	1
SLC35B1	-0.4303358	0.488019	-0.8818	0.379	-0.582636277	count	1
FBXO6	-0.4179928	0.4140603	-1.0095	0.313	-0.582355561	count	1
PILRB	-0.4185486	0.3767703	-1.1109	0.267	-0.582219053	count	1
HNRNPUL1	-0.4448433	0.3479423	-1.2785	0.202	-0.581899614	count	1
ISCA1	-0.4163984	0.2772068	-1.5021	0.134	-0.581738947	count	1
ZNF292	-0.4422014	0.3535379	-1.2508	0.212	-0.581675428	count	1
STAT3	-0.4138842	0.277015	-1.4941	0.1361	-0.581553718	count	1
CRTAM	-0.4090751	0.2814375	-1.4535	0.147	-0.581236516	count	1
FAIM	-0.4326932	0.5110445	-0.8467	0.398	-0.580926868	count	1
PWWP2B	-0.4355037	0.5834462	-0.7464	0.456	-0.580635249	count	1
GOLGB1	-0.4176463	0.2698811	-1.5475	0.123	-0.580487019	count	1
TM9SF2	-0.4389079	0.3603006	-1.2182	0.224	-0.580235204	count	1
PPIG	-0.4062029	0.194521	-2.0882	0.0376	-0.579974665	count	1
RASA3	-0.4855928	0.6996632	-0.694	0.488	-0.579819362	count	1
DDX39A	-0.4162274	0.3785098	-1.0996	0.272	-0.579466123	count	1
KDSR	-0.4278186	0.616137	-0.6944	0.488	-0.579257165	count	1
GTF2H3	-0.467478	0.7402673	-0.6315	0.528	-0.578557954	count	1
TARBP1	-0.7640223	1.1291146	-0.6767	0.499	-0.578208563	count	1
EEF1AKMT4	-0.7640223	1.1291146	-0.6767	0.499	-0.578208563	count	1
TLR2	-0.7640223	1.1291146	-0.6767	0.499	-0.578208563	count	1
AC144652.1	-0.7640223	1.1291146	-0.6767	0.499	-0.578208563	count	1

MTM1	-0.7640223	1.1291146	-0.6767	0.499	-0.578208563	count	1
SLC39A14	-0.7640223	1.3165791	-0.5803	0.5621	-0.578208563	count	1
MDH1	-0.4084455	0.2425117	-1.6842	0.0931	-0.578052934	count	1
TBK1	-0.4318894	0.4342255	-0.9946	0.321	-0.57795083	count	1
EMC9	-0.4669643	0.7602932	-0.6142	0.54	-0.577935906	count	1
SIDT1-AS1	-0.6327033	0.8893754	-0.7114	0.477	-0.577625619	count	1
CCT2	-0.4092551	0.2530154	-1.6175	0.1067	-0.577185605	count	1
CLEC2B	-0.401719	0.1380791	-2.9093	0.00387	-0.577133583	count	1
TPRKB	-0.421754	0.4191983	-1.0061	0.315	-0.577013016	count	1
FAM41C	-0.4661883	1.001378	-0.4655	0.642	-0.576996172	count	1
BZW2	-0.4270963	0.51626	-0.8273	0.409	-0.576811204	count	1
TXN2	-0.4115775	0.352076	-1.169	0.2433	-0.576379518	count	1
METTL5	-0.4123223	0.3859611	-1.0683	0.286	-0.574909755	count	1
PPME1	-0.4640184	0.7313046	-0.6345	0.526	-0.574367985	count	1
ST20	-0.4805833	0.8855933	-0.5427	0.588	-0.573999214	count	1
STX10	-0.4091967	0.4004035	-1.022	0.3076	-0.573657966	count	1
RFFL	-0.4478866	0.8252568	-0.5427	0.588	-0.573489623	count	1
SDHB	-0.411793	0.2892476	-1.4237	0.156	-0.573321176	count	1
DYNC1H1	-0.4335605	0.4026196	-1.0768	0.282	-0.573248663	count	1
ARFIP2	-0.4475181	0.6228433	-0.7185	0.473	-0.573025457	count	1
ZNF649	-0.4475181	0.6556143	-0.6826	0.495	-0.573025457	count	1
RBMX	-0.4095924	0.2421727	-1.6913	0.0917	-0.572624141	count	1
TFDP2	-0.4261562	0.3869143	-1.1014	0.272	-0.572233963	count	1
ACVR1B	-0.4617621	0.7039119	-0.656	0.512	-0.571634463	count	1
GPR137B	-0.4903372	1.0164464	-0.4824	0.63	-0.571288342	count	1
ZNF548	-0.4903372	1.2553082	-0.3906	0.6963	-0.571288342	count	1
LTC4S	-0.4779555	0.5608072	-0.8523	0.395	-0.570944569	count	1
ZNF182	-0.5643997	1.0961706	-0.5149	0.607	-0.570919126	count	1
MED16	-0.5643997	1.1623293	-0.4856	0.628	-0.570919126	count	1
AMN1	-0.5643997	1.2918172	-0.4369	0.662	-0.570919126	count	1
TRMT1L	-0.5288538	0.8837351	-0.5984	0.55	-0.570499535	count	1
MYO1G	-0.4494844	0.5209575	-0.8628	0.389	-0.570165322	count	1
ZNF615	-0.5629518	0.9765921	-0.5764	0.565	-0.569535854	count	1
ATF7IP2	-0.4142084	0.3898968	-1.0624	0.289	-0.569428306	count	1
CLASP2	-0.5275458	0.8032616	-0.6568	0.512	-0.569150126	count	1
MOB1B	-0.5275458	0.8846377	-0.5963	0.551	-0.569150126	count	1
SETX	-0.4479423	0.4066969	-1.1014	0.272	-0.568243329	count	1
RUFY2	-0.4583532	0.6816564	-0.6724	0.502	-0.567503229	count	1
COQ10A	-0.447251	0.5253044	-0.8514	0.395	-0.567381638	count	1
RAB1A	-0.4176033	0.4668998	-0.8944	0.372	-0.566875364	count	1
GLUL	-0.4148933	0.3780583	-1.0974	0.273	-0.566684083	count	1
NEU3	-1.213602	1.290623	-0.9403	0.348	-0.566366799	count	1
TAF5L	-0.7453501	0.8356759	-0.8919	0.373	-0.565777755	count	1
TOR1B	-0.744819	0.9328458	-0.7984	0.425	-0.565422805	count	1
ALMS1	-0.744819	0.9683761	-0.7691	0.442	-0.565422805	count	1

SETBP1	-0.744819	0.9683761	-0.7691	0.442	-0.565422805	count	1
INIP	-0.4223742	0.5376761	-0.7856	0.433	-0.56534276	count	1
BORCS8	-0.473046	0.5328148	-0.8878	0.375	-0.565234615	count	1
LINC00891	-0.7438702	1.2883667	-0.5774	0.5641	-0.564788413	count	1
ZNF347	-0.7438702	1.3124827	-0.5668	0.5713	-0.564788413	count	1
AC022706.1	-0.4450335	0.6703832	-0.6638	0.507	-0.56461718	count	1
WDR36	-0.4449473	0.5898336	-0.7544	0.451	-0.564509708	count	1
NDUFAF2	-0.4110113	0.4434293	-0.9269	0.355	-0.564231143	count	1
TMEM259	-0.406572	0.3026716	-1.3433	0.18	-0.563627306	count	1
STRADA	-1.204677	0.6839903	-1.7612	0.0791	-0.563384279	count	1
SLC25A32	-0.4988523	0.595802	-0.8373	0.403	-0.56287173	count	1
MCFD2	-0.4706258	0.465099	-1.0119	0.312	-0.562418397	count	1
EIF4EBP1	-0.4028911	0.3297107	-1.222	0.223	-0.562207763	count	1
FGR	-0.4248933	0.4529943	-0.938	0.349	-0.56191946	count	1
ANXA4	-0.4350885	0.5684918	-0.7653	0.445	-0.561850723	count	1
GPN2	-0.4970674	0.6171773	-0.8054	0.421	-0.560928686	count	1
RAB22A	-0.4045063	0.4250956	-0.9516	0.342	-0.560778079	count	1
FBXO7	-0.4021424	0.3176353	-1.2661	0.206	-0.560369782	count	1
TOX4	-0.3984129	0.2664986	-1.495	0.136	-0.560120949	count	1
MLYCD	-0.4302151	0.7169724	-0.6	0.549	-0.559550976	count	1
TET1	-0.495341	1.0257737	-0.4829	0.629	-0.559048741	count	1
CERS2	-0.410124	0.4236545	-0.9681	0.334	-0.55801862	count	1
TCOF1	-0.4938716	0.5443557	-0.9073	0.365	-0.5574482	count	1
TATDN2	-0.6079746	1.2232387	-0.497	0.62	-0.556725465	count	1
DUSP7	-0.6079746	1.2232387	-0.497	0.62	-0.556725465	count	1
VIPAS39	-0.6079746	1.2232387	-0.497	0.62	-0.556725465	count	1
CDKN3	-0.6079746	1.2975654	-0.4686	0.64	-0.556725465	count	1
PROX2	-0.6079746	1.2975654	-0.4686	0.64	-0.556725465	count	1
TTLL1	-0.6079746	1.2975654	-0.4686	0.64	-0.556725465	count	1
AL080276.2	-0.6079746	1.5615898	-0.3893	0.697	-0.556725465	count	1
CHD7	-0.6079746	1.5615898	-0.3893	0.697	-0.556725465	count	1
NAV2	-0.6079746	1.5615898	-0.3893	0.697	-0.556725465	count	1
PLCE1	-0.6079746	1.5615898	-0.3893	0.697	-0.556725465	count	1
BMP2	-0.6079746	1.5615898	-0.3893	0.697	-0.556725465	count	1
FITM2	-0.6079746	1.5615898	-0.3893	0.697	-0.556725465	count	1
HAR1A	-0.6079746	1.5615898	-0.3893	0.697	-0.556725465	count	1
ZNF845	-0.6079746	1.5615898	-0.3893	0.697	-0.556725465	count	1
PRDM15	-0.6079746	1.5615898	-0.3893	0.697	-0.556725465	count	1
TNIP1	-0.4435794	0.4700115	-0.9438	0.346	-0.556714434	count	1
AGO2	-0.4054773	0.4234493	-0.9576	0.339	-0.556681684	count	1
CD244	-0.4128919	0.4809243	-0.8585	0.391	-0.556252482	count	1
ZNF92	-0.416896	0.5347301	-0.7796	0.436	-0.556077796	count	1
NCOA4	-0.4245369	0.4603656	-0.9222	0.357	-0.555674357	count	1
FLOT2	-0.437831	0.5914122	-0.7403	0.46	-0.555634121	count	1
GTF2E2	-0.4334038	0.4917329	-0.8814	0.379	-0.555235512	count	1

ANKRA2	-0.4552254	0.7320646	-0.6218	0.534	-0.554995165	count	1
KDM7A	-0.4551148	0.6743016	-0.6749	0.5	-0.554863387	count	1
STAU1	-0.3978824	0.2901236	-1.3714	0.171	-0.554861422	count	1
HLA-DPB1	-0.3921577	0.2359773	-1.6618	0.0975	-0.554800277	count	1
SNHG9	-0.406054	0.4209378	-0.9646	0.335	-0.554694065	count	1
RNMT	-0.3920472	0.2579134	-1.5201	0.1295	-0.5545117	count	1
COMT	-0.3991667	0.3762098	-1.061	0.289	-0.554441708	count	1
ABI3	-0.3923892	0.3123641	-1.2562	0.21	-0.55396668	count	1
HSD17B10	-0.4230625	0.3586002	-1.1798	0.239	-0.553768691	count	1
CCDC174	-0.4098578	0.3651998	-1.1223	0.263	-0.553724543	count	1
OSER1	-0.3929578	0.3090964	-1.2713	0.2045	-0.55352812	count	1
MED18	-0.7268501	0.8585463	-0.8466	0.398	-0.553366503	count	1
DMAC2	-0.4537669	0.7215768	-0.6289	0.53	-0.553257174	count	1
APOOL	-0.4193688	0.6200954	-0.6763	0.499	-0.551997087	count	1
NAGK	-0.4216737	0.5706637	-0.7389	0.46	-0.551973475	count	1
APOPT1	-0.3963486	0.3659792	-1.083	0.28	-0.551021998	count	1
CLTC	-0.4300551	0.5084644	-0.8458	0.398	-0.55101149	count	1
VMP1	-0.3989674	0.3567112	-1.1185	0.264	-0.550751771	count	1
LRRC40	-0.600213	0.7101851	-0.8452	0.399	-0.550132798	count	1
RPAP2	-0.4228348	0.4193881	-1.0082	0.314	-0.550079479	count	1
ATAD5	-0.5997137	0.8920357	-0.6723	0.502	-0.549708161	count	1
MPI	-0.5997137	0.9479657	-0.6326	0.527	-0.549708161	count	1
LMCD1	-0.5997137	0.9849558	-0.6089	0.543	-0.549708161	count	1
VDAC1	-0.3881936	0.2599349	-1.4934	0.1363	-0.549700196	count	1
TPST2	-0.3995251	0.2908177	-1.3738	0.17	-0.549363362	count	1
SUSD3	-0.4374185	0.5537964	-0.7899	0.43	-0.549122944	count	1
RABGAP1	-0.5986609	0.6799153	-0.8805	0.379	-0.548812595	count	1
DENND4B	-0.5412192	0.6637607	-0.8154	0.415	-0.548713929	count	1
GSR	-0.4497566	0.5172693	-0.8695	0.385	-0.548476793	count	1
8-Mar	-0.5978272	0.8389132	-0.7126	0.477	-0.548103194	count	1
BIRC3	-0.3969941	0.3015042	-1.3167	0.189	-0.548042515	count	1
PLEKHA2	-0.4852119	0.477327	-1.0165	0.31	-0.548007342	count	1
RAB27B	-0.540033	0.7396394	-0.7301	0.466	-0.547574257	count	1
CCNDBP1	-0.3863795	0.2564781	-1.5065	0.1329	-0.546514492	count	1
MAPK3	-0.4680531	0.6035903	-0.7754	0.439	-0.546080116	count	1
HSPE1	-0.3811396	0.1831951	-2.0805	0.0383	-0.545570066	count	1
DIAPH1	-0.3976705	0.3501678	-1.1357	0.257	-0.545180802	count	1
IKZF1	-0.3877611	0.2518724	-1.5395	0.1247	-0.544965492	count	1
SAAL1	-0.4187311	0.762293	-0.5493	0.583	-0.544810686	count	1
ERCC1	-0.3884716	0.3611523	-1.0756	0.2829	-0.544704316	count	1
STYXL1	-0.4214382	0.5782515	-0.7288	0.467	-0.544471887	count	1
SLC12A9	-0.4214382	0.6760963	-0.6233	0.533	-0.544471887	count	1
TIGD5	-0.4214382	0.6845066	-0.6157	0.539	-0.544471887	count	1
SASH3	-0.3912331	0.348922	-1.1213	0.263	-0.54394215	count	1
FAM172A	-0.4071218	0.4846338	-0.8401	0.401	-0.543167523	count	1

RC3H2	-0.4277006	0.5345451	-0.8001	0.424	-0.542989014	count	1
ZC3H7A	-0.40854	0.3960204	-1.0316	0.303	-0.542892995	count	1
TMEM256	-0.3891728	0.3654498	-1.0649	0.288	-0.542373032	count	1
CEP85	-1.1416252	1.1136746	-1.0251	0.3061	-0.541816197	count	1
RPTOR	-1.1416252	1.2274959	-0.93	0.353	-0.541816197	count	1
ETHE1	-0.3895288	0.3431997	-1.135	0.257	-0.541115972	count	1
GAN	-0.5330816	0.8876354	-0.6006	0.549	-0.540889006	count	1
EFEMP1	-0.5330816	1.0939565	-0.4873	0.626	-0.540889006	count	1
DPM3	-0.3919661	0.3750407	-1.0451	0.297	-0.540473934	count	1
GABPB1-AS1	-0.3831446	0.2891972	-1.3249	0.1862	-0.540113026	count	1
FUBP1	-0.4123255	0.3225192	-1.2785	0.202	-0.539884968	count	1
CCDC91	-0.3887779	0.3229622	-1.2038	0.23	-0.539589523	count	1
CLTA	-0.3809927	0.2490465	-1.5298	0.127	-0.53940904	count	1
TAPBPL	-0.4341066	0.4487566	-0.9674	0.334	-0.538073379	count	1
SF1	-0.3792621	0.1823135	-2.0803	0.0383	-0.537726495	count	1
MRPL23	-0.3986777	0.4741178	-0.8409	0.401	-0.537262789	count	1
HIST1H1D	-0.3866375	0.3672448	-1.0528	0.293	-0.537117634	count	1
SAP30BP	-0.3923607	0.3266628	-1.2011	0.231	-0.537057729	count	1
PPP1R12C	-0.3985028	0.7167403	-0.556	0.579	-0.537029043	count	1
KNOP1	-0.4100462	0.6088884	-0.6734	0.501	-0.536936296	count	1
IFIT3	-0.3909674	0.7936855	-0.4926	0.6226	-0.536879927	count	1
GTF3C5	-0.4184126	0.6121753	-0.6835	0.495	-0.536316164	count	1
TALDO1	-0.3786561	0.2336131	-1.6209	0.106	-0.536224347	count	1
CORO1B	-0.3937968	0.4400691	-0.8949	0.372	-0.535960944	count	1
INTS12	-0.4090783	0.6053608	-0.6758	0.5	-0.535684001	count	1
CD47	-0.3925575	0.2855202	-1.3749	0.17	-0.535367524	count	1
MAP1S	-0.426262	0.7032934	-0.6061	0.545	-0.535363789	count	1
HEMK1	-0.473517	0.8522251	-0.5556	0.579	-0.535235072	count	1
OGG1	-0.404086	0.5495808	-0.7353	0.463	-0.53469548	count	1
SCOC	-0.3911172	0.4247393	-0.9208	0.358	-0.53442324	count	1
PPP1R3B	-1.11725	1.281588	-0.8718	0.384	-0.533243021	count	1
CRYAB	-1.1172502	1.5335223	-0.7286	0.4668	-0.533242944	count	1
PIK3R6	-1.1172502	1.5335223	-0.7286	0.4668	-0.533242944	count	1
LINS1	-0.436952	0.4880654	-0.8953	0.371	-0.533197814	count	1
APOBEC3F	-0.4924999	0.6457041	-0.7627	0.446	-0.532860407	count	1
DHODH	-0.4450764	0.8052944	-0.5527	0.581	-0.532631944	count	1
GOLIM4	-0.3797747	0.3611107	-1.0517	0.2937	-0.531998815	count	1
NDUFB7	-0.3753487	0.2584178	-1.4525	0.1473	-0.531880577	count	1
SLC6A6	-0.4060579	0.5733875	-0.7082	0.479	-0.531775584	count	1
SSR1	-0.3848495	0.3817449	-1.0081	0.314	-0.531364623	count	1
CHKA	-0.6940016	0.7905894	-0.8778	0.3807	-0.531097885	count	1
SMAD4	-0.4281146	0.6169626	-0.6939	0.488	-0.530788315	count	1
KLHL28	-0.3936804	0.3989061	-0.9869	0.324	-0.530583314	count	1
EIF4G2	-0.3765931	0.2262932	-1.6642	0.097	-0.529957235	count	1
COMMD2	-0.3798572	0.3534202	-1.0748	0.283	-0.529824808	count	1

TCTA	-0.4098323	0.6241179	-0.6567	0.512	-0.529680726	count	1
UBE2H	-0.3915006	0.3811467	-1.0272	0.305	-0.529118098	count	1
CRBN	-0.3833268	0.375354	-1.0212	0.308	-0.528624749	count	1
HAGH	-0.3827742	0.3469216	-1.1033	0.271	-0.528513997	count	1
UPP1	-0.3762992	0.3285878	-1.1452	0.253	-0.528202567	count	1
FCHSD2	-0.4158375	0.5438896	-0.7646	0.445	-0.528165913	count	1
CPSF2	-0.4119117	0.8655174	-0.4759	0.6345	-0.528104214	count	1
WBP2	-0.3917705	0.3945595	-0.9929	0.321	-0.528030068	count	1
SLC25A29	-0.3956382	0.69544	-0.5689	0.57	-0.52799005	count	1
CMPK2	-0.4025209	1.0954532	-0.3674	0.7135	-0.52719762	count	1
DEF6	-0.3787977	0.2752556	-1.3762	0.17	-0.52716046	count	1
CYTOR	-0.3792675	0.2660709	-1.4254	0.155	-0.526449464	count	1
TMED5	-0.3832994	0.3749977	-1.0221	0.307	-0.526411077	count	1
BBS7	-0.4394458	0.8102929	-0.5423	0.588	-0.526053877	count	1
TMEM167A	-0.3783638	0.3473342	-1.0893	0.277	-0.525674256	count	1
MAX	-0.3898737	0.3414019	-1.142	0.254	-0.525494093	count	1
EID3	-0.4181531	0.6384365	-0.655	0.513	-0.525353532	count	1
CAPN7	-0.4034819	0.4775754	-0.8449	0.399	-0.525217699	count	1
RNF114	-0.3875137	0.4129404	-0.9384	0.349	-0.525096075	count	1
NOL8	-0.3895355	0.421742	-0.9236	0.356	-0.5250419	count	1
RPL36A	-0.3746793	0.2487802	-1.5061	0.133	-0.524885279	count	1
CTU2	-0.4384125	0.8729995	-0.5022	0.616	-0.524846167	count	1
RGL2	-0.3981982	0.5343432	-0.7452	0.457	-0.524437713	count	1
UBE2F	-0.3927212	0.4246039	-0.9249	0.356	-0.52413316	count	1
WAC-AS1	-0.3801847	0.4737188	-0.8026	0.423	-0.522918206	count	1
WRAP73	-0.4009822	0.5670269	-0.7072	0.48	-0.522003814	count	1
CHPT1	-0.3896195	0.4345613	-0.8966	0.371	-0.521889856	count	1
ITGB3BP	-0.4036878	0.6141068	-0.6574	0.511	-0.521844292	count	1
CHMP6	-0.3824252	0.4796783	-0.7973	0.426	-0.521639592	count	1
ACSS1	-0.4462749	0.6538453	-0.6825	0.495	-0.521361948	count	1
COX18	-0.5660734	0.9517409	-0.5948	0.5524	-0.520951796	count	1
POMZP3	-0.4343334	0.7778697	-0.5584	0.577	-0.520076971	count	1
RINT1	-0.4192981	0.6798901	-0.6167	0.538	-0.520060624	count	1
EXOC3-AS1	-0.4795731	0.9722222	-0.4933	0.622	-0.519410534	count	1
AC116407.2	-0.4795731	0.9722222	-0.4933	0.622	-0.519410534	count	1
APOM	-0.4795731	1.3220004	-0.3628	0.717	-0.519410534	count	1
IGF1R	-0.4795731	1.3220004	-0.3628	0.717	-0.519410534	count	1
PRKCH	-0.3739862	0.317187	-1.1791	0.239	-0.519151002	count	1
PTBP3	-0.3747772	0.3392205	-1.1048	0.27	-0.51869647	count	1
CARS2	-0.6756436	0.8753133	-0.7719	0.441	-0.518524792	count	1
CSNK1D	-0.3843129	0.3657749	-1.0507	0.294	-0.518058056	count	1
REST	-0.3674778	0.2746725	-1.3379	0.1819	-0.51791228	count	1
MRPS12	-0.3712506	0.3054767	-1.2153	0.225	-0.517866409	count	1
TRGC2	-0.4071387	0.5720685	-0.7117	0.477	-0.517286364	count	1
JHY	-0.6733847	0.9014265	-0.747	0.456	-0.516971398	count	1

AC109826.1	-0.6733847	1.00893	-0.6674	0.505	-0.516971398	count	1
CD74	-0.3592061	0.1482745	-2.4226	0.016	-0.516289867	count	1
TDG	-0.379769	0.3273368	-1.1602	0.247	-0.515879202	count	1
C12orf57	-0.3605717	0.1853549	-1.9453	0.0526	-0.51527331	count	1
ISG15	-0.3614289	0.2589069	-1.396	0.1637	-0.514974198	count	1
WDCP	-0.4752285	0.9636995	-0.4931	0.622	-0.514882467	count	1
SIDT2	-0.4752285	0.9926038	-0.4788	0.632	-0.514882467	count	1
DOCK7	-0.4752285	1.0541472	-0.4508	0.652	-0.514882467	count	1
ZNF101	-0.4212924	0.5980384	-0.7045	0.482	-0.514480316	count	1
EVA1B	-0.3749977	0.4218787	-0.8889	0.375	-0.513433913	count	1
GSKIP	-0.386059	0.5689075	-0.6786	0.498	-0.513303802	count	1
MANBA	-0.5569338	0.4958728	-1.1231	0.262	-0.513089598	count	1
S1PR4	-0.3874357	0.4662171	-0.831	0.407	-0.512884506	count	1
ELOVL1	-0.3718314	0.3954447	-0.9403	0.348	-0.512852998	count	1
HOXB2	-0.3696461	0.4736434	-0.7804	0.4357	-0.512668716	count	1
CARHSP1	-0.3676391	0.3134257	-1.173	0.242	-0.512479299	count	1
BLCAP	-0.3993268	0.5262463	-0.7588	0.449	-0.512194032	count	1
DGUOK	-0.3624189	0.2906538	-1.2469	0.2133	-0.511902578	count	1
TESK1	-0.4190293	0.6580869	-0.6367	0.525	-0.511772435	count	1
KATNA1	-0.3796128	0.4286104	-0.8857	0.376	-0.511771374	count	1
PSMB8	-0.3579611	0.1820703	-1.9661	0.0502	-0.510893945	count	1
RAB6A	-0.3808127	0.4060037	-0.938	0.349	-0.510193317	count	1
MYC	-0.3759747	0.4919174	-0.7643	0.445	-0.509571393	count	1
POLR1D	-0.3585387	0.2449471	-1.4637	0.144	-0.509098336	count	1
OTULIN	-0.3623665	0.3325654	-1.0896	0.2767	-0.508704991	count	1
GINS3	-1.04836	0.8580964	-1.2217	0.223	-0.508295671	count	1
NRDC	-0.3614786	0.297526	-1.2149	0.225	-0.507917987	count	1
TMEM165	-0.3606733	0.2923422	-1.2337	0.218	-0.507418867	count	1
SIPA1L1	-0.4339823	0.6330041	-0.6856	0.493	-0.507374705	count	1
POLR3D	-0.4339823	0.662963	-0.6546	0.513	-0.507374705	count	1
DEDD2	-0.3749986	0.3939729	-0.9518	0.342	-0.50697986	count	1
PRIMPOL	-0.4032264	0.6452768	-0.6249	0.532	-0.506905869	count	1
ATL2	-0.3987864	0.6619656	-0.6024	0.547	-0.506832095	count	1
TTTY15	-0.378142	0.5670127	-0.6669	0.505	-0.506645199	count	1
RHEBL1	-0.6583915	0.6294333	-1.046	0.296	-0.50662628	count	1
ERO1A	-0.3985906	0.5181899	-0.7692	0.442	-0.506586923	count	1
RPGR	-0.422215	0.5825356	-0.7248	0.469	-0.505893425	count	1
NEDD9	-0.3574351	0.2864166	-1.248	0.2129	-0.505782716	count	1
SEL1L	-0.3819738	0.4771532	-0.8005	0.424	-0.50572478	count	1
ZNHIT2	-0.4661883	1.0961925	-0.4253	0.671	-0.505448285	count	1
TTBK2	-0.4661883	1.1326687	-0.4116	0.681	-0.505448285	count	1
AL132639.2	-0.4661883	1.2366528	-0.377	0.706	-0.505448285	count	1
PLXDC1	-0.4661883	1.2366528	-0.377	0.706	-0.505448285	count	1
FCGR3B	-0.4661883	1.376901	-0.3386	0.735	-0.505448285	count	1
NAPG	-0.3834866	0.5491339	-0.6983	0.485	-0.505263669	count	1

MPHOSPH10	-0.3682924	0.4210781	-0.8746	0.382	-0.505134994	count	1
AAAS	-0.5474049	0.5699326	-0.9605	0.338	-0.504870266	count	1
SREK1	-0.3638685	0.3488056	-1.0432	0.298	-0.504191749	count	1
CD320	-0.3843976	0.4737373	-0.8114	0.418	-0.50372276	count	1
IPP	-0.5458695	0.7455499	-0.7322	0.465	-0.503543769	count	1
FKBP4	-0.3866077	0.5976357	-0.6469	0.518	-0.503510875	count	1
MTG2	-0.4305034	0.9904719	-0.4346	0.664	-0.503411671	count	1
TBP	-0.4305034	0.8476173	-0.5079	0.612	-0.503411662	count	1
MTRF1L	-0.3957203	0.6736277	-0.5874	0.557	-0.502992398	count	1
RPS10	-0.3509135	0.1457404	-2.4078	0.0166	-0.50255584	count	1
AC245297.3	-0.3663599	0.3566145	-1.0273	0.305	-0.502499237	count	1
JAK2	-0.4048798	0.7950649	-0.5092	0.611	-0.502494953	count	1
ORAI1	-0.3609769	0.2849749	-1.2667	0.206	-0.502457845	count	1
RAB11A	-0.3608419	0.3098269	-1.1647	0.245	-0.50227067	count	1
SYVN1	-0.4628729	0.8708202	-0.5315	0.595	-0.501984272	count	1
ZNF273	-1.030847	1.1169414	-0.9229	0.3567	-0.501783524	count	1
UBR2	-0.3640861	0.3953077	-0.921	0.358	-0.501576196	count	1
BTBD1	-0.4621599	0.5889194	-0.7848	0.433	-0.501239031	count	1
RGS2	-0.3873441	0.4511229	-0.8586	0.391	-0.500981762	count	1
DDHD2	-0.3783182	0.6179826	-0.6122	0.541	-0.500931497	count	1
EMC10	-0.3779645	0.3347371	-1.1291	0.26	-0.500467661	count	1
AP3M2	-0.4277544	0.7700775	-0.5555	0.579	-0.500278688	count	1
ZCWPW1	-0.4277544	0.9770794	-0.4378	0.662	-0.500278688	count	1
PLEKHO1	-0.3551178	0.3089687	-1.1494	0.2513	-0.500187807	count	1
ING3	-0.3677691	0.4150579	-0.8861	0.376	-0.499686837	count	1
SEC23IP	-0.5411759	1.0374862	-0.5216	0.602	-0.499485105	count	1
NAA80	-0.4602413	0.7910412	-0.5818	0.561	-0.499233148	count	1
RAP2A	-0.4602413	0.9213616	-0.4995	0.618	-0.499233148	count	1
ATP8B2	-0.4598186	0.8225752	-0.559	0.577	-0.498791116	count	1
PLK2	-0.4598186	0.9300084	-0.4944	0.6213	-0.498791116	count	1
EXT1	-0.5400731	1.0354673	-0.5216	0.602	-0.498530715	count	1
ZNF443	-0.5400731	1.0354673	-0.5216	0.602	-0.498530715	count	1
FOXJ2	-0.5400731	1.1517456	-0.4689	0.639	-0.498530715	count	1
CEP112	-0.5400731	1.2668311	-0.4263	0.67	-0.498530715	count	1
DDRGK1	-0.3568756	0.3451906	-1.0339	0.302	-0.498229564	count	1
TXNDC9	-0.3605909	0.3693717	-0.9762	0.33	-0.498031187	count	1
PTGES3	-0.3489012	0.1723394	-2.0245	0.0437	-0.497868136	count	1
RARS	-0.4580262	0.6752582	-0.6783	0.498	-0.496916365	count	1
LYST	-0.3552042	0.3111169	-1.1417	0.254	-0.496847787	count	1
VHL	-0.3735596	0.5182248	-0.7208	0.472	-0.496835198	count	1
RPS27L	-0.3482422	0.2005545	-1.7364	0.0834	-0.496440498	count	1
PAPOLG	-0.4138903	0.5651611	-0.7323	0.464	-0.4961372	count	1
LARS	-0.3549364	0.3353124	-1.0585	0.2906	-0.495858428	count	1
SEC23B	-0.4568744	0.7776924	-0.5875	0.557	-0.495711301	count	1
SPOPL	-0.4568744	0.8078378	-0.5656	0.572	-0.495711301	count	1

CAB39L	-0.4568744	0.8982408	-0.5086	0.611	-0.495711301	count	1
ZNF700	-0.4568744	0.9145467	-0.4996	0.618	-0.495711301	count	1
SDHAF1	-0.3758259	0.4891959	-0.7683	0.443	-0.495271983	count	1
ANKRD54	-0.4560161	0.6456056	-0.7063	0.48	-0.494813147	count	1
MRPL14	-0.3508054	0.2841207	-1.2347	0.2178	-0.494638003	count	1
NR1H2	-0.3537667	0.2934679	-1.2055	0.229	-0.494542762	count	1
ARF5	-0.3493812	0.2534916	-1.3783	0.1691	-0.494409606	count	1
SORL1	-0.4554742	0.5375598	-0.8473	0.397	-0.494246001	count	1
CCDC92	-0.3685324	0.5184873	-0.7108	0.478	-0.493874346	count	1
PML	-0.3976266	0.5557761	-0.7154	0.475	-0.493648321	count	1
DNASE2	-0.3976266	0.5717331	-0.6955	0.487	-0.493648321	count	1
TIA1	-0.4541778	0.4773992	-0.9514	0.342	-0.492888983	count	1
GLIPR2	-0.3441765	0.2061892	-1.6692	0.096	-0.49248855	count	1
SOD3	-0.4028863	0.9606927	-0.4194	0.675	-0.492436056	count	1
RNFT1	-0.3562275	0.4600559	-0.7743	0.439	-0.492032766	count	1
PIEZO1	-0.453194	0.6782149	-0.6682	0.504	-0.49185895	count	1
SEC11A	-0.3447521	0.1730256	-1.9925	0.0472	-0.49184117	count	1
PARP3	-0.401905	0.7294895	-0.5509	0.582	-0.491259473	count	1
AC139530.1	-0.3956382	0.9752144	-0.4057	0.685	-0.491221925	count	1
ALG3	-0.3665111	0.4478906	-0.8183	0.414	-0.49118728	count	1
PKM	-0.3451048	0.2335744	-1.4775	0.1405	-0.490881628	count	1
ECI2	-0.3499413	0.3159333	-1.1076	0.269	-0.490338156	count	1
ARL5B	-0.3604543	0.4252982	-0.8475	0.397	-0.489812305	count	1
MXD4	-0.3465336	0.2832584	-1.2234	0.2221	-0.489780461	count	1
HPS4	-0.4180266	0.553569	-0.7551	0.451	-0.489182187	count	1
PCBD1	-0.3846592	0.7019664	-0.548	0.584	-0.489131788	count	1
PPT1	-0.3559016	0.5370793	-0.6627	0.508	-0.488982199	count	1
TMEM238	-0.353107	0.3149165	-1.1213	0.263	-0.488837133	count	1
LRBA	-0.3885016	0.4778996	-0.8129	0.417	-0.488681498	count	1
COPA	-0.393316	0.3804609	-1.0338	0.302	-0.488387582	count	1
UPF1	-0.6321238	0.9456408	-0.6685	0.504	-0.488357053	count	1
GADD45GIP1	-0.3442457	0.2544967	-1.3527	0.1771	-0.487692143	count	1
MCRIP2	-0.3799388	0.5800605	-0.655	0.513	-0.48765038	count	1
NUBP2	-0.3544086	0.4070927	-0.8706	0.385	-0.487645287	count	1
HAUS1	-0.4156803	0.5463931	-0.7608	0.447	-0.48650346	count	1
P2RX4	-0.4055675	0.6958513	-0.5828	0.56	-0.486372828	count	1
GMCL1	-0.3556602	0.4190711	-0.8487	0.397	-0.486255889	count	1
RNF40	-0.3863222	0.733012	-0.527	0.599	-0.485981945	count	1
SH3BP2	-0.6287241	0.653505	-0.9621	0.3367	-0.485979181	count	1
PARL	-0.3558253	0.444111	-0.8012	0.424	-0.485573194	count	1
TRAF6	-0.3858466	0.704253	-0.5479	0.584	-0.485392763	count	1
LSM2	-0.3394726	0.2183769	-1.5545	0.121	-0.484465176	count	1
ZNF667-AS1	-0.365561	0.4677838	-0.7815	0.435	-0.484195664	count	1
STARD10	-0.3713365	0.7154664	-0.519	0.604	-0.48384311	count	1
SBNO1	-0.5227429	0.4946268	-1.0568	0.291	-0.483493133	count	1

LAX1	-0.3645852	0.49222	-0.7407	0.459	-0.482915008	count	1
SELPLG	-0.3499996	0.3254935	-1.0753	0.283	-0.482883314	count	1
RGS1	-0.3401816	0.2879475	-1.1814	0.2383	-0.482841039	count	1
UBE2J2	-0.3537938	0.4073609	-0.8685	0.386	-0.482817133	count	1
WDR61	-0.4124306	0.4517737	-0.9129	0.362	-0.482791858	count	1
CIP2A	-0.623168	0.7924962	-0.7863	0.432	-0.482086351	count	1
FAM213B	-0.623168	0.8092983	-0.77	0.442	-0.482086351	count	1
SCUBE1	-0.623168	0.8283017	-0.7523	0.452	-0.482086351	count	1
FABP5	-0.3412574	0.3087024	-1.1055	0.2698	-0.482078151	count	1
ZMAT3	-0.3879456	0.7301079	-0.5314	0.5955	-0.481830107	count	1
ATP5MD	-0.3394174	0.2221336	-1.528	0.1275	-0.481423421	count	1
KIN	-0.3459657	0.3690313	-0.9375	0.349	-0.481249032	count	1
PSME2	-0.3356835	0.1531896	-2.1913	0.0291	-0.481211251	count	1
AC012615.1	-0.3870998	0.6007141	-0.6444	0.52	-0.48079702	count	1
FLT3LG	-0.348475	0.3984676	-0.8745	0.382	-0.480789629	count	1
PUM1	-0.3815818	0.5035435	-0.7578	0.449	-0.480108234	count	1
GFOD1	-0.4099411	0.452983	-0.905	0.366	-0.479947357	count	1
SLC25A11	-0.3440651	0.3479492	-0.9888	0.323	-0.479734575	count	1
PFDN2	-0.3397633	0.2357892	-1.441	0.1506	-0.479698545	count	1
UTP3	-0.3636488	0.5393981	-0.6742	0.501	-0.47937955	count	1
CPNE3	-0.3411822	0.3119129	-1.0938	0.275	-0.479266399	count	1
POLM	-0.6188676	0.7806847	-0.7927	0.429	-0.47906788	count	1
CD70	-0.6188676	0.8356069	-0.7406	0.459	-0.47906788	count	1
CYLD	-0.33839	0.2417395	-1.3998	0.1625	-0.478167057	count	1
HOOK3	-0.3664095	0.4799508	-0.7634	0.446	-0.477493036	count	1
SAMD10	-0.5153692	1.005194	-0.5127	0.609	-0.477072596	count	1
NPIPB5	-0.5153692	1.08989	-0.4729	0.637	-0.477072596	count	1
TNFRSF18	-0.3334394	0.2165456	-1.5398	0.1246	-0.476904113	count	1
PTPN2	-0.3386396	0.2704736	-1.252	0.2115	-0.476861851	count	1
TTC5	-0.3788127	0.5725073	-0.6617	0.509	-0.476675898	count	1
DYM	-0.3968567	0.5889131	-0.6739	0.501	-0.476142266	count	1
RELA	-0.3576389	0.4693596	-0.762	0.447	-0.475841598	count	1
DZANK1	-0.5122765	1.1584368	-0.4422	0.659	-0.47437573	count	1
GNPNAT1	-0.4050578	0.6581258	-0.6155	0.539	-0.474364805	count	1
HCFC1R1	-0.3467398	0.4263452	-0.8133	0.417	-0.474127332	count	1
FAM98A	-0.4042438	0.5771249	-0.7004	0.484	-0.473433873	count	1
INPP5B	-0.3801588	0.6914654	-0.5498	0.583	-0.472315691	count	1
VAMP3	-0.3465859	0.505538	-0.6856	0.493	-0.472093667	count	1
SNHG7	-0.3358233	0.2535461	-1.3245	0.186	-0.471965443	count	1
NFKBID	-0.3350619	0.3071292	-1.0909	0.2761	-0.471291028	count	1
KBTBD2	-0.349153	0.3898913	-0.8955	0.371	-0.470994668	count	1
UTP15	-0.38489	0.7976115	-0.4826	0.63	-0.470837595	count	1
MFSD4A	-0.401702	1.0928416	-0.3676	0.713	-0.470526284	count	1
CRK	-0.3735494	0.5865811	-0.6368	0.525	-0.470149494	count	1
HNRNPC	-0.3313094	0.1764096	-1.8781	0.0613	-0.47010897	count	1

DCAF5	-0.3692675	0.4855499	-0.7605	0.448	-0.469822087	count	1
EID1	-0.3291417	0.2133257	-1.5429	0.124	-0.469116011	count	1
ZNF780B	-0.4004076	0.6789413	-0.5898	0.556	-0.469045186	count	1
HNRNPU	-0.3284007	0.1861291	-1.7644	0.0786	-0.468974431	count	1
ARL6IP1	-0.3351456	0.3116271	-1.0755	0.283	-0.468877423	count	1
AKAP9	-0.3309908	0.2341296	-1.4137	0.1584	-0.468647496	count	1
PDE12	-0.3677917	0.686907	-0.5354	0.593	-0.467969259	count	1
LINC02273	-0.3643204	0.9090237	-0.4008	0.689	-0.467850391	count	1
DYNLL2	-0.35886	0.5641285	-0.6361	0.525	-0.467758708	count	1
PSMB5	-0.3393062	0.4386401	-0.7735	0.44	-0.467597551	count	1
FCMR	-0.341001	0.4026413	-0.8469	0.398	-0.467133544	count	1
MRPL36	-0.3398127	0.4226713	-0.804	0.422	-0.466985909	count	1
CKS1B	-0.3816356	0.6224493	-0.6131	0.54	-0.466927113	count	1
RDH11	-0.3413887	0.4546689	-0.7509	0.453	-0.466849779	count	1
CSNK2B	-0.3280988	0.1924818	-1.7046	0.0892	-0.466356652	count	1
PRPF3	-0.388217	0.5313885	-0.7306	0.466	-0.465984206	count	1
SARS	-0.3341499	0.3445896	-0.9697	0.333	-0.465610645	count	1
IFT57	-0.3694542	0.5027933	-0.7348	0.463	-0.4650693	count	1
ZNF264	-0.3591022	0.6090508	-0.5896	0.556	-0.464869371	count	1
C7orf50	-0.3298236	0.2943034	-1.1207	0.2632	-0.464808117	count	1
HLA-DRB1	-0.3271955	0.2344744	-1.3954	0.1638	-0.464438498	count	1
CTCF	-0.3361519	0.3111891	-1.0802	0.281	-0.464424104	count	1
ZSCAN16	-0.5005176	0.8404781	-0.5955	0.552	-0.464100737	count	1
RBM12B	-0.5005176	0.9509669	-0.5263	0.599	-0.464100737	count	1
MEMO1	-0.5005176	1.0356068	-0.4833	0.6292	-0.464100737	count	1
SYNGR3	-0.4085589	0.8493655	-0.481	0.631	-0.463836693	count	1
YIPF1	-0.355798	0.5431265	-0.6551	0.513	-0.463809091	count	1
MTHFD1	-0.3863225	0.7667128	-0.5039	0.615	-0.463755305	count	1
TSPAN2	-0.3456532	0.5264686	-0.6566	0.512	-0.463442802	count	1
CSK	-0.3331322	0.2978831	-1.1183	0.264	-0.462656691	count	1
PRKD3	-0.348867	0.6331385	-0.551	0.582	-0.462275872	count	1
TOR1AIP1	-0.3368078	0.3468264	-0.9711	0.332	-0.462169909	count	1
HINT1	-0.3222901	0.1252137	-2.5739	0.0105	-0.462125332	count	1
OSBPL3	-0.3943379	0.8396989	-0.4696	0.6389	-0.462096535	count	1
BLOC1S5	-0.3503868	0.5218653	-0.6714	0.502	-0.462057302	count	1
RAB24	-0.9275932	1.309575	-0.7083	0.479	-0.461977369	count	1
NFIA	-0.9275932	1.40554	-0.66	0.51	-0.461977369	count	1
ALCAM	-0.9275932	1.40554	-0.66	0.51	-0.461977369	count	1
GALNT1	-0.3370882	0.300719	-1.1209	0.263	-0.461800944	count	1
RNF2	-0.3483887	0.5367263	-0.6491	0.517	-0.461647527	count	1
PITPNM1	-0.3713365	0.8134027	-0.4565	0.648	-0.461526902	count	1
YAE1D1	-0.4970674	0.7978117	-0.623	0.534	-0.461079608	count	1
TIMM10B	-0.3438698	0.4638611	-0.7413	0.459	-0.461069204	count	1
NAA38	-0.3345211	0.3210667	-1.0419	0.298	-0.460409409	count	1
UBTF	-0.328509	0.3053153	-1.076	0.2827	-0.460392936	count	1

CNOT8	-0.3343954	0.4235017	-0.7896	0.43	-0.459576796	count	1
FIBP	-0.328277	0.3442802	-0.9535	0.341	-0.459563703	count	1
SCAPER	-0.3467051	0.4626095	-0.7495	0.454	-0.459435638	count	1
LAT	-0.3243068	0.2864751	-1.1321	0.2584	-0.459098261	count	1
SLC39A3	-0.3822165	0.6480458	-0.5898	0.556	-0.458922773	count	1
THRA	-0.3641914	0.6444462	-0.5651	0.572	-0.458537825	count	1
CASP10	-0.374518	0.6024741	-0.6216	0.535	-0.458369686	count	1
TUT4	-0.325456	0.2535121	-1.2838	0.2001	-0.458340637	count	1
MATR3	-0.5891033	0.8078637	-0.7292	0.466	-0.458042742	count	1
DDX20	-0.3741158	0.6826118	-0.5481	0.584	-0.457885925	count	1
ABI2	-0.3741158	0.6828942	-0.5478	0.584	-0.457885925	count	1
CCNK	-0.3403572	0.4068903	-0.8365	0.403	-0.457860727	count	1
RNF38	-0.3558705	0.781297	-0.4555	0.649	-0.457127805	count	1
KIF5B	-0.3247418	0.2762278	-1.1756	0.2406	-0.456623672	count	1
COX7C	-0.3182114	0.1278731	-2.4885	0.0133	-0.456587822	count	1
MRFAP1	-0.3219429	0.2314364	-1.3911	0.1652	-0.456243823	count	1
UBL7-AS1	-0.3580677	0.7110456	-0.5036	0.615	-0.455755188	count	1
RPUSD2	-0.585289	0.9673892	-0.605	0.546	-0.455331681	count	1
PDE3B	-0.585289	1.082415	-0.5407	0.589	-0.455331681	count	1
COL9A2	-0.4450764	1.0349246	-0.4301	0.667	-0.455310783	count	1
MRPL17	-0.3328841	0.4355459	-0.7643	0.445	-0.455280432	count	1
SPIN2B	-0.4449709	1.0020206	-0.4441	0.657	-0.455207171	count	1
ZSWIM8	-0.4448709	0.7666623	-0.5803	0.562	-0.455108967	count	1
ABCA7	-0.4448709	0.9027655	-0.4928	0.6225	-0.455108967	count	1
RPS6KB1	-0.4447844	0.6170843	-0.7208	0.472	-0.45502401	count	1
FOSL2	-0.3209372	0.2398214	-1.3382	0.1818	-0.45482101	count	1
ODC1	-0.3266089	0.3313653	-0.9856	0.325	-0.454033132	count	1
EXOSC4	-0.3301622	0.4189454	-0.7881	0.431	-0.453786199	count	1
MRPS18B	-0.3362555	0.4214385	-0.7979	0.426	-0.453710942	count	1
PRCP	-0.3706199	0.5851956	-0.6333	0.527	-0.45368018	count	1
ZNF77	-0.3993312	1.0110221	-0.395	0.693	-0.453633234	count	1
RCBTB2	-0.3993312	1.0546166	-0.3787	0.705	-0.453633234	count	1
NOL12	-0.5825833	0.9656388	-0.6033	0.547	-0.453406303	count	1
SIVA1	-0.3187099	0.2315738	-1.3763	0.17	-0.453030166	count	1
PTK2B	-0.3434639	0.5088614	-0.675	0.5	-0.453009233	count	1
NDUFV2-AS1	-0.352579	0.8443323	-0.4176	0.677	-0.452949079	count	1
ACADVL	-0.3471083	0.3617227	-0.9596	0.338	-0.452595827	count	1
HABP4	-0.4155304	0.7198495	-0.5772	0.564	-0.452282792	count	1
SMIM29	-0.3337187	0.3845025	-0.8679	0.386	-0.451526551	count	1
IK	-0.3200683	0.222362	-1.4394	0.151	-0.451090867	count	1
AQP3	-0.3399342	0.5559473	-0.6115	0.541	-0.45053793	count	1
METTL25	-0.3749374	0.8524853	-0.4398	0.66	-0.45034971	count	1
LANCL1	-0.3749374	0.8652142	-0.4333	0.665	-0.45034971	count	1
SMIM37	-0.3285838	0.4454624	-0.7376	0.461	-0.450208043	count	1
ATP11C	-0.3367989	0.7833177	-0.43	0.6675	-0.450071316	count	1

RC3H1	-0.3837636	0.6117633	-0.6273	0.531	-0.449977096	count	1
GNL3	-0.3353755	0.4140955	-0.8099	0.419	-0.44976086	count	1
SCML4	-0.3353426	0.4065396	-0.8249	0.41	-0.449717051	count	1
TSC22D2	-0.3377839	0.484087	-0.6978	0.486	-0.449633576	count	1
DHX30	-0.3416066	0.561404	-0.6085	0.543	-0.448181513	count	1
ZNF107	-0.3941879	0.6175344	-0.6383	0.524	-0.447939677	count	1
MOSMO	-0.359762	0.8429708	-0.4268	0.67	-0.447357903	count	1
IRF2BP2	-0.3168617	0.2559139	-1.2382	0.2166	-0.447162961	count	1
HIPK3	-0.3239241	0.3818822	-0.8482	0.397	-0.447060809	count	1
MMP23B	-0.3548453	0.6814939	-0.5207	0.603	-0.446930918	count	1
MPG	-0.3136774	0.2216099	-1.4154	0.1579	-0.446794486	count	1
MRPL48	-0.3444285	0.4976071	-0.6922	0.489	-0.446076187	count	1
ANXA11	-0.3142613	0.2169686	-1.4484	0.1485	-0.445718049	count	1
NOP10	-0.3134985	0.2293742	-1.3668	0.173	-0.445693845	count	1
RPP38	-0.3246624	0.442963	-0.7329	0.464	-0.445583045	count	1
HOXB-AS1	-0.3494067	0.8594435	-0.4065	0.685	-0.444867818	count	1
SCAF11	-0.3129003	0.2235228	-1.3999	0.1625	-0.444593096	count	1
TMEM120B	-0.3202018	0.3591264	-0.8916	0.373	-0.444349796	count	1
ZC3H15	-0.311662	0.2251759	-1.3841	0.167	-0.443927175	count	1
NCALD	-0.3624767	0.5935461	-0.6107	0.542	-0.443877251	count	1
PEX19	-0.3780719	0.7475765	-0.5057	0.613	-0.443446456	count	1
XRCC4	-0.5680949	0.7859061	-0.7229	0.47	-0.443064105	count	1
IRS2	-0.5680949	0.8883984	-0.6395	0.523	-0.443064105	count	1
2-Sep	-0.3153382	0.2457167	-1.2833	0.2	-0.443052955	count	1
LPP	-0.3280681	0.50278	-0.6525	0.515	-0.442733784	count	1
AC016065.1	-0.8794432	0.8776674	-1.002	0.3171	-0.442584722	count	1
PISD	-0.5673512	0.7082716	-0.801	0.424	-0.442531781	count	1
PIAS2	-0.3681907	0.627435	-0.5868	0.558	-0.442396921	count	1
RIOK1	-0.3173595	0.4087838	-0.7764	0.4381	-0.442290348	count	1
ZBTB41	-0.4057759	0.8597401	-0.472	0.637	-0.441988315	count	1
PAFAH1B2	-0.3301915	0.421916	-0.7826	0.434	-0.441305947	count	1
C18orf21	-0.3499444	0.6248884	-0.56	0.576	-0.440840541	count	1
MAP3K2	-0.3265399	0.3880792	-0.8414	0.401	-0.440684415	count	1
N4BP1	-0.3125668	0.2779271	-1.1246	0.2616	-0.44054127	count	1
DPH7	-0.3666032	0.5631525	-0.651	0.516	-0.440524683	count	1
TPM3	-0.3066785	0.1211654	-2.5311	0.0118	-0.440362045	count	1
SERPINB8	-0.3455383	0.5964118	-0.5794	0.563	-0.440002455	count	1
CPD	-0.3490458	0.4502507	-0.7752	0.439	-0.439723549	count	1
POLR1B	-0.5629518	1.1453275	-0.4915	0.623	-0.439379851	count	1
LRSAM1	-0.5629518	1.1453275	-0.4915	0.623	-0.439379851	count	1
MIGA2	-0.5629518	1.1453275	-0.4915	0.623	-0.439379851	count	1
ZNF529-AS1	-0.5629518	1.1453275	-0.4915	0.623	-0.439379851	count	1
SSB	-0.3120671	0.2200706	-1.418	0.157	-0.439195743	count	1
METTL15	-0.5625402	0.5835171	-0.9641	0.336	-0.439084704	count	1
GATAD2B	-0.3528296	0.428424	-0.8236	0.411	-0.438863746	count	1

WAC	-0.3161231	0.355852	-0.8884	0.375	-0.438282353	count	1
POLB	-0.3332485	0.5555415	-0.5999	0.549	-0.437314753	count	1
DMAP1	-0.3433683	0.5132218	-0.669	0.504	-0.437272516	count	1
RNF146	-0.3433683	0.5395399	-0.6364	0.525	-0.437272516	count	1
METAP2	-0.3113409	0.3534731	-0.8808	0.3791	-0.437253597	count	1
L3MBTL3	-0.3401344	0.687871	-0.4945	0.621	-0.43714028	count	1
NUP160	-0.3722855	0.6170542	-0.6033	0.547	-0.436802012	count	1
RAB10	-0.317729	0.377466	-0.8417	0.401	-0.436774069	count	1
ST20-AS1	-0.3838686	1.0933939	-0.3511	0.726	-0.436502684	count	1
ADCK2	-0.3563409	0.5798644	-0.6145	0.539	-0.43648513	count	1
RBM26	-0.3220435	0.4209553	-0.765	0.445	-0.435824033	count	1
PSIP1	-0.3077521	0.2726415	-1.1288	0.2598	-0.435291716	count	1
EIF1AY	-0.3091045	0.3311918	-0.9333	0.3514	-0.435035589	count	1
PDGFD	-0.317116	0.4510955	-0.703	0.483	-0.434569972	count	1
ARIH1	-0.3987001	0.4736577	-0.8417	0.401	-0.434509543	count	1
TMBIM1	-0.3330236	0.4714622	-0.7064	0.48	-0.434406567	count	1
SRPRA	-0.3131919	0.3531419	-0.8869	0.376	-0.434233156	count	1
PHF12	-0.8578948	0.5892329	-1.456	0.1464	-0.433734506	count	1
KIAA1551	-0.3044824	0.2098131	-1.4512	0.1477	-0.433173626	count	1
CRADD	-0.3973126	0.8600866	-0.4619	0.644	-0.433041912	count	1
PLXNC1	-0.3430887	0.8327499	-0.412	0.681	-0.432316396	count	1
CANX	-0.3100183	0.2564747	-1.2088	0.228	-0.431410732	count	1
IBTK	-0.3215985	0.5317689	-0.6048	0.546	-0.431409515	count	1
POLDIP2	-0.3521288	0.7716849	-0.4563	0.648	-0.431407758	count	1
FTX	-0.3324925	0.4843482	-0.6865	0.493	-0.430774414	count	1
NUDT19	-0.3233688	0.6844969	-0.4724	0.6369	-0.430588042	count	1
PLAC8	-0.3075121	0.3246646	-0.9472	0.344	-0.430573025	count	1
C10orf143	-0.3581415	0.947219	-0.3781	0.706	-0.430539289	count	1
PTPN7	-0.3029573	0.2522667	-1.2009	0.2307	-0.429465899	count	1
NUDCD3	-0.356377	0.5249273	-0.6789	0.498	-0.42845581	count	1
EIF2S1	-0.3085523	0.4132489	-0.7466	0.456	-0.428239812	count	1
SLFN12L	-0.3087974	0.3743708	-0.8248	0.41	-0.428161932	count	1
TNRC6A	-0.3180163	0.5936621	-0.5357	0.593	-0.42799904	count	1
SLC37A1	-0.3439427	1.0732478	-0.3205	0.749	-0.427966305	count	1
NFYB	-0.3212857	0.4961944	-0.6475	0.518	-0.427834574	count	1
AP5Z1	-0.3643994	0.6159013	-0.5917	0.554	-0.427738192	count	1
BAZ1B	-0.3278434	0.4764314	-0.6881	0.492	-0.427712378	count	1
HSP90AA1	-0.2973314	0.1445236	-2.0573	0.0405	-0.427337314	count	1
ATP5PD	-0.3002214	0.2201058	-1.364	0.1735	-0.426724512	count	1
ITFG1	-0.4160311	0.639494	-0.6506	0.516	-0.426696359	count	1
UBL7	-0.3349316	0.6987471	-0.4793	0.632	-0.426654163	count	1
WDR45	-0.308011	0.499684	-0.6164	0.538	-0.426638675	count	1
ZBTB1	-0.305979	0.2922812	-1.0469	0.296	-0.426150512	count	1
ITGAE	-0.3090107	0.3501786	-0.8824	0.378	-0.426019028	count	1
SLF2	-0.3423287	0.5264526	-0.6503	0.516	-0.425986151	count	1

FLOT1	-0.3061667	0.3255573	-0.9404	0.348	-0.42570939	count	1
ARSK	-0.3539433	0.5772085	-0.6132	0.54	-0.42558139	count	1
MX2	-0.3241359	0.7477452	-0.4335	0.665	-0.425460421	count	1
RPLP0	-0.2954044	0.0913659	-3.2332	0.00135	-0.425384438	count	1
KHSRP	-0.8370355	0.778594	-1.0751	0.2831	-0.425066246	count	1
UTRN	-0.3005548	0.2880164	-1.0435	0.2975	-0.424801469	count	1
CTNNBL1	-0.3465307	0.5244246	-0.6608	0.509	-0.424656127	count	1
DDX10	-0.3890484	0.6055676	-0.6425	0.521	-0.424293003	count	1
C9orf16	-0.296117	0.1932704	-1.5321	0.126	-0.424212653	count	1
LGALS1	-0.2949961	0.2068063	-1.4264	0.155	-0.423797007	count	1
SLC39A1	-0.322773	0.4271156	-0.7557	0.45	-0.423686876	count	1
NABP2	-0.3720343	0.7046853	-0.5279	0.598	-0.42336437	count	1
ACSL4	-0.3720343	0.7219865	-0.5153	0.607	-0.42336437	count	1
C14orf93	-0.3870998	0.8059149	-0.4803	0.631	-0.422228251	count	1
IFI27L2	-0.3029005	0.3258103	-0.9297	0.353	-0.422200641	count	1
MAD2L2	-0.3103611	0.3957268	-0.7843	0.433	-0.422108257	count	1
TOP2B	-0.3120577	0.3961314	-0.7878	0.431	-0.421256227	count	1
HSF1	-0.3208817	0.3771396	-0.8508	0.395	-0.421225467	count	1
DXO	-0.3700397	0.906253	-0.4083	0.683	-0.421147665	count	1
FGD3	-0.3225963	0.4534994	-0.7113	0.477	-0.420929367	count	1
TEN1	-0.3125365	0.4612348	-0.6776	0.499	-0.42066972	count	1
COQ4	-0.3203763	0.5003893	-0.6403	0.522	-0.420567673	count	1
IL6ST	-0.5368051	0.5223408	-1.0277	0.305	-0.420545315	count	1
CDCA4	-0.3202876	0.627206	-0.5107	0.61	-0.420452225	count	1
CCT6A	-0.2999937	0.2884378	-1.0401	0.299	-0.420306373	count	1
SAPCD2	-0.8255779	0.9659119	-0.8547	0.393	-0.42026266	count	1
FAM160A2	-0.3488142	0.7332838	-0.4757	0.635	-0.419520808	count	1
STX3	-0.5352781	0.7772208	-0.6887	0.491	-0.419440003	count	1
RBM4B	-0.5352781	0.7937833	-0.6743	0.501	-0.419440003	count	1
FAM89B	-0.2966676	0.2473742	-1.1993	0.2313	-0.419427542	count	1
ZNF493	-0.3369125	0.6702062	-0.5027	0.616	-0.419338946	count	1
LGALS3	-0.2998133	0.39486	-0.7593	0.4482	-0.419067056	count	1
RRP8	-0.3232787	0.5608162	-0.5764	0.565	-0.418953482	count	1
CBX8	-0.8209959	0.7684216	-1.0684	0.286	-0.41833326	count	1
NUP214	-0.3409783	0.7750197	-0.44	0.66	-0.417955638	count	1
KMT2B	-0.3079142	0.4523804	-0.6807	0.497	-0.416810224	count	1
SLC27A3	-0.3128336	0.4580577	-0.683	0.495	-0.416659325	count	1
CERS6	-0.5312739	0.5410749	-0.9819	0.327	-0.416538767	count	1
SUPT16H	-0.3125557	0.3699751	-0.8448	0.399	-0.416291803	count	1
EIF2D	-0.3236348	0.7369185	-0.4392	0.661	-0.416156846	count	1
TRAT1	-0.2992514	0.4459495	-0.671	0.503	-0.416124566	count	1
GTF2I	-0.2998158	0.3736844	-0.8023	0.423	-0.41575116	count	1
ENTPD6	-0.3184401	0.7535373	-0.4226	0.673	-0.415554895	count	1
ACLY	-0.3449582	0.7660255	-0.4503	0.653	-0.414962119	count	1
DNAJC19	-0.2949948	0.3176938	-0.9286	0.354	-0.414888216	count	1

LMNB1	-0.3143053	0.5995063	-0.5243	0.6	-0.414857836	count	1
FCGR3A	-0.3001284	0.5024302	-0.5974	0.551	-0.414841414	count	1
MAP2K4	-0.3225575	0.6345176	-0.5084	0.612	-0.414785864	count	1
NFKBIZ	-0.2943523	0.3263907	-0.9018	0.3678	-0.41477623	count	1
ZMYM6	-0.3524228	0.9046398	-0.3896	0.697	-0.413954847	count	1
PYURF	-0.2917681	0.2232787	-1.3067	0.1922	-0.413785696	count	1
PRRC1	-0.4028675	0.6761039	-0.5959	0.552	-0.413669878	count	1
BBS9	-0.3319653	0.8596247	-0.3862	0.7	-0.413264314	count	1
GFM1	-0.3628536	0.6162699	-0.5888	0.556	-0.413155871	count	1
BTF3L4	-0.2964113	0.3648024	-0.8125	0.417	-0.412865193	count	1
DNAJA2	-0.2936052	0.2870431	-1.0229	0.307	-0.412402101	count	1
H1FX	-0.2876134	0.2163847	-1.3292	0.185	-0.412173137	count	1
LPCAT1	-0.3061629	0.5086376	-0.6019	0.548	-0.412142581	count	1
G3BP2	-0.2907088	0.1944217	-1.4952	0.1358	-0.411423556	count	1
VILL	-0.4407027	0.8530654	-0.5166	0.606	-0.41132552	count	1
AKAP11	-0.3171216	0.5156715	-0.615	0.539	-0.411049868	count	1
APH1B	-0.8037987	0.7621661	-1.0546	0.292	-0.411049057	count	1
USP38	-0.3495859	0.694712	-0.5032	0.615	-0.410686806	count	1
RNF181	-0.2930376	0.3189581	-0.9187	0.359	-0.410357299	count	1
EPB41L4A-AS1	-0.3014247	0.3900537	-0.7728	0.44	-0.410015714	count	1
DCTPP1	-0.3089976	0.4591608	-0.673	0.501	-0.409839831	count	1
RPP14	-0.3597765	0.8187639	-0.4394	0.661	-0.40973115	count	1
NOL9	-0.3597765	0.9348301	-0.3849	0.701	-0.40973115	count	1
CCDC88C	-0.2993706	0.3831846	-0.7813	0.435	-0.409654796	count	1
FZR1	-0.3748609	0.7546515	-0.4967	0.62	-0.409243799	count	1
ARHGAP26	-0.3013254	0.4776141	-0.6309	0.529	-0.408947046	count	1
EEF2	-0.2863598	0.1811659	-1.5806	0.1149	-0.40864534	count	1
RPL7	-0.2842613	0.0924533	-3.0746	0.00229	-0.408618836	count	1
COX17	-0.2887482	0.2793614	-1.0336	0.3021	-0.408032564	count	1
NAIP	-0.5191498	0.656263	-0.7911	0.429	-0.407729704	count	1
ADARB1	-0.3386182	0.7141617	-0.4741	0.636	-0.407462323	count	1
EBPL	-0.3025632	0.3927562	-0.7704	0.442	-0.407325493	count	1
CFD	-0.3269292	1.0208381	-0.3203	0.749	-0.407077573	count	1
AGK	-0.3083194	0.461748	-0.6677	0.505	-0.407017634	count	1
VCP	-0.2962725	0.3161342	-0.9372	0.349	-0.406784379	count	1
NSUN4	-0.4352895	0.9953795	-0.4373	0.6622	-0.406508268	count	1
PSMA4	-0.2871959	0.2570113	-1.1174	0.2646	-0.406363302	count	1
C15orf61	-0.2942527	0.3695014	-0.7964	0.426	-0.406263477	count	1
COQ9	-0.3220301	0.6249626	-0.5153	0.607	-0.406100292	count	1
TRAF7	-0.3091704	0.5864105	-0.5272	0.598	-0.40597748	count	1
USP4	-0.3109146	0.510025	-0.6096	0.543	-0.405819754	count	1
GTPBP4	-0.299583	0.4084978	-0.7334	0.464	-0.405593544	count	1
MCM3	-0.4342267	0.5601463	-0.7752	0.439	-0.4055617	count	1
DBR1	-0.3713365	1.0384519	-0.3576	0.721	-0.405499589	count	1
PSMD5	-0.3713365	1.2678789	-0.2929	0.77	-0.405499589	count	1

DDA1	-0.3084253	0.4622887	-0.6672	0.505	-0.405006994	count	1
RFX5	-0.5153692	1.1008728	-0.4681	0.64	-0.404975284	count	1
TGFBRAP1	-0.5153692	1.210807	-0.4256	0.671	-0.404975284	count	1
ZNF682	-0.5153692	1.280646	-0.4024	0.688	-0.404975284	count	1
ZNF573	-0.5153692	1.280646	-0.4024	0.688	-0.404975284	count	1
ZNF284	-0.5153692	1.280646	-0.4024	0.688	-0.404975284	count	1
USP35	-0.5153692	1.3222633	-0.3898	0.697	-0.404975284	count	1
COPS8	-0.2928902	0.3941734	-0.743	0.458	-0.404872468	count	1
SLC25A51	-0.4333256	0.851146	-0.5091	0.611	-0.404758938	count	1
RAB30	-0.4333256	0.8664014	-0.5001	0.617	-0.404758938	count	1
SUCLG1	-0.2912199	0.3415631	-0.8526	0.395	-0.404261863	count	1
TXNIP	-0.280953	0.1190316	-2.3603	0.0189	-0.403900879	count	1
HERC3	-0.3543936	0.6186234	-0.5729	0.567	-0.403736377	count	1
PRDM2	-0.2898947	0.318537	-0.9101	0.363	-0.403491446	count	1
SPSB2	-0.343225	0.6670603	-0.5145	0.607	-0.403354818	count	1
TCEA1	-0.2838363	0.2190881	-1.2955	0.1961	-0.403104293	count	1
C19orf70	-0.2843654	0.2480123	-1.1466	0.2524	-0.402733367	count	1
CDC16	-0.3190651	0.5288252	-0.6033	0.547	-0.402405234	count	1
ELAC1	-0.3122742	0.8773309	-0.3559	0.722	-0.4016937	count	1
ST3GAL5	-0.3073399	0.5273083	-0.5828	0.56	-0.401193766	count	1
MTURN	-0.3072336	0.512612	-0.5993	0.549	-0.401056189	count	1
PYM1	-0.3145204	0.5598181	-0.5618	0.575	-0.400934485	count	1
INO80E	-0.3145204	0.6049056	-0.5199	0.603	-0.400934485	count	1
FNDC3B	-0.3517566	0.6237668	-0.5639	0.573	-0.400797893	count	1
FYN	-0.2814466	0.2574549	-1.0932	0.275	-0.400764179	count	1
IDH3B	-0.309009	0.393573	-0.7851	0.433	-0.400630846	count	1
AC087190.1	-0.3261412	0.6797986	-0.4798	0.632	-0.400031439	count	1
KLHL20	-0.3211143	0.7595021	-0.4228	0.673	-0.399930412	count	1
ZEB1	-0.3062278	0.4394772	-0.6968	0.486	-0.399754389	count	1
GLCCI1	-0.2860194	0.2873932	-0.9952	0.32	-0.399578207	count	1
PANK3	-0.3885974	0.6231877	-0.6236	0.533	-0.399508161	count	1
SPCS1	-0.2791962	0.1620729	-1.7227	0.0859	-0.399282036	count	1
RHEB	-0.2853717	0.2470167	-1.1553	0.249	-0.399180906	count	1
TOP3B	-0.388217	0.9241688	-0.4201	0.675	-0.39913008	count	1
ZDHHC6	-0.2909	0.4346814	-0.6693	0.504	-0.398797585	count	1
TFRC	-0.339214	0.5605659	-0.6051	0.546	-0.398728409	count	1
PRPF19	-0.3249359	0.5969746	-0.5443	0.587	-0.398574137	count	1
FEZ2	-0.2923028	0.4746732	-0.6158	0.538	-0.398508735	count	1
TRAM1	-0.2820296	0.2262175	-1.2467	0.2134	-0.398444158	count	1
CD96	-0.2794373	0.1827101	-1.5294	0.1271	-0.398343852	count	1
MAPRE2	-0.2967265	0.3767682	-0.7876	0.432	-0.398248018	count	1
NUP50	-0.3194346	0.4241997	-0.753	0.452	-0.397865144	count	1
UHRF1BP1L	-0.424802	0.8138002	-0.522	0.602	-0.397156355	count	1
C11orf71	-0.424802	0.8259058	-0.5143	0.607	-0.397156355	count	1
SMIM12	-0.2843723	0.355694	-0.7995	0.425	-0.397015962	count	1

HAUS8	-0.3481338	0.8395547	-0.4147	0.679	-0.396759051	count	1
DHRS4L2	-0.2927672	0.4643996	-0.6304	0.529	-0.39641419	count	1
PTRHD1	-0.290645	0.3960766	-0.7338	0.464	-0.396259051	count	1
PNN	-0.2779858	0.1858555	-1.4957	0.1357	-0.39612706	count	1
TPR	-0.2778215	0.1970562	-1.4099	0.16	-0.395733957	count	1
RTRAF	-0.2786295	0.2098132	-1.328	0.1851	-0.395591895	count	1
DNAJC3	-0.2857443	0.3216367	-0.8884	0.375	-0.395474813	count	1
CDC5L	-0.2809513	0.2824846	-0.9946	0.321	-0.395337141	count	1
SUB1	-0.2758265	0.1212123	-2.2756	0.0235	-0.395071883	count	1
WASF1	-0.7640223	1.1291146	-0.6767	0.499	-0.393942588	count	1
POLA2	-0.7640223	1.1291146	-0.6767	0.499	-0.393942588	count	1
RIC8B	-0.7640223	1.1291146	-0.6767	0.499	-0.393942588	count	1
LRRC8C-DT	-0.7640223	1.470594	-0.5195	0.604	-0.393942588	count	1
POLR1A	-0.7640223	1.470594	-0.5195	0.604	-0.393942588	count	1
TCF19	-0.7640223	1.470594	-0.5195	0.604	-0.393942588	count	1
LINC01006	-0.7640223	1.470594	-0.5195	0.604	-0.393942588	count	1
SPIN4	-0.7640223	1.470594	-0.5195	0.604	-0.393942588	count	1
HMGN5	-0.7640223	1.470594	-0.5195	0.604	-0.393942588	count	1
TKTL1	-0.7640223	1.470594	-0.5195	0.604	-0.393942588	count	1
RTN4RL2	-0.7640223	1.470594	-0.5195	0.604	-0.393942588	count	1
AGAP4	-0.7640223	1.470594	-0.5195	0.604	-0.393942588	count	1
IL34	-0.7640223	1.470594	-0.5195	0.604	-0.393942588	count	1
CDH13	-0.7640223	1.470594	-0.5195	0.604	-0.393942588	count	1
FAM157C	-0.7640223	1.470594	-0.5195	0.604	-0.393942588	count	1
P2RX5	-0.7640223	1.470594	-0.5195	0.604	-0.393942588	count	1
NGDN	-0.2894055	0.3806631	-0.7603	0.448	-0.393744925	count	1
PYGO2	-0.4996654	0.9292052	-0.5377	0.591	-0.393495939	count	1
TMEM220	-0.3206461	0.858752	-0.3734	0.709	-0.393385982	count	1
APBB1	-0.3206461	0.9684087	-0.3311	0.741	-0.393385982	count	1
BAZ2A	-0.3013049	0.4210535	-0.7156	0.475	-0.393381485	count	1
ADSS	-0.2875559	0.34983	-0.822	0.412	-0.392837103	count	1
RBM33	-0.2833572	0.3177861	-0.8917	0.373	-0.392182014	count	1
MICU2	-0.2868022	0.4323773	-0.6633	0.508	-0.391811981	count	1
CENPV	-0.2901269	0.5150725	-0.5633	0.574	-0.391811778	count	1
RPL13A	-0.2720724	0.0708829	-3.8383	0.000149	-0.39175784	count	1
BCAS4	-0.358319	0.5525439	-0.6485	0.517	-0.39165064	count	1
CBFB	-0.3581149	0.5040219	-0.7105	0.478	-0.391433263	count	1
GOSR1	-0.2819333	0.3250365	-0.8674	0.386	-0.391410204	count	1
SNU13	-0.2740664	0.1848673	-1.4825	0.139	-0.391146669	count	1
ARHGEF7	-0.3426252	0.9543731	-0.359	0.72	-0.390613739	count	1
NDUFA13	-0.2870735	0.4420642	-0.6494	0.517	-0.390587143	count	1
LAGE3	-0.2824763	0.3382563	-0.8351	0.404	-0.390526133	count	1
FKBP1A	-0.2772241	0.2775511	-0.9988	0.319	-0.389615092	count	1
CEP78	-0.3124314	0.5721507	-0.5461	0.585	-0.389250889	count	1
SYAP1	-0.2777294	0.2445475	-1.1357	0.257	-0.389185345	count	1

XRCC5	-0.2725318	0.1826853	-1.4918	0.1367	-0.389015552	count	1
MTHFD2	-0.2945255	0.411238	-0.7162	0.474	-0.388940286	count	1
BRI3	-0.2782938	0.3474753	-0.8009	0.424	-0.388812695	count	1
EPHA1-AS1	-0.3048792	0.605647	-0.5034	0.615	-0.388771165	count	1
RHOQ	-0.2885119	0.5287561	-0.5456	0.586	-0.3885146	count	1
RPIA	-0.286137	0.39596	-0.7226	0.47	-0.388435172	count	1
TIGD1	-0.3074903	0.8967324	-0.3429	0.732	-0.387971388	count	1
IL2RB	-0.2716968	0.2030002	-1.3384	0.182	-0.38796068	count	1
NSRP1	-0.2745237	0.3115984	-0.881	0.379	-0.38751856	count	1
TEX261	-0.3762652	0.9054258	-0.4156	0.678	-0.387236326	count	1
THEMIS2	-0.3762652	0.9094075	-0.4137	0.679	-0.387236326	count	1
AKAP10	-0.3762652	0.9094075	-0.4137	0.679	-0.387236326	count	1
L3MBTL4	-0.3762652	0.9620008	-0.3911	0.696	-0.387236326	count	1
CHMP1A	-0.3153216	0.4158002	-0.7583	0.449	-0.386943262	count	1
PSMB2	-0.2748924	0.2970875	-0.9253	0.3555	-0.386828041	count	1
NELFA	-0.296002	0.6615056	-0.4475	0.655	-0.386514391	count	1
ZDHHC12	-0.2878022	0.443463	-0.649	0.517	-0.386339769	count	1
TMEM243	-0.2737598	0.2616522	-1.0463	0.2962	-0.386323164	count	1
TMEM160	-0.272573	0.2434085	-1.1198	0.2636	-0.386139324	count	1
FCF1	-0.3204394	0.8109441	-0.3951	0.693	-0.385927656	count	1
FGD4	-0.3202876	0.7382121	-0.4339	0.665	-0.385747646	count	1
STK24	-0.2855745	0.4926165	-0.5797	0.563	-0.385696171	count	1
PAXBP1	-0.282305	0.3330596	-0.8476	0.397	-0.385694671	count	1
CD52	-0.2701548	0.207291	-1.3033	0.1934	-0.385480687	count	1
ERH	-0.2718221	0.2284806	-1.1897	0.235	-0.38538971	count	1
DCAF16	-0.302162	0.5611046	-0.5385	0.591	-0.385341472	count	1
FAM136A	-0.2767758	0.3142822	-0.8807	0.379	-0.385283146	count	1
CFAP97	-0.2735746	0.3094504	-0.8841	0.3773	-0.385273298	count	1
FUT11	-0.488303	0.8072017	-0.6049	0.546	-0.385152082	count	1
RCBTB1	-0.4883029	1.0667257	-0.4578	0.6474	-0.385152029	count	1
CDK2	-0.4883029	1.0040523	-0.4863	0.6271	-0.385152012	count	1
ARPP19	-0.2836315	0.2764616	-1.0259	0.306	-0.385050309	count	1
SERF2	-0.2673895	0.0865798	-3.0884	0.00219	-0.385030403	count	1
ATG4A	-0.3516162	0.7782235	-0.4518	0.652	-0.384507791	count	1
ZNF580	-0.2881925	0.4196616	-0.6867	0.493	-0.384050774	count	1
C19orf48	-0.2887205	0.6151625	-0.4693	0.639	-0.383126531	count	1
FUT8	-0.3356193	0.5934469	-0.5655	0.572	-0.382790965	count	1
DHRS11	-0.4085589	1.0149685	-0.4025	0.688	-0.382622931	count	1
UBIAD1	-0.4085589	1.1313827	-0.3611	0.718	-0.382622931	count	1
RAD52	-0.4085589	1.3663303	-0.299	0.7651	-0.382622931	count	1
CD9	-0.2762881	0.3953173	-0.6989	0.485	-0.382429543	count	1
CCT4	-0.2733297	0.2849342	-0.9593	0.338	-0.381894363	count	1
RRP1B	-0.2844184	0.4486523	-0.6339	0.527	-0.381823252	count	1
TMEM141	-0.2825728	0.370918	-0.7618	0.447	-0.381663093	count	1
CCR7	-0.7357194	0.5861966	-1.2551	0.21	-0.381551124	count	1

TWF1	-0.2990897	0.5426081	-0.5512	0.582	-0.38146269	count	1
PTP4A2	-0.2702062	0.2261818	-1.1946	0.2331	-0.381316883	count	1
C12orf65	-0.2700771	0.3293524	-0.82	0.4128	-0.381135003	count	1
CORO7	-0.3337464	0.4469466	-0.7467	0.456	-0.380698358	count	1
NDUFA2	-0.2707943	0.2632223	-1.0288	0.304	-0.380427504	count	1
USE1	-0.282413	0.4802183	-0.5881	0.557	-0.380346117	count	1
U2AF2	-0.2931269	0.4631346	-0.6329	0.527	-0.38021653	count	1
RAB21	-0.2743425	0.352712	-0.7778	0.437	-0.380150597	count	1
HEBP2	-0.2683278	0.2562436	-1.0472	0.2958	-0.380052124	count	1
PPCS	-0.2699624	0.3247991	-0.8312	0.4065	-0.379903739	count	1
FADS3	-0.7307055	0.8300264	-0.8803	0.3793	-0.379336992	count	1
TM2D3	-0.2721866	0.374063	-0.7276	0.467	-0.379214423	count	1
NUDT1	-0.2737781	0.4313847	-0.6346	0.526	-0.378966319	count	1
ATE1	-0.2868432	0.5299291	-0.5413	0.589	-0.378866188	count	1
INO80	-0.2831241	0.3967198	-0.7137	0.476	-0.378780846	count	1
HNRNPDL	-0.2637174	0.1253686	-2.1035	0.0362	-0.378658307	count	1
UQCRFS1	-0.268924	0.2163725	-1.2429	0.215	-0.378592804	count	1
PSMG4	-0.2849725	0.4263372	-0.6684	0.504	-0.378185653	count	1
NOL7	-0.2676545	0.2418478	-1.1067	0.2692	-0.37795086	count	1
MAF1	-0.2703984	0.2465472	-1.0967	0.274	-0.377808751	count	1
AL157938.3	-0.7268501	0.9234616	-0.7871	0.432	-0.377630574	count	1
CLIP4	-0.330419	0.702362	-0.4704	0.638	-0.376979225	count	1
MRPS34	-0.2665817	0.2522426	-1.0568	0.2914	-0.376957557	count	1
JAK3	-0.3203449	0.5624885	-0.5695	0.569	-0.376932196	count	1
ZFAND1	-0.2747754	0.3756648	-0.7314	0.465	-0.376778504	count	1
MTOR	-0.3439427	1.005009	-0.3422	0.732	-0.376320659	count	1
BET1L	-0.3439427	1.014456	-0.339	0.735	-0.376320659	count	1
AC078883.1	-0.3439427	1.1099391	-0.3099	0.757	-0.376320659	count	1
NSUN2	-0.4755719	0.747647	-0.6361	0.525	-0.375765493	count	1
DNAJC24	-0.2808527	0.5483549	-0.5122	0.609	-0.375759785	count	1
AC106795.2	-0.2944869	0.8157778	-0.361	0.718	-0.375649904	count	1
TMED1	-0.2708686	0.4339419	-0.6242	0.533	-0.374951583	count	1
MRPS25	-0.2838361	0.5662299	-0.5013	0.617	-0.374921668	count	1
PTTG1IP	-0.2792478	0.4850947	-0.5757	0.565	-0.374920439	count	1
UBE2L6	-0.2636368	0.2505954	-1.052	0.294	-0.374885237	count	1
MRPS5	-0.2696773	0.3879675	-0.6951	0.487	-0.374787965	count	1
NCOA1	-0.2703995	0.396175	-0.6825	0.495	-0.374703533	count	1
SYNRG	-0.26781	0.2871745	-0.9326	0.3517	-0.374441465	count	1
UAP1	-0.293335	0.5792348	-0.5064	0.613	-0.374194868	count	1
NDUFS8	-0.2627233	0.2281308	-1.1516	0.2503	-0.373961135	count	1
DDX28	-0.3628536	1.024562	-0.3542	0.723	-0.373855785	count	1
AC245060.5	-0.3628536	1.024562	-0.3542	0.723	-0.373855785	count	1
PDS5A	-0.2959745	0.5356573	-0.5525	0.581	-0.373596985	count	1
ESD	-0.2661917	0.3297003	-0.8074	0.42	-0.373250294	count	1
TTI1	-0.3270534	0.7818189	-0.4183	0.676	-0.373215592	count	1

LIMK2	-0.3270534	0.8349452	-0.3917	0.696	-0.373215592	count	1
AC008105.3	-0.2799059	0.4326935	-0.6469	0.518	-0.37307546	count	1
NSMCE4A	-0.2759816	0.4262029	-0.6475	0.518	-0.372805361	count	1
YKT6	-0.2766744	0.6339905	-0.4364	0.663	-0.372658179	count	1
MEA1	-0.2650326	0.2957575	-0.8961	0.371	-0.372514463	count	1
RPS20	-0.2596233	0.1046595	-2.4806	0.0136	-0.372368923	count	1
CALCOCO2	-0.2670506	0.3057271	-0.8735	0.383	-0.372077546	count	1
BUD23	-0.2674522	0.2926866	-0.9138	0.362	-0.372028954	count	1
DGAT1	-0.3395991	0.7441354	-0.4564	0.648	-0.37168168	count	1
ATP13A1	-0.3395991	0.8550084	-0.3972	0.691	-0.37168168	count	1
NSF	-0.3961349	0.7477403	-0.5298	0.597	-0.371466793	count	1
DDX23	-0.395827	0.5856381	-0.6759	0.5	-0.371189879	count	1
PTDSS1	-0.297757	0.5877669	-0.5066	0.613	-0.371182577	count	1
SH3YL1	-0.3249359	0.7687719	-0.4227	0.673	-0.370846748	count	1
DUS1L	-0.2703068	0.3402474	-0.7944	0.428	-0.370674441	count	1
SNX27	-0.3148567	0.8571003	-0.3674	0.714	-0.370582897	count	1
FBXL5	-0.2733578	0.4167235	-0.656	0.512	-0.3702599	count	1
CASK	-0.2680018	0.3549533	-0.755	0.451	-0.370139593	count	1
COPZ1	-0.2633299	0.2882955	-0.9134	0.362	-0.369791921	count	1
ZZEF1	-0.3238913	0.623063	-0.5198	0.604	-0.369677896	count	1
MRPL24	-0.2728522	0.4992351	-0.5465	0.585	-0.369578325	count	1
KPNA4	-0.2714834	0.4377712	-0.6201	0.536	-0.369469255	count	1
TYSND1	-0.2846604	0.6120667	-0.4651	0.642	-0.369324964	count	1
ZNF148	-0.2633719	0.3368854	-0.7818	0.435	-0.369109351	count	1
SPAG7	-0.2644474	0.3539835	-0.7471	0.456	-0.369008864	count	1
DENND1A	-0.3579857	1.0020362	-0.3573	0.721	-0.368990335	count	1
NGLY1	-0.2723335	0.323324	-0.8423	0.4	-0.368879075	count	1
WASHC2C	-0.312926	0.615918	-0.5081	0.612	-0.368348235	count	1
LYSMD3	-0.2886621	0.4162832	-0.6934	0.489	-0.368290903	count	1
RABEP1	-0.2785293	0.540321	-0.5155	0.607	-0.367958921	count	1
ZNF22	-0.2648828	0.3765777	-0.7034	0.482	-0.3678063	count	1
TWF2	-0.2602743	0.2893998	-0.8994	0.3691	-0.367207725	count	1
HIST2H2AC	-0.2988214	0.458236	-0.6521	0.515	-0.366955718	count	1
PCM1	-0.2599761	0.2484857	-1.0462	0.2962	-0.36690337	count	1
NEPRO	-0.2690168	0.438187	-0.6139	0.54	-0.366896355	count	1
HIGD1A	-0.2775416	0.417354	-0.665	0.507	-0.366662789	count	1
ZDHHC2	-0.3344983	0.5904502	-0.5665	0.571	-0.366229772	count	1
SNW1	-0.2595256	0.2448066	-1.0601	0.2899	-0.365912106	count	1
ACADM	-0.2667954	0.3964498	-0.673	0.501	-0.365877276	count	1
PCMT1	-0.2586928	0.246066	-1.0513	0.2939	-0.365818585	count	1
SF3B5	-0.2574503	0.2217109	-1.1612	0.2464	-0.36555716	count	1
AGO3	-0.3201871	0.6230341	-0.5139	0.608	-0.365531681	count	1
SIPA1	-0.2647062	0.40797	-0.6488	0.517	-0.365144675	count	1
ELK4	-0.2681053	0.4291721	-0.6247	0.533	-0.364891746	count	1
INTS6L	-0.3331617	0.8214902	-0.4056	0.685	-0.364800408	count	1

CHRAC1	-0.26797	0.328776	-0.8151	0.416	-0.364708395	count	1
FIS1	-0.2556897	0.1856907	-1.377	0.169	-0.364487067	count	1
AKIRIN1	-0.2623757	0.3060301	-0.8574	0.392	-0.364334748	count	1
CHD4	-0.2612793	0.30158	-0.8664	0.387	-0.36433368	count	1
ELAC2	-0.2966488	0.6789675	-0.4369	0.662	-0.364321472	count	1
TUFM	-0.2563016	0.2258143	-1.135	0.2572	-0.364044058	count	1
AGPAT2	-0.2672057	0.3894957	-0.686	0.493	-0.363672643	count	1
RSBN1L	-0.2553287	0.1994133	-1.2804	0.201	-0.363487624	count	1
TMEM69	-0.2909	0.696878	-0.4175	0.677	-0.362713634	count	1
FARSA	-0.2840716	0.5513114	-0.5153	0.607	-0.362488983	count	1
SENP7	-0.2638437	0.2986005	-0.8836	0.378	-0.362421649	count	1
PSENEN	-0.2567366	0.2942576	-0.8725	0.3836	-0.362107143	count	1
PARP4	-0.2901303	0.4834863	-0.6001	0.549	-0.361782396	count	1
KDM5A	-0.2587323	0.3161737	-0.8183	0.414	-0.361053175	count	1
UTP23	-0.2585435	0.4391048	-0.5888	0.5564	-0.36079034	count	1
COX6C	-0.2519098	0.1541898	-1.6338	0.1033	-0.360598986	count	1
MT-ATP6	-0.2501956	0.071967	-3.4765	0.000577	-0.360565133	count	1
FHIT	-0.3838686	1.0933939	-0.3511	0.726	-0.360418766	count	1
DAGLB	-0.3838686	1.0933939	-0.3511	0.726	-0.360418766	count	1
CNIH2	-0.3838686	1.0933939	-0.3511	0.726	-0.360418766	count	1
RMDN2	-0.3838686	1.3917829	-0.2758	0.783	-0.360418766	count	1
RLN2	-0.3838686	1.3917829	-0.2758	0.783	-0.360418766	count	1
AC106739.1	-0.3838686	1.3917829	-0.2758	0.783	-0.360418766	count	1
RXRA	-0.2723869	0.5326905	-0.5113	0.609	-0.359897262	count	1
FAM120AOS	-0.2607347	0.3748456	-0.6956	0.487	-0.359683967	count	1
SERP1	-0.2517905	0.1726922	-1.458	0.146	-0.359457275	count	1
SNAPIN	-0.258156	0.4544513	-0.5681	0.57	-0.359430311	count	1
SLC16A7	-0.2646048	0.421189	-0.6282	0.53	-0.359334893	count	1
CAST	-0.2522998	0.2199816	-1.1469	0.252	-0.359220305	count	1
RPA2	-0.2561461	0.3206596	-0.7988	0.425	-0.35919315	count	1
UBR3	-0.3481477	0.7488623	-0.4649	0.642	-0.359143076	count	1
NDUFA11	-0.2529697	0.2516312	-1.0053	0.3155	-0.358894669	count	1
STAT2	-0.2712665	0.7368218	-0.3682	0.713	-0.358426483	count	1
SYTL3	-0.2545836	0.2375417	-1.0717	0.2846	-0.357855164	count	1
CASP9	-0.3129969	0.7659126	-0.4087	0.683	-0.357477331	count	1
GPATCH8	-0.2598524	0.2989101	-0.8693	0.385	-0.35695837	count	1
MYLIP	-0.2654668	0.3800495	-0.6985	0.485	-0.356514634	count	1
TRIQK	-0.3793719	0.8811568	-0.4305	0.667	-0.356360409	count	1
COX7A2	-0.2482238	0.1599487	-1.5519	0.122	-0.355678949	count	1
PNPLA2	-0.2578033	0.401353	-0.6423	0.521	-0.35565297	count	1
ERF	-0.2786342	0.4920903	-0.5662	0.572	-0.355614032	count	1
EPN1	-0.3242812	0.662824	-0.4892	0.625	-0.355295673	count	1
AP4S1	-0.6768916	0.8048009	-0.8411	0.4009	-0.355214368	count	1
CCDC122	-0.2944869	0.8372159	-0.3517	0.725	-0.35510789	count	1
HSPD1	-0.2499392	0.2108465	-1.1854	0.2367	-0.354960971	count	1

ARAP2	-0.2562783	0.336205	-0.7623	0.446	-0.353555795	count	1
RPL27	-0.2459461	0.0965868	-2.5464	0.0113	-0.353516311	count	1
TRIB2	-0.2673727	0.6127034	-0.4364	0.663	-0.353314288	count	1
SERINC3	-0.2571701	0.3575711	-0.7192	0.473	-0.353286465	count	1
TRIM56	-0.2832377	0.5601734	-0.5056	0.613	-0.353281462	count	1
NCL	-0.2455199	0.1117107	-2.1978	0.0287	-0.353081078	count	1
ZFPL1	-0.29275	0.6284432	-0.4658	0.642	-0.353042102	count	1
SHLD1	-0.2636632	0.6857879	-0.3845	0.701	-0.35288634	count	1
NDUFS5	-0.2464482	0.1762311	-1.3984	0.1629	-0.352576561	count	1
HMGB2	-0.2477938	0.2020617	-1.2263	0.221	-0.351972825	count	1
YAF2	-0.262686	0.4216835	-0.6229	0.534	-0.351585456	count	1
C20orf27	-0.275165	0.5210064	-0.5281	0.598	-0.351226154	count	1
SEC31A	-0.2623757	0.3909054	-0.6712	0.503	-0.351172359	count	1
RPL36	-0.2437633	0.0816212	-2.9865	0.00304	-0.350560057	count	1
PAXIP1-AS2	-0.2699417	0.6671321	-0.4046	0.686	-0.350375729	count	1
SSNA1	-0.2470778	0.2468757	-1.0008	0.318	-0.350281232	count	1
TNFSF12	-0.247508	0.2558728	-0.9673	0.3341	-0.350023919	count	1
GMPPA	-0.2970075	0.7765788	-0.3825	0.702	-0.349903334	count	1
IL1R1	-0.3386182	0.9275252	-0.3651	0.715	-0.349586626	count	1
HYI	-0.3386182	0.9386931	-0.3607	0.719	-0.349586626	count	1
SLC35E2A	-0.3386182	1.0411808	-0.3252	0.745	-0.349586626	count	1
TSGA10	-0.3386182	1.0411808	-0.3252	0.745	-0.349586626	count	1
SP140L	-0.2504926	0.3226486	-0.7764	0.438	-0.34932792	count	1
PSMF1	-0.2478251	0.2778615	-0.8919	0.373	-0.349321544	count	1
EVI2A	-0.2480754	0.278928	-0.8894	0.3745	-0.349156273	count	1
LPXN	-0.2521394	0.35018	-0.72	0.472	-0.349100262	count	1
GPR65	-0.2473816	0.2124983	-1.1642	0.2452	-0.349045995	count	1
TRIM11	-0.2606469	0.5337322	-0.4883	0.626	-0.348870736	count	1
PDHB	-0.2479345	0.3031801	-0.8178	0.4141	-0.348818662	count	1
MED1	-0.662282	0.8598257	-0.7703	0.4417	-0.348552695	count	1
PMPCB	-0.2545345	0.3757983	-0.6773	0.499	-0.348530553	count	1
GPATCH1	-0.3048792	0.9297223	-0.3279	0.7432	-0.348374295	count	1
ATP5MPL	-0.2447971	0.2467331	-0.9922	0.3219	-0.348075219	count	1
LEPROT	-0.2496923	0.362458	-0.6889	0.491	-0.347950732	count	1
TRIM28	-0.2700918	0.4059859	-0.6653	0.506	-0.347886004	count	1
TRMT11	-0.3043778	0.5663103	-0.5375	0.591	-0.347811695	count	1
TMEM116	-0.3367247	0.6606202	-0.5097	0.611	-0.347685684	count	1
APBA3	-0.3170993	0.9284999	-0.3415	0.733	-0.347598941	count	1
APBB3	-0.3170993	1.0495392	-0.3021	0.7627	-0.347598941	count	1
ATP6V1G1	-0.242908	0.1604399	-1.514	0.131	-0.347416894	count	1
ALDOA	-0.2880208	0.6699086	-0.4299	0.668	-0.347415429	count	1
EPOR	-0.263866	0.553849	-0.4764	0.634	-0.346889538	count	1
GMFG	-0.2417791	0.1488433	-1.6244	0.105	-0.346714884	count	1
DAP3	-0.2558915	0.4401619	-0.5814	0.561	-0.346706391	count	1
ZBED5	-0.2939646	0.5680332	-0.5175	0.605	-0.346373415	count	1

NPC2	-0.245372	0.3146859	-0.7797	0.436	-0.34492974	count	1
ZNF829	-0.4324632	0.8335101	-0.5188	0.604	-0.343690306	count	1
RPS11	-0.2391	0.0956328	-2.5002	0.0129	-0.343628104	count	1
BCCIP	-0.2564423	0.4212541	-0.6088	0.543	-0.3432722	count	1
ZC3H14	-0.2686819	0.5684687	-0.4726	0.637	-0.343023196	count	1
DBI	-0.2396493	0.1852284	-1.2938	0.1967	-0.342298778	count	1
ST13	-0.2427391	0.220484	-1.1009	0.272	-0.342280937	count	1
VAMP8	-0.2389089	0.1541071	-1.5503	0.1221	-0.341716967	count	1
BTAF1	-0.6467153	0.7083161	-0.913	0.362	-0.341401846	count	1
TM2D2	-0.2701405	0.5686252	-0.4751	0.635	-0.34129997	count	1
PPP1R8	-0.2701405	0.7659329	-0.3527	0.725	-0.34129997	count	1
ABRAXAS1	-0.3625237	0.6620229	-0.5476	0.584	-0.341115559	count	1
JAGN1	-0.2499842	0.5083055	-0.4918	0.623	-0.341038545	count	1
SLC25A38	-0.2474024	0.386092	-0.6408	0.522	-0.340897117	count	1
PRMT5	-0.2892374	0.9067817	-0.319	0.75	-0.340887055	count	1
ESRRA	-0.2521526	0.4690152	-0.5376	0.591	-0.340761842	count	1
MTX1	-0.2463465	0.4446512	-0.554	0.58	-0.340721175	count	1
RRAGC	-0.276991	0.705102	-0.3928	0.695	-0.340461082	count	1
STAT5A	-0.2974311	0.5910741	-0.5032	0.615	-0.340013151	count	1
TNFRSF9	-0.2566574	0.442426	-0.5801	0.562	-0.339240482	count	1
MSL1	-0.3092733	0.5414178	-0.5712	0.568	-0.339201833	count	1
NUDT8	-0.249677	0.5532819	-0.4513	0.652	-0.339146143	count	1
PRKACB	-0.242604	0.2894998	-0.838	0.403	-0.33883153	count	1
TMEM167B	-0.2755919	0.5625217	-0.4899	0.625	-0.338761133	count	1
TMEM192	-0.2805489	0.7617089	-0.3683	0.713	-0.338519736	count	1
ZNF605	-0.2803886	0.8375218	-0.3348	0.738	-0.33832881	count	1
CSTB	-0.2357435	0.1601392	-1.4721	0.142	-0.337741891	count	1
MTERF2	-0.326725	0.7496069	-0.4359	0.663	-0.337635255	count	1
SAP130	-0.326725	0.8984723	-0.3636	0.716	-0.337635255	count	1
ZNF470	-0.326725	0.8984723	-0.3636	0.716	-0.337635255	count	1
AC004982.2	-0.326725	0.9317423	-0.3507	0.726	-0.337635255	count	1
PTP4A1	-0.2432372	0.309366	-0.7862	0.432	-0.337504139	count	1
RNF115	-0.241094	0.2859492	-0.8431	0.3998	-0.337372889	count	1
COPS3	-0.242704	0.4038132	-0.601	0.548	-0.337088211	count	1
KMT2C	-0.2517372	0.392494	-0.6414	0.522	-0.337005935	count	1
RABL2A	-0.2944869	0.9565926	-0.3078	0.758	-0.336705678	count	1
DUSP4	-0.2387571	0.2863855	-0.8337	0.4051	-0.336440667	count	1
SPN	-0.2447147	0.3873089	-0.6318	0.528	-0.336231797	count	1
DHX40	-0.4221085	0.7616266	-0.5542	0.58	-0.3359198	count	1
PPAT	-0.3061689	0.9651456	-0.3172	0.751	-0.335867997	count	1
UBA2	-0.241968	0.3291274	-0.7352	0.463	-0.335410386	count	1
LACTB	-0.2448522	0.4479599	-0.5466	0.585	-0.335318789	count	1
PSMB9	-0.2336089	0.1380704	-1.692	0.0916	-0.335162482	count	1
HRAS	-0.2494189	0.4455543	-0.5598	0.576	-0.335066752	count	1
FBXW11	-0.2683272	0.5568339	-0.4819	0.63	-0.334873909	count	1

CD84	-0.2840033	0.7542687	-0.3765	0.707	-0.334808755	count	1
LAIR1	-0.2840033	0.7546776	-0.3763	0.707	-0.334808755	count	1
CHID1	-0.2518662	0.567779	-0.4436	0.658	-0.334500138	count	1
PEF1	-0.2518617	0.4141387	-0.6082	0.544	-0.334494197	count	1
WWP1	-0.2481645	0.4802729	-0.5167	0.606	-0.334435321	count	1
MCRIP1	-0.2430449	0.3166988	-0.7674	0.443	-0.333944915	count	1
NDUFB1	-0.2367923	0.3000002	-0.7893	0.4305	-0.333907955	count	1
NUP37	-0.2493254	0.5261971	-0.4738	0.636	-0.333793366	count	1
HTT	-0.2831257	0.6391684	-0.443	0.658	-0.333789234	count	1
HERC5	-0.2569104	0.5749353	-0.4469	0.655	-0.333583642	count	1
ZNF585B	-0.4186267	0.7768508	-0.5389	0.59	-0.333301277	count	1
PRKCA	-0.4186267	0.8491201	-0.493	0.622	-0.333301277	count	1
TRAPPC4	-0.2357139	0.2668888	-0.8832	0.3778	-0.333191432	count	1
ACD	-0.2446187	0.6070349	-0.403	0.6872	-0.333050389	count	1
EXOSC8	-0.2380255	0.3219196	-0.7394	0.46	-0.33288358	count	1
TULP4	-0.2821785	0.5631306	-0.5011	0.617	-0.332688744	count	1
SSR3	-0.2372124	0.291702	-0.8132	0.417	-0.332335596	count	1
CHMP2A	-0.2347482	0.2649532	-0.886	0.3763	-0.33145096	count	1
PUF60	-0.2353755	0.3239361	-0.7266	0.468	-0.331178095	count	1
RHOG	-0.2329372	0.2221859	-1.0484	0.2952	-0.330620868	count	1
ZDHHC14	-0.623168	0.9003132	-0.6922	0.489	-0.330481839	count	1
RAPGEF2	-0.623168	0.9582367	-0.6503	0.516	-0.330481839	count	1
MBNL3	-0.623168	1.021126	-0.6103	0.542	-0.330481839	count	1
TTC9	-0.623168	1.021126	-0.6103	0.542	-0.330481839	count	1
FES	-0.623168	1.021126	-0.6103	0.542	-0.330481839	count	1
NFS1	-0.623168	1.021126	-0.6103	0.542	-0.330481839	count	1
ZNF585A	-0.623168	1.021126	-0.6103	0.542	-0.330481839	count	1
SLC44A2	-0.2564398	0.7312817	-0.3507	0.726	-0.330437094	count	1
SH2D1A	-0.2320682	0.2434333	-0.9533	0.341	-0.329606297	count	1
TMEM154	-0.3002236	0.7032701	-0.4269	0.67	-0.329478724	count	1
PAG1	-0.3182769	0.7078866	-0.4496	0.653	-0.329129446	count	1
FER	-0.3182769	0.836992	-0.3803	0.704	-0.329129446	count	1
OPA3	-0.3489449	0.6028524	-0.5788	0.563	-0.328784625	count	1
SPAG16	-0.2599772	0.5732812	-0.4535	0.65	-0.328575465	count	1
TMEM42	-0.2485251	0.6727446	-0.3694	0.712	-0.328553827	count	1
TAGLN	-0.2598639	0.4838896	-0.537	0.592	-0.328433555	count	1
BDH1	-0.3171607	0.8166377	-0.3884	0.698	-0.328004617	count	1
DLL1	-0.3171607	0.818793	-0.3874	0.699	-0.328004617	count	1
ACTR8	-0.3171006	0.4647269	-0.6823	0.496	-0.327944041	count	1
UBXN4	-0.2296567	0.2043103	-1.1241	0.262	-0.327809051	count	1
NUCB2	-0.2300061	0.1981747	-1.1606	0.2466	-0.327713749	count	1
FLI1	-0.2463881	0.3946302	-0.6244	0.533	-0.3272643	count	1
ATP5MC1	-0.2323161	0.255181	-0.9104	0.3633	-0.327136855	count	1
CHTOP	-0.2501088	0.4234518	-0.5906	0.555	-0.326988515	count	1
RLF	-0.2771814	0.5491473	-0.5047	0.614	-0.326880873	count	1

IGHMBP2	-0.6150267	0.8978128	-0.685	0.494	-0.326677603	count	1
GPX4	-0.2285863	0.164716	-1.3878	0.166	-0.326628652	count	1
LFNG	-0.236072	0.3535562	-0.6677	0.505	-0.326548949	count	1
CDKN2D	-0.2283251	0.1919954	-1.1892	0.235	-0.326196084	count	1
BEX2	-0.2363105	0.3932862	-0.6009	0.548	-0.325203521	count	1
NFKB2	-0.2572049	0.5243883	-0.4905	0.624	-0.325102741	count	1
ACAP2	-0.2311117	0.2995128	-0.7716	0.441	-0.325054506	count	1
HLA-A	-0.2254337	0.0624949	-3.6072	0.000358	-0.324928492	count	1
PRPF8	-0.246845	0.5224592	-0.4725	0.637	-0.324649557	count	1
11-Sep	-0.2355634	0.3403376	-0.6921	0.489	-0.323697173	count	1
ORC5	-0.2679294	0.6745582	-0.3972	0.691	-0.323479482	count	1
GSTK1	-0.2266789	0.2009893	-1.1278	0.2602	-0.323163021	count	1
NCAM1	-0.2332786	0.3675923	-0.6346	0.526	-0.323054563	count	1
HIGD2A	-0.22592	0.1647878	-1.371	0.171	-0.322762122	count	1
HNRNPA1	-0.2249112	0.1292063	-1.7407	0.0827	-0.322688026	count	1
TTPAL	-0.4038403	0.9780545	-0.4129	0.68	-0.322149394	count	1
C7orf43	-0.4038403	0.9810557	-0.4116	0.681	-0.322149394	count	1
NOTCH2	-0.6053709	0.7267358	-0.833	0.405	-0.322146525	count	1
LLGL2	-0.2382428	0.5456742	-0.4366	0.663	-0.322042517	count	1
USP3	-0.2330519	0.4084761	-0.5705	0.569	-0.322003776	count	1
MTMR14	-0.2413092	0.6063095	-0.398	0.691	-0.32189576	count	1
FASLG	-0.2260112	0.232491	-0.9721	0.332	-0.321748942	count	1
DCTN2	-0.2348653	0.3437097	-0.6833	0.495	-0.321687051	count	1
HSPA14	-0.3105344	0.6852709	-0.4532	0.651	-0.321322238	count	1
FAM207A	-0.2308942	0.4830298	-0.478	0.633	-0.321293509	count	1
TES	-0.2269731	0.2901949	-0.7821	0.4347	-0.321100511	count	1
EMC3	-0.2323693	0.3404349	-0.6826	0.495	-0.32106319	count	1
RPL15	-0.2228044	0.0711773	-3.1303	0.00191	-0.320937069	count	1
TMEM131L	-0.255883	0.6553773	-0.3904	0.696	-0.319492741	count	1
ANKRD46	-0.263866	0.791289	-0.3335	0.739	-0.318632368	count	1
SND1	-0.2783265	0.6114342	-0.4552	0.649	-0.318527844	count	1
PARP14	-0.2308353	0.3771393	-0.6121	0.541	-0.318134591	count	1
BLOC1S1	-0.2243356	0.238661	-0.94	0.3479	-0.317881128	count	1
NAPA	-0.2280599	0.3409308	-0.6689	0.504	-0.31787037	count	1
DGKD	-0.2428977	0.537552	-0.4519	0.652	-0.317620203	count	1
ELAVL1	-0.2335833	0.423674	-0.5513	0.582	-0.317367947	count	1
RPSA	-0.2202959	0.0763583	-2.885	0.00418	-0.317302037	count	1
CEP95	-0.2398232	0.4535685	-0.5287	0.597	-0.317113538	count	1
LRCH1	-0.245962	0.671889	-0.3661	0.715	-0.31703412	count	1
NIF3L1	-0.2381603	0.636221	-0.3743	0.708	-0.316392722	count	1
RPS18	-0.219382	0.058125	-3.7743	0.000191	-0.316254222	count	1
ODR4	-0.2569734	0.9403927	-0.2733	0.785	-0.316117479	count	1
ESYT2	-0.3955123	0.5876004	-0.6731	0.501	-0.315846053	count	1
NIPSNAP2	-0.2297588	0.5033782	-0.4564	0.648	-0.3152463	count	1
BAG1	-0.2255319	0.33499	-0.6732	0.501	-0.315243809	count	1

SPAG9	-0.2314874	0.2967516	-0.7801	0.436	-0.315236208	count	1
SNRPE	-0.223984	0.2566606	-0.8727	0.383	-0.315174415	count	1
RFC1	-0.2250745	0.2800914	-0.8036	0.422	-0.31480566	count	1
IRAK4	-0.2275463	0.3671059	-0.6198	0.536	-0.314786067	count	1
NDUFA10	-0.2253287	0.3224747	-0.6987	0.485	-0.314751868	count	1
BRD7	-0.2240485	0.2799661	-0.8003	0.424	-0.314260857	count	1
ERN1	-0.2458202	0.4019291	-0.6116	0.541	-0.314065197	count	1
TOMM70	-0.240118	0.6868157	-0.3496	0.727	-0.314007882	count	1
HERC1	-0.2596448	0.584173	-0.4445	0.657	-0.313594844	count	1
ZNF138	-0.2595962	0.5841967	-0.4444	0.657	-0.313536835	count	1
GPS1	-0.2392303	0.5706147	-0.4193	0.675	-0.312854159	count	1
GTF2H1	-0.2392303	0.5948436	-0.4022	0.688	-0.312854159	count	1
PNRC2	-0.2212417	0.2252108	-0.9824	0.3266	-0.312674435	count	1
CDKN1C	-0.3900709	0.5478605	-0.712	0.477	-0.311718885	count	1
RSF1	-0.2218965	0.2445076	-0.9075	0.365	-0.311556112	count	1
CNOT1	-0.2323185	0.4297213	-0.5406	0.589	-0.311129758	count	1
LTB	-0.2188731	0.2989812	-0.7321	0.4647	-0.31088275	count	1
FAM173A	-0.2211388	0.2938282	-0.7526	0.452	-0.310780871	count	1
HEMGN	-0.3885974	0.9783416	-0.3972	0.691	-0.310600093	count	1
SNX25	-0.3885974	1.092391	-0.3557	0.722	-0.310600093	count	1
RABL2B	-0.2826814	0.9375739	-0.3015	0.763	-0.310591985	count	1
ZNF397	-0.2826814	1.1846096	-0.2386	0.812	-0.310591985	count	1
DDX27	-0.2262687	0.3554722	-0.6365	0.525	-0.310472136	count	1
FLNA	-0.2335655	0.4184357	-0.5582	0.577	-0.310319567	count	1
PIAS4	-0.2995148	0.7154947	-0.4186	0.676	-0.310191207	count	1
ACER3	-0.2995148	0.8113396	-0.3692	0.712	-0.310191207	count	1
ELOA	-0.2294173	0.387609	-0.5919	0.554	-0.310160081	count	1
RPL4	-0.2164252	0.117258	-1.8457	0.0658	-0.310066875	count	1
ABCA5	-0.2626516	0.683835	-0.3841	0.701	-0.309974272	count	1
DNMT1	-0.2218581	0.2748321	-0.8072	0.42	-0.309699583	count	1
XAF1	-0.2221401	0.3625233	-0.6128	0.54	-0.309636323	count	1
MKNK1	-0.28177	0.7517039	-0.3748	0.708	-0.309609338	count	1
LMAN1	-0.2232693	0.2931193	-0.7617	0.447	-0.309552966	count	1
POLE3	-0.221206	0.3502797	-0.6315	0.528	-0.309404867	count	1
LTA4H	-0.2308859	0.4814835	-0.4795	0.632	-0.309219862	count	1
KLHDC1	-0.2557172	1.0710706	-0.2387	0.811	-0.308905727	count	1
TEX10	-0.2472455	0.6741387	-0.3668	0.714	-0.308807042	count	1
MT-ND4L	-0.2164787	0.1925145	-1.1245	0.262	-0.308767149	count	1
CAV1	-0.2439524	0.8039655	-0.3034	0.762	-0.308491517	count	1
JKAMP	-0.2321776	0.5090928	-0.4561	0.649	-0.308484846	count	1
DERL2	-0.2238047	0.3729093	-0.6002	0.549	-0.308471619	count	1
MAPKAPK5-AS1	-0.2285598	0.4488146	-0.5093	0.611	-0.308124794	count	1
DNAJC4	-0.2208959	0.3477626	-0.6352	0.526	-0.307905593	count	1
COMMD9	-0.2408044	0.5484851	-0.439	0.661	-0.307705434	count	1
CHCHD3	-0.2200695	0.3705328	-0.5939	0.553	-0.307622856	count	1

SMC3	-0.2205914	0.2688456	-0.8205	0.413	-0.307240377	count	1
DHX9	-0.2223209	0.3509188	-0.6335	0.527	-0.307215078	count	1
GCHFR	-0.2174953	0.3406131	-0.6385	0.5236	-0.307212298	count	1
TMEM126B	-0.2300494	0.3942721	-0.5835	0.56	-0.306947136	count	1
NFYA	-0.3249359	0.9941228	-0.3269	0.744	-0.306886727	count	1
DPF3	-0.3249359	1.1289699	-0.2878	0.774	-0.306886727	count	1
GANC	-0.3249359	1.1563685	-0.281	0.779	-0.306886727	count	1
AC002310.1	-0.3249359	1.2867139	-0.2525	0.8008	-0.306886727	count	1
REV3L	-0.2359489	0.4935741	-0.478	0.633	-0.306543376	count	1
SDAD1	-0.2203791	0.3255113	-0.677	0.499	-0.306428471	count	1
MT-ND5	-0.2130046	0.0960895	-2.2167	0.0273	-0.306373521	count	1
FXYD5	-0.2129348	0.1340914	-1.588	0.113	-0.306079658	count	1
APRT	-0.2134592	0.16715	-1.2771	0.202	-0.305911579	count	1
ZMYM2	-0.2291757	0.3870962	-0.592	0.554	-0.305786873	count	1
ZFPM1	-0.266936	0.6890902	-0.3874	0.699	-0.305691817	count	1
HMGN2	-0.2133315	0.1620849	-1.3162	0.189	-0.305470131	count	1
CEP41	-0.2944869	1.0229845	-0.2879	0.774	-0.305104996	count	1
CTPS2	-0.2944869	1.0229845	-0.2879	0.774	-0.305104996	count	1
BEST4	-0.2944869	1.1603475	-0.2538	0.8	-0.305104996	count	1
C8orf58	-0.2944869	1.1603475	-0.2538	0.8	-0.305104996	count	1
TCFL5	-0.2944869	1.1603475	-0.2538	0.8	-0.305104996	count	1
ATP8B3	-0.2944869	1.1603475	-0.2538	0.8	-0.305104996	count	1
ZNF888	-0.2944869	1.1603475	-0.2538	0.8	-0.305104996	count	1
SMIM3	-0.2944869	1.1904567	-0.2474	0.805	-0.305104996	count	1
CAMKK1	-0.2944869	1.1904567	-0.2474	0.805	-0.305104996	count	1
AC127521.1	-0.2944869	1.1904567	-0.2474	0.805	-0.305104996	count	1
ALKBH1	-0.2944869	1.2609403	-0.2335	0.815	-0.305104996	count	1
GRAP2	-0.2944869	1.2609403	-0.2335	0.815	-0.305104996	count	1
XPNPEP3	-0.2944869	1.2609403	-0.2335	0.815	-0.305104996	count	1
KIF27	-0.2944869	1.4520057	-0.2028	0.839	-0.305104996	count	1
TSPAN15	-0.2944869	1.4520057	-0.2028	0.839	-0.305104996	count	1
VAMP1	-0.2944869	1.4520057	-0.2028	0.839	-0.305104996	count	1
AC024909.2	-0.2944869	1.4520057	-0.2028	0.839	-0.305104996	count	1
BEX3	-0.5689046	0.9238843	-0.6158	0.538	-0.304848079	count	1
CCDC25	-0.2189813	0.4045472	-0.5413	0.589	-0.304751656	count	1
CDK13	-0.2197296	0.3504709	-0.627	0.531	-0.304656778	count	1
SRSF4	-0.22159	0.356422	-0.6217	0.535	-0.304551061	count	1
FYTTD1	-0.220752	0.2988875	-0.7386	0.461	-0.304275352	count	1
STAU2	-0.2380569	0.540324	-0.4406	0.66	-0.304220783	count	1
NFATC3	-0.2256253	0.3823866	-0.59	0.556	-0.304184716	count	1
TAOK1	-0.3789335	0.4850416	-0.7812	0.435	-0.303250244	count	1
ZNF683	-0.2173614	0.3895538	-0.558	0.5772	-0.302988723	count	1
EZR	-0.2114679	0.1775945	-1.1907	0.2346	-0.302918647	count	1
SMARCB1	-0.2187719	0.3503706	-0.6244	0.533	-0.302677368	count	1
HIST1H1C	-0.2267171	0.4510179	-0.5027	0.616	-0.302521625	count	1

TBC1D10C	-0.2121982	0.1877429	-1.1303	0.259	-0.30235918	count	1
TXN	-0.2114213	0.1711637	-1.2352	0.218	-0.301861034	count	1
LZTFL1	-0.2338727	0.6024043	-0.3882	0.698	-0.301558011	count	1
ZBTB44	-0.2382857	0.6701837	-0.3556	0.722	-0.30138341	count	1
C11orf49	-0.2552589	0.6136068	-0.416	0.678	-0.301361325	count	1
NFE2L3	-0.2552589	0.7904911	-0.3229	0.747	-0.301361325	count	1
SF3A2	-0.2630351	0.7393053	-0.3558	0.722	-0.301291453	count	1
NDUFB9	-0.2131146	0.2486566	-0.8571	0.392	-0.301032045	count	1
MORC3	-0.2273118	0.4001922	-0.568	0.57	-0.300655788	count	1
TAF1B	-0.2734289	0.5137155	-0.5323	0.595	-0.300609788	count	1
CUEDC2	-0.214671	0.4502063	-0.4768	0.6338	-0.300463332	count	1
HNRNPK	-0.2097726	0.1509104	-1.39	0.1655	-0.300118936	count	1
PSMB6	-0.2110622	0.1929305	-1.094	0.2748	-0.300065346	count	1
RB1CC1	-0.2326717	0.3471942	-0.6701	0.503	-0.300019874	count	1
IL18RAP	-0.2221361	0.3702367	-0.6	0.549	-0.299499244	count	1
AC100810.1	-0.2174219	0.4690681	-0.4635	0.643	-0.299280723	count	1
MAML2	-0.2217345	0.4489923	-0.4938	0.622	-0.298959914	count	1
KPNB1	-0.2224073	0.4170846	-0.5332	0.594	-0.298931944	count	1
GSDMD	-0.2198687	0.3449515	-0.6374	0.524	-0.298799058	count	1
COMMD7	-0.2102394	0.2276335	-0.9236	0.3564	-0.298484983	count	1
APBB1IP	-0.2088954	0.1693014	-1.2339	0.218	-0.298102652	count	1
CENPC	-0.2122483	0.2510712	-0.8454	0.399	-0.297885699	count	1
MACO1	-0.2382136	0.4233886	-0.5626	0.574	-0.297625084	count	1
SDCBP	-0.2080707	0.1719011	-1.2104	0.227	-0.297293851	count	1
ILF3	-0.2186722	0.3179437	-0.6878	0.492	-0.297178622	count	1
WDR18	-0.2235386	0.5381016	-0.4154	0.678	-0.297061788	count	1
ECD	-0.2235386	0.609167	-0.367	0.714	-0.297061788	count	1
PPP1R14B	-0.2074796	0.1810223	-1.1462	0.2526	-0.296991924	count	1
UBE2W	-0.2250043	0.4793166	-0.4694	0.639	-0.296081017	count	1
TUBGCP2	-0.2164292	0.359773	-0.6016	0.548	-0.295978704	count	1
TCF12	-0.2274438	0.6568025	-0.3463	0.729	-0.29556172	count	1
TMOD3	-0.2094086	0.2574124	-0.8135	0.4165	-0.295440467	count	1
SPTLC1	-0.2183714	0.5806617	-0.3761	0.707	-0.295282367	count	1
DNAJA1	-0.2057754	0.1426879	-1.4421	0.15	-0.295181225	count	1
RHOH	-0.2074596	0.2068293	-1.003	0.317	-0.295068058	count	1
MRPL16	-0.223051	0.4077922	-0.547	0.585	-0.295048612	count	1
MRPL11	-0.2156166	0.4081503	-0.5283	0.598	-0.294870805	count	1
CCDC66	-0.2124102	0.3565177	-0.5958	0.552	-0.294531109	count	1
GTF2B	-0.2072078	0.249449	-0.8307	0.4068	-0.294506062	count	1
CFAP20	-0.2109086	0.3863605	-0.5459	0.586	-0.294231817	count	1
FUS	-0.2058575	0.1550823	-1.3274	0.1853	-0.293471805	count	1
TMEM199	-0.2558365	0.7413214	-0.3451	0.73	-0.293165287	count	1
MCUB	-0.2095425	0.2719686	-0.7705	0.442	-0.292933696	count	1
CBX3	-0.2055362	0.2142926	-0.9591	0.338	-0.292930795	count	1
FBXO9	-0.2184695	0.4080673	-0.5354	0.593	-0.292661627	count	1

KRT86	-0.2033484	0.1810982	-1.1229	0.262	-0.292071648	count	1
EAF1	-0.21597	0.4880459	-0.4425	0.658	-0.292047082	count	1
TSN	-0.2207694	0.5845048	-0.3777	0.706	-0.292045545	count	1
BNIP3L	-0.2086114	0.3110217	-0.6707	0.5029	-0.291818887	count	1
IFI35	-0.2102377	0.3447711	-0.6098	0.542	-0.291525329	count	1
GSAP	-0.254381	0.6429832	-0.3956	0.693	-0.291521322	count	1
RABEP2	-0.5406675	0.7490635	-0.7218	0.471	-0.291252323	count	1
RAPGEF6	-0.5406675	0.7742375	-0.6983	0.485	-0.291252323	count	1
HUS1B	-0.3628536	1.184476	-0.3063	0.76	-0.290973637	count	1
FAM200A	-0.3628536	1.184476	-0.3063	0.76	-0.290973637	count	1
IGHM	-0.3628536	1.184476	-0.3063	0.76	-0.290973637	count	1
CASS4	-0.5400105	0.7776775	-0.6944	0.488	-0.290933899	count	1
TTYH3	-0.5400105	0.8205527	-0.6581	0.511	-0.290933899	count	1
EIF5B	-0.2049294	0.2550164	-0.8036	0.4222	-0.290802125	count	1
LINC00996	-0.2115212	0.4128566	-0.5123	0.609	-0.290750098	count	1
PMF1	-0.2074159	0.3502317	-0.5922	0.554	-0.290658635	count	1
AP2B1	-0.2111215	0.4788408	-0.4409	0.66	-0.290630932	count	1
DNAJC15	-0.2059072	0.257054	-0.801	0.4237	-0.290410995	count	1
IGFBP4	-0.2455462	0.8953949	-0.2742	0.784	-0.290034381	count	1
NIP7	-0.2106058	0.431773	-0.4878	0.626	-0.289922862	count	1
TLN1	-0.2024981	0.1493152	-1.3562	0.176	-0.289816964	count	1
TBC1D2B	-0.2111045	0.4102924	-0.5145	0.607	-0.289722932	count	1
SMG1	-0.2097981	0.4033898	-0.5201	0.603	-0.289215133	count	1
ARPC2	-0.2008366	0.1036078	-1.9384	0.0534	-0.288876337	count	1
RRAGA	-0.207499	0.3662028	-0.5666	0.571	-0.288803647	count	1
ERP44	-0.2064065	0.290366	-0.7108	0.478	-0.288739736	count	1
CHPF2	-0.3597932	0.7747358	-0.4644	0.643	-0.288630485	count	1
WDR43	-0.2108518	0.3580918	-0.5888	0.556	-0.288373901	count	1
MCUR1	-0.2306684	0.4904105	-0.4704	0.638	-0.288277275	count	1
XRCC6	-0.203156	0.2246743	-0.9042	0.3665	-0.287647585	count	1
ANP32A	-0.2059719	0.2645232	-0.7787	0.437	-0.287357388	count	1
NLRC3	-0.2610984	0.4681893	-0.5577	0.577	-0.28728516	count	1
GYPC	-0.1999462	0.1379931	-1.449	0.148	-0.286863396	count	1
RHOB	-0.2203342	0.641766	-0.3433	0.732	-0.286377515	count	1
FAM114A2	-0.2757949	0.5925463	-0.4654	0.642	-0.286155677	count	1
KCTD9	-0.2757949	0.7271327	-0.3793	0.705	-0.286155677	count	1
TTC17	-0.2364371	0.4343082	-0.5444	0.587	-0.285860139	count	1
PREB	-0.2417774	0.6386621	-0.3786	0.705	-0.28563586	count	1
RPS6KA5	-0.2257242	0.4966303	-0.4545	0.65	-0.285615717	count	1
EPS8L2	-0.2068547	0.418026	-0.4948	0.621	-0.285540689	count	1
RALGDS	-0.2091182	0.4316479	-0.4845	0.628	-0.285460331	count	1
MSL3	-0.2119324	0.4987399	-0.4249	0.671	-0.284907957	count	1
MTDH	-0.1996469	0.1972362	-1.0122	0.312	-0.28462355	count	1
PIK3R1	-0.1978984	0.1531541	-1.2922	0.197	-0.284180511	count	1
MED7	-0.2306846	0.6321239	-0.3649	0.715	-0.284078272	count	1

AP1B1	-0.5235629	0.754497	-0.6939	0.4882	-0.282932051	count	1
TXNRD1	-0.2071683	0.4272306	-0.4849	0.628	-0.28280655	count	1
S1PR2	-0.2173754	0.7497784	-0.2899	0.772	-0.282554158	count	1
GOLGA1	-0.2463132	0.543223	-0.4534	0.651	-0.282403305	count	1
SPATA5L1	-0.522041	0.7249721	-0.7201	0.472	-0.282188648	count	1
SBK1	-0.2714363	0.9153138	-0.2965	0.767	-0.281727991	count	1
TXK	-0.197893	0.215849	-0.9168	0.36	-0.281614924	count	1
SRSF8	-0.2015253	0.3437031	-0.5863	0.558	-0.281366263	count	1
KLF9	-0.2115979	0.3017301	-0.7013	0.484	-0.281265118	count	1
HSPA8	-0.1955231	0.1150787	-1.699	0.0903	-0.281219612	count	1
OPA1	-0.2553465	0.5819378	-0.4388	0.661	-0.281061132	count	1
GOSR2	-0.2104967	0.566126	-0.3718	0.71	-0.280970145	count	1
ARID4A	-0.2026357	0.2895294	-0.6999	0.485	-0.280712176	count	1
CEBPB	-0.1989376	0.2542228	-0.7825	0.4345	-0.280592814	count	1
FMC1	-0.2174066	0.5067984	-0.429	0.668	-0.280459101	count	1
MED28	-0.2078462	0.4136551	-0.5025	0.616	-0.280303212	count	1
CREB3L2	-0.2273495	0.5521593	-0.4117	0.681	-0.280008171	count	1
PLEKHB2	-0.2102487	0.4116672	-0.5107	0.61	-0.279479657	count	1
SCAMP1	-0.2100714	0.4760519	-0.4413	0.659	-0.279245019	count	1
FAM13B	-0.2233366	0.529796	-0.4216	0.674	-0.279188285	count	1
TIMM13	-0.2027855	0.3546923	-0.5717	0.568	-0.279184137	count	1
EXO5	-0.5153692	1.2108158	-0.4256	0.671	-0.278923846	count	1
PRMT6	-0.5153692	1.280644	-0.4024	0.688	-0.278923846	count	1
DHFR	-0.5153692	1.280644	-0.4024	0.688	-0.278923846	count	1
CAMK4	-0.5153692	1.280644	-0.4024	0.688	-0.278923846	count	1
LINC01431	-0.5153692	1.322215	-0.3898	0.697	-0.278923846	count	1
TSPO	-0.1945524	0.1539082	-1.2641	0.207	-0.278879774	count	1
SRPK1	-0.2066999	0.3807765	-0.5428	0.588	-0.278762882	count	1
CREBBP	-0.2144049	0.5402686	-0.3968	0.692	-0.278714999	count	1
TAF9	-0.1984366	0.3419398	-0.5803	0.562	-0.278664103	count	1
AIMP1	-0.198213	0.3594602	-0.5514	0.5817	-0.278215933	count	1
CYTIP	-0.1938886	0.1445696	-1.3411	0.181	-0.27795815	count	1
EIF4E	-0.2024312	0.314734	-0.6432	0.521	-0.277851026	count	1
SMAD5	-0.2195308	0.5037521	-0.4358	0.663	-0.277836003	count	1
SF3B6	-0.1954955	0.2138025	-0.9144	0.3612	-0.277747809	count	1
RBPJ	-0.2006043	0.3277096	-0.6121	0.541	-0.277274198	count	1
FBXO28	-0.2217202	0.8593237	-0.258	0.797	-0.277183754	count	1
CAMTA1	-0.2033533	0.3714563	-0.5474	0.584	-0.277041509	count	1
SLC5A3	-0.1990265	0.3653129	-0.5448	0.586	-0.276285935	count	1
COX5B	-0.1929565	0.1680297	-1.1483	0.2517	-0.276265278	count	1
MRPL20	-0.1944073	0.2540575	-0.7652	0.4447	-0.276202981	count	1
PPP2R2A	-0.1979338	0.2762823	-0.7164	0.474	-0.276162762	count	1
RALB	-0.2181754	0.5387249	-0.405	0.686	-0.276132962	count	1
NVL	-0.2059579	0.5176191	-0.3979	0.691	-0.275967324	count	1
ARHGAP45	-0.2012536	0.3529467	-0.5702	0.569	-0.275776891	count	1

RALGPS2	-0.2136094	0.656293	-0.3255	0.745	-0.275590347	count	1
EPS15	-0.20184	0.340945	-0.592	0.554	-0.275553968	count	1
GLUD1	-0.2054804	0.3824491	-0.5373	0.591	-0.275330015	count	1
NDE1	-0.2272688	0.5517276	-0.4119	0.681	-0.27488557	count	1
RNF7	-0.1923566	0.1744092	-1.1029	0.2709	-0.274693471	count	1
RAF1	-0.2112632	0.5059529	-0.4176	0.677	-0.274653829	count	1
MMGT1	-0.2112632	0.5075196	-0.4163	0.677	-0.274653829	count	1
ATG101	-0.1952161	0.2914917	-0.6697	0.504	-0.274518986	count	1
KLRC3	-0.2268978	0.9129104	-0.2485	0.804	-0.27444127	count	1
SERAC1	-0.3410616	1.0020928	-0.3403	0.7338	-0.274243226	count	1
ITPA	-0.2033162	0.3130802	-0.6494	0.517	-0.274215662	count	1
YTHDF1	-0.2390524	0.6074	-0.3936	0.694	-0.274189337	count	1
RAB1B	-0.1991193	0.3580152	-0.5562	0.578	-0.274148966	count	1
SAMSN1	-0.1937575	0.2297817	-0.8432	0.3997	-0.274089243	count	1
CEP83	-0.2165299	0.5760571	-0.3759	0.707	-0.274065184	count	1
AKAP5	-0.2164702	0.5806304	-0.3728	0.71	-0.27399016	count	1
CEP63	-0.2007805	0.4283685	-0.4687	0.64	-0.273546867	count	1
MGAT4A	-0.2138215	0.5410861	-0.3952	0.693	-0.273453807	count	1
DNAJB14	-0.1942964	0.2642082	-0.7354	0.463	-0.273227364	count	1
CCDC86	-0.2381603	0.8897059	-0.2677	0.789	-0.273179607	count	1
PPP2R3B	-0.2381603	0.9615241	-0.2477	0.805	-0.273179607	count	1
OFD1	-0.192534	0.2446055	-0.7871	0.4318	-0.273178363	count	1
NUDCD1	-0.5028715	0.592957	-0.8481	0.397	-0.272782262	count	1
SRP9	-0.1923678	0.190915	-1.0076	0.3144	-0.27268083	count	1
UFL1	-0.1977263	0.4657404	-0.4245	0.671	-0.272612774	count	1
SNTA1	-0.2472455	0.9168821	-0.2697	0.788	-0.272286252	count	1
FAM120C	-0.2472455	0.996421	-0.2481	0.804	-0.272286252	count	1
SRM	-0.1934741	0.3093342	-0.6255	0.532	-0.271954068	count	1
DGCR6L	-0.19246	0.2771122	-0.6945	0.4878	-0.271888077	count	1
KTN1	-0.191957	0.210922	-0.9101	0.3634	-0.271806044	count	1
PIM3	-0.1935916	0.2721375	-0.7114	0.477	-0.271738286	count	1
MIA2	-0.1943113	0.2783542	-0.6981	0.486	-0.27167523	count	1
COA4	-0.1949161	0.3162252	-0.6164	0.538	-0.271544384	count	1
BBS4	-0.2168424	0.6193484	-0.3501	0.726	-0.271133107	count	1
BUB3	-0.1908056	0.2195928	-0.8689	0.3855	-0.270916441	count	1
SUMO4	-0.2358451	0.7759673	-0.3039	0.761	-0.270558598	count	1
KLF3	-0.2093638	0.4172836	-0.5017	0.616	-0.270145256	count	1
ARGLU1	-0.1886193	0.1664391	-1.1333	0.2579	-0.269817964	count	1
HTRA2	-0.2006348	0.4996553	-0.4015	0.688	-0.269775703	count	1
NARF	-0.198908	0.4036194	-0.4928	0.622	-0.26975323	count	1
SS18	-0.2153713	0.5829919	-0.3694	0.712	-0.269307817	count	1
FTL	-0.1869013	0.0768537	-2.4319	0.0156	-0.26925849	count	1
EIF3D	-0.1944488	0.265589	-0.7321	0.465	-0.268784546	count	1
P4HA1	-0.2100334	0.6210414	-0.3382	0.735	-0.268640116	count	1
C19orf25	-0.196987	0.3888095	-0.5066	0.613	-0.268393598	count	1

IFI16	-0.1874904	0.1824427	-1.0277	0.3049	-0.267466501	count	1
AC005070.3	-0.4920356	0.895252	-0.5496	0.583	-0.267430098	count	1
PAQR3	-0.4920356	1.043255	-0.4716	0.638	-0.267430098	count	1
TMEM203	-0.2029556	0.4568429	-0.4443	0.657	-0.267206087	count	1
DPF2	-0.2135688	0.5495166	-0.3886	0.698	-0.267071046	count	1
THUMPD3-AS1	-0.1936094	0.3303778	-0.586	0.558	-0.266949577	count	1
UQCRB	-0.1861838	0.1440182	-1.2928	0.197	-0.266764079	count	1
AKAP8	-0.2131031	0.6763806	-0.3151	0.753	-0.266493092	count	1
COX5A	-0.1860697	0.1730797	-1.0751	0.2832	-0.26606707	count	1
FAHD1	-0.2559231	0.8168212	-0.3133	0.754	-0.265941417	count	1
CEP85L	-0.1956069	0.4224362	-0.463	0.644	-0.265290308	count	1
GEMIN7	-0.2791263	0.663269	-0.4208	0.674	-0.264776534	count	1
USP12	-0.2091005	0.4127735	-0.5066	0.613	-0.264726078	count	1
TAF8	-0.2783265	0.8263773	-0.3368	0.736	-0.264037583	count	1
CHST2	-0.2783265	0.9213267	-0.3021	0.763	-0.264037583	count	1
SFMBT1	-0.2783265	0.9658475	-0.2882	0.773	-0.264037583	count	1
EXT2	-0.2783265	1.0281531	-0.2707	0.787	-0.264037583	count	1
GIGYF2	-0.1962971	0.590916	-0.3322	0.74	-0.263963838	count	1
CMTM6	-0.1868957	0.2703057	-0.6914	0.4898	-0.263958294	count	1
PTPA	-0.2139941	0.7229124	-0.296	0.767	-0.263697359	count	1
C17orf67	-0.2139941	0.7923094	-0.2701	0.787	-0.263697359	count	1
HMGB1	-0.1831474	0.0939153	-1.9501	0.052	-0.263588763	count	1
6-Sep	-0.1848622	0.2065383	-0.8951	0.3714	-0.263533745	count	1
PGD	-0.4840364	0.9176875	-0.5275	0.598	-0.263462882	count	1
MAPK11	-0.4840364	1.098382	-0.4407	0.66	-0.263462882	count	1
HSPB1	-0.1884492	0.333865	-0.5644	0.573	-0.263319059	count	1
YIF1B	-0.2010886	0.5601124	-0.359	0.72	-0.263226563	count	1
SMAP2	-0.19083	0.2710258	-0.7041	0.482	-0.263125894	count	1
BCL2L13	-0.1986337	0.529661	-0.375	0.708	-0.262892339	count	1
PDAP1	-0.1866886	0.2414513	-0.7732	0.44	-0.262863068	count	1
RAD23A	-0.186524	0.2353451	-0.7926	0.429	-0.262828913	count	1
PCCB	-0.276991	1.0159662	-0.2726	0.785	-0.262803403	count	1
ICA1L	-0.276991	1.0241058	-0.2705	0.787	-0.262803403	count	1
COQ2	-0.276991	1.0900012	-0.2541	0.8	-0.262803403	count	1
PDCD4-AS1	-0.2383297	0.7956339	-0.2995	0.765	-0.262616807	count	1
FTSJ3	-0.2284078	0.6763409	-0.3377	0.736	-0.262133813	count	1
GAK	-0.2284078	0.7180461	-0.3181	0.751	-0.262133813	count	1
SMC4	-0.1879917	0.4384522	-0.4288	0.668	-0.261914241	count	1
WRB	-0.2045366	0.7345682	-0.2784	0.781	-0.261652904	count	1
GALK1	-0.2091981	0.6869435	-0.3045	0.761	-0.261645982	count	1
C12orf73	-0.2091981	0.6981884	-0.2996	0.765	-0.261645982	count	1
CHERP	-0.2091981	0.7195382	-0.2907	0.771	-0.261645982	count	1
FAM219B	-0.2211916	0.5237574	-0.4223	0.673	-0.26157788	count	1
PA2G4	-0.1836523	0.1972016	-0.9313	0.352	-0.261362865	count	1
ZNF14	-0.1996317	0.5567562	-0.3586	0.72	-0.261328821	count	1

RGS3	-0.1917789	0.4653056	-0.4122	0.68	-0.261317615	count	1
RUNX3	-0.1892211	0.2307784	-0.8199	0.413	-0.261252231	count	1
GOLGA3	-0.2157971	0.5269599	-0.4095	0.682	-0.261139843	count	1
DMTN	-0.478837	0.8161571	-0.5867	0.558	-0.260876854	count	1
ARHGEF19	-0.3237338	0.8085331	-0.4004	0.689	-0.260865036	count	1
MSANTD2	-0.250851	0.7471493	-0.3357	0.737	-0.260770727	count	1
JAK1	-0.1815484	0.1239231	-1.465	0.144	-0.260544629	count	1
MOB4	-0.1886705	0.3267426	-0.5774	0.564	-0.260493659	count	1
NT5C3A	-0.1878536	0.3671477	-0.5117	0.609	-0.26027503	count	1
MBOAT7	-0.2266843	0.5561762	-0.4076	0.684	-0.260180362	count	1
ACAA1	-0.1898465	0.3691891	-0.5142	0.607	-0.259721162	count	1
TRIM25	-0.2192871	1.0543744	-0.208	0.835	-0.259349408	count	1
EIF2AK1	-0.1866366	0.3870366	-0.4822	0.63	-0.259118532	count	1
LRPAP1	-0.1858226	0.2622643	-0.7085	0.479	-0.259099077	count	1
MRPL51	-0.1823181	0.2366716	-0.7703	0.4417	-0.258786683	count	1
CITED4	-0.1988819	0.7427966	-0.2677	0.789	-0.258641564	count	1
SRP54	-0.1896841	0.3344075	-0.5672	0.571	-0.258471174	count	1
TMEM70	-0.186858	0.3817837	-0.4894	0.625	-0.258313538	count	1
NAA50	-0.1878345	0.3621736	-0.5186	0.604	-0.258267877	count	1
SLC39A4	-0.1932607	0.602907	-0.3205	0.749	-0.258051919	count	1
VPS39	-0.2131031	0.6492109	-0.3282	0.743	-0.257909593	count	1
ODF2L	-0.1856377	0.3241949	-0.5726	0.567	-0.257734253	count	1
CD46	-0.1842828	0.3150928	-0.5849	0.559	-0.257506261	count	1
LINC01137	-0.4719778	0.8752918	-0.5392	0.59	-0.257456575	count	1
BCL7C	-0.1807842	0.2391606	-0.7559	0.4503	-0.257049454	count	1
INO80D	-0.1881537	0.4475432	-0.4204	0.674	-0.256919259	count	1
TMCO1	-0.1821224	0.2340964	-0.778	0.437	-0.256633588	count	1
RAB11FIP4	-0.2026176	0.694363	-0.2918	0.771	-0.256572602	count	1
TMEM223	-0.1924364	0.5096732	-0.3776	0.706	-0.255897132	count	1
CLIC3	-0.1776285	0.2139239	-0.8303	0.407	-0.255232749	count	1
MECP2	-0.1849051	0.3482134	-0.531	0.596	-0.254622388	count	1
NOL11	-0.244635	0.5710559	-0.4284	0.669	-0.254427781	count	1
ULK4	-0.4655809	0.8580197	-0.5426	0.5878	-0.254257739	count	1
PHF1	-0.1896301	0.3612265	-0.525	0.6	-0.254167588	count	1
FAM199X	-0.196832	0.5166423	-0.381	0.703	-0.254064377	count	1
FAM98C	-0.1919273	0.5177599	-0.3707	0.711	-0.254053545	count	1
RPN2	-0.1816124	0.2631066	-0.6903	0.491	-0.253946574	count	1
CCDC88B	-0.2005298	0.5832336	-0.3438	0.731	-0.253945977	count	1
YDJC	-0.1917781	0.3739136	-0.5129	0.608	-0.253856874	count	1
ORMDL1	-0.1786199	0.2709049	-0.6593	0.5101	-0.253451574	count	1
SCARB2	-0.1905829	0.5431147	-0.3509	0.726	-0.253442074	count	1
SEC11C	-0.1816708	0.2811059	-0.6463	0.519	-0.25291609	count	1
MAP1LC3A	-0.1893052	0.4779055	-0.3961	0.692	-0.25278993	count	1
RNF169	-0.1893052	0.5222519	-0.3625	0.717	-0.25278993	count	1
ZFC3H1	-0.1825877	0.3825318	-0.4773	0.633	-0.252715089	count	1

SNX10	-0.1793395	0.3239639	-0.5536	0.58	-0.252110064	count	1
CPLANE1	-0.2420896	1.057244	-0.229	0.819	-0.251828451	count	1
CLCF1	-0.3119108	1.083252	-0.2879	0.774	-0.251699268	count	1
SENP5	-0.2126663	0.5015249	-0.424	0.672	-0.251598769	count	1
ADAM28	-0.2077383	0.9236029	-0.2249	0.822	-0.251474407	count	1
IL15RA	-0.2008956	0.9508414	-0.2113	0.833	-0.251335446	count	1
RBM3	-0.1751647	0.172791	-1.0137	0.311	-0.250427085	count	1
NCKAP1L	-0.1865427	0.4660967	-0.4002	0.689	-0.250043823	count	1
YBEY	-0.1897766	0.5591475	-0.3394	0.735	-0.249930921	count	1
RABGAP1L	-0.1805171	0.3294569	-0.5479	0.584	-0.249854728	count	1
MPC1	-0.1795667	0.3766787	-0.4767	0.634	-0.249778419	count	1
SRSF5	-0.174652	0.1713645	-1.0192	0.3089	-0.249772615	count	1
TBC1D15	-0.1886705	0.4967317	-0.3798	0.704	-0.249760161	count	1
YARS	-0.1783746	0.3304766	-0.5397	0.59	-0.248915386	count	1
C11orf58	-0.1742251	0.1709397	-1.0192	0.3089	-0.248829716	count	1
RNASEH2C	-0.1787233	0.3115248	-0.5737	0.567	-0.248607208	count	1
RAB3IP	-0.1986726	0.6533916	-0.3041	0.761	-0.248573646	count	1
ARPC1B	-0.174185	0.2055403	-0.8474	0.3974	-0.248366491	count	1
PPM1K	-0.1757436	0.3006641	-0.5845	0.5593	-0.248072222	count	1
MEF2D	-0.190584	0.6344808	-0.3004	0.764	-0.247903699	count	1
ULK3	-0.1936629	0.6055211	-0.3198	0.749	-0.247823265	count	1
S100A11	-0.1723499	0.1473611	-1.1696	0.243	-0.247803377	count	1
INPP4B	-0.1955679	0.5754161	-0.3399	0.734	-0.24770191	count	1
EIF2AK2	-0.1760374	0.3611988	-0.4874	0.6263	-0.247684657	count	1
CLTB	-0.1744786	0.2653896	-0.6574	0.5114	-0.247579711	count	1
TNK2	-0.2602852	0.661769	-0.3933	0.694	-0.247335527	count	1
DUSP28	-0.208973	0.8378471	-0.2494	0.803	-0.247272812	count	1
SNX16	-0.2241576	0.8177091	-0.2741	0.784	-0.247221267	count	1
AC009061.2	-0.2241576	1.0258205	-0.2185	0.827	-0.247221267	count	1
TMSB15B	-0.2241576	1.073742	-0.2088	0.835	-0.247221267	count	1
SUSD1	-0.2240332	0.8682101	-0.258	0.797	-0.247085994	count	1
JAZF1	-0.1780475	0.4037853	-0.4409	0.66	-0.2469705	count	1
SYNE2	-0.1765154	0.3168898	-0.557	0.5779	-0.246829618	count	1
ILKAP	-0.1808691	0.3656702	-0.4946	0.621	-0.246491107	count	1
TRAF2	-0.2229445	0.6420345	-0.3472	0.729	-0.245902006	count	1
DYRK4	-0.2029556	0.5867636	-0.3459	0.73	-0.245734681	count	1
CPSF4	-0.1992104	0.7408798	-0.2689	0.788	-0.245619914	count	1
RETREG1	-0.2138215	0.6438719	-0.3321	0.74	-0.245588345	count	1
AC108134.3	-0.2225524	0.6970361	-0.3193	0.75	-0.245475542	count	1
ELOB	-0.171451	0.1523836	-1.1251	0.2614	-0.245324364	count	1
RMDN3	-0.1936719	0.563142	-0.3439	0.731	-0.245315388	count	1
MED14	-0.1936719	0.6324358	-0.3062	0.76	-0.245315388	count	1
GIMAP4	-0.1726098	0.2257328	-0.7647	0.445	-0.245255666	count	1
CEP170	-0.2221683	0.5668878	-0.3919	0.695	-0.245057761	count	1
SEC16A	-0.1869931	0.7066436	-0.2646	0.791	-0.244859561	count	1

RABGGTA	-0.2131031	0.812288	-0.2623	0.793	-0.244772692	count	1
TSPAN31	-0.1813246	0.5660125	-0.3204	0.749	-0.244647501	count	1
CAPN12	-0.1720492	0.2317292	-0.7425	0.4583	-0.244532915	count	1
ZNF579	-0.2216286	0.9263609	-0.2392	0.811	-0.244470689	count	1
ZNF800	-0.1752841	0.2940158	-0.5962	0.551	-0.244427451	count	1
BLOC1S2	-0.1762781	0.3190015	-0.5526	0.581	-0.243998464	count	1
HAUS3	-0.1807398	0.4416323	-0.4093	0.683	-0.243860886	count	1
RGCC	-0.16916	0.1544876	-1.095	0.274	-0.243713262	count	1
UBALD1	-0.1923693	0.7048712	-0.2729	0.785	-0.243675602	count	1
GCLC	-0.1923693	0.7497363	-0.2566	0.798	-0.243675602	count	1
GNGT2	-0.2119591	0.9073727	-0.2336	0.815	-0.243473677	count	1
CBX7	-0.2337121	0.8645753	-0.2703	0.7871	-0.243265585	count	1
PPP2R5C	-0.1761504	0.2845577	-0.619	0.536	-0.24292625	count	1
SUMO2	-0.1690125	0.113434	-1.49	0.137	-0.242825396	count	1
BTF3	-0.1686143	0.1013379	-1.6639	0.0971	-0.242561049	count	1
NDUFB4	-0.1705438	0.2222885	-0.7672	0.4435	-0.24235877	count	1
HSP90AB1	-0.16831	0.1062927	-1.5835	0.114	-0.242186735	count	1
DHX15	-0.1827146	0.6070473	-0.301	0.764	-0.24190685	count	1
BCKDK	-0.254381	0.8037889	-0.3165	0.752	-0.241855853	count	1
GTSF1	-0.254381	0.9300945	-0.2735	0.785	-0.241855853	count	1
CBX5	-0.1747933	0.4751795	-0.3678	0.713	-0.241371323	count	1
RANBP2	-0.1832446	0.389944	-0.4699	0.639	-0.241364376	count	1
MPV17L2	-0.1926493	0.9110158	-0.2115	0.833	-0.241088039	count	1
ARHGEF1	-0.1735042	0.252314	-0.6877	0.492	-0.240917172	count	1
CKAP2	-0.1750978	0.4132202	-0.4237	0.672	-0.240792835	count	1
RPS6	-0.1672072	0.0706683	-2.3661	0.0186	-0.24078998	count	1
BAG5	-0.179435	0.3768156	-0.4762	0.634	-0.240548239	count	1
PSMD2	-0.1787662	0.3983284	-0.4488	0.654	-0.240464775	count	1
MTX2	-0.181555	0.4640861	-0.3912	0.696	-0.240377566	count	1
RPUSD1	-0.2306222	0.6849909	-0.3367	0.737	-0.240104285	count	1
VTI1B	-0.1784408	0.3635287	-0.4909	0.624	-0.240028442	count	1
HMBOX1	-0.2304824	0.7084095	-0.3254	0.745	-0.239961212	count	1
SERTAD2	-0.1973869	0.588885	-0.3352	0.738	-0.23904839	count	1
UBA5	-0.1795666	0.4839419	-0.371	0.711	-0.2388461	count	1
C1orf174	-0.1744628	0.4874234	-0.3579	0.721	-0.238724985	count	1
EDF1	-0.1666239	0.1437645	-1.159	0.2473	-0.238567038	count	1
CALR	-0.1665041	0.183575	-0.907	0.365	-0.238148246	count	1
ARNTL	-0.228703	0.5734939	-0.3988	0.69	-0.23813991	count	1
KCNK6	-0.2944869	1.1603475	-0.2538	0.8	-0.238136495	count	1
STIMATE	-0.2944869	1.1904567	-0.2474	0.805	-0.238136495	count	1
SSBP3	-0.2944869	1.4520056	-0.2028	0.839	-0.238136495	count	1
HIST2H2BF	-0.2944869	1.4520056	-0.2028	0.839	-0.238136495	count	1
CHAC2	-0.2944869	1.4520056	-0.2028	0.839	-0.238136495	count	1
AC016727.1	-0.2944869	1.4520056	-0.2028	0.839	-0.238136495	count	1
ACVR2B	-0.2944869	1.4520056	-0.2028	0.839	-0.238136495	count	1

THEMIS	-0.2944869	1.4520056	-0.2028	0.839	-0.238136495	count	1
FAM220A	-0.2944869	1.4520056	-0.2028	0.839	-0.238136495	count	1
CYP51A1	-0.2944869	1.4520056	-0.2028	0.839	-0.238136495	count	1
AC078846.1	-0.2944869	1.4520056	-0.2028	0.839	-0.238136495	count	1
LY96	-0.2944869	1.4520056	-0.2028	0.839	-0.238136495	count	1
AC067735.1	-0.2944869	1.4520056	-0.2028	0.839	-0.238136495	count	1
STK24-AS1	-0.2944869	1.4520056	-0.2028	0.839	-0.238136495	count	1
NOP9	-0.2944869	1.4520056	-0.2028	0.839	-0.238136495	count	1
SAV1	-0.2944869	1.4520056	-0.2028	0.839	-0.238136495	count	1
SORD	-0.2944869	1.4520056	-0.2028	0.839	-0.238136495	count	1
LYSMD4	-0.2944869	1.4520056	-0.2028	0.839	-0.238136495	count	1
AC027682.4	-0.2944869	1.4520056	-0.2028	0.839	-0.238136495	count	1
AC007114.1	-0.2944869	1.4520056	-0.2028	0.839	-0.238136495	count	1
CAPS	-0.2944869	1.4520056	-0.2028	0.839	-0.238136495	count	1
ZNF563	-0.2944869	1.4520056	-0.2028	0.839	-0.238136495	count	1
PTGER1	-0.2944869	1.4520056	-0.2028	0.839	-0.238136495	count	1
FUCA1	-0.1928331	0.5566796	-0.3464	0.729	-0.237814686	count	1
DNAJB4	-0.1928331	0.6405445	-0.301	0.764	-0.237814686	count	1
ELP6	-0.1814849	0.5529737	-0.3282	0.743	-0.237678341	count	1
DHCR7	-0.1850672	0.6682656	-0.2769	0.782	-0.236883802	count	1
SERINC1	-0.1700963	0.3256439	-0.5223	0.602	-0.236827433	count	1
PSMD9	-0.1919343	0.5172836	-0.371	0.711	-0.236714294	count	1
BTG1	-0.1641662	0.0876561	-1.8728	0.062	-0.236675171	count	1
ATAD2B	-0.1749242	0.4326806	-0.4043	0.686	-0.236037382	count	1
USP47	-0.1759971	0.3814475	-0.4614	0.645	-0.235954356	count	1
PLEK	-0.1665365	0.3122215	-0.5334	0.5941	-0.23555513	count	1
BNIP2	-0.1694264	0.2973037	-0.5699	0.569	-0.235485079	count	1
ASF1A	-0.1699194	0.3724331	-0.4562	0.649	-0.235470446	count	1
CALM2	-0.1639066	0.1143498	-1.4334	0.153	-0.235358092	count	1
TET2	-0.1737292	0.4086396	-0.4251	0.671	-0.235090497	count	1
INTS5	-0.2045366	1.0312321	-0.1983	0.843	-0.23504106	count	1
TCTN1	-0.2045366	1.0390753	-0.1968	0.844	-0.23504106	count	1
ACTR3	-0.1646179	0.1703196	-0.9665	0.335	-0.234909908	count	1
EIF2S3	-0.1699121	0.3440673	-0.4938	0.622	-0.234643614	count	1
GPBP1L1	-0.171494	0.4416432	-0.3883	0.698	-0.234225256	count	1
APMAP	-0.1655735	0.2231496	-0.742	0.4586	-0.234133038	count	1
PLXNA3	-0.2893415	0.8066379	-0.3587	0.72	-0.234118917	count	1
CD63	-0.1629361	0.1269188	-1.2838	0.2	-0.234109792	count	1
RHOF	-0.1650574	0.2108226	-0.7829	0.4342	-0.233693571	count	1
HLA-C	-0.1620971	0.0631214	-2.568	0.0107	-0.233550247	count	1
ATG16L1	-0.2449986	0.8041204	-0.3047	0.761	-0.233134317	count	1
C1GALT1	-0.1689298	0.3095845	-0.5457	0.586	-0.23298766	count	1
MLLT6	-0.1768167	0.3890831	-0.4544	0.65	-0.232931592	count	1
GPALPP1	-0.1748856	0.6015594	-0.2907	0.771	-0.232641832	count	1
SNAPC2	-0.1727914	0.4047149	-0.4269	0.67	-0.232452221	count	1

AC079922.2	-0.191891	0.7644358	-0.251	0.802	-0.232446093	count	1
DELE1	-0.1881537	0.6788417	-0.2772	0.782	-0.232084843	count	1
HACD2	-0.1881537	0.7257345	-0.2593	0.796	-0.232084843	count	1
GOT1	-0.1782611	0.5308865	-0.3358	0.737	-0.231947825	count	1
EIF1AD	-0.1782611	0.5860604	-0.3042	0.761	-0.231947825	count	1
PMAIP1	-0.1666911	0.2992457	-0.557	0.578	-0.231896127	count	1
IFNAR2	-0.1759406	0.4977933	-0.3534	0.724	-0.231782022	count	1
SNX3	-0.1629486	0.2354778	-0.692	0.4894	-0.231672917	count	1
GATAD1	-0.1683579	0.3760366	-0.4477	0.655	-0.231543094	count	1
IRF2	-0.1649725	0.2408457	-0.685	0.494	-0.231498214	count	1
PHC1	-0.2855542	0.9314062	-0.3066	0.759	-0.231158176	count	1
DCLRE1B	-0.2855542	1.056844	-0.2702	0.787	-0.231158176	count	1
AL592494.3	-0.2855542	1.0637037	-0.2685	0.789	-0.231158176	count	1
SLC27A1	-0.2855542	1.0637037	-0.2685	0.789	-0.231158176	count	1
ELOVL5	-0.1744736	0.3898353	-0.4476	0.655	-0.231036773	count	1
MRPS21	-0.162666	0.2172221	-0.7488	0.4545	-0.23078756	count	1
TMEM50A	-0.1612952	0.1742091	-0.9259	0.3552	-0.230501443	count	1
PIP5K1C	-0.2000083	0.6916306	-0.2892	0.773	-0.229892832	count	1
FIP1L1	-0.1652589	0.305194	-0.5415	0.589	-0.229701731	count	1
MRPL22	-0.1646802	0.3438396	-0.4789	0.632	-0.229483823	count	1
RAB31	-0.1700445	0.6011109	-0.2829	0.777	-0.229471599	count	1
PAWR	-0.1832144	0.5906167	-0.3102	0.757	-0.229355568	count	1
VPS33A	-0.1832144	0.6195239	-0.2957	0.768	-0.229355568	count	1
TMEM267	-0.1832144	0.6275756	-0.2919	0.771	-0.229355568	count	1
CHRNB1	-0.1832144	0.6735811	-0.272	0.786	-0.229355568	count	1
MMAB	-0.2076154	0.6907854	-0.3005	0.764	-0.229212121	count	1
CSTF3	-0.1890995	0.7264379	-0.2603	0.795	-0.229091306	count	1
DDX46	-0.1612122	0.2041887	-0.7895	0.4304	-0.229071392	count	1
BRK1	-0.1606953	0.200276	-0.8024	0.4229	-0.228405591	count	1
TMEM50B	-0.1783058	0.5251184	-0.3396	0.734	-0.228274464	count	1
ZDHHC18	-0.2189731	0.5120616	-0.4276	0.669	-0.228171406	count	1
WDR77	-0.1925255	0.8071319	-0.2385	0.812	-0.22798732	count	1
ALG1	-0.1925255	0.9293133	-0.2072	0.836	-0.22798732	count	1
AC009053.2	-0.1925255	1.0629353	-0.1811	0.856	-0.22798732	count	1
NCR3	-0.1602458	0.2272296	-0.7052	0.481	-0.227985591	count	1
SPG7	-0.1723771	0.4645859	-0.371	0.711	-0.227105681	count	1
LPGAT1	-0.1652905	0.3842423	-0.4302	0.667	-0.226979762	count	1
BORCS7	-0.1666182	0.4138689	-0.4026	0.688	-0.226076812	count	1
YWHAB	-0.1565984	0.1168182	-1.3405	0.181	-0.224712524	count	1
YIPF3	-0.1647692	0.3460338	-0.4762	0.634	-0.224601705	count	1
GAPDH	-0.155748	0.0935194	-1.6654	0.0968	-0.224438416	count	1
9-Sep	-0.1597235	0.2806074	-0.5692	0.57	-0.224359567	count	1
RBM17	-0.1597722	0.3020412	-0.529	0.597	-0.224320421	count	1
MTRR	-0.1890258	0.7240353	-0.2611	0.794	-0.223879465	count	1
NAA16	-0.1663417	0.5021994	-0.3312	0.741	-0.223800713	count	1

BRD1	-0.165161	0.3261636	-0.5064	0.613	-0.223526632	count	1
PRR13	-0.1562257	0.1853817	-0.8427	0.4	-0.223384246	count	1
NR4A2	-0.1554495	0.1751399	-0.8876	0.375	-0.223268981	count	1
LCLAT1	-0.214089	0.7776396	-0.2753	0.783	-0.223161532	count	1
CBFA2T2	-0.214089	0.7796138	-0.2746	0.784	-0.223161532	count	1
LINC00623	-0.1578945	0.267934	-0.5893	0.5561	-0.223099056	count	1
RASA2	-0.163587	0.2799318	-0.5844	0.559	-0.222993949	count	1
RCL1	-0.2131031	0.8394628	-0.2539	0.8	-0.222149751	count	1
C16orf70	-0.2131031	0.8891469	-0.2397	0.811	-0.222149751	count	1
ZNF525	-0.2131031	0.9967506	-0.2138	0.831	-0.222149751	count	1
SNRNP48	-0.166673	0.5769665	-0.2889	0.773	-0.221753583	count	1
MED30	-0.1582648	0.2778805	-0.5695	0.569	-0.221740092	count	1
TK2	-0.4013238	0.7556402	-0.5311	0.596	-0.221647305	count	1
RILPL2	-0.1569707	0.3617949	-0.4339	0.6647	-0.221526363	count	1
CSNK1A1	-0.1562159	0.2135935	-0.7314	0.4651	-0.221426816	count	1
COP1	-0.4002009	0.7270667	-0.5504	0.582	-0.221069781	count	1
RALGAPA2	-0.2723212	0.7176356	-0.3795	0.705	-0.220789524	count	1
BTN3A2	-0.1563449	0.2613032	-0.5983	0.55	-0.220498403	count	1
MRPL1	-0.162063	0.3964079	-0.4088	0.683	-0.220435355	count	1
KDM2A	-0.1648857	0.3717762	-0.4435	0.658	-0.220284813	count	1
LINC002481	-0.1817414	0.6137232	-0.2961	0.767	-0.220244324	count	1
ABRAXAS2	-0.2308768	0.88664	-0.2604	0.795	-0.219975659	count	1
ZNF511	-0.1633394	0.4748837	-0.344	0.731	-0.219772753	count	1
SLC25A3	-0.1532875	0.1521585	-1.0074	0.314	-0.219761412	count	1
NDUFA9	-0.1580187	0.3470638	-0.4553	0.649	-0.219653123	count	1
DCTN5	-0.1657484	0.5885137	-0.2816	0.778	-0.219523418	count	1
TSR2	-0.1587837	0.3749492	-0.4235	0.672	-0.219019293	count	1
RBM4	-0.1682527	0.5334942	-0.3154	0.753	-0.218980611	count	1
EPC1	-0.1542624	0.2115731	-0.7291	0.4664	-0.218567611	count	1
POLR2J3	-0.1668063	0.3943397	-0.423	0.673	-0.2185311	count	1
KIZ	-0.1898969	0.6245265	-0.3041	0.761	-0.218387222	count	1
BPNT1	-0.2690065	1.0503801	-0.2561	0.798	-0.218186572	count	1
SLC20A1	-0.1614688	0.4756816	-0.3394	0.734	-0.217929873	count	1
PIF1	-0.1637794	0.5622425	-0.2913	0.771	-0.217916319	count	1
COX6B1	-0.1520757	0.1449556	-1.0491	0.295	-0.217822151	count	1
AZI2	-0.1764483	0.4841462	-0.3645	0.716	-0.217741945	count	1
SYF2	-0.1527823	0.1889855	-0.8084	0.419	-0.217695978	count	1
KAT7	-0.1835956	0.405902	-0.4523	0.651	-0.217502706	count	1
THAP7	-0.1711938	0.517379	-0.3309	0.741	-0.216997485	count	1
FPGS	-0.1666308	0.6145184	-0.2712	0.786	-0.216878541	count	1
CTNNBIP1	-0.1562239	0.3993743	-0.3912	0.696	-0.216745806	count	1
MAPK6	-0.1626876	0.643018	-0.253	0.8	-0.216468329	count	1
TMED10	-0.1528169	0.2510359	-0.6087	0.5431	-0.216375708	count	1
HLA-B	-0.1498381	0.0542158	-2.7637	0.00604	-0.216073825	count	1
FIG4	-0.2266843	0.7732118	-0.2932	0.77	-0.216061881	count	1

MNT	-0.2266843	0.9699856	-0.2337	0.815	-0.216061881	count	1
PDCD4	-0.1511654	0.1622343	-0.9318	0.352	-0.21597806	count	1
PFDN1	-0.1567127	0.3299522	-0.475	0.635	-0.215871674	count	1
NAA25	-0.3893172	0.9741112	-0.3997	0.6897	-0.215458713	count	1
IRF5	-0.3893172	0.9869412	-0.3945	0.6935	-0.215458713	count	1
DDOST	-0.1541032	0.3070562	-0.5019	0.616	-0.215089485	count	1
CTSW	-0.1488425	0.0980751	-1.5176	0.13	-0.214471188	count	1
CSNK2A2	-0.160489	0.5608352	-0.2862	0.775	-0.214428729	count	1
SRRM1	-0.1495076	0.1498483	-0.9977	0.319	-0.214221722	count	1
CEP104	-0.1594623	0.5303411	-0.3007	0.764	-0.213850842	count	1
SPTLC2	-0.1762761	0.4056488	-0.4346	0.664	-0.213669272	count	1
SLC27A5	-0.1759406	0.641559	-0.2742	0.784	-0.213265543	count	1
ARHGAP10	-0.1617928	0.4573106	-0.3538	0.724	-0.213211156	count	1
TECR	-0.1521236	0.2678387	-0.568	0.57	-0.213022742	count	1
ATP5F1B	-0.1503098	0.2012623	-0.7468	0.4557	-0.213016557	count	1
HDAC8	-0.1850672	0.7373975	-0.251	0.802	-0.212886769	count	1
ZNF76	-0.1850672	0.9614124	-0.1925	0.847	-0.212886769	count	1
ZNF808	-0.2618311	0.6648812	-0.3938	0.694	-0.212544097	count	1
COTL1	-0.1478217	0.1575118	-0.9385	0.349	-0.212432094	count	1
SUPT4H1	-0.1510092	0.3227598	-0.4679	0.6402	-0.212415246	count	1
KLHL24	-0.1547891	0.3760115	-0.4117	0.681	-0.212235034	count	1
DPY19L3	-0.3830681	0.8337024	-0.4595	0.6462	-0.212226014	count	1
KMT5B	-0.1540441	0.4763013	-0.3234	0.747	-0.212202198	count	1
FAM204A	-0.1511955	0.3256881	-0.4642	0.6428	-0.21218602	count	1
ANKRD40	-0.1561317	0.5547157	-0.2815	0.779	-0.211881953	count	1
LRP12	-0.2607173	0.9052638	-0.288	0.774	-0.211667292	count	1
ANKS1A	-0.2607173	0.9146443	-0.285	0.776	-0.211667292	count	1
MAP1B	-0.2607173	1.0255572	-0.2542	0.799	-0.211667292	count	1
RNF113A	-0.1504255	0.2919015	-0.5153	0.607	-0.211503854	count	1
BUD13	-0.1782611	0.743281	-0.2398	0.811	-0.211234869	count	1
PSEN2	-0.1782611	0.8285401	-0.2152	0.83	-0.211234869	count	1
CLDND2	-0.1782611	0.8349278	-0.2135	0.831	-0.211234869	count	1
TBC1D14	-0.1782611	0.9247325	-0.1928	0.847	-0.211234869	count	1
TOMM5	-0.1665732	0.63064	-0.2641	0.792	-0.211170958	count	1
ZNF224	-0.1564354	0.407894	-0.3835	0.702	-0.211153918	count	1
POLR2J	-0.1508732	0.2928965	-0.5151	0.607	-0.211147243	count	1
ANAPC11	-0.1490694	0.2525381	-0.5903	0.555	-0.210316539	count	1
NBDY	-0.1492588	0.3006802	-0.4964	0.6199	-0.209771529	count	1
AC118549.1	-0.1574088	0.6572791	-0.2395	0.811	-0.209466396	count	1
ICE1	-0.1580861	0.3563729	-0.4436	0.658	-0.209408761	count	1
PBDC1	-0.148048	0.2969382	-0.4986	0.6184	-0.209256051	count	1
REXO2	-0.1500941	0.3384725	-0.4434	0.658	-0.209184464	count	1
UFD1	-0.1483235	0.3196469	-0.464	0.6429	-0.208972367	count	1
ZNF350	-0.1552539	0.5635827	-0.2755	0.783	-0.208922757	count	1
IKBKG	-0.1563297	0.5207498	-0.3002	0.764	-0.208887858	count	1

RNF24	-0.3756854	0.6415416	-0.5856	0.5586	-0.208396603	count	1
MED19	-0.1500099	0.4119462	-0.3641	0.716	-0.208341479	count	1
NXT1	-0.1466622	0.2239794	-0.6548	0.5131	-0.207850638	count	1
EIF3K	-0.1445072	0.1231137	-1.1738	0.241	-0.20754988	count	1
PPP3R1	-0.1620393	0.4984726	-0.3251	0.745	-0.207546852	count	1
PRR5	-0.1635424	0.567573	-0.2881	0.773	-0.207348149	count	1
FAM241A	-0.1800487	0.5871639	-0.3066	0.759	-0.20716803	count	1
RPL29	-0.1436726	0.0609719	-2.3564	0.019	-0.207110928	count	1
ITGB7	-0.1506199	0.3894372	-0.3868	0.699	-0.206871106	count	1
ZNF544	-0.1631541	0.5037821	-0.3239	0.746	-0.206858323	count	1
WIPI2	-0.1521465	0.4065578	-0.3742	0.708	-0.206486155	count	1
MED4	-0.1500159	0.3140628	-0.4777	0.633	-0.206362416	count	1
ARHGEF6	-0.1975017	0.5041626	-0.3917	0.696	-0.206117477	count	1
TROVE2	-0.1482281	0.3374626	-0.4392	0.661	-0.206062459	count	1
STXBP2	-0.1478095	0.3120801	-0.4736	0.636	-0.206004493	count	1
ABRACL	-0.1443079	0.1997442	-0.7225	0.4705	-0.205727069	count	1
POGZ	-0.1580237	0.6278135	-0.2517	0.801	-0.205720126	count	1
C3orf38	-0.156981	0.446297	-0.3517	0.725	-0.205706451	count	1
TMSB4X	-0.1422742	0.0634611	-2.2419	0.0256	-0.205156002	count	1
TIMM10	-0.1503822	0.403761	-0.3725	0.71	-0.205031801	count	1
SZT2	-0.3682373	0.82847	-0.4445	0.657	-0.204522023	count	1
HIST1H4C	-0.1431276	0.2045084	-0.6999	0.485	-0.20444582	count	1
SH3BGRL3	-0.1417906	0.0955975	-1.4832	0.139	-0.204217446	count	1
PRRC2A	-0.1566723	0.6556804	-0.2389	0.811	-0.203967666	count	1
RAP2B	-0.148211	0.4097389	-0.3617	0.718	-0.203568524	count	1
GOLGA2	-0.148211	0.5112075	-0.2899	0.772	-0.203568524	count	1
SZRD1	-0.1464523	0.3952515	-0.3705	0.711	-0.203407335	count	1
CSF3R	-0.1946475	1.258892	-0.1546	0.8772	-0.203180125	count	1
OTUD6B-AS1	-0.1451647	0.3827178	-0.3793	0.705	-0.203166977	count	1
RERE	-0.1673886	0.5234578	-0.3198	0.749	-0.202970167	count	1
ARL8B	-0.1548794	0.3704464	-0.4181	0.676	-0.202962457	count	1
ZDHHC4	-0.1517519	0.6173012	-0.2458	0.806	-0.202788376	count	1
GGCT	-0.1597318	0.4900734	-0.3259	0.745	-0.202540645	count	1
PHACTR2	-0.1469901	0.3661147	-0.4015	0.688	-0.202501359	count	1
NSD3	-0.1422487	0.2048897	-0.6943	0.488	-0.202313832	count	1
CD44	-0.141028	0.1396874	-1.0096	0.313	-0.202259088	count	1
USP11	-0.1752899	0.4606019	-0.3806	0.704	-0.201742182	count	1
ZNF322	-0.1701695	0.5813256	-0.2927	0.77	-0.201721071	count	1
JOSD2	-0.1467603	0.4472553	-0.3281	0.743	-0.201579523	count	1
MFSD1	-0.1546474	0.5652054	-0.2736	0.785	-0.201341587	count	1
IAH1	-0.1425674	0.2770074	-0.5147	0.6071	-0.201150397	count	1
LSP1	-0.1400165	0.1344833	-1.0411	0.299	-0.200794554	count	1
LY6G5C	-0.191891	0.9325687	-0.2058	0.837	-0.200342038	count	1
TAS2R14	-0.191891	0.9325687	-0.2058	0.837	-0.200342038	count	1
MTFP1	-0.191891	0.9325687	-0.2058	0.837	-0.200342038	count	1

PQLC2	-0.191891	1.2001371	-0.1599	0.873	-0.200342038	count	1
PDE5A	-0.191891	1.2001371	-0.1599	0.873	-0.200342038	count	1
RAD50	-0.191891	1.2001371	-0.1599	0.873	-0.200342038	count	1
LINC02328	-0.191891	1.2001371	-0.1599	0.873	-0.200342038	count	1
ZNHIT1	-0.1407274	0.2428706	-0.5794	0.5627	-0.20003904	count	1
PSMC5	-0.1414662	0.2836382	-0.4988	0.6183	-0.199725406	count	1
PPOX	-0.1682527	0.7745332	-0.2172	0.828	-0.199466234	count	1
MIDN	-0.1412239	0.2547735	-0.5543	0.5797	-0.199444542	count	1
STUB1	-0.1408113	0.2314566	-0.6084	0.5434	-0.199293464	count	1
IER3	-0.1428426	0.3615411	-0.3951	0.693	-0.199244052	count	1
RPL26	-0.1382447	0.0687153	-2.0118	0.0451	-0.199150596	count	1
MSI2	-0.1678668	0.541038	-0.3103	0.757	-0.199012227	count	1
POU3F1	-0.244635	0.8538123	-0.2865	0.775	-0.198978634	count	1
MPP7	-0.244635	0.9050929	-0.2703	0.787	-0.198978634	count	1
SERPINA1	-0.244635	0.9050929	-0.2703	0.787	-0.198978634	count	1
MBTD1	-0.244635	0.953481	-0.2566	0.798	-0.198978634	count	1
FAM126A	-0.244635	0.9554148	-0.2561	0.7981	-0.198978634	count	1
NUDT22	-0.1423492	0.3579946	-0.3976	0.691	-0.198977777	count	1
CYC1	-0.1416438	0.3014053	-0.4699	0.639	-0.198586742	count	1
NDUFV2	-0.1395563	0.2371631	-0.5884	0.5566	-0.198375223	count	1
TRAPPC11	-0.1723297	0.7820757	-0.2203	0.826	-0.198365558	count	1
DHRS3	-0.1404049	0.3045678	-0.461	0.6451	-0.198035609	count	1
BBC3	-0.1462014	0.3300353	-0.443	0.658	-0.197925943	count	1
MIA3	-0.1415973	0.3223749	-0.4392	0.661	-0.197509097	count	1
RPF2	-0.1515611	0.4566534	-0.3319	0.74	-0.197338432	count	1
KDM4C	-0.1437514	0.4713189	-0.305	0.761	-0.197128263	count	1
H3F3A	-0.1368761	0.0841027	-1.6275	0.105	-0.197065302	count	1
SAT1	-0.1381987	0.2419045	-0.5713	0.568	-0.197039651	count	1
ASAP1	-0.1454402	0.4875163	-0.2983	0.766	-0.196897765	count	1
RPL5	-0.1363795	0.0672998	-2.0264	0.0435	-0.196556703	count	1
UBL5	-0.1374851	0.1665948	-0.8253	0.41	-0.196426555	count	1
EBLN3P	-0.1464101	0.4143718	-0.3533	0.724	-0.196392571	count	1
FRYL	-0.1507174	0.3692709	-0.4081	0.683	-0.196243974	count	1
PDIA6	-0.1379958	0.2314703	-0.5962	0.5515	-0.196008024	count	1
MSN	-0.1375786	0.1971074	-0.698	0.486	-0.195980567	count	1
AASDHPPT	-0.1440267	0.458998	-0.3138	0.754	-0.195490084	count	1
LITAF	-0.1366259	0.1842657	-0.7415	0.459	-0.195426666	count	1
SLC38A10	-0.1871085	0.6058939	-0.3088	0.758	-0.195415123	count	1
ARL16	-0.1498654	0.4044456	-0.3705	0.711	-0.1951387	count	1
BIN1	-0.1379546	0.2879824	-0.479	0.6322	-0.194888685	count	1
MTHFS	-0.1477834	0.5721238	-0.2583	0.796	-0.194809325	count	1
RPL12	-0.1351029	0.0660776	-2.0446	0.0417	-0.194746354	count	1
MICU3	-0.1553913	0.6547286	-0.2373	0.813	-0.194708278	count	1
KHNYN	-0.1601378	0.9038536	-0.1772	0.859	-0.194235062	count	1
ELF1	-0.1353702	0.1366137	-0.9909	0.322	-0.19417285	count	1

RNH1	-0.1375849	0.242463	-0.5674	0.571	-0.19354848	count	1
EMP3	-0.1348153	0.1558971	-0.8648	0.388	-0.193445447	count	1
TMEM183A	-0.1407496	0.3748516	-0.3755	0.708	-0.192314779	count	1
INPP5K	-0.2360691	0.6033565	-0.3913	0.696	-0.192198764	count	1
SARAF	-0.1338483	0.1242673	-1.0771	0.282	-0.192156625	count	1
PPARA	-0.1499706	0.8227575	-0.1823	0.855	-0.192154531	count	1
IFNAR1	-0.1418575	0.3502588	-0.405	0.686	-0.191522264	count	1
TTF1	-0.1421919	0.527318	-0.2697	0.7876	-0.191387804	count	1
GPSM3	-0.1341453	0.1763901	-0.7605	0.448	-0.191226853	count	1
RANBP1	-0.1369456	0.2725564	-0.5024	0.616	-0.191027989	count	1
GPANK1	-0.1489585	0.5371078	-0.2773	0.782	-0.190863182	count	1
ZNF331	-0.1326102	0.1870636	-0.7089	0.479	-0.190761674	count	1
SRI	-0.1341623	0.2061433	-0.6508	0.5156	-0.190683584	count	1
GGNBP2	-0.1348422	0.2209649	-0.6102	0.5421	-0.190549854	count	1
LIMD2	-0.132773	0.1699878	-0.7811	0.435	-0.190336746	count	1
PRADC1	-0.3410616	0.9393359	-0.3631	0.717	-0.190289809	count	1
KIAA0753	-0.3410616	0.9989457	-0.3414	0.733	-0.190289809	count	1
HMBS	-0.3410616	1.048884	-0.3252	0.745	-0.190289809	count	1
NSD2	-0.1652503	0.5869528	-0.2815	0.778	-0.190285671	count	1
BABAM2	-0.1447602	0.4468175	-0.324	0.746	-0.189746029	count	1
SFXN1	-0.1409099	0.3569361	-0.3948	0.693	-0.18966634	count	1
MATK	-0.1321499	0.1328331	-0.9949	0.321	-0.189621568	count	1
RAB2B	-0.1494366	0.6776211	-0.2205	0.826	-0.189545905	count	1
MSH3	-0.232186	0.543913	-0.4269	0.67	-0.189120469	count	1
VTA1	-0.1441262	0.5674481	-0.254	0.8	-0.188917756	count	1
CREM	-0.1331657	0.2445478	-0.5445	0.5864	-0.188811918	count	1
GIGYF1	-0.1499842	0.6000823	-0.2499	0.803	-0.187966715	count	1
SURF4	-0.1365887	0.4047677	-0.3374	0.736	-0.187921383	count	1
TMUB2	-0.1464541	0.4826119	-0.3035	0.762	-0.187667448	count	1
ARHGAP25	-0.1464541	0.5026304	-0.2914	0.771	-0.187667448	count	1
MS4A1	-0.141543	0.5302817	-0.2669	0.79	-0.187558896	count	1
ERGIC3	-0.1357087	0.3730297	-0.3638	0.7162	-0.187479167	count	1
COX8A	-0.1310438	0.1635226	-0.8014	0.4235	-0.187425911	count	1
MAPRE1	-0.1337428	0.3475267	-0.3848	0.7006	-0.18741646	count	1
PGPEP1	-0.16919	0.7158357	-0.2364	0.813	-0.187222425	count	1
RPS5	-0.1298233	0.0729971	-1.7785	0.0763	-0.187059742	count	1
FUNDC1	-0.1385018	0.5346138	-0.2591	0.796	-0.1870018	count	1
LUZP1	-0.1364649	0.3387318	-0.4029	0.687	-0.186823551	count	1
PKNOX1	-0.1538854	0.6552384	-0.2349	0.814	-0.186698231	count	1
NPM1	-0.1296573	0.1176351	-1.1022	0.271	-0.186169673	count	1
RPUSD3	-0.1467225	0.6248742	-0.2348	0.815	-0.186118646	count	1
DNAJB6	-0.129923	0.170339	-0.7627	0.4462	-0.185877043	count	1
AC004817.3	-0.1944359	0.8222701	-0.2365	0.813	-0.185849049	count	1
CMAS	-0.1359208	0.4670992	-0.291	0.771	-0.185728755	count	1
UTP14A	-0.1611837	0.6992898	-0.2305	0.818	-0.185641452	count	1

FMNL3	-0.1431704	0.5760839	-0.2485	0.804	-0.185066647	count	1
IMPACT	-0.1520964	0.761007	-0.1999	0.842	-0.184540956	count	1
ETF1	-0.1355385	0.3263039	-0.4154	0.678	-0.184427667	count	1
MRPL33	-0.1322826	0.3137387	-0.4216	0.674	-0.184388751	count	1
NAT10	-0.1928331	0.887205	-0.2173	0.828	-0.184342498	count	1
NEIL2	-0.1928331	0.9856874	-0.1956	0.845	-0.184342498	count	1
KIR3DL1	-0.1928331	1.1040453	-0.1747	0.861	-0.184342498	count	1
BSDC1	-0.1390015	0.5437571	-0.2556	0.798	-0.184200684	count	1
ABI1	-0.134736	0.3827484	-0.352	0.725	-0.183739308	count	1
ARHGEF3	-0.1321821	0.3923632	-0.3369	0.736	-0.183243873	count	1
MEI1	-0.1443976	0.5271989	-0.2739	0.784	-0.183182361	count	1
THOC6	-0.138781	0.578268	-0.24	0.81	-0.182977868	count	1
SH2D1B	-0.1296851	0.330357	-0.3926	0.6949	-0.18279939	count	1
LRIG2	-0.143682	0.8514643	-0.1687	0.866	-0.182278478	count	1
FIGNL1	-0.143682	0.9368335	-0.1534	0.878	-0.182278478	count	1
FAM133B	-0.1282653	0.2104052	-0.6096	0.5426	-0.182006605	count	1
YY1AP1	-0.149844	0.7696921	-0.1947	0.846	-0.181824406	count	1
ZDHHC21	-0.1731588	0.5974013	-0.2899	0.772	-0.181023161	count	1
COPS5	-0.1298382	0.3526046	-0.3682	0.713	-0.18098522	count	1
LINC01678	-0.3228701	0.6747352	-0.4785	0.633	-0.180680098	count	1
IARS	-0.1351502	0.6049247	-0.2234	0.823	-0.180658659	count	1
ZFAND6	-0.1289062	0.3003054	-0.4293	0.668	-0.180645065	count	1
ST8SIA6	-0.1440452	0.71646	-0.2011	0.841	-0.180558947	count	1
NME1	-0.135561	0.5549922	-0.2443	0.807	-0.18046999	count	1
GBP4	-0.13356	0.3759919	-0.3552	0.723	-0.180343737	count	1
RPS21	-0.1252435	0.0880284	-1.4228	0.156	-0.180213081	count	1
ARPC4	-0.1264367	0.1916131	-0.6599	0.51	-0.18017143	count	1
COG3	-0.1458206	0.6659778	-0.219	0.827	-0.180148195	count	1
NANS	-0.1484394	0.4375952	-0.3392	0.735	-0.180130093	count	1
ADAL	-0.1882426	0.6658258	-0.2827	0.778	-0.180025103	count	1
AIDA	-0.1433662	0.4715912	-0.304	0.761	-0.179711824	count	1
SP2	-0.2203248	0.5997076	-0.3674	0.714	-0.179698845	count	1
UBA52	-0.1246578	0.0749409	-1.6634	0.0972	-0.179438383	count	1
PCF11	-0.1303209	0.3178563	-0.41	0.682	-0.179034775	count	1
AP2S1	-0.1272702	0.3169173	-0.4016	0.688	-0.178893688	count	1
BPTF	-0.1258013	0.2262225	-0.5561	0.5785	-0.17847908	count	1
IL18R1	-0.1352176	0.4421682	-0.3058	0.76	-0.178293266	count	1
CEMIP2	-0.1269009	0.2672574	-0.4748	0.6352	-0.178293205	count	1
NELFE	-0.1331978	0.4686774	-0.2842	0.776	-0.178055182	count	1
TERF2IP	-0.1244705	0.1652252	-0.7533	0.452	-0.177952643	count	1
ZFAND3	-0.141947	0.5836493	-0.2432	0.808	-0.17794109	count	1
UBE2K	-0.127856	0.3693148	-0.3462	0.729	-0.177774683	count	1
SPOP	-0.1274154	0.4111694	-0.3099	0.757	-0.177611614	count	1
HMOX2	-0.1256651	0.2801522	-0.4486	0.654	-0.177486016	count	1
CTBP2	-0.1347365	0.435839	-0.3091	0.757	-0.176647717	count	1

RPL31	-0.1229919	0.1081402	-1.1373	0.256	-0.176623613	count	1
TUBB	-0.1237444	0.1691662	-0.7315	0.465	-0.176281408	count	1
DCTN4	-0.1842223	0.7063194	-0.2608	0.794	-0.1762409	count	1
AFF1	-0.1423967	0.5782225	-0.2463	0.806	-0.175939856	count	1
DEK	-0.1227981	0.1640491	-0.7485	0.4547	-0.175902279	count	1
UBFD1	-0.3128163	0.8694986	-0.3598	0.7193	-0.175341029	count	1
RAB9A	-0.126441	0.2624483	-0.4818	0.63	-0.175294743	count	1
CCDC127	-0.1353994	0.5151477	-0.2628	0.793	-0.175056825	count	1
IQGAP1	-0.1235918	0.1915221	-0.6453	0.5192	-0.174893613	count	1
NDUFAF8	-0.1256469	0.3133841	-0.4009	0.689	-0.174706544	count	1
ZNF354A	-0.1514641	0.6211738	-0.2438	0.808	-0.174532771	count	1
SKAP1	-0.1216209	0.167706	-0.7252	0.4689	-0.174233776	count	1
MAP3K8	-0.1220674	0.1986165	-0.6146	0.539	-0.174154597	count	1
HNRNPR	-0.12296	0.2078865	-0.5915	0.5546	-0.174124994	count	1
SLC20A2	-0.1302168	0.6952578	-0.1873	0.852	-0.174079703	count	1
SNHG10	-0.2131031	1.1327259	-0.1881	0.851	-0.173948824	count	1
TRIB1	-0.2131031	1.295856	-0.1644	0.869	-0.173948824	count	1
RAB38	-0.2131031	1.295856	-0.1644	0.869	-0.173948824	count	1
GGA3	-0.2131031	1.295856	-0.1644	0.869	-0.173948824	count	1
RPL32	-0.1205689	0.0575783	-2.094	0.037	-0.173823589	count	1
ATF6B	-0.1366393	0.5038078	-0.2712	0.786	-0.1733806	count	1
LENG8	-0.1656292	0.5921734	-0.2797	0.78	-0.173242019	count	1
SKAP2	-0.1231833	0.3047834	-0.4042	0.686	-0.173230222	count	1
NDUFA4	-0.1207597	0.1540068	-0.7841	0.4335	-0.172965198	count	1
SEM1	-0.1208643	0.2050776	-0.5894	0.556	-0.172409753	count	1
RPL39L	-0.1553913	0.9279779	-0.1675	0.867	-0.172091967	count	1
KIAA0825	-0.1553913	0.9279779	-0.1675	0.867	-0.172091967	count	1
FBXO46	-0.1553913	1.0323451	-0.1505	0.88	-0.172091967	count	1
GSTZ1	-0.1553913	1.0924668	-0.1422	0.887	-0.172091967	count	1
FAM131A	-0.1553913	1.1270892	-0.1379	0.89	-0.172091967	count	1
SLC11A2	-0.1553913	1.1824032	-0.1314	0.896	-0.172091967	count	1
SNHG22	-0.1553913	1.2959618	-0.1199	0.905	-0.172091967	count	1
HELZ2	-0.1388544	0.6194911	-0.2241	0.823	-0.171584808	count	1
SETDB2	-0.1388544	0.6625853	-0.2096	0.834	-0.171584808	count	1
FAM111A	-0.1226756	0.3428085	-0.3579	0.721	-0.171011295	count	1
MCTS1	-0.1247831	0.4257838	-0.2931	0.77	-0.170856151	count	1
HNRNPH3	-0.1243314	0.2816067	-0.4415	0.659	-0.170818476	count	1
EIF3A	-0.1234554	0.3273265	-0.3772	0.706	-0.170779829	count	1
CDC123	-0.1215802	0.2770711	-0.4388	0.661	-0.170742543	count	1
POLR2D	-0.1242347	0.5066167	-0.2452	0.806	-0.170685814	count	1
ZBTB7A	-0.1211909	0.2481461	-0.4884	0.626	-0.170503367	count	1
PPP6R1	-0.1263844	0.4184474	-0.302	0.763	-0.170155944	count	1
ACTN4	-0.120008	0.2169675	-0.5531	0.5806	-0.170096054	count	1
ELOF1	-0.1227831	0.3927533	-0.3126	0.755	-0.16919842	count	1
NSA2	-0.1189724	0.1917161	-0.6206	0.5353	-0.168972296	count	1

ATP5F1A	-0.1187114	0.2297477	-0.5167	0.6057	-0.168805604	count	1
AZIN1	-0.1240149	0.4230213	-0.2932	0.77	-0.168774812	count	1
RIC8A	-0.1287023	0.4952867	-0.2599	0.795	-0.168759494	count	1
ABHD3	-0.1363174	0.6229544	-0.2188	0.827	-0.168464984	count	1
SELENOT	-0.1195871	0.2762248	-0.4329	0.665	-0.168176713	count	1
NOP14-AS1	-0.1298913	0.640848	-0.2027	0.84	-0.167959201	count	1
WBP11	-0.1202809	0.2914317	-0.4127	0.68	-0.167676385	count	1
WDHD1	-0.1601378	0.9882328	-0.162	0.871	-0.167561567	count	1
TTF2	-0.1601378	0.9941096	-0.1611	0.872	-0.167561567	count	1
NUP50-DT	-0.1601378	1.072132	-0.1494	0.881	-0.167561567	count	1
ZNF438	-0.1601378	1.0775519	-0.1486	0.882	-0.167561567	count	1
ZNF460	-0.1601378	1.1520952	-0.139	0.89	-0.167561567	count	1
RPS2	-0.1161663	0.0625024	-1.8586	0.064	-0.167465605	count	1
ZNF581	-0.1335344	0.5424546	-0.2462	0.806	-0.167441044	count	1
ZDHHC3	-0.1208655	0.4086669	-0.2958	0.768	-0.167201519	count	1
YY1	-0.1173891	0.1866043	-0.6291	0.53	-0.167157574	count	1
FAM122C	-0.1448329	0.8660083	-0.1672	0.867	-0.166947051	count	1
ALG2	-0.128074	0.4916943	-0.2605	0.795	-0.166852348	count	1
COL18A1	-0.1505235	0.6677075	-0.2254	0.822	-0.166748001	count	1
RANBP9	-0.1349056	0.5511817	-0.2448	0.807	-0.166728585	count	1
SNAP29	-0.1242286	0.4497453	-0.2762	0.783	-0.166703055	count	1
B9D2	-0.1237792	0.570318	-0.217	0.828	-0.166655607	count	1
LUC7L3	-0.1183246	0.2481208	-0.4769	0.634	-0.16660912	count	1
ZDBF2	-0.1739947	0.7923429	-0.2196	0.826	-0.16660106	count	1
RAB40B	-0.1739947	0.8133547	-0.2139	0.831	-0.16660106	count	1
ZNF675	-0.1347371	0.4685586	-0.2876	0.774	-0.166521333	count	1
STX12	-0.1588704	0.5469631	-0.2905	0.772	-0.166249867	count	1
PRELID1	-0.1158003	0.1514902	-0.7644	0.445	-0.165958723	count	1
PDE4D	-0.1216646	0.4150334	-0.2931	0.77	-0.165943189	count	1
NUS1	-0.1732207	0.8812065	-0.1966	0.844	-0.165870797	count	1
ZNF35	-0.1732207	1.0292193	-0.1683	0.866	-0.165870797	count	1
TAF1D	-0.1166247	0.1963481	-0.594	0.553	-0.165612047	count	1
HNRNPUL2	-0.2019977	0.8050425	-0.2509	0.802	-0.165086558	count	1
PSMB3	-0.1156814	0.1877358	-0.6162	0.5382	-0.165046504	count	1
MRPS22	-0.1216504	0.4267489	-0.2851	0.776	-0.164750865	count	1
YES1	-0.1204738	0.3890107	-0.3097	0.757	-0.16465442	count	1
PRPF4B	-0.1156201	0.190113	-0.6082	0.5435	-0.164520882	count	1
PSME1	-0.114144	0.1142371	-0.9992	0.318	-0.164177941	count	1
EIF1B	-0.1149462	0.1976551	-0.5815	0.5613	-0.16368025	count	1
CLPP	-0.1165245	0.2655403	-0.4388	0.661	-0.163567521	count	1
CPT1A	-0.1274519	0.5712276	-0.2231	0.824	-0.163403897	count	1
RPL3	-0.1132798	0.0663359	-1.7077	0.0887	-0.163212564	count	1
DUSP22	-0.1342869	0.6506598	-0.2064	0.837	-0.163047042	count	1
ILK	-0.1155719	0.3099966	-0.3728	0.71	-0.162735764	count	1
NRBF2	-0.1167192	0.2942312	-0.3967	0.692	-0.16183176	count	1

VDAC3	-0.115762	0.3353475	-0.3452	0.73	-0.161506537	count	1
SLC25A39	-0.1156132	0.3236836	-0.3572	0.721	-0.16129913	count	1
CAMK1D	-0.1237792	0.605632	-0.2044	0.838	-0.161273671	count	1
LETM1	-0.1398496	0.570716	-0.245	0.807	-0.161242836	count	1
PTPRE	-0.1150874	0.3099779	-0.3713	0.711	-0.161203484	count	1
PGM3	-0.145425	1.0797421	-0.1347	0.893	-0.16114726	count	1
STAM	-0.1199838	0.4375443	-0.2742	0.784	-0.161018647	count	1
RPL28	-0.1116037	0.0486577	-2.2937	0.0225	-0.160943	count	1
PRRC2C	-0.1125921	0.1578472	-0.7133	0.4762	-0.160861085	count	1
TBCA	-0.1126599	0.1819338	-0.6192	0.536	-0.160653687	count	1
HINT2	-0.1146775	0.344392	-0.333	0.739	-0.160534461	count	1
GPATCH4	-0.1215311	0.5826387	-0.2086	0.835	-0.160293212	count	1
PDIA3	-0.1116743	0.155546	-0.7179	0.473	-0.160087344	count	1
GTF2A2	-0.1132975	0.2452638	-0.4619	0.644	-0.160075799	count	1
KIAA1109	-0.1188264	0.4837692	-0.2456	0.806	-0.160000152	count	1
BOD1L1	-0.1122391	0.2089385	-0.5372	0.592	-0.159930489	count	1
ATP6V0D1	-0.1159821	0.3542475	-0.3274	0.744	-0.159838396	count	1
VASP	-0.1127551	0.2568087	-0.4391	0.6609	-0.15952675	count	1
SHPRH	-0.1156107	0.3766499	-0.3069	0.759	-0.159327206	count	1
RPL10	-0.1103463	0.0413085	-2.6713	0.00794	-0.159160499	count	1
MED29	-0.118523	0.499314	-0.2374	0.813	-0.159062215	count	1
SLC25A6	-0.1106867	0.1251998	-0.8841	0.377	-0.158978693	count	1
CAPZA1	-0.112482	0.2473347	-0.4548	0.6496	-0.158621722	count	1
RTF2	-0.1125192	0.273921	-0.4108	0.6815	-0.15861832	count	1
WDR33	-0.113137	0.255646	-0.4426	0.658	-0.158281887	count	1
AHCTF1	-0.1933455	0.6052851	-0.3194	0.75	-0.158165359	count	1
DPY19L1	-0.1931346	0.7376951	-0.2618	0.794	-0.15799647	count	1
PTPN6	-0.1109185	0.2457006	-0.4514	0.652	-0.157853053	count	1
RSL1D1	-0.1102414	0.2046763	-0.5386	0.5905	-0.156889792	count	1
ARHGDIA	-0.1102258	0.2288473	-0.4817	0.6304	-0.156605104	count	1
CHMP5	-0.1104294	0.2754126	-0.401	0.689	-0.155728905	count	1
NDUFA5	-0.1101672	0.2680072	-0.4111	0.6813	-0.155609606	count	1
STIM2	-0.1118336	0.3150603	-0.355	0.723	-0.155518793	count	1
CEBPG	-0.1143434	0.4699529	-0.2433	0.808	-0.155267661	count	1
RITA1	-0.2752187	0.6938948	-0.3966	0.692	-0.155199727	count	1
PQLC3	-0.1140158	0.4333543	-0.2631	0.793	-0.155188311	count	1
FAM177A1	-0.1086777	0.192465	-0.5647	0.573	-0.155176043	count	1
ABHD15	-0.1129918	0.5032806	-0.2245	0.823	-0.155005372	count	1
UFC1	-0.1086074	0.1986091	-0.5468	0.585	-0.154943881	count	1
NACA	-0.1075261	0.0741034	-1.451	0.148	-0.154929588	count	1
DDX19A	-0.1611837	0.7795713	-0.2068	0.836	-0.154500502	count	1
MLLT3	-0.1125094	0.3995808	-0.2816	0.778	-0.154344492	count	1
DIS3L2	-0.1391045	0.78484	-0.1772	0.859	-0.154199188	count	1
MSANTD4	-0.1391045	0.7828418	-0.1777	0.859	-0.154199188	count	1
GSTO1	-0.1091796	0.2389428	-0.4569	0.648	-0.154120517	count	1

TNFSF9	-0.1101487	0.4274485	-0.2577	0.7968	-0.154104562	count	1
ARPC5	-0.1077195	0.1789046	-0.6021	0.548	-0.154058388	count	1
C2CD2L	-0.2723212	0.8494413	-0.3206	0.749	-0.153636174	count	1
AC097534.2	-0.2723212	0.923874	-0.2948	0.768	-0.153636174	count	1
LRRC37A2	-0.2723212	0.9218811	-0.2954	0.768	-0.153636174	count	1
LRFN1	-0.2723212	0.9744303	-0.2795	0.7801	-0.153636174	count	1
TSPYL4	-0.1876649	0.8547439	-0.2196	0.8264	-0.153613391	count	1
PIGL	-0.1463162	1.0193608	-0.1435	0.886	-0.153243473	count	1
ZSWIM9	-0.1463162	1.0155286	-0.1441	0.886	-0.153243473	count	1
SERPINB6	-0.1110175	0.2682602	-0.4138	0.679	-0.15321234	count	1
CCDC57	-0.1118365	0.4646883	-0.2407	0.81	-0.15315402	count	1
POP5	-0.1115377	0.2938202	-0.3796	0.704	-0.153013254	count	1
BIN2	-0.1069376	0.1795852	-0.5955	0.5519	-0.152587505	count	1
DECR1	-0.1098881	0.2865698	-0.3835	0.702	-0.152526351	count	1
C19orf53	-0.1066107	0.1593491	-0.669	0.504	-0.152433145	count	1
HERPUD2	-0.1081629	0.3103036	-0.3486	0.728	-0.151984897	count	1
ADAM17	-0.1364396	0.5132925	-0.2658	0.791	-0.151268057	count	1
WASHC2A	-0.1243652	0.8857263	-0.1404	0.888	-0.151058608	count	1
MNDA	-0.1243652	1.1850404	-0.1049	0.9165	-0.151058608	count	1
GNAI2	-0.1060859	0.1671257	-0.6348	0.526	-0.150996545	count	1
IFITM2	-0.1047555	0.1028138	-1.0189	0.309	-0.150889978	count	1
ZBTB20	-0.1174004	0.4393658	-0.2672	0.789	-0.150558208	count	1
UBL4A	-0.1217636	0.5601382	-0.2174	0.828	-0.150556022	count	1
NCK1-DT	-0.1163415	0.6061225	-0.1919	0.848	-0.150490014	count	1
SLC3A2	-0.1054871	0.206641	-0.5105	0.61	-0.150126067	count	1
PPFIA1	-0.1193895	0.6022295	-0.1982	0.843	-0.14977239	count	1
TENT2	-0.1155836	0.534561	-0.2162	0.829	-0.149512504	count	1
ENDOD1	-0.1155054	0.584816	-0.1975	0.844	-0.149411642	count	1
CCSER2	-0.1056782	0.2184	-0.4839	0.6288	-0.149400783	count	1
GABPA	-0.1207264	0.5968626	-0.2023	0.84	-0.149278959	count	1
APP	-0.1818506	0.7947369	-0.2288	0.819	-0.1489479	count	1
HHLA3	-0.1818506	1.078733	-0.1686	0.866	-0.1489479	count	1
ICE2	-0.1150074	0.5572022	-0.2064	0.837	-0.148769316	count	1
SSSCA1	-0.1059063	0.3068864	-0.3451	0.73	-0.148173799	count	1
FAM122A	-0.2620462	0.5842385	-0.4485	0.6541	-0.148078619	count	1
URI1	-0.105577	0.2665043	-0.3962	0.692	-0.147976416	count	1
BAG4	-0.1098241	0.5538243	-0.1983	0.843	-0.14790011	count	1
RBM18	-0.1142998	0.4902303	-0.2332	0.816	-0.147856617	count	1
ATP6AP2	-0.1045411	0.2231423	-0.4685	0.64	-0.14757627	count	1
PDSS1	-0.2607173	0.9454565	-0.2758	0.783	-0.147358375	count	1
CHST14	-0.2607173	0.9454565	-0.2758	0.783	-0.147358375	count	1
OGDH	-0.2607173	1.0255572	-0.2542	0.799	-0.147358375	count	1
FBLIM1	-0.2607173	1.1318227	-0.2304	0.818	-0.147358375	count	1
TTC4	-0.2607173	1.1318227	-0.2304	0.818	-0.147358375	count	1
CCDC80	-0.2607173	1.1318227	-0.2304	0.818	-0.147358375	count	1

ADH1B	-0.2607173	1.1318227	-0.2304	0.818	-0.147358375	count	1
SSPN	-0.2607173	1.1318227	-0.2304	0.818	-0.147358375	count	1
TPTEP2-CSNK1E	-0.2607173	1.1318227	-0.2304	0.818	-0.147358375	count	1
DDAH2	-0.1048024	0.3061673	-0.3423	0.732	-0.147196418	count	1
ZNF773	-0.1237792	0.8879281	-0.1394	0.889	-0.147030931	count	1
KIFC3	-0.1237792	0.9203272	-0.1345	0.893	-0.147030931	count	1
RDH5	-0.1237792	1.125375	-0.11	0.912	-0.147030931	count	1
THRAP3	-0.103747	0.2004381	-0.5176	0.6051	-0.147027395	count	1
PNISR	-0.1032249	0.2099011	-0.4918	0.6232	-0.146831998	count	1
TBC1D9B	-0.1530405	0.753924	-0.203	0.839	-0.146794183	count	1
NAP1L1	-0.1033082	0.1969471	-0.5245	0.6003	-0.14675735	count	1
PSMD10	-0.1789818	0.7941753	-0.2254	0.822	-0.146643554	count	1
NMI	-0.1133245	0.4224754	-0.2682	0.789	-0.146598567	count	1
SLC35C2	-0.1141057	0.5183049	-0.2202	0.826	-0.146345981	count	1
TRIM24	-0.1141057	0.6386291	-0.1787	0.858	-0.146345981	count	1
DDIT3	-0.1031634	0.2425759	-0.4253	0.6709	-0.146035115	count	1
CHML	-0.1315365	0.7058521	-0.1864	0.852	-0.145872612	count	1
EPS8	-0.1315365	0.7739824	-0.1699	0.865	-0.145872612	count	1
SMCHD1	-0.1035221	0.2243976	-0.4613	0.645	-0.145835024	count	1
MTF2	-0.1085599	0.4186608	-0.2593	0.796	-0.145715969	count	1
MRPL55	-0.1084716	0.3946591	-0.2748	0.784	-0.145597663	count	1
CCT5	-0.1039009	0.3019357	-0.3441	0.731	-0.14554599	count	1
STAT6	-0.1102382	0.5453245	-0.2022	0.84	-0.145432713	count	1
NPLOC4	-0.1101487	0.8389688	-0.1313	0.896	-0.14531491	count	1
NEAT1	-0.1016817	0.142187	-0.7151	0.475	-0.14523934	count	1
TMEM175	-0.1099394	0.7304416	-0.1505	0.88	-0.145039417	count	1
CLNS1A	-0.1025321	0.29204	-0.3511	0.7257	-0.144914873	count	1
MRPL52	-0.1040485	0.2981966	-0.3489	0.727	-0.144702466	count	1
GPN1	-0.1096284	0.4357333	-0.2516	0.802	-0.144630055	count	1
AC016831.5	-0.2554884	0.4852657	-0.5265	0.599	-0.144521128	count	1
NELFB	-0.1044484	0.4901822	-0.2131	0.831	-0.144340424	count	1
DCAF15	-0.1056801	0.4391405	-0.2407	0.81	-0.143521404	count	1
MED26	-0.1180109	0.6716844	-0.1757	0.861	-0.143375459	count	1
SELENOH	-0.1003361	0.2196811	-0.4567	0.6482	-0.143071817	count	1
CNOT6L	-0.1006638	0.212424	-0.4739	0.6359	-0.142747871	count	1
CFL1	-0.0991163	0.088009	-1.1262	0.261	-0.14269743	count	1
ARF1	-0.0987392	0.135121	-0.7307	0.465	-0.1414816	count	1
ZNF746	-0.2494365	0.7475418	-0.3337	0.7388	-0.141230856	count	1
COA6	-0.1013874	0.3680465	-0.2755	0.783	-0.141004872	count	1
PSMD4	-0.0991401	0.2410896	-0.4112	0.6812	-0.140792528	count	1
IGFLR1	-0.1107122	0.5894891	-0.1878	0.851	-0.140589044	count	1
MFSD14B	-0.1106923	0.6880792	-0.1609	0.872	-0.140563854	count	1
ABR	-0.1106923	0.72466	-0.1528	0.879	-0.140563854	count	1
MYO6	-0.1180364	0.8280395	-0.1425	0.887	-0.140243892	count	1
SNHG8	-0.0980208	0.2064553	-0.4748	0.635	-0.139832807	count	1

AKT1	-0.0997949	0.3413029	-0.2924	0.77	-0.139349235	count	1
TSSC4	-0.1005227	0.3528252	-0.2849	0.776	-0.138920906	count	1
MAP3K13	-0.1001813	0.4861829	-0.2061	0.837	-0.138773966	count	1
MED10	-0.0988144	0.2754242	-0.3588	0.72	-0.138343798	count	1
RPL6	-0.0958011	0.0600191	-1.5962	0.111	-0.1380915	count	1
AKIRIN2	-0.0996834	0.3352827	-0.2973	0.766	-0.137927967	count	1
MTRNR2L12	-0.096051	0.1934281	-0.4966	0.6198	-0.137225297	count	1
EIF4E2	-0.0969825	0.3256951	-0.2978	0.766	-0.136464433	count	1
LSM4	-0.096664	0.2569803	-0.3762	0.707	-0.136373318	count	1
ABCD3	-0.1181308	0.791207	-0.1493	0.881	-0.136346604	count	1
RETREG2	-0.1025917	0.4896026	-0.2095	0.834	-0.136049852	count	1
SIGIRR	-0.0957049	0.2101167	-0.4555	0.6491	-0.136023151	count	1
HNRNPA3	-0.0950834	0.1671097	-0.569	0.5698	-0.135788988	count	1
INTS10	-0.1000932	0.4988052	-0.2007	0.841	-0.135224583	count	1
UBE2I	-0.0950521	0.1780764	-0.5338	0.5939	-0.135210341	count	1
FBXW5	-0.0971913	0.3120326	-0.3115	0.756	-0.135049481	count	1
ZSCAN26	-0.1075787	0.665131	-0.1617	0.872	-0.135006176	count	1
AC005332.1	-0.1075787	0.6673784	-0.1612	0.872	-0.135006176	count	1
MICU1	-0.1134324	0.5925509	-0.1914	0.848	-0.134800138	count	1
SRRM2	-0.0939922	0.1489705	-0.6309	0.5285	-0.134588385	count	1
MDC1	-0.2372225	0.9236582	-0.2568	0.797	-0.134569411	count	1
SLC35A1	-0.2372225	0.9236582	-0.2568	0.797	-0.134569411	count	1
AC006299.1	-0.2372225	0.9236582	-0.2568	0.797	-0.134569411	count	1
LINC00324	-0.1037429	0.5376747	-0.1929	0.847	-0.134235661	count	1
AC124016.1	-0.1162429	0.8754813	-0.1328	0.894	-0.13417982	count	1
ANP32B	-0.0940443	0.1880235	-0.5002	0.617	-0.134172779	count	1
SKP1	-0.0933416	0.1186524	-0.7867	0.432	-0.134088933	count	1
BST2	-0.0935157	0.1591767	-0.5875	0.5573	-0.133998082	count	1
TMEM161A	-0.1066686	0.6739866	-0.1583	0.874	-0.133867854	count	1
KLRK1	-0.1275688	0.7218429	-0.1767	0.86	-0.133776237	count	1
FNBP1	-0.0951253	0.2644434	-0.3597	0.719	-0.133676039	count	1
S100A6	-0.0928405	0.1325335	-0.7005	0.484	-0.133347077	count	1
LNPK	-0.1076327	0.4586736	-0.2347	0.815	-0.133148697	count	1
RPLP1	-0.0921124	0.0511896	-1.7994	0.0729	-0.13284678	count	1
ATP10D	-0.101212	0.4190012	-0.2416	0.809	-0.132793733	count	1
KRIT1	-0.0992936	0.3964054	-0.2505	0.802	-0.132277845	count	1
CEP57	-0.0957799	0.3309162	-0.2894	0.772	-0.13183647	count	1
CHP1	-0.1064362	0.5536533	-0.1922	0.848	-0.131673938	count	1
EAPP	-0.0929411	0.2815712	-0.3301	0.7416	-0.13166224	count	1
SCPEP1	-0.1024969	0.5042914	-0.2032	0.839	-0.131497925	count	1
TAOK3	-0.0951206	0.3050586	-0.3118	0.755	-0.131462432	count	1
NT5DC1	-0.0986305	0.4254029	-0.2319	0.817	-0.131396066	count	1
TMEM134	-0.0933144	0.2842428	-0.3283	0.743	-0.131305874	count	1
ATG4B	-0.1012394	0.5771951	-0.1754	0.861	-0.131004418	count	1
TMBIM4	-0.0919611	0.1947409	-0.4722	0.637	-0.130959501	count	1

BTN2A1	-0.1019571	0.4920662	-0.2072	0.836	-0.130807262	count	1
TSG101	-0.0929886	0.2944221	-0.3158	0.752	-0.130734622	count	1
MYL9	-0.099007	0.5888827	-0.1681	0.867	-0.130646006	count	1
TOPORS	-0.0946346	0.386823	-0.2446	0.807	-0.130625049	count	1
ZMYND11	-0.0973599	0.4826571	-0.2017	0.84	-0.130231144	count	1
ANKRD11	-0.0927249	0.2223578	-0.417	0.677	-0.129974353	count	1
NFKBIE	-0.1238185	0.6390056	-0.1938	0.846	-0.129875638	count	1
SNRPA	-0.0948185	0.4067339	-0.2331	0.816	-0.12963294	count	1
DPY30	-0.0942749	0.3462048	-0.2723	0.786	-0.129568813	count	1
CWF19L2	-0.0923383	0.2990978	-0.3087	0.758	-0.129282932	count	1
MAGT1	-0.096641	0.4672061	-0.2068	0.836	-0.12927114	count	1
HNRNPL	-0.0929106	0.3145547	-0.2954	0.768	-0.128849772	count	1
R3HDM2	-0.0916779	0.2686524	-0.3413	0.733	-0.128773585	count	1
EXOC8	-0.1115317	0.7798348	-0.143	0.886	-0.128770835	count	1
IFT52	-0.1113225	0.5632358	-0.1976	0.843	-0.12853059	count	1
STAM2	-0.1037663	0.7723263	-0.1344	0.893	-0.128382661	count	1
PIGS	-0.0991748	0.6290798	-0.1577	0.875	-0.12833934	count	1
CHST12	-0.0898144	0.1902694	-0.472	0.637	-0.128274751	count	1
RELB	-0.0922511	0.3521472	-0.262	0.794	-0.128190529	count	1
PRKDC	-0.0926358	0.3122707	-0.2967	0.767	-0.128031474	count	1
PRKAR2A	-0.0940174	0.4601837	-0.2043	0.838	-0.128003022	count	1
SOS2	-0.0930746	0.4136795	-0.225	0.822	-0.12748976	count	1
NCOA2	-0.0970419	0.6450209	-0.1504	0.881	-0.1273339	count	1
EP400	-0.1549441	0.8336005	-0.1859	0.853	-0.127274589	count	1
EIF3M	-0.0893255	0.2169847	-0.4117	0.6809	-0.126771201	count	1
SYNCRIP	-0.0906811	0.2757602	-0.3288	0.742	-0.126539792	count	1
RPL10A	-0.0875789	0.0728777	-1.2017	0.23	-0.126140483	count	1
CCNQ	-0.0914531	0.4239991	-0.2157	0.829	-0.125694606	count	1
AP4B1	-0.0939343	0.5387817	-0.1743	0.862	-0.125656441	count	1
PSTPIP1	-0.0898979	0.2791405	-0.3221	0.748	-0.124802293	count	1
RDH14	-0.0910632	0.388285	-0.2345	0.815	-0.124737641	count	1
CNOT7	-0.0906389	0.3071657	-0.2951	0.768	-0.124576696	count	1
DYNC1I2	-0.0901179	0.399151	-0.2258	0.822	-0.124554627	count	1
SLC2A3	-0.0874884	0.1909741	-0.4581	0.6472	-0.124437485	count	1
PDE4DIP	-0.0938134	0.5077659	-0.1848	0.854	-0.12442975	count	1
PRKX	-0.0989812	0.4449073	-0.2225	0.824	-0.124249961	count	1
PPP6C	-0.0914338	0.4228589	-0.2162	0.829	-0.12419867	count	1
MRPS23	-0.0914338	0.4486741	-0.2038	0.839	-0.12419867	count	1
PSMD14	-0.0895381	0.4459689	-0.2008	0.841	-0.124176593	count	1
ABLIM1	-0.0908208	0.4001312	-0.227	0.821	-0.124173586	count	1
POLR2C	-0.0896567	0.3414447	-0.2626	0.793	-0.12406655	count	1
RNF5	-0.0889714	0.3466614	-0.2567	0.798	-0.123962382	count	1
PSMC3	-0.0888146	0.3154802	-0.2815	0.778	-0.123937006	count	1
TM9SF3	-0.0896589	0.323846	-0.2769	0.782	-0.123419453	count	1
PON2	-0.0996648	0.7707161	-0.1293	0.897	-0.123325359	count	1

MLST8	-0.0996648	0.794599	-0.1254	0.9	-0.123325359	count	1
ZNF737	-0.0996648	0.9731313	-0.1024	0.918	-0.123325359	count	1
AC020915.3	-0.0996648	1.0158003	-0.0981	0.922	-0.123325359	count	1
COMMD8	-0.0900356	0.3901979	-0.2307	0.818	-0.12310123	count	1
MAP4K5	-0.0905625	0.4315392	-0.2099	0.834	-0.12301663	count	1
MT-ND4	-0.0851003	0.0735439	-1.1571	0.248	-0.122639977	count	1
RBM38	-0.0860422	0.2088241	-0.412	0.6806	-0.12229395	count	1
FGFR1OP2	-0.0857828	0.2073981	-0.4136	0.679	-0.122106225	count	1
RPS19	-0.084583	0.0747979	-1.1308	0.259	-0.121926459	count	1
PTMA	-0.0845512	0.0507981	-1.6645	0.097	-0.121911585	count	1
CYP2R1	-0.1094297	0.7504121	-0.1458	0.884	-0.121506186	count	1
PBX2	-0.0998878	0.6570867	-0.152	0.879	-0.121440405	count	1
NDRG1	-0.0998878	0.6962532	-0.1435	0.886	-0.121440405	count	1
TMEM273	-0.0945567	0.6360855	-0.1487	0.882	-0.121336497	count	1
SMARCA5	-0.0860079	0.2695691	-0.3191	0.7499	-0.121219207	count	1
RNF145	-0.0905719	0.358772	-0.2524	0.801	-0.121165575	count	1
RBFA	-0.0951441	0.5428087	-0.1753	0.861	-0.12087361	count	1
KLRC4	-0.0867297	0.4486047	-0.1933	0.847	-0.120740448	count	1
CTBP1-DT	-0.1255337	0.8739573	-0.1436	0.886	-0.120680345	count	1
VPS26A	-0.0856179	0.2628717	-0.3257	0.745	-0.120430199	count	1
GMPPB	-0.1083218	0.6646767	-0.163	0.871	-0.120283354	count	1
7-Sep	-0.0833589	0.0899313	-0.9269	0.355	-0.119977281	count	1
FBXL15	-0.0856238	0.3484209	-0.2457	0.806	-0.119958309	count	1
GMDS-DT	-0.1080159	0.6695887	-0.1613	0.872	-0.119945695	count	1
GLI4	-0.0955088	0.5512684	-0.1733	0.863	-0.119903955	count	1
ABHD17A	-0.0838509	0.1631173	-0.5141	0.6076	-0.119828519	count	1
PPIB	-0.0834132	0.1293143	-0.645	0.519	-0.11982016	count	1
LCK	-0.0839106	0.2158014	-0.3888	0.6977	-0.119441976	count	1
ITM2B	-0.0829426	0.0915206	-0.9063	0.365	-0.119330965	count	1
RPS8	-0.0827533	0.0536013	-1.5439	0.124	-0.119313865	count	1
OCIAD2	-0.0836846	0.2143115	-0.3905	0.6964	-0.119012437	count	1
RPS16	-0.0820918	0.0788863	-1.0406	0.299	-0.118166777	count	1
POLD4	-0.0971184	0.7047426	-0.1378	0.89	-0.118085681	count	1
NCOA3	-0.0966663	0.4152151	-0.2328	0.816	-0.117537956	count	1
RHOC	-0.0816526	0.1746956	-0.4674	0.6405	-0.116955596	count	1
FAM162A	-0.0838911	0.3684373	-0.2277	0.82	-0.116351017	count	1
SNX4	-0.0877021	0.5396521	-0.1625	0.871	-0.116337578	count	1
NCOR2	-0.0877021	0.558713	-0.157	0.875	-0.116337578	count	1
MYL6	-0.0806559	0.0937174	-0.8606	0.39	-0.116183439	count	1
DEAF1	-0.0914247	0.6304864	-0.145	0.885	-0.116160604	count	1
ARRDC1-AS1	-0.0884236	0.6625571	-0.1335	0.894	-0.116046815	count	1
ARMC1	-0.0896263	0.5431594	-0.165	0.869	-0.116009965	count	1
UBLCP1	-0.0859876	0.4400145	-0.1954	0.845	-0.115843288	count	1
ERVK3-1	-0.1204461	0.5518472	-0.2183	0.827	-0.115836645	count	1
RPS14	-0.0802412	0.049661	-1.6158	0.107	-0.11571127	count	1

CCDC107	-0.081518	0.2009378	-0.4057	0.6852	-0.115673108	count	1
LRRFIP1	-0.0806377	0.1590808	-0.5069	0.6126	-0.115510053	count	1
CISD3	-0.0825756	0.3393876	-0.2433	0.808	-0.115236303	count	1
RPS13	-0.0795613	0.0615071	-1.2935	0.197	-0.114663688	count	1
LRRC75A	-0.087366	0.4755498	-0.1837	0.854	-0.114661408	count	1
RPL41	-0.0794943	0.041251	-1.9271	0.0548	-0.114642154	count	1
APAF1	-0.0921823	0.5360651	-0.172	0.864	-0.114095302	count	1
RPL34	-0.0787677	0.0576017	-1.3675	0.172	-0.113548779	count	1
SYS1	-0.0813142	0.3539156	-0.2298	0.818	-0.113477089	count	1
DRAM2	-0.0839447	0.4589182	-0.1829	0.855	-0.113435776	count	1
TRIP4	-0.0953371	0.7236524	-0.1317	0.895	-0.113382346	count	1
C8orf33	-0.0875283	0.4699458	-0.1863	0.852	-0.113300133	count	1
RPL9	-0.0785733	0.0712641	-1.1026	0.271	-0.113172924	count	1
PARK7	-0.0783604	0.1707225	-0.459	0.647	-0.112330762	count	1
TRABD	-0.0796084	0.2929755	-0.2717	0.786	-0.112028633	count	1
PRMT1	-0.0795064	0.2402721	-0.3309	0.741	-0.112018337	count	1
CCDC71L	-0.0890827	0.6071744	-0.1467	0.883	-0.111858534	count	1
CAPNS1	-0.078898	0.2885928	-0.2734	0.7847	-0.111554967	count	1
PGLS	-0.0778439	0.1863427	-0.4177	0.6764	-0.11123883	count	1
ACP1	-0.0786449	0.2752109	-0.2858	0.7752	-0.110805074	count	1
PREX1	-0.0783036	0.289845	-0.2702	0.7872	-0.110773766	count	1
POLR2K	-0.0776858	0.2001562	-0.3881	0.6982	-0.110622619	count	1
FAM192A	-0.0848077	0.4618143	-0.1836	0.854	-0.110596995	count	1
VPS37B	-0.0784412	0.2689724	-0.2916	0.7708	-0.110386838	count	1
NLRP2	-0.0906726	1.0336681	-0.0877	0.93	-0.11027468	count	1
ATP5MC3	-0.0767692	0.1736319	-0.4421	0.6587	-0.109886419	count	1
ADGRG1	-0.0886858	0.6312715	-0.1405	0.888	-0.109780495	count	1
STX4	-0.0789161	0.3820044	-0.2066	0.836	-0.109773863	count	1
COX7A2L	-0.0770046	0.2018716	-0.3815	0.7031	-0.109484071	count	1
ARRB1	-0.084244	0.5789239	-0.1455	0.884	-0.10905746	count	1
SSR4	-0.0757477	0.1326582	-0.571	0.568	-0.108759876	count	1
ARIH2OS	-0.0831363	0.7265554	-0.1144	0.909	-0.107626368	count	1
MAT2A	-0.0798733	0.3986631	-0.2004	0.841	-0.107616274	count	1
TXNL4A	-0.0762168	0.232961	-0.3272	0.744	-0.107257909	count	1
RPL24	-0.0743409	0.0633671	-1.1732	0.242	-0.107116613	count	1
MRTO4	-0.079428	0.4644732	-0.171	0.864	-0.107017042	count	1
SH3KBP1	-0.0753544	0.2652917	-0.284	0.7766	-0.106968144	count	1
CASP4	-0.0748713	0.1959949	-0.382	0.703	-0.106841531	count	1
TIMM17B	-0.0759504	0.3023645	-0.2512	0.8018	-0.106742787	count	1
TRAPPC2L	-0.0791854	0.4472593	-0.177	0.86	-0.106690576	count	1
DNAJC2	-0.0764485	0.3518847	-0.2173	0.828	-0.106609843	count	1
NXPE3	-0.1858048	0.6739744	-0.2757	0.783	-0.106223968	count	1
COX15	-0.1009532	0.8353687	-0.1208	0.904	-0.106049537	count	1
ANXA5	-0.0758663	0.2967293	-0.2557	0.798	-0.105955616	count	1
TUBA4A	-0.0738003	0.1470737	-0.5018	0.616	-0.105903877	count	1

TNFSF10	-0.0742733	0.2594656	-0.2863	0.7749	-0.105886187	count	1
TOMM22	-0.0753375	0.257042	-0.2931	0.77	-0.105671847	count	1
HCG18	-0.0795991	0.3800085	-0.2095	0.834	-0.105605059	count	1
UPF3A	-0.0750326	0.2984625	-0.2514	0.802	-0.105547465	count	1
HECTD1	-0.0812985	0.4181719	-0.1944	0.846	-0.105251847	count	1
ING2	-0.0757119	0.3332796	-0.2272	0.82	-0.105223016	count	1
ICK	-0.0819019	0.6299662	-0.13	0.897	-0.105132302	count	1
RASSF5	-0.0747464	0.272964	-0.2738	0.784	-0.104726874	count	1
LINC02001	-0.0790641	0.5890326	-0.1342	0.893	-0.104371978	count	1
CNDP2	-0.076787	0.4427773	-0.1734	0.862	-0.104324255	count	1
H2AFZ	-0.0726308	0.1378036	-0.5271	0.599	-0.104187209	count	1
RNF187	-0.0749897	0.3751054	-0.1999	0.842	-0.104013787	count	1
PITHD1	-0.074711	0.2918008	-0.256	0.798	-0.103732776	count	1
G0S2	-0.0985631	0.9493361	-0.1038	0.917	-0.10355466	count	1
GTF2H2	-0.1255337	0.7682382	-0.1634	0.87	-0.103431758	count	1
BCL11B	-0.1255337	0.7682382	-0.1634	0.87	-0.103431758	count	1
CACFD1	-0.1255337	0.9429318	-0.1331	0.894	-0.103431758	count	1
FBXO31	-0.1255337	0.9818509	-0.1279	0.898	-0.103431758	count	1
CLEC11A	-0.1255337	0.9818509	-0.1279	0.898	-0.103431758	count	1
FRAT1	-0.0792391	0.7148089	-0.1109	0.912	-0.10334798	count	1
RPS12	-0.0714197	0.0532861	-1.3403	0.181	-0.102994596	count	1
ZC3H18	-0.0775661	0.5405279	-0.1435	0.886	-0.102911784	count	1
GSPT1	-0.0730952	0.3003803	-0.2433	0.8079	-0.102866801	count	1
NORAD	-0.0737754	0.3036422	-0.243	0.808	-0.10233062	count	1
RBCK1	-0.0722051	0.2605162	-0.2772	0.7818	-0.102249652	count	1
IWS1	-0.0743105	0.3870649	-0.192	0.848	-0.102153	count	1
RPL36AL	-0.0709256	0.0913063	-0.7768	0.438	-0.10201955	count	1
MPC2	-0.0719787	0.2188249	-0.3289	0.7424	-0.101997931	count	1
RPL18	-0.0706435	0.052687	-1.3408	0.181	-0.101845031	count	1
SRA1	-0.0724507	0.3252299	-0.2228	0.824	-0.101569337	count	1
EIF4EBP2	-0.0742736	0.4167752	-0.1782	0.859	-0.101367102	count	1
EIF2S2	-0.0718183	0.2468456	-0.2909	0.771	-0.101299907	count	1
RPL18A	-0.0702163	0.0572101	-1.2273	0.221	-0.101220265	count	1
TXNL4B	-0.1224604	0.8670692	-0.1412	0.888	-0.100931267	count	1
AHCYL2	-0.1224604	0.9205827	-0.133	0.894	-0.100931267	count	1
ZNF280C	-0.1224604	0.9205827	-0.133	0.894	-0.100931267	count	1
DHX29	-0.072965	0.3831851	-0.1904	0.849	-0.100864125	count	1
CFLAR	-0.0708347	0.2150744	-0.3293	0.7421	-0.100769449	count	1
ITGB1	-0.0705819	0.1815547	-0.3888	0.6977	-0.100542689	count	1
MEX3C	-0.0765291	0.450535	-0.1699	0.865	-0.100461794	count	1
CBX1	-0.0951563	0.507065	-0.1877	0.851	-0.099997114	count	1
NOC2L	-0.0735894	0.4728809	-0.1556	0.876	-0.099984276	count	1
MUTYH	-0.0863939	0.7604806	-0.1136	0.91	-0.099866079	count	1
SUGP1	-0.0863939	0.7646741	-0.113	0.91	-0.099866079	count	1
SLC38A2	-0.0704452	0.2464776	-0.2858	0.775	-0.099053737	count	1

GANAB	-0.0764469	0.4911465	-0.1556	0.876	-0.098982295	count	1
RPL13	-0.0686154	0.0487767	-1.4067	0.16	-0.09895142	count	1
TOX	-0.070393	0.3440749	-0.2046	0.838	-0.098889183	count	1
HNRNPF	-0.0693399	0.1860532	-0.3727	0.7096	-0.098681591	count	1
UGP2	-0.0697107	0.2967677	-0.2349	0.8144	-0.098513882	count	1
LCP1	-0.068471	0.1268568	-0.5398	0.59	-0.098404864	count	1
LINC00987	-0.1020523	0.7826278	-0.1304	0.896	-0.098289678	count	1
ENKD1	-0.1714953	1.0027159	-0.171	0.8643	-0.098250256	count	1
CXXC5	-0.0694699	0.2535445	-0.274	0.7843	-0.098085182	count	1
RPL35A	-0.067965	0.0625453	-1.0867	0.278	-0.0979538	count	1
SP100	-0.0684839	0.1833579	-0.3735	0.709	-0.097808311	count	1
HIST1H1E	-0.0684621	0.2285517	-0.2995	0.7647	-0.097656375	count	1
TMX4	-0.0684174	0.2427155	-0.2819	0.778	-0.097592625	count	1
RPL37A	-0.0678516	0.0960037	-0.7068	0.48	-0.097572023	count	1
MRPL54	-0.0686644	0.235925	-0.291	0.7712	-0.097539725	count	1
ZNF277	-0.1012406	0.7235149	-0.1399	0.889	-0.097514103	count	1
9-Mar	-0.0717546	0.4344326	-0.1652	0.869	-0.097493794	count	1
ARFGAP3	-0.069304	0.32052	-0.2162	0.829	-0.097405642	count	1
MDN1	-0.1179663	0.7015147	-0.1682	0.867	-0.097271772	count	1
RPL39	-0.067085	0.0574838	-1.167	0.244	-0.096681657	count	1
KIF13B	-0.0811833	0.6691946	-0.1213	0.904	-0.096605845	count	1
SMAP1	-0.0680209	0.261974	-0.2596	0.7953	-0.096470741	count	1
ARL6IP4	-0.0675188	0.1721072	-0.3923	0.695	-0.096468443	count	1
PDLIM2	-0.0680658	0.2495724	-0.2727	0.7852	-0.096366942	count	1
STAT5B	-0.0999182	0.7018227	-0.1424	0.887	-0.096250328	count	1
ITPR1	-0.1670928	0.5752715	-0.2905	0.772	-0.095789721	count	1
COPB2	-0.071824	0.3841888	-0.1869	0.852	-0.09573044	count	1
HMGN1	-0.0667343	0.1408084	-0.4739	0.636	-0.095698453	count	1
ARPC3	-0.065915	0.1078951	-0.6109	0.542	-0.094771732	count	1
AGO4	-0.0819019	0.8122975	-0.1008	0.92	-0.094693328	count	1
RBM19	-0.0819019	0.8740133	-0.0937	0.925	-0.094693328	count	1
EZH2	-0.0981699	0.7010169	-0.14	0.889	-0.094579111	count	1
TSC2	-0.0981699	0.7693592	-0.1276	0.899	-0.094579111	count	1
GOLGA8R	-0.0774602	0.5732876	-0.1351	0.893	-0.094251641	count	1
KCTD10	-0.074108	0.6985987	-0.1061	0.916	-0.094204191	count	1
ERRFI1	-0.076787	0.7330679	-0.1047	0.917	-0.093434796	count	1
RPLP2	-0.0648086	0.0556361	-1.1649	0.245	-0.093402574	count	1
PSME3	-0.0886642	0.6098518	-0.1454	0.884	-0.093213216	count	1
ZNF326	-0.0674183	0.4094808	-0.1646	0.869	-0.092685426	count	1
FAM200B	-0.068507	0.4051724	-0.1691	0.866	-0.092595652	count	1
MAML1	-0.0957505	0.9446371	-0.1014	0.919	-0.092265588	count	1
NFATC1	-0.0957505	0.9499341	-0.1008	0.92	-0.092265588	count	1
MEX3D	-0.0957505	0.9499341	-0.1008	0.92	-0.092265588	count	1
LNPEP	-0.0657673	0.3027319	-0.2172	0.828	-0.092253926	count	1
LRMP	-0.0678852	0.4116926	-0.1649	0.869	-0.092008533	count	1

SON	-0.0642758	0.1321558	-0.4864	0.627	-0.092001776	count	1
DERA	-0.0710414	0.5991287	-0.1186	0.906	-0.09199516	count	1
PURB	-0.0731557	0.5618308	-0.1302	0.896	-0.091903722	count	1
GSS	-0.0721668	0.5759918	-0.1253	0.9	-0.091741488	count	1
METAP1	-0.0747378	0.6334559	-0.118	0.906	-0.090948084	count	1
MZT2B	-0.0634663	0.1666709	-0.3808	0.7036	-0.090802923	count	1
RELL1	-0.0695079	0.5853911	-0.1187	0.906	-0.090675664	count	1
RRP36	-0.0660059	0.4140302	-0.1594	0.873	-0.090441074	count	1
GBP2	-0.0635518	0.2686477	-0.2366	0.8131	-0.08991081	count	1
RPL19	-0.0619416	0.0465198	-1.3315	0.184	-0.089322739	count	1
SCAP	-0.0921823	0.8440568	-0.1092	0.913	-0.088851858	count	1
ITGA6	-0.0921823	1.0039462	-0.0918	0.927	-0.088851858	count	1
ZXDC	-0.0921823	1.0882733	-0.0847	0.933	-0.088851858	count	1
TMEM80	-0.0649815	0.5931644	-0.1096	0.913	-0.088299147	count	1
PAIP2	-0.0617713	0.1783271	-0.3464	0.7293	-0.088268805	count	1
PSMD12	-0.0632136	0.3521502	-0.1795	0.858	-0.088092562	count	1
RNF220	-0.0699798	0.6279119	-0.1114	0.911	-0.087922254	count	1
RNF14	-0.0699798	0.6755435	-0.1036	0.918	-0.087922254	count	1
UBE2D2	-0.0614612	0.1571247	-0.3912	0.696	-0.087791865	count	1
VAPB	-0.0736449	0.6657609	-0.1106	0.912	-0.087662206	count	1
RBBP6	-0.0625256	0.3078296	-0.2031	0.839	-0.087560117	count	1
APOBEC3G	-0.0604035	0.1227983	-0.4919	0.623	-0.086914187	count	1
EPB41	-0.0616737	0.3093564	-0.1994	0.842	-0.086685312	count	1
ASB8	-0.062857	0.338223	-0.1858	0.853	-0.086550204	count	1
ATF7IP	-0.0641976	0.3117909	-0.2059	0.837	-0.086232572	count	1
RPS23	-0.0596031	0.0513738	-1.1602	0.247	-0.085935456	count	1
ZNF669	-0.0649815	0.7172182	-0.0906	0.928	-0.085805402	count	1
LEMD2	-0.0889001	0.6062391	-0.1466	0.884	-0.085709988	count	1
HERC2	-0.0622247	0.3896255	-0.1597	0.873	-0.085680132	count	1
PABPC1	-0.0593734	0.1153943	-0.5145	0.607	-0.085201061	count	1
GNG5	-0.059451	0.1764967	-0.3368	0.7365	-0.085081898	count	1
BAD	-0.0628783	0.3652742	-0.1721	0.863	-0.084994723	count	1
KLF12	-0.0807973	0.6798985	-0.1188	0.905	-0.084984842	count	1
UTP25	-0.0732796	0.7016532	-0.1044	0.917	-0.084758014	count	1
RPL11	-0.0585369	0.0500294	-1.17	0.243	-0.08440136	count	1
JADE1	-0.0654777	0.5601749	-0.1169	0.907	-0.084084845	count	1
EIF6	-0.0595114	0.2758983	-0.2157	0.829	-0.084002102	count	1
C1orf56	-0.0595622	0.28588	-0.2083	0.8351	-0.083988739	count	1
ST3GAL1	-0.0638311	0.4141183	-0.1541	0.878	-0.083814932	count	1
CORO1A	-0.0582215	0.1048518	-0.5553	0.579	-0.083772645	count	1
C11orf1	-0.0868564	0.6120355	-0.1419	0.8872	-0.083752819	count	1
LCP2	-0.0590069	0.2946202	-0.2003	0.8414	-0.083503784	count	1
KIF22	-0.06052	0.4148138	-0.1459	0.884	-0.083453139	count	1
KANK3	-0.0649065	0.6955321	-0.0933	0.926	-0.083352526	count	1
CXorf21	-0.0863939	1.1704642	-0.0738	0.941	-0.083309811	count	1

ZBTB20-AS1	-0.0863939	1.311119	-0.0659	0.9475	-0.083309811	count	1
GATA3	-0.0585699	0.2262021	-0.2589	0.7959	-0.083147144	count	1
CBX4	-0.0599778	0.340648	-0.1761	0.86	-0.083114019	count	1
NDUFB3	-0.0595214	0.3023932	-0.1968	0.844	-0.083078096	count	1
HLA-F	-0.0580766	0.1701395	-0.3413	0.7331	-0.08303805	count	1
TMSB10	-0.0576102	0.0702716	-0.8198	0.413	-0.083013909	count	1
DCLRE1C	-0.0745941	0.7097872	-0.1051	0.916	-0.08298198	count	1
TEDC1	-0.0786583	0.7421502	-0.106	0.916	-0.082746078	count	1
TGOLN2	-0.0591774	0.2598085	-0.2278	0.82	-0.08271513	count	1
OASL	-0.0581328	0.2864578	-0.2029	0.8393	-0.082568898	count	1
RAB28	-0.0676996	0.5994786	-0.1129	0.91	-0.082404262	count	1
MT-ND1	-0.0570596	0.0905044	-0.6305	0.529	-0.082158287	count	1
RNASET2	-0.0590017	0.3388068	-0.1741	0.862	-0.08201108	count	1
POLR2G	-0.057725	0.2552784	-0.2261	0.8212	-0.081903313	count	1
UTY	-0.0594131	0.4947776	-0.1201	0.904	-0.08181111	count	1
RBBP4	-0.0587695	0.3410636	-0.1723	0.863	-0.081688485	count	1
TAF7	-0.0579976	0.1927791	-0.3008	0.764	-0.081660208	count	1
DNAJA3	-0.0843914	0.6632999	-0.1272	0.899	-0.081391327	count	1
PIP4P1	-0.0633126	0.4518571	-0.1401	0.889	-0.080505145	count	1
RPS4Y1	-0.0559277	0.1252938	-0.4464	0.656	-0.080428737	count	1
SRSF9	-0.0561422	0.1921615	-0.2922	0.77	-0.080200436	count	1
ZMAT5	-0.0604334	0.4955872	-0.1219	0.903	-0.079806872	count	1
CHM	-0.0594043	0.4413982	-0.1346	0.893	-0.079511879	count	1
LPCAT3	-0.0685457	0.6689604	-0.1025	0.918	-0.079299728	count	1
CCDC50	-0.0685457	0.7620546	-0.0899	0.928	-0.079299728	count	1
MZT2A	-0.0555195	0.232375	-0.2389	0.8113	-0.079217405	count	1
ACOT13	-0.0591571	0.6315927	-0.0937	0.925	-0.079181327	count	1
ARIH2	-0.0684044	0.4836556	-0.1414	0.888	-0.079136768	count	1
RPL23	-0.0551152	0.1128963	-0.4882	0.626	-0.079073456	count	1
TCIRG1	-0.055957	0.3311259	-0.169	0.866	-0.078652867	count	1
MT-ATP8	-0.0584707	0.5582347	-0.1047	0.917	-0.07854709	count	1
RPS15A	-0.0544558	0.0503533	-1.0815	0.28	-0.078516181	count	1
GPX7	-0.0617334	0.6849482	-0.0901	0.928	-0.078500478	count	1
MAGOH	-0.0554235	0.219978	-0.252	0.8012	-0.078414007	count	1
FAM53C	-0.0571972	0.3997908	-0.1431	0.886	-0.078379372	count	1
RPL22	-0.0542627	0.0876323	-0.6192	0.536	-0.078115045	count	1
CROT	-0.0699798	0.8520379	-0.0821	0.935	-0.077867737	count	1
F2R	-0.056086	0.4851986	-0.1156	0.908	-0.077806636	count	1
NHP2	-0.0548502	0.2741678	-0.2001	0.8416	-0.077714321	count	1
RPS9	-0.0531405	0.0712636	-0.7457	0.456	-0.076533772	count	1
PAXX	-0.0535972	0.2080991	-0.2576	0.7969	-0.076481229	count	1
MTPN	-0.0539042	0.2214156	-0.2435	0.8078	-0.076339659	count	1
ERCC5	-0.0914581	0.737219	-0.1241	0.901	-0.075614604	count	1
OAZ1	-0.0525542	0.102009	-0.5152	0.607	-0.075593216	count	1
GNAI3	-0.0536792	0.2699501	-0.1988	0.843	-0.074926984	count	1

MIR4435-2HG	-0.0549886	0.4750266	-0.1158	0.908	-0.074542386	count	1
LSM6	-0.0527189	0.3176882	-0.1659	0.8683	-0.074367421	count	1
HBP1	-0.0537109	0.2907959	-0.1847	0.854	-0.074261688	count	1
CALM1	-0.0515258	0.0753925	-0.6834	0.495	-0.074238227	count	1
ERC1	-0.0577282	0.5496882	-0.105	0.916	-0.07414756	count	1
DHX8	-0.0582109	0.5725313	-0.1017	0.919	-0.074028308	count	1
MBP	-0.0516803	0.1689235	-0.3059	0.7598	-0.073998545	count	1
OST4	-0.0515084	0.157712	-0.3266	0.7442	-0.073692643	count	1
C11orf24	-0.0584707	0.702774	-0.0832	0.934	-0.073487288	count	1
ZNF131	-0.0532332	0.3641237	-0.1462	0.884	-0.073410506	count	1
PMM2	-0.0631517	0.6582843	-0.0959	0.924	-0.073077337	count	1
DNLZ	-0.075387	1.0210028	-0.0738	0.941	-0.072757064	count	1
HAUS5	-0.075387	1.0224858	-0.0737	0.941	-0.072757064	count	1
THOC5	-0.075387	1.0224858	-0.0737	0.941	-0.072757064	count	1
ERLIN1	-0.075387	1.0567974	-0.0713	0.943	-0.072757064	count	1
ZNF468	-0.075387	1.1878685	-0.0635	0.949	-0.072757064	count	1
AIFM1	-0.075387	1.1929599	-0.0632	0.95	-0.072757064	count	1
FAM206A	-0.075387	1.1929599	-0.0632	0.95	-0.072757064	count	1
ZNF317	-0.075387	1.1929599	-0.0632	0.95	-0.072757064	count	1
TRIM62	-0.075387	1.4031558	-0.0537	0.957	-0.072757064	count	1
ZFP69B	-0.075387	1.4031558	-0.0537	0.957	-0.072757064	count	1
ZYG11B	-0.075387	1.4031558	-0.0537	0.957	-0.072757064	count	1
RGS18	-0.075387	1.4031558	-0.0537	0.957	-0.072757064	count	1
TMEM177	-0.075387	1.4031558	-0.0537	0.957	-0.072757064	count	1
RENBP	-0.075387	1.4031558	-0.0537	0.957	-0.072757064	count	1
INVS	-0.075387	1.4031558	-0.0537	0.957	-0.072757064	count	1
AGAP5	-0.075387	1.4031558	-0.0537	0.957	-0.072757064	count	1
IL17D	-0.075387	1.4031558	-0.0537	0.957	-0.072757064	count	1
AL139089.1	-0.075387	1.4031558	-0.0537	0.957	-0.072757064	count	1
ANGEL1	-0.075387	1.4031558	-0.0537	0.957	-0.072757064	count	1
NKD1	-0.075387	1.4031558	-0.0537	0.957	-0.072757064	count	1
GGT1	-0.075387	1.4031558	-0.0537	0.957	-0.072757064	count	1
SLC35E4	-0.075387	1.4031558	-0.0537	0.957	-0.072757064	count	1
PMPCA	-0.0542745	0.4622389	-0.1174	0.907	-0.072651839	count	1
XCL2	-0.0505326	0.159613	-0.3166	0.752	-0.072647169	count	1
PLCXD1	-0.1255337	1.090304	-0.1151	0.908	-0.072395818	count	1
AL353194.1	-0.1255337	1.090304	-0.1151	0.908	-0.072395818	count	1
HIST1H2AE	-0.1255337	1.094848	-0.1147	0.909	-0.072395818	count	1
MOK	-0.1255337	1.094848	-0.1147	0.909	-0.072395818	count	1
MBD6	-0.0623203	0.5439561	-0.1146	0.909	-0.072117972	count	1
METTL7A	-0.0605498	0.6756079	-0.0896	0.929	-0.072112485	count	1
SRSF1	-0.0579763	0.4850286	-0.1195	0.905	-0.071838721	count	1
ATP6V1F	-0.0505039	0.2049839	-0.2464	0.8055	-0.071832172	count	1
MRPS18C	-0.0508478	0.3252037	-0.1564	0.8758	-0.071821117	count	1
ARID1A	-0.0536164	0.4205049	-0.1275	0.899	-0.071771681	count	1

LSM8	-0.050626	0.2280927	-0.222	0.8245	-0.07160926	count	1
PCBP1	-0.0499534	0.1551126	-0.322	0.7476	-0.071564494	count	1
NKIRAS2	-0.0679475	0.6478509	-0.1049	0.917	-0.07152629	count	1
OXSR1	-0.0575227	0.5272956	-0.1091	0.913	-0.0712777	count	1
HLA-E	-0.0494923	0.0775239	-0.6384	0.524	-0.071253546	count	1
GPR155	-0.086101	0.9412103	-0.0915	0.927	-0.071223165	count	1
PAXIP1	-0.086101	0.9698615	-0.0888	0.929	-0.071223165	count	1
AP2M1	-0.0503383	0.2528463	-0.1991	0.8423	-0.071183334	count	1
PSME4	-0.0564719	0.5455424	-0.1035	0.918	-0.07097931	count	1
TBC1D20	-0.0525088	0.4855595	-0.1081	0.914	-0.070776475	count	1
PIP4K2A	-0.0496418	0.179658	-0.2763	0.7825	-0.070576282	count	1
IFNGR1	-0.0496256	0.2243675	-0.2212	0.8251	-0.070313358	count	1
GID8	-0.0509498	0.3439337	-0.1481	0.882	-0.070163401	count	1
DDX3Y	-0.0496036	0.3327247	-0.1491	0.8816	-0.069973951	count	1
RIOK3	-0.0498461	0.2667878	-0.1868	0.852	-0.069927779	count	1
DTYMK	-0.0525804	0.5226521	-0.1006	0.92	-0.069793837	count	1
SMPD4	-0.1204461	0.9883811	-0.1219	0.903	-0.06951158	count	1
AC005921.2	-0.0515661	0.6457499	-0.0799	0.936	-0.069506788	count	1
RTCB	-0.0534419	0.4112095	-0.13	0.897	-0.069233184	count	1
GOLT1B	-0.0510495	0.4471985	-0.1142	0.909	-0.069206287	count	1
BIRC6	-0.0516372	0.4502853	-0.1147	0.909	-0.069124532	count	1
NSD1	-0.0500362	0.2919313	-0.1714	0.864	-0.068571989	count	1
KLHL5	-0.0553026	0.7518607	-0.0736	0.941	-0.068531599	count	1
ENC1	-0.0614954	0.8321373	-0.0739	0.941	-0.068457334	count	1
ETNK1	-0.0485888	0.3441178	-0.1412	0.8878	-0.068234217	count	1
B3GAT2	-0.1179663	0.8241962	-0.1431	0.886	-0.068104152	count	1
CPEB2	-0.1179663	0.8867672	-0.133	0.894	-0.068104152	count	1
TP53INP1	-0.1179663	1.0188727	-0.1158	0.9079	-0.068104152	count	1
TMEM250	-0.0520436	0.5274064	-0.0987	0.921	-0.067918757	count	1
MYCBP2	-0.0477707	0.2218134	-0.2154	0.8296	-0.067906564	count	1
GBP5	-0.0480687	0.2257088	-0.213	0.831	-0.067785738	count	1
TSR3	-0.04854	0.2753087	-0.1763	0.86	-0.06765157	count	1
PPIA	-0.0470194	0.1037047	-0.4534	0.651	-0.067609527	count	1
POLR2F	-0.0478131	0.2716205	-0.176	0.8604	-0.067555861	count	1
FXN	-0.0553835	0.7683803	-0.0721	0.943	-0.067442645	count	1
SNAP23	-0.0503734	0.3901104	-0.1291	0.897	-0.067434128	count	1
MSRB1	-0.0535012	0.6988705	-0.0766	0.939	-0.067251283	count	1
TRIAP1	-0.0482607	0.3826261	-0.1261	0.9	-0.066729569	count	1
NSMCE2	-0.0491071	0.4956861	-0.0991	0.921	-0.06619468	count	1
AMD1	-0.0467269	0.203973	-0.2291	0.8189	-0.06614558	count	1
EMSY	-0.0626767	0.6096955	-0.1028	0.918	-0.065999308	count	1
SLC7A5	-0.0682213	0.5886743	-0.1159	0.908	-0.065877073	count	1
COL4A3BP	-0.0474596	0.3943742	-0.1203	0.904	-0.065844401	count	1
MINDY3	-0.0591397	0.6881012	-0.0859	0.932	-0.065842992	count	1
NDUFB6	-0.0464735	0.298809	-0.1555	0.877	-0.06543847	count	1

CTNNA1	-0.0786583	0.9075032	-0.0867	0.931	-0.065114021	count	1
HEATR3	-0.0584707	1.1054303	-0.0529	0.958	-0.065100418	count	1
RING1	-0.0506633	0.6423133	-0.0789	0.937	-0.065084657	count	1
DHX38	-0.049552	0.5442487	-0.091	0.928	-0.065084403	count	1
NUP153	-0.049552	0.5532122	-0.0896	0.929	-0.065084403	count	1
C1orf122	-0.0467543	0.4636731	-0.1008	0.92	-0.064797276	count	1
C22orf39	-0.0476792	0.4311287	-0.1106	0.912	-0.064271279	count	1
ELK3	-0.0493268	0.6436162	-0.0766	0.939	-0.063908199	count	1
BCAP31	-0.044961	0.2351124	-0.1912	0.8485	-0.063883843	count	1
MOB1A	-0.0451509	0.2570236	-0.1757	0.8607	-0.063795188	count	1
STK26	-0.0460557	0.3486796	-0.1321	0.895	-0.06351702	count	1
GNA13	-0.0480592	0.5052188	-0.0951	0.924	-0.063480882	count	1
PDCL3	-0.0451837	0.2995008	-0.1509	0.88	-0.063388861	count	1
MAD1L1	-0.0453252	0.3510888	-0.1291	0.897	-0.062884341	count	1
ASCC3	-0.045837	0.3752946	-0.1221	0.903	-0.062820185	count	1
ARL13B	-0.0541981	0.6493819	-0.0835	0.934	-0.062741716	count	1
RAB7A	-0.0441772	0.2106678	-0.2097	0.834	-0.062695755	count	1
TM7SF3	-0.0557294	0.6741964	-0.0827	0.934	-0.062057086	count	1
COPE	-0.0433913	0.1855918	-0.2338	0.815	-0.06196177	count	1
PIGC	-0.0444842	0.3959708	-0.1123	0.911	-0.061949571	count	1
TMEM123	-0.0440691	0.2819539	-0.1563	0.876	-0.061470923	count	1
H2AFV	-0.0427871	0.1912082	-0.2238	0.8231	-0.06089776	count	1
PAOX	-0.049876	0.7041259	-0.0708	0.944	-0.060747731	count	1
CDV3	-0.0428736	0.217481	-0.1971	0.8438	-0.060720791	count	1
CHCHD5	-0.0443431	0.4056425	-0.1093	0.913	-0.060661424	count	1
KIAA0040	-0.0507785	0.392862	-0.1293	0.897	-0.060498591	count	1
FXYD7	-0.0507736	0.824044	-0.0616	0.951	-0.060492764	count	1
MVD	-0.0466778	0.5663914	-0.0824	0.934	-0.060479812	count	1
RPS4X	-0.041544	0.0566955	-0.7328	0.464	-0.059884935	count	1
CEP164	-0.0469355	0.5275473	-0.089	0.929	-0.059707122	count	1
TP53I11	-0.0463879	0.8544039	-0.0543	0.957	-0.059598547	count	1
RPL14	-0.041325	0.0572227	-0.7222	0.471	-0.059570179	count	1
DCTN1	-0.055929	0.7791013	-0.0718	0.943	-0.058918214	count	1
C4orf3	-0.0412606	0.2033244	-0.2029	0.839	-0.058838517	count	1
NOP58	-0.0414788	0.249284	-0.1664	0.868	-0.058192538	count	1
RPS24	-0.04026	0.0482482	-0.8344	0.405	-0.058052774	count	1
CNN2	-0.0408193	0.2493026	-0.1637	0.87	-0.057837966	count	1
OARD1	-0.0447557	0.4235394	-0.1057	0.916	-0.057503824	count	1
CNTRL	-0.0440256	0.3407402	-0.1292	0.897	-0.057464877	count	1
PLEKHF2	-0.0471704	0.5351321	-0.0881	0.93	-0.057457823	count	1
PRDX2	-0.0405145	0.2730106	-0.1484	0.8821	-0.057430873	count	1
CCND2	-0.0403408	0.2038379	-0.1979	0.8432	-0.05740921	count	1
ZC3H12A	-0.0410104	0.3340563	-0.1228	0.902	-0.057367437	count	1
TUBA1B	-0.039967	0.1413095	-0.2828	0.7775	-0.057262387	count	1
KMT5C	-0.0690173	0.7337873	-0.0941	0.925	-0.057186659	count	1

RBM25	-0.0398882	0.1671721	-0.2386	0.812	-0.057011705	count	1
AIP	-0.0403462	0.234984	-0.1717	0.864	-0.056973206	count	1
CAPG	-0.0393149	0.1992468	-0.1973	0.844	-0.056298583	count	1
TAF15	-0.0423595	0.3727876	-0.1136	0.91	-0.056237223	count	1
REV1	-0.0415781	0.5341924	-0.0778	0.938	-0.055667921	count	1
LCORL	-0.0447781	0.697813	-0.0642	0.949	-0.055508076	count	1
SMG9	-0.0522253	0.9336796	-0.0559	0.955	-0.05502895	count	1
MAN1B1	-0.0443431	0.8323813	-0.0533	0.958	-0.054969593	count	1
CD48	-0.0381603	0.1265304	-0.3016	0.763	-0.054783035	count	1
AL357060.1	-0.0563348	0.9456188	-0.0596	0.953	-0.05444746	count	1
NCOR1	-0.0378391	0.1872527	-0.2021	0.84	-0.054144999	count	1
TYROBP	-0.0374496	0.0796118	-0.4704	0.638	-0.053949415	count	1
ZNF480	-0.0452342	0.568503	-0.0796	0.937	-0.05390468	count	1
WDR83OS	-0.0375792	0.1455101	-0.2583	0.7964	-0.053763103	count	1
LSS	-0.0648134	1.1301249	-0.0574	0.954	-0.053725154	count	1
TRPM2	-0.0648134	1.2024079	-0.0539	0.957	-0.053725154	count	1
DENND3	-0.0449673	0.5769555	-0.0779	0.938	-0.053587178	count	1
USP9X	-0.0432103	0.5879277	-0.0735	0.941	-0.053567237	count	1
CISD2	-0.038446	0.3379394	-0.1138	0.909	-0.053448154	count	1
CAMK1	-0.0396592	0.5593875	-0.0709	0.944	-0.053292062	count	1
RPL21	-0.0369741	0.0648913	-0.5698	0.569	-0.053275788	count	1
CLPTM1L	-0.0410283	0.4443463	-0.0923	0.926	-0.053166684	count	1
CYTH1	-0.0425697	0.47097	-0.0904	0.928	-0.052774158	count	1
ATP6V1E1	-0.0383806	0.4215193	-0.0911	0.928	-0.052696259	count	1
NDUFAB1	-0.0371314	0.2514877	-0.1476	0.8827	-0.052667903	count	1
ENDOV	-0.0399041	0.5828608	-0.0685	0.945	-0.052422463	count	1
RANGAP1	-0.0429643	0.8279249	-0.0519	0.959	-0.052342087	count	1
RSRC2	-0.0363827	0.1718039	-0.2118	0.8324	-0.05190605	count	1
ATP6V0E1	-0.0359792	0.1515612	-0.2374	0.8125	-0.051552537	count	1
RBM39	-0.0359582	0.1243163	-0.2892	0.773	-0.051409757	count	1
ZNF124	-0.0618212	0.868193	-0.0712	0.943	-0.051259593	count	1
TCEAL4	-0.0378424	0.3654263	-0.1036	0.918	-0.05117107	count	1
MAP3K4	-0.041914	0.6732445	-0.0623	0.95	-0.051064402	count	1
EMILIN2	-0.0439965	0.8637345	-0.0509	0.959	-0.050955027	count	1
TTC14	-0.0373999	0.3558849	-0.1051	0.916	-0.050573028	count	1
SPECC1	-0.03628	0.375567	-0.0966	0.923	-0.050389622	count	1
NOP16	-0.0392057	0.6750494	-0.0581	0.954	-0.05037981	count	1
SLU7	-0.0367248	0.3390553	-0.1083	0.914	-0.050244006	count	1
FAU	-0.0347526	0.055547	-0.6256	0.532	-0.050101275	count	1
MBD5	-0.0365676	0.3872958	-0.0944	0.925	-0.050029028	count	1
INTS3	-0.0859272	0.8061831	-0.1066	0.9152	-0.049827795	count	1
LY6E	-0.0345888	0.1294089	-0.2673	0.789	-0.049719156	count	1
B2M	-0.0344013	0.0341687	-1.0068	0.315	-0.049622409	count	1
SAMD9	-0.0351959	0.274374	-0.1283	0.898	-0.049599983	count	1
PDCD6	-0.0348841	0.2189642	-0.1593	0.8735	-0.049582815	count	1

MANF	-0.0360099	0.3204117	-0.1124	0.911	-0.04926634	count	1
TOP1	-0.0348983	0.2683801	-0.13	0.897	-0.049142697	count	1
RPL8	-0.0339886	0.0594741	-0.5715	0.568	-0.048993842	count	1
SUZ12	-0.0349542	0.2961477	-0.118	0.906	-0.048897721	count	1
VPS36	-0.0353265	0.3510813	-0.1006	0.92	-0.048851463	count	1
SDR39U1	-0.0420955	0.585944	-0.0718	0.943	-0.048757442	count	1
DHRSX	-0.0384133	0.7205141	-0.0533	0.958	-0.04830674	count	1
KIR2DL3	-0.0384133	0.7454623	-0.0515	0.959	-0.04830674	count	1
FUBP3	-0.0830228	0.7172559	-0.1158	0.908	-0.048162639	count	1
SEL1L3	-0.0386212	0.506476	-0.0763	0.939	-0.047885086	count	1
PARVG	-0.0342123	0.3379557	-0.1012	0.919	-0.047687834	count	1
CARS	-0.0424554	0.6738098	-0.063	0.95	-0.047308031	count	1
FAM96A	-0.0349495	0.4354427	-0.0803	0.936	-0.047120515	count	1
AC007728.2	-0.0364232	1.2346711	-0.0295	0.9765	-0.046807429	count	1
CELF2	-0.0331601	0.2192281	-0.1513	0.88	-0.046676523	count	1
PRCC	-0.0354439	0.4765602	-0.0744	0.941	-0.046567179	count	1
MRPL49	-0.0344394	0.4655867	-0.074	0.941	-0.046433119	count	1
STIM1	-0.0411775	0.668107	-0.0616	0.951	-0.045887041	count	1
PSMA7	-0.0317591	0.1269853	-0.2501	0.803	-0.04564923	count	1
RBM8A	-0.0317023	0.1552314	-0.2042	0.8383	-0.045274551	count	1
GPR174	-0.0321786	0.2755509	-0.1168	0.907	-0.045147428	count	1
MOCS2	-0.0356335	0.5845287	-0.061	0.951	-0.044814546	count	1
CFH	-0.0463281	0.935453	-0.0495	0.961	-0.044809148	count	1
UBE4B	-0.0343148	0.6680042	-0.0514	0.959	-0.044473725	count	1
SGTB	-0.0343148	0.6905681	-0.0497	0.96	-0.044473725	count	1
PLA2G16	-0.0313686	0.2318913	-0.1353	0.8925	-0.044407565	count	1
GCC2	-0.0314419	0.2125781	-0.1479	0.8825	-0.044401232	count	1
ZNF414	-0.0357181	0.7867007	-0.0454	0.964	-0.044289647	count	1
TPGS1	-0.0321852	0.3309144	-0.0973	0.923	-0.044116936	count	1
CDC27	-0.0354673	0.52954	-0.067	0.947	-0.043979004	count	1
DEGS1	-0.0319367	0.3707884	-0.0861	0.931	-0.043609089	count	1
PSMD8	-0.0305213	0.1935745	-0.1577	0.8748	-0.04359705	count	1
HDGFL3	-0.0333595	0.5612161	-0.0594	0.953	-0.043552686	count	1
CDC40	-0.0310239	0.2841402	-0.1092	0.913	-0.043428176	count	1
TDRD3	-0.0326401	0.453419	-0.072	0.943	-0.043341046	count	1
PELP1	-0.0386112	0.8554251	-0.0451	0.964	-0.043032812	count	1
RNF144A	-0.0386112	0.9748263	-0.0396	0.968	-0.043032812	count	1
E2F4	-0.0313311	0.4097423	-0.0765	0.939	-0.042867416	count	1
SLC16A8	-0.038025	0.9758953	-0.039	0.969	-0.04238074	count	1
FAM213A	-0.038025	1.046552	-0.0363	0.971	-0.04238074	count	1
ZNF646	-0.038025	1.2344916	-0.0308	0.975	-0.04238074	count	1
ATF1	-0.03148	0.4091024	-0.0769	0.939	-0.042306528	count	1
PSMD7	-0.0297642	0.2485367	-0.1198	0.9048	-0.042175547	count	1
HAVCR2	-0.0305403	0.4342204	-0.0703	0.944	-0.042002644	count	1
PPP1R11	-0.0297828	0.2906898	-0.1025	0.918	-0.041829242	count	1

DPYSL2	-0.0318098	0.5785584	-0.055	0.956	-0.041795601	count	1
PEX16	-0.033534	0.5549357	-0.0604	0.952	-0.041584236	count	1
CNPY3	-0.0294023	0.309264	-0.0951	0.924	-0.041420838	count	1
ACTR10	-0.0296738	0.3115633	-0.0952	0.924	-0.041293324	count	1
NOP53	-0.0286304	0.1526805	-0.1875	0.8514	-0.040876008	count	1
SDF2L1	-0.028759	0.3138134	-0.0916	0.927	-0.040371406	count	1
BRIX1	-0.0296541	0.4245128	-0.0699	0.944	-0.040212751	count	1
EHD4	-0.0319577	0.4794271	-0.0667	0.947	-0.04019586	count	1
SNRPG	-0.0280633	0.2035674	-0.1379	0.8904	-0.039774295	count	1
NFE2L2	-0.0278084	0.2345803	-0.1185	0.906	-0.039641777	count	1
LARP1	-0.0296933	0.4664443	-0.0637	0.949	-0.03943019	count	1
KRT81	-0.027305	0.160136	-0.1705	0.865	-0.039247232	count	1
ATP6V0B	-0.0274014	0.208128	-0.1317	0.8953	-0.038868089	count	1
SLC25A5	-0.0268755	0.1365012	-0.1969	0.844	-0.038572993	count	1
TAF12	-0.0276266	0.3484223	-0.0793	0.937	-0.038569292	count	1
LIPT1	-0.0360756	0.8925219	-0.0404	0.968	-0.038049153	count	1
NNT-AS1	-0.0285242	0.7198009	-0.0396	0.968	-0.037691128	count	1
P4HTM	-0.0282726	0.5807723	-0.0487	0.961	-0.036915392	count	1
CYHR1	-0.0268297	0.380194	-0.0706	0.944	-0.036778205	count	1
APEX1	-0.0259113	0.2480961	-0.1044	0.917	-0.036517353	count	1
CSNK1G3	-0.0263098	0.3391455	-0.0776	0.938	-0.036508213	count	1
FKBP11	-0.025998	0.3301386	-0.0787	0.937	-0.036496158	count	1
RPS6KA4	-0.0345653	0.9237997	-0.0374	0.97	-0.036459501	count	1
NAA20	-0.0259549	0.3371101	-0.077	0.939	-0.036052061	count	1
UNG	-0.0275378	0.5453259	-0.0505	0.96	-0.035956519	count	1
ISY1	-0.0266189	0.4348077	-0.0612	0.951	-0.035506387	count	1
ANAPC16	-0.0247098	0.1595234	-0.1549	0.877	-0.03530325	count	1
TGFB1	-0.0245666	0.1446933	-0.1698	0.865	-0.035271814	count	1
TRDC	-0.024415	0.1235645	-0.1976	0.843	-0.035123106	count	1
LINC00426	-0.0264537	0.5656757	-0.0468	0.963	-0.034761761	count	1
MLEC	-0.0247874	0.2928287	-0.0846	0.933	-0.034720578	count	1
CYTH4	-0.0255613	0.486181	-0.0526	0.958	-0.034570258	count	1
SLC7A6OS	-0.0289598	0.5967907	-0.0485	0.961	-0.034532465	count	1
RPL17	-0.0248559	0.2721552	-0.0913	0.927	-0.034454641	count	1
NDUFAF3	-0.0241899	0.2441603	-0.0991	0.9211	-0.03434474	count	1
PPIP5K2	-0.0254856	0.39716	-0.0642	0.949	-0.033677892	count	1
RNF25	-0.0281769	0.6428162	-0.0438	0.965	-0.033599915	count	1
GALNT2	-0.0288994	0.6446537	-0.0448	0.964	-0.033492214	count	1
TMC6	-0.024751	0.3108069	-0.0796	0.937	-0.033474744	count	1
UBE2V2	-0.0234772	0.2729423	-0.086	0.932	-0.03315497	count	1
FRG1	-0.0235671	0.2938939	-0.0802	0.936	-0.033030726	count	1
PFDN5	-0.0227302	0.0934157	-0.2433	0.808	-0.032691769	count	1
RACK1	-0.0226504	0.0733514	-0.3088	0.758	-0.032635802	count	1
CYBA	-0.0225814	0.0979217	-0.2306	0.818	-0.032485886	count	1
GOLGA8N	-0.0254933	0.8352524	-0.0305	0.976	-0.032448503	count	1

FASTKD2	-0.0307395	0.5956108	-0.0516	0.959	-0.032431388	count	1
PTPRC	-0.0225141	0.0821572	-0.274	0.784	-0.032406924	count	1
CASP8AP2	-0.0249269	0.5184101	-0.0481	0.962	-0.032313316	count	1
NSMCE3	-0.0226685	0.21007	-0.1079	0.914	-0.032291759	count	1
HPCAL1	-0.0231144	0.3638453	-0.0635	0.949	-0.032194852	count	1
IL10RB-DT	-0.0551436	0.7697527	-0.0716	0.943	-0.032109662	count	1
BCL3	-0.0235574	0.3533836	-0.0667	0.947	-0.03210185	count	1
MECR	-0.0302707	0.8996926	-0.0336	0.973	-0.031937669	count	1
B3GLCT	-0.0302707	1.0510681	-0.0288	0.977	-0.031937669	count	1
SAFB	-0.0240774	0.4033881	-0.0597	0.952	-0.031817854	count	1
C1GALT1C1	-0.0237439	0.4392659	-0.0541	0.957	-0.031672991	count	1
PGRMC1	-0.0237354	0.5033412	-0.0472	0.962	-0.031521882	count	1
DNAJB11	-0.0235947	0.3720377	-0.0634	0.949	-0.031474044	count	1
ESYT1	-0.0238926	0.5225751	-0.0457	0.964	-0.031397892	count	1
PPP2R1A	-0.0225256	0.3949108	-0.057	0.955	-0.031347809	count	1
NDUFA3	-0.0220195	0.232492	-0.0947	0.9246	-0.031325569	count	1
DIDO1	-0.0252449	0.6267549	-0.0403	0.968	-0.031313255	count	1
ATG3	-0.0222316	0.2984902	-0.0745	0.941	-0.031209556	count	1
SSR2	-0.0216856	0.1346306	-0.1611	0.872	-0.031101011	count	1
NAP1L4	-0.0218754	0.2578697	-0.0848	0.932	-0.030852386	count	1
SNX18	-0.0231142	0.6207459	-0.0372	0.97	-0.030697227	count	1
DUT	-0.0218007	0.2797979	-0.0779	0.938	-0.030648939	count	1
CD37	-0.0213631	0.1309143	-0.1632	0.87	-0.030639954	count	1
XPC	-0.0231955	0.5579399	-0.0416	0.967	-0.030482229	count	1
ADGRE5	-0.0211083	0.1950084	-0.1082	0.914	-0.030088139	count	1
TMEM59	-0.0210188	0.1674486	-0.1255	0.9	-0.030032025	count	1
EML3	-0.0360756	0.8407618	-0.0429	0.966	-0.029985357	count	1
RABL3	-0.0360756	0.9722677	-0.0371	0.9704	-0.029985357	count	1
ACBD5	-0.0218679	0.4483544	-0.0488	0.961	-0.029978183	count	1
POR	-0.0232133	0.5533263	-0.042	0.967	-0.02984087	count	1
ATP13A3	-0.0256958	0.7926732	-0.0324	0.974	-0.029783599	count	1
ZNF281	-0.02395	0.494336	-0.0484	0.961	-0.029708268	count	1
CDKN1B	-0.0203964	0.1919538	-0.1063	0.9154	-0.028964115	count	1
GNPTAB	-0.0205673	0.2659084	-0.0773	0.938	-0.028940605	count	1
RXYLT1	-0.0232403	0.7675054	-0.0303	0.976	-0.028828563	count	1
ST8SIA4	-0.0206448	0.2774098	-0.0744	0.941	-0.028823476	count	1
IREB2	-0.0217091	0.6146217	-0.0353	0.972	-0.028689435	count	1
POLR2B	-0.0210594	0.3137518	-0.0671	0.947	-0.028632564	count	1
PPA1	-0.0206695	0.310899	-0.0665	0.947	-0.028550988	count	1
POLR1E	-0.0221151	0.6598814	-0.0335	0.973	-0.028151095	count	1
LSM7	-0.0196011	0.2162619	-0.0906	0.9278	-0.027869791	count	1
CATSPERG	-0.0231153	0.7601054	-0.0304	0.976	-0.02756944	count	1
A2M	-0.0231153	0.822691	-0.0281	0.978	-0.02756944	count	1
FUT7	-0.0231153	0.9333818	-0.0248	0.98	-0.02756944	count	1
BRAT1	-0.0204203	0.4932461	-0.0414	0.967	-0.027447757	count	1

IMP4	-0.0202738	0.4110734	-0.0493	0.961	-0.027339833	count	1
PPDPF	-0.0184666	0.148751	-0.1241	0.901	-0.026483866	count	1
POGK	-0.0232787	0.6904989	-0.0337	0.973	-0.025964383	count	1
SETD3	-0.018728	0.4690757	-0.0399	0.968	-0.025463514	count	1
GLS	-0.0180197	0.2440506	-0.0738	0.941	-0.025194188	count	1
PPP2R5D	-0.0302707	0.9673911	-0.0313	0.975	-0.025174021	count	1
ZWILCH	-0.0302707	0.9673911	-0.0313	0.975	-0.025174021	count	1
EEF2K	-0.0302707	0.9673911	-0.0313	0.975	-0.025174021	count	1
SEMA4A	-0.0302707	1.2727098	-0.0238	0.981	-0.025174021	count	1
VSIG8	-0.0302707	1.2727098	-0.0238	0.981	-0.025174021	count	1
SEMA4C	-0.0302707	1.2727098	-0.0238	0.981	-0.025174021	count	1
TRIM66	-0.0302707	1.2727098	-0.0238	0.981	-0.025174021	count	1
AC023509.4	-0.0302707	1.2727098	-0.0238	0.981	-0.025174021	count	1
CIITA	-0.0302707	1.2727098	-0.0238	0.981	-0.025174021	count	1
POLI	-0.0302707	1.2727098	-0.0238	0.981	-0.025174021	count	1
MAFB	-0.0302707	1.2727098	-0.0238	0.981	-0.025174021	count	1
RAB3D	-0.0302707	1.2727098	-0.0238	0.981	-0.025174021	count	1
AC008764.6	-0.0302707	1.2727098	-0.0238	0.981	-0.025174021	count	1
ZNF582-AS1	-0.0302707	1.2727098	-0.0238	0.981	-0.025174021	count	1
ZNF416	-0.0302707	1.2727098	-0.0238	0.981	-0.025174021	count	1
ADORA2A-AS1	-0.0302707	1.2727098	-0.0238	0.981	-0.025174021	count	1
LRRC75B	-0.0302707	1.2727098	-0.0238	0.981	-0.025174021	count	1
SIK1B	-0.0302707	1.2727098	-0.0238	0.981	-0.025174021	count	1
SOAT1	-0.0182297	0.5312598	-0.0343	0.973	-0.024786156	count	1
SCAND1	-0.0171474	0.203575	-0.0842	0.9329	-0.024487534	count	1
DNAJC10	-0.0191998	0.4720394	-0.0407	0.968	-0.024441932	count	1
GADD45B	-0.0169704	0.1626666	-0.1043	0.917	-0.024435874	count	1
TARSL2	-0.0182187	0.3788795	-0.0481	0.962	-0.024401604	count	1
COX6A1	-0.0167042	0.1433219	-0.1166	0.907	-0.023943829	count	1
GRK2	-0.0171244	0.3485583	-0.0491	0.961	-0.023832207	count	1
CD2	-0.0164616	0.1887472	-0.0872	0.9306	-0.023586821	count	1
TNFSF8	-0.0193226	0.6797568	-0.0284	0.977	-0.023559181	count	1
ATP5F1D	-0.01615	0.1455214	-0.111	0.9117	-0.023140359	count	1
AP3B1	-0.0168198	0.3614433	-0.0465	0.963	-0.023135825	count	1
AP3M1	-0.0184295	0.6042871	-0.0305	0.976	-0.022864335	count	1
TEFM	-0.0196184	0.8521947	-0.023	0.982	-0.022745302	count	1
TUBGCP3	-0.0196184	0.9462351	-0.0207	0.983	-0.022745302	count	1
GUK1	-0.0156763	0.1270146	-0.1234	0.902	-0.022512413	count	1
SUDS3	-0.0162268	0.3463257	-0.0469	0.963	-0.022205399	count	1
NDUFB2	-0.0149048	0.1791406	-0.0832	0.934	-0.021288046	count	1
FXR1	-0.0151077	0.3192753	-0.0473	0.962	-0.021123196	count	1
PTEN	-0.0150469	0.3565435	-0.0422	0.966	-0.02095932	count	1
HIVEP1	-0.0355094	0.5734887	-0.0619	0.951	-0.02073031	count	1
KLF13	-0.0143531	0.2075586	-0.0692	0.9449	-0.020334891	count	1
SERPINE2	-0.0146798	0.4155416	-0.0353	0.972	-0.020251584	count	1

PHACTR4	-0.0150482	0.5815029	-0.0259	0.979	-0.02022851	count	1
ZNF12	-0.0340722	0.7893655	-0.0432	0.9656	-0.019895	count	1
BET1	-0.0162409	0.5174774	-0.0314	0.975	-0.019803853	count	1
DCXR	-0.013923	0.2476039	-0.0562	0.955	-0.019591992	count	1
ALKBH3	-0.0146798	0.5377754	-0.0273	0.978	-0.019402424	count	1
RAD21	-0.0137116	0.2503364	-0.0548	0.956	-0.019083076	count	1
ZNF143	-0.0150882	0.5595075	-0.027	0.979	-0.018986642	count	1
SNRNP200	-0.0141841	0.4601171	-0.0308	0.975	-0.018747421	count	1
SPART	-0.0146859	0.4601	-0.0319	0.975	-0.018480603	count	1
AP1S2	-0.0131688	0.4016871	-0.0328	0.974	-0.018256086	count	1
RAB37	-0.0134482	0.4515078	-0.0298	0.976	-0.01824044	count	1
LAS1L	-0.0144707	0.5967063	-0.0243	0.981	-0.018209905	count	1
MAP3K1	-0.0162529	0.8059266	-0.0202	0.984	-0.018134286	count	1
PKN2	-0.0130605	0.2817932	-0.0463	0.963	-0.018041867	count	1
HDAC10	-0.0308198	0.8860365	-0.0348	0.9723	-0.018003511	count	1
CD99	-0.0123134	0.127406	-0.0966	0.923	-0.017710357	count	1
MT-ND3	-0.0122292	0.0859095	-0.1423	0.887	-0.017605274	count	1
DNAJB1	-0.0122074	0.2857062	-0.0427	0.966	-0.017470266	count	1
TPT1	-0.0120471	0.054071	-0.2228	0.824	-0.017370814	count	1
HDGFL2	-0.0125854	0.4521519	-0.0278	0.978	-0.01722306	count	1
CUTA	-0.0119782	0.1722073	-0.0696	0.945	-0.017106999	count	1
ABCB7	-0.0161744	0.7742752	-0.0209	0.983	-0.017079181	count	1
WSB2	-0.0161744	0.899773	-0.018	0.986	-0.017079181	count	1
EHMT2	-0.0124049	0.5104053	-0.0243	0.981	-0.016906522	count	1
RPL35	-0.0117351	0.0722152	-0.1625	0.871	-0.016896339	count	1
NAMPT	-0.0121409	0.3942537	-0.0308	0.975	-0.016849033	count	1
UBE3A	-0.0120832	0.3291669	-0.0367	0.971	-0.016785776	count	1
AHNAK	-0.0118104	0.2843331	-0.0415	0.9669	-0.016724956	count	1
ADH5	-0.0115706	0.2450046	-0.0472	0.9624	-0.016439571	count	1
NUCKS1	-0.0114577	0.1825646	-0.0628	0.95	-0.016337138	count	1
WTAP	-0.0115408	0.2346244	-0.0492	0.961	-0.016283205	count	1
ARPC5L	-0.0111704	0.1267554	-0.0881	0.93	-0.016046004	count	1
LCOR	-0.0123006	0.4465224	-0.0275	0.978	-0.015949987	count	1
C6orf226	-0.0117136	0.5116518	-0.0229	0.982	-0.015888074	count	1
COQ8B	-0.0190843	0.8289907	-0.023	0.9816	-0.015887441	count	1
PPP1CA	-0.0106415	0.1885147	-0.0564	0.955	-0.015199052	count	1
PRDX1	-0.0106958	0.2177958	-0.0491	0.9609	-0.015178378	count	1
DNMT3A	-0.010816	0.4509874	-0.024	0.981	-0.014994664	count	1
SUGP2	-0.0125613	0.5505907	-0.0228	0.982	-0.014987705	count	1
DUSP2	-0.0103756	0.111371	-0.0932	0.926	-0.014955516	count	1
NMRK1	-0.0105473	0.3319725	-0.0318	0.9747	-0.014848276	count	1
RMND5A	-0.0118309	0.5686965	-0.0208	0.983	-0.014680795	count	1
ATP5PB	-0.0102663	0.2377482	-0.0432	0.9656	-0.014531371	count	1
M6PR	-0.0102244	0.2972612	-0.0344	0.973	-0.01436844	count	1
C9orf72	-0.010907	0.73321	-0.0149	0.988	-0.013726698	count	1

MLKL	-0.0118309	0.6386989	-0.0185	0.985	-0.013721118	count	1
PPP1CB	-0.0096681	0.1847843	-0.0523	0.9583	-0.013691317	count	1
MYL12B	-0.0094139	0.0941469	-0.1	0.92	-0.013548076	count	1
ZCRB1	-0.0095055	0.3088877	-0.0308	0.975	-0.013241068	count	1
SHISAL2A	-0.0092807	0.4719022	-0.0197	0.984	-0.012766675	count	1
MORF4L1	-0.0088518	0.1588703	-0.0557	0.956	-0.012660216	count	1
RPL23A	-0.0087561	0.0688501	-0.1272	0.899	-0.012614468	count	1
PIGT	-0.0090042	0.4174199	-0.0216	0.983	-0.012532016	count	1
OCEL1	-0.0104648	0.6676968	-0.0157	0.988	-0.01248721	count	1
TOPBP1	-0.0094575	0.4650594	-0.0203	0.984	-0.012431812	count	1
ASAH1	-0.0085611	0.3334993	-0.0257	0.98	-0.012031101	count	1
THOC7	-0.0084114	0.2677387	-0.0314	0.975	-0.01185093	count	1
CCDC18	-0.0095005	0.7339505	-0.0129	0.99	-0.011789862	count	1
MED20	-0.0118309	1.0192039	-0.0116	0.991	-0.011471467	count	1
RNF217	-0.0095695	0.8663461	-0.011	0.991	-0.011419265	count	1
ZNF862	-0.0095695	0.8663461	-0.011	0.991	-0.011419265	count	1
MYH10	-0.0095695	1.1357001	-0.0084	0.993	-0.011419265	count	1
HCST	-0.0075019	0.0907323	-0.0827	0.934	-0.010798622	count	1
SYNGR1	-0.0077944	0.3331769	-0.0234	0.981	-0.010781213	count	1
TMA16	-0.0076407	0.4079265	-0.0187	0.985	-0.010604086	count	1
JMJD8	-0.007913	0.6303492	-0.0126	0.99	-0.010175887	count	1
PDCD2	-0.0072998	0.339546	-0.0215	0.983	-0.010159968	count	1
ALKBH8	-0.0094282	0.8517869	-0.0111	0.991	-0.009959482	count	1
RASGRP1	-0.0078131	0.6347167	-0.0123	0.99	-0.009696335	count	1
NECAP1	-0.0068696	0.4464388	-0.0154	0.988	-0.00938307	count	1
NEK6	-0.0074222	0.605143	-0.0123	0.99	-0.009341894	count	1
ROCK1	-0.006266	0.235847	-0.0266	0.9788	-0.008857543	count	1
TRIR	-0.0061593	0.1382649	-0.0445	0.964	-0.008827858	count	1
NDUFA12	-0.0062137	0.1900971	-0.0327	0.9739	-0.008825736	count	1
CDC37	-0.0061735	0.222426	-0.0278	0.9779	-0.008733933	count	1
PPIL2	-0.0061749	0.4510466	-0.0137	0.989	-0.008466451	count	1
KRCC1	-0.006072	0.3808655	-0.0159	0.987	-0.00846539	count	1
TMEM126A	-0.0060141	0.4113543	-0.0146	0.988	-0.008197183	count	1
GPR82	-0.0073409	0.7296274	-0.0101	0.992	-0.008194219	count	1
TLNRD1	-0.0060526	0.6136603	-0.0099	0.992	-0.008187971	count	1
LDHB	-0.0057178	0.214069	-0.0267	0.9787	-0.008158692	count	1
NKAPD1	-0.0063895	0.6630038	-0.0096	0.992	-0.00813667	count	1
HAUS7	-0.0094282	0.8670266	-0.0109	0.991	-0.007855763	count	1
UBP1	-0.0094282	1.0402449	-0.0091	0.993	-0.007855763	count	1
PRSS22	-0.0094282	1.0402449	-0.0091	0.993	-0.007855763	count	1
ZNF8	-0.0094282	1.0419348	-0.009	0.993	-0.007855763	count	1
EMID1	-0.0094282	1.171829	-0.008	0.994	-0.007855763	count	1
PRF1	-0.0053178	0.1362138	-0.039	0.969	-0.007627895	count	1
CHD9	-0.0053567	0.2654433	-0.0202	0.984	-0.007479614	count	1
KLF2	-0.0050759	0.2184035	-0.0232	0.9815	-0.007284539	count	1

RMND5B	-0.0054171	0.7712053	-0.007	0.994	-0.006723296	count	1
TSPYL2	-0.0046664	0.256302	-0.0182	0.9855	-0.006569464	count	1
FAM84B	-0.0076968	0.9627934	-0.008	0.994	-0.006414131	count	1
COA1	-0.0045434	0.3135263	-0.0145	0.988	-0.00634403	count	1
HSPB11	-0.0044643	0.2530305	-0.0176	0.9859	-0.006329185	count	1
LAMTOR4	-0.0043331	0.2076822	-0.0209	0.9834	-0.006192579	count	1
CD8B	-0.0063163	0.9155858	-0.0069	0.994	-0.00612678	count	1
GFOD2	-0.0063163	0.8700611	-0.0073	0.9942	-0.00612678	count	1
TRIM41	-0.0063163	0.9262032	-0.0068	0.995	-0.00612678	count	1
DDX21	-0.0038212	0.2180713	-0.0175	0.986	-0.005412712	count	1
MXD1	-0.0045107	0.4833217	-0.0093	0.993	-0.005232982	count	1
POMP	-0.003609	0.1885937	-0.0191	0.9847	-0.005151742	count	1
SERBP1	-0.0035465	0.1390639	-0.0255	0.98	-0.005083938	count	1
SNRPD2	-0.0033654	0.1278026	-0.0263	0.979	-0.004828115	count	1
CCT8	-0.0032732	0.246797	-0.0133	0.9894	-0.004650706	count	1
ZFAND2B	-0.0033709	0.3452169	-0.0098	0.992	-0.004613529	count	1
GCH1	-0.0033517	0.4536867	-0.0074	0.994	-0.004430875	count	1
RSRC1	-0.0032563	0.4270722	-0.0076	0.994	-0.0043624	count	1
ELP5	-0.0032364	0.4504438	-0.0072	0.994	-0.004299622	count	1
DIS3	-0.0032364	0.48932	-0.0066	0.995	-0.004299622	count	1
FAS	-0.0035299	0.8392207	-0.0042	0.997	-0.004213163	count	1
CCDC93	-0.0032725	0.5752433	-0.0057	0.995	-0.004167671	count	1
H3F3B	-0.0028465	0.075535	-0.0377	0.97	-0.004103771	count	1
ABHD14B	-0.0027788	0.3538022	-0.0079	0.994	-0.003852623	count	1
PHAX	-0.0026908	0.3540569	-0.0076	0.994	-0.003675493	count	1
IRAK1	-0.0031202	0.6053798	-0.0052	0.996	-0.003297215	count	1
RNF10	-0.003316	0.7332824	-0.0045	0.996	-0.003217179	count	1
SNRPD1	-0.0019928	0.2529403	-0.0079	0.9937	-0.002825284	count	1
PRPF39	-0.002211	0.5118826	-0.0043	0.997	-0.002783253	count	1
CHIC1	-0.003316	0.8282987	-0.004	0.997	-0.002764481	count	1
SF3B2	-0.0019327	0.2132625	-0.0091	0.9928	-0.00274968	count	1
PSAP	-0.0019231	0.2739101	-0.007	0.994	-0.002703923	count	1
RPS27A	-0.0018093	0.0465813	-0.0388	0.969	-0.002609189	count	1
SMC1A	-0.0018268	0.3735942	-0.0049	0.996	-0.00253543	count	1
THAP3	-0.0017342	0.6297065	-0.0028	0.998	-0.002249224	count	1
BTG3	-0.0009646	0.2727199	-0.0035	0.9972	-0.001361499	count	1
MAN1A2	-0.0010159	0.4090084	-0.0025	0.998	-0.001293866	count	1
FUOM	-0.000857	0.4023536	-0.0021	0.998	-0.001182431	count	1
SCAMP1-AS1	-0.0009108	0.6646993	-0.0014	0.999	-0.001087203	count	1
CAPRIN1	-0.0009108	0.76839	-0.0012	0.999	-0.001087203	count	1
HNRNPA2B1	-0.0007102	0.0979956	-0.0072	0.994	-0.001020552	count	1
USP46	-8.00E-04	0.5715931	-0.0014	0.999	-0.000993036	count	1
FNBP4	-0.0005741	0.2776063	-0.0021	0.998	-0.00079836	count	1
RPS26	-0.0005266	0.0611463	-0.0086	0.993	-0.000758955	count	1
MAEA	-0.0004795	0.516478	-9.00E-04	0.999	-0.000626437	count	1

BFAR	-0.0004043	0.3136096	-0.0013	0.999	-0.000558567	count	1
DUSP11	-0.0003324	0.3878293	-9.00E-04	0.999	-0.000457981	count	1
NEMF	-0.0002783	0.3013051	-9.00E-04	0.999	-0.000387013	count	1
ZNF444	-0.8007583	0.4943966	-1.6197	0.106	-1.49E-07	count	1
POC5	-0.6826825	0.4569712	-1.4939	0.136	-1.32E-07	count	1
DHX16	-0.6577807	0.5142068	-1.2792	0.202	-1.25E-07	count	1
CEP68	-2.5094188	0.7454543	-3.3663	9.00E-04	-1.16E-07	count	1
CHUK	-2.196169	1.1235336	-1.9547	0.0515	-1.09E-07	count	1
LRRC47	-0.5405425	0.4464009	-1.2109	0.227	-1.07E-07	count	1
XYLT1	-2.1186623	1.1510279	-1.8407	0.0666	-1.06E-07	count	1
EIF2AK3	-2.1592044	1.1952813	-1.8064	0.0718	-1.06E-07	count	1
PLK3	-1.8282528	0.9229594	-1.9809	0.0485	-1.00E-07	count	1
C1QTNF6	-1.962458	1.2055933	-1.6278	0.105	-1.00E-07	count	1
MAN2C1	-1.8941043	0.8410377	-2.2521	0.025	-9.86E-08	count	1
TIMM23B	-1.7928628	0.658271	-2.7236	0.0068	-9.75E-08	count	1
HIVEP2	-1.6801747	0.6847498	-2.4537	0.0147	-9.43E-08	count	1
PGAP3	-1.757256	0.9850819	-1.7839	0.0754	-9.38E-08	count	1
KAT2A	-1.7183582	1.1058757	-1.5538	0.121	-9.22E-08	count	1
DPYD	-0.4535077	0.5594151	-0.8107	0.418	-9.22E-08	count	1
SNX11	-1.7183582	0.8788968	-1.9551	0.0514	-9.21E-08	count	1
SIRPG	-1.7183582	0.8788968	-1.9551	0.0514	-9.21E-08	count	1
AC083798.2	-1.5957724	1.0105842	-1.5791	0.115	-8.84E-08	count	1
PTRH1	-1.5957724	1.0105842	-1.5791	0.115	-8.84E-08	count	1
TMOD2	-1.5957724	1.0105842	-1.5791	0.115	-8.84E-08	count	1
GPD1L	-1.4722259	0.8176784	-1.8005	0.0727	-8.59E-08	count	1
ZNF93	-1.3458185	0.7624249	-1.7652	0.0785	-8.41E-08	count	1
NCKAP5L	-1.4624435	0.9499338	-1.5395	0.125	-8.38E-08	count	1
GOLGA8Q	-1.4624435	0.9499338	-1.5395	0.125	-8.38E-08	count	1
SMCR8	-1.4236994	1.149167	-1.2389	0.216	-8.16E-08	count	1
BLMH	-0.406487	0.5568561	-0.73	0.466	-8.16E-08	count	1
RARG	-1.3975997	0.9527039	-1.467	0.143	-8.08E-08	count	1
ACVR2A	-1.3975997	0.9527039	-1.467	0.143	-8.08E-08	count	1
LARS2	-1.3975997	0.9527039	-1.467	0.143	-8.08E-08	count	1
ATRN	-1.3975997	0.9527039	-1.467	0.143	-8.08E-08	count	1
ZNF248	-1.3569881	0.7284359	-1.8629	0.0634	-8.08E-08	count	1
FLYWCH1	-0.3989847	0.5462345	-0.7304	0.466	-8.07E-08	count	1
FAM53B	-1.3975997	1.0347182	-1.3507	0.178	-8.07E-08	count	1
SLC25A25-AS1	-1.3975997	1.0347182	-1.3507	0.178	-8.07E-08	count	1
EPS8L1	-1.3975997	1.0347182	-1.3507	0.178	-8.07E-08	count	1
AC007114.2	-1.3569881	0.9297843	-1.4595	0.145	-8.07E-08	count	1
MBOAT1	-1.3569881	0.9297843	-1.4595	0.145	-8.07E-08	count	1
UTP4	-1.3609766	0.5897792	-2.3076	0.0217	-8.06E-08	count	1
GPM6B	-1.3188725	0.6859048	-1.9228	0.0554	-7.97E-08	count	1
ECE1	-1.3193112	0.8382979	-1.5738	0.117	-7.90E-08	count	1
MFSD13A	-1.3180527	1.1716777	-1.1249	0.261	-7.73E-08	count	1

SREBF2	-1.3180527	1.1091952	-1.1883	0.236	-7.72E-08	count	1
MED24	-1.2373194	0.9687682	-1.2772	0.202	-7.45E-08	count	1
RASSF1-AS1	-1.1904931	0.9303222	-1.2797	0.202	-7.40E-08	count	1
TRIM39	-1.1990289	1.1200553	-1.0705	0.285	-7.27E-08	count	1
MYO19	-1.1516788	0.7088294	-1.6248	0.105	-7.18E-08	count	1
TADA1	-1.137833	0.7607258	-1.4957	0.136	-7.06E-08	count	1
AC005523.2	-1.137833	0.7607258	-1.4957	0.136	-7.06E-08	count	1
TSPOAP1	-1.137833	0.7216878	-1.5766	0.116	-7.03E-08	count	1
ELOVL6	-1.137833	0.7216878	-1.5766	0.116	-7.03E-08	count	1
RAB20	-1.0986123	0.9522617	-1.1537	0.249	-6.93E-08	count	1
RNASEH2A	-1.0625706	1.1822428	-0.8988	0.369	-6.66E-08	count	1
COG1	-1.0381146	0.8175868	-1.2697	0.205	-6.60E-08	count	1
BICD2	-1.0036482	0.6985218	-1.4368	0.152	-6.54E-08	count	1
NMD3	-1.0036482	0.6702904	-1.4973	0.135	-6.48E-08	count	1
MLLT11	-1.0024566	0.7278213	-1.3773	0.169	-6.42E-08	count	1
CNEP1R1	-0.9804745	0.6507727	-1.5066	0.133	-6.42E-08	count	1
AL049840.1	-0.3039416	0.5489617	-0.5537	0.58	-6.29E-08	count	1
FYCO1	-0.3039416	0.5976226	-0.5086	0.611	-6.25E-08	count	1
REPS1	-0.9555114	0.5907378	-1.6175	0.107	-6.22E-08	count	1
LMOD3	-0.9329239	0.7769961	-1.2007	0.231	-6.13E-08	count	1
BUB1	-0.9329239	0.7142027	-1.3062	0.192	-6.10E-08	count	1
TBX19	-0.9329239	0.920362	-1.0136	0.312	-6.10E-08	count	1
KCTD12	-0.9023848	1.1447422	-0.7883	0.431	-5.91E-08	count	1
SLC25A12	-0.878374	0.5870885	-1.4962	0.136	-5.90E-08	count	1
C10orf88	-0.9023848	1.0149039	-0.8891	0.375	-5.89E-08	count	1
MCM4	-0.8795465	0.695953	-1.2638	0.207	-5.87E-08	count	1
CARF	-0.8930322	0.8893244	-1.0042	0.316	-5.87E-08	count	1
ZNF211	-0.8599177	0.7478177	-1.1499	0.251	-5.75E-08	count	1
CCP110	-0.8420815	0.6650804	-1.2661	0.206	-5.68E-08	count	1
CTTNBP2NL	-0.8501509	0.8798732	-0.9662	0.335	-5.66E-08	count	1
ZNF562	-0.8420815	0.7859676	-1.0714	0.285	-5.58E-08	count	1
SLC35F2	-0.8226154	0.9052051	-0.9088	0.364	-5.49E-08	count	1
RFX2	-0.8226154	0.9052051	-0.9088	0.364	-5.49E-08	count	1
CRLS1	-0.8226154	0.8690687	-0.9465	0.345	-5.48E-08	count	1
TRIM23	-0.8007583	0.8472163	-0.9452	0.345	-5.40E-08	count	1
DDX51	-19.4716276	2269.72964	-0.0086	0.993	-5.23E-08	count	1
SNHG25	-19.3353596	2582.609819	-0.0075	0.994	-5.15E-08	count	1
UTP20	-19.6013729	1500.108481	-0.0131	0.99	-5.10E-08	count	1
AL645728.1	-0.7323679	0.762583	-0.9604	0.338	-5.08E-08	count	1
C15orf41	-0.7323679	0.5970607	-1.2266	0.221	-5.01E-08	count	1
ZNF559	-0.7256476	0.5520906	-1.3144	0.19	-5.01E-08	count	1
NUP188	-0.7256476	0.7493889	-0.9683	0.334	-4.98E-08	count	1
WDR7	-0.7256476	0.8935014	-0.8121	0.417	-4.97E-08	count	1
FAM135A	-0.7256476	0.8935014	-0.8121	0.417	-4.97E-08	count	1
GGACT	-0.7256476	0.8268916	-0.8776	0.381	-4.96E-08	count	1

GUCY1A1	-19.0990251	2719.394109	-0.007	0.994	-4.93E-08	count	1
TCAF2	-19.2555692	1974.55557	-0.0098	0.992	-4.93E-08	count	1
CRTC1	-18.9227382	2530.252124	-0.0075	0.994	-4.90E-08	count	1
SLC16A6	-18.9227382	2530.252124	-0.0075	0.994	-4.90E-08	count	1
LINC00672	-0.7078998	0.8396955	-0.843	0.4	-4.90E-08	count	1
PANO1	-0.7078998	0.8396955	-0.843	0.4	-4.90E-08	count	1
SLC2A14	-0.7078998	0.8396955	-0.843	0.4	-4.90E-08	count	1
CES2	-0.7078998	0.6540662	-1.0823	0.28	-4.86E-08	count	1
NEMP1	-19.1113435	1905.300436	-0.01	0.992	-4.86E-08	count	1
TMEM185B	-0.7078998	0.7481441	-0.9462	0.345	-4.85E-08	count	1
TNPO2	-0.7078998	1.0595493	-0.6681	0.505	-4.85E-08	count	1
CDC42EP4	-0.7078998	0.992474	-0.7133	0.476	-4.85E-08	count	1
GPATCH3	-0.7078998	0.992474	-0.7133	0.476	-4.85E-08	count	1
AL139393.2	-0.7078998	1.0171308	-0.696	0.487	-4.85E-08	count	1
NPIPB4	-19.0392679	2086.043791	-0.0091	0.993	-4.83E-08	count	1
ABCC10	-19.1155726	2009.753474	-0.0095	0.992	-4.83E-08	count	1
GRK4	-19.1155726	2009.753474	-0.0095	0.992	-4.83E-08	count	1
POMGNT1	-19.1155726	2009.753474	-0.0095	0.992	-4.83E-08	count	1
ZNF232	-19.1155726	2009.753474	-0.0095	0.992	-4.83E-08	count	1
AC005261.1	-19.0246789	2139.047339	-0.0089	0.993	-4.80E-08	count	1
FBXW8	-18.8461958	2413.214989	-0.0078	0.994	-4.78E-08	count	1
ABHD4	-18.8461958	2413.214989	-0.0078	0.994	-4.78E-08	count	1
SSH3	-18.9474518	1778.79246	-0.0107	0.992	-4.77E-08	count	1
NPW	-18.8864994	2410.446831	-0.0078	0.994	-4.77E-08	count	1
DHX33	-18.8723293	2164.556707	-0.0087	0.993	-4.76E-08	count	1
BACH2	-18.8723293	2164.556707	-0.0087	0.993	-4.76E-08	count	1
ATP10A	-18.601161	2178.384365	-0.0085	0.993	-4.75E-08	count	1
MPDU1	-18.601161	2178.384365	-0.0085	0.993	-4.75E-08	count	1
CCDC142	-18.8848715	2022.383327	-0.0093	0.993	-4.74E-08	count	1
EPOP	-18.8992208	1911.964997	-0.0099	0.992	-4.73E-08	count	1
ZNF687	-18.8992208	1911.964997	-0.0099	0.992	-4.73E-08	count	1
NEFM	-18.315711	1693.279311	-0.0108	0.991	-4.72E-08	count	1
SLC31A1	-18.7473959	2280.955709	-0.0082	0.993	-4.72E-08	count	1
AC104986.2	-18.7473959	2280.955709	-0.0082	0.993	-4.72E-08	count	1
ARL6	-18.7473959	2280.955709	-0.0082	0.993	-4.72E-08	count	1
DHTKD1	-18.7473959	2280.955709	-0.0082	0.993	-4.72E-08	count	1
KCNMA1	-18.7473959	2280.955709	-0.0082	0.993	-4.72E-08	count	1
MAP3K21	-18.7473959	2280.955709	-0.0082	0.993	-4.72E-08	count	1
CAVIN1	-18.7562336	2059.012152	-0.0091	0.993	-4.70E-08	count	1
USP43	-18.7562336	2059.012152	-0.0091	0.993	-4.70E-08	count	1
ITPRIPL1	-18.508602	1733.644922	-0.0107	0.991	-4.65E-08	count	1
KIAA1841	-18.508602	1733.644922	-0.0107	0.991	-4.65E-08	count	1
ATP8B4	-0.2168605	0.3706315	-0.5851	0.559	-4.56E-08	count	1
RTKN2	-0.6427386	0.8223109	-0.7816	0.435	-4.49E-08	count	1
PCDH9	-0.635639	0.9843572	-0.6457	0.519	-4.44E-08	count	1

INPPL1	-0.6295027	0.716308	-0.8788	0.38	-4.44E-08	count	1
PLEKHA8	-0.5948222	0.7889023	-0.754	0.451	-4.21E-08	count	1
ACAD10	-0.5948222	0.8993865	-0.6614	0.509	-4.21E-08	count	1
PAN2	-0.5948222	0.9430664	-0.6307	0.529	-4.20E-08	count	1
FAM239A	-0.5948222	0.9430664	-0.6307	0.529	-4.20E-08	count	1
PIK3R3	-0.5948222	0.8805533	-0.6755	0.5	-4.19E-08	count	1
ZNF501	-0.5500463	0.7272346	-0.7564	0.45	-3.96E-08	count	1
MAP2K5	-2.2364453	0.7846915	-2.8501	0.0046	-3.96E-08	count	1
MEF2C	-0.1877838	0.7001476	-0.2682	0.789	-3.96E-08	count	1
GPR89B	-0.5500463	0.7686701	-0.7156	0.475	-3.94E-08	count	1
ZNF589	-0.5500463	0.7143093	-0.77	0.442	-3.94E-08	count	1
KAT2B	-0.5500463	0.6633596	-0.8292	0.408	-3.93E-08	count	1
FANCM	-0.5500463	0.9127212	-0.6026	0.547	-3.93E-08	count	1
AMIGO2	-0.5274773	0.662997	-0.7956	0.427	-3.81E-08	count	1
PHOSPHO2	-0.5259058	0.6399347	-0.8218	0.412	-3.78E-08	count	1
ZNF445	-0.5259058	0.8571521	-0.6136	0.54	-3.77E-08	count	1
TRMT10A	-2.0541237	0.7745967	-2.6519	0.0084	-3.76E-08	count	1
DTHD1	-0.510784	0.5756921	-0.8873	0.376	-3.69E-08	count	1
ZBTB33	-1.8309802	0.8740074	-2.0949	0.037	-3.53E-08	count	1
THTPA	-1.8309802	0.8169689	-2.2412	0.0257	-3.52E-08	count	1
WDR25	-0.4591644	0.7397176	-0.6207	0.535	-3.37E-08	count	1
FANCD2	-0.4591644	0.8714231	-0.5269	0.599	-3.35E-08	count	1
PPM1F	-0.4591644	0.9673509	-0.4747	0.635	-3.35E-08	count	1
BRPF1	-0.4591644	1.0076009	-0.4557	0.649	-3.34E-08	count	1
TTC22	-0.4591644	1.0076009	-0.4557	0.649	-3.34E-08	count	1
MCM3AP	-0.4591644	1.0076009	-0.4557	0.649	-3.34E-08	count	1
LTBP2	-0.4591644	1.0836252	-0.4237	0.672	-3.34E-08	count	1
LY75	-0.4591644	1.0836252	-0.4237	0.672	-3.34E-08	count	1
CYTH3	-0.4591644	1.0836252	-0.4237	0.672	-3.34E-08	count	1
ANKRD52	-0.4548283	0.7849187	-0.5795	0.563	-3.33E-08	count	1
AACS	-0.4548283	0.7485784	-0.6076	0.544	-3.32E-08	count	1
RBL1	-0.4446858	0.567918	-0.783	0.434	-3.26E-08	count	1
RAB40C	-0.4446858	0.6186405	-0.7188	0.473	-3.26E-08	count	1
PTGR2	-0.4446858	0.8819171	-0.5042	0.614	-3.26E-08	count	1
DCUN1D3	-0.4446858	0.8819171	-0.5042	0.614	-3.26E-08	count	1
KIF18A	-0.4446858	0.6531553	-0.6808	0.496	-3.25E-08	count	1
NDUFA7	-0.4446858	0.6531553	-0.6808	0.496	-3.25E-08	count	1
ADAT3	-0.4446858	0.6531553	-0.6808	0.496	-3.25E-08	count	1
ZNF197	-0.4446858	0.5866309	-0.758	0.449	-3.25E-08	count	1
GLCE	-0.4446858	0.8003771	-0.5556	0.579	-3.25E-08	count	1
CYP2U1	-1.5432981	0.8046504	-1.918	0.056	-3.16E-08	count	1
ATN1	-1.5432981	0.8046504	-1.918	0.056	-3.16E-08	count	1
PRCD	-1.5432981	0.8046504	-1.918	0.056	-3.16E-08	count	1
LINC01115	-1.5432981	0.8046504	-1.918	0.056	-3.16E-08	count	1
NLRX1	-1.3862944	0.7888106	-1.7574	0.0798	-2.97E-08	count	1

TPX2	-1.3975997	0.84564	-1.6527	0.0994	-2.96E-08	count	1
INTS1	-1.3975997	0.937072	-1.4915	0.137	-2.96E-08	count	1
SEC31B	-1.3862944	0.5577733	-2.4854	0.0134	-2.94E-08	count	1
RDH13	-0.3905682	0.5427577	-0.7196	0.472	-2.90E-08	count	1
TNFSF11	-0.1357302	0.5700014	-0.2381	0.812	-2.90E-08	count	1
ZNF335	-0.3778405	0.6070847	-0.6224	0.534	-2.81E-08	count	1
ZNF557	-0.3778405	0.611963	-0.6174	0.537	-2.81E-08	count	1
FGFBP2	-1.2373194	0.9037096	-1.3692	0.172	-2.73E-08	count	1
DIP2B	-0.3577913	1.0163326	-0.352	0.725	-2.67E-08	count	1
AP5B1	-0.3577913	1.0163326	-0.352	0.725	-2.67E-08	count	1
ZNF554	-1.1631508	0.6022079	-1.9315	0.0543	-2.61E-08	count	1
DUSP16	-1.1631508	0.496904	-2.3408	0.0198	-2.61E-08	count	1
BOK	-1.137833	0.9001029	-1.2641	0.207	-2.57E-08	count	1
FAM19A2	-1.137833	0.9001029	-1.2641	0.207	-2.57E-08	count	1
PRMT5-AS1	-1.137833	0.9001029	-1.2641	0.207	-2.57E-08	count	1
C21orf58	-1.137833	0.9001029	-1.2641	0.207	-2.57E-08	count	1
AL050341.2	-1.137833	0.9001029	-1.2641	0.207	-2.57E-08	count	1
AC013400.1	-1.137833	0.9001029	-1.2641	0.207	-2.57E-08	count	1
ZKSCAN8	-1.137833	0.9001029	-1.2641	0.207	-2.57E-08	count	1
IER3-AS1	-1.137833	0.9001029	-1.2641	0.207	-2.57E-08	count	1
POLH	-1.137833	0.9001029	-1.2641	0.207	-2.57E-08	count	1
TRBV28	-1.137833	0.9001029	-1.2641	0.207	-2.57E-08	count	1
AMMECR1	-1.137833	0.9001029	-1.2641	0.207	-2.57E-08	count	1
SERINC2	-1.137833	0.9001029	-1.2641	0.207	-2.57E-08	count	1
HIST2H2BE	-1.137833	0.9001029	-1.2641	0.207	-2.57E-08	count	1
AJM1	-1.137833	0.9001029	-1.2641	0.207	-2.57E-08	count	1
EPG5	-1.137833	0.9001029	-1.2641	0.207	-2.57E-08	count	1
DERL3	-1.137833	0.9001029	-1.2641	0.207	-2.57E-08	count	1
PIGA	-1.137833	0.9001029	-1.2641	0.207	-2.57E-08	count	1
FOLR2	-1.137833	0.7607258	-1.4957	0.136	-2.57E-08	count	1
UBA7	-1.137833	0.7607258	-1.4957	0.136	-2.57E-08	count	1
AMOTL1	-1.137833	0.7607258	-1.4957	0.136	-2.57E-08	count	1
SCMH1	-0.3356409	0.904907	-0.3709	0.711	-2.52E-08	count	1
SETD6	-0.3290647	0.702199	-0.4686	0.64	-2.48E-08	count	1
TRIM47	-0.3208124	0.7712308	-0.416	0.678	-2.42E-08	count	1
NCAPD2	-0.1107711	0.7873221	-0.1407	0.888	-2.38E-08	count	1
YARS2	-0.3134729	0.5404529	-0.58	0.562	-2.37E-08	count	1
SLC35A3	-0.1090663	0.4768609	-0.2287	0.819	-2.34E-08	count	1
LOH12CR2	-0.9329239	1.1052674	-0.8441	0.399	-2.23E-08	count	1
TMEM63B	-0.9329239	1.1052674	-0.8441	0.399	-2.23E-08	count	1
STK33	-0.9329239	1.1052674	-0.8441	0.399	-2.23E-08	count	1
ADAMTSL4	-0.9329239	1.1052674	-0.8441	0.399	-2.23E-08	count	1
LMLN	-0.9329239	1.1052674	-0.8441	0.399	-2.23E-08	count	1
ARHGAP5-AS1	-0.9329239	1.1052674	-0.8441	0.399	-2.23E-08	count	1
TMEM98	-0.9329239	1.1052674	-0.8441	0.399	-2.23E-08	count	1

CDK2AP1	-0.9329239	1.1052674	-0.8441	0.399	-2.23E-08	count	1
Z68871.1	-0.9329239	1.1052674	-0.8441	0.399	-2.23E-08	count	1
GOLGA6L10	-0.9329239	1.1052674	-0.8441	0.399	-2.23E-08	count	1
SEMA4F	-0.9329239	1.1052674	-0.8441	0.399	-2.23E-08	count	1
AC010969.2	-0.9329239	0.920362	-1.0136	0.312	-2.22E-08	count	1
NIPAL2	-0.9329239	0.920362	-1.0136	0.312	-2.22E-08	count	1
PRKAB2	-0.280039	0.8927699	-0.3137	0.754	-2.13E-08	count	1
DAAM1	-0.280039	0.8927699	-0.3137	0.754	-2.13E-08	count	1
ZNF592	-0.280039	0.9267946	-0.3022	0.763	-2.13E-08	count	1
KIF13A	-0.280039	0.9267946	-0.3022	0.763	-2.13E-08	count	1
TFE3	-0.280039	0.9267946	-0.3022	0.763	-2.13E-08	count	1
ZNF490	-0.280039	0.9267946	-0.3022	0.763	-2.13E-08	count	1
CNN3	-0.280039	0.9267946	-0.3022	0.763	-2.13E-08	count	1
AC025181.2	-0.280039	0.9267946	-0.3022	0.763	-2.13E-08	count	1
LINC00299	-0.280039	0.9267946	-0.3022	0.763	-2.13E-08	count	1
SEMA7A	-0.280039	0.9267946	-0.3022	0.763	-2.13E-08	count	1
HCFC1	-0.8754687	0.5367177	-1.6312	0.104	-2.12E-08	count	1
NT5M	-0.8754687	0.6728112	-1.3012	0.194	-2.12E-08	count	1
ZNF165	-0.8501509	0.6341072	-1.3407	0.181	-2.08E-08	count	1
BAIAP3	-0.8501509	0.7763435	-1.0951	0.274	-2.07E-08	count	1
MCM9	-0.2672575	0.6258672	-0.427	0.67	-2.06E-08	count	1
AL731577.1	-0.7078998	0.8396955	-0.843	0.4	-1.78E-08	count	1
ICAM4	-0.7078998	1.1102959	-0.6376	0.524	-1.78E-08	count	1
KHK	-0.7078998	1.1102959	-0.6376	0.524	-1.78E-08	count	1
UBOX5	-0.7078998	1.1102959	-0.6376	0.524	-1.78E-08	count	1
NGFR	-0.7078998	1.1102959	-0.6376	0.524	-1.78E-08	count	1
BRICD5	-0.7078998	1.1102959	-0.6376	0.524	-1.78E-08	count	1
ZC3H11A	-0.7078998	1.1102959	-0.6376	0.524	-1.78E-08	count	1
IGHG2	-20.1297638	2614.358201	-0.0077	0.994	-1.77E-08	count	1
IGHG3	-20.1297638	2614.358201	-0.0077	0.994	-1.77E-08	count	1
NR2F6	-20.1297638	2614.358201	-0.0077	0.994	-1.77E-08	count	1
TSPAN6	-0.6931472	0.6423818	-1.079	0.281	-1.77E-08	count	1
GATA6	-19.927454	3151.898989	-0.0063	0.995	-1.75E-08	count	1
VPS54	-19.9324507	2814.493507	-0.0071	0.994	-1.75E-08	count	1
MS4A7	-19.9324507	2814.493507	-0.0071	0.994	-1.75E-08	count	1
AC087294.1	-19.9324507	2814.493507	-0.0071	0.994	-1.75E-08	count	1
AC040977.1	-19.9324507	2814.493507	-0.0071	0.994	-1.75E-08	count	1
CCDC102B	-19.9324507	2814.493507	-0.0071	0.994	-1.75E-08	count	1
CDHR1	-19.9324507	2814.493507	-0.0071	0.994	-1.75E-08	count	1
LINC02384	-19.9324507	2814.493507	-0.0071	0.994	-1.75E-08	count	1
TCF20	-19.9324507	2814.493507	-0.0071	0.994	-1.75E-08	count	1
TTLL4	-19.9324507	2814.493507	-0.0071	0.994	-1.75E-08	count	1
ZNF738	-19.942652	2432.814453	-0.0082	0.993	-1.74E-08	count	1
TNFRSF11A	-19.3644529	2544.321406	-0.0076	0.994	-1.73E-08	count	1
GOLT1A	-19.6665497	3520.894373	-0.0056	0.996	-1.73E-08	count	1

TTC21A	-19.6665497	3520.894373	-0.0056	0.996	-1.73E-08	count	1
CENPP	-19.6665497	3520.894373	-0.0056	0.996	-1.73E-08	count	1
CTNNAL1	-19.6665497	3520.894373	-0.0056	0.996	-1.73E-08	count	1
STAC3	-19.6665497	3520.894373	-0.0056	0.996	-1.73E-08	count	1
TPSAB1	-19.6665497	3520.894373	-0.0056	0.996	-1.73E-08	count	1
CST6	-19.6665497	3520.894373	-0.0056	0.996	-1.73E-08	count	1
IL17RC	-19.6665497	3520.894373	-0.0056	0.996	-1.73E-08	count	1
AC095055.1	-19.6665497	3520.894373	-0.0056	0.996	-1.73E-08	count	1
MTRNR2L6	-19.6665497	3520.894373	-0.0056	0.996	-1.73E-08	count	1
IL33	-19.6665497	3520.894373	-0.0056	0.996	-1.73E-08	count	1
SNCG	-19.6665497	3520.894373	-0.0056	0.996	-1.73E-08	count	1
GID4	-19.6665497	3520.894373	-0.0056	0.996	-1.73E-08	count	1
GNG11	-19.6665497	3520.894373	-0.0056	0.996	-1.73E-08	count	1
LINC01588	-0.2239144	0.6901196	-0.3245	0.746	-1.72E-08	count	1
CHI3L2	-0.2239144	0.6103719	-0.3668	0.714	-1.72E-08	count	1
SLC25A25	-0.2239144	0.7902177	-0.2834	0.777	-1.72E-08	count	1
AL035661.1	-19.6791397	2619.245003	-0.0075	0.994	-1.72E-08	count	1
FZD8	-19.6791397	2619.245003	-0.0075	0.994	-1.72E-08	count	1
LINC00294	-19.6791397	2619.245003	-0.0075	0.994	-1.72E-08	count	1
KDM4A	-19.6791397	2619.245003	-0.0075	0.994	-1.72E-08	count	1
ADHFE1	-19.6791397	2619.245003	-0.0075	0.994	-1.72E-08	count	1
CALCRL	-19.6791397	2619.245003	-0.0075	0.994	-1.72E-08	count	1
CCL21	-19.6791397	2619.245003	-0.0075	0.994	-1.72E-08	count	1
DENND5A	-19.6791397	2619.245003	-0.0075	0.994	-1.72E-08	count	1
CTHRC1	-19.6791397	2619.245003	-0.0075	0.994	-1.72E-08	count	1
REXO5	-19.6791397	2619.245003	-0.0075	0.994	-1.72E-08	count	1
TM4SF1	-19.6791397	2619.245003	-0.0075	0.994	-1.72E-08	count	1
CXCL12	-19.6791397	2619.245003	-0.0075	0.994	-1.72E-08	count	1
H2AFY2	-19.5854843	3433.783865	-0.0057	0.995	-1.72E-08	count	1
QPRT	-19.5854843	3433.783865	-0.0057	0.995	-1.72E-08	count	1
ZNF703	-19.5854843	3433.783865	-0.0057	0.995	-1.72E-08	count	1
AGAP9	-19.5854843	3433.783865	-0.0057	0.995	-1.72E-08	count	1
ZNF473	-19.5854843	3433.783865	-0.0057	0.995	-1.72E-08	count	1
LRRC8C	-19.5854843	3433.783865	-0.0057	0.995	-1.72E-08	count	1
TTC24	-19.5854843	3433.783865	-0.0057	0.995	-1.72E-08	count	1
SLC45A4	-19.5854843	3433.783865	-0.0057	0.995	-1.72E-08	count	1
LPP-AS2	-19.5854843	3433.783865	-0.0057	0.995	-1.72E-08	count	1
DACT1	-19.5854843	3433.783865	-0.0057	0.995	-1.72E-08	count	1
CABLES2	-19.5854843	3433.783865	-0.0057	0.995	-1.72E-08	count	1
MPV17L	-19.5854843	3433.783865	-0.0057	0.995	-1.72E-08	count	1
ARRDC5	-19.6855591	2009.884566	-0.0098	0.992	-1.71E-08	count	1
AC244090.1	-19.5891294	2678.600699	-0.0073	0.994	-1.71E-08	count	1
AC108718.1	-19.0959903	2843.648266	-0.0067	0.995	-1.71E-08	count	1
AL354707.1	-19.0959903	2843.648266	-0.0067	0.995	-1.71E-08	count	1
AC136475.3	-19.0959903	2843.648266	-0.0067	0.995	-1.71E-08	count	1

A2M-AS1	-19.0959903	2843.648266	-0.0067	0.995	-1.71E-08	count	1
AC048341.2	-19.0959903	2843.648266	-0.0067	0.995	-1.71E-08	count	1
KIF1B	-0.2215423	0.5669117	-0.3908	0.696	-1.71E-08	count	1
PPRC1	-19.3312558	3091.416169	-0.0063	0.995	-1.70E-08	count	1
PFKM	-19.3312558	3091.416169	-0.0063	0.995	-1.70E-08	count	1
DNMBP	-19.3312558	3091.416169	-0.0063	0.995	-1.70E-08	count	1
FLCN	-19.3312558	3091.416169	-0.0063	0.995	-1.70E-08	count	1
ZNF446	-19.1854342	2109.967653	-0.0091	0.993	-1.70E-08	count	1
AL390728.5	-19.2950546	2924.043781	-0.0066	0.995	-1.70E-08	count	1
CD302	-19.2950546	2924.043781	-0.0066	0.995	-1.70E-08	count	1
PDE1C	-19.2950546	2924.043781	-0.0066	0.995	-1.70E-08	count	1
AQP11	-19.2950546	2924.043781	-0.0066	0.995	-1.70E-08	count	1
OTOF	-19.2950546	2924.043781	-0.0066	0.995	-1.70E-08	count	1
LINC02580	-19.2950546	2924.043781	-0.0066	0.995	-1.70E-08	count	1
NCKAP1	-19.2950546	2924.043781	-0.0066	0.995	-1.70E-08	count	1
AL161729.1	-19.2950546	2924.043781	-0.0066	0.995	-1.70E-08	count	1
ITGA7	-19.2950546	2924.043781	-0.0066	0.995	-1.70E-08	count	1
IGHV4-28	-19.2950546	2924.043781	-0.0066	0.995	-1.70E-08	count	1
BAIAP2	-19.2950546	2924.043781	-0.0066	0.995	-1.70E-08	count	1
AL391244.3	-19.2950546	2924.043781	-0.0066	0.995	-1.70E-08	count	1
TMEM201	-19.2950546	2924.043781	-0.0066	0.995	-1.70E-08	count	1
PLA2G2D	-19.2950546	2924.043781	-0.0066	0.995	-1.70E-08	count	1
AMY2B	-19.2950546	2924.043781	-0.0066	0.995	-1.70E-08	count	1
AL355488.1	-19.2950546	2924.043781	-0.0066	0.995	-1.70E-08	count	1
PIFO	-19.2950546	2924.043781	-0.0066	0.995	-1.70E-08	count	1
DNAH6	-19.2950546	2924.043781	-0.0066	0.995	-1.70E-08	count	1
LINC01825	-19.2950546	2924.043781	-0.0066	0.995	-1.70E-08	count	1
HDAC11	-19.2950546	2924.043781	-0.0066	0.995	-1.70E-08	count	1
GAP43	-19.2950546	2924.043781	-0.0066	0.995	-1.70E-08	count	1
LIMCH1	-19.2950546	2924.043781	-0.0066	0.995	-1.70E-08	count	1
EBF1	-19.2950546	2924.043781	-0.0066	0.995	-1.70E-08	count	1
DDR1	-19.2950546	2924.043781	-0.0066	0.995	-1.70E-08	count	1
GRB10	-19.2950546	2924.043781	-0.0066	0.995	-1.70E-08	count	1
SRRM3	-19.2950546	2924.043781	-0.0066	0.995	-1.70E-08	count	1
CLDN15	-19.2950546	2924.043781	-0.0066	0.995	-1.70E-08	count	1
AC009275.1	-19.2950546	2924.043781	-0.0066	0.995	-1.70E-08	count	1
TRMT12	-19.2950546	2924.043781	-0.0066	0.995	-1.70E-08	count	1
PCSK5	-19.2950546	2924.043781	-0.0066	0.995	-1.70E-08	count	1
ANKS6	-19.2950546	2924.043781	-0.0066	0.995	-1.70E-08	count	1
DNAJC25-GNG10	-19.2950546	2924.043781	-0.0066	0.995	-1.70E-08	count	1
AL138781.1	-19.2950546	2924.043781	-0.0066	0.995	-1.70E-08	count	1
EGFL7	-19.2950546	2924.043781	-0.0066	0.995	-1.70E-08	count	1
FRMD4A	-19.2950546	2924.043781	-0.0066	0.995	-1.70E-08	count	1
ARHGAP22	-19.2950546	2924.043781	-0.0066	0.995	-1.70E-08	count	1
DNA2	-19.2950546	2924.043781	-0.0066	0.995	-1.70E-08	count	1

CD163	-19.2950546	2924.043781	-0.0066	0.995	-1.70E-08	count	1
LUM	-19.2950546	2924.043781	-0.0066	0.995	-1.70E-08	count	1
AL359232.1	-19.2950546	2924.043781	-0.0066	0.995	-1.70E-08	count	1
IGHV1-18	-19.2950546	2924.043781	-0.0066	0.995	-1.70E-08	count	1
GOLGA8H	-19.2950546	2924.043781	-0.0066	0.995	-1.70E-08	count	1
MNS1	-19.2950546	2924.043781	-0.0066	0.995	-1.70E-08	count	1
KDM8	-19.2950546	2924.043781	-0.0066	0.995	-1.70E-08	count	1
MMP2	-19.2950546	2924.043781	-0.0066	0.995	-1.70E-08	count	1
NOL3	-19.2950546	2924.043781	-0.0066	0.995	-1.70E-08	count	1
PRELID3A	-19.2950546	2924.043781	-0.0066	0.995	-1.70E-08	count	1
ZNF442	-19.2950546	2924.043781	-0.0066	0.995	-1.70E-08	count	1
AC006213.1	-19.2950546	2924.043781	-0.0066	0.995	-1.70E-08	count	1
AURKC	-19.2950546	2924.043781	-0.0066	0.995	-1.70E-08	count	1
ZNF837	-19.2950546	2924.043781	-0.0066	0.995	-1.70E-08	count	1
RSPH1	-19.2950546	2924.043781	-0.0066	0.995	-1.70E-08	count	1
AC007325.4	-19.2950546	2924.043781	-0.0066	0.995	-1.70E-08	count	1
AL162741.1	-19.2950546	2924.043781	-0.0066	0.995	-1.70E-08	count	1
SMPDL3B	-19.2950546	2924.043781	-0.0066	0.995	-1.70E-08	count	1
F3	-19.2950546	2924.043781	-0.0066	0.995	-1.70E-08	count	1
NBPF26	-19.2950546	2924.043781	-0.0066	0.995	-1.70E-08	count	1
ANKRD35	-19.2950546	2924.043781	-0.0066	0.995	-1.70E-08	count	1
FALEC	-19.2950546	2924.043781	-0.0066	0.995	-1.70E-08	count	1
SLC16A14	-19.2950546	2924.043781	-0.0066	0.995	-1.70E-08	count	1
CACNA2D2	-19.2950546	2924.043781	-0.0066	0.995	-1.70E-08	count	1
SFRP2	-19.2950546	2924.043781	-0.0066	0.995	-1.70E-08	count	1
RAB23	-19.2950546	2924.043781	-0.0066	0.995	-1.70E-08	count	1
NT5E	-19.2950546	2924.043781	-0.0066	0.995	-1.70E-08	count	1
AL023806.1	-19.2950546	2924.043781	-0.0066	0.995	-1.70E-08	count	1
AC124242.1	-19.2950546	2924.043781	-0.0066	0.995	-1.70E-08	count	1
ALDH1A1	-19.2950546	2924.043781	-0.0066	0.995	-1.70E-08	count	1
ZNF79	-19.2950546	2924.043781	-0.0066	0.995	-1.70E-08	count	1
NCR3LG1	-19.2950546	2924.043781	-0.0066	0.995	-1.70E-08	count	1
RIN1	-19.2950546	2924.043781	-0.0066	0.995	-1.70E-08	count	1
PTPN20	-19.2950546	2924.043781	-0.0066	0.995	-1.70E-08	count	1
MORN4	-19.2950546	2924.043781	-0.0066	0.995	-1.70E-08	count	1
TWNK	-19.2950546	2924.043781	-0.0066	0.995	-1.70E-08	count	1
FGF14-AS2	-19.2950546	2924.043781	-0.0066	0.995	-1.70E-08	count	1
EARS2	-19.2950546	2924.043781	-0.0066	0.995	-1.70E-08	count	1
FBXL19-AS1	-19.2950546	2924.043781	-0.0066	0.995	-1.70E-08	count	1
AL034549.1	-19.2950546	2924.043781	-0.0066	0.995	-1.70E-08	count	1
GNA15	-19.2950546	2924.043781	-0.0066	0.995	-1.70E-08	count	1
ZNF730	-19.2950546	2924.043781	-0.0066	0.995	-1.70E-08	count	1
CDC45	-19.2950546	2924.043781	-0.0066	0.995	-1.70E-08	count	1
IGLC7	-19.2950546	2924.043781	-0.0066	0.995	-1.70E-08	count	1
ATP5MGL	-19.2950546	2924.043781	-0.0066	0.995	-1.70E-08	count	1

PINK1-AS	-19.2950546	2924.043781	-0.0066	0.995	-1.70E-08	count	1
LIN9	-19.2950546	2924.043781	-0.0066	0.995	-1.70E-08	count	1
AC012360.1	-19.2950546	2924.043781	-0.0066	0.995	-1.70E-08	count	1
CRYBG3	-19.2950546	2924.043781	-0.0066	0.995	-1.70E-08	count	1
LEKR1	-19.2950546	2924.043781	-0.0066	0.995	-1.70E-08	count	1
AC097460.1	-19.2950546	2924.043781	-0.0066	0.995	-1.70E-08	count	1
AC093297.2	-19.2950546	2924.043781	-0.0066	0.995	-1.70E-08	count	1
ZFP2	-19.2950546	2924.043781	-0.0066	0.995	-1.70E-08	count	1
AC008610.1	-19.2950546	2924.043781	-0.0066	0.995	-1.70E-08	count	1
HIST1H4J	-19.2950546	2924.043781	-0.0066	0.995	-1.70E-08	count	1
LAMB1	-19.2950546	2924.043781	-0.0066	0.995	-1.70E-08	count	1
ATG9B	-19.2950546	2924.043781	-0.0066	0.995	-1.70E-08	count	1
ZNF252P-AS1	-19.2950546	2924.043781	-0.0066	0.995	-1.70E-08	count	1
FRMPD1	-19.2950546	2924.043781	-0.0066	0.995	-1.70E-08	count	1
ANGPTL2	-19.2950546	2924.043781	-0.0066	0.995	-1.70E-08	count	1
FAM129B	-19.2950546	2924.043781	-0.0066	0.995	-1.70E-08	count	1
STKLD1	-19.2950546	2924.043781	-0.0066	0.995	-1.70E-08	count	1
NECTIN1	-19.2950546	2924.043781	-0.0066	0.995	-1.70E-08	count	1
JMJD1C-AS1	-19.2950546	2924.043781	-0.0066	0.995	-1.70E-08	count	1
AC079601.1	-19.2950546	2924.043781	-0.0066	0.995	-1.70E-08	count	1
POSTN	-19.2950546	2924.043781	-0.0066	0.995	-1.70E-08	count	1
ATP11A	-19.2950546	2924.043781	-0.0066	0.995	-1.70E-08	count	1
AC007216.2	-19.2950546	2924.043781	-0.0066	0.995	-1.70E-08	count	1
FGF11	-19.2950546	2924.043781	-0.0066	0.995	-1.70E-08	count	1
ADAM11	-19.2950546	2924.043781	-0.0066	0.995	-1.70E-08	count	1
HM13-AS1	-19.2950546	2924.043781	-0.0066	0.995	-1.70E-08	count	1
ZNF341	-19.2950546	2924.043781	-0.0066	0.995	-1.70E-08	count	1
SLC12A5-AS1	-19.2950546	2924.043781	-0.0066	0.995	-1.70E-08	count	1
AC011498.1	-19.2950546	2924.043781	-0.0066	0.995	-1.70E-08	count	1
CD79A	-19.2950546	2924.043781	-0.0066	0.995	-1.70E-08	count	1
ZNF222	-19.2950546	2924.043781	-0.0066	0.995	-1.70E-08	count	1
TTYH1	-19.2950546	2924.043781	-0.0066	0.995	-1.70E-08	count	1
AL008635.1	-19.2950546	2924.043781	-0.0066	0.995	-1.70E-08	count	1
FP565260.1	-19.2950546	2924.043781	-0.0066	0.995	-1.70E-08	count	1
AC239800.2	-19.2950546	2924.043781	-0.0066	0.995	-1.70E-08	count	1
FCRL5	-19.2950546	2924.043781	-0.0066	0.995	-1.70E-08	count	1
GAREM2	-19.2950546	2924.043781	-0.0066	0.995	-1.70E-08	count	1
AL662797.1	-19.2950546	2924.043781	-0.0066	0.995	-1.70E-08	count	1
TCIM	-19.2950546	2924.043781	-0.0066	0.995	-1.70E-08	count	1
TSPYL5	-19.2950546	2924.043781	-0.0066	0.995	-1.70E-08	count	1
RACGAP1	-19.2950546	2924.043781	-0.0066	0.995	-1.70E-08	count	1
MYRFL	-19.2950546	2924.043781	-0.0066	0.995	-1.70E-08	count	1
AC008149.1	-19.2950546	2924.043781	-0.0066	0.995	-1.70E-08	count	1
LINC00562	-19.2950546	2924.043781	-0.0066	0.995	-1.70E-08	count	1
ZSCAN2	-19.2950546	2924.043781	-0.0066	0.995	-1.70E-08	count	1

AC009021.1	-19.2950546	2924.043781	-0.0066	0.995	-1.70E-08	count	1
AC011446.2	-19.2950546	2924.043781	-0.0066	0.995	-1.70E-08	count	1
ZNF671	-19.2950546	2924.043781	-0.0066	0.995	-1.70E-08	count	1
CENPM	-19.2950546	2924.043781	-0.0066	0.995	-1.70E-08	count	1
AP000692.2	-19.2950546	2924.043781	-0.0066	0.995	-1.70E-08	count	1
AL590666.2	-19.2950546	2924.043781	-0.0066	0.995	-1.70E-08	count	1
C1orf74	-19.2950546	2924.043781	-0.0066	0.995	-1.70E-08	count	1
CENPO	-19.2950546	2924.043781	-0.0066	0.995	-1.70E-08	count	1
CACNB4	-19.2950546	2924.043781	-0.0066	0.995	-1.70E-08	count	1
NAALADL2	-19.2950546	2924.043781	-0.0066	0.995	-1.70E-08	count	1
SNX24	-19.2950546	2924.043781	-0.0066	0.995	-1.70E-08	count	1
IGIP	-19.2950546	2924.043781	-0.0066	0.995	-1.70E-08	count	1
AL137003.2	-19.2950546	2924.043781	-0.0066	0.995	-1.70E-08	count	1
GPR146	-19.2950546	2924.043781	-0.0066	0.995	-1.70E-08	count	1
ZDHHC9	-19.2950546	2924.043781	-0.0066	0.995	-1.70E-08	count	1
MPDZ	-19.2950546	2924.043781	-0.0066	0.995	-1.70E-08	count	1
TMEM268	-19.2950546	2924.043781	-0.0066	0.995	-1.70E-08	count	1
MAMDC4	-19.2950546	2924.043781	-0.0066	0.995	-1.70E-08	count	1
GALNT4	-19.2950546	2924.043781	-0.0066	0.995	-1.70E-08	count	1
AL157955.1	-19.2950546	2924.043781	-0.0066	0.995	-1.70E-08	count	1
FEM1A	-19.2950546	2924.043781	-0.0066	0.995	-1.70E-08	count	1
ZNF471	-19.2950546	2924.043781	-0.0066	0.995	-1.70E-08	count	1
PAXBP1-AS1	-19.2950546	2924.043781	-0.0066	0.995	-1.70E-08	count	1
ARAP3	-19.2950546	2924.043781	-0.0066	0.995	-1.70E-08	count	1
AC007349.2	-19.2950546	2924.043781	-0.0066	0.995	-1.70E-08	count	1
AC025423.1	-19.2950546	2924.043781	-0.0066	0.995	-1.70E-08	count	1
DECR2	-19.2950546	2924.043781	-0.0066	0.995	-1.70E-08	count	1
NETO2	-19.2950546	2924.043781	-0.0066	0.995	-1.70E-08	count	1
AC092747.4	-19.2950546	2924.043781	-0.0066	0.995	-1.70E-08	count	1
ACVRL1	-19.2950546	2924.043781	-0.0066	0.995	-1.70E-08	count	1
INSL3	-19.2950546	2924.043781	-0.0066	0.995	-1.70E-08	count	1
ZNF607	-19.2950546	2924.043781	-0.0066	0.995	-1.70E-08	count	1
TMCO6	-19.2243508	1762.637602	-0.0109	0.991	-1.70E-08	count	1
MRC2	-19.297914	2049.332158	-0.0094	0.992	-1.70E-08	count	1
AC010247.2	-19.297914	2049.332158	-0.0094	0.992	-1.70E-08	count	1
EFNA5	-18.9818636	2685.923031	-0.0071	0.994	-1.70E-08	count	1
ZNF813	-18.9818636	2685.923031	-0.0071	0.994	-1.70E-08	count	1
PLD1	-18.9818636	2685.923031	-0.0071	0.994	-1.70E-08	count	1
UNC119B	-18.9818636	2685.923031	-0.0071	0.994	-1.70E-08	count	1
MAPK8IP1	-19.030773	2153.348379	-0.0088	0.993	-1.69E-08	count	1
AMN	-19.030773	2153.348379	-0.0088	0.993	-1.69E-08	count	1
CXCL9	-19.0871411	2703.28341	-0.0071	0.994	-1.69E-08	count	1
DPPA4	-19.0871411	2703.28341	-0.0071	0.994	-1.69E-08	count	1
PDK4	-19.0871411	2703.28341	-0.0071	0.994	-1.69E-08	count	1
SLC31A2	-19.0871411	2703.28341	-0.0071	0.994	-1.69E-08	count	1

DHX57	-19.0871411	2703.28341	-0.0071	0.994	-1.69E-08	count	1
SETD7	-19.0871411	2703.28341	-0.0071	0.994	-1.69E-08	count	1
ZWINT	-19.0871411	2703.28341	-0.0071	0.994	-1.69E-08	count	1
PKIA	-19.0871411	2703.28341	-0.0071	0.994	-1.69E-08	count	1
NBPF1	-18.7865181	2435.986312	-0.0077	0.994	-1.69E-08	count	1
DNM3	-18.7865181	2435.986312	-0.0077	0.994	-1.69E-08	count	1
CR1	-18.7865181	2435.986312	-0.0077	0.994	-1.69E-08	count	1
RPS6KB2-AS1	-18.7865181	2435.986312	-0.0077	0.994	-1.69E-08	count	1
CHRNA5	-18.7865181	2435.986312	-0.0077	0.994	-1.69E-08	count	1
ZNF710	-18.7865181	2435.986312	-0.0077	0.994	-1.69E-08	count	1
WISP2	-18.7865181	2435.986312	-0.0077	0.994	-1.69E-08	count	1
SCN1B	-18.7865181	2435.986312	-0.0077	0.994	-1.69E-08	count	1
KLHL3	-18.7865181	2435.986312	-0.0077	0.994	-1.69E-08	count	1
KDM4D	-18.7865181	2435.986312	-0.0077	0.994	-1.69E-08	count	1
AL122035.1	-18.7865181	2435.986312	-0.0077	0.994	-1.69E-08	count	1
AL691403.2	-18.7865181	2435.986312	-0.0077	0.994	-1.69E-08	count	1
BMF	-18.7865181	2435.986312	-0.0077	0.994	-1.69E-08	count	1
C20orf194	-18.7865181	2435.986312	-0.0077	0.994	-1.69E-08	count	1
SLCO4A1	-18.7865181	2435.986312	-0.0077	0.994	-1.69E-08	count	1
C7	-18.7865181	2435.986312	-0.0077	0.994	-1.69E-08	count	1
LINC00342	-18.7865181	2435.986312	-0.0077	0.994	-1.69E-08	count	1
STAB1	-18.7865181	2435.986312	-0.0077	0.994	-1.69E-08	count	1
GPX3	-18.7865181	2435.986312	-0.0077	0.994	-1.69E-08	count	1
CD24	-18.7865181	2435.986312	-0.0077	0.994	-1.69E-08	count	1
CLEC7A	-18.7865181	2435.986312	-0.0077	0.994	-1.69E-08	count	1
KIAA0391	-18.8140271	1815.255033	-0.0104	0.992	-1.68E-08	count	1
AL024508.2	-18.8140271	1815.255033	-0.0104	0.992	-1.68E-08	count	1
DNAJC11	-18.8140271	1815.255033	-0.0104	0.992	-1.68E-08	count	1
NDUFB2-AS1	-18.8140271	1815.255033	-0.0104	0.992	-1.68E-08	count	1
AC092718.3	-18.8140271	1815.255033	-0.0104	0.992	-1.68E-08	count	1
MYO1C	-18.8140271	1815.255033	-0.0104	0.992	-1.68E-08	count	1
DUSP3	-18.827657	1385.256784	-0.0136	0.989	-1.68E-08	count	1
ATP13A4	-18.4443613	2052.940853	-0.009	0.993	-1.68E-08	count	1
AC048341.1	-18.4443613	2052.940853	-0.009	0.993	-1.68E-08	count	1
DOCK9	-18.4443613	2052.940853	-0.009	0.993	-1.68E-08	count	1
CCDC78	-18.4443613	2052.940853	-0.009	0.993	-1.68E-08	count	1
EPHA2	-18.4443613	2052.940853	-0.009	0.993	-1.68E-08	count	1
CCDC17	-18.4443613	2052.940853	-0.009	0.993	-1.68E-08	count	1
S100A16	-18.4443613	2052.940853	-0.009	0.993	-1.68E-08	count	1
BGLAP	-18.4443613	2052.940853	-0.009	0.993	-1.68E-08	count	1
PRRX1	-18.4443613	2052.940853	-0.009	0.993	-1.68E-08	count	1
FMO2	-18.4443613	2052.940853	-0.009	0.993	-1.68E-08	count	1
AC092835.1	-18.4443613	2052.940853	-0.009	0.993	-1.68E-08	count	1
AC109587.1	-18.4443613	2052.940853	-0.009	0.993	-1.68E-08	count	1
ALG1L2	-18.4443613	2052.940853	-0.009	0.993	-1.68E-08	count	1

AC123595.1	-18.4443613	2052.940853	-0.009	0.993	-1.68E-08	count	1
ZNF354C	-18.4443613	2052.940853	-0.009	0.993	-1.68E-08	count	1
Z98200.1	-18.4443613	2052.940853	-0.009	0.993	-1.68E-08	count	1
CYBB	-18.4443613	2052.940853	-0.009	0.993	-1.68E-08	count	1
OR1L8	-18.4443613	2052.940853	-0.009	0.993	-1.68E-08	count	1
BX255925.3	-18.4443613	2052.940853	-0.009	0.993	-1.68E-08	count	1
PARVA	-18.4443613	2052.940853	-0.009	0.993	-1.68E-08	count	1
AL450384.2	-18.4443613	2052.940853	-0.009	0.993	-1.68E-08	count	1
AC137590.2	-18.4443613	2052.940853	-0.009	0.993	-1.68E-08	count	1
NEK5	-18.4443613	2052.940853	-0.009	0.993	-1.68E-08	count	1
SLX1A	-18.4443613	2052.940853	-0.009	0.993	-1.68E-08	count	1
SLC25A35	-18.4443613	2052.940853	-0.009	0.993	-1.68E-08	count	1
TOM1L2	-18.4443613	2052.940853	-0.009	0.993	-1.68E-08	count	1
CCL23	-18.4443613	2052.940853	-0.009	0.993	-1.68E-08	count	1
PCGF2	-18.4443613	2052.940853	-0.009	0.993	-1.68E-08	count	1
EFCAB13	-18.4443613	2052.940853	-0.009	0.993	-1.68E-08	count	1
AC005332.6	-18.4443613	2052.940853	-0.009	0.993	-1.68E-08	count	1
AC005332.8	-18.4443613	2052.940853	-0.009	0.993	-1.68E-08	count	1
ANKEF1	-18.4443613	2052.940853	-0.009	0.993	-1.68E-08	count	1
AC119396.1	-18.4443613	2052.940853	-0.009	0.993	-1.68E-08	count	1
Z95115.1	-18.4443613	2052.940853	-0.009	0.993	-1.68E-08	count	1
CDC20	-18.4443613	2052.940853	-0.009	0.993	-1.68E-08	count	1
KCNA2	-18.4443613	2052.940853	-0.009	0.993	-1.68E-08	count	1
ABCB10	-18.4443613	2052.940853	-0.009	0.993	-1.68E-08	count	1
SPEG	-18.4443613	2052.940853	-0.009	0.993	-1.68E-08	count	1
AC134772.1	-18.4443613	2052.940853	-0.009	0.993	-1.68E-08	count	1
WWTR1	-18.4443613	2052.940853	-0.009	0.993	-1.68E-08	count	1
TRMT44	-18.4443613	2052.940853	-0.009	0.993	-1.68E-08	count	1
RPL34-AS1	-18.4443613	2052.940853	-0.009	0.993	-1.68E-08	count	1
SORBS2	-18.4443613	2052.940853	-0.009	0.993	-1.68E-08	count	1
PCDHGB2	-18.4443613	2052.940853	-0.009	0.993	-1.68E-08	count	1
F12	-18.4443613	2052.940853	-0.009	0.993	-1.68E-08	count	1
PACSIN1	-18.4443613	2052.940853	-0.009	0.993	-1.68E-08	count	1
UHRF1BP1	-18.4443613	2052.940853	-0.009	0.993	-1.68E-08	count	1
ZNF275	-18.4443613	2052.940853	-0.009	0.993	-1.68E-08	count	1
SOX17	-18.4443613	2052.940853	-0.009	0.993	-1.68E-08	count	1
AC083843.3	-18.4443613	2052.940853	-0.009	0.993	-1.68E-08	count	1
AL136141.1	-18.4443613	2052.940853	-0.009	0.993	-1.68E-08	count	1
PTGDS	-18.4443613	2052.940853	-0.009	0.993	-1.68E-08	count	1
LBHD1	-18.4443613	2052.940853	-0.009	0.993	-1.68E-08	count	1
IQSEC3	-18.4443613	2052.940853	-0.009	0.993	-1.68E-08	count	1
AC009133.4	-18.4443613	2052.940853	-0.009	0.993	-1.68E-08	count	1
AC092140.1	-18.4443613	2052.940853	-0.009	0.993	-1.68E-08	count	1
CDH20	-18.4443613	2052.940853	-0.009	0.993	-1.68E-08	count	1
ZNF343	-18.4443613	2052.940853	-0.009	0.993	-1.68E-08	count	1

AL121761.2	-18.4443613	2052.940853	-0.009	0.993	-1.68E-08	count	1
AC008764.8	-18.4443613	2052.940853	-0.009	0.993	-1.68E-08	count	1
AC245884.8	-18.4443613	2052.940853	-0.009	0.993	-1.68E-08	count	1
AC003681.1	-18.4443613	2052.940853	-0.009	0.993	-1.68E-08	count	1
LINC02574	-18.4443613	2052.940853	-0.009	0.993	-1.68E-08	count	1
COL24A1	-18.4443613	2052.940853	-0.009	0.993	-1.68E-08	count	1
MOV10	-18.4443613	2052.940853	-0.009	0.993	-1.68E-08	count	1
AL591895.1	-18.4443613	2052.940853	-0.009	0.993	-1.68E-08	count	1
CPA3	-18.4443613	2052.940853	-0.009	0.993	-1.68E-08	count	1
AC107068.1	-18.4443613	2052.940853	-0.009	0.993	-1.68E-08	count	1
NHS	-18.4443613	2052.940853	-0.009	0.993	-1.68E-08	count	1
C5	-18.4443613	2052.940853	-0.009	0.993	-1.68E-08	count	1
AP006621.3	-18.4443613	2052.940853	-0.009	0.993	-1.68E-08	count	1
PHLDB1	-18.4443613	2052.940853	-0.009	0.993	-1.68E-08	count	1
CCDC168	-18.4443613	2052.940853	-0.009	0.993	-1.68E-08	count	1
AC021752.1	-18.4443613	2052.940853	-0.009	0.993	-1.68E-08	count	1
CDH5	-18.4443613	2052.940853	-0.009	0.993	-1.68E-08	count	1
AC004943.2	-18.4443613	2052.940853	-0.009	0.993	-1.68E-08	count	1
PIGU	-18.4443613	2052.940853	-0.009	0.993	-1.68E-08	count	1
BX640514.2	-18.4443613	2052.940853	-0.009	0.993	-1.68E-08	count	1
F2RL3	-18.4443613	2052.940853	-0.009	0.993	-1.68E-08	count	1
RBM11	-18.4443613	2052.940853	-0.009	0.993	-1.68E-08	count	1
ADCY7	-0.0751656	0.4403441	-0.1707	0.865	-1.63E-08	count	1
LSM11	-0.1957076	0.82473	-0.2373	0.813	-1.52E-08	count	1
GFPT1	-0.0598225	0.5638159	-0.1061	0.916	-1.30E-08	count	1
NOP2	-0.4700036	0.5204165	-0.9031	0.367	-1.26E-08	count	1
ZNF512B	-0.4700036	0.4859127	-0.9673	0.334	-1.26E-08	count	1
TFAP4	-0.4700036	0.6123724	-0.7675	0.443	-1.26E-08	count	1
HIRA	-0.4700036	0.6123724	-0.7675	0.443	-1.26E-08	count	1
RGPD2	-0.1570037	0.6467578	-0.2428	0.808	-1.23E-08	count	1
MYLK-AS1	-0.4446858	0.7328281	-0.6068	0.544	-1.20E-08	count	1
ZSCAN9	-0.4446858	0.7328281	-0.6068	0.544	-1.20E-08	count	1
GTF2IRD2B	-0.4446858	0.7328281	-0.6068	0.544	-1.20E-08	count	1
HIST1H3A	-0.4446858	0.7328281	-0.6068	0.544	-1.20E-08	count	1
PLBD1	-0.4446858	0.8819171	-0.5042	0.614	-1.20E-08	count	1
FARP1	-0.4446858	0.8819171	-0.5042	0.614	-1.20E-08	count	1
IQCH-AS1	-0.4446858	0.8819171	-0.5042	0.614	-1.20E-08	count	1
SREBF1	-0.4446858	0.8819171	-0.5042	0.614	-1.20E-08	count	1
TNFAIP1	-0.4446858	0.8819171	-0.5042	0.614	-1.20E-08	count	1
IGLV3-21	-0.4446858	0.8819171	-0.5042	0.614	-1.20E-08	count	1
RELL2	-0.4446858	0.8819171	-0.5042	0.614	-1.20E-08	count	1
TRMT2B	-0.4446858	0.8819171	-0.5042	0.614	-1.20E-08	count	1
TMOD1	-0.4446858	0.8819171	-0.5042	0.614	-1.20E-08	count	1
LRRC20	-0.4446858	0.8819171	-0.5042	0.614	-1.20E-08	count	1
ZNF558	-0.4446858	0.8819171	-0.5042	0.614	-1.20E-08	count	1

TRIT1	-0.4446858	0.8819171	-0.5042	0.614	-1.20E-08	count	1
DTL	-0.4446858	0.8819171	-0.5042	0.614	-1.20E-08	count	1
PTP4A3	-0.4446858	0.8819171	-0.5042	0.614	-1.20E-08	count	1
MPZL2	-0.4446858	0.8819171	-0.5042	0.614	-1.20E-08	count	1
TEX2	-0.4446858	0.8819171	-0.5042	0.614	-1.20E-08	count	1
SPAG4	-0.4446858	0.8819171	-0.5042	0.614	-1.20E-08	count	1
SNAI1	-0.4446858	0.8819171	-0.5042	0.614	-1.20E-08	count	1
KIF5C	-0.4446858	0.8819171	-0.5042	0.614	-1.20E-08	count	1
DHX35	-0.4446858	0.8819171	-0.5042	0.614	-1.20E-08	count	1
CCDC112	-0.4446858	0.7895928	-0.5632	0.574	-1.20E-08	count	1
EVC2	-0.4446858	0.8819171	-0.5042	0.614	-1.20E-08	count	1
POU5F2	-0.4446858	0.8819171	-0.5042	0.614	-1.20E-08	count	1
TBC1D22B	-0.4446858	0.8819171	-0.5042	0.614	-1.20E-08	count	1
FBXO30	-0.4446858	0.8819171	-0.5042	0.614	-1.20E-08	count	1
AC018647.2	-0.4446858	0.8819171	-0.5042	0.614	-1.20E-08	count	1
ZNF711	-0.4446858	0.8819171	-0.5042	0.614	-1.20E-08	count	1
AMOT	-0.4446858	0.8819171	-0.5042	0.614	-1.20E-08	count	1
GINS4	-0.4446858	0.8819171	-0.5042	0.614	-1.20E-08	count	1
EMP1	-0.4446858	0.8819171	-0.5042	0.614	-1.20E-08	count	1
SLC16A13	-0.4446858	0.8819171	-0.5042	0.614	-1.20E-08	count	1
TEPSIN	-0.4446858	0.8819171	-0.5042	0.614	-1.20E-08	count	1
FNBP1L	-0.4446858	0.8819171	-0.5042	0.614	-1.20E-08	count	1
RHOBTB2	-0.4446858	0.8819171	-0.5042	0.614	-1.20E-08	count	1
IL7	-0.4446858	0.8819171	-0.5042	0.614	-1.20E-08	count	1
RAD54B	-0.4446858	0.8819171	-0.5042	0.614	-1.20E-08	count	1
MYO1E	-0.4446858	0.8819171	-0.5042	0.614	-1.20E-08	count	1
MAPK7	-0.4446858	0.8819171	-0.5042	0.614	-1.20E-08	count	1
GPD2	-0.4446858	0.8819171	-0.5042	0.614	-1.20E-08	count	1
SRP68	-0.0544192	0.4128122	-0.1318	0.895	-1.18E-08	count	1
MAP3K7	-0.1503475	0.7159368	-0.21	0.834	-1.18E-08	count	1
TGIF2	-0.0416131	0.5180733	-0.0803	0.936	-9.07E-09	count	1
CLOCK	-0.0416131	0.5336387	-0.078	0.938	-9.06E-09	count	1
TMEM143	-0.1107711	1.0548781	-0.105	0.916	-8.75E-09	count	1
PKD1	-0.1107711	1.0548781	-0.105	0.916	-8.75E-09	count	1
CAVIN3	-0.1107711	1.0548781	-0.105	0.916	-8.75E-09	count	1
ZNF367	-0.1107711	1.0548781	-0.105	0.916	-8.75E-09	count	1
GMEB2	-0.1107711	1.0548781	-0.105	0.916	-8.75E-09	count	1
PARP16	-0.1107711	0.741645	-0.1494	0.881	-8.75E-09	count	1
RBSN	-0.1107711	0.800492	-0.1384	0.89	-8.75E-09	count	1
AC055713.1	-0.1107711	1.0786898	-0.1027	0.918	-8.74E-09	count	1
TSTD3	-0.1107711	1.0786898	-0.1027	0.918	-8.74E-09	count	1
NT5C3B	-0.1107711	1.0786898	-0.1027	0.918	-8.74E-09	count	1
CTH	-0.1107711	1.0786898	-0.1027	0.918	-8.74E-09	count	1
MAGI2	-0.1107711	1.0786898	-0.1027	0.918	-8.74E-09	count	1
AK1	-0.1107711	1.0786898	-0.1027	0.918	-8.74E-09	count	1

ZNF519	-0.1107711	1.0786898	-0.1027	0.918	-8.74E-09	count	1
PNPLA6	-0.1107711	1.0786898	-0.1027	0.918	-8.74E-09	count	1
ZNF790	-0.1107711	0.8865694	-0.1249	0.901	-8.74E-09	count	1
AC008608.2	-0.1107711	0.8595403	-0.1289	0.898	-8.74E-09	count	1
TUBGCP6	-0.1107711	0.8595403	-0.1289	0.898	-8.74E-09	count	1
MCOLN2	-0.1107711	0.8595403	-0.1289	0.898	-8.74E-09	count	1
AC022075.1	-0.1082136	0.5296928	-0.2043	0.838	-8.55E-09	count	1
CPNE8	-0.0361056	0.4465372	-0.0809	0.936	-7.91E-09	count	1
SRGAP2	-0.0784113	0.6250296	-0.1255	0.9	-6.24E-09	count	1
ADAM22	-19.613466	3428.672635	-0.0057	0.995	-6.22E-09	count	1
BMP8B	-19.613466	3428.672635	-0.0057	0.995	-6.22E-09	count	1
AL033527.3	-19.613466	3428.672635	-0.0057	0.995	-6.22E-09	count	1
AL451074.2	-19.613466	3428.672635	-0.0057	0.995	-6.22E-09	count	1
AL353708.3	-19.613466	3428.672635	-0.0057	0.995	-6.22E-09	count	1
AL360091.3	-19.613466	3428.672635	-0.0057	0.995	-6.22E-09	count	1
AC009506.1	-19.613466	3428.672635	-0.0057	0.995	-6.22E-09	count	1
BBS5	-19.613466	3428.672635	-0.0057	0.995	-6.22E-09	count	1
OSBPL6	-19.613466	3428.672635	-0.0057	0.995	-6.22E-09	count	1
BHLHE40-AS1	-19.613466	3428.672635	-0.0057	0.995	-6.22E-09	count	1
AC084048.1	-19.613466	3428.672635	-0.0057	0.995	-6.22E-09	count	1
CXCL10	-19.613466	3428.672635	-0.0057	0.995	-6.22E-09	count	1
RASGRF2-AS1	-19.613466	3428.672635	-0.0057	0.995	-6.22E-09	count	1
SLCO4C1	-19.613466	3428.672635	-0.0057	0.995	-6.22E-09	count	1
PSORS1C1	-19.613466	3428.672635	-0.0057	0.995	-6.22E-09	count	1
AGER	-19.613466	3428.672635	-0.0057	0.995	-6.22E-09	count	1
PPARD	-19.613466	3428.672635	-0.0057	0.995	-6.22E-09	count	1
SNAP91	-19.613466	3428.672635	-0.0057	0.995	-6.22E-09	count	1
PTPRK	-19.613466	3428.672635	-0.0057	0.995	-6.22E-09	count	1
PDE7B	-19.613466	3428.672635	-0.0057	0.995	-6.22E-09	count	1
CD36	-19.613466	3428.672635	-0.0057	0.995	-6.22E-09	count	1
WDR91	-19.613466	3428.672635	-0.0057	0.995	-6.22E-09	count	1
LINC01504	-19.613466	3428.672635	-0.0057	0.995	-6.22E-09	count	1
AKAP2	-19.613466	3428.672635	-0.0057	0.995	-6.22E-09	count	1
MPPED2	-19.613466	3428.672635	-0.0057	0.995	-6.22E-09	count	1
ECHDC3	-19.613466	3428.672635	-0.0057	0.995	-6.22E-09	count	1
AL365203.2	-19.613466	3428.672635	-0.0057	0.995	-6.22E-09	count	1
EIF5AL1	-19.613466	3428.672635	-0.0057	0.995	-6.22E-09	count	1
CUZD1	-19.613466	3428.672635	-0.0057	0.995	-6.22E-09	count	1
EGLN3	-19.613466	3428.672635	-0.0057	0.995	-6.22E-09	count	1
POMT2	-19.613466	3428.672635	-0.0057	0.995	-6.22E-09	count	1
AL136040.1	-19.613466	3428.672635	-0.0057	0.995	-6.22E-09	count	1
WDR90	-19.613466	3428.672635	-0.0057	0.995	-6.22E-09	count	1
FBXL8	-19.613466	3428.672635	-0.0057	0.995	-6.22E-09	count	1
LIG3	-19.613466	3428.672635	-0.0057	0.995	-6.22E-09	count	1
AXIN2	-19.613466	3428.672635	-0.0057	0.995	-6.22E-09	count	1

CYGB	-19.613466	3428.672635	-0.0057	0.995	-6.22E-09	count	1
TSPAN10	-19.613466	3428.672635	-0.0057	0.995	-6.22E-09	count	1
MMP9	-19.613466	3428.672635	-0.0057	0.995	-6.22E-09	count	1
SOX18	-19.613466	3428.672635	-0.0057	0.995	-6.22E-09	count	1
PLVAP	-19.613466	3428.672635	-0.0057	0.995	-6.22E-09	count	1
AC022150.1	-19.613466	3428.672635	-0.0057	0.995	-6.22E-09	count	1
ZNF552	-19.613466	3428.672635	-0.0057	0.995	-6.22E-09	count	1
Z82188.2	-19.613466	3428.672635	-0.0057	0.995	-6.22E-09	count	1
KLHDC7B	-19.613466	3428.672635	-0.0057	0.995	-6.22E-09	count	1
AC078785.1	-19.613466	3428.672635	-0.0057	0.995	-6.22E-09	count	1
NR3C2	-19.613466	3428.672635	-0.0057	0.995	-6.22E-09	count	1
KHDC1	-19.613466	3428.672635	-0.0057	0.995	-6.22E-09	count	1
ZC3H12D	-19.613466	3428.672635	-0.0057	0.995	-6.22E-09	count	1
ZP3	-19.613466	3428.672635	-0.0057	0.995	-6.22E-09	count	1
AL590764.1	-19.613466	3428.672635	-0.0057	0.995	-6.22E-09	count	1
TNFRSF10D	-19.613466	3428.672635	-0.0057	0.995	-6.22E-09	count	1
TLR4	-19.613466	3428.672635	-0.0057	0.995	-6.22E-09	count	1
NAA40	-19.613466	3428.672635	-0.0057	0.995	-6.22E-09	count	1
SCART1	-19.613466	3428.672635	-0.0057	0.995	-6.22E-09	count	1
TRAV17	-19.613466	3428.672635	-0.0057	0.995	-6.22E-09	count	1
XRCC3	-19.613466	3428.672635	-0.0057	0.995	-6.22E-09	count	1
CERNA1	-19.613466	3428.672635	-0.0057	0.995	-6.22E-09	count	1
UBE2Q2L	-19.613466	3428.672635	-0.0057	0.995	-6.22E-09	count	1
IRX5	-19.613466	3428.672635	-0.0057	0.995	-6.22E-09	count	1
AC055811.3	-19.613466	3428.672635	-0.0057	0.995	-6.22E-09	count	1
OPRL1	-19.613466	3428.672635	-0.0057	0.995	-6.22E-09	count	1
RGS9BP	-19.613466	3428.672635	-0.0057	0.995	-6.22E-09	count	1
LENG8-AS1	-19.613466	3428.672635	-0.0057	0.995	-6.22E-09	count	1
BIK	-19.613466	3428.672635	-0.0057	0.995	-6.22E-09	count	1
GATD3B	-19.613466	3428.672635	-0.0057	0.995	-6.22E-09	count	1
SPC25	-19.613466	3428.672635	-0.0057	0.995	-6.22E-09	count	1
XYLB	-19.613466	3428.672635	-0.0057	0.995	-6.22E-09	count	1
CCR9	-19.613466	3428.672635	-0.0057	0.995	-6.22E-09	count	1
EREG	-19.613466	3428.672635	-0.0057	0.995	-6.22E-09	count	1
C5orf17	-19.613466	3428.672635	-0.0057	0.995	-6.22E-09	count	1
TYW1B	-19.613466	3428.672635	-0.0057	0.995	-6.22E-09	count	1
GVQW3	-19.613466	3428.672635	-0.0057	0.995	-6.22E-09	count	1
USP6NL	-19.613466	3428.672635	-0.0057	0.995	-6.22E-09	count	1
SGSM2	-19.613466	3428.672635	-0.0057	0.995	-6.22E-09	count	1
UBE2C	-19.613466	3428.672635	-0.0057	0.995	-6.22E-09	count	1
ATP9A	-19.613466	3428.672635	-0.0057	0.995	-6.22E-09	count	1
NECTIN2	-19.613466	3428.672635	-0.0057	0.995	-6.22E-09	count	1
CHAF1B	-19.613466	3428.672635	-0.0057	0.995	-6.22E-09	count	1
AL121983.2	-19.613466	3428.672635	-0.0057	0.995	-6.22E-09	count	1
ZNF496	-19.613466	3428.672635	-0.0057	0.995	-6.22E-09	count	1

PLEKHM3	-19.613466	3428.672635	-0.0057	0.995	-6.22E-09	count	1
SMAD1	-19.613466	3428.672635	-0.0057	0.995	-6.22E-09	count	1
PXDC1	-19.613466	3428.672635	-0.0057	0.995	-6.22E-09	count	1
FIRRE	-19.613466	3428.672635	-0.0057	0.995	-6.22E-09	count	1
MAMLD1	-19.613466	3428.672635	-0.0057	0.995	-6.22E-09	count	1
AC026979.1	-19.613466	3428.672635	-0.0057	0.995	-6.22E-09	count	1
ZBTB34	-19.613466	3428.672635	-0.0057	0.995	-6.22E-09	count	1
BOLA2-SMG1P6	-19.613466	3428.672635	-0.0057	0.995	-6.22E-09	count	1
FKBP10	-19.613466	3428.672635	-0.0057	0.995	-6.22E-09	count	1
AC008655.2	-19.613466	3428.672635	-0.0057	0.995	-6.22E-09	count	1
STIL	-19.613466	3428.672635	-0.0057	0.995	-6.22E-09	count	1
PALMD	-19.613466	3428.672635	-0.0057	0.995	-6.22E-09	count	1
EPHX1	-19.613466	3428.672635	-0.0057	0.995	-6.22E-09	count	1
SH3BP4	-19.613466	3428.672635	-0.0057	0.995	-6.22E-09	count	1
RBMS3	-19.613466	3428.672635	-0.0057	0.995	-6.22E-09	count	1
P2RY14	-19.613466	3428.672635	-0.0057	0.995	-6.22E-09	count	1
ANKRD50	-19.613466	3428.672635	-0.0057	0.995	-6.22E-09	count	1
TRIM7	-19.613466	3428.672635	-0.0057	0.995	-6.22E-09	count	1
GSTA4	-19.613466	3428.672635	-0.0057	0.995	-6.22E-09	count	1
BICC1	-19.613466	3428.672635	-0.0057	0.995	-6.22E-09	count	1
CC2D2B	-19.613466	3428.672635	-0.0057	0.995	-6.22E-09	count	1
CYP2E1	-19.613466	3428.672635	-0.0057	0.995	-6.22E-09	count	1
SLC8A3	-19.613466	3428.672635	-0.0057	0.995	-6.22E-09	count	1
AC068446.2	-19.613466	3428.672635	-0.0057	0.995	-6.22E-09	count	1
OSBPL1A	-19.613466	3428.672635	-0.0057	0.995	-6.22E-09	count	1
PYGB	-19.613466	3428.672635	-0.0057	0.995	-6.22E-09	count	1
AC104532.1	-19.613466	3428.672635	-0.0057	0.995	-6.22E-09	count	1
AC020922.4	-19.613466	3428.672635	-0.0057	0.995	-6.22E-09	count	1
AC004264.1	-19.613466	3428.672635	-0.0057	0.995	-6.22E-09	count	1
BACE2	-19.613466	3428.672635	-0.0057	0.995	-6.22E-09	count	1
AL031280.1	-19.4516138	2360.280172	-0.0082	0.993	-6.17E-09	count	1
RHBDD1	-19.4516138	2360.280172	-0.0082	0.993	-6.17E-09	count	1
KANTR	-19.4516138	2360.280172	-0.0082	0.993	-6.17E-09	count	1
RBPMS	-19.4516138	2360.280172	-0.0082	0.993	-6.17E-09	count	1
HOXA5	-19.4516138	2360.280172	-0.0082	0.993	-6.17E-09	count	1
ABTB2	-19.4516138	2360.280172	-0.0082	0.993	-6.17E-09	count	1
AL512408.1	-18.7745077	2421.401479	-0.0078	0.994	-6.16E-09	count	1
OSCP1	-18.7745077	2421.401479	-0.0078	0.994	-6.16E-09	count	1
AC008074.3	-18.7745077	2421.401479	-0.0078	0.994	-6.16E-09	count	1
PKP4	-18.7745077	2421.401479	-0.0078	0.994	-6.16E-09	count	1
LINC00880	-18.7745077	2421.401479	-0.0078	0.994	-6.16E-09	count	1
AC004951.1	-18.7745077	2421.401479	-0.0078	0.994	-6.16E-09	count	1
FBXL13	-18.7745077	2421.401479	-0.0078	0.994	-6.16E-09	count	1
AC090114.2	-18.7745077	2421.401479	-0.0078	0.994	-6.16E-09	count	1
PRDM11	-18.7745077	2421.401479	-0.0078	0.994	-6.16E-09	count	1

AP002433.1	-18.7745077	2421.401479	-0.0078	0.994	-6.16E-09	count	1
AC016957.2	-18.7745077	2421.401479	-0.0078	0.994	-6.16E-09	count	1
AC008083.2	-18.7745077	2421.401479	-0.0078	0.994	-6.16E-09	count	1
ADPRHL1	-18.7745077	2421.401479	-0.0078	0.994	-6.16E-09	count	1
AC007601.1	-18.7745077	2421.401479	-0.0078	0.994	-6.16E-09	count	1
AC046158.1	-18.7745077	2421.401479	-0.0078	0.994	-6.16E-09	count	1
KRT17	-18.7745077	2421.401479	-0.0078	0.994	-6.16E-09	count	1
ANKLE1	-18.7745077	2421.401479	-0.0078	0.994	-6.16E-09	count	1
ZNF492	-18.7745077	2421.401479	-0.0078	0.994	-6.16E-09	count	1
KDM4A-AS1	-18.7745077	2421.401479	-0.0078	0.994	-6.16E-09	count	1
MCOLN3	-18.7745077	2421.401479	-0.0078	0.994	-6.16E-09	count	1
FMOD	-18.7745077	2421.401479	-0.0078	0.994	-6.16E-09	count	1
SOX13	-18.7745077	2421.401479	-0.0078	0.994	-6.16E-09	count	1
LINC01119	-18.7745077	2421.401479	-0.0078	0.994	-6.16E-09	count	1
HK2	-18.7745077	2421.401479	-0.0078	0.994	-6.16E-09	count	1
AC009948.3	-18.7745077	2421.401479	-0.0078	0.994	-6.16E-09	count	1
GLB1L	-18.7745077	2421.401479	-0.0078	0.994	-6.16E-09	count	1
AC104078.1	-18.7745077	2421.401479	-0.0078	0.994	-6.16E-09	count	1
DKK2	-18.7745077	2421.401479	-0.0078	0.994	-6.16E-09	count	1
CKMT2	-18.7745077	2421.401479	-0.0078	0.994	-6.16E-09	count	1
PDGFRB	-18.7745077	2421.401479	-0.0078	0.994	-6.16E-09	count	1
RANBP17	-18.7745077	2421.401479	-0.0078	0.994	-6.16E-09	count	1
U91328.3	-18.7745077	2421.401479	-0.0078	0.994	-6.16E-09	count	1
AL031777.3	-18.7745077	2421.401479	-0.0078	0.994	-6.16E-09	count	1
ZSCAN31	-18.7745077	2421.401479	-0.0078	0.994	-6.16E-09	count	1
KLC4	-18.7745077	2421.401479	-0.0078	0.994	-6.16E-09	count	1
PLN	-18.7745077	2421.401479	-0.0078	0.994	-6.16E-09	count	1
INHBA	-18.7745077	2421.401479	-0.0078	0.994	-6.16E-09	count	1
TRBV3-1	-18.7745077	2421.401479	-0.0078	0.994	-6.16E-09	count	1
TCAF2C	-18.7745077	2421.401479	-0.0078	0.994	-6.16E-09	count	1
ZNF775	-18.7745077	2421.401479	-0.0078	0.994	-6.16E-09	count	1
PCAT1	-18.7745077	2421.401479	-0.0078	0.994	-6.16E-09	count	1
INSC	-18.7745077	2421.401479	-0.0078	0.994	-6.16E-09	count	1
AC073592.1	-18.7745077	2421.401479	-0.0078	0.994	-6.16E-09	count	1
ATL1	-18.7745077	2421.401479	-0.0078	0.994	-6.16E-09	count	1
FBN1	-18.7745077	2421.401479	-0.0078	0.994	-6.16E-09	count	1
SHC4	-18.7745077	2421.401479	-0.0078	0.994	-6.16E-09	count	1
AC022087.1	-18.7745077	2421.401479	-0.0078	0.994	-6.16E-09	count	1
SULT1A3	-18.7745077	2421.401479	-0.0078	0.994	-6.16E-09	count	1
AC132938.3	-18.7745077	2421.401479	-0.0078	0.994	-6.16E-09	count	1
SDCBP2-AS1	-18.7745077	2421.401479	-0.0078	0.994	-6.16E-09	count	1
C19orf38	-18.7745077	2421.401479	-0.0078	0.994	-6.16E-09	count	1
CCDC194	-18.7745077	2421.401479	-0.0078	0.994	-6.16E-09	count	1
CCDC106	-18.7745077	2421.401479	-0.0078	0.994	-6.16E-09	count	1
ZNF543	-18.7745077	2421.401479	-0.0078	0.994	-6.16E-09	count	1

CLDN5	-18.7745077	2421.401479	-0.0078	0.994	-6.16E-09	count	1
AGAP1	-18.7745077	2421.401479	-0.0078	0.994	-6.16E-09	count	1
TNIP3	-18.7745077	2421.401479	-0.0078	0.994	-6.16E-09	count	1
AL513548.1	-18.7745077	2421.401479	-0.0078	0.994	-6.16E-09	count	1
C7orf61	-18.7745077	2421.401479	-0.0078	0.994	-6.16E-09	count	1
TRBV6-2	-18.7745077	2421.401479	-0.0078	0.994	-6.16E-09	count	1
ZNF572	-18.7745077	2421.401479	-0.0078	0.994	-6.16E-09	count	1
ACRBP	-18.7745077	2421.401479	-0.0078	0.994	-6.16E-09	count	1
IGFBP6	-18.7745077	2421.401479	-0.0078	0.994	-6.16E-09	count	1
KSR2	-18.7745077	2421.401479	-0.0078	0.994	-6.16E-09	count	1
DGKH	-18.7745077	2421.401479	-0.0078	0.994	-6.16E-09	count	1
AC123768.4	-18.7745077	2421.401479	-0.0078	0.994	-6.16E-09	count	1
TRPV1	-18.7745077	2421.401479	-0.0078	0.994	-6.16E-09	count	1
PLEKHG2	-18.7745077	2421.401479	-0.0078	0.994	-6.16E-09	count	1
AL590822.2	-18.7745077	2421.401479	-0.0078	0.994	-6.16E-09	count	1
RHBDL2	-18.7745077	2421.401479	-0.0078	0.994	-6.16E-09	count	1
NID1	-18.7745077	2421.401479	-0.0078	0.994	-6.16E-09	count	1
AC231981.1	-18.7745077	2421.401479	-0.0078	0.994	-6.16E-09	count	1
TANC1	-18.7745077	2421.401479	-0.0078	0.994	-6.16E-09	count	1
LINC02478	-18.7745077	2421.401479	-0.0078	0.994	-6.16E-09	count	1
AC011405.1	-18.7745077	2421.401479	-0.0078	0.994	-6.16E-09	count	1
ZNF879	-18.7745077	2421.401479	-0.0078	0.994	-6.16E-09	count	1
FAM185A	-18.7745077	2421.401479	-0.0078	0.994	-6.16E-09	count	1
AC008264.2	-18.7745077	2421.401479	-0.0078	0.994	-6.16E-09	count	1
CCDC180	-18.7745077	2421.401479	-0.0078	0.994	-6.16E-09	count	1
SARDH	-18.7745077	2421.401479	-0.0078	0.994	-6.16E-09	count	1
ACCS	-18.7745077	2421.401479	-0.0078	0.994	-6.16E-09	count	1
CCDC15	-18.7745077	2421.401479	-0.0078	0.994	-6.16E-09	count	1
LINC00167	-18.7745077	2421.401479	-0.0078	0.994	-6.16E-09	count	1
AL157895.1	-18.7745077	2421.401479	-0.0078	0.994	-6.16E-09	count	1
VDR	-18.7745077	2421.401479	-0.0078	0.994	-6.16E-09	count	1
NDUFA4L2	-18.7745077	2421.401479	-0.0078	0.994	-6.16E-09	count	1
VSIG10	-18.7745077	2421.401479	-0.0078	0.994	-6.16E-09	count	1
ABCC4	-18.7745077	2421.401479	-0.0078	0.994	-6.16E-09	count	1
CBLN3	-18.7745077	2421.401479	-0.0078	0.994	-6.16E-09	count	1
HEATR5A	-18.7745077	2421.401479	-0.0078	0.994	-6.16E-09	count	1
ITPKA	-18.7745077	2421.401479	-0.0078	0.994	-6.16E-09	count	1
NTRK3	-18.7745077	2421.401479	-0.0078	0.994	-6.16E-09	count	1
CDRT4	-18.7745077	2421.401479	-0.0078	0.994	-6.16E-09	count	1
CACTIN	-18.7745077	2421.401479	-0.0078	0.994	-6.16E-09	count	1
AC092279.1	-18.7745077	2421.401479	-0.0078	0.994	-6.16E-09	count	1
MIF-AS1	-18.7745077	2421.401479	-0.0078	0.994	-6.16E-09	count	1
NEFH	-18.7745077	2421.401479	-0.0078	0.994	-6.16E-09	count	1
GIPC2	-18.7745077	2421.401479	-0.0078	0.994	-6.16E-09	count	1
PTGFRN	-18.7745077	2421.401479	-0.0078	0.994	-6.16E-09	count	1

NUDT17	-18.7745077	2421.401479	-0.0078	0.994	-6.16E-09	count	1
TMEM81	-18.7745077	2421.401479	-0.0078	0.994	-6.16E-09	count	1
STK11IP	-18.7745077	2421.401479	-0.0078	0.994	-6.16E-09	count	1
AC090948.2	-18.7745077	2421.401479	-0.0078	0.994	-6.16E-09	count	1
TRPC1	-18.7745077	2421.401479	-0.0078	0.994	-6.16E-09	count	1
MYOZ2	-18.7745077	2421.401479	-0.0078	0.994	-6.16E-09	count	1
FAM198B	-18.7745077	2421.401479	-0.0078	0.994	-6.16E-09	count	1
PALLD	-18.7745077	2421.401479	-0.0078	0.994	-6.16E-09	count	1
HAPLN1	-18.7745077	2421.401479	-0.0078	0.994	-6.16E-09	count	1
HBEGF	-18.7745077	2421.401479	-0.0078	0.994	-6.16E-09	count	1
U91328.1	-18.7745077	2421.401479	-0.0078	0.994	-6.16E-09	count	1
ZKSCAN4	-18.7745077	2421.401479	-0.0078	0.994	-6.16E-09	count	1
BEND3	-18.7745077	2421.401479	-0.0078	0.994	-6.16E-09	count	1
F8	-18.7745077	2421.401479	-0.0078	0.994	-6.16E-09	count	1
AC246817.2	-18.7745077	2421.401479	-0.0078	0.994	-6.16E-09	count	1
ADAMDEC1	-18.7745077	2421.401479	-0.0078	0.994	-6.16E-09	count	1
AZIN1-AS1	-18.7745077	2421.401479	-0.0078	0.994	-6.16E-09	count	1
TNFRSF11B	-18.7745077	2421.401479	-0.0078	0.994	-6.16E-09	count	1
DNAJB5	-18.7745077	2421.401479	-0.0078	0.994	-6.16E-09	count	1
S1PR3	-18.7745077	2421.401479	-0.0078	0.994	-6.16E-09	count	1
LAYN	-18.7745077	2421.401479	-0.0078	0.994	-6.16E-09	count	1
ZC3H10	-18.7745077	2421.401479	-0.0078	0.994	-6.16E-09	count	1
RASSF9	-18.7745077	2421.401479	-0.0078	0.994	-6.16E-09	count	1
ULK1	-18.7745077	2421.401479	-0.0078	0.994	-6.16E-09	count	1
ALG11	-18.7745077	2421.401479	-0.0078	0.994	-6.16E-09	count	1
EME2	-18.7745077	2421.401479	-0.0078	0.994	-6.16E-09	count	1
NOMO3	-18.7745077	2421.401479	-0.0078	0.994	-6.16E-09	count	1
C16orf86	-18.7745077	2421.401479	-0.0078	0.994	-6.16E-09	count	1
PLA2G15	-18.7745077	2421.401479	-0.0078	0.994	-6.16E-09	count	1
FAM57A	-18.7745077	2421.401479	-0.0078	0.994	-6.16E-09	count	1
TLCD1	-18.7745077	2421.401479	-0.0078	0.994	-6.16E-09	count	1
AC104984.4	-18.7745077	2421.401479	-0.0078	0.994	-6.16E-09	count	1
ACSS2	-18.7745077	2421.401479	-0.0078	0.994	-6.16E-09	count	1
ZSWIM3	-18.7745077	2421.401479	-0.0078	0.994	-6.16E-09	count	1
ZNF304	-18.7745077	2421.401479	-0.0078	0.994	-6.16E-09	count	1
SLC19A1	-18.7745077	2421.401479	-0.0078	0.994	-6.16E-09	count	1
MCM3AP-AS1	-18.7745077	2421.401479	-0.0078	0.994	-6.16E-09	count	1
TLR3	-18.7745077	2421.401479	-0.0078	0.994	-6.16E-09	count	1
PWAR6	-18.7745077	2421.401479	-0.0078	0.994	-6.16E-09	count	1
LPCAT2	-18.7745077	2421.401479	-0.0078	0.994	-6.16E-09	count	1
RASD1	-0.0711948	0.6888352	-0.1034	0.918	-5.67E-09	count	1
SRD5A3	-0.0711948	0.6327446	-0.1125	0.91	-5.67E-09	count	1
FMO5	-0.1823216	0.4212709	-0.4328	0.665	-5.21E-09	count	1
ABAT	-0.0598225	0.6875839	-0.087	0.931	-4.78E-09	count	1
MTFR1L	-0.0598225	0.6901998	-0.0867	0.931	-4.77E-09	count	1

DTWD2	-0.1107711	1.0548781	-0.105	0.916	-3.22E-09	count	1
MTO1	-0.1107711	1.0548781	-0.105	0.916	-3.22E-09	count	1
RAB15	-0.1107711	1.0548781	-0.105	0.916	-3.22E-09	count	1
VEGFA	-0.1107711	1.0548781	-0.105	0.916	-3.22E-09	count	1
ZNF653	-0.1107711	1.0548781	-0.105	0.916	-3.22E-09	count	1
ITGAD	-0.1107711	1.0548781	-0.105	0.916	-3.22E-09	count	1
BX890604.1	-0.1107711	1.0548781	-0.105	0.916	-3.22E-09	count	1
HIC2	-0.1107711	1.0548781	-0.105	0.916	-3.22E-09	count	1
ARL10	-0.1107711	1.0548781	-0.105	0.916	-3.22E-09	count	1
SLC43A2	-0.1107711	1.0548781	-0.105	0.916	-3.22E-09	count	1
AC245297.2	-0.1107711	1.0548781	-0.105	0.916	-3.22E-09	count	1
AC132872.1	-0.1107711	1.0786899	-0.1027	0.918	-3.22E-09	count	1
TIPARP-AS1	-0.1107711	1.0786899	-0.1027	0.918	-3.22E-09	count	1
MCTP1	-0.1107711	1.0786899	-0.1027	0.918	-3.22E-09	count	1
AC009414.2	-0.1107711	1.0786899	-0.1027	0.918	-3.22E-09	count	1
PTCD1	-0.1107711	1.0786899	-0.1027	0.918	-3.22E-09	count	1
LINC01473	-0.1107711	1.0786899	-0.1027	0.918	-3.22E-09	count	1
CHRFAM7A	-0.1107711	1.0786899	-0.1027	0.918	-3.22E-09	count	1
IMPA2	-0.1107711	1.0786899	-0.1027	0.918	-3.22E-09	count	1
NTNG2	-0.1107711	1.0786899	-0.1027	0.918	-3.22E-09	count	1
ACAD11	-0.1107711	1.0786899	-0.1027	0.918	-3.22E-09	count	1
CUL7	-0.1107711	1.0786899	-0.1027	0.918	-3.22E-09	count	1
MRM1	-0.1107711	1.0786899	-0.1027	0.918	-3.22E-09	count	1
GLS2	-0.1107711	1.0786899	-0.1027	0.918	-3.22E-09	count	1
URB1	-0.1107711	1.0786899	-0.1027	0.918	-3.22E-09	count	1
AL662884.4	-0.1107711	1.0786899	-0.1027	0.918	-3.22E-09	count	1
FKBP7	-0.1107711	1.0786899	-0.1027	0.918	-3.22E-09	count	1
PROS1	-0.0392207	0.9198877	-0.0426	0.966	-3.14E-09	count	1
FAM173B	-0.0392207	0.9198877	-0.0426	0.966	-3.14E-09	count	1
ZNF707	-0.0392207	0.9198877	-0.0426	0.966	-3.14E-09	count	1
FOXJ1	-0.0392207	0.9198877	-0.0426	0.966	-3.14E-09	count	1
ZNF565	-0.0392207	0.9198877	-0.0426	0.966	-3.14E-09	count	1
ZNF584	-0.0392207	0.9198877	-0.0426	0.966	-3.14E-09	count	1
SGO2	-0.0392207	0.591434	-0.0663	0.947	-3.14E-09	count	1
IL12RB2	-0.0645385	0.4743959	-0.136	0.892	-1.89E-09	count	1
VWA8	-0.0392207	0.7607258	-0.0516	0.959	-1.16E-09	count	1
POFUT2	-0.0392207	0.7607258	-0.0516	0.959	-1.16E-09	count	1
CACNB3	-0.0392207	0.7607258	-0.0516	0.959	-1.16E-09	count	1
AQR	0.0038238	0.4997515	0.0077	0.994	8.41E-10	count	1
MIR3142HG	0.0584967	0.7436591	0.0787	0.937	1.76E-09	count	1
CUL9	0.2231436	0.5665577	0.3939	0.694	2.55E-09	count	1
MEGF9	0.2231436	0.5665577	0.3939	0.694	2.55E-09	count	1
AC078925.1	0.2231436	0.5665577	0.3939	0.694	2.55E-09	count	1
PROSER1	0.2231436	0.5665577	0.3939	0.694	2.55E-09	count	1
ZNF500	0.2231436	0.5665577	0.3939	0.694	2.55E-09	count	1

CDCP1	0.2231436	0.5665577	0.3939	0.694	2.55E-09	count	1
SEMA3A	0.2231436	0.5665577	0.3939	0.694	2.55E-09	count	1
NME2	0.2231436	0.5665577	0.3939	0.694	2.55E-09	count	1
ARL15	0.038238	0.7187062	0.0532	0.958	3.12E-09	count	1
CASZ1	0.038238	0.7154097	0.0534	0.957	3.12E-09	count	1
FAM66C	0.0584967	0.8182125	0.0715	0.943	4.79E-09	count	1
SUMF1	0.0584967	0.8182125	0.0715	0.943	4.79E-09	count	1
LRRC8A	0.0584967	0.8182125	0.0715	0.943	4.79E-09	count	1
SCD	0.0584967	0.8182125	0.0715	0.943	4.79E-09	count	1
TET3	0.0584967	0.8266191	0.0708	0.944	4.79E-09	count	1
GPR107	0.0807794	0.6537478	0.1236	0.902	6.65E-09	count	1
S100Z	0.2231436	0.5665577	0.3939	0.694	6.94E-09	count	1
SYP	0.2231436	0.5665577	0.3939	0.694	6.94E-09	count	1
MAN2A2	0.2231436	0.5665577	0.3939	0.694	6.94E-09	count	1
FAM184A	0.2231436	0.5665577	0.3939	0.694	6.94E-09	count	1
IPPK	0.2231436	0.496904	0.4491	0.654	6.95E-09	count	1
ZFAND4	0.2231436	0.4714045	0.4734	0.636	6.96E-09	count	1
NIPA1	0.2484614	0.7607258	0.3266	0.744	7.77E-09	count	1
SMN2	0.2484614	0.7607258	0.3266	0.744	7.77E-09	count	1
PRDM12	0.2484614	0.9694404	0.2563	0.798	7.77E-09	count	1
ENO2	0.2484614	0.9694404	0.2563	0.798	7.77E-09	count	1
AL049780.2	0.2484614	0.9694404	0.2563	0.798	7.77E-09	count	1
AL117381.1	0.2484614	0.9694404	0.2563	0.798	7.77E-09	count	1
PIK3C2B	0.2484614	0.9694404	0.2563	0.798	7.77E-09	count	1
GDPD5	0.2484614	0.9694404	0.2563	0.798	7.77E-09	count	1
ZNF578	0.2484614	0.9694404	0.2563	0.798	7.77E-09	count	1
AGO1	0.2484614	0.9694404	0.2563	0.798	7.77E-09	count	1
CCR4	0.2484614	0.9694404	0.2563	0.798	7.77E-09	count	1
CDKL3	0.2484614	0.9694404	0.2563	0.798	7.77E-09	count	1
HCG25	0.2484614	0.9694404	0.2563	0.798	7.77E-09	count	1
SUV39H1	0.2484614	0.9694404	0.2563	0.798	7.77E-09	count	1
AC044839.1	0.2484614	0.9694404	0.2563	0.798	7.77E-09	count	1
ZG16B	0.2484614	0.9694404	0.2563	0.798	7.77E-09	count	1
ALDH3A1	0.2484614	0.9694404	0.2563	0.798	7.77E-09	count	1
AC004825.2	0.0992702	0.8945654	0.111	0.912	8.19E-09	count	1
RANBP10	0.0992702	0.8945654	0.111	0.912	8.19E-09	count	1
LIX1L-AS1	0.0992702	0.8945654	0.111	0.912	8.19E-09	count	1
MAGIX	0.0992702	0.7921787	0.1253	0.9	8.19E-09	count	1
RNF121	0.0992702	0.7886611	0.1259	0.9	8.20E-09	count	1
SESTD1	0.0992702	0.9748727	0.1018	0.919	8.21E-09	count	1
ZNF408	0.0992702	0.656589	0.1512	0.88	8.21E-09	count	1
PITRM1	0.0992702	0.8390727	0.1183	0.906	8.24E-09	count	1
GSTM4	0.040822	0.3966899	0.1029	0.918	9.07E-09	count	1
LINC02446	0.11493	0.5543354	0.2073	0.836	9.54E-09	count	1
ZNF628	0.136249	0.7931025	0.1718	0.864	1.13E-08	count	1

MIOS	0.136249	0.7931025	0.1718	0.864	1.13E-08	count	1
ZNF597	0.136249	0.7109495	0.1916	0.848	1.13E-08	count	1
PRRT2	0.9162907	0.5892556	1.555	0.121	1.17E-08	count	1
AMDHD2	0.9162907	0.5892556	1.555	0.121	1.17E-08	count	1
MELTF	0.9162907	0.5892556	1.555	0.121	1.17E-08	count	1
AC012358.3	0.9162907	0.5892556	1.555	0.121	1.17E-08	count	1
AL158071.4	0.9162907	0.5892556	1.555	0.121	1.17E-08	count	1
GPNMB	0.9162907	0.5892556	1.555	0.121	1.17E-08	count	1
CYB5D1	0.9162907	0.5892556	1.555	0.121	1.17E-08	count	1
CLASP1	0.1431008	0.4630864	0.309	0.758	1.19E-08	count	1
WDR92	0.1538865	0.6903526	0.2229	0.824	1.28E-08	count	1
ZNF37A	0.1791866	0.5775537	0.3103	0.757	1.50E-08	count	1
CTC1	0.4863575	0.9293277	0.5233	0.601	1.59E-08	count	1
GRAP	0.4863575	0.9293277	0.5233	0.601	1.59E-08	count	1
FUT8-AS1	0.4863575	1.1591267	0.4196	0.675	1.59E-08	count	1
ZNF28	0.4863575	1.1591267	0.4196	0.675	1.59E-08	count	1
TOM1	0.4863575	1.1591267	0.4196	0.675	1.59E-08	count	1
D2HGDH	0.4863575	1.1591267	0.4196	0.675	1.59E-08	count	1
SMC5-AS1	0.4863575	1.1591267	0.4196	0.675	1.59E-08	count	1
MYO7A	0.4863575	1.1591267	0.4196	0.675	1.59E-08	count	1
C17orf51	0.4863575	1.1591267	0.4196	0.675	1.59E-08	count	1
CD274	0.4863575	1.1591267	0.4196	0.675	1.59E-08	count	1
TBC1D16	0.4863575	1.1591267	0.4196	0.675	1.59E-08	count	1
CABLES1	0.4863575	1.1591267	0.4196	0.675	1.59E-08	count	1
MFAP4	0.4863575	1.1591267	0.4196	0.675	1.59E-08	count	1
AC124016.2	0.4863575	1.1591267	0.4196	0.675	1.59E-08	count	1
ARG2	0.4863575	1.1591267	0.4196	0.675	1.59E-08	count	1
ZNF316	0.4863575	1.1591267	0.4196	0.675	1.59E-08	count	1
SLC7A1	0.4863575	1.1591267	0.4196	0.675	1.59E-08	count	1
CDA	18.3870749	3631.53755	0.0051	0.996	1.79E-08	count	1
PLA2G4A	18.3870749	3631.53755	0.0051	0.996	1.79E-08	count	1
BATF3	18.3870749	3631.53755	0.0051	0.996	1.79E-08	count	1
SIX3-AS1	18.3870749	3631.537684	0.0051	0.996	1.79E-08	count	1
HMMR	18.3870749	3631.537684	0.0051	0.996	1.79E-08	count	1
TEAD3	18.3870749	3631.53755	0.0051	0.996	1.79E-08	count	1
VGF	18.3870749	3631.537684	0.0051	0.996	1.79E-08	count	1
RIBC1	18.3870749	3631.53755	0.0051	0.996	1.79E-08	count	1
AL132656.2	18.3870749	3631.53755	0.0051	0.996	1.79E-08	count	1
CFAP54	18.3870749	3631.537684	0.0051	0.996	1.79E-08	count	1
MORN3	18.3870749	3631.53755	0.0051	0.996	1.79E-08	count	1
FRY	18.3870749	3631.53755	0.0051	0.996	1.79E-08	count	1
ACP6	18.3870747	3631.537707	0.0051	0.996	1.79E-08	count	1
TMEM79	18.3870748	3631.537483	0.0051	0.996	1.79E-08	count	1
AL591846.2	18.3870748	3631.537483	0.0051	0.996	1.79E-08	count	1
RBKS	18.3870748	3631.537483	0.0051	0.996	1.79E-08	count	1

ATOH8	18.3870747	3631.537707	0.0051	0.996	1.79E-08	count	1
AC053503.5	18.387075	3631.537729	0.0051	0.996	1.79E-08	count	1
LINC02018	18.387075	3631.537729	0.0051	0.996	1.79E-08	count	1
ZBTB20-AS4	18.387075	3631.537729	0.0051	0.996	1.79E-08	count	1
AC111006.1	18.3870747	3631.537707	0.0051	0.996	1.79E-08	count	1
TMEM232	18.387075	3631.537617	0.0051	0.996	1.79E-08	count	1
TSLP	18.3870747	3631.537707	0.0051	0.996	1.79E-08	count	1
NPM2	18.3870748	3631.537483	0.0051	0.996	1.79E-08	count	1
NKX3-1	18.387075	3631.537729	0.0051	0.996	1.79E-08	count	1
STK3	18.387075	3631.537729	0.0051	0.996	1.79E-08	count	1
ANKRD20A2	18.3870747	3631.537707	0.0051	0.996	1.79E-08	count	1
AC084337.2	18.387075	3631.537729	0.0051	0.996	1.79E-08	count	1
AP001267.1	18.387075	3631.537617	0.0051	0.996	1.79E-08	count	1
AP003486.1	18.387075	3631.537729	0.0051	0.996	1.79E-08	count	1
AC073389.1	18.387075	3631.537617	0.0051	0.996	1.79E-08	count	1
AC068888.1	18.387075	3631.537617	0.0051	0.996	1.79E-08	count	1
LINC01465	18.387075	3631.537617	0.0051	0.996	1.79E-08	count	1
ATXN2-AS	18.387075	3631.537617	0.0051	0.996	1.79E-08	count	1
DIABLO	18.3870748	3631.537483	0.0051	0.996	1.79E-08	count	1
ABCB9	18.387075	3631.537617	0.0051	0.996	1.79E-08	count	1
AC073911.1	18.3870748	3631.537483	0.0051	0.996	1.79E-08	count	1
LINC01198	18.387075	3631.537617	0.0051	0.996	1.79E-08	count	1
C15orf53	18.387075	3631.537617	0.0051	0.996	1.79E-08	count	1
AC020659.1	18.387075	3631.537617	0.0051	0.996	1.79E-08	count	1
AC025219.1	18.3870747	3631.537707	0.0051	0.996	1.79E-08	count	1
CRNDE	18.387075	3631.537617	0.0051	0.996	1.79E-08	count	1
PIGW	18.3870748	3631.537483	0.0051	0.996	1.79E-08	count	1
AC142472.1	18.3870748	3631.537483	0.0051	0.996	1.79E-08	count	1
LKAAEAR1	18.3870748	3631.537483	0.0051	0.996	1.79E-08	count	1
C19orf47	18.3870747	3631.537707	0.0051	0.996	1.79E-08	count	1
RNF215	18.387075	3631.537729	0.0051	0.996	1.79E-08	count	1
AL353622.1	18.3870749	3631.537774	0.0051	0.996	1.79E-08	count	1
RWDD3	18.3870748	3631.537595	0.0051	0.996	1.79E-08	count	1
ZNF662	18.3870747	3631.53755	0.0051	0.996	1.79E-08	count	1
MCM2	18.3870749	3631.537774	0.0051	0.996	1.79E-08	count	1
ABCG2	18.3870748	3631.537595	0.0051	0.996	1.79E-08	count	1
AC096711.2	18.3870748	3631.537595	0.0051	0.996	1.79E-08	count	1
HIST1H3H	18.3870747	3631.53755	0.0051	0.996	1.79E-08	count	1
UNC5CL	18.3870747	3631.53755	0.0051	0.996	1.79E-08	count	1
AL135905.1	18.3870748	3631.537595	0.0051	0.996	1.79E-08	count	1
AC072061.1	18.3870748	3631.537595	0.0051	0.996	1.79E-08	count	1
ZNF777	18.3870747	3631.53755	0.0051	0.996	1.79E-08	count	1
MBTPS2	18.3870748	3631.537595	0.0051	0.996	1.79E-08	count	1
IFITM10	18.3870747	3631.53755	0.0051	0.996	1.79E-08	count	1
AC127164.1	18.3870749	3631.537774	0.0051	0.996	1.79E-08	count	1

TRAV36DV7	18.3870747	3631.53755	0.0051	0.996	1.79E-08	count	1
AC025580.3	18.3870749	3631.537774	0.0051	0.996	1.79E-08	count	1
PRSS27	18.3870748	3631.537595	0.0051	0.996	1.79E-08	count	1
AC092117.1	18.3870748	3631.537595	0.0051	0.996	1.79E-08	count	1
GNAO1	18.3870748	3631.537595	0.0051	0.996	1.79E-08	count	1
AL049712.1	18.3870747	3631.53755	0.0051	0.996	1.79E-08	count	1
DLGAP4-AS1	18.3870747	3631.53755	0.0051	0.996	1.79E-08	count	1
AC022098.4	18.3870747	3631.53755	0.0051	0.996	1.79E-08	count	1
AC011462.1	18.3870747	3631.53755	0.0051	0.996	1.79E-08	count	1
ZNF526	18.3870747	3631.53755	0.0051	0.996	1.79E-08	count	1
CNFN	18.3870747	3631.53755	0.0051	0.996	1.79E-08	count	1
CPLANE2	18.3870748	3631.537639	0.0051	0.996	1.79E-08	count	1
ZFP69	18.3870748	3631.537483	0.0051	0.996	1.79E-08	count	1
ACOXL	18.3870748	3631.537639	0.0051	0.996	1.79E-08	count	1
PPM1L	18.3870749	3631.537528	0.0051	0.996	1.79E-08	count	1
HIST1H2AH	18.3870749	3631.537528	0.0051	0.996	1.79E-08	count	1
SLC29A1	18.3870748	3631.537639	0.0051	0.996	1.79E-08	count	1
RPS6KA2	18.3870748	3631.537639	0.0051	0.996	1.79E-08	count	1
RBAK	18.3870748	3631.537639	0.0051	0.996	1.79E-08	count	1
TSPAN13	18.3870748	3631.537639	0.0051	0.996	1.79E-08	count	1
HEPH	18.3870748	3631.537483	0.0051	0.996	1.79E-08	count	1
NUGGC	18.3870748	3631.537639	0.0051	0.996	1.79E-08	count	1
GLIS3	18.3870749	3631.537528	0.0051	0.996	1.79E-08	count	1
TNC	18.3870748	3631.537483	0.0051	0.996	1.79E-08	count	1
AP000787.1	18.3870748	3631.537483	0.0051	0.996	1.79E-08	count	1
AP003392.6	18.3870748	3631.537483	0.0051	0.996	1.79E-08	count	1
AL512598.1	18.3870748	3631.537483	0.0051	0.996	1.79E-08	count	1
TPCN1	18.3870748	3631.537639	0.0051	0.996	1.79E-08	count	1
MMP14	18.3870748	3631.537639	0.0051	0.996	1.79E-08	count	1
HIF1A-AS2	18.3870748	3631.537483	0.0051	0.996	1.79E-08	count	1
VASH1	18.3870748	3631.537483	0.0051	0.996	1.79E-08	count	1
SENP8	18.3870748	3631.537483	0.0051	0.996	1.79E-08	count	1
PRR11	18.3870748	3631.537639	0.0051	0.996	1.79E-08	count	1
AC132872.2	18.3870748	3631.537639	0.0051	0.996	1.79E-08	count	1
AC022726.2	18.3870748	3631.537639	0.0051	0.996	1.79E-08	count	1
APOL4	18.3870748	3631.537639	0.0051	0.996	1.79E-08	count	1
NPTXR	18.3870748	3631.537639	0.0051	0.996	1.79E-08	count	1
AC011043.1	18.3870748	3631.537639	0.0051	0.996	1.79E-08	count	1
WDPCP	18.3870746	3631.537505	0.0051	0.996	1.79E-08	count	1
ABHD18	18.3870748	3631.537505	0.0051	0.996	1.79E-08	count	1
TONSL	18.3870746	3631.537505	0.0051	0.996	1.79E-08	count	1
AL354733.3	18.3870748	3631.537505	0.0051	0.996	1.79E-08	count	1
ZNF365	18.3870748	3631.537505	0.0051	0.996	1.79E-08	count	1
POLE2	18.3870748	3631.537505	0.0051	0.996	1.79E-08	count	1
AC003102.1	18.3870746	3631.537505	0.0051	0.996	1.79E-08	count	1

AL357033.4	18.3870746	3631.537505	0.0051	0.996	1.79E-08	count	1
AC098484.2	18.3870748	3631.537639	0.0051	0.996	1.79E-08	count	1
AC099063.4	18.3870748	3631.537639	0.0051	0.996	1.79E-08	count	1
KIF14	18.3870746	3631.537505	0.0051	0.996	1.79E-08	count	1
AC092803.2	18.3870746	3631.537505	0.0051	0.996	1.79E-08	count	1
ALS2	18.3870743	3631.537729	0.0051	0.996	1.79E-08	count	1
AC099778.1	18.3870746	3631.537505	0.0051	0.996	1.79E-08	count	1
LNP1	18.3870746	3631.537505	0.0051	0.996	1.79E-08	count	1
RAB6B	18.3870743	3631.537729	0.0051	0.996	1.79E-08	count	1
TRIP13	18.3870746	3631.537505	0.0051	0.996	1.79E-08	count	1
HIST1H3E	18.3870746	3631.537505	0.0051	0.996	1.79E-08	count	1
DDO	18.3870746	3631.537505	0.0051	0.996	1.79E-08	count	1
CAHM	18.3870746	3631.537505	0.0051	0.996	1.79E-08	count	1
TTC26	18.3870746	3631.537505	0.0051	0.996	1.79E-08	count	1
SAMD12	18.3870746	3631.537528	0.0051	0.996	1.79E-08	count	1
ADGRB1	18.3870748	3631.537639	0.0051	0.996	1.79E-08	count	1
CDC37L1-DT	18.3870746	3631.537528	0.0051	0.996	1.79E-08	count	1
ANKRD20A4	18.3870748	3631.537639	0.0051	0.996	1.79E-08	count	1
AC011611.4	18.3870746	3631.537505	0.0051	0.996	1.79E-08	count	1
HCAR1	18.3870746	3631.537505	0.0051	0.996	1.79E-08	count	1
RBM26-AS1	18.3870746	3631.537528	0.0051	0.996	1.79E-08	count	1
TMED8	18.3870746	3631.537505	0.0051	0.996	1.79E-08	count	1
SERPINA11	18.3870746	3631.537528	0.0051	0.996	1.79E-08	count	1
HEXA-AS1	18.3870748	3631.537639	0.0051	0.996	1.79E-08	count	1
ARRDC4	18.3870748	3631.537639	0.0051	0.996	1.79E-08	count	1
CDR2L	18.3870746	3631.537528	0.0051	0.996	1.79E-08	count	1
RBFADN	18.3870746	3631.537505	0.0051	0.996	1.79E-08	count	1
ZNF555	18.3870748	3631.537639	0.0051	0.996	1.79E-08	count	1
ZNF233	18.3870743	3631.537729	0.0051	0.996	1.79E-08	count	1
AP001437.1	18.3870748	3631.537639	0.0051	0.996	1.79E-08	count	1
AL606491.1	18.3870746	3631.537416	0.0051	0.996	1.79E-08	count	1
MEIS1	18.3870746	3631.537416	0.0051	0.996	1.79E-08	count	1
FHL2	18.3870744	3631.537639	0.0051	0.996	1.79E-08	count	1
AC005037.1	18.3870745	3631.537662	0.0051	0.996	1.79E-08	count	1
EFCC1	18.3870744	3631.537639	0.0051	0.996	1.79E-08	count	1
TRGV2	18.3870745	3631.537662	0.0051	0.996	1.79E-08	count	1
ZMAT4	18.3870746	3631.537416	0.0051	0.996	1.79E-08	count	1
AL354920.1	18.3870746	3631.537416	0.0051	0.996	1.79E-08	count	1
RNF141	18.3870744	3631.537528	0.0051	0.996	1.79E-08	count	1
CAPN5	18.3870745	3631.537662	0.0051	0.996	1.79E-08	count	1
DIXDC1	18.3870745	3631.537662	0.0051	0.996	1.79E-08	count	1
AC010864.1	18.3870745	3631.537662	0.0051	0.996	1.79E-08	count	1
AC067750.1	18.3870745	3631.537662	0.0051	0.996	1.79E-08	count	1
LAG3	18.3870744	3631.537528	0.0051	0.996	1.79E-08	count	1
RERGL	18.3870744	3631.537639	0.0051	0.996	1.79E-08	count	1

LINC02323	18.3870746	3631.537416	0.0051	0.996	1.79E-08	count	1
ZNF768	18.3870744	3631.537639	0.0051	0.996	1.79E-08	count	1
AC123912.4	18.3870746	3631.537416	0.0051	0.996	1.79E-08	count	1
GCAT	18.3870746	3631.537416	0.0051	0.996	1.79E-08	count	1
WBP2NL	18.3870744	3631.537639	0.0051	0.996	1.79E-08	count	1
MAP3K7CL	18.3870744	3631.537639	0.0051	0.996	1.79E-08	count	1
BCAN	18.3870746	3631.537595	0.0051	0.996	1.79E-08	count	1
LAMP3	18.3870746	3631.537595	0.0051	0.996	1.79E-08	count	1
AL137003.1	18.3870746	3631.537595	0.0051	0.996	1.79E-08	count	1
ACTR3C	18.3870746	3631.537595	0.0051	0.996	1.79E-08	count	1
FAM168A	18.3870746	3631.537595	0.0051	0.996	1.79E-08	count	1
AC005840.4	18.3870746	3631.537595	0.0051	0.996	1.79E-08	count	1
ISLR	18.3870746	3631.537595	0.0051	0.996	1.79E-08	count	1
CEACAM1	18.3870746	3631.537595	0.0051	0.996	1.79E-08	count	1
APOBEC3A	18.3870746	3631.537595	0.0051	0.996	1.79E-08	count	1
CRYBG2	18.3870746	3631.537572	0.0051	0.996	1.79E-08	count	1
NFYC-AS1	18.3870744	3631.537438	0.0051	0.996	1.79E-08	count	1
ECM1	18.3870744	3631.537371	0.0051	0.996	1.79E-08	count	1
C2orf81	18.3870744	3631.537371	0.0051	0.996	1.79E-08	count	1
EPB41L5	18.3870744	3631.537371	0.0051	0.996	1.79E-08	count	1
USP40	18.3870743	3631.537393	0.0051	0.996	1.79E-08	count	1
AC105760.2	18.3870746	3631.537572	0.0051	0.996	1.79E-08	count	1
AC106707.1	18.3870743	3631.537393	0.0051	0.996	1.79E-08	count	1
MCF2L2	18.3870744	3631.537371	0.0051	0.996	1.79E-08	count	1
AL008729.2	18.3870744	3631.537438	0.0051	0.996	1.79E-08	count	1
IGFBP3	18.3870746	3631.537572	0.0051	0.996	1.79E-08	count	1
TRBV10-2	18.3870746	3631.537572	0.0051	0.996	1.79E-08	count	1
OR2A1-AS1	18.3870743	3631.537393	0.0051	0.996	1.79E-08	count	1
GPR173	18.3870745	3631.537729	0.0051	0.996	1.79E-08	count	1
TSIX	18.3870746	3631.537572	0.0051	0.996	1.79E-08	count	1
PYCR3	18.3870743	3631.537393	0.0051	0.996	1.79E-08	count	1
NACC2	18.3870746	3631.537572	0.0051	0.996	1.79E-08	count	1
AC103736.1	18.3870743	3631.537393	0.0051	0.996	1.79E-08	count	1
AP000442.2	18.3870744	3631.537371	0.0051	0.996	1.79E-08	count	1
RHOD	18.3870744	3631.537371	0.0051	0.996	1.79E-08	count	1
AP000802.1	18.3870743	3631.537393	0.0051	0.996	1.79E-08	count	1
TMEM136	18.3870744	3631.537371	0.0051	0.996	1.79E-08	count	1
ASAH2	18.3870744	3631.537371	0.0051	0.996	1.79E-08	count	1
ARHGAP19	18.3870744	3631.537438	0.0051	0.996	1.79E-08	count	1
DPCD	18.3870743	3631.537393	0.0051	0.996	1.79E-08	count	1
SUFU	18.3870743	3631.537393	0.0051	0.996	1.79E-08	count	1
CLEC12A-AS1	18.3870744	3631.537438	0.0051	0.996	1.79E-08	count	1
GLIPR1L2	18.3870746	3631.537572	0.0051	0.996	1.79E-08	count	1
CEP83-DT	18.3870745	3631.537729	0.0051	0.996	1.79E-08	count	1
AMDHD1	18.3870743	3631.537393	0.0051	0.996	1.79E-08	count	1

SLC25A30	18.3870744	3631.537371	0.0051	0.996	1.79E-08	count	1
DNAJA4	18.3870745	3631.537729	0.0051	0.996	1.79E-08	count	1
KRBA2	18.3870744	3631.537371	0.0051	0.996	1.79E-08	count	1
AC087741.1	18.3870746	3631.537572	0.0051	0.996	1.79E-08	count	1
AC027575.2	18.3870744	3631.537371	0.0051	0.996	1.79E-08	count	1
AL035563.1	18.3870743	3631.537393	0.0051	0.996	1.79E-08	count	1
LINC01597	18.3870744	3631.537371	0.0051	0.996	1.79E-08	count	1
AL031663.3	18.3870746	3631.537572	0.0051	0.996	1.79E-08	count	1
ADGRL1	18.3870743	3631.537393	0.0051	0.996	1.79E-08	count	1
SLC5A5	18.3870746	3631.537572	0.0051	0.996	1.79E-08	count	1
AC020928.2	18.3870744	3631.537371	0.0051	0.996	1.79E-08	count	1
SRRM5	18.3870746	3631.537572	0.0051	0.996	1.79E-08	count	1
SPIB	18.3870744	3631.537371	0.0051	0.996	1.79E-08	count	1
Z83847.1	18.3870745	3631.537729	0.0051	0.996	1.79E-08	count	1
AF165147.1	18.3870744	3631.537371	0.0051	0.996	1.79E-08	count	1
CNIH3	18.9784852	1910.358008	0.0099	0.992	1.81E-08	count	1
TTL	18.9784852	1910.358008	0.0099	0.992	1.81E-08	count	1
AC132192.2	18.9784852	1910.358008	0.0099	0.992	1.81E-08	count	1
ANKRD34A	18.9784852	1910.357976	0.0099	0.992	1.81E-08	count	1
RHOU	18.9784852	1910.357976	0.0099	0.992	1.81E-08	count	1
MSH5-SAPCD1	18.9784852	1910.357976	0.0099	0.992	1.81E-08	count	1
TMEM67	18.9784852	1910.357976	0.0099	0.992	1.81E-08	count	1
TTC16	18.9784852	1910.357976	0.0099	0.992	1.81E-08	count	1
MOCOS	18.9784852	1910.357976	0.0099	0.992	1.81E-08	count	1
AC093227.1	18.9784852	1910.357976	0.0099	0.992	1.81E-08	count	1
LEXM	18.9784852	1910.357932	0.0099	0.992	1.81E-08	count	1
AC098936.1	18.978485	1910.357845	0.0099	0.992	1.81E-08	count	1
KIF9	18.9784852	1910.357932	0.0099	0.992	1.81E-08	count	1
CCDC39	18.9784852	1910.357932	0.0099	0.992	1.81E-08	count	1
AL365205.1	18.978485	1910.357845	0.0099	0.992	1.81E-08	count	1
AL359711.2	18.9784852	1910.357932	0.0099	0.992	1.81E-08	count	1
AGBL3	18.9784852	1910.357932	0.0099	0.992	1.81E-08	count	1
GFI1B	18.9784852	1910.357932	0.0099	0.992	1.81E-08	count	1
FCN1	18.9784852	1910.357932	0.0099	0.992	1.81E-08	count	1
CCDC87	18.9784852	1910.357932	0.0099	0.992	1.81E-08	count	1
FAR2	18.9784852	1910.357932	0.0099	0.992	1.81E-08	count	1
DCN	18.9784852	1910.357932	0.0099	0.992	1.81E-08	count	1
KTN1-AS1	18.9784852	1910.357932	0.0099	0.992	1.81E-08	count	1
TTC7B	18.9784852	1910.357932	0.0099	0.992	1.81E-08	count	1
TMEM106A	18.9784852	1910.357932	0.0099	0.992	1.81E-08	count	1
HSF5	18.9784852	1910.357932	0.0099	0.992	1.81E-08	count	1
AC100786.1	18.978485	1910.357845	0.0099	0.992	1.81E-08	count	1
AC144831.1	18.9784852	1910.357932	0.0099	0.992	1.81E-08	count	1
GREM2	18.978485	1910.357987	0.0099	0.992	1.81E-08	count	1
AC012368.1	18.9784851	1910.357856	0.0099	0.992	1.81E-08	count	1

C2orf15	18.978485	1910.357987	0.0099	0.992	1.81E-08	count	1
MARS2	18.978485	1910.357987	0.0099	0.992	1.81E-08	count	1
AL035701.1	18.978485	1910.357987	0.0099	0.992	1.81E-08	count	1
FAM24B	18.978485	1910.357987	0.0099	0.992	1.81E-08	count	1
AL359317.2	18.9784851	1910.357856	0.0099	0.992	1.81E-08	count	1
C15orf65	18.978485	1910.357987	0.0099	0.992	1.81E-08	count	1
TRIM72	18.978485	1910.357987	0.0099	0.992	1.81E-08	count	1
MFSD6L	18.9784851	1910.357856	0.0099	0.992	1.81E-08	count	1
AC103810.2	18.978485	1910.357987	0.0099	0.992	1.81E-08	count	1
C1QTNF12	18.9784848	1910.357976	0.0099	0.992	1.81E-08	count	1
AL139289.2	18.9784848	1910.357976	0.0099	0.992	1.81E-08	count	1
RTL5	18.9784848	1910.357976	0.0099	0.992	1.81E-08	count	1
LINC00630	18.9784848	1910.357976	0.0099	0.992	1.81E-08	count	1
RFX3-AS1	18.9784848	1910.357976	0.0099	0.992	1.81E-08	count	1
GARNL3	18.9784848	1910.357976	0.0099	0.992	1.81E-08	count	1
HARBI1	18.9784848	1910.357976	0.0099	0.992	1.81E-08	count	1
ATP6V0C	18.9784848	1910.357976	0.0099	0.992	1.81E-08	count	1
PRR22	18.9784848	1910.357976	0.0099	0.992	1.81E-08	count	1
AC010487.2	18.9784848	1910.357976	0.0099	0.992	1.81E-08	count	1
C19orf18	18.9784848	1910.357976	0.0099	0.992	1.81E-08	count	1
TCEANC2	18.9784848	1910.358052	0.0099	0.992	1.81E-08	count	1
TPRG1	18.9784848	1910.358052	0.0099	0.992	1.81E-08	count	1
CDC14B	18.9784848	1910.358052	0.0099	0.992	1.81E-08	count	1
EGLN2	18.9784848	1910.358052	0.0099	0.992	1.81E-08	count	1
AC008040.5	18.9784847	1910.357921	0.0099	0.992	1.81E-08	count	1
SCRG1	18.9784847	1910.357921	0.0099	0.992	1.81E-08	count	1
AC008549.2	18.9784847	1910.357921	0.0099	0.992	1.81E-08	count	1
PVRIG	18.9784849	1910.357965	0.0099	0.992	1.81E-08	count	1
FAM131B	18.9784849	1910.357965	0.0099	0.992	1.81E-08	count	1
PDGFRL	18.9784849	1910.357965	0.0099	0.992	1.81E-08	count	1
PLCB3	18.9784849	1910.357965	0.0099	0.992	1.81E-08	count	1
ANK3	18.9784847	1910.357921	0.0099	0.992	1.81E-08	count	1
ENTPD7	18.9784849	1910.357965	0.0099	0.992	1.81E-08	count	1
CLECL1	18.9784849	1910.357965	0.0099	0.992	1.81E-08	count	1
SCARB1	18.9784847	1910.357921	0.0099	0.992	1.81E-08	count	1
CCDC198	18.9784847	1910.357921	0.0099	0.992	1.81E-08	count	1
AC116913.1	18.9784847	1910.357921	0.0099	0.992	1.81E-08	count	1
LINC01569	18.9784847	1910.357921	0.0099	0.992	1.81E-08	count	1
PLEKHG4	18.9784849	1910.357965	0.0099	0.992	1.81E-08	count	1
ZNF418	18.9784849	1910.357965	0.0099	0.992	1.81E-08	count	1
AL137802.2	18.9784848	1910.357867	0.0099	0.992	1.81E-08	count	1
UBXN10-AS1	18.9784847	1910.357899	0.0099	0.992	1.81E-08	count	1
NBPF12	18.9784847	1910.357899	0.0099	0.992	1.81E-08	count	1
RGS13	18.978485	1910.35803	0.0099	0.992	1.81E-08	count	1
TRIM17	18.9784848	1910.357867	0.0099	0.992	1.81E-08	count	1

CD86	18.978485	1910.35803	0.0099	0.992	1.81E-08	count	1
MELTF-AS1	18.9784848	1910.357867	0.0099	0.992	1.81E-08	count	1
CCDC96	18.978485	1910.35803	0.0099	0.992	1.81E-08	count	1
LNX1	18.9784848	1910.357867	0.0099	0.992	1.81E-08	count	1
TNRC18	18.9784848	1910.357867	0.0099	0.992	1.81E-08	count	1
CLIP2	18.9784848	1910.357867	0.0099	0.992	1.81E-08	count	1
ADAM9	18.9784847	1910.357899	0.0099	0.992	1.81E-08	count	1
LACC1	18.9784847	1910.357899	0.0099	0.992	1.81E-08	count	1
AL049840.2	18.9784847	1910.357899	0.0099	0.992	1.81E-08	count	1
ADSSL1	18.9784847	1910.357899	0.0099	0.992	1.81E-08	count	1
LINC00235	18.9784848	1910.357867	0.0099	0.992	1.81E-08	count	1
AC011933.4	18.9784848	1910.357867	0.0099	0.992	1.81E-08	count	1
KCNN1	18.9784847	1910.357899	0.0099	0.992	1.81E-08	count	1
LINC01393	18.9784847	1910.35803	0.0099	0.992	1.81E-08	count	1
PRICKLE3	18.9784847	1910.35803	0.0099	0.992	1.81E-08	count	1
STAM-AS1	18.9784847	1910.35803	0.0099	0.992	1.81E-08	count	1
ZC2HC1C	18.9784847	1910.35803	0.0099	0.992	1.81E-08	count	1
ZNF319	18.9784847	1910.35803	0.0099	0.992	1.81E-08	count	1
SLC12A4	18.9784847	1910.35803	0.0099	0.992	1.81E-08	count	1
LINC01572	18.9784847	1910.35803	0.0099	0.992	1.81E-08	count	1
CRB3	18.9784847	1910.35803	0.0099	0.992	1.81E-08	count	1
FRMPD3	19.6498981	2672.454325	0.0074	0.994	1.81E-08	count	1
PDZD7	19.6498981	2672.454325	0.0074	0.994	1.81E-08	count	1
AC009318.3	19.6498981	2672.454325	0.0074	0.994	1.81E-08	count	1
HSD17B6	19.6498981	2672.454325	0.0074	0.994	1.81E-08	count	1
PDE6A	19.6498978	2672.454354	0.0074	0.994	1.81E-08	count	1
HPSE	19.6498979	2672.454354	0.0074	0.994	1.81E-08	count	1
HIST1H4A	19.6498979	2672.454354	0.0074	0.994	1.81E-08	count	1
FBXL18	19.6498979	2672.454354	0.0074	0.994	1.81E-08	count	1
ATP6V0E2-AS1	19.6498979	2672.454354	0.0074	0.994	1.81E-08	count	1
SNX30	19.6498979	2672.454354	0.0074	0.994	1.81E-08	count	1
PDE2A	19.6498979	2672.454354	0.0074	0.994	1.81E-08	count	1
AL355490.2	19.6498979	2672.454354	0.0074	0.994	1.81E-08	count	1
ADAM21	19.6498979	2672.454354	0.0074	0.994	1.81E-08	count	1
AC009093.2	19.6498979	2672.454354	0.0074	0.994	1.81E-08	count	1
HOXB7	19.6498979	2672.454354	0.0074	0.994	1.81E-08	count	1
AC005837.1	19.6498979	2672.454354	0.0074	0.994	1.81E-08	count	1
ZNF865	19.6498979	2672.454354	0.0074	0.994	1.81E-08	count	1
TMEM237	19.6498976	2672.454175	0.0074	0.994	1.81E-08	count	1
LINC01011	19.6498976	2672.454175	0.0074	0.994	1.81E-08	count	1
MFAP5	19.6498976	2672.454175	0.0074	0.994	1.81E-08	count	1
HOXC4	19.6498976	2672.454175	0.0074	0.994	1.81E-08	count	1
REM2	19.6498976	2672.454175	0.0074	0.994	1.81E-08	count	1
SLC5A2	19.6498976	2672.454175	0.0074	0.994	1.81E-08	count	1
ABCA9	19.6498976	2672.454175	0.0074	0.994	1.81E-08	count	1

AL031731.1	19.649897	2672.454235	0.0074	0.994	1.81E-08	count	1
RN7SL832P	19.649897	2672.454235	0.0074	0.994	1.81E-08	count	1
PSMD6-AS2	19.649897	2672.454235	0.0074	0.994	1.81E-08	count	1
AL163051.1	19.649897	2672.454235	0.0074	0.994	1.81E-08	count	1
GOLGA6L4	19.649897	2672.454235	0.0074	0.994	1.81E-08	count	1
SLPI	19.6498977	2672.453996	0.0074	0.994	1.81E-08	count	1
GIPR	19.6498977	2672.453996	0.0074	0.994	1.81E-08	count	1
KRBA1	19.6498977	2672.453937	0.0074	0.994	1.81E-08	count	1
AC138356.1	19.6498977	2672.453937	0.0074	0.994	1.81E-08	count	1
TMEM9B-AS1	19.6498977	2672.453937	0.0074	0.994	1.81E-08	count	1
AP002807.1	19.6498977	2672.453937	0.0074	0.994	1.81E-08	count	1
AL117336.3	19.6498977	2672.453937	0.0074	0.994	1.81E-08	count	1
AC027020.2	19.6498977	2672.453937	0.0074	0.994	1.81E-08	count	1
TCF3	19.6498977	2672.453937	0.0074	0.994	1.81E-08	count	1
AC090061.1	19.6564336	1854.222492	0.0106	0.992	1.81E-08	count	1
BTBD8	19.6564331	1854.222492	0.0106	0.992	1.81E-08	count	1
SGCE	19.656432	1854.222221	0.0106	0.992	1.81E-08	count	1
AL137779.1	19.6564321	1854.2222	0.0106	0.992	1.81E-08	count	1
ANKDD1B	20.0230381	2358.376491	0.0085	0.993	1.82E-08	count	1
AC000032.1	20.0230385	2358.376261	0.0085	0.993	1.82E-08	count	1
COL4A4	20.0230378	2358.376363	0.0085	0.993	1.82E-08	count	1
SF3B3	0.0867114	0.479071	0.181	0.856	1.94E-08	count	1
GAB1	0.2376221	0.7771019	0.3058	0.76	2.02E-08	count	1
EXOC6B	0.2376221	0.9069862	0.262	0.793	2.02E-08	count	1
SIRT5	0.2376221	0.9069862	0.262	0.793	2.02E-08	count	1
CNTROB	0.2376221	0.9069862	0.262	0.793	2.02E-08	count	1
AC116366.3	0.2376221	0.9069862	0.262	0.793	2.02E-08	count	1
ZNF503	0.2376221	0.9069862	0.262	0.793	2.02E-08	count	1
SLC29A3	0.2376221	0.8209174	0.2895	0.772	2.02E-08	count	1
STYK1	0.2376221	0.8142853	0.2918	0.771	2.02E-08	count	1
MBLAC1	0.2376221	1.1117474	0.2137	0.831	2.02E-08	count	1
MIEF1	0.2415624	0.7124033	0.3391	0.735	2.05E-08	count	1
SPRED2	0.2415624	0.8117844	0.2976	0.766	2.06E-08	count	1
ZNF550	0.2415624	0.8890411	0.2717	0.786	2.06E-08	count	1
SLC36A4	0.2484614	0.6309898	0.3938	0.694	2.11E-08	count	1
FOXP4	0.2484614	0.9694404	0.2563	0.798	2.12E-08	count	1
ALDH18A1	0.6539265	0.8510463	0.7684	0.443	2.19E-08	count	1
AC016876.1	0.6539265	1.0734353	0.6092	0.543	2.20E-08	count	1
GDPD3	0.7113817	1.1568427	0.6149	0.539	2.40E-08	count	1
CHRM3-AS2	0.7113817	1.1568427	0.6149	0.539	2.40E-08	count	1
PROSER3	0.7113817	1.1568427	0.6149	0.539	2.40E-08	count	1
ZNF510	0.7113817	1.1568427	0.6149	0.539	2.40E-08	count	1
CASTOR1	0.7113817	0.8648567	0.8225	0.411	2.40E-08	count	1
ABHD17C	0.7113817	0.8648567	0.8225	0.411	2.40E-08	count	1
PGBD4	0.7113817	0.8648567	0.8225	0.411	2.40E-08	count	1

IL6R	0.7113817	1.0748726	0.6618	0.509	2.41E-08	count	1
AC103724.4	0.3285041	0.808494	0.4063	0.685	2.84E-08	count	1
SGPL1	0.3683362	0.5638287	0.6533	0.514	3.22E-08	count	1
AL078644.2	0.37328	1.0959203	0.3406	0.734	3.25E-08	count	1
MYBBP1A	0.37328	1.0959203	0.3406	0.734	3.25E-08	count	1
C3AR1	0.37328	1.0217972	0.3653	0.715	3.25E-08	count	1
STRN4	0.37328	1.0217972	0.3653	0.715	3.25E-08	count	1
LINC01136	0.37328	0.9202505	0.4056	0.685	3.25E-08	count	1
LRRC8D	0.9416085	0.7905694	1.1911	0.235	3.29E-08	count	1
LNX2	0.9416085	0.7905694	1.1911	0.235	3.29E-08	count	1
AC091271.1	0.9416085	0.8806037	1.0693	0.286	3.29E-08	count	1
NUDT7	0.9416085	0.8806037	1.0693	0.286	3.29E-08	count	1
PIGQ	0.402612	0.6470404	0.6222	0.534	3.52E-08	count	1
GSTM2	0.1551861	0.5875796	0.2641	0.792	3.52E-08	count	1
TMEM68	0.402612	0.5945028	0.6772	0.499	3.53E-08	count	1
UBA6-AS1	0.4140967	0.7322819	0.5655	0.572	3.63E-08	count	1
AC026401.3	0.168851	0.7243147	0.2331	0.816	3.84E-08	count	1
AC245140.2	0.1782168	0.8165819	0.2182	0.827	4.15E-08	count	1
GSTM3	0.4716049	0.6609477	0.7135	0.476	4.18E-08	count	1
NEMP2	0.4716049	0.6609477	0.7135	0.476	4.18E-08	count	1
LIN7B	0.4716049	0.6609477	0.7135	0.476	4.18E-08	count	1
HIF1AN	0.4716049	0.7101121	0.6641	0.507	4.18E-08	count	1
RNF212	0.4716049	0.7994211	0.5899	0.556	4.19E-08	count	1
PHRF1	1.1760574	0.7842112	1.4997	0.135	4.20E-08	count	1
MAFK	1.1760574	0.841074	1.3983	0.163	4.21E-08	count	1
AC026471.1	1.1760574	0.841074	1.3983	0.163	4.21E-08	count	1
DFFB	0.4716049	1.0215275	0.4617	0.645	4.22E-08	count	1
TTLL5	0.4716049	1.0215275	0.4617	0.645	4.22E-08	count	1
CUEDC1	0.4716049	1.0215275	0.4617	0.645	4.22E-08	count	1
PPP1R3D	0.4863575	0.8735424	0.5568	0.578	4.32E-08	count	1
KDELC2	0.4863575	0.7553727	0.6439	0.52	4.32E-08	count	1
C22orf46	0.4863575	0.9293277	0.5233	0.601	4.32E-08	count	1
AC093512.1	0.4863575	0.9293277	0.5233	0.601	4.32E-08	count	1
CCZ1B	0.4863575	0.6173509	0.7878	0.431	4.36E-08	count	1
PGAM5	0.1956044	0.5388461	0.363	0.717	4.49E-08	count	1
ADAMTS1	0.5041053	0.6860124	0.7348	0.463	4.50E-08	count	1
SLC12A6	0.5041053	0.6446763	0.782	0.435	4.53E-08	count	1
RASGRP3	1.3217558	0.6350529	2.0813	0.0382	4.78E-08	count	1
CEP57L1	0.5340895	0.516631	1.0338	0.302	4.84E-08	count	1
TNR	18.0815635	3117.08259	0.0058	0.995	4.86E-08	count	1
FZD5	18.0815635	3117.08259	0.0058	0.995	4.86E-08	count	1
HOXA10-AS	18.0815634	3117.082462	0.0058	0.995	4.86E-08	count	1
WDR86	18.0815634	3117.082462	0.0058	0.995	4.86E-08	count	1
AC103724.3	18.0815634	3117.082462	0.0058	0.995	4.86E-08	count	1
PLAT	18.0815634	3117.082462	0.0058	0.995	4.86E-08	count	1

CD44-AS1	18.0815635	3117.08259	0.0058	0.995	4.86E-08	count	1
LINC02422	18.0815635	3117.08259	0.0058	0.995	4.86E-08	count	1
TRMT5	18.0815634	3117.082462	0.0058	0.995	4.86E-08	count	1
AC016355.1	18.0815635	3117.08259	0.0058	0.995	4.86E-08	count	1
GOLGA6L9	18.0815635	3117.08259	0.0058	0.995	4.86E-08	count	1
SEMA4B	18.0815635	3117.08259	0.0058	0.995	4.86E-08	count	1
PLD2	18.0815634	3117.082462	0.0058	0.995	4.86E-08	count	1
TMEM88	18.0815635	3117.08259	0.0058	0.995	4.86E-08	count	1
AC005332.4	18.0815635	3117.08259	0.0058	0.995	4.86E-08	count	1
ICAM5	18.0815635	3117.08259	0.0058	0.995	4.86E-08	count	1
AC020907.3	18.0815634	3117.082462	0.0058	0.995	4.86E-08	count	1
LINC01358	18.0815633	3117.082292	0.0058	0.995	4.86E-08	count	1
NCKIPSD	18.0815633	3117.082292	0.0058	0.995	4.86E-08	count	1
AC006160.1	18.0815633	3117.082561	0.0058	0.995	4.86E-08	count	1
ULBP3	18.0815633	3117.082561	0.0058	0.995	4.86E-08	count	1
LINC01176	18.0815633	3117.082561	0.0058	0.995	4.86E-08	count	1
VPS37D	18.0815633	3117.082292	0.0058	0.995	4.86E-08	count	1
IGSF22	18.0815633	3117.082561	0.0058	0.995	4.86E-08	count	1
AL022341.1	18.0815633	3117.082292	0.0058	0.995	4.86E-08	count	1
AC093525.4	18.0815633	3117.082292	0.0058	0.995	4.86E-08	count	1
AC007613.1	18.0815633	3117.082561	0.0058	0.995	4.86E-08	count	1
LINC02081	18.0815633	3117.082561	0.0058	0.995	4.86E-08	count	1
ADCYAP1	18.0815633	3117.082292	0.0058	0.995	4.86E-08	count	1
ZNF627	18.0815633	3117.082292	0.0058	0.995	4.86E-08	count	1
CLTCL1	18.0815633	3117.082292	0.0058	0.995	4.86E-08	count	1
LINC01679	18.0815633	3117.082561	0.0058	0.995	4.86E-08	count	1
LAMA2	18.0815633	3117.08259	0.0058	0.995	4.86E-08	count	1
AC104389.4	18.0815633	3117.08259	0.0058	0.995	4.86E-08	count	1
AC010999.2	18.0815633	3117.08259	0.0058	0.995	4.86E-08	count	1
CYB5RL	18.0815633	3117.082519	0.0058	0.995	4.86E-08	count	1
GPSM2	18.0815633	3117.082618	0.0058	0.995	4.86E-08	count	1
AC245014.1	18.0815633	3117.082575	0.0058	0.995	4.86E-08	count	1
EFNA3	18.0815633	3117.082519	0.0058	0.995	4.86E-08	count	1
AC092053.3	18.0815632	3117.082505	0.0058	0.995	4.86E-08	count	1
MST1	18.0815632	3117.082505	0.0058	0.995	4.86E-08	count	1
TRIM60	18.0815633	3117.082575	0.0058	0.995	4.86E-08	count	1
CMYA5	18.0815632	3117.082505	0.0058	0.995	4.86E-08	count	1
SERINC5	18.0815633	3117.082519	0.0058	0.995	4.86E-08	count	1
AC139795.2	18.0815633	3117.082618	0.0058	0.995	4.86E-08	count	1
BX322234.1	18.0815633	3117.082575	0.0058	0.995	4.86E-08	count	1
ETV1	18.0815632	3117.082505	0.0058	0.995	4.86E-08	count	1
MUC12	18.0815632	3117.082505	0.0058	0.995	4.86E-08	count	1
NALT1	18.0815633	3117.082575	0.0058	0.995	4.86E-08	count	1
BMPR1A	18.0815633	3117.082575	0.0058	0.995	4.86E-08	count	1
MAP1A	18.0815633	3117.082575	0.0058	0.995	4.86E-08	count	1

SLC12A5	18.0815633	3117.082519	0.0058	0.995	4.86E-08	count	1
PIK3R2	18.0815633	3117.082519	0.0058	0.995	4.86E-08	count	1
AP001065.1	18.0815633	3117.082618	0.0058	0.995	4.86E-08	count	1
CD34	18.0815631	3117.082476	0.0058	0.995	4.86E-08	count	1
AC020571.1	18.0815631	3117.082476	0.0058	0.995	4.86E-08	count	1
AL513550.1	18.081563	3117.082349	0.0058	0.995	4.86E-08	count	1
AL080317.3	18.081563	3117.082349	0.0058	0.995	4.86E-08	count	1
STC1	18.0815631	3117.082476	0.0058	0.995	4.86E-08	count	1
KANK1	18.081563	3117.082349	0.0058	0.995	4.86E-08	count	1
STXBP1	18.081563	3117.082349	0.0058	0.995	4.86E-08	count	1
KCTD21-AS1	18.081563	3117.082349	0.0058	0.995	4.86E-08	count	1
PSD	18.081563	3117.082349	0.0058	0.995	4.86E-08	count	1
CRY1	18.0815631	3117.082292	0.0058	0.995	4.86E-08	count	1
LRRC3	18.081563	3117.082349	0.0058	0.995	4.86E-08	count	1
AC096536.1	18.0815631	3117.082264	0.0058	0.995	4.86E-08	count	1
LEPR	18.0815632	3117.082321	0.0058	0.995	4.86E-08	count	1
AL121983.1	18.081563	3117.082434	0.0058	0.995	4.86E-08	count	1
IRF6	18.0815632	3117.082377	0.0058	0.995	4.86E-08	count	1
LYG1	18.0815631	3117.082264	0.0058	0.995	4.86E-08	count	1
HECW2	18.081563	3117.082123	0.0058	0.995	4.86E-08	count	1
LINC02019	18.0815632	3117.082377	0.0058	0.995	4.86E-08	count	1
AC107027.1	18.0815629	3117.08225	0.0058	0.995	4.86E-08	count	1
AC116651.1	18.0815629	3117.08225	0.0058	0.995	4.86E-08	count	1
F2RL1	18.081563	3117.082123	0.0058	0.995	4.86E-08	count	1
AC005618.1	18.0815631	3117.082264	0.0058	0.995	4.86E-08	count	1
NOTCH4	18.0815631	3117.082264	0.0058	0.995	4.86E-08	count	1
ELOVL4	18.081563	3117.082123	0.0058	0.995	4.86E-08	count	1
AL137784.2	18.0815629	3117.082094	0.0058	0.995	4.86E-08	count	1
SYNJ2	18.081563	3117.082123	0.0058	0.995	4.86E-08	count	1
TSPAN33	18.0815629	3117.08225	0.0058	0.995	4.86E-08	count	1
AC018643.1	18.0815631	3117.082264	0.0058	0.995	4.86E-08	count	1
ZHX2	18.0815629	3117.082094	0.0058	0.995	4.86E-08	count	1
EPPK1	18.081563	3117.082123	0.0058	0.995	4.86E-08	count	1
ENTPD2	18.0815631	3117.082264	0.0058	0.995	4.86E-08	count	1
AC026250.1	18.0815631	3117.082264	0.0058	0.995	4.86E-08	count	1
AP006333.1	18.081563	3117.082123	0.0058	0.995	4.86E-08	count	1
IDI2-AS1	18.0815631	3117.082264	0.0058	0.995	4.86E-08	count	1
LINC00866	18.0815629	3117.08225	0.0058	0.995	4.86E-08	count	1
ADRA2A	18.0815632	3117.082377	0.0058	0.995	4.86E-08	count	1
AC025263.1	18.0815631	3117.082264	0.0058	0.995	4.86E-08	count	1
THSD1	18.0815632	3117.082377	0.0058	0.995	4.86E-08	count	1
AL357153.1	18.0815631	3117.082264	0.0058	0.995	4.86E-08	count	1
AC141586.3	18.0815631	3117.082264	0.0058	0.995	4.86E-08	count	1
AC092338.2	18.0815629	3117.08225	0.0058	0.995	4.86E-08	count	1
TMEM231	18.081563	3117.082123	0.0058	0.995	4.86E-08	count	1

CDC6	18.0815632	3117.082377	0.0058	0.995	4.86E-08	count	1
C17orf53	18.081563	3117.082123	0.0058	0.995	4.86E-08	count	1
B4GALT6	18.0815631	3117.082264	0.0058	0.995	4.86E-08	count	1
RPL17-C18orf32	18.0815632	3117.082377	0.0058	0.995	4.86E-08	count	1
STARD6	18.0815631	3117.082264	0.0058	0.995	4.86E-08	count	1
AC064801.1	18.0815629	3117.08225	0.0058	0.995	4.86E-08	count	1
FSTL3	18.0815629	3117.08225	0.0058	0.995	4.86E-08	count	1
AC027307.3	18.0815631	3117.082264	0.0058	0.995	4.86E-08	count	1
THAP7-AS1	18.0815631	3117.082264	0.0058	0.995	4.86E-08	count	1
FAM171B	18.0815628	3117.082292	0.0058	0.995	4.86E-08	count	1
ST5	18.0815629	3117.082448	0.0058	0.995	4.86E-08	count	1
AP004609.3	18.0815629	3117.082448	0.0058	0.995	4.86E-08	count	1
MIR9-3HG	18.0815628	3117.082292	0.0058	0.995	4.86E-08	count	1
AL031708.1	18.0815628	3117.082292	0.0058	0.995	4.86E-08	count	1
ORC6	18.0815628	3117.082292	0.0058	0.995	4.86E-08	count	1
CKLF-CMTM1	18.0815629	3117.082448	0.0058	0.995	4.86E-08	count	1
MIRLET7BHG	18.0815629	3117.082448	0.0058	0.995	4.86E-08	count	1
Z97192.1	18.0815628	3117.082292	0.0058	0.995	4.86E-08	count	1
AZIN2	18.0815629	3117.08242	0.0058	0.995	4.86E-08	count	1
ERMAP	18.0815629	3117.082151	0.0058	0.995	4.86E-08	count	1
SLC9B1	18.0815629	3117.08242	0.0058	0.995	4.86E-08	count	1
ZGRF1	18.0815629	3117.082151	0.0058	0.995	4.86E-08	count	1
RSPH4A	18.0815629	3117.08242	0.0058	0.995	4.86E-08	count	1
ZKSCAN5	18.0815629	3117.08242	0.0058	0.995	4.86E-08	count	1
GIMAP5	18.081563	3117.082278	0.0058	0.995	4.86E-08	count	1
AL022157.1	18.0815629	3117.082151	0.0058	0.995	4.86E-08	count	1
TEK	18.0815629	3117.08242	0.0058	0.995	4.86E-08	count	1
B4GALNT4	18.0815629	3117.08242	0.0058	0.995	4.86E-08	count	1
FAM222A	18.0815629	3117.08242	0.0058	0.995	4.86E-08	count	1
MAP3K9	18.0815628	3117.082406	0.0058	0.995	4.86E-08	count	1
CLEC16A	18.081563	3117.082278	0.0058	0.995	4.86E-08	count	1
AC124283.1	18.0815629	3117.08242	0.0058	0.995	4.86E-08	count	1
MYOM1	18.0815629	3117.08242	0.0058	0.995	4.86E-08	count	1
AC005523.1	18.0815629	3117.082151	0.0058	0.995	4.86E-08	count	1
AC022098.2	18.0815628	3117.082406	0.0058	0.995	4.86E-08	count	1
ZNF726	18.0815628	3117.082406	0.0058	0.995	4.86E-08	count	1
AL358075.2	18.0815628	3117.082335	0.0058	0.995	4.86E-08	count	1
AL450468.2	18.0815628	3117.082335	0.0058	0.995	4.86E-08	count	1
NUF2	18.0815629	3117.08259	0.0058	0.995	4.86E-08	count	1
XPR1	18.0815629	3117.082448	0.0058	0.995	4.86E-08	count	1
IPO9-AS1	18.0815628	3117.082335	0.0058	0.995	4.86E-08	count	1
AC104695.3	18.0815628	3117.082335	0.0058	0.995	4.86E-08	count	1
PJVK	18.0815628	3117.082335	0.0058	0.995	4.86E-08	count	1
EGOT	18.0815629	3117.082448	0.0058	0.995	4.86E-08	count	1
ZNF660	18.0815628	3117.082335	0.0058	0.995	4.86E-08	count	1

CCR2	18.0815628	3117.082335	0.0058	0.995	4.86E-08	count	1
PRKAR2A-AS1	18.0815629	3117.082222	0.0058	0.995	4.86E-08	count	1
LINC01949	18.0815629	3117.08259	0.0058	0.995	4.86E-08	count	1
GDF9	18.0815629	3117.082222	0.0058	0.995	4.86E-08	count	1
HAVCR1	18.081563	3117.082519	0.0058	0.995	4.86E-08	count	1
Z84485.1	18.0815628	3117.082335	0.0058	0.995	4.86E-08	count	1
AC105446.1	18.0815629	3117.082448	0.0058	0.995	4.86E-08	count	1
CLCN1	18.0815629	3117.082448	0.0058	0.995	4.86E-08	count	1
NUDT11	18.0815629	3117.08259	0.0058	0.995	4.86E-08	count	1
EXPH5	18.081563	3117.082519	0.0058	0.995	4.86E-08	count	1
DDX47	18.0815629	3117.082448	0.0058	0.995	4.86E-08	count	1
RBMS2	18.0815627	3117.082377	0.0058	0.995	4.86E-08	count	1
RB1-DT	18.0815628	3117.082335	0.0058	0.995	4.86E-08	count	1
EXD1	18.0815628	3117.082335	0.0058	0.995	4.86E-08	count	1
AC009118.2	18.0815628	3117.082335	0.0058	0.995	4.86E-08	count	1
AC005332.3	18.0815627	3117.082377	0.0058	0.995	4.86E-08	count	1
SLC16A5	18.0815627	3117.082377	0.0058	0.995	4.86E-08	count	1
AC090229.1	18.0815629	3117.082222	0.0058	0.995	4.86E-08	count	1
PRX	18.0815629	3117.082222	0.0058	0.995	4.86E-08	count	1
RIMBP3	18.0815628	3117.082335	0.0058	0.995	4.86E-08	count	1
CHD5	18.0815629	3117.08242	0.0058	0.995	4.86E-08	count	1
HIST2H2AB	18.0815628	3117.082406	0.0058	0.995	4.86E-08	count	1
PIGR	18.0815628	3117.082222	0.0058	0.995	4.86E-08	count	1
IGKV4-1	18.0815628	3117.082264	0.0058	0.995	4.86E-08	count	1
FSIP2	18.0815628	3117.082222	0.0058	0.995	4.86E-08	count	1
AC092053.2	18.0815628	3117.082222	0.0058	0.995	4.86E-08	count	1
KALRN	18.0815629	3117.08242	0.0058	0.995	4.86E-08	count	1
LINC01063	18.0815628	3117.082406	0.0058	0.995	4.86E-08	count	1
AC106047.1	18.0815628	3117.082406	0.0058	0.995	4.86E-08	count	1
FHDC1	18.0815628	3117.082278	0.0058	0.995	4.86E-08	count	1
AC025171.4	18.0815629	3117.08242	0.0058	0.995	4.86E-08	count	1
AGGF1	18.0815629	3117.08242	0.0058	0.995	4.86E-08	count	1
EPB41L4A	18.0815626	3117.082222	0.0058	0.995	4.86E-08	count	1
HIST1H2BI	18.0815629	3117.08242	0.0058	0.995	4.86E-08	count	1
RPS10-NUDT3	18.0815629	3117.082476	0.0058	0.995	4.86E-08	count	1
ULBP1	18.0815629	3117.08242	0.0058	0.995	4.86E-08	count	1
TAB3	18.0815629	3117.08242	0.0058	0.995	4.86E-08	count	1
STMN4	18.0815628	3117.082406	0.0058	0.995	4.86E-08	count	1
AC023632.2	18.0815628	3117.082222	0.0058	0.995	4.86E-08	count	1
ZNF623	18.0815628	3117.082406	0.0058	0.995	4.86E-08	count	1
AL158152.1	18.0815626	3117.082222	0.0058	0.995	4.86E-08	count	1
TPH1	18.0815629	3117.08242	0.0058	0.995	4.86E-08	count	1
AC034102.6	18.0815628	3117.082406	0.0058	0.995	4.86E-08	count	1
VASH1-AS1	18.0815628	3117.082278	0.0058	0.995	4.86E-08	count	1
SHF	18.0815628	3117.082278	0.0058	0.995	4.86E-08	count	1

Z69720.1	18.0815628	3117.082278	0.0058	0.995	4.86E-08	count	1
AC020765.2	18.0815628	3117.082406	0.0058	0.995	4.86E-08	count	1
AC027682.1	18.0815628	3117.082406	0.0058	0.995	4.86E-08	count	1
AC087500.1	18.0815629	3117.082476	0.0058	0.995	4.86E-08	count	1
TVP23C	18.0815628	3117.082406	0.0058	0.995	4.86E-08	count	1
TEX14	18.0815628	3117.082222	0.0058	0.995	4.86E-08	count	1
PLPP2	18.0815628	3117.082264	0.0058	0.995	4.86E-08	count	1
SIPA1L3	18.0815628	3117.082222	0.0058	0.995	4.86E-08	count	1
ATP1A3	18.0815628	3117.082278	0.0058	0.995	4.86E-08	count	1
RTN4R	18.0815629	3117.08242	0.0058	0.995	4.86E-08	count	1
PM20D1	18.0815627	3117.082335	0.0058	0.995	4.86E-08	count	1
ARHGEF26	18.0815627	3117.082335	0.0058	0.995	4.86E-08	count	1
TMEM217	18.0815627	3117.082335	0.0058	0.995	4.86E-08	count	1
RMRP	18.0815627	3117.082335	0.0058	0.995	4.86E-08	count	1
ZBTB5	18.0815627	3117.082335	0.0058	0.995	4.86E-08	count	1
HMGA2	18.0815627	3117.082335	0.0058	0.995	4.86E-08	count	1
AC009065.4	18.0815626	3117.082236	0.0058	0.995	4.86E-08	count	1
RARB	18.0815626	3117.082491	0.0058	0.995	4.86E-08	count	1
EDIL3	18.0815626	3117.082491	0.0058	0.995	4.86E-08	count	1
SLC22A5	18.0815626	3117.082491	0.0058	0.995	4.86E-08	count	1
NPIPA5	18.0815626	3117.082491	0.0058	0.995	4.86E-08	count	1
ZNF20	18.0815626	3117.082491	0.0058	0.995	4.86E-08	count	1
LGALS4	18.0815626	3117.082491	0.0058	0.995	4.86E-08	count	1
ATXN7	18.0805403	2188.630976	0.0083	0.993	4.87E-08	count	1
AMMECR1L	18.08054	2188.630777	0.0083	0.993	4.87E-08	count	1
AL365361.1	18.0805399	2188.630906	0.0083	0.993	4.87E-08	count	1
FBXO41	18.0805397	2188.630688	0.0083	0.993	4.87E-08	count	1
ZNF208	18.0805397	2188.630688	0.0083	0.993	4.87E-08	count	1
GPR160	18.0805397	2188.630886	0.0083	0.993	4.87E-08	count	1
IPO13	18.0805397	2188.630638	0.0083	0.993	4.87E-08	count	1
BOLA3-AS1	18.0805395	2188.630896	0.0083	0.993	4.87E-08	count	1
AC018638.7	18.0805396	2188.630728	0.0083	0.993	4.87E-08	count	1
KLHL11	18.0805396	2188.630926	0.0083	0.993	4.87E-08	count	1
TRIP10	18.0805396	2188.630926	0.0083	0.993	4.87E-08	count	1
ZSCAN12	18.0805394	2188.630737	0.0083	0.993	4.87E-08	count	1
HSPA1L	18.0805395	2188.630896	0.0083	0.993	4.87E-08	count	1
ACP2	18.0805396	2188.630896	0.0083	0.993	4.87E-08	count	1
SUV39H2	18.0805392	2188.630708	0.0083	0.993	4.87E-08	count	1
PTDSS2	18.0805393	2188.630906	0.0083	0.993	4.87E-08	count	1
IL1RAP	18.0805395	2188.630747	0.0083	0.993	4.87E-08	count	1
PLXNA4	18.0805393	2188.630876	0.0083	0.993	4.87E-08	count	1
C1S	18.0805394	2188.630737	0.0083	0.993	4.87E-08	count	1
MROH8	18.4814459	2822.783744	0.0065	0.995	4.88E-08	count	1
DDIT4-AS1	18.481446	2822.783527	0.0065	0.995	4.88E-08	count	1
TBC1D12	18.4814459	2822.783744	0.0065	0.995	4.88E-08	count	1

ARHGAP11B	18.481446	2822.783514	0.0065	0.995	4.88E-08	count	1
TEPP	18.4814459	2822.783884	0.0065	0.995	4.88E-08	count	1
EVI5L	18.481446	2822.783769	0.0065	0.995	4.88E-08	count	1
AC240274.1	18.4814456	2822.783884	0.0065	0.995	4.88E-08	count	1
ZSWIM6	18.4814461	2822.78354	0.0065	0.995	4.88E-08	count	1
AFG1L	18.4814457	2822.783476	0.0065	0.995	4.88E-08	count	1
ZNF41	18.4814458	2822.78354	0.0065	0.995	4.88E-08	count	1
C12orf60	18.4814454	2822.783998	0.0065	0.995	4.88E-08	count	1
ERI2	18.4814455	2822.783514	0.0065	0.995	4.88E-08	count	1
FGGY	18.4814457	2822.783756	0.0065	0.995	4.88E-08	count	1
PLCD1	18.4814451	2822.783323	0.0065	0.995	4.88E-08	count	1
SAP25	18.4814455	2822.783629	0.0065	0.995	4.88E-08	count	1
DDN-AS1	18.4814456	2822.783374	0.0065	0.995	4.88E-08	count	1
LMAN2L	18.4814456	2822.783884	0.0065	0.995	4.88E-08	count	1
RORB	18.4814452	2822.783578	0.0065	0.995	4.88E-08	count	1
AL118506.1	18.4814456	2822.783731	0.0065	0.995	4.88E-08	count	1
CXXC4	18.4814455	2822.783578	0.0065	0.995	4.88E-08	count	1
AC006480.2	18.4814456	2822.783502	0.0065	0.995	4.88E-08	count	1
SH2B2	18.4814453	2822.783846	0.0065	0.995	4.88E-08	count	1
LINC02055	18.4814447	2822.783514	0.0065	0.995	4.88E-08	count	1
LINC02021	18.4793167	3802.938881	0.0049	0.996	4.88E-08	count	1
PYGM	18.4793167	3802.938881	0.0049	0.996	4.88E-08	count	1
AC091132.5	18.4793167	3802.938881	0.0049	0.996	4.88E-08	count	1
MINDY4	18.4793166	3802.938401	0.0049	0.996	4.88E-08	count	1
AC005670.2	18.4793166	3802.938401	0.0049	0.996	4.88E-08	count	1
TTC28	18.4793166	3802.938401	0.0049	0.996	4.88E-08	count	1
KLF4	18.4793164	3802.938487	0.0049	0.996	4.88E-08	count	1
SLC11A1	18.4793164	3802.938658	0.0049	0.996	4.88E-08	count	1
IL2	18.479316	3802.938607	0.0049	0.996	4.88E-08	count	1
CA2	18.479316	3802.938607	0.0049	0.996	4.88E-08	count	1
CENPA	18.4793158	3802.938521	0.0049	0.996	4.88E-08	count	1
KLHL6-AS1	18.4793158	3802.938521	0.0049	0.996	4.88E-08	count	1
AC096733.2	18.4793158	3802.938521	0.0049	0.996	4.88E-08	count	1
TNNI2	18.4793158	3802.938521	0.0049	0.996	4.88E-08	count	1
AC090515.2	18.479316	3802.938572	0.0049	0.996	4.88E-08	count	1
CCDC74B	18.479316	3802.93835	0.0049	0.996	4.88E-08	count	1
SAG	18.4793157	3802.938538	0.0049	0.996	4.88E-08	count	1
SETMAR	18.479316	3802.93835	0.0049	0.996	4.88E-08	count	1
AC104041.1	18.479316	3802.93835	0.0049	0.996	4.88E-08	count	1
HEATR9	18.479316	3802.93835	0.0049	0.996	4.88E-08	count	1
LRFN4	18.7628637	2147.9853	0.0087	0.993	4.90E-08	count	1
AC127070.1	18.7610411	2651.909962	0.0071	0.994	4.90E-08	count	1
AC103702.2	18.7610408	2651.909843	0.0071	0.994	4.90E-08	count	1
TMEM121	18.7610404	2651.910057	0.0071	0.994	4.90E-08	count	1
H1FX-AS1	18.7585356	3071.831139	0.0061	0.995	4.91E-08	count	1

POU6F1	18.7585358	3071.831057	0.0061	0.995	4.91E-08	count	1
PDP2	18.7585357	3071.831029	0.0061	0.995	4.91E-08	count	1
RIC3	18.7585356	3071.831469	0.0061	0.995	4.91E-08	count	1
AC026304.1	18.7585355	3071.831414	0.0061	0.995	4.91E-08	count	1
FAM83D	18.7585354	3071.831414	0.0061	0.995	4.91E-08	count	1
RSPH3	18.7585353	3071.831359	0.0061	0.995	4.91E-08	count	1
OSR2	18.7585356	3071.831715	0.0061	0.995	4.91E-08	count	1
LENG9	18.7585354	3071.831304	0.0061	0.995	4.91E-08	count	1
RND1	18.7585355	3071.831139	0.0061	0.995	4.91E-08	count	1
SLAMF8	18.7585353	3071.831112	0.0061	0.995	4.91E-08	count	1
ABHD14A-ACY1	18.7585352	3071.831194	0.0061	0.995	4.91E-08	count	1
HIST1H2AG	18.7585351	3071.831057	0.0061	0.995	4.91E-08	count	1
LCMT2	18.7585352	3071.831084	0.0061	0.995	4.91E-08	count	1
RASL11A	18.7585347	3071.831057	0.0061	0.995	4.91E-08	count	1
AL451085.2	18.757204	3441.146195	0.0055	0.996	4.91E-08	count	1
BAHD1	18.7572038	3441.146041	0.0055	0.996	4.91E-08	count	1
RBBP9	18.7572036	3441.146011	0.0055	0.996	4.91E-08	count	1
AC009163.7	18.7572037	3441.145949	0.0055	0.996	4.91E-08	count	1
TSNARE1	18.5191307	2574.00515	0.0072	0.994	4.92E-08	count	1
FLNB	18.5191318	2574.005714	0.0072	0.994	4.92E-08	count	1
CCDC163	18.5191309	2574.005444	0.0072	0.994	4.92E-08	count	1
CEP120	18.5191312	2574.006855	0.0072	0.994	4.92E-08	count	1
ZNF610	18.5191323	2574.007113	0.0072	0.994	4.92E-08	count	1
C11orf98	18.5191321	2574.005432	0.0072	0.994	4.92E-08	count	1
LINC01534	18.5191311	2574.00488	0.0072	0.994	4.92E-08	count	1
IL10RB	18.519131	2574.004597	0.0072	0.994	4.92E-08	count	1
FNTB	18.5191302	2574.006867	0.0072	0.994	4.92E-08	count	1
AC090948.1	18.5191285	2574.00661	0.0072	0.994	4.92E-08	count	1
SIRT4	18.5191311	2574.006008	0.0072	0.994	4.92E-08	count	1
TUBGCP4	18.5191312	2574.005456	0.0072	0.994	4.92E-08	count	1
ZNF785	18.5191294	2574.006327	0.0072	0.994	4.92E-08	count	1
PLD6	18.5191269	2574.005505	0.0072	0.994	4.92E-08	count	1
BX284668.6	18.5191265	2574.004389	0.0072	0.994	4.92E-08	count	1
ARHGAP31	18.8264065	2580.853401	0.0073	0.994	4.93E-08	count	1
DVL1	18.8264646	3450.997299	0.0055	0.996	4.93E-08	count	1
ANKRD9	18.8264628	3451.000523	0.0055	0.996	4.93E-08	count	1
LINC01004	18.8264603	3450.998552	0.0055	0.996	4.93E-08	count	1
ZNF784	18.8264639	3450.999437	0.0055	0.996	4.93E-08	count	1
CLCN6	18.8264594	3451.00069	0.0055	0.996	4.93E-08	count	1
CCDC134	18.8264618	3451.001592	0.0055	0.996	4.93E-08	count	1
CSRNP2	18.8264577	3450.995528	0.0055	0.996	4.93E-08	count	1
CNNM4	18.8264563	3450.999771	0.0055	0.996	4.93E-08	count	1
CCDC144NL	18.8264569	3450.999738	0.0055	0.996	4.93E-08	count	1
AC008267.5	18.8264572	3450.999771	0.0055	0.996	4.93E-08	count	1
SNHG19	18.826458	3450.995562	0.0055	0.996	4.93E-08	count	1

AC078881.1	18.8264591	3450.993423	0.0055	0.996	4.93E-08	count	1
CCDC184	18.8264584	3450.997633	0.0055	0.996	4.93E-08	count	1
ZNF569	18.8264557	3450.996681	0.0055	0.996	4.93E-08	count	1
AC009779.2	18.8264542	3450.993674	0.0055	0.996	4.93E-08	count	1
ATXN7	18.7506744	4355.55943	0.0043	0.997	4.93E-08	count	1
HIST1H4L	18.7506744	4355.55943	0.0043	0.997	4.93E-08	count	1
PRRG4	18.7506744	4355.55943	0.0043	0.997	4.93E-08	count	1
AP000866.1	18.7506744	4355.55943	0.0043	0.997	4.93E-08	count	1
AL365356.1	18.7506744	4355.55943	0.0043	0.997	4.93E-08	count	1
SLC25A18	18.7506744	4355.55943	0.0043	0.997	4.93E-08	count	1
AL022238.2	18.7506744	4355.55943	0.0043	0.997	4.93E-08	count	1
AC068338.3	18.7506738	4355.559353	0.0043	0.997	4.93E-08	count	1
ZNF527	18.9710989	2883.948415	0.0066	0.995	4.95E-08	count	1
BRD3OS	18.9710995	2883.948236	0.0066	0.995	4.95E-08	count	1
TTN-AS1	18.9710986	2883.948109	0.0066	0.995	4.95E-08	count	1
AL360012.1	18.9710983	2883.94816	0.0066	0.995	4.95E-08	count	1
AC108673.3	18.9897394	2514.38189	0.0076	0.994	4.95E-08	count	1
LONP1	18.9693737	3196.25193	0.0059	0.995	4.95E-08	count	1
AC055822.1	18.9822738	2792.19051	0.0068	0.995	4.95E-08	count	1
SLC51A	18.9822716	2792.191248	0.0068	0.995	4.95E-08	count	1
PUS7	18.9822723	2792.190051	0.0068	0.995	4.95E-08	count	1
ZNF169	18.9822723	2792.190051	0.0068	0.995	4.95E-08	count	1
HECTD3	18.9822702	2792.18958	0.0068	0.995	4.95E-08	count	1
ZNF551	18.9822704	2792.190064	0.0068	0.995	4.95E-08	count	1
AC135068.9	18.9983781	1929.454428	0.0098	0.992	4.95E-08	count	1
AC019129.2	18.9983777	1929.454451	0.0098	0.992	4.95E-08	count	1
AC109779.1	18.9983781	1929.454488	0.0098	0.992	4.95E-08	count	1
AC092953.2	18.9983781	1929.454413	0.0098	0.992	4.95E-08	count	1
MAPK8IP3	18.9983781	1929.454413	0.0098	0.992	4.95E-08	count	1
AC135050.1	18.9983781	1929.454413	0.0098	0.992	4.95E-08	count	1
MPRIP-AS1	19.052502	3177.490514	0.006	0.995	4.96E-08	count	1
GDAP1	19.052497	3177.487159	0.006	0.995	4.96E-08	count	1
COX6A2	19.2016197	2534.595272	0.0076	0.994	4.97E-08	count	1
LDAH	19.2028109	3398.688985	0.0057	0.995	4.98E-08	count	1
VCL	19.1945013	2817.923102	0.0068	0.995	4.98E-08	count	1
AL158071.3	19.1944996	2817.921334	0.0068	0.995	4.98E-08	count	1
AC015802.6	19.2763597	1307.610925	0.0147	0.988	4.98E-08	count	1
ABHD8	19.2755925	2632.6388	0.0073	0.994	4.99E-08	count	1
CLYBL	19.2510715	2943.001664	0.0065	0.995	5.00E-08	count	1
TXNRD2	19.2510687	2943.001689	0.0065	0.995	5.00E-08	count	1
PSTK	0.5774557	0.7629204	0.7569	0.45	5.21E-08	count	1
ARSB	0.6010732	0.9149748	0.6569	0.512	5.44E-08	count	1
PCTP	0.6010732	0.7703953	0.7802	0.436	5.45E-08	count	1
TMEM206	0.6010732	0.6200613	0.9694	0.333	5.46E-08	count	1
MAN1C1	0.2434013	1.0022622	0.2429	0.808	5.70E-08	count	1

MINK1	1.6094379	0.485118	3.3176	0.001	5.90E-08	count	1
RIPOR1	0.6383754	0.6966088	0.9164	0.36	5.92E-08	count	1
ZFP57	0.6539265	0.7508568	0.8709	0.384	5.99E-08	count	1
CIB2	0.6539265	0.8190127	0.7984	0.425	6.00E-08	count	1
MAVS	0.2932661	0.557886	0.5257	0.599	6.88E-08	count	1
C17orf58	0.7374036	0.6587411	1.1194	0.264	6.89E-08	count	1
NAT9	0.7487886	0.8067277	0.9282	0.354	6.97E-08	count	1
HLA-DQA2	0.759287	0.5501901	1.38	0.169	7.12E-08	count	1
SMAD3	0.7809143	0.9129876	0.8553	0.393	7.27E-08	count	1
NINJ2	0.7809143	0.7310621	1.0682	0.286	7.28E-08	count	1
AC022182.2	0.8080771	0.6840837	1.1813	0.238	7.57E-08	count	1
PERP	0.3285041	0.4187482	0.7845	0.433	7.78E-08	count	1
CDKAL1	0.8253689	0.5863472	1.4076	0.16	7.82E-08	count	1
PCYOX1L	0.8410284	0.8713803	0.9652	0.335	7.87E-08	count	1
CCDC157	0.8410284	1.0185403	0.8257	0.41	7.88E-08	count	1
ZXDA	0.8410284	1.0185403	0.8257	0.41	7.88E-08	count	1
AC087623.3	0.8410284	1.0185403	0.8257	0.41	7.88E-08	count	1
USP5	0.8410284	1.0185403	0.8257	0.41	7.88E-08	count	1
BANK1	0.8410284	1.0185403	0.8257	0.41	7.88E-08	count	1
FAM229B	0.8410284	1.0083163	0.8341	0.405	7.88E-08	count	1
CDT1	0.8410284	1.0083163	0.8341	0.405	7.88E-08	count	1
TMEM176A	0.8410284	1.2624049	0.6662	0.506	7.90E-08	count	1
CCDC3	0.8410284	1.2624049	0.6662	0.506	7.90E-08	count	1
RTTN	0.8410284	1.2624049	0.6662	0.506	7.90E-08	count	1
ANAPC2	0.8410284	1.2624049	0.6662	0.506	7.90E-08	count	1
CHSY1	0.8410284	0.9104031	0.9238	0.356	7.97E-08	count	1
MAPK8	0.8410284	0.6365345	1.3213	0.187	8.01E-08	count	1
RRM1	0.8410284	0.5764825	1.4589	0.146	8.05E-08	count	1
MYO18A	0.87707	0.9339348	0.9391	0.348	8.34E-08	count	1
CKB	0.87707	0.7885621	1.1122	0.267	8.35E-08	count	1
PRR14L	0.900806	0.6297587	1.4304	0.154	8.83E-08	count	1
TMEM63A	0.9301366	0.7821192	1.1893	0.235	8.94E-08	count	1
TOE1	0.9416085	0.6610002	1.4245	0.155	8.99E-08	count	1
GNB4	0.9416085	1.1735905	0.8023	0.423	9.01E-08	count	1
CNNM2	0.9774867	0.7748051	1.2616	0.208	9.41E-08	count	1
ZNF383	0.9774867	0.8194263	1.1929	0.234	9.45E-08	count	1
ZNF546	1.0157771	1.0174827	0.9983	0.319	9.71E-08	count	1
TCF4	1.0157771	0.8138038	1.2482	0.213	9.72E-08	count	1
SLX4	1.0157771	1.0324461	0.9839	0.326	9.73E-08	count	1
HKR1	1.0157771	1.0324461	0.9839	0.326	9.73E-08	count	1
SLC25A40	1.0157771	1.0324461	0.9839	0.326	9.73E-08	count	1
IL17RA	1.0157771	0.8876477	1.1443	0.253	9.74E-08	count	1
RAB30-AS1	1.0593916	0.6917024	1.5316	0.127	1.03E-07	count	1
GUF1	1.0580061	0.7704542	1.3732	0.171	1.03E-07	count	1
AGPAT4	1.0965104	0.8657231	1.2666	0.206	1.06E-07	count	1

MARK4	1.0965104	0.8177857	1.3408	0.181	1.06E-07	count	1
AKAP1	1.0965104	0.9668224	1.1341	0.258	1.06E-07	count	1
EPN2	1.0965104	0.9668224	1.1341	0.258	1.06E-07	count	1
AC007365.1	1.0965104	0.9668224	1.1341	0.258	1.06E-07	count	1
HIPK1-AS1	1.0965104	0.9241441	1.1865	0.236	1.06E-07	count	1
AL137077.2	1.0965104	0.9241441	1.1865	0.236	1.06E-07	count	1
COA7	1.0965104	1.0583073	1.0361	0.301	1.06E-07	count	1
CA13	1.0965104	1.0583073	1.0361	0.301	1.06E-07	count	1
KCTD2	1.0965104	1.3642178	0.8038	0.422	1.07E-07	count	1
PTPRF	1.0965104	1.3642178	0.8038	0.422	1.07E-07	count	1
AL391121.1	1.0965104	1.3642178	0.8038	0.422	1.07E-07	count	1
ZNF841	1.0977689	0.7454537	1.4726	0.142	1.07E-07	count	1
ZMAT1	1.0965104	0.8920728	1.2292	0.22	1.08E-07	count	1
AL359513.1	1.0965104	0.8456303	1.2967	0.196	1.09E-07	count	1
SYMPK	1.1354458	0.9665308	1.1748	0.241	1.12E-07	count	1
PRKCI	1.1354458	0.7550564	1.5038	0.134	1.12E-07	count	1
MAN1B1-DT	1.1647521	0.9560722	1.2183	0.224	1.14E-07	count	1
KIR2DL1	1.1647521	1.0701332	1.0884	0.277	1.14E-07	count	1
ZCCHC3	1.17008	0.814326	1.4369	0.152	1.16E-07	count	1
CCDC125	1.1647521	0.5993824	1.9433	0.0529	1.16E-07	count	1
ANAPC7	0.478651	0.5435287	0.8806	0.379	1.16E-07	count	1
PRRG2	1.2021571	0.8568684	1.403	0.162	1.20E-07	count	1
DLAT	1.2409013	0.9337017	1.329	0.185	1.22E-07	count	1
TBCK	1.2409013	1.0677158	1.1622	0.246	1.22E-07	count	1
RPP21	1.2972003	0.8725615	1.4867	0.138	1.28E-07	count	1
AC110769.2	1.2972003	1.0182278	1.274	0.204	1.28E-07	count	1
NOCT	1.2972003	1.0182278	1.274	0.204	1.28E-07	count	1
TXNRD3	1.2972003	1.4613101	0.8877	0.375	1.30E-07	count	1
AMFR	1.3089436	0.7082003	1.8483	0.0655	1.33E-07	count	1
RAP2C-AS1	17.9650267	2107.784756	0.0085	0.993	1.35E-07	count	1
ASAH2B	17.9650266	2107.784736	0.0085	0.993	1.35E-07	count	1
OXCT2	17.9650265	2107.784695	0.0085	0.993	1.35E-07	count	1
CCDC136	17.9650264	2107.784783	0.0085	0.993	1.35E-07	count	1
HIST1H1A	17.9614216	2413.147753	0.0074	0.994	1.35E-07	count	1
AL049597.2	18.0497747	2504.469619	0.0072	0.994	1.35E-07	count	1
AC009126.1	18.1971316	2191.232693	0.0083	0.993	1.36E-07	count	1
ZNF761	18.1971323	2191.232338	0.0083	0.993	1.36E-07	count	1
NRAV	18.1971297	2191.231323	0.0083	0.993	1.36E-07	count	1
MIR210HG	18.1971292	2191.231861	0.0083	0.993	1.36E-07	count	1
TAB1	18.1374087	1826.418727	0.0099	0.992	1.36E-07	count	1
C5orf30	18.1374085	1826.418751	0.0099	0.992	1.36E-07	count	1
RBP7	17.9501801	2918.902261	0.0061	0.995	1.36E-07	count	1
ARRDC3-AS1	17.9501801	2918.902261	0.0061	0.995	1.36E-07	count	1
PYGL	17.9501801	2918.902261	0.0061	0.995	1.36E-07	count	1
CDH4	17.9501801	2918.902261	0.0061	0.995	1.36E-07	count	1

GAB2	17.9501798	2918.902299	0.0061	0.995	1.36E-07	count	1
ZNF771	17.9501798	2918.902299	0.0061	0.995	1.36E-07	count	1
AC011451.1	17.9501798	2918.902299	0.0061	0.995	1.36E-07	count	1
RFPL2	17.9501798	2918.902299	0.0061	0.995	1.36E-07	count	1
CCNE2	18.2289445	2073.806788	0.0088	0.993	1.36E-07	count	1
RYR1	18.2289426	2073.806942	0.0088	0.993	1.36E-07	count	1
AC021054.1	0.5489882	0.9015589	0.6089	0.543	1.36E-07	count	1
MAST4	1.3742301	1.0093309	1.3615	0.174	1.37E-07	count	1
GNA12	18.276986	1873.45867	0.0098	0.992	1.37E-07	count	1
FICD	18.276986	1873.458729	0.0098	0.992	1.37E-07	count	1
GRK5	18.3639929	2048.478886	0.009	0.993	1.37E-07	count	1
PBX4	18.3639943	2048.47885	0.009	0.993	1.37E-07	count	1
ZBTB49	18.3639918	2048.478314	0.009	0.993	1.37E-07	count	1
POPDC2	18.4487295	2105.304886	0.0088	0.993	1.37E-07	count	1
FAM27C	18.4487293	2105.305167	0.0088	0.993	1.37E-07	count	1
MTFMT	18.3758835	1964.715688	0.0094	0.993	1.37E-07	count	1
TRIM61	18.2710113	1993.606505	0.0092	0.993	1.38E-07	count	1
HAR1B	18.3647437	2593.053641	0.0071	0.994	1.38E-07	count	1
GGT7	18.4275251	2250.847632	0.0082	0.993	1.38E-07	count	1
CDAN1	18.4275252	2250.848221	0.0082	0.993	1.38E-07	count	1
NPL	18.427524	2250.84778	0.0082	0.993	1.38E-07	count	1
CBWD2	18.5848797	1966.744498	0.0094	0.992	1.39E-07	count	1
MYO1D	18.6215892	2091.648323	0.0089	0.993	1.39E-07	count	1
P3H4	18.5021127	2400.967661	0.0077	0.994	1.40E-07	count	1
C15orf39	18.3551465	3120.676864	0.0059	0.995	1.40E-07	count	1
SGCB	18.4781968	2326.583155	0.0079	0.994	1.42E-07	count	1
VPS8	1.4208541	0.9322919	1.524	0.128	1.42E-07	count	1
ASB3	18.7469836	2500.074413	0.0075	0.994	1.43E-07	count	1
DLG5	18.8011165	2029.113066	0.0093	0.993	1.43E-07	count	1
IL4I1	18.3199558	3511.685496	0.0052	0.996	1.45E-07	count	1
C1orf159	18.7090844	3003.974011	0.0062	0.995	1.46E-07	count	1
MFSD9	18.8062003	2336.247346	0.008	0.994	1.47E-07	count	1
SSX2IP	1.4628909	1.0541365	1.3878	0.166	1.47E-07	count	1
FAM69A	1.4628909	1.1044031	1.3246	0.186	1.47E-07	count	1
PPP2R1B	1.4628909	1.1986289	1.2205	0.223	1.48E-07	count	1
SHLD3	18.8770806	2273.060156	0.0083	0.993	1.48E-07	count	1
IKZF4	18.9321368	2420.162325	0.0078	0.994	1.48E-07	count	1
KIAA0556	1.4968159	0.8544726	1.7517	0.0808	1.50E-07	count	1
ZNF792	18.8756969	2758.612987	0.0068	0.995	1.50E-07	count	1
AP1S3	18.9465766	2664.824192	0.0071	0.994	1.53E-07	count	1
CKAP4	18.4636791	3773.328201	0.0049	0.996	1.53E-07	count	1
CCR5	1.5357137	0.7515177	2.0435	0.0418	1.54E-07	count	1
FOCAD	1.5357137	1.0296118	1.4915	0.137	1.54E-07	count	1
CCDC51	1.5357137	0.8872723	1.7308	0.0844	1.55E-07	count	1
MLLT1	1.5357137	1.1100444	1.3835	0.167	1.55E-07	count	1

OSBPL7	1.5357137	0.9292966	1.6526	0.0994	1.55E-07	count	1
AC062029.1	1.5357137	1.1439137	1.3425	0.18	1.56E-07	count	1
MAU2	0.618588	0.5588741	1.1068	0.269	1.57E-07	count	1
KCTD17	0.629516	0.6097222	1.0325	0.303	1.58E-07	count	1
SPON2	1.7008996	0.966302	1.7602	0.0793	1.75E-07	count	1
S100A12	18.6659408	4174.89153	0.0045	0.996	1.76E-07	count	1
AKT3	0.7016466	0.5662264	1.2392	0.216	1.77E-07	count	1
TNPO3	1.8766819	1.0525294	1.783	0.0755	1.93E-07	count	1
SLAMF1	1.7525388	1.5951744	1.0987	0.273	1.94E-07	count	1
SYNE3	1.8766819	1.0148075	1.8493	0.0653	1.96E-07	count	1
FBXO5	1.91663	0.8896157	2.1544	0.0319	1.99E-07	count	1
LINC00513	0.7475178	0.6839933	1.0929	0.275	2.00E-07	count	1
RNF19B	1.9919746	1.2450067	1.6	0.111	2.18E-07	count	1
NT5C2	2.0402208	1.0180975	2.004	0.0459	2.20E-07	count	1
BTBD9	2.1820194	0.9300197	2.3462	0.0196	2.26E-07	count	1
CEP19	2.1306723	0.9165492	2.3247	0.0207	2.28E-07	count	1
FOXO4	2.1441725	0.9028459	2.3749	0.0181	2.30E-07	count	1
GRAMD1B	1.018864	0.7246131	1.4061	0.161	2.76E-07	count	1
SESN2	1.1222732	0.8000325	1.4028	0.162	3.11E-07	count	1
RPS6KC1	1.3049155	0.5824385	2.2404	0.0257	3.73E-07	count	1
HSPA2	1.4824758	0.8283735	1.7896	0.0744	4.29E-07	count	1
C6orf120	1.49913	1.0309861	1.4541	0.147	4.46E-07	count	1
PCNX4	1.746981	0.6897025	2.5329	0.0118	5.76E-07	count	1
MYO1F	3.76E-05	0.3543215	1.00E-04	1	5.15E-05	count	1
NUDT5	0.0001201	0.3554876	3.00E-04	1	0.000166134	count	1
TP53I13	0.0001657	0.3672787	5.00E-04	1	0.00023021	count	1
KLRB1	0.0001911	0.1040461	0.0018	0.999	0.000275123	count	1
MTHFR	0.0005205	0.5934903	9.00E-04	0.999	0.000305273	count	1
SYNGR2	0.0002759	0.3615308	8.00E-04	0.999	0.000383677	count	1
TMX3	0.0003172	0.4792302	7.00E-04	0.999	0.00042497	count	1
LARP7	0.0004006	0.3249459	0.0012	0.999	0.00056326	count	1
SERPINB9	0.0006997	0.4744417	0.0015	0.999	0.000583536	count	1
MAF	0.0010667	0.7904468	0.0013	0.9989	0.000625662	count	1
USP15	0.0004466	0.2011997	0.0022	0.9982	0.000634656	count	1
DCP1A	0.0005608	0.5574499	0.001	0.999	0.000669452	count	1
YPEL3	0.0005477	0.1878855	0.0029	0.9977	0.000783476	count	1
WDR13	0.0006405	0.6088766	0.0011	0.999	0.000823827	count	1
SNX13	0.0016003	0.6408857	0.0025	0.998	0.000938703	count	1
KBTBD3	0.00085	0.6436149	0.0013	0.999	0.001014693	count	1
DUSP8	0.001402	0.5849244	0.0024	0.998	0.001169313	count	1
CDKN2A	0.000979	0.4834637	0.002	0.998	0.001311631	count	1
TTC39B	0.0009942	0.4042734	0.0025	0.998	0.001336764	count	1
IRF3	0.0011027	0.3263439	0.0034	0.997	0.001542941	count	1
RPS15	0.0011329	0.0533798	0.0212	0.983	0.001633032	count	1
OCIAD1	0.001328	0.2126246	0.0062	0.995	0.001891679	count	1

MT-CO2	0.0013646	0.0552439	0.0247	0.98	0.001967755	count	1
DNAJC21	0.001549	0.2532474	0.0061	0.995	0.002176965	count	1
UBE3C	0.0017238	0.8669535	0.002	0.998	0.002290294	count	1
UBXN1	0.0021086	0.1682609	0.0125	0.99	0.003013138	count	1
EIF3B	0.0023898	0.455774	0.0052	0.996	0.003099798	count	1
AAGAB	0.0028825	0.6486938	0.0044	0.996	0.003345093	count	1
SMIM24	0.0028825	1.0089237	0.0029	0.998	0.003345093	count	1
NUP107	0.0029144	0.5519121	0.0053	0.996	0.003931431	count	1
ATF4	0.003063	0.2581613	0.0119	0.9905	0.004333531	count	1
MRPL27	0.0031803	0.3863146	0.0082	0.993	0.004393936	count	1
ATP5PF	0.0034525	0.1642636	0.021	0.983	0.004932926	count	1
TMEM230	0.0035165	0.2290664	0.0154	0.988	0.004998065	count	1
S100A10	0.0035288	0.1430479	0.0247	0.98	0.005076928	count	1
CLUAP1	0.0042637	0.6505218	0.0066	0.995	0.005368173	count	1
PITPNA	0.0042637	0.6461366	0.0066	0.995	0.005368173	count	1
SSBP2	0.0042533	0.6629314	0.0064	0.995	0.005417776	count	1
CCND3	0.0039069	0.2056793	0.019	0.9849	0.005546562	count	1
PHB2	0.0039278	0.2339691	0.0168	0.9866	0.0055721	count	1
TSTA3	0.0041664	0.3926998	0.0106	0.992	0.005740714	count	1
MALAT1	0.0040308	0.0430004	0.0937	0.9254	0.00581507	count	1
CTSC	0.0044123	0.1604392	0.0275	0.9781	0.0063219	count	1
UQCR10	0.0044406	0.2386062	0.0186	0.9852	0.006326875	count	1
EVI5	0.0051797	0.8276837	0.0063	0.995	0.006521615	count	1
ISCU	0.0047564	0.205619	0.0231	0.9816	0.006754945	count	1
KARS	0.0056004	0.5178091	0.0108	0.991	0.007051386	count	1
ITGAL	0.0054052	0.4010531	0.0135	0.989	0.007242186	count	1
AKAP17A	0.0052237	0.3122131	0.0167	0.987	0.007271104	count	1
ZNF652	0.005181	0.263144	0.0197	0.984	0.007297232	count	1
TTC32	0.0055168	0.4192861	0.0132	0.99	0.007590106	count	1
RFT1	0.0060636	0.7387478	0.0082	0.993	0.00763469	count	1
LIPA	0.0063664	0.5814575	0.0109	0.991	0.008371084	count	1
TMEM109	0.0063337	0.2507214	0.0253	0.98	0.008930904	count	1
SWI5	0.0087482	0.5801644	0.0151	0.988	0.00925071	count	1
ITGB2-AS1	0.0087482	0.6888289	0.0127	0.99	0.00925071	count	1
POLR2E	0.0066916	0.2870807	0.0233	0.9814	0.009445292	count	1
RAB27A	0.0070794	0.3329156	0.0213	0.983	0.009878507	count	1
ENO1	0.0070339	0.1885902	0.0373	0.97	0.010056147	count	1
MTHFD2L	0.0079829	0.6332219	0.0126	0.99	0.010355813	count	1
CUL1	0.0085365	0.5199021	0.0164	0.987	0.010417751	count	1
RNF103	0.0107622	0.7639336	0.0141	0.989	0.010451646	count	1
DAG1	0.0107622	0.815163	0.0132	0.989	0.010451646	count	1
RPS19BP1	0.0079404	0.235807	0.0337	0.9732	0.011234297	count	1
MKNK2	0.00976	0.322351	0.0303	0.976	0.011329525	count	1
DARS	0.0085112	0.3183191	0.0267	0.979	0.011744199	count	1
CCNI	0.0082508	0.1442571	0.0572	0.954	0.011837034	count	1

TRIM38	0.0086148	0.3382551	0.0255	0.98	0.011917699	count	1
C8orf59	0.0086009	0.2135417	0.0403	0.9679	0.012243265	count	1
ERG28	0.0093305	0.4734288	0.0197	0.984	0.012659071	count	1
NDUFAF6	0.0097866	0.5696903	0.0172	0.986	0.012696146	count	1
ATP5MC2	0.0090658	0.1073862	0.0844	0.933	0.013033963	count	1
SEC61A1	0.0104274	0.5125173	0.0203	0.984	0.01328425	count	1
ZNF330	0.0101359	0.4788111	0.0212	0.983	0.013402634	count	1
KLRF1	0.0094363	0.1888893	0.05	0.9602	0.013511166	count	1
SLC50A1	0.0101284	0.390936	0.0259	0.979	0.013775944	count	1
CSRNP1	0.0098875	0.231645	0.0427	0.966	0.014003084	count	1
C8orf76	0.0144448	0.6706714	0.0215	0.983	0.014031516	count	1
ZDHHC5	0.0110481	0.5370207	0.0206	0.984	0.014213726	count	1
C9orf78	0.0099679	0.1659298	0.0601	0.9521	0.014243213	count	1
AGTPBP1	0.0104612	0.4414172	0.0237	0.981	0.014345932	count	1
GTDC1	0.0110434	0.6051738	0.0182	0.985	0.014522064	count	1
TIMM8A	0.0138038	0.9245496	0.0149	0.988	0.01460084	count	1
TMEM64	0.0138038	1.000574	0.0138	0.989	0.01460084	count	1
C3orf14	0.0138038	1.071627	0.0129	0.99	0.01460084	count	1
ZNF816	0.0138038	1.260988	0.0109	0.991	0.01460084	count	1
REEP3	0.0125854	0.599539	0.021	0.983	0.014610973	count	1
DAPP1	0.0158503	0.8561944	0.0185	0.985	0.015398278	count	1
SLTM	0.0109286	0.1863135	0.0587	0.9533	0.015500997	count	1
SNX6	0.0109535	0.2030201	0.054	0.957	0.015566368	count	1
PSMD1	0.0115724	0.4236374	0.0273	0.978	0.015701187	count	1
PSMB1	0.0109799	0.1658077	0.0662	0.9472	0.015714185	count	1
DET1	0.01899	1.0133365	0.0187	0.9851	0.015862961	count	1
SELENOW	0.0111578	0.1909321	0.0584	0.9534	0.015886935	count	1
NMNAT1	0.0270237	0.6475948	0.0417	0.967	0.015901701	count	1
SRSF11	0.0113124	0.2019295	0.056	0.955	0.016166443	count	1
TRAPPC1	0.0113685	0.1979836	0.0574	0.954	0.016232403	count	1
N4BP2L2	0.0115078	0.171598	0.0671	0.947	0.016466747	count	1
SNRPC	0.0119701	0.2287368	0.0523	0.9583	0.017000099	count	1
WIPF1	0.0120495	0.2466303	0.0489	0.9611	0.017048249	count	1
AC243829.1	0.0131959	0.7196413	0.0183	0.985	0.017120257	count	1
RBM23	0.0127947	0.4850834	0.0264	0.979	0.017144941	count	1
NDUFS3	0.0123214	0.2965549	0.0415	0.967	0.017193748	count	1
ATP6V1C1	0.0150542	0.6514415	0.0231	0.982	0.017478877	count	1
KMT2E	0.0123997	0.1292552	0.0959	0.9236	0.01776281	count	1
IDI1	0.012506	0.2473831	0.0506	0.9597	0.017778182	count	1
NIT1	0.0149712	0.806361	0.0186	0.985	0.018274302	count	1
SC5D	0.0191341	0.5167467	0.037	0.97	0.018592581	count	1
METRNL	0.0129921	0.1596018	0.0814	0.935	0.018649028	count	1
ADA	0.0145122	0.6497524	0.0223	0.982	0.018671861	count	1
ANXA6	0.0132333	0.2600558	0.0509	0.959	0.018697097	count	1
OXLD1	0.0137078	0.3846742	0.0356	0.972	0.018764428	count	1

RPS25	0.0131126	0.0544833	0.2407	0.81	0.018896718	count	1
EXOSC1	0.0140516	0.3965071	0.0354	0.972	0.01895803	count	1
NEDD8	0.0133277	0.1667357	0.0799	0.9363	0.019083846	count	1
PCBD2	0.022895	0.9650169	0.0237	0.981	0.019131464	count	1
FAM221A	0.022895	0.9906623	0.0231	0.982	0.019131464	count	1
CDK17	0.0171237	0.4838664	0.0354	0.972	0.019136495	count	1
FAM49B	0.0133947	0.1727389	0.0775	0.938	0.019152345	count	1
RSRP1	0.0134683	0.1416569	0.0951	0.9243	0.019290229	count	1
DCAF10	0.0161894	0.8139449	0.0199	0.984	0.019336937	count	1
GDF11	0.0174375	0.6032041	0.0289	0.977	0.019487467	count	1
TMEM106B	0.0148296	0.4518338	0.0328	0.974	0.019610687	count	1
ADIRF	0.0169345	0.8996175	0.0188	0.985	0.019663526	count	1
GRPEL1	0.0144082	0.3075537	0.0468	0.963	0.020174844	count	1
TRBC1	0.0141603	0.2084142	0.0679	0.946	0.020233937	count	1
TMEM135	0.0344225	0.8199032	0.042	0.967	0.020273741	count	1
MYNN	0.0148154	0.4554895	0.0325	0.974	0.020280847	count	1
GRASP	0.0163197	0.3795473	0.043	0.9657	0.020553773	count	1
VEZF1	0.015152	0.4812871	0.0315	0.975	0.020558827	count	1
ZBTB48	0.0163853	0.6453003	0.0254	0.98	0.020877501	count	1
PPAN	0.0159563	0.4599855	0.0347	0.972	0.021101049	count	1
NDFIP1	0.0152693	0.2671164	0.0572	0.954	0.021450835	count	1
GPR180	0.0184973	0.6990235	0.0265	0.979	0.021479536	count	1
METTL3	0.0184973	0.7663523	0.0241	0.981	0.021479536	count	1
FTH1	0.0149095	0.0835029	0.1786	0.858	0.021493958	count	1
MLLT10	0.0169345	0.5189212	0.0326	0.974	0.021789657	count	1
RGS14	0.0156667	0.2972106	0.0527	0.958	0.021963057	count	1
AREG	0.0153991	0.2125313	0.0725	0.942	0.022146893	count	1
CARD19	0.0158034	0.358105	0.0441	0.965	0.022222047	count	1
COX11	0.0173528	0.5637404	0.0308	0.975	0.022515348	count	1
SECISBP2	0.0162365	0.3840228	0.0423	0.966	0.022581825	count	1
PPP2R2B	0.016602	0.3630294	0.0457	0.964	0.022682355	count	1
HLA-DMB	0.0176383	0.4894042	0.036	0.971	0.022695596	count	1
C16orf87	0.0170067	0.5147125	0.033	0.974	0.022700761	count	1
TBCB	0.0160971	0.2004841	0.0803	0.9361	0.022975568	count	1
PET100	0.0164365	0.3031015	0.0542	0.957	0.023000825	count	1
NINJ1	0.0169524	0.4021875	0.0422	0.966	0.023393602	count	1
MRPS33	0.0171633	0.4186921	0.041	0.967	0.023745418	count	1
HERPUD1	0.0166208	0.1603055	0.1037	0.917	0.023781035	count	1
LINC02256	0.0199947	0.6907812	0.0289	0.977	0.024410058	count	1
CTSZ	0.0178239	0.3135523	0.0568	0.955	0.024741798	count	1
IVNS1ABP	0.01806	0.2512088	0.0719	0.943	0.02495517	count	1
SRRD	0.0186106	0.4783914	0.0389	0.969	0.02518459	count	1
PRDX5	0.0178177	0.1765717	0.1009	0.9197	0.025373017	count	1
SEC61B	0.0178705	0.1560652	0.1145	0.9089	0.025588832	count	1
UTP18	0.0197718	0.4451658	0.0444	0.965	0.026004082	count	1

IKZF3	0.0183288	0.2223226	0.0824	0.934	0.026115436	count	1
COX7B	0.0183342	0.2015603	0.091	0.928	0.026117456	count	1
RPS7	0.0181376	0.0517986	0.3502	0.726	0.026150159	count	1
TRAC	0.0185454	0.2638018	0.0703	0.944	0.026218312	count	1
SIRT1	0.0270247	0.5316449	0.0508	0.959	0.02627386	count	1
PRIM1	0.0199464	0.5667425	0.0352	0.972	0.026379486	count	1
EGR2	0.0191769	0.5004908	0.0383	0.9695	0.026978171	count	1
MT1F	0.0199615	0.4989734	0.04	0.968	0.0272156	count	1
P4HB	0.0194022	0.2457525	0.0789	0.937	0.027230025	count	1
ARID3A	0.0244028	0.6912556	0.0353	0.972	0.027280475	count	1
SNRNP40	0.0203224	0.3767843	0.0539	0.957	0.027644465	count	1
INPP1	0.0216346	0.5752268	0.0376	0.97	0.027840214	count	1
STMN1	0.0198487	0.2493296	0.0796	0.937	0.027946923	count	1
ETV3	0.021204	0.4802074	0.0442	0.965	0.028043293	count	1
WDR4	0.0265508	0.7653414	0.0347	0.972	0.028103658	count	1
STRIP1	0.0265508	0.777841	0.0341	0.973	0.028103658	count	1
AURKAIP1	0.019763	0.2254167	0.0877	0.9302	0.028152917	count	1
GBP3	0.0213568	0.6295935	0.0339	0.973	0.02850925	count	1
SELENOK	0.0199775	0.1461953	0.1366	0.8914	0.028633913	count	1
MT-CO3	0.019909	0.0660249	0.3015	0.763	0.028705226	count	1
MRPS31	0.0206544	0.3564693	0.0579	0.954	0.028922084	count	1
LPIN2	0.0212444	0.4038847	0.0526	0.958	0.029083506	count	1
FERMT3	0.0206764	0.264105	0.0783	0.938	0.029124114	count	1
ATRX	0.020839	0.2380562	0.0875	0.9303	0.029444016	count	1
SAP18	0.0205816	0.1560533	0.1319	0.8952	0.02950485	count	1
USO1	0.0228865	0.5681021	0.0403	0.968	0.029698772	count	1
ING1	0.0217426	0.3408443	0.0638	0.949	0.029707334	count	1
RUNX1	0.0241789	0.4919942	0.0491	0.961	0.030035007	count	1
SUGT1	0.0214717	0.2867178	0.0749	0.94	0.030134816	count	1
ATP5MG	0.0212029	0.1254248	0.169	0.866	0.030417948	count	1
YRDC	0.0277452	0.484484	0.0573	0.954	0.031021825	count	1
RALGPS1	0.0529462	1.032924	0.0513	0.959	0.031253341	count	1
CDKN2B	0.0529462	1.228266	0.0431	0.9656	0.031253341	count	1
TLE4	0.0227178	0.2520271	0.0901	0.928	0.031260714	count	1
MIEN1	0.0222242	0.289437	0.0768	0.939	0.031304532	count	1
PPP4R3B	0.022519	0.3701822	0.0608	0.952	0.031376007	count	1
EEF1A1	0.0218485	0.0465554	0.4693	0.639	0.031510357	count	1
COMMD1	0.0240814	0.3853909	0.0625	0.95	0.031850363	count	1
SUPT20H	0.0240814	0.4031111	0.0597	0.952	0.031850363	count	1
PRMT2	0.0224093	0.209646	0.1069	0.9149	0.031852597	count	1
PUS3	0.0285355	0.8192942	0.0348	0.972	0.031906621	count	1
GLMP	0.0279203	0.7064208	0.0395	0.968	0.032434056	count	1
CCDC43	0.0246026	0.4998378	0.0492	0.961	0.032539999	count	1
DPM2	0.0237948	0.3854698	0.0617	0.951	0.032960535	count	1
SKA2	0.0245465	0.3900844	0.0629	0.95	0.033122117	count	1

TMEM33	0.025069	0.5959906	0.0421	0.966	0.033320246	count	1
RBL2	0.0243997	0.3825099	0.0638	0.949	0.033338746	count	1
GZMM	0.0237297	0.1504001	0.1578	0.875	0.034110211	count	1
RABAC1	0.0239826	0.1699398	0.1411	0.8879	0.034345021	count	1
CEPT1	0.0411579	0.8250521	0.0499	0.9602	0.034446508	count	1
SMNDC1	0.0248371	0.2812647	0.0883	0.93	0.034513188	count	1
NXF1	0.0355191	0.623383	0.057	0.955	0.034551857	count	1
PSMA3	0.0244631	0.2433442	0.1005	0.92	0.034554665	count	1
FAF1	0.0271277	0.5311686	0.0511	0.959	0.034913201	count	1
MRPL18	0.0250415	0.3683309	0.068	0.946	0.035181017	count	1
SQSTM1	0.0246612	0.1716212	0.1437	0.886	0.035244003	count	1
RAB29	0.0262823	0.4380047	0.06	0.952	0.035465157	count	1
TUBA1C	0.0253743	0.3104352	0.0817	0.935	0.035510123	count	1
ENTR1	0.026761	0.4701165	0.0569	0.955	0.035726296	count	1
CASP8	0.0256815	0.2770005	0.0927	0.926	0.035866808	count	1
SART1	0.0308836	0.6399949	0.0483	0.962	0.03588066	count	1
MYDGF	0.0255589	0.2273631	0.1124	0.9106	0.035972988	count	1
AC242426.2	0.0340086	0.7782228	0.0437	0.965	0.036012282	count	1
AL135925.1	0.0324605	0.6390217	0.0508	0.96	0.036301869	count	1
NKG7	0.025327	0.0781028	0.3243	0.746	0.036515174	count	1
WDR60	0.0272071	0.5392766	0.0505	0.96	0.036713525	count	1
TOMM7	0.0259524	0.1180223	0.2199	0.826	0.037226034	count	1
ZNF776	0.0322519	0.809088	0.0399	0.968	0.037472393	count	1
GTPBP2	0.0322519	1.1116832	0.029	0.977	0.037472393	count	1
CCDC167	0.026736	0.3111032	0.0859	0.932	0.037543429	count	1
STK10	0.0272628	0.3488955	0.0781	0.938	0.037624996	count	1
SNRNP27	0.0274921	0.3826089	0.0719	0.943	0.038083181	count	1
VMA21	0.0280476	0.4194414	0.0669	0.947	0.038156476	count	1
RPS6KA1	0.0320812	0.5112827	0.0627	0.95	0.038339984	count	1
REX1BD	0.0270105	0.2086029	0.1295	0.8971	0.038426289	count	1
ANXA7	0.0273095	0.2970191	0.0919	0.927	0.038468526	count	1
ATXN1	0.0294342	0.5368814	0.0548	0.956	0.038476547	count	1
PPWD1	0.0284156	0.4654839	0.061	0.951	0.038827596	count	1
USP16	0.0276056	0.2506701	0.1101	0.912	0.038943167	count	1
GOLGA4	0.0278565	0.2763944	0.1008	0.92	0.038984437	count	1
ADNP	0.0280425	0.3706628	0.0757	0.94	0.039005092	count	1
IFRD1	0.0280303	0.2873536	0.0975	0.922	0.039023125	count	1
DNAJC1	0.0274402	0.2034237	0.1349	0.8928	0.039114659	count	1
CD82	0.0303205	0.5151724	0.0589	0.953	0.039351563	count	1
RSAD1	0.0333067	0.6945357	0.048	0.962	0.039806275	count	1
DDI2	0.0333067	0.8682006	0.0384	0.969	0.039806275	count	1
AP1G1	0.0316235	0.6647824	0.0476	0.962	0.039843998	count	1
VAC14	0.0359259	0.8149183	0.0441	0.965	0.040183738	count	1
ZNF75D	0.0359259	0.9233113	0.0389	0.969	0.040183738	count	1
PSMA5	0.0282257	0.2323958	0.1215	0.9034	0.040185962	count	1

UBB	0.0280148	0.0807963	0.3467	0.729	0.040336057	count	1
RARRES3	0.028283	0.1300062	0.2176	0.828	0.040601236	count	1
AKAP13	0.028608	0.2064129	0.1386	0.8899	0.040693602	count	1
LYPLA2	0.0296221	0.3547851	0.0835	0.934	0.040764004	count	1
MLXIP	0.0352067	1.0688024	0.0329	0.9737	0.040910266	count	1
YBX1	0.0286852	0.1187491	0.2416	0.809	0.041226954	count	1
C6orf62	0.0306717	0.3914881	0.0783	0.938	0.041390584	count	1
CD59	0.0319676	0.5499319	0.0581	0.954	0.041490655	count	1
ATP5F1C	0.0293366	0.203367	0.1443	0.885	0.04180527	count	1
ACAD8	0.0362082	0.6119879	0.0592	0.953	0.042075677	count	1
RAD23B	0.0320374	0.5001117	0.0641	0.949	0.042145292	count	1
UQCRH	0.0294551	0.1568611	0.1878	0.8512	0.042197485	count	1
IMP3	0.030007	0.2163458	0.1387	0.8898	0.042639723	count	1
RNF125	0.0305087	0.2186417	0.1395	0.889	0.043053812	count	1
MDM4	0.031427	0.3104999	0.1012	0.919	0.043312961	count	1
KLRC1	0.0306849	0.1529258	0.2007	0.841	0.044064067	count	1
LINC00667	0.0315429	0.4178387	0.0755	0.94	0.044144451	count	1
SNRNP70	0.0318427	0.3136594	0.1015	0.919	0.044250373	count	1
UBALD2	0.030975	0.1433405	0.2161	0.8291	0.044345305	count	1
BBS2	0.0529462	0.8631705	0.0613	0.951	0.04435684	count	1
HS1BP3	0.0529462	0.9411381	0.0563	0.955	0.04435684	count	1
ZNF263	0.0529462	0.9441485	0.0561	0.955	0.04435684	count	1
INTS9	0.0529462	1.040638	0.0509	0.959	0.04435684	count	1
PVT1	0.034264	0.5283908	0.0648	0.948	0.044473229	count	1
CCDC12	0.031332	0.2159416	0.1451	0.8847	0.044597938	count	1
NUDT21	0.0328384	0.3560062	0.0922	0.927	0.044676456	count	1
TMEM138	0.0337202	0.6501387	0.0519	0.959	0.045021688	count	1
PRDX4	0.0331912	0.5281215	0.0628	0.95	0.045045042	count	1
WASHC3	0.032844	0.375755	0.0874	0.93	0.045127392	count	1
ARID1B	0.0370082	0.3546258	0.1044	0.917	0.04520477	count	1
RNF157	0.0359259	0.7237224	0.0496	0.96	0.045269815	count	1
MHENCR	0.0338551	0.4890838	0.0692	0.945	0.045379922	count	1
VIM	0.0315501	0.105988	0.2977	0.766	0.045479692	count	1
RPS3A	0.0322306	0.0565732	0.5697	0.569	0.046467034	count	1
LMO4	0.0328262	0.1906179	0.1722	0.8634	0.046667665	count	1
C2CD5	0.0788885	0.7633163	0.1033	0.918	0.046709226	count	1
KDM1B	0.0788885	0.7775426	0.1015	0.919	0.046709226	count	1
SPIN1	0.0404203	0.5711679	0.0708	0.944	0.04697813	count	1
ARFGAP1	0.0395035	0.8424487	0.0469	0.963	0.047222515	count	1
IRF1	0.0328356	0.1248738	0.263	0.793	0.047280827	count	1
NDUFC2	0.0332228	0.21941	0.1514	0.8797	0.047295399	count	1
GMPS	0.03478	0.53934	0.0645	0.949	0.047319054	count	1
EIF3L	0.0344097	0.3078772	0.1118	0.911	0.047986467	count	1
KDELR1	0.0338844	0.2595238	0.1306	0.8962	0.04800391	count	1
RPL30	0.0335004	0.0455149	0.736	0.462	0.048309095	count	1

SRGN	0.0335554	0.0668081	0.5023	0.616	0.048374661	count	1
NUTM2B-AS1	0.0355384	0.325246	0.1093	0.913	0.048461689	count	1
TRMT10C	0.0351488	0.3000092	0.1172	0.907	0.048797461	count	1
EIF3E	0.0344923	0.1923121	0.1794	0.8578	0.048876237	count	1
CHAMP1	0.0380696	0.681884	0.0558	0.956	0.049007128	count	1
VTI1A	0.0395012	0.490042	0.0806	0.936	0.049089628	count	1
TSNAX	0.0349966	0.2828619	0.1237	0.902	0.049215578	count	1
S100A4	0.0341743	0.1251657	0.273	0.785	0.049228365	count	1
UROS	0.0352688	0.3203972	0.1101	0.912	0.049420326	count	1
UNC119	0.0374097	0.5090701	0.0735	0.941	0.049489717	count	1
GSTP1	0.0344261	0.1260251	0.2732	0.785	0.04957653	count	1
ARRB2	0.0364801	0.4312186	0.0846	0.933	0.050204759	count	1
PLEKHA1	0.0474963	0.5085139	0.0934	0.926	0.050331119	count	1
SUCO	0.0369048	0.4962712	0.0744	0.941	0.050431989	count	1
ITGB1BP1	0.0361365	0.2687211	0.1345	0.893	0.050541764	count	1
EFR3A	0.0383792	0.4881838	0.0786	0.937	0.050773105	count	1
MED11	0.0378251	0.5012696	0.0755	0.94	0.050884496	count	1
RNASEH2B	0.0367356	0.3408332	0.1078	0.914	0.051051677	count	1
NUDT14	0.0364896	0.327602	0.1114	0.911	0.051131332	count	1
SLC39A7	0.039721	0.4796524	0.0828	0.934	0.051561891	count	1
TMEM19	0.0395035	0.7254784	0.0545	0.957	0.05164909	count	1
GSE1	0.0395035	0.7639625	0.0517	0.959	0.05164909	count	1
CD2AP	0.0376681	0.495879	0.076	0.939	0.051670275	count	1
H2AFJ	0.0363552	0.2239499	0.1623	0.871	0.051779547	count	1
ZFHX3	0.0492773	0.6662627	0.074	0.941	0.052223366	count	1
SMIM19	0.0373972	0.3266995	0.1145	0.909	0.052305429	count	1
ATG4D	0.0392389	0.4743267	0.0827	0.934	0.052394363	count	1
EOMES	0.0380193	0.4345218	0.0875	0.93	0.052474855	count	1
ACTL6A	0.0429896	0.6700548	0.0642	0.949	0.052520642	count	1
SPARCL1	0.0429896	0.7980207	0.0539	0.957	0.052520642	count	1
APPL1	0.0372934	0.333447	0.1118	0.911	0.052593542	count	1
DNM2	0.0394389	0.3807362	0.1036	0.918	0.052661574	count	1
ACTR6	0.0391282	0.4120793	0.095	0.924	0.05280823	count	1
SSBP1	0.0372447	0.2026452	0.1838	0.8543	0.052980223	count	1
DPH5	0.0446291	0.723426	0.0617	0.951	0.053359096	count	1
RANBP6	0.0446291	0.7285437	0.0613	0.951	0.053359096	count	1
PXK	0.0462251	0.6376136	0.0725	0.942	0.053736898	count	1
FAM110A	0.0386702	0.3951316	0.0979	0.922	0.053885735	count	1
GNL1	0.0450876	0.508418	0.0887	0.929	0.053908132	count	1
CUL2	0.04415	0.5738116	0.0769	0.939	0.053940234	count	1
SLC30A1	0.0509557	0.8560951	0.0595	0.953	0.054006921	count	1
SNHG15	0.0384806	0.2867827	0.1342	0.893	0.054065586	count	1
EEF1D	0.0376778	0.0904626	0.4165	0.677	0.054241849	count	1
SGF29	0.0405464	0.4864623	0.0833	0.934	0.054354253	count	1
ITM2A	0.0380157	0.1952883	0.1947	0.8458	0.05441666	count	1

ICOS	0.0437272	0.7679281	0.0569	0.955	0.055111107	count	1
MRFAP1L1	0.0414127	0.3838373	0.1079	0.914	0.055298764	count	1
CDK2AP2	0.0390046	0.1981084	0.1969	0.844	0.055646998	count	1
UCK2	0.0450394	0.5857303	0.0769	0.939	0.055980792	count	1
TRMT10B	0.0421519	0.6722249	0.0627	0.95	0.056041107	count	1
CCDC61	0.0427085	0.680393	0.0628	0.95	0.056193825	count	1
EIF3H	0.0394906	0.1646365	0.2399	0.811	0.056518193	count	1
GTF3C6	0.0399183	0.2836436	0.1407	0.8882	0.056540308	count	1
MT-CYB	0.0394212	0.0691616	0.57	0.569	0.056843987	count	1
PECAM1	0.06793	0.6063702	0.112	0.911	0.056980972	count	1
BZW1	0.0400148	0.1380283	0.2899	0.7721	0.057299266	count	1
MCF2L-AS1	0.0471656	0.9176632	0.0514	0.959	0.057629869	count	1
RAD51C	0.0433589	0.5874319	0.0738	0.941	0.057646913	count	1
GPRIN3	0.0434547	0.3383271	0.1284	0.898	0.057774369	count	1
SCP2	0.0414617	0.2704792	0.1533	0.878	0.058384141	count	1
PITPNB	0.0421433	0.3283535	0.1283	0.898	0.05838551	count	1
PHPT1	0.0414308	0.2524058	0.1641	0.8697	0.058669055	count	1
NECAP2	0.0420092	0.3542175	0.1186	0.906	0.05871784	count	1
TUBB4B	0.0414008	0.200327	0.2067	0.8364	0.05874	count	1
SLC35D2	0.042745	0.3912059	0.1093	0.913	0.058830073	count	1
HNRNPA0	0.041605	0.214949	0.1936	0.8466	0.059018268	count	1
TRMT112	0.0413757	0.1536284	0.2693	0.7879	0.059238796	count	1
PTPN1	0.0424334	0.276547	0.1534	0.878	0.059268945	count	1
IL18	0.0428267	0.3796094	0.1128	0.91	0.059460234	count	1
MAT2B	0.0431698	0.3192825	0.1352	0.893	0.059503284	count	1
CIAO1	0.0438886	0.4521925	0.0971	0.923	0.059570499	count	1
ITGAM	0.0571397	0.651614	0.0877	0.93	0.060580989	count	1
RP9	0.0456912	0.4431951	0.1031	0.918	0.060749995	count	1
NUPL2	0.0443863	0.3865879	0.1148	0.909	0.060889835	count	1
MZF1	0.0442412	0.4685558	0.0944	0.925	0.060890136	count	1
TDP2	0.1030786	0.5226969	0.1972	0.8438	0.061201489	count	1
CDC26	0.0437229	0.2777597	0.1574	0.875	0.061371155	count	1
INSIG2	0.0465706	0.5658604	0.0823	0.934	0.061618174	count	1
FEN1	0.0485102	0.6222577	0.078	0.938	0.061856877	count	1
PIKFYVE	0.0485102	0.6572492	0.0738	0.941	0.061856877	count	1
CFAP36	0.0472144	0.4871283	0.0969	0.923	0.062127398	count	1
CYTH2	0.0519603	0.5855645	0.0887	0.929	0.062139935	count	1
PTPN18	0.0488327	0.4831728	0.1011	0.92	0.062268571	count	1
RABIF	0.0468304	0.4390344	0.1067	0.915	0.062783823	count	1
TTC7A	0.0644991	0.7297174	0.0884	0.93	0.062861314	count	1
KIAA1328	0.0500158	0.6783459	0.0737	0.941	0.063046877	count	1
ESCO1	0.046267	0.3230583	0.1432	0.886	0.063470979	count	1
RPS3	0.044152	0.0483029	0.9141	0.361	0.063667239	count	1
ANP32E	0.0453284	0.2208611	0.2052	0.838	0.063855668	count	1
C9orf40	0.0769023	0.8881347	0.0866	0.931	0.064554639	count	1

TGFBR1	0.0517669	0.6367584	0.0813	0.935	0.065257074	count	1
ANXA2	0.0459787	0.2165448	0.2123	0.832	0.065423444	count	1
NTHL1	0.0490572	0.5917189	0.0829	0.934	0.065513977	count	1
AK2	0.0473353	0.3706426	0.1277	0.898	0.065653576	count	1
NDUFS1	0.048732	0.4835122	0.1008	0.92	0.065777863	count	1
SPARC	0.0787149	0.9809062	0.0802	0.936	0.06608597	count	1
ITGA8	0.0787149	0.9809062	0.0802	0.936	0.06608597	count	1
PATZ1	0.0787149	0.9809062	0.0802	0.936	0.06608597	count	1
EXOC7	0.0511582	0.4712006	0.1086	0.914	0.066423741	count	1
RPP30	0.0517609	0.4613189	0.1122	0.911	0.066651627	count	1
CDADC1	0.0507279	0.5280518	0.0961	0.924	0.06675476	count	1
ATP13A2	0.112523	1.00786	0.1116	0.911	0.06688	count	1
NATD1	0.112523	1.1129826	0.1011	0.9195	0.06688	count	1
ZNF91	0.0508878	0.4677062	0.1088	0.913	0.066965365	count	1
TOB2	0.0501741	0.42302	0.1186	0.906	0.067006657	count	1
NFU1	0.0487924	0.3841372	0.127	0.899	0.067256579	count	1
MINOS1	0.0476609	0.2902181	0.1642	0.8697	0.067307734	count	1
TRAF1	0.054532	0.5339489	0.1021	0.919	0.06779714	count	1
EVI2B	0.0476397	0.2049818	0.2324	0.8164	0.0680066	count	1
CCL4L2	0.0471938	0.2189905	0.2155	0.83	0.068050829	count	1
GIMAP7	0.047506	0.1930738	0.2461	0.8058	0.068106671	count	1
VPS18	0.0612402	1.1384392	0.0538	0.9571	0.068576049	count	1
ADRM1	0.048711	0.2867136	0.1699	0.865	0.068621939	count	1
B3GNT2	0.0516245	0.3519267	0.1467	0.883	0.068945114	count	1
GALNT10	0.0650095	0.8849478	0.0735	0.941	0.068953108	count	1
ZNF789	0.0650095	0.9533805	0.0682	0.946	0.068953108	count	1
ZCCHC14	0.0650095	1.0138022	0.0641	0.949	0.068953108	count	1
LTA	0.0548104	0.5281959	0.1038	0.917	0.069098946	count	1
SNRNP35	0.0500043	0.4498181	0.1112	0.912	0.069114392	count	1
VAMP7	0.057936	0.6863956	0.0844	0.933	0.069300357	count	1
TPRA1	0.057936	0.697576	0.0831	0.934	0.069300357	count	1
ETAA1	0.057936	0.7839897	0.0739	0.941	0.069300357	count	1
GBP1	0.051342	0.4147383	0.1238	0.902	0.069506477	count	1
RPL22L1	0.0486775	0.1750933	0.278	0.781	0.069631222	count	1
HSBP1	0.049639	0.3379334	0.1469	0.8833	0.069901701	count	1
SDHA	0.0509779	0.3863455	0.1319	0.895	0.069936798	count	1
CAPS2	0.0625912	0.8318983	0.0752	0.94	0.070093049	count	1
AL451085.1	0.0625912	0.8856241	0.0707	0.944	0.070093049	count	1
METTL13	0.0625912	0.9866768	0.0634	0.949	0.070093049	count	1
ZNF251	0.0625912	0.9866768	0.0634	0.949	0.070093049	count	1
RER1	0.0495804	0.2406675	0.206	0.8369	0.070228177	count	1
ITPR2	0.0604865	0.4746289	0.1274	0.899	0.070354477	count	1
MAP4K4	0.0568705	0.5648544	0.1007	0.92	0.070709002	count	1
JMJD6	0.0503045	0.3049917	0.1649	0.869	0.070946769	count	1
B3GALT6	0.0533547	0.5402886	0.0988	0.921	0.070947673	count	1

LAP3	0.0507941	0.3440724	0.1476	0.883	0.071048741	count	1
STRAP	0.0508985	0.2598964	0.1958	0.845	0.071240976	count	1
APOBEC3H	0.0531682	0.4687795	0.1134	0.91	0.072173676	count	1
AC012645.3	0.0541137	0.5829823	0.0928	0.926	0.072812811	count	1
PCID2	0.0541405	0.4199209	0.1289	0.897	0.072848898	count	1
TNFSF14	0.0512752	0.2266677	0.2262	0.821	0.073129256	count	1
ZNF891	0.075045	1.0853602	0.0691	0.945	0.073188403	count	1
SNN	0.075045	1.3082038	0.0574	0.954	0.073188403	count	1
ZNF45	0.075045	1.3082038	0.0574	0.954	0.073188403	count	1
CNPY2	0.0527604	0.3276893	0.161	0.872	0.073527032	count	1
PHB	0.0526975	0.27366	0.1926	0.847	0.07389194	count	1
RNF165	0.0890332	0.8612467	0.1034	0.918	0.07481125	count	1
PPP2R5B	0.0890332	0.9422612	0.0945	0.925	0.07481125	count	1
ETS1	0.0524254	0.164863	0.318	0.7507	0.074861502	count	1
LBR	0.0538328	0.2800777	0.1922	0.848	0.07489141	count	1
RSBN1	0.0534502	0.2957211	0.1807	0.857	0.075329	count	1
PNKD	0.0534684	0.2740564	0.1951	0.845	0.07551104	count	1
COA3	0.0537465	0.3588015	0.1498	0.881	0.0755203	count	1
RPS28	0.0524167	0.0588367	0.8909	0.374	0.075542187	count	1
STK17A	0.0525302	0.1078871	0.4869	0.627	0.075551884	count	1
EEF1B2	0.0527145	0.0846153	0.623	0.534	0.075911985	count	1
RRN3	0.0779078	0.7346794	0.106	0.916	0.075994059	count	1
LRRFIP2	0.0592653	0.5152259	0.115	0.908	0.076327042	count	1
TRNT1	0.0593758	0.5594453	0.1061	0.916	0.076469531	count	1
YBX3	0.0640774	0.4865061	0.1317	0.895	0.0766622	count	1
VPS41	0.0603894	0.5394708	0.1119	0.911	0.077025387	count	1
PCED1B	0.0603894	0.5647412	0.1069	0.915	0.077025387	count	1
ARHGEF9	0.0566167	0.5304566	0.1067	0.915	0.077047502	count	1
CST7	0.0536423	0.1015084	0.5285	0.598	0.07722832	count	1
TNF	0.0541407	0.4001915	0.1353	0.892	0.077338377	count	1
AL592183.1	0.0694756	0.648801	0.1071	0.915	0.077826007	count	1
UBQLN1	0.0624163	0.5107276	0.1222	0.903	0.07870246	count	1
FAM43A	0.0601913	0.3927089	0.1533	0.878	0.078727742	count	1
CEP350	0.0570406	0.3687911	0.1547	0.877	0.078741238	count	1
UBE2B	0.0553751	0.1758823	0.3148	0.7531	0.078774514	count	1
CMTM8	0.132298	0.6388842	0.2071	0.836	0.078805888	count	1
MAP7D3	0.0629781	0.5137167	0.1226	0.903	0.079411948	count	1
RALY	0.0564822	0.2278061	0.2479	0.804	0.079439252	count	1
CTDNEP1	0.0563794	0.2574525	0.219	0.827	0.079487902	count	1
PIM1	0.0564282	0.2755508	0.2048	0.8379	0.079666558	count	1
MKRN2	0.0685747	0.6355611	0.1079	0.914	0.079786655	count	1
ECH1	0.0558878	0.1910412	0.2925	0.7701	0.079921318	count	1
PPP2R3C	0.0598416	0.3607819	0.1659	0.868	0.08024203	count	1
SUN2	0.0573393	0.2691525	0.213	0.831	0.080402688	count	1
CARD16	0.0565377	0.2558729	0.221	0.8253	0.080417532	count	1

TRA2B	0.0566701	0.1787554	0.317	0.7514	0.080582949	count	1
GTF2F2	0.0596945	0.4070701	0.1466	0.884	0.080586117	count	1
GTF2A1	0.0615792	0.5447572	0.113	0.91	0.081049588	count	1
HSPA1B	0.0583417	0.3241886	0.18	0.8573	0.081090566	count	1
CYB561D2	0.0670647	0.8586799	0.0781	0.938	0.081992976	count	1
TCP1	0.0588049	0.2920093	0.2014	0.841	0.082020348	count	1
SUPT5H	0.0608166	0.4887233	0.1244	0.901	0.082102081	count	1
TXLNG	0.0842364	0.6090562	0.1383	0.89	0.082199705	count	1
LAMTOR5	0.0583686	0.2418015	0.2414	0.809	0.082230822	count	1
KHDC4	0.0775588	0.8029931	0.0966	0.9231	0.082316866	count	1
ENY2	0.0579416	0.2179037	0.2659	0.79	0.082391273	count	1
TRAF3IP3	0.0580752	0.212273	0.2736	0.7846	0.082569177	count	1
EIF5A	0.0577691	0.1592591	0.3627	0.717	0.082777133	count	1
TIPARP	0.0580357	0.2010379	0.2887	0.773	0.082873732	count	1
EIF1AX	0.0584694	0.2339755	0.2499	0.8028	0.082912343	count	1
SRSF7	0.0581458	0.1412491	0.4117	0.6809	0.083113015	count	1
RCSD1	0.0597077	0.2559737	0.2333	0.816	0.08328005	count	1
GPAT4	0.0787149	0.7568012	0.104	0.917	0.083548817	count	1
RNF219	0.0615676	0.5499224	0.112	0.911	0.083583653	count	1
KLHDC3	0.0750475	0.5111494	0.1468	0.883	0.084087908	count	1
KIAA2026	0.0607558	0.3743202	0.1623	0.871	0.084364078	count	1
PABPN1	0.0614532	0.4159465	0.1477	0.883	0.084717908	count	1
EBAG9	0.0623332	0.429537	0.1451	0.885	0.084832245	count	1
IGSF8	0.0626756	0.4312433	0.1453	0.885	0.085298556	count	1
ARFRP1	0.0630414	0.4322814	0.1458	0.884	0.085357116	count	1
LINC02361	0.0874857	0.8724695	0.1003	0.92	0.085387664	count	1
LY9	0.0606709	0.2869102	0.2115	0.833	0.085406128	count	1
WDR74	0.0650592	0.3939272	0.1652	0.869	0.085635	count	1
MMD	0.0645517	0.5115943	0.1262	0.9	0.086225762	count	1
ATPAF1	0.0649048	0.593203	0.1094	0.913	0.086321533	count	1
EYA3	0.0775754	0.5396569	0.1437	0.886	0.086929791	count	1
BCLAF1	0.0616377	0.232523	0.2651	0.791	0.086934837	count	1
EED	0.0682409	0.5472599	0.1247	0.901	0.087055232	count	1
CBWD3	0.1035246	0.7146537	0.1449	0.885	0.08708826	count	1
ETFA	0.0620574	0.2883182	0.2152	0.83	0.087159044	count	1
WDR48	0.0658971	0.7553177	0.0872	0.9305	0.087216759	count	1
ATP5F1E	0.0606736	0.0988687	0.6137	0.54	0.087327392	count	1
UTS2	0.075045	0.8733782	0.0859	0.932	0.08733602	count	1
RAD51B	0.075045	0.941949	0.0797	0.937	0.08733602	count	1
FCHSD1	0.075045	1.0781411	0.0696	0.945	0.08733602	count	1
ADAMTS17	0.0730775	0.785339	0.0931	0.926	0.087456006	count	1
GPATCH2L	0.0649341	0.5879386	0.1104	0.912	0.087665198	count	1
ZCCHC10	0.0645272	0.4173188	0.1546	0.877	0.087820288	count	1
RNF167	0.0622701	0.3321605	0.1875	0.8514	0.087827026	count	1
ORMDL2	0.0628424	0.3195221	0.1967	0.844	0.087907736	count	1

SGTA	0.0787973	0.609784	0.1292	0.897	0.088303668	count	1
ELMO1	0.0833724	0.7006036	0.119	0.905	0.088513271	count	1
MALT1	0.0694688	0.4278182	0.1624	0.871	0.088624108	count	1
TMEM99	0.0712987	0.7992025	0.0892	0.929	0.088682523	count	1
ENTPD1-AS1	0.10549	0.760794	0.1387	0.89	0.088755357	count	1
TFEB	0.10549	0.8760844	0.1204	0.904	0.088755357	count	1
PES1	0.10549	0.8848722	0.1192	0.905	0.088755357	count	1
FAM118A	0.0642921	0.3777083	0.1702	0.865	0.088756928	count	1
LYRM1	0.0662152	0.472391	0.1402	0.889	0.088796205	count	1
COPB1	0.0639934	0.2822681	0.2267	0.821	0.089032999	count	1
ECI1	0.06407	0.3988242	0.1606	0.872	0.089294837	count	1
NR3C1	0.0637736	0.2610216	0.2443	0.807	0.089570222	count	1
UQCRC2	0.0639644	0.2665434	0.24	0.81	0.089838289	count	1
GNAS	0.0629544	0.113152	0.5564	0.578	0.090500301	count	1
TPM4	0.0635002	0.1851391	0.343	0.732	0.090515138	count	1
LAMP1	0.0643371	0.231961	0.2774	0.782	0.090605815	count	1
IQGAP2	0.064438	0.233912	0.2755	0.783	0.090632197	count	1
ACIN1	0.0655863	0.3297726	0.1989	0.842	0.090774623	count	1
RPL38	0.0632863	0.1050063	0.6027	0.547	0.090825056	count	1
TMEM14B	0.0642367	0.2436142	0.2637	0.7922	0.09092687	count	1
ZSCAN21	0.0858663	1.0991702	0.0781	0.938	0.091172436	count	1
GPBP1	0.0642024	0.1718672	0.3736	0.709	0.091468223	count	1
AAK1	0.0655084	0.2512299	0.2608	0.794	0.091576736	count	1
NABP1	0.069827	0.4824496	0.1447	0.885	0.091918112	count	1
CDKN2AIPNL	0.0704015	0.5918771	0.1189	0.905	0.092099313	count	1
ID2	0.0644203	0.1504796	0.4281	0.669	0.092320075	count	1
TDRD7	0.0737438	0.7565077	0.0975	0.922	0.093011337	count	1
TIFA	0.0679182	0.4540909	0.1496	0.881	0.093023867	count	1
ABHD5	0.0739156	0.5088366	0.1453	0.885	0.093228411	count	1
PHF21A	0.0731057	0.5544978	0.1318	0.895	0.093271417	count	1
INTS8	0.1108268	1.032662	0.1073	0.915	0.093284558	count	1
PHLDB2	0.1108268	1.1067581	0.1001	0.92	0.093284558	count	1
CATSPER2	0.1108268	1.1067581	0.1001	0.92	0.093284558	count	1
BEND5	0.1108268	1.1193743	0.099	0.921	0.093284558	count	1
CASP2	0.1562884	0.7570026	0.2065	0.8366	0.093337443	count	1
ATP2B4	0.0682428	0.3663369	0.1863	0.852	0.093638069	count	1
ACTR2	0.0660445	0.1955548	0.3377	0.7358	0.094039225	count	1
TMEM147	0.068035	0.3701629	0.1838	0.854	0.094378597	count	1
ZNF227	0.112523	0.7856762	0.1432	0.886	0.094724809	count	1
KLHL36	0.0845287	0.592509	0.1427	0.887	0.094749728	count	1
TRAPPC12	0.0775588	0.6337109	0.1224	0.903	0.094852304	count	1
PACS2	0.0972718	0.7208656	0.1349	0.893	0.094996247	count	1
SLC2A11	0.0972718	0.7616353	0.1277	0.898	0.094996247	count	1
HNRNPAB	0.0667731	0.1962086	0.3403	0.7338	0.095141575	count	1
WDR1	0.0682099	0.3249415	0.2099	0.834	0.095419129	count	1

GLIPR1	0.0670798	0.2332004	0.2876	0.7738	0.095466185	count	1
LINC00662	0.0730775	0.6221484	0.1175	0.907	0.095604649	count	1
SRSF6	0.0702906	0.4132233	0.1701	0.865	0.095670199	count	1
NBAS	0.0769094	0.5349253	0.1438	0.886	0.095675439	count	1
TRADD	0.0714207	0.4297002	0.1662	0.868	0.09578369	count	1
TMEM216	0.0714207	0.4743683	0.1506	0.88	0.09578369	count	1
RPS29	0.0668981	0.0690909	0.9683	0.334	0.096328562	count	1
COA5	0.0697011	0.48295	0.1443	0.885	0.096354221	count	1
PI4KB	0.0860641	0.5694867	0.1511	0.88	0.096477081	count	1
GTF2H5	0.0699136	0.311995	0.2241	0.823	0.096648144	count	1
SH3BP1	0.0685745	0.3308638	0.2073	0.8359	0.096819854	count	1
CALM3	0.0683594	0.2317831	0.2949	0.7682	0.097164373	count	1
EHMT1	0.0711527	0.4645085	0.1532	0.878	0.097267751	count	1
CFL2	0.0707963	0.4682603	0.1512	0.88	0.097308182	count	1
MESD	0.06945	0.2795601	0.2484	0.804	0.097335456	count	1
COMMD6	0.0678071	0.1326409	0.5112	0.61	0.097341586	count	1
MRPS7	0.0707715	0.3621084	0.1954	0.845	0.097427624	count	1
GABPB1	0.0777361	0.4951562	0.157	0.875	0.098056171	count	1
PQBP1	0.0698087	0.3501605	0.1994	0.842	0.098188652	count	1
STARD3NL	0.069133	0.2847874	0.2428	0.8084	0.098362937	count	1
RNF111	0.0762787	0.5648119	0.1351	0.893	0.099088554	count	1
EVL	0.0692146	0.1260073	0.5493	0.583	0.099375457	count	1
FAM49A	0.0735788	0.3919249	0.1877	0.851	0.100149236	count	1
NKTR	0.0717407	0.2778057	0.2582	0.796	0.100360449	count	1
SYPL1	0.0715001	0.2950269	0.2424	0.809	0.100426075	count	1
OAT	0.0759361	0.4499792	0.1688	0.866	0.100519713	count	1
STRN3	0.0759361	0.4898278	0.155	0.877	0.100519713	count	1
IL4R	0.08117	0.575237	0.1411	0.888	0.100986952	count	1
NFKBIL1	0.075045	0.5041664	0.1488	0.882	0.101328224	count	1
RNF213	0.0713717	0.2021709	0.353	0.7243	0.101535061	count	1
SLC39A10	0.0723227	0.2997915	0.2412	0.81	0.101679612	count	1
HOXA9	0.0907138	1.0056103	0.0902	0.928	0.101709356	count	1
RWDD2B	0.0907138	1.0056103	0.0902	0.928	0.101709356	count	1
KNTC1	0.0907138	1.14036	0.0795	0.937	0.101709356	count	1
ATP5PO	0.071857	0.2124667	0.3382	0.735	0.101741398	count	1
C1RL	0.0850546	0.7510943	0.1132	0.91	0.101829452	count	1
DOK2	0.0712725	0.1705425	0.4179	0.676	0.101985793	count	1
UBA1	0.096043	0.7568116	0.1269	0.899	0.102029972	count	1
SARNP	0.096043	0.8385758	0.1145	0.909	0.102029972	count	1
CTDP1	0.0769176	0.4613786	0.1667	0.868	0.102316689	count	1
DBT	0.1218302	0.8169296	0.1491	0.882	0.102633808	count	1
MYL12A	0.0712491	0.0735693	0.9685	0.334	0.102673823	count	1
GDE1	0.0796937	0.6981214	0.1142	0.909	0.102679951	count	1
CPSF3	0.0793902	0.4973575	0.1596	0.873	0.103136595	count	1
WHAMM	0.0773838	0.3384979	0.2286	0.819	0.103385313	count	1

HOPX	0.0722911	0.154446	0.4681	0.64	0.103722209	count	1
UBE2N	0.073036	0.2336528	0.3126	0.7548	0.1038743	count	1
CCDC58	0.079448	0.6466619	0.1229	0.902	0.10395074	count	1
SEC62	0.0732927	0.1748271	0.4192	0.675	0.104772841	count	1
ERP29	0.0737694	0.1990272	0.3706	0.7111	0.104822697	count	1
FGFBP3	0.1247805	0.8897265	0.1402	0.8886	0.105143061	count	1
MIER1	0.0740716	0.197985	0.3741	0.7086	0.105181748	count	1
CCNY	0.0824362	0.4261557	0.1934	0.847	0.105197351	count	1
ZRSR2	0.0783078	0.4886858	0.1602	0.873	0.105400434	count	1
FYB1	0.0741284	0.1678336	0.4417	0.659	0.105499495	count	1
DR1	0.075245	0.3360937	0.2239	0.823	0.105578638	count	1
ZFYVE27	0.0850545	0.5374704	0.1582	0.874	0.105830562	count	1
CFAP298	0.0787493	0.3876926	0.2031	0.839	0.105995279	count	1
AATF	0.0763186	0.3625377	0.2105	0.833	0.106090552	count	1
CSNK1G2	0.0766818	0.3959872	0.1936	0.847	0.106263686	count	1
ELMOD3	0.0947625	0.8750214	0.1083	0.914	0.106266885	count	1
MRPL50	0.0779608	0.4786381	0.1629	0.871	0.10658189	count	1
ADI1	0.0763967	0.3205207	0.2384	0.812	0.106651259	count	1
STOM	0.0752793	0.2138026	0.3521	0.725	0.106818935	count	1
RPL7A	0.0742783	0.0491186	1.5122	0.131	0.10710407	count	1
SPPL2A	0.0762857	0.2772684	0.2751	0.783	0.107253324	count	1
ECPAS	0.0896348	0.6750566	0.1328	0.894	0.107328806	count	1
HSPA1A	0.0751303	0.2844168	0.2642	0.7918	0.107347056	count	1
VCPKMT	0.0828507	0.5339824	0.1552	0.877	0.107639206	count	1
INPP5D	0.0793209	0.4074562	0.1947	0.846	0.107707059	count	1
RNF126	0.0770546	0.3408393	0.2261	0.8213	0.107797557	count	1
DDX1	0.0807364	0.4804785	0.168	0.867	0.107869496	count	1
STAP1	0.0962096	0.6856835	0.1403	0.889	0.107896199	count	1
DENND1C	0.0926763	0.6757592	0.1371	0.891	0.107924986	count	1
CHCHD2	0.075348	0.1261893	0.5971	0.551	0.108229688	count	1
SH2D2A	0.0767712	0.2805152	0.2737	0.785	0.108286423	count	1
PSMG1	0.0801318	0.4650358	0.1723	0.863	0.108519146	count	1
GIPC1	0.080917	0.4515938	0.1792	0.858	0.108533211	count	1
TNFRSF1A	0.0778757	0.347732	0.224	0.823	0.108633303	count	1
ATAD3A	0.1290218	0.7787838	0.1657	0.869	0.108752136	count	1
G6PD	0.0796151	0.3695376	0.2154	0.83	0.108845311	count	1
BAK1	0.0806332	0.4778721	0.1687	0.866	0.108881019	count	1
RCN2	0.0782493	0.2914763	0.2685	0.789	0.108974438	count	1
TFIP11	0.1116909	0.778236	0.1435	0.886	0.109173171	count	1
SRP14	0.0762577	0.1057744	0.7209	0.471	0.109654177	count	1
TNFRSF1B	0.079105	0.3157229	0.2506	0.802	0.109857868	count	1
TMEM189	0.112523	0.6360972	0.1769	0.86	0.109991985	count	1
CNOT2	0.0784098	0.267855	0.2927	0.77	0.110134989	count	1
RFTN1	0.0857519	0.5446645	0.1574	0.875	0.1104991	count	1
IER3IP1	0.0806764	0.3201931	0.252	0.801	0.110711458	count	1

C11orf96	0.0953301	0.7977954	0.1195	0.905	0.111026102	count	1
KIAA1671	0.0953301	0.8006642	0.1191	0.905	0.111026102	count	1
MRPS15	0.0805757	0.396576	0.2032	0.839	0.111097839	count	1
ZNF83	0.0863442	0.447356	0.193	0.847	0.111263661	count	1
PSMB10	0.0785223	0.3019964	0.26	0.795	0.111400238	count	1
GTPBP3	0.0912704	0.7022766	0.13	0.897	0.111665424	count	1
RPL37	0.0776931	0.0637146	1.2194	0.224	0.111924454	count	1
OXA1L	0.0824656	0.4713482	0.175	0.861	0.112509993	count	1
AGAP2	0.086634	0.5198647	0.1666	0.868	0.112562452	count	1
THG1L	0.0905442	0.6050791	0.1496	0.881	0.112677263	count	1
RAB11B	0.0808599	0.3226359	0.2506	0.802	0.112707338	count	1
GLOD4	0.0811858	0.3667209	0.2214	0.825	0.112749262	count	1
SLC44A1	0.1013421	0.5852206	0.1732	0.863	0.113676436	count	1
AC008555.5	0.0893635	1.0049106	0.0889	0.929	0.114054544	count	1
METTL8	0.1355811	0.789068	0.1718	0.864	0.114337834	count	1
PRR7	0.0818951	0.3468961	0.2361	0.814	0.114495453	count	1
SMIM30	0.1358085	0.5825512	0.2331	0.816	0.114531572	count	1
HMGCS1	0.0865852	0.54235	0.1596	0.873	0.114635439	count	1
TNPO1	0.0872323	0.4551392	0.1917	0.848	0.114863217	count	1
HSDL2	0.1175538	0.7442431	0.158	0.875	0.114944058	count	1
DCTN6	0.0837642	0.3342976	0.2506	0.802	0.114952089	count	1
DNAJC17	0.0854863	0.5584782	0.1531	0.878	0.1157777	count	1
ANKAR	0.0884766	0.6047702	0.1463	0.884	0.115782257	count	1
TPI1	0.0806935	0.1283722	0.6286	0.53	0.115950834	count	1
TRMT6	0.0853638	0.4624824	0.1846	0.854	0.11620467	count	1
ATP5ME	0.0825037	0.221301	0.3728	0.7095	0.117308682	count	1
RNF13	0.0841951	0.3390731	0.2483	0.804	0.117358638	count	1
HUWE1	0.0886579	0.5735764	0.1546	0.877	0.117383376	count	1
NUFIP2	0.0846959	0.3209644	0.2639	0.792	0.117505032	count	1
TKT	0.0836326	0.3001104	0.2787	0.781	0.11764025	count	1
RPRD1B	0.1108268	0.7868132	0.1409	0.888	0.117820898	count	1
ERI1	0.1108268	0.9014895	0.1229	0.902	0.117820898	count	1
YWHAE	0.083575	0.2372955	0.3522	0.725	0.117967501	count	1
MMP25-AS1	0.0896403	0.4681832	0.1915	0.848	0.118038612	count	1
DHRS13	0.1206981	1.1164681	0.1081	0.914	0.118040521	count	1
PAFAH1B1	0.083473	0.2335276	0.3574	0.721	0.11816397	count	1
LAPTM5	0.0830274	0.165043	0.5031	0.6153	0.118433531	count	1
AC026979.2	0.0919855	0.5087117	0.1808	0.857	0.118546486	count	1
PPP1R2	0.0833244	0.1706483	0.4883	0.626	0.118898478	count	1
CUL4A	0.1119717	0.6208431	0.1804	0.857	0.119044667	count	1
ATP11B	0.0916213	0.4591303	0.1996	0.842	0.119053464	count	1
RAI1	0.1412428	0.8130535	0.1737	0.8622	0.11916319	count	1
CHORDC1	0.0867439	0.2999895	0.2892	0.773	0.119244913	count	1
FAM50B	0.0907138	0.5796321	0.1565	0.876	0.119454296	count	1
MXRA7	0.0870784	0.4667276	0.1866	0.852	0.119503896	count	1

RNF4	0.089904	0.4315453	0.2083	0.835	0.12013338	count	1
C4orf48	0.0879907	0.471311	0.1867	0.852	0.120305907	count	1
EBNA1BP2	0.0956244	0.5601519	0.1707	0.865	0.120671572	count	1
SH3GLB1	0.085186	0.2440745	0.349	0.7273	0.120808269	count	1
AGTRAP	0.0851279	0.2108667	0.4037	0.6867	0.120885681	count	1
PAK2	0.0849502	0.1825814	0.4653	0.642	0.121084318	count	1
PSMB7	0.0868322	0.3674328	0.2363	0.813	0.121134012	count	1
CLNK	0.0903166	0.425513	0.2123	0.832	0.121155868	count	1
HSPBP1	0.0910735	0.5255079	0.1733	0.863	0.121698078	count	1
PURA	0.0875062	0.2650785	0.3301	0.742	0.122074775	count	1
ZSCAN18	0.0918298	0.4206241	0.2183	0.827	0.122179852	count	1
LRRC61	0.0998771	0.8463176	0.118	0.906	0.122225188	count	1
HMG20A	0.1250548	0.5201529	0.2404	0.81	0.122332665	count	1
THUMPD2	0.1099291	0.7480519	0.147	0.883	0.123352191	count	1
BRAF	0.1030242	0.5199099	0.1982	0.843	0.123413724	count	1
SKI	0.0996467	0.5109118	0.195	0.845	0.124033763	count	1
SHOC2	0.0917458	0.4219046	0.2175	0.828	0.124264124	count	1
LXN	0.0957911	0.5912649	0.162	0.871	0.124481347	count	1
MXI1	0.1272456	0.4760961	0.2673	0.789	0.124491757	count	1
MFSD8	0.1019337	0.4955682	0.2057	0.837	0.124749179	count	1
DHRS7	0.0872489	0.1621838	0.538	0.591	0.124754831	count	1
UBE2L3	0.088492	0.2324546	0.3807	0.7037	0.126158903	count	1
ADAP1	0.0902018	0.3717606	0.2426	0.808	0.126574773	count	1
RFX3	0.1194899	0.9594418	0.1245	0.901	0.127083844	count	1
FASTKD5	0.1194899	1.0901608	0.1096	0.913	0.127083844	count	1
H2AFX	0.0951526	0.3808122	0.2499	0.803	0.127155949	count	1
IL2RG	0.0892671	0.146733	0.6084	0.543	0.127720194	count	1
ZBTB21	0.1204442	0.6016127	0.2002	0.841	0.128104653	count	1
NDUFB11	0.0897084	0.1764236	0.5085	0.6115	0.128398865	count	1
CENPJ	0.0965214	0.563732	0.1712	0.864	0.128430754	count	1
CUL5	0.0929256	0.3408473	0.2726	0.785	0.128483119	count	1
CASD1	0.1049842	0.5926047	0.1772	0.859	0.12849344	count	1
TMEM156	0.0973507	0.4496756	0.2165	0.829	0.128909846	count	1
NHLRC3	0.0972534	0.5138378	0.1893	0.85	0.129406112	count	1
C9orf85	0.1026367	0.5817111	0.1764	0.86	0.129541711	count	1
SLC35D1	0.1026367	0.657719	0.156	0.876	0.129541711	count	1
AC093323.1	0.1043138	0.6899433	0.1512	0.88	0.129858428	count	1
ANAPC1	0.0965435	0.5400638	0.1788	0.858	0.129975037	count	1
GLG1	0.0943236	0.3585972	0.263	0.793	0.130248094	count	1
PNPT1	0.1072739	0.7926633	0.1353	0.892	0.131304257	count	1
COQ8A	0.1072739	1.0001498	0.1073	0.915	0.131304257	count	1
URM1	0.095116	0.3611415	0.2634	0.792	0.13167502	count	1
ZEB2	0.0944008	0.3065091	0.308	0.758	0.13169861	count	1
MRPL2	0.1006807	0.5681828	0.1772	0.859	0.131780575	count	1
ACTG1	0.0915398	0.0851387	1.0752	0.283	0.13192696	count	1

NRF1	0.156278	0.9502708	0.1645	0.869	0.131995071	count	1
DDX49	0.0996896	0.5652433	0.1764	0.86	0.132011659	count	1
SUMO1	0.0931102	0.1978989	0.4705	0.6383	0.132224016	count	1
IRF8	0.0950942	0.2517314	0.3778	0.706	0.132330771	count	1
RNF135	0.1014723	0.6379027	0.1591	0.874	0.132818495	count	1
CEBPZOS	0.0994002	0.4771913	0.2083	0.835	0.13283987	count	1
IFT43	0.0983712	0.4841712	0.2032	0.839	0.132861272	count	1
VPS28	0.0927539	0.1661603	0.5582	0.5771	0.132881957	count	1
CDK5	0.1054367	0.6156679	0.1713	0.864	0.133084278	count	1
SDCCAG8	0.1087862	0.5712915	0.1904	0.849	0.133160924	count	1
PNKP	0.0986815	0.38096	0.259	0.796	0.133280854	count	1
AC099791.2	0.1581641	1.0850246	0.1458	0.8842	0.133606592	count	1
CEBPZ	0.0949222	0.2396477	0.3961	0.692	0.133801169	count	1
ZBTB2	0.1038415	0.5385811	0.1928	0.847	0.133857293	count	1
PJA2	0.0952225	0.2605543	0.3655	0.715	0.134008847	count	1
PLCG2	0.0950743	0.2527709	0.3761	0.707	0.134065273	count	1
STX7	0.1095563	0.5680367	0.1929	0.847	0.134106439	count	1
METTL2A	0.1043504	0.5377136	0.1941	0.846	0.134514631	count	1
PHF20	0.0958483	0.2371406	0.4042	0.686	0.134572721	count	1
CCM2	0.0954152	0.2739446	0.3483	0.7278	0.134773316	count	1
MIF4GD	0.0971477	0.4489664	0.2164	0.829	0.134931854	count	1
GLRX5	0.0957698	0.2914292	0.3286	0.7427	0.135141768	count	1
VPS4B	0.0978157	0.3204337	0.3053	0.76	0.135249485	count	1
THEM6	0.1059584	0.6243106	0.1697	0.865	0.13528259	count	1
DDX3X	0.0953856	0.1773997	0.5377	0.5912	0.135432319	count	1
LMTK2	0.1383853	0.6884444	0.201	0.841	0.13547789	count	1
TRAF5	0.0967566	0.2812385	0.344	0.731	0.135545837	count	1
XRN1	0.0962929	0.3320193	0.29	0.772	0.135682199	count	1
SRSF10	0.0959585	0.2108071	0.4552	0.6493	0.135738319	count	1
TPD52	0.100601	0.5016152	0.2006	0.841	0.135876428	count	1
SIN3A	0.1064715	0.5155011	0.2065	0.836	0.13593916	count	1
SS18L2	0.0963045	0.2370774	0.4062	0.685	0.136029914	count	1
ZFAND2A	0.0995192	0.4596362	0.2165	0.829	0.13608355	count	1
POT1	0.2268603	1.08163	0.2097	0.834	0.136460377	count	1
KBTBD8	0.2268603	1.136601	0.1996	0.842	0.136460377	count	1
NLN	0.2268603	1.136601	0.1996	0.842	0.136460377	count	1
AC087239.1	0.1025676	0.6381035	0.1607	0.872	0.136487612	count	1
RAC1	0.0960597	0.1745168	0.5504	0.582	0.137171265	count	1
DCAF7	0.0987575	0.4035188	0.2447	0.807	0.137303879	count	1
PRKRIP1	0.1102797	0.5389631	0.2046	0.838	0.137305853	count	1
PWP1	0.1020136	0.39828	0.2561	0.798	0.137348601	count	1
YIPF6	0.1403453	0.9051387	0.1551	0.877	0.137412182	count	1
TMEM62	0.1403453	0.9556683	0.1469	0.883	0.137412182	count	1
NAGS	0.1403453	1.1278828	0.1244	0.9011	0.137412182	count	1
FAM210A	0.1146978	0.6767435	0.1695	0.866	0.137447547	count	1

MORC4	0.1146978	0.7720941	0.1486	0.882	0.137447547	count	1
SLC16A3	0.0976232	0.2773667	0.352	0.725	0.137503129	count	1
MPLKIP	0.0991525	0.3873068	0.256	0.798	0.137575937	count	1
OSBPL8	0.0989249	0.2957592	0.3345	0.738	0.137903117	count	1
ZNF791	0.0979297	0.31471	0.3112	0.756	0.137935021	count	1
PTPRN2	0.1054709	0.5335585	0.1977	0.843	0.138061714	count	1
MBIP	0.1026367	0.4497575	0.2282	0.82	0.138188583	count	1
ABT1	0.0989927	0.3143788	0.3149	0.753	0.138594631	count	1
IPCEF1	0.1029641	0.397843	0.2588	0.796	0.138629945	count	1
WLS	0.2310177	1.367445	0.1689	0.866	0.139017124	count	1
AC099568.2	0.2310177	1.371939	0.1684	0.866	0.139017124	count	1
AMIGO1	0.2310177	1.371939	0.1684	0.866	0.139017124	count	1
SMOC2	0.2310177	1.367445	0.1689	0.866	0.139017124	count	1
COPG2	0.2310177	1.371939	0.1684	0.866	0.139017124	count	1
AC005229.4	0.2310177	1.367445	0.1689	0.866	0.139017124	count	1
SPIN2A	0.2310177	1.371939	0.1684	0.866	0.139017124	count	1
PTK2	0.2310177	1.367445	0.1689	0.866	0.139017124	count	1
GOLM1	0.2310177	1.371939	0.1684	0.866	0.139017124	count	1
AL450306.1	0.2310177	1.371939	0.1684	0.866	0.139017124	count	1
EPYC	0.2310177	1.371939	0.1684	0.866	0.139017124	count	1
SAMD4A	0.2310177	1.367445	0.1689	0.866	0.139017124	count	1
AF001548.2	0.2310177	1.371939	0.1684	0.866	0.139017124	count	1
GEMIN4	0.2310177	1.367445	0.1689	0.866	0.139017124	count	1
FASN	0.2310177	1.371939	0.1684	0.866	0.139017124	count	1
MACROD2	0.2310177	1.367445	0.1689	0.866	0.139017124	count	1
ZBTB45	0.2310177	1.367445	0.1689	0.866	0.139017124	count	1
MTMR3	0.2310177	1.371939	0.1684	0.866	0.139017124	count	1
ARL6IP5	0.0970215	0.131514	0.7377	0.461	0.13925503	count	1
EFHD2	0.0973876	0.1964163	0.4958	0.62	0.139340448	count	1
LETMD1	0.1043138	0.4727455	0.2207	0.825	0.139415746	count	1
NDUFB5	0.1010899	0.3658186	0.2763	0.782	0.139598898	count	1
UVRAG	0.1010352	0.4104135	0.2462	0.806	0.139704518	count	1
TMEM179B	0.1001727	0.3348869	0.2991	0.765	0.140063618	count	1
SMARCD1	0.1013826	0.419533	0.2417	0.809	0.14018525	count	1
SRP19	0.0996316	0.2903176	0.3432	0.732	0.140333205	count	1
PPM1M	0.1043755	0.4724819	0.2209	0.825	0.140532666	count	1
DRAP1	0.0988322	0.1923964	0.5137	0.6078	0.14109934	count	1
PPP4R2	0.1016095	0.3334404	0.3047	0.761	0.141761678	count	1
NCAPH2	0.1110656	0.6887293	0.1613	0.872	0.141818422	count	1
GNL3L	0.2357955	0.8610159	0.2739	0.784	0.141957523	count	1
SRGAP2C	0.2357955	0.8976148	0.2627	0.793	0.141957523	count	1
SMAGP	0.2357955	0.9240322	0.2552	0.799	0.141957523	count	1
ZNF586	0.2357955	0.9828576	0.2399	0.8106	0.141957523	count	1
PHYKPL	0.1020723	0.3368523	0.303	0.762	0.142173568	count	1
KHDRBS1	0.1001491	0.2101624	0.4765	0.634	0.142271132	count	1

DALRD3	0.1069513	0.4894823	0.2185	0.827	0.142329901	count	1
ERLEC1	0.1059992	0.3401499	0.3116	0.756	0.142721664	count	1
NTMT1	0.1048514	0.4477972	0.2341	0.815	0.14276195	count	1
EXOC1	0.1048514	0.4677019	0.2242	0.823	0.14276195	count	1
CRLF3	0.1035723	0.3751935	0.2761	0.783	0.142832583	count	1
MAP2K1	0.1132188	0.6512562	0.1738	0.862	0.142932383	count	1
SEC61G	0.1010764	0.2084508	0.4849	0.628	0.14305583	count	1
ACTR5	0.1276488	0.7985375	0.1599	0.873	0.143338056	count	1
SERTAD3	0.1017972	0.2473208	0.4116	0.6809	0.143878579	count	1
NUCB1	0.1027854	0.2978386	0.3451	0.73	0.143996169	count	1
PIN1	0.1014388	0.2289378	0.4431	0.658	0.144079543	count	1
ATF6	0.1058694	0.4949828	0.2139	0.831	0.144149528	count	1
ZNF140	0.1471804	0.8245142	0.1785	0.858	0.144160647	count	1
RHOT1	0.1109921	0.6375776	0.1741	0.862	0.144275119	count	1
FAM214A	0.1160555	0.454747	0.2552	0.799	0.144517891	count	1
OXR1	0.1055219	0.4576075	0.2306	0.818	0.144579108	count	1
LTBP4	0.1121606	0.6080747	0.1845	0.854	0.144604437	count	1
ARHGAP4	0.1065041	0.3896476	0.2733	0.785	0.145014665	count	1
PABPC4	0.1060685	0.3997557	0.2653	0.791	0.145048202	count	1
GMNN	0.1211634	0.437834	0.2767	0.782	0.145224343	count	1
CCR6	0.2414647	0.9648708	0.2503	0.803	0.145449435	count	1
TMEM168	0.2414647	0.9648708	0.2503	0.803	0.145449435	count	1
CD200	0.2414647	1.032592	0.2338	0.815	0.145449435	count	1
PUDP	0.2414647	1.032592	0.2338	0.815	0.145449435	count	1
RDH10	0.2414647	1.032592	0.2338	0.815	0.145449435	count	1
TUT1	0.2414647	1.032592	0.2338	0.815	0.145449435	count	1
FERMT2	0.2414647	1.032592	0.2338	0.815	0.145449435	count	1
CCT6B	0.2414647	1.032592	0.2338	0.815	0.145449435	count	1
DNAJC9	0.1081706	0.4050153	0.2671	0.79	0.145649169	count	1
ATP2A3	0.1122302	0.566001	0.1983	0.843	0.145887729	count	1
ANKMY1	0.117316	0.7666914	0.153	0.878	0.146092083	count	1
MBD2	0.1049184	0.2759175	0.3803	0.704	0.146140442	count	1
SAMD4B	0.1194899	0.8067496	0.1481	0.882	0.146305991	count	1
EIF3G	0.1021557	0.1490626	0.6853	0.494	0.146596849	count	1
WDR5	0.244084	0.7475958	0.3265	0.744	0.147063815	count	1
C5orf22	0.244084	0.785413	0.3108	0.756	0.147063815	count	1
KRBOX4	0.244084	0.785413	0.3108	0.756	0.147063815	count	1
ADM	0.244084	0.8853934	0.2757	0.783	0.147063815	count	1
BCL7B	0.1083624	0.3309994	0.3274	0.744	0.147547707	count	1
TMEM43	0.1121618	0.4686722	0.2393	0.811	0.147748382	count	1
VPS37A	0.1088327	0.5381421	0.2022	0.84	0.147827611	count	1
ZNF428	0.1046356	0.2606668	0.4014	0.688	0.147934799	count	1
FN3KRP	0.118855	0.6930981	0.1715	0.864	0.14801419	count	1
MANEA	0.1271605	0.8182447	0.1554	0.877	0.148263547	count	1
TMEM38B	0.1271605	0.8411639	0.1512	0.88	0.148263547	count	1

NRDE2	0.1163852	0.6259318	0.1859	0.853	0.148627413	count	1
SYNJ2BP	0.1109128	0.588492	0.1885	0.851	0.148824308	count	1
MVP	0.1119739	0.4709231	0.2378	0.812	0.149024477	count	1
SERPINB1	0.1052697	0.2502542	0.4207	0.6743	0.149268948	count	1
PPP1CC	0.1091965	0.3637301	0.3002	0.764	0.149330152	count	1
PAQR6	0.1143845	0.6555964	0.1745	0.862	0.149752114	count	1
MRPS10	0.109378	0.4640936	0.2357	0.814	0.149867762	count	1
TRAPPC6A	0.1066484	0.2851791	0.374	0.709	0.150221071	count	1
COG6	0.1781619	0.818037	0.2178	0.828	0.150716614	count	1
HP1BP3	0.1058292	0.1860208	0.5689	0.57	0.150896102	count	1
RAB2A	0.1068388	0.2440074	0.4379	0.662	0.150915674	count	1
ZMYM5	0.1079012	0.3370055	0.3202	0.749	0.150977361	count	1
DDX52	0.1094477	0.4516681	0.2423	0.809	0.151706151	count	1
CD7	0.1054107	0.0935917	1.1263	0.261	0.151849772	count	1
ZFYVE28	0.109837	0.3738193	0.2938	0.769	0.151885155	count	1
TIMM29	0.1192034	0.535771	0.2225	0.824	0.152235206	count	1
DPP3	0.1444705	0.76259	0.1894	0.85	0.153832673	count	1
MRPL35	0.1175361	0.5949147	0.1976	0.844	0.153886267	count	1
TANK	0.1113664	0.3234498	0.3443	0.731	0.154001811	count	1
LSM10	0.1106297	0.3359272	0.3293	0.742	0.154101197	count	1
GABARAPL2	0.1081373	0.1999062	0.5409	0.5889	0.154110302	count	1
QSOX1	0.1145036	0.5333194	0.2147	0.83	0.154678867	count	1
GAPVD1	0.1200475	0.4807611	0.2497	0.803	0.154796118	count	1
DNAL4	0.1378135	0.7441797	0.1852	0.853	0.154814191	count	1
PDXDC1	0.1146106	0.5166888	0.2218	0.825	0.154823599	count	1
RNGTT	0.1327622	0.5774112	0.2299	0.818	0.154824525	count	1
FKBP2	0.1091673	0.2509565	0.435	0.6639	0.15482944	count	1
USP1	0.1123335	0.4094456	0.2744	0.784	0.154925676	count	1
HMGCL	0.1230475	0.7622157	0.1614	0.872	0.155374902	count	1
CCPG1	0.1101114	0.2545575	0.4326	0.666	0.155440465	count	1
BAZ1A	0.1120726	0.2956549	0.3791	0.705	0.155677339	count	1
VAMP4	0.1175912	0.4442511	0.2647	0.791	0.155759039	count	1
RUNX2	0.1253119	0.5226759	0.2398	0.811	0.156079898	count	1
CNOT10	0.1306743	0.7398904	0.1766	0.86	0.156669004	count	1
USP22	0.1190275	0.4474814	0.266	0.79	0.15680927	count	1
CHMP3	0.1473152	0.5454398	0.2701	0.787	0.156882253	count	1
KDELR2	0.1107423	0.2384701	0.4644	0.6427	0.157297965	count	1
ZNRD1	0.1107423	0.2478963	0.4467	0.6554	0.157297965	count	1
CD247	0.1096681	0.1199019	0.9146	0.361	0.157604003	count	1
SNRPB2	0.1108178	0.2252472	0.492	0.6231	0.157789117	count	1
ZNF394	0.1176477	0.31586	0.3725	0.71	0.157874674	count	1
BAZ2B	0.1165994	0.426384	0.2735	0.785	0.157970645	count	1
ARHGAP18	0.1125465	0.2947046	0.3819	0.703	0.158109907	count	1
PARP11	0.1226409	0.6584667	0.1863	0.852	0.158147985	count	1
YIPF4	0.1168128	0.4742844	0.2463	0.806	0.158260131	count	1

TMEM258	0.1119914	0.225446	0.4968	0.62	0.158468935	count	1
MRPL21	0.1144682	0.3445101	0.3323	0.74	0.15884364	count	1
NNT	0.1277475	0.5822117	0.2194	0.826	0.159122941	count	1
AC073111.5	0.1302631	0.9301885	0.14	0.889	0.159543377	count	1
UFSP2	0.1238733	0.5898431	0.21	0.834	0.159740918	count	1
B3GNT7	0.1136762	0.3024452	0.3759	0.7073	0.161159226	count	1
SFXN4	0.1441514	0.9879421	0.1459	0.884	0.161973904	count	1
EIF4A1	0.1157181	0.2564921	0.4512	0.652	0.162025521	count	1
SMIM20	0.1212161	0.4587862	0.2642	0.792	0.16204209	count	1
DCPS	0.1213236	0.527604	0.23	0.818	0.162186025	count	1
TFAM	0.1191267	0.3181436	0.3744	0.708	0.162222176	count	1
RAN	0.1132222	0.1384108	0.818	0.414	0.162488906	count	1
GLMN	0.1230078	0.5257538	0.234	0.815	0.162946668	count	1
WASHC1	0.1230078	0.6032666	0.2039	0.839	0.162946668	count	1
SMG6	0.117941	0.4007354	0.2943	0.769	0.16348855	count	1
SHMT1	0.1257417	0.5708455	0.2203	0.826	0.163490464	count	1
IER5	0.1164927	0.304268	0.3829	0.702	0.163656725	count	1
GPCPD1	0.1336759	0.3382692	0.3952	0.693	0.163738221	count	1
PYROXD1	0.1336981	0.7436249	0.1798	0.857	0.163765511	count	1
IARS2	0.1255456	0.5421468	0.2316	0.817	0.164394574	count	1
BID	0.1179605	0.3788889	0.3113	0.756	0.164458194	count	1
TAPBP	0.1154014	0.1939444	0.595	0.552	0.164734225	count	1
CTNNB1	0.1176205	0.3330336	0.3532	0.724	0.16498251	count	1
ITM2C	0.1150679	0.1583349	0.7267	0.468	0.165446899	count	1
SNRPB	0.1159551	0.1734633	0.6685	0.504	0.165575377	count	1
NAA10	0.1176514	0.288253	0.4082	0.6834	0.165659811	count	1
PCNX1	0.1558564	0.3546865	0.4394	0.661	0.166042788	count	1
UBC	0.1153667	0.0825823	1.397	0.163	0.166128981	count	1
RANBP3	0.1333604	0.7414971	0.1799	0.857	0.166136924	count	1
ZNF620	0.1333604	0.8282187	0.161	0.872	0.166136924	count	1
CIRBP	0.1159683	0.1432864	0.8093	0.4189	0.166256917	count	1
SLC4A1AP	0.1213992	0.3627081	0.3347	0.738	0.166357012	count	1
CERS5	0.156278	0.6859085	0.2278	0.82	0.166495133	count	1
ANKRD13A	0.1427334	0.7009702	0.2036	0.839	0.166508746	count	1
DBNL	0.1193163	0.2974551	0.4011	0.689	0.166731957	count	1
SELENOM	0.1202031	0.4076614	0.2949	0.768	0.167446321	count	1
GON7	0.1291388	0.595242	0.217	0.828	0.167917371	count	1
LINC01353	0.1582329	0.8781713	0.1802	0.857	0.16859271	count	1
COX4I1	0.1173537	0.0982869	1.194	0.233	0.168894338	count	1
WDSUB1	0.1322332	0.5102623	0.2591	0.796	0.168920488	count	1
KEAP1	0.1246709	0.5418519	0.2301	0.818	0.168920821	count	1
PSMB8-AS1	0.1189431	0.2638165	0.4509	0.6524	0.168950181	count	1
PUM3	0.1235457	0.344179	0.359	0.72	0.168975808	count	1
DERL1	0.1211393	0.3128291	0.3872	0.699	0.169028481	count	1
CCNH	0.1190407	0.2042633	0.5828	0.5604	0.16905987	count	1

KIFAP3	0.1251362	0.4071472	0.3073	0.759	0.169062467	count	1
LTBP3	0.1449173	0.6078932	0.2384	0.812	0.169068754	count	1
CYB5A	0.1260666	0.477518	0.264	0.792	0.169781982	count	1
FARS2	0.1250865	0.5656683	0.2211	0.825	0.169934076	count	1
XCL1	0.1179432	0.1474821	0.7997	0.424	0.170056711	count	1
POLG	0.1597298	0.7228487	0.221	0.825	0.170199095	count	1
VDAC2	0.1195743	0.1981088	0.6036	0.5465	0.170482849	count	1
CTSS	0.1202156	0.2349467	0.5117	0.6092	0.170729001	count	1
AC010642.2	0.1338175	0.5377037	0.2489	0.804	0.170949795	count	1
SIGMAR1	0.142544	0.6809331	0.2093	0.834	0.170960186	count	1
VPS29	0.1210342	0.2178912	0.5555	0.579	0.171129373	count	1
DCAF1	0.2831711	0.7084544	0.3997	0.6896	0.171231404	count	1
UBE2M	0.1201919	0.1834953	0.655	0.5129	0.171459508	count	1
RAB8B	0.120046	0.1825919	0.6575	0.5114	0.171547707	count	1
LSM14B	0.1405021	0.6102445	0.2302	0.818	0.172130663	count	1
HEBP1	0.1302631	0.4713653	0.2764	0.782	0.172575763	count	1
ISOC1	0.1282102	0.4942498	0.2594	0.795	0.172673247	count	1
CAPZA2	0.1230891	0.2763477	0.4454	0.656	0.172751676	count	1
KIAA1143	0.1346357	0.5162623	0.2608	0.794	0.173654588	count	1
STK4	0.1214259	0.1356241	0.8953	0.3713	0.173934257	count	1
DNASE1L1	0.1423495	0.6753563	0.2108	0.833	0.1744024	count	1
RPS27	0.1211431	0.0511761	2.3672	0.0185	0.17463428	count	1
LSM3	0.1250769	0.3012256	0.4152	0.678	0.175026088	count	1
MAPK13	0.1358345	0.5763614	0.2357	0.814	0.175204706	count	1
AL451165.2	0.1562726	0.6903427	0.2264	0.821	0.175675381	count	1
ARL4C	0.1228698	0.1725997	0.7119	0.477	0.175754362	count	1
STK17B	0.1227689	0.1278405	0.9603	0.338	0.175946026	count	1
AC025159.1	0.1329412	0.5949704	0.2234	0.823	0.176130533	count	1
FOXN2	0.1264732	0.3107474	0.407	0.684	0.176187469	count	1
OSBP	0.1357874	0.4683269	0.2899	0.772	0.176582786	count	1
EPHA4	0.2919064	0.6910437	0.4224	0.673	0.176651188	count	1
TIPIN	0.1401855	0.671248	0.2088	0.835	0.177082115	count	1
SH3GL1	0.1324869	0.6087082	0.2177	0.828	0.177134789	count	1
OSTM1	0.1304016	0.4494907	0.2901	0.772	0.177164478	count	1
SLC25A24	0.1332921	0.4733605	0.2816	0.778	0.177448627	count	1
PRPSAP1	0.1357551	0.4604526	0.2948	0.768	0.177792783	count	1
PHF5A	0.127919	0.3320718	0.3852	0.7	0.177881277	count	1
APOBEC3C	0.1249198	0.2195879	0.5689	0.5698	0.178205748	count	1
PARN	0.1374258	0.5720366	0.2402	0.81	0.178718454	count	1
ABHD17B	0.141844	0.579582	0.2447	0.807	0.179183553	count	1
FOPNL	0.144313	0.6271612	0.2301	0.818	0.179828291	count	1
VAV3	0.1336366	0.3284531	0.4069	0.684	0.179992887	count	1
C4orf33	0.1502669	0.6527779	0.2302	0.818	0.180263312	count	1
CBR4	0.1343168	0.5748421	0.2337	0.815	0.18028045	count	1
SAFB2	0.1343174	0.336502	0.3992	0.69	0.180281257	count	1

SPTSSA	0.136873	0.5400752	0.2534	0.8	0.180368574	count	1
LIMK1	0.1370601	0.6367804	0.2152	0.83	0.18061564	count	1
WASF2	0.1277657	0.2300042	0.5555	0.579	0.180799428	count	1
NDUFAF4	0.1294958	0.3668379	0.353	0.724	0.180835118	count	1
ACAP1	0.1267332	0.1726832	0.7339	0.4635	0.180840334	count	1
TUBG1	0.1699701	0.8698534	0.1954	0.845	0.181193344	count	1
ATP5MF	0.1276243	0.2281365	0.5594	0.5763	0.181458458	count	1
NARS	0.135214	0.4620428	0.2926	0.77	0.181486651	count	1
VOPP1	0.1513965	0.5384952	0.2811	0.779	0.181624354	count	1
GON4L	0.1321072	0.3453518	0.3825	0.702	0.18167492	count	1
PRNP	0.1321072	0.3447087	0.3832	0.702	0.18167492	count	1
CHFR	0.151711	0.6820065	0.2224	0.824	0.182003302	count	1
DNAJC30	0.1441514	0.7058623	0.2042	0.838	0.182107411	count	1
ZMAT2	0.13033	0.3102348	0.4201	0.675	0.182133539	count	1
ING4	0.146172	0.5934605	0.2463	0.806	0.182152762	count	1
FBRS	0.1431743	0.5065599	0.2826	0.778	0.182937083	count	1
RAD9A	0.1376815	0.5526688	0.2491	0.803	0.183302953	count	1
AP3S1	0.1317862	0.3503412	0.3762	0.707	0.183433188	count	1
GCA	0.1400722	0.6776483	0.2067	0.836	0.183459402	count	1
CCNC	0.1386752	0.5433801	0.2552	0.799	0.183742354	count	1
ASPSCR1	0.1386752	0.5858974	0.2367	0.813	0.183742354	count	1
XKR8	0.1499859	0.7067705	0.2122	0.832	0.183794859	count	1
YWHAH	0.1305487	0.2840011	0.4597	0.646	0.184046802	count	1
SEC14L1	0.1335246	0.325117	0.4107	0.682	0.184181949	count	1
COPS7A	0.1356137	0.6889433	0.1968	0.844	0.184255435	count	1
RASGEF1B	0.1294753	0.2867516	0.4515	0.6519	0.184317498	count	1
BORCS5	0.1385102	0.5701337	0.2429	0.808	0.184408293	count	1
OSGIN2	0.1581977	0.704009	0.2247	0.822	0.184643239	count	1
UXS1	0.17415	0.6136303	0.2838	0.777	0.185683468	count	1
TRAF3	0.1459137	0.7554735	0.1931	0.847	0.186447348	count	1
TFPT	0.1446264	0.4966283	0.2912	0.771	0.18657498	count	1
HENMT1	0.1446264	0.510659	0.2832	0.777	0.18657498	count	1
KDM6A	0.1598598	0.9935113	0.1609	0.872	0.186593284	count	1
PRKAB1	0.1598598	1.002287	0.1595	0.873	0.186593284	count	1
RAB35	0.1523191	0.5210774	0.2923	0.77	0.186665246	count	1
LBH	0.1309279	0.2046341	0.6398	0.5227	0.186674596	count	1
FRAT2	0.142544	0.5390207	0.2644	0.792	0.186704191	count	1
RRP15	0.1375554	0.4029991	0.3413	0.733	0.187351673	count	1
FAM208B	0.1466956	0.5006233	0.293	0.77	0.187449337	count	1
ARMCX3	0.1417214	0.4078814	0.3475	0.728	0.187786589	count	1
PRPS1	0.1433924	0.3509475	0.4086	0.683	0.187817957	count	1
VEZT	0.1411733	0.5767501	0.2448	0.807	0.187960543	count	1
WDR83	0.1471241	0.6457254	0.2278	0.82	0.18799846	count	1
DMXL1	0.1457798	0.6325281	0.2305	0.818	0.188066872	count	1
ZNF570	0.1457798	0.6938934	0.2101	0.834	0.188066872	count	1

AKTIP	0.1612878	0.5670881	0.2844	0.776	0.188268813	count	1
TTC19	0.1398768	0.4027344	0.3473	0.729	0.188411256	count	1
SIRT7	0.1366036	0.3477775	0.3928	0.695	0.188692107	count	1
SCYL2	0.1515883	0.6952024	0.218	0.828	0.188926246	count	1
TMEM9B	0.1354609	0.2821446	0.4801	0.631	0.189171408	count	1
PSMC6	0.1354609	0.3010456	0.45	0.653	0.189171408	count	1
ZNF598	0.1774404	0.6971998	0.2545	0.799	0.189219044	count	1
SIN3B	0.1456425	0.635919	0.229	0.819	0.189430569	count	1
7-Mar	0.1377745	0.287197	0.4797	0.632	0.189477359	count	1
C19orf66	0.1352745	0.2797507	0.4836	0.629	0.189543075	count	1
CRYZL1	0.1582151	0.6308505	0.2508	0.802	0.189841619	count	1
MT-CO1	0.1316852	0.0459346	2.8668	0.00442	0.189949468	count	1
CCDC59	0.1359066	0.2736364	0.4967	0.62	0.190191402	count	1
CENPL	0.1789332	0.8719428	0.2052	0.838	0.190823368	count	1
NSMAF	0.1440413	0.693017	0.2078	0.835	0.190866769	count	1
MICAL1	0.1515247	0.7548472	0.2007	0.841	0.191452289	count	1
UNKL	0.1702292	0.797891	0.2133	0.831	0.191465066	count	1
VPS50	0.1702574	0.6776178	0.2513	0.802	0.191496982	count	1
ITFG2	0.195167	0.6301468	0.3097	0.757	0.19166496	count	1
AGL	0.1391737	0.4998303	0.2784	0.781	0.191982478	count	1
PGK1	0.1343094	0.1605655	0.8365	0.404	0.192038339	count	1
ANKRD44	0.1414471	0.2910252	0.486	0.627	0.192192436	count	1
VSIR	0.1362561	0.2685154	0.5074	0.612	0.192429521	count	1
DOCK2	0.142725	0.4415321	0.3232	0.747	0.192862948	count	1
DDX58	0.1495549	0.5374006	0.2783	0.781	0.192950239	count	1
SRSF2	0.1358167	0.172163	0.7889	0.4308	0.192958993	count	1
LDLRAD4	0.1375982	0.3583315	0.384	0.701	0.193228812	count	1
PNRC1	0.1343883	0.0811191	1.6567	0.0986	0.193616221	count	1
PEX14	0.2281934	0.9932576	0.2297	0.818	0.193703058	count	1
MAFA	0.2281934	1.042302	0.2189	0.827	0.193703058	count	1
DCP1B	0.2281934	1.0797216	0.2113	0.8327	0.193703058	count	1
EDARADD	0.2281934	1.146405	0.1991	0.842	0.193703058	count	1
SPATA2	0.2281934	1.146405	0.1991	0.842	0.193703058	count	1
HIST1H2BN	0.2281934	1.149862	0.1985	0.843	0.193703058	count	1
MED21	0.1434003	0.3373203	0.4251	0.671	0.193776898	count	1
IDH1	0.1471451	0.6307618	0.2333	0.816	0.193934688	count	1
ARRDC1	0.1421911	0.4025137	0.3533	0.724	0.194108068	count	1
ATG12	0.1393586	0.2753222	0.5062	0.613	0.194152836	count	1
SNAI3	0.1492667	0.5432018	0.2748	0.784	0.194156265	count	1
ACAA2	0.1365895	0.2389602	0.5716	0.568	0.194347363	count	1
DHRS1	0.1430935	0.414911	0.3449	0.73	0.194905013	count	1
NDUFAF1	0.1527886	0.6558474	0.233	0.816	0.195258287	count	1
SMAD7	0.1460786	0.4758361	0.307	0.759	0.195340491	count	1
TERF2	0.153109	0.645729	0.2371	0.813	0.195668965	count	1
CCS	0.1396553	0.331263	0.4216	0.674	0.19611944	count	1

C3orf62	0.2310177	1.1653438	0.1982	0.843	0.19613685	count	1
EFCAB7	0.2310177	1.2148767	0.1902	0.849	0.19613685	count	1
LINC00957	0.2310177	1.2148767	0.1902	0.849	0.19613685	count	1
SLC9A7	0.2310177	1.2148767	0.1902	0.849	0.19613685	count	1
PRKACA	0.2310177	1.2148767	0.1902	0.849	0.19613685	count	1
TINAGL1	0.2310177	1.367445	0.1689	0.866	0.19613685	count	1
MIR4422HG	0.2310177	1.367445	0.1689	0.866	0.19613685	count	1
MAP10	0.2310177	1.371939	0.1684	0.866	0.19613685	count	1
FBXO36	0.2310177	1.367445	0.1689	0.866	0.19613685	count	1
ZNF619	0.2310177	1.367445	0.1689	0.866	0.19613685	count	1
CEP72	0.2310177	1.371939	0.1684	0.866	0.19613685	count	1
MICALL2	0.2310177	1.371939	0.1684	0.866	0.19613685	count	1
HOXA11	0.2310177	1.371939	0.1684	0.866	0.19613685	count	1
FKBP14	0.2310177	1.371939	0.1684	0.866	0.19613685	count	1
P2RY8	0.2310177	1.371939	0.1684	0.866	0.19613685	count	1
AL683807.1	0.2310177	1.371939	0.1684	0.866	0.19613685	count	1
AL034397.3	0.2310177	1.371939	0.1684	0.866	0.19613685	count	1
LACTB2-AS1	0.2310177	1.371939	0.1684	0.866	0.19613685	count	1
AC009812.1	0.2310177	1.371939	0.1684	0.866	0.19613685	count	1
TTLL11	0.2310177	1.367445	0.1689	0.866	0.19613685	count	1
CAMSAP1	0.2310177	1.371939	0.1684	0.866	0.19613685	count	1
AC136475.5	0.2310177	1.367445	0.1689	0.866	0.19613685	count	1
DNHD1	0.2310177	1.367445	0.1689	0.866	0.19613685	count	1
IPO8	0.2310177	1.371939	0.1684	0.866	0.19613685	count	1
FAM216A	0.2310177	1.371939	0.1684	0.866	0.19613685	count	1
HOMEZ	0.2310177	1.371939	0.1684	0.866	0.19613685	count	1
NGRN	0.2310177	1.371939	0.1684	0.866	0.19613685	count	1
MIR762HG	0.2310177	1.371939	0.1684	0.866	0.19613685	count	1
HSD3B7	0.2310177	1.371939	0.1684	0.866	0.19613685	count	1
PSMC3IP	0.2310177	1.371939	0.1684	0.866	0.19613685	count	1
AC137723.1	0.2310177	1.371939	0.1684	0.866	0.19613685	count	1
COLEC12	0.2310177	1.371939	0.1684	0.866	0.19613685	count	1
STK35	0.2310177	1.371939	0.1684	0.866	0.19613685	count	1
ZNF561	0.2310177	1.367445	0.1689	0.866	0.19613685	count	1
ZNF345	0.2310177	1.367445	0.1689	0.866	0.19613685	count	1
AC005498.3	0.2310177	1.371939	0.1684	0.866	0.19613685	count	1
ZNF587B	0.2310177	1.371939	0.1684	0.866	0.19613685	count	1
EMC4	0.1384182	0.2590513	0.5343	0.5935	0.196371774	count	1
SMC5	0.1457867	0.3266048	0.4464	0.656	0.196385	count	1
MAPK1	0.137684	0.2129473	0.6466	0.5184	0.196419535	count	1
BTN3A3	0.2001911	0.6334342	0.316	0.752	0.196650685	count	1
ERGIC1	0.1422263	0.3390233	0.4195	0.675	0.196720287	count	1
ITGA4	0.1384639	0.2056188	0.6734	0.5012	0.196815897	count	1
MTRF1	0.2324951	0.9282906	0.2505	0.802	0.197410261	count	1
AGAP3	0.1853491	0.8420444	0.2201	0.8259	0.197720585	count	1

AKT2	0.1696745	0.5305627	0.3198	0.749	0.198111949	count	1
RRBP1	0.1421984	0.4068182	0.3495	0.727	0.198112528	count	1
NFATC2	0.1590556	0.6792948	0.2341	0.815	0.198267071	count	1
CKLF	0.138787	0.1777681	0.7807	0.436	0.198296538	count	1
TOR3A	0.1617804	0.4948379	0.3269	0.744	0.198307918	count	1
ALOX5AP	0.1379596	0.1269283	1.0869	0.278	0.198409522	count	1
UBE2S	0.1388158	0.1785829	0.7773	0.438	0.198471785	count	1
ZNF566	0.2341011	0.7788877	0.3006	0.764	0.198794752	count	1
YWHAZ	0.1385558	0.1212309	1.1429	0.254	0.198873092	count	1
DENND6A	0.1704987	0.7657095	0.2227	0.824	0.199079524	count	1
AC084033.3	0.1704987	0.8947049	0.1906	0.849	0.199079524	count	1
CD81	0.1384161	0.0941243	1.4706	0.142	0.199277419	count	1
F11R	0.2347606	0.8699854	0.2698	0.787	0.199363347	count	1
HARS2	0.2347606	0.9764656	0.2404	0.81	0.199363347	count	1
FOXN3	0.1442152	0.3293899	0.4378	0.662	0.199474036	count	1
IFT20	0.1509438	0.4672509	0.323	0.747	0.20003238	count	1
MBNL1	0.1403781	0.1918699	0.7316	0.465	0.200112647	count	1
ARMC10	0.1538844	0.5191852	0.2964	0.767	0.200178132	count	1
ZNF121	0.1538848	0.621228	0.2477	0.805	0.200178654	count	1
SPDL1	0.2357955	0.7932319	0.2973	0.766	0.200255686	count	1
WHRN	0.1784672	0.7569075	0.2358	0.814	0.200791627	count	1
LSR	0.1726492	0.471999	0.3658	0.715	0.201604318	count	1
PDE4A	0.1577594	0.5341945	0.2953	0.768	0.201630176	count	1
STOML2	0.1456908	0.3500097	0.4162	0.678	0.202419763	count	1
AIFM2	0.2384761	0.9774451	0.244	0.807	0.202567481	count	1
EDEM3	0.2384761	1.06302	0.2243	0.823	0.202567481	count	1
LDB1	0.1546285	0.5234482	0.2954	0.768	0.202570953	count	1
STAG2	0.1445701	0.2845045	0.5081	0.612	0.202695995	count	1
IGBP1	0.1440144	0.297407	0.4842	0.629	0.20280274	count	1
NIPA2	0.1587605	0.520029	0.3053	0.76	0.202913577	count	1
SPCS2	0.1419133	0.149079	0.9519	0.342	0.2029791	count	1
GHITM	0.1434769	0.2110935	0.6797	0.497	0.203146056	count	1
ZNF567	0.1657351	0.5113885	0.3241	0.746	0.203175825	count	1
SPOCK2	0.1496006	0.2771109	0.5399	0.59	0.203287541	count	1
CARNMT1	0.2394985	0.7475496	0.3204	0.7489	0.203449384	count	1
DDX42	0.1468441	0.4045396	0.363	0.717	0.203593784	count	1
SRRT	0.1522559	0.439295	0.3466	0.729	0.203616595	count	1
THAP2	0.1546124	0.5584235	0.2769	0.782	0.203798812	count	1
AL139246.5	0.1548862	0.655769	0.2362	0.813	0.204160531	count	1
ERICH6-AS1	0.1548862	0.6786723	0.2282	0.82	0.204160531	count	1
EIF2B1	0.1492667	0.3688105	0.4047	0.686	0.204202133	count	1
TRAPPC2B	0.144394	0.270529	0.5337	0.5939	0.204286973	count	1
NCF4	0.1918665	0.7218463	0.2658	0.791	0.204730191	count	1
TRMT13	0.1553181	0.6163053	0.252	0.801	0.204731122	count	1
GPATCH11	0.1525165	0.4399183	0.3467	0.729	0.204752661	count	1

ZBED5-AS1	0.2414647	0.853302	0.283	0.777	0.205145656	count	1
PCNT	0.2415255	0.5863069	0.4119	0.681	0.205198118	count	1
DNAJC25	0.1624106	0.8086514	0.2008	0.841	0.205253612	count	1
CREBRF	0.1473477	0.3217101	0.458	0.647	0.205292775	count	1
HOTAIRM1	0.1582064	0.4071526	0.3886	0.698	0.205815104	count	1
HSPA5	0.1436821	0.1556726	0.923	0.3567	0.205909863	count	1
CLK3	0.153012	0.4607704	0.3321	0.74	0.206134758	count	1
HSD17B11	0.1459626	0.241173	0.6052	0.545	0.206507275	count	1
TCF25	0.1459561	0.2115705	0.6899	0.4908	0.206606055	count	1
RNF130	0.1500242	0.3959831	0.3789	0.705	0.207517317	count	1
TRIM22	0.1477686	0.2858835	0.5169	0.606	0.207624757	count	1
NDUFA1	0.1456446	0.1820224	0.8001	0.424	0.207797485	count	1
FBXL4	0.1780722	0.8495252	0.2096	0.834	0.207972478	count	1
NBN	0.1505625	0.414509	0.3632	0.717	0.208262689	count	1
HSD17B4	0.169938	0.629477	0.27	0.787	0.208350145	count	1
RFXANK	0.155948	0.4478832	0.3482	0.728	0.208563644	count	1
ALDH3A2	0.1605354	0.5690453	0.2821	0.778	0.208852987	count	1
TBC1D5	0.1605354	0.6399596	0.2509	0.802	0.208852987	count	1
ALAS1	0.1788321	0.7828666	0.2284	0.819	0.20886497	count	1
ACBD4	0.1788321	0.7904766	0.2262	0.821	0.20886497	count	1
HIST1H2BC	0.1857963	1.0118846	0.1836	0.8544	0.20909317	count	1
BDP1	0.1476934	0.2472206	0.5974	0.5506	0.209315741	count	1
CDC42	0.145945	0.1068552	1.3658	0.173	0.209722353	count	1
PRPF18	0.1612467	0.4540466	0.3551	0.723	0.209780828	count	1
DAPL1	0.2135741	0.8136566	0.2625	0.793	0.209941902	count	1
TMEM125	0.2135741	1.069354	0.1997	0.842	0.209941902	count	1
CCDC24	0.2135741	1.069354	0.1997	0.842	0.209941902	count	1
TMEM53	0.2135741	1.069354	0.1997	0.842	0.209941902	count	1
ENPP6	0.2135741	1.069354	0.1997	0.842	0.209941902	count	1
GPRIN1	0.2135741	1.069354	0.1997	0.842	0.209941902	count	1
HIST1H4F	0.2135741	1.069354	0.1997	0.842	0.209941902	count	1
PSPH	0.2135741	1.069354	0.1997	0.842	0.209941902	count	1
TPST1	0.2135741	1.069354	0.1997	0.842	0.209941902	count	1
NCS1	0.2135741	1.069354	0.1997	0.842	0.209941902	count	1
ZCCHC24	0.2135741	1.069354	0.1997	0.842	0.209941902	count	1
DOCK9-DT	0.2135741	1.069354	0.1997	0.842	0.209941902	count	1
EMP2	0.2135741	1.069354	0.1997	0.842	0.209941902	count	1
TLDC1	0.2135741	1.069354	0.1997	0.842	0.209941902	count	1
NAPB	0.2135741	1.069354	0.1997	0.842	0.209941902	count	1
PKIG	0.2135741	1.069354	0.1997	0.842	0.209941902	count	1
FCGBP	0.2135741	1.069354	0.1997	0.842	0.209941902	count	1
NAPA-AS1	0.2135741	1.069354	0.1997	0.842	0.209941902	count	1
SYNJ1	0.2135741	1.069354	0.1997	0.842	0.209941902	count	1
MED13	0.1626938	0.4029229	0.4038	0.687	0.209950805	count	1
ZFP36L1	0.1464037	0.1632756	0.8967	0.371	0.21011512	count	1

MAGED2	0.1490467	0.2591553	0.5751	0.566	0.210292142	count	1
PPHLN1	0.1510469	0.3323311	0.4545	0.65	0.210955257	count	1
CGGBP1	0.1491352	0.2513531	0.5933	0.553	0.210998048	count	1
ROGDI	0.156657	0.6463537	0.2424	0.809	0.211053804	count	1
TIAL1	0.1493715	0.2675825	0.5582	0.577	0.2111551	count	1
GABARAPL1	0.1483636	0.1685454	0.8803	0.3794	0.211215433	count	1
VKORC1	0.152078	0.3382512	0.4496	0.653	0.211703219	count	1
NDUFA8	0.1564931	0.4516677	0.3465	0.729	0.212108379	count	1
MFF	0.1535884	0.386586	0.3973	0.691	0.212179531	count	1
AC013264.1	0.3494271	0.683044	0.5116	0.609	0.212495008	count	1
PSMD13	0.1519168	0.2853123	0.5325	0.595	0.212613914	count	1
C2orf74	0.1574433	0.5400178	0.2916	0.771	0.212785275	count	1
GATAD2A	0.1616001	0.422348	0.3826	0.702	0.213031065	count	1
LSM12	0.1547437	0.3292448	0.47	0.639	0.213174481	count	1
STX17	0.1609017	0.4340992	0.3707	0.711	0.213257804	count	1
HIST3H2BB	0.2176737	0.9400361	0.2316	0.817	0.214016375	count	1
ARMCX4	0.2176737	0.9400361	0.2316	0.817	0.214016375	count	1
TMEM86B	0.2176737	0.9640998	0.2258	0.822	0.214016375	count	1
CACNA1C-AS2	0.2176737	1.216585	0.1789	0.858	0.214016375	count	1
MYLPF	0.2176737	1.216585	0.1789	0.858	0.214016375	count	1
ERCC6	0.1748349	0.6142171	0.2846	0.776	0.214380027	count	1
ABCA2	0.1905958	0.7407507	0.2573	0.797	0.214531467	count	1
PHIP	0.1526414	0.3493998	0.4369	0.6625	0.215366828	count	1
CD55	0.1554735	0.255036	0.6096	0.543	0.215570347	count	1
TINF2	0.1571887	0.3858801	0.4074	0.684	0.215851349	count	1
ARID5A	0.153172	0.2534338	0.6044	0.546	0.215960028	count	1
SNX9	0.1848885	0.6631532	0.2788	0.781	0.215979364	count	1
ANKZF1	0.1849153	0.5143065	0.3595	0.719	0.216010849	count	1
CYB5B	0.1594793	0.4350742	0.3666	0.714	0.216162365	count	1
MGAT1	0.1565717	0.288609	0.5425	0.588	0.217094599	count	1
PLRG1	0.1698859	0.4810376	0.3532	0.724	0.217179042	count	1
TRMO	0.16017	0.4674435	0.3427	0.732	0.217672423	count	1
ERV3-1	0.1703626	0.6171043	0.2761	0.783	0.217790399	count	1
NDUFV1	0.1572206	0.366171	0.4294	0.668	0.217995252	count	1
OTUB1	0.1577095	0.3711397	0.4249	0.671	0.218159427	count	1
RPN1	0.1648612	0.4088535	0.4032	0.687	0.218517399	count	1
NAA15	0.1729755	0.4817033	0.3591	0.72	0.21865305	count	1
PSMD11	0.1605359	0.3941156	0.4073	0.684	0.218698951	count	1
MPHOSPH8	0.1539998	0.2099026	0.7337	0.4637	0.21880257	count	1
SNX5	0.1590732	0.3884104	0.4095	0.682	0.218805762	count	1
OLA1	0.1573426	0.3373709	0.4664	0.641	0.218836794	count	1
CCL3	0.1518875	0.1894463	0.8017	0.423	0.218955849	count	1
CCNT2	0.1789332	0.5580225	0.3207	0.749	0.219427483	count	1
ZMYM1	0.1789332	0.5984521	0.299	0.765	0.219427483	count	1
RBM48	0.1827859	0.5720269	0.3195	0.75	0.219474981	count	1

PARP8	0.1563845	0.2511366	0.6227	0.534	0.220045979	count	1
EIF4A2	0.1552378	0.1880638	0.8255	0.4097	0.220525437	count	1
SASS6	0.1665117	0.548809	0.3034	0.762	0.220709978	count	1
GAS7	0.1746957	0.5508607	0.3171	0.751	0.220835238	count	1
KLHL7	0.1961688	0.6395689	0.3067	0.759	0.220848158	count	1
IL32	0.1536104	0.1678314	0.9153	0.361	0.220981193	count	1
HNRNPM	0.1562674	0.2136859	0.7313	0.4651	0.22179088	count	1
BEX4	0.156732	0.2676382	0.5856	0.5585	0.222029844	count	1
GALNT6	0.197253	0.6137817	0.3214	0.748	0.222077276	count	1
HPRT1	0.1584257	0.3248126	0.4877	0.626	0.222261877	count	1
PTBP1	0.1678142	0.3673242	0.4569	0.648	0.222440319	count	1
SBDS	0.1559668	0.1772346	0.88	0.38	0.222566971	count	1
MAP4	0.1599702	0.3038798	0.5264	0.599	0.222700021	count	1
CLIP1	0.1631918	0.5451952	0.2993	0.765	0.223279098	count	1
PRPF38A	0.1615202	0.3806099	0.4244	0.672	0.22370729	count	1
NDUFS4	0.1596641	0.3322875	0.4805	0.631	0.223743724	count	1
HSCB	0.1624457	0.4989654	0.3256	0.745	0.22412349	count	1
RIT1	0.1702393	0.5387638	0.316	0.752	0.224447475	count	1
PMEPA1	0.1641768	0.4179756	0.3928	0.695	0.224628665	count	1
ARL14EP	0.1614364	0.3785701	0.4264	0.6701	0.224743083	count	1
LAMTOR3	0.1654802	0.4697714	0.3523	0.725	0.224900515	count	1
PLAGL1	0.2288792	0.9683051	0.2364	0.813	0.225160138	count	1
RBM22	0.1616881	0.2995204	0.5398	0.59	0.225658508	count	1
CSGALNACT2	0.1635694	0.5351415	0.3057	0.76	0.225675671	count	1
PWWP2A	0.1661549	0.4392239	0.3783	0.705	0.225818941	count	1
ZFAS1	0.1590773	0.1906715	0.8343	0.4047	0.226019571	count	1
ARL8A	0.1648879	0.4643594	0.3551	0.723	0.226035085	count	1
AUH	0.1844788	0.6813611	0.2708	0.787	0.226258776	count	1
MTIF2	0.1709575	0.6440402	0.2654	0.791	0.226616331	count	1
LINC01771	0.2310177	0.8937411	0.2585	0.796	0.227287993	count	1
MBLAC2	0.2310177	1.0189065	0.2267	0.821	0.227287993	count	1
WWOX	0.2310177	1.2333162	0.1873	0.852	0.227287993	count	1
NRSN2	0.2310177	1.2333162	0.1873	0.852	0.227287993	count	1
CTSK	0.2310177	1.371939	0.1684	0.8664	0.227287993	count	1
CMPK1	0.1655897	0.3304105	0.5012	0.617	0.227402859	count	1
PCNP	0.1629425	0.264952	0.615	0.539	0.227410738	count	1
IFI27L1	0.1947625	0.7057908	0.2759	0.783	0.227583018	count	1
EIF1	0.1580274	0.0632458	2.4986	0.013	0.227757269	count	1
RNF20	0.1782619	0.5586912	0.3191	0.75	0.227922432	count	1
CXXC1	0.1899118	0.657359	0.2889	0.773	0.22807542	count	1
RTN4	0.1615823	0.2315501	0.6978	0.486	0.228143624	count	1
IKBIP	0.1637796	0.3629085	0.4513	0.652	0.228208062	count	1
DHX36	0.1603201	0.2272695	0.7054	0.4811	0.228271263	count	1
CCDC85B	0.159572	0.1763038	0.9051	0.3661	0.22839605	count	1
FAM98B	0.1786481	0.5236387	0.3412	0.733	0.228417856	count	1

RNF168	0.1613308	0.2520477	0.6401	0.5226	0.228437742	count	1
DDB2	0.173293	0.5234097	0.3311	0.741	0.228483398	count	1
USB1	0.1661498	0.4262321	0.3898	0.697	0.228552118	count	1
TBX21	0.1656681	0.3669131	0.4515	0.652	0.228574688	count	1
GNAQ	0.1790749	0.6306172	0.284	0.777	0.228965368	count	1
TMF1	0.1645937	0.333941	0.4929	0.622	0.229142692	count	1
FAR1	0.2329721	0.5983736	0.3893	0.6973	0.229232975	count	1
THUMPD1	0.1623364	0.2258088	0.7189	0.473	0.229424747	count	1
MDM2	0.172449	0.4390082	0.3928	0.695	0.229694432	count	1
KAT6B	0.1655876	0.2923081	0.5665	0.571	0.230092236	count	1
CRYBG1	0.1640212	0.2789311	0.588	0.557	0.230117901	count	1
HDHD3	0.2340942	0.9772108	0.2396	0.8108	0.230349805	count	1
SLC7A6	0.378265	0.6999909	0.5404	0.589	0.230557342	count	1
VPS13C	0.1633863	0.2467321	0.6622	0.508	0.230615414	count	1
GPS2	0.1703702	0.4447616	0.3831	0.702	0.230949298	count	1
EMB	0.1634145	0.2243944	0.7282	0.467	0.23121118	count	1
LEPROTL1	0.1614585	0.1423007	1.1346	0.2574	0.231528151	count	1
RIPK2	0.1732913	0.5272195	0.3287	0.743	0.231806997	count	1
PSMA1	0.1635053	0.2222623	0.7356	0.4625	0.232068165	count	1
EHD1	0.2065638	0.4737864	0.436	0.663	0.232635766	count	1
NUDT16L1	0.1667314	0.3017099	0.5526	0.581	0.232879931	count	1
PFDN6	0.170562	0.4209173	0.4052	0.686	0.232897188	count	1
AKT1S1	0.2368175	0.87426	0.2709	0.787	0.23306071	count	1
FCHO1	0.2368175	0.986496	0.2401	0.81	0.23306071	count	1
RAP1A	0.1646684	0.248022	0.6639	0.5072	0.233222858	count	1
GNG10	0.3834854	1.063576	0.3606	0.719	0.23383294	count	1
ZRANB2	0.1659689	0.2639123	0.6289	0.53	0.233925498	count	1
LINC01184	0.1952331	0.6912328	0.2824	0.778	0.234499621	count	1
IL16	0.1659355	0.2534009	0.6548	0.513	0.234583372	count	1
RPS6KA3	0.1716184	0.4495751	0.3817	0.703	0.234825154	count	1
GLRX3	0.1820823	0.5026351	0.3623	0.717	0.235049923	count	1
RNASEH1-AS1	0.2205707	0.9783405	0.2255	0.822	0.2356399	count	1
ZNF691	0.2205707	1.0832936	0.2036	0.839	0.2356399	count	1
MED14OS	0.2205707	1.0832936	0.2036	0.839	0.2356399	count	1
POLE	0.2205707	1.0832936	0.2036	0.839	0.2356399	count	1
AL135999.1	0.2205707	1.171955	0.1882	0.851	0.2356399	count	1
TYK2	0.1844718	0.7613582	0.2423	0.809	0.235889289	count	1
RTCA	0.1771519	0.3596555	0.4926	0.623	0.235972356	count	1
ZBTB10	0.2019675	0.5723216	0.3529	0.724	0.236053765	count	1
PDCD10	0.1663575	0.2492346	0.6675	0.5049	0.236073215	count	1
HMGCR	0.1892958	0.6269712	0.3019	0.763	0.236121839	count	1
ZNF641	0.1892958	0.8276632	0.2287	0.819	0.236121839	count	1
PYROXD2	0.2213671	0.9897572	0.2237	0.823	0.236498334	count	1
POLR3B	0.2213671	1.261406	0.1755	0.861	0.236498334	count	1
ACTB	0.1640211	0.0729204	2.2493	0.0252	0.236552374	count	1

TSEN15	0.1737568	0.438995	0.3958	0.693	0.236738518	count	1
TMEM186	0.221787	0.7771174	0.2854	0.776	0.236950961	count	1
MT-ND2	0.1645979	0.0902075	1.8247	0.069	0.237109423	count	1
KLRD1	0.1649552	0.1086523	1.5182	0.13	0.23733032	count	1
SLC4A7	0.1807367	0.4485632	0.4029	0.687	0.238322576	count	1
FAH	0.391561	0.8753498	0.4473	0.655	0.238903566	count	1
CMTM7	0.1767437	0.4854271	0.3641	0.716	0.238917783	count	1
CDK5RAP2	0.1748345	0.4381375	0.399	0.69	0.239232188	count	1
MSRA	0.1855419	0.500016	0.3711	0.711	0.239529963	count	1
TRGV9	0.1731248	0.3792373	0.4565	0.648	0.240071752	count	1
NUMA1	0.1823159	0.4661834	0.3911	0.696	0.2404102	count	1
ARHGAP9	0.1696377	0.1957088	0.8668	0.387	0.24048419	count	1
ATP5IF1	0.1687481	0.1702724	0.991	0.3224	0.240749414	count	1
TSEN54	0.1726133	0.2837791	0.6083	0.543	0.241102765	count	1
PLD3	0.184104	0.5644214	0.3262	0.744	0.241292856	count	1
CREB1	0.1854966	0.4548519	0.4078	0.684	0.241423833	count	1
NR4A1	0.1697588	0.2126492	0.7983	0.4253	0.242371143	count	1
MOB3A	0.1863104	0.4164091	0.4474	0.655	0.242486089	count	1
S100A13	0.1943872	0.6038164	0.3219	0.748	0.242499393	count	1
FCER1G	0.1687399	0.1138983	1.4815	0.139	0.242808871	count	1
TNIP2	0.1763154	0.3993556	0.4415	0.659	0.242930467	count	1
C6orf47	0.2852376	0.7391219	0.3859	0.7	0.242994306	count	1
AAED1	0.1738014	0.3146508	0.5524	0.581	0.24310755	count	1
SMAD2	0.215877	0.5464806	0.395	0.693	0.243202527	count	1
SELENOO	0.2024511	0.5874765	0.3446	0.731	0.243216017	count	1
CXCR3	0.1718175	0.259202	0.6629	0.5079	0.243467446	count	1
POLDIP3	0.3991004	0.7016423	0.5688	0.57	0.24364107	count	1
SCRN2	0.1932085	0.6636288	0.2911	0.771	0.244327885	count	1
USP33	0.1768076	0.3996308	0.4424	0.658	0.244609321	count	1
SRP72	0.1732923	0.2533548	0.684	0.494	0.244687355	count	1
UCHL3	0.1788017	0.4075583	0.4387	0.661	0.24597933	count	1
LMNB2	0.2310177	0.8497402	0.2719	0.786	0.246904138	count	1
PCAT6	0.2310177	0.9124861	0.2532	0.8	0.246904138	count	1
BCAR3	0.2310177	0.9665421	0.239	0.811	0.246904138	count	1
FAM20B	0.2310177	0.9839011	0.2348	0.815	0.246904138	count	1
HACE1	0.2310177	1.1653438	0.1982	0.843	0.246904138	count	1
POMC	0.1789452	0.3285097	0.5447	0.586	0.24691655	count	1
TSTD1	0.173081	0.2189445	0.7905	0.4298	0.246954569	count	1
CD79B	0.1809567	0.4476141	0.4043	0.686	0.247113077	count	1
GDI2	0.1772864	0.3326157	0.533	0.594	0.247448648	count	1
TMBIM6	0.1724891	0.1279649	1.3479	0.179	0.247503486	count	1
ZNF844	0.4062402	0.6864529	0.5918	0.554	0.248130623	count	1
TGFBR2	0.1799632	0.3695021	0.487	0.627	0.248323	count	1
CBX6	0.1864184	0.4017419	0.464	0.643	0.248344018	count	1
AES	0.1733246	0.1484818	1.1673	0.2439	0.248465449	count	1

SAMD9L	0.1773435	0.3294273	0.5383	0.591	0.248684187	count	1
AP002387.2	0.1809371	0.4208661	0.4299	0.668	0.249306657	count	1
B4GALT5	0.1949546	0.5866253	0.3323	0.74	0.249341339	count	1
UBE2T	0.2135741	0.8136566	0.2625	0.793	0.249705611	count	1
AC112907.3	0.2135741	0.8136566	0.2625	0.793	0.249705611	count	1
ERCC8	0.2135741	0.8136566	0.2625	0.793	0.249705611	count	1
SLC25A14	0.2135741	0.8136566	0.2625	0.793	0.249705611	count	1
AARSD1	0.2135741	0.8136566	0.2625	0.793	0.249705611	count	1
USP32	0.2135741	0.8136566	0.2625	0.793	0.249705611	count	1
NKILA	0.2135741	1.069354	0.1997	0.842	0.249705611	count	1
SHC1	0.2336293	0.8120291	0.2877	0.774	0.249721196	count	1
PLEKHJ1	0.1842278	0.4183176	0.4404	0.66	0.249768044	count	1
USP34	0.1775952	0.265621	0.6686	0.504	0.249800853	count	1
IST1	0.1809798	0.40674	0.445	0.657	0.25038835	count	1
ICAM3	0.1757537	0.1818507	0.9665	0.335	0.250790762	count	1
ATRAID	0.1766767	0.236997	0.7455	0.4565	0.250818017	count	1
USF2	0.1784027	0.2616956	0.6817	0.496	0.251049696	count	1
HSP90B1	0.1751051	0.147246	1.1892	0.235	0.25125774	count	1
ARID4B	0.1768681	0.2131989	0.8296	0.4074	0.25130807	count	1
SCAMP3	0.1820555	0.511187	0.3561	0.722	0.251555999	count	1
CEP295	0.2353923	0.6597381	0.3568	0.721	0.251623149	count	1
MAN1A1	0.2353923	0.7447099	0.3161	0.752	0.251623149	count	1
EMC6	0.1784838	0.3134517	0.5694	0.569	0.251671287	count	1
NADSYN1	0.2233971	0.736698	0.3032	0.762	0.251738724	count	1
MISP3	0.2234586	1.0691107	0.209	0.835	0.25180855	count	1
CNBP	0.1758941	0.1402043	1.2546	0.211	0.251858673	count	1
BCAP29	0.1814515	0.3672329	0.4941	0.622	0.251902674	count	1
SCRIB	0.2235593	1.0012445	0.2233	0.823	0.25192288	count	1
MAIP1	0.2235593	1.0312009	0.2168	0.829	0.25192288	count	1
FGFR1OP	0.2357625	0.8713901	0.2706	0.787	0.252022554	count	1
OSTF1	0.1761801	0.1737081	1.0142	0.3112	0.252172672	count	1
HACD4	0.1861488	0.5046137	0.3689	0.712	0.252377107	count	1
PFN1	0.1751147	0.0726879	2.4091	0.0165	0.252396942	count	1
SPAST	0.236362	0.7003981	0.3375	0.736	0.252669362	count	1
STMN3	0.236362	0.7628856	0.3098	0.757	0.252669362	count	1
APOBEC3D	0.236362	0.9416011	0.251	0.802	0.252669362	count	1
ASH1L-AS1	0.2161831	0.8677849	0.2491	0.803	0.252775384	count	1
CCAR1	0.1882228	0.4533149	0.4152	0.678	0.252790671	count	1
HBS1L	0.1892958	0.4696465	0.4031	0.687	0.253263181	count	1
DAZAP1	0.1814997	0.3003965	0.6042	0.546	0.253334952	count	1
ITCH	0.188368	0.4712766	0.3997	0.69	0.253862695	count	1
BUD31	0.1800561	0.2644058	0.681	0.496	0.253982419	count	1
IDNK	0.1865041	0.464097	0.4019	0.688	0.254135257	count	1
SAMD3	0.1771831	0.1695008	1.0453	0.297	0.254212979	count	1
SLC15A4	0.1842308	0.382981	0.481	0.631	0.254219214	count	1

COMTD1	0.1823158	0.3505167	0.5201	0.603	0.254475131	count	1
BRD2	0.1797555	0.2170707	0.8281	0.4082	0.254540455	count	1
IL10RA	0.1850284	0.2829344	0.654	0.514	0.254557227	count	1
MAGOHB	0.184876	0.3670603	0.5037	0.615	0.254741117	count	1
AC243960.1	0.1791	0.2674057	0.6698	0.5035	0.255305336	count	1
G6PC3	0.1916831	0.8703395	0.2202	0.8258	0.255373919	count	1
RAE1	0.2019781	0.5112539	0.3951	0.693	0.255461363	count	1
RPS17	0.2019781	0.5456895	0.3701	0.712	0.255461363	count	1
ZNF766	0.2087973	0.5105212	0.409	0.683	0.256232913	count	1
EPRS	0.1820876	0.3238719	0.5622	0.574	0.256456213	count	1
POLL	0.2193136	0.6943766	0.3158	0.752	0.256459254	count	1
SET	0.1787795	0.1290524	1.3853	0.167	0.25647552	count	1
DHPS	0.1832565	0.32313	0.5671	0.571	0.256514389	count	1
KLHL6	0.1945714	0.5072473	0.3836	0.702	0.256613876	count	1
GLRX	0.183179	0.2912792	0.6289	0.53	0.256724164	count	1
RARA	0.1918932	0.48395	0.3965	0.692	0.256745959	count	1
GIMAP6	0.2007452	0.5978952	0.3358	0.737	0.256773891	count	1
SPNS1	0.2062974	0.5553713	0.3715	0.711	0.257422666	count	1
IMPDH1	0.2098574	0.4948445	0.4241	0.672	0.257540184	count	1
SPIDR	0.2285837	0.7093817	0.3222	0.747	0.257628129	count	1
THOC1	0.206971	0.4913045	0.4213	0.674	0.258266856	count	1
UPF2	0.1850826	0.3016282	0.6136	0.54	0.258901544	count	1
PRKRA	0.1954688	0.482882	0.4048	0.686	0.259190452	count	1
SLC9A3R1	0.1832946	0.2379065	0.7704	0.442	0.259284899	count	1
AC058791.1	0.2631807	0.3858479	0.6821	0.496	0.259332628	count	1
TMX2	0.2081021	0.5173779	0.4022	0.688	0.259684451	count	1
CDC42SE2	0.1834886	0.2209146	0.8306	0.407	0.259698128	count	1
ABHD10	0.2053504	0.5333015	0.3851	0.7	0.259743486	count	1
TAX1BP1	0.1819786	0.1644975	1.1063	0.269	0.259762018	count	1
CXCR4	0.180709	0.1026138	1.7611	0.0792	0.259940243	count	1
HIST1H4B	0.2310177	1.371939	0.1684	0.8664	0.260392497	count	1
ABHD2	0.2227145	0.546071	0.4078	0.684	0.260461926	count	1
NFX1	0.2645549	0.7349384	0.36	0.7191	0.260703451	count	1
SAP30L	0.2062425	0.5244165	0.3933	0.694	0.260876344	count	1
MRPL41	0.1832934	0.2044148	0.8967	0.3706	0.261025843	count	1
CHURC1	0.1844288	0.2705328	0.6817	0.4959	0.261161606	count	1
TGFBR3	0.2319878	0.6844891	0.3389	0.735	0.261494368	count	1
L3MBTL1	0.2179514	0.9042215	0.241	0.81	0.261943017	count	1
FPGT	0.2179514	0.9108365	0.2393	0.811	0.261943017	count	1
PSTPIP2	0.2048599	0.5083538	0.403	0.687	0.26205608	count	1
MFAP1	0.194064	0.3693055	0.5255	0.6	0.262376518	count	1
PPM1A	0.2016399	0.5078818	0.397	0.692	0.262499817	count	1
ECSIT	0.2245288	0.7665328	0.2929	0.77	0.262597505	count	1
LINC00910	0.4296416	0.9629145	0.4462	0.656	0.26286575	count	1
SMURF1	0.2248048	0.9943309	0.2261	0.821	0.262922399	count	1

ZNHIT3	0.1864707	0.3183795	0.5857	0.5585	0.263306937	count	1
NUP85	0.2190888	0.8418175	0.2603	0.795	0.263317658	count	1
PRDX6	0.1845755	0.1876353	0.9837	0.326	0.263337451	count	1
OTUD3	0.225218	0.829306	0.2716	0.786	0.2634088	count	1
TANGO2	0.225218	0.8475562	0.2657	0.791	0.2634088	count	1
CEP97	0.225218	0.8670266	0.2598	0.795	0.2634088	count	1
C1orf50	0.225218	0.9169979	0.2456	0.806	0.2634088	count	1
NUP43	0.225218	0.9324881	0.2415	0.809	0.2634088	count	1
FDPS	0.189107	0.3170121	0.5965	0.551	0.263754563	count	1
CGRRF1	0.2086884	0.4207839	0.496	0.62	0.263982491	count	1
ATP6V0E2	0.2014536	0.4315084	0.4669	0.641	0.264098047	count	1
ZNHIT6	0.1982867	0.4886529	0.4058	0.685	0.26419266	count	1
WRN	0.2343948	0.7578892	0.3093	0.757	0.264228546	count	1
TMEM107	0.2048874	0.4059969	0.5047	0.614	0.264589355	count	1
B4GALT3	0.1963243	0.4949362	0.3967	0.692	0.264607175	count	1
MOSPD3	0.2006927	0.5499134	0.365	0.715	0.264708818	count	1
OGA	0.1894378	0.3636388	0.521	0.603	0.26481652	count	1
CCT7	0.1877623	0.2528233	0.7427	0.458	0.264953303	count	1
PHLDA1	0.1916832	0.3613024	0.5305	0.596	0.265534906	count	1
ORAI2	0.1972779	0.3914179	0.504	0.615	0.265895047	count	1
FAM107B	0.1872342	0.2088443	0.8965	0.3706	0.265908344	count	1
CANT1	0.3118853	0.8423631	0.3703	0.711	0.266109234	count	1
RNF122	0.3118853	0.8960818	0.3481	0.728	0.266109234	count	1
PCMTD2	0.2082326	0.5177518	0.4022	0.688	0.26638619	count	1
PPP1R16B	0.2706729	0.5622125	0.4814	0.631	0.266807983	count	1
RWDD1	0.1865683	0.1757125	1.0618	0.2891	0.266823645	count	1
EGR1	0.1918121	0.4067287	0.4716	0.638	0.266831343	count	1
NEIL1	0.2139277	0.5057237	0.423	0.673	0.266986445	count	1
FOXK1	0.3130136	0.858108	0.3648	0.716	0.267089081	count	1
PARP1	0.1894957	0.2669132	0.71	0.478	0.267400917	count	1
ACBD6	0.1932085	0.3331965	0.5799	0.562	0.269257232	count	1
DDX24	0.1879964	0.1234649	1.5227	0.129	0.269837801	count	1
IRF2BPL	0.1990425	0.4003076	0.4972	0.619	0.269891064	count	1
MGST3	0.1910294	0.2534628	0.7537	0.4516	0.27015769	count	1
CTU1	0.2310177	0.7570045	0.3052	0.76	0.270236903	count	1
DYRK2	0.1983929	0.4263034	0.4654	0.642	0.270363234	count	1
RNF6	0.2250868	0.6630359	0.3395	0.734	0.270567768	count	1
RNPEPL1	0.1944476	0.2744072	0.7086	0.479	0.270985836	count	1
ZNF253	0.2318882	0.7402571	0.3133	0.754	0.271261914	count	1
SFT2D3	0.2257439	0.8613138	0.2621	0.793	0.271362148	count	1
MFGE8	0.2213671	0.7581813	0.292	0.77	0.271736723	count	1
CHN2	0.2213671	0.8653396	0.2558	0.798	0.271736723	count	1
ZNF136	0.4440783	0.6622707	0.6705	0.503	0.271970491	count	1
KIAA1191	0.2759365	0.6526932	0.4228	0.673	0.272062044	count	1
DNM1L	0.2033354	0.4954573	0.4104	0.682	0.272090445	count	1

PHF11	0.1961934	0.3824864	0.5129	0.608	0.272099557	count	1
POLR2H	0.1978301	0.3807059	0.5196	0.604	0.272194835	count	1
SLC38A1	0.1937669	0.2456835	0.7887	0.431	0.272308367	count	1
PDLIM5	0.2330232	0.6961588	0.3347	0.738	0.27259843	count	1
TTN	0.2764793	0.7825391	0.3533	0.724	0.272603972	count	1
LDHA	0.1899843	0.1226176	1.5494	0.122	0.272740342	count	1
DNTTIP2	0.192114	0.2206867	0.8705	0.3847	0.273193021	count	1
TCEAL3	0.197632	0.3917046	0.5045	0.614	0.273785761	count	1
CSRP1	0.1987658	0.4110338	0.4836	0.629	0.273906692	count	1
SYK	0.205658	0.4936033	0.4166	0.677	0.274037933	count	1
TRBC2	0.1908249	0.1606873	1.1876	0.236	0.274052032	count	1
ANKRD12	0.1917935	0.1891033	1.0142	0.311	0.274072005	count	1
PDE9A	0.2167592	0.8129725	0.2666	0.79	0.274233545	count	1
IDH3G	0.1972879	0.3464758	0.5694	0.569	0.274708776	count	1
RPF1	0.2115213	0.4811707	0.4396	0.661	0.275404644	count	1
APOBR	0.2291107	0.7954454	0.288	0.774	0.275432635	count	1
FKBP8	0.1937744	0.2191024	0.8844	0.3771	0.275472168	count	1
CLIC1	0.1913061	0.086635	2.2082	0.0279	0.275502967	count	1
RASSF2	0.2178415	0.8130133	0.2679	0.789	0.275608402	count	1
WNK1	0.1989441	0.367154	0.5419	0.588	0.275919037	count	1
BABAM1	0.1982619	0.3260439	0.6081	0.544	0.276536362	count	1
AC016831.7	0.2126159	0.4347123	0.4891	0.625	0.276834346	count	1
MITD1	0.1997341	0.3771185	0.5296	0.597	0.277016009	count	1
AGFG1	0.2189556	0.4222566	0.5185	0.604	0.277023701	count	1
CCNJL	0.222445	0.7672683	0.2899	0.772	0.277664797	count	1
AKIP1	0.222445	0.7882094	0.2822	0.778	0.277664797	count	1
RPP25L	0.2264363	0.7091563	0.3193	0.75	0.277991127	count	1
TJAP1	0.2264363	0.7463443	0.3034	0.762	0.277991127	count	1
VAPA	0.1946888	0.18857	1.0324	0.303	0.278017951	count	1
BBX	0.1984669	0.2636516	0.7528	0.452	0.278169064	count	1
CEBPD	0.1937244	0.2179253	0.8889	0.3747	0.278220775	count	1
VPS53	0.23154	0.5511258	0.4201	0.675	0.27837001	count	1
NMT2	0.2383057	0.58615	0.4066	0.685	0.278819685	count	1
TRIP11	0.2043744	0.4010307	0.5096	0.611	0.279146835	count	1
SLC25A26	0.2328147	0.6068689	0.3836	0.702	0.279911414	count	1
AP4E1	0.2189594	0.7610598	0.2877	0.774	0.280160674	count	1
ZNF655	0.2094123	0.5042665	0.4153	0.678	0.280241106	count	1
PEPD	0.201607	0.3539632	0.5696	0.569	0.280472338	count	1
BEX5	0.2397208	0.673926	0.3557	0.722	0.280486505	count	1
UBN1	0.201028	0.3247672	0.619	0.536	0.280831878	count	1
PAIP1	0.2017167	0.388526	0.5192	0.604	0.280882238	count	1
RFC2	0.2091039	0.4537231	0.4609	0.645	0.280897786	count	1
SLC2A4RG	0.2002513	0.2774942	0.7216	0.471	0.281818961	count	1
MLH3	0.2637192	0.6177909	0.4269	0.67	0.282210024	count	1
VRK1	0.2094102	0.4970966	0.4213	0.674	0.282281895	count	1

MARF1	0.2300518	0.88427	0.2602	0.795	0.28245262	count	1
TBC1D19	0.2265286	0.6847952	0.3308	0.741	0.282785523	count	1
ABCB1	0.2044195	0.2924863	0.6989	0.485	0.282860032	count	1
ACSL3	0.2051805	0.4290738	0.4782	0.633	0.283167827	count	1
TRUB1	0.2148221	0.6144469	0.3496	0.727	0.283397832	count	1
CCNL2	0.2214853	0.4044942	0.5476	0.584	0.283404807	count	1
NAGPA	0.2310177	0.7489997	0.3084	0.758	0.283644626	count	1
MBNL2	0.2151037	0.4286447	0.5018	0.616	0.283770362	count	1
RRP7A	0.2017832	0.312845	0.645	0.519	0.283849998	count	1
RUFY3	0.2275386	0.7858962	0.2895	0.772	0.284052139	count	1
DDX5	0.1979048	0.0931408	2.1248	0.0344	0.284378775	count	1
DNAJC7	0.2028131	0.2627178	0.772	0.441	0.285426999	count	1
TMEM11	0.208539	0.4684305	0.4452	0.656	0.285428962	count	1
GCNT1	0.2326454	0.6025105	0.3861	0.7	0.285653438	count	1
CETN2	0.2294501	0.8359707	0.2745	0.784	0.286449411	count	1
ZNF296	0.2218659	0.4183856	0.5303	0.596	0.286592983	count	1
ATP6AP1	0.2153054	0.3942531	0.5461	0.585	0.286925242	count	1
UBE2E1	0.2299025	0.4880805	0.471	0.638	0.287016802	count	1
ELL2	0.2301473	0.6436313	0.3576	0.721	0.287323827	count	1
FAM76B	0.2135176	0.4653839	0.4588	0.647	0.287830388	count	1
NCBP2-AS2	0.207043	0.3966113	0.522	0.602	0.288043352	count	1
CREB3L4	0.227948	0.6333559	0.3599	0.719	0.288448883	count	1
KPNA3	0.2139817	0.4270402	0.5011	0.617	0.288457341	count	1
DKK3	0.2218019	0.6235858	0.3557	0.722	0.288833979	count	1
LINC00847	0.2218019	0.6511567	0.3406	0.734	0.288833979	count	1
BTBD6	0.2122388	0.4058311	0.523	0.601	0.289265936	count	1
EID2B	0.2194071	0.6611265	0.3319	0.74	0.28946359	count	1
THAP4	0.2172613	0.590572	0.3679	0.713	0.289538272	count	1
PPP4R1	0.2290393	0.5570002	0.4112	0.681	0.289835618	count	1
S100PBP	0.2098479	0.3845657	0.5457	0.586	0.290011135	count	1
C17orf49	0.2246543	0.5817884	0.3861	0.7	0.290207549	count	1
YWHAG	0.2162144	0.4344931	0.4976	0.619	0.290471157	count	1
PUSL1	0.3400478	0.9411558	0.3613	0.718	0.290592009	count	1
BTRC	0.3400478	0.994572	0.3419	0.733	0.290592009	count	1
GALC	0.3400478	0.9960114	0.3414	0.733	0.290592021	count	1
ZNF668	0.3400478	1.0422589	0.3263	0.744	0.290592021	count	1
AL441992.1	0.3400478	1.050507	0.3237	0.746	0.290592021	count	1
EPC2	0.2172755	0.4520175	0.4807	0.631	0.290788915	count	1
TFDP1	0.2204237	0.5437197	0.4054	0.685	0.290808583	count	1
HES6	0.225218	0.6389907	0.3525	0.725	0.290938295	count	1
BTG2	0.2025859	0.1344379	1.5069	0.133	0.291340176	count	1
ZBTB17	0.34125	0.9826637	0.3473	0.729	0.29163827	count	1
GRAMD4	0.34125	1.056735	0.3229	0.747	0.29163827	count	1
ZNF181	0.34125	1.15515	0.2954	0.768	0.29163827	count	1
MRPS11	0.2084782	0.3770202	0.553	0.581	0.292036772	count	1

SMIM26	0.2079499	0.3198415	0.6502	0.516	0.293140342	count	1
TUT7	0.2202204	0.376001	0.5857	0.558	0.293491719	count	1
ZNRF1	0.2170614	0.4177733	0.5196	0.604	0.293534231	count	1
PATL2	0.2975711	0.9267444	0.3211	0.748	0.293676511	count	1
KDM4B	0.3439742	0.589449	0.5836	0.56	0.294009459	count	1
LSM5	0.2084716	0.2812086	0.7413	0.459	0.294297308	count	1
NR1D2	0.2328101	0.475642	0.4895	0.625	0.294627569	count	1
RAB8A	0.2106546	0.2987719	0.7051	0.481	0.295088296	count	1
FBL	0.2130991	0.3187103	0.6686	0.504	0.295240513	count	1
DENND6B	0.2993345	0.9070498	0.33	0.742	0.295439573	count	1
ARHGDIB	0.2052491	0.0860155	2.3862	0.0176	0.295604856	count	1
MRPS14	0.2288792	0.4754895	0.4814	0.631	0.295684687	count	1
NFKBIB	0.2255412	0.5021441	0.4492	0.654	0.295774743	count	1
RAD17	0.2769411	0.4986398	0.5554	0.579	0.296503505	count	1
CWC22	0.2192619	0.4042238	0.5424	0.588	0.296516109	count	1
BECN1	0.2208017	0.4809718	0.4591	0.646	0.296647944	count	1
RAC2	0.2063134	0.116685	1.7681	0.078	0.29668383	count	1
EIF4B	0.2125876	0.2618845	0.8118	0.418	0.296996919	count	1
ZNRF2	0.2252763	0.4484798	0.5023	0.616	0.297229089	count	1
RBAK-RBAKDN	0.2282519	0.6050678	0.3772	0.706	0.297261072	count	1
METTL21A	0.2192917	0.5156955	0.4252	0.671	0.297402962	count	1
KLF10	0.216483	0.3275209	0.661	0.509	0.297408212	count	1
TGS1	0.2138781	0.3511217	0.6091	0.543	0.297563473	count	1
TSHZ1	0.3015918	0.7329733	0.4115	0.681	0.297696708	count	1
TOB1	0.2353522	0.4364003	0.5393	0.59	0.297858361	count	1
TEX264	0.2114165	0.33274	0.6354	0.526	0.29791514	count	1
ATXN7L3	0.2783036	0.8173161	0.3405	0.734	0.297976999	count	1
PGAM1	0.2099849	0.2248648	0.9338	0.3511	0.29823495	count	1
APLP2	0.2364173	0.5124928	0.4613	0.645	0.29921208	count	1
TSC22D4	0.2114009	0.2708175	0.7806	0.4356	0.29952163	count	1
TSEN34	0.2208585	0.4005606	0.5514	0.582	0.299532048	count	1
KAT5	0.2230083	0.5573855	0.4001	0.689	0.299619285	count	1
SNX14	0.2217044	0.502224	0.4414	0.659	0.299826028	count	1
CD160	0.2094362	0.2086121	1.004	0.316	0.300371247	count	1
METTL26	0.2121893	0.2445736	0.8676	0.3863	0.300639305	count	1
ZCCHC9	0.2249229	0.5787419	0.3886	0.698	0.301048583	count	1
SNX20	0.2222266	0.5051412	0.4399	0.66	0.301391162	count	1
FBXO33	0.2316738	0.4014949	0.577	0.564	0.301732355	count	1
CCL5	0.2094339	0.0980825	2.1353	0.0335	0.30201647	count	1
B4GALT1	0.2153549	0.3141943	0.6854	0.494	0.302204664	count	1
ZFP36L2	0.2102011	0.0941012	2.2338	0.0262	0.302789495	count	1
RORA	0.2129558	0.2188527	0.9731	0.3313	0.302888709	count	1
GOLGA5	0.4930693	0.7783279	0.6335	0.527	0.302938072	count	1
RBX1	0.2131003	0.2101163	1.0142	0.3112	0.30295914	count	1
CASP7	0.3542565	0.8274963	0.4281	0.669	0.302963359	count	1

PTPN12	0.221813	0.3353054	0.6615	0.509	0.303007929	count	1
HDAC9	0.2685128	0.5564007	0.4826	0.63	0.303018842	count	1
APH1A	0.2160581	0.2752447	0.785	0.433	0.303354768	count	1
CIB1	0.2122856	0.1786262	1.1884	0.2355	0.303838042	count	1
CISH	0.2228136	0.4245548	0.5248	0.6	0.304377196	count	1
TMA7	0.2119092	0.110894	1.9109	0.0569	0.304476784	count	1
ATP8A1	0.2254805	0.5095526	0.4425	0.658	0.304943382	count	1
CST3	0.2186114	0.3650207	0.5989	0.55	0.305421199	count	1
FAM96B	0.2138326	0.235099	0.9095	0.3637	0.305535625	count	1
FAAP24	0.3573372	0.769551	0.4643	0.643	0.305647267	count	1
PHF20L1	0.2181965	0.308163	0.7081	0.479	0.306025271	count	1
PLPBP	0.3099338	0.5942316	0.5216	0.602	0.306040769	count	1
PYHIN1	0.2151542	0.2319885	0.9274	0.3544	0.306060601	count	1
CRIM1	0.2208129	0.4736935	0.4662	0.641	0.306288383	count	1
BCL10	0.2215839	0.3505839	0.632	0.528	0.306643147	count	1
SPRY1	0.2186495	0.2944957	0.7425	0.4583	0.30683204	count	1
MYO5A	0.3588196	0.7111625	0.5046	0.614	0.306938936	count	1
ACADS	0.5000605	0.9308161	0.5372	0.591	0.307365011	count	1
ANKDD1A	0.5000605	0.9308161	0.5372	0.591	0.307365011	count	1
COL6A2	0.5000605	0.9308161	0.5372	0.591	0.307365011	count	1
EIF3C	0.5000605	0.9647492	0.5183	0.605	0.307365011	count	1
RIN3	0.2241361	0.365774	0.6128	0.54	0.307396032	count	1
GZMA	0.2133931	0.0957737	2.2281	0.0266	0.307459038	count	1
TMIGD2	0.2150027	0.2069432	1.0389	0.2996	0.307501156	count	1
SPATA5	0.2872405	0.6057581	0.4742	0.636	0.307644512	count	1
IFNG	0.2159655	0.3292816	0.6559	0.512	0.30764541	count	1
ZNF574	0.359953	0.7296289	0.4933	0.622	0.307926563	count	1
GBF1	0.2875694	0.7368572	0.3903	0.697	0.308000383	count	1
LTB4R	0.2875694	0.7507528	0.383	0.702	0.308000383	count	1
ZNF765	0.2875694	0.8366442	0.3437	0.731	0.308000383	count	1
TRAPPC10	0.2182825	0.2739439	0.7968	0.426	0.308052053	count	1
EXOSC9	0.2229278	0.3875136	0.5753	0.566	0.308505425	count	1
HMGXB4	0.2266058	0.5548895	0.4084	0.683	0.308883645	count	1
RASAL3	0.2225055	0.3001504	0.7413	0.459	0.309283207	count	1
EWSR1	0.22247	0.2909029	0.7648	0.445	0.310339491	count	1
SLC25A53	0.2750334	0.7467442	0.3683	0.713	0.310439387	count	1
CCNT1	0.2486241	0.4597907	0.5407	0.589	0.310503729	count	1
C1D	0.2220923	0.290658	0.7641	0.445	0.310512313	count	1
PCGF5	0.2215589	0.2790395	0.794	0.428	0.310745413	count	1
SHARPIN	0.226968	0.4136127	0.5487	0.584	0.311286296	count	1
TBRG1	0.229687	0.381027	0.6028	0.547	0.311529713	count	1
HSPA6	0.2591517	0.6493819	0.3991	0.69	0.311775106	count	1
BYSL	0.3159413	0.9732518	0.3246	0.746	0.31205227	count	1
PAQR8	0.3159413	1.0659708	0.2964	0.767	0.31205227	count	1
KIF21A	0.2279882	0.3743535	0.609	0.543	0.313239699	count	1

PIM2	0.2241719	0.293439	0.7639	0.445	0.313422811	count	1
DYNLRB1	0.2196009	0.1878165	1.1692	0.2432	0.313509127	count	1
KCTD5	0.2778931	0.7051734	0.3941	0.694	0.313694428	count	1
TRAPPC2	0.229383	0.4112938	0.5577	0.577	0.314008269	count	1
MAZ	0.2215712	0.2495156	0.888	0.3752	0.314346416	count	1
TCERG1	0.2317833	0.3706435	0.6254	0.532	0.314378728	count	1
TRIM8	0.2317833	0.3815108	0.6075	0.544	0.314378728	count	1
ARL1	0.226494	0.3573603	0.6338	0.527	0.315136644	count	1
SNX17	0.2258516	0.2951301	0.7653	0.445	0.315773661	count	1
UBE2D1	0.2290193	0.3654834	0.6266	0.531	0.316116298	count	1
AIMP2	0.2801844	0.6905111	0.4058	0.685	0.316302786	count	1
METTL2B	0.2801844	0.6992473	0.4007	0.689	0.316302786	count	1
GLYCTK	0.2801844	0.7360765	0.3806	0.704	0.316302786	count	1
THAP12	0.2302011	0.3804243	0.6051	0.546	0.316805378	count	1
CCDC124	0.226022	0.3443623	0.6563	0.512	0.317010873	count	1
UNC45A	0.258046	0.632416	0.408	0.684	0.317015247	count	1
BGN	0.2966438	0.8907098	0.333	0.739	0.31782122	count	1
SNF8	0.2262318	0.2807212	0.8059	0.421	0.317972812	count	1
C12orf75	0.2266992	0.2758524	0.8218	0.412	0.318308698	count	1
RGL4	0.2285728	0.351362	0.6505	0.516	0.318322708	count	1
RAB33B	0.2974428	0.5830992	0.5101	0.61	0.318686148	count	1
NEU1	0.2277225	0.3837447	0.5934	0.553	0.319020677	count	1
ELP3	0.2654417	0.683357	0.3884	0.698	0.319389404	count	1
TULP3	0.5200243	1.097944	0.4736	0.636	0.320015326	count	1
GABARAP	0.2987472	0.8831202	0.3383	0.735	0.320098258	count	1
CFDP1	0.230426	0.3503563	0.6577	0.511	0.320306269	count	1
XRCC1	0.2666465	0.5913814	0.4509	0.652	0.320848049	count	1
MRPL43	0.2281046	0.273639	0.8336	0.405	0.321320871	count	1
CPEB4	0.274507	0.6422165	0.4274	0.669	0.321490716	count	1
PPP1R7	0.2287622	0.2942357	0.7775	0.437	0.321685827	count	1
POLR3C	0.2619765	0.6823719	0.3839	0.701	0.321870414	count	1
TOGARAM1	0.3759501	0.7305375	0.5146	0.607	0.321874238	count	1
HK1	0.2578638	0.6581347	0.3918	0.695	0.322099929	count	1
NPAT	0.2677585	0.4034671	0.6636	0.507	0.322194387	count	1
ENPP4	0.2678131	0.6981713	0.3836	0.702	0.322260498	count	1
TRIM44	0.2331913	0.3267072	0.7138	0.476	0.322318618	count	1
TBL1XR1	0.2355445	0.2705189	0.8707	0.385	0.322457633	count	1
USPL1	0.3767419	0.6534184	0.5766	0.5646	0.322564957	count	1
STN1	0.230125	0.2673729	0.8607	0.39	0.322584158	count	1
TMEM87B	0.2522738	0.7427987	0.3396	0.734	0.322964614	count	1
OGFRL1	0.2642408	0.6056272	0.4363	0.663	0.324667662	count	1
NOL10	0.2877958	0.8265155	0.3482	0.728	0.324969202	count	1
LAMTOR1	0.2276971	0.208197	1.0937	0.2749	0.324993318	count	1
HDAC5	0.3798842	0.6675989	0.569	0.57	0.325306437	count	1
FAM117A	0.2421037	0.4302863	0.5627	0.574	0.325336729	count	1

ZHX1	0.3799782	0.7493834	0.5071	0.612	0.325388454	count	1
PARP6	0.3801539	0.9903167	0.3839	0.7013	0.32554175	count	1
NIT2	0.2359174	0.4313357	0.5469	0.585	0.32609202	count	1
AMZ2	0.2405982	0.4319161	0.557	0.578	0.326359621	count	1
EIF2A	0.2366735	0.3025183	0.7823	0.435	0.326697056	count	1
STK16	0.2451715	0.4113626	0.596	0.552	0.326835052	count	1
DDX41	0.2616398	0.5254344	0.4979	0.619	0.32683981	count	1
NONO	0.237132	0.3398289	0.6978	0.486	0.326860326	count	1
SEC22B	0.2333187	0.375829	0.6208	0.535	0.327065294	count	1
REXO1	0.2466931	0.4924712	0.5009	0.617	0.327315551	count	1
FBH1	0.3822506	0.7331618	0.5214	0.602	0.327371319	count	1
PHTF2	0.2720405	0.6834509	0.398	0.691	0.327379229	count	1
GAMT	0.2899403	0.696478	0.4163	0.677	0.32741144	count	1
CD151	0.2326122	0.3302342	0.7044	0.482	0.327540088	count	1
OS9	0.2469326	0.4693556	0.5261	0.599	0.327634217	count	1
IPO5	0.2798919	0.7559669	0.3702	0.711	0.327843066	count	1
MCMBP	0.2798919	0.79098	0.3539	0.724	0.327843066	count	1
TAF5	0.2798919	0.8286462	0.3378	0.736	0.327843066	count	1
AKNA	0.2373347	0.3131937	0.7578	0.449	0.328053849	count	1
ZC3H13	0.2368227	0.291962	0.8111	0.418	0.328153915	count	1
ZNF816-ZNF321P	0.3834854	1.0977883	0.3493	0.7271	0.328448898	count	1
FUNDC2	0.2333108	0.3248661	0.7182	0.473	0.328660448	count	1
HOXA10	0.2417356	0.5073277	0.4765	0.634	0.328759767	count	1
FAM50A	0.2341575	0.3175276	0.7374	0.461	0.32912225	count	1
NIPBL	0.2329626	0.3107215	0.7497	0.454	0.329212143	count	1
NOSIP	0.2389789	0.3677772	0.6498	0.516	0.329409955	count	1
OXCT1	0.2444945	0.5085842	0.4807	0.631	0.329686522	count	1
GNPTG	0.2517134	0.4818833	0.5224	0.602	0.330210604	count	1
EIF2AK4	0.2557015	0.5481659	0.4665	0.641	0.330469285	count	1
DYNLT3	0.2366095	0.3020123	0.7834	0.434	0.330830667	count	1
EIF4H	0.2387555	0.3022361	0.79	0.43	0.330835719	count	1
SMU1	0.2408277	0.3052497	0.789	0.431	0.330909468	count	1
RCAN3	0.2440008	0.3708276	0.658	0.511	0.330984669	count	1
GOLGA8A	0.2523681	0.602336	0.419	0.676	0.331072244	count	1
GMPR2	0.2423232	0.5310695	0.4563	0.648	0.331078229	count	1
LTV1	0.240574	0.4242106	0.5671	0.571	0.331103452	count	1
MIATNB	0.2475327	0.6546657	0.3781	0.706	0.331388952	count	1
REPIN1	0.2411828	0.5780634	0.4172	0.677	0.331398191	count	1
TRIM59	0.2476294	0.6864585	0.3607	0.7185	0.331518737	count	1
PNPLA8	0.2399206	0.3435955	0.6983	0.486	0.33163332	count	1
WDR41	0.2528053	0.5408291	0.4674	0.64	0.331647638	count	1
ZNF26	0.387258	0.910632	0.4253	0.671	0.331741623	count	1
POLD2	0.2478274	0.4502348	0.5504	0.582	0.331784482	count	1
ATG2A	0.2591367	0.6166731	0.4202	0.675	0.331786656	count	1
AL121944.1	0.262151	0.740656	0.3539	0.724	0.331930491	count	1

ZBP1	0.2429486	0.428725	0.5667	0.571	0.331934254	count	1
CBL	0.2658874	0.7020185	0.3787	0.705	0.332172248	count	1
GTPBP1	0.2761408	0.7360626	0.3752	0.708	0.332344729	count	1
RAB12	0.2761408	0.771396	0.358	0.721	0.332344729	count	1
ZNF836	0.2533525	0.6327485	0.4004	0.689	0.332367812	count	1
MAPK1IP1L	0.2396284	0.3189408	0.7513	0.453	0.332422223	count	1
DDX6	0.2335715	0.1941668	1.2029	0.2299	0.332578358	count	1
C6orf203	0.3366402	0.6975629	0.4826	0.63	0.332780308	count	1
MRGBP	0.2557682	0.5270137	0.4853	0.628	0.333224803	count	1
RUNDC1	0.3112564	0.8741081	0.3561	0.722	0.333644747	count	1
RLIM	0.2503227	0.5213966	0.4801	0.631	0.333720526	count	1
EIF4E3	0.2483876	0.6650485	0.3735	0.709	0.333801376	count	1
VAMP2	0.2340762	0.1808395	1.2944	0.196	0.334155471	count	1
KPNA5	0.2588156	0.8089172	0.32	0.749	0.33450913	count	1
ANXA1	0.2326596	0.1536982	1.5137	0.131	0.334780327	count	1
EIF3F	0.2339679	0.1398345	1.6732	0.0953	0.335161703	count	1
MRPL34	0.2374053	0.2825128	0.8403	0.401	0.335693823	count	1
MAD2L1BP	0.2511597	0.457894	0.5485	0.584	0.336257037	count	1
TRAPPC13	0.2795579	0.8695081	0.3215	0.748	0.336483367	count	1
LMAN2	0.2377948	0.2329847	1.0206	0.3082	0.33730801	count	1
UBE2D3	0.2360213	0.1617588	1.4591	0.146	0.337607003	count	1
ASB1	0.2667733	0.6837027	0.3902	0.697	0.337809617	count	1
CYB5R4	0.2575674	0.5545866	0.4644	0.643	0.337915301	count	1
NDUFS7	0.2397505	0.2450677	0.9783	0.329	0.338130923	count	1
ZNF780A	0.2578035	0.6764536	0.3811	0.703	0.33822606	count	1
SEC22C	0.2553279	0.5666796	0.4506	0.653	0.338805357	count	1
SESN1	0.2420557	0.3149905	0.7685	0.443	0.339324766	count	1
HSF2	0.5506668	0.5514885	0.9985	0.319	0.339454303	count	1
LYN	0.2444951	0.3514403	0.6957	0.487	0.339544729	count	1
SNRK	0.2433279	0.3135573	0.776	0.438	0.33999091	count	1
CSE1L	0.2903554	0.8087839	0.359	0.72	0.340189926	count	1
DNAJC27	0.2903554	0.8461894	0.3431	0.732	0.340189926	count	1
YPEL2	0.2903554	0.8633823	0.3363	0.737	0.340189926	count	1
RAB11FIP2	0.2615424	0.6624213	0.3948	0.693	0.340774188	count	1
TVP23B	0.3020663	0.875635	0.345	0.73	0.341224938	count	1
CBR3	0.3020663	0.875635	0.345	0.73	0.341224938	count	1
PLXND1	0.3020663	0.9199476	0.3284	0.743	0.341224938	count	1
CNIH4	0.2450084	0.3737385	0.6556	0.513	0.341816565	count	1
PPP1R18	0.240038	0.1949947	1.231	0.2192	0.342406506	count	1
FCHO2	0.3195293	0.9679464	0.3301	0.742	0.342607858	count	1
PHTF1	0.2742714	0.5175862	0.5299	0.597	0.342699257	count	1
MCL1	0.2390273	0.1519797	1.5728	0.117	0.343146613	count	1
PRMT9	0.2529822	0.3774118	0.6703	0.503	0.343193729	count	1
SPTAN1	0.2498581	0.3887312	0.6428	0.521	0.343338491	count	1
CHST11	0.2538834	0.3503274	0.7247	0.469	0.343443226	count	1

PRDX3	0.2449103	0.3535969	0.6926	0.489	0.343528786	count	1
RBM14	0.3483503	0.9523283	0.3658	0.715	0.34451679	count	1
NOD2	0.3483503	1.304145	0.2671	0.79	0.34451679	count	1
ADAMTS10	0.3483503	1.304145	0.2671	0.79	0.34451679	count	1
C1QBP	0.2436996	0.2805	0.8688	0.386	0.344600131	count	1
CHMP4A	0.2432357	0.2296536	1.0591	0.2903	0.345100005	count	1
NUDCD2	0.2504044	0.3447992	0.7262	0.468	0.345679751	count	1
SNRPD3	0.2428982	0.1879098	1.2926	0.1971	0.346357168	count	1
TENT5C	0.243077	0.1922926	1.2641	0.2071	0.346472242	count	1
ZC3HAV1	0.2538819	0.2911365	0.872	0.384	0.34690031	count	1
PPP4R3A	0.2572434	0.3616822	0.7112	0.477	0.346918119	count	1
BMS1	0.2739966	0.5025846	0.5452	0.586	0.346998251	count	1
CLCN7	0.4048782	1.2707481	0.3186	0.75	0.347129676	count	1
ZNF461	0.4048782	1.2707481	0.3186	0.75	0.347129676	count	1
SAT2	0.2467661	0.3398422	0.7261	0.468	0.347184684	count	1
IFIT5	0.2744402	0.8904463	0.3082	0.758	0.347562597	count	1
MRPL13	0.251437	0.4032061	0.6236	0.533	0.348432632	count	1
EMILIN1	0.5649324	1.0434692	0.5414	0.589	0.348511418	count	1
KLHDC8B	0.5649324	1.0434692	0.5414	0.589	0.348511418	count	1
PCDH7	0.5649324	1.0434692	0.5414	0.589	0.348511418	count	1
BHMT2	0.5649324	1.0434692	0.5414	0.589	0.348511418	count	1
VARS	0.5649324	1.0434692	0.5414	0.589	0.348511418	count	1
SLAIN1	0.5649324	1.0434692	0.5414	0.589	0.348511418	count	1
NAXD	0.5649324	1.0434692	0.5414	0.589	0.348511418	count	1
LARP6	0.5649324	1.0434692	0.5414	0.589	0.348511418	count	1
CASTOR3	0.5649324	1.1741598	0.4811	0.631	0.348511418	count	1
AC069185.1	0.5649324	1.1741598	0.4811	0.631	0.348511418	count	1
NPDC1	0.5649324	1.1741598	0.4811	0.631	0.348511418	count	1
RAD51AP1	0.5649324	1.1741598	0.4811	0.631	0.348511418	count	1
ZDHHC20	0.2584417	0.4532101	0.5702	0.569	0.348537905	count	1
HMG20B	0.2505029	0.334036	0.7499	0.454	0.348901442	count	1
ZMIZ2	0.4069713	0.8529053	0.4772	0.634	0.348958578	count	1
CCL4	0.2419852	0.1206813	2.0052	0.0458	0.349084888	count	1
GSK3B	0.2631291	0.5106947	0.5152	0.607	0.3491873	count	1
UFM1	0.248241	0.278791	0.8904	0.374	0.349416154	count	1
SLC2A1	0.2908403	0.8351976	0.3482	0.728	0.350151185	count	1
XKR6	0.2908403	0.8724181	0.3334	0.739	0.350151185	count	1
UQCRQ	0.2456184	0.2071983	1.1854	0.2367	0.350168041	count	1
CDKN2AIP	0.2518815	0.276024	0.9125	0.362	0.350505023	count	1
RFLNB	0.255797	0.4202858	0.6086	0.543	0.350896127	count	1
CCDC141	0.5686916	0.6332685	0.898	0.37	0.350898757	count	1
IRF9	0.2637523	0.4523003	0.5831	0.56	0.351674047	count	1
PRKD2	0.2616852	0.4422112	0.5918	0.554	0.351716155	count	1
RNF34	0.2600367	0.427049	0.6089	0.543	0.351785802	count	1
NDUFA6	0.2490858	0.2526677	0.9858	0.325	0.351791994	count	1

CWC25	0.2531588	0.3135254	0.8075	0.42	0.351947542	count	1
RHOA	0.2454439	0.1273845	1.9268	0.0549	0.352242193	count	1
SGK3	0.4109933	0.706363	0.5818	0.561	0.352473439	count	1
RASSF1	0.2504437	0.2595387	0.965	0.335	0.352669813	count	1
ADAR	0.2559437	0.313019	0.8177	0.414	0.352831715	count	1
PSEN1	0.2643329	0.4726211	0.5593	0.576	0.353939956	count	1
CIC	0.3581299	0.7749207	0.4622	0.644	0.354323539	count	1
KIAA0513	0.3581299	0.962755	0.372	0.71	0.354323539	count	1
CEP192	0.5742486	0.825477	0.6957	0.487	0.354428215	count	1
ZNF337	0.5742486	0.9184506	0.6252	0.5323	0.354428215	count	1
YTHDF2	0.2567726	0.3391364	0.7571	0.45	0.354484427	count	1
METRN	0.2575921	0.4097084	0.6287	0.53	0.354564356	count	1
CRCP	0.2673513	0.5365732	0.4983	0.619	0.354806787	count	1
C5orf15	0.2771716	0.5230226	0.5299	0.597	0.354976311	count	1
BCL2L1	0.5756162	0.5716436	1.0069	0.315	0.355296903	count	1
RECQL	0.2573649	0.3498322	0.7357	0.462	0.355303365	count	1
TYW3	0.2629722	0.4666233	0.5636	0.573	0.355765938	count	1
NSUN3	0.3598338	0.8478807	0.4244	0.672	0.356032624	count	1
LONRF3	0.3598338	0.8725138	0.4124	0.68	0.356032624	count	1
ZNF540	0.3598338	0.9599929	0.3748	0.708	0.356032624	count	1
GIMAP2	0.2624959	0.4612275	0.5691	0.57	0.356127796	count	1
ADRB1	0.296085	0.6255417	0.4733	0.636	0.356506315	count	1
EXOC4	0.2646377	0.4915246	0.5384	0.591	0.356913653	count	1
MGAT4B	0.2640417	0.4747904	0.5561	0.579	0.357216067	count	1
EML2	0.2668338	0.4831617	0.5523	0.581	0.357297379	count	1
TTTY14	0.3617406	0.6932273	0.5218	0.602	0.357945388	count	1
SCYL1	0.2865522	0.5275726	0.5432	0.587	0.358123114	count	1
PRPF40A	0.2542641	0.2701651	0.9411	0.347	0.358201796	count	1
SSU72	0.2524118	0.2223098	1.1354	0.257	0.358399208	count	1
SENP6	0.2556009	0.2979737	0.8578	0.392	0.358538934	count	1
ZNF644	0.2539639	0.2845061	0.8926	0.3727	0.359124388	count	1
C2orf42	0.30648	0.7278231	0.4211	0.674	0.359225417	count	1
BACH1	0.2694271	0.5022409	0.5364	0.592	0.359261529	count	1
SLC25A4	0.3631748	0.5544464	0.655	0.513	0.359384183	count	1
P2RY10	0.2632524	0.3513063	0.7494	0.454	0.359728482	count	1
MRE11	0.2679464	0.5658213	0.4736	0.636	0.360152453	count	1
LCMT1	0.2666492	0.3616475	0.7373	0.461	0.360751639	count	1
SIKE1	0.260673	0.3988782	0.6535	0.514	0.360818811	count	1
PRPF38B	0.2533513	0.1861198	1.3612	0.1744	0.360842391	count	1
ATP6V1H	0.2695847	0.4761596	0.5662	0.572	0.360990553	count	1
DENND4A	0.2818655	0.4774923	0.5903	0.555	0.361013267	count	1
RARS2	0.5851876	0.6383022	0.9168	0.36	0.361377347	count	1
TNRC6B	0.2568284	0.2661138	0.9651	0.335	0.361668218	count	1
CNOT4	0.2589704	0.3272777	0.7913	0.429	0.362130793	count	1
SF3B4	0.2596738	0.3158435	0.8222	0.412	0.362584866	count	1

CAP1	0.2547792	0.1838389	1.3859	0.167	0.363777908	count	1
PHC3	0.26994	0.4386823	0.6153	0.539	0.364081807	count	1
NFIL3	0.2584335	0.3395388	0.7611	0.4471	0.364501582	count	1
ZCCHC4	0.3400478	0.9045778	0.3759	0.707	0.36485173	count	1
LAMTOR2	0.2584149	0.3146052	0.8214	0.412	0.364977627	count	1
ITGB2	0.2551695	0.1525267	1.6729	0.0953	0.365266475	count	1
BLOC1S3	0.3406194	1.03684	0.3285	0.7427	0.365471663	count	1
WDR46	0.2741614	0.497064	0.5516	0.582	0.365591983	count	1
RELT	0.34125	0.9796159	0.3484	0.728	0.366155586	count	1
TUBA1A	0.255377	0.1626832	1.5698	0.1174	0.366376654	count	1
MRPL32	0.2668128	0.3859464	0.6913	0.49	0.366676992	count	1
DENND2D	0.2618745	0.2996115	0.874	0.383	0.366685974	count	1
UBAC2	0.2626473	0.3449648	0.7614	0.447	0.36701582	count	1
C19orf24	0.2595241	0.3140491	0.8264	0.4092	0.367096399	count	1
AGPS	0.2867115	0.5984692	0.4791	0.632	0.367246444	count	1
ZNF518A	0.2820436	0.6061009	0.4653	0.642	0.367584654	count	1
IMPDH2	0.2735362	0.4735324	0.5777	0.564	0.367684681	count	1
CHCHD10	0.2619262	0.2446227	1.0707	0.285	0.368533017	count	1
TMEM101	0.3149278	0.6936973	0.454	0.65	0.369202176	count	1
ARHGAP30	0.2682751	0.3286342	0.8163	0.415	0.369293604	count	1
MFSD12	0.3270909	0.6565811	0.4982	0.619	0.369751902	count	1
ATXN3	0.327131	0.6267417	0.522	0.602	0.369797635	count	1
TBC1D25	0.3154544	0.6707386	0.4703	0.638	0.369824172	count	1
MTMR2	0.2819574	0.698081	0.4039	0.687	0.370024463	count	1
DPP9	0.2890633	0.5691715	0.5079	0.612	0.370271661	count	1
PIK3IP1	0.2673171	0.2604708	1.0263	0.306	0.370470374	count	1
NUP62	0.2806524	0.5931161	0.4732	0.636	0.370537305	count	1
TAGLN2	0.2575973	0.1049662	2.4541	0.0146	0.370645099	count	1
THAP11	0.2719432	0.3644064	0.7463	0.456	0.370812131	count	1
KIAA2013	0.3753066	0.5228368	0.7178	0.473	0.37155842	count	1
CYB561D2	0.2645821	0.3305575	0.8004	0.424	0.371742984	count	1
ASCC2	0.2816479	0.4952619	0.5687	0.57	0.371855782	count	1
ADAM10	0.283357	0.4460654	0.6352	0.526	0.371867416	count	1
COMMD4	0.2664689	0.3298597	0.8078	0.42	0.3720839	count	1
GADD45A	0.2658506	0.296956	0.8953	0.371	0.37271606	count	1
ABCF1	0.263431	0.2605126	1.0112	0.313	0.372729081	count	1
FHL3	0.2672089	0.3742504	0.714	0.476	0.372826359	count	1
PDE4B	0.261849	0.1743188	1.5021	0.134	0.373037448	count	1
HSD17B12	0.2830163	0.499164	0.567	0.571	0.373668171	count	1
GCLM	0.2918776	0.4618373	0.632	0.528	0.373891995	count	1
TOR1AIP2	0.2712616	0.4859285	0.5582	0.577	0.373982695	count	1
ACYP2	0.2771918	0.5183666	0.5347	0.593	0.37504768	count	1
LMNA	0.2657961	0.2670149	0.9954	0.3203	0.37541047	count	1
RALBP1	0.2667689	0.241191	1.106	0.27	0.376534536	count	1
GABPB2	0.4386356	0.9352981	0.469	0.639	0.376648196	count	1

GTF2H2C	0.4386356	1.0237038	0.4285	0.669	0.376648196	count	1
PHKG1	0.4386356	1.052617	0.4167	0.677	0.376648196	count	1
HHEX	0.2941147	0.5322654	0.5526	0.581	0.376769954	count	1
SELENOS	0.2672992	0.2402807	1.1124	0.267	0.376875762	count	1
MRPS35	0.2725426	0.3483147	0.7825	0.435	0.377272876	count	1
EIF4ENIF1	0.6102879	0.7289768	0.8372	0.403	0.377327766	count	1
VPS13A	0.2745702	0.4106698	0.6686	0.504	0.377356385	count	1
PHC2	0.2946427	0.5831673	0.5052	0.614	0.377449228	count	1
CDK5RAP1	0.3812201	0.8156112	0.4674	0.641	0.377494849	count	1
CDYL	0.3072171	0.4193804	0.7325	0.464	0.377792631	count	1
TIGIT	0.2651898	0.2519359	1.0526	0.2933	0.377798965	count	1
METTL23	0.2691965	0.2784895	0.9666	0.334	0.378598306	count	1
ZFP91	0.3078845	0.7700152	0.3998	0.69	0.378618099	count	1
TNFAIP8	0.266034	0.2210109	1.2037	0.23	0.378630521	count	1
CTSF	0.2871028	0.5548857	0.5174	0.605	0.379080792	count	1
CCDC138	0.3834854	0.7008088	0.5472	0.585	0.379769309	count	1
SELENOF	0.2688561	0.2145254	1.2533	0.211	0.380410375	count	1
APEH	0.2811851	0.4766359	0.5899	0.556	0.380463136	count	1
CHCHD1	0.2804124	0.4279559	0.6552	0.513	0.380489362	count	1
PRKAG2	0.2807149	0.3635709	0.7721	0.441	0.380900719	count	1
ALG13	0.2773979	0.3776147	0.7346	0.463	0.381249373	count	1
RNPEP	0.3171959	0.641954	0.4941	0.622	0.382096419	count	1
ZNF354B	0.3171959	0.7380645	0.4298	0.668	0.382096419	count	1
SBNO2	0.3171959	0.7611935	0.4167	0.677	0.382096419	count	1
MEPCE	0.3380172	0.6007212	0.5627	0.574	0.382215125	count	1
ACSL5	0.3380172	0.6842936	0.494	0.622	0.382215125	count	1
CD200R1	0.3859464	0.8393922	0.4598	0.646	0.38224048	count	1
AASDH	0.2956981	0.4876763	0.6063	0.545	0.382374636	count	1
ERICH1	0.2708139	0.2803796	0.9659	0.335	0.382502969	count	1
MMAA	0.6191356	1.004321	0.6165	0.538	0.382951332	count	1
ZNF212	0.6191356	1.033409	0.5991	0.55	0.382951332	count	1
CEP250	0.2886381	0.8249059	0.3499	0.7266	0.383143467	count	1
ARHGAP15	0.2789436	0.3133696	0.8901	0.374	0.383377429	count	1
CD3E	0.2692946	0.2521559	1.068	0.2863	0.38393679	count	1
QTRT2	0.3033833	0.8710761	0.3483	0.7279	0.384395668	count	1
DSTN	0.2740894	0.2592176	1.0574	0.291	0.384712316	count	1
ANKRD37	0.2815373	0.4711564	0.5975	0.551	0.384763814	count	1
LTN1	0.2788536	0.4279195	0.6516	0.515	0.385017139	count	1
SENCR	0.3889579	1.0254704	0.3793	0.705	0.385264762	count	1
GRK6	0.2732104	0.2907641	0.9396	0.348	0.385360345	count	1
STARD3	0.2816232	0.5397634	0.5218	0.602	0.385663013	count	1
AC004812.2	0.3594374	0.9414345	0.3818	0.703	0.385888024	count	1
GAB3	0.2987472	0.6430367	0.4646	0.643	0.386333149	count	1
SFMBT2	0.287736	0.4783472	0.6015	0.548	0.386821137	count	1
STRN	0.314566	0.5234234	0.601	0.548	0.386882755	count	1

PRIM2	0.3019781	0.6282724	0.4806	0.631	0.386886928	count	1
ITPKB	0.3098431	0.6393835	0.4846	0.628	0.387387056	count	1
TBC1D10A	0.2895251	0.4303567	0.6728	0.502	0.387765122	count	1
PDLIM7	0.3105108	0.70327	0.4415	0.659	0.388226211	count	1
TTC37	0.290079	0.4493496	0.6456	0.519	0.388508955	count	1
TOR1A	0.2914883	0.359818	0.8101	0.418	0.388764011	count	1
MKKS	0.4525728	0.8904145	0.5083	0.612	0.388847719	count	1
ACBD3	0.2824593	0.3248464	0.8695	0.385	0.388852173	count	1
CCSAP	0.3037843	0.6163617	0.4929	0.622	0.389210968	count	1
CLHC1	0.393196	0.9647533	0.4076	0.684	0.389521424	count	1
SAMD8	0.393196	1.0375051	0.379	0.705	0.389521424	count	1
CLSTN3	0.2969633	0.5081591	0.5844	0.559	0.389785852	count	1
C16orf58	0.344734	0.8101147	0.4255	0.671	0.389878877	count	1
SETD1B	0.344734	0.8499626	0.4056	0.685	0.389878877	count	1
IFRD2	0.2991878	0.5523976	0.5416	0.588	0.390012249	count	1
PLOD3	0.4542844	0.7589573	0.5986	0.55	0.390346355	count	1
RRAGB	0.4542844	0.7854528	0.5784	0.563	0.390346355	count	1
POC1B-AS1	0.3641291	0.8686995	0.4192	0.675	0.390980344	count	1
ZNF568	0.3641291	0.8763564	0.4155	0.678	0.390980344	count	1
NFKBIA	0.2717889	0.188262	1.4437	0.15	0.3917279	count	1
GPR108	0.285262	0.3466454	0.8229	0.411	0.392076617	count	1
SPATA13	0.2908571	0.498487	0.5835	0.56	0.392363833	count	1
CMTM3	0.2776582	0.2723698	1.0194	0.3088	0.393276679	count	1
PCGF1	0.3663383	0.8046812	0.4553	0.649	0.39337847	count	1
B3GALT4	0.2979393	0.5338966	0.558	0.577	0.393435398	count	1
CCRL2	0.3104992	0.6887677	0.4508	0.652	0.393454715	count	1
EIF4G1	0.291921	0.4046507	0.7214	0.471	0.39380251	count	1
GMEB1	0.3046031	0.5432845	0.5607	0.575	0.393936172	count	1
ZYX	0.2800235	0.3465265	0.8081	0.42	0.394193103	count	1
UPRT	0.3980405	0.8028385	0.4958	0.62	0.394387951	count	1
SIRT2	0.2958599	0.3912792	0.7561	0.45	0.394611157	count	1
NDUFB8	0.2765614	0.17631	1.5686	0.1177	0.395146428	count	1
SPNS3	0.3118528	0.5965145	0.5228	0.601	0.395178085	count	1
RNASEK	0.2844841	0.370176	0.7685	0.443	0.395556774	count	1
ZSWIM7	0.2857906	0.3629194	0.7875	0.432	0.395639388	count	1
EIF2B4	0.3685621	0.5876276	0.6272	0.531	0.395792622	count	1
XBP1	0.2770954	0.1689488	1.6401	0.102	0.395827885	count	1
SEC24A	0.4605672	0.9974968	0.4617	0.645	0.395848211	count	1
TIMM50	0.2917933	0.4991403	0.5846	0.559	0.395966652	count	1
YWHAQ	0.2796197	0.2381864	1.174	0.241	0.396244272	count	1
KMT2D	0.4004373	0.8285534	0.4833	0.6292	0.396795959	count	1
TNKS2	0.2906584	0.3873816	0.7503	0.454	0.397253818	count	1
VCPIP1	0.3138329	0.4318488	0.7267	0.468	0.397699177	count	1
GSK3A	0.3052737	0.5142944	0.5936	0.553	0.397975131	count	1
DGKZ	0.282048	0.25258	1.1167	0.265	0.397979011	count	1

DNAJB9	0.2885751	0.3095041	0.9324	0.352	0.398461252	count	1
ZBTB38	0.2851261	0.306738	0.9295	0.353	0.398464068	count	1
ZNF274	0.3240301	0.5556302	0.5832	0.56	0.398591496	count	1
IKZF5	0.3057628	0.6346746	0.4818	0.63	0.398615108	count	1
LINC01934	0.3241203	0.5434215	0.5964	0.551	0.398703102	count	1
ZNF593	0.3020663	0.5067308	0.5961	0.552	0.398902805	count	1
PDIA4	0.2990751	0.4342306	0.6887	0.491	0.398911781	count	1
SIAH2	0.2899854	0.2876067	1.0083	0.314	0.399230783	count	1
NUP54	0.3062421	0.4672373	0.6554	0.513	0.399242269	count	1
MCEE	0.3319253	0.7473437	0.4441	0.657	0.399959338	count	1
GTF3C2	0.3319253	0.7873642	0.4216	0.674	0.399959338	count	1
MAP2K2	0.2801372	0.1817154	1.5416	0.124	0.400117542	count	1
COMMD5	0.3004903	0.5084136	0.591	0.555	0.400804794	count	1
COPS9	0.2819014	0.2227831	1.2654	0.2066	0.401116938	count	1
SLC30A5	0.4666474	0.7914108	0.5896	0.556	0.401173747	count	1
CCDC71	0.3133391	0.724945	0.4322	0.666	0.401506389	count	1
NMRAL1	0.3026223	0.5098056	0.5936	0.553	0.401763511	count	1
ITPRIP	0.3213291	0.6858922	0.4685	0.64	0.401824069	count	1
SHISA5	0.2855904	0.3097403	0.922	0.357	0.40203669	count	1
MPP6	0.6511287	0.841395	0.7739	0.44	0.403287313	count	1
APOLD1	0.3118639	0.5628138	0.5541	0.58	0.40336426	count	1
DPP7	0.2864671	0.2457411	1.1657	0.245	0.404219528	count	1
DAD1	0.2827504	0.1690426	1.6727	0.0954	0.404222871	count	1
PLEKHF1	0.2901699	0.3455857	0.8396	0.402	0.404223327	count	1
BIRC2	0.2903754	0.3069975	0.9459	0.345	0.404858115	count	1
PDCL	0.2948859	0.4525495	0.6516	0.515	0.406608152	count	1
ANXA2R	0.2875548	0.320127	0.8983	0.3697	0.406660227	count	1
SPPL2B	0.4729587	0.5729481	0.8255	0.41	0.406702782	count	1
SMYD2	0.3039816	0.4782448	0.6356	0.525	0.407180317	count	1
INPP4A	0.3215639	0.5302606	0.6064	0.545	0.407543266	count	1
SAR1B	0.3004297	0.4197283	0.7158	0.475	0.407712741	count	1
CXorf38	0.3004297	0.4513872	0.6656	0.506	0.407712741	count	1
SLC52A2	0.2966774	0.3792344	0.7823	0.435	0.407794453	count	1
UCKL1	0.3128276	0.6002095	0.5212	0.603	0.407859778	count	1
COPS7B	0.3483503	0.7229176	0.4819	0.63	0.408697578	count	1
CAND1	0.2938522	0.3294838	0.8919	0.373	0.408989482	count	1
C1orf52	0.2931126	0.3290691	0.8907	0.374	0.409013972	count	1
RAB3GAP2	0.3325305	0.5729297	0.5804	0.562	0.409110021	count	1
ST3GAL4	0.3327878	0.7021472	0.474	0.636	0.409428437	count	1
APTX	0.3045224	0.5778907	0.527	0.599	0.409447427	count	1
SNX1	0.3049201	0.4793392	0.6361	0.525	0.409983535	count	1
1-Sep	0.2870403	0.1864511	1.5395	0.1247	0.410178302	count	1
SDHC	0.2951731	0.3082971	0.9574	0.339	0.410439324	count	1
GPI	0.2914119	0.3251411	0.8963	0.371	0.410902341	count	1
RNF166	0.2914417	0.2586592	1.1267	0.2607	0.411096915	count	1

RPUSD4	0.334875	0.7424675	0.451	0.652	0.412011467	count	1
AC007686.3	0.334875	0.7694054	0.4352	0.664	0.412011467	count	1
ST3GAL2	0.6648664	0.6833256	0.973	0.3313	0.41201865	count	1
TAPT1	0.4159296	0.9400222	0.4425	0.658	0.412365435	count	1
HNRNPD	0.2921759	0.2150997	1.3583	0.175	0.412564015	count	1
TMEM173	0.299755	0.3366245	0.8905	0.374	0.412704078	count	1
WASHC4	0.322275	0.5436188	0.5928	0.554	0.413007184	count	1
NAA35	0.3264469	0.5385672	0.6061	0.545	0.413761618	count	1
TMEM208	0.2955533	0.3124001	0.9461	0.345	0.415301569	count	1
XPA	0.2986507	0.3284274	0.9093	0.364	0.415676928	count	1
PPP4C	0.2932585	0.2634035	1.1133	0.2664	0.415678814	count	1
ILF3-DT	0.3024149	0.4042052	0.7482	0.455	0.415694911	count	1
NELFCD	0.3091837	0.4361025	0.709	0.479	0.415731082	count	1
SAR1A	0.2919378	0.1903929	1.5333	0.1262	0.41606091	count	1
CYP20A1	0.3014358	0.3795893	0.7941	0.428	0.416248049	count	1
NPRL3	0.3548263	0.6931628	0.5119	0.609	0.416354269	count	1
STX6	0.3548263	0.7007415	0.5064	0.613	0.416354269	count	1
AL139274.2	0.6719053	0.7757842	0.8661	0.387	0.416491851	count	1
SPG11	0.3220498	0.4974064	0.6475	0.518	0.416592356	count	1
SRF	0.3685133	0.7158454	0.5148	0.607	0.41702274	count	1
MIS18A	0.3686085	0.832611	0.4427	0.658	0.417131445	count	1
UMAD1	0.3686085	0.8635522	0.4269	0.67	0.417131445	count	1
PARVB	0.3686085	0.896301	0.4113	0.681	0.417131445	count	1
WDR82	0.3026342	0.4486836	0.6745	0.5	0.41730986	count	1
AFTPH	0.3078079	0.4945538	0.6224	0.534	0.41774833	count	1
TRPM7	0.3096812	0.4046919	0.7652	0.445	0.417821337	count	1
PUM2	0.3406194	0.5813405	0.5859	0.558	0.419121049	count	1
GORASP2	0.3061263	0.3852029	0.7947	0.427	0.419284204	count	1
DOK1	0.3708961	0.5937487	0.6247	0.533	0.41974365	count	1
PLPP1	0.3711432	0.894992	0.4147	0.679	0.420025824	count	1
CCDC130	0.3185588	0.5169853	0.6162	0.538	0.420754827	count	1
PGGT1B	0.322873	0.524744	0.6153	0.539	0.421006326	count	1
CHD1	0.3010851	0.2742566	1.0978	0.273	0.421378645	count	1
BOLA3	0.3149603	0.5332734	0.5906	0.555	0.421926872	count	1
C18orf32	0.3926663	0.7606169	0.5162	0.606	0.421970665	count	1
C7orf26	0.4256851	0.7006815	0.6075	0.544	0.422173433	count	1
CSDE1	0.2998259	0.2368626	1.2658	0.206	0.42227399	count	1
RREB1	0.4259869	0.6993217	0.6091	0.543	0.422476902	count	1
SLC4A4	0.3240289	0.5116342	0.6333	0.527	0.422519184	count	1
PLAGL2	0.3740078	0.8532271	0.4383	0.661	0.423297142	count	1
TRIP12	0.3111949	0.4800224	0.6483	0.517	0.423445929	count	1
EAF2	0.3521063	0.8791862	0.4005	0.689	0.424443552	count	1
AC004865.2	0.3062658	0.5854895	0.5231	0.601	0.424531983	count	1
ZNF677	0.345328	0.6705935	0.515	0.607	0.424949267	count	1
GNL2	0.3161051	0.5188324	0.6093	0.543	0.425061999	count	1

ABTB1	0.3031475	0.3125338	0.97	0.333	0.425062698	count	1
EMD	0.304392	0.2819351	1.0797	0.281	0.425722651	count	1
LUC7L2	0.3408054	0.4699208	0.7252	0.469	0.426311573	count	1
POLR2L	0.2988046	0.2010775	1.486	0.138	0.426493677	count	1
LMO7	0.3297375	0.7026068	0.4693	0.639	0.426577435	count	1
SCX	0.3297375	0.7211626	0.4572	0.648	0.426577435	count	1
RGS16	0.3214498	0.5031072	0.6389	0.523	0.426837496	count	1
ARMC7	0.3371468	0.6742853	0.5	0.617	0.427389401	count	1
DGKA	0.3419198	0.5496968	0.622	0.534	0.427712968	count	1
MTMR9	0.3419198	0.5524206	0.6189	0.536	0.427712968	count	1
PCBP2	0.3012474	0.2021649	1.4901	0.1372	0.428079076	count	1
SAMHD1	0.3232019	0.3900604	0.8286	0.408	0.429171177	count	1
ESF1	0.3084207	0.3633019	0.8489	0.397	0.429293453	count	1
IFT80	0.3994443	0.8227441	0.4855	0.628	0.429335062	count	1
ZBTB11	0.3207451	0.3833199	0.8368	0.403	0.42969767	count	1
DCAF13	0.3085203	0.3670434	0.8406	0.401	0.429820131	count	1
TIMM22	0.3222948	0.4603524	0.7001	0.484	0.42997497	count	1
CHD3	0.3187086	0.3792859	0.8403	0.401	0.430031545	count	1
CIR1	0.3026307	0.2266596	1.3352	0.1828	0.430116083	count	1
GALT	0.3163866	0.4623063	0.6844	0.494	0.430525669	count	1
CASP3	0.3047682	0.251023	1.2141	0.226	0.430635901	count	1
RBM34	0.3501419	0.6574359	0.5326	0.595	0.430908356	count	1
NBR1	0.3402699	0.490125	0.6943	0.488	0.431367541	count	1
TMED2	0.3097231	0.3308104	0.9363	0.35	0.431497989	count	1
PITPNC1	0.3081829	0.2838231	1.0858	0.278	0.431872671	count	1
SPTBN1	0.3407405	0.5991047	0.5687	0.5699	0.431966999	count	1
DUSP10	0.318265	0.3248178	0.9798	0.328	0.431972849	count	1
MED25	0.358528	0.6974942	0.514	0.608	0.432236766	count	1
CMC2	0.3049979	0.2579828	1.1822	0.238	0.432518685	count	1
TSFM	0.3229914	0.4532332	0.7126	0.477	0.432715276	count	1
COX14	0.3052692	0.2362651	1.2921	0.197	0.43290366	count	1
KMT2A	0.3065866	0.2310508	1.3269	0.185	0.43292885	count	1
OXNAD1	0.3070462	0.2849206	1.0777	0.282	0.433432459	count	1
ETS2	0.3178959	0.5026741	0.6324	0.528	0.43360939	count	1
ISYNA1	0.3265513	0.5588543	0.5843	0.559	0.433632473	count	1
SOD1	0.3034654	0.1705752	1.7791	0.0762	0.433658031	count	1
NAXE	0.3136226	0.3316992	0.9455	0.345	0.433683819	count	1
COQ6	0.3424393	0.916562	0.3736	0.709	0.434130996	count	1
ZNF740	0.7001958	0.9107743	0.7688	0.443	0.434464784	count	1
SLIRP	0.331116	0.4533131	0.7304	0.466	0.434777417	count	1
SGK1	0.331116	0.5394701	0.6138	0.54	0.434777417	count	1
FANCB	0.3476637	0.8152883	0.4264	0.67	0.434936511	count	1
RALGAPB	0.4048782	1.0560385	0.3834	0.702	0.435240008	count	1
PCOLCE	0.4048782	1.2259368	0.3303	0.7414	0.435240008	count	1
PEBP1	0.3055012	0.1912583	1.5973	0.1112	0.435749254	count	1

BCL2A1	0.3082901	0.3498217	0.8813	0.3788	0.436006189	count	1
GRWD1	0.3719501	0.6339148	0.5868	0.558	0.436605537	count	1
PHF3	0.3096812	0.2583707	1.1986	0.232	0.436684996	count	1
ATM	0.3176885	0.329305	0.9647	0.335	0.436727989	count	1
RNPS1	0.3090231	0.2230715	1.3853	0.167	0.437043611	count	1
ATP5S	0.3178819	0.4049906	0.7849	0.433	0.437705359	count	1
MVK	0.7057114	0.7584163	0.9305	0.353	0.43796745	count	1
PSMC2	0.3134518	0.3113515	1.0067	0.315	0.438097377	count	1
C6orf106	0.5095583	0.7472415	0.6819	0.496	0.438784337	count	1
APOO	0.5095583	0.844268	0.6036	0.547	0.438784337	count	1
TBL1X	0.3576256	0.8029487	0.4454	0.656	0.4401734	count	1
MAN2A1	0.3751799	0.8481807	0.4423	0.659	0.440426064	count	1
ALDH9A1	0.3221885	0.3716255	0.867	0.387	0.440436849	count	1
IER2	0.3060206	0.1363288	2.2447	0.0255	0.440614907	count	1
DPH3	0.321469	0.3832429	0.8388	0.402	0.441934397	count	1
PAN3	0.3218209	0.3710756	0.8673	0.386	0.443138557	count	1
CCNL1	0.3121101	0.1754748	1.7787	0.0762	0.443297243	count	1
CCDC90B	0.3163767	0.3382247	0.9354	0.35	0.443632084	count	1
MIF	0.3123256	0.2183539	1.4304	0.1536	0.443973589	count	1
FAM3C	0.3196052	0.3009998	1.0618	0.289	0.444460005	count	1
GINM1	0.3274565	0.4791484	0.6834	0.495	0.444476813	count	1
NSMF	0.4482316	0.9145422	0.4901	0.624	0.444851263	count	1
TAMM41	0.4482316	0.9410505	0.4763	0.634	0.444851263	count	1
TMEM97	0.4482316	1.1990452	0.3738	0.7088	0.444851263	count	1
CRY2	0.4482316	1.2044457	0.3721	0.71	0.444851263	count	1
GCC1	0.3794361	0.9640715	0.3936	0.694	0.445461105	count	1
NEK3	0.3794361	1.054902	0.3597	0.719	0.445461105	count	1
SDF2	0.3180154	0.3506968	0.9068	0.365	0.446187653	count	1
BCKDHB	0.3626239	0.7702889	0.4708	0.638	0.446362116	count	1
SNX2	0.3223792	0.294838	1.0934	0.275	0.446372482	count	1
ATXN7L3B	0.3179939	0.2975648	1.0687	0.286	0.44640282	count	1
FAM227B	0.5182716	0.8044368	0.6443	0.52	0.446425688	count	1
NET1	0.5182716	0.9668224	0.5361	0.592	0.446425688	count	1
CPSF1	0.5182716	1.112074	0.466	0.641	0.446425688	count	1
ZFP82	0.5182716	1.3035372	0.3976	0.6912	0.446425688	count	1
TRIM65	0.3525798	0.759891	0.464	0.643	0.447049477	count	1
NTAN1	0.3314279	0.5613168	0.5904	0.555	0.447236983	count	1
PIGH	0.5200243	0.8264836	0.6292	0.53	0.447962896	count	1
TRGV10	0.5200243	0.9284741	0.5601	0.5758	0.447962896	count	1
SLC35E2B	0.5200243	0.9329862	0.5574	0.578	0.447962896	count	1
WDR27	0.5200243	0.9545734	0.5448	0.586	0.447962896	count	1
PARD6B	0.5200243	1.0005925	0.5197	0.6036	0.447962896	count	1
ANAPC4	0.3411618	0.5941884	0.5742	0.566	0.448015053	count	1
UQCC2	0.3346065	0.4700868	0.7118	0.477	0.448319643	count	1
PAAF1	0.3398555	0.4884234	0.6958	0.487	0.448978584	count	1

HPF1	0.3292454	0.5008552	0.6574	0.511	0.449122805	count	1
ACO2	0.344432	0.4662538	0.7387	0.461	0.449226718	count	1
BLOC1S4	0.3223051	0.3584597	0.8991	0.369	0.449434975	count	1
6-Mar	0.3316788	0.4305615	0.7703	0.442	0.450221062	count	1
GMFB	0.3454465	0.5609618	0.6158	0.538	0.450554865	count	1
GOLGA8B	0.3323415	0.5077918	0.6545	0.513	0.451122651	count	1
EBP	0.3236674	0.3834744	0.844	0.399	0.452392281	count	1
DICER1	0.3856432	0.6594693	0.5848	0.559	0.452804777	count	1
PHKA2	0.4210596	1.0596245	0.3974	0.6914	0.452828649	count	1
DMAC1	0.3231885	0.3121975	1.0352	0.301	0.452923916	count	1
ATP2B1	0.3288347	0.2796483	1.1759	0.241	0.453501098	count	1
TXNDC12	0.3234469	0.2847504	1.1359	0.257	0.454065611	count	1
HEXIM1	0.3460879	0.4426676	0.7818	0.435	0.454506851	count	1
NFYC	0.3772263	0.6172887	0.6111	0.542	0.454933965	count	1
SLAMF6	0.3772263	0.6720037	0.5613	0.575	0.454933965	count	1
ZNF43	0.3772263	0.7630578	0.4944	0.621	0.454933965	count	1
RAP1B	0.318461	0.1574085	2.0232	0.0439	0.45515334	count	1
TSPYL1	0.3224321	0.2862433	1.1264	0.261	0.455322335	count	1
SDF4	0.3297273	0.3288905	1.0025	0.317	0.455381232	count	1
SLC2A13	0.4596214	0.8165925	0.5629	0.574	0.456312015	count	1
MEAF6	0.3213811	0.2189984	1.4675	0.1432	0.456472786	count	1
CXorf56	0.4598506	1.0627402	0.4327	0.666	0.456542657	count	1
TUBD1	0.3788268	0.8083171	0.4687	0.64	0.456877097	count	1
BANF1	0.3207757	0.2081236	1.5413	0.1242	0.457179134	count	1
MID1IP1	0.3369783	0.377911	0.8917	0.373	0.457431044	count	1
RTL8A	0.3656228	0.6480642	0.5642	0.573	0.457526208	count	1
SIRT6	0.3656228	0.650303	0.5622	0.574	0.457526208	count	1
ZNF622	0.3374573	0.4061093	0.831	0.407	0.458082738	count	1
PDCD7	0.3255455	0.288433	1.1287	0.26	0.458136072	count	1
TMEM263	0.3309605	0.3754183	0.8816	0.379	0.458272678	count	1
APPL2	0.4261971	0.9212647	0.4626	0.644	0.45841424	count	1
CCDC97	0.3578871	0.5894143	0.6072	0.544	0.458856495	count	1
JOSD1	0.3329079	0.3908565	0.8517	0.395	0.459127985	count	1
DGLUCY	0.3910082	0.6153809	0.6354	0.526	0.459152836	count	1
BRD4	0.3299116	0.2953581	1.117	0.265	0.459247797	count	1
YTHDF3-AS1	0.533538	1.095022	0.4872	0.626	0.459816314	count	1
ETV7	0.533538	1.10512	0.4828	0.63	0.459816314	count	1
DONSON	0.533538	1.084308	0.4921	0.623	0.459816332	count	1
ALDOC	0.3674946	0.6305231	0.5828	0.56	0.459880977	count	1
ZNF680	0.3674946	0.6671739	0.5508	0.582	0.459880977	count	1
SAP30	0.3329876	0.3861686	0.8623	0.389	0.459891328	count	1
DUSP14	0.3676756	0.6003722	0.6124	0.541	0.460108688	count	1
FRMD4B	0.3375709	0.6062942	0.5568	0.5781	0.460503557	count	1
MTIF3	0.3257409	0.3232049	1.0078	0.3143	0.46057189	count	1
DRG2	0.3376807	0.3997295	0.8448	0.399	0.460653654	count	1

ZBTB24	0.3343732	0.4508241	0.7417	0.459	0.461152241	count	1
FLYWCH2	0.3442058	0.4837029	0.7116	0.477	0.461217101	count	1
UBE2A	0.330597	0.2839194	1.1644	0.245	0.461388268	count	1
HIVEP3	0.368714	0.6543463	0.5635	0.573	0.461415052	count	1
CCDC117	0.3688627	0.6597761	0.5591	0.576	0.461602128	count	1
SMPD3	0.354303	0.5649239	0.6272	0.531	0.46215012	count	1
ASH1L	0.3305778	0.3311673	0.9982	0.319	0.462703091	count	1
RBMS1	0.3280244	0.2639088	1.2429	0.215	0.46290993	count	1
STK38	0.3699419	0.4797502	0.7711	0.441	0.462959846	count	1
SLC40A1	0.5374225	1.0405359	0.5165	0.606	0.463223917	count	1
FOXRED1	0.5374225	1.140076	0.4714	0.638	0.463223917	count	1
IFI30	0.5374225	1.1734248	0.458	0.647	0.463223917	count	1
PADI4	0.5374225	1.226543	0.4382	0.662	0.463223917	count	1
LRRIQ3	0.5374225	1.226543	0.4382	0.662	0.463223917	count	1
AL353708.1	0.5374225	1.226543	0.4382	0.662	0.463223917	count	1
AC018797.2	0.5374225	1.226543	0.4382	0.662	0.463223917	count	1
COQ3	0.5374225	1.226543	0.4382	0.662	0.463223917	count	1
TRAF3IP2	0.5374225	1.226543	0.4382	0.662	0.463223917	count	1
SPRYD3	0.5374225	1.226543	0.4382	0.662	0.463223917	count	1
KNSTRN	0.5374225	1.226543	0.4382	0.662	0.463223917	count	1
RFWD3	0.5374225	1.226543	0.4382	0.662	0.463223917	count	1
FAM171A2	0.5374225	1.226543	0.4382	0.662	0.463223917	count	1
PEX26	0.5374225	1.226543	0.4382	0.662	0.463223917	count	1
PHETA2	0.5374225	1.226543	0.4382	0.662	0.463223917	count	1
ARHGEF35	0.5374225	1.3833575	0.3885	0.6979	0.463223917	count	1
RPH3AL	0.5374225	1.3833575	0.3885	0.6979	0.463223917	count	1
SF3A1	0.3437094	0.4659626	0.7376	0.461	0.463851917	count	1
EIF4A3	0.3302052	0.2482755	1.33	0.184	0.464043153	count	1
WDTC1	0.4099452	0.6851413	0.5983	0.55	0.464355075	count	1
GTF3C3	0.3851332	0.8592431	0.4482	0.654	0.464534092	count	1
HDAC1	0.3322643	0.3002211	1.1067	0.269	0.464751531	count	1
NRM	0.4682343	0.9290152	0.504	0.615	0.464980294	count	1
UBL3	0.3395208	0.4428847	0.7666	0.444	0.465115235	count	1
GLT8D1	0.3786346	0.4917407	0.77	0.442	0.466189332	count	1
STX8	0.3383378	0.3218655	1.0512	0.294	0.466629265	count	1
UNC93B1	0.3389486	0.4471997	0.7579	0.449	0.466764843	count	1
LINC00526	0.4121035	0.9225921	0.4467	0.655	0.466821843	count	1
LYRM9	0.4121035	0.9627246	0.4281	0.669	0.466821843	count	1
TESPA1	0.3979939	0.5612304	0.7091	0.479	0.467419473	count	1
SMPD1	0.3798162	0.6645814	0.5715	0.568	0.467652783	count	1
MRPL3	0.3401743	0.3965867	0.8578	0.392	0.468455683	count	1
ICAM2	0.3385494	0.3589043	0.9433	0.346	0.46935159	count	1
UMPS	0.3892043	0.5548575	0.7014	0.484	0.469477468	count	1
DBP	0.3518518	0.5667461	0.6208	0.535	0.469526788	count	1
ZBTB25	0.3555258	0.5463532	0.6507	0.516	0.469749894	count	1

SWSAP1	0.3815188	0.6474852	0.5892	0.556	0.469761547	count	1
TSPOAP1-AS1	0.5450243	0.6871776	0.7931	0.428	0.469892797	count	1
HIPK1	0.3493605	0.3182111	1.0979	0.273	0.469902637	count	1
SLC9A3R2	0.7565224	1.122504	0.674	0.501	0.470203293	count	1
PIP4K2B	0.7565224	1.122504	0.674	0.501	0.470203293	count	1
AC004585.1	0.7565224	1.122504	0.674	0.501	0.470203293	count	1
AL158071.1	0.7565224	1.221282	0.6194	0.536	0.470203293	count	1
RNF26	0.7565224	1.221282	0.6194	0.536	0.470203293	count	1
LINC00920	0.7565224	1.221282	0.6194	0.536	0.470203293	count	1
CENPS	0.7565224	1.417702	0.5336	0.594	0.470203293	count	1
C5orf34	0.7565224	1.417702	0.5336	0.594	0.470203293	count	1
JARID2-AS1	0.7565224	1.417702	0.5336	0.594	0.470203293	count	1
TRAM2-AS1	0.7565224	1.417702	0.5336	0.594	0.470203293	count	1
RARRES2	0.7565224	1.417702	0.5336	0.594	0.470203293	count	1
SUOX	0.7565224	1.417702	0.5336	0.594	0.470203293	count	1
AL356020.1	0.7565224	1.417702	0.5336	0.594	0.470203293	count	1
KNL1	0.7565224	1.417702	0.5336	0.594	0.470203293	count	1
FANCI	0.7565224	1.417702	0.5336	0.594	0.470203293	count	1
IFT140	0.7565224	1.417702	0.5336	0.594	0.470203293	count	1
SLC38A7	0.7565224	1.417702	0.5336	0.594	0.470203293	count	1
RARA-AS1	0.7565224	1.417702	0.5336	0.594	0.470203293	count	1
REEP6	0.7565224	1.417702	0.5336	0.594	0.470203293	count	1
ETV2	0.7565224	1.417702	0.5336	0.594	0.470203293	count	1
TCTN3	0.3711432	0.6094843	0.6089	0.543	0.470703034	count	1
MAP7D1	0.3413152	0.426765	0.7998	0.424	0.470742563	count	1
CENPH	0.4011721	0.750249	0.5347	0.593	0.471180761	count	1
AKAP7	0.3483503	0.4568252	0.7625	0.446	0.471579074	count	1
JUND	0.327125	0.0849226	3.852	0.000141	0.471645208	count	1
PGAP2	0.4386356	0.7169134	0.6118	0.541	0.471939943	count	1
C11orf54	0.4386356	0.8069753	0.5436	0.587	0.471939943	count	1
TBPL1	0.3360059	0.3144205	1.0687	0.286	0.472656163	count	1
SLC25A33	0.4172189	0.5435219	0.7676	0.443	0.472668736	count	1
GOPC	0.337341	0.2960541	1.1395	0.255	0.47384066	count	1
CRIPT	0.338605	0.3678918	0.9204	0.358	0.473951416	count	1
SUCLG2	0.3475318	0.3417537	1.0169	0.31	0.474120668	count	1
CD8A	0.3855465	0.6037782	0.6386	0.524	0.474750293	count	1
ZFP62	0.419138	0.7489349	0.5596	0.576	0.474862395	count	1
STT3A	0.419138	0.8507359	0.4927	0.623	0.474862395	count	1
CLSTN1	0.3641631	0.664514	0.548	0.584	0.475060606	count	1
NENF	0.342666	0.3868483	0.8858	0.376	0.475067211	count	1
ASNSD1	0.3491694	0.4669221	0.7478	0.455	0.475236757	count	1
USP8	0.3412122	0.3585504	0.9516	0.342	0.475427317	count	1
SERGEF	0.4048782	0.7231792	0.5599	0.576	0.475567044	count	1
LMBRD1	0.3565427	0.4325943	0.8242	0.41	0.475804885	count	1
SFT2D1	0.339553	0.2970088	1.1432	0.254	0.475882878	count	1

CD164	0.335537	0.222176	1.5102	0.132	0.47605237	count	1
IDH2	0.3448229	0.2878635	1.1979	0.232	0.476264173	count	1
SMC6	0.3871046	0.7586357	0.5103	0.61	0.476680243	count	1
HNRNPH1	0.3332077	0.1840368	1.8105	0.0711	0.477169574	count	1
BCAS2	0.336722	0.266632	1.2629	0.2075	0.477328979	count	1
CLEC2D	0.3596022	0.526248	0.6833	0.495	0.477663189	count	1
ATG10	0.381921	0.5917683	0.6454	0.519	0.478031786	count	1
TNFRSF10A	0.3962799	0.6971389	0.5684	0.57	0.478069818	count	1
TMEM30A	0.3535504	0.4031477	0.877	0.381	0.478635553	count	1
POM121C	0.373432	0.4466331	0.8361	0.404	0.478876664	count	1
SPSB3	0.3415305	0.2756029	1.2392	0.216	0.478942134	count	1
AC103591.3	0.4454234	0.675467	0.6594	0.51	0.479322303	count	1
ZNF506	0.3619586	0.5868534	0.6168	0.538	0.480802903	count	1
SIDT1	0.424343	0.6340589	0.6692	0.504	0.480812398	count	1
GGA2	0.424343	0.6847467	0.6197	0.536	0.480812398	count	1
DTX2	0.424343	0.7261684	0.5844	0.559	0.480812398	count	1
RP2	0.3842758	0.6944484	0.5534	0.58	0.480994834	count	1
EGLN1	0.3751101	0.6406269	0.5855	0.559	0.481038087	count	1
TP53TG1	0.354336	0.4866232	0.7282	0.467	0.481048094	count	1
INTS4	0.3987574	0.7115404	0.5604	0.576	0.481078609	count	1
KLRG1	0.3694342	0.5222716	0.7074	0.48	0.481962932	count	1
BLOC1S6	0.3524562	0.4175914	0.844	0.399	0.48287051	count	1
THYN1	0.3447376	0.3202024	1.0766	0.282	0.483156997	count	1
TRIM13	0.36127	0.4320135	0.8362	0.404	0.484146759	count	1
ATP1B3	0.3566257	0.3272736	1.0897	0.277	0.484163679	count	1
FKBPL	0.3686244	0.511984	0.72	0.472	0.48421042	count	1
FSD1	0.3631843	0.6179312	0.5877	0.557	0.48469411	count	1
CHIC2	0.3428601	0.2790582	1.2286	0.22	0.484935588	count	1
CGAS	0.3882656	0.7761102	0.5003	0.617	0.486015394	count	1
CASC3	0.3755724	0.636681	0.5899	0.556	0.486129837	count	1
RETREG3	0.5636077	0.7059873	0.7983	0.4253	0.486196829	count	1
C9orf64	0.5649324	0.9270064	0.6094	0.543	0.487359068	count	1
GRPEL2	0.5649324	1.0434692	0.5414	0.589	0.487359068	count	1
DEPP1	0.5649324	1.0434692	0.5414	0.589	0.487359068	count	1
HTRA1	0.5649324	1.0434692	0.5414	0.589	0.487359068	count	1
NCOA5	0.5649324	1.0434692	0.5414	0.589	0.487359068	count	1
MIAT	0.5649324	1.0434692	0.5414	0.589	0.487359068	count	1
ZNF419	0.5649324	1.1172337	0.5057	0.613	0.487359068	count	1
KLHL15	0.5649324	1.1741598	0.4811	0.631	0.487359068	count	1
FAM111A-DT	0.5649324	1.1741598	0.4811	0.631	0.487359068	count	1
TMTC4	0.5649324	1.1741598	0.4811	0.631	0.487359068	count	1
ZNF223	0.5649324	1.1741598	0.4811	0.631	0.487359068	count	1
C19orf73	0.5649324	1.1741598	0.4811	0.631	0.487359068	count	1
MAP3K6	0.3958957	0.8271126	0.4786	0.633	0.487570124	count	1
KIFC2	0.3958957	0.9787873	0.4045	0.686	0.487570124	count	1

ODF2	0.370045	0.6532698	0.5665	0.571	0.488998036	count	1
CKS2	0.3535738	0.303592	1.1646	0.245	0.489021373	count	1
ZNF429	0.4319202	0.7278057	0.5935	0.553	0.489474988	count	1
HSD17B7	0.3707064	0.5632917	0.6581	0.511	0.489874932	count	1
EXOG	0.3656228	0.5347015	0.6838	0.495	0.489996206	count	1
KRR1	0.3498814	0.3051509	1.1466	0.252	0.490070188	count	1
CAPZB	0.3423082	0.1574674	2.1738	0.0304	0.490224903	count	1
PGS1	0.3794361	0.7312904	0.5189	0.604	0.491151229	count	1
KCTD20	0.3794361	0.7926181	0.4787	0.632	0.491151229	count	1
CDIP1	0.3628703	0.5246401	0.6917	0.49	0.491283152	count	1
ABCD4	0.367177	0.5774896	0.6358	0.525	0.49208484	count	1
CD53	0.343024	0.1299402	2.6399	0.0087	0.492423396	count	1
TRERF1	0.791917	0.8202961	0.9654	0.3351	0.492613035	count	1
DYNLL1	0.3438905	0.1557944	2.2073	0.028	0.492674913	count	1
SFPQ	0.3449948	0.1475441	2.3382	0.02	0.493120952	count	1
CYB5R3	0.3547614	0.3263212	1.0872	0.278	0.4934204	count	1
LRRCC1	0.3891479	0.7778006	0.5003	0.617	0.4936498	count	1
ATF5	0.4204599	0.6851569	0.6137	0.54	0.494011099	count	1
MRPL9	0.3599295	0.3728934	0.9652	0.335	0.494049663	count	1
RIOX2	0.4096501	0.8252349	0.4964	0.62	0.494308391	count	1
FNIP2	0.4096501	0.8533659	0.48	0.632	0.494308391	count	1
SYTL1	0.3518308	0.2819384	1.2479	0.213	0.494938355	count	1
PEX3	0.3728643	0.4685713	0.7957	0.427	0.495334643	count	1
BNIP3	0.4602012	0.6071432	0.758	0.449	0.495397163	count	1
DPP4	0.5742868	0.6557809	0.8757	0.382	0.495566337	count	1
MROH1	0.5742868	0.8555858	0.6712	0.503	0.495566337	count	1
ASNA1	0.366323	0.3408432	1.0748	0.283	0.495968829	count	1
FAM104B	0.4380526	0.940206	0.4659	0.642	0.496486485	count	1
GTF3A	0.3469213	0.1755126	1.9766	0.0489	0.496583661	count	1
TIMM17A	0.3567866	0.3550193	1.005	0.316	0.496710467	count	1
ARF4	0.3508078	0.257018	1.3649	0.173	0.496843206	count	1
PBXIP1	0.3638179	0.3213238	1.1322	0.258	0.497465975	count	1
C21orf91	0.3764939	0.4278506	0.88	0.38	0.497548126	count	1
TAGAP	0.3468792	0.14384	2.4116	0.0164	0.497958708	count	1
SCFD2	0.3978881	0.7562541	0.5261	0.599	0.498124791	count	1
SOCS1	0.3468194	0.1608557	2.1561	0.0318	0.498312886	count	1
BATF	0.3796782	0.4821349	0.7875	0.432	0.498781762	count	1
QSER1	0.4244937	0.7581189	0.5599	0.576	0.498786538	count	1
MTX3	0.8032806	0.9562247	0.8401	0.4015	0.499797676	count	1
ADPGK	0.3830539	0.4155083	0.9219	0.357	0.499798996	count	1
ANKRD13D	0.3551286	0.3477595	1.0212	0.308	0.499808201	count	1
C12orf29	0.3676453	0.4279126	0.8592	0.391	0.500439428	count	1
COPS4	0.3959629	0.4287725	0.9235	0.356	0.502336582	count	1
GTPBP10	0.4079214	0.6567034	0.6212	0.535	0.502468699	count	1
CCDC14	0.5062907	0.6714177	0.7541	0.451	0.503294812	count	1

RIF1	0.3742496	0.4280174	0.8744	0.383	0.503469903	count	1
SLC25A28	0.3790617	0.4809671	0.7881	0.431	0.503593141	count	1
PLP2	0.3510701	0.1470712	2.3871	0.0176	0.503638352	count	1
ELMSAN1	0.3773914	0.4959301	0.761	0.447	0.503710571	count	1
RAP1GDS1	0.3633148	0.3122598	1.1635	0.245	0.504830056	count	1
YPEL1	0.3640664	0.3239329	1.1239	0.262	0.505343872	count	1
TMUB1	0.3622412	0.3391541	1.0681	0.286	0.505608684	count	1
INTS14	0.5858147	0.8357128	0.701	0.484	0.505680309	count	1
SLC17A5	0.5858147	0.9668089	0.6059	0.545	0.505680309	count	1
COQ5	0.3944881	0.6446348	0.612	0.541	0.505999914	count	1
MDH2	0.3587168	0.223421	1.6056	0.109	0.506079983	count	1
TNFRSF10B	0.43101	0.6654462	0.6477	0.518	0.506501399	count	1
GLO1	0.370901	0.4638397	0.7996	0.425	0.507170596	count	1
PPP1R10	0.3572543	0.2314076	1.5438	0.1236	0.507180216	count	1
LIMA1	0.3817806	0.5256757	0.7263	0.468	0.507216386	count	1
SEPSECS-AS1	0.4714636	1.0858536	0.4342	0.664	0.507650145	count	1
C4orf46	0.4714636	1.0858536	0.4342	0.664	0.507650145	count	1
RBM6	0.3751104	0.3626598	1.0343	0.302	0.507894679	count	1
SNHG12	0.3694293	0.3425936	1.0783	0.282	0.50799372	count	1
TIMM8B	0.3656228	0.3610691	1.0126	0.312	0.508041552	count	1
SLC41A3	0.4004715	0.6345964	0.6311	0.528	0.508083802	count	1
CCDC82	0.3640885	0.3909317	0.9313	0.352	0.508190293	count	1
ZNF24	0.3627625	0.2824661	1.2843	0.2	0.509843352	count	1
KANSL2	0.3805481	0.4108504	0.9262	0.355	0.510054647	count	1
CHMP2B	0.367361	0.3455406	1.0631	0.289	0.510460209	count	1
DYNC2H1	0.5921355	0.8992451	0.6585	0.5107	0.511225604	count	1
AC007384.1	0.3787981	0.4755647	0.7965	0.426	0.511329288	count	1
EIF3J-DT	0.3919687	0.5663309	0.6921	0.489	0.511474711	count	1
ARL4A	0.358491	0.2216033	1.6177	0.1067	0.51154983	count	1
AL133453.1	0.51462	0.6590023	0.7809	0.435	0.51168262	count	1
TBCC	0.3641251	0.2518956	1.4455	0.149	0.512009605	count	1
BARD1	0.3809278	0.4401219	0.8655	0.387	0.512477516	count	1
MPP5	0.4762861	0.9556646	0.4984	0.619	0.512897255	count	1
KDM5D	0.4762861	0.979528	0.4862	0.627	0.512897255	count	1
TBC1D23	0.4097021	0.5603233	0.7312	0.465	0.512993723	count	1
RAB4A	0.3814332	0.4008141	0.9516	0.342	0.513159217	count	1
GTF2F1	0.3667727	0.3424697	1.071	0.285	0.513424468	count	1
TOMM20	0.3607568	0.2173577	1.6597	0.0979	0.513521334	count	1
SMARCA4	0.3804927	0.4554345	0.8354	0.404	0.513622451	count	1
TMX1	0.3693525	0.3577551	1.0324	0.303	0.514228075	count	1
CENPX	0.3652361	0.2979693	1.2258	0.221	0.514487883	count	1
PPP3CC	0.3720765	0.374319	0.994	0.321	0.514653386	count	1
ARL4D	0.3945336	0.5898623	0.6689	0.504	0.51483411	count	1
ASB2	0.4386356	0.6501016	0.6747	0.5	0.51553029	count	1
KIAA0586	0.3797293	0.4011037	0.9467	0.344	0.515602927	count	1

RMC1	0.4387969	0.574063	0.7644	0.445	0.515721282	count	1
PPP2CA	0.3668318	0.380321	0.9645	0.3355	0.516061284	count	1
MRPS36	0.3681054	0.327135	1.1252	0.261	0.516250081	count	1
PNO1	0.3868387	0.4233253	0.9138	0.361	0.516356943	count	1
MRPL42	0.3684212	0.3868915	0.9523	0.342	0.516693442	count	1
PRPSAP2	0.388933	0.4370384	0.8899	0.374	0.516748088	count	1
RPA3	0.3731719	0.3416029	1.0924	0.275	0.516816484	count	1
GPR35	0.4560561	0.6578181	0.6933	0.489	0.517073894	count	1
CCDC186	0.3653349	0.2569745	1.4217	0.156	0.517169596	count	1
KIF2A	0.3678217	0.2648315	1.3889	0.166	0.517212521	count	1
CAPN10	0.4286323	0.7114164	0.6025	0.547	0.517367381	count	1
SRSF3	0.3623396	0.1495085	2.4235	0.0159	0.517648733	count	1
UBAC1	0.3900913	0.456572	0.8544	0.394	0.518291747	count	1
NCK1	0.373689	0.3572389	1.046	0.296	0.518720009	count	1
PTPRJ	0.4415382	0.5391079	0.819	0.413	0.518967225	count	1
U2SURP	0.3648829	0.213004	1.713	0.0877	0.520072373	count	1
CDC73	0.3786676	0.3457314	1.0953	0.274	0.52072005	count	1
VPS51	0.3786127	0.3458266	1.0948	0.274	0.521486053	count	1
CALHM2	0.4164626	0.5830129	0.7143	0.476	0.521503116	count	1
GNAI1	0.6039805	1.00463	0.6012	0.548	0.521616502	count	1
DLST	0.3895442	0.5344082	0.7289	0.467	0.522145575	count	1
NFRKB	0.4444849	0.8498789	0.523	0.601	0.522456478	count	1
ZNF528	0.4444849	0.9095132	0.4887	0.625	0.522456478	count	1
EXD3	0.4444849	0.9316016	0.4771	0.634	0.522456478	count	1
ZNF234	0.6057175	0.7899464	0.7668	0.444	0.523140159	count	1
ELOC	0.3695538	0.2318081	1.5942	0.112	0.523539119	count	1
FAM234A	0.4867348	0.757836	0.6423	0.521	0.524266699	count	1
SLC41A2	0.4867348	1.0211	0.4767	0.634	0.524266699	count	1
HLTF	0.3800429	0.3553735	1.0694	0.286	0.524992271	count	1
ITGA5	0.5279743	0.8290933	0.6368	0.525	0.525131478	count	1
BICDL1	0.4000617	0.5121345	0.7812	0.435	0.525654886	count	1
SLC35A4	0.5285267	0.8266499	0.6394	0.523	0.525687794	count	1
HEATR1	0.5285267	0.934024	0.5659	0.572	0.525687794	count	1
FLVCR1-DT	0.5285267	0.9480992	0.5575	0.578	0.525687794	count	1
UQCC3	0.3814894	0.3910316	0.9756	0.33	0.526248924	count	1
CUX1	0.4203157	0.704184	0.5969	0.551	0.526353199	count	1
CMC1	0.3679092	0.2086054	1.7637	0.0787	0.526385656	count	1
MGMT	0.3747045	0.2659699	1.4088	0.16	0.526643788	count	1
CD69	0.3656209	0.1085816	3.3672	0.000851	0.527064396	count	1
CD72	0.4154884	0.7385721	0.5626	0.574	0.527227805	count	1
NUP155	0.4154884	0.7963363	0.5217	0.602	0.527227805	count	1
SF3B1	0.3696651	0.1838402	2.0108	0.0452	0.527360033	count	1
ANAPC15	0.3887636	0.4615982	0.8422	0.4	0.52789775	count	1
CARD8	0.3809243	0.3739864	1.0186	0.309	0.528777886	count	1
ZNF154	0.4382884	0.7247246	0.6048	0.546	0.529098964	count	1

MRPS2	0.4385432	0.555365	0.7896	0.43	0.529408545	count	1
GIT2	0.4230896	0.4603887	0.919	0.359	0.529844935	count	1
TRAPPC8	0.4672977	0.5304353	0.881	0.379	0.529930789	count	1
APEX2	0.4233786	0.7628855	0.555	0.579	0.530208723	count	1
RMI1	0.4518476	0.6188796	0.7301	0.466	0.531175196	count	1
FLAD1	0.3960543	0.4599228	0.8611	0.39	0.532881459	count	1
C1orf43	0.3824538	0.3098421	1.2344	0.218	0.532969891	count	1
KCNA3	0.4116228	0.6215185	0.6623	0.508	0.532988431	count	1
MUS81	0.4417646	0.6656696	0.6636	0.507	0.533322574	count	1
NISCH	0.4537968	0.7711622	0.5885	0.557	0.533483479	count	1
SMPD2	0.4330618	0.7995059	0.5417	0.588	0.533620816	count	1
CDC42EP3	0.388202	0.3250775	1.1942	0.233	0.533854739	count	1
CCDC159	0.4207206	0.5417289	0.7766	0.438	0.533898497	count	1
WRNIP1	0.4422752	0.7171487	0.6167	0.538	0.533942959	count	1
WAS	0.3805994	0.2948446	1.2908	0.198	0.534094597	count	1
ERGIC2	0.3862006	0.3296347	1.1716	0.242	0.534220815	count	1
MAP1LC3B	0.3734525	0.1598518	2.3362	0.0201	0.534345829	count	1
CHD2	0.3831488	0.3209112	1.1939	0.233	0.534394512	count	1
SERTAD1	0.3764125	0.2133267	1.7645	0.0786	0.534494089	count	1
NCK2	0.3975094	0.3781904	1.0511	0.294	0.534844302	count	1
FBXL20	0.6191356	0.8154256	0.7593	0.448	0.534909028	count	1
C19orf12	0.3914858	0.4603875	0.8503	0.396	0.53537573	count	1
ADAM8	0.4035852	0.3774407	1.0693	0.286	0.536275712	count	1
CTSD	0.373939	0.1301592	2.8729	0.00434	0.536648857	count	1
UHMK1	0.3882785	0.3750305	1.0353	0.301	0.537099596	count	1
KRT10	0.3775937	0.2087908	1.8085	0.0715	0.537569609	count	1
ABHD6	0.8637374	0.8583598	1.0063	0.315	0.537919395	count	1
HOOK1	0.4194504	0.6955645	0.603	0.547	0.538161196	count	1
DCLRE1A	0.4305179	0.7191572	0.5986	0.55	0.539195879	count	1
AL138762.1	0.4305179	0.8023976	0.5365	0.592	0.539195879	count	1
DNASE1	0.6243083	0.9416575	0.663	0.508	0.53944529	count	1
SFSWAP	0.392147	0.3809492	1.0294	0.304	0.540160169	count	1
FEM1C	0.4386356	0.66236	0.6622	0.508	0.540528314	count	1
DHX58	0.4598506	0.9703266	0.4739	0.636	0.540652705	count	1
SP3	0.394774	0.3072427	1.2849	0.2	0.542908639	count	1
GLTP	0.3990136	0.3602738	1.1075	0.269	0.543233727	count	1
NKAP	0.3913369	0.2995776	1.3063	0.192	0.543252935	count	1
CCAR2	0.545971	1.0059168	0.5428	0.588	0.543256129	count	1
AL645933.2	0.545971	1.1373872	0.48	0.632	0.543256129	count	1
IFFO1	0.4113128	0.4895921	0.8401	0.401	0.543718081	count	1
ADD1	0.3905471	0.3383231	1.1544	0.249	0.544726243	count	1
IGF2R	0.4081279	0.55211	0.7392	0.46	0.54485758	count	1
ADIPOR2	0.4107714	0.5341866	0.769	0.442	0.545853612	count	1
CAAP1	0.41549	0.5575109	0.7453	0.457	0.545997467	count	1
TADA3	0.3861836	0.2663146	1.4501	0.148	0.54625943	count	1

GNB2	0.3843088	0.2298615	1.6719	0.0955	0.546273919	count	1
AKR1A1	0.3921511	0.3262658	1.2019	0.23	0.546966253	count	1
DAXX	0.3965711	0.399556	0.9925	0.322	0.54708858	count	1
SCFD1	0.4164785	0.5025142	0.8288	0.408	0.547300887	count	1
SOCS3	0.4082729	0.4978505	0.8201	0.413	0.547319075	count	1
ELF4	0.633634	1.1194632	0.566	0.5718	0.547622504	count	1
COPS6	0.3871412	0.2427174	1.595	0.112	0.547767792	count	1
TMEM71	0.4449625	0.6924394	0.6426	0.521	0.54836943	count	1
DUSP5	0.3938821	0.2901206	1.3576	0.176	0.549383628	count	1
ATF3	0.4283813	0.5270451	0.8128	0.4169	0.549669006	count	1
SPCS3	0.3893787	0.2357944	1.6513	0.0996	0.549759802	count	1
CSF1	0.4851508	0.7915408	0.6129	0.54	0.55035128	count	1
KIF21B	0.4851508	0.8166812	0.5941	0.553	0.55035128	count	1
RALGAPA1	0.4053756	0.4260804	0.9514	0.342	0.550506308	count	1
TUBGCP5	0.4682343	0.5815762	0.8051	0.421	0.550581578	count	1
ZNF32	0.3989302	0.4220123	0.9453	0.345	0.551126016	count	1
JUNB	0.3828072	0.1258678	3.0413	0.00255	0.551497453	count	1
ZNF430	0.4116128	0.4613385	0.8922	0.373	0.551808497	count	1
LAT2	0.3875987	0.2111115	1.836	0.0673	0.552092351	count	1
RPL7L1	0.3986763	0.3722686	1.0709	0.285	0.552842508	count	1
TNFAIP3	0.3935916	0.2209916	1.781	0.0758	0.552936666	count	1
AHCYL1	0.513227	0.6618787	0.7754	0.439	0.553096505	count	1
PRRC2B	0.4073912	0.3720527	1.095	0.274	0.553249588	count	1
SLC35B2	0.4276129	0.6333791	0.6751	0.5	0.553776184	count	1
ESS2	0.4276129	0.8161745	0.5239	0.601	0.553776184	count	1
SLC17A9	0.4591335	0.8012519	0.573	0.567	0.554427412	count	1
DSE	0.4591335	0.8721299	0.5265	0.599	0.554427412	count	1
SLC9A9	0.4255291	0.5908624	0.7202	0.472	0.555434988	count	1
INTS6	0.403368	0.2954759	1.3651	0.173	0.556480726	count	1
ZMPSTE24	0.4910551	0.8387935	0.5854	0.559	0.557105062	count	1
SNAP47	0.4076569	0.5049085	0.8074	0.42	0.557534749	count	1
NCOA7	0.4309417	0.4317006	0.9982	0.319	0.55810397	count	1
GMIP	0.4532677	0.6849035	0.6618	0.509	0.558662735	count	1
NUDC	0.3951111	0.2297565	1.7197	0.0864	0.558736331	count	1
SINHCAF	0.3987033	0.347965	1.1458	0.253	0.559208764	count	1
ATG5	0.4098485	0.312673	1.3108	0.191	0.559326879	count	1
UNK	0.4935028	0.7718657	0.6394	0.523	0.559904948	count	1
STK25	0.4047461	0.4195243	0.9648	0.335	0.559914846	count	1
BCL2L12	0.4482316	0.5651783	0.7931	0.428	0.561496086	count	1
RASSF7	0.4188525	0.4497316	0.9313	0.352	0.56154014	count	1
UST	0.4655529	0.9109338	0.5111	0.61	0.562228091	count	1
ZNF692	0.5649324	0.8185919	0.6901	0.491	0.562351467	count	1
NQO1	0.5649324	1.1237013	0.5027	0.615	0.562351467	count	1
GALE	0.4142397	0.510544	0.8114	0.418	0.56257069	count	1
HSPA13	0.428127	0.5894257	0.7263	0.468	0.56266081	count	1

SLC35B3	0.4960182	0.8577639	0.5783	0.563	0.562782309	count	1
ERAP1	0.4283639	0.5632083	0.7606	0.447	0.5629732	count	1
CTDSP1	0.4003606	0.2577337	1.5534	0.121	0.563012232	count	1
CRTAP	0.4503468	0.6480898	0.6949	0.488	0.564159099	count	1
RAB5A	0.4195902	0.4208743	0.9969	0.32	0.564631436	count	1
SLA2	0.4128569	0.4296512	0.9609	0.337	0.564660461	count	1
EIF3J	0.4082147	0.2870614	1.422	0.156	0.564720578	count	1
ADNP2	0.9066608	0.9840893	0.9213	0.3576	0.564859848	count	1
AP003774.4	0.4156119	0.4992631	0.8325	0.406	0.565880084	count	1
YME1L1	0.4069314	0.3357296	1.2121	0.226	0.566085995	count	1
LYRM2	0.4016615	0.3987255	1.0074	0.3145	0.566297048	count	1
A1BG	0.4145048	0.436055	0.9506	0.343	0.566918645	count	1
SLK	0.411475	0.3513322	1.1712	0.242	0.567683407	count	1
TRIM52	0.4383834	0.5178751	0.8465	0.398	0.567779169	count	1
POLR3GL	0.4013233	0.2333936	1.7195	0.0865	0.567848657	count	1
WSB1	0.4108529	0.2946223	1.3945	0.164	0.56837582	count	1
MLX	0.4149517	0.4392927	0.9446	0.346	0.568667177	count	1
GCSAM	0.5012562	0.8056087	0.6222	0.534	0.568774084	count	1
ADPRH	0.5012562	0.8056087	0.6222	0.534	0.568774084	count	1
NDN	0.5012562	0.8056087	0.6222	0.534	0.568774084	count	1
ZFP30	0.5012562	0.8056087	0.6222	0.534	0.568774084	count	1
CHEK2	0.5012562	0.8056087	0.6222	0.534	0.568774084	count	1
GPR157	0.5012562	1.0028252	0.4998	0.618	0.568774084	count	1
TESK2	0.5012562	1.0028252	0.4998	0.618	0.568774084	count	1
MRPS30-DT	0.5012562	1.0028252	0.4998	0.618	0.568774084	count	1
HS6ST2	0.5012562	1.0028252	0.4998	0.618	0.568774084	count	1
COL14A1	0.5012562	1.0028252	0.4998	0.618	0.568774084	count	1
ARHGEF39	0.5012562	1.0028252	0.4998	0.618	0.568774084	count	1
OGN	0.5012562	1.0028252	0.4998	0.618	0.568774084	count	1
ZNF189	0.5012562	1.0028252	0.4998	0.618	0.568774084	count	1
RNASE6	0.5012562	1.0028252	0.4998	0.618	0.568774084	count	1
ASPHD2	0.5012562	1.0028252	0.4998	0.618	0.568774084	count	1
GNG2	0.4061637	0.251045	1.6179	0.107	0.56968334	count	1
UBE2G1	0.4336855	0.3110609	1.3942	0.164	0.569990596	count	1
JADE2	0.4339043	0.5760105	0.7533	0.452	0.570279123	count	1
POLE4	0.4164132	0.3898332	1.0682	0.286	0.570673802	count	1
AC106739.2	0.6614843	0.8892948	0.7438	0.458	0.572032607	count	1
LPAR2	0.6614843	0.9932448	0.666	0.5059	0.572032607	count	1
TSPAN5	0.4114014	0.3370985	1.2204	0.223	0.572851076	count	1
SOCS4	0.4276351	0.534868	0.7995	0.425	0.573346077	count	1
CATSPER1	0.4276351	0.572471	0.747	0.456	0.573346077	count	1
HSPA14	0.4468308	0.6102355	0.7322	0.465	0.573443496	count	1
SYNC	0.4752627	0.7240906	0.6564	0.512	0.574027558	count	1
RUBCN	0.4752627	0.8275551	0.5743	0.566	0.574027558	count	1
ZNF451	0.4267597	0.4609869	0.9258	0.355	0.574303617	count	1

ACADSB	0.6645411	0.971694	0.6839	0.495	0.57471069	count	1
SPECC1L	0.6645411	0.9929239	0.6693	0.504	0.57471069	count	1
RNF139	0.4324501	0.4337087	0.9971	0.319	0.574749108	count	1
MUC20-OT1	0.4478516	0.4212419	1.0632	0.288	0.574758973	count	1
DHRS4	0.4532828	0.670014	0.6765	0.499	0.575417505	count	1
NFXL1	0.4444849	0.8803498	0.5049	0.614	0.575712133	count	1
LINC02084	0.4158187	0.4087402	1.0173	0.31	0.575972609	count	1
ZNF18	0.5350948	0.9029317	0.5926	0.554	0.576894774	count	1
PHYH	0.4201835	0.499708	0.8409	0.401	0.576919356	count	1
GPATCH2	0.4185566	0.4187214	0.9996	0.318	0.577469298	count	1
AC013394.1	0.5799544	0.6759278	0.858	0.392	0.577477801	count	1
MZT1	0.4215774	0.4179074	1.0088	0.314	0.577764255	count	1
ZKSCAN1	0.4783994	0.6894006	0.6939	0.488	0.577839379	count	1
ARL2	0.421316	0.4464198	0.9438	0.346	0.578477064	count	1
ZNF84	0.4466322	0.5943778	0.7514	0.453	0.578504016	count	1
PGP	0.4128139	0.2937876	1.4051	0.161	0.578678583	count	1
IMPA1	0.4359021	0.52721	0.8268	0.409	0.579350465	count	1
IL15	0.5374225	0.7267445	0.7395	0.46	0.579427897	count	1
DOCK5	0.5374225	1.0743911	0.5002	0.617	0.579427897	count	1
MRPL58	0.4260822	0.5330781	0.7993	0.425	0.580166069	count	1
IPO11	0.4633942	0.6160787	0.7522	0.452	0.580586063	count	1
NUP58	0.4633942	0.6796428	0.6818	0.496	0.580586063	count	1
STX11	0.4113438	0.3284038	1.2526	0.211	0.580797155	count	1
IRAK1BP1	0.6717374	1.0782189	0.623	0.534	0.581014415	count	1
RBM27	0.6717374	1.0935923	0.6142	0.539	0.581014415	count	1
CWF19L1	0.6717375	1.195157	0.562	0.574	0.581014489	count	1
BCDIN3D	0.6717375	1.22355	0.549	0.583	0.581014489	count	1
ARHGAP11A	0.6717375	1.31081	0.5125	0.609	0.581014489	count	1
ZNF747	0.6717375	1.31081	0.5125	0.609	0.581014489	count	1
AP001020.1	0.6717375	1.31081	0.5125	0.609	0.581014489	count	1
ZNF486	0.6717375	1.31081	0.5125	0.609	0.581014489	count	1
PIAS3	0.6717375	1.327908	0.5059	0.613	0.581014489	count	1
SLC19A2	0.6717375	1.327908	0.5059	0.613	0.581014489	count	1
MAK16	0.4714636	0.7651452	0.6162	0.538	0.581215638	count	1
FAAP100	0.4943085	0.6854394	0.7212	0.471	0.581463632	count	1
BRF2	0.4454284	0.6541227	0.681	0.496	0.581504152	count	1
CASP1	0.4156817	0.3585718	1.1593	0.247	0.582702815	count	1
PSD4	0.4364267	0.4680422	0.9325	0.352	0.582746156	count	1
DGKE	0.4735284	0.5945042	0.7965	0.426	0.583774933	count	1
DOPEY1	0.5875691	0.9220564	0.6372	0.524	0.585144487	count	1
EPDR1	0.5875691	1.0216157	0.5751	0.566	0.585144487	count	1
ZXDB	0.5875691	1.1179051	0.5256	0.6	0.585144487	count	1
ZNF286A	0.5875691	1.1179051	0.5256	0.6	0.585144487	count	1
ZDHHC8	0.5875691	1.1179051	0.5256	0.6	0.585144487	count	1
ZNF518B	0.5884988	0.6661103	0.8835	0.378	0.586080483	count	1

TMED9	0.4133843	0.2431516	1.7001	0.0901	0.58608133	count	1
HECA	0.4413134	0.4514423	0.9776	0.329	0.58656356	count	1
RXRB	0.4629359	0.7738367	0.5982	0.55	0.587726904	count	1
ZNF787	0.4471462	0.5172583	0.8645	0.388	0.587741258	count	1
OAZ2	0.4537968	0.5418058	0.8376	0.403	0.587819431	count	1
GADD45G	0.4217962	0.3676137	1.1474	0.252	0.58786825	count	1
LRRC57	0.4636295	0.7133684	0.6499	0.516	0.58861138	count	1
CNOT9	0.4359578	0.4449681	0.9798	0.328	0.588687786	count	1
BLM	0.5006466	0.7354202	0.6808	0.497	0.588970654	count	1
GTF3C4	0.5006466	0.7716712	0.6488	0.517	0.588970654	count	1
C1orf131	0.4306262	0.4631305	0.9298	0.353	0.589010911	count	1
GZMK	0.4094028	0.1376635	2.9739	0.00316	0.589085683	count	1
MTREX	0.4483853	0.5386429	0.8324	0.406	0.589375288	count	1
THAP1	0.5194329	0.5801521	0.8953	0.371	0.589566583	count	1
CCDC77	0.5017731	0.6730603	0.7455	0.457	0.590304922	count	1
BIN3	0.5017731	0.7395701	0.6785	0.498	0.590304922	count	1
MYO9B	0.4293538	0.3938304	1.0902	0.276	0.59055002	count	1
MORF4L2	0.4271206	0.3429376	1.2455	0.214	0.590915198	count	1
CAMK2G	0.4656607	0.5712062	0.8152	0.416	0.591201557	count	1
PEX13	0.4661055	0.5405865	0.8622	0.389	0.591768762	count	1
LDLRAP1	0.4289285	0.4719653	0.9088	0.364	0.5918022	count	1
AUTS2	0.4336316	0.4546324	0.9538	0.341	0.591850631	count	1
NPRL2	0.4570504	0.5202078	0.8786	0.38	0.592049809	count	1
NPEPL1	0.4803516	0.7644965	0.6283	0.53	0.592232278	count	1
IGFBP7	0.4174866	0.682986	0.6113	0.541	0.592853558	count	1
PACS1	0.4541421	0.4790441	0.948	0.344	0.592920057	count	1
TMEM214	0.550469	0.9675096	0.569	0.57	0.593625245	count	1
ATG7	0.6865366	1.058215	0.6488	0.517	0.593973259	count	1
BAX	0.4177537	0.2150249	1.9428	0.0529	0.594204221	count	1
DTD1	0.4344414	0.3885989	1.118	0.264	0.594239241	count	1
BCL6	0.5051325	0.7894952	0.6398	0.523	0.594283878	count	1
ASB7	0.4630912	0.7512725	0.6164	0.538	0.594398289	count	1
ATMIN	0.5240295	0.6279255	0.8345	0.405	0.594824592	count	1
ASB6	0.5975332	0.8697242	0.687	0.493	0.595175441	count	1
CHD6	0.4348114	0.4071579	1.0679	0.286	0.595934875	count	1
RAB11FIP1	0.4162502	0.1733534	2.4012	0.0169	0.595955991	count	1
SUCLA2	0.4444849	0.4863432	0.9139	0.361	0.595997031	count	1
LINC00861	0.4508088	0.4174618	1.0799	0.281	0.596098954	count	1
TNKS	0.4836268	0.4957393	0.9756	0.33	0.596291903	count	1
ATP1B1	0.4202099	0.2440175	1.722	0.086	0.597954697	count	1
AP001816.1	0.4955807	0.617447	0.8026	0.423	0.59871901	count	1
MLF2	0.4275981	0.3062622	1.3962	0.164	0.599055982	count	1
APOL2	0.4967813	0.7999693	0.621	0.535	0.600178057	count	1
ARF6	0.4214757	0.1842915	2.287	0.0228	0.600369703	count	1
RPL26L1	0.4348733	0.5066357	0.8584	0.391	0.600862507	count	1

HCLS1	0.422337	0.2262231	1.8669	0.0628	0.601224452	count	1
ZNF720	0.4685539	0.6284457	0.7456	0.456	0.601438233	count	1
GPR171	0.4740543	0.5740763	0.8258	0.41	0.601905123	count	1
GUSB	0.4459341	0.3768829	1.1832	0.238	0.602190762	count	1
NOL4L	0.4746131	0.4979384	0.9532	0.341	0.602617713	count	1
KCMF1	0.4438326	0.4065381	1.0917	0.276	0.602849722	count	1
RCOR1	0.461921	0.5016588	0.9208	0.358	0.603111439	count	1
TPMT	0.4812942	0.6590145	0.7303	0.466	0.603123413	count	1
H2AFY	0.4284306	0.268012	1.5986	0.111	0.603337499	count	1
VCAN	0.5132113	0.9655614	0.5315	0.595	0.60385256	count	1
CXorf40A	0.4819187	0.624843	0.7713	0.441	0.603909708	count	1
TNIK	0.4526294	0.4933109	0.9175	0.36	0.604440846	count	1
KANSL3	0.4491148	0.6126867	0.733	0.464	0.60446333	count	1
MOB2	0.4364987	0.4265178	1.0234	0.307	0.605368584	count	1
CLINT1	0.42826	0.295503	1.4493	0.148	0.605469709	count	1
LLGL1	0.9719013	1.152076	0.8436	0.4	0.605559423	count	1
ZNF614	0.7001958	1.1405915	0.6139	0.5397	0.605927497	count	1
STIP1	0.4461716	0.44306	1.007	0.315	0.607577459	count	1
LHPP	0.6103198	0.8674447	0.7036	0.482	0.608045507	count	1
GPHN	0.4854035	0.8242991	0.5889	0.556	0.608297365	count	1
AAMDC	0.4741547	0.709247	0.6685	0.504	0.608656193	count	1
ACSF3	0.4857254	0.6532044	0.7436	0.458	0.608702668	count	1
ALG6	0.5649324	0.7859712	0.7188	0.473	0.609363009	count	1
NEK1	0.4747949	0.608608	0.7801	0.436	0.60948125	count	1
HAX1	0.4302937	0.2655125	1.6206	0.106	0.609501636	count	1
SPINDOC	0.4714636	0.6697556	0.7039	0.482	0.610790345	count	1
MAP3K12	0.4542391	0.5276124	0.8609	0.39	0.611376782	count	1
ACACA	0.5195769	0.7844524	0.6623	0.508	0.611391981	count	1
PDK1	0.50611	0.6879495	0.7357	0.462	0.611514788	count	1
TBC1D1	0.4560409	0.4638528	0.9832	0.326	0.611532	count	1
RNF8	0.5200243	0.5394032	0.9641	0.336	0.611921882	count	1
PPP2R5E	0.4496727	0.3199633	1.4054	0.161	0.612354682	count	1
LDB2	0.4422505	0.3279659	1.3485	0.178	0.614034773	count	1
LPIN1	0.4436688	0.357767	1.2401	0.216	0.614606625	count	1
PPP3CA	0.4395477	0.2950844	1.4896	0.137	0.615007417	count	1
AL512791.2	0.6174267	1.2170048	0.5073	0.6123	0.615197475	count	1
USP21	0.6174267	1.3933543	0.4431	0.658	0.615197475	count	1
AEBP2	0.5092282	0.6696751	0.7604	0.448	0.615304152	count	1
DDX60	0.4750682	0.6254759	0.7595	0.448	0.615477215	count	1
CNPPD1	0.463015	0.4661632	0.9932	0.321	0.615491841	count	1
SMIM27	0.4462545	0.4231028	1.0547	0.292	0.615745397	count	1
TMEM87A	0.4501372	0.3413704	1.3186	0.188	0.615748959	count	1
NHLRC2	0.5424818	0.7764204	0.6987	0.485	0.615931083	count	1
CREBL2	0.5424818	0.7764204	0.6987	0.485	0.615931083	count	1
TMEM39B	0.5424818	0.7778546	0.6974	0.486	0.615931083	count	1

ARL6IP6	0.4477994	0.396194	1.1303	0.259	0.615964135	count	1
OGT	0.4535136	0.3254526	1.3935	0.164	0.616026999	count	1
ELF2	0.440156	0.2933285	1.5006	0.134	0.616272285	count	1
AHR	0.5112925	0.7504206	0.6813	0.496	0.617812759	count	1
CCDC22	0.5112925	0.7533897	0.6787	0.498	0.617812759	count	1
AC239800.3	0.4527827	0.3751129	1.2071	0.228	0.618041019	count	1
CTR9	0.4567122	0.4138007	1.1037	0.271	0.618660428	count	1
SRBD1	0.4638741	0.63895	0.726	0.468	0.619497282	count	1
CS	0.7161533	0.7703385	0.9297	0.353	0.619884477	count	1
C18orf25	0.5028218	0.7670446	0.6555	0.513	0.620084214	count	1
SACM1L	0.4439107	0.2876853	1.543	0.124	0.620228521	count	1
TARS2	0.9957043	0.9760588	1.0201	0.308	0.620322987	count	1
MED6	0.4510081	0.39782	1.1337	0.258	0.620385089	count	1
FBXL12	0.4892309	0.6469167	0.7563	0.45	0.621258554	count	1
AUP1	0.444408	0.2620586	1.6958	0.0909	0.621379653	count	1
CLP1	0.4796298	0.5897159	0.8133	0.417	0.621408414	count	1
HIP1	0.528917	0.629708	0.8399	0.402	0.622454112	count	1
NDUFAF5	0.528917	0.6342012	0.834	0.405	0.622454112	count	1
ZNF718	0.4902123	0.7297515	0.6718	0.502	0.622510052	count	1
MGA	0.4504642	0.4910524	0.9173	0.36	0.623259275	count	1
CC2D1B	0.5782289	0.9342556	0.6189	0.5364	0.623829081	count	1
PPIL1	0.5786154	0.7881307	0.7342	0.463	0.624249546	count	1
HNRNPH2	0.4677342	0.4250502	1.1004	0.272	0.624665907	count	1
CHST7	1.002758	0.8169999	1.2274	0.221	0.624688309	count	1
PTGDR	0.4373816	0.2151303	2.0331	0.0429	0.624940472	count	1
HIKESHI	0.4497418	0.3712375	1.2115	0.227	0.6257205	count	1
VMAC	0.5183702	0.907927	0.5709	0.568	0.626413709	count	1
ICAM1	0.464311	0.380661	1.2197	0.223	0.627064412	count	1
MAFF	0.4383327	0.1785076	2.4555	0.0146	0.627208014	count	1
GRIPAP1	0.4747973	0.5245791	0.9051	0.366	0.627915425	count	1
ZNF880	0.4538992	0.4675526	0.9708	0.332	0.628018736	count	1
ATXN2L	0.5835057	0.5515104	1.058	0.291	0.629569382	count	1
CITED2	0.441425	0.2357742	1.8722	0.0621	0.629719309	count	1
KANSL1	0.4538758	0.2693291	1.6852	0.0929	0.630197258	count	1
SMIM13	0.5115564	0.647541	0.79	0.43	0.63091059	count	1
OPTN	0.4458347	0.2558497	1.7426	0.0824	0.631831072	count	1
AL662844.4	0.5857218	0.7788363	0.752	0.453	0.631980019	count	1
C1orf21	0.451692	0.3342754	1.3513	0.178	0.63201766	count	1
CDC37L1	0.4997641	0.6510384	0.7676	0.443	0.634690552	count	1
LATS1	0.7331443	0.8719971	0.8408	0.401	0.63473386	count	1
PICALM	0.4708741	0.4497702	1.0469	0.296	0.635947805	count	1
PIP4P2	0.4957975	0.668556	0.7416	0.459	0.636548101	count	1
PCCA	0.5900037	0.8316132	0.7095	0.479	0.636637569	count	1
SMIM1	0.5605986	0.8863075	0.6325	0.527	0.636651342	count	1
SETD5	0.4815803	0.4346545	1.108	0.269	0.636911906	count	1

ARSD	0.639148	1.019399	0.627	0.531	0.637049659	count	1
PAIP2B	0.639148	1.066667	0.5992	0.549	0.637049659	count	1
FAM32A	0.4604764	0.3474505	1.3253	0.186	0.63713198	count	1
MRPL15	0.5021963	0.6633035	0.7571	0.45	0.637792076	count	1
UBN2	0.5619226	0.7168501	0.7839	0.434	0.638165488	count	1
NUP88	0.5026698	0.6239798	0.8056	0.421	0.638395881	count	1
PTPRA	0.4537799	0.2776075	1.6346	0.103	0.638503073	count	1
ADIPOR1	0.4857412	0.432573	1.1229	0.262	0.638638628	count	1
PGM2	0.4621973	0.4023661	1.1487	0.252	0.638673751	count	1
CLK1	0.4500211	0.2510505	1.7926	0.074	0.638963121	count	1
PSPC1	0.4788891	0.4113026	1.1643	0.245	0.639602178	count	1
MLH1	0.5105162	0.568874	0.8974	0.37	0.639915963	count	1
PRKAR1A	0.4555241	0.3177573	1.4336	0.153	0.640030392	count	1
FAM117B	0.5189174	0.7317352	0.7092	0.479	0.640034204	count	1
FO393401.1	0.4711702	0.4111813	1.1459	0.253	0.640060446	count	1
HIBADH	0.5296965	0.7297518	0.7259	0.468	0.640177164	count	1
NIPAL3	0.4798257	0.8269181	0.5803	0.5621	0.640856273	count	1
MAP3K10	0.7407505	1.012306	0.7317	0.465	0.641377171	count	1
FBXO44	0.545971	0.8225956	0.6637	0.507	0.642650936	count	1
SPTY2D1	0.4746295	0.3214159	1.4767	0.141	0.642983482	count	1
PDPK1	0.5321355	0.6360828	0.8366	0.403	0.643140881	count	1
SYT11	0.7428239	0.7916756	0.9383	0.349	0.643187617	count	1
ZNF639	0.4976479	0.7312095	0.6806	0.497	0.644836227	count	1
PNMA1	1.0355134	0.6708851	1.5435	0.124	0.644899021	count	1
LIX1L	0.4742667	0.4022551	1.179	0.239	0.645913244	count	1
ECHDC1	0.462731	0.3101745	1.4918	0.137	0.647026972	count	1
B4GALT4	0.535621	0.4722354	1.1342	0.258	0.647376194	count	1
KLC1	0.4998741	0.5676138	0.8807	0.379	0.647730772	count	1
SACS	0.5006466	0.6349412	0.7885	0.431	0.648735189	count	1
BROX	0.467362	0.3762831	1.242	0.215	0.648946995	count	1
PPP1R12B	1.0422407	1.0564084	0.9866	0.3246	0.649037055	count	1
MRPL12	0.4699659	0.4005223	1.1734	0.242	0.649424082	count	1
BSG	0.4560201	0.1888568	2.4146	0.0163	0.649442844	count	1
HIRIP3	0.4910328	0.6112764	0.8033	0.422	0.64944895	count	1
LRRC14	0.5265294	0.7091989	0.7424	0.458	0.649468631	count	1
UBE2V1	0.5265294	0.8511773	0.6186	0.537	0.649468631	count	1
SLC35F5	0.5265294	0.8819373	0.597	0.551	0.649468631	count	1
ANAPC10	0.5064735	0.5616092	0.9018	0.368	0.650306402	count	1
IMMP1L	0.4921081	0.6264783	0.7855	0.433	0.650875135	count	1
L3MBTL2	0.5072649	0.7868601	0.6447	0.52	0.65132627	count	1
SPATA33	0.7522634	0.8968733	0.8388	0.402	0.651427318	count	1
RNF44	0.492932	0.5107995	0.965	0.335	0.651967878	count	1
TMCO4	0.5140016	0.5532089	0.9291	0.354	0.652845836	count	1
TBRG4	0.6057157	0.907866	0.6672	0.505	0.653725227	count	1
PCNX2	0.6057157	0.9103884	0.6653	0.506	0.653725227	count	1

PDPR	0.5759726	0.890329	0.6469	0.518	0.654231807	count	1
XPOT	0.5759726	0.9214513	0.6251	0.532	0.654231807	count	1
MRPL10	0.4769499	0.3813728	1.2506	0.212	0.655001874	count	1
MON1A	0.7565224	1.063215	0.7115	0.4773	0.655143492	count	1
CCNF	0.7565224	1.122504	0.674	0.501	0.655143492	count	1
TGFB1I1	0.7565224	1.122504	0.674	0.501	0.655143492	count	1
MALINC1	0.7565224	1.221282	0.6194	0.536	0.655143492	count	1
ULBP2	0.7565224	1.221282	0.6194	0.536	0.655143492	count	1
SLC9A1	0.7565224	1.4177025	0.5336	0.594	0.655143492	count	1
NME3	0.4611695	0.2538847	1.8165	0.0702	0.656084579	count	1
PRH1	0.49388	0.6251431	0.79	0.43	0.656635416	count	1
RBM5	0.4866123	0.3832219	1.2698	0.205	0.657249951	count	1
BAALC	0.5119619	0.588433	0.87	0.385	0.657379224	count	1
CRIP2	0.5244808	0.7491834	0.7001	0.484	0.657497457	count	1
DDIT4	0.4571807	0.122598	3.7291	0.000227	0.657988609	count	1
DDX17	0.4671353	0.1974047	2.3664	0.0186	0.658165094	count	1
SPOUT1	0.5083758	0.7604262	0.6685	0.504	0.658784714	count	1
PTAR1	0.4785056	0.5133151	0.9322	0.352	0.659326519	count	1
KLHDC2	0.4820062	0.4293928	1.1225	0.262	0.660736271	count	1
TCEA2	0.48558	0.4276792	1.1354	0.257	0.661350216	count	1
IBA57	0.5361304	0.6086437	0.8809	0.379	0.661367761	count	1
ARRDC3	0.4689883	0.286236	1.6385	0.102	0.661792746	count	1
RAD1	0.4988697	0.570737	0.8741	0.383	0.663286658	count	1
STX2	0.5019604	0.6553256	0.766	0.444	0.663942235	count	1
FHL1	0.5233614	0.8665069	0.604	0.546	0.664780743	count	1
DENND4C	0.5236085	0.6208321	0.8434	0.4	0.665095817	count	1
MYO9A	0.5236085	0.6962499	0.752	0.453	0.665095817	count	1
PELI2	1.07001	1.1435	0.9357	0.35	0.66606923	count	1
AC067852.2	1.07001	1.1435	0.9357	0.35	0.66606923	count	1
CLSPN	1.07001	1.303999	0.8206	0.412	0.66606923	count	1
WDR26	1.07001	1.303999	0.8206	0.412	0.66606923	count	1
FASTKD3	1.07001	1.303999	0.8206	0.412	0.66606923	count	1
AP002748.3	1.07001	1.303999	0.8206	0.412	0.66606923	count	1
ITPRIPL2	1.07001	1.505516	0.7107	0.4778	0.66606923	count	1
GALNT3	0.5313308	0.6586039	0.8068	0.42	0.666121247	count	1
DHDDS	0.5864007	0.7163053	0.8186	0.414	0.666154629	count	1
NADK2	0.5864007	0.7288011	0.8046	0.422	0.666154629	count	1
KDM3A	0.5864007	0.7817264	0.7501	0.454	0.666154629	count	1
XPO1	0.5101746	0.3697659	1.3797	0.169	0.666330076	count	1
FAHD2A	0.7696242	0.8740567	0.8805	0.379	0.666569473	count	1
TSTD2	0.7696242	0.7429705	1.0359	0.301	0.666569499	count	1
ZFP1	0.7696242	0.9743034	0.7899	0.4302	0.666569499	count	1
TXNDC17	0.4747125	0.3600098	1.3186	0.188	0.667014929	count	1
RICTOR	0.4940005	0.537568	0.919	0.359	0.667250001	count	1
LYSMD2	0.4827577	0.3190383	1.5132	0.131	0.668004473	count	1

YPEL5	0.4663841	0.1667503	2.7969	0.0055	0.668697611	count	1
STK39	0.5266649	0.4132991	1.2743	0.203	0.668992999	count	1
API5	0.5088074	0.5496097	0.9258	0.355	0.669056839	count	1
ZNF184	0.6718417	0.7944793	0.8456	0.398	0.669915977	count	1
PNPLA4	0.6218282	0.8605197	0.7226	0.47	0.671243237	count	1
BMI1	0.6218282	0.8605197	0.7226	0.47	0.671243237	count	1
UBQLN4	0.6218282	0.8648851	0.719	0.473	0.671243237	count	1
MTR	0.5143214	0.5933092	0.8669	0.387	0.671762697	count	1
RASSF3	0.5291076	0.6581511	0.8039	0.422	0.672107611	count	1
ZNF831	0.7763873	0.5210961	1.4899	0.137	0.672463828	count	1
HSH2D	0.4817804	0.3433032	1.4034	0.161	0.672682698	count	1
CSNK1E	0.5566781	0.7218398	0.7712	0.441	0.672960778	count	1
IL12RB1	0.6243083	0.8480897	0.7361	0.462	0.673939155	count	1
KLF6	0.468371	0.1316991	3.5564	0.000432	0.674538185	count	1
RWDD4	0.5032027	0.4676883	1.0759	0.283	0.674932819	count	1
UBE2E3	0.4860884	0.3024974	1.6069	0.109	0.67498201	count	1
EXOSC10	0.6254755	0.6935983	0.9018	0.368	0.67520787	count	1
PHF10	0.5020323	0.4626961	1.085	0.279	0.675857037	count	1
TMED7	0.5116306	0.5394309	0.9485	0.344	0.676767542	count	1
TYW1	0.5751103	0.6884865	0.8353	0.404	0.677153664	count	1
LRRC28	0.5751103	0.7589743	0.7577	0.449	0.677153664	count	1
DCK	0.4910328	0.3919316	1.2529	0.211	0.67762619	count	1
TRMT2A	0.5499727	0.6861163	0.8016	0.423	0.678522103	count	1
MRPS26	0.4789511	0.3097058	1.5465	0.123	0.679098922	count	1
HAUS6	0.550469	0.7211123	0.7634	0.446	0.679137118	count	1
PRPF31	0.4945534	0.3796173	1.3028	0.194	0.679215619	count	1
CYREN	0.5620164	0.8025772	0.7003	0.484	0.679446148	count	1
RRM2B	0.5982858	0.8230926	0.7269	0.468	0.679741068	count	1
SCAANT1	0.5982858	1.11043	0.5388	0.5904	0.679741068	count	1
MBTPS1	0.5006811	0.4568137	1.096	0.274	0.680229062	count	1
PMS2	0.6303681	0.7849379	0.8031	0.423	0.680525612	count	1
GGCX	0.536495	0.7303871	0.7345	0.463	0.681526835	count	1
IMPAD1	0.5153962	0.5676685	0.9079	0.365	0.681761655	count	1
CYSTM1	0.4866656	0.3403892	1.4297	0.154	0.681895142	count	1
SH3GLB2	0.5052113	0.4712504	1.0721	0.284	0.682423776	count	1
NFATC2IP	0.5161949	0.5310477	0.972	0.332	0.682820918	count	1
C8orf82	0.5137226	0.4952702	1.0373	0.3	0.683085049	count	1
SPATC1L	0.5382917	0.654352	0.8226	0.411	0.683817638	count	1
FNDC3A	0.6020204	0.5062199	1.1892	0.235	0.684009723	count	1
TRNAU1AP	0.5022007	0.4737668	1.06	0.29	0.684028773	count	1
CASC4	0.4911739	0.4741247	1.036	0.301	0.684075268	count	1
AP2A1	0.545971	0.8374803	0.6519	0.515	0.68455133	count	1
MSANTD3	0.5669953	0.7009061	0.8089	0.419	0.685494584	count	1
UGGT1	0.6874091	0.8630895	0.7965	0.426	0.685553051	count	1
ZNF195	0.5250087	0.5327313	0.9855	0.325	0.68576348	count	1

SUMO3	0.4946054	0.3063489	1.6145	0.107	0.686067297	count	1
GIMAP1	0.4922472	0.3194144	1.5411	0.124	0.68618057	count	1
EDEM1	0.5834019	0.7062326	0.8261	0.409	0.68696937	count	1
OAS1	0.5057596	0.6899579	0.733	0.464	0.687141474	count	1
BAG3	0.7966852	0.9022382	0.883	0.378	0.690138292	count	1
PLEKHG3	0.7966852	0.9853351	0.8085	0.419	0.690138292	count	1
AC079305.1	0.7966852	1.094321	0.728	0.467	0.690138292	count	1
KCTD13	0.7966852	1.094321	0.728	0.467	0.690138292	count	1
MAN2B1	0.7966852	1.094321	0.728	0.467	0.690138292	count	1
FANCL	0.7966852	1.240273	0.6423	0.5211	0.690138292	count	1
UBASH3B	0.5722324	0.6823568	0.8386	0.402	0.691856341	count	1
ADCK5	0.5875691	0.8207821	0.7159	0.475	0.691902142	count	1
ABCC1	0.5875691	0.9458158	0.6212	0.535	0.691902142	count	1
VPS35L	0.5875691	0.9747148	0.6028	0.547	0.691902142	count	1
TCF7L2	0.5875691	1.0216157	0.5751	0.566	0.691902142	count	1
LMF1	0.5875691	1.1179051	0.5256	0.6	0.691902142	count	1
HSD11B1L	0.5875691	1.1179051	0.5256	0.6	0.691902142	count	1
MGRN1	0.5233381	0.5806864	0.9012	0.368	0.692294353	count	1
PAF1	0.5236731	0.4894684	1.0699	0.285	0.692738629	count	1
ARL2BP	0.497401	0.3274762	1.5189	0.13	0.692757443	count	1
FOXJ3	0.5092815	0.384865	1.3233	0.187	0.69369016	count	1
SUPT3H	0.5532832	0.5596753	0.9886	0.324	0.693755724	count	1
NIFK-AS1	0.6106094	0.8514982	0.7171	0.474	0.693825882	count	1
EPS15L1	0.695762	0.7078197	0.983	0.326	0.693939473	count	1
GART	0.5216791	0.5689113	0.917	0.36	0.696895466	count	1
ZNF302	0.7001958	0.6212952	1.127	0.261	0.698389968	count	1
PTTG1	0.5127759	0.40512	1.2657	0.207	0.698458037	count	1
PUS1	0.5931493	0.7302017	0.8123	0.417	0.698507038	count	1
ZNF407	0.5931493	0.778279	0.7621	0.447	0.698507038	count	1
SLC10A7	0.5931493	0.805902	0.736	0.462	0.698507038	count	1
FBRSL1	0.5931493	0.8170019	0.726	0.468	0.698507038	count	1
USF3	0.5498892	0.5694428	0.9657	0.335	0.698603858	count	1
BDH2	0.5443732	0.549337	0.991	0.322	0.699143926	count	1
CDC42SE1	0.4899215	0.1871678	2.6176	0.0093	0.699297393	count	1
MRPL47	0.5113126	0.3868248	1.3218	0.187	0.699584506	count	1
EDC3	1.1254871	1.0790465	1.043	0.2977	0.699844955	count	1
GALNS	0.7019468	0.8356898	0.84	0.402	0.70014732	count	1
PEX10	0.7019468	0.9495329	0.7393	0.46	0.70014732	count	1
UBR1	0.5403903	0.5875003	0.9198	0.358	0.700407029	count	1
COQ10B	0.5061903	0.2739389	1.8478	0.0655	0.700471597	count	1
EMC2	0.5416242	0.5483794	0.9877	0.324	0.702011107	count	1
MDM1	0.7045575	0.8318629	0.847	0.398	0.702767251	count	1
DCAF6	0.7045575	0.8894962	0.7921	0.429	0.702767251	count	1
TBC1D24	0.7045575	0.9253184	0.7614	0.447	0.702767251	count	1
ALKBH5	0.5476459	0.5027489	1.0893	0.277	0.703360666	count	1

STAT4	0.535621	0.332937	1.6088	0.109	0.704414298	count	1
LRMDA	0.5828615	0.775378	0.7517	0.453	0.704766811	count	1
SLC35C1	0.8142363	1.020122	0.7982	0.425	0.705400169	count	1
TCTEX1D2	0.5718453	0.6251586	0.9147	0.361	0.705624118	count	1
AC074032.1	0.6543103	0.9027299	0.7248	0.469	0.706538761	count	1
BCOR	0.6544048	0.6654855	0.9833	0.326	0.706641404	count	1
KCTD11	0.654561	1.182424	0.5536	0.5803	0.706811059	count	1
SLC25A22	0.6548686	0.917004	0.7141	0.476	0.707145156	count	1
GALNT11	0.5189174	0.4663052	1.1128	0.267	0.708484062	count	1
NUDT4	0.526373	0.539236	0.9761	0.33	0.708694458	count	1
CXCR6	0.5062272	0.2482825	2.0389	0.0423	0.709328888	count	1
NDEL1	0.5309997	0.4088956	1.2986	0.195	0.709374305	count	1
HM13	0.5098545	0.315318	1.617	0.107	0.709457841	count	1
RGS19	0.5081134	0.3079988	1.6497	0.1	0.709487858	count	1
PTGER4	0.5052214	0.2551262	1.9803	0.0485	0.70954994	count	1
PDCD6IP	0.5156263	0.3403924	1.5148	0.131	0.710549647	count	1
PFDN4	0.5410273	0.3988961	1.3563	0.176	0.71154273	count	1
PGM1	0.5893853	0.6947632	0.8483	0.397	0.712689983	count	1
C5orf24	0.5328411	0.5516363	0.9659	0.335	0.714773714	count	1
CUL4B	0.5629764	0.6682323	0.8425	0.4	0.715287842	count	1
AL109955.1	0.5797745	0.8400875	0.6901	0.491	0.715447577	count	1
C5orf51	0.8260566	0.6956014	1.1875	0.236	0.715667066	count	1
RAB39B	0.6627558	0.6725901	0.9854	0.325	0.715710609	count	1
NEK7	0.5650043	0.5661636	0.998	0.319	0.717872916	count	1
LRP10	0.5258013	0.3447973	1.525	0.128	0.717897615	count	1
XPO4	0.7203559	0.7243804	0.9944	0.321	0.718615062	count	1
KCNAB2	0.5733809	0.4987284	1.1497	0.251	0.719051165	count	1
FAM91A1	0.5551243	0.6063835	0.9155	0.361	0.71956043	count	1
IRF4	0.6333982	0.890656	0.7112	0.477	0.719862475	count	1
AC147067.1	0.6333982	0.9466476	0.6691	0.504	0.719862475	count	1
KCTD6	0.6333982	0.9878099	0.6412	0.522	0.719862475	count	1
CYSLTR1	0.5251092	0.3797487	1.3828	0.168	0.719917397	count	1
CTDSP2	0.5316782	0.5503059	0.9662	0.335	0.72042478	count	1
FGD5-AS1	0.544563	0.3954383	1.3771	0.169	0.720441491	count	1
C9orf3	1.160136	0.8195849	1.4155	0.158	0.720757029	count	1
ADCK1	1.160136	0.8195849	1.4155	0.158	0.720757029	count	1
PLS1	1.160136	1.196596	0.9695	0.333	0.720757029	count	1
MIPEP	1.160136	1.196596	0.9695	0.333	0.720757029	count	1
SNX29	0.5448859	0.6380617	0.854	0.394	0.720869674	count	1
PTPN9	0.5842061	0.6201715	0.942	0.347	0.720937465	count	1
DHRS7B	0.8321855	0.912337	0.9121	0.362	0.720986656	count	1
MYBL1	0.5083562	0.2590937	1.9621	0.0506	0.721273076	count	1
NEDD1	0.5680853	0.7050418	0.8057	0.421	0.721800331	count	1
POLD3	0.7237997	0.9240372	0.7833	0.434	0.722068095	count	1
CRIP1	0.5074517	0.2047456	2.4784	0.0137	0.722448594	count	1

NUTF2	0.5140512	0.3146921	1.6335	0.103	0.723044892	count	1
FNIP1	0.5262776	0.380654	1.3826	0.168	0.724089569	count	1
TOMM40	0.5257267	0.4705983	1.1171	0.265	0.724486666	count	1
VEGFB	0.5170981	0.3420533	1.5117	0.1316	0.724574274	count	1
SLC39A13	1.166515	0.7462153	1.5632	0.119	0.724590543	count	1
RAMMET	0.5324414	0.4150116	1.283	0.2	0.725289441	count	1
C16orf72	0.5597526	0.4909173	1.1402	0.255	0.725576569	count	1
CCDC47	0.5240882	0.3673477	1.4267	0.155	0.726165758	count	1
TRAF3IP2-AS1	0.6389425	0.8619292	0.7413	0.459	0.726194932	count	1
ZNF106	0.5241306	0.3367263	1.5565	0.121	0.726224573	count	1
ZFP14	0.6005347	0.6546949	0.9173	0.36	0.726229251	count	1
HAUS2	0.5560954	0.49743	1.1179	0.264	0.726484128	count	1
CDK11B	0.5273047	0.3715743	1.4191	0.157	0.726664034	count	1
FBXO45	0.5564896	0.5874471	0.9473	0.344	0.727000444	count	1
NAA60	0.5663619	0.5776225	0.9805	0.328	0.727473472	count	1
LYPLA1	0.5228315	0.3279146	1.5944	0.112	0.727534148	count	1
ARFGAP2	0.5532714	0.4862296	1.1379	0.256	0.727686316	count	1
SPPL3	0.5677355	0.6491473	0.8746	0.382	0.729243005	count	1
RNF11	0.5816665	0.5482288	1.061	0.289	0.729478131	count	1
STX5	0.5399983	0.4617363	1.1695	0.243	0.729504946	count	1
ITSN2	0.5197271	0.3481209	1.493	0.136	0.730682536	count	1
ACYP1	0.5420466	0.4355356	1.2446	0.214	0.732276958	count	1
PDLIM1	0.5229385	0.3635434	1.4384	0.151	0.732297516	count	1
DMWD	0.5841647	0.6681049	0.8744	0.383	0.732621788	count	1
KMT5A	0.5441401	0.4369618	1.2453	0.214	0.732661856	count	1
MALSU1	0.5425387	0.5149417	1.0536	0.293	0.732942924	count	1
IFT88	0.5845463	0.7465882	0.783	0.4342	0.733101974	count	1
HADHA	0.5207964	0.2400572	2.1695	0.0308	0.733206445	count	1
SLFN13	0.5324369	0.577589	0.9218	0.3573	0.733745522	count	1
FBXL3	0.5579651	0.4521604	1.234	0.218	0.733874525	count	1
DRAXIN	0.5553242	0.5565734	0.9978	0.319	0.734711114	count	1
C3orf58	0.6465699	0.6388408	1.0121	0.312	0.734905196	count	1
USP42	0.5955264	0.7564691	0.7872	0.432	0.734959701	count	1
ZNF174	0.6808336	0.8395838	0.8109	0.418	0.735334923	count	1
KMT2E-AS1	0.6808395	0.6491891	1.0488	0.295	0.735341329	count	1
PGBD2	0.6808395	0.7274341	0.9359	0.35	0.735341329	count	1
OSM	0.5307167	0.4365437	1.2157	0.225	0.735360366	count	1
FKBP1B	0.7383038	1.0056691	0.7341	0.463	0.736604708	count	1
GNPDA1	0.7383038	1.0056691	0.7341	0.463	0.736604708	count	1
FRZB	0.5474284	0.8264919	0.6624	0.508	0.73709749	count	1
TPD52L2	0.5577458	0.6502277	0.8578	0.392	0.73792209	count	1
UNC13D	0.564989	0.4998072	1.1304	0.259	0.738132419	count	1
GGA1	0.5482769	0.4062832	1.3495	0.178	0.738242028	count	1
SPRTN	0.5697058	0.5289119	1.0771	0.282	0.738513579	count	1
HERC4	0.5452682	0.4792804	1.1377	0.256	0.738870804	count	1

ZNF839	0.8531766	0.8610911	0.9908	0.323	0.739184947	count	1
FECH	0.570249	0.5212346	1.094	0.275	0.739219568	count	1
ZBTB7B	0.6112918	0.7541947	0.8105	0.418	0.7392899	count	1
TTC39C	0.5214331	0.2766451	1.8848	0.0603	0.739683129	count	1
ACAT1	0.5821631	0.584394	0.9962	0.32	0.73974409	count	1
PRDM1	0.528793	0.2663456	1.9854	0.0479	0.740009461	count	1
NDUFC1	0.5319303	0.3562496	1.4931	0.136	0.740208035	count	1
POP4	0.5262729	0.3820341	1.3776	0.169	0.7402478	count	1
ZNF250	0.5711287	0.5769466	0.9899	0.323	0.740362948	count	1
DPH6	0.5543033	0.6420162	0.8634	0.389	0.740571936	count	1
MPV17	0.5465217	0.4569452	1.196	0.233	0.740572155	count	1
DDX31	0.6519432	0.7454985	0.8745	0.382	0.741040322	count	1
GTPBP6	0.5474501	0.4597381	1.1908	0.235	0.741832248	count	1
HMCES	0.5492413	0.4422339	1.242	0.215	0.742013498	count	1
LYPLAL1	0.6528065	0.7609238	0.8579	0.392	0.742025947	count	1
IFITM1	0.5193832	0.1761473	2.9486	0.0034	0.742480776	count	1
TIMMDC1	0.5397562	0.4038026	1.3367	0.182	0.74265855	count	1
PTCH2	1.1969573	1.0323193	1.1595	0.2471	0.742813767	count	1
WARS	1.1969573	1.0547999	1.1348	0.2573	0.742813767	count	1
CDC34	0.5409585	0.3370123	1.6052	0.109	0.743042497	count	1
HSPBAP1	0.8577227	0.8895384	0.9642	0.336	0.743121717	count	1
PGAP1	0.8577227	0.8987968	0.9543	0.341	0.743121717	count	1
SRPRB	0.5350537	0.4855731	1.1019	0.271	0.743826864	count	1
INO80C	0.6328307	0.7732591	0.8184	0.414	0.745456939	count	1
NFE2L1	0.6328307	0.8740446	0.724	0.47	0.745456939	count	1
PLEKHM2	0.5805392	0.5889134	0.9858	0.325	0.745736247	count	1
AP1M1	0.5690662	0.5223404	1.0895	0.277	0.74850949	count	1
C12orf49	0.5440121	0.4386902	1.2401	0.216	0.748521581	count	1
ZNF433	0.6190392	0.8260484	0.7494	0.454	0.748694881	count	1
EXTL3	0.6190392	0.9286534	0.6666	0.506	0.748694881	count	1
N4BP3	0.5731014	0.5879603	0.9747	0.33	0.748756838	count	1
STX1A	0.6070875	0.7832056	0.7751	0.439	0.749277927	count	1
TSPAN14	0.5834739	0.4815553	1.2116	0.227	0.749516331	count	1
CTSO	0.6952708	0.8164954	0.8515	0.395	0.750998357	count	1
ACSL1	0.867691	0.991747	0.8749	0.382	0.751748257	count	1
TOMM34	0.867691	0.991747	0.8749	0.382	0.751748257	count	1
AL355075.4	0.867691	1.17548	0.7382	0.461	0.751748257	count	1
AMPD2	0.5999824	0.9546947	0.6285	0.53	0.752524032	count	1
ZBTB43	0.6005224	0.5229906	1.1482	0.252	0.753203396	count	1
DAP	0.5639459	0.4635385	1.2166	0.225	0.753479769	count	1
KTI12	0.5732639	0.5156662	1.1117	0.267	0.754043141	count	1
AL355001.2	0.7565224	0.9483539	0.7977	0.426	0.75484858	count	1
COPZ2	0.7565224	1.053465	0.7181	0.473	0.75484858	count	1
ENDOG	0.6640552	0.6676557	0.9946	0.321	0.754866272	count	1
PAFAH2	0.5706688	0.6173209	0.9244	0.356	0.755056728	count	1

COPRS	0.5642036	0.5610625	1.0056	0.315	0.756926825	count	1
PSMD3	0.5587657	0.4384539	1.2744	0.203	0.757190172	count	1
BNIP1	0.5605986	0.4579087	1.2243	0.222	0.75738256	count	1
LASP1	0.5560679	0.3578581	1.5539	0.121	0.75752272	count	1
TBCD	0.5409188	0.3268877	1.6548	0.0989	0.758442437	count	1
POM121	0.6438866	0.5922092	1.0873	0.278	0.758531265	count	1
AP1G2	0.6438866	0.6217705	1.0356	0.301	0.758531265	count	1
NCF1	0.5414187	0.2821767	1.9187	0.0559	0.758680542	count	1
PYCR2	0.5463956	0.3547446	1.5403	0.124	0.758821687	count	1
SLAMF7	0.5547533	0.3839581	1.4448	0.149	0.759110108	count	1
SNIP1	0.6294168	0.581763	1.0819	0.28	0.761290683	count	1
GALNT7	0.6296961	0.7927453	0.7943	0.428	0.761629651	count	1
NPM3	0.6296961	0.7927453	0.7943	0.428	0.761629651	count	1
RFC4	0.5757033	0.4943945	1.1645	0.245	0.761731543	count	1
ZNF146	0.5880963	0.5701305	1.0315	0.303	0.762414229	count	1
HEG1	0.5938974	0.7538513	0.7878	0.431	0.762941478	count	1
PATJ	0.6324784	0.5569471	1.1356	0.257	0.765006221	count	1
PELI1	1.2343612	1.158777	1.0652	0.288	0.765034007	count	1
DBF4	0.554286	0.3743577	1.4806	0.14	0.766100802	count	1
PARP2	0.5963569	0.6203344	0.9613	0.337	0.76610901	count	1
PPP2R5A	0.6335257	0.6009639	1.0542	0.293	0.766277154	count	1
COQ7	0.6740995	0.6118516	1.1017	0.271	0.766328239	count	1
ADRB2	0.5442145	0.268117	2.0298	0.0432	0.767739967	count	1
KPNA6	0.5749029	0.5189197	1.1079	0.269	0.768146118	count	1
RTKN	0.6353502	0.7065015	0.8993	0.369	0.768491197	count	1
CYB5R1	0.5931493	0.5450465	1.0883	0.277	0.768980447	count	1
TRAPPC3	0.556141	0.3524048	1.5781	0.116	0.769675827	count	1
CYCS	0.5401324	0.1800954	2.9991	0.0029	0.770440588	count	1
GZMH	0.5400233	0.2554364	2.1141	0.0353	0.770833492	count	1
DNPEP	0.5593869	0.4192418	1.3343	0.183	0.772079444	count	1
CMTR1	0.6013026	0.7801565	0.7707	0.441	0.772478184	count	1
POLR3H	0.7151939	0.8117597	0.881	0.379	0.772599362	count	1
CKAP5	0.6261591	0.7867592	0.7959	0.427	0.772892059	count	1
SAC3D1	0.7162687	0.9034109	0.7928	0.428	0.773764186	count	1
MGST2	0.568217	0.4845532	1.1727	0.242	0.774096211	count	1
FEZ1	0.5770791	0.5207313	1.1082	0.269	0.774230052	count	1
HTATIP2	0.5647275	0.4787309	1.1796	0.239	0.774307721	count	1
GPN3	0.5665567	0.4755543	1.1914	0.234	0.775282267	count	1
FBXW2	0.5576908	0.4633055	1.2037	0.23	0.775326801	count	1
ZADH2	0.5836977	0.5760326	1.0133	0.312	0.776341948	count	1
MAP3K11	0.6590171	0.6230387	1.0577	0.291	0.776418487	count	1
SBF2	0.5988799	0.8280775	0.7232	0.47	0.776426743	count	1
GRSF1	0.5687236	0.4344678	1.309	0.191	0.776587402	count	1
WNT10A	0.683664	0.9654447	0.7081	0.479	0.777239375	count	1
AKAP8L	0.564221	0.3447778	1.6365	0.103	0.777599029	count	1

TMEM41B	0.5852016	0.5050603	1.1587	0.247	0.778345754	count	1
DEDD	1.259205	0.8121517	1.5505	0.122	0.779684176	count	1
TMEM39A	0.6618946	0.7656166	0.8645	0.388	0.779819492	count	1
GBA	0.6618946	0.7694962	0.8602	0.39	0.779819492	count	1
WDR11	0.7819408	0.9397102	0.8321	0.406	0.780270599	count	1
PCMTD1	0.5526378	0.2562761	2.1564	0.0318	0.780709676	count	1
COPS2	0.56049	0.3665928	1.5289	0.127	0.780714182	count	1
NXT2	0.7827662	1.01546	0.7708	0.441	0.781095449	count	1
TANC2	0.6468942	0.948861	0.6818	0.496	0.782497937	count	1
TYW5	0.6650288	0.8508551	0.7816	0.435	0.783523579	count	1
STMP1	0.5582331	0.2921205	1.911	0.0569	0.784855302	count	1
TGDS	0.6359237	0.6565993	0.9685	0.334	0.784979345	count	1
BOLA1	0.9067213	0.942809	0.9617	0.337	0.785444092	count	1
RND3	0.9067213	0.942809	0.9617	0.337	0.785444092	count	1
GRAMD1C	0.9067213	0.942809	0.9617	0.337	0.785444092	count	1
FGFR1	0.9067213	0.942809	0.9617	0.337	0.785444092	count	1
SGMS1-AS1	0.9067213	0.942809	0.9617	0.337	0.785444092	count	1
DOT1L	0.9067213	0.942809	0.9617	0.337	0.785444092	count	1
FOSB	0.5472365	0.1661292	3.294	0.0011	0.785665936	count	1
ARMC5	0.618239	0.9517989	0.6495	0.516	0.785713416	count	1
NSUN5	0.6022612	0.6711396	0.8974	0.37	0.786939517	count	1
CSAD	0.5917264	0.6240463	0.9482	0.344	0.787039138	count	1
AP3D1	0.6277879	0.6849772	0.9165	0.36	0.787498815	count	1
MED27	0.581311	0.471981	1.2316	0.219	0.787786606	count	1
TNRC6C	0.5770189	0.5158822	1.1185	0.264	0.787928629	count	1
C16orf54	0.5528894	0.2182915	2.5328	0.0118	0.788169653	count	1
DNAJB12	0.584463	0.4413171	1.3244	0.186	0.789673333	count	1
ZNF449	0.9121911	0.8819212	1.0343	0.302	0.790155428	count	1
AC098487.1	0.9121911	1.172756	0.7778	0.437	0.790155428	count	1
BTBD7	0.6305555	0.4616564	1.3659	0.173	0.790979151	count	1
CEP126	0.6717375	0.7921386	0.848	0.397	0.791451072	count	1
MTERF4	0.5835907	0.4353728	1.3404	0.181	0.793063114	count	1
BOP1	0.7354035	0.8198254	0.897	0.37	0.794492368	count	1
MTG1	0.699258	1.054058	0.6634	0.508	0.795021523	count	1
CYFIP1	0.699258	1.054058	0.6634	0.508	0.795021523	count	1
DLGAP4	0.6446756	0.5334766	1.2084	0.228	0.79581111	count	1
PPID	0.6031171	0.5707301	1.0567	0.291	0.798072128	count	1
SVIL	0.6363842	0.7057364	0.9017	0.368	0.798308362	count	1
CIDEB	0.5914899	0.549587	1.0762	0.283	0.799180415	count	1
MOB3C	0.9241973	0.8695966	1.0628	0.289	0.800487001	count	1
RINL	0.5838642	0.4506701	1.2955	0.196	0.800576319	count	1
GNE	1.297061	1.211043	1.071	0.285	0.801833551	count	1
BHLHE40	0.5648428	0.1961731	2.8793	0.0043	0.802909199	count	1
UAP1L1	0.9282238	0.8865019	1.0471	0.296	0.803948759	count	1
C2orf69	0.9282238	1.0366397	0.8954	0.371	0.803948759	count	1

ZNF3	0.9282238	1.0366397	0.8954	0.371	0.803948759	count	1
ATXN7L1	0.9282238	1.12223	0.8271	0.409	0.803948759	count	1
8-Sep	0.9282238	1.4600855	0.6357	0.5254	0.803948759	count	1
ISOC2	0.6033305	0.5231287	1.1533	0.25	0.806191796	count	1
AC092683.1	0.7469682	0.7616178	0.9808	0.327	0.807011209	count	1
PSMA3-AS1	0.5807621	0.3248167	1.788	0.0747	0.807428482	count	1
NAGLU	0.7106368	0.6174101	1.151	0.251	0.807990784	count	1
FCRL6	0.6357904	0.5423277	1.1723	0.242	0.808069563	count	1
FBXO21	0.5962734	0.4797812	1.2428	0.215	0.808089787	count	1
GEMIN2	0.9333181	0.756115	1.2344	0.218	0.808326281	count	1
GOLPH3	0.5934615	0.4361221	1.3608	0.175	0.8085305	count	1
3-Mar	0.65517	0.74636	0.8778	0.381	0.808796912	count	1
TRIM37	0.6366045	0.6324936	1.0065	0.315	0.809106373	count	1
HCG11	0.8111461	0.8105531	1.0007	0.318	0.809429525	count	1
EPM2AIP1	0.6126404	0.5448498	1.1244	0.262	0.810694268	count	1
STARD7	0.5873592	0.3687771	1.5927	0.112	0.811857403	count	1
KRTCAP2	0.5745797	0.2519669	2.2804	0.0232	0.813386981	count	1
HELQ	0.6279808	0.5532997	1.135	0.257	0.814232229	count	1
CUTC	0.5965425	0.4162085	1.4333	0.153	0.81461888	count	1
VPS13B	0.6340375	0.6936325	0.9141	0.361	0.814624326	count	1
CHCHD7	0.5848936	0.4790539	1.2209	0.223	0.814732688	count	1
ANO6	0.7565224	0.6803872	1.1119	0.267	0.817348296	count	1
AC006033.2	0.7565224	0.9523662	0.7944	0.428	0.817348296	count	1
KIF3B	0.6313663	0.639258	0.9877	0.324	0.818629435	count	1
TNFRSF14	0.5971138	0.3678769	1.6231	0.106	0.818762177	count	1
USP10	0.6083962	0.5099723	1.193	0.234	0.819321595	count	1
MPZ	0.8248891	1.177818	0.7004	0.484	0.823130335	count	1
GPC1	0.8248891	1.177818	0.7004	0.484	0.823130335	count	1
TTI2	0.8248891	1.177818	0.7004	0.484	0.823130335	count	1
BRCA2	0.8248891	1.177818	0.7004	0.484	0.823130335	count	1
DPEP2	0.8248891	1.177818	0.7004	0.484	0.823130335	count	1
MTHFSD	0.8248891	1.177818	0.7004	0.484	0.823130335	count	1
POLR2J2	0.8248891	1.299356	0.6348	0.526	0.823130335	count	1
CDC7	0.8248891	1.4207827	0.5806	0.5619	0.823130335	count	1
PRSS21	0.8248891	1.4207827	0.5806	0.5619	0.823130335	count	1
C20orf197	0.8248891	1.4207827	0.5806	0.5619	0.823130335	count	1
EML4	0.5773361	0.1929512	2.9921	0.003	0.823443064	count	1
GZF1	0.6260639	0.6131629	1.021	0.308	0.823628376	count	1
TBCCD1	0.9526674	1.0989739	0.8669	0.3867	0.824929415	count	1
GRINA	0.7019468	0.6573348	1.0679	0.286	0.827129217	count	1
RNF170	0.7019468	0.8335806	0.8421	0.4	0.827129217	count	1
ACTA2	0.6089255	0.5766061	1.0561	0.292	0.827531973	count	1
ABCE1	0.6293411	0.4896594	1.2853	0.2	0.827946054	count	1
DDX59	0.6230221	0.5129083	1.2147	0.225	0.82872888	count	1
MDFIC	0.5866831	0.2672679	2.1951	0.0289	0.828827757	count	1

METTL17	0.652495	0.5803168	1.1244	0.262	0.829340987	count	1
CDKL1	0.8314041	0.8576276	0.9694	0.333	0.829620493	count	1
EXOSC2	0.8314041	0.9130464	0.9106	0.363	0.829620493	count	1
IDS	0.5848573	0.2056854	2.8435	0.00475	0.830658511	count	1
SEC23A	0.7309427	0.7595896	0.9623	0.337	0.831120865	count	1
BRMS1	0.6006071	0.3734215	1.6084	0.109	0.831271075	count	1
FMNL1	0.6011048	0.3143173	1.9124	0.0567	0.831960424	count	1
EXTL2	0.8341701	1.0683029	0.7808	0.4355	0.832374982	count	1
AC020916.1	0.5850129	0.2285749	2.5594	0.0109	0.833612106	count	1
SLC1A5	0.7081422	0.4635581	1.5276	0.128	0.834441944	count	1
COASY	0.6501341	0.5499233	1.1822	0.238	0.835341054	count	1
KATNBL1	0.6255709	0.4950091	1.2638	0.207	0.835950091	count	1
RNF138	0.6287019	0.4162265	1.5105	0.132	0.836293613	count	1
FURIN	0.6593059	0.7680109	0.8585	0.391	0.838012029	count	1
GYG1	0.5988406	0.2771411	2.1608	0.0314	0.838108176	count	1
FIZ1	0.7760078	0.9091096	0.8536	0.394	0.838414444	count	1
ZNF254	0.7760078	0.9619356	0.8067	0.42	0.838414444	count	1
BCL9L	0.776541	0.6794316	1.1429	0.254	0.838990573	count	1
RFK	0.6938449	0.476756	1.4553	0.147	0.83942478	count	1
ORAI3	0.6691484	0.6655304	1.0054	0.315	0.839491971	count	1
CARD8-AS1	1.3629408	1.1510569	1.1841	0.2372	0.839850959	count	1
AFF4	0.6140868	0.5312589	1.1559	0.2486	0.840393852	count	1
TBC1D31	0.7778429	0.5929611	1.3118	0.191	0.840397264	count	1
SOD2	0.7778815	0.6634231	1.1725	0.242	0.840438982	count	1
ATP6V1B2	0.629141	0.5305147	1.1859	0.237	0.840726359	count	1
SARM1	0.9719011	1.152006	0.8437	0.399	0.841394665	count	1
STARD5	0.9719013	1.172142	0.8292	0.408	0.841394837	count	1
NSDHL	0.9719013	1.208813	0.804	0.422	0.841394837	count	1
ALOX12-AS1	0.9719013	1.2701536	0.7652	0.4447	0.841394837	count	1
STRADB	0.9719013	1.304071	0.7453	0.4566	0.841394837	count	1
SPEF2	0.9719013	1.304071	0.7453	0.4566	0.841394837	count	1
RBM15	0.6954983	0.8418389	0.8262	0.409	0.841428178	count	1
KYAT3	0.7144461	0.7320301	0.976	0.33	0.841881159	count	1
GSPT2	0.6716556	0.6895322	0.9741	0.331	0.842642298	count	1
ALDH6A1	0.7151917	0.774889	0.923	0.357	0.842760921	count	1
HOOK2	0.6338374	0.5426901	1.168	0.244	0.843132989	count	1
CDS2	0.6376733	0.4447447	1.4338	0.153	0.843866328	count	1
SLCO3A1	0.6377696	0.4885186	1.3055	0.193	0.84399392	count	1
RPP40	0.7164165	0.8512267	0.8416	0.401	0.844206078	count	1
CXCL2	0.7164165	1.0533107	0.6802	0.497	0.844206078	count	1
TSC22D3	0.5866432	0.097753	6.0013	5.24E-09	0.844916571	count	0.000127421
NOC3L	0.613297	0.4922809	1.2458	0.214	0.845298314	count	1
SOCS2	0.6059554	0.3228704	1.8768	0.0614	0.846221295	count	1
HDDC2	0.6156499	0.3933752	1.565	0.119	0.84719473	count	1
ZNF236	0.6761244	0.6869336	0.9843	0.326	0.848256943	count	1

FBXL6	0.6304571	0.5065205	1.2447	0.214	0.84906418	count	1
ASXL1	0.6110946	0.3326266	1.8372	0.0671	0.849628728	count	1
TATDN3	0.630948	0.7124276	0.8856	0.376	0.849725934	count	1
ANKRD42	1.3816545	1.000183	1.3814	0.168	0.850521916	count	1
XPO7	1.3816545	1.3988795	0.9877	0.324	0.850521916	count	1
PPP1R15A	0.5932012	0.1844146	3.2167	0.0014	0.850630049	count	1
GNB1L	0.748374	0.8722011	0.858	0.392	0.850961021	count	1
HEXA	0.6407504	0.3885525	1.6491	0.1	0.85233895	count	1
C1orf123	0.6308101	0.4059558	1.5539	0.121	0.852368983	count	1
TMC8	0.644242	0.5285196	1.219	0.224	0.852569077	count	1
CRELD2	0.6544405	0.4231583	1.5466	0.123	0.855231891	count	1
NCBP2	0.6596811	0.4987095	1.3228	0.187	0.855396835	count	1
ASPH	0.6820531	0.6020609	1.1329	0.258	0.855704944	count	1
TFCP2	0.682521	0.7212146	0.9463	0.345	0.856292706	count	1
LRRC27	0.682521	1.1679841	0.5844	0.5594	0.856292717	count	1
CRYL1	0.6289429	0.4078364	1.5421	0.124	0.856918112	count	1
GRAMD1A	0.6088077	0.2990695	2.0357	0.0426	0.85718653	count	1
MMS19	0.6835777	0.6926198	0.9869	0.324	0.857620075	count	1
DAZAP2	0.6082982	0.2205136	2.7586	0.00614	0.857867634	count	1
AC044802.2	0.8598084	1.2618668	0.6814	0.4961	0.857878152	count	1
IL21R	0.7966852	0.704335	1.1311	0.259	0.860744305	count	1
ZNF66	0.7966852	0.9616556	0.8285	0.408	0.860744305	count	1
LINC00909	0.6511828	0.5191057	1.2544	0.211	0.861763981	count	1
ZNF329	0.7315828	0.8236766	0.8882	0.375	0.862095626	count	1
PM20D2	0.7315828	0.9388608	0.7792	0.436	0.862095626	count	1
FBXO38	1.402645	0.9335181	1.5025	0.134	0.862421424	count	1
ID3	0.6170878	0.4054418	1.522	0.129	0.862432289	count	1
TCAF1	0.6716744	0.9824991	0.6836	0.4947	0.863054867	count	1
STX18	0.661381	0.6118375	1.081	0.281	0.864311768	count	1
IQCB1	0.6446266	0.4587536	1.4052	0.161	0.864976136	count	1
AC008741.2	0.8006537	1.0903752	0.7343	0.4633	0.865026834	count	1
GAS6	0.8006537	1.0970371	0.7298	0.466	0.865026834	count	1
CPSF7	0.7007272	0.7058684	0.9927	0.322	0.865132655	count	1
MMP25	0.9999835	0.7983533	1.2526	0.211	0.865361979	count	1
TNFAIP8L1	0.8013274	0.6612113	1.2119	0.226	0.865753756	count	1
CCDC7	0.8013274	0.6747968	1.1875	0.236	0.865753756	count	1
KIF1BP	0.7025692	0.7368052	0.9535	0.341	0.867409047	count	1
MRPL28	0.6271906	0.3599015	1.7427	0.0823	0.868088314	count	1
PARD6A	0.6566419	0.5601521	1.1723	0.242	0.868995365	count	1
FMR1	0.6343158	0.4365286	1.4531	0.147	0.869815598	count	1
MAP9	0.7383525	0.4502833	1.6398	0.102	0.870077599	count	1
ISG20L2	0.6717441	0.4106614	1.6358	0.103	0.871055647	count	1
SMC2	0.8077851	0.7255605	1.1133	0.266	0.872719952	count	1
TSR1	0.8749389	0.7690328	1.1377	0.256	0.872903695	count	1
ST6GALNAC4	0.8749389	0.9395257	0.9313	0.352	0.872903695	count	1

ORC4	0.6873214	0.570223	1.2054	0.229	0.873666366	count	1
CLEC12A	0.654014	0.5314185	1.2307	0.219	0.873997257	count	1
CCDC69	0.633292	0.3745215	1.6909	0.0918	0.874173872	count	1
PIGX	0.660741	0.4566735	1.4469	0.149	0.874424866	count	1
COPG1	0.722818	0.8134235	0.8886	0.375	0.874516358	count	1
ZNF654	0.7428269	0.6973386	1.0652	0.288	0.875352089	count	1
TFG	0.6389882	0.346893	1.842	0.0664	0.876226669	count	1
EFTUD2	0.6898537	0.6949321	0.9927	0.322	0.876888072	count	1
RTN3	0.654134	0.3512387	1.8624	0.0635	0.877743716	count	1
VPS16	0.6769238	0.595453	1.1368	0.256	0.877778294	count	1
LPAR6	0.7256436	0.8445396	0.8592	0.391	0.877936904	count	1
ADAM19	1.4317781	1.3734989	1.0424	0.298	0.878811074	count	1
TAF1A	1.4317781	1.5331462	0.9339	0.3511	0.878811074	count	1
CD58	0.6350905	0.3575109	1.7764	0.0766	0.880110095	count	1
SLC35E3	0.6742525	0.7237658	0.9316	0.352	0.881148108	count	1
ITGAV	0.7151889	0.8367093	0.8548	0.393	0.883001685	count	1
EID2	0.7162687	0.5989067	1.196	0.233	0.884335595	count	1
PANX1	1.0238786	1.133269	0.9035	0.367	0.885683591	count	1
FBXO8	0.6789063	0.5424174	1.2516	0.212	0.887234511	count	1
ZNF708	0.6792206	0.4624142	1.4689	0.143	0.88764554	count	1
PIGF	0.6534502	0.5360354	1.219	0.224	0.888092061	count	1
OGFR	0.6481413	0.3902252	1.6609	0.0977	0.888785052	count	1
AC097376.2	0.6855881	0.6485942	1.057	0.291	0.889022082	count	1
MED31	0.6458943	0.4076349	1.5845	0.114	0.890254303	count	1
IRAK2	0.8242288	0.8101745	1.0173	0.31	0.890445647	count	1
FOXO1	0.6590701	0.5135852	1.2833	0.2	0.890584047	count	1
POLR2A	0.7213768	0.5527885	1.305	0.193	0.890645199	count	1
MAP3K14	0.7560957	0.7949774	0.9511	0.342	0.890987957	count	1
TIMM44	0.681778	0.5218125	1.3066	0.192	0.89098997	count	1
SEC13	0.6457319	0.393818	1.6397	0.102	0.891354088	count	1
NDUFS2	0.6874091	0.4084292	1.6831	0.0933	0.891384975	count	1
GFI1	0.7565224	0.9222521	0.8203	0.413	0.891490641	count	1
AAMP	0.6463066	0.4011037	1.6113	0.108	0.893390846	count	1
ARHGEF2	0.6573981	0.4480171	1.4674	0.143	0.893460541	count	1
ATP23	0.7030822	0.6528738	1.0769	0.282	0.893714885	count	1
KLF16	0.6892709	0.5121202	1.3459	0.179	0.89380073	count	1
TESC	0.6340527	0.3143042	2.0173	0.0445	0.893853935	count	1
NIN	0.7125319	0.5629708	1.2657	0.207	0.893977203	count	1
UTP6	0.7241971	0.5673091	1.2765	0.203	0.894128446	count	1
TIMM9	0.6843061	0.4722327	1.4491	0.148	0.894295921	count	1
AC093010.2	0.8283724	0.6614876	1.2523	0.211	0.894909304	count	1
CDC25B	0.7042123	0.7063889	0.9969	0.32	0.895152138	count	1
MTF1	0.7882692	0.7676568	1.0269	0.305	0.896309147	count	1
CLK2	0.7882692	0.9237562	0.8533	0.394	0.896309147	count	1
HIST1H4E	0.7890888	0.8651086	0.9121	0.362	0.897239831	count	1

C11orf21	0.6829479	0.6128478	1.1144	0.266	0.898544058	count	1
FOS	0.6243204	0.1556357	4.0114	7.50E-05	0.900141139	count	1
CORO1C	0.8337364	0.8294052	1.0052	0.316	0.900685767	count	1
TTC31	0.8337364	0.8937911	0.9328	0.352	0.900685767	count	1
MTMR1	0.9032018	0.906752	0.9961	0.32	0.90091684	count	1
TAF11	0.7190302	0.5364203	1.3404	0.181	0.90213316	count	1
VPS35	0.6549982	0.3372507	1.9422	0.053	0.902807833	count	1
RABGGTB	0.702783	0.4508271	1.5589	0.12	0.903058432	count	1
RBBP8	0.6965149	0.6201786	1.1231	0.262	0.903199205	count	1
AC108863.1	1.476417	0.9061021	1.6294	0.104	0.90363347	count	1
TBC1D7	1.476417	0.9803792	1.506	0.133	0.90363347	count	1
DSN1	1.4764173	1.4194699	1.0401	0.2991	0.903633631	count	1
MFSD3	0.7952134	0.9776947	0.8134	0.417	0.904193187	count	1
EIF2B2	0.6875527	0.4820334	1.4264	0.155	0.904605599	count	1
DDX56	0.692883	0.5539617	1.2508	0.212	0.905510633	count	1
PLIN3	0.6682984	0.5250922	1.2727	0.204	0.905787823	count	1
METTL18	0.7693819	0.7276592	1.0573	0.291	0.906635598	count	1
RNF216	0.6763746	0.4226659	1.6003	0.111	0.907604923	count	1
BICRAL	1.0498464	1.0716705	0.9796	0.328	0.907688677	count	1
IVD	0.6760136	0.4485263	1.5072	0.133	0.91046148	count	1
NFAT5	0.7726448	0.581763	1.3281	0.185	0.910477045	count	1
MIR22HG	0.7381845	0.4208574	1.754	0.0804	0.911399249	count	1
KDM5B	0.6596904	0.3544986	1.8609	0.0637	0.911898216	count	1
PPP1R15B	0.6856152	0.4123179	1.6628	0.0973	0.912064239	count	1
MANEA-DT	0.9148697	0.7690343	1.1896	0.235	0.912460185	count	1
MTSS1	0.741069	0.6776814	1.0935	0.275	0.914959874	count	1
TXNDC15	0.6649677	0.4011603	1.6576	0.0984	0.915098448	count	1
MARCKSL1	0.6823253	0.4642326	1.4698	0.143	0.915593092	count	1
SDHD	0.6546313	0.3010956	2.1742	0.0304	0.919514845	count	1
FCRLB	0.8105352	0.8372916	0.968	0.334	0.921578153	count	1
MED15	0.6930998	0.5327312	1.301	0.194	0.92202409	count	1
ZNF44	0.7827663	0.7305599	1.0715	0.285	0.92238957	count	1
MTCH2	0.7064059	0.4875089	1.449	0.148	0.923188713	count	1
SGMS1	0.7263685	0.6585644	1.103	0.271	0.923322017	count	1
CERS4	0.6663768	0.4099706	1.6254	0.105	0.923482678	count	1
E2F3	0.8550916	0.6712208	1.2739	0.204	0.923661774	count	1
SEC24D	1.0692665	0.8837647	1.2099	0.227	0.924088684	count	1
MAST3	1.0692665	1.2494034	0.8558	0.3927	0.924088684	count	1
ENTPD3-AS1	1.513734	1.197349	1.2642	0.207	0.924105921	count	1
AMBRA1	1.513734	1.197349	1.2642	0.207	0.924105921	count	1
ZNF529	0.8555982	0.9289683	0.921	0.358	0.924206421	count	1
NLRP3	1.07001	0.9405409	1.1377	0.256	0.924715558	count	1
SDR42E2	1.07001	1.160414	0.9221	0.357	0.924715558	count	1
MATN1-AS1	1.07001	1.2198777	0.8771	0.3811	0.924715558	count	1
TAF4	1.07001	1.303999	0.8206	0.4125	0.924715558	count	1

ZNF225	1.07001	1.303999	0.8206	0.4125	0.924715558	count	1
RRAS2	0.764785	0.5602164	1.3652	0.173	0.925284414	count	1
ARNT	0.7290691	0.5998636	1.2154	0.225	0.92675451	count	1
MFSD11	0.686469	0.5704421	1.2034	0.23	0.927622861	count	1
RBM15B	0.7675129	0.7039735	1.0903	0.276	0.928581601	count	1
PRR34-AS1	0.8176692	0.6171948	1.3248	0.186	0.929667696	count	1
COMMD10	0.6991909	0.4273597	1.6361	0.103	0.930128699	count	1
SSFA2	0.8615628	0.7461675	1.1547	0.249	0.930617177	count	1
CCDC84	0.7074071	0.5943693	1.1902	0.235	0.930735198	count	1
KIF20B	0.7122787	0.5923818	1.2024	0.23	0.930864557	count	1
MAPKAPK5	0.7078088	0.5662976	1.2499	0.212	0.931263753	count	1
ADA2	1.0796774	0.8316202	1.2983	0.195	0.932859945	count	1
PPP1R37	1.0797222	0.8739832	1.2354	0.218	0.932897669	count	1
RIOX1	1.0797222	0.9513375	1.135	0.2572	0.932897669	count	1
SLC25A1	0.6983756	0.470513	1.4843	0.139	0.933309616	count	1
ME2	0.6992236	0.5182147	1.3493	0.178	0.934443028	count	1
CBWD1	0.7025693	0.599028	1.1728	0.242	0.93462354	count	1
SMIM10L1	0.66669	0.3109491	2.144	0.0328	0.934882954	count	1
HDGF	0.6593246	0.2588103	2.5475	0.0113	0.9351601	count	1
MARK2	0.727778	0.4731012	1.5383	0.125	0.935180083	count	1
GUCD1	0.7219657	0.716878	1.0071	0.315	0.936208027	count	1
UBE3B	0.7367578	0.7377999	0.9986	0.319	0.936525516	count	1
MMP24OS	0.6778107	0.3160207	2.1448	0.0327	0.938177245	count	1
MINDY2	0.7028381	0.4490565	1.5651	0.119	0.939273895	count	1
FARSB	1.544769	0.8314128	1.858	0.0641	0.940932685	count	1
RGS9	0.7203754	0.6500226	1.1082	0.269	0.941445522	count	1
USP53	0.7636078	0.7007212	1.0897	0.277	0.942769771	count	1
SFI1	0.7794053	0.6165061	1.2642	0.207	0.942951627	count	1
MFSD10	0.6781291	0.3180238	2.1323	0.0337	0.945498757	count	1
TMEM184B	0.8332211	0.6274245	1.328	0.185	0.947290939	count	1
PIK3R5	0.8046421	0.7323262	1.0987	0.273	0.948116955	count	1
PLEKHM1	0.8781377	0.6768591	1.2974	0.195	0.948416863	count	1
DCBLD1	0.8781377	0.9452647	0.929	0.354	0.948416863	count	1
EEF1E1	0.6965691	0.4481427	1.5543	0.121	0.949099451	count	1
E2F6	0.8790045	1.0926558	0.8045	0.422	0.949347115	count	1
VIRMA	0.7324654	0.5039657	1.4534	0.147	0.949820247	count	1
AC090152.1	0.6927339	0.3903261	1.7748	0.0769	0.949952523	count	1
TMEM248	0.6898078	0.350281	1.9693	0.0498	0.950798414	count	1
BANP	0.6968213	0.5370286	1.2975	0.195	0.951641812	count	1
SHKBP1	0.6884692	0.4292476	1.6039	0.11	0.951687268	count	1
WDR70	0.8371707	0.6295684	1.3298	0.185	0.951763955	count	1
NBL1	0.6939238	0.4161101	1.6676	0.0964	0.953328257	count	1
STXBP3	0.6998215	0.405608	1.7254	0.0854	0.953531059	count	1
DAB2IP	0.7725276	0.6488398	1.1906	0.235	0.953769455	count	1
SGPP1	0.7725276	0.7723617	1.0002	0.318	0.953769455	count	1

IPO9	0.9574092	0.9897145	0.9674	0.334	0.954431511	count	1
PSMA6	0.7739165	0.7234191	1.0698	0.286	0.955481881	count	1
ARHGAP17	1.1075798	1.0501857	1.0547	0.2924	0.956294394	count	1
AFDN	1.1075798	1.1425195	0.9694	0.3331	0.956294394	count	1
ZNF431	0.7379858	0.5327391	1.3853	0.167	0.956975734	count	1
P3H1	1.1090529	0.8255421	1.3434	0.18	0.95752854	count	1
AVPI1	0.7760078	0.8541335	0.9085	0.364	0.958060169	count	1
LMF2	0.7348415	0.4485348	1.6383	0.102	0.960345531	count	1
STK40	0.8894486	0.9089617	0.9785	0.329	0.960550445	count	1
SLC35G1	0.8894486	0.9938446	0.895	0.371	0.960550445	count	1
PREP	0.7945575	0.5735068	1.3854	0.167	0.961250172	count	1
ZNF484	1.1140764	1.0022284	1.1116	0.267	0.961734906	count	1
TTC21B	0.8165871	0.8613832	0.948	0.344	0.962153294	count	1
POP7	0.7064998	0.4745604	1.4887	0.138	0.962630119	count	1
CDR2	0.7958525	0.8115496	0.9807	0.327	0.96281351	count	1
MRPS18A	0.7493627	0.6248836	1.1992	0.231	0.962902852	count	1
PIGO	0.8475912	0.9275903	0.9138	0.362	0.963560153	count	1
TBCE	0.817906	1.107841	0.7383	0.4609	0.963702573	count	1
LRRC23	0.7693715	0.8139752	0.9452	0.345	0.965262589	count	1
PMVK	0.7043738	0.4452519	1.582	0.115	0.965914311	count	1
TMEM8B	0.8506329	1.1027068	0.7714	0.441	0.96700196	count	1
DGCR6	0.8506329	1.1027068	0.7714	0.441	0.96700196	count	1
POU2F1	0.9706371	0.8739699	1.1106	0.268	0.967444201	count	1
CLU	0.7008435	0.4462163	1.5706	0.117	0.967445439	count	1
ZNF688	0.7461024	0.5388668	1.3846	0.167	0.967494618	count	1
MAP2K6	0.772995	0.6519535	1.1857	0.237	0.969802616	count	1
KLRC2	0.7005072	0.420667	1.6652	0.0968	0.970786057	count	1
SLC37A3	0.899057	0.9442542	0.9521	0.342	0.970849049	count	1
SLC26A2	0.8248891	0.7960162	1.0363	0.301	0.971903694	count	1
IL2RA	0.8248891	0.8776166	0.9399	0.348	0.971903694	count	1
SIRT3	0.8248891	1.0168574	0.8112	0.418	0.971903694	count	1
VPS26C	0.7173006	0.4312651	1.6632	0.0972	0.972211966	count	1
SECISBP2L	0.7078088	0.370149	1.9122	0.0567	0.974060776	count	1
MFSD14C	0.8065646	0.6445655	1.2513	0.212	0.975741891	count	1
SELENOI	0.8289716	0.7827626	1.059	0.29	0.976696849	count	1
DAPK2	0.859325	0.5708547	1.5053	0.133	0.976833653	count	1
SCRN3	1.1336932	1.0271512	1.1037	0.2705	0.978125592	count	1
PRKAR1B	0.9067213	0.7535922	1.2032	0.23	0.979058047	count	1
ATAD3C	0.9067213	0.942809	0.9617	0.337	0.979058047	count	1
FNDC10	0.9067213	0.942809	0.9617	0.337	0.979058047	count	1
PANK4	0.9067213	0.942809	0.9617	0.337	0.979058047	count	1
PIP5K1A	0.9067213	0.942809	0.9617	0.337	0.979058047	count	1
HMCN1	0.9067213	0.942809	0.9617	0.337	0.979058047	count	1
AC009237.14	0.9067213	0.942809	0.9617	0.337	0.979058047	count	1
ITPR1-DT	0.9067213	0.942809	0.9617	0.337	0.979058047	count	1

LINC00685	0.9067213	0.942809	0.9617	0.337	0.979058047	count	1
LY6E-DT	0.9067213	0.942809	0.9617	0.337	0.979058047	count	1
RLN1	0.9067213	0.942809	0.9617	0.337	0.979058047	count	1
AP000866.5	0.9067213	0.942809	0.9617	0.337	0.979058047	count	1
GSTO2	0.9067213	0.942809	0.9617	0.337	0.979058047	count	1
OR4D1	0.9067213	0.942809	0.9617	0.337	0.979058047	count	1
ZNF229	0.9067213	0.942809	0.9617	0.337	0.979058047	count	1
FOXP1	0.6997203	0.3070419	2.2789	0.0233	0.979334186	count	1
ATL3	0.7563169	0.5501351	1.3748	0.17	0.980729224	count	1
TMEM218	0.7949004	0.6117436	1.2994	0.195	0.981342639	count	1
SMYD3	0.7824891	0.6513107	1.2014	0.23	0.981695406	count	1
RASGEF1A	0.7824891	0.6965597	1.1234	0.262	0.981695406	count	1
ATG16L2	0.7475477	0.7387308	1.0119	0.312	0.983531214	count	1
ZNF135	1.625296	1.3950257	1.1651	0.2448	0.983723204	count	1
2-Mar	0.8350316	0.5301418	1.5751	0.116	0.983809796	count	1
WDYHV1	0.7849055	0.684039	1.1475	0.252	0.98472167	count	1
CNNM3	0.9121911	0.7181885	1.2701	0.205	0.984913331	count	1
NSL1	0.7092068	0.3820585	1.8563	0.0643	0.985056428	count	1
APIP	0.7214424	0.497822	1.4492	0.148	0.985262522	count	1
HELB	0.7331106	0.3421466	2.1427	0.0329	0.993633389	count	1
SMIM14	0.7331899	0.4822796	1.5203	0.129	0.993740817	count	1
NUDT15	0.9206661	0.8572418	1.074	0.284	0.993980227	count	1
TUBB2A	0.7282449	0.3187658	2.2846	0.023	0.994549637	count	1
SHISA4	1.1544049	1.2165868	0.9489	0.3434	0.995368715	count	1
CSNK1G1	0.9222768	0.9141173	1.0089	0.314	0.995702659	count	1
C10orf95	0.8234066	0.9254076	0.8898	0.374	0.99605548	count	1
MON1B	0.8078325	1.065624	0.7581	0.449	0.997269459	count	1
GPAA1	0.7305647	0.4709813	1.5512	0.122	0.997716548	count	1
VAT1	1.0021085	0.9965988	1.0055	0.315	0.998325097	count	1
CPPED1	1.0021085	1.1398134	0.8792	0.38	0.998325097	count	1
RAPGEF5	1.0021085	1.253658	0.7993	0.425	0.998325097	count	1
UBA3	0.7869919	0.5411845	1.4542	0.147	1.000309704	count	1
TMEM94	0.828203	0.787641	1.0515	0.294	1.00183756	count	1
DDB1	0.9289124	0.7101224	1.3081	0.192	1.002795994	count	1
CCDC126	0.7539911	0.6659429	1.1322	0.258	1.003004163	count	1
RAD51D	1.0072793	0.9308954	1.0821	0.28	1.003387901	count	1
USP45	1.0072793	0.9667552	1.0419	0.298	1.003387901	count	1
RYBP	0.7582238	0.4715449	1.608	0.109	1.003434801	count	1
B4GALT7	0.8014016	0.7550191	1.0614	0.289	1.005374013	count	1
ZSCAN16-AS1	0.9313702	0.7272527	1.2807	0.201	1.005422273	count	1
CABIN1	0.7287063	0.3612363	2.0173	0.0445	1.005899847	count	1
CTDSPL2	0.752853	0.4659053	1.6159	0.107	1.006089015	count	1
DIMT1	0.7577847	0.5147575	1.4721	0.142	1.008046144	count	1
MIIP	0.7462557	0.6583996	1.1334	0.2579	1.00839601	count	1
RCE1	0.785257	0.5576378	1.4082	0.16	1.008967459	count	1

MTMR4	0.8890907	0.7861875	1.1309	0.259	1.010458383	count	1
PREPL	0.8355893	0.5679779	1.4712	0.142	1.010739088	count	1
FAM45A	0.7258523	0.4108741	1.7666	0.0782	1.011141051	count	1
MIB2	0.7270253	0.3569435	2.0368	0.0425	1.01183479	count	1
TPM1	0.7514421	0.5841531	1.2864	0.199	1.012033626	count	1
ARHGAP21	1.684588	0.7232704	2.3291	0.0205	1.014397494	count	1
ZNF75A	0.7430961	0.476031	1.561	0.119	1.014822262	count	1
SPRY2	0.7157622	0.2532641	2.8261	0.005	1.015208614	count	1
CDK5RAP3	0.7451117	0.4261552	1.7485	0.0813	1.01522225	count	1
CCL3L1	0.7172305	0.3505073	2.0463	0.0415	1.017071157	count	1
KPNA1	0.7923144	0.5361975	1.4777	0.14	1.018018629	count	1
RBM12	0.9431666	0.7709051	1.2235	0.222	1.018019185	count	1
TRAFD1	0.7801657	0.6423998	1.2145	0.225	1.019519623	count	1
RTL8C	0.8136744	0.7569386	1.075	0.283	1.020730612	count	1
LUC7L	0.7607177	0.4376163	1.7383	0.0831	1.020756802	count	1
SLF1	0.7619753	0.4140867	1.8401	0.0667	1.022442691	count	1
ACTR1B	0.7886981	0.5302412	1.4874	0.138	1.022660337	count	1
USP44	0.7731899	0.485402	1.5929	0.112	1.023218722	count	1
TCEAL1	0.9496566	0.8749347	1.0854	0.279	1.024943777	count	1
HGS	0.9496566	0.8769812	1.0829	0.28	1.024943777	count	1
WDR20	0.7905947	0.6068484	1.3028	0.194	1.025115079	count	1
RCC2	1.029715	0.5900888	1.745	0.0819	1.02531792	count	1
KBTBD4	1.191125	0.8433591	1.4124	0.159	1.02577548	count	1
MORC2	1.191125	0.9619295	1.2383	0.217	1.02577548	count	1
ZNF606	1.1911251	1.313826	0.9066	0.3653	1.025775746	count	1
ZNF805	0.9039503	0.9800725	0.9223	0.357	1.027218042	count	1
DLGAP1-AS1	0.8189417	0.70106	1.1681	0.244	1.027319155	count	1
AL445472.1	0.8189417	0.7852244	1.0429	0.298	1.027319155	count	1
DTWD1	0.7926899	0.6677264	1.1871	0.236	1.027826709	count	1
ABCD1	0.9533127	0.878429	1.0852	0.279	1.028842841	count	1
VKORC1L1	0.9533127	0.978175	0.9746	0.33	1.028842841	count	1
HGSNAT	0.8743027	0.7845606	1.1144	0.266	1.029844736	count	1
POLR3E	0.8743027	0.8016253	1.0907	0.276	1.029844736	count	1
RNPC3	0.7541783	0.4891152	1.5419	0.124	1.032146889	count	1
SELENOP	0.7973593	0.91951	0.8672	0.386	1.033869291	count	1
IFI27	0.7973593	0.91951	0.8672	0.386	1.033869291	count	1
TAB2	1.0398336	0.8120481	1.2805	0.201	1.035187929	count	1
MFNG	0.7480393	0.3502213	2.1359	0.0334	1.037840438	count	1
SSBP4	0.7325619	0.2452136	2.9874	0.00303	1.037850861	count	1
SMKR1	0.7807523	0.58798	1.3279	0.185	1.038563031	count	1
ZPR1	0.7644165	0.6563827	1.1646	0.245	1.038887183	count	1
CTBP1	0.7716983	0.4809253	1.6046	0.11	1.039288844	count	1
PTER	1.7399359	1.3592344	1.2801	0.2014	1.042373121	count	1
LINC02352	0.885242	0.9345705	0.9472	0.344	1.042648796	count	1
SP1	0.885242	0.9711888	0.9115	0.363	1.042648796	count	1

CDK6	0.7569898	0.3828147	1.9774	0.0488	1.043371521	count	1
ATF2	0.8125052	0.5264743	1.5433	0.124	1.043902069	count	1
TXLNA	0.864079	0.8459246	1.0215	0.308	1.045041659	count	1
CEP128	0.864079	0.9350901	0.9241	0.356	1.045041659	count	1
GMPR	1.0511499	0.7971778	1.3186	0.188	1.046210959	count	1
ITGBL1	1.0511499	0.9232129	1.1386	0.256	1.046210959	count	1
ZNF318	1.0511499	0.9672923	1.0867	0.278	1.046210959	count	1
RSU1	0.7709456	0.4485921	1.7186	0.0866	1.047752169	count	1
CASP6	0.7971949	0.7221109	1.104	0.27	1.048763855	count	1
F8A1	0.7884899	0.5262466	1.4983	0.135	1.048840377	count	1
ANKH	0.8094174	0.6116186	1.3234	0.187	1.049469445	count	1
LACTB2	0.8265928	0.6259516	1.3205	0.188	1.050517406	count	1
ZNF830	0.7906292	0.481015	1.6437	0.101	1.051681538	count	1
CDKN2C	0.7570535	0.4140533	1.8284	0.0684	1.052577366	count	1
RAPGEF1	0.8708699	0.7046192	1.2359	0.217	1.053210416	count	1
TENT5A	0.7673477	0.4831133	1.5883	0.113	1.054160107	count	1
EOGT	1.2260577	1.1138037	1.1008	0.272	1.054499206	count	1
AL118558.3	1.2260577	1.1138037	1.1008	0.272	1.054499206	count	1
B4GALT2	1.2260577	1.508816	0.8126	0.417	1.054499206	count	1
CLCC1	1.2260577	1.508816	0.8126	0.417	1.054499206	count	1
MAP4K3	1.2260577	1.508816	0.8126	0.417	1.054499206	count	1
LRP2BP	1.2260577	1.508816	0.8126	0.417	1.054499206	count	1
AC106791.1	1.2260577	1.508816	0.8126	0.417	1.054499206	count	1
AL021368.2	1.2260577	1.508816	0.8126	0.417	1.054499206	count	1
AEBP1	1.2260577	1.508816	0.8126	0.417	1.054499206	count	1
CLN3	1.2260577	1.508816	0.8126	0.417	1.054499206	count	1
ZFP28	1.2260577	1.508816	0.8126	0.417	1.054499206	count	1
UBE2Q1	0.7764932	0.4679785	1.6592	0.098	1.055283738	count	1
PIBF1	0.7898181	0.4705441	1.6785	0.0942	1.055429115	count	1
SCNM1	0.7593841	0.3400886	2.2329	0.0262	1.055815868	count	1
NOTCH1	0.8165917	0.9133957	0.894	0.372	1.058748384	count	1
MUT	0.9003194	0.8208256	1.0968	0.274	1.060281669	count	1
GIN1	0.8767996	0.8697146	1.0081	0.314	1.060340732	count	1
EIF2B3	0.8254799	0.5517379	1.4961	0.136	1.060525581	count	1
STAG1	0.8128914	0.4789967	1.6971	0.0906	1.062201122	count	1
CREG1	0.9350819	1.0936244	0.855	0.393	1.062269463	count	1
PELO	0.9023759	0.5613994	1.6074	0.109	1.062685375	count	1
BCAT2	0.8472446	0.680463	1.2451	0.214	1.062697073	count	1
NFIC	0.7820269	0.4716797	1.658	0.0983	1.062795715	count	1
GFER	0.7698017	0.4271291	1.8023	0.0724	1.064075506	count	1
WDR45B	0.8372988	0.6429885	1.3022	0.194	1.064078287	count	1
LMBR1L	1.070443	0.6098034	1.7554	0.0801	1.064965768	count	1
S100A9	0.8819998	0.9890932	0.8917	0.3732	1.066591878	count	1
SREK1IP1	0.7607197	0.3614683	2.1045	0.0361	1.066717149	count	1
TRA2A	0.7687742	0.2425016	3.1702	0.00167	1.067758121	count	1

ACOT4	0.9918561	1.1065707	0.8963	0.371	1.069863568	count	1
PHF6	0.7778119	0.4294157	1.8113	0.071	1.070345298	count	1
MGME1	0.853572	0.6838492	1.2482	0.213	1.070600306	count	1
TAF2	0.8198105	0.6729127	1.2183	0.224	1.071219938	count	1
PLCL2	0.8556816	0.5340519	1.6022	0.11	1.073234795	count	1
SH2D3C	0.8556816	0.6034697	1.4179	0.157	1.073234795	count	1
PIK3CG	0.8460119	0.5951593	1.4215	0.156	1.075110618	count	1
MYD88	0.8915553	0.8124964	1.0973	0.273	1.078073657	count	1
PPIH	0.7846886	0.3646427	2.1519	0.0321	1.079799	count	1
PATL1	1.2578253	1.0412141	1.208	0.2279	1.080439933	count	1
AGFG2	1.2578253	1.0537111	1.1937	0.2335	1.080439933	count	1
TRDMT1	0.8506329	0.7911361	1.0752	0.283	1.080960052	count	1
ALKBH6	0.9183236	0.7557544	1.2151	0.225	1.081314308	count	1
PPP1R9B	1.8194344	1.3667132	1.3312	0.184	1.081411923	count	1
CIZ1	0.9189099	1.1179489	0.822	0.412	1.081998792	count	1
TMEM106C	0.799679	0.4619196	1.7312	0.0844	1.083768059	count	1
TMEM234	0.8364785	0.6916646	1.2094	0.227	1.084457774	count	1
SSRP1	0.8651386	0.6569321	1.3169	0.189	1.085041757	count	1
CNIH1	0.783395	0.4070868	1.9244	0.0552	1.085600223	count	1
OTUD7B	1.2669049	1.037031	1.2217	0.223	1.087821485	count	1
UTP11	0.7933705	0.4975206	1.5946	0.112	1.087877948	count	1
HEXB	0.8469831	0.6301067	1.3442	0.18	1.088059058	count	1
TRAPPC6B	0.8082545	0.4594694	1.7591	0.0795	1.088448783	count	1
SKIL	0.779516	0.2654743	2.9363	0.00356	1.088597785	count	1
EIF5A2	0.8060562	0.5551025	1.4521	0.147	1.089106561	count	1
DUSP6	0.7871868	0.2938894	2.6785	0.00777	1.094443018	count	1
RTP4	0.9639811	0.6456752	1.493	0.136	1.094729067	count	1
TCTE3	0.9639811	0.8428664	1.1437	0.254	1.094729067	count	1
ALKBH2	0.8621215	0.6489953	1.3284	0.185	1.095497949	count	1
CNBD2	0.8165899	0.5637361	1.4485	0.148	1.095614721	count	1
UBE2Q2	0.7967311	0.3954502	2.0147	0.0448	1.096352097	count	1
FNTA	0.824492	0.4309446	1.9132	0.0566	1.09663433	count	1
PUS7L	0.8295518	0.4748698	1.7469	0.0816	1.097659068	count	1
SFT2D2	1.0214217	0.8049603	1.2689	0.205	1.101219755	count	1
ZER1	1.8647839	1.5230989	1.2243	0.2217	1.103064609	count	1
QRICH1	1.2857372	0.7301179	1.761	0.0792	1.103084416	count	1
THOP1	1.0237347	0.9926357	1.0313	0.303	1.103668603	count	1
ZNF230	1.0238788	0.8860294	1.1556	0.249	1.103821165	count	1
DRG1	0.8304263	0.5042531	1.6468	0.101	1.104508106	count	1
CDK9	0.7927036	0.3223159	2.4594	0.0144	1.106115797	count	1
ARMC6	0.8533424	0.614031	1.3897	0.166	1.106245554	count	1
LRIG1	0.974312	1.0080438	0.9665	0.334	1.106313458	count	1
SMIM15	0.8365726	0.4818451	1.7362	0.0835	1.106923995	count	1
TOLLIP	0.7975042	0.4544361	1.7549	0.0802	1.10762718	count	1
CAPN1	0.8157653	0.4338085	1.8805	0.0609	1.108579234	count	1

FDX2	0.8437921	0.6161163	1.3695	0.172	1.109910109	count	1
HIST1H2BH	0.9429965	1.093998	0.862	0.389	1.11009456	count	1
CDK8	1.297061	0.9483635	1.3677	0.172	1.112230588	count	1
BCR	0.888919	0.7319224	1.2145	0.225	1.114708351	count	1
UBAP1	0.8686368	0.6868744	1.2646	0.207	1.115762708	count	1
VAV1	0.861731	0.4837989	1.7812	0.0758	1.117078564	count	1
FTSJ1	1.30316	0.6649205	1.9599	0.0509	1.117147238	count	1
MAFG	0.8405485	0.4782261	1.7576	0.0798	1.117935605	count	1
RAP2C	0.8060884	0.3602176	2.2378	0.0259	1.118311819	count	1
TAZ	0.8628085	0.7341701	1.1752	0.241	1.118469802	count	1
AC020910.4	0.9067213	0.7535922	1.2032	0.23	1.118744962	count	1
MRM3	1.1267717	0.803937	1.4016	0.162	1.119430117	count	1
KATNAL1	0.8725129	0.5883926	1.4829	0.139	1.120719275	count	1
FRA10AC1	0.8476744	0.4760178	1.7808	0.0759	1.121570547	count	1
ATP2C1	0.9302137	0.6679068	1.3927	0.165	1.124457865	count	1
DNAAF2	0.8159876	0.3989512	2.0453	0.0416	1.124603085	count	1
CLPTM1	0.8857703	0.5646617	1.5687	0.118	1.125401157	count	1
TADA2A	0.8776476	0.7109295	1.2345	0.218	1.127284098	count	1
DPAGT1	1.0462586	0.8257233	1.2671	0.206	1.127482526	count	1
TMEM185A	0.872558	0.6197689	1.4079	0.16	1.131055581	count	1
PDE7A	0.82439	0.3197107	2.5786	0.0104	1.132425403	count	1
WASL	0.8575077	0.4986764	1.7196	0.0865	1.134539572	count	1
WDFY2	0.9655603	0.826069	1.1689	0.243	1.136369037	count	1
YTHDC2	0.8386302	0.442706	1.8943	0.0591	1.136456409	count	1
SLFN11	1.1448803	1.0437996	1.0968	0.274	1.136842023	count	1
WDR5B	1.1448803	1.0593198	1.0808	0.281	1.136842023	count	1
NRG2	1.1448803	1.096296	1.0443	0.297	1.136842023	count	1
ZNF853	1.1448803	1.096296	1.0443	0.297	1.136842023	count	1
CARMIL2	1.1448803	1.096296	1.0443	0.297	1.136842023	count	1
XXYLT1	1.1448803	1.1106016	1.0309	0.303	1.136842023	count	1
STAG3	1.1448803	1.199649	0.9543	0.341	1.136842023	count	1
LONRF1	0.942836	0.6994021	1.3481	0.179	1.139578169	count	1
GPR153	1.058174	0.9816403	1.078	0.282	1.140055665	count	1
ATP6V1A	1.058174	1.0145089	1.043	0.298	1.140055665	count	1
RPRD2	0.883894	0.5630956	1.5697	0.117	1.145683571	count	1
SLC4A10	1.3389787	1.088482	1.2301	0.22	1.145878012	count	1
AL121603.2	0.9492762	0.8925954	1.0635	0.288	1.147288052	count	1
AC004687.1	0.8384489	0.3722726	2.2522	0.025	1.147321434	count	1
SMN1	0.9067213	0.6440612	1.4078	0.16	1.151866621	count	1
RBM43	0.8402013	0.5571685	1.508	0.133	1.151990794	count	1
GGPS1	0.8667644	0.5647276	1.5348	0.126	1.152694441	count	1
PRKCQ	0.8768787	0.616759	1.4218	0.156	1.153274614	count	1
SMG7	0.9354181	0.6530056	1.4325	0.153	1.153878442	count	1
HAT1	0.8904154	0.4836102	1.8412	0.0665	1.154095882	count	1
PKN1	0.8264581	0.3563051	2.3195	0.021	1.154970466	count	1

AXIN1	0.9364274	0.7730169	1.2114	0.227	1.15511306	count	1
SETDB1	0.9223209	0.779329	1.1835	0.237	1.156319249	count	1
TRAPPC9	0.8939051	0.6746148	1.3251	0.186	1.158596543	count	1
ATP2B1-AS1	0.8448836	0.443055	1.9069	0.0574	1.160526257	count	1
SLC12A2	0.9050298	0.6986844	1.2953	0.196	1.162269061	count	1
MOAP1	0.9622535	0.6465178	1.4884	0.138	1.162813577	count	1
DCAF11	0.8636464	0.5687886	1.5184	0.13	1.162863155	count	1
INSIG1	0.8499262	0.3474375	2.4463	0.015	1.162994709	count	1
MAGEH1	0.8746387	0.596864	1.4654	0.144	1.163129564	count	1
LIF	1.365998	1.385359	0.986	0.3249	1.167385829	count	1
PTCD3	0.9926032	0.6123643	1.6209	0.106	1.167798656	count	1
IQCE	1.085203	0.9835142	1.1034	0.271	1.16851011	count	1
PIGB	1.085203	0.9835142	1.1034	0.271	1.16851011	count	1
GBP7	1.085203	1.0353084	1.0482	0.295	1.16851011	count	1
RHOBTB3	1.085203	1.0353084	1.0482	0.295	1.16851011	count	1
DNAJC18	1.085203	1.0353084	1.0482	0.295	1.16851011	count	1
ZNF696	1.085203	1.0353084	1.0482	0.295	1.16851011	count	1
GINS2	1.085203	1.0353084	1.0482	0.295	1.16851011	count	1
DDX19B	0.8890005	0.5717556	1.5549	0.121	1.16914999	count	1
GNB1	0.8588268	0.3359287	2.5566	0.011	1.169918909	count	1
ZFP36	0.8135503	0.1458643	5.5774	5.15E-08	1.171667424	count	0.001252223
AL162377.1	0.9238555	0.6702577	1.3784	0.169	1.173491224	count	1
DTX3	2.0222668	1.5107732	1.3386	0.1817	1.174692965	count	1
AC104506.1	1.0917765	1.1626557	0.939	0.3484	1.175415976	count	1
H6PD	1.0367109	0.8693008	1.1926	0.234	1.176051322	count	1
CAMTA2	1.0367109	0.870243	1.1913	0.234	1.176051322	count	1
P2RX7	1.0367109	0.9107069	1.1384	0.256	1.176051322	count	1
KCNQ5	1.1864469	0.8950754	1.3255	0.186	1.176619087	count	1
A1BG-AS1	0.8809812	0.5799331	1.5191	0.13	1.176926678	count	1
TOX2	0.8499334	0.3791436	2.2417	0.0257	1.177684527	count	1
ODF3B	1.0021085	0.8918734	1.1236	0.262	1.178829491	count	1
AC007952.4	1.3812771	0.7619207	1.8129	0.0708	1.17948392	count	1
CDPF1	1.3816545	0.8978663	1.5388	0.125	1.179781994	count	1
MTAP	0.9427315	0.7382515	1.277	0.203	1.181710073	count	1
WARS2	2.038715	1.200323	1.6985	0.09038	1.181850855	count	1
UGCG	0.8990661	0.4725068	1.9028	0.058	1.182327365	count	1
NEK9	1.1924833	1.1035482	1.0806	0.281	1.182372819	count	1
MEN1	1.0454179	0.9730535	1.0744	0.283	1.18574899	count	1
ISCA2	0.8673064	0.5046006	1.7188	0.0866	1.186722322	count	1
ANKRD6	1.3905821	0.9510067	1.4622	0.145	1.186828258	count	1
PLOD1	1.3905821	1.1069648	1.2562	0.21	1.186828258	count	1
AL136987.1	1.3905821	1.1069648	1.2562	0.21	1.186828258	count	1
DOLPP1	1.3905821	1.1069648	1.2562	0.21	1.186828258	count	1
PEX5	1.3905821	1.1069648	1.2562	0.21	1.186828258	count	1
BBS10	1.3905821	1.1069648	1.2562	0.21	1.186828258	count	1

FSIP1	1.3905821	1.1069648	1.2562	0.21	1.186828258	count	1
AC009133.3	1.3905821	1.1069648	1.2562	0.21	1.186828258	count	1
VPS9D1	1.3905821	1.1069648	1.2562	0.21	1.186828258	count	1
DSEL	1.3905821	1.1069648	1.2562	0.21	1.186828258	count	1
ADGRE2	1.3905821	1.1069648	1.2562	0.21	1.186828258	count	1
FKRP	1.3905821	1.1069648	1.2562	0.21	1.186828258	count	1
ZNF582	1.3905821	1.1069648	1.2562	0.21	1.186828258	count	1
RAB3A	1.1979052	0.7318095	1.6369	0.103	1.187535815	count	1
PEX11A	0.9848023	0.7201476	1.3675	0.172	1.189756595	count	1
CCZ1	0.9120246	0.5241438	1.74	0.0828	1.191220327	count	1
NQO2	0.8904402	0.4896164	1.8186	0.0699	1.194408377	count	1
KIDINS220	0.9150693	0.5371066	1.7037	0.0894	1.195175606	count	1
DUSP1	0.8302793	0.1388389	5.9802	5.89E-09	1.195996413	count	0.000143221
GPR68	0.8784487	0.4434771	1.9808	0.0485	1.19657781	count	1
SP4	0.9926422	0.8138105	1.2197	0.223	1.199114025	count	1
PDZD8	0.9928224	0.5296248	1.8746	0.0618	1.199329036	count	1
NOLC1	0.9070377	0.5139882	1.7647	0.0786	1.19980325	count	1
TICAM1	1.0613836	0.9308574	1.1402	0.255	1.203508735	count	1
TBC1D10B	1.0237958	0.6524844	1.5691	0.118	1.203964365	count	1
METTL4	1.0237958	0.7566301	1.3531	0.177	1.203964365	count	1
RASA1	0.9490812	0.6060374	1.566	0.118	1.20529443	count	1
TSC1	1.1213572	0.9138585	1.2271	0.221	1.206421153	count	1
PIGM	1.0262686	1.0409024	0.9859	0.325	1.206827285	count	1
TMEM8A	0.9637119	0.7242468	1.3306	0.184	1.207779627	count	1
ZNF626	0.9679337	0.6071739	1.5942	0.112	1.213021695	count	1
STX16	1.0704951	0.6123002	1.7483	0.0814	1.213630788	count	1
SNUPN	0.9702547	0.6765906	1.434	0.153	1.215903039	count	1
CHRNE	1.2304734	1.0106503	1.2175	0.224	1.218446063	count	1
CENPT	1.0375538	0.6516842	1.5921	0.112	1.219884881	count	1
AP5S1	1.0794705	1.0496024	1.0285	0.305	1.223592013	count	1
KIF3A	1.080859	0.5553828	1.9462	0.0525	1.225132185	count	1
HPS3	0.8995667	0.5306139	1.6953	0.091	1.225256344	count	1
MRPL39	1.014779	0.7861747	1.2908	0.198	1.225506223	count	1
WIPI1	0.9458696	0.5825036	1.6238	0.105	1.225538129	count	1
TRIB3	0.9391	0.5476797	1.7147	0.0874	1.226376307	count	1
IL27RA	0.8790457	0.4224435	2.0809	0.0382	1.22643292	count	1
NUP35	1.0451635	0.7285619	1.4346	0.152	1.22868228	count	1
NME7	1.4446205	1.247729	1.1578	0.248	1.229124876	count	1
LINC02482	1.4446205	1.247729	1.1578	0.248	1.229124876	count	1
JAG1	1.4446205	1.247729	1.1578	0.248	1.229124876	count	1
IGFBP5	1.4446205	1.273192	1.1346	0.257	1.229124876	count	1
SLC9B2	1.4446205	1.273192	1.1346	0.257	1.229124876	count	1
AMER1	1.4446205	1.273192	1.1346	0.257	1.229124876	count	1
NUP210	0.9683738	0.5698711	1.6993	0.0902	1.229589327	count	1
AC005332.5	0.9597916	0.6420181	1.495	0.136	1.232106933	count	1

E2F5	1.002046	0.7953046	1.26	0.209	1.235212737	count	1
MYADM	0.8688225	0.2077321	4.1824	3.72E-05	1.236078928	count	0.9044436
ANKRD28	0.8724422	0.2790305	3.1267	0.00193	1.23677782	count	1
ABHD11	1.4547001	1.423075	1.0222	0.3074	1.236945571	count	1
FANCF	1.0538599	0.6500448	1.6212	0.106	1.238728516	count	1
WDR73	1.0055507	0.7395191	1.3597	0.175	1.239481049	count	1
POLR3A	1.0055507	0.8085935	1.2436	0.215	1.239481049	count	1
NUBP1	0.9686609	0.5455856	1.7755	0.0768	1.243400673	count	1
DCTD	0.9536588	0.4995343	1.9091	0.0571	1.245263908	count	1
FAM3A	1.0322663	0.6789447	1.5204	0.129	1.246322988	count	1
TAX1BP3	0.9952769	0.7633973	1.3037	0.193	1.246940662	count	1
HCCS	0.9234949	0.5092476	1.8134	0.0707	1.247303929	count	1
MCTP2	0.9127413	0.3683395	2.478	0.0137	1.248710415	count	1
AL392172.1	1.26511	0.8828195	1.433	0.153	1.25112	count	1
ZNF398	1.1065949	0.8906867	1.2424	0.215	1.253636055	count	1
SIAH1	1.0043759	0.6319301	1.5894	0.113	1.258215211	count	1
PINK1	1.2734102	0.7981378	1.5955	0.112	1.258918383	count	1
WDR35	1.1766515	0.8498921	1.3845	0.167	1.264048599	count	1
SLC25A13	1.1766515	0.908797	1.2947	0.196	1.264048599	count	1
TRPV2	0.9470009	0.5388163	1.7576	0.0798	1.264717885	count	1
MR1	1.0767917	0.88747	1.2133	0.226	1.265180578	count	1
ZFYVE21	0.932656	0.5782233	1.613	0.108	1.266949924	count	1
ITK	0.945006	0.4915277	1.9226	0.0554	1.267273413	count	1
RBM45	1.4945338	1.3013607	1.1484	0.2516	1.267634354	count	1
JUN	0.8817594	0.2165543	4.0718	5.87E-05	1.268112611	count	1
AL161772.1	0.9801807	0.6892427	1.4221	0.156	1.269653529	count	1
TMEM191C	1.2852748	0.9250265	1.3894	0.166	1.270043974	count	1
SCAF1	1.2852748	0.9669502	1.3292	0.185	1.270044013	count	1
PCYOX1	1.1218177	0.8110515	1.3832	0.168	1.270457015	count	1
ALYREF	0.915025	0.3718418	2.4608	0.0144	1.270554249	count	1
AL355816.2	1.1850084	0.818102	1.4485	0.148	1.272718943	count	1
LRRC43	1.1850084	0.8949272	1.3241	0.186	1.272718943	count	1
SLC26A11	1.1850084	0.9472754	1.251	0.212	1.272718943	count	1
BCO2	0.9177127	0.4425161	2.0739	0.0389	1.272875192	count	1
DCUN1D4	1.0181326	0.7165214	1.4209	0.156	1.275248669	count	1
C6orf89	1.034988	0.6300811	1.6426	0.101	1.275291045	count	1
MED8	0.9405142	0.5167838	1.8199	0.0697	1.277580636	count	1
TCAIM	0.9677255	0.5706001	1.696	0.0909	1.279616275	count	1
MZF1-AS1	0.9881461	0.79972	1.2356	0.2175	1.279884702	count	1
NR4A3	0.9425533	0.4722363	1.9959	0.0468	1.283588123	count	1
FAF2	1.011653	0.5207793	1.9426	0.0529	1.283995665	count	1
ATIC	0.9465476	0.457697	2.0681	0.0394	1.285741211	count	1
FAM120B	1.002069	0.6517771	1.5374	0.125	1.285894605	count	1
PIK3CD	1.3055759	0.9555745	1.3663	0.173	1.289020625	count	1
POP1	1.004534	0.7707998	1.3032	0.193	1.289026907	count	1

KIAA0100	1.1396376	0.7116542	1.6014	0.11	1.290109618	count	1
MRM2	1.0488373	0.7067976	1.4839	0.139	1.292111956	count	1
KRT18	1.0488373	1.0917857	0.9607	0.3374	1.292111956	count	1
NAP1L2	1.1008308	0.9370701	1.1748	0.241	1.292846985	count	1
ADSL	1.3106747	0.9637602	1.36	0.175	1.293774699	count	1
MICA	2.317798	1.075064	2.156	0.0318	1.293896007	count	1
ZRANB3	1.102841	0.8921017	1.2362	0.217	1.295157421	count	1
PPTC7	1.2074972	0.8611089	1.4023	0.162	1.295998022	count	1
ABCB8	1.2074972	0.9081009	1.3297	0.185	1.295998022	count	1
POGLUT1	1.075282	0.697618	1.5414	0.124	1.297402508	count	1
MRI1	1.075282	0.7768106	1.3842	0.167	1.297402508	count	1
PIAS1	1.0753997	0.6572077	1.6363	0.103	1.297542017	count	1
NMUR1	0.9643882	0.4850085	1.9884	0.0476	1.297923817	count	1
TP53RK	0.9738419	0.5717202	1.7034	0.0895	1.300355663	count	1
DDHD1	0.9663238	0.4252968	2.2721	0.0237	1.300514832	count	1
PTPMT1	1.1498096	0.7218989	1.5928	0.112	1.301308904	count	1
YIPF2	1.1498096	0.7357592	1.5628	0.119	1.301308904	count	1
CYSLTR2	0.9976752	0.650509	1.5337	0.126	1.302294288	count	1
FAM208A	1.0405625	0.4815606	2.1608	0.0314	1.302988178	count	1
ETFRF1	0.9518015	0.4768432	1.996	0.0468	1.304543785	count	1
SPATS2L	0.966597	0.4443708	2.1752	0.0303	1.305242067	count	1
AC025164.1	0.9249214	0.3350213	2.7608	0.006095	1.305584647	count	1
TMEM170A	0.9388768	0.3592705	2.6133	0.00939	1.30623365	count	1
MSMO1	0.9783857	0.5454425	1.7937	0.0738	1.306385275	count	1
UBE2J1	1.0010109	0.4807022	2.0824	0.0381	1.306611532	count	1
VPS72	0.9544154	0.4485556	2.1278	0.0341	1.308112424	count	1
TARS	0.9959349	0.5455298	1.8256	0.0688	1.308884044	count	1
PHLDA3	1.2260577	1.0050681	1.2199	0.223	1.315150925	count	1
ZNF33B	1.2260577	1.0050681	1.2199	0.223	1.315150925	count	1
IL11RA	1.2260577	1.072573	1.1431	0.254	1.315150925	count	1
NEO1	1.2260577	1.072573	1.1431	0.254	1.315150925	count	1
RPP25	1.2260577	1.1138036	1.1008	0.272	1.315150925	count	1
GDPGP1	1.2260577	1.1138036	1.1008	0.272	1.315150925	count	1
MORC2-AS1	1.2260577	1.1138036	1.1008	0.272	1.315150925	count	1
KLRC4-KLRK1	1.2260577	1.5088157	0.8126	0.417	1.315150925	count	1
ATXN1L	1.2261553	0.6094526	2.0119	0.0451	1.315251503	count	1
ZNF600	1.559978	0.8490768	1.8373	0.0671	1.317275427	count	1
TELO2	1.093358	0.8054662	1.3574	0.176	1.318810912	count	1
GTF2E1	1.093358	0.8733214	1.252	0.211	1.318810912	count	1
HIBCH	1.1234716	0.7896336	1.4228	0.156	1.318842802	count	1
ETV6	1.0946152	0.6786291	1.613	0.108	1.320298574	count	1
ENOSF1	1.0443521	0.5709436	1.8292	0.0683	1.325009294	count	1
CSKMT	0.9711701	0.4663669	2.0824	0.0381	1.325475443	count	1
GAR1	0.9774477	0.4486887	2.1785	0.0301	1.327514285	count	1
COG2	1.0781601	0.5882375	1.8329	0.0677	1.32766778	count	1

ZNF358	1.061098	0.7832428	1.3547	0.176	1.32834761	count	1
FAXDC2	1.5756762	1.242406	1.2682	0.206	1.329034616	count	1
ZMYM3	1.5756762	1.242406	1.2682	0.206	1.329034616	count	1
TOP3A	1.5756762	1.242406	1.2682	0.206	1.329034616	count	1
AC245452.1	1.5756762	1.242406	1.2682	0.206	1.329034616	count	1
TMEM245	1.5756762	1.4939874	1.0547	0.2924	1.329034616	count	1
SNHG21	1.5756762	1.5831344	0.9953	0.3203	1.329034616	count	1
SENP2	1.133748	0.7063182	1.6052	0.109	1.330621781	count	1
BSPRY	1.13542	0.8760475	1.2961	0.196	1.332536948	count	1
CENPN	1.13542	0.9248979	1.2276	0.22	1.332536948	count	1
RRNAD1	1.13542	1.0204342	1.1127	0.267	1.332536948	count	1
CHMP1B	0.9445214	0.3064354	3.0823	0.00223	1.333205913	count	1
R3HCC1L	1.0055507	0.5514277	1.8235	0.0691	1.336215651	count	1
CCDC28A	1.017185	0.5636871	1.8045	0.0721	1.336577853	count	1
LARP4	1.1826913	0.6244203	1.8941	0.0591	1.337414664	count	1
BBIP1	1.1838357	0.6015218	1.9681	0.0499	1.33866856	count	1
FBXW4	1.1862314	0.5751687	2.0624	0.04	1.341292886	count	1
ZNF571	1.1132428	0.8574297	1.2983	0.195	1.342321252	count	1
ANKRD36	1.0606073	0.5739164	1.848	0.0655	1.34536636	count	1
SH3BP5	1.031866	0.6074575	1.6987	0.0903	1.346512757	count	1
SNX19	1.3693455	0.8892883	1.5398	0.125	1.348118646	count	1
DIS3L	1.262541	0.8562668	1.4745	0.141	1.352635353	count	1
ATG13	1.037766	0.9080315	1.1429	0.254	1.354135574	count	1
TRPC4AP	1.068689	0.6239689	1.7127	0.0877	1.355479372	count	1
ZBTB14	1.613911	1.1711837	1.378	0.169	1.357428535	count	1
CNPY4	1.613911	1.264123	1.2767	0.203	1.357428535	count	1
AC093462.1	1.613911	1.264123	1.2767	0.203	1.357428535	count	1
PRPF4	1.0849718	0.6433534	1.6864	0.0927	1.357782995	count	1
ATPAF2	1.104773	0.7795312	1.4172	0.157	1.359865858	count	1
ULK2	1.2035404	1.099723	1.0944	0.2746	1.360229094	count	1
AL161421.1	1.1293685	0.8436541	1.3387	0.182	1.36135525	count	1
CEP162	1.3859052	1.125678	1.2312	0.219	1.363333742	count	1
BLVRA	0.9790384	0.4283287	2.2857	0.0229	1.364437308	count	1
CCDC32	1.0389446	0.5872479	1.7692	0.0778	1.364907442	count	1
AP005482.1	0.9952769	0.5399234	1.8434	0.0662	1.366397677	count	1
PFKL	1.0475851	0.5792002	1.8087	0.0714	1.366816713	count	1
PJA1	1.1684736	0.8582114	1.3615	0.174	1.370327958	count	1
TRAP1	1.2145815	0.9116275	1.3323	0.184	1.372285367	count	1
PLCB1	1.0065094	0.5161851	1.9499	0.0521	1.373464402	count	1
AC044849.1	0.982783	0.2973064	3.3056	0.00105	1.374940699	count	1
AGPAT3	1.2847776	0.9179935	1.3995	0.163	1.375372413	count	1
PARP10	1.0726862	0.6881397	1.5588	0.12	1.375456633	count	1
DEXI	1.0545749	0.5671143	1.8595	0.0639	1.375839945	count	1
C11orf68	1.1008308	0.7454391	1.4768	0.141	1.377307806	count	1
SLC30A7	1.40477	0.9798008	1.4337	0.153	1.380598527	count	1

HEATR6	1.1787741	0.740902	1.591	0.113	1.382076261	count	1
CCDC137	1.055181	0.5940665	1.7762	0.0766	1.386026892	count	1
FAM160B2	1.4127784	1.3061024	1.0817	0.2802	1.387905099	count	1
ELFN1-AS1	1.0959197	1.0429382	1.0508	0.2941	1.389513948	count	1
RCC1L	1.1856738	0.7266799	1.6316	0.104	1.389937993	count	1
USP20	1.232477	0.9659538	1.2759	0.203	1.391787746	count	1
IQCG	1.1883761	0.8138716	1.4602	0.145	1.393015362	count	1
XYLT2	1.1883761	1.0037067	1.184	0.237	1.393015362	count	1
WDFY1	1.1887864	0.6894928	1.7241	0.0856	1.393482544	count	1
DHRS12	1.2377402	0.8131211	1.5222	0.129	1.397514322	count	1
ARL5A	1.3113866	1.0410704	1.2597	0.209	1.402467079	count	1
PLBD2	1.3113866	1.1202058	1.1707	0.243	1.402467079	count	1
NMB	1.0682214	0.6075794	1.7582	0.0797	1.402976806	count	1
PPP5C	1.0759969	0.5960349	1.8053	0.072	1.403472969	count	1
RSPRY1	1.0622222	0.7192696	1.4768	0.141	1.403503296	count	1
RNF41	1.054371	0.5880239	1.7931	0.0739	1.407062655	count	1
ANKLE2	1.0317455	0.4311345	2.3931	0.0173	1.407704312	count	1
PPP6R3	1.0556225	0.5162028	2.045	0.0417	1.408718256	count	1
DCAF17	1.1126208	0.6553296	1.6978	0.0905	1.410356175	count	1
TBL2	1.4379326	0.906682	1.5859	0.114	1.41076743	count	1
CCL28	1.059452	0.6331294	1.6734	0.0952	1.41378375	count	1
SPATA6	1.2530161	0.8921861	1.4044	0.161	1.414111073	count	1
CENPU	1.6928166	0.9468643	1.7878	0.0747	1.414888097	count	1
TMEM229B	1.6928166	0.9468643	1.7878	0.0747	1.414888097	count	1
ZNF616	1.6928166	0.9468643	1.7878	0.0747	1.414888097	count	1
KIAA1468	1.6928166	1.0287704	1.6455	0.101	1.414888097	count	1
STAMBPL1	1.1330953	0.6026967	1.88	0.061	1.416956691	count	1
NUMB	1.4472004	1.002918	1.443	0.15	1.41915667	count	1
UVSSA	1.2119418	0.8052236	1.5051	0.133	1.419810384	count	1
ZNF672	1.2610738	0.8471898	1.4885	0.138	1.422850865	count	1
TECPR1	1.2610738	0.992468	1.2706	0.205	1.422850865	count	1
AC025171.3	1.138115	0.6239989	1.8239	0.0691	1.423116111	count	1
SPICE1	1.45659	0.987332	1.4753	0.141	1.427637135	count	1
THOC3	1.1268147	0.6297853	1.7892	0.0745	1.428049847	count	1
AC109446.3	1.075683	0.6064071	1.7739	0.077	1.428579901	count	1
TAF6	1.1281493	0.6761298	1.6685	0.0962	1.429712584	count	1
HARS	1.0687372	0.4837095	2.2095	0.0278	1.432000575	count	1
BTBD2	1.1466079	0.9072995	1.2638	0.207	1.433531617	count	1
TPP1	1.1189891	0.7937024	1.4098	0.16	1.433971009	count	1
IPMK	1.3434403	1.0572792	1.2707	0.205	1.434936815	count	1
SLC25A17	1.2260577	0.8575215	1.4298	0.154	1.435824279	count	1
GBA2	1.7242335	1.223346	1.4094	0.16	1.437327282	count	1
DRAM1	1.7242335	1.327128	1.2992	0.1948	1.437327282	count	1
ZNF689	1.7242335	1.360303	1.2675	0.206	1.437327282	count	1
AC243965.1	1.194403	0.7758464	1.5395	0.125	1.437812819	count	1

FBXL17	1.1350064	0.7673364	1.4792	0.14	1.438252987	count	1
ZBTB16	1.060694	0.4865238	2.1801	0.03	1.443549084	count	1
PNP	1.0394719	0.3757829	2.7662	0.006	1.448354602	count	1
CAMK2D	1.4798781	0.8044148	1.8397	0.0667	1.448587112	count	1
SLX4IP	1.480467	0.974984	1.5185	0.13	1.449115477	count	1
PCGF6	1.480467	1.0061003	1.4715	0.142	1.449115477	count	1
VRK2	1.1041905	0.6010981	1.837	0.0671	1.449670287	count	1
SAYSD1	1.2880696	0.8323352	1.5475	0.123	1.452056627	count	1
CHMP4B	1.1146729	0.5279869	2.1112	0.0355	1.453279478	count	1
MPP1	1.2091197	0.8269506	1.4621	0.145	1.455043255	count	1
AC068338.2	1.1850084	0.7628342	1.5534	0.121	1.456493599	count	1
SIK2	1.7533361	1.1726042	1.4952	0.136	1.457885126	count	1
ZNF432	1.7533361	1.1726042	1.4952	0.136	1.457885126	count	1
ARMCX2	1.7533361	1.2110188	1.4478	0.149	1.457885126	count	1
BLZF1	1.139349	0.6918678	1.6468	0.101	1.459643341	count	1
SLC43A1	1.2481701	0.8068465	1.547	0.123	1.460853557	count	1
CSPP1	1.0950906	0.573818	1.9084	0.0572	1.460884423	count	1
PLCB2	1.1221506	0.6129751	1.8307	0.0681	1.46289635	count	1
ZNF7	1.1919909	0.7883564	1.512	0.132	1.464868998	count	1
FAM174A	1.1105686	0.5284229	2.1017	0.0364	1.466677103	count	1
METTL16	1.1949299	0.5491358	2.176	0.0303	1.468392607	count	1
MTA1	1.1595371	0.6984845	1.6601	0.0979	1.468769389	count	1
ERAL1	1.5029723	0.8310003	1.8086	0.0714	1.469243078	count	1
WDR6	1.2563424	0.6570448	1.9121	0.0567	1.470086149	count	1
GPR34	1.151049	0.6677946	1.7237	0.0857	1.474379765	count	1
BTD	1.1425212	0.5869341	1.9466	0.0524	1.47729777	count	1
DHX37	1.312186	0.8379502	1.5659	0.118	1.478046716	count	1
AL117379.1	1.312186	0.8379502	1.5659	0.118	1.478046716	count	1
TBCEL	1.312186	0.9198877	1.4265	0.155	1.478046716	count	1
CLDN7	1.312186	0.9198877	1.4265	0.155	1.478046716	count	1
GRHPR	1.083857	0.369233	2.9354	0.00357	1.481544192	count	1
DVL3	1.206094	0.8783378	1.3732	0.171	1.481768238	count	1
SLC46A3	1.206094	0.8901796	1.3549	0.176	1.481768238	count	1
APBA2	1.189715	0.706164	1.6848	0.093	1.486282698	count	1
RFX1	1.5242695	1.004668	1.5172	0.13	1.488184296	count	1
GLB1	1.5242696	0.9835722	1.5497	0.122	1.488184368	count	1
SLC25A20	1.110773	0.6293413	1.765	0.0785	1.493373189	count	1
SLC30A9	1.3267967	0.9923737	1.337	0.182	1.493745484	count	1
TENT4B	1.2498093	0.7189735	1.7383	0.0831	1.502537319	count	1
NUTM2A-AS1	1.2498093	0.7727591	1.6173	0.107	1.502537319	count	1
ANKMY2	1.3369735	0.8171054	1.6362	0.103	1.504658529	count	1
HECTD4	1.2877746	0.7981049	1.6135	0.108	1.50550466	count	1
PECR	1.5444939	1.2628992	1.223	0.2222	1.506074188	count	1
CRAT	1.4185543	1.0213995	1.3888	0.166	1.510265126	count	1
C16orf74	1.4185543	1.0251455	1.3838	0.167	1.510265126	count	1

HPS6	1.8312402	1.3288467	1.3781	0.1691	1.511811539	count	1
ZNF846	1.2399132	0.62291	1.9905	0.0474	1.522195673	count	1
AC060780.1	1.8492796	1.214796	1.5223	0.129	1.524065319	count	1
UBR5-AS1	1.8492796	1.304429	1.4177	0.1572	1.524065319	count	1
PRDM4	1.8492796	1.4664753	1.261	0.2082	1.524065319	count	1
RCCD1	1.8492796	1.4664753	1.261	0.2082	1.524065319	count	1
CCDC88A	1.3569788	0.8543672	1.5883	0.113	1.526058733	count	1
HIST4H4	1.3063889	0.8854163	1.4755	0.141	1.526409334	count	1
AL096865.1	1.2704042	0.8399157	1.5125	0.131	1.526492106	count	1
EFCAB2	1.2278944	0.758886	1.618	0.107	1.53283658	count	1
FAM168B	1.1995053	0.5099322	2.3523	0.0193	1.535277997	count	1
TACO1	1.4446205	0.9130638	1.5822	0.115	1.536144573	count	1
ZBTB22	1.4446205	1.0408173	1.388	0.166	1.536144573	count	1
HOMER1	1.4446205	1.07578	1.3429	0.18	1.536144573	count	1
AC004854.2	1.4446205	1.07578	1.3429	0.18	1.536144573	count	1
AC005332.7	1.8724918	1.2868589	1.4551	0.1466	1.539701844	count	1
KLHL9	1.5839524	1.125255	1.4076	0.1602	1.540699757	count	1
DCAF8	1.1764002	0.5776922	2.0364	0.0425	1.543128758	count	1
PCYT1A	1.3760058	1.0262981	1.3407	0.181	1.546346768	count	1
SAMD1	1.1886209	0.5207683	2.2824	0.0231	1.548187782	count	1
SLC22A18	1.2147325	0.8457132	1.4363	0.152	1.554368939	count	1
ZNF852	1.599868	0.9254628	1.7287	0.0848	1.554559592	count	1
DUBR	1.599868	0.9254628	1.7287	0.0848	1.554559592	count	1
LMNTD2	1.599868	0.9254628	1.7287	0.0848	1.554559592	count	1
INCENP	1.599868	0.9254628	1.7287	0.0848	1.554559592	count	1
ZNF426	1.4637711	1.026724	1.4257	0.155	1.555068797	count	1
PRR5L	1.2040585	0.6913454	1.7416	0.0825	1.555462522	count	1
NSUN6	1.3370099	0.7485188	1.7862	0.075	1.560680378	count	1
TOR2A	1.3410963	0.7559829	1.774	0.077	1.565242576	count	1
NUDT3	1.3056995	0.6497202	2.0096	0.0453	1.56740826	count	1
NBEAL2	1.2577861	0.8643826	1.4551	0.147	1.569169405	count	1
AP005329.3	1.4057798	0.8463981	1.6609	0.0977	1.577962398	count	1
SERPINH1	1.4889357	1.17743	1.2646	0.207	1.579818491	count	1
LGALS9C	1.9376846	1.227233	1.5789	0.115	1.58281981	count	1
TMEM140	1.201254	0.6475896	1.855	0.0645	1.584737607	count	1
ABL1	1.3591041	0.8400419	1.6179	0.107	1.585314693	count	1
CROCC	1.3634309	0.8014346	1.7012	0.0899	1.590129545	count	1
TMEM14A	1.2450068	0.5127327	2.4282	0.0157	1.592256472	count	1
LINC02132	1.1667092	0.4734501	2.4643	0.0142	1.593847574	count	1
CEP131	1.9577538	1.342665	1.4581	0.146	1.595853733	count	1
USP28	1.2100245	0.5015496	2.4126	0.0164	1.596122941	count	1
ZNF576	1.4233154	1.060517	1.3421	0.181	1.596505593	count	1
ZNF134	1.5065776	0.9176806	1.6417	0.102	1.597088445	count	1
SELENON	1.424885	0.9475326	1.5038	0.134	1.598162523	count	1
KDM1A	1.206094	0.6912631	1.7448	0.082	1.599507525	count	1

PLCH2	1.3058275	0.656812	1.9881	0.0476	1.600576537	count	1
TMEM41A	1.2214341	0.5548961	2.2012	0.0284	1.601208484	count	1
KLHL12	1.5120515	1.0612642	1.4248	0.155	1.60243317	count	1
FAM104A	1.1982757	0.5746552	2.0852	0.0378	1.603554907	count	1
COX16	1.657567	1.1016342	1.5046	0.133	1.604281406	count	1
XPO6	1.657567	1.136693	1.4582	0.146	1.604281456	count	1
REEP4	1.6575671	0.9737178	1.7023	0.0897	1.604281526	count	1
NREP	1.34	0.6966082	1.9236	0.0553	1.606998463	count	1
NAIF1	1.34	0.9316936	1.4382	0.151	1.606998463	count	1
HDX	1.378714	0.815216	1.6912	0.0918	1.607111377	count	1
LMBRD2	1.976884	1.115324	1.7725	0.07726	1.608171927	count	1
NR2C1	1.2292177	0.6762057	1.8178	0.07	1.611229822	count	1
PIGK	1.2771134	0.6881137	1.856	0.0644	1.614188498	count	1
NUFIP1	1.6736336	0.9391389	1.7821	0.0757	1.617977068	count	1
UBQLN2	1.2669834	0.7233465	1.7516	0.0808	1.619700648	count	1
SVBP	1.1977416	0.5265691	2.2746	0.0236	1.624093411	count	1
AL390728.6	1.5345365	0.8602969	1.7837	0.0754	1.624318202	count	1
RNASEH1	1.211344	0.5588432	2.1676	0.0309	1.626967949	count	1
ASRGL1	1.361382	0.9186599	1.4819	0.139	1.631588732	count	1
LINC00893	1.361382	0.9822451	1.386	0.167	1.631588732	count	1
PAK1IP1	1.2767567	0.7390852	1.7275	0.085	1.631889066	count	1
SUSD6	1.457235	0.7438609	1.959	0.051	1.632207696	count	1
WDR37	1.2948347	0.6455635	2.0057	0.0457	1.635975192	count	1
ELP4	1.3672075	0.6958876	1.9647	0.0503	1.638276322	count	1
PARG	1.340367	0.737244	1.8181	0.07	1.641417383	count	1
OMA1	1.4671319	0.7530236	1.9483	0.0522	1.642582441	count	1
PAN3-AS1	1.5550483	0.8440224	1.8424	0.0663	1.644184049	count	1
CCND1	1.3209277	0.8284857	1.5944	0.112	1.645562803	count	1
TNFAIP8L2	1.3794874	0.7926752	1.7403	0.0828	1.652355758	count	1
ARMCX5	1.328603	0.7358847	1.8055	0.0719	1.654814803	count	1
ANKRD36B	1.2379715	0.5963445	2.0759	0.0387	1.655908335	count	1
ZNF333	2.0538338	1.2110753	1.6959	0.0909	1.65666534	count	1
SCAF8	1.5756762	1.126616	1.3986	0.163	1.664065758	count	1
HYLS1	1.3620873	0.8137746	1.6738	0.0951	1.667014628	count	1
CMSS1	1.275611	0.7801561	1.6351	0.103	1.670851391	count	1
INO80B	1.285876	0.7696636	1.6707	0.0957	1.672293856	count	1
MOSPD2	1.4379326	0.8464338	1.6988	0.0903	1.672534154	count	1
AEN	1.7469348	0.8744985	1.9976	0.0466	1.679608413	count	1
AL021453.1	1.5952599	1.141105	1.398	0.163	1.682849941	count	1
KIAA1958	1.753834	1.0920795	1.606	0.109	1.685335761	count	1
SPATA7	1.753834	1.0920795	1.606	0.109	1.685335761	count	1
PLA2G6	1.753834	1.0920795	1.606	0.109	1.685335761	count	1
ZSCAN25	1.7538342	1.5992372	1.0967	0.2736	1.685336055	count	1
DENND2C	1.7538342	1.7030961	1.0298	0.3039	1.685336055	count	1
AL391069.2	1.7538342	1.7030961	1.0298	0.3039	1.685336055	count	1

ADGRG3	1.6001212	0.7030637	2.2759	0.0235	1.687498819	count	1
ST7L	1.413372	0.9952713	1.4201	0.157	1.691081348	count	1
IFIT1	1.243194	0.5973486	2.0812	0.0382	1.693453945	count	1
PARP15	1.5197154	0.8983835	1.6916	0.0917	1.697374643	count	1
MBNL1-AS1	1.5218306	1.4912965	1.0205	0.3083	1.699566816	count	1
MANBAL	1.3957417	0.9346593	1.4933	0.136	1.706540756	count	1
ZNF524	1.2685717	0.5462113	2.3225	0.0208	1.708596045	count	1
OSGEP	1.2648492	0.500349	2.5279	0.0119	1.709017823	count	1
TMCO3	1.3393751	0.5689064	2.3543	0.0192	1.70973146	count	1
CXorf40B	1.4016069	0.7030907	1.9935	0.047	1.713412037	count	1
ABCF3	1.5410242	0.8255303	1.8667	0.0628	1.719416047	count	1
AL627171.1	1.4812228	0.7066949	2.096	0.0369	1.719962268	count	1
CENPB	1.3029311	0.5971599	2.1819	0.0298	1.725617774	count	1
VNN2	1.299644	0.7022821	1.8506	0.0651	1.729605564	count	1
DUSP18	1.342437	0.7563541	1.7749	0.0769	1.729912562	count	1
FAM241B	1.3716747	0.8558368	1.6027	0.11	1.73000651	count	1
MICB	1.6464829	0.7929933	2.0763	0.0387	1.731551946	count	1
YLPM1	1.3982471	0.7201113	1.9417	0.053	1.738405508	count	1
AMPD3	1.5037163	0.7235951	2.0781	0.0385	1.744467602	count	1
ELMOD2	1.3346662	0.6077094	2.1962	0.0288	1.746459239	count	1
KIAA0930	1.4628906	0.8530659	1.7149	0.0873	1.747334317	count	1
WIPF2	1.4366308	0.711924	2.018	0.0444	1.754334078	count	1
AC105020.6	17.8785865	1594.587877	0.0112	0.991	1.757429555	count	1
AURKA	18.0709546	1592.733055	0.0113	0.991	1.757429565	count	1
AL359644.1	18.360456	2355.197545	0.0078	0.994	1.757429577	count	1
CEP70	18.900429	2034.847419	0.0093	0.9926	1.757429592	count	1
SORBS3	19.16758	3011.782619	0.0064	0.995	1.757429597	count	1
ARHGAP33	19.3051864	2173.228641	0.0089	0.993	1.7574296	count	1
STPG1	19.8395885	2787.354173	0.0071	0.994	1.757429605	count	1
PTGS2	19.8395885	2787.354173	0.0071	0.994	1.757429605	count	1
PACRGL	19.8395881	2787.354078	0.0071	0.994	1.757429605	count	1
PTPN13	19.8395881	2787.354078	0.0071	0.994	1.757429605	count	1
TRIM36	19.8395885	2787.354173	0.0071	0.994	1.757429605	count	1
AIRN	19.8395881	2787.354078	0.0071	0.994	1.757429605	count	1
AL022069.1	19.8395885	2787.354173	0.0071	0.994	1.757429605	count	1
FAM86B1	19.8395885	2787.354173	0.0071	0.994	1.757429605	count	1
C8orf48	19.8395881	2787.354078	0.0071	0.994	1.757429605	count	1
PCNX3	19.8395885	2787.354173	0.0071	0.994	1.757429605	count	1
ABCC9	19.8395885	2787.354173	0.0071	0.994	1.757429605	count	1
CENPBD1	19.8395885	2787.354173	0.0071	0.994	1.757429605	count	1
WDR81	19.8395885	2787.354173	0.0071	0.994	1.757429605	count	1
SHPK	19.8395881	2787.354078	0.0071	0.994	1.757429605	count	1
ZNF286B	19.8395885	2787.354173	0.0071	0.994	1.757429605	count	1
AC145343.1	19.8395881	2787.354078	0.0071	0.994	1.757429605	count	1
TAF4B	19.8395885	2787.354173	0.0071	0.994	1.757429605	count	1

CD3EAP	19.8395885	2787.354173	0.0071	0.994	1.757429605	count	1
AC002470.1	19.8395885	2787.354173	0.0071	0.994	1.757429605	count	1
DEPDC5	19.8395881	2787.354078	0.0071	0.994	1.757429605	count	1
RNF32	17.8079955	1792.470512	0.0099	0.992	1.757429652	count	1
FKBP15	17.8109202	1444.419182	0.0123	0.99	1.757429652	count	1
ZNF268	17.8785872	1594.587863	0.0112	0.991	1.757429656	count	1
SLC38A9	18.115192	1450.647897	0.0125	0.99	1.757429669	count	1
AC107375.1	18.1209362	1515.241887	0.012	0.9905	1.757429669	count	1
ANKS3	18.2764243	1528.219655	0.012	0.99	1.757429676	count	1
ZNF346	18.3604553	2355.197622	0.0078	0.994	1.757429679	count	1
SHROOM1	18.3604556	2355.197617	0.0078	0.994	1.757429679	count	1
PXN-AS1	18.3604559	2355.197603	0.0078	0.994	1.757429679	count	1
DROSHA	18.3604565	2355.197892	0.0078	0.994	1.757429679	count	1
NEXN	18.3604571	2355.198201	0.0078	0.994	1.757429679	count	1
HLA-G	18.3604571	2355.19821	0.0078	0.994	1.757429679	count	1
SLC18B1	18.3604572	2355.198186	0.0078	0.994	1.757429679	count	1
ARC	18.3604573	2355.198557	0.0078	0.994	1.757429679	count	1
FAM219A	18.3604573	2355.198186	0.0078	0.994	1.757429679	count	1
PDE8A	18.3604578	2355.198119	0.0078	0.994	1.757429679	count	1
APOC2	18.3614894	1825.270621	0.0101	0.992	1.757429679	count	1
NKIRAS1	18.3614897	1825.270902	0.0101	0.992	1.757429679	count	1
PFKFB2	18.5062942	2765.445702	0.0067	0.995	1.757429684	count	1
SOCS5	18.5104895	2177.942106	0.0085	0.993	1.757429684	count	1
ZBTB6	18.5104898	2177.941567	0.0085	0.993	1.757429684	count	1
MIER2	18.5104911	2177.942283	0.0085	0.993	1.757429684	count	1
AC138696.2	18.5444952	2036.919652	0.0091	0.993	1.757429685	count	1
ARHGEF40	18.6931945	2547.661659	0.0073	0.994	1.757429689	count	1
WIZ	18.6931948	2547.66071	0.0073	0.994	1.757429689	count	1
MED22	18.8117991	2032.458539	0.0093	0.993	1.757429692	count	1
DNAJC16	18.8322926	1941.015534	0.0097	0.9923	1.757429693	count	1
CA11	18.9004301	2034.847591	0.0093	0.9926	1.757429694	count	1
CARD6	19.1675749	3011.781786	0.0064	0.995	1.757429699	count	1
ZNF587	19.1675755	3011.78157	0.0064	0.995	1.757429699	count	1
TCEA3	19.167577	3011.781087	0.0064	0.995	1.757429699	count	1
FBXL19	19.1675778	3011.783135	0.0064	0.995	1.757429699	count	1
LINC00092	19.1675798	3011.782569	0.0064	0.995	1.757429699	count	1
ZFP3	19.1675801	3011.782636	0.0064	0.995	1.757429699	count	1
MEGF6	19.3051837	2173.228941	0.0089	0.993	1.757429701	count	1
GPR89A	19.3051843	2173.229956	0.0089	0.993	1.757429701	count	1
SNX21	19.3051842	2173.22946	0.0089	0.993	1.757429701	count	1
ATAT1	19.3051851	2173.229922	0.0089	0.993	1.757429701	count	1
THAP9	19.3051861	2173.229241	0.0089	0.993	1.757429701	count	1
PRUNE2	19.3051867	2173.22976	0.0089	0.993	1.757429701	count	1
CTNS	19.3051862	2173.22923	0.0089	0.993	1.757429701	count	1
DTNB	19.3051872	2173.230245	0.0089	0.993	1.757429701	count	1

NDC1	19.5478988	2475.505684	0.0079	0.9937	1.757429704	count	1
MAP3K14-AS1	19.547899	2475.506449	0.0079	0.9937	1.757429704	count	1
ZNF280B	19.5478993	2475.505684	0.0079	0.9937	1.757429704	count	1
CPE	19.5479003	2475.50522	0.0079	0.9937	1.757429704	count	1
GSEC	19.5478997	2475.506424	0.0079	0.9937	1.757429704	count	1
TAOK2	19.5479002	2475.505596	0.0079	0.9937	1.757429704	count	1
GOLGA6L7	19.5582816	2117.525281	0.0092	0.993	1.757429704	count	1
PERM1	19.8395882	2787.354078	0.0071	0.994	1.757429707	count	1
HES4	19.8395882	2787.354078	0.0071	0.994	1.757429707	count	1
SLC25A34-AS1	19.8395882	2787.354078	0.0071	0.994	1.757429707	count	1
ARHGEF10L	19.8395882	2787.354078	0.0071	0.994	1.757429707	count	1
HSPG2	19.8395882	2787.354078	0.0071	0.994	1.757429707	count	1
ZNF436	19.8395882	2787.354078	0.0071	0.994	1.757429707	count	1
EFCAB14-AS1	19.8395882	2787.354078	0.0071	0.994	1.757429707	count	1
KIRREL1	19.8395882	2787.354078	0.0071	0.994	1.757429707	count	1
PRELP	19.8395882	2787.354078	0.0071	0.994	1.757429707	count	1
CDC42BPA	19.8395883	2787.353983	0.0071	0.994	1.757429707	count	1
AC073195.1	19.8395882	2787.354078	0.0071	0.994	1.757429707	count	1
LTBP1	19.8395882	2787.354078	0.0071	0.994	1.757429707	count	1
PLEKHH2	19.8395882	2787.354078	0.0071	0.994	1.757429707	count	1
LOXL3	19.8395882	2787.354078	0.0071	0.994	1.757429707	count	1
C2orf40	19.8395882	2787.354078	0.0071	0.994	1.757429707	count	1
STEAP3	19.8395882	2787.354078	0.0071	0.994	1.757429707	count	1
KCNE4	19.8395882	2787.354078	0.0071	0.994	1.757429707	count	1
IQCA1	19.8395881	2787.353964	0.0071	0.994	1.757429707	count	1
SNED1	19.8395882	2787.354078	0.0071	0.994	1.757429707	count	1
AC069277.1	19.8395883	2787.354116	0.0071	0.994	1.757429707	count	1
CAND2	19.8395883	2787.353983	0.0071	0.994	1.757429707	count	1
FGD5	19.8395883	2787.354097	0.0071	0.994	1.757429707	count	1
TGM4	19.8395882	2787.354116	0.0071	0.994	1.757429707	count	1
SHQ1	19.8395883	2787.354097	0.0071	0.994	1.757429707	count	1
TMEM45A	19.8395882	2787.354078	0.0071	0.994	1.757429707	count	1
CCDC191	19.8395883	2787.354097	0.0071	0.994	1.757429707	count	1
ITGB5	19.8395882	2787.354078	0.0071	0.994	1.757429707	count	1
CPNE4	19.8395882	2787.354078	0.0071	0.994	1.757429707	count	1
IGSF10	19.8395882	2787.354078	0.0071	0.994	1.757429707	count	1
SUCNR1	19.8395882	2787.354078	0.0071	0.994	1.757429707	count	1
MYL5	19.8395884	2787.354116	0.0071	0.994	1.757429707	count	1
FGFRL1	19.8395883	2787.354021	0.0071	0.994	1.757429707	count	1
BMP2K	19.8395883	2787.354021	0.0071	0.994	1.757429707	count	1
PDLIM3	19.8395882	2787.354078	0.0071	0.994	1.757429707	count	1
CMBL	19.8395882	2787.354078	0.0071	0.994	1.757429707	count	1
MYO10	19.8395883	2787.354116	0.0071	0.994	1.757429707	count	1
AC010273.1	19.8395883	2787.353983	0.0071	0.994	1.757429707	count	1
AC010226.1	19.8395883	2787.354021	0.0071	0.994	1.757429707	count	1

ALDH7A1	19.8395882	2787.354078	0.0071	0.994	1.757429707	count	1
FOXC1	19.8395882	2787.354078	0.0071	0.994	1.757429707	count	1
TFAP2A	19.8395882	2787.354078	0.0071	0.994	1.757429707	count	1
HIST1H4D	19.8395884	2787.354135	0.0071	0.994	1.757429707	count	1
ABHD16A	19.8395883	2787.354097	0.0071	0.994	1.757429707	count	1
RCAN2	19.8395882	2787.354078	0.0071	0.994	1.757429707	count	1
AL603910.1	19.8395882	2787.354078	0.0071	0.994	1.757429707	count	1
METTL24	19.8395882	2787.354078	0.0071	0.994	1.757429707	count	1
TPD52L1	19.8395883	2787.353945	0.0071	0.994	1.757429707	count	1
AL159163.1	19.8395882	2787.354078	0.0071	0.994	1.757429707	count	1
MACC1	19.8395883	2787.354097	0.0071	0.994	1.757429707	count	1
INMT	19.8395882	2787.354078	0.0071	0.994	1.757429707	count	1
AQP1	19.8395882	2787.354078	0.0071	0.994	1.757429707	count	1
TNS3	19.8395882	2787.354078	0.0071	0.994	1.757429707	count	1
AC211476.2	19.8395884	2787.354116	0.0071	0.994	1.757429707	count	1
FZD1	19.8395882	2787.354078	0.0071	0.994	1.757429707	count	1
LRRC17	19.8395882	2787.354078	0.0071	0.994	1.757429707	count	1
LSMEM1	19.8395884	2787.354135	0.0071	0.994	1.757429707	count	1
AC083862.2	19.8395882	2787.354116	0.0071	0.994	1.757429707	count	1
ACTR3B	19.8395883	2787.354116	0.0071	0.994	1.757429707	count	1
INE2	19.8395882	2787.354078	0.0071	0.994	1.757429707	count	1
AC234772.3	19.8395883	2787.354097	0.0071	0.994	1.757429707	count	1
LINC01560	19.8395883	2787.354116	0.0071	0.994	1.757429707	count	1
PABPC5	19.8395882	2787.354078	0.0071	0.994	1.757429707	count	1
ARHGEF10	19.8395882	2787.354078	0.0071	0.994	1.757429707	count	1
MCPH1-AS1	19.8395882	2787.354078	0.0071	0.994	1.757429707	count	1
NECAB1	19.8395883	2787.354021	0.0071	0.994	1.757429707	count	1
PTPRD	19.8395881	2787.353964	0.0071	0.994	1.757429707	count	1
CCL27	19.8395884	2787.354116	0.0071	0.994	1.757429707	count	1
PRRX2	19.8395882	2787.354078	0.0071	0.994	1.757429707	count	1
DBH-AS1	19.8395883	2787.354021	0.0071	0.994	1.757429707	count	1
COL5A1	19.8395882	2787.354078	0.0071	0.994	1.757429707	count	1
LMO2	19.8395882	2787.354078	0.0071	0.994	1.757429707	count	1
ROM1	19.8395882	2787.354078	0.0071	0.994	1.757429707	count	1
C11orf80	19.8395883	2787.354097	0.0071	0.994	1.757429707	count	1
CACNA1C	19.8395882	2787.354078	0.0071	0.994	1.757429707	count	1
LMO3	19.8395882	2787.354078	0.0071	0.994	1.757429707	count	1
AC025031.2	19.8395883	2787.353983	0.0071	0.994	1.757429707	count	1
ADCY6	19.8395882	2787.354078	0.0071	0.994	1.757429707	count	1
AC025259.3	19.8395884	2787.354116	0.0071	0.994	1.757429707	count	1
AC068790.8	19.8395881	2787.353964	0.0071	0.994	1.757429707	count	1
UGGT2	19.8395882	2787.354078	0.0071	0.994	1.757429707	count	1
EFNB2	19.8395884	2787.354116	0.0071	0.994	1.757429707	count	1
SLC22A17	19.8395882	2787.354078	0.0071	0.994	1.757429707	count	1
EFS	19.8395882	2787.354078	0.0071	0.994	1.757429707	count	1

ARMH4	19.8395882	2787.354078	0.0071	0.994	1.757429707	count	1
TMEM30B	19.8395882	2787.354078	0.0071	0.994	1.757429707	count	1
MEG3	19.8395882	2787.354078	0.0071	0.994	1.757429707	count	1
AC123768.3	19.8395881	2787.353964	0.0071	0.994	1.757429707	count	1
THSD4	19.8395882	2787.354078	0.0071	0.994	1.757429707	count	1
LOXL1	19.8395882	2787.354078	0.0071	0.994	1.757429707	count	1
SOX8	19.8395882	2787.354078	0.0071	0.994	1.757429707	count	1
NOMO1	19.8395881	2787.353964	0.0071	0.994	1.757429707	count	1
AC137932.3	19.8395883	2787.353983	0.0071	0.994	1.757429707	count	1
SOCS7	19.8395882	2787.354078	0.0071	0.994	1.757429707	count	1
C17orf82	19.8395882	2787.354078	0.0071	0.994	1.757429707	count	1
GPRC5C	19.8395882	2787.354078	0.0071	0.994	1.757429707	count	1
MRPL38	19.8395882	2787.354116	0.0071	0.994	1.757429707	count	1
LGALS3BP	19.8395882	2787.354078	0.0071	0.994	1.757429707	count	1
ENGASE	19.8395882	2787.354078	0.0071	0.994	1.757429707	count	1
TWSG1	19.8395882	2787.354078	0.0071	0.994	1.757429707	count	1
GATA6-AS1	19.8395882	2787.354078	0.0071	0.994	1.757429707	count	1
CCDC68	19.8395882	2787.354078	0.0071	0.994	1.757429707	count	1
ATP8B1	19.8395882	2787.354078	0.0071	0.994	1.757429707	count	1
NEDD4L	19.8395881	2787.353964	0.0071	0.994	1.757429707	count	1
CFAP61	19.8395882	2787.354078	0.0071	0.994	1.757429707	count	1
PTGIS	19.8395882	2787.354078	0.0071	0.994	1.757429707	count	1
DOK5	19.8395882	2787.354078	0.0071	0.994	1.757429707	count	1
AC016588.2	19.8395883	2787.354116	0.0071	0.994	1.757429707	count	1
PRSS57	19.8395883	2787.354021	0.0071	0.994	1.757429707	count	1
ZNF823	19.8395884	2787.354116	0.0071	0.994	1.757429707	count	1
CACNA1A	19.8395882	2787.354116	0.0071	0.994	1.757429707	count	1
ZSWIM4	19.8395882	2787.354116	0.0071	0.994	1.757429707	count	1
LRFN3	19.8395883	2787.354021	0.0071	0.994	1.757429707	count	1
BCAM	19.8395882	2787.354078	0.0071	0.994	1.757429707	count	1
DMPK	19.8395884	2787.354116	0.0071	0.994	1.757429707	count	1
PLA2G4C	19.8395882	2787.354078	0.0071	0.994	1.757429707	count	1
ZNF415	19.8395883	2787.354021	0.0071	0.994	1.757429707	count	1
SSC5D	19.8395882	2787.354078	0.0071	0.994	1.757429707	count	1
RTL10	19.8395882	2787.354078	0.0071	0.994	1.757429707	count	1
TIMP3	19.8395882	2787.354078	0.0071	0.994	1.757429707	count	1
ACR	19.8395882	2787.354078	0.0071	0.994	1.757429707	count	1
TRIM69	1.394742	0.6827309	2.0429	0.0419	1.758089313	count	1
RB1	1.3183404	0.5072012	2.5992	0.00977	1.7615503	count	1
INTS2	1.5873809	1.0281776	1.5439	0.124	1.767031267	count	1
USP27X	1.8567149	0.9722783	1.9097	0.0571	1.769194776	count	1
RNF139-AS1	1.8567149	0.9722783	1.9097	0.0571	1.769194776	count	1
AC104794.2	1.8567149	1.0743563	1.7282	0.0849	1.769194776	count	1
UBE3D	1.8567149	1.0743563	1.7282	0.0849	1.769194776	count	1
MTHFD1L	1.8567149	1.0743563	1.7282	0.0849	1.769194776	count	1

FAM169A	1.8567149	1.21579	1.5272	0.128	1.769194776	count	1
CHPF	1.4863183	0.8221858	1.8078	0.0716	1.773801777	count	1
AC245595.1	1.8811598	0.9996251	1.8819	0.0608	1.788683863	count	1
BCS1L	1.8811598	1.0752187	1.7496	0.0811	1.788683863	count	1
SPA17	1.8811598	1.0752187	1.7496	0.0811	1.788683863	count	1
MMS22L	1.8811598	1.1607976	1.6206	0.106	1.788683863	count	1
ZKSCAN7	1.8811598	1.226498	1.5338	0.126	1.788683863	count	1
MIEF2	1.8811598	1.226498	1.5338	0.126	1.788683863	count	1
GOLGA8M	2.2801127	1.248353	1.8265	0.0687	1.789298707	count	1
TSC22D1	1.3318059	0.5265195	2.5295	0.0119	1.792346835	count	1
TMEM102	1.4450467	0.648464	2.2284	0.0265	1.794195119	count	1
MED23	1.5074283	0.9274201	1.6254	0.1051	1.797567706	count	1
ING5	1.3324179	0.5609259	2.3754	0.0181	1.804081649	count	1
TOGARAM2	1.4581445	0.7378014	1.9763	0.049	1.809751225	count	1
RGS5	1.379155	0.7885664	1.7489	0.0812	1.814391837	count	1
NECTIN3	2.338135	1.1461068	2.0401	0.0422	1.82088657	count	1
COLGALT1	1.5285532	0.7356234	2.0779	0.0385	1.821269853	count	1
RRP9	1.4051418	0.7169104	1.96	0.0509	1.823215571	count	1
CRYZ	1.397476	0.6508579	2.1471	0.0325	1.826495569	count	1
CRACR2B	1.375383	0.699743	1.9656	0.0502	1.82828403	count	1
SLC35A2	1.6490146	0.867742	1.9004	0.0583	1.8296004	count	1
MAP3K5	1.5888408	0.8847776	1.7958	0.0735	1.836307483	count	1
ENTPD4	1.5888408	1.001707	1.5861	0.114	1.836307483	count	1
ERCC4	1.4611566	0.7193783	2.0311	0.0431	1.83854424	count	1
FGL2	1.420533	0.7647369	1.8575	0.0641	1.842579343	count	1
ZFY	1.4242295	0.5661895	2.5155	0.0124	1.847225855	count	1
SH2B1	1.6668674	0.998979	1.6686	0.0962	1.84756143	count	1
ZNF621	1.6668674	1.036901	1.6075	0.109	1.84756143	count	1
LEAP2	1.599868	0.8136566	1.9663	0.0501	1.848096639	count	1
IRGQ	1.599868	0.8136566	1.9663	0.0501	1.848096639	count	1
ELP1	1.5549155	0.8307138	1.8718	0.0621	1.850732726	count	1
HIST1H2BE	1.7755847	1.129819	1.5716	0.117	1.851406522	count	1
VPS26B	1.5575652	0.7907891	1.9696	0.0497	1.85368682	count	1
PUS10	2.4024239	1.4272894	1.6832	0.0933	1.854729137	count	1
TECPR2	1.9707157	1.127274	1.7482	0.0814	1.858607123	count	1
RIDA	1.9707157	1.225176	1.6085	0.109	1.858607123	count	1
RABGEF1	1.9707157	1.7029115	1.1573	0.248	1.858607123	count	1
RGPD5	1.7891734	0.9099003	1.9663	0.0501	1.863770355	count	1
GIMAP8	1.7891734	0.976196	1.8328	0.0677	1.863770355	count	1
SBF1	1.6839264	1.186679	1.419	0.157	1.86465386	count	1
ZNF100	1.466337	0.6021163	2.4353	0.0154	1.866117555	count	1
PAM	1.9904502	1.2630921	1.5759	0.116	1.873698062	count	1
FANCA	1.9904502	1.37428	1.4484	0.148	1.873698062	count	1
CD226	1.418141	0.7769001	1.8254	0.0689	1.874595195	count	1
CCDC34	1.8023145	1.2080974	1.4919	0.1367	1.875679947	count	1

TENT4A	2.4785287	1.5410785	1.6083	0.1087	1.893227672	count	1
AC040162.1	1.510154	0.7667357	1.9696	0.0497	1.897499032	count	1
RHBDF2	2.5111279	1.089531	2.3048	0.0218	1.909202519	count	1
CDK5R1	1.8492796	1.0579651	1.748	0.0814	1.917860397	count	1
CYB561A3	1.5109273	0.6910887	2.1863	0.0295	1.920539457	count	1
AC243829.4	1.4015735	0.4636377	3.023	0.0027	1.921497389	count	1
ELOA-AS1	1.51518	0.9342293	1.6219	0.106	1.925715288	count	1
LARP4B	1.8610517	0.8671724	2.1461	0.0326	1.928338092	count	1
FOSL1	1.560198	1.0542784	1.4799	0.1399	1.930042589	count	1
AC012360.3	1.6777933	1.0492187	1.5991	0.111	1.930667322	count	1
MUL1	1.753575	1.0791504	1.625	0.105	1.933710877	count	1
GNS	1.6369333	0.8622161	1.8985	0.0585	1.941542136	count	1
ZNF664	1.5704871	0.8241837	1.9055	0.0576	1.942076319	count	1
SNAPC3	2.0865967	1.1364466	1.8361	0.0673	1.94554352	count	1
GALK2	1.4396493	0.7364404	1.9549	0.0515	1.946493843	count	1
SLC41A1	1.8831368	0.9531661	1.9757	0.049	1.947890424	count	1
SIGLEC7	1.6489299	0.8529063	1.9333	0.0541	1.954712248	count	1
CA5B	1.4571092	0.5271268	2.7642	0.00603	1.963762775	count	1
AC245014.3	1.7115189	0.7205294	2.3754	0.0181	1.9659873	count	1
FSCN1	1.9052827	1.1138492	1.7105	0.08812	1.967358568	count	1
PRKAG2-AS1	1.5226988	0.8077632	1.8851	0.0603	1.970397794	count	1
ZNF487	1.560814	0.7700715	2.0268	0.0435	1.981089703	count	1
C12orf76	1.5073576	0.7166298	2.1034	0.0362	1.989062618	count	1
ARHGAP1	1.5873809	0.7530239	2.108	0.0358	1.989679717	count	1
ZNF699	1.5714886	0.6864334	2.2894	0.0227	1.993998359	count	1
PQLC1	1.5203778	0.5416749	2.8068	0.00531	1.994489594	count	1
UBE2D4	1.8175154	0.9838497	1.8474	0.0656	1.996041945	count	1
RGS17	1.6217913	0.7515782	2.1578	0.0317	2.001808483	count	1
WDR3	2.1726741	1.2763573	1.7022	0.0897	2.007458012	count	1
CSTF2	1.753834	0.9890789	1.7732	0.0771	2.009932721	count	1
PPP1R16A	1.753834	1.0105193	1.7356	0.0836	2.009932721	count	1
AL035071.1	1.753834	1.0105193	1.7356	0.0836	2.009932721	count	1
SEPHS1	1.5760811	0.6906506	2.282	0.0231	2.019451038	count	1
Z93241.1	1.9821756	1.2385429	1.6004	0.1105	2.033860093	count	1
TRMT61B	2.2283092	0.9329865	2.3884	0.0175	2.046243681	count	1
GORAB	1.6009321	0.8447375	1.8952	0.059	2.04981662	count	1
AL355353.1	2.005334	0.9643864	2.0794	0.0384	2.053549957	count	1
LINC00484	2.005334	0.9643864	2.0794	0.0384	2.053549957	count	1
NRSN2-AS1	2.005334	0.9643864	2.0794	0.0384	2.053549957	count	1
RHOT2	1.5431325	0.6372148	2.4217	0.016	2.05409465	count	1
OTUD1	1.6275429	0.7880243	2.0653	0.0397	2.061496588	count	1
TMEM91	2.250755	1.4657288	1.5356	0.1256	2.061614932	count	1
ZNF799	2.0160066	0.936272	2.1532	0.032	2.062570244	count	1
PHF19	1.750688	0.7197824	2.4322	0.0155	2.065204967	count	1
TMEM9	1.644143	0.7374713	2.2294	0.0265	2.081390543	count	1

TADA2B	2.2831021	0.8347597	2.735	0.00658	2.08348552	count	1
JAG2	1.7686507	1.057858	1.6719	0.0955	2.084474852	count	1
PHLDB3	2.3223097	1.4425148	1.6099	0.1084	2.109547768	count	1
GNPDA2	1.7961599	1.0009803	1.7944	0.0737	2.113844266	count	1
TFB2M	1.711668	0.7630582	2.2432	0.0256	2.135966552	count	1
AL355472.1	2.104453	1.013677	2.0761	0.0387	2.136000088	count	1
LINC01943	1.8811598	1.0422574	1.8049	0.072	2.139557132	count	1
SDC4	1.823012	0.7730411	2.3582	0.019	2.142343699	count	1
S100A8	3.0955059	3.2032499	0.9664	0.335	2.145578458	count	1
NF1	1.9835104	0.9874754	2.0087	0.0454	2.152786042	count	1
UEVLD	1.753834	0.9190874	1.9082	0.0572	2.153335007	count	1
SQLE	1.7647232	0.843945	2.091	0.0373	2.165680999	count	1
DDX55	1.6565211	0.6412144	2.5834	0.0102	2.165900792	count	1
HIST3H2A	1.6489777	0.5757037	2.8643	0.00445	2.180062568	count	1
ZDHHC7	1.8128588	1.1823608	1.5333	0.1262	2.180252416	count	1
CREB3	1.7117291	0.7567162	2.262	0.0244	2.184058048	count	1
CSF2	1.5946703	0.5221701	3.0539	0.00245	2.222662801	count	1
PRKCZ	1.8623018	0.9638555	1.9321	0.0542	2.23409309	count	1
MRPL44	1.8274493	0.7233304	2.5264	0.012	2.236326346	count	1
NPC1	2.2319795	1.3327882	1.6747	0.095	2.237607687	count	1
ALKBH4	1.832412	0.9990959	1.8341	0.0676	2.241880444	count	1
BRMS1L	1.9904502	1.1013175	1.8073	0.0716	2.247501422	count	1
SRP14-AS1	1.943872	0.9724205	1.999	0.0464	2.268473752	count	1
ZNF513	2.3220331	0.9665234	2.4025	0.0168	2.306231685	count	1
SIMC1	2.3329316	1.4162221	1.6473	0.1005	2.314358102	count	1
LANCL3	2.3329316	1.4501746	1.6087	0.1086	2.314358102	count	1
PAK4	2.3329316	1.5467454	1.5083	0.1325	2.314358102	count	1
ZNF821	1.809436	0.7115401	2.543	0.0115	2.32142867	count	1
AP001160.1	2.3435506	1.1475081	2.0423	0.0419	2.322238621	count	1
ZNF71	1.9977192	1.4305436	1.3965	0.1635	2.323487598	count	1
FEM1B	2.0701606	0.9262477	2.235	0.0261	2.324173	count	1
GNB5	1.8147572	0.9807554	1.8504	0.0652	2.327821083	count	1
STYX	2.0279798	0.8750801	2.3175	0.0211	2.354070396	count	1
CTGF	2.0299105	1.2715953	1.5963	0.1114	2.356013385	count	1
GMDS	1.88755	0.949266	1.9884	0.04761	2.367586387	count	1
E4F1	2.7676405	1.1509766	2.4046	0.0168	2.37100145	count	1
ASB16-AS1	2.4456581	0.9213296	2.6545	0.00833	2.396120024	count	1
FAM118B	1.927028	0.8494322	2.2686	0.0239	2.412945992	count	1
ZNF395	2.293016	0.8871511	2.5847	0.0102	2.42359992	count	1
KLC2	2.056507	0.8847051	2.3245	0.0207	2.439968514	count	1
TIMELESS	2.056507	0.930749	2.2095	0.0278	2.439968514	count	1
ETFDH	2.1995905	1.0206403	2.1551	0.0319	2.44476597	count	1
ARMH3	2.1248138	1.163566	1.8261	0.0688	2.450267154	count	1
AP1S1	1.9612554	0.7358311	2.6654	0.00808	2.452012554	count	1
ZNF141	1.940743	0.7182819	2.7019	0.00726	2.47770229	count	1

TXNDC16	2.096204	0.8037237	2.6081	0.00953	2.48088161	count	1
LYRM7	2.1036937	0.7157496	2.9391	0.00353	2.488554106	count	1
RUVBL2	2.0811918	0.77895	2.6718	0.00793	2.513196806	count	1
MIR155HG	2.2816216	1.0871243	2.0988	0.0366	2.51858501	count	1
PIP4K2C	18.1805536	1596.82525	0.0114	0.991	2.526545653	count	1
PPP1R21	18.2434034	1632.162045	0.0112	0.991	2.526545658	count	1
MYEF2	18.2553848	1536.360692	0.0119	0.991	2.526545659	count	1
GCFC2	18.3211532	1472.319031	0.0124	0.99	2.526545664	count	1
NAP1L5	18.4766809	1495.394504	0.0124	0.99	2.526545675	count	1
GLA	18.5027303	1658.005694	0.0112	0.991	2.526545677	count	1
AL031963.3	18.7252336	2092.054582	0.009	0.993	2.52654569	count	1
NEK8	18.7252336	2092.054553	0.009	0.993	2.52654569	count	1
VSTM2L	18.7252338	2092.054578	0.009	0.993	2.52654569	count	1
CARM1	18.8558015	2577.050752	0.0073	0.994	2.526545696	count	1
OGFOD2	18.8558016	2577.052307	0.0073	0.994	2.526545696	count	1
LIPE	18.8558022	2577.05214	0.0073	0.994	2.526545696	count	1
SH3TC1	18.8558031	2577.051837	0.0073	0.994	2.526545696	count	1
SLC9A3	18.8568435	2337.72746	0.0081	0.994	2.526545696	count	1
UGDH-AS1	18.9581715	2974.511746	0.0064	0.995	2.5265457	count	1
CPEB3	18.959471	2432.198361	0.0078	0.994	2.5265457	count	1
PLK4	18.9690579	1837.029528	0.0103	0.992	2.526545701	count	1
FAM110B	18.9690595	1837.029048	0.0103	0.992	2.526545701	count	1
YPEL4	18.978224	2296.40595	0.0083	0.993	2.526545701	count	1
ANKRD27	18.9782256	2296.406917	0.0083	0.993	2.526545701	count	1
MEX3B	19.1230234	2471.400513	0.0077	0.994	2.526545706	count	1
AL049795.1	19.5242198	2380.723145	0.0082	0.993	2.526545717	count	1
AC114490.2	19.5242197	2380.723027	0.0082	0.993	2.526545717	count	1
HIST2H4A	19.5242197	2380.723027	0.0082	0.993	2.526545717	count	1
OCLM	19.52422	2380.723098	0.0082	0.993	2.526545717	count	1
TAF1A-AS1	19.52422	2380.723098	0.0082	0.993	2.526545717	count	1
AC098828.2	19.5242198	2380.723145	0.0082	0.993	2.526545717	count	1
MAPRE3	19.52422	2380.723098	0.0082	0.993	2.526545717	count	1
FMNL2	19.5242197	2380.72311	0.0082	0.993	2.526545717	count	1
LINC01963	19.5242197	2380.722932	0.0082	0.993	2.526545717	count	1
ZBTB20-AS2	19.5242197	2380.72311	0.0082	0.993	2.526545717	count	1
EHHADH	19.5242197	2380.723027	0.0082	0.993	2.526545717	count	1
AC025171.5	19.5242197	2380.72311	0.0082	0.993	2.526545717	count	1
AC104109.2	19.5242197	2380.72311	0.0082	0.993	2.526545717	count	1
TIGD6	19.5242197	2380.72311	0.0082	0.993	2.526545717	count	1
AL512329.2	19.52422	2380.723098	0.0082	0.993	2.526545717	count	1
HIST1H2BL	19.5242197	2380.723027	0.0082	0.993	2.526545717	count	1
LRP11	19.5242197	2380.72311	0.0082	0.993	2.526545717	count	1
IYD	19.5242198	2380.723145	0.0082	0.993	2.526545717	count	1
KIAA1324L	19.5242198	2380.723145	0.0082	0.993	2.526545717	count	1
RUNDC3B	19.5242197	2380.72311	0.0082	0.993	2.526545717	count	1

XRCC2	19.5242199	2380.72298	0.0082	0.993	2.526545717	count	1
CHMP4C	19.5242197	2380.72311	0.0082	0.993	2.526545717	count	1
JRK	19.52422	2380.723098	0.0082	0.993	2.526545717	count	1
TBC1D2	19.52422	2380.723098	0.0082	0.993	2.526545717	count	1
STX17-AS1	19.52422	2380.723098	0.0082	0.993	2.526545717	count	1
AL133551.1	19.52422	2380.723098	0.0082	0.993	2.526545717	count	1
KCNH3	19.5242198	2380.723145	0.0082	0.993	2.526545717	count	1
MAP1LC3B2	19.5242197	2380.722932	0.0082	0.993	2.526545717	count	1
AC117503.1	19.5242198	2380.723063	0.0082	0.993	2.526545717	count	1
AL136962.1	19.5242197	2380.72311	0.0082	0.993	2.526545717	count	1
SRRM2-AS1	19.52422	2380.723098	0.0082	0.993	2.526545717	count	1
NPIPB2	19.5242199	2380.72298	0.0082	0.993	2.526545717	count	1
NPIPA1	19.5242197	2380.72311	0.0082	0.993	2.526545717	count	1
COG7	19.5242198	2380.723145	0.0082	0.993	2.526545717	count	1
AC026471.2	19.52422	2380.723098	0.0082	0.993	2.526545717	count	1
TMEM104	19.5242199	2380.72298	0.0082	0.993	2.526545717	count	1
AC015819.1	19.52422	2380.723098	0.0082	0.993	2.526545717	count	1
KAT14	19.5242199	2380.72298	0.0082	0.993	2.526545717	count	1
PXMP4	19.52422	2380.723098	0.0082	0.993	2.526545717	count	1
PCSK4	19.52422	2380.723098	0.0082	0.993	2.526545717	count	1
ZNF599	19.5242198	2380.723145	0.0082	0.993	2.526545717	count	1
AC008537.4	19.5242197	2380.72311	0.0082	0.993	2.526545717	count	1
ZNF155	19.5242198	2380.723145	0.0082	0.993	2.526545717	count	1
ERCC2	19.5242197	2380.722932	0.0082	0.993	2.526545717	count	1
ZNF577	19.52422	2380.723098	0.0082	0.993	2.526545717	count	1
RHBDD3	19.5242197	2380.72311	0.0082	0.993	2.526545717	count	1
USP13	19.5242201	2380.723181	0.0082	0.993	2.526545717	count	1
AKAP12	19.5242201	2380.723181	0.0082	0.993	2.526545717	count	1
AC007349.3	19.5242201	2380.723181	0.0082	0.993	2.526545717	count	1
TCEANC	19.5242201	2380.723181	0.0082	0.993	2.526545717	count	1
NCBP1	19.5242201	2380.723181	0.0082	0.993	2.526545717	count	1
AP005329.1	19.5242201	2380.723181	0.0082	0.993	2.526545717	count	1
KLHL33	19.525939	1664.198982	0.0117	0.991	2.526545717	count	1
HSD17B1	19.8090091	2775.661733	0.0071	0.994	2.526545723	count	1
PLXNB2	19.8090119	2775.660737	0.0071	0.994	2.526545723	count	1
C5orf63	18.0912624	1681.196594	0.0108	0.991	2.526545764	count	1
PARP12	18.0912635	1681.197186	0.0108	0.991	2.526545764	count	1
MCU	18.0912638	1681.19694	0.0108	0.991	2.526545764	count	1
EFCAB11	18.0912638	1681.196476	0.0108	0.991	2.526545764	count	1
XPO5	18.0912641	1681.196909	0.0108	0.991	2.526545764	count	1
ALPL	18.1062216	2178.592053	0.0083	0.993	2.526545765	count	1
ANTXR2	18.234442	1406.093699	0.013	0.99	2.526545777	count	1
SKP2	18.3793459	1745.444956	0.0105	0.992	2.526545788	count	1
PRKCD	18.4295126	1755.023299	0.0105	0.992	2.526545791	count	1
AC091959.3	18.7252337	2092.054595	0.009	0.993	2.526545809	count	1

AC011773.4	18.7252337	2092.054599	0.009	0.993	2.526545809	count	1
PCSK6	18.7252337	2092.054595	0.009	0.993	2.526545809	count	1
PEX7	18.7252338	2092.054624	0.009	0.993	2.526545809	count	1
ISPD	18.7252338	2092.054587	0.009	0.993	2.526545809	count	1
CNR2	18.725234	2092.054703	0.009	0.993	2.526545809	count	1
FAM184B	18.7252341	2092.054553	0.009	0.993	2.526545809	count	1
KBTBD6	18.725234	2092.054703	0.009	0.993	2.526545809	count	1
ADPGK-AS1	18.7252341	2092.054549	0.009	0.993	2.526545809	count	1
CUBN	18.8558015	2577.052254	0.0073	0.994	2.526545815	count	1
ERP27	18.8558016	2577.052307	0.0073	0.994	2.526545815	count	1
TMEM25	18.8558017	2577.050741	0.0073	0.994	2.526545815	count	1
HIST1H4H	18.855802	2577.052192	0.0073	0.994	2.526545815	count	1
AL020996.1	18.8558023	2577.050491	0.0073	0.994	2.526545815	count	1
HIST1H2BJ	18.8558023	2577.052098	0.0073	0.994	2.526545815	count	1
NEDD4	18.8558023	2577.050491	0.0073	0.994	2.526545815	count	1
DCST1-AS1	18.8558028	2577.05047	0.0073	0.994	2.526545815	count	1
RTN4IP1	18.8558026	2577.052046	0.0073	0.994	2.526545815	count	1
ZNF202	18.8558026	2577.050418	0.0073	0.994	2.526545815	count	1
ZFR2	18.8558029	2577.051941	0.0073	0.994	2.526545815	count	1
AC090948.3	18.8558057	2577.05263	0.0073	0.994	2.526545815	count	1
PLPP6	18.8558056	2577.052599	0.0073	0.994	2.526545815	count	1
SLC2A8	18.8558057	2577.05262	0.0073	0.994	2.526545815	count	1
RAD18	18.9581709	2974.511904	0.0064	0.995	2.526545819	count	1
BAMBI	18.9594696	2432.198619	0.0078	0.994	2.526545819	count	1
AFAP1	18.9594708	2432.199382	0.0078	0.994	2.526545819	count	1
ZBED1	18.9594712	2432.199372	0.0078	0.994	2.526545819	count	1
ZNF583	18.9594724	2432.199154	0.0078	0.994	2.526545819	count	1
SEC61A2	18.959473	2432.200055	0.0078	0.994	2.526545819	count	1
KLHL17	18.9690572	1837.028915	0.0103	0.992	2.52654582	count	1
ZNF2	18.9690586	1837.028776	0.0103	0.992	2.52654582	count	1
UTP14C	18.9782216	2296.404246	0.0083	0.993	2.52654582	count	1
MAGEF1	18.9782252	2296.406975	0.0083	0.993	2.52654582	count	1
QPCTL	19.0402558	3308.595311	0.0058	0.995	2.526545823	count	1
FAM89A	19.0454065	2775.307757	0.0069	0.995	2.526545823	count	1
AC020978.5	19.0463108	2570.600195	0.0074	0.994	2.526545823	count	1
MRPS17	19.0463135	2570.60244	0.0074	0.994	2.526545823	count	1
DVL2	19.0463136	2570.601532	0.0074	0.994	2.526545823	count	1
LINC01132	19.0709233	1942.887471	0.0098	0.992	2.526545824	count	1
ZNF507	19.0947113	1745.413495	0.0109	0.991	2.526545824	count	1
MTFR1	19.1186678	2797.635328	0.0068	0.995	2.526545825	count	1
SYNM	19.1186684	2797.636746	0.0068	0.995	2.526545825	count	1
DST	19.1811855	1753.650887	0.0109	0.991	2.526545827	count	1
CLIC4	19.5242196	2380.722992	0.0082	0.993	2.526545837	count	1
AL445524.1	19.5242196	2380.722992	0.0082	0.993	2.526545837	count	1
ZNF717	19.5242196	2380.722992	0.0082	0.993	2.526545837	count	1

WDR49	19.5242196	2380.722992	0.0082	0.993	2.526545837	count	1
AC024243.1	19.5242196	2380.722992	0.0082	0.993	2.526545837	count	1
TGFBI	19.5242196	2380.722992	0.0082	0.993	2.526545837	count	1
SYNPO	19.5242196	2380.722992	0.0082	0.993	2.526545837	count	1
SFRP4	19.5242196	2380.722992	0.0082	0.993	2.526545837	count	1
ELN	19.5242196	2380.722992	0.0082	0.993	2.526545837	count	1
TCEAL9	19.5242196	2380.722992	0.0082	0.993	2.526545837	count	1
CABP4	19.5242196	2380.722992	0.0082	0.993	2.526545837	count	1
NEURL1	19.5242196	2380.722992	0.0082	0.993	2.526545837	count	1
AC091057.2	19.5242196	2380.722992	0.0082	0.993	2.526545837	count	1
THAP10	19.5242196	2380.722992	0.0082	0.993	2.526545837	count	1
COG8	19.5242196	2380.722992	0.0082	0.993	2.526545837	count	1
AC002091.2	19.5242196	2380.722992	0.0082	0.993	2.526545837	count	1
HPCA	19.5242197	2380.722909	0.0082	0.993	2.526545837	count	1
TIE1	19.5242199	2380.723122	0.0082	0.993	2.526545837	count	1
ORC1	19.52422	2380.723145	0.0082	0.993	2.526545837	count	1
CCNT2-AS1	19.52422	2380.723098	0.0082	0.993	2.526545837	count	1
METAP1D	19.5242199	2380.723181	0.0082	0.993	2.526545837	count	1
STAC	19.52422	2380.723145	0.0082	0.993	2.526545837	count	1
SLC35G2	19.5242199	2380.723181	0.0082	0.993	2.526545837	count	1
HES1	19.52422	2380.723098	0.0082	0.993	2.526545837	count	1
EDN1	19.5242199	2380.723181	0.0082	0.993	2.526545837	count	1
ID4	19.52422	2380.723051	0.0082	0.993	2.526545837	count	1
AL356417.1	19.5242198	2380.723003	0.0082	0.993	2.526545837	count	1
AC083973.1	19.5242198	2380.723003	0.0082	0.993	2.526545837	count	1
AC136475.7	19.52422	2380.723051	0.0082	0.993	2.526545837	count	1
AC136475.9	19.5242199	2380.723181	0.0082	0.993	2.526545837	count	1
PCF11-AS1	19.5242199	2380.723122	0.0082	0.993	2.526545837	count	1
MANSC1	19.52422	2380.723051	0.0082	0.993	2.526545837	count	1
CCDC65	19.5242197	2380.722909	0.0082	0.993	2.526545837	count	1
ZNF219	19.52422	2380.723181	0.0082	0.993	2.526545837	count	1
LINC02285	19.52422	2380.723181	0.0082	0.993	2.526545837	count	1
TMEM202-AS1	19.5242198	2380.723003	0.0082	0.993	2.526545837	count	1
AL031714.1	19.52422	2380.723145	0.0082	0.993	2.526545837	count	1
ZNF469	19.5242199	2380.723098	0.0082	0.993	2.526545837	count	1
AC010761.1	19.5242199	2380.723181	0.0082	0.993	2.526545837	count	1
NR1D1	19.5242199	2380.723181	0.0082	0.993	2.526545837	count	1
HEXIM2	19.5242197	2380.722909	0.0082	0.993	2.526545837	count	1
ITCH-AS1	19.5242197	2380.722909	0.0082	0.993	2.526545837	count	1
LINC01841	19.52422	2380.723098	0.0082	0.993	2.526545837	count	1
ZNF781	19.5242199	2380.723098	0.0082	0.993	2.526545837	count	1
CCDC114	19.5242198	2380.723003	0.0082	0.993	2.526545837	count	1
LINC01547	19.52422	2380.723051	0.0082	0.993	2.526545837	count	1
AC108488.1	19.5242201	2380.723134	0.0082	0.993	2.526545837	count	1
PCDHGA3	19.5242201	2380.723134	0.0082	0.993	2.526545837	count	1

C12orf54	19.5242201	2380.723134	0.0082	0.993	2.526545837	count	1
NACA2	19.5242201	2380.723134	0.0082	0.993	2.526545837	count	1
C1orf54	19.5259389	1664.19894	0.0117	0.991	2.526545837	count	1
CTDSPL	19.5259391	1664.198932	0.0117	0.991	2.526545837	count	1
LTBR	19.5259391	1664.199023	0.0117	0.991	2.526545837	count	1
HYPK	19.5259389	1664.19894	0.0117	0.991	2.526545837	count	1
NDST2	19.5259393	1664.199023	0.0117	0.991	2.526545837	count	1
PGBD1	19.809008	2775.659345	0.0071	0.994	2.526545842	count	1
HCN2	19.8090102	2775.661206	0.0071	0.994	2.526545842	count	1
MAPK12	19.8090099	2775.661396	0.0071	0.994	2.526545842	count	1
SCLY	2.4227755	1.262863	1.9185	0.0559	2.528158393	count	1
ZSCAN29	2.432077	1.2723894	1.9114	0.0568	2.535440862	count	1
LRP5L	2.4852779	1.3869534	1.7919	0.0741	2.57653934	count	1
FAM122B	2.226362	1.0092972	2.2059	0.0281	2.612038553	count	1
TRIM35	2.7880046	1.2454293	2.2386	0.0259	2.618461619	count	1
CCDC74A	2.5433774	1.1869908	2.1427	0.0329	2.620337498	count	1
MAP3K3	2.9184731	1.330024	2.1943	0.02892	2.692920428	count	1
TOMM40L	2.516159	0.9443791	2.6644	0.0081	2.717845675	count	1
CEP89	2.5731878	2.0614188	1.2483	0.213	2.763559415	count	1
WDR89	2.832185	1.124569	2.5185	0.0123	2.82100644	count	1
SLC16A1	2.8627182	1.2509728	2.2884	0.0228	2.840552929	count	1
MTMR12	2.4967167	0.9742974	2.5626	0.0108	2.86872464	count	1
ANKRD13C	2.4227755	1.0054298	2.4097	0.0165	2.909626864	count	1
DOK7	2.698481	0.8960935	3.0114	0.00281	2.962126713	count	1
PAGR1	2.698481	0.9237938	2.9211	0.00373	2.962126713	count	1
AL356512.1	2.8476816	1.5954277	1.7849	0.0752	2.968062612	count	1
MIR222HG	2.6175433	1.0168099	2.5743	0.0105	2.976060708	count	1
ROBO3	19.0562933	2305.105152	0.0083	0.9934	3.025534948	count	1
CARMIL1	19.1177883	1958.425855	0.0098	0.992	3.025534952	count	1
PPP1R3F	19.2548509	2886.568372	0.0067	0.995	3.025534958	count	1
RRP12	19.3214304	2773.325606	0.007	0.994	3.025534961	count	1
TRIM14	17.9837054	1147.878849	0.0157	0.988	3.02553497	count	1
AL451007.2	19.9031556	2877.357494	0.0069	0.994	3.02553498	count	1
NOSTRIN	19.9031559	2877.357494	0.0069	0.994	3.02553498	count	1
ZNF391	19.9031559	2877.357494	0.0069	0.994	3.02553498	count	1
ZNF618	19.9031559	2877.357494	0.0069	0.994	3.02553498	count	1
BCL2L2	19.9031556	2877.357494	0.0069	0.994	3.02553498	count	1
AC023908.3	19.9031556	2877.357494	0.0069	0.994	3.02553498	count	1
CCDC183	19.912592	2133.672772	0.0093	0.993	3.02553498	count	1
AC108134.2	20.1276186	2547.611649	0.0079	0.994	3.025534984	count	1
NACC1	18.4277986	1716.012131	0.0107	0.991	3.025535027	count	1
AC025171.2	18.4999582	1626.552722	0.0114	0.991	3.025535034	count	1
SLC39A9	18.6504916	1822.730086	0.0102	0.992	3.025535048	count	1
ZNF362	18.6834526	1603.834479	0.0116	0.991	3.02553505	count	1
UBTD2	19.0562933	2305.10524	0.0083	0.9934	3.025535075	count	1

DUS4L	19.0661532	1965.614269	0.0097	0.9923	3.025535076	count	1
SPRED1	19.1636554	2755.057202	0.007	0.994	3.025535081	count	1
DNAAF5	19.1636595	2755.057626	0.007	0.994	3.025535081	count	1
AC020911.2	19.1636595	2755.057604	0.007	0.994	3.025535081	count	1
PICK1	19.2544857	2328.601295	0.0083	0.993	3.025535085	count	1
AL135791.1	19.3203049	2567.713286	0.0075	0.994	3.025535088	count	1
C1RL-AS1	19.3258016	2248.640915	0.0086	0.993	3.025535088	count	1
SLC26A6	19.9031552	2877.35748	0.0069	0.994	3.025535106	count	1
DLX6-AS1	19.9031553	2877.357257	0.0069	0.994	3.025535106	count	1
MTCP1	19.9031552	2877.35748	0.0069	0.994	3.025535106	count	1
AC084346.2	19.9031552	2877.35748	0.0069	0.994	3.025535106	count	1
LINC01505	19.9031552	2877.35748	0.0069	0.994	3.025535106	count	1
AL117332.1	19.9031552	2877.35748	0.0069	0.994	3.025535106	count	1
P4HA2	19.9125925	2133.672855	0.0093	0.993	3.025535106	count	1
SLC25A15	19.9125924	2133.672824	0.0093	0.993	3.025535106	count	1
RASL12	19.9125925	2133.672897	0.0093	0.993	3.025535106	count	1
NKAPL	19.9125927	2133.672855	0.0093	0.993	3.025535106	count	1
EFEMP2	19.9125927	2133.672855	0.0093	0.993	3.025535106	count	1
SETD1A	19.9125927	2133.672855	0.0093	0.993	3.025535106	count	1
PEAK3	19.9125927	2133.672939	0.0093	0.993	3.025535106	count	1
UBASH3A	3.0214892	1.3007858	2.3228	0.0208	3.084068598	count	1
DNAJC27-AS1	2.573188	1.089311	2.3622	0.0188	3.14313438	count	1
ELL	2.6413238	0.7083506	3.7288	0.000227	3.210944706	count	1
ENTPD1	2.9263991	1.7199329	1.7015	0.0898	3.228224631	count	1
LINC00665	18.6208805	1631.63126	0.0114	0.991	3.395584968	count	1
AVL9	18.7894889	1679.219801	0.0112	0.991	3.395584986	count	1
PIDD1	18.9025407	1829.664666	0.0103	0.992	3.395584996	count	1
NRBP2	19.2983973	2147.062575	0.009	0.9928	3.395585024	count	1
SESN3	19.3773259	2898.500436	0.0067	0.995	3.395585028	count	1
AC133550.2	19.377326	2898.500403	0.0067	0.995	3.395585028	count	1
MICALL1	19.4758234	2827.795063	0.0069	0.995	3.395585033	count	1
HEATR5B	19.590908	2199.969706	0.0089	0.993	3.395585038	count	1
AL606760.3	20.1525032	3259.410282	0.0062	0.995	3.395585056	count	1
AC015712.1	20.1525032	3259.410282	0.0062	0.995	3.395585056	count	1
AP001412.1	20.1525032	3259.410282	0.0062	0.995	3.395585056	count	1
MYLK	20.1734655	2300.455952	0.0088	0.993	3.395585057	count	1
IGHV1-3	20.1734655	2300.455886	0.0088	0.993	3.395585057	count	1
AC022916.1	20.1734655	2300.455886	0.0088	0.993	3.395585057	count	1
AGBL5	20.1734654	2300.455973	0.0088	0.993	3.395585057	count	1
ALAD	20.1734658	2300.456017	0.0088	0.993	3.395585057	count	1
LINC02470	20.1734656	2300.455908	0.0088	0.993	3.395585057	count	1
MAN2B2	18.7025088	1764.528297	0.0106	0.992	3.395585108	count	1
ZC4H2	18.7761047	1608.290043	0.0117	0.991	3.395585115	count	1
SCAMP4	19.3022249	2020.295064	0.0096	0.9924	3.395585154	count	1
IQCN	19.4420387	2310.362236	0.0084	0.993	3.395585162	count	1

AC092343.1	20.1525031	3259.410373	0.0062	0.995	3.395585187	count	1
AC034111.1	20.1525031	3259.410373	0.0062	0.995	3.395585187	count	1
BNC2	20.1525031	3259.410373	0.0062	0.995	3.395585187	count	1
AC015726.1	20.1525031	3259.410161	0.0062	0.995	3.395585187	count	1
ZNF835	20.1525031	3259.410373	0.0062	0.995	3.395585187	count	1
NPHP3	3.1788954	1.5878372	2.002	0.0461	3.409745593	count	1
HMGN4	2.93049	0.982133	2.9838	0.00306	3.63143244	count	1
SGSH	18.8321729	1603.192713	0.0117	0.991	3.689831379	count	1
BRF1	19.0197874	1973.294743	0.0096	0.992	3.689831399	count	1
TGFA	18.7298929	1300.063059	0.0144	0.9885	3.6898315	count	1
TOR4A	19.5565854	2747.947687	0.0071	0.994	3.689831571	count	1
PRKG2	20.3542574	2955.950535	0.0069	0.995	3.689831602	count	1
ZNF547	20.3542578	2955.950481	0.0069	0.995	3.689831602	count	1
SLC4A8	20.3542577	2955.950427	0.0069	0.995	3.689831602	count	1
WBP1L	20.3763606	2139.313989	0.0095	0.992	3.689831602	count	1
MLF1	18.9418697	1738.117368	0.0109	0.9913	3.934111873	count	1
LRWD1	19.5052721	1740.525596	0.0112	0.991	3.934112061	count	1
WDR59	19.8178608	2053.692526	0.0096	0.9923	4.142957852	count	1
PIGN	18.9509546	1947.242207	0.0097	0.9922	4.325358807	count	1
SOST	19.6562065	2543.142288	0.0077	0.994	4.325358888	count	1
ZNF34	19.9177527	2557.976397	0.0078	0.9938	4.325358907	count	1
VCAM1	19.6562066	2543.142316	0.0077	0.994	4.325359025	count	1
TFPI	19.6562066	2543.142316	0.0077	0.994	4.325359025	count	1
RAPH1	19.6562066	2543.142316	0.0077	0.994	4.325359025	count	1
RPS6KL1	19.6562066	2543.142316	0.0077	0.994	4.325359025	count	1
LINC00539	18.9946374	1649.622141	0.0115	0.9908	4.632821981	count	1
