# Supplementary material for: Decoding the transcriptome of calcified atherosclerotic plaque at single-cell resolution
Source: Commun Biol. 2022 Oct 12;5:1084. doi: 10.1038/s42003-022-04056-7 (PMC9556750; doi:10.1038/s42003-022-04056-7)
Supplement: Supplementary file 7 — Supplementary Data 5 [file 42003_2022_4056_MOESM7_ESM.pdf]

Full differential gene expression results for VSMCs.

gene_short_name	estimate	std_err	test_val	p_value	normalized_effect	model_component	q_value
SPP1	-5.1463304	0.8557049	-6.0141	1.99E-09	-5.449429276	count	4.69E-05
ITLN1	-4.0624293	1.1501473	-3.5321	4.00E-04	-5.084395544	count	1
SFRP5	-3.958646	0.5980948	-6.6188	4.15E-11	-4.359312075	count	9.84E-07
MMP9	-4.5782513	4.5209904	-1.0127	0.3113	-4.11167556	count	1
IBSP	-2.8901382	0.5491914	-5.2625	1.50E-07	-3.916537343	count	0.0035097
CRTAC1	-2.3325481	0.2013085	-11.5869	1.62E-30	-3.217381776	count	3.91E-26
IGKC	-2.3527145	0.4223459	-5.5706	2.72E-08	-3.078637789	count	0.000638738
DGKI	-2.564935	0.6210194	-4.1302	3.71E-05	-2.783678264	count	0.8493303
APOC1	-2.0602721	0.2567081	-8.0257	1.35E-15	-2.502924068	count	3.22E-11
GRIA2	-1.9313205	0.2952037	-6.5423	6.90E-11	-2.478049134	count	1.64E-06
ACTC1	-1.7278856	0.1938914	-8.9116	7.77E-19	-2.465308277	count	1.86E-14
LINC01497	-3.352668	1.4024605	-2.3906	0.0169	-2.447618708	count	1
APOE	-1.5760211	0.1441845	-10.9306	2.17E-27	-2.259213563	count	5.23E-23
SOX18	-1.881135	0.4310552	-4.364	1.31E-05	-2.248716092	count	0.3015882
ACP5	-2.4889989	0.5399751	-4.6095	4.17E-06	-2.244141755	count	0.09646461
POSTN	-1.5617143	0.1670525	-9.3486	1.50E-20	-2.205699173	count	3.60E-16
BMX	-2.430146	0.6449786	-3.7678	0.000167	-2.205324674	count	1
RAC2	-3.5960875	0.9825723	-3.6599	0.000256	-2.188751893	count	1
CLDN5	-4.035386	1.2168959	-3.3161	0.000922	-2.188265073	count	1
CRHBP	-2.0698977	0.555014	-3.7295	0.000195	-2.159265097	count	1
KRT25	-19.0812354	1371.940911	-0.0139	0.989	-2.141749698	count	1
IGHA2	-19.775445	1852.612999	-0.0107	0.991	-2.141749153	count	1
IL1RN	-19.028199	1021.085705	-0.0186	0.985	-2.141749139	count	1
RHBDF2	-2.282556	0.5325886	-4.2858	1.87E-05	-2.132505112	count	0.429726
LYZ	-1.9588466	0.3501165	-5.5948	2.37E-08	-2.131146478	count	0.000556737
ITGB7	-3.633791	2.912447	-1.2477	0.212	-2.116092887	count	1
EGFL7	-1.6899552	0.3729948	-4.5308	6.06E-06	-2.106602487	count	0.13991934
TRDC	-4.0884795	1.9625158	-2.0833	0.0373	-2.104535704	count	1
MMP11	-1.8168532	0.2781965	-6.5308	7.44E-11	-2.066451379	count	1.76E-06
KRT19	-1.622397	0.310712	-5.2215	1.87E-07	-2.051846444	count	0.00437206
SERPINE1	-1.4479772	0.180919	-8.0035	1.61E-15	-2.034609213	count	3.85E-11
TM6SF1	-3.624478	0.8407978	-4.3108	1.67E-05	-2.026862054	count	0.3840499
THEMIS2	-18.951456	1153.683444	-0.0164	0.987	-2.025621482	count	1
SEMA3F-AS1	-3.6015481	2.9260245	-1.2309	0.2185	-2.022178875	count	1
PTGER3	-1.6274289	0.1806505	-9.0087	3.28E-19	-2.017505948	count	7.87E-15
PLVAP	-1.599063	0.5812171	-2.7512	0.00597	-2.014511149	count	1
C1QB	-2.3314731	0.5018902	-4.6454	3.51E-06	-1.960364661	count	0.08124597
TFPI2	-1.437435	0.173319	-8.2936	1.53E-16	-1.955316182	count	3.66E-12
C1QA	-2.2970522	0.3261244	-7.0435	2.23E-12	-1.938554504	count	5.30E-08
KRT17	-1.3863283	0.1830464	-7.5736	4.57E-14	-1.933729329	count	1.09E-09
FCER1G	-1.9521839	0.3791022	-5.1495	2.75E-07	-1.922111899	count	0.0064229
IGHG1	-2.2500423	0.5605866	-4.0137	6.10E-05	-1.867165052	count	1
SCGB3A2	-1.9712293	0.6002059	-3.2843	0.00103	-1.866090822	count	1
SCX	-1.373606	0.15561	-8.8272	1.63E-18	-1.864944121	count	3.91E-14

PART1	-2.3012006	0.2448185	-9.3996	9.35E-21	-1.854618613	count	2.25E-16
IL1B	-2.934577	0.755731	-3.8831	0.000105	-1.841817131	count	1
IGFBP3	-1.2878751	0.1290118	-9.9826	3.58E-23	-1.837781294	count	8.62E-19
CCL3	-1.7695448	0.2860887	-6.1853	6.88E-10	-1.834887585	count	1.63E-05
SUGCT	-1.3323637	0.1009718	-13.1954	7.07E-39	-1.813426796	count	1.71E-34
PLEKHS1	-2.836432	0.9735939	-2.9134	0.0036	-1.807071795	count	1
GSTA1	-19.417297	1548.866452	-0.0125	0.99	-1.760893508	count	1
SDS	-3.8052394	1.5402865	-2.4705	0.0135	-1.745630006	count	1
DSP	-1.8182175	0.4029146	-4.5127	6.60E-06	-1.739551854	count	0.152361
PDE4B	-1.5068323	0.2532834	-5.9492	2.95E-09	-1.734406184	count	6.95E-05
KRT14	-2.2642053	0.7339715	-3.0849	0.00205	-1.734122447	count	1
GCNT2	-1.5265094	0.2408196	-6.3388	2.60E-10	-1.723045111	count	6.15E-06
LINC02175	-2.445575	0.9098811	-2.6878	0.00723	-1.713061681	count	1
CCL4L2	-1.3736547	0.429599	-3.1975	0.0014	-1.712485668	count	1
FAM241B	-1.4405319	0.2269387	-6.3477	2.45E-10	-1.706126679	count	5.80E-06
CCL4	-1.2678782	0.2481417	-5.1095	3.39E-07	-1.704956939	count	0.007912938
TMEM255B	-1.6637813	0.4315948	-3.855	0.000118	-1.702215089	count	1
BAALC	-3.310544	1.4303318	-2.3145	0.0207	-1.657968516	count	1
TMEM217	-2.224909	0.8043462	-2.7661	0.0057	-1.657317018	count	1
ALOX5AP	-2.1288663	0.6527412	-3.2614	0.00112	-1.653376591	count	1
IGSF6	-1.608787	0.8525784	-1.887	0.0592	-1.650076962	count	1
F5	-3.264322	1.9190407	-1.701	0.089	-1.647835113	count	1
C5orf46	-1.8052412	0.324361	-5.5655	2.80E-08	-1.621009532	count	0.000657496
IGHG3	-2.0053167	0.7806862	-2.5687	0.0102	-1.620907985	count	1
TRIM14	-2.0714591	0.7255137	-2.8552	0.00433	-1.617599798	count	1
C1QC	-2.5376631	0.5434073	-4.6699	3.12E-06	-1.610088656	count	0.07227168
GOLGA6L7	-19.260122	1215.99814	-0.0158	0.987	-1.607759633	count	1
CCL7	-19.260122	1215.99814	-0.0158	0.987	-1.607759633	count	1
ADRA2C	-1.5986445	0.2070341	-7.7216	1.47E-14	-1.606260264	count	3.51E-10
CXCL8	-1.3502726	0.5481147	-2.4635	0.0138	-1.602316993	count	1
RHOH	-3.6247609	1.0268748	-3.5299	0.000421	-1.59400415	count	1
NRN1	-2.03163	0.4692875	-4.3292	1.54E-05	-1.592253812	count	0.3542616
SLC45A4	-2.0203036	0.6894247	-2.9304	0.00341	-1.584968799	count	1
DMKN	-1.1510549	0.1355945	-8.489	2.99E-17	-1.578774365	count	7.16E-13
PRUNE2	-1.1485675	0.1395805	-8.2287	2.60E-16	-1.571636904	count	6.22E-12
ST6GAL2	-1.3120474	0.1950994	-6.725	2.03E-11	-1.568102974	count	4.82E-07
IGHA1	-1.4830842	0.2511776	-5.9045	3.86E-09	-1.55720873	count	9.10E-05
DEF6	-2.600571	1.7032667	-1.5268	0.127	-1.55082934	count	1
KCNJ6	-1.889239	0.6546662	-2.8858	0.00393	-1.541483488	count	1
C5orf38	-1.82574	0.2307768	-7.9113	3.35E-15	-1.53549376	count	8.00E-11
OCIAD2	-1.099652	0.1230927	-8.9335	6.40E-19	-1.519894324	count	1.54E-14
HTR2B	-1.2473499	0.2329898	-5.3537	9.15E-08	-1.513778579	count	0.002143205
SGIP1	-1.177621	0.1616191	-7.2864	3.88E-13	-1.511762676	count	9.24E-09
ALDH1B1	-1.2307896	0.1347853	-9.1315	1.09E-19	-1.49788601	count	2.62E-15
IFI27	-1.0512711	0.1361526	-7.7213	1.48E-14	-1.494978407	count	3.53E-10

HIST1H1D	-1.2552042	0.2569639	-4.8847	1.08E-06	-1.4816045	count	0.02511432
LINC01088	-1.1994303	0.1558769	-7.6947	1.81E-14	-1.471012265	count	4.32E-10
PLN	-1.0279192	0.0562233	-18.2828	1.56E-71	-1.468519072	count	3.79E-67
IGLC2	-1.3820726	0.1474579	-9.3727	1.20E-20	-1.467888213	count	2.88E-16
MYRFL	-1.9097825	0.4372739	-4.3675	1.29E-05	-1.464913231	count	0.2970096
TBXA2R	-1.1148975	0.1485575	-7.5048	7.68E-14	-1.464299217	count	1.83E-09
PDE4D	-1.2400319	0.1965521	-6.3089	3.14E-10	-1.464004062	count	7.43E-06
SLC25A34-AS1	-1.2627416	0.211029	-5.9837	2.39E-09	-1.461412458	count	5.64E-05
AC006064.4	-1.7724995	0.6591908	-2.6889	0.0072	-1.458149408	count	1
MKX-AS1	-2.595864	0.933622	-2.7804	0.00546	-1.455350755	count	1
JAK3	-1.6335367	0.4291173	-3.8067	0.000143	-1.452965297	count	1
DOCK10	-1.1323378	0.193774	-5.8436	5.55E-09	-1.450500437	count	0.000130675
MEGF10	-2.1903246	0.6743166	-3.2482	0.00117	-1.449028018	count	1
AREG	-1.4613984	0.5523258	-2.6459	0.00818	-1.441431432	count	1
QRFPR	-20.077231	2154.353043	-0.0093	0.993	-1.436419921	count	1
AC004908.1	-20.077231	2154.353043	-0.0093	0.993	-1.436419921	count	1
AC022148.1	-20.077231	2154.353043	-0.0093	0.993	-1.436419921	count	1
AC016027.1	-20.077231	2154.353043	-0.0093	0.993	-1.436419921	count	1
TREM2	-20.075647	1773.687543	-0.0113	0.991	-1.436419921	count	1
TBX5	-20.072861	1132.468752	-0.0177	0.986	-1.436419921	count	1
LINC01013	-19.340453	1732.644027	-0.0112	0.991	-1.436419915	count	1
ASPHD1	-19.260238	918.1223843	-0.021	0.983	-1.436419915	count	1
CD3D	-2.0202555	0.5992529	-3.3713	0.000756	-1.423081243	count	1
MRAP2	-1.068512	0.134905	-7.9205	3.11E-15	-1.419290964	count	7.42E-11
TRAF1	-1.1642027	0.2508233	-4.6415	3.58E-06	-1.41783273	count	0.08285194
KCNMA1	-1.0380001	0.097464	-10.6501	4.18E-26	-1.416312553	count	1.01E-21
KRT16	-1.45255	0.780911	-1.8601	0.063	-1.414406385	count	1
FBXO32	-1.0174939	0.0881978	-11.5365	2.86E-30	-1.413488512	count	6.91E-26
ANGPT2	-1.0336287	0.0852137	-12.1298	3.15E-33	-1.409165254	count	7.61E-29
BRINP1	-2.7649027	0.6359284	-4.3478	1.41E-05	-1.404650006	count	0.3244833
RPS6KA6	-1.8135206	0.3343617	-5.4238	6.21E-08	-1.401060622	count	0.001455562
SRGN	-0.9988801	0.1230998	-8.1144	6.60E-16	-1.395911846	count	1.58E-11
PCDH7	-0.9798717	0.0757438	-12.9367	1.82E-37	-1.391132784	count	4.40E-33
LINC00844	-1.3584738	0.2819035	-4.8189	1.50E-06	-1.391094921	count	0.0348435
SLC22A3	-1.1586746	0.1885484	-6.1452	8.84E-10	-1.387523053	count	2.09E-05
PHYHIPL	-1.078271	0.2110253	-5.1097	3.39E-07	-1.378086818	count	0.007912938
FCGR3A	-2.0545676	0.678401	-3.0285	0.00247	-1.377833374	count	1
TGM2	-0.9897515	0.0977917	-10.121	9.12E-24	-1.37619183	count	2.20E-19
NPTX2	-1.024043	0.1611415	-6.3549	2.34E-10	-1.376181241	count	5.54E-06
LINC00607	-1.508875	0.5785485	-2.608	0.00914	-1.375991913	count	1
ERG	-1.0315834	0.1364947	-7.5577	5.15E-14	-1.373634802	count	1.23E-09
GRP	-3.1336443	0.7767894	-4.0341	5.59E-05	-1.371916191	count	1
MAOB	-1.1215147	0.1680102	-6.6753	2.84E-11	-1.369750586	count	6.74E-07
CD86	-3.118723	1.3887463	-2.2457	0.0248	-1.368798657	count	1
CXCR4	-1.594322	0.4537277	-3.5138	0.000447	-1.359122594	count	1

PTK7	-1.3862116	0.4382828	-3.1628	0.00158	-1.352513679	count	1
SLC16A3	-1.1342019	0.2517757	-4.5048	6.85E-06	-1.349812538	count	0.1581117
CTSC	-0.9721123	0.1000796	-9.7134	4.86E-22	-1.348900924	count	1.17E-17
F2R	-0.9695621	0.101443	-9.5577	2.13E-21	-1.348466793	count	5.12E-17
CCDC68	-0.9638099	0.1530487	-6.2974	3.38E-10	-1.344884884	count	8.00E-06
AL355974.2	-1.1182298	0.2304229	-4.8529	1.27E-06	-1.339518609	count	0.02951226
IGFBPL1	-1.982009	0.710103	-2.7912	0.00528	-1.337933487	count	1
GLDN	-0.999762	0.1215303	-8.2264	2.65E-16	-1.337151792	count	6.34E-12
INSC	-2.294704	0.4956493	-4.6297	3.79E-06	-1.337053181	count	0.08769302
ITGA8	-0.9317047	0.0615741	-15.1314	3.20E-50	-1.336535251	count	7.76E-46
TSPAN2	-0.9589497	0.0962253	-9.9657	4.23E-23	-1.332226994	count	1.02E-18
COL13A1	-1.28551	0.2647195	-4.8561	1.25E-06	-1.331846554	count	0.0290525
CDH2	-1.0041608	0.1706838	-5.8832	4.39E-09	-1.329810679	count	0.00010345
COLEC11	-1.236257	0.2405955	-5.1383	2.92E-07	-1.32749923	count	0.006818784
ANKRD1	-1.353314	0.4087311	-3.311	0.000939	-1.321543253	count	1
MS4A7	-2.5058575	0.5876172	-4.2644	2.05E-05	-1.320685855	count	0.470926
ZNF385D	-0.9858262	0.1310279	-7.5238	6.66E-14	-1.318538444	count	1.59E-09
CRNDE	-0.9402899	0.0969056	-9.7032	5.36E-22	-1.310309353	count	1.29E-17
FAT3	-1.2271921	0.3119511	-3.9339	8.51E-05	-1.307414075	count	1
P2RY6	-1.1253811	0.284288	-3.9586	7.68E-05	-1.304478355	count	1
AQP1	-0.9136025	0.070255	-13.0041	7.86E-38	-1.301892783	count	1.90E-33
MSR1	-2.8173076	0.7939575	-3.5484	0.000392	-1.29782435	count	1
OASL	-1.6641729	0.635563	-2.6184	0.00887	-1.297692714	count	1
AL022341.2	-3.5444174	2.309397	-1.5348	0.125	-1.291341259	count	1
RAMP3	-1.0161939	0.3161247	-3.2145	0.00132	-1.280007528	count	1
SARDH	-1.999435	0.617196	-3.2395	0.00121	-1.277940239	count	1
GAP43	-0.9328598	0.1423128	-6.555	6.34E-11	-1.2729949	count	1.50E-06
ATXN7	-1.565783	0.6956813	-2.2507	0.0245	-1.266377911	count	1
EDIL3	-0.8874642	0.0553005	-16.048	4.59E-56	-1.264933185	count	1.11E-51
HCCAT5	-1.312205	0.3906569	-3.359	0.00079	-1.264636506	count	1
GJD2	-1.755708	0.4967261	-3.5346	0.000413	-1.262339539	count	1
UNC5B	-0.9373248	0.1515962	-6.183	6.98E-10	-1.258025338	count	1.65E-05
SUSD3	-1.0362891	0.2208587	-4.6921	2.80E-06	-1.256357195	count	0.0648844
TSPAN15	-2.3293461	0.4242644	-5.4903	4.28E-08	-1.255304586	count	0.001003874
CCNO	-1.2012603	0.3304428	-3.6353	0.000281	-1.24618428	count	1
THEM6	-1.030551	0.248998	-4.1388	3.57E-05	-1.245971572	count	0.8174586
IGLV11-55	-19.851654	1924.568222	-0.0103	0.992	-1.241954889	count	1
CLDN14	-19.850037	1518.712162	-0.0131	0.99	-1.241954889	count	1
DHRS9	-19.848632	1173.410041	-0.0169	0.987	-1.241954889	count	1
LILRB4	-19.264126	1430.600853	-0.0135	0.989	-1.241954886	count	1
S1PR5	-19.234956	1431.90428	-0.0134	0.989	-1.241954885	count	1
MLC1	-19.160767	1450.971294	-0.0132	0.989	-1.241954885	count	1
CARD16	-0.9558673	0.15261	-6.2635	4.20E-10	-1.235219144	count	9.93E-06
GADD45A	-0.8662438	0.0603848	-14.3454	1.89E-45	-1.22673916	count	4.58E-41
TNFSF15	-1.782384	0.727307	-2.4507	0.0143	-1.221817414	count	1

ITGA10	-0.8634184	0.1128029	-7.6542	2.47E-14	-1.206746918	count	5.89E-10
ABLIM3	-1.4854146	0.4278738	-3.4716	0.000523	-1.205303419	count	1
PRSS35	-0.8973226	0.1408813	-6.3694	2.13E-10	-1.202595583	count	5.04E-06
ENPP1	-0.8902364	0.1211915	-7.3457	2.51E-13	-1.202018495	count	5.98E-09
LINC01238	-2.995007	1.5295245	-1.9581	0.0503	-1.199111882	count	1
TK1	-1.3605115	0.5204086	-2.6143	0.00898	-1.197083861	count	1
AIF1	-2.1801782	0.4461516	-4.8866	1.07E-06	-1.194796597	count	0.02488606
P4HA3	-1.4685477	0.3436632	-4.2732	1.98E-05	-1.192332037	count	0.4549842
LOXL2	-1.5764794	0.3384394	-4.6581	3.30E-06	-1.191633535	count	0.0764148
FNDC4	-0.9047567	0.1405962	-6.4351	1.39E-10	-1.186885687	count	3.29E-06
COL15A1	-0.8820452	0.1291155	-6.8314	9.80E-12	-1.184898926	count	2.33E-07
FXYD5	-0.8285238	0.0635007	-13.0475	4.57E-38	-1.182339781	count	1.11E-33
AP002026.1	-1.3679135	0.3402249	-4.0206	5.92E-05	-1.175751604	count	1
S100A3	-0.9745463	0.1680504	-5.7991	7.23E-09	-1.174979279	count	0.000170165
SPOCD1	-1.621769	0.6257275	-2.5918	0.00958	-1.174974046	count	1
NRP2	-0.8815846	0.1569492	-5.617	2.09E-08	-1.173313178	count	0.000491046
ARHGDIB	-0.825788	0.0682251	-12.1039	4.27E-33	-1.172457293	count	1.03E-28
TMEM130	-0.9389131	0.209875	-4.4737	7.92E-06	-1.170114635	count	0.18265896
TNIP3	-2.112383	0.5331385	-3.9622	7.57E-05	-1.165713707	count	1
IGFBP2	-0.8060286	0.0540258	-14.9193	6.53E-49	-1.161095976	count	1.58E-44
MYO18B	-2.094624	0.568429	-3.6849	0.000232	-1.157933944	count	1
VCAM1	-0.8270859	0.0951275	-8.6945	5.17E-18	-1.157539882	count	1.24E-13
MELTF-AS1	-1.104057	0.3074693	-3.5908	0.000334	-1.15725539	count	1
FBLIM1	-0.8081709	0.0587186	-13.7635	4.61E-42	-1.154441167	count	1.12E-37
ANK3	-0.8510836	0.1637206	-5.1984	2.12E-07	-1.152568348	count	0.0049555
CFAP58-DT	-2.735274	1.3936723	-1.9626	0.0498	-1.140199635	count	1
MSH4	-2.735274	1.4592018	-1.8745	0.0609	-1.140199635	count	1
TTN	-1.0181639	0.5074921	-2.0063	0.0449	-1.140173712	count	1
LINC02185	-1.0980297	0.2848757	-3.8544	0.000118	-1.140058308	count	1
FOLR2	-1.8795133	0.5790518	-3.2458	0.00118	-1.140054819	count	1
JCHAIN	-1.876708	0.4360781	-4.3036	1.72E-05	-1.138595086	count	0.3954624
NRL	-1.3985224	0.5801693	-2.4105	0.016	-1.137945068	count	1
ASS1	-0.8317599	0.1320847	-6.2972	3.39E-10	-1.13526656	count	8.02E-06
SELL	-1.5609131	0.5758717	-2.7105	0.00675	-1.134084043	count	1
AC093390.2	-1.3914002	0.4585741	-3.0342	0.00243	-1.132367013	count	1
TTLL7	-0.8039797	0.078085	-10.2962	1.58E-24	-1.129428794	count	3.81E-20
LGR6	-0.9762055	0.2135135	-4.5721	4.99E-06	-1.127131844	count	0.11533886
TGFB1	-0.8103473	0.0939454	-8.6257	9.33E-18	-1.125429406	count	2.24E-13
FN1	-0.7814435	0.075089	-10.4069	5.12E-25	-1.125267496	count	1.23E-20
ISG15	-0.7975964	0.117367	-6.7957	1.25E-11	-1.121552604	count	2.97E-07
ALDH1A2	-0.9167688	0.1887954	-4.8559	1.25E-06	-1.120175008	count	0.0290525
CCDC144NL-AS1	-0.9166685	0.2227522	-4.1152	3.95E-05	-1.120052167	count	0.9039575
BMP6	-1.6168553	0.6243948	-2.5895	0.00965	-1.118984388	count	1
ITGB2	-1.6149565	0.4952233	-3.2611	0.00112	-1.117773065	count	1
KALRN	-0.8403986	0.2069029	-4.0618	4.97E-05	-1.117757467	count	1

CDC42EP3	-0.7938265	0.1000869	-7.9314	2.85E-15	-1.116637162	count	6.80E-11
SUSD5	-0.8182655	0.1395734	-5.8626	4.96E-09	-1.114590655	count	0.000116848
SV2A	-0.9589668	0.2568458	-3.7336	0.000192	-1.112071465	count	1
SLC13A3	-1.2818394	0.5823702	-2.2011	0.0278	-1.103734763	count	1
PGM2L1	-0.8560359	0.1487378	-5.7553	9.36E-09	-1.102332986	count	0.000220222
ASTN1	-2.582386	0.7603565	-3.3963	0.00069	-1.100261529	count	1
AL121983.1	-2.582386	0.8531806	-3.0268	0.00249	-1.100261529	count	1
AC073655.2	-2.582386	0.8531806	-3.0268	0.00249	-1.100261529	count	1
E2F3	-0.9360071	0.1995304	-4.6911	2.82E-06	-1.098707758	count	0.06534222
EFNB2	-0.8460808	0.1736168	-4.8733	1.14E-06	-1.09665453	count	0.02650386
DUSP26	-0.8151442	0.1333776	-6.1116	1.09E-09	-1.095889179	count	2.57E-05
RNF175	-1.2424885	0.5011694	-2.4792	0.0132	-1.09566972	count	1
ITGA3	-0.8103495	0.1318059	-6.1481	8.68E-10	-1.094871204	count	2.05E-05
AC093390.1	-1.0891516	0.2219842	-4.9064	9.68E-07	-1.09482664	count	0.022522456
CD300A	-3.350815	1.2483092	-2.6843	0.0073	-1.09215888	count	1
CSGALNACT1	-0.8181118	0.1460848	-5.6003	2.30E-08	-1.089952597	count	0.000540316
TSLP	-0.9851882	0.2197433	-4.4834	7.57E-06	-1.089866752	count	0.17463233
LINC00702	-0.8782305	0.1680512	-5.226	1.83E-07	-1.087751243	count	0.004279089
CYFIP2	-0.7943635	0.1662864	-4.7771	1.85E-06	-1.083847678	count	0.04293295
SVIL	-0.7586309	0.0495413	-15.3131	2.35E-51	-1.082753066	count	5.70E-47
SYNJ2	-0.8657693	0.2404493	-3.6006	0.000322	-1.080819978	count	1
FAM198B	-0.8004376	0.1481983	-5.4011	7.04E-08	-1.077493111	count	0.001649542
HDAC9	-0.8452123	0.1555197	-5.4348	5.84E-08	-1.077398541	count	0.00136913
CARMN	-0.7566164	0.1743313	-4.3401	1.46E-05	-1.074960185	count	0.335946
UNC5B-AS1	-1.0822929	0.2826166	-3.8295	0.000131	-1.074651462	count	1
SH3BGR	-0.77071	0.0898672	-8.5761	1.43E-17	-1.073647309	count	3.42E-13
IGLC3	-0.8568293	0.163627	-5.2365	1.73E-07	-1.073554768	count	0.004045778
ERP27	-1.4079455	0.4189312	-3.3608	0.000785	-1.070744674	count	1
GSG1L	-1.1844605	0.3758928	-3.1511	0.00164	-1.067545321	count	1
LOH12CR2	-0.9851052	0.2998528	-3.2853	0.00103	-1.067485373	count	1
AL359504.2	-0.9310995	0.2931282	-3.1764	0.0015	-1.064915224	count	1
GZMA	-1.7384788	0.4913783	-3.538	0.000408	-1.064696207	count	1
IFI6	-0.7452725	0.0816929	-9.1229	1.18E-19	-1.061746232	count	2.83E-15
AC012085.2	-1.022404	0.3980878	-2.5683	0.0103	-1.061636801	count	1
VAMP8	-1.2003581	0.3354123	-3.5788	0.00035	-1.059001242	count	1
IL13RA2	-0.8012507	0.1999497	-4.0073	6.26E-05	-1.054952815	count	1
RGS1	-3.071165	0.6232851	-4.9274	8.70E-07	-1.049675902	count	0.02025273
TPST2	-0.8831965	0.1805857	-4.8907	1.05E-06	-1.047867481	count	0.02442195
PAPPA	-1.0280433	0.4007731	-2.5652	0.0104	-1.045377346	count	1
PPP1R3C	-0.7719598	0.1213094	-6.3636	2.21E-10	-1.042893867	count	5.23E-06
ST6GAL1	-0.9551362	0.2551649	-3.7432	0.000184	-1.042417516	count	1
RAB38	-1.2386647	0.2472268	-5.0102	5.69E-07	-1.04013696	count	0.013269649
PPP1R16A	-0.8000124	0.173304	-4.6162	4.04E-06	-1.039886325	count	0.0934654
TEX9	-0.8156924	0.2012544	-4.053	5.16E-05	-1.039634934	count	1
SPINT1	-1.8394515	0.5868912	-3.1342	0.00174	-1.038954066	count	1

DUSP15	-0.989705	0.2966929	-3.3358	0.000859	-1.037453247	count	1
FAM227B	-0.9806359	0.3179211	-3.0845	0.00205	-1.037217452	count	1
CHSY3	-0.970903	0.2401034	-4.0437	5.37E-05	-1.035662968	count	1
NUPR2	-0.9921964	0.3287418	-3.0182	0.00256	-1.030179788	count	1
AC009309.1	-1.819936	0.738071	-2.4658	0.0137	-1.029320858	count	1
COL4A1	-0.7280891	0.0915358	-7.9541	2.38E-15	-1.02558828	count	5.68E-11
CD3E	-2.014309	1.0768536	-1.8706	0.0615	-1.025095152	count	1
LDHD	-1.2932596	0.3817114	-3.3881	0.000711	-1.021704043	count	1
RGS3	-0.7295604	0.0888441	-8.2117	2.99E-16	-1.020050633	count	7.15E-12
LIMCH1	-0.7195824	0.0618376	-11.6366	9.25E-31	-1.019131305	count	2.23E-26
SYNPO	-0.72312	0.091117	-7.9362	2.75E-15	-1.01815816	count	6.57E-11
MYOM3	-19.56107	1664.310577	-0.0118	0.991	-1.017131669	count	1
SPATA16	-19.56107	1664.310577	-0.0118	0.991	-1.017131669	count	1
AC090971.1	-19.56107	1664.310577	-0.0118	0.991	-1.017131669	count	1
AC023906.3	-19.56107	1664.310577	-0.0118	0.991	-1.017131669	count	1
LINC02351	-19.56107	1664.310577	-0.0118	0.991	-1.017131669	count	1
C20orf204	-19.56107	1664.310577	-0.0118	0.991	-1.017131669	count	1
SOX11	-19.55956	1237.866749	-0.0158	0.987	-1.017131669	count	1
ST14	-19.55956	1237.866749	-0.0158	0.987	-1.017131669	count	1
KRT28	-19.55956	1237.866749	-0.0158	0.987	-1.017131669	count	1
AL157895.1	-19.55848	956.3530192	-0.0205	0.984	-1.017131669	count	1
FCER1A	-18.79408	1404.911048	-0.0134	0.989	-1.017131664	count	1
LINC01411	-18.79408	1404.911048	-0.0134	0.989	-1.017131664	count	1
RAB26	-18.79296	1212.347305	-0.0155	0.988	-1.017131664	count	1
MMP24	-18.79296	1212.347305	-0.0155	0.988	-1.017131664	count	1
ITGAM	-19.1682999	2061.468093	-0.0093	0.993	-1.017131448	count	1
HSPA2	-0.7433058	0.1085818	-6.8456	8.89E-12	-1.016751386	count	2.11E-07
POT1-AS1	-1.1790218	0.442448	-2.6648	0.00774	-1.016356903	count	1
TFPT	-0.8612343	0.2307721	-3.732	0.000193	-1.01438601	count	1
HPD	-0.9484636	0.3256967	-2.9121	0.00361	-1.011604957	count	1
SLC2A1-AS1	-1.375638	0.517691	-2.6573	0.00791	-1.005526627	count	1
LRRC61	-1.012081	0.373364	-2.7107	0.00675	-1.004835114	count	1
LINC00623	-0.7659746	0.1478096	-5.1822	2.31E-07	-1.004414024	count	0.005398008
SEMA3F	-1.2297933	0.4658401	-2.6399	0.00833	-1.003743576	count	1
PDGFC	-0.7633478	0.1498046	-5.0956	3.65E-07	-1.002566649	count	0.00851837
SMC4	-0.7287177	0.1179922	-6.176	7.29E-10	-1.001594927	count	1.72E-05
MEF2C	-0.7069818	0.0576435	-12.2647	6.41E-34	-1.001085945	count	1.55E-29
TES	-0.7233054	0.099454	-7.2728	4.28E-13	-0.998857715	count	1.02E-08
PDZK1IP1	-1.0186221	0.3829418	-2.66	0.00785	-0.998078525	count	1
MYH10	-0.6936175	0.0588619	-11.7838	1.74E-31	-0.997700224	count	4.20E-27
ITGA2	-0.988355	0.3191915	-3.0964	0.00197	-0.99333964	count	1
SMOC1	-1.1250293	0.2194513	-5.1266	3.10E-07	-0.992912015	count	0.00723819
MYH11	-0.6893241	0.0516697	-13.341	1.11E-39	-0.99110804	count	2.69E-35
ADRB2	-1.1180003	0.5558766	-2.0112	0.0444	-0.986713781	count	1
TNFRSF11B	-0.6876403	0.1089501	-6.3115	3.09E-10	-0.984312144	count	7.31E-06

CRLF1	-0.7364385	0.186669	-3.9452	8.12E-05	-0.98355159	count	1
EEPD1	-0.9895532	0.2738932	-3.6129	0.000307	-0.982349407	count	1
QPCT	-1.2879793	0.395277	-3.2584	0.00113	-0.98186788	count	1
FAM53A	-1.7250563	0.6705763	-2.5725	0.0101	-0.981469824	count	1
COL4A2	-0.6894599	0.0756809	-9.1101	1.32E-19	-0.979813319	count	3.17E-15
CCL3L1	-0.9070894	0.4486827	-2.0217	0.0433	-0.975017264	count	1
RGS5	-0.6769104	0.070024	-9.6668	7.58E-22	-0.974908821	count	1.82E-17
ATP6AP1L	-0.8016881	0.1997091	-4.0143	6.08E-05	-0.971314551	count	1
MYH9	-0.678927	0.0546016	-12.4342	8.49E-35	-0.97092395	count	2.05E-30
KCNJ3	-1.1886417	0.8369581	-1.4202	0.156	-0.97043192	count	1
AC018816.1	-1.3256242	0.4751723	-2.7898	0.0053	-0.969892548	count	1
AL592183.1	-0.9634318	0.2712141	-3.5523	0.000387	-0.968124098	count	1
INO80B	-1.0722885	0.3166376	-3.3865	0.000715	-0.966695284	count	1
ARL4C	-0.7650291	0.2172655	-3.5212	0.000435	-0.964587454	count	1
GAS6-DT	-1.454444	0.4427313	-3.2852	0.00103	-0.960253788	count	1
SPRY3	-2.618042	1.4588005	-1.7947	0.0728	-0.958951711	count	1
GMFG	-1.0422892	0.3641935	-2.8619	0.00423	-0.958057844	count	1
KCNT2	-0.8976115	0.191756	-4.681	2.96E-06	-0.956974569	count	0.06857432
DNAJC22	-2.6083564	0.7070486	-3.6891	0.000228	-0.956686179	count	1
CD93	-0.795371	0.3825008	-2.0794	0.0377	-0.952388954	count	1
KCTD11	-0.80859	0.2072446	-3.9016	9.73E-05	-0.951962971	count	1
CDH13	-0.66829	0.0792903	-8.4284	4.97E-17	-0.949250268	count	1.19E-12
LGALS9	-1.8388323	0.7237079	-2.5408	0.0111	-0.948732893	count	1
ARMC9	-0.7818363	0.2089349	-3.742	0.000185	-0.947097077	count	1
PARD6B	-1.0719784	0.3333274	-3.216	0.00131	-0.946012421	count	1
PRKG1	-0.6812163	0.0790248	-8.6203	9.78E-18	-0.945342421	count	2.34E-13
STAC2	-1.4304846	0.5552738	-2.5762	0.01	-0.945039231	count	1
MYLK	-0.6637553	0.0594856	-11.1583	1.86E-28	-0.944746646	count	4.49E-24
SLC16A5	-1.027072	0.4614598	-2.2257	0.0261	-0.94398452	count	1
RGS2	-0.7176295	0.1478513	-4.8537	1.26E-06	-0.943753215	count	0.02928114
STEAP1	-0.7406966	0.1184968	-6.2508	4.55E-10	-0.943608385	count	1.08E-05
MYO1D	-0.6830734	0.0947773	-7.2071	6.90E-13	-0.941855513	count	1.64E-08
LIG1	-1.0239674	0.281324	-3.6398	0.000277	-0.941110803	count	1
PDE8B	-0.9149214	0.3145403	-2.9088	0.00365	-0.939791832	count	1
NXPH4	-1.516429	0.4857199	-3.122	0.00181	-0.938481171	count	1
ASPN	-0.6529601	0.0710133	-9.1949	6.13E-20	-0.93739393	count	1.47E-15
A2M	-0.6531757	0.0515116	-12.6802	4.31E-36	-0.93728937	count	1.04E-31
COL23A1	-1.5128026	0.578703	-2.6141	0.00898	-0.936350586	count	1
BATF3	-0.8065968	0.2003707	-4.0255	5.80E-05	-0.934448596	count	1
GPC1	-0.7084711	0.1278121	-5.5431	3.18E-08	-0.934426981	count	0.0007466
FBXL22	-0.7224611	0.1924741	-3.7536	0.000177	-0.933859566	count	1
SORBS1	-0.6752864	0.09841	-6.862	7.94E-12	-0.932730701	count	1.89E-07
COL18A1	-0.6535381	0.068983	-9.4739	4.68E-21	-0.931170707	count	1.12E-16
SUGP1	-0.8804479	0.243086	-3.622	0.000296	-0.930507007	count	1
ATP2A3	-0.7960217	0.2382116	-3.3417	0.000841	-0.929823872	count	1

SCLY	-1.1750774	0.5382788	-2.183	0.0291	-0.929263267	count	1
RGS4	-0.6886009	0.1436578	-4.7933	1.71E-06	-0.928114464	count	0.03969765
LMOD1	-0.6467875	0.0474095	-13.6426	2.25E-41	-0.927901551	count	5.45E-37
DNASE1L1	-0.7481989	0.1743438	-4.2915	1.82E-05	-0.927840115	count	0.418327
C8orf34	-0.7630766	0.2441631	-3.1253	0.00179	-0.926708423	count	1
C4orf48	-0.7357187	0.1782012	-4.1286	3.73E-05	-0.925885396	count	0.8538716
MAGI1	-0.7661297	0.2929431	-2.6153	0.00895	-0.925342184	count	1
ABRACL	-0.6931541	0.1287024	-5.3857	7.67E-08	-0.921911665	count	0.001796928
PPFIA2	-1.097203	0.2599792	-4.2203	2.50E-05	-0.92181348	count	0.573475
LSM11	-0.8512458	0.4593801	-1.853	0.064	-0.921491284	count	1
EDN1	-0.7613518	0.2815177	-2.7045	0.00687	-0.916877325	count	1
KIF15	-2.445575	0.8900069	-2.7478	0.00603	-0.916442391	count	1
DNAAF1	-2.445575	0.9603513	-2.5465	0.0109	-0.916442391	count	1
HRASLS2	-2.445575	1.7545922	-1.3938	0.163	-0.916442391	count	1
AC005261.2	-2.445575	1.862547	-1.313	0.189	-0.916442391	count	1
ALPK3	-0.7363056	0.2092844	-3.5182	0.00044	-0.914842267	count	1
BGN	-0.6326916	0.0379339	-16.6788	2.94E-60	-0.911525286	count	7.14E-56
SPHK1	-0.8050906	0.2317352	-3.4742	0.000518	-0.910475385	count	1
PNP	-0.7579011	0.2346927	-3.2293	0.00125	-0.909971337	count	1
GPLD1	-1.0310131	0.473422	-2.1778	0.0295	-0.909624135	count	1
PLOD1	-0.7291343	0.170704	-4.2713	1.99E-05	-0.909407815	count	0.4572622
EOGT	-0.7274992	0.1599385	-4.5486	5.57E-06	-0.909056304	count	0.12865029
CCDC102B	-0.6747574	0.1145117	-5.8925	4.15E-09	-0.909014518	count	9.78E-05
TENT5B	-0.7548038	0.1971682	-3.8282	0.000131	-0.908927443	count	1
AKAP6	-0.6958915	0.1932323	-3.6013	0.000321	-0.905726981	count	1
SULF1	-0.6353354	0.0740267	-8.5825	1.35E-17	-0.903019683	count	3.23E-13
ATP8B1	-0.6975376	0.1513895	-4.6076	4.21E-06	-0.900489959	count	0.0973773
AC113383.1	-0.9469371	0.2412329	-3.9254	8.82E-05	-0.900261072	count	1
C5AR1	-1.2275106	0.4800301	-2.5572	0.0106	-0.899017578	count	1
RBPMS-AS1	-1.0419264	0.3781326	-2.7555	0.00589	-0.898004968	count	1
UPP1	-0.6670281	0.1460986	-4.5656	5.14E-06	-0.89582034	count	0.11878026
FAM13C	-0.6698725	0.1111431	-6.0271	1.83E-09	-0.895279178	count	4.32E-05
SMPX	-0.7757773	0.3584403	-2.1643	0.0305	-0.894439708	count	1
BEX5	-0.6887865	0.1521056	-4.5283	6.13E-06	-0.892912535	count	0.14152944
AC068446.2	-1.27759	0.6669822	-1.9155	0.0555	-0.892871647	count	1
ECSCR	-1.2177033	0.7075959	-1.7209	0.0854	-0.891868949	count	1
C3orf80	-1.2760871	0.837063	-1.5245	0.127	-0.89183276	count	1
CTSZ	-0.6246576	0.0448295	-13.9341	4.83E-43	-0.891645561	count	1.17E-38
PSRC1	-0.9378925	0.3508641	-2.6731	0.00755	-0.891558636	count	1
ASB2	-0.9224367	0.343079	-2.6887	0.00721	-0.890276522	count	1
CDKN2A	-0.6843062	0.1571011	-4.3558	1.36E-05	-0.889718671	count	0.313072
AC068620.2	-1.943974	0.7779399	-2.4989	0.0125	-0.888546034	count	1
RAB31	-0.6362685	0.0849589	-7.4891	8.64E-14	-0.888366112	count	2.06E-09
AL606491.1	-0.8955781	0.4668459	-1.9184	0.0551	-0.888191326	count	1
TIMP1	-0.6160313	0.0549614	-11.2084	1.08E-28	-0.888126128	count	2.61E-24

SGCA	-0.6337701	0.0725017	-8.7415	3.44E-18	-0.887200141	count	8.25E-14
DTNA	-0.7758726	0.1855892	-4.1806	2.98E-05	-0.886146098	count	0.6829266
AC231981.1	-1.3387499	0.4640865	-2.8847	0.00394	-0.886023243	count	1
ECT2	-1.0273489	0.3752436	-2.7378	0.00621	-0.885320765	count	1
RERG	-0.6451882	0.0922189	-6.9963	3.11E-12	-0.883745497	count	7.39E-08
HIP1R	-1.0823683	0.4534836	-2.3868	0.017	-0.883556539	count	1
PAIP2B	-0.8227256	0.2689276	-3.0593	0.00223	-0.883450815	count	1
SERPINA1	-2.3202835	0.8926988	-2.5992	0.00938	-0.882642921	count	1
COL21A1	-0.6232017	0.0800018	-7.7898	8.67E-15	-0.881164212	count	2.07E-10
ACTN1	-0.6166113	0.0561582	-10.9799	1.28E-27	-0.88072731	count	3.09E-23
ROM1	-0.7019615	0.1505524	-4.6626	3.23E-06	-0.880096923	count	0.07480357
AC099792.1	-2.294704	0.6762309	-3.3934	0.000698	-0.875438309	count	1
AL445493.3	-2.294704	0.7268375	-3.1571	0.00161	-0.875438309	count	1
SHCBP1	-2.294704	0.7268375	-3.1571	0.00161	-0.875438309	count	1
AC010976.2	-2.294704	0.8187195	-2.8028	0.00509	-0.875438309	count	1
AC092620.3	-2.294704	0.8187195	-2.8028	0.00509	-0.875438309	count	1
C9orf16	-0.612019	0.0443623	-13.7959	3.01E-42	-0.873775559	count	7.29E-38
MARCO	-1.902059	1.0953524	-1.7365	0.0826	-0.872136023	count	1
ESRRG	-1.902059	1.1561964	-1.6451	0.1	-0.872136023	count	1
AQP9	-1.902059	1.5877129	-1.198	0.231	-0.872136023	count	1
ART4	-0.6534533	0.1690022	-3.8665	0.000112	-0.871853191	count	1
ANGPT1	-0.6238675	0.0676606	-9.2205	4.86E-20	-0.871670119	count	1.17E-15
ENG	-0.6105938	0.0703488	-8.6795	5.88E-18	-0.869451018	count	1.41E-13
AC092747.4	-1.008189	0.4550087	-2.2158	0.0268	-0.868625058	count	1
SALL2	-0.8759628	0.420987	-2.0807	0.0375	-0.868477684	count	1
IFI44L	-0.7031966	0.179938	-3.908	9.47E-05	-0.867951016	count	1
ARNTL2	-1.0976852	0.4857693	-2.2597	0.0239	-0.867885932	count	1
MIR497HG	-0.6448347	0.1212853	-5.3167	1.12E-07	-0.864691202	count	0.002622256
SPEG	-0.6275209	0.1555928	-4.0331	5.62E-05	-0.86377056	count	1
ACTR5	-0.8602038	0.2474736	-3.4759	0.000515	-0.863297753	count	1
AJ011932.1	-0.738841	0.2236995	-3.3028	0.000966	-0.862413449	count	1
RILPL2	-0.6111855	0.0701463	-8.713	4.40E-18	-0.862297621	count	1.05E-13
WWTR1	-0.6095298	0.0743474	-8.1984	3.33E-16	-0.861873331	count	7.96E-12
CSPG4	-0.6390523	0.1816206	-3.5186	0.000439	-0.861455404	count	1
PTPRE	-0.8309768	0.2889924	-2.8754	0.00406	-0.861340559	count	1
AC087741.1	-1.175968	0.714751	-1.6453	0.1	-0.861330488	count	1
MALRD1	-1.088619	0.493855	-2.2043	0.0276	-0.860659041	count	1
F2RL1	-1.2995594	0.5077276	-2.5596	0.0105	-0.860469987	count	1
KY	-2.2415358	1.0050729	-2.2302	0.0258	-0.860132225	count	1
OSBPL7	-1.0215327	0.407634	-2.506	0.0123	-0.8577152	count	1
ENHO	-1.050739	0.5886509	-1.785	0.0743	-0.857498165	count	1
GLRB	-0.766278	0.2213261	-3.4622	0.000542	-0.856441129	count	1
LNP1	-0.6318042	0.1421553	-4.4445	9.07E-06	-0.855863194	count	0.2090635
SLC7A7	-1.121516	0.3181328	-3.5253	0.000428	-0.855477711	count	1
ATRNL1	-0.8169832	0.3628465	-2.2516	0.0244	-0.854844528	count	1

CLIC5	-0.7560825	0.3641658	-2.0762	0.0379	-0.854461275	count	1
PRRG4	-0.9475224	0.4035343	-2.3481	0.0189	-0.853210589	count	1
TSC22D1	-0.5976516	0.0484054	-12.3468	2.42E-34	-0.853176204	count	5.85E-30
AL031714.1	-1.634238	0.8850339	-1.8465	0.0649	-0.852870013	count	1
ANKH	-0.6089404	0.1016863	-5.9884	2.32E-09	-0.852521114	count	5.47E-05
LINC00574	-1.2191781	0.7295716	-1.6711	0.0948	-0.852295022	count	1
COL27A1	-0.7928926	0.2171301	-3.6517	0.000264	-0.851009273	count	1
BCAS4	-0.7283713	0.2066261	-3.5251	0.000429	-0.850064286	count	1
CDC42EP5	-0.6131713	0.0976538	-6.279	3.80E-10	-0.849782148	count	8.99E-06
CNTN3	-0.8199119	0.3417033	-2.3995	0.0165	-0.849709661	count	1
NPNT	-0.6524267	0.1416253	-4.6067	4.23E-06	-0.8496574	count	0.09783567
AC093635.1	-1.113106	0.6942021	-1.6034	0.109	-0.849015354	count	1
HCST	-1.8410405	0.5373731	-3.426	0.000619	-0.847731379	count	1
TNC	-0.6235476	0.2725672	-2.2877	0.0222	-0.847193637	count	1
PPP1R3B	-0.6424479	0.1623956	-3.9561	7.76E-05	-0.845715585	count	1
PDE3A	-0.6364401	0.1038475	-6.1286	9.80E-10	-0.845663296	count	2.31E-05
KCNN4	-0.9790534	0.3982558	-2.4584	0.014	-0.843187677	count	1
LINC02544	-0.6315674	0.1712724	-3.6875	0.00023	-0.84252824	count	1
LRRFIP1	-0.5897027	0.0579549	-10.1752	5.31E-24	-0.842525621	count	1.28E-19
MXRA5	-0.7537678	0.2289693	-3.292	0.001	-0.842287583	count	1
FAP	-0.6167977	0.1071742	-5.7551	9.37E-09	-0.841720727	count	0.000220448
MFGE8	-0.5854095	0.0400755	-14.6077	5.12E-47	-0.841074653	count	1.24E-42
MTTP	-2.9412025	1.0333744	-2.8462	0.00445	-0.839410086	count	1
MPP7	-0.6708557	0.1581197	-4.2427	2.26E-05	-0.839312305	count	0.518896
GJA1	-0.5984145	0.1005384	-5.9521	2.90E-09	-0.839272466	count	6.84E-05
TSTD1	-0.6057886	0.1268265	-4.7765	1.85E-06	-0.838664954	count	0.04293295
LINC01137	-0.7332894	0.2372797	-3.0904	0.00201	-0.836977859	count	1
MAST4-AS1	-1.1408947	0.4685631	-2.4349	0.0149	-0.835529564	count	1
HIST1H2BG	-2.1579491	0.7040956	-3.0649	0.00219	-0.835171425	count	1
ALS2CR12	-1.593668	0.8495964	-1.8758	0.0608	-0.833066563	count	1
FAM129B	-0.6689395	0.1537805	-4.35	1.40E-05	-0.832180274	count	0.322196
LINC01615	-0.6523338	0.1620991	-4.0243	5.83E-05	-0.831515234	count	1
SNAP25	-1.2508229	0.5241797	-2.3862	0.0171	-0.828436435	count	1
SDK2	-1.250816	0.5655338	-2.2117	0.027	-0.828431644	count	1
ALCAM	-0.5923927	0.0890488	-6.6524	3.31E-11	-0.826837262	count	7.85E-07
RRM2	-2.8604659	1.493859	-1.9148	0.0556	-0.826609738	count	1
TIPARP-AS1	-1.1818041	0.4692571	-2.5185	0.0118	-0.826137646	count	1
PPP1R14A	-0.5737368	0.0470205	-12.2018	1.35E-33	-0.825707518	count	3.26E-29
AC020915.3	-0.7191692	0.22098	-3.2545	0.00115	-0.824648909	count	1
SLC14A1	-0.6071375	0.1838734	-3.3019	0.000969	-0.823744105	count	1
CUBN	-1.0400659	0.3800853	-2.7364	0.00624	-0.821841379	count	1
HOXB4	-0.7918808	0.1858131	-4.2617	2.08E-05	-0.820228841	count	0.4777552
BTC	-0.6674394	0.1634789	-4.0827	4.55E-05	-0.819764618	count	1
IL17B	-0.7218477	0.4158786	-1.7357	0.0827	-0.819614349	count	1
ARHGAP22	-0.7426929	0.2943933	-2.5228	0.0117	-0.819568685	count	1

ITLN2	-0.8766207	0.3969651	-2.2083	0.0273	-0.818706397	count	1
ASTN2	-0.8069872	0.3412734	-2.3646	0.0181	-0.818575274	count	1
AC073941.1	-1.2356662	0.525028	-2.3535	0.0186	-0.818420136	count	1
DACT3	-0.5990712	0.1023363	-5.8539	5.22E-09	-0.817453271	count	0.000122957
NDC80	-0.8477367	0.2998263	-2.8274	0.00472	-0.817134426	count	1
AL121992.1	-1.766361	0.698538	-2.5287	0.0115	-0.817055818	count	1
CACFD1	-0.7885938	0.3014316	-2.6162	0.00893	-0.81677054	count	1
CHN1	-0.672873	0.1766296	-3.8095	0.000142	-0.816270377	count	1
LNX1	-0.8890116	0.2624251	-3.3877	0.000712	-0.815579212	count	1
ENPP2	-0.5780805	0.1048754	-5.5121	3.79E-08	-0.814463239	count	0.000889437
CCSAP	-0.8694515	0.2983253	-2.9144	0.00358	-0.811891879	count	1
ANKRD44	-0.6639501	0.2133	-3.1128	0.00187	-0.811566251	count	1
AL136295.6	-1.5496906	0.7467552	-2.0752	0.038	-0.811328051	count	1
HHEX	-1.3044538	0.4697342	-2.777	0.00551	-0.810783739	count	1
RAMP1	-0.5640455	0.0535673	-10.5297	1.46E-25	-0.810700158	count	3.52E-21
CD82	-0.6116443	0.1237204	-4.9438	8.01E-07	-0.810547822	count	0.018652086
SPECC1	-0.5819613	0.09119	-6.3819	1.97E-10	-0.808183355	count	4.66E-06
CDH11	-0.5773115	0.11094	-5.2038	2.06E-07	-0.807082894	count	0.004815456
PTN	-0.561449	0.1074901	-5.2233	1.86E-07	-0.805207426	count	0.004348866
LAPTM5	-1.151343	0.4644817	-2.4788	0.0132	-0.80471658	count	1
FAM89A	-0.6667687	0.1781985	-3.7417	0.000186	-0.804323752	count	1
ZNF608	-0.8023089	0.2560508	-3.1334	0.00174	-0.804306235	count	1
FGF1	-0.678901	0.1949877	-3.4818	0.000504	-0.80371449	count	1
MSC	-0.6124293	0.1166763	-5.249	1.62E-07	-0.803506957	count	0.003789504
AC005837.1	-1.2908568	0.5298651	-2.4362	0.0149	-0.80239628	count	1
AC106897.1	-0.7272943	0.287729	-2.5277	0.0115	-0.802342563	count	1
IFIT2	-0.5942476	0.189915	-3.129	0.00177	-0.802117144	count	1
PXMP2	-0.5846281	0.1255473	-4.6566	3.33E-06	-0.801500863	count	0.07710282
RYR2	-0.8732782	0.309868	-2.8182	0.00485	-0.800881797	count	1
BX004987.1	-0.8283672	0.8295256	-0.9986	0.318	-0.798133425	count	1
TMEM102	-1.3851946	0.4138835	-3.3468	0.000826	-0.797783252	count	1
C9orf3	-0.5596844	0.0501938	-11.1505	2.03E-28	-0.797639958	count	4.90E-24
ASAP1	-0.6001056	0.1287447	-4.6612	3.26E-06	-0.797639246	count	0.07549182
USP3-AS1	-1.139898	0.4156279	-2.7426	0.00613	-0.796646056	count	1
PPP2R2B	-0.6772898	0.1530704	-4.4247	9.94E-06	-0.795989794	count	0.22902754
UNC13C	-1.515395	0.4356208	-3.4787	0.00051	-0.794187465	count	1
TM4SF1	-0.5554254	0.0747346	-7.432	1.32E-13	-0.793881045	count	3.14E-09
NECAB1	-0.8499779	0.3279091	-2.5921	0.00958	-0.793372056	count	1
TRIM59	-0.7816271	0.4719149	-1.6563	0.0977	-0.792423234	count	1
EHD3	-0.7577208	0.2780659	-2.725	0.00646	-0.7919219	count	1
AL355001.2	-0.7271876	0.2548635	-2.8532	0.00435	-0.791358102	count	1
CFH	-0.5508368	0.0566565	-9.7224	4.46E-22	-0.790304819	count	1.07E-17
LIMD2	-0.6312639	0.2371005	-2.6624	0.00779	-0.789392044	count	1
CCDC151	-0.5896337	0.1381246	-4.2689	2.01E-05	-0.788821188	count	0.4618176
LRRC15	-1.6949637	0.5804346	-2.9202	0.00352	-0.786933986	count	1

NCAM1	-0.6270815	0.2929234	-2.1408	0.0324	-0.786904869	count	1
MPPED2	-0.6230414	0.1931692	-3.2254	0.00127	-0.785715732	count	1
CASKIN2	-0.7581794	0.2964794	-2.5573	0.0106	-0.784760675	count	1
SFTA1P	-1.6876389	0.3217073	-5.2459	1.64E-07	-0.783801473	count	0.003835796
PDE1A	-0.5740492	0.0937428	-6.1237	1.01E-09	-0.781637257	count	2.39E-05
RHOD	-0.5671514	0.0933552	-6.0752	1.36E-09	-0.781209157	count	3.21E-05
ITPR1	-0.6012314	0.2123949	-2.8307	0.00467	-0.781115396	count	1
SYNGR2	-0.5649786	0.1038993	-5.4377	5.75E-08	-0.780679503	count	0.001348145
AC243964.2	-2.600285	0.7831342	-3.3204	0.000908	-0.780069971	count	1
PCDH11X	-0.987957	0.2992506	-3.3014	0.000971	-0.779991255	count	1
ZNF529-AS1	-0.752937	0.3901299	-1.93	0.0537	-0.779241627	count	1
AKAP1	-0.5919692	0.1262696	-4.6881	2.86E-06	-0.778926197	count	0.0662662
CRISPLD1	-0.5772043	0.0965625	-5.9775	2.48E-09	-0.778088229	count	5.85E-05
NRCAM	-1.9782003	0.7770081	-2.5459	0.0109	-0.777837726	count	1
AC023590.1	-1.9782003	0.9427748	-2.0983	0.0359	-0.777837726	count	1
SNX10	-0.9850601	0.913509	-1.0783	0.281	-0.777659644	count	1
MSH3	-0.6169469	0.1549154	-3.9825	6.95E-05	-0.776703539	count	1
MYEF2	-0.5895135	0.1877089	-3.1406	0.0017	-0.776254457	count	1
SMIM1	-0.6250499	0.1955371	-3.1966	0.0014	-0.775587272	count	1
EMILIN1	-0.555669	0.0776083	-7.1599	9.71E-13	-0.775405814	count	2.31E-08
RASL11B	-0.6506068	0.2774533	-2.3449	0.0191	-0.775083691	count	1
SPRED3	-1.4752105	0.5610665	-2.6293	0.00859	-0.773903252	count	1
ZDHHC13	-0.8158362	0.3102315	-2.6298	0.00858	-0.773742681	count	1
PPP1R12B	-0.5448966	0.0834861	-6.5268	7.64E-11	-0.77352863	count	1.81E-06
ADGRE2	-1.957735	0.4796876	-4.0813	4.57E-05	-0.771000931	count	1
ACSS2	-0.6373672	0.2243576	-2.8409	0.00452	-0.77067339	count	1
ACTN2	-0.9458914	0.3510972	-2.6941	0.00709	-0.770605532	count	1
MYL3	-1.9538146	0.8673817	-2.2525	0.0243	-0.769684403	count	1
CHCHD10	-0.5395608	0.0528407	-10.2111	3.71E-24	-0.769375578	count	8.94E-20
IL34	-0.5691375	0.1030429	-5.5233	3.56E-08	-0.76898099	count	0.000835603
FCGR2B	-2.5368997	1.84454	-1.3754	0.169	-0.767457867	count	1
OSBPL3	-2.5368993	1.1477412	-2.2103	0.0271	-0.767457833	count	1
SCG2	-0.6069419	0.2557361	-2.3733	0.0177	-0.766472085	count	1
CHST1	-0.6573239	0.1826035	-3.5997	0.000323	-0.766236418	count	1
AC026801.2	-0.8074048	0.3617082	-2.2322	0.0257	-0.765585022	count	1
SMOC2	-0.5413892	0.0599446	-9.0315	2.68E-19	-0.765577571	count	6.43E-15
MYBL1	-0.6711865	0.323881	-2.0723	0.0383	-0.765231914	count	1
NSUN7	-1.9397488	0.6855673	-2.8294	0.00469	-0.76494183	count	1
PPP1R26	-1.0949455	0.4097071	-2.6725	0.00756	-0.764840058	count	1
CCDC167	-0.5896043	0.1347675	-4.375	1.25E-05	-0.763617484	count	0.28785
LMO7	-0.536169	0.0970115	-5.5269	3.49E-08	-0.763302671	count	0.000819243
SLC30A3	-0.8873043	0.3688414	-2.4057	0.0162	-0.762768799	count	1
SFTPD	-0.8873043	0.4855845	-1.8273	0.0677	-0.762768799	count	1
AKAP2	-0.9345938	0.3332118	-2.8048	0.00506	-0.761204503	count	1
PURG	-0.9068729	0.5471909	-1.6573	0.0975	-0.759840061	count	1

GRIK5	-0.732557	0.3084166	-2.3752	0.0176	-0.757783086	count	1
LEF1	-1.218903	0.9155823	-1.3313	0.183	-0.757682434	count	1
ATP6V1FNB	-2.4891165	1.4594523	-1.7055	0.0882	-0.757610553	count	1
SYNPO2	-0.5343949	0.0660647	-8.089	8.10E-16	-0.757316912	count	1.94E-11
AC007728.2	-1.9171876	0.9856698	-1.9451	0.0518	-0.757274758	count	1
TRBC2	-1.9165443	0.6679175	-2.8694	0.00414	-0.757055063	count	1
CIB2	-0.5994299	0.1629482	-3.6787	0.000238	-0.756910359	count	1
GPRC5D-AS1	-0.7060084	0.3780288	-1.8676	0.0619	-0.756424358	count	1
MIR34AHG	-0.723625	0.3922448	-1.8448	0.0651	-0.755692377	count	1
VCAN	-0.5238434	0.0598202	-8.757	3.01E-18	-0.753952988	count	7.22E-14
HIF1A	-0.542124	0.0857916	-6.3191	2.95E-10	-0.753307719	count	6.98E-06
AC024901.1	-1.1348773	0.3823679	-2.968	0.00302	-0.751237743	count	1
KCNMB1	-0.5334789	0.0746025	-7.151	1.04E-12	-0.751114892	count	2.47E-08
NRIP3	-0.8966566	0.3973053	-2.2568	0.0241	-0.751086994	count	1
CLEC5A	-20.151112	1577.974186	-0.0128	0.99	-0.750687231	count	1
IL11	-20.151112	1577.974186	-0.0128	0.99	-0.750687231	count	1
DPF1	-19.466711	2102.986181	-0.0093	0.993	-0.750687229	count	1
LILRB2	-19.466711	2102.986181	-0.0093	0.993	-0.750687229	count	1
LILRB1	-19.466711	2102.986181	-0.0093	0.993	-0.750687229	count	1
AC012313.5	-19.466711	2102.986181	-0.0093	0.993	-0.750687229	count	1
AL031283.2	-19.152289	1356.651203	-0.0141	0.989	-0.750687228	count	1
SGO1	-19.152289	1356.651203	-0.0141	0.989	-0.750687228	count	1
PF4	-19.152289	1356.651203	-0.0141	0.989	-0.750687228	count	1
LINC02562	-19.152289	1356.651203	-0.0141	0.989	-0.750687228	count	1
MYB	-19.152289	1356.651203	-0.0141	0.989	-0.750687228	count	1
HCFC1-AS1	-19.152289	1356.651203	-0.0141	0.989	-0.750687228	count	1
AC084082.1	-19.152289	1356.651203	-0.0141	0.989	-0.750687228	count	1
AP002812.5	-19.152289	1356.651203	-0.0141	0.989	-0.750687228	count	1
CYP17A1	-19.152289	1356.651203	-0.0141	0.989	-0.750687228	count	1
AC002563.1	-19.152289	1356.651203	-0.0141	0.989	-0.750687228	count	1
AC138627.1	-19.152289	1356.651203	-0.0141	0.989	-0.750687228	count	1
AQP4	-19.152289	1356.651203	-0.0141	0.989	-0.750687228	count	1
PLIN5	-19.152289	1356.651203	-0.0141	0.989	-0.750687228	count	1
DENND1C	-19.152289	1356.651203	-0.0141	0.989	-0.750687228	count	1
SIGLEC9	-19.152289	1356.651203	-0.0141	0.989	-0.750687228	count	1
Z95114.2	-19.152289	1356.651203	-0.0141	0.989	-0.750687228	count	1
GTSE1	-19.152289	1356.651203	-0.0141	0.989	-0.750687228	count	1
GDF9	-19.7391907	1983.758551	-0.01	0.992	-0.750686996	count	1
CCR6	-19.5191637	1722.275698	-0.0113	0.991	-0.750686995	count	1
AC015967.1	-19.5191637	1722.275698	-0.0113	0.991	-0.750686995	count	1
HIST1H2AL	-19.5184067	1394.947268	-0.014	0.989	-0.750686995	count	1
HOXA11-AS	-19.5184067	1394.947268	-0.014	0.989	-0.750686995	count	1
KCNG1	-19.5184067	1394.947268	-0.014	0.989	-0.750686995	count	1
AC245128.3	-19.0454545	2306.449155	-0.0083	0.993	-0.750686993	count	1
AP003721.4	-18.8091862	1514.391015	-0.0124	0.99	-0.750686992	count	1

SNX20	-18.7687748	1461.142901	-0.0128	0.99	-0.750686992	count	1
SH2D1A	-18.5380059	1426.773436	-0.013	0.99	-0.75068699	count	1
NREP	-0.6342876	0.1694757	-3.7426	0.000185	-0.750319386	count	1
DUSP23	-0.5492984	0.1045319	-5.2548	1.57E-07	-0.750129508	count	0.003672858
DMTN	-0.6355814	0.2789529	-2.2785	0.0228	-0.749183756	count	1
TUBA4A	-0.5459265	0.1093315	-4.9933	6.21E-07	-0.74912858	count	0.014477373
AC110285.6	-1.1315513	0.4824543	-2.3454	0.0191	-0.749005359	count	1
PATJ	-0.5876966	0.2027833	-2.8982	0.00378	-0.74855593	count	1
LRRC1	-0.7303404	0.3009231	-2.427	0.0153	-0.74768309	count	1
CCDC110	-0.6253156	0.1906186	-3.2805	0.00105	-0.74702597	count	1
SPOUT1	-0.6194536	0.2478767	-2.499	0.0125	-0.746675735	count	1
ADAMTS12	-0.9795374	0.3812646	-2.5692	0.0102	-0.745642313	count	1
SLMAP	-0.5407849	0.0925658	-5.8422	5.60E-09	-0.745346502	count	0.000131846
DGKH	-0.6395525	0.1816509	-3.5208	0.000436	-0.745266094	count	1
MSC-AS1	-0.5660636	0.1176981	-4.8095	1.57E-06	-0.745208249	count	0.03646168
KCNS3	-0.6639444	0.1748824	-3.7965	0.000149	-0.744916587	count	1
TPH1	-0.5593848	0.1760736	-3.177	0.0015	-0.744560156	count	1
TPM2	-0.5164719	0.0402046	-12.8461	5.61E-37	-0.744539512	count	1.36E-32
IFNAR2	-0.6061588	0.2292712	-2.6439	0.00823	-0.743826858	count	1
NMRK1	-0.5450736	0.1093695	-4.9838	6.52E-07	-0.743119895	count	0.015198772
NEU3	-0.8449336	0.4045634	-2.0885	0.0368	-0.742962671	count	1
ETV6	-0.5743457	0.1643603	-3.4944	0.000481	-0.7429529	count	1
LINC00982	-0.552347	0.1507042	-3.6651	0.000251	-0.74255581	count	1
IDO1	-1.0623728	0.7931487	-1.3394	0.181	-0.741695828	count	1
NKG7	-0.7955982	0.5833818	-1.3638	0.173	-0.741595979	count	1
TPM4	-0.5158421	0.0342929	-15.0422	1.14E-49	-0.741044937	count	2.77E-45
LINC01106	-1.0131688	0.6982262	-1.4511	0.147	-0.740694019	count	1
HIST1H2BE	-1.1915595	0.6316374	-1.8865	0.0593	-0.740557423	count	1
AL138762.1	-0.8242802	0.3292423	-2.5036	0.0123	-0.740241716	count	1
DMD	-0.527064	0.1105511	-4.7676	1.94E-06	-0.739901671	count	0.04500606
SERTAD4	-0.5964072	0.1652718	-3.6086	0.000312	-0.739733195	count	1
AC046143.1	-2.405396	0.8877461	-2.7096	0.00677	-0.739646763	count	1
MAD2L1	-0.8412018	0.3402176	-2.4725	0.0135	-0.739604631	count	1
PRKCZ	-0.9377482	0.51873	-1.8078	0.0707	-0.739516177	count	1
SCN9A	-1.0580124	0.4565951	-2.3172	0.0205	-0.738591902	count	1
TREM1	-1.188077	0.8424692	-1.4102	0.159	-0.738371435	count	1
VOPP1	-0.5995987	0.1216424	-4.9292	8.62E-07	-0.737388381	count	0.02006736
MECOM	-0.5283658	0.1834844	-2.8796	0.004	-0.735198604	count	1
DLX3	-0.638692	0.4137114	-1.5438	0.123	-0.734671669	count	1
NPY2R	-0.6413918	0.2765343	-2.3194	0.0204	-0.734377332	count	1
ITGA6	-0.6621675	0.3040435	-2.1779	0.0295	-0.734127975	count	1
RPL27A	-0.511674	0.0287694	-17.7854	6.06E-68	-0.733908794	count	1.47E-63
FLNA	-0.5107631	0.0549045	-9.3028	2.29E-20	-0.733870742	count	5.50E-16
LDOC1	-0.520176	0.0721708	-7.2076	6.88E-13	-0.733266106	count	1.64E-08
NUDT10	-0.9635973	0.3635039	-2.6509	0.00806	-0.733229583	count	1

PRR26	-1.2715843	0.4976442	-2.5552	0.0107	-0.732885682	count	1
CST7	-2.37185	0.8033431	-2.9525	0.00317	-0.732193671	count	1
SERTAD4-AS1	-0.5364576	0.0933619	-5.746	9.88E-09	-0.731942258	count	0.000232427
ACTA2	-0.5072385	0.0440577	-11.513	3.72E-30	-0.731434206	count	8.98E-26
SCRG1	-0.5159925	0.1066776	-4.8369	1.37E-06	-0.731270825	count	0.03183195
HIST2H2BE	-1.1765873	0.395464	-2.9752	0.00295	-0.73115213	count	1
CORO7	-1.104163	0.5298963	-2.0837	0.0373	-0.730589429	count	1
HACD4	-0.5896921	0.1575292	-3.7434	0.000184	-0.729826903	count	1
MKX	-0.8711945	0.3593158	-2.4246	0.0154	-0.729254955	count	1
TMEM44	-0.8488624	0.4785407	-1.7739	0.0762	-0.728967861	count	1
THY1	-0.5248322	0.1164427	-4.5072	6.77E-06	-0.728480991	count	0.15627191
LINC00839	-0.7365585	0.6201393	-1.1877	0.235	-0.7280151	count	1
EMB	-0.7566036	0.4961623	-1.5249	0.127	-0.727655156	count	1
COPRS	-0.5107486	0.0518894	-9.843	1.40E-22	-0.727630856	count	3.37E-18
CCL5	-0.6795133	0.2478688	-2.7414	0.00615	-0.727567011	count	1
KIF4A	-2.3505854	1.0069089	-2.3345	0.0196	-0.727393418	count	1
STARD6	-2.3505854	1.0069089	-2.3345	0.0196	-0.727393418	count	1
BX293535.1	-2.3505854	1.797346	-1.3078	0.191	-0.727393418	count	1
TOX2	-0.9556907	0.2828367	-3.3789	0.000735	-0.727067893	count	1
AC098829.1	-1.095546	0.4304249	-2.5453	0.011	-0.724783483	count	1
FAH	-0.5906967	0.2011527	-2.9366	0.00334	-0.724668904	count	1
PTPN6	-1.8232486	0.7375443	-2.4721	0.0135	-0.724571576	count	1
CCDC9B	-0.7763978	0.2860356	-2.7143	0.00667	-0.723299703	count	1
CAMK2G	-0.5272941	0.1176019	-4.4837	7.56E-06	-0.722664516	count	0.1744092
CASTOR1	-0.7627305	0.5283921	-1.4435	0.149	-0.722338211	count	1
CASK	-0.5650077	0.1335525	-4.2306	2.39E-05	-0.722323215	count	0.548505
SLC9A3R2	-0.5113547	0.0796378	-6.421	1.53E-10	-0.722301298	count	3.62E-06
LIMD1	-0.7306429	0.3291237	-2.22	0.0265	-0.72204674	count	1
SOS1	-0.5396594	0.1049615	-5.1415	2.87E-07	-0.722032044	count	0.006702311
TMSB4X	-0.5006112	0.0236112	-21.2023	3.30E-94	-0.721271922	count	8.02E-90
GAPDH	-0.4997753	0.0225266	-22.186	1.94E-102	-0.720431555	count	4.72E-98
CCDC112	-0.5339597	0.1264253	-4.2235	2.46E-05	-0.720140214	count	0.564447
EMCN	-0.6726308	0.3875666	-1.7355	0.0827	-0.720070889	count	1
SNAI1	-0.6349837	0.2342533	-2.7107	0.00675	-0.719731923	count	1
DIAPH3	-1.1581329	0.5989532	-1.9336	0.0532	-0.719533346	count	1
INHBA-AS1	-1.0874926	0.3640744	-2.987	0.00284	-0.719352974	count	1
NRM	-0.6018886	0.1741332	-3.4565	0.000553	-0.718726774	count	1
CPPED1	-0.5494656	0.1348404	-4.0749	4.70E-05	-0.718050431	count	1
IQGAP2	-0.9439188	0.5584724	-1.6902	0.0911	-0.717888593	count	1
HIPK1-AS1	-1.5358444	0.5897445	-2.6043	0.00924	-0.717230857	count	1
AC023043.1	-0.7453626	0.2447366	-3.0456	0.00234	-0.71660828	count	1
RNF150	-0.6748353	0.1887332	-3.5756	0.000354	-0.716553979	count	1
AL354836.1	-2.2990979	1.0143649	-2.2665	0.0235	-0.715526875	count	1
AL135791.1	-0.8330168	0.3456576	-2.4099	0.016	-0.71502271	count	1
MIS18A-AS1	-2.293069	0.9453857	-2.4255	0.0153	-0.714115058	count	1

AC001226.1	-2.293069	1.0755985	-2.1319	0.0331	-0.714115058	count	1
AC130469.1	-2.293069	1.3573634	-1.6894	0.0912	-0.714115058	count	1
AC018742.1	-1.1493178	0.4901538	-2.3448	0.0191	-0.71397365	count	1
CIRBP-AS1	-2.2908437	1.2090352	-1.8948	0.0582	-0.713592441	count	1
JAK2	-0.5522451	0.1551847	-3.5586	0.000378	-0.713415022	count	1
HAPLN3	-0.5515532	0.1474333	-3.741	0.000186	-0.713274356	count	1
UACA	-0.5025381	0.0791702	-6.3476	2.45E-10	-0.712641727	count	5.80E-06
C12orf75	-0.4973668	0.0510455	-9.7436	3.64E-22	-0.712333189	count	8.76E-18
SERINC2	-1.5231677	0.5375606	-2.8335	0.00463	-0.71153691	count	1
ADARB1	-0.5524896	0.1703473	-3.2433	0.00119	-0.711365021	count	1
INHBA	-0.5049839	0.0984794	-5.1278	3.08E-07	-0.711264493	count	0.0071918
SMIM10L2B	-1.3521393	0.4408369	-3.0672	0.00218	-0.710552971	count	1
TCEAL4	-0.4948345	0.040248	-12.2946	4.50E-34	-0.709823289	count	1.09E-29
CALD1	-0.4916902	0.0293639	-16.7447	1.05E-60	-0.708847249	count	2.55E-56
AC027702.1	-1.3482342	0.6124197	-2.2015	0.0278	-0.708515051	count	1
NTN1	-0.6853587	0.217147	-3.1562	0.00161	-0.708076375	count	1
AC103706.1	-0.7475189	0.4885787	-1.53	0.126	-0.707606603	count	1
GMDS	-0.4969645	0.0623301	-7.9731	2.05E-15	-0.707367358	count	4.90E-11
STN1	-0.5578677	0.1564963	-3.5647	0.000369	-0.70724697	count	1
REPS2	-1.0678445	0.4459569	-2.3945	0.0167	-0.706084836	count	1
PRKAR1B	-0.6966049	0.2611571	-2.6674	0.00768	-0.704676616	count	1
SLC29A2	-1.5066735	0.6747008	-2.2331	0.0256	-0.704099395	count	1
GKAP1	-0.5483264	0.1351426	-4.0574	5.07E-05	-0.703477989	count	1
NEXN	-0.489952	0.0547028	-8.9566	5.21E-19	-0.703053785	count	1.25E-14
TSPAN5	-0.638502	0.1958544	-3.2601	0.00112	-0.702986855	count	1
C1QTNF1	-0.4972522	0.0671506	-7.405	1.62E-13	-0.702783113	count	3.86E-09
DLX4	-0.633363	0.2954393	-2.1438	0.0321	-0.701704469	count	1
PSTPIP1	-1.0063068	0.5954673	-1.6899	0.0911	-0.701695351	count	1
AC090409.1	-0.8895276	0.4412268	-2.016	0.0439	-0.700536757	count	1
ST6GALNAC5	-1.7556834	0.6674351	-2.6305	0.00856	-0.700300014	count	1
PTPRC	-1.1265181	0.4652219	-2.4215	0.0155	-0.699566031	count	1
LRIG1	-0.5509723	0.1543731	-3.5691	0.000363	-0.699454954	count	1
FERMT3	-1.0029765	0.3508934	-2.8584	0.00428	-0.699313615	count	1
SLC2A1	-0.6697636	0.3082129	-2.1731	0.0298	-0.698447121	count	1
DUSP27	-1.4922156	0.7070914	-2.1104	0.0349	-0.697553718	count	1
LAMA5	-0.542721	0.1705992	-3.1813	0.00148	-0.697071397	count	1
C1orf122	-0.4921645	0.0571697	-8.6088	1.08E-17	-0.696362641	count	2.59E-13
LDB3	-0.5508318	0.2064201	-2.6685	0.00765	-0.696149601	count	1
RFLNA	-0.8321623	0.4775184	-1.7427	0.0815	-0.695749594	count	1
COL5A2	-0.5049343	0.1077762	-4.685	2.90E-06	-0.695669101	count	0.0671901
INKA1	-0.5727341	0.2233398	-2.5644	0.0104	-0.695490304	count	1
GPR65	-1.2053505	0.6546893	-1.8411	0.0657	-0.694446058	count	1
CMC4	-0.6069401	0.3638356	-1.6682	0.0954	-0.694395604	count	1
NCALD	-0.5160568	0.128208	-4.0252	5.81E-05	-0.694164474	count	1
UBE2E1-AS1	-1.7368628	0.9015314	-1.9266	0.0541	-0.693431671	count	1

HNRNPA1P48	-0.7727787	0.259908	-2.9733	0.00297	-0.692898266	count	1
GPR34	-0.9928451	0.7003901	-1.4176	0.156	-0.692064421	count	1
CCDC88B	-0.743224	0.5173486	-1.4366	0.151	-0.691677609	count	1
ADSSL1	-0.5688174	0.2093664	-2.7169	0.00662	-0.690683322	count	1
LPCAT2	-0.5395881	0.1916347	-2.8157	0.00489	-0.690469905	count	1
CYP4F11	-1.4766121	0.5315501	-2.7779	0.0055	-0.690462298	count	1
IFFO2	-0.7698689	0.3781517	-2.0359	0.0418	-0.690222274	count	1
MYOZ2	-0.5056045	0.1815002	-2.7857	0.00537	-0.68932021	count	1
DUSP2	-0.5689474	0.3291357	-1.7286	0.084	-0.689000391	count	1
TCAF2	-0.9873237	1.106856	-0.892	0.372	-0.68811164	count	1
C4orf47	-1.1944599	0.4825704	-2.4752	0.0134	-0.688088738	count	1
GBP7	-1.1083897	0.3965946	-2.7948	0.00522	-0.68808321	count	1
ELFN1	-2.1847235	0.8088693	-2.701	0.00695	-0.687935137	count	1
AL590705.1	-2.1847235	1.1505997	-1.8988	0.0577	-0.687935137	count	1
NUAK1	-0.5201543	0.248868	-2.0901	0.0367	-0.68781942	count	1
AC079089.1	-0.940414	0.4091189	-2.2986	0.0216	-0.686208856	count	1
TEAD4	-0.7354568	0.2673834	-2.7506	0.00598	-0.684272681	count	1
PDLIM3	-0.4760303	0.0457963	-10.3945	5.81E-25	-0.684254478	count	1.40E-20
RABGAP1	-0.4928059	0.0977444	-5.0418	4.84E-07	-0.683557968	count	0.011289784
KCNMB4	-0.5818516	0.2310588	-2.5182	0.0118	-0.682555572	count	1
CDKN2C	-0.4887765	0.0880958	-5.5482	3.09E-08	-0.682403368	count	0.000725501
GUK1	-0.4747084	0.0316037	-15.0206	1.55E-49	-0.681962926	count	3.76E-45
FYB1	-0.9345769	0.6090501	-1.5345	0.125	-0.681827107	count	1
LPP	-0.4767237	0.0520627	-9.1567	8.67E-20	-0.681509139	count	2.08E-15
CHMP1A	-0.5311201	0.1181789	-4.4942	7.20E-06	-0.681242914	count	0.1661544
RTN1	-0.5206678	0.2051239	-2.5383	0.0112	-0.680189306	count	1
EPAS1	-0.4784074	0.0648684	-7.375	2.02E-13	-0.679688307	count	4.81E-09
MIAT	-1.0282134	0.5129652	-2.0045	0.0451	-0.679249049	count	1
RND1	-1.2912724	0.5823627	-2.2173	0.0267	-0.678615983	count	1
AC010883.1	-1.4492848	0.6600098	-2.1959	0.0282	-0.677976857	count	1
LINC00672	-0.5714243	0.1941658	-2.943	0.00327	-0.677450235	count	1
AC108134.2	-1.1751344	0.4603765	-2.5526	0.0107	-0.676784064	count	1
P2RY2	-0.9700208	0.5738741	-1.6903	0.0911	-0.675715091	count	1
CARTPT	-1.6872908	1.237577	-1.3634	0.173	-0.675125028	count	1
AP003548.1	-0.8568634	0.3591663	-2.3857	0.0171	-0.674088655	count	1
SLX4IP	-0.6125284	0.2061264	-2.9716	0.00298	-0.673929433	count	1
PRKX	-0.7520238	0.3855783	-1.9504	0.0512	-0.673809799	count	1
PPM1L	-0.5385074	0.1620472	-3.3232	0.000899	-0.67368309	count	1
AC009831.1	-2.1261508	0.9939085	-2.1392	0.0325	-0.673152507	count	1
PRMT5-AS1	-1.2800841	0.6624894	-1.9322	0.0534	-0.672706966	count	1
ZNF436-AS1	-0.7505572	0.339625	-2.21	0.0272	-0.672460877	count	1
TMEM25	-0.501034	0.1274186	-3.9322	8.57E-05	-0.67206191	count	1
SEMA3E	-0.9213032	0.3196442	-2.8823	0.00397	-0.671858171	count	1
GSTT2B	-0.4808632	0.0871562	-5.5173	3.68E-08	-0.671141298	count	0.000863659
APLP1	-0.6438734	0.2629702	-2.4485	0.0144	-0.670934121	count	1

SLC35F1	-1.0157387	0.3974758	-2.5555	0.0106	-0.670783619	count	1
LINC00327	-2.116126	0.9526674	-2.2213	0.0264	-0.670578812	count	1
KLRC1	-2.116126	1.682196	-1.258	0.208	-0.670578812	count	1
SESN3	-0.4954106	0.1001974	-4.9443	7.98E-07	-0.670470835	count	0.018584622
ZNF512	-0.5335621	0.1572495	-3.3931	0.000698	-0.669777831	count	1
CKMT2	-0.5503658	0.150955	-3.6459	0.00027	-0.669776761	count	1
DAPK2	-1.2735329	0.5342543	-2.3838	0.0172	-0.669241737	count	1
EPHA4	-0.780932	0.2705635	-2.8863	0.00392	-0.669150456	count	1
TAGLN	-0.4635139	0.0362971	-12.77	1.43E-36	-0.668531607	count	3.46E-32
AC062017.1	-0.7982204	0.3883828	-2.0552	0.0399	-0.666587749	count	1
DOK2	-2.0998946	1.000448	-2.099	0.0359	-0.666384095	count	1
CDKN2D	-0.4887907	0.1134124	-4.3099	1.68E-05	-0.666316136	count	0.386316
LINC01094	-0.9568334	0.7148466	-1.3385	0.181	-0.666258267	count	1
PARP12	-0.652184	0.2813306	-2.3182	0.0205	-0.666170863	count	1
RELB	-0.5371599	0.2420042	-2.2196	0.0265	-0.66559203	count	1
PLS3	-0.4644274	0.0391173	-11.8727	6.26E-32	-0.665482737	count	1.51E-27
AC018647.2	-0.7965506	0.3241354	-2.4575	0.014	-0.665152707	count	1
LINC02447	-0.875121	0.3626579	-2.4131	0.0159	-0.664135678	count	1
MMP28	-0.6570254	0.3042509	-2.1595	0.0309	-0.663823422	count	1
ARHGAP31	-0.6311198	0.3159555	-1.9975	0.0458	-0.663492781	count	1
PLXNA2	-0.5248987	0.1954133	-2.6861	0.00726	-0.663108057	count	1
AL080317.3	-0.9086249	0.6483933	-1.4013	0.161	-0.662330748	count	1
TCAP	-2.0839489	0.642821	-3.2419	0.0012	-0.662231038	count	1
ZNF503-AS1	-1.0029193	0.4208672	-2.383	0.0172	-0.662076048	count	1
LAMB1	-0.5120696	0.1784348	-2.8698	0.00413	-0.661874542	count	1
BOP1	-0.5786297	0.1936927	-2.9874	0.00283	-0.661551452	count	1
ANO4	-0.8710255	0.7071133	-1.2318	0.218	-0.660931169	count	1
CX3CL1	-0.4875177	0.1315594	-3.7057	0.000214	-0.65998069	count	1
AC015909.2	-1.2544974	0.6743348	-1.8603	0.0629	-0.659151644	count	1
PHACTR1	-0.5818166	0.2333181	-2.4937	0.0127	-0.65861485	count	1
PREX1	-0.5568205	0.1901986	-2.9276	0.00344	-0.657633457	count	1
SMTN	-0.4903813	0.1377325	-3.5604	0.000375	-0.657399672	count	1
ADAMTS8	-0.7061204	0.3005499	-2.3494	0.0189	-0.656304422	count	1
CEP170B	-0.6135465	0.3056511	-2.0073	0.0448	-0.655733124	count	1
SFI1	-0.6420258	0.3340278	-1.9221	0.0547	-0.655579288	count	1
FAM193B	-0.6236839	0.265945	-2.3452	0.0191	-0.655524762	count	1
PALLD	-0.4582756	0.0423273	-10.8269	6.52E-27	-0.655523439	count	1.57E-22
NALCN	-0.7474321	0.4140395	-1.8052	0.0711	-0.655142406	count	1
GABBR1	-0.5308501	0.2461594	-2.1565	0.0311	-0.654945887	count	1
FAM178B	-1.245977	0.5107607	-2.4395	0.0148	-0.654625213	count	1
AC093673.1	-0.5158371	0.1589401	-3.2455	0.00118	-0.654502706	count	1
BEX2	-0.4941171	0.2399581	-2.0592	0.0395	-0.654485799	count	1
MSRB3	-0.4592648	0.0515786	-8.9042	8.29E-19	-0.653994201	count	1.99E-14
ENKUR	-1.630852	0.9311253	-1.7515	0.0799	-0.653914905	count	1
GLIS3	-0.5362478	0.1919879	-2.7931	0.00525	-0.652413523	count	1

SYNDIG1L	-0.9369389	0.6751091	-1.3878	0.165	-0.651978754	count	1
TNS3	-0.4849956	0.1623765	-2.9869	0.00284	-0.651342841	count	1
ZNF524	-0.4764252	0.1048482	-4.544	5.70E-06	-0.650464978	count	0.1316472
RPS10	-0.4561329	0.0390863	-11.6699	6.35E-31	-0.650418431	count	1.53E-26
TCEAL3	-0.455914	0.0524596	-8.6908	5.34E-18	-0.650338065	count	1.28E-13
FAM198B-AS1	-0.5840952	0.3334451	-1.7517	0.0799	-0.650236254	count	1
LHPP	-0.5383809	0.1969748	-2.7332	0.0063	-0.649788761	count	1
C1orf115	-1.0481314	0.3345974	-3.1325	0.00175	-0.649761482	count	1
PFKFB3	-0.5206445	0.183673	-2.8346	0.00461	-0.648745465	count	1
GRAMD1A	-0.5067976	0.1605642	-3.1564	0.00161	-0.647368795	count	1
SAA2	-1.3826567	0.9169406	-1.5079	0.132	-0.647203211	count	1
SMAGP	-0.5686123	0.307213	-1.8509	0.0643	-0.64674873	count	1
ADAMDEC1	-3.2217065	1.81446	-1.7756	0.0759	-0.64652334	count	1
HTR4	-1.6110459	0.6261216	-2.5731	0.0101	-0.646382541	count	1
DDIT4L	-0.6944024	0.2087123	-3.3271	0.000886	-0.645133878	count	1
HIST1H2BC	-0.9270996	0.557838	-1.662	0.0966	-0.644911267	count	1
NUDT22	-0.4783201	0.1108488	-4.3151	1.64E-05	-0.644796981	count	0.3771836
PEBP4	-0.6029624	0.2522156	-2.3907	0.0169	-0.64421286	count	1
ZNF460-AS1	-1.6053248	0.7884727	-2.036	0.0418	-0.644198372	count	1
CALM3	-0.4531841	0.0476698	-9.5067	3.44E-21	-0.644189302	count	8.27E-17
STMN1	-0.4575029	0.1384031	-3.3056	0.000957	-0.644025043	count	1
PKIG	-0.4526751	0.0557707	-8.1167	6.47E-16	-0.643610344	count	1.55E-11
LYNX1	-0.5193197	0.1772631	-2.9297	0.00341	-0.64327686	count	1
GCNT1	-1.2241441	0.5986043	-2.045	0.0409	-0.642999623	count	1
MBNL1-AS1	-0.4550569	0.0857663	-5.3058	1.19E-07	-0.642576462	count	0.002785433
AC116407.2	-0.6360255	0.3520604	-1.8066	0.0709	-0.642154126	count	1
AMN1	-0.5160935	0.1579488	-3.2675	0.0011	-0.64179269	count	1
MST1	-1.035415	0.591976	-1.7491	0.0804	-0.64164779	count	1
RAB29	-0.4783853	0.1518652	-3.1501	0.00165	-0.641544605	count	1
AC108718.1	-1.3691085	0.6117788	-2.2379	0.0253	-0.640891366	count	1
DOCK5	-0.4987626	0.1803076	-2.7662	0.0057	-0.640212706	count	1
AL161421.1	-0.6872178	0.3464006	-1.9839	0.0473	-0.638285495	count	1
SHISA4	-0.4622243	0.0840892	-5.4968	4.13E-08	-0.638239774	count	0.000968898
GIN1	-0.5302806	0.2037526	-2.6026	0.00929	-0.63619845	count	1
PMEPA1	-0.4462479	0.0608673	-7.3315	2.79E-13	-0.635939392	count	6.64E-09
MASP1	-0.5860966	0.368791	-1.5892	0.112	-0.635522453	count	1
MRPL49	-0.4680099	0.1116886	-4.1903	2.85E-05	-0.63519092	count	0.6533055
CSF3R	-0.9135597	0.6632925	-1.3773	0.169	-0.635180675	count	1
SEZ6L2	-0.6722439	0.3647944	-1.8428	0.0654	-0.634702311	count	1
PTPA	-0.4770879	0.1301979	-3.6643	0.000251	-0.634505424	count	1
HSPB7	-0.4526062	0.1050033	-4.3104	1.67E-05	-0.634233342	count	0.3840499
FRK	-0.5633733	0.2449667	-2.2998	0.0215	-0.634020156	count	1
METAP1	-0.4868387	0.1314611	-3.7033	0.000216	-0.633440838	count	1
AL450332.1	-0.8350145	0.3816838	-2.1877	0.0288	-0.632739633	count	1
ETV2	-0.8055938	0.3704663	-2.1745	0.0297	-0.632530697	count	1

TMEM47	-0.4462702	0.073065	-6.1078	1.12E-09	-0.632119748	count	2.64E-05
ICAM3	-0.5139589	0.1547559	-3.3211	0.000905	-0.631092903	count	1
COL3A1	-0.4403003	0.1079469	-4.0789	4.62E-05	-0.630226719	count	1
MFAP3L	-0.7046383	0.4269434	-1.6504	0.0989	-0.630226711	count	1
RIPK3	-1.9629149	1.1797973	-1.6638	0.0962	-0.629677349	count	1
PTMS	-0.4388511	0.0334045	-13.1375	1.47E-38	-0.629591819	count	3.56E-34
FAM166B	-1.3448701	0.5387727	-2.4962	0.0126	-0.629556244	count	1
ADIRF	-0.4369308	0.0314947	-13.8732	1.08E-42	-0.629475892	count	2.62E-38
AL606760.3	-1.09354	0.5628983	-1.9427	0.0521	-0.628758981	count	1
PCLAF	-1.09354	0.7808996	-1.4004	0.161	-0.628758981	count	1
C1QL4	-1.196092	0.513598	-2.3288	0.0199	-0.628008065	count	1
ITGA1	-0.4458572	0.1009765	-4.4155	1.04E-05	-0.627865783	count	0.2395744
SPSB2	-0.5089657	0.1787035	-2.8481	0.00442	-0.627682933	count	1
PCDH10	-0.4659081	0.1204413	-3.8683	0.000111	-0.627482129	count	1
CDK6	-0.5262962	0.236648	-2.224	0.0262	-0.627460165	count	1
PGR	-0.4803233	0.1428027	-3.3635	0.000777	-0.627164617	count	1
DENND3	-0.7328252	0.4458326	-1.6437	0.1	-0.626760733	count	1
ESM1	-1.558055	0.7381179	-2.1108	0.0349	-0.62601264	count	1
RPS19	-0.4345889	0.0253837	-17.1208	2.81E-63	-0.625937058	count	6.82E-59
EEF2KMT	-0.6199729	0.2514483	-2.4656	0.0137	-0.625594707	count	1
VIM	-0.4335081	0.0202375	-21.421	5.16E-96	-0.625309266	count	1.25E-91
CD3G	-2.9904324	1.427783	-2.0945	0.0363	-0.622920068	count	1
RAB13	-0.436276	0.0466722	-9.3477	1.51E-20	-0.622899272	count	3.63E-16
AC090825.1	-1.0059326	0.5949132	-1.6909	0.0909	-0.622806183	count	1
MIB1	-0.4733353	0.1397447	-3.3871	0.000714	-0.622018591	count	1
RGS7BP	-0.4447538	0.1107767	-4.0149	6.07E-05	-0.621824715	count	1
PPFIA3	-0.6227824	0.3310928	-1.881	0.0601	-0.621078888	count	1
ANTXR1	-0.4401488	0.0708033	-6.2165	5.65E-10	-0.620952247	count	1.34E-05
TMEM191B	-1.0019925	0.516103	-1.9415	0.0523	-0.620285192	count	1
AP001267.3	-0.9410071	0.4460399	-2.1097	0.035	-0.619922223	count	1
ANO5	-0.851718	0.3857117	-2.2082	0.0273	-0.619513621	count	1
EVI2A	-1.5405237	0.4754094	-3.2404	0.0012	-0.619206083	count	1
PGM5	-0.4767246	0.1332508	-3.5776	0.000351	-0.619016559	count	1
CCDC7	-0.7072742	0.2740361	-2.581	0.00989	-0.618954852	count	1
FAM129A	-0.4367998	0.0727342	-6.0054	2.09E-09	-0.618762064	count	4.93E-05
GATA2	-0.5258773	0.3001312	-1.7522	0.0798	-0.618395255	count	1
FBXO28	-0.4911482	0.208232	-2.3587	0.0184	-0.618148323	count	1
SNRPN	-0.4338216	0.0534628	-8.1145	6.59E-16	-0.616195032	count	1.57E-11
LRRC23	-0.4727288	0.1675069	-2.8221	0.0048	-0.616093253	count	1
JMJD1C-AS1	-0.9952697	0.4710047	-2.1131	0.0347	-0.615982247	count	1
ME1	-0.5605051	0.1885037	-2.9734	0.00296	-0.615763027	count	1
LINC02269	-0.6753644	0.3257002	-2.0736	0.0382	-0.615541373	count	1
DTD2	-0.6424235	0.2945469	-2.1811	0.0292	-0.615451816	count	1
PRR7	-0.623803	0.3831477	-1.6281	0.104	-0.614273991	count	1
4-Sep	-0.4387008	0.0801208	-5.4755	4.66E-08	-0.613132837	count	0.00109291

IRX3	-0.5610073	0.2821988	-1.988	0.0469	-0.612172759	count	1
LEFTY2	-0.520614	0.2381026	-2.1865	0.0288	-0.612125953	count	1
PTGER2	-0.6711837	0.3941486	-1.7029	0.0887	-0.611625403	count	1
AC018809.2	-1.5210613	0.919671	-1.6539	0.0982	-0.611612324	count	1
PWAR6	-0.5879836	0.3158129	-1.8618	0.0627	-0.611574541	count	1
LINC00670	-0.6126212	0.3841591	-1.5947	0.111	-0.610714883	count	1
AMBRA1	-0.7143693	0.3642403	-1.9613	0.0499	-0.610499498	count	1
BARD1	-0.4701982	0.1378702	-3.4104	0.000656	-0.610485793	count	1
P3H2	-0.6467192	0.6714354	-0.9632	0.336	-0.609992968	count	1
S1PR3	-0.5761739	0.1776557	-3.2432	0.00119	-0.609975744	count	1
TIMP4	-0.4653825	0.1914098	-2.4313	0.0151	-0.609583982	count	1
IHH	-1.889239	0.7175362	-2.633	0.0085	-0.608993868	count	1
SYK	-1.889239	0.7175362	-2.633	0.0085	-0.608993868	count	1
CDT1	-1.889239	0.7175362	-2.633	0.0085	-0.608993868	count	1
AC093484.3	-1.889239	0.796667	-2.3714	0.0178	-0.608993868	count	1
ZNF229	-0.9249632	0.5732059	-1.6137	0.107	-0.608976412	count	1
IL17RE	-0.5474078	0.2769883	-1.9763	0.0482	-0.60874791	count	1
RGS12	-0.5250301	0.2836847	-1.8508	0.0643	-0.607650813	count	1
AL020996.1	-1.1580817	0.5345785	-2.1663	0.0303	-0.607604734	count	1
TPX2	-1.0574757	0.5271823	-2.0059	0.0449	-0.607401964	count	1
ABHD17B	-0.7287007	0.248065	-2.9375	0.00333	-0.606830328	count	1
CPLANE2	-0.7101377	0.4688014	-1.5148	0.13	-0.606771516	count	1
REEP2	-0.5121731	0.219103	-2.3376	0.0195	-0.606378467	count	1
ZNF793	-0.6932698	0.2862401	-2.422	0.0155	-0.606337972	count	1
BCAR1	-0.452873	0.1562797	-2.8978	0.00378	-0.606308602	count	1
PAX9	-0.6330582	0.4088935	-1.5482	0.122	-0.606254842	count	1
ZNF587B	-0.7711328	0.4484159	-1.7197	0.0856	-0.604582511	count	1
GPR68	-1.1513606	0.8325385	-1.383	0.167	-0.603987012	count	1
ATG16L1	-0.8699463	0.2924565	-2.9746	0.00295	-0.603805401	count	1
AC068491.3	-0.7695184	0.8609801	-0.8938	0.372	-0.603273158	count	1
RIPK4	-0.7688429	0.4386086	-1.7529	0.0797	-0.602725317	count	1
OLA1	-0.4354776	0.0822984	-5.2914	1.28E-07	-0.602461043	count	0.00299584
TRAF2	-0.8287657	0.3116818	-2.659	0.00787	-0.602227312	count	1
SLC44A5	-1.0486841	0.4676926	-2.2423	0.025	-0.602185612	count	1
TMEM74B	-1.0486841	0.4905849	-2.1376	0.0326	-0.602185612	count	1
AOC3	-0.4383497	0.1015213	-4.3178	1.62E-05	-0.602130764	count	0.3726162
SCN1A	-1.147514	0.553877	-2.0718	0.0384	-0.601915362	count	1
BIRC3	-0.5096692	0.2019186	-2.5241	0.0116	-0.601268444	count	1
AL160272.1	-1.2835605	0.5765107	-2.2264	0.026	-0.600654587	count	1
PDP2	-0.7937956	0.3384317	-2.3455	0.0191	-0.600449695	count	1
TOMM7	-0.4175318	0.0271276	-15.3914	7.56E-52	-0.599647959	count	1.83E-47
PDE5A	-0.4178849	0.0638383	-6.546	6.73E-11	-0.599602659	count	1.60E-06
LTBP1	-0.4179189	0.0521057	-8.0206	1.40E-15	-0.599337127	count	3.34E-11
ZC3H8	-0.4547757	0.1376992	-3.3027	0.000967	-0.599254613	count	1
MIR193BHG	-0.5079366	0.268989	-1.8883	0.0591	-0.599198328	count	1

CEMIP	-1.2793	0.4239719	-3.0174	0.00257	-0.59863474	count	1
SPARCL1	-0.4151542	0.0425648	-9.7535	3.31E-22	-0.598072313	count	7.96E-18
ACOT4	-0.9670122	0.4744275	-2.0383	0.0416	-0.597877046	count	1
FGF13-AS1	-0.9079528	0.3655834	-2.4836	0.0131	-0.597363608	count	1
PLD5	-0.5439135	0.2609354	-2.0845	0.0372	-0.597225652	count	1
TNS1	-0.4223092	0.0721735	-5.8513	5.30E-09	-0.596485817	count	0.000124831
NAA40	-0.6547488	0.4091799	-1.6001	0.11	-0.596234223	count	1
LIMK2	-0.642208	0.2962452	-2.1678	0.0302	-0.595398712	count	1
CYLD	-0.4524394	0.1317359	-3.4344	6.00E-04	-0.595292481	count	1
SLC41A1	-0.4788856	0.1634627	-2.9296	0.00341	-0.595100711	count	1
ALOX5	-2.758931	1.2178003	-2.2655	0.0235	-0.594870745	count	1
AL162231.1	-0.5139792	0.2059532	-2.4956	0.0126	-0.594683464	count	1
MARVELD2	-0.8183239	0.5442256	-1.5036	0.133	-0.594361368	count	1
TRIM68	-0.7345166	0.3433988	-2.139	0.0325	-0.59409664	count	1
GUCY1A1	-0.4157784	0.0515677	-8.0628	1.00E-15	-0.593987921	count	2.39E-11
ARSE	-0.8177903	0.5582943	-1.4648	0.143	-0.593959392	count	1
IRF6	-0.5660766	0.3371817	-1.6788	0.0933	-0.593832961	count	1
STAP2	-0.6943829	0.3561682	-1.9496	0.0513	-0.592893701	count	1
GALNT13	-0.7835002	0.3330746	-2.3523	0.0187	-0.592383113	count	1
COL14A1	-0.4119439	0.0400894	-10.2756	1.94E-24	-0.591771387	count	4.67E-20
TPD52L1	-0.4202074	0.0977083	-4.3006	1.75E-05	-0.591414691	count	0.4023425
AL512791.2	-1.2639758	0.8146438	-1.5516	0.121	-0.591357998	count	1
PPP1R13L	-0.4730086	0.2042704	-2.3156	0.0206	-0.591045964	count	1
TOM1L1	-0.5007478	0.190009	-2.6354	0.00844	-0.590610026	count	1
EHD4	-0.4770965	0.1875733	-2.5435	0.011	-0.590488423	count	1
AC007920.2	-1.1262974	0.4717994	-2.3872	0.017	-0.590472166	count	1
AL512329.2	-0.5999483	0.2800344	-2.1424	0.0322	-0.59023454	count	1
MCM8	-0.6752093	0.5552737	-1.216	0.224	-0.590071018	count	1
CSRP1	-0.4104424	0.0442228	-9.2812	2.79E-20	-0.589724573	count	6.70E-16
SH2D3C	-1.2559411	0.5467529	-2.2971	0.0217	-0.58753566	count	1
CYBB	-2.7035845	1.3954274	-1.9375	0.0528	-0.587462557	count	1
PMAIP1	-1.4591756	0.5402183	-2.7011	0.00694	-0.587214261	count	1
NECTIN3-AS1	-1.254726	0.9474398	-1.3243	0.185	-0.586957018	count	1
TBX18	-0.5114014	0.2071106	-2.4692	0.0136	-0.586398437	count	1
RFX5	-0.4989945	0.2475787	-2.0155	0.0439	-0.586382023	count	1
AC009163.7	-1.2529701	0.4711688	-2.6593	0.00786	-0.586121091	count	1
FDX2	-0.4935832	0.2723226	-1.8125	0.07	-0.586079073	count	1
CHRNB1	-0.4833525	0.1715813	-2.817	0.00487	-0.585861252	count	1
HOPX	-0.5370879	0.2547594	-2.1082	0.0351	-0.585619374	count	1
VSIG8	-0.9471167	0.696781	-1.3593	0.174	-0.585113425	count	1
TC2N	-0.4608057	0.19066	-2.4169	0.0157	-0.584981352	count	1
TGFB1I1	-0.4091918	0.0439166	-9.3175	2.00E-20	-0.584850415	count	4.80E-16
GAL3ST1	-1.2502545	0.7007534	-1.7842	0.0745	-0.58482751	count	1
TSPAN9	-0.4680635	0.1356219	-3.4512	0.000564	-0.584810712	count	1
SMIM10	-0.4156381	0.0777015	-5.3492	9.38E-08	-0.584426684	count	0.00219689

DUSP7	-0.519883	0.2254936	-2.3055	0.0212	-0.584334631	count	1
KCND3	-0.6414627	0.3631543	-1.7664	0.0774	-0.583795951	count	1
SH2B3	-0.5672052	0.3441275	-1.6482	0.0994	-0.583727974	count	1
AC147651.1	-0.58586	0.2677924	-2.1877	0.0288	-0.583432103	count	1
SLC5A3	-0.4487884	0.1975954	-2.2712	0.0232	-0.583068021	count	1
ST20	-0.5547315	0.2936517	-1.8891	0.059	-0.58169377	count	1
CSDC2	-0.4417007	0.1248511	-3.5378	0.000408	-0.581508224	count	1
ASRGL1	-1.4437131	0.6655607	-2.1692	0.0301	-0.581061279	count	1
MXI1	-0.4118196	0.0702323	-5.8637	4.93E-09	-0.580854946	count	0.000116146
AJM1	-1.0118651	0.4195173	-2.412	0.0159	-0.580302201	count	1
DKK3	-0.4052164	0.0456378	-8.879	1.04E-18	-0.579997723	count	2.49E-14
RTN4R	-0.569232	0.416389	-1.3671	0.172	-0.579739132	count	1
MIR29B2CHG	-0.4631315	0.2700375	-1.7151	0.0864	-0.579626456	count	1
DEPTOR	-0.5391793	0.3304702	-1.6316	0.103	-0.579406105	count	1
RNF24	-0.4218424	0.1261215	-3.3447	0.000832	-0.579386954	count	1
SHROOM3	-0.4347735	0.1725315	-2.52	0.0118	-0.578939392	count	1
AL645728.1	-0.9371088	0.5200878	-1.8018	0.0717	-0.578688752	count	1
ADAMTS9-AS2	-0.4890475	0.1877338	-2.605	0.00922	-0.578662496	count	1
RIN3	-1.0088827	0.3495353	-2.8864	0.00392	-0.578527219	count	1
SNHG18	-0.4184092	0.0991282	-4.2209	2.49E-05	-0.578518123	count	0.5712309
PTMA	-0.4008193	0.0229408	-17.4719	1.00E-65	-0.577798848	count	2.43E-61
CCL27	-1.4343215	0.6353845	-2.2574	0.024	-0.577313484	count	1
RNF144B	-1.4343215	0.6398704	-2.2416	0.025	-0.577313484	count	1
FCGR1A	-0.6941874	0.3228935	-2.1499	0.0316	-0.577172276	count	1
FGD6	-0.8329402	0.3049669	-2.7312	0.00634	-0.577157669	count	1
RGS19	-0.5552256	0.2609701	-2.1275	0.0334	-0.576817541	count	1
GPSM3	-0.646172	0.3230508	-2.0002	0.0456	-0.57648327	count	1
ZNF747	-0.5101851	0.2175451	-2.3452	0.0191	-0.576360472	count	1
FANCE	-0.7625734	0.2913521	-2.6174	0.0089	-0.575987123	count	1
ZNF766	-0.4442063	0.1384591	-3.2082	0.00135	-0.575953467	count	1
GRAMD1C	-1.0980525	0.7793649	-1.4089	0.159	-0.575199086	count	1
FOXC2	-0.4069207	0.0748849	-5.4339	5.87E-08	-0.574942181	count	0.001376104
RASSF3	-0.4273067	0.1141537	-3.7433	0.000184	-0.57479407	count	1
RGS14	-0.5773231	0.4107655	-1.4055	0.16	-0.574733196	count	1
TMEM238	-0.5348102	0.2071398	-2.5819	0.00986	-0.574623517	count	1
PGAM2	-0.4550673	0.2705731	-1.6819	0.0927	-0.574194791	count	1
AL024508.2	-0.7914782	0.4749611	-1.6664	0.0957	-0.574135878	count	1
DDO	-0.6727307	0.3123301	-2.1539	0.0313	-0.57382758	count	1
RPS21	-0.3995847	0.0277594	-14.3946	9.66E-46	-0.573750399	count	2.34E-41
LRRC8A	-0.4407241	0.1585066	-2.7805	0.00546	-0.573598143	count	1
HIST1H1A	-0.4336299	0.1979687	-2.1904	0.0286	-0.572825772	count	1
HBEGF	-0.5185111	0.3125744	-1.6588	0.0972	-0.57255257	count	1
WNK3	-0.5277629	0.2709761	-1.9476	0.0515	-0.571183487	count	1
PLA2G5	-0.4906989	0.1660942	-2.9543	0.00315	-0.56977731	count	1
LINC01091	-1.4150781	0.5462992	-2.5903	0.00963	-0.569609784	count	1

TSPAN10	-0.6261138	0.3325011	-1.883	0.0598	-0.569431971	count	1
HK1	-0.4206199	0.1280754	-3.2842	0.00103	-0.569077172	count	1
HLA-DQA1	-0.5220017	0.2320385	-2.2496	0.0245	-0.568881181	count	1
CCND3	-0.4258342	0.1190621	-3.5766	0.000353	-0.568209403	count	1
RPL36A	-0.4067952	0.068359	-5.9509	2.92E-09	-0.567994304	count	6.88E-05
HPSE2	-1.749235	0.527784	-3.3143	0.000928	-0.567981937	count	1
ZCCHC18	-0.819559	0.4970289	-1.6489	0.0993	-0.567518925	count	1
SMCO4	-0.445232	0.1496743	-2.9747	0.00295	-0.567403531	count	1
PRDM8	-1.081969	0.2724261	-3.9716	7.28E-05	-0.566483766	count	1
GPR155	-0.5129448	0.2315856	-2.2149	0.0268	-0.566302154	count	1
KIF13B	-0.4437669	0.1431896	-3.0992	0.00196	-0.56627137	count	1
GALNT3	-1.2113005	0.5072144	-2.3881	0.017	-0.566215635	count	1
ZNF711	-0.5192224	0.2180409	-2.3813	0.0173	-0.565798403	count	1
PFKP	-0.4343735	0.1475942	-2.943	0.00327	-0.5657969	count	1
SHB	-0.5683065	0.2700069	-2.1048	0.0354	-0.565548091	count	1
LINC00310	-0.5395184	0.232991	-2.3156	0.0206	-0.565422517	count	1
TNFAIP6	-0.4143412	0.2417995	-1.7136	0.0867	-0.564964469	count	1
LIX1L	-0.4119315	0.1078046	-3.8211	0.000135	-0.564751418	count	1
ZDHHC14	-0.4804965	0.2355978	-2.0395	0.0415	-0.564366169	count	1
SPATA13	-0.4992962	0.3474231	-1.4371	0.151	-0.563869867	count	1
CHAC1	-1.0758288	0.5084747	-2.1158	0.0344	-0.563153318	count	1
GPM6B	-0.4172386	0.1185084	-3.5208	0.000436	-0.562351886	count	1
NPTXR	-0.5719904	0.4064495	-1.4073	0.159	-0.562080873	count	1
AL121832.2	-0.9106882	0.4992485	-1.8241	0.0682	-0.561716296	count	1
RPL15	-0.3899748	0.0211309	-18.4552	8.51E-73	-0.56168705	count	2.07E-68
GALNT7	-0.4409579	0.1676753	-2.6298	0.00858	-0.56115388	count	1
CSRP2	-0.3939107	0.0673663	-5.8473	5.43E-09	-0.560833529	count	0.000127877
A1BG-AS1	-0.5259419	0.2603813	-2.0199	0.0435	-0.560463234	count	1
NXNL2	-0.7425757	0.3458694	-2.147	0.0319	-0.560321516	count	1
LINC00987	-0.9083623	0.502881	-1.8063	0.071	-0.560221477	count	1
SGCG	-0.422762	0.1979195	-2.136	0.0327	-0.559855651	count	1
CHST11	-0.4717455	0.2055159	-2.2954	0.0218	-0.559835193	count	1
RPS16	-0.388903	0.0219505	-17.7172	1.85E-67	-0.559774433	count	4.49E-63
RPS11	-0.3891273	0.0230673	-16.8693	1.50E-61	-0.55961997	count	3.64E-57
HAAO	-0.4179689	0.1121207	-3.7278	0.000196	-0.558813453	count	1
NAP1L1	-0.3906792	0.0387605	-10.0793	1.38E-23	-0.558507346	count	3.32E-19
HDAC1	-0.4121452	0.1266762	-3.2535	0.00115	-0.558154436	count	1
MT1H	-1.716466	0.8799621	-1.9506	0.0512	-0.558077418	count	1
FGFRL1	-0.4561067	0.2228814	-2.0464	0.0408	-0.558064466	count	1
AL445472.1	-0.5158465	0.2843605	-1.8141	0.0698	-0.55805361	count	1
ADCY5	-0.511539	0.2085439	-2.4529	0.0142	-0.557277496	count	1
PTPN21	-0.4383927	0.193889	-2.2611	0.0238	-0.557091954	count	1
DUS4L	-0.541458	0.2445398	-2.2142	0.0269	-0.556675846	count	1
ABCA7	-1.7109803	0.8449887	-2.0249	0.043	-0.556408569	count	1
CENPM	-1.7109803	1.0267081	-1.6665	0.0957	-0.556408569	count	1

A1BG	-0.4275028	0.1284711	-3.3276	0.000885	-0.556280817	count	1
TBC1D2	-0.7674388	0.384137	-1.9978	0.0458	-0.55602522	count	1
RBP7	-0.5652927	0.4902774	-1.153	0.249	-0.555340187	count	1
TLCD1	-0.6511044	0.5798154	-1.123	0.262	-0.554794345	count	1
LEAP2	-1.0598681	0.5764342	-1.8387	0.066	-0.554488246	count	1
BMF	-1.0598681	0.5907176	-1.7942	0.0729	-0.554488246	count	1
GSDMB	-1.1868138	0.7852547	-1.5114	0.131	-0.554465339	count	1
AL138963.3	-1.1868138	0.8418608	-1.4098	0.159	-0.554465339	count	1
AC010680.2	-1.1868138	0.9529305	-1.2454	0.213	-0.554465339	count	1
AC130343.2	-0.5891798	0.8744834	-0.6737	0.501	-0.554346502	count	1
NLN	-0.6502457	0.3695692	-1.7595	0.0786	-0.554038835	count	1
WFDC1	-0.3993776	0.114693	-3.4821	0.000503	-0.553131431	count	1
CDKL2	-0.4509764	0.3634174	-1.2409	0.215	-0.553012889	count	1
KRBOX1	-0.452757	0.2098043	-2.158	0.031	-0.552593445	count	1
AC012360.3	-0.6199278	0.3450031	-1.7969	0.0724	-0.552383632	count	1
ARG2	-0.5779617	0.2804408	-2.0609	0.0394	-0.552195418	count	1
CDHR3	-0.5867929	0.5217439	-1.1247	0.261	-0.552040316	count	1
MPND	-0.4795312	0.2053413	-2.3353	0.0196	-0.551855318	count	1
C8orf44	-0.5546995	0.2914086	-1.9035	0.0571	-0.551692268	count	1
SCMH1	-0.4481642	0.177885	-2.5194	0.0118	-0.550778959	count	1
BCDIN3D-AS1	-0.7045362	0.5743507	-1.2267	0.22	-0.550585603	count	1
PRR34-AS1	-0.4204755	0.1184822	-3.5489	0.000392	-0.550420552	count	1
TEPP	-2.4511795	1.0974629	-2.2335	0.0256	-0.550060138	count	1
PKIB	-2.4511795	1.752925	-1.3983	0.1621	-0.550060138	count	1
FAM207A	-0.4210864	0.1204716	-3.4953	0.000479	-0.549837801	count	1
ZSCAN16	-0.5671624	0.2864875	-1.9797	0.0478	-0.549643699	count	1
DERL3	-0.8381137	0.4814838	-1.7407	0.0818	-0.549630818	count	1
PTPN3	-0.4976406	0.2679783	-1.857	0.0634	-0.549122901	count	1
HABP4	-0.3961508	0.0854034	-4.6386	3.63E-06	-0.548807905	count	0.08400183
DSTN	-0.3798189	0.0310247	-12.2425	8.35E-34	-0.547535037	count	2.02E-29
NUDT4B	-1.1722174	0.5220238	-2.2455	0.0248	-0.547443966	count	1
DGKA	-0.4463216	0.2455518	-1.8176	0.0692	-0.54724672	count	1
PRDM11	-0.4620303	0.2625699	-1.7596	0.0786	-0.546303505	count	1
CCDC189	-1.3572922	0.6730528	-2.0166	0.0438	-0.546289143	count	1
PALM3	-1.1694956	0.6164319	-1.8972	0.0579	-0.54613332	count	1
MFSD12	-0.4648241	0.1950241	-2.3834	0.0172	-0.545722201	count	1
MYL9	-0.3782799	0.0376732	-10.0411	2.01E-23	-0.545447316	count	4.84E-19
GZMH	-2.4204238	1.094336	-2.2118	0.027	-0.545088676	count	1
POLE4	-0.3888582	0.0646785	-6.0122	2.01E-09	-0.544751919	count	4.74E-05
ZNF33B	-0.4710951	0.174752	-2.6958	0.00705	-0.544398827	count	1
PDGFA	-0.382601	0.0641717	-5.9621	2.72E-09	-0.543599818	count	6.41E-05
DLEU2	-0.448409	0.240087	-1.8677	0.0619	-0.543056355	count	1
CYB5D1	-0.6096717	0.3151483	-1.9346	0.0531	-0.542971365	count	1
LY6G5C	-0.654042	0.3117031	-2.0983	0.0359	-0.542701741	count	1
SORD	-0.4712842	0.2127956	-2.2147	0.0268	-0.542229801	count	1

TSPO	-0.3776687	0.0327631	-11.5273	3.17E-30	-0.541903397	count	7.65E-26
KLRB1	-2.399501	0.6425869	-3.7341	0.000191	-0.541654696	count	1
AC096586.1	-1.6624778	0.8461624	-1.9647	0.0495	-0.541522324	count	1
ARPC5L	-0.3955429	0.1011796	-3.9093	9.42E-05	-0.541460564	count	1
MSRA	-0.4249436	0.1285036	-3.3069	0.000953	-0.541350891	count	1
LINC02478	-0.8780972	0.5874103	-1.4949	0.135	-0.540762689	count	1
ZDHHC23	-1.0337755	0.7784952	-1.3279	0.184	-0.540300071	count	1
TSR2	-0.3857327	0.0715158	-5.3937	7.34E-08	-0.540036964	count	0.001719689
NDUFA4L2	-0.3756698	0.0744737	-5.0443	4.77E-07	-0.539439317	count	0.011126979
ASL	-0.4249886	0.1628936	-2.609	0.00912	-0.539159844	count	1
CTF1	-0.426206	0.1667819	-2.5555	0.0106	-0.539133187	count	1
KLHL21	-0.4285064	0.1873626	-2.287	0.0223	-0.5386708	count	1
FRZB	-0.3740571	0.0545403	-6.8584	8.14E-12	-0.538607781	count	1.93E-07
KIAA1324	-1.652631	0.6931137	-2.3844	0.0172	-0.53847202	count	1
PLA2G6	-1.3376941	0.5493676	-2.435	0.0149	-0.538320066	count	1
KCTD14	-0.6043268	0.4305675	-1.4036	0.161	-0.538067664	count	1
MAP4K4	-0.4000123	0.1113285	-3.5931	0.000331	-0.537692531	count	1
RASGRF2	-0.6479266	0.3737024	-1.7338	0.083	-0.537454438	count	1
AC092807.3	-0.4194498	0.2283623	-1.8368	0.0663	-0.53705765	count	1
FHL2	-0.381791	0.0860694	-4.4358	9.44E-06	-0.537003778	count	0.21753536
RAVER2	-0.5176313	0.3195902	-1.6197	0.105	-0.536974166	count	1
AC009549.1	-0.7420681	0.5528753	-1.3422	0.18	-0.53691656	count	1
RBMXL1	-0.4219728	0.2008788	-2.1006	0.0357	-0.536807646	count	1
S100A16	-0.3798297	0.066293	-5.7296	1.09E-08	-0.536576506	count	0.00025639
HIST1H4A	-1.3330914	0.6795942	-1.9616	0.0499	-0.536444355	count	1
SSFA2	-0.3950361	0.1250673	-3.1586	0.0016	-0.535798832	count	1
AC096733.2	-0.7751754	0.5455587	-1.4209	0.155	-0.535548697	count	1
C11orf1	-0.4060326	0.1446661	-2.8067	0.00503	-0.535438461	count	1
INSIG2	-0.3868894	0.1028456	-3.7618	0.000171	-0.535238643	count	1
SNX25	-0.4609712	0.2054119	-2.2441	0.0249	-0.534798251	count	1
AP000866.2	-0.4690792	0.4322217	-1.0853	0.278	-0.534634998	count	1
MALL	-0.4689801	0.3516367	-1.3337	0.182	-0.534520369	count	1
IRX1	-0.8159644	0.2539351	-3.2133	0.00132	-0.534483984	count	1
PICK1	-0.4586029	0.1935706	-2.3692	0.0179	-0.534187258	count	1
HSD17B1	-0.7088033	0.3313356	-2.1392	0.0325	-0.533876989	count	1
AC022613.1	-2.3522028	0.6903169	-3.4074	0.000663	-0.533736394	count	1
LINC01303	-1.1434304	0.5273975	-2.1681	0.0302	-0.533561712	count	1
RAPGEF5	-0.4143078	0.1366466	-3.032	0.00245	-0.533549666	count	1
FHL3	-0.3917739	0.1137309	-3.4447	0.000578	-0.533331987	count	1
GPR183	-0.7719566	0.4076428	-1.8937	0.0583	-0.533230548	count	1
KCNK6	-0.4016165	0.2177088	-1.8447	0.0652	-0.5330258	count	1
PNMT	-2.3437867	1.0189694	-2.3002	0.0215	-0.532304856	count	1
SLC41A3	-0.3882091	0.0969958	-4.0023	6.40E-05	-0.531886223	count	1
AC073611.1	-1.3218006	0.6462381	-2.0454	0.0409	-0.53183655	count	1
ANKRD28	-0.4087167	0.1573236	-2.5979	0.00942	-0.531681628	count	1

CYTIP	-1.6296851	0.5422071	-3.0057	0.00267	-0.531328161	count	1
WIF1	-0.5478554	0.7544119	-0.7262	0.468	-0.530469293	count	1
CCDC152	-0.4158093	0.1980866	-2.0991	0.0359	-0.530329856	count	1
SVIP	-0.3815078	0.0903494	-4.2226	2.47E-05	-0.530139061	count	0.5667168
SPI1	-1.6251866	0.6477164	-2.5091	0.0121	-0.529921842	count	1
COL8A1	-0.3690857	0.102858	-3.5883	0.000337	-0.529358224	count	1
MGST2	-0.3796179	0.0945287	-4.0159	6.04E-05	-0.529256592	count	1
AP002884.1	-0.378783	0.103754	-3.6508	0.000265	-0.528706602	count	1
C1orf162	-0.6774058	0.4321625	-1.5675	0.117	-0.528608295	count	1
SSX2IP	-0.5138435	0.2477303	-2.0742	0.0381	-0.527689995	count	1
ZFP28	-0.6202797	0.3519632	-1.7623	0.0781	-0.527688504	count	1
MERTK	-0.544726	0.3004913	-1.8128	0.0699	-0.527362851	count	1
C6orf48	-0.3783006	0.0601323	-6.2911	3.52E-10	-0.527146855	count	8.33E-06
RCBTB2	-0.437397	0.1675339	-2.6108	0.00907	-0.526661797	count	1
CLDN7	-0.7623765	0.4825279	-1.58	0.114	-0.526331653	count	1
ZNF561	-0.4324812	0.2000259	-2.1621	0.0307	-0.526284244	count	1
FANCG	-0.5598525	0.3658538	-1.5303	0.126	-0.526025903	count	1
SMYD2	-0.459255	0.2271622	-2.0217	0.0433	-0.525778175	count	1
EHBP1L1	-0.3876667	0.1129134	-3.4333	0.000603	-0.525765849	count	1
ZNF597	-0.568764	0.4134558	-1.3756	0.169	-0.525529117	count	1
EVI2B	-1.1264503	0.6446235	-1.7475	0.0806	-0.5253533	count	1
ARHGEF17	-0.3906289	0.1328566	-2.9402	0.0033	-0.525250742	count	1
RAI14	-0.392104	0.1376059	-2.8495	0.0044	-0.525103494	count	1
SBSPON	-0.3995463	0.1194707	-3.3443	0.000833	-0.52492641	count	1
MED20	-0.6168106	0.3152695	-1.9565	0.0505	-0.52463994	count	1
VGLL4	-0.3702581	0.0633137	-5.848	5.41E-09	-0.52432403	count	0.000127411
TNFAIP8	-0.3949021	0.1214494	-3.2516	0.00116	-0.524068913	count	1
CENPBD1	-0.5888879	0.2945027	-1.9996	0.0456	-0.523909159	count	1
MTRNR2L10	-0.9172865	0.429275	-2.1368	0.0327	-0.523882769	count	1
BX537318.1	-0.5335838	0.4003442	-1.3328	0.183	-0.523451755	count	1
ALDH1L1	-0.5334797	0.4960653	-1.0754	0.282	-0.523347134	count	1
DNAJB5	-0.5047199	0.2148316	-2.3494	0.0189	-0.523303656	count	1
DBF4	-0.4808735	0.2799577	-1.7177	0.0859	-0.523292753	count	1
APOBEC3G	-0.4369944	0.1824273	-2.3954	0.0167	-0.523088506	count	1
GLIPR1	-0.3675081	0.0562182	-6.5372	7.13E-11	-0.522611917	count	1.69E-06
UQCRH	-0.3648807	0.0372159	-9.8044	2.03E-22	-0.522081163	count	4.88E-18
VAV3	-1.1193003	0.553941	-2.0206	0.0434	-0.521892741	count	1
EPB41L4A-AS1	-0.3776427	0.0762517	-4.9526	7.65E-07	-0.521845028	count	0.01781991
GIMAP7	-0.8484718	0.7077351	-1.1989	0.231	-0.521706208	count	1
HEY2	-0.3761683	0.0870992	-4.3188	1.61E-05	-0.521705293	count	0.3703483
PAX8	-0.7216795	0.5556027	-1.2989	0.194	-0.521566984	count	1
MAGEE2	-0.7216795	0.5991444	-1.2045	0.228	-0.521566984	count	1
RPL27	-0.3627569	0.0268549	-13.508	1.29E-40	-0.521055247	count	3.13E-36
AC021097.1	-0.9982825	0.7106015	-1.4048	0.16	-0.520961951	count	1
CD109	-0.404047	0.1379792	-2.9283	0.00343	-0.520820331	count	1

NINJ1	-0.3698319	0.0756716	-4.8873	1.07E-06	-0.520816364	count	0.02488606
RFC4	-0.4692479	0.2319036	-2.0235	0.0431	-0.520497409	count	1
CACNA1C	-0.401465	0.2744729	-1.4627	0.144	-0.520183104	count	1
PLEKHM3	-0.484319	0.245553	-1.9724	0.0486	-0.519406632	count	1
HMG20B	-0.3734495	0.0780484	-4.7848	1.78E-06	-0.519286245	count	0.04131202
GPR63	-1.1131328	0.4213767	-2.6417	0.00829	-0.518905874	count	1
BAX	-0.3681768	0.0686352	-5.3643	8.63E-08	-0.518736983	count	0.002021578
AC100810.1	-0.3854927	0.124558	-3.0949	0.00198	-0.518535127	count	1
AL136454.1	-0.9936611	0.4720078	-2.1052	0.0353	-0.518441244	count	1
TMEM144	-0.4358288	0.2163536	-2.0144	0.044	-0.518414297	count	1
HSPA1L	-1.5871626	0.7161309	-2.2163	0.0267	-0.517960718	count	1
ZNF780B	-0.4525077	0.3763242	-1.2024	0.229	-0.517942103	count	1
CTNNBIP1	-0.415645	0.1782711	-2.3315	0.0198	-0.51780305	count	1
TRMT9B	-0.4393187	0.2250549	-1.9521	0.051	-0.517290628	count	1
SORT1	-0.3773619	0.1034887	-3.6464	0.00027	-0.51721389	count	1
RPS23	-0.3584007	0.0188131	-19.0506	3.11E-77	-0.516711754	count	7.56E-73
NACC2	-0.4246902	0.197783	-2.1473	0.0318	-0.516703677	count	1
NUDT17	-0.5593238	0.3355761	-1.6668	0.0956	-0.516562733	count	1
AL354920.1	-0.9896981	0.4642202	-2.132	0.0331	-0.516279166	count	1
PODXL	-0.6620211	0.3420535	-1.9354	0.053	-0.516154202	count	1
AC110597.1	-2.2497059	1.0449966	-2.1528	0.0314	-0.515839053	count	1
EBF4	-0.6853638	0.3120981	-2.196	0.0282	-0.515536776	count	1
AC092835.1	-0.6608	0.4363639	-1.5143	0.13	-0.515166009	count	1
NRTN	-0.9876	0.5798596	-1.7032	0.0886	-0.515134418	count	1
NPW	-0.9876	0.7486343	-1.3192	0.187	-0.515134418	count	1
AC127496.5	-1.280525	0.6165139	-2.077	0.0379	-0.514916817	count	1
KIF23	-1.1046863	0.4813164	-2.2951	0.0218	-0.514812576	count	1
CTPS1	-0.4402288	0.1576536	-2.7924	0.00526	-0.514525323	count	1
AL136084.3	-0.9851282	0.4653738	-2.1169	0.0343	-0.513785546	count	1
PEX19	-0.3932935	0.1163901	-3.3791	0.000735	-0.513770421	count	1
KCTD15	-0.5239238	0.3162692	-1.6566	0.0977	-0.513745737	count	1
SAMD14	-0.6382659	0.5463272	-1.1683	0.243	-0.513694215	count	1
ABAT	-0.744564	0.4652463	-1.6004	0.11	-0.51350761	count	1
POLR2C	-0.3673831	0.0766012	-4.796	1.68E-06	-0.513140524	count	0.03900456
COL11A1	-0.5231394	0.7672927	-0.6818	0.495	-0.512957799	count	1
CAV1	-0.3565025	0.0375428	-9.4959	3.81E-21	-0.512493594	count	9.16E-17
RPL13A	-0.3559229	0.0219462	-16.218	3.51E-57	-0.512293729	count	8.52E-53
HSPB1	-0.3556455	0.0279862	-12.7079	3.07E-36	-0.512189092	count	7.43E-32
MX1	-0.3710205	0.1071549	-3.4625	0.000541	-0.511461693	count	1
CBL	-0.4074953	0.1982496	-2.0555	0.0399	-0.511182008	count	1
TMEM74	-0.7411901	0.4856689	-1.5261	0.127	-0.511079154	count	1
GYPE	-0.6170732	0.5845521	-1.0556	0.291	-0.510999366	count	1
CFAP221	-0.6170732	0.7282245	-0.8474	0.397	-0.510999366	count	1
DNHD1	-0.8300878	0.478631	-1.7343	0.0829	-0.509879205	count	1
ZBTB40	-0.4621029	0.2144968	-2.1544	0.0313	-0.509267996	count	1

AC011043.1	-0.6327174	0.3574888	-1.7699	0.0768	-0.509066951	count	1
AC092164.1	-0.3812666	0.1371892	-2.7791	0.00548	-0.508468071	count	1
TMEM38A	-0.4189326	0.2141219	-1.9565	0.0505	-0.50832096	count	1
GNG5	-0.3546571	0.0367354	-9.6544	8.54E-22	-0.508296251	count	2.05E-17
EZR	-0.3813652	0.1152118	-3.3101	0.000942	-0.507489133	count	1
FAM177A1	-0.3574934	0.058774	-6.0825	1.30E-09	-0.506939434	count	3.07E-05
ZNF236-DT	-1.5496906	0.9324226	-1.662	0.0966	-0.50604805	count	1
AC092053.3	-1.5496906	0.9498674	-1.6315	0.103	-0.50604805	count	1
ITM2C	-0.3587513	0.0751681	-4.7727	1.89E-06	-0.505838908	count	0.04385178
MTMR11	-0.4147727	0.1609219	-2.5775	0.00999	-0.505768583	count	1
RPL37A	-0.3514318	0.0240876	-14.5898	6.57E-47	-0.505528955	count	1.59E-42
ZNF443	-1.0855361	0.550112	-1.9733	0.0485	-0.505521038	count	1
RPS18	-0.3505701	0.020346	-17.2304	4.89E-64	-0.505211641	count	1.19E-59
TMIE	-1.5465477	0.5556203	-2.7835	0.00541	-0.505043429	count	1
ZBTB47	-0.4390362	0.2208705	-1.9878	0.0469	-0.504617608	count	1
CD163	-2.1879178	1.309791	-1.6704	0.0949	-0.504564567	count	1
DNM1P35	-2.1879178	1.3574289	-1.6118	0.107	-0.504564567	count	1
LTBP2	-0.3545591	0.0856227	-4.1409	3.54E-05	-0.504400877	count	0.8106954
AC083973.1	-1.5444734	0.914238	-1.6894	0.0912	-0.504379925	count	1
ADGRL3	-0.4238089	0.1904463	-2.2253	0.0261	-0.503947359	count	1
CRIP2	-0.3506133	0.0406544	-8.6242	9.45E-18	-0.50390552	count	2.26E-13
RSAD2	-0.5458397	0.333218	-1.6381	0.101	-0.503762407	count	1
AMPD2	-0.423645	0.194443	-2.1788	0.0294	-0.503750134	count	1
SEC14L2	-0.5453295	0.3532275	-1.5438	0.123	-0.50327826	count	1
POLA1	-0.5361396	0.306494	-1.7493	0.0803	-0.503153795	count	1
DDX49	-0.3999616	0.1322746	-3.0237	0.00251	-0.502491037	count	1
AL157395.1	-1.5381621	1.091685	-1.409	0.159	-0.502358961	count	1
IFI27L2	-0.3560238	0.0702841	-5.0655	4.27E-07	-0.502209783	count	0.00996191
AC079610.2	-0.7684848	0.5319969	-1.4445	0.149	-0.502021553	count	1
AP003059.1	-0.7684848	0.5444199	-1.4116	0.158	-0.502021553	count	1
LGALS1	-0.3481979	0.0219261	-15.8805	5.65E-55	-0.501871525	count	1.37E-50
AC092809.4	-1.2480574	0.8085381	-1.5436	0.123	-0.501530612	count	1
DYRK4	-0.3665132	0.1017998	-3.6003	0.000322	-0.50123451	count	1
MYL6B	-0.3568766	0.0744356	-4.7944	1.70E-06	-0.500236894	count	0.0394672
VCL	-0.3517567	0.0638285	-5.511	3.81E-08	-0.500010199	count	0.000894093
AC093157.1	-0.4914326	0.2839357	-1.7308	0.0836	-0.498876411	count	1
SLC38A5	-0.5736608	0.4729175	-1.213	0.225	-0.498768102	count	1
HTRA1	-0.3476526	0.0410534	-8.4683	3.55E-17	-0.498731069	count	8.50E-13
SDC4	-0.3672071	0.1158911	-3.1686	0.00154	-0.498401865	count	1
CFAP36	-0.3536649	0.0647273	-5.4639	4.97E-08	-0.498262072	count	0.001165465
MRVI1	-0.3564561	0.1084691	-3.2862	0.00102	-0.498026472	count	1
PVR	-0.5397889	0.2839408	-1.9011	0.0574	-0.498021275	count	1
DYSF	-0.8737868	0.7371232	-1.1854	0.236	-0.497876773	count	1
INAFM1	-0.3548356	0.0760049	-4.6686	3.14E-06	-0.497511386	count	0.07273182
CLP1	-0.4458353	0.217196	-2.0527	0.0402	-0.497056271	count	1

FAM27C	-0.4484197	0.2303525	-1.9467	0.0517	-0.497021927	count	1
LRRFIP2	-0.3540629	0.0698362	-5.0699	4.18E-07	-0.496927971	count	0.009752776
MIF4GD	-0.3645576	0.0993739	-3.6685	0.000247	-0.496861876	count	1
SZRD1	-0.3559799	0.0854478	-4.1661	3.17E-05	-0.495665151	count	0.7262153
LNX2	-0.5461693	0.3863235	-1.4138	0.158	-0.494748965	count	1
STARD10	-0.3601466	0.1106896	-3.2537	0.00115	-0.494721814	count	1
SLC25A4	-0.3447549	0.0494504	-6.9717	3.70E-12	-0.493942134	count	8.79E-08
ZNF682	-0.6143107	0.3255481	-1.887	0.0592	-0.493724782	count	1
ILK	-0.3453217	0.046158	-7.4813	9.16E-14	-0.493673743	count	2.18E-09
MZF1-AS1	-0.5449329	0.3233015	-1.6855	0.092	-0.49359612	count	1
PCA3	-2.1291472	0.7362279	-2.892	0.00385	-0.493505565	count	1
SSH1	-0.4028569	0.2363598	-1.7044	0.0884	-0.493440273	count	1
APOOL	-0.3655051	0.106491	-3.4323	0.000605	-0.493319174	count	1
CDK18	-0.5256003	0.2445965	-2.1488	0.0317	-0.492996889	count	1
AKAP5	-0.7545177	0.4220125	-1.7879	0.0739	-0.492476904	count	1
HIST1H2BB	-1.058563	0.8821691	-1.2	0.23	-0.492410286	count	1
HMGN1	-0.3465422	0.058972	-5.8764	4.57E-09	-0.492221265	count	0.000107678
TOP1MT	-0.7149501	0.3304177	-2.1638	0.0305	-0.49220001	count	1
MAML2	-0.3637854	0.1172143	-3.1036	0.00193	-0.491899724	count	1
AC105383.1	-0.5937836	0.3001487	-1.9783	0.048	-0.49105398	count	1
TEX41	-0.3706968	0.1302639	-2.8457	0.00446	-0.490877469	count	1
EMX2	-0.385199	0.201778	-1.909	0.0563	-0.490348242	count	1
HCCS	-0.3877573	0.2079929	-1.8643	0.0624	-0.490120084	count	1
CD163L1	-0.750673	0.4398563	-1.7066	0.088	-0.489850152	count	1
SLC26A4	-0.679356	0.3393572	-2.0019	0.0454	-0.489733223	count	1
SMIM4	-0.3612721	0.1111469	-3.2504	0.00116	-0.489676204	count	1
RPRML	-0.798263	0.5693678	-1.402	0.161	-0.489408032	count	1
MFSD6	-0.4876347	0.2984795	-1.6337	0.102	-0.489379275	count	1
CHST6	-1.4968329	0.5849775	-2.5588	0.0105	-0.489044737	count	1
DCT	-1.0515462	0.6224896	-1.6893	0.0913	-0.488995653	count	1
NRGN	-0.3436892	0.0832905	-4.1264	3.77E-05	-0.488790039	count	0.8628776
CASP1	-0.3910115	0.183933	-2.1258	0.0336	-0.488628484	count	1
MGP	-0.3386701	0.0458626	-7.3844	1.88E-13	-0.488547184	count	4.48E-09
RPL26	-0.339295	0.0218462	-15.5311	9.86E-53	-0.488497273	count	2.39E-48
ATG7	-0.4333124	0.1956991	-2.2142	0.0269	-0.488280431	count	1
LTA4H	-0.3645038	0.1107379	-3.2916	0.00101	-0.488212797	count	1
RUBCN	-0.4760606	0.2418001	-1.9688	0.049	-0.48808729	count	1
ARHGAP45	-1.0494677	0.9661422	-1.0862	0.277	-0.48798385	count	1
MS4A4A	-2.1003815	0.6200582	-3.3874	0.000713	-0.487974896	count	1
ARPP21	-1.493295	0.7936513	-1.8815	0.06	-0.487898623	count	1
AC022382.1	-1.493295	0.8157376	-1.8306	0.0672	-0.487898623	count	1
GHRL	-1.493295	0.9668718	-1.5445	0.123	-0.487898623	count	1
AC023509.4	-0.50461	0.3224057	-1.5651	0.118	-0.487582037	count	1
BST2	-0.3405828	0.0568608	-5.9898	2.30E-09	-0.487538334	count	5.42E-05
TTC39B	-0.3795272	0.1464619	-2.5913	0.0096	-0.487341914	count	1

NCK2	-0.3744336	0.1237798	-3.025	0.0025	-0.487262927	count	1
XK	-1.0478654	0.5487756	-1.9095	0.0563	-0.487203776	count	1
COLCA1	-0.6490012	0.2725244	-2.3814	0.0173	-0.48711596	count	1
ZNF746	-0.4747809	0.3312231	-1.4334	0.152	-0.486747236	count	1
MICAL2	-0.3432321	0.0953199	-3.6008	0.000321	-0.486251437	count	1
AC087164.1	-1.2090481	0.6914823	-1.7485	0.0805	-0.485366815	count	1
DLG3	-0.3782792	0.1433886	-2.6381	0.00837	-0.485155142	count	1
ZNF529	-0.5462595	0.2836665	-1.9257	0.0542	-0.484869311	count	1
PDLIM5	-0.3418477	0.0588665	-5.8072	6.89E-09	-0.484706117	count	0.000162184
GPC4	-0.3995807	0.1959803	-2.0389	0.0415	-0.484595594	count	1
CD48	-2.082055	0.4552789	-4.5731	4.96E-06	-0.484411359	count	0.11465536
RAPGEF6	-0.5162908	0.3807295	-1.3561	0.175	-0.484029969	count	1
PRKACB	-0.3652969	0.154514	-2.3642	0.0181	-0.483382611	count	1
TRAF3IP3	-2.0767754	1.169396	-1.7759	0.0758	-0.483379029	count	1
NFXL1	-0.4223106	0.2831016	-1.4917	0.136	-0.482897154	count	1
ANKS1B	-0.9276009	0.3352172	-2.7672	0.00568	-0.482358103	count	1
THRB	-0.3577413	0.154998	-2.308	0.0211	-0.482236942	count	1
NME4	-0.3392254	0.0536682	-6.3208	2.91E-10	-0.482014563	count	6.89E-06
AC040970.1	-1.4743282	0.7290833	-2.0222	0.0432	-0.481738439	count	1
PCP4	-1.4743282	0.8490895	-1.7364	0.0826	-0.481738439	count	1
AC002401.4	-1.4743282	1.235694	-1.1931	0.233	-0.481738439	count	1
IFI30	-0.8467932	0.4173009	-2.0292	0.0425	-0.481733734	count	1
ZNF781	-0.5545339	0.3891813	-1.4249	0.154	-0.481616176	count	1
VPREB3	-2.0671009	0.8961838	-2.3066	0.0211	-0.481480691	count	1
AL355472.1	-0.5134681	0.3076391	-1.6691	0.0952	-0.481312084	count	1
RRAD	-0.3391007	0.0872779	-3.8853	0.000104	-0.480694496	count	1
GAS6	-0.3344638	0.0421137	-7.9419	2.62E-15	-0.480372275	count	6.26E-11
CD53	-1.4694272	0.5733111	-2.5631	0.0104	-0.480142234	count	1
HAGLROS	-1.196092	0.8288684	-1.443	0.149	-0.479980767	count	1
HIST1H1B	-1.196092	0.8288684	-1.443	0.149	-0.479980767	count	1
AC083843.2	-1.196092	0.8288684	-1.443	0.149	-0.479980767	count	1
MYLIP	-0.3566416	0.1018223	-3.5026	0.000466	-0.479971098	count	1
PIN4	-0.3430364	0.0798758	-4.2946	1.80E-05	-0.479968566	count	0.413766
GALNT5	-0.4554691	0.3744206	-1.2165	0.224	-0.47996177	count	1
AC008443.5	-0.6396072	0.3807745	-1.6798	0.0931	-0.479781157	count	1
SCHIP1	-1.0323884	0.5391658	-1.9148	0.0556	-0.479664972	count	1
SGMS1-AS1	-0.7829452	0.3877852	-2.019	0.0436	-0.479558159	count	1
FGF13	-0.4076557	0.1817025	-2.2435	0.0249	-0.479556079	count	1
ZNF439	-0.4678138	0.2619904	-1.7856	0.0742	-0.479453104	count	1
PGM1	-0.3588312	0.1265059	-2.8365	0.00459	-0.479405681	count	1
DGKE	-0.4353028	0.2371767	-1.8354	0.0665	-0.479250936	count	1
AC006333.2	-0.391233	0.215698	-1.8138	0.0698	-0.479062621	count	1
AP1S2	-0.3472347	0.0998956	-3.476	0.000515	-0.478326518	count	1
AL359232.1	-1.4632482	0.7258796	-2.0158	0.0439	-0.478127272	count	1
CCDC141	-0.4243175	0.4786192	-0.8865	0.375	-0.477991433	count	1

ANO1	-0.3497193	0.1266294	-2.7618	0.00578	-0.477716456	count	1
PDK2	-0.3494098	0.1037895	-3.3665	0.000769	-0.477292314	count	1
KIFC3	-0.3863921	0.2502238	-1.5442	0.123	-0.477257585	count	1
SMCO3	-0.9181822	0.4801721	-1.9122	0.0559	-0.477207945	count	1
PAFAH1B3	-0.45262	0.2508385	-1.8044	0.0712	-0.476901022	count	1
TXNDC17	-0.3384945	0.0653173	-5.1823	2.31E-07	-0.47683667	count	0.005398008
OSGIN2	-0.3885084	0.1814815	-2.1408	0.0324	-0.476784624	count	1
TPGS1	-0.3402784	0.0733874	-4.6367	3.66E-06	-0.476216442	count	0.08468874
SPATA6L	-1.4572362	0.8086398	-1.8021	0.0716	-0.476164049	count	1
RPS6	-0.3304291	0.0222042	-14.8814	1.12E-48	-0.475889163	count	2.72E-44
GGCT	-0.3541699	0.1095492	-3.233	0.00124	-0.475822217	count	1
ZNF579	-0.3955413	0.1908427	-2.0726	0.0383	-0.475728372	count	1
MCAM	-0.3344096	0.0743614	-4.4971	7.10E-06	-0.475276578	count	0.1638609
FXYD1	-0.331211	0.0366584	-9.0351	2.59E-19	-0.475258576	count	6.21E-15
KIF1C	-0.3527856	0.1233958	-2.859	0.00427	-0.475149508	count	1
KCTD9	-0.3507815	0.1332937	-2.6316	0.00853	-0.475083506	count	1
AC009228.1	-0.835664	0.5193083	-1.6092	0.108	-0.475078417	count	1
GABRA4	-1.1836642	0.5792797	-2.0433	0.0411	-0.474806354	count	1
TPK1	-0.4311878	0.2606077	-1.6545	0.0981	-0.474645256	count	1
DLGAP4-AS1	-1.18324	0.8752465	-1.3519	0.176	-0.474629624	count	1
C1orf112	-0.9123684	0.4203282	-2.1706	0.03	-0.474028525	count	1
AL592295.4	-1.0202817	0.7964558	-1.281	0.2	-0.473763249	count	1
RPL3	-0.3286249	0.022708	-14.4718	3.35E-46	-0.473291182	count	8.12E-42
RPL9	-0.3284997	0.0214472	-15.3167	2.23E-51	-0.473075261	count	5.41E-47
NCS1	-0.3425066	0.0908877	-3.7685	0.000167	-0.472706488	count	1
LPL	-2.0217244	0.9397739	-2.1513	0.03152	-0.472463133	count	1
ALDOC	-0.3699798	0.1753067	-2.1105	0.0349	-0.472080767	count	1
MRPS18C	-0.3332662	0.0616441	-5.4063	6.84E-08	-0.471755547	count	0.001602954
SAMMSON	-1.4429295	0.6637737	-2.1738	0.0298	-0.471481693	count	1
ASGR1	-1.014925	0.6796136	-1.4934	0.135	-0.471150796	count	1
CCDC24	-0.4471793	0.2871598	-1.5572	0.119	-0.471057418	count	1
ATP5F1E	-0.3275168	0.0261036	-12.5468	2.18E-35	-0.471023668	count	5.27E-31
LMO7-AS1	-1.0146405	0.4569363	-2.2205	0.0264	-0.471012031	count	1
MMS22L	-0.4948982	0.4222191	-1.1721	0.241	-0.470920578	count	1
TMEM204	-0.339912	0.1164638	-2.9186	0.00354	-0.470854025	count	1
KCNAB2	-0.542516	0.4979546	-1.0895	0.276	-0.470848774	count	1
HRCT1	-0.3369407	0.1218554	-2.7651	0.00572	-0.470584083	count	1
COMTD1	-0.3458065	0.1157551	-2.9874	0.00283	-0.470410639	count	1
SRP68	-0.359831	0.1260484	-2.8547	0.00433	-0.470204696	count	1
URB1	-0.486786	0.2580788	-1.8862	0.0593	-0.469933998	count	1
AMY2B	-1.011938	0.5832675	-1.7349	0.0828	-0.469693765	count	1
FAM49B	-0.388162	0.2067801	-1.8772	0.0606	-0.469356917	count	1
SMIM29	-0.3493482	0.1241586	-2.8137	0.00492	-0.469317239	count	1
AC244090.1	-0.3961795	0.1915298	-2.0685	0.0387	-0.469157505	count	1
SPATA24	-0.6254702	0.4086054	-1.5307	0.126	-0.468749772	count	1

CASC15	-0.6507399	0.3952402	-1.6464	0.0998	-0.468240598	count	1
TRIM26	-0.472419	0.2373431	-1.9904	0.0466	-0.468082266	count	1
TLR2	-0.8236949	0.4602437	-1.7897	0.0736	-0.467921612	count	1
NACC1	-0.4847168	0.3024383	-1.6027	0.109	-0.467886419	count	1
TMEM136	-0.3620557	0.1316228	-2.7507	0.00598	-0.467845542	count	1
MT-ATP8	-0.3427748	0.0850273	-4.0314	5.66E-05	-0.467835858	count	1
AL596202.1	-0.8232797	0.6088092	-1.3523	0.176	-0.467673375	count	1
PDLIM1	-0.3279845	0.0489165	-6.705	2.32E-11	-0.467511684	count	5.51E-07
SLC22A2	-0.7178868	0.6120578	-1.1729	0.241	-0.467463205	count	1
CHMP6	-0.3585318	0.1783852	-2.0099	0.0445	-0.466868716	count	1
UFC1	-0.3275276	0.0524233	-6.2477	4.64E-10	-0.466836725	count	1.10E-05
SLFN12L	-1.9926871	1.2438508	-1.602	0.109	-0.466595278	count	1
AC135050.1	-1.9926871	1.558616	-1.2785	0.201	-0.466595278	count	1
SPAG1	-0.3913742	0.2288285	-1.7103	0.0873	-0.466435027	count	1
MRPL23	-0.3372591	0.0823444	-4.0957	4.30E-05	-0.466272746	count	0.983711
CXorf56	-0.3936999	0.2104717	-1.8706	0.0615	-0.466186177	count	1
AC004825.2	-0.5811561	0.4001749	-1.4523	0.147	-0.466127014	count	1
TNK2	-0.51513	0.3530791	-1.459	0.145	-0.465831477	count	1
LTC4S	-0.3362361	0.1224835	-2.7452	0.00608	-0.465815642	count	1
MIR155HG	-0.8971184	0.5686262	-1.5777	0.115	-0.465687578	count	1
E2F5	-0.5494857	0.4720578	-1.164	0.244	-0.465577899	count	1
ALDH5A1	-0.5802733	0.3193403	-1.8171	0.0693	-0.465392915	count	1
Z84485.1	-0.8959274	0.5352294	-1.6739	0.0942	-0.465036125	count	1
ABHD1	-0.8959274	0.6014067	-1.4897	0.136	-0.465036125	count	1
EPSTI1	-0.392252	0.2219152	-1.7676	0.0772	-0.464451279	count	1
RPLP2	-0.322444	0.0214505	-15.032	1.32E-49	-0.464249764	count	3.20E-45
RANBP3L	-0.3883416	0.1759088	-2.2076	0.0273	-0.464219098	count	1
ZNF740	-0.4336189	0.2391041	-1.8135	0.0698	-0.46407973	count	1
PCDHGA6	-0.4953113	0.4450908	-1.1128	0.266	-0.46384018	count	1
PCDHB9	-0.8155538	0.4602684	-1.7719	0.0765	-0.463054386	count	1
KIAA1211	-0.5772844	0.5220796	-1.1057	0.269	-0.462907695	count	1
CSRNP3	-0.5217237	0.2173039	-2.4009	0.0164	-0.462440573	count	1
COX10	-0.4286146	0.3618577	-1.1845	0.236	-0.462127351	count	1
AC136475.5	-1.4137698	0.6959465	-2.0314	0.0423	-0.461893812	count	1
WDFY3-AS1	-1.4137698	0.8637907	-1.6367	0.102	-0.461893812	count	1
DESI1	-0.3665674	0.1680016	-2.1819	0.0292	-0.46169654	count	1
RBM20	-0.6723948	0.3796578	-1.7711	0.0766	-0.461621401	count	1
SRPK3	-0.7090612	0.3835537	-1.8487	0.0646	-0.461441616	count	1
IRX5	-0.4928078	0.4249529	-1.1597	0.246	-0.461432623	count	1
WDR1	-0.330163	0.0689865	-4.7859	1.77E-06	-0.461425338	count	0.04108524
RPL7	-0.3203605	0.0236479	-13.5471	7.79E-41	-0.460904917	count	1.89E-36
BCL2	-0.4210975	0.1792224	-2.3496	0.0188	-0.46032966	count	1
CCL14	-1.147019	0.6831718	-1.679	0.0932	-0.459508686	count	1
MSRB1	-0.3487335	0.1320753	-2.6404	0.00832	-0.459496099	count	1
CALM1	-0.3187679	0.0245638	-12.9771	1.10E-37	-0.459141378	count	2.66E-33

AC027031.2	-0.3518819	0.1519883	-2.3152	0.0207	-0.458970525	count	1
AIG1	-0.3239204	0.0561418	-5.7697	8.60E-09	-0.458942085	count	0.000202367
KLHL3	-0.5718354	0.3578322	-1.5981	0.11	-0.458378188	count	1
PKM	-0.3202674	0.039607	-8.0861	8.29E-16	-0.458305178	count	1.98E-11
ZNF771	-0.3606068	0.1379523	-2.614	0.00899	-0.458187176	count	1
RBPMS	-0.321273	0.0537647	-5.9755	2.51E-09	-0.457664278	count	5.92E-05
ZBTB2	-0.4422559	0.2625112	-1.6847	0.0921	-0.457284568	count	1
FBXW9	-0.4512044	0.3031476	-1.4884	0.137	-0.45717973	count	1
GNB5	-0.3601435	0.128501	-2.8027	0.0051	-0.456953505	count	1
KIF3C	-1.945607	0.6357413	-3.0604	0.00223	-0.456922802	count	1
AC027307.3	-0.5885652	0.4259301	-1.3818	0.167	-0.456810087	count	1
PTPN1	-0.341035	0.1009354	-3.3787	0.000736	-0.456426621	count	1
NXPH3	-0.3463926	0.1176585	-2.9441	0.00326	-0.456395372	count	1
FAM189A2	-0.8037014	0.5303597	-1.5154	0.13	-0.455969578	count	1
ABCG2	-0.8033454	0.6663272	-1.2056	0.228	-0.455756805	count	1
CD200	-0.3338787	0.0871931	-3.8292	0.000131	-0.455534663	count	1
SCG5	-0.568389	0.3631692	-1.5651	0.118	-0.455514164	count	1
TBX15	-0.568388	0.2723335	-2.0871	0.0369	-0.455513343	count	1
TRPC6	-0.5252272	0.2806823	-1.8713	0.0614	-0.455373005	count	1
ADO	-0.4042533	0.2111159	-1.9148	0.0556	-0.455055832	count	1
AC004839.1	-0.6992253	0.4644402	-1.5055	0.132	-0.454733492	count	1
RPL39L	-0.3360587	0.1332027	-2.5229	0.0117	-0.454595932	count	1
AP002387.2	-0.3703547	0.2078662	-1.7817	0.0749	-0.454294939	count	1
KLHL5	-0.3824745	0.1948568	-1.9628	0.0497	-0.454245821	count	1
RAB40C	-0.4434892	0.270926	-1.6369	0.102	-0.454008646	count	1
TSPOAP1-AS1	-0.6980133	0.4368643	-1.5978	0.11	-0.453907116	count	1
MAST4	-0.3446507	0.1426796	-2.4156	0.0158	-0.453765742	count	1
EVA1B	-0.3185636	0.0529711	-6.0139	1.99E-09	-0.453739136	count	4.69E-05
TDRD7	-0.4431772	0.2936192	-1.5094	0.131	-0.453682512	count	1
KRT81	-1.132721	0.8575073	-1.3209	0.187	-0.453525001	count	1
NEDD4	-0.3618132	0.1415082	-2.5568	0.0106	-0.453418836	count	1
PEX11A	-0.3483889	0.1901689	-1.832	0.067	-0.452749713	count	1
SLC37A1	-0.6596653	0.5066105	-1.3021	0.193	-0.452486409	count	1
THAP9-AS1	-0.3592811	0.1562655	-2.2992	0.0216	-0.452446347	count	1
AL022328.3	-1.3851137	1.0419244	-1.3294	0.184	-0.45241618	count	1
CISD1	-0.3246494	0.0761818	-4.2615	2.08E-05	-0.452336924	count	0.4777552
NPM3	-0.3977628	0.224617	-1.7708	0.0767	-0.452257483	count	1
GJC1	-0.4832088	0.2023331	-2.3882	0.017	-0.452205098	count	1
MZT2B	-0.3157912	0.0349826	-9.0271	2.78E-19	-0.451894194	count	6.67E-15
AC079922.2	-0.4331011	0.2906247	-1.4902	0.136	-0.451886032	count	1
CMKLR1	-0.3856542	0.2100331	-1.8362	0.0664	-0.451693281	count	1
LAMA5-AS1	-0.6946937	0.5808668	-1.196	0.232	-0.451643969	count	1
C1orf226	-0.739244	0.5842006	-1.2654	0.206	-0.451478563	count	1
OSGEP	-0.3395564	0.1232447	-2.7551	0.0059	-0.451343113	count	1
TRNP1	-0.3392698	0.1195018	-2.839	0.00455	-0.451217377	count	1

SLC8A1	-0.4156966	0.188632	-2.2037	0.0276	-0.451209313	count	1
DAP	-0.3226668	0.0740714	-4.3562	1.36E-05	-0.451083873	count	0.313072
TBL1X	-0.3403167	0.1465235	-2.3226	0.0203	-0.451015861	count	1
LMOD3	-0.7383649	0.4498585	-1.6413	0.101	-0.45091407	count	1
RPS27L	-0.3135025	0.0306125	-10.241	2.75E-24	-0.450801127	count	6.62E-20
AC245297.3	-0.3321165	0.1052425	-3.1557	0.00161	-0.450465007	count	1
PHKG1	-0.3509223	0.1396289	-2.5132	0.012	-0.450365876	count	1
S100A5	-1.9137791	0.9669174	-1.9793	0.0479	-0.450274725	count	1
SCAMP5	-1.9137791	1.1519686	-1.6613	0.0967	-0.450274725	count	1
WNT7B	-1.9137791	1.1519686	-1.6613	0.0967	-0.450274725	count	1
HCFC1R1	-0.3137991	0.0375503	-8.3568	9.03E-17	-0.450177657	count	2.16E-12
ORMDL3	-0.3889101	0.235676	-1.6502	0.099	-0.450161392	count	1
PLCL1	-0.3376081	0.1292801	-2.6114	0.00905	-0.449987038	count	1
JCAD	-0.3798453	0.1796246	-2.1147	0.0345	-0.44958957	count	1
SKAP1	-0.9705423	0.7766818	-1.2496	0.212	-0.4494828	count	1
GCDH	-0.3618703	0.2015878	-1.7951	0.0727	-0.449396968	count	1
UPK3A	-1.9089572	0.6925562	-2.7564	0.00587	-0.449259984	count	1
TMSB15B	-0.4386948	0.3320696	-1.3211	0.187	-0.448997738	count	1
AC079630.1	-0.5990306	0.3801516	-1.5758	0.115	-0.448142491	count	1
ATP1A2	-0.3512369	0.1586978	-2.2132	0.0269	-0.447996119	count	1
SLC16A10	-0.7903535	0.4486384	-1.7617	0.0782	-0.447993108	count	1
MTHFD2	-0.3214923	0.0759259	-4.2343	2.35E-05	-0.447836162	count	0.5394425
RPL23	-0.3111291	0.0289168	-10.7595	1.33E-26	-0.447094671	count	3.21E-22
AL049775.3	-0.8630606	0.6239092	-1.3833	0.167	-0.447057601	count	1
ADTRP	-1.368833	0.7098946	-1.9282	0.0539	-0.447008309	count	1
TMEM159	-0.3189361	0.0683906	-4.6634	3.22E-06	-0.446471864	count	0.0745752
POMGNT1	-0.359522	0.191262	-1.8797	0.0602	-0.446455045	count	1
CENPB	-0.3355291	0.1326431	-2.5296	0.0115	-0.446219073	count	1
RPL41	-0.3092627	0.0176967	-17.4757	9.44E-66	-0.445883147	count	2.29E-61
SAMSN1	-0.5960795	0.6190925	-0.9628	0.336	-0.445844496	count	1
NEK6	-0.4132347	0.2302641	-1.7946	0.0728	-0.445255839	count	1
MCRIP1	-0.3147698	0.0657474	-4.7876	1.75E-06	-0.444731404	count	0.0406245
ZNF44	-0.3910919	0.2172688	-1.8	0.0719	-0.444565099	count	1
MOCOS	-0.9599159	0.5828386	-1.647	0.0997	-0.444290136	count	1
SLC26A6	-0.5543712	0.4673702	-1.1862	0.236	-0.443871904	count	1
SIVA1	-0.3105096	0.0418964	-7.4114	1.54E-13	-0.443736663	count	3.67E-09
STX11	-0.5120896	0.2763121	-1.8533	0.0639	-0.443624971	count	1
KIAA0355	-0.3384638	0.1747516	-1.9368	0.0528	-0.443571415	count	1
ADAM23	-0.5118714	0.301483	-1.6978	0.0896	-0.443429927	count	1
PQBP1	-0.3172081	0.0768832	-4.1258	3.78E-05	-0.443132869	count	0.8651286
EFTUD2	-0.3898362	0.2147108	-1.8156	0.0695	-0.443117399	count	1
EDF1	-0.30883	0.0315912	-9.7758	2.67E-22	-0.442854034	count	6.42E-18
C9orf50	-0.5111309	0.3860435	-1.324	0.186	-0.44276809	count	1
ACTN4	-0.3095013	0.0517115	-5.9852	2.37E-09	-0.442660439	count	5.59E-05
NSUN6	-0.3466054	0.1953454	-1.7743	0.0761	-0.44261644	count	1

XRCC4	-0.349904	0.2194427	-1.5945	0.111	-0.442578985	count	1
MGAT4B	-0.3274391	0.1208248	-2.71	0.00676	-0.442575711	count	1
ASNS	-0.3791656	0.2197137	-1.7257	0.0845	-0.442303067	count	1
PIM3	-0.3201078	0.1133191	-2.8248	0.00476	-0.442295151	count	1
MRPL36	-0.3169596	0.0731703	-4.3318	1.52E-05	-0.442045842	count	0.3496912
AC005070.3	-1.3531145	0.5911328	-2.289	0.0221	-0.441771957	count	1
RGN	-0.3199444	0.1044143	-3.0642	0.0022	-0.441711224	count	1
HLCS	-0.4721738	0.31408	-1.5034	0.133	-0.441604362	count	1
FBXW2	-0.3342711	0.1339077	-2.4963	0.0126	-0.441549593	count	1
CMSS1	-0.3290092	0.1070404	-3.0737	0.00213	-0.441493007	count	1
UNC119	-0.3415612	0.1527828	-2.2356	0.0254	-0.441196349	count	1
PELI3	-0.3845483	0.2109125	-1.8233	0.0683	-0.441175831	count	1
SP100	-0.3114272	0.0600617	-5.1851	2.28E-07	-0.441008257	count	0.005328132
EML2	-0.3507259	0.1746197	-2.0085	0.0447	-0.440878391	count	1
PEG10	-0.4190086	0.2195161	-1.9088	0.0564	-0.440828474	count	1
B4GALT1-AS1	-0.3637346	0.2133064	-1.7052	0.0882	-0.440690593	count	1
ZNF787	-0.3416635	0.1431017	-2.3876	0.017	-0.440389604	count	1
PERM1	-1.3482342	0.8211598	-1.6419	0.101	-0.440143217	count	1
CDCA5	-1.3482342	0.8211598	-1.6419	0.101	-0.440143217	count	1
ITGB1	-0.3062255	0.0334465	-9.1557	8.76E-20	-0.440069669	count	2.10E-15
ZFAS1	-0.3099509	0.0548927	-5.6465	1.76E-08	-0.439613918	count	0.000413635
AL160006.1	-0.7206901	0.5169214	-1.3942	0.163	-0.439570481	count	1
ASB1	-0.3560765	0.2352566	-1.5136	0.13	-0.439480343	count	1
IFT46	-0.3677585	0.1887565	-1.9483	0.0515	-0.439345838	count	1
BIN2	-1.8617402	0.8002464	-2.3265	0.02	-0.439220243	count	1
POLN	-1.8617402	0.9605255	-1.9383	0.0527	-0.439220243	count	1
SH2D2A	-1.8617402	1.140761	-1.632	0.103	-0.439220243	count	1
ITGB1BP2	-0.6120023	0.3676727	-1.6645	0.0961	-0.439198851	count	1
MCOLN2	-1.3448701	0.6307417	-2.1322	0.0331	-0.439019666	count	1
SLC24A4	-1.3448701	0.7879236	-1.7069	0.0879	-0.439019666	count	1
HMGN4	-0.413713	0.2447156	-1.6906	0.091	-0.438856926	count	1
SYMPK	-0.3772931	0.2366819	-1.5941	0.111	-0.4383459	count	1
SERGEF	-0.3214809	0.1326934	-2.4227	0.0155	-0.438089789	count	1
ATP10A	-0.375556	0.2079733	-1.8058	0.071	-0.438039636	count	1
SEH1L	-0.385421	0.2776105	-1.3884	0.165	-0.438027815	count	1
POPDC2	-0.3893425	0.2805387	-1.3878	0.165	-0.438025517	count	1
RPL13	-0.3034168	0.0176095	-17.2303	4.90E-64	-0.437447456	count	1.19E-59
ACOT7	-0.3528474	0.1962585	-1.7979	0.0723	-0.437237141	count	1
EIF3G	-0.3058147	0.038307	-7.9833	1.89E-15	-0.437076777	count	4.51E-11
RASA4	-0.3947435	0.2809836	-1.4049	0.16	-0.436626349	count	1
NEXN-AS1	-0.7712796	0.442484	-1.7431	0.0814	-0.436600164	count	1
POU3F1	-0.7160475	0.4109699	-1.7423	0.0815	-0.436592359	count	1
TPTEP2-CSNK1E	-0.9441148	0.7544263	-1.2514	0.211	-0.436566494	count	1
PCED1A	-0.3915065	0.2253006	-1.7377	0.0823	-0.43559051	count	1
TLN1	-0.3031453	0.037663	-8.0489	1.12E-15	-0.434994338	count	2.68E-11

RHNO1	-0.3728508	0.195946	-1.9028	0.0571	-0.434844894	count	1
TMEM59L	-1.3301629	0.6375723	-2.0863	0.037	-0.434100294	count	1
CHI3L2	-0.4028182	0.3318023	-1.214	0.225	-0.433837271	count	1
MBD3	-0.3376557	0.1451594	-2.3261	0.0201	-0.433731194	count	1
ERO1A	-0.3354462	0.1355265	-2.4751	0.0134	-0.433697171	count	1
RPL23A	-0.3010691	0.0228218	-13.1922	7.37E-39	-0.433498366	count	1.78E-34
BIRC5	-0.8379337	0.9271205	-0.9038	0.366	-0.433315335	count	1
MRPS6	-0.3048233	0.0638418	-4.7747	1.87E-06	-0.433184516	count	0.04339335
C17orf82	-0.4998846	0.2855604	-1.7505	0.0801	-0.432720668	count	1
FOXL1	-0.4078761	0.2393034	-1.7044	0.0884	-0.432551414	count	1
TIGD7	-0.4320499	0.28657	-1.5077	0.132	-0.432414893	count	1
KLC2	-0.3785743	0.1974757	-1.9171	0.0553	-0.432220339	count	1
BEND5	-0.3328102	0.1470449	-2.2633	0.0237	-0.431975793	count	1
PRPF31	-0.3102797	0.0808536	-3.8375	0.000126	-0.431622765	count	1
TPD52	-0.4035488	0.4517393	-0.8933	0.372	-0.431333778	count	1
INAFM2	-0.3293825	0.2186586	-1.5064	0.132	-0.431261434	count	1
PAWR	-0.3040082	0.0651544	-4.666	3.18E-06	-0.431261364	count	0.07365198
GRK2	-0.3695227	0.2195965	-1.6827	0.0925	-0.430915072	count	1
TXNL4B	-0.4977509	0.2976145	-1.6725	0.0945	-0.430815366	count	1
ARHGEF9	-0.3118559	0.1209349	-2.5787	0.00996	-0.430516923	count	1
RPL35	-0.2993567	0.0240967	-12.4232	9.69E-35	-0.430397601	count	2.34E-30
SERP2	-0.3533748	0.1652908	-2.1379	0.0326	-0.430201113	count	1
LCMT2	-0.4680207	0.4574908	-1.023	0.306	-0.430082184	count	1
ANP32B	-0.3015482	0.0477894	-6.3099	3.12E-10	-0.429909273	count	7.38E-06
NFU1	-0.3098088	0.0832997	-3.7192	0.000203	-0.42953582	count	1
CCDC88A	-0.310468	0.1192814	-2.6028	0.00928	-0.427784835	count	1
COX7C	-0.29755	0.0265378	-11.2123	1.03E-28	-0.427410446	count	2.49E-24
LRMDA	-0.3142686	0.1374993	-2.2856	0.0223	-0.427370185	count	1
NUP93	-0.3966105	0.2658022	-1.4921	0.136	-0.427035661	count	1
DDX41	-0.3348314	0.1979195	-1.6918	0.0908	-0.426925601	count	1
UXT	-0.3004346	0.0511206	-5.877	4.55E-09	-0.426884258	count	0.000107212
C19orf24	-0.3115413	0.0987743	-3.1541	0.00162	-0.426849877	count	1
UQCC2	-0.3106447	0.0877103	-3.5417	0.000402	-0.426542534	count	1
OXLD1	-0.3202177	0.1312679	-2.4394	0.0148	-0.426240997	count	1
TBC1D22A	-0.3337917	0.1885008	-1.7708	0.0767	-0.426140395	count	1
ZNF90	-0.4360069	0.3484147	-1.2514	0.211	-0.425643674	count	1
GSTO1	-0.29816	0.0421494	-7.0739	1.80E-12	-0.425632421	count	4.28E-08
PDZRN4	-0.3982761	0.2731287	-1.4582	0.145	-0.425597737	count	1
MAP1B	-0.2957955	0.043151	-6.8549	8.34E-12	-0.42541637	count	1.98E-07
RPS20	-0.2961834	0.0275158	-10.7641	1.27E-26	-0.425103851	count	3.06E-22
LDLRAD2	-0.9202309	0.5388881	-1.7076	0.0878	-0.424888292	count	1
WDFY4	-0.9202309	0.8289403	-1.1101	0.267	-0.424888292	count	1
SYP	-0.3757357	0.1926655	-1.9502	0.0512	-0.424722236	count	1
HSPB3	-1.7940337	0.9310445	-1.9269	0.0541	-0.424505442	count	1
TANC1	-0.3229216	0.1735053	-1.8612	0.0628	-0.423752516	count	1

FBXO24	-19.0433874	1400.86744	-0.0136	0.989	-0.423628649	count	1
AC062004.1	-19.0433874	1400.86744	-0.0136	0.989	-0.423628649	count	1
NDRG4	-19.0433874	1400.86744	-0.0136	0.989	-0.423628649	count	1
MATK	-19.0433874	1400.86744	-0.0136	0.989	-0.423628649	count	1
LY9	-18.9640981	1866.083827	-0.0102	0.992	-0.423628648	count	1
AC007879.3	-18.9640981	1866.083827	-0.0102	0.992	-0.423628648	count	1
NEIL3	-18.9640981	1866.083827	-0.0102	0.992	-0.423628648	count	1
AC008629.1	-18.9640981	1866.083827	-0.0102	0.992	-0.423628648	count	1
SHROOM2	-18.9640981	1866.083827	-0.0102	0.992	-0.423628648	count	1
PRSS3	-18.9640981	1866.083827	-0.0102	0.992	-0.423628648	count	1
CAVIN4	-18.9640981	1866.083827	-0.0102	0.992	-0.423628648	count	1
C9orf147	-18.9640981	1866.083827	-0.0102	0.992	-0.423628648	count	1
AC044839.3	-18.9640981	1866.083827	-0.0102	0.992	-0.423628648	count	1
AP001189.1	-18.9640981	1866.083827	-0.0102	0.992	-0.423628648	count	1
AL603756.1	-18.9640981	1866.083827	-0.0102	0.992	-0.423628648	count	1
PLK1	-18.9640981	1866.083827	-0.0102	0.992	-0.423628648	count	1
AC055811.3	-18.9640981	1866.083827	-0.0102	0.992	-0.423628648	count	1
IKZF3	-18.9640981	1866.083827	-0.0102	0.992	-0.423628648	count	1
SYT5	-18.9640981	1866.083827	-0.0102	0.992	-0.423628648	count	1
PRR11	-18.6359527	1451.538387	-0.0128	0.99	-0.423628647	count	1
AL592430.1	-18.6157339	1997.672932	-0.0093	0.993	-0.423628647	count	1
LYPLAL1-DT	-18.6153785	1624.944628	-0.0115	0.991	-0.423628647	count	1
NRG1	-18.5980341	1337.344144	-0.0139	0.989	-0.423628647	count	1
CAMK1G	-18.4477282	1139.249904	-0.0162	0.987	-0.423628646	count	1
TAS1R1	-18.3935957	1327.94079	-0.0139	0.989	-0.423628646	count	1
GATA3	-18.3935957	1327.94079	-0.0139	0.989	-0.423628646	count	1
CPA3	-18.3509279	1661.280281	-0.011	0.991	-0.423628646	count	1
EOMES	-17.9769516	1006.967756	-0.0179	0.986	-0.423628643	count	1
MMP7	-19.542027	1498.699749	-0.013	0.99	-0.423628503	count	1
GRIK3	-19.45604	1579.162469	-0.0123	0.99	-0.423628503	count	1
CLCA2	-19.45604	1579.162469	-0.0123	0.99	-0.423628503	count	1
HIST2H2AA4	-19.45604	1579.162469	-0.0123	0.99	-0.423628503	count	1
AL451050.2	-19.45604	1579.162469	-0.0123	0.99	-0.423628503	count	1
LEMD1	-19.45604	1579.162469	-0.0123	0.99	-0.423628503	count	1
NEK2	-19.45604	1579.162469	-0.0123	0.99	-0.423628503	count	1
MTRNR2L11	-19.45604	1579.162469	-0.0123	0.99	-0.423628503	count	1
C2orf48	-19.45604	1579.162469	-0.0123	0.99	-0.423628503	count	1
LINC01954	-19.45604	1579.162469	-0.0123	0.99	-0.423628503	count	1
LINC00276	-19.45604	1579.162469	-0.0123	0.99	-0.423628503	count	1
KRTCAP3	-19.45604	1579.162469	-0.0123	0.99	-0.423628503	count	1
CAPN14	-19.45604	1579.162469	-0.0123	0.99	-0.423628503	count	1
LINC00211	-19.45604	1579.162469	-0.0123	0.99	-0.423628503	count	1
AC011306.1	-19.45604	1579.162469	-0.0123	0.99	-0.423628503	count	1
GPR39	-19.45604	1579.162469	-0.0123	0.99	-0.423628503	count	1
HOXD1	-19.45604	1579.162469	-0.0123	0.99	-0.423628503	count	1

AC020571.1	-19.45604	1579.162469	-0.0123	0.99	-0.423628503	count	1
AC112721.2	-19.45604	1579.162469	-0.0123	0.99	-0.423628503	count	1
AC026202.3	-19.45604	1579.162469	-0.0123	0.99	-0.423628503	count	1
AC123023.1	-19.45604	1579.162469	-0.0123	0.99	-0.423628503	count	1
C3orf67-AS1	-19.45604	1579.162469	-0.0123	0.99	-0.423628503	count	1
AC110491.1	-19.45604	1579.162469	-0.0123	0.99	-0.423628503	count	1
IGSF11	-19.45604	1579.162469	-0.0123	0.99	-0.423628503	count	1
AC079848.1	-19.45604	1579.162469	-0.0123	0.99	-0.423628503	count	1
NPHP3-ACAD11	-19.45604	1579.162469	-0.0123	0.99	-0.423628503	count	1
AC024933.1	-19.45604	1579.162469	-0.0123	0.99	-0.423628503	count	1
AC080013.6	-19.45604	1579.162469	-0.0123	0.99	-0.423628503	count	1
ARL14	-19.45604	1579.162469	-0.0123	0.99	-0.423628503	count	1
MAP6D1	-19.45604	1579.162469	-0.0123	0.99	-0.423628503	count	1
FABP2	-19.45604	1579.162469	-0.0123	0.99	-0.423628503	count	1
C4orf45	-19.45604	1579.162469	-0.0123	0.99	-0.423628503	count	1
GABRB2	-19.45604	1579.162469	-0.0123	0.99	-0.423628503	count	1
HMMR	-19.45604	1579.162469	-0.0123	0.99	-0.423628503	count	1
AL357052.1	-19.45604	1579.162469	-0.0123	0.99	-0.423628503	count	1
NRSN1	-19.45604	1579.162469	-0.0123	0.99	-0.423628503	count	1
HCG20	-19.45604	1579.162469	-0.0123	0.99	-0.423628503	count	1
HLA-DQB2	-19.45604	1579.162469	-0.0123	0.99	-0.423628503	count	1
PNPLA1	-19.45604	1579.162469	-0.0123	0.99	-0.423628503	count	1
AL355297.2	-19.45604	1579.162469	-0.0123	0.99	-0.423628503	count	1
PLG	-19.45604	1579.162469	-0.0123	0.99	-0.423628503	count	1
AC093620.1	-19.45604	1579.162469	-0.0123	0.99	-0.423628503	count	1
AC004895.1	-19.45604	1579.162469	-0.0123	0.99	-0.423628503	count	1
AL683807.1	-19.45604	1579.162469	-0.0123	0.99	-0.423628503	count	1
AL035425.1	-19.45604	1579.162469	-0.0123	0.99	-0.423628503	count	1
SPANXA2-OT1	-19.45604	1579.162469	-0.0123	0.99	-0.423628503	count	1
LINC01605	-19.45604	1579.162469	-0.0123	0.99	-0.423628503	count	1
AC069120.1	-19.45604	1579.162469	-0.0123	0.99	-0.423628503	count	1
AC022182.2	-19.45604	1579.162469	-0.0123	0.99	-0.423628503	count	1
AC090987.1	-19.45604	1579.162469	-0.0123	0.99	-0.423628503	count	1
LINC01410	-19.45604	1579.162469	-0.0123	0.99	-0.423628503	count	1
AL137067.1	-19.45604	1579.162469	-0.0123	0.99	-0.423628503	count	1
AL157935.2	-19.45604	1579.162469	-0.0123	0.99	-0.423628503	count	1
AL136141.1	-19.45604	1579.162469	-0.0123	0.99	-0.423628503	count	1
FIBCD1	-19.45604	1579.162469	-0.0123	0.99	-0.423628503	count	1
AC136475.7	-19.45604	1579.162469	-0.0123	0.99	-0.423628503	count	1
OR5AS1	-19.45604	1579.162469	-0.0123	0.99	-0.423628503	count	1
GAL3ST3	-19.45604	1579.162469	-0.0123	0.99	-0.423628503	count	1
DNAJB13	-19.45604	1579.162469	-0.0123	0.99	-0.423628503	count	1
AC016825.1	-19.45604	1579.162469	-0.0123	0.99	-0.423628503	count	1
AL157832.1	-19.45604	1579.162469	-0.0123	0.99	-0.423628503	count	1
OLR1	-19.45604	1579.162469	-0.0123	0.99	-0.423628503	count	1

KRT7-AS	-19.45604	1579.162469	-0.0123	0.99	-0.423628503	count	1
LRP1-AS	-19.45604	1579.162469	-0.0123	0.99	-0.423628503	count	1
AC012555.1	-19.45604	1579.162469	-0.0123	0.99	-0.423628503	count	1
AC069234.2	-19.45604	1579.162469	-0.0123	0.99	-0.423628503	count	1
PCDH20	-19.45604	1579.162469	-0.0123	0.99	-0.423628503	count	1
ACOT1	-19.45604	1579.162469	-0.0123	0.99	-0.423628503	count	1
AC005479.1	-19.45604	1579.162469	-0.0123	0.99	-0.423628503	count	1
KCNK13	-19.45604	1579.162469	-0.0123	0.99	-0.423628503	count	1
IGHV5-51	-19.45604	1579.162469	-0.0123	0.99	-0.423628503	count	1
AC055855.1	-19.45604	1579.162469	-0.0123	0.99	-0.423628503	count	1
ADPGK-AS1	-19.45604	1579.162469	-0.0123	0.99	-0.423628503	count	1
AC090260.1	-19.45604	1579.162469	-0.0123	0.99	-0.423628503	count	1
AC091078.1	-19.45604	1579.162469	-0.0123	0.99	-0.423628503	count	1
AC036108.2	-19.45604	1579.162469	-0.0123	0.99	-0.423628503	count	1
AC015712.1	-19.45604	1579.162469	-0.0123	0.99	-0.423628503	count	1
AC005606.1	-19.45604	1579.162469	-0.0123	0.99	-0.423628503	count	1
AC130456.5	-19.45604	1579.162469	-0.0123	0.99	-0.423628503	count	1
AC106745.1	-19.45604	1579.162469	-0.0123	0.99	-0.423628503	count	1
AC116552.1	-19.45604	1579.162469	-0.0123	0.99	-0.423628503	count	1
AC138028.2	-19.45604	1579.162469	-0.0123	0.99	-0.423628503	count	1
SLC52A1	-19.45604	1579.162469	-0.0123	0.99	-0.423628503	count	1
ALOX15B	-19.45604	1579.162469	-0.0123	0.99	-0.423628503	count	1
AC114684.1	-19.45604	1579.162469	-0.0123	0.99	-0.423628503	count	1
SPEF1	-19.45604	1579.162469	-0.0123	0.99	-0.423628503	count	1
BANF2	-19.45604	1579.162469	-0.0123	0.99	-0.423628503	count	1
GDAP1L1	-19.45604	1579.162469	-0.0123	0.99	-0.423628503	count	1
SLC12A5-AS1	-19.45604	1579.162469	-0.0123	0.99	-0.423628503	count	1
BIRC7	-19.45604	1579.162469	-0.0123	0.99	-0.423628503	count	1
AC004528.2	-19.45604	1579.162469	-0.0123	0.99	-0.423628503	count	1
FCER2	-19.45604	1579.162469	-0.0123	0.99	-0.423628503	count	1
LINC01842	-19.45604	1579.162469	-0.0123	0.99	-0.423628503	count	1
DLL3	-19.45604	1579.162469	-0.0123	0.99	-0.423628503	count	1
ADM5	-19.45604	1579.162469	-0.0123	0.99	-0.423628503	count	1
AC010320.1	-19.45604	1579.162469	-0.0123	0.99	-0.423628503	count	1
AC246785.3	-19.241104	1879.694245	-0.0102	0.992	-0.423628502	count	1
AC016737.2	-19.241104	1879.694245	-0.0102	0.992	-0.423628502	count	1
AC074183.1	-19.241104	1879.694245	-0.0102	0.992	-0.423628502	count	1
HTR7	-19.241104	1879.694245	-0.0102	0.992	-0.423628502	count	1
SLCO4A1	-19.241104	1879.694245	-0.0102	0.992	-0.423628502	count	1
AC114271.1	-19.241104	1879.694245	-0.0102	0.992	-0.423628502	count	1
ITGB2-AS1	-18.987652	1949.379221	-0.0097	0.992	-0.423628502	count	1
AC022784.1	-18.241229	1484.288138	-0.0123	0.99	-0.423628498	count	1
TOMM20L	-18.241229	1484.288138	-0.0123	0.99	-0.423628498	count	1
DOC2A	-18.241229	1484.288138	-0.0123	0.99	-0.423628498	count	1
MAP6	-0.4896281	0.2983066	-1.6414	0.101	-0.423564857	count	1

SPRY1	-0.3043598	0.0816616	-3.7271	0.000197	-0.423418826	count	1
TNFSF11	-1.787958	0.4443254	-4.024	5.84E-05	-0.423167266	count	1
ARHGAP18	-0.318869	0.1344159	-2.3723	0.0177	-0.422973202	count	1
ADGRE5	-0.3045237	0.1070725	-2.8441	0.00448	-0.422951981	count	1
BNC2-AS1	-0.9152499	0.5912945	-1.5479	0.122	-0.422452516	count	1
NIPSNAP3B	-0.417435	0.2458762	-1.6977	0.0896	-0.422253917	count	1
AP2S1	-0.298474	0.0557701	-5.3519	9.24E-08	-0.422114154	count	0.002164193
AC007114.2	-1.057056	0.4237446	-2.4946	0.0127	-0.421746027	count	1
SYNM	-0.3001563	0.0792525	-3.7873	0.000155	-0.421241468	count	1
C3AR1	-1.7768879	0.8733637	-2.0345	0.042	-0.420721535	count	1
CD72	-1.7768879	0.8808263	-2.0173	0.0437	-0.420721535	count	1
WISP1	-0.5263249	0.459246	-1.1461	0.252	-0.420613647	count	1
CYHR1	-0.3011154	0.0737605	-4.0823	4.55E-05	-0.420385141	count	1
TNFAIP3	-0.3509545	0.2626927	-1.336	0.182	-0.420324208	count	1
NDUFA12	-0.2947138	0.0475627	-6.1963	6.42E-10	-0.420286757	count	1.52E-05
NHP2	-0.2966448	0.053908	-5.5028	3.99E-08	-0.420235703	count	0.000936254
PDLIM7	-0.2949049	0.0616042	-4.7871	1.76E-06	-0.420189332	count	0.04085488
CCNQ	-0.3172045	0.1116253	-2.8417	0.00451	-0.419980395	count	1
COPS9	-0.2954786	0.045766	-6.4563	1.21E-10	-0.419658003	count	2.87E-06
FAM136A	-0.3129007	0.1267044	-2.4695	0.0136	-0.419018564	count	1
AJUBA	-0.4959874	0.3467973	-1.4302	0.153	-0.418820001	count	1
UQCRB	-0.2916817	0.0304232	-9.5875	1.61E-21	-0.41880103	count	3.87E-17
B3GALNT1	-0.3474638	0.1625184	-2.138	0.0326	-0.418506483	count	1
BANF1	-0.2932398	0.0457682	-6.4071	1.67E-10	-0.418230457	count	3.96E-06
SNTA1	-0.3033219	0.0774062	-3.9186	9.07E-05	-0.418209737	count	1
PSME2	-0.2943195	0.0601587	-4.8924	1.04E-06	-0.418204594	count	0.02419144
GALNT18	-0.3284232	0.1776503	-1.8487	0.0646	-0.418145772	count	1
GPR62	-1.2826185	0.7231291	-1.7737	0.0762	-0.418118084	count	1
C9orf170	-1.2826185	0.7835804	-1.6369	0.102	-0.418118084	count	1
UBE2C	-1.2826185	0.8679455	-1.4778	0.14	-0.418118084	count	1
TRIR	-0.2925921	0.0390839	-7.4863	8.83E-14	-0.418105453	count	2.10E-09
AGPAT2	-0.3032935	0.1008481	-3.0074	0.00265	-0.418023514	count	1
LYPD1	-0.6451645	0.4862286	-1.3269	0.185	-0.417926697	count	1
LINC00324	-0.4471091	0.2879221	-1.5529	0.121	-0.417556494	count	1
CHMP4A	-0.2997015	0.0746672	-4.0138	6.09E-05	-0.417467767	count	1
SLC20A1	-0.3148604	0.2249068	-1.4	0.162	-0.417378181	count	1
SURF6	-0.3168796	0.1326178	-2.3894	0.0169	-0.417316524	count	1
JPH1	-1.2800841	0.9135901	-1.4012	0.161	-0.41726295	count	1
WDR62	-1.2800841	0.9135901	-1.4012	0.161	-0.41726295	count	1
CFP	-1.2800841	0.9476061	-1.3509	0.177	-0.41726295	count	1
AC026150.3	-1.2800841	0.9476061	-1.3509	0.177	-0.41726295	count	1
AL441883.1	-1.7601881	0.77755	-2.2638	0.0236	-0.417014343	count	1
NKX6-1	-1.7601881	0.9279774	-1.8968	0.0579	-0.417014343	count	1
NRK	-0.4537575	0.3421858	-1.3261	0.185	-0.416619265	count	1
RPL36	-0.289844	0.0245017	-11.8296	1.03E-31	-0.416596499	count	2.49E-27

CLDND2	-0.581582	0.4141419	-1.4043	0.16	-0.416444467	count	1
AK9	-0.4073878	0.201864	-2.0181	0.0437	-0.416313919	count	1
TMEM160	-0.2942913	0.0734864	-4.0047	6.33E-05	-0.415932251	count	1
LRTOMT	-0.3954753	0.2161841	-1.8293	0.0674	-0.415614304	count	1
PLXNA4	-0.4524955	0.4026925	-1.1237	0.261	-0.415428773	count	1
DLG2	-0.3606056	0.224983	-1.6028	0.109	-0.415200594	count	1
LAYN	-0.3000984	0.1114363	-2.693	0.00711	-0.414382779	count	1
OSBPL6	-0.6398064	0.3082434	-2.0757	0.038	-0.414285455	count	1
LIMS1	-0.2928356	0.05694	-5.1429	2.85E-07	-0.414061121	count	0.00665589
FAM89B	-0.2916471	0.0539823	-5.4026	6.98E-08	-0.413942189	count	0.001635693
RPL21	-0.2873585	0.0208201	-13.8019	2.78E-42	-0.413908681	count	6.74E-38
TMEM158	-0.5547222	0.424676	-1.3062	0.192	-0.413690521	count	1
ZFP69B	-0.7327902	0.5541023	-1.3225	0.186	-0.41363566	count	1
WNK1	-0.3071033	0.1067993	-2.8755	0.00406	-0.41305424	count	1
KCTD21	-0.6377063	0.4372024	-1.4586	0.145	-0.412858649	count	1
EMC10	-0.2979462	0.0818121	-3.6418	0.000274	-0.412358147	count	1
RPS27A	-0.2860211	0.0201243	-14.2127	1.15E-44	-0.412357796	count	2.79E-40
MPP6	-0.3581277	0.1670676	-2.1436	0.0321	-0.412310473	count	1
IPO7	-0.3020613	0.1099379	-2.7476	0.00603	-0.412103147	count	1
CLSPN	-1.7368628	0.889118	-1.9535	0.0508	-0.411801014	count	1
AC098934.4	-1.7368628	0.889118	-1.9535	0.0508	-0.411801014	count	1
POTEE	-1.7368628	0.889118	-1.9535	0.0508	-0.411801014	count	1
AGR2	-1.7368628	0.889118	-1.9535	0.0508	-0.411801014	count	1
COLEC10	-1.7368628	0.889118	-1.9535	0.0508	-0.411801014	count	1
CDK1	-1.7368628	0.889118	-1.9535	0.0508	-0.411801014	count	1
MSMP	-1.7368628	1.0255546	-1.6936	0.0904	-0.411801014	count	1
AP002761.2	-1.7368628	1.0255546	-1.6936	0.0904	-0.411801014	count	1
CHCHD5	-0.3000333	0.0889041	-3.3748	0.000746	-0.411759859	count	1
ZFP91	-0.3331183	0.1803661	-1.8469	0.0648	-0.411750384	count	1
NECAP2	-0.3052694	0.1280786	-2.3835	0.0172	-0.411732781	count	1
NABP2	-0.3043107	0.1064348	-2.8591	0.00427	-0.411646116	count	1
ZNHIT3	-0.2953127	0.0764719	-3.8617	0.000115	-0.411428243	count	1
TRPV2	-0.3987243	0.2685552	-1.4847	0.138	-0.411412501	count	1
LMCD1	-0.2870162	0.0618262	-4.6423	3.57E-06	-0.411182986	count	0.08262408
AC124283.1	-0.676381	0.3829744	-1.7661	0.0775	-0.411177814	count	1
SGK3	-0.3180888	0.1832318	-1.736	0.0826	-0.411120105	count	1
AC090152.1	-0.4998283	0.5736423	-0.8713	0.384	-0.410874899	count	1
AC027288.3	-1.7326871	1.095173	-1.5821	0.114	-0.410863416	count	1
S1PR1	-0.4021492	0.4213678	-0.9544	0.34	-0.410851507	count	1
HS2ST1	-0.3715195	0.2138231	-1.7375	0.0824	-0.41054733	count	1
FHOD1	-0.4991289	0.3529179	-1.4143	0.157	-0.410280098	count	1
KBTBD8	-1.7298899	0.8806206	-1.9644	0.0496	-0.410234681	count	1
PCGF5	-0.2904562	0.0810348	-3.5843	0.000342	-0.41015348	count	1
SRGAP2C	-0.3425242	0.1786615	-1.9172	0.0553	-0.410121124	count	1
TAP1	-0.328056	0.1662287	-1.9735	0.0485	-0.410075161	count	1

BFSP1	-0.6746348	0.4582657	-1.4721	0.141	-0.410060417	count	1
LYRM9	-0.3189774	0.1279753	-2.4925	0.0127	-0.410053954	count	1
AC016065.1	-0.4389482	0.2956588	-1.4846	0.138	-0.409736326	count	1
ARHGEF26	-0.3100517	0.1570985	-1.9736	0.0485	-0.409674829	count	1
ZNF568	-0.3381401	0.195345	-1.731	0.0835	-0.409379071	count	1
PRDM16	-0.3205889	0.2935606	-1.0921	0.275	-0.409170678	count	1
SURF2	-0.304029	0.0998919	-3.0436	0.00235	-0.409073338	count	1
LUZP1	-0.2970093	0.1034963	-2.8698	0.00413	-0.409045791	count	1
MYO18A	-0.3549342	0.365326	-0.9716	0.331	-0.408586264	count	1
GGT5	-0.3036731	0.1042182	-2.9138	0.00359	-0.408251143	count	1
DGCR6L	-0.2939823	0.0788784	-3.727	0.000197	-0.408180023	count	1
EXOSC5	-0.31309	0.1375651	-2.2759	0.0229	-0.408097382	count	1
SIRT7	-0.3282353	0.1699056	-1.9319	0.0535	-0.408063881	count	1
NBPF19	-0.5109325	0.3253547	-1.5704	0.116	-0.407870662	count	1
ESS2	-0.3690703	0.206585	-1.7865	0.0741	-0.407799063	count	1
CTSD	-0.2852305	0.0425315	-6.7063	2.30E-11	-0.407787044	count	5.46E-07
ST6GALNAC4	-0.3225003	0.1478646	-2.1811	0.0292	-0.40766678	count	1
RIMKLB	-0.3141825	0.1275922	-2.4624	0.0138	-0.407657574	count	1
RPS9	-0.2830585	0.0214485	-13.1972	6.92E-39	-0.407534717	count	1.68E-34
TRAPPC1	-0.2871944	0.060787	-4.7246	2.39E-06	-0.407523362	count	0.05540976
GMPR	-0.3101465	0.1530729	-2.0261	0.0428	-0.406901371	count	1
DUSP19	-0.3404678	0.1971198	-1.7272	0.0842	-0.406400035	count	1
FRY	-0.3060247	0.1485973	-2.0594	0.0395	-0.406339584	count	1
C12orf57	-0.2824473	0.027646	-10.2166	3.51E-24	-0.406058546	count	8.45E-20
AC018653.3	-0.8814504	0.6072388	-1.4516	0.147	-0.405923995	count	1
SPINT2	-0.2832793	0.0576394	-4.9147	9.28E-07	-0.405859204	count	0.021595488
AC018797.2	-0.37723	0.2423751	-1.5564	0.12	-0.405817319	count	1
TRDMT1	-0.3893991	0.2245851	-1.7339	0.083	-0.405446699	count	1
HMOX1	-0.327754	0.223678	-1.4653	0.143	-0.405062549	count	1
RNASEH2B	-0.2978507	0.0989765	-3.0093	0.00264	-0.404728627	count	1
TMSB10	-0.2810947	0.0404317	-6.9523	4.23E-12	-0.40469619	count	1.00E-07
EVA1C	-0.3095486	0.1666908	-1.857	0.0634	-0.40449196	count	1
ZNF256	-0.4490676	0.3940336	-1.1397	0.254	-0.404482617	count	1
HIST1H4D	-0.784612	0.7848253	-0.9997	0.318	-0.404178082	count	1
LRRC28	-0.3297936	0.1992162	-1.6555	0.0979	-0.404099488	count	1
LRRC37B	-0.3956481	0.3912336	-1.0113	0.312	-0.404075414	count	1
TCEAL5	-0.3541478	0.3327808	-1.0642	0.287	-0.403964778	count	1
FGGY	-0.3878671	0.2943736	-1.3176	0.188	-0.403821121	count	1
HDDC2	-0.286104	0.0676659	-4.2282	2.41E-05	-0.403657382	count	0.5530468
UBA52	-0.2803943	0.0214867	-13.0497	4.44E-38	-0.403448103	count	1.07E-33
TCEAL2	-0.2863303	0.106522	-2.688	0.00722	-0.40334047	count	1
GALNT1	-0.3003196	0.1028108	-2.9211	0.00351	-0.403203645	count	1
RPL36AL	-0.2804605	0.0251241	-11.163	1.77E-28	-0.403136759	count	4.27E-24
ST8SIA6-AS1	-1.2380979	0.7089141	-1.7465	0.0808	-0.403053578	count	1
SPC24	-1.2380979	0.7685614	-1.6109	0.107	-0.403053578	count	1

DNER	-1.2380979	0.7783833	-1.5906	0.112	-0.403053578	count	1
AL590004.4	-1.2380979	0.8950049	-1.3833	0.167	-0.403053578	count	1
ZNF446	-0.4189524	0.2570353	-1.6299	0.103	-0.402953592	count	1
COX20	-0.2852646	0.0703267	-4.0563	5.09E-05	-0.402766687	count	1
FGD1	-0.5404937	0.3899941	-1.3859	0.166	-0.402652242	count	1
SCUBE1	-0.5898182	0.5923612	-0.9957	0.319	-0.402492458	count	1
PCLO	-0.8743791	0.7957395	-1.0988	0.272	-0.402466522	count	1
NFKB2	-0.3525039	0.2018065	-1.7467	0.0808	-0.402064494	count	1
RAB3IP	-0.4305927	0.3809026	-1.1305	0.258	-0.401734826	count	1
CLEC7A	-1.2334111	0.8207976	-1.5027	0.133	-0.401462675	count	1
RPL4	-0.2802506	0.033366	-8.3993	6.34E-17	-0.401355574	count	1.52E-12
AC009948.1	-0.4233598	0.2955456	-1.4325	0.152	-0.4012403	count	1
CNN2	-0.2872744	0.0944754	-3.0407	0.00238	-0.401209416	count	1
CD151	-0.2786763	0.032625	-8.5418	1.91E-17	-0.400815281	count	4.57E-13
AC010201.2	-1.6872908	1.07342	-1.5719	0.116	-0.400588714	count	1
ASPRV1	-1.6872908	1.188293	-1.4199	0.156	-0.400588714	count	1
MYL6	-0.2777748	0.026908	-10.3231	1.20E-24	-0.400400929	count	2.89E-20
INPP5F	-0.3582261	0.2035285	-1.7601	0.0785	-0.400319967	count	1
RPS3	-0.2778008	0.0223365	-12.4371	8.20E-35	-0.400290894	count	1.98E-30
TIMM8B	-0.2890502	0.0906001	-3.1904	0.00143	-0.400018682	count	1
PARK7	-0.2784128	0.0304647	-9.1389	1.02E-19	-0.399746086	count	2.45E-15
CNKSR3	-0.3248024	0.2745497	-1.183	0.237	-0.399699071	count	1
FAM117A	-0.3429472	0.1937612	-1.7699	0.0768	-0.399557313	count	1
SNCAIP	-0.3709074	0.206923	-1.7925	0.0731	-0.398900709	count	1
ETV7	-0.3588723	0.2353756	-1.5247	0.127	-0.398752671	count	1
RNASEH1-AS1	-0.3260823	0.1731668	-1.8831	0.0598	-0.39858717	count	1
EEF1B2	-0.2771513	0.0280814	-9.8696	1.08E-22	-0.398522819	count	2.60E-18
PANO1	-0.656588	0.5774664	-1.137	0.256	-0.398519847	count	1
PLXNB2	-0.2936511	0.1437912	-2.0422	0.0412	-0.398507955	count	1
MCTS1	-0.2917428	0.0946753	-3.0815	0.00207	-0.398499763	count	1
AP001527.2	-0.8659405	0.5037988	-1.7188	0.0857	-0.398340956	count	1
KCNJ2-AS1	-1.0012349	0.5853001	-1.7106	0.0872	-0.398213015	count	1
MTRF1L	-0.2911102	0.1099295	-2.6482	0.00813	-0.39812568	count	1
NT5C3B	-0.2872641	0.0799456	-3.5932	0.000331	-0.398050046	count	1
PTPN14	-0.30967	0.1765525	-1.754	0.0795	-0.398013678	count	1
CCNI	-0.2767449	0.0281516	-9.8305	1.58E-22	-0.397915847	count	3.80E-18
CCDC9	-0.4193696	0.2609824	-1.6069	0.108	-0.397364467	count	1
PRMT2	-0.2803715	0.0618444	-4.5335	5.99E-06	-0.397314206	count	0.13832108
ABCF2	-0.3423821	0.2448471	-1.3984	0.162	-0.397300478	count	1
UFSP1	-0.4835663	0.3222934	-1.5004	0.134	-0.397054508	count	1
TGFBI	-0.2975206	0.1315165	-2.2622	0.0237	-0.396961482	count	1
CENPU	-0.5554604	0.437536	-1.2695	0.204	-0.39694819	count	1
ZNF620	-0.3847109	0.2438019	-1.578	0.115	-0.396673511	count	1
MTRNR2L8	-0.2848148	0.0720531	-3.9528	7.87E-05	-0.396567083	count	1
DAGLB	-0.5813732	0.4926418	-1.1801	0.238	-0.396465399	count	1

ZCRB1	-0.2798734	0.0579764	-4.8274	1.44E-06	-0.396420277	count	0.03345552
UBXN1	-0.2780676	0.0451666	-6.1565	8.24E-10	-0.396388923	count	1.95E-05
RPS15A	-0.2751477	0.0210293	-13.084	2.89E-38	-0.396347413	count	7.00E-34
CCNJL	-1.2183317	0.635713	-1.9165	0.0554	-0.396338144	count	1
AL138737.1	-0.7695184	0.5497348	-1.3998	0.162	-0.395940189	count	1
LTB4R	-0.531601	0.841464	-0.6318	0.528	-0.395760157	count	1
RASSF1-AS1	-0.9950554	0.7151065	-1.3915	0.164	-0.395604933	count	1
EGLN2	-0.9950554	0.827449	-1.2026	0.229	-0.395604933	count	1
ARRDC3-AS1	-0.7024809	0.618332	-1.1361	0.256	-0.395583188	count	1
MAP7	-1.2159087	0.4688084	-2.5936	0.00954	-0.395513916	count	1
ICK	-0.2905276	0.0995514	-2.9184	0.00354	-0.395446668	count	1
AL139289.2	-0.7678026	0.6869178	-1.1178	0.264	-0.39500409	count	1
CD2AP	-0.3040147	0.1443744	-2.1057	0.0353	-0.39476404	count	1
LRRC32	-0.2915379	0.1176715	-2.4776	0.0133	-0.394584242	count	1
ZNF419	-0.6099503	0.3607162	-1.6909	0.0909	-0.394022253	count	1
PRAF2	-0.2841572	0.0878164	-3.2358	0.00122	-0.393843482	count	1
LINC01943	-0.7656187	0.5079098	-1.5074	0.132	-0.393812729	count	1
KLHL11	-0.4792494	0.3616337	-1.3252	0.185	-0.393389074	count	1
MAST3	-0.3464085	0.3749071	-0.924	0.356	-0.39310717	count	1
PPM1B	-0.3041225	0.1333039	-2.2814	0.0226	-0.392961954	count	1
RNF7	-0.2749907	0.0480514	-5.7228	1.13E-08	-0.392805107	count	0.000265787
FBXO4	-0.3121902	0.1749773	-1.7842	0.0745	-0.392715272	count	1
URM1	-0.2852937	0.0996141	-2.864	0.00421	-0.392503953	count	1
EID3	-0.3170149	0.2342693	-1.3532	0.176	-0.392470992	count	1
PFDN5	-0.2724723	0.0256856	-10.608	6.47E-26	-0.392017875	count	1.56E-21
CHTF18	-0.8529849	0.5048923	-1.6894	0.0912	-0.392008336	count	1
TPM1	-0.2720592	0.034998	-7.7736	9.84E-15	-0.391982481	count	2.35E-10
RNF180	-0.285814	0.1341699	-2.1302	0.0332	-0.391768716	count	1
C1orf54	-0.2803585	0.0797264	-3.5165	0.000443	-0.391638146	count	1
CCDC115	-0.2803204	0.0741267	-3.7816	0.000158	-0.391480762	count	1
KCNC4	-0.4645945	0.3433191	-1.3532	0.176	-0.391472589	count	1
CLIC4	-0.275488	0.0608526	-4.5271	6.17E-06	-0.391455968	count	0.14244679
MYO9B	-0.3000626	0.1398198	-2.1461	0.0319	-0.39136506	count	1
PAQR8	-0.5739412	0.4010826	-1.431	0.153	-0.39116487	count	1
FNDC1	-0.4014572	0.3030363	-1.3248	0.185	-0.391160084	count	1
BET1L	-0.2998812	0.2225727	-1.3473	0.178	-0.391127172	count	1
THNSL1	-0.4640866	0.3198404	-1.451	0.147	-0.391030756	count	1
PIANP	-0.4432424	0.2493254	-1.7778	0.0755	-0.390955603	count	1
CD9	-0.2715301	0.0379813	-7.1491	1.05E-12	-0.390869382	count	2.50E-08
AGAP2-AS1	-0.3256533	0.2058637	-1.5819	0.114	-0.390861738	count	1
NR2C2AP	-0.3138823	0.1617804	-1.9402	0.0524	-0.390806983	count	1
SNRPF	-0.2774024	0.0619762	-4.476	7.84E-06	-0.39071045	count	0.1808296
TRAPPC13	-0.3234667	0.2379077	-1.3596	0.174	-0.390395755	count	1
FOXS1	-0.2849406	0.1277492	-2.2305	0.0258	-0.390215939	count	1
ENPP5	-0.3823071	0.3054003	-1.2518	0.211	-0.390179921	count	1

TMEM203	-0.2889952	0.0974585	-2.9653	0.00304	-0.390148887	count	1
BHLHB9	-0.4117359	0.3283724	-1.2539	0.21	-0.389953013	count	1
MAPK4	-1.640511	0.6020109	-2.7251	0.00646	-0.389849312	count	1
HINT3	-0.3145656	0.1506818	-2.0876	0.0369	-0.389413279	count	1
CLYBL	-0.4175998	0.2738049	-1.5252	0.127	-0.389303139	count	1
LSM7	-0.2743555	0.056355	-4.8683	1.17E-06	-0.388955698	count	0.02719899
HELLS	-0.4170942	0.2952772	-1.4126	0.158	-0.38881964	count	1
HRAS	-0.2808091	0.0899177	-3.123	0.0018	-0.388758102	count	1
MMRN2	-0.5223057	0.335099	-1.5587	0.119	-0.388562023	count	1
EFR3B	-1.634238	0.9884842	-1.6533	0.0984	-0.388397769	count	1
LBX2	-1.634238	0.9884842	-1.6533	0.0984	-0.388397769	count	1
MYRIP	-1.634238	0.9884842	-1.6533	0.0984	-0.388397769	count	1
NSG1	-1.634238	0.9884842	-1.6533	0.0984	-0.388397769	count	1
FSCN3	-1.634238	0.9884842	-1.6533	0.0984	-0.388397769	count	1
MANCR	-1.634238	0.9884842	-1.6533	0.0984	-0.388397769	count	1
AC087564.1	-1.634238	0.9884842	-1.6533	0.0984	-0.388397769	count	1
AC044802.1	-1.634238	0.9884842	-1.6533	0.0984	-0.388397769	count	1
LINC00114	-1.634238	0.9884842	-1.6533	0.0984	-0.388397769	count	1
CAPN11	-1.634238	1.422203	-1.1491	0.251	-0.388397769	count	1
AP4B1-AS1	-1.634238	1.492024	-1.0953	0.273	-0.388397769	count	1
SSSCA1	-0.2797159	0.0908331	-3.0794	0.00209	-0.388283426	count	1
STX4	-0.2906986	0.1083906	-2.682	0.00735	-0.388218826	count	1
PQLC1	-0.2990843	0.1238234	-2.4154	0.0158	-0.387956253	count	1
DACT1	-0.3038753	0.1981104	-1.5339	0.125	-0.387698622	count	1
AL035701.1	-0.9761937	0.8053039	-1.2122	0.226	-0.38764194	count	1
AL592148.3	-0.9761937	0.9017802	-1.0825	0.279	-0.38764194	count	1
TMPPE	-0.9761937	0.9343481	-1.0448	0.296	-0.38764194	count	1
SMIM25	-0.9761937	1.0459848	-0.9333	0.351	-0.38764194	count	1
KIAA0895	-0.6003781	0.3815327	-1.5736	0.116	-0.387535857	count	1
BCL7C	-0.2723374	0.0565719	-4.814	1.54E-06	-0.387440927	count	0.0357665
PDK1	-0.4029193	0.3672138	-1.0972	0.273	-0.387170428	count	1
FADS3	-0.2755527	0.0774425	-3.5582	0.000378	-0.386836325	count	1
TBC1D7	-0.3572898	0.2016673	-1.7717	0.0765	-0.386826261	count	1
SHTN1	-0.3196661	0.2424452	-1.3185	0.187	-0.386799581	count	1
JADE1	-0.2999843	0.173272	-1.7313	0.0835	-0.386767638	count	1
CORO1A	-0.5664895	0.4696199	-1.2063	0.228	-0.385853767	count	1
GCA	-0.2980026	0.1430173	-2.0837	0.0373	-0.385789725	count	1
KXD1	-0.2744432	0.0736294	-3.7274	0.000196	-0.385717918	count	1
HGF	-0.2870418	0.1171615	-2.45	0.0143	-0.385644618	count	1
ERMARD	-0.3819376	0.2642301	-1.4455	0.148	-0.385627055	count	1
GEM	-0.268736	0.057838	-4.6464	3.50E-06	-0.385521941	count	0.0810215
C19orf53	-0.2692769	0.0387629	-6.9468	4.40E-12	-0.385208026	count	1.05E-07
FAU	-0.2672106	0.0203128	-13.1548	1.18E-38	-0.385088855	count	2.86E-34
CST6	-0.2756727	0.1585611	-1.7386	0.0822	-0.384733387	count	1
ACTG1	-0.2667239	0.0190551	-13.9975	2.07E-43	-0.384477625	count	5.02E-39

AC008966.1	-0.5382565	0.5335753	-1.0088	0.313	-0.384131742	count	1
MT-ATP6	-0.2660634	0.0215649	-12.3378	2.69E-34	-0.383554654	count	6.50E-30
GGN	-1.1804511	0.6234198	-1.8935	0.0584	-0.383427711	count	1
TRPC4	-0.7462048	0.3288732	-2.269	0.0233	-0.383227826	count	1
EPS8	-0.2706098	0.0541526	-4.9972	6.09E-07	-0.383083734	count	0.014199444
CMPK2	-0.5361727	0.3254702	-1.6474	0.0996	-0.382580749	count	1
AL137186.2	-0.4542833	0.50227	-0.9045	0.366	-0.382506543	count	1
AC060780.1	-0.4037029	0.2595961	-1.5551	0.12	-0.382158924	count	1
BLVRA	-0.2767583	0.0961553	-2.8782	0.00402	-0.382143532	count	1
AC027237.3	-0.6794169	0.6539614	-1.0389	0.299	-0.381869236	count	1
SARNP	-0.3190888	0.184466	-1.7298	0.0838	-0.381778098	count	1
SLFN13	-1.1754899	0.6103385	-1.926	0.0542	-0.381733235	count	1
RPS4Y1	-0.2655018	0.0412134	-6.4421	1.33E-10	-0.380680596	count	3.15E-06
VIPR1	-0.5114421	0.3965916	-1.2896	0.197	-0.380157171	count	1
SLC47A1	-0.8287087	0.4103379	-2.0196	0.0435	-0.380147835	count	1
VIM-AS1	-0.3688841	0.3574644	-1.0319	0.302	-0.38004489	count	1
PPP1R14B	-0.2663084	0.0468229	-5.6876	1.39E-08	-0.379925496	count	0.000326831
DAAM1	-0.2699503	0.0790464	-3.4151	0.000645	-0.379733747	count	1
NOC4L	-0.3285277	0.1851188	-1.7747	0.076	-0.379451192	count	1
ZNF71	-0.3647437	0.3352155	-1.0881	0.277	-0.379306476	count	1
COX8A	-0.2646176	0.0371353	-7.1258	1.24E-12	-0.378761832	count	2.95E-08
RPS29	-0.2635539	0.0275497	-9.5665	1.96E-21	-0.37853232	count	4.71E-17
DHRS1	-0.2810642	0.1285592	-2.1863	0.0289	-0.378524876	count	1
AC011450.1	-0.9540043	0.6411698	-1.4879	0.137	-0.378270811	count	1
GOLGA6L10	-0.9540043	0.819928	-1.1635	0.245	-0.378270811	count	1
METTL3	-0.3109182	0.16846	-1.8456	0.065	-0.378050448	count	1
RBM45	-0.3467226	0.2663623	-1.3017	0.193	-0.377821031	count	1
LAP3	-0.2746851	0.0877131	-3.1316	0.00175	-0.377579278	count	1
AVEN	-0.2820297	0.1301193	-2.1675	0.0303	-0.37715627	count	1
AC084809.2	-1.1615546	0.9222921	-1.2594	0.208	-0.376969524	count	1
TMCC2	-0.7339045	0.6175017	-1.1885	0.235	-0.376527387	count	1
CCDC90B	-0.2681967	0.0683095	-3.9262	8.79E-05	-0.376471286	count	1
CPEB4	-0.2867054	0.1325185	-2.1635	0.0306	-0.376282897	count	1
METTL26	-0.2647544	0.054873	-4.8249	1.46E-06	-0.376170065	count	0.03391872
RILP	-0.28759	0.1623454	-1.7715	0.0766	-0.375961998	count	1
MCF2	-1.1583788	0.4763779	-2.4316	0.0151	-0.375883071	count	1
AC112229.3	-0.620236	0.6758123	-0.9178	0.359	-0.37532101	count	1
SPHK2	-0.3379991	0.2613421	-1.2933	0.196	-0.37522788	count	1
ITIH4	-0.3292416	0.6047776	-0.5444	0.586	-0.37519297	count	1
PDCD4-AS1	-1.576569	0.831592	-1.8958	0.0581	-0.374936176	count	1
MTX3	-0.3243825	0.2529859	-1.2822	0.2	-0.374605205	count	1
CDK2	-0.3570903	0.327487	-1.0904	0.276	-0.374571318	count	1
HEPH	-0.3121441	0.1757507	-1.7761	0.0758	-0.374487093	count	1
TM4SF18	-1.5744017	1.14159	-1.3791	0.168	-0.37442608	count	1
CYSLTR2	-0.729731	0.5423994	-1.3454	0.179	-0.374255082	count	1

STARD13	-0.3268761	0.2121745	-1.5406	0.124	-0.374207792	count	1
UQCR11	-0.2625661	0.0435184	-6.0334	1.76E-09	-0.373903257	count	4.15E-05
AC092171.4	-0.8155597	0.5099958	-1.5991	0.11	-0.37372764	count	1
RPL24	-0.2593327	0.0212717	-12.1915	1.53E-33	-0.373651148	count	3.70E-29
DDAH1	-0.3064686	0.2279422	-1.3445	0.179	-0.373507522	count	1
MDFIC	-0.2772061	0.1150291	-2.4099	0.016	-0.373459108	count	1
AL645608.3	-1.1511034	0.9303225	-1.2373	0.216	-0.373393053	count	1
RPS25	-0.259187	0.0205488	-12.6133	9.75E-36	-0.373371989	count	2.36E-31
RPL19	-0.258866	0.0180385	-14.3508	1.76E-45	-0.373213841	count	4.27E-41
LFNG	-0.6167902	0.4851492	-1.2713	0.204	-0.373125621	count	1
CBWD5	-0.3117294	0.2369691	-1.3155	0.188	-0.372884051	count	1
ENC1	-0.4549503	0.2713553	-1.6766	0.0937	-0.372784	count	1
RNF25	-0.3022477	0.179266	-1.686	0.0919	-0.372506141	count	1
IFIT3	-0.2686804	0.1617375	-1.6612	0.0968	-0.372399208	count	1
RPS14	-0.2582853	0.0193061	-13.3784	6.86E-40	-0.372325252	count	1.66E-35
GFRA2	-0.615022	0.5670498	-1.0846	0.278	-0.371999331	count	1
MCRIP2	-0.2957159	0.1747528	-1.6922	0.0907	-0.371842084	count	1
CNST	-0.2887701	0.1720023	-1.6789	0.0933	-0.371820061	count	1
AL359258.2	-0.4417065	0.3321232	-1.3299	0.184	-0.371582118	count	1
MRPS12	-0.2707267	0.087435	-3.0963	0.00197	-0.371438086	count	1
HLA-C	-0.2583252	0.0301012	-8.5819	1.36E-17	-0.371310845	count	3.26E-13
FAM120C	-0.3721558	0.2860211	-1.3011	0.193	-0.371270161	count	1
NRAV	-0.4530978	0.3443051	-1.316	0.188	-0.371215077	count	1
POLR2G	-0.2680482	0.0796868	-3.3638	0.000777	-0.371054426	count	1
SERF2	-0.257427	0.0205806	-12.5082	3.48E-35	-0.370829716	count	8.41E-31
METTL5	-0.2611559	0.0617757	-4.2275	2.42E-05	-0.370761713	count	0.5553174
LRCH4	-0.3380675	0.4175909	-0.8096	0.418	-0.370694287	count	1
SAMD3	-0.4654706	0.6941059	-0.6706	0.503	-0.370334594	count	1
DUSP3	-0.2769368	0.1244795	-2.2248	0.0262	-0.370318772	count	1
STX6	-0.289823	0.1807075	-1.6038	0.109	-0.3701259	count	1
CMC2	-0.2730837	0.1163162	-2.3478	0.0189	-0.370027438	count	1
ORC6	-0.9342865	0.6200478	-1.5068	0.132	-0.369942217	count	1
AC010654.1	-0.6590631	0.6546192	-1.0068	0.314	-0.369786029	count	1
GADD45GIP1	-0.2591794	0.0444317	-5.8332	5.91E-09	-0.369780279	count	0.000139133
LMAN2L	-0.3555894	0.2280058	-1.5596	0.119	-0.369612746	count	1
AC108047.1	-0.57382	0.5320771	-1.0785	0.281	-0.369567923	count	1
BEND6	-0.3307242	0.2434246	-1.3586	0.174	-0.369162295	count	1
MTHFD1	-0.3169871	0.1737346	-1.8245	0.0682	-0.368966839	count	1
FDXACB1	-0.4967837	0.463918	-1.0708	0.284	-0.368830419	count	1
CNBD2	-0.3741072	0.2587204	-1.446	0.148	-0.368720578	count	1
COL1A1	-0.2580248	0.1072578	-2.4056	0.0162	-0.368642746	count	1
TBC1D1	-0.2681804	0.0927818	-2.8904	0.00387	-0.368615845	count	1
COPS7B	-0.3234929	0.1941624	-1.6661	0.0958	-0.368557908	count	1
SELENOM	-0.255809	0.0250667	-10.2051	3.94E-24	-0.368346009	count	9.49E-20
MRPL47	-0.2679771	0.0850364	-3.1513	0.00164	-0.368188893	count	1

HOXB3	-0.3649248	0.2384151	-1.5306	0.126	-0.368107366	count	1
APOPT1	-0.2681295	0.096795	-2.7701	0.00563	-0.368097813	count	1
SELENOW	-0.2557325	0.0274368	-9.3208	1.94E-20	-0.368051561	count	4.66E-16
TNIP2	-0.2745934	0.116341	-2.3602	0.0183	-0.368041075	count	1
SLC25A38	-0.2859347	0.1421643	-2.0113	0.0444	-0.36773995	count	1
CAV2	-0.2580863	0.0484854	-5.323	1.08E-07	-0.36771611	count	0.00252882
LINC02298	-0.426717	0.4512209	-0.9457	0.344	-0.367575378	count	1
C1orf198	-0.2643417	0.1044355	-2.5311	0.0114	-0.367304684	count	1
CBX4	-0.3044864	0.2281902	-1.3344	0.182	-0.367271969	count	1
PPP1R18	-0.2805625	0.1187172	-2.3633	0.0182	-0.367027834	count	1
RANGAP1	-0.3530525	0.286461	-1.2325	0.218	-0.366927534	count	1
AC022613.2	-0.7158549	0.4613481	-1.5517	0.121	-0.36670452	count	1
AC026202.2	-0.7158549	0.4959759	-1.4433	0.149	-0.36670452	count	1
AK4	-0.3064982	0.1733905	-1.7677	0.0772	-0.366563947	count	1
EIF3L	-0.2584929	0.051408	-5.0283	5.19E-07	-0.366351323	count	0.012104118
BX284668.6	-0.4353399	0.3774548	-1.1534	0.249	-0.366057046	count	1
ZNF79	-0.4928306	0.483095	-1.0202	0.308	-0.365778654	count	1
NDUFB2	-0.2561613	0.0415346	-6.1674	7.69E-10	-0.365755439	count	1.82E-05
CHKB	-0.4344795	0.3495616	-1.2429	0.214	-0.365310614	count	1
IFIH1	-0.3017422	0.1914686	-1.5759	0.115	-0.364910458	count	1
LINC01198	-1.1262975	0.6965175	-1.617	0.106	-0.364892508	count	1
HDX	-0.3202171	0.1666844	-1.9211	0.0548	-0.364778038	count	1
TOP3B	-0.3857055	0.5446519	-0.7082	0.479	-0.364716176	count	1
SLC12A1	-1.5325413	0.7558163	-2.0277	0.0427	-0.364521197	count	1
LINC02453	-1.5325413	0.9895773	-1.5487	0.122	-0.364521197	count	1
CYP2E1	-1.5325413	1.329332	-1.1529	0.249	-0.364521197	count	1
FZR1	-0.2983125	0.2432232	-1.2265	0.22	-0.364349264	count	1
RCN2	-0.2578219	0.0579886	-4.4461	9.00E-06	-0.364161627	count	0.207459
FAM87B	-0.6021344	0.4888549	-1.2317	0.218	-0.363795764	count	1
MIS18A	-0.3193537	0.1959122	-1.6301	0.103	-0.363781916	count	1
NR1H2	-0.2609983	0.0800765	-3.2594	0.00113	-0.363651128	count	1
RANBP9	-0.2803616	0.1489973	-1.8817	0.06	-0.363537471	count	1
GNAI2	-0.2556672	0.04872	-5.2477	1.63E-07	-0.363481954	count	0.003812733
AL365259.1	-0.4721071	0.4028459	-1.1719	0.241	-0.363335706	count	1
CEP72	-1.5271024	0.7086678	-2.1549	0.0312	-0.363226807	count	1
RAB11FIP1	-0.268537	0.1334089	-2.0129	0.0442	-0.363098365	count	1
STAC	-0.3035244	0.3205507	-0.9469	0.344	-0.362971874	count	1
CNPY2	-0.2570093	0.0672342	-3.8226	0.000134	-0.362901383	count	1
MAPKAPK2	-0.2845372	0.1440075	-1.9759	0.0482	-0.362869736	count	1
SP4	-0.3331786	0.2332187	-1.4286	0.153	-0.362836992	count	1
RSU1	-0.2569509	0.0601631	-4.2709	2.00E-05	-0.362601451	count	0.45954
ARPC4	-0.2576791	0.0635758	-4.0531	5.16E-05	-0.362188454	count	1
AL365434.2	-0.9155418	0.6837318	-1.339	0.181	-0.362024912	count	1
TGFB2-AS1	-0.9155418	0.7519871	-1.2175	0.223	-0.362024912	count	1
LINC02055	-0.9155418	0.7519871	-1.2175	0.223	-0.362024912	count	1

AL591501.1	-0.9155418	0.9255333	-0.9892	0.323	-0.362024912	count	1
GPR85	-0.9155418	1.102495	-0.8304	0.406	-0.362024912	count	1
IRX2	-1.521986	0.3661466	-4.1568	3.30E-05	-0.36200767	count	0.755865
AL669831.5	-0.3207175	0.2366875	-1.355	0.175	-0.361736826	count	1
H2AFZ	-0.2531947	0.043365	-5.8387	5.72E-09	-0.361724057	count	0.000134666
AC090673.1	-1.5197381	0.8612777	-1.7645	0.0777	-0.361471511	count	1
SH3BGRL3	-0.2513337	0.0406923	-6.1764	7.27E-10	-0.361371765	count	1.72E-05
HECTD3	-0.3509649	0.2796933	-1.2548	0.21	-0.361241644	count	1
GATA6-AS1	-0.3080739	0.2623642	-1.1742	0.24	-0.361210888	count	1
CKMT2-AS1	-0.3316917	0.2216019	-1.4968	0.135	-0.361192848	count	1
IL18BP	-0.3665331	0.213805	-1.7143	0.0866	-0.361096218	count	1
KLC4	-0.3619409	0.293723	-1.2323	0.218	-0.360870101	count	1
TNFAIP8L3	-0.2757044	0.1309907	-2.1048	0.0354	-0.360345757	count	1
PLCXD3	-0.3811311	0.2569113	-1.4835	0.138	-0.360287145	count	1
CCHCR1	-0.3404145	0.2322678	-1.4656	0.143	-0.359846156	count	1
MROH6	-0.4089049	0.3025097	-1.3517	0.177	-0.359825679	count	1
AC120036.4	-1.1112669	0.8641516	-1.286	0.199	-0.359734462	count	1
ATP5MC2	-0.2505338	0.0313484	-7.9919	1.76E-15	-0.359631874	count	4.20E-11
MVK	-0.3650366	0.2429431	-1.5026	0.133	-0.359590366	count	1
ZMAT3	-0.2597357	0.1094252	-2.3736	0.0177	-0.359575384	count	1
HAR1B	-0.9088135	0.7919957	-1.1475	0.251	-0.359183364	count	1
TCEA2	-0.2563805	0.0791257	-3.2402	0.00121	-0.359179552	count	1
ST7-AS1	-0.3798481	0.2587215	-1.4682	0.142	-0.35904525	count	1
MIEN1	-0.2572749	0.0735313	-3.4989	0.000473	-0.359014742	count	1
USP27X	-0.4079372	0.3407974	-1.197	0.231	-0.358949842	count	1
UBLCP1	-0.2714249	0.1364223	-1.9896	0.0467	-0.358880012	count	1
Z95114.4	-0.9080273	0.6446864	-1.4085	0.159	-0.358851333	count	1
DEDD	-0.2762193	0.1620478	-1.7046	0.0884	-0.358815755	count	1
RPL18A	-0.2487268	0.0195776	-12.7046	3.20E-36	-0.358425465	count	7.74E-32
XRN2	-0.2535628	0.0608886	-4.1644	3.19E-05	-0.358112318	count	0.7307652
MRFAP1	-0.2504629	0.0370674	-6.757	1.63E-11	-0.357833764	count	3.87E-07
TGFBR3L	-0.5559025	0.4234286	-1.3129	0.189	-0.357471447	count	1
APBB1	-0.2837492	0.1701042	-1.6681	0.0954	-0.35725535	count	1
NUBP2	-0.2610456	0.0877404	-2.9752	0.00295	-0.357241485	count	1
FMO3	-0.4055667	0.238255	-1.7022	0.0888	-0.35680471	count	1
POLR2I	-0.2535781	0.0623283	-4.0684	4.83E-05	-0.356712916	count	1
SCYL2	-0.2772878	0.2227759	-1.2447	0.213	-0.356553731	count	1
C3orf52	-0.5545262	0.5234107	-1.0594	0.289	-0.356543184	count	1
SS18	-0.2700836	0.1182559	-2.2839	0.0224	-0.356154596	count	1
AL022328.4	-0.6964122	0.3005605	-2.317	0.0206	-0.356137455	count	1
ZNF696	-0.4135019	0.339672	-1.2174	0.224	-0.355855278	count	1
IDH3A	-0.2677825	0.1158755	-2.3109	0.0209	-0.355765544	count	1
SMIM30	-0.2631291	0.1282026	-2.0524	0.0402	-0.355762264	count	1
BUD31	-0.2527481	0.06966	-3.6283	0.000289	-0.355656856	count	1
SOCS2	-0.2606349	0.1247826	-2.0887	0.0368	-0.355524385	count	1

RPL38	-0.2474064	0.0294065	-8.4133	5.64E-17	-0.354768811	count	1.35E-12
TTC9	-0.5517887	0.5868341	-0.9403	0.347	-0.354697234	count	1
MYO10	-0.5225853	0.618282	-0.8452	0.398	-0.354638024	count	1
CKAP2	-0.3556631	0.2789078	-1.2752	0.202	-0.354482899	count	1
ZNF85	-0.3694448	0.2647249	-1.3956	0.163	-0.354285511	count	1
ARL6IP4	-0.2476082	0.0368162	-6.7255	2.02E-11	-0.354216789	count	4.79E-07
CDC25B	-0.355313	0.2606671	-1.3631	0.173	-0.354126785	count	1
FOXC1	-0.2480718	0.0594269	-4.1744	3.06E-05	-0.354042307	count	0.7011378
RWDD2A	-0.3042469	0.2184224	-1.3929	0.164	-0.353970137	count	1
IFITM10	-0.6922721	0.6144798	-1.1266	0.26	-0.353889327	count	1
CRELD1	-0.2705503	0.1457878	-1.8558	0.0636	-0.353575545	count	1
C19orf54	-0.5498143	0.5323206	-1.0329	0.302	-0.353366187	count	1
AC099778.1	-0.3937005	0.3136767	-1.2551	0.21	-0.353318159	count	1
FIGN	-0.2878288	0.178247	-1.6148	0.106	-0.353052948	count	1
ATP1B3	-0.2532223	0.0733622	-3.4517	0.000563	-0.353045758	count	1
RAPH1	-0.2904669	0.1535776	-1.8913	0.0587	-0.352964343	count	1
SPARC	-0.2457618	0.0434603	-5.6549	1.68E-08	-0.352864404	count	0.000394867
GCAT	-0.3286578	0.1959264	-1.6775	0.0935	-0.352757175	count	1
DHRS11	-0.3678179	0.2641676	-1.3924	0.164	-0.352689713	count	1
ELOA-AS1	-0.4098685	0.3217357	-1.2739	0.203	-0.352635631	count	1
SLC19A1	-0.6893967	0.5677797	-1.2142	0.225	-0.352328388	count	1
TMEM8A	-0.2984661	0.1741868	-1.7135	0.0867	-0.352315163	count	1
WSCD2	-0.6886254	0.6989688	-0.9852	0.325	-0.351909763	count	1
PAG1	-0.4188403	0.358557	-1.1681	0.243	-0.351754636	count	1
CDNF	-0.6880195	0.4162893	-1.6527	0.0985	-0.351580902	count	1
ABCB4	-0.6880195	0.5625703	-1.223	0.221	-0.351580902	count	1
VAV2	-0.3996637	0.3392188	-1.1782	0.239	-0.351465117	count	1
ZFY-AS1	-0.3613833	0.445178	-0.8118	0.417	-0.35128145	count	1
CD2BP2	-0.2523971	0.0789734	-3.196	0.00141	-0.351237567	count	1
SLC24A3	-0.4292532	0.3814095	-1.1254	0.26	-0.351046739	count	1
TMEM240	-0.4737194	0.5749587	-0.8239	0.41	-0.351042687	count	1
HINT1	-0.244563	0.0300103	-8.1493	4.97E-16	-0.351032408	count	1.19E-11
AC005618.1	-0.6868515	0.512668	-1.3398	0.18	-0.350947022	count	1
9-Sep	-0.2825641	0.1538952	-1.8361	0.0664	-0.350861288	count	1
MT-ND6	-0.2653507	0.120411	-2.2037	0.0276	-0.350813419	count	1
C14orf28	-0.2847798	0.1571298	-1.8124	0.07	-0.350804236	count	1
FAM189B	-0.2968967	0.2032212	-1.461	0.144	-0.350443152	count	1
GNAQ	-0.2574608	0.1242002	-2.0729	0.0382	-0.350410761	count	1
CARD19	-0.2540205	0.0921486	-2.7566	0.00587	-0.350268906	count	1
ECE1	-0.254752	0.1192491	-2.1363	0.0327	-0.350044627	count	1
EIF3D	-0.2491059	0.0619489	-4.0212	5.91E-05	-0.349570396	count	1
C17orf100	-0.4717149	0.5783062	-0.8157	0.415	-0.349498848	count	1
CRYBG1	-0.3430038	0.3084102	-1.1122	0.266	-0.349324711	count	1
ATOX1	-0.2459359	0.0550099	-4.4708	8.03E-06	-0.349114118	count	0.18518786
MYL12A	-0.242499	0.0316237	-7.6683	2.22E-14	-0.348977259	count	5.29E-10

TNIP1	-0.2770805	0.1317113	-2.1037	0.0355	-0.348800844	count	1
RILPL1	-0.2580322	0.1228979	-2.0996	0.0358	-0.34872419	count	1
JPH2	-0.2983059	0.2636464	-1.1315	0.258	-0.348328876	count	1
ZNF774	-1.0779763	0.7178255	-1.5017	0.133	-0.348293693	count	1
GOLGA7B	-1.4632483	0.8329336	-1.7567	0.079	-0.347909741	count	1
AC002398.2	-1.4632483	0.9276172	-1.5774	0.115	-0.347909741	count	1
HTRA2	-0.2569788	0.0952342	-2.6984	0.007	-0.347903509	count	1
KMT5A	-0.252754	0.1046414	-2.4154	0.0158	-0.347830852	count	1
ZNF20	-0.8819037	0.7891869	-1.1175	0.264	-0.347821729	count	1
KIF2C	-1.0758288	0.719858	-1.4945	0.135	-0.347554988	count	1
RPL10A	-0.241246	0.0221628	-10.8852	3.52E-27	-0.347480182	count	8.49E-23
TMEM121	-0.3213539	0.2302328	-1.3958	0.163	-0.347333507	count	1
STEAP2	-0.2719137	0.165201	-1.646	0.0999	-0.347114298	count	1
RPL5	-0.2408094	0.02199	-10.9509	1.75E-27	-0.347093609	count	4.22E-23
HPCAL1	-0.2665403	0.1962349	-1.3583	0.174	-0.346810897	count	1
KANK2	-0.2467915	0.0689765	-3.5779	0.000351	-0.346765183	count	1
TFB1M	-0.2703043	0.1550095	-1.7438	0.0813	-0.34672774	count	1
EMX2OS	-0.3164286	0.3013278	-1.0501	0.294	-0.346624268	count	1
GART	-0.2722174	0.1704094	-1.5974	0.11	-0.346608082	count	1
EIF1AY	-0.2541779	0.0915071	-2.7777	0.0055	-0.346599977	count	1
EXOSC2	-0.2908024	0.2541302	-1.1443	0.253	-0.346551108	count	1
CALCRL	-0.2607203	0.1120604	-2.3266	0.02	-0.34594165	count	1
ACAN	-0.2561299	0.1916648	-1.3363	0.182	-0.345893552	count	1
EIF5B	-0.2425557	0.0522974	-4.638	3.64E-06	-0.34582011	count	0.0842296
LRRC29	-0.3556958	0.3157548	-1.1265	0.26	-0.345632854	count	1
HMGB3	-0.4349628	0.3999037	-1.0877	0.277	-0.345239218	count	1
OCRL	-0.3295426	0.2609685	-1.2628	0.207	-0.345186316	count	1
RSL24D1	-0.2422808	0.0452013	-5.36	8.83E-08	-0.345178632	count	0.002068339
AC009414.2	-0.2687455	0.1660908	-1.6181	0.106	-0.344716372	count	1
TMEM208	-0.2447848	0.0674022	-3.6317	0.000285	-0.344346506	count	1
RPL35A	-0.2388986	0.0199146	-11.9962	1.50E-32	-0.344236008	count	3.62E-28
PLEKHO1	-0.248845	0.1032268	-2.4107	0.016	-0.344165117	count	1
ANO2	-0.7545888	0.7392795	-1.0207	0.307	-0.344009588	count	1
VMAC	-0.2968613	0.2350189	-1.2631	0.207	-0.34389461	count	1
AEN	-0.3099432	0.2164213	-1.4321	0.152	-0.343657532	count	1
BTF3	-0.2386572	0.0230837	-10.3388	1.02E-24	-0.343564907	count	2.46E-20
PGS1	-0.2962356	0.19291	-1.5356	0.125	-0.343161497	count	1
TESK2	-0.3755471	0.2801867	-1.3403	0.18	-0.343080401	count	1
RAET1E	-1.4429524	0.7097969	-2.0329	0.0421	-0.342997282	count	1
GSK3A	-0.2710548	0.131901	-2.055	0.04	-0.342723886	count	1
DGKD	-0.5055131	0.2724118	-1.8557	0.0636	-0.342538064	count	1
CPNE2	-0.2724092	0.1566739	-1.7387	0.0822	-0.342334567	count	1
AL354811.1	-0.6709696	0.4447355	-1.5087	0.131	-0.342334015	count	1
WDR4	-0.4456507	0.3731374	-1.1943	0.232	-0.342241749	count	1
STK26	-0.6116324	0.3362728	-1.8189	0.069	-0.341710149	count	1

TBCB	-0.2400612	0.0469968	-5.108	3.42E-07	-0.341673071	count	0.00798228
AC242426.2	-0.3390393	0.328145	-1.0332	0.302	-0.34149691	count	1
NME1	-0.2447867	0.0784406	-3.1207	0.00182	-0.341430834	count	1
HDGFL3	-0.2430595	0.0683191	-3.5577	0.000379	-0.341392977	count	1
CD7	-2.8713996	1.8558506	-1.5472	0.1219	-0.341244206	count	1
NUDT3	-0.2548331	0.1719896	-1.4817	0.139	-0.34114341	count	1
HDAC11	-0.2933119	0.1779151	-1.6486	0.0993	-0.341107126	count	1
AL162274.2	-0.5656305	0.7037551	-0.8037	0.422	-0.340615343	count	1
AL731571.1	-0.443531	0.3389123	-1.3087	0.191	-0.340554332	count	1
SPIRE1	-0.2648721	0.1604642	-1.6507	0.0989	-0.34049762	count	1
RNF208	-0.4792028	0.3583702	-1.3372	0.181	-0.340303406	count	1
ARVCF	-0.2729538	0.2035003	-1.3413	0.18	-0.340102938	count	1
TBC1D10C	-2.854833	1.233759	-2.3139	0.0207	-0.340073255	count	1
RPS3A	-0.2359139	0.0209229	-11.2754	5.18E-29	-0.340065316	count	1.25E-24
SUN2	-0.243797	0.0744605	-3.2742	0.00107	-0.33971353	count	1
CAMTA1	-0.2381093	0.0495081	-4.8095	1.57E-06	-0.339695816	count	0.03646168
RPS15	-0.2357887	0.0186217	-12.662	5.38E-36	-0.339659469	count	1.30E-31
RPLP1	-0.2354096	0.0206996	-11.3727	1.77E-29	-0.339517776	count	4.27E-25
GNPAT	-0.2642994	0.148417	-1.7808	0.075	-0.338580468	count	1
GNG2	-0.2946309	0.3310799	-0.8899	0.374	-0.338385028	count	1
CCDC163	-0.8584931	0.5102313	-1.6826	0.0925	-0.337943811	count	1
AC007620.2	-0.8584931	0.6569588	-1.3068	0.191	-0.337943811	count	1
SPATA5	-0.3317617	0.2210034	-1.5012	0.133	-0.337662598	count	1
ARHGAP4	-2.8211883	1.077845	-2.6174	0.0089	-0.337653094	count	1
TRIM13	-0.2499177	0.114989	-2.1734	0.0298	-0.337600659	count	1
ZC3H18	-0.2631616	0.177912	-1.4792	0.139	-0.3375122	count	1
MTM1	-0.3763992	0.2567063	-1.4663	0.143	-0.3373849	count	1
ATP5PF	-0.235226	0.0327207	-7.1889	7.88E-13	-0.337307066	count	1.87E-08
COLCA2	-0.4558294	0.3597348	-1.2671	0.205	-0.337276266	count	1
ATP13A3	-0.2449543	0.1433567	-1.7087	0.0876	-0.33713574	count	1
NFATC2	-0.3626303	0.3910047	-0.9274	0.354	-0.336864635	count	1
ARRDC2	-0.2810015	0.2020511	-1.3907	0.164	-0.336780296	count	1
PELO	-0.2454738	0.1114138	-2.2033	0.0276	-0.336779543	count	1
LIFR-AS1	-0.2958431	0.2821461	-1.0485	0.294	-0.336677491	count	1
ARHGAP5-AS1	-0.2943568	0.1965095	-1.4979	0.134	-0.336543208	count	1
DTNBP1	-0.2531092	0.1137637	-2.2249	0.0262	-0.336384933	count	1
PRKD1	-0.3509769	0.2599678	-1.3501	0.177	-0.336184827	count	1
PC	-0.4116326	0.5075274	-0.8111	0.417	-0.336175348	count	1
ATP6V0B	-0.2368	0.0496601	-4.7684	1.93E-06	-0.33613541	count	0.044776
TCEAL1	-0.2394447	0.0742888	-3.2232	0.00128	-0.33568134	count	1
FAM86B1	-0.4232991	0.4148674	-1.0203	0.308	-0.335666586	count	1
MINOS1	-0.2359081	0.0511262	-4.6142	4.08E-06	-0.335630583	count	0.09438672
POMP	-0.2350103	0.0436849	-5.3797	7.93E-08	-0.335564762	count	0.001857761
APPL2	-0.2607075	0.1487099	-1.7531	0.0797	-0.335486492	count	1
IGBP1	-0.2402111	0.0652145	-3.6834	0.000233	-0.335227677	count	1

CAMLG	-0.2374176	0.0563326	-4.2146	2.56E-05	-0.335169978	count	0.587136
NBPF1	-0.2788539	0.1941245	-1.4365	0.151	-0.335143218	count	1
NPAS1	-0.2743291	0.2725446	-1.0065	0.314	-0.334814364	count	1
LONP1	-0.3019865	0.2007718	-1.5041	0.133	-0.33471506	count	1
CBLN4	-0.3664487	0.4671024	-0.7845	0.433	-0.33456007	count	1
FDXR	-0.2888393	0.1777809	-1.6247	0.104	-0.3344976	count	1
AP1G2	-0.6563898	0.5011666	-1.3097	0.19	-0.334437877	count	1
KRT86	-0.4705893	0.6403555	-0.7349	0.462	-0.333934169	count	1
FLI1	-0.4931289	0.475144	-1.0379	0.299	-0.333775319	count	1
KLHL4	-1.404945	0.9165844	-1.5328	0.125	-0.333745409	count	1
AC007786.1	-1.404945	0.9165844	-1.5328	0.125	-0.333745409	count	1
AGAP2	-1.404945	0.9389051	-1.4964	0.135	-0.333745409	count	1
PVT1	-0.5202283	0.7009054	-0.7422	0.458	-0.333454948	count	1
AL359643.3	-0.434545	0.4391183	-0.9896	0.322	-0.333405477	count	1
TGIF2	-0.397611	0.3797494	-1.047	0.295	-0.333388663	count	1
BCL6B	-1.0337475	1.0723203	-0.964	0.3351	-0.333067193	count	1
PHB	-0.2355901	0.0635649	-3.7063	0.000213	-0.333065692	count	1
PFKFB4	-0.6538445	0.654862	-0.9984	0.318	-0.333060482	count	1
SLC25A42	-0.3082849	0.2231716	-1.3814	0.167	-0.332996216	count	1
TRAF6	-0.2812157	0.2082579	-1.3503	0.177	-0.332870153	count	1
NACA	-0.2309208	0.0210476	-10.9714	1.40E-27	-0.332770841	count	3.38E-23
STK38L	-0.2409554	0.1065854	-2.2607	0.0238	-0.33276701	count	1
PPP1CB	-0.2320759	0.0434491	-5.3413	9.79E-08	-0.332513495	count	0.002292818
DEDD2	-0.283773	0.1868306	-1.5189	0.129	-0.332422227	count	1
PPARG	-0.2847465	0.235935	-1.2069	0.228	-0.332326549	count	1
C2orf42	-0.4490291	0.3125877	-1.4365	0.151	-0.332050784	count	1
SUPT4H1	-0.2371015	0.070131	-3.3808	0.00073	-0.331859831	count	1
LAMB3	-0.5177872	0.4418208	-1.1719	0.241	-0.331815093	count	1
HIST4H4	-0.3776748	0.423853	-0.8911	0.373	-0.33160327	count	1
DUSP6	-0.2462342	0.1431583	-1.72	0.0855	-0.331597318	count	1
TMEM106A	-0.4899975	0.2941792	-1.6656	0.0959	-0.331561629	count	1
RIMS3	-0.4061512	0.3286947	-1.2356	0.217	-0.331555064	count	1
TTC12	-0.3857866	0.2993881	-1.2886	0.198	-0.33132683	count	1
DCDC2C	-0.4480795	0.5228768	-0.857	0.392	-0.331321434	count	1
SLC25A3	-0.2306817	0.0291881	-7.9033	3.56E-15	-0.331283787	count	8.50E-11
RPS4X	-0.2298141	0.0204265	-11.2508	6.79E-29	-0.331248399	count	1.64E-24
BCAP29	-0.2323113	0.0531915	-4.3674	1.29E-05	-0.331158901	count	0.2970096
EIF3J	-0.2373528	0.0837183	-2.8351	0.00461	-0.331108399	count	1
AL022069.1	-0.7279944	0.6509963	-1.1183	0.264	-0.331082264	count	1
DMAC1	-0.235671	0.07411	-3.18	0.00148	-0.331061645	count	1
RBP1	-0.2400494	0.1101659	-2.179	0.0294	-0.330962766	count	1
RPL28	-0.2295167	0.0214044	-10.7229	1.95E-26	-0.33065359	count	4.70E-22
CCDC85B	-0.230696	0.0420147	-5.4908	4.27E-08	-0.330271397	count	0.001001571
NSA2	-0.2318354	0.0449528	-5.1573	2.64E-07	-0.330217107	count	0.006166512
AC008393.1	-0.5486817	0.3474498	-1.5792	0.114	-0.329883255	count	1

SMIM27	-0.2826104	0.2336158	-1.2097	0.226	-0.329806769	count	1
AC004130.1	-0.2958365	0.2560596	-1.1553	0.248	-0.32971604	count	1
LINC01759	-0.3309563	0.2627725	-1.2595	0.208	-0.329379268	count	1
FPGT	-0.2800011	0.2334582	-1.1994	0.23	-0.329147067	count	1
RACGAP1	-0.5890508	0.4738284	-1.2432	0.214	-0.328389198	count	1
GYPC	-0.2305278	0.0483819	-4.7648	1.96E-06	-0.328389171	count	0.04546808
ZNF296	-1.0197631	0.70225	-1.4521	0.147	-0.328248515	count	1
PHACTR2	-0.2327514	0.0764196	-3.0457	0.00234	-0.328105973	count	1
SEL1L3	-0.8349703	0.8730292	-0.9564	0.339	-0.32802706	count	1
CARD8	-0.2856898	0.2947462	-0.9693	0.332	-0.327997829	count	1
ERICH2	-0.6444688	0.5776958	-1.1156	0.265	-0.327989801	count	1
C1orf35	-0.2416055	0.1030752	-2.344	0.0191	-0.32798768	count	1
DOPEY2	-0.3424562	0.3558643	-0.9623	0.336	-0.327844208	count	1
KIAA1551	-0.2448557	0.1088527	-2.2494	0.0245	-0.327583351	count	1
CHMP3	-0.2345877	0.0690409	-3.3978	0.000687	-0.327275866	count	1
OGDHL	-0.3466736	0.3690599	-0.9393	0.348	-0.326985436	count	1
TRPS1	-0.2447399	0.1343706	-1.8214	0.0686	-0.326949678	count	1
GPR35	-1.0157387	0.8228217	-1.2345	0.217	-0.326861558	count	1
AL442663.3	-0.8319987	0.7216809	-1.1529	0.249	-0.326775016	count	1
UBL5	-0.2283666	0.0364727	-6.2613	4.26E-10	-0.326679877	count	1.01E-05
PIAS3	-0.2559924	0.1737221	-1.4736	0.141	-0.326669528	count	1
MS4A6A	-2.6728653	1.0451949	-2.5573	0.0106	-0.326294604	count	1
AIP	-0.2317138	0.0616291	-3.7598	0.000173	-0.325989553	count	1
CCT3	-0.2302026	0.0530684	-4.3378	1.48E-05	-0.325762062	count	0.3405332
STX10	-0.2396583	0.099662	-2.4047	0.0162	-0.325733137	count	1
COA4	-0.231988	0.0708141	-3.276	0.00106	-0.32555973	count	1
ITPKC	-0.2789209	0.2186845	-1.2754	0.202	-0.32545533	count	1
ZNF154	-0.3448802	0.5056478	-0.6821	0.495	-0.325255238	count	1
NUP88	-0.2805739	0.2480342	-1.1312	0.258	-0.324820307	count	1
TMEM177	-0.3103972	0.2477427	-1.2529	0.21	-0.324801535	count	1
RNF6	-0.2637794	0.1992815	-1.3237	0.186	-0.324736161	count	1
PLIN3	-0.2322484	0.0755201	-3.0753	0.00212	-0.324613382	count	1
ROGDI	-0.2654211	0.1611593	-1.6469	0.0997	-0.324611193	count	1
BLID	-0.5826035	0.6631562	-0.8785	0.38	-0.324591928	count	1
CNN1	-0.2276236	0.0726851	-3.1316	0.00175	-0.32457569	count	1
KAT14	-0.4232826	0.4045983	-1.0462	0.296	-0.324456173	count	1
RNF152	-0.2650757	0.1549362	-1.7109	0.0872	-0.324185324	count	1
DOK1	-0.2908848	0.1985207	-1.4653	0.143	-0.324124959	count	1
RPL22	-0.2250622	0.020962	-10.7367	1.69E-26	-0.324091372	count	4.08E-22
GOLGA8M	-0.7133233	0.7508981	-0.95	0.342	-0.32396211	count	1
DM1-AS	-0.7133233	0.7749596	-0.9205	0.357	-0.32396211	count	1
MORN3	-0.7133233	0.7835438	-0.9104	0.363	-0.32396211	count	1
NFIA-AS2	-0.7133233	0.8134281	-0.8769	0.381	-0.32396211	count	1
SEC61B	-0.2257305	0.034735	-6.4986	9.20E-11	-0.323786643	count	2.18E-06
LGI2	-0.3066256	0.1841059	-1.6655	0.0959	-0.323564298	count	1

RCN1	-0.2293712	0.0625772	-3.6654	0.00025	-0.323478233	count	1
OST4	-0.225602	0.0319958	-7.051	2.11E-12	-0.323435515	count	5.02E-08
FKBP10	-0.2320512	0.0747429	-3.1047	0.00192	-0.323320083	count	1
RCHY1	-0.2482761	0.1425925	-1.7412	0.0817	-0.323218575	count	1
LUC7L3	-0.2274363	0.0667422	-3.4077	0.000662	-0.323217077	count	1
RPL18	-0.2240099	0.0201064	-11.1412	2.24E-28	-0.322851424	count	5.40E-24
PDAP1	-0.2268307	0.0483188	-4.6945	2.77E-06	-0.322361943	count	0.06419475
KIF5B	-0.2259874	0.0462915	-4.8818	1.10E-06	-0.322244995	count	0.0255761
AMOT	-0.2783651	0.2510582	-1.1088	0.268	-0.322235041	count	1
GTF3C6	-0.2280063	0.0640215	-3.5614	0.000374	-0.321945904	count	1
TRIB2	-0.2516826	0.1842722	-1.3658	0.172	-0.321937061	count	1
TPT1	-0.2232681	0.0183579	-12.1619	2.16E-33	-0.321931523	count	5.22E-29
RNF126	-0.2423304	0.1246006	-1.9449	0.0519	-0.321633504	count	1
CRK	-0.2409712	0.1003926	-2.4003	0.0164	-0.321572709	count	1
AC004854.2	-0.8194087	0.7678991	-1.0671	0.286	-0.321472498	count	1
LSM10	-0.2320114	0.0856234	-2.7097	0.00677	-0.321437454	count	1
ATP10D	-0.2393332	0.1145231	-2.0898	0.0367	-0.321337045	count	1
SP110	-0.2319601	0.0991567	-2.3393	0.0194	-0.321321975	count	1
NCAPD2	-0.3268081	0.3100894	-1.0539	0.292	-0.321190704	count	1
KAT2B	-0.2637732	0.1894378	-1.3924	0.164	-0.321052413	count	1
CTBS	-0.2307729	0.0875275	-2.6366	0.00841	-0.321024184	count	1
GDF7	-0.2436751	0.2096452	-1.1623	0.245	-0.320736448	count	1
MPHOSPH9	-0.3039181	0.292313	-1.0397	0.299	-0.320661292	count	1
AL031777.3	-0.7062743	0.464234	-1.5214	0.128	-0.320544208	count	1
MAP1LC3B2	-0.6303239	0.5160974	-1.2213	0.222	-0.320348897	count	1
WFDC3	-0.8166025	0.7196746	-1.1347	0.257	-0.3202911	count	1
CFAP45	-0.8166025	0.7388526	-1.1052	0.269	-0.3202911	count	1
AC061992.1	-0.8166025	0.7599551	-1.0745	0.283	-0.3202911	count	1
CXorf40A	-0.3011315	0.2295327	-1.3119	0.19	-0.320278939	count	1
IL1RL1	-2.5963558	0.896934	-2.8947	0.0038	-0.319986458	count	1
RPS5	-0.2220418	0.0227131	-9.7759	2.67E-22	-0.319696299	count	6.42E-18
GABRB1	-2.5906501	1.574429	-1.6455	0.1	-0.319503582	count	1
MYO7A	-2.5906501	1.6738963	-1.5477	0.1218	-0.319503582	count	1
ARPC5	-0.2227305	0.0366171	-6.0827	1.30E-09	-0.319421666	count	3.07E-05
VEZT	-0.2394745	0.1271405	-1.8835	0.0597	-0.319403939	count	1
PIN1	-0.22561	0.0520796	-4.332	1.52E-05	-0.319296848	count	0.3496912
CSDE1	-0.2241135	0.0466383	-4.8054	1.61E-06	-0.319189867	count	0.03738581
LINC01123	-1.3448701	0.7790187	-1.7264	0.0844	-0.318994709	count	1
OLAH	-1.3448701	0.7970633	-1.6873	0.0916	-0.318994709	count	1
QPCTL	-0.573065	0.5325401	-1.0761	0.282	-0.318979115	count	1
ANTXR2	-0.2451719	0.1271646	-1.928	0.0539	-0.318871965	count	1
S100A4	-0.2210142	0.0250277	-8.8308	1.58E-18	-0.318721605	count	3.79E-14
MT-ND1	-0.2212393	0.0256592	-8.6222	9.62E-18	-0.318608956	count	2.30E-13
SPTY2D1OS	-0.4497121	0.3786226	-1.1878	0.235	-0.318523763	count	1
PACSIN2	-0.2338874	0.0909991	-2.5702	0.0102	-0.318510602	count	1

RPS2	-0.2208928	0.0236807	-9.328	1.81E-20	-0.318160965	count	4.35E-16
NKIRAS2	-0.2464563	0.1256893	-1.9608	0.05	-0.318071235	count	1
IMP4	-0.2328542	0.0963319	-2.4172	0.0157	-0.317944565	count	1
PCDHB8	-0.9876	0.8207322	-1.2033	0.229	-0.317161725	count	1
CYP11A1	-0.9876	1.0819795	-0.9128	0.361	-0.317161725	count	1
MAPKAPK5-AS1	-0.23826	0.1279403	-1.8623	0.0626	-0.317103743	count	1
TUBD1	-0.2750365	0.1803972	-1.5246	0.127	-0.317007565	count	1
EFR3A	-0.232652	0.0935339	-2.4874	0.0129	-0.316911649	count	1
SNX18	-0.2274294	0.0826863	-2.7505	0.00598	-0.316837806	count	1
AL450306.1	-0.6236129	0.37661	-1.6559	0.0978	-0.31672767	count	1
ATPAF2	-0.2827286	0.2529962	-1.1175	0.264	-0.316678412	count	1
GOLGA8B	-0.2516422	0.2024074	-1.2432	0.214	-0.316571309	count	1
KLF15	-0.2649461	0.219604	-1.2065	0.228	-0.316423359	count	1
FAM228B	-0.2359621	0.1465878	-1.6097	0.108	-0.316376218	count	1
NDUFS5	-0.2202868	0.0319544	-6.8938	6.37E-12	-0.316267809	count	1.51E-07
NUTF2	-0.2254619	0.0717541	-3.1421	0.00169	-0.316266333	count	1
LCMT1	-0.2327716	0.1132934	-2.0546	0.04	-0.316156167	count	1
HGH1	-0.3078495	0.2624822	-1.1728	0.241	-0.316110663	count	1
LYPLA1	-0.2349832	0.1165564	-2.016	0.0439	-0.316011849	count	1
KCNK3	-0.3254178	0.2178155	-1.494	0.135	-0.315612244	count	1
NT5DC1	-0.226779	0.0811217	-2.7955	0.00521	-0.315317028	count	1
ITGA7	-0.2312292	0.11442	-2.0209	0.0434	-0.315310128	count	1
FMO2	-0.2221761	0.076373	-2.9091	0.00365	-0.315283813	count	1
LINC01597	-0.6953036	0.4285312	-1.6225	0.105	-0.315229028	count	1
GAK	-0.2769566	0.2044143	-1.3549	0.176	-0.314933767	count	1
MAP4	-0.2210754	0.0484325	-4.5646	5.17E-06	-0.31491211	count	0.11946836
EIF3K	-0.2195422	0.0310516	-7.0702	1.84E-12	-0.314892808	count	4.37E-08
AP001816.1	-0.255204	0.1986794	-1.2845	0.199	-0.314758861	count	1
C12orf76	-0.2335893	0.1105195	-2.1136	0.0346	-0.314639343	count	1
AC106791.1	-1.3252965	0.7548538	-1.7557	0.0792	-0.314158339	count	1
C17orf113	-0.6188059	0.5753839	-1.0755	0.282	-0.314135453	count	1
REM2	-0.6188059	0.814535	-0.7597	0.447	-0.314135453	count	1
GPR137C	-0.6188059	0.9035756	-0.6848	0.493	-0.314135453	count	1
BCAS3	-0.2592361	0.2261004	-1.1466	0.252	-0.313901596	count	1
LSM2	-0.2221722	0.0623137	-3.5654	0.000368	-0.313889019	count	1
RMDN3	-0.2460291	0.1661032	-1.4812	0.139	-0.313881728	count	1
GLS	-0.2210674	0.0712493	-3.1027	0.00193	-0.3136588	count	1
SEC31B	-0.3505467	0.5404266	-0.6486	0.517	-0.313629963	count	1
NDUFA2	-0.22166	0.0566728	-3.9112	9.35E-05	-0.31361333	count	1
CDKN2B-AS1	-2.5211059	1.0333725	-2.4397	0.0147	-0.313477946	count	1
ATP5IF1	-0.2194775	0.0425368	-5.1597	2.60E-07	-0.313436205	count	0.00607334
AC009812.1	-0.4896372	0.3450987	-1.4188	0.156	-0.312939664	count	1
AKT3	-0.2283426	0.1010279	-2.2602	0.0239	-0.312670499	count	1
COX7B	-0.2192773	0.0446414	-4.912	9.41E-07	-0.312557569	count	0.02189707
TMEM132A	-0.7978839	0.3858178	-2.068	0.0387	-0.312415561	count	1

C16orf87	-0.2448778	0.1612349	-1.5188	0.129	-0.31240436	count	1
LMO2	-0.2179878	0.1001194	-2.1773	0.0295	-0.312260525	count	1
C19orf70	-0.2193021	0.0488084	-4.4931	7.23E-06	-0.312240196	count	0.16683948
IKBKG	-0.2558792	0.1527497	-1.6752	0.094	-0.312117175	count	1
CISD3	-0.2233517	0.0815999	-2.7372	0.00623	-0.312000303	count	1
BRK1	-0.218057	0.0376905	-5.7855	7.84E-09	-0.311950534	count	0.000184507
CETP	-1.3160505	0.6953865	-1.8925	0.0585	-0.311869003	count	1
CXorf36	-1.3160505	1.112199	-1.1833	0.237	-0.311869003	count	1
AC027644.3	-0.2325965	0.108526	-2.1432	0.0322	-0.311848319	count	1
AL590399.1	-0.7963254	0.5581041	-1.4268	0.154	-0.311760226	count	1
SMDT1	-0.2190088	0.0497053	-4.4061	1.08E-05	-0.311729721	count	0.2487564
PTPRD-AS1	-0.237467	0.1632706	-1.4544	0.146	-0.311618941	count	1
HENMT1	-0.3479532	0.3657363	-0.9514	0.341	-0.31125055	count	1
P2RX4	-0.2598296	0.1698724	-1.5296	0.126	-0.311180991	count	1
AC023632.2	-0.7949279	0.6771258	-1.174	0.24	-0.311172654	count	1
OVCH1-AS1	-0.4610979	0.4411642	-1.0452	0.296	-0.311171565	count	1
YBX1	-0.2161026	0.0251711	-8.5854	1.32E-17	-0.311053639	count	3.16E-13
TNFRSF4	-2.4927738	0.8580334	-2.9052	0.00369	-0.310948628	count	1
HSD17B10	-0.2224598	0.0698932	-3.1829	0.00147	-0.310859542	count	1
TRAPPC8	-0.251523	0.2019752	-1.2453	0.213	-0.310820165	count	1
RPL31	-0.2166065	0.0324486	-6.6754	2.84E-11	-0.310635849	count	6.74E-07
ATP5MG	-0.2167384	0.0326323	-6.6418	3.55E-11	-0.310612634	count	8.42E-07
ACACA	-0.3056008	0.2479132	-1.2327	0.218	-0.310568416	count	1
MMADHC	-0.2210572	0.0705703	-3.1324	0.00175	-0.310489481	count	1
B3GNT5	-2.486318	0.7374952	-3.3713	0.000756	-0.310366334	count	1
FBXW5	-0.2213579	0.0752549	-2.9414	0.00329	-0.309981553	count	1
TENT4A	-0.3082701	0.2925757	-1.0536	0.292	-0.309943875	count	1
AC006449.6	-0.3804562	0.3081468	-1.2347	0.217	-0.309935735	count	1
AEBP1	-0.2155095	0.0482172	-4.4696	8.07E-06	-0.309813839	count	0.18610227
PLPP5	-0.2251386	0.0988011	-2.2787	0.0227	-0.309688072	count	1
S100A2	-0.4376687	0.3866653	-1.1319	0.258	-0.309652197	count	1
RGL2	-0.2364741	0.1731179	-1.366	0.172	-0.309592689	count	1
GATD3A	-0.9652953	0.7458149	-1.2943	0.196	-0.30947183	count	1
AC124016.1	-0.3798095	0.3674938	-1.0335	0.301	-0.309392454	count	1
BMPR2	-0.2240393	0.1025806	-2.184	0.029	-0.309309304	count	1
TCTEX1D4	-0.7904739	0.6096167	-1.2967	0.195	-0.309300405	count	1
AL080317.2	-0.7904739	0.7959744	-0.9931	0.321	-0.309300405	count	1
MEGF9	-0.247867	0.2003132	-1.2374	0.216	-0.309187177	count	1
DYNC1LI2	-0.2194251	0.061698	-3.5564	0.000381	-0.309158687	count	1
FHL1	-0.2146726	0.0454159	-4.7268	2.37E-06	-0.30911529	count	0.05494845
FAM131A	-0.2604687	0.2128117	-1.2239	0.221	-0.309092177	count	1
CHURC1	-0.2170494	0.0476882	-4.5514	5.50E-06	-0.309062117	count	0.12705
HDHD5	-0.257212	0.1875891	-1.3711	0.17	-0.308907149	count	1
B3GAT3	-0.2236563	0.0904725	-2.4721	0.0135	-0.30887867	count	1
CFL1	-0.2150133	0.0294211	-7.3081	3.31E-13	-0.308811089	count	7.88E-09

FZD6	-0.2446725	0.1910087	-1.2809	0.2	-0.30869974	count	1
YDJC	-0.2542635	0.2070442	-1.2281	0.22	-0.308617409	count	1
SMIM10L1	-0.2210386	0.0901279	-2.4525	0.0142	-0.308600633	count	1
GMDS-DT	-0.2786337	0.2469121	-1.1285	0.259	-0.308498453	count	1
AC025164.1	-0.3686689	0.2518925	-1.4636	0.143	-0.308420168	count	1
FBL	-0.2288977	0.1049849	-2.1803	0.0293	-0.308177446	count	1
TMEM87B	-0.2403897	0.1636923	-1.4685	0.142	-0.308147642	count	1
B3GNTL1	-0.6806265	0.4977784	-1.3673	0.172	-0.308126679	count	1
EVL	-0.2289161	0.1306001	-1.7528	0.0797	-0.308078401	count	1
PACSIN3	-0.2414804	0.1484712	-1.6264	0.104	-0.308045146	count	1
ANAPC11	-0.2157442	0.0434374	-4.9668	7.12E-07	-0.30783366	count	0.016590312
NOS1AP	-0.2734464	0.3210745	-0.8517	0.394	-0.307792785	count	1
OGN	-0.2135792	0.0400559	-5.332	1.03E-07	-0.307623914	count	0.002411951
BOD1	-0.2241649	0.0812459	-2.7591	0.00582	-0.307543673	count	1
DYNLRB1	-0.2150161	0.0391456	-5.4927	4.23E-08	-0.307439966	count	0.000992231
AC078909.1	-0.3024972	0.2709648	-1.1164	0.264	-0.307358339	count	1
HECA	-0.2435916	0.1704861	-1.4288	0.153	-0.307327393	count	1
ARF4-AS1	-0.5531604	0.4580771	-1.2076	0.227	-0.307287019	count	1
AC016394.1	-0.3435822	0.3187655	-1.0779	0.281	-0.30724194	count	1
ZNF570	-0.2580653	0.189468	-1.3621	0.173	-0.307186482	count	1
IRF7	-0.2401907	0.1983985	-1.2106	0.226	-0.307155104	count	1
GNLY	-0.3769565	0.4799643	-0.7854	0.432	-0.306996274	count	1
WDR66	-0.3167114	0.668865	-0.4735	0.636	-0.306996153	count	1
CSK	-0.2484191	0.1644935	-1.5102	0.131	-0.306956436	count	1
TRMT61A	-0.2478446	0.1545758	-1.6034	0.109	-0.306852705	count	1
TFIP11	-0.2805767	0.2039913	-1.3754	0.169	-0.306826809	count	1
MYO1B	-0.2220858	0.1004768	-2.2103	0.0271	-0.30680258	count	1
HDAC5	-0.2304794	0.15254	-1.5109	0.131	-0.30671014	count	1
DECR2	-0.253309	0.2323495	-1.0902	0.276	-0.306664897	count	1
DUSP5	-0.3163598	0.3286736	-0.9625	0.336	-0.306648356	count	1
MRPS33	-0.2228958	0.0970576	-2.2965	0.0217	-0.306177064	count	1
NTHL1	-0.2301903	0.1189648	-1.9349	0.0531	-0.30615555	count	1
L3HYPDH	-0.2343544	0.1416861	-1.654	0.0982	-0.305803476	count	1
AP4B1	-0.3246124	0.3184235	-1.0194	0.308	-0.305723673	count	1
LINC01971	-0.9537942	0.623423	-1.5299	0.126	-0.305506968	count	1
NUP43	-0.2873689	0.1993635	-1.4414	0.15	-0.305420652	count	1
C16orf91	-0.2308572	0.1251008	-1.8454	0.0651	-0.305420117	count	1
VASP	-0.2230336	0.0990476	-2.2518	0.0244	-0.305383675	count	1
B9D2	-0.2639502	0.2380906	-1.1086	0.268	-0.305372224	count	1
PHC2	-0.2243276	0.0896612	-2.5019	0.0124	-0.305198265	count	1
KLHL35	-0.3192787	0.319032	-1.0008	0.317	-0.30519205	count	1
FAM69C	-0.7806583	0.5937179	-1.3149	0.189	-0.30517641	count	1
MRPL33	-0.2156122	0.0547642	-3.9371	8.40E-05	-0.30513525	count	1
SLC6A8	-0.3069967	0.333692	-0.92	0.358	-0.305088858	count	1
AC024941.2	-2.427714	1.304399	-1.8612	0.0628	-0.304976524	count	1

THAP8	-0.2738153	0.3161065	-0.8662	0.386	-0.304866672	count	1
COX6B1	-0.2125756	0.0327551	-6.4898	9.74E-11	-0.304770969	count	2.31E-06
RASSF7	-0.3854207	0.4305002	-0.8953	0.371	-0.304668726	count	1
DAP3	-0.2303259	0.1214705	-1.8961	0.058	-0.304326013	count	1
BIN3	-0.2367259	0.2197222	-1.0774	0.281	-0.304124647	count	1
IL27RA	-0.9496623	0.611471	-1.5531	0.12	-0.304082674	count	1
AL023581.2	-1.2844415	0.6935323	-1.852	0.0641	-0.304021125	count	1
STKLD1	-1.2844415	0.7238301	-1.7745	0.0761	-0.304021125	count	1
GALNT17	-1.2844414	0.7976141	-1.6104	0.107	-0.304021112	count	1
CAPRIN2	-0.2332828	0.1845993	-1.2637	0.206	-0.303875757	count	1
MRPL51	-0.212749	0.0433252	-4.9105	9.48E-07	-0.303668544	count	0.022059012
SEMA3G	-2.41279	1.384952	-1.7421	0.0816	-0.303574308	count	1
TCF4-AS2	-2.41279	1.384952	-1.7421	0.0816	-0.303574308	count	1
PSMA2	-0.2168519	0.0821049	-2.6412	0.0083	-0.302692084	count	1
GULP1	-0.2183206	0.0811658	-2.6898	0.00718	-0.302599842	count	1
RPL8	-0.2099078	0.0196034	-10.7077	2.29E-26	-0.30256282	count	5.52E-22
NFKBIB	-0.2558	0.1925455	-1.3285	0.184	-0.302506357	count	1
FBLN7	-0.2457527	0.1623225	-1.514	0.13	-0.302380075	count	1
AC137767.1	-0.3614863	0.3963583	-0.912	0.362	-0.302236854	count	1
DDX20	-0.2714009	0.1927024	-1.4084	0.159	-0.302144631	count	1
THBS2	-0.2127062	0.0827895	-2.5692	0.0102	-0.302084314	count	1
PHKA1	-0.3822423	0.4157729	-0.9194	0.358	-0.302074201	count	1
ARHGEF39	-2.3953702	1.34543	-1.7804	0.0751	-0.301922522	count	1
RPS7	-0.2094906	0.0213635	-9.806	2.00E-22	-0.301886248	count	4.81E-18
TNFRSF25	-0.9428747	0.5417182	-1.7405	0.0819	-0.301743119	count	1
TSN	-0.22411	0.1088879	-2.0582	0.0396	-0.30171137	count	1
MRPL40	-0.2129603	0.0696598	-3.0571	0.00225	-0.301425261	count	1
TIMM10B	-0.2273667	0.1192566	-1.9065	0.0567	-0.301157176	count	1
VPS51	-0.2172557	0.0770851	-2.8184	0.00485	-0.301121208	count	1
TMEM222	-0.2221434	0.1196011	-1.8574	0.0633	-0.301111809	count	1
HBS1L	-0.2270403	0.1286223	-1.7652	0.0776	-0.301085687	count	1
NDUFB7	-0.2103693	0.0382639	-5.4979	4.11E-08	-0.300819017	count	0.000964247
NAA10	-0.2137412	0.0701007	-3.0491	0.00231	-0.300755656	count	1
KBTBD12	-0.664999	0.4723734	-1.4078	0.159	-0.300575849	count	1
PM20D2	-0.238532	0.1728843	-1.3797	0.168	-0.300444408	count	1
MAF1	-0.2130405	0.0568202	-3.7494	0.00018	-0.300437309	count	1
MTCP1	-0.9387611	0.6783237	-1.3839	0.166	-0.300325364	count	1
FOXD2	-0.2200152	0.1361696	-1.6157	0.106	-0.300216604	count	1
IFITM3	-0.2083146	0.0236192	-8.8197	1.74E-18	-0.300207909	count	4.17E-14
STEAP3	-0.3056712	0.3729667	-0.8196	0.413	-0.300018244	count	1
RABL6	-0.2196759	0.1060394	-2.0716	0.0384	-0.299674412	count	1
TAF6L	-0.3052579	0.257996	-1.1832	0.237	-0.299604683	count	1
ATF4	-0.2113025	0.0573284	-3.6858	0.000231	-0.299567886	count	1
MSTO1	-0.2840423	0.2561135	-1.109	0.267	-0.29937048	count	1
EXT1	-0.216417	0.1266715	-1.7085	0.0876	-0.299366561	count	1

RAB33A	-0.4688299	0.4967847	-0.9437	0.345	-0.299031289	count	1
SUPT7L	-0.2380893	0.1592225	-1.4953	0.135	-0.298935046	count	1
GJA5	-0.2451059	0.2253351	-1.0877	0.277	-0.298873641	count	1
DUSP28	-0.2885367	0.2617112	-1.1025	0.27	-0.298825048	count	1
NPM1	-0.2078993	0.0300434	-6.92	5.31E-12	-0.298666912	count	1.26E-07
LINC00595	-2.360759	1.7795435	-1.3266	0.1847	-0.298591779	count	1
PRELID1	-0.209607	0.0485268	-4.3194	1.61E-05	-0.298021345	count	0.3703483
SERPINE2	-0.211958	0.1944939	-1.0898	0.276	-0.29781721	count	1
CPTP	-0.2554227	0.1942046	-1.3152	0.189	-0.297764586	count	1
TWSG1	-0.214247	0.0819798	-2.6134	0.009	-0.297656085	count	1
TOMM20	-0.207895	0.0381084	-5.4554	5.21E-08	-0.297419823	count	0.001221693
AL022322.2	-0.6583662	0.9143586	-0.72	0.472	-0.297374798	count	1
TRIM24	-0.2511447	0.1966331	-1.2772	0.202	-0.296949688	count	1
PLPP7	-0.3022366	0.3310194	-0.913	0.361	-0.296582042	count	1
SNRPE	-0.2107969	0.0653436	-3.226	0.00127	-0.296450471	count	1
SATB2	-0.2918202	0.4481528	-0.6512	0.515	-0.296322134	count	1
PTDSS1	-0.2677346	0.2054151	-1.3034	0.193	-0.296278349	count	1
ZNF223	-0.4193861	0.4012776	-1.0451	0.296	-0.296211293	count	1
RAB4A	-0.2137925	0.0742218	-2.8805	0.00399	-0.296192212	count	1
BCR	-0.2618121	0.2310746	-1.133	0.257	-0.296057882	count	1
C8orf33	-0.2180466	0.1009119	-2.1608	0.0308	-0.296011979	count	1
NUDT16	-0.2155667	0.0932581	-2.3115	0.0209	-0.29590668	count	1
FKBP1A	-0.2079501	0.0477759	-4.3526	1.38E-05	-0.295854454	count	0.317607
CHMP4B	-0.2116989	0.0753998	-2.8077	0.00502	-0.295750786	count	1
HOXB2	-0.2153137	0.0957107	-2.2496	0.0245	-0.295739483	count	1
CHSY1	-0.2269639	0.1487775	-1.5255	0.127	-0.295606819	count	1
GRM8	-0.7574907	0.6557288	-1.1552	0.248	-0.295454506	count	1
LINC01521	-0.7574907	0.6770799	-1.1188	0.263	-0.295454506	count	1
SRD5A1	-0.337396	0.3123157	-1.0803	0.28	-0.295343981	count	1
FIBP	-0.2117493	0.082356	-2.5711	0.0102	-0.295311075	count	1
SLCO5A1	-0.3304745	0.3436067	-0.9618	0.336	-0.295232915	count	1
RPL34	-0.2048058	0.0199464	-10.2678	2.10E-24	-0.295204853	count	5.06E-20
DAPK3	-0.2241635	0.1032739	-2.1706	0.03	-0.295157196	count	1
USP13	-0.3135143	0.2985425	-1.0501	0.294	-0.295046336	count	1
DPYSL3	-0.2124743	0.0896497	-2.3701	0.0178	-0.295011336	count	1
COA1	-0.2212353	0.1303349	-1.6974	0.0897	-0.294992415	count	1
FAM225A	-1.2480574	0.8581752	-1.4543	0.146	-0.294950079	count	1
YPEL5	-0.20969	0.0700445	-2.9937	0.00277	-0.294939823	count	1
TMEM183A	-0.2174292	0.1035089	-2.1006	0.0357	-0.294893898	count	1
FAF1	-0.2372416	0.1574201	-1.5071	0.132	-0.294765048	count	1
RHOC	-0.2055959	0.0372502	-5.5193	3.64E-08	-0.294696743	count	0.000854344
ZNF362	-0.2314482	0.1434319	-1.6136	0.107	-0.294409132	count	1
LYL1	-0.2506243	0.2651105	-0.9454	0.345	-0.294287792	count	1
TMEM251	-0.2241313	0.1334013	-1.6801	0.093	-0.294265385	count	1
FSD1L	-0.4167273	0.3830686	-1.0879	0.277	-0.294259361	count	1

COMMD6	-0.205136	0.0330453	-6.2077	5.98E-10	-0.294195679	count	1.41E-05
TEX2	-0.2543792	0.2206663	-1.1528	0.249	-0.294184639	count	1
KLHL17	-0.351724	0.40992	-0.858	0.391	-0.293841458	count	1
UQCRQ	-0.205267	0.0446189	-4.6005	4.36E-06	-0.29335643	count	0.100825
GOLGA8A	-0.2230868	0.219815	-1.0149	0.31	-0.293317238	count	1
AP5Z1	-0.2406619	0.3057666	-0.7871	0.431	-0.292704764	count	1
RNF115	-0.2095992	0.0832765	-2.5169	0.0119	-0.292527152	count	1
SEC11C	-0.208991	0.0716876	-2.9153	0.00357	-0.292508662	count	1
ENO1	-0.2045009	0.0440792	-4.6394	3.62E-06	-0.292447074	count	0.08377404
E2F6	-0.2675826	0.2477824	-1.0799	0.28	-0.292429669	count	1
RNF181	-0.206422	0.0554784	-3.7208	0.000202	-0.292318976	count	1
SERBP1	-0.2045091	0.0391432	-5.2246	1.84E-07	-0.292053005	count	0.004302288
STYX	-0.2350565	0.178421	-1.3174	0.188	-0.292031635	count	1
TMEM141	-0.209867	0.078765	-2.6645	0.00774	-0.291663755	count	1
RPL26L1	-0.2104731	0.0895884	-2.3493	0.0189	-0.291625124	count	1
USP30-AS1	-2.290156	1.10271	-2.0768	0.0379	-0.291597176	count	1
AC073111.5	-0.2933355	0.2544231	-1.1529	0.249	-0.29126414	count	1
FREM1	-0.4872401	0.3371806	-1.445	0.149	-0.291163194	count	1
SRRM4	-0.5251098	0.5223889	-1.0052	0.315	-0.290859879	count	1
FOXO4	-0.5251098	0.5449695	-0.9636	0.335	-0.290859879	count	1
ZNF266	-0.4863972	0.4239379	-1.1473	0.251	-0.29063418	count	1
IQSEC2	-0.2675403	0.2361653	-1.1329	0.257	-0.290428727	count	1
RYR3	-0.5745202	0.7134288	-0.8053	0.421	-0.290322552	count	1
PSMB8-AS1	-0.2801139	0.2607353	-1.0743	0.283	-0.289962071	count	1
KCTD3	-0.2517565	0.1723176	-1.461	0.144	-0.289897046	count	1
YARS	-0.2291008	0.1662485	-1.3781	0.168	-0.289793032	count	1
ARMCX6	-0.2170852	0.123291	-1.7608	0.0784	-0.289736324	count	1
LINC00989	-0.3793671	0.2371111	-1.6	0.11	-0.28967917	count	1
HSD11B1L	-0.2441049	0.1898396	-1.2858	0.199	-0.289500021	count	1
HACD1	-0.2093047	0.1028327	-2.0354	0.0419	-0.289467342	count	1
RMND1	-0.2232727	0.1550006	-1.4405	0.15	-0.289449554	count	1
ARHGEF19	-0.3179873	0.3167747	-1.0038	0.316	-0.289315386	count	1
RPRD1A	-0.2247156	0.1501205	-1.4969	0.135	-0.289256472	count	1
CDK5	-0.2453983	0.220706	-1.1119	0.266	-0.289108381	count	1
SOX4	-0.2057157	0.0951021	-2.1631	0.0306	-0.288647159	count	1
EIF3E	-0.2016109	0.035498	-5.6795	1.46E-08	-0.288524193	count	0.000343231
MTUS2	-0.2565211	0.2869529	-0.8939	0.371	-0.28852145	count	1
GBP3	-0.2124758	0.169971	-1.2501	0.211	-0.28842921	count	1
SND1	-0.2169018	0.1230634	-1.7625	0.0781	-0.288418795	count	1
IGHG4	-2.2578448	0.7065965	-3.1954	0.00141	-0.288307064	count	1
C12orf73	-0.2453785	0.207281	-1.1838	0.237	-0.288069666	count	1
FOSL1	-0.3770511	0.4511489	-0.8358	0.403	-0.287850624	count	1
TMEM219	-0.2023899	0.0526066	-3.8472	0.000121	-0.287659658	count	1
SLC39A4	-0.2314547	0.2262811	-1.0229	0.306	-0.287526656	count	1
LINC01018	-2.249899	0.9759097	-2.3054	0.0212	-0.287489425	count	1

EIF6	-0.2031388	0.0652476	-3.1134	0.00186	-0.287468364	count	1
SET	-0.2011973	0.0433322	-4.6431	3.55E-06	-0.287391814	count	0.0821683
EIF2S3B	-1.2178343	0.8056896	-1.5115	0.131	-0.287388319	count	1
RPL11	-0.1992425	0.0177146	-11.2474	7.05E-29	-0.28723544	count	1.70E-24
HERC5	-0.2194348	0.1315297	-1.6683	0.0953	-0.287189883	count	1
UNK	-0.2338341	0.1724948	-1.3556	0.175	-0.286988091	count	1
FAM227A	-0.7369416	0.5031002	-1.4648	0.143	-0.286846878	count	1
AC009237.14	-0.5679812	0.5143734	-1.1042	0.27	-0.286817613	count	1
MRPL21	-0.2045881	0.0687169	-2.9773	0.00293	-0.286815506	count	1
RPP21	-0.2434304	0.2301978	-1.0575	0.29	-0.28676839	count	1
FOXN3-AS1	-0.25913	0.254	-1.0202	0.308	-0.286638106	count	1
TMCC1	-0.2428827	0.1902424	-1.2767	0.202	-0.286117181	count	1
GYS1	-0.3042185	0.4071097	-0.7473	0.455	-0.286112876	count	1
PKN3	-0.8974853	0.6991531	-1.2837	0.199	-0.286107539	count	1
AL137077.2	-0.8974853	0.7661988	-1.1713	0.242	-0.286107539	count	1
TRIM16L	-0.362558	0.324223	-1.1182	0.264	-0.28602971	count	1
MON1B	-0.2346091	0.2059696	-1.139	0.255	-0.285977237	count	1
ACTA2-AS1	-0.8960137	0.6221916	-1.4401	0.15	-0.285600981	count	1
CCDC124	-0.202744	0.0646621	-3.1354	0.00173	-0.285396524	count	1
ADORA2B	-0.3613392	0.4629715	-0.7805	0.435	-0.285037644	count	1
PYGM	-0.4038959	0.2424032	-1.6662	0.0958	-0.284849475	count	1
LINC02188	-0.8936453	0.8320291	-1.0741	0.283	-0.284785768	count	1
BEX1	-0.4037493	0.4711199	-0.857	0.392	-0.284742065	count	1
SF3B5	-0.199707	0.0508262	-3.9292	8.68E-05	-0.284119929	count	1
PGF	-0.2007597	0.0873946	-2.2972	0.0217	-0.283888765	count	1
PLS1	-0.4025557	0.4805484	-0.8377	0.402	-0.283867613	count	1
HEXIM2	-0.2370369	0.2327081	-1.0186	0.308	-0.28365596	count	1
CORO1C	-0.2144627	0.1264963	-1.6954	0.0901	-0.283649194	count	1
AC138696.2	-0.6292	0.5364355	-1.1729	0.241	-0.283327193	count	1
EFHD1	-0.1995841	0.0621342	-3.2121	0.00133	-0.283272191	count	1
ZNF25	-0.2275778	0.2232377	-1.0194	0.308	-0.283203191	count	1
MSANTD3	-0.2131659	0.1427333	-1.4935	0.135	-0.282951594	count	1
EIF5A	-0.1983829	0.0431209	-4.6006	4.35E-06	-0.282911395	count	0.10060245
STX18-AS1	-0.7271314	0.5800908	-1.2535	0.21	-0.282743205	count	1
DLGAP1-AS2	-0.7271314	0.7632949	-0.9526	0.341	-0.282743205	count	1
FOLR1	-0.3703017	0.512694	-0.7223	0.47	-0.282525107	count	1
KIAA0040	-0.2071605	0.1076145	-1.925	0.0543	-0.282486058	count	1
PSMC5	-0.1986378	0.0474668	-4.1848	2.92E-05	-0.282420674	count	0.6693224
ZNF341	-0.4731443	0.4958238	-0.9543	0.34	-0.282324978	count	1
NDUFC2	-0.1971184	0.0381869	-5.1619	2.57E-07	-0.282209077	count	0.006003777
RARRES3	-0.2008427	0.0736396	-2.7274	0.00641	-0.282181945	count	1
RCL1	-0.2430448	0.1624671	-1.496	0.135	-0.282088496	count	1
AP001437.1	-0.5591485	0.740795	-0.7548	0.45	-0.282088047	count	1
OCLM	-1.196092	0.8288684	-1.443	0.149	-0.281935274	count	1
AC010969.1	-1.196092	0.8288684	-1.443	0.149	-0.281935274	count	1

AC073050.1	-1.196092	0.8288684	-1.443	0.149	-0.281935274	count	1
AC107294.2	-1.196092	0.8288684	-1.443	0.149	-0.281935274	count	1
LINC02432	-1.196092	0.8288684	-1.443	0.149	-0.281935274	count	1
AL031058.1	-1.196092	0.8288684	-1.443	0.149	-0.281935274	count	1
Z98200.1	-1.196092	0.8288684	-1.443	0.149	-0.281935274	count	1
SLC22A16	-1.196092	0.8288684	-1.443	0.149	-0.281935274	count	1
AL049548.1	-1.196092	0.8288684	-1.443	0.149	-0.281935274	count	1
CAMK2B	-1.196092	0.8288684	-1.443	0.149	-0.281935274	count	1
SMKR1	-1.196092	0.8288684	-1.443	0.149	-0.281935274	count	1
SYT7	-1.196092	0.8288684	-1.443	0.149	-0.281935274	count	1
AP000904.1	-1.196092	0.8288684	-1.443	0.149	-0.281935274	count	1
AC004816.2	-1.196092	0.8288684	-1.443	0.149	-0.281935274	count	1
HEATR4	-1.196092	0.8288684	-1.443	0.149	-0.281935274	count	1
LINC00677	-1.196092	0.8288684	-1.443	0.149	-0.281935274	count	1
AC023034.1	-1.196092	0.8288684	-1.443	0.149	-0.281935274	count	1
AC027801.3	-1.196092	0.8288684	-1.443	0.149	-0.281935274	count	1
C17orf64	-1.196092	0.8288684	-1.443	0.149	-0.281935274	count	1
AC011825.4	-1.196092	0.8288684	-1.443	0.149	-0.281935274	count	1
AC091551.1	-1.196092	0.8288684	-1.443	0.149	-0.281935274	count	1
AL031666.1	-1.196092	0.8288684	-1.443	0.149	-0.281935274	count	1
LKAAEAR1	-1.196092	0.8288684	-1.443	0.149	-0.281935274	count	1
TSSK6	-1.196092	0.8288684	-1.443	0.149	-0.281935274	count	1
KRTDAP	-1.196092	0.8288684	-1.443	0.149	-0.281935274	count	1
CCDC188	-1.196092	0.8288684	-1.443	0.149	-0.281935274	count	1
RAD51C	-0.2128361	0.1155663	-1.8417	0.0656	-0.281839909	count	1
MCTP2	-0.3570477	0.9306993	-0.3836	0.701	-0.281545791	count	1
EPDR1	-0.2050309	0.0970592	-2.1124	0.0347	-0.281471666	count	1
ZNF821	-0.2454397	0.2548241	-0.9632	0.336	-0.281313596	count	1
AC016907.2	-2.1908187	1.062447	-2.0621	0.0393	-0.28130498	count	1
SDF2L1	-0.2034384	0.0895406	-2.272	0.0231	-0.281297637	count	1
FBXO22	-0.2112122	0.1671022	-1.264	0.206	-0.281285906	count	1
ATP2A2	-0.2075501	0.1021128	-2.0326	0.0422	-0.281278243	count	1
ANKZF1	-0.2671188	0.194053	-1.3765	0.169	-0.28127119	count	1
MLLT11	-0.224058	0.1856252	-1.207	0.227	-0.281210404	count	1
SLIRP	-0.200061	0.0645331	-3.1001	0.00195	-0.281082434	count	1
ZCCHC8	-0.2283988	0.1594077	-1.4328	0.152	-0.280877343	count	1
TNIK	-0.3146952	0.345392	-0.9111	0.362	-0.280800579	count	1
MANEA-DT	-0.2797174	0.3072127	-0.9105	0.363	-0.28074527	count	1
ALKBH7	-0.1977384	0.0527519	-3.7485	0.000181	-0.280663198	count	1
DOCK9-DT	-0.2940711	0.3816944	-0.7704	0.441	-0.280617677	count	1
RNF11	-0.2015486	0.0791172	-2.5475	0.0109	-0.280463243	count	1
HAX1	-0.1978483	0.0559014	-3.5392	0.000406	-0.280414469	count	1
SYT11	-0.2087454	0.1439087	-1.4505	0.147	-0.280385412	count	1
LDLRAD4	-0.2260838	0.1800774	-1.2555	0.209	-0.280276766	count	1
EML4	-0.2028857	0.114549	-1.7712	0.0766	-0.280229994	count	1

GRSF1	-0.2078361	0.1069425	-1.9434	0.052	-0.2798496	count	1
CHI3L1	-2.1766105	1.3843702	-1.5723	0.116	-0.279790242	count	1
MPZL2	-0.5061407	0.8916389	-0.5677	0.57	-0.279786518	count	1
RPL10	-0.1939588	0.0172096	-11.2704	5.47E-29	-0.279729871	count	1.32E-24
RPL32	-0.1940164	0.0189682	-10.2285	3.12E-24	-0.27968195	count	7.52E-20
SACS-AS1	-0.7197333	0.7539965	-0.9546	0.34	-0.279651086	count	1
SGSM1	-0.3351425	0.4449771	-0.7532	0.451	-0.27960532	count	1
SYNGAP1	-0.3664895	0.3143566	-1.1658	0.244	-0.279519364	count	1
DGKQ	-0.6212729	0.4413104	-1.4078	0.159	-0.279517613	count	1
SSBP1	-0.1958788	0.0429994	-4.5554	5.40E-06	-0.279501327	count	0.1247508
VEGFC	-0.4158518	0.4434187	-0.9378	0.348	-0.279404063	count	1
WDR44	-0.2470534	0.1907562	-1.2951	0.195	-0.279183825	count	1
HOTAIRM1	-0.2143333	0.1544319	-1.3879	0.165	-0.279083707	count	1
UBE2Q2	-0.2019329	0.0894341	-2.2579	0.024	-0.278641682	count	1
GTF2F2	-0.2066572	0.1243726	-1.6616	0.0967	-0.278583456	count	1
CCDC42	-0.3255474	0.3966464	-0.8207	0.412	-0.278277851	count	1
C6orf226	-0.2138958	0.1348349	-1.5864	0.113	-0.278265045	count	1
TANC2	-0.254718	0.2648042	-0.9619	0.336	-0.278190899	count	1
TTTY15	-0.2159147	0.1935219	-1.1157	0.265	-0.278172162	count	1
AL450998.2	-0.242689	0.1790119	-1.3557	0.175	-0.278127913	count	1
PRUNE1	-0.2688042	0.2764864	-0.9722	0.331	-0.278072253	count	1
SPATS2L	-0.1959262	0.0612382	-3.1994	0.00139	-0.277969451	count	1
IL18R1	-0.6179858	0.461466	-1.3392	0.181	-0.27793903	count	1
SLC1A4	-0.224931	0.2953617	-0.7615	0.446	-0.277737605	count	1
AC048382.6	-0.7145233	0.5031529	-1.4201	0.156	-0.277474906	count	1
HCG25	-1.1776931	0.9329357	-1.2624	0.207	-0.277313431	count	1
CD4	-1.1776931	0.9329357	-1.2624	0.207	-0.277313431	count	1
LAT2	-1.1776931	1.036884	-1.1358	0.256	-0.277313431	count	1
LUM	-0.1929612	0.0715489	-2.6969	0.00703	-0.277156026	count	1
TMEM134	-0.206629	0.106844	-1.9339	0.0532	-0.277050178	count	1
TSFM	-0.2072948	0.1273403	-1.6279	0.104	-0.277039046	count	1
ACTR2	-0.1970101	0.0622076	-3.167	0.00155	-0.277010769	count	1
HSP90AB1	-0.1921493	0.0232736	-8.2561	2.08E-16	-0.276773799	count	4.97E-12
SNRNP25	-0.2070461	0.1078426	-1.9199	0.0549	-0.276705585	count	1
ZNRF3	-0.3166115	0.2761525	-1.1465	0.252	-0.276700497	count	1
MCUR1	-0.2007306	0.0875971	-2.2915	0.022	-0.276554471	count	1
NDUFC1	-0.1936041	0.0416708	-4.646	3.50E-06	-0.276348335	count	0.0810215
MGME1	-0.2603038	0.2109309	-1.2341	0.217	-0.276251709	count	1
HK2	-2.1429303	0.8993741	-2.3827	0.0172	-0.276157525	count	1
AC109779.1	-2.1429303	1.285352	-1.6672	0.0956	-0.276157525	count	1
SLC38A4	-2.1429303	1.285352	-1.6672	0.0956	-0.276157525	count	1
ZNF3	-0.2545291	0.285232	-0.8924	0.372	-0.27612013	count	1
NFATC4	-0.2260071	0.3087454	-0.732	0.464	-0.276063577	count	1
CD247	-1.1726359	0.9086806	-1.2905	0.197	-0.276041942	count	1
SUB1	-0.1929746	0.0423483	-4.5568	5.36E-06	-0.275998226	count	0.12383744

AKR1C2	-0.1977563	0.0898397	-2.2012	0.0278	-0.275899165	count	1
RRAS	-0.1919821	0.0333475	-5.757	9.26E-09	-0.275480788	count	0.000217888
BVES	-0.308838	0.3390204	-0.911	0.362	-0.275450322	count	1
IDH3G	-0.196166	0.0639647	-3.0668	0.00218	-0.275302526	count	1
LYPLA2	-0.2148841	0.1417623	-1.5158	0.13	-0.27528804	count	1
ITPRIP	-0.2313729	0.2562985	-0.9027	0.367	-0.275145385	count	1
LACC1	-0.3489949	0.3205665	-1.0887	0.276	-0.274999019	count	1
SSNA1	-0.195131	0.067165	-2.9052	0.00369	-0.274947582	count	1
SBNO2	-0.2358474	0.3039536	-0.7759	0.438	-0.274728633	count	1
AL135905.1	-2.1291472	1.178569	-1.8066	0.0709	-0.274653941	count	1
CAP1	-0.1930294	0.0476419	-4.0517	5.19E-05	-0.274609874	count	1
MED27	-0.2032493	0.1080271	-1.8815	0.06	-0.274587262	count	1
PITPNB	-0.1951699	0.0678918	-2.8747	0.00407	-0.274450585	count	1
LIMS2	-0.1963395	0.0961712	-2.0416	0.0413	-0.274404777	count	1
LRRC4C	-0.4317048	0.266104	-1.6223	0.105	-0.274314981	count	1
LMNB2	-0.265051	0.2256102	-1.1748	0.24	-0.274129358	count	1
PNPLA2	-0.1964901	0.0809297	-2.4279	0.0152	-0.274078686	count	1
AC002467.1	-0.4081492	0.3597419	-1.1346	0.257	-0.274016357	count	1
AK2	-0.1983006	0.0876838	-2.2615	0.0238	-0.274013865	count	1
UFD1	-0.1945341	0.0684121	-2.8436	0.00449	-0.27380028	count	1
AGAP6	-0.328366	0.4282611	-0.7667	0.443	-0.273796111	count	1
RNF207	-0.3132422	0.3816087	-0.8208	0.412	-0.273682783	count	1
EIF4H	-0.1938947	0.0610351	-3.1768	0.0015	-0.273021883	count	1
ALG12	-0.213313	0.1939971	-1.0996	0.272	-0.272939826	count	1
NCF1	-0.7032794	0.6163621	-1.141	0.254	-0.272782316	count	1
SMIM26	-0.1926008	0.0571595	-3.3695	0.000761	-0.272475363	count	1
EME2	-0.326785	0.6895655	-0.4739	0.636	-0.272441535	count	1
TMEM191C	-0.493277	0.4664757	-1.0575	0.29	-0.272294434	count	1
LINC01116	-0.4055409	0.5021321	-0.8076	0.419	-0.272193349	count	1
ROMO1	-0.1929652	0.0565451	-3.4126	0.00065	-0.272170111	count	1
FEZ2	-0.1930286	0.0788722	-2.4474	0.0144	-0.271880001	count	1
SYF2	-0.191109	0.0496023	-3.8528	0.000119	-0.27187628	count	1
ACSM5	-0.8560795	0.5844925	-1.4647	0.143	-0.271867415	count	1
DIS3	-0.1975838	0.1152563	-1.7143	0.0866	-0.271814118	count	1
ADAM12	-0.8557676	0.7074466	-1.2097	0.226	-0.271760266	count	1
AGO1	-0.2224517	0.213794	-1.0405	0.298	-0.271689245	count	1
FRG1	-0.1937722	0.0674719	-2.8719	0.0041	-0.271634658	count	1
CETN2	-0.1965136	0.0875882	-2.2436	0.0249	-0.271581991	count	1
TESK1	-0.2248492	0.1454969	-1.5454	0.122	-0.271231242	count	1
TCTA	-0.2121647	0.1288147	-1.6471	0.0996	-0.271133322	count	1
PHPT1	-0.1894817	0.0381106	-4.9719	6.93E-07	-0.271081055	count	0.016151058
SMAD6	-0.2046934	0.180489	-1.1341	0.257	-0.271018443	count	1
GBA2	-0.2701814	0.2381161	-1.1347	0.257	-0.271012021	count	1
FAM192A	-0.2024243	0.1215079	-1.6659	0.0958	-0.270897944	count	1
ZNF630	-0.4905275	0.3621998	-1.3543	0.176	-0.270694914	count	1

RPS27	-0.1879431	0.0208141	-9.0296	2.72E-19	-0.270638349	count	6.53E-15
NPC2	-0.1887141	0.0421161	-4.4808	7.66E-06	-0.270624878	count	0.17669322
MRPL34	-0.1903777	0.0518487	-3.6718	0.000244	-0.270576171	count	1
SYT12	-0.4899222	0.43944	-1.1149	0.265	-0.27034288	count	1
ARID4A	-0.1968871	0.0913113	-2.1562	0.0311	-0.270324039	count	1
UTP4	-0.2272206	0.2099088	-1.0825	0.279	-0.27016573	count	1
ETNK2	-0.2379672	0.2430289	-0.9792	0.328	-0.270135949	count	1
SENCR	-0.6969147	0.5794387	-1.2027	0.229	-0.270128472	count	1
PGAP3	-0.2874614	0.2520768	-1.1404	0.254	-0.270032461	count	1
MMACHC	-0.3428729	0.4699653	-0.7296	0.466	-0.270026799	count	1
RPL6	-0.1873004	0.0198091	-9.4553	5.57E-21	-0.269943372	count	1.34E-16
AC132192.2	-0.3026868	0.3773031	-0.8022	0.422	-0.269835666	count	1
SH2B1	-0.2355203	0.1715096	-1.3732	0.17	-0.269828556	count	1
IQSEC1	-0.2029496	0.1322822	-1.5342	0.125	-0.269803229	count	1
SH3BGRL	-0.1882261	0.0348407	-5.4025	6.99E-08	-0.269761925	count	0.001637967
HIST1H2AG	-0.600811	0.5636771	-1.0659	0.287	-0.269701881	count	1
TCAF1	-0.2025278	0.1495882	-1.3539	0.176	-0.269681665	count	1
ASPSCR1	-0.2121067	0.1540766	-1.3766	0.169	-0.269675207	count	1
ESAM	-0.1948055	0.0911557	-2.1371	0.0327	-0.269655166	count	1
TPPP	-0.3672007	0.5400553	-0.6799	0.497	-0.269525014	count	1
ATP13A2	-0.4016758	0.3634253	-1.1053	0.269	-0.269493238	count	1
AP000866.1	-0.5349521	0.4179869	-1.2798	0.201	-0.269161548	count	1
MYL10	-2.0767752	0.9687957	-2.1437	0.0321	-0.268851911	count	1
CEP250	-0.2576436	0.2666087	-0.9664	0.334	-0.268806704	count	1
MCCC2	-0.2597987	0.20881	-1.2442	0.214	-0.268614	count	1
SMIM11A	-0.4869069	0.6227358	-0.7819	0.434	-0.268589671	count	1
DEXI	-0.1920702	0.0854674	-2.2473	0.0247	-0.268585001	count	1
TANGO2	-0.2460258	0.2088688	-1.1779	0.239	-0.268578835	count	1
RHBDD1	-0.2573767	0.251017	-1.0253	0.305	-0.268524097	count	1
CREB1	-0.1977702	0.100501	-1.9678	0.0492	-0.268499643	count	1
HMGB1	-0.1864342	0.0231973	-8.0369	1.23E-15	-0.268330362	count	2.94E-11
COPS8	-0.191977	0.0723639	-2.6529	0.00801	-0.268322258	count	1
POMT2	-0.2812994	0.4982874	-0.5645	0.572	-0.268192651	count	1
POP4	-0.1970232	0.1044464	-1.8864	0.0593	-0.268190946	count	1
LPP-AS2	-0.2618649	0.407295	-0.6429	0.52	-0.268167237	count	1
PSMB4	-0.1894174	0.051801	-3.6566	0.000259	-0.268109463	count	1
SFMBT1	-0.5328952	0.3760405	-1.4171	0.157	-0.268064744	count	1
NDUFAF8	-0.1924209	0.0809502	-2.377	0.0175	-0.267997952	count	1
C7orf50	-0.1899246	0.0677806	-2.8021	0.0051	-0.267831722	count	1
HMG20A	-0.2130647	0.2376087	-0.8967	0.37	-0.267762012	count	1
MRPL20	-0.1879699	0.0468639	-4.011	6.17E-05	-0.26773814	count	1
CDKN2AIPNL	-0.2105482	0.1401524	-1.5023	0.133	-0.26768308	count	1
EIF4B	-0.1887926	0.0502094	-3.7601	0.000172	-0.267612153	count	1
NPPC	-0.6907853	0.9850473	-0.7013	0.483	-0.267574545	count	1
RPS8	-0.1855067	0.0195298	-9.4986	3.71E-21	-0.267474785	count	8.92E-17

TMCO4	-0.2279389	0.180615	-1.262	0.207	-0.267412723	count	1
ARSJ	-0.2464605	0.2422058	-1.0176	0.309	-0.267254928	count	1
ZSWIM7	-0.1932366	0.1222741	-1.5804	0.114	-0.267246804	count	1
RRM2B	-0.20346	0.1396223	-1.4572	0.145	-0.267019777	count	1
FAXC	-1.1366081	0.7278302	-1.5616	0.118	-0.266971727	count	1
EIF3H	-0.1866885	0.0401011	-4.6554	3.35E-06	-0.266804905	count	0.07756255
CLIP3	-0.2156498	0.2440268	-0.8837	0.377	-0.266736097	count	1
ORMDL2	-0.1958123	0.1036215	-1.8897	0.0589	-0.266613253	count	1
PCDHGA3	-0.2603058	0.3775306	-0.6895	0.491	-0.266545447	count	1
ZNF319	-0.4475606	0.5647677	-0.7925	0.428	-0.266330531	count	1
PCBP4	-0.1981048	0.1386616	-1.4287	0.153	-0.266275403	count	1
PPP1R12A	-0.1869929	0.0499838	-3.7411	0.000186	-0.266198918	count	1
IL11RA	-0.1908262	0.1028527	-1.8553	0.0636	-0.266039867	count	1
SNRPD2	-0.1860929	0.0386999	-4.8086	1.58E-06	-0.265995862	count	0.03669076
SYNCRIP	-0.1901638	0.076198	-2.4957	0.0126	-0.2659798	count	1
LINC02019	-0.6868516	0.6173215	-1.1126	0.266	-0.265936397	count	1
TMEM86A	-0.4822359	0.5371247	-0.8978	0.369	-0.26587537	count	1
AL117335.1	-0.4822359	0.5489174	-0.8785	0.38	-0.26587537	count	1
SMCR8	-0.2714834	0.3031732	-0.8955	0.371	-0.265869192	count	1
AP005329.3	-0.2310395	0.2479899	-0.9316	0.352	-0.265807639	count	1
RDX	-0.1884864	0.0651325	-2.8939	0.00383	-0.265790229	count	1
PMVK	-0.1894692	0.0738264	-2.5664	0.0103	-0.265667834	count	1
AC022034.2	-0.4182162	0.3599716	-1.1618	0.245	-0.265368381	count	1
CHCHD1	-0.192762	0.0996495	-1.9344	0.0531	-0.265364532	count	1
EPB41L1	-0.202968	0.1794005	-1.1314	0.258	-0.265338766	count	1
LINC01750	-0.3182439	0.4766902	-0.6676	0.504	-0.265128606	count	1
ADGRL2	-0.2181372	0.1523707	-1.4316	0.152	-0.265110661	count	1
STK17B	-0.2001207	0.1361871	-1.4695	0.142	-0.265104224	count	1
SCUBE3	-0.1940309	0.1558437	-1.245	0.213	-0.265083424	count	1
TRAF3IP1	-0.2021304	0.1320215	-1.531	0.126	-0.264451395	count	1
RND2	-0.2512897	0.2443306	-1.0285	0.304	-0.264367555	count	1
SETD9	-0.2047013	0.1558174	-1.3137	0.189	-0.264212834	count	1
PDCL3	-0.1939774	0.0983421	-1.9725	0.0486	-0.264182177	count	1
COG1	-0.2435083	0.2347087	-1.0375	0.3	-0.264012805	count	1
NASP	-0.1937117	0.0942971	-2.0543	0.04	-0.263963629	count	1
MPC2	-0.1865137	0.0562211	-3.3175	0.000917	-0.263907074	count	1
AC055874.1	-0.6817055	0.6057185	-1.1254	0.26	-0.263794524	count	1
PHF10	-0.1951945	0.1126219	-1.7332	0.0831	-0.263770484	count	1
AL022157.1	-0.8321623	0.822956	-1.0112	0.312	-0.263656626	count	1
AC007773.1	-0.8321623	0.822956	-1.0112	0.312	-0.263656626	count	1
AC092329.3	-0.8321623	0.8623311	-0.965	0.335	-0.263656626	count	1
FBRS	-0.2091533	0.1977682	-1.0576	0.29	-0.263637192	count	1
MET	-0.5244559	0.4253434	-1.233	0.218	-0.263568203	count	1
PEX10	-0.238467	0.1895695	-1.2579	0.208	-0.263515143	count	1
TMTC1	-0.2027715	0.1335992	-1.5178	0.129	-0.263493113	count	1

ETHE1	-0.1891984	0.0798571	-2.3692	0.0179	-0.263393485	count	1
ST8SIA4	-0.3085017	0.3752735	-0.8221	0.411	-0.263338244	count	1
FOXP1	-0.1847722	0.0608388	-3.0371	0.0024	-0.263119305	count	1
RPS24	-0.1822869	0.0196817	-9.2617	3.33E-20	-0.26274025	count	8.00E-16
MT-ND2	-0.1822338	0.0256865	-7.0945	1.55E-12	-0.262580495	count	3.69E-08
PLP2	-0.183264	0.0430069	-4.2613	2.08E-05	-0.262408424	count	0.4777552
ARL10	-0.2455195	0.3094981	-0.7933	0.428	-0.262391057	count	1
AL353751.1	-0.8284405	0.8498002	-0.9749	0.33	-0.262380092	count	1
BCL2L1	-0.1980132	0.1384363	-1.4304	0.153	-0.262302581	count	1
GPT2	-0.5850325	0.5955836	-0.9823	0.326	-0.262151116	count	1
ABCC3	-0.2178487	0.28495	-0.7645	0.445	-0.26200618	count	1
DCAF4	-0.3145232	0.2804327	-1.1216	0.262	-0.261945559	count	1
DEPP1	-0.1838563	0.0898077	-2.0472	0.0407	-0.261865184	count	1
AC005332.7	-0.3723505	0.288095	-1.2925	0.196	-0.261789673	count	1
AGRN	-0.261048	0.2881345	-0.906	0.365	-0.261698698	count	1
RASL12	-0.1962348	0.1141398	-1.7192	0.0857	-0.261686383	count	1
ITGA4	-0.3721929	0.4707661	-0.7906	0.429	-0.261674732	count	1
IK	-0.1845533	0.051571	-3.5786	0.00035	-0.261658685	count	1
RRS1	-0.2380248	0.2100071	-1.1334	0.257	-0.261411933	count	1
ZNF623	-0.2463278	0.2337376	-1.0539	0.292	-0.261216932	count	1
EHHADH	-0.3222261	0.3917174	-0.8226	0.411	-0.26119881	count	1
MYLPF	-0.6751062	0.5743579	-1.1754	0.24	-0.261049665	count	1
FIS1	-0.18232	0.0369629	-4.9325	8.48E-07	-0.26102892	count	0.019742288
TTLL1	-0.2502309	0.211367	-1.1839	0.237	-0.260960362	count	1
F2RL2	-2.0073508	1.219707	-1.6458	0.0999	-0.260947971	count	1
SEMA3A	-0.5824292	0.4931866	-1.181	0.238	-0.260906909	count	1
PDE7B	-0.6744662	0.5079859	-1.3277	0.184	-0.260783579	count	1
PAQR4	-0.5189357	0.4701301	-1.1038	0.27	-0.260630071	count	1
ARMCX5	-0.2296572	0.1862978	-1.2327	0.218	-0.26060453	count	1
RTL8A	-0.1848754	0.0666947	-2.772	0.0056	-0.260471369	count	1
UBE4B	-0.2274173	0.2490922	-0.913	0.361	-0.260452753	count	1
HMCN1	-0.1833797	0.1216248	-1.5077	0.132	-0.260409311	count	1
CYB561A3	-0.211834	0.1640923	-1.2909	0.197	-0.260370485	count	1
ST20-AS1	-0.3550857	0.3713427	-0.9562	0.339	-0.260328052	count	1
CCNB1	-0.3884738	0.4550388	-0.8537	0.393	-0.260282525	count	1
COX14	-0.184028	0.0554033	-3.3216	0.000904	-0.260236239	count	1
PRICKLE2	-0.2261435	0.2180314	-1.0372	0.3	-0.260119847	count	1
DHX37	-0.410255	0.3619537	-1.1334	0.257	-0.260096745	count	1
ATP5MD	-0.1821838	0.0461896	-3.9443	8.15E-05	-0.260088671	count	1
POLR2F	-0.1839228	0.0588467	-3.1255	0.00179	-0.260061335	count	1
IMPDH1	-0.2168338	0.1899402	-1.1416	0.254	-0.260043177	count	1
KRT8	-0.1844494	0.115215	-1.6009	0.109	-0.26002406	count	1
SH3GLB1	-0.183382	0.0606603	-3.0231	0.00252	-0.259923858	count	1
EIF1AD	-0.2155412	0.1858708	-1.1596	0.246	-0.259918317	count	1
GLIPR1L2	-0.2727795	0.2958697	-0.922	0.357	-0.259914023	count	1

ANKRD37	-0.1888096	0.1300303	-1.452	0.147	-0.259913245	count	1
HIVEP2	-0.2113999	0.2052854	-1.0298	0.303	-0.259833325	count	1
C4orf3	-0.1812812	0.0359713	-5.0396	4.89E-07	-0.259812624	count	0.011405925
LINC00865	-0.471555	0.4974938	-0.9479	0.343	-0.259676124	count	1
NF2	-0.2222	0.2134189	-1.0411	0.298	-0.259668778	count	1
BORCS8	-0.196307	0.1188852	-1.6512	0.0988	-0.259551928	count	1
MEF2C-AS1	-0.2557991	0.2720162	-0.9404	0.347	-0.259173728	count	1
NDST1-AS1	-1.1051002	0.8218261	-1.3447	0.179	-0.259025367	count	1
SPN	-1.1051002	0.8218261	-1.3447	0.179	-0.259025367	count	1
CDC25A	-1.1051002	0.9060302	-1.2197	0.223	-0.259025367	count	1
AL390208.1	-1.1051002	1.251583	-0.883	0.377	-0.259025367	count	1
AC127070.1	-0.5157325	0.8191812	-0.6296	0.529	-0.258926307	count	1
PPIL1	-0.2066676	0.173132	-1.1937	0.233	-0.258831158	count	1
SNHG8	-0.1830921	0.0591199	-3.097	0.00197	-0.258797568	count	1
ATL2	-0.2134026	0.1558255	-1.3695	0.171	-0.258667673	count	1
AL591895.1	-0.3287943	0.4021955	-0.8175	0.414	-0.258608666	count	1
RPL30	-0.1793817	0.0183381	-9.7819	2.52E-22	-0.258590039	count	6.06E-18
ZNF382	-0.3525535	0.4681222	-0.7531	0.451	-0.258407866	count	1
WFS1	-0.2008607	0.1607548	-1.2495	0.212	-0.25840778	count	1
AP1S1	-0.1934543	0.1191417	-1.6237	0.105	-0.258357771	count	1
RGMB	-0.2549923	0.3211076	-0.7941	0.427	-0.258343234	count	1
EFHB	-0.6683103	0.6748289	-0.9903	0.322	-0.25822531	count	1
FAM96B	-0.1812088	0.0471361	-3.8444	1.00E-04	-0.258053212	count	1
SRP14-AS1	-0.2274306	0.2485087	-0.9152	0.36	-0.258051685	count	1
EMC9	-0.203785	0.1491642	-1.3662	0.172	-0.257970024	count	1
TRPM3	-0.3519605	0.5012103	-0.7022	0.483	-0.257958307	count	1
PLEK	-0.4341006	0.6556876	-0.6621	0.508	-0.257940919	count	1
PARP10	-0.2262624	0.2083272	-1.0861	0.278	-0.257935801	count	1
ZNF580	-0.1849735	0.0780941	-2.3686	0.0179	-0.257894296	count	1
PPP1R9A	-0.2495352	0.5514562	-0.4525	0.651	-0.25784463	count	1
L3MBTL1	-0.366177	0.4696016	-0.7798	0.436	-0.257289606	count	1
CEP68	-0.2127185	0.1699793	-1.2514	0.211	-0.257169699	count	1
MSX2	-0.1927542	0.1825406	-1.056	0.291	-0.257161672	count	1
CTDSPL	-0.1834186	0.0886084	-2.07	0.0385	-0.257121921	count	1
SCARA3	-0.1851344	0.0968259	-1.912	0.0559	-0.256865869	count	1
PPP2R5B	-0.2113895	0.2162154	-0.9777	0.328	-0.256851147	count	1
GAD1	-1.969782	0.5905551	-3.3355	0.00086	-0.256572003	count	1
MEA1	-0.1822101	0.0802113	-2.2716	0.0232	-0.256527924	count	1
ZRSR2	-0.1997782	0.1989189	-1.0043	0.315	-0.256436239	count	1
RPL12	-0.1778902	0.0201753	-8.8172	1.78E-18	-0.256418042	count	4.27E-14
TOMM70	-0.1881244	0.1062427	-1.7707	0.0767	-0.256332701	count	1
RNF121	-0.205638	0.1794059	-1.1462	0.252	-0.256200482	count	1
PEF1	-0.1852898	0.0832518	-2.2257	0.0261	-0.256161336	count	1
EVI5L	-0.3002568	0.3525631	-0.8516	0.394	-0.256124123	count	1
SETDB2	-0.2477678	0.2911159	-0.8511	0.395	-0.255991226	count	1

PMPCB	-0.184294	0.0787605	-2.3399	0.0193	-0.255950156	count	1
POLR2J	-0.180706	0.0513977	-3.5158	0.000444	-0.255912437	count	1
ITPKB	-0.2686224	0.3352965	-0.8011	0.423	-0.255877604	count	1
RAC1	-0.1780133	0.0273187	-6.5162	8.19E-11	-0.255865554	count	1.94E-06
TLN2	-0.1898159	0.1550868	-1.2239	0.221	-0.255819371	count	1
GABPB1	-0.2315035	0.2128011	-1.0879	0.277	-0.255731467	count	1
UBE2F	-0.1893472	0.1214413	-1.5592	0.119	-0.255664062	count	1
RBSN	-0.2041334	0.1695008	-1.2043	0.229	-0.255638809	count	1
HINFP	-0.2580248	0.3146794	-0.82	0.412	-0.255620356	count	1
ARL16	-0.1882772	0.0993458	-1.8952	0.0581	-0.255177491	count	1
RPS19BP1	-0.1798232	0.0578395	-3.109	0.00189	-0.255132238	count	1
ABHD3	-0.2468816	0.2902334	-0.8506	0.395	-0.255062028	count	1
PDXK	-0.1872243	0.1134861	-1.6498	0.0991	-0.254893212	count	1
GIMAP4	-0.5697328	0.6292145	-0.9055	0.365	-0.254845563	count	1
IL7R	-1.955112	0.4365145	-4.4789	7.73E-06	-0.254845025	count	0.17830018
ACAA1	-0.1867394	0.094747	-1.9709	0.0488	-0.254843785	count	1
SLC2A6	-0.4022774	0.3798709	-1.059	0.29	-0.254820944	count	1
RCOR3	-0.190293	0.122774	-1.5499	0.121	-0.254614441	count	1
UBQLN4	-0.2513149	0.3194892	-0.7866	0.432	-0.254558686	count	1
RBKS	-0.2669432	0.3409659	-0.7829	0.434	-0.254247713	count	1
NAGS	-0.6583857	0.6693297	-0.9836	0.325	-0.254104934	count	1
AP003352.1	-0.803538	0.6457038	-1.2444	0.213	-0.253847851	count	1
ZNF572	-1.084171	0.6135563	-1.767	0.0773	-0.253741706	count	1
MTHFS	-0.2047112	0.1930961	-1.0602	0.289	-0.253617305	count	1
SLC25A14	-0.2411826	0.2926328	-0.8242	0.41	-0.253587522	count	1
AC074387.1	-0.4607552	0.4989471	-0.9235	0.356	-0.253418568	count	1
PTPN4	-0.2194471	0.2363778	-0.9284	0.353	-0.253414563	count	1
TEX14	-1.941991	1.109726	-1.75	0.0802	-0.253291807	count	1
CLEC20A	-1.941991	1.147527	-1.6923	0.0907	-0.253291807	count	1
AP002360.3	-1.941991	1.219619	-1.5923	0.111	-0.253291807	count	1
NPIPB11	-1.941991	1.219619	-1.5923	0.111	-0.253291807	count	1
ZMYND8	-0.1954456	0.1676722	-1.1656	0.244	-0.253220934	count	1
CTDSP1	-0.1819138	0.0816438	-2.2281	0.0259	-0.252905328	count	1
LINC01423	-0.360087	0.383363	-0.9393	0.348	-0.252854604	count	1
NOL12	-0.2275287	0.228517	-0.9957	0.319	-0.252771073	count	1
LINC00562	-1.079929	0.9178194	-1.1766	0.239	-0.252670381	count	1
STAB1	-1.0794642	0.7286747	-1.4814	0.139	-0.252552992	count	1
TRIM58	-1.9345168	0.9878416	-1.9583	0.0503	-0.252403331	count	1
CSF1R	-1.9345168	1.149644	-1.6827	0.0925	-0.252403331	count	1
AC097461.1	-1.9345168	1.264511	-1.5299	0.126	-0.252403331	count	1
MPV17L2	-0.2441619	0.2523614	-0.9675	0.333	-0.252210884	count	1
AC109322.1	-0.3443149	0.5823813	-0.5912	0.554	-0.252165632	count	1
IQCD	-0.3113661	0.4010426	-0.7764	0.438	-0.252151785	count	1
NDUFA13	-0.1796431	0.0665752	-2.6983	0.007	-0.252024623	count	1
PRKAA2	-0.2002587	0.2015266	-0.9937	0.32	-0.251975546	count	1

PKNOX2	-0.3438715	0.3981971	-0.8636	0.388	-0.25182989	count	1
AL451085.1	-0.4578357	0.5072276	-0.9026	0.367	-0.251728857	count	1
ALAD	-0.1958841	0.1695837	-1.1551	0.248	-0.251697573	count	1
AC005332.6	-0.6524904	0.6222633	-1.0486	0.294	-0.25165985	count	1
TUBG1	-0.1959471	0.1728836	-1.1334	0.257	-0.251495468	count	1
RTN4IP1	-0.5624634	0.451478	-1.2458	0.213	-0.251380193	count	1
PARS2	-0.3970421	0.407712	-0.9738	0.33	-0.251362397	count	1
RPL37	-0.174493	0.0208942	-8.3512	9.46E-17	-0.251266896	count	2.26E-12
PGBD4	-0.5012472	0.3645372	-1.375	0.169	-0.251232314	count	1
RSPRY1	-0.1999395	0.1428635	-1.3995	0.162	-0.251175355	count	1
DNTTIP1	-0.1908672	0.1252376	-1.524	0.128	-0.251158591	count	1
TNFRSF12A	-0.1755637	0.076373	-2.2988	0.0216	-0.251028474	count	1
RPSA	-0.1745241	0.0258979	-6.7389	1.85E-11	-0.250934823	count	4.39E-07
ETV1	-0.1939827	0.1873474	-1.0354	0.301	-0.250825628	count	1
COX6A1	-0.1746347	0.031602	-5.5261	3.50E-08	-0.250626367	count	0.000821555
UHRF1	-0.7929207	0.6862369	-1.1555	0.248	-0.250215255	count	1
CXCL9	-0.3564244	0.3760941	-0.9477	0.343	-0.250189477	count	1
ZNF514	-0.2359829	0.2488629	-0.9482	0.343	-0.250100923	count	1
SPATC1L	-0.1990517	0.16695	-1.1923	0.233	-0.250053797	count	1
CAB39	-0.1921571	0.140154	-1.371	0.17	-0.249868778	count	1
DDT	-0.1764051	0.0575609	-3.0647	0.00219	-0.249707872	count	1
USE1	-0.1804605	0.0954294	-1.891	0.0587	-0.249548546	count	1
ANOS1	-0.2150215	0.2514902	-0.855	0.393	-0.249271955	count	1
PIP4K2B	-0.1974056	0.2032542	-0.9712	0.332	-0.249124343	count	1
ROPN1B	-0.6461512	0.7510254	-0.8604	0.39	-0.249032738	count	1
MRPS7	-0.1800098	0.0849815	-2.1182	0.0342	-0.248961159	count	1
MIR181A1HG	-0.557109	0.5865508	-0.9498	0.342	-0.248830154	count	1
POGLUT1	-0.1905098	0.1479741	-1.2875	0.198	-0.248785331	count	1
SLC40A1	-0.175861	0.0782982	-2.246	0.0248	-0.248711183	count	1
PDLIM4	-0.17662	0.0791334	-2.2319	0.0257	-0.248588215	count	1
MRPL14	-0.1781198	0.0740268	-2.4062	0.0162	-0.248537529	count	1
SNRPG	-0.175819	0.0565815	-3.1074	0.0019	-0.248217307	count	1
SLC25A40	-0.2689654	0.3689718	-0.729	0.466	-0.248191926	count	1
RPS26	-0.1723332	0.0225024	-7.6584	2.39E-14	-0.248102945	count	5.70E-10
C2orf27B	-0.7861135	0.4985804	-1.5767	0.115	-0.247888009	count	1
CPSF1	-0.2400318	0.2657125	-0.9034	0.366	-0.247882674	count	1
UBOX5	-0.3386349	0.3890916	-0.8703	0.384	-0.247866618	count	1
GOSR2	-0.1868687	0.1275774	-1.4647	0.143	-0.247786972	count	1
SNHG25	-0.3059857	0.4956246	-0.6174	0.537	-0.24767482	count	1
SF3B6	-0.1734215	0.0443777	-3.9079	9.48E-05	-0.247509385	count	1
GSAP	-0.3257464	0.4708409	-0.6918	0.489	-0.247497701	count	1
WDR83OS	-0.1728225	0.0364647	-4.7394	2.22E-06	-0.247449872	count	0.0514818
PSMB9	-0.1756526	0.0814477	-2.1566	0.0311	-0.246937602	count	1
POLR2K	-0.1737115	0.0509757	-3.4077	0.000662	-0.246776432	count	1
AC067735.1	-0.7828368	0.6983964	-1.1209	0.262	-0.246768267	count	1

AP3M2	-0.1995625	0.1588088	-1.2566	0.209	-0.246715101	count	1
OXNAD1	-0.2673402	0.2912562	-0.9179	0.359	-0.246661886	count	1
NDUFAF2	-0.1759576	0.0715819	-2.4581	0.014	-0.246651374	count	1
BPNT1	-0.2143771	0.2039794	-1.051	0.293	-0.246458946	count	1
AC100803.3	-0.3895606	0.324633	-1.2	0.23	-0.246425124	count	1
EDA2R	-0.2433796	0.2298587	-1.0588	0.29	-0.246397194	count	1
RBM41	-0.2193577	0.242579	-0.9043	0.366	-0.246293649	count	1
SLC11A1	-0.3509167	0.3734012	-0.9398	0.347	-0.246184596	count	1
CROT	-0.1933947	0.1588267	-1.2176	0.223	-0.246091605	count	1
KIF20B	-0.2169684	0.1781971	-1.2176	0.223	-0.246062465	count	1
ZFAND2A	-0.1906964	0.1482515	-1.2863	0.198	-0.246057237	count	1
MYOCD	-0.1861453	0.1677566	-1.1096	0.267	-0.245754241	count	1
NIPSNAP2	-0.1764497	0.0811986	-2.1731	0.0298	-0.245516046	count	1
JRKL	-0.1925233	0.1550092	-1.242	0.214	-0.245295218	count	1
VPS28	-0.1714699	0.0377786	-4.5388	5.84E-06	-0.245282666	count	0.13486312
RACK1	-0.1702383	0.0207316	-8.2115	2.99E-16	-0.245221411	count	7.15E-12
EID1	-0.1703518	0.0237303	-7.1787	8.48E-13	-0.245127086	count	2.02E-08
IMPACT	-0.1899656	0.1229414	-1.5452	0.122	-0.245110126	count	1
FILIP1	-0.1763589	0.073691	-2.3932	0.0168	-0.244972331	count	1
RARB	-0.3026998	0.3444111	-0.8789	0.38	-0.244942388	count	1
PPIL3	-0.1890724	0.1472152	-1.2843	0.199	-0.244928885	count	1
AAK1	-0.1784364	0.1226226	-1.4552	0.146	-0.244791393	count	1
TPRKB	-0.1774012	0.1025584	-1.7298	0.0838	-0.244610903	count	1
NDUFS6	-0.1724414	0.0497626	-3.4653	0.000536	-0.244535942	count	1
DES	-0.2064752	0.2083033	-0.9912	0.322	-0.244522199	count	1
CDC37L1	-0.1804902	0.09617	-1.8768	0.0606	-0.244437908	count	1
SCNM1	-0.1797197	0.1154077	-1.5573	0.119	-0.244374971	count	1
NOTCH3	-0.1751697	0.0910904	-1.923	0.0546	-0.244347703	count	1
SLC25A29	-0.1964442	0.2182237	-0.9002	0.368	-0.244234342	count	1
PRKACA	-0.1880975	0.1333785	-1.4103	0.159	-0.244122093	count	1
ITIH2	-1.8617402	0.9742791	-1.9109	0.0561	-0.243618285	count	1
HIST1H2BH	-1.8617402	1.218114	-1.5284	0.127	-0.243618285	count	1
AC090337.1	-1.8617402	1.218114	-1.5284	0.127	-0.243618285	count	1
CTC1	-0.293011	0.4668018	-0.6277	0.53	-0.243574167	count	1
RPL7A	-0.1688483	0.0194596	-8.6769	6.02E-18	-0.243392379	count	1.44E-13
SLCO3A1	-0.1786046	0.1007623	-1.7725	0.0764	-0.24333298	count	1
SMIM37	-0.1732164	0.0760503	-2.2777	0.0228	-0.243300169	count	1
CCT8	-0.1715775	0.059245	-2.8961	0.0038	-0.243030641	count	1
CCDC159	-0.2200984	0.1915214	-1.1492	0.251	-0.242992961	count	1
PARM1	-0.1867923	0.1298756	-1.4382	0.15	-0.24286448	count	1
NARS2	-0.2516	0.2482213	-1.0138	0.311	-0.242820013	count	1
UBTD1	-0.2003621	0.1774199	-1.1293	0.259	-0.242751135	count	1
FAM83D	-1.8516043	1.0528083	-1.7587	0.0787	-0.242375872	count	1
ATP8B3	-1.039182	0.7585154	-1.37	0.171	-0.242375561	count	1
ELOB	-0.1689108	0.0329968	-5.119	3.23E-07	-0.242311329	count	0.007540758

MRPS36	-0.1718537	0.0647158	-2.6555	0.00795	-0.242284207	count	1
AC093827.4	-0.3455235	0.3018143	-1.1448	0.252	-0.242266412	count	1
SAP30L-AS1	-0.7695184	0.714698	-1.0767	0.282	-0.242220545	count	1
AP1S3	-0.7695184	0.8599033	-0.8949	0.371	-0.242220545	count	1
NRG3	-1.0383011	0.6815116	-1.5235	0.128	-0.242152955	count	1
AC130650.2	-1.0383011	0.7145681	-1.453	0.146	-0.242152955	count	1
MTMR2	-0.1901328	0.1491116	-1.2751	0.202	-0.241920435	count	1
MKL2	-0.1925713	0.1742097	-1.1054	0.269	-0.241868567	count	1
STOX2	-1.0370027	0.6367544	-1.6286	0.103	-0.241824839	count	1
KAT7	-0.1815695	0.1152979	-1.5748	0.115	-0.241817656	count	1
CDKN2B	-0.2473179	0.2894059	-0.8546	0.393	-0.241807401	count	1
LIN7B	-0.1893287	0.1986667	-0.953	0.341	-0.241512017	count	1
PPCS	-0.1716035	0.0626183	-2.7405	0.00616	-0.241471783	count	1
NBPF11	-0.2663757	0.3664041	-0.727	0.467	-0.241408567	count	1
EEF1D	-0.1677277	0.0264857	-6.3328	2.70E-10	-0.241310851	count	6.39E-06
LINC00662	-0.1870165	0.1578406	-1.1848	0.236	-0.241288388	count	1
FBXL19	-0.4823183	0.3734248	-1.2916	0.197	-0.241205174	count	1
FBXL2	-0.2136305	0.2054154	-1.04	0.298	-0.241040477	count	1
ISG20	-0.2022508	0.1949841	-1.0373	0.3	-0.240994881	count	1
KDM5C	-0.2022349	0.2545309	-0.7945	0.427	-0.24097579	count	1
PDZD11	-0.1736877	0.0873165	-1.9892	0.0468	-0.240774081	count	1
SCML2	-0.2975462	0.8391908	-0.3546	0.723	-0.240659499	count	1
ST3GAL6	-0.3600004	0.3828899	-0.9402	0.347	-0.240483012	count	1
COX7A2	-0.1677054	0.0337371	-4.9709	6.97E-07	-0.240464185	count	0.016243585
RBX1	-0.1688369	0.0465054	-3.6305	0.000287	-0.24045638	count	1
ACTL10	-0.3804304	0.4360135	-0.8725	0.383	-0.240408172	count	1
SLC25A26	-0.1852424	0.1355587	-1.3665	0.172	-0.240401411	count	1
NDUFA4	-0.1670791	0.0337933	-4.9442	7.99E-07	-0.240338969	count	0.018607112
BX322562.1	-0.2306294	0.2553223	-0.9033	0.366	-0.240239653	count	1
AC104407.1	-0.1847583	0.2140221	-0.8633	0.388	-0.240209255	count	1
GOLGA6L9	-0.5389578	0.9467043	-0.5693	0.569	-0.240201363	count	1
PNMA8A	-0.2426859	0.3395729	-0.7147	0.475	-0.240179	count	1
HPX	-1.83374	1.2548429	-1.4613	0.144	-0.240175212	count	1
STK19	-0.1803881	0.1197715	-1.5061	0.132	-0.239981456	count	1
C2orf68	-0.232426	0.2687355	-0.8649	0.387	-0.239916833	count	1
DAAM2	-0.1943709	0.1655085	-1.1744	0.24	-0.239774244	count	1
RUSC1-AS1	-0.623495	0.5202203	-1.1985	0.231	-0.239661917	count	1
RPL29	-0.1662977	0.0204472	-8.133	5.67E-16	-0.239628893	count	1.36E-11
FYCO1	-0.1831142	0.1766209	-1.0368	0.3	-0.239479179	count	1
SYNC	-0.1818883	0.1281954	-1.4188	0.156	-0.239469131	count	1
NUDT2	-0.1808199	0.1070836	-1.6886	0.0914	-0.239157987	count	1
BTBD10	-0.1862874	0.1258769	-1.4799	0.139	-0.239041365	count	1
ZBTB10	-0.1814396	0.1106864	-1.6392	0.101	-0.239040836	count	1
FAM50B	-0.1824558	0.163456	-1.1162	0.264	-0.238990355	count	1
TMEM35A	-0.2070655	0.2079472	-0.9958	0.319	-0.238988214	count	1

HNRNPUL2	-0.1948433	0.1854395	-1.0507	0.293	-0.238834164	count	1
UBQLN1	-0.1814664	0.1248964	-1.4529	0.146	-0.23874477	count	1
RNASEH2C	-0.167823	0.0541217	-3.1008	0.00194	-0.238696467	count	1
EPB41L4A-DT	-0.5357259	0.6266674	-0.8549	0.393	-0.238667593	count	1
CYTOR	-0.1751499	0.1007378	-1.7387	0.0822	-0.238284386	count	1
RPS12	-0.1652776	0.0197028	-8.3885	6.94E-17	-0.238272086	count	1.66E-12
NDUFB8	-0.1666279	0.0394583	-4.2229	2.47E-05	-0.238239538	count	0.5667168
TBCA	-0.1662883	0.0383807	-4.3326	1.51E-05	-0.237935722	count	0.3474057
LRFN4	-0.226383	0.2714896	-0.8339	0.404	-0.237821659	count	1
MYBBP1A	-0.1972393	0.243565	-0.8098	0.418	-0.23769276	count	1
FAAH	-0.2623233	0.4217841	-0.6219	0.534	-0.237660215	count	1
F11R	-0.3250103	0.5764211	-0.5638	0.573	-0.237570322	count	1
KLF16	-0.2724768	0.3171691	-0.8591	0.39	-0.237275908	count	1
ZMAT2	-0.1700712	0.0784982	-2.1666	0.0303	-0.237226799	count	1
SCAPER	-0.1786354	0.1532077	-1.166	0.244	-0.237111167	count	1
AC008074.1	-0.7545177	0.7290739	-1.0349	0.301	-0.23710553	count	1
CERNA1	-0.7545177	0.777346	-0.9706	0.332	-0.23710553	count	1
SURF1	-0.1693918	0.0821613	-2.0617	0.0393	-0.237059879	count	1
MANF	-0.1705996	0.0786578	-2.1689	0.0302	-0.236961066	count	1
SNF8	-0.1679213	0.0628623	-2.6713	0.00759	-0.236877333	count	1
COX7A2L	-0.1661528	0.0443784	-3.744	0.000184	-0.236858248	count	1
PSMB6	-0.1656668	0.0401448	-4.1267	3.76E-05	-0.236819642	count	0.860664
IL10RB-DT	-0.4737194	0.6367881	-0.7439	0.457	-0.236660587	count	1
TTC38	-0.2144153	0.2575993	-0.8324	0.405	-0.236650114	count	1
LINC01556	-0.6161095	0.7237094	-0.8513	0.395	-0.236613657	count	1
FGF22	-0.6161095	0.7237094	-0.8513	0.395	-0.236613657	count	1
RSPO2	-0.179545	0.2307264	-0.7782	0.437	-0.236536137	count	1
GRWD1	-0.1902308	0.1806931	-1.0528	0.293	-0.236464689	count	1
GPD1L	-0.209567	0.2411942	-0.8689	0.385	-0.236409963	count	1
AL603839.3	-0.3994109	0.8247959	-0.4843	0.628	-0.236403988	count	1
PLLP	-0.3741875	0.4672647	-0.8008	0.423	-0.236299385	count	1
TMPO-AS1	-0.3741875	0.6267264	-0.5971	0.551	-0.236299385	count	1
PGM2	-0.18479	0.1548268	-1.1935	0.233	-0.236277506	count	1
ZNF628	-0.3531024	0.4282844	-0.8245	0.41	-0.235700319	count	1
SNCG	-0.164615	0.0711871	-2.3124	0.0208	-0.235671199	count	1
NUDT6	-0.1990437	0.2267727	-0.8777	0.38	-0.235652611	count	1
CACNA1C-AS2	-0.6130067	0.8721434	-0.7029	0.482	-0.235334	count	1
NCF4	-1.7939495	0.7561129	-2.3726	0.0177	-0.235224397	count	1
SENP8	-0.3970865	0.3778018	-1.051	0.293	-0.234965448	count	1
CAND2	-0.2372124	0.2923518	-0.8114	0.417	-0.234675397	count	1
ENO1-AS1	-0.3716391	0.6434787	-0.5775	0.564	-0.234623416	count	1
RSPH3	-0.1968552	0.1960218	-1.0043	0.315	-0.23451731	count	1
C1orf50	-0.2068743	0.1991959	-1.0385	0.299	-0.234504455	count	1
ARPC3	-0.1636395	0.0369563	-4.4279	9.79E-06	-0.234467931	count	0.22558118
HPS5	-0.1840369	0.1868384	-0.985	0.325	-0.234431328	count	1

MAP3K5	-0.1726703	0.1358679	-1.2709	0.204	-0.234418778	count	1
HNRNPUL1	-0.1725705	0.0976208	-1.7678	0.0772	-0.234138402	count	1
SPOCK2	-0.7456789	0.8648624	-0.8622	0.389	-0.234095372	count	1
VAC14	-0.2308943	0.2911679	-0.793	0.428	-0.23357006	count	1
HEBP2	-0.1638689	0.0524799	-3.1225	0.00181	-0.233544742	count	1
APBB2	-0.1760292	0.1341254	-1.3124	0.189	-0.233368529	count	1
GRAMD2A	-1.7768878	1.128337	-1.5748	0.115	-0.233081263	count	1
IL2RG	-1.7768878	1.164764	-1.5255	0.127	-0.233081263	count	1
TYRP1	-1.7768878	1.164764	-1.5255	0.127	-0.233081263	count	1
AP000426.1	-1.7768878	1.202101	-1.4782	0.139	-0.233081263	count	1
OSMR-AS1	-0.3073177	0.32301	-0.9514	0.341	-0.233078394	count	1
GAREM1	-0.2066362	0.3033779	-0.6811	0.496	-0.233071165	count	1
FAM124A	-0.2677485	0.4057087	-0.66	0.509	-0.233066102	count	1
TUSC3	-0.2448106	0.2464425	-0.9934	0.321	-0.232795518	count	1
GYG1	-0.1670828	0.0742093	-2.2515	0.0244	-0.232614292	count	1
ARHGAP1	-0.1733963	0.1097177	-1.5804	0.114	-0.232578458	count	1
HECW2	-0.7409452	0.5043265	-1.4692	0.142	-0.232484463	count	1
GRHL2	-1.771413	0.8455104	-2.0951	0.0362	-0.232391085	count	1
TSEN34	-0.1664968	0.0784604	-2.122	0.0339	-0.232278248	count	1
OARD1	-0.1727583	0.1155242	-1.4954	0.135	-0.232214872	count	1
THUMPD2	-0.1706197	0.1120924	-1.5221	0.128	-0.232041351	count	1
RAB34	-0.1628622	0.0440634	-3.6961	2.00E-04	-0.232018073	count	1
FAM222B	-0.2291615	0.2751927	-0.8327	0.405	-0.231791201	count	1
NDUFB10	-0.1618425	0.0358601	-4.5132	6.59E-06	-0.231760894	count	0.15213674
ISY1	-0.1785774	0.156412	-1.1417	0.254	-0.23171738	count	1
MRPS15	-0.1660357	0.0786749	-2.1104	0.0349	-0.231612593	count	1
SGTA	-0.1833228	0.1419148	-1.2918	0.197	-0.231604086	count	1
ARPC2	-0.1613534	0.0368561	-4.3779	1.23E-05	-0.231463329	count	0.2832567
FAM173A	-0.1683977	0.0860583	-1.9568	0.0504	-0.231460898	count	1
COQ3	-0.1968209	0.2231314	-0.8821	0.378	-0.231434205	count	1
AGPAT3	-0.2337675	0.2317547	-1.0087	0.313	-0.231213316	count	1
KLHL26	-0.2948682	0.3553367	-0.8298	0.407	-0.231190765	count	1
LRP5	-0.1890073	0.2232734	-0.8465	0.397	-0.23111664	count	1
DCBLD2	-0.1708458	0.1217947	-1.4027	0.161	-0.230952282	count	1
TMEM129	-0.1857967	0.128696	-1.4437	0.149	-0.230921552	count	1
MOAP1	-0.1777987	0.1166004	-1.5249	0.127	-0.230916017	count	1
BRWD3	-0.2027609	0.3342924	-0.6065	0.544	-0.230896814	count	1
URB2	-0.4216621	0.4082222	-1.0329	0.302	-0.230861673	count	1
USP40	-0.1926562	0.2475223	-0.7783	0.436	-0.230841816	count	1
CAVIN1	-0.1610554	0.0362811	-4.4391	9.30E-06	-0.230806326	count	0.2143278
TBCE	-0.2651695	0.2669585	-0.9933	0.321	-0.230771086	count	1
UCK1	-0.1900648	0.1511962	-1.2571	0.209	-0.230770188	count	1
OSBPL2	-0.1863674	0.1599835	-1.1649	0.244	-0.230759661	count	1
HARS	-0.1709103	0.1029919	-1.6595	0.0971	-0.230624681	count	1
WDR24	-0.3900453	0.347239	-1.1233	0.261	-0.230611347	count	1

LINC02001	-0.1774393	0.1293392	-1.3719	0.17	-0.230234739	count	1
PI15	-0.5179001	0.4617863	-1.1215	0.262	-0.230222572	count	1
AC004148.2	-0.7341847	0.356238	-2.0609	0.0394	-0.230185257	count	1
NTPCR	-0.1674133	0.1019252	-1.6425	0.101	-0.23006069	count	1
KCNQ4	-0.3645761	0.4716468	-0.773	0.44	-0.229982289	count	1
NDUFA8	-0.1640703	0.0738483	-2.2217	0.0264	-0.229759016	count	1
CAPNS1	-0.1615288	0.0522184	-3.0933	0.00199	-0.229652724	count	1
MAFA	-0.4194952	0.418168	-1.0032	0.316	-0.229615849	count	1
CCDC136	-0.4600715	0.565227	-0.814	0.416	-0.229461473	count	1
ANAPC15	-0.1697986	0.1201496	-1.4132	0.158	-0.229288452	count	1
HMGN5	-0.1776918	0.1406795	-1.2631	0.207	-0.229207534	count	1
ALDH3A2	-0.1672366	0.1023231	-1.6344	0.102	-0.229101972	count	1
WDR45B	-0.1707855	0.1063846	-1.6054	0.109	-0.228965933	count	1
DYNLT1	-0.1609379	0.0530831	-3.0318	0.00245	-0.22895893	count	1
LSM4	-0.1642265	0.0736718	-2.2292	0.0259	-0.228842839	count	1
COG8	-0.3133528	0.4037399	-0.7761	0.438	-0.228778535	count	1
CBWD3	-0.283106	0.3162034	-0.8953	0.371	-0.228676576	count	1
FLVCR1-DT	-0.3013006	0.3495421	-0.862	0.389	-0.228379474	count	1
SAE1	-0.1741916	0.1308221	-1.3315	0.183	-0.228302264	count	1
TMEM126A	-0.1649666	0.0917612	-1.7978	0.0723	-0.228090248	count	1
DNAAF4	-0.1828088	0.1868262	-0.9785	0.328	-0.228007554	count	1
MRPL43	-0.1605556	0.0520856	-3.0825	0.00207	-0.227911936	count	1
TRIM46	-0.4164628	0.5625791	-0.7403	0.459	-0.227873239	count	1
NCBP2	-0.1664358	0.0981565	-1.6956	0.09	-0.227797681	count	1
CIPC	-0.208944	0.2990247	-0.6988	0.485	-0.227654467	count	1
MAP2	-0.1812634	0.2187915	-0.8285	0.407	-0.227592123	count	1
LYVE1	-0.5122544	0.6076149	-0.8431	0.399	-0.227553209	count	1
FNTB	-0.2130986	0.2523737	-0.8444	0.399	-0.227329835	count	1
CCDC92	-0.1658069	0.1274405	-1.3011	0.193	-0.227288627	count	1
PCM1	-0.1607392	0.063736	-2.522	0.0117	-0.227210685	count	1
NPL	-0.3594298	0.5770682	-0.6229	0.533	-0.226604253	count	1
MHENCR	-0.3103069	0.2969809	-1.0449	0.296	-0.22648418	count	1
LINC00957	-0.323746	0.3593134	-0.901	0.368	-0.226480421	count	1
LLGL2	-0.2884046	0.4050579	-0.712	0.477	-0.225983265	count	1
EHD1	-0.1760952	0.1540238	-1.1433	0.253	-0.225645924	count	1
TAF3	-0.1730694	0.1217044	-1.422	0.155	-0.225547327	count	1
FADS1	-0.1735578	0.1328489	-1.3064	0.191	-0.225386844	count	1
TTC31	-0.2338311	0.3142145	-0.7442	0.457	-0.225377994	count	1
PPIA	-0.1567483	0.0259908	-6.0309	1.79E-09	-0.225359164	count	4.22E-05
DYNLT3	-0.159153	0.0593354	-2.6823	0.00735	-0.225343607	count	1
TENT5A	-0.1613328	0.0712886	-2.2631	0.0237	-0.225328306	count	1
CARNMT1	-0.2406884	0.2845892	-0.8457	0.398	-0.225317192	count	1
AURKA	-0.5881145	0.610403	-0.9635	0.335	-0.22508963	count	1
PA2G4	-0.1581505	0.0512193	-3.0877	0.00203	-0.225086589	count	1
IER3	-0.157691	0.0952324	-1.6559	0.0978	-0.225078041	count	1

ZNF425	-0.4517253	0.4488649	-1.0064	0.314	-0.225067524	count	1
DNAJC30	-0.169893	0.1518878	-1.1185	0.263	-0.224938038	count	1
FAM218A	-0.337396	0.4707549	-0.7167	0.474	-0.224831435	count	1
KIF9	-0.1812577	0.1874296	-0.9671	0.334	-0.224827609	count	1
AL391834.1	-0.5873584	0.7644413	-0.7683	0.442	-0.224779081	count	1
AC016355.1	-0.5873584	0.7644413	-0.7683	0.442	-0.224779081	count	1
TRIP13	-0.5873584	0.7955178	-0.7383	0.46	-0.224779081	count	1
ZBTB20-AS4	-0.5873584	0.7965715	-0.7374	0.461	-0.224779081	count	1
ZC3H13	-0.1593342	0.0620351	-2.5685	0.0103	-0.224773057	count	1
PPP1R3G	-0.2868463	0.3680622	-0.7793	0.436	-0.22472859	count	1
PHF23	-0.1759817	0.1817248	-0.9684	0.333	-0.224689629	count	1
TMEM63A	-0.1932136	0.3608134	-0.5355	0.592	-0.224665453	count	1
TPD52L2	-0.1628642	0.0830606	-1.9608	0.05	-0.224534168	count	1
AL031663.3	-1.709376	0.8750293	-1.9535	0.0508	-0.224486606	count	1
ZNF114	-1.709376	1.210781	-1.4118	0.158	-0.224486606	count	1
IFT20	-0.1626379	0.0865548	-1.879	0.0603	-0.224445535	count	1
ZNF837	-0.3799438	0.4549464	-0.8351	0.404	-0.224374396	count	1
ARMC8	-0.1758107	0.1594802	-1.1024	0.27	-0.224190128	count	1
NFAM1	-0.9664784	0.8132008	-1.1885	0.235	-0.224007468	count	1
AGT	-0.1608117	0.0915385	-1.7568	0.079	-0.223942415	count	1
IKBIP	-0.160357	0.0859749	-1.8652	0.0622	-0.223723237	count	1
MAPKAPK3	-0.2052838	0.2269641	-0.9045	0.366	-0.223622424	count	1
TMED9	-0.1575073	0.0508257	-3.099	0.00196	-0.223446904	count	1
NPIPB2	-0.5034631	0.7307055	-0.689	0.491	-0.223401745	count	1
RFX8	-0.5034631	0.7689852	-0.6547	0.513	-0.223401745	count	1
TAP2	-0.2110208	0.2095755	-1.0069	0.314	-0.22332391	count	1
C19orf25	-0.1666967	0.1334818	-1.2488	0.212	-0.223165999	count	1
ZNF671	-0.2344902	0.5496411	-0.4266	0.67	-0.222812262	count	1
PSMC4	-0.1593199	0.0704074	-2.2628	0.0237	-0.222792213	count	1
SMURF1	-0.1881446	0.2681859	-0.7015	0.483	-0.222652546	count	1
ZNF789	-0.2342283	0.2812581	-0.8328	0.405	-0.222559092	count	1
FIBIN	-0.1574532	0.0826806	-1.9044	0.0569	-0.222337629	count	1
LINC00910	-0.4463264	0.5332015	-0.8371	0.403	-0.222228749	count	1
DIXDC1	-0.1573641	0.0723328	-2.1756	0.0297	-0.222019107	count	1
TP53	-0.1869517	0.2314208	-0.8078	0.419	-0.221942429	count	1
BRD7	-0.157067	0.0678645	-2.3144	0.0207	-0.221914696	count	1
PINX1	-0.1791613	0.2137124	-0.8383	0.402	-0.221786596	count	1
ENTPD3-AS1	-0.2150825	0.259647	-0.8284	0.408	-0.22177603	count	1
S100A6	-0.1537234	0.0184946	-8.3118	1.31E-16	-0.221681474	count	3.13E-12
SHROOM4	-0.4997757	0.6069575	-0.8234	0.41	-0.221662387	count	1
CALCB	-1.6872908	0.9721114	-1.7357	0.0827	-0.221636949	count	1
MPZL3	-1.6872908	1.073708	-1.5715	0.116	-0.221636949	count	1
LINC01828	-1.6872908	1.125487	-1.4992	0.134	-0.221636949	count	1
AC007014.1	-1.6872908	1.125487	-1.4992	0.134	-0.221636949	count	1
AL022328.2	-1.6872908	1.125487	-1.4992	0.134	-0.221636949	count	1

AL050343.2	-1.6872908	1.214319	-1.3895	0.165	-0.221636949	count	1
AC005014.2	-1.6872908	1.214319	-1.3895	0.165	-0.221636949	count	1
JMJD8	-0.1599781	0.0861714	-1.8565	0.0635	-0.221593621	count	1
FBXO46	-0.3035936	0.2925356	-1.0378	0.299	-0.221431445	count	1
ZMYND15	-0.708222	0.6459057	-1.0965	0.273	-0.22137253	count	1
CIC	-0.1763029	0.1872082	-0.9417	0.346	-0.221331959	count	1
RPS13	-0.1534457	0.0198212	-7.7415	1.26E-14	-0.221179425	count	3.01E-10
AC090510.1	-0.7073713	0.7067602	-1.0009	0.317	-0.22108426	count	1
GPR150	-0.7073713	0.7355026	-0.9618	0.336	-0.22108426	count	1
POP7	-0.1610877	0.0888511	-1.813	0.0699	-0.221041412	count	1
LINC01588	-0.4042202	0.6902741	-0.5856	0.558	-0.220847656	count	1
AC112220.3	-0.9537942	0.7549633	-1.2634	0.207	-0.220805748	count	1
FAM111B	-0.9537942	0.8447518	-1.1291	0.259	-0.220805748	count	1
TTC25	-0.3024701	0.4697073	-0.644	0.52	-0.220586412	count	1
POLM	-0.1820406	0.299258	-0.6083	0.543	-0.220410156	count	1
TP53TG1	-0.160746	0.0995584	-1.6146	0.106	-0.220385991	count	1
KHK	-0.2036704	0.2603393	-0.7823	0.434	-0.220346956	count	1
FAM19A2	-0.4968177	0.3866957	-1.2848	0.199	-0.220267902	count	1
LINC01426	-0.4966317	0.4518818	-1.099	0.272	-0.22018025	count	1
AL136040.1	-0.2811938	0.456051	-0.6166	0.538	-0.22018007	count	1
IL15	-0.1848166	0.1869599	-0.9885	0.323	-0.220073047	count	1
PPIE	-0.1588229	0.0851879	-1.8644	0.0623	-0.219991204	count	1
NPIPB15	-0.9505044	0.9279294	-1.0243	0.306	-0.219975586	count	1
AC019205.1	-0.2529998	0.3652445	-0.6927	0.489	-0.219952746	count	1
TIMM10	-0.1612442	0.0855568	-1.8846	0.0596	-0.219868443	count	1
AC131025.2	-0.9500349	0.6072915	-1.5644	0.118	-0.21985711	count	1
INTS4	-0.1911911	0.3028436	-0.6313	0.528	-0.219575457	count	1
THG1L	-0.1729259	0.2107062	-0.8207	0.412	-0.219336951	count	1
MCM3AP-AS1	-0.5735081	0.6575137	-0.8722	0.383	-0.219097028	count	1
LINC01376	-0.7008464	0.5969577	-1.174	0.24	-0.21887413	count	1
PFN1	-0.1521848	0.0315595	-4.8222	1.48E-06	-0.218831786	count	0.0343804
C1GALT1C1L	-0.2637413	0.3687922	-0.7151	0.475	-0.218669455	count	1
YPEL1	-0.2141234	0.285072	-0.7511	0.453	-0.218624535	count	1
SLC35E4	-0.2162047	0.3047894	-0.7094	0.478	-0.21850085	count	1
ATP5F1D	-0.1523266	0.0358015	-4.2547	2.15E-05	-0.218164613	count	0.4937045
TPMT	-0.1710528	0.1620119	-1.0558	0.291	-0.218095911	count	1
FOXP4	-0.180504	0.2220314	-0.813	0.416	-0.21797249	count	1
CREB3	-0.1597093	0.0893305	-1.7878	0.0739	-0.217828245	count	1
ROR1	-0.1702469	0.1933306	-0.8806	0.379	-0.217601841	count	1
MT1F	-0.1667769	0.1588143	-1.0501	0.294	-0.217501745	count	1
SPTBN4	-1.6554446	1.0360119	-1.5979	0.1102	-0.217496442	count	1
SLAIN2	-0.1587641	0.0883762	-1.7965	0.0725	-0.217474306	count	1
AKR1C3	-0.1546184	0.0688721	-2.245	0.0248	-0.217459386	count	1
VAMP5	-0.1527249	0.0558997	-2.7321	0.0063	-0.217432	count	1
C20orf96	-0.1970806	0.2461687	-0.8006	0.423	-0.217322749	count	1

TMEM101	-0.16354	0.1174128	-1.3929	0.164	-0.217262701	count	1
NIFK-AS1	-0.1736838	0.1639611	-1.0593	0.29	-0.216943849	count	1
PDCD1LG2	-0.2355092	0.342076	-0.6885	0.491	-0.216758992	count	1
PTCHD4	-0.3102266	0.3567096	-0.8697	0.385	-0.216709904	count	1
ZNF98	-0.6941281	0.4861985	-1.4277	0.153	-0.216600485	count	1
POLR3K	-0.1663968	0.136624	-1.2179	0.223	-0.216442547	count	1
ZFHX3	-0.1533264	0.0804295	-1.9063	0.0567	-0.21608555	count	1
PCAT6	-0.4344532	0.4015143	-1.082	0.279	-0.215995722	count	1
AC125807.2	-0.9342865	0.8349807	-1.1189	0.263	-0.215884665	count	1
BBOX1	-0.9342865	0.8591729	-1.0874	0.277	-0.215884665	count	1
LINC01534	-0.3243236	0.3970049	-0.8169	0.414	-0.215808113	count	1
NUCKS1	-0.1502268	0.0295875	-5.0774	4.02E-07	-0.215793395	count	0.009380268
METRN	-0.1556696	0.0808713	-1.9249	0.0543	-0.215733694	count	1
ATAD2	-0.1713291	0.1785988	-0.9593	0.337	-0.215731919	count	1
C9orf47	-0.5651986	0.3358458	-1.6829	0.0925	-0.215694329	count	1
TTC1	-0.1533338	0.0677596	-2.2629	0.0237	-0.215651109	count	1
ATP5MC1	-0.15159	0.0469146	-3.2312	0.00124	-0.2156327	count	1
PDE4DIP	-0.156357	0.1032234	-1.5147	0.13	-0.215585448	count	1
STAT1	-0.1548698	0.0947436	-1.6346	0.102	-0.215475546	count	1
MRPL52	-0.1529601	0.0649651	-2.3545	0.0186	-0.215398094	count	1
GDF11	-0.1991437	0.2188609	-0.9099	0.363	-0.215395574	count	1
TRIM38	-0.159128	0.104647	-1.5206	0.128	-0.215299029	count	1
ZBTB18	-0.3082229	0.376765	-0.8181	0.413	-0.215263785	count	1
H2AFV	-0.1525302	0.0676469	-2.2548	0.0242	-0.214972229	count	1
LINC02193	-0.3414854	0.6561644	-0.5204	0.603	-0.214850047	count	1
KRT18	-0.1530498	0.2209989	-0.6925	0.489	-0.214831017	count	1
WDR78	-0.2080411	0.2725856	-0.7632	0.445	-0.214420543	count	1
FOXP4-AS1	-0.3407832	0.7157088	-0.4761	0.634	-0.214390862	count	1
MLKL	-0.2078579	0.2926273	-0.7103	0.478	-0.214229247	count	1
FBXO25	-0.1657213	0.1755515	-0.944	0.345	-0.21414275	count	1
ARID5A	-0.1566742	0.1003239	-1.5617	0.118	-0.214057945	count	1
BABAM1	-0.1537882	0.0775309	-1.9836	0.0474	-0.213991897	count	1
PTPRM	-0.1855709	0.2167781	-0.856	0.392	-0.213975995	count	1
TSPAN1	-0.3919385	0.8144866	-0.4812	0.63	-0.213815692	count	1
STAU2	-0.1770143	0.1764056	-1.0035	0.316	-0.21373113	count	1
CABCOCO1	-0.1962302	0.3770905	-0.5204	0.603	-0.213654996	count	1
FZD10	-0.4296114	0.5392331	-0.7967	0.426	-0.21345792	count	1
IFI44	-0.1809696	0.2414685	-0.7495	0.454	-0.213389006	count	1
PRKN	-0.4292608	0.4665072	-0.9202	0.358	-0.213274258	count	1
ZNF74	-0.3909647	0.416685	-0.9383	0.348	-0.213258833	count	1
MPG	-0.1497306	0.0497747	-3.0082	0.00265	-0.21312642	count	1
C11orf68	-0.1570521	0.1203876	-1.3046	0.192	-0.213105436	count	1
SLN	-0.6832324	0.4213143	-1.6217	0.105	-0.212917426	count	1
C1orf216	-0.184578	0.1959826	-0.9418	0.346	-0.212821615	count	1
OSBPL10	-0.1941798	0.2254878	-0.8612	0.389	-0.212772038	count	1

HAGH	-0.1532101	0.0827608	-1.8512	0.0642	-0.212620885	count	1
ATP5MF	-0.1499871	0.0503099	-2.9813	0.00289	-0.212578929	count	1
C2orf40	-0.1479461	0.0734448	-2.0144	0.044	-0.21238953	count	1
LAMTOR1	-0.1490536	0.0499243	-2.9856	0.00285	-0.212260142	count	1
PCDHGA8	-1.6140093	0.9137748	-1.7663	0.0774	-0.212056104	count	1
AL031280.1	-1.6140093	1.107022	-1.458	0.145	-0.212056104	count	1
EPHA1-AS1	-0.388768	0.4364362	-0.8908	0.373	-0.212003074	count	1
SPRED1	-0.1593464	0.1395691	-1.1417	0.254	-0.211675588	count	1
GPRASP2	-0.2622355	0.2971458	-0.8825	0.378	-0.211404626	count	1
FTL	-0.1465794	0.024147	-6.0703	1.41E-09	-0.21136044	count	3.33E-05
TNKS2-AS1	-0.3587263	0.4878125	-0.7354	0.462	-0.211311789	count	1
IMPDH2	-0.1527382	0.0726991	-2.101	0.0357	-0.211126678	count	1
AC027682.6	-0.1873542	0.2775017	-0.6751	0.5	-0.211125248	count	1
AC011472.1	-0.6775383	0.6139778	-1.1035	0.27	-0.210994857	count	1
GALK1	-0.1593847	0.1193579	-1.3354	0.182	-0.21098385	count	1
FHL5	-0.1627917	0.1083598	-1.5023	0.133	-0.210959901	count	1
RARS	-0.1596052	0.153936	-1.0368	0.3	-0.210881509	count	1
STX17-AS1	-0.3171533	0.5860087	-0.5412	0.588	-0.210867759	count	1
YAP1	-0.1531371	0.0980637	-1.5616	0.118	-0.210768361	count	1
PALB2	-0.2085516	0.3079493	-0.6772	0.498	-0.210659874	count	1
VPS26C	-0.1663102	0.1710408	-0.9723	0.331	-0.210614843	count	1
EMC8	-0.1590555	0.1366787	-1.1637	0.245	-0.210546775	count	1
SLC2A4RG	-0.1501136	0.0658406	-2.28	0.0227	-0.210330834	count	1
EFCAB14	-0.1531269	0.092152	-1.6617	0.0967	-0.210312812	count	1
TMSB15B-AS1	-0.3570286	0.6149261	-0.5806	0.562	-0.210268841	count	1
FAM71E1	-0.475261	0.6393975	-0.7433	0.457	-0.210128189	count	1
PLEC	-0.1507103	0.1064557	-1.4157	0.157	-0.210007509	count	1
CAVIN3	-0.1461613	0.0328684	-4.4469	8.97E-06	-0.20996895	count	0.20679438
BOLA3-AS1	-0.1737422	0.2360205	-0.7361	0.462	-0.209755106	count	1
ZNF540	-0.1731384	0.2243533	-0.7717	0.44	-0.209564185	count	1
NELL1	-0.672753	0.4115935	-1.6345	0.102	-0.209380285	count	1
MRPL55	-0.1499903	0.0929694	-1.6133	0.107	-0.209377157	count	1
SNCA	-0.2767652	0.5593327	-0.4948	0.621	-0.209267049	count	1
MYOM2	-0.2997485	0.4048604	-0.7404	0.459	-0.209153281	count	1
FCHSD2	-0.1538838	0.1150729	-1.3373	0.181	-0.208925534	count	1
EIF3F	-0.1460507	0.0380342	-3.84	1.00E-04	-0.208753836	count	1
TXNL1	-0.147921	0.0629455	-2.35	0.0188	-0.208651739	count	1
EIPR1	-0.222968	0.2427213	-0.9186	0.358	-0.208445175	count	1
PRRG2	-0.4196855	0.3539068	-1.1859	0.236	-0.208262694	count	1
KIAA1958	-0.3132532	0.4059511	-0.7717	0.44	-0.208183306	count	1
NLGN2	-0.1899348	0.2312182	-0.8215	0.411	-0.208073104	count	1
AP2M1	-0.1466427	0.0509158	-2.8801	0.004	-0.208010694	count	1
OTUD7B	-0.1635624	0.2203737	-0.7422	0.458	-0.207966648	count	1
SPRYD7	-0.153455	0.1158211	-1.3249	0.185	-0.207951095	count	1
EXOSC4	-0.1541191	0.1422178	-1.0837	0.279	-0.207838206	count	1

MRPL28	-0.1503528	0.0864365	-1.7395	0.082	-0.207824654	count	1
MRM1	-0.4702516	0.4227252	-1.1124	0.266	-0.207777731	count	1
KHDC4	-0.1675474	0.3247844	-0.5159	0.606	-0.20773177	count	1
NAA80	-0.2015417	0.2511069	-0.8026	0.422	-0.207636267	count	1
HMGN2	-0.1452062	0.0405673	-3.5794	3.00E-04	-0.207553765	count	1
ICAM2	-0.1670808	0.2377138	-0.7029	0.482	-0.207539202	count	1
PSMA7	-0.1446206	0.0316313	-4.5721	4.99E-06	-0.207529927	count	0.11533886
PSMA5	-0.1479981	0.0691053	-2.1416	0.0323	-0.207334542	count	1
NIPAL2	-0.1993084	0.2697464	-0.7389	0.46	-0.207216641	count	1
AP003068.2	-0.417387	0.3337703	-1.2505	0.211	-0.207061138	count	1
UPRT	-0.2071079	0.2430107	-0.8523	0.394	-0.206885217	count	1
FAM24B	-0.3796624	0.5431817	-0.699	0.485	-0.206803417	count	1
TMEM163	-1.5744017	0.8162086	-1.9289	0.0538	-0.206802732	count	1
FAM225B	-1.5744017	0.8162086	-1.9289	0.0538	-0.206802732	count	1
RASL10A	-1.5744017	1.0697243	-1.4718	0.141	-0.206802732	count	1
MEIS1-AS3	-1.5744017	1.14159	-1.3791	0.168	-0.206802732	count	1
AL158850.1	-1.5744017	1.14159	-1.3791	0.168	-0.206802732	count	1
PSPC1	-0.1732112	0.1610396	-1.0756	0.282	-0.206783478	count	1
EFCAB11	-0.2211699	0.2558615	-0.8644	0.387	-0.206735367	count	1
GSTZ1	-0.1753266	0.1990669	-0.8807	0.379	-0.206687114	count	1
AL157938.3	-0.2837286	0.3832995	-0.7402	0.459	-0.206514024	count	1
PPP2R5D	-0.182291	0.2444065	-0.7459	0.456	-0.206395799	count	1
TAF8	-0.1680767	0.1934358	-0.8689	0.385	-0.206290469	count	1
RWDD1	-0.1440066	0.0419677	-3.4314	0.000607	-0.206216249	count	1
CIB1	-0.1449513	0.0523011	-2.7715	0.00561	-0.206119185	count	1
PLCB3	-0.2280518	0.2480826	-0.9193	0.358	-0.206041729	count	1
ZBTB8OS	-0.1496558	0.0956842	-1.5641	0.118	-0.206006861	count	1
GLCCI1	-0.1905444	0.2431864	-0.7835	0.433	-0.2059955	count	1
FCHSD1	-0.2111336	0.3463648	-0.6096	0.542	-0.205904923	count	1
FIZ1	-0.2110678	0.2571947	-0.8207	0.412	-0.20583978	count	1
AL137145.2	-1.566243	0.8258403	-1.8965	0.058	-0.205714444	count	1
AC006160.1	-1.566243	1.065013	-1.4706	0.141	-0.205714444	count	1
DGUOK	-0.1457081	0.0642758	-2.2669	0.0235	-0.20557048	count	1
MRPL41	-0.1448037	0.0554217	-2.6128	0.00902	-0.205356575	count	1
LSM5	-0.145765	0.0618507	-2.3567	0.0185	-0.205267792	count	1
TMOD1	-0.1519916	0.1035212	-1.4682	0.142	-0.205189667	count	1
DDX47	-0.4647221	0.5639724	-0.824	0.41	-0.205185834	count	1
CKS2	-0.2010131	0.2869799	-0.7004	0.484	-0.205064365	count	1
AL162426.1	-0.6598313	0.6400637	-1.0309	0.303	-0.205026184	count	1
GAPLINC	-0.4642347	0.6775647	-0.6852	0.493	-0.204957499	count	1
ATG3	-0.1474653	0.080572	-1.8302	0.0673	-0.204955531	count	1
SOCS6	-0.1952182	0.212923	-0.9168	0.359	-0.204699345	count	1
AC104986.2	-0.2619071	0.2722669	-0.962	0.336	-0.204691619	count	1
TMEM170B	-0.325855	0.4312105	-0.7557	0.45	-0.204643322	count	1
LIX1L-AS1	-0.2356805	0.2806115	-0.8399	0.401	-0.204589505	count	1

MSN	-0.1448312	0.062397	-2.3211	0.0203	-0.204563073	count	1
PPCDC	-0.198593	0.2257998	-0.8795	0.379	-0.204559936	count	1
KARS	-0.1523569	0.1394088	-1.0929	0.275	-0.204467844	count	1
PHB2	-0.1439899	0.0506927	-2.8404	0.00453	-0.2044664	count	1
CCT6A	-0.1444702	0.0582597	-2.4798	0.0132	-0.204450313	count	1
FOXK2	-0.1823945	0.1807584	-1.0091	0.313	-0.204420288	count	1
INTS2	-0.2354871	0.3174436	-0.7418	0.458	-0.204418168	count	1
POLR1D	-0.1449266	0.0621398	-2.3323	0.0197	-0.204337918	count	1
NEK9	-0.1804653	0.2124269	-0.8495	0.396	-0.204310601	count	1
SEPHS1	-0.1610961	0.1357358	-1.1868	0.235	-0.204266076	count	1
JAG1	-0.1445255	0.0749256	-1.9289	0.0538	-0.204241259	count	1
LTN1	-0.1509734	0.107664	-1.4023	0.161	-0.204172446	count	1
CHMP2A	-0.1432104	0.0433733	-3.3018	0.001	-0.204049791	count	1
TOR4A	-0.2117997	0.3650591	-0.5802	0.562	-0.203815335	count	1
PLOD2	-0.1514636	0.1243419	-1.2181	0.223	-0.203773936	count	1
NUDC	-0.1431434	0.0482346	-2.9677	0.00302	-0.203712217	count	1
FNBP1	-0.1495349	0.1200386	-1.2457	0.213	-0.20365367	count	1
GGPS1	-0.1530315	0.1117392	-1.3695	0.171	-0.203600136	count	1
BATF	-1.550417	0.6299792	-2.4611	0.0139	-0.203597787	count	1
ZNF441	-0.1732814	0.2103958	-0.8236	0.41	-0.203556474	count	1
C10orf143	-0.2345061	0.2763639	-0.8485	0.396	-0.203549167	count	1
NBDY	-0.1438546	0.0609015	-2.3621	0.0182	-0.203362668	count	1
SNURF	-0.373507	0.4326075	-0.8634	0.388	-0.203293702	count	1
PCBD2	-0.1973652	0.2202345	-0.8962	0.37	-0.203279286	count	1
ZNF829	-0.3456096	0.3975024	-0.8695	0.385	-0.203262719	count	1
YIPF2	-0.1465671	0.0838483	-1.748	0.0805	-0.203261855	count	1
CCDC191	-0.1728862	0.2559639	-0.6754	0.499	-0.203088854	count	1
ATP9B	-0.1661658	0.2345097	-0.7086	0.479	-0.203027259	count	1
ATP5ME	-0.1423911	0.045197	-3.1505	0.0016	-0.20289995	count	1
DIRAS3	-0.1684943	0.1896725	-0.8883	0.374	-0.202837041	count	1
VWA1	-0.1968561	0.2284762	-0.8616	0.389	-0.20274833	count	1
H3F3A	-0.140759	0.0228847	-6.1508	8.54E-10	-0.202552378	count	2.02E-05
FAM162A	-0.1428614	0.0598112	-2.3885	0.017	-0.20249465	count	1
ECHS1	-0.143112	0.0585296	-2.4451	0.0145	-0.202451517	count	1
PCDHGB4	-0.8806705	0.7048497	-1.2494	0.212	-0.202384849	count	1
AC023509.2	-0.8806705	0.7944324	-1.1086	0.268	-0.202384849	count	1
HAS3	-0.8806705	0.9544646	-0.9227	0.356	-0.202384849	count	1
SLC4A5	-0.8806705	1.089788	-0.8081	0.419	-0.202384849	count	1
RAB5IF	-0.1496234	0.102939	-1.4535	0.146	-0.202272849	count	1
SERAC1	-0.1928917	0.2941084	-0.6559	0.512	-0.202231059	count	1
AC036108.3	-0.531601	0.6657354	-0.7985	0.425	-0.20198585	count	1
KCTD16	-0.531601	0.7472395	-0.7114	0.477	-0.20198585	count	1
TRAPPC12-AS1	-0.531601	0.7517583	-0.7071	0.48	-0.20198585	count	1
PRR16	-0.1767123	0.1755989	-1.0063	0.314	-0.201915442	count	1
MARK4	-0.1893568	0.2486	-0.7617	0.446	-0.201724869	count	1

RAPGEF2	-0.1716419	0.2168953	-0.7914	0.429	-0.201616615	count	1
SYTL1	-0.5305844	0.8366642	-0.6342	0.526	-0.201572351	count	1
TMEM126B	-0.145774	0.0907506	-1.6063	0.108	-0.201394623	count	1
IQCJ-SCHIP1	-0.2190289	0.3175326	-0.6898	0.49	-0.201326688	count	1
FAM110C	-0.5299507	0.782761	-0.677	0.498	-0.20131463	count	1
HS3ST2	-0.529518	0.7394404	-0.7161	0.474	-0.201138639	count	1
KCNA5	-0.1710394	0.1630242	-1.0492	0.294	-0.200903794	count	1
LDHB	-0.1405443	0.0384207	-3.658	3.00E-04	-0.200878179	count	1
CUTA	-0.1406663	0.0413249	-3.4039	7.00E-04	-0.200850344	count	1
HNRNPA3	-0.1409931	0.047343	-2.9781	0.0029	-0.200842247	count	1
MALSU1	-0.1455671	0.0955436	-1.5236	0.128	-0.200652255	count	1
DDX11-AS1	-0.8723258	0.7540047	-1.1569	0.247	-0.200287971	count	1
MCF2L2	-1.525008	0.9288422	-1.6418	0.101	-0.200183881	count	1
KRBOX4	-0.1698428	0.195693	-0.8679	0.386	-0.200176947	count	1
RTL8B	-0.1481258	0.1266272	-1.1698	0.242	-0.200103174	count	1
NLK	-0.453853	0.3596639	-1.2619	0.207	-0.200099189	count	1
ATP5MPL	-0.1408663	0.0475962	-2.9596	0.0031	-0.200093375	count	1
RGP1	-0.1656193	0.2396359	-0.6911	0.49	-0.19988841	count	1
HAR1A	-0.2482301	0.4042965	-0.614	0.539	-0.199846223	count	1
OSTC	-0.1404562	0.0486491	-2.8871	0.00391	-0.199836436	count	1
ZNF581	-0.1565273	0.1433967	-1.0916	0.275	-0.199749371	count	1
TTC33	-0.1546462	0.1344376	-1.1503	0.25	-0.199576529	count	1
COX6C	-0.1391041	0.0329382	-4.2232	2.47E-05	-0.199439353	count	0.5667168
SLC25A6	-0.1389574	0.0335558	-4.1411	3.53E-05	-0.199417149	count	0.8084759
C12orf65	-0.144073	0.0974989	-1.4777	0.14	-0.199309059	count	1
SERINC5	-0.1689889	0.3051679	-0.5538	0.58	-0.199163464	count	1
AC008267.5	-0.1855928	0.2482878	-0.7475	0.455	-0.199159759	count	1
MAGI2	-0.1501108	0.1446882	-1.0375	0.3	-0.199146279	count	1
HADH	-0.1462734	0.1038832	-1.4081	0.159	-0.199093538	count	1
NMD3	-0.1503211	0.1049884	-1.4318	0.152	-0.199072931	count	1
AC015917.2	-0.3383801	0.5321385	-0.6359	0.525	-0.198835129	count	1
AL137802.2	-0.4506367	0.4833184	-0.9324	0.351	-0.198596045	count	1
MDP1	-0.1909552	0.2775811	-0.6879	0.492	-0.198428217	count	1
PCNP	-0.1401425	0.0517311	-2.7091	0.00678	-0.198416524	count	1
CEP57	-0.1456382	0.096503	-1.5092	0.131	-0.198390727	count	1
ROBO2	-0.2033168	0.4204542	-0.4836	0.629	-0.198169874	count	1
AL513548.1	-0.3158867	0.6625822	-0.4768	0.634	-0.198149824	count	1
GPN2	-0.1660152	0.2291074	-0.7246	0.469	-0.198136876	count	1
MAGED2	-0.1386748	0.0433745	-3.1971	0.0014	-0.197982984	count	1
PLEKHN1	-0.8630606	0.9510174	-0.9075	0.364	-0.197961377	count	1
PRKAR2A-AS1	-0.8630606	0.9510174	-0.9075	0.364	-0.197961377	count	1
AC008522.1	-0.8630606	0.9510174	-0.9075	0.364	-0.197961377	count	1
AL022318.1	-0.8630606	0.9510174	-0.9075	0.364	-0.197961377	count	1
HIST1H3E	-0.8630606	0.9811823	-0.8796	0.379	-0.197961377	count	1
TOP2A	-0.8630606	0.9811823	-0.8796	0.379	-0.197961377	count	1

SAMD5	-0.2617624	0.6065759	-0.4315	0.666	-0.197619264	count	1
RNF169	-0.1497139	0.120842	-1.2389	0.215	-0.197518539	count	1
AC012640.2	-0.6367471	0.4597565	-1.385	0.166	-0.197268746	count	1
AL357060.1	-0.6367471	0.5321475	-1.1966	0.232	-0.197268746	count	1
PTPN11	-0.1412425	0.104148	-1.3562	0.175	-0.197239947	count	1
SEC11A	-0.1376152	0.0362364	-3.7977	0.000148	-0.197112853	count	1
PEA15	-0.1404193	0.0790942	-1.7753	0.0759	-0.197012148	count	1
CNTRL	-0.1577951	0.1652891	-0.9547	0.34	-0.197002235	count	1
PRPF40B	-0.1767062	0.2594526	-0.6811	0.496	-0.196915937	count	1
ZRANB3	-0.2967104	0.3568117	-0.8316	0.406	-0.196818506	count	1
SNU13	-0.1380912	0.0452073	-3.0546	0.0023	-0.196680531	count	1
CHCHD2	-0.1368348	0.0257554	-5.3129	1.14E-07	-0.1966449	count	0.00266874
AMDHD2	-0.1807052	0.2289218	-0.7894	0.43	-0.196583092	count	1
ROCK1	-0.1385491	0.0553271	-2.5042	0.0123	-0.196565078	count	1
SRP14	-0.1364505	0.0225596	-6.0484	1.61E-09	-0.196470132	count	3.80E-05
RCOR2	-0.3612671	0.6152741	-0.5872	0.557	-0.196327463	count	1
ZNF121	-0.166044	0.2481134	-0.6692	0.503	-0.19632235	count	1
GPANK1	-0.1471081	0.1252802	-1.1742	0.24	-0.196211535	count	1
EIF3I	-0.1382347	0.0504329	-2.741	0.00616	-0.196154485	count	1
RTF2	-0.1384476	0.0598904	-2.3117	0.0209	-0.195992523	count	1
MIR133A1HG	-0.6326099	0.755268	-0.8376	0.402	-0.19588142	count	1
FRMD8	-0.2010023	0.2609912	-0.7701	0.441	-0.195881036	count	1
PDHA1	-0.1421956	0.0929164	-1.5304	0.126	-0.195792747	count	1
SAP30L	-0.1458171	0.1262666	-1.1548	0.248	-0.19575462	count	1
PTGES3	-0.1366357	0.0347282	-3.9344	8.49E-05	-0.195682425	count	1
CYB5R1	-0.1395483	0.0841881	-1.6576	0.0975	-0.195178791	count	1
TMEM220	-0.1448046	0.1090155	-1.3283	0.184	-0.19517609	count	1
CYTH2	-0.1424386	0.0892639	-1.5957	0.111	-0.195031292	count	1
DNAJC7	-0.1397731	0.0927737	-1.5066	0.132	-0.194975194	count	1
AL391832.2	-0.4428085	0.7537234	-0.5875	0.557	-0.194941628	count	1
SH3BP4	-0.2026092	0.2586356	-0.7834	0.433	-0.194838231	count	1
COL4A5	-0.1477227	0.191152	-0.7728	0.44	-0.194754746	count	1
GOLGB1	-0.1370814	0.0695108	-1.9721	0.0487	-0.194687213	count	1
FLOT2	-0.1613306	0.1789823	-0.9014	0.367	-0.194681161	count	1
PHF7	-0.1949371	0.3205918	-0.6081	0.543	-0.194564488	count	1
2-Mar	-0.1390763	0.0716589	-1.9408	0.0524	-0.19456368	count	1
AL139353.1	-0.2677558	0.3821682	-0.7006	0.484	-0.194557123	count	1
LINC01725	-1.4833929	0.6610983	-2.2438	0.0249	-0.194554165	count	1
TANK	-0.1422686	0.0977747	-1.4551	0.146	-0.194389494	count	1
PSMC3	-0.1372851	0.0549777	-2.4971	0.0126	-0.194374204	count	1
ZNF780A	-0.2048085	0.2541631	-0.8058	0.42	-0.194173663	count	1
NR3C2	-0.1866696	0.2453921	-0.7607	0.447	-0.193922467	count	1
AKNA	-0.1782776	0.2896213	-0.6156	0.538	-0.193915941	count	1
ARSD	-0.1883373	0.2690407	-0.7	0.484	-0.193868251	count	1
CEP95	-0.1541413	0.1599109	-0.9639	0.335	-0.193691516	count	1

SDHAF1	-0.147865	0.1515574	-0.9756	0.329	-0.193691378	count	1
ECSIT	-0.1466284	0.1175304	-1.2476	0.212	-0.193687186	count	1
ENO2	-0.1468025	0.1450259	-1.0122	0.311	-0.19366641	count	1
IDI1	-0.1422886	0.116758	-1.2187	0.223	-0.193659744	count	1
MTHFD1L	-0.144776	0.1443601	-1.0029	0.316	-0.19365926	count	1
RIN2	-0.1419742	0.1115182	-1.2731	0.203	-0.193646611	count	1
NDC1	-0.2341317	0.3039094	-0.7704	0.441	-0.193588752	count	1
BTBD3	-0.1817729	0.2410286	-0.7542	0.451	-0.193558907	count	1
AL359644.1	-0.4394544	0.5098083	-0.862	0.389	-0.193377602	count	1
MDH1B	-0.4394544	0.5726434	-0.7674	0.443	-0.193377602	count	1
WRNIP1	-0.1497123	0.1500865	-0.9975	0.319	-0.19318606	count	1
AC010642.2	-0.1484013	0.148772	-0.9975	0.319	-0.193126879	count	1
FCGR2A	-0.1688512	0.1663016	-1.0153	0.31	-0.19286149	count	1
MFN2	-0.1855434	0.2519515	-0.7364	0.462	-0.192738778	count	1
C7orf31	-0.2031399	0.3573871	-0.5684	0.57	-0.19256703	count	1
ULBP2	-0.8414778	0.6724141	-1.2514	0.211	-0.192548552	count	1
RUBCNL	-0.8414778	0.7324801	-1.1488	0.251	-0.192548552	count	1
EML5	-0.8414778	0.7324801	-1.1488	0.251	-0.192548552	count	1
ZKSCAN5	-0.2465944	0.3739118	-0.6595	0.51	-0.192429992	count	1
NOL3	-0.1401622	0.1044406	-1.342	0.18	-0.192282791	count	1
NHS	-0.1490036	0.2529079	-0.5892	0.556	-0.192268254	count	1
AL391834.2	-0.2760783	0.4343455	-0.6356	0.525	-0.192135483	count	1
DYNLL1	-0.1334666	0.0270359	-4.9366	8.30E-07	-0.192057879	count	0.01932489
BECN1	-0.1415108	0.1441018	-0.982	0.326	-0.191976571	count	1
ZFAND1	-0.137181	0.072366	-1.8957	0.0581	-0.191864564	count	1
MICALL1	-0.506647	0.5123818	-0.9888	0.323	-0.19185852	count	1
FBXL15	-0.1394762	0.1032324	-1.3511	0.177	-0.191831992	count	1
CATSPER3	-1.4632481	1.142382	-1.2809	0.2	-0.191812755	count	1
JMJD7-PLA2G4B	-1.4632481	1.142382	-1.2809	0.2	-0.191812755	count	1
RAB3B	-1.4632481	1.21346	-1.2058	0.228	-0.191812755	count	1
AL590714.1	-1.4632481	1.21346	-1.2058	0.228	-0.191812755	count	1
LINC00570	-1.4632481	1.21346	-1.2058	0.228	-0.191812755	count	1
ABCB6	-1.4632481	1.21346	-1.2058	0.228	-0.191812755	count	1
IL31RA	-1.4632481	1.21346	-1.2058	0.228	-0.191812755	count	1
LINC02542	-1.4632481	1.21346	-1.2058	0.228	-0.191812755	count	1
GIMAP8	-1.4632481	1.21346	-1.2058	0.228	-0.191812755	count	1
AC124242.1	-1.4632481	1.21346	-1.2058	0.228	-0.191812755	count	1
LAMC3	-1.4632481	1.21346	-1.2058	0.228	-0.191812755	count	1
AC022021.1	-1.4632481	1.21346	-1.2058	0.228	-0.191812755	count	1
RNASE6	-1.4632481	1.21346	-1.2058	0.228	-0.191812755	count	1
CHRNA5	-1.4632481	1.21346	-1.2058	0.228	-0.191812755	count	1
AC239799.2	-1.4632481	1.404365	-1.0419	0.298	-0.191812755	count	1
LINC01807	-1.4632481	1.404365	-1.0419	0.298	-0.191812755	count	1
LINC00471	-1.4632481	1.404365	-1.0419	0.298	-0.191812755	count	1
ESPNL	-1.4632481	1.404365	-1.0419	0.298	-0.191812755	count	1

AC016257.1	-1.4632481	1.404365	-1.0419	0.298	-0.191812755	count	1
AC027682.4	-1.4632481	1.404365	-1.0419	0.298	-0.191812755	count	1
MOB2	-0.1376837	0.0856726	-1.6071	0.108	-0.191778178	count	1
AC024610.2	-0.3054841	0.6349227	-0.4811	0.63	-0.191386877	count	1
AURKAIP1	-0.1346411	0.057196	-2.354	0.0186	-0.191345648	count	1
MC1R	-0.3519233	0.4966338	-0.7086	0.479	-0.191021168	count	1
ICOSLG	-0.8352343	0.7647497	-1.0922	0.275	-0.190984644	count	1
ZKSCAN2	-0.2744383	0.6994287	-0.3924	0.695	-0.190959168	count	1
MZT2A	-0.1356474	0.0711639	-1.9061	0.0567	-0.190695032	count	1
APLF	-0.2113242	0.4599977	-0.4594	0.646	-0.190663951	count	1
CSPP1	-0.1489999	0.1442202	-1.0331	0.302	-0.190569266	count	1
COMMD7	-0.1389779	0.0975145	-1.4252	0.154	-0.190453088	count	1
RPA3	-0.1386039	0.1001849	-1.3835	0.167	-0.190258944	count	1
STEAP4	-0.1407211	0.149657	-0.9403	0.347	-0.190146812	count	1
REEP3	-0.1347275	0.0636786	-2.1157	0.0344	-0.190098986	count	1
TIMELESS	-0.6146508	0.6127889	-1.003	0.316	-0.189870033	count	1
DLGAP1	-0.6146508	0.6687775	-0.9191	0.358	-0.189870033	count	1
TFRC	-0.1723253	0.2162276	-0.797	0.426	-0.189774976	count	1
CFAP97	-0.1335536	0.0541378	-2.4669	0.0137	-0.189745621	count	1
ARNTL	-0.613839	0.3761105	-1.6321	0.103	-0.189598708	count	1
MAPKBP1	-0.2510026	0.3194971	-0.7856	0.432	-0.189284407	count	1
TJAP1	-0.1635924	0.2809126	-0.5824	0.56	-0.189222558	count	1
CC2D1A	-0.1680483	0.2439964	-0.6887	0.491	-0.18918857	count	1
SRSF4	-0.1354567	0.0740726	-1.8287	0.0675	-0.189168616	count	1
ARID4B	-0.1344559	0.0640196	-2.1002	0.0358	-0.189150206	count	1
ENAH	-0.1339608	0.0732031	-1.83	0.0673	-0.189106314	count	1
EI24	-0.1353554	0.0748136	-1.8092	0.0705	-0.188910104	count	1
ADGRL4	-0.2715569	0.6104912	-0.4448	0.656	-0.18889331	count	1
AC024940.1	-0.6117198	0.5767808	-1.0606	0.289	-0.188890656	count	1
DEF8	-0.1385004	0.1066136	-1.2991	0.194	-0.188801532	count	1
FAIM	-0.1391752	0.1104638	-1.2599	0.208	-0.188683407	count	1
MAP2K4	-0.1496691	0.1677476	-0.8922	0.372	-0.188626596	count	1
CLTA	-0.1318498	0.0382252	-3.4493	0.000568	-0.188600751	count	1
FANCB	-0.2846366	0.4625454	-0.6154	0.538	-0.188546446	count	1
LHFPL6	-0.1316102	0.0379352	-3.4693	0.000528	-0.188492665	count	1
MXD3	-0.8249596	0.7070016	-1.1668	0.243	-0.188413003	count	1
MACF1	-0.1329277	0.07545	-1.7618	0.0782	-0.188357689	count	1
PPIP5K1	-0.4284739	0.3736412	-1.1468	0.252	-0.188264903	count	1
TMEM256	-0.1350763	0.074134	-1.8221	0.0685	-0.188199118	count	1
ASB8	-0.1378164	0.0922665	-1.4937	0.135	-0.188109714	count	1
NDUFB4	-0.1315183	0.0390656	-3.3666	0.000769	-0.188009428	count	1
ZNF569	-0.179295	0.2633635	-0.6808	0.496	-0.187818157	count	1
MMEL1	-1.4336875	1.032174	-1.389	0.165	-0.187772199	count	1
TSTA3	-0.1384978	0.125435	-1.1041	0.27	-0.187703387	count	1
IMMP1L	-0.1454482	0.1385734	-1.0496	0.294	-0.187664238	count	1

SMIM12	-0.137041	0.1051798	-1.3029	0.193	-0.187628786	count	1
DDTL	-0.2333842	0.3918879	-0.5955	0.552	-0.187623233	count	1
GNG7	-0.2333484	0.2543375	-0.9175	0.359	-0.187593796	count	1
NDUFA6	-0.1328156	0.0597312	-2.2236	0.0262	-0.187567063	count	1
SNHG10	-0.2213863	0.2349597	-0.9422	0.346	-0.18754182	count	1
TPI1	-0.1308193	0.0358846	-3.6456	0.000271	-0.187460425	count	1
HDGF	-0.1339331	0.0804856	-1.6641	0.0962	-0.187447889	count	1
TADA3	-0.1344309	0.0734294	-1.8308	0.0672	-0.187280229	count	1
SLC24A1	-0.3793213	0.5077839	-0.747	0.455	-0.187241518	count	1
C6orf163	-0.4949957	0.7030941	-0.704	0.481	-0.187146633	count	1
ARHGEF37	-0.2480929	0.3617116	-0.6859	0.493	-0.187033211	count	1
VDAC2	-0.1307807	0.0410199	-3.1882	0.00144	-0.1869738	count	1
GRK3	-0.1972758	0.6644751	-0.2969	0.767	-0.186923562	count	1
MRPL13	-0.1334293	0.0718917	-1.856	0.0635	-0.186811777	count	1
UBAP2L	-0.1447606	0.1595885	-0.9071	0.364	-0.186580638	count	1
CHD4	-0.1348172	0.0911227	-1.4795	0.139	-0.18654198	count	1
ZNF329	-0.2152762	0.3273388	-0.6577	0.511	-0.186540817	count	1
TAGAP	-0.8170701	0.7533153	-1.0846	0.278	-0.186440115	count	1
TSC22D1-AS1	-0.3177643	0.4615438	-0.6885	0.491	-0.186244512	count	1
SKA2	-0.1361286	0.1177656	-1.1559	0.248	-0.185940661	count	1
SRSF8	-0.1346839	0.0869706	-1.5486	0.122	-0.185906559	count	1
SLC25A21-AS1	-0.1651093	0.2122799	-0.7778	0.437	-0.185852369	count	1
PCDHGB3	-0.3427903	0.8202017	-0.4179	0.676	-0.185844496	count	1
BTLA	-1.4190992	0.4875451	-2.9107	0.00363	-0.185770792	count	1
RTRAF	-0.1304569	0.0424873	-3.0705	0.00215	-0.185738267	count	1
SNAP47	-0.1474812	0.1532444	-0.9624	0.336	-0.185574574	count	1
CYP26B1	-0.1905104	0.3544479	-0.5375	0.591	-0.185514016	count	1
ARPC1B	-0.1307958	0.0650535	-2.0106	0.0444	-0.185249597	count	1
FILIP1L	-0.1290216	0.0544775	-2.3683	0.0179	-0.185226631	count	1
RCAN3	-0.1877709	0.3503634	-0.5359	0.592	-0.18512322	count	1
NT5C2	-0.1576824	0.2100669	-0.7506	0.453	-0.185109412	count	1
KIAA1211L	-0.8114852	0.7695692	-1.0545	0.292	-0.185044485	count	1
EIF5A2	-0.1477513	0.1659099	-0.8906	0.373	-0.185029281	count	1
ESF1	-0.1325651	0.0968698	-1.3685	0.171	-0.184944468	count	1
ARSB	-0.1737176	0.3113003	-0.558	0.577	-0.184892489	count	1
AIMP1	-0.1317161	0.0703599	-1.872	0.0613	-0.184860972	count	1
AC239800.3	-0.1922508	0.2888474	-0.6656	0.506	-0.184733294	count	1
TPR	-0.1297466	0.0605268	-2.1436	0.0321	-0.184575081	count	1
SNRPD1	-0.1310451	0.0640778	-2.0451	0.0409	-0.184189402	count	1
FAAP20	-0.131489	0.0802265	-1.639	0.101	-0.184146485	count	1
ZNF134	-0.1867763	0.2847662	-0.6559	0.512	-0.184129486	count	1
CCSER2	-0.1302956	0.065536	-1.9882	0.0469	-0.184125824	count	1
SLC23A3	-0.4874905	0.6754914	-0.7217	0.471	-0.184117277	count	1
AC004754.1	-0.4874905	0.7997646	-0.6095	0.542	-0.184117277	count	1
GNB1	-0.1316651	0.0798838	-1.6482	0.0994	-0.183931534	count	1

TFAP2A	-0.1770368	0.2692584	-0.6575	0.511	-0.183802743	count	1
INTS6-AS1	-0.1822705	0.3584915	-0.5084	0.611	-0.183786928	count	1
PLAGL2	-0.2532791	0.3392234	-0.7466	0.455	-0.183750074	count	1
KTN1	-0.1283879	0.042789	-3.0005	0.00271	-0.183743486	count	1
NEDD8	-0.1283295	0.0346553	-3.703	0.000216	-0.183736149	count	1
NEMP2	-0.2169015	0.3189745	-0.68	0.497	-0.183666733	count	1
RNF185	-0.1569185	0.2206596	-0.7111	0.477	-0.183549159	count	1
ARMC2	-0.2283772	0.3212792	-0.7108	0.477	-0.183507739	count	1
AC091057.3	-0.3718536	0.8291607	-0.4485	0.654	-0.183371675	count	1
SLC25A23	-0.1460154	0.1483817	-0.9841	0.325	-0.183144143	count	1
TBC1D10B	-0.1494343	0.2427693	-0.6155	0.538	-0.182889699	count	1
FXN	-0.1473273	0.2361737	-0.6238	0.533	-0.18288709	count	1
PLEKHG3	-0.14907	0.2542052	-0.5864	0.558	-0.182843354	count	1
HCG27	-1.397447	0.6726476	-2.0775	0.0378	-0.182791821	count	1
AC004982.1	-1.397447	1.013931	-1.3782	0.168	-0.182791821	count	1
TNFRSF14-AS1	-1.397447	1.122316	-1.2451	0.2132	-0.182791821	count	1
LINC00624	-1.397447	1.122316	-1.2451	0.2132	-0.182791821	count	1
RPUSD4	-0.1760475	0.1937151	-0.9088	0.364	-0.182764055	count	1
MYO5A	-0.1760449	0.2670084	-0.6593	0.51	-0.182761326	count	1
LRP5L	-0.3371049	0.5865995	-0.5747	0.566	-0.182626995	count	1
AC073896.2	-0.2419676	0.3090966	-0.7828	0.434	-0.182298017	count	1
LINC00941	-0.5915379	0.6901257	-0.8571	0.391	-0.182160594	count	1
ZNF248	-0.1443327	0.1801825	-0.801	0.423	-0.182143936	count	1
NCOA4	-0.1327703	0.0930624	-1.4267	0.154	-0.181893428	count	1
LNPEP	-0.1353125	0.109514	-1.2356	0.217	-0.181850606	count	1
ZNF485	-0.2333482	0.3364676	-0.6935	0.488	-0.181849148	count	1
CENPX	-0.1293309	0.0720766	-1.7944	0.0728	-0.1818283	count	1
RTL6	-0.1822994	0.2944498	-0.6191	0.536	-0.181790449	count	1
SHOX2	-0.1419624	0.1979154	-0.7173	0.473	-0.181749898	count	1
PET100	-0.13138	0.0797272	-1.6479	0.0995	-0.181647742	count	1
SERPINB9P1	-0.1890743	0.433248	-0.4364	0.663	-0.181637306	count	1
TMEM167A	-0.1285522	0.0642368	-2.0012	0.0454	-0.181602387	count	1
DNAJC3-DT	-0.1946661	0.2958111	-0.6581	0.511	-0.181581585	count	1
POLD2	-0.132465	0.0884561	-1.4975	0.134	-0.18122987	count	1
SCAND1	-0.1271801	0.0504338	-2.5217	0.0117	-0.18117762	count	1
ZNF275	-0.1885538	0.3383797	-0.5572	0.577	-0.181130123	count	1
GSTP1	-0.1256216	0.0238976	-5.2567	1.55E-07	-0.180889338	count	0.003626225
UQCRHL	-0.4125701	0.4087771	-1.0093	0.313	-0.180880671	count	1
GNL3L	-0.1756425	0.2261412	-0.7767	0.437	-0.180651123	count	1
YRDC	-0.1414998	0.1864286	-0.759	0.448	-0.180499962	count	1
SOCS7	-0.20842	0.2978834	-0.6997	0.484	-0.180488908	count	1
IRF9	-0.1362035	0.1703865	-0.7994	0.424	-0.180436722	count	1
PUS3	-0.1483462	0.1677407	-0.8844	0.377	-0.180289041	count	1
ACTR1B	-0.1381633	0.1166067	-1.1849	0.236	-0.180222942	count	1
KCTD10	-0.1344716	0.1336966	-1.0058	0.315	-0.18001158	count	1

ETS1	-0.1381913	0.1359933	-1.0162	0.31	-0.179954354	count	1
ASCC3	-0.1317833	0.1070858	-1.2306	0.219	-0.179815761	count	1
HIP1	-0.1314284	0.1172918	-1.1205	0.263	-0.179809412	count	1
APEX1	-0.1276119	0.0675993	-1.8878	0.0591	-0.179788031	count	1
AC109347.2	-1.3754758	0.8585304	-1.6021	0.109	-0.179759343	count	1
SLA	-1.3754758	0.8933086	-1.5398	0.124	-0.179759343	count	1
SOX5	-0.1416089	0.1526052	-0.9279	0.353	-0.179704517	count	1
HIPK2	-0.1462088	0.1962295	-0.7451	0.456	-0.179700818	count	1
CD44	-0.1277868	0.0768483	-1.6628	0.0964	-0.179694688	count	1
AL035563.1	-0.4764045	0.4106329	-1.1602	0.246	-0.179651088	count	1
ZNHIT6	-0.1330996	0.1198083	-1.1109	0.267	-0.179634847	count	1
SCAMP1-AS1	-0.1630456	0.2786592	-0.5851	0.559	-0.179465197	count	1
SERPINH1	-0.1276778	0.0647498	-1.9719	0.0487	-0.17938176	count	1
CCDC117	-0.1555744	0.1996145	-0.7794	0.436	-0.179141748	count	1
AC007365.1	-0.2167753	0.5003728	-0.4332	0.665	-0.178942558	count	1
SLC2A12	-0.1917968	0.3162852	-0.6064	0.544	-0.178864005	count	1
MECP2	-0.1314586	0.1117923	-1.1759	0.24	-0.178843987	count	1
BRAF	-0.1369575	0.1688985	-0.8109	0.417	-0.178793005	count	1
SHISAL2A	-0.7860745	0.6924101	-1.1353	0.256	-0.178705218	count	1
TMEM178A	-0.7860745	0.9607565	-0.8182	0.413	-0.178705218	count	1
ZNF197	-0.1772908	0.2912829	-0.6087	0.543	-0.178704633	count	1
AC084018.2	-0.2464943	0.5467818	-0.4508	0.652	-0.178695139	count	1
ACVR1	-0.1461476	0.1874171	-0.7798	0.436	-0.178442381	count	1
RPS6KC1	-0.1513057	0.2189445	-0.6911	0.49	-0.178190069	count	1
YTHDF3-AS1	-0.2694615	0.477914	-0.5638	0.573	-0.178177307	count	1
HSBP1L1	-0.1389938	0.2159366	-0.6437	0.52	-0.178142308	count	1
CENPV	-0.1411544	0.2118203	-0.6664	0.505	-0.178116518	count	1
MYL5	-0.1713861	0.239621	-0.7152	0.475	-0.177871555	count	1
MARVELD1	-0.1302991	0.0942181	-1.383	0.167	-0.177832315	count	1
LZTS1	-0.2212766	0.3397252	-0.6513	0.515	-0.17767744	count	1
AL590560.2	-0.7814466	0.6679444	-1.1699	0.242	-0.177552674	count	1
SULT2B1	-0.4711403	0.5719015	-0.8238	0.41	-0.177533873	count	1
CDK5RAP2	-0.1296162	0.1038434	-1.2482	0.212	-0.177407408	count	1
TXNL4A	-0.1255922	0.0607309	-2.068	0.0387	-0.177312634	count	1
ARMCX2	-0.1415745	0.1247905	-1.1345	0.257	-0.177260495	count	1
URI1	-0.1278381	0.0796988	-1.604	0.109	-0.177142279	count	1
BCS1L	-0.141817	0.1645364	-0.8619	0.389	-0.176966022	count	1
EIF4A2	-0.1236804	0.0393369	-3.1441	0.0017	-0.176816447	count	1
AC009404.1	-0.5749262	0.6707797	-0.8571	0.391	-0.176639422	count	1
ANP32A	-0.1261149	0.0776145	-1.6249	0.104	-0.17662646	count	1
RFESD	-0.254395	0.3187114	-0.7982	0.425	-0.176612476	count	1
P3H4	-0.1377942	0.1546167	-0.8912	0.373	-0.176599317	count	1
SNAPC5	-0.1300055	0.1025478	-1.2678	0.205	-0.176555519	count	1
TRIM69	-0.1354302	0.1216714	-1.1131	0.266	-0.176498577	count	1
AL441992.1	-0.26697	0.6943367	-0.3845	0.701	-0.176477864	count	1

HLA-F	-0.1275575	0.084523	-1.5091	0.131	-0.176356128	count	1
RBMX	-0.1255487	0.0627188	-2.0018	0.0454	-0.176347231	count	1
FNIP2	-0.1378399	0.1806637	-0.763	0.446	-0.176243896	count	1
AC105760.2	-0.2819886	0.354861	-0.7946	0.427	-0.176163792	count	1
ZNF2	-0.2819886	0.3563387	-0.7913	0.429	-0.176163792	count	1
OXCT1	-0.1357677	0.1269751	-1.0692	0.285	-0.176158855	count	1
MED28	-0.1298998	0.0921938	-1.409	0.159	-0.176141234	count	1
PDCD5	-0.1235638	0.0506008	-2.4419	0.0147	-0.175942936	count	1
ARHGEF25	-0.132103	0.1493234	-0.8847	0.376	-0.175877814	count	1
ADCK1	-0.4013167	0.4982782	-0.8054	0.421	-0.175670875	count	1
NDUFB11	-0.1228028	0.0408483	-3.0063	0.00266	-0.175625416	count	1
SLC7A11	-1.3448701	1.076167	-1.2497	0.211	-0.175520047	count	1
SPRY4-AS1	-1.3448701	1.076167	-1.2497	0.211	-0.175520047	count	1
NOS3	-1.3448701	1.076167	-1.2497	0.211	-0.175520047	count	1
AL135744.1	-1.3448701	1.076167	-1.2497	0.211	-0.175520047	count	1
PAGR1	-1.3448701	1.076167	-1.2497	0.211	-0.175520047	count	1
DOC2B	-1.3448701	1.076167	-1.2497	0.211	-0.175520047	count	1
VWA3B	-1.3448701	1.127783	-1.1925	0.233	-0.175520047	count	1
RGMB-AS1	-1.3448701	1.127783	-1.1925	0.233	-0.175520047	count	1
KLHL32	-1.3448701	1.127783	-1.1925	0.233	-0.175520047	count	1
ARFGEF3	-1.3448701	1.127783	-1.1925	0.233	-0.175520047	count	1
BX649632.1	-1.3448701	1.127783	-1.1925	0.233	-0.175520047	count	1
AC124798.1	-1.3448701	1.127783	-1.1925	0.233	-0.175520047	count	1
FAM53B-AS1	-1.3448701	1.127783	-1.1925	0.233	-0.175520047	count	1
SSTR1	-1.3448701	1.127783	-1.1925	0.233	-0.175520047	count	1
AC090510.2	-1.3448701	1.127783	-1.1925	0.233	-0.175520047	count	1
AC105020.1	-1.3448701	1.127783	-1.1925	0.233	-0.175520047	count	1
AC074050.4	-1.3448701	1.127783	-1.1925	0.233	-0.175520047	count	1
AP005264.1	-1.3448701	1.127783	-1.1925	0.233	-0.175520047	count	1
AL512408.1	-1.3448701	1.286614	-1.0453	0.296	-0.175520047	count	1
CLMAT3	-1.3448701	1.286614	-1.0453	0.296	-0.175520047	count	1
AL031775.2	-1.3448701	1.286614	-1.0453	0.296	-0.175520047	count	1
TSGA13	-1.3448701	1.286614	-1.0453	0.296	-0.175520047	count	1
TRPV6	-1.3448701	1.286614	-1.0453	0.296	-0.175520047	count	1
RBM14-RBM4	-1.3448701	1.286614	-1.0453	0.296	-0.175520047	count	1
TMEM253	-1.3448701	1.286614	-1.0453	0.296	-0.175520047	count	1
AC015845.2	-1.3448701	1.286614	-1.0453	0.296	-0.175520047	count	1
APC2	-1.3448701	1.286614	-1.0453	0.296	-0.175520047	count	1
CCDC91	-0.1281648	0.0963023	-1.3309	0.183	-0.175496759	count	1
SLC39A1	-0.1245241	0.0599779	-2.0762	0.0379	-0.175352298	count	1
RRP9	-0.1558317	0.1990085	-0.783	0.434	-0.175326739	count	1
HAT1	-0.1273677	0.0916255	-1.3901	0.165	-0.175316633	count	1
ATP11C	-0.14354	0.2114039	-0.679	0.497	-0.175242229	count	1
BICD2	-0.1378637	0.1564319	-0.8813	0.378	-0.175167833	count	1
HNRNPA1	-0.1220748	0.0347483	-3.5131	0.000448	-0.174843799	count	1

PRPS2	-0.1405239	0.1720285	-0.8169	0.414	-0.174724254	count	1
MTO1	-0.1443955	0.199177	-0.725	0.469	-0.174588295	count	1
STON1-GTF2A1L	-0.7695184	0.8756485	-0.8788	0.38	-0.174584977	count	1
TTC23L	-0.7695184	0.8756485	-0.8788	0.38	-0.174584977	count	1
AC026691.1	-0.7695184	0.8756485	-0.8788	0.38	-0.174584977	count	1
OTUD6A	-0.7695184	0.8756485	-0.8788	0.38	-0.174584977	count	1
PTK6	-0.7695184	0.8756485	-0.8788	0.38	-0.174584977	count	1
CAPN12	-0.7695184	0.8756485	-0.8788	0.38	-0.174584977	count	1
PASK	-0.7695184	0.9079758	-0.8475	0.397	-0.174584977	count	1
FOCAD-AS1	-0.7695184	0.9079758	-0.8475	0.397	-0.174584977	count	1
AC005944.1	-0.7695184	0.9079758	-0.8475	0.397	-0.174584977	count	1
AC093525.7	-0.7695184	1.0382103	-0.7412	0.459	-0.174584977	count	1
RUNDC3B	-0.7695184	1.106138	-0.6957	0.487	-0.174584977	count	1
IL1R2	-0.7695184	1.238815	-0.6212	0.535	-0.174584977	count	1
ZBED3-AS1	-0.4629092	0.4875951	-0.9494	0.342	-0.174228161	count	1
GPRC5C	-0.1259735	0.0949202	-1.3272	0.185	-0.174214784	count	1
URGCP	-0.1416824	0.1883642	-0.7522	0.452	-0.17411039	count	1
DCBLD1	-0.1708623	0.2853446	-0.5988	0.549	-0.173956747	count	1
AL031963.3	-0.2784923	0.3631015	-0.767	0.443	-0.173904751	count	1
LRRC75A	-0.1278557	0.1115346	-1.1463	0.252	-0.173834856	count	1
STUB1	-0.1224033	0.0530588	-2.3069	0.0211	-0.173667421	count	1
BBC3	-0.1351565	0.1716206	-0.7875	0.431	-0.173598592	count	1
CD274	-0.7653857	0.4938488	-1.5498	0.121	-0.173557813	count	1
CTNNBL1	-0.1310025	0.1134622	-1.1546	0.248	-0.173425042	count	1
TTLL12	-0.2965202	0.340666	-0.8704	0.384	-0.17332623	count	1
GBE1	-0.1342292	0.1396698	-0.961	0.337	-0.173317594	count	1
HCN3	-1.328581	0.7472257	-1.778	0.0755	-0.173257199	count	1
ATP6V1F	-0.1218286	0.0495087	-2.4608	0.0139	-0.173231524	count	1
ZNF277	-0.1412517	0.1430843	-0.9872	0.324	-0.17320654	count	1
SLC39A3	-0.1290795	0.1150988	-1.1215	0.262	-0.173163487	count	1
RAP2C-AS1	-0.1668837	0.315787	-0.5285	0.597	-0.173148424	count	1
CIRBP	-0.1206678	0.03096	-3.8975	9.89E-05	-0.173144759	count	1
MOCS1	-0.1700678	0.2348506	-0.7242	0.469	-0.173138541	count	1
EIF4E2	-0.126178	0.0924411	-1.365	0.172	-0.173070678	count	1
ZSCAN32	-0.1522277	0.2602435	-0.5849	0.559	-0.172932857	count	1
LHFPL2	-0.141619	0.2114692	-0.6697	0.503	-0.172885047	count	1
GPRASP1	-0.1544673	0.2933026	-0.5266	0.598	-0.172873549	count	1
GTDC1	-0.1440664	0.2013336	-0.7156	0.474	-0.17278959	count	1
TIFA	-0.1322015	0.1428512	-0.9254	0.355	-0.172706488	count	1
MCU	-0.1476167	0.2436806	-0.6058	0.545	-0.172598305	count	1
PANK1	-0.2611595	0.426572	-0.6122	0.54	-0.172517861	count	1
ZNF213-AS1	-0.16779	0.3230988	-0.5193	0.604	-0.172485332	count	1
TMEM88	-0.5621739	0.9304922	-0.6042	0.546	-0.172412617	count	1
MDM2	-0.126763	0.1221369	-1.0379	0.299	-0.172396053	count	1
CERK	-0.145377	0.1699042	-0.8556	0.392	-0.172295902	count	1

ZNF667	-0.1794759	0.2889589	-0.6211	0.535	-0.172290247	count	1
AATF	-0.1338288	0.1396756	-0.9581	0.338	-0.172261654	count	1
COG5	-0.1466285	0.1743101	-0.8412	0.4	-0.172050658	count	1
NMI	-0.1315442	0.1470436	-0.8946	0.371	-0.171983002	count	1
SARAF	-0.1203628	0.0391495	-3.0744	0.0021	-0.171917325	count	1
TERF2	-0.138955	0.1574008	-0.8828	0.377	-0.171789298	count	1
NUTM2A-AS1	-0.1390705	0.190391	-0.7304	0.465	-0.171591872	count	1
ESCO2	-1.3156184	0.976409	-1.3474	0.178	-0.17145351	count	1
AC137936.1	-1.3156184	0.9837325	-1.3374	0.181	-0.17145351	count	1
KLHL8	-0.1901475	0.2495387	-0.762	0.446	-0.171250122	count	1
LPGAT1	-0.1266249	0.1181973	-1.0713	0.284	-0.171183456	count	1
PSME3	-0.148573	0.189967	-0.7821	0.434	-0.171023614	count	1
TIMM9	-0.1273964	0.1117639	-1.1399	0.254	-0.1709744	count	1
MRPL54	-0.121267	0.0671348	-1.8063	0.071	-0.170874227	count	1
MPP2	-0.2020581	0.3697764	-0.5464	0.585	-0.170861398	count	1
ZNF839	-0.1662036	0.3344679	-0.4969	0.619	-0.170836565	count	1
RAB27A	-0.1371416	0.170038	-0.8065	0.42	-0.170500146	count	1
LINC01184	-0.1299024	0.1530713	-0.8486	0.396	-0.170354199	count	1
HMGCL	-0.1259794	0.118854	-1.06	0.289	-0.170129711	count	1
MZB1	-1.3060137	0.7532807	-1.7338	0.083	-0.170115407	count	1
TARSL2	-0.1288488	0.1673694	-0.7698	0.441	-0.169692672	count	1
AC105285.1	-0.7495924	0.8465222	-0.8855	0.376	-0.16963749	count	1
KIAA0232	-0.1236624	0.0909962	-1.359	0.174	-0.169615234	count	1
PPP1R21	-0.1356944	0.1701384	-0.7976	0.425	-0.169583435	count	1
MEF2A	-0.1204689	0.073286	-1.6438	0.1	-0.16947733	count	1
PPP1CA	-0.1224238	0.085368	-1.4341	0.152	-0.16939772	count	1
NDRG3	-0.1284472	0.1441718	-0.8909	0.373	-0.169389435	count	1
PDCD2	-0.1227657	0.0844826	-1.4531	0.146	-0.169296701	count	1
TNFSF12	-0.1188382	0.047639	-2.4946	0.0127	-0.169085982	count	1
LINC00476	-0.1481068	0.228823	-0.6473	0.518	-0.168998478	count	1
RPL14	-0.117157	0.0206407	-5.676	1.49E-08	-0.168844065	count	0.000350269
EIF4E	-0.1228029	0.0905308	-1.3565	0.175	-0.168772802	count	1
ADPRHL1	-0.4490699	0.4938053	-0.9094	0.363	-0.168683266	count	1
AC005229.4	-0.1755194	0.2729715	-0.643	0.52	-0.168440915	count	1
VPS4A	-0.1253373	0.1045752	-1.1985	0.231	-0.168417639	count	1
SACS	-0.1254487	0.186964	-0.671	0.502	-0.168355072	count	1
TMC3-AS1	-0.3852462	0.6409887	-0.601	0.548	-0.168253435	count	1
C1QL1	-0.3852462	0.8986874	-0.4287	0.668	-0.168253435	count	1
GSDMD	-0.1229828	0.0943285	-1.3038	0.192	-0.16815912	count	1
EBPL	-0.1208852	0.0762611	-1.5851	0.113	-0.1680133	count	1
TYRO3	-0.1941666	0.3939149	-0.4929	0.622	-0.167928586	count	1
ADPRHL2	-0.1273211	0.1309072	-0.9726	0.331	-0.167900569	count	1
AC011447.3	-0.2542954	0.450892	-0.564	0.573	-0.167845856	count	1
CADM1	-0.1774105	0.4271942	-0.4153	0.678	-0.167839494	count	1
RAB5C	-0.1188493	0.0561993	-2.1148	0.0345	-0.167681286	count	1

AGBL5	-0.2090426	0.3525925	-0.5929	0.553	-0.167648991	count	1
MAN1B1	-0.1262215	0.1532501	-0.8236	0.41	-0.167474936	count	1
CLASP2	-0.1272702	0.1966557	-0.6472	0.518	-0.167376701	count	1
UBR5-AS1	-0.1796025	0.5057286	-0.3551	0.723	-0.167326928	count	1
MLF2	-0.118295	0.0564729	-2.0947	0.0363	-0.167320243	count	1
BRWD1-AS2	-0.7398526	0.8084057	-0.9152	0.36	-0.167223934	count	1
FOXD2-AS1	-0.2085066	0.5788153	-0.3602	0.719	-0.167210115	count	1
NOP10	-0.1176405	0.0523629	-2.2466	0.0247	-0.167196828	count	1
MXD4	-0.1193803	0.0769459	-1.5515	0.121	-0.167088426	count	1
AC006480.2	-0.222242	0.518066	-0.429	0.668	-0.167085109	count	1
ENDOD1	-0.1205342	0.0896362	-1.3447	0.179	-0.167010075	count	1
RAI1	-0.17403	0.4371998	-0.3981	0.691	-0.166992398	count	1
TIMM13	-0.1196971	0.0710917	-1.6837	0.0923	-0.166849261	count	1
SLAIN1	-0.1623194	0.3851196	-0.4215	0.673	-0.166800975	count	1
ACTG2	-0.117648	0.1083686	-1.0856	0.278	-0.166794324	count	1
SRM	-0.1183138	0.0591885	-1.9989	0.0457	-0.166791887	count	1
EIF2A	-0.1183006	0.0630654	-1.8758	0.0608	-0.166582639	count	1
LINC01985	-1.279442	0.665292	-1.9231	0.0545	-0.166406817	count	1
FTH1	-0.1153687	0.021836	-5.2834	1.34E-07	-0.166385307	count	0.003136002
ATRX	-0.1170809	0.0609461	-1.9211	0.0548	-0.166131279	count	1
NSF	-0.1420485	0.2304032	-0.6165	0.538	-0.166046838	count	1
PLXNA1	-0.1460463	0.2434242	-0.6	0.549	-0.165859889	count	1
SART1	-0.1343901	0.1608657	-0.8354	0.404	-0.165790619	count	1
COMMD1	-0.1177088	0.072927	-1.6141	0.107	-0.16578816	count	1
ISCU	-0.1154151	0.0332971	-3.4662	0.000534	-0.165517192	count	1
GOLGA8N	-0.2199488	0.3096855	-0.7102	0.478	-0.165320168	count	1
TWF2	-0.1201291	0.0981105	-1.2244	0.221	-0.165279708	count	1
DCTPP1	-0.1232562	0.1106009	-1.1144	0.265	-0.165263399	count	1
ZSCAN30	-0.141312	0.231218	-0.6112	0.541	-0.165180502	count	1
CTGF	-0.1146092	0.0543483	-2.1088	0.035	-0.165118698	count	1
LRRC73	-0.3364645	0.6096671	-0.5519	0.581	-0.165118316	count	1
PNPLA4	-0.1352709	0.1723994	-0.7846	0.433	-0.165097655	count	1
ERP29	-0.1159056	0.0476421	-2.4328	0.015	-0.165066552	count	1
BLMH	-0.1427722	0.174571	-0.8178	0.413	-0.164982305	count	1
ATF7	-0.1225404	0.1182836	-1.036	0.3	-0.16491831	count	1
RRP36	-0.1207778	0.1053881	-1.146	0.252	-0.164733593	count	1
TRAPPC3	-0.1179821	0.0751722	-1.5695	0.117	-0.164503689	count	1
COL5A1	-0.1210717	0.1509253	-0.8022	0.422	-0.16450022	count	1
CPSF2	-0.1291448	0.1525632	-0.8465	0.397	-0.164476886	count	1
UQCR10	-0.1155432	0.0464133	-2.4894	0.0128	-0.164220822	count	1
ADAMTS9	-0.1792431	0.3603594	-0.4974	0.619	-0.164218762	count	1
NTRK3	-0.1219206	0.1390566	-0.8768	0.381	-0.164147008	count	1
MYH7B	-1.26276	0.7452815	-1.6943	0.0903	-0.164073992	count	1
MTPN	-0.1151095	0.0501438	-2.2956	0.0218	-0.164001749	count	1
SARS2	-0.2630682	0.3807298	-0.691	0.49	-0.163958745	count	1

VPS29	-0.1151359	0.0482326	-2.3871	0.017	-0.163821415	count	1
PSMG4	-0.1195633	0.0960801	-1.2444	0.213	-0.163738217	count	1
ELOF1	-0.1203574	0.0984359	-1.2227	0.222	-0.163670327	count	1
VAT1L	-0.1486976	0.3014806	-0.4932	0.622	-0.16354302	count	1
JUP	-0.1338614	0.1915995	-0.6987	0.485	-0.163369024	count	1
AL365356.5	-0.4352834	0.6207034	-0.7013	0.483	-0.163176332	count	1
CLNS1A	-0.1167613	0.0706884	-1.6518	0.0987	-0.163161931	count	1
NAA16	-0.1356721	0.1850882	-0.733	0.464	-0.163119336	count	1
1-Mar	-0.1979051	0.3085075	-0.6415	0.521	-0.163067304	count	1
SAMD12	-0.2471853	0.3909789	-0.6322	0.527	-0.163013397	count	1
AC120053.1	-1.2542062	0.8028973	-1.5621	0.118	-0.162876509	count	1
AC010531.6	-1.2542062	0.9188955	-1.3649	0.172	-0.162876509	count	1
NKAIN2	-1.2542062	0.9587684	-1.3081	0.191	-0.162876509	count	1
SELPLG	-1.2542062	1.055102	-1.1887	0.235	-0.162876509	count	1
MARF1	-0.1290697	0.1721887	-0.7496	0.454	-0.162809475	count	1
CTU1	-0.1357586	0.1886997	-0.7194	0.472	-0.162771961	count	1
GPR157	-0.2349561	0.4446556	-0.5284	0.597	-0.162752109	count	1
TRAPPC12	-0.1242931	0.1378003	-0.902	0.367	-0.162731436	count	1
HIRIP3	-0.1217388	0.120208	-1.0127	0.311	-0.162624067	count	1
ILF2	-0.1154124	0.0631465	-1.8277	0.0677	-0.162559313	count	1
AP000487.1	-0.2786683	0.6301781	-0.4422	0.658	-0.16251616	count	1
HGS	-0.1270394	0.1733486	-0.7329	0.464	-0.162387335	count	1
DMWD	-0.1234072	0.1756776	-0.7025	0.482	-0.162168466	count	1
CCDC6	-0.1164335	0.0883207	-1.3183	0.187	-0.161946132	count	1
MMP24OS	-0.1143512	0.0555871	-2.0572	0.0397	-0.161804927	count	1
MAP3K13	-0.117278	0.0992905	-1.1812	0.238	-0.161554391	count	1
CLPP	-0.1162413	0.0813271	-1.4293	0.153	-0.161552425	count	1
USF2	-0.1169655	0.0878795	-1.331	0.183	-0.161497446	count	1
NADK2	-0.1339643	0.2026024	-0.6612	0.509	-0.161489306	count	1
PITHD1	-0.1167426	0.0816156	-1.4304	0.153	-0.16145793	count	1
GNAS	-0.1121294	0.023598	-4.7516	2.10E-06	-0.161424684	count	0.0487032
AC245297.2	-0.2990067	0.4402037	-0.6792	0.497	-0.161168596	count	1
LRP4	-0.2326258	0.6575831	-0.3538	0.724	-0.161094189	count	1
DIO2	-0.1256854	0.1607294	-0.782	0.434	-0.160841741	count	1
TYMP	-0.1186444	0.0957185	-1.2395	0.215	-0.160750969	count	1
ITPA	-0.1187469	0.1043449	-1.138	0.255	-0.160732329	count	1
SPRYD4	-0.1331624	0.1833902	-0.7261	0.468	-0.160517657	count	1
NOMO1	-0.169677	0.3993112	-0.4249	0.671	-0.160424546	count	1
NEURL2	-0.156082	0.3149946	-0.4955	0.62	-0.160324412	count	1
SRPK1	-0.1191807	0.1162329	-1.0254	0.305	-0.160259974	count	1
NIPSNAP3A	-0.1183698	0.1231216	-0.9614	0.336	-0.160221012	count	1
PTTG1IP	-0.1122853	0.0530435	-2.1169	0.0343	-0.160182502	count	1
NUDT4	-0.1166431	0.0939832	-1.2411	0.215	-0.160170458	count	1
DOK5	-0.1180906	0.1526985	-0.7734	0.439	-0.159947568	count	1
CHAF1A	-0.5240936	0.3554432	-1.4745	0.14	-0.159854016	count	1

C8orf59	-0.1124263	0.0503595	-2.2325	0.0256	-0.15979485	count	1
PFN2	-0.1117274	0.0638031	-1.7511	0.08	-0.159757959	count	1
RBM17	-0.1146722	0.0773648	-1.4822	0.138	-0.159744178	count	1
CRTAP	-0.1123243	0.0495333	-2.2677	0.0234	-0.159695297	count	1
ELOVL1	-0.1173224	0.1118449	-1.049	0.294	-0.159626871	count	1
NKAPL	-0.1263641	0.2418967	-0.5224	0.601	-0.159617124	count	1
CINP	-0.1184074	0.1087849	-1.0885	0.276	-0.159531424	count	1
GPR161	-0.1398559	0.2347393	-0.5958	0.551	-0.159519224	count	1
AC007405.3	-0.2123228	0.3392542	-0.6259	0.531	-0.159456401	count	1
TRAPPC2L	-0.1132315	0.0742385	-1.5252	0.127	-0.159337855	count	1
FBXW11	-0.1166859	0.122976	-0.9489	0.343	-0.159305237	count	1
AC072061.1	-0.2416607	0.5664958	-0.4266	0.67	-0.159263485	count	1
CNTN1	-0.1597968	0.3886212	-0.4112	0.681	-0.159095313	count	1
THSD7B	-1.226768	0.6161278	-1.9911	0.0465	-0.159030398	count	1
ZNF182	-0.1596664	0.428794	-0.3724	0.71	-0.158963986	count	1
NDOR1	-0.424501	0.4989618	-0.8508	0.395	-0.158881342	count	1
AC092069.1	-0.1412434	0.2222987	-0.6354	0.525	-0.158794215	count	1
NAGK	-0.1149141	0.0904288	-1.2708	0.204	-0.158784417	count	1
TP53RK	-0.1170172	0.1280116	-0.9141	0.361	-0.158742829	count	1
ACOT13	-0.1161288	0.1121672	-1.0353	0.301	-0.15866021	count	1
MYH14	-1.2239959	0.7783247	-1.5726	0.116	-0.158641356	count	1
CD3EAP	-0.1877382	0.2524021	-0.7438	0.457	-0.158537527	count	1
GEMIN2	-0.1479982	0.2121842	-0.6975	0.486	-0.15846455	count	1
NAP1L3	-0.1289763	0.1664448	-0.7749	0.438	-0.158425969	count	1
PHACTR4	-0.1211064	0.1697505	-0.7134	0.476	-0.158299849	count	1
NAA20	-0.1129792	0.0670043	-1.6861	0.0919	-0.158293926	count	1
ZNF143	-0.1226573	0.1640807	-0.7475	0.455	-0.158004413	count	1
ZNF879	-0.2395576	0.4234718	-0.5657	0.572	-0.157837121	count	1
AC130466.1	-0.7016829	0.4896784	-1.4329	0.152	-0.157797943	count	1
HEYL	-0.1340861	0.2405312	-0.5575	0.577	-0.157793894	count	1
RSF1	-0.1110493	0.0570898	-1.9452	0.0518	-0.157787179	count	1
TMSB15B	-0.2101467	0.3804118	-0.5524	0.581	-0.157784726	count	1
CDC37	-0.1115477	0.0609975	-1.8287	0.0675	-0.157704645	count	1
AC092802.1	-0.5174467	0.7544197	-0.6859	0.493	-0.157672029	count	1
INE2	-0.5174467	0.7774493	-0.6656	0.506	-0.157672029	count	1
DKKL1	-0.5174467	0.7780733	-0.665	0.506	-0.157672029	count	1
AC018362.2	-0.5174467	0.8736317	-0.5923	0.554	-0.157672029	count	1
JPT2	-0.1293774	0.2079202	-0.6222	0.534	-0.157502946	count	1
EIF4ENIF1	-0.1367231	0.1789948	-0.7638	0.445	-0.15729471	count	1
ATG16L2	-0.1367231	0.2080448	-0.6572	0.511	-0.15729471	count	1
NME3	-0.110148	0.0431351	-2.5536	0.0107	-0.157246317	count	1
PANX1	-0.156074	0.2386504	-0.654	0.513	-0.157085896	count	1
DNAJC9	-0.1244843	0.1323432	-0.9406	0.347	-0.157004008	count	1
PEBP1	-0.1090362	0.025713	-4.2405	2.29E-05	-0.156847539	count	0.5257611
CYP39A1	-0.1857269	0.3344966	-0.5552	0.579	-0.156808965	count	1

TOMM22	-0.1118807	0.0660738	-1.6933	0.0905	-0.156753645	count	1
SWI5	-0.114256	0.0893819	-1.2783	0.201	-0.156630081	count	1
CYP17A1-AS1	-0.2516	0.3926303	-0.6409	0.522	-0.156579212	count	1
LRRC4B	-0.2514635	0.4351197	-0.5779	0.563	-0.15649729	count	1
BAIAP3	-0.513288	0.9542624	-0.5379	0.591	-0.156308426	count	1
AC010894.2	-0.513288	0.9792892	-0.5241	0.6	-0.156308426	count	1
PRDX2	-0.1093207	0.0371524	-2.9425	0.0033	-0.156307692	count	1
GMCL1	-0.1211156	0.1264482	-0.9578	0.338	-0.156174744	count	1
REXO4	-0.1227894	0.1798127	-0.6829	0.495	-0.156153786	count	1
PPP1R1C	-0.2508305	0.4210882	-0.5957	0.551	-0.156090826	count	1
MTG1	-0.2008294	0.3809922	-0.5271	0.598	-0.155979792	count	1
BCL2L11	-0.1703095	0.2479315	-0.6869	0.492	-0.155916354	count	1
OSBPL11	-0.147861	0.2687107	-0.5503	0.582	-0.155881315	count	1
POLR2H	-0.1141786	0.101143	-1.1289	0.259	-0.155838224	count	1
CKS1B	-0.1122161	0.0991192	-1.1321	0.258	-0.155825645	count	1
ZNF559	-0.1386051	0.2255767	-0.6144	0.539	-0.155806718	count	1
PROB1	-0.6931799	0.7269151	-0.9536	0.34	-0.155705619	count	1
AC079807.1	-0.6931799	0.7786779	-0.8902	0.373	-0.155705619	count	1
CDYL2	-0.6931799	0.8414805	-0.8238	0.41	-0.155705619	count	1
TRDN	-0.6931799	0.9059977	-0.7651	0.444	-0.155705619	count	1
ZNF416	-0.2073792	0.3737616	-0.5548	0.579	-0.155659755	count	1
COPE	-0.1095187	0.0473219	-2.3143	0.0207	-0.155654924	count	1
SPICE1	-0.1476163	0.2196602	-0.672	0.502	-0.155620935	count	1
AL392172.1	-0.158163	0.3109989	-0.5086	0.611	-0.155595896	count	1
SUPT5H	-0.1199527	0.1679621	-0.7142	0.475	-0.155580381	count	1
PALM2-AKAP2	-0.215346	0.5188791	-0.415	0.678	-0.155572769	count	1
FPR1	-0.2671577	0.888796	-0.3006	0.764	-0.155568603	count	1
MANBAL	-0.112125	0.0850029	-1.3191	0.187	-0.155566499	count	1
GZMK	-0.3176938	0.8013269	-0.3965	0.692	-0.155495947	count	1
PRRT3-AS1	-0.2070885	0.3846089	-0.5384	0.59	-0.155436611	count	1
CAP2	-0.1121832	0.1002989	-1.1185	0.263	-0.15542686	count	1
PGAP1	-0.1513074	0.2510995	-0.6026	0.547	-0.155370057	count	1
SLC7A2	-0.1188512	0.1274266	-0.9327	0.351	-0.155344221	count	1
ROCK2	-0.1098576	0.0588518	-1.8667	0.062	-0.155195539	count	1
GPBP1L1	-0.1168531	0.133238	-0.877	0.381	-0.155192848	count	1
IL33	-0.1105583	0.0745604	-1.4828	0.138	-0.155183749	count	1
COA5	-0.1122839	0.0927348	-1.2108	0.226	-0.155145988	count	1
DHDDS	-0.124973	0.1762248	-0.7092	0.478	-0.155023537	count	1
C17orf75	-0.1247073	0.1740925	-0.7163	0.474	-0.154978408	count	1
FRMD4B	-0.1159102	0.2013582	-0.5756	0.565	-0.154896664	count	1
ZNF776	-0.2351254	0.397546	-0.5914	0.554	-0.154833201	count	1
AC240274.1	-0.1399637	0.2845555	-0.4919	0.623	-0.154782899	count	1
PSMD7	-0.1094767	0.0599554	-1.826	0.0679	-0.154759569	count	1
HTATIP2	-0.1155863	0.0960821	-1.203	0.229	-0.154751728	count	1
ZNF816-ZNF321P	-0.2657452	0.3736924	-0.7111	0.477	-0.154717283	count	1

NELFE	-0.1117266	0.0892249	-1.2522	0.211	-0.154709807	count	1
UBE2N	-0.1104131	0.067364	-1.6391	0.101	-0.154618935	count	1
EIF1AX	-0.1090029	0.0499206	-2.1835	0.0291	-0.154603519	count	1
PPFIBP1	-0.109311	0.0669051	-1.6338	0.102	-0.154596573	count	1
AC103736.1	-0.5080082	0.5117279	-0.9927	0.321	-0.154578969	count	1
ASB16-AS1	-0.178968	0.2897074	-0.6178	0.537	-0.154567308	count	1
DYNC1LI1	-0.1112574	0.0757488	-1.4688	0.142	-0.154565706	count	1
NOL7	-0.1082927	0.0471227	-2.2981	0.0216	-0.154545147	count	1
HIST1H2BJ	-0.2058691	0.4753114	-0.4331	0.665	-0.154500738	count	1
PCSK7	-0.1131341	0.1463361	-0.7731	0.44	-0.154449131	count	1
SUDS3	-0.1111734	0.0931428	-1.1936	0.233	-0.15437629	count	1
PCDHGB6	-0.1517824	0.2546428	-0.5961	0.551	-0.154329611	count	1
AC112220.4	-0.6868515	0.8225449	-0.835	0.404	-0.154150274	count	1
PCDHGA4	-0.6868515	0.8225449	-0.835	0.404	-0.154150274	count	1
PROM1	-0.6868515	0.9219685	-0.745	0.456	-0.154150274	count	1
CARD9	-0.6868515	0.9774949	-0.7027	0.482	-0.154150274	count	1
EMILIN3	-0.6868515	1.068788	-0.6426	0.52	-0.154150274	count	1
GREB1L	-0.1784782	0.608579	-0.2933	0.769	-0.154137273	count	1
ZBTB49	-0.2053402	0.352952	-0.5818	0.561	-0.154094878	count	1
AP003486.1	-0.506142	0.4363585	-1.1599	0.246	-0.153968156	count	1
UBE2G1	-0.1160572	0.1199826	-0.9673	0.333	-0.153958774	count	1
POLR3GL	-0.1102701	0.0787861	-1.3996	0.162	-0.153952117	count	1
B4GALT7	-0.1172445	0.1294817	-0.9055	0.365	-0.1539392	count	1
ACP6	-0.1681491	0.280067	-0.6004	0.548	-0.153910285	count	1
HOMER2	-0.1779538	0.4862029	-0.366	0.714	-0.153676902	count	1
ZNF511	-0.1120311	0.0972862	-1.1516	0.25	-0.153672862	count	1
PCGF1	-0.1278207	0.2112641	-0.605	0.545	-0.153632044	count	1
ZDHHC20	-0.1210369	0.1811258	-0.6682	0.504	-0.153508585	count	1
ARF1	-0.1071562	0.0351523	-3.0483	0.00232	-0.153429818	count	1
TTC32	-0.1195761	0.1354143	-0.883	0.377	-0.153352449	count	1
NENF	-0.106937	0.0345993	-3.0907	0.00201	-0.153301389	count	1
KIF13A	-0.1112199	0.1200299	-0.9266	0.354	-0.153252275	count	1
TMEM242	-0.1107472	0.0915938	-1.2091	0.227	-0.153245236	count	1
NARFL	-0.1326286	0.2125749	-0.6239	0.533	-0.153187842	count	1
GNG11	-0.1080837	0.0541417	-1.9963	0.046	-0.153075337	count	1
TIAM2	-0.1734576	0.454238	-0.3819	0.703	-0.152963142	count	1
BCL2L2	-0.120125	0.2486773	-0.4831	0.629	-0.152951054	count	1
AL451007.2	-0.5029448	0.7175362	-0.7009	0.483	-0.152922284	count	1
LINC01612	-0.5029448	0.7175362	-0.7009	0.483	-0.152922284	count	1
CHRM2	-0.5029448	0.7175362	-0.7009	0.483	-0.152922284	count	1
ARX	-0.5029448	0.7175362	-0.7009	0.483	-0.152922284	count	1
MKI67	-0.5029448	0.7175362	-0.7009	0.483	-0.152922284	count	1
C1QTNF8	-0.5029448	0.7175362	-0.7009	0.483	-0.152922284	count	1
PITPNM3	-0.5029448	0.7175362	-0.7009	0.483	-0.152922284	count	1
ICAM5	-0.5029448	0.7175362	-0.7009	0.483	-0.152922284	count	1

AL353596.1	-0.5029448	0.9545277	-0.5269	0.598	-0.152922284	count	1
LYRM7	-0.1236695	0.1647378	-0.7507	0.453	-0.152812991	count	1
PPM1J	-0.1908381	0.3165162	-0.6029	0.547	-0.152766747	count	1
AL353708.3	-1.1820798	0.8099831	-1.4594	0.145	-0.152752419	count	1
OSGEPL1-AS1	-1.1820798	0.9585514	-1.2332	0.218	-0.152752419	count	1
PPP4R3B	-0.1116093	0.1026139	-1.0877	0.277	-0.15265751	count	1
MT-ND3	-0.1059974	0.0246905	-4.2931	1.81E-05	-0.152642707	count	0.4160466
PSMF1	-0.108522	0.0649459	-1.671	0.0948	-0.152528295	count	1
DNAJC21	-0.1081305	0.0666691	-1.6219	0.105	-0.152524798	count	1
MRPS26	-0.1083313	0.0694705	-1.5594	0.119	-0.152342937	count	1
ZNF517	-0.3504856	0.3993879	-0.8776	0.38	-0.152302846	count	1
IL3RA	-0.3503646	0.5090381	-0.6883	0.491	-0.152247558	count	1
RAC3	-0.3112219	0.646523	-0.4814	0.63	-0.152188118	count	1
CHMP7	-0.1421247	0.1791909	-0.7931	0.428	-0.152121495	count	1
MT-ND5	-0.1055322	0.0270895	-3.8957	9.97E-05	-0.151844064	count	1
RPL39	-0.10544	0.0230678	-4.5709	5.02E-06	-0.151829438	count	0.11601722
NAALADL1	-0.1154737	0.1310626	-0.8811	0.378	-0.15171759	count	1
C22orf39	-0.1106121	0.0994783	-1.1119	0.266	-0.151595783	count	1
TRAPPC6B	-0.1182609	0.1320819	-0.8954	0.371	-0.151486996	count	1
FAM84A	-1.172879	0.4630637	-2.5329	0.0114	-0.151458165	count	1
INTS6L	-0.2300768	0.3828219	-0.601	0.548	-0.151414977	count	1
TUSC1	-0.1085299	0.0883205	-1.2288	0.219	-0.151411898	count	1
VPS16	-0.133335	0.179931	-0.741	0.459	-0.151327638	count	1
AC040169.1	-0.2813964	0.5367381	-0.5243	0.6	-0.151311966	count	1
FTSJ1	-0.1222183	0.1713955	-0.7131	0.476	-0.151305796	count	1
RBP5	-0.2427266	0.4090321	-0.5934	0.553	-0.150892228	count	1
TINAGL1	-0.1056163	0.0544291	-1.9404	0.0524	-0.150867594	count	1
C21orf2	-0.1117462	0.1159709	-0.9636	0.335	-0.150766498	count	1
ADSS	-0.1113164	0.1200793	-0.927	0.354	-0.150757365	count	1
CAMKMT	-0.1304212	0.2289462	-0.5697	0.569	-0.150622554	count	1
AL359633.2	-0.3463562	0.7122149	-0.4863	0.627	-0.150416729	count	1
DPH6	-0.117989	0.1731514	-0.6814	0.496	-0.150412378	count	1
2-Sep	-0.1057102	0.048919	-2.1609	0.0308	-0.150316892	count	1
IGSF22	-0.3456142	0.699743	-0.4939	0.621	-0.150078022	count	1
RHOT2	-0.1158063	0.1791386	-0.6465	0.518	-0.14990429	count	1
ZSCAN16-AS1	-0.1123517	0.1099875	-1.0215	0.307	-0.149837116	count	1
PDHB	-0.1069785	0.0716309	-1.4935	0.135	-0.149761864	count	1
RBBP9	-0.1318945	0.2090561	-0.6309	0.528	-0.149681851	count	1
RANGRF	-0.1094232	0.1098886	-0.9958	0.319	-0.149628474	count	1
SDSL	-0.1561338	0.2500865	-0.6243	0.532	-0.149611037	count	1
C3orf14	-0.1129094	0.1329406	-0.8493	0.396	-0.1495993	count	1
NGDN	-0.1103726	0.1053197	-1.048	0.295	-0.149477094	count	1
PRMT5	-0.1279503	0.1851939	-0.6909	0.49	-0.149472352	count	1
MAIP1	-0.1317096	0.1789578	-0.736	0.462	-0.149470619	count	1
ABLIM2	-0.4922956	0.5353499	-0.9196	0.358	-0.149444033	count	1

ZNF607	-0.2779871	0.4434225	-0.6269	0.531	-0.149408431	count	1
DDX28	-0.162552	0.2777762	-0.5852	0.558	-0.148716131	count	1
VSNL1	-1.152967	0.7467063	-1.5441	0.123	-0.148655888	count	1
AC080013.5	-1.152967	0.7899155	-1.4596	0.144	-0.148655888	count	1
LINC02515	-0.398437	0.3814917	-1.0444	0.296	-0.148543573	count	1
RNF112	-0.6639297	0.6300133	-1.0538	0.292	-0.148530445	count	1
MRPS27	-0.1170569	0.1297888	-0.9019	0.367	-0.148443797	count	1
MRM3	-0.1223723	0.207828	-0.5888	0.556	-0.148212077	count	1
DNAJC8	-0.1042217	0.0477954	-2.1806	0.0293	-0.148189374	count	1
SPATA1	-0.3032136	0.4525221	-0.6701	0.503	-0.148102127	count	1
FAM98C	-0.1120778	0.1296808	-0.8643	0.388	-0.14794616	count	1
LRG1	-1.1468879	0.9130326	-1.2561	0.209	-0.147800068	count	1
CSNK2B	-0.1036978	0.0503262	-2.0605	0.0394	-0.147610478	count	1
TPRG1-AS1	-0.4864378	0.6204249	-0.784	0.433	-0.147534307	count	1
AL442128.2	-0.4864378	0.6600538	-0.737	0.461	-0.147534307	count	1
RBM44	-0.4864378	0.8268976	-0.5883	0.556	-0.147534307	count	1
KMT2E	-0.1039505	0.0559272	-1.8587	0.0632	-0.147528419	count	1
ABI2	-0.1092189	0.1167801	-0.9353	0.35	-0.147457757	count	1
AKIRIN1	-0.1085092	0.1040878	-1.0425	0.297	-0.14744907	count	1
SELENOH	-0.1036818	0.0597944	-1.734	0.083	-0.147300894	count	1
ARPP19	-0.1060797	0.0883391	-1.2008	0.23	-0.147189125	count	1
EIF3M	-0.1039657	0.0562414	-1.8486	0.0646	-0.147098072	count	1
KLHL13	-0.14474	0.2678606	-0.5404	0.589	-0.147097032	count	1
NUDT1	-0.1084482	0.1075223	-1.0086	0.313	-0.147054528	count	1
AC106707.1	-0.135445	0.2964011	-0.457	0.648	-0.146964993	count	1
LIN9	-0.1431319	0.291838	-0.4904	0.624	-0.14689352	count	1
REPIN1	-0.1148751	0.1451516	-0.7914	0.429	-0.146789928	count	1
BRSK1	-0.393993	0.5937506	-0.6636	0.507	-0.146787445	count	1
AC092803.2	-0.2362634	0.3167137	-0.746	0.456	-0.146752836	count	1
YY1AP1	-0.1187467	0.1786532	-0.6647	0.506	-0.14670539	count	1
SCRN3	-0.123454	0.202278	-0.6103	0.542	-0.146639381	count	1
PCIF1	-0.1160209	0.1477828	-0.7851	0.432	-0.146507164	count	1
CHCHD7	-0.11156	0.1378293	-0.8094	0.418	-0.146457688	count	1
MORF4L2	-0.1031995	0.0468553	-2.2025	0.0277	-0.146402996	count	1
TBC1D13	-0.1455324	0.3106116	-0.4685	0.639	-0.146366264	count	1
RBAK-RBAKDN	-0.1295398	0.1787834	-0.7246	0.469	-0.14628253	count	1
HACL1	-0.123449	0.175033	-0.7053	0.481	-0.146172335	count	1
PCBD1	-0.1034549	0.0587377	-1.7613	0.0783	-0.146103093	count	1
IPCEF1	-0.22204	1.213834	-0.1829	0.855	-0.145981247	count	1
SMAP1	-0.1046405	0.0833236	-1.2558	0.209	-0.145967814	count	1
LRRC24	-0.2717748	0.4703842	-0.5778	0.563	-0.145943835	count	1
AGTPBP1	-0.120369	0.2280461	-0.5278	0.598	-0.14577458	count	1
TEX26	-0.1822624	0.4280574	-0.4258	0.67	-0.145773153	count	1
WDR13	-0.1053507	0.0808544	-1.303	0.193	-0.145770175	count	1
HS1BP3	-0.1182175	0.1682594	-0.7026	0.482	-0.145758136	count	1

AC006449.2	-0.3359821	0.5340989	-0.6291	0.529	-0.145686721	count	1
SDC2	-0.1030658	0.0604632	-1.7046	0.0884	-0.145475969	count	1
TRIM35	-0.1650618	0.298325	-0.5533	0.58	-0.145448592	count	1
AC093297.2	-0.3352861	0.557364	-0.6016	0.548	-0.145369809	count	1
PAK1	-0.1225904	0.1874948	-0.6538	0.513	-0.145150362	count	1
RMRP	-0.4788946	0.4972886	-0.963	0.336	-0.14507887	count	1
AC104031.1	-0.3344353	0.5071543	-0.6594	0.51	-0.144982499	count	1
ZBTB26	-0.1812723	0.4416532	-0.4104	0.682	-0.144966434	count	1
EPB41L4A	-0.1511868	0.2645933	-0.5714	0.568	-0.144814136	count	1
UVRAG	-0.1068783	0.1066043	-1.0026	0.316	-0.144784315	count	1
COX7A1	-0.1012745	0.04222	-2.3987	0.0165	-0.14477644	count	1
AC120114.1	-0.6480226	0.7193875	-0.9008	0.368	-0.144643615	count	1
ADAMTS3	-0.1609501	0.4034888	-0.3989	0.69	-0.144586266	count	1
RALBP1	-0.101951	0.0604659	-1.6861	0.0919	-0.144576684	count	1
KIAA1671	-0.1125253	0.1989798	-0.5655	0.572	-0.144445278	count	1
CUL4B	-0.115644	0.18555	-0.6232	0.533	-0.144433542	count	1
AC092117.1	-0.6461512	1.003904	-0.6436	0.52	-0.144187081	count	1
CCDC59	-0.1036274	0.0820424	-1.2631	0.207	-0.144180763	count	1
AC114490.3	-0.2192859	0.5254798	-0.4173	0.676	-0.144121359	count	1
PRPF38A	-0.1073137	0.1219365	-0.8801	0.379	-0.144096372	count	1
ATP5PO	-0.1007832	0.0400338	-2.5175	0.0119	-0.144016556	count	1
RNASEH1	-0.1092179	0.1277461	-0.855	0.393	-0.143974161	count	1
PPM1G	-0.1024055	0.0711515	-1.4393	0.15	-0.14397351	count	1
AL133415.1	-0.4753443	0.4828779	-0.9844	0.325	-0.143924674	count	1
LINC01503	-0.1309092	0.2471502	-0.5297	0.596	-0.143835008	count	1
AC068338.3	-0.1461825	0.5388107	-0.2713	0.786	-0.143681043	count	1
FMC1	-0.1074688	0.1193497	-0.9005	0.368	-0.143663029	count	1
PKIA	-0.3314633	0.4940601	-0.6709	0.502	-0.14363018	count	1
HIGD1B	-0.1520955	0.3603205	-0.4221	0.673	-0.143598017	count	1
SERPINF2	-0.3858602	0.7600482	-0.5077	0.612	-0.143578454	count	1
ZFYVE27	-0.1227441	0.1980619	-0.6197	0.535	-0.143356586	count	1
IFITM2	-0.099651	0.0312907	-3.1847	0.00146	-0.143346673	count	1
NRSN2	-0.1149487	0.1795289	-0.6403	0.522	-0.143315182	count	1
NDUFA11	-0.1003932	0.0436617	-2.2993	0.0215	-0.143285169	count	1
9-Mar	-0.116194	0.1708718	-0.68	0.497	-0.143253142	count	1
AC013565.1	-0.6422332	0.4625661	-1.3884	0.165	-0.143231793	count	1
EMG1	-0.1086506	0.1130392	-0.9612	0.337	-0.143224645	count	1
NBPF26	-0.1985554	0.4093658	-0.485	0.628	-0.143167703	count	1
TRIM16	-0.1337424	0.2288916	-0.5843	0.559	-0.143076255	count	1
1-Sep	-0.1090392	0.1241018	-0.8786	0.38	-0.143035804	count	1
ZBTB42	-0.2665265	0.5296878	-0.5032	0.615	-0.143020867	count	1
CHST2	-0.2665265	0.5393374	-0.4942	0.621	-0.143020867	count	1
SLU7	-0.1029031	0.077739	-1.3237	0.186	-0.143014489	count	1
STK17A	-0.1026427	0.113367	-0.9054	0.365	-0.14294344	count	1
PPIB	-0.0994112	0.0290397	-3.4233	0.000625	-0.142915021	count	1

PPDPF	-0.0995897	0.0369301	-2.6967	0.00703	-0.142906714	count	1
MRPS21	-0.1002805	0.0460798	-2.1762	0.0296	-0.142832362	count	1
NDUFA5	-0.1005266	0.0500302	-2.0093	0.0446	-0.142599694	count	1
EMP2	-0.0995503	0.0401373	-2.4802	0.0132	-0.142598269	count	1
NDUFB5	-0.1007585	0.0573827	-1.7559	0.0792	-0.142287896	count	1
TYMS	-0.1248156	0.2192139	-0.5694	0.569	-0.142257956	count	1
UBALD1	-0.1211576	0.1948231	-0.6219	0.534	-0.142003787	count	1
RBM12B	-0.1175627	0.1811577	-0.649	0.516	-0.141998599	count	1
BID	-0.2160568	0.3336747	-0.6475	0.517	-0.141942133	count	1
SETD5	-0.1059129	0.1207848	-0.8769	0.381	-0.141841251	count	1
HMGXB3	-0.10694	0.1287426	-0.8306	0.406	-0.141839149	count	1
KYAT1	-0.1550385	0.3164897	-0.4899	0.624	-0.141750637	count	1
MYCBP	-0.1140472	0.1973154	-0.578	0.563	-0.141680684	count	1
AL731537.1	-0.3270495	0.4314281	-0.7581	0.448	-0.141623683	count	1
ZNF280C	-0.1680159	0.3890692	-0.4318	0.666	-0.14161356	count	1
CLEC4A	-0.2904526	0.6331941	-0.4587	0.646	-0.141607684	count	1
DMPK	-0.1091886	0.205295	-0.5319	0.595	-0.141582339	count	1
TRIAP1	-0.1053363	0.108596	-0.97	0.332	-0.141554015	count	1
NBPF3	-0.2050486	0.4727145	-0.4338	0.664	-0.141534531	count	1
CSNK2A2	-0.1041017	0.1054598	-0.9871	0.324	-0.141367691	count	1
DET1	-0.1601849	0.2727674	-0.5873	0.557	-0.141088356	count	1
PCF11-AS1	-0.4664396	0.6331791	-0.7367	0.461	-0.141033968	count	1
WDR46	-0.1074627	0.1284974	-0.8363	0.403	-0.140962925	count	1
TAF15	-0.1051329	0.1069305	-0.9832	0.326	-0.140920321	count	1
GNA14	-0.2427257	0.5112576	-0.4748	0.635	-0.140882264	count	1
ATAD1	-0.1043986	0.0961021	-1.0863	0.277	-0.140837091	count	1
MIR762HG	-0.1336171	0.2746361	-0.4865	0.627	-0.14073702	count	1
CEMIP2	-0.1358429	0.2661979	-0.5103	0.61	-0.140654222	count	1
RAN	-0.0982682	0.0372436	-2.6385	0.00836	-0.140598644	count	1
AL034549.1	-0.1948141	0.3358614	-0.58	0.562	-0.140409393	count	1
ATP5PD	-0.0980827	0.0421609	-2.3264	0.0201	-0.140195181	count	1
CLTCL1	-0.4636448	0.5852811	-0.7922	0.428	-0.140127953	count	1
SETD1B	-0.1317755	0.2439556	-0.5402	0.589	-0.139891467	count	1
POLR1E	-0.1081844	0.128598	-0.8413	0.4	-0.13987606	count	1
JOSD2	-0.099806	0.0858214	-1.163	0.245	-0.13987273	count	1
BMPR1B-DT	-0.2869928	0.8280816	-0.3466	0.729	-0.13985041	count	1
TRIM5	-0.1086731	0.1528743	-0.7109	0.477	-0.139791436	count	1
PITRM1	-0.1156322	0.2134716	-0.5417	0.588	-0.13965631	count	1
PRDX6	-0.0976358	0.0369483	-2.6425	0.00826	-0.139647256	count	1
ZNF827	-0.1243099	0.2486508	-0.4999	0.617	-0.139632586	count	1
CHD5	-0.4619844	0.9476759	-0.4875	0.626	-0.139589978	count	1
LILRB3	-0.4619844	1.101746	-0.4193	0.675	-0.139589978	count	1
PIK3R3	-0.1269988	0.3415908	-0.3718	0.71	-0.139507481	count	1
ABHD17A	-0.0995132	0.0739837	-1.3451	0.179	-0.139471621	count	1
ATP11B	-0.1099812	0.196891	-0.5586	0.576	-0.139442215	count	1

RALB	-0.1055074	0.160495	-0.6574	0.511	-0.139343519	count	1
CREB3L4	-0.1417681	0.2591787	-0.547	0.584	-0.139295759	count	1
BRMS1	-0.1060062	0.1325883	-0.7995	0.424	-0.139250238	count	1
PEX12	-0.1549906	0.3772893	-0.4108	0.681	-0.139159036	count	1
ABL2	-0.1343755	0.196559	-0.6836	0.494	-0.139121136	count	1
TMEM120A	-0.1002995	0.0926778	-1.0822	0.279	-0.1389651	count	1
UBE2E3	-0.098561	0.059581	-1.6542	0.0982	-0.138912151	count	1
STX17	-0.1048242	0.1112487	-0.9423	0.346	-0.138864779	count	1
LZTFL1	-0.115507	0.1699533	-0.6796	0.497	-0.138763638	count	1
AP1AR	-0.114285	0.192475	-0.5938	0.553	-0.13871707	count	1
RABAC1	-0.0965215	0.0284594	-3.3916	0.000702	-0.138712296	count	1
PRKCB	-0.623495	0.7299677	-0.8541	0.393	-0.138672711	count	1
STRA6	-0.623495	0.7429218	-0.8392	0.401	-0.138672711	count	1
COX6A2	-0.623495	0.7429218	-0.8392	0.401	-0.138672711	count	1
CELF4	-0.623495	0.8169485	-0.7632	0.445	-0.138672711	count	1
AKR7A3	-0.623495	0.9383757	-0.6644	0.506	-0.138672711	count	1
AL162632.3	-0.623495	0.9383757	-0.6644	0.506	-0.138672711	count	1
EMC6	-0.0995894	0.0838215	-1.1881	0.235	-0.138659446	count	1
THTPA	-0.1188909	0.1543127	-0.7705	0.441	-0.138315114	count	1
RALY	-0.0977456	0.063688	-1.5348	0.125	-0.138289146	count	1
CHEK1	-0.2575181	0.4199935	-0.6131	0.54	-0.13801241	count	1
ITGB3	-0.1996997	0.5131156	-0.3892	0.697	-0.137753875	count	1
GATA2-AS1	-0.1153076	0.2606401	-0.4424	0.658	-0.137741951	count	1
MIR181A2HG	-0.1996408	0.4712934	-0.4236	0.672	-0.137712269	count	1
CCR7	-1.074955	0.9278361	-1.1586	0.247	-0.137667679	count	1
LINC01752	-1.074955	0.9278361	-1.1586	0.247	-0.137667679	count	1
GET4	-1.074955	1.041201	-1.0324	0.302	-0.137667679	count	1
POLR3H	-0.1100545	0.1775901	-0.6197	0.535	-0.137659068	count	1
FCF1	-0.1110251	0.1557301	-0.7129	0.476	-0.137657337	count	1
DCTN6	-0.0980323	0.0677453	-1.4471	0.148	-0.137648948	count	1
ST13	-0.0961324	0.0402008	-2.3913	0.0168	-0.137578081	count	1
TMEM150A	-0.1103088	0.1601769	-0.6887	0.491	-0.137509439	count	1
FAM131C	-0.1594106	0.4036805	-0.3949	0.693	-0.137424022	count	1
RNF8	-0.1016252	0.1234235	-0.8234	0.41	-0.13733481	count	1
CASTOR2	-0.1833501	0.4162389	-0.4405	0.66	-0.137257886	count	1
SLC2A11	-0.1155082	0.278783	-0.4143	0.679	-0.13715518	count	1
PSMG1	-0.100693	0.1062467	-0.9477	0.343	-0.136892231	count	1
TBCK	-0.1238438	0.2357944	-0.5252	0.599	-0.136833664	count	1
TMC4	-0.4533804	0.5081813	-0.8922	0.372	-0.136805658	count	1
NOP16	-0.1155713	0.1690738	-0.6836	0.494	-0.136798287	count	1
EHD2	-0.0986282	0.0883409	-1.1165	0.264	-0.136665447	count	1
TBL2	-0.1041125	0.1486465	-0.7004	0.484	-0.136456723	count	1
MYL12B	-0.094722	0.0268657	-3.5258	0.000427	-0.136331626	count	1
MBNL1	-0.0952979	0.0570368	-1.6708	0.0948	-0.136162158	count	1
HSF2	-0.1272393	0.1829424	-0.6955	0.487	-0.136064725	count	1

ABHD17C	-0.2790833	0.4223405	-0.6608	0.509	-0.135838797	count	1
AC005261.3	-0.279034	0.4353112	-0.641	0.522	-0.13581382	count	1
SMIM8	-0.1029544	0.1328608	-0.7749	0.438	-0.135699747	count	1
BCAN	-1.06091	0.6936161	-1.5295	0.126	-0.135689465	count	1
FCGR1B	-1.06091	0.8158307	-1.3004	0.194	-0.135689465	count	1
PSKH1	-0.1165536	0.255394	-0.4564	0.648	-0.135581214	count	1
SYTL3	-0.3135945	0.5457522	-0.5746	0.566	-0.135520856	count	1
AKT1	-0.1002292	0.0885425	-1.132	0.258	-0.135492252	count	1
COMMD3	-0.0969009	0.077213	-1.255	0.21	-0.135272813	count	1
FMR1	-0.1006402	0.1203294	-0.8364	0.403	-0.13523274	count	1
LOXL3	-0.1331555	0.2564276	-0.5193	0.604	-0.135213854	count	1
MVB12B	-0.1455325	0.3771456	-0.3859	0.7	-0.135202953	count	1
TMEM231	-0.121556	0.2638898	-0.4606	0.645	-0.135060128	count	1
SUCLG2	-0.0981274	0.0857438	-1.1444	0.253	-0.135000452	count	1
ATP2B1-AS1	-0.109707	0.190552	-0.5757	0.565	-0.13494801	count	1
NCOA6	-0.116418	0.2046762	-0.5688	0.57	-0.134900111	count	1
ALDH7A1	-0.0995709	0.1174999	-0.8474	0.397	-0.134870707	count	1
SKP1	-0.0936856	0.024957	-3.7539	0.000177	-0.134797264	count	1
ZNF557	-0.1130269	0.2471528	-0.4573	0.647	-0.134605492	count	1
MAGOHB	-0.1008978	0.1159182	-0.8704	0.384	-0.134601166	count	1
C10orf25	-0.2170559	0.6293731	-0.3449	0.73	-0.134487026	count	1
EEF1A1	-0.0929589	0.019807	-4.6932	2.79E-06	-0.134068285	count	0.06465546
FCN1	-0.2753005	0.974856	-0.2824	0.778	-0.13392303	count	1
TRMT112	-0.0934851	0.0343253	-2.7235	0.00649	-0.133905578	count	1
GAMT	-0.0958276	0.0830765	-1.1535	0.249	-0.133831456	count	1
ARHGEF33	-0.203993	0.5438502	-0.3751	0.708	-0.133814408	count	1
UIMC1	-0.1054986	0.1480664	-0.7125	0.476	-0.13374131	count	1
AC141928.1	-1.046231	0.5237244	-1.9977	0.0458	-0.133622478	count	1
SAMD1	-0.10354	0.1490204	-0.6948	0.487	-0.133451695	count	1
STX3	-0.1214546	0.2489746	-0.4878	0.626	-0.133374931	count	1
LRRC8C	-0.1312409	0.3927476	-0.3342	0.738	-0.133251606	count	1
AP000692.2	-0.1850073	0.549007	-0.337	0.736	-0.133189388	count	1
RITA1	-0.1090445	0.1975735	-0.5519	0.581	-0.132960061	count	1
DCAF13	-0.0985471	0.1126364	-0.8749	0.382	-0.132832322	count	1
STAT4	-0.5988679	0.7408152	-0.8084	0.419	-0.132706169	count	1
CCM2	-0.0999189	0.1237117	-0.8077	0.419	-0.132431661	count	1
NDEL1	-0.0998957	0.149084	-0.6701	0.503	-0.132400851	count	1
CWC27	-0.0985524	0.1058173	-0.9313	0.352	-0.132312644	count	1
CCDC3	-0.0954751	0.0741734	-1.2872	0.198	-0.132274751	count	1
CMPK1	-0.0940521	0.0614424	-1.5307	0.126	-0.132265096	count	1
PRRT3	-0.1277827	0.2264177	-0.5644	0.573	-0.132236664	count	1
TLE2	-0.0996698	0.1732504	-0.5753	0.565	-0.13186435	count	1
C2orf74	-0.0956546	0.0920124	-1.0396	0.299	-0.131666408	count	1
DDA1	-0.0968239	0.1125497	-0.8603	0.39	-0.131625062	count	1
TRAPPC9	-0.1239925	0.2559269	-0.4845	0.628	-0.131564364	count	1

CSTF2	-0.1416582	0.2883755	-0.4912	0.623	-0.131560483	count	1
HOXB-AS1	-0.1025543	0.1301187	-0.7882	0.431	-0.131462722	count	1
TNFAIP1	-0.1061519	0.1835671	-0.5783	0.563	-0.131343651	count	1
ZC3H4	-0.1102968	0.1980615	-0.5569	0.578	-0.131339072	count	1
ADRM1	-0.0934188	0.0664826	-1.4052	0.16	-0.131198196	count	1
PHC1	-0.1075179	0.2899531	-0.3708	0.711	-0.131091157	count	1
PTK2	-0.0932384	0.0780566	-1.1945	0.232	-0.130841863	count	1
DPM1	-0.0982461	0.1208335	-0.8131	0.416	-0.130789165	count	1
LINC00960	-0.1288175	0.3861581	-0.3336	0.739	-0.130768602	count	1
AC015982.1	-0.4345503	0.4556785	-0.9536	0.34	-0.130732493	count	1
NACA2	-0.4344154	0.5991491	-0.7251	0.468	-0.130689083	count	1
PLEKHJ1	-0.0949818	0.1055752	-0.8997	0.368	-0.130668206	count	1
AC022916.1	-0.434287	0.6878351	-0.6314	0.528	-0.130647774	count	1
NCBP2-AS2	-0.0955064	0.0919461	-1.0387	0.299	-0.130605826	count	1
AC020928.2	-0.4339366	0.7969765	-0.5445	0.586	-0.130535037	count	1
LINC00242	-0.4339366	0.9272678	-0.468	0.64	-0.130535037	count	1
MESP1	-0.1272884	0.3080174	-0.4133	0.679	-0.13049133	count	1
TRBC1	-1.0238048	0.5921782	-1.7289	0.0839	-0.130466246	count	1
SMAD7	-0.098621	0.1962037	-0.5026	0.615	-0.130393878	count	1
LINC02018	-1.0232841	0.6185961	-1.6542	0.0982	-0.130392998	count	1
ZNHIT1	-0.0916161	0.0524076	-1.7481	0.0805	-0.130348085	count	1
CENPP	-0.154615	0.2203378	-0.7017	0.483	-0.130147494	count	1
SERF1A	-0.2677669	0.5314473	-0.5038	0.614	-0.1301132	count	1
HNRNPR	-0.0917804	0.0539877	-1.7	0.0892	-0.130022448	count	1
UROS	-0.0977561	0.1152122	-0.8485	0.396	-0.129782604	count	1
MAPK8IP3	-0.1625001	0.3703658	-0.4388	0.661	-0.129699406	count	1
WASL	-0.0916355	0.0583066	-1.5716	0.116	-0.129664857	count	1
KANSL1-AS1	-0.1027601	0.1104684	-0.9302	0.352	-0.129519572	count	1
RELA	-0.0957844	0.131414	-0.7289	0.466	-0.129519241	count	1
LINC02104	-0.2236402	0.4761901	-0.4696	0.639	-0.129468331	count	1
FBXW4	-0.1080473	0.1875453	-0.5761	0.565	-0.12940161	count	1
DHRS4-AS1	-0.1000864	0.1270229	-0.7879	0.431	-0.129379494	count	1
TNFRSF10C	-0.3496684	0.4881281	-0.7163	0.474	-0.129377624	count	1
NAPA-AS1	-0.1574992	0.349033	-0.4512	0.652	-0.129251129	count	1
ARMC6	-0.1156809	0.2920681	-0.3961	0.692	-0.129198239	count	1
PGPEP1L	-0.5842695	0.6421088	-0.9099	0.363	-0.129183491	count	1
AC103691.1	-0.5842695	0.6839219	-0.8543	0.393	-0.129183491	count	1
AC018553.1	-1.0139492	0.7556987	-1.3417	0.18	-0.129079961	count	1
C2orf15	-1.0139492	0.8040465	-1.2611	0.207	-0.129079961	count	1
CCDC158	-0.1793546	0.5567781	-0.3221	0.747	-0.129034486	count	1
MITD1	-0.0955848	0.1140483	-0.8381	0.402	-0.128976231	count	1
AL132780.2	-0.2989228	0.4620439	-0.647	0.518	-0.128890235	count	1
BCL2A1	-0.1965532	0.6383858	-0.3079	0.758	-0.128812957	count	1
MED1	-0.1033559	0.1757105	-0.5882	0.556	-0.128812596	count	1
SDHAF4	-0.1187993	0.2181156	-0.5447	0.586	-0.128776271	count	1

SNRNP35	-0.0937874	0.1036856	-0.9045	0.366	-0.128751547	count	1
ADAMTS9-AS1	-1.0114654	0.8207017	-1.2324	0.2179	-0.128730685	count	1
DDR1	-0.1165117	0.3723141	-0.3129	0.754	-0.128679363	count	1
MLIP	-0.4278758	0.5669668	-0.7547	0.45	-0.12858664	count	1
CEP63	-0.0937493	0.1037998	-0.9032	0.366	-0.128374711	count	1
ADCY4	-0.5808483	0.7085419	-0.8198	0.412	-0.128359502	count	1
MTX2	-0.0947945	0.1130522	-0.8385	0.402	-0.128134833	count	1
CRYZL1	-0.0950897	0.1108811	-0.8576	0.391	-0.128018175	count	1
MSRB2	-0.0903522	0.0584214	-1.5466	0.122	-0.127927434	count	1
ZNF337	-0.1145438	0.2485181	-0.4609	0.645	-0.127920336	count	1
TSTD3	-0.115161	0.2302114	-0.5002	0.617	-0.127909395	count	1
RIPOR1	-0.1010428	0.1771635	-0.5703	0.568	-0.127899437	count	1
ODF3B	-0.0972867	0.1172832	-0.8295	0.407	-0.127679782	count	1
APTX	-0.1041684	0.166674	-0.625	0.532	-0.12756431	count	1
TP53TG3D	-0.2623858	0.5513113	-0.4759	0.634	-0.127396424	count	1
GALNT14	-1.0012349	0.8737926	-1.1458	0.252	-0.127292431	count	1
ZNF697	-1.0012349	0.9725637	-1.0295	0.303	-0.127292431	count	1
ALDH1L1-AS2	-1.0012349	1.049725	-0.9538	0.34	-0.127292431	count	1
AC006547.3	-1.0012349	1.049725	-0.9538	0.34	-0.127292431	count	1
OXTR	-0.1702668	0.5562957	-0.3061	0.76	-0.127275728	count	1
GRPEL1	-0.0947938	0.1108409	-0.8552	0.392	-0.127204537	count	1
PLAUR	-0.0943535	0.1393706	-0.677	0.498	-0.127171213	count	1
STOML2	-0.0907225	0.0689395	-1.316	0.188	-0.12692291	count	1
LRRCC1	-0.0953467	0.1357457	-0.7024	0.482	-0.126922447	count	1
SEMA3B-AS1	-0.5746117	0.5638804	-1.019	0.308	-0.12685899	count	1
RNPC3	-0.0926862	0.1400764	-0.6617	0.508	-0.126593918	count	1
DDX60	-0.1035221	0.2064336	-0.5015	0.616	-0.126488639	count	1
PCDHGB5	-0.2185849	0.4648067	-0.4703	0.638	-0.126453849	count	1
GPC6	-0.0937463	0.0949003	-0.9878	0.323	-0.12644556	count	1
TRAPPC11	-0.1046827	0.2104685	-0.4974	0.619	-0.126377438	count	1
UNC45A	-0.0953235	0.1153294	-0.8265	0.409	-0.126329472	count	1
EXOC6B	-0.0997794	0.1861661	-0.536	0.592	-0.126295475	count	1
POU6F2	-0.341468	0.4641026	-0.7358	0.462	-0.126178464	count	1
CDK19	-0.1122991	0.2492571	-0.4505	0.652	-0.126060557	count	1
RPS17	-0.0951568	0.1169445	-0.8137	0.416	-0.125882127	count	1
EDEM2	-0.0981235	0.2081013	-0.4715	0.637	-0.125623368	count	1
ZNF426-DT	-0.3399651	0.5265431	-0.6457	0.519	-0.125592893	count	1
TDRKH	-0.3390676	0.5526259	-0.6136	0.54	-0.125243322	count	1
ITPR1-DT	-0.3390676	0.6260135	-0.5416	0.588	-0.125243322	count	1
PPP3CC	-0.0996991	0.1732431	-0.5755	0.565	-0.125071174	count	1
EFHD2	-0.1050458	0.2244377	-0.468	0.64	-0.125058502	count	1
BTN2A1	-0.0963783	0.146279	-0.6589	0.51	-0.125047603	count	1
IPO8	-0.0997083	0.1554576	-0.6414	0.521	-0.124881641	count	1
ZBED8	-0.1449771	0.3138135	-0.462	0.644	-0.124809115	count	1
CLUAP1	-0.091666	0.1027064	-0.8925	0.372	-0.1247102	count	1

CGRRF1	-0.0935407	0.1110848	-0.8421	0.4	-0.1246435	count	1
HSPBP1	-0.0954628	0.1521756	-0.6273	0.53	-0.124607562	count	1
CD36	-0.1128427	0.354851	-0.318	0.751	-0.124601284	count	1
AC093827.5	-0.337396	0.6524996	-0.5171	0.605	-0.12459246	count	1
GNG8	-0.337396	0.6555459	-0.5147	0.607	-0.12459246	count	1
GIMAP2	-0.337396	0.7011377	-0.4812	0.63	-0.12459246	count	1
AP001830.1	-0.337396	0.7096095	-0.4755	0.634	-0.12459246	count	1
FAM184B	-0.18097	0.3617517	-0.5003	0.617	-0.124550102	count	1
ODC1	-0.0912816	0.0929625	-0.9819	0.326	-0.124486315	count	1
MAP4K3	-0.0984337	0.1500194	-0.6561	0.512	-0.124232727	count	1
PECAM1	-0.1264741	0.73535	-0.172	0.863	-0.124123538	count	1
RHOG	-0.0900264	0.1055254	-0.8531	0.394	-0.12406326	count	1
ZGPAT	-0.3358706	0.7404936	-0.4536	0.65	-0.123998791	count	1
GNRH2	-0.3358706	0.7917011	-0.4242	0.671	-0.123998791	count	1
MRPL57	-0.0874986	0.056667	-1.5441	0.123	-0.123990329	count	1
ANXA2R	-0.255251	0.5884723	-0.4338	0.664	-0.123800108	count	1
GLRX5	-0.0880967	0.0609834	-1.4446	0.149	-0.123704915	count	1
PAPOLA	-0.0880892	0.073554	-1.1976	0.231	-0.123687026	count	1
PPP1R11	-0.0894749	0.0959864	-0.9322	0.351	-0.123563211	count	1
POLR2J2	-0.1273715	0.3522765	-0.3616	0.718	-0.123434934	count	1
HPRT1	-0.0910097	0.1126982	-0.8076	0.419	-0.123415311	count	1
LIPT2	-0.2309641	0.4552723	-0.5073	0.612	-0.123313736	count	1
FAM229A	-0.1790476	0.6315164	-0.2835	0.777	-0.123197953	count	1
BBX	-0.0871065	0.059684	-1.4595	0.145	-0.123131242	count	1
CNOT7	-0.0902553	0.1209138	-0.7464	0.455	-0.122988847	count	1
ZNF577	-0.1053635	0.2448631	-0.4303	0.667	-0.122958971	count	1
AC026979.3	-0.3331866	0.7331535	-0.4545	0.65	-0.122954782	count	1
MBD2	-0.0876967	0.0732329	-1.1975	0.231	-0.12289183	count	1
CALR	-0.0858586	0.0372113	-2.3073	0.0211	-0.122809057	count	1
AC018647.1	-0.1497484	0.2907296	-0.5151	0.607	-0.122792934	count	1
IMPA1	-0.0959975	0.1366243	-0.7026	0.482	-0.122750345	count	1
TIGD5	-0.118646	0.2974478	-0.3989	0.69	-0.12270515	count	1
BORCS7	-0.0876301	0.069124	-1.2677	0.205	-0.122703354	count	1
TRIM66	-0.2850601	0.478738	-0.5954	0.552	-0.122648725	count	1
MTMR6	-0.0944194	0.1172895	-0.805	0.421	-0.122611335	count	1
RBAK	-0.1394013	0.3380939	-0.4123	0.68	-0.122546587	count	1
AC092794.1	-0.9664784	0.7213596	-1.3398	0.18	-0.122411915	count	1
ZFR	-0.0873075	0.0706736	-1.2354	0.217	-0.122403911	count	1
WIPF3	-0.1492576	0.3404755	-0.4384	0.661	-0.122384299	count	1
OSBPL5	-0.1041242	0.2077224	-0.5013	0.616	-0.122371922	count	1
TYW3	-0.0877746	0.0767033	-1.1443	0.253	-0.122355073	count	1
FBXL6	-0.108981	0.236253	-0.4613	0.645	-0.12231401	count	1
AP006623.1	-0.251963	0.494685	-0.5093	0.611	-0.122145039	count	1
VIPAS39	-0.1093894	0.2004839	-0.5456	0.585	-0.122129549	count	1
METTL18	-0.0993119	0.186838	-0.5315	0.595	-0.122115816	count	1

MORF4L2-AS1	-0.2287288	0.5310688	-0.4307	0.667	-0.122080895	count	1
BAG1	-0.0862492	0.0609081	-1.4161	0.157	-0.122076775	count	1
PARD6A	-0.112642	0.2122898	-0.5306	0.596	-0.122056678	count	1
TEAD3	-0.0990214	0.163143	-0.607	0.544	-0.1220076	count	1
TFAM	-0.0887789	0.0958164	-0.9266	0.354	-0.121964824	count	1
CMAS	-0.0896981	0.110136	-0.8144	0.415	-0.121960716	count	1
TUSC2	-0.0897907	0.1034401	-0.868	0.385	-0.121945034	count	1
USO1	-0.0881307	0.0916135	-0.962	0.336	-0.121922553	count	1
TLE6	-0.4070629	0.6528032	-0.6236	0.533	-0.121918834	count	1
UBALD2	-0.0892483	0.0991742	-0.8999	0.368	-0.12189286	count	1
C1D	-0.0874607	0.0662135	-1.3209	0.187	-0.121858053	count	1
ERAP1	-0.0983229	0.1845301	-0.5328	0.594	-0.121855368	count	1
C19orf66	-0.089077	0.1100821	-0.8092	0.418	-0.121803597	count	1
BEX3	-0.0848715	0.0385885	-2.1994	0.0279	-0.12171093	count	1
AC009812.4	-0.4063158	0.686637	-0.5917	0.554	-0.121680156	count	1
AF213884.3	-0.4063158	0.8857935	-0.4587	0.646	-0.121680156	count	1
COG4	-0.0932654	0.1939498	-0.4809	0.631	-0.121633172	count	1
UBE2M	-0.0857238	0.0569199	-1.506	0.132	-0.12151786	count	1
LARS2	-0.210154	0.4579975	-0.4589	0.646	-0.121434806	count	1
ATP5F1A	-0.0852585	0.0534129	-1.5962	0.111	-0.121326402	count	1
AC132872.1	-0.4052065	0.6486255	-0.6247	0.532	-0.121325853	count	1
PLXND1	-0.0942525	0.1658698	-0.5682	0.57	-0.12132479	count	1
AC002456.1	-0.5514368	0.6704378	-0.8225	0.411	-0.121301136	count	1
AC006483.2	-0.5514368	0.7650159	-0.7208	0.471	-0.121301136	count	1
CYTH4	-0.5514368	0.8252858	-0.6682	0.504	-0.121301136	count	1
SOD3	-0.0841421	0.0316908	-2.6551	0.00796	-0.121165752	count	1
SEC61G	-0.0848071	0.0424132	-1.9995	0.0456	-0.12115679	count	1
PLEKHA3	-0.0865473	0.0777635	-1.113	0.266	-0.121077961	count	1
ATF2	-0.0916156	0.1243009	-0.737	0.461	-0.121038666	count	1
PAK2	-0.0856822	0.0664481	-1.2895	0.197	-0.120974606	count	1
ZSCAN5A	-0.1281834	0.4618991	-0.2775	0.781	-0.120783414	count	1
AES	-0.0847452	0.0512062	-1.655	0.098	-0.120715173	count	1
AP4S1	-0.1042342	0.2143012	-0.4864	0.627	-0.120711909	count	1
AC243547.2	-0.9537942	1.0293868	-0.9266	0.354	-0.120633396	count	1
ASPM	-0.9537942	1.0293868	-0.9266	0.354	-0.120633396	count	1
LGALS8-AS1	-0.9537942	1.0293868	-0.9266	0.354	-0.120633396	count	1
MIR3945HG	-0.9537942	1.0293868	-0.9266	0.354	-0.120633396	count	1
GDNF-AS1	-0.9537942	1.0293868	-0.9266	0.354	-0.120633396	count	1
AC016596.1	-0.9537942	1.0293868	-0.9266	0.354	-0.120633396	count	1
AC084346.2	-0.9537942	1.0293868	-0.9266	0.354	-0.120633396	count	1
EPPK1	-0.9537942	1.0293868	-0.9266	0.354	-0.120633396	count	1
SCNN1A	-0.9537942	1.0293868	-0.9266	0.354	-0.120633396	count	1
CYP27B1	-0.9537942	1.0293868	-0.9266	0.354	-0.120633396	count	1
AC107032.2	-0.9537942	1.0293868	-0.9266	0.354	-0.120633396	count	1
AC026367.1	-0.9537942	1.0293868	-0.9266	0.354	-0.120633396	count	1

MED4-AS1	-0.9537942	1.0293868	-0.9266	0.354	-0.120633396	count	1
LINC00565	-0.9537942	1.0293868	-0.9266	0.354	-0.120633396	count	1
AC024337.1	-0.9537942	1.0293868	-0.9266	0.354	-0.120633396	count	1
AC084809.1	-0.9537942	1.0293868	-0.9266	0.354	-0.120633396	count	1
AC020916.2	-0.9537942	1.0293868	-0.9266	0.354	-0.120633396	count	1
ZNF404	-0.9537942	1.0293868	-0.9266	0.354	-0.120633396	count	1
IFNLR1	-0.9537942	1.1056466	-0.8627	0.388	-0.120633396	count	1
NR5A2	-0.9537942	1.1056466	-0.8627	0.388	-0.120633396	count	1
AL512328.1	-0.9537942	1.1056466	-0.8627	0.388	-0.120633396	count	1
RGS7	-0.9537942	1.1056466	-0.8627	0.388	-0.120633396	count	1
C2orf50	-0.9537942	1.1056466	-0.8627	0.388	-0.120633396	count	1
MYCN	-0.9537942	1.1056466	-0.8627	0.388	-0.120633396	count	1
CFLAR-AS1	-0.9537942	1.1056466	-0.8627	0.388	-0.120633396	count	1
AC007370.2	-0.9537942	1.1056466	-0.8627	0.388	-0.120633396	count	1
1-Mar	-0.9537942	1.1056466	-0.8627	0.388	-0.120633396	count	1
AC114947.2	-0.9537942	1.1056466	-0.8627	0.388	-0.120633396	count	1
FAM169A	-0.9537942	1.1056466	-0.8627	0.388	-0.120633396	count	1
LINC01933	-0.9537942	1.1056466	-0.8627	0.388	-0.120633396	count	1
AL096711.2	-0.9537942	1.1056466	-0.8627	0.388	-0.120633396	count	1
AC103724.4	-0.9537942	1.1056466	-0.8627	0.388	-0.120633396	count	1
TMEM229B	-0.9537942	1.1056466	-0.8627	0.388	-0.120633396	count	1
AC025043.1	-0.9537942	1.1056466	-0.8627	0.388	-0.120633396	count	1
PAQR5	-0.9537942	1.1056466	-0.8627	0.388	-0.120633396	count	1
AC046168.2	-0.9537942	1.1056466	-0.8627	0.388	-0.120633396	count	1
HBA2	-0.9537942	1.1056466	-0.8627	0.388	-0.120633396	count	1
BICDL2	-0.9537942	1.1056466	-0.8627	0.388	-0.120633396	count	1
AC087289.5	-0.9537942	1.1056466	-0.8627	0.388	-0.120633396	count	1
AC027601.4	-0.9537942	1.1056466	-0.8627	0.388	-0.120633396	count	1
MYO1F	-0.9537942	1.1056466	-0.8627	0.388	-0.120633396	count	1
NTN5	-0.9537942	1.1056466	-0.8627	0.388	-0.120633396	count	1
AC092070.3	-0.9537942	1.1056466	-0.8627	0.388	-0.120633396	count	1
HPF1	-0.0862594	0.073571	-1.1725	0.241	-0.120550621	count	1
NDUFB1	-0.0846819	0.0511717	-1.6549	0.098	-0.120344501	count	1
NQO2	-0.0870979	0.0818729	-1.0638	0.287	-0.120314666	count	1
AC145285.2	-0.5464984	0.6478946	-0.8435	0.399	-0.12012054	count	1
ASNA1	-0.0870405	0.09224	-0.9436	0.345	-0.120084069	count	1
EDARADD	-0.9498162	0.7085965	-1.3404	0.18	-0.120075951	count	1
BX890604.1	-0.9498162	0.7085965	-1.3404	0.18	-0.120075951	count	1
PFKL	-0.0899819	0.1207792	-0.745	0.456	-0.11995453	count	1
NSRP1	-0.0847469	0.0639005	-1.3262	0.185	-0.119924485	count	1
CMC1	-0.0872885	0.0885826	-0.9854	0.324	-0.119722987	count	1
KBTBD6	-0.1192017	0.2612795	-0.4562	0.648	-0.119655917	count	1
HAUS6	-0.1083607	0.1828074	-0.5928	0.553	-0.119621713	count	1
DCAF11	-0.1004373	0.1779091	-0.5645	0.572	-0.119548563	count	1
AL591846.2	-0.1935136	0.4449749	-0.4349	0.664	-0.119527662	count	1

DHTKD1	-0.1933904	0.3531116	-0.5477	0.584	-0.119449594	count	1
ADAM32	-0.2775538	0.483943	-0.5735	0.566	-0.119278783	count	1
GRASP	-0.0941198	0.1994753	-0.4718	0.637	-0.119276228	count	1
KIAA0319L	-0.101099	0.2450909	-0.4125	0.68	-0.119202286	count	1
TRIP12	-0.0870429	0.105886	-0.822	0.411	-0.119046704	count	1
ATMIN	-0.0898039	0.1412327	-0.6359	0.525	-0.118931201	count	1
RANBP17	-0.2056713	0.6348831	-0.324	0.746	-0.118770455	count	1
TMEM200A	-0.1052122	0.2481	-0.4241	0.672	-0.118659248	count	1
CD2	-0.9391266	0.6665373	-1.409	0.159	-0.118578801	count	1
NHLRC1	-0.1650327	0.4131938	-0.3994	0.69	-0.118529901	count	1
USP27X-AS1	-0.1650327	0.4923802	-0.3352	0.738	-0.118529901	count	1
FANCA	-0.2447543	1.031088	-0.2374	0.812	-0.118521471	count	1
RAB11FIP3	-0.1239755	0.2657217	-0.4666	0.641	-0.118490457	count	1
CNBP	-0.08256	0.0315206	-2.6192	0.00885	-0.118411248	count	1
KLHDC3	-0.0885591	0.1201901	-0.7368	0.461	-0.118288142	count	1
DNAJC4	-0.086709	0.1010569	-0.858	0.391	-0.118243129	count	1
DPY30	-0.0840403	0.0684301	-1.2281	0.219	-0.118107574	count	1
RERE	-0.0859588	0.1139793	-0.7542	0.451	-0.118040426	count	1
SLC20A2	-0.0938721	0.1557463	-0.6027	0.547	-0.117924977	count	1
KDM4B	-0.0964936	0.2312623	-0.4172	0.677	-0.117869922	count	1
PRCC	-0.0911015	0.1371086	-0.6644	0.506	-0.117738039	count	1
CCNG1	-0.0859451	0.1023232	-0.8399	0.401	-0.117624112	count	1
PGAM5	-0.1312253	0.3136329	-0.4184	0.676	-0.117569034	count	1
FANCF	-0.0987328	0.2065261	-0.4781	0.633	-0.117511171	count	1
NEK3	-0.0921194	0.2203468	-0.4181	0.676	-0.117494363	count	1
SPIN4	-0.1635024	0.4062228	-0.4025	0.687	-0.1174094	count	1
NIN	-0.0943987	0.1565489	-0.603	0.547	-0.117406296	count	1
ZNF667-AS1	-0.0855055	0.0940304	-0.9093	0.363	-0.117323146	count	1
UPF3B	-0.086984	0.12475	-0.6973	0.486	-0.117311882	count	1
FAM161B	-0.1089709	0.225701	-0.4828	0.629	-0.117241493	count	1
CDC42SE2	-0.0957299	0.1633073	-0.5862	0.558	-0.117194078	count	1
CFAP57	-0.391523	0.6979172	-0.561	0.575	-0.11696404	count	1
EPM2A	-0.0991962	0.3183322	-0.3116	0.755	-0.116948829	count	1
IPO9-AS1	-0.2192999	0.4247276	-0.5163	0.606	-0.116888344	count	1
PITPNA-AS1	-0.0920857	0.1369654	-0.6723	0.501	-0.116849298	count	1
AL355488.1	-0.9260301	0.8549891	-1.0831	0.279	-0.116746271	count	1
AL139412.1	-0.9260301	0.8549891	-1.0831	0.279	-0.116746271	count	1
LINC01963	-0.9260301	0.8549891	-1.0831	0.279	-0.116746271	count	1
MYH3	-0.9260301	0.8549891	-1.0831	0.279	-0.116746271	count	1
AF129408.1	-0.9260301	0.8549891	-1.0831	0.279	-0.116746271	count	1
AP004609.1	-0.9260301	0.9226737	-1.0036	0.316	-0.116746271	count	1
AC005224.1	-0.9260301	1.044973	-0.8862	0.376	-0.116746271	count	1
PRTFDC1	-0.0889319	0.1401123	-0.6347	0.526	-0.116693336	count	1
VIPR2	-0.1002126	0.2286084	-0.4384	0.661	-0.116482969	count	1
RRM1	-0.0951322	0.1487107	-0.6397	0.522	-0.116459783	count	1

LCP1	-0.3164421	0.6090115	-0.5196	0.603	-0.116458873	count	1
ZBTB7B	-0.111577	0.2927323	-0.3812	0.703	-0.116368976	count	1
PURA	-0.0823603	0.0645723	-1.2755	0.202	-0.116367236	count	1
KIF18A	-0.201529	0.7053083	-0.2857	0.775	-0.116311076	count	1
GFOD2	-0.0993259	0.216824	-0.4581	0.647	-0.116300273	count	1
RABIF	-0.0941508	0.1633036	-0.5765	0.564	-0.116218775	count	1
NT5C	-0.0827358	0.065534	-1.2625	0.207	-0.116179968	count	1
RNF20	-0.0896615	0.1141337	-0.7856	0.432	-0.11587274	count	1
MAN1B1-DT	-0.1500058	0.2903651	-0.5166	0.605	-0.115866657	count	1
PPP6R1	-0.1180108	0.2220149	-0.5315	0.595	-0.115741784	count	1
UBE2V1	-0.1002936	0.199249	-0.5034	0.615	-0.115660382	count	1
GFER	-0.089222	0.1652235	-0.54	0.589	-0.11563517	count	1
HNRNPD	-0.0814661	0.0517493	-1.5742	0.116	-0.115453857	count	1
CLIC3	-0.0835228	0.1392524	-0.5998	0.549	-0.115336256	count	1
PRKRA	-0.0833418	0.0858655	-0.9706	0.332	-0.115308285	count	1
AL034417.3	-0.3134284	0.5036529	-0.6223	0.534	-0.115292904	count	1
EREG	-0.9149993	0.7209173	-1.2692	0.204	-0.115204374	count	1
TOX3	-0.100698	0.4693484	-0.2145	0.83	-0.1151461	count	1
DALRD3	-0.0863743	0.1262746	-0.684	0.494	-0.115137502	count	1
FEM1B	-0.0948568	0.1490788	-0.6363	0.525	-0.115049195	count	1
MAML3	-0.1760003	0.2830806	-0.6217	0.534	-0.115040428	count	1
DR1	-0.084274	0.1067643	-0.7893	0.43	-0.115035005	count	1
FERMT2	-0.0806424	0.0488692	-1.6502	0.099	-0.114978441	count	1
ACSF2	-0.1307643	0.37052	-0.3529	0.724	-0.114860626	count	1
AGER	-0.9123463	0.7534289	-1.2109	0.226	-0.114833756	count	1
MREG	-0.1670608	0.5592259	-0.2987	0.765	-0.114780009	count	1
ARFRP1	-0.0843728	0.1186119	-0.7113	0.477	-0.114776202	count	1
LINC01089	-0.1011711	0.230517	-0.4389	0.661	-0.114633446	count	1
ZNF292	-0.0829158	0.1040307	-0.797	0.425	-0.114443441	count	1
MRPL37	-0.0830743	0.1120525	-0.7414	0.459	-0.11427135	count	1
ZFP62	-0.1061927	0.2321894	-0.4574	0.647	-0.114232768	count	1
AC104984.3	-0.3823192	0.7893847	-0.4843	0.628	-0.114039235	count	1
SRA1	-0.0823403	0.0855768	-0.9622	0.336	-0.114016574	count	1
BAP1	-0.1001232	0.2174106	-0.4605	0.645	-0.11397165	count	1
COA3	-0.080731	0.0625384	-1.2909	0.197	-0.113963711	count	1
EEF1AKMT4	-0.1355234	0.3801672	-0.3565	0.721	-0.113860319	count	1
SOCS4	-0.0857816	0.1660726	-0.5165	0.606	-0.113858417	count	1
FAM213B	-0.1227644	0.409334	-0.2999	0.764	-0.113829021	count	1
FAM3A	-0.083691	0.1102676	-0.759	0.448	-0.113815621	count	1
LINC01023	-0.1388524	0.347887	-0.3991	0.69	-0.113730062	count	1
ZC3HC1	-0.1012935	0.2261135	-0.448	0.654	-0.113638692	count	1
LINC00893	-0.1205229	0.3141423	-0.3837	0.701	-0.113492195	count	1
XAF1	-0.0837241	0.1534898	-0.5455	0.585	-0.113487792	count	1
HSPE1	-0.0797368	0.0506681	-1.5737	0.116	-0.113456234	count	1
LAMTOR4	-0.0795504	0.0436613	-1.822	0.0685	-0.113454733	count	1

FMNL3	-0.1000236	0.3154318	-0.3171	0.751	-0.113326428	count	1
PARG	-0.096783	0.184703	-0.524	0.6	-0.113309509	count	1
TFE3	-0.0933015	0.1934193	-0.4824	0.63	-0.113155936	count	1
MORC2	-0.1013949	0.3426299	-0.2959	0.767	-0.113154069	count	1
RAB8A	-0.0814849	0.092048	-0.8852	0.376	-0.11303706	count	1
PTPN2	-0.0836474	0.1076386	-0.7771	0.437	-0.11300841	count	1
TMEM260	-0.1035872	0.2524659	-0.4103	0.682	-0.112924265	count	1
DIDO1	-0.086098	0.1565042	-0.5501	0.582	-0.112884583	count	1
AL358852.1	-0.164335	0.4584749	-0.3584	0.72	-0.112868947	count	1
ANAPC2	-0.1285119	0.3383787	-0.3798	0.704	-0.112858147	count	1
LANCL1	-0.0884471	0.2505125	-0.3531	0.724	-0.112798706	count	1
ARMCX4	-0.103351	0.2449535	-0.4219	0.673	-0.112665185	count	1
BARX1	-0.0888969	0.2402026	-0.3701	0.711	-0.112639786	count	1
R3HCC1	-0.0826395	0.1064444	-0.7764	0.438	-0.112628933	count	1
TEC	-0.3776057	0.5238665	-0.7208	0.471	-0.112544209	count	1
ZNF704	-0.0808506	0.0897458	-0.9009	0.368	-0.112527807	count	1
TOE1	-0.0993161	0.2353872	-0.4219	0.673	-0.112520647	count	1
FAM107B	-0.0830974	0.1281804	-0.6483	0.517	-0.112455494	count	1
PSMD8	-0.0784838	0.0409653	-1.9159	0.0555	-0.112125872	count	1
PEX26	-0.0994438	0.2084883	-0.477	0.633	-0.112119094	count	1
AC121247.1	-0.1941666	0.6268793	-0.3097	0.757	-0.1119462	count	1
ZNF654	-0.0970812	0.2114462	-0.4591	0.646	-0.111938113	count	1
MAZ	-0.0823103	0.0894802	-0.9199	0.358	-0.111806817	count	1
AIMP2	-0.0850228	0.1300725	-0.6537	0.513	-0.111793367	count	1
TMA7	-0.0780125	0.0341128	-2.2869	0.0223	-0.111762356	count	1
REEP4	-0.1248097	0.4039053	-0.309	0.757	-0.111755395	count	1
GTPBP6	-0.0834095	0.1094831	-0.7618	0.446	-0.111609157	count	1
ZNF185	-0.1624781	0.5355305	-0.3034	0.762	-0.111567749	count	1
BCOR	-0.1005178	0.2744173	-0.3663	0.714	-0.111553613	count	1
SLC6A1	-0.8873819	0.5647996	-1.5711	0.116	-0.111350907	count	1
FXR2	-0.0956778	0.1904487	-0.5024	0.615	-0.111187899	count	1
CCDC25	-0.079169	0.0773494	-1.0235	0.306	-0.111094641	count	1
FAM184A	-0.0995535	0.2378397	-0.4186	0.676	-0.111087771	count	1
MON1A	-0.1197093	0.2735181	-0.4377	0.662	-0.110966894	count	1
TICAM1	-0.1197093	0.2870378	-0.4171	0.677	-0.110966894	count	1
AP001160.3	-0.1615068	0.4055017	-0.3983	0.69	-0.110887347	count	1
KLHL42	-0.082078	0.114914	-0.7143	0.475	-0.110807137	count	1
SLC6A16	-0.1544673	0.3538208	-0.4366	0.662	-0.110801474	count	1
ACTR3	-0.0787698	0.0611274	-1.2886	0.198	-0.11079809	count	1
HACD2	-0.0859705	0.1417328	-0.6066	0.544	-0.110755752	count	1
LSM3	-0.0780134	0.0589077	-1.3243	0.185	-0.110671786	count	1
YWHAB	-0.0771448	0.0322688	-2.3907	0.0169	-0.110627811	count	1
CEBPB-AS1	-0.1692319	0.3530648	-0.4793	0.632	-0.110519367	count	1
AC005332.4	-0.1387268	0.5128011	-0.2705	0.787	-0.110444215	count	1
AC007563.2	-0.1539562	0.4141869	-0.3717	0.71	-0.110428068	count	1

BAZ1B	-0.0805473	0.1003437	-0.8027	0.422	-0.110398278	count	1
FBXO9	-0.0784141	0.0821645	-0.9544	0.34	-0.110239089	count	1
MSI2	-0.0853961	0.1788011	-0.4776	0.633	-0.110238817	count	1
AL359555.4	-0.2571989	0.4567533	-0.5631	0.573	-0.11017529	count	1
DNAJA2	-0.0788906	0.074939	-1.0527	0.293	-0.110143786	count	1
FAM200B	-0.0789592	0.0770616	-1.0246	0.306	-0.110048022	count	1
SMC2	-0.0852039	0.12432	-0.6854	0.493	-0.109990155	count	1
PGLS	-0.0776376	0.057135	-1.3588	0.174	-0.109958896	count	1
HLA-G	-0.5036685	0.7497424	-0.6718	0.502	-0.109938946	count	1
EFNB1	-0.094555	0.2540576	-0.3722	0.71	-0.109877191	count	1
MAP3K4	-0.0854039	0.1371424	-0.6227	0.533	-0.109791472	count	1
ZBED3	-0.0846923	0.171894	-0.4927	0.622	-0.109752055	count	1
SLC9A6	-0.1183231	0.2950194	-0.4011	0.688	-0.109668715	count	1
BOLA3	-0.0794079	0.097417	-0.8151	0.415	-0.109666212	count	1
CGAS	-0.0950321	0.2296573	-0.4138	0.679	-0.10956436	count	1
PABPC1L	-0.5019014	0.5982577	-0.8389	0.402	-0.109521148	count	1
AC093525.6	-0.36804	0.5597162	-0.6575	0.511	-0.109516185	count	1
LITAF	-0.077237	0.0589668	-1.3098	0.19	-0.10949432	count	1
CXCL3	-0.0892415	0.4107275	-0.2173	0.828	-0.109461611	count	1
ZNF768	-0.1010813	0.2417001	-0.4182	0.676	-0.109452852	count	1
AP000911.1	-0.2981412	0.7198339	-0.4142	0.679	-0.109393613	count	1
AL121672.2	-0.2981412	0.8137946	-0.3664	0.714	-0.109393613	count	1
SBF2-AS1	-0.1301775	0.3531213	-0.3686	0.712	-0.109309991	count	1
VPS33A	-0.1008924	0.2288381	-0.4409	0.659	-0.109247047	count	1
ADCK2	-0.0995424	0.2171579	-0.4584	0.647	-0.109173096	count	1
USP4	-0.0921553	0.1827256	-0.5043	0.614	-0.108969722	count	1
SPDL1	-0.1216969	0.278833	-0.4365	0.663	-0.108936959	count	1
CCDC85C	-0.4993458	0.585841	-0.8524	0.394	-0.108917241	count	1
CLBA1	-0.2539641	0.3246124	-0.7824	0.434	-0.108733306	count	1
ELAC1	-0.0921396	0.2391769	-0.3852	0.7	-0.10859496	count	1
ARID3B	-0.1761743	0.4671688	-0.3771	0.706	-0.10856384	count	1
UBR7	-0.0909582	0.2303905	-0.3948	0.693	-0.108221728	count	1
PPAN	-0.101336	0.1795176	-0.5645	0.572	-0.108188556	count	1
AC087190.1	-0.1148282	0.3104258	-0.3699	0.711	-0.108077692	count	1
DZIP1L	-0.0967406	0.2378976	-0.4066	0.684	-0.107932087	count	1
PRDX5	-0.0754312	0.0393707	-1.9159	0.0555	-0.10789748	count	1
ETAA1	-0.0828016	0.1531545	-0.5406	0.589	-0.107869707	count	1
AC019069.1	-0.8619626	0.7962952	-1.0825	0.279	-0.107813892	count	1
SCAF1	-0.1016971	0.2379093	-0.4275	0.669	-0.107752835	count	1
AC053503.5	-0.2938411	0.6120098	-0.4801	0.631	-0.107738832	count	1
AL353708.1	-0.2938411	0.6432623	-0.4568	0.648	-0.107738832	count	1
GRB2	-0.0772707	0.0778865	-0.9921	0.321	-0.107703535	count	1
FMOD	-0.0768018	0.0965382	-0.7956	0.426	-0.107674339	count	1
RAG1	-0.4938185	0.6828052	-0.7232	0.47	-0.107612438	count	1
CCDC175	-0.4938185	0.7710377	-0.6405	0.522	-0.107612438	count	1

C11orf21	-0.4938185	0.7864668	-0.6279	0.53	-0.107612438	count	1
ZNF672	-0.0969129	0.2067608	-0.4687	0.639	-0.107530979	count	1
FAM122B	-0.1179749	0.2765363	-0.4266	0.67	-0.107512541	count	1
SBDS	-0.07486	0.0331793	-2.2562	0.0241	-0.107401554	count	1
MAGOH	-0.0763374	0.0668179	-1.1425	0.253	-0.107375043	count	1
TMEM62	-0.1094421	0.3017196	-0.3627	0.717	-0.107266109	count	1
NTNG2	-0.1081525	0.3085101	-0.3506	0.726	-0.107265252	count	1
VPS72	-0.079033	0.115082	-0.6868	0.492	-0.107188371	count	1
TMEM104	-0.22209	0.3537308	-0.6279	0.53	-0.107174496	count	1
ARL3	-0.0756817	0.0579997	-1.3049	0.192	-0.107055517	count	1
RNF214	-0.0799738	0.1074819	-0.7441	0.457	-0.107054158	count	1
DTD1	-0.0788168	0.1165421	-0.6763	0.499	-0.106927666	count	1
TMEM189	-0.0957727	0.2028252	-0.4722	0.637	-0.106846451	count	1
GMPS	-0.0791438	0.1007491	-0.7856	0.432	-0.106802568	count	1
MT-ND4	-0.0740877	0.0212099	-3.4931	0.000483	-0.106800005	count	1
DERL1	-0.0786793	0.1203602	-0.6537	0.513	-0.106740907	count	1
PRKD2	-0.088824	0.2820523	-0.3149	0.753	-0.106591972	count	1
TOP1	-0.0756466	0.072276	-1.0466	0.295	-0.106564122	count	1
CCDC149	-0.0871712	0.2032141	-0.429	0.668	-0.106444897	count	1
PHF13	-0.0820339	0.1919977	-0.4273	0.669	-0.106398427	count	1
ANKRD34A	-0.1846133	0.5515081	-0.3347	0.738	-0.10629461	count	1
ING4	-0.0812262	0.1205392	-0.6739	0.5	-0.106159607	count	1
CD47	-0.0749891	0.0585402	-1.281	0.2	-0.106010268	count	1
MAP3K7	-0.0849503	0.1654109	-0.5136	0.608	-0.105808601	count	1
GAB1	-0.0845623	0.1576311	-0.5365	0.592	-0.105685032	count	1
RTF1	-0.0746233	0.0586295	-1.2728	0.203	-0.105478784	count	1
MKL1	-0.100332	0.2593098	-0.3869	0.699	-0.105448297	count	1
AC007114.1	-0.1288557	0.2925526	-0.4405	0.66	-0.10543188	count	1
PLEKHF2	-0.0840577	0.166261	-0.5056	0.613	-0.105396376	count	1
ABCG1	-0.1288113	0.2588813	-0.4976	0.619	-0.105395057	count	1
FUNDC2	-0.0742514	0.0540626	-1.3734	0.17	-0.105333279	count	1
CRADD	-0.0960208	0.2428402	-0.3954	0.693	-0.10528893	count	1
C3orf33	-0.1286706	0.3665199	-0.3511	0.726	-0.105278378	count	1
PCDHB6	-0.4838723	0.4706406	-1.0281	0.304	-0.105269116	count	1
EXOC3-AS1	-0.1087135	0.271262	-0.4008	0.689	-0.105196853	count	1
AC124312.1	-0.1533402	0.3453675	-0.444	0.657	-0.105172583	count	1
FAM229B	-0.0763176	0.0913047	-0.8359	0.403	-0.105155552	count	1
HHAT	-0.3540365	0.4675614	-0.7572	0.449	-0.105098023	count	1
DIS3L	-0.0945477	0.2013241	-0.4696	0.639	-0.104892545	count	1
NPTN	-0.0738168	0.0571394	-1.2919	0.196	-0.104818341	count	1
PFDN1	-0.0745605	0.0653485	-1.141	0.254	-0.104737094	count	1
CENPO	-0.2857624	0.7456354	-0.3832	0.702	-0.10463548	count	1
CDCA8	-0.2857624	0.798196	-0.358	0.72	-0.10463548	count	1
SLC44A2	-0.0777359	0.15072	-0.5158	0.606	-0.104584032	count	1
EIF3B	-0.0793166	0.1298523	-0.6108	0.541	-0.104421077	count	1

ERI2	-0.1109549	0.3633873	-0.3053	0.76	-0.10439776	count	1
CREM	-0.075665	0.1174549	-0.6442	0.519	-0.104394093	count	1
HLA-A	-0.0725498	0.030378	-2.3882	0.017	-0.104325939	count	1
FAT4	-0.079929	0.1537032	-0.52	0.603	-0.104293285	count	1
EIF3J-DT	-0.0766285	0.1104018	-0.6941	0.488	-0.104258955	count	1
EBNA1BP2	-0.0753735	0.0807346	-0.9336	0.351	-0.103974766	count	1
ZNF333	-0.1088699	0.2271515	-0.4793	0.632	-0.103924063	count	1
DCXR	-0.0747021	0.0695765	-1.0737	0.283	-0.103697241	count	1
AL359736.1	-0.8317595	0.6765568	-1.2294	0.219	-0.103624917	count	1
ACTN1-AS1	-0.8317595	0.6765568	-1.2294	0.219	-0.103624917	count	1
AC091563.1	-0.8317595	0.8078351	-1.0296	0.303	-0.103624917	count	1
AC125494.2	-0.8317595	0.8078351	-1.0296	0.303	-0.103624917	count	1
ASB12	-0.8317595	0.8638264	-0.9629	0.336	-0.103624917	count	1
PACS1	-0.0856692	0.1518899	-0.564	0.573	-0.103609975	count	1
ANKRD54	-0.0831426	0.1830259	-0.4543	0.65	-0.103550716	count	1
UQCC1	-0.1001793	0.3449306	-0.2904	0.772	-0.103474482	count	1
PCDHGA12	-0.0922296	0.2434169	-0.3789	0.705	-0.103418868	count	1
TASP1	-0.0854495	0.1534505	-0.5569	0.578	-0.103343357	count	1
ATP9A	-0.0855713	0.2110688	-0.4054	0.685	-0.103226246	count	1
DENR	-0.0748482	0.0907673	-0.8246	0.41	-0.103198716	count	1
AC019171.1	-0.15051	0.3625041	-0.4152	0.678	-0.103194608	count	1
LINC00619	-0.8274785	0.7906383	-1.0466	0.295	-0.103032478	count	1
AL512353.1	-0.1939876	0.6023962	-0.322	0.747	-0.103012008	count	1
NOP14-AS1	-0.093907	0.259482	-0.3619	0.717	-0.102958234	count	1
CLASP1	-0.0839323	0.216862	-0.387	0.699	-0.102929235	count	1
TRIM39	-0.133368	0.3660017	-0.3644	0.716	-0.102823676	count	1
TRIM45	-0.1936271	0.570448	-0.3394	0.734	-0.102815056	count	1
TMEM42	-0.080803	0.1536588	-0.5259	0.599	-0.102767815	count	1
SERTAD2	-0.078138	0.1618962	-0.4826	0.629	-0.102725281	count	1
DUSP12	-0.0774086	0.1326275	-0.5837	0.559	-0.102550503	count	1
BUB3	-0.0741015	0.0850349	-0.8714	0.384	-0.102543117	count	1
TTC3	-0.0721703	0.0658002	-1.0968	0.273	-0.102539951	count	1
PSMB8	-0.0728443	0.0645454	-1.1286	0.259	-0.102536998	count	1
LINC02361	-0.2397961	0.4694959	-0.5108	0.61	-0.102433184	count	1
C5orf24	-0.0729295	0.0690135	-1.0567	0.291	-0.102100603	count	1
SGCB	-0.0722244	0.065291	-1.1062	0.269	-0.101996744	count	1
TMF1	-0.0734659	0.0754533	-0.9737	0.33	-0.101955392	count	1
DCUN1D5	-0.074934	0.1026243	-0.7302	0.465	-0.101893852	count	1
URB1-AS1	-0.0947342	0.2938264	-0.3224	0.747	-0.101833813	count	1
TGDS	-0.0815963	0.1799442	-0.4535	0.65	-0.101795747	count	1
VMO1	-0.1914761	0.3844702	-0.498	0.618	-0.101640296	count	1
DOPEY1	-0.0888775	0.2081139	-0.4271	0.669	-0.101568302	count	1
COX5A	-0.0714405	0.0538066	-1.3277	0.184	-0.10148924	count	1
LRCH2	-0.0937193	0.2270277	-0.4128	0.68	-0.101435281	count	1
TMEM230	-0.0711857	0.0438212	-1.6245	0.104	-0.10137158	count	1

LINC01011	-0.1236941	0.3376708	-0.3663	0.714	-0.101153601	count	1
AL162258.2	-0.2366673	0.5247584	-0.451	0.652	-0.101045331	count	1
PYROXD1	-0.0820309	0.182948	-0.4484	0.654	-0.1010113	count	1
JMY	-0.0778337	0.1405157	-0.5539	0.58	-0.100940123	count	1
C8orf88	-0.0773906	0.1267512	-0.6106	0.542	-0.100721604	count	1
SFXN5	-0.0844192	0.3035276	-0.2781	0.781	-0.100712795	count	1
ATP5MC3	-0.0703039	0.0361549	-1.9445	0.0519	-0.100706522	count	1
NONO	-0.0731715	0.0813577	-0.8994	0.369	-0.100675071	count	1
ATP6V1G1	-0.0702588	0.0356937	-1.9684	0.0491	-0.100602277	count	1
EIF2B4	-0.0782006	0.1453449	-0.538	0.591	-0.100511687	count	1
RBM23	-0.0782573	0.1363095	-0.5741	0.566	-0.100475113	count	1
ANKRD13A	-0.0788931	0.189921	-0.4154	0.678	-0.100461091	count	1
RELN	-0.8083895	0.860306	-0.9397	0.347	-0.100394936	count	1
CUEDC2	-0.0714912	0.0723366	-0.9883	0.323	-0.100340221	count	1
CUX1	-0.0713858	0.0866089	-0.8242	0.41	-0.100292614	count	1
NDUFAB1	-0.0703023	0.0430016	-1.6349	0.102	-0.100289754	count	1
SIAE	-0.1009625	0.3641297	-0.2773	0.782	-0.100079009	count	1
LARP1B	-0.0765064	0.170034	-0.4499	0.653	-0.10005851	count	1
THBD	-0.0742236	0.1280518	-0.5796	0.562	-0.099891992	count	1
ZBTB48	-0.0878065	0.225392	-0.3896	0.697	-0.099887397	count	1
R3HDM1	-0.0765534	0.1587735	-0.4822	0.63	-0.099880718	count	1
METAP2	-0.0707829	0.0688906	-1.0275	0.304	-0.099856118	count	1
SAXO2	-0.1616765	0.5606616	-0.2884	0.773	-0.099432741	count	1
SOX8	-0.118513	0.4642485	-0.2553	0.799	-0.099397315	count	1
SNRPC	-0.0706061	0.0683409	-1.0331	0.302	-0.099273269	count	1
AC097534.2	-0.0772352	0.172522	-0.4477	0.654	-0.099268253	count	1
AC010491.1	-0.14469	0.5603056	-0.2582	0.796	-0.099131209	count	1
AC011374.2	-0.4573836	0.5289812	-0.8647	0.387	-0.099058013	count	1
CMTM5	-0.1106618	0.3078338	-0.3595	0.719	-0.098957592	count	1
UBE2G2	-0.0717565	0.0985202	-0.7283	0.466	-0.098948958	count	1
CA3	-0.456719	0.7121164	-0.6414	0.521	-0.098902739	count	1
C5orf51	-0.0758106	0.1374841	-0.5514	0.581	-0.098828334	count	1
C8orf58	-0.1020698	0.2672722	-0.3819	0.703	-0.09871501	count	1
CLPTM1	-0.0736787	0.1217838	-0.605	0.545	-0.098616309	count	1
PRDM1	-0.4554749	0.4236306	-1.0752	0.282	-0.098612147	count	1
AP000808.1	-0.2048634	0.6196686	-0.3306	0.741	-0.098597138	count	1
C11orf54	-0.0722091	0.1397326	-0.5168	0.605	-0.098450556	count	1
IRF2	-0.0708051	0.0836282	-0.8467	0.397	-0.09844258	count	1
SSB	-0.0688602	0.0424019	-1.624	0.1045	-0.098374824	count	1
VPS9D1	-0.1100128	0.3068862	-0.3585	0.72	-0.098371278	count	1
SENP3	-0.1599103	0.4612876	-0.3467	0.729	-0.098322617	count	1
LSM8	-0.0700032	0.0744688	-0.94	0.347	-0.098195606	count	1
SINHCAF	-0.0779013	0.2083769	-0.3738	0.709	-0.098111415	count	1
C3orf70	-0.0912662	0.1874836	-0.4868	0.626	-0.098084483	count	1
APBA3	-0.0889501	0.3488885	-0.255	0.799	-0.098083965	count	1

KNL1	-0.7913919	0.7416808	-1.067	0.286	-0.098052393	count	1
HIF1AN	-0.0796255	0.2069629	-0.3847	0.7	-0.097838825	count	1
POGK	-0.0791353	0.1764397	-0.4485	0.654	-0.097822908	count	1
ARHGEF10L	-0.0811059	0.1454243	-0.5577	0.577	-0.097821796	count	1
LHFPL1	-0.1842655	0.570879	-0.3228	0.747	-0.097707253	count	1
BUD23	-0.0706774	0.0983656	-0.7185	0.472	-0.097688588	count	1
OSBPL8	-0.0693418	0.0627386	-1.1052	0.269	-0.097678873	count	1
AC006059.1	-0.1364466	0.4002122	-0.3409	0.733	-0.09766138	count	1
RNF38	-0.0771583	0.1865807	-0.4135	0.679	-0.097596059	count	1
OTUB1	-0.0704212	0.1229853	-0.5726	0.567	-0.097566149	count	1
ERGIC2	-0.0690805	0.0658688	-1.0488	0.294	-0.097480855	count	1
MOCS2	-0.0691594	0.0689579	-1.0029	0.316	-0.097385431	count	1
SMNDC1	-0.070854	0.0859073	-0.8248	0.41	-0.097356528	count	1
IDH1-AS1	-0.3293331	0.662967	-0.4968	0.619	-0.097347353	count	1
RIIAD1	-0.3293331	0.756747	-0.4352	0.663	-0.097347353	count	1
SLAMF8	-0.3291882	0.6601318	-0.4987	0.618	-0.097302058	count	1
ZNF788P	-0.2281754	0.5630776	-0.4052	0.685	-0.097284889	count	1
MRPS34	-0.0686839	0.0577973	-1.1884	0.235	-0.097249226	count	1
GAR1	-0.0729689	0.1257438	-0.5803	0.562	-0.097143316	count	1
C15orf65	-0.0949063	0.2772645	-0.3423	0.732	-0.097072012	count	1
MOXD1	-0.1578177	0.51519	-0.3063	0.759	-0.097007973	count	1
IPPK	-0.1303307	0.3452315	-0.3775	0.706	-0.096974249	count	1
USP22	-0.0709115	0.1098268	-0.6457	0.519	-0.096895618	count	1
PHTF2	-0.0744873	0.1692033	-0.4402	0.66	-0.096502591	count	1
TBX2	-0.0704719	0.1081	-0.6519	0.514	-0.096339852	count	1
ANLN	-0.2638211	0.8871739	-0.2974	0.766	-0.096243883	count	1
AC002066.1	-0.2637068	0.578775	-0.4556	0.649	-0.096200312	count	1
AL591845.1	-0.2637068	0.602613	-0.4376	0.662	-0.096200312	count	1
NOTCH1	-0.097017	0.3205889	-0.3026	0.762	-0.096138749	count	1
PEAK1	-0.0739696	0.1713436	-0.4317	0.666	-0.095919559	count	1
LIAS	-0.0811154	0.180356	-0.4498	0.653	-0.095868215	count	1
STARD4-AS1	-0.4433145	0.4616365	-0.9603	0.337	-0.095777007	count	1
PTP4A2	-0.0679229	0.0617133	-1.1006	0.271	-0.095764135	count	1
GPAA1	-0.0682621	0.0711717	-0.9591	0.338	-0.095631115	count	1
FAM161A	-0.075944	0.1716024	-0.4426	0.658	-0.095495938	count	1
TMEM273	-0.7722294	0.6605386	-1.1691	0.242	-0.0954187	count	1
AC010618.3	-0.1238923	0.3756026	-0.3298	0.742	-0.095415595	count	1
RPS28	-0.0661291	0.022208	-2.9777	0.00292	-0.095222778	count	1
SOD1	-0.0662491	0.0291534	-2.2724	0.0231	-0.095165763	count	1
AC147651.4	-0.1655416	0.3604894	-0.4592	0.646	-0.095053883	count	1
LINC01762	-0.7695184	1.238814	-0.6212	0.535	-0.095046744	count	1
CNTN2	-0.7695184	1.238814	-0.6212	0.535	-0.095046744	count	1
LINC01740	-0.7695184	1.238814	-0.6212	0.535	-0.095046744	count	1
AC104695.2	-0.7695184	1.238814	-0.6212	0.535	-0.095046744	count	1
LINC01829	-0.7695184	1.238814	-0.6212	0.535	-0.095046744	count	1

AC074099.1	-0.7695184	1.238814	-0.6212	0.535	-0.095046744	count	1
AC010136.1	-0.7695184	1.238814	-0.6212	0.535	-0.095046744	count	1
AC022001.3	-0.7695184	1.238814	-0.6212	0.535	-0.095046744	count	1
BSN	-0.7695184	1.238814	-0.6212	0.535	-0.095046744	count	1
AC063944.3	-0.7695184	1.238814	-0.6212	0.535	-0.095046744	count	1
AC117402.1	-0.7695184	1.238814	-0.6212	0.535	-0.095046744	count	1
TIGIT	-0.7695184	1.238814	-0.6212	0.535	-0.095046744	count	1
EPHB1	-0.7695184	1.238814	-0.6212	0.535	-0.095046744	count	1
NME9	-0.7695184	1.238814	-0.6212	0.535	-0.095046744	count	1
AC022126.1	-0.7695184	1.238814	-0.6212	0.535	-0.095046744	count	1
PTCHD1	-0.7695184	1.238814	-0.6212	0.535	-0.095046744	count	1
AL109797.1	-0.7695184	1.238814	-0.6212	0.535	-0.095046744	count	1
CASC9	-0.7695184	1.238814	-0.6212	0.535	-0.095046744	count	1
AB015752.1	-0.7695184	1.238814	-0.6212	0.535	-0.095046744	count	1
AL162412.1	-0.7695184	1.238814	-0.6212	0.535	-0.095046744	count	1
AL359710.1	-0.7695184	1.238814	-0.6212	0.535	-0.095046744	count	1
BX649601.1	-0.7695184	1.238814	-0.6212	0.535	-0.095046744	count	1
ASCL2	-0.7695184	1.238814	-0.6212	0.535	-0.095046744	count	1
AC068733.3	-0.7695184	1.238814	-0.6212	0.535	-0.095046744	count	1
DDIAS	-0.7695184	1.238814	-0.6212	0.535	-0.095046744	count	1
AP003059.2	-0.7695184	1.238814	-0.6212	0.535	-0.095046744	count	1
LINC00940	-0.7695184	1.238814	-0.6212	0.535	-0.095046744	count	1
PTPRR	-0.7695184	1.238814	-0.6212	0.535	-0.095046744	count	1
TRAV38-2DV8	-0.7695184	1.238814	-0.6212	0.535	-0.095046744	count	1
TRAV39	-0.7695184	1.238814	-0.6212	0.535	-0.095046744	count	1
CPNE6	-0.7695184	1.238814	-0.6212	0.535	-0.095046744	count	1
PLEK2	-0.7695184	1.238814	-0.6212	0.535	-0.095046744	count	1
APBA2	-0.7695184	1.238814	-0.6212	0.535	-0.095046744	count	1
SLC24A5	-0.7695184	1.238814	-0.6212	0.535	-0.095046744	count	1
TVP23C-CDRT4	-0.7695184	1.238814	-0.6212	0.535	-0.095046744	count	1
WNK4	-0.7695184	1.238814	-0.6212	0.535	-0.095046744	count	1
AC005180.2	-0.7695184	1.238814	-0.6212	0.535	-0.095046744	count	1
AC006238.1	-0.7695184	1.238814	-0.6212	0.535	-0.095046744	count	1
AC027307.1	-0.7695184	1.238814	-0.6212	0.535	-0.095046744	count	1
CACTIN-AS1	-0.7695184	1.238814	-0.6212	0.535	-0.095046744	count	1
AC011511.5	-0.7695184	1.238814	-0.6212	0.535	-0.095046744	count	1
ZNF709	-0.7695184	1.238814	-0.6212	0.535	-0.095046744	count	1
MFNG	-0.7695184	1.238814	-0.6212	0.535	-0.095046744	count	1
PRKCZ-AS1	-0.7695184	1.328646	-0.5792	0.563	-0.095046744	count	1
IL10	-0.7695184	1.328646	-0.5792	0.563	-0.095046744	count	1
HOXD4	-0.7695184	1.328646	-0.5792	0.563	-0.095046744	count	1
CPO	-0.7695184	1.328646	-0.5792	0.563	-0.095046744	count	1
CNTN6	-0.7695184	1.328646	-0.5792	0.563	-0.095046744	count	1
ACVR2B-AS1	-0.7695184	1.328646	-0.5792	0.563	-0.095046744	count	1
SLCO2A1	-0.7695184	1.328646	-0.5792	0.563	-0.095046744	count	1

LNCSRLR	-0.7695184	1.328646	-0.5792	0.563	-0.095046744	count	1
AC110813.1	-0.7695184	1.328646	-0.5792	0.563	-0.095046744	count	1
ALDH8A1	-0.7695184	1.328646	-0.5792	0.563	-0.095046744	count	1
THRSP	-0.7695184	1.328646	-0.5792	0.563	-0.095046744	count	1
AC124947.1	-0.7695184	1.328646	-0.5792	0.563	-0.095046744	count	1
LINC00571	-0.7695184	1.328646	-0.5792	0.563	-0.095046744	count	1
AL355974.3	-0.7695184	1.328646	-0.5792	0.563	-0.095046744	count	1
PDIA2	-0.7695184	1.328646	-0.5792	0.563	-0.095046744	count	1
AL121761.2	-0.7695184	1.328646	-0.5792	0.563	-0.095046744	count	1
AC010327.3	-0.7695184	1.328646	-0.5792	0.563	-0.095046744	count	1
SCIN	-0.1107602	0.2406539	-0.4602	0.645	-0.095033336	count	1
ACAT2	-0.0714227	0.1378239	-0.5182	0.604	-0.094981531	count	1
DNAJB6	-0.0673696	0.063793	-1.0561	0.291	-0.094899614	count	1
TMEM147-AS1	-0.197294	0.4909225	-0.4019	0.688	-0.09484136	count	1
AC008124.1	-0.076877	0.1704007	-0.4512	0.652	-0.094837591	count	1
LSM14A	-0.0680897	0.0727477	-0.936	0.349	-0.094736623	count	1
RASAL2	-0.0721458	0.1407351	-0.5126	0.608	-0.094700336	count	1
RBBP7	-0.0671704	0.0626828	-1.0716	0.284	-0.094579035	count	1
ANKS6	-0.0991314	0.3572814	-0.2775	0.781	-0.094551266	count	1
TWIST1	-0.0679092	0.0732263	-0.9274	0.354	-0.094434562	count	1
NFYC	-0.0734813	0.1334267	-0.5507	0.582	-0.094433826	count	1
CPSF6	-0.0710776	0.1323877	-0.5369	0.591	-0.094367104	count	1
SLC44A3	-0.115343	0.3081485	-0.3743	0.708	-0.094240885	count	1
TRIM28	-0.0695712	0.0968305	-0.7185	0.473	-0.094192853	count	1
RHOF	-0.1957296	0.4524635	-0.4326	0.665	-0.094066157	count	1
ZNF496	-0.0886896	0.3600335	-0.2463	0.805	-0.093890645	count	1
PRMT1	-0.0663253	0.0575815	-1.1519	0.249	-0.093867774	count	1
SERPINB8	-0.0825065	0.2641498	-0.3123	0.755	-0.093832077	count	1
SUMO2	-0.0652632	0.0248883	-2.6222	0.00877	-0.093821039	count	1
CBX7	-0.0701631	0.1320647	-0.5313	0.595	-0.093639337	count	1
AQP11	-0.1308797	0.4697766	-0.2786	0.781	-0.093612988	count	1
PPHLN1	-0.0687303	0.094199	-0.7296	0.466	-0.093502361	count	1
GNG10	-0.0923508	0.3100488	-0.2979	0.766	-0.093502173	count	1
TMOD3	-0.0664295	0.0658878	-1.0082	0.313	-0.093378242	count	1
TAX1BP1	-0.0653207	0.0465799	-1.4023	0.1609	-0.093139297	count	1
SPDYE16	-0.7544248	0.7501369	-1.0057	0.315	-0.092978858	count	1
LINC00266-1	-0.7544248	0.7652133	-0.9859	0.324	-0.092978858	count	1
AC078909.2	-0.7544248	0.8256365	-0.9137	0.361	-0.092978858	count	1
FAS	-0.0685152	0.1065975	-0.6427	0.52	-0.092937652	count	1
C1orf43	-0.0656676	0.0569566	-1.1529	0.249	-0.092905766	count	1
XPO5	-0.1081335	0.280768	-0.3851	0.7	-0.092755308	count	1
HYLS1	-0.0915499	0.2639405	-0.3469	0.729	-0.092685799	count	1
AC092140.1	-0.1750111	0.5785908	-0.3025	0.762	-0.0926708	count	1
AL078639.1	-0.0700237	0.1595104	-0.439	0.661	-0.092642351	count	1
AL136295.2	-0.4293775	0.5021361	-0.8551	0.393	-0.092539398	count	1

FBXO5	-0.0996956	0.2507353	-0.3976	0.691	-0.092252668	count	1
SH3KBP1	-0.0700594	0.1289371	-0.5434	0.587	-0.092088156	count	1
TSEN54	-0.0951318	0.2653491	-0.3585	0.72	-0.09195307	count	1
TBPL1	-0.0706405	0.1422157	-0.4967	0.619	-0.091841707	count	1
NDUFB6	-0.0648478	0.0528144	-1.2278	0.22	-0.091826433	count	1
PSMD4	-0.0647143	0.055231	-1.1717	0.241	-0.091778898	count	1
C2orf73	-0.424501	0.6873272	-0.6176	0.537	-0.091409571	count	1
UTP14C	-0.0757807	0.2257219	-0.3357	0.737	-0.091379178	count	1
KANK1	-0.0659724	0.1122624	-0.5877	0.557	-0.091374099	count	1
GIGYF2	-0.0671248	0.1430729	-0.4692	0.639	-0.091212556	count	1
NDUFV2	-0.0638705	0.0431712	-1.4795	0.139	-0.091117469	count	1
NDUFAF3	-0.0638139	0.0449753	-1.4189	0.156	-0.091073226	count	1
LRP10	-0.0643016	0.0552149	-1.1646	0.244	-0.091062923	count	1
MOB4	-0.066107	0.0845594	-0.7818	0.434	-0.090996823	count	1
CEP83-DT	-0.7398526	0.9888716	-0.7482	0.454	-0.090987399	count	1
AC130324.1	-0.7398526	0.9888716	-0.7482	0.454	-0.090987399	count	1
INTS6L-AS1	-0.7398526	1.041304	-0.7105	0.477	-0.090987399	count	1
SPART-AS1	-0.7398526	1.110747	-0.6661	0.505	-0.090987399	count	1
AC022098.4	-0.7398526	1.110747	-0.6661	0.505	-0.090987399	count	1
EEF2	-0.0635392	0.0332373	-1.9117	0.056	-0.090968464	count	1
RSAD1	-0.0780471	0.1717131	-0.4545	0.649	-0.090963428	count	1
UPF2	-0.0655819	0.1066375	-0.615	0.539	-0.090770974	count	1
HINT2	-0.0645214	0.0671496	-0.9609	0.337	-0.090733865	count	1
CAMSAP2	-0.0682642	0.1139506	-0.5991	0.549	-0.090726608	count	1
NALT1	-0.1581283	0.5172591	-0.3057	0.76	-0.090699788	count	1
AL021068.1	-0.2492364	0.6661815	-0.3741	0.708	-0.090696233	count	1
CCDC28B	-0.0750127	0.1857798	-0.4038	0.686	-0.090682894	count	1
ITGAV	-0.0650021	0.0918857	-0.7074	0.479	-0.090389569	count	1
CDC37L1-DT	-0.1574992	0.4717078	-0.3339	0.738	-0.090330693	count	1
RGS5	-0.1879269	0.5206532	-0.3609	0.718	-0.090204856	count	1
COG8	-0.0881793	0.3960203	-0.2227	0.824	-0.090147719	count	1
C2orf92	-0.306189	0.7580441	-0.4039	0.686	-0.090137347	count	1
B3GALT4	-0.0671579	0.143657	-0.4675	0.64	-0.089998445	count	1
SNRNP70	-0.0647697	0.0891247	-0.7267	0.467	-0.08999595	count	1
PANK3	-0.074316	0.1842673	-0.4033	0.687	-0.089838116	count	1
HIST1H1C	-0.065354	0.1107654	-0.59	0.555	-0.089827638	count	1
UBE2Z	-0.0664806	0.1106688	-0.6007	0.548	-0.089759847	count	1
CMTM7	-0.102459	0.3300126	-0.3105	0.756	-0.089754304	count	1
TOX	-0.0941219	0.5098938	-0.1846	0.854	-0.089735485	count	1
PGBD1	-0.0884919	0.3229689	-0.274	0.784	-0.089569564	count	1
ATP5F1B	-0.0631883	0.0481554	-1.3122	0.19	-0.08956388	count	1
MED8	-0.0661994	0.0981075	-0.6748	0.5	-0.089473342	count	1
MPHOSPH6	-0.0706465	0.1686671	-0.4189	0.675	-0.089465553	count	1
SSC4D	-0.4158518	0.8901929	-0.4671	0.64	-0.089409524	count	1
AC090844.2	-0.4158518	0.8901929	-0.4671	0.64	-0.089409524	count	1

AC021016.3	-0.4158518	0.9526101	-0.4365	0.662	-0.089409524	count	1
AL136162.1	-0.4158518	0.9526101	-0.4365	0.662	-0.089409524	count	1
BRIP1	-0.4158518	1.18416	-0.3512	0.725	-0.089409524	count	1
TRNT1	-0.0672918	0.1497492	-0.4494	0.653	-0.089383616	count	1
CYB561D2	-0.1453471	0.3973831	-0.3658	0.715	-0.089188124	count	1
ABHD12	-0.0736795	0.1367176	-0.5389	0.59	-0.089066375	count	1
ZC3H14	-0.0655	0.1271831	-0.515	0.607	-0.089002587	count	1
CHFR	-0.0847169	0.3090085	-0.2742	0.784	-0.088943462	count	1
NR2F2-AS1	-0.0701803	0.177144	-0.3962	0.692	-0.088750636	count	1
NSMCE4A	-0.0655713	0.1108196	-0.5917	0.554	-0.088683773	count	1
DOHH	-0.0697294	0.1513562	-0.4607	0.645	-0.08853952	count	1
PHF1	-0.0645784	0.1080602	-0.5976	0.55	-0.088455557	count	1
COPS6	-0.0622381	0.051051	-1.2191	0.223	-0.088381748	count	1
DFFB	-0.1235695	0.4044881	-0.3055	0.76	-0.088304677	count	1
DTWD2	-0.092406	0.2365793	-0.3906	0.696	-0.088086826	count	1
MFSD14C	-0.0715158	0.1885157	-0.3794	0.704	-0.088029356	count	1
PMM1	-0.0636302	0.0870018	-0.7314	0.465	-0.08787519	count	1
HOOK2	-0.0713846	0.181801	-0.3927	0.695	-0.087867436	count	1
HSD11B2	-0.4091076	0.7722809	-0.5297	0.596	-0.087853459	count	1
SNHG21	-0.0829527	0.3335261	-0.2487	0.804	-0.087783932	count	1
IGFBP7	-0.0607865	0.023324	-2.6062	0.00919	-0.087680495	count	1
DCTN4	-0.0689544	0.1481931	-0.4653	0.642	-0.087553417	count	1
ALDH3B1	-0.0789661	0.3628164	-0.2176	0.828	-0.087527783	count	1
SERPINB6	-0.0616479	0.0545571	-1.13	0.259	-0.087509095	count	1
NKAPD1	-0.0654474	0.1105616	-0.592	0.554	-0.08746421	count	1
POT1	-0.0903093	0.2320493	-0.3892	0.697	-0.087257216	count	1
ADGRF5	-0.0858459	0.2924938	-0.2935	0.769	-0.086874255	count	1
TSC1	-0.0675798	0.2165568	-0.3121	0.755	-0.086835542	count	1
MPHOSPH10	-0.06272	0.0859751	-0.7295	0.466	-0.086669887	count	1
SLC12A8	-0.2384277	0.5599178	-0.4258	0.67	-0.086600814	count	1
KLHDC10	-0.0698165	0.1913747	-0.3648	0.715	-0.086440031	count	1
ARMC1	-0.0645645	0.1062636	-0.6076	0.543	-0.086403018	count	1
GABARAPL2	-0.0602132	0.0356766	-1.6878	0.0915	-0.086391612	count	1
G3BP2	-0.0619381	0.0791192	-0.7828	0.434	-0.086172854	count	1
PFKM	-0.0659051	0.1245225	-0.5293	0.597	-0.086104479	count	1
AC011603.2	-0.7028082	0.7094284	-0.9907	0.322	-0.08594822	count	1
SUSD4	-0.7028082	0.7155326	-0.9822	0.326	-0.08594822	count	1
OSBP	-0.0644798	0.1083711	-0.595	0.552	-0.08582769	count	1
BAD	-0.0607871	0.0590243	-1.0299	0.303	-0.085797444	count	1
TCF19	-0.1397724	0.376015	-0.3717	0.71	-0.085700564	count	1
AC099791.2	-0.0830587	0.3081032	-0.2696	0.787	-0.08568727	count	1
6-Mar	-0.0620672	0.1009124	-0.6151	0.539	-0.085634429	count	1
DDB2	-0.0632532	0.1246574	-0.5074	0.612	-0.085545474	count	1
BRD3OS	-0.0721432	0.2361366	-0.3055	0.76	-0.085501294	count	1
GMEB1	-0.0714799	0.1847878	-0.3868	0.699	-0.085475627	count	1

BCAT2	-0.0667355	0.1515875	-0.4402	0.66	-0.085462553	count	1
FDPS	-0.061056	0.0740811	-0.8242	0.41	-0.085432241	count	1
DNAJB11	-0.0657663	0.1359533	-0.4837	0.629	-0.085418838	count	1
COPS2	-0.0608084	0.0644188	-0.944	0.345	-0.085386524	count	1
PIP4K2A	-0.0634449	0.1026192	-0.6183	0.536	-0.085370257	count	1
SLC25A1	-0.0627495	0.1180244	-0.5317	0.595	-0.085310624	count	1
PSMD13	-0.0613967	0.0742149	-0.8273	0.408	-0.08528702	count	1
AP000317.1	-0.2904526	0.7117026	-0.4081	0.683	-0.085264142	count	1
CACNB2	-0.0851555	0.2314909	-0.3679	0.713	-0.085262057	count	1
SGTB	-0.0648022	0.1329379	-0.4875	0.626	-0.085167939	count	1
5-Mar	-0.065379	0.1394299	-0.4689	0.639	-0.085063809	count	1
PRELID3A	-0.0879721	0.3011187	-0.2922	0.77	-0.084982666	count	1
AKAP9	-0.0598573	0.0641905	-0.9325	0.351	-0.084884837	count	1
WRAP53	-0.1038302	0.2899076	-0.3581	0.72	-0.084729959	count	1
MRPL10	-0.0635618	0.1057866	-0.6008	0.548	-0.084648207	count	1
YAF2	-0.0637744	0.1161679	-0.549	0.583	-0.084464411	count	1
RPL17-C18orf32	-0.393993	0.774781	-0.5085	0.611	-0.084377289	count	1
AL391684.1	-0.393993	0.8665128	-0.4547	0.649	-0.084377289	count	1
LRRC8B	-0.1985197	0.5514195	-0.36	0.719	-0.084226367	count	1
CHMP5	-0.0592196	0.0473661	-1.2503	0.211	-0.084188835	count	1
FNDC3A	-0.060312	0.0807212	-0.7472	0.455	-0.084085641	count	1
AC064807.1	-0.0924663	0.3552822	-0.2603	0.795	-0.084071045	count	1
ANXA7	-0.0592331	0.0510596	-1.1601	0.246	-0.084034482	count	1
AL133342.1	-0.1976113	0.5217383	-0.3788	0.705	-0.083828197	count	1
MRM2	-0.0641599	0.1264634	-0.5073	0.612	-0.083820914	count	1
ZNF641	-0.0750572	0.2027371	-0.3702	0.711	-0.083637915	count	1
STMP1	-0.0590744	0.0625446	-0.9445	0.345	-0.083637043	count	1
MAPK8	-0.0758708	0.2046875	-0.3707	0.711	-0.083597304	count	1
IL13RA1	-0.0601935	0.084507	-0.7123	0.476	-0.083565729	count	1
ZNF174	-0.0809562	0.2696612	-0.3002	0.764	-0.083505728	count	1
NDUFS4	-0.0591086	0.0564115	-1.0478	0.295	-0.083495858	count	1
OLMALINC	-0.0901383	0.4135237	-0.218	0.827	-0.083337908	count	1
PYGB	-0.0636943	0.1634319	-0.3897	0.697	-0.083211715	count	1
NPC1	-0.0735428	0.2370663	-0.3102	0.756	-0.083206145	count	1
LETM2	-0.1048753	0.4497945	-0.2332	0.816	-0.083184523	count	1
LPXN	-0.1574729	0.4590279	-0.3431	0.732	-0.083161625	count	1
TMED7	-0.0598608	0.0911796	-0.6565	0.512	-0.083073648	count	1
CP	-0.0586301	0.0893441	-0.6562	0.512	-0.083064871	count	1
TUFM	-0.058691	0.0552234	-1.0628	0.288	-0.083024821	count	1
DHRS4L2	-0.060107	0.087331	-0.6883	0.491	-0.082967677	count	1
DRAP1	-0.0583899	0.0542858	-1.0756	0.282	-0.082839806	count	1
LY75	-0.1212335	0.3552975	-0.3412	0.733	-0.082811143	count	1
C5orf49	-0.679315	0.6785763	-1.0011	0.317	-0.082770843	count	1
PRDM6	-0.058834	0.1132717	-0.5194	0.604	-0.08258838	count	1
PIDD1	-0.1156103	0.3039833	-0.3803	0.704	-0.082535324	count	1

AC005498.3	-0.1111316	0.6041983	-0.1839	0.854	-0.082500365	count	1
TKT	-0.0582372	0.0603045	-0.9657	0.334	-0.082467706	count	1
MSH2	-0.086486	0.2344562	-0.3689	0.712	-0.082402284	count	1
PRPF40A	-0.0586769	0.0743741	-0.7889	0.43	-0.082397157	count	1
KHDC1	-0.0937972	0.3104328	-0.3021	0.763	-0.082097178	count	1
EEF1G	-0.1554641	0.5731979	-0.2712	0.786	-0.082075462	count	1
ZNF232	-0.0937678	0.2441192	-0.3841	0.701	-0.082071212	count	1
MRPL38	-0.1554419	0.5906253	-0.2632	0.792	-0.082063456	count	1
NANOS3	-0.1263348	0.46312	-0.2728	0.785	-0.08203715	count	1
CBFA2T2	-0.07361	0.2725717	-0.2701	0.787	-0.082018488	count	1
HNRNPC	-0.0579242	0.0530443	-1.092	0.275	-0.081996371	count	1
FAM66C	-0.0884791	0.3106233	-0.2848	0.776	-0.081791728	count	1
CD37	-0.2254895	0.658569	-0.3424	0.732	-0.081716561	count	1
STARD3NL	-0.0627854	0.1311076	-0.4789	0.632	-0.081684068	count	1
AC092903.2	-0.154536	0.5393231	-0.2865	0.774	-0.08157384	count	1
COIL	-0.0599236	0.1181024	-0.5074	0.612	-0.081533827	count	1
AAED1	-0.0586403	0.0812146	-0.722	0.47	-0.081511374	count	1
PAAF1	-0.0656631	0.1540667	-0.4262	0.67	-0.081437735	count	1
SPAG4	-0.1700215	0.5220823	-0.3257	0.745	-0.081377128	count	1
PLA1A	-0.6683698	0.8140483	-0.821	0.412	-0.081295744	count	1
AC105094.2	-0.6683698	0.8140483	-0.821	0.412	-0.081295744	count	1
AHCY	-0.0622243	0.1396336	-0.4456	0.656	-0.081288426	count	1
HIBCH	-0.0590422	0.108042	-0.5465	0.585	-0.081236266	count	1
NDUFB3	-0.0575842	0.0608076	-0.947	0.344	-0.081147487	count	1
ZDHHC2	-0.0648244	0.1813711	-0.3574	0.721	-0.08096334	count	1
SHROOM1	-0.0759145	0.408639	-0.1858	0.853	-0.08091491	count	1
MTA1	-0.0639817	0.1494065	-0.4282	0.669	-0.080896255	count	1
ZNF347	-0.0799447	0.2336555	-0.3421	0.732	-0.080866734	count	1
WDR54	-0.0627632	0.1432293	-0.4382	0.661	-0.080807595	count	1
AL138995.1	-0.1686295	0.4212369	-0.4003	0.689	-0.080692787	count	1
LINC01431	-0.1903662	0.477175	-0.3989	0.69	-0.08065651	count	1
SLC12A2	-0.0668722	0.248292	-0.2693	0.788	-0.080607369	count	1
DPM2	-0.0591515	0.104866	-0.5641	0.573	-0.080526444	count	1
PTPRG	-0.0595931	0.1328103	-0.4487	0.654	-0.080508144	count	1
PLPP1	-0.0563568	0.0539106	-1.0454	0.296	-0.080289914	count	1
PMS2	-0.0691352	0.2354754	-0.2936	0.769	-0.080239227	count	1
ARMCX3	-0.0573421	0.0698854	-0.8205	0.412	-0.080190265	count	1
UCK2	-0.0981874	0.3288461	-0.2986	0.765	-0.080076443	count	1
KATNA1	-0.0599231	0.1197152	-0.5005	0.617	-0.080035201	count	1
RPAIN	-0.0570206	0.0763306	-0.747	0.455	-0.079961849	count	1
UBA5	-0.0601399	0.1158448	-0.5191	0.604	-0.079916114	count	1
TUBB2A	-0.0575118	0.1022017	-0.5627	0.574	-0.079905875	count	1
MAN2B2	-0.0628833	0.1588926	-0.3958	0.692	-0.079830128	count	1
MCM3	-0.0698435	0.1464128	-0.477	0.633	-0.079737534	count	1
CNIH3	-0.0836675	0.3561048	-0.235	0.814	-0.079697789	count	1

MIR210HG	-0.0862061	0.3778408	-0.2282	0.82	-0.079674264	count	1
ANAPC16	-0.0557523	0.042684	-1.3062	0.1916	-0.079672455	count	1
SLC25A15	-0.1508996	0.8005092	-0.1885	0.85	-0.079609728	count	1
ARFIP2	-0.0635151	0.1464657	-0.4337	0.665	-0.079585277	count	1
ZBTB5	-0.1110843	0.4567056	-0.2432	0.808	-0.079259374	count	1
HIVEP3	-0.0999541	0.3244123	-0.3081	0.758	-0.079237554	count	1
CTCF	-0.058397	0.0984767	-0.593	0.553	-0.079224938	count	1
DCK	-0.0707207	0.1857076	-0.3808	0.703	-0.079205686	count	1
TSPAN31	-0.0580203	0.0926313	-0.6264	0.531	-0.079150513	count	1
SLC25A11	-0.0575387	0.0823534	-0.6987	0.485	-0.079122752	count	1
PTEN	-0.0563988	0.06572	-0.8582	0.391	-0.07909973	count	1
DFFA	-0.0597384	0.1279337	-0.4669	0.641	-0.079065412	count	1
COPZ2	-0.0555816	0.0537468	-1.0341	0.301	-0.078981877	count	1
MRPL44	-0.0606499	0.1281818	-0.4732	0.636	-0.078969066	count	1
GMPR2	-0.0578318	0.1066382	-0.5423	0.588	-0.078893159	count	1
AC034111.1	-0.1861888	0.7656057	-0.2432	0.808	-0.078830978	count	1
CD226	-0.1861888	1.162697	-0.1601	0.873	-0.078830978	count	1
RNF138	-0.0942003	0.2435391	-0.3868	0.699	-0.078807558	count	1
DNAJC11	-0.0737952	0.2304553	-0.3202	0.749	-0.078645075	count	1
EXOC7	-0.057692	0.1395428	-0.4134	0.679	-0.078361133	count	1
COX5B	-0.0546419	0.0321498	-1.6996	0.0893	-0.07833182	count	1
AL139147.1	-0.6461512	0.995902	-0.6488	0.517	-0.078311731	count	1
AC097059.1	-0.6461512	0.9978798	-0.6475	0.517	-0.078311731	count	1
AC118553.2	-0.6461512	1.024584	-0.6306	0.528	-0.078311731	count	1
AL121655.1	-0.6461512	1.024584	-0.6306	0.528	-0.078311731	count	1
GPR83	-0.6461512	1.024584	-0.6306	0.528	-0.078311731	count	1
AP005329.1	-0.6461512	1.024584	-0.6306	0.528	-0.078311731	count	1
LINC01535	-0.6461512	1.024584	-0.6306	0.528	-0.078311731	count	1
TDRKH-AS1	-0.6461512	1.026506	-0.6295	0.529	-0.078311731	count	1
ANO7	-0.6461512	1.026506	-0.6295	0.529	-0.078311731	count	1
AC137630.2	-0.6461512	1.026506	-0.6295	0.529	-0.078311731	count	1
HIST1H4I	-0.6461512	1.026506	-0.6295	0.529	-0.078311731	count	1
MCMDC2	-0.6461512	1.026506	-0.6295	0.529	-0.078311731	count	1
TMEM92-AS1	-0.6461512	1.026506	-0.6295	0.529	-0.078311731	count	1
AC009318.4	-0.6461512	1.171371	-0.5516	0.581	-0.078311731	count	1
PIK3CA	-0.0591642	0.1255355	-0.4713	0.637	-0.078257315	count	1
CLEC2D	-0.2162298	0.5117824	-0.4225	0.673	-0.078233196	count	1
MRPS9	-0.0579886	0.1142087	-0.5077	0.612	-0.078227311	count	1
PIGZ	-0.0764128	0.2563433	-0.2981	0.766	-0.078051572	count	1
TRAM1L1	-0.0831003	0.4259087	-0.1951	0.845	-0.078001544	count	1
NOL10	-0.064377	0.1741598	-0.3696	0.712	-0.077986518	count	1
PRPF3	-0.0635579	0.1869738	-0.3399	0.734	-0.077883695	count	1
TRAF5	-0.0588546	0.1695861	-0.347	0.729	-0.077799718	count	1
VWF	-0.127059	0.6277972	-0.2024	0.84	-0.077766048	count	1
ANKRD42	-0.0687294	0.2770996	-0.248	0.804	-0.077739957	count	1

SLC39A14	-0.0612072	0.1364893	-0.4484	0.654	-0.077698374	count	1
RSBN1L	-0.0548006	0.0717949	-0.7633	0.445	-0.077531911	count	1
HIST1H4H	-0.0792741	0.3319025	-0.2388	0.811	-0.077511492	count	1
MARCKS	-0.054302	0.0612272	-0.8869	0.375	-0.077452199	count	1
PREPL	-0.0566167	0.1022165	-0.5539	0.58	-0.077234247	count	1
ARIH2	-0.0574937	0.1277796	-0.4499	0.653	-0.077067766	count	1
AC126118.1	-0.6362398	0.8499179	-0.7486	0.454	-0.076985273	count	1
SLC35A3	-0.0616646	0.1597902	-0.3859	0.7	-0.076877847	count	1
CRBN	-0.0548788	0.0689377	-0.7961	0.426	-0.076869023	count	1
WDR77	-0.0644479	0.1857161	-0.347	0.729	-0.076820263	count	1
WIPI2	-0.0564443	0.1137582	-0.4962	0.62	-0.076795941	count	1
FAM104A	-0.0591011	0.1456542	-0.4058	0.685	-0.07668004	count	1
C1QTNF2	-0.0561352	0.085904	-0.6535	0.513	-0.076537953	count	1
DYNLL2	-0.0566914	0.1181714	-0.4797	0.631	-0.07647574	count	1
ANGPTL2	-0.0579082	0.1442979	-0.4013	0.688	-0.076402811	count	1
ZZEF1	-0.0645448	0.2152881	-0.2998	0.764	-0.076226477	count	1
RBM8A	-0.0537392	0.0575523	-0.9337	0.35	-0.076202989	count	1
ACADM	-0.0550172	0.0864609	-0.6363	0.525	-0.07617171	count	1
HSCB	-0.059423	0.137343	-0.4327	0.665	-0.076168838	count	1
SPRY4	-0.0664323	0.3200046	-0.2076	0.836	-0.076160774	count	1
SPOP	-0.0547691	0.0899203	-0.6091	0.543	-0.076152814	count	1
FANCL	-0.0605625	0.1565456	-0.3869	0.699	-0.076116603	count	1
PRPF6	-0.0544885	0.0812955	-0.6703	0.503	-0.076066176	count	1
CHRM3	-0.2103866	0.5227528	-0.4025	0.687	-0.076040357	count	1
ADPRH	-0.0751286	0.5534025	-0.1358	0.892	-0.075967613	count	1
ABCB8	-0.0628457	0.2469377	-0.2545	0.799	-0.075936499	count	1
POLR1C	-0.0613404	0.1780606	-0.3445	0.73	-0.075922721	count	1
CSNK1A1	-0.0533291	0.0526843	-1.0122	0.311	-0.075896198	count	1
RNPS1	-0.0541294	0.0751316	-0.7205	0.471	-0.07589101	count	1
YJEFN3	-0.1793792	0.6502411	-0.2759	0.783	-0.075860246	count	1
SCAP	-0.0658868	0.2080492	-0.3167	0.751	-0.075851143	count	1
LRRC37A3	-0.1235739	0.4911042	-0.2516	0.801	-0.075595635	count	1
WWOX	-0.0599791	0.2205233	-0.272	0.786	-0.075496375	count	1
FAM111A-DT	-0.0744762	0.2770427	-0.2688	0.788	-0.07530423	count	1
CAPZA2	-0.0529791	0.0528196	-1.003	0.316	-0.075245659	count	1
TMEM11	-0.0561283	0.1176749	-0.477	0.633	-0.075067264	count	1
ZNF846	-0.0662979	0.1907507	-0.3476	0.728	-0.074979742	count	1
RCBTB1	-0.1009739	0.2714919	-0.3719	0.71	-0.074867937	count	1
MRPL11	-0.0532797	0.0738904	-0.7211	0.471	-0.074563628	count	1
PHF11	-0.054243	0.1023616	-0.5299	0.596	-0.074517419	count	1
ZCCHC17	-0.0537032	0.0872672	-0.6154	0.538	-0.074411113	count	1
UNC13D	-0.2549809	0.62002	-0.4112	0.681	-0.074368092	count	1
MRPL1	-0.0545541	0.1142785	-0.4774	0.633	-0.074242609	count	1
TMEM187	-0.059974	0.1779284	-0.3371	0.736	-0.074227841	count	1
PARVA	-0.0534266	0.088365	-0.6046	0.545	-0.074112535	count	1

NAP1L2	-0.0907796	0.2534889	-0.3581	0.72	-0.073975546	count	1
MARS	-0.0612089	0.1825805	-0.3352	0.737	-0.073953774	count	1
CAMK1	-0.054499	0.0934643	-0.5831	0.56	-0.073909691	count	1
B4GALT3	-0.0607187	0.1785018	-0.3402	0.734	-0.073897835	count	1
CBLL1	-0.0608604	0.1963591	-0.3099	0.757	-0.073895605	count	1
ENGASE	-0.0931742	0.253887	-0.367	0.714	-0.073806603	count	1
WDR61	-0.0530619	0.0929456	-0.5709	0.568	-0.073761657	count	1
GPAT4	-0.0798414	0.2238292	-0.3567	0.721	-0.073749492	count	1
NIP7	-0.0569902	0.162288	-0.3512	0.725	-0.07373099	count	1
THAP11	-0.0541864	0.1012521	-0.5352	0.593	-0.073721524	count	1
CYB5R2	-0.0661809	0.26635	-0.2485	0.804	-0.07370932	count	1
SAC3D1	-0.0612651	0.1667065	-0.3675	0.713	-0.073635813	count	1
FAM157C	-0.2035794	0.6559794	-0.3103	0.756	-0.073490944	count	1
CCDC184	-0.077145	0.3315904	-0.2327	0.816	-0.073443873	count	1
C2orf49	-0.0539348	0.0999552	-0.5396	0.59	-0.073439	count	1
BRPF3	-0.080659	0.2812607	-0.2868	0.774	-0.073255638	count	1
KRBA1	-0.1281706	0.4121725	-0.311	0.756	-0.073193457	count	1
PTP4A1	-0.056112	0.1339574	-0.4189	0.675	-0.07317438	count	1
ARHGEF7-AS2	-0.2510238	0.4111467	-0.6105	0.542	-0.073160331	count	1
AL354733.3	-0.1731132	0.5523408	-0.3134	0.754	-0.073132236	count	1
LAG3	-0.1731132	0.9226438	-0.1876	0.851	-0.073132236	count	1
ZNF502	-0.0818887	0.3382719	-0.2421	0.809	-0.073028664	count	1
ZNF470	-0.0873231	0.3567999	-0.2447	0.807	-0.073001347	count	1
EIF3A	-0.0522596	0.0828287	-0.6309	0.528	-0.07296592	count	1
RAD51AP2	-0.6059481	0.8407578	-0.7207	0.471	-0.072949831	count	1
EGOT	-0.6059481	0.8407578	-0.7207	0.471	-0.072949831	count	1
AC003986.2	-0.6059481	0.8407578	-0.7207	0.471	-0.072949831	count	1
PDE2A	-0.6059481	0.8502984	-0.7126	0.476	-0.072949831	count	1
USP6	-0.6059481	0.9140813	-0.6629	0.507	-0.072949831	count	1
AL136366.1	-0.1526895	0.5824483	-0.2622	0.793	-0.072876637	count	1
DPH5	-0.0657698	0.189406	-0.3472	0.728	-0.072844618	count	1
C6orf106	-0.0551497	0.1325634	-0.416	0.677	-0.072711838	count	1
PIGS	-0.0590386	0.2473861	-0.2386	0.811	-0.072637475	count	1
COL24A1	-0.0717837	0.2967094	-0.2419	0.809	-0.072567056	count	1
CD58	-0.0524533	0.0933121	-0.5621	0.574	-0.072507123	count	1
CDKL5	-0.0606295	0.2346555	-0.2584	0.796	-0.07246674	count	1
PSMB2	-0.0511245	0.0551462	-0.9271	0.354	-0.07246062	count	1
SSBP3	-0.0544629	0.1118244	-0.487	0.626	-0.072403274	count	1
C1QBP	-0.0509959	0.05569	-0.9157	0.36	-0.072294532	count	1
CIR1	-0.0510354	0.0596415	-0.8557	0.392	-0.072275084	count	1
PDLIM2	-0.0506231	0.047913	-1.0566	0.2908	-0.072198802	count	1
CACNA2D1	-0.054244	0.129262	-0.4196	0.675	-0.072150495	count	1
AC104596.1	-0.150729	0.6041169	-0.2495	0.803	-0.071917901	count	1
MYZAP	-0.150729	0.6922472	-0.2177	0.828	-0.071917901	count	1
AC083862.2	-0.3390676	0.8457172	-0.4009	0.689	-0.071879134	count	1

AC069544.1	-0.0820959	0.488585	-0.168	0.867	-0.071772707	count	1
RBCK1	-0.0518681	0.0909657	-0.5702	0.569	-0.071749522	count	1
ZNF467	-0.1698997	0.5103824	-0.3329	0.739	-0.071735271	count	1
FKBP2	-0.0501085	0.0405429	-1.2359	0.217	-0.071675152	count	1
DDX54	-0.0571184	0.1283127	-0.4452	0.656	-0.07166856	count	1
PIP5K1B	-0.0857398	0.2210106	-0.3879	0.698	-0.071665746	count	1
SEM1	-0.0500544	0.0406907	-1.2301	0.219	-0.071613757	count	1
PITPNM1	-0.0818767	0.3632398	-0.2254	0.822	-0.071579517	count	1
UBR3	-0.058013	0.1846587	-0.3142	0.753	-0.071517136	count	1
LINC01703	-0.3373959	0.9234101	-0.3654	0.715	-0.071502126	count	1
AC112220.2	-0.3373959	0.9234446	-0.3654	0.715	-0.071502126	count	1
AL080317.1	-0.3373959	0.9234446	-0.3654	0.715	-0.071502126	count	1
AL078581.1	-0.3373959	0.9234446	-0.3654	0.715	-0.071502126	count	1
GLB1L2	-0.3373959	0.9234101	-0.3654	0.715	-0.071502126	count	1
FAM189A1	-0.3373959	0.9234446	-0.3654	0.715	-0.071502126	count	1
AC011471.2	-0.3373959	0.9234446	-0.3654	0.715	-0.071502126	count	1
NCAPG	-0.3373959	0.9320857	-0.362	0.717	-0.071502126	count	1
VPS33B-DT	-0.3373959	0.9320857	-0.362	0.717	-0.071502126	count	1
GTF2H4	-0.3373959	1.078871	-0.3127	0.755	-0.071502126	count	1
AC022390.1	-0.3373959	1.078871	-0.3127	0.755	-0.071502126	count	1
AC106881.1	-0.0635086	0.3684068	-0.1724	0.863	-0.071463096	count	1
NGEF	-0.1101037	0.6013287	-0.1831	0.855	-0.071339675	count	1
DNAJC15	-0.0501201	0.0653365	-0.7671	0.443	-0.071227393	count	1
ZBTB37	-0.0643027	0.2458757	-0.2615	0.794	-0.071213559	count	1
EIF2B1	-0.0529853	0.1158379	-0.4574	0.647	-0.071139571	count	1
COX17	-0.0501407	0.0580304	-0.864	0.388	-0.071080133	count	1
NAA38	-0.0502119	0.0593691	-0.8458	0.398	-0.071029443	count	1
PCYT2	-0.0567778	0.161264	-0.3521	0.725	-0.071011948	count	1
GUCY1B1	-0.0513369	0.082524	-0.6221	0.534	-0.071004199	count	1
ZFP69	-0.3349521	0.5083101	-0.659	0.51	-0.07095136	count	1
TEN1	-0.0562045	0.178201	-0.3154	0.752	-0.070944755	count	1
AC114760.2	-0.5904452	0.5606523	-1.0531	0.292	-0.070895714	count	1
LSS	-0.0615348	0.2168491	-0.2838	0.777	-0.070825201	count	1
PLA2R1	-0.0516758	0.1903057	-0.2715	0.786	-0.070723869	count	1
FLOT1	-0.0504883	0.0790608	-0.6386	0.523	-0.070671283	count	1
BAMBI	-0.0538562	0.1766164	-0.3049	0.76	-0.070611963	count	1
TACR2	-0.1479926	0.6573468	-0.2251	0.822	-0.070580685	count	1
ITIH1	-0.3331866	0.9187006	-0.3627	0.717	-0.070553736	count	1
TMEM151A	-0.3331866	0.9187006	-0.3627	0.717	-0.070553736	count	1
AL109924.2	-0.3331866	0.9337596	-0.3568	0.721	-0.070553736	count	1
CD300C	-0.3331866	0.9337596	-0.3568	0.721	-0.070553736	count	1
SPATA45	-0.3331866	1.189264	-0.2802	0.779	-0.070553736	count	1
AL390955.2	-0.3331866	1.189264	-0.2802	0.779	-0.070553736	count	1
AL390067.1	-0.3331866	1.189264	-0.2802	0.779	-0.070553736	count	1
AL928654.3	-0.3331866	1.189264	-0.2802	0.779	-0.070553736	count	1

ZNF691	-0.0690632	0.2845436	-0.2427	0.808	-0.070506176	count	1
SCOC-AS1	-0.5860484	0.7329593	-0.7996	0.424	-0.070314561	count	1
AC093424.1	-0.5860484	0.7795905	-0.7517	0.452	-0.070314561	count	1
EAF1-AS1	-0.5860484	0.7904078	-0.7415	0.458	-0.070314561	count	1
MEIOC	-0.5860484	0.8418973	-0.6961	0.486	-0.070314561	count	1
IER5	-0.0524753	0.1188352	-0.4416	0.659	-0.070303676	count	1
SF3A2	-0.0587901	0.1761241	-0.3338	0.739	-0.07026259	count	1
LINC00271	-0.1334021	0.5350878	-0.2493	0.803	-0.07018764	count	1
AFTPH	-0.0569244	0.1553481	-0.3664	0.714	-0.07017231	count	1
REP15	-0.1941666	0.7767046	-0.25	0.803	-0.06997499	count	1
AC022784.3	-0.1941666	0.8508702	-0.2282	0.82	-0.06997499	count	1
AC011416.3	-0.1941666	1.0133953	-0.1916	0.848	-0.06997499	count	1
LINC02334	-0.1941666	1.194887	-0.1625	0.871	-0.06997499	count	1
SYNJ2BP	-0.0509081	0.1179553	-0.4316	0.666	-0.069748713	count	1
LIG3	-0.0636392	0.2301289	-0.2765	0.782	-0.069646056	count	1
SLC25A39	-0.0514652	0.1293102	-0.398	0.691	-0.069613377	count	1
THAP7	-0.0513156	0.1052057	-0.4878	0.626	-0.069501727	count	1
REM1	-0.0537531	0.1509466	-0.3561	0.722	-0.069261863	count	1
FOPNL	-0.0528465	0.1199492	-0.4406	0.66	-0.069235154	count	1
ALS2	-0.0649847	0.2846466	-0.2283	0.819	-0.069215271	count	1
CLK3	-0.05339	0.1598525	-0.334	0.738	-0.069196702	count	1
ARL17A	-0.0648616	0.4342802	-0.1494	0.881	-0.069083593	count	1
TINF2	-0.0506172	0.1024465	-0.4941	0.621	-0.069078782	count	1
ACTR1A	-0.0508259	0.1018226	-0.4992	0.618	-0.06906817	count	1
NDUFA1	-0.0484674	0.0421414	-1.1501	0.2502	-0.069061375	count	1
DNAJC19	-0.0489089	0.0697577	-0.7011	0.483	-0.069046035	count	1
TMEM8B	-0.0647849	0.2019908	-0.3207	0.748	-0.069001549	count	1
MPP1	-0.0562608	0.1663912	-0.3381	0.735	-0.068922627	count	1
ZFAND6	-0.0498562	0.0819512	-0.6084	0.543	-0.068874141	count	1
KBTBD4	-0.0581489	0.205839	-0.2825	0.778	-0.068872276	count	1
ZYX	-0.0486901	0.0687516	-0.7082	0.479	-0.068775052	count	1
ABCB10	-0.0722444	0.2987333	-0.2418	0.809	-0.068749464	count	1
AC097532.2	-0.5741764	0.646358	-0.8883	0.374	-0.068748546	count	1
ETFA	-0.0495032	0.0783382	-0.6319	0.527	-0.068725681	count	1
ARMC10	-0.0500157	0.0934643	-0.5351	0.593	-0.068719745	count	1
AC087623.3	-0.1306546	0.5404336	-0.2418	0.809	-0.068712522	count	1
RPE	-0.0527826	0.1495112	-0.353	0.724	-0.0685328	count	1
TRIM56	-0.0501524	0.1420101	-0.3532	0.724	-0.068527059	count	1
ZMYND11	-0.0496872	0.0876399	-0.5669	0.571	-0.068332213	count	1
LAMTOR5	-0.0480797	0.0502789	-0.9563	0.339	-0.068293783	count	1
TADA2B	-0.0588704	0.2044071	-0.288	0.773	-0.068291048	count	1
MAP3K12	-0.0519825	0.1417797	-0.3666	0.714	-0.068202132	count	1
AC026471.2	-0.1115235	0.4536234	-0.2459	0.806	-0.068106634	count	1
MRPS2	-0.0500951	0.107143	-0.4676	0.64	-0.068093819	count	1
MYLK-AS1	-0.2343199	0.5197439	-0.4508	0.652	-0.068079552	count	1

COLQ	-0.3214209	0.6645757	-0.4836	0.629	-0.06790973	count	1
ASF1A	-0.0504734	0.1135081	-0.4447	0.657	-0.067528213	count	1
OGFOD1	-0.052872	0.204423	-0.2586	0.796	-0.067523474	count	1
AL121917.1	-0.1415901	0.8106787	-0.1747	0.861	-0.067456307	count	1
RAB30-AS1	-0.0520286	0.1403194	-0.3708	0.711	-0.067303152	count	1
ZC3H12B	-0.0769455	0.4072498	-0.1889	0.85	-0.067235552	count	1
CFAP300	-0.057477	0.1988759	-0.289	0.773	-0.067166773	count	1
UBE2H	-0.0476364	0.0665285	-0.716	0.474	-0.067072679	count	1
PLA2G16	-0.0474693	0.0646882	-0.7338	0.463	-0.067066883	count	1
UBE2L6	-0.0481147	0.0824316	-0.5837	0.559	-0.067041323	count	1
NOV	-0.0465604	0.074014	-0.6291	0.529	-0.066982235	count	1
C16orf86	-0.0615477	0.203146	-0.303	0.762	-0.066923174	count	1
PILRB	-0.0536385	0.2427625	-0.221	0.825	-0.066853106	count	1
ASMTL	-0.0514683	0.133723	-0.3849	0.7	-0.066702741	count	1
RNF5	-0.0481196	0.0833629	-0.5772	0.564	-0.066589666	count	1
LDLRAP1	-0.0532009	0.1913418	-0.278	0.781	-0.066530258	count	1
BTN3A2	-0.0496624	0.1269417	-0.3912	0.696	-0.0665022	count	1
WDTC1	-0.0596426	0.2136655	-0.2791	0.78	-0.066402134	count	1
MIF-AS1	-0.102541	0.5570855	-0.1841	0.854	-0.066370596	count	1
SMN1	-0.0670131	0.305455	-0.2194	0.826	-0.066250075	count	1
PSMB1	-0.0461781	0.0315331	-1.4644	0.143	-0.066211418	count	1
ERBB4	-0.054239	0.1850799	-0.2931	0.769	-0.066147039	count	1
NSUN3	-0.0602909	0.2030331	-0.297	0.767	-0.065968165	count	1
TXN	-0.0459657	0.0377395	-1.218	0.223	-0.065965957	count	1
D2HGDH	-0.0592398	0.2252777	-0.263	0.793	-0.06595214	count	1
AC092745.1	-0.5514368	0.8607435	-0.6407	0.522	-0.065762237	count	1
FBXO41	-0.5514368	0.9136269	-0.6036	0.546	-0.065762237	count	1
AC091488.1	-0.5514368	0.9136269	-0.6036	0.546	-0.065762237	count	1
CNNM1	-0.5514368	0.9136269	-0.6036	0.546	-0.065762237	count	1
RNASEH2B-AS1	-0.5514368	0.9136269	-0.6036	0.546	-0.065762237	count	1
AL133523.1	-0.5514368	0.9136269	-0.6036	0.546	-0.065762237	count	1
HID1	-0.5514368	0.9136269	-0.6036	0.546	-0.065762237	count	1
LDLRAD4-AS1	-0.5514368	0.9136269	-0.6036	0.546	-0.065762237	count	1
LINC01311	-0.5514368	0.9136269	-0.6036	0.546	-0.065762237	count	1
CCIN	-0.5514368	1.029234	-0.5358	0.592	-0.065762237	count	1
MOBP	-0.5514368	1.063363	-0.5186	0.604	-0.065762237	count	1
CASS4	-0.5514368	1.063363	-0.5186	0.604	-0.065762237	count	1
OXCT2	-0.226612	0.7426154	-0.3052	0.76	-0.065744679	count	1
AL162311.3	-0.226612	0.7425914	-0.3052	0.76	-0.065744679	count	1
DOLPP1	-0.0711984	0.2878274	-0.2474	0.805	-0.065714345	count	1
VPS37A	-0.0492906	0.1196825	-0.4118	0.68	-0.065658061	count	1
TNRC6C	-0.0490617	0.1439228	-0.3409	0.733	-0.065637785	count	1
SLC9A7	-0.137804	0.5485522	-0.2512	0.802	-0.065611613	count	1
FBXL12	-0.0501747	0.1219667	-0.4114	0.681	-0.065528131	count	1
LANCL3	-0.0919265	0.94768	-0.097	0.923	-0.065431837	count	1

TRAM2	-0.0523484	0.1592818	-0.3287	0.742	-0.06524225	count	1
PPP1R35	-0.0486386	0.1187305	-0.4097	0.682	-0.065187476	count	1
PGM5-AS1	-0.0780152	0.3869257	-0.2016	0.84	-0.06515577	count	1
ZNF33A	-0.050418	0.1337388	-0.377	0.706	-0.065153982	count	1
FAM120A	-0.0487138	0.1154144	-0.4221	0.673	-0.065141961	count	1
HAUS1	-0.0494148	0.1564129	-0.3159	0.752	-0.065056175	count	1
UTP6	-0.0489091	0.1197465	-0.4084	0.683	-0.065012791	count	1
RNF19B	-0.0604608	0.2305821	-0.2622	0.793	-0.064849334	count	1
CNNM3	-0.0952342	0.3154935	-0.3019	0.763	-0.064830591	count	1
PPIH	-0.0487093	0.1211653	-0.402	0.688	-0.064815818	count	1
AHCTF1	-0.0500077	0.1385884	-0.3608	0.718	-0.06474693	count	1
HAUS4	-0.0654853	0.2628339	-0.2492	0.803	-0.064731771	count	1
CFAP58	-0.543233	0.8072434	-0.6729	0.501	-0.064689226	count	1
AL137003.2	-0.543233	0.8291265	-0.6552	0.512	-0.064689226	count	1
SPAG5	-0.543233	0.9155067	-0.5934	0.553	-0.064689226	count	1
LINC01684	-0.543233	1.03041	-0.5272	0.598	-0.064689226	count	1
CBY1	-0.0472725	0.099884	-0.4733	0.636	-0.06457412	count	1
COL6A1	-0.0451476	0.0540642	-0.8351	0.404	-0.06444666	count	1
VXN	-0.5413447	0.4046552	-1.3378	0.181	-0.064442583	count	1
PRRC2B	-0.0480014	0.1196476	-0.4012	0.688	-0.064388162	count	1
EP400	-0.0518514	0.2087114	-0.2484	0.804	-0.064276311	count	1
FAM214B	-0.054444	0.26207	-0.2077	0.835	-0.064267643	count	1
AL132639.2	-0.539181	0.7319947	-0.7366	0.461	-0.064160114	count	1
SLCO1C1	-0.5391166	0.3135557	-1.7194	0.0856	-0.06415171	count	1
MPO	-0.1783769	1.055665	-0.169	0.866	-0.064101538	count	1
LAMTOR3	-0.0485666	0.1198774	-0.4051	0.685	-0.064064673	count	1
ARF3	-0.0466422	0.1049646	-0.4444	0.657	-0.063858938	count	1
AL357079.1	-0.5367657	0.684724	-0.7839	0.433	-0.063845005	count	1
STAMBP	-0.0470815	0.1126641	-0.4179	0.676	-0.063783203	count	1
RNF217	-0.0480323	0.1522241	-0.3155	0.752	-0.063705045	count	1
RP9	-0.0469509	0.111082	-0.4227	0.673	-0.063646147	count	1
NCL	-0.0443475	0.0349758	-1.2679	0.205	-0.063592957	count	1
TMUB1	-0.048982	0.1561103	-0.3138	0.754	-0.063534638	count	1
PSMB3	-0.0445359	0.0439403	-1.0136	0.311	-0.0635127	count	1
TSC2	-0.0512877	0.1876946	-0.2733	0.785	-0.06345714	count	1
NEMP1	-0.0801084	0.4343991	-0.1844	0.854	-0.063362751	count	1
GORASP2	-0.0459312	0.0818238	-0.5613	0.575	-0.063265253	count	1
MRPS35	-0.0458543	0.0861698	-0.5321	0.595	-0.063233482	count	1
C10orf88	-0.0619611	0.2704172	-0.2291	0.819	-0.063222356	count	1
PPP1R7	-0.0451191	0.0625928	-0.7208	0.471	-0.063162363	count	1
AP5S1	-0.0664049	0.2787029	-0.2383	0.812	-0.063160627	count	1
PIK3R2	-0.1755194	0.730286	-0.2403	0.81	-0.063041908	count	1
GGH	-0.0534678	0.1995213	-0.268	0.789	-0.062904498	count	1
MCM7	-0.0582449	0.2371119	-0.2456	0.806	-0.062899825	count	1
MIA2	-0.0484822	0.1304581	-0.3716	0.71	-0.062769295	count	1

AL049629.2	-0.2978864	0.6574869	-0.4531	0.651	-0.062651965	count	1
DNPEP	-0.0457924	0.1037454	-0.4414	0.659	-0.062595432	count	1
TMEM268	-0.1098586	0.3525792	-0.3116	0.755	-0.06256414	count	1
TRIM62	-0.1314196	0.5032524	-0.2611	0.794	-0.06250584	count	1
MED13L	-0.0457149	0.104034	-0.4394	0.66	-0.062503952	count	1
NRBP1	-0.0450755	0.0905948	-0.4976	0.619	-0.062400968	count	1
ADNP-AS1	-0.1187623	0.4488359	-0.2646	0.791	-0.06234142	count	1
GPKOW	-0.0499688	0.1973441	-0.2532	0.8	-0.06227138	count	1
GPX4	-0.0432446	0.0289676	-1.4929	0.136	-0.06217668	count	1
SDF4	-0.0439661	0.0580193	-0.7578	0.449	-0.062139793	count	1
PNN	-0.0438563	0.0654095	-0.6705	0.503	-0.061904359	count	1
UBE3A	-0.0451407	0.0951051	-0.4746	0.635	-0.061894466	count	1
BRI3	-0.0431435	0.0372923	-1.1569	0.247	-0.061862282	count	1
ZCWPW2	-0.0680204	0.3099575	-0.2195	0.826	-0.061703962	count	1
TTC23	-0.0517872	0.1939712	-0.267	0.789	-0.061694477	count	1
USP9X	-0.0466973	0.1217186	-0.3837	0.701	-0.061635755	count	1
RNASEH2A	-0.2129626	0.6924401	-0.3076	0.758	-0.061625076	count	1
AC008764.6	-0.2129626	0.7018394	-0.3034	0.762	-0.061625076	count	1
ANKRD7	-0.2129626	1.093022	-0.1948	0.846	-0.061625076	count	1
PDK3	-0.0477666	0.1700939	-0.2808	0.779	-0.061474219	count	1
WDYHV1	-0.0522394	0.179703	-0.2907	0.771	-0.061455735	count	1
PGP	-0.0451025	0.1171666	-0.3849	0.7	-0.061388308	count	1
RAPGEF3	-0.0754192	0.4362586	-0.1729	0.863	-0.061355033	count	1
TMEM254	-0.0585397	0.2367913	-0.2472	0.805	-0.061349683	count	1
AC009133.1	-0.0901383	0.383268	-0.2352	0.814	-0.061320064	count	1
PCDH1	-0.0900648	0.2816059	-0.3198	0.749	-0.061269464	count	1
LYSMD2	-0.044938	0.0975772	-0.4605	0.645	-0.061164264	count	1
ITFG2-AS1	-0.0826715	0.4681775	-0.1766	0.86	-0.06116069	count	1
BLOC1S4	-0.044772	0.1041256	-0.43	0.667	-0.061141643	count	1
ZSWIM8	-0.0507992	0.2145514	-0.2368	0.813	-0.061029454	count	1
FZD3	-0.0698186	0.2948238	-0.2368	0.813	-0.060964598	count	1
EGFL6	-0.0730052	0.4208479	-0.1735	0.862	-0.060939023	count	1
AC023157.3	-0.0534043	0.2099053	-0.2544	0.799	-0.060916442	count	1
SND1-IT1	-0.5142308	0.5130877	-1.0022	0.316	-0.060915003	count	1
NSD2	-0.0563763	0.3127731	-0.1802	0.857	-0.060874591	count	1
IPO4	-0.1160129	0.4220689	-0.2749	0.783	-0.060871683	count	1
SMIM7	-0.0429738	0.0572292	-0.7509	0.453	-0.060842491	count	1
ZFPM1	-0.0448251	0.1135871	-0.3946	0.693	-0.060799914	count	1
IVNS1ABP	-0.0485438	0.1919334	-0.2529	0.8	-0.060696707	count	1
ARHGEF11	-0.0569048	0.2567367	-0.2216	0.825	-0.060576744	count	1
MTSS1L	-0.0765364	0.3974099	-0.1926	0.847	-0.060512728	count	1
NXN	-0.0510839	0.3136939	-0.1628	0.871	-0.060484875	count	1
MOB1A	-0.0428244	0.0716635	-0.5976	0.55	-0.059917917	count	1
AZIN1-AS1	-0.0672059	0.3247676	-0.2069	0.836	-0.059849507	count	1
FBXO31	-0.0553872	0.2953807	-0.1875	0.851	-0.059802769	count	1

TMTC2	-0.0628255	0.4815564	-0.1305	0.896	-0.059737559	count	1
RPP25L	-0.0458678	0.1302808	-0.3521	0.725	-0.059648936	count	1
AL445228.2	-0.2843111	0.7514683	-0.3783	0.705	-0.0596382	count	1
AMT	-0.2843111	0.7514683	-0.3783	0.705	-0.0596382	count	1
NUP85	-0.0530137	0.2328749	-0.2276	0.82	-0.05961861	count	1
LAS1L	-0.047587	0.1846908	-0.2577	0.797	-0.059595343	count	1
YIF1B	-0.0479945	0.1939449	-0.2475	0.805	-0.059261304	count	1
PFDN6	-0.0447416	0.1159051	-0.386	0.7	-0.059198019	count	1
B2M	-0.041033	0.0227857	-1.8008	0.0718	-0.059173563	count	1
CCDC107	-0.0414101	0.050029	-0.8277	0.408	-0.059133572	count	1
DNM2	-0.0477886	0.176098	-0.2714	0.786	-0.05912024	count	1
TBC1D23	-0.0443979	0.1232264	-0.3603	0.719	-0.059073715	count	1
GOLGA8R	-0.2044759	0.5744186	-0.356	0.722	-0.059073455	count	1
NIFK	-0.0422514	0.0860241	-0.4912	0.623	-0.059060945	count	1
SAAL1	-0.0515133	0.2056565	-0.2505	0.802	-0.059010957	count	1
ASB7	-0.066194	0.3028611	-0.2186	0.827	-0.058942562	count	1
AC007389.5	-0.1402651	0.7195604	-0.1949	0.845	-0.05892007	count	1
KIT	-0.1402651	0.8492474	-0.1652	0.869	-0.05892007	count	1
TMLHE	-0.0535427	0.2169075	-0.2468	0.805	-0.058916433	count	1
DDX58	-0.0497574	0.1687302	-0.2949	0.768	-0.058910671	count	1
PFDN2	-0.0415041	0.0557564	-0.7444	0.457	-0.058840301	count	1
MAML1	-0.0548358	0.2481897	-0.2209	0.825	-0.058794391	count	1
HRH1	-0.0447227	0.1306399	-0.3423	0.732	-0.058751188	count	1
NAV2-AS3	-0.0863963	0.4620558	-0.187	0.852	-0.058745122	count	1
METTL24	-0.0742284	0.5844586	-0.127	0.899	-0.058672409	count	1
TTL	-0.0475418	0.2727586	-0.1743	0.862	-0.058585667	count	1
GIPC2	-0.0719736	0.5856922	-0.1229	0.902	-0.058529654	count	1
ZNF513	-0.0563149	0.3077687	-0.183	0.855	-0.058508526	count	1
SLC4A2	-0.0481407	0.1940346	-0.2481	0.804	-0.058421527	count	1
RCSD1	-0.0469891	0.1925705	-0.244	0.807	-0.058345441	count	1
AL359711.2	-0.1022232	0.553689	-0.1846	0.854	-0.058148506	count	1
APCDD1L	-0.1022232	0.5604878	-0.1824	0.855	-0.058148506	count	1
AC005332.1	-0.4922956	0.8463	-0.5817	0.561	-0.058080768	count	1
C9orf78	-0.0409864	0.0653843	-0.6269	0.531	-0.058033088	count	1
TMEM216	-0.0438874	0.1487536	-0.295	0.768	-0.058031645	count	1
PFDN4	-0.0421522	0.0911923	-0.4622	0.644	-0.058027206	count	1
TMEM17	-0.1103848	0.377971	-0.292	0.77	-0.057866881	count	1
LYRM4	-0.0431349	0.1659894	-0.2599	0.795	-0.05785526	count	1
EXD2	-0.0600145	0.2787155	-0.2153	0.83	-0.057839851	count	1
PLXNA3	-0.0516543	0.2502701	-0.2064	0.836	-0.057788989	count	1
FARP2	-0.0555957	0.2306087	-0.2411	0.81	-0.057758339	count	1
RPF1	-0.044666	0.1446729	-0.3087	0.758	-0.057538662	count	1
AC011611.4	-0.1988937	0.6199876	-0.3208	0.748	-0.057399235	count	1
WWP2	-0.0435665	0.2055064	-0.212	0.832	-0.057230794	count	1
FBXO30	-0.0507272	0.2200421	-0.2305	0.818	-0.057039873	count	1

AC006942.1	-0.0667341	0.3119574	-0.2139	0.831	-0.057002629	count	1
HLA-DRA	-0.0405574	0.1145837	-0.354	0.723	-0.057000855	count	1
ID4	-0.0396212	0.0537167	-0.7376	0.461	-0.056940796	count	1
GSS	-0.0457553	0.1819082	-0.2515	0.801	-0.056912639	count	1
RANBP1	-0.0402625	0.0622643	-0.6466	0.518	-0.0568283	count	1
ZCCHC10	-0.0431057	0.1367953	-0.3151	0.753	-0.056817404	count	1
ZC3H15	-0.0400537	0.0553989	-0.723	0.47	-0.056809438	count	1
AL034417.2	-0.0741538	0.3948246	-0.1878	0.851	-0.056779506	count	1
CCNJ	-0.0767396	0.4166629	-0.1842	0.854	-0.056730717	count	1
OAZ1	-0.0394308	0.0262482	-1.5022	0.133	-0.056703237	count	1
CRYAB	-0.0392973	0.0448933	-0.8753	0.381	-0.056576692	count	1
NOA1	-0.0454011	0.1397502	-0.3249	0.745	-0.056570063	count	1
AL603910.1	-0.4804742	0.7443654	-0.6455	0.519	-0.056560793	count	1
FLG-AS1	-0.4804742	0.8037706	-0.5978	0.55	-0.056560793	count	1
AC005480.1	-0.4804742	0.8037706	-0.5978	0.55	-0.056560793	count	1
CAPS2	-0.053938	0.2765224	-0.1951	0.845	-0.056508907	count	1
MRPS28	-0.0445928	0.1370541	-0.3254	0.745	-0.056503008	count	1
MRPL27	-0.040231	0.0725862	-0.5543	0.579	-0.056477311	count	1
AC012358.3	-0.0763995	0.3826017	-0.1997	0.842	-0.056476918	count	1
ABCB1	-0.1077779	0.878713	-0.1227	0.902	-0.056476806	count	1
STOML1	-0.0456948	0.173209	-0.2638	0.792	-0.056416945	count	1
ACSL1	-0.0470486	0.23131	-0.2034	0.839	-0.056359787	count	1
EMC4	-0.0396896	0.0527748	-0.7521	0.452	-0.056357608	count	1
FAM32A	-0.0403398	0.0781204	-0.5164	0.606	-0.056355842	count	1
CTBP2	-0.0413406	0.106179	-0.3893	0.697	-0.056339849	count	1
AC007686.3	-0.0645473	0.3462749	-0.1864	0.852	-0.056331931	count	1
PTCD2	-0.060133	0.2847202	-0.2112	0.833	-0.05632875	count	1
WDR25	-0.0517234	0.2108397	-0.2453	0.806	-0.056206087	count	1
MKKS	-0.0441537	0.1493592	-0.2956	0.768	-0.056166416	count	1
CLIC1	-0.0389957	0.0314882	-1.2384	0.216	-0.056022644	count	1
SSR4	-0.0389353	0.0327091	-1.1904	0.234	-0.055912796	count	1
FN3KRP	-0.0425887	0.1203228	-0.354	0.723	-0.055905358	count	1
AGAP4	-0.0669498	0.4148439	-0.1614	0.872	-0.055848196	count	1
AC073389.1	-0.0914634	0.4033066	-0.2268	0.821	-0.055694265	count	1
ADAR	-0.0410364	0.1252603	-0.3276	0.743	-0.055640663	count	1
TIMM17B	-0.0399895	0.0927505	-0.4312	0.666	-0.055576654	count	1
AC237221.1	-0.0974797	0.5353719	-0.1821	0.856	-0.055410194	count	1
NBPF10	-0.1320151	0.5223206	-0.2527	0.8	-0.05537438	count	1
DAB2IP	-0.0477108	0.2147698	-0.2221	0.824	-0.055314705	count	1
WBP2	-0.0397264	0.0810414	-0.4902	0.624	-0.055259124	count	1
RYR1	-0.1314778	0.6285447	-0.2092	0.834	-0.055143794	count	1
MGAT5	-0.0450994	0.1724908	-0.2615	0.794	-0.05510471	count	1
KAT6A	-0.0425255	0.1392977	-0.3053	0.76	-0.054721124	count	1
ELMO2	-0.0481643	0.3098987	-0.1554	0.876	-0.054674821	count	1
MTCH1	-0.0382247	0.0461221	-0.8288	0.407	-0.054495667	count	1

G6PD	-0.0407181	0.1124919	-0.362	0.717	-0.054441632	count	1
PPP6C	-0.0398955	0.1198701	-0.3328	0.739	-0.054438466	count	1
VRK2	-0.0438907	0.170063	-0.2581	0.796	-0.054392388	count	1
TMEM18	-0.0385201	0.0742573	-0.5187	0.604	-0.054362169	count	1
DPF3	-0.4632961	0.5581464	-0.8301	0.407	-0.054361573	count	1
MAGEF1	-0.039521	0.097993	-0.4033	0.687	-0.054303817	count	1
EIF5AL1	-0.1293029	0.5420791	-0.2385	0.811	-0.054210843	count	1
AC006157.1	-0.4619697	0.6781531	-0.6812	0.496	-0.054192234	count	1
LMNA	-0.037781	0.0384178	-0.9834	0.325	-0.054189461	count	1
POFUT1	-0.0494339	0.2455791	-0.2013	0.84	-0.054052544	count	1
TEX22	-0.2589526	0.8192711	-0.3161	0.752	-0.054046576	count	1
RIPPLY2	-0.2589526	0.8218764	-0.3151	0.753	-0.054046576	count	1
AL157392.5	-0.2589526	0.8218764	-0.3151	0.753	-0.054046576	count	1
ARMS2	-0.2589526	0.8218764	-0.3151	0.753	-0.054046576	count	1
IL1RAPL1	-0.2589526	0.9447966	-0.2741	0.784	-0.054046576	count	1
SLC17A3	-0.2589526	0.9759874	-0.2653	0.791	-0.054046576	count	1
GAS2	-0.2589526	0.9971514	-0.2597	0.795	-0.054046576	count	1
GTF3C5	-0.0454401	0.1988022	-0.2286	0.819	-0.053955459	count	1
QTRT1	-0.0410103	0.1271752	-0.3225	0.747	-0.053944549	count	1
HNRNPH3	-0.0382632	0.0642134	-0.5959	0.551	-0.053935487	count	1
LZIC	-0.0392426	0.0833857	-0.4706	0.638	-0.053879409	count	1
MTREX	-0.0404452	0.1259491	-0.3211	0.748	-0.0538383	count	1
TRIM6	-0.1869178	0.5239133	-0.3568	0.721	-0.053818531	count	1
CCL2	-0.0375138	0.141854	-0.2645	0.791	-0.053816537	count	1
SLC35B1	-0.0404547	0.1345998	-0.3006	0.764	-0.053794365	count	1
AL139089.1	-0.1281487	0.5544396	-0.2311	0.817	-0.053716009	count	1
ARHGDIA	-0.0380713	0.0641952	-0.5931	0.553	-0.053655598	count	1
AC015802.6	-0.1861579	0.5595688	-0.3327	0.739	-0.053591839	count	1
ENY2	-0.0376464	0.0562421	-0.6694	0.503	-0.053494103	count	1
STARD3	-0.0440242	0.2022904	-0.2176	0.828	-0.053416941	count	1
VARS	-0.0468373	0.237381	-0.1973	0.844	-0.053406854	count	1
FAM13A-AS1	-0.1490654	0.5104381	-0.292	0.77	-0.053280834	count	1
CNOT3	-0.0486935	0.19704	-0.2471	0.805	-0.053240516	count	1
P4HB	-0.0373614	0.0501918	-0.7444	0.457	-0.053205614	count	1
DCLRE1B	-0.069372	0.3871944	-0.1792	0.858	-0.053087761	count	1
GLIDR	-0.0693251	0.4399548	-0.1576	0.875	-0.053051573	count	1
ADSL	-0.0550416	0.2589579	-0.2126	0.832	-0.05302474	count	1
UROD	-0.0379696	0.0760674	-0.4992	0.618	-0.052965448	count	1
AL355297.4	-0.0779825	0.4991547	-0.1562	0.876	-0.052964443	count	1
ZNF213	-0.0572903	0.266511	-0.215	0.83	-0.052810014	count	1
AKR7A2	-0.0374052	0.0604541	-0.6187	0.536	-0.052788416	count	1
SNRPD3	-0.0371235	0.050583	-0.7339	0.463	-0.052776412	count	1
MAN2A2	-0.0507465	0.2439916	-0.208	0.835	-0.052702168	count	1
HEY1	-0.0631	0.3386839	-0.1863	0.852	-0.052614957	count	1
ANK1	-0.061529	0.5122979	-0.1201	0.904	-0.052528078	count	1

RAB21	-0.0376834	0.0814481	-0.4627	0.644	-0.052491621	count	1
HOXA10	-0.1822624	0.8880993	-0.2052	0.837	-0.052430724	count	1
HIST2H4B	-0.1822624	0.9635616	-0.1892	0.85	-0.052430724	count	1
LMX1B	-0.1822624	0.9708073	-0.1877	0.851	-0.052430724	count	1
SLC22A17	-0.0391923	0.1263868	-0.3101	0.757	-0.052222708	count	1
FRY-AS1	-0.4462791	0.5018522	-0.8893	0.374	-0.052194293	count	1
ERCC1	-0.0376085	0.0846423	-0.4443	0.657	-0.052193669	count	1
MIR4435-2HG	-0.0393917	0.1272805	-0.3095	0.757	-0.052144701	count	1
EIF5	-0.0364597	0.0436512	-0.8353	0.4036	-0.052071071	count	1
RNF141	-0.0387671	0.1493475	-0.2596	0.795	-0.052058202	count	1
CCDC102A	-0.0388171	0.1465928	-0.2648	0.791	-0.051992119	count	1
RIC8A	-0.0385878	0.1040628	-0.3708	0.711	-0.051961121	count	1
FAM78B	-0.1453413	0.5136042	-0.283	0.777	-0.05191383	count	1
MDH2	-0.036456	0.0467448	-0.7799	0.436	-0.051903968	count	1
AC007388.1	-0.0408544	0.2187882	-0.1867	0.852	-0.0518975	count	1
PLEKHG5	-0.1092762	0.5079143	-0.2151	0.83	-0.051782038	count	1
PIK3C3	-0.0408664	0.1582586	-0.2582	0.796	-0.051775282	count	1
SLC27A5	-0.0399599	0.1564833	-0.2554	0.798	-0.05162476	count	1
SAP18	-0.0359851	0.0321394	-1.1197	0.263	-0.051611188	count	1
MFAP1	-0.0379208	0.0960153	-0.3949	0.693	-0.05144523	count	1
PARP1	-0.037713	0.1068598	-0.3529	0.724	-0.051393416	count	1
MRPL58	-0.0390457	0.1371396	-0.2847	0.776	-0.051286735	count	1
TMEM245	-0.0383533	0.1348175	-0.2845	0.776	-0.051103917	count	1
FKBP8	-0.0358456	0.0482917	-0.7423	0.458	-0.050973289	count	1
AC024909.2	-0.1772669	0.7380821	-0.2402	0.81	-0.050944114	count	1
AC012510.1	-0.1772669	0.7455539	-0.2378	0.812	-0.050944114	count	1
CCDC168	-0.1772669	0.8858339	-0.2001	0.841	-0.050944114	count	1
AL359397.2	-0.2444685	0.8884623	-0.2752	0.783	-0.050875367	count	1
ADAM10	-0.0380866	0.1430604	-0.2662	0.79	-0.050848564	count	1
SHARPIN	-0.0363478	0.090124	-0.4033	0.687	-0.050625574	count	1
CDK2AP2	-0.0359959	0.080727	-0.4459	0.656	-0.050607579	count	1
TRPA1	-0.2427801	0.4377817	-0.5546	0.579	-0.050506781	count	1
LRFN3	-0.0516615	0.2980454	-0.1733	0.862	-0.050398527	count	1
NKRF	-0.048897	0.2462911	-0.1985	0.843	-0.050319763	count	1
ZCCHC14	-0.0403114	0.1859771	-0.2168	0.828	-0.050305109	count	1
TMSB15A	-0.0680115	0.3574993	-0.1902	0.849	-0.050223902	count	1
NSFL1C	-0.0361866	0.0841877	-0.4298	0.667	-0.050131199	count	1
ZNF212	-0.065461	0.3369725	-0.1943	0.846	-0.050071343	count	1
KIAA1755	-0.0432295	0.2906475	-0.1487	0.882	-0.049911633	count	1
SMIM20	-0.0360229	0.0878634	-0.41	0.682	-0.049786292	count	1
DNAJA3	-0.0400361	0.2681569	-0.1493	0.881	-0.049515378	count	1
PSMB7	-0.0352456	0.0687619	-0.5126	0.608	-0.04950309	count	1
AC006504.1	-0.4246899	0.7750081	-0.548	0.584	-0.049461257	count	1
SAMM50	-0.0383885	0.126941	-0.3024	0.762	-0.049443428	count	1
DTWD1	-0.0354377	0.0836547	-0.4236	0.672	-0.049423501	count	1

PIK3C2A	-0.0365709	0.1161283	-0.3149	0.753	-0.04938744	count	1
C1orf56	-0.0367154	0.1213785	-0.3025	0.762	-0.049341474	count	1
PSAT1	-0.1177323	0.821796	-0.1433	0.886	-0.049258857	count	1
GAS6-AS1	-0.4228335	0.4287704	-0.9862	0.324	-0.049227129	count	1
APH1A	-0.0356334	0.0810651	-0.4396	0.66	-0.049108639	count	1
NMB	-0.0392709	0.1612543	-0.2435	0.808	-0.049086715	count	1
ZBTB46	-0.0806524	0.4322677	-0.1866	0.852	-0.049033503	count	1
NR2F6	-0.0359835	0.1007857	-0.357	0.721	-0.049022505	count	1
UBE2D2	-0.0344792	0.0527719	-0.6534	0.514	-0.048993028	count	1
PTBP2	-0.0380933	0.1729712	-0.2202	0.826	-0.048959857	count	1
AGAP9	-0.2355062	0.4369124	-0.539	0.59	-0.048921454	count	1
XKR9	-0.1369898	0.7986615	-0.1715	0.864	-0.0488547	count	1
C5orf17	-0.1167782	0.7065376	-0.1653	0.869	-0.04885138	count	1
SMOX	-0.0560174	0.3831744	-0.1462	0.884	-0.048845529	count	1
DNAJA4	-0.0548949	0.4340778	-0.1265	0.899	-0.048827188	count	1
ZNF605	-0.0405478	0.1912207	-0.212	0.832	-0.048821977	count	1
PTGES2	-0.0358046	0.1098209	-0.326	0.744	-0.048739695	count	1
CHD1L	-0.0403457	0.2195275	-0.1838	0.854	-0.048704098	count	1
SP5	-0.0858258	0.4513972	-0.1901	0.849	-0.048698764	count	1
FAR2	-0.136329	0.4944304	-0.2757	0.783	-0.048613038	count	1
GON7	-0.0391603	0.1605712	-0.2439	0.807	-0.048521799	count	1
SNHG7	-0.0341696	0.0659205	-0.5183	0.604	-0.04847699	count	1
MTHFD2L	-0.0381845	0.1727113	-0.2211	0.825	-0.048438277	count	1
CCDC8	-0.0405595	0.185546	-0.2186	0.827	-0.048435514	count	1
USP35	-0.0632678	0.4010825	-0.1577	0.875	-0.048380999	count	1
AL451085.2	-0.044436	0.2626137	-0.1692	0.866	-0.048264775	count	1
AC097634.1	-0.1352887	0.4646113	-0.2912	0.771	-0.0482327	count	1
SHC1	-0.0373648	0.1685784	-0.2216	0.825	-0.048221158	count	1
ST3GAL2	-0.0488358	0.2466945	-0.198	0.843	-0.048209101	count	1
POLR2E	-0.0340399	0.0560816	-0.607	0.544	-0.048065494	count	1
AL021707.2	-0.0788607	0.7618539	-0.1035	0.918	-0.04793158	count	1
ZNF284	-0.0844002	0.4428075	-0.1906	0.849	-0.047879344	count	1
ICE2	-0.0364928	0.1648126	-0.2214	0.825	-0.047826978	count	1
ZFY	-0.0384771	0.1711388	-0.2248	0.822	-0.047761654	count	1
AL034417.4	-0.2301496	0.6241788	-0.3687	0.712	-0.047756706	count	1
KIF6	-0.410484	0.7948451	-0.5164	0.606	-0.047673209	count	1
GATC	-0.0406528	0.2292784	-0.1773	0.859	-0.047635457	count	1
ANKMY1	-0.0908131	0.5034201	-0.1804	0.857	-0.047457653	count	1
MRPS11	-0.0352216	0.1240751	-0.2839	0.777	-0.047407382	count	1
CAPZB	-0.0332033	0.0426167	-0.7791	0.436	-0.047400977	count	1
MYO5B	-0.1329366	0.715255	-0.1859	0.853	-0.047373266	count	1
PARD6G-AS1	-0.1329366	0.7157393	-0.1857	0.853	-0.047373266	count	1
CPNE1	-0.0348429	0.0953966	-0.3652	0.715	-0.04736436	count	1
FAM168B	-0.0356279	0.1464909	-0.2432	0.808	-0.047294104	count	1
AL136088.1	-0.1649027	0.4888183	-0.3373	0.736	-0.047276219	count	1

LRRC47	-0.038877	0.1366667	-0.2845	0.776	-0.047273889	count	1
ATP6V0D1	-0.034445	0.0923379	-0.373	0.709	-0.047251769	count	1
FARSA	-0.0368437	0.1545494	-0.2384	0.812	-0.047248974	count	1
THOC2	-0.0340964	0.0875329	-0.3895	0.697	-0.047240719	count	1
TMEM173	-0.0339778	0.0942708	-0.3604	0.719	-0.047186718	count	1
NARS	-0.033564	0.0722403	-0.4646	0.642	-0.046906474	count	1
LCP2	-0.1635271	0.6054977	-0.2701	0.787	-0.046869165	count	1
PRICKLE3	-0.0990765	0.4557469	-0.2174	0.828	-0.046867873	count	1
MED24	-0.0690836	0.3183394	-0.217	0.828	-0.046864155	count	1
LMCD1-AS1	-0.0726658	0.8400194	-0.0865	0.931	-0.046838007	count	1
MB21D2	-0.1633914	0.3726023	-0.4385	0.661	-0.04682902	count	1
GTF2A2	-0.0330205	0.0545187	-0.6057	0.545	-0.046807877	count	1
ERI1	-0.0499695	0.275677	-0.1813	0.856	-0.046765422	count	1
NME6	-0.0359952	0.1397421	-0.2576	0.797	-0.046757436	count	1
CENPE	-0.1118009	0.4820075	-0.2319	0.817	-0.046727806	count	1
TDP2	-0.0357527	0.1366874	-0.2616	0.794	-0.046713581	count	1
EEF1E1	-0.0338697	0.0974368	-0.3476	0.728	-0.046637191	count	1
ARFGAP3	-0.0332178	0.0673517	-0.4932	0.622	-0.046433705	count	1
PIGM	-0.0375622	0.1899621	-0.1977	0.843	-0.046362021	count	1
FAM50A	-0.032981	0.0666479	-0.4949	0.621	-0.046319027	count	1
MYSM1	-0.036372	0.1662636	-0.2188	0.827	-0.046197092	count	1
KAT6B	-0.0337665	0.098948	-0.3413	0.733	-0.046159992	count	1
AL355075.4	-0.0974715	0.5273237	-0.1848	0.853	-0.046096068	count	1
STIP1	-0.0342226	0.1185944	-0.2886	0.773	-0.045951904	count	1
NUDCD1	-0.0389141	0.1963512	-0.1982	0.843	-0.045902234	count	1
ACOT8	-0.0360694	0.1685358	-0.214	0.831	-0.045871324	count	1
PGRMC1	-0.0321234	0.0447382	-0.718	0.473	-0.045847546	count	1
EGR2	-0.0562003	0.3129824	-0.1796	0.858	-0.045622259	count	1
KIAA0391	-0.2201659	0.5732491	-0.3841	0.701	-0.045591984	count	1
TLE4	-0.035181	0.1664493	-0.2114	0.833	-0.045489027	count	1
CIART	-0.0377757	0.194793	-0.1939	0.846	-0.045478809	count	1
TMEM258	-0.031918	0.0456026	-0.6999	0.484	-0.045397917	count	1
ARFGAP2	-0.0340801	0.1382072	-0.2466	0.805	-0.045287611	count	1
ZFYVE19	-0.0370011	0.191866	-0.1928	0.847	-0.045093636	count	1
BCL7B	-0.0327551	0.0874521	-0.3745	0.708	-0.045086031	count	1
PEX14	-0.0390576	0.2067917	-0.1889	0.85	-0.045085158	count	1
C6orf52	-0.2177654	0.9032004	-0.2411	0.809	-0.045072697	count	1
CDC42EP1	-0.0325464	0.0912775	-0.3566	0.721	-0.04505022	count	1
LETMD1	-0.0329068	0.0962852	-0.3418	0.733	-0.045045333	count	1
TRPC1	-0.0394336	0.1730655	-0.2279	0.82	-0.044946675	count	1
C11orf74	-0.0335757	0.1282779	-0.2617	0.794	-0.044800533	count	1
SDHB	-0.031987	0.0671028	-0.4767	0.634	-0.044705616	count	1
ZNF22	-0.031937	0.0788216	-0.4052	0.685	-0.044632113	count	1
RPRD2	-0.0333933	0.1357284	-0.246	0.806	-0.044621051	count	1
TMEM14B	-0.0313862	0.0497683	-0.6306	0.528	-0.044545193	count	1

IP6K2	-0.0324623	0.111141	-0.2921	0.77	-0.044512533	count	1
TOM1L2	-0.0345869	0.2301744	-0.1503	0.881	-0.044495989	count	1
CBX8	-0.0939454	0.585693	-0.1604	0.873	-0.044401867	count	1
G6PC3	-0.0349012	0.1519231	-0.2297	0.818	-0.044384071	count	1
TATDN3	-0.036483	0.2250303	-0.1621	0.871	-0.044358497	count	1
TAF11	-0.0326995	0.1160653	-0.2817	0.778	-0.044216973	count	1
SUPT3H	-0.0415031	0.2179455	-0.1904	0.849	-0.044135324	count	1
ZNF710-AS1	-0.3805105	0.8884097	-0.4283	0.6684	-0.043927925	count	1
ZNF473	-0.0722143	0.4507649	-0.1602	0.873	-0.043848843	count	1
ENSA	-0.0310059	0.0579541	-0.535	0.593	-0.043831166	count	1
ME2	-0.0350551	0.1639491	-0.2138	0.831	-0.043810779	count	1
UBE2T	-0.0554739	0.4500936	-0.1232	0.902	-0.043753249	count	1
TUBB	-0.0305664	0.0412177	-0.7416	0.458	-0.043746935	count	1
CTNNAL1	-0.031231	0.0995296	-0.3138	0.754	-0.043743817	count	1
GLOD4	-0.0315655	0.0844631	-0.3737	0.709	-0.043692022	count	1
MAP3K10	-0.0923801	0.3432232	-0.2692	0.788	-0.043650407	count	1
UBE2D4	-0.0328062	0.1211081	-0.2709	0.786	-0.043496932	count	1
INTS3	-0.0350602	0.2031412	-0.1726	0.863	-0.043435034	count	1
C3orf58	-0.0328284	0.1105269	-0.297	0.766	-0.043232441	count	1
ABHD14A-ACY1	-0.082615	0.5640167	-0.1465	0.884	-0.04311613	count	1
CBX3	-0.0306416	0.0663609	-0.4617	0.644	-0.04311178	count	1
AC083798.2	-0.0911996	0.3569331	-0.2555	0.798	-0.043083931	count	1
RTCA-AS1	-0.3730642	0.6899117	-0.5407	0.589	-0.04300336	count	1
XAB2	-0.0357051	0.1786321	-0.1999	0.842	-0.042750076	count	1
TAB3	-0.0470222	0.356544	-0.1319	0.895	-0.042570439	count	1
AC124319.2	-0.2061606	0.6554525	-0.3145	0.753	-0.042568891	count	1
AP002449.1	-0.2061606	0.6545544	-0.315	0.753	-0.042568891	count	1
LINC00886	-0.2061606	0.7920922	-0.2603	0.795	-0.042568891	count	1
SNAP29	-0.0315712	0.1084775	-0.291	0.771	-0.042539717	count	1
AC090517.4	-0.0447825	0.4861792	-0.0921	0.927	-0.042514117	count	1
ARPC1A	-0.0298806	0.0607494	-0.4919	0.623	-0.042349998	count	1
USP42	-0.039244	0.2645865	-0.1483	0.882	-0.042328273	count	1
DSEL	-0.0305709	0.122314	-0.2499	0.803	-0.042236157	count	1
MAPRE3	-0.0364362	0.1933789	-0.1884	0.851	-0.042053499	count	1
ABHD2	-0.0310676	0.1190938	-0.2609	0.794	-0.04205082	count	1
CYS1	-0.0307451	0.1163735	-0.2642	0.792	-0.041957518	count	1
PRRC2C	-0.0294179	0.0526171	-0.5591	0.576	-0.041898485	count	1
NUS1	-0.0333179	0.1763118	-0.189	0.85	-0.041837055	count	1
SORL1	-0.0399229	0.3354323	-0.119	0.905	-0.041784412	count	1
LRRC41	-0.0317608	0.1342549	-0.2366	0.813	-0.041769014	count	1
KLLN	-0.1000854	0.5423374	-0.1845	0.854	-0.041743551	count	1
CFAP298	-0.0302206	0.0985461	-0.3067	0.759	-0.041726684	count	1
CEP290	-0.0312816	0.1339166	-0.2336	0.815	-0.041654123	count	1
LPCAT3	-0.0388559	0.1933024	-0.201	0.841	-0.04161684	count	1
CYB5R3	-0.0289781	0.0348191	-0.8322	0.405	-0.041591674	count	1

KLF12	-0.039416	0.1932641	-0.2039	0.838	-0.041588216	count	1
ATOH8	-0.0302041	0.1431007	-0.2111	0.833	-0.041528354	count	1
HNRNPM	-0.0294206	0.0584103	-0.5037	0.615	-0.041514963	count	1
HIST1H2AH	-0.360795	0.7779136	-0.4638	0.643	-0.041485137	count	1
ZNF395	-0.0348401	0.2562078	-0.136	0.892	-0.041473645	count	1
NNT-AS1	-0.030166	0.1133439	-0.2661	0.79	-0.041345381	count	1
TRMU	-0.0331571	0.2195891	-0.151	0.88	-0.041223871	count	1
SLC15A3	-0.038681	0.3429455	-0.1128	0.91	-0.041126334	count	1
YEATS2	-0.0482245	0.2837687	-0.1699	0.865	-0.041112361	count	1
ETS2	-0.0292062	0.0711925	-0.4102	0.682	-0.041068619	count	1
H2AFY2	-0.0438167	0.2856732	-0.1534	0.878	-0.040984255	count	1
TM4SF1-AS1	-0.1987017	0.674576	-0.2946	0.768	-0.040965393	count	1
MRGBP	-0.0315169	0.1625294	-0.1939	0.846	-0.040707813	count	1
HSPB11	-0.0291123	0.07702	-0.378	0.705	-0.040706315	count	1
BANP	-0.0395517	0.239056	-0.1654	0.869	-0.040674363	count	1
UGDH-AS1	-0.0573654	0.3973822	-0.1444	0.885	-0.040649977	count	1
BRF2	-0.0322353	0.1501639	-0.2147	0.83	-0.040476166	count	1
ATF6B	-0.0307131	0.1321987	-0.2323	0.816	-0.040471559	count	1
TSHZ1	-0.0356351	0.2018193	-0.1766	0.86	-0.040424082	count	1
RIF1	-0.0294515	0.122602	-0.2402	0.81	-0.040348624	count	1
NCOA3	-0.0300551	0.1261057	-0.2383	0.812	-0.04011965	count	1
TMEM56-RWDD3	-0.1941666	1.080613	-0.1797	0.857	-0.039992683	count	1
SULT1B1	-0.1941666	1.080613	-0.1797	0.857	-0.039992683	count	1
FBXO43	-0.1941666	1.080613	-0.1797	0.857	-0.039992683	count	1
AP003419.2	-0.1941666	1.080613	-0.1797	0.857	-0.039992683	count	1
AL158211.3	-0.1941666	1.080613	-0.1797	0.857	-0.039992683	count	1
RASGEF1A	-0.1941666	1.080613	-0.1797	0.857	-0.039992683	count	1
SEMA4G	-0.1941666	1.080613	-0.1797	0.857	-0.039992683	count	1
RAD9B	-0.1941666	1.080613	-0.1797	0.857	-0.039992683	count	1
RORA-AS1	-0.1941666	1.080613	-0.1797	0.857	-0.039992683	count	1
AC103740.1	-0.1941666	1.080613	-0.1797	0.857	-0.039992683	count	1
AC021739.2	-0.1941666	1.080613	-0.1797	0.857	-0.039992683	count	1
CFAP52	-0.1941666	1.080613	-0.1797	0.857	-0.039992683	count	1
MFSD2A	-0.1941666	1.117097	-0.1738	0.862	-0.039992683	count	1
TRIM54	-0.1941666	1.117097	-0.1738	0.862	-0.039992683	count	1
TRABD2A	-0.1941666	1.117097	-0.1738	0.862	-0.039992683	count	1
IRF5	-0.1941666	1.117097	-0.1738	0.862	-0.039992683	count	1
TROAP	-0.1941666	1.117097	-0.1738	0.862	-0.039992683	count	1
AL139385.1	-0.1941666	1.117097	-0.1738	0.862	-0.039992683	count	1
AC015722.2	-0.1941666	1.117097	-0.1738	0.862	-0.039992683	count	1
FAM169B	-0.1941666	1.117097	-0.1738	0.862	-0.039992683	count	1
ZNF730	-0.1941666	1.117097	-0.1738	0.862	-0.039992683	count	1
AC011481.2	-0.1941666	1.117097	-0.1738	0.862	-0.039992683	count	1
LRRC39	-0.1941666	1.315578	-0.1476	0.883	-0.039992683	count	1
HIST2H4A	-0.1941666	1.315578	-0.1476	0.883	-0.039992683	count	1

LINC00487	-0.1941666	1.315578	-0.1476	0.883	-0.039992683	count	1
IL4	-0.1941666	1.315578	-0.1476	0.883	-0.039992683	count	1
C11orf72	-0.1941666	1.315578	-0.1476	0.883	-0.039992683	count	1
TSHR	-0.1941666	1.315578	-0.1476	0.883	-0.039992683	count	1
TBX21	-0.1941666	1.315578	-0.1476	0.883	-0.039992683	count	1
AL121772.1	-0.1941666	1.315578	-0.1476	0.883	-0.039992683	count	1
SDR39U1	-0.0298865	0.1296032	-0.2306	0.818	-0.039875108	count	1
AGPAT1	-0.0307747	0.1516403	-0.2029	0.839	-0.039824904	count	1
AC020910.4	-0.1932657	0.4251115	-0.4546	0.649	-0.039799652	count	1
BX322234.1	-0.1931882	0.5659317	-0.3414	0.733	-0.03978305	count	1
SETD3	-0.030336	0.1268589	-0.2391	0.811	-0.039722521	count	1
CRIPT	-0.0286891	0.0942014	-0.3046	0.761	-0.039709389	count	1
SRI	-0.0278128	0.0442472	-0.6286	0.5297	-0.039697043	count	1
AC008105.3	-0.3458162	0.5524341	-0.626	0.531	-0.039640452	count	1
PINK1	-0.0289344	0.0934297	-0.3097	0.757	-0.039631428	count	1
NOCT	-0.0759763	0.6921809	-0.1098	0.913	-0.039608578	count	1
MDK	-0.0292217	0.1162603	-0.2513	0.802	-0.039443962	count	1
C6orf203	-0.0296084	0.1124897	-0.2632	0.792	-0.039383787	count	1
LARP1	-0.0294105	0.1152629	-0.2552	0.799	-0.039332666	count	1
ZDHHC21	-0.030776	0.1688436	-0.1823	0.855	-0.039325436	count	1
GNE	-0.0393046	0.2622593	-0.1499	0.881	-0.039212938	count	1
ZNF318	-0.0384828	0.2549531	-0.1509	0.88	-0.039196692	count	1
RBM10	-0.0358301	0.1773267	-0.2021	0.84	-0.039144372	count	1
SF3A3	-0.028317	0.0864951	-0.3274	0.743	-0.039022127	count	1
AMOTL1	-0.0307077	0.1463816	-0.2098	0.834	-0.038944585	count	1
PRPSAP1	-0.0290208	0.1044204	-0.2779	0.781	-0.038863753	count	1
MGST3	-0.0269461	0.027085	-0.9949	0.32	-0.038753514	count	1
COQ5	-0.0316207	0.1558729	-0.2029	0.839	-0.03869999	count	1
QSOX1	-0.0279069	0.1162746	-0.24	0.81	-0.038599476	count	1
SMUG1	-0.0321242	0.1726379	-0.1861	0.852	-0.03856241	count	1
PABPC1	-0.0270558	0.0475096	-0.5695	0.569	-0.038513253	count	1
U2AF1L4	-0.0323305	0.1979472	-0.1633	0.87	-0.038481826	count	1
MADD	-0.0424961	0.3348595	-0.1269	0.899	-0.038456054	count	1
DARS	-0.0278504	0.0903517	-0.3082	0.758	-0.038359386	count	1
DUS2	-0.0920924	0.4111864	-0.224	0.823	-0.038354514	count	1
ANKRD12	-0.0268924	0.059378	-0.4529	0.651	-0.038333434	count	1
PGK1	-0.0269272	0.0493947	-0.5451	0.586	-0.038322923	count	1
DHFR	-0.0402729	0.2651556	-0.1519	0.879	-0.038217668	count	1
AC011247.1	-0.3334625	0.6362621	-0.5241	0.6	-0.038126441	count	1
AC105942.1	-0.0303406	0.1821472	-0.1666	0.868	-0.038094654	count	1
DCUN1D2	-0.0592065	0.4966701	-0.1192	0.905	-0.038089728	count	1
EIF2S3	-0.0271904	0.0714041	-0.3808	0.703	-0.038085826	count	1
USP11	-0.0281751	0.1070593	-0.2632	0.792	-0.038057213	count	1
AL133338.1	-0.0805417	0.4663122	-0.1727	0.863	-0.037979587	count	1
AC079305.1	-0.0805417	0.4892951	-0.1646	0.869	-0.037979587	count	1

AL513550.1	-0.3312899	0.7498592	-0.4418	0.659	-0.037860873	count	1
TMEM56	-0.0292201	0.167912	-0.174	0.862	-0.037847006	count	1
PSMC2	-0.0271587	0.0782062	-0.3473	0.728	-0.037820014	count	1
TRAM2-AS1	-0.0304736	0.1768903	-0.1723	0.863	-0.03774647	count	1
EIF4G2	-0.026494	0.0555332	-0.4771	0.633	-0.037680615	count	1
RFLNB	-0.0338202	0.2891239	-0.117	0.907	-0.037595968	count	1
DDX46	-0.0265172	0.0722887	-0.3668	0.714	-0.037534792	count	1
HELQ	-0.0289208	0.1625727	-0.1779	0.859	-0.037459043	count	1
ZC3H11B	-0.3278246	0.7530165	-0.4353	0.663	-0.037437728	count	1
AC099063.4	-0.3278246	0.8081365	-0.4057	0.685	-0.037437728	count	1
AC095055.1	-0.0528634	0.6842204	-0.0773	0.938	-0.03743766	count	1
SMARCA5	-0.0265559	0.0708277	-0.3749	0.708	-0.037327277	count	1
INCENP	-0.0654642	0.4448694	-0.1472	0.883	-0.037028178	count	1
AL022068.1	-0.1298667	0.5193424	-0.2501	0.803	-0.036973294	count	1
ZMAT5	-0.0269202	0.1132056	-0.2378	0.812	-0.036855585	count	1
FGF14	-0.05204	0.3061962	-0.17	0.865	-0.036850543	count	1
TRIM32	-0.0372911	0.2710226	-0.1376	0.891	-0.036777813	count	1
LRRC20	-0.0543	0.4678097	-0.1161	0.908	-0.03676384	count	1
TRMT1	-0.0303829	0.2652168	-0.1146	0.909	-0.036754328	count	1
AL390198.1	-0.3214209	0.9207304	-0.3491	0.727	-0.03665718	count	1
AL390115.1	-0.3214209	0.9567816	-0.3359	0.737	-0.03665718	count	1
ATP2A1-AS1	-0.3214209	0.9567816	-0.3359	0.737	-0.03665718	count	1
ERICH5	-0.3214209	0.9798762	-0.328	0.743	-0.03665718	count	1
XPO7	-0.0324586	0.196045	-0.1656	0.869	-0.03664008	count	1
LYST	-0.0282056	0.1667286	-0.1692	0.866	-0.036565676	count	1
VNN2	-0.1281953	1.084656	-0.1182	0.906	-0.03648518	count	1
PEPD	-0.0261226	0.0668542	-0.3907	0.696	-0.03644243	count	1
BBS5	-0.0351075	0.2737465	-0.1282	0.898	-0.036092042	count	1
ZNF263	-0.0369794	0.2481209	-0.149	0.882	-0.036030994	count	1
ZNF226	-0.0273436	0.1909129	-0.1432	0.886	-0.036004902	count	1
LINC01560	-0.0431072	0.329076	-0.131	0.896	-0.035866136	count	1
PSMD2	-0.0261337	0.0768245	-0.3402	0.734	-0.035793988	count	1
AC087645.2	-0.1257576	0.7023062	-0.1791	0.858	-0.035773834	count	1
SLC25A5	-0.025011	0.0482544	-0.5183	0.604	-0.035598672	count	1
AGA	-0.0271424	0.1420595	-0.1911	0.848	-0.035538027	count	1
EBAG9	-0.0256797	0.0982848	-0.2613	0.794	-0.035465948	count	1
IFRD2	-0.0264304	0.1128902	-0.2341	0.815	-0.035408651	count	1
AGBL3	-0.0444248	0.3283105	-0.1353	0.892	-0.034993273	count	1
ABI3	-0.3064004	0.5638929	-0.5434	0.587	-0.034833529	count	1
KLHDC9	-0.0314464	0.2463144	-0.1277	0.898	-0.034757571	count	1
DNAH5	-0.0471031	0.6456942	-0.0729	0.942	-0.034692502	count	1
GLI4	-0.0269436	0.157793	-0.1708	0.864	-0.034581155	count	1
IKZF4	-0.0359429	0.3468336	-0.1036	0.917	-0.034569054	count	1
CPNE5	-0.0974797	0.6762309	-0.1442	0.885	-0.034505145	count	1
KCNMB2-AS1	-0.0974797	0.8319326	-0.1172	0.907	-0.034505145	count	1

AP000350.5	-0.0974797	0.8319326	-0.1172	0.907	-0.034505145	count	1
HOMER3-AS1	-0.0974797	1.077849	-0.0904	0.928	-0.034505145	count	1
ECI1	-0.0245612	0.0747131	-0.3287	0.742	-0.034459027	count	1
TEK	-0.0467728	0.5704396	-0.082	0.935	-0.034447784	count	1
INPP4B	-0.0269067	0.1289714	-0.2086	0.835	-0.034294698	count	1
CDKN1B	-0.0246808	0.0797699	-0.3094	0.757	-0.034276368	count	1
ACYP2	-0.0256739	0.1386893	-0.1851	0.853	-0.034092478	count	1
H2AFY	-0.0245288	0.0778512	-0.3151	0.753	-0.034028776	count	1
PEX16	-0.0253518	0.1378013	-0.184	0.854	-0.033886394	count	1
TMEM87A	-0.0242833	0.0668554	-0.3632	0.716	-0.033872912	count	1
HEATR3	-0.0335467	0.4295203	-0.0781	0.938	-0.033813031	count	1
TIMM50	-0.0257829	0.1366658	-0.1887	0.85	-0.033756901	count	1
CPED1	-0.0244249	0.078687	-0.3104	0.756	-0.033651426	count	1
AMIGO2	-0.0258479	0.1795086	-0.144	0.886	-0.033625734	count	1
EP300	-0.0256272	0.1416711	-0.1809	0.856	-0.033602643	count	1
ARFGEF1	-0.0255894	0.1242819	-0.2059	0.837	-0.033553049	count	1
IL7	-0.0305062	0.2363312	-0.1291	0.897	-0.033520419	count	1
FAM219A	-0.0364198	0.2962952	-0.1229	0.902	-0.033506267	count	1
MPLKIP	-0.0242111	0.0891264	-0.2716	0.786	-0.033500411	count	1
VARS2	-0.052123	0.4456655	-0.117	0.907	-0.033498666	count	1
AC005838.2	-0.052123	0.5809481	-0.0897	0.929	-0.033498666	count	1
C1orf109	-0.0288035	0.1706598	-0.1688	0.866	-0.033488864	count	1
C17orf51	-0.0295169	0.1839956	-0.1604	0.873	-0.033472254	count	1
UBL4A	-0.0250596	0.1273376	-0.1968	0.844	-0.033448047	count	1
BET1	-0.0254884	0.1332737	-0.1912	0.848	-0.033395991	count	1
LINC01936	-0.0357289	0.3686467	-0.0969	0.923	-0.033394584	count	1
ADGRL1	-0.0470105	0.3777752	-0.1244	0.901	-0.033266978	count	1
GNG12	-0.0235039	0.059486	-0.3951	0.693	-0.033222523	count	1
AC092040.1	-0.0490924	0.2749095	-0.1786	0.858	-0.033211972	count	1
MMP14	-0.0245458	0.1299156	-0.1889	0.85	-0.033152971	count	1
SELENOI	-0.034938	0.3041566	-0.1149	0.909	-0.033139276	count	1
ZNF639	-0.0244785	0.1286045	-0.1903	0.849	-0.033038872	count	1
CLCN6	-0.035282	0.3216608	-0.1097	0.913	-0.032975531	count	1
BRCC3	-0.0261497	0.1632348	-0.1602	0.873	-0.032927457	count	1
TIMM22	-0.0260824	0.1481731	-0.176	0.86	-0.03284264	count	1
XYLT2	-0.0275752	0.2395297	-0.1151	0.908	-0.032814639	count	1
REEP6	-0.2896705	0.6862936	-0.4221	0.673	-0.032814355	count	1
AC137932.3	-0.2896705	0.7972428	-0.3633	0.716	-0.032814355	count	1
TMEM44-AS1	-0.0578557	0.3635856	-0.1591	0.874	-0.032685616	count	1
AC138150.2	-0.0577994	0.6125787	-0.0944	0.925	-0.032653519	count	1
TNFRSF19	-0.0577994	0.6864485	-0.0842	0.933	-0.032653519	count	1
AC006213.1	-0.0577994	0.7003238	-0.0825	0.934	-0.032653519	count	1
RHOJ	-0.0254923	0.1869984	-0.1363	0.892	-0.032606887	count	1
SLTM	-0.0230503	0.079027	-0.2917	0.771	-0.032481458	count	1
C12orf43	-0.0251494	0.125847	-0.1998	0.842	-0.032379823	count	1

CA9	-0.1137885	0.8765256	-0.1298	0.897	-0.032290779	count	1
AC005476.2	-0.1137885	0.8817339	-0.1291	0.897	-0.032290779	count	1
ATP5MGL	-0.1137885	0.8817339	-0.1291	0.897	-0.032290779	count	1
AL035587.1	-0.1137885	0.9752571	-0.1167	0.907	-0.032290779	count	1
ZNF354A	-0.0270384	0.1847738	-0.1463	0.884	-0.032269262	count	1
MINDY2	-0.0231469	0.0822156	-0.2815	0.778	-0.032160331	count	1
C2CD2	-0.0257801	0.2625195	-0.0982	0.922	-0.032099851	count	1
THRA	-0.0228276	0.0796803	-0.2865	0.775	-0.031971519	count	1
BLOC1S1	-0.0224442	0.0498407	-0.4503	0.653	-0.031911901	count	1
MRPL22	-0.0227636	0.0749354	-0.3038	0.761	-0.031890716	count	1
MNAT1	-0.0229704	0.0944269	-0.2433	0.808	-0.03186631	count	1
NAA30	-0.0265643	0.1972975	-0.1346	0.893	-0.031792812	count	1
SPCS1	-0.0221593	0.0343957	-0.6442	0.519	-0.031765674	count	1
RCAN2	-0.0222907	0.0621617	-0.3586	0.72	-0.031732895	count	1
DBNL	-0.0231081	0.0928707	-0.2488	0.804	-0.031675572	count	1
YBEY	-0.0242423	0.1647534	-0.1471	0.883	-0.031395391	count	1
CCDC86	-0.0277187	0.2251272	-0.1231	0.902	-0.031127083	count	1
GADL1	-0.275502	0.6358043	-0.4333	0.665	-0.031114338	count	1
AC012645.1	-0.0439543	0.4427937	-0.0993	0.921	-0.03109168	count	1
KIAA2013	-0.0229458	0.1177562	-0.1949	0.846	-0.031001785	count	1
CNTNAP3	-0.0876385	0.7172442	-0.1222	0.903	-0.03096296	count	1
ARNT2	-0.2738274	0.7549586	-0.3627	0.717	-0.030914024	count	1
HOMER3	-0.0227636	0.1223644	-0.186	0.852	-0.030856004	count	1
PNKD	-0.0215502	0.0539422	-0.3995	0.69	-0.030604258	count	1
TNFAIP8L1	-0.0541397	0.5219981	-0.1037	0.917	-0.03056837	count	1
VDAC1	-0.0215534	0.0524708	-0.4108	0.681	-0.030554973	count	1
GALT	-0.0230313	0.1395774	-0.165	0.869	-0.030476934	count	1
CTTNBP2NL	-0.022801	0.1252776	-0.182	0.856	-0.030387154	count	1
ZNF460	-0.0295581	0.2514931	-0.1175	0.906	-0.03037437	count	1
SNX2	-0.0216	0.0643775	-0.3355	0.737	-0.030366722	count	1
PSME1	-0.0212073	0.0401364	-0.5284	0.597	-0.030334038	count	1
DNAJC2	-0.0221315	0.1182899	-0.1871	0.852	-0.030198951	count	1
MED21	-0.0217173	0.0945173	-0.2298	0.818	-0.030149254	count	1
AC120049.1	-0.085286	0.5963918	-0.143	0.886	-0.030118132	count	1
CR381653.1	-0.085286	0.5961087	-0.1431	0.886	-0.030118132	count	1
PCDHB13	-0.0425771	0.4368643	-0.0975	0.922	-0.030112	count	1
CDK8	-0.0338674	0.2870511	-0.118	0.906	-0.030060986	count	1
TERF2IP	-0.0210576	0.0503223	-0.4185	0.676	-0.030019525	count	1
PCDH11Y	-0.1467866	0.5156942	-0.2846	0.776	-0.029932773	count	1
UBB	-0.0207659	0.0259166	-0.8013	0.423	-0.029875684	count	1
PELI2	-0.0275083	0.2553122	-0.1077	0.914	-0.029846007	count	1
PLK2	-0.02099	0.0939686	-0.2234	0.823	-0.029838482	count	1
AC099522.2	-0.0842308	0.4968682	-0.1695	0.865	-0.029739429	count	1
ADAT3	-0.0402209	0.6644717	-0.0605	0.952	-0.029597671	count	1
ENTPD1	-0.0247978	0.2381633	-0.1041	0.917	-0.029592195	count	1

CDPF1	-0.0307537	0.2868604	-0.1072	0.915	-0.029564849	count	1
TP53BP2	-0.0218543	0.1193064	-0.1832	0.855	-0.029546607	count	1
UBE3B	-0.0256138	0.1938713	-0.1321	0.895	-0.029545935	count	1
FUCA1	-0.0217992	0.1095641	-0.199	0.842	-0.029421263	count	1
GIPC1	-0.0215257	0.0944761	-0.2278	0.82	-0.029407643	count	1
ZNF555	-0.0432834	0.3500929	-0.1236	0.902	-0.029258609	count	1
MINCR	-0.0322971	0.2780452	-0.1162	0.908	-0.029197727	count	1
KIF5C	-0.0823817	0.3749419	-0.2197	0.826	-0.02907616	count	1
ZEB1-AS1	-0.0253063	0.240296	-0.1053	0.916	-0.029071827	count	1
TMEM94	-0.0288515	0.5029694	-0.0574	0.954	-0.029069805	count	1
VPS37D	-0.0427598	0.3816293	-0.112	0.911	-0.028902573	count	1
RAB39B	-0.0476788	0.5402118	-0.0883	0.93	-0.028844804	count	1
PHC3	-0.0219726	0.124335	-0.1767	0.86	-0.028765515	count	1
GRID1	-0.255792	0.5967228	-0.4287	0.668	-0.028764874	count	1
AL928921.2	-0.055351	0.3600404	-0.1537	0.878	-0.028758258	count	1
C11orf58	-0.0199962	0.0340488	-0.5873	0.557	-0.028657873	count	1
TIGD2	-0.254516	0.5896228	-0.4317	0.666	-0.02861342	count	1
PYCARD-AS1	-0.0689196	0.8372189	-0.0823	0.934	-0.028582579	count	1
AC105446.1	-0.1402983	0.4818289	-0.2912	0.771	-0.028569884	count	1
CD83	-0.0307548	0.3044177	-0.101	0.92	-0.028279297	count	1
SNX30	-0.0311419	0.3066273	-0.1016	0.919	-0.028150211	count	1
FAM199X	-0.0223816	0.160356	-0.1396	0.889	-0.02813702	count	1
POMC	-0.0271	0.2846261	-0.0952	0.924	-0.028095955	count	1
OSER1-DT	-0.0272731	0.2447975	-0.1114	0.911	-0.028021445	count	1
DCTN3	-0.0197037	0.0470049	-0.4192	0.675	-0.028019764	count	1
MBTD1	-0.0309841	0.2971229	-0.1043	0.917	-0.028007138	count	1
HYOU1	-0.0224794	0.2024865	-0.111	0.912	-0.027986699	count	1
ZNF132	-0.0982342	0.6356466	-0.1545	0.877	-0.027788527	count	1
SMYD3	-0.0224819	0.1777316	-0.1265	0.899	-0.02773303	count	1
BRAP	-0.0226608	0.202533	-0.1119	0.911	-0.027663347	count	1
GALE	-0.0317963	0.3169395	-0.1003	0.92	-0.027656555	count	1
AC008014.1	-0.0531648	0.6377324	-0.0834	0.934	-0.027612375	count	1
KEAP1	-0.0204118	0.122425	-0.1667	0.868	-0.027604953	count	1
AC011479.1	-0.0586271	0.6349131	-0.0923	0.926	-0.027540934	count	1
COX16	-0.0247852	0.2145561	-0.1155	0.908	-0.027537347	count	1
AC145124.1	-0.0224343	0.1740844	-0.1289	0.897	-0.027505793	count	1
PPM1H	-0.058532	0.7378397	-0.0793	0.937	-0.027495803	count	1
CHAF1B	-0.058532	0.7750005	-0.0755	0.94	-0.027495803	count	1
AL662884.4	-0.0971038	0.429847	-0.2259	0.821	-0.027462398	count	1
AC091948.1	-0.2444685	0.9167473	-0.2667	0.79	-0.02742333	count	1
GFAP	-0.2444685	0.9172351	-0.2665	0.79	-0.02742333	count	1
RNF114	-0.0199023	0.1016542	-0.1958	0.845	-0.027232991	count	1
AC025159.1	-0.0234411	0.2284198	-0.1026	0.918	-0.027036602	count	1
SIL1	-0.0195029	0.0929324	-0.2099	0.834	-0.027034733	count	1
HHIPL1	-0.0367305	0.4131227	-0.0889	0.929	-0.027017107	count	1

CAMSAP1	-0.0296279	0.2338728	-0.1267	0.899	-0.026777692	count	1
PTPN20	-0.0255291	0.4213902	-0.0606	0.952	-0.026691912	count	1
HSD17B12	-0.0187854	0.0636323	-0.2952	0.768	-0.026590271	count	1
TAOK2	-0.0258794	0.2761345	-0.0937	0.925	-0.026586707	count	1
SYNE2	-0.0190078	0.0932829	-0.2038	0.839	-0.026508041	count	1
LINC00346	-0.0251327	0.2987141	-0.0841	0.933	-0.026491204	count	1
WIPF1	-0.0204753	0.1289844	-0.1587	0.874	-0.026489303	count	1
PLEKHG4	-0.0927826	0.5198224	-0.1785	0.858	-0.026217036	count	1
AC022364.1	-0.0927826	0.5207613	-0.1782	0.859	-0.026217036	count	1
CYC1	-0.0184369	0.0579254	-0.3183	0.75	-0.026122686	count	1
ITGB5	-0.0184735	0.0640057	-0.2886	0.773	-0.02595947	count	1
CALM2	-0.0180289	0.0246712	-0.7308	0.465	-0.025951253	count	1
PABPC4	-0.0189799	0.1137476	-0.1669	0.867	-0.025897357	count	1
TRIM27	-0.0200329	0.1366756	-0.1466	0.883	-0.025891892	count	1
OXER1	-0.073426	0.5111982	-0.1436	0.886	-0.025870278	count	1
NAXD	-0.0212978	0.1769586	-0.1204	0.904	-0.02581744	count	1
MAEA	-0.0194065	0.1206439	-0.1609	0.872	-0.025752349	count	1
FAM114A2	-0.0201171	0.1397532	-0.1439	0.886	-0.025666704	count	1
MMGT1	-0.0191478	0.1108392	-0.1728	0.863	-0.025625929	count	1
PTGIR	-0.0186077	0.1080891	-0.1722	0.863	-0.025551407	count	1
RCC1L	-0.0196088	0.1246842	-0.1573	0.875	-0.02554812	count	1
OGFOD3	-0.0192008	0.1059933	-0.1812	0.856	-0.025534624	count	1
TALDO1	-0.01795	0.0488284	-0.3676	0.713	-0.025514868	count	1
TIMM8A	-0.0240996	0.2253709	-0.1069	0.915	-0.025400406	count	1
PEX5	-0.0228641	0.289698	-0.0789	0.937	-0.025258307	count	1
IPO9	-0.0210805	0.1999885	-0.1054	0.916	-0.025223478	count	1
PSMD11	-0.0182577	0.0853912	-0.2138	0.831	-0.025152728	count	1
CDC42BPA	-0.0183997	0.1037792	-0.1773	0.859	-0.025105488	count	1
SEMA4F	-0.0368104	0.5659428	-0.065	0.948	-0.024860681	count	1
TSPAN3	-0.0176153	0.0613213	-0.2873	0.774	-0.02481297	count	1
FAM86C1	-0.0315321	0.3913477	-0.0806	0.936	-0.024799828	count	1
TBC1D8	-0.0337109	0.308827	-0.1092	0.913	-0.024786447	count	1
NDUFA9	-0.0179056	0.0869343	-0.206	0.837	-0.024780799	count	1
OAS2	-0.0268822	0.345266	-0.0779	0.938	-0.024709314	count	1
AC068282.1	-0.2201659	0.7699784	-0.2859	0.775	-0.024564576	count	1
USH1C	-0.2201659	0.7699784	-0.2859	0.775	-0.024564576	count	1
AC064801.1	-0.2201659	0.7699784	-0.2859	0.775	-0.024564576	count	1
AL353719.1	-0.0697594	0.6194756	-0.1126	0.91	-0.024560859	count	1
C16orf95	-0.1205204	0.7383386	-0.1632	0.87	-0.024437705	count	1
AC007663.3	-0.1205204	0.7383386	-0.1632	0.87	-0.024437705	count	1
AC021752.1	-0.1205204	0.741087	-0.1626	0.871	-0.024437705	count	1
CYP51A1-AS1	-0.1205204	0.818635	-0.1472	0.883	-0.024437705	count	1
AC008556.1	-0.1205204	0.8209359	-0.1468	0.883	-0.024437705	count	1
JAGN1	-0.0175876	0.0870216	-0.2021	0.84	-0.024373039	count	1
CDCA4	-0.022172	0.255435	-0.0868	0.931	-0.024350059	count	1

ERVK3-1	-0.0193616	0.2346634	-0.0825	0.934	-0.02418424	count	1
ARL6IP1	-0.0175381	0.0937306	-0.1871	0.852	-0.02411868	count	1
ITPRIPL2	-0.0174656	0.0866691	-0.2015	0.84	-0.023982769	count	1
RIC8B	-0.0242471	0.2380337	-0.1019	0.919	-0.023887545	count	1
TIMM17A	-0.01756	0.1060412	-0.1656	0.868	-0.023698128	count	1
CNN3	-0.0165832	0.0448556	-0.3697	0.712	-0.023659786	count	1
SLC26A11	-0.0232302	0.2488012	-0.0934	0.926	-0.023633403	count	1
RBMX2	-0.01741	0.0981022	-0.1775	0.859	-0.023559682	count	1
DESI2	-0.0168171	0.0731607	-0.2299	0.818	-0.023549022	count	1
AC016588.2	-0.1160492	0.597085	-0.1944	0.846	-0.023508208	count	1
ANKRD52	-0.0298728	0.4662826	-0.0641	0.949	-0.02349015	count	1
AP3D1	-0.0179827	0.1588529	-0.1132	0.91	-0.023447781	count	1
HADHB	-0.0166851	0.0694074	-0.2404	0.81	-0.023383469	count	1
AL035413.1	-0.0286774	0.3316152	-0.0865	0.931	-0.023206921	count	1
NRG4	-0.2071737	0.6779011	-0.3056	0.76	-0.023047875	count	1
ATP7B	-0.2071737	0.7536923	-0.2749	0.783	-0.023047875	count	1
AC008686.1	-0.1136897	0.5532809	-0.2055	0.837	-0.023018399	count	1
ZNF19	-0.0275706	0.4582969	-0.0602	0.952	-0.022899957	count	1
CDK3	-0.0812217	0.6375418	-0.1274	0.899	-0.022895696	count	1
TOR1A	-0.0171611	0.1169097	-0.1468	0.883	-0.022808983	count	1
AC018413.1	-0.0485954	0.643131	-0.0756	0.94	-0.022788311	count	1
PDZD2	-0.0808059	0.5071109	-0.1593	0.873	-0.022776524	count	1
CD320	-0.0163808	0.0846074	-0.1936	0.846	-0.022770698	count	1
AKT2	-0.0181194	0.1315699	-0.1377	0.89	-0.022668746	count	1
TRAPPC4	-0.0162549	0.0756543	-0.2149	0.83	-0.022642575	count	1
ERBIN	-0.0173492	0.1475039	-0.1176	0.906	-0.022621393	count	1
ARL8A	-0.0166717	0.1045167	-0.1595	0.873	-0.022617003	count	1
INO80C	-0.0177178	0.1583472	-0.1119	0.911	-0.022548361	count	1
COMMD4	-0.0163816	0.0888465	-0.1844	0.854	-0.022424357	count	1
ANKRD46	-0.0188211	0.1906989	-0.0987	0.921	-0.022320661	count	1
SLC16A12	-0.053738	0.6003933	-0.0895	0.929	-0.022224053	count	1
SENP6	-0.0157621	0.0814988	-0.1934	0.847	-0.022071523	count	1
SNAP23	-0.0158828	0.0876412	-0.1812	0.856	-0.022045564	count	1
PRKCA	-0.0238647	0.2895562	-0.0824	0.934	-0.021929408	count	1
LARP7	-0.0155104	0.0641454	-0.2418	0.809	-0.021821604	count	1
PLA2G12A	-0.0170781	0.1840051	-0.0928	0.926	-0.021706306	count	1
ADIPOR1	-0.0158473	0.1057825	-0.1498	0.881	-0.021511172	count	1
CCT5	-0.0152371	0.0589495	-0.2585	0.796	-0.02149568	count	1
MBOAT7	-0.0184193	0.2289124	-0.0805	0.936	-0.021483017	count	1
ZNF583	-0.019122	0.2002997	-0.0955	0.924	-0.021352315	count	1
DOK4	-0.0175541	0.2002581	-0.0877	0.93	-0.021326827	count	1
WDR5	-0.0179178	0.22535	-0.0795	0.937	-0.021312804	count	1
GOLGA7	-0.0151683	0.0736596	-0.2059	0.837	-0.021236909	count	1
SNX17	-0.0151556	0.0652516	-0.2323	0.816	-0.021225139	count	1
VAMP1	-0.0210692	0.308694	-0.0683	0.946	-0.021215534	count	1

PDIA5	-0.0157123	0.1558912	-0.1008	0.92	-0.021045442	count	1
CMTM4	-0.024134	0.3969073	-0.0608	0.952	-0.020975095	count	1
MID2	-0.0206776	0.3422409	-0.0604	0.952	-0.020820566	count	1
RNF26	-0.0179005	0.1986932	-0.0901	0.928	-0.020721695	count	1
TSR3	-0.0148445	0.0842528	-0.1762	0.86	-0.020599247	count	1
ALG9	-0.019998	0.3269246	-0.0612	0.951	-0.020535414	count	1
TRPM4	-0.0188956	0.373194	-0.0506	0.96	-0.020489876	count	1
VTN	-0.025321	0.4662193	-0.0543	0.957	-0.020482852	count	1
LGALS8	-0.0159964	0.1543787	-0.1036	0.917	-0.020478513	count	1
GTPBP8	-0.0155293	0.1697089	-0.0915	0.927	-0.020469732	count	1
SRP9	-0.0143442	0.0455863	-0.3147	0.753	-0.020439133	count	1
BTAF1	-0.0161505	0.187721	-0.086	0.931	-0.020417496	count	1
PRR14	-0.0170032	0.2066593	-0.0823	0.934	-0.020397316	count	1
TXNDC11	-0.0172377	0.2107412	-0.0818	0.935	-0.020377681	count	1
KHSRP	-0.0163148	0.1852773	-0.0881	0.93	-0.02034219	count	1
FRA10AC1	-0.014462	0.0909635	-0.159	0.874	-0.02023761	count	1
PARP16	-0.0191674	0.2595068	-0.0739	0.941	-0.020194919	count	1
NSMCE1	-0.0144399	0.0766051	-0.1885	0.85	-0.020131311	count	1
BLOC1S6	-0.014596	0.0888458	-0.1643	0.87	-0.020033808	count	1
FOXJ3	-0.01506	0.1456134	-0.1035	0.918	-0.020025702	count	1
AC025171.2	-0.0211295	0.3118267	-0.0678	0.946	-0.020016827	count	1
FUBP1	-0.0148193	0.1090926	-0.1358	0.892	-0.019954529	count	1
C4orf33	-0.0183537	0.2425271	-0.0757	0.94	-0.019901548	count	1
ATIC	-0.0150384	0.1353501	-0.1111	0.912	-0.019872538	count	1
THAP6	-0.0154107	0.1359817	-0.1133	0.91	-0.019772466	count	1
NOB1	-0.0146592	0.1018375	-0.1439	0.886	-0.019731378	count	1
LTB	-0.0350241	0.731876	-0.0479	0.962	-0.019715395	count	1
ZNF548	-0.0306228	0.2951665	-0.1037	0.917	-0.01961965	count	1
RB1	-0.0147602	0.1466069	-0.1007	0.92	-0.019606366	count	1
MRPL2	-0.0142772	0.0945689	-0.151	0.88	-0.01947454	count	1
DMC1	-0.0689251	0.5464891	-0.1261	0.9	-0.019379848	count	1
DYNLRB2	-0.0689251	0.5699492	-0.1209	0.904	-0.019379848	count	1
VKORC1	-0.0134478	0.0424677	-0.3167	0.7515	-0.019189953	count	1
TARS2	-0.0231073	0.3577817	-0.0646	0.949	-0.019183211	count	1
ELK1	-0.0183112	0.2419228	-0.0757	0.94	-0.019135236	count	1
FOCAD	-0.0196259	0.3186418	-0.0616	0.951	-0.019094319	count	1
RHEB	-0.013417	0.0550907	-0.2435	0.808	-0.019072472	count	1
IKZF2	-0.0259116	0.32767	-0.0791	0.937	-0.019032773	count	1
CCDC130	-0.0143387	0.1369888	-0.1047	0.917	-0.018959171	count	1
RXRB	-0.0154456	0.1507577	-0.1025	0.918	-0.018931993	count	1
WDR73	-0.0145799	0.15346	-0.095	0.924	-0.018877024	count	1
VAMP7	-0.0149669	0.1492073	-0.1003	0.92	-0.018839125	count	1
DNASE1L2	-0.1707256	0.904101	-0.1888	0.85	-0.018836663	count	1
IGLV3-21	-0.1707256	0.904101	-0.1888	0.85	-0.018836663	count	1
DENND2D	-0.1707256	0.9351945	-0.1826	0.855	-0.018836663	count	1

AC053503.2	-0.1707256	0.9351945	-0.1826	0.855	-0.018836663	count	1
LINC02469	-0.1707256	0.9351945	-0.1826	0.855	-0.018836663	count	1
ZNF804B	-0.1707256	0.9351945	-0.1826	0.855	-0.018836663	count	1
SNX15	-0.1707256	0.9351945	-0.1826	0.855	-0.018836663	count	1
AC026369.2	-0.1707256	0.9351945	-0.1826	0.855	-0.018836663	count	1
SLC25A41	-0.1707256	0.9351945	-0.1826	0.855	-0.018836663	count	1
AL078604.2	-0.1707256	1.084979	-0.1574	0.875	-0.018836663	count	1
IGLV2-14	-0.1707256	1.084979	-0.1574	0.875	-0.018836663	count	1
PODNL1	-0.0151202	0.2443771	-0.0619	0.951	-0.018819543	count	1
PCBP2	-0.013212	0.0486713	-0.2715	0.786	-0.018810824	count	1
NDUFA10	-0.013409	0.0695376	-0.1928	0.847	-0.018765307	count	1
NIM1K	-0.0400678	0.5868769	-0.0683	0.946	-0.018761152	count	1
SRSF12	-0.0400678	0.5868982	-0.0683	0.946	-0.018761152	count	1
TTC19	-0.0145587	0.1384622	-0.1051	0.916	-0.018699147	count	1
TRAK2	-0.0141596	0.1475876	-0.0959	0.924	-0.018561103	count	1
SATL1	-0.1680394	0.9154787	-0.1836	0.854	-0.018528876	count	1
LINC00894	-0.1680394	0.9154787	-0.1836	0.854	-0.018528876	count	1
AC107884.1	-0.1680394	0.9154787	-0.1836	0.854	-0.018528876	count	1
AP000941.1	-0.1680394	0.9154787	-0.1836	0.854	-0.018528876	count	1
PPFIA4	-0.1680394	0.9300263	-0.1807	0.857	-0.018528876	count	1
AC012603.1	-0.1680394	0.9300263	-0.1807	0.857	-0.018528876	count	1
APOBEC2	-0.1680394	1.043769	-0.161	0.872	-0.018528876	count	1
TBC1D25	-0.0168602	0.2690228	-0.0627	0.95	-0.018510266	count	1
P2RY1	-0.0262271	0.3205064	-0.0818	0.935	-0.018508258	count	1
ZNF214	-0.0187636	0.3670999	-0.0511	0.959	-0.018476891	count	1
ANXA6	-0.0132039	0.0723883	-0.1824	0.855	-0.018467151	count	1
LSM1	-0.0133	0.0896592	-0.1483	0.882	-0.01842804	count	1
AP001062.1	-0.1669871	0.7484294	-0.2231	0.823	-0.018408399	count	1
AC104794.4	-0.1669871	0.7989405	-0.209	0.834	-0.018408399	count	1
ACTB	-0.012756	0.0219871	-0.5802	0.562	-0.018394573	count	1
ATP5F1C	-0.0126901	0.0424433	-0.299	0.765	-0.018133708	count	1
CCT7	-0.0127609	0.0518751	-0.246	0.806	-0.018067854	count	1
YY1	-0.0125783	0.0539853	-0.233	0.816	-0.017856476	count	1
MOSPD2	-0.0144623	0.1542046	-0.0938	0.925	-0.017726049	count	1
MTMR7	-0.1609339	0.7316451	-0.22	0.826	-0.017716436	count	1
AL590999.1	-0.1609339	0.7485209	-0.215	0.83	-0.017716436	count	1
AAAS	-0.0147489	0.1897099	-0.0777	0.938	-0.017642491	count	1
SAR1B	-0.0126547	0.087057	-0.1454	0.884	-0.017586581	count	1
SLC1A1	-0.1596445	0.5333523	-0.2993	0.765	-0.017569275	count	1
CACNB1	-0.0205806	0.3550225	-0.058	0.954	-0.017493536	count	1
SUMO1	-0.0122329	0.0478899	-0.2554	0.798	-0.017391871	count	1
NECAB3	-0.0147562	0.177595	-0.0831	0.934	-0.017268745	count	1
YIF1A	-0.0119715	0.0579255	-0.2067	0.836	-0.016968865	count	1
FSBP	-0.0410065	0.6216782	-0.066	0.947	-0.016918611	count	1
ACO2	-0.0127477	0.1174475	-0.1085	0.914	-0.016875034	count	1

GSPT1	-0.01206	0.0744153	-0.1621	0.871	-0.016820767	count	1
ZHX1	-0.0122978	0.1016065	-0.121	0.904	-0.016806553	count	1
IER3-AS1	-0.0278224	0.4078286	-0.0682	0.946	-0.01678131	count	1
DMAP1	-0.0124885	0.1245352	-0.1003	0.92	-0.016688551	count	1
NFE2L3	-0.0475633	0.4400353	-0.1081	0.914	-0.016673204	count	1
AC023355.1	-0.0274777	0.5697768	-0.0482	0.962	-0.016572527	count	1
NDUFAF6	-0.0140449	0.2289646	-0.0613	0.951	-0.016547358	count	1
SOX12	-0.0140809	0.3515621	-0.0401	0.968	-0.016359077	count	1
KHDRBS1	-0.0114509	0.0564427	-0.2029	0.839	-0.016245435	count	1
EMD	-0.0115469	0.0781047	-0.1478	0.882	-0.01622525	count	1
RFX1	-0.0150887	0.2253985	-0.0669	0.947	-0.016134889	count	1
ZNF586	-0.0263774	0.362462	-0.0728	0.942	-0.015906223	count	1
WDR11	-0.0130412	0.185598	-0.0703	0.944	-0.015877933	count	1
TMEM14C	-0.0110767	0.0361222	-0.3066	0.759	-0.015868128	count	1
MPDZ	-0.0115656	0.135891	-0.0851	0.932	-0.01583773	count	1
UBE2V2	-0.0112514	0.0637252	-0.1766	0.86	-0.015800162	count	1
RNF187	-0.0113882	0.0816206	-0.1395	0.889	-0.015759813	count	1
BCAP31	-0.0110686	0.0532169	-0.208	0.835	-0.015672947	count	1
VAMP4	-0.0115183	0.1180009	-0.0976	0.922	-0.015569856	count	1
ASH2L	-0.0123578	0.2209793	-0.0559	0.955	-0.015553632	count	1
PAXIP1-AS1	-0.0125604	0.2106359	-0.0596	0.952	-0.015547711	count	1
EPM2AIP1	-0.0116687	0.1229198	-0.0949	0.924	-0.015547528	count	1
SYCP2	-0.0376637	0.5726055	-0.0658	0.948	-0.015529707	count	1
FRYL	-0.0115545	0.1604018	-0.072	0.943	-0.015418143	count	1
C12orf10	-0.0110231	0.0731862	-0.1506	0.88	-0.015416783	count	1
COX4I1	-0.0107191	0.0281083	-0.3814	0.703	-0.015399407	count	1
PIGW	-0.0226959	0.3997677	-0.0568	0.955	-0.015297938	count	1
TBC1D15	-0.0115687	0.1077542	-0.1074	0.915	-0.015237766	count	1
ZNF148	-0.0108832	0.0863863	-0.126	0.9	-0.015214002	count	1
ERGIC3	-0.0107298	0.0717973	-0.1494	0.881	-0.01505412	count	1
MCPH1	-0.0115909	0.1406255	-0.0824	0.934	-0.01504686	count	1
APOM	-0.0156201	0.3267551	-0.0478	0.962	-0.014996315	count	1
AC004943.2	-0.0533974	0.6259924	-0.0853	0.932	-0.014965172	count	1
ZNF426	-0.0116456	0.1996571	-0.0583	0.953	-0.014940064	count	1
AC011498.1	-0.0425771	0.7951711	-0.0535	0.957	-0.014910568	count	1
LTB4R2	-0.0425771	0.9444576	-0.0451	0.964	-0.014910568	count	1
GLTP	-0.0108723	0.1042592	-0.1043	0.917	-0.014833119	count	1
FAM120AOS	-0.0106323	0.0792303	-0.1342	0.893	-0.014827936	count	1
TCF3	-0.0133045	0.2198495	-0.0605	0.952	-0.01477154	count	1
AC068888.1	-0.010817	0.1030735	-0.1049	0.916	-0.014761307	count	1
C11orf98	-0.0230222	0.4368113	-0.0527	0.958	-0.014733635	count	1
DNM3OS	-0.0118547	0.1639694	-0.0723	0.942	-0.014700895	count	1
MED13	-0.010757	0.1254957	-0.0857	0.932	-0.014693612	count	1
AC093677.2	-0.1331058	0.5843862	-0.2278	0.82	-0.014558735	count	1
AC239800.2	-0.0724154	0.7424202	-0.0975	0.922	-0.014528848	count	1

CD81-AS1	-0.0724154	0.7424202	-0.0975	0.922	-0.014528848	count	1
SAMD10	-0.0724154	0.8057406	-0.0899	0.928	-0.014528848	count	1
ADH1A	-0.0724154	0.8620485	-0.084	0.933	-0.014528848	count	1
TEX12	-0.0724154	0.9081478	-0.0797	0.936	-0.014528848	count	1
FDX1	-0.0106541	0.1049209	-0.1015	0.919	-0.014460732	count	1
CMTM2	-0.1313909	0.8506354	-0.1545	0.877	-0.014365409	count	1
LURAP1	-0.0119983	0.1987825	-0.0604	0.952	-0.014350464	count	1
HIST1H2AE	-0.025321	0.5390109	-0.047	0.963	-0.014231257	count	1
AC116667.2	-0.025321	0.5390109	-0.047	0.963	-0.014231257	count	1
TOLLIP-AS1	-0.015424	0.4584203	-0.0336	0.973	-0.013920725	count	1
ASXL3	-0.0267706	0.3107819	-0.0861	0.931	-0.013842563	count	1
FASTKD5	-0.0150544	0.2463419	-0.0611	0.951	-0.013821892	count	1
MTBP	-0.0330775	0.4176206	-0.0792	0.937	-0.013626966	count	1
COTL1	-0.0106127	0.1782232	-0.0595	0.953	-0.013568531	count	1
PPIF	-0.01288	0.2738783	-0.047	0.962	-0.013564381	count	1
LINC00535	-0.0224904	0.8498547	-0.0265	0.979	-0.013554174	count	1
KPNB1	-0.0097865	0.0838353	-0.1167	0.907	-0.013449788	count	1
KIF7	-0.012424	0.4456912	-0.0279	0.978	-0.013283003	count	1
B4GALT2	-0.0100922	0.1326223	-0.0761	0.939	-0.013265679	count	1
LINC00921	-0.0219563	0.4569785	-0.048	0.962	-0.013231204	count	1
ZNF250	-0.0121279	0.2566575	-0.0473	0.962	-0.013229684	count	1
RAP1A	-0.0093115	0.0613657	-0.1517	0.879	-0.013107554	count	1
WDR45	-0.0093473	0.0706587	-0.1323	0.895	-0.013020954	count	1
HECTD4	-0.0100525	0.2093509	-0.048	0.962	-0.012923489	count	1
AC003681.1	-0.1178102	0.5567852	-0.2116	0.832	-0.012839634	count	1
PDZK1	-0.0457422	0.8863286	-0.0516	0.959	-0.012799067	count	1
LINC01600	-0.0457422	0.8863286	-0.0516	0.959	-0.012799067	count	1
AC005899.6	-0.0457422	0.8863286	-0.0516	0.959	-0.012799067	count	1
FGR	-0.0457422	1.071181	-0.0427	0.966	-0.012799067	count	1
MED31	-0.0096223	0.1301942	-0.0739	0.941	-0.012721641	count	1
AP3M1	-0.0102202	0.1609676	-0.0635	0.949	-0.012696063	count	1
NFYA	-0.0179929	0.3544262	-0.0508	0.96	-0.012683353	count	1
FAM13B	-0.009553	0.1567013	-0.061	0.951	-0.012681174	count	1
ARHGAP19	-0.0307537	0.5482982	-0.0561	0.955	-0.012664092	count	1
RTCB	-0.0091971	0.0991644	-0.0927	0.926	-0.012571418	count	1
HYI	-0.0087986	0.0670846	-0.1312	0.896	-0.012405001	count	1
AC091982.3	-0.1130164	0.7363428	-0.1535	0.878	-0.012303278	count	1
PAIP2	-0.008641	0.0581015	-0.1487	0.882	-0.012197367	count	1
MT-CO3	-0.0084141	0.0223265	-0.3769	0.706	-0.012131348	count	1
NCKAP1	-0.0086812	0.0907187	-0.0957	0.924	-0.012116422	count	1
PAPSS1	-0.0088415	0.1081722	-0.0817	0.935	-0.01204995	count	1
C16orf70	-0.0138274	0.282339	-0.049	0.961	-0.01200452	count	1
CLUH	-0.0111359	0.3940294	-0.0283	0.977	-0.011988708	count	1
RFXANK	-0.0092238	0.1247041	-0.074	0.941	-0.011929346	count	1
AC011978.2	-0.0288872	0.6501981	-0.0444	0.965	-0.011891303	count	1

PTPRJ	-0.0227787	0.4882686	-0.0467	0.963	-0.011770466	count	1
PPP4R1	-0.0093512	0.1714656	-0.0545	0.957	-0.011595248	count	1
DHPS	-0.0084252	0.08728	-0.0965	0.923	-0.011578727	count	1
CEP85	-0.0578557	0.6664315	-0.0868	0.931	-0.011569939	count	1
TOMM40L	-0.0178838	0.4216557	-0.0424	0.966	-0.011436536	count	1
SF3A1	-0.0087375	0.1260118	-0.0693	0.945	-0.011408543	count	1
AL645568.1	-0.0132946	0.4306532	-0.0309	0.975	-0.011291477	count	1
CASC10	-0.0160108	0.8355074	-0.0192	0.985	-0.011283124	count	1
DDX19B	-0.0100467	0.193446	-0.0519	0.959	-0.011270414	count	1
RBMS3-AS3	-0.0132287	0.2645114	-0.05	0.96	-0.011235425	count	1
TACC3	-0.1028604	0.8182657	-0.1257	0.9	-0.011170818	count	1
NDUFS3	-0.0079048	0.0712176	-0.111	0.912	-0.011090361	count	1
FKBPL	-0.0097052	0.2739181	-0.0354	0.972	-0.010941183	count	1
TBX6	-0.1007645	0.6411799	-0.1572	0.875	-0.010937764	count	1
AC096642.1	-0.1007645	0.7223404	-0.1395	0.889	-0.010937764	count	1
LATS2-AS1	-0.1007645	0.7971527	-0.1264	0.899	-0.010937764	count	1
USP9Y	-0.0108164	0.3212188	-0.0337	0.973	-0.0108826	count	1
TUBA1B	-0.0075534	0.0332648	-0.2271	0.82	-0.010858236	count	1
AL450384.2	-0.0177683	0.2906046	-0.0611	0.951	-0.010700544	count	1
INTS1	-0.0087133	0.2243504	-0.0388	0.969	-0.010606723	count	1
GLRX	-0.0075632	0.0958474	-0.0789	0.937	-0.010532389	count	1
ZBTB22	-0.0088	0.1914737	-0.046	0.963	-0.010431326	count	1
OR51E1	-0.0222188	0.2867248	-0.0775	0.938	-0.010370646	count	1
GHITM	-0.0073247	0.0548654	-0.1335	0.894	-0.010360824	count	1
AC007406.3	-0.0516536	0.5623905	-0.0918	0.927	-0.010315241	count	1
L3MBTL2	-0.0085485	0.1913308	-0.0447	0.964	-0.01030541	count	1
STARD4	-0.0291409	0.3655649	-0.0797	0.936	-0.010177916	count	1
FBXO33	-0.0079893	0.1854182	-0.0431	0.966	-0.010164405	count	1
NTMT1	-0.0075463	0.1278759	-0.059	0.953	-0.010114927	count	1
SLC26A2	-0.0082699	0.1701238	-0.0486	0.961	-0.010089632	count	1
NDUFS7	-0.0071092	0.0536382	-0.1325	0.895	-0.010075112	count	1
NBPF12	-0.0287452	0.4170445	-0.0689	0.945	-0.010038917	count	1
PLGLB1	-0.0925652	0.8167149	-0.1133	0.91	-0.010028187	count	1
LINC01176	-0.0925652	0.8167149	-0.1133	0.91	-0.010028187	count	1
AC022509.1	-0.0925652	0.8167149	-0.1133	0.91	-0.010028187	count	1
AP003392.5	-0.0925652	0.9178572	-0.1008	0.92	-0.010028187	count	1
MLLT1	-0.0082476	0.1976714	-0.0417	0.967	-0.010016272	count	1
ZNF432	-0.0089891	0.3757994	-0.0239	0.981	-0.009921852	count	1
GATD1	-0.0078103	0.1668477	-0.0468	0.963	-0.009813847	count	1
WDR48	-0.0074191	0.1505805	-0.0493	0.961	-0.009802326	count	1
ELP2	-0.0074748	0.147203	-0.0508	0.96	-0.009751742	count	1
ATP6V1D	-0.0070541	0.0887354	-0.0795	0.937	-0.009728061	count	1
ZBTB16	-0.0068528	0.0786052	-0.0872	0.931	-0.009522984	count	1
FAM221A	-0.01226	0.3167709	-0.0387	0.969	-0.009316604	count	1
KCNB1	-0.0153772	0.4566658	-0.0337	0.973	-0.00925714	count	1

ZNF420	-0.008171	0.2406372	-0.034	0.973	-0.009210777	count	1
PAXX	-0.0066105	0.0916441	-0.0721	0.943	-0.009190298	count	1
KIAA1468	-0.0082756	0.2323461	-0.0356	0.972	-0.009080569	count	1
GLO1	-0.0064546	0.0627124	-0.1029	0.918	-0.009076256	count	1
STRAP	-0.0064853	0.0720683	-0.09	0.928	-0.00907337	count	1
WTAP	-0.0064456	0.0751939	-0.0857	0.932	-0.00904903	count	1
SBNO1	-0.0067842	0.1132814	-0.0599	0.952	-0.00903846	count	1
TRMT10C	-0.0063711	0.0962082	-0.0662	0.947	-0.008830354	count	1
FOXN3	-0.0063158	0.1017309	-0.0621	0.951	-0.008646253	count	1
PSMD1	-0.0062448	0.0849865	-0.0735	0.941	-0.00863423	count	1
FOXN2	-0.0070491	0.1423384	-0.0495	0.961	-0.00861878	count	1
MIIP	-0.0072453	0.1538028	-0.0471	0.962	-0.008613834	count	1
SENP5	-0.0065389	0.1501082	-0.0436	0.965	-0.008600213	count	1
B3GALNT2	-0.0069317	0.2092896	-0.0331	0.974	-0.008528651	count	1
MDFI	-0.0242311	0.4792391	-0.0506	0.96	-0.008454773	count	1
NPLOC4	-0.0069552	0.2404655	-0.0289	0.977	-0.008405333	count	1
CDS2	-0.0061551	0.1045918	-0.0588	0.953	-0.008390514	count	1
CAPG	-0.006199	0.1226149	-0.0506	0.96	-0.008345823	count	1
AC123768.4	-0.0418719	0.5668259	-0.0739	0.941	-0.008343371	count	1
APEH	-0.0065294	0.1484047	-0.044	0.965	-0.008327165	count	1
POLR2A	-0.0063309	0.1210008	-0.0523	0.958	-0.008291158	count	1
YTHDC1	-0.0059023	0.0834309	-0.0707	0.944	-0.008248056	count	1
IMMT	-0.0061264	0.1058792	-0.0579	0.954	-0.008218481	count	1
TMEM250	-0.0066638	0.155674	-0.0428	0.966	-0.008215283	count	1
TJP1	-0.0058418	0.0806559	-0.0724	0.942	-0.008185814	count	1
LAMTOR2	-0.0058556	0.0702428	-0.0834	0.934	-0.008162163	count	1
TOLLIP	-0.0062248	0.1363714	-0.0456	0.964	-0.008079641	count	1
ARHGEF12	-0.005728	0.1154046	-0.0496	0.96	-0.007769318	count	1
KMT2A	-0.005488	0.0624173	-0.0879	0.93	-0.007767386	count	1
AC091959.3	-0.038256	0.5007105	-0.0764	0.939	-0.007616624	count	1
SLC9A3	-0.0079121	0.266058	-0.0297	0.976	-0.007486437	count	1
RAET1G	-0.0160108	0.7001195	-0.0229	0.982	-0.007464743	count	1
ENOX2	-0.0062246	0.1461578	-0.0426	0.966	-0.007399985	count	1
DHX40	-0.0054808	0.1198273	-0.0457	0.964	-0.007355401	count	1
GNB2	-0.0051801	0.0516045	-0.1004	0.92	-0.007348494	count	1
KDM1A	-0.0057259	0.1294347	-0.0442	0.965	-0.007328018	count	1
AL354696.2	-0.0363411	0.8773723	-0.0414	0.967	-0.007232229	count	1
AC025682.1	-0.0363411	0.8790465	-0.0413	0.967	-0.007232229	count	1
RHBDL3	-0.0654728	0.7320924	-0.0894	0.929	-0.007047189	count	1
FBP2	-0.0654728	0.8576551	-0.0763	0.939	-0.007047189	count	1
SFT2D3	-0.0061971	0.19527	-0.0317	0.975	-0.007018265	count	1
DKC1	-0.0052338	0.1369222	-0.0382	0.97	-0.006833161	count	1
PWP1	-0.0049113	0.0803686	-0.0611	0.951	-0.00681782	count	1
PDCD7	-0.0047964	0.0901897	-0.0532	0.958	-0.006662169	count	1
MBD6	-0.0058854	0.2238798	-0.0263	0.979	-0.006600631	count	1

SRSF10	-0.0046439	0.0655946	-0.0708	0.944	-0.006580895	count	1
MANEA	-0.0052475	0.1466221	-0.0358	0.971	-0.006562043	count	1
EPHB4	-0.008244	0.4418585	-0.0187	0.985	-0.006465718	count	1
N4BP2L2	-0.0044882	0.0503939	-0.0891	0.929	-0.006407796	count	1
USP3	-0.0049898	0.1433981	-0.0348	0.972	-0.006347685	count	1
GNAL	-0.0051898	0.1650124	-0.0315	0.975	-0.006301924	count	1
GTF3A	-0.0044433	0.0632854	-0.0702	0.944	-0.006267467	count	1
COMMD9	-0.0048128	0.1646533	-0.0292	0.977	-0.006246663	count	1
AC009041.2	-0.0048587	0.2184073	-0.0222	0.982	-0.006113645	count	1
GEMIN4	-0.0090806	0.3462205	-0.0262	0.979	-0.006108917	count	1
IST1	-0.0044553	0.1194784	-0.0373	0.97	-0.006006984	count	1
MORF4L1	-0.0041498	0.029924	-0.1387	0.89	-0.005958929	count	1
NEK7	-0.0042961	0.1081317	-0.0397	0.968	-0.005826991	count	1
AP002360.1	-0.0078094	0.2955411	-0.0264	0.979	-0.00572272	count	1
OPTN	-0.0040425	0.0588945	-0.0686	0.945	-0.005714762	count	1
LPAR4	-0.0204393	0.4821488	-0.0424	0.966	-0.005688415	count	1
CA2	-0.0528855	0.6559302	-0.0806	0.936	-0.005675049	count	1
KIF5A	-0.0087971	0.6412313	-0.0137	0.989	-0.005618113	count	1
ZNF211	-0.0087283	0.3551002	-0.0246	0.98	-0.005574118	count	1
TMEM30A	-0.0038424	0.0643845	-0.0597	0.952	-0.005421999	count	1
MRPL4	-0.0038958	0.0872095	-0.0447	0.964	-0.005408692	count	1
CCT2	-0.0038186	0.0660477	-0.0578	0.954	-0.005375152	count	1
AC009113.1	-0.0056861	0.2725515	-0.0209	0.983	-0.00521585	count	1
ZCCHC2	-0.0042211	0.184179	-0.0229	0.982	-0.005213492	count	1
GPN3	-0.0037662	0.0835579	-0.0451	0.964	-0.005201747	count	1
CSF2RA	-0.0247941	0.7102379	-0.0349	0.972	-0.004921304	count	1
CKAP5	-0.0038319	0.1467254	-0.0261	0.979	-0.004799287	count	1
APCDD1	-0.0040397	0.1625208	-0.0249	0.98	-0.004757154	count	1
SRD5A3	-0.0036694	0.1555485	-0.0236	0.981	-0.00474499	count	1
THRAP3	-0.0032813	0.050735	-0.0647	0.948	-0.004661966	count	1
DCP2	-0.0034927	0.1424548	-0.0245	0.98	-0.004577217	count	1
LYG2	-0.0230677	0.6792795	-0.034	0.973	-0.00457683	count	1
FEZ1	-0.0032282	0.0612068	-0.0527	0.958	-0.004563139	count	1
SIRT6	-0.0035888	0.2541208	-0.0141	0.989	-0.004501844	count	1
ANKRD16	-0.0041015	0.2291699	-0.0179	0.986	-0.004382565	count	1
ZMYND10	-0.01035	0.6987365	-0.0148	0.988	-0.004245595	count	1
TEAD2	-0.0033849	0.1615991	-0.0209	0.983	-0.004232566	count	1
SPSB3	-0.0030216	0.0858917	-0.0352	0.972	-0.004176994	count	1
SARS	-0.0028928	0.0743605	-0.0389	0.969	-0.00405817	count	1
KPNA4	-0.0028985	0.091297	-0.0317	0.975	-0.004022154	count	1
AL445686.2	-0.0363411	1.24365	-0.0292	0.977	-0.003884025	count	1
AL360219.1	-0.0363411	1.238916	-0.0293	0.977	-0.003884025	count	1
LINC01142	-0.0363411	1.24365	-0.0292	0.977	-0.003884025	count	1
AL359921.1	-0.0363411	1.24365	-0.0292	0.977	-0.003884025	count	1
AC092053.2	-0.0363411	1.238916	-0.0293	0.977	-0.003884025	count	1

CACNA2D3-AS1	-0.0363411	1.24098	-0.0293	0.977	-0.003884025	count	1
AC131235.3	-0.0363411	1.238916	-0.0293	0.977	-0.003884025	count	1
CPA2	-0.0363411	1.24365	-0.0292	0.977	-0.003884025	count	1
FAM156A	-0.0363411	1.24098	-0.0293	0.977	-0.003884025	count	1
AC131902.1	-0.0363411	1.238916	-0.0293	0.977	-0.003884025	count	1
TRPM6	-0.0363411	1.238916	-0.0293	0.977	-0.003884025	count	1
FAM78A	-0.0363411	1.238916	-0.0293	0.977	-0.003884025	count	1
CEND1	-0.0363411	1.238916	-0.0293	0.977	-0.003884025	count	1
AP003774.1	-0.0363411	1.238916	-0.0293	0.977	-0.003884025	count	1
AP000654.1	-0.0363411	1.238916	-0.0293	0.977	-0.003884025	count	1
AC005342.1	-0.0363411	1.238916	-0.0293	0.977	-0.003884025	count	1
STAC3	-0.0363411	1.238916	-0.0293	0.977	-0.003884025	count	1
AC066613.1	-0.0363411	1.238916	-0.0293	0.977	-0.003884025	count	1
HEXA-AS1	-0.0363411	1.24365	-0.0292	0.977	-0.003884025	count	1
AC025287.2	-0.0363411	1.238916	-0.0293	0.977	-0.003884025	count	1
NOS2	-0.0363411	1.24098	-0.0293	0.977	-0.003884025	count	1
TAC4	-0.0363411	1.24365	-0.0292	0.977	-0.003884025	count	1
OGFR-AS1	-0.0363411	1.24365	-0.0292	0.977	-0.003884025	count	1
AC011444.1	-0.0363411	1.238916	-0.0293	0.977	-0.003884025	count	1
MEF2B	-0.0363411	1.24098	-0.0293	0.977	-0.003884025	count	1
NEFH	-0.0363411	1.238916	-0.0293	0.977	-0.003884025	count	1
Z73429.1	-0.0363411	1.24365	-0.0292	0.977	-0.003884025	count	1
MED4	-0.0027488	0.071574	-0.0384	0.969	-0.003835989	count	1
MCFD2	-0.002706	0.068217	-0.0397	0.968	-0.003790393	count	1
FKBP3	-0.0026487	0.071308	-0.0371	0.97	-0.003696295	count	1
CSTB	-0.0025007	0.037496	-0.0667	0.947	-0.003589552	count	1
STAU1	-0.0024489	0.056961	-0.043	0.966	-0.003460044	count	1
RBM3	-0.0023898	0.0438162	-0.0545	0.957	-0.003403319	count	1
PIGC	-0.0024337	0.1139228	-0.0214	0.983	-0.003323146	count	1
TTLL4	-0.0080713	0.461519	-0.0175	0.986	-0.003309428	count	1
BOLA2B	-0.0054741	0.5965216	-0.0092	0.993	-0.003290372	count	1
BDP1	-0.0023223	0.0916262	-0.0253	0.98	-0.003262996	count	1
CCDC181	-0.0037276	0.3350361	-0.0111	0.991	-0.003232722	count	1
B3GNT2	-0.0024352	0.1289336	-0.0189	0.985	-0.003142816	count	1
CBWD6	-0.0075079	0.6226363	-0.0121	0.99	-0.003078089	count	1
RGS17	-0.0021167	0.1544355	-0.0137	0.989	-0.002948978	count	1
ZNF32	-0.0020895	0.0853894	-0.0245	0.98	-0.002871901	count	1
MCM5	-0.0027399	0.2412137	-0.0114	0.991	-0.002725375	count	1
DPH7	-0.0022534	0.1687103	-0.0134	0.989	-0.002693964	count	1
PINK1-AS	-0.0064955	0.8129266	-0.008	0.994	-0.00266251	count	1
LINC01268	-0.0064955	0.8129266	-0.008	0.994	-0.00266251	count	1
BRD8	-0.0020213	0.1110794	-0.0182	0.985	-0.002636653	count	1
FKBP7	-0.0018232	0.086307	-0.0211	0.983	-0.002535149	count	1
TOX4	-0.0017855	0.0673851	-0.0265	0.979	-0.002503015	count	1
ARHGEF7	-0.0019425	0.1389579	-0.014	0.989	-0.002485732	count	1

GTF2H5	-0.0016682	0.0714734	-0.0233	0.981	-0.002340651	count	1
GSTM4	-0.0017261	0.1311516	-0.0132	0.989	-0.002312378	count	1
ACP1	-0.0016171	0.0677867	-0.0239	0.981	-0.002266341	count	1
HTR1B	-0.0062156	0.6592536	-0.0094	0.992	-0.0021609	count	1
RAB1B	-0.0015502	0.1017317	-0.0152	0.988	-0.002132596	count	1
PPP4R3A	-0.0015794	0.1451788	-0.0109	0.991	-0.002079832	count	1
CATSPERE	-0.0184003	0.7369031	-0.025	0.98	-0.001957933	count	1
KIFC2	-0.0055619	0.5386174	-0.0103	0.992	-0.00193338	count	1
ELK4	-0.0014828	0.150035	-0.0099	0.992	-0.001919138	count	1
PRKAR2A	-0.0013356	0.1011849	-0.0132	0.989	-0.001817177	count	1
MZF1	-0.0015936	0.2509757	-0.0063	0.995	-0.0017957	count	1
CCAR1	-0.0013161	0.1330176	-0.0099	0.992	-0.001743808	count	1
CCNT2-AS1	-0.0047912	0.7527485	-0.0064	0.995	-0.001665215	count	1
UTP15	-0.0015702	0.3140391	-0.005	0.996	-0.001543963	count	1
USP1	-0.0010715	0.0931327	-0.0115	0.991	-0.00146925	count	1
FUOM	-0.0010387	0.1555643	-0.0067	0.995	-0.001396671	count	1
PTPRO	-0.0014522	0.7260317	-0.002	0.998	-0.001231793	count	1
TRIQK	-0.0009348	0.1233842	-0.0076	0.994	-0.001229322	count	1
FYTTD1	-0.0008271	0.0850847	-0.0097	0.992	-0.001141802	count	1
ECHDC3	-0.0010081	0.3809911	-0.0026	0.998	-0.000991208	count	1
NQO1	-0.0007031	0.1034747	-0.0068	0.995	-0.000971925	count	1
GPR176	-0.0008186	0.2304492	-0.0036	0.997	-0.000969982	count	1
SFXN4	-0.0007682	0.1724759	-0.0045	0.996	-0.000918329	count	1
HEBP1	-0.0005117	0.0535077	-0.0096	0.992	-0.000727478	count	1
KDM8	-0.0019866	0.5919893	-0.0034	0.997	-0.000690064	count	1
GALM	-0.0004961	0.1574107	-0.0032	0.997	-0.000627713	count	1
CHAMP1	-0.0004445	0.1469677	-0.003	0.998	-0.000537025	count	1
ANKRD50	-0.0003717	0.1767694	-0.0021	0.998	-0.000434675	count	1
TM2D3	-0.0002581	0.082962	-0.0031	0.998	-0.000358104	count	1
COQ8B	-0.0002732	0.1794537	-0.0015	0.999	-0.000271696	count	1
HSPA8	-0.000188	0.0339528	-0.0055	0.996	-0.000270015	count	1
FAM20B	-0.0001932	0.1601948	-0.0012	0.999	-0.000239038	count	1
RIT1	-0.0001741	0.1467111	-0.0012	0.999	-0.000229103	count	1
CLDN12	-0.0001797	0.2347034	-8.00E-04	0.999	-0.000190549	count	1
NXT2	-0.0001246	0.2696831	-5.00E-04	1	-0.000136649	count	1
TMED2	-6.55E-05	0.0538976	-0.0012	0.999	-9.25E-05	count	1
AC067852.2	-9.51E-05	0.2522444	-4.00E-04	1	-8.99E-05	count	1
NELFA	-3.86E-05	0.1774752	-2.00E-04	1	-4.64E-05	count	1
TDO2	-2.09004	0.3324813	-6.2862	3.64E-10	-2.46E-07	count	8.61E-06
AL031846.2	-1.4491529	0.4594266	-3.1543	0.0016	-1.59E-07	count	1
CCL11	-2.9848824	1.5818428	-1.887	0.0592	-1.23E-07	count	1
AC009570.1	-2.8105322	0.8221073	-3.4187	6.00E-04	-1.16E-07	count	1
PPP1R14C	-2.7761606	1.3916415	-1.9949	0.0461	-1.15E-07	count	1
SPINK2	-0.9919213	0.2167123	-4.5771	4.87E-06	-1.01E-07	count	0.11258953
RELL2	-2.1412418	1.0694506	-2.0022	0.0453	-8.95E-08	count	1

BANK1	-2.1235303	0.9110805	-2.3308	0.0198	-8.90E-08	count	1
DLL4	-1.9957372	0.9647027	-2.0688	0.0386	-8.32E-08	count	1
IGHG2	-1.8101086	0.2310031	-7.8359	6.05E-15	-7.52E-08	count	1.44E-10
IFNG	-1.7269117	1.002165	-1.7232	0.0849	-7.08E-08	count	1
LINC02397	-1.6840772	0.8034613	-2.096	0.0361	-6.88E-08	count	1
ITGAX	-18.1050315	1445.678125	-0.0125	0.99	-6.30E-08	count	1
RUNX3	-18.5346051	1614.693832	-0.0115	0.991	-6.28E-08	count	1
TNFRSF10A	-1.3638902	0.6152854	-2.2167	0.0267	-5.38E-08	count	1
AC022144.1	-1.3596971	0.8183939	-1.6614	0.0967	-5.36E-08	count	1
LINC00265	-1.2293615	0.660126	-1.8623	0.0626	-4.76E-08	count	1
IRF8	-1.178367	0.4049013	-2.9103	0.0036	-4.53E-08	count	1
TPSB2	-2.8278514	1.2914431	-2.1897	0.0286	-4.28E-08	count	1
AC005899.5	-2.3407128	1.3955082	-1.6773	0.0936	-3.61E-08	count	1
SERPINA3	-0.9581477	0.6062427	-1.5805	0.114	-3.55E-08	count	1
AC104170.2	-2.2888549	1.1722729	-1.9525	0.051	-3.52E-08	count	1
FEZF1-AS1	-0.9487642	0.9313588	-1.0187	0.308	-3.51E-08	count	1
CRB2	-2.2500773	1.3254832	-1.6976	0.0897	-3.47E-08	count	1
JAML	-2.1479668	0.9988419	-2.1505	0.0316	-3.32E-08	count	1
AC092542.1	-2.1479668	0.843086	-2.5477	0.0109	-3.32E-08	count	1
AC025580.3	-0.9004349	0.7100416	-1.2681	0.205	-3.30E-08	count	1
AC004231.1	-2.1347042	1.0969249	-1.9461	0.0517	-3.28E-08	count	1
TPSAB1	-2.1347042	0.8129831	-2.6258	0.0087	-3.28E-08	count	1
AC119396.2	-2.1347042	1.2462411	-1.7129	0.0868	-3.28E-08	count	1
C20orf202	-2.0305427	0.7590207	-2.6752	0.0075	-3.13E-08	count	1
POU3F3	-2.0202002	1.0505327	-1.923	0.0546	-3.11E-08	count	1
AP001330.5	-2.0202002	1.0061432	-2.0079	0.0447	-3.10E-08	count	1
AC005288.1	-2.0202002	1.1492583	-1.7578	0.0789	-3.10E-08	count	1
PLCB2	-1.9601263	0.9492291	-2.065	0.039	-3.01E-08	count	1
CDH7	-1.9523827	0.8312332	-2.3488	0.0189	-2.98E-08	count	1
AC124068.2	-1.9523827	1.1755413	-1.6608	0.0968	-2.98E-08	count	1
EVA1A	-1.9270331	0.5382989	-3.5799	3.00E-04	-2.95E-08	count	1
SH3RF2	-1.8864505	0.8559163	-2.204	0.0276	-2.89E-08	count	1
C11orf45	-1.8094764	1.0804946	-1.6747	0.0941	-2.75E-08	count	1
PITRM1-AS1	-1.8094764	1.364634	-1.326	0.185	-2.75E-08	count	1
SFXN2	-0.7375616	0.4949434	-1.4902	0.136	-2.62E-08	count	1
AL451074.2	-1.7244152	0.8005191	-2.1541	0.0313	-2.61E-08	count	1
PICSAR	-1.7244152	0.8005191	-2.1541	0.0313	-2.61E-08	count	1
H3F3C	-1.7244152	0.8005191	-2.1541	0.0313	-2.61E-08	count	1
CATSPER1	-1.7244152	0.8005191	-2.1541	0.0313	-2.61E-08	count	1
SLC9C1	-1.7292391	1.1022246	-1.5689	0.117	-2.61E-08	count	1
AC092140.2	-1.7292391	1.1022246	-1.5689	0.117	-2.61E-08	count	1
AC037487.1	-1.7292391	1.1022246	-1.5689	0.117	-2.61E-08	count	1
AC021851.1	-1.6343281	1.0389215	-1.5731	0.116	-2.45E-08	count	1
CSNK2A3	-1.6343281	1.0389215	-1.5731	0.116	-2.45E-08	count	1
AC013472.3	-1.6343281	1.0389215	-1.5731	0.116	-2.45E-08	count	1

AL137784.2	-1.6343281	1.0389215	-1.5731	0.116	-2.45E-08	count	1
AC083805.3	-1.6343281	0.8538688	-1.914	0.0557	-2.45E-08	count	1
AL512343.2	-1.6343281	0.8538688	-1.914	0.0557	-2.45E-08	count	1
LINC02145	-1.6343281	1.1033479	-1.4812	0.139	-2.45E-08	count	1
AL162586.1	-1.5957077	0.8003929	-1.9937	0.0463	-2.37E-08	count	1
AC133919.1	-1.5876611	0.7267621	-2.1846	0.029	-2.37E-08	count	1
CD300E	-18.2085596	1095.079068	-0.0166	0.987	-2.34E-08	count	1
VAV1	-18.5361275	1636.733561	-0.0113	0.991	-2.34E-08	count	1
ZNF831	-18.2090949	1705.929494	-0.0107	0.991	-2.33E-08	count	1
AC135457.1	-18.2090949	1705.929494	-0.0107	0.991	-2.33E-08	count	1
ARMC4	-18.4090335	1093.165393	-0.0168	0.987	-2.33E-08	count	1
ASIC2	-18.4096386	1573.369689	-0.0117	0.991	-2.33E-08	count	1
AL390957.1	-18.4100385	1938.234467	-0.0095	0.992	-2.33E-08	count	1
AC073389.3	-18.5749681	1367.79808	-0.0136	0.989	-2.33E-08	count	1
CDCA3	-18.7846656	2150.508313	-0.0087	0.993	-2.33E-08	count	1
GCKR	-18.8398798	1896.953644	-0.0099	0.992	-2.32E-08	count	1
INPP5J	-18.8398798	1896.953644	-0.0099	0.992	-2.32E-08	count	1
CST1	-18.863377	1435.968965	-0.0131	0.99	-2.31E-08	count	1
SOX3	-18.4218324	881.3165526	-0.0209	0.983	-2.31E-08	count	1
MMP13	-19.4788083	1169.693011	-0.0167	0.987	-2.31E-08	count	1
BTBD16	-18.2406357	1631.602462	-0.0112	0.991	-2.31E-08	count	1
DNAH17	-18.2406357	1631.602462	-0.0112	0.991	-2.31E-08	count	1
TRG-AS1	-18.2406357	1631.602462	-0.0112	0.991	-2.31E-08	count	1
AL137145.1	-18.2413737	1980.045991	-0.0092	0.993	-2.31E-08	count	1
IGHV4-59	-18.2413737	1980.045991	-0.0092	0.993	-2.31E-08	count	1
AC020741.1	-18.2413737	1980.045991	-0.0092	0.993	-2.31E-08	count	1
GCM1	-18.2413737	1980.045991	-0.0092	0.993	-2.31E-08	count	1
AC068831.1	-18.2413737	1980.045991	-0.0092	0.993	-2.31E-08	count	1
SLIT1	-18.2413737	1980.045991	-0.0092	0.993	-2.31E-08	count	1
DAPP1	-18.2413737	1980.045991	-0.0092	0.993	-2.31E-08	count	1
CXCR6	-18.4241098	1843.16645	-0.01	0.992	-2.31E-08	count	1
AC009148.1	-18.4241098	1843.16645	-0.01	0.992	-2.31E-08	count	1
P2RY13	-18.4241098	1843.16645	-0.01	0.992	-2.31E-08	count	1
LCK	-18.5786107	1738.683578	-0.0107	0.991	-2.31E-08	count	1
AC009119.2	-18.5786107	1738.683578	-0.0107	0.991	-2.31E-08	count	1
CHRM3-AS2	-18.4248368	2170.270033	-0.0085	0.993	-2.30E-08	count	1
AC108134.3	-18.4248368	2170.270033	-0.0085	0.993	-2.30E-08	count	1
AC009137.1	-18.4248368	2170.270033	-0.0085	0.993	-2.30E-08	count	1
AC097534.1	-18.4248368	2170.270033	-0.0085	0.993	-2.30E-08	count	1
AC007938.3	-18.4248368	2170.270033	-0.0085	0.993	-2.30E-08	count	1
AL590133.1	-18.5799874	2345.331911	-0.0079	0.994	-2.30E-08	count	1
AC092757.3	-18.5799874	2345.331911	-0.0079	0.994	-2.30E-08	count	1
CASR	-18.5799874	2345.331911	-0.0079	0.994	-2.30E-08	count	1
TAS2R13	-18.714367	2508.328851	-0.0075	0.994	-2.30E-08	count	1
VSIG4	-19.0328975	1944.79818	-0.0098	0.992	-2.30E-08	count	1

JAKMIP1	-18.8328387	2661.400675	-0.0071	0.994	-2.30E-08	count	1
IGKV1-27	-19.0343868	2943.580294	-0.0065	0.995	-2.29E-08	count	1
AL109811.2	-1.5399464	0.9867587	-1.5606	0.119	-2.29E-08	count	1
SELP	-1.5399464	0.9867587	-1.5606	0.119	-2.29E-08	count	1
ADAM11	-1.5399464	0.9153425	-1.6824	0.0926	-2.29E-08	count	1
AC069200.1	-1.5399464	1.087675	-1.4158	0.157	-2.29E-08	count	1
AC126283.1	-1.5399464	1.2655929	-1.2168	0.224	-2.29E-08	count	1
ERC2	-1.5399464	1.2655929	-1.2168	0.224	-2.29E-08	count	1
DPYSL4	-1.5285774	0.7688238	-1.9882	0.0469	-2.27E-08	count	1
LAIR1	-1.441557	0.8290877	-1.7387	0.0822	-2.11E-08	count	1
AC008280.3	-0.5867672	0.7968702	-0.7363	0.462	-2.02E-08	count	1
MYLK4	-1.323774	0.8801098	-1.5041	0.133	-1.91E-08	count	1
DNAJC5G	-1.3152591	0.7798689	-1.6865	0.0918	-1.90E-08	count	1
GZMM	-1.2870266	1.2405674	-1.0374	0.3	-1.85E-08	count	1
AC008040.1	-1.2870266	0.8816535	-1.4598	0.144	-1.85E-08	count	1
AL359555.2	-1.2870266	0.8816535	-1.4598	0.144	-1.85E-08	count	1
AC011352.1	-1.2870266	0.8816535	-1.4598	0.144	-1.85E-08	count	1
AL021807.1	-1.2870266	0.8816535	-1.4598	0.144	-1.85E-08	count	1
DEGS2	-1.2870266	0.8816535	-1.4598	0.144	-1.85E-08	count	1
MIR137HG	-1.2870266	0.8816535	-1.4598	0.144	-1.85E-08	count	1
ACOXL	-1.2870266	1.308674	-0.9835	0.325	-1.85E-08	count	1
ZFHX4-AS1	-1.2870266	1.308674	-0.9835	0.325	-1.85E-08	count	1
LINC02454	-1.2592355	0.8977282	-1.4027	0.161	-1.80E-08	count	1
CCL18	-0.5257874	0.626098	-0.8398	0.401	-1.78E-08	count	1
AC004520.1	-1.226038	0.7109192	-1.7246	0.0847	-1.75E-08	count	1
AC084880.4	-1.226038	0.7420341	-1.6523	0.0986	-1.75E-08	count	1
AL359715.3	-1.1902426	0.7565273	-1.5733	0.116	-1.68E-08	count	1
AC098828.2	-1.163766	1.0014914	-1.162	0.245	-1.65E-08	count	1
AL592166.1	-1.163766	1.0014914	-1.162	0.245	-1.65E-08	count	1
AC073316.3	-1.163766	1.0014914	-1.162	0.245	-1.65E-08	count	1
AP005899.1	-1.163766	0.9862304	-1.18	0.238	-1.65E-08	count	1
AC009118.2	-1.163766	1.0014914	-1.162	0.245	-1.65E-08	count	1
AL138787.2	-1.163766	0.9862304	-1.18	0.238	-1.65E-08	count	1
AL022323.3	-1.163766	1.0014914	-1.162	0.245	-1.65E-08	count	1
FP565260.6	-1.163766	1.0014914	-1.162	0.245	-1.65E-08	count	1
GIPC3	-1.163766	1.0014914	-1.162	0.245	-1.65E-08	count	1
LINC02576	-1.163766	0.9862304	-1.18	0.238	-1.65E-08	count	1
PCSK4	-1.163766	0.9862304	-1.18	0.238	-1.65E-08	count	1
ARHGAP30	-1.163766	1.0014914	-1.162	0.245	-1.65E-08	count	1
AC125611.4	-1.163766	0.9862304	-1.18	0.238	-1.65E-08	count	1
SLC3A1	-1.163766	1.0014914	-1.162	0.245	-1.65E-08	count	1
AP000550.1	-1.163766	1.0014914	-1.162	0.245	-1.65E-08	count	1
LINC02362	-1.163766	1.0014914	-1.162	0.245	-1.65E-08	count	1
AC093525.4	-1.163766	1.0014914	-1.162	0.245	-1.65E-08	count	1
AL589843.1	-1.163766	0.9862304	-1.18	0.238	-1.65E-08	count	1

GHRHR	-1.163766	0.9862304	-1.18	0.238	-1.65E-08	count	1
RASAL3	-1.163766	1.167921	-0.9964	0.319	-1.64E-08	count	1
RGPD8	-1.163766	1.167921	-0.9964	0.319	-1.64E-08	count	1
AL133351.1	-1.163766	1.167921	-0.9964	0.319	-1.64E-08	count	1
ADH6	-1.163766	1.167921	-0.9964	0.319	-1.64E-08	count	1
ZAR1L	-1.163766	1.167921	-0.9964	0.319	-1.64E-08	count	1
AC025257.1	-1.163766	1.167921	-0.9964	0.319	-1.64E-08	count	1
HOXB7	-1.163766	0.7023469	-1.657	0.0976	-1.64E-08	count	1
U2AF1	-0.480907	0.5734295	-0.8387	0.402	-1.61E-08	count	1
SPNS3	-1.1003822	1.017479	-1.0815	0.28	-1.54E-08	count	1
TNFSF8	-1.0457622	0.6931353	-1.5087	0.131	-1.45E-08	count	1
VAX2	-1.0457622	0.9763343	-1.0711	0.284	-1.45E-08	count	1
PSMD6-AS2	-1.0360919	0.8536597	-1.2137	0.225	-1.43E-08	count	1
PAPOLB	-1.0360919	0.6522547	-1.5885	0.112	-1.43E-08	count	1
RHCE	-0.4162561	0.553042	-0.7527	0.452	-1.38E-08	count	1
FAM182B	-0.4039857	0.8134582	-0.4966	0.619	-1.33E-08	count	1
TARID	-0.9729818	0.8315367	-1.1701	0.242	-1.33E-08	count	1
HS3ST3B1	-0.948045	0.7501739	-1.2638	0.206	-1.29E-08	count	1
IGHM	-0.9346399	0.3154293	-2.9631	0.0031	-1.27E-08	count	1
PRPH2	-0.3733942	0.7420614	-0.5032	0.615	-1.22E-08	count	1
RNFT1-DT	-0.8989193	0.8403485	-1.0697	0.285	-1.21E-08	count	1
MS4A2	-0.8537704	0.9403898	-0.9079	0.364	-1.14E-08	count	1
AC017071.1	-0.8537704	0.7835087	-1.0897	0.276	-1.14E-08	count	1
AL359513.1	-0.8239293	0.7661942	-1.0754	0.282	-1.10E-08	count	1
AC009318.3	-0.8239293	0.7661942	-1.0754	0.282	-1.10E-08	count	1
AL512770.1	-0.8239293	0.7661942	-1.0754	0.282	-1.10E-08	count	1
AC012640.4	-0.7484099	0.6000802	-1.2472	0.212	-9.80E-09	count	1
AC090515.2	-0.7484099	0.6000802	-1.2472	0.212	-9.80E-09	count	1
LINC01970	-0.7484099	0.8193575	-0.9134	0.361	-9.80E-09	count	1
LRRC56	-0.7484099	0.6782772	-1.1034	0.27	-9.80E-09	count	1
AL008729.2	-0.7484099	0.6782772	-1.1034	0.27	-9.80E-09	count	1
IL10RA	-0.2902166	0.4484979	-0.6471	0.518	-9.30E-09	count	1
AL117381.1	-18.5959179	1793.901735	-0.0104	0.992	-8.65E-09	count	1
AC133555.3	-18.5959179	1793.901735	-0.0104	0.992	-8.65E-09	count	1
ATP6V1B1	-18.5959179	1793.901735	-0.0104	0.992	-8.65E-09	count	1
AC010271.2	-18.5959179	1793.901735	-0.0104	0.992	-8.65E-09	count	1
LINC02419	-18.5959179	1793.901735	-0.0104	0.992	-8.65E-09	count	1
AC116447.1	-18.5959179	1793.901735	-0.0104	0.992	-8.65E-09	count	1
PCDH12	-18.5959179	1793.901735	-0.0104	0.992	-8.65E-09	count	1
ZFP57	-18.5959179	1793.901735	-0.0104	0.992	-8.65E-09	count	1
XCL1	-18.5959179	1793.901735	-0.0104	0.992	-8.65E-09	count	1
IGKV3-20	-18.5959179	1793.901735	-0.0104	0.992	-8.65E-09	count	1
AC135178.2	-18.5959179	1793.901735	-0.0104	0.992	-8.65E-09	count	1
OR51E2	-18.5959179	1793.901735	-0.0104	0.992	-8.65E-09	count	1
BAIAP2L1	-18.5959179	1793.901735	-0.0104	0.992	-8.65E-09	count	1

CCDC192	-18.5959179	1793.901735	-0.0104	0.992	-8.65E-09	count	1
AC099669.1	-18.5959179	1793.901735	-0.0104	0.992	-8.65E-09	count	1
ZNF556	-18.5959179	1793.901735	-0.0104	0.992	-8.65E-09	count	1
AC010542.5	-18.5959179	1793.901735	-0.0104	0.992	-8.65E-09	count	1
S100A7	-18.5959179	1793.901735	-0.0104	0.992	-8.65E-09	count	1
SALL1	-18.5959179	1793.901735	-0.0104	0.992	-8.65E-09	count	1
CDKL4	-18.5959179	1793.901735	-0.0104	0.992	-8.65E-09	count	1
Z84484.1	-18.5959179	1793.901735	-0.0104	0.992	-8.65E-09	count	1
VCAN-AS1	-19.0330879	1768.661923	-0.0108	0.991	-8.64E-09	count	1
MYO1G	-19.0338739	2010.586316	-0.0095	0.992	-8.63E-09	count	1
CD8A	-18.9530351	1805.813307	-0.0105	0.992	-8.62E-09	count	1
OOEP	-18.9530351	1805.813307	-0.0105	0.992	-8.62E-09	count	1
TMEM169	-18.9530351	1805.813307	-0.0105	0.992	-8.62E-09	count	1
AC092614.1	-18.9530351	1805.813307	-0.0105	0.992	-8.62E-09	count	1
CALHM6	-18.9530351	1805.813307	-0.0105	0.992	-8.62E-09	count	1
AC107871.1	-18.9530351	1805.813307	-0.0105	0.992	-8.62E-09	count	1
AC114811.2	-18.9530351	1805.813307	-0.0105	0.992	-8.62E-09	count	1
HPGDS	-19.2900704	1792.868627	-0.0108	0.991	-8.62E-09	count	1
AC099066.2	-19.3303245	1751.27951	-0.011	0.991	-8.62E-09	count	1
LINC00691	-18.953199	2362.963646	-0.008	0.994	-8.62E-09	count	1
HCG22	-19.0054086	886.7591823	-0.0214	0.983	-8.59E-09	count	1
MS4A1	-19.0064687	1147.979183	-0.0166	0.987	-8.58E-09	count	1
LGALS2	-19.0064687	1147.979183	-0.0166	0.987	-8.58E-09	count	1
LINC02541	-19.0064687	1147.979183	-0.0166	0.987	-8.58E-09	count	1
IGHV3-49	-19.0064687	1147.979183	-0.0166	0.987	-8.58E-09	count	1
AC074327.1	-19.0064687	1147.979183	-0.0166	0.987	-8.58E-09	count	1
GJA3	-19.2944903	885.7166195	-0.0218	0.983	-8.58E-09	count	1
DMBX1	-19.00795	1543.613871	-0.0123	0.99	-8.57E-09	count	1
CIDEA	-19.2964188	1258.505539	-0.0153	0.988	-8.57E-09	count	1
AC016722.2	-19.2983945	1784.875363	-0.0108	0.991	-8.56E-09	count	1
TEX45	-19.2983945	1784.875363	-0.0108	0.991	-8.56E-09	count	1
AC026347.1	-18.320579	1455.558747	-0.0126	0.99	-8.54E-09	count	1
AOAH	-18.320579	1455.558747	-0.0126	0.99	-8.54E-09	count	1
C10orf105	-18.320579	1455.558747	-0.0126	0.99	-8.54E-09	count	1
AC121338.2	-18.320579	1455.558747	-0.0126	0.99	-8.54E-09	count	1
CD33	-18.320579	1455.558747	-0.0126	0.99	-8.54E-09	count	1
AC002470.1	-18.320579	1455.558747	-0.0126	0.99	-8.54E-09	count	1
ALX3	-18.320579	1455.558747	-0.0126	0.99	-8.54E-09	count	1
AC012442.1	-18.320579	1455.558747	-0.0126	0.99	-8.54E-09	count	1
AC078883.1	-18.320579	1455.558747	-0.0126	0.99	-8.54E-09	count	1
AC063944.1	-18.320579	1455.558747	-0.0126	0.99	-8.54E-09	count	1
TRGC1	-18.320579	1455.558747	-0.0126	0.99	-8.54E-09	count	1
COBL	-18.320579	1455.558747	-0.0126	0.99	-8.54E-09	count	1
PRNCR1	-18.320579	1455.558747	-0.0126	0.99	-8.54E-09	count	1
CRTAM	-18.320579	1455.558747	-0.0126	0.99	-8.54E-09	count	1

GRAMD1B	-18.320579	1455.558747	-0.0126	0.99	-8.54E-09	count	1
SNX22	-18.320579	1455.558747	-0.0126	0.99	-8.54E-09	count	1
ASGR2	-18.320579	1455.558747	-0.0126	0.99	-8.54E-09	count	1
AC005695.3	-18.320579	1455.558747	-0.0126	0.99	-8.54E-09	count	1
GDF5	-18.320579	1455.558747	-0.0126	0.99	-8.54E-09	count	1
CATSPERD	-18.320579	1455.558747	-0.0126	0.99	-8.54E-09	count	1
AP001059.3	-18.320579	1455.558747	-0.0126	0.99	-8.54E-09	count	1
IGSF21	-18.320579	1455.558747	-0.0126	0.99	-8.54E-09	count	1
ACVR1C	-18.320579	1455.558747	-0.0126	0.99	-8.54E-09	count	1
ST3GAL6-AS1	-18.320579	1455.558747	-0.0126	0.99	-8.54E-09	count	1
TNF	-18.320579	1455.558747	-0.0126	0.99	-8.54E-09	count	1
GJB7	-18.320579	1455.558747	-0.0126	0.99	-8.54E-09	count	1
ARL11	-18.320579	1455.558747	-0.0126	0.99	-8.54E-09	count	1
LINC00652	-18.320579	1455.558747	-0.0126	0.99	-8.54E-09	count	1
DNAJB7	-18.320579	1455.558747	-0.0126	0.99	-8.54E-09	count	1
AC036214.1	-19.5238571	1997.866581	-0.0098	0.992	-8.54E-09	count	1
LINC00840	-19.5238571	1997.866581	-0.0098	0.992	-8.54E-09	count	1
AC098484.3	-18.321061	2060.531032	-0.0089	0.993	-8.54E-09	count	1
AP001267.1	-18.321061	2060.531032	-0.0089	0.993	-8.54E-09	count	1
AL022310.1	-18.321061	2060.531032	-0.0089	0.993	-8.54E-09	count	1
PROM2	-18.321061	2060.531032	-0.0089	0.993	-8.54E-09	count	1
GRK7	-18.321061	2060.531032	-0.0089	0.993	-8.54E-09	count	1
AC007370.1	-18.321061	2060.531032	-0.0089	0.993	-8.54E-09	count	1
AC016687.2	-18.321061	2060.531032	-0.0089	0.993	-8.54E-09	count	1
UPK3BL1	-18.321061	2060.531032	-0.0089	0.993	-8.54E-09	count	1
AC005586.1	-18.321061	2060.531032	-0.0089	0.993	-8.54E-09	count	1
FOXH1	-18.321061	2060.531032	-0.0089	0.993	-8.54E-09	count	1
AL935212.1	-18.321061	2060.531032	-0.0089	0.993	-8.54E-09	count	1
LMO1	-18.321061	2060.531032	-0.0089	0.993	-8.54E-09	count	1
PHOX2A	-18.321061	2060.531032	-0.0089	0.993	-8.54E-09	count	1
IGHV4-61	-18.321061	2060.531032	-0.0089	0.993	-8.54E-09	count	1
NPIPB3	-18.321061	2060.531032	-0.0089	0.993	-8.54E-09	count	1
RASGRP4	-18.321061	2060.531032	-0.0089	0.993	-8.54E-09	count	1
BACH1-AS1	-18.321061	2060.531032	-0.0089	0.993	-8.54E-09	count	1
PTAFR	-18.321061	2060.531032	-0.0089	0.993	-8.54E-09	count	1
AL035409.1	-18.321061	2060.531032	-0.0089	0.993	-8.54E-09	count	1
LINC02022	-18.321061	2060.531032	-0.0089	0.993	-8.54E-09	count	1
AC069213.1	-18.321061	2060.531032	-0.0089	0.993	-8.54E-09	count	1
AC096564.2	-18.321061	2060.531032	-0.0089	0.993	-8.54E-09	count	1
ZCCHC23	-18.321061	2060.531032	-0.0089	0.993	-8.54E-09	count	1
AL355297.3	-18.321061	2060.531032	-0.0089	0.993	-8.54E-09	count	1
AC004862.1	-18.321061	2060.531032	-0.0089	0.993	-8.54E-09	count	1
TREH	-18.321061	2060.531032	-0.0089	0.993	-8.54E-09	count	1
AL606489.1	-18.321061	2060.531032	-0.0089	0.993	-8.54E-09	count	1
AL355472.3	-18.321061	2060.531032	-0.0089	0.993	-8.54E-09	count	1

YIPF7	-18.321061	2060.531032	-0.0089	0.993	-8.54E-09	count	1
RAB3C	-18.321061	2060.531032	-0.0089	0.993	-8.54E-09	count	1
MYOT	-18.321061	2060.531032	-0.0089	0.993	-8.54E-09	count	1
FGD2	-18.321061	2060.531032	-0.0089	0.993	-8.54E-09	count	1
PCAT5	-18.321061	2060.531032	-0.0089	0.993	-8.54E-09	count	1
AL356124.1	-18.321061	2060.531032	-0.0089	0.993	-8.54E-09	count	1
ECT2L	-18.321061	2060.531032	-0.0089	0.993	-8.54E-09	count	1
AL138895.1	-18.321061	2060.531032	-0.0089	0.993	-8.54E-09	count	1
AL138921.2	-18.321061	2060.531032	-0.0089	0.993	-8.54E-09	count	1
HCAR2	-18.321061	2060.531032	-0.0089	0.993	-8.54E-09	count	1
AC004585.1	-18.321061	2060.531032	-0.0089	0.993	-8.54E-09	count	1
ARHGAP9	-18.7268099	1455.009215	-0.0129	0.99	-8.53E-09	count	1
MYLK3	-18.7268099	1455.009215	-0.0129	0.99	-8.53E-09	count	1
LCN12	-18.7268099	1455.009215	-0.0129	0.99	-8.53E-09	count	1
KCNMA1-AS1	-18.7268099	1455.009215	-0.0129	0.99	-8.53E-09	count	1
TNFAIP8L2	-18.7268099	1455.009215	-0.0129	0.99	-8.53E-09	count	1
IKZF1	-18.7268099	1455.009215	-0.0129	0.99	-8.53E-09	count	1
P2RX1	-18.7268099	1455.009215	-0.0129	0.99	-8.53E-09	count	1
AC097652.1	-18.7279145	2525.377262	-0.0074	0.994	-8.52E-09	count	1
JSRP1	-18.7279145	2525.377262	-0.0074	0.994	-8.52E-09	count	1
LINC02535	-18.7279145	2525.377262	-0.0074	0.994	-8.52E-09	count	1
DBH-AS1	-18.7279145	2525.377262	-0.0074	0.994	-8.52E-09	count	1
PLD4	-18.7279145	2525.377262	-0.0074	0.994	-8.52E-09	count	1
AC135050.3	-18.7279145	2525.377262	-0.0074	0.994	-8.52E-09	count	1
AC091588.2	-18.7279145	2525.377262	-0.0074	0.994	-8.52E-09	count	1
GPR42	-18.7279145	2525.377262	-0.0074	0.994	-8.52E-09	count	1
ADORA2A	-18.7279145	2525.377262	-0.0074	0.994	-8.52E-09	count	1
KLHL40	-18.7279145	2525.377262	-0.0074	0.994	-8.52E-09	count	1
GNA15	-19.0156278	1783.479694	-0.0107	0.991	-8.52E-09	count	1
SLC8A1-AS1	-19.0156278	1783.479694	-0.0107	0.991	-8.52E-09	count	1
AC067747.1	-19.2393718	1722.335877	-0.0112	0.991	-8.51E-09	count	1
MUM1L1	-19.0162411	2305.036456	-0.0082	0.993	-8.51E-09	count	1
AC006146.1	-19.0169584	2918.04075	-0.0065	0.995	-8.50E-09	count	1
AC018682.1	-19.0169584	2918.04075	-0.0065	0.995	-8.50E-09	count	1
AC084819.1	-19.0169584	2918.04075	-0.0065	0.995	-8.50E-09	count	1
AC020651.1	-19.0169584	2918.04075	-0.0065	0.995	-8.50E-09	count	1
TM4SF19	-19.0169584	2918.04075	-0.0065	0.995	-8.50E-09	count	1
AC233280.1	-0.6306268	0.5017463	-1.2569	0.209	-8.05E-09	count	1
AC004865.2	-0.6197487	0.8889773	-0.6971	0.486	-7.90E-09	count	1
LINC01480	-0.6197487	0.6027033	-1.0283	0.304	-7.89E-09	count	1
AC026785.2	-1.441557	0.7592876	-1.8986	0.0577	-7.78E-09	count	1
AL008733.1	-1.441557	0.7592876	-1.8986	0.0577	-7.78E-09	count	1
ACTA1	-1.441557	0.7592876	-1.8986	0.0577	-7.78E-09	count	1
MRC1	-1.441557	0.7592876	-1.8986	0.0577	-7.78E-09	count	1
DNAJC9-AS1	-1.441557	0.8581721	-1.6798	0.0931	-7.78E-09	count	1

AC011815.1	-1.441557	0.8581721	-1.6798	0.0931	-7.78E-09	count	1
AL358781.2	-1.441557	0.8581721	-1.6798	0.0931	-7.78E-09	count	1
AC024592.3	-1.4046435	0.5780188	-2.4301	0.0151	-7.55E-09	count	1
INKA2	-1.4046435	0.5780188	-2.4301	0.0151	-7.55E-09	count	1
SPINK1	-1.4046435	0.5780188	-2.4301	0.0151	-7.55E-09	count	1
AP001189.5	-1.4046435	0.5780188	-2.4301	0.0151	-7.55E-09	count	1
AC091153.3	-1.4046435	0.5780188	-2.4301	0.0151	-7.55E-09	count	1
KCNS2	-1.4046435	0.6667234	-2.1068	0.0352	-7.55E-09	count	1
TRIM9	-0.5889548	0.6694886	-0.8797	0.379	-7.45E-09	count	1
AC138207.2	-0.2263375	0.5206362	-0.4347	0.664	-7.14E-09	count	1
SLCO2B1	-0.5660883	0.7582156	-0.7466	0.455	-7.12E-09	count	1
STC2	-0.5272205	0.8166481	-0.6456	0.519	-6.58E-09	count	1
AL022341.1	-0.5272205	0.8166481	-0.6456	0.519	-6.58E-09	count	1
LINC00887	-0.5272205	0.8166481	-0.6456	0.519	-6.58E-09	count	1
AC079447.1	-0.5272205	0.8166481	-0.6456	0.519	-6.58E-09	count	1
TIE1	-0.5272205	0.8166481	-0.6456	0.519	-6.58E-09	count	1
PATL2	-0.5272205	0.8166481	-0.6456	0.519	-6.58E-09	count	1
STX19	-0.5272205	1.1034833	-0.4778	0.633	-6.58E-09	count	1
SLC25A34	-0.5272205	0.9698871	-0.5436	0.587	-6.58E-09	count	1
FP565260.1	-0.2000193	0.7148074	-0.2798	0.78	-6.27E-09	count	1
ARMCX3-AS1	-0.4654863	0.8563153	-0.5436	0.587	-5.73E-09	count	1
AC010761.1	-0.4654863	0.6632594	-0.7018	0.483	-5.73E-09	count	1
PTPN7	-0.4654863	0.6635308	-0.7015	0.483	-5.73E-09	count	1
LCMT1-AS2	-0.4346924	0.9996011	-0.4349	0.664	-5.31E-09	count	1
LRRC4	-0.4346924	0.9996011	-0.4349	0.664	-5.31E-09	count	1
AC025031.4	-0.4346924	0.8205706	-0.5297	0.596	-5.31E-09	count	1
AC107214.1	-0.4346924	0.8205706	-0.5297	0.596	-5.31E-09	count	1
AC145212.1	-0.4346924	0.801657	-0.5422	0.588	-5.31E-09	count	1
AC011815.2	-0.4346924	0.8205706	-0.5297	0.596	-5.31E-09	count	1
ARHGEF18	-0.4346924	0.8205706	-0.5297	0.596	-5.31E-09	count	1
C6orf47-AS1	-0.4346924	0.801657	-0.5422	0.588	-5.31E-09	count	1
TF	-0.4346924	0.9508882	-0.4571	0.648	-5.31E-09	count	1
LHFPL4	-0.4346924	0.9508882	-0.4571	0.648	-5.31E-09	count	1
CLEC12A	-0.4346924	0.9508882	-0.4571	0.648	-5.31E-09	count	1
CACNA1C-AS1	-0.4346924	0.9508882	-0.4571	0.648	-5.31E-09	count	1
UTS2	-1.0360919	0.8055999	-1.2861	0.198	-5.27E-09	count	1
AL133245.1	-1.0360919	0.8055999	-1.2861	0.198	-5.27E-09	count	1
AC117498.2	-1.0360919	0.8055999	-1.2861	0.198	-5.27E-09	count	1
AL358334.2	-1.0360919	0.8055999	-1.2861	0.198	-5.27E-09	count	1
S1PR4	-1.0360919	0.8055999	-1.2861	0.198	-5.27E-09	count	1
PLB1	-1.0360919	0.8055999	-1.2861	0.198	-5.27E-09	count	1
OR4D9	-1.0360919	0.8055999	-1.2861	0.198	-5.27E-09	count	1
AC089984.1	-1.0360919	0.8055999	-1.2861	0.198	-5.27E-09	count	1
LINC02574	-1.0360919	0.8055999	-1.2861	0.198	-5.27E-09	count	1
AL645939.4	-1.0360919	0.8055999	-1.2861	0.198	-5.27E-09	count	1

SIGLEC1	-1.0360919	0.8055999	-1.2861	0.198	-5.27E-09	count	1
ALDH3A1	-1.0360919	0.8055999	-1.2861	0.198	-5.27E-09	count	1
AC060766.4	-1.0360919	0.9585891	-1.0809	0.28	-5.27E-09	count	1
GPR160	-1.0360919	0.9585891	-1.0809	0.28	-5.27E-09	count	1
AC106886.5	-1.0360919	0.9585891	-1.0809	0.28	-5.27E-09	count	1
CRTC3-AS1	-1.0360919	0.9585891	-1.0809	0.28	-5.27E-09	count	1
NMU	-0.9991784	0.5020104	-1.9904	0.0466	-5.05E-09	count	1
TMEM232	-0.1597089	0.6300549	-0.2535	0.8	-4.95E-09	count	1
PCSK1	-0.3703282	0.5844772	-0.6336	0.526	-4.45E-09	count	1
AL590617.2	-0.3429448	0.5365588	-0.6392	0.523	-4.10E-09	count	1
SYCP3	-0.3429448	0.6065197	-0.5654	0.572	-4.10E-09	count	1
AL162431.2	-0.3429448	0.8003089	-0.4285	0.668	-4.10E-09	count	1
AL590648.3	-0.7114963	0.4716615	-1.5085	0.132	-3.40E-09	count	1
IGSF1	-18.9031012	1464.775442	-0.0129	0.99	-3.17E-09	count	1
AC010319.4	-18.9031012	1464.775442	-0.0129	0.99	-3.17E-09	count	1
C5orf64-AS1	-18.9031012	1464.775442	-0.0129	0.99	-3.17E-09	count	1
AC008609.1	-18.9031012	1464.775442	-0.0129	0.99	-3.17E-09	count	1
SOD2	-18.9031012	1464.775442	-0.0129	0.99	-3.17E-09	count	1
HECW1	-18.9031012	1464.775442	-0.0129	0.99	-3.17E-09	count	1
PAEP	-18.9031012	1464.775442	-0.0129	0.99	-3.17E-09	count	1
AL118558.4	-18.9031012	1464.775442	-0.0129	0.99	-3.17E-09	count	1
MAST1	-18.9031012	1464.775442	-0.0129	0.99	-3.17E-09	count	1
IGLV3-9	-18.9031012	1464.775442	-0.0129	0.99	-3.17E-09	count	1
LINC01136	-18.9031012	1464.775442	-0.0129	0.99	-3.17E-09	count	1
TREX1	-18.9031012	1464.775442	-0.0129	0.99	-3.17E-09	count	1
MEPE	-18.9031012	1464.775442	-0.0129	0.99	-3.17E-09	count	1
AC123595.1	-18.9031012	1464.775442	-0.0129	0.99	-3.17E-09	count	1
AL357497.1	-18.9031012	1464.775442	-0.0129	0.99	-3.17E-09	count	1
PTCRA	-18.9031012	1464.775442	-0.0129	0.99	-3.17E-09	count	1
LEP	-18.9031012	1464.775442	-0.0129	0.99	-3.17E-09	count	1
AC073878.1	-18.9031012	1464.775442	-0.0129	0.99	-3.17E-09	count	1
AC135371.1	-18.9031012	1464.775442	-0.0129	0.99	-3.17E-09	count	1
E2F7	-18.9031012	1464.775442	-0.0129	0.99	-3.17E-09	count	1
AC138123.1	-18.9031012	1464.775442	-0.0129	0.99	-3.17E-09	count	1
LINC02345	-18.9031012	1464.775442	-0.0129	0.99	-3.17E-09	count	1
AC087294.1	-18.9031012	1464.775442	-0.0129	0.99	-3.17E-09	count	1
AL031432.1	-18.9031012	1464.775442	-0.0129	0.99	-3.17E-09	count	1
SMPDL3B	-18.9031012	1464.775442	-0.0129	0.99	-3.17E-09	count	1
TMIGD3	-18.9031012	1464.775442	-0.0129	0.99	-3.17E-09	count	1
LINC01348	-18.9031012	1464.775442	-0.0129	0.99	-3.17E-09	count	1
TPO	-18.9031012	1464.775442	-0.0129	0.99	-3.17E-09	count	1
ADGRF3	-18.9031012	1464.775442	-0.0129	0.99	-3.17E-09	count	1
AC073257.2	-18.9031012	1464.775442	-0.0129	0.99	-3.17E-09	count	1
AC009495.3	-18.9031012	1464.775442	-0.0129	0.99	-3.17E-09	count	1
DAZL	-18.9031012	1464.775442	-0.0129	0.99	-3.17E-09	count	1

P3H2-AS1	-18.9031012	1464.775442	-0.0129	0.99	-3.17E-09	count	1
NRROS	-18.9031012	1464.775442	-0.0129	0.99	-3.17E-09	count	1
AC079766.1	-18.9031012	1464.775442	-0.0129	0.99	-3.17E-09	count	1
BTNL9	-18.9031012	1464.775442	-0.0129	0.99	-3.17E-09	count	1
AL357054.2	-18.9031012	1464.775442	-0.0129	0.99	-3.17E-09	count	1
AL022724.1	-18.9031012	1464.775442	-0.0129	0.99	-3.17E-09	count	1
HIST1H3C	-18.9031012	1464.775442	-0.0129	0.99	-3.17E-09	count	1
NCR3	-18.9031012	1464.775442	-0.0129	0.99	-3.17E-09	count	1
HDAC2-AS2	-18.9031012	1464.775442	-0.0129	0.99	-3.17E-09	count	1
TMEM244	-18.9031012	1464.775442	-0.0129	0.99	-3.17E-09	count	1
CRHR2	-18.9031012	1464.775442	-0.0129	0.99	-3.17E-09	count	1
AC009542.1	-18.9031012	1464.775442	-0.0129	0.99	-3.17E-09	count	1
CTAGE6	-18.9031012	1464.775442	-0.0129	0.99	-3.17E-09	count	1
ADAM28	-18.9031012	1464.775442	-0.0129	0.99	-3.17E-09	count	1
AC137579.1	-18.9031012	1464.775442	-0.0129	0.99	-3.17E-09	count	1
DCSTAMP	-18.9031012	1464.775442	-0.0129	0.99	-3.17E-09	count	1
AL353770.4	-18.9031012	1464.775442	-0.0129	0.99	-3.17E-09	count	1
AC136475.9	-18.9031012	1464.775442	-0.0129	0.99	-3.17E-09	count	1
LINC01219	-18.9031012	1464.775442	-0.0129	0.99	-3.17E-09	count	1
AP001107.6	-18.9031012	1464.775442	-0.0129	0.99	-3.17E-09	count	1
CYP26A1	-18.9031012	1464.775442	-0.0129	0.99	-3.17E-09	count	1
AC089983.1	-18.9031012	1464.775442	-0.0129	0.99	-3.17E-09	count	1
AC243965.1	-18.9031012	1464.775442	-0.0129	0.99	-3.17E-09	count	1
AL121790.1	-18.9031012	1464.775442	-0.0129	0.99	-3.17E-09	count	1
SERPINA6	-18.9031012	1464.775442	-0.0129	0.99	-3.17E-09	count	1
IGHGP	-18.9031012	1464.775442	-0.0129	0.99	-3.17E-09	count	1
AC027237.2	-18.9031012	1464.775442	-0.0129	0.99	-3.17E-09	count	1
AC100835.2	-18.9031012	1464.775442	-0.0129	0.99	-3.17E-09	count	1
ACSM2B	-18.9031012	1464.775442	-0.0129	0.99	-3.17E-09	count	1
CPNE7	-18.9031012	1464.775442	-0.0129	0.99	-3.17E-09	count	1
AC244100.2	-18.9031012	1464.775442	-0.0129	0.99	-3.17E-09	count	1
PHOSPHO1	-18.9031012	1464.775442	-0.0129	0.99	-3.17E-09	count	1
ASIP	-18.9031012	1464.775442	-0.0129	0.99	-3.17E-09	count	1
AC004156.1	-18.9031012	1464.775442	-0.0129	0.99	-3.17E-09	count	1
AC020917.3	-18.9031012	1464.775442	-0.0129	0.99	-3.17E-09	count	1
AC022154.1	-18.9031012	1464.775442	-0.0129	0.99	-3.17E-09	count	1
VSTM1	-18.9031012	1464.775442	-0.0129	0.99	-3.17E-09	count	1
FASLG	-18.9031012	1464.775442	-0.0129	0.99	-3.17E-09	count	1
MYBPH	-18.9031012	1464.775442	-0.0129	0.99	-3.17E-09	count	1
IGKV1-39	-18.9031012	1464.775442	-0.0129	0.99	-3.17E-09	count	1
AC007879.2	-18.9031012	1464.775442	-0.0129	0.99	-3.17E-09	count	1
AC112907.1	-18.9031012	1464.775442	-0.0129	0.99	-3.17E-09	count	1
AC010343.3	-18.9031012	1464.775442	-0.0129	0.99	-3.17E-09	count	1
LINC01844	-18.9031012	1464.775442	-0.0129	0.99	-3.17E-09	count	1
KIF4B	-18.9031012	1464.775442	-0.0129	0.99	-3.17E-09	count	1

PPP1R2B	-18.9031012	1464.775442	-0.0129	0.99	-3.17E-09	count	1
AL359382.1	-18.9031012	1464.775442	-0.0129	0.99	-3.17E-09	count	1
AC020743.1	-18.9031012	1464.775442	-0.0129	0.99	-3.17E-09	count	1
MUC3A	-18.9031012	1464.775442	-0.0129	0.99	-3.17E-09	count	1
AL353732.1	-18.9031012	1464.775442	-0.0129	0.99	-3.17E-09	count	1
AL355574.1	-18.9031012	1464.775442	-0.0129	0.99	-3.17E-09	count	1
C8G	-18.9031012	1464.775442	-0.0129	0.99	-3.17E-09	count	1
AC079329.1	-18.9031012	1464.775442	-0.0129	0.99	-3.17E-09	count	1
AC018511.1	-18.9031012	1464.775442	-0.0129	0.99	-3.17E-09	count	1
OPN4	-18.9031012	1464.775442	-0.0129	0.99	-3.17E-09	count	1
GRIN2B	-18.9031012	1464.775442	-0.0129	0.99	-3.17E-09	count	1
TESPA1	-18.9031012	1464.775442	-0.0129	0.99	-3.17E-09	count	1
APOF	-18.9031012	1464.775442	-0.0129	0.99	-3.17E-09	count	1
PPFIA2-AS1	-18.9031012	1464.775442	-0.0129	0.99	-3.17E-09	count	1
AL161756.1	-18.9031012	1464.775442	-0.0129	0.99	-3.17E-09	count	1
ACSBG1	-18.9031012	1464.775442	-0.0129	0.99	-3.17E-09	count	1
AC126755.3	-18.9031012	1464.775442	-0.0129	0.99	-3.17E-09	count	1
AC026464.4	-18.9031012	1464.775442	-0.0129	0.99	-3.17E-09	count	1
HSH2D	-18.9031012	1464.775442	-0.0129	0.99	-3.17E-09	count	1
KIR3DX1	-18.9031012	1464.775442	-0.0129	0.99	-3.17E-09	count	1
CELSR1	-18.9031012	1464.775442	-0.0129	0.99	-3.17E-09	count	1
AL109761.1	-18.9031012	1464.775442	-0.0129	0.99	-3.17E-09	count	1
KCNE1	-18.9031012	1464.775442	-0.0129	0.99	-3.17E-09	count	1
AL844908.1	-18.9031012	1464.775442	-0.0129	0.99	-3.17E-09	count	1
DCST1-AS1	-18.9031012	1464.775442	-0.0129	0.99	-3.17E-09	count	1
SDC1	-18.9031012	1464.775442	-0.0129	0.99	-3.17E-09	count	1
TSPYL6	-18.9031012	1464.775442	-0.0129	0.99	-3.17E-09	count	1
EMX1	-18.9031012	1464.775442	-0.0129	0.99	-3.17E-09	count	1
CIDEC	-18.9031012	1464.775442	-0.0129	0.99	-3.17E-09	count	1
CCK	-18.9031012	1464.775442	-0.0129	0.99	-3.17E-09	count	1
AC010245.1	-18.9031012	1464.775442	-0.0129	0.99	-3.17E-09	count	1
LINC01366	-18.9031012	1464.775442	-0.0129	0.99	-3.17E-09	count	1
AL136307.1	-18.9031012	1464.775442	-0.0129	0.99	-3.17E-09	count	1
OPRM1	-18.9031012	1464.775442	-0.0129	0.99	-3.17E-09	count	1
TRBV28	-18.9031012	1464.775442	-0.0129	0.99	-3.17E-09	count	1
GUCY2F	-18.9031012	1464.775442	-0.0129	0.99	-3.17E-09	count	1
C8orf37-AS1	-18.9031012	1464.775442	-0.0129	0.99	-3.17E-09	count	1
PLPP4	-18.9031012	1464.775442	-0.0129	0.99	-3.17E-09	count	1
PABPC3	-18.9031012	1464.775442	-0.0129	0.99	-3.17E-09	count	1
ST20-MTHFS	-18.9031012	1464.775442	-0.0129	0.99	-3.17E-09	count	1
AC138207.7	-18.9031012	1464.775442	-0.0129	0.99	-3.17E-09	count	1
AC015961.1	-18.9031012	1464.775442	-0.0129	0.99	-3.17E-09	count	1
SLA2	-18.9031012	1464.775442	-0.0129	0.99	-3.17E-09	count	1
AC008759.3	-18.9031012	1464.775442	-0.0129	0.99	-3.17E-09	count	1
PDE4C	-18.9031012	1464.775442	-0.0129	0.99	-3.17E-09	count	1

TMEM145	-18.9031012	1464.775442	-0.0129	0.99	-3.17E-09	count	1
FPR3	-18.9031012	1464.775442	-0.0129	0.99	-3.17E-09	count	1
SPECC1L-ADORA2A	-18.9031012	1464.775442	-0.0129	0.99	-3.17E-09	count	1
TNFRSF13C	-18.9031012	1464.775442	-0.0129	0.99	-3.17E-09	count	1
BX539320.1	-18.9031012	1464.775442	-0.0129	0.99	-3.17E-09	count	1
AL033528.2	-18.9031012	1464.775442	-0.0129	0.99	-3.17E-09	count	1
BMP8A	-18.9031012	1464.775442	-0.0129	0.99	-3.17E-09	count	1
PKN2-AS1	-18.9031012	1464.775442	-0.0129	0.99	-3.17E-09	count	1
SLAMF7	-18.9031012	1464.775442	-0.0129	0.99	-3.17E-09	count	1
AL359551.1	-18.9031012	1464.775442	-0.0129	0.99	-3.17E-09	count	1
MYOSLID	-18.9031012	1464.775442	-0.0129	0.99	-3.17E-09	count	1
SSUH2	-18.9031012	1464.775442	-0.0129	0.99	-3.17E-09	count	1
SERPINI2	-18.9031012	1464.775442	-0.0129	0.99	-3.17E-09	count	1
MSH5-SAPCD1	-18.9031012	1464.775442	-0.0129	0.99	-3.17E-09	count	1
ATP5MF-PTCD1	-18.9031012	1464.775442	-0.0129	0.99	-3.17E-09	count	1
AL035427.1	-18.9031012	1464.775442	-0.0129	0.99	-3.17E-09	count	1
SIT1	-18.9031012	1464.775442	-0.0129	0.99	-3.17E-09	count	1
OBP2B	-18.9031012	1464.775442	-0.0129	0.99	-3.17E-09	count	1
SUCLA2-AS1	-18.9031012	1464.775442	-0.0129	0.99	-3.17E-09	count	1
IGHV4-34	-18.9031012	1464.775442	-0.0129	0.99	-3.17E-09	count	1
LINC00920	-18.9031012	1464.775442	-0.0129	0.99	-3.17E-09	count	1
AC009060.1	-18.9031012	1464.775442	-0.0129	0.99	-3.17E-09	count	1
CLEC3A	-18.9031012	1464.775442	-0.0129	0.99	-3.17E-09	count	1
AC243571.2	-18.9031012	1464.775442	-0.0129	0.99	-3.17E-09	count	1
WDR49	-19.5982072	1463.482942	-0.0134	0.989	-3.16E-09	count	1
CCDC88C	-19.5982072	1463.482942	-0.0134	0.989	-3.16E-09	count	1
AC109479.1	-19.5982072	1463.482942	-0.0134	0.989	-3.16E-09	count	1
LINC02502	-19.5982072	1463.482942	-0.0134	0.989	-3.16E-09	count	1
CXCR3	-19.5982072	1463.482942	-0.0134	0.989	-3.16E-09	count	1
LINC01641	-19.5993524	2074.72277	-0.0094	0.992	-3.16E-09	count	1
TNR	-19.5993524	2074.72277	-0.0094	0.992	-3.16E-09	count	1
MYPN	-19.5993524	2074.72277	-0.0094	0.992	-3.16E-09	count	1
KIF14	-19.5993524	2074.72277	-0.0094	0.992	-3.16E-09	count	1
CST2	-19.5993524	2074.72277	-0.0094	0.992	-3.16E-09	count	1
EDRF1-AS1	-19.5993524	2074.72277	-0.0094	0.992	-3.16E-09	count	1
CCDC62	-19.5993524	2074.72277	-0.0094	0.992	-3.16E-09	count	1
MGAM2	-19.5993524	2074.72277	-0.0094	0.992	-3.16E-09	count	1
AC104170.1	-18.6265588	2400.585336	-0.0078	0.994	-3.15E-09	count	1
DNASE2B	-18.6265588	2400.585336	-0.0078	0.994	-3.15E-09	count	1
AL606534.2	-18.6265588	2400.585336	-0.0078	0.994	-3.15E-09	count	1
AC017083.2	-18.6265588	2400.585336	-0.0078	0.994	-3.15E-09	count	1
VIL1	-18.6265588	2400.585336	-0.0078	0.994	-3.15E-09	count	1
CELSR3	-18.6265588	2400.585336	-0.0078	0.994	-3.15E-09	count	1
FAM19A1	-18.6265588	2400.585336	-0.0078	0.994	-3.15E-09	count	1
FAM160A1-DT	-18.6265588	2400.585336	-0.0078	0.994	-3.15E-09	count	1

LINC02102	-18.6265588	2400.585336	-0.0078	0.994	-3.15E-09	count	1
LINC02236	-18.6265588	2400.585336	-0.0078	0.994	-3.15E-09	count	1
AC093673.2	-18.6265588	2400.585336	-0.0078	0.994	-3.15E-09	count	1
LINC01545	-18.6265588	2400.585336	-0.0078	0.994	-3.15E-09	count	1
FAAH2	-18.6265588	2400.585336	-0.0078	0.994	-3.15E-09	count	1
AC138356.1	-18.6265588	2400.585336	-0.0078	0.994	-3.15E-09	count	1
TNNI2	-18.6265588	2400.585336	-0.0078	0.994	-3.15E-09	count	1
CXCR5	-18.6265588	2400.585336	-0.0078	0.994	-3.15E-09	count	1
AL157786.1	-18.6265588	2400.585336	-0.0078	0.994	-3.15E-09	count	1
AL359094.1	-18.6265588	2400.585336	-0.0078	0.994	-3.15E-09	count	1
AC131238.1	-18.6265588	2400.585336	-0.0078	0.994	-3.15E-09	count	1
AC243965.2	-18.6265588	2400.585336	-0.0078	0.994	-3.15E-09	count	1
PAK6	-18.6265588	2400.585336	-0.0078	0.994	-3.15E-09	count	1
AC007493.2	-18.6265588	2400.585336	-0.0078	0.994	-3.15E-09	count	1
AC005304.3	-18.6265588	2400.585336	-0.0078	0.994	-3.15E-09	count	1
AC090772.3	-18.6265588	2400.585336	-0.0078	0.994	-3.15E-09	count	1
AC017100.1	-18.6265588	2400.585336	-0.0078	0.994	-3.15E-09	count	1
AC011509.2	-18.6265588	2400.585336	-0.0078	0.994	-3.15E-09	count	1
AC003956.1	-18.6265588	2400.585336	-0.0078	0.994	-3.15E-09	count	1
PARVG	-18.6265588	2400.585336	-0.0078	0.994	-3.15E-09	count	1
AL121672.1	-18.6265588	2400.585336	-0.0078	0.994	-3.15E-09	count	1
AP000688.3	-18.6265588	2400.585336	-0.0078	0.994	-3.15E-09	count	1
LINC01160	-18.6265588	2400.585336	-0.0078	0.994	-3.15E-09	count	1
GPR17	-18.6265588	2400.585336	-0.0078	0.994	-3.15E-09	count	1
LINC02483	-18.6265588	2400.585336	-0.0078	0.994	-3.15E-09	count	1
AC104123.1	-18.6265588	2400.585336	-0.0078	0.994	-3.15E-09	count	1
PCDHA9	-18.6265588	2400.585336	-0.0078	0.994	-3.15E-09	count	1
ITK	-18.6265588	2400.585336	-0.0078	0.994	-3.15E-09	count	1
LINC01847	-18.6265588	2400.585336	-0.0078	0.994	-3.15E-09	count	1
HIST1H3G	-18.6265588	2400.585336	-0.0078	0.994	-3.15E-09	count	1
MPIG6B	-18.6265588	2400.585336	-0.0078	0.994	-3.15E-09	count	1
AL109947.1	-18.6265588	2400.585336	-0.0078	0.994	-3.15E-09	count	1
AC002480.3	-18.6265588	2400.585336	-0.0078	0.994	-3.15E-09	count	1
AC093668.3	-18.6265588	2400.585336	-0.0078	0.994	-3.15E-09	count	1
AC091736.1	-18.6265588	2400.585336	-0.0078	0.994	-3.15E-09	count	1
CSMD1	-18.6265588	2400.585336	-0.0078	0.994	-3.15E-09	count	1
AC011632.1	-18.6265588	2400.585336	-0.0078	0.994	-3.15E-09	count	1
AC090921.1	-18.6265588	2400.585336	-0.0078	0.994	-3.15E-09	count	1
SLURP1	-18.6265588	2400.585336	-0.0078	0.994	-3.15E-09	count	1
AL159169.2	-18.6265588	2400.585336	-0.0078	0.994	-3.15E-09	count	1
AL133417.1	-18.6265588	2400.585336	-0.0078	0.994	-3.15E-09	count	1
SPX	-18.6265588	2400.585336	-0.0078	0.994	-3.15E-09	count	1
AC127164.1	-18.6265588	2400.585336	-0.0078	0.994	-3.15E-09	count	1
CSNK1A1L	-18.6265588	2400.585336	-0.0078	0.994	-3.15E-09	count	1
DIAPH3-AS1	-18.6265588	2400.585336	-0.0078	0.994	-3.15E-09	count	1

AL133279.1	-18.6265588	2400.585336	-0.0078	0.994	-3.15E-09	count	1
AC130456.3	-18.6265588	2400.585336	-0.0078	0.994	-3.15E-09	count	1
C16orf54	-18.6265588	2400.585336	-0.0078	0.994	-3.15E-09	count	1
10-Mar	-18.6265588	2400.585336	-0.0078	0.994	-3.15E-09	count	1
HM13-AS1	-18.6265588	2400.585336	-0.0078	0.994	-3.15E-09	count	1
AC245884.10	-18.6265588	2400.585336	-0.0078	0.994	-3.15E-09	count	1
AL096855.1	-18.6265588	2400.585336	-0.0078	0.994	-3.15E-09	count	1
LINC01144	-18.6265588	2400.585336	-0.0078	0.994	-3.15E-09	count	1
AL513218.1	-18.6265588	2400.585336	-0.0078	0.994	-3.15E-09	count	1
AL513285.1	-18.6265588	2400.585336	-0.0078	0.994	-3.15E-09	count	1
AC092807.2	-18.6265588	2400.585336	-0.0078	0.994	-3.15E-09	count	1
KCNA3	-18.6265588	2400.585336	-0.0078	0.994	-3.15E-09	count	1
CD160	-18.6265588	2400.585336	-0.0078	0.994	-3.15E-09	count	1
TNN	-18.6265588	2400.585336	-0.0078	0.994	-3.15E-09	count	1
IBA57-DT	-18.6265588	2400.585336	-0.0078	0.994	-3.15E-09	count	1
SNORC	-18.6265588	2400.585336	-0.0078	0.994	-3.15E-09	count	1
CCR5	-18.6265588	2400.585336	-0.0078	0.994	-3.15E-09	count	1
AC093895.1	-18.6265588	2400.585336	-0.0078	0.994	-3.15E-09	count	1
AC034229.1	-18.6265588	2400.585336	-0.0078	0.994	-3.15E-09	count	1
PCDHA2	-18.6265588	2400.585336	-0.0078	0.994	-3.15E-09	count	1
AL121936.1	-18.6265588	2400.585336	-0.0078	0.994	-3.15E-09	count	1
LRFN2	-18.6265588	2400.585336	-0.0078	0.994	-3.15E-09	count	1
AC004014.1	-18.6265588	2400.585336	-0.0078	0.994	-3.15E-09	count	1
ERCC6L	-18.6265588	2400.585336	-0.0078	0.994	-3.15E-09	count	1
AC067817.2	-18.6265588	2400.585336	-0.0078	0.994	-3.15E-09	count	1
LINC01592	-18.6265588	2400.585336	-0.0078	0.994	-3.15E-09	count	1
AL590369.1	-18.6265588	2400.585336	-0.0078	0.994	-3.15E-09	count	1
AC015689.1	-18.6265588	2400.585336	-0.0078	0.994	-3.15E-09	count	1
FOLR3	-18.6265588	2400.585336	-0.0078	0.994	-3.15E-09	count	1
AP001189.6	-18.6265588	2400.585336	-0.0078	0.994	-3.15E-09	count	1
TMPRSS5	-18.6265588	2400.585336	-0.0078	0.994	-3.15E-09	count	1
RPEL1	-18.6265588	2400.585336	-0.0078	0.994	-3.15E-09	count	1
AL451069.1	-18.6265588	2400.585336	-0.0078	0.994	-3.15E-09	count	1
AC092490.1	-18.6265588	2400.585336	-0.0078	0.994	-3.15E-09	count	1
SCN8A	-18.6265588	2400.585336	-0.0078	0.994	-3.15E-09	count	1
AC121757.1	-18.6265588	2400.585336	-0.0078	0.994	-3.15E-09	count	1
AC126178.1	-18.6265588	2400.585336	-0.0078	0.994	-3.15E-09	count	1
RNF219-AS1	-18.6265588	2400.585336	-0.0078	0.994	-3.15E-09	count	1
AC022929.2	-18.6265588	2400.585336	-0.0078	0.994	-3.15E-09	count	1
AC104590.1	-18.6265588	2400.585336	-0.0078	0.994	-3.15E-09	count	1
AC027228.1	-18.6265588	2400.585336	-0.0078	0.994	-3.15E-09	count	1
AC105339.3	-18.6265588	2400.585336	-0.0078	0.994	-3.15E-09	count	1
AC243562.3	-18.6265588	2400.585336	-0.0078	0.994	-3.15E-09	count	1
Z69666.1	-18.6265588	2400.585336	-0.0078	0.994	-3.15E-09	count	1
CCDC154	-18.6265588	2400.585336	-0.0078	0.994	-3.15E-09	count	1

GLIS2-AS1	-18.6265588	2400.585336	-0.0078	0.994	-3.15E-09	count	1
AC133550.1	-18.6265588	2400.585336	-0.0078	0.994	-3.15E-09	count	1
AC007496.2	-18.6265588	2400.585336	-0.0078	0.994	-3.15E-09	count	1
AC092723.4	-18.6265588	2400.585336	-0.0078	0.994	-3.15E-09	count	1
AC090617.2	-18.6265588	2400.585336	-0.0078	0.994	-3.15E-09	count	1
AC027796.3	-18.6265588	2400.585336	-0.0078	0.994	-3.15E-09	count	1
CDRT4	-18.6265588	2400.585336	-0.0078	0.994	-3.15E-09	count	1
SLC47A2	-18.6265588	2400.585336	-0.0078	0.994	-3.15E-09	count	1
HAP1	-18.6265588	2400.585336	-0.0078	0.994	-3.15E-09	count	1
AC064805.1	-18.6265588	2400.585336	-0.0078	0.994	-3.15E-09	count	1
AC145207.6	-18.6265588	2400.585336	-0.0078	0.994	-3.15E-09	count	1
AC024361.2	-18.6265588	2400.585336	-0.0078	0.994	-3.15E-09	count	1
AC091043.1	-18.6265588	2400.585336	-0.0078	0.994	-3.15E-09	count	1
SIRPB2	-18.6265588	2400.585336	-0.0078	0.994	-3.15E-09	count	1
AL135937.1	-18.6265588	2400.585336	-0.0078	0.994	-3.15E-09	count	1
AL118523.1	-18.6265588	2400.585336	-0.0078	0.994	-3.15E-09	count	1
AC010300.1	-18.6265588	2400.585336	-0.0078	0.994	-3.15E-09	count	1
FFAR3	-18.6265588	2400.585336	-0.0078	0.994	-3.15E-09	count	1
AC002116.2	-18.6265588	2400.585336	-0.0078	0.994	-3.15E-09	count	1
MAP4K1	-18.6265588	2400.585336	-0.0078	0.994	-3.15E-09	count	1
FCGBP	-18.6265588	2400.585336	-0.0078	0.994	-3.15E-09	count	1
SRRM5	-18.6265588	2400.585336	-0.0078	0.994	-3.15E-09	count	1
AC011453.1	-18.6265588	2400.585336	-0.0078	0.994	-3.15E-09	count	1
AC005498.1	-18.6265588	2400.585336	-0.0078	0.994	-3.15E-09	count	1
AC012313.6	-18.6265588	2400.585336	-0.0078	0.994	-3.15E-09	count	1
Z98885.2	-18.6265588	2400.585336	-0.0078	0.994	-3.15E-09	count	1
PLAC4	-18.6265588	2400.585336	-0.0078	0.994	-3.15E-09	count	1
COL18A1-AS1	-18.6265588	2400.585336	-0.0078	0.994	-3.15E-09	count	1
HES2	-18.6265588	2400.585336	-0.0078	0.994	-3.15E-09	count	1
AC244453.2	-18.6265588	2400.585336	-0.0078	0.994	-3.15E-09	count	1
FCRL6	-18.6265588	2400.585336	-0.0078	0.994	-3.15E-09	count	1
Z99943.1	-18.6265588	2400.585336	-0.0078	0.994	-3.15E-09	count	1
AL121983.2	-18.6265588	2400.585336	-0.0078	0.994	-3.15E-09	count	1
AL118511.1	-18.6265588	2400.585336	-0.0078	0.994	-3.15E-09	count	1
AL451007.3	-18.6265588	2400.585336	-0.0078	0.994	-3.15E-09	count	1
GCSAML	-18.6265588	2400.585336	-0.0078	0.994	-3.15E-09	count	1
AC009486.1	-18.6265588	2400.585336	-0.0078	0.994	-3.15E-09	count	1
AC007391.1	-18.6265588	2400.585336	-0.0078	0.994	-3.15E-09	count	1
AC019129.2	-18.6265588	2400.585336	-0.0078	0.994	-3.15E-09	count	1
AC093110.1	-18.6265588	2400.585336	-0.0078	0.994	-3.15E-09	count	1
AC096669.1	-18.6265588	2400.585336	-0.0078	0.994	-3.15E-09	count	1
AC012508.2	-18.6265588	2400.585336	-0.0078	0.994	-3.15E-09	count	1
ERMN	-18.6265588	2400.585336	-0.0078	0.994	-3.15E-09	count	1
LINC01825	-18.6265588	2400.585336	-0.0078	0.994	-3.15E-09	count	1
TM4SF20	-18.6265588	2400.585336	-0.0078	0.994	-3.15E-09	count	1

PLS1-AS1	-18.6265588	2400.585336	-0.0078	0.994	-3.15E-09	count	1
GPR171	-18.6265588	2400.585336	-0.0078	0.994	-3.15E-09	count	1
AC024560.3	-18.6265588	2400.585336	-0.0078	0.994	-3.15E-09	count	1
ODAPH	-18.6265588	2400.585336	-0.0078	0.994	-3.15E-09	count	1
AC104785.1	-18.6265588	2400.585336	-0.0078	0.994	-3.15E-09	count	1
AC092611.2	-18.6265588	2400.585336	-0.0078	0.994	-3.15E-09	count	1
LINC02226	-18.6265588	2400.585336	-0.0078	0.994	-3.15E-09	count	1
AC025178.1	-18.6265588	2400.585336	-0.0078	0.994	-3.15E-09	count	1
AC092354.2	-18.6265588	2400.585336	-0.0078	0.994	-3.15E-09	count	1
POU3F2	-18.6265588	2400.585336	-0.0078	0.994	-3.15E-09	count	1
LINC00222	-18.6265588	2400.585336	-0.0078	0.994	-3.15E-09	count	1
AC005537.1	-18.6265588	2400.585336	-0.0078	0.994	-3.15E-09	count	1
LINC01447	-18.6265588	2400.585336	-0.0078	0.994	-3.15E-09	count	1
AC005009.2	-18.6265588	2400.585336	-0.0078	0.994	-3.15E-09	count	1
AC004522.2	-18.6265588	2400.585336	-0.0078	0.994	-3.15E-09	count	1
OR9A4	-18.6265588	2400.585336	-0.0078	0.994	-3.15E-09	count	1
AC092681.3	-18.6265588	2400.585336	-0.0078	0.994	-3.15E-09	count	1
MAGEA8-AS1	-18.6265588	2400.585336	-0.0078	0.994	-3.15E-09	count	1
AC246817.2	-18.6265588	2400.585336	-0.0078	0.994	-3.15E-09	count	1
AC084024.3	-18.6265588	2400.585336	-0.0078	0.994	-3.15E-09	count	1
OPRK1	-18.6265588	2400.585336	-0.0078	0.994	-3.15E-09	count	1
AC083967.1	-18.6265588	2400.585336	-0.0078	0.994	-3.15E-09	count	1
AL138781.1	-18.6265588	2400.585336	-0.0078	0.994	-3.15E-09	count	1
AP001775.2	-18.6265588	2400.585336	-0.0078	0.994	-3.15E-09	count	1
AL732437.1	-18.6265588	2400.585336	-0.0078	0.994	-3.15E-09	count	1
PRF1	-18.6265588	2400.585336	-0.0078	0.994	-3.15E-09	count	1
AC092112.1	-18.6265588	2400.585336	-0.0078	0.994	-3.15E-09	count	1
AC084816.1	-18.6265588	2400.585336	-0.0078	0.994	-3.15E-09	count	1
AMDHD1	-18.6265588	2400.585336	-0.0078	0.994	-3.15E-09	count	1
AC007569.1	-18.6265588	2400.585336	-0.0078	0.994	-3.15E-09	count	1
ENOX1-AS1	-18.6265588	2400.585336	-0.0078	0.994	-3.15E-09	count	1
AL138960.1	-18.6265588	2400.585336	-0.0078	0.994	-3.15E-09	count	1
GPR18	-18.6265588	2400.585336	-0.0078	0.994	-3.15E-09	count	1
AC013451.1	-18.6265588	2400.585336	-0.0078	0.994	-3.15E-09	count	1
AC087457.1	-18.6265588	2400.585336	-0.0078	0.994	-3.15E-09	count	1
AC021483.1	-18.6265588	2400.585336	-0.0078	0.994	-3.15E-09	count	1
CEMP1	-18.6265588	2400.585336	-0.0078	0.994	-3.15E-09	count	1
AC022167.3	-18.6265588	2400.585336	-0.0078	0.994	-3.15E-09	count	1
AC109446.3	-18.6265588	2400.585336	-0.0078	0.994	-3.15E-09	count	1
AC099518.3	-18.6265588	2400.585336	-0.0078	0.994	-3.15E-09	count	1
AC009093.5	-18.6265588	2400.585336	-0.0078	0.994	-3.15E-09	count	1
AC009088.1	-18.6265588	2400.585336	-0.0078	0.994	-3.15E-09	count	1
AC138028.1	-18.6265588	2400.585336	-0.0078	0.994	-3.15E-09	count	1
AC098850.2	-18.6265588	2400.585336	-0.0078	0.994	-3.15E-09	count	1
RASL10B	-18.6265588	2400.585336	-0.0078	0.994	-3.15E-09	count	1

AC005920.1	-18.6265588	2400.585336	-0.0078	0.994	-3.15E-09	count	1
AC005920.3	-18.6265588	2400.585336	-0.0078	0.994	-3.15E-09	count	1
LINC01929	-18.6265588	2400.585336	-0.0078	0.994	-3.15E-09	count	1
AL161938.1	-18.6265588	2400.585336	-0.0078	0.994	-3.15E-09	count	1
LINC01775	-18.6265588	2400.585336	-0.0078	0.994	-3.15E-09	count	1
AC004637.1	-18.6265588	2400.585336	-0.0078	0.994	-3.15E-09	count	1
AC020908.3	-18.6265588	2400.585336	-0.0078	0.994	-3.15E-09	count	1
AC020928.3	-18.6265588	2400.585336	-0.0078	0.994	-3.15E-09	count	1
AC020909.3	-18.6265588	2400.585336	-0.0078	0.994	-3.15E-09	count	1
Z82243.1	-18.6265588	2400.585336	-0.0078	0.994	-3.15E-09	count	1
SMC1B	-18.6265588	2400.585336	-0.0078	0.994	-3.15E-09	count	1
U62317.5	-18.6265588	2400.585336	-0.0078	0.994	-3.15E-09	count	1
AC233755.1	-18.6265588	2400.585336	-0.0078	0.994	-3.15E-09	count	1
AL035706.1	-18.6265588	2400.585336	-0.0078	0.994	-3.15E-09	count	1
AC104836.1	-18.6265588	2400.585336	-0.0078	0.994	-3.15E-09	count	1
AL359962.2	-18.6265588	2400.585336	-0.0078	0.994	-3.15E-09	count	1
ATP2B2	-18.6265588	2400.585336	-0.0078	0.994	-3.15E-09	count	1
LINC01998	-18.6265588	2400.585336	-0.0078	0.994	-3.15E-09	count	1
AC055764.2	-18.6265588	2400.585336	-0.0078	0.994	-3.15E-09	count	1
AC112250.2	-18.6265588	2400.585336	-0.0078	0.994	-3.15E-09	count	1
FABP6	-18.6265588	2400.585336	-0.0078	0.994	-3.15E-09	count	1
AL023284.4	-18.6265588	2400.585336	-0.0078	0.994	-3.15E-09	count	1
MAS1	-18.6265588	2400.585336	-0.0078	0.994	-3.15E-09	count	1
AC073957.3	-18.6265588	2400.585336	-0.0078	0.994	-3.15E-09	count	1
TFEC	-18.6265588	2400.585336	-0.0078	0.994	-3.15E-09	count	1
WEE2	-18.6265588	2400.585336	-0.0078	0.994	-3.15E-09	count	1
AF196972.1	-18.6265588	2400.585336	-0.0078	0.994	-3.15E-09	count	1
P2RY10	-18.6265588	2400.585336	-0.0078	0.994	-3.15E-09	count	1
SASH3	-18.6265588	2400.585336	-0.0078	0.994	-3.15E-09	count	1
AP000943.3	-18.6265588	2400.585336	-0.0078	0.994	-3.15E-09	count	1
CARMIL3	-18.6265588	2400.585336	-0.0078	0.994	-3.15E-09	count	1
AL356756.1	-18.6265588	2400.585336	-0.0078	0.994	-3.15E-09	count	1
AL049870.2	-18.6265588	2400.585336	-0.0078	0.994	-3.15E-09	count	1
CA5A	-18.6265588	2400.585336	-0.0078	0.994	-3.15E-09	count	1
PIK3R5	-18.6265588	2400.585336	-0.0078	0.994	-3.15E-09	count	1
ABCA9-AS1	-18.6265588	2400.585336	-0.0078	0.994	-3.15E-09	count	1
QRICH2	-18.6265588	2400.585336	-0.0078	0.994	-3.15E-09	count	1
AC015819.2	-18.6265588	2400.585336	-0.0078	0.994	-3.15E-09	count	1
ZNF341-AS1	-18.6265588	2400.585336	-0.0078	0.994	-3.15E-09	count	1
FO393401.1	-18.6265588	2400.585336	-0.0078	0.994	-3.15E-09	count	1
AC012615.2	-18.6265588	2400.585336	-0.0078	0.994	-3.15E-09	count	1
KCNN1	-18.6265588	2400.585336	-0.0078	0.994	-3.15E-09	count	1
SLC5A9	-18.6265588	2400.585336	-0.0078	0.994	-3.15E-09	count	1
ZRANB2-AS1	-18.6265588	2400.585336	-0.0078	0.994	-3.15E-09	count	1
SETSIP	-18.6265588	2400.585336	-0.0078	0.994	-3.15E-09	count	1

LINC01731	-18.6265588	2400.585336	-0.0078	0.994	-3.15E-09	count	1
AC012073.1	-18.6265588	2400.585336	-0.0078	0.994	-3.15E-09	count	1
AC106869.1	-18.6265588	2400.585336	-0.0078	0.994	-3.15E-09	count	1
RPRM	-18.6265588	2400.585336	-0.0078	0.994	-3.15E-09	count	1
HJURP	-18.6265588	2400.585336	-0.0078	0.994	-3.15E-09	count	1
LINC01967	-18.6265588	2400.585336	-0.0078	0.994	-3.15E-09	count	1
CD80	-18.6265588	2400.585336	-0.0078	0.994	-3.15E-09	count	1
LINC02014	-18.6265588	2400.585336	-0.0078	0.994	-3.15E-09	count	1
LINC02067	-18.6265588	2400.585336	-0.0078	0.994	-3.15E-09	count	1
AC093591.2	-18.6265588	2400.585336	-0.0078	0.994	-3.15E-09	count	1
STMN4	-18.6265588	2400.585336	-0.0078	0.994	-3.15E-09	count	1
CNGB3	-18.6265588	2400.585336	-0.0078	0.994	-3.15E-09	count	1
AC016405.2	-18.6265588	2400.585336	-0.0078	0.994	-3.15E-09	count	1
AL391987.2	-18.6265588	2400.585336	-0.0078	0.994	-3.15E-09	count	1
AL451065.1	-18.6265588	2400.585336	-0.0078	0.994	-3.15E-09	count	1
STPG3-AS1	-18.6265588	2400.585336	-0.0078	0.994	-3.15E-09	count	1
AC068385.1	-18.6265588	2400.585336	-0.0078	0.994	-3.15E-09	count	1
AP002360.2	-18.6265588	2400.585336	-0.0078	0.994	-3.15E-09	count	1
CFAP43	-18.6265588	2400.585336	-0.0078	0.994	-3.15E-09	count	1
TMEM52B	-18.6265588	2400.585336	-0.0078	0.994	-3.15E-09	count	1
AC025031.2	-18.6265588	2400.585336	-0.0078	0.994	-3.15E-09	count	1
AL590096.1	-18.6265588	2400.585336	-0.0078	0.994	-3.15E-09	count	1
C13orf42	-18.6265588	2400.585336	-0.0078	0.994	-3.15E-09	count	1
LINC01500	-18.6265588	2400.585336	-0.0078	0.994	-3.15E-09	count	1
AC084756.1	-18.6265588	2400.585336	-0.0078	0.994	-3.15E-09	count	1
AC090617.5	-18.6265588	2400.585336	-0.0078	0.994	-3.15E-09	count	1
AC068418.1	-18.6265588	2400.585336	-0.0078	0.994	-3.15E-09	count	1
AC104564.4	-18.6265588	2400.585336	-0.0078	0.994	-3.15E-09	count	1
AC139530.3	-18.6265588	2400.585336	-0.0078	0.994	-3.15E-09	count	1
AC132938.1	-18.6265588	2400.585336	-0.0078	0.994	-3.15E-09	count	1
CEACAM4	-18.6265588	2400.585336	-0.0078	0.994	-3.15E-09	count	1
FP236383.2	-18.6265588	2400.585336	-0.0078	0.994	-3.15E-09	count	1
LINC00165	-18.6265588	2400.585336	-0.0078	0.994	-3.15E-09	count	1
AL031432.3	-18.6265588	2400.585336	-0.0078	0.994	-3.15E-09	count	1
AL049795.1	-18.6265588	2400.585336	-0.0078	0.994	-3.15E-09	count	1
AC104458.1	-18.6265588	2400.585336	-0.0078	0.994	-3.15E-09	count	1
AC097059.2	-18.6265588	2400.585336	-0.0078	0.994	-3.15E-09	count	1
AC092894.1	-18.6265588	2400.585336	-0.0078	0.994	-3.15E-09	count	1
GPR156	-18.6265588	2400.585336	-0.0078	0.994	-3.15E-09	count	1
LINC02021	-18.6265588	2400.585336	-0.0078	0.994	-3.15E-09	count	1
AC110751.1	-18.6265588	2400.585336	-0.0078	0.994	-3.15E-09	count	1
AC034199.1	-18.6265588	2400.585336	-0.0078	0.994	-3.15E-09	count	1
AC018635.2	-18.6265588	2400.585336	-0.0078	0.994	-3.15E-09	count	1
CYSRT1	-18.6265588	2400.585336	-0.0078	0.994	-3.15E-09	count	1
AC131571.1	-18.6265588	2400.585336	-0.0078	0.994	-3.15E-09	count	1

AL731575.1	-18.6265588	2400.585336	-0.0078	0.994	-3.15E-09	count	1
KCNMA1-AS3	-18.6265588	2400.585336	-0.0078	0.994	-3.15E-09	count	1
FOXM1	-18.6265588	2400.585336	-0.0078	0.994	-3.15E-09	count	1
FAM186B	-18.6265588	2400.585336	-0.0078	0.994	-3.15E-09	count	1
NR1H4	-18.6265588	2400.585336	-0.0078	0.994	-3.15E-09	count	1
AC069503.1	-18.6265588	2400.585336	-0.0078	0.994	-3.15E-09	count	1
AL356259.1	-18.6265588	2400.585336	-0.0078	0.994	-3.15E-09	count	1
ITGAL	-18.6265588	2400.585336	-0.0078	0.994	-3.15E-09	count	1
AL121890.4	-18.6265588	2400.585336	-0.0078	0.994	-3.15E-09	count	1
TLDC2	-18.6265588	2400.585336	-0.0078	0.994	-3.15E-09	count	1
AL353803.1	-18.6265588	2400.585336	-0.0078	0.994	-3.15E-09	count	1
CCNA1	-18.6265588	2400.585336	-0.0078	0.994	-3.15E-09	count	1
AC010547.1	-18.6265588	2400.585336	-0.0078	0.994	-3.15E-09	count	1
AC127537.1	-18.6265588	2400.585336	-0.0078	0.994	-3.15E-09	count	1
TMPRSS2	-18.6265588	2400.585336	-0.0078	0.994	-3.15E-09	count	1
LRP4-AS1	-0.2305118	0.7638701	-0.3018	0.763	-2.68E-09	count	1
AP003555.2	-0.0814593	0.5812796	-0.1401	0.889	-2.48E-09	count	1
AC009154.2	-0.2106951	0.6167365	-0.3416	0.733	-2.44E-09	count	1
ATXN7	-0.2106951	0.7026068	-0.2999	0.764	-2.44E-09	count	1
RNF148	-0.5272205	1.2050645	-0.4375	0.662	-2.42E-09	count	1
AL354760.1	-0.5272205	1.1034833	-0.4778	0.633	-2.42E-09	count	1
HSD17B2	-0.5272205	1.2050645	-0.4375	0.662	-2.42E-09	count	1
SYTL5	-0.5272205	1.1034833	-0.4778	0.633	-2.42E-09	count	1
CATSPERB	-0.5272205	1.1034833	-0.4778	0.633	-2.42E-09	count	1
PGLYRP2	-0.5272205	1.1034833	-0.4778	0.633	-2.42E-09	count	1
IL12RB1	-0.5272205	1.2050645	-0.4375	0.662	-2.42E-09	count	1
AL590666.3	-0.5272205	1.2050645	-0.4375	0.662	-2.42E-09	count	1
AC021242.3	-0.5272205	1.2050645	-0.4375	0.662	-2.42E-09	count	1
ANKRD61	-0.5272205	1.1034833	-0.4778	0.633	-2.42E-09	count	1
NETO2	-0.5272205	1.2050645	-0.4375	0.662	-2.42E-09	count	1
TMEM81	-0.5272205	1.1034833	-0.4778	0.633	-2.42E-09	count	1
AC112715.1	-0.5272205	1.2050645	-0.4375	0.662	-2.42E-09	count	1
AC100803.1	-0.5272205	1.2050645	-0.4375	0.662	-2.42E-09	count	1
ERFE	-0.5272205	1.2050645	-0.4375	0.662	-2.42E-09	count	1
AC034243.1	-0.5272205	1.2050645	-0.4375	0.662	-2.42E-09	count	1
CORO2A	-0.5272205	1.1034833	-0.4778	0.633	-2.42E-09	count	1
AC027319.1	-0.5272205	1.1034833	-0.4778	0.633	-2.42E-09	count	1
SMIM11B	-0.5272205	1.1034833	-0.4778	0.633	-2.42E-09	count	1
AOC2	-0.5272205	1.2050645	-0.4375	0.662	-2.42E-09	count	1
AL353622.1	-0.5272205	1.2050645	-0.4375	0.662	-2.42E-09	count	1
FCRL5	-0.5272205	1.1034833	-0.4778	0.633	-2.42E-09	count	1
ABCA4	-0.5272205	1.2050645	-0.4375	0.662	-2.42E-09	count	1
FAM239A	-0.5272205	1.2050645	-0.4375	0.662	-2.42E-09	count	1
OSBP2	-0.5272205	1.2050645	-0.4375	0.662	-2.42E-09	count	1
AC008735.2	-0.5272205	1.2050645	-0.4375	0.662	-2.42E-09	count	1

CHRNA10	-0.5272205	1.2050645	-0.4375	0.662	-2.42E-09	count	1
AC006518.7	-0.5272205	1.1034833	-0.4778	0.633	-2.42E-09	count	1
POU2AF1	-0.5272205	1.1034833	-0.4778	0.633	-2.42E-09	count	1
AC120114.3	-0.5272205	1.1034833	-0.4778	0.633	-2.42E-09	count	1
OSM	-0.5272205	1.1034833	-0.4778	0.633	-2.42E-09	count	1
AC010904.2	-0.5272205	1.1034833	-0.4778	0.633	-2.42E-09	count	1
AC135012.1	-0.5272205	1.2050645	-0.4375	0.662	-2.42E-09	count	1
CTSG	-0.5272205	1.2050645	-0.4375	0.662	-2.42E-09	count	1
CDH3	-0.5272205	1.1034833	-0.4778	0.633	-2.42E-09	count	1
AC117490.2	-0.5272205	1.2050645	-0.4375	0.662	-2.42E-09	count	1
GPT	-0.5272205	1.1034833	-0.4778	0.633	-2.42E-09	count	1
KIAA1614-AS1	-0.5272205	1.1034833	-0.4778	0.633	-2.42E-09	count	1
AL136304.1	-0.5272205	1.2050645	-0.4375	0.662	-2.42E-09	count	1
AL139158.2	-0.5272205	1.1034833	-0.4778	0.633	-2.42E-09	count	1
AC015961.2	-0.5272205	1.2050645	-0.4375	0.662	-2.42E-09	count	1
FAM71D	-0.5272205	1.1034833	-0.4778	0.633	-2.42E-09	count	1
ROPN1	-0.5272205	1.2050645	-0.4375	0.662	-2.42E-09	count	1
AL137779.2	-0.5272205	1.1034833	-0.4778	0.633	-2.42E-09	count	1
FAM95B1	-0.5272205	1.2050645	-0.4375	0.662	-2.42E-09	count	1
TRAT1	-0.5272205	1.2050645	-0.4375	0.662	-2.42E-09	count	1
AC005614.1	-0.5272205	1.2050645	-0.4375	0.662	-2.42E-09	count	1
ZBTB8B	-0.5272205	1.1034833	-0.4778	0.633	-2.42E-09	count	1
AC025887.2	-0.5272205	1.1034833	-0.4778	0.633	-2.42E-09	count	1
PPEF1	-0.5272205	1.2050645	-0.4375	0.662	-2.42E-09	count	1
AL031666.2	-0.5272205	1.2050645	-0.4375	0.662	-2.42E-09	count	1
AC017002.3	-0.5272205	1.1034833	-0.4778	0.633	-2.42E-09	count	1
AL031665.2	-0.5272205	1.2050645	-0.4375	0.662	-2.42E-09	count	1
VWC2	-0.5272205	1.2050645	-0.4375	0.662	-2.42E-09	count	1
RTEL1	-0.5272205	1.1034833	-0.4778	0.633	-2.42E-09	count	1
HIST1H4J	-0.5272205	1.1034833	-0.4778	0.633	-2.42E-09	count	1
AL356020.1	-0.5272205	1.2050645	-0.4375	0.662	-2.42E-09	count	1
DSG2	-0.5272205	1.2050645	-0.4375	0.662	-2.42E-09	count	1
KLHL10	-0.5272205	1.1034833	-0.4778	0.633	-2.42E-09	count	1
TRIM72	-0.5272205	1.2050645	-0.4375	0.662	-2.42E-09	count	1
LY86	-0.5272205	1.1034833	-0.4778	0.633	-2.42E-09	count	1
AL157400.3	-0.5272205	1.2050645	-0.4375	0.662	-2.42E-09	count	1
AC074044.1	-0.5272205	1.1034833	-0.4778	0.633	-2.42E-09	count	1
PCYT1B	-0.5272205	1.1034833	-0.4778	0.633	-2.42E-09	count	1
AC090912.1	-0.5272205	1.1034833	-0.4778	0.633	-2.42E-09	count	1
UXT-AS1	-0.5272205	1.1034833	-0.4778	0.633	-2.42E-09	count	1
AC087393.2	-0.5272205	1.2050645	-0.4375	0.662	-2.42E-09	count	1
KCNC1	-0.5272205	1.2050645	-0.4375	0.662	-2.42E-09	count	1
SCML4	-0.5272205	1.1034833	-0.4778	0.633	-2.42E-09	count	1
AL132821.1	-0.5272205	1.2050645	-0.4375	0.662	-2.42E-09	count	1
AC073335.2	-0.1555218	0.8253019	-0.1884	0.851	-1.77E-09	count	1

AC002454.1	-0.1555218	0.7409474	-0.2099	0.834	-1.77E-09	count	1
LEKR1	-0.1555218	0.7409474	-0.2099	0.834	-1.77E-09	count	1
NFE4	-0.1555218	0.7409474	-0.2099	0.834	-1.77E-09	count	1
PCDHA7	-0.3429448	0.8282103	-0.4141	0.679	-1.51E-09	count	1
LINC00664	-0.3429448	0.8282103	-0.4141	0.679	-1.51E-09	count	1
IL1RL2	-0.3429448	0.9305197	-0.3686	0.712	-1.51E-09	count	1
FRMPD3	-0.3429448	0.9305197	-0.3686	0.712	-1.51E-09	count	1
DEPDC4	-0.3429448	0.9305197	-0.3686	0.712	-1.51E-09	count	1
AC006213.3	-0.3429448	0.9305197	-0.3686	0.712	-1.51E-09	count	1
AL603840.1	-0.3429448	0.9305197	-0.3686	0.712	-1.51E-09	count	1
KCNK12	-0.3429448	0.9305197	-0.3686	0.712	-1.51E-09	count	1
SH2D6	-0.3429448	0.9305197	-0.3686	0.712	-1.51E-09	count	1
ZAP70	-0.3429448	0.9305197	-0.3686	0.712	-1.51E-09	count	1
LINC01886	-0.3429448	0.9305197	-0.3686	0.712	-1.51E-09	count	1
AC010127.1	-0.3429448	0.9305197	-0.3686	0.712	-1.51E-09	count	1
LINC01208	-0.3429448	0.9305197	-0.3686	0.712	-1.51E-09	count	1
AC106047.1	-0.3429448	0.9305197	-0.3686	0.712	-1.51E-09	count	1
AC096586.2	-0.3429448	0.9305197	-0.3686	0.712	-1.51E-09	count	1
SPINK9	-0.3429448	0.9305197	-0.3686	0.712	-1.51E-09	count	1
AL133255.1	-0.3429448	0.9305197	-0.3686	0.712	-1.51E-09	count	1
AL391863.1	-0.3429448	0.9305197	-0.3686	0.712	-1.51E-09	count	1
ASIC3	-0.3429448	0.9305197	-0.3686	0.712	-1.51E-09	count	1
FGF20	-0.3429448	0.9305197	-0.3686	0.712	-1.51E-09	count	1
AC021785.1	-0.3429448	0.9305197	-0.3686	0.712	-1.51E-09	count	1
KCNK9	-0.3429448	0.9305197	-0.3686	0.712	-1.51E-09	count	1
LYNX1-SLURP2	-0.3429448	0.9305197	-0.3686	0.712	-1.51E-09	count	1
ANO9	-0.3429448	0.9305197	-0.3686	0.712	-1.51E-09	count	1
NAV2-AS2	-0.3429448	0.9305197	-0.3686	0.712	-1.51E-09	count	1
MPEG1	-0.3429448	0.9305197	-0.3686	0.712	-1.51E-09	count	1
LINC00167	-0.3429448	0.9305197	-0.3686	0.712	-1.51E-09	count	1
AL138820.1	-0.3429448	0.9305197	-0.3686	0.712	-1.51E-09	count	1
MAGEL2	-0.3429448	0.9305197	-0.3686	0.712	-1.51E-09	count	1
CPEB1-AS1	-0.3429448	0.9305197	-0.3686	0.712	-1.51E-09	count	1
SH3GL3	-0.3429448	0.9305197	-0.3686	0.712	-1.51E-09	count	1
AC019254.1	-0.3429448	0.9305197	-0.3686	0.712	-1.51E-09	count	1
AC008731.1	-0.3429448	0.9305197	-0.3686	0.712	-1.51E-09	count	1
AC106782.1	-0.3429448	0.9305197	-0.3686	0.712	-1.51E-09	count	1
AC106738.1	-0.3429448	0.9305197	-0.3686	0.712	-1.51E-09	count	1
AC080038.2	-0.3429448	0.9305197	-0.3686	0.712	-1.51E-09	count	1
AC087222.1	-0.3429448	0.9305197	-0.3686	0.712	-1.51E-09	count	1
MTRNR2L3	-0.3429448	0.9305197	-0.3686	0.712	-1.51E-09	count	1
AC010336.4	-0.3429448	0.9305197	-0.3686	0.712	-1.51E-09	count	1
OR7A17	-0.3429448	0.9305197	-0.3686	0.712	-1.51E-09	count	1
UNC13A	-0.3429448	0.9305197	-0.3686	0.712	-1.51E-09	count	1
CEACAM1	-0.3429448	0.9305197	-0.3686	0.712	-1.51E-09	count	1

IGFL4	-0.3429448	0.9305197	-0.3686	0.712	-1.51E-09	count	1
PTPRH	-0.3429448	0.9305197	-0.3686	0.712	-1.51E-09	count	1
AC008735.4	-0.3429448	0.9305197	-0.3686	0.712	-1.51E-09	count	1
AL354956.1	-0.3429448	0.9305197	-0.3686	0.712	-1.51E-09	count	1
DAB1	-0.3429448	0.9305197	-0.3686	0.712	-1.51E-09	count	1
AL355472.4	-0.3429448	0.9305197	-0.3686	0.712	-1.51E-09	count	1
AC016722.3	-0.3429448	0.9305197	-0.3686	0.712	-1.51E-09	count	1
LINC01816	-0.3429448	0.9305197	-0.3686	0.712	-1.51E-09	count	1
AC012511.1	-0.3429448	0.9305197	-0.3686	0.712	-1.51E-09	count	1
CATIP	-0.3429448	0.9305197	-0.3686	0.712	-1.51E-09	count	1
AC026191.1	-0.3429448	0.9305197	-0.3686	0.712	-1.51E-09	count	1
AC105345.1	-0.3429448	0.9305197	-0.3686	0.712	-1.51E-09	count	1
ANKRD55	-0.3429448	0.9305197	-0.3686	0.712	-1.51E-09	count	1
LINC02147	-0.3429448	0.9305197	-0.3686	0.712	-1.51E-09	count	1
SNHG4	-0.3429448	0.9305197	-0.3686	0.712	-1.51E-09	count	1
LRRD1	-0.3429448	0.9305197	-0.3686	0.712	-1.51E-09	count	1
AC005064.1	-0.3429448	0.9305197	-0.3686	0.712	-1.51E-09	count	1
AC232271.1	-0.3429448	0.9305197	-0.3686	0.712	-1.51E-09	count	1
AC037459.4	-0.3429448	0.9305197	-0.3686	0.712	-1.51E-09	count	1
AGBL2	-0.3429448	0.9305197	-0.3686	0.712	-1.51E-09	count	1
ITGB1-DT	-0.3429448	0.9305197	-0.3686	0.712	-1.51E-09	count	1
BLNK	-0.3429448	0.9305197	-0.3686	0.712	-1.51E-09	count	1
KCNIP2	-0.3429448	0.9305197	-0.3686	0.712	-1.51E-09	count	1
CLIP1-AS1	-0.3429448	0.9305197	-0.3686	0.712	-1.51E-09	count	1
ELL3	-0.3429448	0.9305197	-0.3686	0.712	-1.51E-09	count	1
LINC02206	-0.3429448	0.9305197	-0.3686	0.712	-1.51E-09	count	1
PRC1-AS1	-0.3429448	0.9305197	-0.3686	0.712	-1.51E-09	count	1
ASB16	-0.3429448	0.9305197	-0.3686	0.712	-1.51E-09	count	1
AC005597.1	-0.3429448	0.9305197	-0.3686	0.712	-1.51E-09	count	1
AL022323.1	-0.3429448	0.9305197	-0.3686	0.712	-1.51E-09	count	1
AL359198.1	-0.3429448	0.6575623	-0.5215	0.602	-1.51E-09	count	1
AC092119.2	-0.3429448	0.6575623	-0.5215	0.602	-1.51E-09	count	1
ADGRG1	-0.3429448	0.6575623	-0.5215	0.602	-1.51E-09	count	1
AC092718.8	-0.3429448	0.6575623	-0.5215	0.602	-1.51E-09	count	1
AC097634.3	-0.3429448	0.6575623	-0.5215	0.602	-1.51E-09	count	1
AC080013.4	-0.3429448	0.9305197	-0.3686	0.712	-1.51E-09	count	1
AC239804.1	-0.3429448	0.9305197	-0.3686	0.712	-1.51E-09	count	1
BTK	-0.3429448	0.7825106	-0.4383	0.661	-1.51E-09	count	1
AL929472.2	-0.3429448	0.7825106	-0.4383	0.661	-1.51E-09	count	1
AP000704.1	-0.3429448	0.7825106	-0.4383	0.661	-1.51E-09	count	1
AC010457.1	-0.3060312	0.704564	-0.4344	0.664	-1.33E-09	count	1
NXPH2	-0.7114963	0.667847	-1.0654	0.287	-1.25E-09	count	1
NUP210	-0.7114963	0.667847	-1.0654	0.287	-1.25E-09	count	1
GPR21	-0.7114963	0.667847	-1.0654	0.287	-1.25E-09	count	1
ADAMTS14	-0.7114963	0.667847	-1.0654	0.287	-1.25E-09	count	1

KNDC1	-0.7114963	0.667847	-1.0654	0.287	-1.25E-09	count	1
AC079313.2	-0.7114963	0.667847	-1.0654	0.287	-1.25E-09	count	1
SCG3	-0.7114963	0.667847	-1.0654	0.287	-1.25E-09	count	1
AL451042.2	-0.7114963	0.667847	-1.0654	0.287	-1.25E-09	count	1
KIF26B	-0.7114963	0.667847	-1.0654	0.287	-1.25E-09	count	1
SATB2-AS1	-0.7114963	0.667847	-1.0654	0.287	-1.25E-09	count	1
CD28	-0.7114963	0.667847	-1.0654	0.287	-1.25E-09	count	1
ASIC4	-0.7114963	0.667847	-1.0654	0.287	-1.25E-09	count	1
AL138828.1	-0.7114963	0.667847	-1.0654	0.287	-1.25E-09	count	1
AC078845.1	-0.7114963	0.667847	-1.0654	0.287	-1.25E-09	count	1
AP003696.1	-0.7114963	0.667847	-1.0654	0.287	-1.25E-09	count	1
DDN	-0.7114963	0.667847	-1.0654	0.287	-1.25E-09	count	1
AC005759.1	-0.7114963	0.667847	-1.0654	0.287	-1.25E-09	count	1
AC010325.1	-0.7114963	0.667847	-1.0654	0.287	-1.25E-09	count	1
IGLV1-51	-0.7114963	0.667847	-1.0654	0.287	-1.25E-09	count	1
ADM2	-0.7114963	0.667847	-1.0654	0.287	-1.25E-09	count	1
AF127577.1	-0.7114963	0.667847	-1.0654	0.287	-1.25E-09	count	1
IGKV3-15	-0.7114963	0.667847	-1.0654	0.287	-1.25E-09	count	1
NEURL3	-0.7114963	0.667847	-1.0654	0.287	-1.25E-09	count	1
UBD	-0.7114963	0.667847	-1.0654	0.287	-1.25E-09	count	1
C10orf142	-0.7114963	0.667847	-1.0654	0.287	-1.25E-09	count	1
AL160313.1	-0.7114963	0.667847	-1.0654	0.287	-1.25E-09	count	1
ZNF385C	-0.7114963	0.667847	-1.0654	0.287	-1.25E-09	count	1
GHET1	-0.0814593	0.8613961	-0.0946	0.925	-9.11E-10	count	1
MTCL1	-0.0814593	0.8552563	-0.0952	0.924	-9.11E-10	count	1
C17orf53	-0.0814593	0.8835152	-0.0922	0.927	-9.11E-10	count	1
AC090630.1	-0.0814593	0.7409615	-0.1099	0.912	-9.11E-10	count	1
TTTY10	-0.0814593	0.9867007	-0.0826	0.934	-9.11E-10	count	1
LINC01483	-0.0086789	1.0464446	-0.0083	0.993	-9.52E-11	count	1
CD96	-0.0086789	0.7411999	-0.0117	0.991	-9.52E-11	count	1
AL390728.5	-0.0086789	0.7411999	-0.0117	0.991	-9.52E-11	count	1
AC004540.1	-0.0086789	0.7406361	-0.0117	0.991	-9.52E-11	count	1
AC026367.3	-0.0086789	0.7411999	-0.0117	0.991	-9.52E-11	count	1
AC083837.1	-0.0183491	0.5780188	-0.0317	0.975	-7.42E-11	count	1
LYPD5	-0.0183491	0.5780188	-0.0317	0.975	-7.42E-11	count	1
AL360091.3	-0.0183491	0.5780188	-0.0317	0.975	-7.42E-11	count	1
C2CD4A	-0.0183491	0.5780188	-0.0317	0.975	-7.42E-11	count	1
MCHR1	-0.0183491	0.747679	-0.0245	0.98	-7.42E-11	count	1
APOA1	0.0459411	0.7447707	0.0617	0.951	4.96E-10	count	1
ITPKA	0.0625204	0.6000802	0.1042	0.917	6.73E-10	count	1
DRAXIN	0.0625204	0.8067835	0.0775	0.938	6.73E-10	count	1
NT5C1A	18.1629337	2944.099178	0.0062	0.995	7.62E-10	count	1
LINC01771	18.1629336	2944.099178	0.0062	0.995	7.62E-10	count	1
GBP6	18.1629337	2944.099197	0.0062	0.995	7.62E-10	count	1
BX005019.1	18.1629338	2944.099367	0.0062	0.995	7.62E-10	count	1

TNFSF18	18.1629339	2944.0995	0.0062	0.995	7.62E-10	count	1
AL449106.1	18.1629337	2944.099254	0.0062	0.995	7.62E-10	count	1
LINC01873	18.1629336	2944.099538	0.0062	0.995	7.62E-10	count	1
AC079354.1	18.1629335	2944.099121	0.0062	0.995	7.62E-10	count	1
WNT10A	18.1629338	2944.0995	0.0062	0.995	7.62E-10	count	1
AC009502.1	18.1629339	2944.099254	0.0062	0.995	7.62E-10	count	1
AC008945.1	18.1629339	2944.099348	0.0062	0.995	7.62E-10	count	1
AC008438.1	18.1629338	2944.099348	0.0062	0.995	7.62E-10	count	1
AC104117.5	18.1629338	2944.099348	0.0062	0.995	7.62E-10	count	1
C4A	18.1629338	2944.09931	0.0062	0.995	7.62E-10	count	1
RIMS1	18.1629338	2944.099367	0.0062	0.995	7.62E-10	count	1
AC004492.1	18.1629339	2944.099254	0.0062	0.995	7.62E-10	count	1
AWAT2	18.1629337	2944.099254	0.0062	0.995	7.62E-10	count	1
TEX11	18.1629336	2944.099348	0.0062	0.995	7.62E-10	count	1
AC019257.1	18.1629338	2944.099386	0.0062	0.995	7.62E-10	count	1
IGHEP2	18.1629336	2944.099538	0.0062	0.995	7.62E-10	count	1
RLN2	18.1629338	2944.099462	0.0062	0.995	7.62E-10	count	1
GLDC	18.1629339	2944.099254	0.0062	0.995	7.62E-10	count	1
PDZD3	18.1629337	2944.099291	0.0062	0.995	7.62E-10	count	1
AC129102.1	18.1629339	2944.099273	0.0062	0.995	7.62E-10	count	1
AC079906.1	18.1629335	2944.099159	0.0062	0.995	7.62E-10	count	1
AC023794.1	18.1629339	2944.0995	0.0062	0.995	7.62E-10	count	1
GZMB	18.1629338	2944.099386	0.0062	0.995	7.62E-10	count	1
G2E3-AS1	18.1629335	2944.099159	0.0062	0.995	7.62E-10	count	1
SERPINA11	18.1629338	2944.099367	0.0062	0.995	7.62E-10	count	1
EXD1	18.1629336	2944.099083	0.0062	0.995	7.62E-10	count	1
AC005736.3	18.1629337	2944.099291	0.0062	0.995	7.62E-10	count	1
LINC00922	18.1629336	2944.099216	0.0062	0.995	7.62E-10	count	1
AC141424.1	18.1629335	2944.099159	0.0062	0.995	7.62E-10	count	1
LPO	18.1629336	2944.099083	0.0062	0.995	7.62E-10	count	1
ALPK2	18.1629337	2944.09931	0.0062	0.995	7.62E-10	count	1
AL132655.1	18.1629338	2944.099367	0.0062	0.995	7.62E-10	count	1
PLPPR3	18.1629335	2944.099121	0.0062	0.995	7.62E-10	count	1
EXOC3L2	18.1629338	2944.099348	0.0062	0.995	7.62E-10	count	1
IGLC6	18.1629335	2944.09914	0.0062	0.995	7.62E-10	count	1
Z83844.1	18.1629339	2944.09931	0.0062	0.995	7.62E-10	count	1
CACNA1I	18.1629335	2944.099159	0.0062	0.995	7.62E-10	count	1
AP001434.1	18.1629338	2944.09931	0.0062	0.995	7.62E-10	count	1
PLCH2	18.1629336	2944.099216	0.0062	0.995	7.62E-10	count	1
CELA2B	18.1629334	2944.098932	0.0062	0.995	7.62E-10	count	1
AL136115.2	18.1629332	2944.099348	0.0062	0.995	7.62E-10	count	1
LRRC53	18.1629332	2944.099424	0.0062	0.995	7.62E-10	count	1
HIST2H3D	18.1629335	2944.099273	0.0062	0.995	7.62E-10	count	1
HORMAD1	18.1629336	2944.099235	0.0062	0.995	7.62E-10	count	1
AL391069.4	18.1629336	2944.099216	0.0062	0.995	7.62E-10	count	1

AL589765.7	18.1629336	2944.098932	0.0062	0.995	7.62E-10	count	1
MAEL	18.1629337	2944.099159	0.0062	0.995	7.62E-10	count	1
AL031733.2	18.1629336	2944.099216	0.0062	0.995	7.62E-10	count	1
AL359265.3	18.1629333	2944.09914	0.0062	0.995	7.62E-10	count	1
CR1L	18.1629332	2944.099159	0.0062	0.995	7.62E-10	count	1
MIR3681HG	18.1629336	2944.099216	0.0062	0.995	7.62E-10	count	1
LINC01812	18.1629338	2944.099329	0.0062	0.995	7.62E-10	count	1
MYO7B	18.1629335	2944.099064	0.0062	0.995	7.62E-10	count	1
ABCB11	18.1629336	2944.09931	0.0062	0.995	7.62E-10	count	1
AC078883.2	18.1629332	2944.099008	0.0062	0.995	7.62E-10	count	1
LINC01802	18.1629334	2944.099159	0.0062	0.995	7.62E-10	count	1
AC022384.1	18.1629336	2944.099216	0.0062	0.995	7.62E-10	count	1
HDAC11-AS1	18.1629337	2944.0995	0.0062	0.995	7.62E-10	count	1
KCNH8	18.1629332	2944.099008	0.0062	0.995	7.62E-10	count	1
CCRL2	18.1629332	2944.099197	0.0062	0.995	7.62E-10	count	1
AC128688.2	18.1629333	2944.09931	0.0062	0.995	7.62E-10	count	1
UROC1	18.1629331	2944.099367	0.0062	0.995	7.62E-10	count	1
LINC02015	18.1629333	2944.099424	0.0062	0.995	7.62E-10	count	1
AC092953.2	18.1629332	2944.099367	0.0062	0.995	7.62E-10	count	1
AC024559.1	18.1629335	2944.099064	0.0062	0.995	7.62E-10	count	1
AC005699.1	18.1629336	2944.099216	0.0062	0.995	7.62E-10	count	1
PHOX2B	18.1629334	2944.099008	0.0062	0.995	7.62E-10	count	1
ALB	18.1629336	2944.099216	0.0062	0.995	7.62E-10	count	1
AP001961.1	18.1629335	2944.099102	0.0062	0.995	7.62E-10	count	1
AC021205.3	18.1629335	2944.099254	0.0062	0.995	7.62E-10	count	1
PLK4	18.1629332	2944.099121	0.0062	0.995	7.62E-10	count	1
PLEKHG4B	18.1629333	2944.099178	0.0062	0.995	7.62E-10	count	1
AC091965.4	18.1629334	2944.099121	0.0062	0.995	7.62E-10	count	1
AC008937.3	18.1629336	2944.099216	0.0062	0.995	7.62E-10	count	1
DEPDC1B	18.1629336	2944.098932	0.0062	0.995	7.62E-10	count	1
AC104113.1	18.1629333	2944.099329	0.0062	0.995	7.62E-10	count	1
AC106732.1	18.1629336	2944.099329	0.0062	0.995	7.62E-10	count	1
GRM6	18.1629336	2944.099329	0.0062	0.995	7.62E-10	count	1
FO393415.1	18.1629336	2944.099045	0.0062	0.995	7.62E-10	count	1
AL023806.2	18.1629336	2944.099386	0.0062	0.995	7.62E-10	count	1
GRM1	18.1629337	2944.099254	0.0062	0.995	7.62E-10	count	1
AC092171.2	18.1629332	2944.099083	0.0062	0.995	7.62E-10	count	1
GRID2IP	18.1629338	2944.099254	0.0062	0.995	7.62E-10	count	1
AC005162.2	18.1629336	2944.099216	0.0062	0.995	7.62E-10	count	1
ZNF716	18.1629335	2944.099254	0.0062	0.995	7.62E-10	count	1
AC092167.1	18.1629334	2944.099008	0.0062	0.995	7.62E-10	count	1
NLRP2B	18.1629336	2944.09931	0.0062	0.995	7.62E-10	count	1
ZIC3	18.1629336	2944.099216	0.0062	0.995	7.62E-10	count	1
GPR50	18.1629334	2944.099008	0.0062	0.995	7.62E-10	count	1
KC877982.1	18.1629334	2944.099008	0.0062	0.995	7.62E-10	count	1

CSAG1	18.1629336	2944.099216	0.0062	0.995	7.62E-10	count	1
AF106564.1	18.1629334	2944.099273	0.0062	0.995	7.62E-10	count	1
AC013643.2	18.1629332	2944.09931	0.0062	0.995	7.62E-10	count	1
MIR124-2HG	18.1629336	2944.099291	0.0062	0.995	7.62E-10	count	1
HNF4G	18.1629337	2944.099121	0.0062	0.995	7.62E-10	count	1
AC018781.1	18.1629336	2944.099216	0.0062	0.995	7.62E-10	count	1
LY6D	18.1629335	2944.098989	0.0062	0.995	7.62E-10	count	1
AF186192.3	18.1629334	2944.099159	0.0062	0.995	7.62E-10	count	1
DMRT2	18.1629336	2944.099216	0.0062	0.995	7.62E-10	count	1
LINC01504	18.1629337	2944.099064	0.0062	0.995	7.62E-10	count	1
AL354861.2	18.1629336	2944.099216	0.0062	0.995	7.62E-10	count	1
C9orf84	18.1629336	2944.099216	0.0062	0.995	7.62E-10	count	1
HMGA1P4	18.1629336	2944.099216	0.0062	0.995	7.62E-10	count	1
PRDM12	18.1629338	2944.099291	0.0062	0.995	7.62E-10	count	1
B4GALNT4	18.1629332	2944.099026	0.0062	0.995	7.62E-10	count	1
AC091053.1	18.1629332	2944.099045	0.0062	0.995	7.62E-10	count	1
AC116535.1	18.1629337	2944.09914	0.0062	0.995	7.62E-10	count	1
FLRT1	18.1629335	2944.099405	0.0062	0.995	7.62E-10	count	1
TMEM262	18.1629336	2944.099159	0.0062	0.995	7.62E-10	count	1
ACY3	18.1629334	2944.099159	0.0062	0.995	7.62E-10	count	1
LINC01488	18.1629336	2944.098932	0.0062	0.995	7.62E-10	count	1
ZNF488	18.1629334	2944.098913	0.0062	0.995	7.62E-10	count	1
MRLN	18.1629335	2944.099329	0.0062	0.995	7.62E-10	count	1
ACSM6	18.1629336	2944.099197	0.0062	0.995	7.62E-10	count	1
AL157834.2	18.1629337	2944.099159	0.0062	0.995	7.62E-10	count	1
AL365273.2	18.1629336	2944.099273	0.0062	0.995	7.62E-10	count	1
AL157888.1	18.1629336	2944.099216	0.0062	0.995	7.62E-10	count	1
STYK1	18.1629335	2944.099178	0.0062	0.995	7.62E-10	count	1
TAS2R10	18.1629338	2944.099329	0.0062	0.995	7.62E-10	count	1
PDE6H	18.1629336	2944.099216	0.0062	0.995	7.62E-10	count	1
AC024896.1	18.1629335	2944.099178	0.0062	0.995	7.62E-10	count	1
AC012150.1	18.1629332	2944.099159	0.0062	0.995	7.62E-10	count	1
AC090115.1	18.1629335	2944.099159	0.0062	0.995	7.62E-10	count	1
CISTR	18.1629333	2944.099273	0.0062	0.995	7.62E-10	count	1
HOXC9	18.1629332	2944.099083	0.0062	0.995	7.62E-10	count	1
AC025569.1	18.1629334	2944.099159	0.0062	0.995	7.62E-10	count	1
AC126175.1	18.1629336	2944.099216	0.0062	0.995	7.62E-10	count	1
RPGRIP1	18.1629336	2944.099329	0.0062	0.995	7.62E-10	count	1
IGHV3-11	18.1629336	2944.099235	0.0062	0.995	7.62E-10	count	1
AC036103.1	18.1629333	2944.099329	0.0062	0.995	7.62E-10	count	1
AC087632.1	18.1629333	2944.099329	0.0062	0.995	7.62E-10	count	1
AC021422.1	18.1629332	2944.099367	0.0062	0.995	7.62E-10	count	1
AC012184.1	18.1629332	2944.099367	0.0062	0.995	7.62E-10	count	1
AC005224.4	18.1629332	2944.099045	0.0062	0.995	7.62E-10	count	1
AC003101.1	18.1629334	2944.098932	0.0062	0.995	7.62E-10	count	1

AC091180.2	18.1629335	2944.099064	0.0062	0.995	7.62E-10	count	1
LINC00483	18.1629336	2944.09931	0.0062	0.995	7.62E-10	count	1
AC007638.2	18.1629332	2944.099083	0.0062	0.995	7.62E-10	count	1
AC021683.3	18.1629336	2944.098932	0.0062	0.995	7.62E-10	count	1
CARD14	18.1629336	2944.099254	0.0062	0.995	7.62E-10	count	1
AC132872.2	18.1629333	2944.099273	0.0062	0.995	7.62E-10	count	1
CLUL1	18.1629336	2944.099197	0.0062	0.995	7.62E-10	count	1
AP001020.1	18.1629337	2944.099083	0.0062	0.995	7.62E-10	count	1
AP001033.1	18.1629334	2944.098913	0.0062	0.995	7.62E-10	count	1
LINC00907	18.1629335	2944.099064	0.0062	0.995	7.62E-10	count	1
LINC01926	18.1629332	2944.099424	0.0062	0.995	7.62E-10	count	1
AL133396.2	18.1629334	2944.099159	0.0062	0.995	7.62E-10	count	1
AL035448.1	18.1629336	2944.099159	0.0062	0.995	7.62E-10	count	1
AL121906.1	18.1629336	2944.099216	0.0062	0.995	7.62E-10	count	1
PHACTR3	18.1629333	2944.099102	0.0062	0.995	7.62E-10	count	1
OR1I1	18.1629336	2944.099216	0.0062	0.995	7.62E-10	count	1
INSL3	18.1629336	2944.099216	0.0062	0.995	7.62E-10	count	1
AC010636.2	18.1629337	2944.099216	0.0062	0.995	7.62E-10	count	1
AC008555.1	18.1629336	2944.098932	0.0062	0.995	7.62E-10	count	1
AC020907.3	18.1629336	2944.099216	0.0062	0.995	7.62E-10	count	1
CYP2S1	18.1629337	2944.099159	0.0062	0.995	7.62E-10	count	1
CCDC114	18.1629334	2944.098913	0.0062	0.995	7.62E-10	count	1
AC018766.1	18.1629332	2944.099348	0.0062	0.995	7.62E-10	count	1
AC006116.8	18.1629335	2944.099102	0.0062	0.995	7.62E-10	count	1
AC010722.1	18.1629332	2944.09931	0.0062	0.995	7.62E-10	count	1
Z83847.1	18.1629338	2944.099254	0.0062	0.995	7.62E-10	count	1
AL671710.1	18.1629333	2944.099008	0.0062	0.995	7.62E-10	count	1
ACR	18.1629332	2944.099008	0.0062	0.995	7.62E-10	count	1
AP001059.2	18.1629334	2944.099235	0.0062	0.995	7.62E-10	count	1
AL391845.2	18.1629329	2944.098932	0.0062	0.995	7.62E-10	count	1
ZNF683	18.1629334	2944.099216	0.0062	0.995	7.62E-10	count	1
C1orf94	18.1629333	2944.099064	0.0062	0.995	7.62E-10	count	1
AC093151.3	18.1629333	2944.099367	0.0062	0.995	7.62E-10	count	1
AL358075.1	18.1629331	2944.099121	0.0062	0.995	7.62E-10	count	1
AL121987.2	18.1629331	2944.098989	0.0062	0.995	7.62E-10	count	1
SERPINC1	18.162933	2944.099102	0.0062	0.995	7.62E-10	count	1
IL24	18.1629334	2944.099178	0.0062	0.995	7.62E-10	count	1
NLRP3	18.1629332	2944.099008	0.0062	0.995	7.62E-10	count	1
AC017076.1	18.1629332	2944.099121	0.0062	0.995	7.62E-10	count	1
AC007681.1	18.1629332	2944.099159	0.0062	0.995	7.62E-10	count	1
KCNH7	18.1629332	2944.098894	0.0062	0.995	7.62E-10	count	1
AC079610.1	18.1629331	2944.099159	0.0062	0.995	7.62E-10	count	1
SGPP2	18.1629333	2944.099008	0.0062	0.995	7.62E-10	count	1
AC034198.2	18.162933	2944.099045	0.0062	0.995	7.62E-10	count	1
BSN-DT	18.1629333	2944.099291	0.0062	0.995	7.62E-10	count	1

AC078785.2	18.1629331	2944.099121	0.0062	0.995	7.62E-10	count	1
LINC01096	18.162933	2944.099064	0.0062	0.995	7.62E-10	count	1
KLB	18.1629333	2944.099367	0.0062	0.995	7.62E-10	count	1
CXCL6	18.1629332	2944.099121	0.0062	0.995	7.62E-10	count	1
CDC20B	18.1629332	2944.099216	0.0062	0.995	7.62E-10	count	1
LINC02056	18.1629329	2944.098932	0.0062	0.995	7.62E-10	count	1
HIST1H2BF	18.1629333	2944.099367	0.0062	0.995	7.62E-10	count	1
HIST1H4L	18.1629333	2944.099367	0.0062	0.995	7.62E-10	count	1
CRIP3	18.1629333	2944.099367	0.0062	0.995	7.62E-10	count	1
Z97205.2	18.1629333	2944.099367	0.0062	0.995	7.62E-10	count	1
AC005014.3	18.1629334	2944.099216	0.0062	0.995	7.62E-10	count	1
AC018638.6	18.1629329	2944.099045	0.0062	0.995	7.62E-10	count	1
AC073878.2	18.1629334	2944.099291	0.0062	0.995	7.62E-10	count	1
ZNF157	18.1629333	2944.099291	0.0062	0.995	7.62E-10	count	1
SPIN4-AS1	18.1629332	2944.098894	0.0062	0.995	7.62E-10	count	1
AL353804.1	18.1629332	2944.09897	0.0062	0.995	7.62E-10	count	1
AC234781.1	18.1629332	2944.099291	0.0062	0.995	7.62E-10	count	1
FAM66E	18.1629329	2944.098932	0.0062	0.995	7.62E-10	count	1
CLVS1	18.1629333	2944.099367	0.0062	0.995	7.62E-10	count	1
LINC01607	18.1629335	2944.099235	0.0062	0.995	7.62E-10	count	1
LINC00861	18.1629333	2944.099216	0.0062	0.995	7.62E-10	count	1
GRIN3A	18.1629332	2944.098856	0.0062	0.995	7.62E-10	count	1
AC011092.2	18.162933	2944.099064	0.0062	0.995	7.62E-10	count	1
TIGD3	18.1629332	2944.09897	0.0062	0.995	7.62E-10	count	1
CCDC83	18.1629332	2944.099159	0.0062	0.995	7.62E-10	count	1
DDI1	18.1629335	2944.09931	0.0062	0.995	7.62E-10	count	1
AP000936.1	18.1629331	2944.099026	0.0062	0.995	7.62E-10	count	1
AL132657.1	18.162933	2944.099064	0.0062	0.995	7.62E-10	count	1
BX248123.1	18.1629334	2944.099216	0.0062	0.995	7.62E-10	count	1
AL390763.1	18.162933	2944.099045	0.0062	0.995	7.62E-10	count	1
LINC02367	18.1629334	2944.099102	0.0062	0.995	7.62E-10	count	1
CLECL1	18.1629334	2944.099102	0.0062	0.995	7.62E-10	count	1
GSG1	18.1629329	2944.098932	0.0062	0.995	7.62E-10	count	1
C12orf54	18.162933	2944.099064	0.0062	0.995	7.62E-10	count	1
GALNT6	18.1629332	2944.099121	0.0062	0.995	7.62E-10	count	1
AC034102.4	18.162933	2944.099064	0.0062	0.995	7.62E-10	count	1
DAO	18.1629333	2944.099367	0.0062	0.995	7.62E-10	count	1
ATXN2-AS	18.1629335	2944.09931	0.0062	0.995	7.62E-10	count	1
PLA2G1B	18.1629335	2944.099273	0.0062	0.995	7.62E-10	count	1
LINC00539	18.1629333	2944.099367	0.0062	0.995	7.62E-10	count	1
AL355916.2	18.1629333	2944.099367	0.0062	0.995	7.62E-10	count	1
PLEKHH1	18.1629334	2944.099254	0.0062	0.995	7.62E-10	count	1
AL139193.2	18.1629331	2944.09914	0.0062	0.995	7.62E-10	count	1
GPR132	18.1629334	2944.099102	0.0062	0.995	7.62E-10	count	1
FAM30A	18.1629332	2944.099121	0.0062	0.995	7.62E-10	count	1

RAD51	18.162933	2944.099045	0.0062	0.995	7.62E-10	count	1
AC084759.2	18.1629335	2944.099273	0.0062	0.995	7.62E-10	count	1
AL031600.3	18.1629333	2944.099367	0.0062	0.995	7.62E-10	count	1
RBFOX1	18.1629332	2944.099159	0.0062	0.995	7.62E-10	count	1
AC007216.4	18.1629332	2944.09897	0.0062	0.995	7.62E-10	count	1
AC093249.2	18.162933	2944.09914	0.0062	0.995	7.62E-10	count	1
AC004943.1	18.1629332	2944.099083	0.0062	0.995	7.62E-10	count	1
AC005725.1	18.1629333	2944.099367	0.0062	0.995	7.62E-10	count	1
AC015849.2	18.1629332	2944.099102	0.0062	0.995	7.62E-10	count	1
AC127496.6	18.1629334	2944.099197	0.0062	0.995	7.62E-10	count	1
AC011825.2	18.1629333	2944.099197	0.0062	0.995	7.62E-10	count	1
AL121772.3	18.1629333	2944.099367	0.0062	0.995	7.62E-10	count	1
BPIFA2	18.1629332	2944.099159	0.0062	0.995	7.62E-10	count	1
AL161937.2	18.1629334	2944.099102	0.0062	0.995	7.62E-10	count	1
CLEC17A	18.1629334	2944.099102	0.0062	0.995	7.62E-10	count	1
AC024075.3	18.162933	2944.099045	0.0062	0.995	7.62E-10	count	1
FAM129C	18.1629331	2944.099291	0.0062	0.995	7.62E-10	count	1
LINC01233	18.1629333	2944.099443	0.0062	0.995	7.62E-10	count	1
UPK1A	18.1629331	2944.099083	0.0062	0.995	7.62E-10	count	1
ATP1A3	18.1629332	2944.099121	0.0062	0.995	7.62E-10	count	1
AC010247.2	18.1629335	2944.099216	0.0062	0.995	7.62E-10	count	1
DPRX	18.1629333	2944.099367	0.0062	0.995	7.62E-10	count	1
AC245052.1	18.162933	2944.099026	0.0062	0.995	7.62E-10	count	1
LINC01694	18.1629333	2944.099008	0.0062	0.995	7.62E-10	count	1
AL139423.1	18.1629333	2944.099216	0.0062	0.995	7.62E-10	count	1
MYCL	18.1629331	2944.099026	0.0062	0.995	7.62E-10	count	1
LINC01353	18.162933	2944.099026	0.0062	0.995	7.62E-10	count	1
FLNB-AS1	18.1629331	2944.099026	0.0062	0.995	7.62E-10	count	1
NADK2-AS1	18.1629327	2944.098856	0.0062	0.995	7.62E-10	count	1
AC136604.2	18.1629333	2944.099216	0.0062	0.995	7.62E-10	count	1
GUCA1B	18.162933	2944.098989	0.0062	0.995	7.62E-10	count	1
AL591468.1	18.1629332	2944.099064	0.0062	0.995	7.62E-10	count	1
AMZ1	18.1629331	2944.099102	0.0062	0.995	7.62E-10	count	1
AC073934.1	18.162933	2944.098989	0.0062	0.995	7.62E-10	count	1
AF131216.4	18.1629331	2944.099462	0.0062	0.995	7.62E-10	count	1
AC107909.2	18.1629332	2944.099386	0.0062	0.995	7.62E-10	count	1
AC103853.1	18.1629328	2944.099102	0.0062	0.995	7.62E-10	count	1
AL591441.1	18.162933	2944.098951	0.0062	0.995	7.62E-10	count	1
KC877392.1	18.1629332	2944.099178	0.0062	0.995	7.62E-10	count	1
AC025822.2	18.162933	2944.09914	0.0062	0.995	7.62E-10	count	1
NUTM2D	18.1629332	2944.099178	0.0062	0.995	7.62E-10	count	1
SH3PXD2A-AS1	18.162933	2944.099008	0.0062	0.995	7.62E-10	count	1
AC010168.1	18.1629331	2944.099235	0.0062	0.995	7.62E-10	count	1
DENND5B-AS1	18.162933	2944.098932	0.0062	0.995	7.62E-10	count	1
AC024257.4	18.1629333	2944.099083	0.0062	0.995	7.62E-10	count	1

AC011595.1	18.1629329	2944.099064	0.0062	0.995	7.62E-10	count	1
TRAV41	18.1629329	2944.099064	0.0062	0.995	7.62E-10	count	1
AL139353.2	18.1629333	2944.099083	0.0062	0.995	7.62E-10	count	1
SLC10A1	18.162933	2944.09914	0.0062	0.995	7.62E-10	count	1
AC010999.2	18.1629331	2944.099008	0.0062	0.995	7.62E-10	count	1
LINC02259	18.1629331	2944.099462	0.0062	0.995	7.62E-10	count	1
LINC02126	18.1629331	2944.099291	0.0062	0.995	7.62E-10	count	1
LINC02182	18.1629332	2944.099178	0.0062	0.995	7.62E-10	count	1
AC006449.3	18.162933	2944.098989	0.0062	0.995	7.62E-10	count	1
CELF5	18.1629329	2944.099064	0.0062	0.995	7.62E-10	count	1
FCHO1	18.1629332	2944.099178	0.0062	0.995	7.62E-10	count	1
AC011477.4	18.1629331	2944.099008	0.0062	0.995	7.62E-10	count	1
BCKDHA	18.162933	2944.09914	0.0062	0.995	7.62E-10	count	1
SYCE3	18.1629331	2944.099008	0.0062	0.995	7.62E-10	count	1
MUSK	0.0271347	0.522232	0.052	0.959	8.01E-10	count	1
AC005332.8	0.0271347	0.7469202	0.0363	0.971	8.01E-10	count	1
WWTR1-AS1	0.0338551	0.5931213	0.0571	0.954	9.98E-10	count	1
AL157392.4	0.1093249	0.9850733	0.111	0.912	1.16E-09	count	1
MED12L	0.1093249	0.9850733	0.111	0.912	1.16E-09	count	1
AC005291.1	0.1093249	0.9850733	0.111	0.912	1.16E-09	count	1
AARD	0.1093249	0.9850733	0.111	0.912	1.16E-09	count	1
PRAG1	0.1093249	0.9850733	0.111	0.912	1.16E-09	count	1
PYDC1	0.1093249	1.0118885	0.108	0.914	1.16E-09	count	1
MDS2	0.1093249	0.9850733	0.111	0.912	1.16E-09	count	1
AL160269.1	0.1093249	0.9850733	0.111	0.912	1.16E-09	count	1
SHBG	0.1093249	1.0118885	0.108	0.914	1.16E-09	count	1
AL353135.1	0.1093249	1.0118885	0.108	0.914	1.16E-09	count	1
AC092652.1	0.1093249	1.0118885	0.108	0.914	1.16E-09	count	1
GINS1	0.1093249	1.0118885	0.108	0.914	1.16E-09	count	1
ATP6V0E2-AS1	0.1093249	0.9850733	0.111	0.912	1.16E-09	count	1
KCNQ3	0.1093249	0.9850733	0.111	0.912	1.16E-09	count	1
AC112229.2	0.3502024	1.0145479	0.3452	0.73	1.28E-09	count	1
FUT2	0.3502024	1.0145479	0.3452	0.73	1.28E-09	count	1
AC090527.3	0.3502024	1.0145479	0.3452	0.73	1.28E-09	count	1
ITCH-AS1	0.3502024	1.0145479	0.3452	0.73	1.28E-09	count	1
AGBL4	0.3502024	0.8055999	0.4347	0.664	1.28E-09	count	1
MAP3K21	0.3502024	0.8055999	0.4347	0.664	1.28E-09	count	1
AC137630.4	0.3502024	0.8055999	0.4347	0.664	1.28E-09	count	1
AC009812.3	0.3502024	0.8055999	0.4347	0.664	1.28E-09	count	1
AL928970.1	0.3502024	0.8055999	0.4347	0.664	1.28E-09	count	1
PCAT7	0.3502024	0.8055999	0.4347	0.664	1.28E-09	count	1
AP001107.4	0.3502024	0.8055999	0.4347	0.664	1.28E-09	count	1
LINC01479	0.3502024	0.8055999	0.4347	0.664	1.28E-09	count	1
WASF3-AS1	0.3502024	0.8055999	0.4347	0.664	1.28E-09	count	1
BCL11B	0.3502024	0.8055999	0.4347	0.664	1.28E-09	count	1

MMP15	0.3502024	0.8055999	0.4347	0.664	1.28E-09	count	1
PAK5	0.3502024	0.8055999	0.4347	0.664	1.28E-09	count	1
AL121583.1	0.3502024	0.8055999	0.4347	0.664	1.28E-09	count	1
AMH	0.3502024	0.8055999	0.4347	0.664	1.28E-09	count	1
Z97055.2	0.3502024	0.8055999	0.4347	0.664	1.28E-09	count	1
AL357568.1	0.3502024	0.8055999	0.4347	0.664	1.28E-09	count	1
AC022893.2	0.3502024	0.8055999	0.4347	0.664	1.28E-09	count	1
LINC01117	0.1715969	0.9032599	0.19	0.849	1.79E-09	count	1
TAS2R30	0.1715969	0.8634446	0.1987	0.842	1.79E-09	count	1
RPL34-AS1	18.5575131	2833.387336	0.0065	0.995	2.07E-09	count	1
GUCA1A	18.2683959	3103.330176	0.0059	0.995	2.07E-09	count	1
IGDCC3	18.2683959	3103.330427	0.0059	0.995	2.07E-09	count	1
VNN3	18.2683959	3103.330249	0.0059	0.995	2.07E-09	count	1
GSN-AS1	18.2683954	3103.330043	0.0059	0.995	2.07E-09	count	1
SPIC	18.2683955	3103.330205	0.0059	0.995	2.07E-09	count	1
KCNK10	18.2683954	3103.329924	0.0059	0.995	2.07E-09	count	1
AC092718.5	18.2668265	2309.586501	0.0079	0.994	2.07E-09	count	1
MIR4500HG	18.2668265	2309.586666	0.0079	0.994	2.07E-09	count	1
FOXL2	18.2668262	2309.586666	0.0079	0.994	2.07E-09	count	1
AL031772.1	18.2668257	2309.586468	0.0079	0.994	2.07E-09	count	1
AC037198.2	18.2668261	2309.586446	0.0079	0.994	2.07E-09	count	1
IGLL5	18.266826	2309.586567	0.0079	0.994	2.07E-09	count	1
SOX30	18.266826	2309.586677	0.0079	0.994	2.07E-09	count	1
AC019186.1	17.8595768	2529.61333	0.0071	0.994	2.07E-09	count	1
AC092652.2	17.8595766	2529.61279	0.0071	0.994	2.07E-09	count	1
SLC17A4	17.8595765	2529.613078	0.0071	0.994	2.07E-09	count	1
FOXD3-AS1	17.8595765	2529.612982	0.0071	0.994	2.07E-09	count	1
GPR78	17.8595764	2529.61297	0.0071	0.994	2.07E-09	count	1
LINC01179	17.8595765	2529.612982	0.0071	0.994	2.07E-09	count	1
AC091167.6	17.8595764	2529.61297	0.0071	0.994	2.07E-09	count	1
GALR2	17.8595765	2529.612982	0.0071	0.994	2.07E-09	count	1
KIAA1257	17.8595765	2529.612958	0.0071	0.994	2.07E-09	count	1
AC026726.1	17.8595764	2529.612862	0.0071	0.994	2.07E-09	count	1
MACC1	17.8595765	2529.612958	0.0071	0.994	2.07E-09	count	1
AL354710.2	17.8595762	2529.613174	0.0071	0.994	2.07E-09	count	1
PLCE1-AS1	17.8595764	2529.612994	0.0071	0.994	2.07E-09	count	1
AL162274.1	17.8595764	2529.61291	0.0071	0.994	2.07E-09	count	1
AC025263.1	17.8595764	2529.612934	0.0071	0.994	2.07E-09	count	1
AC073534.2	17.8595764	2529.61291	0.0071	0.994	2.07E-09	count	1
ADCYAP1	17.8595763	2529.613078	0.0071	0.994	2.07E-09	count	1
TAL1	17.859576	2529.612898	0.0071	0.994	2.07E-09	count	1
AC011451.1	18.2657067	1786.038645	0.0102	0.992	2.07E-09	count	1
C9orf129	18.2657061	1786.038586	0.0102	0.992	2.07E-09	count	1
COL19A1	18.2657061	1786.038458	0.0102	0.992	2.07E-09	count	1
AC078795.2	18.2657056	1786.038467	0.0102	0.992	2.07E-09	count	1

AC009021.1	18.2657054	1786.038704	0.0102	0.992	2.07E-09	count	1
AC099062.1	17.8583763	1786.004203	0.01	0.992	2.07E-09	count	1
HHIPL2	17.8583763	1786.004203	0.01	0.992	2.07E-09	count	1
ARSD-AS1	17.858376	1786.004347	0.01	0.992	2.07E-09	count	1
LINC01993	17.8583763	1786.004203	0.01	0.992	2.07E-09	count	1
CAMK2A	17.8583759	1786.004398	0.01	0.992	2.07E-09	count	1
AP002373.1	17.8583757	1786.004127	0.01	0.992	2.07E-09	count	1
PXT1	17.8583759	1786.004304	0.01	0.992	2.07E-09	count	1
AC005180.1	17.8583756	1786.00433	0.01	0.992	2.07E-09	count	1
RTEL1-TNFRSF6B	17.8583757	1786.004237	0.01	0.992	2.07E-09	count	1
IGLV4-69	17.8583757	1786.004254	0.01	0.992	2.07E-09	count	1
SLC5A7	17.8583756	1786.004372	0.01	0.992	2.07E-09	count	1
AC008440.1	17.8583757	1786.004415	0.01	0.992	2.07E-09	count	1
RSPH14	17.8583754	1786.004194	0.01	0.992	2.07E-09	count	1
WWC3-AS1	17.8583754	1786.004321	0.01	0.992	2.07E-09	count	1
IL1RAPL2	17.8583753	1786.00422	0.01	0.992	2.07E-09	count	1
AC068389.4	17.8583753	1786.004237	0.01	0.992	2.07E-09	count	1
MAPK15	17.8583755	1786.004262	0.01	0.992	2.07E-09	count	1
AL158152.2	17.8583755	1786.00411	0.01	0.992	2.07E-09	count	1
TTC36	17.8583754	1786.004254	0.01	0.992	2.07E-09	count	1
POU2F3	17.8583754	1786.00433	0.01	0.992	2.07E-09	count	1
AC121761.1	17.8583755	1786.004211	0.01	0.992	2.07E-09	count	1
AC020558.1	17.8583756	1786.004415	0.01	0.992	2.07E-09	count	1
AP000919.3	17.8583755	1786.004042	0.01	0.992	2.07E-09	count	1
LINC00028	17.8583754	1786.004355	0.01	0.992	2.07E-09	count	1
XCL2	17.8583754	1786.004254	0.01	0.992	2.07E-09	count	1
ANKRD23	17.8583754	1786.004262	0.01	0.992	2.07E-09	count	1
NFE2	17.8583754	1786.004279	0.01	0.992	2.07E-09	count	1
KLHDC7B	17.8583752	1786.004144	0.01	0.992	2.07E-09	count	1
SMCR5	18.7844431	2506.55172	0.0075	0.994	2.08E-09	count	1
AC145207.2	18.7844434	2506.552121	0.0075	0.994	2.08E-09	count	1
GRIP1	18.7844427	2506.551609	0.0075	0.994	2.08E-09	count	1
AC122129.1	18.7844428	2506.551789	0.0075	0.994	2.08E-09	count	1
AL139124.1	18.7844426	2506.551803	0.0075	0.994	2.08E-09	count	1
LINC00485	18.7844426	2506.551817	0.0075	0.994	2.08E-09	count	1
SEC23A-AS1	18.7844426	2506.551858	0.0075	0.994	2.08E-09	count	1
LINC00648	18.7844429	2506.551969	0.0075	0.994	2.08E-09	count	1
AL049874.3	18.7844429	2506.551886	0.0075	0.994	2.08E-09	count	1
IMPG1	18.7844428	2506.552024	0.0075	0.994	2.08E-09	count	1
AC055822.1	18.7844422	2506.551927	0.0075	0.994	2.08E-09	count	1
SYN3	18.7844424	2506.55219	0.0075	0.994	2.08E-09	count	1
AF230666.1	18.7844426	2506.552052	0.0075	0.994	2.08E-09	count	1
ADGRB1	18.7844427	2506.5519	0.0075	0.994	2.08E-09	count	1
MYH15	18.784442	2506.551969	0.0075	0.994	2.08E-09	count	1
AC002553.2	18.7844422	2506.55183	0.0075	0.994	2.08E-09	count	1

OR2A1	18.3785249	3547.818308	0.0052	0.996	2.08E-09	count	1
PTH2	18.378525	3547.818485	0.0052	0.996	2.08E-09	count	1
C6orf58	18.3785247	3547.81823	0.0052	0.996	2.08E-09	count	1
AC005091.1	18.3785247	3547.81825	0.0052	0.996	2.08E-09	count	1
AC023794.3	18.3785247	3547.818269	0.0052	0.996	2.08E-09	count	1
HAL	18.3785245	3547.818308	0.0052	0.996	2.08E-09	count	1
LINC00638	18.3785248	3547.818269	0.0052	0.996	2.08E-09	count	1
AC136285.1	18.3785248	3547.818269	0.0052	0.996	2.08E-09	count	1
NEUROD2	18.3785247	3547.81825	0.0052	0.996	2.08E-09	count	1
RGS9BP	18.3785249	3547.818485	0.0052	0.996	2.08E-09	count	1
SPACA6P-AS	18.3785247	3547.81825	0.0052	0.996	2.08E-09	count	1
LINC01460	18.3785246	3547.818269	0.0052	0.996	2.08E-09	count	1
FAM153A	18.3785246	3547.818034	0.0052	0.996	2.08E-09	count	1
AC138230.1	18.3785246	3547.818269	0.0052	0.996	2.08E-09	count	1
AL513190.1	18.3785244	3547.818015	0.0052	0.996	2.08E-09	count	1
AC004846.2	18.3785243	3547.818113	0.0052	0.996	2.08E-09	count	1
LINC02554	18.3785247	3547.818211	0.0052	0.996	2.08E-09	count	1
LRRC70	18.3785246	3547.818426	0.0052	0.996	2.08E-09	count	1
FUT9	18.3785246	3547.818426	0.0052	0.996	2.08E-09	count	1
UBQLNL	18.3785245	3547.81823	0.0052	0.996	2.08E-09	count	1
AL354809.1	18.3785245	3547.81823	0.0052	0.996	2.08E-09	count	1
LINC00605	18.3785246	3547.818054	0.0052	0.996	2.08E-09	count	1
AC004477.2	18.3785248	3547.81823	0.0052	0.996	2.08E-09	count	1
AC080162.1	18.3785244	3547.81825	0.0052	0.996	2.08E-09	count	1
AC007250.1	18.3785244	3547.81825	0.0052	0.996	2.08E-09	count	1
CPNE9	18.3785243	3547.818015	0.0052	0.996	2.08E-09	count	1
EPHA5-AS1	18.3785245	3547.818406	0.0052	0.996	2.08E-09	count	1
AC079340.2	18.3785245	3547.818367	0.0052	0.996	2.08E-09	count	1
H3.Y	18.3785246	3547.818269	0.0052	0.996	2.08E-09	count	1
AL356277.3	18.3785245	3547.817995	0.0052	0.996	2.08E-09	count	1
AC093799.1	18.3785245	3547.818406	0.0052	0.996	2.08E-09	count	1
AC092111.1	18.3785245	3547.818152	0.0052	0.996	2.08E-09	count	1
SLITRK1	18.3785243	3547.818015	0.0052	0.996	2.08E-09	count	1
CHRNA7	18.3785243	3547.817995	0.0052	0.996	2.08E-09	count	1
AC108861.1	18.3785245	3547.818152	0.0052	0.996	2.08E-09	count	1
AC138904.1	18.3785244	3547.818054	0.0052	0.996	2.08E-09	count	1
DRC7	18.3785242	3547.81825	0.0052	0.996	2.08E-09	count	1
TMED6	18.3785244	3547.818152	0.0052	0.996	2.08E-09	count	1
AC004702.1	18.3785243	3547.818015	0.0052	0.996	2.08E-09	count	1
ZBP1	18.3785247	3547.818289	0.0052	0.996	2.08E-09	count	1
ZNF723	18.3785243	3547.818152	0.0052	0.996	2.08E-09	count	1
Z97192.1	18.3785245	3547.818367	0.0052	0.996	2.08E-09	count	1
LAX1	18.3785241	3547.818171	0.0052	0.996	2.08E-09	count	1
AC016831.6	18.3785241	3547.818171	0.0052	0.996	2.08E-09	count	1
LCTL	18.3785241	3547.818113	0.0052	0.996	2.08E-09	count	1

Z99916.1	18.3785241	3547.818113	0.0052	0.996	2.08E-09	count	1
AC073365.1	18.378524	3547.818093	0.0052	0.996	2.08E-09	count	1
AC021224.1	18.378524	3547.818093	0.0052	0.996	2.08E-09	count	1
HOXB-AS3	18.3777687	2506.387922	0.0073	0.994	2.08E-09	count	1
LINC002481	18.3777685	2506.387867	0.0073	0.994	2.08E-09	count	1
AC016877.3	18.3777681	2506.387604	0.0073	0.994	2.08E-09	count	1
AL359636.1	18.3777684	2506.387908	0.0073	0.994	2.08E-09	count	1
AL121749.1	18.3777682	2506.387674	0.0073	0.994	2.08E-09	count	1
AC024941.1	18.3777683	2506.387687	0.0073	0.994	2.08E-09	count	1
LINC00426	18.3777686	2506.387729	0.0073	0.994	2.08E-09	count	1
TDRD9	18.3777683	2506.38777	0.0073	0.994	2.08E-09	count	1
AC021755.3	18.3777683	2506.387729	0.0073	0.994	2.08E-09	count	1
AP000253.1	18.3777683	2506.387853	0.0073	0.994	2.08E-09	count	1
AL162591.2	18.377768	2506.387687	0.0073	0.994	2.08E-09	count	1
AL139011.1	18.3777683	2506.387632	0.0073	0.994	2.08E-09	count	1
TDGF1	18.3777682	2506.387618	0.0073	0.994	2.08E-09	count	1
PRKG2	18.3777683	2506.387563	0.0073	0.994	2.08E-09	count	1
AP000439.1	18.377768	2506.387508	0.0073	0.994	2.08E-09	count	1
AC092490.2	18.3777683	2506.387632	0.0073	0.994	2.08E-09	count	1
SLC28A1	18.3777682	2506.387591	0.0073	0.994	2.08E-09	count	1
APOBR	18.3777687	2506.387867	0.0073	0.994	2.08E-09	count	1
AC010632.1	18.3777683	2506.38777	0.0073	0.994	2.08E-09	count	1
Z98885.3	18.3777682	2506.387674	0.0073	0.994	2.08E-09	count	1
CAMP	18.3777681	2506.387756	0.0073	0.994	2.08E-09	count	1
AC104118.1	18.3777682	2506.387784	0.0073	0.994	2.08E-09	count	1
STK32A-AS1	18.3777681	2506.38777	0.0073	0.994	2.08E-09	count	1
AC008610.1	18.3777681	2506.387812	0.0073	0.994	2.08E-09	count	1
RNF182	18.3777685	2506.387798	0.0073	0.994	2.08E-09	count	1
AC136475.2	18.3777682	2506.387922	0.0073	0.994	2.08E-09	count	1
TLL2	18.377768	2506.387729	0.0073	0.994	2.08E-09	count	1
AL731566.1	18.3777681	2506.387715	0.0073	0.994	2.08E-09	count	1
HOXC8	18.3777684	2506.387743	0.0073	0.994	2.08E-09	count	1
TRPV4	18.3777684	2506.387784	0.0073	0.994	2.08E-09	count	1
ATP8A2	18.377768	2506.387895	0.0073	0.994	2.08E-09	count	1
AC141586.2	18.3777681	2506.387743	0.0073	0.994	2.08E-09	count	1
AC007608.1	18.3777683	2506.387743	0.0073	0.994	2.08E-09	count	1
GNGT2	18.3777681	2506.387867	0.0073	0.994	2.08E-09	count	1
LRRC25	18.3777683	2506.387687	0.0073	0.994	2.08E-09	count	1
AL391244.3	18.3777681	2506.387756	0.0073	0.994	2.08E-09	count	1
AL445231.1	18.377768	2506.387729	0.0073	0.994	2.08E-09	count	1
AC096677.1	18.377768	2506.387784	0.0073	0.994	2.08E-09	count	1
LINC01819	18.3777681	2506.387674	0.0073	0.994	2.08E-09	count	1
PRKAG3	18.3777682	2506.387715	0.0073	0.994	2.08E-09	count	1
SOX2	18.3777681	2506.387812	0.0073	0.994	2.08E-09	count	1
AC104806.2	18.3777681	2506.387674	0.0073	0.994	2.08E-09	count	1

CDS1	18.3777681	2506.387715	0.0073	0.994	2.08E-09	count	1
AC244517.1	18.3777679	2506.387632	0.0073	0.994	2.08E-09	count	1
SAPCD1-AS1	18.377768	2506.387784	0.0073	0.994	2.08E-09	count	1
RAET1E-AS1	18.377768	2506.387604	0.0073	0.994	2.08E-09	count	1
AC093627.5	18.3777681	2506.387784	0.0073	0.994	2.08E-09	count	1
AL158055.1	18.3777678	2506.387729	0.0073	0.994	2.08E-09	count	1
AC019270.1	18.3777684	2506.38777	0.0073	0.994	2.08E-09	count	1
RDH10-AS1	18.3777681	2506.387687	0.0073	0.994	2.08E-09	count	1
GLIS3-AS1	18.3777681	2506.387908	0.0073	0.994	2.08E-09	count	1
IZUMO1R	18.377768	2506.387674	0.0073	0.994	2.08E-09	count	1
SLC39A12	18.377768	2506.387563	0.0073	0.994	2.08E-09	count	1
AC073912.2	18.3777682	2506.387784	0.0073	0.994	2.08E-09	count	1
LINC00377	18.377768	2506.387798	0.0073	0.994	2.08E-09	count	1
GPC5-AS1	18.377768	2506.387729	0.0073	0.994	2.08E-09	count	1
F7	18.3777683	2506.387743	0.0073	0.994	2.08E-09	count	1
AC022167.4	18.3777683	2506.387743	0.0073	0.994	2.08E-09	count	1
AC027796.4	18.377768	2506.387743	0.0073	0.994	2.08E-09	count	1
AC004223.2	18.3777683	2506.387812	0.0073	0.994	2.08E-09	count	1
AP005205.2	18.3777679	2506.387604	0.0073	0.994	2.08E-09	count	1
KIZ-AS1	18.3777681	2506.387826	0.0073	0.994	2.08E-09	count	1
ISL1	18.377768	2506.387701	0.0073	0.994	2.08E-09	count	1
LRRC19	18.3777679	2506.387646	0.0073	0.994	2.08E-09	count	1
MCM10	18.3777682	2506.387853	0.0073	0.994	2.08E-09	count	1
LINC00621	18.3777676	2506.387466	0.0073	0.994	2.08E-09	count	1
AP002884.3	18.3777678	2506.387632	0.0073	0.994	2.08E-09	count	1
AC025035.1	18.3777679	2506.387522	0.0073	0.994	2.08E-09	count	1
LRRC18	18.3777678	2506.387701	0.0073	0.994	2.08E-09	count	1
AC025423.4	18.3777676	2506.387549	0.0073	0.994	2.08E-09	count	1
HPCAL4	17.6832648	2506.037739	0.0071	0.994	2.08E-09	count	1
LINC01063	17.6832648	2506.037753	0.0071	0.994	2.08E-09	count	1
TMPRSS11A	17.6832648	2506.037739	0.0071	0.994	2.08E-09	count	1
AL049697.1	17.6832648	2506.037753	0.0071	0.994	2.08E-09	count	1
AC087292.1	17.6832649	2506.037822	0.0071	0.994	2.08E-09	count	1
RDM1	17.6832649	2506.037788	0.0071	0.994	2.08E-09	count	1
PPP1R16B	17.6832648	2506.037739	0.0071	0.994	2.08E-09	count	1
FAM83F	17.6832649	2506.037788	0.0071	0.994	2.08E-09	count	1
AC254562.2	17.6832649	2506.037822	0.0071	0.994	2.08E-09	count	1
CLCNKA	17.6832649	2506.037746	0.0071	0.994	2.08E-09	count	1
RAD54L	17.6832649	2506.03785	0.0071	0.994	2.08E-09	count	1
AC090948.1	17.6832648	2506.037753	0.0071	0.994	2.08E-09	count	1
AC097382.2	17.6832648	2506.03785	0.0071	0.994	2.08E-09	count	1
AC110609.1	17.6832648	2506.037767	0.0071	0.994	2.08E-09	count	1
LINC01513	17.6832648	2506.037781	0.0071	0.994	2.08E-09	count	1
AL031963.1	17.6832648	2506.037808	0.0071	0.994	2.08E-09	count	1
SNAP91	17.6832648	2506.03785	0.0071	0.994	2.08E-09	count	1

AL731684.2	17.6832648	2506.037788	0.0071	0.994	2.08E-09	count	1
AC019117.1	17.6832648	2506.037788	0.0071	0.994	2.08E-09	count	1
URGCP-MRPS24	17.6832649	2506.03785	0.0071	0.994	2.08E-09	count	1
CYP3A43	17.6832648	2506.037739	0.0071	0.994	2.08E-09	count	1
PIK3CG	17.6832648	2506.037822	0.0071	0.994	2.08E-09	count	1
AL391294.1	17.6832648	2506.037781	0.0071	0.994	2.08E-09	count	1
AP000786.1	17.6832648	2506.037829	0.0071	0.994	2.08E-09	count	1
AL022344.2	17.6832648	2506.037739	0.0071	0.994	2.08E-09	count	1
NRAP	17.6832648	2506.037808	0.0071	0.994	2.08E-09	count	1
AC009509.4	17.6832649	2506.03776	0.0071	0.994	2.08E-09	count	1
HCAR3	17.6832648	2506.03785	0.0071	0.994	2.08E-09	count	1
AC090970.2	17.6832648	2506.037815	0.0071	0.994	2.08E-09	count	1
LINC00923	17.6832649	2506.037726	0.0071	0.994	2.08E-09	count	1
OR2C1	17.6832648	2506.037836	0.0071	0.994	2.08E-09	count	1
AC126763.1	17.6832649	2506.037815	0.0071	0.994	2.08E-09	count	1
CKLF-CMTM1	17.6832648	2506.037739	0.0071	0.994	2.08E-09	count	1
LINC00854	17.6832649	2506.03776	0.0071	0.994	2.08E-09	count	1
MYADML2	17.6832648	2506.037912	0.0071	0.994	2.08E-09	count	1
AL354813.1	17.6832648	2506.037767	0.0071	0.994	2.08E-09	count	1
LINC00160	17.6832648	2506.037836	0.0071	0.994	2.08E-09	count	1
LINC01424	17.6832648	2506.037836	0.0071	0.994	2.08E-09	count	1
TMEM51-AS1	17.6832648	2506.037822	0.0071	0.994	2.08E-09	count	1
GRHL3	17.6832648	2506.037698	0.0071	0.994	2.08E-09	count	1
TMEM269	17.6832648	2506.037822	0.0071	0.994	2.08E-09	count	1
STIL	17.6832647	2506.037705	0.0071	0.994	2.08E-09	count	1
TSACC	17.6832647	2506.037719	0.0071	0.994	2.08E-09	count	1
AIM2	17.6832646	2506.03776	0.0071	0.994	2.08E-09	count	1
ELF3-AS1	17.6832648	2506.037843	0.0071	0.994	2.08E-09	count	1
AC144450.1	17.6832648	2506.037753	0.0071	0.994	2.08E-09	count	1
LINC01814	17.6832647	2506.037732	0.0071	0.994	2.08E-09	count	1
SIX3-AS1	17.6832648	2506.037801	0.0071	0.994	2.08E-09	count	1
AC013270.1	17.6832647	2506.037801	0.0071	0.994	2.08E-09	count	1
AC018690.1	17.6832648	2506.037705	0.0071	0.994	2.08E-09	count	1
AC092667.1	17.6832648	2506.037795	0.0071	0.994	2.08E-09	count	1
AC092620.1	17.6832645	2506.037726	0.0071	0.994	2.08E-09	count	1
AC016737.1	17.6832649	2506.037788	0.0071	0.994	2.08E-09	count	1
AC073254.1	17.6832647	2506.037767	0.0071	0.994	2.08E-09	count	1
TMEM110-MUSTN1	17.6832648	2506.03776	0.0071	0.994	2.08E-09	count	1
MSANTD1	17.6832648	2506.03776	0.0071	0.994	2.08E-09	count	1
AC025754.2	17.6832647	2506.037836	0.0071	0.994	2.08E-09	count	1
AC092343.1	17.6832647	2506.037774	0.0071	0.994	2.08E-09	count	1
AC025188.1	17.6832648	2506.037774	0.0071	0.994	2.08E-09	count	1
TMED7-TICAM2	17.6832647	2506.037829	0.0071	0.994	2.08E-09	count	1
ARL14EPL	17.6832648	2506.037781	0.0071	0.994	2.08E-09	count	1
AC091979.2	17.6832647	2506.037746	0.0071	0.994	2.08E-09	count	1

ZBTB12	17.6832648	2506.037767	0.0071	0.994	2.08E-09	count	1
AL121574.1	17.6832648	2506.037864	0.0071	0.994	2.08E-09	count	1
ADGRF1	17.6832648	2506.037719	0.0071	0.994	2.08E-09	count	1
HCRTR2	17.6832648	2506.037815	0.0071	0.994	2.08E-09	count	1
WISP3	17.6832646	2506.037753	0.0071	0.994	2.08E-09	count	1
ATP1B4	17.6832648	2506.037698	0.0071	0.994	2.08E-09	count	1
FAM87A	17.6832647	2506.037705	0.0071	0.994	2.08E-09	count	1
RALYL	17.6832647	2506.037808	0.0071	0.994	2.08E-09	count	1
AC083836.1	17.6832648	2506.037795	0.0071	0.994	2.08E-09	count	1
MLANA	17.6832646	2506.037753	0.0071	0.994	2.08E-09	count	1
AL161729.2	17.6832647	2506.037712	0.0071	0.994	2.08E-09	count	1
DDIT4-AS1	17.6832648	2506.037774	0.0071	0.994	2.08E-09	count	1
AC022400.7	17.6832647	2506.03776	0.0071	0.994	2.08E-09	count	1
AC010997.3	17.6832647	2506.037801	0.0071	0.994	2.08E-09	count	1
DNMBP-AS1	17.6832648	2506.037774	0.0071	0.994	2.08E-09	count	1
AL138921.1	17.6832645	2506.037726	0.0071	0.994	2.08E-09	count	1
AL360182.2	17.6832649	2506.03787	0.0071	0.994	2.08E-09	count	1
OVCH1	17.6832646	2506.037808	0.0071	0.994	2.08E-09	count	1
AC008083.2	17.6832646	2506.037753	0.0071	0.994	2.08E-09	count	1
AC025259.1	17.6832648	2506.037864	0.0071	0.994	2.08E-09	count	1
AC025262.1	17.6832649	2506.037767	0.0071	0.994	2.08E-09	count	1
AC008149.1	17.6832649	2506.037815	0.0071	0.994	2.08E-09	count	1
AC026765.2	17.6832648	2506.037795	0.0071	0.994	2.08E-09	count	1
AC084018.1	17.6832648	2506.037815	0.0071	0.994	2.08E-09	count	1
GALNT9	17.6832647	2506.03776	0.0071	0.994	2.08E-09	count	1
OR11H4	17.6832646	2506.037732	0.0071	0.994	2.08E-09	count	1
AL161668.3	17.6832648	2506.03776	0.0071	0.994	2.08E-09	count	1
AL121594.1	17.6832646	2506.037795	0.0071	0.994	2.08E-09	count	1
AL359237.1	17.6832647	2506.037732	0.0071	0.994	2.08E-09	count	1
LINC02254	17.6832646	2506.037767	0.0071	0.994	2.08E-09	count	1
AC015660.2	17.6832648	2506.037781	0.0071	0.994	2.08E-09	count	1
AC092368.3	17.6832648	2506.037815	0.0071	0.994	2.08E-09	count	1
CMTM1	17.6832647	2506.037705	0.0071	0.994	2.08E-09	count	1
AC025287.4	17.6832648	2506.037801	0.0071	0.994	2.08E-09	count	1
AC046158.1	17.6832647	2506.037705	0.0071	0.994	2.08E-09	count	1
AC105411.1	17.6832647	2506.037705	0.0071	0.994	2.08E-09	count	1
AC138028.4	17.6832649	2506.037788	0.0071	0.994	2.08E-09	count	1
AC006435.3	17.6832649	2506.037767	0.0071	0.994	2.08E-09	count	1
AC005696.2	17.6832645	2506.037726	0.0071	0.994	2.08E-09	count	1
EFNB3	17.6832648	2506.037767	0.0071	0.994	2.08E-09	count	1
AC015908.4	17.6832648	2506.037864	0.0071	0.994	2.08E-09	count	1
ARL5C	17.6832648	2506.037801	0.0071	0.994	2.08E-09	count	1
ITGA2B	17.6832646	2506.037753	0.0071	0.994	2.08E-09	count	1
ANKFN1	17.6832648	2506.03776	0.0071	0.994	2.08E-09	count	1
SCN4A	17.6832647	2506.037795	0.0071	0.994	2.08E-09	count	1

AC037487.2	17.6832648	2506.037767	0.0071	0.994	2.08E-09	count	1
KIF19	17.6832647	2506.037774	0.0071	0.994	2.08E-09	count	1
AC027601.1	17.6832647	2506.037705	0.0071	0.994	2.08E-09	count	1
AC139099.2	17.6832648	2506.037801	0.0071	0.994	2.08E-09	count	1
AC011731.1	17.6832646	2506.037753	0.0071	0.994	2.08E-09	count	1
ONECUT2	17.6832648	2506.037795	0.0071	0.994	2.08E-09	count	1
LINC01730	17.6832648	2506.037836	0.0071	0.994	2.08E-09	count	1
AL121782.1	17.6832646	2506.037753	0.0071	0.994	2.08E-09	count	1
C19orf71	17.6832645	2506.037732	0.0071	0.994	2.08E-09	count	1
TUBB4A	17.6832648	2506.037898	0.0071	0.994	2.08E-09	count	1
AC008764.4	17.6832648	2506.037801	0.0071	0.994	2.08E-09	count	1
AC004784.1	17.6832647	2506.037705	0.0071	0.994	2.08E-09	count	1
AC020922.4	17.6832647	2506.037712	0.0071	0.994	2.08E-09	count	1
AC000068.1	17.6832647	2506.037808	0.0071	0.994	2.08E-09	count	1
5-Sep	17.6832648	2506.037822	0.0071	0.994	2.08E-09	count	1
AL008582.1	17.6832647	2506.037705	0.0071	0.994	2.08E-09	count	1
SHISA8	17.6832648	2506.037781	0.0071	0.994	2.08E-09	count	1
RSPH1	17.6832648	2506.03776	0.0071	0.994	2.08E-09	count	1
LINC01786	17.6832647	2506.037698	0.0071	0.994	2.08E-09	count	1
AL590822.1	17.6832647	2506.037643	0.0071	0.994	2.08E-09	count	1
AL513320.1	17.6832646	2506.037726	0.0071	0.994	2.08E-09	count	1
AL365330.1	17.6832648	2506.037822	0.0071	0.994	2.08E-09	count	1
AGMAT	17.6832646	2506.037767	0.0071	0.994	2.08E-09	count	1
AL031728.1	17.6832647	2506.037829	0.0071	0.994	2.08E-09	count	1
SLC30A2	17.6832646	2506.037746	0.0071	0.994	2.08E-09	count	1
GUCA2B	17.6832646	2506.037712	0.0071	0.994	2.08E-09	count	1
AL357673.1	17.6832647	2506.037774	0.0071	0.994	2.08E-09	count	1
AL353771.1	17.6832647	2506.037795	0.0071	0.994	2.08E-09	count	1
AL450992.2	17.6832647	2506.037836	0.0071	0.994	2.08E-09	count	1
SPRR2E	17.6832646	2506.037822	0.0071	0.994	2.08E-09	count	1
LOR	17.6832646	2506.037677	0.0071	0.994	2.08E-09	count	1
SLAMF9	17.6832647	2506.037801	0.0071	0.994	2.08E-09	count	1
KCNJ9	17.6832648	2506.037815	0.0071	0.994	2.08E-09	count	1
CASQ1	17.6832648	2506.037843	0.0071	0.994	2.08E-09	count	1
AL139011.2	17.6832646	2506.037732	0.0071	0.994	2.08E-09	count	1
CFAP126	17.6832648	2506.037815	0.0071	0.994	2.08E-09	count	1
ILDR2	17.6832648	2506.037801	0.0071	0.994	2.08E-09	count	1
AL031599.1	17.6832647	2506.03785	0.0071	0.994	2.08E-09	count	1
AL590723.1	17.6832647	2506.03776	0.0071	0.994	2.08E-09	count	1
AC104461.1	17.6832646	2506.037732	0.0071	0.994	2.08E-09	count	1
AL513283.1	17.6832647	2506.037788	0.0071	0.994	2.08E-09	count	1
PGBD5	17.6832646	2506.037712	0.0071	0.994	2.08E-09	count	1
AL357556.4	17.6832649	2506.037836	0.0071	0.994	2.08E-09	count	1
EXO1	17.6832647	2506.037767	0.0071	0.994	2.08E-09	count	1
ATP6V1C2	17.6832648	2506.037781	0.0071	0.994	2.08E-09	count	1

AC018467.1	17.6832647	2506.03776	0.0071	0.994	2.08E-09	count	1
MFSD2B	17.6832647	2506.037746	0.0071	0.994	2.08E-09	count	1
LINC01381	17.6832647	2506.037857	0.0071	0.994	2.08E-09	count	1
AC013403.2	17.6832648	2506.037801	0.0071	0.994	2.08E-09	count	1
C2orf91	17.6832647	2506.037767	0.0071	0.994	2.08E-09	count	1
AC018682.2	17.6832646	2506.03776	0.0071	0.994	2.08E-09	count	1
AC018462.1	17.6832646	2506.037705	0.0071	0.994	2.08E-09	count	1
AC007040.2	17.6832647	2506.037857	0.0071	0.994	2.08E-09	count	1
RAB6C	17.6832647	2506.037788	0.0071	0.994	2.08E-09	count	1
LINC01124	17.6832648	2506.037815	0.0071	0.994	2.08E-09	count	1
AC009948.4	17.6832646	2506.037767	0.0071	0.994	2.08E-09	count	1
AC005037.1	17.6832647	2506.037753	0.0071	0.994	2.08E-09	count	1
AC008269.1	17.6832646	2506.037712	0.0071	0.994	2.08E-09	count	1
MOGAT1	17.6832647	2506.037795	0.0071	0.994	2.08E-09	count	1
AC009950.1	17.6832645	2506.037808	0.0071	0.994	2.08E-09	count	1
SYN2	17.6832647	2506.037746	0.0071	0.994	2.08E-09	count	1
CYP8B1	17.6832647	2506.03787	0.0071	0.994	2.08E-09	count	1
SPATA12	17.6832647	2506.037698	0.0071	0.994	2.08E-09	count	1
AC073352.1	17.6832646	2506.037691	0.0071	0.994	2.08E-09	count	1
AC107027.1	17.6832645	2506.037753	0.0071	0.994	2.08E-09	count	1
AC117395.1	17.6832647	2506.037788	0.0071	0.994	2.08E-09	count	1
YEATS2-AS1	17.6832646	2506.037726	0.0071	0.994	2.08E-09	count	1
HRASLS	17.6832647	2506.037719	0.0071	0.994	2.08E-09	count	1
LINC02026	17.6832646	2506.037836	0.0071	0.994	2.08E-09	count	1
AC092574.1	17.6832646	2506.037712	0.0071	0.994	2.08E-09	count	1
ERVMER34-1	17.6832646	2506.037705	0.0071	0.994	2.08E-09	count	1
AC021146.12	17.6832647	2506.037774	0.0071	0.994	2.08E-09	count	1
AC107072.2	17.6832648	2506.037788	0.0071	0.994	2.08E-09	count	1
AC114781.2	17.6832645	2506.037677	0.0071	0.994	2.08E-09	count	1
LINC02428	17.6832647	2506.037857	0.0071	0.994	2.08E-09	count	1
AC004069.1	17.6832646	2506.037719	0.0071	0.994	2.08E-09	count	1
AC096564.1	17.6832647	2506.037753	0.0071	0.994	2.08E-09	count	1
AC079298.1	17.6832648	2506.037746	0.0071	0.994	2.08E-09	count	1
AC091891.2	17.6832649	2506.037781	0.0071	0.994	2.08E-09	count	1
AC008966.2	17.6832647	2506.037808	0.0071	0.994	2.08E-09	count	1
AC025470.2	17.6832646	2506.037774	0.0071	0.994	2.08E-09	count	1
LINC02197	17.6832646	2506.037726	0.0071	0.994	2.08E-09	count	1
LIX1-AS1	17.6832647	2506.037774	0.0071	0.994	2.08E-09	count	1
AC106786.2	17.6832648	2506.037836	0.0071	0.994	2.08E-09	count	1
CTXN3	17.6832647	2506.037843	0.0071	0.994	2.08E-09	count	1
PSD2	17.6832646	2506.037829	0.0071	0.994	2.08E-09	count	1
SLC36A2	17.6832647	2506.037739	0.0071	0.994	2.08E-09	count	1
AC025437.2	17.6832647	2506.037719	0.0071	0.994	2.08E-09	count	1
LINC02227	17.6832647	2506.037801	0.0071	0.994	2.08E-09	count	1
AC011365.2	17.6832647	2506.037746	0.0071	0.994	2.08E-09	count	1

FGFR4	17.6832647	2506.037795	0.0071	0.994	2.08E-09	count	1
AL031123.2	17.6832647	2506.037677	0.0071	0.994	2.08E-09	count	1
AL157373.2	17.6832647	2506.037808	0.0071	0.994	2.08E-09	count	1
AL008729.1	17.6832646	2506.037767	0.0071	0.994	2.08E-09	count	1
HIST1H2BI	17.6832647	2506.037767	0.0071	0.994	2.08E-09	count	1
LINC00243	17.6832645	2506.037753	0.0071	0.994	2.08E-09	count	1
SFTA2	17.6832648	2506.037815	0.0071	0.994	2.08E-09	count	1
AL021368.3	17.6832649	2506.037864	0.0071	0.994	2.08E-09	count	1
AL445250.1	17.6832648	2506.037815	0.0071	0.994	2.08E-09	count	1
EYS	17.6832647	2506.037746	0.0071	0.994	2.08E-09	count	1
KHDC1L	17.6832648	2506.03787	0.0071	0.994	2.08E-09	count	1
TRDN-AS1	17.6832648	2506.037884	0.0071	0.994	2.08E-09	count	1
ULBP3	17.6832647	2506.037739	0.0071	0.994	2.08E-09	count	1
AC073957.1	17.6832647	2506.037739	0.0071	0.994	2.08E-09	count	1
AC073316.2	17.6832647	2506.037719	0.0071	0.994	2.08E-09	count	1
AC011284.1	17.6832647	2506.037788	0.0071	0.994	2.08E-09	count	1
AC005082.1	17.6832645	2506.037746	0.0071	0.994	2.08E-09	count	1
AC006027.1	17.6832647	2506.037739	0.0071	0.994	2.08E-09	count	1
AC005154.4	17.6832647	2506.037774	0.0071	0.994	2.08E-09	count	1
AC010132.4	17.6832647	2506.037808	0.0071	0.994	2.08E-09	count	1
UPK3B	17.6832646	2506.037767	0.0071	0.994	2.08E-09	count	1
AC079760.2	17.6832647	2506.037698	0.0071	0.994	2.08E-09	count	1
AC005096.1	17.6832646	2506.037857	0.0071	0.994	2.08E-09	count	1
AC073130.2	17.6832646	2506.037726	0.0071	0.994	2.08E-09	count	1
LRGUK	17.6832647	2506.037746	0.0071	0.994	2.08E-09	count	1
SVOPL	17.6832646	2506.037815	0.0071	0.994	2.08E-09	count	1
LINC01204	17.6832647	2506.037719	0.0071	0.994	2.08E-09	count	1
FOXP3	17.6832647	2506.03776	0.0071	0.994	2.08E-09	count	1
IGBP1-AS2	17.6832648	2506.037801	0.0071	0.994	2.08E-09	count	1
ALG13-AS1	17.6832647	2506.03785	0.0071	0.994	2.08E-09	count	1
FIRRE	17.6832646	2506.037767	0.0071	0.994	2.08E-09	count	1
LINC00629	17.6832647	2506.037781	0.0071	0.994	2.08E-09	count	1
PNMA5	17.6832646	2506.037767	0.0071	0.994	2.08E-09	count	1
FAM167A	17.6832646	2506.037712	0.0071	0.994	2.08E-09	count	1
CDCA2	17.6832647	2506.037795	0.0071	0.994	2.08E-09	count	1
AC090103.1	17.6832645	2506.037705	0.0071	0.994	2.08E-09	count	1
ADRA1A	17.6832646	2506.037836	0.0071	0.994	2.08E-09	count	1
AC124067.4	17.6832647	2506.037781	0.0071	0.994	2.08E-09	count	1
AC091173.1	17.6832647	2506.037753	0.0071	0.994	2.08E-09	count	1
TRIM55	17.6832647	2506.037801	0.0071	0.994	2.08E-09	count	1
AC018442.2	17.6832647	2506.037767	0.0071	0.994	2.08E-09	count	1
AP003354.2	17.6832647	2506.037698	0.0071	0.994	2.08E-09	count	1
AC012213.3	17.6832647	2506.037753	0.0071	0.994	2.08E-09	count	1
TG	17.6832649	2506.037864	0.0071	0.994	2.08E-09	count	1
RHPN1-AS1	17.6832646	2506.037739	0.0071	0.994	2.08E-09	count	1

FAM83H	17.6832647	2506.037753	0.0071	0.994	2.08E-09	count	1
C9orf92	17.6832647	2506.03776	0.0071	0.994	2.08E-09	count	1
AL161909.2	17.6832647	2506.037795	0.0071	0.994	2.08E-09	count	1
EQTN	17.6832648	2506.037829	0.0071	0.994	2.08E-09	count	1
BANCR	17.6832646	2506.037705	0.0071	0.994	2.08E-09	count	1
AL353768.1	17.6832647	2506.037815	0.0071	0.994	2.08E-09	count	1
AL358937.1	17.6832647	2506.037781	0.0071	0.994	2.08E-09	count	1
ACTL7B	17.6832646	2506.037795	0.0071	0.994	2.08E-09	count	1
AL135787.1	17.6832647	2506.037774	0.0071	0.994	2.08E-09	count	1
1-Dec	17.6832646	2506.037739	0.0071	0.994	2.08E-09	count	1
AL161908.1	17.6832647	2506.037698	0.0071	0.994	2.08E-09	count	1
AL360268.2	17.6832647	2506.037801	0.0071	0.994	2.08E-09	count	1
CEL	17.6832648	2506.037877	0.0071	0.994	2.08E-09	count	1
TH	17.6832647	2506.037774	0.0071	0.994	2.08E-09	count	1
RASSF10	17.6832647	2506.037719	0.0071	0.994	2.08E-09	count	1
AC090589.2	17.6832645	2506.03765	0.0071	0.994	2.08E-09	count	1
AP000781.1	17.6832646	2506.037836	0.0071	0.994	2.08E-09	count	1
ZP1	17.6832647	2506.03776	0.0071	0.994	2.08E-09	count	1
KCNK4	17.6832647	2506.037801	0.0071	0.994	2.08E-09	count	1
ARL2-SNX15	17.6832646	2506.037857	0.0071	0.994	2.08E-09	count	1
AP003716.1	17.6832648	2506.037788	0.0071	0.994	2.08E-09	count	1
AP000446.1	17.6832648	2506.037746	0.0071	0.994	2.08E-09	count	1
MMP1	17.6832647	2506.037719	0.0071	0.994	2.08E-09	count	1
AP003063.1	17.6832647	2506.037739	0.0071	0.994	2.08E-09	count	1
NXPE1	17.6832646	2506.037808	0.0071	0.994	2.08E-09	count	1
TMPRSS4	17.6832647	2506.037795	0.0071	0.994	2.08E-09	count	1
AP003393.1	17.6832647	2506.037829	0.0071	0.994	2.08E-09	count	1
AP000866.5	17.6832646	2506.037774	0.0071	0.994	2.08E-09	count	1
HEPACAM	17.6832645	2506.037663	0.0071	0.994	2.08E-09	count	1
AP000842.1	17.6832645	2506.037712	0.0071	0.994	2.08E-09	count	1
KCNJ5	17.6832648	2506.037781	0.0071	0.994	2.08E-09	count	1
C10orf67	17.6832647	2506.037795	0.0071	0.994	2.08E-09	count	1
ANTXRL	17.6832648	2506.037822	0.0071	0.994	2.08E-09	count	1
GPRIN2	17.6832648	2506.037815	0.0071	0.994	2.08E-09	count	1
NPFFR1	17.6832647	2506.037781	0.0071	0.994	2.08E-09	count	1
SFTPD-AS1	17.6832646	2506.03785	0.0071	0.994	2.08E-09	count	1
RGR	17.6832647	2506.03776	0.0071	0.994	2.08E-09	count	1
AL121928.1	17.6832646	2506.037781	0.0071	0.994	2.08E-09	count	1
AL731566.2	17.6832647	2506.037726	0.0071	0.994	2.08E-09	count	1
AL160290.2	17.6832648	2506.037767	0.0071	0.994	2.08E-09	count	1
AL354950.1	17.6832645	2506.037732	0.0071	0.994	2.08E-09	count	1
LINC02398	17.6832647	2506.037753	0.0071	0.994	2.08E-09	count	1
ESPL1	17.6832645	2506.037705	0.0071	0.994	2.08E-09	count	1
AC073896.5	17.6832649	2506.037781	0.0071	0.994	2.08E-09	count	1
MYF5	17.6832645	2506.037746	0.0071	0.994	2.08E-09	count	1

C12orf50	17.6832647	2506.037698	0.0071	0.994	2.08E-09	count	1
AC079907.2	17.6832646	2506.037726	0.0071	0.994	2.08E-09	count	1
AC084880.3	17.6832647	2506.037753	0.0071	0.994	2.08E-09	count	1
AC004263.1	17.6832646	2506.037836	0.0071	0.994	2.08E-09	count	1
MSI1	17.6832647	2506.037795	0.0071	0.994	2.08E-09	count	1
AC026333.4	17.6832647	2506.037739	0.0071	0.994	2.08E-09	count	1
DNAH10OS	17.6832648	2506.037808	0.0071	0.994	2.08E-09	count	1
AC073592.1	17.6832646	2506.037753	0.0071	0.994	2.08E-09	count	1
GJB2	17.6832647	2506.037753	0.0071	0.994	2.08E-09	count	1
USP12-AS2	17.6832645	2506.037753	0.0071	0.994	2.08E-09	count	1
LINC00543	17.6832647	2506.037857	0.0071	0.994	2.08E-09	count	1
VWA8-AS1	17.6832647	2506.037795	0.0071	0.994	2.08E-09	count	1
AL138689.2	17.6832646	2506.037808	0.0071	0.994	2.08E-09	count	1
LINC00551	17.6832648	2506.037815	0.0071	0.994	2.08E-09	count	1
OR4K13	17.6832647	2506.037781	0.0071	0.994	2.08E-09	count	1
LMLN2	17.6832647	2506.03776	0.0071	0.994	2.08E-09	count	1
AL122125.1	17.6832646	2506.037739	0.0071	0.994	2.08E-09	count	1
HIF1A-AS1	17.6832648	2506.037795	0.0071	0.994	2.08E-09	count	1
AL359317.1	17.6832646	2506.037732	0.0071	0.994	2.08E-09	count	1
AL049839.2	17.6832647	2506.037877	0.0071	0.994	2.08E-09	count	1
MKRN3	17.6832647	2506.037795	0.0071	0.994	2.08E-09	count	1
PLA2G4E	17.6832645	2506.037726	0.0071	0.994	2.08E-09	count	1
AC025430.1	17.6832647	2506.037857	0.0071	0.994	2.08E-09	count	1
AC100839.2	17.6832647	2506.037781	0.0071	0.994	2.08E-09	count	1
EWSAT1	17.6832647	2506.037643	0.0071	0.994	2.08E-09	count	1
LINC02204	17.6832646	2506.037739	0.0071	0.994	2.08E-09	count	1
AC010931.3	17.6832648	2506.037808	0.0071	0.994	2.08E-09	count	1
TMEM266	17.6832646	2506.037815	0.0071	0.994	2.08E-09	count	1
AC104758.4	17.6832647	2506.037843	0.0071	0.994	2.08E-09	count	1
SH2D7	17.6832647	2506.037801	0.0071	0.994	2.08E-09	count	1
KIAA1024	17.6832647	2506.037739	0.0071	0.994	2.08E-09	count	1
AC087286.1	17.6832647	2506.03776	0.0071	0.994	2.08E-09	count	1
TTLL13P	17.6832647	2506.037719	0.0071	0.994	2.08E-09	count	1
HS3ST6	17.6832645	2506.037726	0.0071	0.994	2.08E-09	count	1
AC009065.6	17.6832646	2506.037781	0.0071	0.994	2.08E-09	count	1
MEFV	17.6832647	2506.037753	0.0071	0.994	2.08E-09	count	1
NLRC3	17.6832648	2506.037829	0.0071	0.994	2.08E-09	count	1
AC022167.2	17.6832645	2506.037746	0.0071	0.994	2.08E-09	count	1
AF001548.1	17.6832646	2506.037753	0.0071	0.994	2.08E-09	count	1
AC099518.4	17.6832648	2506.037815	0.0071	0.994	2.08E-09	count	1
CHP2	17.6832647	2506.037836	0.0071	0.994	2.08E-09	count	1
LINC01567	17.6832648	2506.037746	0.0071	0.994	2.08E-09	count	1
AC023813.4	17.6832645	2506.037801	0.0071	0.994	2.08E-09	count	1
CES5A	17.6832646	2506.037829	0.0071	0.994	2.08E-09	count	1
KCTD19	17.6832647	2506.037864	0.0071	0.994	2.08E-09	count	1

AC027682.2	17.6832647	2506.037808	0.0071	0.994	2.08E-09	count	1
AC027682.1	17.6832646	2506.037712	0.0071	0.994	2.08E-09	count	1
AC020978.5	17.6832646	2506.037774	0.0071	0.994	2.08E-09	count	1
LINC01572	17.6832646	2506.037753	0.0071	0.994	2.08E-09	count	1
LINC01228	17.6832646	2506.03785	0.0071	0.994	2.08E-09	count	1
AC134312.1	17.6832646	2506.037781	0.0071	0.994	2.08E-09	count	1
AC134312.6	17.6832647	2506.037719	0.0071	0.994	2.08E-09	count	1
AC137932.1	17.6832646	2506.037767	0.0071	0.994	2.08E-09	count	1
AC118754.1	17.6832646	2506.037781	0.0071	0.994	2.08E-09	count	1
AIPL1	17.6832646	2506.037746	0.0071	0.994	2.08E-09	count	1
SLC5A10	17.6832649	2506.037864	0.0071	0.994	2.08E-09	count	1
LINC02094	17.6832647	2506.037691	0.0071	0.994	2.08E-09	count	1
AC130324.3	17.6832646	2506.037795	0.0071	0.994	2.08E-09	count	1
AC099811.4	17.6832647	2506.037843	0.0071	0.994	2.08E-09	count	1
PPY	17.6832647	2506.037781	0.0071	0.994	2.08E-09	count	1
AC091152.2	17.6832647	2506.037788	0.0071	0.994	2.08E-09	count	1
AC068234.3	17.6832646	2506.037719	0.0071	0.994	2.08E-09	count	1
CACNA1G-AS1	17.6832649	2506.037864	0.0071	0.994	2.08E-09	count	1
EFCAB3	17.6832647	2506.037801	0.0071	0.994	2.08E-09	count	1
AC022966.1	17.6832647	2506.037746	0.0071	0.994	2.08E-09	count	1
LINC02078	17.6832647	2506.037698	0.0071	0.994	2.08E-09	count	1
NPTX1	17.6832646	2506.037829	0.0071	0.994	2.08E-09	count	1
AC145207.4	17.6832647	2506.037829	0.0071	0.994	2.08E-09	count	1
AP005482.1	17.6832647	2506.037753	0.0071	0.994	2.08E-09	count	1
HRH4	17.6832646	2506.037739	0.0071	0.994	2.08E-09	count	1
AC006305.1	17.6832647	2506.037836	0.0071	0.994	2.08E-09	count	1
SRXN1	17.6832645	2506.037726	0.0071	0.994	2.08E-09	count	1
AL136531.2	17.6832646	2506.03776	0.0071	0.994	2.08E-09	count	1
NKX2-2	17.6832646	2506.037822	0.0071	0.994	2.08E-09	count	1
GGTLC1	17.6832645	2506.037739	0.0071	0.994	2.08E-09	count	1
XKR7	17.6832647	2506.037753	0.0071	0.994	2.08E-09	count	1
AL035420.3	17.6832647	2506.037788	0.0071	0.994	2.08E-09	count	1
LIME1	17.6832646	2506.037822	0.0071	0.994	2.08E-09	count	1
PRTN3	17.6832646	2506.037739	0.0071	0.994	2.08E-09	count	1
AC011446.1	17.6832646	2506.03776	0.0071	0.994	2.08E-09	count	1
AC123912.4	17.6832646	2506.037719	0.0071	0.994	2.08E-09	count	1
FXYD7	17.6832648	2506.037774	0.0071	0.994	2.08E-09	count	1
AC002511.1	17.6832646	2506.037781	0.0071	0.994	2.08E-09	count	1
MIA-RAB4B	17.6832647	2506.037691	0.0071	0.994	2.08E-09	count	1
PSG2	17.6832647	2506.037864	0.0071	0.994	2.08E-09	count	1
CD177	17.6832647	2506.037677	0.0071	0.994	2.08E-09	count	1
AC005757.1	17.6832647	2506.037808	0.0071	0.994	2.08E-09	count	1
FOXA3	17.6832647	2506.03776	0.0071	0.994	2.08E-09	count	1
AC026803.2	17.6832647	2506.037843	0.0071	0.994	2.08E-09	count	1
AC010522.1	17.6832646	2506.037739	0.0071	0.994	2.08E-09	count	1

AC012313.7	17.6832647	2506.037746	0.0071	0.994	2.08E-09	count	1
Z82246.1	17.6832646	2506.037712	0.0071	0.994	2.08E-09	count	1
AL021707.4	17.6832647	2506.037801	0.0071	0.994	2.08E-09	count	1
AF130359.1	17.6832647	2506.037767	0.0071	0.994	2.08E-09	count	1
IGSF5	17.6832646	2506.037719	0.0071	0.994	2.08E-09	count	1
UBASH3A	17.6832647	2506.03776	0.0071	0.994	2.08E-09	count	1
CENPS-CORT	17.6832646	2506.037753	0.0071	0.994	2.08E-09	count	1
AL109811.4	17.6832646	2506.037705	0.0071	0.994	2.08E-09	count	1
TNFRSF8	17.6832645	2506.037732	0.0071	0.994	2.08E-09	count	1
UBXN10-AS1	17.6832645	2506.037705	0.0071	0.994	2.08E-09	count	1
AC103923.1	17.6832645	2506.037767	0.0071	0.994	2.08E-09	count	1
SPAG17	17.6832646	2506.037788	0.0071	0.994	2.08E-09	count	1
AL355388.2	17.6832647	2506.037843	0.0071	0.994	2.08E-09	count	1
LINC01732	17.6832645	2506.037801	0.0071	0.994	2.08E-09	count	1
AL445470.1	17.6832645	2506.037753	0.0071	0.994	2.08E-09	count	1
AC020594.1	17.6832646	2506.037829	0.0071	0.994	2.08E-09	count	1
AC009229.3	17.6832646	2506.037829	0.0071	0.994	2.08E-09	count	1
AC073082.1	17.6832646	2506.037726	0.0071	0.994	2.08E-09	count	1
BHLHE40-AS1	17.6832644	2506.037753	0.0071	0.994	2.08E-09	count	1
AC034187.1	17.6832645	2506.037739	0.0071	0.994	2.08E-09	count	1
XIRP1	17.6832646	2506.037732	0.0071	0.994	2.08E-09	count	1
XCR1	17.6832646	2506.037739	0.0071	0.994	2.08E-09	count	1
AC096887.1	17.6832645	2506.037691	0.0071	0.994	2.08E-09	count	1
OR5H14	17.6832646	2506.037753	0.0071	0.994	2.08E-09	count	1
AC112503.1	17.6832646	2506.037788	0.0071	0.994	2.08E-09	count	1
GP9	17.6832645	2506.037726	0.0071	0.994	2.08E-09	count	1
AC139887.1	17.6832645	2506.037774	0.0071	0.994	2.08E-09	count	1
AC097515.1	17.6832646	2506.037739	0.0071	0.994	2.08E-09	count	1
GABRG1	17.6832646	2506.037663	0.0071	0.994	2.08E-09	count	1
CSN1S1	17.6832647	2506.037808	0.0071	0.994	2.08E-09	count	1
C4orf36	17.6832645	2506.037753	0.0071	0.994	2.08E-09	count	1
LINC01019	17.6832645	2506.037746	0.0071	0.994	2.08E-09	count	1
AC018754.1	17.6832646	2506.037732	0.0071	0.994	2.08E-09	count	1
AC011365.1	17.6832645	2506.037767	0.0071	0.994	2.08E-09	count	1
AC011389.1	17.6832644	2506.037705	0.0071	0.994	2.08E-09	count	1
AL031118.1	17.6832644	2506.037705	0.0071	0.994	2.08E-09	count	1
MOG	17.6832646	2506.037684	0.0071	0.994	2.08E-09	count	1
C6orf141	17.6832645	2506.037705	0.0071	0.994	2.08E-09	count	1
TFAP2B	17.6832644	2506.037753	0.0071	0.994	2.08E-09	count	1
AL646090.1	17.6832646	2506.037753	0.0071	0.994	2.08E-09	count	1
AL132996.1	17.6832646	2506.037705	0.0071	0.994	2.08E-09	count	1
AL589740.1	17.6832645	2506.037746	0.0071	0.994	2.08E-09	count	1
AL450344.2	17.6832644	2506.037732	0.0071	0.994	2.08E-09	count	1
AC007029.1	17.6832647	2506.037795	0.0071	0.994	2.08E-09	count	1
AC005550.2	17.6832644	2506.037719	0.0071	0.994	2.08E-09	count	1

GRM3	17.6832645	2506.037726	0.0071	0.994	2.08E-09	count	1
AC069294.1	17.6832645	2506.037753	0.0071	0.994	2.08E-09	count	1
LINC01393	17.6832646	2506.037788	0.0071	0.994	2.08E-09	count	1
AC234772.2	17.6832646	2506.03776	0.0071	0.994	2.08E-09	count	1
AF131215.7	17.6832645	2506.037767	0.0071	0.994	2.08E-09	count	1
PBK	17.6832644	2506.03767	0.0071	0.994	2.08E-09	count	1
AC087362.1	17.6832644	2506.037712	0.0071	0.994	2.08E-09	count	1
IDO2	17.6832644	2506.037712	0.0071	0.994	2.08E-09	count	1
AC108860.2	17.6832644	2506.037691	0.0071	0.994	2.08E-09	count	1
AP003550.1	17.6832645	2506.037691	0.0071	0.994	2.08E-09	count	1
CASC11	17.6832644	2506.037726	0.0071	0.994	2.08E-09	count	1
AC067930.2	17.6832644	2506.037691	0.0071	0.994	2.08E-09	count	1
DNAI1	17.6832646	2506.03776	0.0071	0.994	2.08E-09	count	1
AL138752.2	17.6832644	2506.037788	0.0071	0.994	2.08E-09	count	1
LCNL1	17.6832645	2506.037795	0.0071	0.994	2.08E-09	count	1
AC124657.1	17.6832645	2506.03776	0.0071	0.994	2.08E-09	count	1
KRTAP5-7	17.6832644	2506.037788	0.0071	0.994	2.08E-09	count	1
CHRDL2	17.6832645	2506.037726	0.0071	0.994	2.08E-09	count	1
GDPD4	17.6832646	2506.037815	0.0071	0.994	2.08E-09	count	1
AP003086.1	17.6832645	2506.037767	0.0071	0.994	2.08E-09	count	1
C11orf53	17.6832646	2506.037705	0.0071	0.994	2.08E-09	count	1
AP002986.1	17.6832645	2506.03767	0.0071	0.994	2.08E-09	count	1
C11orf44	17.6832646	2506.037788	0.0071	0.994	2.08E-09	count	1
AC007406.4	17.6832645	2506.037753	0.0071	0.994	2.08E-09	count	1
AC005833.1	17.6832646	2506.037726	0.0071	0.994	2.08E-09	count	1
GPR19	17.6832646	2506.037788	0.0071	0.994	2.08E-09	count	1
SP7	17.6832646	2506.03776	0.0071	0.994	2.08E-09	count	1
AC069234.4	17.6832645	2506.037795	0.0071	0.994	2.08E-09	count	1
LBHD2	17.6832647	2506.037795	0.0071	0.994	2.08E-09	count	1
DUOX2	17.6832645	2506.037732	0.0071	0.994	2.08E-09	count	1
AC068722.2	17.6832646	2506.037788	0.0071	0.994	2.08E-09	count	1
AC068870.1	17.6832644	2506.037788	0.0071	0.994	2.08E-09	count	1
AP3B2	17.6832645	2506.037753	0.0071	0.994	2.08E-09	count	1
AC118658.1	17.6832645	2506.03776	0.0071	0.994	2.08E-09	count	1
LMF1-AS1	17.6832645	2506.037774	0.0071	0.994	2.08E-09	count	1
AC012645.4	17.6832645	2506.037712	0.0071	0.994	2.08E-09	count	1
AC007493.1	17.6832645	2506.03776	0.0071	0.994	2.08E-09	count	1
AC134312.3	17.6832645	2506.037732	0.0071	0.994	2.08E-09	count	1
AC132825.3	17.6832645	2506.037691	0.0071	0.994	2.08E-09	count	1
AC061975.1	17.6832645	2506.037691	0.0071	0.994	2.08E-09	count	1
AC138207.5	17.6832646	2506.037781	0.0071	0.994	2.08E-09	count	1
CCL23	17.6832645	2506.037705	0.0071	0.994	2.08E-09	count	1
KRTAP9-6	17.6832645	2506.037732	0.0071	0.994	2.08E-09	count	1
AC015909.4	17.6832645	2506.037795	0.0071	0.994	2.08E-09	count	1
AC015845.1	17.6832646	2506.037732	0.0071	0.994	2.08E-09	count	1

AC004687.1	17.6832646	2506.037781	0.0071	0.994	2.08E-09	count	1
AC116025.1	17.6832647	2506.037788	0.0071	0.994	2.08E-09	count	1
FAM83C-AS1	17.6832646	2506.037726	0.0071	0.994	2.08E-09	count	1
AL354993.1	17.6832646	2506.037705	0.0071	0.994	2.08E-09	count	1
LRRC8E	17.6832646	2506.037767	0.0071	0.994	2.08E-09	count	1
AC010422.6	17.6832647	2506.037836	0.0071	0.994	2.08E-09	count	1
AC138474.1	17.6832645	2506.037726	0.0071	0.994	2.08E-09	count	1
AD000671.3	17.6832645	2506.037808	0.0071	0.994	2.08E-09	count	1
AC007785.3	17.6832645	2506.037732	0.0071	0.994	2.08E-09	count	1
AC010524.1	17.6832644	2506.037719	0.0071	0.994	2.08E-09	count	1
SRY	17.6832645	2506.037746	0.0071	0.994	2.08E-09	count	1
CCT8L2	17.6832646	2506.037753	0.0071	0.994	2.08E-09	count	1
Z99774.1	17.6832645	2506.037726	0.0071	0.994	2.08E-09	count	1
LINC01422	17.6832645	2506.037684	0.0071	0.994	2.08E-09	count	1
AL049536.1	17.6832644	2506.037712	0.0071	0.994	2.08E-09	count	1
BX324167.1	17.6832645	2506.037677	0.0071	0.994	2.08E-09	count	1
AP001056.2	17.6832644	2506.037691	0.0071	0.994	2.08E-09	count	1
AF064858.2	17.6832644	2506.037643	0.0071	0.994	2.08E-09	count	1
AC084757.3	0.0792323	0.6534026	0.1213	0.903	2.31E-09	count	1
AC112487.1	0.2325855	1.261577	0.1844	0.854	2.39E-09	count	1
ELOVL3	0.2325855	1.261577	0.1844	0.854	2.39E-09	count	1
SLC4A8	0.2325855	1.261577	0.1844	0.854	2.39E-09	count	1
AC004257.1	0.2325855	1.261577	0.1844	0.854	2.39E-09	count	1
AC084782.1	0.2325855	1.261577	0.1844	0.854	2.39E-09	count	1
PPP4R4	0.2325855	0.9947438	0.2338	0.815	2.39E-09	count	1
AL031775.1	0.2325855	0.9967705	0.2333	0.816	2.39E-09	count	1
AC009005.1	0.2325855	1.261577	0.1844	0.854	2.39E-09	count	1
LINC02181	0.2325855	0.9947438	0.2338	0.815	2.39E-09	count	1
AL365181.1	0.2325855	0.9947438	0.2338	0.815	2.39E-09	count	1
CD79A	0.2325855	0.9947438	0.2338	0.815	2.39E-09	count	1
GAGE10	0.2325855	0.9947438	0.2338	0.815	2.39E-09	count	1
AC010273.2	0.2325855	0.9967705	0.2333	0.816	2.39E-09	count	1
AC006012.1	0.2325855	0.9947438	0.2338	0.815	2.39E-09	count	1
AL451062.3	0.2325855	0.9947438	0.2338	0.815	2.39E-09	count	1
AL121906.2	0.2325855	0.9967705	0.2333	0.816	2.39E-09	count	1
XRCC2	0.2325855	0.9947438	0.2338	0.815	2.39E-09	count	1
GYG2	0.2325855	0.9947438	0.2338	0.815	2.39E-09	count	1
AP006287.2	0.2325855	0.9947438	0.2338	0.815	2.39E-09	count	1
STXBP2	0.2325855	0.9947438	0.2338	0.815	2.39E-09	count	1
JAKMIP3	0.2325855	0.9947438	0.2338	0.815	2.39E-09	count	1
DMRT3	0.2325855	0.9967705	0.2333	0.816	2.39E-09	count	1
AC126614.1	0.2325855	0.9947438	0.2338	0.815	2.39E-09	count	1
PIK3AP1	0.2325855	0.9947438	0.2338	0.815	2.39E-09	count	1
AC107081.2	0.2325855	0.9947438	0.2338	0.815	2.39E-09	count	1
AC018557.1	0.2325855	0.9967705	0.2333	0.816	2.39E-09	count	1

LINC00842	0.2325855	0.9947438	0.2338	0.815	2.39E-09	count	1
AC112907.3	0.2325855	0.9947438	0.2338	0.815	2.39E-09	count	1
AC016745.2	0.7556675	1.1192119	0.6752	0.5	2.45E-09	count	1
KCNA1	0.7556675	0.8954832	0.8439	0.399	2.45E-09	count	1
TAF7L	0.7556675	0.7592876	0.9952	0.32	2.46E-09	count	1
RNF165	0.7556675	0.7592876	0.9952	0.32	2.46E-09	count	1
NPHP3-AS1	0.7556675	0.7592876	0.9952	0.32	2.46E-09	count	1
SCNN1D	0.2921547	0.6962087	0.4196	0.675	2.95E-09	count	1
AC107375.1	0.2921547	0.6962087	0.4196	0.675	2.95E-09	count	1
AL139398.1	0.1093249	0.6074612	0.18	0.857	3.16E-09	count	1
ZNF215	0.1201131	0.5094821	0.2358	0.814	3.46E-09	count	1
AC018645.2	0.1201131	0.5084862	0.2362	0.813	3.46E-09	count	1
CCDC178	0.3502024	0.780461	0.4487	0.654	3.48E-09	count	1
AC119674.1	0.3502024	0.6472722	0.541	0.589	3.48E-09	count	1
IGFLR1	0.1406114	0.5534234	0.2541	0.799	4.02E-09	count	1
C1orf220	0.4741363	0.8264256	0.5737	0.566	4.54E-09	count	1
LDHC	0.4855053	0.8960811	0.5418	0.588	4.64E-09	count	1
AC012640.1	0.4855053	0.8960811	0.5418	0.588	4.64E-09	count	1
TNFRSF18	0.4855053	0.8960811	0.5418	0.588	4.64E-09	count	1
C8orf34-AS1	0.5043531	0.8500337	0.5933	0.553	4.79E-09	count	1
GOLGA8Q	0.5043531	0.8829945	0.5712	0.568	4.79E-09	count	1
OSBPL10-AS1	0.5243956	0.6673942	0.7857	0.432	4.95E-09	count	1
LRRN2	0.53322	0.6661911	0.8004	0.424	5.02E-09	count	1
AC012358.1	0.573346	1.0059935	0.5699	0.569	5.34E-09	count	1
AC109454.2	0.573346	0.6108122	0.9387	0.348	5.34E-09	count	1
AC005046.1	0.5798871	0.9117136	0.636	0.525	5.39E-09	count	1
AC083829.2	0.5798871	0.9577211	0.6055	0.545	5.39E-09	count	1
AC113349.2	0.5798871	0.9577211	0.6055	0.545	5.39E-09	count	1
AL132656.2	0.5798871	0.9117136	0.636	0.525	5.39E-09	count	1
AL009176.1	0.5798871	0.9577211	0.6055	0.545	5.39E-09	count	1
NAGPA-AS1	0.5798871	0.9577211	0.6055	0.545	5.39E-09	count	1
GCC2-AS1	0.5798871	0.9577211	0.6055	0.545	5.39E-09	count	1
ACP7	18.3774856	3277.447611	0.0056	0.996	5.61E-09	count	1
AC007998.3	18.2594744	3089.654953	0.0059	0.995	5.61E-09	count	1
LTF	18.1250491	2888.789923	0.0063	0.995	5.61E-09	count	1
ANKRD20A4	18.2573883	2376.774573	0.0077	0.994	5.61E-09	count	1
GNA14-AS1	17.7848644	2436.945185	0.0073	0.994	5.62E-09	count	1
TMX2-CTNND1	17.7848644	2436.945185	0.0073	0.994	5.62E-09	count	1
AL031595.2	17.7832305	2007.054752	0.0089	0.993	5.62E-09	count	1
TMEM184A	17.559039	2176.749364	0.0081	0.994	5.62E-09	count	1
AC116096.1	17.5590385	2176.74928	0.0081	0.994	5.62E-09	count	1
PACSIN1	18.6398744	4043.083036	0.0046	0.996	5.63E-09	count	1
C7orf61	18.6398746	4043.083086	0.0046	0.996	5.63E-09	count	1
CD40LG	18.6398744	4043.083384	0.0046	0.996	5.63E-09	count	1
SHISA9	18.6398741	4043.0834	0.0046	0.996	5.63E-09	count	1

ZNF536	18.6398741	4043.0834	0.0046	0.996	5.63E-09	count	1
NOD2	18.639007	3511.166017	0.0053	0.996	5.63E-09	count	1
AC073283.1	18.6390067	3511.166103	0.0053	0.996	5.63E-09	count	1
AC005393.1	18.6390069	3511.165773	0.0053	0.996	5.63E-09	count	1
CXCL13	18.0330336	2820.803575	0.0064	0.995	5.63E-09	count	1
TTC39A	18.4845575	3740.986243	0.0049	0.996	5.63E-09	count	1
C1orf143	18.4845575	3740.986243	0.0049	0.996	5.63E-09	count	1
AC099508.1	18.4845577	3740.986013	0.0049	0.996	5.63E-09	count	1
AP001020.3	18.4845575	3740.986243	0.0049	0.996	5.63E-09	count	1
AC011337.1	18.4845574	3740.986365	0.0049	0.996	5.63E-09	count	1
LRRC74B	18.4845572	3740.986105	0.0049	0.996	5.63E-09	count	1
AC008121.2	18.484557	3740.986151	0.0049	0.996	5.63E-09	count	1
SYS1-DBNDD2	18.484557	3740.986151	0.0049	0.996	5.63E-09	count	1
LINC01372	18.6381202	2997.265084	0.0062	0.995	5.63E-09	count	1
NEFM	18.0315675	2269.017112	0.0079	0.994	5.63E-09	count	1
PLIN1	18.0309607	2108.559905	0.0086	0.993	5.64E-09	count	1
SYNJ2BP-COX16	18.0308661	2108.544521	0.0086	0.993	5.64E-09	count	1
DOK3	18.4835792	3177.014559	0.0058	0.995	5.64E-09	count	1
AL391988.1	18.3007573	3412.514095	0.0054	0.996	5.64E-09	count	1
Z69720.1	18.3007573	3412.514095	0.0054	0.996	5.64E-09	count	1
KRT23	18.3007573	3412.514095	0.0054	0.996	5.64E-09	count	1
AC104452.1	18.3007572	3412.513955	0.0054	0.996	5.64E-09	count	1
XIST	18.3007571	3412.513677	0.0054	0.996	5.64E-09	count	1
LINC00958	18.3007571	3412.513677	0.0054	0.996	5.64E-09	count	1
HFM1	18.0300013	1935.025237	0.0093	0.993	5.64E-09	count	1
IGFL2	17.8457773	2581.051395	0.0069	0.994	5.64E-09	count	1
AL162457.2	18.9931353	1518.128306	0.0125	0.99	5.64E-09	count	1
AC145343.1	18.3067323	2466.086329	0.0074	0.994	5.64E-09	count	1
LHCGR	18.7697189	1700.404407	0.011	0.991	5.64E-09	count	1
NTRK1	18.2997083	2811.843358	0.0065	0.995	5.64E-09	count	1
LYPD2	18.2997085	2811.843243	0.0065	0.995	5.64E-09	count	1
SV2B	17.8446986	2181.792203	0.0082	0.993	5.64E-09	count	1
AC116351.1	17.8446779	2181.812907	0.0082	0.993	5.64E-09	count	1
FSIP2-AS1	17.8446288	2181.796022	0.0082	0.993	5.64E-09	count	1
ZIC4	18.2992969	2457.780568	0.0074	0.994	5.64E-09	count	1
AC010834.3	18.0758466	3049.549378	0.0059	0.995	5.64E-09	count	1
AC138028.6	18.0758465	3049.549254	0.0059	0.995	5.64E-09	count	1
AL391069.3	17.8439048	1951.949475	0.0091	0.993	5.64E-09	count	1
CTNND2	17.8438505	1951.925751	0.0091	0.993	5.64E-09	count	1
IGFN1	18.2987251	2259.756603	0.0081	0.994	5.64E-09	count	1
DRD4	18.2987249	2259.756539	0.0081	0.994	5.64E-09	count	1
AC233263.6	18.2987244	2259.756438	0.0081	0.994	5.64E-09	count	1
AL121894.1	18.4809191	1756.993522	0.0105	0.992	5.64E-09	count	1
AC015727.1	18.1617105	2625.327689	0.0069	0.994	5.64E-09	count	1
CDH4	18.4803516	1519.935821	0.0122	0.99	5.64E-09	count	1

HTRA4	18.4803511	1519.93597	0.0122	0.99	5.64E-09	count	1
AC023794.4	18.0747844	2408.821746	0.0075	0.994	5.64E-09	count	1
MYO5C	18.0747841	2408.821736	0.0075	0.994	5.64E-09	count	1
CAGE1	18.0747841	2408.821736	0.0075	0.994	5.64E-09	count	1
GLYATL2	18.297869	1800.629365	0.0102	0.992	5.64E-09	count	1
DOCK3	18.297869	1800.629218	0.0102	0.992	5.64E-09	count	1
PSG5	17.6110657	2312.664472	0.0076	0.994	5.65E-09	count	1
AC007529.2	18.2972559	1520.169728	0.012	0.99	5.65E-09	count	1
RXFP2	18.2972558	1520.169653	0.012	0.99	5.65E-09	count	1
GRIN2C	18.1604563	1985.758548	0.0091	0.993	5.65E-09	count	1
LINC02518	18.0738632	1863.934274	0.0097	0.992	5.65E-09	count	1
AL392046.1	18.0738629	1863.934168	0.0097	0.992	5.65E-09	count	1
PPT2-EGFL8	18.0738628	1863.934221	0.0097	0.992	5.65E-09	count	1
YBX2	18.0738625	1863.934183	0.0097	0.992	5.65E-09	count	1
ANKHD1-EIF4EBP3	18.0738626	1863.934092	0.0097	0.992	5.65E-09	count	1
AL034397.3	17.7861415	2638.317113	0.0067	0.995	5.65E-09	count	1
SPDYE3	17.7861415	2638.317119	0.0067	0.995	5.65E-09	count	1
TEKT2	17.7861414	2638.317097	0.0067	0.995	5.65E-09	count	1
AC021086.1	17.7861412	2638.31706	0.0067	0.995	5.65E-09	count	1
PCDHA11	17.7861414	2638.317113	0.0067	0.995	5.65E-09	count	1
AC005486.1	17.7861412	2638.31706	0.0067	0.995	5.65E-09	count	1
MAP3K9	17.7861413	2638.317087	0.0067	0.995	5.65E-09	count	1
ZNF32-AS2	17.7861412	2638.317129	0.0067	0.995	5.65E-09	count	1
P2RY12	17.6109407	2312.645203	0.0076	0.994	5.65E-09	count	1
AP002807.1	18.456213	2296.620311	0.008	0.994	5.65E-09	count	1
CLEC1A	17.6102641	1973.420137	0.0089	0.993	5.65E-09	count	1
AC090136.3	17.610279	1973.419819	0.0089	0.993	5.65E-09	count	1
MEX3A	17.6103041	1973.432825	0.0089	0.993	5.65E-09	count	1
AC024243.1	17.6102419	1973.421065	0.0089	0.993	5.65E-09	count	1
AC068733.1	17.6102298	1973.400766	0.0089	0.993	5.65E-09	count	1
AC120498.10	17.610274	1973.405995	0.0089	0.993	5.65E-09	count	1
KCNT1	17.6102355	1973.414505	0.0089	0.993	5.65E-09	count	1
MST1R	17.6102621	1973.42029	0.0089	0.993	5.65E-09	count	1
AL359317.2	17.6102579	1973.413547	0.0089	0.993	5.65E-09	count	1
AC103746.1	18.0732041	1520.3005	0.0119	0.991	5.65E-09	count	1
AP000962.1	18.0732041	1520.300481	0.0119	0.991	5.65E-09	count	1
LRRTM1	18.0732038	1520.300469	0.0119	0.991	5.65E-09	count	1
SERPINB2	18.0732038	1520.300481	0.0119	0.991	5.65E-09	count	1
AC009802.1	17.6102376	1973.414331	0.0089	0.993	5.65E-09	count	1
AC022893.1	18.2506818	2346.888515	0.0078	0.994	5.65E-09	count	1
TNFRSF9	17.7851519	1964.66618	0.0091	0.993	5.65E-09	count	1
AC100858.3	17.7851518	1964.666228	0.0091	0.993	5.65E-09	count	1
AC006946.2	17.7851519	1964.666192	0.0091	0.993	5.65E-09	count	1
Z97353.2	17.785152	1964.666216	0.0091	0.993	5.65E-09	count	1
AC093151.2	17.7851518	1964.666192	0.0091	0.993	5.65E-09	count	1

AC114939.1	17.7851518	1964.666224	0.0091	0.993	5.65E-09	count	1
AC009120.5	17.785152	1964.666308	0.0091	0.993	5.65E-09	count	1
SLC37A2	17.7851517	1964.666196	0.0091	0.993	5.65E-09	count	1
CCDC194	18.0739081	3004.747805	0.006	0.995	5.65E-09	count	1
OR56B1	18.073913	3004.73424	0.006	0.995	5.65E-09	count	1
LUCAT1	18.07388	3004.718305	0.006	0.995	5.65E-09	count	1
AC011997.1	17.9864123	2341.218246	0.0077	0.994	5.65E-09	count	1
AC004466.1	17.9860224	1997.903278	0.009	0.993	5.65E-09	count	1
TMEM200C	18.2503805	2002.751078	0.0091	0.993	5.65E-09	count	1
AC000403.1	18.2504028	2002.755123	0.0091	0.993	5.65E-09	count	1
AL359715.1	17.9859811	1997.903991	0.009	0.993	5.65E-09	count	1
TSPAN33	18.2503808	2002.755371	0.0091	0.993	5.65E-09	count	1
SLC14A2-AS1	18.3072979	1991.657708	0.0092	0.993	5.65E-09	count	1
AP000851.2	17.9859775	1997.894134	0.009	0.993	5.65E-09	count	1
AL596325.1	18.3072675	1991.656137	0.0092	0.993	5.65E-09	count	1
SLC10A6	17.9859421	1997.899634	0.009	0.993	5.65E-09	count	1
AC008549.2	18.0733368	2381.020616	0.0076	0.994	5.65E-09	count	1
CHGB	18.0733171	2381.014353	0.0076	0.994	5.65E-09	count	1
AC100814.1	18.0732957	2380.999478	0.0076	0.994	5.65E-09	count	1
AC024575.1	18.073296	2381.001607	0.0076	0.994	5.65E-09	count	1
AC006064.2	18.0729318	1997.641004	0.009	0.993	5.65E-09	count	1
MEG9	18.0729079	1997.644725	0.009	0.993	5.65E-09	count	1
EME1	18.0728926	1997.625027	0.009	0.993	5.65E-09	count	1
MCTP1	18.0728934	1997.646608	0.009	0.993	5.65E-09	count	1
HSD17B13	18.0728786	1997.644875	0.009	0.993	5.65E-09	count	1
VSIG10L2	18.072881	1997.641268	0.009	0.993	5.65E-09	count	1
COL11A2	17.7618829	2557.261943	0.0069	0.994	5.65E-09	count	1
AC021188.1	17.7618742	2557.249398	0.0069	0.994	5.65E-09	count	1
AL162377.3	17.761897	2557.254163	0.0069	0.994	5.65E-09	count	1
ADGRG3	17.7618976	2557.249104	0.0069	0.994	5.65E-09	count	1
AC108058.1	17.7615388	2002.84211	0.0089	0.993	5.66E-09	count	1
SMUG1-AS1	17.2917524	1998.158836	0.0087	0.993	5.66E-09	count	1
AC002310.2	17.2917498	1998.148918	0.0087	0.993	5.66E-09	count	1
AL357078.3	17.7615566	2002.847918	0.0089	0.993	5.66E-09	count	1
AC015802.4	17.2917422	1998.148989	0.0087	0.993	5.66E-09	count	1
LINC01798	17.2917415	1998.156537	0.0087	0.993	5.66E-09	count	1
CFAP206	17.2917536	1998.158779	0.0087	0.993	5.66E-09	count	1
AC099560.1	17.2917406	1998.151565	0.0087	0.993	5.66E-09	count	1
NUF2	17.7615305	2002.83624	0.0089	0.993	5.66E-09	count	1
DNA2	17.7615173	2002.844281	0.0089	0.993	5.66E-09	count	1
AL161431.1	17.2917527	1998.156308	0.0087	0.993	5.66E-09	count	1
ASB15	17.2917533	1998.161307	0.0087	0.993	5.66E-09	count	1
AL512506.3	17.2917405	1998.149072	0.0087	0.993	5.66E-09	count	1
UPB1	17.2917526	1998.156317	0.0087	0.993	5.66E-09	count	1
ACY1	17.7615095	2002.844317	0.0089	0.993	5.66E-09	count	1

LINC01351	17.7615474	2002.847985	0.0089	0.993	5.66E-09	count	1
AL109976.1	17.2917532	1998.158792	0.0087	0.993	5.66E-09	count	1
ACSL6	17.2917299	1998.154282	0.0087	0.993	5.66E-09	count	1
ZBED9	17.2917516	1998.161373	0.0087	0.993	5.66E-09	count	1
RB1-DT	17.2917307	1998.151732	0.0087	0.993	5.66E-09	count	1
ATP11A-AS1	17.2917307	1998.156757	0.0087	0.993	5.66E-09	count	1
SPINT1-AS1	17.2917307	1998.156757	0.0087	0.993	5.66E-09	count	1
PEAK3	17.7615036	2002.826547	0.0089	0.993	5.66E-09	count	1
GVQW2	17.7615345	2002.832209	0.0089	0.993	5.66E-09	count	1
PCDHA6	17.2917409	1998.154057	0.0087	0.993	5.66E-09	count	1
SMTNL2	17.2917387	1998.144144	0.0087	0.993	5.66E-09	count	1
NPIPB6	17.7615053	2002.828501	0.0089	0.993	5.66E-09	count	1
LINC01209	17.2917624	1998.163659	0.0087	0.993	5.66E-09	count	1
ROPN1L	17.2917422	1998.151503	0.0087	0.993	5.66E-09	count	1
KLHL41	17.7615262	2002.846195	0.0089	0.993	5.66E-09	count	1
FOXA1	17.2917107	1998.159606	0.0087	0.993	5.66E-09	count	1
IL17RB	17.2917528	1998.156304	0.0087	0.993	5.66E-09	count	1
AC132938.3	17.2917409	1998.14905	0.0087	0.993	5.66E-09	count	1
AC007786.2	17.2917499	1998.158929	0.0087	0.993	5.66E-09	count	1
ADGRG6	17.7615387	2002.846075	0.0089	0.993	5.66E-09	count	1
AL358790.1	17.2917145	1998.161958	0.0087	0.993	5.66E-09	count	1
AC090061.1	17.2917301	1998.15677	0.0087	0.993	5.66E-09	count	1
AC025423.1	17.2917499	1998.151429	0.0087	0.993	5.66E-09	count	1
LINC01843	17.2917105	1998.154603	0.0087	0.993	5.66E-09	count	1
SMILR	17.291743	1998.151464	0.0087	0.993	5.66E-09	count	1
LINC01909	17.291731	1998.149226	0.0087	0.993	5.66E-09	count	1
AC093157.2	17.2917118	1998.159562	0.0087	0.993	5.66E-09	count	1
AC007494.2	17.2917308	1998.154238	0.0087	0.993	5.66E-09	count	1
AP001432.1	17.2917024	1998.147191	0.0087	0.993	5.66E-09	count	1
AC007448.3	17.2917312	1998.154225	0.0087	0.993	5.66E-09	count	1
LINC02029	17.2917214	1998.14687	0.0087	0.993	5.66E-09	count	1
AC006115.1	17.2917528	1998.166336	0.0087	0.993	5.66E-09	count	1
AC016876.3	17.2917386	1998.146641	0.0087	0.993	5.66E-09	count	1
ASCL1	17.2917141	1998.159465	0.0087	0.993	5.66E-09	count	1
AC008747.1	17.2917032	1998.159676	0.0087	0.993	5.66E-09	count	1
ELAVL4	17.2917207	1998.161931	0.0087	0.993	5.66E-09	count	1
AL645504.1	17.2917002	1998.144768	0.0087	0.993	5.66E-09	count	1
LINC00598	17.7614936	2002.832559	0.0089	0.993	5.66E-09	count	1
MYL4	17.2917096	1998.149644	0.0087	0.993	5.66E-09	count	1
NUP62CL	17.291713	1998.154497	0.0087	0.993	5.66E-09	count	1
AC019080.4	17.2917515	1998.156361	0.0087	0.993	5.66E-09	count	1
AC064807.2	17.2917123	1998.154528	0.0087	0.993	5.66E-09	count	1
AL353803.4	17.2917333	1998.15914	0.0087	0.993	5.66E-09	count	1
AC068305.2	17.2917199	1998.149437	0.0087	0.993	5.66E-09	count	1
HIST1H4B	17.761496	2002.842491	0.0089	0.993	5.66E-09	count	1

AC079465.1	17.29173	1998.154269	0.0087	0.993	5.66E-09	count	1
TMEM35B	17.7615112	2002.840338	0.0089	0.993	5.66E-09	count	1
AL354872.2	17.2917422	1998.1465	0.0087	0.993	5.66E-09	count	1
FER1L5	17.2917032	1998.154664	0.0087	0.993	5.66E-09	count	1
JPH4	17.2917237	1998.156796	0.0087	0.993	5.66E-09	count	1
SEPT4-AS1	17.2917124	1998.159535	0.0087	0.993	5.66E-09	count	1
NOL4	17.2917109	1998.157095	0.0087	0.993	5.66E-09	count	1
CDC20	17.7615125	2002.83045	0.0089	0.993	5.66E-09	count	1
ASB9	17.2917232	1998.16183	0.0087	0.993	5.66E-09	count	1
AC080112.1	17.2917127	1998.164525	0.0087	0.993	5.66E-09	count	1
AC023509.3	17.2917307	1998.154255	0.0087	0.993	5.66E-09	count	1
DDX39B-AS1	17.2917238	1998.161813	0.0087	0.993	5.66E-09	count	1
AC093797.1	17.2917218	1998.154365	0.0087	0.993	5.66E-09	count	1
AP000919.4	17.2917191	1998.149481	0.0087	0.993	5.66E-09	count	1
AP003392.1	17.2917124	1998.15954	0.0087	0.993	5.66E-09	count	1
SRRM2-AS1	17.2917332	1998.156647	0.0087	0.993	5.66E-09	count	1
AL732314.4	17.2916916	1998.147402	0.0087	0.993	5.66E-09	count	1
AL138976.2	17.2917316	1998.154211	0.0087	0.993	5.66E-09	count	1
LINC00484	17.2917224	1998.151842	0.0087	0.993	5.66E-09	count	1
LINC01797	17.2917223	1998.151842	0.0087	0.993	5.66E-09	count	1
LINC01679	17.2916927	1998.149855	0.0087	0.993	5.66E-09	count	1
AC009034.1	17.7615026	2002.828554	0.0089	0.993	5.66E-09	count	1
IGLV5-52	17.2916893	1998.144992	0.0087	0.993	5.66E-09	count	1
ENDOU	17.7614943	2002.824646	0.0089	0.993	5.66E-09	count	1
AC010680.5	17.2917292	1998.149292	0.0087	0.993	5.66E-09	count	1
RPS6KB2-AS1	17.2916922	1998.147384	0.0087	0.993	5.66E-09	count	1
PITX2	17.2917305	1998.159267	0.0087	0.993	5.66E-09	count	1
LINC01415	17.2917316	1998.161716	0.0087	0.993	5.66E-09	count	1
SLC10A5	17.2917119	1998.154541	0.0087	0.993	5.66E-09	count	1
AC104984.6	17.2917313	1998.159223	0.0087	0.993	5.66E-09	count	1
AC022075.1	17.7614892	2002.838575	0.0089	0.993	5.66E-09	count	1
ESR2	17.7614747	2002.826773	0.0089	0.993	5.66E-09	count	1
AC025265.1	17.2916992	1998.149828	0.0087	0.993	5.66E-09	count	1
SLC15A2	17.2917029	1998.154677	0.0087	0.993	5.66E-09	count	1
AC090515.4	17.291721	1998.14439	0.0087	0.993	5.66E-09	count	1
AC005856.1	17.2916922	1998.142372	0.0087	0.993	5.66E-09	count	1
DAND5	17.2917033	1998.159667	0.0087	0.993	5.66E-09	count	1
ERVMER61-1	17.2916919	1998.144878	0.0087	0.993	5.66E-09	count	1
VSTM2A	17.291693	1998.149846	0.0087	0.993	5.66E-09	count	1
LINC00513	0.2045122	0.3681196	0.5556	0.579	5.75E-09	count	1
DLK2	0.6699741	1.0809014	0.6198	0.535	6.07E-09	count	1
AC117382.2	0.6699741	1.1228825	0.5967	0.551	6.07E-09	count	1
PARD3-AS1	0.6699741	1.1228825	0.5967	0.551	6.07E-09	count	1
UCKL1-AS1	0.6699741	1.0809014	0.6198	0.535	6.07E-09	count	1
AC092718.1	0.6699741	0.9272541	0.7225	0.47	6.07E-09	count	1

FBLL1	0.6699741	0.9272541	0.7225	0.47	6.07E-09	count	1
GPR174	0.6699741	0.9272541	0.7225	0.47	6.07E-09	count	1
ARHGAP11B	0.6699741	0.9272541	0.7225	0.47	6.07E-09	count	1
DSC2	0.6699741	0.9272541	0.7225	0.47	6.07E-09	count	1
AC127024.5	0.6699741	0.9272541	0.7225	0.47	6.07E-09	count	1
CLEC4E	0.6699741	0.9272541	0.7225	0.47	6.07E-09	count	1
AC004923.4	0.6699741	0.9272541	0.7225	0.47	6.07E-09	count	1
AL354977.2	0.6699741	0.9272541	0.7225	0.47	6.07E-09	count	1
Z99289.3	0.2325855	0.5739893	0.4052	0.685	6.49E-09	count	1
SLC13A4	0.7550353	1.3518219	0.5585	0.577	6.67E-09	count	1
AC087430.1	0.7550353	1.3518219	0.5585	0.577	6.67E-09	count	1
CX3CR1	0.7550353	1.2165704	0.6206	0.535	6.67E-09	count	1
AC087071.1	0.2518823	0.5957537	0.4228	0.672	6.99E-09	count	1
WNT3	0.8320094	1.0330409	0.8054	0.421	7.18E-09	count	1
Z69706.1	0.9056853	1.2727904	0.7116	0.477	7.65E-09	count	1
AC080188.1	0.9056853	0.9139171	0.991	0.322	7.65E-09	count	1
TKTL1	0.9657591	0.8182782	1.1802	0.238	8.01E-09	count	1
ANKRD34C	0.9657591	1.1092708	0.8706	0.384	8.01E-09	count	1
AL117339.4	0.9761016	1.1812369	0.8263	0.409	8.08E-09	count	1
DCDC1	0.9761016	0.9959903	0.98	0.327	8.08E-09	count	1
AC008763.2	0.9761016	0.9959903	0.98	0.327	8.08E-09	count	1
AC009159.3	0.9761016	1.0926466	0.8933	0.372	8.08E-09	count	1
Z99289.1	0.9761016	1.0926466	0.8933	0.372	8.08E-09	count	1
CXorf21	1.0433496	1.2210385	0.8545	0.393	8.46E-09	count	1
AC004908.3	1.0433496	1.2210385	0.8545	0.393	8.46E-09	count	1
CPB2-AS1	1.0433496	1.0077399	1.0353	0.301	8.46E-09	count	1
ADAM20	1.0433496	1.0077399	1.0353	0.301	8.46E-09	count	1
AL512604.3	1.0935258	1.057704	1.0339	0.301	8.74E-09	count	1
BDH1	1.0935258	0.8521515	1.2833	0.199	8.74E-09	count	1
GPR173	0.3213738	0.5639853	0.5698	0.569	8.74E-09	count	1
LIN37	1.1535831	0.8849726	1.3035	0.192	9.06E-09	count	1
LINC00545	1.1535831	0.8849726	1.3035	0.192	9.06E-09	count	1
TBC1D3L	1.1611326	1.2698741	0.9144	0.361	9.09E-09	count	1
STMN2	0.3407593	0.6259968	0.5443	0.586	9.22E-09	count	1
TRIM17	1.1956362	0.8565988	1.3958	0.163	9.27E-09	count	1
AC110048.2	1.2112041	1.5933951	0.7601	0.447	9.34E-09	count	1
AL158071.4	1.2112041	1.5933951	0.7601	0.447	9.34E-09	count	1
AC012313.1	1.2112041	1.2785822	0.9473	0.344	9.35E-09	count	1
AL354732.1	1.2112041	0.8650259	1.4002	0.162	9.35E-09	count	1
NYAP1	1.2112041	1.0302444	1.1756	0.24	9.35E-09	count	1
AL355916.1	1.2501532	0.9232185	1.3541	0.176	9.54E-09	count	1
AC027020.2	1.2664932	0.9398485	1.3476	0.178	9.62E-09	count	1
AC005696.1	1.2664932	0.7999455	1.5832	0.113	9.62E-09	count	1
ZNF804A	1.2664932	0.7198691	1.7593	0.0786	9.62E-09	count	1
NIPAL1	1.2862717	1.2268155	1.0485	0.294	9.70E-09	count	1

RHPN1	1.3531987	0.942626	1.4356	0.151	1.00E-08	count	1
AC008914.1	1.3531987	0.8649299	1.5645	0.118	1.00E-08	count	1
CILP2	0.375814	0.5297954	0.7094	0.478	1.01E-08	count	1
AC239868.3	1.4018921	1.0334168	1.3566	0.175	1.02E-08	count	1
LINC02210-CRHR1	0.3877741	0.515371	0.7524	0.452	1.04E-08	count	1
SLC25A18	1.4488147	1.408015	1.029	0.304	1.04E-08	count	1
CORIN	0.3996647	0.5604569	0.7131	0.476	1.06E-08	count	1
AFF2	1.5200873	0.9290075	1.6362	0.102	1.07E-08	count	1
LRRTM2	0.414868	0.9843027	0.4215	0.673	1.10E-08	count	1
SOD2-OT1	1.6029654	1.4938207	1.0731	0.283	1.10E-08	count	1
LINC01118	1.7364968	1.275089	1.3619	0.173	1.15E-08	count	1
CERKL	1.8542798	1.652979	1.1218	0.262	1.18E-08	count	1
SIRPB1	1.9596403	1.7273144	1.1345	0.257	1.21E-08	count	1
AC009948.3	1.9596403	1.4266604	1.3736	0.17	1.21E-08	count	1
S100P	1.9596403	1.0690577	1.8331	0.0669	1.22E-08	count	1
HLA-DQA2	0.5206474	0.1904248	2.7341	0.0063	1.34E-08	count	1
RAB40AL	17.4823145	2094.870082	0.0083	0.993	1.52E-08	count	1
GSTM1	17.8134066	1799.249177	0.0099	0.992	1.52E-08	count	1
MMP3	17.4409628	2081.778825	0.0084	0.993	1.53E-08	count	1
CFAP161	18.0929958	3075.810695	0.0059	0.995	1.53E-08	count	1
CYP4Z1	17.8924919	2782.407442	0.0064	0.995	1.53E-08	count	1
LINC02033	17.8924919	2782.407442	0.0064	0.995	1.53E-08	count	1
ZNF559-ZNF177	17.8924919	2782.407442	0.0064	0.995	1.53E-08	count	1
SLCO6A1	18.1793658	2955.772972	0.0062	0.995	1.53E-08	count	1
AC084782.2	18.1793657	2955.773026	0.0062	0.995	1.53E-08	count	1
INPP5D	18.0925088	2546.203835	0.0071	0.994	1.53E-08	count	1
PSG4	18.0925085	2546.203635	0.0071	0.994	1.53E-08	count	1
AL117190.1	17.9971007	2381.155166	0.0076	0.994	1.53E-08	count	1
AC005154.6	18.0011734	2276.468857	0.0079	0.994	1.53E-08	count	1
TFAP2C	18.2586889	2850.107273	0.0064	0.995	1.53E-08	count	1
SLC5A2	17.8915684	2249.013605	0.008	0.994	1.53E-08	count	1
TCHH	18.1787147	2035.912596	0.0089	0.993	1.53E-08	count	1
AC010680.4	17.9965599	2112.538261	0.0085	0.993	1.53E-08	count	1
GNMT	17.7735089	2316.932843	0.0077	0.994	1.53E-08	count	1
AC133540.1	17.7735088	2316.932745	0.0077	0.994	1.53E-08	count	1
CDHR4	18.0916781	2032.912037	0.0089	0.993	1.53E-08	count	1
AL358113.1	17.8907096	1826.071785	0.0098	0.992	1.53E-08	count	1
ZNF451-AS1	17.7727354	2018.634844	0.0088	0.993	1.53E-08	count	1
KANK4	17.798086	1424.485769	0.0125	0.99	1.53E-08	count	1
SMIM5	17.911208	2491.320619	0.0072	0.994	1.53E-08	count	1
AC079148.1	17.9112047	2491.318494	0.0072	0.994	1.53E-08	count	1
ATP13A4	17.7923761	2629.163175	0.0068	0.995	1.53E-08	count	1
AHRR	17.656318	2453.806794	0.0072	0.994	1.53E-08	count	1
UGT8	18.5791772	1617.802838	0.0115	0.991	1.53E-08	count	1
HAVCR2	17.4980695	2264.097691	0.0077	0.994	1.53E-08	count	1

SPATA17	18.0809545	1353.951719	0.0134	0.989	1.53E-08	count	1
TMEM178B	17.5130888	1314.709299	0.0133	0.989	1.53E-08	count	1
TM4SF4	17.497362	1911.806413	0.0092	0.993	1.53E-08	count	1
AC079341.2	18.5188584	3805.699828	0.0049	0.996	1.53E-08	count	1
ARHGAP25	17.4966984	1597.697257	0.011	0.991	1.53E-08	count	1
KCNK5	17.6537635	1322.445742	0.0133	0.989	1.53E-08	count	1
HOXB5	17.7676149	1210.922499	0.0147	0.988	1.53E-08	count	1
BCO1	0.6699741	1.1061953	0.6057	0.545	1.65E-08	count	1
SLC25A25-AS1	0.727591	0.9553472	0.7616	0.446	1.76E-08	count	1
AC104463.2	0.7554482	0.6911005	1.0931	0.274	1.81E-08	count	1
AC005523.1	0.7953638	0.8220001	0.9676	0.333	1.89E-08	count	1
CSF2RB	0.8725921	1.0928567	0.7985	0.425	2.02E-08	count	1
NBEAL2	0.2868009	0.5565156	0.5154	0.606	2.14E-08	count	1
AC005837.3	0.9761016	1.2461711	0.7833	0.434	2.19E-08	count	1
LY6G5B	1.0101144	0.8348039	1.21	0.226	2.25E-08	count	1
AC022306.2	1.1956362	1.3351257	0.8955	0.371	2.52E-08	count	1
KCNA2	1.2391332	1.0331773	1.1993	0.23	2.58E-08	count	1
LINC01678	1.384703	1.0520049	1.3163	0.188	2.76E-08	count	1
HCK	1.4018921	1.3711573	1.0224	0.307	2.78E-08	count	1
CBX2	1.4143603	1.2601186	1.1224	0.262	2.79E-08	count	1
AC013565.3	1.5448519	1.1962644	1.2914	0.197	2.94E-08	count	1
CNTNAP4	1.5622184	1.6315481	0.9575	0.338	2.95E-08	count	1
RAB11FIP4	1.7004932	1.4607677	1.1641	0.244	3.09E-08	count	1
LINC01914	1.7004932	0.9195294	1.8493	0.0645	3.09E-08	count	1
AC010173.1	1.8057603	1.4935597	1.209	0.227	3.18E-08	count	1
TIAF1	1.8690991	1.6775064	1.1142	0.265	3.23E-08	count	1
AC005899.8	1.8839263	2.0604137	0.9143	0.361	3.24E-08	count	1
AL359715.2	2.0282739	1.3454602	1.5075	0.132	3.35E-08	count	1
FCGR3B	2.1174071	1.1377874	1.861	0.0628	3.41E-08	count	1
AL358072.1	2.2961126	1.4984051	1.5324	0.126	3.52E-08	count	1
AC108673.2	2.4110179	2.4286212	0.9928	0.321	3.59E-08	count	1
RBM47	2.4902686	1.2612622	1.9744	0.0484	3.63E-08	count	1
SLITRK5	2.4967849	1.8745516	1.3319	0.183	3.63E-08	count	1
ZSCAN23	2.5139297	1.5475754	1.6244	0.104	3.64E-08	count	1
AL731577.1	0.6117388	0.3718271	1.6452	0.1	4.16E-08	count	1
AC244197.2	17.5940168	2389.827531	0.0074	0.994	4.17E-08	count	1
S100A12	17.822499	2564.845638	0.0069	0.994	4.20E-08	count	1
GPR82	0.6699741	0.5583292	1.2	0.23	4.48E-08	count	1
AL356019.2	0.9483213	0.7204774	1.3162	0.188	5.85E-08	count	1
SLC2A14	1.3612624	0.585752	2.324	0.0202	7.44E-08	count	1
ITPK1-AS1	1.9451332	1.5220263	1.278	0.201	8.99E-08	count	1
FAM204A	1.73E-05	0.0656841	3.00E-04	1	2.44E-05	count	1
CCNA2	0.0012386	0.8191563	0.0015	0.999	0.000343107	count	1
DUT	0.0002765	0.045775	0.006	0.995	0.000395013	count	1
ATF7IP2	0.0003302	0.237584	0.0014	0.999	0.000397908	count	1

WAC-AS1	0.0003146	0.1067464	0.0029	0.998	0.000417297	count	1
CTDP1	0.000598	0.3449396	0.0017	0.999	0.000437729	count	1
CS	0.0004073	0.2167987	0.0019	0.999	0.000476287	count	1
AP001269.4	0.0018847	0.5781338	0.0033	0.997	0.000522011	count	1
PIGK	0.0003822	0.1064741	0.0036	0.997	0.00052363	count	1
AC012360.1	0.0016574	0.9355368	0.0018	0.999	0.000575287	count	1
AL096865.1	0.0016574	0.9156616	0.0018	0.999	0.000575287	count	1
CLOCK	0.0004766	0.1659037	0.0029	0.998	0.000585032	count	1
POLL	0.0005272	0.1672133	0.0032	0.997	0.000614296	count	1
AL355338.1	0.0006841	0.3073838	0.0022	0.998	0.000664399	count	1
TMBIM1	0.0004695	0.0647879	0.0072	0.994	0.000665002	count	1
ZNF317	0.0008257	0.3077727	0.0027	0.998	0.000715732	count	1
TMEM170A	0.000553	0.1329828	0.0042	0.997	0.000717007	count	1
LINC00504	0.0021576	0.6344151	0.0034	0.997	0.000748831	count	1
EMG1	0.0021576	0.7013983	0.0031	0.998	0.000748831	count	1
XXYLT1	0.0007629	0.3167439	0.0024	0.998	0.000820662	count	1
TUT7	0.0006851	0.178555	0.0038	0.997	0.000881414	count	1
SCART1	0.0016574	0.8577459	0.0019	0.998	0.000927439	count	1
AC008040.5	0.0049313	0.5401573	0.0091	0.993	0.000972127	count	1
ZNF181	0.0007779	0.1893283	0.0041	0.997	0.000988153	count	1
MT-CO2	0.000719	0.0220535	0.0326	0.974	0.0010366	count	1
ZNF428	0.0007587	0.0573613	0.0132	0.989	0.001075532	count	1
MKLN1	0.0008232	0.1515217	0.0054	0.996	0.00109371	count	1
SNRPB	0.0007784	0.0605262	0.0129	0.99	0.001097799	count	1
KIN	0.0008509	0.1183514	0.0072	0.994	0.001170541	count	1
DCTD	0.0009012	0.1070801	0.0084	0.993	0.00120563	count	1
FBXL17	0.001035	0.2039983	0.0051	0.996	0.001222458	count	1
PYCR3	0.001264	0.2567351	0.0049	0.996	0.001242579	count	1
FKBP11	0.001037	0.1172857	0.0088	0.993	0.001396479	count	1
APEX2	0.0015342	0.2341949	0.0066	0.995	0.0014504	count	1
SNX12	0.0013105	0.1935763	0.0068	0.995	0.001476445	count	1
STK10	0.0154886	0.6045277	0.0256	0.98	0.001634312	count	1
LINC01355	0.005918	0.5256291	0.0113	0.991	0.001637694	count	1
UFSP2	0.0012479	0.0907453	0.0138	0.989	0.001720766	count	1
AUP1	0.0012598	0.0814805	0.0155	0.988	0.001756585	count	1
SPRYD3	0.0013209	0.1038054	0.0127	0.99	0.001804413	count	1
SLC25A43	0.0019665	0.1914218	0.0103	0.992	0.001859013	count	1
EIF4A1	0.0014688	0.0760424	0.0193	0.985	0.002061451	count	1
SDK1	0.001782	0.212293	0.0084	0.993	0.002068608	count	1
GPR88	0.0026437	0.3332091	0.0079	0.994	0.002131578	count	1
AC093901.1	0.0079307	0.7169286	0.0111	0.991	0.002193711	count	1
AC022007.1	0.0079307	0.7169286	0.0111	0.991	0.002193711	count	1
TMCC1-AS1	0.0079307	0.7169471	0.0111	0.991	0.002193711	count	1
ARHGAP39	0.0079307	0.7169187	0.0111	0.991	0.002193711	count	1
AL356056.2	0.0079307	0.7169187	0.0111	0.991	0.002193711	count	1

AC104653.1	0.0079307	0.7912319	0.01	0.992	0.002193711	count	1
AC145285.6	0.0079307	0.7912487	0.01	0.992	0.002193711	count	1
RSPH9	0.0024937	0.3283614	0.0076	0.994	0.002246629	count	1
DDX42	0.001643	0.0864579	0.019	0.985	0.002293538	count	1
SLC39A13	0.0017233	0.1153585	0.0149	0.988	0.002328159	count	1
RAB3GAP2	0.0018096	0.1397871	0.0129	0.99	0.002367658	count	1
CBWD1	0.001838	0.1524375	0.0121	0.99	0.002433738	count	1
NUDT13	0.005967	0.4814552	0.0124	0.99	0.002440039	count	1
ATP5PB	0.0017672	0.052323	0.0338	0.973	0.002503322	count	1
SNRPA1	0.0018754	0.0940478	0.0199	0.984	0.002573539	count	1
RFX3-AS1	0.0135932	0.7134636	0.0191	0.985	0.002674297	count	1
LRRIQ3	0.0135932	0.811841	0.0167	0.987	0.002674297	count	1
LINC00884	0.0053089	0.5463765	0.0097	0.992	0.00273011	count	1
NUB1	0.0020595	0.0702328	0.0293	0.977	0.002873688	count	1
MTRNR2L1	0.0281128	0.73773	0.0381	0.97	0.002957018	count	1
INHBB	0.0281128	0.788486	0.0357	0.972	0.002957018	count	1
SERF1B	0.0281128	0.7872338	0.0357	0.972	0.002957018	count	1
AC090568.2	0.0281128	0.7872338	0.0357	0.972	0.002957018	count	1
XIAP	0.0022075	0.1149526	0.0192	0.985	0.002992907	count	1
PDE3B	0.0051108	0.4948225	0.0103	0.992	0.003066921	count	1
CDK2AP1	0.0025521	0.2208765	0.0116	0.991	0.003105216	count	1
PPP1R9B	0.0028558	0.2750685	0.0104	0.992	0.003112201	count	1
FAM208B	0.0024197	0.1302881	0.0186	0.985	0.00324271	count	1
SRSF9	0.0023188	0.0465502	0.0498	0.96	0.003298364	count	1
AC107959.1	0.0073967	0.4750069	0.0156	0.988	0.003433995	count	1
ATP6V0A1	0.0026999	0.1832761	0.0147	0.988	0.003529742	count	1
FKBP4	0.0026604	0.1177127	0.0226	0.982	0.003545492	count	1
EPN1	0.0027678	0.1475193	0.0188	0.985	0.003591671	count	1
CDC73	0.0025813	0.083053	0.0311	0.975	0.003594694	count	1
VAMP3	0.0025752	0.0608581	0.0423	0.966	0.003636589	count	1
LMNB1	0.0136496	0.5289853	0.0258	0.979	0.00377092	count	1
CRIM1	0.0027438	0.074267	0.0369	0.971	0.003908455	count	1
NCAPH	0.0380437	0.8748169	0.0435	0.965	0.003991605	count	1
AC079322.1	0.0380437	0.8771772	0.0434	0.965	0.003991605	count	1
FGF11	0.0380437	0.872655	0.0436	0.965	0.003991605	count	1
PLAC8	0.0380437	0.9347921	0.0407	0.968	0.003991605	count	1
RAB12	0.0030628	0.1225374	0.025	0.98	0.004004147	count	1
MRPS5	0.0029271	0.0933221	0.0314	0.975	0.004069045	count	1
AGPAT5	0.0033964	0.1811391	0.0188	0.985	0.004132354	count	1
FKBP15	0.0037172	0.1999314	0.0186	0.985	0.004166551	count	1
SPATA2L	0.0056757	0.3414737	0.0166	0.987	0.004303286	count	1
PHLDA1	0.0032106	0.1093791	0.0294	0.977	0.004394699	count	1
MAPK11	0.0420199	0.6152663	0.0683	0.946	0.004404375	count	1
ZNF860	0.0079036	0.7143389	0.0111	0.991	0.004418111	count	1
SOX17	0.0079036	1.344198	0.0059	0.995	0.004418111	count	1

AL691432.2	0.0065956	0.3604122	0.0183	0.985	0.004427216	count	1
SAP30	0.0032043	0.0854581	0.0375	0.97	0.004446225	count	1
HOXD8	0.0234776	0.7368449	0.0319	0.975	0.004608297	count	1
PEX7	0.0040173	0.2087628	0.0192	0.985	0.004696877	count	1
CELF1	0.0036442	0.1754282	0.0208	0.983	0.004745142	count	1
SNRNP40	0.0034842	0.0949637	0.0367	0.971	0.004771258	count	1
PLGRKT	0.003585	0.1266723	0.0283	0.977	0.004784625	count	1
KAT2A	0.0059427	0.2918961	0.0204	0.984	0.004789643	count	1
VSIG10	0.0039102	0.1672934	0.0234	0.981	0.00483716	count	1
PKN1	0.0036022	0.1174572	0.0307	0.976	0.004861308	count	1
AIDA	0.0035858	0.0916857	0.0391	0.969	0.004972533	count	1
DBI	0.0035666	0.0441026	0.0809	0.9356	0.005085423	count	1
AC078802.1	0.0185424	0.5105601	0.0363	0.971	0.005117168	count	1
CCNY	0.0038685	0.1163712	0.0332	0.973	0.005133785	count	1
PCDHA3	0.0497617	0.8374784	0.0594	0.953	0.005205642	count	1
NOP53	0.0036716	0.0427248	0.0859	0.932	0.005230327	count	1
KDM4A	0.0051894	0.2667739	0.0195	0.984	0.005367115	count	1
PRKAG1	0.003991	0.1149683	0.0347	0.972	0.005404054	count	1
HSPA9	0.0038965	0.0791742	0.0492	0.961	0.005408602	count	1
HS3ST1	0.0063899	0.3028107	0.0211	0.983	0.005415378	count	1
ZNF235	0.0065237	0.4332817	0.0151	0.988	0.00552869	count	1
RAB11FIP5	0.0048959	0.2337743	0.0209	0.983	0.005541102	count	1
DMGDH	0.0079307	0.4937095	0.0161	0.987	0.005570638	count	1
ZNF384	0.0051209	0.2127775	0.0241	0.981	0.005579836	count	1
ERMAP	0.0054159	0.3911713	0.0138	0.989	0.005649748	count	1
MAPK9	0.0044738	0.1869281	0.0239	0.981	0.005710485	count	1
MPV17	0.0041635	0.0969302	0.043	0.966	0.005720972	count	1
AC005726.5	0.011182	0.668935	0.0167	0.987	0.005744514	count	1
RMI1	0.008653	0.3801973	0.0228	0.982	0.005806501	count	1
FOXC2-AS1	0.0299214	0.8783813	0.0341	0.973	0.005864265	count	1
LINC01358	0.0073274	0.4438958	0.0165	0.987	0.005904699	count	1
PCDHB12	0.0127578	0.4594713	0.0278	0.978	0.005917143	count	1
FAM219B	0.004571	0.137218	0.0333	0.973	0.006010374	count	1
ATG5	0.0045133	0.1091201	0.0414	0.967	0.006050755	count	1
RTCA	0.0046823	0.1602578	0.0292	0.977	0.006075731	count	1
FAM174A	0.0045071	0.1041267	0.0433	0.965	0.006136626	count	1
PCDHGA7	0.0133437	0.5865629	0.0227	0.982	0.006188223	count	1
PSMG2	0.0043768	0.0538106	0.0813	0.935	0.006206083	count	1
DNAJC18	0.0051076	0.1614551	0.0316	0.975	0.006255322	count	1
MTURN	0.0045628	0.1011938	0.0451	0.964	0.006275394	count	1
NDUFAF1	0.0053617	0.1614232	0.0332	0.974	0.006351343	count	1
ZPR1	0.0049252	0.1527051	0.0323	0.974	0.006379314	count	1
GCFC2	0.0050639	0.1663634	0.0304	0.976	0.00644794	count	1
CRNKL1	0.0048897	0.1195125	0.0409	0.967	0.006474128	count	1
CIAO1	0.0049239	0.1230074	0.04	0.968	0.006474345	count	1

CDK5R1	0.0621488	0.7402387	0.084	0.933	0.006481063	count	1
MAP3K20	0.0046541	0.0868228	0.0536	0.957	0.006488584	count	1
AC003102.1	0.0159528	0.5169255	0.0309	0.975	0.006510886	count	1
RCOR1	0.0054249	0.174925	0.031	0.975	0.006552441	count	1
XPA	0.0047057	0.0808155	0.0582	0.954	0.006611883	count	1
PTCD1	0.007647	0.3638815	0.021	0.983	0.006623708	count	1
RASGEF1B	0.0060435	0.2371402	0.0255	0.98	0.006701897	count	1
SSH3	0.0056768	0.2384249	0.0238	0.981	0.006724501	count	1
LINC00847	0.0060313	0.2322509	0.026	0.979	0.00679314	count	1
ZNF70	0.0067862	0.2727806	0.0249	0.98	0.006818	count	1
TXNDC12	0.0049004	0.082882	0.0591	0.953	0.006820678	count	1
KCNQ5	0.0079121	0.6681592	0.0118	0.991	0.006853137	count	1
GEMIN7	0.0060314	0.2676503	0.0225	0.982	0.006857244	count	1
ZNF48	0.0108255	0.3402468	0.0318	0.975	0.006893258	count	1
ELL	0.0064846	0.2330361	0.0278	0.978	0.007190854	count	1
SPRED2	0.006449	0.2556918	0.0252	0.98	0.007227398	count	1
AC006441.1	0.0694756	0.9916614	0.0701	0.9442	0.007231609	count	1
C19orf48	0.0062876	0.1821558	0.0345	0.972	0.007240499	count	1
AC099518.5	0.0061365	0.180216	0.0341	0.973	0.007246133	count	1
RALA	0.0052552	0.090981	0.0578	0.954	0.007275176	count	1
PHKA2	0.0123274	0.4120899	0.0299	0.976	0.00738911	count	1
RBM42	0.0053384	0.0771319	0.0692	0.945	0.007432534	count	1
ERH	0.0052042	0.0393233	0.1323	0.895	0.007440475	count	1
DIABLO	0.0380437	0.6164857	0.0617	0.951	0.007441935	count	1
C18orf25	0.0069007	0.2730378	0.0253	0.98	0.007470133	count	1
VPS54	0.0069083	0.2332186	0.0296	0.976	0.007526537	count	1
ARHGEF28	0.006655	0.2550259	0.0261	0.979	0.00756596	count	1
DPP9	0.0064208	0.2410032	0.0266	0.979	0.00765118	count	1
FUT8-AS1	0.0739881	0.6547982	0.113	0.91	0.007692438	count	1
SMAD4	0.0059039	0.1439467	0.041	0.967	0.007740971	count	1
ARHGAP27	0.0193336	0.8782221	0.022	0.982	0.007885537	count	1
ETV5	0.0081514	0.2410639	0.0338	0.973	0.007911463	count	1
AC132192.1	0.0766231	0.5221419	0.1467	0.883	0.007961028	count	1
RAB9B	0.0070286	0.2452415	0.0287	0.977	0.008060272	count	1
REST	0.0058197	0.0777636	0.0748	0.94	0.008108173	count	1
ETFB	0.0058775	0.0568505	0.1034	0.918	0.008324026	count	1
CHERP	0.0071672	0.2292476	0.0313	0.975	0.008378276	count	1
TUBA1C	0.0059685	0.0676889	0.0882	0.93	0.008446471	count	1
ZFP1	0.0075867	0.2021527	0.0375	0.97	0.008457926	count	1
RAB40B	0.0065897	0.1372994	0.048	0.962	0.008542649	count	1
SMARCA1	0.0062329	0.0956063	0.0652	0.948	0.00858857	count	1
MIS12	0.0077183	0.2013263	0.0383	0.969	0.008649243	count	1
THOP1	0.0082369	0.2308709	0.0357	0.972	0.008661329	count	1
ANAPC7	0.0068622	0.1515437	0.0453	0.964	0.008853874	count	1
CDH26	0.0456408	0.8478349	0.0538	0.957	0.008912072	count	1

AC004803.1	0.0134142	0.4049292	0.0331	0.974	0.008995269	count	1
ALAS1	0.0078198	0.2649735	0.0295	0.976	0.009108234	count	1
TIPRL	0.0066237	0.0805165	0.0823	0.934	0.009183843	count	1
VSTM4	0.0068299	0.1464151	0.0466	0.963	0.009241711	count	1
NOC3L	0.0068858	0.1152841	0.0597	0.952	0.009292122	count	1
FAM72A	0.0340114	0.6885535	0.0494	0.961	0.009354372	count	1
AC092647.5	0.0344028	0.8750766	0.0393	0.969	0.009461206	count	1
AL132780.1	0.0344028	0.8750766	0.0393	0.969	0.009461206	count	1
DHDH	0.0344028	0.8721848	0.0394	0.969	0.009461206	count	1
AC245123.1	0.0344028	1.0353676	0.0332	0.973	0.009461206	count	1
MLST8	0.0074835	0.1683661	0.0444	0.965	0.009479177	count	1
NBEAL1	0.006877	0.0860763	0.0799	0.936	0.009590126	count	1
CARS	0.0076762	0.1489128	0.0515	0.959	0.009654716	count	1
ZNF257	0.0281128	0.6524686	0.0431	0.966	0.009705211	count	1
CAMK2D	0.0072355	0.112133	0.0645	0.949	0.009711971	count	1
AL627309.1	0.0952007	1.094661	0.087	0.931	0.009844143	count	1
NPPA-AS1	0.0952007	1.094661	0.087	0.931	0.009844143	count	1
DLGAP3	0.0952007	1.094661	0.087	0.931	0.009844143	count	1
AL109659.2	0.0952007	1.094661	0.087	0.931	0.009844143	count	1
AL357078.1	0.0952007	1.094661	0.087	0.931	0.009844143	count	1
LINC01799	0.0952007	1.094661	0.087	0.931	0.009844143	count	1
MUSTN1	0.0952007	1.094661	0.087	0.931	0.009844143	count	1
LRTM1	0.0952007	1.094661	0.087	0.931	0.009844143	count	1
SCAANT1	0.0952007	1.094661	0.087	0.931	0.009844143	count	1
CTBP1-AS	0.0952007	1.103682	0.0863	0.931	0.009844143	count	1
PDE6A	0.0952007	1.094661	0.087	0.931	0.009844143	count	1
AL024498.1	0.0952007	1.094661	0.087	0.931	0.009844143	count	1
HCG14	0.0952007	1.094661	0.087	0.931	0.009844143	count	1
SYNE1-AS1	0.0952007	1.094661	0.087	0.931	0.009844143	count	1
FAM66D	0.0952007	1.094661	0.087	0.931	0.009844143	count	1
LACTB2-AS1	0.0952007	1.094661	0.087	0.931	0.009844143	count	1
RASEF	0.0952007	1.094661	0.087	0.931	0.009844143	count	1
KIF12	0.0952007	1.103682	0.0863	0.931	0.009844143	count	1
AL592211.1	0.0952007	1.103682	0.0863	0.931	0.009844143	count	1
UBASH3B	0.0952007	1.094661	0.087	0.931	0.009844143	count	1
AC137834.2	0.0952007	1.094661	0.087	0.931	0.009844143	count	1
AC131212.1	0.0952007	1.094661	0.087	0.931	0.009844143	count	1
AL691403.2	0.0952007	1.094661	0.087	0.931	0.009844143	count	1
CHRFAM7A	0.0952007	1.103682	0.0863	0.931	0.009844143	count	1
AC003965.1	0.0952007	1.094661	0.087	0.931	0.009844143	count	1
AC135048.3	0.0952007	1.094661	0.087	0.931	0.009844143	count	1
SPIRE2	0.0952007	1.094661	0.087	0.931	0.009844143	count	1
RAPGEFL1	0.0952007	1.094661	0.087	0.931	0.009844143	count	1
AC091588.3	0.0952007	1.094661	0.087	0.931	0.009844143	count	1
AC100778.3	0.0952007	1.094661	0.087	0.931	0.009844143	count	1

AC005790.1	0.0952007	1.103682	0.0863	0.931	0.009844143	count	1
PRR22	0.0952007	1.094661	0.087	0.931	0.009844143	count	1
ANGPTL6	0.0952007	1.094661	0.087	0.931	0.009844143	count	1
ANGPTL8	0.0952007	1.103682	0.0863	0.931	0.009844143	count	1
AL022328.1	0.0952007	1.094661	0.087	0.931	0.009844143	count	1
DSCR9	0.0952007	1.094661	0.087	0.931	0.009844143	count	1
CRB1	0.0952007	1.365266	0.0697	0.944	0.009844143	count	1
MTRF1	0.0119926	0.3439275	0.0349	0.972	0.009916407	count	1
MIER1	0.0070347	0.0566763	0.1241	0.901	0.009964196	count	1
ARID5B	0.0069732	0.0497394	0.1402	0.889	0.01000433	count	1
GHDC	0.0079639	0.1928295	0.0413	0.967	0.010016471	count	1
ZFP3	0.0124819	0.4146526	0.0301	0.976	0.01005222	count	1
MALINC1	0.0219227	0.4060317	0.054	0.957	0.010150788	count	1
RABL3	0.008231	0.1716611	0.0479	0.962	0.010217949	count	1
PHF14	0.0073355	0.0864801	0.0848	0.932	0.010276417	count	1
SPAG16	0.0074681	0.1134534	0.0658	0.948	0.01027992	count	1
CDC42	0.0071882	0.0370167	0.1942	0.846	0.010296366	count	1
AC144652.1	0.0299214	0.6208614	0.0482	0.962	0.01032572	count	1
ZNF582	0.0115164	0.2790859	0.0413	0.967	0.010365909	count	1
RSRC2	0.0073386	0.0563849	0.1302	0.896	0.010419733	count	1
HSF1	0.0076696	0.1084565	0.0707	0.944	0.010424923	count	1
ESD	0.007296	0.0356153	0.2049	0.838	0.01044408	count	1
PPME1	0.0080554	0.1551834	0.0519	0.959	0.010451695	count	1
HOGA1	0.0257723	0.548846	0.047	0.963	0.010498518	count	1
ZNF784	0.0259054	0.4135019	0.0626	0.95	0.010552464	count	1
RBM14	0.0099208	0.3119025	0.0318	0.975	0.010590288	count	1
PTGS2	0.0082368	0.2272135	0.0363	0.971	0.01062702	count	1
YPEL2	0.0076682	0.1031627	0.0743	0.941	0.010661603	count	1
PTPRU	0.010863	0.3368388	0.0322	0.974	0.01079314	count	1
SLC26A4-AS1	0.104701	0.7585703	0.138	0.89	0.0108	count	1
ABCC5	0.0099971	0.2521875	0.0396	0.968	0.010819785	count	1
ADH5	0.0075916	0.038716	0.1961	0.845	0.010867842	count	1
YWHAG	0.0079371	0.1009842	0.0786	0.937	0.010960981	count	1
KDM3B	0.0086228	0.1796357	0.048	0.962	0.011004967	count	1
CROCC	0.0090092	0.1974787	0.0456	0.964	0.01100811	count	1
PALM2	0.0184228	0.6287002	0.0293	0.977	0.011032091	count	1
BAHCC1	0.0092688	0.2378405	0.039	0.969	0.01104345	count	1
RNF31	0.0570238	0.7139058	0.0799	0.936	0.011104814	count	1
AIFM3	0.0570238	0.7165881	0.0796	0.937	0.011104814	count	1
SCYL1	0.0085204	0.1251331	0.0681	0.946	0.011137594	count	1
RNF41	0.0086656	0.1343313	0.0645	0.949	0.011157647	count	1
AC105277.1	0.0200486	0.6820557	0.0294	0.977	0.011184658	count	1
DHRS12	0.0088421	0.2072656	0.0427	0.966	0.011229056	count	1
OCIAD1	0.0080365	0.0451488	0.178	0.859	0.011475355	count	1
NOP2	0.0591146	0.6150223	0.0961	0.923	0.011506262	count	1

IMPAD1	0.0087286	0.0995702	0.0877	0.93	0.011659255	count	1
MYOZ1	0.0087936	0.1614155	0.0545	0.957	0.011826678	count	1
LRRC69	0.1150625	0.5683492	0.2025	0.84	0.01183698	count	1
GLYCTK	0.0257302	0.4528937	0.0568	0.955	0.011905405	count	1
IFI35	0.0088768	0.0948556	0.0936	0.925	0.01208857	count	1
GALNT2	0.0088443	0.1114604	0.0793	0.937	0.012133158	count	1
AL160153.1	0.0155324	0.7116274	0.0218	0.983	0.012146463	count	1
PSPN	0.0262944	0.6193417	0.0425	0.966	0.012165194	count	1
RPLP0	0.0084625	0.0268701	0.3149	0.753	0.012185566	count	1
MICALL2	0.0103302	0.2690224	0.0384	0.969	0.012233976	count	1
FUBP3	0.0098173	0.1339682	0.0733	0.942	0.012249778	count	1
CXorf38	0.0098612	0.2100568	0.0469	0.963	0.012324842	count	1
HIKESHI	0.0088176	0.0779437	0.1131	0.91	0.012328281	count	1
AC008906.1	0.02675	0.9326616	0.0287	0.977	0.012374938	count	1
PUS1	0.0159291	0.3168557	0.0503	0.96	0.012456073	count	1
P3H1	0.010174	0.1564241	0.065	0.948	0.012509458	count	1
INTS14	0.0110288	0.2256831	0.0489	0.961	0.012535324	count	1
SRP72	0.0089373	0.059959	0.1491	0.882	0.01262224	count	1
NAB2	0.0109143	0.1864582	0.0585	0.953	0.012710554	count	1
MOV10	0.0182645	0.3938059	0.0464	0.963	0.012810873	count	1
PPP2R1A	0.0091931	0.0819499	0.1122	0.911	0.012817357	count	1
TRIT1	0.0130232	0.3414276	0.0381	0.97	0.013077537	count	1
HAUS3	0.0114081	0.2820687	0.0404	0.968	0.01307943	count	1
SGSM3	0.0477089	0.4703406	0.1014	0.919	0.013082028	count	1
ZNF136	0.0107222	0.1723449	0.0622	0.95	0.013183209	count	1
MRGPRF-AS1	0.0226029	0.4284864	0.0528	0.958	0.013526279	count	1
RNASE4	0.0134219	0.2812578	0.0477	0.962	0.01361542	count	1
TBX3	0.0134219	0.3027784	0.0443	0.965	0.01361542	count	1
CDC26	0.0097176	0.0826609	0.1176	0.906	0.013621489	count	1
RCN3	0.0097198	0.0670871	0.1449	0.885	0.013640852	count	1
SMN2	0.0396426	0.3768809	0.1052	0.916	0.01365291	count	1
MATN1-AS1	0.0703333	0.619866	0.1135	0.91	0.013653385	count	1
SOX7	0.0711791	0.928008	0.0767	0.939	0.013814783	count	1
C5orf58	0.0272065	0.7239351	0.0376	0.97	0.013937735	count	1
GM2A	0.0119307	0.2150835	0.0555	0.956	0.013943282	count	1
SPATA5L1	0.0148551	0.3141905	0.0473	0.962	0.014026309	count	1
TCEA1	0.0098466	0.0479843	0.2052	0.8374	0.014039306	count	1
FAM49A	0.0132586	0.2916537	0.0455	0.964	0.014045572	count	1
POLDIP3	0.0112249	0.1560345	0.0719	0.943	0.014051185	count	1
CCDC34	0.0109212	0.1719161	0.0635	0.949	0.014156168	count	1
LRRC66	0.0518025	0.7961463	0.0651	0.948	0.014191615	count	1
Z97832.2	0.0518025	0.9314596	0.0556	0.956	0.014191615	count	1
NFATC2IP	0.0114181	0.145184	0.0786	0.937	0.014197801	count	1
AC008608.2	0.0136575	0.4148096	0.0329	0.974	0.014238425	count	1
AC005165.1	0.0415957	0.4693939	0.0886	0.929	0.014319737	count	1

MRPS18B	0.0102968	0.0819252	0.1257	0.9	0.014375148	count	1
RMDN1	0.0109367	0.1289245	0.0848	0.932	0.014503504	count	1
TAX1BP3	0.0105519	0.0929331	0.1135	0.91	0.01451914	count	1
MPP3	0.0360347	0.5007207	0.072	0.943	0.014649571	count	1
TRIM47	0.0106092	0.0873501	0.1215	0.903	0.014674149	count	1
COA7	0.0146368	0.2715526	0.0539	0.957	0.014695917	count	1
PDXDC1	0.0119792	0.1529311	0.0783	0.938	0.014841938	count	1
C16orf46	0.07694	0.6296071	0.1222	0.903	0.014912332	count	1
TBKBP1	0.0185567	0.4421829	0.042	0.967	0.014933663	count	1
RYK	0.0109705	0.1095385	0.1002	0.92	0.014987687	count	1
AAMP	0.0108791	0.0809995	0.1343	0.893	0.015014099	count	1
CNOT6L	0.0113913	0.1433459	0.0795	0.937	0.01503475	count	1
ST8SIA1	0.0165018	0.3303763	0.0499	0.96	0.015104156	count	1
FBXO21	0.0111395	0.1067071	0.1044	0.917	0.01512763	count	1
BCAS2	0.0108619	0.0820327	0.1324	0.895	0.015138178	count	1
MNDA	0.019447	0.3357204	0.0579	0.954	0.015200325	count	1
DDX21	0.0108759	0.0672651	0.1617	0.872	0.015296299	count	1
AC109347.1	0.079496	0.5557066	0.1431	0.886	0.015398292	count	1
AC124016.2	0.079496	0.5910212	0.1345	0.893	0.015398292	count	1
ROBO1	0.0135195	0.1704861	0.0793	0.937	0.015742372	count	1
INF2	0.0119755	0.1586769	0.0755	0.94	0.015775286	count	1
NHLRC2	0.0120788	0.1475976	0.0818	0.935	0.015787594	count	1
OSER1	0.0115099	0.1138203	0.1011	0.919	0.015825739	count	1
SH2B2	0.030922	0.5860793	0.0528	0.958	0.015830831	count	1
CASC3	0.012299	0.1321198	0.0931	0.926	0.015834264	count	1
AC100786.1	0.0462609	0.6021073	0.0768	0.939	0.015910306	count	1
AGL	0.0115794	0.1246241	0.0929	0.926	0.015918366	count	1
RPUSD1	0.0134342	0.1915927	0.0701	0.944	0.015956098	count	1
BEST1	0.03127	0.3599341	0.0869	0.931	0.016008014	count	1
FNDC5	0.0130806	0.2191231	0.0597	0.952	0.016048451	count	1
ENTR1	0.0132972	0.1652906	0.0804	0.936	0.016055401	count	1
PHRF1	0.0145333	0.242848	0.0598	0.952	0.016108045	count	1
INTS9	0.0190611	0.3456456	0.0551	0.956	0.016131264	count	1
ALKBH3	0.0125864	0.135539	0.0929	0.926	0.016153011	count	1
WDFY1	0.0123147	0.1479909	0.0832	0.934	0.016200484	count	1
RAB18	0.0117029	0.0813203	0.1439	0.886	0.016239245	count	1
TAOK3	0.0120788	0.100797	0.1198	0.905	0.01655623	count	1
MPHOSPH8	0.0117044	0.0505316	0.2316	0.817	0.016728307	count	1
ZNF337-AS1	0.0221532	0.4825427	0.0459	0.963	0.016760937	count	1
AP001528.1	0.0239414	0.3999629	0.0599	0.952	0.016779398	count	1
MAP3K1	0.0186716	0.244724	0.0763	0.939	0.016794053	count	1
AC068620.1	0.1655115	0.8678805	0.1907	0.849	0.016803435	count	1
SLC2A5	0.1655115	0.8766229	0.1888	0.85	0.016803435	count	1
CFHR3	0.1655115	0.9610876	0.1722	0.863	0.016803435	count	1
GPX7	0.0124796	0.1299249	0.0961	0.923	0.016879338	count	1

MRPL24	0.0122939	0.089048	0.1381	0.89	0.016939026	count	1
RASGRP2	0.0131843	0.1407736	0.0937	0.925	0.017133966	count	1
UNC13B	0.014473	0.3164622	0.0457	0.964	0.017136818	count	1
SDAD1	0.0124908	0.0826955	0.151	0.88	0.017308813	count	1
PDS5B	0.0130157	0.1154364	0.1128	0.91	0.017454068	count	1
LRRC59	0.0131613	0.1048549	0.1255	0.9	0.017527341	count	1
CANX	0.0124703	0.0474535	0.2628	0.793	0.017705602	count	1
CHRAC1	0.0130419	0.0934185	0.1396	0.889	0.017725744	count	1
GPR137	0.0156108	0.2175726	0.0717	0.943	0.017738548	count	1
ZFYVE9	0.0147009	0.1577551	0.0932	0.926	0.017749164	count	1
ZMIZ2	0.0160408	0.2421391	0.0662	0.947	0.017777204	count	1
LINC00278	0.0652735	0.8887023	0.0734	0.941	0.017828464	count	1
POLI	0.0141892	0.1751445	0.081	0.935	0.01786876	count	1
ETNK1	0.0134627	0.157251	0.0856	0.932	0.01790089	count	1
AC005363.2	0.0323182	0.7371179	0.0438	0.965	0.017992704	count	1
DACT3-AS1	0.0352089	0.4626513	0.0761	0.939	0.018011953	count	1
INTS12	0.0141556	0.1515838	0.0934	0.926	0.018278206	count	1
COL26A1	0.0952007	0.7736334	0.1231	0.902	0.018370775	count	1
SMIM2-AS1	0.0952007	0.7736334	0.1231	0.902	0.018370775	count	1
FAM83G	0.0952007	0.7736334	0.1231	0.902	0.018370775	count	1
AL136038.5	0.0952007	0.9269506	0.1027	0.918	0.018370775	count	1
RNF43	0.0398301	0.5474698	0.0728	0.942	0.018381362	count	1
TERF1	0.0134685	0.0857917	0.157	0.875	0.018518289	count	1
MXRA7	0.0129937	0.039684	0.3274	0.743	0.018575234	count	1
TPP2	0.0146232	0.1448647	0.1009	0.92	0.018614289	count	1
AKAP8L	0.0143127	0.1354675	0.1057	0.916	0.018631444	count	1
CC2D1B	0.0234101	0.3366301	0.0695	0.945	0.018828491	count	1
MRPS22	0.0136996	0.0961227	0.1425	0.887	0.018829044	count	1
WAS	0.1882233	0.8141833	0.2312	0.817	0.018994508	count	1
CASP8	0.0199135	0.3038622	0.0655	0.948	0.019057197	count	1
PARP15	0.0699854	0.9035895	0.0775	0.938	0.019095291	count	1
PANX2	0.0699854	0.9887756	0.0708	0.944	0.019095291	count	1
ULK2	0.0161961	0.229388	0.0706	0.944	0.019175454	count	1
PFAS	0.0262916	0.5133645	0.0512	0.959	0.019179331	count	1
GLUD2	0.0999938	0.7138968	0.1401	0.889	0.019273369	count	1
ZNF492	0.0999938	0.8975967	0.1114	0.911	0.019273369	count	1
NFATC1	0.0188874	0.2826323	0.0668	0.947	0.019336649	count	1
DNAJB2	0.0151393	0.135855	0.1114	0.911	0.019340608	count	1
MRPS31	0.0143871	0.1055284	0.1363	0.892	0.019445775	count	1
LYPLAL1	0.0144238	0.1103999	0.1307	0.896	0.019514718	count	1
LEO1	0.0150742	0.1133878	0.1329	0.894	0.019554683	count	1
ISOC2	0.0142836	0.0955328	0.1495	0.881	0.019609196	count	1
SNX31	0.1952013	0.6463593	0.302	0.763	0.019662104	count	1
CSNK1G1	0.0172387	0.2588152	0.0666	0.947	0.019673567	count	1
SF3B2	0.0139628	0.0517239	0.2699	0.787	0.019800929	count	1

SZT2	0.0175107	0.4012316	0.0436	0.965	0.019803754	count	1
PGAM1	0.0139204	0.0443503	0.3139	0.754	0.019814161	count	1
PYGO1	0.0205357	0.283441	0.0725	0.942	0.019909344	count	1
CDIPT	0.0143604	0.0723456	0.1985	0.843	0.020033568	count	1
AC008622.2	0.1994457	0.6295775	0.3168	0.751	0.020066884	count	1
UBE2O	0.018173	0.3679642	0.0494	0.961	0.020137509	count	1
PDZRN3	0.0177382	0.1712778	0.1036	0.918	0.020153438	count	1
ARHGAP29	0.0144778	0.0633358	0.2286	0.819	0.020305032	count	1
CAHM	0.0340114	0.4379804	0.0777	0.938	0.020316471	count	1
MRNIP	0.0150656	0.1496464	0.1007	0.92	0.020327536	count	1
SDF2	0.0145008	0.0753177	0.1925	0.847	0.02034237	count	1
BRSK2	0.0340603	0.418626	0.0814	0.935	0.020345522	count	1
SLC7A5	0.0341536	0.5641609	0.0605	0.952	0.020400949	count	1
AC093462.1	0.0341536	0.5954626	0.0574	0.954	0.020400949	count	1
SCOC	0.0145588	0.0636679	0.2287	0.819	0.020408139	count	1
CCNC	0.0155626	0.1181304	0.1317	0.895	0.02041492	count	1
RAB7B	0.0192986	0.2404182	0.0803	0.936	0.020587307	count	1
NPRL2	0.0167214	0.1575899	0.1061	0.916	0.020595612	count	1
USP28	0.0176821	0.2128648	0.0831	0.934	0.020658757	count	1
OCA2	0.0297339	0.5333388	0.0558	0.956	0.020822174	count	1
TMEM120B	0.0253773	0.3259267	0.0779	0.938	0.020951368	count	1
PDGFRB	0.0148445	0.0756258	0.1963	0.844	0.020986503	count	1
CHUK	0.0178599	0.2720276	0.0657	0.948	0.021143603	count	1
AC008741.2	0.0250705	0.5832035	0.043	0.966	0.021202632	count	1
NET1	0.0152758	0.0721934	0.2116	0.832	0.02132347	count	1
CYFIP1	0.0157429	0.1236859	0.1273	0.899	0.021458912	count	1
ZNF763	0.0626992	0.6235517	0.1006	0.92	0.021489701	count	1
DISC1	0.0626992	0.7072303	0.0887	0.929	0.021489701	count	1
TMEM31	0.0628541	0.8454402	0.0743	0.941	0.021542088	count	1
ANKRD2	0.0628541	0.8489386	0.074	0.941	0.021542088	count	1
RNF32	0.0628541	0.9858339	0.0638	0.949	0.021542088	count	1
CD1D	0.0628541	1.043079	0.0603	0.952	0.021542088	count	1
PCOLCE-AS1	0.0628541	1.046091	0.0601	0.952	0.021542088	count	1
HSP90AA1	0.0150786	0.0278198	0.542	0.588	0.021680678	count	1
CTNNA1	0.0154227	0.0652695	0.2363	0.813	0.021701325	count	1
COPS3	0.0159701	0.0982974	0.1625	0.871	0.021779507	count	1
ZNF75D	0.0311968	0.3390444	0.092	0.927	0.021842127	count	1
PABPC4L	0.0258755	0.5088086	0.0509	0.959	0.021881449	count	1
ABCF1	0.0159171	0.088412	0.18	0.857	0.021888255	count	1
BX284668.5	0.0176102	0.2394088	0.0736	0.941	0.022073992	count	1
MED23	0.0185475	0.201405	0.0921	0.927	0.022089008	count	1
CEP70	0.0184737	0.190005	0.0972	0.923	0.022125739	count	1
CWC15	0.0157766	0.0714111	0.2209	0.825	0.022161938	count	1
CDK10	0.018307	0.1991938	0.0919	0.927	0.022207691	count	1
TARDBP	0.0173905	0.1220138	0.1425	0.887	0.022214982	count	1

PHF8	0.02324	0.3053857	0.0761	0.939	0.022233872	count	1
ZNF793-AS1	0.035098	0.429214	0.0818	0.935	0.022266752	count	1
PHLDB1	0.0164956	0.1444983	0.1142	0.909	0.022386301	count	1
MYORG	0.0827327	0.635025	0.1303	0.896	0.022508749	count	1
ABTB2	0.0336954	0.3580626	0.0941	0.925	0.022528738	count	1
RHOBTB2	0.0193178	0.2812915	0.0687	0.945	0.022567906	count	1
RABEP1	0.0166751	0.1116131	0.1494	0.881	0.022609512	count	1
ARRDC1-AS1	0.0197311	0.2390637	0.0825	0.934	0.022611286	count	1
MT-CYB	0.0156846	0.020398	0.7689	0.442	0.022620546	count	1
P4HTM	0.0164764	0.096833	0.1702	0.865	0.022669412	count	1
AL021408.1	0.2273245	0.7634514	0.2978	0.766	0.022701434	count	1
AMPD3	0.2273245	0.8674333	0.2621	0.793	0.022701434	count	1
MED15	0.0187443	0.2003134	0.0936	0.925	0.022791088	count	1
TEX261	0.0180917	0.1431905	0.1263	0.899	0.02281317	count	1
ABHD4	0.0201755	0.2136944	0.0944	0.925	0.022813939	count	1
PIH1D1	0.0164216	0.0794753	0.2066	0.836	0.02286559	count	1
PPP1R13B	0.0327893	0.3332939	0.0984	0.922	0.022951956	count	1
AC022706.1	0.0279782	0.4947687	0.0565	0.955	0.023091645	count	1
SARM1	0.0273277	0.340005	0.0804	0.936	0.023105702	count	1
AC009403.1	0.0296155	0.3356074	0.0882	0.93	0.023118931	count	1
FAHD2B	0.0191854	0.1926046	0.0996	0.921	0.023216369	count	1
ALMS1	0.0268765	0.2640709	0.1018	0.919	0.023231454	count	1
WARS2	0.0181307	0.1445758	0.1254	0.9	0.023239085	count	1
TAF12	0.0169555	0.0922055	0.1839	0.854	0.02326736	count	1
RAD9A	0.0199253	0.2312987	0.0861	0.931	0.023276881	count	1
CRACR2B	0.030922	0.4469017	0.0692	0.945	0.023368741	count	1
TBC1D14	0.0208755	0.203446	0.1026	0.918	0.023374757	count	1
DPP3	0.0368687	0.2918303	0.1263	0.899	0.02338376	count	1
PSMD6	0.0169024	0.0862418	0.196	0.845	0.023414918	count	1
SELENOK	0.0164366	0.0439874	0.3737	0.709	0.02345711	count	1
ZNF853	0.0245631	0.2476706	0.0992	0.921	0.023805332	count	1
TOR1AIP2	0.0174451	0.0917725	0.1901	0.849	0.024085022	count	1
BRD2	0.0174509	0.1043875	0.1672	0.867	0.0241455	count	1
SRRM1	0.0169648	0.0451324	0.3759	0.707	0.02418359	count	1
YWHAQ	0.0170213	0.0446695	0.3811	0.703	0.024248808	count	1
AC068338.2	0.0363711	0.5184423	0.0702	0.944	0.024308151	count	1
PLCD4	0.2448162	0.7472089	0.3276	0.743	0.024332956	count	1
SMAP2	0.0202502	0.185783	0.109	0.913	0.024380882	count	1
TRAPPC6A	0.019047	0.1526395	0.1248	0.901	0.024517091	count	1
RCC1	0.038723	0.4138595	0.0936	0.925	0.024552844	count	1
FKTN	0.0233935	0.3386279	0.0691	0.945	0.024571471	count	1
PPP4C	0.0176912	0.0932242	0.1898	0.849	0.024620691	count	1
TAF1	0.0211325	0.2075822	0.1018	0.919	0.024685596	count	1
SCUBE2	0.248714	0.6862635	0.3624	0.717	0.024694278	count	1
GMIP	0.1291051	0.6316326	0.2044	0.838	0.024708872	count	1

RHBDL2	0.1291051	0.6576483	0.1963	0.844	0.024708872	count	1
PSIP1	0.0173	0.0446876	0.3871	0.6987	0.024721169	count	1
CAMKK1	0.0724728	0.5609197	0.1292	0.897	0.024788359	count	1
GTF3C2	0.0240398	0.269815	0.0891	0.929	0.024827071	count	1
SSU72	0.0177206	0.071147	0.2491	0.803	0.024973501	count	1
MALAT1	0.0173277	0.0274896	0.6303	0.5285	0.024997742	count	1
POP5	0.0181265	0.0948756	0.1911	0.848	0.025033162	count	1
AL137026.1	0.0624398	0.544807	0.1146	0.909	0.025252299	count	1
UBE2Q1	0.0190033	0.1346164	0.1412	0.888	0.025265052	count	1
HLA-DQB1	0.0242797	0.3135134	0.0774	0.938	0.025292152	count	1
LINC01139	0.2562621	0.8407535	0.3048	0.761	0.025391611	count	1
KYAT3	0.0204673	0.1589641	0.1288	0.898	0.025485443	count	1
CADM4	0.05561	0.4620393	0.1204	0.904	0.025588072	count	1
KATNBL1	0.0187591	0.1187109	0.158	0.874	0.025619395	count	1
AL121672.3	0.0952007	0.7187951	0.1324	0.895	0.025827926	count	1
TMEM161B-AS1	0.0219546	0.2079287	0.1056	0.916	0.025904338	count	1
RRP8	0.0205471	0.156819	0.131	0.896	0.025907182	count	1
AC093772.1	0.1359728	0.8314621	0.1635	0.87	0.0259795	count	1
AL078459.1	0.1359728	0.9781247	0.139	0.889	0.0259795	count	1
PHF5A	0.0187401	0.0993578	0.1886	0.85	0.026014665	count	1
SEMA5A	0.0187226	0.1261648	0.1484	0.882	0.026076662	count	1
LPAR6	0.0195546	0.1402073	0.1395	0.889	0.026126637	count	1
ZNF700	0.0568691	0.4774228	0.1191	0.905	0.026161235	count	1
TRADD	0.019192	0.1041008	0.1844	0.854	0.026414135	count	1
CCDC93	0.0207508	0.147142	0.141	0.888	0.026504717	count	1
TMC7	0.0577167	0.7940703	0.0727	0.942	0.026546918	count	1
AC123768.3	0.0481259	0.7621218	0.0631	0.95	0.026722151	count	1
ITPR2	0.0199638	0.1551237	0.1287	0.898	0.026833624	count	1
RPS6KA5	0.0274412	0.2459958	0.1116	0.911	0.026915152	count	1
TMEM139	0.0529611	0.7503018	0.0706	0.944	0.027008362	count	1
MGAT3	0.0529611	0.7503018	0.0706	0.944	0.027008362	count	1
MTCH2	0.0198665	0.1197597	0.1659	0.868	0.027042819	count	1
IRF2BP1	0.0224807	0.1708433	0.1316	0.895	0.027063638	count	1
RBM22	0.0195553	0.0818579	0.2389	0.811	0.027092718	count	1
AL359091.4	0.1424397	0.7630067	0.1867	0.852	0.02717189	count	1
MRGPRF	0.0195649	0.0856932	0.2283	0.819	0.027175063	count	1
SCML1	0.0213536	0.1704782	0.1253	0.9	0.027175745	count	1
ZNF599	0.0235116	0.236334	0.0995	0.921	0.027363004	count	1
MED10	0.0195046	0.065892	0.296	0.767	0.027462663	count	1
SAT1	0.0193451	0.071995	0.2687	0.788	0.027519441	count	1
LLPH	0.0202722	0.1051872	0.1927	0.847	0.027525977	count	1
XKR6	0.2797692	0.5999643	0.4663	0.641	0.027543639	count	1
ZNF316	0.0284833	0.3446382	0.0826	0.934	0.027594886	count	1
DHX36	0.0195584	0.0679661	0.2878	0.774	0.027650991	count	1
MAP9	0.0197069	0.0838136	0.2351	0.814	0.027697417	count	1

CBR4	0.0225587	0.1798903	0.1254	0.9	0.027778899	count	1
ENPP4	0.0216655	0.14651	0.1479	0.882	0.027797267	count	1
GFM2	0.0262926	0.2176799	0.1208	0.904	0.027826943	count	1
SREK1IP1	0.0198934	0.0961365	0.2069	0.836	0.027871912	count	1
AC008079.2	0.0692225	0.665676	0.104	0.917	0.027957572	count	1
PAN3-AS1	0.146744	0.5057995	0.2901	0.772	0.027963333	count	1
SCRN1	0.0208513	0.1306401	0.1596	0.873	0.0279923	count	1
RAB6A	0.020425	0.1053834	0.1938	0.846	0.028027122	count	1
CNTD1	0.2867706	0.7923017	0.3619	0.717	0.028178836	count	1
PORCN	0.0299214	0.2651748	0.1128	0.91	0.028211952	count	1
TAF9	0.0207823	0.0970709	0.2141	0.83	0.028446285	count	1
TRIM4	0.0214688	0.1303882	0.1647	0.869	0.028479773	count	1
ZNF677	0.022197	0.1383565	0.1604	0.873	0.028509373	count	1
FAM213A	0.0206605	0.1044135	0.1979	0.843	0.028659454	count	1
ORMDL1	0.0203797	0.0652988	0.3121	0.755	0.028720467	count	1
GTF2I	0.0204684	0.0740616	0.2764	0.782	0.028735212	count	1
P2RY11	0.0564122	0.573168	0.0984	0.922	0.028750587	count	1
RINL	0.0296821	0.2639074	0.1125	0.91	0.028753193	count	1
KIAA0586	0.0230008	0.147467	0.156	0.876	0.029158397	count	1
CENPK	0.0856665	0.6063025	0.1413	0.888	0.029219134	count	1
AP001160.2	0.0856665	0.6057489	0.1414	0.888	0.029219134	count	1
GDI1	0.0218831	0.1233083	0.1775	0.859	0.029289485	count	1
CSAD	0.0215559	0.1416771	0.1521	0.879	0.029317938	count	1
RNF113A	0.0224868	0.1779157	0.1264	0.899	0.029360661	count	1
RASL11A	0.0211676	0.0896081	0.2362	0.813	0.029369816	count	1
ARRB2	0.0278516	0.283953	0.0981	0.922	0.029693369	count	1
LRMP	0.0425121	0.7629073	0.0557	0.956	0.029716886	count	1
POLR3E	0.0299708	0.2143795	0.1398	0.889	0.029729815	count	1
PSMC1	0.0211151	0.0664409	0.3178	0.751	0.029731334	count	1
MAP4K2	0.0381541	0.5160749	0.0739	0.941	0.029752484	count	1
GRTP1	0.0355081	0.2836762	0.1252	0.9	0.029994427	count	1
ABCD3	0.0240912	0.1911078	0.1261	0.9	0.030044604	count	1
NGLY1	0.0233264	0.1754842	0.1329	0.894	0.030052296	count	1
IDH3B	0.0225073	0.1139126	0.1976	0.843	0.030083812	count	1
PARL	0.0220596	0.0967393	0.228	0.82	0.030096361	count	1
IDE	0.025403	0.2224488	0.1142	0.909	0.030154309	count	1
POLR3F	0.0243082	0.212077	0.1146	0.909	0.030157737	count	1
RNASET2	0.0215871	0.1140859	0.1892	0.85	0.030291025	count	1
KIAA0895L	0.040138	0.3126647	0.1284	0.898	0.030297061	count	1
IL6R-AS1	0.1595126	0.8705814	0.1832	0.855	0.030300774	count	1
LINC00309	0.1595126	0.8705814	0.1832	0.855	0.030300774	count	1
EVX1	0.1595126	0.8705814	0.1832	0.855	0.030300774	count	1
GNAS-AS1	0.1595126	0.8705814	0.1832	0.855	0.030300774	count	1
AL365356.4	0.1595126	0.8785495	0.1816	0.856	0.030300774	count	1
AC110285.5	0.1595126	0.8785495	0.1816	0.856	0.030300774	count	1

AC073352.2	0.1595126	1.0980881	0.1453	0.885	0.030300774	count	1
PSD4	0.3119224	0.8258884	0.3777	0.706	0.030438913	count	1
AC012409.3	0.3119224	0.9382947	0.3324	0.74	0.030438913	count	1
VILL	0.0391525	0.3143188	0.1246	0.901	0.030527185	count	1
PTPRB	0.3139016	0.9304082	0.3374	0.736	0.030615311	count	1
RPP38	0.023011	0.1514919	0.1519	0.879	0.030668896	count	1
MEI1	0.0903312	0.5455982	0.1656	0.869	0.030779534	count	1
MRPL32	0.0218692	0.0630187	0.347	0.729	0.030849982	count	1
THUMPD3	0.0228266	0.1260414	0.1811	0.856	0.030974972	count	1
PMPCA	0.0239408	0.1443496	0.1659	0.868	0.031233219	count	1
BPTF	0.022198	0.0857916	0.2587	0.796	0.031258799	count	1
RTTN	0.1159875	0.4584094	0.253	0.8	0.031318634	count	1
PPP2CB	0.0223966	0.0710606	0.3152	0.753	0.0313432	count	1
VBP1	0.022726	0.0880312	0.2582	0.796	0.031354543	count	1
OBSCN	0.1655115	0.7664602	0.2159	0.829	0.031393594	count	1
DMAC2	0.0251523	0.2607534	0.0965	0.923	0.031469511	count	1
MORN2	0.0235503	0.1240522	0.1898	0.849	0.031533813	count	1
RFC1	0.0223508	0.0727473	0.3072	0.759	0.031586857	count	1
TPGS2	0.0229733	0.0890642	0.2579	0.796	0.03165992	count	1
AL590428.1	0.1675572	0.6157075	0.2721	0.786	0.031765474	count	1
AL139246.5	0.0436582	0.3345569	0.1305	0.896	0.03177308	count	1
TMEM147	0.0226159	0.0666399	0.3394	0.734	0.031899353	count	1
KIAA0825	0.0322605	0.3140246	0.1027	0.918	0.031994824	count	1
PSMA3-AS1	0.0239976	0.1449539	0.1656	0.869	0.032103973	count	1
RNF144A	0.0459918	0.3977357	0.1156	0.908	0.032133386	count	1
RANBP3	0.02693	0.1661147	0.1621	0.871	0.032151411	count	1
GPATCH2	0.0239645	0.1198203	0.2	0.841	0.032169783	count	1
C16orf72	0.0246282	0.1491688	0.1651	0.869	0.032228953	count	1
HSF2BP	0.1198271	0.6026809	0.1988	0.842	0.03232693	count	1
ARAP2	0.1198271	0.6082234	0.197	0.844	0.03232693	count	1
LINC00472	0.0463113	0.3671514	0.1261	0.9	0.032355141	count	1
GPC2	0.0952007	0.7938405	0.1199	0.905	0.032405022	count	1
GRIA3	0.0344995	0.5872196	0.0588	0.953	0.032514342	count	1
PSMB5	0.0228679	0.0461038	0.496	0.62	0.032575256	count	1
LAMA3	0.0315637	0.3550063	0.0889	0.929	0.032578283	count	1
LINC00937	0.0549082	0.5617544	0.0977	0.922	0.032688638	count	1
NXPE3	0.0264148	0.2343668	0.1127	0.91	0.032709099	count	1
MRPL17	0.0236335	0.0911368	0.2593	0.795	0.032762233	count	1
HNRNPK	0.0229455	0.0408103	0.5622	0.574	0.032762897	count	1
AKT1S1	0.0252336	0.1285568	0.1963	0.844	0.03278222	count	1
KIAA1191	0.0246277	0.1367975	0.18	0.857	0.032885964	count	1
NDUFS1	0.0239983	0.0964611	0.2488	0.804	0.032935316	count	1
UBAC2	0.0245593	0.102211	0.2403	0.81	0.032981276	count	1
SPAG7	0.0237663	0.0825609	0.2879	0.773	0.033005506	count	1
ZNF749	0.0821122	0.6624398	0.124	0.901	0.033077889	count	1

MYO15A	0.0821122	0.6894392	0.1191	0.905	0.033077889	count	1
ELOC	0.0233422	0.0542543	0.4302	0.667	0.033102913	count	1
MAPRE2	0.0246205	0.1190911	0.2067	0.836	0.033128512	count	1
CEBPZ	0.0238019	0.0911027	0.2613	0.794	0.033233847	count	1
HECTD1	0.0246432	0.1040987	0.2367	0.813	0.033256389	count	1
ASH1L-AS1	0.0414477	0.3787075	0.1094	0.913	0.033263047	count	1
RAB42	0.0498868	0.5228195	0.0954	0.924	0.033274651	count	1
UBP1	0.029177	0.1997233	0.1461	0.884	0.033275381	count	1
H2AFJ	0.0232383	0.0401506	0.5788	0.563	0.033293969	count	1
THOC5	0.02784	0.1788771	0.1556	0.876	0.033329147	count	1
MGA	0.0269904	0.1739587	0.1552	0.877	0.033358912	count	1
DTX2	0.0354023	0.3595519	0.0985	0.922	0.033362323	count	1
COX11	0.0247418	0.0986762	0.2507	0.802	0.033468617	count	1
CNPY3	0.0248639	0.1058737	0.2348	0.814	0.033542258	count	1
UBL7	0.0259974	0.1446459	0.1797	0.857	0.033831359	count	1
PUF60	0.0241846	0.0694086	0.3484	0.728	0.033837482	count	1
ASCC1	0.0272084	0.2062589	0.1319	0.895	0.033870759	count	1
PSMG3	0.0253472	0.1094553	0.2316	0.817	0.033877755	count	1
AFAP1	0.02623	0.1408768	0.1862	0.852	0.03398517	count	1
VWA8	0.0323824	0.2816006	0.115	0.908	0.033990224	count	1
TNRC18	0.0263666	0.1579851	0.1669	0.867	0.034033557	count	1
RSL1D1	0.0239383	0.0475655	0.5033	0.615	0.034053447	count	1
AK1	0.0259632	0.1516048	0.1713	0.864	0.034073794	count	1
C12orf4	0.030875	0.2199726	0.1404	0.888	0.034370907	count	1
POLE3	0.02562	0.1145217	0.2237	0.823	0.034446106	count	1
AC108463.3	0.3585553	0.6656273	0.5387	0.59	0.034539135	count	1
STARD7	0.0275283	0.1535759	0.1792	0.858	0.034547264	count	1
DEK	0.0242152	0.0432408	0.56	0.5755	0.034596306	count	1
UBE2I	0.0244482	0.057157	0.4277	0.669	0.034630908	count	1
DCTN1	0.0267299	0.1325745	0.2016	0.84	0.03472472	count	1
CDK15	0.3612359	0.6832558	0.5287	0.597	0.034771279	count	1
TRIM33	0.0265977	0.1300274	0.2046	0.838	0.034804533	count	1
EMID1	0.129783	0.843018	0.154	0.878	0.034932766	count	1
AC111182.1	0.129783	0.9329663	0.1391	0.889	0.034932766	count	1
MT1X	0.0244315	0.0711891	0.3432	0.7315	0.034968512	count	1
ZCCHC3	0.0306552	0.1930119	0.1588	0.874	0.035108389	count	1
ACHE	0.0483224	0.4687	0.1031	0.918	0.035145109	count	1
ZSCAN22	0.1307675	0.7872989	0.1661	0.868	0.03518977	count	1
DGKZ	0.0312998	0.2000551	0.1565	0.876	0.035200178	count	1
AL023881.1	0.3664516	0.8361961	0.4382	0.661	0.035221862	count	1
SPIN1	0.0260917	0.0976522	0.2672	0.789	0.035282247	count	1
ELOVL4	0.0345433	0.2880198	0.1199	0.905	0.035320928	count	1
RRAGA	0.0249467	0.054226	0.4601	0.646	0.035353337	count	1
AMIGO1	0.0531171	0.3568392	0.1489	0.882	0.035412254	count	1
ZNF552	0.0471569	0.4396305	0.1073	0.915	0.035562242	count	1

SLC33A1	0.0316561	0.2276916	0.139	0.889	0.035600109	count	1
MTMR12	0.029701	0.2280337	0.1302	0.896	0.035650329	count	1
DYNC1I2	0.0252159	0.0518548	0.4863	0.627	0.035754787	count	1
FGFR1OP2	0.0255815	0.077006	0.3322	0.74	0.035767149	count	1
PWWP2B	0.0474936	0.4670466	0.1017	0.919	0.035814566	count	1
NFE2L1	0.0263686	0.1023684	0.2576	0.797	0.035870721	count	1
MAB21L2	0.0266609	0.1942279	0.1373	0.891	0.035872653	count	1
SEC23B	0.0319004	0.2245554	0.1421	0.887	0.035874315	count	1
GPHN	0.0313259	0.288924	0.1084	0.914	0.035875156	count	1
C5orf34	0.0345088	0.4989247	0.0692	0.945	0.035919769	count	1
SORBS2	0.025359	0.0852615	0.2974	0.766	0.035920895	count	1
MCM4	0.0367739	0.347775	0.1057	0.916	0.036039353	count	1
EFEMP2	0.0254767	0.0513438	0.4962	0.62	0.036196892	count	1
ZNF790	0.032717	0.2793799	0.1171	0.907	0.036220245	count	1
NR2F2	0.0253983	0.0490631	0.5177	0.605	0.036311003	count	1
AC026461.3	0.3810401	1.129112	0.3375	0.736	0.036474429	count	1
MRO	0.3810401	1.129112	0.3375	0.736	0.036474429	count	1
DEFB124	0.3810401	1.129112	0.3375	0.736	0.036474429	count	1
LINC02586	0.3810401	1.203576	0.3166	0.752	0.036474429	count	1
RASGRP3	0.3810401	1.203576	0.3166	0.752	0.036474429	count	1
LINC02388	0.3810401	1.203576	0.3166	0.752	0.036474429	count	1
NETO1	0.3810401	1.297554	0.2937	0.769	0.036474429	count	1
C3orf38	0.027647	0.1315003	0.2102	0.833	0.036475958	count	1
C4orf46	0.0433112	0.3358488	0.129	0.897	0.036553281	count	1
AC116913.1	0.0911153	0.4990992	0.1826	0.855	0.036638099	count	1
APC	0.0274016	0.1154673	0.2373	0.812	0.036655353	count	1
ZKSCAN3	0.1369004	0.5145557	0.2661	0.79	0.036788017	count	1
PSEN2	0.0356795	0.2463656	0.1448	0.885	0.036814519	count	1
SYNJ1	0.0348262	0.2718797	0.1281	0.898	0.036835527	count	1
LRP2BP	0.0506904	0.4546034	0.1115	0.911	0.036855396	count	1
SEC16A	0.0334191	0.2072725	0.1612	0.872	0.036995861	count	1
GORASP1	0.0389649	0.221801	0.1757	0.861	0.037223819	count	1
DDX10	0.0310376	0.1754908	0.1769	0.86	0.037252359	count	1
CDH19	0.0377089	0.4415341	0.0854	0.932	0.037380829	count	1
ACSF3	0.0351098	0.1844958	0.1903	0.849	0.037412054	count	1
ZNF528	0.0363882	0.2670739	0.1362	0.892	0.037543676	count	1
PRKCE	0.0445158	0.3701312	0.1203	0.904	0.037564737	count	1
AC002553.1	0.3975927	0.7050897	0.5639	0.573	0.037881863	count	1
ETV4	0.0568675	0.6254194	0.0909	0.928	0.037891387	count	1
CTU2	0.034016	0.1997781	0.1703	0.865	0.03805774	count	1
NLGN1	0.0321754	0.2522846	0.1275	0.899	0.038064337	count	1
CLEC18A	0.1420166	0.45687	0.3108	0.756	0.038117683	count	1
KLHL2	0.0394191	0.266418	0.148	0.882	0.038151875	count	1
ATXN7L3	0.0338627	0.2475446	0.1368	0.891	0.038260006	count	1
JPT1	0.0286259	0.1377064	0.2079	0.835	0.03834292	count	1

AIFM2	0.0344694	0.2325845	0.1482	0.882	0.038363627	count	1
MRPL39	0.0292587	0.1241776	0.2356	0.814	0.03836832	count	1
TAF4	0.0696361	0.3578591	0.1946	0.846	0.038524179	count	1
ACVR1B	0.0380537	0.243832	0.1561	0.876	0.038525131	count	1
VAPA	0.0270326	0.043049	0.6279	0.5301	0.038567054	count	1
WHRN	0.2066206	0.7980221	0.2589	0.796	0.038790061	count	1
SERP1	0.0273277	0.0519164	0.5264	0.599	0.038882299	count	1
GATAD2A	0.0312294	0.1769734	0.1765	0.86	0.038936443	count	1
KCNJ15	0.410681	0.6533083	0.6286	0.53	0.03898439	count	1
NUCB1	0.0275192	0.054581	0.5042	0.614	0.039037363	count	1
RNF111	0.0302525	0.1485424	0.2037	0.839	0.03904502	count	1
UNC5C	0.0328403	0.2521704	0.1302	0.896	0.039084174	count	1
FASTK	0.0292968	0.1214727	0.2412	0.809	0.039189093	count	1
AC068473.5	0.0774138	0.4208337	0.184	0.854	0.03930531	count	1
SLC48A1	0.0301018	0.1223913	0.2459	0.806	0.039444576	count	1
FAM41C	0.0479455	0.3948076	0.1214	0.903	0.039478533	count	1
AC092171.3	0.1165285	0.7883414	0.1478	0.882	0.039483086	count	1
SMC3	0.0280904	0.0648025	0.4335	0.665	0.039510844	count	1
DHODH	0.0449057	0.2739419	0.1639	0.87	0.039533065	count	1
TUBGCP5	0.0317482	0.16828	0.1887	0.85	0.039648175	count	1
AL021154.1	0.148254	1.0209364	0.1452	0.885	0.039734257	count	1
HOXA11	0.148254	1.0209364	0.1452	0.885	0.039734257	count	1
TMEM229A	0.148254	1.0209364	0.1452	0.885	0.039734257	count	1
CNIH2	0.148254	1.0209364	0.1452	0.885	0.039734257	count	1
NOX5	0.148254	1.0209364	0.1452	0.885	0.039734257	count	1
AC092115.3	0.148254	1.0209364	0.1452	0.885	0.039734257	count	1
AC107982.3	0.148254	1.0209364	0.1452	0.885	0.039734257	count	1
AL109741.1	0.148254	1.0524685	0.1409	0.888	0.039734257	count	1
AC010680.1	0.148254	1.0524685	0.1409	0.888	0.039734257	count	1
AC007336.1	0.148254	1.160805	0.1277	0.898	0.039734257	count	1
AC026367.2	0.148254	1.188633	0.1247	0.901	0.039734257	count	1
CALML4	0.148254	1.188633	0.1247	0.901	0.039734257	count	1
AC083964.1	0.148254	1.42238	0.1042	0.917	0.039734257	count	1
CCDC150	0.4197626	0.9141005	0.4592	0.646	0.039744044	count	1
AC093503.2	0.4197626	0.9141005	0.4592	0.646	0.039744044	count	1
ANP32E	0.0283946	0.0705912	0.4022	0.688	0.039771814	count	1
OTUD4	0.0361526	0.2029798	0.1781	0.859	0.039788984	count	1
GPR162	0.2129579	0.6015357	0.354	0.723	0.039915912	count	1
AC013400.1	0.2129579	0.7904884	0.2694	0.788	0.039915912	count	1
PML	0.0319522	0.1526703	0.2093	0.834	0.039967344	count	1
GIMAP1	0.0673133	0.7537615	0.0893	0.929	0.03999276	count	1
PCGF3	0.0367871	0.2275242	0.1617	0.872	0.039999154	count	1
ATP6AP2	0.0280088	0.0384319	0.7288	0.4662	0.040030252	count	1
DENND6A	0.0323703	0.1708388	0.1895	0.85	0.040074417	count	1
MEMO1	0.0551586	0.3649977	0.1511	0.88	0.040079449	count	1

PRELID2	0.0359002	0.209632	0.1713	0.864	0.040161135	count	1
TIGAR	0.0477089	0.2398281	0.1989	0.842	0.040244482	count	1
PRSS27	0.118864	0.7571422	0.157	0.875	0.040254063	count	1
AC010245.2	0.118864	0.8141639	0.146	0.884	0.040254063	count	1
MMRN1	0.118864	0.9358112	0.127	0.899	0.040254063	count	1
DUSP22	0.0303662	0.105326	0.2883	0.773	0.040297106	count	1
SIX5	0.0330241	0.1760611	0.1876	0.851	0.040310749	count	1
TBX1	0.0605413	0.281542	0.215	0.83	0.040317128	count	1
RAP1GDS1	0.0323368	0.1600931	0.202	0.84	0.040382357	count	1
ZNF507	0.0342473	0.1794309	0.1909	0.849	0.040383709	count	1
TRIM52	0.0331638	0.236855	0.14	0.889	0.040390936	count	1
UTP25	0.0341625	0.1946096	0.1755	0.861	0.040534858	count	1
AC005332.5	0.1517119	0.5521338	0.2748	0.784	0.040628361	count	1
XPO1	0.0302809	0.1024201	0.2957	0.768	0.040643971	count	1
EIF2S1	0.0295616	0.0869648	0.3399	0.734	0.040666212	count	1
SUCNR1	0.0284301	0.1375448	0.2067	0.836	0.040668838	count	1
ZNF483	0.0346357	0.1833417	0.1889	0.85	0.040708116	count	1
WARS	0.0316075	0.1613967	0.1958	0.845	0.040831725	count	1
IQCB1	0.0323004	0.1512416	0.2136	0.831	0.040880316	count	1
AC079834.2	0.4342593	0.8696014	0.4994	0.618	0.040947586	count	1
OXA1L	0.030025	0.1116151	0.269	0.788	0.041036891	count	1
TOMM34	0.0449864	0.2881368	0.1561	0.876	0.041058635	count	1
BYSL	0.05123	0.3551417	0.1443	0.885	0.041064308	count	1
SMPD4	0.0335381	0.2396913	0.1399	0.889	0.041113061	count	1
SLC49A3	0.0372908	0.3418592	0.1091	0.913	0.041271685	count	1
CUL3	0.0310007	0.111093	0.2791	0.78	0.04144816	count	1
ALG13	0.0304063	0.1070871	0.2839	0.776	0.041488941	count	1
NPIPA1	0.038694	0.4793653	0.0807	0.936	0.041514124	count	1
CCDC106	0.0336894	0.1683257	0.2001	0.841	0.041705311	count	1
AP000880.1	0.1234411	0.7501635	0.1646	0.869	0.041762658	count	1
SKIDA1	0.1234411	0.7501635	0.1646	0.869	0.041762658	count	1
BX088651.4	0.1234411	0.8272644	0.1492	0.881	0.041762658	count	1
C3orf49	0.1234411	1.0083033	0.1224	0.903	0.041762658	count	1
ADAL	0.0384751	0.2355846	0.1633	0.87	0.041829739	count	1
DUBR	0.0350812	0.1974491	0.1777	0.859	0.041866831	count	1
AC008946.1	0.091682	0.4855018	0.1888	0.85	0.041898006	count	1
SLC25A35	0.091682	0.5337271	0.1718	0.864	0.041898006	count	1
MOGS	0.0387928	0.2009236	0.1931	0.847	0.041902492	count	1
SH3RF3-AS1	0.4472376	0.6533146	0.6846	0.494	0.042015617	count	1
SLC45A1	0.0435287	0.3441534	0.1265	0.899	0.042113614	count	1
SAMD9	0.0320268	0.1598561	0.2003	0.841	0.042196168	count	1
NUP98	0.0344375	0.188178	0.183	0.855	0.042386237	count	1
ADI1	0.0296759	0.0393062	0.755	0.45	0.04246562	count	1
LLGL1	0.1255781	0.3641552	0.3448	0.73	0.042465934	count	1
TRMT5	0.0347009	0.1600998	0.2167	0.828	0.042624222	count	1

GOLPH3L	0.0339288	0.1924456	0.1763	0.86	0.042634558	count	1
RANBP6	0.0347349	0.2106614	0.1649	0.869	0.042665926	count	1
MEIS3	0.039019	0.2108842	0.185	0.853	0.042682456	count	1
PARP9	0.0328346	0.1411595	0.2326	0.816	0.042684862	count	1
CFB	0.0453601	0.2768873	0.1638	0.87	0.042705572	count	1
LEPROTL1	0.0311582	0.1004503	0.3102	0.756	0.042714572	count	1
CIITA	0.2289056	0.9203386	0.2487	0.804	0.042732067	count	1
GTPBP3	0.0375784	0.2341273	0.1605	0.872	0.042836111	count	1
ZNF786	0.0679688	0.3362147	0.2022	0.84	0.042901208	count	1
ZNF727	0.0591466	0.4023876	0.147	0.883	0.042953576	count	1
FAM228A	0.072505	0.6666591	0.1088	0.913	0.043040612	count	1
RNF166	0.0365596	0.216327	0.169	0.866	0.043241546	count	1
RTKN2	0.0595854	0.7065923	0.0843	0.933	0.043269619	count	1
PTRHD1	0.0318472	0.1020728	0.312	0.755	0.043432679	count	1
PPID	0.0323612	0.098966	0.327	0.744	0.043554491	count	1
RING1	0.0328321	0.1149063	0.2857	0.775	0.043567075	count	1
SDC3	0.0351494	0.2136447	0.1645	0.869	0.04358955	count	1
AC005034.3	0.1633421	0.6974084	0.2342	0.815	0.043624403	count	1
AC005498.2	0.1633421	0.6993159	0.2336	0.815	0.043624403	count	1
TM2D1	0.0309929	0.0644048	0.4812	0.63	0.043628824	count	1
PIP4K2C	0.043136	0.2445691	0.1764	0.86	0.043652095	count	1
CARS2	0.0343047	0.1596376	0.2149	0.83	0.043746338	count	1
NDE1	0.0358796	0.2003277	0.1791	0.858	0.043886192	count	1
RXRA	0.033449	0.1435532	0.233	0.816	0.043889897	count	1
MAN1A2	0.03159	0.0837494	0.3772	0.706	0.043925416	count	1
TRMT2A	0.0368767	0.1893954	0.1947	0.846	0.044005857	count	1
FRRS1L	0.4717911	0.7300773	0.6462	0.518	0.044011877	count	1
AP001318.2	0.4717911	0.7300773	0.6462	0.518	0.044011877	count	1
AP003108.2	0.0743442	0.545065	0.1364	0.892	0.044119039	count	1
NT5M	0.1655115	0.5002382	0.3309	0.741	0.044181361	count	1
TRMT2B	0.0429294	0.3885937	0.1105	0.912	0.04426978	count	1
DNAJC25-GNG10	0.0970567	0.7231515	0.1342	0.893	0.044308397	count	1
APOBEC3A	0.0970567	1.023311	0.0948	0.924	0.044308397	count	1
TENT5C	0.044312	0.218207	0.2031	0.839	0.044380808	count	1
GPR135	0.041406	0.3434472	0.1206	0.904	0.04441524	count	1
MRPL45	0.0332739	0.1258616	0.2644	0.792	0.044423574	count	1
SHC4	0.0378814	0.299158	0.1266	0.899	0.044515463	count	1
LRSAM1	0.0402971	0.3269213	0.1233	0.902	0.044590284	count	1
PCED1B	0.0489133	0.4253432	0.115	0.908	0.044624828	count	1
BSG	0.03122	0.0348695	0.8953	0.371	0.044700884	count	1
POLDIP2	0.0350011	0.1382289	0.2532	0.8	0.04473834	count	1
TCP10L	0.2404492	0.5689789	0.4226	0.673	0.0447553	count	1
AC136604.3	0.4812007	1.157761	0.4156	0.678	0.044768491	count	1
PCDH19	0.4812007	1.157761	0.4156	0.678	0.044768491	count	1
PPP1R42	0.4812007	1.157761	0.4156	0.678	0.044768491	count	1

AC001226.2	0.4812007	1.157761	0.4156	0.678	0.044768491	count	1
XRCC3	0.4812007	1.157761	0.4156	0.678	0.044768491	count	1
WSCD1	0.4812007	1.157761	0.4156	0.678	0.044768491	count	1
AC090579.1	0.4812007	1.162495	0.4139	0.679	0.044768491	count	1
AL158151.3	0.4812007	1.162495	0.4139	0.679	0.044768491	count	1
AC068768.1	0.4812007	1.162495	0.4139	0.679	0.044768491	count	1
AC005696.4	0.4812007	1.162495	0.4139	0.679	0.044768491	count	1
METTL7B	0.4812007	1.204446	0.3995	0.69	0.044768491	count	1
UBL7-AS1	0.0385057	0.2337213	0.1648	0.869	0.044777517	count	1
FPGT-TNNI3K	0.075584	0.5125502	0.1475	0.883	0.04484563	count	1
TSSC4	0.0325168	0.0830077	0.3917	0.695	0.044938841	count	1
POMZP3	0.0410943	0.247898	0.1658	0.868	0.044946365	count	1
SEMA4D	0.0816679	0.5526416	0.1478	0.883	0.045086782	count	1
LENG8-AS1	0.0599616	0.4111857	0.1458	0.884	0.045141929	count	1
PJA1	0.0371223	0.1557617	0.2383	0.812	0.04530531	count	1
SEMA6B	0.1131876	0.8016882	0.1412	0.888	0.045309679	count	1
RPP14	0.0416855	0.2291044	0.1819	0.856	0.045310135	count	1
TICAM2	0.0823552	0.6133193	0.1343	0.893	0.045460811	count	1
DGCR2	0.0366252	0.166802	0.2196	0.826	0.045498016	count	1
UCP2	0.0491022	0.3149008	0.1559	0.876	0.045523934	count	1
ISCA1	0.0326773	0.0767854	0.4256	0.67	0.045575023	count	1
ST6GALNAC6	0.0999706	0.5970898	0.1674	0.867	0.045613034	count	1
AC022098.1	0.0999938	0.4511995	0.2216	0.825	0.045623415	count	1
MTRNR2L6	0.2454984	0.6940438	0.3537	0.724	0.04563624	count	1
GPR37	0.2454984	0.7539383	0.3256	0.745	0.04563624	count	1
PJA2	0.0321543	0.0498122	0.6455	0.519	0.045654977	count	1
PBXIP1	0.0327528	0.0771573	0.4245	0.671	0.045731932	count	1
CDKL3	0.0396783	0.2428496	0.1634	0.87	0.045789844	count	1
GABARAP	0.0394596	0.1993182	0.198	0.843	0.045884439	count	1
GTPBP1	0.0428209	0.2942467	0.1455	0.884	0.045928354	count	1
UPF3A	0.0326079	0.0744892	0.4378	0.662	0.045943679	count	1
POLD4	0.0475981	0.3271967	0.1455	0.884	0.046033608	count	1
KCNMB3	0.0497466	0.8187007	0.0608	0.952	0.046118425	count	1
SPA17	0.037507	0.1718967	0.2182	0.827	0.046158476	count	1
PCDHB15	0.0536992	0.3468307	0.1548	0.877	0.046278769	count	1
PSMA1	0.0326057	0.0518532	0.6288	0.53	0.04629489	count	1
CCDC40	0.1157364	0.5235177	0.2211	0.825	0.046305817	count	1
PUM3	0.0335901	0.103481	0.3246	0.746	0.046461204	count	1
CD59	0.0324518	0.0390568	0.8309	0.406	0.046517868	count	1
ACSS3	0.046472	0.2334423	0.1991	0.842	0.046535638	count	1
PRRT1	0.0403383	0.2823895	0.1428	0.886	0.0465498	count	1
SEPHS2	0.0340897	0.1088527	0.3132	0.754	0.046631781	count	1
VDR	0.2516069	0.847054	0.297	0.766	0.046698724	count	1
POPDC3	0.2516069	1.041231	0.2416	0.809	0.046698724	count	1
TMEM14A	0.0333242	0.0776656	0.4291	0.668	0.046718313	count	1

TRPT1	0.035374	0.1181292	0.2995	0.765	0.046748473	count	1
PPP2R5E	0.0341066	0.0999034	0.3414	0.733	0.046754619	count	1
ZNF438	0.0416177	0.1791152	0.2324	0.816	0.046774422	count	1
AL138724.1	0.1175984	0.4676149	0.2515	0.801	0.047032843	count	1
THRIL	0.5097876	0.7157533	0.7122	0.476	0.047038661	count	1
FRAS1	0.5097876	0.7364252	0.6922	0.489	0.047038661	count	1
CPSF3	0.0403707	0.2845731	0.1419	0.887	0.047110863	count	1
LUC7L2	0.0353731	0.1022759	0.3459	0.729	0.047132785	count	1
GSTA4	0.0344587	0.113946	0.3024	0.762	0.047157097	count	1
ATP5S	0.0361176	0.1249659	0.289	0.773	0.047214526	count	1
TRAF4	0.0471727	0.3027243	0.1558	0.876	0.047234485	count	1
FBXL3	0.0353127	0.0882642	0.4001	0.689	0.047333935	count	1
LINC01852	0.0750863	0.4099222	0.1832	0.855	0.047340664	count	1
MCM9	0.0498265	0.3086153	0.1615	0.872	0.04755171	count	1
UEVLD	0.0457684	0.2576814	0.1776	0.859	0.047598425	count	1
ACBD6	0.0344907	0.0865112	0.3987	0.69	0.047686261	count	1
THAP5	0.0350385	0.098647	0.3552	0.722	0.047770674	count	1
GTF2A1	0.037001	0.1229176	0.301	0.763	0.047791797	count	1
KCTD21-AS1	0.1050628	0.4279081	0.2455	0.806	0.047889303	count	1
OTUD6B	0.0415427	0.2348096	0.1769	0.86	0.047936458	count	1
SP2	0.0424667	0.228061	0.1862	0.852	0.04795654	count	1
CSTF1	0.0398842	0.1939884	0.2056	0.837	0.04797692	count	1
CAPN10-DT	0.1424397	0.6317836	0.2255	0.822	0.047991021	count	1
RPL22L1	0.0342456	0.0768446	0.4456	0.656	0.048166455	count	1
GNL1	0.035851	0.1077168	0.3328	0.739	0.048284474	count	1
RAB36	0.0875989	0.5204846	0.1683	0.866	0.048311411	count	1
HNRNPA1L2	0.0875989	0.5833079	0.1502	0.881	0.048311411	count	1
CSNK1G2	0.0380568	0.1522924	0.2499	0.803	0.048525276	count	1
COPA	0.0352787	0.1008377	0.3499	0.726	0.048561459	count	1
CRLS1	0.0373244	0.1215495	0.3071	0.759	0.048676293	count	1
RBM7	0.0360572	0.097554	0.3696	0.712	0.04870048	count	1
B3GNT10	0.2638866	0.5705488	0.4625	0.644	0.048823757	count	1
ERCC2	0.0483157	0.2854641	0.1693	0.866	0.048872786	count	1
C6orf47	0.0391323	0.1461981	0.2677	0.789	0.04901336	count	1
AC004846.1	0.0476035	0.3481277	0.1367	0.891	0.049071659	count	1
ELP3	0.0454733	0.3082987	0.1475	0.883	0.04909566	count	1
GNA13	0.0406618	0.1731286	0.2349	0.814	0.049158179	count	1
SNAPC2	0.0373703	0.1308185	0.2857	0.775	0.049197924	count	1
AC015922.4	0.0503799	0.320393	0.1572	0.875	0.049313905	count	1
HSPD1	0.034695	0.0495068	0.7008	0.483	0.049340007	count	1
DIRAS1	0.5396547	0.8443051	0.6392	0.523	0.049364989	count	1
PCDHB10	0.0977596	0.3199517	0.3055	0.76	0.049451827	count	1
NEB	0.2687108	1.152558	0.2331	0.816	0.049654627	count	1
C3orf67	0.2687108	1.152558	0.2331	0.816	0.049654627	count	1
AC107204.1	0.2687108	1.193605	0.2251	0.822	0.049654627	count	1

VEPH1	0.2687108	1.193605	0.2251	0.822	0.049654627	count	1
PLS3-AS1	0.2687108	1.193605	0.2251	0.822	0.049654627	count	1
AC100812.1	0.2687108	1.193605	0.2251	0.822	0.049654627	count	1
AC069503.2	0.2687108	1.193605	0.2251	0.822	0.049654627	count	1
AC021504.1	0.2687108	1.193605	0.2251	0.822	0.049654627	count	1
CAMTA1-DT	0.2687108	1.338447	0.2008	0.841	0.049654627	count	1
AC003991.1	0.2687108	1.338447	0.2008	0.841	0.049654627	count	1
SALRNA2	0.2687108	1.338447	0.2008	0.841	0.049654627	count	1
LINC01481	0.2687108	1.367249	0.1965	0.844	0.049654627	count	1
CRYBB2	0.2687108	1.367249	0.1965	0.844	0.049654627	count	1
PPIP5K2	0.0366821	0.1041025	0.3524	0.725	0.049673998	count	1
ISOC1	0.04167	0.1736931	0.2399	0.81	0.049714405	count	1
FAM181B	0.049727	0.2204218	0.2256	0.822	0.049781316	count	1
TSPAN11	0.1094853	0.4205708	0.2603	0.795	0.049862428	count	1
INCA1	0.1097083	0.5474834	0.2004	0.841	0.049961826	count	1
AC073263.2	0.5478141	0.724575	0.756	0.45	0.049992477	count	1
KBTBD7	0.0427206	0.1901601	0.2247	0.822	0.050020797	count	1
R3HCC1L	0.0433832	0.212531	0.2041	0.838	0.050055108	count	1
MEIG1	0.1887951	0.9619558	0.1963	0.844	0.05012136	count	1
LINC01562	0.1887951	0.9767795	0.1933	0.847	0.05012136	count	1
KIF11	0.1887951	0.9767795	0.1933	0.847	0.05012136	count	1
COMMD5	0.0373324	0.1105164	0.3378	0.736	0.050162596	count	1
C7orf43	0.0846821	0.4232657	0.2001	0.841	0.050168235	count	1
MBTPS1	0.0359944	0.0919161	0.3916	0.695	0.050181588	count	1
PROCA1	0.0610428	0.4819687	0.1267	0.899	0.050184322	count	1
NUP50-DT	0.0584359	0.4101165	0.1425	0.887	0.050334169	count	1
AC034236.2	0.0588733	0.3237777	0.1818	0.856	0.050708431	count	1
PRX	0.5591479	0.6064416	0.922	0.357	0.050858384	count	1
AGO2	0.0385906	0.1428154	0.2702	0.787	0.050868885	count	1
RECQL	0.0370505	0.0870302	0.4257	0.67	0.050868983	count	1
TSPYL4	0.0398047	0.1566606	0.2541	0.799	0.050870574	count	1
PPIG	0.0357714	0.0487943	0.7331	0.4635	0.051026117	count	1
YPEL3	0.0358865	0.0517037	0.6941	0.488	0.051059782	count	1
VPS25	0.0370994	0.0910749	0.4073	0.684	0.051084051	count	1
TMEM205	0.0365736	0.0763386	0.4791	0.632	0.051235128	count	1
WDR70	0.0393949	0.181714	0.2168	0.828	0.051247424	count	1
UGCG	0.0382456	0.1009896	0.3787	0.705	0.051368244	count	1
TTN-AS1	0.0554677	0.2535865	0.2187	0.827	0.051392998	count	1
CACYBP	0.036559	0.0583782	0.6262	0.531	0.051616899	count	1
RANBP10	0.0542688	0.2914414	0.1862	0.852	0.051769517	count	1
RFNG	0.0376613	0.0968645	0.3888	0.697	0.051775074	count	1
MCRS1	0.0397776	0.1320105	0.3013	0.763	0.051830368	count	1
MRPS23	0.0376294	0.0844727	0.4455	0.656	0.051907096	count	1
GLRX2	0.0385431	0.1154755	0.3338	0.739	0.051926833	count	1
EXO5	0.0691065	0.4584267	0.1507	0.88	0.051963086	count	1

ZNF845	0.0603419	0.4296845	0.1404	0.888	0.051964769	count	1
RPS6KB2	0.0410974	0.1646834	0.2496	0.803	0.052136406	count	1
CYSTM1	0.0365441	0.0461753	0.7914	0.429	0.05219149	count	1
GOLGA4	0.0369407	0.075705	0.488	0.626	0.052196175	count	1
MARCKSL1	0.0396774	0.1934265	0.2051	0.837	0.052197683	count	1
CRYGS	0.5770893	1.013155	0.5696	0.569	0.052215608	count	1
AC108865.2	0.5770893	1.013155	0.5696	0.569	0.052215608	count	1
AP001453.1	0.5770893	1.013155	0.5696	0.569	0.052215608	count	1
AL139383.1	0.5770893	1.013155	0.5696	0.569	0.052215608	count	1
AC092287.1	0.5770893	1.013155	0.5696	0.569	0.052215608	count	1
AC005697.2	0.5770893	1.013155	0.5696	0.569	0.052215608	count	1
HOXB6	0.5770893	1.013155	0.5696	0.569	0.052215608	count	1
MIR646HG	0.5770893	1.013155	0.5696	0.569	0.052215608	count	1
AL021707.1	0.5770893	1.013155	0.5696	0.569	0.052215608	count	1
ZNF705E	0.5770893	1.108662	0.5205	0.603	0.052215608	count	1
AC006441.4	0.5770893	1.108662	0.5205	0.603	0.052215608	count	1
HIST1H4F	0.5770893	1.161013	0.4971	0.619	0.052215608	count	1
ASMTL-AS1	0.5770893	1.161013	0.4971	0.619	0.052215608	count	1
AC023813.3	0.5770893	1.161013	0.4971	0.619	0.052215608	count	1
ZNRF3-AS1	0.5770893	1.161013	0.4971	0.619	0.052215608	count	1
RPS6KA1	0.5770893	1.24523	0.4634	0.643	0.052215608	count	1
SORCS3	0.5770893	1.24523	0.4634	0.643	0.052215608	count	1
SCARF1	0.2838564	0.7424226	0.3823	0.702	0.05224861	count	1
SPDYA	0.2838564	0.7588799	0.374	0.708	0.05224861	count	1
PAM16	0.2842273	0.591524	0.4805	0.631	0.052311858	count	1
PDHX	0.0435019	0.1998608	0.2177	0.828	0.052319936	count	1
METTL16	0.0405944	0.1570185	0.2585	0.796	0.05232519	count	1
PNMA8C	0.0885309	0.4484955	0.1974	0.844	0.052414887	count	1
ALDH16A1	0.0456283	0.1847367	0.247	0.805	0.052428825	count	1
RAB22A	0.0386818	0.0970664	0.3985	0.69	0.052476323	count	1
DTX4	0.581812	0.8940836	0.6507	0.515	0.052570149	count	1
ZNF674	0.0837214	0.3732062	0.2243	0.823	0.052712902	count	1
TCF7	0.1571686	0.3851067	0.4081	0.683	0.052782316	count	1
RBM4	0.0443952	0.165529	0.2682	0.789	0.052806381	count	1
AC010864.1	0.1325963	0.5757998	0.2303	0.818	0.052867691	count	1
MTERF4	0.0400082	0.1347175	0.297	0.766	0.052930209	count	1
GID8	0.0390624	0.1025114	0.3811	0.703	0.05294445	count	1
TCEAL9	0.0373372	0.0572326	0.6524	0.514	0.053034528	count	1
SRSF2	0.0375783	0.0592133	0.6346	0.526	0.05309327	count	1
ZNF254	0.0440895	0.1839277	0.2397	0.811	0.053293883	count	1
PRPSAP2	0.0418636	0.1595113	0.2624	0.793	0.053372679	count	1
POC1A	0.159357	0.8141958	0.1957	0.845	0.053491399	count	1
DCTN2	0.0378842	0.0558659	0.6781	0.498	0.053530842	count	1
HDAC2	0.0387886	0.093112	0.4166	0.677	0.053640972	count	1
YKT6	0.044166	0.2266166	0.1949	0.845	0.053641989	count	1

GATA6	0.0387172	0.0973477	0.3977	0.691	0.053676879	count	1
TMEM181	0.0467878	0.2334069	0.2005	0.841	0.053757607	count	1
ZNF239	0.0908575	0.4240037	0.2143	0.83	0.053771544	count	1
GABRR1	0.2037583	0.5398446	0.3774	0.706	0.053902197	count	1
SIRT4	0.2037583	0.5564189	0.3662	0.714	0.053902197	count	1
MVB12A	0.0386886	0.0807739	0.479	0.632	0.053911206	count	1
BTBD2	0.043674	0.2100369	0.2079	0.835	0.053943388	count	1
ICE1	0.0399124	0.1058768	0.377	0.706	0.054028316	count	1
PTS	0.0391729	0.0942318	0.4157	0.678	0.054077145	count	1
SRRM3	0.0605278	0.3201628	0.1891	0.85	0.05420453	count	1
EMC3	0.0388178	0.0841724	0.4612	0.645	0.05421993	count	1
RPTOR	0.0548324	0.3533867	0.1552	0.877	0.054274497	count	1
MEGF8	0.05373	0.2641088	0.2034	0.839	0.054325013	count	1
AL023806.1	0.086723	0.4991231	0.1738	0.862	0.054576755	count	1
COPZ1	0.0391739	0.0792165	0.4945	0.621	0.054739317	count	1
GGA2	0.0451913	0.2033344	0.2223	0.824	0.054755531	count	1
HNMT	0.0396107	0.0847951	0.4671	0.64	0.054894407	count	1
HMGN3	0.0384635	0.0496019	0.7754	0.438	0.054913283	count	1
SIK1	0.2999463	0.8057957	0.3722	0.71	0.054980133	count	1
TMEM185B	0.0492279	0.2336706	0.2107	0.833	0.055024926	count	1
ARID1A	0.0426523	0.1506567	0.2831	0.777	0.055082164	count	1
PDCD11	0.0551027	0.2778335	0.1983	0.843	0.05513758	count	1
AZIN2	0.1007236	0.4756508	0.2118	0.832	0.0554227	count	1
TIGD6	0.0590707	0.3839337	0.1539	0.878	0.055540042	count	1
DDX11	0.0609864	0.2883689	0.2115	0.833	0.055570571	count	1
CHMP2B	0.0394832	0.0667458	0.5915	0.554	0.055580113	count	1
MIER3	0.0555578	0.2324717	0.239	0.811	0.055590804	count	1
CFL2	0.0394371	0.063602	0.6201	0.535	0.055658751	count	1
GOLGA8H	0.6244141	0.7985789	0.7819	0.434	0.055716759	count	1
PSMD6-AS1	0.6244141	0.9749295	0.6405	0.522	0.055716759	count	1
IRF3	0.0409467	0.1169491	0.3501	0.726	0.055734294	count	1
ZNF394	0.0436072	0.1679664	0.2596	0.795	0.055787203	count	1
RAD1	0.0433663	0.1816171	0.2388	0.811	0.055837346	count	1
TRIM23	0.0445314	0.1310733	0.3397	0.734	0.056019707	count	1
PUM2	0.0429034	0.1602296	0.2678	0.789	0.056075302	count	1
NDUFV1	0.0401667	0.0825238	0.4867	0.626	0.056075336	count	1
VSIG10L	0.1111865	0.4582754	0.2426	0.808	0.056105015	count	1
PYGO2	0.0568186	0.2320168	0.2449	0.807	0.056230691	count	1
TSR1	0.0452437	0.1882075	0.2404	0.81	0.056283685	count	1
SMG8	0.0749566	0.4462595	0.168	0.867	0.056317635	count	1
AC114490.2	0.3079026	0.8387598	0.3671	0.714	0.056321642	count	1
UBE2L5	0.3079026	0.8387598	0.3671	0.714	0.056321642	count	1
HMSD	0.3079026	0.8387598	0.3671	0.714	0.056321642	count	1
MTFR2	0.3079026	1.049516	0.2934	0.769	0.056321642	count	1
CENPJ	0.0494787	0.2125354	0.2328	0.816	0.056362277	count	1

PSMC3IP	0.0686296	0.2809139	0.2443	0.807	0.056370009	count	1
UCHL5	0.0426127	0.1160877	0.3671	0.714	0.056405516	count	1
ZNF417	0.6340655	0.7075559	0.8961	0.37	0.05641685	count	1
FKBP1B	0.0442741	0.1983805	0.2232	0.823	0.056508564	count	1
AC136475.1	0.1120457	0.3892722	0.2878	0.773	0.056529591	count	1
ATXN1	0.0427539	0.1456965	0.2934	0.769	0.05687188	count	1
FCRLB	0.1258627	0.5818353	0.2163	0.829	0.057138537	count	1
EDDM13	0.6444166	1.1814993	0.5454	0.5855	0.057162482	count	1
AL118506.1	0.1440189	0.6073148	0.2371	0.813	0.057286217	count	1
TMX1	0.0423175	0.0889251	0.4759	0.634	0.05748844	count	1
RAB5B	0.0441171	0.1308862	0.3371	0.736	0.057570272	count	1
MED29	0.0420137	0.124459	0.3376	0.736	0.057657016	count	1
JTB	0.0406857	0.05161	0.7883	0.431	0.0577766	count	1
TWISTNB	0.0421149	0.0956336	0.4404	0.66	0.057784454	count	1
C1orf123	0.0408871	0.057558	0.7104	0.478	0.057796056	count	1
MRPS17	0.054373	0.2821016	0.1927	0.847	0.057857344	count	1
RGPD5	0.0567041	0.3121283	0.1817	0.856	0.05787665	count	1
C20orf27	0.043368	0.1183487	0.3664	0.714	0.058072214	count	1
RFC5	0.0522561	0.2415491	0.2163	0.829	0.058094127	count	1
NSD3	0.0410382	0.0713186	0.5754	0.565	0.058133479	count	1
API5	0.0447714	0.126791	0.3531	0.724	0.05818358	count	1
MT-ND4L	0.040796	0.0396988	1.0276	0.304	0.058295156	count	1
CUL4A	0.0474035	0.1577654	0.3005	0.764	0.058314138	count	1
IVD	0.0428889	0.1295227	0.3311	0.741	0.058422011	count	1
TMEM123	0.0417437	0.0820142	0.509	0.611	0.058555038	count	1
NAPA	0.0421081	0.088294	0.4769	0.633	0.058556952	count	1
WDPCP	0.0614619	0.2903736	0.2117	0.832	0.058591399	count	1
TMED1	0.0434028	0.1034914	0.4194	0.675	0.058594004	count	1
AC005253.1	0.32166	0.6861212	0.4688	0.639	0.058626882	count	1
WEE2-AS1	0.32166	0.7373088	0.4363	0.663	0.058626882	count	1
TNFSF13	0.32166	0.7425798	0.4332	0.665	0.058626882	count	1
MBLAC1	0.0570204	0.3070338	0.1857	0.853	0.058734899	count	1
AC004241.1	0.129783	0.6345855	0.2045	0.838	0.058873028	count	1
ISPD	0.0996282	0.3791051	0.2628	0.793	0.058875982	count	1
MCAT	0.0483882	0.1653219	0.2927	0.77	0.058894247	count	1
MRPS30	0.0448154	0.1315258	0.3407	0.733	0.058909877	count	1
ZKSCAN7	0.1482416	0.459041	0.3229	0.747	0.058914082	count	1
PNPLA7	0.6692065	0.8365578	0.8	0.424	0.058926401	count	1
AC068790.9	0.6692065	1.079592	0.6199	0.535	0.058926401	count	1
DYM	0.0463188	0.1524719	0.3038	0.761	0.058972658	count	1
PBLD	0.0785553	0.3195439	0.2458	0.806	0.058992818	count	1
SMC6	0.0450719	0.1510563	0.2984	0.765	0.059165157	count	1
HAPLN1	0.0444751	0.1610816	0.2761	0.782	0.059189607	count	1
ORC4	0.045039	0.129879	0.3468	0.729	0.059203471	count	1
LINC00630	0.0703766	0.5015451	0.1403	0.888	0.059209647	count	1

TP63	0.673415	1.064351	0.6327	0.527	0.059222816	count	1
CFAP74	0.673415	1.215035	0.5542	0.579	0.059222816	count	1
AL451064.1	0.673415	1.215035	0.5542	0.579	0.059222816	count	1
HTR2A	0.0674395	0.3248768	0.2076	0.836	0.059225433	count	1
HSP90B1	0.0412173	0.0323675	1.2734	0.203	0.059284525	count	1
TAGLN2	0.0412579	0.0336334	1.2267	0.22	0.059304877	count	1
AKR1E2	0.1775119	0.6427107	0.2762	0.782	0.059346026	count	1
ARHGAP10	0.042285	0.0706319	0.5987	0.549	0.059523053	count	1
RAB23	0.0446366	0.1439326	0.3101	0.756	0.059579742	count	1
TTLL11	0.082379	0.2949892	0.2793	0.78	0.059632054	count	1
ZNF773	0.0679808	0.3137523	0.2167	0.828	0.05969725	count	1
ARHGAP28	0.1786434	0.5077517	0.3518	0.725	0.059709258	count	1
ACTR10	0.0423535	0.0659445	0.6423	0.521	0.059717	count	1
MFF	0.0429706	0.0836129	0.5139	0.607	0.05978704	count	1
RAP2A	0.0447189	0.1475142	0.3031	0.762	0.059853299	count	1
AC104187.1	0.329512	0.881733	0.3737	0.709	0.059934427	count	1
PGM5P4-AS1	0.329512	0.9694193	0.3399	0.734	0.059934427	count	1
TSPYL1	0.0425709	0.0696421	0.6113	0.541	0.060028379	count	1
TCEA3	0.0430715	0.0765269	0.5628	0.574	0.060044148	count	1
DCAF17	0.05347	0.2199788	0.2431	0.808	0.060051448	count	1
YJU2	0.0488568	0.1666662	0.2931	0.769	0.060098345	count	1
WDR59	0.055	0.2266138	0.2427	0.808	0.060099172	count	1
METTL1	0.053666	0.206443	0.26	0.795	0.060270842	count	1
PES1	0.0513276	0.2709625	0.1894	0.85	0.060274591	count	1
C22orf46	0.0609771	0.2827342	0.2157	0.829	0.060324097	count	1
SSRP1	0.0458623	0.1136059	0.4037	0.686	0.060325083	count	1
SLC30A5	0.0477553	0.1418633	0.3366	0.736	0.060326054	count	1
AC010331.1	0.0960396	0.5829925	0.1647	0.869	0.060350176	count	1
RBFOX2	0.0432269	0.0828948	0.5215	0.602	0.060376114	count	1
MRPL15	0.044897	0.1145159	0.3921	0.695	0.060457602	count	1
TBC1D5	0.045336	0.117723	0.3851	0.7	0.060540887	count	1
CCDC58	0.0480053	0.201987	0.2377	0.812	0.060556693	count	1
PIAS4	0.0584127	0.2380902	0.2453	0.806	0.060688457	count	1
DNM3	0.134092	0.3977597	0.3371	0.736	0.060776265	count	1
ZNF367	0.1822803	0.7117826	0.2561	0.798	0.060875447	count	1
ZNF81	0.1344907	0.4293772	0.3132	0.754	0.060952192	count	1
TCEAL6	0.0485579	0.2534737	0.1916	0.848	0.060985307	count	1
AC004771.3	0.3359506	0.7115411	0.4721	0.637	0.061002182	count	1
DPF2	0.0462062	0.1277789	0.3616	0.718	0.061011077	count	1
ABCF3	0.0538166	0.1874578	0.2871	0.774	0.06101462	count	1
MPZ	0.6996507	1.2030919	0.5815	0.5609	0.061050875	count	1
KIAA1522	0.1538579	0.5057527	0.3042	0.761	0.061074499	count	1
SNRNP27	0.0445238	0.0995937	0.4471	0.655	0.061111566	count	1
WDR35	0.0515553	0.2628514	0.1961	0.845	0.061119412	count	1
TMEM39B	0.0520951	0.1688764	0.3085	0.758	0.061173433	count	1

TMEM167B	0.0460725	0.1205002	0.3823	0.702	0.06121779	count	1
ZNF169	0.1352412	0.4271792	0.3166	0.752	0.061283276	count	1
HMGN3-AS1	0.0613485	0.2666225	0.2301	0.818	0.061354439	count	1
ATAT1	0.0663822	0.3355287	0.1978	0.843	0.061438447	count	1
WDR18	0.04637	0.1266278	0.3662	0.714	0.06164515	count	1
BDNF-AS	0.0795228	0.3040182	0.2616	0.794	0.061683272	count	1
NUBP1	0.0463786	0.1543162	0.3005	0.764	0.061688648	count	1
LSMEM1	0.1363641	0.4179066	0.3263	0.744	0.061778456	count	1
TMEM135	0.0478689	0.1853439	0.2583	0.796	0.06186862	count	1
MRPL16	0.0445242	0.0810538	0.5493	0.583	0.061954039	count	1
GAL3ST4	0.0619732	0.2706004	0.229	0.819	0.061975866	count	1
SLC35E1	0.0513197	0.1644761	0.312	0.755	0.062013205	count	1
YES1	0.0479838	0.1428853	0.3358	0.737	0.062016916	count	1
PPP2R2D	0.047256	0.1574657	0.3001	0.764	0.062278353	count	1
F13A1	0.1869246	0.4652058	0.4018	0.688	0.062361747	count	1
ARL9	0.1869991	0.4985908	0.3751	0.708	0.062385565	count	1
METTL23	0.0456768	0.0904133	0.5052	0.613	0.062457499	count	1
RBFA	0.0465272	0.1139437	0.4083	0.683	0.062506425	count	1
AC093495.1	0.2384441	0.5681258	0.4197	0.675	0.062556102	count	1
NHLRC4	0.1577499	0.6273304	0.2515	0.801	0.062568497	count	1
POLR3C	0.0517978	0.1806263	0.2868	0.774	0.062589567	count	1
DNAJC28	0.1060357	0.4680403	0.2266	0.821	0.062595219	count	1
C14orf93	0.0783633	0.3043183	0.2575	0.797	0.062601517	count	1
RBM4B	0.0573384	0.1890812	0.3032	0.762	0.062644415	count	1
IFI16	0.0443628	0.0569776	0.7786	0.436	0.062797517	count	1
RASAL2-AS1	0.1884594	0.5870519	0.321	0.748	0.062852205	count	1
SLC31A1	0.0530515	0.2560707	0.2072	0.836	0.063075942	count	1
PCBP1	0.0441661	0.0408352	1.0816	0.28	0.063164079	count	1
PAQR3	0.0663474	0.2871441	0.2311	0.817	0.063219297	count	1
CCDC190	0.0706989	0.7289188	0.097	0.923	0.063244507	count	1
EDC4	0.2422342	0.6926299	0.3497	0.727	0.063492338	count	1
NUDT9	0.046487	0.0949644	0.4895	0.625	0.063521147	count	1
ARL2	0.0446089	0.04267	1.0454	0.296	0.063683566	count	1
PTPN12	0.0462727	0.1072751	0.4313	0.666	0.063685855	count	1
PPP2R3C	0.0475147	0.1239555	0.3833	0.702	0.063701391	count	1
CHRNE	0.1266315	0.7684275	0.1648	0.869	0.063715649	count	1
TMEM218	0.0507274	0.1351735	0.3753	0.707	0.063800023	count	1
CHCHD3	0.0465208	0.0926747	0.502	0.616	0.063813138	count	1
NPIPB4	0.2438284	0.6288524	0.3877	0.698	0.063885582	count	1
TRPV1	0.741614	0.6039965	1.2278	0.22	0.063904579	count	1
NAPEPLD	0.0510037	0.2421366	0.2106	0.833	0.063953927	count	1
CAST	0.0446419	0.0324678	1.375	0.169	0.06400885	count	1
DCAF15	0.0522624	0.246586	0.2119	0.832	0.064016264	count	1
PIP5K1A	0.0563449	0.2013939	0.2798	0.78	0.064157768	count	1
RGS16	0.0454449	0.1216017	0.3737	0.709	0.064186996	count	1

AC044839.1	0.0705918	0.3024306	0.2334	0.815	0.064259043	count	1
ZNF37A	0.0499699	0.1470816	0.3397	0.734	0.064261303	count	1
TMUB2	0.0506098	0.1303403	0.3883	0.698	0.064266017	count	1
AC016575.1	0.1623249	0.66304	0.2448	0.807	0.064321375	count	1
TIMM29	0.0508672	0.1441081	0.353	0.724	0.064592314	count	1
TMX4	0.0458261	0.067362	0.6803	0.496	0.064611827	count	1
AC009283.1	0.2468488	0.8472325	0.2914	0.771	0.06462974	count	1
LYG1	0.2468488	0.8618485	0.2864	0.775	0.06462974	count	1
AL358472.5	0.2468488	0.9488804	0.2601	0.795	0.06462974	count	1
LINC01301	0.2468488	0.9488804	0.2601	0.795	0.06462974	count	1
PSMA4	0.0457649	0.0593553	0.771	0.441	0.064726298	count	1
ADGRB2	0.7545388	1.133455	0.6657	0.506	0.06476628	count	1
RAVER1	0.7545388	1.133455	0.6657	0.506	0.06476628	count	1
CEACAM19	0.7545388	1.133455	0.6657	0.506	0.06476628	count	1
HDHD5-AS1	0.7545388	1.133455	0.6657	0.506	0.06476628	count	1
AL356056.1	0.7545388	1.214153	0.6215	0.534	0.06476628	count	1
AC124045.1	0.7545388	1.232336	0.6123	0.54	0.06476628	count	1
AC055713.1	0.3588804	0.6789567	0.5286	0.597	0.064772362	count	1
EPOP	0.755089	0.8181255	0.923	0.356	0.064802761	count	1
GINS4	0.755089	0.9196459	0.8211	0.412	0.064802761	count	1
FAM214A	0.0542287	0.2168716	0.25	0.803	0.064839797	count	1
SLC25A30	0.1182629	0.3327952	0.3554	0.722	0.064872848	count	1
SUPV3L1	0.0573817	0.2323233	0.247	0.805	0.065042634	count	1
NOL9	0.0522559	0.1491209	0.3504	0.726	0.065100262	count	1
WDR17	0.248769	0.506938	0.4907	0.624	0.065102218	count	1
C19orf47	0.1038469	0.3853393	0.2695	0.788	0.065174529	count	1
ACAD8	0.0552441	0.1838432	0.3005	0.764	0.065279659	count	1
VPS26A	0.0470259	0.0819786	0.5736	0.566	0.0653065	count	1
FEM1A	0.0871087	0.3029604	0.2875	0.774	0.065340389	count	1
AP001469.3	0.3630226	0.7398824	0.4906	0.624	0.06544804	count	1
SCGB1B2P	0.3630226	0.8100198	0.4482	0.654	0.06544804	count	1
AC015726.1	0.3630226	0.8388739	0.4327	0.665	0.06544804	count	1
CYP4F3	0.3630226	1.02072	0.3557	0.722	0.06544804	count	1
JADE3	0.3630226	1.107288	0.3278	0.743	0.06544804	count	1
ZNF75A	0.0552331	0.1888987	0.2924	0.77	0.065662708	count	1
ODF2	0.0638348	0.2225504	0.2868	0.774	0.065718225	count	1
NBPF15	0.0824054	0.3742832	0.2202	0.826	0.065797048	count	1
ST3GAL4	0.0483528	0.1084033	0.446	0.656	0.065892954	count	1
ORC2	0.0584248	0.2556214	0.2286	0.819	0.065913695	count	1
AC015712.2	0.3664516	0.7539237	0.4861	0.627	0.066006137	count	1
TSPYL2	0.0480514	0.0955596	0.5028	0.615	0.066049902	count	1
USP19	0.0786979	0.2976233	0.2644	0.791	0.066145832	count	1
ROR2	0.0855267	0.4955	0.1726	0.863	0.066288241	count	1
BRD3	0.0508788	0.1324887	0.384	0.701	0.066329048	count	1
IGSF9B	0.1320533	1.079501	0.1223	0.903	0.066376418	count	1

BICD1	0.0525988	0.1880519	0.2797	0.78	0.066613662	count	1
ZNF593	0.0485947	0.1012108	0.4801	0.631	0.066708361	count	1
RSBN1	0.0488382	0.0990828	0.4929	0.622	0.066716093	count	1
UCHL3	0.048695	0.1053755	0.4621	0.644	0.066793514	count	1
DOCK2	0.3714836	0.9050357	0.4105	0.681	0.066823081	count	1
MPRIP	0.0483631	0.0964825	0.5013	0.616	0.066938659	count	1
ZBTB11	0.0542335	0.1758154	0.3085	0.758	0.066957364	count	1
KLRD1	0.148254	0.9152901	0.162	0.871	0.067007574	count	1
MYPOP	0.0636021	0.2336459	0.2722	0.785	0.0671276	count	1
PRSS36	0.2571864	0.5728463	0.449	0.653	0.067167781	count	1
APMAP	0.0504856	0.1126509	0.4482	0.654	0.067281023	count	1
PPP1R26-AS1	0.7938802	0.7789855	1.0191	0.308	0.067339885	count	1
OAZ2	0.0491567	0.092364	0.5322	0.595	0.067343836	count	1
PCDHGB1	0.3747423	1.0285641	0.3643	0.716	0.067350835	count	1
AP001922.5	0.3747423	1.0285641	0.3643	0.716	0.067350835	count	1
AL731563.3	0.3747423	1.0285641	0.3643	0.716	0.067350835	count	1
LBP	0.3747423	1.0285641	0.3643	0.716	0.067350835	count	1
AL445423.1	0.3747423	1.0373804	0.3612	0.718	0.067350835	count	1
NHEJ1	0.3747423	1.0373804	0.3612	0.718	0.067350835	count	1
POLE2	0.3747423	1.0373804	0.3612	0.718	0.067350835	count	1
P2RX6	0.3747423	1.0373804	0.3612	0.718	0.067350835	count	1
NHSL2	0.0566872	0.2797626	0.2026	0.839	0.067386544	count	1
ABHD15	0.0869792	0.427762	0.2033	0.839	0.067401177	count	1
JRK	0.1492796	0.3394879	0.4397	0.66	0.067457398	count	1
OCLN	0.2589115	0.7697882	0.3363	0.737	0.067589978	count	1
HNRNPDL	0.0472098	0.0380171	1.2418	0.214	0.067625817	count	1
DDX18	0.0478335	0.0646699	0.7397	0.46	0.067632121	count	1
AASDHPPT	0.0495288	0.1033586	0.4792	0.632	0.067642971	count	1
IFT80	0.0651522	0.266727	0.2443	0.807	0.067654651	count	1
PRPS1	0.051289	0.1292343	0.3969	0.691	0.067672361	count	1
CITED2	0.0480166	0.0776376	0.6185	0.536	0.067679524	count	1
NCAPD3	0.0641574	0.2274182	0.2821	0.778	0.067710841	count	1
AL139220.2	0.0905423	0.3533182	0.2563	0.798	0.067884192	count	1
KLHL30	0.0808707	0.3942686	0.2051	0.837	0.067954641	count	1
MSL3	0.0539986	0.1562744	0.3455	0.73	0.068005405	count	1
VMA21	0.0506639	0.1138625	0.445	0.656	0.068031161	count	1
REL	0.0513197	0.158257	0.3243	0.746	0.068181975	count	1
CCDC89	0.1510816	0.5252529	0.2876	0.774	0.068247305	count	1
DPH1	0.2619976	0.8224325	0.3186	0.75	0.068344298	count	1
ILVBL	0.0523116	0.1413147	0.3702	0.711	0.068355021	count	1
DHRS4	0.0555169	0.1601205	0.3467	0.729	0.068406716	count	1
EEA1	0.0483039	0.0528432	0.9141	0.361	0.068414299	count	1
EIF3C	0.0780397	0.437003	0.1786	0.858	0.0684544	count	1
EPC2	0.0530356	0.1305576	0.4062	0.685	0.068536037	count	1
TSPOAP1	0.0767556	0.4669448	0.1644	0.869	0.068618008	count	1

AR	0.0498859	0.0851327	0.586	0.558	0.068655563	count	1
LINC01354	0.1253455	0.3832635	0.327	0.744	0.068671597	count	1
ABHD14B	0.0496153	0.0879136	0.5644	0.573	0.068725238	count	1
ZNF689	0.0953211	0.2541326	0.3751	0.708	0.068874475	count	1
RNF220	0.0555082	0.1907991	0.2909	0.771	0.068905096	count	1
PIGP	0.0500145	0.0820399	0.6096	0.542	0.068947907	count	1
PLEKHA4	0.0504734	0.1030848	0.4896	0.624	0.068964205	count	1
SAMD13	0.1745823	0.5906458	0.2956	0.768	0.069000147	count	1
LINC00240	0.3850642	0.7541841	0.5106	0.61	0.069015752	count	1
NSMF	0.055055	0.1600466	0.344	0.731	0.069023617	count	1
ARPIN	0.054637	0.1367311	0.3996	0.689	0.069097296	count	1
PRKAR1A	0.0491606	0.0643015	0.7645	0.445	0.069112219	count	1
SESN1	0.0539592	0.1395566	0.3866	0.699	0.06923695	count	1
SPATA18	0.0761512	0.3196725	0.2382	0.812	0.069279569	count	1
FAM208A	0.0525833	0.1127213	0.4665	0.641	0.069290216	count	1
GRHPR	0.0493138	0.0616032	0.8005	0.423	0.069309191	count	1
SSR2	0.0485277	0.0380397	1.2757	0.202	0.069310505	count	1
SDCBP2-AS1	0.1759319	0.5896255	0.2984	0.765	0.069513736	count	1
ZNF264	0.0562647	0.1731446	0.325	0.745	0.069589805	count	1
AL356417.2	0.829803	0.7499451	1.1065	0.269	0.069625773	count	1
GPI	0.0514663	0.104123	0.4943	0.621	0.069741826	count	1
MUC20	0.8321307	0.7874477	1.0567	0.291	0.069771804	count	1
TBX22	0.832253	1.100492	0.7563	0.45	0.069779469	count	1
PIPOX	0.832253	1.100492	0.7563	0.45	0.069779469	count	1
AC006449.5	0.832253	1.100492	0.7563	0.45	0.069779469	count	1
DNMT3B	0.832253	1.100492	0.7563	0.45	0.069779469	count	1
HSPB9	0.832253	1.268517	0.6561	0.512	0.069779469	count	1
FST	0.0525765	0.1587894	0.3311	0.741	0.069994574	count	1
ZNF18	0.1550746	0.3632293	0.4269	0.669	0.069995505	count	1
MYNN	0.0555064	0.1510628	0.3674	0.713	0.070000521	count	1
ZNF792	0.2691492	0.6803782	0.3956	0.692	0.070087612	count	1
AL138885.3	0.1562053	0.4750029	0.3289	0.742	0.070490013	count	1
ENOPH1	0.054911	0.1381342	0.3975	0.691	0.070677432	count	1
ITSN1	0.0534102	0.1232903	0.4332	0.665	0.070680303	count	1
CXCL11	0.1409082	0.7714039	0.1827	0.855	0.070709791	count	1
SPEN	0.053089	0.1283809	0.4135	0.679	0.070815413	count	1
CAMK2N2	0.2729651	0.8537323	0.3197	0.749	0.071015088	count	1
TTYH3	0.0756986	0.6511888	0.1162	0.907	0.071058063	count	1
AC092295.2	0.2144421	0.8685724	0.2469	0.805	0.071100433	count	1
RNF40	0.0656825	0.217575	0.3019	0.763	0.071275439	count	1
HADHA	0.0502127	0.0500748	1.0028	0.316	0.071289902	count	1
TAPBP	0.0509492	0.0822081	0.6198	0.535	0.071345636	count	1
ATP6V1A	0.0597391	0.1772396	0.3371	0.736	0.071409492	count	1
PSMA3	0.0505373	0.0575256	0.8785	0.38	0.071460746	count	1
MGAT4A	0.0651159	0.3476375	0.1873	0.851	0.071527955	count	1

UBA6-AS1	0.0689198	0.308089	0.2237	0.823	0.071545647	count	1
TGFBRAP1	0.0648346	0.2179923	0.2974	0.766	0.071627161	count	1
NES	0.0559807	0.172359	0.3248	0.745	0.071826293	count	1
PPP1R8	0.0596231	0.1815595	0.3284	0.743	0.071839638	count	1
MRPS14	0.0527964	0.0956327	0.5521	0.581	0.072264908	count	1
ARL5A	0.0569269	0.1901332	0.2994	0.765	0.072272314	count	1
LARP4	0.0550118	0.1327364	0.4144	0.679	0.072294685	count	1
C5orf22	0.067151	0.2525106	0.2659	0.79	0.072389275	count	1
CPS1	0.4065733	0.565824	0.7186	0.472	0.072452292	count	1
RPL17	0.0531421	0.0898213	0.5916	0.554	0.072504389	count	1
TAF10	0.0516253	0.0648426	0.7962	0.426	0.072509033	count	1
CHN2	0.8767336	0.7608607	1.1523	0.249	0.072521583	count	1
ATP2C1	0.0579572	0.1737655	0.3335	0.739	0.072541201	count	1
LIPE-AS1	0.1233718	0.4285595	0.2879	0.773	0.072615989	count	1
TNK1	0.1844199	0.4726199	0.3902	0.696	0.072736675	count	1
MIS18BP1	0.0546752	0.1323839	0.413	0.68	0.072821771	count	1
FKRP	0.0737205	0.2617536	0.2816	0.778	0.072848699	count	1
EIF2AK2	0.0521629	0.0804126	0.6487	0.517	0.07297111	count	1
TEFM	0.0595472	0.1636237	0.3639	0.716	0.073068238	count	1
ZNF665	0.0717148	0.3719393	0.1928	0.847	0.073107266	count	1
UTP11	0.0541346	0.0975943	0.5547	0.579	0.073117979	count	1
ZNF444	0.0595446	0.1888874	0.3152	0.753	0.073212923	count	1
CEP112	0.0579806	0.2067758	0.2804	0.779	0.073317305	count	1
MTIF3	0.0522669	0.0727349	0.7186	0.472	0.073354729	count	1
PPP1R1A	0.0603081	0.1960944	0.3075	0.758	0.073363782	count	1
ETF1	0.0547516	0.109005	0.5023	0.615	0.073484462	count	1
ASTE1	0.0702666	0.2226119	0.3156	0.752	0.0735427	count	1
KANTR	0.0736279	0.3055605	0.241	0.81	0.07355681	count	1
KDELR3	0.0537989	0.0980318	0.5488	0.583	0.073603896	count	1
YIPF6	0.0531276	0.0860132	0.6177	0.537	0.073661886	count	1
LINC01099	0.8986992	1.221639	0.7357	0.462	0.073842265	count	1
HEXIM1	0.0553523	0.1097002	0.5046	0.614	0.073935485	count	1
IDS	0.0523224	0.0702871	0.7444	0.457	0.074031592	count	1
MYO15B	0.9038917	0.9495319	0.9519	0.341	0.074151269	count	1
FXR1	0.0528258	0.0711915	0.742	0.458	0.074219211	count	1
UBAP1L	0.9061101	0.7944927	1.1405	0.254	0.074282916	count	1
SLC1A5	0.0621562	0.1810103	0.3434	0.731	0.074290107	count	1
PLEKHA7	0.9065383	1.070439	0.8469	0.397	0.0743083	count	1
GRB7	0.9065383	1.070439	0.8469	0.397	0.0743083	count	1
AC097359.2	0.9065383	1.606333	0.5644	0.573	0.0743083	count	1
AC102953.2	0.9079474	0.8713905	1.042	0.298	0.074391779	count	1
FRMD3	0.0695103	0.2348933	0.2959	0.767	0.074411398	count	1
TECPR2	0.0612182	0.2018063	0.3034	0.762	0.074467906	count	1
RAB11B	0.0534479	0.0766109	0.6977	0.485	0.074502137	count	1
TRMT6	0.0575978	0.1654538	0.3481	0.728	0.074628469	count	1

ANKLE2	0.0570057	0.1249886	0.4561	0.648	0.074701544	count	1
ZFPL1	0.0549198	0.1121312	0.4898	0.624	0.074717128	count	1
NADSYN1	0.0693383	0.2174694	0.3188	0.75	0.074735519	count	1
HLA-B	0.051947	0.0367537	1.4134	0.158	0.07473722	count	1
TRIM37	0.0596622	0.1579093	0.3778	0.706	0.074787052	count	1
STYXL1	0.0636349	0.2579549	0.2467	0.805	0.074924286	count	1
GPR108	0.0551176	0.1045248	0.5273	0.598	0.074945314	count	1
C3orf18	0.084019	0.2861519	0.2936	0.769	0.075052522	count	1
B3GLCT	0.073648	0.2374407	0.3102	0.756	0.07506595	count	1
ZNF107	0.0723602	0.2326975	0.311	0.756	0.075096631	count	1
PUM1	0.0554693	0.1088725	0.5095	0.61	0.075229388	count	1
C17orf49	0.076183	0.2901772	0.2625	0.793	0.075265537	count	1
IFT172	0.0771101	0.3474052	0.222	0.824	0.075296084	count	1
USP47	0.0556796	0.1108903	0.5021	0.616	0.075302786	count	1
ACSL4	0.0577636	0.1331209	0.4339	0.664	0.075350603	count	1
DBNDD2	0.1915384	0.5986604	0.3199	0.749	0.075430077	count	1
TCEAL8	0.053345	0.0623597	0.8554	0.392	0.075443841	count	1
ZNF76	0.0660969	0.237091	0.2788	0.78	0.075544006	count	1
BCAR3	0.0598777	0.1562734	0.3832	0.702	0.075711214	count	1
RRP7A	0.0544787	0.0987764	0.5515	0.581	0.07582866	count	1
GPR89B	0.1096448	0.4422781	0.2479	0.804	0.075899511	count	1
RHOT1	0.0576109	0.1377355	0.4183	0.676	0.076002127	count	1
SRP19	0.0546917	0.0911275	0.6002	0.548	0.076013775	count	1
KLF5	0.0720771	0.2788071	0.2585	0.796	0.076023493	count	1
FITM2	0.0822952	0.3324485	0.2475	0.805	0.076043505	count	1
MIR4458HG	0.0601449	0.2063417	0.2915	0.771	0.076048362	count	1
10-Sep	0.0540655	0.0625805	0.8639	0.388	0.076066836	count	1
CCDC12	0.0548164	0.0872951	0.6279	0.53	0.076119205	count	1
AC112206.2	0.9418512	0.8762443	1.0749	0.283	0.076373505	count	1
DDX50	0.0554632	0.10635	0.5215	0.602	0.076490797	count	1
EXOSC7	0.0560865	0.0972068	0.577	0.564	0.076609438	count	1
NME7	0.0578461	0.1244017	0.465	0.642	0.076630779	count	1
HERC3	0.0645016	0.1813394	0.3557	0.722	0.076646128	count	1
SPAG8	0.0991113	0.4409637	0.2248	0.822	0.076679756	count	1
FAM185A	0.0717224	0.3239159	0.2214	0.825	0.076767068	count	1
ZNRF2	0.0669824	0.1892252	0.354	0.723	0.076871582	count	1
ARL13B	0.057793	0.1242868	0.465	0.642	0.076891407	count	1
SYTL2	0.0575409	0.1576703	0.3649	0.715	0.076925794	count	1
C18orf54	0.1541539	0.4988975	0.309	0.757	0.077163529	count	1
YIPF5	0.0554348	0.075568	0.7336	0.463	0.077284464	count	1
CCDC30	0.0812667	0.3291898	0.2469	0.805	0.077324242	count	1
ZNF532	0.0574312	0.1321071	0.4347	0.664	0.077346539	count	1
RNF34	0.061909	0.1476416	0.4193	0.675	0.077352431	count	1
PNO1	0.0597079	0.1308713	0.4562	0.648	0.077357796	count	1
MAD2L2	0.0608328	0.1478392	0.4115	0.681	0.077414621	count	1

LINC01473	0.0946363	0.3449684	0.2743	0.784	0.077484561	count	1
NCOR1	0.0548353	0.0678403	0.8083	0.419	0.077632749	count	1
AC069185.1	0.1422261	0.7261546	0.1959	0.845	0.077684934	count	1
MAPT	0.1727503	0.4550358	0.3796	0.704	0.077698888	count	1
KCNAB3	0.9663716	0.9221078	1.048	0.295	0.077775012	count	1
WBP11	0.0564431	0.0972585	0.5803	0.562	0.077791205	count	1
AC008440.2	0.9669883	1.038452	0.9312	0.352	0.07780992	count	1
AP001029.2	0.9669883	1.131061	0.8549	0.393	0.07780992	count	1
COL6A2	0.0542396	0.0373386	1.4526	0.146	0.077842376	count	1
MAFIP	0.441038	0.6914958	0.6378	0.524	0.077866356	count	1
GMPPA	0.0632359	0.1637141	0.3863	0.699	0.077893154	count	1
ZDHHC11B	0.4413583	0.4710904	0.9369	0.349	0.077916135	count	1
ZC3H7A	0.0568346	0.100448	0.5658	0.572	0.077918196	count	1
LIMK1	0.1246278	0.3532746	0.3528	0.724	0.077953905	count	1
TAF1D	0.0555723	0.0726994	0.7644	0.445	0.077963598	count	1
CCDC84	0.0614484	0.1893386	0.3245	0.746	0.078000704	count	1
SLC38A1	0.0712115	0.1751206	0.4066	0.684	0.07819162	count	1
TUBB6	0.0557545	0.066556	0.8377	0.402	0.078353552	count	1
CELSR2	0.1994698	0.8568404	0.2328	0.816	0.078420791	count	1
SLC25A27	0.1088462	0.4624208	0.2354	0.814	0.078495484	count	1
VPS26B	0.0613595	0.15554	0.3945	0.693	0.078542043	count	1
PAOX	0.0711939	0.3066669	0.2322	0.816	0.078619569	count	1
OBSL1	0.0614233	0.1635204	0.3756	0.707	0.078623549	count	1
TMEM65	0.0712343	0.2014453	0.3536	0.724	0.078663972	count	1
LINC01715	0.1192491	0.4097993	0.291	0.771	0.078703865	count	1
FUT8	0.0650928	0.1609288	0.4045	0.686	0.078798283	count	1
NAP1L4	0.0565407	0.0813893	0.6947	0.487	0.078803627	count	1
SECISBP2L	0.0572977	0.0912628	0.6278	0.53	0.078929129	count	1
NDRG2	0.0574463	0.0949481	0.605	0.545	0.079198491	count	1
TBL3	0.0706242	0.2045461	0.3453	0.73	0.079232119	count	1
POC1B	0.0778068	0.2205101	0.3528	0.724	0.079277358	count	1
SNRPB2	0.0561093	0.0664097	0.8449	0.398	0.079371484	count	1
TVP23A	0.120463	0.5378249	0.224	0.823	0.079489976	count	1
CYP19A1	0.4526087	0.6749011	0.6706	0.502	0.07965853	count	1
MOSPD1	0.0670499	0.2147385	0.3122	0.755	0.079664097	count	1
BRD4	0.0569896	0.0872599	0.6531	0.514	0.079708025	count	1
U2AF1L5	0.1276862	0.6321277	0.202	0.84	0.079827059	count	1
SPART	0.0573036	0.0683532	0.8383	0.402	0.079873741	count	1
AL355353.1	0.3099229	0.6673844	0.4644	0.642	0.079900321	count	1
HIST1H2AB	1.005042	1.094653	0.9181	0.359	0.079932013	count	1
CPM	0.0896811	0.2631834	0.3408	0.733	0.08006119	count	1
SEC16B	1.00756	0.6114883	1.6477	0.0995	0.080070258	count	1
GTPBP2	0.1113042	0.4016015	0.2772	0.782	0.080239796	count	1
CEBPZOS	0.0582425	0.0917326	0.6349	0.526	0.080256214	count	1
DUSP8	0.0645848	0.191136	0.3379	0.735	0.08028403	count	1

PIGV	0.0796153	0.1990705	0.3999	0.689	0.080321107	count	1
COQ9	0.0620223	0.1564384	0.3965	0.692	0.08049379	count	1
IGIP	0.070647	0.3701835	0.1908	0.849	0.080722824	count	1
CNOT9	0.0639978	0.1480248	0.4323	0.666	0.08090915	count	1
ZBTB38	0.0573024	0.0606976	0.9441	0.345	0.080940385	count	1
TMEM184C	0.0711611	0.1960711	0.3629	0.717	0.080956959	count	1
EIF2D	0.0602565	0.1275272	0.4725	0.637	0.080993279	count	1
CBX1	0.0581666	0.0835022	0.6966	0.486	0.081105663	count	1
ATG10	0.0693332	0.1982858	0.3497	0.727	0.081341869	count	1
AC005162.3	1.031748	0.8685649	1.1879	0.235	0.081384229	count	1
CNR1	1.032559	1.088325	0.9488	0.343	0.081427788	count	1
AC012651.1	1.032559	1.088325	0.9488	0.343	0.081427788	count	1
AC010487.2	1.032559	1.088325	0.9488	0.343	0.081427788	count	1
AC087286.2	1.032559	1.36055	0.7589	0.448	0.081427788	count	1
RNASE1	0.0672853	0.4492444	0.1498	0.881	0.081444282	count	1
RHEBL1	0.2479682	0.5354506	0.4631	0.643	0.081590077	count	1
PCNX1	0.073958	0.2019265	0.3663	0.714	0.081656945	count	1
THAP7-AS1	0.0794311	0.2624834	0.3026	0.762	0.081671267	count	1
KIF16B	0.0721315	0.2068331	0.3487	0.727	0.081688623	count	1
ZNF227	0.087228	0.2819303	0.3094	0.757	0.081786958	count	1
PIGG	0.0664206	0.384453	0.1728	0.863	0.081805244	count	1
SRR	0.0900542	0.3273414	0.2751	0.783	0.081808417	count	1
PAICS	0.0644259	0.1465418	0.4396	0.66	0.081875591	count	1
STIMATE	0.0790443	0.4335826	0.1823	0.855	0.081989815	count	1
TRIM44	0.0593033	0.1057221	0.5609	0.575	0.082081587	count	1
ZNF646	0.0979022	0.4022558	0.2434	0.808	0.082099457	count	1
LAGE3	0.0619104	0.1266068	0.489	0.625	0.082189008	count	1
MTAP	0.0620207	0.1128449	0.5496	0.583	0.082199493	count	1
SP1	0.0652515	0.1741142	0.3748	0.708	0.082377271	count	1
GZF1	0.0713082	0.2353566	0.303	0.762	0.082464781	count	1
GTF2F1	0.0598392	0.0890538	0.6719	0.502	0.082508222	count	1
LARS	0.0595912	0.0798889	0.7459	0.456	0.082555516	count	1
HDDC3	0.0666194	0.167419	0.3979	0.691	0.082806596	count	1
NOSIP	0.0592247	0.0792372	0.7474	0.455	0.082826298	count	1
BMI1	0.0641927	0.1545706	0.4153	0.678	0.08300491	count	1
TRUB2	0.0652647	0.1436893	0.4542	0.65	0.083042285	count	1
LAMP5	1.063444	0.7694516	1.3821	0.167	0.083068648	count	1
KAT5	0.0639861	0.1499341	0.4268	0.67	0.083109794	count	1
AC008771.1	0.0788455	0.2966231	0.2658	0.79	0.083119491	count	1
CYB561D1	0.1154799	0.3841645	0.3006	0.764	0.083200104	count	1
METTL4	0.0688033	0.2560435	0.2687	0.788	0.083275947	count	1
UQCC3	0.0602454	0.0986878	0.6105	0.542	0.083406862	count	1
CXXC5	0.0595311	0.0912202	0.6526	0.514	0.083535133	count	1
DUSP1	0.0580542	0.0377617	1.5374	0.124	0.083543826	count	1
WDR33	0.0609066	0.1048202	0.5811	0.561	0.083659278	count	1

CAMK4	0.3258825	0.8339037	0.3908	0.696	0.083682479	count	1
AC093512.1	0.3258825	0.8832556	0.369	0.712	0.083682479	count	1
FAM66B	0.3258825	0.8936007	0.3647	0.715	0.083682479	count	1
C1QTNF1-AS1	0.3258825	1.268706	0.2569	0.797	0.083682479	count	1
GBF1	0.0708841	0.1715558	0.4132	0.679	0.083693809	count	1
FGFBP3	0.0938164	0.2885567	0.3251	0.745	0.083715198	count	1
SPOPL	0.065081	0.1459163	0.446	0.656	0.083741555	count	1
SDHC	0.0594875	0.0623555	0.954	0.34	0.083823568	count	1
KLHL29	0.1430825	0.4659655	0.3071	0.759	0.083934166	count	1
TRAPPC2B	0.0621741	0.1244276	0.4997	0.617	0.083935832	count	1
KAZALD1	0.0619634	0.1385952	0.4471	0.655	0.083951306	count	1
FBXO45	0.0769899	0.2023183	0.3805	0.704	0.084000961	count	1
ZNF564	0.4812007	0.9409927	0.5114	0.609	0.084032398	count	1
CHST8	1.0820754	0.9970211	1.0853	0.278	0.084039311	count	1
TFR2	1.0820754	1.439933	0.7515	0.452	0.084039311	count	1
NFKBIL1	0.0617975	0.113333	0.5453	0.586	0.084042028	count	1
E2F4	0.0646347	0.1270621	0.5087	0.611	0.084162249	count	1
ZBED4	0.0982066	0.36138	0.2718	0.786	0.084207281	count	1
PBDC1	0.0607089	0.0845243	0.7182	0.473	0.084257461	count	1
PPOX	0.0731935	0.237161	0.3086	0.758	0.084307949	count	1
BAG6	0.0653066	0.1397223	0.4674	0.64	0.084362915	count	1
AL035071.1	0.0875597	0.321871	0.272	0.786	0.084367077	count	1
RNF167	0.0620372	0.0985927	0.6292	0.529	0.084435567	count	1
LST1	0.2155229	0.6011021	0.3585	0.72	0.084440736	count	1
ZNF706	0.0594749	0.0522031	1.1393	0.255	0.084514967	count	1
ZNF613	0.2157496	0.4295571	0.5023	0.616	0.084525429	count	1
HOXD-AS2	0.2575033	0.6840172	0.3765	0.707	0.08454169	count	1
SLC45A3	0.2575033	0.7421672	0.347	0.729	0.08454169	count	1
BACE1-AS	0.1034612	0.3452584	0.2997	0.764	0.084617544	count	1
ZNF611	0.0815974	0.27116	0.3009	0.763	0.084620761	count	1
CCNE2	0.1887951	0.7431253	0.2541	0.799	0.084641292	count	1
CAPN2	0.0602654	0.0627081	0.961	0.337	0.084694611	count	1
AL359921.2	1.0954617	1.064096	1.0295	0.303	0.084727896	count	1
AL356258.1	1.0954617	1.064096	1.0295	0.303	0.084727896	count	1
MSS51	1.0954617	1.064096	1.0295	0.303	0.084727896	count	1
AC010359.1	1.0954617	1.150013	0.9526	0.341	0.084727896	count	1
TATDN1	0.0618878	0.0928264	0.6667	0.505	0.084839921	count	1
PHF2	0.0703813	0.1591331	0.4423	0.658	0.084972316	count	1
DNASE1	0.0882395	0.366564	0.2407	0.81	0.085016608	count	1
ZDHHC3	0.0629169	0.1159998	0.5424	0.588	0.085023733	count	1
UQCRC1	0.0610279	0.0781897	0.7805	0.435	0.085131112	count	1
INTS5	0.1069621	0.2732096	0.3915	0.695	0.085137423	count	1
PPP1CC	0.0610585	0.06812	0.8963	0.37	0.085247045	count	1
CHKB-DT	0.1566109	0.4525796	0.346	0.729	0.085320209	count	1
UBE2E1	0.0634577	0.1137889	0.5577	0.577	0.085389255	count	1

ZDHHC12	0.0651789	0.1739171	0.3748	0.708	0.085393778	count	1
PSMC6	0.0608562	0.0688939	0.8833	0.377	0.085403869	count	1
THAP9	0.1019749	0.3990917	0.2555	0.798	0.085472986	count	1
AL031316.1	0.333499	0.6814396	0.4894	0.625	0.085475798	count	1
AC026904.3	0.333499	0.6826541	0.4885	0.625	0.085475798	count	1
SAMD15	0.333499	0.6814396	0.4894	0.625	0.085475798	count	1
AC079174.2	0.1713997	0.5010714	0.3421	0.732	0.085514901	count	1
ATPAF1	0.0633518	0.1110097	0.5707	0.568	0.085553151	count	1
IFNGR2	0.0622845	0.0946347	0.6582	0.51	0.085613694	count	1
ZNF850	0.2186793	0.4525825	0.4832	0.629	0.085619145	count	1
SMC5-AS1	0.2186793	0.4697864	0.4655	0.642	0.085619145	count	1
CCDC14	0.0648645	0.1548788	0.4188	0.675	0.08581534	count	1
MIGA2	0.1149146	0.2798163	0.4107	0.681	0.085868967	count	1
BTF3L4	0.061666	0.0747485	0.825	0.409	0.086020718	count	1
HIST1H2BN	0.0918136	0.360456	0.2547	0.799	0.086047043	count	1
SUSD2	0.0832761	0.3136331	0.2655	0.791	0.086350034	count	1
LRRC27	0.1199289	0.5385485	0.2227	0.824	0.086350061	count	1
POLH	0.082635	0.3213259	0.2572	0.797	0.086404287	count	1
NDUFV2-AS1	0.0841311	0.3741265	0.2249	0.822	0.086470525	count	1
WDR75	0.0673958	0.1685328	0.3999	0.689	0.086533911	count	1
FSIP1	0.4978077	1.1206532	0.4442	0.657	0.086537178	count	1
ARHGAP24	0.0673454	0.1560411	0.4316	0.666	0.086649201	count	1
AMFR	0.0680352	0.1375982	0.4944	0.621	0.086664949	count	1
EIF2AK4	0.0629244	0.1036681	0.607	0.544	0.086800039	count	1
ZP3	0.265025	0.3250056	0.8154	0.415	0.086860148	count	1
RAB3GAP1	0.0634976	0.0990809	0.6409	0.522	0.086936058	count	1
VCPIP1	0.0738785	0.2060166	0.3586	0.72	0.086938704	count	1
AC009053.2	0.1942502	0.528031	0.3679	0.713	0.086990715	count	1
CES2	0.0678384	0.179946	0.377	0.706	0.087008043	count	1
CENPI	1.141097	1.139152	1.0017	0.317	0.087020796	count	1
MAK	1.141097	1.266344	0.9011	0.368	0.087020796	count	1
ZNF777	0.0943059	0.3204847	0.2943	0.769	0.087034423	count	1
THAP4	0.0720561	0.158391	0.4549	0.649	0.087199982	count	1
DAG1	0.0664976	0.1420689	0.4681	0.64	0.087303631	count	1
ZNF576	0.0685062	0.1368204	0.5007	0.617	0.087367914	count	1
DCAKD	0.0717019	0.1848337	0.3879	0.698	0.087376219	count	1
NDUFV3	0.0631691	0.0805925	0.7838	0.433	0.087380814	count	1
SMAD1	0.0796771	0.1978538	0.4027	0.687	0.087436686	count	1
FBXW7	0.0697002	0.1615392	0.4315	0.666	0.087470872	count	1
USP12	0.066116	0.1213617	0.5448	0.586	0.087519284	count	1
TFDP2	0.0629258	0.0817115	0.7701	0.441	0.087569939	count	1
ARMCX1	0.0637707	0.0868236	0.7345	0.463	0.087606311	count	1
LINC02268	1.155775	0.9421319	1.2268	0.22	0.087740569	count	1
ZNF491	0.2679433	0.7653961	0.3501	0.726	0.087757279	count	1
ZNF14	0.0783345	0.258932	0.3025	0.762	0.08783932	count	1

SLK	0.0637104	0.0836309	0.7618	0.446	0.087990612	count	1
ZNF527	0.1076891	0.3844276	0.2801	0.779	0.088029084	count	1
BOD1L1	0.0621209	0.056713	1.0954	0.273	0.088076791	count	1
TCP1	0.0629751	0.0713791	0.8823	0.378	0.088195345	count	1
DHRS13	0.1506456	0.5166052	0.2916	0.771	0.088255578	count	1
BUD13	0.0750482	0.1796125	0.4178	0.676	0.088309761	count	1
ARV1	0.0674621	0.1307119	0.5161	0.606	0.0883154	count	1
OXR1	0.0631281	0.0829602	0.7609	0.447	0.088391162	count	1
ZFP90	0.068401	0.1796444	0.3808	0.703	0.088435526	count	1
CHCHD6	0.0717648	0.14817	0.4843	0.628	0.088538308	count	1
RBBP8	0.0828291	0.2606547	0.3178	0.751	0.088582637	count	1
SACM1L	0.0690624	0.1710825	0.4037	0.686	0.088669147	count	1
AC016394.2	1.176176	1.062028	1.1075	0.268	0.088726975	count	1
CCR10	0.0791511	0.2143526	0.3693	0.712	0.088750399	count	1
NNMT	0.0618346	0.040704	1.5191	0.129	0.088788505	count	1
PELI1	0.0849531	0.2406955	0.3529	0.724	0.088811941	count	1
PRPF4	0.0762701	0.1770569	0.4308	0.667	0.08883175	count	1
CDR2	0.069956	0.1689515	0.4141	0.679	0.08888688	count	1
PHF20	0.0639954	0.0843968	0.7583	0.448	0.088906709	count	1
TBC1D31	0.3484067	0.4805567	0.725	0.468	0.088964011	count	1
GEMIN8	0.071623	0.2171107	0.3299	0.742	0.089008444	count	1
ZNF707	0.5149272	0.6642107	0.7752	0.438	0.089091904	count	1
AC044802.2	0.5149272	0.7611952	0.6765	0.499	0.089091904	count	1
GHRLOS	1.1838394	0.8986818	1.3173	0.188	0.089093317	count	1
ILF3	0.0644258	0.0832732	0.7737	0.439	0.089142277	count	1
FARS2	0.0777405	0.1930172	0.4028	0.687	0.089162191	count	1
WDR74	0.0689308	0.1364785	0.5051	0.614	0.089201813	count	1
SLC9A1	0.0847215	0.3153649	0.2686	0.788	0.089273691	count	1
DIP2B	0.0940314	0.4733491	0.1987	0.843	0.089358663	count	1
NOP56	0.0653985	0.1033059	0.6331	0.527	0.089516969	count	1
ACSS1	0.0857117	0.3212277	0.2668	0.79	0.089599647	count	1
ZNF138	0.0779449	0.2583708	0.3017	0.763	0.08975635	count	1
ZNF625-ZNF20	1.197918	1.02869	1.1645	0.244	0.089760348	count	1
PTPN22	1.197918	1.086894	1.1021	0.27	0.089760348	count	1
TCF23	1.197918	1.147352	1.0441	0.297	0.089760348	count	1
SUGT1	0.0635573	0.0565836	1.1232	0.261	0.0898151	count	1
MOSPD3	0.067382	0.1269095	0.5309	0.595	0.089853415	count	1
PNISR	0.0630341	0.0537558	1.1726	0.241	0.08993473	count	1
SELENOT	0.0642765	0.0675232	0.9519	0.341	0.090047534	count	1
MBIP	0.0660291	0.1083576	0.6094	0.542	0.090134104	count	1
AKIP1	0.0675326	0.1156567	0.5839	0.559	0.090578249	count	1
FAM47E	0.154879	0.4860048	0.3187	0.75	0.090669235	count	1
NPIPB5	0.0836753	0.3508916	0.2385	0.812	0.090685219	count	1
ZNF782	0.1822436	0.3676165	0.4957	0.62	0.090736176	count	1
DPAGT1	0.0791637	0.1789494	0.4424	0.658	0.090786927	count	1

FUT10	0.0783823	0.1949781	0.402	0.688	0.090950426	count	1
UBA2	0.0643909	0.0626285	1.0281	0.304	0.090968038	count	1
MCF2L	1.225336	0.8106763	1.5115	0.131	0.09103777	count	1
ELP4	0.0745632	0.1787038	0.4172	0.677	0.091050824	count	1
FAM160A2	0.0787842	0.2362006	0.3335	0.739	0.091071945	count	1
IFT22	0.0665345	0.0930779	0.7148	0.475	0.091109314	count	1
NUDT18	0.0778049	0.1887868	0.4121	0.68	0.091239758	count	1
AC004918.1	0.1324093	0.4166636	0.3178	0.751	0.091345046	count	1
RAD17	0.0720171	0.1526422	0.4718	0.637	0.091383316	count	1
DDX56	0.0718417	0.1570921	0.4573	0.647	0.091390626	count	1
CTDNEP1	0.0654746	0.0733627	0.8925	0.372	0.091393485	count	1
POLR3D	0.0709972	0.1802235	0.3939	0.694	0.091429993	count	1
NKIRAS1	0.0709456	0.1673405	0.424	0.672	0.0914546	count	1
NANP	0.0844397	0.3514842	0.2402	0.81	0.091508678	count	1
LRRC7	0.3600621	0.6430787	0.5599	0.576	0.091671098	count	1
CHD6	0.0676921	0.1211648	0.5587	0.576	0.091703764	count	1
FRAT2	0.0929855	0.2904458	0.3201	0.749	0.091726838	count	1
NAB1	0.0697106	0.1495511	0.4661	0.641	0.091820639	count	1
NANOS1	0.1689857	0.476556	0.3546	0.723	0.091854874	count	1
SNAPIN	0.0662659	0.0852314	0.7775	0.437	0.091876261	count	1
EIF2S2	0.0646355	0.0572256	1.1295	0.259	0.091893879	count	1
MKRN2	0.0742171	0.1450455	0.5117	0.609	0.091895995	count	1
THAP3	0.0704632	0.1476574	0.4772	0.633	0.092099231	count	1
GNRH1	0.3619542	0.8064092	0.4488	0.654	0.092108886	count	1
WDR60	0.0675278	0.1101342	0.6131	0.54	0.092221359	count	1
OFD1	0.0688009	0.1151955	0.5973	0.55	0.092238627	count	1
SMIM10L2A	1.252605	1.058265	1.1836	0.237	0.092280128	count	1
AL591848.4	1.252605	1.287744	0.9727	0.331	0.092280128	count	1
FABP5	0.0690076	0.1612959	0.4278	0.669	0.092397254	count	1
ZNF596	0.1405447	0.5280523	0.2662	0.79	0.092449082	count	1
ZFAND2B	0.0680268	0.1055859	0.6443	0.519	0.092453403	count	1
RBM26-AS1	0.170128	0.4177221	0.4073	0.684	0.092456501	count	1
GARNL3	0.0892482	0.3704841	0.2409	0.81	0.092498113	count	1
SGCD	0.0692119	0.1447705	0.4781	0.633	0.092506146	count	1
USP16	0.0660385	0.0835171	0.7907	0.429	0.092539289	count	1
NFKBIE	0.105867	0.3476316	0.3045	0.761	0.092574435	count	1
SLC39A11	0.0880662	0.2587512	0.3404	0.734	0.092774157	count	1
PLK3	0.0689195	0.1536082	0.4487	0.654	0.092798495	count	1
SPATA33	0.0774989	0.2021323	0.3834	0.701	0.092812265	count	1
LRR1	0.0964225	0.2734074	0.3527	0.724	0.092828376	count	1
SRCAP	0.2082709	0.5712514	0.3646	0.715	0.093003602	count	1
MAP7D3	0.0667586	0.0824329	0.8099	0.418	0.093111904	count	1
SDHAF3	0.0703127	0.1271809	0.5529	0.58	0.093118767	count	1
TROVE2	0.0663762	0.0702655	0.9446	0.345	0.093135347	count	1
CAPN15	0.0980684	0.2966227	0.3306	0.741	0.093158194	count	1

TXNDC15	0.0667696	0.0684911	0.9749	0.33	0.093371782	count	1
SFN	0.1251595	0.5120473	0.2444	0.807	0.093390984	count	1
ALG5	0.0682186	0.0942168	0.7241	0.469	0.093393293	count	1
PTX3	0.0705237	0.253334	0.2784	0.781	0.093397745	count	1
SLC4A1AP	0.069757	0.1084428	0.6433	0.52	0.093439468	count	1
WASHC3	0.0665505	0.0698917	0.9522	0.341	0.093572321	count	1
HMCES	0.0720835	0.1322267	0.5452	0.586	0.093605781	count	1
ACOT2	0.2098829	0.6680624	0.3142	0.753	0.093692555	count	1
GTPBP4	0.0700715	0.1193954	0.5869	0.557	0.09373748	count	1
TRMT1L	0.0840635	0.1912976	0.4394	0.66	0.093753889	count	1
OSCAR	1.2861864	0.7747779	1.6601	0.097	0.093772083	count	1
DND1	1.2861864	0.9285504	1.3852	0.166	0.093772083	count	1
AC105020.6	0.3692773	0.7530879	0.4904	0.624	0.093798902	count	1
L3MBTL4	0.3694921	0.5338684	0.6921	0.489	0.093848373	count	1
TIPIN	0.0798026	0.2056436	0.3881	0.698	0.093880797	count	1
TMED4	0.0662223	0.0531384	1.2462	0.213	0.093974006	count	1
ITGB1BP1	0.0665019	0.0557995	1.1918	0.233	0.094035434	count	1
NLRP1	0.0690936	0.1428393	0.4837	0.629	0.094053785	count	1
WLS	0.0689241	0.1018393	0.6768	0.499	0.094169988	count	1
LGALS3BP	0.0662071	0.0556717	1.1892	0.234	0.094177643	count	1
SHF	0.080085	0.1811848	0.442	0.659	0.094211611	count	1
PARP14	0.0685964	0.1169374	0.5866	0.558	0.094366326	count	1
NAT10	0.0946412	0.2504212	0.3779	0.706	0.094375404	count	1
ANKRD36C	0.071484	0.1534642	0.4658	0.641	0.094388799	count	1
PHAX	0.067843	0.0783511	0.8659	0.387	0.094389003	count	1
B9D1	0.0780534	0.2297595	0.3397	0.734	0.09443167	count	1
SEMA6C	0.118864	0.4357433	0.2728	0.785	0.094465322	count	1
ID1	0.0661356	0.0877504	0.7537	0.451	0.09447296	count	1
DEAF1	0.0723538	0.1414472	0.5115	0.609	0.094493292	count	1
SPG7	0.0703067	0.1831515	0.3839	0.701	0.094556907	count	1
TSNAXIP1	1.3052329	1.039459	1.2557	0.209	0.094600054	count	1
APOBEC3D	1.3052329	1.25249	1.0421	0.297	0.094600054	count	1
KCNC3	1.3052329	1.390615	0.9386	0.348	0.094600054	count	1
MCM3AP	0.081551	0.2566786	0.3177	0.751	0.094610429	count	1
TMX2	0.0686577	0.1104643	0.6215	0.534	0.09467206	count	1
FAM135A	0.0860398	0.3033592	0.2836	0.777	0.09491953	count	1
PPP1R3E	0.116313	0.2643579	0.44	0.66	0.094976092	count	1
LRRN3	0.1749706	0.285247	0.6134	0.54	0.095004013	count	1
SLC17A9	0.1322214	0.4279398	0.309	0.757	0.095031123	count	1
CNRIP1	0.0696328	0.1008814	0.6902	0.49	0.0950707	count	1
ZNF431	0.0833907	0.2055127	0.4058	0.685	0.095212001	count	1
DBR1	0.088468	0.2121012	0.4171	0.677	0.09522269	count	1
LRRC8C-DT	0.1112326	0.3374192	0.3297	0.742	0.095231462	count	1
ATG4C	0.0794969	0.1817248	0.4375	0.662	0.095449296	count	1
EAPP	0.0678351	0.0642351	1.056	0.291	0.095728271	count	1

KLRG1	0.2460408	0.5713841	0.4306	0.667	0.095761652	count	1
DLD	0.0719032	0.1194589	0.6019	0.547	0.095779393	count	1
LINC02352	0.3779198	0.8219145	0.4598	0.646	0.095784435	count	1
B3GALT1	0.3779198	0.8304104	0.4551	0.649	0.095784435	count	1
PSD	0.1539457	0.9167462	0.1679	0.867	0.095828523	count	1
AL355312.3	0.2944914	0.6895686	0.4271	0.669	0.095857875	count	1
ZNF23	0.2944914	0.7555764	0.3898	0.697	0.095857875	count	1
FAM210B	0.0690393	0.0960485	0.7188	0.472	0.095908932	count	1
ALKBH4	0.0760578	0.1586091	0.4795	0.632	0.095982838	count	1
ZNF274	0.0808459	0.2344441	0.3448	0.73	0.0959889	count	1
TFB2M	0.0892457	0.2393634	0.3728	0.709	0.096054328	count	1
EXT2	0.0749241	0.1327363	0.5645	0.572	0.096177273	count	1
CCDC113	0.0937317	0.2904581	0.3227	0.747	0.096261863	count	1
SNRNP200	0.0710497	0.1155584	0.6148	0.539	0.096419834	count	1
VPS37C	0.0930985	0.2438371	0.3818	0.703	0.096458587	count	1
OSGEPL1	0.0890502	0.215822	0.4126	0.68	0.09647342	count	1
M6PR	0.0695185	0.0934085	0.7442	0.457	0.096486326	count	1
STON1	0.0904525	0.234723	0.3854	0.7	0.096680917	count	1
C9orf153	1.355888	0.9239322	1.4675	0.142	0.096739153	count	1
C1orf229	1.355888	0.9854343	1.3759	0.169	0.096739153	count	1
SBK1	1.355888	1.424062	0.9521	0.341	0.096739153	count	1
CTIF	0.0769046	0.2056945	0.3739	0.709	0.096772977	count	1
AMER1	0.1472825	0.4291359	0.3432	0.731	0.096777706	count	1
KCTD12	0.0763869	0.1709846	0.4467	0.655	0.096913633	count	1
SPCS2	0.067637	0.0358079	1.8889	0.059	0.096918796	count	1
OPA1	0.0783149	0.1650654	0.4744	0.635	0.096953815	count	1
ZC3H3	0.1786953	0.369495	0.4836	0.629	0.096960157	count	1
AC022730.4	0.2983924	0.8356761	0.3571	0.721	0.097038929	count	1
DIO3	0.2983924	0.9497041	0.3142	0.753	0.097038929	count	1
UBE2L3	0.0686952	0.0558727	1.2295	0.219	0.097053088	count	1
PDCD2L	0.0846955	0.2273261	0.3726	0.709	0.097099393	count	1
WDR34	0.0741972	0.138588	0.5354	0.592	0.097115117	count	1
ERI3	0.0708564	0.093747	0.7558	0.45	0.097142034	count	1
MFSD11	0.0778779	0.1626644	0.4788	0.632	0.097245562	count	1
C20orf194	0.0829637	0.2601993	0.3188	0.75	0.097262469	count	1
ATM	0.0744424	0.1228517	0.606	0.545	0.097363172	count	1
FAM173B	0.0893961	0.222971	0.4009	0.688	0.097452549	count	1
ZNF326	0.072318	0.1210324	0.5975	0.55	0.097509697	count	1
TEKT3	0.3001042	0.4518603	0.6642	0.507	0.097556455	count	1
KANSL3	0.0880121	0.2403685	0.3662	0.714	0.097616831	count	1
DGAT1	0.0864291	0.2582422	0.3347	0.738	0.097794621	count	1
NCAPH2	0.0821414	0.1898611	0.4326	0.665	0.097804542	count	1
ZSCAN21	0.0915551	0.2068624	0.4426	0.658	0.097851406	count	1
HDGFL2	0.0732229	0.1044178	0.7012	0.483	0.097858861	count	1
VRK1	0.0903774	0.2518721	0.3588	0.72	0.097901959	count	1

ZNF627	0.1313298	0.3745746	0.3506	0.726	0.097910405	count	1
STK32B	0.5761956	0.5903013	0.9761	0.329	0.098008674	count	1
EGFLAM	0.0814534	0.167178	0.4872	0.626	0.098042349	count	1
PCNA	0.0733275	0.1012066	0.7245	0.469	0.098042733	count	1
SLC36A4	0.0830943	0.2280845	0.3643	0.716	0.098048418	count	1
PIEZO1	0.1122842	0.2792452	0.4021	0.688	0.098114271	count	1
HOOK1	0.5770893	0.9973476	0.5786	0.563	0.09813613	count	1
AC084871.2	0.5770893	1.212531	0.4759	0.634	0.09813613	count	1
MARK2	0.0821881	0.2298944	0.3575	0.721	0.098136391	count	1
IGSF3	0.2203878	0.487442	0.4521	0.651	0.098170224	count	1
ZFP64	0.0950552	0.2819089	0.3372	0.736	0.098470289	count	1
CCDC71L	0.07162	0.1011802	0.7078	0.479	0.098504451	count	1
ACTR6	0.0720229	0.094762	0.76	0.447	0.098617028	count	1
SEPSECS	0.1179683	0.2988139	0.3948	0.693	0.098687204	count	1
ZRANB1	0.0761598	0.1747289	0.4359	0.663	0.098714469	count	1
HSPH1	0.0725498	0.1222108	0.5936	0.553	0.098726974	count	1
LCLAT1	0.0854655	0.2164721	0.3948	0.693	0.098757736	count	1
MTMR8	0.2544405	0.6976481	0.3647	0.715	0.098849074	count	1
MTDH	0.0690192	0.0432618	1.5954	0.111	0.098851995	count	1
ALKBH6	0.0855829	0.2308102	0.3708	0.711	0.098892732	count	1
STK11	0.0780772	0.1570511	0.4971	0.619	0.098924053	count	1
CEP19	0.0963562	0.3473344	0.2774	0.781	0.098935679	count	1
RAB11A	0.0711279	0.0818605	0.8689	0.385	0.099015787	count	1
NAPRT	0.0742391	0.1532276	0.4845	0.628	0.099259695	count	1
CHCHD4	0.0895682	0.2020045	0.4434	0.658	0.099332482	count	1
PLPBP	0.0726015	0.104776	0.6929	0.488	0.099344405	count	1
PDSS2	0.0863676	0.2105516	0.4102	0.682	0.099406974	count	1
AC008758.4	0.5865114	1.086248	0.5399	0.589	0.099475407	count	1
SYDE1	0.0774214	0.1557161	0.4972	0.619	0.099582375	count	1
MOB1B	0.0903942	0.2501933	0.3613	0.718	0.099693985	count	1
PANK2	0.0763457	0.1709817	0.4465	0.655	0.099772203	count	1
PUDP	0.1295211	0.4254682	0.3044	0.761	0.099799656	count	1
AKAP11	0.0752638	0.1333617	0.5644	0.573	0.099939645	count	1
TXLNB	0.1713123	0.3953171	0.4334	0.665	0.100002906	count	1
MEIS2	0.072882	0.1103511	0.6605	0.509	0.100053895	count	1
NDUFAF5	0.1145492	0.2879325	0.3978	0.691	0.100067562	count	1
FAM98A	0.0738592	0.1057279	0.6986	0.485	0.100138995	count	1
PEX2	0.0730791	0.1108093	0.6595	0.51	0.100206305	count	1
ANXA5	0.0698289	0.0299687	2.3301	0.0199	0.100279854	count	1
NDUFA3	0.0713241	0.0669681	1.065	0.287	0.100468098	count	1
MIB2	0.0857307	0.195688	0.4381	0.661	0.100491405	count	1
CNOT2	0.0731891	0.0965162	0.7583	0.448	0.100695923	count	1
CA13	0.1620209	0.6769809	0.2393	0.811	0.100719624	count	1
CYP20A1	0.0725322	0.0782175	0.9273	0.354	0.100801796	count	1
FAAP100	0.120552	0.3251908	0.3707	0.711	0.100816873	count	1

TMEM202-AS1	0.4000241	0.6154138	0.65	0.516	0.10081845	count	1
FGFR3	0.4009709	0.8441156	0.475	0.635	0.101032661	count	1
AC226118.1	0.4009709	0.8441156	0.475	0.635	0.101032661	count	1
GEMIN7-AS1	0.4009709	0.8441156	0.475	0.635	0.101032661	count	1
TEDC2	0.4009709	0.9898277	0.4051	0.685	0.101032661	count	1
SDE2	0.0927145	0.1902995	0.4872	0.626	0.101046435	count	1
AAR2	0.0868278	0.1913936	0.4537	0.65	0.101069362	count	1
WDR76	0.1274241	0.3254671	0.3915	0.695	0.101155405	count	1
NPIPA5	1.477906	0.7987051	1.8504	0.0643	0.101532089	count	1
TDRD6	1.479311	1.175916	1.258	0.208	0.10158446	count	1
MRFAP1L1	0.0773869	0.118556	0.6527	0.514	0.101641588	count	1
GGT7	0.0859521	0.212797	0.4039	0.686	0.101719521	count	1
PLEKHH3	0.0749532	0.1122435	0.6678	0.504	0.101798084	count	1
TXN2	0.072411	0.0631533	1.1466	0.252	0.10183408	count	1
TACO1	0.0790978	0.1517383	0.5213	0.602	0.101836576	count	1
NUDT16L1	0.0764637	0.1201581	0.6364	0.525	0.101844059	count	1
CENPH	0.1218032	0.3202986	0.3803	0.704	0.101847692	count	1
PAQR7	0.0941076	0.257751	0.3651	0.715	0.101915464	count	1
GOT1	0.0860111	0.1705221	0.5044	0.614	0.102094784	count	1
COLGALT1	0.0834688	0.1517266	0.5501	0.582	0.102103978	count	1
AHSA1	0.0741802	0.0829294	0.8945	0.371	0.102344178	count	1
AL157756.1	0.2643695	0.7899276	0.3347	0.738	0.102482656	count	1
AL117379.1	0.2643695	0.9339838	0.2831	0.777	0.102482656	count	1
RDH11	0.0744533	0.0946523	0.7866	0.432	0.102538781	count	1
OPRL1	0.6088401	0.9137636	0.6663	0.505	0.102616287	count	1
FSCN1	0.0858295	0.2424306	0.354	0.723	0.102747228	count	1
STARD8	1.512793	1.153281	1.3117	0.19	0.102813315	count	1
ZNF606	0.1116286	0.3394746	0.3288	0.742	0.10283614	count	1
SEMA4B	0.1230335	0.3554148	0.3462	0.729	0.102860976	count	1
IRS2	0.0789706	0.1725593	0.4576	0.647	0.102872141	count	1
IQGAP1	0.0726682	0.0569568	1.2758	0.202	0.10301838	count	1
AC022211.2	0.6121198	0.7801994	0.7846	0.433	0.10307374	count	1
ERLIN2	0.080124	0.1362202	0.5882	0.556	0.103154517	count	1
GINS2	0.2326122	0.7215406	0.3224	0.747	0.103354558	count	1
RHOA	0.0719799	0.0310124	2.321	0.0203	0.10340712	count	1
IFT52	0.0759925	0.1116709	0.6805	0.496	0.103487341	count	1
MRPS30-DT	0.0911239	0.2300684	0.3961	0.692	0.103542507	count	1
XRCC5	0.0730281	0.0499978	1.4606	0.144	0.10379931	count	1
MTRNR2L12	0.072559	0.0435973	1.6643	0.0961	0.103906685	count	1
KANSL1L	0.0817794	0.1522772	0.537	0.591	0.103997471	count	1
TSEN15	0.0781845	0.1324275	0.5904	0.555	0.104027643	count	1
ZNF582-AS1	0.1023981	0.2848864	0.3594	0.719	0.104117159	count	1
UBC	0.0724163	0.0226624	3.1954	0.00141	0.104289318	count	1
LOXL1-AS1	0.0983601	0.2834245	0.347	0.729	0.104318926	count	1
NR2C2	0.0869286	0.2516708	0.3454	0.73	0.104334931	count	1

HIST1H2AC	0.0817773	0.1890173	0.4326	0.665	0.104369685	count	1
SLC44A1	0.0771753	0.115778	0.6666	0.505	0.104533535	count	1
UTP23	0.0751241	0.0957408	0.7847	0.433	0.104543239	count	1
HSD17B4	0.0780683	0.1044414	0.7475	0.455	0.104643426	count	1
PMF1	0.0781374	0.1169073	0.6684	0.504	0.104647047	count	1
ILF3-DT	0.0765443	0.1091582	0.7012	0.483	0.104687485	count	1
PCDHB16	0.0980471	0.2565824	0.3821	0.702	0.104739051	count	1
PIP5K1C	0.0858161	0.1667834	0.5145	0.607	0.104740066	count	1
AC021054.1	0.1938237	0.3848021	0.5037	0.615	0.104875867	count	1
ME3	0.0937234	0.2514931	0.3727	0.709	0.104992023	count	1
FAM193A	0.0875698	0.2785631	0.3144	0.753	0.105101263	count	1
RAD21	0.075435	0.0735915	1.0251	0.305	0.10512814	count	1
ALDH4A1	0.1466484	0.3447177	0.4254	0.671	0.105177709	count	1
CDH8	0.6276306	0.7981091	0.7864	0.432	0.105223676	count	1
TSPAN32	0.6276306	0.8586843	0.7309	0.465	0.105223676	count	1
FAM84B	0.6276306	0.9227112	0.6802	0.496	0.105223676	count	1
RINT1	0.0961831	0.27596	0.3485	0.727	0.105430382	count	1
ARMC7	0.1145292	0.2826241	0.4052	0.685	0.105476203	count	1
PUS7	0.1027927	0.2457278	0.4183	0.676	0.105487948	count	1
LRCH1	0.1097684	0.2135388	0.514	0.607	0.105541355	count	1
CD302	0.0753254	0.0689243	1.0929	0.275	0.105626493	count	1
AC090204.1	1.5938509	1.8642704	0.8549	0.3926	0.105645322	count	1
AC139887.2	0.1236105	0.3234267	0.3822	0.702	0.105674349	count	1
IFT74	0.0779164	0.1225662	0.6357	0.525	0.10593507	count	1
TSG101	0.0756926	0.0721451	1.0492	0.294	0.10616748	count	1
ABCD2	1.610801	1.068195	1.508	0.132	0.106212697	count	1
COPS5	0.076061	0.0771241	0.9862	0.324	0.106248215	count	1
MID1	0.0822569	0.1879307	0.4377	0.662	0.106305974	count	1
C16orf58	0.0881579	0.1850514	0.4764	0.634	0.106345787	count	1
SSR1	0.0762988	0.0790622	0.965	0.335	0.10635072	count	1
NTAN1	0.0762966	0.0793313	0.9617	0.336	0.10635741	count	1
NAF1	0.0843048	0.1517192	0.5557	0.578	0.106505678	count	1
EYA3	0.0937959	0.2558493	0.3666	0.714	0.106561213	count	1
AC027097.2	1.6279463	1.00853	1.6142	0.107	0.106778206	count	1
CDH15	1.6279463	2.0201667	0.8058	0.4204	0.106778206	count	1
SLC25A12	0.0830408	0.1488883	0.5577	0.577	0.106791567	count	1
ZNF547	0.3310149	0.7331699	0.4515	0.652	0.106822461	count	1
CLIP2	0.109735	0.3448206	0.3182	0.75	0.106829	count	1
DCAF7	0.0833962	0.1502986	0.5549	0.579	0.106909238	count	1
NIPSNAP1	0.089477	0.1756	0.5096	0.61	0.107094531	count	1
SMAD2	0.0801268	0.1137226	0.7046	0.481	0.107166387	count	1
NIPA1	0.1394645	0.324531	0.4297	0.667	0.107316155	count	1
CASC2	0.4289829	0.8227634	0.5214	0.602	0.107317299	count	1
AC003101.2	0.4289829	0.8227634	0.5214	0.602	0.107317299	count	1
ACE2	0.4289829	0.950389	0.4514	0.652	0.107317299	count	1

ANKIB1	0.0802885	0.1318761	0.6088	0.543	0.107334256	count	1
SCARF2	0.0892178	0.1938067	0.4603	0.645	0.107618968	count	1
AC027117.2	1.666221	1.274313	1.3075	0.191	0.108010365	count	1
ABT1	0.0806996	0.1721455	0.4688	0.639	0.108072817	count	1
TTYH2	0.6486624	0.604963	1.0722	0.284	0.108103478	count	1
DCAF12	0.0925782	0.2421053	0.3824	0.702	0.108108606	count	1
SH3BP5-AS1	0.2187074	0.5555894	0.3936	0.694	0.10812202	count	1
ZNF888	0.6503105	0.6030978	1.0783	0.281	0.108327431	count	1
STAT5A	0.0927762	0.2062899	0.4497	0.653	0.108338641	count	1
CLTB	0.0760306	0.0454273	1.6737	0.0943	0.108342349	count	1
ECD	0.08598	0.1835975	0.4683	0.64	0.108469855	count	1
VPS37B	0.0917009	0.2588851	0.3542	0.723	0.108490602	count	1
POLRMT	0.1145265	0.2274715	0.5035	0.615	0.108615391	count	1
CSE1L	0.0874572	0.2141182	0.4085	0.683	0.108617722	count	1
PEMT	0.0844898	0.1554013	0.5437	0.587	0.108760503	count	1
ATP23	0.0981402	0.193936	0.506	0.613	0.108776705	count	1
TRIP6	0.0774685	0.0693411	1.1172	0.264	0.10892037	count	1
EXOSC9	0.0836472	0.1455831	0.5746	0.566	0.109037522	count	1
MBD4	0.0790226	0.0958904	0.8241	0.41	0.109134097	count	1
C9orf106	1.705752	1.22324	1.3945	0.163	0.109240107	count	1
DECR1	0.0782298	0.0804919	0.9719	0.331	0.109293192	count	1
SIKE1	0.0821883	0.1220102	0.6736	0.501	0.109400354	count	1
PHIP	0.0791054	0.101503	0.7793	0.436	0.109579561	count	1
KIZ	0.0860877	0.134025	0.6423	0.521	0.109594232	count	1
ATL3	0.0782939	0.07747	1.0106	0.312	0.109758712	count	1
PRIMPOL	0.0930798	0.2196381	0.4238	0.672	0.109773432	count	1
VAMP2	0.0768439	0.0445917	1.7233	0.0849	0.109795614	count	1
PRC1	0.1887951	0.5404742	0.3493	0.727	0.109869845	count	1
ZDHHC4	0.0813588	0.114016	0.7136	0.476	0.109908728	count	1
NAA35	0.0887865	0.1565419	0.5672	0.571	0.110068853	count	1
PRELID3B	0.0853566	0.1542052	0.5535	0.58	0.110091013	count	1
CD63	0.0765029	0.0209817	3.6462	0.00027	0.11020265	count	1
GRK6	0.1039567	0.232638	0.4469	0.655	0.110207281	count	1
PPM1K	0.080346	0.1016637	0.7903	0.429	0.110246541	count	1
SYDE2	0.0893406	0.2633664	0.3392	0.734	0.110349967	count	1
ARMH3	0.1096675	0.277666	0.395	0.693	0.110351926	count	1
TST	0.0897085	0.1990692	0.4506	0.652	0.110379267	count	1
WDR20	0.0853313	0.1605792	0.5314	0.595	0.110472937	count	1
ZRANB2	0.0777309	0.0649972	1.1959	0.232	0.110473819	count	1
MTUS1	0.0792363	0.0761136	1.041	0.298	0.110598137	count	1
ZMPSTE24	0.0808128	0.1048407	0.7708	0.441	0.110661769	count	1
AC084033.3	0.0851119	0.169124	0.5033	0.615	0.110762684	count	1
RPS6KA2	0.0954374	0.3112679	0.3066	0.759	0.111037811	count	1
RPA4	0.6706708	0.6489159	1.0335	0.301	0.111073599	count	1
NUAK2	0.6706708	0.683493	0.9812	0.327	0.111073599	count	1

ATR	0.1026695	0.2306484	0.4451	0.656	0.111119088	count	1
C17orf107	0.3458168	0.5884858	0.5876	0.557	0.111206565	count	1
PIK3CD	0.3458168	0.6367502	0.5431	0.587	0.111206565	count	1
NANOG	0.6719999	1.131627	0.5938	0.553	0.111251553	count	1
AC092718.3	0.6719999	1.131627	0.5938	0.553	0.111251553	count	1
C19orf57	0.6719999	1.131627	0.5938	0.553	0.111251553	count	1
AL049840.2	0.6719999	1.139617	0.5897	0.555	0.111251553	count	1
TMEM92	0.6719999	1.139617	0.5897	0.555	0.111251553	count	1
MB	0.6719999	1.139617	0.5897	0.555	0.111251553	count	1
KCNK7	0.6719999	1.292406	0.52	0.603	0.111251553	count	1
LINC02572	0.6719999	1.302785	0.5158	0.606	0.111251553	count	1
MRPS10	0.0798689	0.0907056	0.8805	0.379	0.111260643	count	1
GBP2	0.0800181	0.0766981	1.0433	0.297	0.111436269	count	1
SMG7	0.089798	0.1992282	0.4507	0.652	0.111514402	count	1
CHD2	0.0833969	0.1402981	0.5944	0.552	0.111532352	count	1
ULK3	0.1087866	0.3013162	0.361	0.718	0.111583005	count	1
HAPLN2	0.448268	0.6705931	0.6685	0.504	0.111584695	count	1
RPP30	0.0832558	0.1056525	0.788	0.431	0.111723327	count	1
BRCA2	0.3476177	0.4813906	0.7221	0.47	0.111737628	count	1
DNAH14	1.791649	0.9317961	1.9228	0.0546	0.111768303	count	1
LINC01474	0.2899991	0.8347096	0.3474	0.728	0.111781802	count	1
GANAB	0.0829393	0.1143883	0.7251	0.468	0.111808563	count	1
SETD1A	0.1136211	0.271924	0.4178	0.676	0.111871628	count	1
CARD8-AS1	1.800954	1.162774	1.5488	0.122	0.112030806	count	1
BTBD1	0.0914621	0.2061071	0.4438	0.657	0.112076591	count	1
PHLDB2	0.0822281	0.098015	0.8389	0.402	0.11210958	count	1
AL513314.2	1.8088187	1.597038	1.1326	0.257	0.112250911	count	1
TRAPPC3L	0.4513644	0.5801361	0.778	0.437	0.112265366	count	1
SNX11	0.1019352	0.2592131	0.3932	0.694	0.112334015	count	1
PCMTD1	0.0795471	0.0609121	1.3059	0.192	0.112555202	count	1
KATNAL1	0.093347	0.1932109	0.4831	0.629	0.112577802	count	1
CAPN6	1.823408	1.30466	1.3976	0.162	0.112655254	count	1
SPTSSB	1.824832	1.110211	1.6437	0.1	0.112694405	count	1
WDR5B	0.1385049	0.2835194	0.4885	0.625	0.112779856	count	1
CANT1	0.0950948	0.2115578	0.4495	0.653	0.112824636	count	1
DDX39A	0.0871217	0.1493495	0.5833	0.56	0.112886538	count	1
BNIP1	0.0958252	0.1971665	0.486	0.627	0.11299483	count	1
SYCP2L	1.840688	1.651126	1.1148	0.265	0.113127207	count	1
DZANK1	0.1826519	0.3925468	0.4653	0.642	0.11315177	count	1
CITED1	0.2294644	0.6692262	0.3429	0.732	0.113200071	count	1
TWNK	0.1948535	0.4707628	0.4139	0.679	0.113273858	count	1
LRP12	0.1034106	0.220733	0.4685	0.639	0.113295766	count	1
DDX55	0.09817	0.180748	0.5431	0.587	0.113355324	count	1
BTN2A2	0.1151494	0.1894218	0.6079	0.543	0.113360384	count	1
AC073610.3	0.2301897	0.5938145	0.3876	0.698	0.113541622	count	1

AC073195.1	0.2301897	0.6191824	0.3718	0.71	0.113541622	count	1
CARNS1	0.2301897	0.6433952	0.3578	0.721	0.113541622	count	1
AP4E1	0.1302701	0.3581345	0.3637	0.716	0.113595524	count	1
FRMD4A	0.0887341	0.1809546	0.4904	0.624	0.113608336	count	1
FAM120B	0.0947268	0.2890348	0.3277	0.743	0.113651582	count	1
TRIP4	0.0967008	0.1826636	0.5294	0.597	0.113657785	count	1
ARHGEF2	0.0875362	0.2152279	0.4067	0.684	0.113719279	count	1
ZNF397	0.0859171	0.1726253	0.4977	0.619	0.113807269	count	1
MEAF6	0.0805133	0.0613264	1.3129	0.189	0.113957761	count	1
LINC00908	1.8724403	1.057345	1.7709	0.0767	0.113975732	count	1
ARMH1	1.8724403	2.0622754	0.9079	0.364	0.113975732	count	1
LINC00909	0.0898884	0.1738583	0.517	0.605	0.113989793	count	1
APLNR	0.4595148	0.6566327	0.6998	0.484	0.114051077	count	1
BAG2	0.0824535	0.0894396	0.9219	0.357	0.114062012	count	1
CCL13	1.878178	0.8773201	2.1408	0.0324	0.114126436	count	1
ZNF841	0.1744847	0.3190627	0.5469	0.585	0.114153047	count	1
AFMID	0.0897044	0.1847965	0.4854	0.627	0.114184457	count	1
GPATCH11	0.0851856	0.1141258	0.7464	0.455	0.114214637	count	1
INPP5K	0.0975317	0.2013073	0.4845	0.628	0.114251183	count	1
TMX3	0.0852117	0.1093366	0.7794	0.436	0.114343497	count	1
RRP12	0.1974834	0.3743553	0.5275	0.598	0.114749063	count	1
LYPD3	0.1166093	0.3130615	0.3725	0.71	0.114782097	count	1
ZNF510	0.1084909	0.2628983	0.4127	0.68	0.114973947	count	1
WIPF2	0.0931171	0.2032109	0.4582	0.647	0.114996481	count	1
SYN1	0.260523	0.5113168	0.5095	0.61	0.11508489	count	1
AL356481.1	0.185958	0.4722501	0.3938	0.694	0.115135414	count	1
KIF22	0.0853368	0.112082	0.7614	0.446	0.1151962	count	1
ZNF778	0.2994941	0.3931338	0.7618	0.446	0.115197407	count	1
ENDOG	0.0888409	0.1384281	0.6418	0.521	0.115210428	count	1
LRWD1	0.1106985	0.2345701	0.4719	0.637	0.115489761	count	1
IAH1	0.0817675	0.0654643	1.249	0.212	0.115578102	count	1
BMP2K	0.1099022	0.2594626	0.4236	0.672	0.115580261	count	1
UNC119B	0.1161677	0.2904912	0.3999	0.689	0.115618355	count	1
PIAS1	0.086509	0.0996072	0.8685	0.385	0.115738035	count	1
E4F1	0.1069929	0.2469307	0.4333	0.665	0.115761979	count	1
PIK3IP1	0.0872828	0.1067084	0.818	0.413	0.115867226	count	1
C1orf53	0.3014302	0.4855379	0.6208	0.535	0.115891924	count	1
SQOR	0.0846644	0.0977442	0.8662	0.386	0.11590487	count	1
PIBF1	0.085853	0.1212409	0.7081	0.479	0.115970433	count	1
AC007878.1	1.953283	1.075153	1.8167	0.0693	0.116029426	count	1
SLF2	0.0881015	0.1712398	0.5145	0.607	0.116065759	count	1
ANKRD10	0.0840097	0.0944035	0.8899	0.374	0.116098878	count	1
OGFOD2	0.1511888	0.356339	0.4243	0.671	0.116151097	count	1
TBRG4	0.0944508	0.1842814	0.5125	0.608	0.116416455	count	1
NSMAF	0.1012804	0.2597414	0.3899	0.697	0.116469607	count	1

TMEM70	0.0873179	0.1125012	0.7762	0.438	0.116498785	count	1
DDX24	0.0814725	0.0433316	1.8802	0.0602	0.116562225	count	1
NUBPL	0.1046588	0.2103699	0.4975	0.619	0.116565666	count	1
AC009779.3	1.9755683	1.072154	1.8426	0.0655	0.116569636	count	1
THOC6	0.0960028	0.1885384	0.5092	0.611	0.116598246	count	1
CCDC13	0.7134148	0.8328366	0.8566	0.392	0.116716312	count	1
EPHA6	0.7134148	1.092133	0.6532	0.514	0.116716312	count	1
DMTF1	0.0928357	0.2940954	0.3157	0.752	0.116751322	count	1
SNW1	0.0831014	0.075017	1.1078	0.268	0.116823275	count	1
IDH2	0.0969686	0.1612765	0.6013	0.548	0.116925368	count	1
EMC2	0.0838002	0.0791766	1.0584	0.29	0.117041165	count	1
ZNF304	0.1576484	0.4634121	0.3402	0.734	0.117093949	count	1
EXOC3L1	0.3051487	0.4732262	0.6448	0.519	0.117223935	count	1
ZC4H2	0.1193272	0.3210941	0.3716	0.71	0.117427816	count	1
LAMC2	2.016044	1.160953	1.7365	0.0826	0.11752305	count	1
EEF1AKMT2	0.089031	0.1357198	0.656	0.512	0.117579344	count	1
TP53INP1	0.1067719	0.2327108	0.4588	0.646	0.117625076	count	1
MAT2B	0.0924281	0.1418548	0.6516	0.515	0.117640527	count	1
TBL1XR1	0.0842639	0.0763424	1.1038	0.27	0.117659346	count	1
LINC02315	0.1902024	0.6789677	0.2801	0.779	0.117678524	count	1
TMED10	0.082524	0.0460665	1.7914	0.0733	0.117689047	count	1
CUL9	0.1011973	0.5060749	0.2	0.842	0.11770117	count	1
SUV39H2	0.2028015	0.3728473	0.5439	0.587	0.117727627	count	1
AL049597.2	0.4765997	0.5896172	0.8083	0.419	0.117766323	count	1
JAKMIP2	0.4765997	0.9451159	0.5043	0.614	0.117766323	count	1
RSRC1	0.0846848	0.0837844	1.0107	0.312	0.117836474	count	1
HAUS7	0.1121093	0.4853515	0.231	0.817	0.117880849	count	1
AP003392.6	0.3070508	0.8963671	0.3426	0.732	0.117904356	count	1
TNRC6A	0.0847784	0.1456487	0.5821	0.561	0.117942605	count	1
CDC7	0.7232624	0.7228533	1.0006	0.317	0.117992944	count	1
DDX59	0.0920937	0.1443718	0.6379	0.524	0.118277444	count	1
CEBPA-DT	0.4789962	0.7246285	0.661	0.509	0.118284431	count	1
CDAN1	0.1593739	0.4282693	0.3721	0.71	0.118346329	count	1
RAB2A	0.0828772	0.0411607	2.0135	0.0441	0.118411506	count	1
MMP25-AS1	0.1496079	0.3990833	0.3749	0.708	0.118419123	count	1
CENPN	0.1915384	0.4421222	0.4332	0.665	0.118478194	count	1
MRPS24	0.2687108	0.8818089	0.3047	0.761	0.118497786	count	1
GBP5	0.2687108	1.102286	0.2438	0.807	0.118497786	count	1
CUL5	0.0860624	0.1028865	0.8365	0.403	0.118509093	count	1
GLS2	0.7274162	1.171749	0.6208	0.535	0.118528828	count	1
APOL2	0.0925676	0.1391242	0.6654	0.506	0.118631396	count	1
COMMD10	0.0888493	0.1478906	0.6008	0.548	0.1187034	count	1
AP001453.2	2.0691148	1.401183	1.4767	0.14	0.118721043	count	1
NXF1	0.1017231	0.2181824	0.4662	0.641	0.118727589	count	1
TBXAS1	0.4812007	0.7360837	0.6537	0.513	0.11876037	count	1

NMRAL1	0.0904077	0.1362102	0.6637	0.507	0.118784302	count	1
GATAD2B	0.0927052	0.1441256	0.6432	0.52	0.11880721	count	1
EXD3	0.1067513	0.2475025	0.4313	0.666	0.118879727	count	1
ELMOD2	0.0923604	0.1617562	0.571	0.568	0.118981792	count	1
ALKBH8	0.1426694	0.2599771	0.5488	0.583	0.118989373	count	1
GLRX3	0.0869723	0.1047115	0.8306	0.406	0.119060305	count	1
ATP6V1C1	0.0919666	0.1496727	0.6145	0.539	0.119147214	count	1
CWC25	0.0880604	0.1081375	0.8143	0.416	0.119254434	count	1
NTS	2.0947539	2.3355517	0.8969	0.3698	0.119279211	count	1
REEP5	0.0835394	0.0422561	1.977	0.0481	0.119308396	count	1
RAD50	0.103383	0.2074468	0.4984	0.618	0.119338609	count	1
KPNA6	0.0909013	0.1468171	0.6191	0.536	0.119431304	count	1
SPATA2	0.1280455	0.3492475	0.3666	0.714	0.119561596	count	1
UBE2D1	0.0871367	0.1023736	0.8512	0.395	0.119576154	count	1
RAB1A	0.0850471	0.0621565	1.3683	0.171	0.119670682	count	1
ADAT1	0.1374257	0.302168	0.4548	0.649	0.119735781	count	1
RAB3A	0.132467	0.2869487	0.4616	0.644	0.119789916	count	1
HTT	0.0957258	0.1992123	0.4805	0.631	0.119830075	count	1
ALDH6A1	0.0905953	0.1130855	0.8011	0.423	0.119853433	count	1
POM121	0.1076487	0.198761	0.5416	0.588	0.119871935	count	1
ZNF165	0.1749176	0.5002187	0.3497	0.727	0.119885254	count	1
NUP54	0.0903448	0.1155098	0.7821	0.434	0.11998744	count	1
CCT4	0.084987	0.0598932	1.419	0.156	0.120008306	count	1
CEP104	0.0984036	0.1681423	0.5852	0.558	0.120035934	count	1
TET3	0.4872654	0.7970849	0.6113	0.541	0.120066467	count	1
SLC10A7	0.1441168	0.3573518	0.4033	0.687	0.120174941	count	1
RNF4	0.1023066	0.1765022	0.5796	0.562	0.120210314	count	1
LRRC45	0.1380422	0.3070496	0.4496	0.653	0.120264293	count	1
KIF1B	0.0920164	0.1459953	0.6303	0.529	0.120291176	count	1
BTG1	0.0841281	0.0555137	1.5154	0.13	0.120334076	count	1
ZNF575	0.1521578	0.3389099	0.449	0.653	0.120396591	count	1
FAHD1	0.0943274	0.1378653	0.6842	0.494	0.120475981	count	1
GCNT4	0.1845599	0.404906	0.4558	0.649	0.120547314	count	1
EXOC3L4	0.4898388	0.8443964	0.5801	0.562	0.120619236	count	1
ARL14EP	0.0881552	0.0986096	0.894	0.371	0.120677516	count	1
AC010226.1	0.1095646	0.204638	0.5354	0.592	0.120678435	count	1
ZEB1	0.0863218	0.0848853	1.0169	0.309	0.12068387	count	1
AGO4	0.1123432	0.2883327	0.3896	0.697	0.120708362	count	1
ZNF585A	0.1335072	0.3183922	0.4193	0.675	0.120716816	count	1
BCL10	0.0889682	0.1159238	0.7675	0.443	0.120838158	count	1
THOC7	0.0853924	0.0658241	1.2973	0.195	0.120846589	count	1
EFCAB13	0.1276105	0.3388344	0.3766	0.706	0.120865676	count	1
PYM1	0.0912641	0.1417912	0.6437	0.52	0.120874676	count	1
THADA	0.1039439	0.209486	0.4962	0.62	0.120876984	count	1
EEF1AKMT1	0.0923353	0.1288878	0.7164	0.474	0.120885712	count	1

TMEM186	0.105164	0.2144632	0.4904	0.624	0.120907918	count	1
RNF219	0.0966475	0.1492635	0.6475	0.517	0.120979579	count	1
UBE2R2	0.0870645	0.0855365	1.0179	0.309	0.121018708	count	1
CALCOCO2	0.0855571	0.056719	1.5084	0.132	0.121185633	count	1
ZNF578	0.2465281	0.3988423	0.6181	0.537	0.121207354	count	1
MAGIX	0.176979	0.3518437	0.503	0.615	0.121259145	count	1
GALK2	0.0975541	0.1835014	0.5316	0.595	0.121317184	count	1
AC087289.2	2.1943323	1.8261671	1.2016	0.2296	0.121326201	count	1
PCGF2	0.0908562	0.1338897	0.6786	0.497	0.121379412	count	1
CCDC171	0.1026751	0.1978678	0.5189	0.604	0.12140449	count	1
IMMP2L	0.0899753	0.1116374	0.806	0.42	0.121405411	count	1
NCOA5	0.1423534	0.3142896	0.4529	0.651	0.121425539	count	1
OXT	0.4939601	1.093741	0.4516	0.652	0.121502708	count	1
LINC00852	0.4939601	1.186454	0.4163	0.677	0.121502708	count	1
AC079601.1	0.4939601	1.186454	0.4163	0.677	0.121502708	count	1
KCNJ14	0.4939601	1.186454	0.4163	0.677	0.121502708	count	1
KIF26A	0.4939601	1.209452	0.4084	0.683	0.121502708	count	1
AL049780.1	0.4939601	1.438641	0.3434	0.731	0.121502708	count	1
ZNF197-AS1	0.4939601	1.485662	0.3325	0.74	0.121502708	count	1
UGP2	0.0859283	0.0619654	1.3867	0.166	0.121536475	count	1
ABTB1	0.0905597	0.1077362	0.8406	0.401	0.121555865	count	1
RRP1	0.104537	0.227378	0.4597	0.646	0.121562632	count	1
ZNF77	0.2765504	0.4300655	0.643	0.52	0.121753478	count	1
TOP2B	0.0923131	0.1186826	0.7778	0.437	0.121754667	count	1
KDM2B	0.1866068	0.399734	0.4668	0.641	0.12184364	count	1
WARS2-IT1	0.7540321	0.9900006	0.7616	0.446	0.121926047	count	1
ANXA9	0.7540321	0.9900006	0.7616	0.446	0.121926047	count	1
AC099550.1	0.7540321	0.9900006	0.7616	0.446	0.121926047	count	1
BX005266.2	0.7540321	0.9900006	0.7616	0.446	0.121926047	count	1
MYCBPAP	0.7540321	0.9900006	0.7616	0.446	0.121926047	count	1
AC004922.1	0.7540321	1.0173864	0.7411	0.459	0.121926047	count	1
PRSS53	0.7540321	1.1505457	0.6554	0.512	0.121926047	count	1
AC004076.2	0.7540321	1.168959	0.645	0.519	0.121926047	count	1
RLF	0.0974619	0.1451726	0.6714	0.502	0.121995192	count	1
AC245060.6	0.7548334	0.7833745	0.9636	0.335	0.12202734	count	1
RGS18	0.7548334	1.5436632	0.489	0.6249	0.12202734	count	1
ARHGAP42	0.0895006	0.1276096	0.7014	0.483	0.122070795	count	1
OXSM	0.1203306	0.2732928	0.4403	0.66	0.122162144	count	1
AL121658.1	0.277576	0.5646041	0.4916	0.623	0.122178527	count	1
AC110769.2	0.277576	0.5820926	0.4769	0.633	0.122178527	count	1
ALG3	0.0914608	0.124672	0.7336	0.463	0.122185542	count	1
C21orf62-AS1	0.1503189	0.3682323	0.4082	0.683	0.122214376	count	1
GCLM	0.0964838	0.1462963	0.6595	0.51	0.122481518	count	1
C8orf82	0.0929577	0.1177477	0.7895	0.43	0.122678605	count	1
SQLE	0.0981744	0.1751587	0.5605	0.575	0.122883673	count	1

SIPA1	0.109321	0.2167934	0.5043	0.614	0.122940338	count	1
ZMYM5	0.0925961	0.15062	0.6148	0.539	0.123035776	count	1
RER1	0.0871124	0.0586232	1.486	0.137	0.12318012	count	1
SUPT16H	0.0882802	0.0733232	1.204	0.229	0.123200665	count	1
ZNF622	0.0938393	0.1475412	0.636	0.525	0.123282026	count	1
ARHGAP5	0.0897057	0.098972	0.9064	0.365	0.123414308	count	1
BORCS5	0.1021276	0.2102225	0.4858	0.627	0.1234186	count	1
CDYL	0.0986054	0.1641677	0.6006	0.548	0.123421102	count	1
GOLM1	0.0984598	0.1786632	0.5511	0.582	0.123429565	count	1
ZC2HC1C	0.1481714	0.6139015	0.2414	0.809	0.123493666	count	1
7-Sep	0.0858654	0.0273753	3.1366	0.00172	0.123509228	count	1
ZNF440	0.1804167	0.493936	0.3653	0.715	0.12354821	count	1
MT1E	0.0870858	0.0650434	1.3389	0.181	0.123673699	count	1
COBLL1	0.0901846	0.0984203	0.9163	0.36	0.123801009	count	1
USP7	0.0913709	0.0993938	0.9193	0.358	0.123843775	count	1
TMEM165	0.0876013	0.0599883	1.4603	0.144	0.124002678	count	1
GRM7	0.7705595	0.7772777	0.9914	0.322	0.124003921	count	1
TCEANC2	0.2141148	0.4351018	0.4921	0.623	0.124043685	count	1
SPIN2B	0.0994708	0.1829286	0.5438	0.587	0.124104124	count	1
SLITRK2	2.351122	1.08828	2.1604	0.0308	0.124189231	count	1
TFG	0.0884394	0.0672977	1.3142	0.189	0.124208595	count	1
RFFL	0.1619719	0.3191796	0.5075	0.612	0.124250004	count	1
AC009961.1	2.355256	1.160644	2.0293	0.0425	0.124259129	count	1
COQ7	0.0982019	0.1499642	0.6548	0.513	0.124333884	count	1
AC015912.3	0.7740161	0.6669352	1.1606	0.246	0.124435439	count	1
OTUB2	0.3913874	0.9600572	0.4077	0.684	0.12448763	count	1
BTBD9	0.1204667	0.2634137	0.4573	0.647	0.124534756	count	1
TRIM22	0.0920414	0.10981	0.8382	0.402	0.124558796	count	1
CASP7	0.1037181	0.1517167	0.6836	0.494	0.124708118	count	1
SYBU	0.254059	0.3757336	0.6762	0.499	0.12472251	count	1
TELO2	0.1161173	0.2491332	0.4661	0.641	0.124728359	count	1
TM9SF3	0.0883677	0.0617834	1.4303	0.153	0.124836129	count	1
RAMMET	0.0914511	0.1127954	0.8108	0.418	0.124846915	count	1
RPF2	0.0899059	0.0932498	0.9641	0.335	0.124868778	count	1
FRMD6-AS1	0.1100792	0.35949	0.3062	0.759	0.124934795	count	1
NANS	0.0909806	0.0897494	1.0137	0.311	0.124939651	count	1
FAHD2A	0.0971715	0.1462084	0.6646	0.506	0.125035617	count	1
AC048341.1	0.1384469	0.4782922	0.2895	0.772	0.125115421	count	1
AKR1B10	2.408795	1.114202	2.1619	0.0307	0.125141084	count	1
CXCL12	0.0876027	0.0686082	1.2769	0.202	0.125161449	count	1
EXOC3	0.1016136	0.1593499	0.6377	0.524	0.12520705	count	1
JAM3	0.0903399	0.0869454	1.039	0.299	0.125220461	count	1
APRT	0.0882083	0.0518947	1.6998	0.0893	0.125259916	count	1
ESYT3	2.4202467	2.3314626	1.0381	0.2993	0.125324024	count	1
SLC9B1	0.7816111	0.6911271	1.1309	0.258	0.125379912	count	1

MRPL42	0.0907593	0.0852668	1.0644	0.287	0.125452035	count	1
AL445183.2	0.3954801	0.9667214	0.4091	0.682	0.125664397	count	1
SLC26A5	0.3954801	0.9667214	0.4091	0.682	0.125664397	count	1
AMN	0.3954801	0.9667214	0.4091	0.682	0.125664397	count	1
AC138894.1	0.3954801	0.9667214	0.4091	0.682	0.125664397	count	1
CKAP2L	0.3954801	1.066826	0.3707	0.711	0.125664397	count	1
SEPT7-AS1	0.3954801	1.066826	0.3707	0.711	0.125664397	count	1
PRR36	0.3954801	1.31477	0.3008	0.764	0.125664397	count	1
R3HDM4	0.1024028	0.1726311	0.5932	0.553	0.125679588	count	1
U62317.3	0.5136567	0.7791404	0.6593	0.51	0.125694662	count	1
KLF6	0.0882038	0.0580798	1.5187	0.129	0.125869125	count	1
RUSC1	0.095699	0.1671932	0.5724	0.567	0.125887139	count	1
SAMD9L	0.0973259	0.1544524	0.6301	0.529	0.125956572	count	1
TMEM97	0.1180992	0.3170036	0.3725	0.71	0.125968554	count	1
ARIH2OS	0.1281765	0.2907237	0.4409	0.659	0.126032309	count	1
LRPAP1	0.0884402	0.0412151	2.1458	0.032	0.126143106	count	1
AC100793.2	0.0979578	0.2359223	0.4152	0.678	0.126294787	count	1
MAP2K2	0.0896659	0.0608381	1.4738	0.141	0.126333233	count	1
AL121761.1	0.3307909	0.654733	0.5052	0.613	0.126342469	count	1
LRP11	0.1038916	0.2208706	0.4704	0.638	0.126419751	count	1
SLC39A6	0.0928332	0.0927915	1.0004	0.317	0.126547155	count	1
NDUFB9	0.0887585	0.044576	1.9912	0.0465	0.12666629	count	1
SHMT2	0.0968772	0.1515164	0.6394	0.523	0.126723316	count	1
SEC14L6	2.5158079	2.8454586	0.8841	0.3767	0.126776036	count	1
RPA1	0.1003366	0.182213	0.5507	0.582	0.126858109	count	1
ULK4	0.3323192	0.3138334	1.0589	0.29	0.126882242	count	1
ARHGAP6	0.0959116	0.1355658	0.7075	0.479	0.126943646	count	1
GTF2E1	0.1239518	0.3887941	0.3188	0.75	0.126975187	count	1
CNIH4	0.090263	0.0655684	1.3766	0.169	0.126992962	count	1
ZNRD1	0.0931494	0.1158904	0.8038	0.422	0.127040074	count	1
DNAJB14	0.0905021	0.0700806	1.2914	0.197	0.127060414	count	1
ZFX	0.0969246	0.1189897	0.8146	0.415	0.127060853	count	1
ARHGAP35	0.0980104	0.1582249	0.6194	0.536	0.127067354	count	1
MYO1C	0.0916729	0.1102968	0.8311	0.406	0.127158893	count	1
MAP4K5	0.0922737	0.1006477	0.9168	0.359	0.127218061	count	1
SMARCC1	0.0997588	0.1397838	0.7137	0.475	0.127389918	count	1
ZNF322	0.0925313	0.0961726	0.9621	0.336	0.127448542	count	1
PDSS1	0.1720934	0.377661	0.4557	0.649	0.12755774	count	1
CHID1	0.0921058	0.0783385	1.1757	0.24	0.127681367	count	1
ZNF330	0.0960004	0.1177523	0.8153	0.415	0.127809971	count	1
SLC25A16	0.1116816	0.224088	0.4984	0.618	0.127831711	count	1
LENG1	0.0943545	0.1168472	0.8075	0.419	0.127842311	count	1
LATS1	0.0999872	0.1937926	0.5159	0.606	0.127968816	count	1
DSTYK	0.1022706	0.1853358	0.5518	0.581	0.127990602	count	1
MACROD1	0.0951637	0.106424	0.8942	0.371	0.128022849	count	1

DAPL1	0.0952919	0.2166273	0.4399	0.66	0.128047632	count	1
DNMT3A	0.1100959	0.2216396	0.4967	0.619	0.128440063	count	1
AC009126.1	0.4054521	0.7415136	0.5468	0.585	0.12852059	count	1
GNAI3	0.0925526	0.088549	1.0452	0.296	0.128541044	count	1
TIMM23B	0.1293412	0.228016	0.5672	0.571	0.128575461	count	1
SNHG12	0.1049109	0.2182829	0.4806	0.631	0.128743723	count	1
CHD3	0.0940856	0.1091064	0.8623	0.389	0.128784412	count	1
PCCB	0.1112529	0.1720722	0.6465	0.518	0.128850878	count	1
ZBTB41	0.1017922	0.168846	0.6029	0.547	0.128863065	count	1
PRDX4	0.09038	0.0501645	1.8017	0.0717	0.128891928	count	1
RPRD1B	0.114662	0.2195604	0.5222	0.602	0.12890231	count	1
NLE1	0.1311611	0.2620068	0.5006	0.617	0.12893094	count	1
SC5D	0.1136647	0.1849728	0.6145	0.539	0.128975307	count	1
CES4A	0.1519132	0.442318	0.3434	0.731	0.129430449	count	1
FSIP2	0.8150673	0.6239503	1.3063	0.192	0.129480199	count	1
C15orf40	0.0940948	0.1095128	0.8592	0.39	0.129534156	count	1
MAP2K7	0.1096576	0.2231979	0.4913	0.623	0.129612999	count	1
CRTC3	0.0981716	0.1368222	0.7175	0.473	0.12977676	count	1
GDI2	0.0919141	0.0540783	1.6996	0.0893	0.12983886	count	1
SMARCD3	0.0945184	0.1203974	0.7851	0.432	0.129887794	count	1
EEF1AKMT3	0.1394222	0.3472744	0.4015	0.688	0.130030832	count	1
KLHL25	0.2658265	0.7109875	0.3739	0.709	0.130191991	count	1
AURKC	0.2658265	0.7615476	0.3491	0.727	0.130191991	count	1
S100A11	0.090447	0.0271793	3.3278	0.000884	0.130278387	count	1
LINC01569	0.3421646	0.7348976	0.4656	0.642	0.130349534	count	1
ANKRD39	0.1024501	0.1598531	0.6409	0.522	0.130351489	count	1
IFT81	0.0997731	0.1287828	0.7747	0.439	0.130501105	count	1
PIGB	0.1286394	0.3819159	0.3368	0.736	0.130503023	count	1
SH3RF3	0.1167721	0.2492541	0.4685	0.639	0.130615565	count	1
OSBPL9	0.0946676	0.0994853	0.9516	0.341	0.130810721	count	1
PAIP1	0.0935429	0.0659278	1.4189	0.156	0.130925196	count	1
PRKCSH	0.0971559	0.1128754	0.8607	0.389	0.130938158	count	1
AL603832.2	0.827882	1.0397854	0.7962	0.426	0.131024945	count	1
PREP	0.1385266	0.25598	0.5412	0.588	0.131060066	count	1
BBS7	0.1009607	0.141306	0.7145	0.475	0.13110822	count	1
MAPKAP1	0.0988528	0.1129988	0.8748	0.382	0.13119104	count	1
NEK5	0.2130618	0.3837894	0.5552	0.579	0.131307052	count	1
TMTC4	0.5406289	0.6423399	0.8417	0.4	0.131353777	count	1
NCBP3	0.0983035	0.1057372	0.9297	0.353	0.131367311	count	1
GFOD1	0.3452516	0.374274	0.9225	0.356	0.131433137	count	1
STX1A	0.2276136	0.5087821	0.4474	0.655	0.131543551	count	1
PRDX1	0.0914966	0.0261256	3.5022	0.000467	0.131546064	count	1
CASC1	0.832253	0.7776012	1.0703	0.285	0.131548594	count	1
UMAD1	0.1084668	0.1572624	0.6897	0.49	0.131660396	count	1
PRPF8	0.0957038	0.1103158	0.8675	0.386	0.131701309	count	1

PURPL	2.931751	1.427437	2.0539	0.0401	0.131761934	count	1
AAGAB	0.1156682	0.2101467	0.5504	0.582	0.131806645	count	1
RFX3	0.2024287	0.3558028	0.5689	0.569	0.131832339	count	1
ATP2B1	0.0974707	0.1147212	0.8496	0.396	0.131893286	count	1
SNHG19	0.1359003	0.2032776	0.6685	0.504	0.131971691	count	1
PAN2	0.1376556	0.3724798	0.3696	0.712	0.131994094	count	1
CCNYL1	0.1187051	0.2471086	0.4804	0.631	0.132086161	count	1
INIP	0.106509	0.137607	0.774	0.439	0.132177103	count	1
SRFBP1	0.097317	0.0982713	0.9903	0.322	0.132376644	count	1
AC090360.1	0.5456229	0.8886764	0.614	0.539	0.132391302	count	1
HELZ2	0.1592896	0.3467477	0.4594	0.646	0.132575629	count	1
KDELR1	0.0928456	0.0418498	2.2185	0.0266	0.132849115	count	1
BCL7A	0.1229374	0.2155275	0.5704	0.568	0.13285804	count	1
PBRM1	0.0971325	0.1245886	0.7796	0.436	0.132862747	count	1
GABRE	0.1338372	0.3245556	0.4124	0.68	0.13299005	count	1
AC005520.2	0.1798509	0.4316682	0.4166	0.677	0.133157879	count	1
THYN1	0.0947486	0.0691266	1.3707	0.171	0.133192209	count	1
CRKL	0.1085949	0.1992711	0.545	0.586	0.13324316	count	1
PPM1N	0.3044551	0.717233	0.4245	0.671	0.133245743	count	1
DCP1B	0.1241682	0.223525	0.5555	0.579	0.133295778	count	1
DCAF10	0.10592	0.1757866	0.6025	0.547	0.133333911	count	1
ADA2	0.1280362	0.3832101	0.3341	0.738	0.133389935	count	1
SNAPC4	0.3513579	0.3870215	0.9079	0.364	0.133571556	count	1
RP2	0.1214414	0.2464794	0.4927	0.622	0.133649851	count	1
ARIH1	0.1016396	0.1631613	0.6229	0.533	0.133768773	count	1
FABP3	0.1010203	0.1494375	0.676	0.499	0.133840403	count	1
LRCH3	0.1018406	0.1857597	0.5482	0.584	0.133856007	count	1
MANEAL	0.4244632	0.5812248	0.7303	0.465	0.133922256	count	1
AC013468.1	0.4244632	0.6198557	0.6848	0.494	0.133922256	count	1
FBXL4	0.1136837	0.1858203	0.6118	0.541	0.133925492	count	1
SH3BP1	0.8533706	0.8525328	1.001	0.317	0.134055384	count	1
RRAS2	0.1092604	0.1929318	0.5663	0.571	0.134055803	count	1
CNTROB	0.3065253	0.4231513	0.7244	0.469	0.134092329	count	1
BRIX1	0.0977787	0.0964252	1.014	0.311	0.134159685	count	1
THNSL2	0.1513831	0.2731652	0.5542	0.579	0.13421802	count	1
GNA12	0.1119697	0.1816266	0.6165	0.538	0.134225472	count	1
TTBK2	0.1165706	0.1899381	0.6137	0.539	0.134457721	count	1
TAPT1	0.1017975	0.1733252	0.5873	0.557	0.13455804	count	1
RYBP	0.105776	0.1693332	0.6247	0.532	0.134567705	count	1
PDCD10	0.0964545	0.0662869	1.4551	0.146	0.134597948	count	1
THAP2	0.1058096	0.210777	0.502	0.616	0.134610294	count	1
GSKIP	0.1192387	0.1945951	0.6128	0.54	0.134640621	count	1
NRXN3	0.1238545	0.3860224	0.3208	0.748	0.134684929	count	1
PRSS23	0.0942057	0.0611511	1.5405	0.1235	0.134751498	count	1
DONSON	0.2188812	0.4724498	0.4633	0.643	0.134758059	count	1

ZNF92	0.1256438	0.2295009	0.5475	0.584	0.134864855	count	1
GNB4	0.0980751	0.1132551	0.866	0.387	0.134960224	count	1
PPP3CB	0.1014584	0.1146659	0.8848	0.376	0.134992101	count	1
PIGBOS1	0.1022414	0.1167151	0.876	0.381	0.135063065	count	1
SLC12A6	0.1286807	0.2550238	0.5046	0.614	0.135126956	count	1
POLR1A	0.1767075	0.3116713	0.567	0.571	0.135275646	count	1
CYB5D2	0.1023052	0.139837	0.7316	0.464	0.135384999	count	1
NUPL2	0.1022788	0.1479235	0.6914	0.489	0.135427409	count	1
PICALM	0.0991766	0.1102325	0.8997	0.368	0.135471923	count	1
MLXIP	0.1107071	0.1799835	0.6151	0.539	0.135544451	count	1
GON4L	0.1015667	0.1280122	0.7934	0.428	0.135594252	count	1
AC012467.2	0.8666032	1.041857	0.8318	0.406	0.135606746	count	1
LMBR1	0.1213822	0.2186624	0.5551	0.579	0.135730867	count	1
RBFADN	0.8679208	0.9571724	0.9068	0.365	0.135760389	count	1
FBP1	0.1533219	0.548003	0.2798	0.78	0.135906849	count	1
ZNF449	0.1294314	0.291855	0.4435	0.657	0.135907078	count	1
SIRT5	0.1368908	0.280267	0.4884	0.625	0.135986149	count	1
SLC7A8	0.1439707	0.2746694	0.5242	0.6	0.136135255	count	1
RASA1	0.1176408	0.1909782	0.616	0.538	0.136198842	count	1
BEND7	0.115691	0.2092224	0.553	0.58	0.136275459	count	1
PPP2R5C	0.100408	0.104035	0.9651	0.335	0.136500524	count	1
ZNF614	0.1390089	0.2498698	0.5563	0.578	0.136544454	count	1
PROSER3	0.1444512	0.2734997	0.5282	0.597	0.136582912	count	1
NELFB	0.1072539	0.1409316	0.761	0.447	0.136605682	count	1
MED6	0.1020193	0.1136783	0.8974	0.37	0.136622004	count	1
NOL11	0.1070769	0.1518802	0.705	0.481	0.136701349	count	1
CBARP	0.2802331	0.7221138	0.3881	0.698	0.136849518	count	1
ERICD	0.2802331	0.8754405	0.3201	0.749	0.136849518	count	1
ARMC5	0.1921651	0.313053	0.6138	0.539	0.136887988	count	1
KATNAL2	0.1609182	0.4768824	0.3374	0.736	0.136952695	count	1
TBC1D8-AS1	0.3137563	0.4616615	0.6796	0.497	0.137042817	count	1
RAD23A	0.0964338	0.0528624	1.8242	0.0682	0.137088553	count	1
ZNF799	0.2228426	0.4654367	0.4788	0.632	0.137102924	count	1
GPS2	0.1042422	0.142225	0.7329	0.464	0.137273523	count	1
PYCR1	0.1195609	0.2727441	0.4384	0.661	0.137342347	count	1
DACH1	0.1856733	0.5391453	0.3444	0.731	0.137352159	count	1
ARID2	0.1121171	0.1846634	0.6071	0.544	0.137543552	count	1
SF3B3	0.1147664	0.1984483	0.5783	0.563	0.137558953	count	1
SPTB	0.362808	0.6479854	0.5599	0.576	0.137563341	count	1
PKNOX1	0.1133564	0.1729075	0.6556	0.512	0.13756423	count	1
REX1BD	0.0970162	0.057142	1.6978	0.0896	0.137570182	count	1
LMAN2	0.0974648	0.0566065	1.7218	0.0852	0.137582492	count	1
RFTN2	0.1087355	0.1469694	0.7399	0.459	0.13761859	count	1
H1FX-AS1	0.1553821	0.3665545	0.4239	0.672	0.137700544	count	1
CABYR	0.2018019	0.5977755	0.3376	0.736	0.137728405	count	1

ZFP37	0.1387804	0.254062	0.5462	0.585	0.137839267	count	1
PPP2R5A	0.1053282	0.1170847	0.8996	0.368	0.137847636	count	1
FAM215B	0.17475	0.2261843	0.7726	0.44	0.137854118	count	1
TMEM106C	0.0974835	0.0651681	1.4959	0.135	0.137862541	count	1
ZSWIM1	0.3641731	0.4954879	0.735	0.462	0.138037725	count	1
NKX3-1	0.3641731	0.6307761	0.5773	0.564	0.138037725	count	1
PIGX	0.1063469	0.1512522	0.7031	0.482	0.138081016	count	1
MAT2A	0.1047429	0.1306247	0.8019	0.423	0.138275698	count	1
PLBD1-AS1	0.4400058	0.8175171	0.5382	0.59	0.138296038	count	1
COG6	0.1148539	0.1767734	0.6497	0.516	0.138372588	count	1
P4HA2	0.0997543	0.0850459	1.1729	0.241	0.138469417	count	1
RMDN2	0.1118248	0.1981613	0.5643	0.573	0.13849832	count	1
KRI1	0.1300042	0.2624282	0.4954	0.62	0.138540175	count	1
MLEC	0.098051	0.0607077	1.6151	0.106	0.138576957	count	1
CSTF3	0.1156376	0.1937549	0.5968	0.551	0.138597165	count	1
TCERG1	0.1034088	0.120346	0.8593	0.39	0.138766609	count	1
PTOV1	0.1004355	0.0868951	1.1558	0.248	0.138770864	count	1
ZSWIM9	0.1223733	0.343471	0.3563	0.722	0.138781059	count	1
NAT14	0.1107864	0.1834615	0.6039	0.546	0.138816551	count	1
B3GAT2	0.2845889	0.5088163	0.5593	0.576	0.138854015	count	1
SLC25A53	0.1632246	0.3127438	0.5219	0.602	0.138876485	count	1
TAB1	0.1490655	0.2514292	0.5929	0.553	0.138884242	count	1
ASPHD2	0.3668071	0.61447	0.5969	0.551	0.138952047	count	1
AC008543.1	0.2413462	1.065427	0.2265	0.821	0.139132502	count	1
ALOX12-AS1	0.1601728	0.2721961	0.5884	0.556	0.139182719	count	1
ZBTB24	0.1072311	0.1594929	0.6723	0.501	0.13922545	count	1
OGA	0.1019989	0.1113995	0.9156	0.36	0.139256235	count	1
HTATSF1	0.1023088	0.1043361	0.9806	0.327	0.139301423	count	1
HBZ	0.8986991	0.7235127	1.2421	0.214	0.139307893	count	1
PTGR1	0.100019	0.0826499	1.2102	0.226	0.13938107	count	1
WDR63	0.8994684	1.239206	0.7258	0.468	0.139395542	count	1
AP000442.2	0.8994684	1.276525	0.7046	0.481	0.139395542	count	1
LDHA	0.097052	0.032311	3.0037	0.00269	0.139481605	count	1
TSNARE1	0.3201472	0.4491897	0.7127	0.476	0.13964207	count	1
FURIN	0.1238221	0.2144344	0.5774	0.564	0.139775032	count	1
AC016910.1	0.5819643	0.9530323	0.6106	0.541	0.139844878	count	1
NAT9	0.1107154	0.1493904	0.7411	0.459	0.139927656	count	1
PRPF39	0.1170989	0.2094919	0.559	0.576	0.139962349	count	1
ZNF550	0.1966671	0.3941535	0.499	0.618	0.139999097	count	1
SWT1	0.1611915	0.2751288	0.5859	0.558	0.140051022	count	1
LMTK3	0.4466099	0.7190024	0.6212	0.535	0.14014293	count	1
LINC00654	0.4466099	0.8370809	0.5335	0.594	0.14014293	count	1
KMT5B	0.1059939	0.1288018	0.8229	0.411	0.140172702	count	1
NARF	0.1115482	0.1358775	0.8209	0.412	0.14018591	count	1
TMSB4Y	0.2280903	0.4134975	0.5516	0.581	0.140203801	count	1

NT5DC2	0.1016323	0.0984134	1.0327	0.302	0.140213564	count	1
AL139161.1	0.5839553	1.0158787	0.5748	0.565	0.140248334	count	1
BEAN1	0.5839553	1.0158787	0.5748	0.565	0.140248334	count	1
KCNE2	0.5839553	1.351645	0.432	0.666	0.140248334	count	1
WAC	0.1010045	0.087838	1.1499	0.25	0.140282133	count	1
GOLT1B	0.1045463	0.1213773	0.8613	0.389	0.140289777	count	1
MRPL30	0.1110125	0.1740112	0.638	0.524	0.140301625	count	1
SEMA6D	0.1689344	0.3451478	0.4895	0.625	0.140432075	count	1
HTR1F	0.585246	0.6240021	0.9379	0.348	0.140509609	count	1
LINC01119	0.585246	0.6885886	0.8499	0.395	0.140509609	count	1
ZNF772	0.1691005	0.6765646	0.2499	0.803	0.140567197	count	1
KLHDC1	0.1210557	0.2083002	0.5812	0.561	0.140639326	count	1
SCRIB	0.1417153	0.3162875	0.4481	0.654	0.140716138	count	1
STK32A	0.4493205	0.4884727	0.9198	0.358	0.140898991	count	1
GORAB	0.1124584	0.1522063	0.7389	0.46	0.140902477	count	1
ARHGEF1	0.1148895	0.2280234	0.5038	0.614	0.140927491	count	1
C12orf66	0.1906898	0.3439155	0.5545	0.579	0.140959741	count	1
DNAJC12	0.1419972	0.2260925	0.628	0.53	0.140992373	count	1
SNX32	0.5877647	0.7780298	0.7555	0.45	0.141018868	count	1
C19orf33	0.2449793	0.6708447	0.3652	0.715	0.141133324	count	1
MUS81	0.1120204	0.1686809	0.6641	0.507	0.141181232	count	1
PPP1R3F	0.1423451	0.3398344	0.4189	0.675	0.14133327	count	1
ZNF358	0.1020789	0.0869391	1.1741	0.24	0.14136555	count	1
FTX	0.10308	0.1181259	0.8726	0.383	0.141531116	count	1
TESC	0.1598421	0.3029619	0.5276	0.598	0.141580493	count	1
TNFRSF10D	0.1915541	0.362324	0.5287	0.597	0.141580716	count	1
USP46-AS1	0.159912	0.2987729	0.5352	0.593	0.141641267	count	1
AC021321.1	16.5947037	1621.382029	0.0102	0.992	0.141693261	count	1
CYP4A22-AS1	16.5947075	1621.381723	0.0102	0.992	0.141693261	count	1
AC016705.2	16.5947109	1621.387919	0.0102	0.992	0.141693261	count	1
AP000915.1	16.8485482	1597.874705	0.0105	0.992	0.141693263	count	1
LINC01987	16.9794687	1619.454502	0.0105	0.992	0.141693264	count	1
AC117503.1	16.9992196	1122.653879	0.0151	0.988	0.141693264	count	1
SLC26A7	17.0649909	1130.975395	0.0151	0.988	0.141693265	count	1
AC135507.1	17.1340487	2278.67923	0.0075	0.994	0.141693265	count	1
AC116651.1	17.1340487	2278.67923	0.0075	0.994	0.141693265	count	1
LINC02242	17.1340487	2278.679235	0.0075	0.994	0.141693265	count	1
AIRN	17.1340487	2278.679235	0.0075	0.994	0.141693265	count	1
AC007285.1	17.1340487	2278.67923	0.0075	0.994	0.141693265	count	1
BMS1P14	17.1340487	2278.679245	0.0075	0.994	0.141693265	count	1
AC106782.6	17.1340487	2278.679245	0.0075	0.994	0.141693265	count	1
PNMA3	17.1340488	2278.67924	0.0075	0.994	0.141693265	count	1
AC092123.1	17.1340488	2278.67924	0.0075	0.994	0.141693265	count	1
PDZRN3-AS1	17.1340499	2278.684147	0.0075	0.994	0.141693265	count	1
TAS2R31	17.1340499	2278.684137	0.0075	0.994	0.141693265	count	1

RDH16	17.1340499	2278.684137	0.0075	0.994	0.141693265	count	1
CCDC169	17.1340539	2278.676746	0.0075	0.994	0.141693265	count	1
FSTL4	17.1340597	2278.676629	0.0075	0.994	0.141693265	count	1
UNC5CL	17.1340597	2278.676629	0.0075	0.994	0.141693265	count	1
HCRTR1	17.1340719	2278.678823	0.0075	0.994	0.141693265	count	1
AC012360.2	17.1340719	2278.678823	0.0075	0.994	0.141693265	count	1
AC115618.1	17.134072	2278.678834	0.0075	0.994	0.141693265	count	1
AL139424.2	17.1701602	1893.203474	0.0091	0.993	0.141693265	count	1
ZNF726	17.1704502	1783.653997	0.0096	0.992	0.141693265	count	1
LINC01389	17.2318599	2216.836339	0.0078	0.994	0.141693266	count	1
TMEM225B	17.4520931	1600.30099	0.0109	0.991	0.141693266	count	1
C2CD4D	17.6207636	1592.147468	0.0111	0.9912	0.141693267	count	1
CCSER1	17.6563212	2267.768629	0.0078	0.994	0.141693267	count	1
DACH2	17.6563213	2267.764546	0.0078	0.994	0.141693267	count	1
AL121894.2	17.6563393	2267.772476	0.0078	0.994	0.141693267	count	1
TTC34	17.6563451	2267.770382	0.0078	0.994	0.141693267	count	1
AL161935.3	17.6563451	2267.770382	0.0078	0.994	0.141693267	count	1
LINC01270	17.6563451	2267.770382	0.0078	0.994	0.141693267	count	1
AC233296.1	17.6978425	3947.637847	0.0045	0.996	0.141693267	count	1
AC097724.1	17.6978428	3947.637951	0.0045	0.996	0.141693267	count	1
FABP1	17.6978428	3947.637951	0.0045	0.996	0.141693267	count	1
POTEF	17.6978428	3947.637951	0.0045	0.996	0.141693267	count	1
LINC01980	17.6978428	3947.637951	0.0045	0.996	0.141693267	count	1
LINC00971	17.6978428	3947.637951	0.0045	0.996	0.141693267	count	1
AC095050.1	17.6978427	3947.63808	0.0045	0.996	0.141693267	count	1
ADAMTS19	17.6978428	3947.637951	0.0045	0.996	0.141693267	count	1
STK31	17.6978428	3947.637951	0.0045	0.996	0.141693267	count	1
AC073133.2	17.6978427	3947.637899	0.0045	0.996	0.141693267	count	1
AC025154.2	17.6978428	3947.637951	0.0045	0.996	0.141693267	count	1
AL390816.2	17.6978428	3947.637951	0.0045	0.996	0.141693267	count	1
VAC14-AS1	17.6978427	3947.638028	0.0045	0.996	0.141693267	count	1
TTLL9	17.6978427	3947.638028	0.0045	0.996	0.141693267	count	1
AC008403.3	17.6978428	3947.637951	0.0045	0.996	0.141693267	count	1
CU633980.1	17.6978428	3947.637951	0.0045	0.996	0.141693267	count	1
AC133644.2	17.6978431	3947.638183	0.0045	0.996	0.141693267	count	1
HOXD3	17.697843	3947.638132	0.0045	0.996	0.141693267	count	1
AC006058.1	17.6978429	3947.638132	0.0045	0.996	0.141693267	count	1
RAB43	17.697843	3947.638183	0.0045	0.996	0.141693267	count	1
LINC02068	17.697843	3947.638002	0.0045	0.996	0.141693267	count	1
AC025175.1	17.6978429	3947.638132	0.0045	0.996	0.141693267	count	1
SOWAHA	17.697843	3947.638132	0.0045	0.996	0.141693267	count	1
KIAA0319	17.6978431	3947.638183	0.0045	0.996	0.141693267	count	1
AL356234.3	17.697843	3947.638132	0.0045	0.996	0.141693267	count	1
AP003355.2	17.6978431	3947.638183	0.0045	0.996	0.141693267	count	1
AL162724.2	17.6978431	3947.638183	0.0045	0.996	0.141693267	count	1

AP000438.1	17.6978432	3947.638183	0.0045	0.996	0.141693267	count	1
TECTA	17.6978432	3947.638183	0.0045	0.996	0.141693267	count	1
AP000755.2	17.6978429	3947.638132	0.0045	0.996	0.141693267	count	1
CEP55	17.697843	3947.638002	0.0045	0.996	0.141693267	count	1
AC006064.1	17.697843	3947.638183	0.0045	0.996	0.141693267	count	1
SKA3	17.697843	3947.638002	0.0045	0.996	0.141693267	count	1
AL139384.2	17.697843	3947.638132	0.0045	0.996	0.141693267	count	1
BCL2L2-PABPN1	17.697843	3947.638132	0.0045	0.996	0.141693267	count	1
COX8C	17.6978432	3947.638183	0.0045	0.996	0.141693267	count	1
PKMYT1	17.697843	3947.638002	0.0045	0.996	0.141693267	count	1
FENDRR	17.697843	3947.638132	0.0045	0.996	0.141693267	count	1
PIMREG	17.697843	3947.638002	0.0045	0.996	0.141693267	count	1
AC003070.2	17.697843	3947.638132	0.0045	0.996	0.141693267	count	1
AC005821.1	17.697843	3947.638132	0.0045	0.996	0.141693267	count	1
ZACN	17.6978432	3947.638183	0.0045	0.996	0.141693267	count	1
FOXJ1	17.697843	3947.638132	0.0045	0.996	0.141693267	count	1
DLGAP1-AS5	17.6978431	3947.638002	0.0045	0.996	0.141693267	count	1
AL157838.1	17.697843	3947.638183	0.0045	0.996	0.141693267	count	1
PRSS57	17.697843	3947.638132	0.0045	0.996	0.141693267	count	1
ZFR2	17.6978429	3947.637951	0.0045	0.996	0.141693267	count	1
ASF1B	17.697843	3947.638002	0.0045	0.996	0.141693267	count	1
AP001626.1	17.697843	3947.638132	0.0045	0.996	0.141693267	count	1
AC079209.1	17.7655369	3156.015954	0.0056	0.996	0.141693268	count	1
AC018904.1	17.765537	3156.015903	0.0056	0.996	0.141693268	count	1
AL353653.1	17.765537	3156.016021	0.0056	0.996	0.141693268	count	1
C4B	17.7655372	3156.016055	0.0056	0.996	0.141693268	count	1
NPC1L1	17.7655372	3156.016055	0.0056	0.996	0.141693268	count	1
AP000879.1	17.7655372	3156.016004	0.0056	0.996	0.141693268	count	1
AC104035.1	17.7655372	3156.016004	0.0056	0.996	0.141693268	count	1
AC092375.2	17.7655371	3156.015987	0.0056	0.996	0.141693268	count	1
AC011921.1	17.7655371	3156.016038	0.0056	0.996	0.141693268	count	1
AC107029.2	17.7655383	3156.019734	0.0056	0.996	0.141693268	count	1
AC005323.2	17.7655474	3156.011616	0.0056	0.996	0.141693268	count	1
AL358472.3	17.7655503	3156.022839	0.0056	0.996	0.141693268	count	1
AC021683.2	17.7655506	3156.02289	0.0056	0.996	0.141693268	count	1
AC093484.2	17.7655508	3156.022924	0.0056	0.996	0.141693268	count	1
P2RX2	17.8197598	1234.749396	0.0144	0.9885	0.141693268	count	1
GP6	17.8706869	2851.054535	0.0063	0.995	0.141693268	count	1
SPSB4	17.9916271	2256.049821	0.008	0.994	0.141693268	count	1
AC002429.2	17.9916339	2256.046011	0.008	0.994	0.141693268	count	1
GPR22	17.9916461	2256.044077	0.008	0.994	0.141693268	count	1
AC007495.1	17.9916504	2256.05527	0.008	0.994	0.141693268	count	1
AL160408.2	18.240166	2246.916566	0.0081	0.9935	0.141693269	count	1
GPD1	16.5946934	1621.382359	0.0102	0.992	0.141693275	count	1
DENND6A-AS1	16.5946946	1621.378267	0.0102	0.992	0.141693275	count	1

RAD21-AS1	16.5946946	1621.37827	0.0102	0.992	0.141693275	count	1
AD000090.1	16.5947037	1621.382029	0.0102	0.992	0.141693275	count	1
APOL5	16.5947037	1621.382028	0.0102	0.992	0.141693275	count	1
GDNF	16.5947038	1621.382032	0.0102	0.992	0.141693275	count	1
AL162171.1	16.5947038	1621.382032	0.0102	0.992	0.141693275	count	1
ST8SIA5	16.5947038	1621.382029	0.0102	0.992	0.141693275	count	1
AC003975.1	16.5947047	1621.382001	0.0102	0.992	0.141693275	count	1
UCP3	16.5947047	1621.381998	0.0102	0.992	0.141693275	count	1
AC023794.2	16.5947076	1621.381723	0.0102	0.992	0.141693275	count	1
AC055876.5	16.5947081	1621.387978	0.0102	0.992	0.141693275	count	1
GP1BA	16.5947102	1621.383849	0.0102	0.992	0.141693275	count	1
AL109811.1	16.5947109	1621.387915	0.0102	0.992	0.141693275	count	1
NEK10	16.5947109	1621.387919	0.0102	0.992	0.141693275	count	1
EIF4EBP3	16.5947109	1621.387915	0.0102	0.992	0.141693275	count	1
ADCYAP1R1	16.594711	1621.387919	0.0102	0.992	0.141693275	count	1
AC091057.2	16.594711	1621.387921	0.0102	0.992	0.141693275	count	1
AL365181.4	16.7645929	1130.31624	0.0148	0.9882	0.141693276	count	1
AC007292.2	16.7650892	1277.061672	0.0131	0.99	0.141693276	count	1
VSIG2	16.7655758	1449.864148	0.0116	0.991	0.141693276	count	1
AC090617.3	16.848528	1597.870755	0.0105	0.992	0.141693277	count	1
Z95114.1	16.8485313	1597.873321	0.0105	0.992	0.141693277	count	1
AL513122.2	16.8485367	1597.87522	0.0105	0.992	0.141693277	count	1
PCDHA12	16.848546	1597.872143	0.0105	0.992	0.141693277	count	1
FGF17	16.8485464	1597.874729	0.0105	0.992	0.141693277	count	1
ADAMTS17	16.8485467	1597.874483	0.0105	0.992	0.141693277	count	1
TRPC3	16.8485483	1597.874713	0.0105	0.992	0.141693277	count	1
BX323046.1	16.8485486	1597.874466	0.0105	0.992	0.141693277	count	1
EFCAB1	16.8485499	1597.879883	0.0105	0.992	0.141693277	count	1
AC090229.1	16.8490673	1768.949303	0.0095	0.992	0.141693277	count	1
AC126768.2	16.8698743	1027.322916	0.0164	0.9869	0.141693277	count	1
AC012065.4	16.8903305	1556.099661	0.0109	0.991	0.141693277	count	1
FRG1-DT	16.9794654	1619.450361	0.0105	0.992	0.141693278	count	1
LIF	16.9794786	1619.451391	0.0105	0.992	0.141693278	count	1
GAS5-AS1	16.9794794	1619.452921	0.0105	0.992	0.141693278	count	1
AC053527.1	16.9794852	1619.451352	0.0105	0.992	0.141693278	count	1
RNF183	16.9794852	1619.45135	0.0105	0.992	0.141693278	count	1
AKAP3	16.979487	1619.452867	0.0105	0.992	0.141693278	count	1
LINC02076	16.9794871	1619.452867	0.0105	0.992	0.141693278	count	1
AC007064.2	16.9796278	1902.822003	0.0089	0.993	0.141693278	count	1
AL606760.2	16.9796408	1902.823735	0.0089	0.993	0.141693278	count	1
C19orf38	17.0420478	1576.432186	0.0108	0.9914	0.141693278	count	1
FLRT3	17.0420516	1576.439022	0.0108	0.9914	0.141693278	count	1
ZNF843	17.0420569	1576.4392	0.0108	0.9914	0.141693278	count	1
CYP21A2	17.0420618	1576.438158	0.0108	0.9914	0.141693278	count	1
PLPP2	17.0426398	1691.082884	0.0101	0.992	0.141693278	count	1

AL355073.2	17.0434739	1899.706854	0.009	0.993	0.141693278	count	1
AL445183.1	17.1340476	2278.674439	0.0075	0.994	0.141693279	count	1
LINC01956	17.1340476	2278.674439	0.0075	0.994	0.141693279	count	1
RHOXF1	17.1340476	2278.674455	0.0075	0.994	0.141693279	count	1
AC022400.6	17.1340476	2278.674455	0.0075	0.994	0.141693279	count	1
AC079328.2	17.1340476	2278.674445	0.0075	0.994	0.141693279	count	1
NKAIN1	17.1340488	2278.679235	0.0075	0.994	0.141693279	count	1
AL451062.1	17.1340488	2278.679245	0.0075	0.994	0.141693279	count	1
FOXE3	17.1340488	2278.67925	0.0075	0.994	0.141693279	count	1
BGLAP	17.1340487	2278.679225	0.0075	0.994	0.141693279	count	1
CD1C	17.1340488	2278.67924	0.0075	0.994	0.141693279	count	1
AL356441.1	17.1340488	2278.67924	0.0075	0.994	0.141693279	count	1
AC116614.1	17.1340488	2278.679245	0.0075	0.994	0.141693279	count	1
AC010967.1	17.1340488	2278.679245	0.0075	0.994	0.141693279	count	1
AC009237.15	17.1340488	2278.67924	0.0075	0.994	0.141693279	count	1
IL1A	17.1340488	2278.679245	0.0075	0.994	0.141693279	count	1
UBE2E2-AS1	17.1340488	2278.67925	0.0075	0.994	0.141693279	count	1
SNRK-AS1	17.1340488	2278.679235	0.0075	0.994	0.141693279	count	1
BFSP2	17.1340488	2278.67924	0.0075	0.994	0.141693279	count	1
AC105254.1	17.1340487	2278.679235	0.0075	0.994	0.141693279	count	1
AC026741.1	17.1340487	2278.67923	0.0075	0.994	0.141693279	count	1
HCG9	17.1340488	2278.679235	0.0075	0.994	0.141693279	count	1
RFPL4B	17.1340488	2278.67924	0.0075	0.994	0.141693279	count	1
HOXA6	17.1340488	2278.679235	0.0075	0.994	0.141693279	count	1
AL672277.1	17.1340488	2278.679225	0.0075	0.994	0.141693279	count	1
TREX2	17.1340488	2278.67924	0.0075	0.994	0.141693279	count	1
SMC2-AS1	17.1340488	2278.679245	0.0075	0.994	0.141693279	count	1
ABCC8	17.1340488	2278.679245	0.0075	0.994	0.141693279	count	1
AL133215.2	17.1340488	2278.679235	0.0075	0.994	0.141693279	count	1
AC005840.2	17.1340488	2278.67924	0.0075	0.994	0.141693279	count	1
GALNT4	17.1340488	2278.679235	0.0075	0.994	0.141693279	count	1
LRRC43	17.1340487	2278.679235	0.0075	0.994	0.141693279	count	1
AC068790.8	17.1340487	2278.679235	0.0075	0.994	0.141693279	count	1
OXGR1	17.1340488	2278.67925	0.0075	0.994	0.141693279	count	1
TPM1-AS	17.1340487	2278.679225	0.0075	0.994	0.141693279	count	1
LINC01579	17.1340488	2278.67923	0.0075	0.994	0.141693279	count	1
BRICD5	17.1340487	2278.67924	0.0075	0.994	0.141693279	count	1
AC009133.4	17.1340488	2278.679235	0.0075	0.994	0.141693279	count	1
SMPD3	17.1340488	2278.67924	0.0075	0.994	0.141693279	count	1
LINC01229	17.1340488	2278.679235	0.0075	0.994	0.141693279	count	1
FAM209B	17.1340488	2278.679245	0.0075	0.994	0.141693279	count	1
CRB3	17.1340488	2278.67924	0.0075	0.994	0.141693279	count	1
HPN	17.1340488	2278.67924	0.0075	0.994	0.141693279	count	1
AL138831.2	17.1340489	2278.679235	0.0075	0.994	0.141693279	count	1
AC006042.4	17.1340489	2278.67925	0.0075	0.994	0.141693279	count	1

LINC01772	17.13405	2278.684137	0.0075	0.994	0.141693279	count	1
KIF21B	17.13405	2278.684137	0.0075	0.994	0.141693279	count	1
GNG4	17.13405	2278.684142	0.0075	0.994	0.141693279	count	1
AC104667.2	17.13405	2278.684147	0.0075	0.994	0.141693279	count	1
AC114730.2	17.13405	2278.684142	0.0075	0.994	0.141693279	count	1
RBMS3-AS2	17.1340499	2278.684142	0.0075	0.994	0.141693279	count	1
LINC02024	17.13405	2278.684142	0.0075	0.994	0.141693279	count	1
ZFP92	17.13405	2278.684147	0.0075	0.994	0.141693279	count	1
AP000640.1	17.13405	2278.684152	0.0075	0.994	0.141693279	count	1
AP001107.7	17.13405	2278.684137	0.0075	0.994	0.141693279	count	1
CFAP46	17.13405	2278.684147	0.0075	0.994	0.141693279	count	1
SOAT2	17.13405	2278.684147	0.0075	0.994	0.141693279	count	1
HOXC6	17.13405	2278.684147	0.0075	0.994	0.141693279	count	1
CLDN10	17.13405	2278.684147	0.0075	0.994	0.141693279	count	1
AK7	17.13405	2278.684142	0.0075	0.994	0.141693279	count	1
AC084855.1	17.13405	2278.684147	0.0075	0.994	0.141693279	count	1
AC022167.1	17.13405	2278.684147	0.0075	0.994	0.141693279	count	1
ELMO3	17.13405	2278.684152	0.0075	0.994	0.141693279	count	1
DNAH9	17.13405	2278.684142	0.0075	0.994	0.141693279	count	1
AC110285.1	17.13405	2278.684142	0.0075	0.994	0.141693279	count	1
LINC01441	17.13405	2278.684147	0.0075	0.994	0.141693279	count	1
AC136469.1	17.1340499	2278.684142	0.0075	0.994	0.141693279	count	1
AL357873.1	17.1340501	2278.684142	0.0075	0.994	0.141693279	count	1
TFAP2E	17.1340501	2278.684147	0.0075	0.994	0.141693279	count	1
AC098820.3	17.1340501	2278.684142	0.0075	0.994	0.141693279	count	1
DLEC1	17.1340501	2278.684152	0.0075	0.994	0.141693279	count	1
AL034374.1	17.1340501	2278.684152	0.0075	0.994	0.141693279	count	1
NPAS4	17.1340501	2278.684142	0.0075	0.994	0.141693279	count	1
C14orf178	17.1340501	2278.684147	0.0075	0.994	0.141693279	count	1
AC007613.1	17.1340501	2278.684157	0.0075	0.994	0.141693279	count	1
ERN2	17.1340501	2278.684157	0.0075	0.994	0.141693279	count	1
AL031673.1	17.1340501	2278.684147	0.0075	0.994	0.141693279	count	1
LINC01719	17.1340539	2278.676751	0.0075	0.994	0.141693279	count	1
AC022784.8	17.134054	2278.676746	0.0075	0.994	0.141693279	count	1
AC100771.2	17.134054	2278.676746	0.0075	0.994	0.141693279	count	1
GPR158	17.134054	2278.676751	0.0075	0.994	0.141693279	count	1
AC138331.1	17.134054	2278.676751	0.0075	0.994	0.141693279	count	1
ENOX1	17.134054	2278.676746	0.0075	0.994	0.141693279	count	1
LINC02125	17.134054	2278.676746	0.0075	0.994	0.141693279	count	1
AC245407.2	17.1340598	2278.676634	0.0075	0.994	0.141693279	count	1
HIST1H2BL	17.1340598	2278.676629	0.0075	0.994	0.141693279	count	1
PRODH	17.1340597	2278.676634	0.0075	0.994	0.141693279	count	1
Z99289.2	17.134072	2278.678828	0.0075	0.994	0.141693279	count	1
AC073073.2	17.134072	2278.678828	0.0075	0.994	0.141693279	count	1
AC091979.1	17.1340721	2278.678834	0.0075	0.994	0.141693279	count	1

GREB1	17.1664024	1160.991147	0.0148	0.9882	0.141693279	count	1
HLA-DOB	17.1701521	1611.283955	0.0107	0.991	0.141693279	count	1
RGPD2	17.2021469	1804.602414	0.0095	0.9924	0.141693279	count	1
ERVK9-11	17.2031134	2020.552174	0.0085	0.9932	0.141693279	count	1
AC233992.3	17.2309582	1898.120821	0.0091	0.993	0.141693279	count	1
NR0B1	17.2309597	1898.124738	0.0091	0.993	0.141693279	count	1
GCOM1	17.2309619	1898.12472	0.0091	0.993	0.141693279	count	1
AL445483.1	17.230962	1898.124731	0.0091	0.993	0.141693279	count	1
AL023583.1	17.230968	1898.125307	0.0091	0.993	0.141693279	count	1
AC010319.3	17.2318579	2216.836365	0.0078	0.994	0.141693279	count	1
ADGRV1	17.2318602	2216.83941	0.0078	0.994	0.141693279	count	1
NR6A1	17.2406462	1521.317746	0.0113	0.991	0.141693279	count	1
ZNF724	17.2406471	1521.318636	0.0113	0.991	0.141693279	count	1
LINC02456	17.2413154	1796.946731	0.0096	0.992	0.141693279	count	1
AC007611.1	17.2413193	1796.94882	0.0096	0.992	0.141693279	count	1
AC078881.1	17.246022	1609.731345	0.0107	0.991	0.141693279	count	1
TNNI3K	17.2460385	1609.738924	0.0107	0.991	0.141693279	count	1
SYCE2	17.2460417	1609.73745	0.0107	0.991	0.141693279	count	1
BCAS1	17.2460464	1609.737439	0.0107	0.991	0.141693279	count	1
MCPH1-AS1	17.2463151	1802.627261	0.0096	0.992	0.141693279	count	1
AC087241.3	17.3211474	1977.427597	0.0088	0.993	0.141693279	count	1
SEMA7A	17.3380316	1920.009401	0.009	0.9928	0.14169328	count	1
PCDHGA1	17.4024558	1803.126086	0.0097	0.992	0.14169328	count	1
MAPK8IP2	17.4026341	2137.333892	0.0081	0.994	0.14169328	count	1
EYA1	17.4384901	1486.345135	0.0117	0.9906	0.14169328	count	1
AL353593.1	17.4384999	1486.345959	0.0117	0.9906	0.14169328	count	1
PI3	17.4389572	1604.136135	0.0109	0.9913	0.14169328	count	1
GRIA4	17.4399088	2007.266488	0.0087	0.993	0.14169328	count	1
SMIM33	17.4406995	2342.399751	0.0074	0.994	0.14169328	count	1
LINC01412	17.4482688	1672.187848	0.0104	0.9917	0.14169328	count	1
BHMT	17.4488856	1834.206928	0.0095	0.9924	0.14169328	count	1
LRRC37A	17.4524141	1746.24587	0.01	0.992	0.14169328	count	1
PCDH9-AS1	17.4524253	1746.247128	0.01	0.992	0.14169328	count	1
GALR1	17.519654	1070.097779	0.0164	0.9869	0.14169328	count	1
AL355922.2	17.5288133	2093.445807	0.0084	0.993	0.14169328	count	1
AC027306.1	17.5425953	1886.042078	0.0093	0.9926	0.14169328	count	1
AC107959.4	17.542602	1886.042028	0.0093	0.9926	0.14169328	count	1
PCDHA13	17.5435061	2102.873351	0.0083	0.9933	0.14169328	count	1
FSD1	17.5447535	2480.561185	0.0071	0.9944	0.14169328	count	1
C9orf135	17.5447658	2480.564969	0.0071	0.9944	0.14169328	count	1
AC073896.3	17.6025631	1355.012965	0.013	0.9896	0.141693281	count	1
GAL	17.6034624	1562.026791	0.0113	0.991	0.141693281	count	1
SULT1A3	17.6036598	1655.764986	0.0106	0.9915	0.141693281	count	1
LIPE	17.6207516	1592.140977	0.0111	0.9912	0.141693281	count	1
CACNA1D	17.6210876	1709.395346	0.0103	0.9918	0.141693281	count	1

HUS1B	17.6215218	1922.557101	0.0092	0.993	0.141693281	count	1
PCSK9	17.6563211	2267.768619	0.0078	0.994	0.141693281	count	1
AL360270.3	17.6563213	2267.764556	0.0078	0.994	0.141693281	count	1
CADM2	17.6563213	2267.768629	0.0078	0.994	0.141693281	count	1
COL6A6	17.6563211	2267.768614	0.0078	0.994	0.141693281	count	1
AL033397.2	17.6563213	2267.768639	0.0078	0.994	0.141693281	count	1
AC073316.1	17.6563213	2267.768634	0.0078	0.994	0.141693281	count	1
DGAT2	17.6563213	2267.764546	0.0078	0.994	0.141693281	count	1
FAM90A1	17.6563213	2267.764556	0.0078	0.994	0.141693281	count	1
RSPO1	17.6563214	2267.764551	0.0078	0.994	0.141693281	count	1
AC097468.3	17.6563212	2267.768629	0.0078	0.994	0.141693281	count	1
CCDC39	17.6563212	2267.768629	0.0078	0.994	0.141693281	count	1
ZNF366	17.6563214	2267.764556	0.0078	0.994	0.141693281	count	1
TFAP2A-AS1	17.6563212	2267.768629	0.0078	0.994	0.141693281	count	1
TAS2R4	17.6563214	2267.764551	0.0078	0.994	0.141693281	count	1
AC011773.4	17.6563214	2267.764551	0.0078	0.994	0.141693281	count	1
HS6ST3	17.6563212	2267.768629	0.0078	0.994	0.141693281	count	1
ATCAY	17.6563212	2267.768614	0.0078	0.994	0.141693281	count	1
NMRK2	17.6563212	2267.768624	0.0078	0.994	0.141693281	count	1
LPAR2	17.6563212	2267.768624	0.0078	0.994	0.141693281	count	1
KLK1	17.6563214	2267.764561	0.0078	0.994	0.141693281	count	1
LHX8	17.6563215	2267.764556	0.0078	0.994	0.141693281	count	1
AC113349.1	17.6563215	2267.764556	0.0078	0.994	0.141693281	count	1
KLF14	17.6563215	2267.764556	0.0078	0.994	0.141693281	count	1
AC010761.2	17.6563393	2267.762361	0.0078	0.994	0.141693281	count	1
CD244	17.6563451	2267.770392	0.0078	0.994	0.141693281	count	1
GPR37L1	17.6563451	2267.770377	0.0078	0.994	0.141693281	count	1
AFP	17.6563451	2267.770377	0.0078	0.994	0.141693281	count	1
PCDHA4	17.6563451	2267.770392	0.0078	0.994	0.141693281	count	1
TEX29	17.6563451	2267.770387	0.0078	0.994	0.141693281	count	1
AC099518.6	17.6563451	2267.770392	0.0078	0.994	0.141693281	count	1
BOLA2	17.6563452	2267.770387	0.0078	0.994	0.141693281	count	1
AL136531.1	17.6563451	2267.770382	0.0078	0.994	0.141693281	count	1
GATA5	17.6563451	2267.770397	0.0078	0.994	0.141693281	count	1
ZIM2-AS1	17.6563451	2267.770372	0.0078	0.994	0.141693281	count	1
AC064807.4	17.6563453	2267.770387	0.0078	0.994	0.141693281	count	1
SPAAR	17.6563453	2267.770387	0.0078	0.994	0.141693281	count	1
C15orf62	17.6563453	2267.770387	0.0078	0.994	0.141693281	count	1
AC135178.5	17.6567511	2769.239659	0.0064	0.995	0.141693281	count	1
AL137796.1	17.656757	2769.23964	0.0064	0.995	0.141693281	count	1
EPHA1	17.6567571	2769.239647	0.0064	0.995	0.141693281	count	1
PKP3	17.6567571	2769.239665	0.0064	0.995	0.141693281	count	1
PPIAL4G	17.656763	2769.249458	0.0064	0.995	0.141693281	count	1
AL121929.2	17.6567811	2769.241819	0.0064	0.995	0.141693281	count	1
CNKSR1	17.6978427	3947.637873	0.0045	0.996	0.141693281	count	1

AL009181.1	17.6978427	3947.638054	0.0045	0.996	0.141693281	count	1
CDCP2	17.6978425	3947.637899	0.0045	0.996	0.141693281	count	1
AL121985.1	17.6978427	3947.638054	0.0045	0.996	0.141693281	count	1
LINC01657	17.6978427	3947.637951	0.0045	0.996	0.141693281	count	1
SNTG2	17.6978427	3947.638106	0.0045	0.996	0.141693281	count	1
AC034195.1	17.6978427	3947.638054	0.0045	0.996	0.141693281	count	1
IQCJ	17.6978427	3947.638002	0.0045	0.996	0.141693281	count	1
LINC02054	17.6978426	3947.637795	0.0045	0.996	0.141693281	count	1
AC017007.5	17.6978427	3947.637899	0.0045	0.996	0.141693281	count	1
AC244517.5	17.6978427	3947.637951	0.0045	0.996	0.141693281	count	1
CDX1	17.6978427	3947.637951	0.0045	0.996	0.141693281	count	1
AL031768.1	17.6978427	3947.637976	0.0045	0.996	0.141693281	count	1
AL034376.1	17.6978426	3947.637976	0.0045	0.996	0.141693281	count	1
KHDC3L	17.6978426	3947.637976	0.0045	0.996	0.141693281	count	1
HOXA9	17.6978427	3947.638183	0.0045	0.996	0.141693281	count	1
MYL7	17.6978427	3947.637899	0.0045	0.996	0.141693281	count	1
AC092634.3	17.6978427	3947.638054	0.0045	0.996	0.141693281	count	1
MAP3K15	17.6978427	3947.638054	0.0045	0.996	0.141693281	count	1
CXorf58	17.6978425	3947.637899	0.0045	0.996	0.141693281	count	1
PNCK	17.6978427	3947.637976	0.0045	0.996	0.141693281	count	1
NAT2	17.6978427	3947.638054	0.0045	0.996	0.141693281	count	1
WDR97	17.6978427	3947.637925	0.0045	0.996	0.141693281	count	1
AC131934.1	17.6978426	3947.637821	0.0045	0.996	0.141693281	count	1
AC090791.1	17.6978426	3947.637821	0.0045	0.996	0.141693281	count	1
MIR194-2HG	17.6978426	3947.637795	0.0045	0.996	0.141693281	count	1
C11orf87	17.6978427	3947.637899	0.0045	0.996	0.141693281	count	1
SCN3B	17.6978426	3947.637769	0.0045	0.996	0.141693281	count	1
AC233309.1	17.6978427	3947.637951	0.0045	0.996	0.141693281	count	1
LINC02395	17.6978426	3947.637821	0.0045	0.996	0.141693281	count	1
ADAM21	17.6978426	3947.638028	0.0045	0.996	0.141693281	count	1
AC104041.1	17.6978427	3947.637899	0.0045	0.996	0.141693281	count	1
FSD2	17.6978427	3947.637925	0.0045	0.996	0.141693281	count	1
AC106820.5	17.6978427	3947.638183	0.0045	0.996	0.141693281	count	1
AC106886.3	17.6978427	3947.638054	0.0045	0.996	0.141693281	count	1
CES3	17.6978426	3947.637976	0.0045	0.996	0.141693281	count	1
TUBB3	17.6978425	3947.637899	0.0045	0.996	0.141693281	count	1
GRAP	17.6978427	3947.638183	0.0045	0.996	0.141693281	count	1
KRT9	17.6978427	3947.637795	0.0045	0.996	0.141693281	count	1
MGAT5B	17.6978427	3947.637899	0.0045	0.996	0.141693281	count	1
AC127496.1	17.6978427	3947.637951	0.0045	0.996	0.141693281	count	1
FUT6	17.6978427	3947.637951	0.0045	0.996	0.141693281	count	1
ANKLE1	17.6978426	3947.638028	0.0045	0.996	0.141693281	count	1
KLC3	17.6978427	3947.637821	0.0045	0.996	0.141693281	count	1
NOP53-AS1	17.6978427	3947.637951	0.0045	0.996	0.141693281	count	1
CRX	17.6978427	3947.638028	0.0045	0.996	0.141693281	count	1

PTOV1-AS2	17.6978427	3947.637925	0.0045	0.996	0.141693281	count	1
NAPSA	17.6978425	3947.637899	0.0045	0.996	0.141693281	count	1
CRYBB3	17.6978427	3947.637951	0.0045	0.996	0.141693281	count	1
AL022238.4	17.6978427	3947.637847	0.0045	0.996	0.141693281	count	1
AIRE	17.6978427	3947.637925	0.0045	0.996	0.141693281	count	1
AL645608.7	17.697843	3947.638209	0.0045	0.996	0.141693281	count	1
AL391244.2	17.6978429	3947.638002	0.0045	0.996	0.141693281	count	1
LINC01672	17.697843	3947.637951	0.0045	0.996	0.141693281	count	1
AL450998.3	17.697843	3947.637795	0.0045	0.996	0.141693281	count	1
TRIM63	17.6978428	3947.637951	0.0045	0.996	0.141693281	count	1
AL391650.1	17.6978429	3947.637976	0.0045	0.996	0.141693281	count	1
AC114488.2	17.697843	3947.637976	0.0045	0.996	0.141693281	count	1
AL139286.2	17.697843	3947.637795	0.0045	0.996	0.141693281	count	1
SLFNL1-AS1	17.697843	3947.63808	0.0045	0.996	0.141693281	count	1
SLFNL1	17.697843	3947.638132	0.0045	0.996	0.141693281	count	1
RIMKLA	17.697843	3947.637795	0.0045	0.996	0.141693281	count	1
FOXD3	17.697843	3947.638106	0.0045	0.996	0.141693281	count	1
AL583808.1	17.697843	3947.638054	0.0045	0.996	0.141693281	count	1
AC000032.1	17.697843	3947.637873	0.0045	0.996	0.141693281	count	1
AL157904.1	17.6978428	3947.63808	0.0045	0.996	0.141693281	count	1
FAM72B	17.697843	3947.638106	0.0045	0.996	0.141693281	count	1
SRGAP2-AS1	17.697843	3947.637795	0.0045	0.996	0.141693281	count	1
AC245100.7	17.697843	3947.638028	0.0045	0.996	0.141693281	count	1
AL589765.6	17.6978428	3947.637821	0.0045	0.996	0.141693281	count	1
DCST2	17.697843	3947.638054	0.0045	0.996	0.141693281	count	1
AL590560.1	17.697843	3947.637795	0.0045	0.996	0.141693281	count	1
ANKRD45	17.697843	3947.637795	0.0045	0.996	0.141693281	count	1
AL513329.1	17.697843	3947.637795	0.0045	0.996	0.141693281	count	1
AL133553.1	17.697843	3947.637795	0.0045	0.996	0.141693281	count	1
C4BPB	17.697843	3947.637795	0.0045	0.996	0.141693281	count	1
OBSCN-AS1	17.697843	3947.637795	0.0045	0.996	0.141693281	count	1
SLC35F3	17.6978428	3947.63808	0.0045	0.996	0.141693281	count	1
AC108488.2	17.697843	3947.637795	0.0045	0.996	0.141693281	count	1
AC010096.1	17.697843	3947.638209	0.0045	0.996	0.141693281	count	1
AC079145.1	17.6978429	3947.638054	0.0045	0.996	0.141693281	count	1
TDRD15	17.6978429	3947.638106	0.0045	0.996	0.141693281	count	1
AL121652.1	17.697843	3947.637795	0.0045	0.996	0.141693281	count	1
LINC02590	17.6978428	3947.638002	0.0045	0.996	0.141693281	count	1
AC009975.1	17.6978429	3947.638028	0.0045	0.996	0.141693281	count	1
EML6	17.6978428	3947.637976	0.0045	0.996	0.141693281	count	1
AC007743.1	17.697843	3947.637951	0.0045	0.996	0.141693281	count	1
LIMS4	17.6978428	3947.638106	0.0045	0.996	0.141693281	count	1
AC017074.1	17.697843	3947.638028	0.0045	0.996	0.141693281	count	1
AC114763.1	17.697843	3947.637795	0.0045	0.996	0.141693281	count	1
AC009480.1	17.6978428	3947.637951	0.0045	0.996	0.141693281	count	1

HOXD9	17.6978428	3947.637951	0.0045	0.996	0.141693281	count	1
AC074286.1	17.6978429	3947.638158	0.0045	0.996	0.141693281	count	1
PTPRN	17.697843	3947.638028	0.0045	0.996	0.141693281	count	1
AC010735.2	17.697843	3947.638002	0.0045	0.996	0.141693281	count	1
CROCC2	17.6978429	3947.638002	0.0045	0.996	0.141693281	count	1
SLC6A11	17.6978429	3947.638002	0.0045	0.996	0.141693281	count	1
TMEM40	17.697843	3947.637795	0.0045	0.996	0.141693281	count	1
SLC38A3	17.697843	3947.637795	0.0045	0.996	0.141693281	count	1
AC012557.2	17.697843	3947.638054	0.0045	0.996	0.141693281	count	1
AC107029.1	17.697843	3947.638054	0.0045	0.996	0.141693281	count	1
NR1I2	17.6978429	3947.637976	0.0045	0.996	0.141693281	count	1
PLSCR2	17.697843	3947.637795	0.0045	0.996	0.141693281	count	1
LINC01213	17.697843	3947.637795	0.0045	0.996	0.141693281	count	1
LINC02066	17.6978428	3947.638002	0.0045	0.996	0.141693281	count	1
KLHL6	17.697843	3947.637951	0.0045	0.996	0.141693281	count	1
AC131235.4	17.697843	3947.637795	0.0045	0.996	0.141693281	count	1
FETUB	17.6978429	3947.638106	0.0045	0.996	0.141693281	count	1
OSTN	17.697843	3947.637795	0.0045	0.996	0.141693281	count	1
AC092535.1	17.697843	3947.637795	0.0045	0.996	0.141693281	count	1
CFAP99	17.697843	3947.63808	0.0045	0.996	0.141693281	count	1
NKX3-2	17.697843	3947.637795	0.0045	0.996	0.141693281	count	1
TAPT1-AS1	17.697843	3947.637795	0.0045	0.996	0.141693281	count	1
EXOC1L	17.697843	3947.637976	0.0045	0.996	0.141693281	count	1
ADH7	17.6978428	3947.638054	0.0045	0.996	0.141693281	count	1
ETNPPL	17.697843	3947.638054	0.0045	0.996	0.141693281	count	1
AC004067.1	17.697843	3947.637795	0.0045	0.996	0.141693281	count	1
NDST4	17.697843	3947.637951	0.0045	0.996	0.141693281	count	1
AC079341.1	17.697843	3947.637795	0.0045	0.996	0.141693281	count	1
LINC02516	17.697843	3947.637795	0.0045	0.996	0.141693281	count	1
AC116563.1	17.6978428	3947.637899	0.0045	0.996	0.141693281	count	1
AC112236.2	17.6978428	3947.637821	0.0045	0.996	0.141693281	count	1
RBM46	17.6978429	3947.638106	0.0045	0.996	0.141693281	count	1
AC021087.1	17.697843	3947.637795	0.0045	0.996	0.141693281	count	1
NKD2	17.697843	3947.637795	0.0045	0.996	0.141693281	count	1
AC091906.1	17.6978429	3947.638054	0.0045	0.996	0.141693281	count	1
AC113386.1	17.697843	3947.637795	0.0045	0.996	0.141693281	count	1
C1QTNF3-AMACR	17.6978429	3947.637976	0.0045	0.996	0.141693281	count	1
AC008957.1	17.697843	3947.638132	0.0045	0.996	0.141693281	count	1
LINC00603	17.697843	3947.63808	0.0045	0.996	0.141693281	count	1
SHISAL2B	17.6978429	3947.638002	0.0045	0.996	0.141693281	count	1
KCNN2	17.697843	3947.638209	0.0045	0.996	0.141693281	count	1
SLC27A6	17.6978429	3947.638002	0.0045	0.996	0.141693281	count	1
CCNI2	17.697843	3947.637795	0.0045	0.996	0.141693281	count	1
C5orf66	17.6978429	3947.637847	0.0045	0.996	0.141693281	count	1
GFRA3	17.6978428	3947.638106	0.0045	0.996	0.141693281	count	1

PCDHAC2	17.697843	3947.637795	0.0045	0.996	0.141693281	count	1
AC034213.1	17.6978429	3947.638002	0.0045	0.996	0.141693281	count	1
AL590004.3	17.6978428	3947.63808	0.0045	0.996	0.141693281	count	1
HIST1H3F	17.697843	3947.637795	0.0045	0.996	0.141693281	count	1
HIST1H2AM	17.697843	3947.637795	0.0045	0.996	0.141693281	count	1
HCG15	17.6978428	3947.63808	0.0045	0.996	0.141693281	count	1
LINC02569	17.697843	3947.638028	0.0045	0.996	0.141693281	count	1
KIFC1	17.697843	3947.637795	0.0045	0.996	0.141693281	count	1
ARMC12	17.697843	3947.638054	0.0045	0.996	0.141693281	count	1
AL136164.2	17.697843	3947.637795	0.0045	0.996	0.141693281	count	1
NMBR	17.697843	3947.637795	0.0045	0.996	0.141693281	count	1
AL355304.1	17.697843	3947.637795	0.0045	0.996	0.141693281	count	1
AL590867.1	17.697843	3947.638028	0.0045	0.996	0.141693281	count	1
LINC01558	17.697843	3947.637795	0.0045	0.996	0.141693281	count	1
AC004080.1	17.697843	3947.637795	0.0045	0.996	0.141693281	count	1
LINC01449	17.697843	3947.637795	0.0045	0.996	0.141693281	count	1
CLDN4	17.697843	3947.637795	0.0045	0.996	0.141693281	count	1
AC004023.1	17.697843	3947.637795	0.0045	0.996	0.141693281	count	1
AC092849.1	17.6978429	3947.637976	0.0045	0.996	0.141693281	count	1
EFCAB10	17.697843	3947.638235	0.0045	0.996	0.141693281	count	1
CFTR	17.6978429	3947.638106	0.0045	0.996	0.141693281	count	1
MESTIT1	17.697843	3947.637873	0.0045	0.996	0.141693281	count	1
OR2A42	17.6978428	3947.637769	0.0045	0.996	0.141693281	count	1
CNPY1	17.697843	3947.637795	0.0045	0.996	0.141693281	count	1
LINC00106	17.697843	3947.637795	0.0045	0.996	0.141693281	count	1
NXF3	17.697843	3947.637795	0.0045	0.996	0.141693281	count	1
AL606763.1	17.697843	3947.638054	0.0045	0.996	0.141693281	count	1
RTL9	17.6978428	3947.637821	0.0045	0.996	0.141693281	count	1
HS6ST2	17.6978428	3947.637847	0.0045	0.996	0.141693281	count	1
PLAC1	17.697843	3947.637795	0.0045	0.996	0.141693281	count	1
AVPR2	17.6978428	3947.63808	0.0045	0.996	0.141693281	count	1
AC245140.3	17.6978429	3947.637925	0.0045	0.996	0.141693281	count	1
AC104964.4	17.697843	3947.637795	0.0045	0.996	0.141693281	count	1
FAM86B2	17.6978429	3947.638002	0.0045	0.996	0.141693281	count	1
STAR	17.697843	3947.637795	0.0045	0.996	0.141693281	count	1
AC087623.1	17.697843	3947.638028	0.0045	0.996	0.141693281	count	1
AC100821.2	17.6978429	3947.638158	0.0045	0.996	0.141693281	count	1
TTPA	17.6978429	3947.638158	0.0045	0.996	0.141693281	count	1
DNAJC5B	17.697843	3947.637795	0.0045	0.996	0.141693281	count	1
AP003465.2	17.6978428	3947.63808	0.0045	0.996	0.141693281	count	1
AP002907.1	17.697843	3947.638209	0.0045	0.996	0.141693281	count	1
AP002852.1	17.697843	3947.637795	0.0045	0.996	0.141693281	count	1
AC090922.1	17.697843	3947.63808	0.0045	0.996	0.141693281	count	1
CCDC26	17.697843	3947.638339	0.0045	0.996	0.141693281	count	1
AC011676.1	17.697843	3947.637976	0.0045	0.996	0.141693281	count	1

PSCA	17.6978428	3947.637976	0.0045	0.996	0.141693281	count	1
AC108002.1	17.697843	3947.637795	0.0045	0.996	0.141693281	count	1
SH3GL2	17.697843	3947.637795	0.0045	0.996	0.141693281	count	1
FAM205C	17.697843	3947.637795	0.0045	0.996	0.141693281	count	1
BX255923.1	17.6978428	3947.638132	0.0045	0.996	0.141693281	count	1
AL391987.4	17.697843	3947.637795	0.0045	0.996	0.141693281	count	1
LINC01506	17.697843	3947.637795	0.0045	0.996	0.141693281	count	1
AL359182.2	17.697843	3947.637795	0.0045	0.996	0.141693281	count	1
AMBP	17.697843	3947.638209	0.0045	0.996	0.141693281	count	1
OR1L8	17.697843	3947.637795	0.0045	0.996	0.141693281	count	1
AL590226.1	17.697843	3947.638028	0.0045	0.996	0.141693281	count	1
CCDC183-AS1	17.6978428	3947.638132	0.0045	0.996	0.141693281	count	1
KCNQ1-AS1	17.6978429	3947.638158	0.0045	0.996	0.141693281	count	1
SLC22A18AS	17.697843	3947.638054	0.0045	0.996	0.141693281	count	1
OR51B5	17.6978429	3947.637821	0.0045	0.996	0.141693281	count	1
AC091564.2	17.697843	3947.637795	0.0045	0.996	0.141693281	count	1
NLRP14	17.6978428	3947.638235	0.0045	0.996	0.141693281	count	1
SYT9	17.6978429	3947.638054	0.0045	0.996	0.141693281	count	1
AC124276.2	17.697843	3947.638209	0.0045	0.996	0.141693281	count	1
AC103974.1	17.697843	3947.637795	0.0045	0.996	0.141693281	count	1
PTPN5	17.6978429	3947.638002	0.0045	0.996	0.141693281	count	1
AP000808.2	17.6978428	3947.637925	0.0045	0.996	0.141693281	count	1
AP005233.2	17.697843	3947.637795	0.0045	0.996	0.141693281	count	1
AP000487.2	17.697843	3947.637925	0.0045	0.996	0.141693281	count	1
KRTAP5-8	17.6978429	3947.638002	0.0045	0.996	0.141693281	count	1
AP006216.1	17.697843	3947.637795	0.0045	0.996	0.141693281	count	1
PKNOX2-AS1	17.6978428	3947.638002	0.0045	0.996	0.141693281	count	1
AP001783.1	17.6978428	3947.638132	0.0045	0.996	0.141693281	count	1
AP003025.2	17.6978428	3947.63808	0.0045	0.996	0.141693281	count	1
AP001999.1	17.697843	3947.637795	0.0045	0.996	0.141693281	count	1
AKR1C4	17.697843	3947.637795	0.0045	0.996	0.141693281	count	1
NEBL	17.6978429	3947.638002	0.0045	0.996	0.141693281	count	1
AL022344.1	17.6978429	3947.638106	0.0045	0.996	0.141693281	count	1
AL358394.1	17.6978429	3947.638002	0.0045	0.996	0.141693281	count	1
TMEM26-AS1	17.697843	3947.637795	0.0045	0.996	0.141693281	count	1
AL451049.1	17.697843	3947.638106	0.0045	0.996	0.141693281	count	1
ATOH7	17.697843	3947.63808	0.0045	0.996	0.141693281	count	1
AC007848.1	17.697843	3947.637795	0.0045	0.996	0.141693281	count	1
AC006581.2	17.697843	3947.637795	0.0045	0.996	0.141693281	count	1
AC018630.2	17.697843	3947.637795	0.0045	0.996	0.141693281	count	1
AC009318.1	17.697843	3947.638028	0.0045	0.996	0.141693281	count	1
GPR84	17.6978429	3947.638209	0.0045	0.996	0.141693281	count	1
AC135279.3	17.697843	3947.637795	0.0045	0.996	0.141693281	count	1
AC133794.1	17.6978428	3947.638002	0.0045	0.996	0.141693281	count	1
AC012464.3	17.697843	3947.637951	0.0045	0.996	0.141693281	count	1

AC010205.1	17.697843	3947.637795	0.0045	0.996	0.141693281	count	1
FAM222A	17.697843	3947.63808	0.0045	0.996	0.141693281	count	1
AC127002.1	17.6978429	3947.637873	0.0045	0.996	0.141693281	count	1
AC027290.1	17.697843	3947.637795	0.0045	0.996	0.141693281	count	1
LINC02405	17.6978428	3947.638028	0.0045	0.996	0.141693281	count	1
AC073911.1	17.697843	3947.63808	0.0045	0.996	0.141693281	count	1
SLC25A30-AS1	17.697843	3947.638002	0.0045	0.996	0.141693281	count	1
LMO7DN	17.697843	3947.637795	0.0045	0.996	0.141693281	count	1
AL355922.5	17.697843	3947.638054	0.0045	0.996	0.141693281	count	1
SLC39A2	17.697843	3947.63808	0.0045	0.996	0.141693281	count	1
TRAV22	17.6978429	3947.638028	0.0045	0.996	0.141693281	count	1
LINC02289	17.697843	3947.637795	0.0045	0.996	0.141693281	count	1
AC008056.2	17.697843	3947.637795	0.0045	0.996	0.141693281	count	1
LINC02328	17.697843	3947.638054	0.0045	0.996	0.141693281	count	1
LINC02321	17.697843	3947.637795	0.0045	0.996	0.141693281	count	1
AL137779.1	17.697843	3947.637795	0.0045	0.996	0.141693281	count	1
TGM7	17.697843	3947.637795	0.0045	0.996	0.141693281	count	1
CCDC33	17.697843	3947.638028	0.0045	0.996	0.141693281	count	1
AC091100.1	17.6978429	3947.637925	0.0045	0.996	0.141693281	count	1
AC103982.1	17.6978429	3947.637847	0.0045	0.996	0.141693281	count	1
LINC00235	17.697843	3947.637976	0.0045	0.996	0.141693281	count	1
AC093525.3	17.697843	3947.637795	0.0045	0.996	0.141693281	count	1
AC092338.1	17.697843	3947.63808	0.0045	0.996	0.141693281	count	1
AC009133.3	17.6978429	3947.638028	0.0045	0.996	0.141693281	count	1
NPIPB13	17.697843	3947.637795	0.0045	0.996	0.141693281	count	1
AC136944.2	17.6978428	3947.637951	0.0045	0.996	0.141693281	count	1
CTRB1	17.697843	3947.638054	0.0045	0.996	0.141693281	count	1
AC092718.2	17.6978428	3947.637821	0.0045	0.996	0.141693281	count	1
LINC01996	17.697843	3947.638235	0.0045	0.996	0.141693281	count	1
AC004771.5	17.697843	3947.638054	0.0045	0.996	0.141693281	count	1
AC107926.1	17.6978429	3947.638054	0.0045	0.996	0.141693281	count	1
SP6	17.697843	3947.637795	0.0045	0.996	0.141693281	count	1
FLJ45513	17.697843	3947.638209	0.0045	0.996	0.141693281	count	1
WFIKKN2	17.697843	3947.637795	0.0045	0.996	0.141693281	count	1
AC015813.2	17.697843	3947.63808	0.0045	0.996	0.141693281	count	1
CA4	17.697843	3947.638132	0.0045	0.996	0.141693281	count	1
AC120024.1	17.6978429	3947.638106	0.0045	0.996	0.141693281	count	1
PPP1R27	17.697843	3947.637795	0.0045	0.996	0.141693281	count	1
PPP4R1-AS1	17.6978428	3947.638054	0.0045	0.996	0.141693281	count	1
AC091198.1	17.697843	3947.637795	0.0045	0.996	0.141693281	count	1
AC118757.1	17.697843	3947.637795	0.0045	0.996	0.141693281	count	1
PRND	17.697843	3947.637795	0.0045	0.996	0.141693281	count	1
AL158042.1	17.6978428	3947.637976	0.0045	0.996	0.141693281	count	1
C20orf203	17.6978429	3947.638002	0.0045	0.996	0.141693281	count	1
C20orf144	17.6978428	3947.637925	0.0045	0.996	0.141693281	count	1

AL391097.2	17.697843	3947.638028	0.0045	0.996	0.141693281	count	1
STX16-NPEPL1	17.6978429	3947.638261	0.0045	0.996	0.141693281	count	1
AL121845.1	17.697843	3947.63808	0.0045	0.996	0.141693281	count	1
AC006273.1	17.697843	3947.637847	0.0045	0.996	0.141693281	count	1
AZU1	17.697843	3947.637847	0.0045	0.996	0.141693281	count	1
AC010336.1	17.697843	3947.638054	0.0045	0.996	0.141693281	count	1
AC010422.3	17.697843	3947.638054	0.0045	0.996	0.141693281	count	1
HAPLN4	17.6978429	3947.638158	0.0045	0.996	0.141693281	count	1
AC025809.1	17.6978428	3947.637976	0.0045	0.996	0.141693281	count	1
HPN-AS1	17.697843	3947.638054	0.0045	0.996	0.141693281	count	1
AC016590.1	17.697843	3947.638028	0.0045	0.996	0.141693281	count	1
AC016590.3	17.6978429	3947.638002	0.0045	0.996	0.141693281	count	1
AC011445.2	17.697843	3947.637795	0.0045	0.996	0.141693281	count	1
HIPK4	17.697843	3947.63808	0.0045	0.996	0.141693281	count	1
AC011481.1	17.697843	3947.637795	0.0045	0.996	0.141693281	count	1
SLC8A2	17.697843	3947.637795	0.0045	0.996	0.141693281	count	1
LHB	17.697843	3947.637795	0.0045	0.996	0.141693281	count	1
IL4I1	17.6978428	3947.638054	0.0045	0.996	0.141693281	count	1
ASPDH	17.697843	3947.638028	0.0045	0.996	0.141693281	count	1
KLK13	17.697843	3947.638028	0.0045	0.996	0.141693281	count	1
ZSCAN5B	17.697843	3947.638054	0.0045	0.996	0.141693281	count	1
SMIM17	17.697843	3947.637795	0.0045	0.996	0.141693281	count	1
ZIM2	17.697843	3947.638054	0.0045	0.996	0.141693281	count	1
AL008638.6	17.6978428	3947.638002	0.0045	0.996	0.141693281	count	1
FO393418.1	17.697843	3947.638002	0.0045	0.996	0.141693281	count	1
AL022476.1	17.6978429	3947.638158	0.0045	0.996	0.141693281	count	1
PHF21B	17.6978429	3947.638209	0.0045	0.996	0.141693281	count	1
CU633967.1	17.697843	3947.637795	0.0045	0.996	0.141693281	count	1
AP001605.1	17.6978428	3947.638002	0.0045	0.996	0.141693281	count	1
BACH1-IT2	17.697843	3947.637795	0.0045	0.996	0.141693281	count	1
AP000569.1	17.6978429	3947.638106	0.0045	0.996	0.141693281	count	1
AP000322.1	17.697843	3947.637795	0.0045	0.996	0.141693281	count	1
AF064858.1	17.697843	3947.637951	0.0045	0.996	0.141693281	count	1
TSPEAR-AS2	17.697843	3947.637795	0.0045	0.996	0.141693281	count	1
AP001468.1	17.697843	3947.637795	0.0045	0.996	0.141693281	count	1
AL591163.1	17.6978431	3947.638028	0.0045	0.996	0.141693281	count	1
UBXN10	17.6978433	3947.638183	0.0045	0.996	0.141693281	count	1
GPR3	17.6978433	3947.638132	0.0045	0.996	0.141693281	count	1
AL513327.1	17.6978431	3947.638106	0.0045	0.996	0.141693281	count	1
AL354864.1	17.6978431	3947.637976	0.0045	0.996	0.141693281	count	1
AL358075.2	17.6978432	3947.638209	0.0045	0.996	0.141693281	count	1
LINC01738	17.6978431	3947.637976	0.0045	0.996	0.141693281	count	1
LINC01135	17.6978431	3947.638106	0.0045	0.996	0.141693281	count	1
LEMD1-AS1	17.6978431	3947.638158	0.0045	0.996	0.141693281	count	1
AL451060.1	17.6978431	3947.638106	0.0045	0.996	0.141693281	count	1

LINC01705	17.6978432	3947.638002	0.0045	0.996	0.141693281	count	1
ALLC	17.6978431	3947.638028	0.0045	0.996	0.141693281	count	1
AGBL5-AS1	17.6978431	3947.638158	0.0045	0.996	0.141693281	count	1
TGFA	17.6978432	3947.638106	0.0045	0.996	0.141693281	count	1
DCTN1-AS1	17.6978431	3947.638106	0.0045	0.996	0.141693281	count	1
AC103563.7	17.6978432	3947.637899	0.0045	0.996	0.141693281	count	1
AC009974.1	17.6978431	3947.637976	0.0045	0.996	0.141693281	count	1
AC005237.1	17.6978431	3947.638054	0.0045	0.996	0.141693281	count	1
AC099329.3	17.6978432	3947.638209	0.0045	0.996	0.141693281	count	1
CSPG5	17.6978431	3947.638106	0.0045	0.996	0.141693281	count	1
LINC02585	17.6978431	3947.63808	0.0045	0.996	0.141693281	count	1
DENND6A-DT	17.6978431	3947.637899	0.0045	0.996	0.141693281	count	1
AC078785.1	17.6978431	3947.638158	0.0045	0.996	0.141693281	count	1
IQCJ-SCHIP1-AS1	17.6978431	3947.638028	0.0045	0.996	0.141693281	count	1
AC107067.1	17.6978431	3947.638261	0.0045	0.996	0.141693281	count	1
AC008808.1	17.6978431	3947.638028	0.0045	0.996	0.141693281	count	1
ELOVL7	17.6978431	3947.638028	0.0045	0.996	0.141693281	count	1
AC093206.1	17.6978432	3947.638209	0.0045	0.996	0.141693281	count	1
AC011373.1	17.6978432	3947.638209	0.0045	0.996	0.141693281	count	1
AC131025.1	17.6978432	3947.638028	0.0045	0.996	0.141693281	count	1
AC008676.1	17.6978431	3947.638132	0.0045	0.996	0.141693281	count	1
AC008637.1	17.6978432	3947.637976	0.0045	0.996	0.141693281	count	1
AL445309.1	17.6978431	3947.638028	0.0045	0.996	0.141693281	count	1
RPS10-NUDT3	17.6978432	3947.637976	0.0045	0.996	0.141693281	count	1
AC003986.3	17.6978431	3947.637847	0.0045	0.996	0.141693281	count	1
MIR503HG	17.6978431	3947.638106	0.0045	0.996	0.141693281	count	1
KBTBD11-OT1	17.6978431	3947.638028	0.0045	0.996	0.141693281	count	1
AC019257.2	17.6978431	3947.638235	0.0045	0.996	0.141693281	count	1
NUGGC	17.6978431	3947.638028	0.0045	0.996	0.141693281	count	1
AC091182.2	17.6978431	3947.638132	0.0045	0.996	0.141693281	count	1
AP003465.1	17.6978431	3947.638287	0.0045	0.996	0.141693281	count	1
AC104958.2	17.6978432	3947.638002	0.0045	0.996	0.141693281	count	1
FAM221B	17.6978432	3947.638158	0.0045	0.996	0.141693281	count	1
AL158154.2	17.6978433	3947.638132	0.0045	0.996	0.141693281	count	1
AL161785.1	17.6978432	3947.638002	0.0045	0.996	0.141693281	count	1
AL157938.2	17.6978431	3947.637847	0.0045	0.996	0.141693281	count	1
AP002990.1	17.6978431	3947.638158	0.0045	0.996	0.141693281	count	1
AP000721.2	17.6978432	3947.638209	0.0045	0.996	0.141693281	count	1
AP003390.1	17.6978431	3947.638339	0.0045	0.996	0.141693281	count	1
PROSER2-AS1	17.6978431	3947.638209	0.0045	0.996	0.141693281	count	1
ANXA8	17.6978431	3947.638158	0.0045	0.996	0.141693281	count	1
C10orf82	17.6978431	3947.638054	0.0045	0.996	0.141693281	count	1
AC068896.1	17.6978432	3947.638028	0.0045	0.996	0.141693281	count	1
MMP21	17.6978431	3947.638028	0.0045	0.996	0.141693281	count	1
DPPA3	17.6978433	3947.638132	0.0045	0.996	0.141693281	count	1

LINC00592	17.6978431	3947.638054	0.0045	0.996	0.141693281	count	1
IL23A	17.6978431	3947.638106	0.0045	0.996	0.141693281	count	1
POC1B-GALNT4	17.6978431	3947.638132	0.0045	0.996	0.141693281	count	1
LINC02344	17.6978431	3947.638106	0.0045	0.996	0.141693281	count	1
AL158196.1	17.6978431	3947.63808	0.0045	0.996	0.141693281	count	1
TGM1	17.6978431	3947.638158	0.0045	0.996	0.141693281	count	1
AL358335.2	17.6978431	3947.63808	0.0045	0.996	0.141693281	count	1
AL627171.2	17.6978432	3947.638002	0.0045	0.996	0.141693281	count	1
SYT16	17.6978431	3947.638158	0.0045	0.996	0.141693281	count	1
AC013451.2	17.6978432	3947.638106	0.0045	0.996	0.141693281	count	1
AL583810.1	17.6978432	3947.638002	0.0045	0.996	0.141693281	count	1
AC025040.1	17.6978431	3947.63808	0.0045	0.996	0.141693281	count	1
AC066613.2	17.6978431	3947.638132	0.0045	0.996	0.141693281	count	1
AC025271.4	17.6978431	3947.638261	0.0045	0.996	0.141693281	count	1
AC090907.2	17.6978432	3947.638158	0.0045	0.996	0.141693281	count	1
NPIPB9	17.6978431	3947.638158	0.0045	0.996	0.141693281	count	1
ATP2A1	17.6978431	3947.637976	0.0045	0.996	0.141693281	count	1
AC009097.4	17.6978431	3947.638183	0.0045	0.996	0.141693281	count	1
AC106729.1	17.6978431	3947.638158	0.0045	0.996	0.141693281	count	1
LINC02170	17.6978431	3947.638106	0.0045	0.996	0.141693281	count	1
ATP2C2	17.6978431	3947.638106	0.0045	0.996	0.141693281	count	1
UNC45B	17.6978433	3947.638132	0.0045	0.996	0.141693281	count	1
B4GALNT2	17.6978432	3947.638002	0.0045	0.996	0.141693281	count	1
AC007423.1	17.6978432	3947.638209	0.0045	0.996	0.141693281	count	1
LINC02080	17.6978431	3947.638158	0.0045	0.996	0.141693281	count	1
C17orf99	17.6978431	3947.638028	0.0045	0.996	0.141693281	count	1
LINC01254	17.6978431	3947.638054	0.0045	0.996	0.141693281	count	1
AP001269.2	17.6978431	3947.63808	0.0045	0.996	0.141693281	count	1
AC090125.1	17.6978431	3947.638235	0.0045	0.996	0.141693281	count	1
AL121895.1	17.6978432	3947.638106	0.0045	0.996	0.141693281	count	1
STK4-AS1	17.6978431	3947.638158	0.0045	0.996	0.141693281	count	1
CDH22	17.6978431	3947.638054	0.0045	0.996	0.141693281	count	1
TUBB1	17.6978431	3947.63808	0.0045	0.996	0.141693281	count	1
SMIM24	17.6978431	3947.638158	0.0045	0.996	0.141693281	count	1
TINCR	17.6978431	3947.638158	0.0045	0.996	0.141693281	count	1
RTBDN	17.6978431	3947.638261	0.0045	0.996	0.141693281	count	1
AF038458.2	17.6978432	3947.638028	0.0045	0.996	0.141693281	count	1
DNAAF3	17.6978432	3947.638028	0.0045	0.996	0.141693281	count	1
AC012313.8	17.6978432	3947.638132	0.0045	0.996	0.141693281	count	1
AL031590.1	17.6978432	3947.637899	0.0045	0.996	0.141693281	count	1
TPTE	17.6978431	3947.638028	0.0045	0.996	0.141693281	count	1
AL391335.1	17.6978434	3947.638287	0.0045	0.996	0.141693281	count	1
SLC26A8	17.6978434	3947.638313	0.0045	0.996	0.141693281	count	1
AC134312.5	17.7386971	1600.443676	0.0111	0.9912	0.141693281	count	1
AL353613.1	17.7439174	1444.32835	0.0123	0.9902	0.141693281	count	1

AC026316.5	17.7472113	2692.623307	0.0066	0.995	0.141693281	count	1
AC100847.1	17.747214	2692.629275	0.0066	0.995	0.141693281	count	1
SERPIND1	17.7641609	1683.124257	0.0106	0.9916	0.141693281	count	1
SRCIN1	17.7655308	3156.012359	0.0056	0.996	0.141693281	count	1
ODF3L2	17.765531	3156.016055	0.0056	0.996	0.141693281	count	1
OR52N4	17.7655314	3156.016072	0.0056	0.996	0.141693281	count	1
LINC01095	17.765537	3156.016038	0.0056	0.996	0.141693281	count	1
AL158070.1	17.765537	3156.016038	0.0056	0.996	0.141693281	count	1
LRRTM3	17.765537	3156.01597	0.0056	0.996	0.141693281	count	1
ELN-AS1	17.7655373	3156.015987	0.0056	0.996	0.141693281	count	1
PRR4	17.7655373	3156.016038	0.0056	0.996	0.141693281	count	1
AC004801.6	17.7655372	3156.015903	0.0056	0.996	0.141693281	count	1
AC126773.2	17.7655371	3156.016055	0.0056	0.996	0.141693281	count	1
ERICH4	17.7655369	3156.015903	0.0056	0.996	0.141693281	count	1
WBP2NL	17.7655373	3156.016055	0.0056	0.996	0.141693281	count	1
SHANK3	17.7655371	3156.016004	0.0056	0.996	0.141693281	count	1
AC119403.1	17.765538	3156.019667	0.0056	0.996	0.141693281	count	1
AC135803.1	17.7655381	3156.019582	0.0056	0.996	0.141693281	count	1
AP003680.1	17.7655382	3156.019734	0.0056	0.996	0.141693281	count	1
LINC01894	17.7655381	3156.019633	0.0056	0.996	0.141693281	count	1
AC026904.4	17.7655385	3156.0197	0.0056	0.996	0.141693281	count	1
SYT8	17.7655383	3156.019683	0.0056	0.996	0.141693281	count	1
AL078644.2	17.7655392	3156.02338	0.0056	0.996	0.141693281	count	1
KCNH1	17.7655394	3156.023312	0.0056	0.996	0.141693281	count	1
AC097381.1	17.7655392	3156.023396	0.0056	0.996	0.141693281	count	1
BLM	17.7655431	3156.015768	0.0056	0.996	0.141693281	count	1
SNAP25-AS1	17.7655449	3156.023278	0.0056	0.996	0.141693281	count	1
AC087752.4	17.7655452	3156.023295	0.0056	0.996	0.141693281	count	1
LINC00316	17.7655451	3156.023278	0.0056	0.996	0.141693281	count	1
AC073862.1	17.7655476	3156.011616	0.0056	0.996	0.141693281	count	1
AC093726.2	17.7655504	3156.022991	0.0056	0.996	0.141693281	count	1
BX284668.2	17.7655506	3156.022856	0.0056	0.996	0.141693281	count	1
AC006252.1	17.7655505	3156.02289	0.0056	0.996	0.141693281	count	1
AC008494.3	17.7655506	3156.022975	0.0056	0.996	0.141693281	count	1
WWC1	17.7655506	3156.022856	0.0056	0.996	0.141693281	count	1
AC022087.1	17.7655505	3156.022941	0.0056	0.996	0.141693281	count	1
AC005962.1	17.765551	3156.022991	0.0056	0.996	0.141693281	count	1
ZNF497	17.7655505	3156.022958	0.0056	0.996	0.141693281	count	1
AC005540.1	17.7655509	3156.022924	0.0056	0.996	0.141693281	count	1
LINC02043	17.7655509	3156.022907	0.0056	0.996	0.141693281	count	1
AC098484.2	17.7655518	3156.026603	0.0056	0.996	0.141693281	count	1
LINC00449	17.7655524	3156.007802	0.0056	0.996	0.141693281	count	1
MSTN	17.7655536	3156.01138	0.0056	0.996	0.141693281	count	1
AC078927.1	17.7655533	3156.011363	0.0056	0.996	0.141693281	count	1
AP000229.1	17.7655533	3156.011363	0.0056	0.996	0.141693281	count	1

C19orf81	17.7655573	3156.026401	0.0056	0.996	0.141693281	count	1
DPYS	17.7655592	3156.011329	0.0056	0.996	0.141693281	count	1
CYP2D6	17.7655592	3156.011279	0.0056	0.996	0.141693281	count	1
AC004477.3	17.7655593	3156.011312	0.0056	0.996	0.141693281	count	1
LINC00843	17.8258007	2590.071115	0.0069	0.9945	0.141693281	count	1
ADRB1	17.867124	1430.594729	0.0125	0.99	0.141693281	count	1
PROK1	17.8695621	2279.883101	0.0078	0.9937	0.141693281	count	1
OVGP1	17.869649	2179.174816	0.0082	0.9935	0.141693281	count	1
SYT10	17.8701555	2559.321824	0.007	0.994	0.141693281	count	1
RCVRN	17.8706832	2851.053762	0.0063	0.995	0.141693281	count	1
GRHL1	17.9592217	1221.297426	0.0147	0.9883	0.141693282	count	1
ADAMTS16	17.9762381	1347.560189	0.0133	0.9894	0.141693282	count	1
SEC14L5	17.9788263	2192.812747	0.0082	0.9935	0.141693282	count	1
AL356356.1	17.9801938	3000.617614	0.006	0.995	0.141693282	count	1
IGF2-AS	17.9916161	2256.042409	0.008	0.994	0.141693282	count	1
VSTM5	17.991616	2256.042389	0.008	0.994	0.141693282	count	1
LINC00683	17.9916336	2256.047887	0.008	0.994	0.141693282	count	1
WDR11-AS1	17.9916341	2256.04228	0.008	0.994	0.141693282	count	1
AC068234.2	17.9916387	2256.053454	0.008	0.994	0.141693282	count	1
AC040170.1	17.9916407	2256.03849	0.008	0.994	0.141693282	count	1
AC016957.2	17.9916452	2256.049663	0.008	0.994	0.141693282	count	1
TSSK3	17.9916461	2256.044077	0.008	0.994	0.141693282	count	1
B3GNT4	17.9916462	2256.044077	0.008	0.994	0.141693282	count	1
AC007566.1	17.9916504	2256.05527	0.008	0.994	0.141693282	count	1
AC123768.2	17.9916513	2256.049663	0.008	0.994	0.141693282	count	1
AC084824.5	17.9921098	2599.664381	0.0069	0.994	0.141693282	count	1
AL035461.2	17.99211	2599.664393	0.0069	0.994	0.141693282	count	1
FAM110D	17.992115	2599.670811	0.0069	0.994	0.141693282	count	1
FGL1	17.9921217	2599.666441	0.0069	0.994	0.141693282	count	1
UPK1B	17.9921218	2599.666452	0.0069	0.994	0.141693282	count	1
ST3GAL5-AS1	17.9921226	2599.659966	0.0069	0.994	0.141693282	count	1
CCM2L	17.9927969	3177.609495	0.0057	0.995	0.141693282	count	1
AL954642.1	18.0018115	1278.919059	0.0141	0.9888	0.141693282	count	1
AC016877.1	18.0765972	1897.710039	0.0095	0.9924	0.141693282	count	1
COL7A1	18.0770925	2120.649208	0.0085	0.9932	0.141693282	count	1
CAV3	18.1261928	1864.947218	0.0097	0.9922	0.141693282	count	1
AL031847.1	18.1304908	2779.99488	0.0065	0.9948	0.141693282	count	1
AC007216.2	18.2401427	2246.913122	0.0081	0.9935	0.141693282	count	1
DDX25	18.2401591	2246.920195	0.0081	0.9935	0.141693282	count	1
AL583839.1	18.2401605	2246.9148	0.0081	0.9935	0.141693282	count	1
JMJD6	0.1126471	0.1397725	0.8059	0.42	0.141766391	count	1
RAB5A	0.1041709	0.1088782	0.9568	0.339	0.141868901	count	1
ERBB2	0.1079921	0.1422264	0.7593	0.448	0.141918553	count	1
GOLIM4	0.0997209	0.0531359	1.8767	0.0606	0.142068041	count	1
AARS2	0.3760279	0.519169	0.7243	0.469	0.142143001	count	1

CTNS	0.1526498	0.2735509	0.558	0.577	0.142170069	count	1
INO80E	0.1121226	0.1744599	0.6427	0.52	0.142255905	count	1
COL5A3	0.1860949	0.3308168	0.5625	0.574	0.14227413	count	1
MBOAT2	0.1234689	0.2467999	0.5003	0.617	0.14235649	count	1
CBX5	0.1018111	0.0811669	1.2543	0.21	0.142507458	count	1
AC026471.4	0.9272049	0.7194633	1.2887	0.198	0.142522593	count	1
CDC34	0.1051321	0.1277215	0.8231	0.41	0.142546236	count	1
DIP2C	0.1181673	0.2421802	0.4879	0.626	0.142693026	count	1
STX12	0.1035169	0.0915746	1.1304	0.258	0.142698267	count	1
BACH1	0.1082338	0.1440561	0.7513	0.453	0.142784408	count	1
STX18	0.1159942	0.1703327	0.681	0.496	0.142838637	count	1
ZNF84	0.1158307	0.1858427	0.6233	0.533	0.142910044	count	1
ADCY6	0.108542	0.1533438	0.7078	0.479	0.142920061	count	1
SAFB	0.1066041	0.1255872	0.8488	0.396	0.142928218	count	1
LSM14B	0.1226414	0.1898738	0.6459	0.518	0.142974847	count	1
DDX52	0.1065381	0.1279693	0.8325	0.405	0.143071014	count	1
ZNF324	0.1267925	0.2625713	0.4829	0.629	0.143100816	count	1
HNRNPAB	0.1014943	0.0676835	1.4995	0.134	0.143175153	count	1
RPL7L1	0.1020243	0.0780382	1.3074	0.191	0.143207513	count	1
HCLS1	0.1817488	0.4006561	0.4536	0.65	0.143239115	count	1
SUZ12	0.105891	0.1152422	0.9189	0.358	0.143265333	count	1
AP002761.3	0.379487	0.5808543	0.6533	0.514	0.14333611	count	1
FAM81A	0.2687108	0.5824901	0.4613	0.645	0.143352545	count	1
SPIDR	0.1200435	0.1965339	0.6108	0.541	0.143460516	count	1
ZC3H10	0.162101	0.2806177	0.5777	0.564	0.143543979	count	1
SATB1	0.1541707	0.2389863	0.6451	0.519	0.143563515	count	1
CCDC122	0.1147994	0.2400281	0.4783	0.632	0.14360022	count	1
AC013553.3	0.6009784	0.976888	0.6152	0.538	0.143677259	count	1
CBWD2	0.1449165	0.1827129	0.7931	0.428	0.143852144	count	1
NORAD	0.1007293	0.0502637	2.004	0.0451	0.143879314	count	1
TUBB2B	0.2499776	0.521772	0.4791	0.632	0.143881228	count	1
C18orf32	0.1248541	0.173029	0.7216	0.471	0.143941763	count	1
SMARCB1	0.1055404	0.0875131	1.206	0.228	0.143945843	count	1
DHX32	0.1205434	0.2023681	0.5957	0.551	0.144054282	count	1
MRPS16	0.1053981	0.0899892	1.1712	0.242	0.144086963	count	1
ZNF155	0.1887951	0.4221118	0.4473	0.655	0.144283491	count	1
LYAR	0.1079641	0.10114	1.0675	0.286	0.144312089	count	1
CCDC82	0.103235	0.0860273	1.2	0.23	0.144329664	count	1
LCORL	0.152789	0.2343975	0.6518	0.515	0.144343222	count	1
KLC1	0.1081801	0.1237358	0.8743	0.382	0.144469054	count	1
AC067750.1	0.1347866	0.3223675	0.4181	0.676	0.14457855	count	1
AC093627.6	0.332389	0.3648204	0.9111	0.362	0.144598684	count	1
PCNT	0.1288426	0.1902025	0.6774	0.498	0.144710392	count	1
MYD88	0.1237166	0.2752658	0.4494	0.653	0.144715539	count	1
NDUFS8	0.1019023	0.0577891	1.7633	0.0779	0.144735645	count	1

NRF1	0.1959537	0.3193293	0.6136	0.539	0.144739076	count	1
WDR26	0.1180253	0.2226441	0.5301	0.596	0.144753929	count	1
DPM3	0.1049391	0.0742509	1.4133	0.158	0.144791764	count	1
DERL2	0.1043428	0.0882124	1.1829	0.237	0.144847933	count	1
HMGB2	0.1037537	0.0860745	1.2054	0.228	0.144964241	count	1
CRELD2	0.1056375	0.088573	1.1927	0.233	0.144982187	count	1
PUSL1	0.1207201	0.2118526	0.5698	0.569	0.145030088	count	1
KCTD7	0.146141	0.2826243	0.5171	0.605	0.145051178	count	1
ARHGEF40	0.1183166	0.1965668	0.6019	0.547	0.145109329	count	1
MEX3C	0.1292048	0.2475366	0.522	0.602	0.145113755	count	1
PDCD6	0.1022361	0.0489191	2.0899	0.0367	0.145275755	count	1
RCAN1	0.1100815	0.1357228	0.8111	0.417	0.145304041	count	1
NFAT5	0.1074793	0.1117717	0.9616	0.336	0.145316771	count	1
ADAM22	0.2990474	0.4735358	0.6315	0.528	0.145479623	count	1
ITGAE	0.1057573	0.1048329	1.0088	0.313	0.145491268	count	1
TCP11L1	0.1283512	0.2144329	0.5986	0.55	0.145505472	count	1
PTPN18	0.1130246	0.150838	0.7493	0.454	0.145651549	count	1
HOMEZ	0.1645706	0.3204997	0.5135	0.608	0.145689355	count	1
ANKRD36	0.1117869	0.1827009	0.6119	0.541	0.145719209	count	1
C1orf74	0.335226	0.7900511	0.4243	0.671	0.145743191	count	1
C22orf15	0.335226	0.8126104	0.4125	0.68	0.145743191	count	1
TBC1D30	0.335226	1.058425	0.3167	0.751	0.145743191	count	1
LINC02550	0.611364	0.7447439	0.8209	0.412	0.145751056	count	1
AC092587.1	0.611364	0.7583495	0.8062	0.42	0.145751056	count	1
MAMDC4	0.611364	1.220886	0.5008	0.6166	0.145751056	count	1
ACAP1	0.2734658	0.348551	0.7846	0.433	0.145755364	count	1
TMEM116	0.1326617	0.3555011	0.3732	0.709	0.145883336	count	1
ZNF66	0.3357614	0.4933477	0.6806	0.496	0.14595902	count	1
TEX264	0.1057182	0.0873588	1.2102	0.226	0.145996605	count	1
POLR2M	0.1148067	0.1413642	0.8121	0.417	0.146010659	count	1
NEK4	0.1428119	0.2237641	0.6382	0.523	0.146058788	count	1
EXOSC1	0.1086284	0.1168027	0.93	0.352	0.146152836	count	1
TCTN2	0.1684341	0.2896772	0.5815	0.561	0.146217856	count	1
CERS5	0.1117981	0.1819607	0.6144	0.539	0.146289221	count	1
BCCIP	0.1094996	0.1073983	1.0196	0.308	0.146359679	count	1
AP000254.1	0.3884785	0.9125675	0.4257	0.67	0.146427438	count	1
BX470102.1	0.3884785	1.080722	0.3595	0.719	0.146427438	count	1
DARS-AS1	0.3884785	1.1489	0.3381	0.735	0.146427438	count	1
KCTD17	0.1189406	0.1739352	0.6838	0.494	0.146448274	count	1
ING1	0.1090387	0.118538	0.9199	0.358	0.14647924	count	1
SAFB2	0.1104078	0.1409428	0.7834	0.433	0.146486261	count	1
LACTB	0.1089139	0.1321274	0.8243	0.41	0.146536129	count	1
RBM26	0.1088979	0.1275478	0.8538	0.393	0.146569239	count	1
PLEKHG2	0.128269	0.2893072	0.4434	0.658	0.146669479	count	1
BICRAL	0.1212383	0.2075818	0.5841	0.559	0.146731691	count	1

CYTH3	0.1125098	0.1420249	0.7922	0.428	0.146773633	count	1
KIF3B	0.1148692	0.16118	0.7127	0.476	0.146778361	count	1
ALG1L2	0.1727244	0.406085	0.4253	0.671	0.14678805	count	1
RETREG3	0.124301	0.213859	0.5812	0.561	0.146806656	count	1
PTHLH	0.1139452	0.2249338	0.5066	0.612	0.146833644	count	1
NDUFS2	0.1084525	0.0915202	1.185	0.236	0.146906924	count	1
KCNK15-AS1	0.2396153	0.4286342	0.559	0.576	0.146992286	count	1
AC010997.5	0.1815369	0.4480714	0.4052	0.685	0.146996767	count	1
PI4K2B	0.1924482	0.3644589	0.528	0.598	0.146999306	count	1
LINC02062	0.9682344	1.087174	0.8906	0.373	0.147031719	count	1
AC007249.2	0.9682344	1.258894	0.7691	0.442	0.147031719	count	1
NFATC3	0.1209451	0.2336136	0.5177	0.605	0.147055605	count	1
U91328.1	0.2562461	0.4067888	0.6299	0.529	0.147319659	count	1
NUDT8	0.117176	0.1631777	0.7181	0.473	0.147440865	count	1
SLC51A	0.972121	0.7633301	1.2735	0.203	0.147451754	count	1
XRCC6	0.1044401	0.0530247	1.9697	0.049	0.147532047	count	1
SLC35A2	0.1196101	0.1713467	0.6981	0.485	0.147549164	count	1
UQCRFS1	0.1051972	0.0679458	1.5483	0.122	0.147614198	count	1
PLCD3	0.1128828	0.1895925	0.5954	0.552	0.147811595	count	1
UBE2W	0.1136221	0.125467	0.9056	0.365	0.147985732	count	1
PRPF18	0.1242094	0.1711829	0.7256	0.468	0.147997627	count	1
LSM12	0.1073514	0.0892665	1.2026	0.229	0.148023631	count	1
RANBP2	0.1114896	0.1292731	0.8624	0.389	0.148152776	count	1
TENM4	0.3416026	0.5065855	0.6743	0.5	0.148309919	count	1
SYVN1	0.1309079	0.1961727	0.6673	0.505	0.148379797	count	1
TMEM45A	0.1081986	0.0930921	1.1623	0.245	0.148405293	count	1
AC016831.1	0.2289329	0.4879623	0.4692	0.639	0.148438934	count	1
SLC12A4	0.1161862	0.1506316	0.7713	0.441	0.148454609	count	1
MRPL18	0.1053299	0.0628468	1.676	0.0938	0.148553096	count	1
DVL1	0.1234131	0.189266	0.6521	0.514	0.148622498	count	1
SMAD5-AS1	0.2792124	0.6730594	0.4148	0.678	0.148652778	count	1
BBIP1	0.1099265	0.1129034	0.9736	0.33	0.148714171	count	1
TMEM199	0.1203435	0.1921639	0.6263	0.531	0.148725592	count	1
NOP58	0.1097536	0.1021338	1.0746	0.283	0.148755868	count	1
S100A9	0.1681772	0.4322758	0.3891	0.697	0.1488201	count	1
CISD2	0.1096485	0.1056686	1.0377	0.299	0.148873638	count	1
RMND5B	0.1130493	0.1353527	0.8352	0.404	0.148932701	count	1
HSPA4	0.1086342	0.093688	1.1595	0.246	0.14900182	count	1
CEP350	0.1107186	0.128911	0.8589	0.39	0.149230919	count	1
ZNF346	0.1560906	0.4046473	0.3857	0.7	0.149395951	count	1
RAB14	0.1054547	0.0562294	1.8754	0.0608	0.149459026	count	1
SNRPA	0.108828	0.0960203	1.1334	0.257	0.149522303	count	1
C12orf45	0.1102962	0.1260628	0.8749	0.382	0.149534434	count	1
EIF4EBP2	0.1121341	0.1160677	0.9661	0.334	0.149597298	count	1
CASP6	0.1196946	0.1916245	0.6246	0.532	0.149694745	count	1

SUCLA2	0.1079255	0.0865801	1.2465	0.213	0.149746171	count	1
TMCO3	0.1084873	0.1039253	1.0439	0.297	0.14974874	count	1
AL135999.1	0.398589	0.7669717	0.5197	0.603	0.149886119	count	1
CXXC1	0.1277445	0.2145231	0.5955	0.552	0.149891319	count	1
CDC123	0.1077727	0.079871	1.3493	0.177	0.149987014	count	1
CHMP4C	0.1215201	0.1858768	0.6538	0.513	0.150172189	count	1
CA5B	0.1187577	0.1527097	0.7777	0.437	0.150248429	count	1
EPHA3	0.1290488	0.1384811	0.9319	0.351	0.150389786	count	1
WASHC4	0.1146342	0.1217887	0.9413	0.347	0.150416075	count	1
NTN4	0.1129984	0.0975963	1.1578	0.247	0.150530897	count	1
DCAF1	0.1473472	0.2995951	0.4918	0.623	0.150637969	count	1
DCAF8	0.1206463	0.177825	0.6785	0.498	0.150640561	count	1
GUCD1	0.1237121	0.1489515	0.8306	0.406	0.150734538	count	1
UBAC1	0.1107393	0.1168692	0.9475	0.343	0.15087458	count	1
PFKFB2	0.2218261	0.4421422	0.5017	0.616	0.150912164	count	1
UBA3	0.1127586	0.1195933	0.9429	0.346	0.150969575	count	1
SLC22A23	1.0057916	0.8959685	1.1226	0.262	0.151039211	count	1
FBXL19-AS1	1.0057916	0.9129272	1.1017	0.271	0.151039211	count	1
DNAAF2	0.1186776	0.1692549	0.7012	0.483	0.151096002	count	1
ZNF644	0.109732	0.0851979	1.288	0.198	0.151130551	count	1
DYRK1B	0.1420792	0.2926285	0.4855	0.627	0.151266221	count	1
XYLB	1.008044	0.7344109	1.3726	0.17	0.15127594	count	1
ADAM19	0.1582762	0.3238256	0.4888	0.625	0.151454511	count	1
TPM3	0.107752	0.0730157	1.4757	0.14	0.151534271	count	1
CHDH	0.6407154	0.4872494	1.315	0.189	0.151537808	count	1
SP3	0.1145457	0.1346622	0.8506	0.395	0.151538182	count	1
CCDC126	0.1204747	0.1785032	0.6749	0.5	0.151572666	count	1
UCN	0.6409956	0.6674373	0.9604	0.337	0.151592524	count	1
AC087672.2	0.6409956	0.7077946	0.9056	0.365	0.151592524	count	1
GABRB3	0.6409956	0.7331627	0.8743	0.382	0.151592524	count	1
PDIA6	0.1064199	0.0465416	2.2866	0.0223	0.151624611	count	1
PTP4A3	0.112535	0.1045118	1.0768	0.282	0.151673786	count	1
TUBG2	0.1182183	0.1736005	0.681	0.496	0.151701797	count	1
IGHMBP2	0.1656375	0.2761228	0.5999	0.549	0.151711379	count	1
SLC25A28	0.1193482	0.1275979	0.9353	0.35	0.151762379	count	1
C19orf73	0.2232118	0.5252766	0.4249	0.671	0.1518211	count	1
CNIH1	0.1078036	0.0637278	1.6916	0.0908	0.15186911	count	1
TMEM209	0.1280148	0.2278117	0.5619	0.574	0.152067528	count	1
PSMD10	0.1179179	0.1448426	0.8141	0.416	0.152081726	count	1
USP30	0.1790991	0.263855	0.6788	0.497	0.152085842	count	1
TIMMDC1	0.1092124	0.0819676	1.3324	0.183	0.152192548	count	1
SHKBP1	0.1188905	0.1484125	0.8011	0.423	0.152234286	count	1
AC062029.1	0.4055363	0.7707077	0.5262	0.599	0.152252045	count	1
MYOZ3	0.286395	0.4919386	0.5822	0.56	0.152264202	count	1
TXNDC9	0.1114903	0.1016288	1.097	0.273	0.152297697	count	1

ANKRA2	0.1205504	0.1561852	0.7718	0.44	0.15230276	count	1
KCND1	0.3141244	0.5227254	0.6009	0.548	0.152342579	count	1
ERICH6-AS1	0.3516814	0.5544719	0.6343	0.526	0.152350616	count	1
ZCWPW1	0.1386808	0.2182681	0.6354	0.525	0.152437479	count	1
PPP2R3A	0.123624	0.2121201	0.5828	0.56	0.15247438	count	1
CASP10	0.3520175	0.603292	0.5835	0.56	0.152485014	count	1
C12orf60	0.3520175	0.610625	0.5765	0.564	0.152485014	count	1
AP002433.1	0.3520175	0.7471432	0.4712	0.638	0.152485014	count	1
NDP	0.6456334	0.7633152	0.8458	0.398	0.15249673	count	1
UBE2D3	0.1072942	0.050097	2.1417	0.0323	0.152558202	count	1
GID4	0.1414439	0.2231614	0.6338	0.526	0.152647594	count	1
GNAI1	0.1194992	0.133491	0.8952	0.371	0.152670618	count	1
ZNF681	0.2246337	0.3814885	0.5888	0.556	0.15275332	count	1
AC233266.2	1.0223256	1.0589875	0.9654	0.334	0.152767621	count	1
ZNF398	0.1799261	0.2783954	0.6463	0.518	0.152772472	count	1
RCE1	0.125714	0.2205762	0.5699	0.569	0.152820103	count	1
MRPL48	0.1154586	0.1446728	0.7981	0.425	0.152829977	count	1
AXIN2	0.2664077	0.4117901	0.647	0.518	0.152875078	count	1
AL021368.2	0.1391547	0.3800465	0.3662	0.714	0.15295325	count	1
KCMF1	0.1145975	0.1127631	1.0163	0.31	0.153087549	count	1
ZNF791	0.114666	0.1403074	0.8172	0.414	0.153108851	count	1
ELOA	0.114066	0.1310106	0.8707	0.384	0.153156766	count	1
OAS1	0.1214403	0.1860896	0.6526	0.514	0.153212761	count	1
DAD1	0.1067849	0.0327801	3.2576	0.00113	0.153257585	count	1
RNF145	0.1164187	0.1495475	0.7785	0.436	0.153260387	count	1
HELB	0.1733044	0.3592159	0.4825	0.63	0.15326597	count	1
AZI2	0.1117948	0.0981468	1.1391	0.255	0.153269097	count	1
TXNDC16	0.1167346	0.177269	0.6585	0.51	0.153372292	count	1
CASP9	0.1807864	0.3276448	0.5518	0.581	0.153486589	count	1
PPIL4	0.1093059	0.064259	1.701	0.089	0.153532237	count	1
TGIF1	0.1171909	0.140437	0.8345	0.404	0.153653274	count	1
ZNF189	0.1629834	0.2874902	0.5669	0.571	0.153812193	count	1
AC024060.1	1.032559	0.9837984	1.0496	0.294	0.153826434	count	1
LIN52	0.1775119	0.2925302	0.6068	0.544	0.153931132	count	1
NMT2	0.1263709	0.1775004	0.7119	0.477	0.153955226	count	1
TLL1	1.0341975	0.958566	1.0789	0.281	0.153995299	count	1
AC011405.1	1.0341975	1.1296772	0.9155	0.36	0.153995299	count	1
RASGRP1	1.0341975	1.1296772	0.9155	0.36	0.153995299	count	1
SAP25	1.0341975	1.2249558	0.8443	0.399	0.153995299	count	1
NAPG	0.1115145	0.096584	1.1546	0.248	0.154101515	count	1
AL359915.2	0.2382038	0.5243504	0.4543	0.65	0.154210102	count	1
ALKBH5	0.1165145	0.1175836	0.9909	0.322	0.154223673	count	1
COL10A1	0.4978077	0.7218837	0.6896	0.49	0.154227217	count	1
TMEM9	0.1101898	0.0761463	1.4471	0.148	0.154309492	count	1
COG2	0.1228442	0.1796138	0.6839	0.494	0.154315472	count	1

LYRM2	0.111541	0.0913637	1.2208	0.222	0.154491686	count	1
NBAS	0.121989	0.1341562	0.9093	0.363	0.154520811	count	1
NEMF	0.1110787	0.0810265	1.3709	0.17	0.154688463	count	1
FDFT1	0.1137332	0.1232955	0.9224	0.356	0.154705544	count	1
TRMT13	0.1229115	0.1800717	0.6826	0.495	0.154844398	count	1
OAS3	0.1579283	0.4103543	0.3849	0.7	0.15484943	count	1
NFIC	0.108655	0.0457178	2.3766	0.0175	0.15491345	count	1
CLK4	0.1188273	0.1317051	0.9022	0.367	0.15498733	count	1
AGFG2	0.1869431	0.3543009	0.5276	0.598	0.155046328	count	1
CCDC97	0.1357525	0.1981505	0.6851	0.493	0.155154924	count	1
MUC12	0.1919529	0.404708	0.4743	0.635	0.155217045	count	1
ZNHIT2	0.126697	0.2417817	0.524	0.6	0.155329634	count	1
ZEB2	0.1107806	0.0682824	1.6224	0.105	0.155366852	count	1
RUVBL1	0.1192484	0.1308992	0.911	0.362	0.155412517	count	1
FBXO42	0.1230421	0.1881061	0.6541	0.513	0.155436488	count	1
RGCC	0.1121719	0.1642771	0.6828	0.495	0.155481198	count	1
YOD1	0.1648269	0.3906361	0.4219	0.673	0.155522219	count	1
AXIN1	0.1728732	0.2637423	0.6555	0.512	0.155628132	count	1
RFC3	0.1674699	0.3032804	0.5522	0.581	0.155727726	count	1
TTI2	0.1341733	0.2328099	0.5763	0.564	0.155760925	count	1
ZNF625	0.2935924	0.4172545	0.7036	0.482	0.155871863	count	1
GATB	0.1465213	0.361989	0.4048	0.686	0.155941391	count	1
TAB2	0.1314139	0.2148031	0.6118	0.541	0.156077435	count	1
ZNF546	0.1801412	0.2851152	0.6318	0.528	0.156161836	count	1
ZNF34	0.1246074	0.1780289	0.6999	0.484	0.156287487	count	1
AFG1L	0.4176141	0.5588452	0.7473	0.455	0.156344501	count	1
JPX	0.1155817	0.1141812	1.0123	0.311	0.156543796	count	1
AC051619.5	0.2208837	0.3936192	0.5612	0.575	0.156654573	count	1
LACTB2	0.1172764	0.1437826	0.8157	0.415	0.156657013	count	1
GPALPP1	0.1179991	0.1323055	0.8919	0.373	0.15677908	count	1
RMC1	0.1453408	0.2272657	0.6395	0.523	0.156807225	count	1
STK16	0.1240014	0.132386	0.9367	0.349	0.156852574	count	1
OAZ3	0.2955543	0.5831938	0.5068	0.612	0.156853317	count	1
CREBBP	0.1168392	0.1352873	0.8636	0.388	0.156871421	count	1
HECTD2	0.1242226	0.1722124	0.7213	0.471	0.156920949	count	1
ZBED1	0.1261355	0.1558641	0.8093	0.418	0.156944336	count	1
PCDHGA11	1.06434	0.8471399	1.2564	0.209	0.157062633	count	1
RNF130	0.1124279	0.0704872	1.595	0.111	0.157071107	count	1
ZSWIM3	0.6700929	0.5939994	1.1281	0.259	0.157220594	count	1
CCDC120	0.6701223	0.5960313	1.1243	0.261	0.157226235	count	1
GLIPR1L1	0.6702679	0.7952202	0.8429	0.399	0.157254127	count	1
BRD9	0.1280934	0.1422221	0.9007	0.368	0.157347003	count	1
GLIPR2	0.113462	0.0829495	1.3678	0.171	0.157491111	count	1
MSH6	0.1202529	0.1218861	0.9866	0.324	0.157543474	count	1
AC040977.1	0.671831	0.7558498	0.8888	0.374	0.157553407	count	1

ADAP1	0.671831	0.8640853	0.7775	0.437	0.157553407	count	1
AL365205.1	0.3647455	0.436779	0.8351	0.404	0.15755848	count	1
SMIM3	0.1172025	0.1206204	0.9717	0.331	0.157602311	count	1
COMMD2	0.1137333	0.0901301	1.2619	0.207	0.157700255	count	1
MZT1	0.1193821	0.1253198	0.9526	0.341	0.157733089	count	1
PLCB4	0.1951702	0.2440751	0.7996	0.424	0.157751163	count	1
CRAMP1	0.2007032	0.3191389	0.6289	0.529	0.157767282	count	1
TATDN2	0.297406	0.462507	0.643	0.52	0.157778875	count	1
POLR1B	0.3261764	0.3190201	1.0224	0.307	0.157794608	count	1
HIST1H4C	0.1106502	0.0555859	1.9906	0.0466	0.157808129	count	1
LINC00667	0.1139177	0.0981304	1.1609	0.246	0.15781919	count	1
SART3	0.1332979	0.1657188	0.8044	0.421	0.157834221	count	1
ZDHHC17	0.1225867	0.14612	0.8389	0.402	0.157926187	count	1
IRAK4	0.1210009	0.1506949	0.803	0.422	0.157934844	count	1
MAPRE1	0.1155471	0.1036157	1.1152	0.265	0.158041268	count	1
AC015813.1	0.2584847	0.8558598	0.302	0.763	0.158042175	count	1
ZNF852	0.2981444	0.403086	0.7397	0.46	0.158147742	count	1
AC012485.3	0.512373	0.5888637	0.8701	0.384	0.1581585	count	1
PSMD3	0.1163395	0.0840627	1.384	0.166	0.158158857	count	1
AC008011.2	0.4230772	0.516526	0.8191	0.413	0.158186973	count	1
PRADC1	0.119536	0.1167484	1.0239	0.306	0.158476645	count	1
STK25	0.1154741	0.1090686	1.0587	0.29	0.158550348	count	1
CCND1	0.110886	0.0764257	1.4509	0.147	0.15864114	count	1
TRMT10B	0.1329274	0.2542787	0.5228	0.601	0.158753412	count	1
HVCN1	0.1408223	0.2202173	0.6395	0.523	0.158790798	count	1
FAM71F2	0.5150465	0.6760445	0.7619	0.446	0.158876471	count	1
ERLEC1	0.1117381	0.0555296	2.0122	0.0443	0.158896871	count	1
PRKRIP1	0.1231045	0.1348885	0.9126	0.361	0.158897087	count	1
XPC	0.1182738	0.107203	1.1033	0.27	0.158917458	count	1
AASS	0.1355298	0.1971301	0.6875	0.492	0.158957583	count	1
SPPL3	0.1206151	0.1325446	0.91	0.363	0.158969286	count	1
CEP192	0.1397232	0.2291554	0.6097	0.542	0.158979245	count	1
TMEM52	1.0839071	1.132379	0.9572	0.339	0.159016018	count	1
RBBP4	0.1169906	0.1098538	1.065	0.287	0.159252989	count	1
HSBP1	0.111942	0.0493607	2.2678	0.0234	0.15929098	count	1
NUFIP1	0.1377554	0.175962	0.7829	0.434	0.15929856	count	1
AL162377.1	0.1972637	0.3043666	0.6481	0.517	0.159398856	count	1
ZNF493	0.125247	0.1761945	0.7108	0.477	0.159423385	count	1
AC027449.1	0.5171132	0.5391697	0.9591	0.338	0.159430701	count	1
CDC40	0.1151804	0.0795265	1.4483	0.148	0.159484616	count	1
AC008035.1	1.088674	0.8848818	1.2303	0.219	0.159487435	count	1
TBCEL	0.1744058	0.4958084	0.3518	0.725	0.159588795	count	1
AKTIP	0.1191399	0.1063989	1.1197	0.263	0.159693579	count	1
AC067838.1	0.5185851	0.852813	0.6081	0.543	0.159825019	count	1
ALKBH2	0.1238288	0.1364617	0.9074	0.364	0.159828352	count	1

ATP1B1	0.1123676	0.1025087	1.0962	0.273	0.159884638	count	1
SRPRB	0.1183809	0.1309268	0.9042	0.366	0.159971533	count	1
ZNF584	0.1633799	0.3485131	0.4688	0.639	0.160110818	count	1
AL109811.3	1.0954617	0.812558	1.3482	0.178	0.16015569	count	1
TPRG1L	0.1208908	0.1119457	1.0799	0.28	0.160267564	count	1
UFM1	0.1141596	0.0853833	1.337	0.181	0.160378749	count	1
AK5	0.1816264	0.3408577	0.5329	0.594	0.160469926	count	1
CENPQ	0.1469523	0.2022559	0.7266	0.468	0.160500977	count	1
ZER1	0.1411155	0.2485061	0.5679	0.57	0.160549318	count	1
MAX	0.1181108	0.109119	1.0824	0.279	0.160561999	count	1
EXTL2	0.1188997	0.1162852	1.0225	0.307	0.16056455	count	1
FAM216A	0.1438081	0.1901531	0.7563	0.45	0.160566725	count	1
LINC01003	0.1418787	0.2312372	0.6136	0.54	0.16070217	count	1
NACAD	0.2268706	0.3544563	0.6401	0.522	0.160751371	count	1
HSPB2	0.1165728	0.106355	1.0961	0.273	0.1609169	count	1
SETDB1	0.1456734	0.2502388	0.5821	0.561	0.160936405	count	1
CDR1	0.3332801	0.5005346	0.6658	0.506	0.160993951	count	1
TLK1	0.1170619	0.0928779	1.2604	0.208	0.161101466	count	1
SPATA20	0.1309342	0.1439882	0.9093	0.363	0.161131496	count	1
MDH1	0.1133081	0.050956	2.2236	0.0262	0.161198278	count	1
GBP1	0.1171691	0.1191146	0.9837	0.325	0.161276184	count	1
BABAM2	0.1212856	0.1362069	0.8905	0.373	0.161380362	count	1
CD164	0.1136665	0.0516667	2.2	0.0279	0.161433028	count	1
CBX6	0.1147068	0.067754	1.693	0.0905	0.161455046	count	1
GGNBP2	0.1151697	0.0783353	1.4702	0.142	0.161526499	count	1
KYNU	0.2823213	0.5953381	0.4742	0.635	0.161528993	count	1
CCZ1B	0.1542167	0.2042094	0.7552	0.45	0.161606995	count	1
TAS2R14	0.2122378	0.4832935	0.4391	0.661	0.161658411	count	1
PET117	0.1365846	0.1937247	0.705	0.481	0.161697489	count	1
ECM2	0.1152015	0.0763258	1.5093	0.131	0.161787898	count	1
GCK	0.2003586	0.3657107	0.5479	0.584	0.16183287	count	1
RUSC2	0.1321128	0.2387679	0.5533	0.58	0.161930135	count	1
TRAIP	1.115171	0.6576398	1.6957	0.09	0.162076179	count	1
EXOSC3	0.1247159	0.1568293	0.7952	0.427	0.162117085	count	1
MED19	0.1182439	0.111526	1.0602	0.289	0.162127602	count	1
ELL2	0.1321012	0.1583478	0.8342	0.404	0.162241313	count	1
UBE2B	0.1136738	0.0430891	2.6381	0.0084	0.162280083	count	1
MINDY4	0.1526892	0.2585228	0.5906	0.555	0.162427134	count	1
TIMM21	0.1300249	0.1783589	0.729	0.466	0.162547745	count	1
TIMM44	0.1270886	0.1351243	0.9405	0.347	0.16268702	count	1
TMEM131	0.1228917	0.1303954	0.9425	0.346	0.162731516	count	1
MICAL3	0.1566455	0.2720614	0.5758	0.565	0.162808984	count	1
MITF	0.1321596	0.139757	0.9456	0.344	0.162941871	count	1
HSD17B6	0.4375258	0.8357934	0.5235	0.601	0.163033977	count	1
LINC01004	1.1259362	0.8794734	1.2802	0.201	0.16311278	count	1

COL4A3BP	0.1181786	0.1022785	1.1555	0.248	0.163297006	count	1
FAM114A1	0.1152346	0.0582327	1.9789	0.0479	0.163385273	count	1
GPAM	0.1709735	0.2415874	0.7077	0.479	0.163394444	count	1
STRIP1	0.166843	0.2787218	0.5986	0.549	0.163449998	count	1
SERINC1	0.1152158	0.05513	2.0899	0.0367	0.163492022	count	1
TDRD1	0.3390937	0.5631014	0.6022	0.547	0.163604427	count	1
ACOT11	0.1405133	0.4081614	0.3443	0.731	0.163642601	count	1
MAPK6	0.1302025	0.1767089	0.7368	0.461	0.163750846	count	1
TMEM54	0.1227222	0.1195497	1.0265	0.305	0.163760862	count	1
AC142472.1	0.3808377	0.4369438	0.8716	0.383	0.163927315	count	1
ZNF571	0.162092	0.3962471	0.4091	0.683	0.163953519	count	1
AC078883.3	0.5346794	1.162632	0.4599	0.646	0.164114356	count	1
PKD2	0.11699	0.0980005	1.1938	0.233	0.164447356	count	1
DGCR6	0.1940697	0.3613211	0.5371	0.591	0.164491858	count	1
NEDD9	0.1339738	0.1754463	0.7636	0.445	0.164527486	count	1
DDRGK1	0.1180172	0.0827396	1.4264	0.154	0.164695531	count	1
TMEM80	0.1198726	0.1013101	1.1832	0.237	0.164701288	count	1
RAD51D	0.1628601	0.3130014	0.5203	0.603	0.164719059	count	1
OTOA	1.1431876	1.0838064	1.0548	0.292	0.164755787	count	1
RHPN2	1.1431876	1.0838064	1.0548	0.292	0.164755787	count	1
AC107952.2	1.1431876	1.1745916	0.9733	0.33	0.164755787	count	1
SIAH1	0.122466	0.1068301	1.1464	0.252	0.16484746	count	1
RPN2	0.1164956	0.0551158	2.1137	0.0346	0.164849002	count	1
NOMO3	0.4430755	0.8232412	0.5382	0.59	0.164885681	count	1
SETD6	0.1601142	0.2621104	0.6109	0.541	0.164967591	count	1
TRPM7	0.1248052	0.1532134	0.8146	0.415	0.164973191	count	1
COA6	0.1221628	0.1063659	1.1485	0.251	0.16501588	count	1
PHKG2	0.1383147	0.1989359	0.6953	0.487	0.165141635	count	1
TCF4	0.1159051	0.0544911	2.127	0.0335	0.165168333	count	1
AK6	0.119044	0.0872593	1.3643	0.173	0.16539492	count	1
CTSB	0.1169489	0.0687265	1.7017	0.0889	0.165412279	count	1
ARHGAP11A	0.7139304	0.7904548	0.9032	0.366	0.165499115	count	1
ZNF415	0.1556418	0.3258041	0.4777	0.633	0.165529496	count	1
STX5	0.1231517	0.1335954	0.9218	0.357	0.165706868	count	1
AL356512.1	0.3439247	0.5181011	0.6638	0.507	0.165768298	count	1
UBE2J2	0.1217107	0.1036825	1.1739	0.241	0.165792295	count	1
SMARCAL1	0.150145	0.2463613	0.6095	0.542	0.165824452	count	1
AP004609.3	0.3135776	0.4339843	0.7226	0.47	0.16583038	count	1
AC012368.1	1.1550423	0.8720979	1.3244	0.185	0.165871971	count	1
ARSI	0.3138205	0.735612	0.4266	0.67	0.165950885	count	1
GINM1	0.1167568	0.0484319	2.4107	0.016	0.166076984	count	1
UQCRC2	0.1182989	0.0688092	1.7192	0.0857	0.16612454	count	1
DNAH11	1.158444	0.6086561	1.9033	0.0571	0.166190292	count	1
SLC9A5	0.5425404	0.9001431	0.6027	0.547	0.166194601	count	1
METTL22	0.1660689	0.2656047	0.6252	0.532	0.166258922	count	1

FAM234A	0.1306275	0.1738092	0.7516	0.452	0.166438825	count	1
VTA1	0.1271015	0.1300173	0.9776	0.328	0.166484624	count	1
MAP1LC3B	0.1174644	0.0635438	1.8486	0.0646	0.166485858	count	1
UBAP2	0.1370602	0.2164173	0.6333	0.527	0.166524007	count	1
TRIP11	0.1188399	0.0728328	1.6317	0.103	0.166892837	count	1
TAF9B	0.1307677	0.149364	0.8755	0.381	0.167002952	count	1
CAMTA2	0.1512753	0.2322018	0.6515	0.515	0.167059492	count	1
DMXL1	0.1250101	0.1514089	0.8256	0.409	0.167109558	count	1
CDKAL1	0.159546	0.203539	0.7839	0.433	0.167118415	count	1
CCNB1IP1	0.1219976	0.1129219	1.0804	0.28	0.167130193	count	1
HDAC8	0.1308883	0.1934249	0.6767	0.499	0.167156277	count	1
ANAPC10	0.1246583	0.1194014	1.044	0.297	0.167275718	count	1
HYAL2	0.138046	0.1694703	0.8146	0.415	0.167331109	count	1
CCDC157	0.2931042	0.5371255	0.5457	0.585	0.167360622	count	1
WBP1L	0.1318785	0.1475065	0.8941	0.371	0.167414132	count	1
ESYT1	0.1294482	0.149981	0.8631	0.388	0.167516955	count	1
GAS8	0.1502991	0.2152868	0.6981	0.485	0.167740328	count	1
CERS4	0.137807	0.1879984	0.733	0.464	0.167798483	count	1
WDCP	0.1677451	0.2907464	0.5769	0.564	0.167911061	count	1
POLR2L	0.1174955	0.0427194	2.7504	0.006	0.167923775	count	1
CTDSP2	0.12299	0.120426	1.0213	0.307	0.167937205	count	1
GEMIN6	0.1304477	0.1629693	0.8004	0.424	0.168175171	count	1
ANKHD1	0.1633528	0.3142449	0.5198	0.603	0.168257447	count	1
MCM2	0.453504	0.8277938	0.5478	0.584	0.168350197	count	1
SLC25A44	0.2089151	0.3932054	0.5313	0.595	0.168550829	count	1
USPL1	0.1518687	0.2578985	0.5889	0.556	0.168606326	count	1
B4GALT6	0.3502888	0.652951	0.5365	0.592	0.168611407	count	1
LINC00853	0.3503399	0.7095358	0.4938	0.622	0.168634193	count	1
UBN2	0.1308955	0.1550078	0.8444	0.398	0.168750079	count	1
PTPRD	0.1276334	0.1720529	0.7418	0.458	0.168797965	count	1
SREK1	0.121908	0.1140766	1.0687	0.285	0.16893629	count	1
DICER1	0.1249539	0.1311029	0.9531	0.341	0.168996983	count	1
NOXA1	0.2776943	0.4202592	0.6608	0.509	0.169208171	count	1
PHF19	0.1290431	0.1646373	0.7838	0.433	0.169256849	count	1
RCC2	0.1918404	0.2568095	0.747	0.455	0.169290967	count	1
GCHFR	0.1256869	0.1564035	0.8036	0.422	0.169295476	count	1
ZNF710	0.4564892	0.6037394	0.7561	0.45	0.169338326	count	1
SIPA1L3	0.2304391	0.4151596	0.5551	0.579	0.169341163	count	1
CYTL1	0.1199559	0.4044151	0.2966	0.7668	0.16946225	count	1
FAT1	0.1253165	0.1152647	1.0872	0.277	0.169486244	count	1
KLHL24	0.1276583	0.1467849	0.8697	0.385	0.169662037	count	1
AC020765.2	0.2051443	0.4598531	0.4461	0.656	0.169742768	count	1
AC116366.1	0.2975698	0.4622411	0.6438	0.52	0.169768062	count	1
ACSL3	0.1218644	0.0829197	1.4697	0.142	0.169786164	count	1
CPNE3	0.1198119	0.0532737	2.249	0.0246	0.169863311	count	1

CSNK1G3	0.1233585	0.1169019	1.0552	0.291	0.169978036	count	1
AC079949.2	1.2002195	1.0570567	1.1354	0.256	0.170030987	count	1
AC021546.1	1.2002195	1.14096	1.0519	0.293	0.170030987	count	1
ZHX1-C8orf76	1.2002195	1.14096	1.0519	0.293	0.170030987	count	1
PDE6D	0.1324866	0.1632627	0.8115	0.417	0.170109493	count	1
SSBP2	0.1224696	0.0748677	1.6358	0.102	0.170274993	count	1
LAT	0.2414104	0.609176	0.3963	0.692	0.170666156	count	1
HRH2	0.1433891	0.1773886	0.8083	0.419	0.170680231	count	1
PLEKHA1	0.1236928	0.1190939	1.0386	0.299	0.17070405	count	1
USP32	0.2064011	0.3206159	0.6438	0.52	0.170754784	count	1
EFCAB6	0.5600335	0.787978	0.7107	0.477	0.170788892	count	1
AC005076.1	0.3551782	0.4700837	0.7556	0.45	0.170789915	count	1
EXOC1	0.128852	0.145048	0.8883	0.374	0.170882185	count	1
ZSCAN18	0.1253089	0.1055324	1.1874	0.235	0.170935855	count	1
FLII	0.1321516	0.1188794	1.1116	0.266	0.171001143	count	1
CABIN1	0.1276122	0.1118005	1.1414	0.254	0.171019093	count	1
H3F3B	0.1187154	0.021717	5.4665	4.90E-08	0.171053772	count	0.001149099
SAMD4A	0.1396697	0.2112714	0.6611	0.509	0.171134363	count	1
USP39	0.1340852	0.1756783	0.7632	0.445	0.171220212	count	1
SNX3	0.1195138	0.0344751	3.4667	0.000533	0.171224733	count	1
MRPL46	0.1268766	0.1154596	1.0989	0.272	0.171252874	count	1
CFDP1	0.1215124	0.0702807	1.729	0.0839	0.171309562	count	1
SON	0.1200132	0.0511865	2.3446	0.0191	0.171804857	count	1
RNF217-AS1	0.3578859	0.5475764	0.6536	0.513	0.171994199	count	1
BAK1	0.1586769	0.3567135	0.4448	0.656	0.172110428	count	1
CYB5RL	0.565103	0.6242079	0.9053	0.365	0.172111319	count	1
EDNRB	0.1672986	0.2489296	0.6721	0.502	0.172263018	count	1
PUS10	0.219795	0.3706478	0.593	0.553	0.172317632	count	1
TADA2A	0.1445599	0.2195405	0.6585	0.51	0.172542391	count	1
LRP8	0.3272232	0.4902821	0.6674	0.505	0.172579848	count	1
AP001627.1	0.753023	1.0683528	0.7048	0.481	0.172680048	count	1
AC139795.2	0.753023	1.1689638	0.6442	0.519	0.172680048	count	1
AC104109.2	0.753023	1.1727489	0.6421	0.521	0.172680048	count	1
AC092687.3	0.753023	1.21119	0.6217	0.534	0.172680048	count	1
CPA1	0.753023	1.21119	0.6217	0.534	0.172680048	count	1
C11orf91	0.753023	1.30855	0.5755	0.565	0.172680048	count	1
SCN2A	0.3595891	0.6298129	0.5709	0.568	0.172750935	count	1
IFFO1	0.1452228	0.1563528	0.9288	0.353	0.172846284	count	1
NSL1	0.1250996	0.0908415	1.3771	0.169	0.172894332	count	1
MED16	0.1470775	0.1826526	0.8052	0.421	0.172948911	count	1
CPNE8	0.1291538	0.1050776	1.2291	0.219	0.173007027	count	1
GCH1	0.7549867	0.6286829	1.2009	0.23	0.173035792	count	1
NAA15	0.1374607	0.1304052	1.0541	0.292	0.173078117	count	1
NXT1	0.1257676	0.0895149	1.405	0.16	0.173207087	count	1
AC018638.7	0.4045468	0.5915995	0.6838	0.494	0.173217597	count	1

SYT1	1.2391253	1.1875238	1.0435	0.297	0.173494662	count	1
WDR41	0.1317701	0.1416475	0.9303	0.352	0.173621435	count	1
GRAMD2B	0.1309107	0.1510111	0.8669	0.386	0.173697332	count	1
LPAR1	0.1359196	0.2419089	0.5619	0.574	0.173747448	count	1
ITFG1	0.12791	0.0951554	1.3442	0.179	0.174476663	count	1
RBM15	0.1880684	0.4132887	0.4551	0.649	0.174494818	count	1
SMG6	0.1287648	0.1313209	0.9805	0.327	0.174567248	count	1
NUMA1	0.127599	0.1029536	1.2394	0.215	0.174568653	count	1
MRPL9	0.1276529	0.0953287	1.3391	0.181	0.174642247	count	1
RTL5	0.1764709	0.3097695	0.5697	0.569	0.174656407	count	1
PCSK1N	1.252675	0.7167504	1.7477	0.0806	0.174675785	count	1
YWHAZ	0.1224765	0.044734	2.7379	0.00621	0.174694019	count	1
RFT1	0.1945406	0.2450519	0.7939	0.427	0.174702091	count	1
HAUS5	0.2475547	0.3228544	0.7668	0.443	0.174841149	count	1
KCNN3	1.2550742	0.9370972	1.3393	0.181	0.174883584	count	1
CPXM1	1.2550742	1.1255899	1.115	0.265	0.174883584	count	1
AC010997.4	1.2550742	1.284468	0.9771	0.329	0.174883584	count	1
THUMPD1	0.1254519	0.0761553	1.6473	0.0996	0.17498286	count	1
SNRK	0.1356715	0.2071945	0.6548	0.513	0.17521393	count	1
CWC22	0.1293416	0.0920779	1.4047	0.16	0.175242767	count	1
KDELR2	0.1230845	0.0489585	2.5141	0.012	0.175333612	count	1
WDR6	0.1428711	0.2024297	0.7058	0.48	0.175384229	count	1
NIT1	0.1352034	0.1297774	1.0418	0.298	0.175397528	count	1
DNAJC27-AS1	0.3082139	0.3397734	0.9071	0.364	0.175488273	count	1
CYBC1	0.131764	0.1154628	1.1412	0.254	0.175539793	count	1
MAPK8IP1	0.2596559	0.3269794	0.7941	0.427	0.175566841	count	1
RMND5A	0.1498254	0.2004447	0.7475	0.455	0.175583356	count	1
PRPF38B	0.1259117	0.1030145	1.2223	0.222	0.175653838	count	1
FAM174B	0.3336284	0.5095474	0.6548	0.513	0.175733941	count	1
NUP155	0.1552971	0.2454055	0.6328	0.527	0.175748381	count	1
BCL9	0.1818247	0.2801843	0.6489	0.516	0.175773189	count	1
LINC00092	0.2246346	0.3895104	0.5767	0.564	0.17599261	count	1
TRMT12	0.1778731	0.2570949	0.6919	0.489	0.176020687	count	1
GTF2E2	0.1371715	0.1608545	0.8528	0.394	0.176097567	count	1
ZNF225	0.1899536	0.4913898	0.3866	0.699	0.176207894	count	1
USP38	0.1962861	0.290537	0.6756	0.499	0.176234205	count	1
ZBTB25	0.1451466	0.2292766	0.6331	0.527	0.176281153	count	1
TOPORS	0.1296773	0.113854	1.139	0.255	0.176345542	count	1
FAM133A	0.1565788	0.4200983	0.3727	0.709	0.176375165	count	1
ACD	0.1416212	0.1599204	0.8856	0.376	0.176393519	count	1
CA11	0.1616895	0.2445192	0.6613	0.508	0.176406582	count	1
NFRKB	0.1449619	0.2089843	0.6936	0.488	0.176451363	count	1
PRDX3	0.1244599	0.0598642	2.079	0.0377	0.176456987	count	1
NFASC	0.1527455	0.2450751	0.6233	0.533	0.176475112	count	1
MRPL12	0.1291001	0.1052066	1.2271	0.22	0.176541475	count	1

ANGEL2	0.1415262	0.1529628	0.9252	0.355	0.176563077	count	1
EZH2	1.2750123	0.8561842	1.4892	0.137	0.176594962	count	1
AC006206.2	0.4133459	0.8895614	0.4647	0.642	0.176637174	count	1
AC008073.3	0.4133459	1.0824665	0.3819	0.703	0.176637174	count	1
DIMT1	0.1329413	0.1135906	1.1704	0.242	0.176841302	count	1
CREBRF	0.1266885	0.089131	1.4214	0.155	0.176956425	count	1
TMEM108	0.241218	0.3255402	0.741	0.459	0.176974201	count	1
AC107068.1	0.4801029	0.7433657	0.6459	0.518	0.177097813	count	1
UMPS	0.1465748	0.1854055	0.7906	0.429	0.177179257	count	1
GGA1	0.1449517	0.1916076	0.7565	0.449	0.177197574	count	1
GJA4	0.127615	0.1164791	1.0956	0.273	0.177208145	count	1
TARS	0.1318782	0.1401741	0.9408	0.347	0.177216023	count	1
FAM122C	0.1835485	0.4028266	0.4557	0.649	0.177408962	count	1
PJVK	0.4812007	0.5534454	0.8695	0.385	0.177456094	count	1
CERCAM	0.1294651	0.0855233	1.5138	0.13	0.177588563	count	1
APOBEC3H	0.5865114	0.9018653	0.6503	0.516	0.177651519	count	1
CDH5	0.4161484	0.477061	0.8723	0.383	0.177723077	count	1
MKRN1	0.1370079	0.1185977	1.1552	0.248	0.177728774	count	1
SYPL1	0.1255849	0.056588	2.2193	0.0265	0.177771792	count	1
B3GALT6	0.148997	0.1757836	0.8476	0.397	0.177797363	count	1
STX16	0.1390614	0.1880361	0.7395	0.46	0.177941509	count	1
PPP6R2	0.1572743	0.182287	0.8628	0.388	0.177963061	count	1
AC009318.2	1.2912672	1.1459325	1.1268	0.26	0.177969899	count	1
MAGT1	0.1313365	0.0969394	1.3548	0.176	0.177992539	count	1
AKR1A1	0.1259825	0.0576989	2.1834	0.0291	0.178034533	count	1
SRSF1	0.1319544	0.1132982	1.1647	0.244	0.178267966	count	1
RAB24	0.2157902	0.4264634	0.506	0.613	0.178303797	count	1
LSM6	0.1282832	0.0813532	1.5769	0.115	0.17830907	count	1
NUP62	0.1475501	0.1924546	0.7667	0.443	0.178349702	count	1
CTSO	0.1339207	0.1102077	1.2152	0.224	0.178404168	count	1
SNAPC1	0.1340315	0.1144233	1.1714	0.242	0.178464234	count	1
MVD	0.1530514	0.1916424	0.7986	0.425	0.178731894	count	1
NEIL2	0.1620548	0.18899	0.8575	0.391	0.178827258	count	1
DPH6-DT	0.4191788	0.6987682	0.5999	0.549	0.178895544	count	1
TXNRD2	0.1460503	0.1985804	0.7355	0.462	0.178900626	count	1
AC092171.5	0.4857122	0.7234592	0.6714	0.502	0.178926209	count	1
CERS3-AS1	0.4857122	0.8960433	0.5421	0.588	0.178926209	count	1
CNPY4	0.1347026	0.1143746	1.1777	0.239	0.178994352	count	1
SLC52A2	0.1359018	0.1347808	1.0083	0.313	0.179046146	count	1
ACBD3	0.1271321	0.0792975	1.6032	0.109	0.179097397	count	1
TBCE	0.2538685	0.5192174	0.4889	0.625	0.179122044	count	1
TCIRG1	0.1431453	0.1532062	0.9343	0.35	0.179134828	count	1
RNASEK	0.1463033	0.1788393	0.8181	0.413	0.17920847	count	1
DLG4	0.1933079	0.4818813	0.4012	0.688	0.179254052	count	1
SCAI	0.1687832	0.2879001	0.5863	0.558	0.179318492	count	1

IQCG	0.1776143	0.248426	0.715	0.475	0.179401918	count	1
GSTCD	0.279059	0.3276289	0.8518	0.394	0.179406807	count	1
ZNF365	0.1881615	0.4414106	0.4263	0.67	0.179504164	count	1
APOBEC3F	0.2122709	0.4184675	0.5073	0.612	0.179507181	count	1
LARP6	0.1282183	0.0785572	1.6322	0.103	0.179515921	count	1
UBR4	0.1398734	0.1415114	0.9884	0.323	0.179550136	count	1
HNRNPH2	0.132659	0.1033086	1.2841	0.199	0.179561663	count	1
LINC00685	0.1533084	0.2927239	0.5237	0.6	0.179629726	count	1
AC016876.1	0.1489953	0.1825807	0.8161	0.415	0.179648981	count	1
NUDT15	0.1420637	0.1642989	0.8647	0.387	0.179817008	count	1
DCLRE1A	0.4887958	0.4569628	1.0697	0.285	0.179928918	count	1
HSD17B8	0.1521274	0.2041293	0.7453	0.456	0.179947059	count	1
AC092279.1	0.4889095	0.5405695	0.9044	0.366	0.179965859	count	1
C11orf80	0.4221363	0.4398142	0.9598	0.337	0.18003804	count	1
PIK3CB	0.1542264	0.2880515	0.5354	0.592	0.180091869	count	1
TAF1A-AS1	0.3763383	0.6552146	0.5744	0.566	0.180159983	count	1
PHTF1	0.1616222	0.2215445	0.7295	0.466	0.180237862	count	1
NPAS2	0.4228661	0.452253	0.935	0.35	0.180319697	count	1
CARMIL1	0.1360748	0.1122409	1.2123	0.225	0.180330819	count	1
CLN6	0.4229162	0.6610419	0.6398	0.522	0.180339031	count	1
MTFR1L	0.1323825	0.0988772	1.3389	0.181	0.180342546	count	1
11-Sep	0.1273507	0.063548	2.004	0.0451	0.180347396	count	1
FAM69B	1.322376	0.7430719	1.7796	0.0752	0.180551105	count	1
NIT2	0.1310237	0.0995284	1.3164	0.188	0.180636729	count	1
ACTR8	0.1518872	0.2609175	0.5821	0.561	0.180714801	count	1
CCDC137	0.1402186	0.1603805	0.8743	0.382	0.180715546	count	1
NDUFAF4	0.1361073	0.134985	1.0083	0.313	0.180854904	count	1
YLPM1	0.1479969	0.2289559	0.6464	0.518	0.180894856	count	1
MAP2K3	0.1416134	0.1851442	0.7649	0.444	0.180990576	count	1
RNF135	0.1516996	0.1831787	0.8282	0.408	0.180996844	count	1
TBRG1	0.1320193	0.0992925	1.3296	0.184	0.181014338	count	1
VPS33B	0.378584	0.4298635	0.8807	0.379	0.181148886	count	1
ZNF30	0.6003822	0.5884194	1.0203	0.308	0.181202786	count	1
SPRTN	0.1568918	0.185992	0.8435	0.399	0.181220342	count	1
CDK16	0.155224	0.2075638	0.7478	0.455	0.181246365	count	1
ZNF471	0.1652223	0.3523528	0.4689	0.639	0.181266427	count	1
MIF	0.1274621	0.0498548	2.5567	0.0106	0.181273691	count	1
KRIT1	0.1309623	0.1123639	1.1655	0.244	0.181302595	count	1
DENND4B	0.2145114	0.3526697	0.6083	0.543	0.181350311	count	1
EIF1B	0.1270698	0.043548	2.9179	0.003545	0.181352215	count	1
SEC24B	0.1475083	0.1901431	0.7758	0.438	0.181395237	count	1
ADAT2	0.2254806	0.362921	0.6213	0.534	0.181509012	count	1
NPM2	0.2476884	0.4914298	0.504	0.614	0.181543052	count	1
ZC2HC1A	0.1318831	0.0855371	1.5418	0.123	0.181614224	count	1
THSD1	0.2690228	0.4449062	0.6047	0.545	0.181619891	count	1

NUDT7	0.1391129	0.1733008	0.8027	0.422	0.181620518	count	1
PATL1	0.1675664	0.2859451	0.586	0.558	0.181631196	count	1
ZNF200	0.1858367	0.3779879	0.4916	0.623	0.181720988	count	1
KDM5D	0.145461	0.2551123	0.5702	0.569	0.18173232	count	1
C21orf58	0.2828826	0.4986732	0.5673	0.571	0.181745086	count	1
GCN1	0.180094	0.2685099	0.6707	0.502	0.181865454	count	1
PRDM10	0.6030212	0.6218401	0.9697	0.332	0.181875002	count	1
AC068870.2	0.6030212	0.7244465	0.8324	0.405	0.181875002	count	1
TYK2	0.2260902	0.3505038	0.645	0.519	0.181984653	count	1
AC079315.1	1.3415695	1.1554045	1.1611	0.246	0.182111202	count	1
CADM3-AS1	1.3415695	1.2145885	1.1045	0.269	0.182111202	count	1
UBA7	0.1642101	0.250859	0.6546	0.513	0.182151249	count	1
IFT57	0.1303752	0.0694395	1.8775	0.0605	0.182213912	count	1
LINC01554	0.4958979	1.0091784	0.4914	0.623	0.182231785	count	1
LRRC37A2	0.4280116	0.3073393	1.3926	0.164	0.182302551	count	1
GPN1	0.1492055	0.1970576	0.7572	0.449	0.182361951	count	1
MISP3	0.1718726	0.2876521	0.5975	0.55	0.182555607	count	1
MAPKAPK5	0.1494125	0.1818886	0.8215	0.411	0.182613205	count	1
CLU	0.126701	0.0568155	2.23	0.0258	0.182640163	count	1
DTX3	0.1401302	0.1601701	0.8749	0.382	0.182660982	count	1
SLC26A10	0.4289829	0.8630915	0.497	0.619	0.182676264	count	1
WWC3	0.1410579	0.1526322	0.9242	0.355	0.182800942	count	1
ATG4D	0.1709094	0.252225	0.6776	0.498	0.182814648	count	1
AL139274.2	0.1709284	0.6034509	0.2833	0.777	0.1828347	count	1
EIF4G1	0.1341311	0.1254573	1.0691	0.285	0.182987793	count	1
C7orf25	0.3485833	0.6112547	0.5703	0.569	0.183063012	count	1
ZFP2	0.3485833	0.6221139	0.5603	0.575	0.183063012	count	1
SRF	0.1518541	0.1704213	0.8911	0.373	0.183069977	count	1
TGFBR1	0.1347192	0.1438818	0.9363	0.349	0.183234749	count	1
EXOC2	0.1834291	0.2576424	0.712	0.477	0.183343061	count	1
LTBP3	0.1297744	0.0654444	1.983	0.0474	0.183388854	count	1
OPHN1	0.1726736	0.2254249	0.766	0.444	0.183394623	count	1
PPARD	0.1835262	0.2984232	0.615	0.539	0.183438453	count	1
PCID2	0.1349933	0.0961005	1.4047	0.16	0.183458804	count	1
BRCA1	0.2083148	0.4184102	0.4979	0.619	0.183469986	count	1
ZSWIM6	0.171547	0.3252253	0.5275	0.598	0.183487471	count	1
SAT2	0.1303316	0.06902	1.8883	0.0591	0.18353591	count	1
DCLK2	0.1415257	0.2495747	0.5671	0.571	0.18356415	count	1
C6orf89	0.1332911	0.0950449	1.4024	0.161	0.18366942	count	1
WDR91	0.1952915	0.3388732	0.5763	0.564	0.183678176	count	1
TIGD4	0.2724904	0.5992942	0.4547	0.649	0.18385547	count	1
KIF2A	0.133206	0.099751	1.3354	0.182	0.184026592	count	1
CWF19L2	0.1331732	0.1049541	1.2689	0.205	0.184111033	count	1
MAP1S	0.1535875	0.2587409	0.5936	0.553	0.18421196	count	1
MTA2	0.1807278	0.2679298	0.6745	0.5	0.184221476	count	1

PYCARD	0.1364204	0.1245057	1.0957	0.273	0.184224642	count	1
KRR1	0.1314865	0.0694109	1.8943	0.0583	0.184351609	count	1
RETREG2	0.1378282	0.1269214	1.0859	0.278	0.184434982	count	1
GOLGA1	0.1533804	0.1777612	0.8628	0.388	0.184436631	count	1
SLC3A2	0.1317536	0.075027	1.7561	0.0792	0.184459836	count	1
PHF3	0.1315826	0.0750697	1.7528	0.0797	0.184463104	count	1
UBA1	0.1434365	0.1314515	1.0912	0.275	0.18447904	count	1
TRABD	0.1468456	0.147672	0.9944	0.32	0.184563722	count	1
PAK1IP1	0.136466	0.1035103	1.3184	0.187	0.184701586	count	1
TRAK1	0.1868331	0.2366906	0.7894	0.43	0.184729025	count	1
SLC25A51	0.2139773	0.3316162	0.6453	0.519	0.18473124	count	1
CYB5B	0.1365414	0.1298213	1.0518	0.293	0.184803377	count	1
BORCS6	0.1486718	0.1599613	0.9294	0.353	0.184812921	count	1
Z97989.1	0.5045933	0.6085604	0.8292	0.407	0.185038854	count	1
TTC7B	0.1871615	0.3130792	0.5978	0.55	0.185047896	count	1
RBM48	0.146868	0.1682643	0.8728	0.383	0.185115692	count	1
POMK	0.2627362	0.5906478	0.4448	0.656	0.185118666	count	1
SAYSD1	0.1489281	0.1704542	0.8737	0.382	0.18512954	count	1
AC007541.1	0.1968798	0.2706205	0.7275	0.467	0.185140732	count	1
MICB	0.6165337	0.5344937	1.1535	0.249	0.185300048	count	1
APOO	0.1874584	0.2889344	0.6488	0.517	0.185336152	count	1
PGAP2	0.1662659	0.2489738	0.6678	0.504	0.185357155	count	1
GADD45G	0.1366061	0.1241091	1.1007	0.271	0.185386844	count	1
PSMD12	0.1327221	0.0724653	1.8315	0.0671	0.185432179	count	1
NUDT14	0.1612281	0.2955228	0.5456	0.585	0.185468689	count	1
AC027277.1	0.2035975	0.3108343	0.655	0.513	0.185695483	count	1
SF3B4	0.1336369	0.0879271	1.5199	0.129	0.185778789	count	1
LEFTY1	0.8280816	1.1212265	0.7385	0.46	0.185942834	count	1
LINC01220	0.8280816	1.1212265	0.7385	0.46	0.185942834	count	1
AL136369.2	0.8280816	1.235648	0.6702	0.503	0.185942834	count	1
AC093388.1	0.8280816	1.410754	0.587	0.557	0.185942834	count	1
PITPNA	0.1432729	0.1378336	1.0395	0.299	0.185979621	count	1
CHTF8	0.1471554	0.1581993	0.9302	0.352	0.185981159	count	1
VIRMA	0.1388902	0.1236637	1.1231	0.261	0.186010397	count	1
DDHD2	0.1486869	0.1574543	0.9443	0.345	0.186028426	count	1
ANKS3	0.1483316	0.1738637	0.8531	0.394	0.186148095	count	1
AP003392.4	0.390105	0.5074968	0.7687	0.442	0.186205367	count	1
ZNF429	0.1566175	0.1843381	0.8496	0.396	0.186296137	count	1
MTA3	0.1464725	0.1865916	0.785	0.433	0.186300752	count	1
GABRD	0.5085595	0.8069674	0.6302	0.529	0.186314682	count	1
ZNF805	0.1864962	0.2274076	0.8201	0.412	0.186355162	count	1
IRAK1	0.1672449	0.187249	0.8932	0.372	0.186435965	count	1
HIST1H4E	0.2647312	0.430743	0.6146	0.539	0.186465177	count	1
TTYH1	0.3555686	0.6601754	0.5386	0.59	0.186469448	count	1
ZBTB11-AS1	0.1983284	0.3096014	0.6406	0.522	0.186474176	count	1

CHIC2	0.1385935	0.1200995	1.154	0.249	0.186495648	count	1
ABR	0.15174	0.1769182	0.8577	0.391	0.186563848	count	1
IZUMO4	0.5094556	0.3678276	1.385	0.166	0.186602553	count	1
AC137630.3	0.832253	1.080506	0.7702	0.441	0.186659928	count	1
SMC1A	0.1377351	0.1057578	1.3024	0.193	0.186753022	count	1
FBXL20	0.1659389	0.2032017	0.8166	0.414	0.186802461	count	1
SRSF3	0.1311852	0.0464324	2.8253	0.00475	0.186979566	count	1
SCAMP1	0.1354654	0.0921329	1.4703	0.142	0.187088595	count	1
ELAVL1	0.137288	0.1142423	1.2017	0.23	0.187194738	count	1
ZNF268	0.2169225	0.3346486	0.6482	0.517	0.187205837	count	1
EMSY	0.1565214	0.263138	0.5948	0.552	0.187209203	count	1
ZNF490	0.3579578	0.6200024	0.5773	0.564	0.187632078	count	1
RPS6KA3	0.1486948	0.1949044	0.7629	0.446	0.187662943	count	1
ATXN10	0.1350651	0.0795696	1.6974	0.0897	0.187780947	count	1
ATXN7L3B	0.1348303	0.084024	1.6047	0.109	0.187808064	count	1
GOLGA5	0.1415232	0.1254553	1.1281	0.259	0.187830388	count	1
BROX	0.1381812	0.1028525	1.3435	0.179	0.187882211	count	1
APH1B	0.145682	0.1446284	1.0073	0.314	0.187904618	count	1
TMCO1	0.1326501	0.0574943	2.3072	0.0211	0.187909938	count	1
TCTE3	0.2480184	0.5218544	0.4753	0.635	0.18793217	count	1
MRTO4	0.13952	0.1420806	0.982	0.326	0.188010916	count	1
FES	0.168678	0.2191133	0.7698	0.441	0.188014894	count	1
RTN4RL1	0.5142936	0.723495	0.7108	0.477	0.188154192	count	1
ATP6V0E2	0.147703	0.1461749	1.0105	0.312	0.188303442	count	1
METTL9	0.1360184	0.0835177	1.6286	0.103	0.188387427	count	1
CBR1	0.1330924	0.054678	2.4341	0.015	0.188391035	count	1
ANKRD27	0.184907	0.3609758	0.5122	0.609	0.188410956	count	1
SERINC3	0.1340531	0.0739055	1.8138	0.0698	0.188426947	count	1
UBE2A	0.1353424	0.0874242	1.5481	0.122	0.188484739	count	1
PPA1	0.1331384	0.0505605	2.6333	0.00849	0.188522592	count	1
PTH1R	0.1868576	0.4311361	0.4334	0.665	0.188578801	count	1
ACSM1	0.6296761	0.8032902	0.7839	0.433	0.188603917	count	1
ST7L	0.1472856	0.1432486	1.0282	0.304	0.188617676	count	1
SPTLC2	0.1586878	0.1963028	0.8084	0.419	0.188737955	count	1
SF3B1	0.1333186	0.0604837	2.2042	0.0276	0.188782584	count	1
ZNF688	0.1413009	0.1078962	1.3096	0.19	0.188818631	count	1
HSPA13	0.14719	0.1449057	1.0158	0.31	0.188896161	count	1
ZFYVE21	0.133378	0.0621935	2.1446	0.0321	0.18892097	count	1
NEO1	0.1406167	0.1309089	1.0742	0.283	0.18899714	count	1
TOM1	0.1547321	0.1730944	0.8939	0.371	0.189068323	count	1
MFN1	0.14474	0.1283331	1.1278	0.259	0.18907937	count	1
RAD54L2	0.176864	0.3537971	0.4999	0.617	0.18909538	count	1
COL25A1	0.8465201	0.7339681	1.1533	0.249	0.18909678	count	1
SLC25A24	0.1385918	0.117079	1.1837	0.237	0.189193222	count	1
MAOA	0.1408261	0.0935034	1.5061	0.132	0.189205098	count	1

SETMAR	0.1698001	0.2347492	0.7233	0.47	0.189250938	count	1
POU2F2	0.2294835	0.4530728	0.5065	0.613	0.189277091	count	1
AC239868.2	0.2586937	0.4983935	0.5191	0.604	0.189291349	count	1
CHORDC1	0.1395166	0.1142302	1.2214	0.222	0.189326789	count	1
AF165147.1	1.436928	0.9216808	1.559	0.119	0.189506298	count	1
RBM43	0.138083	0.0985646	1.4009	0.161	0.189566825	count	1
MT2A	0.1319903	0.0655445	2.0138	0.0441	0.189593371	count	1
LURAP1L	0.144927	0.1317772	1.0998	0.271	0.189602622	count	1
DUSP11	0.1413585	0.1186947	1.1909	0.234	0.189618683	count	1
FO704657.1	0.3982478	0.9711311	0.4101	0.682	0.189762228	count	1
DOCK11	0.147175	0.1386068	1.0618	0.288	0.189821244	count	1
XRCC1	0.1605663	0.1708549	0.9398	0.347	0.189841718	count	1
FAM104B	0.1426195	0.1361	1.0479	0.295	0.189954195	count	1
GRPEL2	0.259717	0.3778947	0.6873	0.492	0.190010344	count	1
CPT2	0.1818	0.2590451	0.7018	0.483	0.190076198	count	1
RRN3	0.1681001	0.2240972	0.7501	0.453	0.190078195	count	1
AL133467.1	0.399056	0.5215115	0.7652	0.444	0.190114483	count	1
AC011468.5	0.399056	0.5225773	0.7636	0.445	0.190114483	count	1
MAN2A1	0.1502999	0.2179495	0.6896	0.49	0.19018388	count	1
ZNF616	0.2367521	0.3268821	0.7243	0.469	0.190289582	count	1
ARFGEF2	0.1587271	0.1927986	0.8233	0.41	0.190326337	count	1
AC025283.2	0.363839	0.5259601	0.6918	0.489	0.190488604	count	1
HDAC3	0.1426016	0.1247444	1.1432	0.253	0.190551216	count	1
KIF27	0.1809546	0.3182092	0.5687	0.57	0.190662732	count	1
PPP1R37	0.1679109	0.3350693	0.5011	0.616	0.190707809	count	1
AL117336.3	0.2373125	0.4856494	0.4886	0.625	0.190725359	count	1
AC084036.1	0.8573943	0.6766712	1.2671	0.205	0.190937785	count	1
TMEM221	0.8573943	0.6804662	1.26	0.208	0.190937785	count	1
RIDA	0.1674127	0.2073235	0.8075	0.419	0.190960208	count	1
GSTO2	0.173304	0.3019876	0.5739	0.566	0.191087016	count	1
CUL1	0.1426081	0.1157249	1.2323	0.218	0.191133745	count	1
WASF2	0.1338922	0.042096	3.1806	0.0015	0.191217663	count	1
NUDT21	0.1414003	0.1107507	1.2767	0.202	0.191242094	count	1
CLCN2	0.640399	0.4827589	1.3265	0.185	0.191279682	count	1
GPATCH4	0.1437608	0.1530208	0.9395	0.348	0.191280834	count	1
BMPR1A	0.1475112	0.1477154	0.9986	0.318	0.19129261	count	1
ADAMTS7	0.1816917	0.3665878	0.4956	0.62	0.191427752	count	1
SNAI3-AS1	0.4519139	0.849873	0.5317	0.595	0.191444436	count	1
PRTG	1.4647812	1.1896019	1.2313	0.218	0.191557858	count	1
TM7SF2	0.203947	0.3246697	0.6282	0.53	0.191641839	count	1
ANO10	0.1492391	0.1838957	0.8115	0.417	0.191710711	count	1
PPP2CA	0.1412726	0.097789	1.4447	0.149	0.191758183	count	1
TMEM50A	0.134119	0.039047	3.4348	6.00E-04	0.191906297	count	1
ZFPM2	0.145618	0.1588914	0.9165	0.359	0.191918208	count	1
ANO3	1.470627	1.20471	1.2207	0.222	0.191982536	count	1

CCDC125	0.1706332	0.2405488	0.7093	0.478	0.192026687	count	1
CENPF	0.5266323	0.5706322	0.9229	0.356	0.192092257	count	1
FUNDC1	0.1465848	0.1655694	0.8853	0.376	0.192172701	count	1
CACNB3	0.1825349	0.2921893	0.6247	0.532	0.192302771	count	1
KMT2D	0.2077189	0.3472913	0.5981	0.55	0.192313551	count	1
ARL1	0.1355789	0.0590928	2.2943	0.0218	0.192420117	count	1
ZMYND12	0.8668685	0.7796392	1.1119	0.266	0.192530321	count	1
LINC00857	0.8668685	0.830423	1.0439	0.297	0.192530321	count	1
SPOCK3	0.8668685	1.362241	0.6364	0.525	0.192530321	count	1
HIST2H2BF	0.2740334	0.3754254	0.7299	0.465	0.192731156	count	1
PIGQ	0.1578421	0.2099547	0.7518	0.452	0.192840588	count	1
IL17RA	0.173991	0.2113794	0.8231	0.41	0.192868026	count	1
SNRNP48	0.1485091	0.1714986	0.8659	0.387	0.192908702	count	1
KIAA0753	0.1817748	0.2841689	0.6397	0.522	0.192919553	count	1
ZNF652	0.1420651	0.1032771	1.3756	0.169	0.192939342	count	1
USP5	0.1723271	0.2075411	0.8303	0.406	0.192988191	count	1
TOMM40	0.1453552	0.1199668	1.2116	0.226	0.193000075	count	1
DHX30	0.1534518	0.1553328	0.9879	0.323	0.193093833	count	1
KRAS	0.1381206	0.0888392	1.5547	0.12	0.193112956	count	1
DNAJB4	0.1380087	0.0820485	1.682	0.0926	0.193125465	count	1
FAM92A	0.1394181	0.0914553	1.5244	0.127	0.193136893	count	1
TMEM140	0.1478699	0.1276383	1.1585	0.247	0.193150773	count	1
RASA2	0.150264	0.1677467	0.8958	0.37	0.193216795	count	1
BRF1	0.1978922	0.2925402	0.6765	0.499	0.193279541	count	1
HERC6	0.1834828	0.3734941	0.4913	0.623	0.19328629	count	1
KLF7	0.1585518	0.1467409	1.0805	0.28	0.193290117	count	1
RAP1B	0.135932	0.0483921	2.809	0.005	0.193403499	count	1
CCDC138	0.3700159	0.4461906	0.8293	0.407	0.1934805	count	1
ZSCAN26	0.154742	0.1715498	0.902	0.367	0.193557026	count	1
PARP8	0.1610448	0.2561374	0.6287	0.53	0.193577889	count	1
FIP1L1	0.1437878	0.1054632	1.3634	0.173	0.193606586	count	1
ZNF202	0.3024824	0.6705298	0.4511	0.652	0.193677179	count	1
LY6E	0.1356448	0.0596005	2.2759	0.0229	0.193715032	count	1
ZNF101	0.6502838	0.5089453	1.2777	0.201	0.193730441	count	1
SLFN12	0.1549332	0.1882779	0.8229	0.411	0.193794689	count	1
NIPBL	0.1407307	0.0987003	1.4258	0.154	0.193806592	count	1
SMAD5	0.1413164	0.092046	1.5353	0.125	0.19384868	count	1
ARFIP1	0.1427055	0.0931483	1.532	0.126	0.19386049	count	1
APOBEC3C	0.1439483	0.122284	1.1772	0.239	0.193891964	count	1
ZBTB3	0.3431995	0.5232664	0.6559	0.512	0.194108701	count	1
KTN1-AS1	0.5330956	0.61195	0.8711	0.384	0.19414413	count	1
LINC00663	0.5330956	0.6834497	0.78	0.435	0.19414413	count	1
MINDY3	0.1593129	0.1565573	1.0176	0.309	0.194211008	count	1
BLZF1	0.1433867	0.1302099	1.1012	0.271	0.194280601	count	1
MTMR1	0.276463	0.3482967	0.7938	0.427	0.19436434	count	1

DGKB	1.504616	0.6199273	2.4271	0.0153	0.194409798	count	1
PYCR2	0.1434018	0.1096199	1.3082	0.191	0.194416367	count	1
KANSL1	0.1415833	0.1013359	1.3972	0.162	0.194680248	count	1
ZNF286B	0.8798103	0.9737145	0.9036	0.366	0.194688574	count	1
TMEM263	0.1411279	0.086939	1.6233	0.105	0.194808806	count	1
ADAP2	0.2771246	0.3695315	0.7499	0.453	0.194808836	count	1
SLC27A4	0.2259865	0.3466875	0.6518	0.515	0.194809126	count	1
NLGN4X	0.4608044	0.4453203	1.0348	0.301	0.194815752	count	1
PTTG1	0.1895744	0.41586	0.4559	0.649	0.194820505	count	1
MED17	0.1836385	0.4294023	0.4277	0.669	0.194868151	count	1
PARP11	0.1913766	0.2732291	0.7004	0.484	0.194889743	count	1
AC026401.1	1.516333	1.477126	1.0265	0.305	0.195230487	count	1
METTL25	0.1662082	0.153138	1.0853	0.278	0.195235087	count	1
ABHD8	0.1647738	0.2159728	0.7629	0.446	0.195349723	count	1
APPL1	0.1383689	0.0653104	2.1186	0.0342	0.195425108	count	1
SOST	0.1365578	0.1454901	0.9386	0.348	0.195618538	count	1
LINC01686	0.3057572	0.5523397	0.5536	0.58	0.195661993	count	1
EPHA2	0.2149312	0.3561922	0.6034	0.546	0.195781337	count	1
MYDGF	0.137466	0.0487697	2.8187	0.00485	0.195849873	count	1
PYURF	0.1379575	0.0498533	2.7673	0.00568	0.19587192	count	1
UBE2K	0.1432559	0.0933525	1.5346	0.125	0.195941029	count	1
DICER1-AS1	0.2590573	0.3643065	0.7111	0.477	0.19597719	count	1
PSTPIP2	0.5391356	0.3801726	1.4181	0.156	0.19605479	count	1
NRG2	0.2192377	0.413202	0.5306	0.596	0.19631734	count	1
ATG4A	0.1656345	0.285697	0.5798	0.562	0.196360957	count	1
HIST2H2AB	1.5332787	1.0166779	1.5081	0.132	0.196403143	count	1
TOR1AIP1	0.1445309	0.1144039	1.2633	0.207	0.196441123	count	1
ZNF219	0.1896861	0.2318686	0.8181	0.413	0.196595804	count	1
MUT	0.1552043	0.143993	1.0779	0.281	0.196608442	count	1
COPS7A	0.1422012	0.0972846	1.4617	0.144	0.196730642	count	1
SMARCA2	0.1435024	0.0913062	1.5717	0.116	0.196802599	count	1
ZNF280D	0.1482162	0.1188789	1.2468	0.213	0.197089301	count	1
AC026471.1	0.2288907	0.3352534	0.6827	0.495	0.197241316	count	1
ZDHHC6	0.1616308	0.1632289	0.9902	0.322	0.197434486	count	1
PLEKHH2	0.1452715	0.1392271	1.0434	0.297	0.197445153	count	1
ZNF230	0.204762	0.3026532	0.6766	0.499	0.197488565	count	1
PRDM4	0.2169638	0.3250158	0.6675	0.504	0.197587154	count	1
NFX1	0.1568369	0.1701685	0.9217	0.357	0.197605147	count	1
APOB	0.2170233	0.4700863	0.4617	0.644	0.197639998	count	1
LCOR	0.1534213	0.1549455	0.9902	0.322	0.197646983	count	1
NMT1	0.1457586	0.1122655	1.2983	0.194	0.197660938	count	1
CASZ1	0.2401958	0.3564505	0.6739	0.5	0.197831182	count	1
AMPH	0.2138996	0.3853795	0.555	0.579	0.197900773	count	1
RAB11FIP2	0.1493995	0.1224043	1.2205	0.222	0.197924131	count	1
GCC2	0.1407981	0.0720057	1.9554	0.0506	0.197948207	count	1

IARS	0.1559487	0.1610144	0.9685	0.333	0.198043892	count	1
ALDH1L1-AS1	0.9002497	1.0965845	0.821	0.412	0.198056974	count	1
UTS2B	0.9002497	1.0965845	0.821	0.412	0.198056974	count	1
AL031708.1	0.9002497	1.099609	0.8187	0.413	0.198056974	count	1
AC007881.3	0.9002497	1.1077486	0.8127	0.416	0.198056974	count	1
AC007950.2	0.9002497	1.1077486	0.8127	0.416	0.198056974	count	1
AC110792.3	0.9002497	1.212498	0.7425	0.458	0.198056974	count	1
TGOLN2	0.1394509	0.0542618	2.57	0.0102	0.198196758	count	1
KLHDC2	0.1418975	0.0908657	1.5616	0.118	0.1983567	count	1
ANAPC1	0.1856864	0.2246043	0.8267	0.408	0.198389196	count	1
LINC00882	0.1810464	0.2795387	0.6477	0.517	0.198397513	count	1
HUS1	0.1597394	0.1916844	0.8333	0.405	0.198478617	count	1
MAP3K3	0.1531166	0.2124691	0.7207	0.471	0.198527515	count	1
WAPL	0.1532345	0.1316716	1.1638	0.245	0.198679658	count	1
HNRNPA0	0.1404655	0.0553147	2.5394	0.0111	0.198705779	count	1
APIP	0.1468581	0.103317	1.4214	0.155	0.198728209	count	1
RPAP2	0.1482463	0.1400241	1.0587	0.29	0.198828834	count	1
NR1D2	0.1434449	0.0976273	1.4693	0.142	0.199017812	count	1
ATXN7L2	0.9061955	0.5512421	1.6439	0.1	0.199027653	count	1
CACNA2D3	0.5486378	0.6971448	0.787	0.431	0.199047279	count	1
AL080250.1	0.6719999	0.7957461	0.8445	0.398	0.199061352	count	1
ZNF285	0.6719999	0.7985917	0.8415	0.4	0.199061352	count	1
AP007216.2	0.6719999	0.8547756	0.7862	0.432	0.199061352	count	1
HIST1H2BD	0.6719999	0.858391	0.7829	0.434	0.199061352	count	1
AL158151.1	0.6719999	0.9043275	0.7431	0.457	0.199061352	count	1
MRE11	0.1578082	0.1536498	1.0271	0.304	0.199095625	count	1
MAATS1	0.2189028	0.3488306	0.6275	0.53	0.199308949	count	1
CYREN	0.1625407	0.1862204	0.8728	0.383	0.19935247	count	1
TPRA1	0.1542438	0.1294983	1.1911	0.234	0.19945041	count	1
STX1B	0.6738472	1.054313	0.6391	0.523	0.199511464	count	1
Z98752.4	0.6738472	1.139268	0.5915	0.554	0.199511464	count	1
LTK	0.6738472	1.194952	0.5639	0.573	0.199511464	count	1
TTC13	0.3122371	0.4088372	0.7637	0.445	0.199581873	count	1
MTF1	0.1927091	0.2864197	0.6728	0.501	0.199676726	count	1
RAB10	0.1429964	0.0735556	1.9441	0.052	0.199751489	count	1
CALCOCO1	0.1460768	0.1117672	1.307	0.191	0.199790485	count	1
LCA5	0.1549916	0.1302889	1.1896	0.234	0.199852505	count	1
PAFAH1B1	0.1417352	0.0683823	2.0727	0.0383	0.2001752	count	1
GDPGP1	0.2649712	0.3746445	0.7073	0.479	0.200275222	count	1
PPIL6	0.2568371	0.3832429	0.6702	0.503	0.200305939	count	1
CLPX	0.1532812	0.1319084	1.162	0.245	0.2003374	count	1
S100PBP	0.1659382	0.2141601	0.7748	0.438	0.200394376	count	1
ZBTB17	0.2054198	0.3165023	0.649	0.516	0.200481589	count	1
SNHG15	0.1496961	0.1162392	1.2878	0.198	0.200519547	count	1
CASP4	0.1451059	0.0910781	1.5932	0.111	0.200605474	count	1

CNP	0.1542229	0.1163733	1.3252	0.185	0.200626912	count	1
HLX-AS1	0.9163396	0.4281345	2.1403	0.0324	0.200674184	count	1
PLA2G15	0.2242471	0.2890356	0.7758	0.438	0.200685045	count	1
TMPO	0.1480414	0.119801	1.2357	0.217	0.200748249	count	1
SPACA9	0.1676479	0.2149187	0.7801	0.435	0.200930776	count	1
EID2B	0.165342	0.2547178	0.6491	0.516	0.201063825	count	1
DELE1	0.1696464	0.1865463	0.9094	0.363	0.201073173	count	1
STBD1	0.555332	0.4694198	1.183	0.237	0.20114567	count	1
GASAL1	0.2995272	0.4351546	0.6883	0.491	0.201188353	count	1
SRGAP3	0.9195633	0.8876855	1.0359	0.3	0.201194929	count	1
DPP8	0.159066	0.1453504	1.0944	0.274	0.201210201	count	1
TGS1	0.1497509	0.1034338	1.4478	0.148	0.201235899	count	1
NEXMIF	0.2998142	0.4140672	0.7241	0.469	0.201371405	count	1
CDC27	0.148627	0.0881446	1.6862	0.0918	0.201540183	count	1
ATP6V1E1	0.1431167	0.0732291	1.9544	0.0507	0.201653443	count	1
RNF14	0.156105	0.151583	1.0298	0.303	0.20166011	count	1
SUV39H1	0.4789962	0.6052624	0.7914	0.429	0.201665036	count	1
HPGD	0.4789962	0.6305637	0.7596	0.448	0.201665036	count	1
ARRDC1	0.1706991	0.2171149	0.7862	0.432	0.201709406	count	1
KPTN	0.2342992	0.4653272	0.5035	0.615	0.201765652	count	1
ACSM3	0.4259892	0.5510766	0.773	0.44	0.201774145	count	1
FRAT1	0.4794813	0.450727	1.0638	0.287	0.201846773	count	1
TSNAX	0.1496613	0.0934723	1.6011	0.109	0.20198561	count	1
C9orf85	0.1563824	0.1405107	1.113	0.266	0.202016675	count	1
C1GALT1	0.148081	0.1113122	1.3303	0.183	0.202115737	count	1
NUDT5	0.1481566	0.1069608	1.3851	0.166	0.202171276	count	1
CLPTM1L	0.1571569	0.1194235	1.316	0.188	0.202235988	count	1
USP21	0.2349117	0.5454427	0.4307	0.667	0.202277597	count	1
AL355596.1	0.2677678	0.5055773	0.5296	0.596	0.202304784	count	1
ZXDA	0.2885497	0.3904278	0.7391	0.46	0.202468043	count	1
RNF10	0.1507468	0.1461921	1.0312	0.303	0.202491994	count	1
AC091271.1	0.1606038	0.2231803	0.7196	0.472	0.202600959	count	1
TAF5L	0.2265847	0.2944054	0.7696	0.442	0.20272128	count	1
ZFC3H1	0.1476664	0.1174463	1.2573	0.209	0.202807989	count	1
PXDC1	0.1432605	0.071446	2.0052	0.045	0.202918252	count	1
AL645933.2	0.2603449	0.3420574	0.7611	0.447	0.202939554	count	1
BCLAF1	0.1445389	0.0686981	2.104	0.0354	0.203063689	count	1
CAAP1	0.1544598	0.1546777	0.9986	0.318	0.203135669	count	1
LINC01990	0.3606781	0.5322481	0.6777	0.498	0.20330655	count	1
C2orf88	0.3374581	0.4822082	0.6998	0.484	0.203402766	count	1
PGPEP1	0.1608732	0.1984924	0.8105	0.418	0.203482386	count	1
VKORC1L1	0.1606914	0.1425721	1.1271	0.26	0.203517823	count	1
CCPG1	0.1424773	0.0435802	3.2693	0.0011	0.203539632	count	1
SHISA5	0.1458413	0.0706201	2.0652	0.039	0.203554549	count	1
UFL1	0.1455579	0.069508	2.0941	0.0363	0.203602466	count	1

ZNF414	0.1751843	0.1886565	0.9286	0.353	0.203606552	count	1
AL122035.2	0.4842345	0.838685	0.5774	0.564	0.203625025	count	1
THAP12	0.1507344	0.1153802	1.3064	0.191	0.203632795	count	1
AC026979.2	0.2696638	0.4598354	0.5864	0.558	0.203679689	count	1
CCDC17	0.4845517	0.6061656	0.7994	0.424	0.203743533	count	1
OPA3	0.202219	0.2557186	0.7908	0.429	0.203792306	count	1
SEC24D	0.1535483	0.1361732	1.1276	0.26	0.203944665	count	1
USF1	0.178965	0.2509033	0.7133	0.476	0.20398641	count	1
CAPN1	0.1532771	0.1034828	1.4812	0.139	0.203998133	count	1
AC017033.1	0.9382389	0.8314536	1.1284	0.259	0.204188052	count	1
DRAM2	0.1514987	0.11732	1.2913	0.197	0.204247657	count	1
VPS53	0.1693201	0.187074	0.9051	0.365	0.204444055	count	1
MUC1	0.2049733	0.3443336	0.5953	0.552	0.204460856	count	1
AC073332.1	0.1928476	0.2265877	0.8511	0.395	0.20448734	count	1
SNX29	0.157502	0.159604	0.9868	0.324	0.204534391	count	1
MIA3	0.1457577	0.07444	1.9581	0.0503	0.204639114	count	1
APLP2	0.1431255	0.043852	3.2638	0.00111	0.204673144	count	1
NRIP1	0.1660134	0.1414751	1.1734	0.241	0.204760314	count	1
ZNF562	0.1851622	0.2875969	0.6438	0.52	0.205088467	count	1
DDX6	0.1463315	0.077665	1.8841	0.0596	0.205100369	count	1
POLK	0.1481166	0.1092047	1.3563	0.175	0.205113484	count	1
USF3	0.1587965	0.1679839	0.9453	0.345	0.205119434	count	1
DLGAP1-AS1	0.1747517	0.1999613	0.8739	0.382	0.205170972	count	1
DDX27	0.1503663	0.116129	1.2948	0.195	0.205369296	count	1
BBS12	0.2562445	0.3161677	0.8105	0.418	0.205403853	count	1
INTS11	0.1504227	0.1302571	1.1548	0.248	0.20544614	count	1
TMEM198	0.4346187	0.5773725	0.7528	0.452	0.205477248	count	1
HDAC6	0.1819203	0.2913802	0.6243	0.532	0.205517164	count	1
AMZ2	0.1569218	0.1331571	1.1785	0.239	0.205520709	count	1
EBLN2	0.4348474	0.6920539	0.6283	0.53	0.205575166	count	1
STS	0.2192428	0.3978854	0.551	0.582	0.205675657	count	1
NUP214	0.1630624	0.1640574	0.9939	0.32	0.205683062	count	1
EMC7	0.1453544	0.0606693	2.3958	0.0166	0.205750399	count	1
NBR1	0.1522321	0.1340604	1.1355	0.256	0.205783719	count	1
MT-CO1	0.1427505	0.0225077	6.3423	2.54E-10	0.205875781	count	6.01E-06
ZMYM3	0.191816	0.2560949	0.749	0.454	0.206210849	count	1
MEF2D	0.1578238	0.1495524	1.0553	0.291	0.206247658	count	1
LRRC40	0.1872697	0.1991217	0.9405	0.347	0.20627739	count	1
GPD2	0.2574538	0.2379552	1.0819	0.279	0.20633857	count	1
AC092155.1	1.6891705	1.3087664	1.2907	0.197	0.206429457	count	1
MROH7	1.6891705	1.546291	1.0924	0.275	0.206429457	count	1
PIGU	0.1777254	0.2350088	0.7562	0.45	0.206528631	count	1
NUMB	0.158537	0.1479767	1.0714	0.284	0.206542833	count	1
HGSNAT	0.1559141	0.1765319	0.8832	0.377	0.206634664	count	1
PNPO	0.2146576	0.2196629	0.9772	0.329	0.206822895	count	1

QDPR	0.1532077	0.1065746	1.4376	0.151	0.206827036	count	1
TMEM109	0.1458443	0.0510615	2.8562	0.00431	0.206860073	count	1
ZDHHC5	0.1603172	0.1708757	0.9382	0.348	0.20688032	count	1
SHQ1	0.2457701	0.2758829	0.8908	0.373	0.2069446	count	1
HCG18	0.1575992	0.1973503	0.7986	0.425	0.207111104	count	1
ZSCAN2	0.7054819	0.5276727	1.337	0.181	0.207137935	count	1
TENT4B	0.17082	0.2026981	0.8427	0.399	0.207205081	count	1
PEX1	0.1578894	0.1512913	1.0436	0.297	0.207213253	count	1
VLDLR-AS1	0.4939601	0.8176814	0.6041	0.546	0.207249473	count	1
MOB3B	0.2019742	0.2199714	0.9182	0.359	0.20733552	count	1
SSH2	0.1661505	0.2263001	0.7342	0.463	0.207405543	count	1
AL445623.2	0.9595679	1.060129	0.9051	0.365	0.207557275	count	1
AC118553.1	0.9595679	1.2451205	0.7707	0.441	0.207557275	count	1
AC083805.1	0.9595679	1.3002172	0.738	0.461	0.207557275	count	1
TSEN2	0.2246337	0.3989622	0.563	0.573	0.207584243	count	1
SEL1L	0.1504038	0.0978104	1.5377	0.124	0.20761847	count	1
RNF123	0.2849022	0.3831096	0.7437	0.457	0.207627088	count	1
TRA2A	0.1504041	0.0927783	1.6211	0.105	0.207769888	count	1
SP140L	0.1636617	0.1768932	0.9252	0.355	0.208034908	count	1
ATP6V0E1	0.1451526	0.033423	4.3429	1.44E-05	0.208136769	count	0.3313584
MTF2	0.1658264	0.184476	0.8989	0.369	0.208264544	count	1
NUP58	0.1844795	0.2208098	0.8355	0.404	0.208372743	count	1
NSD1	0.1525069	0.1038202	1.469	0.142	0.208379845	count	1
MAP3K2	0.1516429	0.1074146	1.4118	0.158	0.208482475	count	1
AC116366.3	0.206993	0.310243	0.6672	0.505	0.208510703	count	1
AC008429.1	1.725285	0.9857874	1.7502	0.0802	0.208565521	count	1
ADAM8	1.725285	1.464535	1.178	0.239	0.208565521	count	1
AC096992.2	1.725285	1.673489	1.031	0.303	0.208565521	count	1
HMGA2	1.725285	1.738777	0.9922	0.321	0.208565521	count	1
NEURL4	0.2767774	0.4324838	0.64	0.522	0.208830502	count	1
STRN4	0.1930651	0.2873388	0.6719	0.502	0.208863594	count	1
RUNDC1	0.1970629	0.2810982	0.701	0.483	0.208885101	count	1
CDK11B	0.1584787	0.1365791	1.1603	0.246	0.208926747	count	1
SLC16A1-AS1	0.1919985	0.2954872	0.6498	0.516	0.20900083	count	1
AL358781.1	0.7134575	0.9550069	0.7471	0.455	0.209036357	count	1
AL683813.1	0.4991747	0.501196	0.996	0.319	0.209185003	count	1
FNTA	0.1514444	0.0893266	1.6954	0.0901	0.209409026	count	1
LEPROT	0.1462406	0.0362348	4.0359	5.55E-05	0.209461842	count	1
C11orf24	0.1605424	0.1560657	1.0287	0.304	0.209468137	count	1
LONP2	0.151648	0.1035357	1.4647	0.143	0.209484848	count	1
ZNF775	0.2148521	0.2703858	0.7946	0.427	0.209489268	count	1
ZNF486	0.2203913	0.2678009	0.823	0.411	0.209544211	count	1
CCDC186	0.1506926	0.0855653	1.7611	0.0783	0.209580002	count	1
AP000787.1	0.3485777	0.5285654	0.6595	0.51	0.209672872	count	1
ORC3	0.1629985	0.1428512	1.141	0.254	0.209712729	count	1

ADGRA3	0.199419	0.2259489	0.8826	0.378	0.209796028	count	1
CSNK2A1	0.1527423	0.0993029	1.5381	0.124	0.209802241	count	1
TCEANC	0.3492717	0.374421	0.9328	0.351	0.210063236	count	1
INO80	0.1576033	0.1354278	1.1637	0.245	0.210334991	count	1
SNX19	0.168253	0.2479876	0.6785	0.498	0.210341514	count	1
ZNF17	0.4052071	0.425267	0.9528	0.341	0.21036379	count	1
LINC02166	0.5852417	0.8079355	0.7244	0.469	0.210422367	count	1
PMEL	0.5852417	0.8282374	0.7066	0.48	0.210422367	count	1
CATSPERG	0.300522	0.3590464	0.837	0.403	0.21046037	count	1
CTR9	0.1596902	0.130937	1.2196	0.223	0.210517174	count	1
UBTF	0.1529497	0.093757	1.6313	0.103	0.21063298	count	1
HDHD2	0.178383	0.179996	0.991	0.322	0.210699288	count	1
PLSCR1	0.1496647	0.0711416	2.1038	0.0355	0.21072216	count	1
SPNS1	0.1644931	0.1583399	1.0389	0.299	0.210756295	count	1
FIG4	0.2059007	0.2709683	0.7599	0.447	0.211292218	count	1
ZNRF1	0.1779513	0.2061925	0.863	0.388	0.211430223	count	1
DVL2	0.1931791	0.183547	1.0525	0.293	0.211502965	count	1
PPT1	0.1526029	0.0908073	1.6805	0.0929	0.211525012	count	1
HSPB6	0.1512484	0.0756275	1.9999	0.0456	0.211535571	count	1
AC010889.1	1.777956	1.2632975	1.4074	0.159	0.211562523	count	1
AC087500.1	0.4078829	0.4838823	0.8429	0.399	0.211636199	count	1
NKAP	0.1531628	0.0849394	1.8032	0.0714	0.211838073	count	1
USP46	0.1579243	0.1404229	1.1246	0.261	0.211850384	count	1
ERC1	0.155504	0.1226848	1.2675	0.205	0.211863705	count	1
SEL1L2	0.5901105	1.074838	0.549	0.583	0.211917186	count	1
ZNF252P-AS1	0.5901105	1.268135	0.4653	0.642	0.211917186	count	1
SLC35G5	0.5901105	1.435639	0.411	0.681	0.211917186	count	1
AL390066.1	0.5901105	1.6324559	0.3615	0.7178	0.211917186	count	1
ARCN1	0.1520973	0.0849099	1.7913	0.0733	0.212035188	count	1
IBTK	0.165149	0.1387163	1.1906	0.234	0.212035403	count	1
AC006254.1	0.9891457	0.6417627	1.5413	0.123	0.212143609	count	1
EPN2	0.1552649	0.1003472	1.5473	0.122	0.21223324	count	1
ATG101	0.1565551	0.1054742	1.4843	0.138	0.212259908	count	1
ACTR3C	0.7272463	0.664977	1.0936	0.274	0.212295559	count	1
MTMR10	0.2130041	0.3295866	0.6463	0.518	0.212308582	count	1
AC009065.4	0.7276107	0.6393406	1.1381	0.255	0.212381288	count	1
AC074032.1	0.1918596	0.2183342	0.8787	0.38	0.21240475	count	1
AC005523.2	0.2180339	0.427374	0.5102	0.61	0.212523619	count	1
LY96	0.1680965	0.1428229	1.177	0.239	0.21256087	count	1
CEP83	0.1818252	0.2160734	0.8415	0.4	0.212693591	count	1
ZNF830	0.1593422	0.126831	1.2563	0.209	0.212744462	count	1
MMP16	0.1831655	0.2628684	0.6968	0.486	0.212781093	count	1
COX19	0.1646471	0.1502855	1.0956	0.273	0.21283251	count	1
SHOC2	0.1560788	0.1002598	1.5567	0.12	0.212903383	count	1
ZBTB1	0.1615875	0.1431544	1.1288	0.259	0.213007679	count	1

BLOC1S3	0.2533082	0.2730318	0.9278	0.354	0.213082711	count	1
UCKL1	0.1770917	0.1669092	1.061	0.289	0.213224782	count	1
USP6NL	0.1880383	0.2655603	0.7081	0.479	0.213287166	count	1
MAGED1	0.1599936	0.1151248	1.3897	0.165	0.213414566	count	1
PREB	0.1703514	0.1819025	0.9365	0.349	0.213594587	count	1
PDE8A	0.1805236	0.2301724	0.7843	0.433	0.213837792	count	1
ZNF720	0.1751717	0.1676229	1.045	0.296	0.213838729	count	1
STXBP5	0.2084759	0.2889089	0.7216	0.471	0.213885574	count	1
TRAPPC5	1.0008351	0.8927822	1.121	0.262	0.213928901	count	1
SMIM15	0.154963	0.0845398	1.833	0.0669	0.214054929	count	1
USP51	0.1774723	0.1817056	0.9767	0.329	0.214199854	count	1
ZNF728	0.249241	0.3616689	0.6891	0.491	0.214229666	count	1
GXYLT1	0.1851383	0.201225	0.9201	0.358	0.214274471	count	1
RBBP6	0.1544126	0.1001555	1.5417	0.123	0.214496383	count	1
RAPGEF4	1.8329016	1.6210346	1.1307	0.2583	0.214544286	count	1
SHPRH	0.1573019	0.1100938	1.4288	0.153	0.214618268	count	1
SFXN3	0.1660525	0.1478301	1.1233	0.261	0.214639582	count	1
DYNC1I1	0.1617297	0.1806181	0.8954	0.371	0.214768613	count	1
JMJD1C	0.1531252	0.0725299	2.1112	0.0348	0.21519161	count	1
ITPRIPL1	0.218438	0.3928771	0.556	0.578	0.215315308	count	1
CGGBP1	0.1537906	0.0807923	1.9035	0.057	0.215328788	count	1
YBX3	0.1500994	0.0340052	4.414	1.04E-05	0.21539449	count	0.2395744
AC073349.1	0.3079473	0.5656677	0.5444	0.586	0.215399832	count	1
AVPI1	0.1640507	0.1332378	1.2313	0.218	0.215408006	count	1
AC026401.3	0.2860678	0.3935544	0.7269	0.467	0.215539098	count	1
SETBP1	0.1628717	0.1374868	1.1846	0.236	0.215579138	count	1
SHISAL1	0.4584988	0.7007621	0.6543	0.513	0.215641669	count	1
KIAA1147	0.1982227	0.2905441	0.6822	0.495	0.215674331	count	1
BOLA1	0.1663302	0.1343501	1.238	0.216	0.21575744	count	1
NCLN	0.1903664	0.2066266	0.9213	0.357	0.21589469	count	1
CFHR1	1.863643	1.013809	1.8383	0.0661	0.216150342	count	1
ESYT2	0.1566022	0.103167	1.5179	0.129	0.216188995	count	1
AC108488.1	0.2170585	0.3066635	0.7078	0.479	0.216265527	count	1
EDA	0.2171088	0.2611012	0.8315	0.406	0.2163146	count	1
GLE1	0.2343379	0.2564302	0.9138	0.361	0.216316694	count	1
MTMR4	0.184982	0.2261882	0.8178	0.414	0.216346475	count	1
HP1BP3	0.1521997	0.0532395	2.8588	0.00428	0.216358303	count	1
GOT2	0.1776776	0.2108161	0.8428	0.399	0.216409463	count	1
HLTF	0.1557732	0.0929783	1.6754	0.0939	0.21647604	count	1
ZFYVE16	0.1579237	0.1136672	1.3894	0.165	0.216490561	count	1
METTL14	0.1658746	0.172677	0.9606	0.337	0.216556031	count	1
FAM53B	0.2876224	0.4295204	0.6696	0.503	0.216659635	count	1
TRO	0.3409908	0.4766646	0.7154	0.474	0.216855071	count	1
RIOX1	0.2154588	0.2592716	0.831	0.406	0.216866559	count	1
INTS8	0.2047332	0.2280424	0.8978	0.369	0.216878886	count	1

DIS3L2	0.2006211	0.2476809	0.81	0.418	0.216911051	count	1
ZNF880	0.1636234	0.1634258	1.0012	0.317	0.216928101	count	1
ZNF658	0.271195	0.2992501	0.9062	0.365	0.216935219	count	1
NATD1	0.2202426	0.383503	0.5743	0.566	0.217055528	count	1
DIAPH1	0.1674016	0.1362674	1.2285	0.219	0.217140011	count	1
C17orf67	0.1962	0.2593356	0.7565	0.449	0.217142132	count	1
AC008764.8	0.5208962	0.4338555	1.2006	0.23	0.217189022	count	1
WDR92	0.1962806	0.2806662	0.6993	0.484	0.217230066	count	1
ASH1L	0.1552281	0.079186	1.9603	0.05	0.21733867	count	1
SCFD1	0.1605782	0.1001251	1.6038	0.109	0.217372356	count	1
SEC31A	0.1534098	0.0613775	2.4994	0.0125	0.217406486	count	1
TMEM91	0.1690491	0.1324038	1.2768	0.202	0.217453664	count	1
CYP46A1	0.3251958	0.6330878	0.5137	0.608	0.217481967	count	1
N4BP2	0.2357254	0.222354	1.0601	0.289	0.217563533	count	1
LINC01290	0.2720918	0.6301221	0.4318	0.666	0.217625226	count	1
PPIC	0.1529182	0.050366	3.0361	0.00241	0.217725895	count	1
SLC35E2B	0.1928708	0.2892195	0.6669	0.505	0.217728358	count	1
PDRG1	0.166923	0.1299565	1.2845	0.199	0.217752458	count	1
VPS52	0.2236762	0.308658	0.7247	0.469	0.217899163	count	1
CRCP	0.1624528	0.1358136	1.1961	0.232	0.217903981	count	1
MRPL50	0.159325	0.1107858	1.4381	0.15	0.21791144	count	1
MROH8	0.5229716	0.5327945	0.9816	0.326	0.217948829	count	1
MAP2K5	0.2029347	0.2505488	0.81	0.418	0.217972779	count	1
CEP135	0.2126169	0.2390103	0.8896	0.374	0.218053012	count	1
KSR1	0.3891779	0.5389908	0.722	0.47	0.218152968	count	1
PMF1-BGLAP	0.7525205	1.0098983	0.7451	0.456	0.21819413	count	1
SLC30A4	0.2240233	0.2829774	0.7917	0.429	0.218229642	count	1
BIRC6	0.1622015	0.1338091	1.2122	0.226	0.218238215	count	1
SRP54	0.1594811	0.0873954	1.8248	0.0681	0.218309624	count	1
STXBP3	0.1595281	0.116448	1.37	0.171	0.21832802	count	1
ZNF354B	0.1943488	0.1862742	1.0433	0.297	0.218364598	count	1
POLR3B	0.2405137	0.4200581	0.5726	0.567	0.218442204	count	1
FAM20C	0.1690188	0.1384007	1.2212	0.222	0.218453095	count	1
AAMDC	0.1545213	0.0606819	2.5464	0.0109	0.218505696	count	1
PLEKHM2	0.1606405	0.1088282	1.4761	0.14	0.218564662	count	1
TMEM241	0.2406848	0.3845674	0.6259	0.531	0.218593272	count	1
ZNF343	0.2903338	0.5461721	0.5316	0.595	0.218612569	count	1
FAM71A	1.033376	0.789695	1.3086	0.191	0.218818199	count	1
FGF12	1.033376	0.8596402	1.2021	0.229	0.218818199	count	1
NAIF1	0.2907042	0.3481373	0.835	0.404	0.218879217	count	1
RMI2	0.6130426	0.8596477	0.7131	0.476	0.218900263	count	1
CDK7	0.1828057	0.2345042	0.7795	0.436	0.21892494	count	1
SCO1	0.1689699	0.1587549	1.0643	0.287	0.218974722	count	1
SIAH2	0.1682122	0.1576517	1.067	0.286	0.219084628	count	1
LEMD3	0.1850137	0.1899659	0.9739	0.33	0.219102168	count	1

SGK1	0.170711	0.1925358	0.8866	0.375	0.219135223	count	1
MAGEH1	0.1572068	0.0812366	1.9352	0.053	0.219262276	count	1
AL022323.4	1.0366488	0.6155845	1.684	0.0923	0.219303442	count	1
PPP3CA	0.1593691	0.0910454	1.7504	0.0801	0.21938113	count	1
YIPF3	0.1553297	0.0645493	2.4064	0.0162	0.219430463	count	1
LINC01001	1.0383045	1.070923	0.9695	0.332	0.219548462	count	1
CDC42SE1	0.1651855	0.1334732	1.2376	0.216	0.21956473	count	1
SHPK	0.3287614	0.5944864	0.553	0.58	0.219732704	count	1
KRT10	0.1541156	0.044691	3.4485	6.00E-04	0.219972985	count	1
CCDC142	0.4689396	0.391571	1.1976	0.231	0.220047331	count	1
ALG10	0.4257715	0.3462855	1.2295	0.219	0.220101351	count	1
IP6K1	0.1890573	0.1974342	0.9576	0.338	0.220314752	count	1
NSUN5	0.1732142	0.201333	0.8603	0.39	0.220365781	count	1
ZSCAN20	0.6179361	0.5979147	1.0335	0.301	0.220378126	count	1
AC005746.2	0.3938964	0.4887802	0.8059	0.42	0.220592802	count	1
SGK494	0.3681701	0.8010697	0.4596	0.646	0.220649463	count	1
CCNG2	0.1712755	0.1175695	1.4568	0.145	0.2207324	count	1
CIAPIN1	0.1797868	0.1567553	1.1469	0.251	0.220766395	count	1
IER3IP1	0.1599573	0.0825207	1.9384	0.0527	0.221002262	count	1
COL12A1	0.1614424	0.1309167	1.2332	0.218	0.221214074	count	1
HAUS8	0.2634119	0.2794852	0.9425	0.346	0.22128894	count	1
ANKRD13C	0.190617	0.1835075	1.0387	0.299	0.221338085	count	1
RPUSD3	0.1698899	0.1494916	1.1365	0.256	0.221432264	count	1
FICD	0.1810739	0.2132332	0.8492	0.396	0.221442801	count	1
AGPAT4	0.1971712	0.2632021	0.7491	0.454	0.221492901	count	1
FAM20A	0.2701668	0.2644473	1.0216	0.307	0.221620471	count	1
TMEM179B	0.1568418	0.0619123	2.5333	0.0113	0.221687394	count	1
LYN	0.2369462	0.3710324	0.6386	0.523	0.221854642	count	1
BMS1	0.1702245	0.1318058	1.2915	0.197	0.221866169	count	1
HOMER1	0.2532945	0.4367417	0.58	0.562	0.221870617	count	1
AC093323.1	0.1869582	0.2252586	0.83	0.407	0.222022648	count	1
DLG5	0.1996595	0.2373064	0.8414	0.4	0.222065138	count	1
DNAJA1	0.1555966	0.0530409	2.9335	0.0034	0.222110636	count	1
NOL8	0.1673495	0.117305	1.4266	0.154	0.222200864	count	1
GOSR1	0.1615087	0.0918752	1.7579	0.0788	0.222319715	count	1
C17orf80	0.189569	0.2122491	0.8931	0.372	0.222378083	count	1
FLYWCH2	0.1612969	0.090289	1.7864	0.0741	0.222421175	count	1
AC099850.1	0.318588	0.342712	0.9296	0.353	0.222454846	count	1
AP001505.1	1.0581053	0.6122118	1.7283	0.084	0.22245539	count	1
GPATCH8	0.1614856	0.1081466	1.4932	0.135	0.222470198	count	1
TMEM254-AS1	0.6249995	0.8223366	0.76	0.447	0.222503698	count	1
SRL	1.0588056	0.7801811	1.3571	0.175	0.222557413	count	1
POLA2	0.3977727	0.5278246	0.7536	0.451	0.222593272	count	1
FNDC3B	0.1616014	0.1132181	1.4273	0.154	0.222629373	count	1
CALHM5	0.187478	0.1616206	1.16	0.246	0.2226336	count	1

KRT7	0.1599901	0.2025148	0.79	0.43	0.2227093	count	1
SLC7A6OS	0.1785156	0.1824442	0.9785	0.328	0.222726555	count	1
USP14	0.1614121	0.0949564	1.6999	0.0892	0.222880085	count	1
TECR	0.1577782	0.065054	2.4253	0.0153	0.222885911	count	1
PCGF6	0.1834748	0.1844673	0.9946	0.32	0.222918486	count	1
EIF2B3	0.1655781	0.1203927	1.3753	0.169	0.222928294	count	1
RFC2	0.1812219	0.1523512	1.1895	0.234	0.222944941	count	1
TBC1D22B	0.3339518	0.5480925	0.6093	0.542	0.223003515	count	1
HERPUD2	0.1690617	0.1598544	1.0576	0.29	0.22308772	count	1
ZNF530	0.6271211	0.6656404	0.9421	0.346	0.223140416	count	1
HIST1H3A	0.7742893	0.7748234	0.9993	0.318	0.223196784	count	1
SLC30A7	0.1922812	0.1674228	1.1485	0.251	0.223248029	count	1
IARS2	0.1658069	0.0963005	1.7218	0.0852	0.223394815	count	1
LINC00665	0.2245153	0.2761301	0.8131	0.416	0.223534224	count	1
AC073508.3	0.3733583	0.5262376	0.7095	0.478	0.223540864	count	1
LIN7C	0.1688615	0.1187799	1.4216	0.155	0.223598195	count	1
TTF2	0.2069584	0.259233	0.7983	0.425	0.223652567	count	1
ARF5	0.1590065	0.0703883	2.259	0.0239	0.223692803	count	1
A2M-AS1	0.2426046	0.3243342	0.748	0.455	0.223739005	count	1
MSANTD4	0.1874553	0.1734341	1.0808	0.28	0.223843661	count	1
CCDC50	0.1610172	0.0829727	1.9406	0.0524	0.223844444	count	1
DENND5B	0.1908887	0.2954974	0.646	0.518	0.223909077	count	1
DDX17	0.1573621	0.06234	2.5243	0.0116	0.223967162	count	1
NAA50	0.1661629	0.1506338	1.1031	0.27	0.224106759	count	1
YTHDF2	0.1649042	0.1009346	1.6338	0.102	0.224111446	count	1
NEK8	0.3357269	0.3764826	0.8917	0.373	0.22412063	count	1
ZFAND3	0.1672853	0.1258203	1.3296	0.184	0.224271872	count	1
KCTD18	0.1830643	0.2288048	0.8001	0.424	0.224311599	count	1
FBF1	0.5406289	0.5162389	1.0472	0.295	0.224378349	count	1
TCTEX1D2	0.1919367	0.1838527	1.044	0.297	0.224388879	count	1
PHLDB3	0.19981	0.2778532	0.7191	0.472	0.224416508	count	1
PTGR2	0.2333703	0.2763721	0.8444	0.398	0.224417119	count	1
TTC28	0.1851914	0.1662885	1.1137	0.265	0.224479061	count	1
KCNK2	0.780746	0.7035214	1.1098	0.267	0.224666799	count	1
PHF20L1	0.1620132	0.0924055	1.7533	0.0796	0.224834493	count	1
AP3S1	0.1579073	0.056143	2.8126	0.00494	0.224877342	count	1
MFSD10	0.1628419	0.085097	1.9136	0.0557	0.224979267	count	1
BICRA	0.3103776	0.4968877	0.6246	0.532	0.225290838	count	1
FAM85B	2.0571899	1.298665	1.5841	0.113	0.225307824	count	1
SPINDOC	0.2343211	0.3340353	0.7015	0.483	0.225309076	count	1
PKP4	0.2485012	0.3307324	0.7514	0.452	0.225487309	count	1
ABHD16A	0.3379314	0.7304825	0.4626	0.644	0.225506888	count	1
HDLBP	0.1592146	0.0583997	2.7263	0.00644	0.225519158	count	1
HEXDC	0.1769785	0.1721682	1.0279	0.304	0.225652273	count	1
PI4KB	0.1838145	0.1839426	0.9993	0.318	0.225670285	count	1

CLIP1	0.1629357	0.0913056	1.7845	0.0744	0.225708	count	1
PAFAH1B2	0.1694815	0.1072048	1.5809	0.114	0.225807217	count	1
AKAP10	0.2064559	0.242565	0.8511	0.395	0.225814615	count	1
YPEL4	0.257984	0.2994882	0.8614	0.389	0.225847416	count	1
ITGB4	0.4380407	0.478048	0.9163	0.36	0.225865667	count	1
STX8	0.1635945	0.0878381	1.8625	0.0626	0.225984696	count	1
7-Mar	0.1655457	0.0961864	1.7211	0.0853	0.22604503	count	1
KNSTRN	0.3117793	0.3373205	0.9243	0.355	0.226258129	count	1
ZNF221	0.4838907	0.539486	0.8969	0.37	0.226315406	count	1
ATG13	0.1923893	0.2391943	0.8043	0.421	0.226366839	count	1
GPATCH1	0.197259	0.2393941	0.824	0.41	0.226406593	count	1
HYAL1	2.0829904	1.2527209	1.6628	0.0964	0.226412108	count	1
RAP1GAP2	1.0857584	1.0809956	1.0044	0.315	0.226443424	count	1
AC011444.2	1.0857584	1.0809956	1.0044	0.315	0.226443424	count	1
AC016708.1	1.0857584	1.345782	0.8068	0.42	0.226443424	count	1
ACAT1	0.1621481	0.0786405	2.0619	0.0393	0.22660374	count	1
ITGA9	0.1945598	0.2686137	0.7243	0.469	0.226652622	count	1
PPA2	0.1696689	0.1288918	1.3164	0.188	0.226682549	count	1
SEC24C	0.1947055	0.2513715	0.7746	0.439	0.226820382	count	1
LAMP1	0.1598086	0.0485484	3.2917	0.00101	0.22691598	count	1
ZNF283	0.2214576	0.3937457	0.5624	0.574	0.226938733	count	1
AL121601.1	1.0896181	0.8001934	1.3617	0.173	0.226993461	count	1
YWHAE	0.1591551	0.0443286	3.5904	0.000335	0.227030898	count	1
MUTYH	0.236287	0.2738458	0.8628	0.388	0.22715269	count	1
FBXO36	0.2648663	0.3284962	0.8063	0.42	0.227208135	count	1
GSTM2	0.1875921	0.2757819	0.6802	0.496	0.227362025	count	1
STAG3	0.6413081	0.6130149	1.0462	0.296	0.227377166	count	1
AC010680.3	2.1082229	1.35027	1.5613	0.119	0.227467289	count	1
ACAD10	0.3588631	0.4374612	0.8203	0.412	0.227491346	count	1
MYO3A	0.642159	0.6897431	0.931	0.352	0.22763012	count	1
RLIM	0.1827831	0.1949	0.9378	0.348	0.227643551	count	1
PLA2G4C	0.196134	0.1830171	1.0717	0.284	0.227668147	count	1
RARS2	0.1739552	0.1399446	1.243	0.214	0.227719252	count	1
DCAF6	0.1795323	0.1610892	1.1145	0.265	0.227795242	count	1
PWWP2A	0.168044	0.1295253	1.2974	0.195	0.227854719	count	1
SEC13	0.1642743	0.0847174	1.9391	0.0526	0.22788759	count	1
ERGIC1	0.1662899	0.0958305	1.7353	0.0828	0.228108062	count	1
IKBKE	0.359936	0.5334749	0.6747	0.5	0.228127398	count	1
BSCL2	0.6444684	0.7317276	0.8807	0.379	0.228316024	count	1
CPSF4	0.1953559	0.1979876	0.9867	0.324	0.228340203	count	1
FLVCR2	2.132908	0.9336709	2.2844	0.0224	0.228476212	count	1
KDM1B	0.1969608	0.2064346	0.9541	0.34	0.228616399	count	1
DDOST	0.1638564	0.0666116	2.4599	0.0139	0.228619036	count	1
WDR47	0.2000633	0.1750465	1.1429	0.253	0.228667584	count	1
SPG21	0.1703976	0.1285692	1.3253	0.185	0.228703238	count	1

NPAS3	0.3430495	0.4168282	0.823	0.411	0.228720746	count	1
AL118558.3	0.3609489	0.3464486	1.0419	0.298	0.228727625	count	1
CD8B	0.4898922	0.766563	0.6391	0.523	0.228817952	count	1
AL133453.1	0.2094196	0.2262567	0.9256	0.355	0.229005048	count	1
SAP130	0.2795703	0.3169379	0.8821	0.378	0.229040361	count	1
RTP4	0.2302068	0.2505472	0.9188	0.358	0.229074368	count	1
KBTBD3	0.1850812	0.175761	1.053	0.292	0.22932157	count	1
PRDM2	0.1732382	0.1400925	1.2366	0.216	0.229368123	count	1
STK36	0.3838762	0.4725094	0.8124	0.417	0.229382821	count	1
CDC23	0.2009293	0.1951011	1.0299	0.303	0.229644542	count	1
NRDC	0.164978	0.0934166	1.766	0.0775	0.229728221	count	1
ASAH1	0.1624171	0.0589964	2.753	0.00593	0.229795888	count	1
MAP3K7CL	0.1821191	0.1449759	1.2562	0.209	0.229863134	count	1
ADPRM	0.2030075	0.2133998	0.9513	0.342	0.230037624	count	1
WDFY3	0.1822982	0.184714	0.9869	0.324	0.230087589	count	1
SLFN5	0.1759442	0.1390532	1.2653	0.206	0.230144557	count	1
SLBP	0.1663749	0.0951918	1.7478	0.0806	0.23034588	count	1
NUP107	0.1860896	0.1516048	1.2275	0.22	0.230560828	count	1
C1QTNF7	0.1698169	0.1356804	1.2516	0.211	0.230578257	count	1
MRPL3	0.1664114	0.0778984	2.1363	0.0327	0.230792442	count	1
SIMC1	0.2345774	0.2373047	0.9885	0.323	0.230854444	count	1
QARS	0.1820486	0.1507325	1.2078	0.227	0.230966277	count	1
ATG12	0.1668267	0.0904609	1.8442	0.0652	0.230970096	count	1
ZNF300	0.2374768	0.3766195	0.6305	0.528	0.231018771	count	1
UBR5	0.175204	0.1542597	1.1358	0.256	0.231155407	count	1
KMT2B	0.1794296	0.1502635	1.1941	0.233	0.231178814	count	1
PRR13	0.165165	0.0672641	2.4555	0.0141	0.231198998	count	1
C1QTNF6	0.3470406	0.3414361	1.0164	0.309	0.23122245	count	1
PHF21A	0.1846538	0.2128732	0.8674	0.386	0.231393549	count	1
LARGE1	0.2985809	0.3793809	0.787	0.431	0.231454604	count	1
IGFBP7-AS1	0.3878562	0.3889374	0.9972	0.319	0.231586539	count	1
CEP41	0.2478258	0.4264392	0.5812	0.561	0.231763037	count	1
FBXO8	0.1789301	0.1375481	1.3009	0.193	0.231809883	count	1
MICU3	0.170812	0.1183256	1.4436	0.149	0.231925207	count	1
CHD7	0.8131466	0.8566098	0.9493	0.343	0.231948825	count	1
ALG14	0.1786314	0.147235	1.2132	0.225	0.232016573	count	1
SSBP4	0.165671	0.0795605	2.0823	0.0374	0.232047017	count	1
AP4M1	0.182939	0.1984449	0.9219	0.357	0.232088202	count	1
FOXK1	0.1884086	0.2054981	0.9168	0.359	0.232149152	count	1
MFSD4A	0.3889248	0.8498231	0.4577	0.647	0.232177569	count	1
METTL6	0.2015822	0.2148486	0.9383	0.348	0.232201071	count	1
CLK1	0.1683422	0.0976368	1.7242	0.0848	0.232255836	count	1
ATL1	0.1805024	0.1208966	1.493	0.136	0.232326869	count	1
NAGA	0.1963375	0.2078047	0.9448	0.345	0.23236592	count	1
NUDT11	0.2839776	0.3399831	0.8353	0.404	0.232510638	count	1

ERAL1	0.1891038	0.1731209	1.0923	0.275	0.232557733	count	1
FAM149B1	0.3210221	0.3957984	0.8111	0.417	0.232624367	count	1
TMEM138	0.182153	0.1638435	1.1117	0.266	0.232732417	count	1
PKP2	2.244948	1.0246826	2.1909	0.0285	0.232779691	count	1
CDK5RAP3	0.1741032	0.1119081	1.5558	0.12	0.23278843	count	1
ICA1	1.13111	1.187196	0.9528	0.341	0.232805754	count	1
OTUD1	0.1905299	0.1512281	1.2599	0.208	0.23290281	count	1
GTF2H1	0.1835922	0.1486119	1.2354	0.217	0.232911198	count	1
ERG28	0.1719334	0.1005603	1.7098	0.0874	0.23337794	count	1
CPNE4	0.1950234	0.3862602	0.5049	0.614	0.233406143	count	1
EXOC8	0.2079696	0.248167	0.838	0.402	0.233449393	count	1
PSMD14	0.1731733	0.111392	1.5546	0.12	0.23368769	count	1
VPS35	0.1664676	0.0658886	2.5265	0.0116	0.233714619	count	1
PRKAB1	0.2014864	0.2540857	0.793	0.428	0.233804909	count	1
PACRGL	0.2038073	0.2403751	0.8479	0.397	0.233824935	count	1
RGL3	0.1869094	0.2525323	0.7401	0.459	0.23384523	count	1
WWC2	0.1792217	0.1768344	1.0135	0.311	0.233889399	count	1
FTSJ3	0.1919043	0.1613943	1.189	0.235	0.234077933	count	1
RNFT2	0.33652	0.5647635	0.5959	0.551	0.234281415	count	1
TRPV3	2.287959	1.616762	1.4151	0.157	0.234317402	count	1
GIMAP6	2.2879591	2.0474147	1.1175	0.2639	0.234317426	count	1
AL512625.3	0.4569717	0.7112074	0.6425	0.521	0.234693068	count	1
SLC35C2	0.1793189	0.1546475	1.1595	0.246	0.234704556	count	1
NRXN2	0.2943841	0.293325	1.0036	0.316	0.234714639	count	1
SRRM2	0.1638824	0.0429499	3.8157	0.000138	0.234740618	count	1
ENO3	1.1452983	0.9536321	1.201	0.23	0.234751275	count	1
AC011511.1	1.1452983	0.9536321	1.201	0.23	0.234751275	count	1
GPM6A	1.1452983	0.9784685	1.1705	0.242	0.234751275	count	1
AC087482.1	1.1452983	1.0460125	1.0949	0.274	0.234751275	count	1
BATF2	0.2869569	0.360027	0.797	0.425	0.234853871	count	1
TIGD1	0.4574658	0.4177889	1.095	0.274	0.23492238	count	1
FAM220A	0.2316878	0.2612795	0.8867	0.375	0.235070516	count	1
SIPA1L1	0.1771259	0.1664079	1.0644	0.287	0.23512494	count	1
CLCF1	0.2640311	0.3823349	0.6906	0.49	0.235170227	count	1
PTCD3	0.1864095	0.167829	1.1107	0.267	0.235239062	count	1
MINDY1	0.2128674	0.3113771	0.6836	0.494	0.235303658	count	1
TMEM59	0.1638978	0.0315884	5.1885	2.23E-07	0.235305657	count	0.00521151
DEGS1	0.1670543	0.0651428	2.5644	0.0104	0.235331305	count	1
TEX10	0.2421772	0.3126524	0.7746	0.439	0.235477911	count	1
AL627171.1	0.2520141	0.3735031	0.6747	0.5	0.235570406	count	1
NGRN	0.2169069	0.2154656	1.0067	0.314	0.235665526	count	1
NBEA	0.2054732	0.3021594	0.68	0.497	0.235711091	count	1
AC080038.1	0.2063525	0.2381565	0.8665	0.386	0.235759854	count	1
SRSF5	0.1653402	0.0469479	3.5218	0.000434	0.235902495	count	1
PITPNM2	0.6705094	0.5002331	1.3404	0.18	0.235984064	count	1

SRSF11	0.1658989	0.0541705	3.0625	0.00221	0.236041531	count	1
C15orf39	0.2651194	0.3501031	0.7573	0.449	0.236108457	count	1
AP001107.9	0.5075491	0.6154438	0.8247	0.41	0.236135613	count	1
REC8	0.6718735	0.6819696	0.9852	0.325	0.23638241	count	1
CDK4	0.1709376	0.0758904	2.2524	0.0244	0.236432416	count	1
SLC35F2	0.253012	0.304761	0.8302	0.406	0.236476973	count	1
PRDM15	0.6724752	0.6594738	1.0197	0.308	0.236557992	count	1
TAF5	0.3267874	0.3203624	1.0201	0.308	0.236584701	count	1
GALNS	0.2191483	0.2457791	0.8916	0.373	0.236599353	count	1
WNT9A	2.357348	1.398688	1.6854	0.092	0.236672443	count	1
SMARCD1	0.1875578	0.1501765	1.2489	0.212	0.236677562	count	1
MXD1	0.2063945	0.2407695	0.8572	0.391	0.236754006	count	1
PLRG1	0.1730763	0.1036139	1.6704	0.0949	0.236818947	count	1
ADCK5	0.249947	0.3822359	0.6539	0.513	0.236894664	count	1
MYOM1	0.207569	0.2009521	1.0329	0.302	0.237130951	count	1
MAGEE1	0.4261778	0.4615943	0.9233	0.356	0.237145439	count	1
KLHL12	0.2664056	0.2530842	1.0526	0.293	0.237216947	count	1
NOL4L	0.4268279	0.5555177	0.7683	0.442	0.237476282	count	1
CERS2	0.1721116	0.0881244	1.9531	0.0509	0.237479918	count	1
ADRA1B	0.3573304	0.6165943	0.5795	0.562	0.237654327	count	1
RUFY1	0.1869303	0.1348349	1.3864	0.166	0.237696033	count	1
ARHGEF6	0.2021404	0.2478679	0.8155	0.415	0.237706761	count	1
PHF12	0.2191212	0.2281827	0.9603	0.337	0.238030534	count	1
PRCD	0.512285	0.6277499	0.8161	0.415	0.238086925	count	1
RAF1	0.1826016	0.154773	1.1798	0.238	0.238094495	count	1
TM2D2	0.1753738	0.1043452	1.6807	0.0929	0.238099304	count	1
C2CD2L	0.3424919	0.361713	0.9469	0.344	0.238202442	count	1
VTI1B	0.1713119	0.075818	2.2595	0.0239	0.238370122	count	1
ZBED6	0.4286464	0.5613882	0.7635	0.445	0.238401187	count	1
ST5	0.175012	0.1476401	1.1854	0.236	0.238421356	count	1
MAFTRR	0.358616	0.5345625	0.6709	0.502	0.238456083	count	1
STK40	0.203457	0.2175845	0.9351	0.35	0.238476732	count	1
MTX1	0.173603	0.1085792	1.5989	0.11	0.23859765	count	1
YTHDF3	0.1847502	0.1641614	1.1254	0.26	0.23866432	count	1
ARSK	0.1866308	0.1542568	1.2099	0.226	0.238678364	count	1
DNASE2	0.1751379	0.0893656	1.9598	0.0501	0.238824754	count	1
AC091132.5	0.581085	0.4490208	1.2941	0.196	0.238874119	count	1
ACADL	0.1898373	0.2052803	0.9248	0.355	0.238874591	count	1
DDX23	0.1901569	0.1936926	0.9817	0.326	0.238934488	count	1
CEP44	0.1842237	0.1321888	1.3936	0.164	0.239038166	count	1
SLC25A25	0.2053853	0.1994278	1.0299	0.303	0.239108245	count	1
LMO4	0.1687537	0.0703332	2.3993	0.0165	0.239148564	count	1
AC009501.1	1.1782836	0.6833384	1.7243	0.0847	0.239193182	count	1
DNTTIP2	0.1715544	0.0922918	1.8588	0.0631	0.239279514	count	1
ABRAXAS1	0.1816549	0.1217599	1.4919	0.136	0.239332556	count	1

XPR1	0.2190705	0.3927486	0.5578	0.577	0.239383359	count	1
AL080276.2	0.5826373	0.6876268	0.8473	0.397	0.239423792	count	1
TIMM23	0.1872328	0.155416	1.2047	0.228	0.239702334	count	1
EBLN3P	0.1755512	0.1017543	1.7252	0.0846	0.239722053	count	1
RTL8C	0.1688554	0.056447	2.9914	0.0028	0.239849864	count	1
U2AF2	0.1915035	0.1622102	1.1806	0.238	0.2399098	count	1
SFSWAP	0.1784543	0.1213279	1.4708	0.141	0.239935286	count	1
SPACA6	0.2237896	0.2694021	0.8307	0.406	0.239972707	count	1
SQSTM1	0.1675206	0.0378737	4.4231	1.00E-05	0.240232693	count	0.2304
AC020928.1	0.2256731	0.349115	0.6464	0.518	0.240332558	count	1
AC027013.1	1.1872929	1.0193286	1.1648	0.244	0.240386812	count	1
STK24-AS1	1.1872929	1.1945697	0.9939	0.32	0.240386812	count	1
F8	0.3323532	0.3997776	0.8313	0.406	0.240400194	count	1
STX7	0.1743248	0.0976586	1.785	0.0743	0.240490318	count	1
FOXO6	0.3211671	0.5538364	0.5799	0.562	0.240694286	count	1
ATRAID	0.1686541	0.0415221	4.0618	4.97E-05	0.240754002	count	1
AC005884.1	0.5865114	0.6956742	0.8431	0.399	0.240793508	count	1
MATR3	0.2261782	0.2660518	0.8501	0.395	0.240860457	count	1
SETD2	0.1773348	0.1343112	1.3203	0.187	0.24088578	count	1
AC120042.3	0.4047274	0.742477	0.5451	0.586	0.240885867	count	1
FCHO2	0.1781038	0.1006852	1.7689	0.077	0.240941076	count	1
ZNF24	0.1737706	0.084409	2.0587	0.0396	0.241117422	count	1
PCMTD2	0.1879574	0.1306645	1.4385	0.15	0.241131369	count	1
C15orf41	0.2625415	0.2606644	1.0072	0.314	0.241576123	count	1
AL031848.2	0.5209092	0.8115921	0.6418	0.521	0.241627857	count	1
SNUPN	0.1836608	0.1189797	1.5436	0.123	0.241811237	count	1
ADCY7	0.2519713	0.4668957	0.5397	0.589	0.241831196	count	1
FMN2	0.5895636	0.760124	0.7756	0.438	0.241870524	count	1
DPH2	0.2770479	0.3020104	0.9173	0.359	0.241961052	count	1
CDK17	0.2110088	0.240716	0.8766	0.381	0.241975384	count	1
POLR2B	0.1805035	0.1129811	1.5976	0.11	0.242020886	count	1
HIST1H1E	0.1766875	0.1304172	1.3548	0.176	0.242142946	count	1
EID2	0.1840442	0.1424074	1.2924	0.196	0.242160167	count	1
PIKFYVE	0.232689	0.2207601	1.054	0.292	0.242220918	count	1
TMEM86B	0.6920301	1.143588	0.6051	0.545	0.242229507	count	1
FASTKD2	0.1928007	0.1597284	1.2071	0.227	0.242230664	count	1
BACE2	0.1749482	0.0801951	2.1815	0.0292	0.242282042	count	1
ORAI2	0.1968397	0.1938701	1.0153	0.31	0.242444518	count	1
AL024507.2	0.6928893	0.5900115	1.1744	0.24	0.242477152	count	1
ATXN1L	0.2194726	0.2157689	1.0172	0.309	0.242487537	count	1
P3H3	0.1823291	0.1161975	1.5691	0.117	0.242494793	count	1
BAG3	0.1776567	0.1036495	1.714	0.0866	0.242533243	count	1
PLCD1	0.2528435	0.2457387	1.0289	0.304	0.24264588	count	1
XYLT1	0.3357142	0.525554	0.6388	0.523	0.242700514	count	1
TMEM168	0.1954476	0.1935533	1.0098	0.313	0.242883623	count	1

TMEM143	0.5924471	0.533132	1.1113	0.267	0.2428863	count	1
PIP4P1	0.1817123	0.1295662	1.4025	0.161	0.242920256	count	1
WDR83	0.1992691	0.1676254	1.1888	0.235	0.242974882	count	1
TCF7L1	0.1844595	0.1471298	1.2537	0.21	0.243009229	count	1
LINC01637	0.8640268	0.8114144	1.0648	0.287	0.243067947	count	1
TDRD10	2.5791773	2.1056259	1.2249	0.2207	0.243254281	count	1
PCMT1	0.172137	0.0606208	2.8396	0.00454	0.243267385	count	1
YARS2	0.2083159	0.2072995	1.0049	0.315	0.24330121	count	1
HAS2-AS1	0.865192	0.5454559	1.5862	0.113	0.243318127	count	1
TMEM50B	0.1752305	0.0775763	2.2588	0.024	0.243329859	count	1
BTRC	0.2214775	0.2291072	0.9667	0.334	0.243341737	count	1
CLEC11A	0.188083	0.1204619	1.5613	0.119	0.243596935	count	1
AC233723.1	0.5945318	0.5084628	1.1693	0.242	0.243619647	count	1
CCT6B	0.5945318	0.5170565	1.1498	0.25	0.243619647	count	1
UAP1L1	0.3508157	0.4672006	0.7509	0.453	0.243652951	count	1
YEATS4	0.188166	0.1492731	1.2605	0.208	0.243703789	count	1
SP2-AS1	0.337204	0.4580348	0.7362	0.462	0.243719272	count	1
KHDRBS3	0.1921739	0.1268674	1.5148	0.13	0.243719544	count	1
NCKAP5	0.2848644	0.3801025	0.7494	0.454	0.243734522	count	1
AP003469.4	0.8671361	0.7722294	1.1229	0.262	0.243735023	count	1
ANAPC5	0.1746939	0.0853236	2.0474	0.0407	0.244031114	count	1
ZNF543	0.2917612	0.4382066	0.6658	0.506	0.244184462	count	1
MTFMT	0.1924137	0.1518258	1.2673	0.205	0.244322875	count	1
TMEM248	0.1757934	0.078227	2.2472	0.0247	0.244421333	count	1
KRCC1	0.1769002	0.0956484	1.8495	0.0645	0.244425867	count	1
MRPS18A	0.1805255	0.1029271	1.7539	0.0795	0.244504562	count	1
NSMCE2	0.1888706	0.1426225	1.3243	0.185	0.244823017	count	1
CDK5RAP1	0.204219	0.1956569	1.0438	0.297	0.244925474	count	1
GARS	0.1823761	0.1356042	1.3449	0.179	0.245002408	count	1
CCDC173	1.226056	1.0021999	1.2234	0.221	0.245428066	count	1
TRIB3	0.3279607	0.5910314	0.5549	0.579	0.245528103	count	1
TUBA1A	0.1710819	0.0472898	3.6177	0.000301	0.245614004	count	1
GSK3B	0.1870499	0.1377901	1.3575	0.175	0.245614198	count	1
CYP2R1	0.2380571	0.3172114	0.7505	0.453	0.245679386	count	1
CD99L2	0.1974746	0.1495871	1.3201	0.187	0.245786699	count	1
HCFC1	0.1981669	0.185722	1.067	0.286	0.245818162	count	1
HKR1	0.2214409	0.2998746	0.7384	0.46	0.245906097	count	1
SCAMP3	0.1864771	0.1535894	1.2141	0.225	0.246102316	count	1
ZNF484	0.2253466	0.2498454	0.9019	0.367	0.246123488	count	1
NVL	0.2123151	0.2317897	0.916	0.36	0.246206996	count	1
MCCC1	0.2217571	0.1897132	1.1689	0.243	0.246251584	count	1
TVP23B	0.1918564	0.1824578	1.0515	0.293	0.246353059	count	1
TEDC1	0.2480184	0.3229859	0.7679	0.443	0.24636838	count	1
BASP1	0.1776735	0.0872686	2.0359	0.0418	0.246378243	count	1
CACNA1A	0.3550452	0.3660869	0.9698	0.332	0.246415894	count	1

INPP1	0.1895069	0.1338109	1.4162	0.157	0.246463069	count	1
SH3GL1	0.1883728	0.1748202	1.0775	0.281	0.246490353	count	1
C2orf76	0.1983661	0.172743	1.1483	0.251	0.246886844	count	1
ABCF2	0.4834475	0.5256103	0.9198	0.358	0.246902259	count	1
AP5M1	0.1987618	0.1773221	1.1209	0.262	0.246966677	count	1
EPHB3	0.604408	0.6115417	0.9883	0.323	0.247082007	count	1
TCF25	0.1742437	0.0606912	2.871	0.00412	0.247131328	count	1
TRAP1	0.1875032	0.1419316	1.3211	0.187	0.247150967	count	1
HMBS	0.2780464	0.3351961	0.8295	0.407	0.247231745	count	1
ZNF69	0.3727995	0.4252923	0.8766	0.381	0.2472745	count	1
ZNF703	0.194088	0.1127202	1.7219	0.0852	0.247313984	count	1
SGMS1	0.2037994	0.1603871	1.2707	0.204	0.247364571	count	1
ZMIZ1-AS1	1.2415079	1.0616051	1.1695	0.242	0.247395392	count	1
CRMP1	1.2415079	1.0751455	1.1547	0.248	0.247395392	count	1
AC008397.2	1.2415079	1.1812049	1.0511	0.293	0.247395392	count	1
WDR3	0.2111906	0.1886305	1.1196	0.263	0.247428884	count	1
IMP3	0.174893	0.0653038	2.6781	0.00744	0.247492279	count	1
RAB7A	0.1748839	0.0521933	3.3507	0.000814	0.247520751	count	1
AEBP2	0.1809194	0.1126662	1.6058	0.108	0.247571645	count	1
GNPDA2	0.1848677	0.1211834	1.5255	0.127	0.247646211	count	1
CCDC66	0.1799551	0.0974555	1.8465	0.0649	0.247930242	count	1
AL158835.1	0.6069496	0.4564126	1.3298	0.184	0.247969858	count	1
PROSER1	0.3575209	0.3159461	1.1316	0.258	0.248031052	count	1
GAB2	0.203059	0.314183	0.6463	0.518	0.248069813	count	1
AFF1	0.1939284	0.1248365	1.5535	0.12	0.24821579	count	1
AC104794.2	0.2527942	0.2726461	0.9272	0.354	0.248327252	count	1
RAE1	0.1974397	0.1478707	1.3352	0.182	0.248365299	count	1
CCDC153	0.4870384	0.5091891	0.9565	0.339	0.248545909	count	1
MASTL	0.4870384	0.5975805	0.815	0.415	0.248545909	count	1
PCCA	0.2335965	0.2159373	1.0818	0.279	0.248607954	count	1
MAP3K6	0.2353765	0.2236757	1.0523	0.293	0.248703082	count	1
CCDC174	0.182417	0.1029677	1.7716	0.0765	0.248719887	count	1
LINC00461	0.2797971	0.6734329	0.4155	0.678	0.248735152	count	1
PLAU	0.2064203	0.1920066	1.0751	0.282	0.248771098	count	1
ST7	0.2111408	0.1895259	1.114	0.265	0.248929646	count	1
ARHGAP17	0.196074	0.1779165	1.1021	0.271	0.248936462	count	1
AC002074.1	1.2538372	0.9413657	1.3319	0.183	0.248948074	count	1
ABCD4	0.1979412	0.1887718	1.0486	0.294	0.248991137	count	1
CALU	0.175559	0.0541871	3.2399	0.00121	0.249197741	count	1
GCLC	0.2713827	0.284661	0.9534	0.34	0.24945718	count	1
FNBP4	0.1812132	0.1065202	1.7012	0.089	0.249619145	count	1
PABPC5	0.2441084	0.2085236	1.1707	0.242	0.249633039	count	1
NBPF14	0.2133405	0.2073421	1.0289	0.304	0.249915934	count	1
KAT8	0.1875743	0.1142053	1.6424	0.101	0.249924635	count	1
EVI5	0.1794931	0.0890518	2.0156	0.0439	0.250110167	count	1

CDV3	0.1771065	0.066556	2.661	0.00782	0.250133394	count	1
ZBTB6	0.235064	0.2135432	1.1008	0.271	0.25013933	count	1
AHR	0.191695	0.1582344	1.2115	0.226	0.250263115	count	1
GRINA	0.1838416	0.1092939	1.6821	0.0926	0.250286248	count	1
LASP1	0.1879461	0.1189034	1.5807	0.114	0.250298216	count	1
DHX29	0.1782401	0.0765244	2.3292	0.0199	0.250301425	count	1
NEPRO	0.194791	0.150141	1.2974	0.195	0.250348654	count	1
KMT2C	0.1859412	0.122729	1.5151	0.13	0.250506959	count	1
FAM210A	0.2073794	0.1978836	1.048	0.295	0.250512188	count	1
MORC2-AS1	0.3151626	0.3960496	0.7958	0.426	0.250534459	count	1
SH3GLB2	0.1819737	0.1007958	1.8054	0.0711	0.250541821	count	1
WBP1	0.4528947	0.7911375	0.5725	0.567	0.250659786	count	1
ADNP	0.1825603	0.1005749	1.8152	0.0696	0.250919704	count	1
INTS10	0.1892034	0.1046164	1.8085	0.0706	0.251081528	count	1
MORC3	0.1882891	0.1134519	1.6596	0.0971	0.251108292	count	1
TM7SF3	0.1958605	0.118438	1.6537	0.0983	0.251201466	count	1
SUMO3	0.1804332	0.0709502	2.5431	0.011	0.251552566	count	1
AC025171.3	1.2758589	0.8066255	1.5817	0.114	0.25168398	count	1
RHOU	0.1960502	0.1457261	1.3453	0.179	0.251701735	count	1
VLDLR	0.2739218	0.3122002	0.8774	0.38	0.251717211	count	1
GLIS1	0.3367513	0.6146097	0.5479	0.584	0.251765808	count	1
SMARCC2	0.1821768	0.1003905	1.8147	0.0697	0.251838911	count	1
MRI1	0.2180928	0.1542224	1.4141	0.157	0.251900707	count	1
BVES-AS1	1.27767	1.067468	1.1969	0.231	0.251906811	count	1
HOXA2	0.2181088	0.2242512	0.9726	0.331	0.251918933	count	1
ACADVL	0.1799739	0.0825221	2.1809	0.0293	0.251980144	count	1
ATP2B4	0.1812658	0.0899766	2.0146	0.044	0.2519885	count	1
PKD1	0.1882265	0.1558628	1.2076	0.227	0.252126678	count	1
AC106782.2	0.7273468	0.7127387	1.0205	0.308	0.252299768	count	1
BFAR	0.187481	0.1100895	1.703	0.0887	0.252484023	count	1
MFSD3	0.3502579	0.3197005	1.0956	0.273	0.252622063	count	1
GMEB2	0.3502699	0.3736343	0.9375	0.349	0.252630226	count	1
ZNF699	0.2328267	0.2644032	0.8806	0.379	0.252648794	count	1
ZEB2-AS1	0.3503222	0.4360614	0.8034	0.422	0.252665811	count	1
AC139795.3	0.3503637	0.6431673	0.5447	0.586	0.252694047	count	1
GOLPH3	0.1868592	0.1035599	1.8044	0.0713	0.252820969	count	1
USP34	0.1808146	0.103308	1.7502	0.0802	0.253103564	count	1
PPP3R1	0.2051938	0.1810544	1.1333	0.257	0.253106707	count	1
THAP1	0.1932564	0.1347723	1.4339	0.152	0.253201885	count	1
ZC3H12C	0.2415624	0.3301504	0.7317	0.464	0.253222205	count	1
VANGL1	0.2169496	0.2409599	0.9004	0.368	0.25325381	count	1
TVP23C	0.4975378	0.500053	0.995	0.32	0.253334893	count	1
TSPYL5	0.2249366	0.2335771	0.963	0.336	0.253371677	count	1
MMAB	0.1884531	0.1262783	1.4924	0.136	0.253419487	count	1
TUT4	0.1809636	0.0841147	2.1514	0.0315	0.253563661	count	1

ZC3H6	0.1853069	0.0982122	1.8868	0.0593	0.253819319	count	1
BTBD6	0.1867939	0.1055881	1.7691	0.077	0.253828617	count	1
NAXE	0.1816509	0.0739041	2.4579	0.014	0.253830344	count	1
AL445426.1	1.2937901	1.0819619	1.1958	0.232	0.253876689	count	1
SCN2B	1.2937901	1.0819619	1.1958	0.232	0.253876689	count	1
FANCD2	1.2937901	1.2276883	1.0538	0.292	0.253876689	count	1
NCR3LG1	1.2937901	1.3319098	0.9714	0.331	0.253876689	count	1
KCTD2	0.2483593	0.2892033	0.8588	0.391	0.253880452	count	1
MRAS	0.1867545	0.1371884	1.3613	0.174	0.254043592	count	1
GTF2IRD1	0.6248057	0.6270722	0.9964	0.319	0.254171165	count	1
GANC	0.2237244	0.2130906	1.0499	0.294	0.254254777	count	1
VDAC3	0.1805046	0.0659596	2.7366	0.00624	0.254297284	count	1
MSANTD2	0.2280777	0.2285296	0.998	0.318	0.254428756	count	1
MPC1	0.1799443	0.0601313	2.9925	0.00279	0.254582088	count	1
ZNF445	0.2205732	0.2479506	0.8896	0.374	0.254725857	count	1
ZNF131	0.1925261	0.1335079	1.4421	0.149	0.254779081	count	1
FADD	0.2078456	0.1589136	1.3079	0.191	0.254887385	count	1
PEX11G	0.3209188	0.3222173	0.996	0.319	0.254898156	count	1
HIST1H2BK	0.430631	0.7206562	0.5976	0.55	0.255030197	count	1
EIF1	0.1770647	0.0202569	8.7409	3.46E-18	0.255040584	count	8.29E-14
CFAP20	0.1931096	0.1307767	1.4766	0.14	0.255110781	count	1
METTL13	0.2287212	0.2799124	0.8171	0.414	0.255134829	count	1
RPH3AL	0.2987576	0.4857737	0.615	0.539	0.255159529	count	1
INTS7	0.3212868	0.3974543	0.8084	0.419	0.255176841	count	1
WASHC1	0.2012915	0.151832	1.3258	0.185	0.255194403	count	1
NUCB2	0.1786959	0.0500404	3.571	0.00036	0.255212084	count	1
FAM111A	0.1903325	0.1509955	1.2605	0.208	0.25524698	count	1
RFXAP	0.241791	0.2057985	1.1749	0.24	0.255341861	count	1
TUT1	0.3215743	0.3974851	0.809	0.419	0.255394561	count	1
CETN3	0.2043253	0.138002	1.4806	0.139	0.255449286	count	1
SUMF2	0.1839407	0.0693914	2.6508	0.00807	0.255510329	count	1
POLD1	0.4067809	0.6921778	0.5877	0.557	0.255624763	count	1
ORAOV1	0.2524626	0.2367134	1.0665	0.286	0.255648964	count	1
SCARB2	0.1818029	0.0780215	2.3302	0.0199	0.255815413	count	1
SPAG9	0.180745	0.0648174	2.7885	0.00532	0.255870174	count	1
TULP4	0.1947978	0.1265234	1.5396	0.124	0.255901488	count	1
SMC5	0.1891625	0.1059779	1.7849	0.0744	0.256004686	count	1
CPEB2	0.2407485	0.2338717	1.0294	0.303	0.25606734	count	1
PARD3	0.207277	0.1648667	1.2572	0.209	0.256116304	count	1
PINLYP	0.2391814	0.2522566	0.9482	0.343	0.256157304	count	1
CASC4	0.1823742	0.068567	2.6598	0.00785	0.256167618	count	1
FARP1	0.1928732	0.1530822	1.2599	0.208	0.256193121	count	1
CD81	0.1778885	0.0213497	8.3321	1.11E-16	0.25627111	count	2.66E-12
DRG1	0.1918307	0.1221882	1.57	0.117	0.256274425	count	1
ENTPD5	0.2530993	0.318029	0.7958	0.426	0.256278233	count	1

CDK11A	0.1969676	0.1521268	1.2948	0.195	0.256312715	count	1
ZBTB45	0.2711027	0.2767911	0.9794	0.327	0.256353127	count	1
AC005726.1	0.9278723	0.834251	1.1122	0.266	0.256481942	count	1
AC007614.1	0.6316223	0.7702509	0.82	0.412	0.256521693	count	1
EFCAB5	0.6316223	0.8822446	0.7159	0.474	0.256521693	count	1
RAB6B	0.2888891	0.2409732	1.1988	0.231	0.256531266	count	1
CEP170	0.1913971	0.108762	1.7598	0.0785	0.256565335	count	1
BTBD7	0.190647	0.1395664	1.366	0.172	0.256730775	count	1
AGGF1	0.2397546	0.2567719	0.9337	0.351	0.256759154	count	1
TMEM63B	0.2841628	0.3477125	0.8172	0.414	0.256762726	count	1
RIOK2	0.1981649	0.1624031	1.2202	0.222	0.256794719	count	1
AC005339.1	0.5585281	0.8095769	0.6899	0.49	0.256885484	count	1
FTO	0.2117528	0.1853469	1.1425	0.253	0.256916045	count	1
ARAP3	0.3009262	0.7531208	0.3996	0.689	0.256938675	count	1
SLC35A4	0.2369072	0.2132302	1.111	0.267	0.25699435	count	1
EIF2B2	0.1936031	0.1237052	1.565	0.118	0.257025636	count	1
PRR14L	0.2062842	0.1882895	1.0956	0.273	0.257069979	count	1
COL6A3	0.1822415	0.0991847	1.8374	0.0662	0.257090856	count	1
KIAA1328	0.2565241	0.2274224	1.128	0.259	0.257189059	count	1
AL022238.2	1.3223328	0.9830871	1.3451	0.179	0.257302953	count	1
GSDME	0.2296439	0.2258393	1.0168	0.309	0.257388534	count	1
MFHAS1	0.2239253	0.3485671	0.6424	0.521	0.257572466	count	1
AGAP3	0.2348339	0.2187988	1.0733	0.283	0.257757013	count	1
CDC14B	0.1961239	0.1656344	1.1841	0.236	0.257802261	count	1
CKLF	0.1875899	0.105631	1.7759	0.0758	0.257813408	count	1
ZNF692	0.2960003	0.3053943	0.9692	0.332	0.25789519	count	1
AC108471.2	1.3275772	1.1944274	1.1115	0.266	0.257924033	count	1
ILKAP	0.1968926	0.1286889	1.53	0.126	0.257938921	count	1
PCNX3	0.3581022	0.3561418	1.0055	0.315	0.257951395	count	1
CD46	0.1819626	0.0604593	3.0097	0.00263	0.258074235	count	1
ODF2L	0.1909001	0.1158243	1.6482	0.0994	0.258267962	count	1
APAF1	0.223879	0.2943679	0.7605	0.447	0.25848961	count	1
AL357055.3	0.9377042	0.7522419	1.2465	0.213	0.258495091	count	1
GAS2L3	0.9377042	0.8592427	1.0913	0.275	0.258495091	count	1
CRACR2A	0.4117426	1.3256103	0.3106	0.7561	0.258505634	count	1
NECTIN2	0.1951727	0.1254191	1.5562	0.12	0.258964152	count	1
AL731569.1	0.9400333	0.6461712	1.4548	0.146	0.258969958	count	1
TMEM223	0.1900114	0.1274441	1.4909	0.136	0.25897824	count	1
FLAD1	0.217957	0.2071975	1.0519	0.293	0.259123086	count	1
RBL1	0.3104176	0.3541086	0.8766	0.381	0.259145945	count	1
PIK3CD-AS2	0.3265733	0.358171	0.9118	0.362	0.259176753	count	1
TOR3A	0.2035951	0.1322593	1.5394	0.124	0.259337753	count	1
ECH1	0.1823747	0.0523384	3.4845	0.000499	0.259498802	count	1
RRAGB	0.2049794	0.1725956	1.1876	0.235	0.259505341	count	1
ATP6V0A2	0.2518489	0.2505272	1.0053	0.315	0.259588951	count	1

RBM25	0.18346	0.0606488	3.025	0.0025	0.259667122	count	1
TMEM206	0.3606597	0.2970538	1.2141	0.225	0.259685593	count	1
PDCL	0.1912167	0.1067245	1.7917	0.0733	0.259817671	count	1
ACOT9	0.1933058	0.1030862	1.8752	0.0608	0.259821135	count	1
JADE2	0.225946	0.1962324	1.1514	0.25	0.259863106	count	1
AC092376.2	1.3442031	0.9945135	1.3516	0.177	0.259875746	count	1
OCEL1	0.1934341	0.115035	1.6815	0.0927	0.259894747	count	1
MUL1	0.2342736	0.1753118	1.3363	0.182	0.259913422	count	1
SCN3A	0.5661639	0.3894085	1.4539	0.146	0.259945069	count	1
SETD7	0.1959261	0.1343767	1.458	0.145	0.259958853	count	1
SEC61A1	0.192857	0.1248222	1.5451	0.122	0.26005764	count	1
AC017083.1	0.3048063	0.5812024	0.5244	0.6	0.260119094	count	1
AL121944.1	0.3194584	0.3601243	0.8871	0.375	0.260275867	count	1
RCCD1	0.2415157	0.2247995	1.0744	0.283	0.260283898	count	1
WDSUB1	0.2238556	0.2408336	0.9295	0.353	0.260318349	count	1
U2SURP	0.1840836	0.0691432	2.6624	0.00779	0.260439234	count	1
CNPPD1	0.1977643	0.130924	1.5105	0.131	0.26044269	count	1
HYAL3	0.3490535	0.5119146	0.6819	0.495	0.260462848	count	1
AC083843.3	0.3620079	0.7038467	0.5143	0.607	0.260599106	count	1
USP31	0.2349214	0.31773	0.7394	0.46	0.260619751	count	1
TAOK1	0.194792	0.1115323	1.7465	0.0808	0.260664105	count	1
HARS2	0.2377324	0.3487999	0.6816	0.496	0.260881043	count	1
RECQL5	0.4738238	0.5087642	0.9313	0.352	0.26112866	count	1
AF001548.2	0.951268	0.7106869	1.3385	0.181	0.261249537	count	1
GNL2	0.2039945	0.1317614	1.5482	0.122	0.261285262	count	1
KIAA2026	0.1953662	0.1224016	1.5961	0.111	0.26142906	count	1
KMT5C	0.4745652	0.4198746	1.1303	0.258	0.261497604	count	1
ANKRD11	0.1858032	0.0859269	2.1623	0.0307	0.261532927	count	1
DHX9	0.1913209	0.1026726	1.8634	0.0625	0.26153699	count	1
POM121C	0.2084	0.1889892	1.1027	0.27	0.261663404	count	1
SLC35B3	0.2183495	0.1978091	1.1038	0.27	0.261675759	count	1
BCL2L13	0.2214671	0.1923757	1.1512	0.25	0.26173444	count	1
MAP2K6	0.2950626	0.313314	0.9417	0.346	0.261813698	count	1
RABGGTB	0.1977924	0.1281018	1.544	0.123	0.262001723	count	1
AC087239.1	0.3641286	0.321643	1.1321	0.258	0.262035143	count	1
LTV1	0.2011572	0.133898	1.5023	0.133	0.262142232	count	1
PPP5C	0.2207102	0.1814138	1.2166	0.224	0.262356079	count	1
DCLRE1C	0.2266475	0.2517181	0.9004	0.368	0.262593751	count	1
PRRX2	0.1857265	0.0744446	2.4948	0.0126	0.262768489	count	1
AF127577.4	0.6502024	0.7747094	0.8393	0.401	0.26288145	count	1
CSGALNACT2	0.2036669	0.1342991	1.5165	0.129	0.262937857	count	1
MPP4	0.9607345	1.0416213	0.9223	0.356	0.263156472	count	1
VN1R1	0.9607345	1.0416213	0.9223	0.356	0.263156472	count	1
WDR93	0.9607345	1.195073	0.8039	0.421	0.263156472	count	1
KIAA1109	0.1907093	0.1036888	1.8392	0.066	0.263307403	count	1

EXOC6	0.2361923	0.2341118	1.0089	0.313	0.263327112	count	1
STAG2	0.1902433	0.0869287	2.1885	0.0287	0.263346192	count	1
ABO	1.375823	1.0823945	1.2711	0.204	0.26351617	count	1
AC004812.2	0.3161165	0.2999334	1.054	0.292	0.263699233	count	1
TRAF3	0.2342592	0.3555476	0.6589	0.51	0.263701837	count	1
HIPK1	0.2055207	0.1428241	1.439	0.15	0.26377391	count	1
MMD	0.235449	0.2597331	0.9065	0.365	0.26378652	count	1
FAM3C	0.188181	0.0643604	2.9239	0.00348	0.263841308	count	1
PAXIP1-AS2	0.2185397	0.2035377	1.0737	0.283	0.263842352	count	1
ZNF558	0.2834486	0.3952082	0.7172	0.473	0.264019319	count	1
OSTM1	0.1936943	0.1020474	1.8981	0.0578	0.26404477	count	1
LINC00115	0.4213598	0.4498985	0.9366	0.349	0.264072261	count	1
MED30	0.1912864	0.0924674	2.0687	0.0386	0.264102129	count	1
PTPRZ1	0.7697729	0.5637939	1.3653	0.172	0.264104972	count	1
TM9SF1	0.2369277	0.2566038	0.9233	0.356	0.26413297	count	1
ARHGAP26	0.2307707	0.2665229	0.8659	0.387	0.26429837	count	1
SGO2	0.4479597	0.3487748	1.2844	0.199	0.264401451	count	1
HIBADH	0.1954847	0.1103333	1.7718	0.0765	0.264609159	count	1
MINK1	0.3103805	0.3479212	0.8921	0.372	0.264681724	count	1
ZNF480	0.2256697	0.2484661	0.9083	0.364	0.265011744	count	1
MDM1	0.2530931	0.2266208	1.1168	0.264	0.265043289	count	1
AL391422.3	0.2989232	0.3033746	0.9853	0.325	0.265112458	count	1
AP000894.4	0.9708151	1.000989	0.9699	0.332	0.265172993	count	1
SAMD8	0.2082295	0.176922	1.177	0.239	0.265195582	count	1
ZBED5	0.1959411	0.1250463	1.5669	0.117	0.265304893	count	1
VCPKMT	0.2481508	0.2543034	0.9758	0.329	0.265567899	count	1
SKI	0.1949719	0.1185417	1.6448	0.1	0.265586212	count	1
DYRK3	0.2499439	0.2399651	1.0416	0.298	0.265643415	count	1
GNPDA1	0.1987778	0.1129864	1.7593	0.0786	0.265745483	count	1
SLC35A1	0.2155464	0.1602725	1.3449	0.179	0.265750576	count	1
OTULIN	0.2127003	0.1719582	1.2369	0.216	0.265825948	count	1
ZBTB34	0.4834282	0.4968098	0.9731	0.331	0.26589804	count	1
TOMM5	0.2098054	0.1728294	1.2139	0.225	0.265901549	count	1
ZNF883	0.4248634	0.5174927	0.821	0.412	0.266094516	count	1
GFM1	0.2221314	0.1538005	1.4443	0.149	0.266154075	count	1
RNF216	0.1975712	0.1276559	1.5477	0.122	0.266204399	count	1
PPM1F	0.2524356	0.2814095	0.897	0.37	0.266340867	count	1
MDC1	0.2489453	0.2582306	0.964	0.335	0.266400734	count	1
PEX6	0.2346466	0.200069	1.1728	0.241	0.266471348	count	1
EFNA4	0.3063846	0.3413057	0.8977	0.369	0.266589267	count	1
TRAPPC10	0.2038218	0.1389625	1.4667	0.143	0.266775299	count	1
EGLN1	0.2002172	0.1423465	1.4065	0.16	0.266815273	count	1
MAPK3	0.1935072	0.090886	2.1291	0.0333	0.266842494	count	1
REV1	0.2016315	0.131586	1.5323	0.126	0.26691391	count	1
KPNA3	0.1999439	0.1223942	1.6336	0.102	0.267062653	count	1

LINC02084	0.7806664	0.7057595	1.1061	0.269	0.267084941	count	1
MAPK7	0.2668349	0.2666506	1.0007	0.317	0.267257738	count	1
AP3B1	0.201052	0.1145144	1.7557	0.0792	0.267272977	count	1
CCND2	0.1897686	0.0718505	2.6412	0.0083	0.267386673	count	1
SETX	0.1981855	0.1280763	1.5474	0.122	0.26739479	count	1
AL158212.2	0.2872575	0.3121091	0.9204	0.357	0.267451106	count	1
DAZAP2	0.1904892	0.0673267	2.8293	0.00469	0.267576858	count	1
NMNAT3	0.2518274	0.2776213	0.9071	0.364	0.267602856	count	1
PRPF4B	0.1891498	0.0652322	2.8996	0.00376	0.267696575	count	1
RRAGC	0.2009481	0.1353252	1.4849	0.138	0.267784577	count	1
CD99	0.1862928	0.0307612	6.0561	1.54E-09	0.267803692	count	3.63E-05
EAF1	0.2194729	0.187777	1.1688	0.243	0.267909044	count	1
PRNP	0.1901844	0.0604645	3.1454	0.00167	0.2681035	count	1
EPS15	0.1976089	0.0990066	1.9959	0.046	0.268251474	count	1
DIAPH2	0.1942583	0.1025547	1.8942	0.0583	0.268309975	count	1
TXNRD1	0.191245	0.075936	2.5185	0.0118	0.268337833	count	1
RIOK3	0.1934721	0.0886586	2.1822	0.0292	0.268360041	count	1
ZNF615	0.3086261	0.3474776	0.8882	0.374	0.268462509	count	1
ZNF571-AS1	0.4289774	1.35144	0.3174	0.751	0.268465203	count	1
USP37	0.258513	0.226191	1.1429	0.253	0.268487426	count	1
ITGB3BP	0.2273051	0.177032	1.284	0.199	0.268543922	count	1
ANKMY2	0.2051954	0.1446437	1.4186	0.156	0.268562339	count	1
DNAJC16	0.2494688	0.2095155	1.1907	0.234	0.268682638	count	1
MIR1-1HG-AS1	1.4224608	1.1842915	1.2011	0.23	0.268717942	count	1
GOLGA6L4	0.2632633	0.484744	0.5431	0.587	0.268742754	count	1
MAP7D1	0.1970376	0.1228886	1.6034	0.109	0.268841275	count	1
NDFIP1	0.1886734	0.0470237	4.0123	6.13E-05	0.268841653	count	1
USP8	0.1936452	0.0955338	2.027	0.0427	0.269046655	count	1
GPBP1	0.1911271	0.076058	2.5129	0.012	0.269076615	count	1
HR	0.3900222	0.4347984	0.897	0.37	0.269093037	count	1
SOS2	0.2033102	0.1554708	1.3077	0.191	0.269124353	count	1
C1orf131	0.2001515	0.1185716	1.688	0.0915	0.269281682	count	1
COPS4	0.1964189	0.0928171	2.1162	0.0344	0.269415968	count	1
TIRAP	0.2555563	0.4968651	0.5143	0.607	0.269561232	count	1
CNOT4	0.1942038	0.0762085	2.5483	0.0109	0.269705101	count	1
SPATS2	0.2146809	0.1634703	1.3133	0.189	0.269865209	count	1
AL118516.1	0.2042338	0.1400115	1.4587	0.145	0.269883068	count	1
N4BP1	0.2046249	0.1261044	1.6227	0.105	0.269921221	count	1
CMTM8	0.3239657	0.3335792	0.9712	0.332	0.26995746	count	1
ABHD10	0.2226474	0.177101	1.2572	0.209	0.269986012	count	1
RBM19	0.2524822	0.2231743	1.1313	0.258	0.270106813	count	1
PLD6	0.4319014	0.5182745	0.8333	0.405	0.270147585	count	1
TAF6	0.2204669	0.1551663	1.4208	0.155	0.27020326	count	1
CYB5R4	0.2391705	0.2007195	1.1916	0.234	0.270350798	count	1
POGZ	0.2061575	0.1326149	1.5546	0.12	0.270375653	count	1

DHX57	0.2426364	0.3292613	0.7369	0.461	0.270385366	count	1
ERCC6	0.2000976	0.1673269	1.1958	0.232	0.270406675	count	1
FUK	0.3414856	0.4782588	0.714	0.475	0.270422219	count	1
SLC22A15	0.3414856	0.6577489	0.5192	0.604	0.270422219	count	1
PARP3	0.2403692	0.2777997	0.8653	0.387	0.270464092	count	1
VCP	0.1940101	0.0722321	2.6859	0.00727	0.270675286	count	1
WDR89	0.2829686	0.268897	1.0523	0.293	0.270680773	count	1
GRIN2D	16.6459795	1084.858678	0.0153	0.988	0.270706325	count	1
AL050403.2	16.7047921	1150.713701	0.0145	0.988	0.270706326	count	1
SPATA41	16.7051664	1045.335029	0.016	0.987	0.270706326	count	1
LINC02489	16.7748	1912.462852	0.0088	0.993	0.270706327	count	1
AC130371.2	16.8402356	1287.816896	0.0131	0.99	0.270706328	count	1
AL669970.3	16.9396125	1079.9775	0.0157	0.987	0.270706329	count	1
AL138899.1	16.9973174	2042.012911	0.0083	0.993	0.27070633	count	1
CCNE1	17.0942834	1919.2197	0.0089	0.993	0.270706331	count	1
AL158163.1	17.0942835	1919.219685	0.0089	0.993	0.270706331	count	1
AP001412.1	17.0942921	1919.221531	0.0089	0.993	0.270706331	count	1
PKD2L2	17.0942965	1919.221508	0.0089	0.993	0.270706331	count	1
AC131011.1	17.0942964	1919.221504	0.0089	0.993	0.270706331	count	1
AC099687.1	17.0942964	1919.221497	0.0089	0.993	0.270706331	count	1
TMEM72-AS1	17.0942965	1919.221508	0.0089	0.993	0.270706331	count	1
AC012377.1	17.0946843	2457.679804	0.007	0.994	0.270706331	count	1
AP003031.2	17.0946962	2457.678865	0.007	0.994	0.270706331	count	1
AC100830.1	17.0959134	975.6636031	0.0175	0.986	0.270706331	count	1
TMEM9B-AS1	17.1250334	1379.103207	0.0124	0.99	0.270706331	count	1
SLC25A10	17.1617533	1016.718678	0.0169	0.9865	0.270706332	count	1
AC090739.1	17.2209353	1632.687449	0.0105	0.992	0.270706332	count	1
CLGN	17.3510818	1535.154086	0.0113	0.991	0.270706333	count	1
AC138393.3	17.3873791	2268.869257	0.0077	0.994	0.270706334	count	1
B3GALT5-AS1	17.3873792	2268.869292	0.0077	0.994	0.270706334	count	1
LINC01277	17.3874143	2268.89334	0.0077	0.994	0.270706334	count	1
AC005041.1	17.3874144	2268.893335	0.0077	0.994	0.270706334	count	1
SCT	17.3874145	2268.893345	0.0077	0.994	0.270706334	count	1
AC007216.1	17.3874145	2268.89335	0.0077	0.994	0.270706334	count	1
PKDREJ	17.3874145	2268.893345	0.0077	0.994	0.270706334	count	1
C10orf95	17.3874184	2268.880219	0.0077	0.994	0.270706334	count	1
SLX1A	17.3874184	2268.880254	0.0077	0.994	0.270706334	count	1
AC091132.1	17.3877151	2789.470477	0.0062	0.995	0.270706334	count	1
CTRB2	17.3877402	2789.476894	0.0062	0.995	0.270706334	count	1
SPRR2A	17.3877408	2789.477264	0.0062	0.995	0.270706334	count	1
4-Mar	17.3924551	2394.788528	0.0073	0.994	0.270706334	count	1
LMNTD2	17.3924551	2394.78847	0.0073	0.994	0.270706334	count	1
AC008555.5	17.3924551	2394.788505	0.0073	0.994	0.270706334	count	1
LINC01191	17.3924553	2394.788505	0.0073	0.994	0.270706334	count	1
C7orf57	17.3924554	2394.788563	0.0073	0.994	0.270706334	count	1

CLEC2L	17.3924554	2394.788528	0.0073	0.994	0.270706334	count	1
MRVI1-AS1	17.3924553	2394.788366	0.0073	0.994	0.270706334	count	1
IDI2	17.3924554	2394.788621	0.0073	0.994	0.270706334	count	1
AC036108.4	17.3924554	2394.788459	0.0073	0.994	0.270706334	count	1
SYNE4	17.3924553	2394.788459	0.0073	0.994	0.270706334	count	1
AL137003.1	17.3924555	2394.788528	0.0073	0.994	0.270706334	count	1
AC009630.1	17.3924555	2394.788598	0.0073	0.994	0.270706334	count	1
AL158211.1	17.3924555	2394.788494	0.0073	0.994	0.270706334	count	1
BAIAP2L2	17.3924555	2394.788598	0.0073	0.994	0.270706334	count	1
AL390719.2	17.3931978	3389.75149	0.0051	0.996	0.270706334	count	1
DDR1-DT	17.393198	3389.751703	0.0051	0.996	0.270706334	count	1
AC007938.2	17.393198	3389.751703	0.0051	0.996	0.270706334	count	1
CA8	17.3931981	3389.751605	0.0051	0.996	0.270706334	count	1
AP000842.2	17.393198	3389.751703	0.0051	0.996	0.270706334	count	1
LINC02551	17.3931981	3389.751605	0.0051	0.996	0.270706334	count	1
FITM1	17.3931981	3389.751654	0.0051	0.996	0.270706334	count	1
DPEP3	17.393198	3389.751769	0.0051	0.996	0.270706334	count	1
PPM1E	17.393198	3389.751687	0.0051	0.996	0.270706334	count	1
AL121890.2	17.393198	3389.751703	0.0051	0.996	0.270706334	count	1
AC010616.1	17.393198	3389.751703	0.0051	0.996	0.270706334	count	1
MRAP	17.393198	3389.751703	0.0051	0.996	0.270706334	count	1
HTR3A	17.3931982	3389.751802	0.0051	0.996	0.270706334	count	1
SHISA2	17.4605024	2451.735991	0.0071	0.994	0.270706334	count	1
FXYD3	17.4605025	2451.735972	0.0071	0.994	0.270706334	count	1
TAL2	17.4611933	2710.540333	0.0064	0.995	0.270706334	count	1
TERC	17.5018941	1815.976216	0.0096	0.992	0.270706335	count	1
DUOXA1	17.5335346	1500.526534	0.0117	0.991	0.270706335	count	1
CD19	17.5477289	1612.548982	0.0109	0.991	0.270706335	count	1
NRXN1	17.5730266	2334.176175	0.0075	0.994	0.270706335	count	1
PRR34	17.6890424	2547.653407	0.0069	0.994	0.270706336	count	1
MFSD14A	17.7859804	1653.711607	0.0108	0.9914	0.270706337	count	1
ACER2	17.8279181	2278.259543	0.0078	0.994	0.270706337	count	1
COL28A1	17.8279449	2278.273713	0.0078	0.994	0.270706337	count	1
C22orf31	17.827945	2278.273734	0.0078	0.994	0.270706337	count	1
EPB41L4B	17.8279937	2800.961392	0.0064	0.995	0.270706337	count	1
SLC29A4	17.8280343	2800.990113	0.0064	0.995	0.270706337	count	1
SPDYE5	17.8280356	2800.996037	0.0064	0.995	0.270706337	count	1
BDKRB1	17.9350457	1675.563962	0.0107	0.9915	0.270706337	count	1
PRLR	17.9364741	1797.37405	0.01	0.992	0.270706337	count	1
FTCDNL1	18.1335465	1485.786071	0.0122	0.9903	0.270706338	count	1
PGM5P3-AS1	16.5428028	1184.150921	0.014	0.989	0.270706347	count	1
AC008736.1	16.6184776	979.7591828	0.017	0.986	0.270706349	count	1
C9orf43	16.6184844	979.7617215	0.017	0.986	0.270706349	count	1
ALX1	16.7051682	1045.335375	0.016	0.987	0.27070635	count	1
LINC01436	16.7746691	1618.348491	0.0104	0.992	0.270706351	count	1

MYBPC1	16.7746818	1618.348335	0.0104	0.992	0.270706351	count	1
AC079907.1	16.774687	1618.350291	0.0104	0.992	0.270706351	count	1
AL050343.1	16.7748001	1912.462857	0.0088	0.993	0.270706351	count	1
AC015987.1	16.774807	1912.461942	0.0088	0.993	0.270706351	count	1
TLR6	16.8253541	1061.676934	0.0158	0.9874	0.270706352	count	1
AC109630.1	16.887869	964.2016019	0.0175	0.986	0.270706353	count	1
LINC02356	16.9972423	1774.843708	0.0096	0.992	0.270706354	count	1
NPFFR2	17.0942834	1919.219674	0.0089	0.993	0.270706356	count	1
FKBP1C	17.0942835	1919.219719	0.0089	0.993	0.270706356	count	1
AC092119.3	17.0942836	1919.219731	0.0089	0.993	0.270706356	count	1
MTFP1	17.0942837	1919.219715	0.0089	0.993	0.270706356	count	1
SPATA9	17.0942966	1919.221508	0.0089	0.993	0.270706356	count	1
AC211476.2	17.0942966	1919.221516	0.0089	0.993	0.270706356	count	1
AP002490.1	17.0946907	2457.673236	0.007	0.994	0.270706356	count	1
MMP25	17.0946907	2457.673207	0.007	0.994	0.270706356	count	1
AL138963.1	17.0947058	2457.684402	0.007	0.994	0.270706356	count	1
PRR7-AS1	17.1153262	1920.803992	0.0089	0.993	0.270706356	count	1
TM4SF19-AS1	17.1153313	1625.426694	0.0105	0.992	0.270706356	count	1
HCG17	17.1153314	1625.426694	0.0105	0.992	0.270706356	count	1
AL391262.1	17.1153398	1625.423102	0.0105	0.992	0.270706356	count	1
AC108474.1	17.1153405	1625.426679	0.0105	0.992	0.270706356	count	1
SLC26A1	17.1153449	1625.429607	0.0105	0.992	0.270706356	count	1
CCR1	17.115345	1625.429619	0.0105	0.992	0.270706356	count	1
ATG9A	17.1250253	1379.107164	0.0124	0.99	0.270706356	count	1
ENTPD7	17.1250253	1379.107164	0.0124	0.99	0.270706356	count	1
SIX3	17.1250325	1379.104199	0.0124	0.99	0.270706356	count	1
LINC01960	17.1250334	1379.103207	0.0124	0.99	0.270706356	count	1
DIRAS2	17.125038	1695.587448	0.0101	0.992	0.270706356	count	1
ADAMTS13	17.12504	1695.585015	0.0101	0.992	0.270706356	count	1
AC010207.1	17.1253902	1870.522914	0.0092	0.993	0.270706356	count	1
MIA	17.1258402	1942.357586	0.0088	0.993	0.270706356	count	1
ZNF878	17.1694247	1586.035198	0.0108	0.991	0.270706356	count	1
AP000769.2	17.1694343	1874.362397	0.0092	0.993	0.270706356	count	1
DLGAP2	17.2175464	1311.598464	0.0131	0.99	0.270706357	count	1
MAMDC2-AS1	17.2351765	1613.859231	0.0107	0.991	0.270706357	count	1
PAQR6	17.2424394	1717.74904	0.01	0.992	0.270706357	count	1
KCTD8	17.3197228	1210.545775	0.0143	0.9886	0.270706358	count	1
NXPE2	17.3507214	1375.228905	0.0126	0.99	0.270706358	count	1
KCNJ16	17.3510526	1823.468404	0.0095	0.992	0.270706358	count	1
AF129075.2	17.3510817	1535.154078	0.0113	0.991	0.270706358	count	1
OIP5	17.3510818	1535.154081	0.0113	0.991	0.270706358	count	1
SLC28A2	17.3510939	1535.155016	0.0113	0.991	0.270706358	count	1
KLK10	17.3576077	1619.993819	0.0107	0.991	0.270706358	count	1
GCNA	17.3577082	2081.277982	0.0083	0.993	0.270706358	count	1
PITX1	17.3577487	1814.072969	0.0096	0.992	0.270706358	count	1

AL021368.1	17.3577523	1814.08423	0.0096	0.992	0.270706358	count	1
AL139424.3	17.3873753	2268.882472	0.0077	0.994	0.270706358	count	1
AL513217.1	17.3873793	2268.869282	0.0077	0.994	0.270706358	count	1
CDH10	17.3873793	2268.869257	0.0077	0.994	0.270706358	count	1
KIF24	17.3873793	2268.869257	0.0077	0.994	0.270706358	count	1
AICDA	17.3873792	2268.869292	0.0077	0.994	0.270706358	count	1
OPLAH	17.3873794	2268.869287	0.0077	0.994	0.270706358	count	1
AKR1B15	17.3874002	2268.88759	0.0077	0.994	0.270706358	count	1
SLC44A4	17.3874047	2268.87467	0.0077	0.994	0.270706358	count	1
MORN5	17.3874047	2268.87468	0.0077	0.994	0.270706358	count	1
AC026250.1	17.3874047	2268.874715	0.0077	0.994	0.270706358	count	1
ZGLP1	17.3874143	2268.89334	0.0077	0.994	0.270706358	count	1
AC104184.1	17.3874144	2268.89334	0.0077	0.994	0.270706358	count	1
HOXA1	17.3874144	2268.893345	0.0077	0.994	0.270706358	count	1
AL139300.2	17.3874145	2268.893355	0.0077	0.994	0.270706358	count	1
AL033527.5	17.3874146	2268.893335	0.0077	0.994	0.270706358	count	1
APLN	17.3874146	2268.89334	0.0077	0.994	0.270706358	count	1
MBOAT4	17.3874185	2268.880219	0.0077	0.994	0.270706358	count	1
AC105053.1	17.3877151	2789.470471	0.0062	0.995	0.270706358	count	1
AC008875.1	17.3877152	2789.470421	0.0062	0.995	0.270706358	count	1
LINC00304	17.3877152	2789.470477	0.0062	0.995	0.270706358	count	1
LRRC46	17.3877152	2789.470434	0.0062	0.995	0.270706358	count	1
PLIN4	17.3877152	2789.470452	0.0062	0.995	0.270706358	count	1
AC005013.1	17.3877405	2789.477233	0.0062	0.995	0.270706358	count	1
LRRC3B	17.3877406	2789.477258	0.0062	0.995	0.270706358	count	1
RET	17.3877408	2789.477283	0.0062	0.995	0.270706358	count	1
AL122035.1	17.3877498	2789.4997	0.0062	0.995	0.270706358	count	1
ITPKB-AS1	17.3924551	2394.788378	0.0073	0.994	0.270706359	count	1
AC009962.1	17.3924551	2394.788459	0.0073	0.994	0.270706359	count	1
STRIP2	17.3924551	2394.788494	0.0073	0.994	0.270706359	count	1
OR52N1	17.3924551	2394.788401	0.0073	0.994	0.270706359	count	1
AP001363.2	17.3924551	2394.788424	0.0073	0.994	0.270706359	count	1
GLT1D1	17.3924551	2394.788505	0.0073	0.994	0.270706359	count	1
GJB6	17.3924551	2394.788401	0.0073	0.994	0.270706359	count	1
WFIKKN1	17.392455	2394.788517	0.0073	0.994	0.270706359	count	1
VWA3A	17.3924551	2394.788505	0.0073	0.994	0.270706359	count	1
EIF3CL	17.3924551	2394.788528	0.0073	0.994	0.270706359	count	1
LINC02087	17.3924551	2394.788459	0.0073	0.994	0.270706359	count	1
CTAGE1	17.392455	2394.788401	0.0073	0.994	0.270706359	count	1
ADORA2A-AS1	17.3924551	2394.788366	0.0073	0.994	0.270706359	count	1
AC105935.1	17.3924552	2394.788447	0.0073	0.994	0.270706359	count	1
AC097358.2	17.3924552	2394.788598	0.0073	0.994	0.270706359	count	1
AC067930.4	17.3924552	2394.788505	0.0073	0.994	0.270706359	count	1
AP006333.1	17.3924552	2394.788459	0.0073	0.994	0.270706359	count	1
LINC01435	17.3924552	2394.788355	0.0073	0.994	0.270706359	count	1

KLRF1	17.3924552	2394.788389	0.0073	0.994	0.270706359	count	1
AL079303.1	17.3924552	2394.788482	0.0073	0.994	0.270706359	count	1
AC243829.1	17.3924552	2394.788505	0.0073	0.994	0.270706359	count	1
AC139099.3	17.3924552	2394.788482	0.0073	0.994	0.270706359	count	1
AL031432.5	17.3924554	2394.788494	0.0073	0.994	0.270706359	count	1
AC096541.1	17.3924554	2394.788528	0.0073	0.994	0.270706359	count	1
AC079354.3	17.3924553	2394.788621	0.0073	0.994	0.270706359	count	1
ANKDD1B	17.3924553	2394.788482	0.0073	0.994	0.270706359	count	1
LINC00491	17.3924554	2394.788366	0.0073	0.994	0.270706359	count	1
SMIM32	17.3924553	2394.78847	0.0073	0.994	0.270706359	count	1
SLC12A9-AS1	17.3924554	2394.788494	0.0073	0.994	0.270706359	count	1
AL590708.1	17.3924553	2394.788482	0.0073	0.994	0.270706359	count	1
AP001453.4	17.3924553	2394.788517	0.0073	0.994	0.270706359	count	1
AF111167.2	17.3924553	2394.788551	0.0073	0.994	0.270706359	count	1
AURKB	17.3924553	2394.788459	0.0073	0.994	0.270706359	count	1
CSF3	17.3924553	2394.78847	0.0073	0.994	0.270706359	count	1
AC018521.2	17.3924553	2394.788482	0.0073	0.994	0.270706359	count	1
C18orf65	17.3924553	2394.788413	0.0073	0.994	0.270706359	count	1
AL008635.1	17.3924554	2394.788389	0.0073	0.994	0.270706359	count	1
AL008726.1	17.3924555	2394.78854	0.0073	0.994	0.270706359	count	1
TMC1	17.3924556	2394.788528	0.0073	0.994	0.270706359	count	1
AC174065.1	17.3924557	2394.788575	0.0073	0.994	0.270706359	count	1
GNG3	17.3924556	2394.788494	0.0073	0.994	0.270706359	count	1
COL9A1	17.3931978	3389.751474	0.0051	0.996	0.270706359	count	1
CD27	17.3931978	3389.751539	0.0051	0.996	0.270706359	count	1
TAS2R19	17.3931978	3389.751507	0.0051	0.996	0.270706359	count	1
AC010536.1	17.3931978	3389.751474	0.0051	0.996	0.270706359	count	1
SPRR2D	17.3931979	3389.751589	0.0051	0.996	0.270706359	count	1
AL159169.3	17.3931979	3389.751572	0.0051	0.996	0.270706359	count	1
C1QTNF9	17.3931979	3389.751654	0.0051	0.996	0.270706359	count	1
AC004147.2	17.3931979	3389.751589	0.0051	0.996	0.270706359	count	1
ST6GALNAC1	17.3931979	3389.751654	0.0051	0.996	0.270706359	count	1
AC008397.1	17.3931979	3389.751605	0.0051	0.996	0.270706359	count	1
FUT1	17.3931979	3389.751654	0.0051	0.996	0.270706359	count	1
AC092958.1	17.3931981	3389.751802	0.0051	0.996	0.270706359	count	1
TESMIN	17.3931981	3389.751671	0.0051	0.996	0.270706359	count	1
AL133353.1	17.3931981	3389.751802	0.0051	0.996	0.270706359	count	1
AC115676.1	17.393198	3389.751736	0.0051	0.996	0.270706359	count	1
AC010542.4	17.393198	3389.751671	0.0051	0.996	0.270706359	count	1
CECR2	17.3931981	3389.751736	0.0051	0.996	0.270706359	count	1
FTCD	17.393198	3389.751589	0.0051	0.996	0.270706359	count	1
AL606760.1	17.3931982	3389.751818	0.0051	0.996	0.270706359	count	1
AC025034.1	17.3931982	3389.751736	0.0051	0.996	0.270706359	count	1
AC019077.1	17.3931983	3389.751638	0.0051	0.996	0.270706359	count	1
VCX3A	17.3931984	3389.751785	0.0051	0.996	0.270706359	count	1

OR52I1	17.3931983	3389.751703	0.0051	0.996	0.270706359	count	1
AP000911.2	17.3931983	3389.751802	0.0051	0.996	0.270706359	count	1
AL161935.1	17.3931984	3389.751785	0.0051	0.996	0.270706359	count	1
LINC02448	17.3931983	3389.751736	0.0051	0.996	0.270706359	count	1
PCDH8	17.3931983	3389.751867	0.0051	0.996	0.270706359	count	1
AC092755.2	17.3931984	3389.751785	0.0051	0.996	0.270706359	count	1
UPK1A-AS1	17.3931983	3389.751736	0.0051	0.996	0.270706359	count	1
AC011476.3	17.3931983	3389.751736	0.0051	0.996	0.270706359	count	1
AL035530.2	17.4256452	1609.176384	0.0108	0.991	0.270706359	count	1
NDST2	17.4602586	1914.486684	0.0091	0.993	0.270706359	count	1
HCAR1	17.5330667	1686.410657	0.0104	0.992	0.27070636	count	1
KRT24	17.5331355	1371.456699	0.0128	0.99	0.27070636	count	1
CILP	17.7887112	949.191206	0.0187	0.985	0.270706361	count	1
LINC02308	17.8186384	1678.761769	0.0106	0.9915	0.270706361	count	1
BARX2	17.8217028	2717.73417	0.0066	0.995	0.270706361	count	1
IRX6	17.8279179	2278.259533	0.0078	0.994	0.270706361	count	1
SLC6A12	17.8279181	2278.259533	0.0078	0.994	0.270706361	count	1
DGUOK-AS1	17.8279205	2278.269353	0.0078	0.994	0.270706361	count	1
KLKB1	17.8279208	2278.269353	0.0078	0.994	0.270706361	count	1
AL596094.1	17.8279297	2278.259513	0.0078	0.994	0.270706361	count	1
AC109460.1	17.8279296	2278.259513	0.0078	0.994	0.270706361	count	1
AC109460.2	17.8279298	2278.259513	0.0078	0.994	0.270706361	count	1
DAGLA	17.8279449	2278.273744	0.0078	0.994	0.270706361	count	1
LINC02106	17.827945	2278.273754	0.0078	0.994	0.270706361	count	1
C16orf71	17.8279936	2800.961379	0.0064	0.995	0.270706361	count	1
OTOGL	17.8279938	2800.961392	0.0064	0.995	0.270706361	count	1
CHRNA3	17.8280345	2800.990101	0.0064	0.995	0.270706361	count	1
SLCO4C1	17.8280356	2800.996062	0.0064	0.995	0.270706361	count	1
AC108062.1	17.8281429	2782.041475	0.0064	0.995	0.270706361	count	1
KCNIP2-AS1	17.8281429	2782.041438	0.0064	0.995	0.270706361	count	1
RNF157	17.8281699	2782.058758	0.0064	0.995	0.270706361	count	1
AL138966.2	17.9268481	2432.029114	0.0074	0.9941	0.270706362	count	1
AC025165.5	17.9374636	2300.931005	0.0078	0.994	0.270706362	count	1
NECAB2	18.1339141	1603.758872	0.0113	0.991	0.270706363	count	1
PTPRQ	18.2194228	1580.606722	0.0115	0.9908	0.270706363	count	1
SNX9	0.192271	0.0628844	3.0575	0.00225	0.270861911	count	1
CLDN23	0.5936976	0.4306235	1.3787	0.168	0.270872629	count	1
DST	0.1898399	0.0805986	2.3554	0.0186	0.270947482	count	1
ETFRF1	0.1930889	0.0823453	2.3449	0.0191	0.271029205	count	1
CEP164	0.2074208	0.1486165	1.3957	0.163	0.271067091	count	1
ZNF16	0.2736415	0.3894514	0.7026	0.482	0.27112832	count	1
DDX1	0.1950543	0.0874701	2.23	0.0258	0.271280101	count	1
PURB	0.2072648	0.1647372	1.2582	0.208	0.271445194	count	1
IDH1	0.2212867	0.1472127	1.5032	0.133	0.271728265	count	1
DROSHA	0.2540656	0.2781924	0.9133	0.361	0.271765169	count	1

PIR	0.2004428	0.1192986	1.6802	0.093	0.271776651	count	1
SEC14L1	0.1967315	0.1153268	1.7059	0.0881	0.271793686	count	1
PCDHB14	0.246548	0.3086489	0.7988	0.424	0.271853831	count	1
SS18L2	0.203294	0.1187732	1.7116	0.0871	0.271872129	count	1
NCBP1	0.2525423	0.2999307	0.842	0.4	0.271925137	count	1
PPP1R2	0.1939561	0.0718464	2.6996	0.00697	0.272055604	count	1
HEATR6	0.2124676	0.1700977	1.2491	0.212	0.272056107	count	1
FAM160B1	0.2109857	0.1693995	1.2455	0.213	0.272066662	count	1
TTF1	0.2040279	0.1203111	1.6958	0.09	0.272121924	count	1
EDEM1	0.2513302	0.2253497	1.1153	0.265	0.27232928	count	1
SMCHD1	0.1980381	0.1018426	1.9446	0.0519	0.272459913	count	1
GAN	0.2500308	0.2161297	1.1569	0.247	0.27256279	count	1
SH3BP5L	0.29745	0.2995291	0.9931	0.321	0.272588388	count	1
USB1	0.2243366	0.1772027	1.266	0.206	0.272614845	count	1
FGFR2	0.4362705	0.6271004	0.6957	0.487	0.27265746	count	1
LINC00323	0.4364412	0.5053251	0.8637	0.388	0.272755439	count	1
SIK3	0.2240325	0.1889058	1.1859	0.236	0.272838428	count	1
PDIA4	0.1987696	0.0954898	2.0816	0.0375	0.272935987	count	1
CCDC183	0.5991192	0.5116714	1.1709	0.242	0.273004989	count	1
AC009118.3	1.4634017	0.9598005	1.5247	0.127	0.273123491	count	1
YAE1D1	0.2093293	0.1549569	1.3509	0.177	0.273140495	count	1
ZNF28	0.3806396	0.5356174	0.7107	0.477	0.273176727	count	1
ZDHHC24	0.2213071	0.1413176	1.566	0.117	0.273277724	count	1
TAF7	0.1931544	0.0571998	3.3768	0.000741	0.273319472	count	1
ZNF573	0.2824901	0.3004134	0.9403	0.347	0.273523558	count	1
AL139339.1	1.467549	1.0341104	1.4191	0.156	0.273561537	count	1
AC116407.1	1.0140572	0.8919521	1.1369	0.256	0.273660234	count	1
WSB2	0.2087552	0.1361567	1.5332	0.125	0.273946659	count	1
MED14	0.2349479	0.2956649	0.7946	0.427	0.273964686	count	1
PARP2	0.2196969	0.1667381	1.3176	0.188	0.274061749	count	1
SMARCE1	0.2062928	0.0999201	2.0646	0.039	0.274066038	count	1
CEBPG	0.2038128	0.142231	1.433	0.152	0.274085566	count	1
ARGLU1	0.192145	0.0585399	3.2823	0.001	0.274263637	count	1
AL135925.1	0.2091161	0.1895551	1.1032	0.27	0.274417424	count	1
UHRF1BP1	0.3983451	0.4061006	0.9809	0.327	0.274443766	count	1
MIOS	0.2690378	0.2864596	0.9392	0.348	0.274488667	count	1
AGFG1	0.206426	0.1642792	1.2566	0.209	0.274517003	count	1
HNRNPA2B1	0.1914363	0.0342574	5.5882	2.46E-08	0.274650521	count	0.000577854
ZMAT1	0.2355548	0.2558508	0.9207	0.357	0.274662172	count	1
GAS1RR	1.01936	0.6645201	1.534	0.125	0.274682961	count	1
HES1	0.1944693	0.0872868	2.2279	0.0259	0.274725494	count	1
GLIS2	0.2078156	0.1485476	1.399	0.162	0.274747221	count	1
DBNDD1	0.3988463	0.5155629	0.7736	0.439	0.274765398	count	1
CRTC1	0.3228188	0.3460242	0.9329	0.351	0.274835442	count	1
OGDH	0.2307066	0.1599455	1.4424	0.149	0.274842843	count	1

C11orf49	0.2072397	0.1463608	1.416	0.157	0.275034822	count	1
DDX51	0.274938	0.2261947	1.2155	0.224	0.275154616	count	1
ZNF653	0.5022118	0.526902	0.9531	0.341	0.27516218	count	1
FAM122A	0.2089049	0.1105953	1.8889	0.059	0.275200225	count	1
OS9	0.1954726	0.059045	3.3106	0.00094	0.275353884	count	1
WDR27	0.2593455	0.3684597	0.7039	0.482	0.275417179	count	1
EWSR1	0.1984204	0.0916953	2.1639	0.0305	0.27545659	count	1
ERCC4	0.2215606	0.1440565	1.538	0.124	0.275476797	count	1
MPDU1	0.2347566	0.2059334	1.14	0.254	0.275534416	count	1
UHRF2	0.2248678	0.2119094	1.0612	0.289	0.27553893	count	1
C19orf12	0.2094299	0.131242	1.5958	0.111	0.275543418	count	1
CDKL1	0.3057514	0.3710424	0.824	0.41	0.2755517	count	1
CDR2L	0.3392381	0.3519869	0.9638	0.335	0.275619611	count	1
SDHAF2	0.2066212	0.1074633	1.9227	0.0546	0.275814703	count	1
AC021092.1	1.025412	1.1748906	0.8728	0.383	0.275845485	count	1
IRGQ	0.3116626	0.3308308	0.9421	0.346	0.275972547	count	1
TCF20	0.3317	0.3173603	1.0452	0.296	0.276109174	count	1
MTR	0.2097597	0.1522128	1.3781	0.168	0.276148406	count	1
ZBTB14	0.2519197	0.2290745	1.0997	0.272	0.276150044	count	1
ZKSCAN1	0.2036046	0.1152662	1.7664	0.0774	0.276282477	count	1
TMTC3	0.216289	0.1431474	1.511	0.131	0.276313491	count	1
STAM2	0.2089072	0.1466158	1.4249	0.154	0.276492708	count	1
CRLF3	0.2738715	0.2628461	1.0419	0.298	0.276761182	count	1
BOLA2-SMG1P6	0.5055031	0.5342223	0.9462	0.344	0.276776793	count	1
AC138150.1	0.4710827	0.5155647	0.9137	0.361	0.276792028	count	1
GSTK1	0.1954931	0.0579788	3.3718	0.000755	0.27683407	count	1
MACROD2	0.3723856	0.4135449	0.9005	0.368	0.276852747	count	1
AC010969.2	0.4210148	0.6204627	0.6785	0.497	0.276876959	count	1
NCK1-DT	0.4713703	0.4216205	1.118	0.264	0.276945305	count	1
PPM1D	0.266937	0.2242534	1.1903	0.234	0.277026611	count	1
NR1D1	0.2150742	0.1449441	1.4838	0.138	0.277037687	count	1
UBXN2B	0.240202	0.2148321	1.1181	0.264	0.277048167	count	1
MFSD8	0.2420874	0.1837495	1.3175	0.188	0.277052921	count	1
ZMYM4	0.2040641	0.118072	1.7283	0.084	0.277057476	count	1
RBM18	0.2174472	0.1576867	1.379	0.168	0.277159246	count	1
CMIP	0.3257393	0.3638327	0.8953	0.371	0.277214026	count	1
PAN3	0.2233774	0.1457125	1.533	0.125	0.277251945	count	1
WDR55	0.2237915	0.1730126	1.2935	0.196	0.277288655	count	1
PTPRK	0.2387729	0.2034603	1.1736	0.241	0.277409583	count	1
MED11	0.2047499	0.1129738	1.8124	0.07	0.27743229	count	1
AP000802.1	1.0341975	1.1056877	0.9353	0.35	0.277523917	count	1
DDX19A	0.2372976	0.1981021	1.1979	0.231	0.277582363	count	1
ARAF	0.2350982	0.1715587	1.3704	0.171	0.277625917	count	1
POLG2	0.2580381	0.2503617	1.0307	0.303	0.27771861	count	1
CCDC47	0.1965893	0.0713704	2.7545	0.00591	0.2777257	count	1

CSTF2T	0.2352175	0.1922224	1.2237	0.221	0.277764877	count	1
IL1R1	0.1996654	0.0957942	2.0843	0.0372	0.277870565	count	1
ZMYM1	0.2229448	0.1912457	1.1658	0.244	0.278074437	count	1
DXO	0.2567567	0.2329519	1.1022	0.27	0.278088737	count	1
ZNF865	0.3086865	0.5282701	0.5843	0.559	0.278097639	count	1
LINGO1	0.2780244	0.2424084	1.1469	0.251	0.278158752	count	1
AL691447.2	0.6125842	0.6550295	0.9352	0.35	0.278273345	count	1
SLC35D2	0.2151094	0.1677716	1.2822	0.2	0.278356932	count	1
YTHDC2	0.2121759	0.140846	1.5064	0.132	0.278408252	count	1
ANKRD40	0.2095161	0.123639	1.6946	0.0902	0.278466201	count	1
AZIN1	0.2102943	0.1272813	1.6522	0.0986	0.27862332	count	1
LPIN2	0.2055776	0.1096805	1.8743	0.061	0.278630806	count	1
ZNF708	0.2172711	0.1512961	1.4361	0.151	0.278740056	count	1
CHPF2	0.2399547	0.1880601	1.2759	0.202	0.2787621	count	1
UHMK1	0.218484	0.1377578	1.586	0.113	0.278785372	count	1
SAR1A	0.1978391	0.0650072	3.0433	0.00236	0.278799454	count	1
TXLNA	0.2151496	0.1487699	1.4462	0.148	0.278891446	count	1
AARS	0.2392772	0.1927979	1.2411	0.215	0.278938938	count	1
CASP8AP2	0.2096647	0.1378651	1.5208	0.128	0.279208783	count	1
NAP1L5	0.2296616	0.1890515	1.2148	0.225	0.279615611	count	1
UTP14A	0.2218999	0.1493758	1.4855	0.137	0.27963247	count	1
FAM151B	0.4484755	0.5056271	0.887	0.375	0.279643556	count	1
MIPEP	0.2825688	0.3903282	0.7239	0.469	0.279721511	count	1
CLASRP	0.2524898	0.2473497	1.0208	0.307	0.279746714	count	1
RPS6KA4	0.245597	0.2067496	1.1879	0.235	0.279869554	count	1
ERCC5	0.3010788	0.3425107	0.879	0.379	0.279875769	count	1
SYAP1	0.2021246	0.0867571	2.3298	0.0199	0.27989259	count	1
CDC5L	0.1991885	0.0772472	2.5786	0.00996	0.279908881	count	1
ZNF500	0.2929399	0.4320515	0.678	0.498	0.279915297	count	1
CCNF	0.617061	0.727618	0.8481	0.396	0.280016241	count	1
ARL6IP6	0.2109316	0.1372696	1.5366	0.124	0.28005301	count	1
SNX8	0.2722738	0.2390287	1.1391	0.255	0.280116721	count	1
SNAPC3	0.2119192	0.1573623	1.3467	0.178	0.280141928	count	1
AC245884.8	0.830245	0.8465413	0.9807	0.327	0.280385161	count	1
SGF29	0.2173555	0.1170703	1.8566	0.0634	0.280481886	count	1
AL449266.1	1.5360623	1.0260451	1.4971	0.134	0.280584385	count	1
MADCAM1	0.4501958	0.7624746	0.5904	0.555	0.28062525	count	1
ANO8	1.0517026	0.6034383	1.7429	0.0814	0.280836442	count	1
SRGAP2B	0.248905	0.2223344	1.1195	0.263	0.281164633	count	1
ZNF282	0.3240806	0.3571717	0.9074	0.364	0.281344756	count	1
C14orf119	0.2027667	0.0815209	2.4873	0.0129	0.281381311	count	1
RPA2	0.2052035	0.0942317	2.1776	0.0295	0.281480315	count	1
GTF2IRD2	0.2616954	0.2914043	0.898	0.369	0.281570784	count	1
BCL9L	0.2212044	0.1599739	1.3828	0.167	0.281583832	count	1
TDRD3	0.2205639	0.1462302	1.5083	0.132	0.281730832	count	1

BRWD1	0.2116661	0.1608043	1.3163	0.188	0.282126657	count	1
DHX16	0.2404783	0.2363184	1.0176	0.309	0.282153686	count	1
BAALC-AS1	0.5621599	1.0048963	0.5594	0.576	0.282255032	count	1
ZNF26	0.3134945	0.3574371	0.8771	0.381	0.282263705	count	1
L2HGDH	0.3135432	0.6177096	0.5076	0.612	0.282305873	count	1
ABRA	1.0596793	0.8721076	1.2151	0.224	0.282331902	count	1
TNFRSF10B	0.2358356	0.1956113	1.2056	0.228	0.282364862	count	1
SEC62	0.196908	0.035684	5.5181	3.66E-08	0.282445656	count	0.000859002
CHML	0.2449608	0.2321077	1.0554	0.291	0.28245061	count	1
CORO1B	0.2055242	0.0838755	2.4503	0.0143	0.282962906	count	1
CAND1	0.209971	0.1167805	1.798	0.0723	0.28303462	count	1
TRMT44	0.4311863	0.3141297	1.3726	0.17	0.283047224	count	1
ARF4	0.1983421	0.0489989	4.0479	5.27E-05	0.283091188	count	1
ARL8B	0.205352	0.0945668	2.1715	0.03	0.283342806	count	1
OTUD6B-AS1	0.2013405	0.0701759	2.8691	0.00414	0.283407713	count	1
SUSD1	0.3412663	0.3450777	0.989	0.323	0.283697469	count	1
TRNAU1AP	0.2170848	0.1359471	1.5968	0.11	0.283820403	count	1
NLGN3	1.5696365	1.187622	1.3217	0.186	0.283882016	count	1
ANKRD49	0.2247816	0.155998	1.4409	0.15	0.283987608	count	1
RNGTT	0.2538245	0.1960657	1.2946	0.196	0.283999939	count	1
SMG5	0.2936596	0.2365191	1.2416	0.214	0.284001076	count	1
EFNA3	0.7134984	0.7793579	0.9155	0.36	0.284030296	count	1
USP20	0.3156282	0.3100171	1.0181	0.309	0.284110745	count	1
RAB2B	0.2255545	0.1604303	1.4059	0.16	0.284195949	count	1
LMAN1	0.2001004	0.0519754	3.8499	0.00012	0.284250377	count	1
HLA-DMA	0.2113298	0.1592588	1.327	0.185	0.28445857	count	1
AL162231.2	0.4856093	0.4221635	1.1503	0.25	0.284509111	count	1
TAMM41	0.2582611	0.205036	1.2596	0.208	0.284516895	count	1
TMED3	0.2033686	0.0674454	3.0153	0.00258	0.284692001	count	1
AC009779.2	0.2599965	0.2989461	0.8697	0.385	0.284826068	count	1
MLLT10	0.2209672	0.1536981	1.4377	0.151	0.284841639	count	1
ATG14	0.2154199	0.1563826	1.3775	0.168	0.284904276	count	1
SWSAP1	0.272621	0.2530985	1.0771	0.281	0.285002176	count	1
IDNK	0.224762	0.188209	1.1942	0.232	0.285047054	count	1
OPCML	1.074624	1.2661587	0.8487	0.396	0.285110335	count	1
RBM38	0.2602802	0.2688809	0.968	0.333	0.2851306	count	1
TOB2	0.212599	0.1312182	1.6202	0.105	0.285415493	count	1
TCHP	0.2202551	0.1615952	1.363	0.173	0.285462355	count	1
CSNK1D	0.2221593	0.1359783	1.6338	0.102	0.285537522	count	1
ARL6IP5	0.1985859	0.0307986	6.4479	1.28E-10	0.28554186	count	3.03E-06
AP003119.2	1.587739	0.6551659	2.4234	0.0154	0.285621819	count	1
AL356599.1	0.3074882	0.3528209	0.8715	0.384	0.285622387	count	1
STRADB	0.2714378	0.2252987	1.2048	0.228	0.285919883	count	1
TRIB1	0.2957727	0.3758831	0.7869	0.431	0.285980103	count	1
MARS2	1.599399	1.078353	1.4832	0.138	0.286728453	count	1

PCDHGA2	0.7217502	0.5149335	1.4016	0.161	0.28672876	count	1
FBXO16	0.7217502	0.9044017	0.798	0.425	0.28672876	count	1
ZFAND4	0.4372801	0.3506212	1.2472	0.212	0.286731293	count	1
PDE4A	0.2402897	0.2066921	1.1625	0.245	0.286876762	count	1
POU2F1	0.2312352	0.2369117	0.976	0.329	0.28690422	count	1
MAP1LC3A	0.2051561	0.0835295	2.4561	0.0141	0.287030897	count	1
TMEM161A	0.2309921	0.1877853	1.2301	0.219	0.287083835	count	1
ZCCHC7	0.21212	0.1112541	1.9066	0.0566	0.287120407	count	1
PDIA3	0.2004547	0.0350492	5.7192	1.16E-08	0.287139852	count	0.000272832
DCP1A	0.2236819	0.1452963	1.5395	0.124	0.287191741	count	1
ANXA4	0.2021608	0.0492889	4.1015	4.19E-05	0.287289618	count	0.958672
UPF1	0.2362172	0.1773142	1.3322	0.183	0.287502918	count	1
STAT5B	0.2252117	0.1905334	1.182	0.237	0.287618592	count	1
ZNF598	0.2731357	0.2130789	1.2819	0.2	0.287665809	count	1
AL121603.2	0.2977247	0.3008189	0.9897	0.322	0.287807354	count	1
ZNF8	0.5283063	0.3756648	1.4063	0.16	0.287892226	count	1
HYPK	0.3552622	0.3295073	1.0782	0.281	0.287978246	count	1
TBX19	1.0905673	0.8224192	1.326	0.185	0.28804091	count	1
NFYB	0.2092586	0.0918333	2.2787	0.0227	0.28813561	count	1
SNX14	0.2283266	0.1494386	1.5279	0.127	0.288425678	count	1
ACBD4	0.2444981	0.2003516	1.2203	0.222	0.288568297	count	1
MND1	0.727444	0.6856835	1.0609	0.289	0.288582853	count	1
CTSW	0.727444	0.8268963	0.8797	0.379	0.288582853	count	1
PKD1L1	1.6210006	1.8846257	0.8601	0.3898	0.28874994	count	1
DCTN5	0.2432945	0.2210065	1.1008	0.271	0.288834258	count	1
ANGPT4	0.4939131	0.5415122	0.9121	0.362	0.288897056	count	1
EML3	0.2669706	0.2184985	1.2218	0.222	0.288914145	count	1
ZSCAN9	0.3270239	0.280987	1.1638	0.245	0.289015665	count	1
PSENEN	0.2068309	0.0764183	2.7066	0.00683	0.289053475	count	1
TCFL5	0.3028514	0.2495871	1.2134	0.225	0.289071978	count	1
BEND3	0.8642776	0.7817064	1.1056	0.269	0.289267907	count	1
ACIN1	0.210749	0.1010364	2.0859	0.0371	0.289278813	count	1
KLHL28	0.2314031	0.1434177	1.6135	0.107	0.289411121	count	1
DCHS1	0.2643164	0.3039403	0.8696	0.385	0.289461475	count	1
SAMD4B	0.2173478	0.1832856	1.1858	0.236	0.289523506	count	1
UBE3D	0.3487078	0.3491595	0.9987	0.318	0.289584484	count	1
PLCG1	0.3076615	0.3415285	0.9008	0.368	0.28973959	count	1
AL353194.1	0.3077417	0.3701722	0.8313	0.406	0.289812504	count	1
AP000766.1	1.63262	0.8423259	1.9382	0.0527	0.289822184	count	1
ALG1L	0.4664481	0.598251	0.7797	0.436	0.289863086	count	1
IQCH-AS1	0.406146	0.4228514	0.9605	0.337	0.290252014	count	1
CHMP1B	0.2109971	0.1236282	1.7067	0.088	0.29028228	count	1
RIBC1	1.6378051	1.0680021	1.5335	0.125	0.290297127	count	1
AC139887.4	1.6378051	1.1993922	1.3655	0.172	0.290297127	count	1
MED7	0.2227739	0.1388032	1.605	0.109	0.290343369	count	1

DNAJC13	0.2273197	0.1764496	1.2883	0.198	0.29060596	count	1
MAD1L1	0.2686059	0.2316787	1.1594	0.246	0.290645501	count	1
SNX4	0.2179035	0.1203864	1.81	0.0704	0.290664962	count	1
FAM76B	0.2170005	0.1991688	1.0895	0.276	0.290718656	count	1
CNTN5	0.7340497	0.5971568	1.2292	0.219	0.290725845	count	1
ITGA11	0.2217597	0.1402319	1.5814	0.114	0.29090313	count	1
AC133550.2	0.6455306	0.6703474	0.963	0.336	0.290998313	count	1
SLC39A9	0.2743693	0.2455411	1.1174	0.264	0.290999501	count	1
PELP1	0.2458796	0.2067638	1.1892	0.234	0.291028957	count	1
IL1RAP	0.4447294	0.4410649	1.0083	0.313	0.291221999	count	1
HMOX2	0.2129617	0.1110433	1.9178	0.0552	0.291266106	count	1
C12orf29	0.2183616	0.1113051	1.9618	0.0499	0.291405154	count	1
SENP1	0.2581388	0.2046948	1.2611	0.207	0.291407124	count	1
STK11IP	0.3695992	0.5066561	0.7295	0.466	0.291470604	count	1
SIGMAR1	0.2225653	0.1232555	1.8057	0.071	0.291555322	count	1
COQ8A	0.2607081	0.2046148	1.2741	0.203	0.291556669	count	1
LRRC14	0.2982578	0.3121368	0.9555	0.339	0.291619155	count	1
NAV2	0.217609	0.1971908	1.1035	0.27	0.291880942	count	1
EHMT2	0.2301383	0.1894116	1.215	0.224	0.292161866	count	1
FALEC	0.5848148	0.868314	0.6735	0.501	0.292166759	count	1
APBA1	1.6590794	0.9237515	1.796	0.0726	0.292224401	count	1
CAPRIN1	0.2131	0.0898036	2.3725	0.0177	0.292277868	count	1
FAM107A	0.2737203	0.3462747	0.7905	0.429	0.29230963	count	1
UTP3	0.2202534	0.1999883	1.1013	0.271	0.292360117	count	1
BLOC1S2	0.2155893	0.110944	1.9432	0.0521	0.292560638	count	1
CXXC4	0.8772335	0.555557	1.579	0.114	0.292597075	count	1
LRBA	0.2499517	0.2165048	1.1545	0.248	0.293102052	count	1
CIP2A	0.3958012	0.4145056	0.9549	0.34	0.293161949	count	1
CNOT1	0.2297165	0.1842134	1.247	0.212	0.293323063	count	1
RBM28	0.2320362	0.1570792	1.4772	0.14	0.293448812	count	1
SPNS2	0.5399194	0.4251096	1.2701	0.204	0.293505114	count	1
CRYM	1.1208368	0.8532944	1.3135	0.189	0.293510543	count	1
DPY19L1	0.2446495	0.2385083	1.0257	0.305	0.293523085	count	1
SEC22B	0.2102277	0.0861145	2.4413	0.0147	0.293586738	count	1
MOSMO	0.2157383	0.1289772	1.6727	0.0945	0.293618054	count	1
TMEM68	0.2558667	0.2051528	1.2472	0.212	0.293702877	count	1
RUNX1	0.245462	0.2014621	1.2184	0.223	0.293735904	count	1
CFAP70	0.4733873	0.6956844	0.6805	0.496	0.293787084	count	1
EEFSEC	0.2432592	0.1826964	1.3315	0.183	0.293996421	count	1
SSPN	0.2056462	0.0450385	4.566	5.13E-06	0.294090898	count	0.1185543
ISYNA1	0.2074746	0.074999	2.7664	0.0057	0.294249346	count	1
BBOF1	0.312652	0.2942635	1.0625	0.288	0.29427312	count	1
TFCP2	0.2819382	0.2403426	1.1731	0.241	0.294497712	count	1
LINC00342	0.5045903	0.5540215	0.9108	0.362	0.294514176	count	1
DCUN1D4	0.2262318	0.1436101	1.5753	0.115	0.294590671	count	1

RPS6KB1	0.2277977	0.2020416	1.1275	0.26	0.294652579	count	1
RXRG	1.6877637	0.9037081	1.8676	0.0619	0.294768115	count	1
PAF1	0.2223018	0.1303779	1.7051	0.0883	0.294910531	count	1
YME1L1	0.2110319	0.0988807	2.1342	0.0329	0.29493435	count	1
DNAH7	0.8866282	0.7586507	1.1687	0.243	0.29499316	count	1
HPS1	0.2251823	0.1415525	1.5908	0.112	0.295163485	count	1
MYO19	0.4311821	0.4196067	1.0276	0.304	0.295382178	count	1
PRR5L	1.1314159	0.7051751	1.6044	0.109	0.295393337	count	1
ZNF106	0.2098801	0.0730132	2.8745	0.00407	0.295814196	count	1
SEC61A2	0.6582933	0.4858106	1.355	0.175	0.295864482	count	1
FBXL5	0.2173463	0.106533	2.0402	0.0414	0.296092171	count	1
FAM110A	0.3241785	0.4197474	0.7723	0.44	0.296140533	count	1
FRS2	0.2348131	0.1755308	1.3377	0.181	0.296150203	count	1
ZNF350-AS1	0.7511197	1.09815	0.684	0.494	0.296223653	count	1
C11orf96	0.2057291	0.0595055	3.4573	0.000552	0.296427687	count	1
GGT1	1.7113163	1.0219117	1.6746	0.0941	0.296810514	count	1
LGR5	1.7113163	1.2957984	1.3207	0.187	0.296810514	count	1
SESN2	0.2781223	0.3307344	0.8409	0.4	0.296900505	count	1
PIWIL2	0.7532616	0.7390596	1.0192	0.308	0.296909423	count	1
ARHGAP23	0.2494951	0.3280396	0.7606	0.447	0.296913125	count	1
E2F1	0.8943033	0.9930085	0.9006	0.368	0.296939446	count	1
TSC22D4	0.2320861	0.1505899	1.5412	0.123	0.296966958	count	1
SCRN2	0.2310718	0.1489958	1.5509	0.121	0.297192725	count	1
CNOT11	0.2633682	0.2039139	1.2916	0.197	0.297201189	count	1
ERBB3	1.7161737	0.6640257	2.5845	0.00979	0.297226616	count	1
ZNF430	0.261214	0.2099076	1.2444	0.213	0.297351034	count	1
FPGS	0.2520857	0.2608008	0.9666	0.334	0.29739119	count	1
EDNRA	0.2304097	0.1492882	1.5434	0.123	0.297470055	count	1
GDPD1	0.5973489	0.4107542	1.4543	0.146	0.297599745	count	1
APBB3	0.3082529	0.2773917	1.1113	0.267	0.29764779	count	1
SRPRA	0.2114395	0.0688136	3.0726	0.00214	0.297706374	count	1
GPATCH2L	0.2237509	0.1536885	1.4559	0.146	0.297720204	count	1
LOXL1	0.2140079	0.0928196	2.3056	0.0212	0.29781142	count	1
VEZF1	0.2170736	0.096161	2.2574	0.024	0.297985723	count	1
TRPC4AP	0.2485589	0.180503	1.377	0.169	0.298150177	count	1
ZNF207	0.2150842	0.1000716	2.1493	0.0317	0.298153163	count	1
ZNF664	0.2608554	0.1981538	1.3164	0.188	0.298158464	count	1
ATP6V1H	0.2294187	0.1599645	1.4342	0.152	0.298238114	count	1
PNPLA8	0.2187751	0.0961632	2.275	0.023	0.298246903	count	1
DHRS7	0.2096782	0.0498847	4.2033	2.69E-05	0.298247224	count	0.6167901
LBH	0.2098899	0.0746152	2.813	0.00493	0.298267446	count	1
AC027307.2	0.8998576	0.7926368	1.1353	0.256	0.298341696	count	1
RIOK1	0.227331	0.1278249	1.7785	0.0754	0.29836247	count	1
TRABD2B	0.2299663	0.1955149	1.1762	0.24	0.298456861	count	1
VPS45	0.2424409	0.1832661	1.3229	0.186	0.298534668	count	1

HBP1	0.2172799	0.094848	2.2908	0.022	0.298536996	count	1
TACC2	0.2429079	0.1785065	1.3608	0.174	0.298542754	count	1
TM9SF2	0.2129676	0.0806771	2.6398	0.00833	0.298654275	count	1
CCL26	0.3271169	0.4903101	0.6672	0.505	0.298719353	count	1
AC110285.2	1.1504973	1.1526825	0.9981	0.318	0.298751708	count	1
EXOSC10	0.2426897	0.1650773	1.4702	0.142	0.298837522	count	1
NUDCD2	0.2157101	0.0878222	2.4562	0.0141	0.298838454	count	1
NPEPPS	0.2198903	0.1275458	1.724	0.0848	0.298859516	count	1
HERC1	0.2331507	0.1733096	1.3453	0.179	0.298943542	count	1
PHYH	0.2141235	0.0809637	2.6447	0.00821	0.29896793	count	1
SRBD1	0.2353382	0.1798955	1.3082	0.191	0.299062578	count	1
FANCC	0.9037114	0.5286421	1.7095	0.0874	0.299311556	count	1
LZTS2	0.2174218	0.1116981	1.9465	0.0517	0.299436829	count	1
CHPF	0.2146399	0.1064367	2.0166	0.0438	0.299467634	count	1
PDZD4	0.5140967	0.8017181	0.6412	0.521	0.299491625	count	1
AGAP5	0.3918715	0.966651	0.4054	0.685	0.299509406	count	1
CTNNA3	0.5142278	0.3902895	1.3176	0.188	0.299560115	count	1
IFT140	0.3335397	0.3158643	1.056	0.291	0.299572645	count	1
KDM5A	0.2206585	0.1280847	1.7228	0.085	0.299658243	count	1
LIN54	0.306768	0.2925709	1.0485	0.294	0.299671824	count	1
LNPK	0.2242128	0.1079341	2.0773	0.0378	0.299701092	count	1
ATXN7L1	0.3282571	0.2745153	1.1958	0.232	0.299719466	count	1
HERC2	0.2233084	0.1298769	1.7194	0.0856	0.29972149	count	1
NELFCD	0.2269669	0.129394	1.7541	0.0795	0.299740261	count	1
VPS41	0.2235005	0.1361607	1.6414	0.101	0.299743452	count	1
SLC19A3	1.7465089	1.2122162	1.4408	0.15	0.299786148	count	1
C1QTNF12	1.7465089	1.3218393	1.3213	0.186	0.299786148	count	1
DNAJC24	0.2477309	0.1896591	1.3062	0.192	0.300016832	count	1
ZNF592	0.3072191	0.2933227	1.0474	0.295	0.300098217	count	1
TRIM3	0.2812594	0.2623308	1.0722	0.284	0.300169861	count	1
KITLG	0.235159	0.1357172	1.7327	0.0832	0.300212135	count	1
THEM4	0.2429298	0.2201824	1.1033	0.27	0.300215076	count	1
CFLAR	0.2156221	0.1023984	2.1057	0.0353	0.300279834	count	1
ALPK1	0.2538398	0.2343232	1.0833	0.279	0.300312232	count	1
IL6ST	0.2116361	0.0547375	3.8664	0.000112	0.300393865	count	1
PBX2	0.2394607	0.2346568	1.0205	0.308	0.300706966	count	1
RFK	0.2270751	0.1160959	1.9559	0.0505	0.300724516	count	1
ABCB9	0.6711466	0.5994833	1.1195	0.263	0.300729553	count	1
DENND4A	0.270459	0.2456589	1.101	0.271	0.300774467	count	1
ADAMTS6	0.2764993	0.3648651	0.7578	0.449	0.300786855	count	1
LRRC3	0.6715208	0.907712	0.7398	0.459	0.300870658	count	1
ARC	0.4222532	0.4843457	0.8718	0.383	0.30094895	count	1
PDPR	0.2820434	0.2500859	1.1278	0.259	0.300986605	count	1
NOP9	0.7660839	0.4493541	1.7049	0.0883	0.300995932	count	1
EXOC5	0.224818	0.1426703	1.5758	0.115	0.301014201	count	1

TMBIM4	0.210466	0.0389921	5.3977	7.18E-08	0.30104878	count	0.001682274
SAXO1	0.672246	0.7889098	0.8521	0.394	0.301144023	count	1
CCDC81	0.672246	0.973443	0.6906	0.49	0.301144023	count	1
AC002310.1	0.6731	0.6293086	1.0696	0.285	0.301465805	count	1
MTIF2	0.2359432	0.1587591	1.4862	0.137	0.301534986	count	1
PLCXD1	0.7681855	0.5183298	1.482	0.138	0.301662617	count	1
TWF1	0.2221908	0.1024675	2.1684	0.0302	0.301730418	count	1
TMEM259	0.2207939	0.105173	2.0993	0.0359	0.301743822	count	1
ID3	0.2095723	0.0445144	4.708	2.59E-06	0.301890552	count	0.0600362
FAM8A1	0.2397729	0.155812	1.5389	0.124	0.301936037	count	1
MAN2C1	0.2493838	0.2577157	0.9677	0.333	0.301992698	count	1
PDP1	0.2212124	0.1329366	1.664	0.0962	0.302045988	count	1
KATNB1	0.3838456	0.3534794	1.0859	0.278	0.302059953	count	1
NCOA1	0.226789	0.1298204	1.7469	0.0807	0.302172184	count	1
WDR43	0.2255224	0.1092488	2.0643	0.0391	0.30219941	count	1
AL139393.2	0.9153877	0.4451568	2.0563	0.0398	0.302234593	count	1
HIGD1A	0.2135681	0.0587634	3.6344	0.000282	0.302444462	count	1
AC007383.2	0.4890573	0.5303571	0.9221	0.357	0.302603558	count	1
CEP76	0.3653349	0.3359605	1.0874	0.277	0.302687933	count	1
SH3PXD2A	0.2401374	0.1999119	1.2012	0.23	0.302799921	count	1
EPC1	0.2167619	0.0750426	2.8885	0.00389	0.302809701	count	1
SMU1	0.2215961	0.0950624	2.3311	0.0198	0.302836017	count	1
MAPK1IP1L	0.2212636	0.0972981	2.2741	0.023	0.302956025	count	1
AL163051.1	1.174816	0.8774929	1.3388	0.181	0.302962528	count	1
RAP2B	0.2447537	0.1483302	1.6501	0.099	0.302973321	count	1
SORBS3	0.216487	0.0833877	2.5961	0.00947	0.303059478	count	1
SPTSSA	0.2183322	0.0773059	2.8243	0.00476	0.303225305	count	1
TACSTD2	0.4103729	0.3832831	1.0707	0.284	0.303240102	count	1
SCAF11	0.2149982	0.0734105	2.9287	0.00342	0.303254101	count	1
ZNF567	0.2350773	0.1750726	1.3427	0.179	0.303448192	count	1
SAP30BP	0.2229184	0.0988846	2.2543	0.0242	0.303502907	count	1
DBP	0.2240042	0.1104015	2.029	0.0425	0.303759748	count	1
TBX2-AS1	0.2373413	0.1346113	1.7632	0.078	0.303954686	count	1
TLE1	0.2317891	0.1306297	1.7744	0.0761	0.303970669	count	1
GGA3	0.3079642	0.2734327	1.1263	0.26	0.304070424	count	1
C9orf24	0.4664228	1.3881033	0.336	0.7369	0.304218927	count	1
ZNF345	0.3119312	0.310208	1.0056	0.315	0.304549458	count	1
ZNF112	0.377059	0.3736991	1.009	0.313	0.304684744	count	1
CAPZA1	0.220949	0.0937475	2.3569	0.0185	0.304705534	count	1
TIA1	0.2359218	0.140067	1.6843	0.0922	0.304806098	count	1
USP18	0.3200352	0.2833724	1.1294	0.259	0.304893548	count	1
ETFBKMT	0.2970972	0.2772227	1.0717	0.284	0.304948041	count	1
C5	0.3398076	0.337722	1.0062	0.314	0.304964956	count	1
TAF13	0.2425703	0.1590144	1.5255	0.127	0.305001972	count	1
TLE3	0.2482527	0.1933063	1.2842	0.199	0.30503368	count	1

CDKN3	0.4466449	0.5893502	0.7579	0.449	0.305146147	count	1
KLHDC4	0.2731828	0.3187443	0.8571	0.391	0.305229621	count	1
FAM217B	0.2343672	0.1440069	1.6275	0.104	0.305342937	count	1
MAPK12	1.1895192	1.0793044	1.1021	0.27	0.305471247	count	1
AL021707.6	1.1895192	1.1848582	1.0039	0.315	0.305471247	count	1
HES6	0.3094619	0.3959115	0.7816	0.434	0.30550193	count	1
POLG	0.3690982	0.2919543	1.2642	0.206	0.305643997	count	1
FBH1	0.2479197	0.1750722	1.4161	0.157	0.305762283	count	1
STAM-AS1	0.7811759	0.6334028	1.2333	0.218	0.305764325	count	1
HIST1H2AK	0.9303005	0.7943036	1.1712	0.242	0.305934303	count	1
SYT17	1.1925702	0.7531151	1.5835	0.113	0.305988309	count	1
HUWE1	0.228382	0.1195947	1.9096	0.0563	0.306011203	count	1
ERCC6L2	0.2301319	0.1929393	1.1928	0.233	0.30601201	count	1
LINC01550	0.3176965	0.3058814	1.0386	0.299	0.30645292	count	1
HES7	1.1954437	0.9632533	1.241	0.215	0.306474168	count	1
DKK2	1.8302507	1.053483	1.7373	0.0824	0.306512226	count	1
KIF1BP	0.2289977	0.121432	1.8858	0.0594	0.306706995	count	1
FAM98B	0.2619118	0.1852204	1.4141	0.157	0.306904273	count	1
AL662844.4	0.4967829	0.3275518	1.5167	0.129	0.306927382	count	1
AC108866.1	0.9347191	0.7709417	1.2124	0.225	0.307023301	count	1
NDUFB2-AS1	0.9347191	0.9956229	0.9388	0.348	0.307023301	count	1
ZDHHC16	0.2901079	0.2091646	1.387	0.166	0.307274832	count	1
FOXO3	0.2266141	0.1272019	1.7815	0.0749	0.307283374	count	1
AC118549.1	0.2415858	0.1651461	1.4629	0.144	0.307291122	count	1
TBC1D10A	0.2614745	0.1843558	1.4183	0.156	0.30736102	count	1
MTG2	0.3152055	0.224095	1.4066	0.16	0.307639527	count	1
FBXO7	0.225115	0.0971493	2.3172	0.0205	0.307693441	count	1
USP33	0.2278955	0.1151624	1.9789	0.0479	0.307698686	count	1
AC104260.1	1.8460448	0.9846016	1.8749	0.0609	0.307726647	count	1
KMT2E-AS1	0.2588523	0.2079946	1.2445	0.213	0.307885248	count	1
AC097376.2	0.2367852	0.1917578	1.2348	0.217	0.307988155	count	1
HYKK	0.2834738	0.2426656	1.1682	0.243	0.30820174	count	1
PCDHB2	0.3197626	0.3925553	0.8146	0.415	0.308376594	count	1
TLCD2	0.2608811	0.2467075	1.0575	0.29	0.308515843	count	1
RETSAT	0.3093845	0.2861238	1.0813	0.28	0.308565242	count	1
AL139384.1	0.790909	0.7566002	1.0453	0.296	0.308815787	count	1
ZNF462	0.275182	0.2017705	1.3638	0.173	0.308868704	count	1
ZBTB7A	0.2202484	0.0856088	2.5727	0.0101	0.308917787	count	1
AF186192.1	1.8619237	0.9985979	1.8645	0.0623	0.308930765	count	1
EFL1	0.3649784	0.2662032	1.3711	0.17	0.308966543	count	1
SNX6	0.217553	0.0526953	4.1285	3.73E-05	0.308999169	count	0.8538716
CREB3L2	0.2270615	0.1226723	1.851	0.0643	0.309120499	count	1
ELP1	0.2941839	0.369252	0.7967	0.426	0.309260715	count	1
DOCK7	0.2299551	0.1331063	1.7276	0.0841	0.309625496	count	1
TLR1	0.6955934	0.4537591	1.533	0.125	0.30988417	count	1

CTSL	0.218785	0.0572108	3.8242	0.000133	0.310044922	count	1
PCNX4	0.2417476	0.1649094	1.4659	0.143	0.310187872	count	1
PPAT	0.2853583	0.2373458	1.2023	0.229	0.310203624	count	1
CCDC32	0.239348	0.1448007	1.6529	0.0984	0.310277988	count	1
TXNRD3	0.326189	0.2826541	1.154	0.249	0.310542819	count	1
SLC37A4	0.3116187	0.2727523	1.1425	0.253	0.310723238	count	1
NFIA	0.2175322	0.0483049	4.5033	6.90E-06	0.310725966	count	0.1592589
GALNTL6	0.4076663	0.8054534	0.5061	0.613	0.310812405	count	1
STK39	0.2600394	0.2552546	1.0187	0.308	0.310929581	count	1
PTBP3	0.239705	0.1646356	1.456	0.145	0.311254579	count	1
AGK	0.2714888	0.2428662	1.1179	0.264	0.31131211	count	1
AKAP7	0.256621	0.2338142	1.0975	0.272	0.311334949	count	1
POLR3A	0.2774302	0.256868	1.08	0.28	0.311341462	count	1
IL4R	0.2685281	0.2067239	1.299	0.194	0.311394909	count	1
EMP3	0.2169903	0.0449423	4.8282	1.43E-06	0.31141022	count	0.03322462
ATE1	0.2379038	0.155525	1.5297	0.126	0.31150658	count	1
AKAP13	0.2227433	0.078345	2.8431	0.00449	0.311861776	count	1
UBAP1	0.2495852	0.1731029	1.4418	0.149	0.311907014	count	1
PHETA1	0.2888452	0.4106915	0.7033	0.482	0.312030757	count	1
TRIM65	0.2947365	0.2636185	1.118	0.264	0.31205175	count	1
RAD23B	0.2295793	0.1026363	2.2368	0.0254	0.312054054	count	1
GTF2H3	0.2433041	0.1327393	1.8329	0.0669	0.312167619	count	1
STXBP4	0.257322	0.198736	1.2948	0.195	0.312174103	count	1
NFS1	0.2948788	0.2382154	1.2379	0.216	0.312198537	count	1
LINC02158	1.9073181	1.566967	1.2172	0.224	0.312281715	count	1
OTUD5	0.2507892	0.1674672	1.4975	0.134	0.312423599	count	1
DENND1B	0.3241885	0.2791241	1.1614	0.246	0.312494098	count	1
ANKRD13D	0.2349846	0.1540258	1.5256	0.127	0.312579545	count	1
SYT15	1.9117347	1.414704	1.3513	0.177	0.312600619	count	1
HMGXB4	0.2293897	0.110157	2.0824	0.0374	0.31265842	count	1
ZBED5-AS1	0.2660996	0.2062193	1.2904	0.197	0.312710648	count	1
CEP126	0.2282503	0.1160848	1.9662	0.0493	0.312859169	count	1
RBM27	0.3434638	0.2359152	1.4559	0.146	0.313027819	count	1
ACAD9	0.2681237	0.1903291	1.4087	0.159	0.313049782	count	1
NMUR2	0.3079854	0.4248774	0.7249	0.469	0.313058833	count	1
CENPT	0.2539392	0.2189787	1.1597	0.246	0.313097452	count	1
SUSD6	0.2593803	0.2051472	1.2644	0.206	0.31321418	count	1
CCNK	0.2462954	0.1578795	1.56	0.119	0.313225051	count	1
ZNF566	0.2865211	0.2145848	1.3352	0.182	0.31323243	count	1
CCBE1	0.805588	0.737807	1.0919	0.275	0.313382789	count	1
AC012615.1	0.4251205	0.4523402	0.9398	0.347	0.313383868	count	1
THOC1	0.2481037	0.1720451	1.4421	0.149	0.313567696	count	1
ZNF286A	0.4414397	0.4871873	0.9061	0.365	0.313603165	count	1
SCGN	0.6348519	0.7053619	0.9	0.368	0.313639354	count	1
ANKFY1	0.2564552	0.2144964	1.1956	0.232	0.313761314	count	1

PAXBP1	0.2380845	0.1494039	1.5936	0.111	0.31376843	count	1
SLC35E2A	0.371069	0.4136306	0.8971	0.37	0.313860456	count	1
BAG4	0.2330268	0.1312726	1.7751	0.076	0.313960072	count	1
ZNF619	0.6359918	0.5313644	1.1969	0.231	0.314121801	count	1
C8orf48	0.2891092	0.3035097	0.9526	0.341	0.314186122	count	1
WEE1	0.2348704	0.1508165	1.5573	0.119	0.314269135	count	1
HMGCR	0.2909762	0.2424636	1.2001	0.23	0.314277767	count	1
MLYCD	0.2701671	0.2101002	1.2859	0.199	0.314344197	count	1
SH3YL1	0.2457914	0.1755194	1.4004	0.161	0.314345982	count	1
AGMO	1.9368173	1.1714918	1.6533	0.0984	0.314388414	count	1
GCSH	0.2257118	0.0821094	2.7489	0.00601	0.314439888	count	1
PTGER4	0.274272	0.1997698	1.3729	0.17	0.314445009	count	1
SGSH	0.2659851	0.2257684	1.1781	0.239	0.314457617	count	1
CRYGN	1.2439083	1.0363822	1.2002	0.23	0.314510262	count	1
GIT1	0.284579	0.2562538	1.1105	0.267	0.314537695	count	1
UTP20	0.3264506	0.2280391	1.4316	0.152	0.314596812	count	1
PIM2	0.4844158	0.4687597	1.0334	0.301	0.314907258	count	1
ZNF621	0.2834817	0.2156283	1.3147	0.189	0.314950165	count	1
AC024257.3	1.2470245	0.9556087	1.305	0.192	0.315016813	count	1
GLYR1	0.2487199	0.1458586	1.7052	0.0882	0.315135629	count	1
MAP3K8	0.2335681	0.1122096	2.0815	0.0375	0.315217306	count	1
RABEPK	0.2434688	0.1594681	1.5268	0.127	0.315308229	count	1
CYR61	0.2192383	0.0503455	4.3547	1.37E-05	0.315362165	count	0.3153192
ACCS	0.3584268	0.4555484	0.7868	0.431	0.315499896	count	1
TMEM127	0.2621268	0.1836036	1.4277	0.153	0.315738779	count	1
RRAGD	0.2501785	0.2075576	1.2053	0.228	0.315751523	count	1
USP25	0.2642651	0.1967632	1.3431	0.179	0.315907854	count	1
PNRC1	0.2197568	0.0331866	6.6218	4.06E-11	0.316008578	count	9.63E-07
CATSPER2	0.328017	0.5329195	0.6155	0.538	0.316052162	count	1
AL662796.1	1.961717	0.8102061	2.4213	0.0155	0.316124234	count	1
FMO4	0.2988168	0.313605	0.9528	0.341	0.316259183	count	1
UNKL	0.3174847	0.2228031	1.425	0.154	0.31638386	count	1
KCTD20	0.2408447	0.1193623	2.0178	0.0437	0.316389365	count	1
USP36	0.2730263	0.2498467	1.0928	0.275	0.316520065	count	1
IGSF8	0.2327099	0.106907	2.1767	0.0296	0.316618047	count	1
AGO3	0.239859	0.1815154	1.3214	0.186	0.316654269	count	1
JMJD4	0.2741659	0.2108347	1.3004	0.194	0.316687716	count	1
PTOV1-AS1	0.713991	0.7643382	0.9341	0.35	0.316688365	count	1
SCD	0.713991	0.801011	0.8914	0.373	0.316688365	count	1
C2orf69	0.2836831	0.2340006	1.2123	0.225	0.316716694	count	1
DDX3Y	0.2315331	0.1087297	2.1294	0.0333	0.31683353	count	1
LINC01006	0.374906	0.4103979	0.9135	0.361	0.316938709	count	1
ACO1	0.2450096	0.1359165	1.8026	0.0715	0.317012519	count	1
AC004982.2	0.3672771	0.4956071	0.7411	0.459	0.317039549	count	1
RNF168	0.2367757	0.1272571	1.8606	0.0629	0.317067244	count	1

DAXX	0.2510312	0.1547517	1.6222	0.105	0.317230233	count	1
MTPAP	0.2727097	0.2143516	1.2723	0.203	0.31725161	count	1
EPG5	0.3068366	0.2610449	1.1754	0.24	0.317272221	count	1
HIGD2A	0.2242149	0.0633987	3.5366	0.00041	0.317550508	count	1
TTC8	0.2549566	0.1819965	1.4009	0.161	0.317556448	count	1
EPHX3	0.9782293	0.805526	1.2144	0.225	0.317571935	count	1
ZDHHC1	0.2831516	0.2043472	1.3856	0.166	0.317630237	count	1
WTIP	0.2257931	0.0970855	2.3257	0.0201	0.317908897	count	1
BNIP3L	0.2235885	0.0531036	4.2104	2.61E-05	0.318036983	count	0.5984991
HEATR5B	0.2965152	0.2612465	1.135	0.256	0.318115302	count	1
ARRDC4	0.3689625	0.3011101	1.2253	0.221	0.318422817	count	1
MBD1	0.2620293	0.1782139	1.4703	0.142	0.318501849	count	1
METTL17	0.2779232	0.1682313	1.652	0.0986	0.31855296	count	1
ZNF503-AS2	0.3556682	0.3235679	1.0992	0.272	0.31856696	count	1
OAT	0.2268369	0.0662782	3.4225	0.000627	0.318587189	count	1
PABPN1	0.233807	0.105894	2.2079	0.0273	0.318654023	count	1
HRC	0.3108933	0.2554316	1.2171	0.224	0.318691979	count	1
OTUD7A	1.271209	0.7117406	1.7861	0.0742	0.318906922	count	1
NUP160	0.3171177	0.2035553	1.5579	0.119	0.319108236	count	1
AC074117.1	0.9858038	0.666339	1.4794	0.139	0.319376066	count	1
BAZ1A	0.2560656	0.1567423	1.6337	0.102	0.319423605	count	1
RNF213	0.2348519	0.1224828	1.9174	0.0553	0.319519759	count	1
AL354822.1	0.9866608	0.622896	1.584	0.113	0.319579603	count	1
SEC22A	0.2768074	0.195286	1.4174	0.156	0.319683866	count	1
NFKBID	0.470017	0.4996986	0.9406	0.347	0.31978689	count	1
ANXA1	0.2222178	0.0314961	7.0554	2.05E-12	0.319865012	count	4.87E-08
VAPB	0.2466085	0.1294144	1.9056	0.0568	0.319882304	count	1
ZNF12	0.2644374	0.1876213	1.4094	0.159	0.319970084	count	1
ZNF141	0.263855	0.1824731	1.446	0.148	0.319991316	count	1
ZNF224	0.2543821	0.1841199	1.3816	0.167	0.320139607	count	1
SLC16A9	0.2607225	0.2413918	1.0801	0.28	0.320162917	count	1
SPPL2A	0.2286748	0.0711965	3.2119	0.00133	0.320212008	count	1
AC007228.2	0.8280816	0.8298464	0.9979	0.318	0.320299377	count	1
AC018529.1	1.2801133	1.19169	1.0742	0.283	0.320320839	count	1
SPIB	1.2801133	1.341889	0.954	0.34	0.320320839	count	1
ELMOD1	1.2801133	1.417355	0.9032	0.366	0.320320839	count	1
MRPL35	0.2389723	0.1293268	1.8478	0.0647	0.320380195	count	1
DDAH2	0.2240677	0.0421624	5.3144	1.13E-07	0.320446048	count	0.002645556
PARN	0.264856	0.1566789	1.6904	0.091	0.320469534	count	1
NUDCD3	0.2473367	0.1414619	1.7484	0.0805	0.320549744	count	1
STX2	0.2351481	0.1065974	2.2059	0.0274	0.320626348	count	1
DNAJC1	0.2290076	0.0755231	3.0323	0.00244	0.320698095	count	1
SIX2	0.3290853	0.2914528	1.1291	0.259	0.320711187	count	1
TMEM79	2.0308025	0.9576585	2.1206	0.034	0.320743574	count	1
CCDC144A	0.2535377	0.3410671	0.7434	0.457	0.320775623	count	1

BCL2L12	0.2846932	0.2032794	1.4005	0.161	0.320778877	count	1
VPS8	0.2677582	0.2395822	1.1176	0.264	0.320841911	count	1
SBF1	0.3885311	0.3915594	0.9923	0.321	0.320850996	count	1
AC007255.1	2.033306	1.409957	1.4421	0.149	0.320905661	count	1
RNF2	0.2848572	0.18845	1.5116	0.131	0.320959888	count	1
ATP13A1	0.308057	0.285806	1.0779	0.281	0.321020748	count	1
LGMN	0.2305779	0.0816722	2.8232	0.00478	0.321083577	count	1
ZNF135	0.3133484	0.548961	0.5708	0.568	0.321133511	count	1
GOLGA2	0.229424	0.081712	2.8077	0.00502	0.321151789	count	1
CREBL2	0.2430881	0.1356918	1.7915	0.0733	0.321257559	count	1
YIPF4	0.2331433	0.104146	2.2386	0.0252	0.321268991	count	1
CHIC1	0.2959147	0.2406076	1.2299	0.219	0.32140483	count	1
MMS19	0.3476539	0.3081115	1.1283	0.259	0.321413088	count	1
CABLES1	0.3728947	0.3883591	0.9602	0.337	0.321647317	count	1
PSPH	0.3383272	0.3370784	1.0037	0.316	0.321659844	count	1
CCDC127	0.2410679	0.1191882	2.0226	0.0432	0.321961081	count	1
SNTB1	0.3201985	0.3085361	1.0378	0.299	0.32210936	count	1
CIZ1	0.2625186	0.176015	1.4915	0.136	0.322340378	count	1
UTY	0.273692	0.226327	1.2093	0.227	0.322466454	count	1
GLUD1	0.2338383	0.0794271	2.9441	0.00326	0.322566734	count	1
AC106739.1	2.0596686	1.0146783	2.0299	0.0424	0.322590659	count	1
PEX3	0.2519567	0.152756	1.6494	0.0991	0.32283705	count	1
RASA3	0.2731924	0.2189298	1.2479	0.212	0.322841069	count	1
SLC4A7	0.238782	0.1249559	1.9109	0.0561	0.322938373	count	1
GNB1L	0.3355264	0.3825568	0.8771	0.381	0.323021226	count	1
AC007325.4	0.5258506	0.4489622	1.1713	0.242	0.323059925	count	1
KLHL7	0.2541326	0.2010835	1.2638	0.206	0.323094291	count	1
RRNAD1	0.2718509	0.2268281	1.1985	0.231	0.323105372	count	1
SMARCA4	0.249593	0.1857949	1.3434	0.179	0.323173578	count	1
NAAA	0.2349748	0.1115472	2.1065	0.0352	0.323262423	count	1
SPPL2B	0.2810183	0.1993257	1.4098	0.159	0.323263474	count	1
HLA-DMB	0.3749111	0.5108302	0.7339	0.463	0.323299288	count	1
XPO4	0.3129145	0.2728313	1.1469	0.251	0.323373421	count	1
FAM91A1	0.2692983	0.1720816	1.5649	0.118	0.32346546	count	1
PPTC7	0.332096	0.3090111	1.0747	0.283	0.323540748	count	1
KLHL18	0.3158583	0.2691783	1.1734	0.241	0.323628192	count	1
INTS13	0.3250351	0.3049808	1.0658	0.287	0.323658571	count	1
UBXN11	0.2849274	0.2266763	1.257	0.209	0.32381535	count	1
CFAP69	0.3755441	0.3628294	1.035	0.301	0.323817675	count	1
SVBP	0.2345329	0.0917403	2.5565	0.0106	0.323942176	count	1
CENPC	0.2307871	0.0849981	2.7152	0.00665	0.32400115	count	1
CHD8	0.2511458	0.14117	1.779	0.0753	0.324012024	count	1
MIEF2	0.3021743	0.2240982	1.3484	0.178	0.32403204	count	1
GIGYF1	0.2603204	0.1803758	1.4432	0.149	0.324159709	count	1
MAP3K14	0.6600281	0.5014793	1.3162	0.188	0.324224866	count	1

HMGA1	0.3509289	0.3148298	1.1147	0.265	0.324314303	count	1
PRRG1	0.29525	0.230562	1.2806	0.2	0.324338406	count	1
ABCD1	0.4268096	0.3877826	1.1006	0.271	0.324424659	count	1
FHIT	0.3331119	0.3601187	0.925	0.355	0.324495055	count	1
DHX8	0.2664546	0.2122773	1.2552	0.209	0.324497999	count	1
HSDL2	0.2361115	0.0939431	2.5133	0.012	0.324600671	count	1
ZC3H7B	0.2576587	0.2235176	1.1527	0.249	0.324660075	count	1
AL136418.1	2.0965907	1.0527934	1.9915	0.0465	0.324884433	count	1
ATAD3A	0.2847555	0.3319579	0.8578	0.391	0.324948677	count	1
SNIP1	0.282563	0.1773475	1.5933	0.111	0.325007028	count	1
PRPF19	0.261011	0.1382719	1.8877	0.0591	0.325009633	count	1
SRPK2	0.2341159	0.0832512	2.8122	0.00495	0.325048806	count	1
IFT43	0.2330998	0.091031	2.5607	0.0105	0.325159117	count	1
ZBTB44	0.2469937	0.158059	1.5627	0.118	0.325229579	count	1
NIPA2	0.256313	0.157415	1.6283	0.104	0.325452308	count	1
DBF4B	0.7382338	0.6513522	1.1334	0.257	0.325542839	count	1
CFAP53	1.0120107	1.094519	0.9246	0.355	0.325545027	count	1
DSCC1	0.3342317	0.4199403	0.7959	0.426	0.325546692	count	1
KCTD13	0.3237309	0.3989563	0.8114	0.417	0.325547815	count	1
ZNF678	0.3237446	0.2520857	1.2843	0.199	0.32556115	count	1
NNT	0.2772856	0.1977683	1.4021	0.161	0.325634995	count	1
HIPK3	0.2370875	0.0931633	2.5449	0.011	0.325652599	count	1
SIDT2	0.3106372	0.2219947	1.3993	0.162	0.326077989	count	1
CPLX1	1.0155107	1.380009	0.7359	0.462	0.326360348	count	1
JAK1	0.2308641	0.0567741	4.0664	4.88E-05	0.326603739	count	1
NF1	0.242077	0.132455	1.8276	0.0677	0.326749673	count	1
FBXL14	0.4448209	0.3606908	1.2332	0.218	0.326845628	count	1
DTYMK	0.2522578	0.1462152	1.7252	0.0846	0.32687419	count	1
CTDSPL2	0.2459101	0.1409783	1.7443	0.0812	0.327022176	count	1
CENPL	0.4619719	0.4470792	1.0333	0.302	0.327038486	count	1
FASTKD3	0.3094688	0.3155727	0.9807	0.327	0.327226995	count	1
TSPAN12	0.2964409	0.1954244	1.5169	0.129	0.32734988	count	1
NBL1	0.228559	0.0506816	4.5097	6.69E-06	0.327357555	count	0.15443196
HEMK1	0.3034054	0.2688813	1.1284	0.259	0.327365757	count	1
MED22	0.3225216	0.274195	1.1762	0.24	0.327370265	count	1
EED	0.2609483	0.2065145	1.2636	0.206	0.327393442	count	1
ZNF160	0.2629488	0.1593964	1.6497	0.0991	0.327394177	count	1
KIDINS220	0.2366592	0.0871204	2.7165	0.00663	0.327484057	count	1
MIEF1	0.312071	0.2785865	1.1202	0.263	0.327540833	count	1
WASHC2A	0.2618569	0.1924224	1.3608	0.174	0.327561504	count	1
ALYREF	0.2379822	0.1028761	2.3133	0.0208	0.327653826	count	1
MCM6	0.2985272	0.2183985	1.3669	0.172	0.327854104	count	1
CMBL	0.2378527	0.0990762	2.4007	0.0164	0.328036621	count	1
ZBTB43	0.2718383	0.1945289	1.3974	0.162	0.328039941	count	1
NUFIP2	0.2396212	0.1072503	2.2342	0.0255	0.328320295	count	1

ZNF43	0.2583137	0.1531976	1.6861	0.0919	0.328356723	count	1
ABCE1	0.241593	0.1079887	2.2372	0.0253	0.328565985	count	1
CHST14	0.2592697	0.1889642	1.3721	0.17	0.328770218	count	1
PHKB	0.249658	0.1223715	2.0402	0.0414	0.32891373	count	1
CBLN3	0.6713057	0.4545477	1.4769	0.14	0.328919156	count	1
LRIF1	0.2622203	0.1455231	1.8019	0.0716	0.328971487	count	1
ADNP2	0.2995862	0.2086113	1.4361	0.151	0.328989728	count	1
WBP4	0.2365366	0.0910238	2.5986	0.0094	0.329077036	count	1
MED12	0.4482321	0.2663565	1.6828	0.0925	0.329166233	count	1
COPB2	0.2359932	0.0711707	3.3159	0.000922	0.329348225	count	1
WDR7	0.338432	0.2874781	1.1772	0.239	0.32948872	count	1
CTH	0.5092069	0.4583378	1.111	0.267	0.32949689	count	1
CXorf40B	0.2902736	0.2851982	1.0178	0.309	0.329768175	count	1
B4GALNT3	0.6180139	0.821856	0.752	0.452	0.330406267	count	1
TRMT10A	0.299297	0.2591492	1.1549	0.248	0.330430812	count	1
ZNF461	0.5392968	0.4736649	1.1386	0.255	0.330449633	count	1
KIAA1586	0.2586155	0.1444901	1.7898	0.0736	0.330589775	count	1
ATF1	0.2505262	0.1189419	2.1063	0.0352	0.330639107	count	1
GRB10	0.3581942	0.3416984	1.0483	0.295	0.330741098	count	1
MORN4	0.3531217	0.3209104	1.1004	0.271	0.330820088	count	1
STMN3	0.2570055	0.137526	1.8688	0.0617	0.330884668	count	1
ERCC8	0.3261385	0.3749186	0.8699	0.384	0.330924039	count	1
AC015908.2	0.7536847	0.6849713	1.1003	0.271	0.331120061	count	1
ATP6AP1	0.2396665	0.0814817	2.9414	0.00329	0.331237943	count	1
PLAG1	0.8645304	1.0187453	0.8486	0.396	0.331298226	count	1
XPNPEP3	0.3329961	0.2580569	1.2904	0.197	0.331314994	count	1
R3HDM2	0.2385249	0.0847793	2.8135	0.00493	0.331503225	count	1
TBK1	0.2602194	0.1685321	1.544	0.123	0.331892961	count	1
NAA60	0.2602334	0.2806306	0.9273	0.354	0.331910643	count	1
C1orf159	0.6212915	0.428483	1.45	0.147	0.331922763	count	1
TAF2	0.2764687	0.2045017	1.3519	0.176	0.331947365	count	1
SLC25A22	0.413051	0.602117	0.686	0.493	0.332004359	count	1
AC098487.1	0.5136567	0.3715497	1.3825	0.167	0.332098732	count	1
HPS6	0.354581	0.2956523	1.1993	0.23	0.332130645	count	1
FBXL8	0.3412729	0.282384	1.2085	0.227	0.332152613	count	1
ITSN2	0.2399631	0.1001437	2.3962	0.0166	0.332209995	count	1
LZTR1	0.2993924	0.2483167	1.2057	0.228	0.33222695	count	1
SERTAD3	0.2406557	0.1098706	2.1904	0.0286	0.332229542	count	1
NT5DC3	0.306163	0.227929	1.3432	0.179	0.332258299	count	1
SOCS5	0.2591138	0.1624793	1.5947	0.111	0.332262189	count	1
MTERF1	0.345603	0.2767296	1.2489	0.212	0.33235205	count	1
FCMR	1.358692	0.7799877	1.7419	0.0816	0.332381125	count	1
PGBD2	0.321949	0.2446142	1.3161	0.188	0.332427934	count	1
MYO1E	0.2650411	0.1678952	1.5786	0.115	0.332470281	count	1
LIFR	0.2725339	0.174535	1.5615	0.118	0.332490072	count	1

SHLD2	0.2744006	0.1565008	1.7533	0.0796	0.332597243	count	1
AC108673.3	0.3418207	0.3139224	1.0889	0.276	0.332666055	count	1
AC087477.2	0.3381161	0.4365369	0.7745	0.439	0.332791909	count	1
CNTLN	0.2454452	0.1455852	1.6859	0.0919	0.332884262	count	1
SLC7A1	0.4712354	0.3456623	1.3633	0.173	0.333063832	count	1
TYSND1	0.3254059	0.2207295	1.4742	0.141	0.333105396	count	1
CHST15	0.3665711	0.298736	1.2271	0.22	0.333142664	count	1
BCORL1	0.4541054	0.330463	1.3741	0.169	0.333154598	count	1
SCCPDH	0.2462247	0.098701	2.4947	0.0127	0.33315735	count	1
DPH3	0.2462667	0.1150956	2.1397	0.0324	0.333314393	count	1
MTOR	0.3257261	0.2792051	1.1666	0.243	0.333422892	count	1
LINC01273	0.544846	0.5595979	0.9736	0.33	0.333485827	count	1
SLC50A1	0.2679021	0.1569093	1.7074	0.0878	0.33348729	count	1
AL049840.1	0.2962559	0.2607874	1.136	0.256	0.333529014	count	1
CA12	0.3670327	0.3046781	1.2047	0.228	0.33354314	count	1
PIP4P2	0.2394685	0.0835655	2.8656	0.00419	0.333643512	count	1
DRG2	0.2512872	0.1350003	1.8614	0.0628	0.333796038	count	1
VEGFB	0.2330016	0.0423242	5.5052	3.94E-08	0.333859277	count	0.00092456
AL050341.2	0.2980056	0.2192804	1.359	0.174	0.333929523	count	1
BNIP3	0.2411373	0.1028924	2.3436	0.0192	0.333954955	count	1
RTN2	0.275132	0.1925293	1.429	0.153	0.33420228	count	1
SUCLG1	0.2393255	0.0725184	3.3002	0.000975	0.334355543	count	1
HOXA-AS2	0.3013573	0.2577167	1.1693	0.242	0.334357263	count	1
CNGA1	0.6266645	0.4103768	1.527	0.127	0.334403125	count	1
CLDND1	0.2380679	0.0696245	3.4193	0.000635	0.334417204	count	1
NFYC-AS1	1.0507598	0.6611025	1.5894	0.112	0.334460296	count	1
TYW5	0.343843	0.3065988	1.1215	0.262	0.334560948	count	1
SUPT6H	0.2518737	0.1347534	1.8691	0.0617	0.334570022	count	1
LRRC17	0.2376333	0.0764719	3.1075	0.0019	0.334621681	count	1
FNBP1L	0.2575137	0.1561633	1.649	0.0992	0.334726957	count	1
FBXO10	1.05214	1.0706073	0.9828	0.326	0.334773325	count	1
N6AMT1	0.2685319	0.2057083	1.3054	0.192	0.334789343	count	1
CMTM6	0.2455194	0.0956931	2.5657	0.0103	0.335168754	count	1
LRPPRC	0.2532628	0.1428023	1.7735	0.0762	0.335171289	count	1
HDHD3	0.2716015	0.2089533	1.2998	0.194	0.335197088	count	1
TOGARAM1	0.2628627	0.2002451	1.3127	0.189	0.335230641	count	1
AC234772.3	1.3784774	1.2632074	1.0913	0.275	0.335301542	count	1
TMEM60	0.2540216	0.1296952	1.9586	0.0502	0.335414119	count	1
TNPO3	0.2867682	0.2206414	1.2997	0.194	0.335516381	count	1
PRKCH	0.7660286	0.5550627	1.3801	0.168	0.335538904	count	1
AQR	0.2788565	0.1645493	1.6947	0.0902	0.335585941	count	1
STT3B	0.2498299	0.1016387	2.458	0.014	0.335628928	count	1
TSTD2	0.286153	0.1882603	1.52	0.129	0.33586627	count	1
ALS2CL	0.4960582	0.5678723	0.8735	0.382	0.335931718	count	1
KDM7A	0.298464	0.1983296	1.5049	0.132	0.335961126	count	1

FAM241A	0.267941	0.1667729	1.6066	0.108	0.336066195	count	1
MILR1	0.4433163	0.5660578	0.7832	0.434	0.336084924	count	1
KDM4A-AS1	2.2986208	1.9739205	1.1645	0.2443	0.336161457	count	1
STPG1	0.3417353	0.3156804	1.0825	0.279	0.336225469	count	1
SMURF2	0.2701638	0.1477004	1.8291	0.0675	0.336268415	count	1
USP48	0.245195	0.1107912	2.2131	0.027	0.336377354	count	1
PDE12	0.2857771	0.2068174	1.3818	0.167	0.336459973	count	1
ANXA11	0.2365008	0.0516119	4.5823	4.75E-06	0.336462798	count	0.10982475
PPM1M	0.2810467	0.2169685	1.2953	0.195	0.33651588	count	1
CCP110	0.2712982	0.1915482	1.4163	0.157	0.336563708	count	1
MLH1	0.3019023	0.1849831	1.6321	0.103	0.336600058	count	1
ZNF684	0.31861	0.2383305	1.3368	0.181	0.336620541	count	1
PHLPP1	0.4192999	0.2510276	1.6703	0.0949	0.336713289	count	1
HEATR1	0.302094	0.2003355	1.5079	0.132	0.336808931	count	1
KCNE4	0.23979	0.0952948	2.5163	0.0119	0.336866552	count	1
PRKCD	0.3105225	0.265642	1.169	0.242	0.336868966	count	1
ZHX2	0.3069556	0.2613501	1.1745	0.24	0.3368864	count	1
LRRC34	0.3166509	0.2700772	1.1724	0.241	0.336916388	count	1
STAT6	0.25183	0.1489274	1.691	0.0909	0.336967854	count	1
DAZAP1	0.2509608	0.1270168	1.9758	0.0483	0.337012912	count	1
SASH1	0.2482654	0.1253645	1.9803	0.0477	0.337154177	count	1
AKAP8	0.310866	0.1879331	1.6541	0.0982	0.337232086	count	1
COX15	0.3040205	0.2332898	1.3032	0.193	0.337243509	count	1
ZNF670	0.5226356	0.4052922	1.2895	0.197	0.337333078	count	1
ITGBL1	0.2358561	0.0625376	3.7714	0.000165	0.337438105	count	1
LARP4B	0.273451	0.2451368	1.1155	0.265	0.337449987	count	1
CCDC148	2.3274046	1.2966371	1.795	0.0727	0.337605144	count	1
UBE2S	0.2421206	0.0922998	2.6232	0.00875	0.337623866	count	1
NPHP3	0.3014171	0.2335398	1.2906	0.197	0.337667258	count	1
MARK1	0.3245874	0.2528513	1.2837	0.199	0.33773295	count	1
INPP5A	0.2769288	0.1746275	1.5858	0.113	0.337776451	count	1
DRC3	0.7725355	0.4197937	1.8403	0.0658	0.337855082	count	1
FAM133B	0.2395493	0.0807039	2.9682	0.00301	0.337912391	count	1
C17orf97	0.5529844	0.3475644	1.591	0.112	0.337924403	count	1
AMD1	0.2422968	0.0904368	2.6792	0.00741	0.338067255	count	1
RTL10	0.3200729	0.3745274	0.8546	0.393	0.338122212	count	1
SNX27	0.2645933	0.1487903	1.7783	0.0754	0.338155654	count	1
HSPA14	0.3667431	0.324605	1.1298	0.259	0.338286848	count	1
BCKDK	0.257591	0.1240989	2.0757	0.038	0.338441475	count	1
TFEB	0.2994024	0.2052598	1.4587	0.145	0.338480705	count	1
HIC2	0.6356801	0.4445678	1.4299	0.153	0.338549334	count	1
ASAP2	0.2442049	0.0989564	2.4678	0.0136	0.338555381	count	1
HNRNPLL	0.2614743	0.1638601	1.5957	0.111	0.338712679	count	1
SLC2A8	0.282248	0.2201055	1.2823	0.2	0.338778214	count	1
TAF1A	0.3444846	0.2445289	1.4088	0.159	0.338831727	count	1

C2CD5	0.3070992	0.3078006	0.9977	0.318	0.338839317	count	1
ISG20L2	0.2680218	0.1928047	1.3901	0.165	0.338902814	count	1
CHTOP	0.2537402	0.1152826	2.201	0.0278	0.338930007	count	1
TTC26	0.4803447	0.3517925	1.3654	0.172	0.338966799	count	1
UBXN6	0.2575931	0.1295471	1.9884	0.0468	0.339083607	count	1
DNM1L	0.2501278	0.111606	2.2412	0.0251	0.339107909	count	1
DDX39B	0.2609431	0.1708137	1.5276	0.127	0.339146842	count	1
TUBGCP3	0.300011	0.2148974	1.3961	0.163	0.339153969	count	1
SLX4	0.7776378	0.4850882	1.6031	0.109	0.339664909	count	1
SEC23IP	0.3078946	0.1919662	1.6039	0.109	0.339695876	count	1
PPARA	0.2863627	0.194316	1.4737	0.141	0.340066874	count	1
CXCL16	0.2677277	0.1751209	1.5288	0.126	0.340198324	count	1
AL158071.1	1.076393	1.1400328	0.9442	0.345	0.340224273	count	1
SIX4	0.5033432	0.5002337	1.0062	0.314	0.340416334	count	1
COG3	0.2988105	0.255521	1.1694	0.242	0.340657041	count	1
AC008467.1	0.6999583	1.0748255	0.6512	0.515	0.340713746	count	1
DEPDC5	0.5038356	0.4845026	1.0399	0.298	0.340718935	count	1
GTF3C3	0.2846437	0.2129204	1.3369	0.181	0.340754057	count	1
NR2C1	0.2705994	0.1626705	1.6635	0.0963	0.340782474	count	1
AHCYL1	0.2494759	0.0846184	2.9482	0.00322	0.340922122	count	1
ACTRT3	0.483486	0.426344	1.134	0.257	0.340997311	count	1
RIOX2	0.2829838	0.2019619	1.4012	0.161	0.341284798	count	1
DNAJC3	0.2411488	0.0529831	4.5514	5.50E-06	0.341347752	count	0.12705
TNFRSF1A	0.2430406	0.0631112	3.851	0.00012	0.341423595	count	1
XPOT	0.2779089	0.2348595	1.1833	0.237	0.341626952	count	1
PRKCI	0.2607505	0.142086	1.8352	0.0666	0.341656539	count	1
SCFD2	0.3259412	0.2627955	1.2403	0.215	0.341669769	count	1
ZNF222	0.5055404	0.4872487	1.0375	0.3	0.341766168	count	1
TTC21B	0.2862963	0.1843299	1.5532	0.12	0.341819487	count	1
PNPT1	0.2748111	0.1625347	1.6908	0.091	0.341981001	count	1
ZNF717	0.3343658	0.3107891	1.0759	0.282	0.341981129	count	1
IP6K3	1.4251868	0.9349852	1.5243	0.128	0.342016382	count	1
CUL7	0.3712372	0.4443063	0.8355	0.403	0.342246416	count	1
LINC00467	0.3660213	0.2563568	1.4278	0.153	0.34238693	count	1
HNRNPL	0.2505598	0.111302	2.2512	0.0244	0.342396904	count	1
MED9	0.3266581	0.2392513	1.3653	0.172	0.342398938	count	1
GGACT	1.0871052	0.5388092	2.0176	0.0437	0.342601859	count	1
TFAP4	0.3840069	0.2835546	1.3543	0.176	0.342714973	count	1
EXOC4	0.2710794	0.1512238	1.7926	0.0731	0.342726401	count	1
HSPA14	0.2842852	0.2369121	1.2	0.23	0.342830146	count	1
ACTL6A	0.2601109	0.1153965	2.2541	0.0243	0.342994776	count	1
FUZ	0.2837956	0.1652665	1.7172	0.086	0.343040871	count	1
AC064836.3	0.3914999	0.4856324	0.8062	0.42	0.343127031	count	1
GIT2	0.2946071	0.1980769	1.4873	0.137	0.343398762	count	1
GOPC	0.2461776	0.0828444	2.9716	0.00298	0.343439223	count	1

SOWAHC	0.2978174	0.213074	1.3977	0.162	0.343472876	count	1
PSMD5	0.2769581	0.2022758	1.3692	0.171	0.343495427	count	1
PCDHGB2	1.0915215	0.7249338	1.5057	0.132	0.343576732	count	1
PPP1R36	1.0915215	0.7874092	1.3862	0.166	0.343576732	count	1
TTC4	0.3921821	0.3515212	1.1157	0.265	0.34369398	count	1
PARD6G	0.4878556	0.3188724	1.5299	0.126	0.343817434	count	1
MSL1	0.2638087	0.1478316	1.7845	0.0744	0.343904836	count	1
KIAA1217	0.3629585	0.2698703	1.3449	0.179	0.344111579	count	1
AC037459.2	0.790842	0.8975763	0.8811	0.378	0.344322645	count	1
ARHGAP44	0.3795851	0.3505531	1.0828	0.279	0.344413342	count	1
SNX7	0.2508409	0.0876394	2.8622	0.00423	0.344469289	count	1
CLSTN1	0.2498581	0.0995433	2.51	0.0121	0.34473882	count	1
CXorf57	0.7930474	0.7551342	1.0502	0.294	0.345096964	count	1
ELF2	0.2471382	0.0872868	2.8313	0.00466	0.345347012	count	1
ATP6V0C	0.4907398	0.5523734	0.8884	0.374	0.345676104	count	1
AL451165.2	0.3102972	0.3669517	0.8456	0.398	0.345740988	count	1
AC093249.6	0.411131	0.6775831	0.6068	0.544	0.345814772	count	1
LINC01140	2.510935	0.9522125	2.6369	0.0084	0.345959036	count	1
OGFR	0.2737189	0.1563854	1.7503	0.0802	0.346026278	count	1
STEAP1B	2.5152371	1.2542845	2.0053	0.045	0.346138206	count	1
Z68871.1	0.7134575	0.6152205	1.1597	0.246	0.346204931	count	1
TNKS	0.2742369	0.1478326	1.855	0.0637	0.346227727	count	1
FGD5-AS1	0.248685	0.0697889	3.5634	0.000371	0.346354503	count	1
HLA-E	0.2418397	0.0418315	5.7813	8.03E-09	0.346459642	count	0.00018897
PPIL2	0.305362	0.1961624	1.5567	0.12	0.34654131	count	1
KNTC1	0.9177225	0.6373818	1.4398	0.15	0.346889887	count	1
PSMA6	0.316311	0.2568456	1.2315	0.218	0.346896028	count	1
DHX34	0.9185578	0.6815959	1.3477	0.178	0.347130417	count	1
MTFR1	0.2824522	0.1737616	1.6255	0.104	0.347134839	count	1
CEP295	0.2820061	0.2299539	1.2264	0.22	0.347236442	count	1
RARA-AS1	0.2933584	0.1948641	1.5055	0.132	0.347256204	count	1
MEX3D	0.3073602	0.1981723	1.551	0.121	0.347278739	count	1
STAM	0.2622371	0.1314529	1.9949	0.0461	0.347339591	count	1
FAM172A	0.2566309	0.1268692	2.0228	0.0432	0.347372999	count	1
HIST1H3H	0.4131051	0.5446276	0.7585	0.448	0.347378662	count	1
SEC23A	0.2553517	0.1117641	2.2847	0.0224	0.347453372	count	1
WHAMM	0.2660031	0.1297507	2.0501	0.0404	0.347508636	count	1
RNF103	0.2946555	0.2234869	1.3184	0.187	0.347758592	count	1
AC005921.2	0.2724922	0.1374064	1.9831	0.0474	0.347768427	count	1
SRC	0.383484	0.3805592	1.0077	0.314	0.347781749	count	1
FAM160B2	0.3067211	0.256334	1.1966	0.232	0.348050158	count	1
BACH2	1.112376	0.6565857	1.6942	0.0903	0.348138276	count	1
PDE9A	0.6568351	0.5021438	1.3081	0.191	0.348200922	count	1
LINC01465	0.922704	0.63466	1.4539	0.146	0.348322335	count	1
COQ6	0.2869514	0.1800265	1.5939	0.111	0.348344248	count	1

MAFG	0.259777	0.1251676	2.0754	0.038	0.348379528	count	1
LMTK2	0.3632339	0.2818894	1.2886	0.198	0.348620427	count	1
TRIM21	0.3002228	0.2676859	1.1215	0.262	0.348644234	count	1
ZFP41	1.1148235	1.1281819	0.9882	0.323	0.348669247	count	1
GDE1	0.2735289	0.128223	2.1332	0.033	0.34907773	count	1
ZNF140	0.3553482	0.2638794	1.3466	0.178	0.349113027	count	1
TCOF1	0.3134436	0.2997395	1.0457	0.296	0.349163599	count	1
DLGAP4	0.2547534	0.1089662	2.3379	0.0194	0.349238081	count	1
ZNF334	0.5434271	0.5780603	0.9401	0.347	0.349372889	count	1
CCDC51	0.2808569	0.1649689	1.7025	0.0887	0.349408577	count	1
GSPT2	0.2715404	0.1613845	1.6826	0.0925	0.349417759	count	1
AC104532.2	0.7216964	0.6627622	1.0889	0.276	0.349535696	count	1
GPR27	1.1193128	0.5893711	1.8992	0.0576	0.349640533	count	1
TBC1D9B	0.2789185	0.180789	1.5428	0.123	0.349669244	count	1
LINC02482	0.3365774	0.2676302	1.2576	0.209	0.349818311	count	1
FLYWCH1	0.30024	0.2107524	1.4246	0.154	0.349838997	count	1
CBFB	0.2842754	0.1955156	1.454	0.146	0.349992136	count	1
EIF4G3	0.2651294	0.116421	2.2773	0.0228	0.350173662	count	1
GTF2H2C	0.3649281	0.2322914	1.571	0.116	0.350179813	count	1
NUP188	0.3213305	0.3280488	0.9795	0.327	0.350306337	count	1
RNF170	0.2772825	0.2064677	1.343	0.179	0.350480181	count	1
TSPAN14	0.3322178	0.2476926	1.3413	0.18	0.350571717	count	1
PPT2	0.4088051	0.3326042	1.2291	0.219	0.350912568	count	1
TPRN	0.3535726	0.3528273	1.0021	0.316	0.35103751	count	1
IGF1R	0.2604607	0.1420239	1.8339	0.0667	0.351075258	count	1
CPAMD8	0.3435843	0.3758115	0.9142	0.361	0.351094585	count	1
PREX2	0.2831701	0.166674	1.6989	0.0894	0.351098558	count	1
HNRNPH1	0.2474138	0.0592988	4.1723	3.08E-05	0.351286868	count	0.7056896
MAST2	0.3438388	0.3082564	1.1154	0.265	0.351345915	count	1
ATXN2	0.2594402	0.1091729	2.3764	0.0175	0.351567427	count	1
GFPT1	0.2609536	0.1207827	2.1605	0.0308	0.351735883	count	1
TONSL	1.496708	1.262561	1.1855	0.236	0.351820686	count	1
COQ4	0.2622474	0.1104954	2.3734	0.0177	0.352079053	count	1
ESCO1	0.2658431	0.1191304	2.2315	0.0257	0.352081512	count	1
GPCPD1	0.2636579	0.1227836	2.1473	0.0318	0.35224526	count	1
TNFRSF1B	0.4526437	0.36513	1.2397	0.215	0.352455797	count	1
ZNF501	0.523103	0.3250245	1.6094	0.108	0.352509801	count	1
GTF2B	0.2579584	0.0988803	2.6088	0.00912	0.352538085	count	1
PRH1	0.2772838	0.1934999	1.433	0.152	0.352621592	count	1
CRY1	0.3552689	0.2641294	1.3451	0.179	0.352659081	count	1
RAB20	0.2836414	0.1825568	1.5537	0.12	0.352827917	count	1
COMT	0.2480569	0.0524399	4.7303	2.33E-06	0.352889842	count	0.05402571
PCDHGB7	0.3202776	0.3077238	1.0408	0.298	0.353015296	count	1
NPEPL1	0.2903449	0.1769	1.6413	0.101	0.353157617	count	1
NCKIPSD	0.3640219	0.3096963	1.1754	0.24	0.353416142	count	1

NOC2L	0.2651596	0.127392	2.0814	0.0375	0.353451103	count	1
GPR4	0.4417594	0.3328984	1.327	0.185	0.353553127	count	1
TK2	0.2683799	0.1296586	2.0699	0.0385	0.353604077	count	1
ELAC2	0.2891724	0.1784652	1.6203	0.105	0.353913735	count	1
LENG9	0.2903992	0.2150589	1.3503	0.177	0.353962478	count	1
ELK3	0.2731501	0.1651846	1.6536	0.0983	0.353997796	count	1
TMEM106B	0.2541247	0.07469	3.4024	0.000675	0.354005067	count	1
MTERF3	0.2975958	0.2229989	1.3345	0.182	0.354139361	count	1
PDPK1	0.2767341	0.1466403	1.8872	0.0592	0.354260991	count	1
AC093702.1	0.7340246	0.5582913	1.3148	0.189	0.354490408	count	1
SASS6	0.3015154	0.2216577	1.3603	0.174	0.354648679	count	1
ADORA1	0.8205054	1.51244	0.5425	0.588	0.354650424	count	1
SELENBP1	0.2547049	0.0725099	3.5127	0.000449	0.354746704	count	1
PPP4R2	0.2565999	0.0932525	2.7517	0.00596	0.354749727	count	1
MAMLD1	0.4702349	0.4946053	0.9507	0.342	0.354945333	count	1
DHX33	0.3296955	0.3975169	0.8294	0.407	0.35494766	count	1
ANO6	0.2548579	0.0797784	3.1946	0.00141	0.355023486	count	1
AL139317.3	1.5218061	1.3652063	1.1147	0.265	0.355128154	count	1
ZNF675	0.3132129	0.2304274	1.3593	0.174	0.355252683	count	1
PKN2	0.2547353	0.0792639	3.2138	0.00132	0.3553153	count	1
IPP	0.2904721	0.1829092	1.5881	0.112	0.355481154	count	1
NT5C3A	0.2705022	0.144009	1.8784	0.0604	0.355497414	count	1
Z95115.1	1.1473281	0.6590851	1.7408	0.0818	0.355630278	count	1
ALDOA	0.2798413	0.1747772	1.6011	0.109	0.35583822	count	1
ZNF100	0.3180817	0.2665774	1.1932	0.233	0.355894471	count	1
DOCK1	0.2692272	0.1517661	1.774	0.0762	0.355946606	count	1
DENND6B	0.4882969	0.4300862	1.1353	0.256	0.356191094	count	1
ECPAS	0.2820297	0.158091	1.784	0.0745	0.356411086	count	1
ANAPC4	0.291416	0.1920996	1.517	0.129	0.356619333	count	1
Z93241.1	0.3767966	0.3151609	1.1956	0.232	0.356661268	count	1
SLC22A4	0.9531452	0.6548479	1.4555	0.146	0.35697351	count	1
ZNF142	0.4733117	0.4377893	1.0811	0.28	0.357088794	count	1
ID2-AS1	0.953584	1.231418	0.7744	0.439	0.3570968	count	1
GNPNAT1	0.2722716	0.1962304	1.3875	0.165	0.357107679	count	1
PIGH	0.270448	0.1273012	2.1245	0.0337	0.357145182	count	1
WNT6	0.8278326	0.6198481	1.3355	0.182	0.357172612	count	1
DCAF12L2	1.5388554	1.1179576	1.3765	0.169	0.357336444	count	1
RNF215	0.318086	0.2530521	1.257	0.209	0.357534562	count	1
AL357054.4	0.9557191	0.5120453	1.8665	0.0621	0.357696925	count	1
AC036176.1	0.3538273	0.3044399	1.1622	0.245	0.358033203	count	1
C18orf21	0.3026528	0.1881769	1.6083	0.108	0.358059471	count	1
PHOSPHO2	0.318566	0.238076	1.3381	0.181	0.358061414	count	1
UBXN4	0.2510556	0.0437928	5.7328	1.07E-08	0.358321426	count	0.000251696
HSPA5	0.2510092	0.0527066	4.7624	1.99E-06	0.358432604	count	0.04615805
MR1	0.2781307	0.1746798	1.5922	0.111	0.358488333	count	1

ELF1	0.2616944	0.1022319	2.5598	0.0105	0.358503781	count	1
AL021453.1	0.510788	0.49256	1.037	0.3	0.358534602	count	1
TMEM131L	0.3150706	0.1962006	1.6059	0.108	0.358786356	count	1
ZADH2	0.3051315	0.1966663	1.5515	0.121	0.358822145	count	1
IER5L	0.2535876	0.0774203	3.2755	0.00106	0.358974998	count	1
LONRF1	0.2934729	0.1501368	1.9547	0.0507	0.359099174	count	1
GAREM2	0.7457742	0.4228356	1.7637	0.0779	0.359180042	count	1
TSPAN6	0.2879633	0.1652653	1.7424	0.0815	0.359258657	count	1
MED25	0.3055996	0.210804	1.4497	0.147	0.359362241	count	1
PNRC2	0.2532082	0.054804	4.6203	3.96E-06	0.359455353	count	0.09161856
MPP5	0.3066508	0.232673	1.3179	0.188	0.359468177	count	1
TMED8	0.3552996	0.2881062	1.2332	0.218	0.359469861	count	1
STIM1	0.3299848	0.2157247	1.5297	0.126	0.35948669	count	1
GLMP	0.2676861	0.1159517	2.3086	0.021	0.359606091	count	1
AP1G1	0.2808003	0.1525964	1.8402	0.0658	0.359783799	count	1
SNX16	0.3033666	0.18662	1.6256	0.104	0.359899631	count	1
LINC01836	0.5940943	0.6212587	0.9563	0.339	0.360084557	count	1
DNAH6	0.9644397	0.8337092	1.1568	0.247	0.360138762	count	1
AL136295.5	0.9644397	0.8512522	1.133	0.257	0.360138762	count	1
BISPR	0.3527834	0.3155605	1.118	0.264	0.360170074	count	1
EFEMP1	0.2502982	0.0459608	5.4459	5.49E-08	0.360312062	count	0.001287295
PIGL	0.3084407	0.2366779	1.3032	0.193	0.360381646	count	1
REXO2	0.2525522	0.0486646	5.1896	2.22E-07	0.36057161	count	0.005188362
ERCC3	0.2915807	0.1680324	1.7353	0.0828	0.360772712	count	1
SPCS3	0.2555441	0.0748057	3.4161	0.000642	0.360867902	count	1
CU638689.4	0.635246	0.6151426	1.0327	0.302	0.360949563	count	1
ANKEF1	1.1730887	0.879586	1.3337	0.182	0.361030342	count	1
AC012065.3	2.964113	2.4654399	1.2023	0.2293	0.361354322	count	1
AL133346.1	1.5710542	1.0240883	1.5341	0.125	0.361423261	count	1
RPP40	0.4136365	0.3801432	1.0881	0.277	0.361463347	count	1
UNC93B1	0.2973383	0.2072022	1.435	0.151	0.361531891	count	1
ZNF208	0.3339994	0.2483139	1.3451	0.179	0.36163327	count	1
NTF3	0.2968251	0.1667121	1.7805	0.0751	0.361674856	count	1
DANT2	0.7523071	0.8881935	0.847	0.397	0.361773751	count	1
AL445524.1	0.5386238	0.4766988	1.1299	0.259	0.36193631	count	1
CUL2	0.2775578	0.1292724	2.1471	0.0319	0.361940978	count	1
DCUN1D1	0.2732354	0.13871	1.9698	0.0489	0.361992253	count	1
BZW2	0.26759	0.1478416	1.81	0.0704	0.362017156	count	1
POU5F2	2.989077	1.670967	1.7888	0.0737	0.362024888	count	1
ZCCHC4	0.3691257	0.3265202	1.1305	0.258	0.362112465	count	1
AC093627.4	0.7531883	0.910386	0.8273	0.408	0.362122866	count	1
ATXN3	0.2669768	0.1212598	2.2017	0.0277	0.362135379	count	1
TRIM41	0.4661517	0.3780387	1.2331	0.218	0.362203958	count	1
ETV3	0.3518131	0.2478937	1.4192	0.156	0.362232263	count	1
TMEM164	0.972121	0.5899656	1.6478	0.0995	0.362277708	count	1

UNC50	0.2588023	0.0740618	3.4944	0.000481	0.362293801	count	1
IFIT5	0.2805995	0.1459839	1.9221	0.0547	0.36229901	count	1
HSD17B14	0.2956332	0.2242428	1.3184	0.187	0.362408358	count	1
DNAL1	0.3001047	0.2065547	1.4529	0.146	0.362435715	count	1
PSME4	0.35527	0.3197074	1.1112	0.267	0.362620014	count	1
PATZ1	0.3739115	0.2803528	1.3337	0.182	0.362621887	count	1
GALNT8	0.843842	0.6068324	1.3906	0.164	0.362643652	count	1
LSG1	0.2943071	0.1323323	2.224	0.0262	0.36282371	count	1
FAM149A	0.3214792	0.2186987	1.47	0.142	0.362860133	count	1
CPSF7	0.3052109	0.1710372	1.7845	0.0744	0.363039829	count	1
CCDC22	0.3070084	0.1895365	1.6198	0.105	0.363117437	count	1
ZNF610	0.5674616	0.5718877	0.9923	0.321	0.363149329	count	1
ZNF468	0.540639	0.5192469	1.0412	0.298	0.363155532	count	1
ZNF518A	0.276581	0.1587247	1.7425	0.0815	0.363187957	count	1
SPR	0.2857295	0.1678853	1.7019	0.0889	0.363241145	count	1
ANAPC13	0.2609488	0.1047431	2.4913	0.0128	0.363249187	count	1
DDX60L	0.3317356	0.2595684	1.278	0.201	0.363361924	count	1
BBS1	0.5679066	0.5526944	1.0275	0.304	0.363402964	count	1
AC009506.1	0.5186033	0.4052737	1.2796	0.201	0.363518064	count	1
RXYLT1	0.2781812	0.1357126	2.0498	0.0405	0.363798008	count	1
CTNNB1	0.2598865	0.0820976	3.1656	0.00156	0.363831839	count	1
TTC37	0.2662148	0.0964131	2.7612	0.00579	0.363847388	count	1
PCDHGA5	0.3710435	0.2867488	1.294	0.196	0.363918423	count	1
C16orf45	0.262759	0.0968674	2.7126	0.00671	0.363973612	count	1
RABEP2	0.3305265	0.2007796	1.6462	0.0998	0.36401677	count	1
VPS18	0.3481578	0.2835808	1.2277	0.22	0.364215673	count	1
DARS2	0.5693613	0.5215139	1.0917	0.275	0.364231744	count	1
TTC30A	0.4836064	0.4356006	1.1102	0.267	0.364242104	count	1
SYTL4	0.3907051	0.2986469	1.3083	0.191	0.364407285	count	1
BPGM	0.276008	0.1389395	1.9865	0.047	0.364430598	count	1
ANKAR	0.2766814	0.167984	1.6471	0.0996	0.364454619	count	1
ZFP82	0.3484011	0.3172776	1.0981	0.272	0.364461984	count	1
SMIM19	0.2586707	0.072493	3.5682	0.000364	0.364462297	count	1
TBC1D20	0.2900646	0.1507644	1.924	0.0544	0.364495762	count	1
TENM1	1.596949	1.0750574	1.4855	0.138	0.364631966	count	1
COPG2	0.4350859	0.2775175	1.5678	0.117	0.364723112	count	1
ZBTB8A	0.3608833	0.2396511	1.5059	0.132	0.364913934	count	1
UHRF1BP1L	0.2881121	0.1646346	1.75	0.0802	0.364921688	count	1
ASB3	0.6032217	0.3617002	1.6677	0.0955	0.364945412	count	1
PIF1	0.6034169	0.5802638	1.0399	0.298	0.365049131	count	1
DVL3	0.2865375	0.1801163	1.5908	0.112	0.36509053	count	1
MMAA	0.5018332	0.3335438	1.5045	0.133	0.365224854	count	1
AC147067.1	0.3520369	0.2893213	1.2168	0.224	0.365354876	count	1
AC140912.1	1.605091	0.8792411	1.8255	0.068	0.365626556	count	1
METTL2A	0.2927803	0.1598554	1.8315	0.0671	0.365745092	count	1

BBS10	0.3923202	0.273961	1.432	0.152	0.365842879	count	1
VASH1-AS1	1.608353	0.9587316	1.6776	0.0935	0.366023116	count	1
RENBP	0.763084	0.5846992	1.3051	0.192	0.366031008	count	1
UBXN7	0.3017585	0.1897026	1.5907	0.112	0.366032835	count	1
SSR3	0.2568141	0.0493233	5.2068	2.03E-07	0.366215825	count	0.004745531
SLC30A9	0.2766691	0.1374242	2.0132	0.0442	0.366311472	count	1
UBE3C	0.2957727	0.1838662	1.6086	0.108	0.366508034	count	1
LPIN1	0.2864486	0.167037	1.7149	0.0865	0.366563527	count	1
CRYZ	0.2813294	0.1633332	1.7224	0.0851	0.366814976	count	1
SLC25A17	0.3130525	0.222553	1.4066	0.16	0.366825232	count	1
HSDL1	0.3265852	0.2141445	1.5251	0.127	0.36685692	count	1
DNAJC17	0.2912273	0.1948451	1.4947	0.135	0.366934517	count	1
ZNF814	0.3935736	0.5948127	0.6617	0.508	0.366956532	count	1
PSMD9	0.2776622	0.1624918	1.7088	0.0876	0.367012586	count	1
SMAD9	0.2651052	0.0946698	2.8003	0.00513	0.367212174	count	1
MIPOL1	0.3021576	0.183874	1.6433	0.1	0.367298711	count	1
GLMN	0.3394158	0.2093389	1.6214	0.105	0.367330922	count	1
MSL2	0.3271415	0.2696743	1.2131	0.225	0.367466637	count	1
DDX5	0.2563481	0.0402066	6.3758	2.05E-10	0.367586505	count	4.85E-06
ZNF350	0.3288593	0.2205284	1.4912	0.136	0.367654997	count	1
GDAP1	0.5058356	0.3325902	1.5209	0.128	0.367886511	count	1
PDGFD	0.2619325	0.0905179	2.8937	0.00383	0.367998282	count	1
PCDHB7	0.5491017	0.3247348	1.6909	0.0909	0.368263765	count	1
LMBRD2	0.3310446	0.2569779	1.2882	0.198	0.368275106	count	1
MGRN1	0.3448243	0.2520207	1.3682	0.171	0.368414553	count	1
AREL1	0.4620487	0.3721286	1.2416	0.214	0.36865089	count	1
AL731661.1	0.526812	0.4593277	1.1469	0.251	0.368734838	count	1
HARBI1	0.6516695	0.5827939	1.1182	0.264	0.368997159	count	1
TBCCD1	0.44069	0.3337925	1.3203	0.187	0.369124865	count	1
PITPNC1	0.368898	0.2648412	1.3929	0.164	0.369266051	count	1
ARF6	0.2704693	0.1032447	2.6197	0.00884	0.369312989	count	1
MVP	0.2696945	0.0859551	3.1376	0.00172	0.369413891	count	1
SS18L1	0.476395	0.3998047	1.1916	0.234	0.369563545	count	1
FAM200A	0.3209484	0.2032312	1.5792	0.114	0.369576027	count	1
AC008972.2	0.4764866	0.8095517	0.5886	0.556	0.369629217	count	1
ZIC1	1.6405991	1.3155232	1.2471	0.212	0.369886309	count	1
ADPGK	0.32055	0.1945715	1.6475	0.0995	0.370460463	count	1
FBXL18	0.8670019	0.6169194	1.4054	0.16	0.370462149	count	1
TOPBP1	0.3069258	0.2164763	1.4178	0.156	0.370535803	count	1
KCNAB1	0.274922	0.1101094	2.4968	0.0126	0.370576129	count	1
UBE2QL1	1.2212258	1.1646995	1.0485	0.294	0.370848742	count	1
RBM34	0.3233409	0.3193616	1.0125	0.311	0.370885475	count	1
MDM4	0.2752026	0.1350536	2.0377	0.0416	0.37095208	count	1
ZNF7	0.3187829	0.1803399	1.7677	0.0772	0.371002053	count	1
MAMSTR	0.4253812	0.2911053	1.4613	0.144	0.371140552	count	1

IQCE	0.3354406	0.2150528	1.5598	0.119	0.371193053	count	1
SOD2	0.2602997	0.0968143	2.6886	0.0072	0.371341465	count	1
RAB3D	0.3987525	0.3343023	1.1928	0.233	0.371553869	count	1
CNNM4	0.511819	0.4296802	1.1912	0.234	0.371857498	count	1
NKILA	0.6575513	0.4880512	1.3473	0.178	0.371862714	count	1
NBPF20	0.494986	1.03841	0.4767	0.634	0.372116155	count	1
ABRAXAS2	0.2856676	0.1526617	1.8712	0.0614	0.372139553	count	1
UBE2E2	0.2678043	0.1049291	2.5522	0.0107	0.37221673	count	1
DLST	0.2909486	0.1618197	1.798	0.0723	0.372258897	count	1
LINC00526	0.4671612	0.3042593	1.5354	0.125	0.37243788	count	1
AL117332.1	0.8734243	0.5338102	1.6362	0.102	0.372610161	count	1
MCOLN1	0.299292	0.1845184	1.622	0.105	0.37262046	count	1
PACRG	0.6177451	0.6775383	0.9117	0.362	0.372635383	count	1
C10orf55	1.230666	1.077058	1.1426	0.253	0.372733171	count	1
EPRS	0.2683095	0.0794532	3.377	0.000741	0.372763293	count	1
XBP1	0.2643335	0.0701979	3.7655	0.000169	0.372805403	count	1
PEG3	0.3849525	0.2725661	1.4123	0.158	0.372871855	count	1
KIAA1324L	0.400316	0.3657681	1.0945	0.274	0.372940484	count	1
CCZ1	0.2752634	0.1350796	2.0378	0.0416	0.372997692	count	1
SELENOF	0.2637337	0.0528085	4.9942	6.18E-07	0.373099991	count	0.01440867
CDC42BPB	0.2788887	0.1402504	1.9885	0.0468	0.373394094	count	1
NOP14	0.2922966	0.2058424	1.42	0.156	0.373569666	count	1
SLC37A3	0.3160627	0.2082822	1.5175	0.129	0.373622085	count	1
SCAMP4	0.3284331	0.2003111	1.6396	0.101	0.373649485	count	1
GLG1	0.2640282	0.0699051	3.777	0.000161	0.373700366	count	1
SKIV2L	0.3476775	0.2950136	1.1785	0.239	0.373732294	count	1
ZFP14	0.3104148	0.1802187	1.7224	0.0851	0.373804951	count	1
AC037459.3	0.5868148	0.8441783	0.6951	0.487	0.374131815	count	1
ZNF835	0.5153096	0.4259223	1.2099	0.226	0.374169611	count	1
RAB15	1.2383351	0.6533236	1.8954	0.0581	0.374254201	count	1
ZC3H12D	1.0169818	1.6568435	0.6138	0.5394	0.374549505	count	1
FBXO11	0.2864815	0.1436841	1.9938	0.0462	0.374555893	count	1
TMEM41B	0.2973997	0.1603653	1.8545	0.0637	0.374613517	count	1
AFF4	0.2707793	0.0947017	2.8593	0.00427	0.374670442	count	1
BOK	0.5882851	0.4355281	1.3507	0.177	0.374962125	count	1
CLCC1	0.3100922	0.1569224	1.9761	0.0482	0.375143719	count	1
KDM2A	0.2828134	0.1779467	1.5893	0.112	0.375550956	count	1
SH3RF1	0.2978692	0.1881822	1.5829	0.114	0.37569368	count	1
H1FX	0.2630599	0.0538746	4.8828	1.09E-06	0.37572479	count	0.02534577
HPS3	0.3105871	0.2045671	1.5183	0.129	0.375732368	count	1
SENP2	0.3092402	0.1716295	1.8018	0.0717	0.375767736	count	1
ZSWIM5	1.0223256	1.1021132	0.9276	0.354	0.375986384	count	1
RRP15	0.2746294	0.123324	2.2269	0.026	0.376216004	count	1
RBMS3	0.2696611	0.0801646	3.3638	0.000777	0.376259201	count	1
ORC5	0.3458539	0.2538116	1.3626	0.173	0.376281442	count	1

MBOAT1	0.562629	0.3774752	1.4905	0.136	0.376389237	count	1
AQP7	1.0238597	0.8201746	1.2483	0.212	0.376397911	count	1
PAM	0.2676534	0.0759713	3.5231	0.000432	0.376426696	count	1
HIST2H2AC	0.2963018	0.1889829	1.5679	0.117	0.376523274	count	1
CEP162	0.30155	0.1922589	1.5685	0.117	0.376552016	count	1
ING5	0.2986183	0.154628	1.9312	0.0535	0.376626615	count	1
EXOSC8	0.2753816	0.0936595	2.9402	0.0033	0.376942185	count	1
THSD7A	1.0261973	0.7261203	1.4133	0.158	0.377024152	count	1
LIPT1	0.3131368	0.1977183	1.5838	0.113	0.377905373	count	1
NKTR	0.2701574	0.1046256	2.5821	0.00986	0.377993755	count	1
TRARG1	0.367755	0.5717498	0.6432	0.52	0.378061862	count	1
CTNND1	0.2942521	0.1662495	1.7699	0.0768	0.378333105	count	1
TAF4B	0.6712566	0.4123468	1.6279	0.104	0.378505869	count	1
COASY	0.3193702	0.1671855	1.9103	0.0562	0.378524014	count	1
DYNC2LI1	0.2778538	0.1314115	2.1144	0.0346	0.378561654	count	1
SMS	0.2717814	0.0829736	3.2755	0.00106	0.378694299	count	1
ENOSF1	0.2942621	0.1683313	1.7481	0.0805	0.37870697	count	1
RBBP5	0.3224108	0.2009822	1.6042	0.109	0.37873531	count	1
INPPL1	0.3227679	0.2835791	1.1382	0.255	0.379146328	count	1
KIAA1143	0.2824363	0.1039315	2.7175	0.00661	0.379150399	count	1
GTF2H2	0.3688744	0.2480344	1.4872	0.137	0.379171202	count	1
ZC3HAV1	0.2915379	0.1442941	2.0204	0.0434	0.379424155	count	1
RUNX2	0.4540586	0.5166437	0.8789	0.38	0.379591696	count	1
MKNK2	0.2829156	0.1665577	1.6986	0.0895	0.379645453	count	1
DYRK1A	0.3027616	0.1712377	1.7681	0.0771	0.379708217	count	1
YWHAH	0.2675696	0.0547483	4.8873	1.07E-06	0.379942419	count	0.02488606
TBCD	0.4773864	0.2542775	1.8774	0.0605	0.379990956	count	1
NECTIN3	0.2862607	0.1287558	2.2233	0.0263	0.380654947	count	1
CSKMT	0.2992869	0.1987848	1.5056	0.132	0.380711779	count	1
STRBP	0.5458246	0.3341996	1.6332	0.103	0.380747649	count	1
ERO1B	0.3322375	0.2022877	1.6424	0.101	0.380856102	count	1
ZNF260	0.3445058	0.2223983	1.549	0.121	0.380952573	count	1
RDH13	0.3365951	0.2587691	1.3008	0.193	0.381131225	count	1
AC016727.1	0.5708171	0.4633567	1.2319	0.218	0.381283755	count	1
MLLT6	0.2870408	0.1559155	1.841	0.0657	0.38131142	count	1
PCDHGA9	0.5713466	0.4057957	1.408	0.159	0.381599661	count	1
AC048382.5	0.5713466	0.5011619	1.14	0.254	0.381599661	count	1
DNMT1	0.2927827	0.1292579	2.2651	0.0236	0.381607704	count	1
NUP50	0.3194693	0.1622776	1.9687	0.0491	0.381685735	count	1
DNAJC10	0.2751598	0.0882587	3.1177	0.00184	0.381796674	count	1
ZNF91	0.2861606	0.1370988	2.0873	0.0369	0.381929215	count	1
NRDE2	0.3102008	0.1812629	1.7113	0.0871	0.382120935	count	1
PHLDA2	0.2715701	0.1041528	2.6074	0.00916	0.382339777	count	1
NOTCH2	0.2861372	0.1542923	1.8545	0.0637	0.382394505	count	1
PPP6R3	0.2901461	0.1404725	2.0655	0.0389	0.382498419	count	1

TM9SF4	0.3163829	0.1602366	1.9745	0.0484	0.382623297	count	1
SNX1	0.2833757	0.1128143	2.5119	0.0121	0.382652194	count	1
FBXO3	0.2919825	0.1271917	2.2956	0.0218	0.382730954	count	1
ICA1L	0.3395847	0.2830057	1.1999	0.23	0.382787091	count	1
SECTM1	0.9042283	0.8425838	1.0732	0.283	0.382792103	count	1
RAB17	1.2825732	1.0806983	1.1868	0.235	0.382858123	count	1
AC087623.4	1.2825732	1.195665	1.0727	0.283	0.382858123	count	1
CU638689.5	1.2825732	1.3292067	0.9649	0.335	0.382858123	count	1
DGKG	0.6804309	0.3997642	1.7021	0.0888	0.382926225	count	1
PCYOX1L	0.905376	0.4777565	1.8951	0.0582	0.383167581	count	1
PPP5D1	0.6378388	0.5521328	1.1552	0.248	0.383184422	count	1
TYW1B	0.682	0.5106944	1.3354	0.182	0.38368011	count	1
KPNA5	0.3073759	0.1887603	1.6284	0.104	0.383725888	count	1
MEST	0.4328991	0.3475214	1.2457	0.213	0.383899586	count	1
AFDN-DT	1.7670534	1.1083789	1.5943	0.111	0.384062078	count	1
ANKRD26	0.296098	0.2035721	1.4545	0.146	0.384077514	count	1
MID1IP1	0.2843191	0.1138439	2.4974	0.0126	0.384160609	count	1
AKNAD1	0.7381291	0.4994973	1.4777	0.14	0.384278334	count	1
KRTCAP2	0.2704864	0.0527844	5.1244	3.14E-07	0.38437482	count	0.007331272
ZNF45	0.6835337	0.4402612	1.5526	0.121	0.384416378	count	1
DHX58	0.4264824	0.3285947	1.2979	0.194	0.384675422	count	1
MGMT	0.2744818	0.0682338	4.0227	5.87E-05	0.384675973	count	1
DENND4C	0.3081543	0.1989882	1.5486	0.122	0.38468406	count	1
AC011472.4	1.2925265	0.7536757	1.715	0.0864	0.38475438	count	1
RBM11	1.2925265	0.7544253	1.7133	0.0867	0.38475438	count	1
C5AR2	1.292906	0.6612032	1.9554	0.0506	0.38482649	count	1
PXDN	0.2871511	0.1632376	1.7591	0.0786	0.384993402	count	1
ELOVL6	0.7400508	0.3657329	2.0235	0.0431	0.385111742	count	1
BNIP2	0.2778398	0.0795831	3.4912	0.000487	0.385241264	count	1
SLC12A9	0.3784879	0.258834	1.4623	0.144	0.385428405	count	1
SLC36A1	0.7409313	0.4725293	1.568	0.117	0.385493281	count	1
VPS4B	0.2781198	0.0854578	3.2545	0.00115	0.385494188	count	1
BCL6	0.3031208	0.2054912	1.4751	0.14	0.38552996	count	1
POMT1	0.3988726	0.2575607	1.5487	0.122	0.385752828	count	1
GNA11	0.2859067	0.1322413	2.162	0.0307	0.385800596	count	1
LINC01547	1.7842484	1.2743305	1.4001	0.162	0.385874525	count	1
8-Sep	0.2887709	0.1244717	2.32	0.0204	0.385889069	count	1
TTC14	0.2813354	0.1105786	2.5442	0.011	0.386087126	count	1
MRPL19	0.2801371	0.0928432	3.0173	0.00257	0.386288428	count	1
STRADA	0.3949463	0.2984316	1.3234	0.186	0.386354114	count	1
EFNA1	0.3647253	0.2471309	1.4758	0.14	0.386417117	count	1
SH3D21	1.3013937	0.9651662	1.3484	0.178	0.386431622	count	1
NCF2	1.301933	0.6248002	2.0838	0.0373	0.386533265	count	1
PISD	0.3648608	0.2205105	1.6546	0.0981	0.386555944	count	1
HSPA4L	0.3263474	0.1869765	1.7454	0.081	0.386630988	count	1

GRIPAP1	0.2964138	0.1738378	1.7051	0.0883	0.386865522	count	1
SUPT20H	0.3053418	0.1501408	2.0337	0.0421	0.3869461	count	1
PPWD1	0.2932042	0.1683667	1.7415	0.0817	0.38694673	count	1
BMP8B	1.7947114	0.8007961	2.2412	0.0251	0.386964381	count	1
AKIRIN2	0.3029189	0.1308972	2.3142	0.0207	0.386987825	count	1
SECISBP2	0.2868553	0.1130892	2.5365	0.0112	0.38707266	count	1
COPB1	0.2784031	0.079556	3.4995	0.000472	0.387244461	count	1
IFNAR1	0.2780055	0.0825177	3.369	0.000762	0.387270338	count	1
LMF2	0.2856203	0.1180836	2.4188	0.0156	0.387279539	count	1
TIAL1	0.2769163	0.0924598	2.995	0.00276	0.387397417	count	1
DHX38	0.3164097	0.1748576	1.8095	0.0705	0.387470531	count	1
DZIP3	0.317138	0.1754928	1.8071	0.0708	0.387587639	count	1
C9orf72	0.3685347	0.2210144	1.6675	0.0955	0.387612598	count	1
C5orf63	0.8195831	0.5260548	1.558	0.119	0.387913894	count	1
BTBD19	0.3972082	0.3228396	1.2304	0.219	0.38847011	count	1
CCL19	0.2747103	0.2890595	0.9504	0.342	0.388578712	count	1
SYNE1	0.275197	0.0848776	3.2423	0.0012	0.388846354	count	1
ZSCAN12	0.5193764	0.3403932	1.5258	0.127	0.388874711	count	1
FH	0.2958296	0.1268624	2.3319	0.0198	0.388980804	count	1
ZNF205	0.3107207	0.2086618	1.4891	0.137	0.388994116	count	1
PEAR1	0.3357727	0.2456604	1.3668	0.172	0.389017867	count	1
RICTOR	0.3098472	0.153498	2.0186	0.0436	0.389020758	count	1
DNAJB9	0.2842169	0.1005855	2.8256	0.00474	0.389055269	count	1
RAD54B	16.8808857	982.1746968	0.0172	0.986	0.389123451	count	1
AC097662.1	16.9175065	1808.455165	0.0094	0.993	0.389123452	count	1
GATD3B	17.1167034	1599.838083	0.0107	0.991	0.389123456	count	1
KCNH3	17.1170038	1771.056369	0.0097	0.992	0.389123456	count	1
AC027575.2	17.2339363	1381.505271	0.0125	0.99	0.389123458	count	1
AC090198.1	17.2340564	1600.041939	0.0108	0.991	0.389123458	count	1
JAZF1-AS1	17.2341739	1591.934458	0.0108	0.991	0.389123458	count	1
AC008758.6	17.3358118	2288.893607	0.0076	0.994	0.389123459	count	1
AC146944.4	17.4307775	1032.130606	0.0169	0.987	0.38912346	count	1
AC011484.1	17.4490585	1823.015451	0.0096	0.992	0.38912346	count	1
NOVA2	17.5386185	948.0343016	0.0185	0.9852	0.389123461	count	1
LINC01068	17.580595	2252.594165	0.0078	0.994	0.389123462	count	1
AC092111.2	17.5805951	2252.594214	0.0078	0.994	0.389123462	count	1
AL158071.3	17.5809849	2608.961002	0.0067	0.995	0.389123462	count	1
HHATL	17.580986	2608.961844	0.0067	0.995	0.389123462	count	1
AP005329.2	17.5809861	2608.961935	0.0067	0.995	0.389123462	count	1
EPCAM	17.5809863	2608.961913	0.0067	0.995	0.389123462	count	1
FAM171A2	17.6125986	1377.403694	0.0128	0.99	0.389123462	count	1
HOXA7	17.6126084	1377.402704	0.0128	0.99	0.389123462	count	1
CD8B2	17.7991681	2395.002873	0.0074	0.994	0.389123464	count	1
RFPL1S	17.7991684	2395.003082	0.0074	0.994	0.389123464	count	1
AC098614.4	17.7998778	3094.953374	0.0058	0.995	0.389123464	count	1

TMEM125	17.799878	3094.953269	0.0058	0.995	0.389123464	count	1
LINC01242	17.799878	3094.953209	0.0058	0.995	0.389123464	count	1
PCDH15	17.799878	3094.953209	0.0058	0.995	0.389123464	count	1
AC011446.2	17.868826	2812.321882	0.0064	0.995	0.389123464	count	1
MAP1LC3C	18.1831322	1430.870193	0.0127	0.9899	0.389123467	count	1
AC074386.1	18.4116127	1409.698062	0.0131	0.9896	0.389123468	count	1
ZNF311	16.4491719	1211.697662	0.0136	0.989	0.389123474	count	1
C15orf59	16.5838427	1319.651861	0.0126	0.99	0.389123478	count	1
CSMD2	16.5839165	1108.036559	0.015	0.988	0.389123478	count	1
AC018362.1	16.7880924	1095.066621	0.0153	0.988	0.389123483	count	1
PROX1	16.8420009	1054.564431	0.016	0.987	0.389123485	count	1
RNASEK-C17orf49	16.917493	1808.449493	0.0094	0.993	0.389123486	count	1
AL445248.1	16.9650055	1380.014802	0.0123	0.99	0.389123487	count	1
WNT4	16.9650211	1380.019411	0.0123	0.99	0.389123487	count	1
LINC01915	16.9650211	1380.019407	0.0123	0.99	0.389123487	count	1
SERTM1	16.9650218	1380.016988	0.0123	0.99	0.389123487	count	1
AC078864.2	16.9652149	1598.294527	0.0106	0.992	0.389123487	count	1
TMC3	16.9655007	1963.417738	0.0086	0.993	0.389123487	count	1
PPARGC1B	16.9986868	979.5798124	0.0174	0.986	0.389123488	count	1
AC013553.4	17.0160028	1830.418192	0.0093	0.993	0.389123488	count	1
AL031055.1	17.0786245	1478.556064	0.0116	0.991	0.389123489	count	1
POU3F4	17.2337043	1548.230018	0.0111	0.991	0.389123492	count	1
LINC02302	17.2339364	1381.505275	0.0125	0.99	0.389123492	count	1
CARD10	17.233951	1381.509448	0.0125	0.99	0.389123492	count	1
B3GALT2	17.2342831	1784.957733	0.0097	0.992	0.389123492	count	1
AC019197.1	17.2738483	1586.844866	0.0109	0.991	0.389123492	count	1
LINC00997	17.2811723	971.7063938	0.0178	0.986	0.389123492	count	1
PEX5L	17.3358101	2288.889937	0.0076	0.994	0.389123493	count	1
AC027271.1	17.3364265	2894.236339	0.006	0.995	0.389123493	count	1
AC022081.1	17.342405	1282.803741	0.0135	0.9892	0.389123493	count	1
C6	17.3801188	1139.295059	0.0153	0.988	0.389123494	count	1
KIAA1549	17.3843884	1620.608472	0.0107	0.991	0.389123494	count	1
WNK2	17.3843895	1620.604282	0.0107	0.991	0.389123494	count	1
PDE11A	17.3844358	1822.434844	0.0095	0.992	0.389123494	count	1
AL035681.1	17.4125475	1458.418821	0.0119	0.9905	0.389123494	count	1
AL157714.2	17.4421858	1379.897832	0.0126	0.99	0.389123494	count	1
CYYR1	17.4425473	2079.444575	0.0084	0.993	0.389123494	count	1
DEFB1	17.4729468	1062.241282	0.0164	0.9869	0.389123495	count	1
AC013731.1	17.580595	2252.594214	0.0078	0.994	0.389123496	count	1
TMEM132C	17.580595	2252.594135	0.0078	0.994	0.389123496	count	1
IRF4	17.5805951	2252.594175	0.0078	0.994	0.389123496	count	1
NELL2	17.5805952	2252.594224	0.0078	0.994	0.389123496	count	1
C1orf87	17.5806155	2252.62426	0.0078	0.994	0.389123496	count	1
AC010907.1	17.5806155	2252.624299	0.0078	0.994	0.389123496	count	1
SLC6A13	17.580935	2608.948184	0.0067	0.995	0.389123496	count	1

AP002884.4	17.5809847	2608.960956	0.0067	0.995	0.389123496	count	1
WNT5A-AS1	17.5809848	2608.961013	0.0067	0.995	0.389123496	count	1
AL662797.1	17.5809848	2608.960979	0.0067	0.995	0.389123496	count	1
GRIK2	17.5809848	2608.960956	0.0067	0.995	0.389123496	count	1
AC002546.1	17.6110132	1022.026793	0.0172	0.9862	0.389123496	count	1
LINC01028	17.6125795	1595.415064	0.011	0.991	0.389123496	count	1
EN1	17.6125891	1377.396252	0.0128	0.99	0.389123496	count	1
SEMA6A-AS1	17.6399354	1918.886566	0.0092	0.993	0.389123497	count	1
AQP5	17.6500725	843.4476811	0.0209	0.9833	0.389123497	count	1
ZYG11A	17.7991682	2395.00285	0.0074	0.994	0.389123498	count	1
SIK1B	17.7991683	2395.002838	0.0074	0.994	0.389123498	count	1
AC137723.1	17.7991684	2395.002966	0.0074	0.994	0.389123498	count	1
AL732292.2	17.7991686	2395.002827	0.0074	0.994	0.389123498	count	1
AL133551.1	17.7991685	2395.003024	0.0074	0.994	0.389123498	count	1
PPP1R1B	17.7991686	2395.002827	0.0074	0.994	0.389123498	count	1
AC026254.2	17.7991689	2395.003105	0.0074	0.994	0.389123498	count	1
ZNF295-AS1	17.7991689	2395.003035	0.0074	0.994	0.389123498	count	1
TMC5	17.7998779	3094.953135	0.0058	0.995	0.389123498	count	1
AL109917.1	17.7998782	3094.953135	0.0058	0.995	0.389123498	count	1
Z94721.1	17.7998782	3094.953329	0.0058	0.995	0.389123498	count	1
ATAD3C	17.7998783	3094.953314	0.0058	0.995	0.389123498	count	1
GCGR	17.7998783	3094.953194	0.0058	0.995	0.389123498	count	1
AC005740.4	17.7998784	3094.953464	0.0058	0.995	0.389123498	count	1
RRM1-AS1	17.8008903	4156.13498	0.0043	0.997	0.389123498	count	1
COX6B2	17.8008903	4156.135181	0.0043	0.997	0.389123498	count	1
COL22A1	17.8008904	4156.135121	0.0043	0.997	0.389123498	count	1
GDA	17.8008904	4156.135302	0.0043	0.997	0.389123498	count	1
AC009120.4	17.8008904	4156.135262	0.0043	0.997	0.389123498	count	1
PSMA8	17.8008904	4156.135121	0.0043	0.997	0.389123498	count	1
ZNF30-AS1	17.8008905	4156.135181	0.0043	0.997	0.389123498	count	1
ALPL	17.8671008	1914.569597	0.0093	0.993	0.389123499	count	1
PCDHA10	17.8671247	1914.577561	0.0093	0.993	0.389123499	count	1
AL049870.3	17.8834089	1894.488013	0.0094	0.992	0.389123499	count	1
REXO5	17.9939237	1529.383325	0.0118	0.9906	0.3891235	count	1
CACNA1G	18.0934206	1576.377076	0.0115	0.9908	0.3891235	count	1
ATG4B	0.3137845	0.2172019	1.4447	0.149	0.389143763	count	1
FANCI	1.0722228	0.8602449	1.2464	0.213	0.389149974	count	1
C11orf71	0.3295197	0.172824	1.9067	0.0566	0.389210176	count	1
ZBTB20	0.2742548	0.0647057	4.2385	2.31E-05	0.389236504	count	0.5303067
CEP89	0.3118739	0.1577457	1.9771	0.0481	0.389261644	count	1
ZIK1	0.4664641	0.4662374	1.0005	0.317	0.389261953	count	1
SHLD1	0.4393467	0.3192783	1.3761	0.169	0.389284821	count	1
IKBKB	0.3522734	0.2604741	1.3524	0.176	0.38930229	count	1
THOC3	0.3861281	0.2350284	1.6429	0.1	0.389438571	count	1
FADS2	0.3247496	0.1943952	1.6706	0.0949	0.389817872	count	1

EIF2AK1	0.2935295	0.1128053	2.6021	0.0093	0.389863816	count	1
AL158152.1	0.5604069	0.3414751	1.6411	0.101	0.389895056	count	1
MGAT4C	1.075597	0.6063409	1.7739	0.0762	0.390023974	count	1
FAR1	0.3057677	0.1966315	1.555	0.12	0.390162901	count	1
LINC00649	0.9269943	0.7637008	1.2138	0.225	0.390189251	count	1
TMEM53	0.3392909	0.2081624	1.6299	0.103	0.390208844	count	1
CCS	0.280871	0.0864033	3.2507	0.00116	0.390235265	count	1
SCD5	0.4331134	0.2721057	1.5917	0.112	0.390322995	count	1
AL022322.1	0.6962517	0.5050754	1.3785	0.168	0.390498919	count	1
ABHD13	0.3035607	0.1740627	1.744	0.0812	0.390909724	count	1
TANGO6	0.5408494	0.3499632	1.5454	0.122	0.390986292	count	1
OGG1	0.3302116	0.1808902	1.8255	0.068	0.391117511	count	1
JKAMP	0.286177	0.0986075	2.9022	0.00373	0.391324557	count	1
ZNF862	1.0807853	0.539714	2.0025	0.0453	0.391363231	count	1
SLC35B2	0.2960956	0.137444	2.1543	0.0313	0.391393213	count	1
MED14OS	0.6178136	0.4196343	1.4723	0.141	0.391516105	count	1
SUFU	0.4214807	0.347813	1.2118	0.226	0.391650521	count	1
SPTAN1	0.2780842	0.0952873	2.9184	0.00354	0.391658139	count	1
ZNF737	0.3652672	0.312053	1.1705	0.242	0.39204228	count	1
CLEC10A	1.3327153	1.4884847	0.8954	0.371	0.392265553	count	1
CAPN10	0.4288964	0.2896351	1.4808	0.139	0.392601125	count	1
CDK12	0.2880886	0.1336458	2.1556	0.0312	0.392617417	count	1
SUMF1	0.3137382	0.1432965	2.1894	0.0286	0.392718822	count	1
ADCY2	0.8322882	0.6338452	1.3131	0.189	0.392734147	count	1
ACVR2B	0.4709379	0.3262003	1.4437	0.149	0.39273925	count	1
DGCR8	0.3974934	0.3292737	1.2072	0.227	0.392807071	count	1
ASB6	0.3524224	0.3028374	1.1637	0.245	0.393290079	count	1
CHD9	0.2774381	0.0683295	4.0603	5.00E-05	0.393293589	count	1
POC5	0.3665412	0.2325289	1.5763	0.115	0.393365936	count	1
GAPVD1	0.3126084	0.1611117	1.9403	0.0524	0.393514742	count	1
KDM3A	0.3263822	0.2225169	1.4668	0.143	0.393602191	count	1
RTN3	0.2789626	0.0573194	4.8668	1.18E-06	0.393710072	count	0.02743028
COQ10B	0.2860582	0.0915085	3.126	0.00179	0.393997448	count	1
KTI12	0.3310559	0.1865305	1.7748	0.076	0.394233656	count	1
PPP1R3D	0.3629068	0.2275459	1.5949	0.111	0.394272131	count	1
RARA	0.3046586	0.1506814	2.0219	0.0433	0.39440763	count	1
UBE2J1	0.2936217	0.1221534	2.4037	0.0163	0.39464832	count	1
MEIS1-AS2	1.3463065	0.6142152	2.1919	0.0284	0.394753515	count	1
SLC25A32	0.3091798	0.1795848	1.7216	0.0852	0.394890749	count	1
SLC5A6	0.7628237	0.6181401	1.2341	0.217	0.394919593	count	1
LINC01564	0.9419969	0.4357066	2.162	0.0307	0.395004812	count	1
TADA1	0.4637805	0.2895867	1.6015	0.109	0.39506671	count	1
ABHD11	0.3707088	0.2209142	1.6781	0.0934	0.395166202	count	1
CAPN7	0.2931489	0.1327561	2.2082	0.0273	0.395250819	count	1
USP10	0.3211279	0.1483253	2.165	0.0305	0.396075356	count	1

ERRFI1	0.2863595	0.1030312	2.7793	0.00547	0.39639347	count	1
COL1A2	0.2760379	0.0586047	4.7102	2.57E-06	0.396610842	count	0.05957517
PIAS2	0.3323592	0.2332484	1.4249	0.154	0.396788012	count	1
ELOVL5	0.3111054	0.1433416	2.1704	0.03	0.396891134	count	1
ATP1A1	0.2849301	0.093341	3.0526	0.00229	0.396955466	count	1
CHAC2	1.3589393	0.795713	1.7078	0.0878	0.397042636	count	1
FLVCR1	1.104391	0.5219916	2.1157	0.0344	0.39739631	count	1
PON2	0.2945733	0.1306452	2.2548	0.0242	0.397420202	count	1
PDS5A	0.3067562	0.1735165	1.7679	0.0772	0.397428661	count	1
JAZF1	0.3017726	0.1683154	1.7929	0.0731	0.397454082	count	1
PHYHD1	0.302344	0.1332448	2.2691	0.0233	0.397715786	count	1
RORA	0.2922373	0.1234939	2.3664	0.018	0.397858871	count	1
SEC24B-AS1	0.7697874	0.5813444	1.3242	0.186	0.397893572	count	1
ADH4	1.9052516	0.9135724	2.0855	0.0371	0.397896478	count	1
UBXN8	0.3462183	0.2217893	1.561	0.119	0.397985672	count	1
AC012306.2	0.3234227	0.1924237	1.6808	0.0929	0.398147874	count	1
MYMX	1.9099568	1.1354112	1.6822	0.0926	0.398338948	count	1
AC005899.7	1.9099568	1.8734548	1.0195	0.308	0.398338948	count	1
BEX4	0.2825834	0.0695876	4.0608	4.99E-05	0.398351926	count	1
FBXO34	0.3170252	0.1799216	1.762	0.0781	0.398458104	count	1
TNPO2	0.3418397	0.2257815	1.514	0.13	0.398547335	count	1
PPARGC1A	0.9534365	0.62666	1.5215	0.128	0.398645263	count	1
FAM168A	0.3419259	0.1999609	1.71	0.0874	0.398645596	count	1
MED18	0.3741459	0.2547513	1.4687	0.142	0.398707744	count	1
AC013394.1	0.3295984	0.2105931	1.5651	0.118	0.399203821	count	1
PLEKHB1	0.5534475	0.4756513	1.1636	0.245	0.399216028	count	1
KCNJ8	0.3402739	0.1610795	2.1125	0.0347	0.399269274	count	1
DNMBP	0.380048	0.32875	1.156	0.248	0.399295911	count	1
LDLRAD3	0.4599315	0.377111	1.2196	0.223	0.399400003	count	1
TFF3	1.9240927	1.1685277	1.6466	0.0997	0.39965727	count	1
SLC41A2	0.3136837	0.1380676	2.272	0.0231	0.399699472	count	1
CKAP4	0.2897485	0.1016984	2.8491	0.00441	0.39989283	count	1
RNPEPL1	0.3146717	0.1766831	1.781	0.075	0.400036093	count	1
CPE	0.2786271	0.0392757	7.0941	1.55E-12	0.400043131	count	3.69E-08
ARNT	0.3310876	0.2011229	1.6462	0.0998	0.40008398	count	1
FXYD2	1.11582	1.0307213	1.0826	0.279	0.400281335	count	1
COL9A2	0.3198752	0.3013981	1.0613	0.289	0.400290561	count	1
MYO6	0.3017923	0.1547106	1.9507	0.0512	0.400549302	count	1
WDR53	0.4532762	0.3407016	1.3304	0.183	0.400882148	count	1
TCF15	1.118808	0.5604888	1.9961	0.046	0.401031752	count	1
LAMC1	0.2942039	0.0906837	3.2443	0.00119	0.401093429	count	1
SMO	0.4458556	0.3268221	1.3642	0.173	0.401143529	count	1
NOM1	0.3363001	0.2232716	1.5062	0.132	0.401400087	count	1
WDR31	0.5571292	0.4573088	1.2183	0.223	0.401612896	count	1
CEP78	0.3481879	0.3031064	1.1487	0.251	0.401651147	count	1

PER3	0.2886321	0.1120712	2.5754	0.0101	0.401914962	count	1
S100A1	1.1233313	1.0509605	1.0689	0.285	0.402164711	count	1
FOXJ2	0.3752797	0.3115069	1.2047	0.228	0.402435768	count	1
ADCY1	0.6375858	0.4629255	1.3773	0.169	0.402470904	count	1
COG7	0.3924671	0.2706072	1.4503	0.147	0.402486915	count	1
ZFP30	0.4637545	0.3198446	1.4499	0.147	0.402507582	count	1
BBS2	0.3217543	0.1843451	1.7454	0.081	0.402608005	count	1
KLF8	0.5396199	0.3159644	1.7078	0.0877	0.402660803	count	1
SOX15	0.3172247	0.2000602	1.5856	0.113	0.402771058	count	1
MFSD13A	0.7812943	0.3937511	1.9842	0.0473	0.402782091	count	1
LBX2-AS1	1.3914569	0.8200629	1.6968	0.0898	0.402832478	count	1
AP1B1	0.3807802	0.264196	1.4413	0.15	0.402838834	count	1
RUVBL2	0.3104212	0.1385179	2.241	0.0251	0.403112996	count	1
TRMT11	0.295631	0.1007875	2.9332	0.00338	0.403396735	count	1
PIGO	0.4565089	0.2964737	1.5398	0.124	0.403566315	count	1
RASSF9	0.3971546	0.2285397	1.7378	0.0823	0.403676711	count	1
FIGNL1	0.4009329	0.4333954	0.9251	0.355	0.403752431	count	1
TNRC6B	0.2869266	0.0752169	3.8147	0.000139	0.403838367	count	1
ZWILCH	0.5247731	0.4356243	1.2046	0.228	0.403938429	count	1
TMEM38B	0.3194073	0.1600768	1.9953	0.0461	0.404038199	count	1
FGFR1OP	0.4239152	0.3159987	1.3415	0.18	0.404039019	count	1
FGF14-AS2	0.3876691	0.2273052	1.7055	0.0882	0.404047851	count	1
NAA25	0.3680188	0.1997221	1.8427	0.0655	0.404085455	count	1
PRKD3	0.3037675	0.1277682	2.3775	0.0175	0.404700948	count	1
TXLNG	0.3156835	0.1458497	2.1644	0.0305	0.404770406	count	1
ACADSB	0.4147645	0.2212368	1.8748	0.0609	0.404851986	count	1
SLC46A1	0.4761889	0.3426374	1.3898	0.165	0.404922049	count	1
BZW1	0.2840657	0.0504948	5.6256	1.99E-08	0.404980141	count	0.00046761
SELENOS	0.2842253	0.0509455	5.579	2.59E-08	0.405101198	count	0.000608287
ALDH9A1	0.3012448	0.0871991	3.4547	0.000557	0.405115024	count	1
KANSL2	0.3253634	0.1820457	1.7873	0.074	0.405224909	count	1
DNALI1	0.3287029	0.2294691	1.4324	0.152	0.405266869	count	1
BAZ2B	0.3013121	0.1523437	1.9778	0.048	0.40534884	count	1
FBXO38	0.3570435	0.2377306	1.5019	0.133	0.405363108	count	1
SDHD	0.2859753	0.0558527	5.1202	3.21E-07	0.405375528	count	0.007494387
MANSC1	0.3125133	0.1439375	2.1712	0.03	0.405474456	count	1
KDM6A	0.3864157	0.237212	1.629	0.103	0.405745151	count	1
DHRS7B	0.3089557	0.1253864	2.464	0.0138	0.406080176	count	1
MMP19	0.3929845	0.2576696	1.5251	0.127	0.406251869	count	1
BBS4	0.3238247	0.1490411	2.1727	0.0299	0.406319704	count	1
ZDHHC18	0.3964624	0.2716565	1.4594	0.145	0.406422768	count	1
ADAMTS1	0.2893762	0.0757913	3.8181	0.000137	0.406519672	count	1
PI4K2A	0.4323437	0.2806996	1.5402	0.124	0.406563278	count	1
DDIT3	0.2911946	0.0936628	3.109	0.00189	0.406565411	count	1
ADD1	0.2891674	0.0706958	4.0903	4.40E-05	0.406609749	count	1

GINS3	1.4132131	0.8764448	1.6124	0.107	0.406624714	count	1
EIF4EBP1	0.3019133	0.1194715	2.5271	0.0115	0.40671522	count	1
MIGA1	0.3069535	0.1138006	2.6973	0.00702	0.40671579	count	1
PCDHB11	0.5877248	0.2551901	2.3031	0.0213	0.406875684	count	1
FXYD6	0.3516559	0.1787122	1.9677	0.0492	0.406985459	count	1
KLHL9	0.3188421	0.1975478	1.614	0.107	0.40707848	count	1
RBM39	0.2858198	0.0550651	5.1906	2.21E-07	0.40709934	count	0.005165212
UBE2Q2L	2.0088642	1.025341	1.9592	0.0502	0.407229097	count	1
MORC4	0.3061854	0.1332929	2.2971	0.0217	0.407326231	count	1
FAM126A	0.3138249	0.1756235	1.7869	0.074	0.407486004	count	1
ACTR3B	0.4397482	0.348034	1.2635	0.206	0.407708375	count	1
PGGT1B	0.3076951	0.1541327	1.9963	0.046	0.40790191	count	1
IFNGR1	0.2921384	0.0699443	4.1767	3.03E-05	0.407910625	count	0.6943548
CUTC	0.3106282	0.1251302	2.4824	0.0131	0.408000466	count	1
ZNF638	0.2925842	0.1020601	2.8668	0.00417	0.408531109	count	1
SUCO	0.3129827	0.1512545	2.0692	0.0386	0.408568279	count	1
COQ2	0.3989138	0.2361858	1.689	0.0913	0.40883588	count	1
FANCM	0.4812528	0.3601891	1.3361	0.182	0.408932179	count	1
AC013271.1	0.4193	0.3843508	1.0909	0.275	0.409071915	count	1
MICU1	0.3060978	0.1128712	2.7119	0.00672	0.409212975	count	1
CLN5	0.3006688	0.0958647	3.1364	0.00172	0.409271877	count	1
DISP2	1.15232	1.153928	0.9986	0.318	0.409339291	count	1
ZNF10	0.354016	0.4599021	0.7698	0.441	0.409651483	count	1
AC025181.2	0.430155	0.2741958	1.5688	0.117	0.409686258	count	1
ANGEL1	0.8778521	0.5584949	1.5718	0.116	0.409718455	count	1
SUN1	0.3029378	0.1126234	2.6898	0.00718	0.409782342	count	1
CNEP1R1	0.356767	0.2265377	1.5749	0.115	0.409811236	count	1
AHCYL2	0.4363042	0.3851677	1.1328	0.257	0.410091824	count	1
KCND2	0.737909	0.7396486	0.9976	0.319	0.410135046	count	1
KCTD6	0.3359641	0.1821091	1.8449	0.0651	0.410193867	count	1
RTKN	0.5341673	0.3216838	1.6605	0.0969	0.410539342	count	1
TP53I13	0.2995295	0.1059158	2.828	0.00471	0.410549493	count	1
WASHC2C	0.3833005	0.2385901	1.6065	0.108	0.410746404	count	1
DPP7	0.2893834	0.06079	4.7604	2.01E-06	0.41094522	count	0.04661793
SPSB1	0.3114124	0.1290979	2.4122	0.0159	0.410996568	count	1
GABPA	0.3271632	0.1401381	2.3346	0.0196	0.411017499	count	1
AC020915.1	1.4390626	1.3711115	1.0496	0.294	0.411046721	count	1
BPI	1.1594438	0.7319806	1.584	0.113	0.411079689	count	1
CPOX	0.5352566	0.2987883	1.7914	0.0733	0.411303169	count	1
GABPB1-AS1	0.3003402	0.1758201	1.7082	0.0877	0.411576579	count	1
PTPN23	0.3922059	0.2605943	1.505	0.132	0.411601727	count	1
RB1CC1	0.2992017	0.0974368	3.0707	0.00215	0.411688828	count	1
LY6E-DT	0.5956675	0.5937814	1.0032	0.316	0.411774533	count	1
LGR4	0.3348443	0.2676492	1.2511	0.211	0.411973038	count	1
DUSP10	0.6549158	0.4371161	1.4983	0.134	0.41198671	count	1

PQLC2	0.4754608	0.2837462	1.6757	0.0939	0.411999045	count	1
PTRH2	0.3227828	0.1590547	2.0294	0.0425	0.412046118	count	1
ZNF551	1.1639959	0.914323	1.2731	0.203	0.412187164	count	1
MBD5	0.3212248	0.1904683	1.6865	0.0918	0.412198833	count	1
DTNB	0.6232565	0.4533434	1.3748	0.169	0.412201471	count	1
HERC4	0.314284	0.1648005	1.9071	0.0566	0.412224748	count	1
CCDC28A	0.3297118	0.1829248	1.8024	0.0716	0.412416748	count	1
RGS9	0.3497579	0.1901676	1.8392	0.066	0.412597402	count	1
GBA	0.3255213	0.1772968	1.836	0.0664	0.412674188	count	1
VPS11	0.445528	0.2229359	1.9985	0.0457	0.412771295	count	1
SRSF7	0.2912499	0.0545808	5.3361	1.01E-07	0.413553957	count	0.002365319
RIPK1	0.3288067	0.1748749	1.8802	0.0602	0.413614518	count	1
NAGLU	0.3068709	0.1016941	3.0176	0.00257	0.413626088	count	1
HNRNPU	0.2901089	0.0632586	4.5861	4.67E-06	0.413734152	count	0.10797974
MIER2	0.4690026	0.5147726	0.9111	0.362	0.413914195	count	1
GNG12-AS1	0.8894073	0.7860921	1.1314	0.258	0.413950818	count	1
THAP10	0.478209	0.4210251	1.1358	0.256	0.414221981	count	1
CLN3	2.0956763	0.8239258	2.5435	0.011	0.414416595	count	1
RAB4B	1.0040371	0.911831	1.1011	0.271	0.414423971	count	1
GK	0.3894428	0.4364025	0.8924	0.372	0.41443868	count	1
PTDSS2	0.3345503	0.2406681	1.3901	0.165	0.414484937	count	1
ZNF714	0.6001804	0.4043032	1.4845	0.138	0.414550256	count	1
SLC18B1	1.4605037	0.6843897	2.134	0.0329	0.414646563	count	1
UBFD1	0.3442264	0.2182019	1.5776	0.115	0.414706758	count	1
FMO5	0.4619653	0.3443275	1.3416	0.18	0.414763177	count	1
SHLD3	0.8104414	0.6049474	1.3397	0.18	0.415021093	count	1
PRRC1	0.3092606	0.1299432	2.38	0.0174	0.4150551	count	1
C3orf62	0.413115	0.2969085	1.3914	0.164	0.415492027	count	1
PIGF	0.3217501	0.1550667	2.0749	0.0381	0.415565313	count	1
SIGLEC15	1.179103	1.1289796	1.0444	0.296	0.415836488	count	1
STK4	0.3154436	0.1402876	2.2485	0.0246	0.416028543	count	1
PROCR	0.2982645	0.0958294	3.1125	0.00187	0.416173014	count	1
CIDEB	0.8955092	0.4453691	2.0107	0.0444	0.416173633	count	1
WASHC5	0.3472245	0.1851277	1.8756	0.0608	0.416258981	count	1
RBM33	0.3098567	0.1718567	1.803	0.0715	0.416463688	count	1
AARSD1	0.3797888	0.2629898	1.4441	0.149	0.416605962	count	1
DSC3	0.8972352	0.8279687	1.0837	0.279	0.41680086	count	1
TRA2B	0.297414	0.0827041	3.5961	0.000327	0.416849526	count	1
MESD	0.295038	0.0645215	4.5727	4.97E-06	0.417068349	count	0.11488155
SLC35C1	0.4817872	0.2568785	1.8755	0.0608	0.417113254	count	1
NR3C1	0.2959757	0.0616627	4.7999	1.65E-06	0.417171058	count	0.0383097
SLC38A6	0.3432657	0.2032147	1.6892	0.0913	0.417236179	count	1
ENTPD1-AS1	0.355969	0.2702426	1.3172	0.188	0.417266678	count	1
AC106786.1	2.1343961	1.1404934	1.8715	0.0614	0.417447239	count	1
BTBD8	0.4388456	0.2815421	1.5587	0.119	0.417535294	count	1

MAFG-DT	0.6051906	0.3391851	1.7842	0.0745	0.417625301	count	1
RNF122	0.7544131	0.4036097	1.8692	0.0617	0.417792921	count	1
PCYOX1	0.2984569	0.0714914	4.1747	3.05E-05	0.417942075	count	0.698877
ZNF407	0.3589035	0.2579642	1.3913	0.164	0.417972745	count	1
MFSD4B	0.4118556	0.3098164	1.3294	0.184	0.417991406	count	1
PALMD	0.3337669	0.226663	1.4725	0.141	0.418016408	count	1
BTG3	0.3024563	0.0855509	3.5354	0.000412	0.418044255	count	1
ENPEP	0.5038116	0.4134361	1.2186	0.223	0.418125008	count	1
TMEM267	0.3369843	0.1772716	1.9009	0.0574	0.418137831	count	1
TMEM128	0.3267703	0.1421884	2.2982	0.0216	0.418385493	count	1
SLC25A46	0.3318765	0.1656054	2.004	0.0451	0.418525101	count	1
CCNT1	0.3451753	0.1980424	1.7429	0.0814	0.41862382	count	1
CHP1	0.3061001	0.1050601	2.9136	0.00359	0.418935557	count	1
IPO5	0.31683	0.1141016	2.7767	0.00552	0.419015123	count	1
NUP205	0.5466866	0.3149161	1.736	0.0827	0.41929828	count	1
DHX15	0.3200283	0.1363751	2.3467	0.019	0.419408024	count	1
LINC02384	2.1621608	1.1781037	1.8353	0.0665	0.419556538	count	1
PDZD7	1.1948339	0.8580319	1.3925	0.164	0.419594275	count	1
NUTM2B-AS1	0.311535	0.1193299	2.6107	0.00907	0.419728014	count	1
GPS1	0.3109326	0.162603	1.9122	0.0559	0.419878917	count	1
SEMA4C	0.3632163	0.2101931	1.728	0.0841	0.420034882	count	1
NOD1	0.5857562	0.4015669	1.4587	0.145	0.420122546	count	1
SFR1	0.3350981	0.1542279	2.1727	0.0299	0.420253997	count	1
SPTY2D1	0.3294945	0.1322917	2.4907	0.0128	0.420502784	count	1
DIRC2	0.4480505	0.245631	1.8241	0.0682	0.420533648	count	1
RC3H2	0.337331	0.2067537	1.6316	0.103	0.420542631	count	1
IFI27L1	0.3526988	0.1775703	1.9862	0.0471	0.420564992	count	1
GPR89A	0.5662269	0.3350761	1.6898	0.0911	0.420609097	count	1
ZNF469	1.0246337	0.8675259	1.1811	0.238	0.420696283	count	1
AC005670.2	1.0246337	1.1625115	0.8814	0.378	0.420696283	count	1
KCTD1	0.3613406	0.2692743	1.3419	0.18	0.420742992	count	1
AC023908.3	1.4994796	1.0661655	1.4064	0.16	0.421034879	count	1
AC103810.2	1.4994796	1.1794941	1.2713	0.204	0.421034879	count	1
AC008074.3	1.4994796	1.1839631	1.2665	0.205	0.421034879	count	1
BHLHE22	1.02672	0.6452246	1.5913	0.112	0.421326822	count	1
GMPPB	0.3685484	0.1697506	2.1711	0.03	0.421402287	count	1
SFPQ	0.2972765	0.0636084	4.6735	3.07E-06	0.421606647	count	0.07111655
AP2B1	0.314073	0.1287714	2.439	0.0148	0.421623748	count	1
UXS1	0.320541	0.1406025	2.2798	0.0227	0.421681888	count	1
MEX3B	0.7132835	0.4919642	1.4499	0.147	0.421851619	count	1
CEPT1	0.3471381	0.207526	1.6727	0.0945	0.421854776	count	1
SOX6	0.3705021	0.5049691	0.7337	0.463	0.421927805	count	1
SLC35A5	0.3476202	0.1680088	2.0691	0.0386	0.422429613	count	1
U47924.2	0.5512765	0.4289732	1.2851	0.199	0.42249868	count	1
ZFAT	0.5898302	0.4502953	1.3099	0.19	0.422738292	count	1

QRICH1	0.3494425	0.2205913	1.5841	0.113	0.422777268	count	1
TMEM234	0.3645946	0.2082901	1.7504	0.0801	0.423034395	count	1
SMIM13	0.390586	0.2264851	1.7246	0.0847	0.423345637	count	1
CAD	0.5368804	0.3520286	1.5251	0.127	0.423374107	count	1
EARS2	0.5909165	0.5618497	1.0517	0.293	0.423434993	count	1
TARBP2	0.5910147	0.3620811	1.6323	0.103	0.423497961	count	1
PRKDC	0.3022975	0.0769612	3.9279	8.73E-05	0.423544359	count	1
TMEM192	0.3342262	0.1758278	1.9009	0.0574	0.423556591	count	1
SDHA	0.3168246	0.0970027	3.2661	0.0011	0.423616671	count	1
TMBIM6	0.2955726	0.0333481	8.8632	1.19E-18	0.423636489	count	2.85E-14
LINS1	0.390869	0.2393573	1.633	0.103	0.423642059	count	1
RFX2	0.3640044	0.32358	1.1249	0.261	0.423769743	count	1
WDFY2	0.3304361	0.204028	1.6196	0.105	0.423871177	count	1
GALNT10	0.3460109	0.21672	1.5966	0.11	0.42388909	count	1
CMTR1	0.4043807	0.2480888	1.63	0.103	0.42389175	count	1
ZNF276	0.3807089	0.3062896	1.243	0.214	0.423921581	count	1
NAIP	0.4811607	0.3956029	1.2163	0.224	0.423944358	count	1
ARHGAP33	0.8323234	0.6685803	1.2449	0.213	0.424074376	count	1
TJP2	0.6161432	0.7933529	0.7766	0.437	0.424323517	count	1
NOTCH4	0.4185951	0.3311574	1.264	0.206	0.424536912	count	1
PTPN9	0.3832185	0.2241839	1.7094	0.0875	0.424573488	count	1
MEN1	0.5726389	0.4320925	1.3253	0.185	0.424905156	count	1
AC006213.2	1.5245573	1.0222044	1.4914	0.136	0.425040874	count	1
STRN3	0.3095024	0.1052678	2.9401	0.0033	0.425075774	count	1
TMEM41A	0.3396629	0.1516794	2.2393	0.0252	0.425287232	count	1
KLHDC8B	0.3235596	0.1432796	2.2582	0.024	0.425351951	count	1
SFT2D2	0.3159601	0.1337513	2.3623	0.0182	0.425502909	count	1
LMO3	0.3018891	0.0975563	3.0945	0.00199	0.425601999	count	1
AC098818.2	0.5739188	0.457308	1.255	0.21	0.42576133	count	1
CCDC77	0.442455	0.2525833	1.7517	0.0799	0.425775251	count	1
ARMT1	0.3368619	0.1488017	2.2638	0.0236	0.425804098	count	1
ZBTB39	0.5948307	0.4819885	1.2341	0.217	0.425942654	count	1
ANKRD17	0.3331636	0.1443895	2.3074	0.0211	0.42602588	count	1
ZNF655	0.322691	0.1712589	1.8842	0.0596	0.426224357	count	1
SLC2A9	0.7220451	0.5692411	1.2684	0.205	0.426245531	count	1
TSGA10	0.5032873	0.4765439	1.0561	0.291	0.426300833	count	1
PRRC2A	0.3449637	0.1650177	2.0905	0.0366	0.426452349	count	1
PTRH1	0.3383058	0.1680449	2.0132	0.0442	0.426514587	count	1
ZCCHC9	0.3169854	0.1367214	2.3185	0.0205	0.426579633	count	1
DENND5A	0.3417619	0.2236789	1.5279	0.127	0.42662267	count	1
PPM1A	0.3331534	0.1283904	2.5948	0.0095	0.426886376	count	1
CNOT6	0.3655761	0.2631373	1.3893	0.165	0.426926863	count	1
CEP120	0.3421931	0.2785529	1.2285	0.219	0.427152409	count	1
HMBOX1	0.368322	0.2209642	1.6669	0.0956	0.427251737	count	1
FER	0.3253085	0.1343548	2.4213	0.0155	0.427361133	count	1

PIK3C2B	0.5284017	0.3600998	1.4674	0.142	0.427375984	count	1
LRRC49	0.3839848	0.215579	1.7812	0.075	0.427458846	count	1
CLIC6	1.049003	0.465972	2.2512	0.0244	0.428006474	count	1
DENND2C	0.4565143	0.2525566	1.8076	0.0708	0.428035666	count	1
HIVEP1	0.3256654	0.1414458	2.3024	0.0214	0.428092807	count	1
ZNF83	0.3200126	0.1429241	2.239	0.0252	0.428197464	count	1
AC108863.1	1.0498237	0.6534116	1.6067	0.108	0.428250333	count	1
DUS1L	0.3279939	0.1366615	2.4	0.0164	0.428262325	count	1
RASSF8	0.305787	0.078783	3.8814	0.000106	0.428284613	count	1
RALGAPA1	0.3260378	0.1344814	2.4244	0.0154	0.428309652	count	1
TTC21A	1.232297	0.5309577	2.3209	0.0203	0.428371736	count	1
ZNF195	0.3587042	0.2255896	1.5901	0.112	0.428666366	count	1
BSDC1	0.3272063	0.1550486	2.1103	0.0349	0.428718442	count	1
PDE6B	1.5484868	0.9588094	1.615	0.106	0.42878861	count	1
DYRK2	0.3638513	0.1976385	1.841	0.0657	0.428843339	count	1
LRIG2	0.3685745	0.2205745	1.671	0.0948	0.42895961	count	1
FAF2	0.3229213	0.106423	3.0343	0.00243	0.428984565	count	1
MSMO1	0.3335074	0.1403746	2.3758	0.0176	0.429001648	count	1
HAUS2	0.3599788	0.1979861	1.8182	0.0691	0.429059122	count	1
RABL2B	0.3565306	0.2512086	1.4193	0.156	0.429230457	count	1
RBMS2	0.3424645	0.2042325	1.6768	0.0937	0.429350321	count	1
EIF4A3	0.3094267	0.0945767	3.2717	0.00108	0.429482377	count	1
CYP1B1	0.3269438	0.1466357	2.2296	0.0258	0.429487908	count	1
FBXO6	0.4041563	0.2375133	1.7016	0.0889	0.429521713	count	1
DNPH1	0.3070931	0.0872102	3.5213	0.000435	0.429569401	count	1
VMP1	0.307576	0.0824801	3.7291	0.000195	0.429940119	count	1
CRAT	0.3378201	0.1900621	1.7774	0.0756	0.430039001	count	1
HOOK3	0.3088314	0.0780142	3.9587	7.68E-05	0.430248182	count	1
IL12A	1.2415079	1.412842	0.8787	0.38	0.430492864	count	1
SUGP2	0.3429368	0.1982619	1.7297	0.0838	0.430532703	count	1
PDGFB	0.7311572	0.4814151	1.5188	0.129	0.430793883	count	1
AC010542.2	0.6896281	0.5057443	1.3636	0.173	0.43080495	count	1
SLC35F5	0.3312314	0.1633551	2.0277	0.0427	0.430845453	count	1
JAM2	0.3301484	0.1912191	1.7265	0.0843	0.431045183	count	1
TNFSF4	1.2443747	0.7574111	1.6429	0.1	0.431150146	count	1
NAE1	0.3199915	0.1138731	2.8101	0.00498	0.431183881	count	1
AC245140.2	0.8497809	0.4571456	1.8589	0.0631	0.431214148	count	1
ZNF626	0.3465892	0.1500747	2.3094	0.021	0.431230849	count	1
DNLZ	1.0603421	0.4625727	2.2923	0.0219	0.431366708	count	1
ANKRD35	0.3136315	0.0990228	3.1673	0.00155	0.431374614	count	1
NEURL1	1.5653792	1.2413716	1.261	0.207	0.431390761	count	1
LAPTM4B	0.3095126	0.0847553	3.6518	0.000264	0.431600326	count	1
AC090114.2	0.4905083	0.2589488	1.8942	0.0583	0.431629104	count	1
N4BP2L1	0.3362754	0.1473342	2.2824	0.0225	0.431689571	count	1
PXDNL	2.3375401	1.4473759	1.615	0.106	0.431723237	count	1

NAALADL2	0.3353343	0.1676865	1.9998	0.0456	0.431725536	count	1
GUF1	0.4122205	0.2466419	1.6713	0.0947	0.431788169	count	1
ERP44	0.3113861	0.0704506	4.4199	1.02E-05	0.431810131	count	0.2349774
SLC16A1	0.3843868	0.2190166	1.7551	0.0793	0.431829706	count	1
BAIAP2-DT	0.7853773	0.5697625	1.3784	0.168	0.431973909	count	1
CDC42EP4	0.3382898	0.1546379	2.1876	0.0288	0.432039341	count	1
ACAA2	0.313214	0.1020054	3.0706	0.00215	0.432493985	count	1
DUSP4	1.0642195	1.1852011	0.8979	0.369	0.432509876	count	1
MACO1	0.3251963	0.1223417	2.6581	0.00789	0.432602999	count	1
METTL8	0.3650786	0.2022919	1.8047	0.0712	0.43269033	count	1
RFX7	0.4232499	0.3545317	1.1938	0.233	0.432716554	count	1
AC109587.1	0.6055196	0.6060385	0.9991	0.318	0.432768915	count	1
CACNA2D4	2.3573723	1.0368764	2.2735	0.0231	0.432980742	count	1
SIN3A	0.3403146	0.2265312	1.5023	0.133	0.43317071	count	1
AL512625.1	0.788195	0.7066896	1.1153	0.265	0.433252301	count	1
ERN1	0.3461489	0.2211497	1.5652	0.118	0.43328043	count	1
AL136982.7	1.067358	1.362792	0.7832	0.434	0.43343279	count	1
TCF7L2	0.3177518	0.0992964	3.2	0.00139	0.433501301	count	1
CDIP1	0.3492872	0.2357251	1.4818	0.138	0.433846348	count	1
SNED1	0.3580222	0.1896703	1.8876	0.0592	0.433898081	count	1
RNH1	0.3032487	0.0388284	7.81	7.41E-15	0.433899997	count	1.77E-10
CCNT2	0.3380315	0.1360829	2.484	0.013	0.433915054	count	1
DUOX1	1.5840623	0.9342665	1.6955	0.0901	0.434227418	count	1
PDE1B	0.552678	0.3334847	1.6573	0.0975	0.434730221	count	1
COP1	0.3836842	0.214465	1.789	0.0737	0.434755792	count	1
MPST	0.3185947	0.1046597	3.0441	0.00235	0.434838123	count	1
SAV1	0.3203833	0.0905868	3.5368	0.00041	0.434939261	count	1
CSNK1E	0.343024	0.1595726	2.1496	0.0316	0.435065298	count	1
MAD2L1BP	0.3457233	0.1889286	1.8299	0.0673	0.435153319	count	1
BRMS1L	0.3493438	0.1599549	2.184	0.029	0.435273963	count	1
INTU	0.3251953	0.1571898	2.0688	0.0386	0.435427345	count	1
RDH14	0.3149125	0.0900917	3.4955	0.000479	0.435524663	count	1
LRP6	0.3338626	0.1745487	1.9127	0.0559	0.435527934	count	1
CHD1	0.32279	0.1086886	2.9699	0.003	0.435639133	count	1
PERP	0.3185491	0.1013058	3.1444	0.00168	0.435965469	count	1
EXTL3	0.4230421	0.3154676	1.341	0.18	0.436031256	count	1
SLC38A2	0.3073349	0.0699745	4.3921	1.15E-05	0.436044641	count	0.264845
BRD1	0.3280019	0.128018	2.5622	0.0104	0.436095202	count	1
PUS7L	0.3312719	0.1440054	2.3004	0.0215	0.436185217	count	1
INPP5B	0.3902147	0.3489804	1.1182	0.264	0.436211865	count	1
TMEM201	0.6997357	0.4951498	1.4132	0.158	0.436223589	count	1
PIGA	0.5160692	0.3797463	1.359	0.174	0.436315675	count	1
PXN-AS1	0.4727214	0.3283509	1.4397	0.15	0.436476258	count	1
SCP2	0.3068956	0.048	6.3937	1.82E-10	0.436537123	count	4.31E-06
RC3H1	0.3434507	0.2122684	1.618	0.106	0.436612675	count	1

BEST4	1.2691883	1.0071032	1.2602	0.208	0.436781432	count	1
KCNK15	0.3246009	0.1119478	2.8996	0.00376	0.436902974	count	1
ZW10	0.4493679	0.2752611	1.6325	0.103	0.436919818	count	1
ALG6	0.4357435	0.3653227	1.1928	0.233	0.437205032	count	1
TUBGCP2	0.3228941	0.1245854	2.5917	0.00959	0.437234926	count	1
ZNF764	0.5726279	0.3944355	1.4518	0.147	0.437309374	count	1
CYP2U1	0.3345821	0.1421705	2.3534	0.0187	0.437386614	count	1
KPNA2	0.3814925	0.2454641	1.5542	0.12	0.43745005	count	1
DOCK6	0.5071624	0.3453642	1.4685	0.142	0.437518075	count	1
HLA-DOA	0.955988	0.8777134	1.0892	0.276	0.437751525	count	1
VHL	0.3902687	0.2062699	1.892	0.0586	0.438239489	count	1
TUBB4B	0.3084377	0.0581145	5.3074	1.18E-07	0.438264612	count	0.002762262
CASP2	0.4220586	0.2463372	1.7133	0.0867	0.438433194	count	1
AL928654.1	0.8002719	0.6245991	1.2813	0.2	0.438708802	count	1
ANKRD6	0.3516318	0.2674549	1.3147	0.189	0.438741937	count	1
RBL2	0.3284937	0.1225756	2.6799	0.0074	0.439090121	count	1
CARHSP1	0.310717	0.0729597	4.2587	2.11E-05	0.439131414	count	0.4845404
NUP153	0.3762987	0.1952349	1.9274	0.054	0.439138081	count	1
CYCS	0.3103888	0.0796308	3.8978	9.88E-05	0.439138964	count	1
C7orf26	0.4341107	0.2371356	1.8306	0.0672	0.439565117	count	1
POU6F1	0.8022306	0.4419044	1.8154	0.0695	0.439590274	count	1
CHST3	0.3474445	0.2433923	1.4275	0.154	0.44006387	count	1
SLC46A3	0.3694892	0.2035138	1.8155	0.0695	0.440142612	count	1
ATN1	0.3469432	0.1691782	2.0508	0.0404	0.440481111	count	1
RASSF8-AS1	0.3407082	0.1465477	2.3249	0.0201	0.440517046	count	1
KIRREL1	0.3587781	0.214342	1.6739	0.0942	0.440883648	count	1
MKS1	0.5466846	0.382224	1.4303	0.153	0.440908595	count	1
TTC9C	0.3571603	0.1828136	1.9537	0.0508	0.441263864	count	1
FASN	0.4400205	0.3025491	1.4544	0.146	0.441295185	count	1
RNF227	0.5619989	0.3824422	1.4695	0.142	0.441397986	count	1
BAG5	0.3229938	0.0910966	3.5456	0.000397	0.441654748	count	1
RABGGTA	0.4082453	0.2121773	1.9241	0.0544	0.441810295	count	1
NSMCE3	0.3227801	0.0982773	3.2844	0.00103	0.441811011	count	1
SENP7	0.3291361	0.1296568	2.5385	0.0112	0.441850921	count	1
L3MBTL3	0.3812477	0.200875	1.8979	0.0578	0.441857158	count	1
PLCL2	0.454892	0.252825	1.7992	0.0721	0.442011652	count	1
DUSP16	0.4553944	0.3044265	1.4959	0.135	0.442474366	count	1
WDHD1	0.9697779	0.4831489	2.0072	0.0448	0.442556917	count	1
RALGAPB	0.3875865	0.3270263	1.1852	0.236	0.442563782	count	1
DCPS	0.3329301	0.1394863	2.3868	0.017	0.442588519	count	1
AL391807.1	0.3806846	0.2056634	1.851	0.0642	0.442694094	count	1
SCYL3	0.5040531	0.3094011	1.6291	0.103	0.442722766	count	1
ELMOD3	0.3864041	0.2204601	1.7527	0.0797	0.442926916	count	1
PRRT4	1.6433998	0.9887364	1.6621	0.0966	0.442954006	count	1
CTTN	0.3127906	0.0617389	5.0663	4.26E-07	0.443001635	count	0.009939006

ANKRD29	0.3700467	0.1725655	2.1444	0.0321	0.443027011	count	1
QTRT2	0.3674044	0.2023267	1.8159	0.0695	0.443066134	count	1
AL139260.1	0.8104529	0.5506386	1.4718	0.141	0.443279975	count	1
ARRDC3	0.3385336	0.1192853	2.838	0.00456	0.443400882	count	1
NRSN2-AS1	0.5251679	0.4604031	1.1407	0.254	0.443417448	count	1
HLA-DPB1	0.3174204	0.104072	3.05	0.0023	0.44342648	count	1
CCDC57	0.5050021	0.2931608	1.7226	0.085	0.443498172	count	1
TBC1D17	0.3815913	0.1765681	2.1612	0.0307	0.443721361	count	1
PLD2	0.4886869	0.4672041	1.046	0.296	0.44391851	count	1
TOP3A	0.4106918	0.3703631	1.1089	0.268	0.444363114	count	1
C14orf39	2.5564416	1.8196638	1.4049	0.16	0.444408315	count	1
EPB41	0.3811239	0.2188386	1.7416	0.0817	0.444626556	count	1
PRKAB2	0.35358	0.1756063	2.0135	0.0441	0.444888072	count	1
POLR2J3	0.3208258	0.1360918	2.3574	0.0185	0.445141274	count	1
LINC02580	0.8147265	0.4990303	1.6326	0.103	0.445190954	count	1
NSDHL	0.4255692	0.2726265	1.561	0.119	0.445201314	count	1
ULK1	0.4196808	0.2436312	1.7226	0.085	0.445384503	count	1
ZYG11B	0.3454853	0.1552929	2.2247	0.0262	0.445440844	count	1
COMMD8	0.329315	0.1165244	2.8261	0.00474	0.445609182	count	1
AF117829.1	0.4097064	0.262713	1.5595	0.119	0.445861435	count	1
AC048341.2	0.6824282	0.4228567	1.6139	0.107	0.446190499	count	1
SLC39A7	0.3240219	0.0844164	3.8384	0.000126	0.446301465	count	1
CLK2	0.43749	0.3055	1.432	0.152	0.446625966	count	1
NPHP4	0.5293491	0.7483372	0.7074	0.479	0.446673345	count	1
IFIT1	0.3239617	0.1658269	1.9536	0.0508	0.446884222	count	1
NCAPG2	0.7641478	0.4061203	1.8816	0.06	0.447078901	count	1
RIPOR2	1.114578	0.4363515	2.5543	0.0107	0.447084134	count	1
ZNF180	0.5097885	0.316405	1.6112	0.107	0.447405316	count	1
XRN1	0.3334454	0.1060299	3.1448	0.00168	0.447422414	count	1
NPRL3	0.3594389	0.1922202	1.8699	0.0616	0.447638655	count	1
NUDT12	0.4027156	0.1988534	2.0252	0.0429	0.447642744	count	1
AC024337.2	0.8910022	0.632076	1.4096	0.159	0.447781718	count	1
PPP1R32	0.6550651	0.7652594	0.856	0.392	0.447858558	count	1
TMEM115	0.3402295	0.1071882	3.1741	0.00152	0.447858587	count	1
ARL15	0.4390113	0.3513274	1.2496	0.212	0.448109118	count	1
TRAF3IP2	0.4353543	0.2642842	1.6473	0.0996	0.448169238	count	1
NDUFAF7	0.3692678	0.247175	1.494	0.135	0.448205355	count	1
PBX1	0.325574	0.0998608	3.2603	0.00112	0.448359134	count	1
GK5	0.3719812	0.2213495	1.6805	0.0929	0.448468388	count	1
SDR42E2	0.6303857	0.3332336	1.8917	0.0586	0.448526036	count	1
MAPK13	0.7674212	0.494292	1.5526	0.121	0.448679143	count	1
MEIS1	0.3544388	0.1574516	2.2511	0.0244	0.448789856	count	1
FAM167B	0.6566915	0.6604445	0.9943	0.32	0.448832877	count	1
CEP97	0.4039566	0.1998559	2.0212	0.0433	0.44897753	count	1
MGAT2	0.391946	0.1718166	2.2812	0.0226	0.449101203	count	1

WDR12	0.406112	0.2035746	1.9949	0.0461	0.44910523	count	1
GLT8D2	0.3215956	0.0675476	4.761	2.00E-06	0.449171213	count	0.046388
COL4A4	0.3596811	0.2065491	1.7414	0.0817	0.449284046	count	1
P4HA1	0.3259304	0.0912837	3.5705	0.000361	0.44932375	count	1
REEP1	0.5448107	0.332299	1.6395	0.101	0.449371833	count	1
PPFIA1	0.3306714	0.1206723	2.7402	0.00617	0.449511982	count	1
AADAT	0.688458	0.4179806	1.6471	0.0996	0.449600261	count	1
ACAP2	0.3237132	0.0983902	3.2901	0.00101	0.449608618	count	1
CLEC2B	0.389914	0.2265179	1.7213	0.0853	0.45007932	count	1
ZNF526	0.6106015	0.4301746	1.4194	0.156	0.450105367	count	1
CAPN3	1.1252841	1.7461331	0.6444	0.5193	0.450118024	count	1
IL6R	0.6896545	0.3636986	1.8962	0.058	0.450275678	count	1
CDO1	0.3169515	0.0840653	3.7703	0.000166	0.450401817	count	1
ZNF624	0.4696127	0.3172474	1.4803	0.139	0.45047488	count	1
CLPB	0.4342374	0.2774458	1.5651	0.118	0.450545775	count	1
PTBP1	0.3380574	0.1159643	2.9152	0.00358	0.450592416	count	1
ZDHHC15	0.6337927	0.3810239	1.6634	0.0963	0.450671512	count	1
RGS20	1.7001896	1.1514243	1.4766	0.14	0.450914741	count	1
CEP295NL	1.7001896	1.256	1.3537	0.176	0.450914741	count	1
CCDC121	0.3857505	0.2271666	1.6981	0.0896	0.451300184	count	1
PTPRA	0.3216038	0.0701912	4.5818	4.76E-06	0.451447324	count	0.1100512
DYNC2H1	0.3506806	0.1817203	1.9298	0.0537	0.451642005	count	1
CCDC15	0.9008422	0.6270884	1.4365	0.151	0.451676241	count	1
CCDC43	0.3496526	0.1496155	2.337	0.0195	0.451936234	count	1
PIGT	0.3212337	0.0648527	4.9533	7.63E-07	0.452054763	count	0.017774085
DCAF16	0.3829419	0.1851965	2.0678	0.0387	0.452093541	count	1
AC078846.1	0.5255395	0.5409513	0.9715	0.331	0.452186396	count	1
OSGIN1	0.5772595	0.2580542	2.237	0.0253	0.452262332	count	1
SERPINI1	0.3472642	0.1397696	2.4845	0.013	0.452477233	count	1
TTC17	0.3491911	0.144048	2.4241	0.0154	0.4524855	count	1
ZMYM6	0.4475721	0.2401909	1.8634	0.0625	0.452557186	count	1
XPNPEP1	0.3812554	0.2041796	1.8673	0.0619	0.452637031	count	1
NIF3L1	0.3699707	0.1678871	2.2037	0.0276	0.452673546	count	1
SURF4	0.3298442	0.1058645	3.1157	0.00185	0.45286092	count	1
MROH1	0.7762833	0.5988025	1.2964	0.195	0.452997358	count	1
LIPC	1.3442031	0.7006857	1.9184	0.0551	0.4531837	count	1
FUS	0.3182714	0.0636403	5.0011	5.97E-07	0.453374399	count	0.013920846
POR	0.3498969	0.1314993	2.6608	0.00783	0.453388776	count	1
TCAIM	0.3678146	0.1725135	2.1321	0.0331	0.453399287	count	1
BTN3A1	0.4146294	0.2873422	1.443	0.149	0.453500682	count	1
KIF3A	0.3493517	0.1341365	2.6044	0.00924	0.453781458	count	1
FAAP24	0.6167397	0.4933926	1.25	0.211	0.454142066	count	1
HAS1	0.7338173	0.6561896	1.1183	0.264	0.454291734	count	1
CCDC85A	1.7276475	0.9623277	1.7953	0.0727	0.454630748	count	1
PSD3	0.3579549	0.1612883	2.2193	0.0265	0.454779042	count	1

AC083880.1	1.3519046	0.809197	1.6707	0.0949	0.454815649	count	1
SIRT3	0.4262268	0.2399666	1.7762	0.0758	0.454987696	count	1
FKBP14	0.341015	0.1175028	2.9022	0.00373	0.455099934	count	1
CLINT1	0.3255625	0.0803474	4.0519	5.18E-05	0.455157296	count	1
WWC2-AS2	1.1438889	0.7164489	1.5966	0.11	0.455336344	count	1
HNRNPF	0.3236389	0.0614268	5.2687	1.45E-07	0.455348592	count	0.003392855
CEP57L1	0.3536358	0.1486754	2.3786	0.0174	0.45539783	count	1
SEC63	0.3276337	0.0783505	4.1816	2.96E-05	0.455445371	count	0.6784024
DHFR2	0.4358796	0.2257485	1.9308	0.0536	0.455533696	count	1
LYSMD3	0.3573532	0.1594809	2.2407	0.0251	0.455548518	count	1
FMNL1	1.355888	0.6149625	2.2048	0.0275	0.455656023	count	1
DOT1L	0.7364579	0.5564048	1.3236	0.186	0.455678537	count	1
EAF2	0.4645877	0.443414	1.0478	0.295	0.455722123	count	1
FAM96A	0.3587675	0.1474735	2.4328	0.015	0.455796066	count	1
CORT	1.008893	0.8756619	1.1521	0.249	0.455957635	count	1
ZNF267	0.4149297	0.247248	1.6782	0.0934	0.456210361	count	1
AC009275.1	1.009681	0.6224354	1.6221	0.105	0.456224055	count	1
CD14	0.3595322	0.167558	2.1457	0.032	0.456224715	count	1
UBE4A	0.347324	0.1504075	2.3092	0.021	0.456542642	count	1
RWDD4	0.3292556	0.0904276	3.6411	0.000275	0.457507801	count	1
HIF1A-AS2	0.6715208	0.8430949	0.7965	0.426	0.457682411	count	1
TAF1C	0.477586	0.3868753	1.2345	0.217	0.457690913	count	1
AC091729.3	0.6718146	0.5342108	1.2576	0.209	0.457857142	count	1
PDZD8	0.3555885	0.1397022	2.5453	0.011	0.457879023	count	1
AC026304.1	0.6027219	0.6644882	0.907	0.364	0.457967963	count	1
MESP2	1.3670319	1.0532176	1.298	0.194	0.457993374	count	1
MYO9A	0.3373938	0.1373559	2.4563	0.0141	0.457994685	count	1
ERF	0.3744104	0.1800413	2.0796	0.0376	0.457997935	count	1
ADHFE1	0.4050698	0.3349911	1.2092	0.227	0.458252151	count	1
CCNDBP1	0.3260217	0.0692709	4.7065	2.61E-06	0.458328796	count	0.06049458
MINPP1	0.4037395	0.2436373	1.6571	0.0976	0.458656172	count	1
AP000845.1	0.7881129	1.5855386	0.4971	0.6192	0.458729333	count	1
BLVRB	0.3228583	0.0527254	6.1234	1.01E-09	0.458885766	count	2.39E-05
GATAD1	0.335977	0.11189	3.0027	0.00269	0.459011175	count	1
ZSCAN31	0.4990078	0.319362	1.5625	0.118	0.459207049	count	1
ZNF594	0.8473835	0.4190912	2.022	0.0433	0.459641354	count	1
SEMA5B	0.9220351	0.5355037	1.7218	0.0852	0.459985448	count	1
GSR	0.4371299	0.2125543	2.0566	0.0398	0.460026776	count	1
SYPL2	1.769804	0.9134494	1.9375	0.0528	0.460171824	count	1
PIM1	0.3849055	0.2124178	1.812	0.0701	0.460409654	count	1
ZNF57	1.1623713	0.542659	2.142	0.0323	0.460453365	count	1
SLC16A4	0.3439307	0.1160647	2.9633	0.00306	0.46048237	count	1
RIPK2	0.4007294	0.1847062	2.1695	0.0301	0.46056791	count	1
FLT3LG	0.3871915	0.1883233	2.056	0.0399	0.460733241	count	1
SKP2	0.606785	0.446294	1.3596	0.174	0.460737612	count	1

8-Mar	0.414937	0.2676202	1.5505	0.121	0.460773942	count	1
TLK2	0.3510275	0.1638167	2.1428	0.0322	0.460780819	count	1
NME2	0.4871977	0.2784749	1.7495	0.0803	0.460854685	count	1
SAMD11	0.3327061	0.1689658	1.9691	0.049	0.4610849	count	1
FAM126B	0.4322658	0.2940338	1.4701	0.142	0.461179248	count	1
EMC1	0.4270286	0.2067183	2.0658	0.0389	0.461376857	count	1
AC016773.1	1.025412	0.7910761	1.2962	0.195	0.46151542	count	1
WRB	0.3338651	0.1096009	3.0462	0.00233	0.461610768	count	1
BACE1	0.35606	0.1524838	2.3351	0.0196	0.461650438	count	1
RRP1B	0.3596952	0.1649426	2.1807	0.0293	0.461789662	count	1
UBA6	0.3791426	0.1806699	2.0985	0.0359	0.461845521	count	1
AC104695.3	0.8525983	0.8315524	1.0253	0.305	0.461923883	count	1
QSOX2	0.6517717	0.5393298	1.2085	0.227	0.461939419	count	1
GALNT11	0.3524058	0.1253959	2.8103	0.00498	0.461975454	count	1
METTL21A	0.408649	0.2270163	1.8001	0.0719	0.462175925	count	1
OSBPL1A	0.3329215	0.0743892	4.4754	7.86E-06	0.462294922	count	0.18128304
LUC7L	0.346717	0.1250248	2.7732	0.00558	0.462438939	count	1
KIFAP3	0.3325354	0.0882961	3.7661	0.000168	0.462762588	count	1
CD40	0.3407901	0.113032	3.015	0.00259	0.462936033	count	1
ALG10B	0.4963509	0.4191227	1.1843	0.236	0.463096813	count	1
SLC35G1	0.4469113	0.294522	1.5174	0.129	0.463114235	count	1
INO80D	0.3515991	0.1637423	2.1473	0.0318	0.463484185	count	1
MRRF	0.4901662	0.2697178	1.8173	0.0692	0.463494606	count	1
PTPMT1	0.3529626	0.1422735	2.4809	0.0132	0.463584915	count	1
RBM6	0.3399721	0.1099802	3.0912	0.00201	0.463829789	count	1
CADPS2	0.3644041	0.1532352	2.3781	0.0175	0.463894031	count	1
PMM2	0.3717369	0.1861383	1.9971	0.0459	0.464081058	count	1
PIH1D2	0.7535154	0.5116646	1.4727	0.141	0.464591556	count	1
GEN1	0.9351877	0.5421373	1.725	0.0846	0.46508831	count	1
LIPA	0.3553948	0.1252898	2.8366	0.00458	0.46523352	count	1
LETM1	0.4115481	0.2010182	2.0473	0.0407	0.465352279	count	1
NEDD1	0.4021821	0.1940149	2.0729	0.0382	0.465447787	count	1
SNAI2	0.3707327	0.1477026	2.51	0.0121	0.465484457	count	1
PTGES3L	0.5423328	0.7783377	0.6968	0.486	0.46550955	count	1
AC245014.3	1.8127357	1.1260593	1.6098	0.108	0.465615608	count	1
ZNF506	0.3647196	0.1793673	2.0334	0.0421	0.465795866	count	1
ITM2B	0.3233716	0.026022	12.4268	9.27E-35	0.465930631	count	2.24E-30
VTI1A	0.4026309	0.2106092	1.9117	0.056	0.465952655	count	1
ZNF701	0.424603	0.2952654	1.438	0.151	0.466468021	count	1
MAPK1	0.342537	0.1127908	3.0369	0.00241	0.466557555	count	1
TMA16	0.3393045	0.0959025	3.538	0.000408	0.46656894	count	1
RPAP3	0.3627164	0.1553174	2.3353	0.0196	0.46693249	count	1
CREG1	0.332989	0.0656276	5.0739	4.09E-07	0.467200188	count	0.009543197
DLC1	0.3395719	0.0916455	3.7053	0.000214	0.467312116	count	1
IRF2BP2	0.3336265	0.0778196	4.2872	1.86E-05	0.467662524	count	0.4274652

IFT27	0.3599965	0.1300465	2.7682	0.00566	0.468160256	count	1
LSR	0.4159698	0.2400429	1.7329	0.0832	0.468210995	count	1
ASPH	0.3289067	0.0562743	5.8447	5.52E-09	0.468211123	count	0.000129979
LZTS3	0.7611143	0.4011498	1.8973	0.0579	0.468536842	count	1
STT3A	0.360504	0.1303268	2.7662	0.0057	0.468812043	count	1
CRY2	0.4067729	0.1892164	2.1498	0.0316	0.468984241	count	1
SNX5	0.339912	0.0930742	3.6521	0.000264	0.469238005	count	1
MCMBP	0.3792375	0.196916	1.9259	0.0542	0.469597546	count	1
LMBR1L	0.4006283	0.2326805	1.7218	0.0852	0.469679177	count	1
PTAR1	0.375845	0.1890031	1.9886	0.0468	0.469806367	count	1
CITED4	0.3400585	0.1107773	3.0697	0.00216	0.469822629	count	1
TCF21	1.425432	0.7021156	2.0302	0.0424	0.469921138	count	1
PIGN	0.4158234	0.3263677	1.2741	0.203	0.470033419	count	1
LTBR	0.3446726	0.1169988	2.946	0.00324	0.47020089	count	1
RABGEF1	0.4507646	0.3222388	1.3989	0.162	0.470407249	count	1
HEATR5A	0.4357378	0.2407627	1.8098	0.0704	0.470423258	count	1
AC104506.1	0.4711179	0.4400153	1.0707	0.284	0.470900362	count	1
HMGCS1	0.3838743	0.2385672	1.6091	0.108	0.471109439	count	1
SLC30A6	0.4019003	0.3578067	1.1232	0.261	0.471130905	count	1
PTER	0.5610014	0.3704614	1.5143	0.13	0.471164009	count	1
RDH5	0.4338952	0.2559357	1.6953	0.0901	0.471196818	count	1
MICA	0.3679226	0.1994945	1.8443	0.0652	0.471274548	count	1
SPTLC1	0.3462434	0.09858	3.5123	0.00045	0.471347469	count	1
TBCC	0.3534055	0.1313306	2.691	0.00716	0.471482778	count	1
BAZ2A	0.366313	0.181152	2.0221	0.0432	0.471498533	count	1
FBXO44	0.3987255	0.1997526	1.9961	0.046	0.471575323	count	1
APTR	0.4521718	0.3539924	1.2773	0.202	0.471810711	count	1
WDR86	1.8667004	1.051093	1.776	0.0758	0.472182173	count	1
CNTNAP1	0.8765441	0.5395146	1.6247	0.104	0.472316956	count	1
AC139530.1	0.4775291	0.3270091	1.4603	0.144	0.472450754	count	1
MFSD9	1.4381802	0.6194917	2.3215	0.0203	0.472454053	count	1
PRKAA1	0.3551116	0.1258285	2.8222	0.0048	0.472460313	count	1
EHMT1	0.3585165	0.1363681	2.629	0.0086	0.472776538	count	1
FGF2	0.3446515	0.1056191	3.2632	0.00111	0.472988809	count	1
UGGT1	0.3821358	0.1808391	2.1131	0.0347	0.473118195	count	1
GPATCH3	0.4574704	0.325504	1.4054	0.16	0.473556798	count	1
PGRMC2	0.3401845	0.0743583	4.5749	4.92E-06	0.473623069	count	0.11373564
CACUL1	0.36811	0.1638311	2.2469	0.0247	0.473779361	count	1
LINC02256	0.4055138	0.260194	1.5585	0.119	0.473798973	count	1
CTBP1-DT	0.4390456	0.2829172	1.5519	0.121	0.473854771	count	1
SLC16A11	1.062888	0.8271144	1.2851	0.199	0.473902689	count	1
THUMPD3-AS1	0.3470977	0.1129796	3.0722	0.00214	0.473903395	count	1
CLTC	0.3435567	0.0947617	3.6255	0.000292	0.474044054	count	1
HMGCLL1	1.8835887	0.7781167	2.4207	0.0155	0.474175624	count	1
EGR3	0.3625664	0.20902	1.7346	0.0829	0.474190192	count	1

AC080013.1	1.2135456	0.7640211	1.5884	0.112	0.474277189	count	1
HPS4	0.4166985	0.2224496	1.8732	0.0611	0.474789947	count	1
SLITRK6	0.6724787	0.8519956	0.7893	0.43	0.474804301	count	1
RHOQ	0.3352652	0.0619002	5.4162	6.48E-08	0.47485725	count	0.001518718
TTLL3	0.4106127	0.2399357	1.7113	0.0871	0.474925355	count	1
B4GALT4	0.3661343	0.1452994	2.5199	0.0118	0.474925362	count	1
ZNF589	0.6094411	0.4271546	1.4267	0.154	0.474958131	count	1
GDAP2	0.4108918	0.2687779	1.5287	0.126	0.475238889	count	1
ATP8B2	0.3803013	0.1997629	1.9038	0.057	0.47527739	count	1
FLNC	0.4245146	0.287347	1.4774	0.14	0.475422464	count	1
DISP1	0.6101896	0.5071896	1.2031	0.229	0.475482507	count	1
TENT2	0.3830082	0.1698692	2.2547	0.0242	0.47570919	count	1
AL358472.2	1.0690812	0.7279977	1.4685	0.142	0.475920283	count	1
NINL	0.6743308	0.4042977	1.6679	0.0954	0.475949094	count	1
SH2D3A	0.8242543	0.8393105	0.9821	0.326	0.476013753	count	1
SLC25A36	0.3409093	0.0860496	3.9618	7.58E-05	0.476116301	count	1
EPHX2	0.3903285	0.1695555	2.3021	0.0214	0.476132371	count	1
UTP18	0.3974668	0.1964395	2.0234	0.0431	0.476240998	count	1
MFSD1	0.3630777	0.1254878	2.8933	0.00383	0.476417494	count	1
ZNF41	0.8253301	0.4809202	1.7161	0.0862	0.476522987	count	1
RAP2C	0.4030729	0.2636035	1.5291	0.126	0.476585408	count	1
RND3	0.341549	0.0884497	3.8615	0.000115	0.476676884	count	1
NEIL1	0.4823767	0.288204	1.6737	0.0943	0.476988126	count	1
AC040162.1	0.7063718	0.6862061	1.0294	0.303	0.478238496	count	1
NFKBIA	0.333122	0.05756	5.7874	7.75E-09	0.478267703	count	0.000182396
MLF1	0.3671677	0.1626172	2.2579	0.024	0.478441175	count	1
NFIB	0.3370402	0.0504919	6.6751	2.84E-11	0.478761161	count	6.74E-07
SMYD5	0.6788933	0.4377385	1.5509	0.121	0.478765036	count	1
SF1	0.3393645	0.0689615	4.9211	8.99E-07	0.478892154	count	0.020924225
ZDBF2	0.4629321	0.2887758	1.6031	0.109	0.478947902	count	1
HS3ST3A1	1.9253098	1.1077888	1.738	0.0823	0.47897779	count	1
PTPN13	0.3711891	0.1661842	2.2336	0.0256	0.478978272	count	1
AP001462.1	0.8305711	0.5868922	1.4152	0.157	0.478999475	count	1
GAA	0.3632741	0.1100352	3.3014	0.000971	0.479254656	count	1
PHEX	1.2325216	0.6793874	1.8142	0.0697	0.479276077	count	1
INSR	0.3606627	0.1230835	2.9302	0.00341	0.479548169	count	1
ENKD1	0.4227441	0.2193039	1.9277	0.054	0.479561169	count	1
NAMPT	0.3449993	0.0954122	3.6159	0.000303	0.479667924	count	1
ADCY9	0.3858333	0.3548619	1.0873	0.277	0.479901303	count	1
CEP85L	0.3886448	0.1799326	2.1599	0.0308	0.480218937	count	1
REXO1	0.4402519	0.2041262	2.1568	0.0311	0.480470636	count	1
PACS2	0.3875763	0.1861611	2.0819	0.0374	0.4805071	count	1
LEMD2	0.4334064	0.2349037	1.845	0.0651	0.480560004	count	1
TUFT1	1.9404585	1.3472106	1.4404	0.15	0.48067906	count	1
TMEM107	0.3671266	0.1305446	2.8123	0.00495	0.481046332	count	1

TDP1	0.6021955	0.4926763	1.2223	0.222	0.481423974	count	1
IREB2	0.3788716	0.17029	2.2249	0.0262	0.481482084	count	1
HYDIN	1.4851905	1.3514431	1.099	0.272	0.481579656	count	1
DIP2A	0.397494	0.2768157	1.436	0.151	0.481705926	count	1
NUSAP1	0.5882166	0.9779009	0.6015	0.548	0.481943706	count	1
ZNF629	0.5882231	0.2775597	2.1193	0.0341	0.481948558	count	1
KDM5B	0.3688538	0.1301725	2.8336	0.00463	0.481982179	count	1
PPP2R1B	0.7468443	0.3178644	2.3496	0.0188	0.482098915	count	1
CYTH1	0.4561018	0.3362353	1.3565	0.175	0.482387283	count	1
HERPUD1	0.3387204	0.0545508	6.2093	5.92E-10	0.482482775	count	1.40E-05
ANXA2	0.3357167	0.0335874	9.9953	3.16E-23	0.482542078	count	7.61E-19
IQSEC3	0.9812652	0.6517544	1.5056	0.132	0.482640926	count	1
NPAT	0.3993136	0.2083677	1.9164	0.0554	0.482818607	count	1
MICU2	0.362795	0.1303337	2.7836	0.0054	0.48302695	count	1
MED26	0.6044483	0.4529345	1.3345	0.182	0.483049859	count	1
ABCB7	0.4075869	0.1913467	2.1301	0.0332	0.483103832	count	1
PIWIL4	1.493947	0.8726368	1.712	0.087	0.483242604	count	1
PAXBP1-AS1	0.8396172	0.382724	2.1938	0.0283	0.483257012	count	1
PDCD6IP	0.3507441	0.0977132	3.5895	0.000336	0.483700394	count	1
ZXDC	0.4646978	0.2745164	1.6928	0.0906	0.484283036	count	1
TAZ	0.4147505	0.2446459	1.6953	0.0901	0.484290137	count	1
MALT1	0.439233	0.2646315	1.6598	0.097	0.484405052	count	1
PSEN1	0.3909012	0.2035262	1.9206	0.0549	0.48454926	count	1
CXCL1	0.4651294	0.9258225	0.5024	0.615	0.484712132	count	1
COL4A6	0.4496536	0.3648458	1.2324	0.218	0.484843275	count	1
BDH2	0.3425512	0.06915	4.9537	7.61E-07	0.484899778	count	0.017728256
DNAJB12	0.3562206	0.1215664	2.9303	0.00341	0.48529327	count	1
BRI3BP	0.4697013	0.2299484	2.0426	0.0412	0.485619779	count	1
TECPR1	0.5934121	0.3793556	1.5643	0.118	0.485806899	count	1
RAPGEF1	0.4164724	0.2511334	1.6584	0.0973	0.486244179	count	1
MBNL2	0.3492322	0.0786848	4.4384	9.33E-06	0.486296974	count	0.21500985
ZNF335	0.7547413	0.3631545	2.0783	0.0378	0.486422172	count	1
GLCE	0.4792692	0.314953	1.5217	0.128	0.487155931	count	1
TNFRSF14	0.3691507	0.1570656	2.3503	0.0188	0.487195778	count	1
CCAR2	0.4676878	0.2345488	1.994	0.0462	0.487254786	count	1
UBQLN2	0.3800119	0.1801803	2.1091	0.035	0.487489059	count	1
ZNF410	0.849183	0.517739	1.6402	0.101	0.487735749	count	1
STK24	0.3809132	0.1275968	2.9853	0.00285	0.488149777	count	1
TMEM214	0.3990156	0.1715931	2.3254	0.0201	0.488386289	count	1
ZNF234	0.4946673	0.2965925	1.6678	0.0954	0.488465672	count	1
PCTP	0.5184669	0.312716	1.6579	0.0974	0.488546856	count	1
NOTCH2NL	0.7241057	0.3145982	2.3017	0.0214	0.488567768	count	1
ARHGEF4	0.9149429	0.5633924	1.624	0.104	0.488681509	count	1
TMEM26	0.6484064	0.626414	1.0351	0.301	0.488839566	count	1
CTSS	0.3741984	0.1524873	2.454	0.0142	0.48887916	count	1

ALDH18A1	0.453636	0.2692317	1.6849	0.0921	0.488962089	count	1
PRR12	0.7249838	0.3693575	1.9628	0.0497	0.489076926	count	1
NID2	0.5978188	0.2781106	2.1496	0.0317	0.489077562	count	1
TDG	0.3705518	0.1289374	2.8739	0.00408	0.489296695	count	1
AC002070.1	1.271585	0.9863729	1.2892	0.197	0.489353375	count	1
FBXO15	1.271585	1.359271	0.9355	0.35	0.489353375	count	1
EPHX4	1.5273182	1.63891	0.9319	0.351	0.489475687	count	1
ARHGEF5	0.6712426	0.5326704	1.2601	0.208	0.489517768	count	1
AGAP1	0.4820545	0.2547193	1.8925	0.0585	0.489842925	count	1
SLC39A10	0.3761521	0.1468898	2.5608	0.0105	0.490708325	count	1
RNF139-AS1	0.85579	0.6132146	1.3956	0.163	0.490815121	count	1
CCDC170	2.03678	0.8306374	2.4521	0.0143	0.49098703	count	1
ST3GAL3	0.5538326	0.288708	1.9183	0.0551	0.491108718	count	1
S100A13	0.3430671	0.0476807	7.1951	7.53E-13	0.491197501	count	1.79E-08
ZNF595	0.4754029	0.2291373	2.0748	0.0381	0.491230948	count	1
NCDN	0.9211294	0.9364103	0.9837	0.325	0.491283394	count	1
RIC3	0.3990363	0.2022846	1.9726	0.0486	0.491956846	count	1
PI4KA	0.4657949	0.298903	1.5583	0.119	0.492184327	count	1
ABCA3	0.4658752	0.3042669	1.5311	0.126	0.49226541	count	1
WASF1	0.5102461	0.3090687	1.6509	0.0988	0.492607285	count	1
GUSB	0.3623329	0.0993086	3.6486	0.000267	0.492731822	count	1
NAGPA	0.4347908	0.2373885	1.8316	0.0671	0.492775723	count	1
SIK2	0.4406416	0.2340035	1.8831	0.0598	0.49285057	count	1
PDPN	0.4117002	0.2790876	1.4752	0.14	0.492873795	count	1
ZNF133	0.4699719	0.3636741	1.2923	0.196	0.49302543	count	1
AL358472.4	1.5480831	0.8997163	1.7206	0.0854	0.493271706	count	1
INPP4A	0.4738476	0.3509483	1.3502	0.177	0.493370329	count	1
AC145207.5	1.0101549	0.7319675	1.3801	0.168	0.493390157	count	1
NEURL1B	0.8614524	0.2834752	3.0389	0.00239	0.49344517	count	1
MMP17	2.065633	0.8635565	2.392	0.0168	0.49390943	count	1
AC007262.2	2.065633	1.4083957	1.4667	0.143	0.49390943	count	1
COQ10A	0.4376878	0.2534586	1.7269	0.0843	0.493916585	count	1
CEP128	1.2910363	0.544436	2.3713	0.0178	0.494265306	count	1
AL390728.6	0.6052885	0.335388	1.8047	0.0712	0.494608966	count	1
PLPPR2	0.4297694	0.1969344	2.1823	0.0292	0.494685784	count	1
MPI	0.4330722	0.2298143	1.8844	0.0596	0.494737993	count	1
NOMO2	1.5566271	0.7396603	2.1045	0.0354	0.494815455	count	1
IRF1	0.3494898	0.1088796	3.2099	0.00134	0.494922975	count	1
C2CD6	0.7704038	0.4215842	1.8274	0.0677	0.49494562	count	1
H1F0	0.3531654	0.0945105	3.7368	0.000189	0.494984274	count	1
RWDD3	1.0154262	0.5689116	1.7849	0.0744	0.495330289	count	1
DHCR7	0.6388598	0.2812373	2.2716	0.0232	0.495448031	count	1
ZNF891	0.4761496	0.2380771	2	0.0456	0.495653494	count	1
PDCD4	0.3605669	0.10164	3.5475	0.000394	0.495714305	count	1
TEF	0.4122566	0.2107635	1.956	0.0505	0.495863278	count	1

GOLGA3	0.3662707	0.124598	2.9396	0.00331	0.495937034	count	1
FEM1C	0.4481108	0.2267485	1.9762	0.0482	0.496261838	count	1
WDR82	0.3698624	0.1466617	2.5219	0.0117	0.496400351	count	1
ZNF770	0.3791244	0.1361533	2.7845	0.00539	0.496573842	count	1
CDADC1	0.4084786	0.2002078	2.0403	0.0414	0.496776829	count	1
PCYT1A	0.4183793	0.1819422	2.2995	0.0215	0.496881394	count	1
C22orf23	1.5681652	0.9684419	1.6193	0.105	0.496883578	count	1
ZMYND19	0.5091692	0.2687088	1.8949	0.0582	0.496907171	count	1
IL17RC	0.3936566	0.1759258	2.2376	0.0253	0.496989529	count	1
TTC5	0.4124592	0.1715654	2.4041	0.0163	0.497231504	count	1
GAB3	0.8176233	0.5076979	1.6105	0.107	0.497385594	count	1
DDX31	0.4993534	0.5561776	0.8978	0.369	0.497574119	count	1
RHBDD2	0.3701634	0.1277826	2.8968	0.00379	0.497694293	count	1
C5orf15	0.3652544	0.1024064	3.5667	0.000366	0.497742651	count	1
FAN1	0.4297686	0.3393837	1.2663	0.205	0.498089916	count	1
DAPK1	0.47509	0.3800284	1.2501	0.211	0.498145386	count	1
TPCN2	0.7763565	0.4756541	1.6322	0.103	0.498167227	count	1
FHOD3	0.5839565	0.3358878	1.7385	0.0822	0.49819463	count	1
RNASEL	0.4042321	0.1631106	2.4783	0.0132	0.498228844	count	1
PLAA	0.4456546	0.2142327	2.0802	0.0376	0.498257129	count	1
SLC27A2	16.4680609	980.890216	0.0168	0.987	0.498553813	count	1
PPDPFL	16.4680786	1317.777797	0.0125	0.99	0.498553813	count	1
RECQL4	16.5883384	1254.965498	0.0132	0.989	0.498553818	count	1
CCDC39	16.7392869	1520.977775	0.011	0.991	0.498553823	count	1
AC019131.2	16.7392921	1520.986317	0.011	0.991	0.498553823	count	1
SPRR2B	16.7398804	1804.936134	0.0093	0.993	0.498553823	count	1
AC090409.2	16.7398876	1804.935562	0.0093	0.993	0.498553823	count	1
LINC02552	16.7405892	2162.305167	0.0077	0.994	0.498553823	count	1
AC010201.1	16.7405892	2162.305173	0.0077	0.994	0.498553823	count	1
AC012313.2	16.7405893	2162.30516	0.0077	0.994	0.498553823	count	1
AC138969.1	16.7597367	1262.235542	0.0133	0.989	0.498553824	count	1
AC104389.4	16.9586883	975.2573971	0.0174	0.986	0.498553829	count	1
AC127070.2	16.9590172	1371.718128	0.0124	0.99	0.498553829	count	1
AP000695.2	17.0391157	2364.820583	0.0072	0.994	0.498553831	count	1
AL021391.1	17.0816022	1375.7978	0.0124	0.99	0.498553832	count	1
AC009093.1	17.0816255	1375.791472	0.0124	0.99	0.498553832	count	1
DEPDC1	17.0878499	1452.694639	0.0118	0.991	0.498553833	count	1
AC008752.2	17.0878499	1452.694608	0.0118	0.991	0.498553833	count	1
SELE	17.0885267	1781.011555	0.0096	0.992	0.498553833	count	1
SPATA22	17.0885268	1781.011523	0.0096	0.992	0.498553833	count	1
AC008264.2	17.0891921	2057.928755	0.0083	0.993	0.498553833	count	1
DLEU7	17.0894844	2301.646618	0.0074	0.994	0.498553833	count	1
NRN1L	17.0894845	2301.646668	0.0074	0.994	0.498553833	count	1
AL161752.1	17.0906056	2913.914013	0.0059	0.995	0.498553833	count	1
LINC01132	17.0906057	2913.914034	0.0059	0.995	0.498553833	count	1

SHOX	17.0906058	2913.914065	0.0059	0.995	0.498553833	count	1
CLEC4M	17.0906058	2913.914065	0.0059	0.995	0.498553833	count	1
STUM	17.1607747	1073.162621	0.016	0.987	0.498553834	count	1
ART3	17.2196545	1593.816457	0.0108	0.991	0.498553835	count	1
TRIM29	17.3282899	1547.458496	0.0112	0.991	0.498553837	count	1
AC007529.1	17.3286805	2199.45009	0.0079	0.994	0.498553837	count	1
AC119674.2	17.4493758	998.5787747	0.0175	0.9861	0.498553839	count	1
LRRC2	17.508991	1265.511916	0.0138	0.989	0.49855384	count	1
KIF9-AS1	17.5226062	1821.065743	0.0096	0.992	0.49855384	count	1
MGAM	17.5269911	1911.425389	0.0092	0.993	0.498553841	count	1
FRMD1	17.5269914	1911.425411	0.0092	0.993	0.498553841	count	1
AC010273.1	17.5279656	2448.996263	0.0072	0.994	0.498553841	count	1
AC100793.4	17.6280854	1362.025188	0.0129	0.99	0.498553842	count	1
VSTM2L	17.6282626	946.6754544	0.0186	0.9851	0.498553842	count	1
AL109936.2	17.7902392	2688.499043	0.0066	0.995	0.498553844	count	1
PDF	17.7907931	3282.569499	0.0054	0.996	0.498553844	count	1
AC068580.4	17.9412033	3229.336941	0.0056	0.996	0.498553845	count	1
AC092338.2	16.3205858	1451.061208	0.0112	0.991	0.498553849	count	1
HPR	16.4875744	1007.359496	0.0164	0.987	0.498553856	count	1
UBAC2-AS1	16.5286554	981.7635319	0.0168	0.987	0.498553858	count	1
ELAVL2	16.7392955	1520.990898	0.011	0.991	0.498553865	count	1
LINC01736	16.7405894	2162.305191	0.0077	0.994	0.498553865	count	1
AC067863.1	16.7405894	2162.30518	0.0077	0.994	0.498553865	count	1
TMEM132E	16.7818254	1389.860829	0.0121	0.99	0.498553867	count	1
PIP5KL1	16.8371324	1049.967288	0.016	0.987	0.498553868	count	1
LINC00517	16.9586506	1067.759771	0.0159	0.987	0.498553872	count	1
CC2D2B	17.0380315	1597.149386	0.0107	0.991	0.498553874	count	1
LINC01336	17.0885267	1781.011497	0.0096	0.992	0.498553875	count	1
HEPN1	17.089192	2057.928791	0.0083	0.993	0.498553875	count	1
AC116345.1	17.0891922	2057.928799	0.0083	0.993	0.498553875	count	1
AC009226.1	17.0894846	2301.646626	0.0074	0.994	0.498553875	count	1
PCAT19	17.0894845	2301.646643	0.0074	0.994	0.498553875	count	1
PROK2	17.0906056	2913.914024	0.0059	0.995	0.498553875	count	1
AADAC	17.0906057	2913.914013	0.0059	0.995	0.498553875	count	1
VENTX	17.0906058	2913.914034	0.0059	0.995	0.498553875	count	1
AC106028.4	17.0906058	2913.914034	0.0059	0.995	0.498553875	count	1
AL606970.4	17.1510705	979.1692324	0.0175	0.986	0.498553876	count	1
CLCNKB	17.3072669	1927.473606	0.009	0.993	0.498553879	count	1
HOXA5	17.4571928	975.5622049	0.0179	0.986	0.498553882	count	1
SFMBT2	17.470735	858.5730351	0.0203	0.9838	0.498553882	count	1
KL	17.5223016	1541.763436	0.0114	0.991	0.498553883	count	1
POU5F1	17.5274577	2196.589249	0.008	0.994	0.498553883	count	1
RXFP1	17.5274579	2196.589378	0.008	0.994	0.498553883	count	1
PON3	17.7389114	2237.276508	0.0079	0.9937	0.498553885	count	1
AC004771.4	17.7894197	1919.232068	0.0093	0.993	0.498553886	count	1

AC087203.3	17.8212444	2837.387515	0.0063	0.995	0.498553886	count	1
AL121821.1	17.8883917	1001.227825	0.0179	0.9857	0.498553887	count	1
AC004889.1	17.9395382	1558.180335	0.0115	0.991	0.498553888	count	1
SORCS2	17.9406203	2508.607557	0.0072	0.994	0.498553888	count	1
DYNC1H1	0.3570857	0.0850831	4.1969	2.77E-05	0.498913605	count	0.6350779
RNMT	0.3570276	0.0869263	4.1072	4.09E-05	0.498941018	count	0.9358738
RALGPS1	1.0256107	0.7654399	1.3399	0.18	0.499060417	count	1
CPQ	0.3516196	0.0518129	6.7863	1.34E-11	0.49922808	count	3.18E-07
DMRTA1	0.5980428	0.509704	1.1733	0.241	0.499468472	count	1
GNB3	0.9409202	0.7400721	1.2714	0.204	0.499542474	count	1
AGPS	0.4165147	0.163182	2.5525	0.0107	0.499689304	count	1
C2CD3	0.8224054	0.467956	1.7574	0.0789	0.499787231	count	1
MUM1	0.3621617	0.1149988	3.1493	0.00165	0.499853599	count	1
FBXO17	0.3660506	0.113814	3.2162	0.00131	0.500134354	count	1
AC104825.1	0.688081	0.6095082	1.1289	0.259	0.500275922	count	1
LRRC58	0.3845437	0.136718	2.8127	0.00494	0.500413629	count	1
ACBD5	0.4109391	0.191357	2.1475	0.0318	0.500707378	count	1
SERHL2	0.5997245	0.3895129	1.5397	0.124	0.500744313	count	1
CCDC134	0.8772964	0.6331159	1.3857	0.166	0.500759565	count	1
AC009093.2	2.1367709	1.2903756	1.6559	0.0978	0.500804422	count	1
GRIA1	0.399967	0.4766827	0.8391	0.401	0.500820272	count	1
IDUA	0.4307631	0.1786183	2.4116	0.0159	0.50084585	count	1
AL391069.2	1.5908316	1.2034716	1.3219	0.186	0.500890946	count	1
TNFSF10	0.3593972	0.1047869	3.4298	0.000611	0.500953269	count	1
B3GNT8	0.4893712	0.3207523	1.5257	0.127	0.500997738	count	1
AC245060.5	0.4114318	0.4766326	0.8632	0.388	0.501294043	count	1
ZNF785	0.6146807	0.2710956	2.2674	0.0234	0.501541438	count	1
SWAP70	0.3696563	0.1056844	3.4977	0.000475	0.501718515	count	1
SLC9A3R1	0.4787959	0.2735296	1.7504	0.0801	0.501848801	count	1
H2AFX	0.3906254	0.1467506	2.6618	0.00781	0.501856297	count	1
KCNJ2	1.033376	0.5578031	1.8526	0.064	0.50188817	count	1
SLC16A2	0.4582325	0.2122248	2.1592	0.0309	0.501974351	count	1
NECAP1	0.4232517	0.1565014	2.7045	0.00687	0.502513689	count	1
ZNF146	0.3996872	0.1985265	2.0133	0.0442	0.502525977	count	1
COL16A1	0.3742495	0.1335489	2.8023	0.0051	0.502782013	count	1
BBS9	0.6024493	0.313562	1.9213	0.0548	0.502809811	count	1
CYP4F12	2.158537	0.6288632	3.4324	0.000605	0.502828611	count	1
SMIM14	0.3541001	0.0599492	5.9067	3.81E-09	0.502842277	count	8.98E-05
P2RX7	0.7489418	0.4283384	1.7485	0.0805	0.502885092	count	1
GUCY1A2	0.7489865	0.240056	3.12	0.00182	0.502910706	count	1
CENPS	0.4957132	0.2922769	1.696	0.09	0.502992045	count	1
TMEM64	0.467574	0.2683822	1.7422	0.0816	0.503349682	count	1
ZNF618	0.4020576	0.220322	1.8249	0.0681	0.503388605	count	1
TRAM1	0.3538647	0.0514118	6.8829	6.87E-12	0.503738974	count	1.63E-07
ATP7A	0.4333815	0.227598	1.9042	0.057	0.503798796	count	1

NCOA2	0.4139652	0.176128	2.3504	0.0188	0.50430999	count	1
ICMT	0.3842083	0.1369766	2.8049	0.00506	0.504440751	count	1
PLAGL1	0.3740913	0.129645	2.8855	0.00393	0.504612003	count	1
NEK1	0.3856056	0.1379315	2.7956	0.00521	0.504621058	count	1
MAP10	2.178338	0.8191215	2.6594	0.00786	0.504636317	count	1
ATAD2B	0.406802	0.2196085	1.8524	0.064	0.504668364	count	1
BMP3	1.3335208	0.9108019	1.4641	0.143	0.504752387	count	1
FZD1	0.365517	0.1373032	2.6621	0.0078	0.50487157	count	1
SIGIRR	0.3680762	0.0968539	3.8003	0.000147	0.505022608	count	1
HESX1	1.1615683	0.944584	1.2297	0.219	0.505069763	count	1
AL161729.1	0.6959181	0.5692798	1.2225	0.222	0.50525539	count	1
DSN1	0.4666396	0.2602196	1.7933	0.073	0.505290801	count	1
STK32C	0.6057938	0.31128	1.9461	0.0517	0.505342158	count	1
TMEM69	0.4203606	0.1773566	2.3701	0.0178	0.505367914	count	1
AIFM1	0.4294995	0.2454337	1.75	0.0802	0.505536401	count	1
TP53TG5	0.790246	0.6594261	1.1984	0.231	0.505645983	count	1
NDN	0.356695	0.0559934	6.3703	2.12E-10	0.505664151	count	5.02E-06
6-Sep	0.4105298	0.1740112	2.3592	0.0184	0.505825626	count	1
NOLC1	0.3796743	0.1212323	3.1318	0.00175	0.505950984	count	1
PCDHB3	1.0447574	0.8121581	1.2864	0.198	0.506007337	count	1
ARID1B	0.3731497	0.1314876	2.8379	0.00457	0.506017631	count	1
LINC01315	0.464685	0.2400439	1.9358	0.053	0.506056679	count	1
FAM102A	0.4219653	0.2244303	1.8802	0.0602	0.50606254	count	1
ZFAND5	0.358898	0.0851547	4.2147	2.56E-05	0.506181382	count	0.587136
RBMS1	0.3599589	0.0708593	5.0799	3.96E-07	0.506265949	count	0.009241452
AC007038.2	0.7915223	0.5201084	1.5218	0.128	0.506330548	count	1
SLC6A9	0.6071507	0.3573412	1.6991	0.0894	0.506368662	count	1
CDK9	0.4051998	0.1730569	2.3414	0.0193	0.506528311	count	1
PXMP4	0.4267646	0.2111519	2.0211	0.0433	0.506571901	count	1
SNN	0.4405154	0.2942768	1.4969	0.134	0.506661317	count	1
ZMYM2	0.3800658	0.1693394	2.2444	0.0249	0.506690678	count	1
MAP2K1	0.4331586	0.1893574	2.2875	0.0222	0.506714507	count	1
ARL2BP	0.3606784	0.0740532	4.8705	1.16E-06	0.506792442	count	0.02696768
CPLANE1	0.4031711	0.221266	1.8221	0.0685	0.506825467	count	1
AGTRAP	0.3695075	0.1021439	3.6175	0.000301	0.50724183	count	1
STAT3	0.3569386	0.0648617	5.5031	3.99E-08	0.507371567	count	0.000936254
TOR1B	0.4521404	0.2810196	1.6089	0.108	0.507481162	count	1
ZNF823	0.6995065	0.3726239	1.8772	0.0606	0.507529469	count	1
ZNF451	0.3812435	0.1656801	2.3011	0.0214	0.507564907	count	1
SNX13	0.3845632	0.1532794	2.5089	0.0122	0.507584234	count	1
SELENON	0.4315976	0.2126121	2.03	0.0424	0.507935751	count	1
ING3	0.4058883	0.1504879	2.6972	0.00703	0.508093075	count	1
OXSR1	0.3948242	0.1729155	2.2833	0.0225	0.508110682	count	1
CCNL2	0.3803656	0.1305771	2.913	0.0036	0.508169194	count	1
ARSG	0.4851529	0.2077171	2.3356	0.0196	0.508193968	count	1

ZSCAN29	0.7272248	0.5066346	1.4354	0.151	0.508231972	count	1
MPV17L	0.5059296	0.2888551	1.7515	0.0799	0.508369892	count	1
BORA	1.0514573	0.9094287	1.1562	0.248	0.508418063	count	1
TEX30	0.4474716	0.2123821	2.1069	0.0352	0.508675974	count	1
RBM5	0.3917863	0.1414385	2.77	0.00563	0.508935696	count	1
ECI2	0.3653502	0.0847732	4.3097	1.68E-05	0.509085186	count	0.386316
METTL2B	0.4147405	0.1700199	2.4394	0.0148	0.509090292	count	1
IL10RB	0.8957565	0.7336382	1.221	0.222	0.509198747	count	1
SMARCD2	0.3940178	0.1665794	2.3653	0.0181	0.509320767	count	1
LINC01266	0.55774	0.4596728	1.2133	0.225	0.509328312	count	1
TFPI	0.3569113	0.054447	6.5552	6.33E-11	0.509344051	count	1.50E-06
B4GALNT1	1.0543417	0.488792	2.157	0.0311	0.509452707	count	1
ZNF669	0.4901785	0.2465535	1.9881	0.0469	0.509540314	count	1
IL32	0.3647991	0.105986	3.442	0.000584	0.509830703	count	1
SNPH	2.2387829	0.9729307	2.3011	0.0214	0.509960265	count	1
CREBZF	0.3745502	0.120885	3.0984	0.00196	0.510079795	count	1
SNTB2	0.3711105	0.1014694	3.6574	0.000258	0.510203991	count	1
EFCAB2	0.4349289	0.2545526	1.7086	0.0876	0.510253558	count	1
ZNF408	0.588032	0.3629997	1.6199	0.105	0.510383866	count	1
PER2	0.377072	0.1255231	3.004	0.00268	0.510441875	count	1
LINC01197	0.4051368	0.1566798	2.5858	0.00975	0.510576383	count	1
SLC25A45	0.4717474	0.2736413	1.724	0.0848	0.510587741	count	1
AKR1B1	0.3740228	0.1001844	3.7333	0.000192	0.510625023	count	1
FUCA2	0.3735741	0.0940106	3.9737	7.21E-05	0.510866826	count	1
USP2-AS1	1.6499727	0.9724229	1.6968	0.0898	0.511008239	count	1
TSC22D2	0.3999562	0.1646555	2.429	0.0152	0.511132246	count	1
EGFL8	1.1829562	0.8048357	1.4698	0.142	0.511552161	count	1
VASH1	0.4922295	0.4061007	1.2121	0.226	0.511566614	count	1
CHM	0.3818995	0.111843	3.4146	0.000646	0.51162915	count	1
RAB11B-AS1	0.4185218	0.2112733	1.981	0.0477	0.511721289	count	1
LRRK2	0.4169941	0.1497183	2.7852	0.00538	0.511795077	count	1
PCBP3	0.7332947	1.136205	0.6454	0.519	0.511885655	count	1
IRS1	0.4758797	0.2539133	1.8742	0.061	0.511902553	count	1
FECH	0.4789587	0.2330391	2.0553	0.0399	0.511977124	count	1
RWDD2B	0.4164128	0.2269703	1.8347	0.0666	0.51201647	count	1
NISCH	0.4366071	0.1983789	2.2009	0.0278	0.512164871	count	1
RAB35	0.4380175	0.1960543	2.2342	0.0255	0.512229809	count	1
CNOT10	0.4340861	0.2032766	2.1354	0.0328	0.51223023	count	1
IRAK2	0.8492147	0.6766475	1.255	0.21	0.513136486	count	1
AL391121.1	0.4690715	0.3039197	1.5434	0.123	0.513366286	count	1
C9orf116	0.6166474	0.4999544	1.2334	0.218	0.513538209	count	1
PGM3	0.3932057	0.1722785	2.2824	0.0225	0.513739609	count	1
ZNF687	0.9756559	0.4505746	2.1654	0.0304	0.513801755	count	1
FOXRED2	0.7681238	0.350844	2.1894	0.0286	0.513823898	count	1
TBC1D32	0.5281749	0.3521395	1.4999	0.134	0.514310065	count	1

PSMB10	0.3994048	0.1567623	2.5478	0.0109	0.514381692	count	1
GPR107	0.4093986	0.1966275	2.0821	0.0374	0.514506771	count	1
DNAJC27	0.4326149	0.3177118	1.3617	0.173	0.514666279	count	1
AASDH	0.4079702	0.160191	2.5468	0.0109	0.514731882	count	1
PLOD3	0.4125212	0.1670778	2.469	0.0136	0.514752919	count	1
ZNF721	0.4003612	0.182339	2.1957	0.0282	0.515126482	count	1
AP1M1	0.4123635	0.166388	2.4783	0.0132	0.515307244	count	1
ZNF544	0.4555017	0.5018712	0.9076	0.364	0.515426689	count	1
USP54	0.5567166	0.2726292	2.042	0.0412	0.515426914	count	1
NFIX	0.3648843	0.0747888	4.8789	1.11E-06	0.515626931	count	0.0258075
NRAS	0.4425984	0.2647458	1.6718	0.0947	0.51582578	count	1
SIRT2	0.4009363	0.1364386	2.9386	0.00332	0.51585497	count	1
ZNF442	1.0723915	0.5729559	1.8717	0.0613	0.515883339	count	1
TMEM161B	0.4828794	0.2596457	1.8598	0.063	0.515979078	count	1
AL359220.1	0.7403718	0.4982551	1.4859	0.137	0.516132305	count	1
OSTF1	0.3787723	0.1034687	3.6607	0.000255	0.516262815	count	1
BLOC1S5	0.396091	0.1658327	2.3885	0.017	0.516368259	count	1
B4GALT5	0.439051	0.2304766	1.905	0.0569	0.516452959	count	1
PDIK1L	0.4934546	0.2637877	1.8707	0.0615	0.516465837	count	1
TCP11L2	0.427946	0.1802779	2.3738	0.0177	0.516582051	count	1
EXOSC6	0.3870453	0.1244786	3.1093	0.00189	0.516786028	count	1
ZNF236	0.5104303	0.2842665	1.7956	0.0726	0.517108472	count	1
UVSSA	0.4232136	0.2667776	1.5864	0.113	0.51732527	count	1
DDN-AS1	0.9851827	0.7229039	1.3628	0.173	0.517660121	count	1
KLHL22	0.5517871	0.2522635	2.1873	0.0288	0.517770261	count	1
STK35	0.5447274	0.3640074	1.4965	0.135	0.517800936	count	1
PEX11B	0.4200379	0.2558323	1.6418	0.101	0.518174552	count	1
PIK3R4	0.4763609	0.3501111	1.3606	0.174	0.518237284	count	1
AC104051.2	0.6721649	0.5716349	1.1759	0.24	0.518345116	count	1
ODR4	0.3979716	0.1413904	2.8147	0.00491	0.518410888	count	1
C15orf61	0.3763341	0.1024931	3.6718	0.000244	0.51855372	count	1
FBXO48	0.6543601	0.3059681	2.1387	0.0325	0.518699399	count	1
PQLC3	0.3723715	0.0765657	4.8634	1.20E-06	0.518824496	count	0.0278928
AC004951.1	0.9885167	0.4656562	2.1228	0.0338	0.519005055	count	1
CEBPA	1.3937131	0.6034684	2.3095	0.021	0.519054114	count	1
ZNF554	0.7779169	0.5513255	1.411	0.158	0.519368463	count	1
SDCCAG8	0.3823014	0.1137015	3.3623	0.000781	0.519931299	count	1
PCDHGC3	0.4934777	0.2235856	2.2071	0.0274	0.520043951	count	1
GPR137B	0.4120253	0.1890122	2.1799	0.0293	0.52039974	count	1
NRBF2	0.3776453	0.0947529	3.9856	6.86E-05	0.520426632	count	1
AL117339.5	0.6126427	0.3712311	1.6503	0.099	0.520436782	count	1
SEC22C	0.3853266	0.1147886	3.3568	0.000796	0.520601924	count	1
FN3K	0.4604154	0.3117364	1.4769	0.14	0.52078797	count	1
RASSF1	0.3893186	0.1207859	3.2232	0.00128	0.520855915	count	1
BPHL	0.4516429	0.1907073	2.3683	0.0179	0.520860359	count	1

WRN	0.4347362	0.2216002	1.9618	0.0499	0.520975656	count	1
USP15	0.379123	0.0897046	4.2264	2.43E-05	0.521063623	count	0.5575878
NCKAP5L	0.4691568	0.2247652	2.0873	0.0369	0.521133576	count	1
UGGT2	0.3938235	0.1427276	2.7593	0.00582	0.521338581	count	1
BDNF	0.3991805	0.3197539	1.2484	0.212	0.521440774	count	1
SLITRK3	1.2169636	0.8108424	1.5009	0.133	0.521662746	count	1
DMXL2	0.5818326	0.3156073	1.8435	0.0653	0.521962866	count	1
DDX43	0.6589955	0.6806645	0.9682	0.333	0.521973843	count	1
SELENOO	0.4723539	0.2211125	2.1363	0.0327	0.522049453	count	1
PGD	0.477901	0.2435504	1.9622	0.0498	0.522627209	count	1
ZC3H11A	1.7217418	1.1635919	1.4797	0.139	0.522648111	count	1
MCOLN3	0.5260965	0.4785891	1.0993	0.272	0.52265292	count	1
AL136038.3	0.7517425	1.0265176	0.7323	0.464	0.522925344	count	1
SNX24	0.4334945	0.200703	2.1599	0.0308	0.523109399	count	1
NEDD4L	1.222343	1.0780575	1.1338	0.257	0.523240085	count	1
GAS2L1	0.4100453	0.1855527	2.2099	0.0272	0.523265517	count	1
SHMT1	0.5584958	0.2576945	2.1673	0.0303	0.523618041	count	1
EGLN3	0.469356	0.2257148	2.0794	0.0376	0.523748558	count	1
SPECC1L	0.4790307	0.2385701	2.0079	0.0447	0.523810872	count	1
DLX6-AS1	0.3672534	0.150966	2.4327	0.015	0.524091069	count	1
PHLDA3	0.372094	0.0749081	4.9673	7.10E-07	0.524259261	count	0.01654513
CCDC74A	0.4411984	0.2265369	1.9476	0.0515	0.524593546	count	1
RAD51AP1	0.8727529	0.6179079	1.4124	0.158	0.524696396	count	1
DOK6	0.6629844	0.330626	2.0052	0.045	0.524786627	count	1
ELP6	0.4045583	0.1252352	3.2304	0.00125	0.524877857	count	1
AC093227.1	1.2280641	0.8428623	1.457	0.145	0.524911173	count	1
RNFT1	0.4605492	0.1865715	2.4685	0.0136	0.525045398	count	1
BTN3A3	0.4986668	0.2388599	2.0877	0.0369	0.525246191	count	1
LYRM1	0.3906632	0.1153907	3.3856	0.000718	0.525543189	count	1
SFXN1	0.4044889	0.1409064	2.8706	0.00412	0.525602441	count	1
TAF1B	0.4164404	0.1414762	2.9435	0.00327	0.525872323	count	1
GEMIN5	0.6077617	0.2383664	2.5497	0.0108	0.525994622	count	1
MFAP4	0.3656507	0.0493015	7.4166	1.48E-13	0.526090909	count	3.53E-09
DNAAF5	0.4673272	0.2221564	2.1036	0.0355	0.526138442	count	1
PTGER1	0.9335655	0.5331227	1.7511	0.08	0.526205208	count	1
TUBE1	0.4167546	0.1968394	2.1172	0.0343	0.526261679	count	1
ZNF281	0.4239761	0.2417125	1.7541	0.0795	0.526351728	count	1
ZNF273	0.4963644	0.2917438	1.7014	0.089	0.526388212	count	1
UTRN	0.3759223	0.0903683	4.1599	3.26E-05	0.526403128	count	0.7467356
GPRIN3	0.8301702	0.508346	1.6331	0.103	0.526844771	count	1
PLEKHG1	1.0081493	0.5377127	1.8749	0.0609	0.52686899	count	1
CCDC65	1.236055	0.9957667	1.2413	0.215	0.527233915	count	1
MOV10L1	0.7923005	0.6602676	1.2	0.23	0.527462838	count	1
PNMA1	0.4025368	0.1267643	3.1755	0.00151	0.527521549	count	1
KCNE3	2.4737546	1.0627668	2.3277	0.02	0.528101738	count	1

ZNF836	0.4917194	0.2478801	1.9837	0.0474	0.528170219	count	1
NLRX1	0.6866036	0.4118668	1.6671	0.0956	0.528171997	count	1
CASP3	0.437469	0.1733552	2.5235	0.0117	0.528934354	count	1
LAPTM4A	0.3680568	0.0284877	12.9199	2.25E-37	0.529273068	count	5.44E-33
MRPS25	0.4037168	0.1392738	2.8987	0.00377	0.529386385	count	1
DBT	0.4519633	0.2313828	1.9533	0.0509	0.529630143	count	1
EPOR	0.4820537	0.2448025	1.9692	0.049	0.529697671	count	1
PARD3B	0.4276841	0.1782707	2.3991	0.0165	0.530016689	count	1
BCL3	0.4055267	0.1621676	2.5007	0.0124	0.530338806	count	1
IQCN	0.7358284	0.7788625	0.9447	0.345	0.530339032	count	1
PCOLCE	0.3707518	0.0498315	7.4401	1.25E-13	0.530508986	count	2.98E-09
CYP27A1	0.4046414	0.1140936	3.5466	0.000395	0.530582746	count	1
FEN1	0.6714431	0.3147224	2.1334	0.033	0.530736026	count	1
NLGN4Y	0.535232	0.3701629	1.4459	0.148	0.531178033	count	1
ZDHHC7	0.4266777	0.1850037	2.3063	0.0211	0.531260876	count	1
SERPINB1	0.3777294	0.0719911	5.2469	1.63E-07	0.531391612	count	0.003812733
C1orf174	0.4086295	0.1405091	2.9082	0.00366	0.531704528	count	1
ABCC4	1.7834368	1.0366915	1.7203	0.0855	0.532117322	count	1
ALKBH1	0.5764344	0.362583	1.5898	0.112	0.532306511	count	1
BIRC2	0.4118271	0.1309836	3.1441	0.00168	0.532436395	count	1
SLC12A7	1.022851	0.6098468	1.6772	0.0936	0.532695132	count	1
AC027097.1	0.7397711	0.3478081	2.127	0.0335	0.532792045	count	1
KDR	1.7896193	1.1651358	1.536	0.125	0.533039696	count	1
TMEM33	0.4087739	0.132822	3.0776	0.0021	0.533050222	count	1
CEBPB	0.3741239	0.0594795	6.29	3.55E-10	0.533086035	count	8.40E-06
MYOF	0.3889759	0.1011183	3.8467	0.000122	0.533115065	count	1
GTF2IRD2B	0.5374069	0.3065311	1.7532	0.0797	0.53320446	count	1
C9orf40	0.5434607	0.2532457	2.146	0.0319	0.533650905	count	1
NEU1	0.3961886	0.1246467	3.1785	0.00149	0.533798205	count	1
CALHM2	0.388877	0.0918414	4.2342	2.35E-05	0.53392993	count	0.5394425
RABL2A	0.7168346	0.3969901	1.8057	0.0711	0.533961189	count	1
C1GALT1C1	0.3866179	0.0870874	4.4394	9.29E-06	0.533988668	count	0.21410663
LSMEM2	1.4599871	0.6279348	2.3251	0.0201	0.534066239	count	1
AC044849.1	1.1250123	0.4694804	2.3963	0.0166	0.534202217	count	1
AMMECR1	0.5445377	0.3506364	1.553	0.121	0.534641317	count	1
LRIG3	0.4269541	0.1603891	2.662	0.0078	0.534675948	count	1
SYNE3	0.4550945	0.1808379	2.5166	0.0119	0.53475211	count	1
VWCE	0.771713	0.3913466	1.9719	0.0487	0.534766345	count	1
EIF4E3	0.4565446	0.2484598	1.8375	0.0662	0.534832251	count	1
GVQW3	0.6314422	0.4793064	1.3174	0.188	0.534885982	count	1
SLC38A10	0.4108646	0.1471387	2.7924	0.00526	0.534964958	count	1
RAB33B	0.4158873	0.1494661	2.7825	0.00542	0.535268425	count	1
SULF2	0.4268573	0.1897293	2.2498	0.0245	0.535291762	count	1
STAT2	0.4094025	0.1473845	2.7778	0.0055	0.53533766	count	1
XRRA1	0.4283901	0.3059189	1.4003	0.161	0.535686483	count	1

ATAD5	0.697846	0.4184549	1.6677	0.0955	0.535781506	count	1
LYSMD4	0.5806513	0.321412	1.8066	0.0709	0.535902546	count	1
PPP2R3B	0.5350553	0.4882219	1.0959	0.273	0.535907961	count	1
EDEM3	0.4138618	0.1556012	2.6598	0.00785	0.535909139	count	1
SMYD4	0.5255946	0.2153858	2.4402	0.0147	0.536027448	count	1
MTMR9	0.4537416	0.210862	2.1518	0.0315	0.536216666	count	1
TMEM243	0.3905696	0.0983717	3.9703	7.31E-05	0.536421256	count	1
AP002748.3	0.4968599	0.4973596	0.999	0.318	0.536544924	count	1
CERS6	0.4411691	0.2381094	1.8528	0.064	0.536631527	count	1
TBC1D19	0.5103521	0.2684408	1.9012	0.0574	0.536937674	count	1
ZBTB4	0.4101948	0.1396787	2.9367	0.00334	0.537421523	count	1
OGFRL1	0.4319709	0.209607	2.0609	0.0394	0.53771072	count	1
MORN1	0.6352451	0.6565595	0.9675	0.333	0.537796527	count	1
CFAP54	1.8223195	0.9628817	1.8926	0.0585	0.537839646	count	1
CNDP2	0.4725016	0.1918276	2.4632	0.0138	0.538182106	count	1
SLC25A19	0.5921096	0.3057128	1.9368	0.0528	0.538222253	count	1
LGALSL	0.4028199	0.1180885	3.4112	0.000654	0.538292629	count	1
TRIM36	0.7239083	0.56604	1.2789	0.201	0.538548264	count	1
CLMP	0.4120521	0.152141	2.7084	0.00679	0.538754117	count	1
CWF19L1	0.4660684	0.3564726	1.3074	0.191	0.538768098	count	1
AFAP1L1	1.8302507	0.8536705	2.144	0.0321	0.538984103	count	1
HM13	0.3824555	0.0678671	5.6354	1.88E-08	0.539000639	count	0.000441819
LDAH	0.5385566	0.2869952	1.8765	0.0607	0.539203885	count	1
NBN	0.3988773	0.1012233	3.9406	8.28E-05	0.539385925	count	1
DEPDC7	1.1404846	0.7044674	1.6189	0.106	0.539468192	count	1
NMNAT1	0.5692622	0.2773682	2.0524	0.0402	0.539468361	count	1
SYNGAP1-AS1	0.8546534	1.0367734	0.8243	0.41	0.539625566	count	1
ZNF660	0.5342136	0.396404	1.3476	0.178	0.539806585	count	1
BMT2	0.5450759	0.3099395	1.7587	0.0787	0.540340114	count	1
MLX	0.406053	0.1163353	3.4904	0.000488	0.540468622	count	1
POFUT2	0.4851552	0.2077331	2.3355	0.0196	0.540675271	count	1
EXOG	0.4428909	0.1941489	2.2812	0.0226	0.54079069	count	1
OMA1	0.4853306	0.187317	2.591	0.00961	0.540862899	count	1
STIM2	0.4185169	0.1982073	2.1115	0.0348	0.540949766	count	1
SULT1E1	1.1452983	1.208546	0.9477	0.343	0.541095412	count	1
PLPPR4	0.9066959	0.4741613	1.9122	0.0559	0.541102165	count	1
TMEM43	0.3861216	0.0771864	5.0025	5.93E-07	0.541216744	count	0.013828167
UBR2	0.4052791	0.1298546	3.121	0.00182	0.541320508	count	1
PHF6	0.4089019	0.1413829	2.8922	0.00385	0.541336977	count	1
AL356275.1	0.7829173	0.4676872	1.674	0.0942	0.541359477	count	1
WRAP73	0.4394343	0.1623642	2.7065	0.00683	0.541552414	count	1
RPIA	0.4961788	0.2205568	2.2497	0.0245	0.541743315	count	1
SDCBP2	0.7294078	0.5985654	1.2186	0.223	0.542104411	count	1
ZUP1	0.5233179	0.2961907	1.7668	0.0773	0.542155965	count	1
PLEKHM1	0.5527957	0.2561544	2.1581	0.031	0.542225313	count	1

ANKRD13B	1.2887656	0.669565	1.9248	0.0543	0.542229321	count	1
SBF2	0.4459493	0.1763899	2.5282	0.0115	0.542298755	count	1
MTERF2	0.4635291	0.1974276	2.3478	0.0189	0.542755713	count	1
SLC10A3	0.4234845	0.1509531	2.8054	0.00505	0.542845964	count	1
ALG8	0.4558232	0.1805594	2.5245	0.0116	0.542864115	count	1
TULP3	0.415147	0.1343347	3.0904	0.00201	0.543820359	count	1
CASD1	0.4269835	0.1744811	2.4472	0.0144	0.543917448	count	1
ZWINT	0.8631336	0.537791	1.605	0.109	0.54401354	count	1
C9orf64	0.4960695	0.2328844	2.1301	0.0332	0.544435919	count	1
ELP5	0.4261421	0.1753053	2.4309	0.0151	0.544568938	count	1
MTMR3	0.6319501	0.32596	1.9387	0.0526	0.544982177	count	1
ESRRA	0.4361202	0.1880235	2.3195	0.0204	0.545149352	count	1
AL356488.3	0.7894007	0.4535807	1.7404	0.0819	0.545158112	count	1
MAFK	0.4458977	0.2301452	1.9375	0.0528	0.54540419	count	1
ZNF563	0.866171	0.7763616	1.1157	0.265	0.545580239	count	1
EPS15L1	0.4594762	0.2055772	2.2351	0.0255	0.54568967	count	1
PSAP	0.3809415	0.0364764	10.4435	3.53E-25	0.545987246	count	8.51E-21
ENDOV	0.4492777	0.2132214	2.1071	0.0352	0.546242603	count	1
TRMT61B	0.4514175	0.1847725	2.4431	0.0146	0.546519177	count	1
KLHL20	0.429754	0.1699023	2.5294	0.0115	0.546790314	count	1
TMEM117	0.69445	0.3758022	1.8479	0.0647	0.546812417	count	1
CTXN1	0.7626685	0.39036	1.9538	0.0508	0.546949034	count	1
TRAFD1	0.4845028	0.2268013	2.1362	0.0327	0.546997884	count	1
PNKP	0.4358009	0.2505107	1.7396	0.082	0.547014099	count	1
HSD17B11	0.3954088	0.0841569	4.6985	2.72E-06	0.547036896	count	0.06304144
PDE1C	1.059867	0.5111957	2.0733	0.0382	0.547129267	count	1
VPS36	0.395528	0.0964663	4.1002	4.22E-05	0.54720084	count	0.9654938
RASD2	1.3078867	1.0119903	1.2924	0.196	0.547530503	count	1
ZNF418	1.3078867	1.378982	0.9484	0.343	0.547530503	count	1
C6orf62	0.4145652	0.1389612	2.9833	0.00287	0.547584707	count	1
HLF	0.4872416	0.1900755	2.5634	0.0104	0.547686213	count	1
ZSWIM4	1.1658288	0.595763	1.9569	0.0504	0.547976721	count	1
CDC16	0.4239972	0.1508216	2.8112	0.00496	0.548376908	count	1
TMEM98	0.3888652	0.0650283	5.9799	2.45E-09	0.548414101	count	5.78E-05
GBP4	0.4376261	0.2172255	2.0146	0.044	0.548516121	count	1
RREB1	0.4881499	0.3153003	1.5482	0.122	0.548667029	count	1
TENM2	1.8997097	1.2033332	1.5787	0.114	0.548685861	count	1
DHX35	0.5874736	0.363489	1.6162	0.106	0.548736379	count	1
IGF2R	0.4165076	0.1374549	3.0301	0.00246	0.548898496	count	1
CLDN15	0.8727472	0.5556272	1.5707	0.116	0.548963792	count	1
ABCC6	1.3135171	0.8926037	1.4716	0.141	0.549077609	count	1
PLEKHG6	1.3135171	0.9371014	1.4017	0.161	0.549077609	count	1
FZD8	0.4153083	0.2289238	1.8142	0.0697	0.549140288	count	1
ZNF391	0.7180096	0.4584627	1.5661	0.117	0.549337013	count	1
GMNN	0.4682686	0.1990426	2.3526	0.0187	0.54974313	count	1

UBIAD1	0.520077	0.2261656	2.2995	0.0215	0.550270168	count	1
POLD3	0.4622658	0.1633774	2.8294	0.00469	0.550311808	count	1
FAM45A	0.4118001	0.1540173	2.6737	0.00753	0.550385922	count	1
DERA	0.421319	0.1360256	3.0974	0.00197	0.55106687	count	1
ARFGAP1	0.5210533	0.2482428	2.099	0.0359	0.551250583	count	1
DCAF5	0.4275228	0.1693474	2.5245	0.0116	0.551460196	count	1
THSD4	0.3973105	0.1624912	2.4451	0.0145	0.5519123	count	1
TUBGCP6	0.8793986	0.3952988	2.2246	0.0262	0.552373683	count	1
ASIC1	2.902624	1.551115	1.8713	0.0614	0.552590912	count	1
S100A10	0.3836479	0.0491421	7.8069	7.59E-15	0.552651505	count	1.81E-10
FBXL7	0.5708039	0.2552746	2.236	0.0254	0.552999841	count	1
AC005225.2	0.8809237	0.6318162	1.3943	0.163	0.553153825	count	1
RUFY2	0.4105637	0.1495938	2.7445	0.00609	0.553336238	count	1
PLEKHO2	0.6016264	0.4179324	1.4395	0.15	0.553716444	count	1
C15orf48	0.5932655	0.4539461	1.3069	0.191	0.553729292	count	1
BAHD1	0.7476827	0.4416311	1.693	0.0905	0.553858159	count	1
TSPAN13	0.3973821	0.2349915	1.691	0.0909	0.554147727	count	1
LMBRD1	0.4018004	0.0922248	4.3567	1.36E-05	0.554219828	count	0.313072
CD24	1.0796118	0.5780151	1.8678	0.0619	0.554691513	count	1
NSUN4	0.5287936	0.3148673	1.6794	0.0932	0.555322486	count	1
C21orf91	0.4579822	0.1887628	2.4262	0.0153	0.555413439	count	1
B3GNT9	0.4542926	0.193437	2.3485	0.0189	0.555414122	count	1
HEXA	0.3993568	0.0813475	4.9093	9.54E-07	0.555489905	count	0.022197672
COX18	0.7275257	0.4152514	1.752	0.0799	0.555693119	count	1
TBC1D4	0.8075667	0.3185989	2.5347	0.0113	0.555737132	count	1
RPGR	0.5739864	0.2732962	2.1002	0.0358	0.555868906	count	1
LRRC57	0.4697344	0.1995807	2.3536	0.0186	0.55603584	count	1
LINC00954	0.8872033	0.9373282	0.9465	0.344	0.556359168	count	1
AJAP1	0.8087393	0.616617	1.3116	0.19	0.556416684	count	1
KCTD5	0.5518209	0.3253126	1.6963	0.0899	0.556518209	count	1
VPS50	0.5132598	0.2135737	2.4032	0.0163	0.556530575	count	1
AC098850.3	0.4702076	0.4535032	1.0368	0.3	0.556578714	count	1
PLEKHB2	0.4448878	0.1893822	2.3492	0.0189	0.556655341	count	1
UBN1	0.4202116	0.1490628	2.819	0.00484	0.556968304	count	1
TLR4	0.4400775	0.2387603	1.8432	0.0654	0.557142514	count	1
ZNF302	0.4100393	0.1177025	3.4837	5.00E-04	0.557196257	count	1
CCDC71	0.5478366	0.2691484	2.0354	0.0419	0.557374439	count	1
SPTBN1	0.3915439	0.0552521	7.0865	1.64E-12	0.558217513	count	3.90E-08
NUDT19	0.5590023	0.317785	1.7591	0.0787	0.558387263	count	1
GLA	0.4577411	0.1731812	2.6431	0.00825	0.558459081	count	1
C6orf120	0.438715	0.1393392	3.1485	0.00165	0.558582786	count	1
ITPK1	0.4811508	0.2388118	2.0148	0.044	0.559244112	count	1
HES4	0.3935046	0.0834691	4.7144	2.51E-06	0.559283819	count	0.05818682
MNT	0.5410475	0.2409049	2.2459	0.0248	0.559495102	count	1
ABCA1	0.4362944	0.1503949	2.901	0.00374	0.559515225	count	1

SLC38A11	1.0931182	0.6304229	1.7339	0.083	0.559809648	count	1
CGREF1	0.5297867	0.2228081	2.3778	0.0175	0.560010645	count	1
UBR1	0.4352573	0.1871913	2.3252	0.0201	0.560279525	count	1
NPB	0.4454929	0.2589376	1.7205	0.0854	0.560396223	count	1
TCTN3	0.436596	0.172215	2.5352	0.0113	0.560949851	count	1
NR2E3	0.8962488	1.1703008	0.7658	0.444	0.560957017	count	1
PAK4	0.6295036	0.2863788	2.1982	0.028	0.560976568	count	1
TTI1	0.6196719	0.3530239	1.7553	0.0793	0.5611556	count	1
ZNF738	0.7358496	0.3359614	2.1903	0.0286	0.561231095	count	1
SSTR2	0.7596503	0.3673387	2.068	0.0387	0.561502358	count	1
WDR36	0.4641603	0.200813	2.3114	0.0209	0.561526294	count	1
NMUR1	1.9991376	1.3510166	1.4797	0.139	0.56161063	count	1
LINC01126	1.5935998	1.3668473	1.1659	0.244	0.56209198	count	1
PSTK	0.6672102	0.3241307	2.0585	0.0396	0.562095901	count	1
MAN2B1	0.8985398	0.5996158	1.4985	0.134	0.562117967	count	1
TRMO	0.5579413	0.24754	2.2539	0.0243	0.562308678	count	1
LGALS3	0.3908131	0.0322264	12.1271	3.25E-33	0.562345677	count	7.85E-29
PCF11	0.4173462	0.1300581	3.2089	0.00134	0.562386103	count	1
RARRES2	0.3930581	0.0493944	7.9575	2.32E-15	0.562786923	count	5.54E-11
CARM1	0.4876131	0.2289179	2.1301	0.0332	0.562791194	count	1
U73166.1	1.598213	0.9845913	1.6232	0.105	0.563008294	count	1
UNC79	1.598213	1.1007039	1.452	0.147	0.563008294	count	1
NIPAL3	0.5329548	0.4084084	1.305	0.192	0.563183912	count	1
PCDHB4	0.4586548	0.1691616	2.7113	0.00673	0.563679847	count	1
CAPN5	0.8586858	0.4101778	2.0934	0.0364	0.564060676	count	1
POC1B-AS1	0.6337839	0.7212943	0.8787	0.38	0.56444804	count	1
HIRA	0.6852671	0.4046285	1.6936	0.0904	0.564570315	count	1
ATF7IP	0.4266053	0.1498585	2.8467	0.00444	0.564765099	count	1
TRIM61	0.9569691	0.7488887	1.2779	0.201	0.564828769	count	1
AC058791.1	0.4845638	0.2398919	2.0199	0.0435	0.564841266	count	1
KCNB2	2.026218	1.0372434	1.9535	0.0508	0.564943223	count	1
NLRC5	0.6344132	0.4475611	1.4175	0.156	0.56495801	count	1
ATG2B	0.5511528	0.309365	1.7816	0.0749	0.565028945	count	1
POLB	0.4863169	0.2138826	2.2738	0.023	0.565040675	count	1
DPCD	0.43775	0.1645484	2.6603	0.00784	0.565397014	count	1
DDX3X	0.4017932	0.0730293	5.5018	4.02E-08	0.565731617	count	0.000943172
KCNRG	1.0248927	0.8862629	1.1564	0.248	0.565749884	count	1
TOR2A	0.5731595	0.2690953	2.13	0.0332	0.566339843	count	1
GABRA2	2.0382398	1.1346195	1.7964	0.0725	0.566397777	count	1
PRMT3	0.5857538	0.2857769	2.0497	0.0405	0.566453751	count	1
ZNF668	0.5860569	0.3093197	1.8947	0.0582	0.566725887	count	1
CACNA2D2	0.9619809	0.5631703	1.7082	0.0877	0.567156788	count	1
CRIP1	0.39894	0.0655982	6.0816	1.31E-09	0.567398516	count	3.09E-05
CCDC61	0.499492	0.2084252	2.3965	0.0166	0.567740163	count	1
C8orf76	0.4957485	0.2135621	2.3213	0.0203	0.567857864	count	1

GPX8	0.4064363	0.0769142	5.2843	1.34E-07	0.568195314	count	0.003136002
PXK	0.42386	0.1094867	3.8713	0.00011	0.568726496	count	1
PLEKHA8	0.5508772	0.3206099	1.7182	0.0858	0.569074726	count	1
MAFB	0.4174943	0.105362	3.9625	7.56E-05	0.569114631	count	1
PAPOLG	0.482651	0.198591	2.4304	0.0151	0.569289826	count	1
CARD6	0.5655476	0.2632506	2.1483	0.0318	0.569491511	count	1
GNL3	0.4372661	0.1183321	3.6952	0.000223	0.569636116	count	1
JOSD1	0.4379722	0.1498685	2.9224	0.00349	0.570129374	count	1
TGFB2	0.4345735	0.2455937	1.7695	0.0769	0.570342499	count	1
ENTPD4	0.5331097	0.2884326	1.8483	0.0646	0.570405585	count	1
ZXDB	0.9692361	0.4790226	2.0234	0.0431	0.570514835	count	1
HSPBAP1	0.7743069	0.4006438	1.9327	0.0534	0.570806757	count	1
ATP6V1E2	0.5725716	0.3007467	1.9038	0.057	0.571058942	count	1
MDN1	0.4781402	0.2561681	1.8665	0.062	0.571451487	count	1
AC097103.2	1.3976753	0.6637917	2.1056	0.0353	0.57145963	count	1
TBC1D8B	0.4706576	0.1763657	2.6686	0.00765	0.571532654	count	1
ZKSCAN4	0.730789	0.4366143	1.6738	0.0943	0.571888787	count	1
DNAL4	0.4836126	0.2382363	2.03	0.0424	0.57194057	count	1
CTTNBP2	0.5282932	0.2278896	2.3182	0.0205	0.572043239	count	1
GSEC	0.5922652	0.4239054	1.3972	0.162	0.572294742	count	1
SYS1	0.4208477	0.1182459	3.5591	0.000377	0.572315525	count	1
RPS6KL1	0.7527227	0.9175064	0.8204	0.412	0.572394346	count	1
SYCE1L	0.874442	0.4831136	1.81	0.0704	0.572563129	count	1
ARL6	0.5387077	0.2562613	2.1022	0.0356	0.572576015	count	1
VPS13B	0.4804051	0.1888617	2.5437	0.011	0.572675561	count	1
KHNYN	0.5802254	0.2631569	2.2049	0.0275	0.572848777	count	1
ENO4	1.1281798	1.05811	1.0662	0.286	0.572889515	count	1
EXPH5	0.624742	0.4203907	1.4861	0.137	0.573206066	count	1
ASXL1	0.4241893	0.1154237	3.6751	0.000241	0.573451409	count	1
FUT11	0.4660397	0.199409	2.3371	0.0195	0.573525637	count	1
PACERR	1.6527233	1.1521037	1.4345	0.152	0.573581469	count	1
C6orf136	0.511427	0.2225174	2.2984	0.0216	0.573742997	count	1
APPBP2	0.4339303	0.1680258	2.5825	0.00985	0.574039767	count	1
IWS1	0.4246207	0.1460272	2.9078	0.00366	0.574196136	count	1
TXNDC5	0.9228617	0.9238117	0.999	0.318	0.574352597	count	1
FAM234B	0.8400568	0.4351192	1.9306	0.0536	0.574420277	count	1
ITGB8	0.6017366	0.2854391	2.1081	0.0351	0.574474907	count	1
RAB37	1.6579842	0.8037984	2.0627	0.0392	0.574577738	count	1
ZNF680	0.4462827	0.1669267	2.6735	0.00754	0.574735001	count	1
MAK16	0.5101391	0.2085412	2.4462	0.0145	0.574760619	count	1
LBR	0.4403733	0.1432255	3.0747	0.00212	0.575226128	count	1
NHLRC3	0.4372924	0.1573415	2.7793	0.00548	0.575243975	count	1
BCLAF3	0.4358132	0.1846919	2.3597	0.0183	0.575273373	count	1
SLC6A6	0.5201576	0.3319351	1.567	0.117	0.575297446	count	1
AC124066.1	2.114732	0.8950409	2.3627	0.0182	0.575304379	count	1

FAM110B	0.4671421	0.2113414	2.2104	0.0271	0.5758304	count	1
AK3	0.4163659	0.0943769	4.4117	1.05E-05	0.575991601	count	0.241857
POLR2D	0.4962833	0.1916265	2.5898	0.00964	0.576208719	count	1
CTBP1	0.4319493	0.1483558	2.9116	0.00362	0.576526777	count	1
DNAJC14	0.759181	0.3200509	2.3721	0.0177	0.576644903	count	1
LINC02035	0.9825685	0.7447059	1.3194	0.187	0.57664881	count	1
TP53BP1	0.4496958	0.1857348	2.4212	0.0155	0.576936448	count	1
AFAP1L2	0.7844219	0.3413217	2.2982	0.0216	0.577191214	count	1
TPP1	0.4342387	0.1366015	3.1789	0.00149	0.577259424	count	1
LYSMD1	0.6297333	0.2878837	2.1875	0.0288	0.577394697	count	1
FAM69A	0.5224344	0.2019868	2.5865	0.00973	0.577702172	count	1
TPCN1	0.4619615	0.1664654	2.7751	0.00555	0.578340483	count	1
TIMP2	0.4036345	0.0430288	9.3806	1.12E-20	0.578383141	count	2.69E-16
SYNDIG1	0.7041746	0.3282538	2.1452	0.032	0.578390463	count	1
RFTN1	0.4203569	0.1364069	3.0816	0.00207	0.578796546	count	1
SLC35B4	0.4730485	0.2151938	2.1982	0.028	0.578818217	count	1
HAND2	0.4186575	0.1340405	3.1234	0.0018	0.578999214	count	1
LCAT	0.607044	0.2945154	2.0612	0.0394	0.579154813	count	1
ZNF217	0.4993622	0.1804284	2.7676	0.00567	0.579654914	count	1
TMED5	0.4285853	0.1216104	3.5242	0.00043	0.580647693	count	1
FARSB	0.497136	0.1783727	2.7871	0.00535	0.58075218	count	1
SRGAP1	0.4550071	0.1899715	2.3951	0.0167	0.580753662	count	1
EZH1	0.4289364	0.1239951	3.4593	0.000548	0.580798832	count	1
GABPB2	0.4766721	0.2091445	2.2792	0.0227	0.580931069	count	1
NUP37	0.4767127	0.179698	2.6529	0.00802	0.580979208	count	1
AC092683.1	0.4973386	0.257069	1.9347	0.0531	0.580980593	count	1
ABHD18	0.6351561	0.3798862	1.672	0.0946	0.581937427	count	1
CLCN5	1.0643931	0.3810696	2.7932	0.00525	0.582186526	count	1
OSCP1	0.5322336	0.3135731	1.6973	0.0897	0.582261739	count	1
CLN8	0.5795811	0.2607215	2.223	0.0263	0.582704371	count	1
AL360012.1	2.1829053	1.0631274	2.0533	0.0401	0.582753538	count	1
BLCAP	0.4543573	0.1540839	2.9488	0.00321	0.58280633	count	1
BCAT1	0.513324	0.189084	2.7148	0.00666	0.582829868	count	1
TRAPPC2	0.4665192	0.184125	2.5337	0.0113	0.583105685	count	1
LXN	0.4250853	0.1431695	2.9691	0.00301	0.583169781	count	1
RAD52	0.6279559	0.4005815	1.5676	0.117	0.583439168	count	1
HSPA1A	0.4057176	0.072217	5.618	2.08E-08	0.583475697	count	0.000488717
C2	0.5919141	0.2541502	2.329	0.0199	0.583587044	count	1
PLXNB1	0.490534	0.4008657	1.2237	0.221	0.584370401	count	1
ZNF765	0.6207558	0.3052146	2.0338	0.042	0.584410945	count	1
AVL9	0.6133177	0.3138727	1.954	0.0508	0.584676785	count	1
PECR	0.6211446	0.2991778	2.0762	0.0379	0.584747507	count	1
HSPG2	0.4179955	0.0981026	4.2608	2.09E-05	0.584866415	count	0.4799894
NAV1	0.429015	0.1562561	2.7456	0.00607	0.585451001	count	1
AFG3L2	0.4499448	0.1522628	2.9551	0.00315	0.585463017	count	1

YTHDF1	0.4903274	0.1794465	2.7324	0.00632	0.585565046	count	1
CDK13	0.4456214	0.1353155	3.2932	0.001	0.586042313	count	1
TMEM67	0.5002893	0.2196444	2.2777	0.0228	0.586044725	count	1
MPPE1	0.4677024	0.1917309	2.4394	0.0148	0.586162834	count	1
BTD	0.4582165	0.1549196	2.9578	0.00312	0.586533248	count	1
AC002451.1	1.7244901	1.2429319	1.3874	0.165	0.586813728	count	1
WSB1	0.4149775	0.0804039	5.1612	2.58E-07	0.58681443	count	0.00602688
EPB41L5	0.6411782	0.3694473	1.7355	0.0827	0.586972461	count	1
FRS3	0.9016656	0.4498528	2.0044	0.0451	0.58708716	count	1
CCDC160	1.726395	0.8105212	2.13	0.0332	0.587154543	count	1
ZNF175	1.0067736	0.4566305	2.2048	0.0275	0.587663163	count	1
ZBTB21	0.4988754	0.2038254	2.4476	0.0144	0.587783473	count	1
CAMKK2	0.4449626	0.1336224	3.33	0.000877	0.587816581	count	1
FOXF1	1.1693494	0.5514506	2.1205	0.034	0.587870604	count	1
AC053513.1	1.7304303	1.2733739	1.3589	0.174	0.587874799	count	1
BCDIN3D	0.495544	0.2182814	2.2702	0.0233	0.588663618	count	1
AP2A1	0.4513636	0.1418662	3.1816	0.00148	0.58895182	count	1
RNF13	0.4207989	0.0668088	6.2986	3.36E-10	0.58896006	count	7.95E-06
SPIN3	0.736244	0.3304375	2.2281	0.0259	0.58896374	count	1
FKBP5	0.4271975	0.0965883	4.4229	1.00E-05	0.589093746	count	0.2304
AP001347.1	1.1730887	0.5433007	2.1592	0.0309	0.589211255	count	1
RRBP1	0.4146733	0.0630859	6.5732	5.62E-11	0.5892186	count	1.33E-06
TTC27	0.5099036	0.2038795	2.501	0.0124	0.589520456	count	1
CFAP44	0.611961	0.6889181	0.8883	0.374	0.589892596	count	1
MKNK1	0.4609815	0.1573161	2.9303	0.00341	0.590004713	count	1
RNF149	0.4405867	0.136238	3.234	0.00123	0.590257485	count	1
APOL1	0.4746424	0.1540558	3.081	0.00208	0.590447229	count	1
NPDC1	0.419856	0.0660962	6.3522	2.38E-10	0.590535678	count	5.63E-06
MXRA8	0.4144575	0.0535741	7.7361	1.32E-14	0.590838323	count	3.15E-10
TUB	0.6789702	0.6103843	1.1124	0.266	0.591335244	count	1
PSORS1C1	0.7827172	0.4529009	1.7282	0.084	0.592030911	count	1
DPT	0.4145936	0.1114151	3.7212	0.000201	0.592546277	count	1
FOXF2	1.3065988	0.8122099	1.6087	0.108	0.592638396	count	1
CLHC1	0.608302	0.3816615	1.5938	0.111	0.59273181	count	1
TM6SF2	1.758915	0.9068588	1.9396	0.0525	0.592892057	count	1
AC073320.1	1.758915	1.7299214	1.0168	0.3093	0.592892057	count	1
DTX3L	0.4797897	0.1929841	2.4862	0.013	0.592983898	count	1
FAM53C	0.493201	0.2336676	2.1107	0.0349	0.593021562	count	1
NRBP2	0.5167255	0.2522903	2.0481	0.0406	0.59305148	count	1
RALGAPA2	0.6822	0.3518152	1.9391	0.0526	0.593862603	count	1
KDELC2	0.4431057	0.1028181	4.3096	1.68E-05	0.59403682	count	0.386316
ADAMTS10	0.514016	0.2763832	1.8598	0.063	0.594094833	count	1
TSHZ3	0.6167954	0.4629195	1.3324	0.183	0.594195876	count	1
FAM206A	0.538126	0.2575845	2.0891	0.0368	0.594243916	count	1
ASNSD1	0.4421128	0.1326828	3.3321	0.00087	0.594409436	count	1

MRS2	0.4871773	0.2452718	1.9863	0.0471	0.5945162	count	1
ACKR2	2.3005938	0.7818467	2.9425	0.00328	0.594599643	count	1
LRRC42	0.4630445	0.1510595	3.0653	0.00219	0.594848219	count	1
CRYL1	0.4351033	0.0937231	4.6424	3.56E-06	0.595378674	count	0.0823962
ZNF124	0.6722412	0.4025469	1.67	0.095	0.595402452	count	1
C11orf95	0.6183395	0.260893	2.3701	0.0178	0.595568986	count	1
MOB3C	0.5079016	0.3151768	1.6115	0.107	0.596369673	count	1
TACC1	0.4216968	0.0631238	6.6805	2.74E-11	0.596781685	count	6.50E-07
C16orf74	1.1003802	0.59431	1.8515	0.0642	0.596813946	count	1
ARTN	1.7853543	0.6157828	2.8993	0.00376	0.597445231	count	1
SGCE	0.4260832	0.0740243	5.756	9.32E-09	0.597981434	count	0.00021929
PTPDC1	0.536614	0.3734148	1.437	0.151	0.598129259	count	1
NADK	0.493381	0.1970122	2.5043	0.0123	0.598328771	count	1
PLPP6	0.6650901	0.3341536	1.9904	0.0466	0.598476317	count	1
ADAM17	0.4697633	0.1772471	2.6503	0.00808	0.59858725	count	1
MTRR	0.581427	0.2507987	2.3183	0.0205	0.598692368	count	1
RFWD3	0.7934563	0.5343624	1.4849	0.138	0.598996559	count	1
KDM4C	0.6560143	0.2759639	2.3772	0.0175	0.599332738	count	1
PRR5	0.6568351	0.2873497	2.2858	0.0223	0.600014733	count	1
C11orf65	1.3314249	1.1067207	1.203	0.229	0.600068262	count	1
AATK	1.3314249	1.1670102	1.1409	0.254	0.600068262	count	1
LATS2	0.4731371	0.1689234	2.8009	0.00512	0.600180567	count	1
ZNF287	0.5784938	0.2975182	1.9444	0.0519	0.600236954	count	1
CCDC116	16.3114776	1256.179975	0.013	0.99	0.600265764	count	1
BEGAIN	16.4046319	1186.016491	0.0138	0.989	0.600265769	count	1
AC007608.3	16.5619272	1400.582545	0.0118	0.991	0.600265777	count	1
CDCA7	16.7532734	1045.330177	0.016	0.987	0.600265785	count	1
FGF10-AS1	16.8223997	1024.3082	0.0164	0.9869	0.600265788	count	1
FREM3	16.8736651	1348.813718	0.0125	0.99	0.60026579	count	1
AL136979.1	16.8740907	1480.80187	0.0114	0.9909	0.60026579	count	1
CPZ	16.8930152	1052.619238	0.016	0.987	0.600265791	count	1
THPO	16.989836	1374.875966	0.0124	0.99	0.600265794	count	1
SLC4A3	16.9899959	1313.143674	0.0129	0.99	0.600265794	count	1
AC137630.1	17.0095847	975.7422585	0.0174	0.986	0.600265794	count	1
ASAH2B	17.0314403	1121.367146	0.0152	0.988	0.600265795	count	1
KBTBD11	17.051137	1242.101447	0.0137	0.989	0.600265796	count	1
ZNF99	17.1438428	1708.284786	0.01	0.992	0.600265798	count	1
RIBC2	17.1838233	1674.226682	0.0103	0.992	0.600265799	count	1
PPP2R2C	17.184164	1785.792055	0.0096	0.992	0.600265799	count	1
AL356488.2	17.1844078	1628.121941	0.0106	0.992	0.600265799	count	1
AC104984.1	17.184973	2047.912978	0.0084	0.993	0.600265799	count	1
ERVW-1	17.1849915	2047.921855	0.0084	0.993	0.600265799	count	1
AL512638.2	17.1950236	1055.362348	0.0163	0.987	0.6002658	count	1
KLHL33	17.2057313	1088.035983	0.0158	0.987	0.6002658	count	1
RASA4B	17.31266	1720.659911	0.0101	0.992	0.600265802	count	1

FAM66A	17.313572	2159.395996	0.008	0.994	0.600265802	count	1
AC007383.3	17.3146391	2687.028392	0.0064	0.995	0.600265802	count	1
GH1	17.3146392	2687.028411	0.0064	0.995	0.600265802	count	1
JAG2	17.3158005	3261.186301	0.0053	0.996	0.600265802	count	1
AC007220.1	17.3158006	3261.186429	0.0053	0.996	0.600265802	count	1
AC022509.3	17.3820734	1696.698888	0.0102	0.992	0.600265804	count	1
SLC7A4	17.4123989	1751.881355	0.0099	0.992	0.600265804	count	1
AADACL2	17.5652695	1141.981234	0.0154	0.9877	0.600265807	count	1
TSPAN19	17.5938371	1380.547798	0.0127	0.99	0.600265808	count	1
RSPO4	17.5939835	1515.606861	0.0116	0.991	0.600265808	count	1
ASPG	17.5939986	1515.611757	0.0116	0.991	0.600265808	count	1
FMN1	17.5945107	2024.134275	0.0087	0.993	0.600265808	count	1
LINC02449	17.5945797	2118.614297	0.0083	0.993	0.600265808	count	1
AL365203.2	17.6873995	2346.023715	0.0075	0.994	0.600265809	count	1
RTL3	17.6875795	2537.208382	0.007	0.994	0.600265809	count	1
VNN1	17.6891752	3603.53122	0.0049	0.996	0.600265809	count	1
SLC6A4	17.7319233	1196.841613	0.0148	0.9882	0.60026581	count	1
HMX1	17.7456247	1380.648053	0.0129	0.99	0.60026581	count	1
PTH2R	17.8764202	2136.029461	0.0084	0.993	0.600265812	count	1
IQCH	17.9183207	2150.081195	0.0083	0.993	0.600265813	count	1
DTX1	17.289978	1081.008061	0.016	0.987	0.600265851	count	1
SUCLG2-AS1	0.7038828	0.4331955	1.6249	0.104	0.600449618	count	1
SLC2A13	0.6043011	0.2851185	2.1195	0.0341	0.600500537	count	1
LY6K	0.5049861	0.2755187	1.8329	0.0669	0.601024736	count	1
SYNRG	0.464535	0.1410716	3.2929	0.001	0.601832112	count	1
FLT1	0.8543081	0.5819607	1.468	0.142	0.602077348	count	1
ZKSCAN8	0.618845	0.349008	1.7732	0.0763	0.602231417	count	1
PEX13	0.472828	0.1852854	2.5519	0.0108	0.602412456	count	1
DKK1	0.7758306	0.7117958	1.09	0.276	0.602428562	count	1
TTLL5	1.0405398	0.4268345	2.4378	0.0148	0.602766214	count	1
CLEC14A	0.6129069	0.4033985	1.5194	0.129	0.602781117	count	1
OGT	0.44546	0.1394472	3.1945	0.00141	0.60294646	count	1
XPO6	0.5493582	0.2854318	1.9247	0.0543	0.603107404	count	1
SRRT	0.4866698	0.1564238	3.1112	0.00188	0.603187955	count	1
ASCC2	0.503195	0.1796542	2.8009	0.00512	0.603287137	count	1
ZNF674-AS1	0.6725961	0.6179689	1.0884	0.276	0.604587082	count	1
FMNL2	0.4968177	0.1615525	3.0753	0.00212	0.604780199	count	1
RAB28	0.4872312	0.1520463	3.2045	0.00136	0.604800419	count	1
ERV3-1	0.5099724	0.2919251	1.7469	0.0807	0.60522927	count	1
MATN3	0.9363694	0.6624473	1.4135	0.158	0.605296203	count	1
CTPS2	0.6038798	0.4978015	1.2131	0.225	0.605460341	count	1
RAB9A	0.4504141	0.1029725	4.3741	1.25E-05	0.605854858	count	0.28785
EDRF1	0.5149089	0.25751	1.9996	0.0456	0.606010943	count	1
VPS13C	0.4349734	0.087844	4.9517	7.69E-07	0.606581408	count	0.017912317
SCPEP1	0.4295047	0.0585957	7.33	2.82E-13	0.606629955	count	6.71E-09

TIAM1	1.3545281	0.6433652	2.1054	0.0353	0.606865117	count	1
C5orf30	0.8058704	0.3386961	2.3793	0.0174	0.607005903	count	1
ZGRF1	1.5433971	0.7912641	1.9505	0.0512	0.607039398	count	1
ZNF331	0.446391	0.1588503	2.8101	0.00498	0.607162598	count	1
ANG	0.4360955	0.0886287	4.9205	9.01E-07	0.60717734	count	0.020969874
TAPBPL	0.4885813	0.1314848	3.7159	0.000206	0.607353594	count	1
ATF6	0.4570061	0.1388332	3.2918	0.00101	0.6074045	count	1
ANKRD36B	0.5139081	0.2081237	2.4692	0.0136	0.608158107	count	1
KIAA0408	1.8549785	0.6634576	2.7959	0.0052	0.608969926	count	1
SLC25A20	0.5970569	0.2662982	2.2421	0.025	0.609050696	count	1
CASQ2	0.5037938	0.153502	3.282	0.00104	0.609316764	count	1
TMEM237	0.4485949	0.1198441	3.7432	0.000185	0.609838586	count	1
MFAP2	0.44353	0.0986527	4.4959	7.14E-06	0.610278562	count	0.16477692
NCEH1	0.6508625	0.2911694	2.2353	0.0255	0.61034662	count	1
CCDC96	1.2335643	0.7818323	1.5778	0.115	0.610434359	count	1
SUOX	0.6429591	0.3490403	1.8421	0.0655	0.610619033	count	1
ZFYVE1	0.5848768	0.3311762	1.7661	0.0775	0.61072291	count	1
QKI	0.4337463	0.0712292	6.0894	1.25E-09	0.610994581	count	2.95E-05
AC106739.2	1.2360077	0.486555	2.5403	0.0111	0.611273816	count	1
MBTPS2	0.7045645	0.2645145	2.6636	0.00776	0.611279112	count	1
RHBDD3	0.5419244	0.3091	1.7532	0.0796	0.611481256	count	1
ZNFX1	0.4888137	0.1726874	2.8306	0.00467	0.611990113	count	1
DNAJC5	0.5380977	0.3551992	1.5149	0.13	0.612091504	count	1
CPT1C	1.3732054	0.7071356	1.9419	0.0522	0.612277907	count	1
DDB1	0.4698856	0.1401597	3.3525	0.000809	0.612717014	count	1
RHOBTB1	0.5309255	0.3427583	1.549	0.121	0.612868163	count	1
ST3GAL1	0.494469	0.2027011	2.4394	0.0148	0.613553605	count	1
LENG8	0.5064403	0.1979809	2.558	0.0106	0.613689206	count	1
DOLK	0.5633528	0.2842828	1.9817	0.0476	0.614572369	count	1
XKR8	0.5698583	0.2431965	2.3432	0.0192	0.614659354	count	1
ALG11	0.709183	0.3282302	2.1606	0.0308	0.614857492	count	1
AL450326.1	0.6034617	0.3055233	1.9752	0.0483	0.615149704	count	1
HPSE	0.8186188	0.4486366	1.8247	0.0681	0.615183049	count	1
ITCH	0.5110731	0.1620917	3.153	0.00163	0.6151986	count	1
DNAJC6	1.5795573	1.281994	1.2321	0.218	0.615271478	count	1
DLG1	0.4668725	0.1585474	2.9447	0.00325	0.615327181	count	1
ZNF808	0.6406943	0.3215486	1.9925	0.0464	0.61537452	count	1
CNNM2	0.5855034	0.2781381	2.1051	0.0354	0.615461524	count	1
AC025171.4	2.5497638	1.2792214	1.9932	0.0463	0.61589526	count	1
CDK14	0.5302208	0.2121031	2.4998	0.0125	0.616043654	count	1
TTTY14	0.6671586	0.3672404	1.8167	0.0693	0.616606108	count	1
TGFB3	0.5020449	0.2077678	2.4164	0.0157	0.61661834	count	1
IRF2BPL	0.4591338	0.1158912	3.9618	7.58E-05	0.61765558	count	1
ABI1	0.4765995	0.1450409	3.286	0.00103	0.617665235	count	1
PCDHGA10	0.5222819	0.2380149	2.1943	0.0283	0.617719695	count	1

PGGHG	0.5577622	0.3132701	1.7805	0.0751	0.617766995	count	1
RASSF5	0.8498963	0.672427	1.2639	0.206	0.617786274	count	1
LMLN	0.8812524	0.4391902	2.0065	0.0449	0.617817936	count	1
SLC30A1	0.555309	0.2255526	2.462	0.0139	0.617981443	count	1
SULT1C4	0.759917	0.7093731	1.0713	0.284	0.618518601	count	1
DENND1A	0.882794	0.3619132	2.4392	0.0148	0.618712063	count	1
AC069224.1	0.7142416	0.3767058	1.896	0.058	0.618769618	count	1
DPY19L4	0.4807737	0.1660474	2.8954	0.00381	0.618851321	count	1
NDUFA7	0.7436307	0.3038391	2.4474	0.0144	0.618976516	count	1
ZFYVE26	0.8256288	0.5104982	1.6173	0.106	0.619658744	count	1
STK33	1.9234725	1.339826	1.4356	0.151	0.619672267	count	1
FOXRED1	0.6137575	0.3034188	2.0228	0.0432	0.619898333	count	1
ABCA5	0.598753	0.2030867	2.9483	0.00322	0.619928505	count	1
CAPS	0.578572	0.2862649	2.0211	0.0433	0.619995808	count	1
G2E3	0.4927208	0.1648988	2.988	0.00283	0.620005529	count	1
SCAMP2	0.4502317	0.0943279	4.7731	1.89E-06	0.620083532	count	0.04385178
MICAL1	0.519464	0.2896458	1.7934	0.073	0.620692194	count	1
ALG2	0.4742601	0.1300108	3.6479	0.000268	0.62081136	count	1
SCAF8	0.5105969	0.2017022	2.5314	0.0114	0.621053492	count	1
PLXDC2	0.4424992	0.0757055	5.845	5.51E-09	0.621144319	count	0.000129749
STAG1	0.479979	0.1552838	3.091	0.00201	0.621475591	count	1
ICAM1	0.477586	0.2608065	1.8312	0.0672	0.621706095	count	1
TMEM39A	0.5237648	0.2173884	2.4094	0.016	0.622607907	count	1
EFCC1	1.9456272	1.5185696	1.2812	0.2	0.623003844	count	1
AC007364.1	1.6180575	1.1416726	1.4173	0.156	0.623785209	count	1
AC026150.1	1.6180575	1.6716576	0.9679	0.3331	0.623785209	count	1
MAVS	0.5151261	0.2072701	2.4853	0.013	0.623889325	count	1
DGLUCY	0.60777	0.2589441	2.3471	0.019	0.624040532	count	1
GTF3C4	0.4944269	0.2458886	2.0108	0.0444	0.624399573	count	1
ZNF433	0.7879221	0.3872782	2.0345	0.042	0.624807452	count	1
ZNF383	0.5479031	0.2973597	1.8426	0.0655	0.6250769	count	1
RGS22	0.7692553	0.7179477	1.0715	0.284	0.625150401	count	1
HILPDA	0.4816634	0.1623584	2.9667	0.00303	0.62600815	count	1
LAMA4	0.4599653	0.0965745	4.7628	1.98E-06	0.626322063	count	0.04593006
NPR2	0.5814031	0.2661398	2.1846	0.029	0.626423432	count	1
PRMT7	0.6792156	0.2888011	2.3518	0.0187	0.626718685	count	1
TRIM2	0.5088807	0.1714268	2.9685	0.00301	0.626917774	count	1
LIPH	2.71695	0.9732551	2.7916	0.00527	0.62769009	count	1
PLXDC1	0.5114805	0.2278044	2.2453	0.0248	0.627876886	count	1
RBM15B	0.5596845	0.1811151	3.0902	0.00202	0.628017378	count	1
AP2A2	0.4864453	0.201767	2.4109	0.016	0.628158098	count	1
TNPO1	0.4616956	0.102958	4.4843	7.54E-06	0.628219922	count	0.17395534
TTPAL	0.7136608	0.3381691	2.1104	0.0349	0.628260744	count	1
ZNF253	0.6482435	0.2660499	2.4365	0.0149	0.628559702	count	1
TRIM8	0.4618614	0.1135436	4.0677	4.85E-05	0.628589632	count	1

AKAP17A	0.5151505	0.1797568	2.8658	0.00418	0.628827345	count	1
ZNF718	0.6821033	0.4086506	1.6692	0.0952	0.629134462	count	1
ZNF585B	0.6564571	0.4709472	1.3939	0.163	0.629256349	count	1
CHST10	1.183612	0.4618275	2.5629	0.0104	0.62939015	count	1
NEGR1	0.501687	0.1593706	3.1479	0.00166	0.629390948	count	1
AHNAK	0.4397085	0.0548238	8.0204	1.41E-15	0.629757226	count	3.37E-11
HACD3	0.4540189	0.0909121	4.994	6.19E-07	0.629934274	count	0.014431366
C9orf66	2.0004525	0.8713708	2.2958	0.0217	0.630983902	count	1
SFT2D1	0.4553744	0.0891814	5.1062	3.45E-07	0.631056352	count	0.008051955
ORAI1	0.5651745	0.2227071	2.5377	0.0112	0.631196369	count	1
SLC27A3	0.4708958	0.1060223	4.4415	9.20E-06	0.631296453	count	0.2120416
SLC35D1	0.5389008	0.2596309	2.0756	0.038	0.631398141	count	1
ZFP36L1	0.4425663	0.0579474	7.6374	2.81E-14	0.631771775	count	6.70E-10
AC016405.3	1.6554804	1.223422	1.3532	0.176	0.631817621	count	1
LRRK1	0.7981891	0.3129232	2.5508	0.0108	0.631833209	count	1
PDK4	0.4704582	0.0898489	5.2361	1.73E-07	0.631844416	count	0.004045778
FAM118B	0.5609242	0.242725	2.3109	0.0209	0.631933063	count	1
PYGL	0.5231278	0.1626098	3.2171	0.00131	0.631958008	count	1
PRR3	0.6385567	0.2974037	2.1471	0.0319	0.631984229	count	1
PPP1R12C	0.4872119	0.1605764	3.0341	0.00243	0.632139307	count	1
PPRC1	0.8210195	0.4486222	1.8301	0.0673	0.63245939	count	1
SNHG9	0.4872231	0.1535144	3.1738	0.00152	0.632631565	count	1
BMP2	0.6529346	0.3007826	2.1708	0.03	0.632738797	count	1
SLC25A33	0.6038228	0.204113	2.9583	0.00311	0.633525672	count	1
USP45	0.5960809	0.2661452	2.2397	0.0252	0.633881366	count	1
WWP1	0.5075155	0.2040086	2.4877	0.0129	0.633939083	count	1
CASTOR3	0.7342685	0.2769084	2.6517	0.00804	0.634183235	count	1
SIN3B	0.4935752	0.1760066	2.8043	0.00507	0.63443419	count	1
SGSM2	0.6887431	0.3917584	1.7581	0.0788	0.634680071	count	1
FZD5	0.8246556	0.4458331	1.8497	0.0644	0.634849111	count	1
MTMR14	0.580019	0.2242928	2.586	0.00975	0.635008683	count	1
PRIM2	0.6012167	0.2435627	2.4684	0.0136	0.63505829	count	1
VPS13A	0.4640122	0.2410776	1.9247	0.0543	0.635545022	count	1
SCLT1	0.5367395	0.2243316	2.3926	0.0168	0.635858942	count	1
TRAF7	0.5465261	0.1799817	3.0366	0.00241	0.636197164	count	1
B4GALT1	0.4639558	0.1006516	4.6095	4.17E-06	0.63626717	count	0.09646461
ERAP2	0.516007	0.1810003	2.8509	0.00438	0.636504252	count	1
SMG9	0.6572305	0.2301693	2.8554	0.00432	0.636560462	count	1
KIAA0930	0.4849569	0.1431936	3.3867	0.000715	0.63656118	count	1
SPTLC3	0.724452	0.31614	2.2916	0.022	0.636738638	count	1
SLC38A9	0.7858041	0.3344391	2.3496	0.0188	0.636838461	count	1
ADAMTS15	1.4618195	0.8540317	1.7117	0.087	0.63697766	count	1
GRIN2A	0.7690529	0.2944547	2.6118	0.00904	0.637513742	count	1
CRTC2	0.5828907	0.2936946	1.9847	0.0473	0.637985366	count	1
ZNF574	0.7393533	0.3626376	2.0388	0.0415	0.638077757	count	1

FAM76A	0.4752453	0.1191245	3.9895	6.75E-05	0.63819451	count	1
SLC8B1	0.6282565	0.2650525	2.3703	0.0178	0.63865941	count	1
DCUN1D3	0.5809565	0.2420516	2.4001	0.0164	0.63911086	count	1
USP49	0.6850835	0.7222583	0.9485	0.343	0.639515246	count	1
RTN4RL2	1.210438	0.4791073	2.5264	0.0116	0.639520739	count	1
ERICH1	0.4626625	0.1173425	3.9428	8.20E-05	0.639662039	count	1
THBS3	0.5069679	0.1610256	3.1484	0.00166	0.639860878	count	1
TMEM61	1.6944795	0.8734907	1.9399	0.0525	0.639938886	count	1
PNMA6A	1.0593172	0.6484596	1.6336	0.102	0.639953892	count	1
ZNF324B	1.0593172	0.6840033	1.5487	0.122	0.639953892	count	1
MBLAC2	0.5536872	0.2648818	2.0903	0.0367	0.640150235	count	1
TMCC3	1.0047789	0.5113764	1.9649	0.0495	0.640187968	count	1
AP000547.3	1.0599717	0.469965	2.2554	0.0242	0.640256173	count	1
NCOR2	0.4876617	0.164364	2.967	0.00303	0.640817629	count	1
HCFC2	0.5072145	0.1428374	3.551	0.000389	0.640926928	count	1
CISH	0.9603758	0.3415561	2.8118	0.00495	0.640945752	count	1
FJX1	0.7434077	0.4621498	1.6086	0.108	0.64117756	count	1
MAPK14	0.5018954	0.187243	2.6804	0.00739	0.64125783	count	1
INTS6	0.4676671	0.1243777	3.7601	0.000173	0.641312688	count	1
NPHP1	0.5249629	0.2115682	2.4813	0.0131	0.641630213	count	1
ZNF525	0.8605716	0.6964057	1.2357	0.217	0.641749087	count	1
PANK4	0.7592452	0.3432473	2.2119	0.027	0.642102363	count	1
MIDN	0.4661263	0.1244183	3.7464	0.000182	0.643339676	count	1
CCDC144NL	0.5769337	0.4013752	1.4374	0.151	0.643681056	count	1
TRAF3IP2-AS1	0.5749922	0.2022279	2.8433	0.00449	0.644363033	count	1
EIF2B5	0.5365289	0.1776995	3.0193	0.00255	0.6448137	count	1
MOB3A	0.6828319	0.2934348	2.327	0.02	0.645127124	count	1
DRD1	1.3392125	0.6740184	1.9869	0.047	0.645473375	count	1
ENTPD6	0.6543824	0.3303272	1.981	0.0477	0.646421797	count	1
TLDC1	0.6264627	0.3981347	1.5735	0.116	0.646691424	count	1
TMEM246	0.5137378	0.1945765	2.6403	0.00832	0.647411411	count	1
MFSD5	0.5578013	0.2360523	2.363	0.0182	0.648786692	count	1
ETFDH	0.5162423	0.184344	2.8004	0.00513	0.648866988	count	1
FAM118A	0.4608104	0.1251394	3.6824	0.000234	0.649115916	count	1
MX2	0.5620828	0.3085273	1.8218	0.0686	0.649446029	count	1
RGS11	0.6796951	0.2694607	2.5224	0.0117	0.649594219	count	1
TMEM220-AS1	1.0246336	0.7654621	1.3386	0.181	0.650066053	count	1
MTHFR	0.557674	0.2292561	2.4325	0.015	0.650606811	count	1
MTHFSD	0.6212411	0.3080804	2.0165	0.0438	0.650622674	count	1
TMEM200B	0.6260617	0.2650489	2.3621	0.0182	0.650904172	count	1
MLLT3	0.5091535	0.2137677	2.3818	0.0173	0.650969389	count	1
NUPR1	0.4534277	0.0388746	11.6639	6.80E-31	0.651035246	count	1.64E-26
CUEDC1	0.5264185	0.2123507	2.479	0.0132	0.651075471	count	1
ZNF662	0.9055859	0.4228816	2.1415	0.0323	0.65131184	count	1
EPHB6	0.5149296	0.178746	2.8808	0.00399	0.651953307	count	1

ATRIP	0.7894384	0.4610061	1.7124	0.0869	0.652237821	count	1
STOX1	2.1619515	1.0864803	1.9899	0.0467	0.652419322	count	1
GPR180	0.5650713	0.2430822	2.3246	0.0201	0.652751445	count	1
SLF1	0.6074659	0.22423	2.7091	0.00678	0.65286291	count	1
SLC9A8	0.6761301	0.3988278	1.6953	0.0901	0.653312437	count	1
BIVM	0.6388587	0.3766732	1.6961	0.09	0.653723303	count	1
SNX21	0.4903414	0.1125653	4.3561	1.36E-05	0.654211949	count	0.313072
SYCE1	1.250304	1.369505	0.913	0.361	0.654248224	count	1
FKBP9	0.4761009	0.0866504	5.4945	4.18E-08	0.65431007	count	0.000980586
VRK3	0.5965543	0.2141815	2.7853	0.00538	0.65534506	count	1
CC2D2A	0.5077984	0.1286359	3.9476	8.04E-05	0.656258447	count	1
EGFR	0.5001471	0.1332798	3.7526	0.000178	0.657334235	count	1
PMS1	0.5283682	0.1802174	2.9318	0.00339	0.65740196	count	1
ANKRD9	0.5149323	0.1603678	3.2109	0.00133	0.657543312	count	1
PRIM1	0.6483558	0.2826384	2.2939	0.0219	0.657597619	count	1
TMEM175	0.5235276	0.1569643	3.3353	0.00086	0.657789887	count	1
G0S2	0.4736328	0.128012	3.6999	0.000219	0.6581475	count	1
TCF12	0.4922619	0.1309252	3.7599	0.000173	0.658571525	count	1
MPRIP-AS1	0.817012	0.4610253	1.7722	0.0765	0.658654809	count	1
FUT4	0.7091572	0.3056616	2.3201	0.0204	0.659833416	count	1
AC016831.7	0.6074934	0.2388752	2.5431	0.011	0.660013925	count	1
ADIPOR2	0.5268094	0.1756882	2.9985	0.00273	0.660088057	count	1
SMG1	0.5027	0.1667356	3.015	0.00259	0.660243709	count	1
GSE1	0.5685576	0.2988428	1.9025	0.0572	0.66077281	count	1
GPR20	0.7693512	0.2760163	2.7873	0.00534	0.660896127	count	1
CYB561	0.5587339	0.2047269	2.7292	0.00638	0.660925728	count	1
MARK3	0.4907506	0.1630111	3.0105	0.00263	0.660956977	count	1
ANPEP	0.6579659	0.2293416	2.8689	0.00414	0.661187578	count	1
CYP1B1-AS1	1.5555371	0.8319108	1.8698	0.0616	0.661389561	count	1
IFRD1	0.4740014	0.0920159	5.1513	2.72E-07	0.661893469	count	0.006353104
APOL3	0.5757031	0.1431629	4.0213	5.90E-05	0.662298386	count	1
KBTBD2	0.5295708	0.2382365	2.2229	0.0263	0.662569041	count	1
KDSR	0.4749604	0.0835526	5.6846	1.41E-08	0.66327271	count	0.000331505
ZNF512B	0.745938	0.3015818	2.4734	0.0134	0.663430234	count	1
C8orf37	0.6802635	0.2753487	2.4706	0.0135	0.663555334	count	1
TMEM182	1.0032413	0.6281915	1.597	0.11	0.663631789	count	1
GALC	0.6350545	0.2543892	2.4964	0.0126	0.664126169	count	1
SCN4B	2.2698643	1.0483567	2.1652	0.0304	0.665148597	count	1
KDM6B	0.5641555	0.214956	2.6245	0.00871	0.665332421	count	1
ACP2	0.620158	0.20739	2.9903	0.00281	0.665678375	count	1
FLCN	0.6469368	0.2676764	2.4169	0.0157	0.666337668	count	1
SMPD2	0.9315719	0.3377246	2.7584	0.00584	0.666638646	count	1
ZNF396	0.7925737	0.3970969	1.9959	0.046	0.666734632	count	1
SOX9	0.577833	0.254083	2.2742	0.023	0.666845573	count	1
AC008915.2	0.9320874	0.4867071	1.9151	0.0556	0.66694063	count	1

CLCN7	0.588859	0.3285228	1.7924	0.0731	0.66709685	count	1
ZNF676	0.6021771	0.2737833	2.1995	0.0279	0.667403737	count	1
INMT	0.4718988	0.0718447	6.5683	5.80E-11	0.667655644	count	1.38E-06
CARF	0.5791505	0.3178869	1.8219	0.0686	0.668298679	count	1
KLHL36	0.5329908	0.1913112	2.786	0.00536	0.669368525	count	1
GALNT16	0.7111933	0.2175246	3.2695	0.00109	0.66939681	count	1
SMARCAD1	0.5177642	0.173743	2.9801	0.0029	0.66941513	count	1
QSER1	0.5234689	0.1702215	3.0752	0.00212	0.669510398	count	1
NDFIP2	0.5428209	0.1699329	3.1943	0.00141	0.669686989	count	1
STARD9	0.5681457	0.2819852	2.0148	0.044	0.669851817	count	1
HDAC10	1.8505594	0.7509689	2.4642	0.0138	0.670004605	count	1
ALG1	0.6879021	0.2612216	2.6334	0.00849	0.67036801	count	1
PTGS1	0.6884406	0.3646755	1.8878	0.0591	0.670847681	count	1
YIPF1	0.6219111	0.2639045	2.3566	0.0185	0.671159657	count	1
RNF146	0.490862	0.1005102	4.8837	1.09E-06	0.671209818	count	0.02534577
A4GALT	0.4868431	0.0937471	5.1932	2.18E-07	0.671703992	count	0.005095532
GRK4	0.8363382	0.3872855	2.1595	0.0309	0.672016802	count	1
RPUSD2	0.6266494	0.2954379	2.1211	0.034	0.672217494	count	1
TRIM7	0.639055	0.2364285	2.703	0.0069	0.672408122	count	1
DHRSX	0.5479515	0.1680666	3.2603	0.00112	0.672432026	count	1
GDPD3	1.3015459	0.8988701	1.448	0.148	0.672608811	count	1
FBXO2	0.5571563	0.2303985	2.4182	0.0156	0.673060985	count	1
ZNF251	0.6359219	0.2973196	2.1388	0.0325	0.673537924	count	1
IBA57	0.861303	0.3538861	2.4338	0.015	0.674321553	count	1
RNLS	0.6557242	0.3090102	2.122	0.0339	0.67473594	count	1
VASN	0.4773023	0.0704664	6.7735	1.46E-11	0.674951459	count	3.47E-07
RGS10	0.4857824	0.1015101	4.7856	1.77E-06	0.675972106	count	0.04108524
NRIP2	0.750155	0.2356948	3.1827	0.00147	0.676278309	count	1
EVC	0.5791334	0.3101961	1.867	0.062	0.676578269	count	1
QRSL1	0.6577595	0.2506151	2.6246	0.00871	0.6766782	count	1
TCTN1	0.5099508	0.1349665	3.7784	0.00016	0.677328438	count	1
TMEM71	4.083091	1.3295262	3.0711	0.00215	0.677573028	count	1
LIN7A	0.7771819	0.2956626	2.6286	0.00861	0.677666066	count	1
AC108734.4	1.3166795	0.6453411	2.0403	0.0414	0.677910346	count	1
NUP133	0.7651693	0.2534851	3.0186	0.00256	0.678596348	count	1
SLC29A1	0.4889991	0.087518	5.5874	2.47E-08	0.678956154	count	0.000580178
APOL6	0.5031811	0.1372381	3.6665	0.000249	0.679162375	count	1
AP001160.1	0.6078125	0.3300801	1.8414	0.0656	0.679231312	count	1
FAM102B	0.567798	0.2528661	2.2454	0.0248	0.679489058	count	1
DUS3L	0.6561955	0.312741	2.0982	0.036	0.680026664	count	1
ZNF565	0.7441259	0.3385023	2.1983	0.028	0.680436069	count	1
ANKDD1A	0.5969805	0.2711294	2.2018	0.0277	0.680875318	count	1
NFE2L2	0.4780719	0.0522305	9.1531	8.96E-20	0.681459589	count	2.15E-15
FBXO27	0.6321356	0.2599025	2.4322	0.0151	0.681517686	count	1
MGARP	0.5384744	0.2527759	2.1302	0.0332	0.682554352	count	1

PRRT2	0.5397387	0.1805795	2.9889	0.00282	0.682571422	count	1
PLSCR4	0.4896421	0.0851153	5.7527	9.50E-09	0.68299138	count	0.000223497
HCG11	0.5549891	0.1499099	3.7022	0.000217	0.683111432	count	1
MLH3	0.5191342	0.1400699	3.7063	0.000213	0.683415983	count	1
AC103591.3	1.1566978	0.4485714	2.5786	0.00996	0.683642141	count	1
GRN	0.4862133	0.0680826	7.1415	1.11E-12	0.68365386	count	2.64E-08
ZNF800	0.5246367	0.1390592	3.7728	0.000164	0.683695679	count	1
EPHB2	1.649412	0.5161335	3.1957	0.00141	0.684157021	count	1
NSUN2	0.6562952	0.2833637	2.3161	0.0206	0.684794209	count	1
SERPINB9	0.5849077	0.3010442	1.9429	0.0521	0.685005267	count	1
C12orf49	0.5418023	0.1760444	3.0776	0.0021	0.685114249	count	1
REV3L	0.5234533	0.1434823	3.6482	0.000268	0.686182357	count	1
CYBA	0.4821695	0.0581207	8.296	1.49E-16	0.686543868	count	3.56E-12
RPP25	0.5815218	0.2544496	2.2854	0.0223	0.686798287	count	1
MRC2	0.4922008	0.1015474	4.847	1.31E-06	0.68695232	count	0.03044047
LDLR	0.7635411	0.3216448	2.3739	0.0177	0.687007478	count	1
CACTIN	0.7765516	0.4145097	1.8734	0.0611	0.687520396	count	1
CPD	0.6010379	0.2317833	2.5931	0.00955	0.687711181	count	1
FOXD1	0.5612954	0.1447822	3.8768	0.000108	0.68828041	count	1
SPAST	0.6351894	0.2021151	3.1427	0.00169	0.688287377	count	1
TEP1	0.7541597	0.5574725	1.3528	0.176	0.688629251	count	1
FNDC10	1.9615537	0.9954792	1.9705	0.0489	0.689157814	count	1
NNAT	1.9615537	1.1125016	1.7632	0.078	0.689157814	count	1
ZHX3	0.5238384	0.1610038	3.2536	0.00115	0.68988453	count	1
VWA5A	0.5609028	0.2131414	2.6316	0.00853	0.690160011	count	1
CTSA	0.4940248	0.0851161	5.8041	7.02E-09	0.690919812	count	0.000165237
ERMP1	1.6816969	0.8484422	1.9821	0.0475	0.691611453	count	1
AC116667.1	0.826782	0.5943162	1.3911	0.164	0.691666728	count	1
CTSF	0.4868495	0.0558164	8.7223	4.06E-18	0.691756193	count	9.73E-14
LRRC8D	1.2552273	0.4267565	2.9413	0.00329	0.691762215	count	1
GHR	0.5732942	0.1855514	3.0897	0.00202	0.691857226	count	1
HAGHL	1.4924642	0.6285628	2.3744	0.0176	0.691869484	count	1
AP3S2	0.6852146	0.3044183	2.2509	0.0245	0.692044978	count	1
AC006504.5	1.0150443	0.5131639	1.978	0.048	0.692806584	count	1
MAP3K11	0.6802597	0.3624179	1.877	0.0606	0.692854328	count	1
NCOA7	0.4977638	0.0861702	5.7765	8.26E-09	0.693013297	count	0.000194374
METTL15	0.5575077	0.1836747	3.0353	0.00242	0.693601998	count	1
RELT	1.989399	1.2156579	1.6365	0.102	0.69369063	count	1
OMG	1.989399	1.4179318	1.403	0.161	0.69369063	count	1
ZBTB7C	0.6875736	0.2760721	2.4906	0.0128	0.694237081	count	1
MCEE	0.5427883	0.1458814	3.7208	0.000202	0.694306173	count	1
BRAT1	0.6117823	0.2055009	2.977	0.00293	0.69437265	count	1
KIAA0513	1.0638279	0.560106	1.8993	0.0576	0.694788652	count	1
AC099489.1	16.239958	1654.685696	0.0098	0.992	0.695276671	count	1
NWD1	16.6655261	1397.84909	0.0119	0.99	0.695276698	count	1

TDRD5	16.9944744	2535.949642	0.0067	0.995	0.695276713	count	1
FHDC1	17.1542634	638.5080727	0.0269	0.9786	0.695276718	count	1
ZFHX2	17.156172	1036.326636	0.0166	0.987	0.695276718	count	1
CYP27C1	17.2355929	1249.469498	0.0138	0.989	0.695276721	count	1
FAM160A1	17.2379625	1678.635392	0.0103	0.992	0.695276721	count	1
CALCA	17.2773533	2580.637426	0.0067	0.995	0.695276722	count	1
AC009242.1	17.4973324	2225.105991	0.0079	0.994	0.695276727	count	1
SDR42E1	17.4977576	2524.315464	0.0069	0.994	0.695276727	count	1
AC022762.2	17.4983247	2661.891768	0.0066	0.995	0.695276727	count	1
ALKAL1	17.5000062	3575.783997	0.0049	0.996	0.695276727	count	1
ST6GALNAC2	17.5000062	3575.78383	0.0049	0.996	0.695276727	count	1
LINC02511	18.3068447	1375.236876	0.0133	0.989	0.69527674	count	1
DUXA	16.4805364	1033.942769	0.0159	0.987	0.695276743	count	1
SHISA6	16.6111579	1037.279852	0.016	0.987	0.695276751	count	1
AP003501.2	16.8258407	2046.218037	0.0082	0.993	0.695276761	count	1
ICAM4	16.8938307	1213.529946	0.0139	0.989	0.695276764	count	1
LRP1B	17.0145798	746.6779371	0.0228	0.9818	0.695276769	count	1
FERMT1	17.1793545	646.6870008	0.0266	0.9788	0.695276774	count	1
AL139022.2	17.4895543	2935.208151	0.006	0.995	0.695276782	count	1
RIPPLY1	17.5000062	3575.783894	0.0049	0.996	0.695276783	count	1
LINC02406	17.5000062	3575.783894	0.0049	0.996	0.695276783	count	1
ADGRG4	17.9347044	1317.032464	0.0136	0.9891	0.695276791	count	1
SLC17A7	17.9383846	978.9728135	0.0183	0.9854	0.695276791	count	1
DSCAML1	18.2255323	1787.536681	0.0102	0.992	0.695276794	count	1
ABCC10	1.1200513	0.4864678	2.3024	0.0214	0.695985901	count	1
AMMECR1L	0.7876308	0.3162506	2.4905	0.0128	0.696169317	count	1
ARHGEF3	1.066989	0.3076884	3.4678	0.000531	0.69638518	count	1
TSC22D3	0.4892472	0.049748	9.8345	1.52E-22	0.696447669	count	3.66E-18
TTC39C	0.6469699	0.2563403	2.5239	0.0116	0.696499753	count	1
TET2	0.547276	0.1816386	3.013	0.0026	0.697139378	count	1
CDKN2AIP	0.5242462	0.1340558	3.9107	9.37E-05	0.697267484	count	1
SLC11A2	0.6171551	0.2049957	3.0106	0.00263	0.697513679	count	1
ZCCHC24	0.5370734	0.1464251	3.6679	0.000248	0.699091828	count	1
COL8A2	0.5533302	0.1754472	3.1538	0.00162	0.699307887	count	1
FBXW8	0.8766265	0.350147	2.5036	0.0123	0.699504798	count	1
SESTD1	0.5116437	0.0832882	6.1431	8.96E-10	0.699874618	count	2.12E-05
ARSA	0.5457114	0.1623012	3.3623	0.000781	0.699920629	count	1
ACVR2A	0.8389281	0.2640331	3.1774	0.0015	0.700433217	count	1
PDGFRA	0.4998447	0.0837582	5.9677	2.63E-09	0.700538909	count	6.20E-05
SEMA3D	0.5760628	0.2024681	2.8452	0.00446	0.700650164	count	1
PRRX1	0.4908536	0.0506376	9.6935	5.88E-22	0.700912123	count	1.41E-17
MECR	0.6155064	0.2305707	2.6695	0.00763	0.700952028	count	1
MIATNB	1.0768711	0.5149993	2.091	0.0366	0.701357421	count	1
TGFBR2	0.4989134	0.0746704	6.6815	2.72E-11	0.701642179	count	6.45E-07
INSIG1	0.5781416	0.2608421	2.2164	0.0267	0.701734357	count	1

ARHGAP20	0.6192131	0.2383053	2.5984	0.0094	0.702375445	count	1
ZC3HAV1L	0.7957071	0.4646717	1.7124	0.0869	0.702450692	count	1
ZNF813	1.3890356	0.5983845	2.3213	0.0203	0.702507135	count	1
AHI1	0.4997214	0.0953202	5.2426	1.67E-07	0.702648995	count	0.003905629
PLBD2	0.5746892	0.1693574	3.3934	0.000698	0.70291583	count	1
TBC1D2B	0.5226275	0.1369257	3.8169	0.000137	0.70331738	count	1
AC016831.5	0.7106425	0.3848175	1.8467	0.0649	0.703561217	count	1
LDB1	0.5766132	0.2632466	2.1904	0.0286	0.703917649	count	1
WIZ	0.6816182	0.2450096	2.782	0.00543	0.70441046	count	1
GPR153	1.542107	0.3931301	3.9226	8.92E-05	0.705820312	count	1
WDR90	1.0870396	0.6038872	1.8001	0.0719	0.706444443	count	1
TNFRSF21	1.2939908	0.4825954	2.6813	0.00737	0.706562977	count	1
GTPBP10	0.5709317	0.2083303	2.7405	0.00616	0.706620121	count	1
STOM	0.503645	0.0701247	7.1821	8.27E-13	0.706822543	count	1.97E-08
AUH	0.5702611	0.1822949	3.1282	0.00177	0.706892639	count	1
OSMR	0.5445119	0.1245214	4.3728	1.26E-05	0.707565249	count	0.290115
SMPD1	0.5637819	0.1625171	3.4691	0.000528	0.707855091	count	1
MOCS3	0.8892072	0.3466515	2.5651	0.0104	0.707986232	count	1
NAPB	1.2139576	0.5819865	2.0859	0.0371	0.708132649	count	1
SLC27A1	0.7231167	0.2840593	2.5457	0.0109	0.708321491	count	1
ARHGEF35	0.9136342	0.4193395	2.1787	0.0294	0.708629718	count	1
BCKDHB	0.5891002	0.1792286	3.2869	0.00102	0.708714294	count	1
NECTIN1	1.0926639	0.5417967	2.0167	0.0438	0.709245328	count	1
AACS	1.0458193	0.3186179	3.2824	0.00104	0.709308052	count	1
NEAT1	0.4933459	0.1026763	4.8049	1.61E-06	0.709311831	count	0.03738581
KCP	2.0916554	1.5649773	1.3365	0.181	0.709446733	count	1
TMCO6	0.9150117	0.4487573	2.039	0.0415	0.709521453	count	1
TMEM9B	0.5211804	0.108962	4.7831	1.79E-06	0.709867668	count	0.04154232
CAMK1D	0.561221	0.2308973	2.4306	0.0151	0.71062869	count	1
ARL17B	0.7702771	0.4332329	1.778	0.0755	0.710658394	count	1
FNIP1	0.5235096	0.1168094	4.4817	7.63E-06	0.710852497	count	0.17600884
C1orf52	0.5255848	0.1185863	4.4321	9.61E-06	0.711242902	count	0.22144323
PCDHB5	0.8372146	0.4515102	1.8543	0.0638	0.711513365	count	1
NCSTN	0.5529545	0.1464217	3.7765	0.000162	0.711517275	count	1
ARMH4	0.6188758	0.2975938	2.0796	0.0376	0.711940462	count	1
Z93930.2	0.6586109	0.3672007	1.7936	0.073	0.712050307	count	1
G3BP1	0.5064649	0.1207458	4.1945	2.80E-05	0.712083943	count	0.641928
MFAP3	1.0119245	0.4745846	2.1322	0.0331	0.712751557	count	1
ANKRD53	2.1163697	1.1485461	1.8427	0.0655	0.713052746	count	1
ATXN2L	0.7079755	0.2853749	2.4809	0.0132	0.713131608	count	1
PTPRF	0.5741633	0.2033294	2.8238	0.00477	0.713689441	count	1
CGNL1	0.5480945	0.1615738	3.3922	0.000701	0.714132365	count	1
TTC30B	0.858152	0.386334	2.2213	0.0264	0.714215493	count	1
RIC1	0.6411999	0.281988	2.2739	0.023	0.714448671	count	1
CPEB3	0.7986787	0.4407909	1.8119	0.0701	0.714915207	count	1

NGF	0.5811532	0.1744355	3.3316	0.000872	0.715438416	count	1
MAN1A1	0.5363682	0.1090486	4.9186	9.10E-07	0.715552082	count	0.02117843
INMT-MINDY4	1.0579376	0.5448071	1.9419	0.0522	0.715729218	count	1
TNS2	0.5252968	0.1115927	4.7073	2.60E-06	0.716033987	count	0.0602654
FGL2	0.4991753	0.0770303	6.4802	1.04E-10	0.71662876	count	2.46E-06
SEMA4A	0.6797388	0.3272746	2.077	0.0379	0.716686322	count	1
AL359541.1	1.7983824	1.2508037	1.4378	0.151	0.717022745	count	1
PARP6	0.9039868	0.3449292	2.6208	0.00881	0.717887805	count	1
VASH2	2.8818891	1.3725615	2.0996	0.0358	0.717992745	count	1
MGLL	0.513436	0.0803334	6.3913	1.85E-10	0.718675518	count	4.38E-06
MCL1	0.5111772	0.0773693	6.607	4.49E-11	0.718835047	count	1.06E-06
IKZF5	0.6348668	0.3045131	2.0849	0.0372	0.719193619	count	1
TPBG	0.5346202	0.1278498	4.1816	2.96E-05	0.719231999	count	0.6784024
IL20RB	2.905401	1.2371811	2.3484	0.0189	0.719487677	count	1
AL049775.1	2.1642806	1.2280584	1.7624	0.0781	0.719828345	count	1
CD79B	1.0658977	0.5910963	1.8033	0.0714	0.719923519	count	1
GCC1	0.7816605	0.4766411	1.6399	0.101	0.720002265	count	1
SYNGR1	0.5237628	0.1155747	4.5318	6.03E-06	0.720209581	count	0.13923873
SLC22A18	0.7621299	0.2879056	2.6472	0.00815	0.720494328	count	1
ADGRB3	0.6474882	0.2919855	2.2175	0.0266	0.721052519	count	1
CMTM3	0.538499	0.1064566	5.0584	4.44E-07	0.721132224	count	0.010358076
TNNC2	0.7538562	0.3293829	2.2887	0.0222	0.72113942	count	1
NABP1	0.5963007	0.2301711	2.5907	0.00962	0.721530805	count	1
EML1	0.5578846	0.1544984	3.6109	0.000309	0.721847694	count	1
TKFC	0.8355157	0.3372078	2.4777	0.0133	0.721965307	count	1
AC068987.5	2.9470676	1.2331414	2.3899	0.0169	0.722057318	count	1
ZNF35	0.7640657	0.3058039	2.4985	0.0125	0.722135506	count	1
SGPL1	0.8358375	0.3563616	2.3455	0.0191	0.722206803	count	1
AC005261.1	0.5949067	0.2250633	2.6433	0.00825	0.722737769	count	1
KDM4D	1.606888	1.1547982	1.3915	0.164	0.723263129	count	1
ABCA2	0.6591105	0.2789784	2.3626	0.0182	0.723364378	count	1
AC007038.1	1.8304125	1.6923684	1.0816	0.2795	0.723592884	count	1
AC007906.2	1.3400007	0.5814485	2.3046	0.0212	0.723640532	count	1
LONRF2	1.3403016	0.4620797	2.9006	0.00375	0.72375045	count	1
ISCA2	0.5595189	0.1609154	3.4771	0.000513	0.724475682	count	1
KNOP1	0.5451561	0.1603439	3.3999	0.000681	0.725879379	count	1
SPEF2	0.6942078	0.2642872	2.6267	0.00866	0.726189042	count	1
GSTM3	0.5167893	0.0736072	7.0209	2.61E-12	0.726295886	count	6.20E-08
SLC25A21	1.845981	1.033172	1.7867	0.0741	0.72672556	count	1
ATP6V1B2	0.5712297	0.1449602	3.9406	8.28E-05	0.726859956	count	1
SCAF4	0.6138627	0.243622	2.5197	0.0118	0.727076695	count	1
ELMSAN1	0.5611442	0.1459129	3.8457	0.000122	0.727636018	count	1
LPCAT4	0.6003145	0.2114607	2.8389	0.00455	0.727649256	count	1
PPFIBP2	0.7243469	0.2724262	2.6589	0.00787	0.728210116	count	1
LAMP2	0.5221707	0.0740983	7.047	2.17E-12	0.728229316	count	5.16E-08

PNPLA6	0.745688	0.3194236	2.3345	0.0196	0.728357422	count	1
FAM180A	0.5513137	0.1740121	3.1682	0.00155	0.728865371	count	1
MEGF6	0.532232	0.1823851	2.9182	0.00354	0.729209815	count	1
LINC00294	0.9215865	0.3956898	2.3291	0.0199	0.729590648	count	1
CLCN3	0.5567771	0.1381563	4.0301	5.69E-05	0.730333089	count	1
ACADS	0.588162	0.1932507	3.0435	0.00235	0.730537953	count	1
AL645608.1	0.7409663	0.5019833	1.4761	0.14	0.730848424	count	1
GLB1	0.5797578	0.1528922	3.7919	0.000152	0.730913073	count	1
SPG11	0.640905	0.2110516	3.0367	0.00241	0.730968395	count	1
SLC43A1	1.089146	0.3762469	2.8948	0.00382	0.73206687	count	1
PLA2G4A	0.76673	0.3062909	2.5033	0.0123	0.732205037	count	1
HAND2-AS1	0.6084902	0.2429052	2.5051	0.0123	0.732685311	count	1
UBL3	0.5430467	0.0951832	5.7053	1.25E-08	0.732895498	count	0.000293975
CNTNAP3B	0.6339474	0.3262666	1.943	0.0521	0.733122113	count	1
SLC38A7	1.2749557	0.5066272	2.5166	0.0119	0.733260515	count	1
AFDN	0.547175	0.1536925	3.5602	0.000375	0.733371882	count	1
RN7SL832P	0.9523823	0.4421406	2.154	0.0313	0.73349042	count	1
NAT1	1.0927115	0.5542999	1.9713	0.0488	0.733914948	count	1
FAM57A	0.8228741	0.3620671	2.2727	0.0231	0.733915132	count	1
ZNF93	0.9075385	0.9307605	0.9751	0.33	0.735044661	count	1
EFS	0.5947853	0.1904613	3.1229	0.0018	0.735169342	count	1
CYP4V2	0.6178548	0.2159803	2.8607	0.00425	0.735194732	count	1
THBS4	1.3719893	0.8737388	1.5703	0.116	0.735203975	count	1
HSPA1B	0.5137595	0.0733058	7.0084	2.86E-12	0.735904567	count	6.80E-08
RABGAP1L	0.5835702	0.1405634	4.1516	3.38E-05	0.73644788	count	0.7741552
PLD3	0.5209033	0.0626644	8.3126	1.30E-16	0.736581238	count	3.11E-12
DPY19L3	0.7104172	0.3295137	2.156	0.0312	0.736920814	count	1
GPHA2	2.295035	1.3314382	1.7237	0.0848	0.736945226	count	1
GNPTG	0.5335825	0.0834347	6.3952	1.81E-10	0.73705549	count	4.29E-06
TMEM185A	0.63778	0.2095717	3.0433	0.00236	0.737334485	count	1
ARL4A	0.5659989	0.1981546	2.8564	0.00431	0.737965905	count	1
HSD17B7	0.7064485	0.2046945	3.4512	0.000564	0.738016775	count	1
DZIP1	0.6343383	0.2537281	2.5001	0.0125	0.738035015	count	1
SRRD	0.6638855	0.2253601	2.9459	0.00324	0.73822866	count	1
SLC35E3	0.5670163	0.1476803	3.8395	0.000125	0.738258192	count	1
TDRP	1.2883001	0.4658668	2.7654	0.00571	0.738626871	count	1
C2orf72	1.288815	0.9197142	1.4013	0.161	0.738832948	count	1
STARD5	0.8748731	0.3904306	2.2408	0.0251	0.738993117	count	1
DPY19L2	1.6693467	0.7422027	2.2492	0.0246	0.739287322	count	1
PHYKPL	0.5618295	0.1349555	4.1631	3.21E-05	0.73937689	count	0.7353147
TNKS1BP1	0.5860315	0.2107147	2.7812	0.00544	0.739464788	count	1
FOSL2	0.5741715	0.1337734	4.2921	1.82E-05	0.74065174	count	0.418327
L1TD1	0.8773139	0.8307045	1.0561	0.291	0.740759076	count	1
ZNF454	0.8775875	0.4523289	1.9402	0.0524	0.740956896	count	1
SLC39A8	0.6960801	0.2575679	2.7025	0.00691	0.741495154	count	1

TLR3	0.6466193	0.2116476	3.0552	0.00227	0.742217207	count	1
FBLN5	0.5207013	0.0620732	8.3885	6.94E-17	0.742906358	count	1.66E-12
CXCL2	0.5392408	0.253523	2.127	0.0335	0.743130988	count	1
DPYD	0.5905282	0.1688786	3.4968	0.000477	0.743202362	count	1
ZNF816	1.2999108	0.617776	2.1042	0.0354	0.743258088	count	1
NYNRIN	0.7632565	0.3260712	2.3408	0.0193	0.743851119	count	1
WDR19	0.6339609	0.3072926	2.0631	0.0392	0.743936755	count	1
CHKA	0.9016374	0.5190471	1.7371	0.0825	0.744970488	count	1
IL15RA	0.590656	0.1567189	3.7689	0.000167	0.745130743	count	1
SIRT1	0.6329028	0.2115329	2.992	0.00279	0.746713062	count	1
SLITRK4	0.7014812	0.3481604	2.0148	0.044	0.746832571	count	1
KIAA1614	0.8535256	0.5732123	1.489	0.137	0.746838774	count	1
INPP5E	1.0361001	0.45516	2.2763	0.0229	0.747135653	count	1
LPCAT1	0.6463773	0.2549395	2.5354	0.0113	0.749090936	count	1
ABHD6	0.9514014	0.379058	2.5099	0.0121	0.749196809	count	1
GNPTAB	0.5673805	0.1590691	3.5669	0.000366	0.750088941	count	1
FOXO1	0.5793579	0.1382063	4.192	2.83E-05	0.750711146	count	0.6487492
SERTAD1	0.5301334	0.0726143	7.3007	3.49E-13	0.751088235	count	8.31E-09
NOL6	0.7986905	0.4575619	1.7455	0.081	0.751303094	count	1
ZBTB20-AS2	1.1274239	0.4571466	2.4662	0.0137	0.751714167	count	1
FANK1	1.419498	1.0496933	1.3523	0.176	0.751915529	count	1
SOAT1	0.5939816	0.1739472	3.4147	0.000645	0.752602224	count	1
LRRN1	1.246042	1.0931343	1.1399	0.254	0.752724346	count	1
FGD4	0.5829579	0.1372199	4.2483	2.21E-05	0.753525286	count	0.5074381
AL161457.2	1.1311362	0.5887839	1.9211	0.0548	0.753596806	count	1
KLF10	0.5465753	0.1096902	4.9829	6.55E-07	0.753760179	count	0.01526805
ACLY	0.6258125	0.1707944	3.6641	0.000252	0.7542754	count	1
COPG1	0.5868801	0.1754219	3.3455	0.000829	0.75473912	count	1
RTN4	0.5275609	0.0404155	13.0534	4.24E-38	0.755022465	count	1.03E-33
POLR2J3	0.8376133	0.8545102	0.9802	0.327	0.755604431	count	1
C2orf81	1.3317387	0.6170664	2.1582	0.031	0.755773402	count	1
PROS1	0.539129	0.0840391	6.4152	1.59E-10	0.756017174	count	3.77E-06
PPP2R2A	0.5518962	0.0970196	5.6885	1.38E-08	0.756390422	count	0.000324507
DPYSL2	0.541653	0.0784241	6.9067	5.82E-12	0.757396147	count	1.38E-07
CORO2B	1.7450618	0.5598402	3.1171	0.00184	0.757703583	count	1
CCNH	0.5584807	0.1130897	4.9384	8.23E-07	0.757727483	count	0.019162732
TEPSIN	0.8403389	0.422014	1.9913	0.0465	0.757756233	count	1
IRAK3	0.5877734	0.1684812	3.4887	0.000491	0.757768296	count	1
TNKS2	0.5775268	0.1353481	4.267	2.03E-05	0.758329983	count	0.4663925
ZNF354C	0.8424228	0.4004171	2.1039	0.0355	0.759399913	count	1
EBF1	0.5469306	0.0838483	6.5229	7.84E-11	0.760207918	count	1.86E-06
NBPF9	0.96836	0.6209641	1.5594	0.119	0.760225461	count	1
STRN	0.5958276	0.1924288	3.0964	0.00197	0.760353238	count	1
GPR1	2.5025544	0.9661941	2.5901	0.00963	0.760359411	count	1
SLC15A4	0.5803118	0.1509378	3.8447	0.000123	0.760564788	count	1

AP001528.2	0.5501496	0.1163347	4.729	2.34E-06	0.760571075	count	0.05425524
ATP6V1G2	2.0297636	0.9767462	2.0781	0.0378	0.760827355	count	1
PARPBP	1.7616112	0.7480038	2.3551	0.0186	0.761585692	count	1
SLC43A2	0.9704855	0.5820583	1.6673	0.0955	0.761601418	count	1
ASB13	0.6776249	0.4193482	1.6159	0.106	0.761906324	count	1
CD74	0.5331431	0.1109668	4.8045	1.61E-06	0.762078886	count	0.03738581
RNF44	1.2030326	0.4358006	2.7605	0.0058	0.762374375	count	1
PER1	0.5868293	0.1437575	4.0821	4.56E-05	0.762399243	count	1
EPS8L1	1.0644707	0.5523079	1.9273	0.054	0.763291164	count	1
DRAM1	0.5914568	0.1430989	4.1332	3.66E-05	0.76363776	count	0.837957
MAU2	0.67405	0.2499414	2.6968	0.00703	0.763892231	count	1
PXYLP1	0.76356	0.3266156	2.3378	0.0195	0.76402073	count	1
ZNF649	0.679776	0.2634871	2.5799	0.00992	0.764181279	count	1
DLX5	0.537139	0.1218844	4.407	1.08E-05	0.764882064	count	0.2487564
ARL5B	0.6750843	0.2118774	3.1862	0.00145	0.764997179	count	1
CABP1	1.460105	0.4084886	3.5744	0.000356	0.765767624	count	1
MON2	0.5948798	0.1700199	3.4989	0.000473	0.767328986	count	1
FAM171B	0.7904805	0.2251785	3.5105	0.000453	0.767682926	count	1
PRKAR2B	0.683502	0.1795751	3.8062	0.000143	0.768119415	count	1
BRPF1	0.898049	0.3917268	2.2925	0.0219	0.768293128	count	1
AC007952.4	0.7114631	0.3494152	2.0362	0.0418	0.769213964	count	1
PLCG2	0.5395573	0.1358385	3.972	7.26E-05	0.770541277	count	1
ASXL2	0.6780888	0.2648156	2.5606	0.0105	0.770993895	count	1
CDC6	2.6180527	1.1271017	2.3228	0.0202	0.771618571	count	1
LRRC6	1.3753393	0.5160178	2.6653	0.00773	0.772493424	count	1
UBTD2	0.5990055	0.1754529	3.4141	0.000647	0.772517166	count	1
PLEKHA5	0.5606365	0.0943948	5.9393	3.13E-09	0.772646424	count	7.38E-05
PRMT6	0.8163812	0.4761448	1.7146	0.0865	0.774424507	count	1
CLEC16A	1.2975794	0.5437131	2.3865	0.0171	0.774677164	count	1
RAD51B	0.9665367	0.4345124	2.2244	0.0262	0.774795979	count	1
HELZ	0.6004053	0.184136	3.2607	0.00112	0.775520443	count	1
SETD4	0.6623326	0.2755292	2.4039	0.0163	0.775599296	count	1
DDI2	0.7355668	0.3708976	1.9832	0.0474	0.775715202	count	1
CLSTN3	0.9471186	0.3465328	2.7331	0.0063	0.776507429	count	1
IPO11	0.994839	0.4023503	2.4726	0.0135	0.777266208	count	1
ANKS1A	0.6420161	0.2030419	3.162	0.00158	0.777673876	count	1
RBM12	0.6874286	0.2304612	2.9828	0.00287	0.778165594	count	1
PRELP	0.5422746	0.0553906	9.79	2.33E-22	0.778463995	count	5.61E-18
SEPSECS-AS1	1.180986	0.8066719	1.464	0.143	0.778487041	count	1
DNAJB1	0.5463763	0.0896483	6.0947	1.21E-09	0.779183166	count	2.86E-05
CREB3L1	0.7804544	0.2352304	3.3178	0.000916	0.779314612	count	1
AL645608.8	0.9733827	0.4782711	2.0352	0.0419	0.779338162	count	1
DLX6	0.5661053	0.1648924	3.4332	0.000603	0.779782509	count	1
2-Mar	0.6118015	0.170914	3.5796	0.000349	0.780926803	count	1
AP002495.2	1.1380756	0.5122713	2.2216	0.0264	0.781244032	count	1

CMTR2	0.7053108	0.2822276	2.4991	0.0125	0.781334038	count	1
IFT88	0.605035	0.1656708	3.652	0.000264	0.781350197	count	1
USP24	0.7123402	0.244437	2.9142	0.00359	0.781531755	count	1
PTPRN2	0.825179	0.4339475	1.9016	0.0573	0.781828833	count	1
TFDP1	0.6606928	0.1910768	3.4577	0.000551	0.781906925	count	1
KAZN	0.5917748	0.1806324	3.2761	0.00106	0.782486441	count	1
LIMA1	0.5513044	0.0686379	8.0321	1.28E-15	0.782499956	count	3.06E-11
EFHC1	0.6165651	0.1586102	3.8873	0.000103	0.782885029	count	1
CFI	0.6072723	0.1336671	4.5432	5.72E-06	0.783540528	count	0.1321034
ADK	0.592922	0.1209699	4.9014	9.93E-07	0.78397356	count	0.023101152
EPS8L2	0.7545406	0.2471806	3.0526	0.00229	0.784096201	count	1
AC107464.3	1.406532	0.927652	1.5162	0.13	0.784157261	count	1
CD276	0.6518814	0.1713493	3.8044	0.000144	0.784356284	count	1
AC004494.1	16.5518505	1780.936528	0.0093	0.993	0.784415294	count	1
LINC00926	16.67405	1076.122051	0.0155	0.9876	0.784415302	count	1
HIST3H2BB	16.8347217	1682.931755	0.01	0.992	0.784415311	count	1
LINC01186	16.943737	1346.603083	0.0126	0.99	0.784415316	count	1
EPHA7	16.9675926	1035.20757	0.0164	0.987	0.784415317	count	1
AL118505.1	17.0995988	2701.147041	0.0063	0.995	0.784415322	count	1
ART5	17.1316645	1160.505294	0.0148	0.988	0.784415324	count	1
BMP7	17.2641603	1123.776564	0.0154	0.988	0.784415328	count	1
AK8	17.2739691	1389.991078	0.0124	0.99	0.784415329	count	1
ODF3L1	17.2852362	1686.983251	0.0102	0.992	0.784415329	count	1
CATSPERZ	17.5579028	1646.529224	0.0107	0.991	0.784415337	count	1
AC024132.3	17.650794	1648.709668	0.0107	0.991	0.784415339	count	1
AL133410.1	17.6521685	2132.344	0.0083	0.993	0.784415339	count	1
LENEP	17.6557875	3865.490443	0.0046	0.996	0.784415339	count	1
TOGARAM2	17.7207549	2782.407811	0.0064	0.995	0.78441534	count	1
DBX2	17.8574635	1509.029557	0.0118	0.991	0.784415343	count	1
EPHA5	16.831616	814.7437406	0.0207	0.984	0.784415371	count	1
SLC16A14	16.8692732	1029.20123	0.0164	0.987	0.784415373	count	1
LMX1A	16.9175912	1037.457865	0.0163	0.987	0.784415375	count	1
LINC01748	17.6546894	3356.5735	0.0053	0.996	0.7844154	count	1
PLAC9	0.5455401	0.0397604	13.7207	8.09E-42	0.784454682	count	1.96E-37
PHETA2	0.7024425	0.194675	3.6083	0.000312	0.784936434	count	1
CCDC36	1.5194548	0.5821482	2.6101	0.00909	0.785310838	count	1
KLF13	0.5609361	0.0845466	6.6346	3.73E-11	0.78606308	count	8.85E-07
AP006621.3	0.8051366	0.3271731	2.4609	0.0139	0.78772294	count	1
TMC6	1.5295891	0.8717964	1.7545	0.0794	0.788565786	count	1
INVS	0.7848279	0.3043999	2.5783	0.00997	0.789597427	count	1
CEP131	0.8964195	0.3863466	2.3202	0.0204	0.790565703	count	1
BHMT2	0.6115791	0.1791109	3.4145	0.000646	0.790822758	count	1
SHE	1.3368196	0.5416003	2.4683	0.0136	0.790910677	count	1
CYP7B1	0.7420889	0.1759988	4.2164	2.54E-05	0.79123803	count	0.5825744
PTCH1	0.8092328	0.3166479	2.5556	0.0106	0.791312703	count	1

SUMO4	1.8970743	1.0000245	1.897	0.0579	0.791512731	count	1
RIPOR3	2.867646	1.085198	2.6425	0.00826	0.792241333	count	1
TMEM99	0.6138748	0.1516698	4.0474	5.28E-05	0.792473449	count	1
AHDC1	0.6419442	0.203981	3.1471	0.00166	0.792563715	count	1
PTK2B	0.9316033	0.5329342	1.7481	0.0805	0.79265008	count	1
SOGA1	0.775213	0.2663826	2.9101	0.00363	0.792672752	count	1
RHBDL1	1.3413125	0.6704282	2.0007	0.0455	0.792743041	count	1
AIF1L	0.6357331	0.2133257	2.9801	0.0029	0.793134298	count	1
IFT122	0.6997449	0.3113242	2.2476	0.0247	0.794159085	count	1
RAB3IL1	0.6670713	0.1430844	4.6621	3.24E-06	0.794789261	count	0.07503192
FGFR1	0.5655488	0.0725799	7.7921	8.52E-15	0.796318698	count	2.03E-10
H6PD	0.6128684	0.1650169	3.714	0.000207	0.796539169	count	1
CYSLTR1	0.904921	0.4205818	2.1516	0.0315	0.797006099	count	1
CIT	0.8348292	0.5572323	1.4982	0.134	0.798141958	count	1
MIR222HG	0.7948278	0.3770822	2.1078	0.0351	0.798687623	count	1
RNF139	0.6432054	0.1829571	3.5156	0.000444	0.79879648	count	1
GPSM1	0.879972	0.5834561	1.5082	0.132	0.799069607	count	1
ZDHHC8	0.7194384	0.2665761	2.6988	0.00699	0.799364202	count	1
CYP3A5	1.2868961	0.6088794	2.1135	0.0346	0.800434525	count	1
IQUB	1.5681652	0.7420865	2.1132	0.0347	0.800739663	count	1
ITFG2	0.6451464	0.190795	3.3814	0.000729	0.80112044	count	1
GSC	0.9837046	0.3579943	2.7478	0.00603	0.801405708	count	1
HHLA3	0.6992245	0.2726431	2.5646	0.0104	0.801825249	count	1
USP44	1.1788715	0.3656722	3.2238	0.00128	0.80237673	count	1
IPO13	1.2307782	0.4518083	2.7241	0.00648	0.802626891	count	1
VPS35L	0.7077971	0.2074021	3.4127	0.00065	0.80274458	count	1
RSRP1	0.5669392	0.0689689	8.2202	2.79E-16	0.803181412	count	6.67E-12
AC087386.1	1.368857	0.6737581	2.0317	0.0423	0.803858918	count	1
TP53I11	0.658619	0.216858	3.0371	0.00241	0.804358063	count	1
ACKR1	0.7472964	0.6588452	1.1343	0.257	0.8050479	count	1
EDC3	0.9028281	0.3861675	2.3379	0.0194	0.806462168	count	1
TRRAP	0.7300651	0.3285447	2.2221	0.0263	0.806891918	count	1
SIRPA	0.8779332	0.2702732	3.2483	0.00117	0.807283429	count	1
SIPA1L2	0.7378996	0.3412674	2.1622	0.0307	0.807584654	count	1
SPIN2A	0.9351263	0.3550442	2.6338	0.00848	0.807727039	count	1
METTL7A	0.5823508	0.0756737	7.6956	1.80E-14	0.808947009	count	4.29E-10
AUTS2	0.6330557	0.2145865	2.9501	0.0032	0.809568416	count	1
NUP35	0.6843145	0.1961759	3.4883	0.000492	0.810483328	count	1
AP001157.1	0.7945387	0.5165055	1.5383	0.124	0.810594437	count	1
SRGAP2	0.660489	0.2121668	3.1131	0.00187	0.810680055	count	1
STK38	0.7003724	0.2391979	2.928	0.00343	0.81069558	count	1
LAMB2	0.580207	0.0911167	6.3677	2.16E-10	0.811443347	count	5.11E-06
WDR37	0.850721	0.2990109	2.8451	0.00446	0.811580391	count	1
ZNF736	0.9581474	0.433693	2.2093	0.0272	0.811667877	count	1
CSRNP2	0.7836459	0.4840157	1.6191	0.106	0.811676115	count	1

PAPSS2	0.7629617	0.2285501	3.3383	0.000851	0.811711341	count	1
CYP4X1	0.591341	0.145701	4.0586	5.04E-05	0.812107578	count	1
DDHD1	0.6877371	0.2303098	2.9861	0.00284	0.812325481	count	1
DNAH1	2.0069855	2.1898953	0.9165	0.3595	0.813494683	count	1
MAF	0.6655113	0.1559983	4.2661	2.04E-05	0.813846213	count	0.4686696
EFCAB7	0.8370249	0.2622376	3.1919	0.00143	0.815537725	count	1
ARHGAP32	1.4011946	0.4872124	2.8759	0.00405	0.816653707	count	1
CBLN1	0.8779682	0.4298285	2.0426	0.0412	0.816729133	count	1
AC114284.1	1.6209074	0.9548658	1.6975	0.0897	0.816838561	count	1
FAM13A	0.6181346	0.1356411	4.5571	5.35E-06	0.817024438	count	0.12361175
RBPJ	0.5825339	0.0775774	7.5091	7.44E-14	0.81798309	count	1.77E-09
FZD2	0.6386489	0.1911449	3.3412	0.000843	0.818009273	count	1
PTCH2	1.0908354	0.4787763	2.2784	0.0228	0.818522711	count	1
ATP1B2	0.7422258	0.2359218	3.1461	0.00167	0.819391534	count	1
EBP	0.6595707	0.1745025	3.7797	0.00016	0.819535993	count	1
FZD7	0.6552781	0.1973219	3.3209	0.000906	0.819888741	count	1
LIG4	0.8955336	0.2679114	3.3426	0.000838	0.821346425	count	1
PLEKHF1	0.6521232	0.1574705	4.1412	3.53E-05	0.822055088	count	0.8084759
FAM162B	0.7607042	0.2146458	3.544	0.000399	0.822601226	count	1
ALDH2	0.5784908	0.0489229	11.8245	1.09E-31	0.822789786	count	2.63E-27
F8A1	0.6400741	0.1502279	4.2607	2.09E-05	0.823352371	count	0.4799894
ACOX1	0.6590836	0.1627758	4.049	5.25E-05	0.823413882	count	1
KLF9	0.5884062	0.0654416	8.9913	3.83E-19	0.823681361	count	9.19E-15
MAGI3	0.8090809	0.2489441	3.2501	0.00116	0.82401023	count	1
ITIH5	0.607611	0.1279204	4.7499	2.11E-06	0.824034726	count	0.04893301
ATRN	0.8160693	0.2791463	2.9234	0.00348	0.824280262	count	1
SCN1B	0.6619965	0.1881268	3.5189	0.000439	0.824719263	count	1
CLTRN	1.8199314	1.8296202	0.9947	0.3199	0.824995349	count	1
CENPW	1.1039729	0.3392871	3.2538	0.00115	0.826281776	count	1
SLC19A2	1.1396245	0.4141138	2.752	0.00595	0.826543798	count	1
TRIM73	0.8265994	0.2838397	2.9122	0.00361	0.827377387	count	1
PDE10A	1.106031	0.7477669	1.4791	0.139	0.827492408	count	1
GLT8D1	0.6041479	0.0959671	6.2954	3.43E-10	0.828823585	count	8.11E-06
PAXIP1	0.9051499	0.3305857	2.738	0.00621	0.828989466	count	1
RAD18	0.8932133	0.332256	2.6883	0.00721	0.829090616	count	1
GNS	0.6312335	0.1347242	4.6854	2.90E-06	0.830970262	count	0.0671901
AC234582.1	1.1126709	0.6307952	1.7639	0.0778	0.831389241	count	1
AC144831.1	1.5407867	0.9108053	1.6917	0.0908	0.831590276	count	1
CLIP4	0.7112458	0.1905898	3.7318	0.000193	0.832075921	count	1
FAM117B	0.8655701	0.2797887	3.0937	0.00199	0.832280673	count	1
CDCA7L	0.8249847	0.3282066	2.5136	0.012	0.832382661	count	1
SH3BP2	1.0889713	0.3605667	3.0202	0.00254	0.836069486	count	1
WIPI1	0.6738888	0.1441023	4.6765	3.02E-06	0.8366537	count	0.06996132
TRIL	3.7842059	1.3351704	2.8342	0.00462	0.837268278	count	1
AL161772.1	0.7848476	0.2713677	2.8922	0.00385	0.837717729	count	1

YY2	1.0917417	0.9723157	1.1228	0.262	0.837757984	count	1
MNS1	0.6954509	0.1973073	3.5247	0.000429	0.837801178	count	1
TOB1	0.5985643	0.0813941	7.3539	2.36E-13	0.838428446	count	5.62E-09
HDAC7	0.6707797	0.1687292	3.9755	7.16E-05	0.838585518	count	1
GREM2	0.6295949	0.2680392	2.3489	0.0189	0.838593191	count	1
CPT1A	0.6411132	0.1680386	3.8153	0.000138	0.838877974	count	1
NR4A3	0.7450436	0.3090506	2.4107	0.016	0.839151907	count	1
AKR1C1	0.615867	0.1042503	5.9076	3.79E-09	0.839596649	count	8.93E-05
CLMN	0.8481079	0.227718	3.7244	0.000199	0.839730496	count	1
RPN1	0.6124205	0.098104	6.2426	4.80E-10	0.839896701	count	1.13E-05
CELF2	0.6109908	0.1005725	6.0751	1.37E-09	0.840063338	count	3.23E-05
EPB41L2	0.5929748	0.0617673	9.6001	1.43E-21	0.841265171	count	3.44E-17
LINC00891	1.0442464	0.5649541	1.8484	0.0646	0.841678673	count	1
FAM43B	2.6349549	1.078415	2.4434	0.0146	0.841749876	count	1
TRHDE	1.4669945	0.6490712	2.2601	0.0239	0.841844462	count	1
GTF3C1	0.7056604	0.207269	3.4046	0.00067	0.842317276	count	1
SH3BGRL2	0.7059553	0.3712091	1.9018	0.0573	0.842651868	count	1
LCA5L	1.1690534	0.5612726	2.0829	0.0373	0.842898489	count	1
AC079298.3	2.6551941	1.1722387	2.2651	0.0236	0.843775742	count	1
BMPER	0.8066817	0.3722704	2.1669	0.0303	0.844117723	count	1
AHNAK2	0.6633954	0.1425215	4.6547	3.36E-06	0.844746358	count	0.07779072
OLFML2B	0.6111734	0.0854466	7.1527	1.02E-12	0.844877309	count	2.43E-08
AL583785.1	2.6707685	1.1157144	2.3938	0.0167	0.845309411	count	1
SLC1A3	2.191236	0.96056	2.2812	0.0226	0.846122658	count	1
ITGA5	0.6655946	0.23116	2.8794	0.00401	0.848285127	count	1
SMAD3	0.6685015	0.1602173	4.1725	3.08E-05	0.848470868	count	0.7056896
ZNF600	1.222423	0.427661	2.8584	0.00428	0.848772532	count	1
MPZL1	0.6203295	0.0995358	6.2322	5.12E-10	0.849747817	count	1.21E-05
CD68	0.6376825	0.1285579	4.9603	7.36E-07	0.849838677	count	0.017147328
GJC2	1.4928556	0.6440704	2.3178	0.0205	0.851439797	count	1
HACE1	0.9101838	0.374426	2.4309	0.0151	0.852159852	count	1
SPATA7	0.674511	0.3240943	2.0812	0.0375	0.852193652	count	1
GNAO1	1.0605347	0.8108802	1.3079	0.191	0.852315382	count	1
MAMDC2	0.8223577	0.2171329	3.7873	0.000155	0.853591904	count	1
RUNX1T1	0.6590934	0.1416877	4.6517	3.41E-06	0.853789709	count	0.07894491
C1RL-AS1	0.8643929	0.399592	2.1632	0.0306	0.85407231	count	1
HOXA3	0.6534827	0.180196	3.6265	0.000291	0.854662985	count	1
TRIM11	0.767197	0.2921217	2.6263	0.00867	0.855697315	count	1
LPIN3	0.7366009	0.2171975	3.3914	0.000703	0.857664863	count	1
TMEM30B	0.6608697	0.1657106	3.9881	6.79E-05	0.857873123	count	1
KIAA0100	0.6916651	0.1687674	4.0983	4.25E-05	0.859068147	count	0.972315
ACYP1	0.6389712	0.1335209	4.7856	1.77E-06	0.859929647	count	0.04108524
GBGT1	0.909	0.4354922	2.0872	0.0369	0.860124449	count	1
TRIOBP	0.6373791	0.1174666	5.426	6.13E-08	0.860832838	count	0.001436872
DLAT	0.8426685	0.2615831	3.2214	0.00129	0.860924283	count	1

AC073115.1	1.5229974	2.3848979	0.6386	0.5231	0.862409069	count	1
CHST12	0.6324122	0.1194608	5.2939	1.27E-07	0.862536721	count	0.002972562
TCN2	0.7561834	0.2261536	3.3437	0.000835	0.863011034	count	1
PID1	0.6218386	0.0789386	7.8775	4.37E-15	0.863608295	count	1.04E-10
TMEM19	0.7430612	0.2173923	3.4181	0.000638	0.86474595	count	1
ZNF609	0.7563081	0.3657497	2.0678	0.0387	0.865994142	count	1
BHLHE41	0.6321672	0.1245007	5.0776	4.01E-07	0.866197114	count	0.009357335
ANGPTL4	0.6375306	0.1519915	4.1945	2.80E-05	0.86625076	count	0.641928
AVIL	1.5340549	0.7131646	2.1511	0.0315	0.866375732	count	1
VPS13D	0.6894265	0.2233213	3.0872	0.00204	0.866387053	count	1
POMGNT2	0.8646235	0.2236207	3.8665	0.000112	0.868126224	count	1
LINC01239	16.7953874	684.2781242	0.0245	0.98	0.868365397	count	1
SPHKAP	16.937459	884.4362315	0.0192	0.985	0.868365405	count	1
HOXC4	16.9812537	618.1637046	0.0275	0.9781	0.868365408	count	1
HCN1	17.0374698	1089.729154	0.0156	0.988	0.86836541	count	1
TMEM132B	17.0493633	1149.225854	0.0148	0.9882	0.868365411	count	1
ZNF233	17.1897054	1330.123259	0.0129	0.9897	0.868365417	count	1
GDF6	17.4628123	1063.909642	0.0164	0.987	0.868365427	count	1
AC005730.2	17.7844845	1452.21149	0.0122	0.99	0.868365435	count	1
SPRR2G	17.7872161	2417.890934	0.0074	0.994	0.868365435	count	1
TMEM45B	17.7897291	3652.766654	0.0049	0.996	0.868365435	count	1
AC123912.1	17.7897293	3652.766733	0.0049	0.996	0.868365435	count	1
SLC6A15	18.0227151	1568.084074	0.0115	0.991	0.86836544	count	1
GNAZ	16.6151217	1097.80668	0.0151	0.9879	0.868365451	count	1
S100A14	17.4368886	2910.634387	0.006	0.995	0.868365491	count	1
KCNIP4	17.786334	2059.910786	0.0086	0.993	0.868365501	count	1
KHDRBS2	17.7867129	2304.441638	0.0077	0.994	0.868365501	count	1
KPNA1	0.6815569	0.2014842	3.3827	0.000725	0.868806843	count	1
SH3PXD2B	0.7427437	0.2296311	3.2345	0.00123	0.869165751	count	1
ADAMTS4	0.9314033	0.3023134	3.0809	0.00208	0.869369677	count	1
ANGPTL1	0.6440616	0.1044955	6.1635	7.88E-10	0.869706491	count	1.86E-05
NPR1	0.6657992	0.1340344	4.9674	7.10E-07	0.869825886	count	0.01654513
CLCN4	1.8127788	0.4747368	3.8185	0.000136	0.870317794	count	1
METTL27	1.3809691	0.6429018	2.148	0.0318	0.871133772	count	1
NKD1	0.8307379	0.3597442	2.3092	0.021	0.872242798	count	1
PAX6	2.3732854	1.9717729	1.2036	0.2288	0.873696646	count	1
NDST1	1.0699106	0.4024258	2.6587	0.00788	0.874206699	count	1
SOCS3	0.6160087	0.0748482	8.2301	2.57E-16	0.874422502	count	6.14E-12
MRPL53	0.9263057	0.4613917	2.0076	0.0448	0.874443403	count	1
GMFB	0.7194022	0.1900007	3.7863	0.000155	0.875239701	count	1
Z97200.1	1.0966618	0.4432513	2.4741	0.0134	0.875606116	count	1
TUBGCP4	0.7775117	0.2557446	3.0402	0.00238	0.876430517	count	1
ZNF713	1.1925384	0.9238816	1.2908	0.197	0.877188049	count	1
PPP1R10	0.6273918	0.0940624	6.67	2.94E-11	0.87850672	count	6.97E-07
CAT	0.6287503	0.0793534	7.9234	3.04E-15	0.878629301	count	7.26E-11

ALDH1A3	1.1293039	0.4416977	2.5567	0.0106	0.878791254	count	1
HFE	1.0548784	0.3451179	3.0566	0.00225	0.879080741	count	1
ZNF487	0.862801	0.3267009	2.641	0.0083	0.879366054	count	1
TMEM51	1.4010842	0.3634901	3.8545	0.000118	0.879825133	count	1
PRKAG2	0.6857932	0.1389435	4.9358	8.34E-07	0.880372166	count	0.019417188
POLE	1.1631713	0.5351881	2.1734	0.0298	0.880460879	count	1
B3GNT7	1.057349	0.8905707	1.1873	0.235	0.880763295	count	1
SKIL	0.6305256	0.0899468	7.01	2.82E-12	0.881485083	count	6.70E-08
ZNF587	0.8298742	0.5450442	1.5226	0.128	0.881518104	count	1
PSMG3-AS1	0.9594868	0.5241946	1.8304	0.0673	0.881988906	count	1
RPAP1	1.0595934	0.4757825	2.2271	0.026	0.882290305	count	1
MFSD14B	0.7043596	0.210178	3.3513	0.000813	0.882304952	count	1
LMF1	0.7476805	0.2497193	2.9941	0.00277	0.883582771	count	1
C1RL	0.6638264	0.1330719	4.9885	6.37E-07	0.883957196	count	0.014849744
DHCR24	1.1691859	0.4236215	2.76	0.00581	0.883983411	count	1
TEAD1	0.6694542	0.1558012	4.2968	1.78E-05	0.884172917	count	0.4092042
ZNF184	0.8692067	0.2888338	3.0094	0.00264	0.885209061	count	1
PCDH18	0.6885068	0.1529714	4.5009	6.98E-06	0.885232094	count	0.1610984
RIN1	1.0246856	0.4299163	2.3835	0.0172	0.885314394	count	1
AC245452.1	1.5896036	0.6364786	2.4975	0.0126	0.885842498	count	1
B4GAT1	0.6826726	0.1215634	5.6158	2.10E-08	0.886619891	count	0.000493374
ADH1B	0.6169338	0.0588407	10.4848	2.31E-25	0.886907724	count	5.57E-21
ANKRD33B	0.8943709	0.3264329	2.7398	0.00618	0.887585216	count	1
AL161669.3	2.4790546	1.0942535	2.2655	0.0235	0.887816615	count	1
CES1	0.6368672	0.123295	5.1654	2.53E-07	0.88789766	count	0.005911092
KLHL23	0.7238604	0.170955	4.2342	2.35E-05	0.887943389	count	0.5394425
DAB2	0.6477327	0.0924443	7.0067	2.89E-12	0.888130491	count	6.87E-08
PALD1	1.1767	0.8244013	1.4273	0.154	0.888368222	count	1
CCDC180	1.3025473	1.0711522	1.216	0.224	0.889261687	count	1
SLC35F6	0.6869205	0.1627959	4.2195	2.51E-05	0.889450622	count	0.5757187
ZNF761	0.8494422	0.3793458	2.2392	0.0252	0.890010416	count	1
MYCBP2	0.628568	0.0873693	7.1944	7.57E-13	0.890620698	count	1.80E-08
CDK20	1.2582177	0.5341253	2.3557	0.0185	0.89083123	count	1
TSPAN17	1.0727334	0.4888413	2.1944	0.0283	0.891197265	count	1
GDF15	0.6676169	0.2207173	3.0248	0.00251	0.891333946	count	1
KRBA2	1.3082406	0.557597	2.3462	0.019	0.892065828	count	1
PPP1R15B	0.672163	0.2174325	3.0914	0.00201	0.892962426	count	1
RHBDF1	0.734825	0.1596421	4.603	4.31E-06	0.893112589	count	0.09968168
CHST7	0.8005346	0.2497877	3.2049	0.00136	0.893661344	count	1
ADAM9	0.6713042	0.1262254	5.3183	1.11E-07	0.89370268	count	0.002598954
TP53INP2	1.5137856	0.5243235	2.8871	0.00391	0.893706426	count	1
CCDC18	1.0062887	0.4877408	2.0632	0.0392	0.896261169	count	1
TYW1	0.8071483	0.2017938	3.9999	6.46E-05	0.8968906	count	1
C7	0.6452645	0.1727418	3.7354	0.00019	0.896956148	count	1
UBXN2A	0.6612441	0.1194067	5.5377	3.28E-08	0.897332469	count	0.000770046

ZSCAN25	1.0089239	0.5206765	1.9377	0.0527	0.898234969	count	1
SAA1	2.565278	1.064494	2.4099	0.016	0.89838724	count	1
WDR81	1.2732492	0.3457361	3.6827	0.000234	0.898673053	count	1
PRDM5	0.8336577	0.2999026	2.7798	0.00547	0.899267925	count	1
ITIH3	1.1341959	0.369569	3.069	0.00216	0.899363257	count	1
ZNF783	1.232591	0.5762911	2.1388	0.0325	0.899410133	count	1
KCNK17	0.6643788	0.1391411	4.7749	1.87E-06	0.899532584	count	0.04339335
BMP1	0.8303333	0.2594886	3.1999	0.00139	0.900395329	count	1
CRABP2	0.7234573	0.1776674	4.072	4.76E-05	0.900623396	count	1
RNPEP	0.7106876	0.1596513	4.4515	8.78E-06	0.901062599	count	0.2024229
NDRG1	0.6798423	0.1389604	4.8923	1.04E-06	0.901760693	count	0.02419144
LINC00640	2.1908355	1.8444752	1.1878	0.235	0.901971075	count	1
AL512598.1	1.7744116	0.9770056	1.8162	0.0694	0.903962416	count	1
HEXB	0.6486007	0.0764878	8.4798	3.23E-17	0.905530595	count	7.73E-13
RNF19A	0.6559443	0.1084854	6.0464	1.63E-09	0.906389406	count	3.85E-05
GRAMD4	1.3984635	0.6370279	2.1953	0.0282	0.907717097	count	1
AFF3	1.210301	0.5257447	2.3021	0.0214	0.907758991	count	1
SOX13	1.1225037	0.3317891	3.3832	0.000724	0.908597587	count	1
AC069148.1	1.040562	0.9428446	1.1036	0.27	0.909487497	count	1
GRK5	0.6874031	0.1766553	3.8912	0.000102	0.909488978	count	1
PAFAH2	0.9476428	0.4366	2.1705	0.03	0.909651938	count	1
SLC25A13	0.7538203	0.2272454	3.3172	0.000918	0.909940793	count	1
BMPR1B	0.7215603	0.2096347	3.442	0.000584	0.910445386	count	1
LANCL2	1.560331	0.7231667	2.1576	0.031	0.911148254	count	1
ELOVL2	1.6710411	1.2102141	1.3808	0.167	0.913021102	count	1
TARBP1	1.0637235	0.5294449	2.0091	0.0446	0.913110273	count	1
CST3	0.6338976	0.033356	19.004	6.98E-77	0.91376413	count	1.70E-72
OMD	0.6381175	0.0608835	10.481	2.40E-25	0.91416701	count	5.78E-21
ORAI3	0.7088493	0.1422795	4.9821	6.58E-07	0.914221394	count	0.015337322
ZNF436	0.925151	0.4601098	2.0107	0.0444	0.914476606	count	1
SH3D19	0.6724323	0.1114286	6.0346	1.75E-09	0.914748079	count	4.13E-05
NUMBL	0.7337073	0.1646565	4.456	8.60E-06	0.915260547	count	0.1982902
SRSF6	0.6729948	0.1086421	6.1946	6.49E-10	0.915302059	count	1.53E-05
VGLL3	0.7258532	0.1964683	3.6945	0.000224	0.915657294	count	1
RBM24	1.052583	0.2746185	3.8329	0.000129	0.918191996	count	1
BCO2	1.0358662	0.3830656	2.7041	0.00688	0.918287363	count	1
RORB	1.2298136	0.3969403	3.0982	0.00196	0.918857004	count	1
TCEAL7	0.7102026	0.1581042	4.492	7.27E-06	0.920675129	count	0.16775525
PHLPP2	1.4997481	0.688771	2.1774	0.0295	0.920838466	count	1
VEGFA	0.7655458	0.1721739	4.4464	8.99E-06	0.921549704	count	0.20724647
NFKB1	0.7160284	0.1737919	4.12	3.87E-05	0.921731334	count	0.8856882
LAMA2	0.6959737	0.1408614	4.9408	8.13E-07	0.921830775	count	0.018930705
ECHDC1	0.6712471	0.106191	6.3211	2.91E-10	0.921967756	count	6.89E-06
RAB8B	0.6707722	0.1079697	6.2126	5.79E-10	0.922414315	count	1.37E-05
ZBTB33	1.059015	0.4104989	2.5798	0.00992	0.922830331	count	1

MANBA	0.854031	0.2375926	3.5945	0.000329	0.923824297	count	1
ZNF503	0.6563538	0.0774612	8.4733	3.41E-17	0.92406019	count	8.16E-13
PARP4	0.7906549	0.2251073	3.5123	0.00045	0.92423122	count	1
NR2F1-AS1	1.5083421	0.42131	3.5801	0.000348	0.924285271	count	1
TSPAN18	1.375458	0.484197	2.8407	0.00453	0.924448102	count	1
AXL	0.6629293	0.0716698	9.2498	3.72E-20	0.925420502	count	8.93E-16
GPR146	1.4400612	0.4476765	3.2167	0.00131	0.926102764	count	1
ATG2A	1.1263595	0.4262445	2.6425	0.00826	0.926971764	count	1
ATAD3B	1.3316344	0.714187	1.8645	0.0623	0.92848289	count	1
CSTA	2.3545436	0.7472183	3.1511	0.00164	0.929062529	count	1
IQCK	0.7407558	0.1813888	4.0838	4.53E-05	0.929441403	count	1
PRMT9	0.8079803	0.2317296	3.4867	0.000495	0.929856069	count	1
TRUB1	0.8330555	0.2513019	3.315	0.000925	0.930747937	count	1
AC098650.1	2.3681791	0.8606345	2.7517	0.00596	0.931151554	count	1
IL6	0.7064362	0.4114149	1.7171	0.086	0.931335559	count	1
MDGA1	1.0897749	0.7789251	1.3991	0.162	0.931386085	count	1
CBFA2T3	2.3736941	1.044592	2.2724	0.0231	0.93198944	count	1
ZNF844	0.8255722	0.3504997	2.3554	0.0186	0.93326701	count	1
CXCL10	0.9229643	0.8078047	1.1426	0.2533	0.933767994	count	1
S100B	1.1907624	0.5968066	1.9952	0.0461	0.934316856	count	1
VPS39	0.8912895	0.3084774	2.8893	0.00388	0.934964509	count	1
TPPP3	0.6803361	0.1390377	4.8932	1.03E-06	0.93498137	count	0.02395986
EIF2AK3	0.9431567	0.387789	2.4321	0.0151	0.937564598	count	1
C14orf180	1.1682766	0.4261693	2.7413	0.00615	0.937805913	count	1
TAC1	0.769819	0.4188959	1.8377	0.0662	0.938449362	count	1
ACOX3	0.9360691	0.3957444	2.3653	0.0181	0.938546026	count	1
MBP	0.6975637	0.1226182	5.6889	1.38E-08	0.940338312	count	0.000324507
ID2	0.6641149	0.0757806	8.7637	2.84E-18	0.942273914	count	6.81E-14
SLC2A4	0.9881539	0.3748323	2.6363	0.00842	0.943176136	count	1
KCNIP3	1.5563875	0.3889231	4.0018	6.41E-05	0.943189746	count	1
ZNF516	1.4164186	0.4119939	3.438	0.000593	0.943530904	count	1
LDB2	0.7834928	0.203682	3.8466	0.000122	0.943839872	count	1
ARHGEF10	0.8298154	0.2829919	2.9323	0.00339	0.944138696	count	1
SLC35G2	0.827366	0.2686109	3.0802	0.00208	0.944644282	count	1
HEG1	0.7162667	0.1660169	4.3144	1.64E-05	0.945366041	count	0.3771836
SULT1A2	1.929369	1.518018	1.271	0.204	0.945388282	count	1
CPXM2	0.6821799	0.0969075	7.0395	2.29E-12	0.945405821	count	5.44E-08
PIFO	1.3679027	0.6029721	2.2686	0.0234	0.946483402	count	1
ATP8A1	0.8681509	0.2896297	2.9975	0.00274	0.94658707	count	1
PLIN2	0.7128426	0.1365242	5.2214	1.87E-07	0.946939828	count	0.00437206
TNNC1	0.8187029	0.3994992	2.0493	0.0405	0.946945711	count	1
ADRA1D	16.9527772	592.2914296	0.0286	0.9772	0.947697895	count	1
FOLH1	17.298475	746.8643461	0.0232	0.9815	0.947697912	count	1
PCP4L1	16.8114113	682.9167956	0.0246	0.98	0.947697955	count	1
RIMS2	16.8911272	740.5095462	0.0228	0.982	0.94769796	count	1

TNNT2	16.9096567	2661.872746	0.0064	0.995	0.947697962	count	1
VANGL2	16.9608401	1084.169333	0.0156	0.9875	0.947697965	count	1
AC092691.1	17.1327846	1170.032482	0.0146	0.988	0.947697974	count	1
OR2L2	17.1505058	2849.322841	0.006	0.995	0.947697975	count	1
HRASLS5	17.4778218	1084.750828	0.0161	0.987	0.947697988	count	1
EYA4	17.7314291	1030.46919	0.0172	0.9863	0.947697996	count	1
AC138932.5	17.9078244	3478.118687	0.0051	0.996	0.947698	count	1
AC009061.2	1.3720737	0.9507991	1.4431	0.149	0.948528262	count	1
RELL1	0.8443541	0.2250504	3.7518	0.000178	0.949458436	count	1
HDAC4	1.033272	0.2680448	3.8548	0.000118	0.950017444	count	1
KLF3	0.6763554	0.0785922	8.6059	1.11E-17	0.953097735	count	2.66E-13
ADAMTSL2	1.801238	0.4768767	3.7772	0.000161	0.953238789	count	1
TBP	0.9612397	0.2734251	3.5156	0.000444	0.953289197	count	1
PLCE1	0.8711645	0.2081984	4.1843	2.93E-05	0.953848859	count	0.6715853
TMEFF2	0.8278051	0.3838338	2.1567	0.0311	0.953940884	count	1
PTGFR	2.186663	0.6185133	3.5354	0.000412	0.953961405	count	1
AP5B1	1.0027409	0.4169256	2.4051	0.0162	0.955121242	count	1
HUNK	1.807812	1.001614	1.8049	0.0712	0.955167126	count	1
PFN4	2.5381676	1.6594757	1.5295	0.126	0.955208221	count	1
SERPING1	0.6650218	0.0352949	18.8419	1.15E-75	0.955536854	count	2.79E-71
PAK3	0.9210191	0.2354145	3.9123	9.31E-05	0.957080892	count	1
HLA-DPA1	0.6809095	0.122998	5.5359	3.31E-08	0.957325719	count	0.000777056
DIRC3	1.390802	0.6192273	2.246	0.0248	0.957645965	count	1
DDR2	0.6860718	0.1203342	5.7014	1.28E-08	0.957880077	count	0.000301005
SLC23A2	0.8943974	0.2808003	3.1852	0.00146	0.958582454	count	1
PRR15	1.0211102	0.334331	3.0542	0.00227	0.96060678	count	1
ZMIZ1	0.7898828	0.1830283	4.3156	1.63E-05	0.961646954	count	0.3749
FZD4	0.7259347	0.1669229	4.3489	1.41E-05	0.962249692	count	0.3244833
ATP11A	1.012862	0.3194436	3.1707	0.00153	0.963369763	count	1
REPS1	0.8945381	0.4171971	2.1442	0.0321	0.963516447	count	1
MGAT1	0.7051348	0.0961727	7.332	2.78E-13	0.964006464	count	6.62E-09
SPON1	0.7615257	0.2563809	2.9703	0.00299	0.964019138	count	1
SOBP	0.7510588	0.2432917	3.0871	0.00204	0.964500254	count	1
NPY5R	1.0828355	0.3442964	3.1451	0.00167	0.964958112	count	1
RARG	0.7528863	0.1563576	4.8152	1.53E-06	0.965152659	count	0.03553578
DOCK9	0.9943173	0.2785378	3.5698	0.000362	0.965616992	count	1
ATF3	0.675095	0.0804154	8.3951	6.56E-17	0.965944866	count	1.57E-12
TSPAN4	0.6853831	0.0680171	10.0766	1.42E-23	0.966728853	count	3.42E-19
P2RY14	2.2739505	0.9869174	2.3041	0.0213	0.970055364	count	1
ABL1	0.7930815	0.1714754	4.625	3.87E-06	0.970197125	count	0.08954019
MEOX2	0.7327305	0.1471219	4.9804	6.64E-07	0.971031327	count	0.015475848
FBXL13	1.0913175	0.2795042	3.9045	9.61E-05	0.971195463	count	1
ELN	0.6824886	0.10855	6.2873	3.61E-10	0.972447999	count	8.54E-06
NRARP	0.8205476	0.2743014	2.9914	0.0028	0.973500576	count	1
TBC1D24	1.1745682	0.3501668	3.3543	0.000804	0.974183481	count	1

PODXL2	0.9396582	0.2148181	4.3742	1.25E-05	0.974302381	count	0.28785
RAB30	0.8027815	0.1602254	5.0103	5.69E-07	0.974812055	count	0.013269649
SLC9B2	0.8724203	0.2428163	3.5929	0.000331	0.974812361	count	1
BX255925.3	1.3316547	0.8004751	1.6636	0.0963	0.974849378	count	1
TMEM184B	0.8396419	0.2270267	3.6984	0.00022	0.974975299	count	1
UNG	0.8476336	0.2049611	4.1356	3.62E-05	0.975143221	count	0.8288352
WFDC2	0.9492307	0.3397082	2.7943	0.00523	0.976937965	count	1
TBC1D9	0.9718691	0.3044542	3.1922	0.00142	0.977192169	count	1
ZNF518B	0.9231189	0.2721672	3.3917	0.000702	0.981147661	count	1
MIR22HG	0.7031254	0.1122505	6.2639	4.19E-10	0.982424809	count	9.91E-06
CYB561D2	0.8372928	0.203451	4.1155	3.95E-05	0.985131602	count	0.9039575
AMACR	1.3986576	1.0613844	1.3178	0.188	0.986279213	count	1
ACOX2	0.8931736	0.2531069	3.5288	0.000423	0.988236351	count	1
CNKSR2	1.927334	0.5370948	3.5884	0.000337	0.988594178	count	1
DNAJC25	0.8988215	0.2309473	3.8919	0.000101	0.989851227	count	1
SGPP1	1.52057	0.3786422	4.0158	6.04E-05	0.989871308	count	1
MT1M	0.7010439	0.0811626	8.6375	8.44E-18	0.991249807	count	2.02E-13
SPATA6	0.735971	0.1261754	5.8329	5.92E-09	0.99190294	count	0.000139363
FAM171A1	0.8895807	0.3607744	2.4658	0.0137	0.996076088	count	1
PYROXD2	1.0534441	0.3362164	3.1332	0.00174	0.996116482	count	1
SEC24A	0.9396406	0.2743507	3.425	0.000622	0.996863171	count	1
CHODL	1.53719	0.4058175	3.7879	0.000154	0.99698011	count	1
CLSTN2	2.16696	0.5478952	3.9551	7.80E-05	0.999798275	count	1
TRANK1	0.9614805	0.3365107	2.8572	0.0043	1.000400083	count	1
SLC4A4	1.1498185	0.4270354	2.6926	0.00712	1.000503352	count	1
POP1	1.2161179	0.4583569	2.6532	0.00801	1.001181532	count	1
AL355312.2	1.9877066	2.0837947	0.9539	0.3402	1.004336318	count	1
ABHD5	0.7547566	0.1653645	4.5642	5.18E-06	1.004490572	count	0.11969426
APP	0.7076602	0.0559557	12.6468	6.48E-36	1.00506955	count	1.57E-31
GATM	1.313987	0.4686039	2.804	0.00507	1.006921988	count	1
RAB32	0.7214373	0.0840574	8.5827	1.35E-17	1.007826556	count	3.23E-13
ACACB	1.0355439	0.2597791	3.9862	6.84E-05	1.008528754	count	1
RASIP1	1.502975	0.4834137	3.1091	0.00189	1.010085852	count	1
TNFSF13B	0.7464255	0.1459797	5.1132	3.33E-07	1.011950988	count	0.007773219
ATP8B4	1.8669019	0.7580283	2.4628	0.0138	1.012618836	count	1
KLHL15	1.1486619	0.4503677	2.5505	0.0108	1.012749058	count	1
IQCC	2.5460642	2.288556	1.1125	0.266	1.013155338	count	1
SIX1	0.7893825	0.1615584	4.886	1.07E-06	1.013556008	count	0.02488606
MELTF	1.1037152	0.5432338	2.0317	0.0423	1.014955745	count	1
ZNF117	1.0674766	0.397791	2.6835	0.00732	1.016887554	count	1
AQP3	0.9796389	0.2578641	3.7991	0.000148	1.017092316	count	1
ARRB1	1.0465688	0.3295048	3.1762	0.0015	1.017712405	count	1
FRMD6	0.8155997	0.1558142	5.2344	1.75E-07	1.017824983	count	0.0040922
ARID3A	2.0435254	0.6983331	2.9263	0.00345	1.018238113	count	1
RHOBTB3	0.762851	0.1078646	7.0723	1.82E-12	1.018455588	count	4.33E-08

MEG8	1.8898939	0.617026	3.0629	0.00221	1.019340572	count	1
PTGDR	1.7683441	0.6577722	2.6884	0.00721	1.019432902	count	1
MIRLET7BHG	0.9696128	0.3760491	2.5784	0.00996	1.019583564	count	1
PDE7A	1.1601208	0.2942536	3.9426	8.21E-05	1.020923545	count	1
CLDN1	2.2751529	1.3105299	1.7361	0.0826	1.021273996	count	1
UCHL1	0.753031	0.134984	5.5787	2.60E-08	1.022143023	count	0.00061061
ADAM15	0.9118709	0.1981966	4.6008	4.35E-06	1.022591987	count	0.10060245
DOK7	17.3540937	1387.377187	0.0125	0.99	1.022894438	count	1
CDH23	17.451809	1479.496629	0.0118	0.991	1.022894442	count	1
LINC00473	16.9792216	1334.524122	0.0127	0.99	1.022894491	count	1
ECEL1	17.2525544	1270.557728	0.0136	0.9892	1.022894506	count	1
ADRA2B	17.4397206	1027.297903	0.017	0.986	1.022894515	count	1
LUZP2	17.5070504	950.9390575	0.0184	0.985	1.022894517	count	1
CD70	18.302633	630.1996241	0.029	0.977	1.022894904	count	1
GLUL	0.7319226	0.0722458	10.131	8.26E-24	1.02400583	count	1.99E-19
PNMA2	0.9020785	0.2534924	3.5586	0.000378	1.026419883	count	1
ARAP1	0.9550864	0.2286886	4.1764	3.03E-05	1.027021015	count	0.6943548
FRRS1	1.2853947	0.3400299	3.7802	0.000159	1.027598648	count	1
HAS2	1.0703531	0.2780496	3.8495	0.00012	1.028460601	count	1
CTHRC1	0.7291646	0.1276034	5.7143	1.19E-08	1.029250972	count	0.000279876
KDELC1	1.0623059	0.2823723	3.7621	0.000171	1.030754452	count	1
MYADM	0.7251348	0.0634865	11.4219	1.03E-29	1.031695879	count	2.49E-25
BCAM	0.7405962	0.1042649	7.103	1.46E-12	1.032279985	count	3.47E-08
NCK1	0.7829486	0.1266115	6.1839	6.94E-10	1.033854559	count	1.64E-05
CDC14A	1.4984549	0.4193103	3.5736	0.000357	1.034459365	count	1
IGFBP4	0.7247367	0.0673361	10.763	1.28E-26	1.036077287	count	3.09E-22
SDCBP	0.7302416	0.0637167	11.4608	6.67E-30	1.036133696	count	1.61E-25
ADA	1.9494284	0.5762196	3.3831	0.000724	1.036205199	count	1
CCDC74B	1.1867536	0.5021227	2.3635	0.0182	1.039756071	count	1
IGF2BP2	1.122616	0.4449625	2.5229	0.0117	1.04022125	count	1
ECHDC2	0.7528921	0.1182133	6.3689	2.14E-10	1.040697178	count	5.07E-06
IMPA2	0.9763234	0.2252803	4.3338	1.50E-05	1.04225322	count	0.34512
RALGDS	1.1916826	0.3233743	3.6851	0.000232	1.043215839	count	1
GLI3	1.0394073	0.2633072	3.9475	8.04E-05	1.043497182	count	1
STK3	0.8165564	0.1407056	5.8033	7.05E-09	1.043570382	count	0.000165936
ARHGAP15	1.4667205	0.345067	4.2505	2.19E-05	1.044158476	count	0.5028678
GLI2	1.8453383	0.5435577	3.3949	0.000694	1.044367461	count	1
LINC01638	3.6024445	1.1995878	3.0031	0.00269	1.044472453	count	1
ABCC1	0.9298473	0.3209546	2.8971	0.00379	1.044683935	count	1
TRIP10	0.7821379	0.1402115	5.5783	2.60E-08	1.045054594	count	0.00061061
ZFYVE28	1.3828039	0.548027	2.5232	0.0117	1.045366913	count	1
SCARB1	2.4120064	1.200012	2.01	0.0445	1.045776564	count	1
SLC2A10	0.9277808	0.2381807	3.8953	9.98E-05	1.04619507	count	1
DUSP14	0.793741	0.1546334	5.133	3.00E-07	1.046694603	count	0.0070053
MCC	1.1077736	0.3835681	2.8881	0.0039	1.0490967	count	1

NEK11	1.0014909	0.3006788	3.3308	0.000875	1.049189763	count	1
OTUD3	0.9732707	0.4143309	2.349	0.0189	1.049488781	count	1
HSD3B7	0.8189017	0.1692415	4.8387	1.36E-06	1.049981726	count	0.03160096
CACNB4	1.1092408	0.3185953	3.4817	0.000504	1.050259508	count	1
PNMA8B	1.3987556	0.4970765	2.814	0.00492	1.054064592	count	1
PHGDH	0.7692216	0.0886706	8.6751	6.11E-18	1.054719824	count	1.46E-13
NR1H3	1.1279058	0.3253799	3.4664	0.000533	1.054824785	count	1
PPP1R15A	0.7383897	0.0581207	12.7044	3.20E-36	1.05539023	count	7.74E-32
CORO6	2.2093404	0.6157327	3.5881	0.000337	1.056033467	count	1
SLC7A6	1.4917608	0.4725537	3.1568	0.00161	1.056396099	count	1
ANXA3	0.9249709	0.3897554	2.3732	0.0177	1.05691254	count	1
EVC2	0.9711478	0.2858013	3.398	0.000686	1.057005755	count	1
OR2A1-AS1	1.2115895	0.5204519	2.328	0.02	1.057107367	count	1
GGCX	0.9010182	0.2387958	3.7732	0.000164	1.059864527	count	1
CNOT8	0.8525191	0.1598399	5.3336	1.02E-07	1.060252469	count	0.002388636
TENM3	2.9535405	1.1491591	2.5702	0.0102	1.061080526	count	1
LGI4	0.8859452	0.1730759	5.1188	3.23E-07	1.061573412	count	0.007540758
IRAK1BP1	0.9235655	0.2536766	3.6407	0.000276	1.061765878	count	1
OPN3	1.1251773	0.6312394	1.7825	0.0748	1.06284488	count	1
NGFR	1.0240656	0.4038047	2.536	0.0113	1.06383816	count	1
DUSP18	0.9841926	0.3765092	2.614	0.00899	1.064926393	count	1
ZNF549	1.7077218	0.8462754	2.0179	0.0437	1.065511417	count	1
CRYBG3	0.8281764	0.1533007	5.4023	7.00E-08	1.065624494	count	0.00164024
GRIP2	1.1419646	0.3299853	3.4607	0.000545	1.065707121	count	1
RALGPS2	1.3512705	0.3464908	3.8999	9.80E-05	1.066881232	count	1
SPOCK1	0.8934407	0.1961389	4.5551	5.41E-06	1.069964015	count	0.12497641
KLF11	0.9110758	0.2605824	3.4963	0.000477	1.070830682	count	1
ZC3H12A	0.9264953	0.3557653	2.6042	0.00925	1.070925164	count	1
TBC1D16	0.9520263	0.3012597	3.1602	0.00159	1.071069448	count	1
SGMS2	0.9024944	0.1811845	4.9811	6.61E-07	1.07115396	count	0.015406588
NT5E	0.7771069	0.1044132	7.4426	1.22E-13	1.072586152	count	2.91E-09
ARHGAP12	0.8266066	0.1353175	6.1086	1.11E-09	1.074182264	count	2.62E-05
AC020916.1	0.7918141	0.2033818	3.8932	0.000101	1.076310412	count	1
GPBAR1	1.4418824	0.4004245	3.6009	0.000321	1.077178544	count	1
AC025259.3	2.108275	0.9244349	2.2806	0.0226	1.077548901	count	1
ITPR3	1.9553761	0.5116192	3.8219	0.000135	1.077599138	count	1
CDKN1A	0.7681269	0.1007154	7.6267	3.05E-14	1.07818822	count	7.27E-10
FCGRT	0.7588105	0.0587106	12.9246	2.12E-37	1.079178544	count	5.13E-33
CHPT1	0.7725971	0.0822049	9.3984	9.46E-21	1.079947211	count	2.27E-16
CPEB1	1.449198	0.4043372	3.5841	0.000343	1.081041476	count	1
ERLIN1	0.8952601	0.1918215	4.6672	3.16E-06	1.082129446	count	0.07319192
ACER3	0.971473	0.3080535	3.1536	0.00163	1.083143707	count	1
FBN2	1.329637	0.5334113	2.4927	0.0127	1.089264855	count	1
RADIL	1.69167	0.4021487	4.2066	2.65E-05	1.090130692	count	0.607645
DOCK8	1.5122312	0.52077	2.9038	0.00371	1.09048536	count	1

MUC20-OT1	0.8151076	0.2097381	3.8863	0.000104	1.090945902	count	1
MCUB	0.86534	0.1600244	5.4075	6.80E-08	1.091652798	count	0.001593648
RGMA	0.9574288	0.1766875	5.4188	6.39E-08	1.094121484	count	0.001497688
ENPP3	16.7854449	2127.533857	0.0079	0.994	1.094364944	count	1
C19orf18	16.9296977	1180.143743	0.0143	0.9886	1.094364955	count	1
MBNL3	17.0381473	656.1949951	0.026	0.979	1.094364962	count	1
AC023024.1	17.4310643	3270.270023	0.0053	0.996	1.094364984	count	1
HMCN2	17.5488938	1364.550564	0.0129	0.99	1.094364989	count	1
ENTPD2	16.4759274	870.1050571	0.0189	0.985	1.09436499	count	1
LINC00968	17.7085999	831.520021	0.0213	0.983	1.094364994	count	1
HOXC5	17.8075961	832.3034791	0.0214	0.983	1.094364997	count	1
ADAMTSL5	18.3784477	1132.404735	0.0162	0.9871	1.094365011	count	1
AL512274.1	17.111768	2944.93154	0.0058	0.995	1.094365044	count	1
LINC02144	17.4311159	3270.289707	0.0053	0.996	1.09436506	count	1
SCN7A	17.4669779	1096.357666	0.0159	0.987	1.094365062	count	1
MMP27	17.5658853	600.4317792	0.0293	0.977	1.094365066	count	1
HLA-DRB5	0.7960593	0.1497684	5.3153	1.13E-07	1.095462385	count	0.002645556
C6orf132	3.4245096	1.7850649	1.9184	0.0551	1.098163842	count	1
EYA2	2.7747059	0.6821204	4.0678	4.85E-05	1.098242099	count	1
MLPH	2.1978075	1.0738676	2.0466	0.0408	1.098643253	count	1
AC020911.2	1.3743711	0.534403	2.5718	0.0102	1.098858306	count	1
ZNF519	1.3468754	0.4370703	3.0816	0.00207	1.099885228	count	1
NMNAT2	1.4868754	0.4081304	3.6431	0.000273	1.100672115	count	1
ELF4	2.4419769	1.0397122	2.3487	0.0189	1.101028547	count	1
FMO1	2.2165512	0.6866394	3.2281	0.00126	1.102868266	count	1
LINC02202	1.1933768	0.3724176	3.2044	0.00137	1.104951015	count	1
LINC01252	1.417602	0.9370255	1.5129	0.13	1.105020058	count	1
JHY	1.1048628	0.309075	3.5747	0.000355	1.107450254	count	1
CABLES2	2.4889113	1.566475	1.5889	0.112	1.109077773	count	1
BHLHE40	0.8367774	0.1471414	5.6869	1.39E-08	1.110080121	count	0.000326831
LRP3	0.9225602	0.1730831	5.3302	1.04E-07	1.112957516	count	0.002435264
SLC22A5	1.2535846	0.3816248	3.2849	0.00103	1.112964959	count	1
PKDCC	0.8413085	0.1295002	6.4966	9.32E-11	1.113075936	count	2.21E-06
HSPA6	1.0678691	0.6453241	1.6548	0.0981	1.116111242	count	1
ZDHHC9	1.0439755	0.4219654	2.4741	0.0134	1.116932121	count	1
PAPLN	1.1957067	0.5909719	2.0233	0.0431	1.117546575	count	1
PBX3	0.8879231	0.1546185	5.7427	1.01E-08	1.117560642	count	0.000237592
AC007032.1	1.4838721	0.6347646	2.3377	0.0195	1.120881592	count	1
PLP1	3.8914324	1.1695592	3.3273	0.000886	1.121752031	count	1
TRIM25	0.9878677	0.2472444	3.9955	6.58E-05	1.122289813	count	1
ARHGAP21	0.8029608	0.1024193	7.8399	5.86E-15	1.12297998	count	1.40E-10
TSPAN7	0.8973201	0.199823	4.4906	7.32E-06	1.125091491	count	0.16889436
HIF3A	1.361722	0.3211488	4.2402	2.29E-05	1.125842214	count	0.5257611
GPAT3	1.337709	0.6110197	2.1893	0.0286	1.126764043	count	1
TMEM255A	4.033295	1.0219284	3.9467	8.07E-05	1.127058441	count	1

OSR1	0.8001164	0.1079019	7.4152	1.50E-13	1.128970996	count	3.57E-09
MAPK10	0.8496167	0.1606509	5.2886	1.30E-07	1.129312753	count	0.00304252
MN1	0.9557303	0.2130131	4.4867	7.45E-06	1.129614143	count	0.1718864
SRPX2	0.9766191	0.2306532	4.2341	2.35E-05	1.13309279	count	0.5394425
Z82217.1	1.6018565	1.2053183	1.329	0.184	1.133477056	count	1
EEF2K	0.9403516	0.2126238	4.4226	1.00E-05	1.136995124	count	0.2304
HSPB8	0.8312655	0.120002	6.9271	5.05E-12	1.139479557	count	1.20E-07
CNFN	1.7525558	0.9510351	1.8428	0.0654	1.144017189	count	1
WASF3	0.9817839	0.1807021	5.4332	5.90E-08	1.144411807	count	0.001383019
GPER1	1.1485265	0.3509665	3.2725	0.00108	1.14449067	count	1
RERGL	0.8121741	0.1386273	5.8587	5.08E-09	1.144613834	count	0.00011967
CYP51A1	1.4551072	0.5475801	2.6573	0.00791	1.145553025	count	1
RAI2	0.9032403	0.1545507	5.8443	5.53E-09	1.146125422	count	0.000130209
C1orf167	0.9858279	0.2205771	4.4693	8.08E-06	1.148730244	count	0.1863248
SLC29A3	1.6969489	0.7570497	2.2415	0.0251	1.14965688	count	1
TMEM154	2.7621039	0.9425443	2.9305	0.00341	1.149922883	count	1
SNX33	1.0067503	0.2516699	4.0003	6.45E-05	1.152135503	count	1
ADAMTSL3	0.846138	0.1271264	6.6559	3.23E-11	1.152304247	count	7.66E-07
MEPCE	1.0141279	0.2729282	3.7157	0.000206	1.152862836	count	1
ADGRA2	0.9620375	0.1812639	5.3074	1.18E-07	1.152935669	count	0.002762262
COX4I2	0.9139479	0.185501	4.9269	8.72E-07	1.153416465	count	0.020298416
HSF4	1.2752719	0.3449853	3.6966	0.000222	1.153606619	count	1
NME5	1.078561	0.4180275	2.5801	0.00992	1.154874839	count	1
COMP	1.51359	0.7129626	2.123	0.0338	1.157714425	count	1
CBR3	0.8554822	0.1136526	7.5272	6.49E-14	1.157792551	count	1.55E-09
VAT1	0.8494018	0.1029336	8.2519	2.15E-16	1.162021793	count	5.14E-12
NOG	16.8623175	1029.498094	0.0164	0.9869	1.162461367	count	1
FGF16	16.9383102	707.0598131	0.024	0.981	1.162461373	count	1
NKAIN4	17.197602	2374.818809	0.0072	0.994	1.162461391	count	1
AC104066.2	17.1990299	3076.26621	0.0056	0.996	1.162461391	count	1
TSKS	17.442071	776.5308423	0.0225	0.9821	1.162461404	count	1
AC092354.1	16.4981507	2059.435617	0.008	0.994	1.16246141	count	1
HSPA12B	17.563312	652.2020067	0.0269	0.979	1.16246141	count	1
SOSTDC1	16.6837514	1299.924822	0.0128	0.99	1.16246143	count	1
AC004540.2	18.4090871	1271.006731	0.0145	0.9884	1.162461434	count	1
GREM1	16.9439762	1166.19922	0.0145	0.9884	1.162461454	count	1
TMEM150C	17.1951123	1316.884497	0.0131	0.9896	1.162461471	count	1
IGF2	17.5757466	1160.937817	0.0151	0.9879	1.16246149	count	1
GPC5	17.6400569	1143.505876	0.0154	0.9877	1.162461493	count	1
TBC1D12	0.9479804	0.1807598	5.2444	1.66E-07	1.165619161	count	0.003882408
CAB39L	0.8729328	0.1233717	7.0756	1.77E-12	1.167327538	count	4.21E-08
PARVB	1.4299271	0.4078236	3.5062	0.00046	1.167424646	count	1
DOCK4	1.295108	0.3355826	3.8593	0.000116	1.167771393	count	1
TRERF1	1.4949405	0.4234752	3.5302	0.00042	1.167815596	count	1
RUFY3	0.9388976	0.1897765	4.9474	7.86E-07	1.168850223	count	0.01830594

RPGRIP1L	1.2646281	0.3384737	3.7363	0.00019	1.169414205	count	1
ALDH1L2	1.0961002	0.223744	4.8989	1.01E-06	1.171302731	count	0.02349563
SH3BP5	0.8505758	0.0997649	8.5258	2.19E-17	1.173562809	count	5.24E-13
KIAA0556	1.4206956	0.5130205	2.7693	0.00565	1.178812807	count	1
HLA-DRB1	0.8282597	0.1200881	6.8971	6.22E-12	1.179096302	count	1.48E-07
OLFML2A	1.3310137	0.2432453	5.4719	4.75E-08	1.179993985	count	0.00111397
PLEKHA6	2.0541446	0.8254449	2.4885	0.0129	1.181576924	count	1
MT1G	1.0101494	0.4337244	2.329	0.0199	1.183222423	count	1
GRB14	1.3565687	1.429593	0.9489	0.3427	1.18362512	count	1
C14orf132	0.9368521	0.2881223	3.2516	0.00116	1.185483252	count	1
FLNB	1.0189806	0.2153154	4.7325	2.30E-06	1.18693792	count	0.0533347
ZFP36L2	0.8300564	0.0535973	15.4869	1.88E-52	1.187612398	count	4.56E-48
COLGALT2	3.117231	0.6407623	4.8649	1.19E-06	1.190083316	count	0.02766155
SREBF2	1.0669809	0.2898104	3.6817	0.000235	1.191031259	count	1
SAMHD1	0.8693823	0.1071634	8.1127	6.69E-16	1.193706002	count	1.60E-11
LONRF3	3.1791862	1.8647847	1.7049	0.0883	1.195848687	count	1
TMEM119	1.1105494	0.4159936	2.6696	0.00763	1.20092928	count	1
TPRG1	1.4874844	0.7243933	2.0534	0.0401	1.201326827	count	1
ATF5	0.9708374	0.2014786	4.8186	1.50E-06	1.201678583	count	0.0348435
CLIC2	0.9763383	0.2023486	4.825	1.46E-06	1.203261446	count	0.03391872
RNF125	2.7818093	1.4670061	1.8962	0.058	1.203413563	count	1
SOCS1	0.9103894	0.1604649	5.6734	1.51E-08	1.205006967	count	0.000354956
LRRC75B	1.0553811	0.2523485	4.1822	2.95E-05	1.205861343	count	0.67614
CDH6	1.073023	0.2039424	5.2614	1.51E-07	1.20912501	count	0.003532796
PLEKHA2	1.1149475	0.2782666	4.0068	6.28E-05	1.210227436	count	1
PLD1	1.2094318	0.3070418	3.939	8.34E-05	1.211679642	count	1
OLFML1	0.8981403	0.1089315	8.245	2.28E-16	1.213480806	count	5.45E-12
KIAA1841	1.1146617	0.3364822	3.3127	0.000933	1.219838879	count	1
ROBO4	2.3627943	0.7845474	3.0117	0.00262	1.221177225	count	1
FAM43A	1.8772933	0.4446933	4.2215	2.49E-05	1.221701532	count	0.5712309
MOK	1.094831	0.2119802	5.1648	2.54E-07	1.22288368	count	0.005934202
ADGRG2	2.93449	0.7902165	3.7135	0.000207	1.223078222	count	1
SLFN11	1.0553031	0.1874657	5.6293	1.94E-08	1.225364869	count	0.000455881
DACT2	3.569735	1.137253	3.1389	0.00171	1.225428446	count	1
FSTL3	0.9095687	0.1280247	7.1046	1.44E-12	1.226033504	count	3.42E-08
GPSM2	1.082368	0.2306844	4.692	2.80E-06	1.226215726	count	0.0648844
FABP4	0.8859249	0.2865203	3.092	0.002	1.226914144	count	1
LRFN5	17.618497	1333.680038	0.0132	0.9895	1.227487957	count	1
PRKCQ	17.2790001	3201.763199	0.0054	0.996	1.227488023	count	1
CTSK	0.8633852	0.0748174	11.5399	2.75E-30	1.227671395	count	6.64E-26
PCSK5	1.0813163	0.2556635	4.2295	2.40E-05	1.228857469	count	0.550776
SLC17A5	0.9913505	0.1879359	5.2749	1.40E-07	1.231631809	count	0.003276
SEMA6A	2.2415121	1.0242816	2.1884	0.0287	1.232745737	count	1
EPHX1	0.8746263	0.0686049	12.7487	1.86E-36	1.233070141	count	4.50E-32
AC239809.3	2.4238087	1.4096428	1.7194	0.0856	1.234224459	count	1

SLC31A2	1.0794066	0.2427632	4.4463	8.99E-06	1.237534644	count	0.20724647
C19orf44	1.2137754	0.4769439	2.5449	0.011	1.238425395	count	1
JUND	0.8625495	0.0391283	22.0441	3.10E-101	1.241347066	count	7.54E-97
STON2	3.1172622	0.8061176	3.867	0.000112	1.243310995	count	1
CBLB	1.0077226	0.1951544	5.1637	2.55E-07	1.244358845	count	0.00595731
ABHD14A	0.9102024	0.1004942	9.0573	2.13E-19	1.245039589	count	5.11E-15
SLC16A8	1.2838073	0.3622154	3.5443	0.000399	1.246047369	count	1
ACAD11	2.4838365	2.1678611	1.1458	0.252	1.246427249	count	1
SHC3	2.7582405	0.7077383	3.8973	9.90E-05	1.24856836	count	1
GABARAPL1	0.9055135	0.0962272	9.4102	8.48E-21	1.24942805	count	2.04E-16
S100A8	1.4832521	0.9901439	1.498	0.1342	1.249907721	count	1
OLFM2	1.095169	0.3586232	3.0538	0.00228	1.250234705	count	1
NKAIN3	1.360134	0.5646249	2.4089	0.016	1.250707418	count	1
NR4A2	0.9164523	0.1627372	5.6315	1.92E-08	1.250901212	count	0.0004512
BMP4	0.9785935	0.1763373	5.5496	3.07E-08	1.253764767	count	0.000720867
PRKAG2-AS1	1.348995	0.3475713	3.8812	0.000106	1.254218665	count	1
CAMK2N1	0.920773	0.1172335	7.8542	5.24E-15	1.254422388	count	1.25E-10
HSPA12A	1.094083	0.2804098	3.9017	9.72E-05	1.256094469	count	1
FGD5	2.075333	0.5664971	3.6634	0.000252	1.257976515	count	1
COCH	1.829165	1.0675886	1.7134	0.0867	1.259263451	count	1
TIPARP	0.9460251	0.1435145	6.5918	4.96E-11	1.260657589	count	1.18E-06
METRNL	0.9129283	0.0977679	9.3377	1.66E-20	1.260992104	count	3.99E-16
EFNA5	1.2929465	0.3239014	3.9918	6.69E-05	1.262597491	count	1
SLC9A9	1.1214826	0.2038188	5.5024	4.00E-08	1.265675998	count	0.00093852
NPR3	0.9341946	0.2351482	3.9728	7.24E-05	1.269073492	count	1
AKAP12	0.8941945	0.0830711	10.7642	1.26E-26	1.26973728	count	3.04E-22
FBRSL1	1.0662123	0.2352668	4.5319	6.03E-06	1.272386225	count	0.13923873
VSIR	0.9135138	0.0940045	9.7178	4.66E-22	1.273166306	count	1.12E-17
PLA2G7	2.6358152	1.2773551	2.0635	0.0391	1.274681811	count	1
METAP1D	1.0826547	0.3029372	3.5739	0.000356	1.274718551	count	1
FOS	0.8842898	0.0433957	20.3773	1.57E-87	1.2749019	count	3.82E-83
DLX2	1.009293	0.245919	4.1042	4.15E-05	1.275616388	count	0.9495615
CYB5A	0.9003117	0.0663521	13.5687	5.89E-41	1.276128191	count	1.43E-36
IGFBP5	0.8868238	0.0540396	16.4106	1.85E-58	1.277492668	count	4.49E-54
ING2	0.9509003	0.1242614	7.6524	2.51E-14	1.278410167	count	5.98E-10
PRIMA1	2.9846819	1.2830792	2.3262	0.0201	1.27905231	count	1
FAM155A	1.8142431	0.8447147	2.1478	0.0318	1.279125412	count	1
IL20RA	2.4437715	0.8031042	3.0429	0.00236	1.279997163	count	1
IL17RD	2.6801596	0.7167349	3.7394	0.000187	1.282254171	count	1
RHOB	0.9017667	0.0611795	14.7397	8.15E-48	1.284524813	count	1.98E-43
COL4A3	1.3479966	0.5337834	2.5254	0.0116	1.28587248	count	1
MMP23B	0.9339274	0.0905747	10.3111	1.36E-24	1.287894711	count	3.28E-20
ISLR	0.9136174	0.0772777	11.8225	1.11E-31	1.288326071	count	2.68E-27
PNLIPRP3	16.9731086	791.3205124	0.0214	0.9829	1.289709524	count	1
ABCB5	17.0477792	859.6060323	0.0198	0.984	1.28970953	count	1

PBX4	17.3520175	2640.567625	0.0066	0.995	1.289709552	count	1
SLC1A2	17.352074	2370.9017	0.0073	0.994	1.289709552	count	1
STRC	17.3527069	3321.960784	0.0052	0.996	1.289709552	count	1
TNMD	18.194325	1843.016395	0.0099	0.992	1.289709587	count	1
KCNE5	17.0938382	1071.364909	0.016	0.9873	1.289709619	count	1
FASTKD1	1.908493	0.8990976	2.1227	0.0338	1.291150268	count	1
IPMK	1.5252717	0.3074484	4.9611	7.33E-07	1.291702789	count	0.017078167
FAM3B	2.0865369	0.6359292	3.2811	0.00104	1.294474443	count	1
PCK2	1.2111363	0.2143655	5.6499	1.73E-08	1.294663992	count	0.000406602
GXYLT2	0.9624165	0.1216272	7.9128	3.30E-15	1.29518096	count	7.88E-11
SLC25A37	0.9311812	0.1114151	8.3578	8.96E-17	1.297777012	count	2.14E-12
BICC1	0.9352284	0.0956668	9.7759	2.67E-22	1.299968765	count	6.42E-18
PIK3R1	0.923422	0.0742198	12.4417	7.76E-35	1.302284228	count	1.88E-30
RGL1	1.1760521	0.1898624	6.1942	6.50E-10	1.302570149	count	1.54E-05
PCNX2	1.87726	0.5180028	3.624	0.000294	1.305623601	count	1
GADD45B	0.9089211	0.0876276	10.3725	7.27E-25	1.306869014	count	1.75E-20
FAM19A5	1.4223234	0.4071184	3.4936	0.000482	1.306969534	count	1
JDP2	0.9514932	0.1085335	8.7668	2.76E-18	1.30729721	count	6.62E-14
OTULINL	1.6835437	0.502194	3.3524	0.000809	1.308735017	count	1
NFIL3	1.0245046	0.1557913	6.5761	5.51E-11	1.31148877	count	1.31E-06
CD38	2.2768078	0.8420689	2.7038	0.00689	1.316332943	count	1
PROSER2	1.9765199	0.4633759	4.2655	2.05E-05	1.317115505	count	0.470926
EHBP1	1.0223213	0.1399491	7.305	3.39E-13	1.320906095	count	8.07E-09
RNF212	1.9911017	0.5382528	3.6992	0.000219	1.322519099	count	1
PCDH9	1.0366746	0.1969969	5.2624	1.50E-07	1.324157012	count	0.0035097
TLNRD1	1.1012137	0.1946731	5.6567	1.66E-08	1.329239613	count	0.0003902
MAP1A	0.9743597	0.1045649	9.3182	1.98E-20	1.32961047	count	4.76E-16
BAIAP2	2.2090342	0.6146392	3.594	0.00033	1.332084426	count	1
FAXDC2	1.0019515	0.1264061	7.9264	2.97E-15	1.33299349	count	7.09E-11
ACAP3	1.1879472	0.324927	3.656	0.00026	1.335907767	count	1
DDIT4	0.9749153	0.114954	8.4809	3.20E-17	1.338237357	count	7.66E-13
NOSTRIN	3.6391412	1.5232118	2.3891	0.0169	1.338740095	count	1
MEDAG	0.9878553	0.1160954	8.509	2.52E-17	1.338875477	count	6.03E-13
RGS6	2.7679195	0.7746428	3.5732	0.000357	1.340858625	count	1
LRP1	0.9427544	0.0725734	12.9904	9.33E-38	1.341932067	count	2.26E-33
HHIP	3.1159256	1.1554193	2.6968	0.00703	1.342831538	count	1
TMEM37	1.2083151	0.349811	3.4542	0.000558	1.342910587	count	1
PQLC2L	2.3922915	0.6199263	3.859	0.000116	1.345828611	count	1
ADRA2A	1.3645181	0.3239365	4.2123	2.59E-05	1.346656183	count	0.5939647
LOX	1.0167706	0.1412229	7.1998	7.28E-13	1.346935981	count	1.73E-08
TP53I3	1.0223087	0.1528264	6.6893	2.58E-11	1.348439817	count	6.12E-07
AQP2	16.9639994	1210.269017	0.014	0.989	1.349358179	count	1
LRRIQ1	17.1030185	789.4522109	0.0217	0.983	1.349358192	count	1
ENTPD3	17.7263664	706.4741693	0.0251	0.98	1.34935823	count	1
BDKRB2	17.7798506	741.3100704	0.024	0.9809	1.349358233	count	1

KCNJ12	18.2015939	663.8196858	0.0274	0.978	1.349358247	count	1
PMP22	0.9453757	0.0531181	17.7976	4.95E-68	1.350651744	count	1.20E-63
LRRC10B	2.070412	0.4853697	4.2656	2.04E-05	1.350930023	count	0.4686696
CAVIN2	0.9764623	0.2197913	4.4427	9.15E-06	1.352670544	count	0.21089835
C1R	0.9420705	0.0465612	20.233	2.20E-86	1.354693251	count	5.35E-82
ACKR4	1.0004285	0.1389459	7.2001	7.26E-13	1.354848715	count	1.73E-08
CDKN1C	0.9526254	0.0723257	13.1713	9.59E-39	1.355246152	count	2.32E-34
DSE	1.0557541	0.1449286	7.2847	3.93E-13	1.355317471	count	9.36E-09
QPRT	1.270139	0.4099575	3.0982	0.00196	1.360076712	count	1
TEX26-AS1	1.5648616	0.8795076	1.7792	0.0753	1.362235624	count	1
CHEK2	1.3372689	0.311241	4.2966	1.78E-05	1.365644832	count	0.4092042
SSC5D	1.2531671	0.2531358	4.9506	7.73E-07	1.36631999	count	0.018003943
CCNL1	0.9680691	0.1003887	9.6432	9.49E-22	1.367663503	count	2.28E-17
AC254633.1	2.223787	0.5461272	4.0719	4.76E-05	1.369515949	count	1
DLL1	2.3587355	1.0893376	2.1653	0.0304	1.373439225	count	1
PPL	1.1884762	0.251873	4.7186	2.46E-06	1.37363219	count	0.05703018
PXN	1.2453579	0.2291761	5.4341	5.87E-08	1.373875003	count	0.001376104
TET1	1.1688926	0.2374393	4.9229	8.90E-07	1.374918228	count	0.02071564
NAALAD2	2.3659623	0.7591049	3.1168	0.00184	1.375313936	count	1
FOXP2	1.2422367	0.3396785	3.6571	0.000259	1.375617343	count	1
GDPD5	1.7003891	0.3698418	4.5976	4.42E-06	1.375761811	count	0.10220808
GPX3	0.9723748	0.0823723	11.8046	1.37E-31	1.376532903	count	3.31E-27
XG	1.0670998	0.1823167	5.853	5.25E-09	1.377528146	count	0.000123659
CYBRD1	0.9781737	0.0767683	12.7419	2.02E-36	1.378487638	count	4.89E-32
S1PR2	1.8254697	0.3684304	4.9547	7.57E-07	1.378868298	count	0.017635829
NTNG1	1.940153	0.5846867	3.3183	0.000915	1.382893717	count	1
UST	1.7233652	0.3576752	4.8182	1.51E-06	1.38817454	count	0.03507277
CCDC69	1.2331456	0.2088189	5.9053	3.84E-09	1.388767582	count	9.05E-05
CCDC146	1.1382018	0.2191886	5.1928	2.18E-07	1.39105793	count	0.005095532
OLFM1	1.196769	0.2058905	5.8126	6.67E-09	1.392008936	count	0.000157012
KIF21A	1.1218741	0.23882	4.6976	2.73E-06	1.392009652	count	0.06327048
CCL28	1.2562289	0.2631197	4.7744	1.87E-06	1.39368785	count	0.04339335
NCAM2	1.9684824	0.6008942	3.2759	0.00106	1.394571832	count	1
KLF2	0.9734089	0.061778	15.7566	3.56E-54	1.398467983	count	8.64E-50
WNT2	3.811211	1.0932597	3.4861	0.000496	1.401039163	count	1
HS6ST1	1.6283124	0.3657646	4.4518	8.77E-06	1.401521786	count	0.20220112
IL17D	1.2017473	0.2305926	5.2116	1.98E-07	1.403464001	count	0.004628844
IQCA1	3.2398939	1.115629	2.9041	0.00371	1.403598498	count	1
POLR3G	3.248232	1.3126272	2.4746	0.0134	1.404483859	count	1
HBB	16.8619924	1709.898609	0.0099	0.9921	1.406638197	count	1
AC092910.3	16.952786	2315.450746	0.0073	0.994	1.406638207	count	1
DPP4	17.4930811	800.5015502	0.0219	0.9826	1.40663825	count	1
PAPPA2	3.2753194	1.6777232	1.9522	0.051	1.407313349	count	1
PTGDS	0.9848936	0.2824053	3.4875	5.00E-04	1.408594669	count	1
SLC43A3	1.0915483	0.1817688	6.0051	2.10E-09	1.410972337	count	4.95E-05

LGI1	2.5146365	0.6263223	4.0149	6.07E-05	1.411543425	count	1
LOXL4	2.2687138	0.59551	3.8097	0.000141	1.415046601	count	1
SPESP1	1.306055	0.3586114	3.642	0.000274	1.415395295	count	1
TTC7A	1.4301492	0.3395191	4.2123	2.59E-05	1.416386592	count	0.5939647
PTGIS	0.9934794	0.0746368	13.3108	1.63E-39	1.417409583	count	3.95E-35
LSAMP	1.1979021	0.3988287	3.0036	0.00269	1.422341488	count	1
IGDCC4	2.569751	0.7237906	3.5504	0.000389	1.423888998	count	1
NID1	1.2453758	0.2244187	5.5493	3.07E-08	1.42444145	count	0.000720867
GDF10	1.1376803	0.296343	3.8391	0.000126	1.425525033	count	1
CSRNP1	1.0633418	0.1266836	8.3937	6.64E-17	1.426739524	count	1.59E-12
CACNA1H	2.3089488	0.6210737	3.7177	0.000204	1.426911642	count	1
HOXA4	1.392622	0.3509945	3.9676	7.40E-05	1.427062669	count	1
APOL4	1.5647614	0.3509785	4.4583	8.51E-06	1.43004811	count	0.19622358
TRAC	1.5053138	0.3373968	4.4616	8.38E-06	1.434396416	count	0.19323442
CHADL	1.7422423	0.4210346	4.138	3.58E-05	1.434658532	count	0.8197126
CSF1	1.0667587	0.139441	7.6503	2.55E-14	1.438530949	count	6.08E-10
KCNK1	2.1581757	0.4383646	4.9232	8.89E-07	1.439268761	count	0.020693253
AVPR1A	1.6215236	0.5110652	3.1728	0.00152	1.441030162	count	1
ST3GAL5	1.4456237	0.3135965	4.6098	4.17E-06	1.454543853	count	0.09646461
JUN	1.0116744	0.0491815	20.5702	4.50E-89	1.457639471	count	1.09E-84
STAMBPL1	1.9066777	0.9372413	2.0344	0.042	1.460273401	count	1
MAL	1.6521136	0.4877659	3.3871	0.000714	1.460418695	count	1
ADAMTSL1	1.7279639	0.3788584	4.561	5.26E-06	1.460492041	count	0.12153756
SNHG22	17.0526949	2126.548124	0.008	0.994	1.461730606	count	1
BMP5	17.4576598	736.685249	0.0237	0.9811	1.461730639	count	1
NRP1	1.0475198	0.0994317	10.5351	1.38E-25	1.468833513	count	3.33E-21
CEBPD	1.0233029	0.0593797	17.2332	4.67E-64	1.471665959	count	1.13E-59
SULT1A1	1.2291752	0.2442744	5.0319	5.09E-07	1.473803036	count	0.011871916
RECK	1.1309732	0.1422477	7.9507	2.45E-15	1.475021514	count	5.85E-11
SELENOP	1.0413624	0.07443	13.9912	2.26E-43	1.478425549	count	5.48E-39
UGDH	1.1158643	0.120318	9.2743	2.97E-20	1.481892045	count	7.13E-16
NINJ2	1.6874745	0.31381	5.3774	8.03E-08	1.4824346	count	0.001881108
SLC2A3	1.0873501	0.1497579	7.2607	4.68E-13	1.482919476	count	1.11E-08
CD55	1.0543757	0.1294296	8.1463	5.09E-16	1.483838374	count	1.22E-11
CNTN4	1.131322	0.1703976	6.6393	3.62E-11	1.486776291	count	8.59E-07
PTGFRN	1.3552869	0.3323579	4.0778	4.64E-05	1.497218102	count	1
JARID2	1.4424539	0.4897862	2.9451	0.00325	1.498125746	count	1
ZDHHC11	1.795084	1.0945285	1.6401	0.101	1.498347048	count	1
HIST3H2A	1.5411812	0.3112775	4.9511	7.71E-07	1.501594304	count	0.017958132
DLX1	1.242794	0.2556191	4.8619	1.21E-06	1.504932665	count	0.02812403
RIMS4	2.1984858	0.5948584	3.6958	0.000222	1.508343478	count	1
SPRY2	1.2665195	0.1707841	7.4159	1.49E-13	1.508785872	count	3.55E-09
EBF2	1.8846127	0.3429967	5.4946	4.18E-08	1.509796375	count	0.000980586
PIEZO2	1.9677297	0.6092805	3.2296	0.00125	1.509798336	count	1
AMOTL2	1.1549903	0.2104766	5.4875	4.35E-08	1.512151974	count	0.001020249

AC116036.2	2.2913348	2.3609817	0.9705	0.332	1.512224586	count	1
CDA	2.087075	0.6117994	3.4114	0.000653	1.51645459	count	1
PTPRS	1.3880667	0.3360414	4.1306	3.70E-05	1.517536595	count	0.847078
USP2	2.4146237	0.7149525	3.3773	0.00074	1.520033316	count	1
CHRD	1.4662239	0.2607945	5.6221	2.03E-08	1.525212988	count	0.000476989
3-Mar	1.484147	0.3516575	4.2204	2.50E-05	1.525473202	count	0.573475
FGF9	4.355423	1.5787252	2.7588	0.00583	1.526553382	count	1
MATN2	1.1389526	0.1996566	5.7046	1.26E-08	1.527603945	count	0.000296314
TRIO	1.2449329	0.2987674	4.1669	3.16E-05	1.529847283	count	0.723956
IFITM1	1.1067608	0.1177874	9.3963	9.65E-21	1.531227682	count	2.32E-16
NOVA1	1.2218048	0.1657318	7.3722	2.06E-13	1.533295224	count	4.91E-09
NTRK2	1.1328495	0.1023096	11.0728	4.71E-28	1.533366653	count	1.14E-23
PCK1	1.591875	0.8807359	1.8074	0.0708	1.539090794	count	1
KCNQ1OT1	1.1441508	0.2668875	4.287	1.86E-05	1.543436782	count	0.4274652
ADAMTS2	1.4164866	0.2601252	5.4454	5.51E-08	1.543522511	count	0.00129193
SLC16A7	1.1591707	0.153607	7.5463	5.61E-14	1.545537413	count	1.34E-09
TNFSF9	2.1679168	0.5476222	3.9588	7.68E-05	1.548292561	count	1
CCL21	1.1123815	0.4348972	2.5578	0.0106	1.549433595	count	1
IER2	1.0802535	0.0596742	18.1025	3.19E-70	1.552745801	count	7.75E-66
ESR1	1.4879415	0.4148741	3.5865	0.00034	1.564253679	count	1
SPRR2F	17.6674479	1457.394285	0.0121	0.99	1.565979273	count	1
LTBP4	1.0974004	0.0604581	18.1514	1.41E-70	1.567532475	count	3.43E-66
SKAP2	1.7724459	0.4404846	4.0239	5.84E-05	1.577218836	count	1
ECM1	1.1675876	0.1268645	9.2034	5.68E-20	1.584039822	count	1.36E-15
TPST1	1.2503984	0.14824	8.435	4.70E-17	1.585555289	count	1.12E-12
ZNF385A	1.3812148	0.2238446	6.1704	7.55E-10	1.586064959	count	1.78E-05
XPNPEP2	3.7676514	1.1191973	3.3664	0.000769	1.586318817	count	1
PRICKLE1	1.726339	0.3555816	4.855	1.25E-06	1.587357793	count	0.0290525
CACHD1	1.5626963	0.364225	4.2905	1.83E-05	1.588974253	count	0.4205889
MAFF	1.1563015	0.1115557	10.3652	7.83E-25	1.589553597	count	1.89E-20
RBPMS2	1.6828736	0.2655492	6.3373	2.62E-10	1.593245424	count	6.20E-06
MATN4	3.3874071	1.2783389	2.6499	0.00809	1.593675374	count	1
PRRG3	3.959481	1.407424	2.8133	0.00493	1.599422981	count	1
OLFM4	1.9833615	0.6912876	2.8691	0.00414	1.615077837	count	1
KRT27	16.811538	608.9671725	0.0276	0.978	1.615408411	count	1
SREBF1	1.558752	0.2652075	5.8775	4.54E-09	1.616397463	count	0.000106981
FSTL1	1.1348367	0.0773288	14.6755	2.00E-47	1.616769545	count	4.85E-43
KANK3	2.814547	0.9558393	2.9446	0.00325	1.617346977	count	1
FOSB	1.135995	0.089904	12.6356	7.42E-36	1.623059848	count	1.79E-31
TMOD2	1.3596652	0.2222384	6.118	1.05E-09	1.623421734	count	2.48E-05
JUNB	1.1274071	0.0510253	22.0951	1.15E-101	1.623676441	count	2.80E-97
C2CD4B	2.481163	1.4680727	1.6901	0.0911	1.627554824	count	1
LINC00632	1.6347247	0.3947845	4.1408	3.54E-05	1.627944392	count	0.8106954
HHIP-AS1	2.713788	0.6042859	4.4909	7.31E-06	1.628275502	count	0.16867094
ABCC9	1.1936121	0.1273365	9.3737	1.19E-20	1.631967471	count	2.86E-16

KREMEN1	1.9374633	0.3299221	5.8725	4.67E-09	1.64248608	count	0.00011003
IL18	1.9092826	0.3893642	4.9036	9.82E-07	1.642784997	count	0.022847212
COLEC12	1.2446003	0.1420685	8.7606	2.92E-18	1.64672083	count	7.00E-14
VEGFD	3.2175689	1.2423048	2.59	0.00964	1.650505197	count	1
ANKRD65	2.3837995	0.6236199	3.8225	0.000134	1.650767744	count	1
THBS1	1.2602583	0.1776539	7.0939	1.56E-12	1.651123792	count	3.71E-08
CPB1	17.2446579	1017.237102	0.017	0.986	1.663199948	count	1
STC1	16.921628	1676.574863	0.0101	0.992	1.663200008	count	1
ACVRL1	1.6135613	0.2326954	6.9342	4.81E-12	1.66836337	count	1.14E-07
TMEM233	1.35475	0.3224147	4.2019	2.71E-05	1.673027677	count	0.6213488
FAM198A	1.8443375	0.4289537	4.2996	1.76E-05	1.675248776	count	0.404624
NAV3	1.9708784	0.5410999	3.6424	0.000274	1.676605278	count	1
GPRC5A	1.2435482	0.1760083	7.0653	1.91E-12	1.677341985	count	4.54E-08
NHSL1	1.8711084	0.5904822	3.1688	0.00154	1.678419598	count	1
LINC00924	2.07796	0.5839086	3.5587	0.000377	1.679701174	count	1
WNT5B	1.9502547	0.5983311	3.2595	0.00113	1.680997445	count	1
ADD3	1.2116066	0.0965198	12.5529	2.03E-35	1.686103938	count	4.91E-31
AC245595.1	1.2944945	0.1379764	9.382	1.10E-20	1.687809377	count	2.64E-16
BNC2	1.8523839	0.3766664	4.9178	9.13E-07	1.693068962	count	0.021247336
HIC1	1.3778294	0.1961551	7.0242	2.55E-12	1.694893701	count	6.06E-08
GPAT2	1.783847	0.6723631	2.6531	0.00801	1.695450914	count	1
MYC	1.2813513	0.1271719	10.0757	1.43E-23	1.699235267	count	3.44E-19
MIR99AHG	1.4939687	0.2052775	7.2778	4.13E-13	1.704088572	count	9.83E-09
GRID2	1.9939309	0.5032629	3.962	7.57E-05	1.704870569	count	1
GLB1L	1.912356	0.5821617	3.2849	0.00103	1.716700106	count	1
GLI1	3.4561882	1.4168976	2.4393	0.0148	1.718221962	count	1
ASAP3	1.9953004	0.4384406	4.5509	5.51E-06	1.721105358	count	0.12726998
PILRA	3.2101083	0.8170022	3.9291	8.68E-05	1.721613893	count	1
NFKBIZ	1.2362854	0.1299557	9.5131	3.24E-21	1.723013131	count	7.79E-17
ELMO1	2.0075391	0.5244394	3.828	0.000131	1.727773033	count	1
MTSS1	1.5856884	0.3493278	4.5393	5.83E-06	1.727936326	count	0.13463802
SERPINA5	4.020789	1.2680137	3.1709	0.00153	1.729811167	count	1
SMPDL3A	1.7038135	0.386895	4.4038	1.09E-05	1.731801176	count	0.2510379
MME	3.0945904	1.0995279	2.8145	0.00491	1.737119272	count	1
SH2D4A	1.6547489	0.2604282	6.354	2.36E-10	1.744366959	count	5.59E-06
ACE	3.402237	1.6167203	2.1044	0.0354	1.74815373	count	1
EBF3	2.7273367	0.5986294	4.556	5.38E-06	1.751640266	count	0.12429414
PLTP	1.2534078	0.0942523	13.2984	1.91E-39	1.752777916	count	4.63E-35
ZNF521	18.1775563	726.0104149	0.025	0.98	1.754280759	count	1
CERS1	18.351613	739.6153586	0.0248	0.98	1.754281172	count	1
PLCB1	2.6487957	0.5357381	4.9442	7.99E-07	1.75817986	count	0.018607112
SHC2	1.8640501	0.375532	4.9638	7.23E-07	1.771165227	count	0.0168459
RASSF4	1.4312828	0.1984085	7.2138	6.58E-13	1.778043615	count	1.57E-08
IL16	2.9024689	0.8711622	3.3317	0.000872	1.793295877	count	1
PRCP	1.3272563	0.120418	11.0221	8.13E-28	1.793469459	count	1.96E-23

NPY1R	1.3289259	0.1570543	8.4616	3.76E-17	1.795627512	count	9.00E-13
COL9A3	17.0952575	1159.355595	0.0147	0.988	1.797751704	count	1
CHL1	17.2637407	474.7691765	0.0364	0.971	1.797751725	count	1
TNFSF14	17.3819606	1452.504149	0.012	0.99	1.797751738	count	1
USP53	1.2896746	0.1288547	10.0087	2.77E-23	1.799221517	count	6.67E-19
ADCY3	1.5620745	0.1788347	8.7347	3.65E-18	1.800705037	count	8.75E-14
GPNMB	1.2709012	0.0756058	16.8096	3.82E-61	1.801542693	count	9.27E-57
HES5	1.638244	0.526547	3.1113	0.00188	1.806423818	count	1
GALNT12	1.9479539	0.348745	5.5856	2.50E-08	1.814046372	count	0.0005872
CKB	1.3275715	0.1105998	12.0034	1.38E-32	1.815099242	count	3.33E-28
WISP2	1.2721705	0.1038565	12.2493	7.70E-34	1.816033087	count	1.86E-29
EGR1	1.2757311	0.0609211	20.9407	4.55E-92	1.828512892	count	1.11E-87
ARL4D	1.372093	0.1530574	8.9646	4.86E-19	1.832084483	count	1.17E-14
BRINP3	17.8858483	700.6803305	0.0255	0.9796	1.839951193	count	1
ADAM33	1.598242	0.2611755	6.1194	1.04E-09	1.846809128	count	2.46E-05
C1S	1.2906154	0.0599587	21.5251	7.06E-97	1.847435412	count	1.72E-92
BIN1	1.684282	0.2019029	8.342	1.02E-16	1.852396768	count	2.44E-12
ZNF423	2.3013896	0.4447978	5.174	2.41E-07	1.855533319	count	0.005631206
MEG3	1.3560309	0.2073679	6.5393	7.04E-11	1.866525231	count	1.67E-06
ACSL5	3.5703581	1.2165482	2.9348	0.00336	1.872507815	count	1
PLPP3	1.3681781	0.1056179	12.954	1.47E-37	1.880569516	count	3.56E-33
SLC18A3	17.9631979	970.5016174	0.0185	0.985	1.880951242	count	1
CEP152	2.3600508	0.7293052	3.236	0.00122	1.880952198	count	1
DENND2A	3.3927299	1.2353056	2.7465	0.00605	1.881720765	count	1
ZFP36	1.3138634	0.0588561	22.3233	1.30E-103	1.890414552	count	3.16E-99
AGTR1	1.8501291	0.3019453	6.1274	9.88E-10	1.891336389	count	2.33E-05
GFPT2	2.0016737	0.2902735	6.8958	6.28E-12	1.893599966	count	1.49E-07
TWIST2	1.4650186	0.1860465	7.8745	4.47E-15	1.897359755	count	1.07E-10
CCL8	1.8008113	0.4855653	3.7087	0.000211	1.903560866	count	1
STXBP1	1.6547247	0.2550646	6.4875	9.90E-11	1.904909725	count	2.35E-06
OLFML3	1.3750508	0.1027233	13.386	6.23E-40	1.905716932	count	1.51E-35
TSPAN8	1.6493115	0.264391	6.2382	4.93E-10	1.917701245	count	1.17E-05
MLXIPL	17.332179	492.4946874	0.0352	0.972	1.920817945	count	1
CH25H	2.331666	0.6955048	3.3525	0.000809	1.921471515	count	1
DIO3OS	1.9667871	0.5209108	3.7757	0.000162	1.922335141	count	1
C1orf21	1.5345577	0.1599524	9.5938	1.52E-21	1.933830959	count	3.65E-17
TCIM	1.5258892	0.2732158	5.5849	2.51E-08	1.942784342	count	0.000589524
ANK2	1.514555	0.2563456	5.9083	3.77E-09	1.944805987	count	8.89E-05
ALKAL2	3.9550429	1.3277249	2.9788	0.00291	1.945250235	count	1
PCDH17	2.7080214	0.7718057	3.5087	0.000456	1.947064574	count	1
TNFAIP2	1.481966	0.1700832	8.7132	4.40E-18	1.954953262	count	1.05E-13
FAIM2	3.2950586	0.8642221	3.8127	0.00014	1.957206935	count	1
DBN1	1.4504148	0.1250392	11.5997	1.40E-30	1.957875664	count	3.38E-26
CMYA5	2.0076353	0.5705877	3.5185	0.000439	1.959550806	count	1
CYGB	1.491391	0.1852979	8.0486	1.12E-15	1.961754822	count	2.68E-11

CRABP1	2.362194	0.6086498	3.881	0.000106	1.968551139	count	1
ZFHX4	1.8749733	0.3570627	5.2511	1.60E-07	1.969255196	count	0.00374288
HLX	2.0614051	0.4003562	5.1489	2.76E-07	1.974621236	count	0.00644598
WNT2B	2.2570439	0.4846313	4.6572	3.32E-06	1.978001819	count	0.0768746
MSX1	1.4728223	0.1379296	10.6781	3.12E-26	1.978534825	count	7.52E-22
RAMP2-AS1	18.0596369	833.3956701	0.0217	0.9827	1.997392185	count	1
SLIT3	1.4238747	0.1107794	12.8532	5.13E-37	2.00405214	count	1.24E-32
ASPA	3.4504671	0.971055	3.5533	0.000385	2.011104165	count	1
NDNF	1.7182123	0.3160346	5.4368	5.78E-08	2.011710434	count	0.001355121
GPC3	1.4924323	0.2344062	6.3669	2.17E-10	2.01690816	count	5.14E-06
ANGPTL5	2.4838857	0.37993	6.5377	7.11E-11	2.022033231	count	1.69E-06
ELANE	3.5777117	1.4323682	2.4978	0.0125	2.029455576	count	1
RASSF2	2.242562	0.4905025	4.572	4.99E-06	2.036768402	count	0.11533886
TLR5	2.048359	0.3620965	5.6569	1.66E-08	2.042533528	count	0.0003902
MMP2	1.4639275	0.1013512	14.4441	4.90E-46	2.047907191	count	1.19E-41
PCOLCE2	1.4503315	0.1083549	13.385	6.31E-40	2.055339775	count	1.53E-35
LSP1	1.6073884	0.1738453	9.2461	3.85E-20	2.057405413	count	9.24E-16
GRIK1	18.1351693	977.4654396	0.0186	0.985	2.070105656	count	1
KRT222	18.2277714	648.415276	0.0281	0.9776	2.070105662	count	1
WNT10B	18.2345027	638.5505402	0.0286	0.977	2.070105662	count	1
RETREG1	2.132802	0.3121624	6.8323	9.74E-12	2.080597947	count	2.31E-07
MAN1C1	1.7737027	0.2224505	7.9735	2.04E-15	2.088177457	count	4.87E-11
BST1	1.6191395	0.1651034	9.8068	1.98E-22	2.090538277	count	4.76E-18
ABCC2	3.1876478	1.1063388	2.8813	0.00398	2.095590489	count	1
WNT5A	3.4946957	1.0217564	3.4203	0.000632	2.101240879	count	1
ALDH1A1	1.5833387	0.1297568	12.2024	1.34E-33	2.103979879	count	3.24E-29
MT1A	1.7035695	0.203648	8.3653	8.42E-17	2.111235173	count	2.01E-12
FYN	1.6838001	0.2021804	8.3282	1.15E-16	2.120663375	count	2.75E-12
PRPH	3.6272342	0.8761747	4.1399	3.55E-05	2.120950865	count	0.8129145
FGF7	1.5132285	0.1020244	14.832	2.23E-48	2.136911164	count	5.41E-44
FGF10	4.3743153	1.5321521	2.855	0.00433	2.137136204	count	1
PDGFRL	1.5284118	0.0957198	15.9676	1.54E-55	2.14578273	count	3.74E-51
STXBP6	2.458165	0.3826947	6.4233	1.50E-10	2.148387858	count	3.55E-06
DCLK1	1.8590983	0.2252033	8.2552	2.09E-16	2.160267318	count	5.00E-12
ADH1C	3.8127513	1.6297252	2.3395	0.0194	2.172391204	count	1
TMEM100	2.2162611	0.5198309	4.2634	2.06E-05	2.181275695	count	0.473182
BTG2	1.5402272	0.0829629	18.5653	1.31E-73	2.199817099	count	3.18E-69
PYHIN1	17.31015	482.3974028	0.0359	0.9714	2.205383373	count	1
NOX4	1.9381534	0.2928542	6.6182	4.17E-11	2.21065621	count	9.89E-07
SLIT2	1.6285121	0.1503612	10.8307	6.27E-27	2.216404437	count	1.51E-22
ISM1	2.366947	0.3275688	7.2258	6.03E-13	2.233498979	count	1.44E-08
NR4A1	1.5667212	0.1044923	14.9937	2.28E-49	2.234153933	count	5.53E-45
ADGRD1	3.4133608	0.8433023	4.0476	5.28E-05	2.236349124	count	1
TNNT3	17.237641	539.0423273	0.032	0.9745	2.237309667	count	1
PCSK6	17.5465169	626.9873373	0.028	0.9777	2.237309713	count	1

TGFBR3	1.8604569	0.2017	9.2239	4.71E-20	2.240977847	count	1.13E-15
FGFBP2	2.4651915	0.8653854	2.8487	0.00441	2.241890464	count	1
ROBO3	2.4455715	0.3936473	6.2126	5.79E-10	2.243428493	count	1.37E-05
CLDN11	2.371617	0.5011001	4.7328	2.30E-06	2.257052312	count	0.0533347
APOLD1	2.2836282	0.4076219	5.6023	2.27E-08	2.269061828	count	0.000533291
CCDC80	1.5847816	0.0794454	19.9481	3.88E-84	2.270896345	count	9.43E-80
ABI3BP	1.6121855	0.1048357	15.3782	9.15E-52	2.283267365	count	2.22E-47
ADAMTSL4	2.8408319	0.4106682	6.9176	5.40E-12	2.294937383	count	1.28E-07
CDON	2.77325	0.4741317	5.8491	5.37E-09	2.300613358	count	0.000126474
RDH10	2.3962418	0.3570436	6.7113	2.23E-11	2.301519761	count	5.29E-07
NR2F1	2.311536	0.4068796	5.6811	1.44E-08	2.320800509	count	0.000338544
CLEC3B	2.2257916	0.291283	7.6413	2.73E-14	2.321962614	count	6.51E-10
SLPI	1.642216	0.2118983	7.75	1.18E-14	2.347930726	count	2.81E-10
TXNIP	1.6471446	0.0726361	22.6767	1.19E-106	2.35599163	count	2.89E-102
LINC02381	2.4108354	0.3732361	6.4593	1.19E-10	2.357388852	count	2.82E-06
SHISA3	2.2658915	0.2984731	7.5916	3.98E-14	2.370974795	count	9.49E-10
CDC42EP2	2.2471903	0.2973708	7.5569	5.18E-14	2.383197012	count	1.23E-09
GSN	1.6722388	0.0646671	25.8592	1.38E-135	2.409951533	count	3.36E-131
GPRC5B	2.3501555	0.5052316	4.6516	3.41E-06	2.42289836	count	0.07894491
EMP1	1.7524051	0.1318236	13.2936	2.03E-39	2.42624867	count	4.92E-35
CREB5	2.0064508	0.2773968	7.2331	5.72E-13	2.430655275	count	1.36E-08
PLXNC1	2.5855177	0.4310369	5.9984	2.19E-09	2.447867211	count	5.17E-05
IGFBP6	1.7043083	0.0805756	21.1517	8.58E-94	2.454404852	count	2.09E-89
CRISPLD2	1.9365391	0.2128049	9.1001	1.45E-19	2.455551893	count	3.48E-15
SEMA3B	2.000187	0.228255	8.7629	2.86E-18	2.464404313	count	6.86E-14
SPON2	2.0648945	0.4880047	4.2313	2.38E-05	2.465600124	count	0.5462338
RASD1	1.8335904	0.3125076	5.8673	4.82E-09	2.469563034	count	0.000113559
ABCA6	1.8793891	0.1783927	10.5351	1.38E-25	2.483096843	count	3.33E-21
ZNF385B	2.5761472	0.3904854	6.5973	4.79E-11	2.490618558	count	1.14E-06
ADAMTS5	3.7300417	0.8805524	4.236	2.33E-05	2.508175675	count	0.5348748
IGSF10	2.0965919	0.4127166	5.08	3.96E-07	2.509274691	count	0.009241452
TIMP3	1.7489714	0.094718	18.465	7.20E-73	2.511170836	count	1.75E-68
PTGES	3.6036757	0.7873217	4.5771	4.87E-06	2.523209922	count	0.11258953
PAMR1	2.0855961	0.4172563	4.9984	6.05E-07	2.527758084	count	0.014106785
BCHE	17.1295296	460.2578363	0.0372	0.9703	2.548525456	count	1
CTSH	1.9436606	0.171421	11.3385	2.59E-29	2.563348055	count	6.25E-25
MAB21L1	3.0424474	0.6631406	4.5879	4.63E-06	2.573552189	count	0.10705949
SLC1A7	17.424422	448.1755742	0.0389	0.969	2.5737525	count	1
UAP1	1.9157942	0.1437438	13.3278	1.31E-39	2.583498055	count	3.17E-35
FLRT2	2.0906035	0.2317264	9.0219	2.92E-19	2.592891097	count	7.01E-15
SRPX	1.9132956	0.1565415	12.2223	1.06E-33	2.597418918	count	2.56E-29
LVRN	17.5522825	420.707172	0.0417	0.967	2.59854586	count	1
OAF	2.6662061	0.2978004	8.953	5.38E-19	2.660867415	count	1.29E-14
DNM1	3.3107855	0.5664196	5.8451	5.50E-09	2.663630324	count	0.00012952
CNTFR	17.6848117	384.7048313	0.046	0.963	2.670468027	count	1

KLF4	1.9083049	0.1378417	13.8442	1.59E-42	2.677678042	count	3.85E-38
PHYHIP	4.405796	1.096394	4.0184	5.98E-05	2.678676943	count	1
C1QTNF4	2.5865155	0.4678074	5.529	3.44E-08	2.689332959	count	0.00080754
DHRS3	1.9745701	0.1378761	14.3213	2.63E-45	2.703397159	count	6.38E-41
MYOC	2.4167954	0.587609	4.1129	3.99E-05	2.716241617	count	0.9130317
ADM	2.101534	0.2894037	7.2616	4.65E-13	2.747484136	count	1.11E-08
ABLIM1	2.1310977	0.2721646	7.8302	6.33E-15	2.763760992	count	1.51E-10
FBN1	1.968854	0.1375381	14.315	2.86E-45	2.766187799	count	6.93E-41
HP	17.9505654	1259.111223	0.0143	0.9886	2.782901674	count	1
ENPP6	17.8252278	490.58199	0.0363	0.971	2.782901779	count	1
C17orf58	2.3343966	0.2320771	10.0587	1.69E-23	2.834642606	count	4.07E-19
GALNT15	2.3333441	0.2759552	8.4555	3.96E-17	2.83787944	count	9.48E-13
GSTM5	2.1810017	0.1970653	11.0674	4.99E-28	2.861245787	count	1.20E-23
GAS7	2.885704	0.479258	6.0212	1.90E-09	2.862175015	count	4.48E-05
NTM	3.4402988	0.6725421	5.1154	3.29E-07	2.874380627	count	0.007680176
BOC	2.4609308	0.3807328	6.4637	1.16E-10	2.902597132	count	2.75E-06
GFRA1	4.1301122	0.6907967	5.9788	2.46E-09	2.922746584	count	5.80E-05
ABCA8	2.1426923	0.163138	13.1342	1.53E-38	2.936597881	count	3.70E-34
LINC01133	2.8210516	0.3615106	7.8035	7.79E-15	2.944564575	count	1.86E-10
DNASE1L3	2.8470294	0.7043075	4.0423	5.40E-05	2.960672274	count	1
RSPO3	2.5068999	0.3488937	7.1853	8.09E-13	2.967713868	count	1.92E-08
APBB1IP	3.6468104	0.705127	5.1718	2.44E-07	2.971260586	count	0.00570106
RARRES1	2.1306254	0.256781	8.2974	1.48E-16	2.976142569	count	3.54E-12
HSD11B1	2.6283486	0.2841056	9.2513	3.67E-20	3.010092819	count	8.81E-16
F10	2.1978724	0.134178	16.3803	2.95E-58	3.031734759	count	7.16E-54
PENK	16.3274951	1267.085044	0.0129	0.9897	3.039829243	count	1
F3	2.3619777	0.2046712	11.5404	2.74E-30	3.080164608	count	6.62E-26
PLBD1	2.4305052	0.2305158	10.5438	1.26E-25	3.099030725	count	3.04E-21
SERPINF1	2.2156957	0.1183502	18.7215	9.10E-75	3.159232235	count	2.21E-70
WNT11	4.3372574	1.1749967	3.6913	0.000226	3.167274663	count	1
EPB41L3	18.1480363	568.0425831	0.0319	0.975	3.177843588	count	1
AOX1	3.178167	0.4058586	7.8307	6.30E-15	3.192275369	count	1.50E-10
FBLN2	2.3143485	0.1172751	19.7344	1.80E-82	3.217885205	count	4.37E-78
SFRP2	2.2407518	0.1520596	14.736	8.58E-48	3.22045297	count	2.08E-43
EMILIN2	3.7112701	0.7031441	5.2781	1.38E-07	3.256601061	count	0.003229338
CD34	2.4392054	0.1901184	12.8299	6.85E-37	3.266036966	count	1.66E-32
FGF18	4.4177704	0.8888703	4.9701	7.00E-07	3.26803496	count	0.0163128
SFRP4	2.2998089	0.2483415	9.2607	3.37E-20	3.276482885	count	8.09E-16
TNXB	2.3827731	0.1680944	14.1752	1.91E-44	3.307863426	count	4.63E-40
CADM3	4.1661857	0.8285282	5.0284	5.18E-07	3.314325159	count	0.012081314
RAMP2	2.4921753	0.2900249	8.593	1.24E-17	3.358135605	count	2.97E-13
SVEP1	3.0777107	0.4181911	7.3596	2.27E-13	3.365128397	count	5.41E-09
ABCA9	3.1491843	0.3359037	9.3753	1.17E-20	3.415501122	count	2.81E-16
ANGPTL7	3.5667701	1.4766002	2.4155	0.0158	3.420521519	count	1
TSKU	3.0986985	0.3388034	9.146	9.56E-20	3.459586263	count	2.29E-15

OSR2	3.1002546	0.333461	9.2972	2.41E-20	3.484899939	count	5.79E-16
CD248	2.9460813	0.2573666	11.447	7.77E-30	3.523157198	count	1.88E-25
CPVL	2.827419	0.3547533	7.9701	2.10E-15	3.569091651	count	5.01E-11
C1QTNF3	4.571466	0.7806711	5.8558	5.16E-09	3.574614218	count	0.000121549
RBP4	3.7311954	0.6055515	6.1616	7.98E-10	3.624331742	count	1.89E-05
SEMA3C	3.0151223	0.2897532	10.4058	5.18E-25	3.647141972	count	1.25E-20
DCN	2.5791901	0.1234321	20.8956	1.06E-91	3.71009239	count	2.58E-87
CYP4B1	4.0614464	0.8037187	5.0533	4.55E-07	3.750637978	count	0.01061424
PLAT	2.7244949	0.2102211	12.9601	1.36E-37	3.753173708	count	3.29E-33
ITM2A	2.7128943	0.1612881	16.8202	3.23E-61	3.760594449	count	7.84E-57
PALM	18.6548742	575.6296069	0.0324	0.974	3.764505134	count	1
PODN	2.972112	0.2302913	12.9059	2.67E-37	3.839110124	count	6.46E-33
ABCA10	4.9645252	1.1373716	4.3649	1.31E-05	3.850825381	count	0.3015882
HTRA3	4.0016345	0.6544229	6.1148	1.07E-09	3.953377734	count	2.53E-05
C16orf89	3.3958323	0.3994363	8.5016	2.68E-17	3.986324997	count	6.42E-13
TMEM176B	3.4078144	0.3432418	9.9283	6.09E-23	3.99740245	count	1.47E-18
TMEM176A	3.7909773	0.4487241	8.4483	4.20E-17	4.096512227	count	1.01E-12
GAS1	3.1928241	0.2368663	13.4794	1.87E-40	4.197124555	count	4.53E-36
MGST1	3.0849197	0.2022863	15.2503	5.81E-51	4.207686918	count	1.41E-46
SFRP1	3.0650539	0.2523575	12.1457	2.62E-33	4.274088538	count	6.33E-29
FAM180B	17.9793929	340.2329313	0.0528	0.958	4.298588268	count	1
VIT	17.9066117	268.2458291	0.0668	0.947	4.306134182	count	1
FBLN1	3.0628256	0.1399379	21.887	6.58E-100	4.360825206	count	1.60E-95
TSHZ2	4.616681	0.6247751	7.3893	1.82E-13	4.516771141	count	4.34E-09
LEPR	3.5291465	0.3521802	10.0209	2.46E-23	4.543205066	count	5.92E-19
ACKR3	3.351995	0.2145628	15.6224	2.58E-53	4.622257338	count	6.26E-49
LRRN4CL	4.7688425	0.5630937	8.469	3.53E-17	4.976059759	count	8.45E-13
CHRDL1	4.9746471	0.5189488	9.586	1.63E-21	5.370602589	count	3.92E-17
IGF1	4.3197059	0.4856388	8.8949	9.00E-19	5.384570694	count	2.16E-14
SCARA5	5.6515375	0.8469896	6.6725	2.89E-11	5.597021801	count	6.86E-07
PRG4	17.9003612	651.239353	0.0275	0.9781	5.665107328	count	1
PI16	4.6699163	0.5365278	8.704	4.76E-18	6.017549243	count	1.14E-13
MFAP5	4.4980391	0.4403886	10.2138	3.61E-24	6.05555333	count	8.70E-20
C3	5.5110554	0.8017343	6.8739	7.31E-12	6.477500165	count	1.74E-07
PLA2G2A	6.280764	1.3186367	4.7631	1.98E-06	7.253301604	count	0.04593006
CXCL14	6.2946866	2.1989508	2.8626	0.00423	7.600449595	count	1
APOD	5.5784369	1.137787	4.9029	9.85E-07	7.730358282	count	0.022916025
CFD	8.0189793	1.9432039	4.1267	3.76E-05	10.72399151	count	0.860664
