# Supplementary material for: Decoding the transcriptome of calcified atherosclerotic plaque at single-cell resolution
Source: Commun Biol. 2022 Oct 12;5:1084. doi: 10.1038/s42003-022-04056-7 (PMC9556750; doi:10.1038/s42003-022-04056-7)
Supplement: Supplementary file 8 — Supplementary Data 6 [file 42003_2022_4056_MOESM8_ESM.pdf]

Full differential gene expression results for ECs.

gene_short_name	estimate	std_err	test_val	p_value	normalized_effect	model_component	q_value
ITLN1	-4.1722926	0.2581334	-16.1633	3.21E-56	-6.009543464	count	7.79E-52
DKK2	-4.468834	0.516515	-8.6519	8.45E-18	-5.522560972	count	2.03E-13
F5	-4.6694793	0.5147427	-9.0715	2.17E-19	-5.267558637	count	5.22E-15
FN1	-3.4973426	0.2216463	-15.7789	9.04E-54	-4.963365584	count	2.19E-49
OGN	-3.5126207	0.3248464	-10.8132	1.01E-26	-4.895360242	count	2.44E-22
OMD	-3.4606343	0.2385148	-14.5091	5.01E-46	-4.833601486	count	1.22E-41
BGN	-3.3998031	0.1679278	-20.2456	4.32E-85	-4.833525893	count	1.05E-80
LY6H	-4.2879663	1.0018638	-4.28	1.93E-05	-4.809078951	count	0.4465827
DCN	-3.3349059	0.2937627	-11.3524	3.17E-29	-4.680054458	count	7.67E-25
TSTD1	-3.804856	0.4719314	-8.0623	1.10E-15	-4.613688085	count	2.64E-11
HMCN1	-3.6566695	0.2651927	-13.7887	7.04E-42	-4.574409394	count	1.71E-37
S100A4	-3.2257799	0.1788366	-18.0376	7.58E-69	-4.570422918	count	1.84E-64
PDE3A	-4.4939056	0.4589135	-9.7925	2.78E-22	-4.486600091	count	6.70E-18
CP	-3.3168571	0.2431467	-13.6414	4.72E-41	-4.463685936	count	1.14E-36
SPP1	-3.995772	0.6345706	-6.2968	3.52E-10	-4.428021726	count	8.35E-06
SCX	-3.5164863	0.3153682	-11.1504	2.83E-28	-4.401471123	count	6.84E-24
AC004540.2	-3.4143943	0.289533	-11.7928	2.37E-31	-4.31330964	count	5.73E-27
APOC1	-4.565626	0.9686085	-4.7136	2.55E-06	-4.2562491	count	0.05946855
SULF1	-2.9993749	0.1914052	-15.6703	4.36E-53	-4.236805178	count	1.06E-48
MMP2	-3.265662	0.1872163	-17.4433	1.01E-64	-4.186725609	count	2.45E-60
HHIP	-3.9336704	0.5324486	-7.3879	1.97E-13	-4.184504438	count	4.71E-09
SELL	-3.1072681	0.19413	-16.0061	3.27E-55	-4.138996429	count	7.94E-51
LTBP2	-3.1150906	0.2376807	-13.1062	4.06E-38	-4.133489873	count	9.84E-34
ESM1	-3.1807946	0.598616	-5.3136	1.16E-07	-4.080086606	count	0.002726348
BMP6	-3.2024981	0.248665	-12.8788	6.69E-37	-4.046753687	count	1.62E-32
PTPRJ	-3.5248748	0.4069547	-8.6616	7.78E-18	-3.949115578	count	1.87E-13
GDF15	-3.0819417	0.4019117	-7.6682	2.40E-14	-3.8535757	count	5.74E-10
AL355596.1	-5.7305828	0.9820376	-5.8354	5.99E-09	-3.830187933	count	0.000141628
ALDH1A1	-2.9071955	0.1970737	-14.7518	1.83E-47	-3.806677219	count	4.44E-43
ECM2	-4.0259635	0.4335427	-9.2862	3.14E-20	-3.731479412	count	7.56E-16
FEZ1	-2.9402872	0.2636017	-11.1543	2.72E-28	-3.731344688	count	6.58E-24
GXYLT2	-3.7889773	0.4212042	-8.9956	4.26E-19	-3.720884727	count	1.02E-14
NRG1	-3.3628329	0.3810812	-8.8245	1.91E-18	-3.710656447	count	4.59E-14
PRELP	-2.8168656	0.2519711	-11.1793	2.08E-28	-3.700686539	count	5.03E-24
MRC2	-3.57128	0.4247559	-8.4078	6.60E-17	-3.649379941	count	1.58E-12
HHIP-AS1	-3.276318	0.3448036	-9.502	4.31E-21	-3.639227689	count	1.04E-16
EFEMP1	-2.542567	0.1330019	-19.1168	1.31E-76	-3.634045968	count	3.18E-72
HMCN2	-4.226826	0.5801133	-7.2862	4.14E-13	-3.629570655	count	9.89E-09
TBX1	-2.961094	0.2654432	-11.1553	2.69E-28	-3.616656268	count	6.50E-24
SERPINE1	-2.5416989	0.1570204	-16.1871	2.25E-56	-3.567700562	count	5.46E-52
COL8A1	-2.5449686	0.1367094	-18.6159	5.80E-73	-3.562809696	count	1.41E-68
GJA5	-2.6409719	0.2567556	-10.2859	2.23E-24	-3.557606113	count	5.38E-20
S100B	-4.4388197	0.5661862	-7.8399	6.39E-15	-3.526642929	count	1.53E-10
APOE	-3.1880842	0.3971453	-8.0275	1.46E-15	-3.526273284	count	3.50E-11

IRF6	-3.6566533	0.3494825	-10.4631	3.75E-25	-3.496982677	count	9.05E-21
SRPX2	-3.184806	0.5781522	-5.5086	3.95E-08	-3.439190041	count	0.000930976
THBS1	-2.4906816	0.1778782	-14.0022	4.34E-43	-3.417790568	count	1.05E-38
SLC7A2	-3.697397	0.4670118	-7.9171	3.49E-15	-3.389645952	count	8.36E-11
CTSK	-2.6574582	0.2436366	-10.9075	3.77E-27	-3.385333275	count	9.11E-23
LINC01133	-4.261055	0.5772385	-7.3818	2.06E-13	-3.33742112	count	4.92E-09
GLIPR1	-2.7630375	0.3327724	-8.3031	1.57E-16	-3.325265516	count	3.77E-12
LINC01013	-3.340772	0.563859	-5.9248	3.51E-09	-3.297026885	count	8.31E-05
RAB34	-2.4820034	0.1709566	-14.5183	4.42E-46	-3.296196648	count	1.07E-41
IQCA1	-3.0857738	0.3401921	-9.0707	2.19E-19	-3.259879901	count	5.27E-15
RCN3	-2.724501	0.2621096	-10.3945	7.50E-25	-3.253744762	count	1.81E-20
PAPSS2	-2.7395883	0.29542	-9.2735	3.52E-20	-3.247767063	count	8.47E-16
SPINT2	-2.4277331	0.1795519	-13.5211	2.20E-40	-3.244652419	count	5.33E-36
GDF7	-2.4916911	0.2272333	-10.9653	2.04E-27	-3.244611422	count	4.93E-23
EDN1	-2.2604229	0.1076221	-21.0033	5.50E-91	-3.24453513	count	1.34E-86
TMEM98	-2.402663	0.1981331	-12.1265	5.19E-33	-3.227638272	count	1.26E-28
TPM2	-2.3512545	0.1900899	-12.3692	3.04E-34	-3.195076037	count	7.36E-30
PROCR	-2.2303528	0.0839716	-26.5608	1.25E-138	-3.188340724	count	3.04E-134
FIBIN	-3.2767462	0.3991253	-8.2098	3.36E-16	-3.176806128	count	8.06E-12
C1S	-2.3084087	0.1442296	-16.0051	3.31E-55	-3.175458045	count	8.03E-51
MEIS2	-2.421175	0.1760127	-13.7557	1.08E-41	-3.144012624	count	2.62E-37
SEMA3C	-3.4166613	0.4567518	-7.4803	9.90E-14	-3.133378961	count	2.37E-09
GDF6	-3.313812	0.3737294	-8.8669	1.32E-18	-3.116443326	count	3.17E-14
FBLIM1	-2.2658386	0.1437712	-15.76	1.19E-53	-3.114372128	count	2.89E-49
SERPINE2	-2.5192205	0.3023396	-8.3324	1.23E-16	-3.091262344	count	2.95E-12
KCNT2	-2.5981023	0.2968902	-8.7511	3.61E-18	-3.07501882	count	8.68E-14
C19orf33	-2.4788113	0.2493228	-9.9422	6.59E-23	-3.073908127	count	1.59E-18
MFAP2	-2.5964284	0.2768515	-9.3784	1.35E-20	-3.061649221	count	3.25E-16
ELN	-2.1557909	0.1087295	-19.8271	6.64E-82	-3.058691392	count	1.61E-77
CYP4X1	-2.7419692	0.3070893	-8.9289	7.67E-19	-3.043462393	count	1.84E-14
LUM	-3.132798	0.6514422	-4.809	1.60E-06	-3.035394418	count	0.03736
MGP	-2.1031129	0.0694017	-30.3035	2.12E-174	-3.033378146	count	5.16E-170
FGF18	-2.3869094	0.3779922	-6.3147	3.14E-10	-3.028699581	count	7.45E-06
CES1	-2.970769	0.3568475	-8.325	1.31E-16	-3.020439117	count	3.14E-12
TFAP2A	-3.6375822	0.4761366	-7.6398	2.98E-14	-3.020171181	count	7.13E-10
NOG	-4.2548885	1.3666125	-3.1135	0.0019	-3.0092441	count	1
AQP5	-3.431695	0.5228487	-6.5635	6.26E-11	-2.977775504	count	1.49E-06
WIPF3	-2.5213793	0.3590315	-7.0227	2.73E-12	-2.969305468	count	6.51E-08
KCNMB4	-2.4448898	0.2361913	-10.3513	1.16E-24	-2.956950096	count	2.80E-20
EMX2	-4.9057979	0.9548239	-5.1379	2.97E-07	-2.932205196	count	0.006963759
CDH11	-2.1542002	0.165986	-12.9782	1.98E-37	-2.92829155	count	4.80E-33
PDZK1IP1	-2.630117	0.3582255	-7.3421	2.75E-13	-2.915649582	count	6.57E-09
PLA2G2A	-2.8435438	0.9756882	-2.9144	0.0036	-2.915168029	count	1
KCNK15	-2.378899	0.2368967	-10.0419	2.49E-23	-2.914797147	count	6.01E-19
COL3A1	-2.2072614	0.189858	-11.6259	1.55E-30	-2.913023329	count	3.75E-26

PTGFRN	-3.3097425	0.5595318	-5.9152	3.72E-09	-2.907426195	count	8.80E-05
GSG1L	-3.6958154	0.6251108	-5.9123	3.79E-09	-2.898906183	count	8.97E-05
PTGIS	-2.0626957	0.1068542	-19.3038	5.47E-78	-2.884318715	count	1.33E-73
POU4F1	-3.6599816	0.6629067	-5.5211	3.68E-08	-2.883149535	count	0.000867413
SLC6A1	-4.1454325	0.7533314	-5.5028	4.08E-08	-2.879828868	count	0.000961452
SLPI	-2.0143869	0.1223893	-16.4588	3.88E-58	-2.842995914	count	9.42E-54
HEY2	-2.2595826	0.384566	-5.8757	4.72E-09	-2.838612637	count	0.000111633
FBP2	-4.0052164	0.6050407	-6.6197	4.31E-11	-2.836553916	count	1.03E-06
SFRP1	-2.1597975	0.2369035	-9.1168	1.45E-19	-2.829536619	count	3.49E-15
IGFBP3	-1.9802505	0.1889308	-10.4814	3.11E-25	-2.821420923	count	7.51E-21
PLA2G5	-3.0166381	0.5428997	-5.5565	3.01E-08	-2.820499183	count	0.000709848
KAZALD1	-3.519033	0.6248147	-5.6321	1.96E-08	-2.817550327	count	0.000462678
FIGN	-3.916105	0.6253522	-6.2622	4.39E-10	-2.80661849	count	1.04E-05
BMX	-1.9642601	0.0922182	-21.3001	2.45E-93	-2.79412841	count	5.95E-89
COL1A2	-2.6258713	0.4100876	-6.4032	1.78E-10	-2.778195659	count	4.23E-06
PCDH7	-2.250575	0.2169287	-10.3747	9.15E-25	-2.757546075	count	2.21E-20
FMOD	-2.2061265	0.2275738	-9.6941	7.10E-22	-2.751263918	count	1.71E-17
TRDC	-3.240022	0.4860304	-6.6663	3.16E-11	-2.740278992	count	7.52E-07
ATP13A3	-2.1675634	0.2168576	-9.9953	3.93E-23	-2.737003284	count	9.48E-19
FOXC2	-2.0683227	0.2254204	-9.1754	8.56E-20	-2.733214963	count	2.06E-15
TRPM3	-4.447476	0.8728935	-5.0951	3.72E-07	-2.72765721	count	0.008718564
NEXN	-2.261298	0.3985396	-5.674	1.54E-08	-2.711327094	count	0.000363671
HSPA6	-2.4497817	0.4827787	-5.0743	4.15E-07	-2.69709797	count	0.00972428
ART4	-2.505224	0.3439518	-7.2836	4.22E-13	-2.664032311	count	1.01E-08
PCDH10	-2.0962465	0.2075273	-10.1011	1.40E-23	-2.663046526	count	3.38E-19
NEGR1	-4.1210939	0.6955533	-5.9249	3.51E-09	-2.649444068	count	8.31E-05
LYPD2	-20.350832	561.4359024	-0.0362	0.971	-2.646890214	count	1
MPZL2	-1.8725487	0.080725	-23.1966	6.62E-109	-2.643660454	count	1.61E-104
FERMT3	-3.739341	0.499789	-7.4818	9.79E-14	-2.640540249	count	2.34E-09
ABCC3	-2.5557128	0.3350261	-7.6284	3.25E-14	-2.630593967	count	7.77E-10
NTN1	-2.5045875	0.3504588	-7.1466	1.13E-12	-2.61154709	count	2.70E-08
RNF212B	-3.2503933	0.477344	-6.8093	1.20E-11	-2.599537674	count	2.86E-07
CALHM4	-4.45776	0.8854814	-5.0343	5.10E-07	-2.59567183	count	0.0119442
FAM107B	-1.8550601	0.1066267	-17.3977	2.06E-64	-2.593625451	count	5.00E-60
C1QB	-2.836142	0.3695808	-7.6739	2.30E-14	-2.589495859	count	5.50E-10
SVEP1	-2.4223306	0.4564247	-5.3072	1.20E-07	-2.583456134	count	0.00281988
BCO2	-2.4691197	0.3486225	-7.0825	1.79E-12	-2.57798537	count	4.27E-08
LSAMP	-3.5790408	0.657826	-5.4407	5.77E-08	-2.576751721	count	0.001358489
SNHG18	-2.6469628	0.5900226	-4.4862	7.55E-06	-2.574505771	count	0.17539405
SULT1A1	-2.6024016	0.4707225	-5.5285	3.53E-08	-2.574067308	count	0.000832198
MSMP	-2.302339	0.4743732	-4.8534	1.28E-06	-2.563591705	count	0.0299136
ITGBL1	-2.4491755	0.4395167	-5.5724	2.75E-08	-2.558975431	count	0.000648725
CCL4L2	-2.0702902	0.2618458	-7.9065	3.79E-15	-2.556414619	count	9.08E-11
HMGCLL1	-3.4628144	1.0441549	-3.3164	0.000924	-2.526143983	count	1
SV2A	-3.720804	0.6075381	-6.1244	1.04E-09	-2.522587366	count	2.46E-05

SEMA5A	-3.6895007	0.5429494	-6.7953	1.32E-11	-2.511047696	count	3.14E-07
RGS10	-2.1172541	0.4026412	-5.2584	1.56E-07	-2.506746932	count	0.003663816
C1R	-1.7544493	0.0807171	-21.7358	7.85E-97	-2.494592783	count	1.91E-92
C4orf48	-1.8783522	0.1347397	-13.9406	9.73E-43	-2.492219918	count	2.36E-38
TMC7	-2.1899652	0.2849066	-7.6866	2.08E-14	-2.491016287	count	4.98E-10
GRB14	-2.110691	0.2995207	-7.0469	2.30E-12	-2.488859575	count	5.49E-08
TWIST2	-3.3784146	0.6165496	-5.4796	4.65E-08	-2.487029323	count	0.00109554
KCNMB1	-2.427506	0.4390251	-5.5293	3.52E-08	-2.481775168	count	0.000829875
SSTR1	-3.030388	0.5582994	-5.4279	6.20E-08	-2.471166988	count	0.001459418
PRKAR2B	-2.522862	0.4568233	-5.5226	3.65E-08	-2.467685161	count	0.000860378
CTSG	-3.572042	1.2425261	-2.8748	0.00407	-2.465503529	count	1
PI15	-20.9581863	725.6798665	-0.0289	0.977	-2.462386899	count	1
RIMS4	-19.898375	621.574901	-0.032	0.974	-2.462386535	count	1
NLGN1	-3.011334	0.5674678	-5.3066	1.21E-07	-2.459363682	count	0.002843137
TNFRSF21	-2.7984342	0.4119734	-6.7928	1.34E-11	-2.450680662	count	3.19E-07
SGCE	-1.7977701	0.1774086	-10.1335	1.01E-23	-2.442865193	count	2.44E-19
PKHD1L1	-1.918999	0.2425872	-7.9106	3.67E-15	-2.436918282	count	8.79E-11
RARRES2	-2.2798383	0.3056983	-7.4578	1.17E-13	-2.418161116	count	2.80E-09
SLIT3	-3.0689179	0.6894397	-4.4513	8.87E-06	-2.414496279	count	0.20595253
BICC1	-2.7410118	0.5938989	-4.6153	4.10E-06	-2.408708738	count	0.0954808
CRYAB	-1.9670263	0.2758129	-7.1317	1.26E-12	-2.397935525	count	3.01E-08
IL11RA	-1.8058516	0.2040241	-8.8512	1.51E-18	-2.392781413	count	3.63E-14
KLHL13	-2.3479907	0.3715226	-6.3199	3.04E-10	-2.376415869	count	7.22E-06
MEDAG	-1.7694661	0.1691926	-10.4583	3.93E-25	-2.36976495	count	9.49E-21
PTPRR	-2.3098995	0.3628566	-6.3659	2.27E-10	-2.369719115	count	5.39E-06
PDGFRL	-2.2374657	0.4111712	-5.4417	5.74E-08	-2.351468629	count	0.001351483
IL1RN	-4.031509	0.8325606	-4.8423	1.35E-06	-2.351269731	count	0.0315441
EMP3	-1.6634411	0.1076864	-15.4471	1.08E-51	-2.348291386	count	2.62E-47
PLA2G4A	-1.8303015	0.1991106	-9.1924	7.35E-20	-2.347013377	count	1.77E-15
NCKAP5	-4.008553	0.6975506	-5.7466	1.01E-08	-2.345240711	count	0.000238602
TMEM120A	-1.6787876	0.0979087	-17.1465	1.05E-62	-2.340506905	count	2.55E-58
BMPER	-2.4067231	0.3554316	-6.7713	1.55E-11	-2.326745553	count	3.69E-07
SMAD6	-1.7291379	0.1630807	-10.603	8.97E-26	-2.311605604	count	2.17E-21
FGFRL1	-1.9940843	0.2558384	-7.7943	9.10E-15	-2.310904426	count	2.18E-10
LGR4	-3.8504755	0.7060771	-5.4533	5.38E-08	-2.300757332	count	0.001266936
EMILIN1	-3.1889735	0.8931941	-3.5703	0.000363	-2.291169379	count	1
TRAPPC3L	-2.4595287	0.4487895	-5.4804	4.63E-08	-2.288722171	count	0.001090874
PDGFD	-1.7337517	0.1822259	-9.5143	3.84E-21	-2.282228605	count	9.25E-17
NAV1	-1.7063665	0.1405483	-12.1408	4.40E-33	-2.279717446	count	1.07E-28
SPI1	-2.637457	0.8023531	-3.2872	0.00102	-2.271221901	count	1
GPR183	-3.7452737	0.9832866	-3.8089	0.000143	-2.268157275	count	1
LYNX1	-2.1792324	0.3358865	-6.488	1.03E-10	-2.267937172	count	2.45E-06
EXT1	-1.8388759	0.1899961	-9.6785	8.23E-22	-2.26777311	count	1.98E-17
COL12A1	-2.8290923	0.463207	-6.1076	1.15E-09	-2.265673205	count	2.72E-05
ANXA3	-1.6445891	0.14987	-10.9734	1.88E-27	-2.263379008	count	4.54E-23

LOX	-2.0431359	0.3064847	-6.6664	3.15E-11	-2.251815053	count	7.50E-07
TREM2	-21.240949	1459.032613	-0.0146	0.988	-2.250779497	count	1
AC105383.1	-20.9605677	723.4811214	-0.029	0.977	-2.250779268	count	1
CCL3	-1.9416289	0.2831096	-6.8582	8.57E-12	-2.250729833	count	2.04E-07
PTGDS	-1.5940984	0.1584373	-10.0614	2.06E-23	-2.243874019	count	4.97E-19
BAMBI	-1.71907	0.2846958	-6.0383	1.77E-09	-2.227944502	count	4.19E-05
APLN	-2.2972575	0.3752517	-6.1219	1.06E-09	-2.227611225	count	2.51E-05
PCDH9	-1.8079946	0.4331614	-4.174	3.09E-05	-2.217719798	count	0.713481
CLIP3	-4.1371882	0.8768279	-4.7184	2.50E-06	-2.211886886	count	0.0583075
GRIK5	-4.1371882	0.8768279	-4.7184	2.50E-06	-2.211886886	count	0.0583075
PLXNA4	-1.7267798	0.2153416	-8.0188	1.56E-15	-2.210912412	count	3.74E-11
ACSM3	-2.4774223	0.3660696	-6.7676	1.59E-11	-2.204267125	count	3.79E-07
DTX3	-3.5378686	0.7538083	-4.6933	2.82E-06	-2.19640555	count	0.06573984
SOBP	-2.3502266	0.7298305	-3.2202	0.0013	-2.194424148	count	1
CRTAC1	-1.5223366	0.0658954	-23.1023	4.14E-108	-2.182220579	count	1.01E-103
PGF	-1.6138843	0.1803067	-8.9508	6.33E-19	-2.180050007	count	1.52E-14
ALCAM	-1.7402245	0.2167457	-8.0289	1.44E-15	-2.176617283	count	3.45E-11
TENT5A	-1.6218876	0.1403348	-11.5573	3.32E-30	-2.173009577	count	8.03E-26
GLIPR2	-1.9316377	0.3346643	-5.7719	8.71E-09	-2.161295902	count	0.000205791
CREB5	-1.7130348	0.2135694	-8.021	1.53E-15	-2.159945668	count	3.67E-11
MAP1B	-1.742131	0.1609522	-10.8239	9.07E-27	-2.153735548	count	2.19E-22
PCDH11X	-2.0223113	0.7985915	-2.5323	0.0114	-2.153014513	count	1
CKB	-2.0377954	0.3228351	-6.3122	3.19E-10	-2.147313161	count	7.57E-06
PLXNA2	-1.5829161	0.1567733	-10.0968	1.45E-23	-2.144565621	count	3.50E-19
HOMER3	-1.554818	0.1248211	-12.4564	1.08E-34	-2.136389547	count	2.62E-30
SELP	-1.5081398	0.0806405	-18.702	1.39E-73	-2.127371617	count	3.38E-69
DDR2	-1.7601593	0.2797685	-6.2915	3.64E-10	-2.126113475	count	8.64E-06
EGFLAM	-2.617515	0.4504461	-5.8109	6.92E-09	-2.120846715	count	0.000163582
NAV2	-1.8279851	0.37184	-4.9161	9.35E-07	-2.119170376	count	0.021864975
FGL2	-1.5756694	0.1742622	-9.0419	2.83E-19	-2.11778303	count	6.81E-15
OTC	-1.6085987	0.1923355	-8.3635	9.53E-17	-2.116848064	count	2.29E-12
HTR4	-3.0627751	0.6923268	-4.4239	1.01E-05	-2.115338975	count	0.2343806
SYNDIG1	-3.7250909	1.3650701	-2.7289	0.0064	-2.103421778	count	1
PLTP	-1.5891851	0.1831048	-8.6791	6.70E-18	-2.101729256	count	1.61E-13
BCAT1	-2.5865762	0.7369432	-3.5099	0.000455	-2.098659349	count	1
SHROOM3	-3.0258831	0.554655	-5.4554	5.32E-08	-2.096003681	count	0.00125286
PRDM6	-3.2820071	0.5317142	-6.1725	7.71E-10	-2.093367921	count	1.83E-05
GATA6-AS1	-1.8232111	0.292027	-6.2433	4.94E-10	-2.091135369	count	1.17E-05
SMIM6	-3.015883	0.7571398	-3.9833	6.97E-05	-2.090698751	count	1
CRISPLD1	-1.8612968	0.2754686	-6.7568	1.71E-11	-2.082556929	count	4.07E-07
CLIC3	-1.7241849	0.2030633	-8.4909	3.30E-17	-2.082336194	count	7.92E-13
SLC25A34-AS1	-2.4663637	0.4778502	-5.1614	2.63E-07	-2.077665033	count	0.006169454
MOV10L1	-2.6649652	0.5667698	-4.702	2.70E-06	-2.074994844	count	0.0629559
CDKN2C	-1.6952004	0.2043304	-8.2964	1.66E-16	-2.053750734	count	3.98E-12
LTBP4	-1.4724491	0.0964138	-15.2722	1.29E-50	-2.052745346	count	3.13E-46

BMP4	-1.4530176	0.1183649	-12.2757	9.13E-34	-2.047635029	count	2.21E-29
LEPR	-1.4900906	0.1222959	-12.1843	2.65E-33	-2.046032045	count	6.42E-29
BMP2	-1.5105951	0.1953777	-7.7317	1.48E-14	-2.044342506	count	3.54E-10
PDZK1	-3.1717136	0.5879778	-5.3943	7.46E-08	-2.043795677	count	0.001755114
ITGB5	-1.7523043	0.2309804	-7.5864	4.47E-14	-2.040934576	count	1.07E-09
SNCAIP	-1.6194368	0.2200588	-7.3591	2.43E-13	-2.037011319	count	5.81E-09
CYR61	-1.4169194	0.1184065	-11.9666	3.28E-32	-2.026211311	count	7.94E-28
FAM3B	-2.151047	0.5420765	-3.9682	7.43E-05	-2.015655032	count	1
LYZ	-1.9722924	0.4639756	-4.2509	2.20E-05	-2.006546446	count	0.508838
GRAMD1B	-20.7783954	859.920683	-0.0242	0.981	-2.002709248	count	1
LINC00607	-20.4605516	852.7165775	-0.024	0.981	-2.002709246	count	1
LILRA2	-21.01748	1585.113289	-0.0133	0.989	-2.002709032	count	1
TRDN-AS1	-21.015837	1115.673802	-0.0188	0.985	-2.002709032	count	1
EREG	-20.220267	804.8441952	-0.0251	0.98	-2.002709028	count	1
APCDD1L	-20.219666	722.0252961	-0.028	0.978	-2.002709028	count	1
CTGF	-1.3951578	0.0988434	-14.1148	9.83E-44	-1.997731347	count	2.38E-39
ANKRD1	-2.3634557	0.9633201	-2.4534	0.0142	-1.996795286	count	1
LRP1	-2.1762824	0.5678437	-3.8325	0.00013	-1.996006377	count	1
MTMR11	-2.048593	0.6007306	-3.4102	0.000659	-1.992958901	count	1
ANGPT2	-1.4154441	0.1705336	-8.3001	1.61E-16	-1.984177143	count	3.86E-12
CXCR4	-2.038732	0.4759503	-4.2835	1.90E-05	-1.983414689	count	0.439736
B3GNT9	-2.2688089	0.3977471	-5.7041	1.29E-08	-1.977608145	count	0.000304672
SCRN1	-2.7903232	0.6843118	-4.0776	4.68E-05	-1.963939763	count	1
EFNA5	-2.1912529	0.4467056	-4.9054	9.87E-07	-1.962726995	count	0.023078034
PLEKHA4	-1.7527286	0.270322	-6.4839	1.06E-10	-1.95987603	count	2.52E-06
LGALS1	-1.3725742	0.0920425	-14.9124	1.99E-48	-1.959075615	count	4.83E-44
QPCT	-1.877865	0.2706056	-6.9395	4.88E-12	-1.957309364	count	1.16E-07
FMO2	-1.5511653	0.2401024	-6.4604	1.23E-10	-1.946136583	count	2.92E-06
SSX2IP	-1.714651	0.2935115	-5.8419	5.77E-09	-1.942053521	count	0.000136432
FBLN7	-1.9878581	0.3428821	-5.7975	7.49E-09	-1.933921411	count	0.000177019
SLC16A5	-1.6964664	0.2363106	-7.179	8.99E-13	-1.932853951	count	2.15E-08
XXYLT1-AS2	-3.759386	0.8730197	-4.3062	1.72E-05	-1.930048769	count	0.39818
RAC2	-2.1537246	0.4946556	-4.354	1.39E-05	-1.929748002	count	0.3220352
PTN	-2.732709	0.9861435	-2.7711	0.00562	-1.929420033	count	1
SEMA7A	-3.241309	1.1498047	-2.819	0.0049	-1.929025707	count	1
C15orf48	-3.746252	0.9030276	-4.1485	3.45E-05	-1.926420044	count	0.7960185
PHLDB1	-1.7731914	0.2585246	-6.8589	8.53E-12	-1.922101456	count	2.03E-07
INMT	-1.3955999	0.1286127	-10.8512	6.81E-27	-1.918899095	count	1.65E-22
RGS9	-2.090841	0.5454994	-3.8329	0.000129	-1.918737508	count	1
OSGIN2	-1.486556	0.2284713	-6.5065	9.10E-11	-1.916950443	count	2.16E-06
SEZ6L2	-2.3283793	0.5259514	-4.427	9.93E-06	-1.904257707	count	0.23046537
FBLN5	-1.5785588	0.3086264	-5.1148	3.36E-07	-1.904118696	count	0.007876176
PMEPA1	-1.3686934	0.1187115	-11.5296	4.51E-30	-1.901327379	count	1.09E-25
RAB31	-2.177879	0.2792223	-7.7998	8.72E-15	-1.900407137	count	2.09E-10
PDE4B	-1.4665384	0.2151005	-6.8179	1.13E-11	-1.900375786	count	2.69E-07

VASN	-3.6465281	0.7695462	-4.7385	2.26E-06	-1.897737005	count	0.05273032
SUCNR1	-3.133635	1.1578938	-2.7063	0.00685	-1.882593905	count	1
PRRT1	-2.1386296	0.4560531	-4.6894	2.87E-06	-1.866580793	count	0.06689683
VAT1L	-3.074796	0.6545497	-4.6976	2.76E-06	-1.856016493	count	0.0643494
PTX3	-2.3579101	0.6548993	-3.6004	0.000323	-1.85529623	count	1
COLEC12	-1.8248426	0.4307272	-4.2367	2.34E-05	-1.85487724	count	0.541008
PTPRG	-1.4153815	0.1439662	-9.8313	1.92E-22	-1.851443986	count	4.63E-18
ACKR3	-1.3206812	0.1535003	-8.6038	1.27E-17	-1.845545867	count	3.05E-13
SLC6A6	-2.010193	0.2892002	-6.9509	4.51E-12	-1.844611037	count	1.08E-07
PLCD3	-1.6366208	0.4502925	-3.6346	0.000284	-1.839919855	count	1
COL4A1	-1.3241151	0.1221307	-10.8418	7.52E-27	-1.836222663	count	1.82E-22
ABI3BP	-1.4101733	0.1700865	-8.2909	1.73E-16	-1.832775636	count	4.15E-12
LYVE1	-1.8216057	0.4171937	-4.3663	1.31E-05	-1.826026469	count	0.3035532
SDC3	-1.6135619	0.2906186	-5.5522	3.09E-08	-1.825127235	count	0.000728591
NPTX2	-2.424066	0.7383259	-3.2832	0.00104	-1.822576267	count	1
NFE2L3	-1.4014695	0.1893459	-7.4016	1.78E-13	-1.812656074	count	4.25E-09
IGKC	-1.407747	0.1070071	-13.1556	2.20E-38	-1.81144068	count	5.33E-34
MYOZ2	-4.1994822	1.4395299	-2.9173	0.0036	-1.806433217	count	1
ALDH3A1	-1.8535423	0.5334573	-3.4746	0.00052	-1.801384037	count	1
EFEMP2	-1.3941486	0.1953366	-7.1372	1.21E-12	-1.798453448	count	2.89E-08
AC245297.3	-1.7048632	0.3243039	-5.257	1.58E-07	-1.793176525	count	0.003710472
CAP2	-1.785707	0.4218189	-4.2333	2.38E-05	-1.789101664	count	0.5501846
NOX4	-2.676319	0.9142616	-2.9273	0.00345	-1.782487058	count	1
CDA	-1.2801882	0.1058376	-12.0958	7.41E-33	-1.781959348	count	1.79E-28
CD44	-2.1029616	0.4911429	-4.2818	1.92E-05	-1.781811441	count	0.4443072
COL16A1	-1.9808962	0.4221621	-4.6923	2.83E-06	-1.774593067	count	0.06597013
PRSS2	-1.5784636	1.1725883	-1.3461	0.1784	-1.77277099	count	1
CCDC152	-1.6231595	0.3538431	-4.5872	4.69E-06	-1.77071587	count	0.10916444
RASGEF1B	-1.622143	0.2252248	-7.2023	7.60E-13	-1.769572176	count	1.81E-08
HSPA1A	-1.2269705	0.0703516	-17.4405	1.05E-64	-1.76339899	count	2.55E-60
PDGFRB	-2.6426659	0.5776419	-4.5749	4.97E-06	-1.762516548	count	0.11567178
RGS1	-3.2247081	0.9321509	-3.4594	0.00055	-1.752886643	count	1
NRTN	-2.847616	0.6309145	-4.5135	6.64E-06	-1.745448494	count	0.15432688
PTGS2	-1.2270202	0.0980262	-12.5173	5.26E-35	-1.743987602	count	1.27E-30
PPM1L	-1.6575725	0.3213712	-5.1578	2.68E-07	-1.741858805	count	0.00628594
TAGLN	-1.2163466	0.1806686	-6.7325	2.02E-11	-1.729872102	count	4.81E-07
CACNG8	-1.7530192	0.3692476	-4.7475	2.16E-06	-1.728975518	count	0.05040576
SFRP4	-1.7808461	0.5641809	-3.1565	0.00161	-1.728671287	count	1
ABCA8	-1.3911056	0.390191	-3.5652	0.00037	-1.722335122	count	1
FCGR2B	-20.727858	1371.417147	-0.0151	0.988	-1.702940616	count	1
DPP10	-20.727858	1371.417147	-0.0151	0.988	-1.702940616	count	1
NLRC3	-20.726558	1018.429566	-0.0204	0.984	-1.702940616	count	1
IGSF23	-20.725615	784.8861939	-0.0264	0.979	-1.702940616	count	1
SCML4	-19.980423	835.9104646	-0.0239	0.981	-1.702940612	count	1
TNS3	-2.0730361	0.3232597	-6.4129	1.67E-10	-1.697811063	count	3.97E-06

PAWR	-1.2427748	0.135041	-9.2029	6.68E-20	-1.697541495	count	1.61E-15
GLT8D2	-1.3515144	0.1855873	-7.2824	4.26E-13	-1.691265263	count	1.02E-08
LRRC17	-2.1447051	0.4035399	-5.3147	1.15E-07	-1.690414125	count	0.00270296
AMHR2	-3.6628464	0.9114699	-4.0186	6.01E-05	-1.68844754	count	1
CPAMD8	-1.3704482	0.1922754	-7.1275	1.30E-12	-1.687607609	count	3.10E-08
KCNN4	-1.427625	0.2036442	-7.0104	2.98E-12	-1.685660501	count	7.11E-08
CLDN15	-1.2288444	0.1406228	-8.7386	4.02E-18	-1.683672561	count	9.66E-14
SFTPD-AS1	-2.352406	0.7164336	-3.2835	0.00104	-1.68138958	count	1
PI16	-2.226699	0.3334375	-6.678	2.92E-11	-1.678381703	count	6.95E-07
CXCL1	-1.2480303	0.407622	-3.0617	0.0022	-1.66803423	count	1
CHST1	-1.4612546	0.2489223	-5.8703	4.87E-09	-1.667911476	count	0.000115171
PAPSS1	-1.2121338	0.1125022	-10.7743	1.52E-26	-1.665285406	count	3.67E-22
DDAH1	-1.7154692	0.4137678	-4.146	3.49E-05	-1.66278981	count	0.8051779
ZNF483	-1.7154692	0.4297524	-3.9918	6.73E-05	-1.66278981	count	1
SLC4A4	-1.9647781	0.3537938	-5.5535	3.07E-08	-1.662729149	count	0.000723906
LAPTM5	-1.2582135	0.1944271	-6.4714	1.14E-10	-1.659552674	count	2.71E-06
LINC01235	-1.550682	0.2925525	-5.3005	1.25E-07	-1.658894477	count	0.002937
CREB3L2	-1.2575712	0.1561271	-8.0548	1.17E-15	-1.657313219	count	2.80E-11
TIMP1	-1.1494269	0.0639013	-17.9875	1.70E-68	-1.656290552	count	4.13E-64
AL365259.1	-2.9893537	0.8549569	-3.4965	0.000479	-1.654666567	count	1
CPE	-1.1584356	0.1137061	-10.188	5.92E-24	-1.652284751	count	1.43E-19
ABHD2	-1.2074203	0.1289827	-9.3611	1.58E-20	-1.649804689	count	3.80E-16
UHRF1	-3.515772	0.9080753	-3.8717	0.000111	-1.647214151	count	1
AC009414.2	-1.2149731	0.1497418	-8.1138	7.30E-16	-1.645160212	count	1.75E-11
RAMP1	-2.0082365	0.7094327	-2.8308	0.00468	-1.643573594	count	1
AL442663.3	-3.5010092	1.2261894	-2.8552	0.00433	-1.64284425	count	1
IL1RL1	-5.2401023	1.3679991	-3.8305	1.00E-04	-1.638420147	count	1
MEI1	-1.8772595	0.4355488	-4.3101	1.69E-05	-1.634710809	count	0.3912519
SFRP5	-2.639255	0.6472011	-4.078	4.67E-05	-1.633244359	count	1
SH3TC1	-1.3413307	0.2467701	-5.4355	5.94E-08	-1.632409237	count	0.001398395
SH3RF2	-1.9298797	0.4749739	-4.0631	4.98E-05	-1.63220031	count	1
DHH	-1.4157568	0.2730534	-5.1849	2.32E-07	-1.632185728	count	0.005444576
HSPG2	-1.1374995	0.0553456	-20.5527	1.84E-87	-1.629802462	count	4.47E-83
ITGAV	-1.2215	0.1584344	-7.7098	1.75E-14	-1.627022286	count	4.19E-10
AC144831.1	-1.987827	0.4373254	-4.5454	5.72E-06	-1.626358101	count	0.13307008
OSBPL3	-1.819256	0.4718389	-3.8557	0.000118	-1.625377572	count	1
PIK3AP1	-3.424037	0.9409679	-3.6388	0.000279	-1.619360941	count	1
RGS7BP	-1.4659233	0.2475027	-5.9229	3.56E-09	-1.618888671	count	8.43E-05
RGS3	-1.1460624	0.1241314	-9.2327	5.11E-20	-1.608210811	count	1.23E-15
TBXAS1	-2.239749	0.566536	-3.9534	7.90E-05	-1.601891061	count	1
PDGFC	-1.6528626	0.343084	-4.8177	1.53E-06	-1.599330434	count	0.03573009
VIPR1	-3.3610102	0.8057722	-4.1712	3.12E-05	-1.599247944	count	0.7203456
AATK	-2.867099	0.5602646	-5.1174	3.31E-07	-1.598673737	count	0.007759633
PDZRN4	-1.7462034	0.4114651	-4.2439	2.27E-05	-1.59537016	count	0.5248921
FBXO3-DT	-3.338362	0.8908579	-3.7474	0.000182	-1.5918238	count	1

COL4A2	-1.141326	0.1120548	-10.1854	6.07E-24	-1.591516388	count	1.46E-19
SGCG	-1.699364	0.455841	-3.728	0.000197	-1.584908942	count	1
DSE	-1.4153683	0.2618896	-5.4044	7.06E-08	-1.583443014	count	0.001661359
SLC26A4	-1.8199215	0.4575058	-3.9779	7.13E-05	-1.5825631	count	1
CRIM1	-1.1062453	0.0637747	-17.3461	4.64E-64	-1.582315229	count	1.13E-59
UNC5B	-1.5651051	0.291712	-5.3652	8.75E-08	-1.581863677	count	0.002058088
SULT1B1	-3.3025925	0.8973558	-3.6804	0.000237	-1.57988589	count	1
RHOB	-1.1039064	0.0691842	-15.956	6.81E-55	-1.579205527	count	1.65E-50
CLCN4	-2.0063816	0.4954424	-4.0497	5.27E-05	-1.579052686	count	1
B3GALNT1	-1.2349327	0.2195544	-5.6247	2.04E-08	-1.575943107	count	0.000481542
PLEKHA6	-2.3462098	0.5869118	-3.9976	6.57E-05	-1.57564408	count	1
TRPV2	-1.9994153	0.5561648	-3.595	0.00033	-1.573366282	count	1
CST6	-1.998206	0.64007	-3.1219	0.00182	-1.572378548	count	1
SYNM	-1.3456977	0.2973576	-4.5255	6.28E-06	-1.571861076	count	0.14604768
DZIP1	-3.273292	0.9373113	-3.4922	0.000487	-1.56991106	count	1
PRKACB	-1.279413	0.1959847	-6.5281	7.90E-11	-1.568425305	count	1.88E-06
SLC16A4	-1.2737262	0.2363225	-5.3898	7.65E-08	-1.565465831	count	0.001799663
SPEG	-2.324351	0.547397	-4.2462	2.25E-05	-1.561145313	count	0.5203125
IL7	-1.4082356	0.3112162	-4.5249	6.30E-06	-1.55255347	count	0.1465002
MAP2	-1.602934	0.2607121	-6.1483	8.96E-10	-1.548503517	count	2.12E-05
EVI5	-1.1186052	0.117757	-9.4993	4.42E-21	-1.547866471	count	1.06E-16
SCN8A	-3.206442	0.7641621	-4.196	2.80E-05	-1.5464927	count	0.646828
ZNF771	-1.2376697	0.2009641	-6.1587	8.40E-10	-1.542149118	count	1.99E-05
AR	-1.194764	0.1944475	-6.1444	9.18E-10	-1.540827946	count	2.18E-05
C8orf34	-3.1599133	0.7091559	-4.4559	8.69E-06	-1.529647875	count	0.20179049
ARHGAP18	-1.0829937	0.0998576	-10.8454	7.24E-27	-1.525650474	count	1.75E-22
DHRS3	-1.1027312	0.1281873	-8.6025	1.29E-17	-1.52464418	count	3.10E-13
GJA4	-1.0848014	0.1546296	-7.0155	2.87E-12	-1.523087003	count	6.85E-08
RBP1	-1.1065122	0.1293398	-8.5551	1.92E-17	-1.519685887	count	4.61E-13
ITGA10	-1.0871525	0.0936101	-11.6136	1.77E-30	-1.519616854	count	4.28E-26
MYO1D	-1.2340815	0.1888037	-6.5363	7.48E-11	-1.519345979	count	1.78E-06
FHDC1	-1.8577637	0.5687801	-3.2662	0.0011	-1.515326774	count	1
TOR4A	-1.4209137	0.3382841	-4.2004	2.75E-05	-1.514045148	count	0.635305
SLC44A5	-1.744434	0.7775505	-2.2435	0.0249	-1.513368591	count	1
PPP1R14A	-1.1914572	0.2017743	-5.9049	3.96E-09	-1.511923349	count	9.37E-05
ARL4D	-1.1100497	0.139259	-7.9711	2.28E-15	-1.511624101	count	5.46E-11
FAM20C	-2.1126892	0.6053911	-3.4898	0.000491	-1.509476951	count	1
CCDC80	-1.0782396	0.1295583	-8.3224	1.34E-16	-1.508402954	count	3.22E-12
TCEAL2	-1.2469186	0.230241	-5.4157	6.63E-08	-1.50468975	count	0.001560371
ZNF396	-2.668741	0.743958	-3.5872	0.00034	-1.500817549	count	1
FUT8	-1.1972622	0.179569	-6.6674	3.13E-11	-1.499989459	count	7.45E-07
PLD1	-1.2821804	0.292844	-4.3784	1.24E-05	-1.495005399	count	0.2873948
P4HA2	-1.1840906	0.2187412	-5.4132	6.72E-08	-1.494506162	count	0.001581485
VAMP8	-1.067116	0.1229189	-8.6815	6.56E-18	-1.493989387	count	1.58E-13
OSBPL6	-1.4151567	0.3325512	-4.2555	2.16E-05	-1.492483363	count	0.499608

HOXB5	-1.546602	0.509282	-3.0368	0.00241	-1.490967352	count	1
SC5D	-1.213036	0.2183901	-5.5544	3.05E-08	-1.488772458	count	0.000719251
NOS1	-2.645214	0.6445617	-4.1039	4.18E-05	-1.488656435	count	0.9637408
PLLP	-1.1148959	0.1750633	-6.3685	2.23E-10	-1.485910437	count	5.29E-06
GATA5	-3.9229826	1.1054286	-3.5488	0.000393	-1.483880679	count	1
CDYL2	-2.209636	0.5055182	-4.371	1.28E-05	-1.483627918	count	0.2966528
ANOS1	-1.5148287	0.3151879	-4.8061	1.62E-06	-1.483404321	count	0.03782214
KLF15	-2.0762466	0.4721346	-4.3976	1.14E-05	-1.482490056	count	0.2643546
HEPH	-3.0254209	0.9607206	-3.1491	0.00166	-1.478435672	count	1
PTGS1	-1.168275	0.2645998	-4.4153	1.05E-05	-1.476702678	count	0.2436
CD68	-1.197231	0.2151042	-5.5658	2.86E-08	-1.476438675	count	0.000674588
RND2	-1.809626	0.5176166	-3.4961	0.00048	-1.473723432	count	1
CDH13	-1.0536171	0.1263687	-8.3376	1.18E-16	-1.472757015	count	2.83E-12
NPAS2	-1.9546037	0.5020915	-3.8929	0.000101	-1.468032949	count	1
SEMA3F	-1.094125	0.1419779	-7.7063	1.79E-14	-1.465580549	count	4.28E-10
THY1	-1.3120516	0.2620913	-5.0061	5.90E-07	-1.463033465	count	0.01381072
RGS11	-1.330123	0.2993719	-4.443	9.22E-06	-1.462588855	count	0.21405152
NETO2	-2.1713252	0.6553762	-3.3131	0.000935	-1.457238263	count	1
CYP1B1	-1.029275	0.0968053	-10.6324	6.62E-26	-1.45334574	count	1.60E-21
CUBN	-1.2943677	0.2851373	-4.5395	5.88E-06	-1.452190628	count	0.13678056
AP000695.2	-1.9344541	0.5208045	-3.7144	0.000208	-1.452028436	count	1
TMSB15B-AS1	-1.675482	0.6889091	-2.4321	0.0151	-1.44971385	count	1
DOCK8	-1.5917663	0.6425059	-2.4774	0.0133	-1.446189402	count	1
CALD1	-1.0086474	0.0585079	-17.2395	2.46E-63	-1.444806424	count	5.97E-59
TRIP6	-1.069748	0.1355925	-7.8894	4.34E-15	-1.443847136	count	1.04E-10
CITED4	-1.0794339	0.2046999	-5.2732	1.44E-07	-1.441483476	count	0.003382848
PDE5A	-1.451988	0.4906395	-2.9594	0.00311	-1.44051106	count	1
SHTN1	-1.3539939	0.361006	-3.7506	0.00018	-1.439127908	count	1
DYRK4	-1.055914	0.1416441	-7.4547	1.20E-13	-1.438068816	count	2.87E-09
SRGN	-1.0000637	0.0708783	-14.1096	1.05E-43	-1.435300685	count	2.55E-39
GPR176	-3.6836884	1.057541	-3.4833	0.000503	-1.433536883	count	1
KANK1	-1.274205	0.2715202	-4.6929	2.83E-06	-1.428542414	count	0.06597013
PHLDA1	-1.0667477	0.2300411	-4.6372	3.70E-06	-1.428392171	count	0.0861878
MIR503HG	-3.6573447	0.8686299	-4.2105	2.63E-05	-1.427444251	count	0.6077141
PLEKHO1	-1.119862	0.1655276	-6.7654	1.62E-11	-1.42589903	count	3.86E-07
UNC5B-AS1	-2.8935492	1.3485387	-2.1457	0.032	-1.424606521	count	1
ASS1	-1.0042159	0.076812	-13.0737	6.08E-38	-1.423147151	count	1.47E-33
MIR4435-2HG	-1.0832293	0.2446722	-4.4273	9.91E-06	-1.414799129	count	0.2300111
ALDH3A2	-1.0539064	0.1451742	-7.2596	5.02E-13	-1.41399577	count	1.20E-08
ID4	-1.1254739	0.2919984	-3.8544	0.000119	-1.413352722	count	1
ZDHHC4	-1.119358	0.2350397	-4.7624	2.01E-06	-1.408162958	count	0.0469134
PTPRF	-1.3519849	0.2990306	-4.5212	6.41E-06	-1.406929898	count	0.14901968
CEBPB	-1.0024916	0.1002001	-10.0049	3.58E-23	-1.403998469	count	8.63E-19
SETBP1	-1.1023911	0.2184279	-5.0469	4.78E-07	-1.400886862	count	0.011196194
PTPRS	-2.2527824	0.8793432	-2.5619	0.0105	-1.400697873	count	1

CGNL1	-1.8689093	0.3970303	-4.7072	2.63E-06	-1.399640445	count	0.06132634
ABAT	-1.7869853	0.4401547	-4.0599	5.05E-05	-1.397045729	count	1
CYBA	-0.9897616	0.0880895	-11.2359	1.13E-28	-1.394049751	count	2.73E-24
ZEB2	-1.1913179	0.2817383	-4.2285	2.43E-05	-1.391493147	count	0.5616702
MSI2	-1.0881854	0.1730035	-6.29	3.68E-10	-1.388878443	count	8.73E-06
C1QC	-1.7063482	0.4364341	-3.9097	9.46E-05	-1.383726469	count	1
TMTC2	-1.1574977	0.247686	-4.6732	3.11E-06	-1.383700508	count	0.07246611
GDF11	-1.8489011	0.4596519	-4.0224	5.92E-05	-1.38355655	count	1
TMEM45A	-1.2702062	0.2576173	-4.9306	8.68E-07	-1.382383089	count	0.020300784
SMOC1	-1.4905561	0.4586591	-3.2498	0.00117	-1.378537865	count	1
MT1G	-2.440593	0.8473609	-2.8802	0.004	-1.378276021	count	1
HSPA1B	-0.9615668	0.0737835	-13.0323	1.01E-37	-1.374251683	count	2.45E-33
PLEKHH2	-1.6373151	0.4027009	-4.0658	4.92E-05	-1.370899802	count	1
GLRB	-1.482124	0.42293	-3.5044	0.000465	-1.370159119	count	1
CAPS	-1.3849097	0.3433626	-4.0334	5.65E-05	-1.369752634	count	1
IGLC2	-1.509475	0.136166	-11.0855	5.68E-28	-1.366095855	count	1.37E-23
SCD5	-1.2978323	0.2957823	-4.3878	1.19E-05	-1.362155475	count	0.2758777
C2orf15	-1.680134	0.4726334	-3.5548	0.000385	-1.360748753	count	1
ARHGDIG	-3.3960076	0.9636599	-3.5241	0.000432	-1.360710914	count	1
FAM241B	-1.419213	0.4827369	-2.9399	0.00331	-1.360338459	count	1
ABCA1	-1.146433	0.2345363	-4.8881	1.08E-06	-1.359784663	count	0.02524824
MFAP5	-1.9121826	0.6158144	-3.1051	0.00192	-1.358748539	count	1
MUM1L1	-3.38799	0.9041542	-3.7471	0.000183	-1.358477742	count	1
IL18	-3.38799	0.9133764	-3.7093	0.000212	-1.358477742	count	1
PRDM16	-2.403357	0.5483814	-4.3826	1.22E-05	-1.357351327	count	0.2828082
PAPLN	-2.3982415	0.7848245	-3.0558	0.00227	-1.35445776	count	1
IGHM	-2.7248038	0.9843849	-2.768	0.00568	-1.350639633	count	1
RGS5	-1.241749	0.3289333	-3.7751	0.000163	-1.349791308	count	1
RCAN2	-1.6668382	0.317151	-5.2557	1.59E-07	-1.349077509	count	0.003733797
ADAM9	-1.0890198	0.2109195	-5.1632	2.60E-07	-1.348991397	count	0.0060996
APOBEC3C	-1.3637338	0.3130772	-4.3559	1.37E-05	-1.347398987	count	0.3174153
NFIX	-1.0211601	0.1728381	-5.9082	3.88E-09	-1.344370029	count	9.18E-05
EGFR	-1.356479	0.3543446	-3.8281	0.000132	-1.33973998	count	1
ALPK3	-1.3764647	0.2783123	-4.9458	8.04E-07	-1.339399894	count	0.018806364
PRR5L	-1.3361113	0.3682725	-3.6281	0.000291	-1.337771955	count	1
COL6A2	-1.2622402	0.218419	-5.779	8.36E-09	-1.336320488	count	0.000197547
ANGPTL2	-1.021947	0.1738908	-5.8769	4.68E-09	-1.331897098	count	0.000110691
MCOLN2	-3.294001	1.623057	-2.0295	0.0425	-1.331454457	count	1
FAP	-1.4416404	0.3129053	-4.6073	4.26E-06	-1.329905776	count	0.09918132
ENOX1	-20.6587087	1208.571483	-0.0171	0.986	-1.324095971	count	1
EMX2OS	-20.3632701	724.3797045	-0.0281	0.978	-1.32409597	count	1
OLR1	-20.0179877	1057.504351	-0.0189	0.985	-1.324095968	count	1
TSPOAP1	-19.8602969	912.0209946	-0.0218	0.983	-1.324095968	count	1
NRK	-19.8601425	736.6406331	-0.027	0.978	-1.324095968	count	1
CD163	-19.8227155	1083.562295	-0.0183	0.985	-1.324095968	count	1

COL8A2	-20.454014	825.0360395	-0.0248	0.98	-1.324095883	count	1
LEMD1	-20.319953	1118.392656	-0.0182	0.986	-1.324095883	count	1
CDS1	-20.319953	1118.392656	-0.0182	0.986	-1.324095883	count	1
HOXA10	-20.319953	1118.392656	-0.0182	0.986	-1.324095883	count	1
PRSS1	-20.319953	1118.392656	-0.0182	0.986	-1.324095883	count	1
AC108002.1	-20.319953	1118.392656	-0.0182	0.986	-1.324095883	count	1
ARHGEF39	-20.319953	1118.392656	-0.0182	0.986	-1.324095883	count	1
USP2-AS1	-20.319953	1118.392656	-0.0182	0.986	-1.324095883	count	1
AC068790.9	-20.319953	1118.392656	-0.0182	0.986	-1.324095883	count	1
AC091057.2	-20.319953	1118.392656	-0.0182	0.986	-1.324095883	count	1
SLAMF9	-20.318901	787.4075016	-0.0258	0.979	-1.324095883	count	1
IGFBP1	-20.318901	787.4075016	-0.0258	0.979	-1.324095883	count	1
PCDH9-AS1	-20.318901	787.4075016	-0.0258	0.979	-1.324095883	count	1
RHBDL1	-20.318901	787.4075016	-0.0258	0.979	-1.324095883	count	1
WNK4	-20.318901	787.4075016	-0.0258	0.979	-1.324095883	count	1
C22orf15	-20.318901	787.4075016	-0.0258	0.979	-1.324095883	count	1
ACVR1	-1.1638772	0.2113176	-5.5077	3.97E-08	-1.323678207	count	0.00093565
TSKU	-1.584833	0.4459012	-3.5542	0.000385	-1.323308915	count	1
ANTXR2	-1.3221776	0.3387543	-3.9031	9.73E-05	-1.32288479	count	1
PSORS1C1	-1.3385675	0.296005	-4.5221	6.38E-06	-1.320828403	count	0.148335
LPCAT2	-0.9581358	0.1156066	-8.2879	1.78E-16	-1.318949989	count	4.27E-12
ETNK2	-1.5334153	0.385257	-3.9802	7.06E-05	-1.317580852	count	1
PXDC1	-0.9449698	0.1016524	-9.2961	2.87E-20	-1.316685158	count	6.91E-16
LDLRAD4-AS1	-1.969463	0.5261582	-3.7431	0.000185	-1.31465142	count	1
POF1B	-1.2808889	0.3547813	-3.6104	0.000311	-1.31301188	count	1
C16orf74	-3.2304392	0.7063464	-4.5734	5.01E-06	-1.312289649	count	0.11659773
CFAP70	-1.7605231	0.6230625	-2.8256	0.00475	-1.312069233	count	1
ADAMTS17	-2.633779	0.7651594	-3.4421	0.000586	-1.308469371	count	1
PRR26	-2.633779	1.0917388	-2.4125	0.0159	-1.308469371	count	1
SPATA18	-2.633779	1.0996833	-2.395	0.0167	-1.308469371	count	1
CHCHD10	-0.9571453	0.1242392	-7.7041	1.82E-14	-1.306635925	count	4.36E-10
SKAP1	-1.952067	0.5388872	-3.6224	0.000297	-1.302122645	count	1
ACVR1C	-3.195823	0.9365276	-3.4124	0.000653	-1.301548729	count	1
HCN1	-3.195823	0.9365276	-3.4124	0.000653	-1.301548729	count	1
BTK	-3.195823	0.9365276	-3.4124	0.000653	-1.301548729	count	1
PCOLCE2	-2.610521	0.7070311	-3.6922	0.000227	-1.297450122	count	1
CRABP2	-3.1804051	0.9275028	-3.429	0.000615	-1.296695562	count	1
TXNRD2	-1.0886489	0.2218092	-4.908	9.73E-07	-1.293660751	count	0.022751659
BEST1	-1.9398669	0.5750635	-3.3733	0.000753	-1.293316529	count	1
SLC9A7	-1.4291638	0.3572934	-4	6.50E-05	-1.287706528	count	1
AC055874.1	-1.104859	0.3621716	-3.0507	0.00231	-1.286506441	count	1
CARF	-1.1922581	0.3211424	-3.7126	0.000209	-1.282011872	count	1
RND3	-0.9371806	0.1777382	-5.2728	1.45E-07	-1.28094852	count	0.00340605
SYNGR1	-1.4543991	0.5325815	-2.7308	0.00636	-1.279521746	count	1
KCNJ2	-1.0070846	0.3706921	-2.7168	0.00663	-1.278950102	count	1

MERTK	-1.2593588	0.3457386	-3.6425	0.000275	-1.273178475	count	1
CALHM5	-1.1727174	0.2475627	-4.7371	2.28E-06	-1.270764389	count	0.05319012
PCDH15	-1.5203313	0.527436	-2.8825	0.00398	-1.264657116	count	1
RHOQ	-1.0224448	0.209498	-4.8805	1.12E-06	-1.264112192	count	0.02618112
ZMAT3	-1.1663539	0.2481901	-4.6994	2.74E-06	-1.263483709	count	0.06388584
SIPA1L2	-1.628108	0.2895765	-5.6224	2.07E-08	-1.262434841	count	0.000488561
NCAM1	-1.073139	0.3421809	-3.1362	0.00173	-1.259222514	count	1
H2AFJ	-0.8984749	0.092871	-9.6744	8.55E-22	-1.258800279	count	2.06E-17
TNFRSF12A	-0.9192213	0.1392146	-6.6029	4.82E-11	-1.25822128	count	1.15E-06
S100A6	-0.8723059	0.0319665	-27.2881	2.25E-145	-1.257343894	count	5.47E-141
LINC00926	-2.523596	1.0278005	-2.4553	0.0141	-1.255419726	count	1
MECOM	-0.9046268	0.1174315	-7.7034	1.83E-14	-1.249807736	count	4.38E-10
ACTC1	-1.682142	0.8715078	-1.9302	0.0537	-1.248171329	count	1
BEX1	-1.1343589	0.391885	-2.8946	0.00383	-1.246650244	count	1
DNAJB1	-0.8739837	0.0713183	-12.2547	1.17E-33	-1.24626592	count	2.83E-29
LAMB1	-0.8951089	0.1282604	-6.9788	3.71E-12	-1.242347944	count	8.85E-08
FBXO17	-1.6040437	0.3048156	-5.2623	1.53E-07	-1.241918751	count	0.003593511
ZNF439	-1.3033616	0.4227292	-3.0832	0.00207	-1.241271751	count	1
IPO13	-1.3516778	0.421498	-3.2068	0.00136	-1.240367854	count	1
TGFB2	-1.0479615	0.3876072	-2.7037	0.0069	-1.238663506	count	1
ATG2A	-1.0235459	0.2542048	-4.0265	5.81E-05	-1.234732148	count	1
ME1	-1.4070529	0.4617031	-3.0475	0.00233	-1.234207729	count	1
NDRG4	-0.9701655	0.2017656	-4.8084	1.60E-06	-1.232862242	count	0.03736
PCDHGA12	-1.2201639	0.4421763	-2.7595	0.00583	-1.230867324	count	1
HTRA1	-0.8644155	0.067012	-12.8994	5.20E-37	-1.228871133	count	1.26E-32
SDC4	-1.4807836	0.3406363	-4.3471	1.43E-05	-1.228637336	count	0.3312738
SH3RF1	-1.0017197	0.2101808	-4.766	1.98E-06	-1.228524993	count	0.04621716
ACOT7	-0.9740233	0.175154	-5.561	2.94E-08	-1.227537755	count	0.00069337
MINPP1	-0.9490059	0.180213	-5.266	1.50E-07	-1.226841241	count	0.0035232
IGSF6	-2.4598105	0.7803336	-3.1523	0.00164	-1.223763365	count	1
SKIL	-0.8655223	0.0718949	-12.0387	1.43E-32	-1.223686114	count	3.46E-28
STMN3	-0.9164618	0.1556922	-5.8864	4.42E-09	-1.223684425	count	0.000104564
CCSER2	-0.8783169	0.0969332	-9.061	2.38E-19	-1.222301509	count	5.73E-15
C1QA	-1.5221439	0.3780857	-4.0259	5.83E-05	-1.221492146	count	1
BPGM	-0.9211123	0.1588168	-5.7998	7.39E-09	-1.218021429	count	0.000174663
SNX18	-0.9522284	0.1783872	-5.338	1.02E-07	-1.215043965	count	0.00239802
CPNE5	-1.0556275	0.2768167	-3.8135	0.00014	-1.214859133	count	1
AC022007.1	-2.932897	0.8653981	-3.3891	0.000711	-1.212979067	count	1
SERPINA5	-2.932897	0.8653981	-3.3891	0.000711	-1.212979067	count	1
SMIM5	-2.932897	0.8653981	-3.3891	0.000711	-1.212979067	count	1
L3MBTL4-AS1	-2.932897	0.8653981	-3.3891	0.000711	-1.212979067	count	1
PPP4R1-AS1	-2.932897	0.8653981	-3.3891	0.000711	-1.212979067	count	1
AP000695.1	-2.932897	0.8653981	-3.3891	0.000711	-1.212979067	count	1
KIAA1958	-2.932897	0.90617	-3.2366	0.00122	-1.212979067	count	1
AL132780.1	-2.932897	0.90617	-3.2366	0.00122	-1.212979067	count	1

P3H3	-1.0427895	0.2507612	-4.1585	3.30E-05	-1.211236312	count	0.76164
STAB1	-0.8841901	0.1416222	-6.2433	4.94E-10	-1.209122631	count	1.17E-05
LMO7	-1.4172834	0.4390101	-3.2284	0.00126	-1.209025644	count	1
C1orf54	-0.8735088	0.1067716	-8.1811	4.24E-16	-1.207104273	count	1.02E-11
RTKN2	-1.8176727	0.874275	-2.0791	0.0377	-1.204333567	count	1
TTLL3	-1.0818671	0.267183	-4.0492	5.28E-05	-1.203133045	count	1
GJA1	-0.857056	0.086359	-9.9243	7.83E-23	-1.202605484	count	1.89E-18
ARL5A	-0.9264438	0.2172948	-4.2635	2.08E-05	-1.202468168	count	0.4811456
HOXA5	-1.3396126	0.3918232	-3.4189	0.000638	-1.200205859	count	1
PODXL	-0.8518121	0.0912957	-9.3303	2.10E-20	-1.199571726	count	5.06E-16
MAP3K5	-1.2622981	0.2916614	-4.328	1.56E-05	-1.199084545	count	0.3612492
DANT2	-2.133355	0.6567664	-3.2483	0.00117	-1.199022106	count	1
QPCTL	-2.133355	0.6567664	-3.2483	0.00117	-1.199022106	count	1
WIPI1	-1.0845472	0.2452369	-4.4224	1.01E-05	-1.197841926	count	0.2343806
TNFRSF4	-0.9216606	0.2468717	-3.7334	0.000193	-1.197415035	count	1
SRRM3	-1.9438334	0.5673827	-3.426	0.000622	-1.197065147	count	1
KISS1	-1.544087	0.8248706	-1.8719	0.0613	-1.190711968	count	1
SMAD7	-0.9641776	0.1799488	-5.3581	9.10E-08	-1.189914932	count	0.002140047
LTC4S	-0.8279616	0.0585566	-14.1395	7.09E-44	-1.186515492	count	1.72E-39
HIPK2	-0.907216	0.1641357	-5.5272	3.56E-08	-1.185387676	count	0.000839199
GPRIN3	-2.3835878	0.5051394	-4.7187	2.49E-06	-1.185085424	count	0.05807676
ETV4	-1.7910959	0.5952117	-3.0092	0.00264	-1.184818786	count	1
AC007998.3	-1.2941003	0.3985946	-3.2467	0.00118	-1.183068739	count	1
MAP1A	-1.9228761	0.7228042	-2.6603	0.00785	-1.182850367	count	1
PKIA	-1.2237605	0.4569983	-2.6778	0.00745	-1.180288039	count	1
NMT2	-0.9859914	0.2308083	-4.2719	2.00E-05	-1.179984151	count	0.46274
MAST4	-0.860159	0.1300813	-6.6125	4.52E-11	-1.179885573	count	1.08E-06
SERPINB9	-0.9019413	0.1579977	-5.7086	1.26E-08	-1.179468177	count	0.000297599
SAMD14	-1.4721491	0.4465776	-3.2965	0.000991	-1.177259436	count	1
SEC31B	-1.7760587	0.6133619	-2.8956	0.00381	-1.173756176	count	1
SAMD11	-1.5906974	0.613557	-2.5926	0.00958	-1.173213701	count	1
BMPR2	-0.82425	0.0725506	-11.361	2.89E-29	-1.171028692	count	6.99E-25
IKZF4	-1.5202739	0.4959507	-3.0654	0.0022	-1.170348416	count	1
IDUA	-0.9978446	0.2540496	-3.9278	8.78E-05	-1.167346292	count	1
KIAA1147	-1.0440687	0.237859	-4.3894	1.18E-05	-1.166736534	count	0.2735948
AXL	-2.8060277	0.7570103	-3.7067	0.000214	-1.165903814	count	1
GPR161	-1.1327661	0.2665952	-4.249	2.22E-05	-1.165629052	count	0.5134416
TBC1D16	-1.1198035	0.3283984	-3.4099	0.000659	-1.16450766	count	1
THSD4	-1.003232	0.2820808	-3.5565	0.000382	-1.163332279	count	1
ERCC6	-0.9312613	0.1931208	-4.8222	1.50E-06	-1.160933678	count	0.0350355
DACH1	-1.0600453	0.4224044	-2.5096	0.0121	-1.160686407	count	1
CNNM2	-1.3650456	0.4044046	-3.3754	0.000747	-1.160159316	count	1
ST8SIA6	-0.8368421	0.1503622	-5.5655	2.86E-08	-1.158938003	count	0.000674588
RGN	-1.7551323	0.491695	-3.5696	0.000364	-1.158337733	count	1
ARHGAP4	-1.1134188	0.2721021	-4.0919	4.40E-05	-1.157435247	count	1

CCL3L1	-1.4467413	0.516423	-2.8015	0.00512	-1.154769824	count	1
LAMA2	-1.358203	0.5909296	-2.2984	0.0216	-1.153759925	count	1
FCHSD1	-1.2628526	0.496921	-2.5414	0.0111	-1.151995812	count	1
MICAL1	-1.3950844	0.497562	-2.8038	0.00509	-1.150522222	count	1
ORMDL3	-1.057743	0.3080003	-3.4342	0.000603	-1.148948974	count	1
ABL2	-0.8604713	0.1350647	-6.3708	2.20E-10	-1.14781115	count	5.22E-06
AL359504.2	-2.0474636	0.5954686	-3.4384	0.000594	-1.146604148	count	1
LTB	-1.4921447	0.7936783	-1.88	0.0602	-1.146282501	count	1
CFH	-0.8010207	0.0604349	-13.2543	6.41E-39	-1.144450484	count	1.55E-34
AAK1	-0.8254537	0.1163949	-7.0918	1.67E-12	-1.140554554	count	3.99E-08
PLEKHA2	-3.8896914	1.3282948	-2.9283	0.00344	-1.140517784	count	1
PREX1	-1.1851425	0.3877767	-3.0563	0.00226	-1.140120271	count	1
SMIM3	-1.4298462	0.4410271	-3.2421	0.0012	-1.139814498	count	1
HSPA4L	-1.0202144	0.3186418	-3.2018	0.00138	-1.138729777	count	1
FADS1	-1.1179202	0.2801731	-3.9901	6.78E-05	-1.135443881	count	1
COL23A1	-2.0264754	0.4203491	-4.8209	1.51E-06	-1.133674385	count	0.03526605
CACNB2	-2.71843	1.091867	-2.4897	0.0128	-1.13182184	count	1
TNIK	-2.71843	1.1108005	-2.4473	0.0145	-1.13182184	count	1
AP001189.5	-1.373936	0.5875441	-2.3384	0.0194	-1.1312477	count	1
FKBP5	-0.8246642	0.098347	-8.3853	7.96E-17	-1.128422271	count	1.91E-12
ATP10A	-2.7075019	0.8049438	-3.3636	0.00078	-1.127482142	count	1
ANGPTL4	-0.8785971	0.1878321	-4.6776	3.04E-06	-1.126790893	count	0.0708472
EVA1A	-1.8402547	0.5150772	-3.5728	0.000359	-1.126453554	count	1
SLC46A1	-1.2130161	0.4812961	-2.5203	0.0118	-1.12636073	count	1
EFHB	-1.612532	0.6925476	-2.3284	0.02	-1.126140804	count	1
TNFSF13B	-1.2116753	0.4984273	-2.431	0.0151	-1.125007032	count	1
NQO1	-0.8143971	0.1211223	-6.7238	2.14E-11	-1.124545202	count	5.09E-07
DMD	-1.7091035	0.4987554	-3.4267	0.00062	-1.124339813	count	1
FAM171B	-0.8720441	0.1630476	-5.3484	9.60E-08	-1.121107911	count	0.002257152
ARSE	-1.6052808	0.5980372	-2.6842	0.00731	-1.120452026	count	1
SPDYA	-2.2566785	0.719227	-3.1376	0.00172	-1.118813959	count	1
PLA2R1	-0.9144658	0.2942765	-3.1075	0.00191	-1.118185552	count	1
C2orf88	-2.0006555	0.6082236	-3.2893	0.00102	-1.117708591	count	1
UQCC2	-0.8178307	0.1197099	-6.8318	1.03E-11	-1.116503702	count	2.45E-07
ANO8	-1.4552057	0.5279959	-2.7561	0.00589	-1.11467054	count	1
TMEM106C	-0.8387613	0.1601354	-5.2378	1.75E-07	-1.113551604	count	0.00410865
NPEPPS	-0.8142268	0.1427315	-5.7046	1.29E-08	-1.11309311	count	0.000304672
ARMCX2	-0.9075581	0.2634812	-3.4445	0.000581	-1.112365108	count	1
ALDOC	-0.897957	0.2529095	-3.5505	0.000391	-1.110935361	count	1
GOLT1A	-2.239749	0.7063383	-3.1709	0.00154	-1.109811703	count	1
DLX6-AS1	-2.239749	0.73964	-3.0282	0.00248	-1.109811703	count	1
HOXA7	-2.239749	0.975255	-2.2966	0.0217	-1.109811703	count	1
GPR34	-2.239749	0.9996378	-2.2406	0.0251	-1.109811703	count	1
LST1	-1.6877288	0.4991653	-3.3811	0.000732	-1.108518245	count	1
FILIP1	-0.7932544	0.1131526	-7.0105	2.97E-12	-1.107503533	count	7.08E-08

SOX5	-1.5101293	0.5032362	-3.0008	0.00272	-1.106963962	count	1
WWTR1	-0.7742585	0.0564629	-13.7127	1.88E-41	-1.106289852	count	4.56E-37
LINC00092	-2.6481246	0.6915824	-3.8291	0.000131	-1.103573388	count	1
POMC	-1.5053117	0.5297857	-2.8414	0.00453	-1.102999717	count	1
FRMD6	-1.0254258	0.2686129	-3.8175	0.000138	-1.10258395	count	1
PFN2	-0.8594213	0.2216518	-3.8773	0.000108	-1.10163321	count	1
AC016394.1	-1.2112088	0.4031385	-3.0044	0.00268	-1.100703548	count	1
KIAA1549L	-3.659084	1.2473433	-2.9335	0.00338	-1.100312954	count	1
LRP3	-1.675861	0.4778869	-3.5068	0.000461	-1.099725787	count	1
IL13RA2	-1.4361519	0.4598117	-3.1233	0.00181	-1.09836475	count	1
TGM2	-0.7791687	0.075382	-10.3363	1.35E-24	-1.098050234	count	3.26E-20
NID2	-1.4353588	0.5017536	-2.8607	0.00426	-1.097686076	count	1
FBXO38	-0.8882729	0.2560563	-3.4691	0.00053	-1.096047731	count	1
APOD	-0.7844744	0.2841828	-2.7605	0.0058	-1.095168045	count	1
MIR155HG	-1.0569349	0.3086854	-3.424	0.000626	-1.094947327	count	1
P4HB	-0.7722206	0.0646269	-11.9489	4.02E-32	-1.094562019	count	9.73E-28
CACNB4	-2.6251887	0.8037821	-3.266	0.0011	-1.094191344	count	1
TPPP3	-0.9593608	0.2931663	-3.2724	0.00108	-1.093421013	count	1
RFLNB	-0.8061959	0.1623806	-4.9649	7.29E-07	-1.091765939	count	0.017057871
MYOM3	-2.2017886	0.6712697	-3.28	0.00105	-1.089498961	count	1
EBF4	-1.488551	0.5082151	-2.929	0.00343	-1.089207169	count	1
SYNPO	-0.78058	0.0983344	-7.938	2.96E-15	-1.089136571	count	7.09E-11
HOXA2	-1.3723858	0.4591794	-2.9888	0.00283	-1.08896645	count	1
ALPL	-1.1544014	0.4585265	-2.5176	0.0119	-1.088454092	count	1
ATP2A3	-1.0047934	0.245046	-4.1004	4.24E-05	-1.088231991	count	0.9774472
INSIG2	-0.8092595	0.1499008	-5.3986	7.29E-08	-1.085548856	count	0.001715337
ROBO3	-0.8461744	0.1870559	-4.5236	6.33E-06	-1.08426153	count	0.14718516
OAZ1	-0.7541141	0.0375455	-20.0853	7.27E-84	-1.083807217	count	1.77E-79
METTL25	-0.8349636	0.1886183	-4.4267	9.94E-06	-1.082546263	count	0.23068752
LRIG1	-0.7990371	0.15921	-5.0188	5.53E-07	-1.081952913	count	0.012946836
AC010737.1	-1.3607036	0.5292531	-2.571	0.0102	-1.078634652	count	1
FLRT3	-1.93739	0.6941354	-2.7911	0.00529	-1.0783364	count	1
CPEB4	-0.7881083	0.1218369	-6.4686	1.17E-10	-1.078183917	count	2.78E-06
TXNDC5	-1.6437881	0.543684	-3.0234	0.00252	-1.075940445	count	1
AL121655.1	-1.643455	0.7733203	-2.1252	0.0337	-1.075693241	count	1
EFS	-1.643455	0.9971897	-1.6481	0.0994	-1.075693241	count	1
IGHG3	-1.932608	0.3066238	-6.3029	3.39E-10	-1.075346725	count	8.04E-06
TDRD9	-1.470908	0.866491	-1.6975	0.0897	-1.074688769	count	1
GSTT2B	-0.7893691	0.1510407	-5.2262	1.86E-07	-1.074292329	count	0.00436635
LIMCH1	-0.7620506	0.0974807	-7.8175	7.60E-15	-1.072839604	count	1.82E-10
MYO9B	-0.8574069	0.1905419	-4.4998	7.08E-06	-1.072781411	count	0.1645038
ARSJ	-1.4652121	0.4228582	-3.465	0.000538	-1.070001267	count	1
TRAM2	-0.8690881	0.2155738	-4.0315	5.69E-05	-1.069083457	count	1
ITSN2	-0.7679895	0.1106183	-6.9427	4.78E-12	-1.068283907	count	1.14E-07
ZSCAN31	-0.8624728	0.2691179	-3.2048	0.00137	-1.068141262	count	1

GFPT1	-0.8725186	0.2312253	-3.7735	0.000164	-1.068057032	count	1
RAB15	-1.2321409	0.4964862	-2.4817	0.0131	-1.066953142	count	1
VASH1	-0.8444872	0.1917321	-4.4045	1.10E-05	-1.065823334	count	0.255101
MEGF6	-0.8932502	0.218626	-4.0857	4.52E-05	-1.065037553	count	1
FOLR2	-2.1559992	0.7706584	-2.7976	0.00518	-1.064775705	count	1
LAIR1	-2.1559992	0.8749808	-2.4641	0.0138	-1.064775705	count	1
FUT10	-0.8895364	0.2762975	-3.2195	0.0013	-1.064105405	count	1
TMEM87B	-0.7931426	0.1582037	-5.0134	5.68E-07	-1.063627382	count	0.01329688
ARHGEF40	-0.9553095	0.3316785	-2.8802	0.004	-1.062667834	count	1
TP53TG3D	-1.2984865	0.5444649	-2.3849	0.0172	-1.062551323	count	1
FZD8	-0.8209321	0.1934082	-4.2446	2.26E-05	-1.061618714	count	0.5226024
SLC22A23	-1.1487319	0.4423896	-2.5967	0.00946	-1.061544771	count	1
AP001527.2	-1.6242765	0.5949209	-2.7302	0.00637	-1.061456429	count	1
HACD1	-0.8480563	0.2155505	-3.9344	8.55E-05	-1.060756807	count	1
SDSL	-0.9641191	0.3241556	-2.9742	0.00296	-1.058073578	count	1
SELENBP1	-0.80016	0.1802999	-4.4379	9.44E-06	-1.057836574	count	0.21914016
ANK1	-1.1916643	0.3973761	-2.9988	0.00273	-1.055924306	count	1
HEG1	-0.7415028	0.073966	-10.0249	2.94E-23	-1.055373515	count	7.09E-19
MICAL2	-0.9205919	0.2731859	-3.3698	0.000762	-1.052841661	count	1
GATA6	-0.7721017	0.1307792	-5.9039	3.98E-09	-1.051850223	count	9.42E-05
FSTL1	-0.7611862	0.1290676	-5.8976	4.14E-09	-1.049481307	count	9.79E-05
IGIP	-1.051082	0.4357839	-2.4119	0.0159	-1.048920056	count	1
CEMIP	-2.5161593	0.5755053	-4.3721	1.28E-05	-1.048523004	count	0.2966528
DYSF	-0.9307028	0.2743374	-3.3925	0.000702	-1.047112692	count	1
SEL1L3	-0.8987252	0.2397574	-3.7485	0.000182	-1.046758485	count	1
CSGALNACT1	-0.7462964	0.1075288	-6.9404	4.85E-12	-1.044556389	count	1.16E-07
KCNK15-AS1	-2.1186365	0.8490478	-2.4953	0.0126	-1.044435266	count	1
IFI6	-0.7313753	0.0819072	-8.9293	7.65E-19	-1.04426154	count	1.84E-14
FHL2	-0.8678843	0.2122036	-4.0899	4.44E-05	-1.044089543	count	1
CAPS2	-1.1538997	0.4407373	-2.6181	0.00889	-1.043913765	count	1
IFI27L2	-0.772272	0.1289412	-5.9893	2.38E-09	-1.043646168	count	5.64E-05
CERCAM	-0.8782226	0.2464698	-3.5632	0.000373	-1.042704059	count	1
CHST3	-0.925624	0.2680035	-3.4538	0.000561	-1.041116821	count	1
SMOX	-1.0280676	0.2734143	-3.7601	0.000173	-1.037982337	count	1
UCHL1	-1.0865869	0.4078459	-2.6642	0.00776	-1.037900992	count	1
SOX8	-1.7039841	0.6661391	-2.558	0.0106	-1.032494808	count	1
DIRAS3	-1.5849161	0.6022819	-2.6315	0.00855	-1.032215143	count	1
TMEM158	-1.58412	0.608591	-2.6029	0.00929	-1.031623526	count	1
SLC6A8	-1.2624907	0.4503645	-2.8033	0.00509	-1.029838959	count	1
COL5A2	-1.581514	0.319729	-4.9464	8.01E-07	-1.029686501	count	0.018736992
TSPAN15	-0.7993719	0.1881304	-4.249	2.22E-05	-1.029516306	count	0.5134416
DKK3	-0.7966297	0.1639681	-4.8584	1.25E-06	-1.027140293	count	0.02921375
PCDH17	-0.75066	0.1237775	-6.0646	1.50E-09	-1.025323363	count	3.55E-05
OGFOD2	-1.0141755	0.3165195	-3.2041	0.00137	-1.022956663	count	1
ALOX5AP	-1.2971569	0.7091812	-1.8291	0.0675	-1.022498765	count	1

TOX	-1.57092	0.4415472	-3.5578	0.00038	-1.021812511	count	1
EPB41L1	-1.0880229	0.4043829	-2.6906	0.00718	-1.020655668	count	1
ITGB3	-0.9878321	0.3685489	-2.6803	0.0074	-1.018737698	count	1
ITPR2	-0.7243848	0.0930046	-7.7887	9.50E-15	-1.018619102	count	2.27E-10
CDC45	-1.6817738	0.69668	-2.414	0.0158	-1.017106418	count	1
C1QTNF4	-2.4418076	0.7227356	-3.3786	0.000739	-1.016421647	count	1
LINC01943	-2.0665611	0.6423058	-3.2174	0.00131	-1.015856302	count	1
FXYD6	-0.7315508	0.1063955	-6.8758	7.59E-12	-1.015796872	count	1.81E-07
ITLN2	-3.2660388	1.0997539	-2.9698	0.00301	-1.015541484	count	1
ZNF365	-3.2660388	1.0997539	-2.9698	0.00301	-1.015541484	count	1
MMP19	-3.2660388	1.1705155	-2.7903	0.0053	-1.015541484	count	1
IDS	-0.7336527	0.1089355	-6.7347	1.99E-11	-1.015456186	count	4.74E-07
FAM102B	-0.8766989	0.3347496	-2.619	0.00887	-1.015400233	count	1
NCS1	-2.4391696	0.8787126	-2.7758	0.00554	-1.015269264	count	1
FCN1	-1.83566	0.7180233	-2.5565	0.0106	-1.014401045	count	1
PRRX2	-1.83566	0.7328722	-2.5047	0.0123	-1.014401045	count	1
NUDT4	-0.7395233	0.1191165	-6.2084	6.16E-10	-1.013887698	count	1.46E-05
SHF	-0.8712391	0.2733956	-3.1867	0.00145	-1.01341185	count	1
AF127577.4	-1.468688	0.6395331	-2.2965	0.0217	-1.013159225	count	1
KIAA1211L	-0.7687669	0.1892064	-4.0631	4.98E-05	-1.011020733	count	1
C21orf2	-0.8458257	0.2251117	-3.7574	0.000175	-1.009932955	count	1
GPRC5A	-0.752438	0.1738133	-4.329	1.55E-05	-1.009797158	count	0.358949
PCDHGB7	-1.1188848	0.431945	-2.5903	0.00964	-1.009303037	count	1
SHISA3	-1.1714344	0.5465594	-2.1433	0.0322	-1.009139611	count	1
S100A3	-1.0578905	0.3331409	-3.1755	0.00151	-1.00824149	count	1
CALU	-0.7150672	0.0877502	-8.1489	5.50E-16	-1.005934062	count	1.32E-11
ANKRD44	-1.1145791	0.3924224	-2.8403	0.00454	-1.005052315	count	1
ZDHHC2	-0.7407348	0.1384771	-5.3492	9.56E-08	-1.003213139	count	0.002247843
ZNF155	-1.0905408	0.3631334	-3.0031	0.0027	-1.003061041	count	1
GLIS2	-1.1645223	0.3774905	-3.0849	0.00206	-1.002569641	count	1
AL031666.1	-1.1642356	0.5970156	-1.9501	0.0513	-1.00229718	count	1
SMARCD3	-1.380999	0.6303786	-2.1907	0.0286	-1.000753076	count	1
CLIP4	-2.396028	0.8046472	-2.9777	0.00293	-0.996296942	count	1
AC092171.4	-2.396028	0.8046472	-2.9777	0.00293	-0.996296942	count	1
XYLT1	-2.396028	0.8494419	-2.8207	0.00483	-0.996296942	count	1
AC080038.1	-2.396028	0.9200335	-2.6043	0.00926	-0.996296942	count	1
DEPP1	-0.6955023	0.0948788	-7.3304	3.00E-13	-0.993545947	count	7.17E-09
SGO1	-3.176456	1.165887	-2.7245	0.00648	-0.993204936	count	1
PDE3B	-2.0244748	0.8794555	-2.302	0.0214	-0.992582926	count	1
PCDHGA11	-2.0244748	1.1536089	-1.7549	0.0794	-0.992582926	count	1
CSRNP3	-2.0241682	0.7787193	-2.5994	0.00939	-0.992412874	count	1
AC018797.2	-1.2627257	0.5092514	-2.4796	0.0132	-0.992145709	count	1
KANK2	-0.8401352	0.3351745	-2.5066	0.0122	-0.992055648	count	1
LINC00167	-1.7979539	0.8143654	-2.2078	0.0273	-0.990551433	count	1
PDE4DIP	-0.862376	0.2733268	-3.1551	0.00162	-0.988425834	count	1

POMT2	-1.0385761	0.4418157	-2.3507	0.0188	-0.988310768	count	1
TUBA1C	-0.7038795	0.0902981	-7.7951	9.05E-15	-0.988114825	count	2.17E-10
SLC39A7	-0.7155957	0.1159259	-6.1729	7.69E-10	-0.988109298	count	1.82E-05
HNRNPA1P48	-0.8460971	0.2667936	-3.1714	0.00153	-0.987280212	count	1
FCN3	-1.78915	0.508464	-3.5187	0.000441	-0.984973602	count	1
Z99289.1	-3.1379686	1.469539	-2.1353	0.0328	-0.983260508	count	1
IL1B	-2.3601976	0.7306727	-3.2302	0.00125	-0.980366485	count	1
TTC9	-0.9632896	0.3219767	-2.9918	0.0028	-0.980236786	count	1
SPARC	-0.6867082	0.0702816	-9.7708	3.42E-22	-0.978664802	count	8.24E-18
PTPN3	-2.3551017	0.6414156	-3.6717	0.000245	-0.978088394	count	1
CACNA2D2	-2.3551017	0.6545596	-3.598	0.000326	-0.978088394	count	1
WFDC1	-2.3551017	0.7518422	-3.1324	0.00175	-0.978088394	count	1
FADS3	-0.7239299	0.1436724	-5.0388	4.99E-07	-0.977529415	count	0.011687079
RETREG1	-0.9602518	0.4152353	-2.3125	0.0208	-0.976930731	count	1
AC244090.1	-0.7827528	0.232302	-3.3695	0.000763	-0.976892388	count	1
N4BP2	-0.8994971	0.2879715	-3.1236	0.00181	-0.975884045	count	1
S1PR2	-1.5063475	0.5572471	-2.7032	0.00691	-0.973811599	count	1
PRKG1	-1.50617	0.620876	-2.4259	0.0153	-0.973679584	count	1
KCTD11	-1.1333411	0.4770386	-2.3758	0.0176	-0.972969189	count	1
PKD1	-0.7931206	0.1921509	-4.1276	3.77E-05	-0.972847818	count	0.8695882
FAM149B1	-1.0218985	0.3802674	-2.6873	0.00725	-0.971123188	count	1
CFB	-1.1035644	0.3958087	-2.7881	0.00534	-0.970469968	count	1
TMED3	-0.7041181	0.1356466	-5.1908	2.25E-07	-0.967134316	count	0.005280525
ACVRL1	-0.691048	0.0837697	-8.2494	2.43E-16	-0.966550582	count	5.83E-12
LY75	-0.9212629	0.3431894	-2.6844	0.00731	-0.966001796	count	1
TNFRSF9	-3.0643495	1.206017	-2.5409	0.0111	-0.963658549	count	1
DOK5	-0.8162344	0.259889	-3.1407	0.0017	-0.96275082	count	1
CXCL2	-0.6670304	0.1221032	-5.4628	5.10E-08	-0.960149548	count	0.001201254
GPX3	-0.7270991	0.1508024	-4.8215	1.50E-06	-0.959705316	count	0.0350355
AAMDC	-0.6792418	0.0784316	-8.6603	7.86E-18	-0.959594929	count	1.89E-13
BNIP3L	-0.6771566	0.0763242	-8.8721	1.26E-18	-0.959478343	count	3.03E-14
DAG1	-0.7493236	0.1797903	-4.1678	3.17E-05	-0.95746838	count	0.7318579
FAM198B	-0.6750793	0.0824648	-8.1863	4.07E-16	-0.956886004	count	9.76E-12
SULT1A2	-3.039434	1.0347314	-2.9374	0.00334	-0.956852525	count	1
SRGAP3	-3.039434	1.0611723	-2.8642	0.00421	-0.956852525	count	1
C3	-1.1153631	0.5987498	-1.8628	0.0626	-0.955932631	count	1
AL118508.1	-1.958514	0.4897108	-3.9993	6.52E-05	-0.955831101	count	1
AC090152.1	-1.958318	0.5819206	-3.3653	0.000775	-0.955721855	count	1
MLLT11	-1.0050571	0.3820889	-2.6304	0.00858	-0.953788907	count	1
C6orf48	-0.6859038	0.0899047	-7.6292	3.23E-14	-0.953595601	count	7.73E-10
ROM1	-0.718906	0.1493251	-4.8144	1.56E-06	-0.953324886	count	0.03642912
LRRC61	-3.0254208	1.0711582	-2.8244	0.00477	-0.952987364	count	1
LTBP3	-0.6870995	0.1167307	-5.8862	4.43E-09	-0.952709673	count	0.000104792
CLMP	-1.2655203	0.7753168	-1.6323	0.103	-0.952687369	count	1
CHD3	-0.7491807	0.1798944	-4.1646	3.22E-05	-0.951799372	count	0.7432726

FUT8-AS1	-1.476162	0.5044867	-2.9261	0.00346	-0.951383318	count	1
MLEC	-0.6887146	0.1123357	-6.1309	9.99E-10	-0.951361779	count	2.37E-05
GAS6	-0.6642353	0.0640808	-10.3656	1.00E-24	-0.951300975	count	2.41E-20
TRNP1	-3.011875	0.7757211	-3.8827	0.000106	-0.9492247	count	1
SLC2A6	-0.9984442	0.3285482	-3.039	0.0024	-0.946988812	count	1
AL136038.3	-1.2104055	0.5077291	-2.384	0.0172	-0.946138017	count	1
LRRK2	-0.7942152	0.2618406	-3.0332	0.00244	-0.94611507	count	1
AC112229.3	-1.104815	0.5834631	-1.8935	0.0584	-0.945947751	count	1
NDUFA4L2	-1.3139369	0.6755627	-1.945	0.0519	-0.945747806	count	1
LDLRAP1	-0.9212537	0.2451718	-3.7576	0.000175	-0.945625089	count	1
RBMS3	-0.6765346	0.0987258	-6.8527	8.90E-12	-0.945011401	count	2.12E-07
AEBP1	-0.8385586	0.2576542	-3.2546	0.00115	-0.944263456	count	1
CALCRL	-0.6567321	0.0485112	-13.5377	1.78E-40	-0.943794268	count	4.31E-36
PTK7	-1.1653939	0.4328598	-2.6923	0.00714	-0.941919242	count	1
MAPK10	-2.2730182	0.7472289	-3.0419	0.00237	-0.940991526	count	1
SNX24	-0.7592742	0.2108242	-3.6015	0.000322	-0.94093622	count	1
CCL4	-0.738671	0.2194906	-3.3654	0.000775	-0.940898066	count	1
NKG7	-1.2490732	0.5555509	-2.2484	0.0246	-0.938707534	count	1
RNASET2	-0.712627	0.1794557	-3.971	7.34E-05	-0.93816962	count	1
COL13A1	-1.5664803	0.5382291	-2.9104	0.00364	-0.937081062	count	1
MXD4	-0.6725574	0.1063967	-6.3212	3.02E-10	-0.936805132	count	7.17E-06
AC012510.1	-2.2599244	0.8421278	-2.6836	0.00733	-0.935007394	count	1
AC006273.1	-2.2599244	1.0402659	-2.1724	0.0299	-0.935007394	count	1
EDIL3	-0.7935784	0.2032354	-3.9047	9.66E-05	-0.935007362	count	1
GZMA	-1.7096357	0.5328626	-3.2084	0.00135	-0.934471619	count	1
PTPRB	-0.6562239	0.069424	-9.4524	6.82E-21	-0.933391585	count	1.64E-16
GINS4	-1.1557013	0.4793139	-2.4112	0.016	-0.933175018	count	1
PIGG	-0.7716381	0.2374544	-3.2496	0.00117	-0.933011548	count	1
NLGN2	-1.0413246	0.3760252	-2.7693	0.00566	-0.932936405	count	1
PANK1	-1.4502248	0.7427684	-1.9525	0.051	-0.932125064	count	1
PLXNA1	-1.4498463	0.5408869	-2.6805	0.00739	-0.931844106	count	1
RBMS3-AS3	-0.8821674	0.2886596	-3.0561	0.00226	-0.931523422	count	1
LINC01759	-1.5580992	0.6636454	-2.3478	0.019	-0.931261024	count	1
CDCA7L	-1.5580992	0.6636454	-2.3478	0.019	-0.931261024	count	1
LINC01063	-1.5580992	0.761531	-2.046	0.0409	-0.931261024	count	1
AC048382.5	-1.914284	0.6543471	-2.9255	0.00347	-0.931026098	count	1
RNF166	-0.7721652	0.2269057	-3.403	0.000676	-0.930898524	count	1
CXCL12	-0.6591128	0.1220928	-5.3985	7.29E-08	-0.927509589	count	0.001715337
NUCB2	-0.6473234	0.053079	-12.1955	2.33E-33	-0.92586969	count	5.64E-29
NME9	-1.546602	0.6112628	-2.5302	0.0115	-0.923277912	count	1
AC092807.3	-1.546602	0.9393492	-1.6465	0.0998	-0.923277912	count	1
HNMT	-0.8461215	0.4113867	-2.0568	0.0398	-0.921975725	count	1
RASD1	-0.6972618	0.2187419	-3.1876	0.00145	-0.921019528	count	1
TCTEX1D4	-1.896283	0.8748868	-2.1675	0.0303	-0.920898839	count	1
EDDM13	-1.896283	0.9255849	-2.0487	0.0406	-0.920898839	count	1

PDGFA	-0.6748297	0.1325706	-5.0903	3.81E-07	-0.920477033	count	0.008929116
GATB	-0.838437	0.3234159	-2.5924	0.00958	-0.919910405	count	1
MIAT	-1.225546	0.3668177	-3.341	0.000846	-0.918737651	count	1
ISG15	-0.6470306	0.0968118	-6.6834	2.81E-11	-0.917658916	count	6.69E-07
GALNT2	-0.6820767	0.1555089	-4.3861	1.20E-05	-0.915915931	count	0.278184
MS4A4A	-2.8935491	1.0825087	-2.673	0.00756	-0.915291661	count	1
LINC01637	-2.8935491	1.3850298	-2.0892	0.0368	-0.915291661	count	1
ARFGAP1	-0.8398488	0.26609	-3.1563	0.00162	-0.91477075	count	1
CDKL5	-0.7929415	0.2899372	-2.7349	0.00628	-0.914437482	count	1
AC004846.1	-1.5320307	0.6564508	-2.3338	0.0197	-0.913162075	count	1
ACTA2	-0.7490515	0.3477033	-2.1543	0.0313	-0.912438121	count	1
ENGASE	-0.9989545	0.3937513	-2.537	0.0112	-0.911501006	count	1
C19orf18	-1.1698731	0.5710618	-2.0486	0.0406	-0.910613299	count	1
MON1A	-0.8626196	0.2925778	-2.9483	0.00322	-0.909637875	count	1
B3GNT7	-1.2685055	0.6080415	-2.0862	0.0371	-0.908600777	count	1
SLFN12	-0.7185466	0.1750895	-4.1039	4.18E-05	-0.907683573	count	0.9637408
HIP1R	-0.7395474	0.2055585	-3.5977	0.000327	-0.907419307	count	1
GNG2	-1.8701451	0.9112707	-2.0522	0.0402	-0.906166046	count	1
JAG1	-0.6541201	0.1310124	-4.9928	6.32E-07	-0.905988876	count	0.01479196
PIH1D2	-0.867158	0.3678185	-2.3576	0.0185	-0.905809011	count	1
MTUS1	-0.630347	0.0471296	-13.3748	1.41E-39	-0.904786879	count	3.42E-35
KCTD2	-0.7848863	0.2880607	-2.7247	0.00648	-0.904754486	count	1
CNR1	-0.9711322	0.3738367	-2.5977	0.00943	-0.902009332	count	1
AC008915.2	-0.9252394	0.4583934	-2.0184	0.0436	-0.901296391	count	1
NOS3	-0.6636381	0.123364	-5.3795	8.09E-08	-0.900228978	count	0.001903092
ADAMTS6	-0.7842625	0.3075879	-2.5497	0.0108	-0.899630492	count	1
COL27A1	-1.857404	0.6688916	-2.7768	0.00553	-0.89897303	count	1
PKIG	-0.6339859	0.078786	-8.0469	1.25E-15	-0.898811468	count	3.00E-11
NIPAL3	-0.7327075	0.2096982	-3.4941	0.000483	-0.898774859	count	1
ASPHD2	-1.2553099	0.58127	-2.1596	0.0309	-0.897833466	count	1
RRAD	-0.8436989	0.4486008	-1.8807	0.0601	-0.896744129	count	1
ZNF550	-1.0796482	0.405417	-2.6631	0.00779	-0.894749328	count	1
AGPS	-0.6884403	0.2069593	-3.3265	0.000891	-0.894704275	count	1
GPR173	-1.3974783	0.659286	-2.1197	0.0341	-0.893019466	count	1
NGRN	-0.7818953	0.2511223	-3.1136	0.00187	-0.892262865	count	1
NAGLU	-0.7326796	0.2249438	-3.2572	0.00114	-0.891871914	count	1
CHPF2	-0.7493975	0.2335141	-3.2092	0.00135	-0.890840258	count	1
ASPHD1	-1.640394	0.8270227	-1.9835	0.0474	-0.890382147	count	1
ZNF703	-2.80984	0.6978189	-4.0266	5.81E-05	-0.890156	count	1
TMEM47	-0.6578879	0.13041	-5.0448	4.84E-07	-0.890101721	count	0.011336248
MFGE8	-0.6438726	0.1111869	-5.7909	7.79E-09	-0.889856115	count	0.000184101
SCN2A	-2.1621765	0.7655635	-2.8243	0.00477	-0.88981593	count	1
PCDH11Y	-2.1621765	0.81843	-2.6419	0.00829	-0.88981593	count	1
AC139795.3	-1.3913671	0.6382535	-2.18	0.0293	-0.888495156	count	1
TRAF1	-1.4954633	0.5769412	-2.5921	0.00959	-0.887790305	count	1

BST2	-0.6167706	0.0532571	-11.581	2.55E-30	-0.88673104	count	6.17E-26
FTL	-0.6139408	0.0261994	-23.4334	6.53E-111	-0.884983876	count	1.59E-106
SLC11A2	-0.7645686	0.2558726	-2.9881	0.00283	-0.884474881	count	1
PDGFB	-0.6677254	0.1515135	-4.407	1.09E-05	-0.883248071	count	0.2527928
ARSA	-0.69934	0.1997851	-3.5005	0.000472	-0.881373751	count	1
AIF1L	-0.6726299	0.1489807	-4.5149	6.60E-06	-0.879537612	count	0.1534038
SFXN1	-0.7240866	0.2231811	-3.2444	0.00119	-0.878685194	count	1
AP001453.2	-1.177834	0.530956	-2.2183	0.0266	-0.878354194	count	1
AC093677.2	-0.9031551	0.4080103	-2.2136	0.0269	-0.878142747	count	1
ITPR3	-0.7559924	0.2757276	-2.7418	0.00615	-0.878078209	count	1
EHD1	-0.7060819	0.2111073	-3.3447	0.000835	-0.876930865	count	1
AC009962.1	-1.294215	0.5141651	-2.5171	0.0119	-0.876538813	count	1
EPB41L2	-0.6411471	0.1395342	-4.5949	4.52E-06	-0.873827153	count	0.10521656
SLC7A8	-1.3712387	0.3647299	-3.7596	0.000174	-0.873604802	count	1
CCDC91	-0.6767167	0.1652912	-4.0941	4.36E-05	-0.871587766	count	1
PHF19	-0.7280464	0.2371967	-3.0694	0.00217	-0.870444155	count	1
FTH1	-0.6033872	0.0291338	-20.7109	1.08E-88	-0.870088963	count	2.62E-84
HMOX2	-0.632198	0.1105757	-5.7173	1.20E-08	-0.869919839	count	0.00028344
TSPAN3	-0.6207172	0.0853581	-7.2719	4.59E-13	-0.869267325	count	1.10E-08
IFT80	-0.7840159	0.2828143	-2.7722	0.00561	-0.869092075	count	1
ZNF540	-0.8420519	0.3395183	-2.4801	0.0132	-0.868811485	count	1
NOMO1	-0.788582	0.3819231	-2.0648	0.039	-0.868549412	count	1
DUSP10	-1.0502041	0.3958703	-2.6529	0.00803	-0.86766973	count	1
RNF10	-0.6294833	0.1198939	-5.2503	1.63E-07	-0.866840552	count	0.003827566
KCNE4	-2.732709	1.0375939	-2.6337	0.00849	-0.866199892	count	1
ATP2B2	-2.732709	1.0375939	-2.6337	0.00849	-0.866199892	count	1
HSPB2	-2.732709	1.0375939	-2.6337	0.00849	-0.866199892	count	1
PTGIR	-2.732709	1.0375939	-2.6337	0.00849	-0.866199892	count	1
ZNF320	-2.732709	1.0375939	-2.6337	0.00849	-0.866199892	count	1
AL136162.1	-2.1069323	0.7950863	-2.6499	0.0081	-0.863913012	count	1
NFIA-AS2	-2.1069323	0.8114048	-2.5966	0.00946	-0.863913012	count	1
SLC31A1	-0.7504774	0.3100542	-2.4205	0.0156	-0.863450863	count	1
KLF5	-0.9694013	0.3254298	-2.9788	0.00292	-0.862570965	count	1
ZNF500	-0.9319717	0.3895587	-2.3924	0.0168	-0.86252907	count	1
LIG4	-0.9486608	0.3735937	-2.5393	0.0112	-0.861534518	count	1
PPP2R5B	-0.6874741	0.2199462	-3.1256	0.00179	-0.859939253	count	1
C15orf39	-0.8613384	0.3840269	-2.2429	0.025	-0.85865562	count	1
FBXL17	-0.7180831	0.2455783	-2.924	0.00348	-0.858123027	count	1
DLGAP1	-1.3490912	0.6858335	-1.9671	0.0493	-0.857242986	count	1
ERCC6L2	-0.6511239	0.210652	-3.091	0.00201	-0.857227808	count	1
FUK	-0.9258538	0.3563301	-2.5983	0.00942	-0.856375237	count	1
ANKRD33B	-2.70126	0.7094179	-3.8077	0.000143	-0.856220507	count	1
ZMYND8	-0.6382715	0.1876663	-3.4011	0.000681	-0.855533166	count	1
ITIH5	-0.7400341	0.319303	-2.3177	0.0205	-0.854941256	count	1
CEP83-DT	-1.3450901	0.6098391	-2.2056	0.0275	-0.854289762	count	1

ZNF268	-0.7273988	0.2667028	-2.7274	0.00642	-0.854181009	count	1
ATXN1	-0.669948	0.2021081	-3.3148	0.000929	-0.853884941	count	1
TM6SF1	-0.6313674	0.1679588	-3.7591	0.000174	-0.853495445	count	1
AL137186.2	-0.7751533	0.3024334	-2.5631	0.0104	-0.853013352	count	1
CYP11A1	-2.079157	0.8311374	-2.5016	0.0124	-0.850803988	count	1
PSTPIP1	-2.079157	0.8311374	-2.5016	0.0124	-0.850803988	count	1
TNFSF13	-2.079157	0.8311374	-2.5016	0.0124	-0.850803988	count	1
AL358113.1	-2.079157	1.0266924	-2.0251	0.043	-0.850803988	count	1
BCAS3	-0.7635226	0.3001564	-2.5437	0.011	-0.850717268	count	1
TMEM164	-0.957067	0.3768232	-2.5398	0.0111	-0.850554541	count	1
POR	-0.6379207	0.1827032	-3.4916	0.000488	-0.849633398	count	1
THEMIS2	-2.07568	0.7852212	-2.6434	0.00825	-0.84915922	count	1
SORT1	-0.7151889	0.2311003	-3.0947	0.00199	-0.848749143	count	1
RHEBL1	-0.976079	0.4563633	-2.1388	0.0325	-0.847856207	count	1
GSTM2	-1.2562333	0.5230038	-2.402	0.0164	-0.84698972	count	1
MYL12B	-0.5883275	0.0311846	-18.866	8.97E-75	-0.846954334	count	2.18E-70
IL32	-0.5975659	0.1425606	-4.1917	2.86E-05	-0.845582465	count	0.6605742
TSPAN2	-0.6141331	0.1299715	-4.7251	2.41E-06	-0.845532308	count	0.05621566
NOTCH2	-1.2513962	0.4405344	-2.8406	0.00454	-0.843233479	count	1
MTHFD1L	-0.6587877	0.2072321	-3.179	0.00149	-0.842772266	count	1
N4BP3	-1.2502103	0.4557495	-2.7432	0.00612	-0.842312816	count	1
AC013400.1	-1.250055	0.6750451	-1.8518	0.0642	-0.842192571	count	1
INAFM2	-0.9287705	0.2917465	-3.1835	0.00147	-0.841844158	count	1
DPP7	-0.593166	0.0689027	-8.6088	1.22E-17	-0.841669597	count	2.93E-13
LDLRAD2	-1.3267668	0.5722606	-2.3185	0.0205	-0.840776742	count	1
RTCA-AS1	-2.0571271	0.7644469	-2.691	0.00717	-0.84036957	count	1
AC096677.1	-2.0571271	0.9168355	-2.2437	0.0249	-0.84036957	count	1
C12orf57	-0.5888094	0.0608551	-9.6756	8.46E-22	-0.840052303	count	2.04E-17
TIMP2	-0.5928886	0.0723765	-8.1917	3.89E-16	-0.839716636	count	9.33E-12
DUSP14	-0.6564117	0.1811069	-3.6244	0.000295	-0.838553875	count	1
MARVELD1	-0.6476904	0.1952517	-3.3172	0.000921	-0.838162431	count	1
PRADC1	-0.6306972	0.1608779	-3.9203	9.06E-05	-0.837883736	count	1
DIXDC1	-0.5987272	0.1074808	-5.5705	2.78E-08	-0.83755772	count	0.000655746
ANAPC15	-0.6206752	0.1529062	-4.0592	5.06E-05	-0.836541862	count	1
ST7	-0.7125219	0.2359273	-3.0201	0.00255	-0.836058939	count	1
HPS5	-0.6808124	0.238351	-2.8563	0.00432	-0.835323726	count	1
STEAP1	-1.4193226	0.6149833	-2.3079	0.0211	-0.835075712	count	1
CDR2	-0.6935076	0.241832	-2.8677	0.00417	-0.833062708	count	1
ELL2	-0.7515227	0.2456808	-3.0589	0.00224	-0.831332417	count	1
SOX17	-0.5908487	0.139077	-4.2484	2.22E-05	-0.829891774	count	0.5134416
NOTCH4	-0.6264096	0.1573084	-3.982	7.01E-05	-0.828909789	count	1
ELMO3	-2.0325312	0.6359113	-3.1962	0.00141	-0.828683824	count	1
NINL	-2.030457	0.7056716	-2.8773	0.00404	-0.827696487	count	1
METRNL	-0.5808547	0.0695336	-8.3536	1.03E-16	-0.827040685	count	2.47E-12
CYB5R2	-1.54078	0.7073406	-2.1783	0.0295	-0.826948408	count	1

MLLT1	-0.6361334	0.1757764	-3.619	0.000301	-0.826328468	count	1
NSUN5	-0.7225555	0.3219526	-2.2443	0.0249	-0.82593166	count	1
FBXO2	-1.003739	0.5696906	-1.7619	0.0782	-0.825106717	count	1
C2orf81	-1.003507	0.5217397	-1.9234	0.0545	-0.82489503	count	1
EIF4EBP1	-0.5995206	0.1166906	-5.1377	2.98E-07	-0.824593826	count	0.006986908
PIGB	-0.8924958	0.4065087	-2.1955	0.0282	-0.822891868	count	1
WSB1	-0.5756909	0.0584517	-9.849	1.62E-22	-0.82121841	count	3.91E-18
PGRMC2	-0.5867939	0.0919058	-6.3847	2.01E-10	-0.820846847	count	4.77E-06
TEX9	-0.9261351	0.358953	-2.5801	0.00993	-0.820491334	count	1
KIF26A	-0.6722707	0.1953307	-3.4417	0.000587	-0.820463447	count	1
CTNNAL1	-0.5785511	0.082017	-7.054	2.19E-12	-0.820392839	count	5.23E-08
MIR193BHG	-0.9060015	0.4452406	-2.0349	0.042	-0.819356781	count	1
CTSB	-0.5799783	0.0855708	-6.7778	1.49E-11	-0.818601816	count	3.55E-07
OCIAD2	-0.575818	0.0682026	-8.4428	4.93E-17	-0.818475371	count	1.18E-12
MIR99AHG	-0.5925957	0.1337121	-4.4319	9.71E-06	-0.818339148	count	0.22537881
AGER	-1.7099544	0.7997239	-2.1382	0.0326	-0.815369742	count	1
CCDC88B	-1.2137541	0.6763566	-1.7945	0.0728	-0.814062056	count	1
NOP9	-0.9398999	0.4067605	-2.3107	0.0209	-0.813347813	count	1
ST20	-0.7996952	0.3613731	-2.2129	0.027	-0.813147141	count	1
TTC13	-0.8402376	0.3727479	-2.2542	0.0243	-0.812454837	count	1
NES	-0.5737708	0.089779	-6.3909	1.93E-10	-0.810818021	count	4.58E-06
LARP1B	-0.6608119	0.2395749	-2.7583	0.00585	-0.810085888	count	1
SPAG8	-1.2083788	0.5722648	-2.1116	0.0348	-0.809905442	count	1
SGCB	-0.6047927	0.1552284	-3.8961	1.00E-04	-0.809696985	count	1
ZNF497	-1.699364	0.7527873	-2.2574	0.0241	-0.809350818	count	1
HOXB-AS3	-1.699364	0.9083128	-1.8709	0.0615	-0.809350818	count	1
CERS1	-1.699364	1.0880277	-1.5619	0.118	-0.809350818	count	1
SKI	-0.6031905	0.1494815	-4.0352	5.60E-05	-0.809096964	count	1
FZD7	-1.6986905	0.5010853	-3.39	0.000709	-0.808968045	count	1
AL008733.1	-20.623999	1302.018173	-0.0158	0.987	-0.808903787	count	1
IL22RA1	-20.623999	1302.018173	-0.0158	0.987	-0.808903787	count	1
SPOCD1	-20.623999	1302.018173	-0.0158	0.987	-0.808903787	count	1
TMEM125	-20.623999	1302.018173	-0.0158	0.987	-0.808903787	count	1
AC135803.1	-20.623999	1302.018173	-0.0158	0.987	-0.808903787	count	1
NEXN-AS1	-20.623999	1302.018173	-0.0158	0.987	-0.808903787	count	1
COL24A1	-20.623999	1302.018173	-0.0158	0.987	-0.808903787	count	1
KIAA1324	-20.623999	1302.018173	-0.0158	0.987	-0.808903787	count	1
BCAN	-20.623999	1302.018173	-0.0158	0.987	-0.808903787	count	1
AL590560.1	-20.623999	1302.018173	-0.0158	0.987	-0.808903787	count	1
AL121987.1	-20.623999	1302.018173	-0.0158	0.987	-0.808903787	count	1
MTRNR2L11	-20.623999	1302.018173	-0.0158	0.987	-0.808903787	count	1
FMN2	-20.623999	1302.018173	-0.0158	0.987	-0.808903787	count	1
NLRP3	-20.623999	1302.018173	-0.0158	0.987	-0.808903787	count	1
KCNF1	-20.623999	1302.018173	-0.0158	0.987	-0.808903787	count	1
NLRC4	-20.623999	1302.018173	-0.0158	0.987	-0.808903787	count	1

GPR17	-20.623999	1302.018173	-0.0158	0.987	-0.808903787	count	1
CCDC74A	-20.623999	1302.018173	-0.0158	0.987	-0.808903787	count	1
LINC01087	-20.623999	1302.018173	-0.0158	0.987	-0.808903787	count	1
TNFAIP6	-20.623999	1302.018173	-0.0158	0.987	-0.808903787	count	1
AC091488.1	-20.623999	1302.018173	-0.0158	0.987	-0.808903787	count	1
KCNH7	-20.623999	1302.018173	-0.0158	0.987	-0.808903787	count	1
AC010680.1	-20.623999	1302.018173	-0.0158	0.987	-0.808903787	count	1
AC096667.1	-20.623999	1302.018173	-0.0158	0.987	-0.808903787	count	1
SLC4A3	-20.623999	1302.018173	-0.0158	0.987	-0.808903787	count	1
LINC01907	-20.623999	1302.018173	-0.0158	0.987	-0.808903787	count	1
ARPC4-TTLL3	-20.623999	1302.018173	-0.0158	0.987	-0.808903787	count	1
ACVR2B-AS1	-20.623999	1302.018173	-0.0158	0.987	-0.808903787	count	1
CSPG5	-20.623999	1302.018173	-0.0158	0.987	-0.808903787	count	1
TMPRSS7	-20.623999	1302.018173	-0.0158	0.987	-0.808903787	count	1
GAP43	-20.623999	1302.018173	-0.0158	0.987	-0.808903787	count	1
ZIC4	-20.623999	1302.018173	-0.0158	0.987	-0.808903787	count	1
MED12L	-20.623999	1302.018173	-0.0158	0.987	-0.808903787	count	1
CNGA1	-20.623999	1302.018173	-0.0158	0.987	-0.808903787	count	1
TMPRSS11E	-20.623999	1302.018173	-0.0158	0.987	-0.808903787	count	1
ETNPPL	-20.623999	1302.018173	-0.0158	0.987	-0.808903787	count	1
AC115622.1	-20.623999	1302.018173	-0.0158	0.987	-0.808903787	count	1
AC019193.2	-20.623999	1302.018173	-0.0158	0.987	-0.808903787	count	1
AF250324.1	-20.623999	1302.018173	-0.0158	0.987	-0.808903787	count	1
AC010457.1	-20.623999	1302.018173	-0.0158	0.987	-0.808903787	count	1
HCG17	-20.623999	1302.018173	-0.0158	0.987	-0.808903787	count	1
RHAG	-20.623999	1302.018173	-0.0158	0.987	-0.808903787	count	1
AL023806.2	-20.623999	1302.018173	-0.0158	0.987	-0.808903787	count	1
TMEM184A	-20.623999	1302.018173	-0.0158	0.987	-0.808903787	count	1
AC004947.1	-20.623999	1302.018173	-0.0158	0.987	-0.808903787	count	1
AC006159.2	-20.623999	1302.018173	-0.0158	0.987	-0.808903787	count	1
CHRM2	-20.623999	1302.018173	-0.0158	0.987	-0.808903787	count	1
MBOAT4	-20.623999	1302.018173	-0.0158	0.987	-0.808903787	count	1
AL354707.1	-20.623999	1302.018173	-0.0158	0.987	-0.808903787	count	1
AL136366.1	-20.623999	1302.018173	-0.0158	0.987	-0.808903787	count	1
CA9	-20.623999	1302.018173	-0.0158	0.987	-0.808903787	count	1
PAPPA-AS1	-20.623999	1302.018173	-0.0158	0.987	-0.808903787	count	1
PAEP	-20.623999	1302.018173	-0.0158	0.987	-0.808903787	count	1
AC174065.1	-20.623999	1302.018173	-0.0158	0.987	-0.808903787	count	1
OVCH2	-20.623999	1302.018173	-0.0158	0.987	-0.808903787	count	1
AC044810.3	-20.623999	1302.018173	-0.0158	0.987	-0.808903787	count	1
AL078612.1	-20.623999	1302.018173	-0.0158	0.987	-0.808903787	count	1
AP001257.1	-20.623999	1302.018173	-0.0158	0.987	-0.808903787	count	1
AP001107.1	-20.623999	1302.018173	-0.0158	0.987	-0.808903787	count	1
TMEM151A	-20.623999	1302.018173	-0.0158	0.987	-0.808903787	count	1
GAL	-20.623999	1302.018173	-0.0158	0.987	-0.808903787	count	1

AP002989.1	-20.623999	1302.018173	-0.0158	0.987	-0.808903787	count	1
PRF1	-20.623999	1302.018173	-0.0158	0.987	-0.808903787	count	1
CNNM1	-20.623999	1302.018173	-0.0158	0.987	-0.808903787	count	1
NKX2-3	-20.623999	1302.018173	-0.0158	0.987	-0.808903787	count	1
MMP21	-20.623999	1302.018173	-0.0158	0.987	-0.808903787	count	1
AC005833.1	-20.623999	1302.018173	-0.0158	0.987	-0.808903787	count	1
GALNT8	-20.623999	1302.018173	-0.0158	0.987	-0.808903787	count	1
AC117498.2	-20.623999	1302.018173	-0.0158	0.987	-0.808903787	count	1
KRT86	-20.623999	1302.018173	-0.0158	0.987	-0.808903787	count	1
MIP	-20.623999	1302.018173	-0.0158	0.987	-0.808903787	count	1
HMGA2	-20.623999	1302.018173	-0.0158	0.987	-0.808903787	count	1
OTOGL	-20.623999	1302.018173	-0.0158	0.987	-0.808903787	count	1
TEX26	-20.623999	1302.018173	-0.0158	0.987	-0.808903787	count	1
AL355974.2	-20.623999	1302.018173	-0.0158	0.987	-0.808903787	count	1
AL139385.1	-20.623999	1302.018173	-0.0158	0.987	-0.808903787	count	1
DHRS2	-20.623999	1302.018173	-0.0158	0.987	-0.808903787	count	1
AL139353.2	-20.623999	1302.018173	-0.0158	0.987	-0.808903787	count	1
AL133163.2	-20.623999	1302.018173	-0.0158	0.987	-0.808903787	count	1
PLEK2	-20.623999	1302.018173	-0.0158	0.987	-0.808903787	count	1
GALNT16	-20.623999	1302.018173	-0.0158	0.987	-0.808903787	count	1
GPR132	-20.623999	1302.018173	-0.0158	0.987	-0.808903787	count	1
IGHV4-4	-20.623999	1302.018173	-0.0158	0.987	-0.808903787	count	1
AC087457.1	-20.623999	1302.018173	-0.0158	0.987	-0.808903787	count	1
C15orf53	-20.623999	1302.018173	-0.0158	0.987	-0.808903787	count	1
AC012377.1	-20.623999	1302.018173	-0.0158	0.987	-0.808903787	count	1
LINC01169	-20.623999	1302.018173	-0.0158	0.987	-0.808903787	count	1
AC068870.1	-20.623999	1302.018173	-0.0158	0.987	-0.808903787	count	1
SRRM2-AS1	-20.623999	1302.018173	-0.0158	0.987	-0.808903787	count	1
LINC00922	-20.623999	1302.018173	-0.0158	0.987	-0.808903787	count	1
AC012184.1	-20.623999	1302.018173	-0.0158	0.987	-0.808903787	count	1
TAT-AS1	-20.623999	1302.018173	-0.0158	0.987	-0.808903787	count	1
NECAB2	-20.623999	1302.018173	-0.0158	0.987	-0.808903787	count	1
FOXL1	-20.623999	1302.018173	-0.0158	0.987	-0.808903787	count	1
ZNF469	-20.623999	1302.018173	-0.0158	0.987	-0.808903787	count	1
SNAI3	-20.623999	1302.018173	-0.0158	0.987	-0.808903787	count	1
AC129492.4	-20.623999	1302.018173	-0.0158	0.987	-0.808903787	count	1
AC005703.4	-20.623999	1302.018173	-0.0158	0.987	-0.808903787	count	1
AC098850.2	-20.623999	1302.018173	-0.0158	0.987	-0.808903787	count	1
CCL7	-20.623999	1302.018173	-0.0158	0.987	-0.808903787	count	1
KRTAP4-11	-20.623999	1302.018173	-0.0158	0.987	-0.808903787	count	1
GFAP	-20.623999	1302.018173	-0.0158	0.987	-0.808903787	count	1
IGF2BP1	-20.623999	1302.018173	-0.0158	0.987	-0.808903787	count	1
DLX4	-20.623999	1302.018173	-0.0158	0.987	-0.808903787	count	1
LINC01152	-20.623999	1302.018173	-0.0158	0.987	-0.808903787	count	1
SOX9	-20.623999	1302.018173	-0.0158	0.987	-0.808903787	count	1

TMEM200C	-20.623999	1302.018173	-0.0158	0.987	-0.808903787	count	1
AC091588.3	-20.623999	1302.018173	-0.0158	0.987	-0.808903787	count	1
HMSD	-20.623999	1302.018173	-0.0158	0.987	-0.808903787	count	1
AL139351.1	-20.623999	1302.018173	-0.0158	0.987	-0.808903787	count	1
SMIM25	-20.623999	1302.018173	-0.0158	0.987	-0.808903787	count	1
LAMA5-AS1	-20.623999	1302.018173	-0.0158	0.987	-0.808903787	count	1
FUT5	-20.623999	1302.018173	-0.0158	0.987	-0.808903787	count	1
RETN	-20.623999	1302.018173	-0.0158	0.987	-0.808903787	count	1
HOMER3-AS1	-20.623999	1302.018173	-0.0158	0.987	-0.808903787	count	1
KRTDAP	-20.623999	1302.018173	-0.0158	0.987	-0.808903787	count	1
RYR1	-20.623999	1302.018173	-0.0158	0.987	-0.808903787	count	1
NPAS1	-20.623999	1302.018173	-0.0158	0.987	-0.808903787	count	1
LINC01869	-20.623999	1302.018173	-0.0158	0.987	-0.808903787	count	1
CACNG6	-20.623999	1302.018173	-0.0158	0.987	-0.808903787	count	1
RAB36	-20.623999	1302.018173	-0.0158	0.987	-0.808903787	count	1
SH3BP1	-20.623999	1302.018173	-0.0158	0.987	-0.808903787	count	1
ARNT2	-20.569872	983.1629617	-0.0209	0.983	-0.808903787	count	1
CAMP	-19.842798	723.8455375	-0.0274	0.978	-0.808903786	count	1
JAML	-19.842798	723.8455375	-0.0274	0.978	-0.808903786	count	1
FGFR2	-20.4551929	1212.738474	-0.0169	0.987	-0.808903725	count	1
NALCN	-20.4551929	1212.738474	-0.0169	0.987	-0.808903725	count	1
LBP	-20.4551929	1212.738474	-0.0169	0.987	-0.808903725	count	1
ITGAM	-20.1986477	972.4960885	-0.0208	0.983	-0.808903725	count	1
CHD5	-20.1663165	1218.493905	-0.0166	0.987	-0.808903725	count	1
AC239803.3	-20.1663165	1218.493905	-0.0166	0.987	-0.808903725	count	1
C5orf58	-20.1663165	1218.493905	-0.0166	0.987	-0.808903725	count	1
IGF2BP3	-20.1663165	1218.493905	-0.0166	0.987	-0.808903725	count	1
PALM2-AKAP2	-20.1663165	1218.493905	-0.0166	0.987	-0.808903725	count	1
SIX4	-20.1663165	1218.493905	-0.0166	0.987	-0.808903725	count	1
SPINT1	-20.1663165	1218.493905	-0.0166	0.987	-0.808903725	count	1
NWD1	-20.1663165	1218.493905	-0.0166	0.987	-0.808903725	count	1
FCGR1A	-20.0576209	1539.941243	-0.013	0.99	-0.808903725	count	1
FNDC5	-20.0572905	1230.679754	-0.0163	0.987	-0.808903725	count	1
XG	-20.0572905	1230.679754	-0.0163	0.987	-0.808903725	count	1
BMP7	-20.0572905	1230.679754	-0.0163	0.987	-0.808903725	count	1
GRM2	-19.891083	1232.224367	-0.0161	0.987	-0.808903724	count	1
AC011632.1	-19.891083	1232.224367	-0.0161	0.987	-0.808903724	count	1
C11orf21	-19.891083	1232.224367	-0.0161	0.987	-0.808903724	count	1
SPTBN5	-19.891083	1232.224367	-0.0161	0.987	-0.808903724	count	1
SMTNL2	-19.6703593	849.510942	-0.0232	0.982	-0.808903724	count	1
DNASE2B	-19.6698758	729.5964702	-0.027	0.978	-0.808903724	count	1
SUSD4	-19.6698758	729.5964702	-0.027	0.978	-0.808903724	count	1
KIF7	-19.6005298	952.758042	-0.0206	0.984	-0.808903723	count	1
SPPL2B	-0.6559327	0.2211547	-2.9659	0.00304	-0.807714352	count	1
MMEL1	-1.0510821	0.4517777	-2.3265	0.0201	-0.807267401	count	1

AC073254.1	-1.5093965	0.6535256	-2.3096	0.021	-0.806997459	count	1
AC015819.2	-1.5093965	0.8129752	-1.8566	0.0635	-0.806997459	count	1
BTC	-0.7773555	0.3397488	-2.288	0.0222	-0.806507088	count	1
ADAMTS7	-1.0917773	0.4557832	-2.3954	0.0167	-0.80598405	count	1
GLG1	-0.5792834	0.1065696	-5.4357	5.93E-08	-0.80594624	count	0.0013961
FAM129A	-0.6514517	0.221547	-2.9405	0.0033	-0.805607564	count	1
NAXE	-0.5782341	0.1020091	-5.6685	1.59E-08	-0.80503061	count	0.000375447
CKAP4	-0.6072986	0.1540712	-3.9417	8.29E-05	-0.804839631	count	1
PXDN	-0.6079949	0.2150279	-2.8275	0.00472	-0.803636834	count	1
TCTN1	-0.6360596	0.1896209	-3.3544	0.000806	-0.802444656	count	1
DISP1	-0.843116	0.3462786	-2.4348	0.015	-0.80240274	count	1
KIF13B	-0.6022074	0.1482703	-4.0616	5.01E-05	-0.80234519	count	1
GABRD	-0.9516945	0.353012	-2.6959	0.00706	-0.802118619	count	1
HYOU1	-0.6645496	0.2082257	-3.1915	0.00143	-0.801965021	count	1
ATP6V1C2	-1.5011862	0.6659387	-2.2542	0.0243	-0.801782707	count	1
C1orf122	-0.5718532	0.0865831	-6.6047	4.76E-11	-0.801666171	count	1.13E-06
SLC7A6	-0.8183278	0.2854945	-2.8664	0.00418	-0.801606369	count	1
ACOX2	-1.1359829	0.5809838	-1.9553	0.0507	-0.801041996	count	1
FGF2	-0.5914099	0.1511045	-3.9139	9.30E-05	-0.800963569	count	1
PLXNA3	-0.9047349	0.4100444	-2.2064	0.0274	-0.799755015	count	1
FHL1	-0.5725906	0.1223687	-4.6792	3.02E-06	-0.798816819	count	0.07038412
IL12A	-1.9696008	0.8908087	-2.211	0.0271	-0.79863261	count	1
AP003419.2	-2.5247664	1.0223595	-2.4695	0.0136	-0.79809216	count	1
CCDC169	-2.5247664	1.0223595	-2.4695	0.0136	-0.79809216	count	1
MPZ	-2.5247664	1.2616677	-2.0011	0.0455	-0.79809216	count	1
OXT	-2.5247664	1.3152073	-1.9197	0.055	-0.79809216	count	1
ECM1	-1.0813294	0.4169175	-2.5936	0.00955	-0.797245211	count	1
PCBP4	-0.7015904	0.2702379	-2.5962	0.00948	-0.796840772	count	1
SLC1A4	-0.9222349	0.4304243	-2.1426	0.0322	-0.796552108	count	1
CALR	-0.5559934	0.0470865	-11.8079	2.00E-31	-0.795402364	count	4.84E-27
MTRF1L	-0.5834945	0.1227062	-4.7552	2.08E-06	-0.795207366	count	0.04854304
GZMK	-2.5161594	0.7846065	-3.2069	0.00136	-0.795170865	count	1
CCDC151	-2.5134947	0.8242454	-3.0494	0.00231	-0.794264934	count	1
FAM95B1	-2.5134947	0.9415496	-2.6695	0.00764	-0.794264934	count	1
ITGA4	-2.5134947	1.014402	-2.4778	0.0133	-0.794264934	count	1
PTBP2	-0.6256803	0.1781452	-3.5122	0.000452	-0.792777604	count	1
ERRFI1	-0.6742524	0.2128415	-3.1679	0.00155	-0.792620753	count	1
CLDN10	-0.6353097	0.3810475	-1.6673	0.0956	-0.791577164	count	1
GMDS	-0.563088	0.0947675	-5.9418	3.17E-09	-0.791175944	count	7.50E-05
GLDN	-1.2587599	0.5574266	-2.2582	0.024	-0.790809175	count	1
WFDC2	-1.2587599	0.6691241	-1.8812	0.06	-0.790809175	count	1
VSIG4	-1.6663915	0.7258384	-2.2958	0.0218	-0.790609955	count	1
CEP170B	-0.8314955	0.3505796	-2.3718	0.0178	-0.79047648	count	1
DST	-0.5661814	0.1117804	-5.0651	4.35E-07	-0.789180126	count	0.010190745
VWF	-0.5483955	0.0541909	-10.1197	1.16E-23	-0.789066564	count	2.80E-19

ECE1	-0.5556318	0.0812455	-6.8389	9.79E-12	-0.788969752	count	2.33E-07
SSR4	-0.5492697	0.0395384	-13.892	1.84E-42	-0.788501248	count	4.46E-38
CAMK2N1	-1.9479988	0.7983817	-2.4399	0.0148	-0.788273327	count	1
TOP2B	-0.5916523	0.1585828	-3.7309	0.000195	-0.787187772	count	1
E2F3	-0.6297204	0.2511587	-2.5073	0.0122	-0.78594311	count	1
BAHD1	-0.7314581	0.2711933	-2.6972	0.00704	-0.784557062	count	1
PRSS36	-1.4734663	0.6699073	-2.1995	0.0279	-0.784193274	count	1
SOX15	-1.4734663	0.7038106	-2.0936	0.0364	-0.784193274	count	1
CYBRD1	-0.5853659	0.1328941	-4.4048	1.10E-05	-0.784139945	count	0.255101
SIPA1L1	-0.6720402	0.2271854	-2.9581	0.00312	-0.783650631	count	1
STK38L	-0.5922257	0.1430657	-4.1395	3.58E-05	-0.78354855	count	0.825906
PDE4A	-0.6828945	0.3447638	-1.9808	0.0477	-0.782624559	count	1
CD276	-0.9569388	0.3081024	-3.1059	0.00192	-0.782471193	count	1
NMRAL1	-0.5928931	0.1636443	-3.6231	0.000296	-0.781727414	count	1
SESTD1	-0.5973841	0.1849382	-3.2302	0.00125	-0.781589097	count	1
ACTR5	-0.7791353	0.343719	-2.2668	0.0235	-0.781345778	count	1
PLPPR2	-0.687897	0.2467509	-2.7878	0.00534	-0.780629323	count	1
CDC25A	-1.6485127	0.6155067	-2.6783	0.00744	-0.780449498	count	1
TTL	-0.6641589	0.2275413	-2.9188	0.00354	-0.780329801	count	1
KDELR3	-0.5822045	0.163121	-3.5692	0.000364	-0.778686054	count	1
SLC22A17	-0.5966044	0.1948599	-3.0617	0.00222	-0.778505239	count	1
TBCEL	-0.8479306	0.363455	-2.333	0.0197	-0.778357465	count	1
GDE1	-0.6081674	0.1810619	-3.3589	0.000793	-0.777730385	count	1
LINC01836	-1.3355754	0.7078468	-1.8868	0.0593	-0.777388901	count	1
MRPS30-DT	-0.7357674	0.3340556	-2.2025	0.0277	-0.775837719	count	1
RNF121	-0.653502	0.2398496	-2.7246	0.00648	-0.775811779	count	1
ARFGEF2	-0.5929409	0.1943137	-3.0515	0.0023	-0.775680353	count	1
SMC6	-0.5853312	0.1527331	-3.8324	0.00013	-0.773773303	count	1
HAS2	-0.6205984	0.200324	-3.098	0.00197	-0.772789505	count	1
LINC01550	-1.914284	0.7301906	-2.6216	0.0088	-0.772068275	count	1
AKAP6	-1.914284	0.7652304	-2.5016	0.0124	-0.772068275	count	1
FAS	-0.8010223	0.3094753	-2.5883	0.00969	-0.77173879	count	1
BMPR1A	-0.8754418	0.4528875	-1.933	0.0533	-0.771459133	count	1
CIART	-0.7040875	0.2840348	-2.4789	0.0132	-0.7710899	count	1
SNX30	-0.7891808	0.3900398	-2.0233	0.0431	-0.771020696	count	1
PINK1	-0.5545811	0.0959714	-5.7786	8.37E-09	-0.770914528	count	0.000197775
FARP1	-0.5719916	0.1463398	-3.9087	9.50E-05	-0.769538046	count	1
KLRG1	-0.9701107	0.3810364	-2.546	0.011	-0.767341316	count	1
CELF2	-0.5736337	0.180719	-3.1742	0.00152	-0.766685278	count	1
MEX3D	-0.7261784	0.2273396	-3.1942	0.00142	-0.765156258	count	1
TEN1	-0.5956097	0.172508	-3.4527	0.000563	-0.764242549	count	1
SSFA2	-0.5376641	0.0785457	-6.8452	9.37E-12	-0.76386689	count	2.23E-07
CPQ	-0.5478361	0.1129684	-4.8495	1.31E-06	-0.763862578	count	0.03061339
RFK	-0.5439971	0.0964085	-5.6426	1.84E-08	-0.763602846	count	0.000434369
CMTM7	-0.6480152	0.2302705	-2.8141	0.00493	-0.763579302	count	1

AC004540.1	-1.1477052	0.4020961	-2.8543	0.00435	-0.763163429	count	1
PIK3CD	-1.1467415	0.7064154	-1.6233	0.105	-0.762423772	count	1
ARRDC3-AS1	-1.144616	0.6300657	-1.8167	0.0694	-0.760792727	count	1
AP001107.9	-1.144616	0.7200031	-1.5897	0.112	-0.760792727	count	1
SEC24A	-0.6478665	0.2735796	-2.3681	0.0179	-0.76050991	count	1
POC1B	-0.6472851	0.2469862	-2.6207	0.00882	-0.759803083	count	1
PLOD1	-0.5949877	0.1941657	-3.0643	0.0022	-0.759532491	count	1
DOK4	-0.6422945	0.243362	-2.6393	0.00836	-0.759376386	count	1
ATAD1	-0.5696461	0.1546446	-3.6836	0.000234	-0.758827247	count	1
TMEM81	-1.886634	0.8334428	-2.2637	0.0237	-0.758749299	count	1
PRND	-1.886634	0.8334428	-2.2637	0.0237	-0.758749299	count	1
AC006213.1	-1.886634	0.8334428	-2.2637	0.0237	-0.758749299	count	1
AP001189.6	-1.886634	1.0174854	-1.8542	0.0638	-0.758749299	count	1
MGAT4B	-0.554665	0.1146783	-4.8367	1.39E-06	-0.758619056	count	0.03247457
AC018816.1	-1.0831276	0.6084623	-1.7801	0.0752	-0.758575237	count	1
CRYZ	-0.6179921	0.3083578	-2.0041	0.0452	-0.757974921	count	1
CMTR1	-0.6507356	0.2727402	-2.3859	0.0171	-0.757896147	count	1
ZNF319	-0.9929787	0.5086745	-1.9521	0.051	-0.757233724	count	1
CYP27A1	-0.6297459	0.1922431	-3.2758	0.00107	-0.756432759	count	1
LMO2	-0.527216	0.0443031	-11.9002	7.00E-32	-0.7563928	count	1.69E-27
ATP5MC3	-0.5304909	0.0622453	-8.5226	2.53E-17	-0.756366753	count	6.07E-13
KLHL29	-1.8800107	0.6664819	-2.8208	0.00482	-0.755555781	count	1
TMEM38A	-0.690291	0.3355081	-2.0574	0.0397	-0.755246664	count	1
AL691432.2	-1.0308133	0.5086156	-2.0267	0.0428	-0.755156987	count	1
LMLN	-0.7164773	0.3348995	-2.1394	0.0325	-0.754360745	count	1
SLC25A4	-0.5439904	0.120545	-4.5128	6.67E-06	-0.753923887	count	0.1550108
ASPM	-4.7605636	2.7698575	-1.7187	0.0858	-0.753507781	count	1
ERGIC1	-0.5639641	0.1498412	-3.7637	0.000171	-0.751579895	count	1
TRERF1	-1.2952048	0.4709612	-2.7501	0.006	-0.749734265	count	1
AC100793.2	-1.0708062	0.5521368	-1.9394	0.0526	-0.748717818	count	1
DDR1	-1.0687252	0.4382226	-2.4388	0.0148	-0.747054675	count	1
ARL17A	-0.815025	0.4070859	-2.0021	0.0454	-0.745631321	count	1
PLAU	-0.7288842	0.444812	-1.6386	0.101	-0.745212198	count	1
NAT14	-0.6453859	0.3119583	-2.0688	0.0387	-0.744938466	count	1
SAMD8	-0.5819438	0.1903641	-3.057	0.00226	-0.743530126	count	1
ZNF529-AS1	-0.7199042	0.3677712	-1.9575	0.0504	-0.743435552	count	1
CCNJ	-0.7124618	0.3464741	-2.0563	0.0398	-0.742782094	count	1
PGM2L1	-0.537758	0.1254965	-4.285	1.89E-05	-0.742467336	count	0.4374594
ERN1	-0.566081	0.2140057	-2.6452	0.00821	-0.741234905	count	1
PHYHD1	-0.810582	0.312786	-2.5915	0.00961	-0.741223278	count	1
RGL3	-2.3600349	0.7843049	-3.0091	0.00264	-0.740948596	count	1
AGO3	-0.5554871	0.1785985	-3.1103	0.00189	-0.740547136	count	1
SLC4A7	-0.5735451	0.2246922	-2.5526	0.0107	-0.739709314	count	1
LSR	-0.5433295	0.148314	-3.6634	0.000254	-0.739536453	count	1
CYTOR	-0.5612299	0.1596852	-3.5146	0.000447	-0.738277766	count	1

DCHS1	-0.5585872	0.1706314	-3.2736	0.00107	-0.737919819	count	1
IRAK1	-0.5855814	0.1904305	-3.075	0.00213	-0.737363144	count	1
NPLOC4	-0.5950243	0.1936106	-3.0733	0.00214	-0.737190149	count	1
ARMC7	-0.5987835	0.2217704	-2.7	0.00698	-0.737183114	count	1
STMP1	-0.5279965	0.1012941	-5.2125	2.00E-07	-0.736496283	count	0.0046948
SMPDL3B	-1.2756826	0.4814354	-2.6497	0.0081	-0.736404177	count	1
ATP10D	-0.5384358	0.159501	-3.3758	0.000746	-0.734891675	count	1
ST3GAL1	-0.5518988	0.1452335	-3.8001	0.000148	-0.734464068	count	1
TMEM37	-0.7540186	0.4222605	-1.7857	0.0743	-0.734262416	count	1
IREB2	-0.5654588	0.1896097	-2.9822	0.00289	-0.729090256	count	1
POGK	-0.5963742	0.2536448	-2.3512	0.0188	-0.728982734	count	1
CAPN5	-0.9981553	0.4185163	-2.385	0.0171	-0.728103354	count	1
AL162231.1	-1.1725507	0.5795092	-2.0234	0.0431	-0.727988583	count	1
CHDH	-1.1725507	0.5938392	-1.9745	0.0484	-0.727988583	count	1
AC010331.1	-1.1725507	0.6676624	-1.7562	0.0792	-0.727988583	count	1
ABCA7	-1.1725507	0.8392211	-1.3972	0.162	-0.727988583	count	1
PIF1	-4.349977	2.3181409	-1.8765	0.0607	-0.727929831	count	1
SREBF2	-0.6388567	0.276934	-2.3069	0.0211	-0.726530603	count	1
PLAUR	-0.5338051	0.1483	-3.5995	0.000324	-0.725569273	count	1
ZBTB4	-0.5565292	0.1575504	-3.5324	0.000419	-0.724704746	count	1
PRTFDC1	-0.7550833	0.3659272	-2.0635	0.0392	-0.724284619	count	1
COG6	-0.5869585	0.2266803	-2.5894	0.00967	-0.723852785	count	1
RNASEH2A	-0.9928962	0.6148478	-1.6149	0.106	-0.723758949	count	1
SIRT4	-1.1653829	0.6444586	-1.8083	0.0707	-0.722796217	count	1
RGCC	-0.5175958	0.1984854	-2.6077	0.00916	-0.722195842	count	1
MGAT4A	-0.5213865	0.1098894	-4.7446	2.19E-06	-0.721661941	count	0.05110365
ATG9B	-1.1633282	0.6060127	-1.9196	0.055	-0.721308763	count	1
CLIP2	-0.7892551	0.7638217	-1.0333	0.302	-0.720101089	count	1
VAX2	-1.5419922	0.8211721	-1.8778	0.0605	-0.720018342	count	1
AC099524.1	-1.5419922	0.9403071	-1.6399	0.101	-0.720018342	count	1
PPP1CB	-0.5068683	0.0667599	-7.5924	4.27E-14	-0.719981029	count	1.02E-09
RCN2	-0.5096728	0.0732019	-6.9626	4.16E-12	-0.719978068	count	9.92E-08
ZNF134	-0.6735639	0.2815817	-2.3921	0.0168	-0.719307913	count	1
BRCA1	-0.7875449	0.3524287	-2.2346	0.0255	-0.718409991	count	1
SYT11	-0.5789546	0.2052791	-2.8203	0.00483	-0.718257887	count	1
AC245060.5	-0.9859953	0.5411045	-1.8222	0.0685	-0.718063577	count	1
RGS5	-1.2472518	0.5776478	-2.1592	0.0309	-0.717046299	count	1
NPW	-2.2923766	0.5743048	-3.9916	6.73E-05	-0.716800993	count	1
PYCARD-AS1	-0.945448	0.5195039	-1.8199	0.0689	-0.716604178	count	1
POU2F1	-0.598442	0.2440806	-2.4518	0.0143	-0.715469983	count	1
IKBKE	-1.7949522	0.7248511	-2.4763	0.0133	-0.714460472	count	1
DPY19L3	-0.6634885	0.3210851	-2.0664	0.0389	-0.713760602	count	1
PGPEP1	-0.6304607	0.2810691	-2.2431	0.025	-0.712839248	count	1
AGA	-0.5548498	0.1852416	-2.9953	0.00277	-0.711838111	count	1
SORBS2	-0.5253024	0.1304368	-4.0273	5.80E-05	-0.711807815	count	1

C7orf26	-0.5972291	0.2296929	-2.6001	0.00937	-0.711683608	count	1
EDC3	-0.6520492	0.2901788	-2.2471	0.0247	-0.711443528	count	1
ANO5	-1.3563147	0.7931946	-1.7099	0.0874	-0.710253612	count	1
CEP131	-0.6765766	0.3629548	-1.8641	0.0624	-0.710077328	count	1
MT-ND6	-0.5298141	0.1438823	-3.6823	0.000236	-0.70921377	count	1
MAPK8IP3	-1.019881	0.3769065	-2.7059	0.00685	-0.708162086	count	1
AC147651.1	-1.5206628	0.757291	-2.008	0.0447	-0.70795498	count	1
APOA1	-1.7809392	0.8741651	-2.0373	0.0417	-0.707681115	count	1
C3orf36	-1.7809392	1.0372553	-1.717	0.0861	-0.707681115	count	1
TGFB1	-0.5046358	0.090329	-5.5866	2.54E-08	-0.707635996	count	0.000599262
HSP90B1	-0.4921327	0.0412663	-11.9258	5.23E-32	-0.707594099	count	1.27E-27
CTSD	-0.5011767	0.0771563	-6.4956	9.77E-11	-0.70719171	count	2.32E-06
ARHGEF5	-1.0183565	0.5485932	-1.8563	0.0635	-0.706952819	count	1
SELENOM	-0.49242	0.0456881	-10.7779	1.47E-26	-0.706777434	count	3.55E-22
CYB561D1	-0.8729258	0.5137414	-1.6992	0.0894	-0.706605932	count	1
VANGL2	-2.2639044	0.8342168	-2.7138	0.00669	-0.70653752	count	1
AC099518.2	-2.2639044	0.8342168	-2.7138	0.00669	-0.70653752	count	1
LINC00278	-2.2639044	0.8611973	-2.6288	0.00862	-0.70653752	count	1
THSD7B	-2.2639044	1.277191	-1.7726	0.0764	-0.70653752	count	1
PRKCB	-2.2639044	1.329829	-1.7024	0.0888	-0.70653752	count	1
ITPRIPL2	-0.5129207	0.1297607	-3.9528	7.92E-05	-0.70588889	count	1
ACVR1B	-0.5821839	0.2342231	-2.4856	0.013	-0.70568836	count	1
CITED2	-0.5050539	0.1158088	-4.3611	1.34E-05	-0.705077796	count	0.310478
UHRF1BP1L	-0.6230766	0.2673147	-2.3309	0.0198	-0.704149577	count	1
FAM104B	-0.5254983	0.1596268	-3.292	0.00101	-0.703711794	count	1
ABHD17C	-0.6535014	0.2831129	-2.3083	0.0211	-0.702479743	count	1
SNHG21	-0.6638842	0.3800671	-1.7468	0.0808	-0.702385597	count	1
RRM2B	-0.5517883	0.2330131	-2.3681	0.018	-0.702356518	count	1
COL21A1	-0.6385228	0.3215245	-1.9859	0.0471	-0.70085647	count	1
HSD17B12	-0.4926513	0.0721205	-6.8309	1.03E-11	-0.700363527	count	2.45E-07
PYGB	-0.5938352	0.2347499	-2.5297	0.0115	-0.700226836	count	1
GTF2I	-0.4961203	0.091516	-5.4211	6.43E-08	-0.69991239	count	0.001513429
ABCA6	-0.5972308	0.4333325	-1.3782	0.168	-0.699072074	count	1
AL157392.5	-1.220508	0.8887653	-1.3733	0.17	-0.698901445	count	1
PAMR1	-1.220508	0.9547654	-1.2783	0.201	-0.698901445	count	1
ACTG2	-1.220508	1.0280918	-1.1872	0.235	-0.698901445	count	1
METTL27	-0.8399089	0.53649	-1.5656	0.118	-0.698776304	count	1
LINC01963	-1.0631983	0.6169417	-1.7233	0.0849	-0.698677184	count	1
HCST	-1.0626251	0.4875627	-2.1795	0.0294	-0.698242548	count	1
PRRT2	-0.8912908	0.3932243	-2.2666	0.0235	-0.698076312	count	1
ITPK1	-0.6034382	0.2263982	-2.6654	0.00773	-0.697868958	count	1
GBP6	-2.239749	0.9996379	-2.2406	0.0251	-0.697786863	count	1
AC019069.1	-2.239749	0.9996379	-2.2406	0.0251	-0.697786863	count	1
AC010680.2	-2.239749	0.9996379	-2.2406	0.0251	-0.697786863	count	1
4-Mar	-2.239749	0.9996379	-2.2406	0.0251	-0.697786863	count	1

AC004492.1	-2.239749	0.9996379	-2.2406	0.0251	-0.697786863	count	1
OR2A42	-2.239749	0.9996379	-2.2406	0.0251	-0.697786863	count	1
AC107959.1	-2.239749	0.9996379	-2.2406	0.0251	-0.697786863	count	1
CA3	-2.239749	0.9996379	-2.2406	0.0251	-0.697786863	count	1
AC087752.4	-2.239749	0.9996379	-2.2406	0.0251	-0.697786863	count	1
SYK	-2.239749	0.9996379	-2.2406	0.0251	-0.697786863	count	1
UBQLNL	-2.239749	0.9996379	-2.2406	0.0251	-0.697786863	count	1
MYEOV	-2.239749	0.9996379	-2.2406	0.0251	-0.697786863	count	1
CACNA1C-AS2	-2.239749	0.9996379	-2.2406	0.0251	-0.697786863	count	1
HP	-2.239749	0.9996379	-2.2406	0.0251	-0.697786863	count	1
RAC3	-2.239749	0.9996379	-2.2406	0.0251	-0.697786863	count	1
AP1M2	-2.239749	0.9996379	-2.2406	0.0251	-0.697786863	count	1
DHDH	-2.239749	0.9996379	-2.2406	0.0251	-0.697786863	count	1
ZKSCAN8	-0.8909246	0.4133987	-2.1551	0.0312	-0.697756424	count	1
IGFBP6	-0.5010784	0.2085445	-2.4027	0.0163	-0.69750669	count	1
11-Sep	-0.4965406	0.0907803	-5.4697	4.91E-08	-0.697430129	count	0.001156698
WLS	-0.5025122	0.1144654	-4.3901	1.18E-05	-0.696577053	count	0.2735948
ATIC	-0.8155446	0.5230162	-1.5593	0.119	-0.695946068	count	1
INKA1	-0.575675	0.2626441	-2.1918	0.0285	-0.695663225	count	1
BCL7A	-1.1277945	0.5825383	-1.936	0.053	-0.695654191	count	1
SLC35F2	-0.5655904	0.2214912	-2.5536	0.0107	-0.69528135	count	1
MT1E	-0.5799409	0.2631802	-2.2036	0.0276	-0.694843962	count	1
DNAJC3	-0.4902561	0.0737383	-6.6486	3.55E-11	-0.694752745	count	8.45E-07
AGPAT1	-0.5239216	0.1527893	-3.429	0.000615	-0.694679805	count	1
NPNT	-0.8595606	0.4814638	-1.7853	0.0743	-0.694624189	count	1
CA2	-1.055594	0.4878485	-2.1638	0.0306	-0.692914318	count	1
ADGRL1	-0.6199885	0.2726058	-2.2743	0.023	-0.692657239	count	1
SYDE2	-0.7612304	0.5040933	-1.5101	0.131	-0.692440964	count	1
AMFR	-0.5517854	0.1710631	-3.2256	0.00127	-0.691530949	count	1
HSDL1	-0.6181212	0.2855255	-2.1649	0.0305	-0.690482325	count	1
RPP25	-0.584207	0.2273606	-2.5695	0.0102	-0.688501762	count	1
PEMT	-0.5052814	0.1428409	-3.5374	0.000411	-0.688099705	count	1
WBP1L	-0.5212277	0.1611879	-3.2337	0.00124	-0.687895504	count	1
FAM69A	-1.2040507	0.7869601	-1.53	0.126	-0.687768614	count	1
ABCA9	-1.2040507	0.8074213	-1.4912	0.136	-0.687768614	count	1
RUNX1T1	-0.491935	0.1087583	-4.5232	6.35E-06	-0.687542075	count	0.14764385
RNF32	-0.9937942	0.9305273	-1.068	0.286	-0.687510257	count	1
TMX4	-0.4892445	0.1033659	-4.7331	2.32E-06	-0.686792854	count	0.05412096
VCL	-0.5035107	0.1293809	-3.8917	0.000102	-0.686252602	count	1
DENND5B	-0.6389004	0.3192087	-2.0015	0.0454	-0.686009409	count	1
RPA4	-1.4810266	0.6542855	-2.2636	0.0237	-0.6855867	count	1
LYPD1	-3.867807	0.7042203	-5.4923	4.33E-08	-0.684711802	count	0.001020321
DIAPH3	-3.867807	0.935149	-4.136	3.64E-05	-0.684711802	count	0.8396752
JCHAIN	-2.2035633	0.3164656	-6.963	4.15E-12	-0.684608011	count	9.90E-08
PRKCE	-0.6587984	0.2972269	-2.2165	0.0267	-0.683777641	count	1

KLHL6	-0.7521673	0.6077413	-1.2376	0.216	-0.683519769	count	1
SLC39A14	-0.5819599	0.2288107	-2.5434	0.011	-0.683222901	count	1
MYBL1	-1.7303837	0.7030259	-2.4613	0.0139	-0.683218595	count	1
PDLIM4	-0.4898346	0.0940245	-5.2097	2.03E-07	-0.683199181	count	0.004765019
SNX29	-0.5319294	0.2112327	-2.5182	0.0119	-0.682720611	count	1
WDR66	-1.1959694	0.6469142	-1.8487	0.0646	-0.682311525	count	1
AC078883.3	-1.3115209	0.6182591	-2.1213	0.034	-0.682209022	count	1
LRRC49	-0.5661244	0.2427698	-2.3319	0.0198	-0.681854458	count	1
CA11	-0.6214482	0.3612987	-1.72	0.0855	-0.681266081	count	1
SUGP2	-0.5369629	0.2175578	-2.4681	0.0136	-0.6811807	count	1
TMIE	-1.1942142	0.6369146	-1.875	0.0609	-0.681127108	count	1
HNRNPUL2	-0.5542054	0.1970971	-2.8118	0.00496	-0.680930618	count	1
BACE1	-0.5890706	0.2527754	-2.3304	0.0199	-0.680656587	count	1
TYMS	-0.7984377	0.4295934	-1.8586	0.0632	-0.679959931	count	1
OSBPL11	-0.6111725	0.2837904	-2.1536	0.0314	-0.678270895	count	1
SLC30A1	-0.5386421	0.2227093	-2.4186	0.0156	-0.678124293	count	1
TBC1D1	-0.4979402	0.1292007	-3.854	0.000119	-0.677817773	count	1
DGKE	-0.5085193	0.1610731	-3.1571	0.00161	-0.677555844	count	1
THEM6	-0.5056969	0.1460509	-3.4625	0.000543	-0.677545701	count	1
CAPG	-0.5332257	0.2034659	-2.6207	0.00882	-0.677316344	count	1
DDIT4	-0.4811252	0.0936243	-5.1389	2.96E-07	-0.677055398	count	0.006940608
AP003486.1	-1.100947	0.5984501	-1.8397	0.0659	-0.676362311	count	1
RNF11	-0.488969	0.1058298	-4.6203	4.01E-06	-0.676203618	count	0.0933929
ARNTL	-1.1863917	0.6548038	-1.8118	0.0701	-0.675852421	count	1
CTSF	-0.4818965	0.10283	-4.6863	2.92E-06	-0.675614258	count	0.06805936
DRAM1	-0.5238348	0.2031034	-2.5792	0.00996	-0.675248937	count	1
AL356481.1	-1.0312785	0.492539	-2.0938	0.0364	-0.674533987	count	1
SNRPN	-0.4799485	0.0865118	-5.5478	3.17E-08	-0.674362256	count	0.000747423
AL355312.4	-1.459925	0.6908862	-2.1131	0.0347	-0.67370832	count	1
SAR1B	-0.4908355	0.1198167	-4.0966	4.31E-05	-0.673476297	count	0.9935412
TTLL7	-0.7417335	0.4299087	-1.7253	0.0846	-0.67326424	count	1
ARSG	-0.69511	0.3402398	-2.043	0.0411	-0.673047274	count	1
WASF1	-0.8623807	0.5815965	-1.4828	0.138	-0.672882975	count	1
MEIS1-AS2	-3.7581563	1.8388239	-2.0438	0.0411	-0.67250577	count	1
NUP160	-0.5538065	0.2125974	-2.605	0.00924	-0.672145861	count	1
CCSAP	-0.9742389	0.5666654	-1.7192	0.0857	-0.672087409	count	1
ZNF182	-0.6746002	0.3714504	-1.8161	0.0695	-0.670146911	count	1
OPHN1	-0.5598136	0.2416689	-2.3164	0.0206	-0.670019898	count	1
AJUBA	-1.4531581	0.70163	-2.0711	0.0384	-0.669904052	count	1
RNASE6	-1.4531581	0.8081938	-1.798	0.0723	-0.669904052	count	1
ABHD15	-0.8896846	0.4872822	-1.8258	0.068	-0.669319883	count	1
KLHL35	-1.0904039	0.6398419	-1.7042	0.0885	-0.668809019	count	1
PPIL2	-0.5756981	0.2760226	-2.0857	0.0371	-0.667542523	count	1
WDFY3	-0.5219771	0.2323917	-2.2461	0.0248	-0.667233599	count	1
BAX	-0.4722738	0.0792881	-5.9564	2.90E-09	-0.666555652	count	6.87E-05

NORAD	-0.4674296	0.069218	-6.753	1.76E-11	-0.664952524	count	4.19E-07
POFUT1	-0.6478678	0.3247111	-1.9952	0.0461	-0.664928471	count	1
PLEKHG4	-1.0840929	0.6757247	-1.6043	0.109	-0.664294029	count	1
SLC2A9	-1.0840929	0.6934394	-1.5634	0.118	-0.664294029	count	1
NF1	-0.5147331	0.1704875	-3.0192	0.00256	-0.66404389	count	1
PDZD8	-0.5193448	0.1796424	-2.891	0.00387	-0.663806235	count	1
PIK3R1	-0.5014429	0.1568453	-3.1971	0.0014	-0.663220931	count	1
DLG5	-1.0816026	0.4748024	-2.278	0.0228	-0.662513718	count	1
GADD45GIP1	-0.4655738	0.0596648	-7.8032	8.50E-15	-0.662417762	count	2.04E-10
MYL6B	-0.4749735	0.098935	-4.8009	1.66E-06	-0.661762464	count	0.03875436
SOX4	-0.4645962	0.088476	-5.2511	1.63E-07	-0.661490542	count	0.003827566
AC067838.1	-2.1404101	1.087337	-1.9685	0.0491	-0.661427704	count	1
IGFBP5	-0.4707252	0.2630334	-1.7896	0.0736	-0.661110219	count	1
FNDC4	-0.5998977	0.2667893	-2.2486	0.0246	-0.660998927	count	1
HCFC1R1	-0.4691831	0.0859092	-5.4614	5.14E-08	-0.66096327	count	0.001210624
PRR15	-0.5635204	0.296552	-1.9002	0.0575	-0.660869057	count	1
RHOBTB2	-0.6034225	0.3437273	-1.7555	0.0793	-0.660623052	count	1
4-Sep	-0.5623096	0.2242599	-2.5074	0.0122	-0.659402471	count	1
TAB2	-0.5640823	0.2438377	-2.3134	0.0208	-0.658991699	count	1
TBCD	-0.5040924	0.2491786	-2.023	0.0432	-0.658887516	count	1
BEX3	-0.4623064	0.0656614	-7.0408	2.40E-12	-0.658617261	count	5.73E-08
TMEM30B	-1.43254	0.5933886	-2.4142	0.0158	-0.658328556	count	1
B9D1	-0.6295428	0.3653272	-1.7232	0.085	-0.658135922	count	1
HEY1	-0.6239552	0.251538	-2.4806	0.0132	-0.657994242	count	1
MX2	-0.5481167	0.2806151	-1.9533	0.0509	-0.657611651	count	1
PQLC2	-0.6135738	0.4090527	-1.5	0.134	-0.657504222	count	1
LINC00311	-2.129204	0.7923739	-2.6871	0.00725	-0.657292838	count	1
SEC61A1	-0.4848044	0.1538126	-3.1519	0.00164	-0.657095377	count	1
A1BG	-2.1277519	0.6849202	-3.1066	0.00191	-0.656756823	count	1
SH3BGRL3	-0.4564406	0.0332727	-13.7182	1.75E-41	-0.656449295	count	4.24E-37
TNFRSF8	-0.7236765	0.4748489	-1.524	0.128	-0.655554099	count	1
THBS3	-0.560774	0.2900308	-1.9335	0.0533	-0.654997939	count	1
CNPY2	-0.4659846	0.0855331	-5.448	5.54E-08	-0.654861561	count	0.001304559
RNF170	-0.5070066	0.2208539	-2.2957	0.0218	-0.654614233	count	1
ERP27	-1.2671955	0.5603158	-2.2616	0.0238	-0.654614131	count	1
PRNCR1	-0.9515304	0.593966	-1.602	0.109	-0.654243007	count	1
CLDN14	-1.068865	0.7238531	-1.4766	0.14	-0.653419595	count	1
AL645465.1	-2.1186365	0.9878091	-2.1448	0.0321	-0.653388596	count	1
TMEM215	-2.1186365	1.0314456	-2.054	0.0401	-0.653388596	count	1
NANOS3	-2.1186365	1.187274	-1.7845	0.0745	-0.653388596	count	1
TOM1L2	-0.5611162	0.2729385	-2.0558	0.0399	-0.652777757	count	1
ZNF175	-0.6852804	0.3798759	-1.804	0.0713	-0.65272858	count	1
AL357055.3	-1.263277	0.8456213	-1.4939	0.135	-0.652182599	count	1
MAP3K7CL	-0.8683564	0.4297685	-2.0205	0.0434	-0.651352297	count	1
UBA6-AS1	-0.6411539	0.3927242	-1.6326	0.103	-0.650467685	count	1

MXD1	-0.6169344	0.3019355	-2.0433	0.0411	-0.650210528	count	1
HRCT1	-0.4683764	0.1201171	-3.8993	9.88E-05	-0.650029171	count	1
HMOX1	-0.4893955	0.21601	-2.2656	0.0236	-0.649912395	count	1
SDC2	-1.0621755	0.6022486	-1.7637	0.0779	-0.648651539	count	1
CD81	-0.4499622	0.0303603	-14.8207	7.08E-48	-0.647798548	count	1.72E-43
CU638689.4	-0.8329671	0.6078769	-1.3703	0.171	-0.647377309	count	1
DUSP8	-0.7152257	0.4007799	-1.7846	0.0744	-0.647282706	count	1
CAPRIN2	-0.5180592	0.2898729	-1.7872	0.074	-0.647204934	count	1
MATN2	-0.4900142	0.1961029	-2.4988	0.0125	-0.647046564	count	1
PYCR1	-1.0596323	0.5090422	-2.0816	0.0375	-0.646840352	count	1
MCAM	-0.4597285	0.1011747	-4.5439	5.76E-06	-0.646661639	count	0.13399488
SMCR5	-2.1000152	0.7867807	-2.6691	0.00765	-0.646496323	count	1
TNFRSF18	-1.4111429	0.7537537	-1.8722	0.0613	-0.646342521	count	1
MSRB1	-0.4972211	0.1854429	-2.6813	0.00738	-0.645687504	count	1
ZNF418	-0.7131325	0.5509598	-1.2943	0.196	-0.645235634	count	1
POLR2J	-0.4577256	0.0887663	-5.1565	2.69E-07	-0.645224796	count	0.006309126
CCDC34	-0.503592	0.2162219	-2.3291	0.0199	-0.64492607	count	1
C9orf3	-0.4602465	0.0838934	-5.4861	4.48E-08	-0.64396883	count	0.001055622
RASGRF2	-0.5235202	0.2145599	-2.44	0.0148	-0.643763264	count	1
KLHL4	-0.7114963	0.4066345	-1.7497	0.0803	-0.643635983	count	1
CALM1	-0.4465775	0.0337998	-13.2124	1.08E-38	-0.643113307	count	2.62E-34
TMEM64	-0.6860109	0.3007053	-2.2813	0.0226	-0.642655379	count	1
ARHGAP32	-0.7782441	0.4059973	-1.9169	0.0554	-0.642512554	count	1
ADM	-0.4721043	0.1679784	-2.8105	0.00498	-0.641775105	count	1
TUBA1A	-0.4485137	0.0588073	-7.6268	3.29E-14	-0.64141031	count	7.87E-10
CHID1	-0.4643263	0.1119509	-4.1476	3.46E-05	-0.640987018	count	0.7982912
SLC35E2B	-0.6467245	0.3183066	-2.0318	0.0423	-0.64074521	count	1
PSAP	-0.4477566	0.050775	-8.8184	2.01E-18	-0.640148312	count	4.83E-14
TOR1AIP2	-0.4605658	0.1042278	-4.4188	1.03E-05	-0.639927915	count	0.2389909
DMKN	-0.5014693	0.2161189	-2.3203	0.0204	-0.639713311	count	1
CAB39L	-0.8544623	0.3706722	-2.3052	0.0212	-0.639684193	count	1
NRGN	-0.4658621	0.1178593	-3.9527	7.92E-05	-0.639375952	count	1
FRMD4B	-0.4506733	0.0728622	-6.1853	7.12E-10	-0.639360499	count	1.69E-05
AL132780.2	-1.04893	0.8677341	-1.2088	0.227	-0.639227165	count	1
SLC27A5	-0.4916632	0.1825292	-2.6936	0.00711	-0.638965006	count	1
GRIP2	-3.4929455	1.5774516	-2.2143	0.0269	-0.638913493	count	1
USP53	-0.4715395	0.1303456	-3.6176	0.000303	-0.638736226	count	1
EFNA4	-0.5642747	0.2814256	-2.0051	0.0451	-0.638580642	count	1
ARFGEF3	-0.5448013	0.2443331	-2.2297	0.0258	-0.638213005	count	1
GEN1	-0.9304467	0.4364556	-2.1318	0.0331	-0.637740533	count	1
MRPL17	-0.4518559	0.0873795	-5.1712	2.49E-07	-0.637125509	count	0.005842785
ZNF618	-0.6287036	0.3468812	-1.8124	0.07	-0.637107353	count	1
ST3GAL4	-0.4893377	0.214667	-2.2795	0.0227	-0.637075872	count	1
VMAC	-0.5790723	0.3195426	-1.8122	0.0701	-0.637072734	count	1
ITGB1	-0.4443406	0.052178	-8.5159	2.67E-17	-0.636124273	count	6.41E-13

PROCA1	-1.12597	0.6299722	-1.7873	0.074	-0.635329204	count	1
SEC14L2	-0.8492104	0.4244218	-2.0009	0.0455	-0.635281454	count	1
TSPAN5	-0.5043826	0.195783	-2.5762	0.01	-0.635148529	count	1
SDF4	-0.4512885	0.0755203	-5.9757	2.58E-09	-0.635054914	count	6.11E-05
AK1	-0.526669	0.3039414	-1.7328	0.0832	-0.634853831	count	1
THTPA	-0.5295532	0.2359383	-2.2445	0.0249	-0.634713114	count	1
CHRNA10	-1.390123	0.8205724	-1.6941	0.0904	-0.634596529	count	1
CAMK1D	-0.5769046	0.2598819	-2.2199	0.0265	-0.634585425	count	1
ATP6V0E2	-0.5732403	0.2695462	-2.1267	0.0335	-0.634439403	count	1
CDCA8	-2.066743	0.7985485	-2.5881	0.0097	-0.634145637	count	1
TMEM44-AS1	-0.8174852	0.4169036	-1.9608	0.05	-0.634005605	count	1
PTPN14	-0.4702112	0.1266235	-3.7135	0.000208	-0.633639021	count	1
PCNX2	-1.6276512	0.618426	-2.6319	0.00854	-0.633583641	count	1
AC009041.2	-0.882614	0.4716618	-1.8713	0.0614	-0.6335137	count	1
WDR26	-0.490417	0.1966473	-2.4939	0.0127	-0.632843711	count	1
FSD1L	-1.229839	0.5342797	-2.3019	0.0214	-0.631493827	count	1
ELMOD2	-0.489347	0.180444	-2.7119	0.00673	-0.631440191	count	1
NAPEPLD	-0.654684	0.3675343	-1.7813	0.075	-0.631324739	count	1
MGAT5	-0.550066	0.3465479	-1.5873	0.113	-0.631170864	count	1
ZNF266	-0.7118858	0.3537017	-2.0127	0.0442	-0.630328712	count	1
LY6K	-0.6367584	0.4486798	-1.4192	0.156	-0.630261104	count	1
CHSY1	-0.4596763	0.1195808	-3.8441	0.000124	-0.629995458	count	1
RAB30	-0.6218667	0.2819519	-2.2056	0.0275	-0.629780238	count	1
EMC1	-0.5657681	0.2405612	-2.3519	0.0187	-0.629673707	count	1
UBR3	-0.5110646	0.2148393	-2.3788	0.0174	-0.629467707	count	1
GBF1	-0.500228	0.2461543	-2.0322	0.0422	-0.628786995	count	1
MYDGF	-0.4414435	0.0596333	-7.4026	1.76E-13	-0.62818221	count	4.21E-09
TMEM189	-0.5068621	0.2391527	-2.1194	0.0341	-0.628100851	count	1
SLC12A2	-0.4872664	0.1855616	-2.6259	0.00869	-0.628028585	count	1
IQCK	-0.4592254	0.1365959	-3.3619	0.000785	-0.626774624	count	1
FAM214A	-0.4890848	0.1640473	-2.9814	0.00289	-0.626021734	count	1
PMPCA	-0.477008	0.1926695	-2.4758	0.0134	-0.625529617	count	1
GAB2	-0.6114703	0.3432465	-1.7814	0.075	-0.625528403	count	1
CACNA1A	-0.6174748	0.3804156	-1.6232	0.105	-0.625077036	count	1
TMEM217	-1.0286191	0.6560329	-1.5679	0.117	-0.624820259	count	1
INHBA	-1.0286191	0.7741381	-1.3287	0.184	-0.624820259	count	1
ZNF382	-0.9136282	0.4919531	-1.8571	0.0634	-0.624623045	count	1
ZNF329	-0.6783311	0.3932658	-1.7249	0.0847	-0.623514229	count	1
KYNU	-0.5322047	0.3036207	-1.7529	0.0797	-0.622988479	count	1
TSNAXIP1	-2.036639	0.6829459	-2.9821	0.00289	-0.622936002	count	1
FBXL22	-1.2159356	0.6755649	-1.7999	0.072	-0.622923239	count	1
DUSP3	-0.475499	0.2236333	-2.1262	0.0336	-0.622530181	count	1
CCDC30	-0.6650179	0.3487986	-1.9066	0.0567	-0.621576915	count	1
ENG	-0.4335326	0.0492577	-8.8013	2.34E-18	-0.621527557	count	5.62E-14
CCL8	-0.5038434	0.9181949	-0.5487	0.5832	-0.620364215	count	1

ALDH18A1	-0.6196385	0.3197586	-1.9378	0.0527	-0.620048786	count	1
SLC20A1	-0.4506698	0.1224583	-3.6802	0.000238	-0.619650355	count	1
ZDHHC11	-1.1023964	0.7364862	-1.4968	0.135	-0.619631388	count	1
STX3	-0.5116169	0.2180509	-2.3463	0.019	-0.619623776	count	1
ADRB2	-0.4636659	0.1537023	-3.0166	0.00258	-0.619412981	count	1
TTN-AS1	-0.5376408	0.3503786	-1.5345	0.125	-0.619229782	count	1
SSR3	-0.4356006	0.0615653	-7.0754	1.88E-12	-0.618962204	count	4.49E-08
GUF1	-0.5058417	0.2754521	-1.8364	0.0664	-0.617159446	count	1
CHRM3	-0.6163851	0.2789778	-2.2094	0.0272	-0.616600713	count	1
PLIN5	-0.7491724	0.5092743	-1.4711	0.141	-0.616191754	count	1
AES	-0.4327408	0.0569902	-7.5932	4.24E-14	-0.616161725	count	1.01E-09
SLC12A4	-0.5643264	0.2610614	-2.1617	0.0307	-0.615991644	count	1
AC048341.1	-0.9529177	0.4611211	-2.0665	0.0389	-0.615831242	count	1
PRKCA	-0.7961332	0.4580158	-1.7382	0.0823	-0.615626168	count	1
TSHZ3	-0.5120334	0.250055	-2.0477	0.0407	-0.614962331	count	1
ZNF90	-0.7281268	0.38219	-1.9051	0.0569	-0.614724334	count	1
ST5	-0.6812942	0.3879602	-1.7561	0.0792	-0.614184163	count	1
AC010976.2	-0.8579602	0.5238266	-1.6379	0.102	-0.61358151	count	1
TMEM91	-0.4510995	0.1610991	-2.8001	0.00514	-0.61353536	count	1
KIAA0556	-0.6459898	0.3797984	-1.7009	0.0891	-0.612767313	count	1
TMEM178A	-0.9474082	0.4493433	-2.1084	0.0351	-0.61173628	count	1
DPY19L1	-0.499973	0.2077995	-2.406	0.0162	-0.611294412	count	1
SFT2D2	-0.4522917	0.14783	-3.0595	0.00224	-0.611130654	count	1
NBPF14	-1.346889	0.4814019	-2.7978	0.00518	-0.610535393	count	1
LINC01715	-1.088439	0.7603762	-1.4314	0.152	-0.610368785	count	1
INPP5D	-0.5013753	0.3141552	-1.5959	0.111	-0.610058946	count	1
PTP4A2	-0.4326624	0.0861925	-5.0197	5.50E-07	-0.609724979	count	0.01287715
SPATA20	-0.4783204	0.1892316	-2.5277	0.0115	-0.609657951	count	1
P2RX4	-0.5423598	0.2586353	-2.097	0.0361	-0.6095717	count	1
VAV2	-0.6241791	0.3411393	-1.8297	0.0674	-0.608777238	count	1
DDHD1	-0.4924359	0.3074705	-1.6016	0.109	-0.608579842	count	1
CD34	-0.4260743	0.0674014	-6.3214	3.01E-10	-0.608339725	count	7.14E-06
TOLLIP-AS1	-0.6887726	0.4094464	-1.6822	0.0926	-0.608189029	count	1
DUSP18	-0.4846798	0.2485162	-1.9503	0.0512	-0.607833154	count	1
MAFK	-0.5061936	0.2314442	-2.1871	0.0288	-0.607757931	count	1
COPS7A	-0.4510058	0.142338	-3.1686	0.00155	-0.60775258	count	1
ASAP2	-0.4458969	0.1516294	-2.9407	0.0033	-0.607279531	count	1
KLHL42	-0.5230393	0.2426104	-2.1559	0.0312	-0.607055652	count	1
USP2	-1.5718895	0.7043161	-2.2318	0.0257	-0.606750132	count	1
ZFY-AS1	-1.5718895	0.7199559	-2.1833	0.0291	-0.606750132	count	1
ZNF221	-1.001749	0.5393592	-1.8573	0.0634	-0.605845455	count	1
HELLS	-0.5927845	0.3036287	-1.9523	0.051	-0.605375315	count	1
TXNRD1	-0.4434364	0.1386099	-3.1992	0.00139	-0.605058054	count	1
NECTIN3-AS1	-1.0004768	0.6445396	-1.5522	0.121	-0.604949561	count	1
MAP3K2	-0.4438809	0.1545049	-2.8729	0.0041	-0.604340044	count	1

SFXN3	-0.4616639	0.1610873	-2.8659	0.00419	-0.603671654	count	1
ELOB	-0.421145	0.0438975	-9.5938	1.83E-21	-0.603451711	count	4.41E-17
JAZF1	-0.4820448	0.249836	-1.9294	0.0538	-0.603425033	count	1
IFT122	-0.5532367	0.2910261	-1.901	0.0574	-0.603368304	count	1
SH3D21	-1.5646636	0.8329595	-1.8784	0.0604	-0.603281067	count	1
LINC01315	-1.5646636	0.8450751	-1.8515	0.0642	-0.603281067	count	1
AL358472.3	-1.5646636	0.8749496	-1.7883	0.0738	-0.603281067	count	1
SCGB1B2P	-1.5646636	0.8918765	-1.7543	0.0795	-0.603281067	count	1
NUSAP1	-1.9827716	0.7221919	-2.7455	0.00608	-0.602809402	count	1
GAA	-0.4530237	0.1662993	-2.7241	0.00649	-0.602451541	count	1
HIVEP3	-0.7144901	0.3554797	-2.0099	0.0445	-0.602163793	count	1
RAB32	-0.435119	0.1112012	-3.9129	9.34E-05	-0.602035109	count	1
HCLS1	-0.5108373	0.2542216	-2.0094	0.0446	-0.601664384	count	1
JPT1	-0.4312217	0.0957302	-4.5046	6.93E-06	-0.601250152	count	0.16104627
CAND2	-0.6664877	0.5266912	-1.2654	0.206	-0.599799237	count	1
DNAJC5	-0.4813218	0.2225595	-2.1627	0.0307	-0.599249639	count	1
PLPP1	-0.421837	0.1207419	-3.4937	0.000484	-0.599009463	count	1
PEPD	-0.4276864	0.0832112	-5.1398	2.94E-07	-0.598738974	count	0.0068943
ABCC1	-0.5702436	0.2879717	-1.9802	0.0478	-0.598619301	count	1
FAM114A1	-0.4282958	0.1031436	-4.1524	3.39E-05	-0.59805362	count	0.7823103
NDUFC2	-0.4176992	0.04742	-8.8085	2.20E-18	-0.597770315	count	5.29E-14
UFD1	-0.4244773	0.0767893	-5.5278	3.55E-08	-0.597448654	count	0.000836877
FAM229B	-0.4453368	0.145215	-3.0667	0.00219	-0.596694754	count	1
IL10RA	-3.2101602	1.4659925	-2.1898	0.0286	-0.596456229	count	1
LDHD	-0.6041323	0.3777232	-1.5994	0.11	-0.596044448	count	1
SKIV2L	-0.5504079	0.4570278	-1.2043	0.229	-0.595891116	count	1
ARMCX6	-0.4395061	0.1328032	-3.3095	0.000947	-0.595170689	count	1
ID2-AS1	-1.546602	0.8653981	-1.7872	0.074	-0.594619605	count	1
SLC15A2	-1.546602	0.8653981	-1.7872	0.074	-0.594619605	count	1
ART3	-1.546602	0.8653981	-1.7872	0.074	-0.594619605	count	1
AC093627.4	-1.546602	0.8653981	-1.7872	0.074	-0.594619605	count	1
ODF3L1	-1.546602	0.8653981	-1.7872	0.074	-0.594619605	count	1
AC015917.2	-1.546602	0.8653981	-1.7872	0.074	-0.594619605	count	1
Z98885.3	-1.546602	0.8653981	-1.7872	0.074	-0.594619605	count	1
MTF1	-0.518417	0.2438709	-2.1258	0.0336	-0.593623108	count	1
TAS2R31	-1.9571313	0.8978666	-2.1798	0.0294	-0.593205052	count	1
ENOSF1	-0.4709569	0.1894948	-2.4853	0.013	-0.593193452	count	1
AP3D1	-0.4610872	0.1721462	-2.6785	0.00744	-0.593087686	count	1
PROSER1	-0.5604037	0.3272097	-1.7127	0.0869	-0.592981719	count	1
KCNK7	-1.3150733	0.759669	-1.7311	0.0835	-0.592921977	count	1
AL358472.2	-1.3150733	0.8499399	-1.5473	0.122	-0.592921977	count	1
AC027682.6	-0.7446275	0.4991559	-1.4918	0.136	-0.592702507	count	1
FZD2	-0.870333	0.6497836	-1.3394	0.181	-0.591053197	count	1
GPSM1	-0.7425854	0.5038211	-1.4739	0.141	-0.59091086	count	1
SERF2	-0.4100864	0.0241232	-16.9997	1.02E-61	-0.590637727	count	2.48E-57

DGKD	-0.6147772	0.4157974	-1.4785	0.139	-0.590383005	count	1
ZNF668	-0.5454489	0.3109273	-1.7543	0.0795	-0.590288227	count	1
SH3KBP1	-0.4649815	0.1802901	-2.5791	0.00996	-0.589927048	count	1
FASTK	-0.4507973	0.1670528	-2.6985	0.00701	-0.58926736	count	1
NEAT1	-0.4096789	0.0623298	-6.5728	5.88E-11	-0.589122446	count	1.40E-06
AGL	-0.4447329	0.1750977	-2.5399	0.0111	-0.588465058	count	1
ARMC2	-0.7930367	0.4866186	-1.6297	0.103	-0.588464875	count	1
RNF207	-0.7927401	0.4266132	-1.8582	0.0632	-0.588219027	count	1
MYL9	-0.4149115	0.0995867	-4.1663	3.19E-05	-0.588110419	count	0.7363796
CBR4	-0.4724078	0.2348	-2.012	0.0443	-0.587918557	count	1
TP53INP2	-0.9135146	0.514307	-1.7762	0.0758	-0.586644784	count	1
AP001627.1	-0.666159	0.3495676	-1.9057	0.0568	-0.586613205	count	1
RHBDF1	-0.6528332	0.3623785	-1.8015	0.0717	-0.586565711	count	1
USB1	-0.4587614	0.2027771	-2.2624	0.0238	-0.586557348	count	1
PCDHB14	-0.5749869	0.5509606	-1.0436	0.297	-0.586228589	count	1
STAM	-0.4492105	0.1734707	-2.5895	0.00966	-0.586173404	count	1
FASTKD5	-0.5688069	0.3284973	-1.7315	0.0835	-0.585701977	count	1
HIST2H2AC	-0.4817744	0.2004734	-2.4032	0.0163	-0.585626963	count	1
CD58	-0.4292427	0.1245146	-3.4473	0.000575	-0.58554387	count	1
AC087741.1	-0.9110704	0.5997575	-1.5191	0.129	-0.584842128	count	1
PCDHB2	-0.600849	0.4070729	-1.476	0.14	-0.584622273	count	1
C7orf50	-0.4173655	0.086803	-4.8082	1.60E-06	-0.584276459	count	0.03736
FXYD2	-1.52479	0.6496101	-2.3472	0.019	-0.58417907	count	1
ZDHHC1	-0.5396844	0.3849014	-1.4021	0.161	-0.583779542	count	1
FUCA2	-0.4316022	0.1449851	-2.9769	0.00294	-0.583350573	count	1
NUDT14	-0.4209991	0.1038372	-4.0544	5.16E-05	-0.583145482	count	1
COPA	-0.4273861	0.1193054	-3.5823	0.000346	-0.582836364	count	1
ATF7IP2	-0.591277	0.3098505	-1.9083	0.0565	-0.582607811	count	1
RAB11B-AS1	-0.4681919	0.2190123	-2.1377	0.0326	-0.582561969	count	1
BTNL9	-0.550953	0.2964758	-1.8583	0.0632	-0.582510879	count	1
IL34	-0.5385057	0.2926464	-1.8401	0.0659	-0.582449252	count	1
TLX1	-3.1255765	1.4430967	-2.1659	0.0304	-0.582410889	count	1
METTL8	-0.4911328	0.2684905	-1.8292	0.0675	-0.581819057	count	1
RNASE1	-0.4036808	0.0358253	-11.268	7.95E-29	-0.58146245	count	1.92E-24
DGCR8	-0.6348106	0.3637257	-1.7453	0.081	-0.580627788	count	1
DENND3	-0.4881839	0.2406353	-2.0287	0.0426	-0.580144155	count	1
RASSF4	-0.5327729	0.3525486	-1.5112	0.131	-0.580118251	count	1
C21orf58	-0.8558979	0.5360407	-1.5967	0.11	-0.57992641	count	1
EOGT	-0.4961122	0.2279076	-2.1768	0.0296	-0.579464741	count	1
ALDH1A3	-0.4315719	0.1726679	-2.4994	0.0125	-0.579376719	count	1
ZHX1-C8orf76	-0.9034381	0.7534402	-1.1991	0.231	-0.579219071	count	1
RARG	-0.4789708	0.2382534	-2.0103	0.0445	-0.57910198	count	1
ARHGAP22	-3.1055984	1.4692748	-2.1137	0.0346	-0.579004253	count	1
POLD4	-0.6124609	0.4185724	-1.4632	0.144	-0.578859017	count	1
GPX7	-0.4384674	0.1939415	-2.2608	0.0238	-0.578553713	count	1

GOLIM4	-0.4100597	0.0891675	-4.5988	4.44E-06	-0.578320461	count	0.10335876
MYO19	-0.5464471	0.2753281	-1.9847	0.0473	-0.577523158	count	1
LRP6	-0.4676888	0.1698845	-2.753	0.00594	-0.577329677	count	1
AC006160.1	-1.914284	0.9074346	-2.1096	0.035	-0.57713073	count	1
GPLD1	-1.914284	0.9074346	-2.1096	0.035	-0.57713073	count	1
SHOX2	-1.914284	0.9341073	-2.0493	0.0405	-0.57713073	count	1
GALNT5	-1.914284	0.9445724	-2.0266	0.0428	-0.57713073	count	1
MCF2L2	-1.914284	0.9445724	-2.0266	0.0428	-0.57713073	count	1
AC019117.2	-1.914284	1.180214	-1.622	0.105	-0.57713073	count	1
PTPRD-AS1	-1.914284	1.180214	-1.622	0.105	-0.57713073	count	1
PCSK7	-0.4561103	0.182817	-2.4949	0.0127	-0.576792792	count	1
CCND1	-0.4057896	0.0963173	-4.213	2.60E-05	-0.576728948	count	0.600808
SMCR8	-0.6008868	0.3618376	-1.6607	0.0969	-0.576192042	count	1
LURAP1L	-0.8985128	0.4422666	-2.0316	0.0423	-0.57559526	count	1
VPS33A	-0.5220241	0.3012098	-1.7331	0.0832	-0.575548559	count	1
TCIM	-0.4222629	0.1405201	-3.005	0.00268	-0.575484536	count	1
CEACAM19	-1.0354877	0.6455796	-1.604	0.109	-0.575455014	count	1
HHIPL1	-1.0354877	0.9361045	-1.1062	0.269	-0.575455014	count	1
PLA2G4C	-0.4625603	0.2009798	-2.3015	0.0214	-0.575409139	count	1
PBX3	-0.4963295	0.268562	-1.8481	0.0647	-0.575082471	count	1
NT5E	-0.4231229	0.1534505	-2.7574	0.00586	-0.575010103	count	1
DPP9	-0.4557023	0.2771302	-1.6444	0.1	-0.574519227	count	1
CCDC167	-0.443532	0.1768688	-2.5077	0.0122	-0.573824083	count	1
CDK2AP2	-0.4141479	0.1137735	-3.6401	0.000278	-0.573603978	count	1
GSTO1	-0.4021294	0.0548016	-7.3379	2.84E-13	-0.573519639	count	6.79E-09
LINC01503	-1.13435	0.6882769	-1.6481	0.0994	-0.573046112	count	1
BICRA	-0.6386998	0.4714966	-1.3546	0.176	-0.572901203	count	1
ATP8B2	-0.4749188	0.2672029	-1.7774	0.0756	-0.572510878	count	1
TMEM181	-0.4640546	0.2237144	-2.0743	0.0381	-0.571533464	count	1
ZFYVE27	-0.4932308	0.2545563	-1.9376	0.0528	-0.571378633	count	1
C8orf37	-0.6253675	0.4122194	-1.5171	0.129	-0.571363815	count	1
GSTM4	-0.4872349	0.228967	-2.128	0.0334	-0.570955382	count	1
WWC2	-0.4388652	0.1956204	-2.2435	0.0249	-0.570448195	count	1
POLR3H	-0.4705924	0.2616214	-1.7988	0.0722	-0.570233498	count	1
KDM8	-1.895012	0.6757798	-2.8042	0.00508	-0.569893716	count	1
AP003306.2	-1.1289798	0.7039115	-1.6039	0.109	-0.569789661	count	1
AP1S3	-3.0515224	1.1467216	-2.6611	0.0078	-0.569614332	count	1
BX088651.4	-3.0515224	1.1467216	-2.6611	0.0078	-0.569614332	count	1
AC034236.2	-1.1285085	0.5838604	-1.9328	0.0534	-0.569504059	count	1
POC1A	-1.2721497	0.7318921	-1.7382	0.0823	-0.569297788	count	1
SH2D4A	-0.6344811	0.2959184	-2.1441	0.0321	-0.568829088	count	1
CERS6	-0.4787464	0.245891	-1.947	0.0516	-0.568620182	count	1
UQCRHL	-0.7168458	0.4984719	-1.4381	0.151	-0.568389698	count	1
NRSN2	-0.5775066	0.3418846	-1.6892	0.0913	-0.568243946	count	1
AC011603.2	-0.9474128	0.6158896	-1.5383	0.124	-0.567787855	count	1

S100A2	-0.9474128	0.6352465	-1.4914	0.136	-0.567787855	count	1
AC007388.1	-0.503621	0.2551159	-1.9741	0.0485	-0.567542711	count	1
FNDC3B	-0.4197865	0.1509414	-2.7811	0.00545	-0.567445346	count	1
TRABD2A	-0.8395183	0.395293	-2.1238	0.0338	-0.567342074	count	1
TRAPPC2L	-0.4031163	0.0834442	-4.831	1.43E-06	-0.56731514	count	0.03340766
COX7A1	-0.4000751	0.0725862	-5.5117	3.88E-08	-0.567237564	count	0.000914516
GABARAPL2	-0.3953285	0.0422116	-9.3654	1.52E-20	-0.56681882	count	3.66E-16
NUDT4B	-1.1235526	0.6335512	-1.7734	0.0763	-0.566502187	count	1
AC051619.5	-0.5620724	0.5333006	-1.054	0.292	-0.565997441	count	1
OTUD7B	-0.4590225	0.2285862	-2.0081	0.0447	-0.565199443	count	1
TMEM143	-0.7368805	0.5269803	-1.3983	0.162	-0.56501836	count	1
PREPL	-0.4390101	0.1768674	-2.4821	0.0131	-0.564881456	count	1
GANAB	-0.4180343	0.1731119	-2.4148	0.0158	-0.564647203	count	1
HOXB7	-0.8357731	0.7126611	-1.1727	0.241	-0.564470946	count	1
CABCOCO1	-1.1199424	0.7544538	-1.4844	0.138	-0.564317373	count	1
ST3GAL6-AS1	-1.1199424	1.4957914	-0.7487	0.4541	-0.564317373	count	1
PLS3	-0.3993628	0.0834829	-4.7838	1.81E-06	-0.563862302	count	0.04225083
GATA2	-0.4122054	0.1225304	-3.3641	0.000778	-0.563836405	count	1
KCNJ2-AS1	-0.7631853	0.6133484	-1.2443	0.213	-0.563799467	count	1
GATA2-AS1	-0.4119418	0.1369096	-3.0089	0.00265	-0.563740208	count	1
GBA	-0.4577175	0.2714828	-1.686	0.0919	-0.563557199	count	1
PTTG1IP	-0.3930619	0.0507914	-7.7387	1.40E-14	-0.562657311	count	3.35E-10
AC240274.1	-0.7615439	0.398539	-1.9108	0.0561	-0.562447689	count	1
INPP5A	-0.528332	0.3033296	-1.7418	0.0817	-0.562199063	count	1
GDPD5	-0.5583379	0.4884583	-1.1431	0.253	-0.562032229	count	1
JADE1	-0.4443416	0.171201	-2.5954	0.0095	-0.561641958	count	1
ATP1B3	-0.3968942	0.0727423	-5.4562	5.30E-08	-0.561480565	count	0.001248203
AC025164.1	-0.5236625	0.3013178	-1.7379	0.0823	-0.561458734	count	1
IMPA2	-0.5516748	0.3238732	-1.7034	0.0886	-0.56122278	count	1
PON2	-0.3992981	0.0853642	-4.6776	3.04E-06	-0.561221925	count	0.0708472
SRR	-0.5445881	0.3627829	-1.5011	0.133	-0.55951023	count	1
RPS27L	-0.3905096	0.0478376	-8.1632	4.90E-16	-0.559159566	count	1.17E-11
APLP1	-0.8287817	0.4728014	-1.7529	0.0797	-0.559117508	count	1
TMEM30A	-0.4006954	0.0979944	-4.089	4.46E-05	-0.559091873	count	1
TP53I11	-0.4554378	0.2311124	-1.9706	0.0489	-0.558213303	count	1
AC025031.2	-2.9868714	1.4540932	-2.0541	0.0401	-0.558068925	count	1
SNIP1	-0.4453534	0.2030643	-2.1932	0.0284	-0.557574035	count	1
AIFM2	-0.8734283	0.5356907	-1.6305	0.103	-0.55719927	count	1
SETD6	-0.5057989	0.2866224	-1.7647	0.0777	-0.556965297	count	1
NXPH3	-1.0067765	0.7347346	-1.3703	0.171	-0.556682318	count	1
DHX57	-0.4996325	0.4524581	-1.1043	0.27	-0.556656276	count	1
EFCAB14	-0.3981886	0.1161364	-3.4286	0.000616	-0.555885956	count	1
AL157832.1	-1.2472518	0.7443145	-1.6757	0.0939	-0.555673494	count	1
GINS2	-1.1050011	0.7548845	-1.4638	0.143	-0.555292163	count	1
TMTC4	-0.5581378	0.3085438	-1.8089	0.0706	-0.555144925	count	1

PRDM1	-0.5039705	0.3209555	-1.5702	0.116	-0.55487345	count	1
AHR	-0.4005275	0.1091158	-3.6707	0.000247	-0.554794465	count	1
GPX4	-0.3859629	0.0438418	-8.8035	2.29E-18	-0.553803088	count	5.51E-14
CS	-0.452767	0.2509039	-1.8045	0.0713	-0.553587016	count	1
CLTB	-0.3897496	0.0588272	-6.6253	4.15E-11	-0.55334089	count	9.87E-07
GYG1	-0.4064307	0.1262584	-3.219	0.0013	-0.553283274	count	1
RB1CC1	-0.3987533	0.1088029	-3.6649	0.000252	-0.553280161	count	1
PAM	-0.4038356	0.1427324	-2.8293	0.0047	-0.553081687	count	1
TSNARE1	-0.6604882	0.5002144	-1.3204	0.187	-0.552735443	count	1
COL17A1	-0.8192592	0.3982944	-2.0569	0.0398	-0.551839252	count	1
ZNF710	-0.7805544	0.5347496	-1.4597	0.144	-0.551640831	count	1
FAM198B-AS1	-0.5228572	0.3219141	-1.6242	0.104	-0.551459907	count	1
TTC39B	-0.4784972	0.2770775	-1.7269	0.0843	-0.551419926	count	1
DEF8	-0.4136998	0.1630581	-2.5371	0.0112	-0.551122229	count	1
RRAS	-0.3871086	0.0691862	-5.5952	2.42E-08	-0.550790084	count	0.000570999
COL6A1	-0.4407303	0.199554	-2.2086	0.0273	-0.550717265	count	1
LAMP2	-0.3939589	0.0992329	-3.97	7.37E-05	-0.550298694	count	1
RHOD	-0.4169039	0.1667783	-2.4997	0.0125	-0.550137761	count	1
AC012629.2	-1.8417174	0.8304793	-2.2177	0.0267	-0.549870932	count	1
LPCAT1	-0.4353763	0.2381972	-1.8278	0.0677	-0.549277529	count	1
AL136454.1	-0.6948472	0.5328367	-1.3041	0.192	-0.549234212	count	1
AL512625.1	-0.9933676	0.5115183	-1.942	0.0522	-0.547955013	count	1
DPF2	-0.4244697	0.1782349	-2.3815	0.0173	-0.547669237	count	1
HYAL2	-0.3813806	0.0581535	-6.5582	6.48E-11	-0.547125573	count	1.54E-06
PCDHB6	-1.4456392	1.015101	-1.4241	0.155	-0.546504469	count	1
SESN1	-0.5020591	0.2790249	-1.7993	0.0721	-0.545332252	count	1
HDLBP	-0.386387	0.0858008	-4.5033	6.97E-06	-0.545071115	count	0.16196886
LRRC75B	-0.6096042	0.3799545	-1.6044	0.109	-0.544879682	count	1
IQSEC1	-0.4556618	0.2196108	-2.0749	0.0381	-0.543920307	count	1
FNBP4	-0.3933805	0.1192513	-3.2988	0.000983	-0.543560882	count	1
PRX	-0.6680954	0.4523253	-1.477	0.14	-0.543538316	count	1
NANOS1	-0.6680954	0.4977137	-1.3423	0.18	-0.543538316	count	1
NOD1	-0.4718554	0.3112252	-1.5161	0.13	-0.543526535	count	1
MANEA-DT	-0.6340611	0.460713	-1.3763	0.169	-0.54294386	count	1
DUS2	-0.5342025	0.6282458	-0.8503	0.395	-0.542537111	count	1
PTCH2	-1.4368152	0.6960172	-2.0643	0.0391	-0.54232783	count	1
NISCH	-0.4381248	0.2251212	-1.9462	0.0517	-0.542291982	count	1
PPIC	-0.3803395	0.0579479	-6.5635	6.26E-11	-0.542139304	count	1.49E-06
SCARF2	-0.6663166	0.5625326	-1.1845	0.236	-0.541957329	count	1
RBM38	-0.4583166	0.245906	-1.8638	0.0625	-0.541895655	count	1
ATAD2	-0.4923255	0.3819347	-1.289	0.197	-0.541561544	count	1
FAM49A	-0.5451174	0.4209253	-1.295	0.195	-0.541481473	count	1
CHMP4C	-1.8187514	0.6161668	-2.9517	0.00319	-0.541243101	count	1
ID1	-0.3762435	0.0580926	-6.4766	1.11E-10	-0.540850623	count	2.64E-06
ZNF385A	-0.5941105	0.3923962	-1.5141	0.13	-0.540809419	count	1

TTC3	-0.3845575	0.0893886	-4.3021	1.75E-05	-0.540618916	count	0.40509
DEAF1	-0.4053417	0.1544373	-2.6246	0.00872	-0.540160412	count	1
SDF2L1	-0.3906865	0.1141136	-3.4237	0.000627	-0.539302046	count	1
NDUFA7	-0.4930984	0.3080232	-1.6008	0.11	-0.538902675	count	1
CDK8	-0.5812234	0.4015371	-1.4475	0.148	-0.538155621	count	1
RRAS2	-0.4635144	0.2001389	-2.316	0.0206	-0.538138687	count	1
MTSS1L	-0.7317767	0.4857516	-1.5065	0.132	-0.538015866	count	1
VAV3	-0.5554302	0.4496165	-1.2353	0.217	-0.537851336	count	1
KCNK1	-1.0759494	0.6026841	-1.7853	0.0743	-0.537824647	count	1
TWIST1	-1.808835	0.4774213	-3.7888	0.000155	-0.537518559	count	1
PLEKHM3	-0.523976	0.3537832	-1.4811	0.139	-0.537290937	count	1
KTN1-AS1	-0.6605968	0.470068	-1.4053	0.16	-0.536877624	count	1
TPM1	-0.3787138	0.0817228	-4.6341	3.75E-06	-0.536857407	count	0.08734875
FNIP2	-0.3819529	0.1134631	-3.3663	0.000772	-0.536824131	count	1
TPRG1L	-0.4006631	0.1474876	-2.7166	0.00664	-0.536522006	count	1
HYAL3	-0.5538615	0.4414566	-1.2546	0.21	-0.53624211	count	1
FAM201A	-2.869324	0.8376097	-3.4256	0.000622	-0.536214045	count	1
SLC47A1	-2.869324	0.8376097	-3.4256	0.000622	-0.536214045	count	1
CCL26	-2.869324	1.361883	-2.1069	0.0352	-0.536214045	count	1
FMN1	-2.869324	1.384276	-2.0728	0.0383	-0.536214045	count	1
TMEM245	-0.39082	0.1628024	-2.4006	0.0164	-0.535967657	count	1
MAP3K7	-0.451565	0.2334537	-1.9343	0.0532	-0.535485448	count	1
IGLC3	-0.5273732	0.2055737	-2.5654	0.0104	-0.535246865	count	1
ZSCAN20	-0.8999738	0.6656124	-1.3521	0.176	-0.534926271	count	1
WDR35	-0.8999738	0.6760989	-1.3311	0.183	-0.534926271	count	1
PGM1	-0.4780023	0.2319499	-2.0608	0.0394	-0.534744351	count	1
TMED10	-0.3764478	0.0711084	-5.294	1.29E-07	-0.533878596	count	0.003030855
LINC01358	-0.6571336	0.6261087	-1.0496	0.294	-0.53380491	count	1
CTBS	-0.3826886	0.1117587	-3.4242	0.000626	-0.533709378	count	1
LRRC8A	-0.3966303	0.1466627	-2.7044	0.00689	-0.533626272	count	1
JUP	-0.4044484	0.1764137	-2.2926	0.0219	-0.533512521	count	1
KLHL24	-0.3919385	0.1504238	-2.6056	0.00922	-0.533409021	count	1
TSPAN6	-0.3839673	0.1190563	-3.2251	0.00127	-0.533195344	count	1
ZNF821	-0.566962	0.3291764	-1.7224	0.0851	-0.533144871	count	1
NPR2	-0.5197983	0.3477063	-1.4949	0.135	-0.532795724	count	1
DNAL1	-0.4305168	0.2432699	-1.7697	0.0769	-0.532683565	count	1
DEPDC4	-0.9696437	0.7330244	-1.3228	0.186	-0.532578637	count	1
PCDHB9	-1.0663384	0.6550631	-1.6278	0.104	-0.53207019	count	1
PCGF5	-0.3933164	0.1319705	-2.9803	0.0029	-0.531934699	count	1
GMPR	-0.3963755	0.1871167	-2.1183	0.0342	-0.531693013	count	1
MICALL2	-0.9665058	0.5323055	-1.8157	0.0695	-0.530551126	count	1
UQCR11	-0.3726135	0.0568573	-6.5535	6.68E-11	-0.530534644	count	1.59E-06
AC113383.1	-2.838283	0.6569331	-4.3205	1.61E-05	-0.53026327	count	0.3727794
EPHB2	-2.838283	1.396509	-2.0324	0.0422	-0.53026327	count	1
FIBP	-0.3781519	0.0935588	-4.0419	5.45E-05	-0.529256609	count	1

CTDSPL	-0.4062366	0.1805349	-2.2502	0.0245	-0.528909126	count	1
NFIC	-0.3717594	0.0720712	-5.1582	2.67E-07	-0.527392199	count	0.006262752
TMEM63A	-0.5098645	0.3963652	-1.2864	0.198	-0.52737269	count	1
FEM1C	-0.4276971	0.189421	-2.2579	0.024	-0.526951865	count	1
EXOC3L1	-0.4792997	0.2697593	-1.7768	0.0757	-0.52669398	count	1
CIB2	-0.4639117	0.2917096	-1.5903	0.112	-0.526693329	count	1
PDIA3	-0.3676618	0.0457158	-8.0423	1.29E-15	-0.52652418	count	3.09E-11
TTYH1	-1.7791982	0.8182048	-2.1745	0.0298	-0.526392873	count	1
PLEKHA5	-0.4129196	0.2166354	-1.9061	0.0567	-0.526357824	count	1
KDM4B	-0.439475	0.256905	-1.7107	0.0873	-0.525706009	count	1
TEP1	-0.6013465	0.4875064	-1.2335	0.217	-0.525265512	count	1
ABLIM2	-0.5680146	0.3761009	-1.5103	0.131	-0.525115395	count	1
STK17B	-0.5233907	0.3480633	-1.5037	0.133	-0.525035181	count	1
BATF2	-0.7154374	0.4976275	-1.4377	0.151	-0.524673309	count	1
ERLEC1	-0.3712084	0.0806295	-4.6039	4.33E-06	-0.524480054	count	0.1008024
ZNF841	-0.5771865	0.3668782	-1.5732	0.116	-0.524337836	count	1
GLIS3	-0.3985354	0.1821611	-2.1878	0.0288	-0.523783201	count	1
RNASEH2C	-0.3714269	0.0726584	-5.112	3.41E-07	-0.523774467	count	0.007992699
COL18A1	-0.3726163	0.0913793	-4.0777	4.68E-05	-0.523477588	count	1
AC098850.3	-0.6650507	0.751582	-0.8849	0.376	-0.523428804	count	1
SULF2	-0.3712796	0.0887278	-4.1845	2.95E-05	-0.523335764	count	0.681273
PARVA	-0.3844832	0.1247181	-3.0828	0.00207	-0.523188493	count	1
SPESP1	-0.4427447	0.358631	-1.2345	0.217	-0.522994746	count	1
CCDC24	-0.6124903	0.4396112	-1.3933	0.164	-0.522990344	count	1
P2RY6	-0.6868518	0.3398545	-2.021	0.0434	-0.522768847	count	1
ATG4D	-0.7808446	0.4448732	-1.7552	0.0793	-0.522637924	count	1
ZNF510	-0.5054865	0.3034819	-1.6656	0.0959	-0.522630513	count	1
HAGHL	-1.3940247	0.8422996	-1.655	0.098	-0.522150338	count	1
SUV39H2	-0.6632719	0.4263199	-1.5558	0.12	-0.521893498	count	1
HIST1H2BN	-0.5746657	0.4587477	-1.2527	0.21	-0.521888832	count	1
ZNF530	-0.8238328	0.445254	-1.8503	0.0644	-0.521134491	count	1
FAM92A	-0.4072579	0.1703104	-2.3913	0.0169	-0.520343379	count	1
NCOA3	-0.3828219	0.1449295	-2.6414	0.0083	-0.520305251	count	1
AC025569.1	-0.8786076	0.4692667	-1.8723	0.0613	-0.520243302	count	1
BCL2L11	-0.4680408	0.2333333	-2.0059	0.045	-0.520218374	count	1
C3orf14	-0.404594	0.2064485	-1.9598	0.0501	-0.519950923	count	1
POLR3A	-0.4827656	0.3323577	-1.4525	0.146	-0.519768404	count	1
RGP1	-0.4675051	0.2563709	-1.8235	0.0683	-0.519601705	count	1
SUSD3	-0.9491987	0.6472945	-1.4664	0.143	-0.519395333	count	1
TM9SF2	-0.3701474	0.0955625	-3.8734	0.00011	-0.519359656	count	1
SACS	-0.4080356	0.3225449	-1.2651	0.206	-0.519353516	count	1
CDO1	-0.3833071	0.1862923	-2.0576	0.0397	-0.51934499	count	1
ANKRD13B	-0.6602273	0.5422157	-1.2176	0.223	-0.519267022	count	1
AC097103.2	-0.7082749	0.5186835	-1.3655	0.172	-0.518839988	count	1
SP2	-0.4455819	0.2581709	-1.7259	0.0845	-0.518787769	count	1

P3H4	-0.4279301	0.2458922	-1.7403	0.0819	-0.518713088	count	1
PPP1R32	-1.0433204	0.6492598	-1.6069	0.108	-0.518339045	count	1
STK4	-0.3862405	0.1421449	-2.7172	0.00662	-0.51820349	count	1
ZNF224	-0.3955513	0.1754318	-2.2547	0.0242	-0.517845295	count	1
TGOLN2	-0.3640945	0.0763973	-4.7658	1.98E-06	-0.516967931	count	0.04621716
ZDHHC6	-0.4017756	0.1754237	-2.2903	0.0221	-0.516851325	count	1
CABIN1	-0.4077788	0.2684649	-1.5189	0.129	-0.516165223	count	1
CSNK2A3	-1.380999	0.8812415	-1.5671	0.117	-0.516034806	count	1
ANTXR1	-1.380999	0.9758142	-1.4152	0.157	-0.516034806	count	1
SLC7A11	-1.380999	1.080311	-1.2783	0.201	-0.516034806	count	1
CABLES1	-0.5338199	0.4382782	-1.218	0.223	-0.51572	count	1
FAM107A	-0.373343	0.1082744	-3.4481	0.000573	-0.515481626	count	1
PPIB	-0.3586155	0.0357529	-10.0304	2.79E-23	-0.515297372	count	6.73E-19
PGR	-1.749411	0.6215346	-2.8147	0.00492	-0.515222402	count	1
FDPS	-0.3642073	0.0720438	-5.0554	4.58E-07	-0.514835823	count	0.010728192
NFIA	-0.3614083	0.0727821	-4.9656	7.27E-07	-0.514679181	count	0.0170118
MACF1	-0.3613528	0.0762149	-4.7412	2.23E-06	-0.514670983	count	0.05203259
MPP3	-1.1709118	0.5349172	-2.189	0.0287	-0.514299651	count	1
FOXC1	-0.3600917	0.0677692	-5.3135	1.16E-07	-0.514282743	count	0.002726348
LIN54	-0.5025577	0.3243863	-1.5493	0.121	-0.514274722	count	1
TKT	-0.3633667	0.077704	-4.6763	3.06E-06	-0.513818621	count	0.07130718
RAB3GAP2	-0.3890313	0.2033124	-1.9135	0.0558	-0.513292494	count	1
C20orf202	-0.9392876	0.643714	-1.4592	0.145	-0.513027655	count	1
FMNL1	-0.9392876	0.6858466	-1.3695	0.171	-0.513027655	count	1
ALDH2	-0.3643417	0.0755303	-4.8238	1.49E-06	-0.513023634	count	0.0348064
GLIPR1L2	-0.6525832	0.5204823	-1.2538	0.21	-0.512680397	count	1
ABR	-0.4073704	0.2474198	-1.6465	0.0998	-0.512540523	count	1
ACE	-0.3921217	0.2058049	-1.9053	0.0568	-0.511624446	count	1
AIF1	-0.6150338	0.6475766	-0.9497	0.342	-0.511535878	count	1
SPRY3	-0.6979772	0.5152574	-1.3546	0.176	-0.510470146	count	1
LUCAT1	-0.7638941	0.751752	-1.0162	0.31	-0.509836394	count	1
REEP5	-0.3573863	0.0578447	-6.1784	7.43E-10	-0.509664444	count	1.76E-05
PSMB5	-0.3572814	0.0538225	-6.6381	3.81E-11	-0.509358224	count	9.06E-07
Z97989.1	-1.161365	0.5971532	-1.9448	0.0519	-0.509170981	count	1
LGALS9	-0.3652053	0.1098689	-3.324	0.000899	-0.509127858	count	1
CCDC3	-0.3747655	0.1405083	-2.6672	0.00769	-0.509122967	count	1
ZNF428	-0.3628306	0.0880415	-4.1211	3.88E-05	-0.509058766	count	0.8948832
CAMSAP1	-0.4800356	0.3036886	-1.5807	0.114	-0.508668963	count	1
PTEN	-0.3653719	0.0983441	-3.7152	0.000207	-0.508310841	count	1
CPEB3	-0.5338806	0.4360621	-1.2243	0.221	-0.508192082	count	1
ASH2L	-0.4072374	0.2156551	-1.8884	0.0591	-0.508101482	count	1
PSME4	-0.4418885	0.2899945	-1.5238	0.128	-0.507982522	count	1
PIAS2	-0.4006896	0.2055469	-1.9494	0.0514	-0.507767658	count	1
CD164	-0.3624392	0.0897376	-4.0389	5.52E-05	-0.507262479	count	1
EXOC3-AS1	-0.5122006	0.3833718	-1.336	0.182	-0.507065222	count	1

GNPTG	-0.3613182	0.0978552	-3.6924	0.000226	-0.506764538	count	1
MRPL34	-0.358845	0.0817682	-4.3886	1.18E-05	-0.506344739	count	0.2735948
ZBTB49	-0.5396007	0.427343	-1.2627	0.207	-0.505827854	count	1
KAT2B	-0.4256804	0.2185593	-1.9477	0.0516	-0.505655968	count	1
WDR92	-0.4085601	0.2842717	-1.4372	0.151	-0.504985434	count	1
HIVEP2	-0.4474935	0.2536288	-1.7644	0.0778	-0.504894764	count	1
SPINDOC	-0.5474238	0.381415	-1.4352	0.151	-0.504849944	count	1
KCNQ1OT1	-0.38614	0.346296	-1.1151	0.265	-0.504562017	count	1
MAN1C1	-0.3952538	0.2224578	-1.7768	0.0757	-0.504137178	count	1
CD63	-0.350099	0.0292147	-11.9837	2.70E-32	-0.503678007	count	6.53E-28
PRKAA2	-0.4791103	0.4996612	-0.9589	0.338	-0.503351213	count	1
ETFBKMT	-0.4970503	0.3712124	-1.339	0.181	-0.50296988	count	1
TFEC	-2.6990201	1.26887	-2.1271	0.0335	-0.502700878	count	1
CCDC18	-0.5652484	0.4350431	-1.2993	0.194	-0.502453555	count	1
AC016394.2	-2.6961697	1.078254	-2.5005	0.0125	-0.5021226	count	1
DDI2	-0.4407347	0.2762406	-1.5955	0.111	-0.501991369	count	1
KIF15	-2.6932144	1.1154196	-2.4145	0.0158	-0.501522506	count	1
PRKCD	-1.1467415	0.9145693	-1.2539	0.21	-0.501336024	count	1
PTCHD4	-0.9206147	0.5804236	-1.5861	0.113	-0.501072718	count	1
MKL1	-0.4950762	0.3708775	-1.3349	0.182	-0.50087388	count	1
ATXN2-AS	-1.7099544	1.0781574	-1.586	0.113	-0.500451802	count	1
APOBEC3A	-1.7099544	1.0781574	-1.586	0.113	-0.500451802	count	1
AC019131.2	-1.7099544	1.121006	-1.5254	0.127	-0.500451802	count	1
AL157832.3	-1.7099544	1.121006	-1.5254	0.127	-0.500451802	count	1
AL139317.3	-1.7099544	1.121006	-1.5254	0.127	-0.500451802	count	1
AC087222.1	-1.7099544	1.182766	-1.4457	0.148	-0.500451802	count	1
ZNF653	-0.6382819	0.4271335	-1.4943	0.135	-0.500387503	count	1
RNF41	-0.3948998	0.1932203	-2.0438	0.0411	-0.500311547	count	1
PELI2	-0.4269276	0.2729944	-1.5639	0.118	-0.50026345	count	1
KCNC4	-0.6023913	0.4296121	-1.4022	0.161	-0.500145653	count	1
ANKRD36B	-0.4297894	0.3120474	-1.3773	0.169	-0.499888593	count	1
TIMM8B	-0.360144	0.095556	-3.7689	0.000167	-0.499523596	count	1
FAM110A	-0.4402363	0.2801136	-1.5716	0.116	-0.498969554	count	1
SLC26A2	-0.3898022	0.2311422	-1.6864	0.0918	-0.498923714	count	1
FKBP2	-0.3491097	0.0593072	-5.8865	4.42E-09	-0.498907316	count	0.000104564
PPARGC1B	-1.141137	0.8156092	-1.3991	0.162	-0.498340059	count	1
AC237221.1	-1.141137	0.8156092	-1.3991	0.162	-0.498340059	count	1
DLGAP2	-1.141137	0.8156092	-1.3991	0.162	-0.498340059	count	1
CTSV	-1.141137	0.8156092	-1.3991	0.162	-0.498340059	count	1
AC120049.1	-1.141137	0.8156092	-1.3991	0.162	-0.498340059	count	1
VN1R1	-1.141137	0.8156092	-1.3991	0.162	-0.498340059	count	1
AC083805.1	-1.141137	1.033045	-1.1046	0.269	-0.498340059	count	1
PRKX	-0.4238023	0.249677	-1.6974	0.0897	-0.498300051	count	1
RALA	-0.3540665	0.0886207	-3.9953	6.63E-05	-0.498289624	count	1
SEPSECS	-0.5235287	0.3467896	-1.5096	0.131	-0.497756654	count	1

TLR5	-0.6349168	0.5689142	-1.116	0.265	-0.497500683	count	1
AL928654.1	-0.8443641	0.6860497	-1.2308	0.219	-0.496870164	count	1
TRIB3	-0.5985686	0.4832142	-1.2387	0.216	-0.49670765	count	1
FAM98B	-0.4025704	0.2187192	-1.8406	0.0658	-0.496421803	count	1
AC087286.2	-1.137368	0.7222732	-1.5747	0.115	-0.49632706	count	1
CHPF	-0.7892721	0.6591235	-1.1975	0.231	-0.496254624	count	1
ZFP1	-0.406798	0.2816184	-1.4445	0.149	-0.496211285	count	1
NFATC1	-0.4023842	0.2142501	-1.8781	0.0605	-0.496187648	count	1
NAA38	-0.3499975	0.0706136	-4.9565	7.61E-07	-0.496167235	count	0.017802834
CCPG1	-0.3483736	0.0658099	-5.2936	1.29E-07	-0.496046258	count	0.003030855
SIL1	-0.3695624	0.161294	-2.2912	0.022	-0.495630794	count	1
ZDHHC20	-0.3862306	0.182164	-2.1202	0.0341	-0.495449002	count	1
RTTN	-0.5378192	0.3792991	-1.4179	0.156	-0.495423611	count	1
ANKRD45	-0.613465	0.5171767	-1.1862	0.236	-0.495253361	count	1
SIN3B	-0.3770029	0.1695322	-2.2238	0.0262	-0.495162159	count	1
PRDX2	-0.3459544	0.0503072	-6.8768	7.54E-12	-0.494154401	count	1.80E-07
ZHX3	-0.3991454	0.2383515	-1.6746	0.0941	-0.494107859	count	1
RTL10	-0.4477434	0.282032	-1.5876	0.112	-0.493890947	count	1
ZNF430	-0.3974456	0.2117016	-1.8774	0.0606	-0.493855979	count	1
GPSM3	-0.3819839	0.2289565	-1.6684	0.0954	-0.493642316	count	1
GLT8D1	-0.3674837	0.1328408	-2.7663	0.00571	-0.493461747	count	1
PEX12	-0.59465	0.4472199	-1.3297	0.184	-0.493186303	count	1
8-Mar	-0.3898447	0.2008339	-1.9411	0.0523	-0.493097148	count	1
ZNF503	-0.3920761	0.1944685	-2.0161	0.0439	-0.492971188	count	1
MBTPS2	-0.5666184	0.3732252	-1.5182	0.129	-0.492708646	count	1
TTF2	-0.4386633	0.2953485	-1.4852	0.138	-0.491997468	count	1
ADI1	-0.34908	0.0853246	-4.0912	4.41E-05	-0.49159207	count	1
RFWD3	-0.578342	0.3825639	-1.5118	0.131	-0.491580188	count	1
PIK3R4	-0.4113325	0.3327064	-1.2363	0.216	-0.491244365	count	1
FCER1G	-0.9044084	0.5797756	-1.5599	0.119	-0.490742284	count	1
SRPRB	-0.3611064	0.1312003	-2.7523	0.00596	-0.489983169	count	1
TRIM33	-0.3676171	0.1791353	-2.0522	0.0402	-0.489928362	count	1
HMGB3	-0.4140571	0.2202202	-1.8802	0.0602	-0.489903726	count	1
EXOG	-0.4193877	0.2976865	-1.4088	0.159	-0.489355888	count	1
MBD3	-0.3837279	0.2009014	-1.91	0.0562	-0.489214273	count	1
IGFBP7	-0.3391973	0.0366292	-9.2603	3.97E-20	-0.489134246	count	9.56E-16
AL138995.1	-0.540678	0.3347067	-1.6154	0.106	-0.488983409	count	1
AC040970.1	-0.832351	0.5624129	-1.48	0.139	-0.488718284	count	1
NAALADL1	-0.3826138	0.2401068	-1.5935	0.111	-0.488389277	count	1
DEXI	-0.3658156	0.1344045	-2.7218	0.00653	-0.488007292	count	1
ZNF124	-0.3949452	0.2979741	-1.3254	0.185	-0.487834576	count	1
RPP14	-0.4191196	0.2488906	-1.684	0.0923	-0.487137846	count	1
SNHG9	-0.365403	0.1672771	-2.1844	0.029	-0.486949587	count	1
TSPYL4	-0.4032039	0.2495449	-1.6158	0.106	-0.486806921	count	1
RPL27A	-0.3387849	0.0320495	-10.5707	1.25E-25	-0.486741817	count	3.02E-21

NDUFB2	-0.340311	0.0512393	-6.6416	3.72E-11	-0.485990727	count	8.85E-07
MRPL33	-0.3419061	0.0674477	-5.0692	4.26E-07	-0.48588839	count	0.00998118
AC004148.2	-0.6203155	0.6001806	-1.0335	0.301	-0.485000335	count	1
DOCK6	-0.3687094	0.175699	-2.0985	0.0359	-0.484848624	count	1
TLE1	-0.362308	0.156845	-2.31	0.021	-0.484707483	count	1
VARS	-0.4422452	0.2694925	-1.641	0.101	-0.484535202	count	1
SEMA3F-AS1	-1.6671	0.7332483	-2.2736	0.0231	-0.484454173	count	1
AL023581.2	-1.6671	0.8581036	-1.9428	0.0521	-0.484454173	count	1
LINC01752	-1.6671	0.9451294	-1.7639	0.0779	-0.484454173	count	1
FHL3	-0.3696929	0.1769628	-2.0891	0.0368	-0.484357133	count	1
PTPRU	-1.3125111	0.7913588	-1.6586	0.0973	-0.484106514	count	1
AL391069.2	-1.3125111	0.8122316	-1.6159	0.106	-0.484106514	count	1
SGIP1	-0.824595	0.602605	-1.3684	0.171	-0.483468618	count	1
AC008736.1	-0.5835462	0.4996204	-1.168	0.243	-0.483224429	count	1
BCL9	-0.4377725	0.3268046	-1.3396	0.181	-0.482509677	count	1
AP001528.1	-1.3085588	0.7137048	-1.8335	0.0668	-0.482276361	count	1
PLIN3	-0.3539029	0.1336259	-2.6485	0.00813	-0.481011258	count	1
AC005332.4	-0.4586645	0.377287	-1.2157	0.224	-0.480970908	count	1
BICRAL	-0.4288509	0.270977	-1.5826	0.114	-0.480636689	count	1
MKLN1	-0.354066	0.1656335	-2.1376	0.0326	-0.48036133	count	1
PIGL	-0.4358868	0.2926772	-1.4893	0.137	-0.480358921	count	1
RAD50	-0.4756316	0.4301883	-1.1056	0.269	-0.480264127	count	1
PANK3	-0.3779565	0.2162027	-1.7482	0.0805	-0.479842622	count	1
SMIM14	-0.3422621	0.1020669	-3.3533	0.000809	-0.478887425	count	1
C1orf216	-0.5301697	0.3551528	-1.4928	0.136	-0.478853541	count	1
RAP2C-AS1	-0.5642411	0.4965542	-1.1363	0.256	-0.478675276	count	1
BAIAP2-DT	-0.519894	0.3117352	-1.6677	0.0955	-0.477877054	count	1
LAMTOR5	-0.3358308	0.0603563	-5.5641	2.89E-08	-0.477754169	count	0.000681607
FAM136A	-0.3610264	0.1699817	-2.1239	0.0338	-0.477499844	count	1
SYNC	-0.3844648	0.2055617	-1.8703	0.0615	-0.477433438	count	1
AL133342.1	-0.8827633	0.6160892	-1.4328	0.152	-0.477012201	count	1
PDIA6	-0.3337103	0.0511861	-6.5196	8.35E-11	-0.476925683	count	1.98E-06
ZNF844	-0.4354918	0.3087523	-1.4105	0.159	-0.476873563	count	1
KIT	-0.7199042	0.6785707	-1.0609	0.289	-0.476861841	count	1
TERF1	-0.3447784	0.1406002	-2.4522	0.0143	-0.476585352	count	1
ZNF407	-0.4028141	0.2190997	-1.8385	0.0661	-0.476273606	count	1
MCRIP2	-0.3955979	0.2371144	-1.6684	0.0954	-0.476127188	count	1
SMARCD2	-0.3782794	0.177066	-2.1364	0.0327	-0.476084632	count	1
PTPN21	-0.6545557	0.4315291	-1.5168	0.129	-0.475400947	count	1
DPP3	-0.4661512	0.3262943	-1.4286	0.153	-0.475326363	count	1
BCAP29	-0.3328313	0.0624646	-5.3283	1.07E-07	-0.475026834	count	0.002515249
PURG	-0.7585889	0.6476293	-1.1713	0.242	-0.474347428	count	1
FAM189A1	-1.095859	0.668798	-1.6385	0.101	-0.474277782	count	1
OS9	-0.335795	0.0734471	-4.5719	5.05E-06	-0.474058582	count	0.11751855
SLC35B2	-0.3917281	0.2768298	-1.4151	0.157	-0.473898734	count	1

AKT1	-0.3464997	0.1296538	-2.6725	0.00757	-0.473753051	count	1
DDX28	-0.5248019	0.4157982	-1.2622	0.207	-0.473687164	count	1
APLP2	-0.3323969	0.0720308	-4.6146	4.12E-06	-0.473046977	count	0.09594244
DNAJC10	-0.3404067	0.1209463	-2.8145	0.00492	-0.47184	count	1
GRK6	-0.446481	0.3053081	-1.4624	0.144	-0.471692477	count	1
PPP1R14B	-0.3296968	0.0539289	-6.1135	1.11E-09	-0.471675935	count	2.63E-05
ARHGAP45	-0.556567	0.5146258	-1.0815	0.28	-0.471668422	count	1
PMEL	-0.9638559	0.7947216	-1.2128	0.225	-0.471513633	count	1
ADCY1	-0.9638559	0.8742779	-1.1025	0.27	-0.471513633	count	1
CCNO	-0.9638559	1.0091502	-0.9551	0.34	-0.471513633	count	1
CDH5	-0.3327841	0.0759804	-4.3799	1.23E-05	-0.471193276	count	0.2850894
AC091959.3	-0.7538696	0.6471272	-1.1649	0.244	-0.470993467	count	1
RALGAPB	-0.3822385	0.2212775	-1.7274	0.0842	-0.47087524	count	1
ZDHHC9	-0.3890342	0.2401047	-1.6203	0.105	-0.470569823	count	1
MAPRE2	-0.361279	0.1706634	-2.1169	0.0344	-0.47009079	count	1
MYOZ3	-0.623456	0.5533561	-1.1267	0.26	-0.469907368	count	1
MAPK1	-0.3464327	0.1370255	-2.5282	0.0115	-0.469784168	count	1
HSPH1	-0.3415327	0.1085174	-3.1473	0.00167	-0.469153082	count	1
PPP1R9A	-0.4697991	0.5693945	-0.8251	0.409	-0.468698875	count	1
DUSP19	-0.4282749	0.3343822	-1.2808	0.2	-0.468693767	count	1
SEC61B	-0.3263439	0.0436123	-7.4828	9.72E-14	-0.467560524	count	2.32E-09
NUDT3	-0.3422746	0.1373909	-2.4912	0.0128	-0.46628221	count	1
TAF5L	-0.5079372	0.3373472	-1.5057	0.132	-0.466206586	count	1
ADAL	-0.4138452	0.261568	-1.5822	0.114	-0.465768472	count	1
TRIM13	-0.3503953	0.144564	-2.4238	0.0154	-0.465761451	count	1
CCDC85B	-0.3237778	0.0352828	-9.1767	8.47E-20	-0.465574137	count	2.04E-15
AC112220.2	-0.797633	0.686505	-1.1619	0.245	-0.465302852	count	1
CYFIP2	-0.797633	0.7606738	-1.0486	0.294	-0.465302852	count	1
DRAP1	-0.3312118	0.0863203	-3.837	0.000127	-0.46505289	count	1
HSPA5	-0.325925	0.0642785	-5.0705	4.23E-07	-0.464523638	count	0.009911313
RDX	-0.3244672	0.0607	-5.3454	9.76E-08	-0.464179222	count	0.002294674
SLC38A6	-0.4123736	0.3066102	-1.3449	0.179	-0.464060973	count	1
GPR153	-2.5134947	0.9429517	-2.6656	0.00773	-0.464008433	count	1
HAVCR2	-2.5134947	0.9429517	-2.6656	0.00773	-0.464008433	count	1
CXCR3	-2.5134947	0.9429517	-2.6656	0.00773	-0.464008433	count	1
TMEM249	-2.5134947	0.9429517	-2.6656	0.00773	-0.464008433	count	1
PLK1	-2.5134947	0.9429517	-2.6656	0.00773	-0.464008433	count	1
PTOV1-AS2	-2.5134947	0.9429517	-2.6656	0.00773	-0.464008433	count	1
APOB	-2.5134947	1.31061	-1.9178	0.0552	-0.464008433	count	1
AL022068.1	-2.5134947	1.31061	-1.9178	0.0552	-0.464008433	count	1
VTN	-2.5134947	1.31061	-1.9178	0.0552	-0.464008433	count	1
AL021707.2	-2.5134947	1.31061	-1.9178	0.0552	-0.464008433	count	1
PANX2	-2.5134947	1.31061	-1.9178	0.0552	-0.464008433	count	1
AC010680.4	-2.5134947	1.353401	-1.8572	0.0634	-0.464008433	count	1
AC092903.2	-2.5134947	1.353401	-1.8572	0.0634	-0.464008433	count	1

TSPAN32	-2.5134947	1.353401	-1.8572	0.0634	-0.464008433	count	1
ZW10	-0.4506771	0.3249352	-1.387	0.166	-0.463528209	count	1
ZNF154	-0.5137972	0.4282359	-1.1998	0.23	-0.463112824	count	1
SELENOT	-0.3293378	0.083625	-3.9383	8.41E-05	-0.462922089	count	1
AL391422.3	-0.4957609	0.3652102	-1.3575	0.175	-0.462343857	count	1
UQCRQ	-0.3242592	0.0565333	-5.7357	1.08E-08	-0.462078272	count	0.000255118
TOLLIP	-0.3534647	0.1718309	-2.057	0.0398	-0.461756924	count	1
SLC9A3R2	-0.3205229	0.0623677	-5.1392	2.95E-07	-0.460839878	count	0.006917455
AC007038.2	-0.5323526	0.4743053	-1.1224	0.262	-0.460811716	count	1
UBB	-0.3199699	0.0323687	-9.8852	1.14E-22	-0.460119199	count	2.75E-18
RECK	-0.3825793	0.2359987	-1.6211	0.105	-0.460118166	count	1
SPEN	-0.3437567	0.1420923	-2.4192	0.0156	-0.459892429	count	1
RPN2	-0.3247199	0.0703191	-4.6178	4.06E-06	-0.459363327	count	0.09455334
SLC29A3	-0.6344238	0.4062501	-1.5617	0.118	-0.459267226	count	1
HSP90AA1	-0.3194451	0.0368387	-8.6715	7.15E-18	-0.459137017	count	1.72E-13
CCL28	-0.3738746	0.2893169	-1.2923	0.196	-0.458406777	count	1
MPZL1	-0.3362873	0.1375172	-2.4454	0.0145	-0.458196477	count	1
CABP4	-0.9408694	0.690267	-1.3631	0.173	-0.458145143	count	1
UNC119B	-0.48377	0.394452	-1.2264	0.22	-0.457859401	count	1
RPUSD2	-0.4272846	0.3547537	-1.2045	0.229	-0.457844757	count	1
SFT2D3	-0.3850902	0.2652652	-1.4517	0.147	-0.457777983	count	1
PGM3	-0.3541338	0.1818118	-1.9478	0.0515	-0.457657284	count	1
ARPC5	-0.3198708	0.0520193	-6.1491	8.92E-10	-0.457065254	count	2.11E-05
ZNRF1	-0.3311307	0.1112208	-2.9772	0.00293	-0.456612051	count	1
LSM10	-0.3318162	0.1289967	-2.5723	0.0102	-0.456518019	count	1
NMD3	-0.3452378	0.160422	-2.1521	0.0315	-0.456410911	count	1
ATPAF1	-0.3627178	0.2089729	-1.7357	0.0827	-0.456187913	count	1
WDR37	-0.3962262	0.2852329	-1.3891	0.165	-0.456042014	count	1
SLC5A3	-0.3675288	0.3052382	-1.2041	0.229	-0.456032471	count	1
MAP3K12	-0.3413545	0.1589257	-2.1479	0.0318	-0.45577358	count	1
FAM222B	-0.3936313	0.3493481	-1.1268	0.26	-0.454887533	count	1
MAGED1	-0.3579051	0.1782839	-2.0075	0.0448	-0.454625942	count	1
AHNAK2	-0.3283311	0.130573	-2.5145	0.012	-0.454470303	count	1
GPR107	-0.3654693	0.228579	-1.5989	0.11	-0.454279013	count	1
GCLM	-0.3457696	0.1765893	-1.958	0.0503	-0.454036338	count	1
ZNF341	-0.6041324	0.5284474	-1.1432	0.253	-0.45395259	count	1
ADNP2	-0.3960761	0.2580147	-1.5351	0.125	-0.453873497	count	1
UBL5	-0.3176921	0.0466566	-6.8092	1.20E-11	-0.453829422	count	2.86E-07
PHIP	-0.3272713	0.1190907	-2.7481	0.00603	-0.453611912	count	1
BLOC1S6	-0.3332271	0.1367765	-2.4363	0.0149	-0.453503329	count	1
AC009560.1	-1.0562048	0.7422713	-1.4229	0.155	-0.453421282	count	1
EXTL2	-0.393587	0.2567323	-1.5331	0.125	-0.452921387	count	1
C11orf49	-0.3579054	0.2318484	-1.5437	0.123	-0.452740651	count	1
CNOT8	-0.3415971	0.1744899	-1.9577	0.0504	-0.452656269	count	1
RASSF8-AS1	-0.3900209	0.2439549	-1.5987	0.11	-0.452439519	count	1

GSTP1	-0.3143597	0.0346616	-9.0694	2.21E-19	-0.452096549	count	5.32E-15
DDX24	-0.3158005	0.0501034	-6.303	3.39E-10	-0.451596232	count	8.04E-06
LASP1	-0.3385691	0.1652147	-2.0493	0.0405	-0.451570335	count	1
PLEKHA3	-0.3218797	0.0919196	-3.5018	0.00047	-0.450907329	count	1
NDUFA4	-0.3141369	0.0378642	-8.2964	1.66E-16	-0.450904181	count	3.98E-12
WRNIP1	-0.3441458	0.1736607	-1.9817	0.0476	-0.450869258	count	1
ZNF318	-0.4308385	0.2917082	-1.477	0.14	-0.450622621	count	1
ANAPC11	-0.3166772	0.0615702	-5.1433	2.89E-07	-0.450493726	count	0.006777628
BCL2A1	-0.7245838	1.0316702	-0.7023	0.483	-0.450274891	count	1
ANKRD9	-0.3585511	0.1963787	-1.8258	0.068	-0.450151681	count	1
ZCRB1	-0.3183981	0.085332	-3.7313	0.000194	-0.449968703	count	1
DAPL1	-0.8395946	0.882304	-0.9516	0.341	-0.449865633	count	1
PTPA	-0.3434438	0.1679821	-2.0445	0.041	-0.449592528	count	1
AC116407.2	-0.4623157	0.380759	-1.2142	0.225	-0.449347012	count	1
DIP2B	-0.4085392	0.2797066	-1.4606	0.144	-0.449227627	count	1
FAM199X	-0.3484394	0.1905496	-1.8286	0.0676	-0.44881539	count	1
NSD3	-0.3173288	0.0787764	-4.0282	5.77E-05	-0.448592302	count	1
TBC1D2B	-0.3520763	0.2100417	-1.6762	0.0938	-0.448292843	count	1
ARHGEF9	-0.3485678	0.2171258	-1.6054	0.109	-0.448013888	count	1
PCMTD1	-0.3156745	0.0690544	-4.5714	5.06E-06	-0.4480118	count	0.1177462
NEK5	-0.5765667	0.5457124	-1.0565	0.291	-0.447800629	count	1
SMG6	-0.3349951	0.1583048	-2.1161	0.0344	-0.447645825	count	1
COL14A1	-0.5962676	0.5810318	-1.0262	0.305	-0.447480717	count	1
AC092747.4	-0.5070823	0.4006753	-1.2656	0.206	-0.447380955	count	1
MIB1	-0.358496	0.254969	-1.406	0.16	-0.447071268	count	1
WASHC4	-0.3391084	0.1643138	-2.0638	0.0391	-0.44706728	count	1
GTF2H2	-0.4441709	0.555831	-0.7991	0.424	-0.447057583	count	1
SLC26A11	-0.4310189	0.285737	-1.5084	0.132	-0.446736592	count	1
GOLGB1	-0.3160871	0.0758407	-4.1678	3.17E-05	-0.446472849	count	0.7318579
PWWP2A	-0.3386757	0.1805761	-1.8755	0.0608	-0.44588628	count	1
TAS2R14	-0.8329054	0.8149238	-1.0221	0.307	-0.445688107	count	1
GALNS	-0.426063	0.4415335	-0.965	0.335	-0.445427343	count	1
CYB5D2	-0.3265345	0.1248314	-2.6158	0.00895	-0.445409963	count	1
CSNK1G1	-0.3642457	0.2916253	-1.249	0.212	-0.445378693	count	1
FAM111A-DT	-0.4582612	0.3824941	-1.1981	0.231	-0.445205257	count	1
SH3BP4	-0.3336432	0.1583996	-2.1063	0.0353	-0.445167294	count	1
SSBP4	-0.3166718	0.0863855	-3.6658	0.000251	-0.445018475	count	1
ARHGAP6	-0.441962	0.3050566	-1.4488	0.148	-0.444732757	count	1
R3HDM1	-0.3398601	0.181297	-1.8746	0.061	-0.444501046	count	1
ACAA1	-0.3226281	0.1223403	-2.6371	0.00841	-0.444371685	count	1
PHPT1	-0.3122213	0.0642534	-4.8592	1.24E-06	-0.443922995	count	0.02898128
AC009118.2	-0.6413457	0.695993	-0.9215	0.357	-0.442957236	count	1
BHMT2	-0.4759816	0.4654609	-1.0226	0.307	-0.44284392	count	1
GPRC5D-AS1	-0.3628338	0.3250722	-1.1162	0.264	-0.442597933	count	1
TGFBR1	-0.4307413	0.298635	-1.4424	0.149	-0.442157194	count	1

AL136295.2	-0.511736	0.5249666	-0.9748	0.33	-0.441732383	count	1
GCN1	-0.4064923	0.3004166	-1.3531	0.176	-0.441086417	count	1
TXNDC16	-0.4536359	0.3019534	-1.5023	0.133	-0.440484155	count	1
LINC00211	-0.7104701	0.6131494	-1.1587	0.247	-0.440349118	count	1
AL928921.2	-0.7104701	0.6323939	-1.1235	0.261	-0.440349118	count	1
GOLGA3	-0.3293392	0.2260327	-1.457	0.145	-0.440235345	count	1
NDRG3	-0.3507361	0.2313842	-1.5158	0.13	-0.440185505	count	1
ZBED4	-0.5502954	0.4370414	-1.2591	0.208	-0.440146461	count	1
ANO6	-0.3269836	0.1406561	-2.3247	0.0202	-0.439928999	count	1
STIMATE	-0.610034	0.4972121	-1.2269	0.22	-0.439831146	count	1
CHEK1	-0.610034	0.5238437	-1.1645	0.244	-0.439831146	count	1
TBC1D5	-0.3228611	0.1259176	-2.5641	0.0104	-0.439670016	count	1
YAF2	-0.3301022	0.1647634	-2.0035	0.0452	-0.439496516	count	1
TRAF3	-0.3950143	0.3522102	-1.1215	0.262	-0.439102049	count	1
CSTB	-0.3064787	0.0507964	-6.0335	1.82E-09	-0.438947703	count	4.31E-05
FAM126B	-0.3615898	0.2161725	-1.6727	0.0945	-0.438934564	count	1
LXN	-0.3088083	0.0861973	-3.5826	0.000346	-0.43890715	count	1
CPXM2	-0.3124811	0.0926404	-3.3731	0.000754	-0.438729745	count	1
KIRREL1	-0.5851953	0.4638629	-1.2616	0.207	-0.438391027	count	1
NAPA	-0.3158692	0.1076539	-2.9341	0.00337	-0.438352882	count	1
HAGH	-0.3169515	0.110278	-2.8741	0.00408	-0.438334386	count	1
ZNF816-ZNF321P	-0.6678483	0.5612908	-1.1898	0.234	-0.43832257	count	1
FGFR1OP	-0.4512286	0.4365509	-1.0336	0.301	-0.438028602	count	1
TMEM154	-0.4455618	0.406356	-1.0965	0.273	-0.437970665	count	1
EVI5L	-0.6347178	0.4604839	-1.3784	0.168	-0.437877149	count	1
MPC2	-0.3103723	0.0801589	-3.872	0.00011	-0.437312118	count	1
ANKRD37	-0.328621	0.1642408	-2.0008	0.0455	-0.437274456	count	1
CTTNBP2NL	-0.312812	0.0910733	-3.4347	0.000602	-0.437088594	count	1
MTFR1L	-0.3451735	0.1913326	-1.804	0.0713	-0.437018228	count	1
ASPRV1	-0.5465609	0.5866743	-0.9316	0.352	-0.436914033	count	1
MASTL	-0.5830902	0.4330738	-1.3464	0.178	-0.436665757	count	1
MAMLD1	-0.5830902	0.4394224	-1.3269	0.185	-0.436665757	count	1
KCTD17	-0.3553891	0.2364974	-1.5027	0.133	-0.436276338	count	1
KCND1	-1.02254	0.761744	-1.3424	0.18	-0.435883138	count	1
PTPN20	-1.02254	0.761744	-1.3424	0.18	-0.435883138	count	1
LINC01750	-1.02254	0.8914027	-1.1471	0.251	-0.435883138	count	1
AC011043.1	-0.4765774	0.4006587	-1.1895	0.234	-0.435729042	count	1
PYGL	-0.4041067	0.2800651	-1.4429	0.149	-0.435331595	count	1
DBNDD1	-0.7029915	0.7057507	-0.9961	0.319	-0.435105452	count	1
LRPAP1	-0.3070524	0.0684933	-4.483	7.66E-06	-0.434979363	count	0.1779418
AC044849.1	-0.4009366	0.3644323	-1.1002	0.271	-0.434851304	count	1
ATL2	-0.3260258	0.2021273	-1.613	0.107	-0.434697378	count	1
CDC14B	-0.6304226	0.3017472	-2.0892	0.0368	-0.434589819	count	1
COX7A2	-0.3036968	0.0487023	-6.2358	5.18E-10	-0.434552165	count	1.23E-05
CES4A	-1.2033793	0.7853008	-1.5324	0.126	-0.434129521	count	1

ZNF774	-1.2033793	0.8049665	-1.4949	0.135	-0.434129521	count	1
SIRPB2	-1.2033793	0.8049665	-1.4949	0.135	-0.434129521	count	1
FAM85B	-1.2033793	0.8243397	-1.4598	0.144	-0.434129521	count	1
VCP	-0.3098741	0.1005453	-3.0819	0.00208	-0.434067292	count	1
TMED7	-0.3240855	0.1372396	-2.3615	0.0183	-0.433763885	count	1
GNRH1	-0.8133464	0.8460619	-0.9613	0.336	-0.433518692	count	1
LYL1	-0.3091204	0.0904782	-3.4165	0.000643	-0.433277928	count	1
KIF1C	-0.3306425	0.1841589	-1.7954	0.0727	-0.432991559	count	1
SMIM20	-0.3218798	0.1561578	-2.0612	0.0394	-0.432836432	count	1
GSDME	-0.6997119	0.7194217	-0.9726	0.331	-0.432809451	count	1
FLOT2	-0.3235096	0.1726746	-1.8735	0.0611	-0.43258355	count	1
JMJD4	-0.3491518	0.2472738	-1.412	0.158	-0.432012268	count	1
SPAG4	-0.4577614	0.3939257	-1.1621	0.245	-0.431921173	count	1
DNAJC16	-0.371272	0.2557983	-1.4514	0.147	-0.431840623	count	1
MYO5A	-0.3355113	0.2136734	-1.5702	0.116	-0.431491033	count	1
ACADVL	-0.3124808	0.1070292	-2.9196	0.00353	-0.431127256	count	1
ADAMTSL3	-0.5757696	0.3443687	-1.672	0.0946	-0.430673212	count	1
TRMT2B	-0.4333113	0.2913063	-1.4875	0.137	-0.430632508	count	1
TWNK	-0.4240971	0.4323082	-0.981	0.327	-0.4306202	count	1
AC005339.1	-1.5203921	0.8386156	-1.813	0.0699	-0.430221002	count	1
GPRC5B	-0.3163149	0.1406607	-2.2488	0.0246	-0.429850956	count	1
STARD5	-0.4884138	0.4756758	-1.0268	0.305	-0.429846853	count	1
USP27X	-0.3847829	0.3326881	-1.1566	0.248	-0.429788155	count	1
HOXB6	-0.7440843	0.6381263	-1.166	0.244	-0.429620601	count	1
NCBP2-AS2	-0.312772	0.1189862	-2.6286	0.00862	-0.429524908	count	1
MYO1C	-0.3121422	0.1197203	-2.6073	0.00918	-0.428740001	count	1
ARPC2	-0.2991625	0.0467914	-6.3935	1.90E-10	-0.428582183	count	4.51E-06
AC011444.2	-1.1905394	0.8255896	-1.442	0.149	-0.428330841	count	1
ZNF700	-0.4612056	0.4181242	-1.103	0.27	-0.428326233	count	1
THEM4	-0.3358882	0.1943838	-1.728	0.0841	-0.427946357	count	1
MCM5	-0.3493161	0.2968593	-1.1767	0.239	-0.427747807	count	1
ADD1	-0.306044	0.1002705	-3.0522	0.00229	-0.427706126	count	1
NPTN	-0.3096984	0.1143084	-2.7093	0.00678	-0.427408087	count	1
ZNF827	-0.5519572	0.4767232	-1.1578	0.247	-0.427047421	count	1
UBASH3B	-1.1876391	0.5461036	-2.1748	0.0297	-0.427023531	count	1
SLC22A5	-0.6517513	0.4474524	-1.4566	0.145	-0.4265149	count	1
AL683813.1	-0.8858441	0.5093391	-1.7392	0.0821	-0.426486123	count	1
NBPF3	-0.6195613	0.5290981	-1.171	0.242	-0.426294158	count	1
TNPO1	-0.311	0.1235767	-2.5167	0.0119	-0.425730911	count	1
THNSL2	-0.8837253	0.5197609	-1.7003	0.0892	-0.425277008	count	1
IDH2	-0.3540032	0.2095539	-1.6893	0.0913	-0.425048435	count	1
MIEN1	-0.3034055	0.0956432	-3.1723	0.00153	-0.424978227	count	1
NCKAP5L	-0.3976449	0.2628821	-1.5126	0.13	-0.424964993	count	1
KIF26B	-1.182855	0.8049512	-1.4695	0.142	-0.424869181	count	1
GPR84	-1.182855	0.8049512	-1.4695	0.142	-0.424869181	count	1

HIST2H2AB	-1.182855	0.8213331	-1.4402	0.15	-0.424869181	count	1
QSOX2	-0.504968	0.4552225	-1.1093	0.267	-0.424863308	count	1
CHMP1B	-0.3101052	0.1136884	-2.7277	0.00642	-0.424013033	count	1
RASSF2	-0.5478233	0.503171	-1.0887	0.276	-0.423573754	count	1
ZNF579	-0.3629064	0.280167	-1.2953	0.195	-0.423482382	count	1
ASB3	-0.5033887	0.4010853	-1.2551	0.21	-0.423439352	count	1
SEC61G	-0.2974757	0.0560441	-5.3079	1.20E-07	-0.423305317	count	0.00281988
TULP4	-0.3127328	0.1379816	-2.2665	0.0235	-0.423233573	count	1
SLC30A5	-0.327665	0.1943562	-1.6859	0.0919	-0.423044039	count	1
L3MBTL3	-0.3595782	0.2522294	-1.4256	0.154	-0.422551584	count	1
FBP1	-0.5022275	0.5005632	-1.0033	0.316	-0.422392697	count	1
ERGIC3	-0.3009287	0.092026	-3.27	0.00109	-0.422361295	count	1
SSUH2	-1.497249	0.5479652	-2.7324	0.00633	-0.421764251	count	1
AC011447.3	-0.4542798	0.3868026	-1.1744	0.24	-0.421536318	count	1
PLEKHH3	-0.3584845	0.2394505	-1.4971	0.134	-0.421236574	count	1
ZNF786	-0.6444973	0.4116714	-1.5656	0.118	-0.421211203	count	1
TMBIM1	-0.3015168	0.0925457	-3.258	0.00114	-0.421147077	count	1
PNKP	-0.321789	0.1598546	-2.013	0.0442	-0.420950012	count	1
ARIH2OS	-0.4460633	0.2979936	-1.4969	0.135	-0.420297441	count	1
AC025682.1	-0.5625293	0.5487393	-1.0251	0.305	-0.419863546	count	1
RPE	-0.3270534	0.1909256	-1.713	0.0868	-0.419547473	count	1
USP46	-0.3232821	0.2102452	-1.5376	0.124	-0.419340653	count	1
MYL4	-1.1698418	0.7392376	-1.5825	0.114	-0.41902227	count	1
ARMH3	-0.4383949	0.3485304	-1.2578	0.209	-0.41900054	count	1
AL132656.2	-0.8725438	0.7427086	-1.1748	0.24	-0.418908648	count	1
AC012467.2	-0.8725438	0.7889167	-1.106	0.269	-0.418908648	count	1
ZSCAN16-AS1	-0.3444932	0.202707	-1.6995	0.0893	-0.418811361	count	1
ZNF680	-0.338522	0.2235615	-1.5142	0.13	-0.418638597	count	1
FAM120C	-0.3857523	0.4240687	-0.9096	0.363	-0.417835588	count	1
CCDC189	-1.1670912	0.6434155	-1.8139	0.0698	-0.41778887	count	1
AC068491.3	-1.4862854	0.7378794	-2.0143	0.0441	-0.417768955	count	1
AL162274.2	-1.4862854	0.7558652	-1.9663	0.0494	-0.417768955	count	1
NUPR1	-0.2927579	0.0755697	-3.874	0.00011	-0.41755039	count	1
ANKFY1	-0.3541673	0.2486722	-1.4242	0.154	-0.417481139	count	1
TIMM8A	-0.3321598	0.2506997	-1.3249	0.185	-0.417184756	count	1
ZKSCAN2	-0.9861547	0.6517742	-1.513	0.13	-0.417108891	count	1
USP42	-0.353562	0.2847425	-1.2417	0.214	-0.416751518	count	1
PRDM8	-2.297496	0.7559495	-3.0392	0.00239	-0.416726636	count	1
CDR2L	-0.4359764	0.2767313	-1.5755	0.115	-0.41657154	count	1
ZNF442	-0.6380341	0.4061623	-1.5709	0.116	-0.416494844	count	1
FMC1	-0.3275884	0.187497	-1.7472	0.0807	-0.416147591	count	1
AL451007.3	-1.4810266	0.9305496	-1.5916	0.112	-0.415855262	count	1
OIT3	-1.4810266	0.9305496	-1.5916	0.112	-0.415855262	count	1
AC008378.1	-1.4810266	0.9845423	-1.5043	0.133	-0.415855262	count	1
AC010542.4	-1.4810266	1.276758	-1.16	0.246	-0.415855262	count	1

EXOSC4	-0.3137474	0.1625376	-1.9303	0.0537	-0.415422219	count	1
SNRK	-0.2961079	0.0901253	-3.2855	0.00103	-0.415420637	count	1
ZNF169	-0.5214639	0.4674527	-1.1155	0.265	-0.415266965	count	1
ZNF503-AS2	-1.4791	0.686312	-2.1551	0.0312	-0.415154661	count	1
PKNOX2	-1.4791	0.7424611	-1.9922	0.0465	-0.415154661	count	1
ANK2	-1.4791	1.069681	-1.3827	0.167	-0.415154661	count	1
AP001830.1	-0.635065	0.4903023	-1.2953	0.195	-0.414331114	count	1
CIB1	-0.2927356	0.0734794	-3.9839	6.95E-05	-0.413448241	count	1
CACNA2D1	-0.3306343	0.2120996	-1.5589	0.119	-0.413210406	count	1
PRMT2	-0.292602	0.0869591	-3.3648	0.000776	-0.413198392	count	1
LTB4R2	-0.5759973	0.5159003	-1.1165	0.264	-0.412913989	count	1
OST4	-0.2882829	0.042389	-6.8009	1.27E-11	-0.412779645	count	3.03E-07
AC108463.3	-2.2792187	0.6818174	-3.3429	0.00084	-0.412642717	count	1
RMND5B	-0.3410811	0.2585394	-1.3193	0.187	-0.412525716	count	1
PTCD2	-0.5754247	0.3981482	-1.4453	0.149	-0.412463263	count	1
DAPK2	-0.601076	0.5668361	-1.0604	0.289	-0.412232102	count	1
SMAD5-AS1	-0.632081	0.6527746	-0.9683	0.333	-0.412158295	count	1
RASSF8	-0.3040958	0.1272778	-2.3892	0.017	-0.4121373	count	1
COBL	-2.2762461	1.205097	-1.8888	0.059	-0.411977509	count	1
LOXL4	-2.2762461	1.205097	-1.8888	0.059	-0.411977509	count	1
AC034243.1	-2.2762461	1.239098	-1.837	0.0663	-0.411977509	count	1
DNA2	-2.2762461	1.239098	-1.837	0.0663	-0.411977509	count	1
DACT3	-2.2762461	1.239098	-1.837	0.0663	-0.411977509	count	1
AL357033.4	-2.2762461	1.31997	-1.7245	0.0847	-0.411977509	count	1
C11orf24	-0.4252512	0.3426692	-1.241	0.215	-0.411600433	count	1
ELFN1	-0.3947709	0.3347226	-1.1794	0.238	-0.411481642	count	1
RRAGD	-0.5739582	0.5281337	-1.0868	0.277	-0.411309181	count	1
ZNF225	-0.5739582	0.5533593	-1.0372	0.3	-0.411309181	count	1
TRAC	-0.8588753	0.7764475	-1.1062	0.269	-0.411152591	count	1
AC078883.1	-0.4299541	0.4082322	-1.0532	0.292	-0.410528015	count	1
CHAC1	-0.6673968	0.6854788	-0.9736	0.33	-0.410301171	count	1
FLNA	-0.2908278	0.0985731	-2.9504	0.0032	-0.410257029	count	1
FAM3D	-0.775259	0.6587989	-1.1768	0.239	-0.410020364	count	1
AC068987.5	-0.9708764	0.5515021	-1.7604	0.0784	-0.409283595	count	1
GRN	-0.2872583	0.064154	-4.4776	7.85E-06	-0.409126797	count	0.1823398
CLTC	-0.2950539	0.1047002	-2.8181	0.00487	-0.408936861	count	1
ABCA3	-0.4759742	0.3583542	-1.3282	0.184	-0.40884299	count	1
NFE2L2	-0.2869037	0.0641796	-4.4703	8.12E-06	-0.40875936	count	0.18857888
AP002884.1	-0.4119438	0.4067441	-1.0128	0.311	-0.408454365	count	1
CNTN1	-0.853455	0.7894236	-1.0811	0.28	-0.408085815	count	1
GOLGA6L10	-0.853455	0.7894236	-1.0811	0.28	-0.408085815	count	1
ENPP6	-0.853455	0.9517458	-0.8967	0.37	-0.408085815	count	1
LINC01242	-0.853455	0.9517458	-0.8967	0.37	-0.408085815	count	1
HOXA1	-0.6264045	0.6821229	-0.9183	0.359	-0.408030297	count	1
AL606491.1	-0.5696505	0.6780219	-0.8402	0.401	-0.407921914	count	1

GOLGA4	-0.2880953	0.0912171	-3.1583	0.0016	-0.407450598	count	1
CSNK2A2	-0.2943851	0.1105	-2.6641	0.00776	-0.407400703	count	1
AC099778.1	-0.5943638	0.4776506	-1.2443	0.213	-0.407143901	count	1
SMC4	-0.2967821	0.1267989	-2.3406	0.0193	-0.407126503	count	1
LRRC69	-0.7097845	0.6706234	-1.0584	0.29	-0.407052492	count	1
NHLRC2	-0.3159819	0.1994737	-1.5841	0.113	-0.406943387	count	1
UQCRC1	-0.2988342	0.1322587	-2.2595	0.0239	-0.40686877	count	1
NBPF1	-0.3903906	0.3242291	-1.2041	0.229	-0.406743543	count	1
AC008764.8	-0.9658931	0.6120787	-1.5781	0.115	-0.406738785	count	1
MICA	-0.3085886	0.1686325	-1.8299	0.0674	-0.406455896	count	1
NEDD4L	-0.3370045	0.2475316	-1.3615	0.173	-0.406441364	count	1
ZNF728	-0.4630776	0.5619051	-0.8241	0.41	-0.406164107	count	1
AC005332.7	-0.3508232	0.2568115	-1.3661	0.172	-0.405879436	count	1
ARHGEF17	-0.7682233	0.5463164	-1.4062	0.16	-0.405709014	count	1
PRR11	-0.7682233	0.5804192	-1.3236	0.186	-0.405709014	count	1
FBXO9	-0.2936606	0.1210143	-2.4267	0.0153	-0.405558583	count	1
PACERR	-0.7070226	0.723788	-0.9768	0.329	-0.405245309	count	1
BACE2	-0.2852713	0.0753122	-3.7879	0.000155	-0.405116959	count	1
KCNK6	-0.4618945	0.4378293	-1.055	0.292	-0.405061483	count	1
NLRX1	-0.5095644	0.5079496	-1.0032	0.316	-0.405049984	count	1
ZCCHC24	-0.3004772	0.1572601	-1.9107	0.0561	-0.404956808	count	1
ITGA3	-0.3188325	0.2063837	-1.5449	0.122	-0.404875775	count	1
TMED9	-0.2852557	0.0664665	-4.2917	1.83E-05	-0.404867542	count	0.4235901
ZNF461	-0.4370829	0.4761211	-0.918	0.359	-0.404718248	count	1
AC087386.1	-0.5087219	0.591992	-0.8593	0.39	-0.404327742	count	1
LINC02478	-0.5087219	0.7048999	-0.7217	0.471	-0.404327742	count	1
MSANTD2	-0.3283916	0.2800113	-1.1728	0.241	-0.404275128	count	1
AP4E1	-0.3761881	0.4115309	-0.9141	0.361	-0.404258125	count	1
BRPF3	-0.3914526	0.3687186	-1.0617	0.288	-0.404160003	count	1
FHOD1	-0.3913816	0.2755184	-1.4205	0.156	-0.404083844	count	1
SMIM7	-0.2872281	0.0848039	-3.387	0.000717	-0.403930223	count	1
LCLAT1	-0.3307641	0.2325766	-1.4222	0.155	-0.403716923	count	1
SLAIN1	-0.9598613	0.5212985	-1.8413	0.0657	-0.403663581	count	1
LY6G5C	-0.4118659	0.3193229	-1.2898	0.197	-0.403343828	count	1
TAOK2	-0.308979	0.2083098	-1.4833	0.138	-0.403047371	count	1
SLC25A45	-0.5230106	0.4061483	-1.2877	0.198	-0.402800583	count	1
SEMA4A	-0.3329907	0.2023976	-1.6452	0.1	-0.402555705	count	1
ERCC4	-0.3124678	0.177551	-1.7599	0.0785	-0.40236425	count	1
ARL6IP5	-0.2804748	0.0440395	-6.3687	2.23E-10	-0.402289397	count	5.29E-06
PCYOX1	-0.2963867	0.1457599	-2.0334	0.0421	-0.402246129	count	1
ATF6	-0.2987984	0.1314161	-2.2737	0.0231	-0.401900276	count	1
ENO1	-0.2815823	0.0527657	-5.3365	1.02E-07	-0.401757892	count	0.00239802
S100A13	-0.2799967	0.0465715	-6.0122	2.07E-09	-0.401697983	count	4.90E-05
FYCO1	-0.3623076	0.2917742	-1.2417	0.214	-0.401690617	count	1
ZNF208	-0.3263223	0.2676933	-1.219	0.223	-0.40168477	count	1

MPP6	-0.4492175	0.4194772	-1.0709	0.284	-0.401542582	count	1
CXCL8	-0.3063394	0.3683118	-0.8317	0.406	-0.40146083	count	1
ATAD3A	-0.3641593	0.2917035	-1.2484	0.212	-0.401427253	count	1
SNX8	-0.3455513	0.3059951	-1.1293	0.259	-0.401222959	count	1
ZNF460	-0.3732821	0.3351091	-1.1139	0.265	-0.401031212	count	1
CPM	-0.8409197	0.5183862	-1.6222	0.105	-0.401012717	count	1
SEC11C	-0.2829123	0.075847	-3.73	0.000195	-0.400983289	count	1
CKAP2	-0.3495258	0.2528529	-1.3823	0.167	-0.400966782	count	1
FAM120AOS	-0.290788	0.1219252	-2.385	0.0171	-0.400931883	count	1
ZNF69	-0.5047196	0.4339589	-1.1631	0.245	-0.400898921	count	1
CYP2U1	-0.3263623	0.2458607	-1.3274	0.184	-0.40089521	count	1
KRT19	-0.7002113	0.6404349	-1.0933	0.274	-0.400794937	count	1
KCNE3	-0.5604385	0.695332	-0.806	0.42	-0.400691612	count	1
YPEL5	-0.2849343	0.0856379	-3.3272	0.000889	-0.400568002	count	1
FAM24B	-0.5041281	0.5931681	-0.8499	0.395	-0.400392473	count	1
CISD2	-0.2977294	0.1515496	-1.9646	0.0496	-0.399965973	count	1
ATXN2	-0.2951905	0.1404393	-2.1019	0.0357	-0.399734978	count	1
MMD	-0.4126977	0.3175752	-1.2995	0.194	-0.398875759	count	1
RNFT1	-0.3326055	0.2754314	-1.2076	0.227	-0.398853388	count	1
NTPCR	-0.287748	0.1175425	-2.448	0.0144	-0.398343507	count	1
KCNQ4	-1.43254	0.8074241	-1.7742	0.0761	-0.398295791	count	1
AC011374.2	-1.43254	0.8074241	-1.7742	0.0761	-0.398295791	count	1
LINC00884	-1.43254	0.8715477	-1.6437	0.1	-0.398295791	count	1
GPATCH2L	-0.3069797	0.2125254	-1.4444	0.149	-0.39796351	count	1
MIR29B2CHG	-0.612512	0.4764629	-1.2855	0.199	-0.397955959	count	1
MAMSTR	-0.5171427	0.4828714	-1.071	0.284	-0.397907359	count	1
AL050341.2	-0.3788307	0.3063906	-1.2364	0.216	-0.397710613	count	1
SURF4	-0.2899957	0.1250228	-2.3195	0.0204	-0.397604835	count	1
COPE	-0.2803485	0.0642454	-4.3637	1.33E-05	-0.397519925	count	0.3081743
CASC2	-0.5563635	0.5792817	-0.9604	0.337	-0.397499081	count	1
PUSL1	-0.3447515	0.2811446	-1.2262	0.22	-0.397047806	count	1
SERTAD4	-0.3508411	0.2449628	-1.4322	0.152	-0.396981077	count	1
HIVEP1	-0.296713	0.154042	-1.9262	0.0542	-0.396851041	count	1
SH3BP5L	-0.4441751	0.3644254	-1.2188	0.223	-0.396770936	count	1
ZDHHC14	-0.3403693	0.2387598	-1.4256	0.154	-0.396583177	count	1
PSMB6	-0.2770097	0.0524459	-5.2818	1.38E-07	-0.394991476	count	0.003242034
ORC6	-0.9427019	0.7359128	-1.281	0.2	-0.394945622	count	1
ITM2C	-0.9427019	0.8254096	-1.1421	0.254	-0.394945622	count	1
AC009163.7	-0.9427019	0.8272306	-1.1396	0.255	-0.394945622	count	1
MAP4K4	-0.2831066	0.1114168	-2.541	0.0111	-0.394638331	count	1
SLC2A13	-0.5130701	0.4721364	-1.0867	0.277	-0.394515657	count	1
DNAJB2	-0.3156932	0.1971426	-1.6013	0.109	-0.394259947	count	1
C1QTNF6	-0.3726469	0.4655496	-0.8004	0.424	-0.394238474	count	1
SCAMP4	-0.3222603	0.2141284	-1.505	0.132	-0.394049494	count	1
ZNF629	-0.4079226	0.3283423	-1.2424	0.214	-0.394043626	count	1

NRSN2-AS1	-0.3931372	0.3745792	-1.0495	0.294	-0.393572501	count	1
PDIA4	-0.2862571	0.1171178	-2.4442	0.0146	-0.393320074	count	1
BSG	-0.2751223	0.0537671	-5.1169	3.32E-07	-0.393103171	count	0.007782744
SEC63	-0.2839135	0.1103624	-2.5726	0.0101	-0.392849675	count	1
TM9SF3	-0.2779672	0.0801341	-3.4688	0.000531	-0.392838776	count	1
GPR162	-0.4948881	0.5119048	-0.9668	0.334	-0.392490873	count	1
XPO6	-0.3469485	0.352781	-0.9835	0.325	-0.392460566	count	1
ZNF416	-0.4810019	0.4622078	-1.0407	0.298	-0.392407766	count	1
PDE6B	-0.5498118	0.6090646	-0.9027	0.367	-0.392373781	count	1
TMEM121	-0.3035504	0.1863193	-1.6292	0.103	-0.392342917	count	1
PHETA1	-0.5100675	0.4189209	-1.2176	0.223	-0.392017389	count	1
SYCE1L	-0.3449363	0.2706519	-1.2745	0.203	-0.392003614	count	1
PXMP2	-0.2976606	0.1774448	-1.6775	0.0936	-0.391930332	count	1
HECW2	-0.3060072	0.2601533	-1.1763	0.24	-0.391776019	count	1
CERNA1	-0.6851701	0.8253906	-0.8301	0.407	-0.39100023	count	1
AC004466.1	-0.6851701	0.8594246	-0.7972	0.425	-0.39100023	count	1
NAP1L1	-0.2737063	0.048463	-5.6477	1.79E-08	-0.390969543	count	0.000422583
GUSB	-0.2824693	0.109912	-2.57	0.0102	-0.39095232	count	1
FRAT2	-0.3242639	0.2103074	-1.5419	0.123	-0.390787535	count	1
GABRE	-0.5725782	0.5413494	-1.0577	0.29	-0.390695684	count	1
CHD4	-0.2812133	0.1036098	-2.7142	0.00669	-0.39068215	count	1
PRDX5	-0.2734958	0.0551503	-4.9591	7.51E-07	-0.390618359	count	0.017571147
C1orf53	-0.6019756	0.4614592	-1.3045	0.192	-0.390342621	count	1
ZNF641	-0.52597	0.3896457	-1.3499	0.177	-0.3902108	count	1
NEMP2	-0.4221872	0.4403519	-0.9587	0.338	-0.390199081	count	1
SCRIB	-0.3989288	0.349019	-1.143	0.253	-0.39010706	count	1
TMEM233	-0.3282379	0.3313716	-0.9905	0.322	-0.390048575	count	1
SLC35E4	-0.3216353	0.2165016	-1.4856	0.137	-0.389564067	count	1
SNHG25	-0.4655657	0.4076629	-1.142	0.254	-0.389494195	count	1
RAB11FIP3	-0.4147078	0.3377227	-1.228	0.22	-0.38927505	count	1
TTC7B	-0.4031908	0.3483796	-1.1573	0.247	-0.389259768	count	1
PPP1R26	-0.4282817	0.4244156	-1.0091	0.313	-0.389175862	count	1
PPM1H	-0.9301505	0.6141685	-1.5145	0.13	-0.388597636	count	1
NECTIN3	-0.3133674	0.2119533	-1.4785	0.139	-0.388528981	count	1
ZNF630	-0.5441815	0.4779503	-1.1386	0.255	-0.38797676	count	1
METTL4	-0.2946614	0.2138774	-1.3777	0.168	-0.38794527	count	1
SGK1	-0.2815007	0.102626	-2.743	0.00613	-0.387741366	count	1
AHCYL1	-0.2849439	0.1280692	-2.2249	0.0262	-0.387196312	count	1
DBN1	-0.2865927	0.1274427	-2.2488	0.0246	-0.386829757	count	1
TIGD6	-0.4421046	0.5142138	-0.8598	0.39	-0.386661199	count	1
RCSD1	-0.4879468	0.4288144	-1.1379	0.255	-0.386567279	count	1
ZNF621	-0.3553497	0.3118771	-1.1394	0.255	-0.386499331	count	1
SPRYD4	-0.3383423	0.3050511	-1.1091	0.267	-0.386102048	count	1
ZC3H11A	-0.6318686	0.7048816	-0.8964	0.37	-0.38580144	count	1
DSCC1	-0.4173743	0.3619315	-1.1532	0.249	-0.385517553	count	1

ZNF423	-0.3590871	0.2638686	-1.3609	0.174	-0.38528928	count	1
USP40	-0.3738287	0.32365	-1.155	0.248	-0.385285346	count	1
AL445472.1	-0.4404747	0.3649009	-1.2071	0.227	-0.385149449	count	1
NADSYN1	-0.3306721	0.2411271	-1.3714	0.17	-0.385030672	count	1
CNPY4	-0.3008195	0.2002964	-1.5019	0.133	-0.384603186	count	1
RPS6KA1	-0.7332612	0.5859007	-1.2515	0.211	-0.384424026	count	1
ZNF624	-0.3512697	0.4001533	-0.8778	0.38	-0.384413885	count	1
LBH	-0.2807712	0.1473067	-1.906	0.0567	-0.384397747	count	1
SYNJ1	-0.3300616	0.2509779	-1.3151	0.189	-0.384303803	count	1
NOVA1	-0.2979073	0.1943436	-1.5329	0.125	-0.384199	count	1
GPX8	-0.2829441	0.1696873	-1.6674	0.0955	-0.384009736	count	1
DEGS1	-0.2768248	0.1097745	-2.5218	0.0117	-0.383370696	count	1
VASH2	-2.1493314	1.013404	-2.1209	0.034	-0.383363703	count	1
F12	-0.5916887	0.5485935	-1.0786	0.281	-0.382932544	count	1
LINC00882	-0.808476	0.5968105	-1.3547	0.176	-0.382834393	count	1
ABCB1	-0.3299165	0.2141426	-1.5406	0.124	-0.382656503	count	1
TAOK3	-0.282574	0.1452477	-1.9455	0.0518	-0.382545048	count	1
PLPP3	-0.277414	0.1353784	-2.0492	0.0405	-0.382538167	count	1
FDXR	-0.3366535	0.3179695	-1.0588	0.29	-0.382353031	count	1
DOCK9-DT	-0.4468227	0.3621299	-1.2339	0.217	-0.382231642	count	1
HEMK1	-0.381884	0.3083759	-1.2384	0.216	-0.381844302	count	1
SGMS1-AS1	-0.4821576	0.4980153	-0.9682	0.333	-0.381634974	count	1
B3GNT8	-0.446143	0.4407494	-1.0122	0.312	-0.381613345	count	1
KCNAB2	-0.446143	0.4779097	-0.9335	0.351	-0.381613345	count	1
SMARCD1	-0.2848405	0.1443061	-1.9739	0.0485	-0.381548192	count	1
8-Sep	-0.310511	0.2382074	-1.3035	0.193	-0.381105922	count	1
P4HTM	-0.2922301	0.180192	-1.6218	0.105	-0.380982296	count	1
CDNF	-0.5589179	0.594339	-0.9404	0.347	-0.380434572	count	1
MFSD9	-0.5589179	0.6200573	-0.9014	0.367	-0.380434572	count	1
ZFYVE1	-0.3544612	0.3001853	-1.1808	0.238	-0.380166708	count	1
TLCD2	-0.3034698	0.2077027	-1.4611	0.144	-0.380038065	count	1
AL669831.5	-0.5132393	0.4220645	-1.216	0.224	-0.379953548	count	1
CYTH3	-0.3568514	0.2944515	-1.2119	0.226	-0.379953413	count	1
IGFBP7-AS1	-0.802551	0.7275375	-1.1031	0.27	-0.379534843	count	1
LAMA4	-0.2691935	0.0907337	-2.9669	0.00303	-0.379196861	count	1
CRELD1	-0.2857282	0.168794	-1.6928	0.0906	-0.379120847	count	1
AC114811.2	-2.129204	0.982021	-2.1682	0.0302	-0.378795378	count	1
AC026367.3	-2.129204	1.017725	-2.0921	0.0365	-0.378795378	count	1
MAP4K2	-0.5566231	0.4514119	-1.2331	0.218	-0.378714788	count	1
PLAGL2	-0.4428731	0.4945637	-0.8955	0.371	-0.378640219	count	1
CASC15	-0.5115157	0.2843816	-1.7987	0.0722	-0.378567599	count	1
ZNF331	-0.3071897	0.2620975	-1.172	0.241	-0.378532685	count	1
AC008050.1	-0.909681	0.6681768	-1.3614	0.173	-0.378298013	count	1
PEBP1	-0.2638883	0.0450621	-5.8561	5.30E-09	-0.378084879	count	0.000125334
KANSL3	-0.3741407	0.2674861	-1.3987	0.162	-0.377928791	count	1

TADA3	-0.2794617	0.1553729	-1.7987	0.0722	-0.377672956	count	1
AC008267.5	-0.3698945	0.3900961	-0.9482	0.343	-0.377368296	count	1
ENC1	-0.4088585	0.3225248	-1.2677	0.205	-0.377245929	count	1
RAB5IF	-0.2766036	0.1171483	-2.3611	0.0183	-0.377132934	count	1
NCKAP1	-0.2703707	0.1044521	-2.5885	0.00969	-0.37703989	count	1
CAVIN3	-0.2651297	0.0686855	-3.8601	0.000116	-0.376788532	count	1
WASHC5	-0.3120074	0.2648783	-1.1779	0.239	-0.376734533	count	1
NDUFS5	-0.2624766	0.0451583	-5.8124	6.87E-09	-0.376016995	count	0.000162407
PTGR2	-0.5528877	0.3350376	-1.6502	0.099	-0.375917904	count	1
LOXL1	-0.5523171	0.8192153	-0.6742	0.5	-0.375490936	count	1
PPM1M	-0.3094619	0.2419071	-1.2793	0.201	-0.375489144	count	1
GPI	-0.2805258	0.1356185	-2.0685	0.0387	-0.375389253	count	1
ERBB2	-0.3277387	0.3198871	-1.0245	0.306	-0.37537977	count	1
ARHGEF2	-0.2879788	0.1813788	-1.5877	0.112	-0.374426735	count	1
MADCAM1	-1.3658211	0.8660261	-1.5771	0.115	-0.374406419	count	1
PSMB4	-0.2639544	0.0642586	-4.1077	4.11E-05	-0.37439565	count	0.9477249
AC021092.1	-1.0687252	0.7920203	-1.3494	0.177	-0.374277952	count	1
AC100803.3	-0.5503957	0.5899667	-0.9329	0.351	-0.374053738	count	1
PPARA	-0.3081465	0.3010953	-1.0234	0.306	-0.37386529	count	1
ST6GALNAC4	-0.2867551	0.1552114	-1.8475	0.0648	-0.373774283	count	1
UBE2S	-0.2669842	0.0983352	-2.715	0.00667	-0.37368646	count	1
NSUN4	-0.3276498	0.2917482	-1.1231	0.262	-0.37360409	count	1
HSPA13	-0.3175971	0.256612	-1.2377	0.216	-0.373487737	count	1
HIST1H2BC	-0.4472177	0.5890552	-0.7592	0.448	-0.373138237	count	1
KIF5B	-0.2629636	0.067424	-3.9001	9.84E-05	-0.37309798	count	1
PCK2	-0.3454636	0.3470759	-0.9954	0.32	-0.37287263	count	1
DUSP16	-0.3815975	0.4272264	-0.8932	0.372	-0.372425656	count	1
UBXN6	-0.2846732	0.150836	-1.8873	0.0592	-0.372231602	count	1
LIMA1	-0.2605419	0.0578647	-4.5026	6.99E-06	-0.372066054	count	0.16242663
URB2	-0.4858862	0.4701196	-1.0335	0.301	-0.371970139	count	1
HCFC2	-0.2903388	0.2003844	-1.4489	0.147	-0.37191177	count	1
TRIM37	-0.2961084	0.2265802	-1.3069	0.191	-0.371875307	count	1
MANF	-0.2695215	0.1036587	-2.6001	0.00937	-0.371730129	count	1
NCBP1	-0.3421249	0.3066744	-1.1156	0.265	-0.371686664	count	1
MYO1B	-0.3171234	0.2691299	-1.1783	0.239	-0.371624256	count	1
NME4	-0.2671723	0.1022192	-2.6137	0.009	-0.371521548	count	1
RDH10	-0.3966723	0.3376027	-1.175	0.24	-0.371521265	count	1
JCAD	-0.2683352	0.1347931	-1.9907	0.0466	-0.371157091	count	1
TBX3	-0.7874296	0.2983991	-2.6388	0.00837	-0.371142747	count	1
DXO	-0.3394427	0.300553	-1.1294	0.259	-0.371095263	count	1
NDUFS8	-0.2621168	0.0776815	-3.3742	0.00075	-0.370983539	count	1
TOP1MT	-0.331549	0.2766345	-1.1985	0.231	-0.3707771	count	1
DCTD	-0.2767801	0.1279269	-2.1636	0.0306	-0.370674931	count	1
KIAA0930	-0.3538764	0.2603974	-1.359	0.174	-0.370621652	count	1
SPSB2	-0.3264	0.3448477	-0.9466	0.344	-0.370396717	count	1

C2CD4D	-0.6532429	0.6960741	-0.9385	0.348	-0.370361782	count	1
SYT17	-0.7854356	0.5549028	-1.4154	0.157	-0.370039205	count	1
SRPX	-0.2704442	0.1305819	-2.0711	0.0384	-0.370024218	count	1
TMEM205	-0.2615851	0.0800061	-3.2696	0.00109	-0.369959384	count	1
ILF3	-0.2664082	0.1063813	-2.5043	0.0123	-0.36978098	count	1
TEAD1	-0.2747283	0.1607029	-1.7095	0.0875	-0.369747425	count	1
USP48	-0.2707914	0.1270138	-2.132	0.0331	-0.369721071	count	1
SMIM30	-0.2721404	0.1267077	-2.1478	0.0318	-0.36959227	count	1
TIGD7	-0.5443872	0.5392681	-1.0095	0.313	-0.369564626	count	1
AC083964.1	-1.3520913	0.7327361	-1.8453	0.0651	-0.369533041	count	1
ANKRD35	-1.3520913	0.8056203	-1.6783	0.0934	-0.369533041	count	1
CTH	-0.4546787	0.4831652	-0.941	0.347	-0.369455071	count	1
BBOF1	-0.3888066	0.3591753	-1.0825	0.279	-0.36942853	count	1
PFDN2	-0.2596576	0.060189	-4.314	1.66E-05	-0.36938786	count	0.3843398
JMY	-0.2869436	0.199477	-1.4385	0.15	-0.369136275	count	1
FRS2	-0.3117082	0.2890307	-1.0785	0.281	-0.368857128	count	1
HECTD4	-0.2945193	0.2986312	-0.9862	0.324	-0.368674078	count	1
IMMP2L	-0.2721836	0.1477264	-1.8425	0.0655	-0.368633051	count	1
MMP25-AS1	-0.3491905	0.3787872	-0.9219	0.357	-0.368606205	count	1
UGGT1	-0.2958686	0.2267101	-1.3051	0.192	-0.36851348	count	1
ZNF852	-0.5191296	0.5456857	-0.9513	0.342	-0.368497158	count	1
IFI44	-0.2665215	0.1273975	-2.092	0.0365	-0.368425917	count	1
NPRL3	-0.298919	0.2089938	-1.4303	0.153	-0.368182887	count	1
PET100	-0.2662292	0.106398	-2.5022	0.0124	-0.36785716	count	1
DOLK	-0.303417	0.2328542	-1.303	0.193	-0.367115973	count	1
CKAP5	-0.2978503	0.2033234	-1.4649	0.143	-0.366846103	count	1
NAP1L3	-0.4047246	0.4229394	-0.9569	0.339	-0.366643472	count	1
PTK2	-0.2652693	0.106988	-2.4794	0.0132	-0.366017441	count	1
PGAM1	-0.2582336	0.0671169	-3.8475	0.000122	-0.365814946	count	1
SIX5	-0.3223692	0.2656469	-1.2135	0.225	-0.365734347	count	1
ARL2BP	-0.2586989	0.0794667	-3.2554	0.00115	-0.365692254	count	1
TARBP1	-0.3119281	0.2797787	-1.1149	0.265	-0.365409129	count	1
PCCB	-0.3551356	0.2933639	-1.2106	0.226	-0.365327986	count	1
AP006621.3	-0.3902069	0.463868	-0.8412	0.4	-0.365173196	count	1
ETAA1	-0.2934963	0.2425715	-1.2099	0.226	-0.364868903	count	1
WSB2	-0.2839877	0.1715097	-1.6558	0.0979	-0.364502124	count	1
PRRC2A	-0.2803981	0.1766309	-1.5875	0.113	-0.364151114	count	1
SREBF1	-0.3891609	0.3516839	-1.1066	0.269	-0.364146991	count	1
MEN1	-0.3572116	0.4038971	-0.8844	0.377	-0.363949724	count	1
UROD	-0.2625953	0.1153103	-2.2773	0.0228	-0.363879413	count	1
UNC119	-0.2852693	0.2131797	-1.3382	0.181	-0.363152995	count	1
ZNF518A	-0.2766377	0.1759677	-1.5721	0.116	-0.362989919	count	1
SYNJ2	-0.299932	0.2662897	-1.1263	0.26	-0.362826515	count	1
LCP1	-0.8777272	0.5403577	-1.6243	0.104	-0.362354361	count	1
DNPH1	-0.264779	0.141633	-1.8695	0.0617	-0.362235097	count	1

SLC22A4	-0.3714967	0.3354743	-1.1074	0.268	-0.362148274	count	1
OBSCN	-0.8771209	0.7250577	-1.2097	0.226	-0.362053447	count	1
LINC01132	-0.8771209	0.7263714	-1.2075	0.227	-0.362053447	count	1
AP001347.1	-0.5625293	0.6271503	-0.897	0.37	-0.362052565	count	1
RNF180	-0.2964098	0.2354491	-1.2589	0.208	-0.361931596	count	1
LTB4R	-0.3757712	0.3524711	-1.0661	0.286	-0.361626484	count	1
HOXD4	-0.4585336	0.7128849	-0.6432	0.52	-0.361584806	count	1
FXYD1	-0.5962701	0.6274762	-0.9503	0.342	-0.361519742	count	1
RETREG3	-0.3109009	0.2705444	-1.1492	0.251	-0.361517862	count	1
AC016065.1	-0.3808342	0.398507	-0.9557	0.339	-0.361505086	count	1
SLC26A6	-0.6950641	0.6627075	-1.0488	0.294	-0.361440389	count	1
ARHGAP33	-0.7697117	0.9715947	-0.7922	0.428	-0.361362765	count	1
LINC00476	-0.317081	0.2967929	-1.0684	0.285	-0.361267665	count	1
COMT	-0.2547423	0.0733316	-3.4738	0.000521	-0.361172365	count	1
MSANTD4	-0.3117802	0.2912875	-1.0704	0.285	-0.361163198	count	1
KDELR2	-0.253992	0.0699096	-3.6331	0.000285	-0.360475854	count	1
NATD1	-0.5598787	0.462583	-1.2103	0.226	-0.36016384	count	1
MFSD12	-0.2855947	0.2390647	-1.1946	0.232	-0.360127662	count	1
MAN2B2	-0.3239169	0.2844689	-1.1387	0.255	-0.360045167	count	1
GPATCH8	-0.263641	0.143212	-1.8409	0.0657	-0.359908923	count	1
IZUMO4	-1.0354877	0.8843408	-1.1709	0.242	-0.359852811	count	1
BDH1	-1.0354877	0.8870855	-1.1673	0.243	-0.359852811	count	1
AL365356.5	-1.0354877	0.8870855	-1.1673	0.243	-0.359852811	count	1
ABHD1	-1.0354877	0.9139577	-1.133	0.257	-0.359852811	count	1
AC010680.3	-1.0354877	0.9139577	-1.133	0.257	-0.359852811	count	1
ANKH	-0.2916566	0.1973986	-1.4775	0.14	-0.359823099	count	1
MPC1	-0.2584395	0.1082237	-2.388	0.017	-0.359643046	count	1
AC006449.2	-0.4706186	0.6239976	-0.7542	0.451	-0.359379928	count	1
TPR	-0.2519155	0.0629012	-4.0049	6.37E-05	-0.359111122	count	1
MYL5	-0.3329054	0.2825115	-1.1784	0.239	-0.358913793	count	1
ZER1	-0.3025244	0.2475061	-1.2223	0.222	-0.358912224	count	1
TBX19	-0.6904826	0.5041434	-1.3696	0.171	-0.358703011	count	1
WDR59	-0.3427823	0.320629	-1.0691	0.285	-0.358614617	count	1
MAN2A1	-0.280125	0.2195089	-1.2761	0.202	-0.358266177	count	1
ADPRHL2	-0.2767269	0.1921886	-1.4399	0.15	-0.357666976	count	1
JAM3	-0.2764333	0.17373	-1.5912	0.112	-0.357628173	count	1
TACR2	-0.8678991	0.9329099	-0.9303	0.352	-0.357484018	count	1
DYNLT3	-0.2583033	0.1162978	-2.2211	0.0264	-0.35722809	count	1
ATP6V0E1	-0.2487886	0.041071	-6.0575	1.57E-09	-0.356724202	count	3.72E-05
ZNF28	-0.3759939	0.3595384	-1.0458	0.296	-0.356700887	count	1
KDM2B	-0.5270492	0.482505	-1.0923	0.275	-0.356656037	count	1
APOL4	-0.3707108	0.4310386	-0.86	0.39	-0.356543267	count	1
BAD	-0.2533634	0.0827907	-3.0603	0.00223	-0.356526631	count	1
AL162431.2	-1.3150733	1.0751091	-1.2232	0.221	-0.356471233	count	1
LINC01018	-1.3150733	1.0751091	-1.2232	0.221	-0.356471233	count	1

ELOVL4	-1.3150733	1.0751091	-1.2232	0.221	-0.356471233	count	1
AC011287.1	-1.3150733	1.0751091	-1.2232	0.221	-0.356471233	count	1
VIPR2	-1.3150733	1.0751091	-1.2232	0.221	-0.356471233	count	1
TMEM35A	-1.3150733	1.0751091	-1.2232	0.221	-0.356471233	count	1
AC083837.1	-1.3150733	1.0751091	-1.2232	0.221	-0.356471233	count	1
AC023632.2	-1.3150733	1.0751091	-1.2232	0.221	-0.356471233	count	1
OPLAH	-1.3150733	1.0751091	-1.2232	0.221	-0.356471233	count	1
RDH16	-1.3150733	1.0751091	-1.2232	0.221	-0.356471233	count	1
AC026401.1	-1.3150733	1.0751091	-1.2232	0.221	-0.356471233	count	1
JPH2	-1.3150733	1.0751091	-1.2232	0.221	-0.356471233	count	1
LINC01480	-1.3150733	1.0751091	-1.2232	0.221	-0.356471233	count	1
OSM	-1.3150733	1.0751091	-1.2232	0.221	-0.356471233	count	1
AL627309.1	-1.3150733	1.147367	-1.1462	0.252	-0.356471233	count	1
AL360270.3	-1.3150733	1.147367	-1.1462	0.252	-0.356471233	count	1
AL355304.1	-1.3150733	1.147367	-1.1462	0.252	-0.356471233	count	1
JAZF1-AS1	-1.3150733	1.147367	-1.1462	0.252	-0.356471233	count	1
AC010834.2	-1.3150733	1.147367	-1.1462	0.252	-0.356471233	count	1
Z95114.4	-1.3150733	1.147367	-1.1462	0.252	-0.356471233	count	1
IFNLR1	-1.3150733	1.457547	-0.9023	0.367	-0.356471233	count	1
AL133523.1	-1.3150733	1.457547	-0.9023	0.367	-0.356471233	count	1
GSEC	-0.3704937	0.4678229	-0.792	0.428	-0.356325303	count	1
MOB1B	-0.3237848	0.266789	-1.2136	0.225	-0.355713755	count	1
PARK7	-0.2481371	0.045696	-5.4302	6.12E-08	-0.355581807	count	0.001440648
NOL6	-0.4084423	0.4277407	-0.9549	0.34	-0.3555553	count	1
TSC22D1-AS1	-0.8633369	0.5647504	-1.5287	0.126	-0.355228653	count	1
EVI2B	-1.024651	0.6262015	-1.6363	0.102	-0.355181508	count	1
RETREG2	-0.263965	0.1408135	-1.8746	0.061	-0.355030421	count	1
EEF1A2	-1.3106704	0.5955635	-2.2007	0.0278	-0.354925451	count	1
GEMIN5	-0.3061621	0.2588548	-1.1828	0.237	-0.354514867	count	1
DLGAP1-AS2	-0.5238525	0.6685059	-0.7836	0.433	-0.354283386	count	1
WWTR1-AS1	-0.5238525	0.6702653	-0.7816	0.435	-0.354283386	count	1
C7orf43	-0.4069633	0.4098514	-0.993	0.321	-0.354194279	count	1
EPC2	-0.265759	0.1545082	-1.72	0.0855	-0.353946826	count	1
LATS2	-0.2806415	0.1964884	-1.4283	0.153	-0.353802649	count	1
CASTOR3	-1.3074454	0.7074534	-1.8481	0.0647	-0.353794256	count	1
PRPF40B	-0.4065248	0.3705015	-1.0972	0.273	-0.353790841	count	1
BTBD2	-0.2804294	0.2206885	-1.2707	0.204	-0.353531862	count	1
AC026471.1	-0.3629151	0.327711	-1.1074	0.268	-0.353432773	count	1
P3H1	-0.3131633	0.2482171	-1.2617	0.207	-0.353323265	count	1
AHCTF1	-0.2673566	0.1570391	-1.7025	0.0888	-0.353298318	count	1
CCDC9B	-1.0202334	0.6447268	-1.5824	0.114	-0.353281832	count	1
SENP3	-0.479844	0.4636063	-1.035	0.301	-0.353218713	count	1
LUC7L3	-0.2505052	0.0975416	-2.5682	0.0103	-0.353143939	count	1
AC096992.2	-1.019685	0.6385315	-1.5969	0.11	-0.353046158	count	1
IER5L	-0.2504883	0.1025128	-2.4435	0.0146	-0.352626445	count	1

LAT	-0.4790486	0.5961243	-0.8036	0.422	-0.352585021	count	1
CD3D	-2.013772	0.8139441	-2.4741	0.0134	-0.352510177	count	1
ATP6V1D	-0.2603303	0.1315643	-1.9787	0.0479	-0.352487588	count	1
ZNF587	-0.3342182	0.4248906	-0.7866	0.432	-0.352296459	count	1
TANC2	-0.2873792	0.2375683	-1.2097	0.227	-0.352279464	count	1
NUBP1	-0.2607594	0.1553953	-1.678	0.0935	-0.352248488	count	1
MAGOHB	-0.2620206	0.1452332	-1.8041	0.0713	-0.351975283	count	1
2-Sep	-0.2476658	0.0616279	-4.0187	6.01E-05	-0.351955938	count	1
MEGF8	-0.4338804	0.4188612	-1.0359	0.3	-0.351428699	count	1
AC099791.2	-0.4774255	0.5242421	-0.9107	0.363	-0.351292354	count	1
LRP8	-0.4774255	0.5648466	-0.8452	0.398	-0.351292354	count	1
CD9	-0.2441994	0.0497319	-4.9103	9.62E-07	-0.351237028	count	0.022495408
KAT14	-0.3607137	0.3633738	-0.9927	0.321	-0.351199423	count	1
KCNQ1	-0.8551209	0.5772737	-1.4813	0.139	-0.35117571	count	1
SLC27A3	-0.2682636	0.1700214	-1.5778	0.115	-0.351123066	count	1
STX6	-0.2645699	0.1614751	-1.6385	0.101	-0.351031201	count	1
DECR2	-0.2802443	0.2253145	-1.2438	0.214	-0.350567303	count	1
NDUFS6	-0.2477966	0.0667779	-3.7108	0.000211	-0.350512006	count	1
MIR181A1HG	-0.4594699	0.4017155	-1.1438	0.253	-0.350219643	count	1
EIF5	-0.2467362	0.0617012	-3.9989	6.53E-05	-0.350148182	count	1
CPOX	-0.4592025	0.3630214	-1.2649	0.206	-0.350000271	count	1
ERV3-1	-0.3047153	0.2931238	-1.0395	0.299	-0.349928033	count	1
SLC7A7	-0.3058713	0.2798039	-1.0932	0.274	-0.349769485	count	1
MORN1	-0.5786062	0.5116703	-1.1308	0.258	-0.349572542	count	1
PCDHB12	-0.5447195	0.6275286	-0.868	0.385	-0.349391784	count	1
SPRED3	-0.5447195	0.6282154	-0.8671	0.386	-0.349391784	count	1
TP53I13	-0.26199	0.1542642	-1.6983	0.0896	-0.349244211	count	1
CUEDC2	-0.2489585	0.0882173	-2.8221	0.00481	-0.349154897	count	1
SAXO2	-0.4740984	0.9013656	-0.526	0.599	-0.3486445	count	1
ARF4-AS1	-0.5771859	0.6591357	-0.8757	0.381	-0.348614873	count	1
ALKBH5	-0.2643175	0.1708464	-1.5471	0.122	-0.348589053	count	1
ZMAT1	-0.279883	0.1915251	-1.4613	0.144	-0.348329744	count	1
PRKD3	-0.2544939	0.1272032	-2.0007	0.0455	-0.348254232	count	1
AC009053.2	-0.6186372	0.6049663	-1.0226	0.307	-0.348230651	count	1
MYH11	-0.3624153	0.5763614	-0.6288	0.53	-0.348221688	count	1
CTSZ	-0.2439264	0.0646234	-3.7746	0.000164	-0.348202862	count	1
DMPK	-0.3728709	0.4383474	-0.8506	0.395	-0.348194838	count	1
MOGS	-0.3132218	0.2362171	-1.326	0.185	-0.347851384	count	1
CBX1	-0.2529683	0.1380057	-1.833	0.0669	-0.347778566	count	1
MZB1	-1.992409	0.8599656	-2.3168	0.0206	-0.347637732	count	1
PCSK4	-1.992409	0.8599656	-2.3168	0.0206	-0.347637732	count	1
CD2	-1.992409	1.224583	-1.627	0.104	-0.347637732	count	1
GHRLOS	-1.992409	1.224583	-1.627	0.104	-0.347637732	count	1
UNC13A	-1.992409	1.224583	-1.627	0.104	-0.347637732	count	1
SYT5	-1.992409	1.224583	-1.627	0.104	-0.347637732	count	1

WDR63	-1.992409	1.277794	-1.5593	0.119	-0.347637732	count	1
TMEM130	-1.992409	1.277794	-1.5593	0.119	-0.347637732	count	1
COPRS	-0.2478717	0.0862648	-2.8734	0.00409	-0.34751497	count	1
PARD6A	-0.2903977	0.2423605	-1.1982	0.231	-0.347352266	count	1
ARHGAP44	-0.5749294	0.581601	-0.9885	0.323	-0.347094256	count	1
LDOC1	-0.2652101	0.17121	-1.549	0.121	-0.347091045	count	1
NUMB	-0.2571795	0.1413981	-1.8188	0.069	-0.347017593	count	1
ACP6	-0.4174659	0.473079	-0.8824	0.378	-0.346774572	count	1
OSBPL7	-0.3772318	0.3884636	-0.9711	0.332	-0.346659203	count	1
CD59	-0.2409885	0.0354099	-6.8057	1.23E-11	-0.346571339	count	2.93E-07
AC074032.1	-0.3604929	0.2919296	-1.2349	0.217	-0.346295269	count	1
EVA1B	-0.245343	0.0930361	-2.6371	0.00841	-0.346010926	count	1
ZFHX3	-0.2761228	0.2146914	-1.2861	0.199	-0.345904535	count	1
MAPK6	-0.2813611	0.2079958	-1.3527	0.176	-0.345522363	count	1
TMEM115	-0.259345	0.1476687	-1.7563	0.0792	-0.34551928	count	1
PDLIM7	-0.2492854	0.1115938	-2.2339	0.0256	-0.344710143	count	1
CBWD5	-0.315686	0.2281872	-1.3835	0.167	-0.344413432	count	1
CUL9	-0.425586	0.5002778	-0.8507	0.395	-0.344266946	count	1
ERCC1	-0.2508283	0.1331636	-1.8836	0.0597	-0.344228524	count	1
PRNP	-0.2529515	0.1388128	-1.8222	0.0685	-0.343887655	count	1
KANSL1L	-0.3007504	0.235218	-1.2786	0.201	-0.343782559	count	1
FBXO34	-0.2537621	0.1345757	-1.8856	0.0594	-0.343763646	count	1
SNX7	-0.2555533	0.1433825	-1.7823	0.0748	-0.34364621	count	1
FAM173A	-0.2517895	0.1431109	-1.7594	0.0786	-0.343566326	count	1
BOP1	-0.3093975	0.2423109	-1.2769	0.202	-0.343495753	count	1
TMEM160	-0.2448303	0.0959826	-2.5508	0.0108	-0.343329051	count	1
FAM120A	-0.2518596	0.1553077	-1.6217	0.105	-0.343227917	count	1
RNF44	-0.2823477	0.2145668	-1.3159	0.188	-0.342891842	count	1
AC005332.5	-0.4856693	0.5683348	-0.8545	0.393	-0.342699255	count	1
GNG5	-0.238253	0.0352684	-6.7554	1.73E-11	-0.342224786	count	4.12E-07
CLCF1	-0.3364112	0.3132027	-1.0741	0.283	-0.342009171	count	1
MLYCD	-0.3003516	0.2695998	-1.1141	0.265	-0.341775236	count	1
TMEM54	-0.2539068	0.1315058	-1.9308	0.0536	-0.341552473	count	1
ID2	-0.2402604	0.0952148	-2.5234	0.0117	-0.341497852	count	1
LAMP1	-0.2414731	0.0748894	-3.2244	0.00128	-0.341411505	count	1
C5	-0.9923407	0.5630175	-1.7625	0.0781	-0.341349059	count	1
DUSP26	-0.7330188	0.6084498	-1.2047	0.228	-0.341294659	count	1
TMEM117	-0.6611177	0.5027272	-1.3151	0.189	-0.341258359	count	1
AL022328.4	-0.5329151	0.5619482	-0.9483	0.343	-0.341039249	count	1
ARID5A	-0.2426986	0.1007714	-2.4084	0.0161	-0.340809655	count	1
ZSCAN30	-0.3235859	0.2627102	-1.2317	0.218	-0.340739148	count	1
FAXDC2	-0.2547188	0.2135258	-1.1929	0.233	-0.34062578	count	1
TM2D1	-0.2428125	0.0924969	-2.6251	0.00871	-0.340579028	count	1
NAALADL2	-0.3383283	0.2816114	-1.2014	0.23	-0.34044617	count	1
EVC2	-0.6595349	0.5084291	-1.2972	0.195	-0.340323032	count	1

POFUT2	-0.3418015	0.2934741	-1.1647	0.244	-0.340269279	count	1
METTL26	-0.2397885	0.0762196	-3.146	0.00167	-0.339751864	count	1
DNAH1	-0.6581622	0.7040365	-0.9348	0.35	-0.339512293	count	1
TMED2	-0.2415016	0.0808664	-2.9864	0.00285	-0.339336104	count	1
PRKCSH	-0.2502309	0.1221468	-2.0486	0.0406	-0.339270793	count	1
AC069224.1	-0.729197	0.528642	-1.3794	0.168	-0.339219054	count	1
GGCX	-0.2687978	0.1811942	-1.4835	0.138	-0.339182293	count	1
PRDM4	-0.2994847	0.2686641	-1.1147	0.265	-0.339175673	count	1
TRAF7	-0.2566583	0.161924	-1.5851	0.113	-0.339051056	count	1
LMOD1	-0.6039668	0.7284085	-0.8292	0.407	-0.338925093	count	1
AC008764.6	-0.7285972	0.8196359	-0.8889	0.374	-0.338893545	count	1
ROR1	-0.7285972	0.8509527	-0.8562	0.392	-0.338893545	count	1
ABCC10	-0.3902856	0.4743021	-0.8229	0.411	-0.338880411	count	1
ZHX2	-0.3442959	0.3614141	-0.9526	0.341	-0.338819304	count	1
ZNF229	-0.9858975	0.6936576	-1.4213	0.155	-0.338607985	count	1
ACACA	-0.3482164	0.3284395	-1.0602	0.289	-0.338539421	count	1
RABGGTB	-0.2506766	0.1345594	-1.8629	0.0626	-0.338312222	count	1
WWC3	-0.2659503	0.2198973	-1.2094	0.227	-0.338282502	count	1
LMF1	-0.2889764	0.3120255	-0.9261	0.354	-0.337997574	count	1
RBM14-RBM4	-0.8280173	0.7211175	-1.1482	0.251	-0.337886017	count	1
BEND3	-0.8280173	0.7534022	-1.099	0.272	-0.337886017	count	1
ANXA5	-0.2353054	0.0462378	-5.089	3.84E-07	-0.336805614	count	0.00899904
RPS6KB1	-0.2559833	0.2027255	-1.2627	0.207	-0.336612254	count	1
DYNC1I1	-0.3604461	0.6280819	-0.5739	0.566	-0.336065414	count	1
CDC42EP2	-0.2464944	0.1344679	-1.8331	0.0669	-0.335880259	count	1
EIF3A	-0.2380314	0.0914788	-2.602	0.00932	-0.335867587	count	1
CBX6	-0.2465685	0.126014	-1.9567	0.0505	-0.335805963	count	1
MARS	-0.3005032	0.2614539	-1.1494	0.251	-0.335216604	count	1
DCUN1D3	-0.2689718	0.1991071	-1.3509	0.177	-0.335153393	count	1
TNRC6A	-0.2485608	0.1583959	-1.5692	0.117	-0.33507764	count	1
PLN	-0.8218915	0.7408458	-1.1094	0.267	-0.334899664	count	1
ZNF287	-0.3489996	0.378214	-0.9228	0.356	-0.334793903	count	1
ZNF276	-0.2862214	0.217027	-1.3188	0.187	-0.334712287	count	1
SERGEF	-0.2583059	0.176952	-1.4598	0.144	-0.334582079	count	1
SARAF	-0.2350522	0.057975	-4.0544	5.17E-05	-0.334405677	count	1
AK3	-0.2399686	0.1174319	-2.0435	0.0411	-0.33423232	count	1
TALDO1	-0.2348303	0.0644928	-3.6412	0.000276	-0.333490385	count	1
ARHGEF10	-0.2974043	0.3076721	-0.9666	0.334	-0.333433497	count	1
SS18L1	-0.3697601	0.3310958	-1.1168	0.264	-0.333418421	count	1
PDE4D	-0.2496547	0.1950228	-1.2801	0.201	-0.333017841	count	1
AP001453.4	-1.2472518	0.9189287	-1.3573	0.175	-0.332853504	count	1
SCNN1D	-1.2472518	0.9584843	-1.3013	0.193	-0.332853504	count	1
AC073610.3	-1.2472518	0.9584843	-1.3013	0.193	-0.332853504	count	1
AC010889.1	-1.2472518	0.9584843	-1.3013	0.193	-0.332853504	count	1
LINC00654	-1.2472518	1.237323	-1.008	0.314	-0.332853504	count	1

VPS37B	-0.2537492	0.1911132	-1.3277	0.184	-0.332463475	count	1
WDR24	-0.3754933	0.4806358	-0.7812	0.435	-0.332316654	count	1
WWP2	-0.2804555	0.2344069	-1.1964	0.232	-0.332260015	count	1
SLC38A7	-0.3913299	0.4410054	-0.8874	0.375	-0.332086114	count	1
SNRPD3	-0.2337867	0.0641028	-3.6471	0.00027	-0.331906522	count	1
MRPL57	-0.2340434	0.0701331	-3.3371	0.000858	-0.331846586	count	1
COX8A	-0.2322738	0.0526418	-4.4123	1.06E-05	-0.331663439	count	0.2458988
SORD	-0.3232153	0.3155045	-1.0244	0.306	-0.331400793	count	1
PIAS3	-0.2683594	0.2147244	-1.2498	0.211	-0.331330519	count	1
9-Mar	-0.270689	0.2173856	-1.2452	0.213	-0.330795891	count	1
ABHD5	-0.2394534	0.1310516	-1.8272	0.0678	-0.33062447	count	1
BET1L	-0.2765625	0.2895278	-0.9552	0.34	-0.330521357	count	1
LARP4B	-0.259151	0.2242864	-1.1554	0.248	-0.330362309	count	1
COX5A	-0.231598	0.0546617	-4.2369	2.34E-05	-0.33031771	count	0.541008
REEP3	-0.2354609	0.1055489	-2.2308	0.0258	-0.330007981	count	1
AC254633.1	-1.914284	1.229147	-1.5574	0.119	-0.329830055	count	1
LINC01635	-1.914284	1.229147	-1.5574	0.119	-0.329830055	count	1
MAEL	-1.914284	1.229147	-1.5574	0.119	-0.329830055	count	1
CFHR3	-1.914284	1.229147	-1.5574	0.119	-0.329830055	count	1
AC013472.3	-1.914284	1.229147	-1.5574	0.119	-0.329830055	count	1
CREG2	-1.914284	1.229147	-1.5574	0.119	-0.329830055	count	1
TMEM163	-1.914284	1.229147	-1.5574	0.119	-0.329830055	count	1
POLQ	-1.914284	1.229147	-1.5574	0.119	-0.329830055	count	1
HCG9	-1.914284	1.229147	-1.5574	0.119	-0.329830055	count	1
LINC01186	-1.914284	1.229147	-1.5574	0.119	-0.329830055	count	1
NEFM	-1.914284	1.229147	-1.5574	0.119	-0.329830055	count	1
RECQL4	-1.914284	1.229147	-1.5574	0.119	-0.329830055	count	1
AF186192.1	-1.914284	1.229147	-1.5574	0.119	-0.329830055	count	1
BMS1P14	-1.914284	1.229147	-1.5574	0.119	-0.329830055	count	1
LBHD1	-1.914284	1.229147	-1.5574	0.119	-0.329830055	count	1
AP000904.1	-1.914284	1.229147	-1.5574	0.119	-0.329830055	count	1
AL356020.1	-1.914284	1.229147	-1.5574	0.119	-0.329830055	count	1
ARHGAP11A	-1.914284	1.229147	-1.5574	0.119	-0.329830055	count	1
AC004494.1	-1.914284	1.229147	-1.5574	0.119	-0.329830055	count	1
SPIRE2	-1.914284	1.229147	-1.5574	0.119	-0.329830055	count	1
GP1BA	-1.914284	1.229147	-1.5574	0.119	-0.329830055	count	1
AC006441.1	-1.914284	1.229147	-1.5574	0.119	-0.329830055	count	1
ELAVL3	-1.914284	1.229147	-1.5574	0.119	-0.329830055	count	1
ZNF20	-1.914284	1.229147	-1.5574	0.119	-0.329830055	count	1
A1BG-AS1	-1.914284	1.229147	-1.5574	0.119	-0.329830055	count	1
AC006547.3	-1.914284	1.229147	-1.5574	0.119	-0.329830055	count	1
TMEM240	-1.914284	1.336383	-1.4324	0.152	-0.329830055	count	1
PIK3CD-AS2	-1.914284	1.336383	-1.4324	0.152	-0.329830055	count	1
FHAD1	-1.914284	1.336383	-1.4324	0.152	-0.329830055	count	1
AGMAT	-1.914284	1.336383	-1.4324	0.152	-0.329830055	count	1

AC005034.4	-1.914284	1.336383	-1.4324	0.152	-0.329830055	count	1
AC093388.1	-1.914284	1.336383	-1.4324	0.152	-0.329830055	count	1
TXK	-1.914284	1.336383	-1.4324	0.152	-0.329830055	count	1
TLR2	-1.914284	1.336383	-1.4324	0.152	-0.329830055	count	1
LINC02363	-1.914284	1.336383	-1.4324	0.152	-0.329830055	count	1
HIST1H4J	-1.914284	1.336383	-1.4324	0.152	-0.329830055	count	1
LINC01564	-1.914284	1.336383	-1.4324	0.152	-0.329830055	count	1
BMP5	-1.914284	1.336383	-1.4324	0.152	-0.329830055	count	1
MCF2	-1.914284	1.336383	-1.4324	0.152	-0.329830055	count	1
TEX15	-1.914284	1.336383	-1.4324	0.152	-0.329830055	count	1
SLC26A7	-1.914284	1.336383	-1.4324	0.152	-0.329830055	count	1
AP003696.1	-1.914284	1.336383	-1.4324	0.152	-0.329830055	count	1
RIMS2	-1.914284	1.336383	-1.4324	0.152	-0.329830055	count	1
ZNF252P-AS1	-1.914284	1.336383	-1.4324	0.152	-0.329830055	count	1
GNA14-AS1	-1.914284	1.336383	-1.4324	0.152	-0.329830055	count	1
AC107884.1	-1.914284	1.336383	-1.4324	0.152	-0.329830055	count	1
AL391684.1	-1.914284	1.336383	-1.4324	0.152	-0.329830055	count	1
FAM71D	-1.914284	1.336383	-1.4324	0.152	-0.329830055	count	1
AC012651.1	-1.914284	1.336383	-1.4324	0.152	-0.329830055	count	1
AC073941.1	-1.914284	1.336383	-1.4324	0.152	-0.329830055	count	1
WDR93	-1.914284	1.336383	-1.4324	0.152	-0.329830055	count	1
PRC1-AS1	-1.914284	1.336383	-1.4324	0.152	-0.329830055	count	1
ZFR2	-1.914284	1.336383	-1.4324	0.152	-0.329830055	count	1
AC008752.1	-1.914284	1.336383	-1.4324	0.152	-0.329830055	count	1
AC020928.3	-1.914284	1.336383	-1.4324	0.152	-0.329830055	count	1
SPIB	-1.914284	1.336383	-1.4324	0.152	-0.329830055	count	1
ZRANB3	-0.4902088	0.4485079	-1.093	0.274	-0.329453179	count	1
U73166.1	-0.6409406	0.4882054	-1.3129	0.189	-0.329373759	count	1
ARID1A	-0.2557926	0.1669844	-1.5318	0.126	-0.329353899	count	1
XRN1	-0.2389087	0.1182554	-2.0203	0.0435	-0.329347742	count	1
COX7B	-0.2315354	0.0576744	-4.0145	6.12E-05	-0.329324124	count	1
SLIT2	-0.6405228	0.6056671	-1.0575	0.29	-0.329128569	count	1
TFDP2	-0.2390121	0.1278294	-1.8698	0.0616	-0.329102947	count	1
REST	-0.2368883	0.1048986	-2.2583	0.024	-0.329021235	count	1
NUP205	-0.387454	0.3743825	-1.0349	0.301	-0.328609432	count	1
AXIN2	-0.9622729	0.6203769	-1.5511	0.121	-0.328607837	count	1
CRY2	-0.3033127	0.2823456	-1.0743	0.283	-0.3283871	count	1
FSTL3	-0.4189721	0.4389262	-0.9545	0.34	-0.328291123	count	1
VOPP1	-0.2530845	0.144869	-1.747	0.0808	-0.328057804	count	1
SH3GLB1	-0.2301085	0.0594504	-3.8706	1.00E-04	-0.327774798	count	1
TRMT9B	-0.5861921	0.4783935	-1.2253	0.221	-0.327712421	count	1
DMGDH	-0.6376612	0.6837305	-0.9326	0.351	-0.32745009	count	1
AL121983.1	-0.6376612	0.7484497	-0.852	0.394	-0.32745009	count	1
CANX	-0.2296446	0.060747	-3.7803	2.00E-04	-0.327358209	count	1
AC114490.2	-0.9592445	0.9947243	-0.9643	0.335	-0.32733171	count	1

PSRC1	-0.9592445	0.9947243	-0.9643	0.335	-0.32733171	count	1
FAM72D	-0.9592445	0.9947243	-0.9643	0.335	-0.32733171	count	1
AC009950.1	-0.9592445	0.9947243	-0.9643	0.335	-0.32733171	count	1
FRK	-0.9592445	0.9947243	-0.9643	0.335	-0.32733171	count	1
GET4	-0.9592445	0.9947243	-0.9643	0.335	-0.32733171	count	1
OR52I1	-0.9592445	0.9947243	-0.9643	0.335	-0.32733171	count	1
LINC02550	-0.9592445	0.9947243	-0.9643	0.335	-0.32733171	count	1
C16orf95	-0.9592445	0.9947243	-0.9643	0.335	-0.32733171	count	1
AC092070.3	-0.9592445	0.9947243	-0.9643	0.335	-0.32733171	count	1
ADGRF3	-0.9592445	1.078244	-0.8896	0.374	-0.32733171	count	1
AP002026.1	-0.9592445	1.078244	-0.8896	0.374	-0.32733171	count	1
SLC26A4-AS1	-0.9592445	1.078244	-0.8896	0.374	-0.32733171	count	1
ERICH5	-0.9592445	1.078244	-0.8896	0.374	-0.32733171	count	1
LINC00937	-0.9592445	1.078244	-0.8896	0.374	-0.32733171	count	1
TECTA	-0.9592445	1.229632	-0.7801	0.435	-0.32733171	count	1
GFRA1	-0.9592445	1.229632	-0.7801	0.435	-0.32733171	count	1
DGUOK-AS1	-0.9592445	1.298125	-0.7389	0.46	-0.32733171	count	1
AC114763.1	-0.9592445	1.298125	-0.7389	0.46	-0.32733171	count	1
AC021321.1	-0.9592445	1.298125	-0.7389	0.46	-0.32733171	count	1
GDF10	-0.9592445	1.298125	-0.7389	0.46	-0.32733171	count	1
PELI3	-0.395244	0.4985692	-0.7928	0.428	-0.327213378	count	1
TBC1D7	-0.2877412	0.234259	-1.2283	0.219	-0.327110846	count	1
CARD6	-0.241522	0.149664	-1.6138	0.107	-0.326884137	count	1
TNFRSF10C	-0.3948467	0.4308181	-0.9165	0.359	-0.326864685	count	1
AL355075.4	-0.3948467	0.6261517	-0.6306	0.528	-0.326864685	count	1
CTSS	-0.2327235	0.1054065	-2.2079	0.0273	-0.326713249	count	1
NOMO3	-1.22917	0.6309278	-1.9482	0.0515	-0.326629225	count	1
PCDHGA3	-1.22917	0.7151977	-1.7186	0.0858	-0.326629225	count	1
AC078909.1	-1.22917	0.7988263	-1.5387	0.124	-0.326629225	count	1
BTBD19	-0.5843278	0.4459908	-1.3102	0.19	-0.32654036	count	1
AGPAT5	-0.2408652	0.1683271	-1.4309	0.153	-0.326505705	count	1
C9orf116	-0.5121786	0.5582873	-0.9174	0.359	-0.326442869	count	1
MRPS28	-0.2484687	0.1655927	-1.5005	0.134	-0.326425716	count	1
COX14	-0.2322175	0.0858562	-2.7047	0.00688	-0.326244145	count	1
CTF1	-0.5836559	0.5377987	-1.0853	0.278	-0.32611812	count	1
BNC2-AS1	-0.8030025	0.654892	-1.2262	0.22	-0.325731836	count	1
RGS12	-0.2593351	0.2071901	-1.2517	0.211	-0.325639991	count	1
MLXIP	-0.2458757	0.1519774	-1.6178	0.106	-0.324900546	count	1
VPS11	-0.2867458	0.2727747	-1.0512	0.293	-0.324427207	count	1
VSTM4	-0.3668743	0.5044762	-0.7272	0.467	-0.324300953	count	1
TOR2A	-0.3109148	0.3855031	-0.8065	0.42	-0.324251244	count	1
PPP4R3A	-0.2422522	0.1526844	-1.5866	0.113	-0.324142999	count	1
CBFA2T2	-0.3105695	0.361859	-0.8583	0.391	-0.323879942	count	1
WDFY2	-0.2953932	0.282523	-1.0456	0.296	-0.323730731	count	1
RHBDD2	-0.2388734	0.1324729	-1.8032	0.0715	-0.323589981	count	1

GPR137	-0.2475688	0.2016283	-1.2278	0.22	-0.323561198	count	1
NFATC4	-1.219705	0.9341136	-1.3057	0.192	-0.32338389	count	1
NPC2	-0.2250586	0.0448167	-5.0218	5.45E-07	-0.323274996	count	0.01276063
CD99	-0.2248461	0.0362383	-6.2047	6.30E-10	-0.32320768	count	1.49E-05
AC009061.2	-0.4263776	0.5354135	-0.7964	0.426	-0.323197849	count	1
AL360012.1	-0.4600046	0.6710352	-0.6855	0.493	-0.323085861	count	1
DPY19L2	-0.4600046	0.7295981	-0.6305	0.528	-0.323085861	count	1
BCL2L1	-0.2464496	0.1602975	-1.5375	0.124	-0.323058757	count	1
PRKCZ	-0.3324321	0.3135364	-1.0603	0.289	-0.322595522	count	1
ZNF93	-0.4592197	0.4385891	-1.047	0.295	-0.322488436	count	1
KIF3B	-0.2559534	0.2028977	-1.2615	0.207	-0.322314692	count	1
ACOX3	-0.2977517	0.3277982	-0.9083	0.364	-0.322204695	count	1
PSMD6-AS2	-0.944851	0.7507907	-1.2585	0.208	-0.321284538	count	1
AC022144.1	-0.944851	0.7753899	-1.2185	0.223	-0.321284538	count	1
AC007541.1	-0.3351642	0.3503634	-0.9566	0.339	-0.320985402	count	1
AMPD2	-0.3447656	0.4245528	-0.8121	0.417	-0.320804876	count	1
DYNLL1	-0.2230837	0.0354736	-6.2887	3.71E-10	-0.320585479	count	8.80E-06
MOAP1	-0.2573777	0.1964472	-1.3102	0.19	-0.3205227	count	1
AL590226.1	-0.2448855	0.1934229	-1.2661	0.206	-0.320515458	count	1
NDUFAF8	-0.2337931	0.1104434	-2.1169	0.0344	-0.320492413	count	1
SERPINB9P1	-0.2731992	0.4035704	-0.677	0.498	-0.320325293	count	1
PIK3R3	-0.2349541	0.123621	-1.9006	0.0575	-0.319912474	count	1
SND1-IT1	-0.9408823	0.7443451	-1.264	0.206	-0.319622444	count	1
RPL13A	-0.2219082	0.0229045	-9.6884	7.49E-22	-0.319576904	count	1.81E-17
DMXL1	-0.2393996	0.1933329	-1.2383	0.216	-0.319090931	count	1
AC090515.2	-0.7891344	0.7511606	-1.0506	0.294	-0.31904038	count	1
ZNF582	-0.274034	0.2799654	-0.9788	0.328	-0.31902706	count	1
ATP2A2	-0.2416051	0.1683529	-1.4351	0.151	-0.319018272	count	1
HRASLS	-1.206603	0.6774988	-1.781	0.075	-0.318906039	count	1
AL133551.1	-1.206603	0.7836413	-1.5397	0.124	-0.318906039	count	1
CLCN7	-0.3209953	0.3037693	-1.0567	0.291	-0.31881405	count	1
TNFSF12	-0.2266896	0.0931954	-2.4324	0.0151	-0.318496581	count	1
LRRC58	-0.2595322	0.2583922	-1.0044	0.315	-0.318340426	count	1
PGLS	-0.2242478	0.0693984	-3.2313	0.00125	-0.318280431	count	1
BMP1	-0.2732257	0.2566218	-1.0647	0.287	-0.318068038	count	1
CPNE1	-0.2362076	0.1172945	-2.0138	0.0441	-0.317353262	count	1
ZBTB38	-0.2248771	0.0893258	-2.5175	0.0119	-0.316780839	count	1
NDUFB11	-0.2216725	0.0528792	-4.1921	2.85E-05	-0.316711612	count	0.658293
IGHMBP2	-0.2891314	0.3866761	-0.7477	0.455	-0.316695444	count	1
SIPA1	-0.2666818	0.2379416	-1.1208	0.262	-0.316639135	count	1
PGM2	-0.2339227	0.1364828	-1.7139	0.0867	-0.316337207	count	1
BRD3OS	-0.3453557	0.3424929	-1.0084	0.313	-0.31604833	count	1
ARHGEF12	-0.2280369	0.1253452	-1.8193	0.069	-0.316039337	count	1
CCL21	-0.2940852	0.3323559	-0.8849	0.376	-0.315938947	count	1
CLCN6	-0.4327274	0.4191192	-1.0325	0.302	-0.315933447	count	1

ATP6AP1	-0.2299078	0.1159962	-1.982	0.0476	-0.315831522	count	1
PCNX1	-0.2596487	0.2162254	-1.2008	0.23	-0.315664665	count	1
AKR1B1	-0.2301245	0.1314496	-1.7507	0.0801	-0.315376834	count	1
TRAPPC10	-0.2418453	0.1734812	-1.3941	0.163	-0.315263669	count	1
SAT1	-0.2195056	0.0755609	-2.905	0.0037	-0.315153007	count	1
KPNA1	-0.2526817	0.2128465	-1.1872	0.235	-0.314601055	count	1
B4GAT1	-0.2395371	0.1765071	-1.3571	0.175	-0.314378567	count	1
NOS1AP	-0.3381147	0.4318081	-0.783	0.434	-0.314348183	count	1
FAM171A2	-1.1931329	0.7175918	-1.6627	0.0965	-0.314320325	count	1
TMIGD3	-1.1931329	0.8110659	-1.4711	0.141	-0.314320325	count	1
CLIC5	-0.3041331	0.3326293	-0.9143	0.361	-0.314154153	count	1
PCDHB16	-0.401909	0.4387852	-0.916	0.36	-0.314043108	count	1
FAM214B	-0.2698276	0.3045548	-0.886	0.376	-0.314037347	count	1
ATM	-0.2406816	0.178863	-1.3456	0.179	-0.313988299	count	1
NDFIP1	-0.2220824	0.0735209	-3.0207	0.00255	-0.313847421	count	1
CEP63	-0.235572	0.1581174	-1.4899	0.136	-0.313478183	count	1
TIAM2	-0.4935203	0.5680052	-0.8689	0.385	-0.313393447	count	1
PSMD8	-0.2199229	0.0579583	-3.7945	2.00E-04	-0.31334582	count	1
SEMA3G	-0.3192913	0.316203	-1.0098	0.313	-0.313311707	count	1
ARMC9	-0.3231907	0.396738	-0.8146	0.415	-0.313284743	count	1
BBC3	-0.2446294	0.1879752	-1.3014	0.193	-0.313137177	count	1
TRIM2	-0.2558379	0.3016661	-0.8481	0.396	-0.313075615	count	1
COX6B1	-0.2186111	0.0451276	-4.8443	1.34E-06	-0.313065797	count	0.03131178
LRCH3	-0.2531984	0.2210284	-1.1455	0.252	-0.312967652	count	1
SEPSECS-AS1	-0.3419223	0.4788328	-0.7141	0.475	-0.312764465	count	1
LSM1	-0.2270887	0.1135721	-1.9995	0.0457	-0.312234102	count	1
JTB	-0.2198687	0.0667221	-3.2953	0.000996	-0.312216272	count	1
DAP	-0.2225408	0.091964	-2.4199	0.0156	-0.312041315	count	1
EPOR	-0.3261628	0.3822784	-0.8532	0.394	-0.312023222	count	1
TMEM17	-0.3470787	0.5577629	-0.6223	0.534	-0.312008478	count	1
HNRNPLL	-0.245106	0.1900715	-1.2895	0.197	-0.31188436	count	1
SCFD2	-0.299107	0.3412512	-0.8765	0.381	-0.311567199	count	1
SH3BP2	-0.2296089	0.1832708	-1.2528	0.21	-0.311454655	count	1
BTBD6	-0.2292623	0.1266628	-1.81	0.0704	-0.311438384	count	1
B4GALT1-AS1	-0.3771705	0.5271117	-0.7155	0.474	-0.311387523	count	1
MCM9	-0.3211775	0.3799443	-0.8453	0.398	-0.311258798	count	1
LINC00982	-0.5208874	0.6087615	-0.8557	0.392	-0.311015019	count	1
CSNK1A1	-0.2177211	0.0531775	-4.0942	4.36E-05	-0.310968503	count	1
LSS	-0.4261727	0.5089639	-0.8373	0.402	-0.310787619	count	1
OOEP	-0.6090503	0.916421	-0.6646	0.506	-0.310762301	count	1
NRDE2	-0.257384	0.2604866	-0.9881	0.323	-0.310580701	count	1
MMP14	-0.2689098	0.2332042	-1.1531	0.249	-0.310549687	count	1
HERC2	-0.2236694	0.1226685	-1.8234	0.0684	-0.310460908	count	1
DNASE2	-0.2270239	0.1413933	-1.6056	0.108	-0.310291578	count	1
TAF1C	-0.2909909	0.2548482	-1.1418	0.254	-0.310262756	count	1

RBAK-RBAKDN	-0.2636445	0.2633175	-1.0012	0.317	-0.309978768	count	1
FBXO3	-0.2294296	0.1338142	-1.7145	0.0865	-0.309915701	count	1
UBA5	-0.2333666	0.1535677	-1.5196	0.129	-0.309864805	count	1
NBPF9	-0.5575398	0.6888491	-0.8094	0.418	-0.309782823	count	1
AC009126.1	-0.4245145	0.5460639	-0.7774	0.437	-0.309487446	count	1
EGFL7	-0.2156426	0.0512502	-4.2076	2.66E-05	-0.309226451	count	0.614593
MZT1	-0.2320681	0.1540035	-1.5069	0.132	-0.308784845	count	1
ABCC4	-0.2663479	0.2411685	-1.1044	0.27	-0.308741454	count	1
C8orf33	-0.2255373	0.1183519	-1.9057	0.0568	-0.308321781	count	1
SLC25A20	-0.3142604	0.3063054	-1.026	0.305	-0.308195197	count	1
PPM1A	-0.245399	0.1957694	-1.2535	0.21	-0.307937968	count	1
LINC00839	-0.2885945	0.3032511	-0.9517	0.341	-0.307637704	count	1
HOXB-AS1	-0.4068069	0.5374815	-0.7569	0.449	-0.307337706	count	1
C3orf62	-0.3004292	0.3572836	-0.8409	0.4	-0.307302263	count	1
POLR2B	-0.2274969	0.1362706	-1.6694	0.0951	-0.30729117	count	1
BSDC1	-0.2362253	0.2061895	-1.1457	0.252	-0.307103018	count	1
TET1	-0.3305647	0.3218238	-1.0272	0.304	-0.307030325	count	1
CLDN5	-0.2137902	0.0893148	-2.3937	0.0167	-0.306991181	count	1
PPP6C	-0.2239228	0.1240807	-1.8047	0.0712	-0.30697009	count	1
AL159169.2	-0.6686547	0.8205292	-0.8149	0.415	-0.306715049	count	1
EVC	-0.6686547	0.824043	-0.8114	0.417	-0.306715049	count	1
AC005253.1	-0.6686547	0.824043	-0.8114	0.417	-0.306715049	count	1
PPT2	-0.3353746	0.3244539	-1.0337	0.301	-0.30650918	count	1
SH2B3	-0.2390479	0.1756172	-1.3612	0.174	-0.30626804	count	1
PI4K2A	-0.371237	0.4217866	-0.8802	0.379	-0.306208504	count	1
WEE2-AS1	-1.8097986	0.8231737	-2.1986	0.028	-0.306111537	count	1
AC104984.3	-1.8097986	1.060884	-1.7059	0.0881	-0.306111537	count	1
ADA2	-0.3543569	0.524008	-0.6762	0.499	-0.306102029	count	1
ZNF254	-0.2425171	0.2347082	-1.0333	0.302	-0.306092214	count	1
CH25H	-0.2550005	0.3759518	-0.6783	0.498	-0.306045845	count	1
MPZL3	-0.4373101	0.3814299	-1.1465	0.252	-0.305870775	count	1
MAGT1	-0.2214536	0.1281686	-1.7278	0.0841	-0.304889706	count	1
ALOX5	-0.5114689	0.5623444	-0.9095	0.363	-0.304794535	count	1
UBE3A	-0.2206409	0.108152	-2.0401	0.0414	-0.304605934	count	1
NBEAL1	-0.224255	0.1305532	-1.7177	0.086	-0.304247792	count	1
MN1	-0.3788481	0.424313	-0.8929	0.372	-0.304207649	count	1
MRAS	-0.2257663	0.1587207	-1.4224	0.155	-0.303487357	count	1
PCGF3	-0.2486029	0.2184324	-1.1381	0.255	-0.303431539	count	1
HECA	-0.2322387	0.1792284	-1.2958	0.195	-0.303365547	count	1
ZNF14	-0.2889	0.3196967	-0.9037	0.366	-0.303181499	count	1
PLBD2	-0.2723899	0.3023687	-0.9009	0.368	-0.303151752	count	1
NRCAM	-0.5466649	0.7686137	-0.7112	0.477	-0.303025084	count	1
ZNF71	-0.3128401	0.3565264	-0.8775	0.38	-0.302877751	count	1
MUC12	-0.7552588	0.6772521	-1.1152	0.265	-0.302837757	count	1
GRAMD1A	-0.2179528	0.1080925	-2.0164	0.0439	-0.302537635	count	1

CASC4	-0.2201956	0.1139683	-1.9321	0.0535	-0.30249552	count	1
SFSWAP	-0.2215386	0.1475379	-1.5016	0.133	-0.302405006	count	1
ZNF660	-0.3209311	0.357634	-0.8974	0.37	-0.302395935	count	1
TTC14	-0.2233405	0.1700721	-1.3132	0.189	-0.302347376	count	1
ZNF609	-0.2563966	0.2442526	-1.0497	0.294	-0.302309748	count	1
LDHB	-0.2120415	0.0568213	-3.7317	2.00E-04	-0.302206907	count	1
PTP4A3	-0.2216121	0.1428921	-1.5509	0.121	-0.302132777	count	1
NNT	-0.2686842	0.2666758	-1.0075	0.314	-0.30207361	count	1
ABCB8	-0.291973	0.332354	-0.8785	0.38	-0.301214615	count	1
ZFC3H1	-0.2192759	0.1266185	-1.7318	0.0834	-0.301112562	count	1
NEU3	-0.39903	0.528765	-0.7546	0.451	-0.301060532	count	1
TPP1	-0.2224741	0.1648166	-1.3498	0.177	-0.300974287	count	1
COPZ1	-0.2151919	0.0916927	-2.3469	0.019	-0.300674781	count	1
ZNF273	-0.2510343	0.3268624	-0.768	0.443	-0.300384154	count	1
TMEM126B	-0.216791	0.1187223	-1.826	0.068	-0.300327501	count	1
ARPC5L	-0.2121684	0.0796557	-2.6636	0.00778	-0.300239103	count	1
PLXNB1	-0.5033887	0.6345033	-0.7934	0.428	-0.299474135	count	1
PRELID1	-0.2099255	0.056988	-3.6837	2.00E-04	-0.29935169	count	1
GIN1	-0.292881	0.3450768	-0.8487	0.396	-0.299341836	count	1
MFAP4	-0.3468946	0.5543237	-0.6258	0.531	-0.299331011	count	1
ZBTB43	-0.2441982	0.2398117	-1.0183	0.309	-0.299280512	count	1
COX6C	-0.2085531	0.0417012	-5.0011	6.06E-07	-0.298788458	count	0.014184642
MRPS6	-0.2143993	0.1137165	-1.8854	0.0595	-0.298551842	count	1
TMEM167B	-0.2290869	0.1876323	-1.2209	0.222	-0.298497672	count	1
RAD51B	-0.272683	0.3437874	-0.7932	0.428	-0.298247878	count	1
PUS10	-0.2554566	0.2639432	-0.9678	0.333	-0.298100942	count	1
B4GALT1	-0.213552	0.1079834	-1.9776	0.0481	-0.298080296	count	1
RXYLT1	-0.2216519	0.1640385	-1.3512	0.177	-0.298042909	count	1
LYSMD3	-0.2321734	0.1766551	-1.3143	0.189	-0.297707152	count	1
SUB1	-0.2082688	0.0492943	-4.225	2.47E-05	-0.297656427	count	0.5708664
MEF2D	-0.2370839	0.2212419	-1.0716	0.284	-0.297385905	count	1
FLVCR1-DT	-0.2968703	0.4708555	-0.6305	0.528	-0.297370781	count	1
CDC27	-0.2123656	0.0927574	-2.2895	0.0221	-0.297236809	count	1
FGFR3	-0.8867854	0.4834825	-1.8342	0.0667	-0.297199275	count	1
PSPH	-0.2935357	0.4112229	-0.7138	0.475	-0.297050362	count	1
SEMA4B	-1.766913	0.7218803	-2.4477	0.0144	-0.296434616	count	1
ANXA7	-0.2096316	0.0724112	-2.895	0.00382	-0.296264017	count	1
SLC43A3	-0.2304147	0.1911475	-1.2054	0.228	-0.296070248	count	1
ALKBH8	-0.2562647	0.3235374	-0.7921	0.428	-0.295673681	count	1
TEX261	-0.2370735	0.2279926	-1.0398	0.299	-0.295445231	count	1
SLC35B4	-0.2539897	0.2718335	-0.9344	0.35	-0.295273836	count	1
NR3C1	-0.2097248	0.0831071	-2.5235	0.0117	-0.295247568	count	1
SPIRE1	-0.2409425	0.2181568	-1.1044	0.269	-0.295237444	count	1
IER3	-0.205074	0.0750053	-2.7341	0.00629	-0.295222809	count	1
ANKRD11	-0.2108805	0.0915683	-2.303	0.0214	-0.295110626	count	1

RNF7	-0.2067391	0.0600667	-3.4418	0.000586	-0.295040898	count	1
MTURN	-0.2178181	0.1270546	-1.7144	0.0866	-0.295021448	count	1
UBFD1	-0.2516318	0.2558623	-0.9835	0.325	-0.294602195	count	1
CPPED1	-0.2291195	0.1930021	-1.1871	0.235	-0.294390695	count	1
NPPC	-0.3221971	0.3767994	-0.8551	0.393	-0.293949145	count	1
TSPYL1	-0.2131959	0.1185389	-1.7985	0.0722	-0.293680235	count	1
SCPEP1	-0.2165807	0.1315822	-1.646	0.0999	-0.293428911	count	1
ORAI2	-0.2439808	0.1967042	-1.2403	0.215	-0.293406573	count	1
TMED4	-0.2107045	0.1160386	-1.8158	0.0695	-0.293305396	count	1
SDHAF1	-0.2337638	0.2076913	-1.1255	0.26	-0.293174732	count	1
MCL1	-0.2058378	0.0637532	-3.2287	0.00126	-0.292848874	count	1
KRBA1	-0.4396858	0.7713374	-0.57	0.569	-0.292659088	count	1
KDM4A-AS1	-1.749411	1.000808	-1.748	0.0806	-0.292498364	count	1
GALNT13	-1.749411	1.000808	-1.748	0.0806	-0.292498364	count	1
ARHGAP9	-1.749411	1.000808	-1.748	0.0806	-0.292498364	count	1
AC027020.2	-1.749411	1.000808	-1.748	0.0806	-0.292498364	count	1
FAM133A	-1.749411	1.033734	-1.6923	0.0907	-0.292498364	count	1
ZNF594	-0.8741258	0.7042651	-1.2412	0.215	-0.292015523	count	1
MS4A7	-0.8741258	0.7357283	-1.1881	0.235	-0.292015523	count	1
CYB5R3	-0.2040607	0.0501454	-4.0694	4.85E-05	-0.291810733	count	1
CMBL	-0.2230994	0.1687034	-1.3224	0.186	-0.291784604	count	1
LBX2-AS1	-0.4618945	0.5866671	-0.7873	0.431	-0.291459652	count	1
LCA5L	-0.3635817	0.4660662	-0.7801	0.435	-0.291233748	count	1
CLCN3	-0.2119883	0.1254865	-1.6893	0.0913	-0.291233647	count	1
PLCL2	-0.5269271	0.6742465	-0.7815	0.435	-0.290827317	count	1
TAX1BP3	-0.2152157	0.1212635	-1.7748	0.076	-0.290816751	count	1
AC239800.3	-1.741519	0.6366095	-2.7356	0.00627	-0.290726028	count	1
PDE6A	-1.741519	0.6741043	-2.5835	0.00983	-0.290726028	count	1
KCTD21	-0.4171633	0.4996637	-0.8349	0.404	-0.29069093	count	1
KPNA6	-0.2231348	0.1743086	-1.2801	0.201	-0.290439837	count	1
MEF2A	-0.2067533	0.0842905	-2.4529	0.0142	-0.290398163	count	1
CCDC191	-0.2448646	0.2541901	-0.9633	0.335	-0.290323988	count	1
SGSM1	-0.2769064	0.3889488	-0.7119	0.477	-0.290247881	count	1
MAFA	-1.1209862	0.7346991	-1.5258	0.127	-0.290080359	count	1
PTHLH	-0.2495437	0.2857939	-0.8732	0.383	-0.290013348	count	1
VCPIP1	-0.2332296	0.2515083	-0.9273	0.354	-0.289587132	count	1
HEXB	-0.2083494	0.1054414	-1.976	0.0483	-0.289369017	count	1
DBI	-0.2029183	0.0609743	-3.3279	0.000886	-0.289335348	count	1
ZNF773	-0.3290443	0.412896	-0.7969	0.426	-0.289317378	count	1
ITPR1-DT	-0.4150922	0.7676468	-0.5407	0.589	-0.289135949	count	1
GDI1	-0.2264144	0.1893021	-1.196	0.232	-0.288927486	count	1
PRKD1	-0.5711196	0.6621637	-0.8625	0.388	-0.288905778	count	1
ATP5F1E	-0.2004445	0.029214	-6.8612	8.39E-12	-0.288325444	count	2.00E-07
ZFP69B	-0.4861867	0.6221275	-0.7815	0.435	-0.288197652	count	1
AL121761.1	-0.4861867	0.6259358	-0.7767	0.437	-0.288197652	count	1

ATP6V0B	-0.2026275	0.0612774	-3.3067	0.000956	-0.288173099	count	1
CRELD2	-0.2097606	0.1184743	-1.7705	0.0768	-0.287997477	count	1
NCOA4	-0.2079005	0.1080625	-1.9239	0.0545	-0.287936228	count	1
ITPA	-0.2166279	0.1646304	-1.3158	0.188	-0.28780963	count	1
AVPI1	-0.2495377	0.2785203	-0.8959	0.37	-0.287769939	count	1
AGAP6	-0.4559766	0.6216673	-0.7335	0.463	-0.287381472	count	1
SMCO4	-0.2153567	0.1709898	-1.2595	0.208	-0.287275513	count	1
TMEM191C	-0.4847659	0.735908	-0.6587	0.51	-0.287269343	count	1
AL024507.2	-0.568206	0.4735031	-1.2	0.23	-0.287239635	count	1
F13A1	-0.8622166	0.8756904	-0.9846	0.325	-0.287161465	count	1
AC109587.1	-0.8622166	0.9285881	-0.9285	0.353	-0.287161465	count	1
FOXQ1	-0.8622166	0.9915979	-0.8695	0.385	-0.287161465	count	1
ADAM17	-0.2125031	0.1427825	-1.4883	0.137	-0.287133103	count	1
PHLDB3	-0.3205667	0.4480416	-0.7155	0.474	-0.287128918	count	1
POLN	-1.111599	0.6439135	-1.7263	0.0844	-0.286967629	count	1
RASGRP3	-0.2213882	0.170816	-1.2961	0.195	-0.28689575	count	1
PRSS27	-1.11112	0.6703513	-1.6575	0.0975	-0.286809032	count	1
ADGRA3	-0.8613157	0.5470359	-1.5745	0.115	-0.286795162	count	1
DCAKD	-0.2621785	0.30044	-0.8726	0.383	-0.286491649	count	1
TMX3	-0.2104656	0.1327525	-1.5854	0.113	-0.285870788	count	1
C1RL	-0.2182457	0.19249	-1.1338	0.257	-0.285816885	count	1
ZNF316	-0.2800022	0.4238834	-0.6606	0.509	-0.285785928	count	1
ARSK	-0.2727216	0.3161961	-0.8625	0.388	-0.285741607	count	1
CD46	-0.2015374	0.0816122	-2.4695	0.0136	-0.285549265	count	1
FLNB	-0.2041879	0.1057749	-1.9304	0.0537	-0.285444137	count	1
AZIN1	-0.216319	0.1560343	-1.3864	0.166	-0.285405296	count	1
TIA1	-0.2196272	0.1731485	-1.2684	0.205	-0.285352533	count	1
ARHGEF37	-0.2627342	0.2746932	-0.9565	0.339	-0.285294132	count	1
H2AFV	-0.2023247	0.0818896	-2.4707	0.0135	-0.285156277	count	1
ZBED3-AS1	-0.5174158	0.5836842	-0.8865	0.375	-0.284980711	count	1
KCTD5	-0.2550487	0.3081457	-0.8277	0.408	-0.284960423	count	1
ZNF652	-0.2106917	0.1480119	-1.4235	0.155	-0.284957483	count	1
ZNF25	-0.2658836	0.3098037	-0.8582	0.391	-0.284889628	count	1
GPAA1	-0.2061916	0.109397	-1.8848	0.0596	-0.28484961	count	1
TMCC1-AS1	-0.4809497	0.5537323	-0.8686	0.385	-0.284778277	count	1
ARAF	-0.2377206	0.2125896	-1.1182	0.264	-0.284215906	count	1
VAMP4	-0.2140094	0.1905802	-1.1229	0.262	-0.28415605	count	1
C10orf88	-0.2783494	0.3605079	-0.7721	0.44	-0.284048647	count	1
BIK	-0.5625293	0.7614264	-0.7388	0.46	-0.283998759	count	1
RAB2A	-0.1988639	0.0589609	-3.3728	8.00E-04	-0.283543226	count	1
ZFPM1	-0.2235028	0.2189013	-1.021	0.307	-0.283397275	count	1
RPS11	-0.1968743	0.0273931	-7.187	8.48E-13	-0.283291541	count	2.02E-08
CDCA3	-0.7137689	0.7902766	-0.9032	0.367	-0.283274209	count	1
MCRIP1	-0.2020522	0.0877875	-2.3016	0.0214	-0.283252664	count	1
SPDL1	-0.2520237	0.3052706	-0.8256	0.409	-0.282960543	count	1

FBH1	-0.21971	0.1876622	-1.1708	0.242	-0.282784461	count	1
CD2AP	-0.2020041	0.1068815	-1.89	0.0589	-0.282577708	count	1
GORASP1	-0.2516862	0.3445085	-0.7306	0.465	-0.282573836	count	1
UBA52	-0.1960748	0.0245163	-7.9977	1.85E-15	-0.282294993	count	4.43E-11
CDC42BPA	-0.2079891	0.1284949	-1.6187	0.106	-0.282163141	count	1
MED16	-0.2215832	0.2098755	-1.0558	0.291	-0.282017925	count	1
SAMD13	-0.3525623	0.5314904	-0.6633	0.507	-0.281903715	count	1
RSPH4A	-0.5587175	0.4013527	-1.3921	0.164	-0.281826491	count	1
TBC1D10C	-0.7104701	0.8416429	-0.8441	0.399	-0.28173222	count	1
AL358472.5	-0.7104701	0.8941338	-0.7946	0.427	-0.28173222	count	1
AC048382.6	-0.7104701	0.9482578	-0.7492	0.454	-0.28173222	count	1
ZNF219	-0.2648757	0.2964927	-0.8934	0.372	-0.281713425	count	1
C17orf97	-0.5118183	0.5689575	-0.8996	0.368	-0.281549473	count	1
FBXW2	-0.2164677	0.1742764	-1.2421	0.214	-0.281456367	count	1
TADA2B	-0.2812697	0.3725205	-0.755	0.45	-0.281252049	count	1
DDX19A	-0.2352056	0.2675936	-0.879	0.379	-0.281164415	count	1
POLR2L	-0.1962137	0.0480585	-4.0828	4.58E-05	-0.281092596	count	1
EME2	-0.4747535	0.4922727	-0.9644	0.335	-0.280740879	count	1
JOSD1	-0.2062183	0.1422267	-1.4499	0.147	-0.280467336	count	1
ATF5	-0.2118305	0.1773727	-1.1943	0.232	-0.280294427	count	1
MRGBP	-0.2228425	0.1944415	-1.1461	0.252	-0.280185016	count	1
C17orf100	-0.4222445	0.62303	-0.6777	0.498	-0.280097984	count	1
TIMMDC1	-0.1990007	0.087654	-2.2703	0.0233	-0.279687507	count	1
YIPF1	-0.2237023	0.2241191	-0.9981	0.318	-0.279526826	count	1
NCOR2	-0.2233406	0.1744173	-1.2805	0.2	-0.279520587	count	1
ARL8B	-0.2051917	0.1359002	-1.5099	0.131	-0.279509893	count	1
U2AF1L5	-0.3182523	0.4620188	-0.6888	0.491	-0.279397597	count	1
ZNF805	-0.2476516	0.3250241	-0.7619	0.446	-0.279329649	count	1
CACUL1	-0.2090809	0.1603308	-1.3041	0.192	-0.279122041	count	1
RGS2	-0.2105505	0.2102741	-1.0013	0.317	-0.279071455	count	1
MMP24OS	-0.1999912	0.1074245	-1.8617	0.0628	-0.278885023	count	1
PTRHD1	-0.2016976	0.1138025	-1.7723	0.0764	-0.278743731	count	1
ZC3H7B	-0.2280421	0.2219233	-1.0276	0.304	-0.27862835	count	1
AC005498.3	-0.384815	0.5648608	-0.6813	0.496	-0.278556601	count	1
C2	-0.7029915	0.9615876	-0.7311	0.465	-0.278243852	count	1
FAM66B	-0.7029915	0.9615876	-0.7311	0.465	-0.278243852	count	1
AC011603.1	-0.7029915	1.0355057	-0.6789	0.497	-0.278243852	count	1
AC092119.2	-0.7029915	1.0355057	-0.6789	0.497	-0.278243852	count	1
LINC01547	-0.7029915	1.0355057	-0.6789	0.497	-0.278243852	count	1
C19orf57	-0.7029915	1.128126	-0.6231	0.533	-0.278243852	count	1
AC018904.1	-0.7029915	1.191759	-0.5899	0.555	-0.278243852	count	1
TEX41	-0.7029915	1.7357289	-0.405	0.6855	-0.278243852	count	1
LGI2	-0.3586471	0.3993388	-0.8981	0.369	-0.278228197	count	1
AL049629.2	-0.348104	0.8323731	-0.4182	0.676	-0.278137197	count	1
COX18	-0.2635097	0.3553569	-0.7415	0.458	-0.27807601	count	1

TSPO	-0.1934894	0.0395158	-4.8965	1.03E-06	-0.277882604	count	0.0240814
REXO2	-0.1951538	0.0682553	-2.8592	0.00428	-0.277671241	count	1
UQCR10	-0.1959089	0.0738713	-2.652	0.00805	-0.277641139	count	1
FAM219B	-0.2274459	0.2354462	-0.966	0.334	-0.277275571	count	1
MOB3A	-0.2495391	0.3159763	-0.7897	0.43	-0.27718762	count	1
TIE1	-0.1976819	0.0972291	-2.0332	0.0421	-0.27713122	count	1
ICAM2	-0.1951801	0.0765138	-2.5509	0.0108	-0.27710737	count	1
NDUFB8	-0.1944374	0.0598465	-3.2489	0.0012	-0.277025646	count	1
PTPN6	-0.4689854	0.5088098	-0.9217	0.357	-0.276990484	count	1
ACOT2	-0.4178798	0.7667365	-0.545	0.586	-0.276966042	count	1
PTDSS2	-0.230487	0.2368095	-0.9733	0.33	-0.276949951	count	1
NGEF	-0.5041281	0.7982321	-0.6316	0.528	-0.276847079	count	1
MIR181A2HG	-0.5041281	0.8656052	-0.5824	0.56	-0.276847079	count	1
RPRML	-0.4405784	0.7565761	-0.5823	0.56	-0.276809244	count	1
DND1	-0.4405784	1.2478863	-0.3531	0.7241	-0.276809244	count	1
TMEM204	-0.195988	0.0891567	-2.1982	0.028	-0.276742023	count	1
DGUOK	-0.198305	0.0996502	-1.99	0.0467	-0.276527882	count	1
RPS6KA2	-0.2046361	0.1470071	-1.392	0.164	-0.27645231	count	1
CDH23	-0.2346328	0.211415	-1.1098	0.267	-0.276243925	count	1
PHACTR2	-0.1941455	0.0692028	-2.8055	0.0051	-0.276154802	count	1
DCTN3	-0.1947863	0.0682305	-2.8548	0.00434	-0.276148914	count	1
TST	-0.2051502	0.1643902	-1.2479	0.212	-0.275847527	count	1
TFPT	-0.2018222	0.1590097	-1.2692	0.204	-0.275775378	count	1
SNF8	-0.1958302	0.0824404	-2.3754	0.0176	-0.275208417	count	1
HLTF	-0.2009865	0.1341923	-1.4977	0.134	-0.275119973	count	1
LINC01006	-0.2438625	0.3030422	-0.8047	0.421	-0.274972072	count	1
GOSR2	-0.2064022	0.1650879	-1.2503	0.211	-0.274854651	count	1
MAML2	-0.21313	0.1904518	-1.1191	0.263	-0.274798604	count	1
AP5S1	-0.2474057	0.3132346	-0.7898	0.43	-0.274768148	count	1
CCDC107	-0.1987435	0.1109561	-1.7912	0.0734	-0.27476666	count	1
YWHAH	-0.1934387	0.070028	-2.7623	0.00578	-0.274576401	count	1
LPXN	-0.3193868	0.5354188	-0.5965	0.551	-0.274483416	count	1
ZDHHC5	-0.2090148	0.1599546	-1.3067	0.191	-0.274429064	count	1
ADAMTS1	-0.1914485	0.0866761	-2.2088	0.0273	-0.274201372	count	1
PHF21A	-0.2280676	0.2387818	-0.9551	0.34	-0.274001941	count	1
PBXIP1	-0.2086787	0.1776118	-1.1749	0.24	-0.273792409	count	1
GSTO2	-0.5443741	0.3854154	-1.4124	0.158	-0.273680818	count	1
LHPP	-0.2636079	0.3356986	-0.7853	0.432	-0.273595988	count	1
GRASP	-0.192317	0.063816	-3.0136	0.0026	-0.27354274	count	1
LIMK2	-0.2343884	0.2947123	-0.7953	0.427	-0.273110113	count	1
ATOX1	-0.1924218	0.0656636	-2.9304	0.00341	-0.27301596	count	1
GPR182	-0.6912505	0.5657647	-1.2218	0.222	-0.272788209	count	1
TSEN34	-0.1972691	0.1105337	-1.7847	0.0744	-0.272238676	count	1
POLH	-0.4337324	0.4405727	-0.9845	0.325	-0.272127065	count	1
RTN4RL1	-0.3628002	0.4877865	-0.7438	0.457	-0.272009468	count	1

AC136475.1	-0.6022657	0.5900401	-1.0207	0.307	-0.271908292	count	1
PANK4	-0.3097177	0.3585362	-0.8638	0.388	-0.271571979	count	1
TNKS1BP1	-0.2123145	0.1928423	-1.101	0.271	-0.271407439	count	1
SLC52A3	-0.3229613	0.3763157	-0.8582	0.391	-0.271269649	count	1
FUCA1	-0.2045854	0.17928	-1.1412	0.254	-0.271268786	count	1
MSANTD3	-0.2151613	0.2058973	-1.045	0.296	-0.271222227	count	1
ABRACL	-0.1940381	0.1005941	-1.9289	0.0538	-0.270962123	count	1
JAG2	-0.2273321	0.1995302	-1.1393	0.255	-0.270841905	count	1
LINC01515	-0.3611621	0.5277308	-0.6844	0.494	-0.270703488	count	1
THAP8	-0.4591954	0.6670718	-0.6884	0.491	-0.27064303	count	1
RPAP1	-0.2494524	0.3786234	-0.6588	0.51	-0.270544073	count	1
LBX2	-0.8209166	0.737993	-1.1124	0.266	-0.270498951	count	1
CDKN2B	-0.8209166	0.8059278	-1.0186	0.308	-0.270498951	count	1
TRAPPC2	-0.2199665	0.2381229	-0.9238	0.356	-0.270342811	count	1
TNFAIP8	-0.2303708	0.2475146	-0.9307	0.352	-0.2702433	count	1
MAPK8IP1	-0.5985444	0.5591161	-1.0705	0.284	-0.269983806	count	1
GALNT10	-0.2161876	0.2036399	-1.0616	0.289	-0.269589929	count	1
CMTM8	-0.1956005	0.1147397	-1.7047	0.0884	-0.269225059	count	1
NME1	-0.192255	0.0942025	-2.0409	0.0414	-0.268910474	count	1
ARRB2	-0.2590926	0.3373286	-0.7681	0.443	-0.268784	count	1
JMJD8	-0.1993881	0.1414185	-1.4099	0.159	-0.268667406	count	1
CHADL	-0.4906308	0.6284601	-0.7807	0.435	-0.268626407	count	1
ZNF521	-0.1965128	0.1242653	-1.5814	0.114	-0.268615705	count	1
PEX26	-0.2185469	0.270054	-0.8093	0.418	-0.268577207	count	1
AL022323.1	-0.8155789	0.7334117	-1.112	0.266	-0.268365045	count	1
GNL3L	-0.2230591	0.2532385	-0.8808	0.378	-0.267901679	count	1
DUSP12	-0.2053829	0.1787662	-1.1489	0.251	-0.267820038	count	1
MED22	-0.2897903	0.340264	-0.8517	0.394	-0.267728224	count	1
COL15A1	-0.1894864	0.0940114	-2.0156	0.0439	-0.267614667	count	1
GABRP	-1.05235	0.9318463	-1.1293	0.259	-0.267549163	count	1
IGHG4	-1.05235	0.9318463	-1.1293	0.259	-0.267549163	count	1
AC007319.1	-1.05235	1.063933	-0.9891	0.323	-0.267549163	count	1
AC138028.4	-1.0523504	1.060513	-0.9923	0.321	-0.267549161	count	1
SYMPK	-0.2296846	0.2472415	-0.929	0.353	-0.267539722	count	1
TM4SF18	-0.1896718	0.1061812	-1.7863	0.0742	-0.267536728	count	1
XYLB	-1.0518244	0.6019995	-1.7472	0.0807	-0.267378565	count	1
NDUFB7	-0.1876088	0.061058	-3.0726	0.0021	-0.267031009	count	1
ZNF684	-0.2291308	0.2706333	-0.8466	0.397	-0.266884122	count	1
MED9	-0.2725306	0.3723298	-0.732	0.464	-0.265960846	count	1
CST7	-1.630004	1.096845	-1.4861	0.137	-0.26589276	count	1
GTF2E2	-0.2077564	0.175017	-1.1871	0.235	-0.265832808	count	1
KRT10	-0.185967	0.0537579	-3.4593	6.00E-04	-0.265739796	count	1
COPZ2	-0.1943801	0.1211646	-1.6043	0.109	-0.265689453	count	1
NPIPB5	-0.2434651	0.2362561	-1.0305	0.303	-0.265597355	count	1
ARMCX3	-0.1912133	0.0980318	-1.9505	0.0512	-0.265536581	count	1

WDR54	-0.1951396	0.1586594	-1.2299	0.219	-0.265041411	count	1
CYLD	-0.2045553	0.1835801	-1.1143	0.265	-0.264913983	count	1
MARVELD2	-0.2713377	0.4445527	-0.6104	0.542	-0.264758999	count	1
RNF168	-0.1921968	0.1326313	-1.4491	0.147	-0.264749539	count	1
TCF19	-0.4228172	0.4812893	-0.8785	0.38	-0.264685198	count	1
PDE12	-0.2198394	0.2190094	-1.0038	0.316	-0.264675004	count	1
ZSWIM9	-0.2473762	0.2956331	-0.8368	0.403	-0.264593655	count	1
TMEM258	-0.1857583	0.0569662	-3.2609	0.0011	-0.264576558	count	1
PIGK	-0.1977829	0.1508866	-1.3108	0.19	-0.264564754	count	1
VKORC1L1	-0.2030942	0.1807558	-1.1236	0.261	-0.264382979	count	1
ZKSCAN1	-0.1931507	0.1695829	-1.139	0.255	-0.264180805	count	1
PIGP	-0.1921454	0.1140378	-1.6849	0.0921	-0.264125497	count	1
PHF23	-0.2012355	0.1820432	-1.1054	0.269	-0.26396073	count	1
NDUFA1	-0.1850962	0.0566807	-3.2656	0.0011	-0.263652827	count	1
ARFGAP3	-0.1891775	0.1058927	-1.7865	0.0741	-0.263549713	count	1
GIGYF2	-0.1957295	0.1528795	-1.2803	0.201	-0.263519386	count	1
ARHGEF19	-0.2297722	0.2803032	-0.8197	0.412	-0.263503358	count	1
AC008124.1	-0.2234505	0.2513241	-0.8891	0.374	-0.262878232	count	1
KCTD9	-0.2257257	0.2216971	-1.0182	0.309	-0.2628541	count	1
SFXN5	-0.313387	0.375217	-0.8352	0.404	-0.262840988	count	1
CSTF3	-0.2159289	0.2415949	-0.8938	0.372	-0.262461299	count	1
B4GALT2	-0.2070648	0.2073063	-0.9988	0.318	-0.262360576	count	1
CFAP53	-0.3790057	0.8543989	-0.4436	0.657	-0.262209945	count	1
APBB1	-0.2295969	0.3052917	-0.7521	0.452	-0.262179325	count	1
BX539320.1	-0.5829592	0.7090467	-0.8222	0.411	-0.261954916	count	1
ABL1	-0.2303106	0.2515889	-0.9154	0.36	-0.261848367	count	1
STS	-0.5824715	0.643374	-0.9053	0.365	-0.2617045	count	1
ENTPD7	-0.6672102	0.7246803	-0.9207	0.357	-0.261697654	count	1
TMEM52	-0.6672102	0.7515606	-0.8878	0.375	-0.261697654	count	1
SLC27A1	-0.2399581	0.3232443	-0.7423	0.458	-0.261688703	count	1
XRCC3	-0.3964878	0.5528068	-0.7172	0.473	-0.261682937	count	1
ZNF674-AS1	-0.4180239	0.6837792	-0.6113	0.541	-0.261426272	count	1
XKR8	-0.2343874	0.33152	-0.707	0.48	-0.261424959	count	1
DNAJC13	-0.2057948	0.2106548	-0.9769	0.329	-0.261413673	count	1
AC015922.4	-0.3957655	0.5241539	-0.7551	0.45	-0.26116886	count	1
GNA12	-0.2201317	0.2478467	-0.8882	0.375	-0.260573894	count	1
RANBP17	-0.7959654	0.494481	-1.6097	0.108	-0.26056295	count	1
NCAPD2	-0.3184273	0.5066745	-0.6285	0.53	-0.260481552	count	1
PTCH1	-0.2453182	0.3225136	-0.7606	0.447	-0.26041738	count	1
ZYX	-0.1891776	0.1744402	-1.0845	0.278	-0.259741431	count	1
STAU2	-0.2010689	0.1780963	-1.129	0.259	-0.259632541	count	1
MORC4	-0.2315933	0.2946289	-0.7861	0.432	-0.259586493	count	1
TXNL4A	-0.1839145	0.0804176	-2.287	0.0223	-0.259298764	count	1
CTDNEP1	-0.1852929	0.1031687	-1.796	0.0726	-0.259228922	count	1
CD48	-1.0264623	0.6383764	-1.6079	0.108	-0.259191556	count	1

INTS8	-0.2102852	0.3024886	-0.6952	0.487	-0.258826197	count	1
SMARCA2	-0.1865986	0.1178812	-1.5829	0.114	-0.258681894	count	1
CXXC5	-0.190995	0.1326743	-1.4396	0.15	-0.258615129	count	1
GCNT1	-0.2226372	0.3124314	-0.7126	0.476	-0.258242379	count	1
SOS1	-0.1939917	0.1479899	-1.3108	0.19	-0.257972535	count	1
ELOVL5	-0.1946138	0.149372	-1.3029	0.193	-0.257969642	count	1
IVNS1ABP	-0.191937	0.1855768	-1.0343	0.301	-0.257687868	count	1
VPS13D	-0.2074407	0.2304073	-0.9003	0.368	-0.257681574	count	1
RAB11B	-0.1851597	0.1038987	-1.7821	0.0748	-0.257608105	count	1
AAGAB	-0.2047258	0.2095936	-0.9768	0.329	-0.257566623	count	1
TEFM	-0.2069759	0.2534179	-0.8167	0.414	-0.257545318	count	1
MTIF2	-0.1968649	0.1779638	-1.1062	0.269	-0.257434805	count	1
AGGF1	-0.2150266	0.2913419	-0.7381	0.461	-0.257428617	count	1
ZNF667	-0.255407	0.3496627	-0.7304	0.465	-0.257380904	count	1
EMC7	-0.1832672	0.0840187	-2.1813	0.0292	-0.257365638	count	1
NBPF15	-0.3226781	0.4333749	-0.7446	0.457	-0.25674903	count	1
TP53INP1	-0.2477788	0.3581624	-0.6918	0.489	-0.256744667	count	1
MIB2	-0.2168662	0.265539	-0.8167	0.414	-0.256652596	count	1
ZBTB14	-0.2121585	0.2658589	-0.798	0.425	-0.25659621	count	1
KIAA0100	-0.1952191	0.1851434	-1.0544	0.292	-0.25637577	count	1
POMP	-0.1788877	0.0482756	-3.7056	0.000215	-0.255982305	count	1
RHOBTB3	-0.2295575	0.2721311	-0.8436	0.399	-0.255933831	count	1
GLRX5	-0.1819036	0.08934	-2.0361	0.0418	-0.255790352	count	1
CMKLR1	-0.2123426	0.2743781	-0.7739	0.439	-0.255531469	count	1
SPTY2D1OS	-0.435433	0.495074	-0.8795	0.379	-0.255330791	count	1
UHMK1	-0.1932798	0.1650869	-1.1708	0.242	-0.255296011	count	1
COG5	-0.2007707	0.3130082	-0.6414	0.521	-0.254974799	count	1
TSTA3	-0.1859135	0.127796	-1.4548	0.146	-0.254842441	count	1
HAGLROS	-0.2073522	0.2216783	-0.9354	0.35	-0.254662766	count	1
G6PD	-0.2056571	0.2062331	-0.9972	0.319	-0.254523554	count	1
DLEU2	-0.217012	0.2485227	-0.8732	0.383	-0.254336473	count	1
OTULIN	-0.206598	0.2121912	-0.9736	0.33	-0.254236675	count	1
TMEM136	-0.2176603	0.2788648	-0.7805	0.435	-0.254225143	count	1
NUDT19	-0.2217974	0.2749054	-0.8068	0.42	-0.254203395	count	1
UBE2F	-0.2057444	0.1928933	-1.0666	0.286	-0.254157606	count	1
NTMT1	-0.1928256	0.1647268	-1.1706	0.242	-0.254056808	count	1
ATP5PF	-0.1771393	0.0422802	-4.1897	2.88E-05	-0.2539961	count	0.6651648
ETF1	-0.1856038	0.1200185	-1.5465	0.122	-0.25398897	count	1
HSD11B1L	-0.2244058	0.2900333	-0.7737	0.439	-0.253845752	count	1
CYB561D2	-0.2042351	0.2262383	-0.9027	0.367	-0.25365756	count	1
RANBP2	-0.1857585	0.1461506	-1.271	0.204	-0.253626523	count	1
TMEM25	-0.1963215	0.2099049	-0.9353	0.35	-0.253455401	count	1
SKP1	-0.1764832	0.0362254	-4.8718	1.17E-06	-0.253423545	count	0.02734641
PKD2	-0.1969406	0.2638303	-0.7465	0.455	-0.2532471	count	1
ZFAND2B	-0.1861644	0.1296842	-1.4355	0.151	-0.253014993	count	1

CEP126	-0.2151746	0.3141633	-0.6849	0.493	-0.25299841	count	1
SERP2	-0.7767613	0.8397848	-0.925	0.355	-0.2529836	count	1
AC007878.1	-0.7767613	0.8848454	-0.8778	0.38	-0.2529836	count	1
AL049780.1	-0.7767613	1.0025431	-0.7748	0.439	-0.2529836	count	1
MRPL53	-0.3020392	0.3663527	-0.8244	0.41	-0.252879688	count	1
CNTNAP1	-0.6478665	0.8255277	-0.7848	0.433	-0.252852711	count	1
PISD	-0.2280319	0.2731883	-0.8347	0.404	-0.252832911	count	1
AL162258.2	-0.5069775	0.6935675	-0.731	0.465	-0.252655897	count	1
SLC44A3	-0.5069775	0.6956185	-0.7288	0.466	-0.252655897	count	1
GDPD1	-0.5069775	0.697385	-0.727	0.467	-0.252655897	count	1
NDUFA11	-0.1771377	0.062409	-2.8383	0.0046	-0.252351378	count	1
INO80B	-0.3173733	0.4179244	-0.7594	0.448	-0.252306625	count	1
MRPL27	-0.1813111	0.1050376	-1.7262	0.0844	-0.252292199	count	1
APEX1	-0.1782637	0.073549	-2.4237	0.0154	-0.252287927	count	1
TMCO1	-0.1776098	0.0700162	-2.5367	0.0112	-0.25188168	count	1
TGFB1I1	-0.1836839	0.1308865	-1.4034	0.161	-0.251877529	count	1
AL034417.4	-1.564617	0.832937	-1.8784	0.0604	-0.251549241	count	1
AC137767.1	-0.2614477	0.5862038	-0.446	0.656	-0.251546228	count	1
ULK1	-0.2230516	0.334745	-0.6663	0.505	-0.251082122	count	1
EML3	-0.2081549	0.234891	-0.8862	0.376	-0.251068477	count	1
EIF4G3	-0.1837181	0.13016	-1.4115	0.158	-0.250710888	count	1
VWA1	-0.1767413	0.0816805	-2.1638	0.0306	-0.250688235	count	1
TMEM220	-0.1901242	0.1973784	-0.9632	0.336	-0.250635683	count	1
KHDRBS3	-0.2082619	0.2485477	-0.8379	0.402	-0.250557652	count	1
YIPF2	-0.1844666	0.1461872	-1.2619	0.207	-0.250486848	count	1
LEAP2	-0.3244601	0.3876257	-0.837	0.403	-0.250247361	count	1
ITFG1	-0.1841144	0.1363938	-1.3499	0.177	-0.250006666	count	1
UBE2H	-0.1791654	0.1159253	-1.5455	0.122	-0.249891116	count	1
KIAA0513	-0.3144372	0.4087765	-0.7692	0.442	-0.249850839	count	1
TEPSIN	-0.2564902	0.3671047	-0.6987	0.485	-0.249826012	count	1
SLMAP	-0.1986189	0.2207837	-0.8996	0.368	-0.249810785	count	1
EMB	-1.5561777	0.7780849	-2	0.0456	-0.249711621	count	1
ARHGEF25	-1.5561777	0.8019676	-1.9404	0.0524	-0.249711621	count	1
PPP1R8	-0.2041302	0.2312124	-0.8829	0.377	-0.249614729	count	1
STK40	-0.2136584	0.2855772	-0.7482	0.454	-0.249480202	count	1
DCTN1	-0.1901181	0.2008264	-0.9467	0.344	-0.249460451	count	1
LPP-AS2	-0.2662742	0.3846364	-0.6923	0.489	-0.249144436	count	1
AGFG1	-0.1887437	0.1544509	-1.222	0.222	-0.249116202	count	1
ZNF362	-0.1998679	0.2249222	-0.8886	0.374	-0.249033661	count	1
RNF139-AS1	-0.5573316	0.6268227	-0.8891	0.374	-0.248862398	count	1
RWDD1	-0.1738508	0.0565072	-3.0766	0.0021	-0.248514238	count	1
MLIP	-0.3604461	0.653337	-0.5517	0.581	-0.248486492	count	1
CD3E	-1.5502348	0.5362099	-2.8911	0.00387	-0.248419531	count	1
ZNF226	-0.1872259	0.1858592	-1.0074	0.314	-0.248396182	count	1
CTDSP1	-0.1792378	0.1069982	-1.6751	0.094	-0.248326175	count	1

AL022238.2	-0.9917125	0.7655582	-1.2954	0.195	-0.248098393	count	1
TVP23C	-0.3039697	0.4485069	-0.6777	0.498	-0.248080426	count	1
SH3D19	-0.1883132	0.1607545	-1.1714	0.242	-0.24807529	count	1
SLC25A13	-0.1895459	0.1904416	-0.9953	0.32	-0.247800651	count	1
ATXN7L3	-0.2005176	0.2282046	-0.8787	0.38	-0.247631551	count	1
DYNC1H1	-0.1772782	0.1040623	-1.7036	0.0886	-0.247569778	count	1
SYNCRIP	-0.1752259	0.0774752	-2.2617	0.0238	-0.247464099	count	1
SBNO2	-0.1908409	0.2030599	-0.9398	0.347	-0.247248427	count	1
LRFN3	-0.2727918	0.4262585	-0.64	0.522	-0.247210214	count	1
RABAC1	-0.1723211	0.0424196	-4.0623	4.99E-05	-0.247198118	count	1
AC093525.4	-0.7619861	0.7828613	-0.9733	0.33	-0.24719294	count	1
NDUFC1	-0.1733191	0.0590448	-2.9354	0.0034	-0.247018784	count	1
CIDEB	-0.2252147	0.2991508	-0.7528	0.452	-0.246792495	count	1
ZBTB20	-0.1733432	0.0805087	-2.1531	0.0314	-0.246417954	count	1
MRPL51	-0.1732056	0.0600184	-2.8859	0.00393	-0.246365726	count	1
VDAC2	-0.172404	0.0550744	-3.1304	0.0018	-0.2459551	count	1
AL161772.1	-1.5387039	0.8905381	-1.7278	0.0841	-0.245917176	count	1
AL022323.3	-1.5387039	0.8905381	-1.7278	0.0841	-0.245917176	count	1
CAGE1	-1.5387039	1.071509	-1.436	0.151	-0.245917176	count	1
C1QL1	-1.5387039	1.18188	-1.3019	0.193	-0.245917176	count	1
XPOT	-0.202351	0.229117	-0.8832	0.377	-0.245761466	count	1
HERC1	-0.1885511	0.1866291	-1.0103	0.312	-0.245722987	count	1
ADPRH	-0.2096281	0.2891417	-0.725	0.469	-0.245555777	count	1
COQ7	-0.1888749	0.2313851	-0.8163	0.414	-0.2451179	count	1
MARK3	-0.1905908	0.1615868	-1.1795	0.238	-0.245018745	count	1
PHETA2	-0.2235495	0.3553472	-0.6291	0.529	-0.244931218	count	1
CCNL2	-0.1818177	0.1401045	-1.2977	0.194	-0.244885991	count	1
AL359915.2	-0.3283228	0.672934	-0.4879	0.626	-0.244662527	count	1
ARCN1	-0.1776151	0.1164663	-1.525	0.127	-0.244603174	count	1
RBBP9	-0.2193895	0.2908875	-0.7542	0.451	-0.244387129	count	1
PRRT3	-0.2425625	0.4464848	-0.5433	0.587	-0.244085419	count	1
HSPA8	-0.169465	0.03676	-4.61	4.21E-06	-0.243712709	count	0.09802985
ATP2C1	-0.1850084	0.1694929	-1.0915	0.275	-0.243695559	count	1
LMOD3	-0.3708074	0.5385644	-0.6885	0.491	-0.24348463	count	1
AC010642.2	-0.1824289	0.148492	-1.2285	0.219	-0.243476364	count	1
PPFIBP2	-0.6271051	0.6826167	-0.9187	0.358	-0.243438651	count	1
NUP188	-0.2310999	0.3197722	-0.7227	0.47	-0.243086858	count	1
TMC4	-0.4479899	0.8698608	-0.515	0.607	-0.24293196	count	1
C1orf162	-0.4894778	0.4873707	-1.0043	0.315	-0.242924127	count	1
PCDH1	-0.1823314	0.154996	-1.1764	0.24	-0.242876193	count	1
CDK2AP1	-0.2127537	0.3128342	-0.6801	0.497	-0.242628141	count	1
FKBP15	-0.2093678	0.2557902	-0.8185	0.413	-0.242615491	count	1
NMT1	-0.1784425	0.1524463	-1.1705	0.242	-0.242274666	count	1
NCOA1	-0.1816739	0.1734079	-1.0477	0.295	-0.242232449	count	1
STN1	-0.1807634	0.1544766	-1.1702	0.242	-0.241686676	count	1

BDH2	-0.1724383	0.1017172	-1.6953	0.0901	-0.241400856	count	1
ZC3H12C	-0.2107199	0.3345614	-0.6298	0.529	-0.241302334	count	1
AL021368.2	-0.4133343	0.5232823	-0.7899	0.43	-0.241211823	count	1
ATAT1	-0.2449866	0.3386696	-0.7234	0.47	-0.241196737	count	1
TGFB2-AS1	-0.9696437	1.079353	-0.8984	0.369	-0.241129401	count	1
FOXP4-AS1	-0.9696437	1.079353	-0.8984	0.369	-0.241129401	count	1
AC084346.2	-0.9696437	1.079353	-0.8984	0.369	-0.241129401	count	1
ST6GALNAC2	-0.9696437	1.079353	-0.8984	0.369	-0.241129401	count	1
KIF2C	-0.9696437	1.08045	-0.8974	0.37	-0.241129401	count	1
PAQR6	-0.9696437	1.08045	-0.8974	0.37	-0.241129401	count	1
AL353593.1	-0.9696437	1.08045	-0.8974	0.37	-0.241129401	count	1
COLEC11	-0.9696437	1.08045	-0.8974	0.37	-0.241129401	count	1
KIF5C	-0.9696437	1.08045	-0.8974	0.37	-0.241129401	count	1
AC017048.3	-0.9696437	1.08045	-0.8974	0.37	-0.241129401	count	1
AL024498.1	-0.9696437	1.08045	-0.8974	0.37	-0.241129401	count	1
SYCP2L	-0.9696437	1.08045	-0.8974	0.37	-0.241129401	count	1
HIST1H1B	-0.9696437	1.08045	-0.8974	0.37	-0.241129401	count	1
CASC8	-0.9696437	1.08045	-0.8974	0.37	-0.241129401	count	1
C9orf163	-0.9696437	1.08045	-0.8974	0.37	-0.241129401	count	1
AC087393.2	-0.9696437	1.08045	-0.8974	0.37	-0.241129401	count	1
AC090912.2	-0.9696437	1.08045	-0.8974	0.37	-0.241129401	count	1
PHACTR3	-0.9696437	1.08045	-0.8974	0.37	-0.241129401	count	1
CACTIN-AS1	-0.9696437	1.08045	-0.8974	0.37	-0.241129401	count	1
CEBPA-DT	-0.9696437	1.08045	-0.8974	0.37	-0.241129401	count	1
CD1C	-0.9696437	1.466782	-0.6611	0.509	-0.241129401	count	1
AC010336.1	-0.9696437	1.466782	-0.6611	0.509	-0.241129401	count	1
FAM20B	-0.1926437	0.2193969	-0.8781	0.38	-0.241120479	count	1
RNF8	-0.1781597	0.1364208	-1.306	0.192	-0.241088192	count	1
CSRP1	-0.1697446	0.0736259	-2.3055	0.0212	-0.24082259	count	1
AC012615.1	-0.2211576	0.336918	-0.6564	0.512	-0.240773566	count	1
IMMT	-0.1806736	0.1409568	-1.2818	0.2	-0.24041537	count	1
TCEANC2	-0.2414752	0.3897516	-0.6196	0.536	-0.240366711	count	1
ROGDI	-0.198389	0.268051	-0.7401	0.459	-0.240325372	count	1
CLPP	-0.1716698	0.0929914	-1.8461	0.065	-0.240306078	count	1
SLC16A2	-0.2652221	0.3659958	-0.7247	0.469	-0.240098987	count	1
UBL4A	-0.1876961	0.1952772	-0.9612	0.337	-0.239685875	count	1
ADGRB1	-0.9649831	0.697988	-1.3825	0.167	-0.239665303	count	1
C5AR1	-0.9649831	0.9433235	-1.023	0.306	-0.239665303	count	1
BISPR	-0.2075148	0.3161825	-0.6563	0.512	-0.239511367	count	1
ENO3	-0.4104964	0.7950175	-0.5163	0.606	-0.239407218	count	1
ANKRD23	-0.4104964	1.10741	-0.3707	0.711	-0.239407218	count	1
CREB3L1	-0.2160212	0.2529011	-0.8542	0.393	-0.239267609	count	1
DCAF7	-0.1836061	0.1646787	-1.1149	0.265	-0.239235797	count	1
FAM192A	-0.1773151	0.1342237	-1.321	0.187	-0.239222526	count	1
ABHD4	-0.2489958	0.3478513	-0.7158	0.474	-0.239196327	count	1

XPO1	-0.1741963	0.1277818	-1.3632	0.173	-0.238873469	count	1
INPP5B	-0.2143815	0.314439	-0.6818	0.495	-0.238706781	count	1
ARL8A	-0.1762634	0.1230601	-1.4323	0.152	-0.238661951	count	1
MTERF4	-0.1842829	0.1675022	-1.1002	0.271	-0.238474786	count	1
RPL27	-0.1658747	0.0293238	-5.6567	1.70E-08	-0.238436194	count	0.000401387
ALDH1L1-AS1	-0.5363445	0.8106689	-0.6616	0.508	-0.238243264	count	1
CEP72	-0.5363445	0.8106689	-0.6616	0.508	-0.238243264	count	1
RNASE4	-0.5363445	0.8336127	-0.6434	0.52	-0.238243264	count	1
ATG9A	-0.5363445	0.9132832	-0.5873	0.557	-0.238243264	count	1
AL009178.2	-0.5363445	0.9132832	-0.5873	0.557	-0.238243264	count	1
TRMU	-0.203956	0.2695261	-0.7567	0.449	-0.237986594	count	1
KIF1B	-0.1821735	0.1824774	-0.9983	0.318	-0.237734322	count	1
PCSK5	-0.2415461	0.4698732	-0.5141	0.607	-0.237712613	count	1
SAMSN1	-1.5006011	0.8367005	-1.7935	0.073	-0.237694332	count	1
ECPAS	-0.1808052	0.1932786	-0.9355	0.35	-0.237656106	count	1
ZMYM5	-0.1804982	0.1777293	-1.0156	0.31	-0.237565354	count	1
NUCKS1	-0.1655662	0.0469719	-3.5248	0.000431	-0.237486588	count	1
KAT5	-0.194237	0.2117458	-0.9173	0.359	-0.237383257	count	1
ACSL4	-0.17796	0.1573	-1.1313	0.258	-0.237255561	count	1
TMEM230	-0.1668917	0.0628109	-2.6571	0.00793	-0.237061575	count	1
ZNF337-AS1	-0.3447656	0.6257235	-0.551	0.582	-0.236958944	count	1
EMC6	-0.1695526	0.0987986	-1.7161	0.0862	-0.236797795	count	1
ALG2	-0.1809009	0.1698727	-1.0649	0.287	-0.236426914	count	1
PRDX1	-0.1643056	0.0338955	-4.8474	1.32E-06	-0.236261567	count	0.03084576
FAM96B	-0.1661396	0.0633205	-2.6238	0.00874	-0.236242498	count	1
FLCN	-0.2899964	0.3631301	-0.7986	0.425	-0.236143615	count	1
TMEM147	-0.1670095	0.0764565	-2.1844	0.029	-0.235935135	count	1
ZCCHC17	-0.169043	0.1051615	-1.6075	0.108	-0.235658439	count	1
NDUFAB1	-0.1658227	0.0653546	-2.5373	0.0112	-0.235648304	count	1
HECTD1	-0.1703284	0.1145193	-1.4873	0.137	-0.235511007	count	1
RAPGEF6	-0.252033	0.4242964	-0.594	0.553	-0.235380122	count	1
TMOD1	-0.1728127	0.1420897	-1.2162	0.224	-0.235305701	count	1
DLG3	-0.4351009	0.5901292	-0.7373	0.461	-0.235248932	count	1
OPA3	-0.191252	0.2429184	-0.7873	0.431	-0.235153537	count	1
STRN4	-0.1911353	0.21541	-0.8873	0.375	-0.235008531	count	1
TOPBP1	-0.180343	0.2016788	-0.8942	0.371	-0.234765002	count	1
MANBAL	-0.1764721	0.1647283	-1.0713	0.284	-0.234554331	count	1
ACOT4	-0.4740798	0.8497431	-0.5579	0.577	-0.234418085	count	1
CCL15	-0.4740798	0.9127618	-0.5194	0.604	-0.234418085	count	1
SUCLG2-AS1	-0.4740798	0.91966	-0.5155	0.606	-0.234418085	count	1
AC073389.1	-0.4740678	0.6993972	-0.6778	0.498	-0.234411484	count	1
LINC01569	-0.4335308	0.7003783	-0.619	0.536	-0.234315687	count	1
IQSEC3	-0.9474081	0.7722123	-1.2269	0.22	-0.234168498	count	1
ADAM28	-0.9474081	0.8354633	-1.134	0.257	-0.234168498	count	1
PSMB3	-0.1638334	0.0524656	-3.1227	0.0018	-0.233790659	count	1

UBL3	-0.1730831	0.138654	-1.2483	0.212	-0.233729858	count	1
AKR1E2	-0.376896	0.6414969	-0.5875	0.557	-0.233694489	count	1
GALK1	-0.1762026	0.1839525	-0.9579	0.338	-0.233693752	count	1
ARC	-0.3143642	0.5693038	-0.5522	0.581	-0.233675244	count	1
SEC13	-0.1696941	0.1217492	-1.3938	0.163	-0.233503862	count	1
CFAP74	-1.4810266	1.1204	-1.3219	0.186	-0.233498549	count	1
CD84	-1.4810266	1.1204	-1.3219	0.186	-0.233498549	count	1
CACNA2D3	-1.4810266	1.1204	-1.3219	0.186	-0.233498549	count	1
PCDHA12	-1.4810266	1.1204	-1.3219	0.186	-0.233498549	count	1
LINC00574	-1.4810266	1.1204	-1.3219	0.186	-0.233498549	count	1
NPM2	-1.4810266	1.1204	-1.3219	0.186	-0.233498549	count	1
CARD9	-1.4810266	1.1204	-1.3219	0.186	-0.233498549	count	1
ADAM8	-1.4810266	1.1204	-1.3219	0.186	-0.233498549	count	1
SERINC4	-1.4810266	1.1204	-1.3219	0.186	-0.233498549	count	1
ACSM2B	-1.4810266	1.1204	-1.3219	0.186	-0.233498549	count	1
AC007333.2	-1.4810266	1.1204	-1.3219	0.186	-0.233498549	count	1
PDF	-1.4810266	1.1204	-1.3219	0.186	-0.233498549	count	1
AC135178.2	-1.4810266	1.1204	-1.3219	0.186	-0.233498549	count	1
NOS2	-1.4810266	1.1204	-1.3219	0.186	-0.233498549	count	1
AP000350.7	-1.4810266	1.1204	-1.3219	0.186	-0.233498549	count	1
MCHR1	-1.4810266	1.1204	-1.3219	0.186	-0.233498549	count	1
TMEM56-RWDD3	-1.4810266	1.316629	-1.1249	0.261	-0.233498549	count	1
FP700111.1	-1.4810266	1.316629	-1.1249	0.261	-0.233498549	count	1
LINC01139	-1.4810266	1.316629	-1.1249	0.261	-0.233498549	count	1
PKP4-AS1	-1.4810266	1.316629	-1.1249	0.261	-0.233498549	count	1
AC007563.2	-1.4810266	1.316629	-1.1249	0.261	-0.233498549	count	1
AC090948.2	-1.4810266	1.316629	-1.1249	0.261	-0.233498549	count	1
ADAMTS9-AS1	-1.4810266	1.316629	-1.1249	0.261	-0.233498549	count	1
CNTN3	-1.4810266	1.316629	-1.1249	0.261	-0.233498549	count	1
ROBO2	-1.4810266	1.316629	-1.1249	0.261	-0.233498549	count	1
AC126283.1	-1.4810266	1.316629	-1.1249	0.261	-0.233498549	count	1
AC092354.1	-1.4810266	1.316629	-1.1249	0.261	-0.233498549	count	1
HIST1H2AM	-1.4810266	1.316629	-1.1249	0.261	-0.233498549	count	1
LINC01556	-1.4810266	1.316629	-1.1249	0.261	-0.233498549	count	1
HCG21	-1.4810266	1.316629	-1.1249	0.261	-0.233498549	count	1
AL033504.1	-1.4810266	1.316629	-1.1249	0.261	-0.233498549	count	1
AL078604.2	-1.4810266	1.316629	-1.1249	0.261	-0.233498549	count	1
LINC01952	-1.4810266	1.316629	-1.1249	0.261	-0.233498549	count	1
ASMTL-AS1	-1.4810266	1.316629	-1.1249	0.261	-0.233498549	count	1
FAM87A	-1.4810266	1.316629	-1.1249	0.261	-0.233498549	count	1
FAM66A	-1.4810266	1.316629	-1.1249	0.261	-0.233498549	count	1
LINC01410	-1.4810266	1.316629	-1.1249	0.261	-0.233498549	count	1
AC124798.1	-1.4810266	1.316629	-1.1249	0.261	-0.233498549	count	1
IDI2	-1.4810266	1.316629	-1.1249	0.261	-0.233498549	count	1
PITX3	-1.4810266	1.316629	-1.1249	0.261	-0.233498549	count	1

SMIM2-AS1	-1.4810266	1.316629	-1.1249	0.261	-0.233498549	count	1
AL928654.3	-1.4810266	1.316629	-1.1249	0.261	-0.233498549	count	1
AC130343.2	-1.4810266	1.316629	-1.1249	0.261	-0.233498549	count	1
AC005224.3	-1.4810266	1.316629	-1.1249	0.261	-0.233498549	count	1
ZNF177	-1.4810266	1.316629	-1.1249	0.261	-0.233498549	count	1
AC005625.1	-1.4810266	1.316629	-1.1249	0.261	-0.233498549	count	1
SULT2B1	-1.4810266	1.316629	-1.1249	0.261	-0.233498549	count	1
SMIM17	-1.4810266	1.316629	-1.1249	0.261	-0.233498549	count	1
TM2D2	-0.16815	0.1167957	-1.4397	0.15	-0.233486311	count	1
TRIM3	-0.2275529	0.366472	-0.6209	0.535	-0.233157307	count	1
ZNF2	-0.2428425	0.4088824	-0.5939	0.553	-0.23310625	count	1
SMG8	-0.2792015	0.4757016	-0.5869	0.557	-0.232927539	count	1
METTL15	-0.1792307	0.2232129	-0.803	0.422	-0.232918564	count	1
STX2	-0.1736512	0.1662438	-1.0446	0.296	-0.232640344	count	1
SF3B5	-0.1634656	0.0625121	-2.6149	0.00897	-0.232459299	count	1
MTA1	-0.1771866	0.1630777	-1.0865	0.277	-0.232390641	count	1
ISOC2	-0.1717429	0.1422544	-1.2073	0.227	-0.232370203	count	1
CHMP1A	-0.1771536	0.1450789	-1.2211	0.222	-0.232347098	count	1
IFI27	-0.1611935	0.0401786	-4.0119	6.18E-05	-0.232280614	count	1
BCOR	-0.2085743	0.3113054	-0.67	0.503	-0.232125506	count	1
CLIC4	-0.1639565	0.0817867	-2.0047	0.0451	-0.231920588	count	1
ANKRD28	-0.1681441	0.1145005	-1.4685	0.142	-0.231898937	count	1
APRT	-0.1623574	0.0588772	-2.7576	0.00586	-0.231773756	count	1
ZNF470	-0.2778359	0.5238666	-0.5304	0.596	-0.231738556	count	1
BMI1	-0.1848854	0.2156675	-0.8573	0.391	-0.231682532	count	1
FASN	-0.292358	0.4187333	-0.6982	0.485	-0.231452203	count	1
IFT20	-0.1671046	0.1108761	-1.5071	0.132	-0.231444701	count	1
AC087500.1	-0.5227935	0.6437959	-0.812	0.417	-0.231436217	count	1
PAM16	-0.3735097	0.4385474	-0.8517	0.394	-0.231429749	count	1
AL445524.1	-0.1904294	0.2349629	-0.8105	0.418	-0.231119011	count	1
FGFR1	-0.172205	0.1544821	-1.1147	0.265	-0.230887921	count	1
IMPA1	-0.1780919	0.1768625	-1.007	0.314	-0.230826026	count	1
GABARAPL1	-0.166137	0.1093355	-1.5195	0.129	-0.230787761	count	1
MESD	-0.1626757	0.0803269	-2.0252	0.0429	-0.230763354	count	1
CYB561D2	-0.3223259	0.6828393	-0.472	0.637	-0.230653486	count	1
OVCH1-AS1	-0.3223259	0.7496435	-0.43	0.667	-0.230653486	count	1
SERINC3	-0.1631374	0.0791589	-2.0609	0.0394	-0.230465886	count	1
TMEM170B	-0.4665507	0.500057	-0.933	0.351	-0.230278433	count	1
PLK3	-0.1723942	0.1839309	-0.9373	0.349	-0.230233734	count	1
MIPOL1	-0.1848253	0.2252264	-0.8206	0.412	-0.230113744	count	1
TECPR2	-0.1971177	0.2748023	-0.7173	0.473	-0.229894638	count	1
ULK4	-0.2463434	0.3534741	-0.6969	0.486	-0.229894096	count	1
CCDC88A	-0.1692103	0.1519783	-1.1134	0.266	-0.229823624	count	1
TMEM80	-0.1739601	0.195519	-0.8897	0.374	-0.229776295	count	1
GOLGA6L4	-0.3346163	0.7611576	-0.4396	0.66	-0.229530666	count	1

CLPTM1L	-0.1720268	0.1513741	-1.1364	0.256	-0.229526218	count	1
CDK16	-0.1833307	0.2980565	-0.6151	0.539	-0.229357841	count	1
AZI2	-0.1695462	0.1485495	-1.1413	0.254	-0.229238515	count	1
PRUNE2	-0.3202492	0.4127595	-0.7759	0.438	-0.22907833	count	1
CHMP2A	-0.1602908	0.0532817	-3.0084	0.00265	-0.228901537	count	1
RTL5	-0.9301505	0.8900199	-1.0451	0.296	-0.228808531	count	1
SOCS1	-0.180964	0.2464756	-0.7342	0.463	-0.228708226	count	1
NPL	-0.2812521	0.5157478	-0.5453	0.586	-0.228698384	count	1
ZNF277	-0.1741493	0.1667999	-1.0441	0.297	-0.228543298	count	1
NINJ1	-0.166158	0.1386008	-1.1988	0.231	-0.228339645	count	1
ALS2CR12	-1.456546	0.8496667	-1.7143	0.0866	-0.228279682	count	1
C19orf70	-0.1605211	0.0675945	-2.3748	0.0176	-0.228103446	count	1
NRG4	-0.7125031	0.71161	-1.0013	0.317	-0.22806098	count	1
KANSL1	-0.1652997	0.1243258	-1.3296	0.184	-0.227856929	count	1
PSMC5	-0.1605589	0.0620765	-2.5865	0.00975	-0.227816699	count	1
REX1BD	-0.1603764	0.0641391	-2.5004	0.0125	-0.227724443	count	1
AC079807.1	-0.5919561	0.6582558	-0.8993	0.369	-0.227689449	count	1
PCM1	-0.1613031	0.0824473	-1.9564	0.0505	-0.227609673	count	1
VMP1	-0.1628643	0.0993592	-1.6391	0.101	-0.227401282	count	1
EXOC6B	-0.1771534	0.2211205	-0.8012	0.423	-0.227379792	count	1
ELP6	-0.1690158	0.1418012	-1.1919	0.233	-0.227315188	count	1
PHF3	-0.1617215	0.0919299	-1.7592	0.0787	-0.227235571	count	1
TECPR1	-0.1759786	0.2046916	-0.8597	0.39	-0.227228609	count	1
ARHGAP23	-0.1701841	0.1807044	-0.9418	0.346	-0.227055783	count	1
DEPTOR	-0.2145274	0.2950178	-0.7272	0.467	-0.22703948	count	1
EIF4G2	-0.1592159	0.0600528	-2.6513	0.0081	-0.226932514	count	1
CHMP3	-0.1609604	0.0757215	-2.1257	0.0336	-0.226863209	count	1
C7orf25	-0.7091837	0.9316804	-0.7612	0.447	-0.226792115	count	1
AMN1	-0.1848581	0.2335208	-0.7916	0.429	-0.22675167	count	1
NAPB	-0.2719756	0.5269536	-0.5161	0.606	-0.226641397	count	1
LCAT	-0.2719756	0.6159355	-0.4416	0.659	-0.226641397	count	1
CREBRF	-0.1630781	0.1084209	-1.5041	0.133	-0.226503721	count	1
SLC25A32	-0.1689832	0.1638285	-1.0315	0.302	-0.226358276	count	1
LINC01353	-0.255018	0.5549598	-0.4595	0.646	-0.226314519	count	1
MTSS1	-0.1734169	0.1730629	-1.002	0.316	-0.226236017	count	1
ATRX	-0.1588703	0.0705338	-2.2524	0.0244	-0.226135236	count	1
ALG12	-0.203121	0.2704781	-0.751	0.453	-0.225950779	count	1
MIR210HG	-0.2779145	0.7677184	-0.362	0.717	-0.225861658	count	1
DHRSX	-0.1836367	0.2267964	-0.8097	0.418	-0.225694789	count	1
ALDH7A1	-0.1705356	0.1832634	-0.9305	0.352	-0.225628684	count	1
MAGED2	-0.1599297	0.0787546	-2.0307	0.0424	-0.225555535	count	1
NOC2L	-0.1722256	0.1622229	-1.0617	0.288	-0.225522421	count	1
MYO10	-0.175174	0.1901618	-0.9212	0.357	-0.225518653	count	1
NR1D2	-0.1645988	0.1374315	-1.1977	0.231	-0.22544187	count	1
ING2	-0.1659268	0.1363296	-1.2171	0.224	-0.225219461	count	1

SLK	-0.1604257	0.0821754	-1.9522	0.051	-0.225185726	count	1
PCYT1A	-0.1759479	0.2275643	-0.7732	0.439	-0.225083476	count	1
AC120114.1	-1.44139	0.8881284	-1.623	0.105	-0.225065145	count	1
GCHFR	-0.1613161	0.1195968	-1.3488	0.178	-0.225044667	count	1
ODR4	-0.1663215	0.1492303	-1.1145	0.265	-0.22500647	count	1
DNAJC15	-0.1580351	0.0690018	-2.2903	0.0221	-0.224951742	count	1
CCDC126	-0.1876402	0.2575535	-0.7285	0.466	-0.224861676	count	1
DNAJC28	-0.417071	0.5811013	-0.7177	0.473	-0.224567321	count	1
TMED5	-0.1674759	0.1534698	-1.0913	0.275	-0.224519058	count	1
APEH	-0.2138082	0.2787606	-0.767	0.443	-0.224503846	count	1
PIP5K1A	-0.197933	0.2432275	-0.8138	0.416	-0.224459159	count	1
RAB7A	-0.1582091	0.0658761	-2.4016	0.0164	-0.224168314	count	1
TXLNA	-0.1726705	0.1771307	-0.9748	0.33	-0.224146268	count	1
ATXN1L	-0.1928384	0.2556101	-0.7544	0.451	-0.224024042	count	1
STK24	-0.1686805	0.1459326	-1.1559	0.248	-0.223909404	count	1
LMAN2	-0.1580711	0.0698672	-2.2624	0.0237	-0.223625834	count	1
LRRC41	-0.1769631	0.1898688	-0.932	0.351	-0.223610052	count	1
NEK4	-0.2205047	0.2733283	-0.8067	0.42	-0.223600656	count	1
AP000547.3	-0.3424205	0.5853359	-0.585	0.559	-0.223559651	count	1
AC005261.1	-0.1815589	0.1851653	-0.9805	0.327	-0.223556898	count	1
PLCD4	-1.434101	0.6767716	-2.119	0.0342	-0.223523782	count	1
EMG1	-1.434101	0.6863808	-2.0894	0.0368	-0.223523782	count	1
XPR1	-0.1832551	0.2453322	-0.747	0.455	-0.22333143	count	1
AC117503.1	-0.9123507	0.672077	-1.3575	0.175	-0.223319541	count	1
TMEM86A	-0.4148079	0.6673614	-0.6216	0.534	-0.223232024	count	1
C2CD2	-0.1907829	0.2806834	-0.6797	0.497	-0.223183637	count	1
SLAIN2	-0.163208	0.126108	-1.2942	0.196	-0.223146251	count	1
MAGI3	-0.2038799	0.2604438	-0.7828	0.434	-0.222984207	count	1
ATG7	-0.1975045	0.2536968	-0.7785	0.436	-0.222929055	count	1
STX16	-0.1700109	0.1726307	-0.9848	0.325	-0.222924594	count	1
TDG	-0.163634	0.1293137	-1.2654	0.206	-0.222879279	count	1
TMEM208	-0.1592204	0.0989955	-1.6084	0.108	-0.222863916	count	1
PIGT	-0.1597145	0.1074489	-1.4864	0.137	-0.22284446	count	1
AP2S1	-0.1564909	0.0616091	-2.5401	0.0111	-0.222639393	count	1
EIF4G1	-0.1621817	0.131734	-1.2311	0.218	-0.222523782	count	1
PDXK	-0.1807214	0.2028666	-0.8908	0.373	-0.2225154	count	1
PTS	-0.1628675	0.128795	-1.2645	0.206	-0.222374521	count	1
CYB561	-0.1669866	0.1806523	-0.9244	0.355	-0.222337596	count	1
EZR	-0.1676365	0.1747621	-0.9592	0.338	-0.222274906	count	1
SHISA4	-0.1681373	0.1853776	-0.907	0.364	-0.221909052	count	1
MRPS34	-0.1572854	0.0850801	-1.8487	0.0646	-0.221899175	count	1
FAM8A1	-0.178823	0.2144887	-0.8337	0.405	-0.221801267	count	1
PDZD2	-0.225733	0.4387658	-0.5145	0.607	-0.221732418	count	1
DLGAP4	-0.1655085	0.1440188	-1.1492	0.251	-0.221683074	count	1
SERPINH1	-0.1571345	0.0873636	-1.7986	0.0722	-0.221536818	count	1

GLTP	-0.1610623	0.1169426	-1.3773	0.169	-0.221235292	count	1
AC015802.6	-0.3390755	0.6032715	-0.5621	0.574	-0.221225136	count	1
SEC23A	-0.1654916	0.1546526	-1.0701	0.285	-0.221174675	count	1
ZFP41	-0.5017417	1.0070283	-0.4982	0.618	-0.220939008	count	1
AC092287.1	-0.5017417	1.0070283	-0.4982	0.618	-0.220939008	count	1
PARVG	-0.5017417	1.0070283	-0.4982	0.618	-0.220939008	count	1
RASA3	-0.2072669	0.3389896	-0.6114	0.541	-0.220832627	count	1
ZNF324	-0.2333001	0.3505546	-0.6655	0.506	-0.220598769	count	1
RFXAP	-0.1808797	0.2126503	-0.8506	0.395	-0.220405958	count	1
CLN5	-0.1604841	0.1367957	-1.1732	0.241	-0.220272626	count	1
SLC25A27	-0.6919396	0.5018585	-1.3788	0.168	-0.220230224	count	1
ADAMTSL1	-0.5749025	0.2978217	-1.9304	0.0537	-0.220134642	count	1
DNASE1	-0.23276	0.6855098	-0.3395	0.734	-0.220072708	count	1
DIO3OS	-0.296849	0.5471672	-0.5425	0.588	-0.219958238	count	1
SAP30L	-0.1644093	0.1820927	-0.9029	0.367	-0.219917933	count	1
TNPO2	-0.1938432	0.2981733	-0.6501	0.516	-0.219748943	count	1
ZZEF1	-0.1810371	0.2659508	-0.6807	0.496	-0.219596617	count	1
TMF1	-0.1584954	0.10442	-1.5179	0.129	-0.219435311	count	1
BAG1	-0.1548503	0.0727512	-2.1285	0.0334	-0.219387664	count	1
AC135178.3	-0.6890349	0.7479264	-0.9213	0.357	-0.219129821	count	1
LDLRAD4	-0.1738657	0.1982963	-0.8768	0.381	-0.219052842	count	1
CBLL1	-0.1828279	0.2152186	-0.8495	0.396	-0.219027592	count	1
MARK4	-0.1979925	0.2483107	-0.7974	0.425	-0.218954069	count	1
LRRC6	-0.2010058	0.2840468	-0.7077	0.479	-0.21842826	count	1
ZNHIT2	-0.2155208	0.4004555	-0.5382	0.59	-0.218425247	count	1
ZNF91	-0.1610071	0.1872574	-0.8598	0.39	-0.218390899	count	1
LINC00623	-0.4962376	0.8104814	-0.6123	0.54	-0.218210127	count	1
PPM1B	-0.1885432	0.2301066	-0.8194	0.413	-0.218147589	count	1
TTLL4	-0.6862543	0.4786979	-1.4336	0.152	-0.218077763	count	1
MALAT1	-0.1511337	0.0269972	-5.5981	2.38E-08	-0.218031072	count	0.000561585
SPCS1	-0.1522383	0.0458195	-3.3226	0.000904	-0.21800933	count	1
CHCHD1	-0.1587076	0.1172549	-1.3535	0.176	-0.21757113	count	1
C12orf73	-0.180114	0.2746115	-0.6559	0.512	-0.21741591	count	1
FAM172A	-0.1614295	0.1517579	-1.0637	0.288	-0.217314734	count	1
PSMA5	-0.1548278	0.0867725	-1.7843	0.0745	-0.217264813	count	1
ZNF417	-0.5681025	0.6268605	-0.9063	0.365	-0.217137987	count	1
TXNL4B	-0.22115	0.3293075	-0.6716	0.502	-0.217111326	count	1
YIPF4	-0.1583166	0.1293449	-1.224	0.221	-0.216720218	count	1
SIGMAR1	-0.1724934	0.2021159	-0.8534	0.393	-0.216678401	count	1
NACC2	-0.1737788	0.2668945	-0.6511	0.515	-0.216600254	count	1
U2AF1L4	-0.1894555	0.273891	-0.6917	0.489	-0.216594179	count	1
TIRAP	-0.292286	0.3952775	-0.7394	0.46	-0.216397589	count	1
NDUFA6	-0.1539332	0.085596	-1.7984	0.0722	-0.216168846	count	1
MROH1	-0.2355332	0.408751	-0.5762	0.565	-0.216018643	count	1
ACAP3	-0.2175143	0.4663992	-0.4664	0.641	-0.215912764	count	1

ZNF707	-0.4021642	0.6240855	-0.6444	0.519	-0.215794361	count	1
RAB6B	-0.3729029	0.7849295	-0.4751	0.635	-0.215686735	count	1
CD151	-0.1500348	0.0414362	-3.6209	0.000299	-0.215556902	count	1
RWDD3	-0.3308751	0.5321268	-0.6218	0.534	-0.215513978	count	1
MIDN	-0.1523913	0.0990191	-1.539	0.124	-0.215317508	count	1
BBS1	-0.5635598	0.7275313	-0.7746	0.439	-0.21514118	count	1
RAB40C	-0.1933938	0.2766548	-0.699	0.485	-0.214950003	count	1
CUX1	-0.1573514	0.1561371	-1.0078	0.314	-0.214717449	count	1
ABHD17A	-0.1515875	0.0778498	-1.9472	0.0516	-0.214705167	count	1
LINC00240	-0.4378376	0.884602	-0.495	0.621	-0.214609917	count	1
BIRC2	-0.1591773	0.1334973	-1.1924	0.233	-0.214427527	count	1
NKRF	-0.209268	0.3400113	-0.6155	0.538	-0.213995296	count	1
SULT1A3	-0.2462434	0.4730312	-0.5206	0.603	-0.213914612	count	1
HKR1	-0.202238	0.2983504	-0.6779	0.498	-0.213769746	count	1
TMCC3	-0.1637265	0.206636	-0.7923	0.428	-0.213686218	count	1
AC010618.3	-0.3696429	0.5500888	-0.672	0.502	-0.213646106	count	1
CSDE1	-0.1500033	0.0599189	-2.5034	0.0124	-0.213622097	count	1
REXO5	-0.4867534	0.607107	-0.8018	0.423	-0.213523242	count	1
UBE2O	-0.1941964	0.3622314	-0.5361	0.592	-0.213468206	count	1
NOM1	-0.1753803	0.2521851	-0.6954	0.487	-0.213155274	count	1
JMJD6	-0.161958	0.1766	-0.9171	0.359	-0.213032701	count	1
SPNS3	-0.4346239	0.5992417	-0.7253	0.468	-0.212867972	count	1
PTPRC	-0.4346231	0.6068007	-0.7163	0.474	-0.212867537	count	1
FBRS	-0.1732278	0.2075396	-0.8347	0.404	-0.212778212	count	1
KRBA2	-0.1902781	0.3487691	-0.5456	0.585	-0.212539436	count	1
ALKBH7	-0.1494524	0.0668821	-2.2346	0.0255	-0.21249139	count	1
TNXB	-0.1524246	0.1407091	-1.0833	0.279	-0.212347891	count	1
DNAJC25	-0.1931411	0.2723984	-0.709	0.478	-0.212288076	count	1
CLCC1	-0.1797618	0.2257993	-0.7961	0.426	-0.212207676	count	1
SH3BGR	-0.2396352	0.4269407	-0.5613	0.575	-0.212190888	count	1
GGT7	-0.2864537	0.5113405	-0.5602	0.575	-0.211854233	count	1
RACGAP1	-0.4325604	0.5838164	-0.7409	0.459	-0.211750734	count	1
AL031663.3	-0.3437489	0.763759	-0.4501	0.653	-0.211648888	count	1
RUNDC1	-0.207018	0.3697172	-0.5599	0.576	-0.211642236	count	1
SDCBP2	-0.1671808	0.2143024	-0.7801	0.435	-0.211438516	count	1
RAB30-AS1	-0.1639154	0.2030941	-0.8071	0.42	-0.211346757	count	1
KLHL21	-0.194481	0.3291995	-0.5908	0.555	-0.211209694	count	1
KIAA1191	-0.1621824	0.1705487	-0.9509	0.342	-0.211155699	count	1
PTGR1	-0.1579776	0.1473102	-1.0724	0.284	-0.211088321	count	1
ZFPL1	-0.1574733	0.1533718	-1.0267	0.305	-0.211053096	count	1
MAP4K3	-0.1699301	0.2062633	-0.8239	0.41	-0.211042812	count	1
TRIM8	-0.1520407	0.1203647	-1.2632	0.207	-0.210872891	count	1
SDF2	-0.1508548	0.0963177	-1.5662	0.117	-0.210854546	count	1
PIP5K1C	-0.1686381	0.1838822	-0.9171	0.359	-0.210821183	count	1
LSM7	-0.1485717	0.0701274	-2.1186	0.0342	-0.210810293	count	1

CEBPA	-0.4812251	0.5745721	-0.8375	0.402	-0.210800252	count	1
PRR19	-0.8706638	0.7337543	-1.1866	0.235	-0.210623162	count	1
EPHA3	-0.8706638	0.8044356	-1.0823	0.279	-0.210623162	count	1
LINC01303	-0.8706638	0.8079733	-1.0776	0.281	-0.210623162	count	1
DDX17	-0.147856	0.0784652	-1.8844	0.0596	-0.210548242	count	1
NDN	-0.1532832	0.1387746	-1.1045	0.269	-0.210480947	count	1
BLOC1S2	-0.1542813	0.1332658	-1.1577	0.247	-0.210152405	count	1
VPS37A	-0.1587128	0.1802829	-0.8804	0.379	-0.210149985	count	1
ANKIB1	-0.1606226	0.1810963	-0.8869	0.375	-0.210089095	count	1
C6orf132	-0.6645576	0.9958158	-0.6673	0.505	-0.209913593	count	1
ARL11	-0.6645576	0.9958158	-0.6673	0.505	-0.209913593	count	1
AKAP5	-0.6645576	0.9958158	-0.6673	0.505	-0.209913593	count	1
RTEL1-TNFRSF6B	-0.6645576	0.9958158	-0.6673	0.505	-0.209913593	count	1
CD53	-0.6645576	1.018276	-0.6526	0.514	-0.209913593	count	1
INE2	-0.6645576	1.018276	-0.6526	0.514	-0.209913593	count	1
AC073611.1	-0.6645576	1.018276	-0.6526	0.514	-0.209913593	count	1
SEPT4-AS1	-0.6645576	1.018276	-0.6526	0.514	-0.209913593	count	1
AC123912.1	-0.6645576	1.018276	-0.6526	0.514	-0.209913593	count	1
MFSD14C	-0.166779	0.2435662	-0.6847	0.494	-0.209750455	count	1
COX17	-0.1485261	0.0769806	-1.9294	0.0538	-0.209697521	count	1
AC008741.2	-0.307288	0.4892746	-0.628	0.53	-0.209659263	count	1
GSTM3	-0.1687961	0.1838638	-0.918	0.359	-0.209621956	count	1
IL13RA1	-0.1504075	0.1088821	-1.3814	0.167	-0.209512577	count	1
NAT9	-0.1739966	0.2343847	-0.7424	0.458	-0.209425416	count	1
MRPL41	-0.1483894	0.0759265	-1.9544	0.0508	-0.209333083	count	1
TMEM251	-0.1623591	0.223844	-0.7253	0.468	-0.209327032	count	1
SLC25A53	-0.2216964	0.3837999	-0.5776	0.564	-0.209311555	count	1
KIAA2013	-0.1564398	0.1642041	-0.9527	0.341	-0.209303908	count	1
CEP192	-0.1757496	0.2364729	-0.7432	0.457	-0.209286427	count	1
CSAD	-0.1587994	0.1867534	-0.8503	0.395	-0.208992767	count	1
SMURF2	-0.1557869	0.1534295	-1.0154	0.31	-0.208956066	count	1
WDPCP	-0.2062948	0.3419437	-0.6033	0.546	-0.208858759	count	1
INO80D	-0.1579883	0.1799424	-0.878	0.38	-0.208573522	count	1
CEP295	-0.1691189	0.2495847	-0.6776	0.498	-0.208095981	count	1
GFOD1	-0.1516562	0.1452914	-1.0438	0.297	-0.208041576	count	1
PSMD7	-0.1470217	0.0828165	-1.7753	0.076	-0.207810476	count	1
MRFAP1	-0.1460204	0.0571434	-2.5553	0.0107	-0.207520589	count	1
SULT1C4	-0.1684016	0.2059951	-0.8175	0.414	-0.207205098	count	1
TMEM38B	-0.2007924	0.304751	-0.6589	0.51	-0.207023936	count	1
NRL	-0.2909469	0.5662706	-0.5138	0.607	-0.206970428	count	1
AC008494.3	-0.3034293	0.8168773	-0.3715	0.71	-0.206868921	count	1
PHACTR1	-0.2296495	0.3008518	-0.7633	0.445	-0.206862989	count	1
MGMT	-0.1460225	0.0737207	-1.9808	0.0477	-0.206698399	count	1
IGF1R	-0.1531146	0.1785236	-0.8577	0.391	-0.206664733	count	1
ZNF638	-0.1487748	0.1038898	-1.432	0.152	-0.206478615	count	1

CTPS1	-0.2219676	0.4691449	-0.4731	0.636	-0.206475871	count	1
ACOX1	-0.1603184	0.2400126	-0.668	0.504	-0.206475631	count	1
C11orf58	-0.1442519	0.0470214	-3.0678	0.00218	-0.206473078	count	1
SUOX	-0.2291861	0.3473235	-0.6599	0.509	-0.206432035	count	1
ATP6V1G1	-0.1441617	0.0471829	-3.0554	0.00227	-0.206421931	count	1
IQGAP1	-0.1454207	0.0747612	-1.9451	0.0519	-0.206290912	count	1
ARHGEF11	-0.2037649	0.4038734	-0.5045	0.614	-0.206238689	count	1
SLC41A1	-0.1669864	0.2555016	-0.6536	0.513	-0.206235933	count	1
PDE7A	-0.2215291	0.3903478	-0.5675	0.57	-0.206055878	count	1
EIF3F	-0.1438072	0.0422127	-3.4067	0.000667	-0.206006527	count	1
REM1	-0.8551209	0.827195	-1.0338	0.301	-0.20594697	count	1
GCSH	-0.1513856	0.1474966	-1.0264	0.305	-0.205927784	count	1
LPP	-0.1467905	0.0938946	-1.5634	0.118	-0.205907595	count	1
FOXJ2	-0.1773679	0.2502171	-0.7089	0.478	-0.205817291	count	1
C9orf16	-0.1438619	0.0514129	-2.7982	0.00517	-0.205810912	count	1
ARPC3	-0.1434567	0.040143	-3.5736	0.000358	-0.205781415	count	1
CBX5	-0.1492203	0.1167316	-1.2783	0.201	-0.205636608	count	1
GNA11	-0.1526559	0.1657322	-0.9211	0.357	-0.205304267	count	1
LRP10	-0.146559	0.0989696	-1.4808	0.139	-0.205281689	count	1
IFT22	-0.1518147	0.140259	-1.0824	0.279	-0.205110278	count	1
IKBKG	-0.1654221	0.2270694	-0.7285	0.466	-0.205033825	count	1
GDAP2	-0.1876263	0.2923385	-0.6418	0.521	-0.204903152	count	1
LIMD2	-0.1544276	0.175696	-0.8789	0.38	-0.204790455	count	1
FAM57A	-0.2596957	0.3862223	-0.6724	0.501	-0.204459824	count	1
SGSH	-0.172688	0.3148771	-0.5484	0.583	-0.204388743	count	1
ZNF284	-0.3546881	0.6572402	-0.5397	0.589	-0.204318633	count	1
AC098650.1	-0.3546881	0.7068328	-0.5018	0.616	-0.204318633	count	1
STAG3	-0.3546881	0.7321201	-0.4845	0.628	-0.204318633	count	1
TUBG1	-0.1555619	0.1759125	-0.8843	0.377	-0.204302048	count	1
OSBPL9	-0.1523918	0.1362492	-1.1185	0.263	-0.204299709	count	1
MSX1	-0.1503588	0.1715697	-0.8764	0.381	-0.204133979	count	1
TSEN54	-0.1717507	0.2569367	-0.6685	0.504	-0.203876693	count	1
FCGRT	-0.1419726	0.0448144	-3.168	0.00155	-0.203854444	count	1
TFRC	-0.1977645	0.2926304	-0.6758	0.499	-0.203835524	count	1
RMRP	-0.4666649	0.5912334	-0.7893	0.43	-0.20366021	count	1
CCNB1IP1	-0.1491677	0.1284192	-1.1616	0.246	-0.203467146	count	1
RNF145	-0.1528483	0.1826851	-0.8367	0.403	-0.203428925	count	1
VANGL1	-0.173396	0.2261886	-0.7666	0.443	-0.20328179	count	1
CDC42EP5	-0.1443616	0.0864148	-1.6706	0.0949	-0.203214347	count	1
ITGA5	-0.1459241	0.1019912	-1.4308	0.153	-0.203057976	count	1
NXF1	-0.167447	0.2529506	-0.662	0.508	-0.202945734	count	1
SEMA6B	-0.154114	0.1471505	-1.0473	0.295	-0.202925959	count	1
MSH3	-0.1665081	0.2407779	-0.6915	0.489	-0.202722496	count	1
AURKAIP1	-0.1427713	0.0682285	-2.0925	0.0365	-0.202576986	count	1
ZMYND11	-0.14855	0.1302936	-1.1401	0.254	-0.202423075	count	1

TET2	-0.164578	0.2183993	-0.7536	0.451	-0.202055195	count	1
SMIM29	-0.149556	0.1395921	-1.0714	0.284	-0.201912564	count	1
NONO	-0.1448797	0.1021465	-1.4184	0.156	-0.201873541	count	1
D2HGDH	-0.1746335	0.3782536	-0.4617	0.644	-0.20184342	count	1
BAG2	-0.3781044	0.4862508	-0.7776	0.437	-0.201747056	count	1
TNFRSF11B	-0.181582	0.3027652	-0.5997	0.549	-0.201614741	count	1
LONP1	-0.1788019	0.330128	-0.5416	0.588	-0.201508522	count	1
CREG1	-0.1453017	0.1102752	-1.3176	0.188	-0.201506344	count	1
ZNF793-AS1	-0.273078	0.5926366	-0.4608	0.645	-0.201467677	count	1
AC110285.6	-0.3776073	0.8839828	-0.4272	0.669	-0.201458301	count	1
PLPPR4	-0.3776073	1.3396409	-0.2819	0.7781	-0.201458301	count	1
INSIG1	-0.1448036	0.1328623	-1.0899	0.276	-0.201393663	count	1
NLK	-0.1763223	0.3704357	-0.476	0.634	-0.201371936	count	1
NUDT12	-0.1711055	0.3324242	-0.5147	0.607	-0.201225649	count	1
IL20RA	-0.8392143	0.7438283	-1.1282	0.259	-0.201194151	count	1
SOD2-OT1	-0.8392143	0.8104659	-1.0355	0.301	-0.201194151	count	1
AC008686.1	-0.8392143	0.8104659	-1.0355	0.301	-0.201194151	count	1
DDAH2	-0.1420407	0.0720378	-1.9718	0.0487	-0.201056347	count	1
BBS2	-0.1682586	0.2799821	-0.601	0.548	-0.200833428	count	1
GMPPA	-0.1615021	0.2281317	-0.7079	0.479	-0.200832537	count	1
MELTF-AS1	-0.5304238	0.786997	-0.674	0.5	-0.200698828	count	1
RNF212	-0.5304238	0.824382	-0.6434	0.52	-0.200698828	count	1
MPG	-0.1422198	0.0796865	-1.7847	0.0744	-0.200441975	count	1
VPS52	-0.1945222	0.4017298	-0.4842	0.628	-0.200423498	count	1
NEK1	-0.1579954	0.188553	-0.8379	0.402	-0.200262781	count	1
MIRLET7BHG	-0.1999717	0.3222761	-0.6205	0.535	-0.200251499	count	1
VSIG2	-0.529361	0.676994	-0.7819	0.434	-0.200239217	count	1
ADSS	-0.1447703	0.1158634	-1.2495	0.212	-0.199889693	count	1
SLC4A2	-0.1728871	0.2510088	-0.6888	0.491	-0.199798628	count	1
TRIM73	-0.2812868	0.5614299	-0.501	0.616	-0.199730562	count	1
FKBP3	-0.1437233	0.1154395	-1.245	0.213	-0.199611186	count	1
SAMD15	-0.4582894	0.6932929	-0.661	0.509	-0.199573911	count	1
MILR1	-0.2255596	0.4168568	-0.5411	0.588	-0.199317963	count	1
TTC37	-0.1463882	0.1348002	-1.086	0.278	-0.199316629	count	1
SNX14	-0.1567863	0.1900505	-0.825	0.409	-0.199223202	count	1
CENPN	-0.2345676	0.4758784	-0.4929	0.622	-0.199010055	count	1
ASXL3	-0.2603245	0.540927	-0.4813	0.63	-0.198544566	count	1
LPIN1	-0.1618084	0.2237321	-0.7232	0.47	-0.198218964	count	1
ARL9	-0.6328256	0.6621495	-0.9557	0.339	-0.198117715	count	1
ASB7	-0.1999067	0.3648944	-0.5478	0.584	-0.198023151	count	1
HES2	-1.3106704	0.9683344	-1.3535	0.176	-0.197902891	count	1
IGSF3	-1.3106704	0.9683344	-1.3535	0.176	-0.197902891	count	1
EPHX3	-1.3106704	0.9683344	-1.3535	0.176	-0.197902891	count	1
FAM162B	-1.3106704	1.141671	-1.148	0.251	-0.197902891	count	1
AP001160.2	-1.3106704	1.141671	-1.148	0.251	-0.197902891	count	1

ZNF625-ZNF20	-1.3106704	1.186717	-1.1045	0.269	-0.197902891	count	1
CTTN	-0.1405567	0.0893396	-1.5733	0.116	-0.197851344	count	1
KIAA1109	-0.1429205	0.1239251	-1.1533	0.249	-0.19785075	count	1
IZUMO1	-0.4065725	0.5666165	-0.7175	0.473	-0.197764209	count	1
IFI44L	-0.1392856	0.0989428	-1.4077	0.159	-0.197759616	count	1
ATP5MG	-0.1379635	0.0436343	-3.1618	0.00158	-0.19759878	count	1
PRPS1	-0.1499151	0.174802	-0.8576	0.391	-0.197369696	count	1
CENPW	-0.1667343	0.2566831	-0.6496	0.516	-0.197262118	count	1
ADGRF5	-0.1434379	0.130918	-1.0956	0.273	-0.196999977	count	1
PINK1-AS	-1.304972	0.8812506	-1.4808	0.139	-0.196743195	count	1
AC018557.1	-1.304972	0.910491	-1.4333	0.152	-0.196743195	count	1
PTPRE	-0.1408281	0.1219055	-1.1552	0.248	-0.19661261	count	1
FIG4	-0.1908719	0.3809975	-0.501	0.616	-0.196584762	count	1
ATP5ME	-0.1384146	0.0653913	-2.1167	0.0344	-0.196418848	count	1
ERMAP	-0.2576033	0.4576071	-0.5629	0.574	-0.19637414	count	1
TBC1D14	-0.1680429	0.3740788	-0.4492	0.653	-0.196261624	count	1
IRGQ	-0.2112546	0.4197991	-0.5032	0.615	-0.196227974	count	1
RHEB	-0.138345	0.0683097	-2.0253	0.0429	-0.196188038	count	1
PSMA2	-0.1412171	0.1002631	-1.4085	0.159	-0.196057693	count	1
AC112220.4	-0.5194878	0.6026616	-0.862	0.389	-0.195980104	count	1
PHC3	-0.1500793	0.2368299	-0.6337	0.526	-0.19562443	count	1
ZNF664	-0.1679349	0.2606152	-0.6444	0.519	-0.195444858	count	1
CCDC84	-0.1553154	0.2512879	-0.6181	0.537	-0.194935838	count	1
ANKRD20A8P	-0.6239297	0.8728168	-0.7148	0.475	-0.19484178	count	1
AL139099.1	-0.6239297	0.8728168	-0.7148	0.475	-0.19484178	count	1
AC007566.1	-0.6239297	0.8848642	-0.7051	0.481	-0.19484178	count	1
KIZ-AS1	-0.6239297	0.8848642	-0.7051	0.481	-0.19484178	count	1
ZNF726	-0.6239297	0.8848642	-0.7051	0.481	-0.19484178	count	1
AL391845.2	-0.6239297	1.018209	-0.6128	0.54	-0.19484178	count	1
PGS1	-0.1612045	0.2117825	-0.7612	0.447	-0.194840032	count	1
LIMK1	-0.2747086	0.4746587	-0.5787	0.563	-0.194814328	count	1
TTC38	-0.2206143	0.3394236	-0.65	0.516	-0.194806764	count	1
DHDDS	-0.157777	0.2300982	-0.6857	0.493	-0.194763896	count	1
LRRC20	-0.4008646	0.6100475	-0.6571	0.511	-0.194713225	count	1
CYB5A	-0.1367386	0.0665432	-2.0549	0.04	-0.194632941	count	1
FBXO42	-0.1794493	0.3948607	-0.4545	0.65	-0.194610732	count	1
NDUFB10	-0.1361317	0.0506819	-2.686	0.00727	-0.194584589	count	1
SGTB	-0.1575746	0.2284283	-0.6898	0.49	-0.194511889	count	1
SERPINA1	-0.8161453	0.8287573	-0.9848	0.325	-0.194360618	count	1
ZFAND2A	-0.1491026	0.1786473	-0.8346	0.404	-0.194344368	count	1
RPS10	-0.1357373	0.0434086	-3.127	0.00178	-0.194066455	count	1
ST3GAL3	-0.2402424	0.3756393	-0.6396	0.523	-0.194038119	count	1
ZNF260	-0.1648173	0.2880422	-0.5722	0.567	-0.19374503	count	1
SETMAR	-0.2118737	0.3616505	-0.5859	0.558	-0.193687098	count	1
ATMIN	-0.1505965	0.198347	-0.7593	0.448	-0.193683081	count	1

IPO4	-0.2850439	0.5422492	-0.5257	0.599	-0.193626516	count	1
TMEM104	-0.2850439	0.5859353	-0.4865	0.627	-0.193626516	count	1
ELAVL1	-0.1432493	0.1363546	-1.0506	0.294	-0.193625098	count	1
ZDHHC21	-0.1548532	0.2234536	-0.693	0.488	-0.193452961	count	1
PFDN6	-0.147569	0.1508027	-0.9786	0.328	-0.192926058	count	1
TIMM10B	-0.1481094	0.1866675	-0.7934	0.428	-0.192888856	count	1
LINC01094	-0.3147826	0.6703185	-0.4696	0.639	-0.192610448	count	1
AL118558.3	-0.2836085	0.6833988	-0.415	0.678	-0.19259633	count	1
ICK	-0.1513987	0.2036868	-0.7433	0.457	-0.192567499	count	1
TCEAL4	-0.1350335	0.058097	-2.3243	0.0202	-0.192528447	count	1
KPNA4	-0.1394304	0.0998203	-1.3968	0.163	-0.192528281	count	1
LUC7L2	-0.1433925	0.1435416	-0.999	0.318	-0.192188536	count	1
HSD17B10	-0.1388001	0.114632	-1.2108	0.226	-0.192184434	count	1
ANKS6	-0.1850244	0.3861777	-0.4791	0.632	-0.192108532	count	1
IQGAP2	-0.8084281	0.743361	-1.0875	0.277	-0.192090397	count	1
HEBP1	-0.1351388	0.0696967	-1.939	0.0526	-0.191882073	count	1
NPIPB15	-1.280559	0.5336739	-2.3995	0.0165	-0.191799754	count	1
FYTTD1	-0.1377343	0.1014575	-1.3576	0.175	-0.191678639	count	1
TUSC3	-0.145316	0.1824024	-0.7967	0.426	-0.191528603	count	1
TTBK2	-0.167071	0.2291308	-0.7292	0.466	-0.191466544	count	1
ZNF212	-0.2699418	0.4585944	-0.5886	0.556	-0.191258887	count	1
LNPEP	-0.1422786	0.15356	-0.9265	0.354	-0.191075237	count	1
TRIM22	-0.1385173	0.1178776	-1.1751	0.24	-0.191063285	count	1
ANKRD39	-0.1559589	0.2233019	-0.6984	0.485	-0.190989125	count	1
GCC1	-0.1889771	0.3270847	-0.5778	0.563	-0.190951543	count	1
ACTR6	-0.1403033	0.1325718	-1.0583	0.29	-0.190907641	count	1
ZNF527	-0.2591954	0.4369639	-0.5932	0.553	-0.190736549	count	1
CEP350	-0.1401103	0.1363086	-1.0279	0.304	-0.190693963	count	1
RAD52	-0.2160763	0.4768929	-0.4531	0.651	-0.190672424	count	1
SUMF2	-0.1366194	0.099803	-1.3689	0.171	-0.190343299	count	1
CDON	-0.1754349	0.3515435	-0.499	0.618	-0.190185155	count	1
DEF6	-0.392237	0.6807198	-0.5762	0.565	-0.190115986	count	1
NQO2	-0.1419563	0.1475529	-0.9621	0.336	-0.189933201	count	1
NPR1	-0.1412656	0.1484155	-0.9518	0.341	-0.189929857	count	1
ABHD8	-0.2015943	0.3976351	-0.507	0.612	-0.189832091	count	1
DIS3L2	-0.1631619	0.2880185	-0.5665	0.571	-0.189823405	count	1
SZRD1	-0.1374893	0.1347815	-1.0201	0.308	-0.189785785	count	1
TOMM5	-0.1389219	0.136051	-1.0211	0.307	-0.189763463	count	1
AC027097.2	-0.6100206	0.72303	-0.8437	0.399	-0.189747098	count	1
DLX2	-0.6100206	0.7246651	-0.8418	0.4	-0.189747098	count	1
ATP6V0D1	-0.1400626	0.1243295	-1.1265	0.26	-0.189720588	count	1
LIN9	-0.219149	0.3714105	-0.59	0.555	-0.189602473	count	1
KCTD20	-0.1391538	0.1404622	-0.9907	0.322	-0.189581652	count	1
KMT5A	-0.1402146	0.1301717	-1.0772	0.282	-0.189570789	count	1
SOCS4	-0.1408408	0.1548778	-0.9094	0.363	-0.189499179	count	1

BAG4	-0.1458428	0.1934812	-0.7538	0.451	-0.18945216	count	1
FAM3A	-0.1424847	0.1666761	-0.8549	0.393	-0.189385412	count	1
SARS2	-0.2787995	0.4472324	-0.6234	0.533	-0.189148836	count	1
RBM34	-0.179204	0.3558842	-0.5035	0.615	-0.188980248	count	1
ATP6V0A1	-0.1503681	0.2609898	-0.5761	0.565	-0.188959929	count	1
AC106707.1	-0.2406788	0.3174116	-0.7583	0.448	-0.188869815	count	1
MRPS11	-0.1388649	0.1330964	-1.0433	0.297	-0.188844649	count	1
SYTL4	-0.1647792	0.258122	-0.6384	0.523	-0.188806268	count	1
YDJC	-0.1495195	0.2109373	-0.7088	0.478	-0.188687951	count	1
C3orf18	-1.2619061	0.7930028	-1.5913	0.112	-0.188049736	count	1
CASP9	-0.1900007	0.4114239	-0.4618	0.644	-0.187988817	count	1
APTX	-0.148015	0.2077776	-0.7124	0.476	-0.187766645	count	1
TLN1	-0.1317554	0.071261	-1.8489	0.0646	-0.187535121	count	1
TPI1	-0.1309913	0.0450475	-2.9078	0.00367	-0.187523867	count	1
RPL15	-0.1300813	0.0215766	-6.0288	1.87E-09	-0.187419215	count	4.43E-05
MPST	-0.1348392	0.1114506	-1.2099	0.226	-0.187371499	count	1
ZNF865	-0.232252	0.4018204	-0.578	0.563	-0.187334771	count	1
YLPM1	-0.1462923	0.1881435	-0.7776	0.437	-0.187323373	count	1
TOB2	-0.1397642	0.1595964	-0.8757	0.381	-0.187228905	count	1
STK11	-0.1439606	0.2063734	-0.6976	0.486	-0.187150507	count	1
CFAP161	-1.257234	0.7223707	-1.7404	0.0819	-0.187114156	count	1
ZNF23	-1.257234	0.9173073	-1.3706	0.171	-0.187114156	count	1
AC006064.2	-1.257234	0.9908143	-1.2689	0.205	-0.187114156	count	1
IMPDH1	-0.1518377	0.1734006	-0.8756	0.381	-0.187015294	count	1
CDKN2D	-0.1350608	0.1194449	-1.1307	0.258	-0.186971991	count	1
LLGL1	-0.4982355	0.6055536	-0.8228	0.411	-0.186878264	count	1
UAP1L1	-0.3263353	0.5769752	-0.5656	0.572	-0.186787498	count	1
NDUFB1	-0.1319563	0.0723401	-1.8241	0.0682	-0.186699619	count	1
AL139246.5	-0.1886751	0.3963227	-0.4761	0.634	-0.186647719	count	1
COX7C	-0.1300339	0.0359888	-3.6132	0.000308	-0.186638843	count	1
AC005261.3	-0.2115187	0.4250405	-0.4976	0.619	-0.186525368	count	1
ZFHX4	-0.7891344	1.0312802	-0.7652	0.444	-0.186449496	count	1
NEURL3	-0.7891344	1.0632278	-0.7422	0.458	-0.186449496	count	1
GPR1	-0.7891344	1.0632278	-0.7422	0.458	-0.186449496	count	1
AC121247.1	-0.7891344	1.0632278	-0.7422	0.458	-0.186449496	count	1
DENND6A-AS1	-0.7891344	1.0632278	-0.7422	0.458	-0.186449496	count	1
STBD1	-0.7891344	1.0632278	-0.7422	0.458	-0.186449496	count	1
RBM24	-0.7891344	1.0632278	-0.7422	0.458	-0.186449496	count	1
AC008149.1	-0.7891344	1.0632278	-0.7422	0.458	-0.186449496	count	1
AL122035.2	-0.7891344	1.0632278	-0.7422	0.458	-0.186449496	count	1
PLS1	-0.7891344	1.37454	-0.5741	0.566	-0.186449496	count	1
LILRB5	-0.7891344	1.37454	-0.5741	0.566	-0.186449496	count	1
ZNF181	-0.147734	0.2464118	-0.5995	0.549	-0.186418838	count	1
RETSAT	-0.176772	0.4178637	-0.423	0.672	-0.186369198	count	1
ARF4	-0.1316115	0.0690046	-1.9073	0.0566	-0.186341204	count	1

REV1	-0.1401322	0.1544833	-0.9071	0.364	-0.18624542	count	1
MZF1	-0.176513	0.2982553	-0.5918	0.554	-0.186091207	count	1
SETD5	-0.1378477	0.1537762	-0.8964	0.37	-0.185988726	count	1
PRRC1	-0.1385612	0.1767414	-0.784	0.433	-0.185916779	count	1
NNMT	-0.1293499	0.0519071	-2.4919	0.0128	-0.185826572	count	1
XPA	-0.1337713	0.1178045	-1.1355	0.256	-0.185506468	count	1
DCP2	-0.1354884	0.1339943	-1.0111	0.312	-0.18550578	count	1
CYP4V2	-0.1721727	0.3343298	-0.515	0.607	-0.185377931	count	1
SCMH1	-0.1817858	0.3259769	-0.5577	0.577	-0.185328229	count	1
CCDC92	-0.1448674	0.199556	-0.7259	0.468	-0.185283186	count	1
NFS1	-0.1661958	0.2582366	-0.6436	0.52	-0.185256383	count	1
CHTF18	-0.2732265	0.594437	-0.4596	0.646	-0.18516119	count	1
GIT2	-0.154762	0.2227975	-0.6946	0.487	-0.18506932	count	1
RARA-AS1	-0.1432463	0.196828	-0.7278	0.467	-0.184722031	count	1
PECAM1	-0.1283652	0.0407374	-3.151	0.00164	-0.184635108	count	1
ASPSCR1	-0.148795	0.2346231	-0.6342	0.526	-0.184587556	count	1
TMEM59	-0.1284883	0.0395848	-3.2459	0.00118	-0.18449425	count	1
KAZN	-0.1417518	0.2093448	-0.6771	0.498	-0.184415781	count	1
PTBP1	-0.135248	0.134793	-1.0034	0.316	-0.184336897	count	1
CTNND1	-0.1344571	0.1353535	-0.9934	0.321	-0.184241577	count	1
USP1	-0.1329747	0.123407	-1.0775	0.281	-0.18421927	count	1
HM13	-0.1312773	0.0901623	-1.456	0.146	-0.183906995	count	1
ZNF326	-0.1328907	0.1185753	-1.1207	0.263	-0.183532181	count	1
TANGO2	-0.2010043	0.3764439	-0.534	0.593	-0.183472965	count	1
FMNL3	-0.1492579	0.2501976	-0.5966	0.551	-0.183087177	count	1
HIF1A	-0.131038	0.0941736	-1.3915	0.164	-0.183084342	count	1
CAMKMT	-0.1831317	0.3254734	-0.5627	0.574	-0.183029146	count	1
CASD1	-0.13889	0.1954135	-0.7107	0.477	-0.183020286	count	1
HSPB1	-0.1271131	0.0335391	-3.79	0.000154	-0.183015327	count	1
METTL17	-0.1565787	0.2554108	-0.613	0.54	-0.182720274	count	1
DDB2	-0.139175	0.1717842	-0.8102	0.418	-0.182425433	count	1
NCSTN	-0.1369734	0.1595669	-0.8584	0.391	-0.182306276	count	1
IGHA1	-1.232694	0.2294966	-5.3713	8.47E-08	-0.182225324	count	0.001992313
TUSC2	-0.1327171	0.1258262	-1.0548	0.292	-0.182206025	count	1
RBBP6	-0.1306169	0.1056691	-1.2361	0.217	-0.181983001	count	1
ACBD5	-0.139227	0.1652209	-0.8427	0.399	-0.18196574	count	1
ATP5PD	-0.1274219	0.052517	-2.4263	0.0153	-0.181959343	count	1
TOX2	-0.1708843	0.2816148	-0.6068	0.544	-0.181411324	count	1
HOMEZ	-0.1696295	0.3121587	-0.5434	0.587	-0.181351054	count	1
ADPRM	-0.1717135	0.3477247	-0.4938	0.621	-0.180942185	count	1
BCL9L	-0.1439489	0.2592522	-0.5552	0.579	-0.18083614	count	1
PPM1J	-0.2668434	0.5671835	-0.4705	0.638	-0.180603891	count	1
ATRIP	-0.1868516	0.3440074	-0.5432	0.587	-0.1804418	count	1
RCC1L	-0.1410722	0.1914418	-0.7369	0.461	-0.180399538	count	1
ETHE1	-0.1275393	0.0855157	-1.4914	0.136	-0.180143804	count	1

AF165147.1	-1.222128	0.7438854	-1.6429	0.101	-0.180133611	count	1
LYSMD4	-0.2129357	0.410011	-0.5193	0.604	-0.180047002	count	1
PLA2G15	-0.1864259	0.4152379	-0.449	0.653	-0.180021054	count	1
MRPL43	-0.1270333	0.076727	-1.6557	0.0979	-0.17977401	count	1
TERF2IP	-0.1262996	0.0668467	-1.8894	0.0589	-0.179627289	count	1
USP18	-0.1588455	0.2923529	-0.5433	0.587	-0.179560772	count	1
ZNF26	-0.1628261	0.3323515	-0.4899	0.624	-0.179502704	count	1
FSIP2	-0.3718157	0.7055304	-0.527	0.598	-0.179303803	count	1
AMOT	-0.1934791	0.4189977	-0.4618	0.644	-0.179284602	count	1
SLC35A5	-0.140227	0.2074865	-0.6758	0.499	-0.179110626	count	1
ATP5F1B	-0.1262751	0.0590548	-2.1383	0.0326	-0.179101592	count	1
SAR1A	-0.1276145	0.0906354	-1.408	0.159	-0.178972534	count	1
RPAIN	-0.1275787	0.0964931	-1.3222	0.186	-0.178943963	count	1
TRABD	-0.1405182	0.1933919	-0.7266	0.468	-0.178857298	count	1
ALG13	-0.1293187	0.1144532	-1.1299	0.259	-0.178791108	count	1
PHACTR4	-0.131565	0.1237682	-1.063	0.288	-0.178644109	count	1
RIDA	-0.1428431	0.216591	-0.6595	0.51	-0.17862233	count	1
CD7	-0.7619861	0.8888581	-0.8573	0.391	-0.178597015	count	1
INMT-MINDY4	-0.7619861	0.9870292	-0.772	0.44	-0.178597015	count	1
ADRM1	-0.1264504	0.0752356	-1.6807	0.0929	-0.178585836	count	1
CLHC1	-0.2527224	0.496057	-0.5095	0.61	-0.178464922	count	1
COPS8	-0.1282926	0.0997073	-1.2867	0.198	-0.178408012	count	1
PNP	-0.126212	0.0816641	-1.5455	0.122	-0.178402228	count	1
PRRC2C	-0.1248847	0.0562273	-2.2211	0.0264	-0.178286286	count	1
CTU2	-0.1474606	0.2562694	-0.5754	0.565	-0.178076901	count	1
ANKRD50	-0.1492839	0.2170477	-0.6878	0.492	-0.177962711	count	1
RAB21	-0.1282952	0.1011963	-1.2678	0.205	-0.177898088	count	1
AL135925.1	-0.1392857	0.2027813	-0.6869	0.492	-0.17769722	count	1
SOX18	-0.1236169	0.0630223	-1.9615	0.0499	-0.177543242	count	1
XPNPEP1	-0.1447686	0.2083115	-0.695	0.487	-0.177534803	count	1
ZBTB3	-0.3684436	0.5356403	-0.6879	0.492	-0.177527821	count	1
HSD17B14	-0.1423058	0.2339818	-0.6082	0.543	-0.177376139	count	1
HECTD2	-0.150071	0.2812168	-0.5336	0.594	-0.1773449	count	1
POGLUT1	-0.1313881	0.1541313	-0.8524	0.394	-0.177121886	count	1
YKT6	-0.1477837	0.2398298	-0.6162	0.538	-0.177118368	count	1
UBL7	-0.1320332	0.1658788	-0.796	0.426	-0.177055062	count	1
VPS53	-0.1449677	0.2921304	-0.4962	0.62	-0.177043679	count	1
YWHAB	-0.1230491	0.0381639	-3.2242	0.00128	-0.176627477	count	1
SELENOS	-0.1241994	0.0684402	-1.8147	0.0697	-0.17662174	count	1
S100A16	-0.1235002	0.0506052	-2.4405	0.0147	-0.176489443	count	1
CBX7	-0.1381922	0.2123066	-0.6509	0.515	-0.176087804	count	1
SCARB2	-0.1249662	0.088254	-1.416	0.157	-0.175745208	count	1
AC026691.1	-0.5711196	0.8295056	-0.6885	0.491	-0.175676402	count	1
AL391832.2	-0.5711196	0.8395697	-0.6803	0.496	-0.175676402	count	1
LINC02574	-0.5711196	0.8558111	-0.6673	0.505	-0.175676402	count	1

ANO1	-0.5711196	0.8958585	-0.6375	0.524	-0.175676402	count	1
C2CD5	-0.16124	0.2814175	-0.573	0.567	-0.175657135	count	1
SHROOM4	-0.1286436	0.1402364	-0.9173	0.359	-0.175605734	count	1
PRAG1	-0.2312673	0.6557557	-0.3527	0.724	-0.175468383	count	1
DAD1	-0.1223005	0.0418645	-2.9213	0.00351	-0.175431552	count	1
SMC1A	-0.1295002	0.1488164	-0.8702	0.384	-0.175423374	count	1
TMEM9	-0.1273404	0.1319144	-0.9653	0.334	-0.175390208	count	1
PTRH2	-0.1310669	0.1502113	-0.8725	0.383	-0.175310543	count	1
LIPG	-0.750476	0.7910433	-0.9487	0.343	-0.175297927	count	1
DNAJC27	-0.1814005	0.3460276	-0.5242	0.6	-0.175057335	count	1
OGFOD1	-0.1436253	0.2423425	-0.5927	0.553	-0.17501203	count	1
SLC23A2	-0.1637756	0.2958871	-0.5535	0.58	-0.174991361	count	1
VAPB	-0.135744	0.1784053	-0.7609	0.447	-0.174823552	count	1
ZNF473	-0.2117969	0.4479612	-0.4728	0.636	-0.174798938	count	1
QSOX1	-0.1333619	0.173044	-0.7707	0.441	-0.174770316	count	1
VKORC1	-0.1230026	0.0710706	-1.7307	0.0836	-0.174654097	count	1
TTYH2	-0.2583515	0.5270876	-0.4901	0.624	-0.174557545	count	1
AGAP4	-0.2583515	0.5527623	-0.4674	0.64	-0.174557545	count	1
FAM131A	-0.1610786	0.2718534	-0.5925	0.554	-0.174384115	count	1
MCU	-0.1450869	0.2728757	-0.5317	0.595	-0.174310115	count	1
ZNF770	-0.1312319	0.1678132	-0.782	0.434	-0.174094503	count	1
FAM53A	-0.4051626	0.8281502	-0.4892	0.625	-0.174011815	count	1
AL590399.1	-0.4051626	0.8361251	-0.4846	0.628	-0.174011815	count	1
PBLD	-0.4051626	0.836677	-0.4843	0.628	-0.174011815	count	1
AC009065.4	-0.4669879	0.8124191	-0.5748	0.565	-0.173660181	count	1
SEMA6D	-0.1903984	0.3692597	-0.5156	0.606	-0.173534185	count	1
FASTKD1	-0.2054439	0.3933954	-0.5222	0.602	-0.173507254	count	1
R3HCC1L	-0.1429547	0.2208242	-0.6474	0.517	-0.173406981	count	1
SGSM3	-0.2850127	0.5202665	-0.5478	0.584	-0.173267048	count	1
BAG3	-0.124508	0.1077858	-1.1551	0.248	-0.172857124	count	1
HK1	-0.1295663	0.1561325	-0.8298	0.407	-0.172745179	count	1
RPS16	-0.1199728	0.0238564	-5.0289	5.25E-07	-0.172729203	count	0.012293925
AL445228.2	-0.5625293	1.016648	-0.5533	0.58	-0.1726048	count	1
AC099522.2	-0.5625293	1.016648	-0.5533	0.58	-0.1726048	count	1
HCG27	-0.5625293	1.0161469	-0.5536	0.58	-0.1726048	count	1
FZD9	-0.5625293	1.0161469	-0.5536	0.58	-0.1726048	count	1
XRCC2	-0.5625293	1.016648	-0.5533	0.58	-0.1726048	count	1
AL158151.1	-0.5625293	1.0161469	-0.5536	0.58	-0.1726048	count	1
GOLGA8H	-0.5625293	1.016648	-0.5533	0.58	-0.1726048	count	1
AC011825.2	-0.5625293	1.0161469	-0.5536	0.58	-0.1726048	count	1
AC020928.2	-0.5625293	1.0161469	-0.5536	0.58	-0.1726048	count	1
DM1-AS	-0.5625293	1.016648	-0.5533	0.58	-0.1726048	count	1
ZNF460-AS1	-0.5625293	1.0161469	-0.5536	0.58	-0.1726048	count	1
MYO6	-0.1292158	0.1533714	-0.8425	0.4	-0.172437612	count	1
EMC9	-0.146386	0.2309361	-0.6339	0.526	-0.172407961	count	1

KCNN3	-0.1237204	0.1228621	-1.007	0.314	-0.172390649	count	1
TACC3	-0.2835893	0.6749756	-0.4201	0.674	-0.172347897	count	1
ST6GALNAC6	-0.2269287	0.5907152	-0.3842	0.701	-0.172041817	count	1
STUB1	-0.121541	0.0810828	-1.499	0.134	-0.172018703	count	1
AC002451.1	-0.2138737	0.5772842	-0.3705	0.711	-0.171979417	count	1
ZSWIM7	-0.1242465	0.1298822	-0.9566	0.339	-0.171811776	count	1
TMEM8A	-0.1428626	0.2559873	-0.5581	0.577	-0.171613377	count	1
PSMA4	-0.1211693	0.0698735	-1.7341	0.083	-0.171472937	count	1
RPL7L1	-0.121323	0.0893182	-1.3583	0.174	-0.17102953	count	1
VGF	-0.3245593	0.7013446	-0.4628	0.644	-0.170988892	count	1
RCC2	-0.1410298	0.2070097	-0.6813	0.496	-0.170652298	count	1
THUMPD2	-0.1420589	0.2111701	-0.6727	0.501	-0.17063915	count	1
C17orf67	-0.1361339	0.2265862	-0.6008	0.548	-0.170438972	count	1
HMGN1	-0.1193375	0.0600405	-1.9876	0.047	-0.170293262	count	1
TAB1	-0.1688762	0.3863733	-0.4371	0.662	-0.170248612	count	1
SPRYD7	-0.1284044	0.1624928	-0.7902	0.429	-0.170236645	count	1
ARL6IP4	-0.1188771	0.0475727	-2.4989	0.0125	-0.170007662	count	1
GOLGA7	-0.1209862	0.0862208	-1.4032	0.161	-0.169993208	count	1
CXCL3	-0.1198066	0.2400097	-0.4992	0.618	-0.169885751	count	1
WDR83OS	-0.1185297	0.0460337	-2.5748	0.0101	-0.169849185	count	1
LRMDA	-0.1221536	0.1123052	-1.0877	0.277	-0.169767771	count	1
RNH1	-0.1189698	0.0558167	-2.1314	0.0331	-0.169733724	count	1
ARL10	-0.2238732	0.5645017	-0.3966	0.692	-0.169631599	count	1
CCDC71L	-0.122629	0.1423513	-0.8615	0.389	-0.169570688	count	1
ANG	-0.1403535	0.2571656	-0.5458	0.585	-0.169419084	count	1
GAPDH	-0.117414	0.0227454	-5.1621	2.62E-07	-0.169253984	count	0.006146258
IQCG	-0.1563089	0.3165252	-0.4938	0.621	-0.16914338	count	1
PHF20L1	-0.1220596	0.1084831	-1.1251	0.261	-0.169048379	count	1
GPS1	-0.1291655	0.1617313	-0.7986	0.425	-0.169003721	count	1
HAUS1	-0.124838	0.1316807	-0.948	0.343	-0.168986102	count	1
CUTA	-0.1190389	0.0711821	-1.6723	0.0946	-0.168962026	count	1
C17orf51	-0.1920876	0.4145429	-0.4634	0.643	-0.16890274	count	1
GCDH	-0.1487973	0.3145086	-0.4731	0.636	-0.168825603	count	1
RAB20	-0.1917306	0.4576883	-0.4189	0.675	-0.168579846	count	1
TMEM184B	-0.1306682	0.1823091	-0.7167	0.474	-0.168251361	count	1
AL355472.1	-0.1790051	0.362548	-0.4937	0.622	-0.168056631	count	1
SCAMP1	-0.1238156	0.138064	-0.8968	0.37	-0.167801234	count	1
CCDC171	-0.1550866	0.3712136	-0.4178	0.676	-0.167801092	count	1
NFIB	-0.117411	0.0584382	-2.0091	0.0446	-0.167767612	count	1
DHFR2	-0.1841227	0.3936665	-0.4677	0.64	-0.167666209	count	1
MNAT1	-0.12298	0.126019	-0.9759	0.329	-0.167613977	count	1
RAB18	-0.1220364	0.114176	-1.0688	0.285	-0.167572026	count	1
EDF1	-0.1168456	0.0392635	-2.9759	0.00295	-0.167561328	count	1
KIAA1755	-0.3915344	0.5816192	-0.6732	0.501	-0.167554921	count	1
YIPF6	-0.1226571	0.1264291	-0.9702	0.332	-0.167528978	count	1

ADCY6	-0.1628079	0.3282356	-0.496	0.62	-0.167166975	count	1
RAB4A	-0.1218217	0.1135906	-1.0725	0.284	-0.166987835	count	1
ZNF185	-0.2938392	1.4300808	-0.2055	0.8372	-0.166943081	count	1
LIN7C	-0.126705	0.1736626	-0.7296	0.466	-0.166896166	count	1
AC008966.1	-0.2596313	0.760123	-0.3416	0.733	-0.166623642	count	1
SGSM2	-0.1728269	0.3716164	-0.4651	0.642	-0.166602573	count	1
C1orf226	-1.152249	0.594239	-1.939	0.0526	-0.166502809	count	1
SAAL1	-0.1487313	0.2835191	-0.5246	0.6	-0.166381765	count	1
SAMD1	-0.1266256	0.1837791	-0.689	0.491	-0.166359212	count	1
AL161457.2	-0.1970774	1.1068706	-0.178	0.8587	-0.166220951	count	1
DSTN	-0.1155916	0.0375222	-3.0806	0.00209	-0.166126096	count	1
ZSCAN26	-0.1354824	0.239049	-0.5668	0.571	-0.166058168	count	1
PPP6R3	-0.1236948	0.1642772	-0.753	0.452	-0.166036071	count	1
INCENP	-0.2733705	0.5914243	-0.4622	0.644	-0.165764531	count	1
ANKRD6	-0.2580307	0.4187104	-0.6163	0.538	-0.165540414	count	1
STX1B	-0.3140914	0.7384339	-0.4253	0.671	-0.165057919	count	1
SARM1	-0.1957395	0.5825891	-0.336	0.737	-0.165057458	count	1
PRPF18	-0.125532	0.1722207	-0.7289	0.466	-0.165025526	count	1
SIGIRR	-0.1164308	0.0721872	-1.6129	0.107	-0.165018457	count	1
IPO7	-0.1212124	0.1362609	-0.8896	0.374	-0.16490575	count	1
FUT11	-0.131889	0.256123	-0.5149	0.607	-0.164832205	count	1
AVL9	-0.1555082	0.4024509	-0.3864	0.699	-0.164831486	count	1
SRL	-0.2901485	0.7564995	-0.3835	0.701	-0.164706196	count	1
KPNA2	-0.1261215	0.1798752	-0.7012	0.483	-0.164634858	count	1
HIST1H1E	-0.1201413	0.1585182	-0.7579	0.449	-0.164478155	count	1
NDUFB3	-0.1170365	0.0834445	-1.4026	0.161	-0.164329812	count	1
PTRH1	-0.1279686	0.186449	-0.6863	0.493	-0.164084537	count	1
MTBP	-0.4440167	0.527931	-0.8411	0.4	-0.164068772	count	1
HIST1H4I	-0.4435272	0.9539729	-0.4649	0.642	-0.163865553	count	1
PLPP7	-0.4435272	0.9539729	-0.4649	0.642	-0.163865553	count	1
LINC01198	-0.4435272	0.9539729	-0.4649	0.642	-0.163865553	count	1
LINC01588	-0.4435272	0.9539729	-0.4649	0.642	-0.163865553	count	1
AC126773.2	-0.4435272	0.9539729	-0.4649	0.642	-0.163865553	count	1
AC007786.1	-0.4435272	0.9539729	-0.4649	0.642	-0.163865553	count	1
AC211476.2	-0.4435272	0.9766226	-0.4541	0.65	-0.163865553	count	1
TCEAL7	-0.4435272	0.9766226	-0.4541	0.65	-0.163865553	count	1
FAM83G	-0.4435272	0.9766226	-0.4541	0.65	-0.163865553	count	1
ZNF592	-0.1596105	0.3534335	-0.4516	0.652	-0.163826099	count	1
AC003102.1	-0.1504397	0.4004214	-0.3757	0.707	-0.163725133	count	1
NLGN4Y	-0.232632	0.4677966	-0.4973	0.619	-0.163636661	count	1
SNX11	-0.1430177	0.3002404	-0.4763	0.634	-0.163590727	count	1
TRAPPC6B	-0.1253794	0.2016492	-0.6218	0.534	-0.16340875	count	1
PRRX1	-0.2696444	0.7733364	-0.3487	0.727	-0.163370777	count	1
ST20-AS1	-0.2322629	0.6738714	-0.3447	0.73	-0.163365237	count	1
FAM71E1	-0.2322629	0.6992822	-0.3321	0.74	-0.163365237	count	1

ATP5PB	-0.1151479	0.0668205	-1.7232	0.085	-0.163339767	count	1
RABEPK	-0.1293757	0.2057452	-0.6288	0.53	-0.163333204	count	1
METTL9	-0.116026	0.0824377	-1.4074	0.159	-0.163314221	count	1
DCUN1D1	-0.1264317	0.1800167	-0.7023	0.483	-0.163238508	count	1
MRPL28	-0.1176209	0.1070904	-1.0983	0.272	-0.162971468	count	1
COMMD8	-0.1190425	0.1303252	-0.9134	0.361	-0.162867229	count	1
TUBGCP5	-0.1494378	0.2720729	-0.5493	0.583	-0.16261941	count	1
AL645939.4	-1.131205	0.8459315	-1.3372	0.181	-0.162469029	count	1
TMEM129	-0.1327631	0.2379593	-0.5579	0.577	-0.162364834	count	1
SLC35B1	-0.1201447	0.1391375	-0.8635	0.388	-0.16225113	count	1
AIG1	-0.1154109	0.0935715	-1.2334	0.218	-0.162234979	count	1
COMMD3	-0.1172008	0.118	-0.9932	0.321	-0.162050577	count	1
PSMB1	-0.1130075	0.0462944	-2.4411	0.0147	-0.161849473	count	1
GIPC1	-0.1199612	0.1280265	-0.937	0.349	-0.161727614	count	1
EIF2B2	-0.1188201	0.1542571	-0.7703	0.441	-0.161726497	count	1
AL358852.1	-0.2520062	0.5427452	-0.4643	0.642	-0.161469313	count	1
ZFAND4	-0.2295494	0.5174431	-0.4436	0.657	-0.161370949	count	1
GNAI2	-0.1123975	0.0444471	-2.5288	0.0115	-0.161202571	count	1
HMGXB3	-0.1204149	0.1689228	-0.7128	0.476	-0.161154289	count	1
ZFHX2	-0.6990189	0.8340857	-0.8381	0.402	-0.160770394	count	1
SMARCA4	-0.1212236	0.1534771	-0.7898	0.43	-0.160592387	count	1
AP3M2	-0.1262023	0.2154267	-0.5858	0.558	-0.160531531	count	1
AP4B1-AS1	-1.1209862	0.7959762	-1.4083	0.159	-0.160522468	count	1
AL034417.3	-1.1209862	0.8578082	-1.3068	0.191	-0.160522468	count	1
GLS2	-1.1209862	0.9516402	-1.178	0.239	-0.160522468	count	1
RASGRP1	-1.1209862	0.9516402	-1.178	0.239	-0.160522468	count	1
CLPTM1	-0.1207746	0.1577712	-0.7655	0.444	-0.16050654	count	1
CENPX	-0.1144089	0.0896308	-1.2764	0.202	-0.160468629	count	1
ANKRD53	-0.3356225	0.9867705	-0.3401	0.734	-0.160382109	count	1
LINC02298	-0.3356225	0.9867705	-0.3401	0.734	-0.160382109	count	1
RBFADN	-0.3356225	0.9867705	-0.3401	0.734	-0.160382109	count	1
GOLGA8A	-0.1309904	0.2296383	-0.5704	0.568	-0.16018027	count	1
GPR89A	-0.1900955	0.3758876	-0.5057	0.613	-0.160154287	count	1
RBPJ	-0.1164151	0.1204881	-0.9662	0.334	-0.16012312	count	1
GNE	-0.1705971	0.3692223	-0.462	0.644	-0.159982747	count	1
TCN2	-0.1132086	0.0897412	-1.2615	0.207	-0.15984501	count	1
ANKRD27	-0.1702997	0.4336107	-0.3927	0.695	-0.159697475	count	1
SRM	-0.1138067	0.0897662	-1.2678	0.205	-0.159670496	count	1
CDKN2A	-1.116497	0.7909203	-1.4116	0.158	-0.159669739	count	1
IFFO2	-0.2815447	0.4767238	-0.5906	0.555	-0.159505046	count	1
PANO1	-0.3744162	0.7997743	-0.4682	0.64	-0.159502982	count	1
AGAP5	-0.3744162	0.7997743	-0.4682	0.64	-0.159502982	count	1
ADNP-AS1	-0.3744162	0.8112599	-0.4615	0.644	-0.159502982	count	1
AC027307.2	-0.3744162	0.8137644	-0.4601	0.645	-0.159502982	count	1
AP003352.1	-0.3744162	0.8615684	-0.4346	0.664	-0.159502982	count	1

TDRD6	-0.3744162	0.8672277	-0.4317	0.666	-0.159502982	count	1
ZDHHC3	-0.1160328	0.1301333	-0.8916	0.373	-0.15942729	count	1
HSPB11	-0.1158984	0.1232219	-0.9406	0.347	-0.159410949	count	1
LRRC27	-0.2486686	0.4563124	-0.545	0.586	-0.159218024	count	1
TMEM14A	-0.1159912	0.1367912	-0.8479	0.397	-0.159040696	count	1
OAT	-0.1225974	0.1815972	-0.6751	0.5	-0.158978114	count	1
LZTR1	-0.1452047	0.2951604	-0.492	0.623	-0.158902974	count	1
CAD	-0.2036575	0.4906656	-0.4151	0.678	-0.158789977	count	1
AC023509.4	-0.1884842	0.4229203	-0.4457	0.656	-0.158755997	count	1
TC2N	-0.2098452	0.7682279	-0.2732	0.785	-0.158597892	count	1
NUDCD2	-0.1136245	0.0972598	-1.1683	0.243	-0.158592834	count	1
BABAM1	-0.113817	0.1090642	-1.0436	0.297	-0.158585312	count	1
SLC25A10	-0.6904826	0.8476291	-0.8146	0.415	-0.158395617	count	1
DOPEY1	-0.1336248	0.376533	-0.3549	0.723	-0.15820877	count	1
RGS17	-0.6895237	0.6558764	-1.0513	0.293	-0.158129479	count	1
ADIPOR1	-0.1169502	0.138496	-0.8444	0.399	-0.157976785	count	1
HACD2	-0.1286002	0.2070929	-0.621	0.535	-0.157877389	count	1
IL7R	-0.5207547	0.6020665	-0.8649	0.387	-0.1578523	count	1
GARS	-0.1205752	0.1642295	-0.7342	0.463	-0.157829651	count	1
ENTPD1-AS1	-0.1918501	0.7306673	-0.2626	0.793	-0.15781998	count	1
TP53BP1	-0.1294708	0.2056273	-0.6296	0.529	-0.157631077	count	1
WWOX	-0.1322823	0.2594261	-0.5099	0.61	-0.157516182	count	1
ARF1	-0.1100838	0.0477866	-2.3037	0.0213	-0.157504852	count	1
ANKRD13A	-0.1272619	0.2253958	-0.5646	0.572	-0.157410709	count	1
HESX1	-0.2459605	0.6501972	-0.3783	0.705	-0.1573935	count	1
PCDHGC3	-0.1634436	0.3590405	-0.4552	0.649	-0.15736911	count	1
POLR1A	-0.1677142	0.4306101	-0.3895	0.697	-0.157218341	count	1
NAMPT	-0.11053	0.0693424	-1.594	0.111	-0.157130448	count	1
LINC01546	-1.103028	0.9197374	-1.1993	0.231	-0.157121125	count	1
WASHC3	-0.1131238	0.1074091	-1.0532	0.292	-0.157078893	count	1
BOLA2-SMG1P6	-0.2774631	0.5866596	-0.473	0.636	-0.157044261	count	1
ZNF582-AS1	-0.1329748	0.2649072	-0.502	0.616	-0.156955074	count	1
FBXL14	-0.1863931	0.4299209	-0.4336	0.665	-0.156942338	count	1
NDUFA13	-0.1133852	0.1236102	-0.9173	0.359	-0.15676527	count	1
MAPKAPK3	-0.1669272	0.3859164	-0.4325	0.665	-0.156464033	count	1
YAP1	-0.1186013	0.1771299	-0.6696	0.503	-0.156374452	count	1
MIGA1	-0.1187876	0.1736374	-0.6841	0.494	-0.15622553	count	1
LINC00504	-0.2982575	0.6519033	-0.4575	0.647	-0.156138134	count	1
MMRN2	-0.1097129	0.0626514	-1.7512	0.08	-0.156008326	count	1
PDK2	-0.1182614	0.1945461	-0.6079	0.543	-0.155924543	count	1
ZNF620	-0.1401819	0.3100258	-0.4522	0.651	-0.155904534	count	1
C2orf42	-0.1944596	0.3963513	-0.4906	0.624	-0.155854397	count	1
LINC01772	-1.095859	0.9721537	-1.1272	0.26	-0.155770121	count	1
MATN3	-1.095859	0.9721537	-1.1272	0.26	-0.155770121	count	1
HAAO	-1.095859	0.9721537	-1.1272	0.26	-0.155770121	count	1

AL031123.1	-1.095859	0.9721537	-1.1272	0.26	-0.155770121	count	1
AL445623.2	-1.095859	0.9721537	-1.1272	0.26	-0.155770121	count	1
SORL1	-1.095859	0.9721537	-1.1272	0.26	-0.155770121	count	1
LINC02308	-1.095859	0.9721537	-1.1272	0.26	-0.155770121	count	1
AC103691.2	-1.095859	0.9721537	-1.1272	0.26	-0.155770121	count	1
TSSK3	-1.095859	1.156854	-0.9473	0.344	-0.155770121	count	1
AL358072.1	-1.095859	1.156854	-0.9473	0.344	-0.155770121	count	1
NOTCH2NL	-1.095859	1.156854	-0.9473	0.344	-0.155770121	count	1
AC010207.1	-1.095859	1.156854	-0.9473	0.344	-0.155770121	count	1
AC110792.3	-1.095859	1.156854	-0.9473	0.344	-0.155770121	count	1
PCSK1	-1.095859	1.156854	-0.9473	0.344	-0.155770121	count	1
PCDHGA7	-1.095859	1.156854	-0.9473	0.344	-0.155770121	count	1
AL357497.1	-1.095859	1.156854	-0.9473	0.344	-0.155770121	count	1
AC092171.5	-1.095859	1.156854	-0.9473	0.344	-0.155770121	count	1
AC113133.1	-1.095859	1.156854	-0.9473	0.344	-0.155770121	count	1
AL158071.5	-1.095859	1.156854	-0.9473	0.344	-0.155770121	count	1
BCDIN3D-AS1	-1.095859	1.156854	-0.9473	0.344	-0.155770121	count	1
LINC00571	-1.095859	1.156854	-0.9473	0.344	-0.155770121	count	1
DPH6-DT	-1.095859	1.156854	-0.9473	0.344	-0.155770121	count	1
AC007496.2	-1.095859	1.156854	-0.9473	0.344	-0.155770121	count	1
DNAAF1	-1.095859	1.156854	-0.9473	0.344	-0.155770121	count	1
SHISA6	-1.095859	1.156854	-0.9473	0.344	-0.155770121	count	1
MPP2	-1.095859	1.156854	-0.9473	0.344	-0.155770121	count	1
HRH4	-1.095859	1.156854	-0.9473	0.344	-0.155770121	count	1
PODNL1	-1.095859	1.156854	-0.9473	0.344	-0.155770121	count	1
AF129408.1	-1.095859	1.156854	-0.9473	0.344	-0.155770121	count	1
AC007405.3	-1.095859	1.259801	-0.8699	0.384	-0.155770121	count	1
PABPN1	-0.1139896	0.1367827	-0.8334	0.405	-0.155733504	count	1
NACC1	-0.1412291	0.2789037	-0.5064	0.613	-0.155389412	count	1
GNB1	-0.1108159	0.0893294	-1.2405	0.215	-0.155366246	count	1
PALMD	-0.1082576	0.0487005	-2.2229	0.0263	-0.155354503	count	1
NKIRAS2	-0.1175079	0.1604501	-0.7324	0.464	-0.155207615	count	1
AGRN	-0.1158971	0.1741222	-0.6656	0.506	-0.155021637	count	1
ABCA2	-0.1443351	0.4223208	-0.3418	0.733	-0.154984399	count	1
WNT3	-0.3647016	0.8372077	-0.4356	0.663	-0.154962556	count	1
KMT2C	-0.1143925	0.1411478	-0.8104	0.418	-0.154950079	count	1
TMEM106B	-0.1112061	0.1226411	-0.9068	0.365	-0.154445793	count	1
PIGM	-0.1296093	0.264361	-0.4903	0.624	-0.154305571	count	1
AC013271.1	-0.1402433	0.3793812	-0.3697	0.712	-0.154290885	count	1
FAM89B	-0.1090186	0.0720214	-1.5137	0.13	-0.154237627	count	1
ULK2	-0.1601788	0.4605873	-0.3478	0.728	-0.154161315	count	1
RAB5B	-0.121516	0.242806	-0.5005	0.617	-0.153961474	count	1
HIGD1A	-0.1124192	0.1249186	-0.8999	0.368	-0.153850658	count	1
AC023590.1	-0.5092074	0.7550372	-0.6744	0.5	-0.153828673	count	1
AC091814.1	-0.5092074	0.8677562	-0.5868	0.557	-0.153828673	count	1

ZNF713	-0.4187115	0.5658162	-0.74	0.459	-0.153626818	count	1
HIST1H1A	-0.1439886	0.3747752	-0.3842	0.701	-0.153546142	count	1
PGBD4	-0.181649	0.4504941	-0.4032	0.687	-0.152831885	count	1
ZMYM6	-0.1503987	0.3250184	-0.4627	0.644	-0.152788247	count	1
PSMB7	-0.1091958	0.089219	-1.2239	0.221	-0.152744983	count	1
USP34	-0.1096613	0.1081692	-1.0138	0.311	-0.152671999	count	1
MDM2	-0.1152926	0.1799776	-0.6406	0.522	-0.152270612	count	1
CCR10	-0.269258	0.7850428	-0.343	0.732	-0.152110317	count	1
NDUFB4	-0.1063706	0.051315	-2.0729	0.0383	-0.152011108	count	1
AC099518.5	-0.1309393	0.2940858	-0.4452	0.656	-0.151970526	count	1
FP565260.6	-1.075419	0.8412272	-1.2784	0.201	-0.151940633	count	1
AC107419.1	-1.075419	0.9165697	-1.1733	0.241	-0.151940633	count	1
AC090971.2	-1.075419	0.9165697	-1.1733	0.241	-0.151940633	count	1
OSTC	-0.1065402	0.0635991	-1.6752	0.094	-0.151710664	count	1
MED21	-0.1125065	0.1348561	-0.8343	0.404	-0.151701632	count	1
PSMA1	-0.1063621	0.0610869	-1.7412	0.0818	-0.151089754	count	1
EIF4E2	-0.1097171	0.1161516	-0.9446	0.345	-0.151070577	count	1
NEU1	-0.1117287	0.1393548	-0.8018	0.423	-0.150854345	count	1
C1orf198	-0.1414768	0.3846086	-0.3678	0.713	-0.150829573	count	1
KAT6B	-0.1149705	0.1655807	-0.6943	0.488	-0.150781353	count	1
KCTD10	-0.1156199	0.2081546	-0.5555	0.579	-0.150634326	count	1
MAP7D1	-0.1123345	0.1354112	-0.8296	0.407	-0.150558768	count	1
NCAPG2	-0.1716724	0.4700168	-0.3652	0.715	-0.150490087	count	1
EMC10	-0.1098539	0.1290772	-0.8511	0.395	-0.150457129	count	1
GPR180	-0.1331567	0.3086338	-0.4314	0.666	-0.150200149	count	1
KNTC1	-0.2660693	0.6055061	-0.4394	0.66	-0.15019752	count	1
TPTEP2-CSNK1E	-0.3157003	0.6498251	-0.4858	0.627	-0.150099321	count	1
MSL2	-0.1301856	0.2728144	-0.4772	0.633	-0.149959487	count	1
ATP5MPL	-0.1057993	0.0655825	-1.6132	0.107	-0.149823297	count	1
ASH1L	-0.1079248	0.1047089	-1.0307	0.303	-0.149747071	count	1
ARL3	-0.1072024	0.1004341	-1.0674	0.286	-0.149738212	count	1
AC079313.2	-0.1780571	0.5663484	-0.3144	0.753	-0.149723622	count	1
CREB3	-0.1106063	0.1433212	-0.7717	0.44	-0.149716989	count	1
DPH7	-0.1210592	0.2033322	-0.5954	0.552	-0.14968752	count	1
LCP2	-0.3147826	0.8742914	-0.36	0.719	-0.149627932	count	1
POMK	-0.3147826	0.8920386	-0.3529	0.724	-0.149627932	count	1
MAPK8	-0.1228531	0.2149483	-0.5715	0.568	-0.149514484	count	1
NDUFS4	-0.1066071	0.0872069	-1.2225	0.222	-0.149315174	count	1
CYB561A3	-0.1243639	0.2351314	-0.5289	0.597	-0.149213824	count	1
SIN3A	-0.1118441	0.1764323	-0.6339	0.526	-0.148968181	count	1
UBA6	-0.1146771	0.1738726	-0.6595	0.51	-0.148915396	count	1
TUFM	-0.104789	0.0633227	-1.6548	0.0981	-0.148792873	count	1
CCDC25	-0.1054914	0.0887303	-1.1889	0.235	-0.148738839	count	1
RHBDD3	-0.138438	0.2875971	-0.4814	0.63	-0.148565884	count	1
LSM3	-0.1046383	0.0728063	-1.4372	0.151	-0.148238536	count	1

HDHD5	-0.1166178	0.2103153	-0.5545	0.579	-0.148093858	count	1
PDAP1	-0.1046679	0.0723882	-1.4459	0.148	-0.14800746	count	1
ARL1	-0.1054645	0.0957855	-1.101	0.271	-0.147722688	count	1
FCRLB	-0.2202669	0.6704953	-0.3285	0.743	-0.1476756	count	1
AL118506.1	-0.3489546	0.6774304	-0.5151	0.607	-0.14764753	count	1
PATL1	-0.1349392	0.2852943	-0.473	0.636	-0.147528084	count	1
CDH24	-0.1481953	0.3956123	-0.3746	0.708	-0.147502029	count	1
MVB12A	-0.1056885	0.1065135	-0.9923	0.321	-0.147388664	count	1
ATP6AP2	-0.103785	0.0690028	-1.5041	0.133	-0.147377562	count	1
STX1A	-0.3483707	0.7253716	-0.4803	0.631	-0.14737736	count	1
SLC52A2	-0.1102493	0.1598141	-0.6899	0.49	-0.147181781	count	1
XBP1	-0.1050048	0.1006373	-1.0434	0.297	-0.147172856	count	1
DECR1	-0.1042074	0.0809598	-1.2871	0.198	-0.14707219	count	1
AC093525.6	-0.3473882	0.7128654	-0.4873	0.626	-0.14692292	count	1
CCNYL1	-0.1154099	0.1947133	-0.5927	0.553	-0.146731131	count	1
VDAC1	-0.10425	0.0796116	-1.3095	0.19	-0.146715696	count	1
MDK	-0.1026163	0.0674556	-1.5212	0.128	-0.146612313	count	1
ZNF496	-0.1706271	0.3882423	-0.4395	0.66	-0.146525271	count	1
FER	-0.1166044	0.2105347	-0.5538	0.58	-0.146285146	count	1
EIF5A2	-0.1251715	0.3849159	-0.3252	0.745	-0.146235892	count	1
RASSF5	-0.2088213	0.78559	-0.2658	0.79	-0.146201734	count	1
HINT2	-0.1043642	0.1053409	-0.9907	0.322	-0.146170049	count	1
GALNT3	-0.1777223	0.6344774	-0.2801	0.779	-0.145857093	count	1
PPP1R2	-0.1034703	0.0867319	-1.193	0.233	-0.145534362	count	1
THUMPD3	-0.108833	0.1658857	-0.6561	0.512	-0.145480292	count	1
WEE1	-0.1157862	0.2489196	-0.4652	0.642	-0.145465889	count	1
AP2B1	-0.1045439	0.1187204	-0.8806	0.379	-0.145437729	count	1
DCUN1D5	-0.1076925	0.1403887	-0.7671	0.443	-0.145390201	count	1
RPL26L1	-0.1040876	0.0986305	-1.0553	0.291	-0.145239932	count	1
NDUFS1	-0.1076998	0.1488014	-0.7238	0.469	-0.145152193	count	1
RNF181	-0.102615	0.0794733	-1.2912	0.197	-0.144954384	count	1
TCF7	-0.227346	0.3886529	-0.585	0.559	-0.144905014	count	1
MTMR9	-0.1213451	0.2353423	-0.5156	0.606	-0.144788839	count	1
TELO2	-0.1235104	0.2687963	-0.4595	0.646	-0.144760878	count	1
PPRC1	-0.1546817	0.3326408	-0.465	0.642	-0.1447467	count	1
ZNF699	-0.1399667	0.4459487	-0.3139	0.754	-0.144620784	count	1
DR1	-0.1066955	0.1427135	-0.7476	0.455	-0.144497884	count	1
GRPEL2	-0.1502136	0.3800243	-0.3953	0.693	-0.144385767	count	1
C12orf4	-0.118561	0.2504062	-0.4735	0.636	-0.144253524	count	1
NMB	-0.1252637	0.2486724	-0.5037	0.614	-0.144234766	count	1
BIVM	-0.1420161	0.3896822	-0.3644	0.716	-0.144134563	count	1
C21orf62-AS1	-0.1910947	0.4898732	-0.3901	0.696	-0.143931625	count	1
HNRNPAB	-0.1017929	0.0705255	-1.4433	0.149	-0.143925689	count	1
ZC2HC1C	-0.4799623	0.735216	-0.6528	0.514	-0.14374391	count	1
CLK1	-0.1034234	0.0951953	-1.0864	0.277	-0.143178321	count	1

TIGD2	-0.302143	0.6218734	-0.4859	0.627	-0.14315586	count	1
TAX1BP1	-0.1004629	0.066616	-1.5081	0.1316	-0.143008949	count	1
UTP11	-0.1032361	0.1087858	-0.949	0.343	-0.143008916	count	1
REPIN1	-0.1108147	0.1915837	-0.5784	0.563	-0.142984468	count	1
SPTAN1	-0.1011109	0.0863072	-1.1715	0.241	-0.142916221	count	1
SDHA	-0.1058764	0.144752	-0.7314	0.465	-0.142884117	count	1
ZFP82	-0.160017	0.4797187	-0.3336	0.739	-0.142697835	count	1
TNRC6C	-0.1114625	0.203065	-0.5489	0.583	-0.142665291	count	1
AC019205.1	-0.6328602	0.8173747	-0.7743	0.439	-0.142629613	count	1
AC107959.4	-0.1482957	0.4642434	-0.3194	0.749	-0.142507081	count	1
MBD2	-0.1012748	0.0895347	-1.1311	0.258	-0.142321656	count	1
PIK3C2B	-0.1060543	0.159621	-0.6644	0.506	-0.142232759	count	1
FAM122B	-0.1255476	0.3181307	-0.3946	0.693	-0.142173814	count	1
SRSF2	-0.1006271	0.075791	-1.3277	0.184	-0.142129635	count	1
AVEN	-0.1091099	0.1878906	-0.5807	0.561	-0.142118172	count	1
TSC22D2	-0.1061042	0.132208	-0.8026	0.422	-0.142065947	count	1
IFNAR1	-0.1009106	0.0916725	-1.1008	0.271	-0.141984926	count	1
GOLGA2	-0.1029711	0.115956	-0.888	0.375	-0.1419693	count	1
LANCL1	-0.1143326	0.2420395	-0.4724	0.637	-0.141819259	count	1
AC008556.1	-0.4740678	0.8953495	-0.5295	0.597	-0.14172966	count	1
TRIP11	-0.1008231	0.1103344	-0.9138	0.361	-0.141435009	count	1
MNS1	-0.1768629	0.4063155	-0.4353	0.663	-0.141324779	count	1
AASDHPPT	-0.1020175	0.1239059	-0.8233	0.41	-0.141246978	count	1
LZIC	-0.1037796	0.1530677	-0.678	0.498	-0.141208389	count	1
ZNF778	-0.2345676	0.674597	-0.3477	0.728	-0.141014031	count	1
REP15	-0.2345676	0.8132869	-0.2884	0.773	-0.141014031	count	1
ABI1	-0.1056318	0.1443088	-0.732	0.464	-0.140599437	count	1
TBC1D9	-0.1103754	0.1959199	-0.5634	0.573	-0.140466629	count	1
TBL1XR1	-0.101037	0.1125458	-0.8977	0.369	-0.140378125	count	1
AKAP13	-0.0989604	0.085758	-1.154	0.249	-0.140377902	count	1
FZD1	-0.2203538	0.3927282	-0.5611	0.575	-0.140237748	count	1
2-Mar	-0.1000462	0.0825523	-1.2119	0.226	-0.140232944	count	1
POLA2	-0.1709416	0.3970977	-0.4305	0.667	-0.140134123	count	1
IGFBP2	-0.0980548	0.0706221	-1.3884	0.1651	-0.139836706	count	1
YEATS2	-0.1178598	0.3489572	-0.3377	0.736	-0.139803979	count	1
TAL1	-0.1174135	0.2530229	-0.464	0.643	-0.139670831	count	1
NDUFAF5	-0.1515134	0.3634247	-0.4169	0.677	-0.139587785	count	1
KLF3	-0.0981828	0.0763943	-1.2852	0.199	-0.139542231	count	1
MZT2B	-0.0975949	0.0507529	-1.9229	0.0546	-0.139540043	count	1
ADGRG1	-0.1030156	0.145815	-0.7065	0.48	-0.139459668	count	1
ZEB1	-0.098899	0.0837529	-1.1808	0.238	-0.139402068	count	1
UNK	-0.1205573	0.2510554	-0.4802	0.631	-0.139295848	count	1
SH3YL1	-0.1083324	0.2069062	-0.5236	0.601	-0.139222289	count	1
PSMC4	-0.09946	0.0936679	-1.0618	0.288	-0.139130474	count	1
LAMTOR4	-0.097418	0.0568477	-1.7137	0.0867	-0.139006634	count	1

OAZ2	-0.1008109	0.134053	-0.752	0.452	-0.138964182	count	1
USF2	-0.1008566	0.1118944	-0.9014	0.367	-0.138755758	count	1
ACTN4	-0.0976709	0.0743344	-1.3139	0.189	-0.138687585	count	1
SELENON	-0.1019197	0.1293489	-0.7879	0.431	-0.138563029	count	1
EPHB1	-0.3815236	0.9978679	-0.3823	0.702	-0.138518707	count	1
CASTOR2	-0.3815236	0.9978679	-0.3823	0.702	-0.138518707	count	1
LOXL1-AS1	-0.3815236	0.9978679	-0.3823	0.702	-0.138518707	count	1
AC092329.3	-0.3815236	0.9978679	-0.3823	0.702	-0.138518707	count	1
ZNF350-AS1	-0.3815236	0.9978679	-0.3823	0.702	-0.138518707	count	1
NALT1	-0.3815236	1.139772	-0.3347	0.738	-0.138518707	count	1
PRDM5	-0.125838	0.2981463	-0.4221	0.673	-0.138259801	count	1
MUL1	-0.1232106	0.2520689	-0.4888	0.625	-0.138207293	count	1
C1orf159	-0.1578719	0.4467096	-0.3534	0.724	-0.138103495	count	1
ARIH2	-0.1053333	0.1667053	-0.6319	0.528	-0.138095535	count	1
SMAP2	-0.1256254	0.3022533	-0.4156	0.678	-0.138023509	count	1
YARS	-0.1115404	0.2231281	-0.4999	0.617	-0.137845133	count	1
AC243960.1	-0.1453179	0.5680721	-0.2558	0.798	-0.137751381	count	1
PI4K2B	-0.1572035	0.3734897	-0.4209	0.674	-0.137504818	count	1
FUBP3	-0.1143042	0.2365243	-0.4833	0.629	-0.137405784	count	1
KHSRP	-0.1106248	0.197766	-0.5594	0.576	-0.137192466	count	1
TMED1	-0.1015957	0.1456226	-0.6977	0.485	-0.137092484	count	1
GLYR1	-0.1060344	0.1825862	-0.5807	0.561	-0.137044061	count	1
ASAH2B	-0.2899852	0.6721459	-0.4314	0.666	-0.136966576	count	1
CABP1	-0.2899852	0.8169698	-0.355	0.723	-0.136966576	count	1
GADD45G	-0.1025152	0.2087241	-0.4912	0.623	-0.136763438	count	1
ARHGAP17	-0.1045062	0.1728682	-0.6045	0.546	-0.136716954	count	1
MB21D2	-0.1506742	0.4825047	-0.3123	0.755	-0.136555621	count	1
TIMM13	-0.0970548	0.0874192	-1.1102	0.267	-0.136554634	count	1
UBA1	-0.1098038	0.2592842	-0.4235	0.672	-0.136401777	count	1
EFNB2	-0.0971945	0.0906573	-1.0721	0.284	-0.136240012	count	1
DHX30	-0.1042959	0.1693098	-0.616	0.538	-0.13603504	count	1
CAAP1	-0.1022979	0.1744733	-0.5863	0.558	-0.135804418	count	1
EIF1AY	-0.0975904	0.1342071	-0.7272	0.467	-0.135751211	count	1
EID1	-0.094429	0.0364432	-2.5911	0.00962	-0.135674246	count	1
AC104031.1	-0.1522067	0.3813303	-0.3991	0.69	-0.135576529	count	1
PIN4	-0.0970802	0.1005538	-0.9655	0.334	-0.135472685	count	1
SLC2A4RG	-0.0965116	0.0966524	-0.9985	0.318	-0.135380569	count	1
MBOAT7	-0.1065046	0.2238177	-0.4759	0.634	-0.134846476	count	1
C19orf12	-0.1098335	0.2262536	-0.4854	0.627	-0.134692349	count	1
MRC1	-0.3204664	0.4781387	-0.6702	0.503	-0.134555186	count	1
SETD7	-0.1031612	0.1877002	-0.5496	0.583	-0.134549356	count	1
CSNK1D	-0.0990242	0.134858	-0.7343	0.463	-0.134508994	count	1
CCS	-0.0975825	0.1193765	-0.8174	0.414	-0.134421168	count	1
YIF1A	-0.0961474	0.1031197	-0.9324	0.351	-0.134331109	count	1
TM7SF3	-0.1001039	0.1394235	-0.718	0.473	-0.134284529	count	1

MRPL40	-0.0955337	0.0936036	-1.0206	0.308	-0.134092918	count	1
MLH3	-0.1032871	0.1975892	-0.5227	0.601	-0.133836213	count	1
SERINC1	-0.0942846	0.0822834	-1.1459	0.252	-0.133703067	count	1
MEX3A	-0.2382541	0.4917774	-0.4845	0.628	-0.133623133	count	1
CSGALNACT2	-0.1065499	0.2361478	-0.4512	0.652	-0.133603471	count	1
MTR	-0.1012618	0.3009514	-0.3365	0.737	-0.133598325	count	1
PIR	-0.0957319	0.1298508	-0.7372	0.461	-0.133566511	count	1
RABEP1	-0.0987728	0.1490902	-0.6625	0.508	-0.133362855	count	1
SRPRA	-0.0947984	0.0865006	-1.0959	0.273	-0.133351614	count	1
DEPDC5	-0.1356632	0.4928321	-0.2753	0.783	-0.133344578	count	1
UBAC2-AS1	-0.1837838	0.6439011	-0.2854	0.775	-0.133333335	count	1
RFTN2	-0.1009263	0.1904695	-0.5299	0.596	-0.133312536	count	1
MRPL42	-0.0962818	0.1067985	-0.9015	0.367	-0.133293744	count	1
HRAS	-0.0966647	0.119513	-0.8088	0.419	-0.133218346	count	1
CACYBP	-0.0945723	0.082557	-1.1455	0.252	-0.13320891	count	1
JOSD2	-0.0962569	0.1261865	-0.7628	0.446	-0.13306535	count	1
SDR39U1	-0.1045246	0.1752955	-0.5963	0.551	-0.1329852	count	1
ZNF593	-0.0956504	0.1137617	-0.8408	0.401	-0.13288387	count	1
LINC01088	-0.5962758	0.8553768	-0.6971	0.486	-0.132860316	count	1
AL137802.2	-0.5962758	0.9341562	-0.6383	0.523	-0.132860316	count	1
LINC01948	-0.5962758	1.178177	-0.5061	0.613	-0.132860316	count	1
ARF3	-0.0975875	0.1447086	-0.6744	0.5	-0.132730405	count	1
SLC25A46	-0.1082402	0.2194434	-0.4932	0.622	-0.13272614	count	1
TMEM203	-0.1001819	0.1653729	-0.6058	0.545	-0.132702086	count	1
TUBGCP3	-0.1036723	0.242674	-0.4272	0.669	-0.132649118	count	1
CHD8	-0.101889	0.1777097	-0.5733	0.566	-0.132570866	count	1
IDH1	-0.1063315	0.1956383	-0.5435	0.587	-0.132502498	count	1
TPCN2	-0.2210412	0.5096895	-0.4337	0.665	-0.132479137	count	1
AC002070.1	-0.1539539	0.5088023	-0.3026	0.762	-0.131862687	count	1
PCDH12	-0.1044583	0.1750363	-0.5968	0.551	-0.131716762	count	1
CMTM6	-0.0950371	0.132955	-0.7148	0.475	-0.131567999	count	1
MAFTRR	-0.253997	0.8158243	-0.3113	0.756	-0.131535881	count	1
CMC2	-0.0982403	0.1430075	-0.687	0.492	-0.131395748	count	1
TBCB	-0.0925304	0.072275	-1.2803	0.201	-0.131343427	count	1
ZMIZ2	-0.1128046	0.282184	-0.3998	0.689	-0.131212325	count	1
AC026801.2	-0.1963822	0.6751225	-0.2909	0.771	-0.131014538	count	1
FKBP7	-0.0986962	0.1818583	-0.5427	0.587	-0.130939569	count	1
NUP58	-0.1034775	0.2116606	-0.4889	0.625	-0.130473769	count	1
NDUFAF3	-0.0916057	0.0638565	-1.4346	0.152	-0.130313418	count	1
DDX39B	-0.1017412	0.1899312	-0.5357	0.592	-0.130307698	count	1
EMC4	-0.0919728	0.0710477	-1.2945	0.196	-0.130293856	count	1
AL049840.1	-0.1223834	0.3158004	-0.3875	0.698	-0.130222291	count	1
CISD1	-0.0937734	0.0933666	-1.0044	0.315	-0.129945715	count	1
AC139530.1	-0.1391436	0.3790485	-0.3671	0.714	-0.129931639	count	1
AKR7A2	-0.0929726	0.0987409	-0.9416	0.346	-0.129930306	count	1

ERAP2	-0.0951936	0.1637074	-0.5815	0.561	-0.129853973	count	1
ZNF576	-0.1021919	0.194588	-0.5252	0.6	-0.129687372	count	1
PARP8	-0.216567	0.6227495	-0.3478	0.728	-0.12966664	count	1
SNPH	-0.9515088	0.6814452	-1.3963	0.163	-0.129441433	count	1
VAMP2	-0.0906999	0.0638651	-1.4202	0.156	-0.129350077	count	1
ZNF814	-0.1148382	0.2866697	-0.4006	0.689	-0.129336496	count	1
GHDC	-0.0993805	0.1861492	-0.5339	0.593	-0.129294629	count	1
TCEAL9	-0.0914304	0.0801443	-1.1408	0.254	-0.129263705	count	1
NCF1	-0.2747086	0.660623	-0.4158	0.678	-0.129239875	count	1
AC022098.1	-0.178294	0.5017409	-0.3554	0.722	-0.12921411	count	1
PIDD1	-0.3078196	0.5716632	-0.5385	0.59	-0.128801678	count	1
ZNF570	-0.1052355	0.2584663	-0.4072	0.684	-0.128490473	count	1
MRPS16	-0.09207	0.1085983	-0.8478	0.397	-0.128418643	count	1
CYP3A5	-0.4346239	0.8483618	-0.5123	0.608	-0.128410137	count	1
AC007228.2	-0.4346239	0.9850866	-0.4412	0.659	-0.128410137	count	1
GPR160	-0.1395508	0.4028813	-0.3464	0.729	-0.128351602	count	1
HFE	-0.1254818	0.4210321	-0.298	0.766	-0.128305697	count	1
ZNF706	-0.0901837	0.0621476	-1.4511	0.147	-0.128297155	count	1
EPS15L1	-0.1119203	0.2557905	-0.4375	0.662	-0.128226401	count	1
MAPKAPK5	-0.1078546	0.2417577	-0.4461	0.656	-0.128216456	count	1
IFT57	-0.0925799	0.1059879	-0.8735	0.382	-0.128177702	count	1
ZNF738	-0.1242108	0.3748044	-0.3314	0.74	-0.128118713	count	1
PI4KA	-0.1194064	0.3271119	-0.365	0.715	-0.127900018	count	1
ZNF554	-0.2136654	0.5667107	-0.377	0.706	-0.127845521	count	1
COG8	-0.1701825	0.4189332	-0.4062	0.685	-0.127686119	count	1
SLC25A44	-0.1258326	0.2932402	-0.4291	0.668	-0.127472299	count	1
KIAA0232	-0.0962814	0.1722973	-0.5588	0.576	-0.127231969	count	1
SUPV3L1	-0.1075676	0.2340519	-0.4596	0.646	-0.127127023	count	1
AL645728.1	-0.1551398	0.5032592	-0.3083	0.758	-0.126844578	count	1
METAP1	-0.1007206	0.2033862	-0.4952	0.62	-0.126804741	count	1
DCTN5	-0.1029972	0.2809175	-0.3666	0.714	-0.126753312	count	1
NPY1R	-0.2696444	0.7445299	-0.3622	0.717	-0.126690858	count	1
TRAPPC1	-0.0891861	0.0790544	-1.1282	0.259	-0.126383814	count	1
SCAND1	-0.0885983	0.0633171	-1.3993	0.162	-0.126060848	count	1
GXYLT1	-0.0963075	0.1995036	-0.4827	0.629	-0.126044502	count	1
KIAA1468	-0.1202091	0.2950802	-0.4074	0.684	-0.125990164	count	1
ABCC6	-0.9317854	0.8384882	-1.1113	0.267	-0.125976306	count	1
LINC02166	-0.9317854	0.969316	-0.9613	0.336	-0.125976306	count	1
AL137784.2	-0.9317854	1.151865	-0.8089	0.419	-0.125976306	count	1
SOX13	-0.1090192	0.2654131	-0.4108	0.681	-0.125852064	count	1
FDX1	-0.0909586	0.1172351	-0.7759	0.438	-0.125730172	count	1
TMEM165	-0.0889343	0.0731614	-1.2156	0.224	-0.125598029	count	1
CDK5RAP3	-0.0926441	0.1362061	-0.6802	0.496	-0.125538685	count	1
KNOP1	-0.0916704	0.1506486	-0.6085	0.543	-0.125403059	count	1
USP38	-0.1042625	0.2473942	-0.4214	0.673	-0.125253114	count	1

MAPRE3	-0.1289794	0.3587117	-0.3596	0.719	-0.125190456	count	1
PGGT1B	-0.0938622	0.1867644	-0.5026	0.615	-0.125187959	count	1
VPS13C	-0.0890934	0.1044036	-0.8534	0.394	-0.125168043	count	1
ZNF607	-0.2093499	0.6465841	-0.3238	0.746	-0.12514114	count	1
AC005726.5	-0.2093499	0.7304867	-0.2866	0.774	-0.12514114	count	1
ZNF613	-0.1728269	0.5907828	-0.2925	0.77	-0.125120059	count	1
BRCA2	-0.1727195	0.4198156	-0.4114	0.681	-0.125039716	count	1
AL121832.2	-0.2663293	0.6201406	-0.4295	0.668	-0.125025582	count	1
OSGEP	-0.0943437	0.1501991	-0.6281	0.53	-0.125013891	count	1
RPS6KB2	-0.0998057	0.2207562	-0.4521	0.651	-0.124915688	count	1
AP002495.2	-0.1875815	0.5129296	-0.3657	0.715	-0.124914354	count	1
TRIM56	-0.0912821	0.1316461	-0.6934	0.488	-0.124680158	count	1
DERL1	-0.0909138	0.1351337	-0.6728	0.501	-0.124668882	count	1
BLCAP	-0.0911776	0.1484486	-0.6142	0.539	-0.124537154	count	1
SPRY4	-0.114124	0.2481542	-0.4599	0.646	-0.124527081	count	1
WDR1	-0.0897655	0.1016133	-0.8834	0.377	-0.124414327	count	1
LAMTOR1	-0.08727	0.0571233	-1.5277	0.1267	-0.124363849	count	1
ZNF619	-0.1560639	0.5532202	-0.2821	0.778	-0.124257507	count	1
ZNF432	-0.1203123	0.3649042	-0.3297	0.742	-0.124044021	count	1
PLPP5	-0.091514	0.1260349	-0.7261	0.468	-0.124004018	count	1
HGS	-0.0993672	0.2361007	-0.4209	0.674	-0.123977624	count	1
VAPA	-0.0868639	0.0536586	-1.6188	0.106	-0.123941329	count	1
TOMM7	-0.0862452	0.0363849	-2.3704	0.0178	-0.123716986	count	1
RPL23	-0.0860196	0.0312218	-2.7551	0.00591	-0.123711802	count	1
AC024909.2	-0.9182065	0.7731912	-1.1876	0.235	-0.123609587	count	1
RFLNA	-0.9182065	1.05275	-0.8722	0.383	-0.123609587	count	1
SLFN13	-0.1855725	0.6612403	-0.2806	0.779	-0.12352476	count	1
ZNF829	-0.1551073	0.3993891	-0.3884	0.698	-0.123475338	count	1
AP000866.2	-0.1285561	0.4303683	-0.2987	0.765	-0.123222415	count	1
BCL2L2	-0.1025257	0.274307	-0.3738	0.709	-0.123152743	count	1
ZDBF2	-0.1336374	0.4232775	-0.3157	0.752	-0.122810525	count	1
AGAP2-AS1	-0.0943807	0.1924887	-0.4903	0.624	-0.122766418	count	1
RAB13	-0.0854513	0.0455007	-1.878	0.0605	-0.122638908	count	1
ATP2B4	-0.0887325	0.1162911	-0.763	0.446	-0.122631727	count	1
RIC1	-0.1178197	0.3296369	-0.3574	0.721	-0.122468854	count	1
CAB39	-0.0951305	0.1879581	-0.5061	0.613	-0.122310589	count	1
YWHAQ	-0.0864185	0.0742244	-1.1643	0.244	-0.122158007	count	1
CHMP7	-0.1257935	0.2704318	-0.4652	0.642	-0.122048706	count	1
LDHC	-0.3397221	0.830396	-0.4091	0.682	-0.121874967	count	1
SFR1	-0.0919214	0.1651122	-0.5567	0.578	-0.12186061	count	1
TXNDC17	-0.0863065	0.0754918	-1.1433	0.253	-0.121762439	count	1
SECISBP2L	-0.08859	0.1276125	-0.6942	0.488	-0.1217035	count	1
ISCU	-0.0851135	0.0554137	-1.536	0.125	-0.121611084	count	1
C19orf47	-0.2177582	0.4879345	-0.4463	0.655	-0.121538239	count	1
CCDC28B	-0.1065173	0.2651369	-0.4017	0.688	-0.121482521	count	1

PSMD13	-0.0868248	0.0942553	-0.9212	0.357	-0.121393255	count	1
MTHFS	-0.0961812	0.3048049	-0.3156	0.752	-0.12089122	count	1
YAE1D1	-0.0990453	0.2593514	-0.3819	0.703	-0.12088762	count	1
RIOK3	-0.0869244	0.0989556	-0.8784	0.38	-0.12080989	count	1
MYEF2	-0.1042365	0.2736415	-0.3809	0.703	-0.120733556	count	1
RABIF	-0.0991236	0.2145153	-0.4621	0.644	-0.120725582	count	1
FAM200B	-0.0872283	0.1191206	-0.7323	0.464	-0.120680066	count	1
PLCB1	-0.0886358	0.1243358	-0.7129	0.476	-0.12062462	count	1
RRP1B	-0.0900394	0.1583991	-0.5684	0.57	-0.120450897	count	1
AC009902.2	-0.2333734	0.8079703	-0.2888	0.773	-0.120240078	count	1
PHYHIPL	-0.2333734	0.8706977	-0.268	0.789	-0.120240078	count	1
ZNF682	-0.1350734	0.4303793	-0.3138	0.754	-0.120009319	count	1
MRM2	-0.0924193	0.2192897	-0.4214	0.673	-0.119806277	count	1
ZNF425	-0.2557338	0.6547615	-0.3906	0.696	-0.119720919	count	1
LRRC34	-0.1503795	0.7819529	-0.1923	0.848	-0.119613238	count	1
SMS	-0.0909755	0.178659	-0.5092	0.611	-0.119611556	count	1
ZNF280D	-0.0909468	0.195502	-0.4652	0.642	-0.119573704	count	1
SUN2	-0.0886802	0.1502643	-0.5902	0.555	-0.119502267	count	1
PHKG1	-0.0976765	0.2473826	-0.3948	0.693	-0.119207179	count	1
RAP2C	-0.0999084	0.2750396	-0.3633	0.716	-0.11903882	count	1
PHAX	-0.0855585	0.1157269	-0.7393	0.46	-0.118946164	count	1
VPS13B	-0.103762	0.2734258	-0.3795	0.704	-0.118801821	count	1
MGA	-0.1029355	0.2390331	-0.4306	0.667	-0.11877282	count	1
LSM11	-0.1877982	0.6242036	-0.3009	0.764	-0.118679488	count	1
FAM86C1	-0.1419356	0.5000045	-0.2839	0.777	-0.118654033	count	1
CDKN1C	-0.0836221	0.102234	-0.8179	0.413	-0.11840225	count	1
TP53RK	-0.0901859	0.2170628	-0.4155	0.678	-0.118173171	count	1
REXO1	-0.0939748	0.1936733	-0.4852	0.628	-0.118104903	count	1
CCNQ	-0.0880965	0.146556	-0.6011	0.548	-0.118090858	count	1
NUP62	-0.0928948	0.2153254	-0.4314	0.666	-0.11798282	count	1
TNKS2-AS1	-0.1774099	0.8763781	-0.2024	0.84	-0.117890136	count	1
FRZB	-0.1695475	0.7677949	-0.2208	0.825	-0.117778461	count	1
PIGQ	-0.1001865	0.2539317	-0.3945	0.693	-0.117607384	count	1
MRPL54	-0.0834873	0.0872409	-0.957	0.339	-0.117410453	count	1
RPLP2	-0.0814539	0.0212767	-3.8283	0.000132	-0.117346011	count	1
MYL6	-0.0814488	0.0276668	-2.9439	0.00327	-0.117319916	count	1
SKA1	-0.4008646	0.9446038	-0.4244	0.671	-0.117230731	count	1
CENPM	-0.4008646	0.9702395	-0.4132	0.68	-0.117230731	count	1
TTC33	-0.0935318	0.2111596	-0.4429	0.658	-0.117202241	count	1
FMNL2	-0.0935318	0.2330346	-0.4014	0.688	-0.117202241	count	1
MAGEH1	-0.087183	0.1631391	-0.5344	0.593	-0.117093924	count	1
PMF1	-0.0855745	0.1174285	-0.7287	0.466	-0.116708694	count	1
PDCD6	-0.0819033	0.0599162	-1.367	0.1718	-0.11661833	count	1
ASRGL1	-0.089817	0.1905211	-0.4714	0.637	-0.1166182	count	1
GAS2L3	-0.2805801	0.7652314	-0.3667	0.714	-0.116532195	count	1

PRDX6	-0.0815051	0.0506273	-1.6099	0.1075	-0.116481652	count	1
ZNF264	-0.0949659	0.2253343	-0.4214	0.673	-0.116358654	count	1
OAZ3	-0.122706	0.4891262	-0.2509	0.802	-0.115966543	count	1
PYCARD	-0.0827784	0.1046481	-0.791	0.429	-0.115916746	count	1
MED11	-0.0882027	0.1868943	-0.4719	0.637	-0.115879235	count	1
GID4	-0.0989883	0.280102	-0.3534	0.724	-0.115812058	count	1
WIPI2	-0.0844166	0.1303987	-0.6474	0.517	-0.115630569	count	1
THUMPD1	-0.0821259	0.0985371	-0.8335	0.405	-0.115522242	count	1
FAM102A	-0.1661021	0.3608779	-0.4603	0.645	-0.115305009	count	1
GNPDA1	-0.0855844	0.1672743	-0.5116	0.609	-0.115280823	count	1
UBE2B	-0.0808403	0.0567697	-1.424	0.1546	-0.115275745	count	1
YIPF3	-0.0812794	0.0735559	-1.105	0.269	-0.115186891	count	1
C1RL-AS1	-0.1448541	0.7723944	-0.1875	0.851	-0.115107243	count	1
RPL17-C18orf32	-0.2773761	0.8423506	-0.3293	0.742	-0.115100066	count	1
LMCD1-AS1	-0.2773761	0.8458065	-0.3279	0.743	-0.115100066	count	1
H3F3A	-0.0798701	0.0295031	-2.7072	0.00683	-0.115002726	count	1
FAT4	-0.0899457	0.2139632	-0.4204	0.674	-0.114889509	count	1
ARG2	-0.123215	0.4251171	-0.2898	0.772	-0.114806271	count	1
CTXN1	-0.8666206	0.8041358	-1.0777	0.281	-0.114760132	count	1
RAB33B	-0.0915009	0.202835	-0.4511	0.652	-0.114472399	count	1
ALG9	-0.2451768	0.4582131	-0.5351	0.593	-0.114462506	count	1
RAD18	-0.1084999	0.3111022	-0.3488	0.727	-0.114448305	count	1
ZFR	-0.0817365	0.1037475	-0.7878	0.431	-0.114402295	count	1
ERICH6-AS1	-0.133942	0.4730904	-0.2831	0.777	-0.114360045	count	1
KDM5D	-0.101601	0.3215117	-0.316	0.752	-0.114298735	count	1
CPSF2	-0.0905677	0.2120683	-0.4271	0.669	-0.11427906	count	1
TMTC3	-0.0920323	0.2230971	-0.4125	0.68	-0.114215501	count	1
PAFAH2	-0.1399187	0.5532388	-0.2529	0.8	-0.114106477	count	1
DKKL1	-0.5236463	0.6905279	-0.7583	0.448	-0.114023297	count	1
SLC39A11	-0.0957159	0.3291478	-0.2908	0.771	-0.11401109	count	1
LINC00840	-0.2440181	0.4366607	-0.5588	0.576	-0.11388701	count	1
ZBED3	-0.0894474	0.1993364	-0.4487	0.654	-0.11385889	count	1
SERHL2	-0.1574512	0.4698714	-0.3351	0.738	-0.113649718	count	1
SLC24A1	-0.1637857	0.4161072	-0.3936	0.694	-0.113643882	count	1
DFFA	-0.0841987	0.1425598	-0.5906	0.555	-0.113643524	count	1
SWI5	-0.0823216	0.1325133	-0.6212	0.534	-0.113613581	count	1
HSBP1	-0.0796307	0.0554804	-1.4353	0.1513	-0.113608765	count	1
DHX29	-0.0814922	0.1182913	-0.6889	0.491	-0.113569528	count	1
IFT140	-0.1391604	0.4538435	-0.3066	0.759	-0.11347351	count	1
NR1H3	-0.096929	0.2989826	-0.3242	0.746	-0.113385554	count	1
STK19	-0.0842727	0.1495936	-0.5633	0.573	-0.113346589	count	1
SLC9A6	-0.1425098	0.5248312	-0.2715	0.786	-0.113197956	count	1
ZSCAN18	-0.0833975	0.1290503	-0.6462	0.518	-0.113115508	count	1
SSU72	-0.0801261	0.0835977	-0.9585	0.338	-0.113099029	count	1
CCDC12	-0.0804558	0.0945774	-0.8507	0.395	-0.113058543	count	1

ABHD14A	-0.0848004	0.1806996	-0.4693	0.639	-0.112965353	count	1
TAOK1	-0.0828498	0.1402845	-0.5906	0.555	-0.112904818	count	1
CAMTA1	-0.0794526	0.0658687	-1.2062	0.228	-0.112839795	count	1
ATP6V1E1	-0.0807347	0.1015769	-0.7948	0.427	-0.112722606	count	1
MTPN	-0.0791025	0.0689446	-1.1473	0.251	-0.112682158	count	1
TBRG1	-0.082521	0.1321401	-0.6245	0.532	-0.112641852	count	1
MRPS15	-0.080574	0.095289	-0.8456	0.398	-0.112542559	count	1
AL138689.2	-0.8530056	0.6535951	-1.3051	0.192	-0.112462177	count	1
PC	-0.8530056	0.7716602	-1.1054	0.269	-0.112462177	count	1
ZNF148	-0.0813152	0.1295472	-0.6277	0.53	-0.112443769	count	1
UQCRB	-0.0782887	0.0351829	-2.2252	0.0261	-0.112425734	count	1
ATRAID	-0.0787568	0.0573433	-1.3734	0.17	-0.112373955	count	1
SELENOK	-0.0789006	0.0616041	-1.2808	0.2004	-0.112348434	count	1
SERPINB1	-0.0784575	0.0667932	-1.1746	0.24	-0.112191621	count	1
CISD3	-0.0810039	0.1155027	-0.7013	0.483	-0.112086823	count	1
SPPL2A	-0.080334	0.0968895	-0.8291	0.407	-0.112032313	count	1
SMPD1	-0.0887467	0.212799	-0.417	0.677	-0.111971332	count	1
ARMCX4	-0.1129677	0.3955192	-0.2856	0.775	-0.111961959	count	1
ZBED1	-0.0869311	0.177404	-0.49	0.624	-0.111949707	count	1
PRSS23	-0.079083	0.0911505	-0.8676	0.386	-0.111882597	count	1
ZNF408	-0.1200946	0.3780501	-0.3177	0.751	-0.111850636	count	1
CDC42EP1	-0.0832955	0.1363423	-0.6109	0.541	-0.111817784	count	1
MT-ATP6	-0.0775603	0.0308888	-2.511	0.0121	-0.111783449	count	1
AC005332.1	-0.313757	0.7928294	-0.3957	0.692	-0.111717954	count	1
UBE2G2	-0.0803748	0.1057252	-0.7602	0.447	-0.11155156	count	1
ZNF577	-0.0948527	0.2626721	-0.3611	0.718	-0.111302954	count	1
SDR16C5	-0.2688481	0.9306012	-0.2889	0.773	-0.111299563	count	1
AJM1	-0.2688481	0.9306012	-0.2889	0.773	-0.111299563	count	1
RPRD1A	-0.0886624	0.2039324	-0.4348	0.664	-0.111237314	count	1
PM20D2	-0.1541517	0.468226	-0.3292	0.742	-0.111196726	count	1
ZNF519	-0.1132906	0.4019883	-0.2818	0.778	-0.111045382	count	1
KIAA1328	-0.0961201	0.282591	-0.3401	0.734	-0.110849798	count	1
UBQLN1	-0.0858742	0.1662168	-0.5166	0.605	-0.110794341	count	1
DCXR	-0.0806894	0.1249257	-0.6459	0.518	-0.110426537	count	1
FARP2	-0.1153912	0.4020271	-0.287	0.774	-0.110413369	count	1
ZNF76	-0.1091727	0.2995034	-0.3645	0.716	-0.110381565	count	1
AL355312.2	-0.214849	0.8986657	-0.2391	0.811	-0.110185667	count	1
EFL1	-0.1052777	0.3904297	-0.2696	0.787	-0.11016508	count	1
SCAMP2	-0.078907	0.1145091	-0.6891	0.491	-0.110096143	count	1
ERC2	-0.5069775	1.183515	-0.4284	0.668	-0.10980511	count	1
AC008083.2	-0.5069775	1.183515	-0.4284	0.668	-0.10980511	count	1
C1QTNF12	-0.5069775	1.212713	-0.4181	0.676	-0.10980511	count	1
AC092634.3	-0.5069775	1.212713	-0.4181	0.676	-0.10980511	count	1
AC026904.3	-0.5069775	1.212713	-0.4181	0.676	-0.10980511	count	1
C8G	-0.5069775	1.212713	-0.4181	0.676	-0.10980511	count	1

AP000654.1	-0.5069775	1.212713	-0.4181	0.676	-0.10980511	count	1
AC126614.1	-0.5069775	1.212713	-0.4181	0.676	-0.10980511	count	1
EWSAT1	-0.5069775	1.212713	-0.4181	0.676	-0.10980511	count	1
AP005329.1	-0.5069775	1.212713	-0.4181	0.676	-0.10980511	count	1
AC138969.1	-0.5069775	1.496755	-0.3387	0.735	-0.10980511	count	1
QKI	-0.0772152	0.0738978	-1.0449	0.296	-0.109692464	count	1
TMEM11	-0.083613	0.1526105	-0.5479	0.584	-0.109685261	count	1
CCL2	-0.0759582	0.1634073	-0.4648	0.642	-0.109362729	count	1
ABI2	-0.0827816	0.1508159	-0.5489	0.583	-0.109344092	count	1
COPG2	-0.1062025	0.3616629	-0.2937	0.769	-0.109324737	count	1
AP3M1	-0.0870551	0.2250784	-0.3868	0.699	-0.109211832	count	1
SZT2	-0.0985168	0.4436806	-0.222	0.824	-0.109159348	count	1
NR2C2	-0.0952276	0.2881381	-0.3305	0.741	-0.108955462	count	1
PLD3	-0.0783645	0.120629	-0.6496	0.516	-0.108944893	count	1
CTNNB1	-0.0768339	0.0872689	-0.8804	0.379	-0.108904066	count	1
ZFP14	-0.0912066	0.2636268	-0.346	0.729	-0.108903633	count	1
C8orf82	-0.0834282	0.1874571	-0.4451	0.656	-0.108897618	count	1
AL732292.2	-0.8309538	0.8104542	-1.0253	0.305	-0.10877385	count	1
AC021097.1	-0.8309538	0.8876173	-0.9362	0.349	-0.10877385	count	1
RORC	-0.8309538	0.929176	-0.8943	0.371	-0.10877385	count	1
RAB5A	-0.0784443	0.1102245	-0.7117	0.477	-0.108704334	count	1
TTC4	-0.1108757	0.4048799	-0.2738	0.784	-0.108645513	count	1
HCFC1	-0.0905158	0.2270962	-0.3986	0.69	-0.10864128	count	1
NUP88	-0.1007597	0.2794919	-0.3605	0.718	-0.108444734	count	1
LNX2	-0.124534	0.3753514	-0.3318	0.74	-0.108384441	count	1
DOT1L	-0.1718483	0.530579	-0.3239	0.746	-0.108221824	count	1
CHST14	-0.0876184	0.2267706	-0.3864	0.699	-0.108136688	count	1
GPR137C	-0.1716649	0.6376644	-0.2692	0.788	-0.108101979	count	1
SENP6	-0.0772674	0.1120436	-0.6896	0.49	-0.108065498	count	1
AP003392.4	-0.2107312	0.6371334	-0.3307	0.741	-0.107962434	count	1
KNSTRN	-0.117566	0.3916888	-0.3002	0.764	-0.107795583	count	1
VEZT	-0.0789135	0.1317782	-0.5988	0.549	-0.107659582	count	1
NBPF20	-0.3714406	0.763956	-0.4862	0.627	-0.107653267	count	1
ZNF502	-0.1098225	0.386915	-0.2838	0.777	-0.107599303	count	1
MCM3AP	-0.0885925	0.326192	-0.2716	0.786	-0.107593434	count	1
MOCS2	-0.0772385	0.1104901	-0.6991	0.485	-0.107546503	count	1
C11orf54	-0.0806024	0.1662846	-0.4847	0.628	-0.107510504	count	1
PLEKHA1	-0.0773938	0.1164368	-0.6647	0.506	-0.107285491	count	1
RBM44	-0.2597302	0.5178121	-0.5016	0.616	-0.107254454	count	1
DMAC1	-0.0764981	0.1043309	-0.7332	0.463	-0.107142844	count	1
AFMID	-0.0832464	0.1936995	-0.4298	0.667	-0.106976978	count	1
PTOV1-AS1	-0.1801531	0.7383863	-0.244	0.807	-0.106974741	count	1
FKBP1A	-0.0742928	0.0337445	-2.2016	0.0278	-0.106868421	count	1
ARHGEF6	-0.1148049	0.432174	-0.2656	0.791	-0.106845824	count	1
PAQR4	-0.1148049	0.4632679	-0.2478	0.804	-0.106845824	count	1

SMIM4	-0.0787235	0.1404618	-0.5605	0.575	-0.106671601	count	1
SLC35A1	-0.0839218	0.2039825	-0.4114	0.681	-0.106671095	count	1
TMEM248	-0.0774971	0.1187795	-0.6524	0.514	-0.106554942	count	1
ZFP30	-0.1100196	0.3972905	-0.2769	0.782	-0.106529365	count	1
FBXO48	-0.0961607	0.3225857	-0.2981	0.766	-0.106525974	count	1
MGST3	-0.0742122	0.0411792	-1.8022	0.0716	-0.106455382	count	1
NBEA	-0.104318	0.3336199	-0.3127	0.755	-0.106409267	count	1
PTGES2	-0.0798607	0.1715067	-0.4656	0.642	-0.106318265	count	1
NECTIN1	-0.366219	0.6883394	-0.532	0.595	-0.105969838	count	1
PKDCC	-0.366219	0.7859143	-0.466	0.641	-0.105969838	count	1
UCHL3	-0.0780388	0.1428868	-0.5462	0.585	-0.105952777	count	1
IMMP1L	-0.0819775	0.1906676	-0.43	0.667	-0.105944834	count	1
1-Sep	-0.1047213	0.3415224	-0.3066	0.759	-0.105825806	count	1
RPL18A	-0.0734087	0.0208461	-3.5215	0.000436	-0.105818371	count	1
ZC2HC1A	-0.0782977	0.1449507	-0.5402	0.589	-0.105801251	count	1
PIP4K2C	-0.1416404	0.5110244	-0.2772	0.782	-0.105705399	count	1
CLU	-0.0731682	0.0441162	-1.6585	0.0973	-0.105490614	count	1
DOCK1	-0.0787262	0.1412766	-0.5572	0.577	-0.105191245	count	1
GNA13	-0.0833834	0.2187656	-0.3812	0.703	-0.105176806	count	1
C2orf27B	-0.2548921	0.8956462	-0.2846	0.776	-0.105115727	count	1
AC007364.1	-0.2548921	0.8956794	-0.2846	0.776	-0.105115727	count	1
ZNF354C	-0.1112849	0.4073127	-0.2732	0.785	-0.105011045	count	1
DYRK1B	-0.0910596	0.2907112	-0.3132	0.754	-0.104972141	count	1
FIS1	-0.0730061	0.0436957	-1.6708	0.0949	-0.10463392	count	1
PDLIM1	-0.0727736	0.0426006	-1.7083	0.0877	-0.104597843	count	1
B9D2	-0.0844121	0.2341937	-0.3604	0.719	-0.104534089	count	1
PSMA7	-0.0727016	0.0369546	-1.9673	0.0492	-0.104404531	count	1
RHOT2	-0.0814499	0.1978311	-0.4117	0.681	-0.104337499	count	1
SCARA3	-0.0791053	0.1742367	-0.454	0.65	-0.104225491	count	1
GMCL1	-0.0818084	0.2301121	-0.3555	0.722	-0.104220238	count	1
MRPS18A	-0.0768612	0.1452561	-0.5291	0.597	-0.104204186	count	1
SESN2	-0.1052239	0.4366699	-0.241	0.81	-0.104188365	count	1
PFDN4	-0.0753804	0.109592	-0.6878	0.492	-0.104184514	count	1
SNHG10	-0.0992967	0.3753049	-0.2646	0.791	-0.103839713	count	1
IRAK1BP1	-0.0826519	0.2353555	-0.3512	0.725	-0.10381534	count	1
UBE2M	-0.0734463	0.0735639	-0.9984	0.318	-0.103634507	count	1
FBXL8	-0.0962454	0.4100605	-0.2347	0.814	-0.103539696	count	1
SNU13	-0.0724462	0.0492858	-1.4699	0.1417	-0.103460523	count	1
SLC39A9	-0.0905683	0.280973	-0.3223	0.747	-0.103157922	count	1
TRIM5	-0.0800419	0.2163634	-0.3699	0.711	-0.103047983	count	1
KLHL20	-0.0794522	0.1940085	-0.4095	0.682	-0.103031829	count	1
Z82217.1	-0.1142657	0.4405226	-0.2594	0.795	-0.103012849	count	1
AC112907.3	-0.2500644	0.8808535	-0.2839	0.777	-0.1029869	count	1
PRICKLE1	-0.095601	0.2919167	-0.3275	0.743	-0.102839865	count	1
HDAC8	-0.0829591	0.2219774	-0.3737	0.709	-0.102726456	count	1

DUS3L	-0.0918032	0.3305442	-0.2777	0.781	-0.102696268	count	1
PIEZO2	-0.7939494	0.3618657	-2.194	0.0283	-0.102678358	count	1
TLR1	-0.1332057	0.5623396	-0.2369	0.813	-0.102557334	count	1
STOML1	-0.0941313	0.3023909	-0.3113	0.756	-0.102516487	count	1
DHX35	-0.11175	0.3983441	-0.2805	0.779	-0.102378061	count	1
PJA2	-0.0728568	0.087053	-0.8369	0.403	-0.102295886	count	1
AC104389.4	-0.1845267	0.7014321	-0.2631	0.793	-0.102176948	count	1
AC074117.1	-0.1324172	0.4605491	-0.2875	0.774	-0.101935613	count	1
MRPL52	-0.0723905	0.0809215	-0.8946	0.371	-0.101803507	count	1
DCAF6	-0.0791532	0.2100243	-0.3769	0.706	-0.101800404	count	1
AC002467.1	-0.1051697	0.4041196	-0.2602	0.795	-0.101769827	count	1
FAM133B	-0.0719227	0.0831217	-0.8653	0.387	-0.101747045	count	1
PDCD4	-0.0720358	0.093293	-0.7721	0.44	-0.101650467	count	1
NEMP1	-0.1611287	0.4986167	-0.3232	0.747	-0.101232259	count	1
GABPB2	-0.0991947	0.3419891	-0.2901	0.772	-0.101123861	count	1
HACD4	-0.1103931	0.4900247	-0.2253	0.822	-0.101115369	count	1
SKA2	-0.0779673	0.1863327	-0.4184	0.676	-0.101100298	count	1
CNOT6	-0.0876898	0.2728091	-0.3214	0.748	-0.101060695	count	1
PNKD	-0.0715389	0.0846618	-0.845	0.398	-0.100911405	count	1
P2RY2	-0.7830262	0.6991284	-1.12	0.263	-0.100901593	count	1
CFL1	-0.0700923	0.0330731	-2.1193	0.0342	-0.1007254	count	1
RBSN	-0.0862332	0.2477158	-0.3481	0.728	-0.100453521	count	1
EID2	-0.0757984	0.1626252	-0.4661	0.641	-0.100380279	count	1
G3BP2	-0.0713543	0.087378	-0.8166	0.414	-0.10024915	count	1
LUC7L	-0.0741903	0.1566223	-0.4737	0.636	-0.100143051	count	1
TFG	-0.0716664	0.0849332	-0.8438	0.399	-0.100089659	count	1
AXIN1	-0.0963942	0.5564432	-0.1732	0.862	-0.099963442	count	1
ZFP3	-0.1685378	0.638013	-0.2642	0.792	-0.09981105	count	1
GORASP2	-0.073174	0.131417	-0.5568	0.578	-0.099722252	count	1
PRKDC	-0.0707971	0.0942253	-0.7514	0.452	-0.099526787	count	1
NKAPL	-0.0898493	0.3915229	-0.2295	0.819	-0.09947718	count	1
ARL6	-0.0937412	0.3392925	-0.2763	0.782	-0.099452594	count	1
TSPYL2	-0.0749717	0.1604104	-0.4674	0.64	-0.099442885	count	1
MAP2K3	-0.0752889	0.1670662	-0.4507	0.652	-0.099304947	count	1
ARHGAP35	-0.0801396	0.2352025	-0.3407	0.733	-0.099219602	count	1
MFN1	-0.0782428	0.1809291	-0.4325	0.665	-0.099182606	count	1
DGCR2	-0.0784105	0.2727929	-0.2874	0.774	-0.099142728	count	1
CCDC85C	-0.1000524	0.4194565	-0.2385	0.811	-0.099004865	count	1
SOST	-0.1939796	1.0299468	-0.1883	0.851	-0.098962681	count	1
GLUD2	-0.1939796	1.054516	-0.184	0.854	-0.098962681	count	1
SMARCAL1	-0.0902378	0.3125355	-0.2887	0.773	-0.098816702	count	1
NACA2	-0.1573546	0.446271	-0.3526	0.724	-0.09877886	count	1
AP2M1	-0.0694778	0.0620938	-1.1189	0.263	-0.098733809	count	1
GLYCTK	-0.0944599	0.3164903	-0.2985	0.765	-0.098730158	count	1
SSR1	-0.0709181	0.1110111	-0.6388	0.523	-0.098345275	count	1

AP002490.1	-0.46044	0.8182519	-0.5627	0.574	-0.098236183	count	1
PROSER3	-0.2121372	0.5107045	-0.4154	0.678	-0.098180154	count	1
CBX3	-0.0690736	0.0628426	-1.0992	0.272	-0.098123402	count	1
ANAPC13	-0.0706912	0.1054531	-0.6704	0.503	-0.098018834	count	1
PRR34-AS1	-0.0800185	0.2203576	-0.3631	0.717	-0.097957605	count	1
UBXN4	-0.0685462	0.0580238	-1.1813	0.2376	-0.097918719	count	1
MAP10	-0.1360337	0.4307719	-0.3158	0.752	-0.09778049	count	1
PCNP	-0.0693201	0.0858228	-0.8077	0.419	-0.097565187	count	1
CD96	-0.7619861	1.205026	-0.6323	0.527	-0.097508302	count	1
RASL11B	-0.7619861	1.205026	-0.6323	0.527	-0.097508302	count	1
SLITRK4	-0.7619861	1.205026	-0.6323	0.527	-0.097508302	count	1
KCNMA1	-0.7619861	1.205026	-0.6323	0.527	-0.097508302	count	1
TMEM121B	-0.7619861	1.205026	-0.6323	0.527	-0.097508302	count	1
TRIM46	-0.7619861	1.268579	-0.6007	0.548	-0.097508302	count	1
DCAF12L1	-0.7619861	1.268579	-0.6007	0.548	-0.097508302	count	1
AC011595.1	-0.7619861	1.268579	-0.6007	0.548	-0.097508302	count	1
SLC24A4	-0.7619861	1.268579	-0.6007	0.548	-0.097508302	count	1
TPX2	-0.7619861	1.268579	-0.6007	0.548	-0.097508302	count	1
PABPC1L	-0.7619861	1.268579	-0.6007	0.548	-0.097508302	count	1
LILRB4	-0.7619861	1.268579	-0.6007	0.548	-0.097508302	count	1
AF130417.1	-0.7619861	1.268579	-0.6007	0.548	-0.097508302	count	1
TEKT2	-0.7619861	1.308295	-0.5824	0.56	-0.097508302	count	1
LINC01816	-0.7619861	1.308295	-0.5824	0.56	-0.097508302	count	1
GCC2-AS1	-0.7619861	1.308295	-0.5824	0.56	-0.097508302	count	1
LINC02009	-0.7619861	1.308295	-0.5824	0.56	-0.097508302	count	1
NSUN7	-0.7619861	1.308295	-0.5824	0.56	-0.097508302	count	1
AC108062.1	-0.7619861	1.308295	-0.5824	0.56	-0.097508302	count	1
AC026741.1	-0.7619861	1.308295	-0.5824	0.56	-0.097508302	count	1
AC010245.1	-0.7619861	1.308295	-0.5824	0.56	-0.097508302	count	1
AC116312.1	-0.7619861	1.308295	-0.5824	0.56	-0.097508302	count	1
PRR7-AS1	-0.7619861	1.308295	-0.5824	0.56	-0.097508302	count	1
AC005162.3	-0.7619861	1.308295	-0.5824	0.56	-0.097508302	count	1
AC018638.6	-0.7619861	1.308295	-0.5824	0.56	-0.097508302	count	1
LINC01607	-0.7619861	1.308295	-0.5824	0.56	-0.097508302	count	1
AL731571.1	-0.7619861	1.308295	-0.5824	0.56	-0.097508302	count	1
AC129507.2	-0.7619861	1.308295	-0.5824	0.56	-0.097508302	count	1
SHBG	-0.7619861	1.308295	-0.5824	0.56	-0.097508302	count	1
AC008763.2	-0.7619861	1.308295	-0.5824	0.56	-0.097508302	count	1
GP6	-0.7619861	1.308295	-0.5824	0.56	-0.097508302	count	1
MIS18A-AS1	-0.7619861	1.308295	-0.5824	0.56	-0.097508302	count	1
YIPF5	-0.0694998	0.0948428	-0.7328	0.464	-0.097461501	count	1
LRRC8D	-0.0910386	0.4017927	-0.2266	0.821	-0.097235827	count	1
INTS10	-0.0708748	0.139073	-0.5096	0.61	-0.097035772	count	1
ATP5F1D	-0.0676597	0.0474337	-1.4264	0.154	-0.096886189	count	1
DBF4B	-0.2748703	0.7402007	-0.3713	0.71	-0.096766901	count	1

MFN2	-0.0847096	0.3188281	-0.2657	0.79	-0.096438195	count	1
RITA1	-0.0795782	0.2431712	-0.3273	0.744	-0.096373362	count	1
CD22	-0.7537716	0.650378	-1.159	0.247	-0.096193891	count	1
WRN	-0.0810655	0.2905208	-0.279	0.78	-0.096192005	count	1
RRAGB	-0.0810546	0.2216441	-0.3657	0.715	-0.096178998	count	1
CCDC88C	-0.0852072	0.2672459	-0.3188	0.75	-0.096163929	count	1
TRMT11	-0.0744245	0.1897658	-0.3922	0.695	-0.096153926	count	1
ZNF81	-0.1291452	0.6557108	-0.197	0.844	-0.096153105	count	1
GATAD2A	-0.0747457	0.2357871	-0.317	0.751	-0.096113806	count	1
FAM89A	-0.0723931	0.14902	-0.4858	0.627	-0.095912299	count	1
DCLK2	-0.1102949	0.4754597	-0.232	0.817	-0.095779746	count	1
SOD1	-0.0667645	0.0489625	-1.3636	0.173	-0.09565657	count	1
CNOT2	-0.0686397	0.1067373	-0.6431	0.52	-0.095650152	count	1
ME3	-0.0867456	0.3219205	-0.2695	0.788	-0.095496783	count	1
UNC13D	-0.1729369	0.5014737	-0.3449	0.73	-0.09549263	count	1
SPCS2	-0.0667433	0.0521459	-1.2799	0.201	-0.09547598	count	1
SEC22C	-0.0707987	0.1490497	-0.475	0.635	-0.095395021	count	1
NKTR	-0.0674284	0.0927712	-0.7268	0.467	-0.095225476	count	1
NCEH1	-0.0789348	0.2738636	-0.2882	0.773	-0.09514094	count	1
GABPB1-AS1	-0.0697224	0.1689715	-0.4126	0.68	-0.095123943	count	1
SPAG16	-0.0714532	0.1678526	-0.4257	0.67	-0.095101171	count	1
GTPBP8	-0.0723242	0.1829413	-0.3953	0.693	-0.095027803	count	1
MIR497HG	-0.1606378	0.6313518	-0.2544	0.799	-0.094959474	count	1
KLKB1	-0.7441327	0.8721605	-0.8532	0.394	-0.094659012	count	1
LPAR4	-0.7441327	0.8721605	-0.8532	0.394	-0.094659012	count	1
GSDMC	-0.7441327	0.9911182	-0.7508	0.453	-0.094659012	count	1
AC012485.3	-0.7441327	1.027152	-0.7245	0.469	-0.094659012	count	1
TMEM50B	-0.0678425	0.1031125	-0.6579	0.511	-0.094612695	count	1
OSBP2	-0.3302475	0.7149135	-0.4619	0.644	-0.094505493	count	1
MTFP1	-0.3302475	0.7328925	-0.4506	0.652	-0.094505493	count	1
MCFD2	-0.0671523	0.0892434	-0.7525	0.452	-0.094361109	count	1
NDUFA12	-0.0662343	0.058881	-1.1249	0.2607	-0.094336113	count	1
AC107464.3	-0.2302597	0.9823443	-0.2344	0.815	-0.094309361	count	1
MBNL1	-0.0663297	0.0710307	-0.9338	0.35	-0.094255144	count	1
ZNF585A	-0.0999957	0.4769511	-0.2097	0.834	-0.094213968	count	1
EMD	-0.0681952	0.1119298	-0.6093	0.542	-0.094122487	count	1
ZBTB5	-0.1310711	0.5644241	-0.2322	0.816	-0.094121564	count	1
USP6NL	-0.080806	0.2389645	-0.3382	0.735	-0.094092768	count	1
FKBPL	-0.0733774	0.1823782	-0.4023	0.687	-0.094062047	count	1
FCHSD2	-0.0684071	0.1530932	-0.4468	0.655	-0.093690538	count	1
RPL37A	-0.06497	0.0248307	-2.6165	0.00893	-0.093513374	count	1
NDUFS7	-0.0661798	0.0810028	-0.817	0.414	-0.093412595	count	1
SPAG9	-0.0656518	0.0650361	-1.0095	0.313	-0.093359607	count	1
LPGAT1	-0.0670114	0.1185737	-0.5651	0.572	-0.093230532	count	1
AIMP1	-0.0665705	0.1058631	-0.6288	0.53	-0.093196747	count	1

MOB3C	-0.0877763	0.2798733	-0.3136	0.754	-0.093066622	count	1
UVRAG	-0.0666542	0.1114095	-0.5983	0.55	-0.093054181	count	1
NUDCD3	-0.0714247	0.1861713	-0.3837	0.701	-0.092972665	count	1
NARS	-0.0669924	0.1034876	-0.6473	0.517	-0.092749438	count	1
PABPC5	-0.1207058	0.4826027	-0.2501	0.803	-0.092721431	count	1
PJA1	-0.1048168	0.4055925	-0.2584	0.796	-0.092703921	count	1
ICMT	-0.0709113	0.2050414	-0.3458	0.729	-0.092585388	count	1
CLSTN1	-0.0699887	0.1614726	-0.4334	0.665	-0.092352689	count	1
AMOTL2	-0.0772053	0.2293055	-0.3367	0.736	-0.092098362	count	1
CPT1A	-0.0678701	0.1529907	-0.4436	0.657	-0.092073758	count	1
ZNF235	-0.1060752	0.5212798	-0.2035	0.839	-0.092054707	count	1
IGHG1	-0.4348681	0.2099134	-2.0717	0.0384	-0.09200943	count	1
RILPL1	-0.0720058	0.1721173	-0.4184	0.676	-0.092002331	count	1
STAG1	-0.0695557	0.1699423	-0.4093	0.682	-0.091992269	count	1
MTCH2	-0.0685787	0.1525013	-0.4497	0.653	-0.091952057	count	1
MAGEE1	-0.1990044	0.5705453	-0.3488	0.727	-0.091781837	count	1
PPP2CB	-0.0670574	0.1138019	-0.5892	0.556	-0.091764496	count	1
ANKRD36C	-0.0736867	0.2729751	-0.2699	0.787	-0.09166286	count	1
BRWD1	-0.069192	0.1632613	-0.4238	0.672	-0.091561333	count	1
AP001107.2	-0.4325604	0.9914033	-0.4363	0.663	-0.091452053	count	1
CNTN4	-0.4325604	1.024322	-0.4223	0.673	-0.091452053	count	1
CEBPB-AS1	-0.4325604	1.024322	-0.4223	0.673	-0.091452053	count	1
PDE11A	-0.4325604	1.1281294	-0.3834	0.701	-0.091452053	count	1
CDKL4	-0.4325604	1.157165	-0.3738	0.709	-0.091452053	count	1
MYLK4	-0.4325604	1.157165	-0.3738	0.709	-0.091452053	count	1
AC006504.1	-0.4325604	1.310327	-0.3301	0.741	-0.091452053	count	1
MIA3	-0.0651958	0.0968648	-0.6731	0.501	-0.091376922	count	1
MTRF1	-0.0982972	0.3734679	-0.2632	0.792	-0.091272558	count	1
TMEM175	-0.0731314	0.2103747	-0.3476	0.728	-0.091261769	count	1
AC018645.2	-0.4314758	0.6747304	-0.6395	0.523	-0.091190346	count	1
PAQR7	-0.0920513	0.3813393	-0.2414	0.809	-0.090997735	count	1
AC090517.4	-0.1150714	0.4857116	-0.2369	0.813	-0.090962486	count	1
MSL3	-0.0672984	0.1352872	-0.4974	0.619	-0.090824443	count	1
ASF1A	-0.0673976	0.1357158	-0.4966	0.62	-0.090740492	count	1
SELENOI	-0.104503	0.4406851	-0.2371	0.813	-0.090668019	count	1
BCORL1	-0.1089315	0.4443204	-0.2452	0.806	-0.090569459	count	1
ZEB1-AS1	-0.0854145	0.3178102	-0.2688	0.788	-0.090540215	count	1
TTC26	-0.1115503	0.4412439	-0.2528	0.8	-0.090532748	count	1
SIPA1L3	-0.1115503	0.4711821	-0.2367	0.813	-0.090532748	count	1
GSE1	-0.0712923	0.2120504	-0.3362	0.737	-0.090227717	count	1
USP5	-0.0799316	0.2904845	-0.2752	0.783	-0.09016926	count	1
TOMM20	-0.0630036	0.0505295	-1.2469	0.213	-0.090025843	count	1
TRAM1	-0.0632658	0.0678214	-0.9328	0.351	-0.089969566	count	1
PLCL1	-0.4264047	0.3800471	-1.122	0.262	-0.089968936	count	1
SELENOF	-0.0633317	0.0639191	-0.9908	0.322	-0.089924008	count	1

RAC1	-0.0625198	0.0319442	-1.9572	0.0504	-0.089912892	count	1
LINC01023	-0.1631447	0.4588662	-0.3555	0.722	-0.089872638	count	1
NDUFA5	-0.0636505	0.0747516	-0.8515	0.395	-0.089856404	count	1
AL357079.1	-0.3147826	1.1612	-0.2711	0.786	-0.089647995	count	1
TMEM232	-0.3147826	1.1612	-0.2711	0.786	-0.089647995	count	1
UNC5CL	-0.3147826	1.1612	-0.2711	0.786	-0.089647995	count	1
AL121748.2	-0.3147826	1.1612	-0.2711	0.786	-0.089647995	count	1
CCBE1	-0.3147826	1.1612	-0.2711	0.786	-0.089647995	count	1
AC022098.3	-0.3147826	1.1612	-0.2711	0.786	-0.089647995	count	1
C7orf61	-0.3147826	1.166985	-0.2697	0.787	-0.089647995	count	1
PLEKHB1	-0.3147826	1.166985	-0.2697	0.787	-0.089647995	count	1
CSPG4	-0.3147826	1.166985	-0.2697	0.787	-0.089647995	count	1
AL031775.1	-0.3147826	1.346355	-0.2338	0.815	-0.089647995	count	1
SHC4	-0.3147826	1.346355	-0.2338	0.815	-0.089647995	count	1
AL391095.1	-0.3147826	1.346355	-0.2338	0.815	-0.089647995	count	1
CTPS2	-0.0937591	0.3635554	-0.2579	0.797	-0.089458325	count	1
BBX	-0.0635267	0.0968192	-0.6561	0.512	-0.08944627	count	1
BTBD7	-0.0650758	0.1213234	-0.5364	0.592	-0.089344397	count	1
PARL	-0.0659734	0.1530287	-0.4311	0.666	-0.089287625	count	1
GOLGA7B	-0.1355232	0.4927141	-0.2751	0.783	-0.089262835	count	1
TCEAL3	-0.0633618	0.0973264	-0.651	0.515	-0.089017443	count	1
ERP44	-0.0633811	0.0966653	-0.6557	0.512	-0.088942655	count	1
RNF103	-0.0765829	0.245544	-0.3119	0.755	-0.088834809	count	1
EPB41	-0.0681904	0.2172847	-0.3138	0.754	-0.088751588	count	1
FAM45A	-0.0652323	0.1516826	-0.4301	0.667	-0.088611733	count	1
ENKD1	-0.0758488	0.2554558	-0.2969	0.767	-0.088587503	count	1
DNAAF4	-0.0700906	0.2274342	-0.3082	0.758	-0.088587191	count	1
AL024508.2	-0.2167215	0.6996435	-0.3098	0.757	-0.088428987	count	1
SMAD4	-0.0665144	0.2004312	-0.3319	0.74	-0.088427682	count	1
TNKS	-0.0699413	0.2094448	-0.3339	0.738	-0.088397849	count	1
CD47	-0.0624985	0.0889472	-0.7026	0.482	-0.088172043	count	1
CARD16	-0.0619955	0.083925	-0.7387	0.46	-0.08777149	count	1
CCDC180	-0.1592068	0.8002399	-0.1989	0.842	-0.087619714	count	1
PPP4R2	-0.0631355	0.1075833	-0.5869	0.557	-0.087602716	count	1
NUBP2	-0.0641991	0.1368714	-0.469	0.639	-0.087578039	count	1
SLC2A10	-0.1106723	0.4813997	-0.2299	0.818	-0.087416763	count	1
SF3B6	-0.0612642	0.0545237	-1.1236	0.2613	-0.087414448	count	1
ZNF135	-0.1218761	0.5373364	-0.2268	0.821	-0.087360191	count	1
ZNF106	-0.0623854	0.0964218	-0.647	0.518	-0.087339334	count	1
ERBIN	-0.0635687	0.1248312	-0.5092	0.611	-0.087304398	count	1
CCDC159	-0.0692566	0.2227857	-0.3109	0.756	-0.08729682	count	1
RAET1G	-0.0926523	0.5013418	-0.1848	0.853	-0.087207827	count	1
SLC46A3	-0.0754585	0.3293975	-0.2291	0.819	-0.08720639	count	1
RAMP2	-0.0605304	0.0366897	-1.6498	0.0991	-0.087192706	count	1
UTY	-0.0713758	0.3090702	-0.2309	0.817	-0.087153488	count	1

DZIP3	-0.0716789	0.3043333	-0.2355	0.814	-0.087153319	count	1
MRPL35	-0.0662682	0.1702423	-0.3893	0.697	-0.087108696	count	1
STMN1	-0.0618911	0.0937817	-0.6599	0.509	-0.087041697	count	1
NOL12	-0.0809322	0.3284493	-0.2464	0.805	-0.086932781	count	1
VPS35L	-0.0714794	0.2210038	-0.3234	0.746	-0.086909677	count	1
RIN3	-0.0837499	0.3042573	-0.2753	0.783	-0.086729682	count	1
NFATC2IP	-0.068902	0.2130133	-0.3235	0.746	-0.08672925	count	1
PAXBP1-AS1	-0.1096648	0.5421546	-0.2023	0.84	-0.086605456	count	1
C19orf53	-0.0605288	0.0506052	-1.1961	0.232	-0.086523468	count	1
BTN2A1	-0.0697199	0.2158637	-0.323	0.747	-0.08641897	count	1
SPTSSA	-0.0620487	0.1045963	-0.5932	0.553	-0.086259691	count	1
MRPS21	-0.0604828	0.0624232	-0.9689	0.333	-0.086089807	count	1
ANO7	-0.1696091	0.6798737	-0.2495	0.803	-0.085997409	count	1
LEPROTL1	-0.0627691	0.1253858	-0.5006	0.617	-0.085993435	count	1
SEC24D	-0.0712999	0.2708466	-0.2632	0.792	-0.085896063	count	1
CTBP1	-0.0645338	0.1593651	-0.4049	0.686	-0.085524147	count	1
ANXA2	-0.0593785	0.0338345	-1.755	0.0794	-0.085372619	count	1
BICD1	-0.0646422	0.2062769	-0.3134	0.754	-0.0853328	count	1
ATP6AP1L	-0.0948501	0.5390028	-0.176	0.86	-0.085264919	count	1
PPIL4	-0.0608925	0.1003899	-0.6066	0.544	-0.085207193	count	1
ROCK2	-0.0608063	0.0974841	-0.6238	0.533	-0.085177371	count	1
CNOT6L	-0.0614137	0.1143623	-0.537	0.591	-0.08514277	count	1
UBE2W	-0.0643613	0.155036	-0.4151	0.678	-0.085059557	count	1
COQ9	-0.0643999	0.1811062	-0.3556	0.722	-0.085012256	count	1
BZW1	-0.0594293	0.054504	-1.0904	0.2756	-0.084854015	count	1
TCEA1	-0.0597356	0.072446	-0.8246	0.41	-0.084764567	count	1
FAM124B	-0.0888129	0.2869292	-0.3095	0.757	-0.084683207	count	1
LTF	-0.1539352	0.5113661	-0.301	0.763	-0.084610173	count	1
CEP120	-0.0724159	0.2637554	-0.2746	0.784	-0.084556296	count	1
MNT	-0.0712065	0.2454305	-0.2901	0.772	-0.084435194	count	1
ZNF511	-0.0637892	0.1794839	-0.3554	0.722	-0.084396988	count	1
RNF219	-0.0645645	0.1827534	-0.3533	0.724	-0.084340928	count	1
MFSD4B	-0.0852443	0.390505	-0.2183	0.827	-0.08419765	count	1
OTUD5	-0.0692077	0.2108056	-0.3283	0.743	-0.084135756	count	1
CTNNA1	-0.0595826	0.0816704	-0.7295	0.466	-0.084065518	count	1
DLGAP1-AS1	-0.0715008	0.2497001	-0.2863	0.775	-0.084025196	count	1
LONP2	-0.0621255	0.1472875	-0.4218	0.673	-0.084020472	count	1
KIF3A	-0.0649379	0.1811952	-0.3584	0.72	-0.084015383	count	1
AC147651.4	-0.0969376	0.3986452	-0.2432	0.808	-0.084004517	count	1
ZNF614	-0.0849702	0.390052	-0.2178	0.828	-0.083924061	count	1
YWHAG	-0.060906	0.1125044	-0.5414	0.588	-0.083884733	count	1
RAB22A	-0.061238	0.1325749	-0.4619	0.644	-0.083738192	count	1
CSNK1G3	-0.0609572	0.1283208	-0.475	0.635	-0.083636911	count	1
PROSER2	-0.0846453	0.3642349	-0.2324	0.816	-0.083599789	count	1
CDC123	-0.0597541	0.0905679	-0.6598	0.509	-0.08354788	count	1

COX10	-0.0836177	0.3409668	-0.2452	0.806	-0.083451881	count	1
RCBTB1	-0.0836177	0.3478939	-0.2404	0.81	-0.083451881	count	1
SYAP1	-0.0606578	0.1132369	-0.5357	0.592	-0.083375729	count	1
SON	-0.0581411	0.0527753	-1.1017	0.271	-0.083191546	count	1
DUSP28	-0.0779859	0.3316821	-0.2351	0.814	-0.083183584	count	1
SRRT	-0.0640366	0.1829626	-0.35	0.726	-0.083130003	count	1
WDR33	-0.0599339	0.1112808	-0.5386	0.59	-0.083118385	count	1
ZNF649	-0.0727609	0.3109407	-0.234	0.815	-0.083097861	count	1
PDCD7	-0.0598328	0.1166987	-0.5127	0.608	-0.083025324	count	1
RALGAPA1	-0.0630448	0.1990259	-0.3168	0.751	-0.08296768	count	1
NAA35	-0.0686844	0.2271059	-0.3024	0.762	-0.082929492	count	1
COX6A1	-0.0578437	0.0428535	-1.3498	0.177	-0.082908869	count	1
CES2	-0.0788256	0.289021	-0.2727	0.785	-0.082886639	count	1
ABLIM1	-0.0586397	0.0885588	-0.6622	0.508	-0.082854156	count	1
ATP6V1B2	-0.06939	0.2400365	-0.2891	0.773	-0.082731392	count	1
PURB	-0.0606911	0.1294257	-0.4689	0.639	-0.082727771	count	1
CACTIN	-0.0954303	0.3621941	-0.2635	0.792	-0.082678731	count	1
AC007611.1	-0.2372494	0.7530561	-0.315	0.753	-0.08260051	count	1
CYTH2	-0.0599892	0.1105314	-0.5427	0.587	-0.082553586	count	1
GTF2IRD2	-0.0971411	0.5247153	-0.1851	0.853	-0.082451071	count	1
SETD3	-0.0619573	0.1476227	-0.4197	0.675	-0.082316412	count	1
LRP5L	-0.1623468	0.5977135	-0.2716	0.786	-0.082163175	count	1
C17orf75	-0.0639173	0.1994466	-0.3205	0.749	-0.081986074	count	1
DNAJB5	-0.1246581	0.7069039	-0.1763	0.86	-0.08191617	count	1
EIF2AK1	-0.0605847	0.1596033	-0.3796	0.704	-0.081908023	count	1
UBE2Z	-0.0599134	0.1228048	-0.4879	0.626	-0.081889492	count	1
GPR89B	-0.1190778	0.4835235	-0.2463	0.805	-0.081873452	count	1
RPSA	-0.0568221	0.0273844	-2.075	0.0381	-0.081799629	count	1
RNF157	-0.1780571	0.8717014	-0.2043	0.838	-0.081663335	count	1
SEH1L	-0.0680784	0.2693142	-0.2528	0.8	-0.081589665	count	1
NOTCH1	-0.0601255	0.1495457	-0.4021	0.688	-0.081386171	count	1
ABHD13	-0.063335	0.20537	-0.3084	0.758	-0.081319854	count	1
UBXN7	-0.0648933	0.2059551	-0.3151	0.753	-0.08119568	count	1
ASAH1	-0.0584097	0.1103354	-0.5294	0.597	-0.081048864	count	1
SNX13	-0.0619317	0.1942787	-0.3188	0.75	-0.080952679	count	1
INPPL1	-0.0974445	0.3690792	-0.264	0.792	-0.080863741	count	1
POGZ	-0.0600078	0.1696515	-0.3537	0.724	-0.080860605	count	1
UTP14C	-0.0794019	0.3492698	-0.2273	0.82	-0.080761302	count	1
PCGF2	-0.057793	0.1003765	-0.5758	0.565	-0.080634675	count	1
DZIP1L	-0.1366671	0.5170522	-0.2643	0.792	-0.080341585	count	1
AC007325.4	-0.1364776	0.6539136	-0.2087	0.835	-0.080226642	count	1
CEP104	-0.0674227	0.2294542	-0.2938	0.769	-0.08015405	count	1
ZCWPW2	-0.2306646	0.5207843	-0.4429	0.658	-0.080151127	count	1
ZNF324B	-0.2306646	0.5851758	-0.3942	0.693	-0.080151127	count	1
RTF2	-0.0566644	0.0721246	-0.7856	0.432	-0.080099706	count	1

IL1RAP	-0.1079776	0.4608455	-0.2343	0.815	-0.080069792	count	1
SLC41A2	-0.0923119	0.4218341	-0.2188	0.827	-0.079937796	count	1
WDR61	-0.0576931	0.1169801	-0.4932	0.622	-0.079922525	count	1
DNAJC8	-0.0562144	0.0665041	-0.8453	0.398	-0.079901844	count	1
TRMT13	-0.064324	0.2020194	-0.3184	0.75	-0.079842505	count	1
CAPZA2	-0.0559166	0.0552404	-1.0122	0.312	-0.079767699	count	1
STIP1	-0.0597803	0.1429427	-0.4182	0.676	-0.079687863	count	1
CYC1	-0.0565901	0.0815458	-0.694	0.488	-0.079663636	count	1
ELOA	-0.058528	0.1355766	-0.4317	0.666	-0.079427102	count	1
PAXIP1	-0.1105292	0.4841919	-0.2283	0.819	-0.079048973	count	1
BRK1	-0.0552942	0.0520261	-1.0628	0.288	-0.078977693	count	1
ZNF662	-0.1001314	0.4549577	-0.2201	0.826	-0.078942424	count	1
STOM	-0.0551563	0.0528593	-1.0435	0.297	-0.078844643	count	1
GNB2	-0.0555272	0.0679963	-0.8166	0.414	-0.078744207	count	1
THADA	-0.0659499	0.259017	-0.2546	0.799	-0.078611365	count	1
FKBP10	-0.0585326	0.1528234	-0.383	0.702	-0.07858312	count	1
ZFPM2	-0.0646082	0.2815386	-0.2295	0.819	-0.078521741	count	1
TCF25	-0.0553213	0.0767725	-0.7206	0.471	-0.078391243	count	1
FAM43A	-0.0581566	0.1789557	-0.325	0.745	-0.078389292	count	1
EEF1G	-0.0993008	0.6099214	-0.1628	0.871	-0.078275971	count	1
AC239868.2	-0.1137699	0.5772104	-0.1971	0.844	-0.078138422	count	1
RUSC1-AS1	-0.2775474	0.8271481	-0.3355	0.737	-0.078128153	count	1
KLHDC9	-0.0678937	0.3259319	-0.2083	0.835	-0.078123394	count	1
RBM12B	-0.0639039	0.2858164	-0.2236	0.823	-0.077994609	count	1
FAM78A	-0.1251195	0.5665465	-0.2208	0.825	-0.077982984	count	1
CHD2	-0.0573261	0.1469899	-0.39	0.697	-0.077980691	count	1
TRIM66	-0.0841381	0.5156012	-0.1632	0.87	-0.07797014	count	1
INO80	-0.0582598	0.1706989	-0.3413	0.733	-0.077966008	count	1
RAN	-0.0542993	0.0475606	-1.1417	0.2537	-0.077673342	count	1
SLC10A7	-0.0959011	0.3648762	-0.2628	0.793	-0.077622478	count	1
DVL3	-0.0777195	0.2675765	-0.2905	0.771	-0.077509807	count	1
AC116667.1	-0.6327451	0.9350967	-0.6767	0.499	-0.077507776	count	1
AC011450.1	-0.6327451	0.9350967	-0.6767	0.499	-0.077507776	count	1
AL139246.3	-0.6327451	1.068577	-0.5921	0.554	-0.077507776	count	1
KIAA2012	-0.6327451	1.068577	-0.5921	0.554	-0.077507776	count	1
PFKFB4	-0.6327451	1.068577	-0.5921	0.554	-0.077507776	count	1
GJB7	-0.6327451	1.068577	-0.5921	0.554	-0.077507776	count	1
FATE1	-0.6327451	1.068577	-0.5921	0.554	-0.077507776	count	1
TMPRSS5	-0.6327451	1.068577	-0.5921	0.554	-0.077507776	count	1
ACSM2A	-0.6327451	1.068577	-0.5921	0.554	-0.077507776	count	1
AC009088.1	-0.6327451	1.068577	-0.5921	0.554	-0.077507776	count	1
APCDD1	-0.6327451	1.068577	-0.5921	0.554	-0.077507776	count	1
FCGBP	-0.6327451	1.068577	-0.5921	0.554	-0.077507776	count	1
EPB41L4B	-0.6327451	1.095066	-0.5778	0.563	-0.077507776	count	1
FOXN2	-0.0577939	0.1580816	-0.3656	0.715	-0.077341489	count	1

RILPL2	-0.0545967	0.0767039	-0.7118	0.477	-0.077333902	count	1
PPP2R1B	-0.066932	0.2635804	-0.2539	0.8	-0.077010879	count	1
FOXP4	-0.1038447	0.4576064	-0.2269	0.82	-0.076944166	count	1
CASC3	-0.0586335	0.1908977	-0.3071	0.759	-0.076847946	count	1
TOR1A	-0.0580543	0.1664642	-0.3487	0.727	-0.076708237	count	1
EEF1E1	-0.0555311	0.1264283	-0.4392	0.661	-0.076534785	count	1
GNAZ	-0.0900044	0.4306843	-0.209	0.834	-0.076305188	count	1
STAG2	-0.0547594	0.1079977	-0.507	0.612	-0.076258808	count	1
RPS29	-0.0530009	0.0286094	-1.8526	0.0641	-0.076234962	count	1
SRGAP2C	-0.0624563	0.2307333	-0.2707	0.787	-0.076221109	count	1
LINC01560	-0.0789541	0.4377576	-0.1804	0.857	-0.076142222	count	1
TMEM254	-0.0833435	0.402167	-0.2072	0.836	-0.076042549	count	1
SMYD5	-0.093881	0.4560871	-0.2058	0.837	-0.075960848	count	1
ATP5MC1	-0.0536361	0.072393	-0.7409	0.459	-0.075884179	count	1
MCCC1	-0.0654473	0.2891448	-0.2263	0.821	-0.075852388	count	1
U62317.3	-0.366219	0.8417156	-0.4351	0.664	-0.075749584	count	1
HES7	-0.366219	0.9096423	-0.4026	0.687	-0.075749584	count	1
AC027575.2	-0.366219	0.965499	-0.3793	0.704	-0.075749584	count	1
PMVK	-0.0562203	0.1451169	-0.3874	0.698	-0.075749471	count	1
MAATS1	-0.2696444	0.9885599	-0.2728	0.785	-0.07571503	count	1
DRAIC	-0.2696444	0.9885599	-0.2728	0.785	-0.07571503	count	1
ANKRD7	-0.2696444	0.9974871	-0.2703	0.787	-0.07571503	count	1
AC009034.1	-0.2696444	1.188738	-0.2268	0.821	-0.07571503	count	1
AL512408.1	-0.2696444	1.2966049	-0.208	0.835	-0.07571503	count	1
YIF1B	-0.0560577	0.1627296	-0.3445	0.731	-0.075706927	count	1
PDP2	-0.1016642	0.6167577	-0.1648	0.869	-0.075297027	count	1
COMMD2	-0.0544583	0.125369	-0.4344	0.664	-0.075210129	count	1
LYPLA1	-0.0537648	0.0955203	-0.5629	0.574	-0.07518452	count	1
TTLL12	-0.1092692	0.4126804	-0.2648	0.791	-0.074977576	count	1
SYNRG	-0.0570122	0.1656822	-0.3441	0.731	-0.074868752	count	1
AC083880.1	-0.1367356	0.5082261	-0.269	0.788	-0.074842156	count	1
AC022613.2	-0.1202119	0.4987456	-0.241	0.81	-0.074842	count	1
AL603832.2	-0.3620297	0.7443086	-0.4864	0.627	-0.074778798	count	1
PPP1CA	-0.053052	0.087081	-0.6092	0.542	-0.074567314	count	1
LRRK1	-0.0670936	0.3123068	-0.2148	0.83	-0.074515877	count	1
SRRD	-0.058413	0.1999611	-0.2921	0.77	-0.074505822	count	1
SREK1	-0.0535398	0.1117477	-0.4791	0.632	-0.074444225	count	1
CHCHD2	-0.0517532	0.0361262	-1.4326	0.152	-0.074276458	count	1
PIGH	-0.0569907	0.181871	-0.3134	0.754	-0.074258597	count	1
AC010999.2	-0.1625471	0.894651	-0.1817	0.856	-0.074240387	count	1
FAM69C	-0.6089485	0.9083283	-0.6704	0.503	-0.073983587	count	1
VPS29	-0.0520509	0.0664341	-0.7835	0.433	-0.073841082	count	1
MYCBP2	-0.0520896	0.0818829	-0.6361	0.525	-0.073824149	count	1
CRIP2	-0.0513321	0.0375442	-1.3672	0.172	-0.073823411	count	1
SRP14	-0.0511751	0.0354528	-1.4435	0.149	-0.073711427	count	1

LARP1	-0.0543085	0.1392156	-0.3901	0.696	-0.073501823	count	1
TLDC1	-0.0735318	0.3512665	-0.2093	0.834	-0.073295989	count	1
MCTS1	-0.0527185	0.1134222	-0.4648	0.642	-0.07306947	count	1
RSRC2	-0.0514767	0.0685707	-0.7507	0.453	-0.073054571	count	1
PEX7	-0.0637482	0.317974	-0.2005	0.841	-0.073044295	count	1
ZFAND3	-0.056021	0.1712705	-0.3271	0.744	-0.072761296	count	1
LATS1	-0.0596312	0.2692918	-0.2214	0.825	-0.072760871	count	1
ARPC1B	-0.0508301	0.052134	-0.975	0.3297	-0.072678792	count	1
TBCE	-0.1799446	0.6435301	-0.2796	0.78	-0.072666073	count	1
ITSN1	-0.0566406	0.2234938	-0.2534	0.8	-0.07262917	count	1
SENP2	-0.0580509	0.2735902	-0.2122	0.832	-0.072608296	count	1
MBTPS1	-0.0529583	0.135168	-0.3918	0.695	-0.072567182	count	1
TUBE1	-0.0555571	0.1990085	-0.2792	0.78	-0.072550111	count	1
CLIP1	-0.0524814	0.1180663	-0.4445	0.657	-0.07236095	count	1
IMPAD1	-0.0549751	0.1705426	-0.3224	0.747	-0.072327614	count	1
SPAAR	-0.0975536	0.4041615	-0.2414	0.809	-0.072195529	count	1
GORAB	-0.0634899	0.2819166	-0.2252	0.822	-0.072152898	count	1
SLA	-0.5957913	0.8043092	-0.7407	0.459	-0.072056192	count	1
CLDN7	-0.1007714	0.6025658	-0.1672	0.867	-0.071930547	count	1
AP3S1	-0.0518066	0.1015679	-0.5101	0.61	-0.071846539	count	1
FAAP20	-0.0511943	0.1047605	-0.4887	0.625	-0.071667264	count	1
KIAA0391	-0.1093526	0.6278148	-0.1742	0.862	-0.071622707	count	1
ATP5IF1	-0.0501477	0.0570551	-0.8789	0.3795	-0.071501206	count	1
LINC01843	-0.2557338	0.7880105	-0.3245	0.746	-0.071494646	count	1
SMUG1	-0.0612273	0.2804193	-0.2183	0.827	-0.071431731	count	1
CAPN15	-0.0641234	0.2914708	-0.22	0.826	-0.071197861	count	1
UPF3A	-0.0503878	0.095331	-0.5286	0.597	-0.071152267	count	1
ILK	-0.0515533	0.1051506	-0.4903	0.624	-0.071101569	count	1
ATP11B	-0.057923	0.2195714	-0.2638	0.792	-0.071096658	count	1
IKBKB	-0.0542158	0.2231611	-0.2429	0.808	-0.071094813	count	1
ORC3	-0.0542772	0.2101615	-0.2583	0.796	-0.070978153	count	1
SEC31A	-0.0507718	0.1024526	-0.4956	0.62	-0.070837872	count	1
SLC30A6	-0.0634365	0.3093918	-0.205	0.838	-0.070785339	count	1
NIT2	-0.0544749	0.192644	-0.2828	0.777	-0.070748875	count	1
C1GALT1C1L	-0.0955962	0.7611262	-0.1256	0.9	-0.070720306	count	1
MSC-AS1	-0.1079807	0.6637353	-0.1627	0.871	-0.070703249	count	1
ERC1	-0.0525686	0.1635627	-0.3214	0.748	-0.070644712	count	1
ATP6V0A2	-0.0621922	0.2898209	-0.2146	0.83	-0.070365498	count	1
GMEB2	-0.0712809	0.5798937	-0.1229	0.902	-0.070283327	count	1
TPGS1	-0.0503443	0.1069673	-0.4707	0.638	-0.070275374	count	1
ZNF37A	-0.0551017	0.2082094	-0.2646	0.791	-0.070271597	count	1
GNAQ	-0.0502098	0.1059951	-0.4737	0.636	-0.070147823	count	1
BACH1	-0.0524887	0.1750696	-0.2998	0.764	-0.070141448	count	1
LRRC37A3	-0.1391057	0.6468566	-0.215	0.83	-0.069983589	count	1
MMACHC	-0.1391057	0.6915009	-0.2012	0.841	-0.069983589	count	1

CDK2	-0.0652385	0.28778	-0.2267	0.821	-0.069964581	count	1
MCTP2	-0.0676126	0.3498748	-0.1932	0.847	-0.06989236	count	1
MTX3	-0.0625821	0.3383027	-0.185	0.853	-0.069826617	count	1
CPSF6	-0.0534743	0.1618261	-0.3304	0.741	-0.069773064	count	1
GABARAP	-0.0732888	0.2894424	-0.2532	0.8	-0.069735786	count	1
SLC35A3	-0.0554217	0.2007114	-0.2761	0.782	-0.069615766	count	1
DDX6	-0.0496843	0.0924515	-0.5374	0.591	-0.069439329	count	1
EDRF1	-0.0587583	0.3936501	-0.1493	0.881	-0.069409913	count	1
PEG3	-0.3378264	0.9014372	-0.3748	0.708	-0.069218387	count	1
CABLES2	-0.3378264	0.9372514	-0.3604	0.719	-0.069218387	count	1
ALG5	-0.0497861	0.1140494	-0.4365	0.662	-0.06917866	count	1
SLC36A4	-0.0603915	0.2931708	-0.206	0.837	-0.068900928	count	1
PTPN4	-0.0565672	0.2272612	-0.2489	0.803	-0.068863699	count	1
RAPGEF2	-0.0534342	0.2042872	-0.2616	0.794	-0.068781441	count	1
HPS4	-0.0555567	0.2582224	-0.2152	0.83	-0.068689166	count	1
TMEM214	-0.0561656	0.2610365	-0.2152	0.83	-0.068659079	count	1
RFX7	-0.0603915	0.2866748	-0.2107	0.833	-0.068613883	count	1
IRAK3	-0.0498859	0.1251856	-0.3985	0.69	-0.068482385	count	1
EHD3	-0.0763744	0.4638596	-0.1646	0.869	-0.068467646	count	1
DERL3	-0.5705888	0.7889911	-0.7232	0.47	-0.068406263	count	1
EFNA3	-0.0709247	0.4475928	-0.1585	0.874	-0.068326837	count	1
GALNT7	-0.0552693	0.2237179	-0.247	0.805	-0.068210779	count	1
TNFAIP2	-0.052137	0.1801222	-0.2895	0.772	-0.068174018	count	1
SLC30A7	-0.0526647	0.2051935	-0.2567	0.797	-0.068162311	count	1
KDELC2	-0.0559333	0.2257645	-0.2478	0.804	-0.068089337	count	1
MZT2A	-0.0482939	0.0947388	-0.5098	0.61	-0.067932579	count	1
CLTA	-0.0473521	0.0455924	-1.0386	0.299	-0.067778389	count	1
FOSL1	-0.0531288	0.2094244	-0.2537	0.8	-0.067749407	count	1
TMEM144	-0.0637006	0.3755568	-0.1696	0.865	-0.067369671	count	1
SNX21	-0.0567721	0.2541879	-0.2233	0.823	-0.067250773	count	1
FGF12	-0.3284974	0.6129216	-0.536	0.592	-0.067097072	count	1
BRAT1	-0.0537984	0.1997155	-0.2694	0.788	-0.066959498	count	1
FNDC3A	-0.0479713	0.1161014	-0.4132	0.68	-0.066922562	count	1
SPG21	-0.0500791	0.154731	-0.3237	0.746	-0.066797968	count	1
ZNF263	-0.0646203	0.2970107	-0.2176	0.828	-0.066776754	count	1
NCDN	-0.1074707	0.7022764	-0.153	0.878	-0.066718367	count	1
DBP	-0.0523879	0.2178692	-0.2405	0.81	-0.066649074	count	1
MAP7	-0.1463467	0.5108578	-0.2865	0.775	-0.066549889	count	1
OSTM1	-0.0500477	0.171289	-0.2922	0.77	-0.066295339	count	1
ACLY	-0.0512458	0.1858506	-0.2757	0.783	-0.066083374	count	1
RIPOR1	-0.0487122	0.1389359	-0.3506	0.726	-0.066035129	count	1
DDIAS	-0.3226714	0.6873043	-0.4695	0.639	-0.065778481	count	1
AC104986.2	-0.1205665	0.544495	-0.2214	0.825	-0.065731005	count	1
MRE11	-0.0501755	0.1811028	-0.2771	0.782	-0.065651009	count	1
KNL1	-0.2361796	0.8317617	-0.284	0.776	-0.065620444	count	1

RHOBTB1	-0.0592776	0.4102922	-0.1445	0.885	-0.065445219	count	1
XRCC6	-0.0461259	0.0746941	-0.6175	0.537	-0.065140537	count	1
ECHS1	-0.0462185	0.0827208	-0.5587	0.576	-0.065129025	count	1
SCRN3	-0.0506225	0.23309	-0.2172	0.828	-0.065091098	count	1
CHPT1	-0.0495108	0.1770415	-0.2797	0.78	-0.064957556	count	1
SPACA9	-0.0536871	0.2729879	-0.1967	0.844	-0.064911017	count	1
SCHIP1	-0.0910791	0.4783128	-0.1904	0.849	-0.064886409	count	1
ZC3H8	-0.0493113	0.1815772	-0.2716	0.786	-0.06482211	count	1
ADAR	-0.0465195	0.1092215	-0.4259	0.67	-0.0647399	count	1
BRWD1-AS2	-0.1611287	0.6588192	-0.2446	0.807	-0.064720997	count	1
AC069544.1	-0.1611287	0.6615681	-0.2436	0.808	-0.064720997	count	1
ENOPH1	-0.0500494	0.170436	-0.2937	0.769	-0.064713551	count	1
PHB	-0.0454653	0.0729064	-0.6236	0.533	-0.064374125	count	1
MPHOSPH9	-0.0628117	0.3872011	-0.1622	0.871	-0.064341187	count	1
BAK1	-0.0572463	0.3226561	-0.1774	0.859	-0.063842567	count	1
DHX33	-0.0620822	0.3532077	-0.1758	0.86	-0.063588597	count	1
PGD	-0.0534311	0.2820749	-0.1894	0.85	-0.063458532	count	1
ELOVL3	-0.0970352	0.6646014	-0.146	0.884	-0.063386324	count	1
C11orf98	-0.1263226	0.4766385	-0.265	0.791	-0.063343723	count	1
SEMA4C	-0.0493586	0.1857225	-0.2658	0.79	-0.063205455	count	1
USE1	-0.0463476	0.1359116	-0.341	0.733	-0.063141181	count	1
ST3GAL6	-0.055132	0.2467361	-0.2234	0.823	-0.06312729	count	1
USP30	-0.0745876	0.4801764	-0.1553	0.877	-0.063075999	count	1
KRT18	-0.045337	0.1433638	-0.3162	0.752	-0.062979252	count	1
ZNF557	-0.058353	0.3290562	-0.1773	0.859	-0.06294111	count	1
FAM111A	-0.0461831	0.14437	-0.3199	0.749	-0.062886121	count	1
UBE2L3	-0.0443557	0.0734046	-0.6043	0.546	-0.062818592	count	1
DGLUCY	-0.0488069	0.1917022	-0.2546	0.799	-0.062812689	count	1
SQOR	-0.0453411	0.1170599	-0.3873	0.699	-0.062517602	count	1
EIF4EBP2	-0.0468726	0.1754074	-0.2672	0.789	-0.062486383	count	1
GTF2F2	-0.046723	0.1611474	-0.2899	0.772	-0.062343383	count	1
PWAR6	-0.0872147	0.4916708	-0.1774	0.859	-0.062085242	count	1
FBXO7	-0.0447325	0.1054475	-0.4242	0.671	-0.061955486	count	1
NFAT5	-0.0444984	0.1299699	-0.3424	0.732	-0.061631024	count	1
RC3H1	-0.0480712	0.216195	-0.2224	0.824	-0.061553362	count	1
HSPE1	-0.0431036	0.0600624	-0.7176	0.473	-0.061465583	count	1
HIST1H2BD	-0.0990187	0.7194405	-0.1376	0.891	-0.061354283	count	1
STEAP2	-0.519753	0.4962311	-1.0474	0.295	-0.061211456	count	1
C4orf46	-0.0778622	0.5167642	-0.1507	0.88	-0.061140659	count	1
MRPL20	-0.0429982	0.0681229	-0.6312	0.528	-0.061104807	count	1
CHM	-0.0451686	0.1371571	-0.3293	0.742	-0.061080693	count	1
POLR2I	-0.0434007	0.0824737	-0.5262	0.599	-0.061046604	count	1
RYR2	-0.51813	0.6797833	-0.7622	0.446	-0.060985442	count	1
GTF2A2	-0.0429506	0.0646091	-0.6648	0.506	-0.060951164	count	1
WWP1	-0.0437327	0.1145553	-0.3818	0.703	-0.060926969	count	1

XXYLT1	-0.0658115	0.3868057	-0.1701	0.865	-0.06082886	count	1
SNX27	-0.0478893	0.2032412	-0.2356	0.814	-0.060461101	count	1
HRH1	-0.045574	0.1583354	-0.2878	0.773	-0.060424839	count	1
ETFA	-0.0434986	0.1097926	-0.3962	0.692	-0.060267308	count	1
VPS26B	-0.0491213	0.2378532	-0.2065	0.836	-0.060261768	count	1
PSMF1	-0.0429865	0.0947037	-0.4539	0.65	-0.060179346	count	1
CEP57L1	-0.0490912	0.2053847	-0.239	0.811	-0.060106382	count	1
UBA3	-0.044619	0.1398211	-0.3191	0.75	-0.059992957	count	1
HLX-AS1	-0.5104434	0.898281	-0.5682	0.57	-0.059918065	count	1
AGT	-0.5104434	0.9547641	-0.5346	0.593	-0.059918065	count	1
PFKFB3	-0.0602033	0.2662694	-0.2261	0.821	-0.059912329	count	1
ROBO1	-0.0622387	0.3043371	-0.2045	0.838	-0.059890447	count	1
DNASE1L1	-0.0445876	0.1413014	-0.3155	0.752	-0.05988391	count	1
PCDHB15	-0.1097946	0.5572014	-0.197	0.844	-0.05969976	count	1
MIA2	-0.0447945	0.1647657	-0.2719	0.786	-0.059684642	count	1
NUFIP2	-0.0425107	0.1266896	-0.3355	0.737	-0.059425711	count	1
CCDC15	-0.0910644	0.6098559	-0.1493	0.881	-0.059409073	count	1
CAVIN1	-0.0414016	0.0499348	-0.8291	0.407	-0.059336633	count	1
KCNN2	-0.0662329	0.3864851	-0.1714	0.864	-0.059285753	count	1
SGMS2	-0.051248	0.2993571	-0.1712	0.864	-0.059113658	count	1
TRAK2	-0.0464142	0.2083897	-0.2227	0.824	-0.059032668	count	1
TBCA	-0.0413408	0.054467	-0.759	0.4479	-0.058948403	count	1
COQ8B	-0.0508796	0.246125	-0.2067	0.836	-0.058901701	count	1
GLYATL2	-0.5028284	0.9866301	-0.5096	0.61	-0.058865648	count	1
TAF5	-0.0559304	0.2941901	-0.1901	0.849	-0.058667329	count	1
TNFAIP8L1	-0.0426279	0.1340091	-0.3181	0.75	-0.058542306	count	1
TBXA2R	-0.0516994	0.2859282	-0.1808	0.857	-0.058441233	count	1
KYAT3	-0.0484635	0.2403932	-0.2016	0.84	-0.058438314	count	1
AFAP1L2	-0.0531783	0.2560569	-0.2077	0.835	-0.058358828	count	1
CBR1	-0.0434322	0.1782801	-0.2436	0.808	-0.0583522	count	1
MAP2K2	-0.0412282	0.0753066	-0.5475	0.584	-0.058286446	count	1
AC073111.5	-0.0579199	0.3695544	-0.1567	0.875	-0.058206546	count	1
DDX51	-0.0527192	0.3699483	-0.1425	0.887	-0.058168907	count	1
MRPL14	-0.0417669	0.1030784	-0.4052	0.685	-0.058088037	count	1
MAN1A2	-0.0419699	0.114112	-0.3678	0.713	-0.058060711	count	1
OBSL1	-0.0473976	0.265245	-0.1787	0.858	-0.057790386	count	1
TRIM35	-0.0550178	0.343961	-0.16	0.873	-0.05770437	count	1
TNIP1	-0.0432657	0.1664351	-0.26	0.795	-0.05764498	count	1
ZMPSTE24	-0.0433968	0.16481	-0.2633	0.792	-0.057177683	count	1
CDH2	-0.1430425	0.4823065	-0.2966	0.767	-0.057160415	count	1
PDLIM5	-0.0403761	0.0799595	-0.505	0.614	-0.057129378	count	1
DESI2	-0.041579	0.1210603	-0.3435	0.731	-0.05712095	count	1
MRM1	-0.0916875	0.496288	-0.1847	0.853	-0.056717578	count	1
BOK	-0.0475919	0.2275274	-0.2092	0.834	-0.056500334	count	1
GPNMB	-0.1657216	0.8598652	-0.1927	0.847	-0.056475137	count	1

RAB14	-0.039808	0.0720357	-0.5526	0.581	-0.056417125	count	1
ALAS1	-0.043836	0.2020172	-0.217	0.828	-0.056403307	count	1
MRPS14	-0.0410427	0.1420368	-0.289	0.773	-0.05605032	count	1
SLC26A1	-0.1237706	0.9256593	-0.1337	0.894	-0.055940113	count	1
HSPB7	-0.1237706	1.0250306	-0.1207	0.904	-0.055940113	count	1
AP002770.1	-0.1237706	1.714501	-0.0722	0.9425	-0.055940113	count	1
OCRL	-0.0516428	0.3557293	-0.1452	0.885	-0.055665876	count	1
DTL	-0.1392041	0.823161	-0.1691	0.866	-0.05556548	count	1
PPP4R1	-0.0426978	0.1750944	-0.2439	0.807	-0.055561199	count	1
APMAP	-0.0410169	0.1453285	-0.2822	0.778	-0.055494675	count	1
ACBD6	-0.040133	0.1178351	-0.3406	0.733	-0.055404697	count	1
MLST8	-0.0426956	0.1856935	-0.2299	0.818	-0.055378867	count	1
VILL	-0.0655483	0.3782655	-0.1733	0.862	-0.05534956	count	1
AC004854.2	-0.0778984	0.5348378	-0.1456	0.884	-0.055349545	count	1
PMPCB	-0.0399054	0.1183201	-0.3373	0.736	-0.055159064	count	1
CKLF	-0.0397932	0.1236767	-0.3218	0.748	-0.055089633	count	1
TMEM139	-0.0772906	0.5746616	-0.1345	0.893	-0.054910962	count	1
MUC1	-0.0772906	0.5982718	-0.1292	0.897	-0.054910962	count	1
NRAS	-0.0420697	0.1785526	-0.2356	0.814	-0.054785168	count	1
FTX	-0.0401939	0.1446336	-0.2779	0.781	-0.054763531	count	1
SYVN1	-0.0517776	0.2949156	-0.1756	0.861	-0.054690768	count	1
SLC12A6	-0.0430316	0.219706	-0.1959	0.845	-0.054455537	count	1
FAM185A	-0.0693415	0.5174209	-0.134	0.893	-0.054365984	count	1
TMEM39A	-0.0517746	0.3549233	-0.1459	0.884	-0.054283709	count	1
BMF	-0.1975231	0.6895538	-0.2865	0.775	-0.054208205	count	1
AC015712.1	-0.1592068	1.119536	-0.1422	0.887	-0.054148256	count	1
AL139260.1	-0.1592068	1.130039	-0.1409	0.888	-0.054148256	count	1
HIST1H4D	-0.1592068	1.130039	-0.1409	0.888	-0.054148256	count	1
TTK	-0.1592068	1.130039	-0.1409	0.888	-0.054148256	count	1
MYCNOS	-0.1592068	1.135903	-0.1402	0.889	-0.054148256	count	1
AC109347.1	-0.1592068	1.135903	-0.1402	0.889	-0.054148256	count	1
GATD3B	-0.1592068	1.135903	-0.1402	0.889	-0.054148256	count	1
STAB2	-0.4664433	0.4056823	-1.1498	0.25	-0.053905893	count	1
SMOC2	-0.4663117	0.9615589	-0.485	0.628	-0.053888161	count	1
HAUS3	-0.0461753	0.2599565	-0.1776	0.859	-0.05380951	count	1
BPNT1	-0.0463315	0.265454	-0.1745	0.861	-0.053807466	count	1
LEKR1	-0.1077387	0.9529203	-0.1131	0.91	-0.053765851	count	1
ACAT2	-0.0423722	0.197096	-0.215	0.83	-0.053686663	count	1
YY1	-0.0377323	0.0633464	-0.5957	0.551	-0.053654213	count	1
GHR	-0.0821143	0.5018893	-0.1636	0.87	-0.053466014	count	1
BX284668.5	-0.0431731	0.2218815	-0.1946	0.846	-0.053434376	count	1
RGL1	-0.0597069	0.3310894	-0.1803	0.857	-0.053391709	count	1
VPS28	-0.03733	0.0482988	-0.7729	0.4397	-0.053375187	count	1
CUL3	-0.039325	0.1584164	-0.2482	0.804	-0.053372504	count	1
TIMM17A	-0.0399804	0.1726316	-0.2316	0.817	-0.053237487	count	1

PSMD11	-0.0381638	0.1000755	-0.3813	0.703	-0.05319282	count	1
AC012360.3	-0.062879	0.4616572	-0.1362	0.892	-0.05307224	count	1
RALBP1	-0.0373509	0.0773617	-0.4828	0.629	-0.052967313	count	1
SGCD	-0.0601373	0.6217831	-0.0967	0.923	-0.052823972	count	1
CLPB	-0.0495952	0.3155525	-0.1572	0.875	-0.052745424	count	1
ZNF639	-0.0397816	0.1634853	-0.2433	0.808	-0.052439554	count	1
UQCRFS1	-0.0375195	0.0933169	-0.4021	0.688	-0.052409923	count	1
GLRX3	-0.0381173	0.1129537	-0.3375	0.736	-0.052333834	count	1
PGP	-0.0384465	0.1218393	-0.3156	0.752	-0.052105929	count	1
DMXL2	-0.0631455	0.5180093	-0.1219	0.903	-0.052098649	count	1
EPHA2	-0.0442767	0.2450219	-0.1807	0.857	-0.05209055	count	1
RNGTT	-0.0456867	0.282173	-0.1619	0.871	-0.052060482	count	1
AGBL5	-0.066391	0.382127	-0.1737	0.862	-0.052024848	count	1
TSLP	-0.0442897	0.2754866	-0.1608	0.872	-0.051943441	count	1
GYS1	-0.0553976	0.5498747	-0.1007	0.92	-0.051874854	count	1
ARL4C	-0.0389889	0.1812732	-0.2151	0.83	-0.051789379	count	1
GINM1	-0.0363688	0.0662348	-0.5491	0.583	-0.051671168	count	1
NFU1	-0.0378537	0.127712	-0.2964	0.767	-0.051521859	count	1
MRPS7	-0.0369402	0.1004875	-0.3676	0.713	-0.051508732	count	1
AC007114.1	-0.0535221	0.5013036	-0.1068	0.915	-0.051443342	count	1
NAGK	-0.0374186	0.1399989	-0.2673	0.789	-0.051267768	count	1
CETN2	-0.0382677	0.146869	-0.2606	0.794	-0.051266678	count	1
COBLL1	-0.0372532	0.1476235	-0.2524	0.801	-0.050937228	count	1
DNAJB4	-0.0356638	0.0866444	-0.4116	0.681	-0.050472457	count	1
ZCCHC14	-0.0413823	0.2389854	-0.1732	0.863	-0.050437434	count	1
RNASEH1-AS1	-0.0433365	0.3089061	-0.1403	0.888	-0.05031751	count	1
ECSCR	-0.0349563	0.0381372	-0.9166	0.359	-0.050236898	count	1
TLNRD1	-0.0364998	0.1348497	-0.2707	0.787	-0.050164623	count	1
BLVRB	-0.0357938	0.0962233	-0.372	0.71	-0.050102261	count	1
H2AFY	-0.0357611	0.099967	-0.3577	0.721	-0.050066473	count	1
WDFY1	-0.0385132	0.1787512	-0.2155	0.829	-0.050027881	count	1
AC107214.1	-0.4372868	0.9568322	-0.457	0.648	-0.050013263	count	1
LINC00303	-0.4372868	1.075515	-0.4066	0.684	-0.050013263	count	1
HIST1H2BB	-0.4372868	1.075515	-0.4066	0.684	-0.050013263	count	1
IKZF1	-0.4372868	1.075515	-0.4066	0.684	-0.050013263	count	1
C9orf170	-0.4372868	1.075515	-0.4066	0.684	-0.050013263	count	1
MRVI1-AS1	-0.4372868	1.075515	-0.4066	0.684	-0.050013263	count	1
SPTBN2	-0.4372868	1.075515	-0.4066	0.684	-0.050013263	count	1
PRKCQ	-0.4372868	1.075515	-0.4066	0.684	-0.050013263	count	1
TEX26-AS1	-0.4372868	1.075515	-0.4066	0.684	-0.050013263	count	1
GZMB	-0.4372868	1.075515	-0.4066	0.684	-0.050013263	count	1
AC025580.1	-0.4372868	1.075515	-0.4066	0.684	-0.050013263	count	1
AC092140.2	-0.4372868	1.075515	-0.4066	0.684	-0.050013263	count	1
LINC00887	-0.4372868	1.129831	-0.387	0.699	-0.050013263	count	1
ARHGAP24	-0.0559393	0.3703647	-0.151	0.88	-0.049994127	count	1

DNAJB11	-0.0372936	0.1299279	-0.287	0.774	-0.04991956	count	1
PTOV1	-0.0365429	0.145102	-0.2518	0.801	-0.049911391	count	1
HP1BP3	-0.0350829	0.0774215	-0.4531	0.65	-0.049815297	count	1
RBBP4	-0.0365758	0.1277736	-0.2863	0.775	-0.049805724	count	1
AC245452.1	-0.1251195	0.802852	-0.1558	0.876	-0.049741925	count	1
GATA3	-0.072968	0.6390123	-0.1142	0.909	-0.049691519	count	1
CEP170	-0.0372236	0.1447021	-0.2572	0.797	-0.04963152	count	1
NBDY	-0.0349607	0.0694193	-0.5036	0.615	-0.049597994	count	1
MRPS12	-0.0354347	0.1055288	-0.3358	0.737	-0.049267131	count	1
PPIA	-0.0342405	0.0316585	-1.0816	0.28	-0.049246787	count	1
NEDD8	-0.0343659	0.0465331	-0.7385	0.46	-0.049202938	count	1
BRD1	-0.0371135	0.1648531	-0.2251	0.822	-0.049167867	count	1
BLM	-0.1449918	0.6825577	-0.2124	0.832	-0.049101573	count	1
LRRC56	-0.1449918	0.7052685	-0.2056	0.837	-0.049101573	count	1
C19orf44	-0.0558545	0.5426097	-0.1029	0.918	-0.049029326	count	1
OGT	-0.0365093	0.2135906	-0.1709	0.864	-0.04900472	count	1
ZBED5-AS1	-0.0431937	0.3069127	-0.1407	0.888	-0.049003305	count	1
MEG8	-0.4290383	0.8771626	-0.4891	0.625	-0.048925169	count	1
XKR6	-0.0689555	0.5424448	-0.1271	0.899	-0.048906973	count	1
GTF2H2C	-0.0466267	0.4536076	-0.1028	0.918	-0.04885898	count	1
IP6K2	-0.0360279	0.1286412	-0.2801	0.779	-0.048738685	count	1
EPCAM	-0.0749139	0.8484897	-0.0883	0.93	-0.048701156	count	1
SREK1IP1	-0.0346204	0.0982286	-0.3524	0.725	-0.048518423	count	1
AC016876.1	-0.0404526	0.2712751	-0.1491	0.881	-0.048514675	count	1
PRDM2	-0.0357667	0.1777228	-0.2012	0.841	-0.048181733	count	1
CAMLG	-0.0341541	0.0792244	-0.4311	0.666	-0.048159331	count	1
ZNF777	-0.0583472	0.4685381	-0.1245	0.901	-0.048100455	count	1
HDAC5	-0.0374305	0.1956403	-0.1913	0.848	-0.048053454	count	1
MCTP1	-0.03402	0.085699	-0.397	0.691	-0.047975684	count	1
ZC3H11B	-0.1755017	0.8552502	-0.2052	0.837	-0.047825673	count	1
AL034549.1	-0.0822433	0.5524001	-0.1489	0.882	-0.047732553	count	1
AL353751.1	-0.0958398	0.7783771	-0.1231	0.902	-0.047680128	count	1
LINC01004	-0.0958398	0.8846576	-0.1083	0.914	-0.047680128	count	1
CHORDC1	-0.0349434	0.1276379	-0.2738	0.784	-0.047557595	count	1
RPLP1	-0.0329083	0.0178654	-1.842	0.0656	-0.047456403	count	1
KCTD21-AS1	-0.1054028	0.7733301	-0.1363	0.892	-0.047400379	count	1
RIC3	-0.0451817	0.4150347	-0.1089	0.913	-0.047337348	count	1
FKBP1C	-0.2387405	0.8447349	-0.2826	0.777	-0.047306598	count	1
AL139412.1	-0.2387405	0.8484338	-0.2814	0.778	-0.047306598	count	1
PHF13	-0.0396199	0.2264629	-0.175	0.861	-0.047267928	count	1
SAMD4A	-0.0440975	0.3357885	-0.1313	0.896	-0.047190072	count	1
MTF2	-0.0347788	0.1347736	-0.2581	0.796	-0.047171104	count	1
ZNF568	-0.0392338	0.2828915	-0.1387	0.89	-0.047166187	count	1
AP001157.1	-0.0602194	0.6472465	-0.093	0.926	-0.047135794	count	1
DUSP11	-0.0345051	0.136281	-0.2532	0.8	-0.047105136	count	1

NUAK2	-0.0763829	0.6527037	-0.117	0.907	-0.047086221	count	1
LINC01252	-0.0763829	0.6941139	-0.11	0.912	-0.047086221	count	1
IPO11	-0.0335707	0.0962191	-0.3489	0.727	-0.047035043	count	1
MIR3681HG	-0.2372494	1.0091923	-0.2351	0.814	-0.046987248	count	1
AC012360.1	-0.2372494	1.0091923	-0.2351	0.814	-0.046987248	count	1
GABRB2	-0.2372494	1.0091923	-0.2351	0.814	-0.046987248	count	1
HUS1B	-0.2372494	1.0091923	-0.2351	0.814	-0.046987248	count	1
AL008729.1	-0.2372494	1.331098	-0.1782	0.859	-0.046987248	count	1
TNF	-0.2372494	1.331098	-0.1782	0.859	-0.046987248	count	1
FADS2	-0.2372494	1.331098	-0.1782	0.859	-0.046987248	count	1
RPUSD3	-0.0366418	0.2110047	-0.1737	0.862	-0.046894447	count	1
PPP6R2	-0.0372682	0.293106	-0.1271	0.899	-0.046769242	count	1
DPY30	-0.0333888	0.0991967	-0.3366	0.736	-0.046740783	count	1
MZF1-AS1	-0.0513151	0.416478	-0.1232	0.902	-0.046601089	count	1
FAM27C	-0.0454927	0.3521472	-0.1292	0.897	-0.046507411	count	1
NHLRC4	-0.0858734	0.5925542	-0.1449	0.885	-0.046416956	count	1
ROCK1	-0.0328963	0.0995702	-0.3304	0.741	-0.046411318	count	1
AC005921.2	-0.0355192	0.1657381	-0.2143	0.83	-0.04638231	count	1
EEF2	-0.032347	0.0410341	-0.7883	0.431	-0.046379525	count	1
GLIS2-AS1	-0.4075918	0.7139099	-0.5709	0.568	-0.046123098	count	1
LAGE3	-0.0329803	0.1112153	-0.2965	0.767	-0.046000237	count	1
TROVE2	-0.0326606	0.0898657	-0.3634	0.716	-0.045998871	count	1
CRLS1	-0.0345664	0.1654083	-0.209	0.834	-0.045789679	count	1
ATF7	-0.0341168	0.1744295	-0.1956	0.845	-0.045663065	count	1
PITRM1	-0.0394666	0.3256051	-0.1212	0.904	-0.045648228	count	1
THAP7	-0.0344669	0.2232822	-0.1544	0.877	-0.045557683	count	1
SRD5A1	-0.0510051	0.512025	-0.0996	0.921	-0.045550265	count	1
SMARCB1	-0.0331455	0.1245329	-0.2662	0.79	-0.045519683	count	1
KDELR1	-0.0317863	0.0521934	-0.609	0.543	-0.045470884	count	1
KLF16	-0.0359684	0.187977	-0.1913	0.848	-0.045261767	count	1
AL354733.3	-0.2291403	0.6965517	-0.329	0.742	-0.045255866	count	1
CTU1	-0.0370282	0.2688127	-0.1377	0.89	-0.04511844	count	1
UBE2J1	-0.0323922	0.1048564	-0.3089	0.757	-0.045048415	count	1
TUBG2	-0.0426716	0.3388553	-0.1259	0.9	-0.045028675	count	1
AC246817.2	-0.1000506	1.0653319	-0.0939	0.925	-0.044927577	count	1
AC090527.3	-0.1000506	1.9624622	-0.051	0.9593	-0.044927577	count	1
AP3B1	-0.0332821	0.1385164	-0.2403	0.81	-0.044800487	count	1
ARMC10	-0.0331284	0.136979	-0.2418	0.809	-0.044757447	count	1
AL117332.1	-0.0423794	0.462631	-0.0916	0.927	-0.044718936	count	1
WDR81	-0.0450089	0.424214	-0.1061	0.916	-0.04470737	count	1
MFSD1	-0.0333637	0.1744372	-0.1913	0.848	-0.044597891	count	1
AL158152.1	-0.1122577	0.8286076	-0.1355	0.892	-0.044463631	count	1
AL136982.7	-0.1317083	0.9787023	-0.1346	0.893	-0.044423336	count	1
AL078581.1	-0.1317083	1.1226979	-0.1173	0.907	-0.044423336	count	1
WNT5B	-0.1317083	1.12142	-0.1174	0.907	-0.044423336	count	1

FAM157C	-0.1317083	1.2291655	-0.1072	0.915	-0.044423336	count	1
AC127496.5	-0.1317083	1.2291655	-0.1072	0.915	-0.044423336	count	1
SPECC1L	-0.0415077	0.3833784	-0.1083	0.914	-0.044406816	count	1
HMGN2	-0.0311136	0.057844	-0.5379	0.591	-0.044381803	count	1
C1QBP	-0.0311828	0.0674862	-0.4621	0.644	-0.044257755	count	1
TYRO3	-0.0975005	0.6346322	-0.1536	0.878	-0.043751872	count	1
RHOF	-0.1104249	0.5652901	-0.1953	0.845	-0.043714564	count	1
TINAGL1	-0.0306312	0.0684447	-0.4475	0.655	-0.043422646	count	1
SERP1	-0.0303282	0.0519035	-0.5843	0.559	-0.043367858	count	1
CCNC	-0.0320059	0.131495	-0.2434	0.808	-0.043357801	count	1
PKM	-0.0303836	0.0657809	-0.4619	0.644	-0.043259197	count	1
SLC33A1	-0.0475617	0.3760107	-0.1265	0.899	-0.043168563	count	1
SOCS2	-0.0302717	0.0796558	-0.38	0.704	-0.043028667	count	1
AC120053.1	-0.2184218	0.8632019	-0.253	0.8	-0.042981215	count	1
LINC01852	-0.2184218	0.8722956	-0.2504	0.802	-0.042981215	count	1
LINGO1	-0.1585353	0.8225126	-0.1927	0.847	-0.042966797	count	1
AC116407.1	-0.1585353	0.8225126	-0.1927	0.847	-0.042966797	count	1
PLEKHM2	-0.0323837	0.1589798	-0.2037	0.839	-0.042940588	count	1
SEC22A	-0.0368647	0.2712618	-0.1359	0.892	-0.042928943	count	1
PEX11A	-0.0468459	0.4645499	-0.1008	0.92	-0.04251438	count	1
AL450384.2	-0.2158395	0.8844389	-0.244	0.807	-0.042435561	count	1
COPS6	-0.029969	0.080784	-0.371	0.711	-0.042328763	count	1
AL591895.1	-0.1255932	0.6582394	-0.1908	0.849	-0.042281938	count	1
NUDT1	-0.034362	0.2357102	-0.1458	0.884	-0.0421994	count	1
ZNF675	-0.0336134	0.2517051	-0.1335	0.894	-0.042113725	count	1
DMRTA1	-0.0848733	0.6920194	-0.1226	0.902	-0.042103686	count	1
PUM1	-0.0308509	0.172096	-0.1793	0.858	-0.041908231	count	1
IER3-AS1	-0.0520805	0.5651681	-0.0922	0.927	-0.041832306	count	1
CDKL3	-0.042087	0.3943902	-0.1067	0.915	-0.041789877	count	1
TSTD3	-0.0419953	0.4500279	-0.0933	0.926	-0.041698349	count	1
SAYSD1	-0.0330962	0.2277762	-0.1453	0.884	-0.041698096	count	1
GBE1	-0.0378299	0.2597687	-0.1456	0.884	-0.041682234	count	1
STAMBP	-0.0313167	0.1631846	-0.1919	0.848	-0.041670028	count	1
POLR2K	-0.0291802	0.0688071	-0.4241	0.672	-0.04135775	count	1
COMMD5	-0.0308679	0.1647086	-0.1874	0.851	-0.041344562	count	1
COMTD1	-0.0322171	0.2083831	-0.1546	0.877	-0.041309181	count	1
GLB1	-0.033158	0.2425012	-0.1367	0.891	-0.041289466	count	1
AL359232.1	-0.0562114	0.7524948	-0.0747	0.94	-0.041267498	count	1
PPIL1	-0.0346425	0.2467637	-0.1404	0.888	-0.041203928	count	1
BOD1	-0.0295756	0.1063622	-0.2781	0.781	-0.041066177	count	1
OPA1	-0.0327055	0.271334	-0.1205	0.904	-0.040852088	count	1
SLCO5A1	-0.366219	1.134027	-0.3229	0.747	-0.040827329	count	1
AL035461.2	-0.366219	1.134027	-0.3229	0.747	-0.040827329	count	1
AC008752.2	-0.366219	1.134027	-0.3229	0.747	-0.040827329	count	1
EAF1-AS1	-0.366219	1.1912	-0.3074	0.759	-0.040827329	count	1

SLC51A	-0.366219	1.1912	-0.3074	0.759	-0.040827329	count	1
AC008522.1	-0.366219	1.1912	-0.3074	0.759	-0.040827329	count	1
AL138831.2	-0.366219	1.1912	-0.3074	0.759	-0.040827329	count	1
AL731533.2	-0.366219	1.1912	-0.3074	0.759	-0.040827329	count	1
FAM222A	-0.366219	1.1912	-0.3074	0.759	-0.040827329	count	1
NOTCH3	-0.366219	1.1912	-0.3074	0.759	-0.040827329	count	1
AC023509.2	-0.366219	1.199435	-0.3053	0.76	-0.040827329	count	1
LINC00639	-0.366219	1.199435	-0.3053	0.76	-0.040827329	count	1
ANKLE1	-0.366219	1.199435	-0.3053	0.76	-0.040827329	count	1
COA6	-0.029852	0.1380325	-0.2163	0.829	-0.040802399	count	1
ZSCAN12	-0.0554867	0.640131	-0.0867	0.931	-0.04072968	count	1
YWHAZ	-0.0286416	0.0668433	-0.4285	0.668	-0.040689389	count	1
ACTN1	-0.0285274	0.0664811	-0.4291	0.6679	-0.040647309	count	1
RAPGEF5	-0.0291114	0.1022121	-0.2848	0.776	-0.040559844	count	1
TOMM40L	-0.0596668	0.4465454	-0.1336	0.894	-0.04051976	count	1
CEP162	-0.0320979	0.2216348	-0.1448	0.885	-0.040091837	count	1
SENP8	-0.0566052	0.4943694	-0.1145	0.909	-0.040047094	count	1
AGO1	-0.0357889	0.3207141	-0.1116	0.911	-0.040029857	count	1
IFNG	-0.3596709	0.917387	-0.3921	0.695	-0.040002344	count	1
CEACAM21	-0.0614515	0.6923892	-0.0888	0.929	-0.039831694	count	1
NAA50	-0.0302334	0.1554618	-0.1945	0.846	-0.039741813	count	1
VPS8	-0.0373803	0.3085797	-0.1211	0.904	-0.039703772	count	1
H1FX	-0.0278174	0.074726	-0.3723	0.7097	-0.039696965	count	1
AC074386.1	-0.1004436	0.7787919	-0.129	0.897	-0.039648665	count	1
TMEM167A	-0.0284821	0.1054698	-0.27	0.787	-0.039631468	count	1
CLDND2	-0.0583472	0.6035608	-0.0967	0.923	-0.039612582	count	1
TMEM192	-0.0304621	0.190594	-0.1598	0.873	-0.039557002	count	1
SLC37A4	-0.0346208	0.2889689	-0.1198	0.905	-0.039414186	count	1
AC092835.1	-0.0640717	0.5707291	-0.1123	0.911	-0.039386107	count	1
GNPDA2	-0.0311038	0.2035779	-0.1528	0.879	-0.039236277	count	1
SSSCA1	-0.0282269	0.1175655	-0.2401	0.81	-0.039184233	count	1
MED12	-0.0328415	0.3030817	-0.1084	0.914	-0.039162725	count	1
PLXDC2	-0.0282357	0.1201034	-0.2351	0.814	-0.03913907	count	1
BFSP1	-0.038971	0.4815112	-0.0809	0.935	-0.039074146	count	1
RGPD5	-0.0412003	0.4111341	-0.1002	0.92	-0.039032498	count	1
COX20	-0.0280518	0.1015175	-0.2763	0.782	-0.039032488	count	1
COPB2	-0.0281648	0.1033694	-0.2725	0.785	-0.038912429	count	1
YY1AP1	-0.033692	0.2462413	-0.1368	0.891	-0.03880859	count	1
ALG11	-0.0482209	0.5363216	-0.0899	0.928	-0.038705754	count	1
SGK3	-0.0318251	0.2261101	-0.1408	0.888	-0.038683513	count	1
ISPD	-0.0668925	0.5695901	-0.1174	0.907	-0.03868144	count	1
AL080276.2	-0.1977645	1.1439943	-0.1729	0.863	-0.038641777	count	1
FBXL13	-0.1977645	1.149677	-0.172	0.863	-0.038641777	count	1
RAD54B	-0.1977645	1.149677	-0.172	0.863	-0.038641777	count	1
AP003086.1	-0.1977645	1.149677	-0.172	0.863	-0.038641777	count	1

AC103740.1	-0.1977645	1.149677	-0.172	0.863	-0.038641777	count	1
WDR62	-0.1977645	1.149677	-0.172	0.863	-0.038641777	count	1
AC137936.1	-0.1977645	1.153572	-0.1714	0.864	-0.038641777	count	1
AL589843.1	-0.1977645	1.153572	-0.1714	0.864	-0.038641777	count	1
PIWIL4	-0.1977645	1.153572	-0.1714	0.864	-0.038641777	count	1
TEX14	-0.1977645	1.153572	-0.1714	0.864	-0.038641777	count	1
SPOCK1	-0.1977645	1.416541	-0.1396	0.889	-0.038641777	count	1
AC010325.1	-0.1977645	1.416541	-0.1396	0.889	-0.038641777	count	1
SRSF10	-0.0273844	0.0805194	-0.3401	0.734	-0.038630208	count	1
SLC25A6	-0.0268322	0.0412932	-0.6498	0.516	-0.03852171	count	1
SIAH2	-0.0296352	0.1796048	-0.165	0.869	-0.038512988	count	1
BTG1	-0.026786	0.0564303	-0.4747	0.635	-0.038470668	count	1
CLOCK	-0.0297787	0.1877021	-0.1586	0.874	-0.038365841	count	1
ULBP2	-0.1141701	0.4942101	-0.231	0.817	-0.038302428	count	1
MAML3	-0.0323748	0.3372814	-0.096	0.924	-0.03828362	count	1
APP	-0.0267463	0.062987	-0.4246	0.671	-0.038195575	count	1
FAM151B	-0.0704117	1.031471	-0.0683	0.946	-0.037913008	count	1
ASPN	-0.0704117	1.16149	-0.0606	0.952	-0.037913008	count	1
STX18	-0.0297316	0.2138028	-0.1391	0.889	-0.037872411	count	1
PLOD2	-0.0288247	0.1802985	-0.1599	0.873	-0.037863966	count	1
PTMA	-0.0262048	0.0210613	-1.2442	0.214	-0.037785631	count	1
AL139384.1	-0.0763177	0.6598356	-0.1157	0.908	-0.037774785	count	1
GARNL3	-0.0763177	0.7851946	-0.0972	0.923	-0.037774785	count	1
COPS5	-0.0269682	0.0932658	-0.2892	0.772	-0.03774215	count	1
SLC37A1	-0.0416051	0.4047332	-0.1028	0.918	-0.037728874	count	1
MRPS27	-0.029	0.1656236	-0.1751	0.861	-0.037716494	count	1
ARHGEF28	-0.0339053	0.2718327	-0.1247	0.901	-0.037541492	count	1
FAM213B	-0.0311914	0.2157731	-0.1446	0.885	-0.037384421	count	1
MRPL47	-0.027133	0.1291318	-0.2101	0.834	-0.03718931	count	1
RPS20	-0.025781	0.0298952	-0.8624	0.389	-0.037055226	count	1
DNAJC25-GNG10	-0.0505277	0.5782526	-0.0874	0.93	-0.037053511	count	1
CLEC14A	-0.0257408	0.0451996	-0.5695	0.569	-0.03701932	count	1
PRKCI	-0.0290959	0.1994518	-0.1459	0.884	-0.037019053	count	1
TNS1	-0.0278692	0.1833472	-0.152	0.879	-0.037005931	count	1
AKAP9	-0.0261616	0.0843505	-0.3102	0.756	-0.036929945	count	1
IER3IP1	-0.0264993	0.1030577	-0.2571	0.797	-0.036888897	count	1
ATG101	-0.0273637	0.1232835	-0.222	0.824	-0.036866456	count	1
APOO	-0.0297039	0.2384475	-0.1246	0.901	-0.036799058	count	1
GNLY	-0.0398643	0.4315156	-0.0924	0.926	-0.03670928	count	1
ITGB2	-0.3325403	0.6197684	-0.5366	0.592	-0.036622345	count	1
CKS1B	-0.0278589	0.163291	-0.1706	0.865	-0.036617446	count	1
NSUN3	-0.0316625	0.2989732	-0.1059	0.916	-0.036464924	count	1
COMMD4	-0.0261732	0.1114272	-0.2349	0.814	-0.036363083	count	1
POLR2H	-0.0261652	0.1097159	-0.2385	0.812	-0.036305114	count	1
CHCHD4	-0.0295634	0.2454109	-0.1205	0.904	-0.036154209	count	1

DPT	-0.3284974	0.9236259	-0.3557	0.722	-0.036123915	count	1
BNIP3	-0.0256286	0.1031544	-0.2484	0.804	-0.035854539	count	1
CCDC163	-0.0552367	0.683571	-0.0808	0.936	-0.035754488	count	1
COX5B	-0.0248744	0.0445684	-0.5581	0.577	-0.035644256	count	1
CRNDE	-0.0273303	0.1618762	-0.1688	0.866	-0.035624903	count	1
HABP4	-0.0276301	0.1544775	-0.1789	0.858	-0.0354917	count	1
SRA1	-0.0254312	0.107073	-0.2375	0.812	-0.035374501	count	1
KLHL11	-0.054519	0.6535088	-0.0834	0.934	-0.035284348	count	1
AQP11	-0.054519	0.7369157	-0.074	0.941	-0.035284348	count	1
MRPL18	-0.0250694	0.0926554	-0.2706	0.787	-0.035247202	count	1
TMEM92	-0.0608756	0.4812728	-0.1265	0.899	-0.035151453	count	1
RANBP1	-0.0248126	0.0719998	-0.3446	0.73	-0.035120187	count	1
NSD1	-0.0259068	0.1410038	-0.1837	0.854	-0.035069627	count	1
PITPNA	-0.0260973	0.1383553	-0.1886	0.85	-0.035045661	count	1
RFNG	-0.0261198	0.168567	-0.155	0.877	-0.034994291	count	1
CCDC130	-0.0264368	0.1592546	-0.166	0.868	-0.034973193	count	1
KBTBD7	-0.0304452	0.2959194	-0.1029	0.918	-0.034926568	count	1
ZNF133	-0.0406339	0.4562575	-0.0891	0.929	-0.034898108	count	1
FOSL2	-0.0250622	0.1165081	-0.2151	0.83	-0.034811692	count	1
PPTC7	-0.032726	0.2784918	-0.1175	0.906	-0.034743125	count	1
L3MBTL1	-0.1293071	0.9205128	-0.1405	0.888	-0.034715466	count	1
AC006480.2	-0.1035619	0.5989849	-0.1729	0.863	-0.034630896	count	1
TMEM185A	-0.0288791	0.3009845	-0.0959	0.924	-0.034607604	count	1
CALM3	-0.0244765	0.0829425	-0.2951	0.768	-0.034335264	count	1
MGAT3	-0.0468133	0.5388589	-0.0869	0.931	-0.034304589	count	1
FDFT1	-0.0260464	0.1582635	-0.1646	0.869	-0.034296942	count	1
SLC6A9	-0.3127957	0.9463668	-0.3305	0.741	-0.034200972	count	1
TMSB15B	-0.3127957	1.021165	-0.3063	0.759	-0.034200972	count	1
AC092111.1	-0.3127957	1.021165	-0.3063	0.759	-0.034200972	count	1
AL049775.1	-0.3127957	1.021165	-0.3063	0.759	-0.034200972	count	1
GOLGA8R	-0.3127957	1.021165	-0.3063	0.759	-0.034200972	count	1
AL390208.1	-0.3127957	1.119343	-0.2794	0.78	-0.034200972	count	1
TNK2	-0.0314475	0.3769324	-0.0834	0.934	-0.034039417	count	1
NREP	-0.0263198	0.1973273	-0.1334	0.894	-0.034028044	count	1
FCGR2A	-0.0864781	0.805555	-0.1074	0.915	-0.03399806	count	1
ROMO1	-0.0239774	0.0817071	-0.2935	0.769	-0.033810354	count	1
SPATS2	-0.0251319	0.1435313	-0.1751	0.861	-0.033748339	count	1
LGALS3BP	-0.308464	0.6379206	-0.4835	0.629	-0.03367406	count	1
LMNA	-0.0234267	0.0476957	-0.4912	0.623	-0.033616463	count	1
RAD23A	-0.0236476	0.0723258	-0.327	0.744	-0.03354859	count	1
SAP130	-0.0492132	0.5052309	-0.0974	0.922	-0.033346909	count	1
RECQL5	-0.0426144	0.5326167	-0.08	0.936	-0.03324859	count	1
NUP98	-0.0266927	0.2217026	-0.1204	0.904	-0.033173364	count	1
B4GALT7	-0.0259615	0.1886905	-0.1376	0.891	-0.033138173	count	1
VPS25	-0.0241785	0.1268919	-0.1905	0.849	-0.033029518	count	1

UQCC3	-0.0237893	0.1220548	-0.1949	0.845	-0.032855733	count	1
ZNF367	-0.0983966	0.8372761	-0.1175	0.906	-0.032851554	count	1
TCP10L	-0.0983966	0.8730016	-0.1127	0.91	-0.032851554	count	1
CCNY	-0.0240503	0.1145101	-0.21	0.834	-0.03274039	count	1
DUSP7	-0.0273162	0.2245696	-0.1216	0.903	-0.03273122	count	1
DTD2	-0.0407005	0.4483505	-0.0908	0.928	-0.032625772	count	1
MAP1S	-0.0268324	0.2660587	-0.1009	0.92	-0.032460384	count	1
AL035413.1	-0.0351461	0.4451225	-0.079	0.937	-0.032342423	count	1
RHOJ	-0.0234261	0.1164931	-0.2011	0.841	-0.032335688	count	1
AC003681.1	-0.2971874	0.7351415	-0.4043	0.686	-0.032309645	count	1
UBTF	-0.0234284	0.1265597	-0.1851	0.853	-0.032291221	count	1
STOML2	-0.0230482	0.0969461	-0.2377	0.812	-0.032166684	count	1
NAP1L4	-0.023091	0.1020723	-0.2262	0.821	-0.032127552	count	1
OR51E1	-0.2949269	0.631297	-0.4672	0.64	-0.032037392	count	1
AC006157.1	-0.1657216	0.979928	-0.1691	0.866	-0.032025737	count	1
AC009404.1	-0.1657216	0.996209	-0.1664	0.868	-0.032025737	count	1
AC005480.1	-0.1657216	0.996209	-0.1664	0.868	-0.032025737	count	1
AMH	-0.1657216	0.996209	-0.1664	0.868	-0.032025737	count	1
UQCRH	-0.0222295	0.0423271	-0.5252	0.599	-0.031853218	count	1
ANAPC1	-0.0268052	0.2679646	-0.1	0.92	-0.031775893	count	1
APOL1	-0.0231306	0.111024	-0.2083	0.835	-0.031771086	count	1
PRR7	-0.0247965	0.1902174	-0.1304	0.896	-0.031751108	count	1
DHCR7	-0.0280574	0.4894704	-0.0573	0.954	-0.031651381	count	1
EMC3	-0.0225734	0.0967796	-0.2332	0.816	-0.031628947	count	1
ZNF605	-0.0260677	0.2900214	-0.0899	0.928	-0.031604629	count	1
EFHC1	-0.0254705	0.2230331	-0.1142	0.909	-0.031600136	count	1
ABCB10	-0.0353665	0.4150302	-0.0852	0.932	-0.031509011	count	1
BCR	-0.023187	0.1452849	-0.1596	0.873	-0.031491858	count	1
AC145207.5	-0.1621322	0.5788358	-0.2801	0.779	-0.031293299	count	1
AC018638.7	-0.1621322	0.5822164	-0.2785	0.781	-0.031293299	count	1
BACH2	-0.0688938	0.8098955	-0.0851	0.932	-0.030672547	count	1
AC111182.1	-0.0688938	0.8711875	-0.0791	0.937	-0.030672547	count	1
ZNF704	-0.0260977	0.2390614	-0.1092	0.913	-0.03056603	count	1
TMEM51	-0.028407	0.3196376	-0.0889	0.929	-0.030548094	count	1
CBFB	-0.0253991	0.2230449	-0.1139	0.909	-0.030506194	count	1
IFRD2	-0.0225564	0.1313913	-0.1717	0.864	-0.030485333	count	1
CUL4B	-0.0237987	0.2125928	-0.1119	0.911	-0.030407553	count	1
SLC25A3	-0.021024	0.0406256	-0.5175	0.605	-0.030154024	count	1
PKN2	-0.0215116	0.102908	-0.209	0.834	-0.030115713	count	1
NDUFV2	-0.0210638	0.0595915	-0.3535	0.724	-0.030004374	count	1
ZCCHC18	-0.2777079	0.8229983	-0.3374	0.736	-0.029977319	count	1
PRPF4	-0.0252027	0.233192	-0.1081	0.914	-0.029956321	count	1
ZNHIT1	-0.0209067	0.0529426	-0.3949	0.693	-0.029865196	count	1
HMGB1	-0.0207334	0.0304491	-0.6809	0.496	-0.029844209	count	1
RIMKLB	-0.0233273	0.2296421	-0.1016	0.919	-0.029836499	count	1

LYN	-0.0241001	0.1805087	-0.1335	0.894	-0.029689656	count	1
WDR82	-0.0222192	0.1634445	-0.1359	0.892	-0.02952775	count	1
H2AFZ	-0.0202559	0.0531107	-0.3814	0.703	-0.029001241	count	1
SNX3	-0.0201387	0.0460399	-0.4374	0.662	-0.028856312	count	1
ZNF443	-0.0391455	0.5319146	-0.0736	0.941	-0.028642375	count	1
HEATR5A	-0.0321439	0.460154	-0.0699	0.944	-0.028623781	count	1
CCNF	-0.0858734	0.9002923	-0.0954	0.924	-0.028560344	count	1
PHKB	-0.0207651	0.1344438	-0.1545	0.877	-0.028429737	count	1
TMEM50A	-0.0195882	0.0535556	-0.3658	0.715	-0.028002966	count	1
SLC25A37	-0.0198547	0.0961322	-0.2065	0.836	-0.027957363	count	1
EXD2	-0.0260575	0.3208478	-0.0812	0.935	-0.027832966	count	1
DESI1	-0.0213965	0.1728237	-0.1238	0.901	-0.02779713	count	1
FAM204A	-0.0195295	0.0785183	-0.2487	0.804	-0.027557682	count	1
ARHGEF7	-0.0215909	0.2049765	-0.1053	0.916	-0.027492212	count	1
CFLAR-AS1	-0.2565265	0.7190757	-0.3567	0.721	-0.027476408	count	1
LRRN2	-0.061531	0.4778717	-0.1288	0.898	-0.027338759	count	1
EPN1	-0.0200686	0.1231149	-0.163	0.871	-0.027178634	count	1
ZFP90	-0.0209329	0.2140732	-0.0978	0.922	-0.027150051	count	1
HAUS7	-0.0468864	0.5980702	-0.0784	0.938	-0.026982883	count	1
AL117336.3	-0.0468864	0.6845044	-0.0685	0.945	-0.026982883	count	1
PITPNM2	-0.0503075	0.5188527	-0.097	0.923	-0.02695168	count	1
CERS2	-0.0195436	0.1080535	-0.1809	0.856	-0.026934954	count	1
STK39	-0.0417177	0.6659411	-0.0626	0.95	-0.026923238	count	1
ALKBH6	-0.0253555	0.3109908	-0.0815	0.935	-0.026897352	count	1
EI24	-0.0191008	0.0946238	-0.2019	0.84	-0.026658352	count	1
FBXW4	-0.0266171	0.3239697	-0.0822	0.935	-0.026647304	count	1
ZNF214	-0.037796	0.7124925	-0.053	0.958	-0.026637521	count	1
ZNF486	-0.0251029	0.3314718	-0.0757	0.94	-0.026628678	count	1
HNRNPU	-0.018486	0.0578184	-0.3197	0.749	-0.026404117	count	1
SEC16A	-0.0230839	0.2622342	-0.088	0.93	-0.026361656	count	1
HPRT1	-0.0192009	0.1176296	-0.1632	0.87	-0.026328204	count	1
EMC2	-0.018813	0.0951688	-0.1977	0.843	-0.026250225	count	1
BLOC1S1	-0.0186329	0.0816307	-0.2283	0.819	-0.026249899	count	1
AC022730.4	-0.0425248	0.7429232	-0.0572	0.954	-0.026011701	count	1
AC106791.1	-0.0425248	0.8804815	-0.0483	0.961	-0.026011701	count	1
LRP12	-0.0259398	0.3625043	-0.0716	0.943	-0.025967079	count	1
SPATA2L	-0.0241042	0.3271661	-0.0737	0.941	-0.025909609	count	1
SF3A2	-0.02055	0.2561298	-0.0802	0.936	-0.025803201	count	1
POP7	-0.0186193	0.12378	-0.1504	0.88	-0.02566066	count	1
NDUFB6	-0.0181783	0.0825281	-0.2203	0.826	-0.025628783	count	1
SP4	-0.024494	0.2949413	-0.083	0.934	-0.025404551	count	1
TRABD2B	-0.0308889	0.5653633	-0.0546	0.956	-0.025344551	count	1
ERG	-0.0179692	0.0800294	-0.2245	0.822	-0.025341665	count	1
PTPN7	-0.1323599	0.7574564	-0.1747	0.861	-0.025285127	count	1
METRN	-0.0181536	0.1195037	-0.1519	0.879	-0.025272835	count	1

NOL4L	-0.0205902	0.2049226	-0.1005	0.92	-0.025216789	count	1
C6orf203	-0.0195547	0.20579	-0.095	0.924	-0.025084622	count	1
SCFD1	-0.0186736	0.1383644	-0.135	0.893	-0.025071328	count	1
HNRNPC	-0.0176181	0.0691748	-0.2547	0.799	-0.02506442	count	1
TIMM21	-0.0209973	0.2476339	-0.0848	0.932	-0.02495031	count	1
SNRNP27	-0.0182443	0.1336536	-0.1365	0.891	-0.024801959	count	1
DCBLD1	-0.0212343	0.26314	-0.0807	0.936	-0.024780297	count	1
CCDC66	-0.0176911	0.1171324	-0.151	0.88	-0.024467085	count	1
KRT8	-0.0187257	0.193547	-0.0968	0.923	-0.024437118	count	1
SPHK2	-0.0268832	0.4988548	-0.0539	0.957	-0.024325208	count	1
DYNC1LI2	-0.017115	0.079684	-0.2148	0.83	-0.024161141	count	1
BAG6	-0.0181612	0.1655006	-0.1097	0.913	-0.024119878	count	1
LCMT1	-0.0177207	0.1619859	-0.1094	0.913	-0.023773196	count	1
TPGS2	-0.017301	0.1377833	-0.1256	0.9	-0.02368532	count	1
LMF2	-0.017486	0.1403422	-0.1246	0.901	-0.023665593	count	1
CTBP2	-0.0173317	0.1348674	-0.1285	0.898	-0.023651869	count	1
ERH	-0.0165453	0.0548207	-0.3018	0.763	-0.023610311	count	1
TMA7	-0.0164173	0.0413324	-0.3972	0.691	-0.023543576	count	1
MSN	-0.0165445	0.0709232	-0.2333	0.816	-0.023504473	count	1
GSTZ1	-0.0197496	0.257516	-0.0767	0.939	-0.023465644	count	1
MGST2	-0.0162809	0.0474667	-0.343	0.732	-0.02333566	count	1
BRD2	-0.0166459	0.0893214	-0.1864	0.852	-0.023316098	count	1
RTF1	-0.0164055	0.0780768	-0.2101	0.834	-0.023222232	count	1
SUMO2	-0.0160915	0.0326868	-0.4923	0.623	-0.023126477	count	1
CCDC102A	-0.0188717	0.236233	-0.0799	0.936	-0.023109738	count	1
ANKRD12	-0.0161939	0.0694043	-0.2333	0.8155	-0.023081162	count	1
AC103724.4	-0.0295214	0.5253766	-0.0562	0.955	-0.022977725	count	1
AC005840.2	-0.0428447	0.8374482	-0.0512	0.959	-0.022910444	count	1
ACADM	-0.0166236	0.1207584	-0.1377	0.891	-0.022895233	count	1
XRCC5	-0.0160735	0.0645853	-0.2489	0.803	-0.02287225	count	1
NEK7	-0.0177828	0.1980748	-0.0898	0.928	-0.022763094	count	1
SLC14A1	-0.0395749	0.4957253	-0.0798	0.936	-0.022735061	count	1
MID1IP1	-0.016631	0.16406	-0.1014	0.919	-0.022647567	count	1
AL392172.1	-0.0213579	0.3731802	-0.0572	0.954	-0.022647048	count	1
PPP6R1	-0.0205708	0.3283958	-0.0626	0.95	-0.022628559	count	1
MAP2K5	-0.0191849	0.2757567	-0.0696	0.945	-0.022385143	count	1
LIMS2	-0.0157658	0.0738015	-0.2136	0.831	-0.02227886	count	1
AC004908.1	-0.1172356	1.032709	-0.1135	0.91	-0.022278616	count	1
RMND5A	-0.0174169	0.2150307	-0.081	0.935	-0.022198094	count	1
PIGC	-0.0165477	0.1646182	-0.1005	0.92	-0.022145873	count	1
PPDPF	-0.0154506	0.0507336	-0.3045	0.761	-0.022113207	count	1
TCAF1	-0.0165522	0.1711339	-0.0967	0.923	-0.021970788	count	1
C20orf96	-0.0207194	0.3466257	-0.0598	0.952	-0.021968517	count	1
ACTR2	-0.0154485	0.0777567	-0.1987	0.843	-0.021846869	count	1
ZBTB1	-0.0167887	0.1650412	-0.1017	0.919	-0.021789268	count	1

PPP1R3C	-0.0220315	0.4710929	-0.0468	0.963	-0.021587834	count	1
AC022916.1	-0.0486137	0.8809339	-0.0552	0.956	-0.021522224	count	1
SLFN11	-0.0164001	0.1967336	-0.0834	0.934	-0.021461489	count	1
GZMM	-0.0648119	0.8200359	-0.079	0.937	-0.021416028	count	1
DCAF1	-0.0205962	0.5007871	-0.0411	0.967	-0.021352465	count	1
RAB43	-0.0803795	0.9703604	-0.0828	0.934	-0.02123835	count	1
GPRIN1	-0.0803795	0.9703604	-0.0828	0.934	-0.02123835	count	1
Z84485.1	-0.0803795	0.9703604	-0.0828	0.934	-0.02123835	count	1
DPEP1	-0.0803795	0.9703604	-0.0828	0.934	-0.02123835	count	1
ASPA	-0.0803795	1.1841324	-0.0679	0.946	-0.02123835	count	1
HDAC3	-0.0160246	0.1636881	-0.0979	0.922	-0.021237245	count	1
AKR1A1	-0.0149337	0.0830807	-0.1797	0.857	-0.021151591	count	1
AC108673.2	-0.0638827	0.7813973	-0.0818	0.935	-0.021102928	count	1
KIAA1217	-0.0165162	0.181407	-0.091	0.927	-0.021095666	count	1
PEA15	-0.0148856	0.0819625	-0.1816	0.856	-0.021014948	count	1
SNRPG	-0.0148114	0.0697532	-0.2123	0.832	-0.020983463	count	1
NDUFB9	-0.0146999	0.0615495	-0.2388	0.811	-0.020969773	count	1
KIAA0895L	-0.0287112	0.43631	-0.0658	0.948	-0.02096441	count	1
CYP1B1-AS1	-0.0226893	0.408131	-0.0556	0.956	-0.020841573	count	1
RAB10	-0.0146843	0.0934297	-0.1572	0.875	-0.020626048	count	1
SCAMP5	-0.0301997	0.8005981	-0.0377	0.97	-0.020380737	count	1
SMAP1	-0.014703	0.1204264	-0.1221	0.903	-0.020306181	count	1
IPO9	-0.0178593	0.2594269	-0.0688	0.945	-0.020303174	count	1
TRIM69	-0.015115	0.155259	-0.0974	0.922	-0.020210823	count	1
ATP5MD	-0.0141212	0.0621175	-0.2273	0.82	-0.020056162	count	1
VPS36	-0.0143168	0.1010891	-0.1416	0.887	-0.019980231	count	1
DYNC2H1	-0.0162849	0.2779053	-0.0586	0.953	-0.019858751	count	1
AC093323.1	-0.0180523	0.3175128	-0.0569	0.955	-0.019853358	count	1
RAB11FIP2	-0.015816	0.2209302	-0.0716	0.943	-0.019797018	count	1
DPCD	-0.0154723	0.203329	-0.0761	0.939	-0.019553273	count	1
UGDH	-0.0177357	0.2664257	-0.0666	0.947	-0.019504582	count	1
ZMAT5	-0.0146604	0.1780582	-0.0823	0.934	-0.01944859	count	1
ZNF250	-0.017257	0.32943	-0.0524	0.958	-0.019360867	count	1
HBP1	-0.0141127	0.1200104	-0.1176	0.906	-0.019295454	count	1
FKBP8	-0.0134718	0.0808902	-0.1665	0.868	-0.019031385	count	1
EEF1D	-0.0132199	0.0310432	-0.4259	0.67	-0.019022171	count	1
ACOT9	-0.0140836	0.1372268	-0.1026	0.918	-0.019012037	count	1
AP001462.1	-0.0429698	0.6579649	-0.0653	0.948	-0.018993718	count	1
ADAMTSL2	-0.0429698	0.6919563	-0.0621	0.95	-0.018993718	count	1
KLHL17	-0.0429698	0.9100417	-0.0472	0.962	-0.018993718	count	1
SCD	-0.0486529	0.5203425	-0.0935	0.926	-0.018917485	count	1
FRRS1	-0.0713743	0.6779538	-0.1053	0.916	-0.018803386	count	1
HNRNPR	-0.013298	0.077512	-0.1716	0.864	-0.018788831	count	1
COQ5	-0.0148918	0.2091831	-0.0712	0.943	-0.018639273	count	1
FAM66C	-0.0262513	0.6518487	-0.0403	0.968	-0.018457346	count	1

B3GAT3	-0.0135937	0.1528596	-0.0889	0.929	-0.018275457	count	1
BPTF	-0.0128867	0.090133	-0.143	0.886	-0.01818375	count	1
MYORG	-0.0338488	0.7758919	-0.0436	0.965	-0.018058954	count	1
ACR	-0.0338488	0.9092005	-0.0372	0.97	-0.018058954	count	1
CASP10	-0.0136696	0.1929808	-0.0708	0.944	-0.017922991	count	1
USP44	-0.0237148	0.5976634	-0.0397	0.968	-0.017888547	count	1
ENO2	-0.0212183	0.4449355	-0.0477	0.962	-0.017785204	count	1
LINC01558	-0.0215385	0.5379216	-0.04	0.968	-0.017643889	count	1
NME3	-0.0123727	0.0661367	-0.1871	0.852	-0.01763372	count	1
IL10RB-DT	-0.0250845	0.5321849	-0.0471	0.962	-0.01763273	count	1
ANO2	-0.0185638	0.3741676	-0.0496	0.96	-0.017532247	count	1
MAP1LC3A	-0.0124424	0.1055264	-0.1179	0.906	-0.017385422	count	1
PRAF2	-0.0125685	0.1148807	-0.1094	0.913	-0.017322355	count	1
MAP2K4	-0.0142232	0.2681011	-0.0531	0.958	-0.017269111	count	1
AL022069.1	-0.0911088	0.9252774	-0.0985	0.922	-0.0171569	count	1
MPV17	-0.0124774	0.1319402	-0.0946	0.925	-0.017029435	count	1
TOE1	-0.014979	0.267009	-0.0561	0.955	-0.017024556	count	1
ATP9A	-0.018238	0.4628736	-0.0394	0.969	-0.016989054	count	1
KIDINS220	-0.012426	0.1462208	-0.085	0.932	-0.016907229	count	1
DIS3L	-0.015856	0.3419454	-0.0464	0.963	-0.016803212	count	1
PORCN	-0.0154433	0.3838549	-0.0402	0.968	-0.016789638	count	1
OTUD7A	-0.0236987	0.5870136	-0.0404	0.968	-0.01665385	count	1
APOBEC3F	-0.0203052	0.5537301	-0.0367	0.971	-0.016630034	count	1
EXOC6	-0.0118156	0.1619866	-0.0729	0.942	-0.016269313	count	1
ARHGEF35	-0.0366402	0.6490245	-0.0565	0.955	-0.016167343	count	1
ZNF292	-0.0118692	0.1734595	-0.0684	0.945	-0.016161364	count	1
TRAF5	-0.0133793	0.2670762	-0.0501	0.96	-0.015975133	count	1
SLC25A22	-0.0184608	0.393644	-0.0469	0.963	-0.015797859	count	1
CHIC1	-0.0128612	0.2737033	-0.047	0.963	-0.015774342	count	1
AL645933.2	-0.0212021	0.4442975	-0.0477	0.962	-0.015458345	count	1
PAIP2	-0.0108032	0.078916	-0.1369	0.891	-0.015306755	count	1
AL353708.1	-0.0583472	1.1238618	-0.0519	0.959	-0.015305839	count	1
MELTF	-0.0583472	1.1238618	-0.0519	0.959	-0.015305839	count	1
PKIB	-0.0583472	1.1238618	-0.0519	0.959	-0.015305839	count	1
ZNF239	-0.0583472	1.1248922	-0.0519	0.959	-0.015305839	count	1
CYP17A1-AS1	-0.0583472	1.1245989	-0.0519	0.959	-0.015305839	count	1
SNX22	-0.0583472	1.1248922	-0.0519	0.959	-0.015305839	count	1
HSPB9	-0.0583472	1.1248922	-0.0519	0.959	-0.015305839	count	1
AC100793.4	-0.0583472	1.1248922	-0.0519	0.959	-0.015305839	count	1
ZNF491	-0.0583472	1.1238618	-0.0519	0.959	-0.015305839	count	1
ZNRF3-AS1	-0.0583472	1.29907	-0.0449	0.964	-0.015305839	count	1
CAND1	-0.0112139	0.1407446	-0.0797	0.937	-0.015146639	count	1
TESK1	-0.0126621	0.2414645	-0.0524	0.958	-0.015037015	count	1
GTF2H5	-0.0106925	0.0951383	-0.1124	0.911	-0.014976061	count	1
CNPY3	-0.0109564	0.1263039	-0.0867	0.931	-0.014949683	count	1

CDV3	-0.010461	0.0669007	-0.1564	0.876	-0.014887963	count	1
ACSL3	-0.0107676	0.1395217	-0.0772	0.938	-0.014505506	count	1
TP53TG1	-0.0106037	0.1320319	-0.0803	0.936	-0.014465041	count	1
FEM1B	-0.0113459	0.2163198	-0.0524	0.958	-0.014456402	count	1
DCAF8	-0.0120918	0.2290261	-0.0528	0.958	-0.014359167	count	1
POLR3K	-0.0106951	0.1467601	-0.0729	0.942	-0.014280591	count	1
FAM162A	-0.0101711	0.0915738	-0.1111	0.912	-0.014250184	count	1
CPLANE2	-0.0202497	0.53674	-0.0377	0.97	-0.014220009	count	1
ZNF74	-0.0202497	0.5303556	-0.0382	0.97	-0.014220009	count	1
TXNDC15	-0.0103178	0.1177868	-0.0876	0.93	-0.014217615	count	1
ACSF3	-0.0127263	0.2437361	-0.0522	0.958	-0.013911839	count	1
MPHOSPH10	-0.0099895	0.1069272	-0.0934	0.926	-0.013909297	count	1
MAPK7	-0.0132003	0.361939	-0.0365	0.971	-0.013885207	count	1
CCDC144NL	-0.0150766	0.4877563	-0.0309	0.975	-0.01383343	count	1
UBR1	-0.0108326	0.1923334	-0.0563	0.955	-0.013832543	count	1
POLR2D	-0.0112539	0.2339776	-0.0481	0.962	-0.013827886	count	1
CFLAR	-0.0097216	0.0841109	-0.1156	0.908	-0.013792902	count	1
PFN1	-0.0095426	0.0370436	-0.2576	0.797	-0.013713609	count	1
GTF3A	-0.0096208	0.0779636	-0.1234	0.902	-0.013601247	count	1
AC124068.2	-0.0254871	0.6870195	-0.0371	0.97	-0.013569056	count	1
WDR44	-0.0116516	0.2643567	-0.0441	0.965	-0.013541794	count	1
ZNF213-AS1	-0.0176785	0.5490726	-0.0322	0.974	-0.013319811	count	1
FAM13B	-0.0105332	0.2007655	-0.0525	0.958	-0.013218898	count	1
SSR2	-0.0092401	0.0537582	-0.1719	0.8635	-0.013185065	count	1
ZBTB8OS	-0.0095572	0.1299519	-0.0735	0.941	-0.013092222	count	1
C22orf39	-0.0096849	0.141435	-0.0685	0.945	-0.012989829	count	1
PPP1R15B	-0.0093529	0.1310453	-0.0714	0.943	-0.012670516	count	1
HDGF	-0.0090983	0.0961158	-0.0947	0.925	-0.01265877	count	1
CHRNA5	-0.0237681	0.560774	-0.0424	0.966	-0.01264836	count	1
RPL6	-0.00874	0.0205613	-0.4251	0.671	-0.012599111	count	1
YTHDF3	-0.0098544	0.2017229	-0.0489	0.961	-0.012555124	count	1
SLX4	-0.0381536	0.5897759	-0.0647	0.948	-0.012503499	count	1
JADE2	-0.0107321	0.2394384	-0.0448	0.964	-0.012384653	count	1
CASK	-0.009967	0.2023296	-0.0493	0.961	-0.012375326	count	1
CEP76	-0.0128879	0.3662338	-0.0352	0.972	-0.012319976	count	1
COA5	-0.008854	0.1228366	-0.0721	0.943	-0.012258942	count	1
CDADC1	-0.0100282	0.2510114	-0.04	0.968	-0.01211834	count	1
AC025259.3	-0.0647061	0.6923839	-0.0935	0.926	-0.012073066	count	1
DNAJC4	-0.0085907	0.1017923	-0.0844	0.933	-0.012006509	count	1
GOT1	-0.0094956	0.2073102	-0.0458	0.963	-0.01199658	count	1
PGGHG	-0.0115301	0.3067667	-0.0376	0.97	-0.011941208	count	1
AL021068.1	-0.1172356	1.170115	-0.1002	0.92	-0.011925448	count	1
LINC00702	-0.1172356	1.170115	-0.1002	0.92	-0.011925448	count	1
AC087482.1	-0.1172356	1.170115	-0.1002	0.92	-0.011925448	count	1
AC074044.1	-0.1172356	1.182523	-0.0991	0.921	-0.011925448	count	1

LINC02289	-0.1172356	1.197251	-0.0979	0.922	-0.011925448	count	1
ISM2	-0.1172356	1.197251	-0.0979	0.922	-0.011925448	count	1
SLC19A1	-0.0195521	0.8093364	-0.0242	0.981	-0.011896206	count	1
SMC5-AS1	-0.0449422	0.9922244	-0.0453	0.964	-0.011737516	count	1
THBS2	-0.0449422	1.054433	-0.0426	0.966	-0.011737516	count	1
LGMN	-0.0086945	0.150862	-0.0576	0.954	-0.011723929	count	1
BTD	-0.0088642	0.1747408	-0.0507	0.96	-0.011657801	count	1
CCZ1	-0.0089758	0.1765637	-0.0508	0.959	-0.011655058	count	1
CRK	-0.0084531	0.1071583	-0.0789	0.937	-0.01165477	count	1
SIRT1	-0.0092547	0.2266699	-0.0408	0.967	-0.011629914	count	1
CERS5	-0.0092668	0.1853823	-0.05	0.96	-0.011628827	count	1
TOPORS	-0.008449	0.1085899	-0.0778	0.938	-0.011627419	count	1
ATG13	-0.0100899	0.4360983	-0.0231	0.982	-0.01155568	count	1
UCP2	-0.0153409	0.5701408	-0.0269	0.979	-0.01155336	count	1
ICAM3	-0.0087923	0.1725276	-0.051	0.959	-0.011526023	count	1
TSPAN13	-0.00847	0.1447411	-0.0585	0.953	-0.011477476	count	1
STAT1	-0.0081593	0.1156066	-0.0706	0.944	-0.011376649	count	1
ANKMY1	-0.0258168	0.5620315	-0.0459	0.963	-0.011357182	count	1
ZDHHC7	-0.00964	0.2423909	-0.0398	0.968	-0.011082326	count	1
PARVB	-0.0078626	0.0890578	-0.0883	0.93	-0.011055428	count	1
DIP2A	-0.0088614	0.2416292	-0.0367	0.971	-0.010946064	count	1
DPY19L4	-0.0086502	0.209787	-0.0412	0.967	-0.010941936	count	1
MORF4L1	-0.0075886	0.043276	-0.1754	0.861	-0.010875683	count	1
PSMD1	-0.0078724	0.1181155	-0.0666	0.947	-0.010850753	count	1
C1QTNF3	-0.0278676	1.110021	-0.0251	0.98	-0.010769647	count	1
LINC01545	-0.0278676	1.108084	-0.0251	0.98	-0.010769647	count	1
LINC00671	-0.0278676	1.403862	-0.0199	0.984	-0.010769647	count	1
UBAC2	-0.0079958	0.1460359	-0.0548	0.956	-0.010759492	count	1
SUCLG1	-0.007666	0.1035466	-0.074	0.941	-0.010675673	count	1
ARMCX5	-0.0094327	0.2849564	-0.0331	0.974	-0.010623594	count	1
GOLGA8B	-0.0103374	0.4035495	-0.0256	0.98	-0.010616405	count	1
PPARG	-0.0077864	0.1455057	-0.0535	0.957	-0.010529236	count	1
FSCN1	-0.0075593	0.1189588	-0.0635	0.949	-0.010471211	count	1
MR1	-0.0101414	0.3734986	-0.0272	0.978	-0.010414878	count	1
PAFAH1B2	-0.007563	0.124629	-0.0607	0.952	-0.010386891	count	1
AC107068.1	-0.0395908	0.9793573	-0.0404	0.968	-0.010321678	count	1
AL662797.1	-0.0395908	0.9793573	-0.0404	0.968	-0.010321678	count	1
SSPO	-0.0395908	1.1620129	-0.0341	0.973	-0.010321678	count	1
AP006623.1	-0.0395733	0.5024265	-0.0788	0.937	-0.010317057	count	1
MDH2	-0.0071234	0.068705	-0.1037	0.917	-0.010115739	count	1
WDR53	-0.0098204	0.3427815	-0.0286	0.977	-0.009906415	count	1
POMT1	-0.0104961	0.3565687	-0.0294	0.977	-0.009901741	count	1
TAF9	-0.0070702	0.1132743	-0.0624	0.95	-0.009815856	count	1
PCDHB10	-0.0152159	0.4985643	-0.0305	0.976	-0.009762129	count	1
CEP290	-0.0071823	0.1497493	-0.048	0.962	-0.009718046	count	1

ATXN10	-0.0069362	0.11714	-0.0592	0.953	-0.009592411	count	1
GRK5	-0.0076776	0.203726	-0.0377	0.97	-0.009546997	count	1
SNRNP70	-0.0068906	0.1118216	-0.0616	0.951	-0.009507178	count	1
APBA3	-0.0094874	0.3695975	-0.0257	0.98	-0.009381967	count	1
SNED1	-0.007642	0.2298665	-0.0332	0.973	-0.009333139	count	1
ARHGDIA	-0.0066197	0.082651	-0.0801	0.936	-0.009314639	count	1
TUBGCP2	-0.0068583	0.1453752	-0.0472	0.962	-0.009218639	count	1
ATP13A2	-0.0082369	0.3053692	-0.027	0.978	-0.009190034	count	1
NCAPH	-0.0911088	1.139917	-0.0799	0.936	-0.009177763	count	1
SH3TC2-DT	-0.0911088	1.139917	-0.0799	0.936	-0.009177763	count	1
LINC00924	-0.0911088	1.139917	-0.0799	0.936	-0.009177763	count	1
HPN	-0.0911088	1.139917	-0.0799	0.936	-0.009177763	count	1
TRO	-0.0911088	1.144219	-0.0796	0.937	-0.009177763	count	1
AC011484.1	-0.0911088	1.144219	-0.0796	0.937	-0.009177763	count	1
SDHB	-0.00662	0.1087005	-0.0609	0.951	-0.009165491	count	1
AC103706.1	-0.0150464	0.5845025	-0.0257	0.979	-0.009145177	count	1
PHLPP2	-0.0207595	0.5288535	-0.0393	0.969	-0.009119488	count	1
AC147067.2	-0.0349898	0.8127889	-0.043	0.966	-0.009108326	count	1
AC103691.1	-0.0349898	0.8125675	-0.0431	0.966	-0.009108326	count	1
MICU1	-0.0067348	0.1710336	-0.0394	0.969	-0.008950084	count	1
ATG3	-0.0063008	0.0979998	-0.0643	0.949	-0.008807136	count	1
TEX264	-0.0062064	0.084706	-0.0733	0.942	-0.008712287	count	1
EFCC1	-0.008701	0.3341808	-0.026	0.979	-0.008511111	count	1
GLI3	-0.0156405	0.4732455	-0.033	0.974	-0.008306026	count	1
CDAN1	-0.0445722	0.6530412	-0.0683	0.946	-0.00825795	count	1
ZMYM1	-0.0063644	0.2474485	-0.0257	0.979	-0.008167593	count	1
ZNF526	-0.0090971	0.5265229	-0.0173	0.986	-0.008072139	count	1
RAD1	-0.0064665	0.2521975	-0.0256	0.98	-0.008053255	count	1
PARD3	-0.0071172	0.328843	-0.0216	0.983	-0.008014119	count	1
ROBO4	-0.0056571	0.137053	-0.0413	0.967	-0.007911276	count	1
ERGIC2	-0.0055605	0.0985373	-0.0564	0.955	-0.007768855	count	1
AL136040.1	-0.0113674	0.563695	-0.0202	0.984	-0.007640561	count	1
AC026202.2	-0.0193389	0.8060949	-0.024	0.981	-0.007454893	count	1
CCDC136	-0.0193389	0.9286452	-0.0208	0.983	-0.007454893	count	1
RBPMS2	-0.0740338	0.8676966	-0.0853	0.932	-0.007410223	count	1
TIPARP	-0.0052374	0.1053693	-0.0497	0.96	-0.007302922	count	1
DHX36	-0.0051045	0.0890985	-0.0573	0.954	-0.007197133	count	1
SURF1	-0.0050564	0.1381717	-0.0366	0.971	-0.00699433	count	1
IGFLR1	-0.026812	0.6656144	-0.0403	0.968	-0.006960712	count	1
MEIS1	-0.026812	0.7717727	-0.0347	0.972	-0.006960712	count	1
AC012313.1	-0.069182	0.9919556	-0.0697	0.944	-0.006912021	count	1
AC093827.5	-0.0112274	0.6364461	-0.0176	0.986	-0.006817921	count	1
PPP3CB	-0.0052238	0.2095565	-0.0249	0.98	-0.006741699	count	1
HTRA2	-0.0049091	0.1253735	-0.0392	0.969	-0.006725552	count	1
AC104532.2	-0.0258168	0.8379016	-0.0308	0.975	-0.006700146	count	1

MAZ	-0.0048959	0.1194332	-0.041	0.967	-0.006670131	count	1
LRP11	-0.005365	0.2261497	-0.0237	0.981	-0.006625786	count	1
RPA1	-0.0051732	0.2118565	-0.0244	0.981	-0.006508462	count	1
ZNF207	-0.0046633	0.1126521	-0.0414	0.967	-0.006483371	count	1
NDUFV1	-0.0046251	0.1068524	-0.0433	0.965	-0.006427678	count	1
GLO1	-0.0045929	0.1076175	-0.0427	0.966	-0.006373513	count	1
EBNA1BP2	-0.004621	0.1167838	-0.0396	0.968	-0.006371749	count	1
GAN	-0.0056595	0.2685635	-0.0211	0.983	-0.006281927	count	1
AL442128.2	-0.01629	0.6639119	-0.0245	0.98	-0.006273931	count	1
MYO7B	-0.0621183	0.8261147	-0.0752	0.94	-0.006189874	count	1
ZNF236-DT	-0.0621183	0.8752415	-0.071	0.943	-0.006189874	count	1
ZXDC	-0.005619	0.3026914	-0.0186	0.985	-0.006102904	count	1
CREB1	-0.0044782	0.1388022	-0.0323	0.974	-0.00607451	count	1
WDR45	-0.0044239	0.1348458	-0.0328	0.974	-0.006027019	count	1
RPL3	-0.00406	0.0203791	-0.1992	0.842	-0.005851593	count	1
KIAA2026	-0.0041091	0.1707238	-0.0241	0.981	-0.005518804	count	1
RBM25	-0.003816	0.064666	-0.059	0.953	-0.005426911	count	1
ILF2	-0.003748	0.0653068	-0.0574	0.954	-0.005305676	count	1
MAST2	-0.0052341	0.5229854	-0.01	0.992	-0.005226235	count	1
FAH	-0.0056912	0.5520413	-0.0103	0.992	-0.005214735	count	1
XKR9	-0.0512394	0.8696892	-0.0589	0.953	-0.005085041	count	1
NUDT18	-0.0038436	0.2439934	-0.0158	0.987	-0.004854564	count	1
DNMBP	-0.0043105	0.3152468	-0.0137	0.989	-0.004830401	count	1
RAB1A	-0.0033931	0.0862485	-0.0393	0.969	-0.004756141	count	1
AC064807.1	-0.0050439	0.3823643	-0.0132	0.989	-0.004754662	count	1
IFI35	-0.0032882	0.1061387	-0.031	0.975	-0.004575038	count	1
CHCHD5	-0.0032815	0.1116085	-0.0294	0.977	-0.004552603	count	1
HAS2-AS1	-0.0118033	0.9686619	-0.0122	0.99	-0.004539899	count	1
RAB37	-0.0118033	0.9686292	-0.0122	0.99	-0.004539899	count	1
AL445426.1	-0.0118033	1.0816451	-0.0109	0.991	-0.004539899	count	1
ZNF572	-0.0118033	1.0816743	-0.0109	0.991	-0.004539899	count	1
FRMD6-AS1	-0.0074669	0.5730836	-0.013	0.99	-0.00453035	count	1
MID2	-0.0046174	0.4194089	-0.011	0.991	-0.004514237	count	1
ANAPC16	-0.0031493	0.053708	-0.0586	0.9532	-0.004498096	count	1
C2CD3	-0.0043355	0.5353126	-0.0081	0.994	-0.004449411	count	1
TPRKB	-0.0031489	0.0977336	-0.0322	0.974	-0.004405033	count	1
CWC15	-0.0029085	0.0973829	-0.0299	0.976	-0.004087163	count	1
EXPH5	-0.0060882	0.6294966	-0.0097	0.992	-0.003692669	count	1
TARSL2	-0.0029346	0.2214125	-0.0133	0.989	-0.003686552	count	1
TPP2	-0.0029604	0.2114469	-0.014	0.989	-0.003655589	count	1
ATR	-0.0032757	0.3021721	-0.0108	0.991	-0.003653091	count	1
SLC35G2	-0.0027098	0.1568868	-0.0173	0.986	-0.003648449	count	1
NOXA1	-0.0044097	0.5764163	-0.0077	0.994	-0.003511933	count	1
RRP36	-0.0025717	0.131278	-0.0196	0.984	-0.00349835	count	1
SLC35A2	-0.0026288	0.1937675	-0.0136	0.989	-0.003366211	count	1

ZDHHC15	-0.0039287	0.3948198	-0.01	0.992	-0.003283434	count	1
AGAP1	-0.0029365	0.3197678	-0.0092	0.993	-0.00327471	count	1
RC3H2	-0.002334	0.2320921	-0.0101	0.992	-0.003029883	count	1
AC108047.1	-0.0060232	0.6852722	-0.0088	0.993	-0.002926305	count	1
COL6A3	-0.0060232	0.7499227	-0.008	0.994	-0.002926305	count	1
TMBIM6	-0.0018672	0.0445233	-0.0419	0.967	-0.00267773	count	1
TMEM231	-0.0022756	0.2734275	-0.0083	0.993	-0.00262421	count	1
INPP5E	-0.0032602	0.3722018	-0.0088	0.993	-0.002595919	count	1
ZNF101	-0.0024462	0.3544927	-0.0069	0.994	-0.002570101	count	1
ABCD1	-0.0036488	0.7204385	-0.0051	0.996	-0.002448453	count	1
HUWE1	-0.0017915	0.1480598	-0.0121	0.99	-0.002429272	count	1
AC008393.1	-0.0032976	0.5281328	-0.0062	0.995	-0.002395689	count	1
COX4I1	-0.0016531	0.033094	-0.05	0.96	-0.002377544	count	1
EIF2AK3	-0.0029602	0.416012	-0.0071	0.994	-0.002356918	count	1
TMEM8B	-0.0030911	0.4623216	-0.0067	0.995	-0.002322441	count	1
PAXBP1	-0.001699	0.1518652	-0.0112	0.991	-0.002281721	count	1
AC010883.1	-0.0042389	0.9019296	-0.0047	0.996	-0.00205844	count	1
EPHB3	-0.0042389	0.8009761	-0.0053	0.996	-0.00205844	count	1
THOC2	-0.0014404	0.1223333	-0.0118	0.991	-0.001982324	count	1
CDK3	-0.0051584	0.6481844	-0.008	0.994	-0.001980176	count	1
NEMF	-0.0013954	0.1036562	-0.0135	0.989	-0.001936497	count	1
RBM39	-0.0011968	0.0555551	-0.0215	0.983	-0.001705875	count	1
PIGS	-0.0012786	0.2500847	-0.0051	0.996	-0.001525388	count	1
ETV2	-0.0020776	0.5567419	-0.0037	0.997	-0.001508997	count	1
TRIM38	-0.0010686	0.1399005	-0.0076	0.994	-0.001451734	count	1
C4orf3	-0.0010111	0.0532738	-0.019	0.9849	-0.001443194	count	1
PSMC3	-0.0009113	0.0793305	-0.0115	0.991	-0.001285844	count	1
TRIM58	-0.0030911	1.1563776	-0.0027	0.998	-0.001185867	count	1
GTF3C1	-0.0009416	0.2259399	-0.0042	0.997	-0.001179397	count	1
CYB5RL	-0.0016524	0.4989849	-0.0033	0.997	-0.001108332	count	1
DDX42	-0.0006568	0.1306314	-0.005	0.996	-0.000917863	count	1
OAF	-0.0006475	0.1732269	-0.0037	0.997	-0.000855359	count	1
TRIP10	-0.0006495	0.1865183	-0.0035	0.997	-0.000851189	count	1
ZSCAN29	-0.0011813	0.611766	-0.0019	0.998	-0.000715671	count	1
RHOA	-0.0004868	0.0364711	-0.0133	0.989	-0.000699077	count	1
CAV1	-0.0003557	0.0415941	-0.0086	0.993	-0.000512396	count	1
MRPL13	-0.0002505	0.105725	-0.0024	0.998	-0.000348247	count	1
PDCD4-AS1	-0.0006401	0.652929	-0.001	0.999	-0.000310538	count	1
RARRES1	-0.0002181	0.3406967	-6.00E-04	0.999	-0.00024652	count	1
ICE2	-0.0001447	0.1500457	-0.001	0.999	-0.000195329	count	1
SPCS3	-7.78E-05	0.071056	-0.0011	0.999	-0.000110354	count	1
PRPF4B	-5.27E-05	0.0788541	-7.00E-04	0.999	-7.47E-05	count	1
VEPH1	-2.5444956	0.6769205	-3.7589	2.00E-04	-1.85E-07	count	1
FOXM1	-3.66826	0.8766987	-4.1842	2.95E-05	-1.10E-07	count	0.681273
MDGA1	-1.693682	0.3341342	-5.0689	4.27E-07	-9.75E-08	count	0.010004183

MOBP	-3.286604	1.4635746	-2.2456	0.0248	-9.66E-08	count	1
AC083967.1	-2.8881016	0.4659016	-6.199	6.53E-10	-8.12E-08	count	1.55E-05
FAM19A5	-1.5027613	0.4370598	-3.4383	6.00E-04	-8.04E-08	count	1
OLFML2B	-2.5999291	0.9336513	-2.7847	0.0054	-6.92E-08	count	1
AC073534.2	-2.5051094	0.7412536	-3.3796	7.00E-04	-6.54E-08	count	1
KLRB1	-20.2356123	1035.306322	-0.0195	0.984	-6.09E-08	count	1
MST1R	-19.8041464	1161.825649	-0.017	0.986	-6.08E-08	count	1
ACTR3C	-19.9850832	1377.575607	-0.0145	0.988	-6.07E-08	count	1
SLC10A5	-19.9850832	1377.575607	-0.0145	0.988	-6.07E-08	count	1
FAM95C	-19.9850832	1377.575607	-0.0145	0.988	-6.07E-08	count	1
ANKRD18A	-19.9850832	1377.575607	-0.0145	0.988	-6.07E-08	count	1
AURKB	-19.9850832	1377.575607	-0.0145	0.988	-6.07E-08	count	1
GTSE1	-19.9850832	1377.575607	-0.0145	0.988	-6.07E-08	count	1
PEAK3	-19.6976694	1193.175564	-0.0165	0.987	-6.07E-08	count	1
AC113349.1	-19.8047141	1328.437406	-0.0149	0.988	-6.07E-08	count	1
ACOT11	-2.3642253	0.7054568	-3.3513	8.00E-04	-6.00E-08	count	1
DKK1	-2.2819658	0.5037423	-4.53	6.15E-06	-5.67E-08	count	0.14304285
SLC6A3	-2.157497	0.8122681	-2.6561	0.008	-5.20E-08	count	1
MIR34AHG	-2.0709834	1.0215306	-2.0273	0.0427	-4.86E-08	count	1
AC008629.1	-2.0709834	1.055563	-1.962	0.0499	-4.85E-08	count	1
AC114760.2	-2.0709834	1.1205312	-1.8482	0.0647	-4.85E-08	count	1
PTK6	-2.0045168	0.7648181	-2.6209	0.0088	-4.62E-08	count	1
AC090229.1	-1.9166133	0.7215687	-2.6562	0.008	-4.30E-08	count	1
GPC1	-0.9697203	0.4109536	-2.3597	0.0184	-4.26E-08	count	1
PITPNM1	-1.6681279	0.6998545	-2.3835	0.0172	-3.47E-08	count	1
IGHG2	-1.5696016	0.2298761	-6.828	1.05E-11	-3.16E-08	count	2.50E-07
LINC00862	-1.4902631	0.8156022	-1.8272	0.0678	-2.91E-08	count	1
AC018643.1	-2.6874309	0.6454505	-4.1637	3.23E-05	-2.69E-08	count	0.7455486
AC009974.1	-2.6874309	0.7473503	-3.5959	3.00E-04	-2.68E-08	count	1
CAVIN4	-2.6874309	0.7473503	-3.5959	3.00E-04	-2.68E-08	count	1
GBGT1	-1.3772117	0.7902148	-1.7428	0.0815	-2.59E-08	count	1
AC022762.2	-1.3772117	0.8405353	-1.6385	0.101	-2.59E-08	count	1
AC011815.2	-1.3772117	0.5395572	-2.5525	0.0107	-2.59E-08	count	1
OXTR	-0.6263138	0.4275164	-1.465	0.143	-2.40E-08	count	1
AC074011.1	-19.593386	1132.555494	-0.0173	0.986	-2.26E-08	count	1
KCNA3	-19.593386	1132.555494	-0.0173	0.986	-2.26E-08	count	1
DOCK2	-19.593386	1132.555494	-0.0173	0.986	-2.26E-08	count	1
EFHC2	-19.593386	1132.555494	-0.0173	0.986	-2.26E-08	count	1
RUBCNL	-19.593386	1132.555494	-0.0173	0.986	-2.26E-08	count	1
AC133540.1	-19.593386	1132.555494	-0.0173	0.986	-2.26E-08	count	1
MCOLN3	-19.593386	1132.555494	-0.0173	0.986	-2.26E-08	count	1
AC024560.3	-19.593386	1132.555494	-0.0173	0.986	-2.26E-08	count	1
AL121845.1	-19.593386	1132.555494	-0.0173	0.986	-2.26E-08	count	1
SPTA1	-19.593386	1132.555494	-0.0173	0.986	-2.26E-08	count	1
NECTIN4	-19.593386	1132.555494	-0.0173	0.986	-2.26E-08	count	1

IL11	-19.593386	1132.555494	-0.0173	0.986	-2.26E-08	count	1
SRC	-19.9990524	795.4136066	-0.0251	0.98	-2.26E-08	count	1
C1QTNF8	-19.9990524	795.4136066	-0.0251	0.98	-2.26E-08	count	1
P2RX6	-19.9990524	795.4136066	-0.0251	0.98	-2.26E-08	count	1
OR52H1	-20.000999	1388.581995	-0.0144	0.989	-2.25E-08	count	1
ZIC2	-20.000999	1388.581995	-0.0144	0.989	-2.25E-08	count	1
DPP6	-20.000999	1388.581995	-0.0144	0.989	-2.25E-08	count	1
SLC9C2	-20.000999	1388.581995	-0.0144	0.989	-2.25E-08	count	1
ANKUB1	-20.000999	1388.581995	-0.0144	0.989	-2.25E-08	count	1
SULT1E1	-20.000999	1388.581995	-0.0144	0.989	-2.25E-08	count	1
AC092691.1	-20.5118914	940.2228856	-0.0218	0.983	-2.25E-08	count	1
RGPD8	-19.8459164	1209.335099	-0.0164	0.987	-2.25E-08	count	1
MANCR	-20.1854816	1379.772764	-0.0146	0.988	-2.24E-08	count	1
GPC6	-20.5127604	1184.470362	-0.0173	0.986	-2.24E-08	count	1
CHL1	-19.3203885	1215.793224	-0.0159	0.987	-2.24E-08	count	1
GZMH	-19.3203885	1215.793224	-0.0159	0.987	-2.24E-08	count	1
SLC16A12	-19.3203885	1215.793224	-0.0159	0.987	-2.24E-08	count	1
AC060766.4	-19.3203885	1215.793224	-0.0159	0.987	-2.24E-08	count	1
EPN3	-19.3203885	1215.793224	-0.0159	0.987	-2.24E-08	count	1
AATBC	-19.3203885	1215.793224	-0.0159	0.987	-2.24E-08	count	1
CCDC129	-19.3203885	1215.793224	-0.0159	0.987	-2.24E-08	count	1
PDCD1	-20.5147011	1795.231728	-0.0114	0.991	-2.24E-08	count	1
PDE8B	-20.5147011	1795.231728	-0.0114	0.991	-2.24E-08	count	1
HAR1A	-20.1530927	1210.906108	-0.0166	0.987	-2.22E-08	count	1
P2RY13	-19.5788905	1505.747932	-0.013	0.99	-2.22E-08	count	1
AP000355.1	-19.5788905	1505.747932	-0.013	0.99	-2.22E-08	count	1
DSCAM	-19.0713188	828.2707621	-0.023	0.982	-2.17E-08	count	1
AC017002.3	-19.3602847	1016.090372	-0.0191	0.985	-2.16E-08	count	1
AC104452.1	-19.0729482	1444.399666	-0.0132	0.989	-2.16E-08	count	1
AL360181.2	-19.0729482	1444.399666	-0.0132	0.989	-2.16E-08	count	1
CD4	-2.2819658	0.6850001	-3.3313	9.00E-04	-2.09E-08	count	1
ADRA2B	-2.2819658	0.6850001	-3.3313	9.00E-04	-2.09E-08	count	1
CORO2B	-2.2819658	0.6850001	-3.3313	9.00E-04	-2.09E-08	count	1
AL357518.1	-2.2819658	0.6850001	-3.3313	9.00E-04	-2.09E-08	count	1
ADRA2A	-2.2819658	0.6850001	-3.3313	9.00E-04	-2.09E-08	count	1
PART1	-2.2819658	0.4835266	-4.7194	2.48E-06	-2.09E-08	count	0.057846
LEFTY2	-1.175441	0.803926	-1.4621	0.144	-2.05E-08	count	1
CD33	-1.1555612	0.7445391	-1.552	0.121	-2.00E-08	count	1
GAPLINC	-1.1555612	0.7445391	-1.552	0.121	-2.00E-08	count	1
HK2	-1.1555612	0.6897983	-1.6752	0.094	-2.00E-08	count	1
NTRK2	-1.1356813	0.692874	-1.6391	0.101	-1.95E-08	count	1
FIRRE	-1.0471456	0.6661399	-1.572	0.116	-1.74E-08	count	1
SALRNA2	-2.0234718	0.8371546	-2.4171	0.0157	-1.73E-08	count	1
CD247	-2.0234718	0.8371546	-2.4171	0.0157	-1.73E-08	count	1
ULBP1	-2.0234718	0.8371546	-2.4171	0.0157	-1.73E-08	count	1

RUNX3	-2.0234718	1.0874784	-1.8607	0.0629	-1.73E-08	count	1
AL731577.1	-0.4398255	0.3424471	-1.2844	0.199	-1.56E-08	count	1
ADCY3	-0.9339107	0.7627211	-1.2244	0.221	-1.49E-08	count	1
AC087627.1	-0.9339107	0.7627211	-1.2244	0.221	-1.49E-08	count	1
AL109811.2	-0.9339107	0.8112289	-1.1512	0.25	-1.49E-08	count	1
AC079193.2	-1.8209161	0.5495776	-3.3133	9.00E-04	-1.46E-08	count	1
DNAH7	-0.8956714	0.5758066	-1.5555	0.12	-1.41E-08	count	1
PPP2R2B	-1.7294147	1.0019231	-1.7261	0.0844	-1.35E-08	count	1
TICAM2	-1.7294147	1.0019231	-1.7261	0.0844	-1.35E-08	count	1
TRAF3IP3	-1.7294147	0.7656726	-2.2587	0.024	-1.35E-08	count	1
AL137002.2	-1.7294147	1.0650048	-1.6239	0.105	-1.35E-08	count	1
AC026150.1	-0.8208593	0.8751388	-0.938	0.348	-1.25E-08	count	1
AL078590.3	-1.6385945	0.4663165	-3.5139	4.00E-04	-1.24E-08	count	1
LGR6	-1.5888186	0.7914276	-2.0075	0.0448	-1.19E-08	count	1
AC098934.4	-1.5888186	0.7914276	-2.0075	0.0448	-1.19E-08	count	1
AL445493.3	-1.5888186	0.7914276	-2.0075	0.0448	-1.19E-08	count	1
AL391832.3	-1.5888186	0.7914276	-2.0075	0.0448	-1.19E-08	count	1
KRTCAP3	-1.5888186	0.7914276	-2.0075	0.0448	-1.19E-08	count	1
AC005740.4	-1.5888186	0.7914276	-2.0075	0.0448	-1.19E-08	count	1
AC114939.1	-1.5888186	0.7914276	-2.0075	0.0448	-1.19E-08	count	1
TCEAL6	-1.5888186	0.7914276	-2.0075	0.0448	-1.19E-08	count	1
AC087521.4	-1.5888186	0.7914276	-2.0075	0.0448	-1.19E-08	count	1
RERG	-1.5888186	0.7914276	-2.0075	0.0448	-1.19E-08	count	1
AC034102.4	-1.5888186	0.7914276	-2.0075	0.0448	-1.19E-08	count	1
AC025423.1	-1.5888186	0.7914276	-2.0075	0.0448	-1.19E-08	count	1
CPB2-AS1	-1.5888186	0.7914276	-2.0075	0.0448	-1.19E-08	count	1
AC001226.1	-1.5888186	0.7914276	-2.0075	0.0448	-1.19E-08	count	1
AL356019.2	-1.5888186	0.7914276	-2.0075	0.0448	-1.19E-08	count	1
AL357093.2	-1.5888186	0.7914276	-2.0075	0.0448	-1.19E-08	count	1
AC008731.1	-1.5888186	0.7914276	-2.0075	0.0448	-1.19E-08	count	1
AC087164.1	-1.5888186	0.7914276	-2.0075	0.0448	-1.19E-08	count	1
VGLL3	-1.5888186	0.7914276	-2.0075	0.0448	-1.19E-08	count	1
AC019257.2	-1.5888186	0.7914276	-2.0075	0.0448	-1.19E-08	count	1
EFCAB1	-1.5888186	0.7914276	-2.0075	0.0448	-1.19E-08	count	1
LCNL1	-1.5888186	0.7914276	-2.0075	0.0448	-1.19E-08	count	1
DRD4	-1.5888186	0.7914276	-2.0075	0.0448	-1.19E-08	count	1
PRR4	-1.5888186	0.7914276	-2.0075	0.0448	-1.19E-08	count	1
AC012085.2	-1.5888186	0.7914276	-2.0075	0.0448	-1.19E-08	count	1
RNFT2	-1.5888186	0.7914276	-2.0075	0.0448	-1.19E-08	count	1
UBE2Q2L	-1.5888186	0.7914276	-2.0075	0.0448	-1.19E-08	count	1
ADGRE2	-1.5888186	0.7914276	-2.0075	0.0448	-1.19E-08	count	1
LMTK3	-1.5888186	0.7914276	-2.0075	0.0448	-1.19E-08	count	1
SIGLEC9	-1.5888186	0.7914276	-2.0075	0.0448	-1.19E-08	count	1
UPB1	-1.5888186	0.7914276	-2.0075	0.0448	-1.19E-08	count	1
C3AR1	-1.5888186	0.5589765	-2.8424	0.0045	-1.19E-08	count	1

FGF14	-1.5888186	0.6047124	-2.6274	0.0086	-1.18E-08	count	1
AC093901.1	-1.5888186	0.6047124	-2.6274	0.0086	-1.18E-08	count	1
MAG	-0.3360557	0.3454363	-0.9728	0.331	-1.15E-08	count	1
DNHD1	-0.737823	0.7681139	-0.9606	0.337	-1.09E-08	count	1
SLC9C1	-1.4154509	0.5283422	-2.679	0.0074	-9.91E-09	count	1
HTRA3	-1.4154509	0.5283422	-2.679	0.0074	-9.91E-09	count	1
KHDC1	-1.4154509	0.5283422	-2.679	0.0074	-9.91E-09	count	1
LINC00605	-1.4154509	0.6090376	-2.3241	0.0202	-9.91E-09	count	1
AP002884.4	-0.6725279	0.5393965	-1.2468	0.213	-9.66E-09	count	1
AL031714.1	-0.6725279	0.4668597	-1.4405	0.15	-9.66E-09	count	1
DOCK10	-0.6429945	0.8916008	-0.7212	0.471	-9.13E-09	count	1
AC004771.5	-0.6429945	0.6444463	-0.9977	0.318	-9.12E-09	count	1
S100A5	-19.8977787	1318.734964	-0.0151	0.988	-8.33E-09	count	1
PAX6	-19.8977787	1318.734964	-0.0151	0.988	-8.33E-09	count	1
SGCA	-19.8977787	1318.734964	-0.0151	0.988	-8.33E-09	count	1
AC011472.1	-19.8977787	1318.734964	-0.0151	0.988	-8.33E-09	count	1
AL139147.1	-19.8977787	1318.734964	-0.0151	0.988	-8.33E-09	count	1
PM20D1	-19.8977787	1318.734964	-0.0151	0.988	-8.33E-09	count	1
AC009242.1	-19.8977787	1318.734964	-0.0151	0.988	-8.33E-09	count	1
CCL20	-19.8977787	1318.734964	-0.0151	0.988	-8.33E-09	count	1
VIPR1-AS1	-19.8977787	1318.734964	-0.0151	0.988	-8.33E-09	count	1
AC107072.2	-19.8977787	1318.734964	-0.0151	0.988	-8.33E-09	count	1
AC026785.2	-19.8977787	1318.734964	-0.0151	0.988	-8.33E-09	count	1
NIM1K	-19.8977787	1318.734964	-0.0151	0.988	-8.33E-09	count	1
ADRA1B	-19.8977787	1318.734964	-0.0151	0.988	-8.33E-09	count	1
MPIG6B	-19.8977787	1318.734964	-0.0151	0.988	-8.33E-09	count	1
IMPG1	-19.8977787	1318.734964	-0.0151	0.988	-8.33E-09	count	1
AL359715.3	-19.8977787	1318.734964	-0.0151	0.988	-8.33E-09	count	1
ESR1	-19.8977787	1318.734964	-0.0151	0.988	-8.33E-09	count	1
AL158070.1	-19.8977787	1318.734964	-0.0151	0.988	-8.33E-09	count	1
FOLR3	-19.8977787	1318.734964	-0.0151	0.988	-8.33E-09	count	1
C10orf105	-19.8977787	1318.734964	-0.0151	0.988	-8.33E-09	count	1
AC067750.1	-19.8977787	1318.734964	-0.0151	0.988	-8.33E-09	count	1
CYP2C9	-19.8977787	1318.734964	-0.0151	0.988	-8.33E-09	count	1
NELL2	-19.8977787	1318.734964	-0.0151	0.988	-8.33E-09	count	1
AC079313.1	-19.8977787	1318.734964	-0.0151	0.988	-8.33E-09	count	1
CA12	-19.8977787	1318.734964	-0.0151	0.988	-8.33E-09	count	1
LINC01597	-19.8977787	1318.734964	-0.0151	0.988	-8.33E-09	count	1
AC004156.1	-19.8977787	1318.734964	-0.0151	0.988	-8.33E-09	count	1
CRLF1	-19.8977787	1318.734964	-0.0151	0.988	-8.33E-09	count	1
CEACAM16	-19.8977787	1318.734964	-0.0151	0.988	-8.33E-09	count	1
CALML6	-19.8977787	1318.734964	-0.0151	0.988	-8.33E-09	count	1
SETSIP	-19.8977787	1318.734964	-0.0151	0.988	-8.33E-09	count	1
COL11A1	-19.8977787	1318.734964	-0.0151	0.988	-8.33E-09	count	1
AC020594.1	-19.8977787	1318.734964	-0.0151	0.988	-8.33E-09	count	1

AC018682.1	-19.8977787	1318.734964	-0.0151	0.988	-8.33E-09	count	1
AC073263.1	-19.8977787	1318.734964	-0.0151	0.988	-8.33E-09	count	1
LINC01856	-19.8977787	1318.734964	-0.0151	0.988	-8.33E-09	count	1
AC019068.1	-19.8977787	1318.734964	-0.0151	0.988	-8.33E-09	count	1
HTD2	-19.8977787	1318.734964	-0.0151	0.988	-8.33E-09	count	1
AC007370.2	-19.8977787	1318.734964	-0.0151	0.988	-8.33E-09	count	1
AC078845.1	-19.8977787	1318.734964	-0.0151	0.988	-8.33E-09	count	1
PHF24	-19.8977787	1318.734964	-0.0151	0.988	-8.33E-09	count	1
AL807752.6	-19.8977787	1318.734964	-0.0151	0.988	-8.33E-09	count	1
AC068733.3	-19.8977787	1318.734964	-0.0151	0.988	-8.33E-09	count	1
MRGPRF-AS1	-19.8977787	1318.734964	-0.0151	0.988	-8.33E-09	count	1
SCN2B	-19.8977787	1318.734964	-0.0151	0.988	-8.33E-09	count	1
AL136369.2	-19.8977787	1318.734964	-0.0151	0.988	-8.33E-09	count	1
ADGRA1	-19.8977787	1318.734964	-0.0151	0.988	-8.33E-09	count	1
GRIN2B	-19.8977787	1318.734964	-0.0151	0.988	-8.33E-09	count	1
C1QL4	-19.8977787	1318.734964	-0.0151	0.988	-8.33E-09	count	1
AC068987.4	-19.8977787	1318.734964	-0.0151	0.988	-8.33E-09	count	1
LIPC	-19.8977787	1318.734964	-0.0151	0.988	-8.33E-09	count	1
SLX1A	-19.8977787	1318.734964	-0.0151	0.988	-8.33E-09	count	1
AC027682.2	-19.8977787	1318.734964	-0.0151	0.988	-8.33E-09	count	1
HCRT	-19.8977787	1318.734964	-0.0151	0.988	-8.33E-09	count	1
TUBB1	-19.8977787	1318.734964	-0.0151	0.988	-8.33E-09	count	1
CDH4	-19.8977787	1318.734964	-0.0151	0.988	-8.33E-09	count	1
CCER2	-19.8977787	1318.734964	-0.0151	0.988	-8.33E-09	count	1
AC011462.1	-19.8977787	1318.734964	-0.0151	0.988	-8.33E-09	count	1
FPR3	-19.8977787	1318.734964	-0.0151	0.988	-8.33E-09	count	1
FASLG	-19.8977787	1318.734964	-0.0151	0.988	-8.33E-09	count	1
CTSE	-19.8977787	1318.734964	-0.0151	0.988	-8.33E-09	count	1
AC097724.1	-19.8977787	1318.734964	-0.0151	0.988	-8.33E-09	count	1
AC019080.4	-19.8977787	1318.734964	-0.0151	0.988	-8.33E-09	count	1
STX19	-19.8977787	1318.734964	-0.0151	0.988	-8.33E-09	count	1
AC016924.1	-19.8977787	1318.734964	-0.0151	0.988	-8.33E-09	count	1
LINC02100	-19.8977787	1318.734964	-0.0151	0.988	-8.33E-09	count	1
AC106786.1	-19.8977787	1318.734964	-0.0151	0.988	-8.33E-09	count	1
PCDHA8	-19.8977787	1318.734964	-0.0151	0.988	-8.33E-09	count	1
GFPT2	-19.8977787	1318.734964	-0.0151	0.988	-8.33E-09	count	1
AC138035.1	-19.8977787	1318.734964	-0.0151	0.988	-8.33E-09	count	1
AC008080.1	-19.8977787	1318.734964	-0.0151	0.988	-8.33E-09	count	1
AC211486.5	-19.8977787	1318.734964	-0.0151	0.988	-8.33E-09	count	1
TSGA13	-19.8977787	1318.734964	-0.0151	0.988	-8.33E-09	count	1
LINC00102	-19.8977787	1318.734964	-0.0151	0.988	-8.33E-09	count	1
AF233439.1	-19.8977787	1318.734964	-0.0151	0.988	-8.33E-09	count	1
LINC01239	-19.8977787	1318.734964	-0.0151	0.988	-8.33E-09	count	1
ADAMTS13	-19.8977787	1318.734964	-0.0151	0.988	-8.33E-09	count	1
GPHA2	-19.8977787	1318.734964	-0.0151	0.988	-8.33E-09	count	1

ST8SIA6-AS1	-19.8977787	1318.734964	-0.0151	0.988	-8.33E-09	count	1
PRKG1-AS1	-19.8977787	1318.734964	-0.0151	0.988	-8.33E-09	count	1
AL356753.1	-19.8977787	1318.734964	-0.0151	0.988	-8.33E-09	count	1
TSPAN8	-19.8977787	1318.734964	-0.0151	0.988	-8.33E-09	count	1
GPR18	-19.8977787	1318.734964	-0.0151	0.988	-8.33E-09	count	1
ZIC5	-19.8977787	1318.734964	-0.0151	0.988	-8.33E-09	count	1
JPH4	-19.8977787	1318.734964	-0.0151	0.988	-8.33E-09	count	1
AL049875.1	-19.8977787	1318.734964	-0.0151	0.988	-8.33E-09	count	1
FAM81A	-19.8977787	1318.734964	-0.0151	0.988	-8.33E-09	count	1
AC099489.1	-19.8977787	1318.734964	-0.0151	0.988	-8.33E-09	count	1
AC009123.1	-19.8977787	1318.734964	-0.0151	0.988	-8.33E-09	count	1
P2RX5	-19.8977787	1318.734964	-0.0151	0.988	-8.33E-09	count	1
LINC01497	-19.8977787	1318.734964	-0.0151	0.988	-8.33E-09	count	1
NETO1	-19.8977787	1318.734964	-0.0151	0.988	-8.33E-09	count	1
EMILIN3	-19.8977787	1318.734964	-0.0151	0.988	-8.33E-09	count	1
AC016590.3	-19.8977787	1318.734964	-0.0151	0.988	-8.33E-09	count	1
AC006946.2	-19.8977787	1318.734964	-0.0151	0.988	-8.33E-09	count	1
ABCA4	-19.8977787	1318.734964	-0.0151	0.988	-8.33E-09	count	1
LHX9	-19.8977787	1318.734964	-0.0151	0.988	-8.33E-09	count	1
MAL	-19.8977787	1318.734964	-0.0151	0.988	-8.33E-09	count	1
TFCP2L1	-19.8977787	1318.734964	-0.0151	0.988	-8.33E-09	count	1
GPR39	-19.8977787	1318.734964	-0.0151	0.988	-8.33E-09	count	1
AC107029.2	-19.8977787	1318.734964	-0.0151	0.988	-8.33E-09	count	1
AC005674.1	-19.8977787	1318.734964	-0.0151	0.988	-8.33E-09	count	1
PROM1	-19.8977787	1318.734964	-0.0151	0.988	-8.33E-09	count	1
AC097480.1	-19.8977787	1318.734964	-0.0151	0.988	-8.33E-09	count	1
CLDN22	-19.8977787	1318.734964	-0.0151	0.988	-8.33E-09	count	1
NCR3	-19.8977787	1318.734964	-0.0151	0.988	-8.33E-09	count	1
AC004948.1	-19.8977787	1318.734964	-0.0151	0.988	-8.33E-09	count	1
TLR7	-19.8977787	1318.734964	-0.0151	0.988	-8.33E-09	count	1
AL359851.1	-19.8977787	1318.734964	-0.0151	0.988	-8.33E-09	count	1
AC107959.2	-19.8977787	1318.734964	-0.0151	0.988	-8.33E-09	count	1
LYNX1-SLURP2	-19.8977787	1318.734964	-0.0151	0.988	-8.33E-09	count	1
C9orf47	-19.8977787	1318.734964	-0.0151	0.988	-8.33E-09	count	1
CALML3-AS1	-19.8977787	1318.734964	-0.0151	0.988	-8.33E-09	count	1
ADRB1	-19.8977787	1318.734964	-0.0151	0.988	-8.33E-09	count	1
AL157756.1	-19.8977787	1318.734964	-0.0151	0.988	-8.33E-09	count	1
ATP8B4	-19.8977787	1318.734964	-0.0151	0.988	-8.33E-09	count	1
CCL22	-19.8977787	1318.734964	-0.0151	0.988	-8.33E-09	count	1
CNDP1	-19.8977787	1318.734964	-0.0151	0.988	-8.33E-09	count	1
AL050327.1	-19.8977787	1318.734964	-0.0151	0.988	-8.33E-09	count	1
CD209	-19.8977787	1318.734964	-0.0151	0.988	-8.33E-09	count	1
FBXO27	-19.8977787	1318.734964	-0.0151	0.988	-8.33E-09	count	1
LINC00853	-19.8977787	1318.734964	-0.0151	0.988	-8.33E-09	count	1
AL136304.1	-19.8977787	1318.734964	-0.0151	0.988	-8.33E-09	count	1

FIBCD1	-19.8977787	1318.734964	-0.0151	0.988	-8.33E-09	count	1
SDS	-19.8977787	1318.734964	-0.0151	0.988	-8.33E-09	count	1
APOBR	-19.8977787	1318.734964	-0.0151	0.988	-8.33E-09	count	1
GRIA3	-20.5924504	1315.213111	-0.0157	0.988	-8.31E-09	count	1
GFRA2	-20.5924504	1315.213111	-0.0157	0.988	-8.31E-09	count	1
LINC02024	-20.5924504	1315.213111	-0.0157	0.988	-8.31E-09	count	1
AL596220.1	-20.5924504	1315.213111	-0.0157	0.988	-8.31E-09	count	1
TPSAB1	-20.5924504	1315.213111	-0.0157	0.988	-8.31E-09	count	1
RNF43	-20.5924504	1315.213111	-0.0157	0.988	-8.31E-09	count	1
SIX2	-20.5924504	1315.213111	-0.0157	0.988	-8.31E-09	count	1
DOK7	-20.5924504	1315.213111	-0.0157	0.988	-8.31E-09	count	1
CLDN10-AS1	-20.5924504	1315.213111	-0.0157	0.988	-8.31E-09	count	1
TRBC1	-0.5877367	0.4538616	-1.295	0.195	-8.16E-09	count	1
ERVW-1	-0.5817077	1.1466035	-0.5073	0.612	-8.06E-09	count	1
AC108863.1	-0.5817077	1.1466035	-0.5073	0.612	-8.06E-09	count	1
AC006042.4	-0.5817077	1.1466035	-0.5073	0.612	-8.05E-09	count	1
FOXS1	-0.5817077	1.1466035	-0.5073	0.612	-8.05E-09	count	1
PTPRH	-0.5817077	1.1466035	-0.5073	0.612	-8.05E-09	count	1
TRIM72	-0.5817077	0.8713277	-0.6676	0.504	-8.05E-09	count	1
LINC02145	-0.5817077	0.8713277	-0.6676	0.504	-8.05E-09	count	1
AL021707.7	-0.5817077	0.8713277	-0.6676	0.504	-8.05E-09	count	1
LINC00598	-0.5817077	0.8937593	-0.6509	0.515	-8.05E-09	count	1
SRRM5	-0.5817077	0.8937593	-0.6509	0.515	-8.05E-09	count	1
AP005717.1	-0.5817077	0.8713277	-0.6676	0.504	-8.05E-09	count	1
ARHGAP30	-0.5817077	0.8713277	-0.6676	0.504	-8.05E-09	count	1
PQLC2L	-0.5817077	0.8713277	-0.6676	0.504	-8.05E-09	count	1
AL355916.1	-0.5817077	0.8937593	-0.6509	0.515	-8.05E-09	count	1
FBXL16	-0.5817077	0.8713277	-0.6676	0.504	-8.05E-09	count	1
TF	-0.5817077	0.8713277	-0.6676	0.504	-8.05E-09	count	1
AC103739.1	-18.9704541	1372.243144	-0.0138	0.989	-7.99E-09	count	1
HBZ	-18.9704541	1372.243144	-0.0138	0.989	-7.99E-09	count	1
FP671120.1	-18.9704541	1372.243144	-0.0138	0.989	-7.99E-09	count	1
SLC6A17	-18.9704541	1372.243144	-0.0138	0.989	-7.99E-09	count	1
ZSWIM2	-18.9704541	1372.243144	-0.0138	0.989	-7.99E-09	count	1
ERVMER34-1	-18.9704541	1372.243144	-0.0138	0.989	-7.99E-09	count	1
AC106789.1	-18.9704541	1372.243144	-0.0138	0.989	-7.99E-09	count	1
GPR63	-18.9704541	1372.243144	-0.0138	0.989	-7.99E-09	count	1
SMIM10L2B	-18.9704541	1372.243144	-0.0138	0.989	-7.99E-09	count	1
AC022784.1	-18.9704541	1372.243144	-0.0138	0.989	-7.99E-09	count	1
PPP1R42	-18.9704541	1372.243144	-0.0138	0.989	-7.99E-09	count	1
AL158071.3	-18.9704541	1372.243144	-0.0138	0.989	-7.99E-09	count	1
AC004466.3	-18.9704541	1372.243144	-0.0138	0.989	-7.99E-09	count	1
AC015909.2	-18.9704541	1372.243144	-0.0138	0.989	-7.99E-09	count	1
AP002478.1	-18.9704541	1372.243144	-0.0138	0.989	-7.99E-09	count	1
ITCH-AS1	-18.9704541	1372.243144	-0.0138	0.989	-7.99E-09	count	1

AL109917.1	-18.9704541	1372.243144	-0.0138	0.989	-7.99E-09	count	1
BX284668.2	-18.9704541	1372.243144	-0.0138	0.989	-7.99E-09	count	1
TMEM269	-18.9704541	1372.243144	-0.0138	0.989	-7.99E-09	count	1
AMPD1	-18.9704541	1372.243144	-0.0138	0.989	-7.99E-09	count	1
SLAMF7	-18.9704541	1372.243144	-0.0138	0.989	-7.99E-09	count	1
DLG1-AS1	-18.9704541	1372.243144	-0.0138	0.989	-7.99E-09	count	1
SYNE1-AS1	-18.9704541	1372.243144	-0.0138	0.989	-7.99E-09	count	1
SLC26A5	-18.9704541	1372.243144	-0.0138	0.989	-7.99E-09	count	1
STAR	-18.9704541	1372.243144	-0.0138	0.989	-7.99E-09	count	1
AC090739.1	-18.9704541	1372.243144	-0.0138	0.989	-7.99E-09	count	1
AP000924.1	-18.9704541	1372.243144	-0.0138	0.989	-7.99E-09	count	1
DPYSL4	-18.9704541	1372.243144	-0.0138	0.989	-7.99E-09	count	1
AC124947.1	-18.9704541	1372.243144	-0.0138	0.989	-7.99E-09	count	1
AC073655.2	-18.9704541	1372.243144	-0.0138	0.989	-7.99E-09	count	1
AL132639.3	-18.9704541	1372.243144	-0.0138	0.989	-7.99E-09	count	1
IGHV1-24	-18.9704541	1372.243144	-0.0138	0.989	-7.99E-09	count	1
RASGRF1	-18.9704541	1372.243144	-0.0138	0.989	-7.99E-09	count	1
AC107958.2	-18.9704541	1372.243144	-0.0138	0.989	-7.99E-09	count	1
KRT28	-18.9704541	1372.243144	-0.0138	0.989	-7.99E-09	count	1
HSF5	-18.9704541	1372.243144	-0.0138	0.989	-7.99E-09	count	1
CDH7	-18.9704541	1372.243144	-0.0138	0.989	-7.99E-09	count	1
APOC2	-18.9704541	1372.243144	-0.0138	0.989	-7.99E-09	count	1
AC007326.4	-18.9704541	1372.243144	-0.0138	0.989	-7.99E-09	count	1
IGLV4-60	-18.9704541	1372.243144	-0.0138	0.989	-7.99E-09	count	1
KMO	-18.9704541	1372.243144	-0.0138	0.989	-7.99E-09	count	1
SCG2	-18.9704541	1372.243144	-0.0138	0.989	-7.99E-09	count	1
SP140	-18.9704541	1372.243144	-0.0138	0.989	-7.99E-09	count	1
CXCR6	-18.9704541	1372.243144	-0.0138	0.989	-7.99E-09	count	1
AC112487.1	-18.9704541	1372.243144	-0.0138	0.989	-7.99E-09	count	1
SLC12A8	-18.9704541	1372.243144	-0.0138	0.989	-7.99E-09	count	1
CXCL5	-18.9704541	1372.243144	-0.0138	0.989	-7.99E-09	count	1
LINC02273	-18.9704541	1372.243144	-0.0138	0.989	-7.99E-09	count	1
LINC02365	-18.9704541	1372.243144	-0.0138	0.989	-7.99E-09	count	1
AC026740.1	-18.9704541	1372.243144	-0.0138	0.989	-7.99E-09	count	1
AL035604.1	-18.9704541	1372.243144	-0.0138	0.989	-7.99E-09	count	1
WDR86	-18.9704541	1372.243144	-0.0138	0.989	-7.99E-09	count	1
AC112493.1	-18.9704541	1372.243144	-0.0138	0.989	-7.99E-09	count	1
XPNPEP2	-18.9704541	1372.243144	-0.0138	0.989	-7.99E-09	count	1
AC100797.4	-18.9704541	1372.243144	-0.0138	0.989	-7.99E-09	count	1
SIT1	-18.9704541	1372.243144	-0.0138	0.989	-7.99E-09	count	1
AL354861.2	-18.9704541	1372.243144	-0.0138	0.989	-7.99E-09	count	1
BX248123.1	-18.9704541	1372.243144	-0.0138	0.989	-7.99E-09	count	1
HTR7	-18.9704541	1372.243144	-0.0138	0.989	-7.99E-09	count	1
VDR	-18.9704541	1372.243144	-0.0138	0.989	-7.99E-09	count	1
AC008147.1	-18.9704541	1372.243144	-0.0138	0.989	-7.99E-09	count	1

C12orf56	-18.9704541	1372.243144	-0.0138	0.989	-7.99E-09	count	1
AC025035.1	-18.9704541	1372.243144	-0.0138	0.989	-7.99E-09	count	1
AC108704.1	-18.9704541	1372.243144	-0.0138	0.989	-7.99E-09	count	1
LINC00545	-18.9704541	1372.243144	-0.0138	0.989	-7.99E-09	count	1
AL162377.3	-18.9704541	1372.243144	-0.0138	0.989	-7.99E-09	count	1
ADAM21	-18.9704541	1372.243144	-0.0138	0.989	-7.99E-09	count	1
ACOT1	-18.9704541	1372.243144	-0.0138	0.989	-7.99E-09	count	1
AC012645.4	-18.9704541	1372.243144	-0.0138	0.989	-7.99E-09	count	1
AC118754.1	-18.9704541	1372.243144	-0.0138	0.989	-7.99E-09	count	1
AC005324.3	-18.9704541	1372.243144	-0.0138	0.989	-7.99E-09	count	1
SLC4A11	-18.9704541	1372.243144	-0.0138	0.989	-7.99E-09	count	1
EDN3	-18.9704541	1372.243144	-0.0138	0.989	-7.99E-09	count	1
AC004221.1	-18.9704541	1372.243144	-0.0138	0.989	-7.99E-09	count	1
IGLV6-57	-18.9704541	1372.243144	-0.0138	0.989	-7.99E-09	count	1
AC114488.1	-18.9704541	1372.243144	-0.0138	0.989	-7.99E-09	count	1
TNFSF4	-18.9704541	1372.243144	-0.0138	0.989	-7.99E-09	count	1
GAS5-AS1	-18.9704541	1372.243144	-0.0138	0.989	-7.99E-09	count	1
B3GALT2	-18.9704541	1372.243144	-0.0138	0.989	-7.99E-09	count	1
LINC01934	-18.9704541	1372.243144	-0.0138	0.989	-7.99E-09	count	1
PPP1R1C	-18.9704541	1372.243144	-0.0138	0.989	-7.99E-09	count	1
PRICKLE2-AS3	-18.9704541	1372.243144	-0.0138	0.989	-7.99E-09	count	1
AC139792.1	-18.9704541	1372.243144	-0.0138	0.989	-7.99E-09	count	1
SOX30	-18.9704541	1372.243144	-0.0138	0.989	-7.99E-09	count	1
AL138831.1	-18.9704541	1372.243144	-0.0138	0.989	-7.99E-09	count	1
AL008729.2	-18.9704541	1372.243144	-0.0138	0.989	-7.99E-09	count	1
AL512274.1	-18.9704541	1372.243144	-0.0138	0.989	-7.99E-09	count	1
DOK2	-18.9704541	1372.243144	-0.0138	0.989	-7.99E-09	count	1
AC022733.2	-18.9704541	1372.243144	-0.0138	0.989	-7.99E-09	count	1
AC103718.1	-18.9704541	1372.243144	-0.0138	0.989	-7.99E-09	count	1
FGD3	-18.9704541	1372.243144	-0.0138	0.989	-7.99E-09	count	1
AC090589.2	-18.9704541	1372.243144	-0.0138	0.989	-7.99E-09	count	1
CATSPER1	-18.9704541	1372.243144	-0.0138	0.989	-7.99E-09	count	1
B3GNT6	-18.9704541	1372.243144	-0.0138	0.989	-7.99E-09	count	1
AC105411.1	-18.9704541	1372.243144	-0.0138	0.989	-7.99E-09	count	1
LINC02073	-18.9704541	1372.243144	-0.0138	0.989	-7.99E-09	count	1
LINC01982	-18.9704541	1372.243144	-0.0138	0.989	-7.99E-09	count	1
CD300LB	-18.9704541	1372.243144	-0.0138	0.989	-7.99E-09	count	1
ATCAY	-18.9704541	1372.243144	-0.0138	0.989	-7.99E-09	count	1
IL12RB1	-18.9704541	1372.243144	-0.0138	0.989	-7.99E-09	count	1
CRYBA4	-18.9704541	1372.243144	-0.0138	0.989	-7.99E-09	count	1
AP001626.1	-18.9704541	1372.243144	-0.0138	0.989	-7.99E-09	count	1
AL391244.3	-18.9704541	1372.243144	-0.0138	0.989	-7.99E-09	count	1
MFSD2A	-18.9704541	1372.243144	-0.0138	0.989	-7.99E-09	count	1
GUCA2A	-18.9704541	1372.243144	-0.0138	0.989	-7.99E-09	count	1
LINC01776	-18.9704541	1372.243144	-0.0138	0.989	-7.99E-09	count	1

AL139161.1	-18.9704541	1372.243144	-0.0138	0.989	-7.99E-09	count	1
BCL11A	-18.9704541	1372.243144	-0.0138	0.989	-7.99E-09	count	1
IL1A	-18.9704541	1372.243144	-0.0138	0.989	-7.99E-09	count	1
CDCA7	-18.9704541	1372.243144	-0.0138	0.989	-7.99E-09	count	1
WNT5A-AS1	-18.9704541	1372.243144	-0.0138	0.989	-7.99E-09	count	1
ESYT3	-18.9704541	1372.243144	-0.0138	0.989	-7.99E-09	count	1
ANKRD31	-18.9704541	1372.243144	-0.0138	0.989	-7.99E-09	count	1
AL139095.4	-18.9704541	1372.243144	-0.0138	0.989	-7.99E-09	count	1
HOXA6	-18.9704541	1372.243144	-0.0138	0.989	-7.99E-09	count	1
HOXA9	-18.9704541	1372.243144	-0.0138	0.989	-7.99E-09	count	1
AL732314.6	-18.9704541	1372.243144	-0.0138	0.989	-7.99E-09	count	1
LINC01402	-18.9704541	1372.243144	-0.0138	0.989	-7.99E-09	count	1
AL590399.3	-18.9704541	1372.243144	-0.0138	0.989	-7.99E-09	count	1
AL162727.2	-18.9704541	1372.243144	-0.0138	0.989	-7.99E-09	count	1
NLRP6	-18.9704541	1372.243144	-0.0138	0.989	-7.99E-09	count	1
AP001972.3	-18.9704541	1372.243144	-0.0138	0.989	-7.99E-09	count	1
SPX	-18.9704541	1372.243144	-0.0138	0.989	-7.99E-09	count	1
AC004816.2	-18.9704541	1372.243144	-0.0138	0.989	-7.99E-09	count	1
DIO2	-18.9704541	1372.243144	-0.0138	0.989	-7.99E-09	count	1
CYP19A1	-18.9704541	1372.243144	-0.0138	0.989	-7.99E-09	count	1
ITGAX	-18.9704541	1372.243144	-0.0138	0.989	-7.99E-09	count	1
CD300E	-18.9704541	1372.243144	-0.0138	0.989	-7.99E-09	count	1
ZBP1	-18.9704541	1372.243144	-0.0138	0.989	-7.99E-09	count	1
AL390036.1	-18.9704541	1372.243144	-0.0138	0.989	-7.99E-09	count	1
SUSD5	-18.9704541	1372.243144	-0.0138	0.989	-7.99E-09	count	1
AC124944.1	-18.9704541	1372.243144	-0.0138	0.989	-7.99E-09	count	1
C6orf15	-18.9704541	1372.243144	-0.0138	0.989	-7.99E-09	count	1
MYH8	-18.9704541	1372.243144	-0.0138	0.989	-7.99E-09	count	1
Z97055.2	-18.9704541	1372.243144	-0.0138	0.989	-7.99E-09	count	1
LCK	-18.9704541	1372.243144	-0.0138	0.989	-7.99E-09	count	1
TENT5B	-19.664885	1369.031422	-0.0144	0.989	-7.97E-09	count	1
AC087645.4	-19.664885	1369.031422	-0.0144	0.989	-7.97E-09	count	1
BX470102.1	-19.6656654	1942.648764	-0.0101	0.992	-7.97E-09	count	1
HIST1H3J	-19.6656654	1942.648764	-0.0101	0.992	-7.97E-09	count	1
AL161785.3	-19.6656654	1942.648764	-0.0101	0.992	-7.97E-09	count	1
FCRL5	-19.6656654	1942.648764	-0.0101	0.992	-7.97E-09	count	1
C4orf45	-19.6656654	1942.648764	-0.0101	0.992	-7.97E-09	count	1
TMEM110-MUSTN1	-19.6656654	1942.648764	-0.0101	0.992	-7.97E-09	count	1
AC004522.2	-19.6656654	1942.648764	-0.0101	0.992	-7.97E-09	count	1
PAGE1	-19.6656654	1942.648764	-0.0101	0.992	-7.97E-09	count	1
AC010271.1	-19.6656654	1942.648764	-0.0101	0.992	-7.97E-09	count	1
AL049775.2	-19.6656654	1942.648764	-0.0101	0.992	-7.97E-09	count	1
AC131159.2	-1.1555612	1.0536576	-1.0967	0.273	-7.37E-09	count	1
AC002553.2	-1.1555612	1.0536576	-1.0967	0.273	-7.37E-09	count	1
KCNJ8	-1.1555612	1.0536576	-1.0967	0.273	-7.37E-09	count	1

CRTAM	-1.1555612	1.0536576	-1.0967	0.273	-7.37E-09	count	1
FCGR1B	-1.1555612	1.0536576	-1.0967	0.273	-7.37E-09	count	1
LINC02345	-1.1555612	1.0536576	-1.0967	0.273	-7.37E-09	count	1
MS4A1	-1.1555612	0.9762994	-1.1836	0.237	-7.37E-09	count	1
COCH	-1.1555612	1.0536576	-1.0967	0.273	-7.37E-09	count	1
SYNJ2BP-COX16	-1.1555612	1.0536576	-1.0967	0.273	-7.37E-09	count	1
DBH-AS1	-1.1555612	1.0536576	-1.0967	0.273	-7.37E-09	count	1
AC138331.1	-1.1555612	1.0536576	-1.0967	0.273	-7.37E-09	count	1
AFF3	-1.1555612	1.0536576	-1.0967	0.273	-7.37E-09	count	1
AC090061.1	-1.1555612	1.0536576	-1.0967	0.273	-7.37E-09	count	1
AC007376.2	-1.1555612	1.0536576	-1.0967	0.273	-7.37E-09	count	1
CHI3L1	-1.1555612	0.9762994	-1.1836	0.237	-7.37E-09	count	1
AC067817.2	-1.1555612	1.0536576	-1.0967	0.273	-7.37E-09	count	1
NODAL	-1.1555612	1.0536576	-1.0967	0.273	-7.37E-09	count	1
AC073592.1	-1.1555612	1.0536576	-1.0967	0.273	-7.37E-09	count	1
AC145207.6	-1.1555612	1.0536576	-1.0967	0.273	-7.37E-09	count	1
ABCG8	-1.1555612	1.0536576	-1.0967	0.273	-7.37E-09	count	1
STAT4	-1.1555612	1.0536576	-1.0967	0.273	-7.37E-09	count	1
AL008723.1	-1.1555612	1.0536576	-1.0967	0.273	-7.37E-09	count	1
AC126755.3	-1.1555612	1.0536576	-1.0967	0.273	-7.37E-09	count	1
AC135178.4	-1.1555612	1.0536576	-1.0967	0.273	-7.37E-09	count	1
AC004816.1	-1.1555612	1.0536576	-1.0967	0.273	-7.37E-09	count	1
AC009902.3	-1.1555612	1.0536576	-1.0967	0.273	-7.37E-09	count	1
AC087071.1	-1.1555612	0.9762994	-1.1836	0.237	-7.37E-09	count	1
NAV2-AS3	-1.1555612	1.0536576	-1.0967	0.273	-7.37E-09	count	1
ADRA1D	-1.1555612	1.0536576	-1.0967	0.273	-7.37E-09	count	1
ANK3	-1.1555612	0.9762994	-1.1836	0.237	-7.37E-09	count	1
AC025165.5	-1.1555612	1.0536576	-1.0967	0.273	-7.37E-09	count	1
KRT17	-1.1555612	0.9762994	-1.1836	0.237	-7.37E-09	count	1
LY6G6C	-1.1555612	1.0536576	-1.0967	0.273	-7.37E-09	count	1
AL358075.1	-1.1555612	1.0536576	-1.0967	0.273	-7.37E-09	count	1
ARSD-AS1	-1.1555612	1.0536576	-1.0967	0.273	-7.37E-09	count	1
AC092118.1	-1.1555612	1.0536576	-1.0967	0.273	-7.37E-09	count	1
AL355488.1	-1.1555612	0.9762994	-1.1836	0.237	-7.37E-09	count	1
HAND2-AS1	-1.1555612	1.0536576	-1.0967	0.273	-7.37E-09	count	1
RIPOR3	-1.1555612	1.0536576	-1.0967	0.273	-7.37E-09	count	1
AC011498.4	-1.1555612	0.9762994	-1.1836	0.237	-7.37E-09	count	1
AC110048.2	-1.1555612	0.9762994	-1.1836	0.237	-7.37E-09	count	1
C1QTNF5	-1.1555612	1.0536576	-1.0967	0.273	-7.37E-09	count	1
LINC02091	-0.5319467	0.6164839	-0.8629	0.388	-7.22E-09	count	1
CCL18	-0.5319467	0.6164839	-0.8629	0.388	-7.22E-09	count	1
RP1	-0.4740531	0.5966732	-0.7945	0.427	-6.28E-09	count	1
PLB1	-0.430808	0.7000874	-0.6154	0.538	-5.61E-09	count	1
AP001020.3	-0.8956714	0.9274946	-0.9657	0.334	-5.17E-09	count	1
KCNA5	-0.8956714	0.9274946	-0.9657	0.334	-5.17E-09	count	1

TEAD3	-0.8956714	0.6850001	-1.3075	0.191	-5.17E-09	count	1
AC002066.1	-0.8956714	0.6850001	-1.3075	0.191	-5.17E-09	count	1
AL591845.1	-0.8956714	0.6850001	-1.3075	0.191	-5.17E-09	count	1
AC022893.1	-0.8956714	0.6850001	-1.3075	0.191	-5.17E-09	count	1
HM13-AS1	-0.8956714	0.6850001	-1.3075	0.191	-5.17E-09	count	1
AC110799.1	-0.8956714	0.6850001	-1.3075	0.191	-5.17E-09	count	1
OR51M1	-0.8956714	0.6850001	-1.3075	0.191	-5.17E-09	count	1
FITM1	-0.8956714	0.6850001	-1.3075	0.191	-5.17E-09	count	1
AC127521.1	-0.8956714	0.6850001	-1.3075	0.191	-5.17E-09	count	1
RSPH1	-0.8956714	0.6850001	-1.3075	0.191	-5.17E-09	count	1
BNIPL	-0.3945091	0.5919581	-0.6664	0.505	-5.06E-09	count	1
TUFT1	-0.3360557	0.4975742	-0.6754	0.499	-4.21E-09	count	1
AL121899.1	-0.7223038	0.6106702	-1.1828	0.237	-3.89E-09	count	1
LINC01060	-0.7223038	0.6106702	-1.1828	0.237	-3.89E-09	count	1
TREM1	-0.7223038	0.6106702	-1.1828	0.237	-3.89E-09	count	1
AL359715.1	-0.7223038	0.6106702	-1.1828	0.237	-3.89E-09	count	1
LAT2	-0.7223038	0.6106702	-1.1828	0.237	-3.89E-09	count	1
MSR1	-0.7223038	0.6106702	-1.1828	0.237	-3.89E-09	count	1
AP003472.1	-0.7223038	0.6106702	-1.1828	0.237	-3.89E-09	count	1
AL512598.1	-0.7223038	0.6106702	-1.1828	0.237	-3.89E-09	count	1
GNB3	-0.7223038	0.6106702	-1.1828	0.237	-3.89E-09	count	1
IGHA2	-0.7223038	0.6106702	-1.1828	0.237	-3.89E-09	count	1
FENDRR	-0.7223038	0.6106702	-1.1828	0.237	-3.89E-09	count	1
AC005759.1	-0.7223038	0.6106702	-1.1828	0.237	-3.89E-09	count	1
GPR171	-0.7223038	0.6106702	-1.1828	0.237	-3.89E-09	count	1
SAP25	-0.7223038	0.6106702	-1.1828	0.237	-3.89E-09	count	1
AC026333.4	-0.7223038	0.6106702	-1.1828	0.237	-3.89E-09	count	1
FAM239B	-0.2623742	0.6038418	-0.4345	0.664	-3.19E-09	count	1
UPK3B	-0.2401389	1.2544861	-0.1914	0.848	-2.90E-09	count	1
AC137630.3	-0.2401389	0.8186954	-0.2933	0.769	-2.90E-09	count	1
AL359091.4	-0.2401389	0.8186954	-0.2933	0.769	-2.90E-09	count	1
EDNRA	-0.2401389	0.8186954	-0.2933	0.769	-2.90E-09	count	1
AL162426.1	-0.2401389	0.8186954	-0.2933	0.769	-2.90E-09	count	1
AL137779.2	-0.2401389	0.8186954	-0.2933	0.769	-2.90E-09	count	1
AL365194.1	-0.4902063	0.6454505	-0.7595	0.448	-2.41E-09	count	1
AL023584.2	-0.4902063	0.6454505	-0.7595	0.448	-2.41E-09	count	1
ST8SIA1	-0.4902063	0.6454505	-0.7595	0.448	-2.41E-09	count	1
AC092653.1	-0.4902063	0.6454505	-0.7595	0.448	-2.41E-09	count	1
VXN	-0.4902063	0.6454505	-0.7595	0.448	-2.41E-09	count	1
CD6	-0.4902063	0.6454505	-0.7595	0.448	-2.41E-09	count	1
NR2E3	-0.4902063	0.6454505	-0.7595	0.448	-2.41E-09	count	1
AC127002.1	-0.2025243	0.6245925	-0.3243	0.746	-2.40E-09	count	1
IMPG2	-0.3168387	0.5248472	-0.6037	0.546	-1.45E-09	count	1
AC091588.2	-0.2876506	0.8399163	-0.3425	0.732	-1.30E-09	count	1
AL021368.3	-0.2876506	0.890913	-0.3229	0.747	-1.30E-09	count	1

SLC5A4	-0.2876506	0.890913	-0.3229	0.747	-1.30E-09	count	1
SERPINA3	-0.2876506	0.8399163	-0.3425	0.732	-1.30E-09	count	1
SYN1	-0.2876506	0.8399163	-0.3425	0.732	-1.30E-09	count	1
TGM1	-0.2876506	0.890913	-0.3229	0.747	-1.30E-09	count	1
GPR37	-0.2876506	0.8399163	-0.3425	0.732	-1.30E-09	count	1
SLC4A8	-0.2876506	0.890913	-0.3229	0.747	-1.30E-09	count	1
AL136141.1	-0.2876506	0.890913	-0.3229	0.747	-1.30E-09	count	1
LINC01176	-0.2876506	0.890913	-0.3229	0.747	-1.30E-09	count	1
GJC1	-0.0098542	0.4780007	-0.0206	0.984	-2.94E-10	count	1
GSTM1	-0.0291566	0.3725025	-0.0783	0.938	-1.19E-10	count	1
FOXO6	-0.0291566	0.5283423	-0.0552	0.956	-1.19E-10	count	1
AC009171.2	-0.0291566	0.5283423	-0.0552	0.956	-1.19E-10	count	1
PZP	-0.0098542	0.5862813	-0.0168	0.987	-1.08E-10	count	1
STEAP4	-0.0098542	0.8303307	-0.0119	0.991	-1.08E-10	count	1
C8orf89	-0.0098542	0.8303307	-0.0119	0.991	-1.08E-10	count	1
AADAT	-0.0098542	0.8303307	-0.0119	0.991	-1.08E-10	count	1
GTF2H4	-0.0098542	0.8303307	-0.0119	0.991	-1.08E-10	count	1
LINC01504	-0.0098542	0.8303307	-0.0119	0.991	-1.08E-10	count	1
PMF1-BGLAP	0.0065064	0.7997091	0.0081	0.994	7.10E-11	count	1
LINC01068	0.0065064	0.8002759	0.0081	0.994	7.10E-11	count	1
LINC02576	0.0065064	0.8002759	0.0081	0.994	7.10E-11	count	1
AC003965.1	0.0065064	0.9167602	0.0071	0.994	7.10E-11	count	1
EVI2A	0.0065064	1.3586498	0.0048	0.996	7.10E-11	count	1
AC032044.1	0.0206193	0.6116181	0.0337	0.973	2.24E-10	count	1
GPR65	0.0206193	1.0605833	0.0194	0.984	2.24E-10	count	1
C9orf66	0.3763085	0.5997022	0.6275	0.53	1.30E-09	count	1
FAM43B	0.3763085	0.5997022	0.6275	0.53	1.30E-09	count	1
AL445985.1	18.0416002	4003.863848	0.0045	0.996	1.69E-09	count	1
PDE1C	18.0416	4003.863136	0.0045	0.996	1.69E-09	count	1
AL354694.1	18.0416002	4003.86319	0.0045	0.996	1.69E-09	count	1
AP002990.1	18.0415995	4003.863465	0.0045	0.996	1.69E-09	count	1
CCDC81	18.0416	4003.863574	0.0045	0.996	1.69E-09	count	1
AL157392.4	18.0415995	4003.863574	0.0045	0.996	1.69E-09	count	1
AC068722.2	18.0415998	4003.863739	0.0045	0.996	1.69E-09	count	1
AC011731.1	18.0416003	4003.863794	0.0045	0.996	1.69E-09	count	1
AL031281.2	18.0415998	4003.864342	0.0045	0.996	1.69E-09	count	1
AL445490.1	18.0416	4003.863574	0.0045	0.996	1.69E-09	count	1
KANK4	18.0416001	4003.863519	0.0045	0.996	1.69E-09	count	1
SPATA42	18.0415996	4003.862861	0.0045	0.996	1.69E-09	count	1
LINC01936	18.0415999	4003.863848	0.0045	0.996	1.69E-09	count	1
SATB2	18.0416001	4003.863136	0.0045	0.996	1.69E-09	count	1
AC055764.2	18.0415994	4003.863519	0.0045	0.996	1.69E-09	count	1
DEPDC1B	18.0415995	4003.863245	0.0045	0.996	1.69E-09	count	1
BEND6	18.0415993	4003.863794	0.0045	0.996	1.69E-09	count	1
AL365275.1	18.0415999	4003.863629	0.0045	0.996	1.69E-09	count	1

MLXIPL	18.0415993	4003.863245	0.0045	0.996	1.69E-09	count	1
AC103724.3	18.0415999	4003.864177	0.0045	0.996	1.69E-09	count	1
LINC01603	18.0415993	4003.863629	0.0045	0.996	1.69E-09	count	1
PGM5P3-AS1	18.0416001	4003.86341	0.0045	0.996	1.69E-09	count	1
BAAT	18.0415997	4003.862861	0.0045	0.996	1.69E-09	count	1
LCN10	18.0415995	4003.863574	0.0045	0.996	1.69E-09	count	1
EHF	18.0415997	4003.862916	0.0045	0.996	1.69E-09	count	1
IL2RA	18.0415989	4003.862533	0.0045	0.996	1.69E-09	count	1
AL591767.1	18.0415998	4003.863136	0.0045	0.996	1.69E-09	count	1
SDK2	18.0415999	4003.863574	0.0045	0.996	1.69E-09	count	1
MYOM1	18.0416001	4003.863739	0.0045	0.996	1.69E-09	count	1
AL132821.1	18.0416	4003.863903	0.0045	0.996	1.69E-09	count	1
DLGAP4-AS1	18.0415995	4003.863245	0.0045	0.996	1.69E-09	count	1
AC005005.3	18.0416	4003.863903	0.0045	0.996	1.69E-09	count	1
AL109811.3	18.0415987	4003.863629	0.0045	0.996	1.69E-09	count	1
AL109659.2	18.0415994	4003.863739	0.0045	0.996	1.69E-09	count	1
AL356488.2	18.0415996	4003.862752	0.0045	0.996	1.69E-09	count	1
PHGDH	18.0415991	4003.862258	0.0045	0.996	1.69E-09	count	1
FAM72C	18.0415988	4003.863136	0.0045	0.996	1.69E-09	count	1
AL359921.2	18.0415999	4003.863136	0.0045	0.996	1.69E-09	count	1
AC098828.2	18.0415999	4003.863465	0.0045	0.996	1.69E-09	count	1
AC018690.1	18.0415998	4003.863465	0.0045	0.996	1.69E-09	count	1
ARHGEF4	18.0415988	4003.863136	0.0045	0.996	1.69E-09	count	1
AC011997.1	18.0415996	4003.863355	0.0045	0.996	1.69E-09	count	1
CCR1	18.0415989	4003.863519	0.0045	0.996	1.69E-09	count	1
AC012557.1	18.0415993	4003.862697	0.0045	0.996	1.69E-09	count	1
KCNAB1	18.0415995	4003.864013	0.0045	0.996	1.69E-09	count	1
SLC7A14	18.041599	4003.863081	0.0045	0.996	1.69E-09	count	1
C1QTNF7	18.0415992	4003.86341	0.0045	0.996	1.69E-09	count	1
AC026726.1	18.0415987	4003.86319	0.0045	0.996	1.69E-09	count	1
PCDHGB2	18.0415996	4003.862971	0.0045	0.996	1.69E-09	count	1
CSF1R	18.0415991	4003.8633	0.0045	0.996	1.69E-09	count	1
FSCN3	18.0415993	4003.863081	0.0045	0.996	1.69E-09	count	1
KEL	18.0415992	4003.863794	0.0045	0.996	1.69E-09	count	1
AL732314.4	18.0415992	4003.863519	0.0045	0.996	1.69E-09	count	1
PHKA1-AS1	18.0415992	4003.863465	0.0045	0.996	1.69E-09	count	1
LINC01285	18.0415991	4003.86341	0.0045	0.996	1.69E-09	count	1
TKTL1	18.0415991	4003.863245	0.0045	0.996	1.69E-09	count	1
TNFRSF10A-AS1	18.0415993	4003.863355	0.0045	0.996	1.69E-09	count	1
ERICD	18.0415992	4003.863848	0.0045	0.996	1.69E-09	count	1
RASEF	18.0415989	4003.863026	0.0045	0.996	1.69E-09	count	1
SHC3	18.0415993	4003.86341	0.0045	0.996	1.69E-09	count	1
LCN8	18.0415985	4003.863629	0.0045	0.996	1.69E-09	count	1
FAM180B	18.0415992	4003.863245	0.0045	0.996	1.69E-09	count	1
AP001267.2	18.041599	4003.862916	0.0045	0.996	1.69E-09	count	1

DDX25	18.0415991	4003.86341	0.0045	0.996	1.69E-09	count	1
WDFY4	18.0415996	4003.863958	0.0045	0.996	1.69E-09	count	1
RBM20	18.041599	4003.863574	0.0045	0.996	1.69E-09	count	1
AC005383.1	18.0415994	4003.863574	0.0045	0.996	1.69E-09	count	1
FAM53B-AS1	18.0415994	4003.863355	0.0045	0.996	1.69E-09	count	1
DDX11-AS1	18.0415993	4003.863355	0.0045	0.996	1.69E-09	count	1
AC083805.3	18.041599	4003.863026	0.0045	0.996	1.69E-09	count	1
F10	18.0415992	4003.862916	0.0045	0.996	1.69E-09	count	1
AC004846.2	18.0415997	4003.863794	0.0045	0.996	1.69E-09	count	1
AC079322.1	18.0415991	4003.863465	0.0045	0.996	1.69E-09	count	1
AC002310.2	18.0415992	4003.863355	0.0045	0.996	1.69E-09	count	1
CAPNS2	18.0415987	4003.86319	0.0045	0.996	1.69E-09	count	1
AC026461.3	18.0415993	4003.863794	0.0045	0.996	1.69E-09	count	1
AC005899.6	18.0415999	4003.864013	0.0045	0.996	1.69E-09	count	1
MEIOC	18.0415993	4003.863629	0.0045	0.996	1.69E-09	count	1
AP005671.1	18.0415996	4003.863136	0.0045	0.996	1.69E-09	count	1
AC007998.4	18.0415997	4003.863848	0.0045	0.996	1.69E-09	count	1
AC100847.1	18.0415986	4003.863519	0.0045	0.996	1.69E-09	count	1
PLCG1-AS1	18.0415993	4003.8633	0.0045	0.996	1.69E-09	count	1
OSCAR	18.0415992	4003.8633	0.0045	0.996	1.69E-09	count	1
AC008735.2	18.0415992	4003.863355	0.0045	0.996	1.69E-09	count	1
BAIAP2L2	18.0415994	4003.863684	0.0045	0.996	1.69E-09	count	1
LINC01990	18.0415988	4003.8633	0.0045	0.996	1.69E-09	count	1
AC012640.4	18.0415984	4003.863739	0.0045	0.996	1.69E-09	count	1
AC010343.3	18.041599	4003.862752	0.0045	0.996	1.69E-09	count	1
AL645929.2	18.0415986	4003.863794	0.0045	0.996	1.69E-09	count	1
CA3-AS1	18.0415992	4003.863519	0.0045	0.996	1.69E-09	count	1
CASC18	18.0415987	4003.864013	0.0045	0.996	1.69E-09	count	1
AC026367.1	18.041599	4003.86341	0.0045	0.996	1.69E-09	count	1
AL162574.2	18.0415987	4003.864397	0.0045	0.996	1.69E-09	count	1
FSD2	18.041599	4003.862587	0.0045	0.996	1.69E-09	count	1
CA5A	18.041599	4003.863958	0.0045	0.996	1.69E-09	count	1
AC138028.1	18.0415993	4003.863629	0.0045	0.996	1.69E-09	count	1
LINC00470	18.0415987	4003.863465	0.0045	0.996	1.69E-09	count	1
FAM209B	18.0415988	4003.863465	0.0045	0.996	1.69E-09	count	1
LINC01783	18.0415984	4003.86319	0.0045	0.996	1.69E-09	count	1
FOXD3-AS1	18.0415985	4003.863574	0.0045	0.996	1.69E-09	count	1
NTNG1	18.0415983	4003.863355	0.0045	0.996	1.69E-09	count	1
OR5H14	18.041599	4003.86319	0.0045	0.996	1.69E-09	count	1
CDKL2	18.0415984	4003.862861	0.0045	0.996	1.69E-09	count	1
OSR2	18.0415991	4003.86341	0.0045	0.996	1.69E-09	count	1
AP000781.1	18.0415986	4003.863245	0.0045	0.996	1.69E-09	count	1
LINC02259	18.0415986	4003.863519	0.0045	0.996	1.69E-09	count	1
AC103876.1	18.0415982	4003.862752	0.0045	0.996	1.69E-09	count	1
SEC14L5	18.041599	4003.86319	0.0045	0.996	1.69E-09	count	1

AC093484.3	18.0415987	4003.863465	0.0045	0.996	1.69E-09	count	1
GAS2L2	18.0415982	4003.863465	0.0045	0.996	1.69E-09	count	1
TBC1D3L	18.0415981	4003.863245	0.0045	0.996	1.69E-09	count	1
CSDC2	18.0415982	4003.862697	0.0045	0.996	1.69E-09	count	1
U62317.4	18.0415984	4003.863794	0.0045	0.996	1.69E-09	count	1
AC009975.1	17.3463251	4002.390864	0.0043	0.997	1.69E-09	count	1
AC020634.1	17.3463256	4002.391438	0.0043	0.997	1.69E-09	count	1
MAS1	17.3463256	4002.391438	0.0043	0.997	1.69E-09	count	1
KCNQ1-AS1	17.3463254	4002.391411	0.0043	0.997	1.69E-09	count	1
ANKK1	17.3463256	4002.391438	0.0043	0.997	1.69E-09	count	1
AC016395.1	17.3463254	4002.391137	0.0043	0.997	1.69E-09	count	1
PLA2G4E-AS1	17.3463256	4002.391438	0.0043	0.997	1.69E-09	count	1
BCAR4	17.3463256	4002.391438	0.0043	0.997	1.69E-09	count	1
AC109460.2	17.3463253	4002.391082	0.0043	0.997	1.69E-09	count	1
FAM57B	17.3463256	4002.39152	0.0043	0.997	1.69E-09	count	1
RNFT1-DT	17.3463253	4002.391082	0.0043	0.997	1.69E-09	count	1
DLGAP1-AS4	17.3463254	4002.391411	0.0043	0.997	1.69E-09	count	1
AL049794.1	17.3463256	4002.391438	0.0043	0.997	1.69E-09	count	1
MYLK2	17.3463254	4002.391411	0.0043	0.997	1.69E-09	count	1
FO393400.1	17.3463256	4002.39152	0.0043	0.997	1.69E-09	count	1
AZU1	17.3463256	4002.391438	0.0043	0.997	1.69E-09	count	1
AL591163.1	17.3463246	4002.39111	0.0043	0.997	1.69E-09	count	1
AL139423.1	17.3463254	4002.39152	0.0043	0.997	1.69E-09	count	1
CD164L2	17.3463247	4002.390973	0.0043	0.997	1.69E-09	count	1
GRIK3	17.3463248	4002.391465	0.0043	0.997	1.69E-09	count	1
AC098484.3	17.3463254	4002.391246	0.0043	0.997	1.69E-09	count	1
AL358075.2	17.346325	4002.390946	0.0043	0.997	1.69E-09	count	1
ELAVL4	17.3463249	4002.391274	0.0043	0.997	1.69E-09	count	1
LINC01135	17.3463253	4002.391356	0.0043	0.997	1.69E-09	count	1
AL354872.2	17.3463254	4002.39152	0.0043	0.997	1.69E-09	count	1
AL355816.1	17.3463253	4002.391274	0.0043	0.997	1.69E-09	count	1
C1orf143	17.3463249	4002.391274	0.0043	0.997	1.69E-09	count	1
LYPLAL1-DT	17.3463251	4002.390946	0.0043	0.997	1.69E-09	count	1
AC096642.1	17.3463252	4002.391219	0.0043	0.997	1.69E-09	count	1
AL672291.1	17.3463253	4002.391164	0.0043	0.997	1.69E-09	count	1
ST6GAL2	17.3463248	4002.391465	0.0043	0.997	1.69E-09	count	1
RPRM	17.3463252	4002.391164	0.0043	0.997	1.69E-09	count	1
TMEFF2	17.3463248	4002.391465	0.0043	0.997	1.69E-09	count	1
CCK	17.3463254	4002.39111	0.0043	0.997	1.69E-09	count	1
ZBTB20-AS3	17.3463246	4002.39111	0.0043	0.997	1.69E-09	count	1
IL12A-AS1	17.346325	4002.391328	0.0043	0.997	1.69E-09	count	1
AC009570.1	17.3463253	4002.391356	0.0043	0.997	1.69E-09	count	1
MTTP	17.3463248	4002.391465	0.0043	0.997	1.69E-09	count	1
AC079341.2	17.3463253	4002.391301	0.0043	0.997	1.69E-09	count	1
TRPC3	17.3463248	4002.391465	0.0043	0.997	1.69E-09	count	1

AC107214.2	17.3463246	4002.39111	0.0043	0.997	1.69E-09	count	1
BHMT	17.3463252	4002.391219	0.0043	0.997	1.69E-09	count	1
AC011405.1	17.3463255	4002.391137	0.0043	0.997	1.69E-09	count	1
SLC4A9	17.3463254	4002.391164	0.0043	0.997	1.69E-09	count	1
AC244517.1	17.3463253	4002.391055	0.0043	0.997	1.69E-09	count	1
LINC01962	17.3463251	4002.391274	0.0043	0.997	1.69E-09	count	1
AL031963.1	17.3463252	4002.390891	0.0043	0.997	1.69E-09	count	1
VWDE	17.3463253	4002.391356	0.0043	0.997	1.69E-09	count	1
AC002064.1	17.3463249	4002.391274	0.0043	0.997	1.69E-09	count	1
OR2AE1	17.3463251	4002.391164	0.0043	0.997	1.69E-09	count	1
DOCK4-AS1	17.3463251	4002.391055	0.0043	0.997	1.69E-09	count	1
TRBC2	17.3463253	4002.391301	0.0043	0.997	1.69E-09	count	1
NDP	17.3463249	4002.391274	0.0043	0.997	1.69E-09	count	1
AC231533.1	17.3463248	4002.391465	0.0043	0.997	1.69E-09	count	1
CT55	17.3463252	4002.391219	0.0043	0.997	1.69E-09	count	1
RP1L1	17.3463252	4002.391219	0.0043	0.997	1.69E-09	count	1
AC037459.2	17.3463251	4002.391164	0.0043	0.997	1.69E-09	count	1
AC009686.2	17.3463255	4002.391438	0.0043	0.997	1.69E-09	count	1
AC016405.2	17.346325	4002.391	0.0043	0.997	1.69E-09	count	1
AC087045.2	17.3463252	4002.391164	0.0043	0.997	1.69E-09	count	1
MELK	17.346325	4002.390946	0.0043	0.997	1.69E-09	count	1
FRMPD1	17.3463254	4002.39152	0.0043	0.997	1.69E-09	count	1
ACTL7B	17.3463254	4002.391137	0.0043	0.997	1.69E-09	count	1
AL157938.2	17.3463254	4002.391137	0.0043	0.997	1.69E-09	count	1
OR56B1	17.3463252	4002.391219	0.0043	0.997	1.69E-09	count	1
TUB	17.3463249	4002.391274	0.0043	0.997	1.69E-09	count	1
AP000753.1	17.346325	4002.390946	0.0043	0.997	1.69E-09	count	1
MIR194-2HG	17.3463253	4002.391301	0.0043	0.997	1.69E-09	count	1
TRPC6	17.3463248	4002.391465	0.0043	0.997	1.69E-09	count	1
TP53AIP1	17.3463255	4002.391301	0.0043	0.997	1.69E-09	count	1
OLAH	17.346325	4002.390946	0.0043	0.997	1.69E-09	count	1
SH2D4B	17.346325	4002.391328	0.0043	0.997	1.69E-09	count	1
AL139124.1	17.3463252	4002.391383	0.0043	0.997	1.69E-09	count	1
AL158163.1	17.3463246	4002.39111	0.0043	0.997	1.69E-09	count	1
AC005832.4	17.3463252	4002.391602	0.0043	0.997	1.69E-09	count	1
PLBD1-AS1	17.3463252	4002.391219	0.0043	0.997	1.69E-09	count	1
AC022509.1	17.3463252	4002.391246	0.0043	0.997	1.69E-09	count	1
DENND5B-AS1	17.3463251	4002.390946	0.0043	0.997	1.69E-09	count	1
PKP2	17.3463253	4002.391055	0.0043	0.997	1.69E-09	count	1
LINC02156	17.3463252	4002.391301	0.0043	0.997	1.69E-09	count	1
AC024257.5	17.3463251	4002.391164	0.0043	0.997	1.69E-09	count	1
CYP27B1	17.3463252	4002.390891	0.0043	0.997	1.69E-09	count	1
WSCD2	17.346325	4002.391219	0.0043	0.997	1.69E-09	count	1
MYO1H	17.3463246	4002.39111	0.0043	0.997	1.69E-09	count	1
ACOD1	17.3463246	4002.39111	0.0043	0.997	1.69E-09	count	1

AL136964.1	17.3463254	4002.391602	0.0043	0.997	1.69E-09	count	1
AL161431.1	17.3463255	4002.391137	0.0043	0.997	1.69E-09	count	1
BCL2L2-PABPN1	17.3463251	4002.391164	0.0043	0.997	1.69E-09	count	1
TRIM9	17.346325	4002.390946	0.0043	0.997	1.69E-09	count	1
PWRN4	17.3463254	4002.391575	0.0043	0.997	1.69E-09	count	1
AC090970.2	17.3463251	4002.390946	0.0043	0.997	1.69E-09	count	1
AC009690.2	17.3463248	4002.391465	0.0043	0.997	1.69E-09	count	1
AC140725.1	17.3463254	4002.39152	0.0043	0.997	1.69E-09	count	1
Z69706.1	17.3463254	4002.39111	0.0043	0.997	1.69E-09	count	1
AC009065.3	17.3463255	4002.391438	0.0043	0.997	1.69E-09	count	1
TMC5	17.3463252	4002.391602	0.0043	0.997	1.69E-09	count	1
LCMT1-AS2	17.3463253	4002.391274	0.0043	0.997	1.69E-09	count	1
ATP2A1	17.3463253	4002.391164	0.0043	0.997	1.69E-09	count	1
AC092337.1	17.3463254	4002.391246	0.0043	0.997	1.69E-09	count	1
BEAN1	17.3463252	4002.391219	0.0043	0.997	1.69E-09	count	1
SCIMP	17.3463252	4002.391301	0.0043	0.997	1.69E-09	count	1
AC129492.2	17.3463253	4002.391411	0.0043	0.997	1.69E-09	count	1
AC005224.1	17.3463248	4002.391465	0.0043	0.997	1.69E-09	count	1
CCDC144NL-AS1	17.346325	4002.390946	0.0043	0.997	1.69E-09	count	1
CRYBA1	17.3463254	4002.39111	0.0043	0.997	1.69E-09	count	1
AC004231.1	17.3463254	4002.39111	0.0043	0.997	1.69E-09	count	1
AC007922.1	17.3463253	4002.391575	0.0043	0.997	1.69E-09	count	1
AL021396.1	17.3463252	4002.391219	0.0043	0.997	1.69E-09	count	1
AL050320.1	17.3463252	4002.390864	0.0043	0.997	1.69E-09	count	1
C20orf144	17.3463253	4002.391301	0.0043	0.997	1.69E-09	count	1
TLDC2	17.3463252	4002.391219	0.0043	0.997	1.69E-09	count	1
GTSF1L	17.346325	4002.391164	0.0043	0.997	1.69E-09	count	1
FAM209A	17.346325	4002.391164	0.0043	0.997	1.69E-09	count	1
AC012617.1	17.3463253	4002.391411	0.0043	0.997	1.69E-09	count	1
AC006213.3	17.3463251	4002.391192	0.0043	0.997	1.69E-09	count	1
NAPSA	17.3463251	4002.391192	0.0043	0.997	1.69E-09	count	1
SIGLEC14	17.3463253	4002.391164	0.0043	0.997	1.69E-09	count	1
AC245128.3	17.346325	4002.391219	0.0043	0.997	1.69E-09	count	1
AC000068.1	17.3463255	4002.391301	0.0043	0.997	1.69E-09	count	1
PWP2	17.3463252	4002.391055	0.0043	0.997	1.69E-09	count	1
FAM87B	17.3463249	4002.391438	0.0043	0.997	1.69E-09	count	1
PLCH2	17.3463249	4002.391055	0.0043	0.997	1.69E-09	count	1
Z97987.1	17.3463252	4002.391192	0.0043	0.997	1.69E-09	count	1
AL031731.1	17.3463245	4002.390973	0.0043	0.997	1.69E-09	count	1
SPATA21	17.3463244	4002.391028	0.0043	0.997	1.69E-09	count	1
AKR7A3	17.3463248	4002.391164	0.0043	0.997	1.69E-09	count	1
RHD	17.346325	4002.391438	0.0043	0.997	1.69E-09	count	1
AL513327.2	17.346325	4002.391137	0.0043	0.997	1.69E-09	count	1
ZMYM4-AS1	17.3463248	4002.391055	0.0043	0.997	1.69E-09	count	1
ORC1	17.3463248	4002.391055	0.0043	0.997	1.69E-09	count	1

ST6GALNAC5	17.3463251	4002.391028	0.0043	0.997	1.69E-09	count	1
LINC01362	17.346325	4002.391383	0.0043	0.997	1.69E-09	count	1
AC239798.2	17.3463249	4002.390918	0.0043	0.997	1.69E-09	count	1
AC239804.1	17.346325	4002.391356	0.0043	0.997	1.69E-09	count	1
THEM5	17.3463248	4002.391328	0.0043	0.997	1.69E-09	count	1
AL365181.3	17.346325	4002.391192	0.0043	0.997	1.69E-09	count	1
ATP1A2	17.346325	4002.391356	0.0043	0.997	1.69E-09	count	1
AL831711.1	17.3463249	4002.391328	0.0043	0.997	1.69E-09	count	1
CCDC181	17.346325	4002.391465	0.0043	0.997	1.69E-09	count	1
C1orf105	17.3463248	4002.391055	0.0043	0.997	1.69E-09	count	1
PAPPA2	17.346325	4002.391383	0.0043	0.997	1.69E-09	count	1
BRINP3	17.346325	4002.391246	0.0043	0.997	1.69E-09	count	1
TNNT2	17.3463248	4002.391055	0.0043	0.997	1.69E-09	count	1
ELF3	17.3463248	4002.391055	0.0043	0.997	1.69E-09	count	1
IL10	17.3463247	4002.39111	0.0043	0.997	1.69E-09	count	1
LINC01814	17.3463248	4002.391465	0.0043	0.997	1.69E-09	count	1
OSR1	17.3463248	4002.391055	0.0043	0.997	1.69E-09	count	1
AC012354.1	17.3463252	4002.39152	0.0043	0.997	1.69E-09	count	1
STPG4	17.3463248	4002.391164	0.0043	0.997	1.69E-09	count	1
AC011306.1	17.3463248	4002.390918	0.0043	0.997	1.69E-09	count	1
AC016747.2	17.3463249	4002.391055	0.0043	0.997	1.69E-09	count	1
AC008074.1	17.3463247	4002.391164	0.0043	0.997	1.69E-09	count	1
FAM178B	17.3463252	4002.391192	0.0043	0.997	1.69E-09	count	1
ZAP70	17.346325	4002.391246	0.0043	0.997	1.69E-09	count	1
LINC01114	17.3463252	4002.391192	0.0043	0.997	1.69E-09	count	1
AC016910.1	17.3463248	4002.391055	0.0043	0.997	1.69E-09	count	1
KCNJ3	17.3463248	4002.391055	0.0043	0.997	1.69E-09	count	1
KLHL41	17.3463249	4002.390918	0.0043	0.997	1.69E-09	count	1
CHRNA1	17.346325	4002.390864	0.0043	0.997	1.69E-09	count	1
AC009948.3	17.3463246	4002.391	0.0043	0.997	1.69E-09	count	1
ZNF804A	17.3463248	4002.391328	0.0043	0.997	1.69E-09	count	1
LINC01792	17.3463248	4002.391	0.0043	0.997	1.69E-09	count	1
ABCB6	17.3463249	4002.39111	0.0043	0.997	1.69E-09	count	1
AC053503.2	17.346325	4002.391192	0.0043	0.997	1.69E-09	count	1
PID1	17.346325	4002.391	0.0043	0.997	1.69E-09	count	1
KCNJ13	17.346325	4002.391192	0.0043	0.997	1.69E-09	count	1
SAG	17.3463252	4002.39111	0.0043	0.997	1.69E-09	count	1
GBX2	17.3463248	4002.391465	0.0043	0.997	1.69E-09	count	1
GPR35	17.3463246	4002.391028	0.0043	0.997	1.69E-09	count	1
AC090948.1	17.3463252	4002.391192	0.0043	0.997	1.69E-09	count	1
LINC02084	17.346325	4002.391246	0.0043	0.997	1.69E-09	count	1
AC112220.3	17.3463248	4002.390782	0.0043	0.997	1.69E-09	count	1
ARPP21	17.3463248	4002.391328	0.0043	0.997	1.69E-09	count	1
CDHR4	17.346325	4002.39152	0.0043	0.997	1.69E-09	count	1
IL17RB	17.3463251	4002.391	0.0043	0.997	1.69E-09	count	1

LINC02044	17.3463249	4002.390809	0.0043	0.997	1.69E-09	count	1
AC112503.1	17.3463248	4002.391055	0.0043	0.997	1.69E-09	count	1
AGTR1	17.3463252	4002.39111	0.0043	0.997	1.69E-09	count	1
AC078795.1	17.3463248	4002.391082	0.0043	0.997	1.69E-09	count	1
AC078795.3	17.3463252	4002.391411	0.0043	0.997	1.69E-09	count	1
AC007823.1	17.3463252	4002.391274	0.0043	0.997	1.69E-09	count	1
AC090425.2	17.346325	4002.391219	0.0043	0.997	1.69E-09	count	1
AC069431.1	17.3463252	4002.391246	0.0043	0.997	1.69E-09	count	1
HTR3C	17.3463248	4002.391411	0.0043	0.997	1.69E-09	count	1
LINC01840	17.3463247	4002.391219	0.0043	0.997	1.69E-09	count	1
AC139887.1	17.3463248	4002.390918	0.0043	0.997	1.69E-09	count	1
CFAP99	17.3463246	4002.391055	0.0043	0.997	1.69E-09	count	1
HGFAC	17.3463246	4002.391055	0.0043	0.997	1.69E-09	count	1
AC025539.1	17.3463248	4002.391055	0.0043	0.997	1.69E-09	count	1
NCAPG	17.3463244	4002.390918	0.0043	0.997	1.69E-09	count	1
RHOH	17.346325	4002.390973	0.0043	0.997	1.69E-09	count	1
AC106052.1	17.3463247	4002.391328	0.0043	0.997	1.69E-09	count	1
AC108067.1	17.3463252	4002.39111	0.0043	0.997	1.69E-09	count	1
CXXC4-AS1	17.3463252	4002.39111	0.0043	0.997	1.69E-09	count	1
LRIT3	17.3463247	4002.390918	0.0043	0.997	1.69E-09	count	1
LINC01091	17.3463253	4002.391219	0.0043	0.997	1.69E-09	count	1
AC097658.2	17.3463249	4002.390918	0.0043	0.997	1.69E-09	count	1
SMARCA5-AS1	17.346325	4002.390836	0.0043	0.997	1.69E-09	count	1
AC110813.1	17.3463252	4002.39111	0.0043	0.997	1.69E-09	count	1
AC079298.3	17.3463249	4002.391356	0.0043	0.997	1.69E-09	count	1
LINC02269	17.3463252	4002.390973	0.0043	0.997	1.69E-09	count	1
HPGD	17.3463248	4002.391164	0.0043	0.997	1.69E-09	count	1
AC093801.1	17.3463251	4002.391602	0.0043	0.997	1.69E-09	count	1
CDH18	17.3463248	4002.391164	0.0043	0.997	1.69E-09	count	1
PURPL	17.3463246	4002.391055	0.0043	0.997	1.69E-09	count	1
AC114956.1	17.3463248	4002.391164	0.0043	0.997	1.69E-09	count	1
AC008966.2	17.3463249	4002.391028	0.0043	0.997	1.69E-09	count	1
LINC02101	17.3463253	4002.391164	0.0043	0.997	1.69E-09	count	1
GAPT	17.3463248	4002.390864	0.0043	0.997	1.69E-09	count	1
AC026725.1	17.3463252	4002.391	0.0043	0.997	1.69E-09	count	1
LINC01949	17.3463248	4002.391055	0.0043	0.997	1.69E-09	count	1
AC010395.1	17.3463247	4002.391028	0.0043	0.997	1.69E-09	count	1
CATSPER3	17.3463252	4002.391192	0.0043	0.997	1.69E-09	count	1
AC008438.1	17.3463248	4002.390918	0.0043	0.997	1.69E-09	count	1
PCDHA3	17.346325	4002.391328	0.0043	0.997	1.69E-09	count	1
AL445309.1	17.3463247	4002.391246	0.0043	0.997	1.69E-09	count	1
ELOVL2	17.3463248	4002.391055	0.0043	0.997	1.69E-09	count	1
AL031775.2	17.346325	4002.39152	0.0043	0.997	1.69E-09	count	1
POM121L2	17.3463252	4002.391274	0.0043	0.997	1.69E-09	count	1
DDX39B-AS1	17.346325	4002.390973	0.0043	0.997	1.69E-09	count	1

TCP11	17.3463252	4002.39111	0.0043	0.997	1.69E-09	count	1
ARMC12	17.346325	4002.391164	0.0043	0.997	1.69E-09	count	1
CRIP3	17.346325	4002.391137	0.0043	0.997	1.69E-09	count	1
POLH-AS1	17.3463252	4002.39111	0.0043	0.997	1.69E-09	count	1
AL359715.2	17.346325	4002.391137	0.0043	0.997	1.69E-09	count	1
SLC16A10	17.3463249	4002.391137	0.0043	0.997	1.69E-09	count	1
WISP3	17.3463248	4002.390864	0.0043	0.997	1.69E-09	count	1
AL034349.1	17.3463246	4002.390727	0.0043	0.997	1.69E-09	count	1
ULBP3	17.3463248	4002.391055	0.0043	0.997	1.69E-09	count	1
C6orf99	17.3463252	4002.391192	0.0043	0.997	1.69E-09	count	1
CCR6	17.3463252	4002.39111	0.0043	0.997	1.69E-09	count	1
KIF25-AS1	17.3463248	4002.391055	0.0043	0.997	1.69E-09	count	1
AC073957.1	17.3463248	4002.390973	0.0043	0.997	1.69E-09	count	1
ANKRD61	17.3463249	4002.391438	0.0043	0.997	1.69E-09	count	1
GRID2IP	17.3463251	4002.391465	0.0043	0.997	1.69E-09	count	1
AC010082.1	17.3463248	4002.391164	0.0043	0.997	1.69E-09	count	1
ANLN	17.3463249	4002.391301	0.0043	0.997	1.69E-09	count	1
GCK	17.3463248	4002.391055	0.0043	0.997	1.69E-09	count	1
DDC	17.346325	4002.391	0.0043	0.997	1.69E-09	count	1
AC068533.4	17.3463248	4002.390864	0.0043	0.997	1.69E-09	count	1
SPDYE5	17.3463248	4002.391246	0.0043	0.997	1.69E-09	count	1
HEPACAM2	17.346325	4002.391	0.0043	0.997	1.69E-09	count	1
ASB4	17.3463251	4002.391465	0.0043	0.997	1.69E-09	count	1
EFCAB10	17.3463248	4002.391055	0.0043	0.997	1.69E-09	count	1
LINC01393	17.346325	4002.391219	0.0043	0.997	1.69E-09	count	1
AC006333.1	17.3463251	4002.391301	0.0043	0.997	1.69E-09	count	1
STRIP2	17.3463249	4002.391192	0.0043	0.997	1.69E-09	count	1
AC091736.1	17.3463252	4002.391383	0.0043	0.997	1.69E-09	count	1
AC115618.1	17.3463252	4002.391192	0.0043	0.997	1.69E-09	count	1
CCNB3	17.346325	4002.391137	0.0043	0.997	1.69E-09	count	1
LINC00891	17.3463249	4002.391	0.0043	0.997	1.69E-09	count	1
NCBP2L	17.3463248	4002.391465	0.0043	0.997	1.69E-09	count	1
LHFPL1	17.3463252	4002.391164	0.0043	0.997	1.69E-09	count	1
SH2D1A	17.3463248	4002.391055	0.0043	0.997	1.69E-09	count	1
SASH3	17.3463252	4002.391192	0.0043	0.997	1.69E-09	count	1
ARHGAP36	17.3463252	4002.39111	0.0043	0.997	1.69E-09	count	1
LINC00632	17.346325	4002.391383	0.0043	0.997	1.69E-09	count	1
PNMA6A	17.3463248	4002.391328	0.0043	0.997	1.69E-09	count	1
AC245140.1	17.346325	4002.391192	0.0043	0.997	1.69E-09	count	1
SMIM9	17.3463251	4002.391	0.0043	0.997	1.69E-09	count	1
AC129915.3	17.3463248	4002.391274	0.0043	0.997	1.69E-09	count	1
KBTBD11-OT1	17.3463251	4002.39111	0.0043	0.997	1.69E-09	count	1
FGF20	17.3463252	4002.391192	0.0043	0.997	1.69E-09	count	1
AC027117.2	17.3463248	4002.391246	0.0043	0.997	1.69E-09	count	1
FGF17	17.3463246	4002.390918	0.0043	0.997	1.69E-09	count	1

LGI3	17.3463248	4002.391438	0.0043	0.997	1.69E-09	count	1
SCARA5	17.3463248	4002.391246	0.0043	0.997	1.69E-09	count	1
AC022915.2	17.3463252	4002.391575	0.0043	0.997	1.69E-09	count	1
AC079089.1	17.3463252	4002.39111	0.0043	0.997	1.69E-09	count	1
AC034114.2	17.3463247	4002.390973	0.0043	0.997	1.69E-09	count	1
SAMD12-AS1	17.3463249	4002.391055	0.0043	0.997	1.69E-09	count	1
MAPK15	17.346325	4002.391055	0.0043	0.997	1.69E-09	count	1
LINC01388	17.3463246	4002.390918	0.0043	0.997	1.69E-09	count	1
AL359076.1	17.3463249	4002.391137	0.0043	0.997	1.69E-09	count	1
ANKRD18B	17.3463249	4002.391055	0.0043	0.997	1.69E-09	count	1
AL354989.1	17.3463252	4002.391328	0.0043	0.997	1.69E-09	count	1
KIF24	17.3463248	4002.391164	0.0043	0.997	1.69E-09	count	1
CNTFR	17.3463248	4002.391246	0.0043	0.997	1.69E-09	count	1
AL591368.1	17.3463248	4002.391246	0.0043	0.997	1.69E-09	count	1
AL353764.1	17.3463249	4002.390918	0.0043	0.997	1.69E-09	count	1
RNF183	17.346325	4002.391356	0.0043	0.997	1.69E-09	count	1
CFAP157	17.3463247	4002.391192	0.0043	0.997	1.69E-09	count	1
AL158151.4	17.3463248	4002.391	0.0043	0.997	1.69E-09	count	1
BX649632.1	17.3463249	4002.391137	0.0043	0.997	1.69E-09	count	1
AC136475.3	17.3463249	4002.391301	0.0043	0.997	1.69E-09	count	1
IFITM10	17.3463251	4002.391219	0.0043	0.997	1.69E-09	count	1
OLFML1	17.346325	4002.391383	0.0043	0.997	1.69E-09	count	1
AMPD3	17.3463248	4002.391	0.0043	0.997	1.69E-09	count	1
LINC02547	17.3463249	4002.391356	0.0043	0.997	1.69E-09	count	1
ABTB2	17.3463252	4002.39111	0.0043	0.997	1.69E-09	count	1
F2	17.3463248	4002.391411	0.0043	0.997	1.69E-09	count	1
MYBPC3	17.3463246	4002.391137	0.0043	0.997	1.69E-09	count	1
CCDC87	17.346325	4002.390973	0.0043	0.997	1.69E-09	count	1
AC004923.4	17.3463248	4002.391301	0.0043	0.997	1.69E-09	count	1
DNAJB13	17.3463248	4002.391	0.0043	0.997	1.69E-09	count	1
AP002812.2	17.3463251	4002.391356	0.0043	0.997	1.69E-09	count	1
CNTN5	17.3463249	4002.391164	0.0043	0.997	1.69E-09	count	1
APOA1-AS	17.3463252	4002.391192	0.0043	0.997	1.69E-09	count	1
SCN4B	17.346325	4002.391383	0.0043	0.997	1.69E-09	count	1
AP004147.1	17.346325	4002.391219	0.0043	0.997	1.69E-09	count	1
AP003501.1	17.3463254	4002.391246	0.0043	0.997	1.69E-09	count	1
GATA3-AS1	17.346325	4002.391383	0.0043	0.997	1.69E-09	count	1
MKX	17.3463249	4002.391356	0.0043	0.997	1.69E-09	count	1
ANTXRL	17.3463252	4002.390864	0.0043	0.997	1.69E-09	count	1
LINC00843	17.3463249	4002.391	0.0043	0.997	1.69E-09	count	1
ATOH7	17.3463248	4002.390918	0.0043	0.997	1.69E-09	count	1
AL450311.2	17.3463246	4002.391438	0.0043	0.997	1.69E-09	count	1
MSS51	17.3463251	4002.390891	0.0043	0.997	1.69E-09	count	1
AL138767.1	17.346325	4002.391219	0.0043	0.997	1.69E-09	count	1
AL389885.1	17.3463248	4002.391164	0.0043	0.997	1.69E-09	count	1

CYP2C8	17.3463248	4002.391246	0.0043	0.997	1.69E-09	count	1
HOGA1	17.3463248	4002.391192	0.0043	0.997	1.69E-09	count	1
HPSE2	17.346325	4002.391356	0.0043	0.997	1.69E-09	count	1
EDRF1-AS1	17.3463251	4002.390891	0.0043	0.997	1.69E-09	count	1
AL162274.1	17.3463252	4002.391	0.0043	0.997	1.69E-09	count	1
AL451069.2	17.3463248	4002.391055	0.0043	0.997	1.69E-09	count	1
AC006064.1	17.3463252	4002.391192	0.0043	0.997	1.69E-09	count	1
PIANP	17.346325	4002.391356	0.0043	0.997	1.69E-09	count	1
AC125494.2	17.346325	4002.391137	0.0043	0.997	1.69E-09	count	1
AC092111.2	17.3463247	4002.391383	0.0043	0.997	1.69E-09	count	1
TAS2R43	17.346325	4002.391246	0.0043	0.997	1.69E-09	count	1
AC010168.1	17.3463252	4002.39111	0.0043	0.997	1.69E-09	count	1
AC092794.1	17.3463252	4002.39111	0.0043	0.997	1.69E-09	count	1
C12orf54	17.3463251	4002.391082	0.0043	0.997	1.69E-09	count	1
HIGD1C	17.3463252	4002.39111	0.0043	0.997	1.69E-09	count	1
AC021072.1	17.3463248	4002.390918	0.0043	0.997	1.69E-09	count	1
PPP1R1A	17.346325	4002.391137	0.0043	0.997	1.69E-09	count	1
AC009779.3	17.3463247	4002.391301	0.0043	0.997	1.69E-09	count	1
SLC39A5	17.3463252	4002.39111	0.0043	0.997	1.69E-09	count	1
NXPH4	17.3463252	4002.391192	0.0043	0.997	1.69E-09	count	1
TPH2	17.3463248	4002.390864	0.0043	0.997	1.69E-09	count	1
AC011611.4	17.346325	4002.390836	0.0043	0.997	1.69E-09	count	1
AC107032.2	17.3463249	4002.391	0.0043	0.997	1.69E-09	count	1
AC124784.1	17.346325	4002.390891	0.0043	0.997	1.69E-09	count	1
C12orf50	17.3463247	4002.391137	0.0043	0.997	1.69E-09	count	1
AC090001.1	17.3463246	4002.391	0.0043	0.997	1.69E-09	count	1
AC026367.2	17.3463249	4002.391164	0.0043	0.997	1.69E-09	count	1
LINC00507	17.3463251	4002.391356	0.0043	0.997	1.69E-09	count	1
AC131212.1	17.3463248	4002.39111	0.0043	0.997	1.69E-09	count	1
PSPC1-AS2	17.3463251	4002.391	0.0043	0.997	1.69E-09	count	1
GJB6	17.3463251	4002.391028	0.0043	0.997	1.69E-09	count	1
AL138966.2	17.3463249	4002.391246	0.0043	0.997	1.69E-09	count	1
SERPINE3	17.3463247	4002.391246	0.0043	0.997	1.69E-09	count	1
AL445209.1	17.3463252	4002.391082	0.0043	0.997	1.69E-09	count	1
ATP11A-AS1	17.3463252	4002.391219	0.0043	0.997	1.69E-09	count	1
RNASE2	17.3463252	4002.391328	0.0043	0.997	1.69E-09	count	1
TRAV22	17.346325	4002.39111	0.0043	0.997	1.69E-09	count	1
AL136018.1	17.346325	4002.391192	0.0043	0.997	1.69E-09	count	1
AL121790.1	17.346325	4002.391356	0.0043	0.997	1.69E-09	count	1
AL049870.3	17.3463246	4002.391137	0.0043	0.997	1.69E-09	count	1
LINC00640	17.3463252	4002.39111	0.0043	0.997	1.69E-09	count	1
ADAM20	17.3463252	4002.391028	0.0043	0.997	1.69E-09	count	1
RGS6	17.346325	4002.391028	0.0043	0.997	1.69E-09	count	1
AF111169.3	17.3463251	4002.391411	0.0043	0.997	1.69E-09	count	1
DIO2-AS1	17.3463252	4002.391164	0.0043	0.997	1.69E-09	count	1

BCL11B	17.3463249	4002.391164	0.0043	0.997	1.69E-09	count	1
PLD4	17.3463252	4002.39111	0.0043	0.997	1.69E-09	count	1
AC012414.5	17.3463249	4002.390809	0.0043	0.997	1.69E-09	count	1
AC087473.1	17.3463248	4002.390918	0.0043	0.997	1.69E-09	count	1
C15orf62	17.3463248	4002.391055	0.0043	0.997	1.69E-09	count	1
AC020661.1	17.3463252	4002.39111	0.0043	0.997	1.69E-09	count	1
JMJD7-PLA2G4B	17.3463248	4002.391	0.0043	0.997	1.69E-09	count	1
AC084757.4	17.3463252	4002.391192	0.0043	0.997	1.69E-09	count	1
PYGO1	17.3463252	4002.39111	0.0043	0.997	1.69E-09	count	1
IGDCC4	17.3463248	4002.391192	0.0043	0.997	1.69E-09	count	1
LINC02204	17.3463252	4002.39111	0.0043	0.997	1.69E-09	count	1
AC090826.1	17.346325	4002.391055	0.0043	0.997	1.69E-09	count	1
CYP1A1	17.3463249	4002.391055	0.0043	0.997	1.69E-09	count	1
AC105020.1	17.3463252	4002.39111	0.0043	0.997	1.69E-09	count	1
TMEM266	17.3463248	4002.391055	0.0043	0.997	1.69E-09	count	1
AC021483.2	17.3463248	4002.391055	0.0043	0.997	1.69E-09	count	1
SH3GL3	17.3463254	4002.391246	0.0043	0.997	1.69E-09	count	1
AC087286.1	17.3463246	4002.390782	0.0043	0.997	1.69E-09	count	1
TTLL13P	17.3463252	4002.391	0.0043	0.997	1.69E-09	count	1
AC021422.1	17.3463252	4002.391192	0.0043	0.997	1.69E-09	count	1
AC090907.1	17.3463252	4002.391192	0.0043	0.997	1.69E-09	count	1
AL022341.1	17.3463246	4002.390918	0.0043	0.997	1.69E-09	count	1
AC093525.3	17.3463248	4002.391055	0.0043	0.997	1.69E-09	count	1
AC141586.2	17.3463249	4002.391137	0.0043	0.997	1.69E-09	count	1
AC004233.4	17.3463248	4002.391055	0.0043	0.997	1.69E-09	count	1
ZSCAN10	17.3463248	4002.391055	0.0043	0.997	1.69E-09	count	1
LINC02177	17.3463251	4002.391356	0.0043	0.997	1.69E-09	count	1
NPIPB6	17.3463245	4002.391246	0.0043	0.997	1.69E-09	count	1
MYLK3	17.3463246	4002.391	0.0043	0.997	1.69E-09	count	1
AC018845.3	17.3463249	4002.391356	0.0043	0.997	1.69E-09	count	1
ITFG1-AS1	17.346325	4002.391328	0.0043	0.997	1.69E-09	count	1
NKD1	17.3463248	4002.391246	0.0043	0.997	1.69E-09	count	1
AC007728.2	17.3463248	4002.391028	0.0043	0.997	1.69E-09	count	1
AC106779.1	17.3463247	4002.391301	0.0043	0.997	1.69E-09	count	1
AC018552.3	17.3463249	4002.390727	0.0043	0.997	1.69E-09	count	1
LINC01572	17.3463248	4002.391164	0.0043	0.997	1.69E-09	count	1
AC009108.3	17.3463247	4002.391137	0.0043	0.997	1.69E-09	count	1
AC010538.1	17.3463249	4002.391301	0.0043	0.997	1.69E-09	count	1
AC116914.2	17.3463251	4002.391246	0.0043	0.997	1.69E-09	count	1
TEKT1	17.3463252	4002.39111	0.0043	0.997	1.69E-09	count	1
AC135178.1	17.3463253	4002.391192	0.0043	0.997	1.69E-09	count	1
AC104982.1	17.3463248	4002.391055	0.0043	0.997	1.69E-09	count	1
AC104984.1	17.3463249	4002.391274	0.0043	0.997	1.69E-09	count	1
TMEM132E	17.3463248	4002.39111	0.0043	0.997	1.69E-09	count	1
AC004223.4	17.3463248	4002.391055	0.0043	0.997	1.69E-09	count	1

UNC45B	17.3463248	4002.391055	0.0043	0.997	1.69E-09	count	1
RDM1	17.3463244	4002.390782	0.0043	0.997	1.69E-09	count	1
CCL15-CCL14	17.3463248	4002.391192	0.0043	0.997	1.69E-09	count	1
IKZF3	17.3463248	4002.391055	0.0043	0.997	1.69E-09	count	1
HAP1	17.346325	4002.391328	0.0043	0.997	1.69E-09	count	1
ASB16	17.3463246	4002.391055	0.0043	0.997	1.69E-09	count	1
SPATA32	17.3463248	4002.391055	0.0043	0.997	1.69E-09	count	1
CR936218.1	17.3463244	4002.390918	0.0043	0.997	1.69E-09	count	1
AC015795.1	17.3463253	4002.391465	0.0043	0.997	1.69E-09	count	1
AC004707.1	17.3463252	4002.39111	0.0043	0.997	1.69E-09	count	1
AC015845.2	17.3463248	4002.391082	0.0043	0.997	1.69E-09	count	1
RBFOX3	17.3463251	4002.391	0.0043	0.997	1.69E-09	count	1
AC110285.3	17.3463251	4002.391219	0.0043	0.997	1.69E-09	count	1
AC129510.2	17.3463248	4002.391055	0.0043	0.997	1.69E-09	count	1
AP000919.4	17.3463248	4002.391164	0.0043	0.997	1.69E-09	count	1
AP001496.1	17.3463248	4002.391164	0.0043	0.997	1.69E-09	count	1
AP001793.2	17.3463251	4002.391082	0.0043	0.997	1.69E-09	count	1
AC090772.3	17.3463248	4002.391055	0.0043	0.997	1.69E-09	count	1
DSCAS	17.3463246	4002.391055	0.0043	0.997	1.69E-09	count	1
DTNA	17.346325	4002.391356	0.0043	0.997	1.69E-09	count	1
DCC	17.3463246	4002.391055	0.0043	0.997	1.69E-09	count	1
AL049712.1	17.3463246	4002.390918	0.0043	0.997	1.69E-09	count	1
AL121890.3	17.3463248	4002.390918	0.0043	0.997	1.69E-09	count	1
AL121890.2	17.3463248	4002.391028	0.0043	0.997	1.69E-09	count	1
SLC24A3	17.3463249	4002.391164	0.0043	0.997	1.69E-09	count	1
FAM182B	17.3463252	4002.391164	0.0043	0.997	1.69E-09	count	1
AL110115.2	17.3463249	4002.391219	0.0043	0.997	1.69E-09	count	1
MYH7B	17.3463249	4002.390918	0.0043	0.997	1.69E-09	count	1
AL035420.2	17.3463246	4002.391055	0.0043	0.997	1.69E-09	count	1
VSTM2L	17.3463247	4002.391328	0.0043	0.997	1.69E-09	count	1
HNF4A	17.3463246	4002.390782	0.0043	0.997	1.69E-09	count	1
STK4-AS1	17.3463248	4002.391164	0.0043	0.997	1.69E-09	count	1
TNNC2	17.3463249	4002.390918	0.0043	0.997	1.69E-09	count	1
AL096828.3	17.3463249	4002.391246	0.0043	0.997	1.69E-09	count	1
AL121829.2	17.3463248	4002.391055	0.0043	0.997	1.69E-09	count	1
LINC00266-1	17.3463247	4002.391	0.0043	0.997	1.69E-09	count	1
ZNF556	17.346325	4002.391602	0.0043	0.997	1.69E-09	count	1
AC007292.1	17.3463248	4002.391055	0.0043	0.997	1.69E-09	count	1
AC011444.1	17.3463246	4002.391192	0.0043	0.997	1.69E-09	count	1
AC011471.2	17.346325	4002.391602	0.0043	0.997	1.69E-09	count	1
MCEMP1	17.3463248	4002.391	0.0043	0.997	1.69E-09	count	1
OR7C1	17.346325	4002.390809	0.0043	0.997	1.69E-09	count	1
CCDC194	17.3463246	4002.39111	0.0043	0.997	1.69E-09	count	1
AC010319.3	17.346325	4002.391328	0.0043	0.997	1.69E-09	count	1
AC007192.2	17.3463248	4002.391219	0.0043	0.997	1.69E-09	count	1

TM6SF2	17.3463252	4002.39111	0.0043	0.997	1.69E-09	count	1
AC010300.1	17.3463247	4002.391028	0.0043	0.997	1.69E-09	count	1
AC011443.1	17.3463249	4002.391328	0.0043	0.997	1.69E-09	count	1
GNG8	17.346325	4002.391356	0.0043	0.997	1.69E-09	count	1
AC010519.1	17.3463249	4002.391493	0.0043	0.997	1.69E-09	count	1
NOP53-AS1	17.3463249	4002.390727	0.0043	0.997	1.69E-09	count	1
SLC17A7	17.3463248	4002.391328	0.0043	0.997	1.69E-09	count	1
ZNF534	17.3463248	4002.391055	0.0043	0.997	1.69E-09	count	1
COX6B2	17.3463251	4002.391082	0.0043	0.997	1.69E-09	count	1
AC010522.1	17.3463248	4002.391055	0.0043	0.997	1.69E-09	count	1
LINC00266-4P	17.346325	4002.391246	0.0043	0.997	1.69E-09	count	1
AC016027.1	17.3463248	4002.391028	0.0043	0.997	1.69E-09	count	1
PRODH	17.346325	4002.391055	0.0043	0.997	1.69E-09	count	1
AC007663.3	17.3463252	4002.39111	0.0043	0.997	1.69E-09	count	1
AC245060.6	17.3463252	4002.391192	0.0043	0.997	1.69E-09	count	1
LINC02556	17.346325	4002.391	0.0043	0.997	1.69E-09	count	1
VPREB3	17.3463249	4002.391055	0.0043	0.997	1.69E-09	count	1
AC253536.6	17.3463252	4002.391328	0.0043	0.997	1.69E-09	count	1
SUSD2	17.3463248	4002.391411	0.0043	0.997	1.69E-09	count	1
GAL3ST1	17.346325	4002.391246	0.0043	0.997	1.69E-09	count	1
IL2RB	17.3463244	4002.391028	0.0043	0.997	1.69E-09	count	1
Z83847.1	17.3463252	4002.39111	0.0043	0.997	1.69E-09	count	1
AL022238.4	17.3463252	4002.39111	0.0043	0.997	1.69E-09	count	1
PHF21B	17.3463247	4002.390918	0.0043	0.997	1.69E-09	count	1
AL023802.1	17.3463248	4002.391055	0.0043	0.997	1.69E-09	count	1
FP236383.2	17.3463249	4002.391055	0.0043	0.997	1.69E-09	count	1
LINC01668	17.3463252	4002.39111	0.0043	0.997	1.69E-09	count	1
LINC01679	17.3463252	4002.39111	0.0043	0.997	1.69E-09	count	1
AP001059.3	17.3463252	4002.39111	0.0043	0.997	1.69E-09	count	1
ADGRB2	17.3463246	4002.390836	0.0043	0.997	1.69E-09	count	1
AL139158.2	17.3463243	4002.390946	0.0043	0.997	1.69E-09	count	1
AL450468.2	17.3463247	4002.390809	0.0043	0.997	1.69E-09	count	1
LINC01719	17.3463247	4002.391301	0.0043	0.997	1.69E-09	count	1
CGN	17.3463244	4002.390918	0.0043	0.997	1.69E-09	count	1
LRRC71	17.3463244	4002.3907	0.0043	0.997	1.69E-09	count	1
CCDC190	17.3463249	4002.390918	0.0043	0.997	1.69E-09	count	1
LAX1	17.3463247	4002.391274	0.0043	0.997	1.69E-09	count	1
AL359921.1	17.3463247	4002.390809	0.0043	0.997	1.69E-09	count	1
VSNL1	17.3463246	4002.39111	0.0043	0.997	1.69E-09	count	1
ABCG5	17.3463245	4002.390918	0.0043	0.997	1.69E-09	count	1
AC007389.5	17.3463247	4002.390946	0.0043	0.997	1.69E-09	count	1
DQX1	17.3463246	4002.3907	0.0043	0.997	1.69E-09	count	1
SH2D6	17.3463245	4002.390782	0.0043	0.997	1.69E-09	count	1
POU3F3	17.3463243	4002.390946	0.0043	0.997	1.69E-09	count	1
AC105760.1	17.3463242	4002.39111	0.0043	0.997	1.69E-09	count	1

ERFE	17.3463246	4002.390836	0.0043	0.997	1.69E-09	count	1
AC098479.1	17.3463247	4002.39111	0.0043	0.997	1.69E-09	count	1
LINC00870	17.3463243	4002.390946	0.0043	0.997	1.69E-09	count	1
PLCXD2	17.3463247	4002.391137	0.0043	0.997	1.69E-09	count	1
TIGIT	17.3463247	4002.390809	0.0043	0.997	1.69E-09	count	1
TP63	17.3463247	4002.390809	0.0043	0.997	1.69E-09	count	1
AC027607.1	17.3463248	4002.391164	0.0043	0.997	1.69E-09	count	1
AC096734.2	17.3463246	4002.391055	0.0043	0.997	1.69E-09	count	1
GYPA	17.3463247	4002.390836	0.0043	0.997	1.69E-09	count	1
C5orf67	17.3463246	4002.391055	0.0043	0.997	1.69E-09	count	1
LINC01170	17.3463247	4002.391082	0.0043	0.997	1.69E-09	count	1
MATR3	17.3463246	4002.391137	0.0043	0.997	1.69E-09	count	1
LTA	17.3463246	4002.390782	0.0043	0.997	1.69E-09	count	1
GUCA1B	17.3463245	4002.390973	0.0043	0.997	1.69E-09	count	1
AL356234.2	17.3463244	4002.391219	0.0043	0.997	1.69E-09	count	1
ECT2L	17.3463249	4002.391301	0.0043	0.997	1.69E-09	count	1
LINC01447	17.3463245	4002.391028	0.0043	0.997	1.69E-09	count	1
AC073842.2	17.3463247	4002.391082	0.0043	0.997	1.69E-09	count	1
AC092849.1	17.3463247	4002.39111	0.0043	0.997	1.69E-09	count	1
AC073314.1	17.3463244	4002.391082	0.0043	0.997	1.69E-09	count	1
VCX3B	17.3463244	4002.391082	0.0043	0.997	1.69E-09	count	1
MAP3K15	17.3463248	4002.391164	0.0043	0.997	1.69E-09	count	1
CENPI	17.3463247	4002.391246	0.0043	0.997	1.69E-09	count	1
ARMCX3-AS1	17.3463248	4002.391164	0.0043	0.997	1.69E-09	count	1
LINC00894	17.3463246	4002.391246	0.0043	0.997	1.69E-09	count	1
AC037459.4	17.3463246	4002.391055	0.0043	0.997	1.69E-09	count	1
AC026979.1	17.3463245	4002.390946	0.0043	0.997	1.69E-09	count	1
AC068389.4	17.3463248	4002.391028	0.0043	0.997	1.69E-09	count	1
CLVS1	17.3463248	4002.391246	0.0043	0.997	1.69E-09	count	1
GPT	17.3463246	4002.390782	0.0043	0.997	1.69E-09	count	1
ORM1	17.3463247	4002.390809	0.0043	0.997	1.69E-09	count	1
LCN12	17.3463244	4002.390891	0.0043	0.997	1.69E-09	count	1
SLC1A2	17.3463246	4002.391137	0.0043	0.997	1.69E-09	count	1
AP003086.3	17.3463245	4002.391219	0.0043	0.997	1.69E-09	count	1
AP001825.1	17.3463246	4002.391055	0.0043	0.997	1.69E-09	count	1
AP000936.1	17.3463244	4002.390918	0.0043	0.997	1.69E-09	count	1
NEBL-AS1	17.3463248	4002.391246	0.0043	0.997	1.69E-09	count	1
AL645634.2	17.3463244	4002.391	0.0043	0.997	1.69E-09	count	1
LINC00595	17.3463249	4002.39111	0.0043	0.997	1.69E-09	count	1
DNMBP-AS1	17.3463248	4002.391082	0.0043	0.997	1.69E-09	count	1
BCL2L14	17.3463245	4002.391465	0.0043	0.997	1.69E-09	count	1
AC087257.1	17.3463247	4002.391082	0.0043	0.997	1.69E-09	count	1
AC084032.1	17.3463244	4002.390891	0.0043	0.997	1.69E-09	count	1
AC010205.1	17.3463248	4002.391465	0.0043	0.997	1.69E-09	count	1
AL590096.1	17.3463247	4002.39111	0.0043	0.997	1.69E-09	count	1

AL161668.3	17.3463246	4002.3907	0.0043	0.997	1.69E-09	count	1
AL121594.1	17.3463246	4002.391055	0.0043	0.997	1.69E-09	count	1
AL162311.1	17.3463248	4002.391164	0.0043	0.997	1.69E-09	count	1
GOLGA8S	17.3463244	4002.390809	0.0043	0.997	1.69E-09	count	1
CCNB2	17.3463248	4002.391356	0.0043	0.997	1.69E-09	count	1
AC036108.2	17.3463246	4002.390918	0.0043	0.997	1.69E-09	count	1
AL031705.1	17.3463246	4002.391	0.0043	0.997	1.69E-09	count	1
AC009065.8	17.3463245	4002.390946	0.0043	0.997	1.69E-09	count	1
MTRNR2L4	17.3463248	4002.391356	0.0043	0.997	1.69E-09	count	1
AC138932.5	17.3463244	4002.391164	0.0043	0.997	1.69E-09	count	1
LINC02137	17.3463247	4002.390946	0.0043	0.997	1.69E-09	count	1
AC009095.1	17.3463246	4002.391137	0.0043	0.997	1.69E-09	count	1
P2RX1	17.3463241	4002.390645	0.0043	0.997	1.69E-09	count	1
CLEC10A	17.3463242	4002.39111	0.0043	0.997	1.69E-09	count	1
AC005695.3	17.3463247	4002.39111	0.0043	0.997	1.69E-09	count	1
AC127537.1	17.3463244	4002.391137	0.0043	0.997	1.69E-09	count	1
AC006441.4	17.3463247	4002.390918	0.0043	0.997	1.69E-09	count	1
AC005180.1	17.3463248	4002.391246	0.0043	0.997	1.69E-09	count	1
AC004687.1	17.3463245	4002.390864	0.0043	0.997	1.69E-09	count	1
GPR142	17.3463244	4002.391055	0.0043	0.997	1.69E-09	count	1
LINC01254	17.3463246	4002.391	0.0043	0.997	1.69E-09	count	1
CIDEA	17.3463247	4002.391301	0.0043	0.997	1.69E-09	count	1
AL121753.1	17.3463246	4002.390782	0.0043	0.997	1.69E-09	count	1
FO393401.1	17.3463247	4002.390946	0.0043	0.997	1.69E-09	count	1
BIRC7	17.3463246	4002.3907	0.0043	0.997	1.69E-09	count	1
MATK	17.3463246	4002.390809	0.0043	0.997	1.69E-09	count	1
AC022098.2	17.346325	4002.391137	0.0043	0.997	1.69E-09	count	1
LINC00664	17.3463246	4002.391055	0.0043	0.997	1.69E-09	count	1
AC010271.2	17.3463249	4002.391137	0.0043	0.997	1.69E-09	count	1
AC011466.1	17.3463244	4002.391164	0.0043	0.997	1.69E-09	count	1
KIR2DL4	17.3463249	4002.39111	0.0043	0.997	1.69E-09	count	1
BRSK1	17.3463244	4002.390891	0.0043	0.997	1.69E-09	count	1
AL022313.2	17.3463249	4002.391356	0.0043	0.997	1.69E-09	count	1
AP001432.1	17.3463246	4002.390782	0.0043	0.997	1.69E-09	count	1
LINC00313	17.3463249	4002.390918	0.0043	0.997	1.69E-09	count	1
LINC00844	18.92534	3970.265366	0.0048	0.996	1.69E-09	count	1
LHFPL4	18.9253393	3970.26426	0.0048	0.996	1.69E-09	count	1
NANOGNB	18.9253395	3970.264813	0.0048	0.996	1.69E-09	count	1
DNAH10OS	18.9253395	3970.264813	0.0048	0.996	1.69E-09	count	1
AC106886.5	18.9253395	3970.264813	0.0048	0.996	1.69E-09	count	1
AC090617.3	18.9253393	3970.264979	0.0048	0.996	1.69E-09	count	1
AD000671.3	18.9253388	3970.264094	0.0048	0.996	1.69E-09	count	1
KCNK17	18.9253389	3970.265919	0.0048	0.996	1.69E-09	count	1
AL138899.1	18.9253384	3970.2652	0.0048	0.996	1.69E-09	count	1
AK5	18.9253383	3970.264758	0.0048	0.996	1.69E-09	count	1

IGKV1-5	18.9253379	3970.264371	0.0048	0.996	1.69E-09	count	1
C12orf42	18.9253379	3970.264371	0.0048	0.996	1.69E-09	count	1
AC010422.2	18.9253381	3970.264758	0.0048	0.996	1.69E-09	count	1
LINC01562	18.9231442	2800.276759	0.0068	0.995	1.69E-09	count	1
DSCAML1	18.9231441	2800.276837	0.0068	0.995	1.69E-09	count	1
IGLV1-51	18.9231441	2800.276837	0.0068	0.995	1.69E-09	count	1
AC009226.1	18.9231426	2800.276214	0.0068	0.995	1.69E-09	count	1
SIDT1	18.9231442	2800.276681	0.0068	0.995	1.69E-09	count	1
ANKRD20A4	18.9231439	2800.276603	0.0068	0.995	1.69E-09	count	1
AC091057.3	18.9231432	2800.275669	0.0068	0.995	1.69E-09	count	1
AL096701.3	18.9231437	2800.276097	0.0068	0.995	1.69E-09	count	1
GPRIN2	18.9231432	2800.276487	0.0068	0.995	1.69E-09	count	1
DISP3	18.9231424	2800.276409	0.0068	0.995	1.69E-09	count	1
AC084035.1	18.9231423	2800.275397	0.0068	0.995	1.69E-09	count	1
RNF133	18.9231424	2800.275786	0.0068	0.995	1.69E-09	count	1
AFF2	18.9231435	2800.275708	0.0068	0.995	1.69E-09	count	1
MYH3	18.9231425	2800.276136	0.0068	0.995	1.69E-09	count	1
STARD6	18.9231424	2800.276954	0.0068	0.995	1.69E-09	count	1
HAPLN1	18.9231414	2800.276058	0.0068	0.995	1.69E-09	count	1
PLXNB3	18.9231419	2800.276214	0.0068	0.995	1.69E-09	count	1
AL031847.1	18.2260961	2798.965808	0.0065	0.995	1.69E-09	count	1
BEST4	18.2260967	2798.966389	0.0065	0.995	1.69E-09	count	1
ZRANB2-AS1	18.2260965	2798.966176	0.0065	0.995	1.69E-09	count	1
LINC01708	18.2260962	2798.966389	0.0065	0.995	1.69E-09	count	1
AL139011.2	18.2260962	2798.966292	0.0065	0.995	1.69E-09	count	1
Z99943.1	18.2260958	2798.966137	0.0065	0.995	1.69E-09	count	1
AL590723.1	18.2260963	2798.965905	0.0065	0.995	1.69E-09	count	1
ACTA1	18.2260965	2798.966079	0.0065	0.995	1.69E-09	count	1
BX323046.1	18.2260961	2798.966079	0.0065	0.995	1.69E-09	count	1
AC007378.1	18.2260963	2798.966157	0.0065	0.995	1.69E-09	count	1
LINC01829	18.2260961	2798.966312	0.0065	0.995	1.69E-09	count	1
LRRTM4	18.2260962	2798.966157	0.0065	0.995	1.69E-09	count	1
ST3GAL5-AS1	18.2260962	2798.966079	0.0065	0.995	1.69E-09	count	1
AC005237.1	18.2260964	2798.966312	0.0065	0.995	1.69E-09	count	1
ZNF197-AS1	18.2260961	2798.965808	0.0065	0.995	1.69E-09	count	1
AC096887.1	18.226096	2798.966118	0.0065	0.995	1.69E-09	count	1
YEATS2-AS1	18.2260961	2798.966137	0.0065	0.995	1.69E-09	count	1
ATP13A4	18.226096	2798.966137	0.0065	0.995	1.69E-09	count	1
AC046143.2	18.2260963	2798.966234	0.0065	0.995	1.69E-09	count	1
RBM47	18.2260963	2798.966137	0.0065	0.995	1.69E-09	count	1
AC095060.1	18.2260963	2798.966002	0.0065	0.995	1.69E-09	count	1
DAPP1	18.2260963	2798.966002	0.0065	0.995	1.69E-09	count	1
PITX2	18.2260963	2798.965963	0.0065	0.995	1.69E-09	count	1
TTC29	18.2260963	2798.965905	0.0065	0.995	1.69E-09	count	1
C5orf66	18.2260961	2798.966273	0.0065	0.995	1.69E-09	count	1

NBAT1	18.2260961	2798.966312	0.0065	0.995	1.69E-09	count	1
TBX18	18.2260963	2798.966079	0.0065	0.995	1.69E-09	count	1
AL589826.2	18.2260961	2798.966292	0.0065	0.995	1.69E-09	count	1
AL023284.4	18.2260963	2798.966176	0.0065	0.995	1.69E-09	count	1
LINC02528	18.2260961	2798.966157	0.0065	0.995	1.69E-09	count	1
AL596442.2	18.2260963	2798.966195	0.0065	0.995	1.69E-09	count	1
AL078605.1	18.2260963	2798.966157	0.0065	0.995	1.69E-09	count	1
SCIN	18.2260963	2798.966079	0.0065	0.995	1.69E-09	count	1
AC105446.1	18.2260965	2798.96606	0.0065	0.995	1.69E-09	count	1
SSMEM1	18.2260965	2798.966195	0.0065	0.995	1.69E-09	count	1
AC092198.1	18.2260964	2798.965982	0.0065	0.995	1.69E-09	count	1
TMEM255A	18.226096	2798.966021	0.0065	0.995	1.69E-09	count	1
AF131215.7	18.2260965	2798.966118	0.0065	0.995	1.69E-09	count	1
LZTS1	18.2260963	2798.966079	0.0065	0.995	1.69E-09	count	1
AC010834.3	18.2260961	2798.966157	0.0065	0.995	1.69E-09	count	1
KCNS2	18.2260963	2798.965905	0.0065	0.995	1.69E-09	count	1
AC103853.1	18.2260963	2798.966176	0.0065	0.995	1.69E-09	count	1
RSPO2	18.2260963	2798.966195	0.0065	0.995	1.69E-09	count	1
AC084116.3	18.2260963	2798.966002	0.0065	0.995	1.69E-09	count	1
EQTN	18.2260961	2798.965808	0.0065	0.995	1.69E-09	count	1
DNM1	18.2260959	2798.966079	0.0065	0.995	1.69E-09	count	1
CFAP77	18.2260963	2798.966002	0.0065	0.995	1.69E-09	count	1
BX649601.1	18.2260963	2798.96604	0.0065	0.995	1.69E-09	count	1
AC069287.2	18.2260967	2798.966195	0.0065	0.995	1.69E-09	count	1
HBG2	18.2260962	2798.96604	0.0065	0.995	1.69E-09	count	1
GIF	18.2260962	2798.966157	0.0065	0.995	1.69E-09	count	1
MYRF	18.2260962	2798.966079	0.0065	0.995	1.69E-09	count	1
HRASLS2	18.2260963	2798.966079	0.0065	0.995	1.69E-09	count	1
AP000769.3	18.2260961	2798.966137	0.0065	0.995	1.69E-09	count	1
OR2AT4	18.226096	2798.966292	0.0065	0.995	1.69E-09	count	1
AP002360.3	18.2260965	2798.966137	0.0065	0.995	1.69E-09	count	1
TENM4	18.2260959	2798.966118	0.0065	0.995	1.69E-09	count	1
NAALAD2	18.2260962	2798.966234	0.0065	0.995	1.69E-09	count	1
ST14	18.2260962	2798.966254	0.0065	0.995	1.69E-09	count	1
ADAMTS8	18.2260963	2798.966079	0.0065	0.995	1.69E-09	count	1
AL157834.2	18.2260963	2798.965905	0.0065	0.995	1.69E-09	count	1
AL133353.1	18.2260962	2798.965866	0.0065	0.995	1.69E-09	count	1
WDR11-AS1	18.226096	2798.966137	0.0065	0.995	1.69E-09	count	1
CLEC4C	18.2260958	2798.965963	0.0065	0.995	1.69E-09	count	1
PRH2	18.2260963	2798.965963	0.0065	0.995	1.69E-09	count	1
AC008011.2	18.226096	2798.96637	0.0065	0.995	1.69E-09	count	1
AC025423.4	18.2260959	2798.965963	0.0065	0.995	1.69E-09	count	1
PPFIA2	18.2260963	2798.966079	0.0065	0.995	1.69E-09	count	1
AC133794.1	18.2260965	2798.966118	0.0065	0.995	1.69E-09	count	1
POC1B-GALNT4	18.2260957	2798.965982	0.0065	0.995	1.69E-09	count	1

FZD10	18.2260958	2798.965885	0.0065	0.995	1.69E-09	count	1
AL590787.1	18.2260963	2798.966079	0.0065	0.995	1.69E-09	count	1
AL355916.2	18.2260959	2798.96604	0.0065	0.995	1.69E-09	count	1
AL162171.2	18.226096	2798.96637	0.0065	0.995	1.69E-09	count	1
COX8C	18.2260963	2798.965905	0.0065	0.995	1.69E-09	count	1
AL355102.3	18.2260962	2798.96635	0.0065	0.995	1.69E-09	count	1
AL160313.1	18.2260963	2798.966002	0.0065	0.995	1.69E-09	count	1
LINC02323	18.2260963	2798.965905	0.0065	0.995	1.69E-09	count	1
IGHV3-64D	18.2260965	2798.966254	0.0065	0.995	1.69E-09	count	1
IGHV3-20	18.226096	2798.966157	0.0065	0.995	1.69E-09	count	1
GREM1	18.2260967	2798.96637	0.0065	0.995	1.69E-09	count	1
AC022929.2	18.2260959	2798.965905	0.0065	0.995	1.69E-09	count	1
C15orf54	18.226096	2798.966002	0.0065	0.995	1.69E-09	count	1
AC079328.2	18.2260963	2798.966002	0.0065	0.995	1.69E-09	count	1
AC011944.1	18.2260963	2798.966195	0.0065	0.995	1.69E-09	count	1
AC120498.9	18.2260961	2798.966079	0.0065	0.995	1.69E-09	count	1
RBFOX1	18.2260964	2798.966312	0.0065	0.995	1.69E-09	count	1
TEKT5	18.2260963	2798.966331	0.0065	0.995	1.69E-09	count	1
IL21R	18.2260964	2798.96637	0.0065	0.995	1.69E-09	count	1
AC138904.3	18.2260967	2798.966215	0.0065	0.995	1.69E-09	count	1
NPIPB13	18.2260958	2798.966137	0.0065	0.995	1.69E-09	count	1
PRSS53	18.2260961	2798.966137	0.0065	0.995	1.69E-09	count	1
AC140912.1	18.2260963	2798.966176	0.0065	0.995	1.69E-09	count	1
AC091153.3	18.2260965	2798.966195	0.0065	0.995	1.69E-09	count	1
PIMREG	18.226096	2798.96604	0.0065	0.995	1.69E-09	count	1
PIK3R5	18.226096	2798.966292	0.0065	0.995	1.69E-09	count	1
MYH13	18.2260958	2798.965982	0.0065	0.995	1.69E-09	count	1
AC015908.3	18.2260963	2798.966331	0.0065	0.995	1.69E-09	count	1
LINC00670	18.2260963	2798.966079	0.0065	0.995	1.69E-09	count	1
AC130324.1	18.226096	2798.966002	0.0065	0.995	1.69E-09	count	1
RASL10B	18.226096	2798.966176	0.0065	0.995	1.69E-09	count	1
AC243829.1	18.226096	2798.96637	0.0065	0.995	1.69E-09	count	1
AC004585.1	18.2260963	2798.966002	0.0065	0.995	1.69E-09	count	1
AC002558.2	18.2260963	2798.965905	0.0065	0.995	1.69E-09	count	1
BRIP1	18.2260963	2798.966176	0.0065	0.995	1.69E-09	count	1
GRIN2C	18.2260961	2798.966079	0.0065	0.995	1.69E-09	count	1
AC132938.1	18.2260963	2798.965905	0.0065	0.995	1.69E-09	count	1
GACAT2	18.2260961	2798.965905	0.0065	0.995	1.69E-09	count	1
KLHL14	18.2260967	2798.966215	0.0065	0.995	1.69E-09	count	1
AC105094.2	18.226096	2798.966002	0.0065	0.995	1.69E-09	count	1
AC018529.2	18.2260964	2798.966137	0.0065	0.995	1.69E-09	count	1
BCAS1	18.2260961	2798.966157	0.0065	0.995	1.69E-09	count	1
OGFR-AS1	18.2260965	2798.966118	0.0065	0.995	1.69E-09	count	1
ZBTB46-AS1	18.2260967	2798.966195	0.0065	0.995	1.69E-09	count	1
AC010422.3	18.2260965	2798.96606	0.0065	0.995	1.69E-09	count	1

AC022148.2	18.226096	2798.965924	0.0065	0.995	1.69E-09	count	1
AC007193.2	18.2260963	2798.966157	0.0065	0.995	1.69E-09	count	1
AC000068.2	18.226096	2798.966021	0.0065	0.995	1.69E-09	count	1
AP000350.6	18.2260966	2798.966389	0.0065	0.995	1.69E-09	count	1
SCO2	18.2260965	2798.966254	0.0065	0.995	1.69E-09	count	1
SMIM11B	18.2260963	2798.966099	0.0065	0.995	1.69E-09	count	1
AP001628.2	18.2260961	2798.966195	0.0065	0.995	1.69E-09	count	1
FRGCA	18.2260961	2798.966312	0.0065	0.995	1.69E-09	count	1
SLFNL1-AS1	18.2260956	2798.965982	0.0065	0.995	1.69E-09	count	1
AL050343.2	18.2260962	2798.966118	0.0065	0.995	1.69E-09	count	1
AC239803.2	18.226096	2798.966079	0.0065	0.995	1.69E-09	count	1
UBE2Q1-AS1	18.2260961	2798.966137	0.0065	0.995	1.69E-09	count	1
AL355388.2	18.2260962	2798.966118	0.0065	0.995	1.69E-09	count	1
LY9	18.2260961	2798.966176	0.0065	0.995	1.69E-09	count	1
RGS4	18.2260955	2798.965963	0.0065	0.995	1.69E-09	count	1
AL606489.1	18.2260962	2798.966118	0.0065	0.995	1.69E-09	count	1
AL591848.2	18.2260959	2798.965944	0.0065	0.995	1.69E-09	count	1
GREB1	18.2260963	2798.965963	0.0065	0.995	1.69E-09	count	1
AC010096.1	18.2260958	2798.966137	0.0065	0.995	1.69E-09	count	1
AC012511.1	18.2260961	2798.966099	0.0065	0.995	1.69E-09	count	1
RNF103-CHMP3	18.2260956	2798.966234	0.0065	0.995	1.69E-09	count	1
CD8B	18.2260956	2798.966079	0.0065	0.995	1.69E-09	count	1
AC074387.1	18.2260961	2798.966157	0.0065	0.995	1.69E-09	count	1
AC017002.1	18.2260955	2798.965963	0.0065	0.995	1.69E-09	count	1
NMUR1	18.2260961	2798.966157	0.0065	0.995	1.69E-09	count	1
HDAC11-AS1	18.2260962	2798.966079	0.0065	0.995	1.69E-09	count	1
BSN	18.2260958	2798.96606	0.0065	0.995	1.69E-09	count	1
OR5H1	18.2260958	2798.966079	0.0065	0.995	1.69E-09	count	1
STXBP5L	18.2260961	2798.966176	0.0065	0.995	1.69E-09	count	1
LINC02029	18.2260958	2798.966118	0.0065	0.995	1.69E-09	count	1
AC026353.1	18.2260959	2798.96604	0.0065	0.995	1.69E-09	count	1
AC092953.2	18.2260956	2798.965866	0.0065	0.995	1.69E-09	count	1
AC131235.4	18.2260959	2798.966099	0.0065	0.995	1.69E-09	count	1
STPG2	18.2260961	2798.966409	0.0065	0.995	1.69E-09	count	1
LINC02435	18.226096	2798.966099	0.0065	0.995	1.69E-09	count	1
AC078850.1	18.2260962	2798.966137	0.0065	0.995	1.69E-09	count	1
AC084871.2	18.2260957	2798.966021	0.0065	0.995	1.69E-09	count	1
AC021087.1	18.226096	2798.966099	0.0065	0.995	1.69E-09	count	1
ADGRV1	18.2260963	2798.965924	0.0065	0.995	1.69E-09	count	1
ARSI	18.2260962	2798.966118	0.0065	0.995	1.69E-09	count	1
AL021997.3	18.2260959	2798.966176	0.0065	0.995	1.69E-09	count	1
CLPSL1	18.2260956	2798.966176	0.0065	0.995	1.69E-09	count	1
Z84484.1	18.226096	2798.965905	0.0065	0.995	1.69E-09	count	1
TRDN	18.2260956	2798.966176	0.0065	0.995	1.69E-09	count	1
MOXD1	18.2260956	2798.966079	0.0065	0.995	1.69E-09	count	1

AL450344.2	18.2260959	2798.966099	0.0065	0.995	1.69E-09	count	1
AC104073.4	18.2260959	2798.966157	0.0065	0.995	1.69E-09	count	1
AC114737.1	18.2260962	2798.966118	0.0065	0.995	1.69E-09	count	1
AC003991.2	18.2260963	2798.966176	0.0065	0.995	1.69E-09	count	1
MUC3A	18.2260956	2798.965827	0.0065	0.995	1.69E-09	count	1
AC093668.3	18.2260959	2798.965944	0.0065	0.995	1.69E-09	count	1
AC007384.1	18.2260961	2798.966273	0.0065	0.995	1.69E-09	count	1
LINC01510	18.2260958	2798.965924	0.0065	0.995	1.69E-09	count	1
NLGN4X	18.2260961	2798.966079	0.0065	0.995	1.69E-09	count	1
WWC3-AS1	18.2260956	2798.966234	0.0065	0.995	1.69E-09	count	1
ALG13-AS1	18.2260959	2798.965827	0.0065	0.995	1.69E-09	count	1
PNCK	18.2260959	2798.966157	0.0065	0.995	1.69E-09	count	1
LINC00535	18.2260961	2798.966157	0.0065	0.995	1.69E-09	count	1
CCDC26	18.2260956	2798.96606	0.0065	0.995	1.69E-09	count	1
TG	18.2260959	2798.966157	0.0065	0.995	1.69E-09	count	1
FOXH1	18.2260959	2798.966176	0.0065	0.995	1.69E-09	count	1
AL136980.1	18.2260959	2798.966176	0.0065	0.995	1.69E-09	count	1
TAL2	18.2260962	2798.966118	0.0065	0.995	1.69E-09	count	1
AL162724.2	18.2260961	2798.966176	0.0065	0.995	1.69E-09	count	1
AC090587.1	18.2260961	2798.966157	0.0065	0.995	1.69E-09	count	1
AC080023.1	18.2260963	2798.965963	0.0065	0.995	1.69E-09	count	1
OR8J3	18.2260962	2798.966118	0.0065	0.995	1.69E-09	count	1
AP002812.5	18.226096	2798.965905	0.0065	0.995	1.69E-09	count	1
LINC00838	18.2260961	2798.966273	0.0065	0.995	1.69E-09	count	1
RPEL1	18.2260962	2798.966118	0.0065	0.995	1.69E-09	count	1
PNLIPRP1	18.226096	2798.965944	0.0065	0.995	1.69E-09	count	1
AC010198.1	18.2260959	2798.966176	0.0065	0.995	1.69E-09	count	1
KRT2	18.2260959	2798.96604	0.0065	0.995	1.69E-09	count	1
LINC02399	18.2260959	2798.966137	0.0065	0.995	1.69E-09	count	1
AC016257.1	18.2260958	2798.966157	0.0065	0.995	1.69E-09	count	1
IL17D	18.226096	2798.965944	0.0065	0.995	1.69E-09	count	1
AL589745.1	18.226096	2798.966157	0.0065	0.995	1.69E-09	count	1
RPGRIP1	18.2260962	2798.966137	0.0065	0.995	1.69E-09	count	1
AL355102.4	18.226096	2798.965944	0.0065	0.995	1.69E-09	count	1
IGHV4-39	18.2260962	2798.966118	0.0065	0.995	1.69E-09	count	1
ITPKA	18.2260959	2798.966157	0.0065	0.995	1.69E-09	count	1
JMJD7	18.2260957	2798.966118	0.0065	0.995	1.69E-09	count	1
CPEB1-AS1	18.226096	2798.965944	0.0065	0.995	1.69E-09	count	1
AC036108.4	18.2260961	2798.966157	0.0065	0.995	1.69E-09	count	1
AL023881.1	18.2260961	2798.966099	0.0065	0.995	1.69E-09	count	1
AL031709.1	18.226096	2798.965982	0.0065	0.995	1.69E-09	count	1
TEDC2	18.2260962	2798.966118	0.0065	0.995	1.69E-09	count	1
C16orf71	18.2260955	2798.965827	0.0065	0.995	1.69E-09	count	1
AC040162.3	18.2260961	2798.966118	0.0065	0.995	1.69E-09	count	1
CDRT1	18.2260962	2798.966118	0.0065	0.995	1.69E-09	count	1

AC120024.1	18.2260954	2798.965866	0.0065	0.995	1.69E-09	count	1
NOL4	18.2260961	2798.966118	0.0065	0.995	1.69E-09	count	1
ZNF559-ZNF177	18.2260958	2798.96606	0.0065	0.995	1.69E-09	count	1
AC008747.1	18.2260956	2798.965866	0.0065	0.995	1.69E-09	count	1
TSKS	18.2260962	2798.966137	0.0065	0.995	1.69E-09	count	1
IZUMO2	18.2260959	2798.96604	0.0065	0.995	1.69E-09	count	1
C19orf81	18.2260961	2798.966137	0.0065	0.995	1.69E-09	count	1
AC006946.3	18.2260959	2798.966176	0.0065	0.995	1.69E-09	count	1
IGLC7	18.2260962	2798.966118	0.0065	0.995	1.69E-09	count	1
FO393418.1	18.2260963	2798.966176	0.0065	0.995	1.69E-09	count	1
PVALB	18.226096	2798.965944	0.0065	0.995	1.69E-09	count	1
AC105053.1	18.2260954	2798.965866	0.0065	0.995	1.69E-09	count	1
AC021151.1	18.2260958	2798.965944	0.0065	0.995	1.69E-09	count	1
COL10A1	18.2260958	2798.965944	0.0065	0.995	1.69E-09	count	1
ACE2	18.2260955	2798.965944	0.0065	0.995	1.69E-09	count	1
CERS3	18.2260955	2798.965944	0.0065	0.995	1.69E-09	count	1
TNFRSF17	18.2260958	2798.965769	0.0065	0.995	1.69E-09	count	1
CTRL	18.2260956	2798.966021	0.0065	0.995	1.69E-09	count	1
KCNH4	18.2260955	2798.965982	0.0065	0.995	1.69E-09	count	1
ACTN2	0.193987	0.3595193	0.5396	0.59	1.96E-09	count	1
HPSE	0.1982341	0.952364	0.2081	0.835	2.00E-09	count	1
CD200R1	0.8871341	0.4711826	1.8828	0.0598	2.52E-09	count	1
ACAP1	0.104721	0.9876369	0.106	0.916	2.99E-09	count	1
LGALS4	0.3659891	0.7857752	0.4658	0.641	3.46E-09	count	1
AL512625.3	0.3659891	0.7857752	0.4658	0.641	3.46E-09	count	1
TNNI2	0.3709531	0.7915377	0.4686	0.639	3.50E-09	count	1
CCL13	0.4986664	1.1088655	0.4497	0.653	4.47E-09	count	1
PNMA8A	0.4986664	0.7460722	0.6684	0.504	4.47E-09	count	1
PLAC4	0.5071505	0.8839757	0.5737	0.566	4.53E-09	count	1
LRRC25	17.9676782	5460.656503	0.0033	0.997	4.58E-09	count	1
PILRA	17.9661851	4498.137886	0.004	0.997	4.58E-09	count	1
RELN	17.7421641	4878.372601	0.0036	0.997	4.58E-09	count	1
IYD	17.7421639	4878.37275	0.0036	0.997	4.58E-09	count	1
TMEM26	17.7421639	4878.37275	0.0036	0.997	4.58E-09	count	1
OR7D4	17.7421641	4878.373245	0.0036	0.997	4.58E-09	count	1
GREM2	18.1467225	2808.392145	0.0065	0.995	4.58E-09	count	1
LPL	17.9643639	3266.922759	0.0055	0.996	4.58E-09	count	1
GHRL	18.1458905	2428.578444	0.0075	0.994	4.58E-09	count	1
ESR2	18.1458901	2428.578887	0.0075	0.994	4.58E-09	count	1
SLC23A3	17.9625533	2429.044982	0.0074	0.994	4.58E-09	count	1
AL358781.1	17.9625543	2429.045007	0.0074	0.994	4.58E-09	count	1
ZNF571-AS1	17.9625545	2429.044908	0.0074	0.994	4.58E-09	count	1
C1orf220	17.962553	2429.044613	0.0074	0.994	4.58E-09	count	1
AL034397.3	17.4515781	4218.663467	0.0041	0.997	4.58E-09	count	1
CMTM2	17.4515781	4218.663467	0.0041	0.997	4.58E-09	count	1

LONRF2	17.4515773	4218.663659	0.0041	0.997	4.58E-09	count	1
AC073869.3	17.451578	4218.663339	0.0041	0.997	4.58E-09	count	1
NBPF19	17.7381415	2429.201477	0.0073	0.994	4.58E-09	count	1
AC015967.1	17.7381419	2429.201329	0.0073	0.994	4.58E-09	count	1
KCTD16	17.7381414	2429.201329	0.0073	0.994	4.58E-09	count	1
FUT2	17.7381416	2429.201698	0.0073	0.994	4.58E-09	count	1
AC011773.4	17.738141	2429.201501	0.0073	0.994	4.58E-09	count	1
AIRN	17.7381402	2429.201378	0.0073	0.994	4.58E-09	count	1
AOAH	17.450034	3140.231052	0.0056	0.996	4.58E-09	count	1
TOMM20L	17.4500336	3140.231258	0.0056	0.996	4.58E-09	count	1
TPM1-AS	17.4500335	3140.230989	0.0056	0.996	4.58E-09	count	1
GEMIN7-AS1	17.4500342	3140.231179	0.0056	0.996	4.58E-09	count	1
AL358933.1	17.4500335	3140.231195	0.0056	0.996	4.58E-09	count	1
TRPC4	17.4500335	3140.230941	0.0056	0.996	4.58E-09	count	1
AC130469.1	17.4500334	3140.230973	0.0056	0.996	4.58E-09	count	1
AC010636.2	17.4500338	3140.230973	0.0056	0.996	4.58E-09	count	1
SBK3	17.4500332	3140.230751	0.0056	0.996	4.58E-09	count	1
MRGPRF	17.4489229	2429.029628	0.0072	0.994	4.58E-09	count	1
UCN	17.4489229	2429.029665	0.0072	0.994	4.58E-09	count	1
LINC02043	17.4489225	2429.029591	0.0072	0.994	4.58E-09	count	1
ADORA3	17.4489228	2429.029628	0.0072	0.994	4.58E-09	count	1
AC144450.1	17.4489225	2429.029444	0.0072	0.994	4.58E-09	count	1
IL20RB	17.4489227	2429.029346	0.0072	0.994	4.58E-09	count	1
AC083843.2	17.4489226	2429.029493	0.0072	0.994	4.58E-09	count	1
AC073912.2	17.4489227	2429.02964	0.0072	0.994	4.58E-09	count	1
RAB11FIP4	17.4489228	2429.029506	0.0072	0.994	4.58E-09	count	1
AC080038.2	17.4489231	2429.029604	0.0072	0.994	4.58E-09	count	1
AC010336.4	17.4489223	2429.029616	0.0072	0.994	4.58E-09	count	1
AL160408.2	17.4489225	2429.029702	0.0072	0.994	4.58E-09	count	1
AC135507.1	17.4489226	2429.02953	0.0072	0.994	4.58E-09	count	1
AC012640.1	17.4489223	2429.02953	0.0072	0.994	4.58E-09	count	1
AL355073.2	17.4489225	2429.02942	0.0072	0.994	4.58E-09	count	1
FPR1	17.4489225	2429.029616	0.0072	0.994	4.58E-09	count	1
AL008635.1	17.448922	2429.029444	0.0072	0.994	4.58E-09	count	1
AL513327.1	17.4489224	2429.029665	0.0072	0.994	4.58E-09	count	1
CSMD2	17.4489221	2429.029457	0.0072	0.994	4.58E-09	count	1
AL133245.1	17.4489224	2429.029591	0.0072	0.994	4.58E-09	count	1
FSIP2-AS1	17.4489224	2429.029738	0.0072	0.994	4.58E-09	count	1
AL645940.1	17.448922	2429.02926	0.0072	0.994	4.58E-09	count	1
AP003392.1	17.4489224	2429.029591	0.0072	0.994	4.58E-09	count	1
AC109460.1	17.4489218	2429.029371	0.0072	0.994	4.58E-09	count	1
AC138207.7	17.4489223	2429.029738	0.0072	0.994	4.58E-09	count	1
MYO5B	17.4489221	2429.029751	0.0072	0.994	4.58E-09	count	1
AL359555.4	17.4489219	2429.029542	0.0072	0.994	4.58E-09	count	1
AP000345.2	17.4489222	2429.029628	0.0072	0.994	4.58E-09	count	1

DCDC2	17.448922	2429.029628	0.0072	0.994	4.58E-09	count	1
AC022021.1	17.4489221	2429.029628	0.0072	0.994	4.58E-09	count	1
AC079601.1	17.4489222	2429.029775	0.0072	0.994	4.58E-09	count	1
AC092718.8	17.4489221	2429.029407	0.0072	0.994	4.58E-09	count	1
TJP3	17.448922	2429.02964	0.0072	0.994	4.58E-09	count	1
AL021707.1	17.4489223	2429.029689	0.0072	0.994	4.58E-09	count	1
ARHGEF10L	19.0295889	4182.795173	0.0045	0.996	4.58E-09	count	1
PTGER2	19.0295889	4182.795173	0.0045	0.996	4.58E-09	count	1
AP005436.1	19.0295881	4182.795518	0.0045	0.996	4.58E-09	count	1
FPR2	19.0295881	4182.795518	0.0045	0.996	4.58E-09	count	1
AC010680.5	19.0295874	4182.795086	0.0045	0.996	4.58E-09	count	1
ZBBX	17.0428118	3438.843885	0.005	0.996	4.58E-09	count	1
BRSK2	17.0428118	3438.843677	0.005	0.996	4.58E-09	count	1
SLC1A7	17.0428117	3438.843937	0.005	0.996	4.58E-09	count	1
C1orf229	17.0428116	3438.843937	0.005	0.996	4.58E-09	count	1
ADGRG4	17.0428115	3438.84385	0.005	0.996	4.58E-09	count	1
ZNF32-AS3	17.0428113	3438.843607	0.005	0.996	4.58E-09	count	1
ADGRD1	17.0428117	3438.843937	0.005	0.996	4.58E-09	count	1
AC107081.2	17.0428113	3438.843972	0.005	0.996	4.58E-09	count	1
FLJ45513	17.0428116	3438.843798	0.005	0.996	4.58E-09	count	1
RGS9BP	17.0428116	3438.843954	0.005	0.996	4.58E-09	count	1
AC000067.1	17.0428115	3438.843607	0.005	0.996	4.58E-09	count	1
ZNF280A	17.0428114	3438.843711	0.005	0.996	4.58E-09	count	1
FOXE3	17.0428114	3438.843746	0.005	0.996	4.58E-09	count	1
CD160	17.0428112	3438.84392	0.005	0.996	4.58E-09	count	1
XKR4	17.0428114	3438.843816	0.005	0.996	4.58E-09	count	1
KLK11	17.0428114	3438.843816	0.005	0.996	4.58E-09	count	1
TNNT1	17.0428115	3438.843902	0.005	0.996	4.58E-09	count	1
AC004882.1	17.0428111	3438.843781	0.005	0.996	4.58E-09	count	1
OMG	18.6257965	3418.096954	0.0054	0.996	4.58E-09	count	1
AC008735.4	18.6257965	3418.096954	0.0054	0.996	4.58E-09	count	1
IGHD	18.6257963	3418.096389	0.0054	0.996	4.58E-09	count	1
SPN	18.3329806	2198.183688	0.0083	0.993	4.58E-09	count	1
AL121601.1	18.3311538	1698.200872	0.0108	0.991	4.58E-09	count	1
IGHGP	18.3311536	1698.20068	0.0108	0.991	4.58E-09	count	1
CENPS-CORT	18.1885895	3853.339405	0.0047	0.996	4.59E-09	count	1
AL731563.3	18.4091039	3670.842421	0.005	0.996	4.59E-09	count	1
AC107952.2	18.1880874	3198.013202	0.0057	0.995	4.59E-09	count	1
WDR97	18.1880899	3198.010638	0.0057	0.995	4.59E-09	count	1
CD248	18.1881117	3198.020027	0.0057	0.995	4.59E-09	count	1
SPC25	18.223756	3626.254255	0.005	0.996	4.59E-09	count	1
AC009486.1	18.2237549	3626.25469	0.005	0.996	4.59E-09	count	1
COL4A6	17.9845626	4146.665794	0.0043	0.997	4.59E-09	count	1
FAM169A	17.9845577	4146.661972	0.0043	0.997	4.59E-09	count	1
HIST1H2AB	18.4079427	3184.74675	0.0058	0.995	4.59E-09	count	1

VSTM5	18.2222592	3145.792453	0.0058	0.995	4.59E-09	count	1
AC021739.2	18.2222811	3145.804231	0.0058	0.995	4.59E-09	count	1
WAS	18.2222396	3145.795697	0.0058	0.995	4.59E-09	count	1
AC135048.3	17.9842115	3202.389221	0.0056	0.996	4.59E-09	count	1
AL137003.1	18.2222325	3145.779707	0.0058	0.995	4.59E-09	count	1
ADAMTS14	18.2222324	3145.779707	0.0058	0.995	4.59E-09	count	1
LINC01460	17.9841738	3202.378498	0.0056	0.996	4.59E-09	count	1
RAD21L1	18.2222324	3145.779599	0.0058	0.995	4.59E-09	count	1
AL390860.1	17.9841731	3202.372201	0.0056	0.996	4.59E-09	count	1
AL596325.1	17.9841803	3202.374867	0.0056	0.996	4.59E-09	count	1
PRMT8	17.9841857	3202.387292	0.0056	0.996	4.59E-09	count	1
ADCY2	17.9842041	3202.383321	0.0056	0.996	4.59E-09	count	1
PCDHA10	17.9841752	3202.38455	0.0056	0.996	4.59E-09	count	1
AC016738.1	17.9841928	3202.380711	0.0056	0.996	4.59E-09	count	1
OCLM	17.984186	3202.377893	0.0056	0.996	4.59E-09	count	1
AL353759.1	17.9842042	3202.373789	0.0056	0.996	4.59E-09	count	1
AC025178.1	17.9841845	3202.371709	0.0056	0.996	4.59E-09	count	1
AL136295.6	18.2222798	3145.804446	0.0058	0.995	4.59E-09	count	1
AC234582.2	18.2222516	3145.792955	0.0058	0.995	4.59E-09	count	1
SELPLG	17.9841862	3202.371577	0.0056	0.996	4.59E-09	count	1
CCNA2	18.222281	3145.804213	0.0058	0.995	4.59E-09	count	1
CTRC	17.9841993	3202.380352	0.0056	0.996	4.59E-09	count	1
AC008764.3	17.984186	3202.381014	0.0056	0.996	4.59E-09	count	1
AC005840.4	17.9841927	3202.386971	0.0056	0.996	4.59E-09	count	1
AP003068.1	17.9841814	3202.384342	0.0056	0.996	4.59E-09	count	1
AC009509.4	17.9841988	3202.383491	0.0056	0.996	4.59E-09	count	1
AC064836.2	17.9841875	3202.377704	0.0056	0.996	4.59E-09	count	1
AC112236.2	17.9841875	3202.390337	0.0056	0.996	4.59E-09	count	1
DNAJC9-AS1	18.222281	3145.803944	0.0058	0.995	4.59E-09	count	1
AC002480.4	18.2222805	3145.804051	0.0058	0.995	4.59E-09	count	1
ADCY5	17.9841784	3202.368891	0.0056	0.996	4.59E-09	count	1
LINC00958	17.9841793	3202.381392	0.0056	0.996	4.59E-09	count	1
AC025263.1	17.7127668	3184.440297	0.0056	0.996	4.59E-09	count	1
SLC16A9	17.7127695	3184.444793	0.0056	0.996	4.59E-09	count	1
AL449106.1	17.7127476	3184.451965	0.0056	0.996	4.59E-09	count	1
GRIP1	17.7127536	3184.436749	0.0056	0.996	4.59E-09	count	1
AC092117.1	17.7127442	3184.442453	0.0056	0.996	4.59E-09	count	1
PTGES3L	17.7127744	3184.454027	0.0056	0.996	4.59E-09	count	1
SLC25A21	17.7127665	3184.440279	0.0056	0.996	4.59E-09	count	1
AC012368.2	17.7127574	3184.446094	0.0056	0.996	4.59E-09	count	1
AC009802.1	17.7127457	3184.432587	0.0056	0.996	4.59E-09	count	1
SEMA4G	17.7127613	3184.440799	0.0056	0.996	4.59E-09	count	1
RFX8	17.7127537	3184.436675	0.0056	0.996	4.59E-09	count	1
AL606760.2	17.7127421	3184.452596	0.0056	0.996	4.59E-09	count	1
RBM5-AS1	17.7127374	3184.438272	0.0056	0.996	4.59E-09	count	1

AC005962.1	17.7127665	3184.440372	0.0056	0.996	4.59E-09	count	1
AC012615.2	17.7127664	3184.440335	0.0056	0.996	4.59E-09	count	1
AL135744.1	17.7127542	3184.436526	0.0056	0.996	4.59E-09	count	1
AL606468.1	17.7127392	3184.44288	0.0056	0.996	4.59E-09	count	1
AL662890.1	17.7127385	3184.443103	0.0056	0.996	4.59E-09	count	1
EFCAB3	17.7127739	3184.454064	0.0056	0.996	4.59E-09	count	1
AC137630.4	17.7127388	3184.442954	0.0056	0.996	4.59E-09	count	1
AC064807.4	17.7127624	3184.440576	0.0056	0.996	4.59E-09	count	1
SLC16A6	17.7127574	3184.446168	0.0056	0.996	4.59E-09	count	1
AC239800.2	17.7127382	3184.443158	0.0056	0.996	4.59E-09	count	1
AC005534.1	17.7127494	3184.456684	0.0056	0.996	4.59E-09	count	1
LRRC73	17.7127548	3184.436489	0.0056	0.996	4.59E-09	count	1
AC099518.6	17.7127515	3184.446781	0.0056	0.996	4.59E-09	count	1
LINC01993	17.7127554	3184.456126	0.0056	0.996	4.59E-09	count	1
TMEM238L	17.7127398	3184.442694	0.0056	0.996	4.59E-09	count	1
TMEM145	17.7127534	3184.436768	0.0056	0.996	4.59E-09	count	1
AP001781.1	17.7127405	3184.433033	0.0056	0.996	4.59E-09	count	1
ZSCAN1	17.7127523	3184.446484	0.0056	0.996	4.59E-09	count	1
AC121761.1	17.7127546	3184.436489	0.0056	0.996	4.59E-09	count	1
GREB1L	17.7127399	3184.442675	0.0056	0.996	4.59E-09	count	1
AC011472.4	17.7127549	3184.436415	0.0056	0.996	4.59E-09	count	1
AC092809.4	17.7127415	3184.432866	0.0056	0.996	4.59E-09	count	1
CHRNA7	17.7127614	3184.440818	0.0056	0.996	4.59E-09	count	1
LRRC66	17.7127395	3184.44275	0.0056	0.996	4.59E-09	count	1
AC211433.2	17.7127703	3184.449865	0.0056	0.996	4.59E-09	count	1
AL606763.1	17.7127733	3184.454157	0.0056	0.996	4.59E-09	count	1
AC090844.2	17.7127401	3184.442731	0.0056	0.996	4.59E-09	count	1
Z82244.2	17.7127569	3184.446224	0.0056	0.996	4.59E-09	count	1
BIRC5	17.7127662	3184.440335	0.0056	0.996	4.59E-09	count	1
AL137847.2	17.7127605	3184.440892	0.0056	0.996	4.59E-09	count	1
LIMS4	17.7127436	3184.442601	0.0056	0.996	4.59E-09	count	1
AC010273.1	17.7127615	3184.440836	0.0056	0.996	4.59E-09	count	1
AC136475.4	17.7127441	3184.442545	0.0056	0.996	4.59E-09	count	1
AC010531.6	17.7127439	3184.442657	0.0056	0.996	4.59E-09	count	1
RB1-DT	17.7127663	3184.440409	0.0056	0.996	4.59E-09	count	1
PLEKHG3	17.7127359	3184.43844	0.0056	0.996	4.59E-09	count	1
AC008440.2	17.7127628	3184.440465	0.0056	0.996	4.59E-09	count	1
ENO4	17.7127567	3184.446224	0.0056	0.996	4.59E-09	count	1
AC018521.2	17.712742	3184.432736	0.0056	0.996	4.59E-09	count	1
KIF20A	17.7127547	3184.436452	0.0056	0.996	4.59E-09	count	1
AC104806.2	17.7127444	3184.442527	0.0056	0.996	4.59E-09	count	1
AC093620.1	17.7127358	3184.433368	0.0056	0.996	4.59E-09	count	1
AC138393.3	17.7127732	3184.454306	0.0056	0.996	4.59E-09	count	1
AL031673.1	17.7127445	3184.442434	0.0056	0.996	4.59E-09	count	1
CD8A	17.7127438	3184.442675	0.0056	0.996	4.59E-09	count	1

AC009630.1	17.7127604	3184.450646	0.0056	0.996	4.59E-09	count	1
AC007952.5	17.7127489	3184.437083	0.0056	0.996	4.59E-09	count	1
ARHGAP15	17.7127405	3184.433052	0.0056	0.996	4.59E-09	count	1
AC078788.1	17.7127597	3184.450701	0.0056	0.996	4.59E-09	count	1
AP001412.1	17.7127439	3184.442527	0.0056	0.996	4.59E-09	count	1
SIRPB1	17.7127447	3184.442471	0.0056	0.996	4.59E-09	count	1
LINC00239	17.7127437	3184.452206	0.0056	0.996	4.59E-09	count	1
AC005050.1	17.7127531	3184.44628	0.0056	0.996	4.59E-09	count	1
VSIG8	17.7127323	3184.438514	0.0056	0.996	4.59E-09	count	1
AC004477.3	17.7127397	3184.442694	0.0056	0.996	4.59E-09	count	1
AL031587.3	17.7127375	3184.438161	0.0056	0.996	4.59E-09	count	1
GGN	0.5255445	0.7802413	0.6736	0.501	4.66E-09	count	1
TMPO-AS1	0.6186588	0.7361235	0.8404	0.401	5.30E-09	count	1
AP000866.5	0.6519882	0.8267036	0.7887	0.43	5.51E-09	count	1
AL162311.3	0.8599741	0.7508324	1.1454	0.252	6.71E-09	count	1
CXADR	1.0694557	0.5142197	2.0798	0.0376	7.72E-09	count	1
KCNJ1	0.4986664	1.6385299	0.3043	0.761	1.22E-08	count	1
COMP	17.7550746	4909.964995	0.0036	0.997	1.24E-08	count	1
RPS6KB2-AS1	17.3069183	3924.302224	0.0044	0.996	1.24E-08	count	1
CYBB	17.439264	3229.208782	0.0054	0.996	1.24E-08	count	1
SOSTDC1	17.7560724	2784.830354	0.0064	0.995	1.24E-08	count	1
NDST3	17.4383524	2769.513823	0.0063	0.995	1.24E-08	count	1
IRX6	17.5552154	2146.97053	0.0082	0.993	1.24E-08	count	1
CLCN2	17.6583643	1470.086163	0.012	0.99	1.24E-08	count	1
LRRC37A	18.1804394	2376.189647	0.0077	0.994	1.25E-08	count	1
LINC002481	18.1573199	3032.288211	0.006	0.995	1.25E-08	count	1
AC068282.1	18.1573195	3032.288041	0.006	0.995	1.25E-08	count	1
P3H2-AS1	17.7217948	1914.569994	0.0093	0.993	1.25E-08	count	1
OR3A3	17.8021437	2653.842023	0.0067	0.995	1.25E-08	count	1
AC012557.2	17.9206756	2690.156047	0.0067	0.995	1.25E-08	count	1
AC137723.1	17.6145819	1920.785411	0.0092	0.993	1.25E-08	count	1
LINC02193	17.4937884	2173.483223	0.008	0.994	1.25E-08	count	1
AC011446.2	17.7874072	2188.146187	0.0081	0.994	1.25E-08	count	1
LKAAEAR1	18.0175411	2444.171227	0.0074	0.994	1.25E-08	count	1
CHRDL1	17.3534999	1933.825113	0.009	0.993	1.25E-08	count	1
NUDT11	17.3534955	1933.825172	0.009	0.993	1.25E-08	count	1
OTUD6A	17.1891934	2977.256761	0.0058	0.995	1.25E-08	count	1
AF213884.3	18.5056191	3489.560438	0.0053	0.996	1.25E-08	count	1
AC093512.1	17.6791958	1941.415286	0.0091	0.993	1.25E-08	count	1
AC074099.1	17.6971063	1918.756474	0.0092	0.993	1.25E-08	count	1
DCAF4L1	0.6461573	0.7977921	0.8099	0.418	1.49E-08	count	1
SLC2A14	0.8091383	0.4021	2.0123	0.0443	1.75E-08	count	1
LAMC2	0.8468141	1.1062048	0.7655	0.444	1.81E-08	count	1
ADAM33	0.9889465	1.8818467	0.5255	0.599	2.00E-08	count	1
AC009336.2	17.0480871	3447.926631	0.0049	0.996	3.38E-08	count	1

MRPL55	7.19E-05	0.1338088	5.00E-04	1	9.93E-05	count	1
CACNB3	0.0004214	0.414713	0.001	0.999	0.000366632	count	1
CCDC125	0.0004713	0.2706615	0.0017	0.999	0.000567817	count	1
SELENOO	0.0005482	0.3853076	0.0014	0.999	0.000598491	count	1
TMEM199	0.0005728	0.2234433	0.0026	0.998	0.000726017	count	1
RPL23A	0.0005259	0.0248994	0.0211	0.983	0.000757311	count	1
C15orf40	0.0005706	0.1620101	0.0035	0.997	0.000778193	count	1
NECAB3	0.0014259	0.4463892	0.0032	0.997	0.001305154	count	1
UQCC1	0.0016346	0.3668189	0.0045	0.996	0.001421879	count	1
USP13	0.0013302	0.2959479	0.0045	0.996	0.001569123	count	1
B3GNT10	0.0034986	0.8417283	0.0042	0.997	0.001695426	count	1
AC119674.2	0.0058797	0.5813996	0.0101	0.992	0.001900562	count	1
AP1M1	0.0015657	0.2156155	0.0073	0.994	0.001957979	count	1
CCNDBP1	0.0016972	0.1025554	0.0165	0.987	0.002379693	count	1
THAP9	0.0034986	0.70182	0.005	0.996	0.002538252	count	1
CCM2	0.0019634	0.1569105	0.0125	0.99	0.002645878	count	1
APLNR	0.0019417	0.1682405	0.0115	0.991	0.002676232	count	1
ZNF776	0.0032183	0.5266976	0.0061	0.995	0.002686447	count	1
NDUFA2	0.0020402	0.078307	0.0261	0.979	0.002885379	count	1
TM9SF4	0.0023782	0.2493753	0.0095	0.992	0.002998888	count	1
LPIN2	0.0024348	0.2265948	0.0107	0.991	0.003082014	count	1
SMARCE1	0.002316	0.1151955	0.0201	0.984	0.003183982	count	1
SIX1	0.0335104	0.9564235	0.035	0.972	0.003221222	count	1
RABL6	0.0024324	0.1663551	0.0146	0.988	0.003236232	count	1
RFX3	0.0028637	0.2791669	0.0103	0.992	0.003387489	count	1
GM2A	0.0036811	0.4013499	0.0092	0.993	0.003465772	count	1
ZNF506	0.0026684	0.1991217	0.0134	0.989	0.003479401	count	1
KIF1BP	0.0026606	0.1714263	0.0155	0.988	0.003536423	count	1
C19orf24	0.0025332	0.101649	0.0249	0.98	0.003536461	count	1
LINC01184	0.0027479	0.1664009	0.0165	0.987	0.003625466	count	1
SLC7A1	0.0038241	0.3829913	0.01	0.992	0.003691951	count	1
PPP1R26-AS1	0.021041	1.156281	0.0182	0.985	0.003809233	count	1
FOLH1	0.021041	1.156281	0.0182	0.985	0.003809233	count	1
AC009318.2	0.021041	1.156281	0.0182	0.985	0.003809233	count	1
ASTN2	0.021041	1.15821	0.0182	0.986	0.003809233	count	1
ERCC8	0.0048471	0.3764697	0.0129	0.99	0.003952385	count	1
KHDC4	0.0037617	0.2308119	0.0163	0.987	0.004212339	count	1
DLST	0.0033886	0.219666	0.0154	0.988	0.004217799	count	1
S100A12	0.0134238	0.8976758	0.015	0.988	0.004328869	count	1
LYAR	0.0031721	0.1234569	0.0257	0.98	0.004349264	count	1
SEC61A2	0.0093629	0.6383859	0.0147	0.988	0.004530133	count	1
BECN1	0.0033098	0.1248476	0.0265	0.979	0.004538899	count	1
UVSSA	0.0035846	0.3597323	0.01	0.992	0.004624454	count	1
POLR3F	0.0039671	0.2524215	0.0157	0.987	0.004718484	count	1
RPL4	0.0032919	0.0358562	0.0918	0.927	0.004723743	count	1

AC020765.2	0.0084386	0.6676835	0.0126	0.99	0.004791532	count	1
SAE1	0.0037635	0.155527	0.0242	0.981	0.004914289	count	1
AP2A2	0.0037061	0.1586251	0.0234	0.981	0.004930652	count	1
NAIF1	0.0068893	0.4312007	0.016	0.987	0.004994817	count	1
GABPA	0.0037742	0.1467151	0.0257	0.979	0.00505011	count	1
CAV2	0.0035449	0.0564223	0.0628	0.95	0.005064604	count	1
FAM206A	0.0043627	0.2765157	0.0158	0.987	0.005188866	count	1
GNG12-AS1	0.0070559	0.4326026	0.0163	0.987	0.005290906	count	1
ETFB	0.003763	0.0759859	0.0495	0.961	0.005351939	count	1
LINC01615	0.0063709	0.4883196	0.013	0.99	0.005429683	count	1
COPS4	0.0040354	0.1347514	0.0299	0.976	0.005471223	count	1
SLC25A12	0.004989	0.2670731	0.0187	0.985	0.005660599	count	1
UBP1	0.0046615	0.2312711	0.0202	0.984	0.005700131	count	1
POLDIP2	0.0044759	0.167897	0.0267	0.979	0.005856719	count	1
BTBD1	0.0045456	0.1911739	0.0238	0.981	0.005863989	count	1
CELF4	0.0103524	0.8388856	0.0123	0.99	0.005875451	count	1
C6orf89	0.0042995	0.1315871	0.0327	0.974	0.005893799	count	1
TXNL1	0.0041971	0.0787213	0.0533	0.957	0.005896631	count	1
POLR2J3	0.0044097	0.1759569	0.0251	0.98	0.006028029	count	1
S100A11	0.0043076	0.0345762	0.1246	0.901	0.006195894	count	1
TERC	0.0346753	1.118725	0.031	0.975	0.00624736	count	1
AC008906.1	0.0346753	1.118725	0.031	0.975	0.00624736	count	1
AL137003.2	0.0346753	1.118725	0.031	0.975	0.00624736	count	1
FSBP	0.0346753	1.118725	0.031	0.975	0.00624736	count	1
C9orf152	0.0346753	1.118725	0.031	0.975	0.00624736	count	1
AC084824.5	0.0346753	1.1171246	0.031	0.975	0.00624736	count	1
HEXA-AS1	0.0346753	1.1171246	0.031	0.975	0.00624736	count	1
GAREM1	0.0346753	1.23073	0.0282	0.978	0.00624736	count	1
CLNS1A	0.0044939	0.0965234	0.0466	0.963	0.006307957	count	1
PPA1	0.0044913	0.0718371	0.0625	0.95	0.006384528	count	1
COMMD9	0.0049192	0.1610635	0.0305	0.976	0.006589938	count	1
MAPRE1	0.0048994	0.1211544	0.0404	0.968	0.006714795	count	1
HLA-C	0.0046647	0.0296225	0.1575	0.8749	0.006718864	count	1
ABCB9	0.021041	0.9476197	0.0222	0.982	0.006769025	count	1
OTUD6B-AS1	0.0048381	0.0997072	0.0485	0.961	0.006796102	count	1
PRMT1	0.0047804	0.0680324	0.0703	0.944	0.006801529	count	1
FPGT	0.0057129	0.258949	0.0221	0.982	0.006880437	count	1
TXN	0.0048097	0.0475503	0.1012	0.919	0.006904977	count	1
ARAP3	0.0054849	0.2100412	0.0261	0.979	0.007028094	count	1
TMEM263	0.0053306	0.1545729	0.0345	0.972	0.007132342	count	1
MST1	0.0755387	0.6911327	0.1093	0.913	0.007146877	count	1
AC139887.4	0.0755387	0.8514546	0.0887	0.929	0.007146877	count	1
RDH5	0.0083566	0.4886886	0.0171	0.986	0.007261258	count	1
SHMT2	0.006353	0.2351367	0.027	0.978	0.007422374	count	1
ATF1	0.0056334	0.1461996	0.0385	0.969	0.007615228	count	1

SLC38A2	0.0055154	0.0782705	0.0705	0.944	0.007825969	count	1
RAD51AP1	0.0139144	0.7113959	0.0196	0.984	0.007890143	count	1
SPICE1	0.0074962	0.3068134	0.0244	0.981	0.007924111	count	1
AARS	0.0067087	0.2472071	0.0271	0.978	0.00793354	count	1
MED27	0.0063065	0.1912746	0.033	0.974	0.007940303	count	1
AL355338.1	0.0067379	0.323984	0.0208	0.983	0.008012506	count	1
ATP5PO	0.0056344	0.0531413	0.106	0.916	0.008047475	count	1
FAM160B1	0.00652	0.2135443	0.0305	0.976	0.008139177	count	1
DDX52	0.0060402	0.1428923	0.0423	0.966	0.008200127	count	1
LRRFIP2	0.0059639	0.0978565	0.0609	0.951	0.008348772	count	1
AL590560.2	0.0465609	0.976254	0.0477	0.962	0.008353511	count	1
PRKAR2A-AS1	0.0465609	0.976254	0.0477	0.962	0.008353511	count	1
ATP5F1C	0.0059075	0.0531727	0.1111	0.912	0.008437157	count	1
RNPEPL1	0.006331	0.1556237	0.0407	0.968	0.00856584	count	1
TCTE3	0.0119883	0.6474144	0.0185	0.985	0.008682727	count	1
NRBP2	0.0082791	0.3022553	0.0274	0.978	0.008811363	count	1
MEA1	0.0063612	0.0972597	0.0654	0.948	0.00888146	count	1
CLN8	0.0068973	0.2469106	0.0279	0.978	0.008928128	count	1
AL050343.1	0.028103	0.8027265	0.035	0.972	0.009020852	count	1
RPS6KA5	0.007612	0.24339	0.0313	0.975	0.009229185	count	1
RAF1	0.0072218	0.1941157	0.0372	0.97	0.009233814	count	1
VPS16	0.0081579	0.2684821	0.0304	0.976	0.009399676	count	1
RAI1	0.0089031	0.374162	0.0238	0.981	0.009474852	count	1
PLEC	0.0067535	0.0932591	0.0724	0.942	0.009485991	count	1
VCPKMT	0.0083764	0.3384201	0.0248	0.98	0.009501214	count	1
ITPRIPL1	0.0137309	0.5299116	0.0259	0.979	0.009574454	count	1
GRIA2	0.1051825	0.9767123	0.1077	0.914	0.009840543	count	1
AC067930.1	0.1051825	0.9767123	0.1077	0.914	0.009840543	count	1
KCNQ3	0.1051825	1.236186	0.0851	0.932	0.009840543	count	1
PTPN11	0.0072559	0.1131003	0.0642	0.949	0.010008692	count	1
TBC1D8B	0.0092256	0.3031888	0.0304	0.976	0.010063332	count	1
HNRNPUL1	0.0074512	0.1265469	0.0589	0.953	0.010095857	count	1
BROX	0.0075664	0.1364755	0.0554	0.956	0.010224607	count	1
CCT3	0.0072469	0.067077	0.108	0.914	0.010255424	count	1
HEBP2	0.0073287	0.071828	0.102	0.9187	0.010438238	count	1
DCLRE1A	0.0185285	0.6700667	0.0277	0.978	0.010494651	count	1
AL136115.2	0.113463	1.141585	0.0994	0.921	0.010582002	count	1
AC006116.8	0.113463	1.141585	0.0994	0.921	0.010582002	count	1
VPS54	0.0094201	0.2728487	0.0345	0.972	0.010812556	count	1
TRMT112	0.0076196	0.047237	0.1613	0.872	0.010905223	count	1
PPP4C	0.0078249	0.0864109	0.0906	0.928	0.010989617	count	1
MCM6	0.0117101	0.3446986	0.034	0.973	0.011012662	count	1
ATAD3B	0.0159134	0.6170082	0.0258	0.979	0.011091227	count	1
ATG12	0.0080537	0.1129845	0.0713	0.943	0.011115387	count	1
MUS81	0.0093051	0.241423	0.0385	0.969	0.011149838	count	1

CCNJL	0.1207569	1.107196	0.1091	0.913	0.011231188	count	1
HR	0.1207569	1.107196	0.1091	0.913	0.011231188	count	1
ZNF763	0.1207569	1.107196	0.1091	0.913	0.011231188	count	1
SLC25A34	0.1207569	1.114578	0.1083	0.914	0.011231188	count	1
AC137932.3	0.1207569	1.114578	0.1083	0.914	0.011231188	count	1
MTCL1	0.0631868	0.5871395	0.1076	0.914	0.011269702	count	1
DPH5	0.0092384	0.2373324	0.0389	0.969	0.011270983	count	1
COPB1	0.0082066	0.1110472	0.0739	0.941	0.011362204	count	1
GNPTAB	0.0088217	0.178909	0.0493	0.961	0.011465016	count	1
RPS19	0.0079556	0.0233843	0.3402	0.734	0.011465372	count	1
HOOK3	0.0083178	0.1163251	0.0715	0.943	0.011506086	count	1
GOT2	0.0096023	0.2567848	0.0374	0.97	0.011561699	count	1
LOXL2	0.009443	0.235124	0.0402	0.968	0.011588379	count	1
PHKG2	0.0099162	0.2709	0.0366	0.971	0.01175706	count	1
ZNF644	0.0085599	0.1114642	0.0768	0.939	0.011830149	count	1
PSMD2	0.0087827	0.1319028	0.0666	0.947	0.012156722	count	1
RNF111	0.0096219	0.2094672	0.0459	0.963	0.012247603	count	1
ZNHIT6	0.0092214	0.1952078	0.0472	0.962	0.012277693	count	1
RAB1B	0.0090913	0.1331252	0.0683	0.946	0.012429108	count	1
FLAD1	0.0103257	0.2778815	0.0372	0.97	0.012461148	count	1
CEP95	0.0108089	0.271246	0.0398	0.968	0.012703307	count	1
IL4R	0.0099552	0.2084788	0.0478	0.962	0.012873712	count	1
HMGN4	0.0164325	0.4079379	0.0403	0.968	0.013037596	count	1
ING5	0.0105421	0.2015123	0.0523	0.958	0.013116558	count	1
EIF5AL1	0.0303453	0.6511322	0.0466	0.963	0.013139808	count	1
MBOAT1	0.0162481	0.4751397	0.0342	0.973	0.013222309	count	1
CHD6	0.0097568	0.1352864	0.0721	0.943	0.0132585	count	1
NEK9	0.0115296	0.2628763	0.0439	0.965	0.013280954	count	1
ENDOG	0.0102	0.1859175	0.0549	0.956	0.013286227	count	1
MAP4	0.0094307	0.0738636	0.1277	0.898	0.013340481	count	1
TRMT61A	0.0114764	0.2791993	0.0411	0.967	0.013402881	count	1
RUVBL2	0.0101041	0.1596101	0.0633	0.95	0.013482993	count	1
SNRPB	0.0094987	0.062936	0.1509	0.88	0.013488391	count	1
ATL3	0.0098493	0.1177038	0.0837	0.933	0.013644406	count	1
FAM168A	0.0121602	0.2934064	0.0414	0.967	0.01366918	count	1
ZNF584	0.0196767	0.4545534	0.0433	0.965	0.013703339	count	1
SNUPN	0.0107811	0.2146111	0.0502	0.96	0.013941253	count	1
PRG4	0.0788469	0.6037999	0.1306	0.896	0.013984731	count	1
PTPRA	0.0101548	0.0962979	0.1055	0.916	0.014196359	count	1
MGAT1	0.0102411	0.1093169	0.0937	0.925	0.014267117	count	1
R3HCC1	0.0108695	0.17123	0.0635	0.949	0.014319912	count	1
PTPN9	0.0139354	0.4461677	0.0312	0.975	0.014504811	count	1
DYNLT1	0.0103357	0.0750037	0.1378	0.89	0.014657002	count	1
TMEM14B	0.0103467	0.0675963	0.1531	0.878	0.014693551	count	1
ZFAT	0.0231498	0.640342	0.0362	0.971	0.014724574	count	1

TIGD1	0.0231498	0.6730098	0.0344	0.973	0.014724574	count	1
SUGCT	0.0231498	0.6798747	0.0341	0.973	0.014724574	count	1
AFG1L	0.0181018	0.5764071	0.0314	0.975	0.014725978	count	1
RANGAP1	0.0152013	0.3369128	0.0451	0.964	0.014823223	count	1
E2F6	0.0121895	0.2338413	0.0521	0.958	0.014838053	count	1
ENDOU	0.0346753	0.6667201	0.052	0.959	0.014996298	count	1
PTPMT1	0.0114787	0.1977135	0.0581	0.954	0.015015922	count	1
FBXO25	0.0116511	0.1829992	0.0637	0.949	0.01503928	count	1
NDUFAF7	0.0128738	0.2679207	0.0481	0.962	0.015127761	count	1
WAC	0.0108609	0.0959551	0.1132	0.91	0.015144722	count	1
DPM3	0.0108958	0.1008916	0.108	0.914	0.015147932	count	1
EXOC3	0.0122057	0.2340421	0.0522	0.958	0.015232132	count	1
YPEL1	0.0127897	0.2966585	0.0431	0.966	0.015432204	count	1
MOSPD3	0.0116529	0.1731113	0.0673	0.946	0.015506643	count	1
ARHGDIB	0.0108904	0.0587327	0.1854	0.8529	0.015538007	count	1
BAHCC1	0.0165508	0.604926	0.0274	0.978	0.015757861	count	1
USP19	0.0170138	0.3819366	0.0445	0.964	0.01576861	count	1
HES6	0.0626157	0.8550986	0.0732	0.942	0.01577936	count	1
SMIM37	0.0113113	0.0966943	0.117	0.907	0.015856279	count	1
CRIPT	0.011931	0.1656114	0.072	0.943	0.015861467	count	1
LINC01876	0.0498223	0.9452322	0.0527	0.958	0.015883399	count	1
HSPA12A	0.0498223	0.9463739	0.0526	0.958	0.015883399	count	1
KATNAL1	0.0135266	0.3178183	0.0426	0.966	0.015894073	count	1
PNISR	0.0112215	0.0743825	0.1509	0.88	0.015897271	count	1
ZNF77	0.0153436	0.3673286	0.0418	0.967	0.01596801	count	1
GPN2	0.0135009	0.2372632	0.0569	0.955	0.016003083	count	1
GPBP1L1	0.0119887	0.1470827	0.0815	0.935	0.01600389	count	1
ADCY9	0.0188598	0.440782	0.0428	0.966	0.016040221	count	1
FAM229A	0.0267317	0.5084316	0.0526	0.958	0.016088926	count	1
PAFAH1B1	0.0115231	0.0996325	0.1157	0.908	0.01619678	count	1
AC005076.1	0.0185849	0.4562196	0.0407	0.968	0.016419827	count	1
CDIPT	0.0118058	0.1037919	0.1137	0.909	0.016423991	count	1
C3orf52	0.0314467	0.6126772	0.0513	0.959	0.01649968	count	1
ZNF257	0.0223465	0.5027394	0.0444	0.965	0.016706798	count	1
CRY1	0.0158768	0.2956507	0.0537	0.957	0.01676786	count	1
AC004130.1	0.0208274	0.5628713	0.037	0.97	0.016935108	count	1
KMT2A	0.0119813	0.0877449	0.1365	0.891	0.016935385	count	1
WDR11	0.012971	0.2059478	0.063	0.95	0.01700161	count	1
DHX38	0.0136307	0.2058939	0.0662	0.947	0.017059773	count	1
ZFAND6	0.01223	0.0998	0.1225	0.902	0.017113616	count	1
NEDD4	0.0125644	0.1294923	0.097	0.923	0.017242795	count	1
IFI27L1	0.0130776	0.1837799	0.0712	0.943	0.017296371	count	1
RCOR1	0.0136441	0.2293829	0.0595	0.953	0.017324038	count	1
SNX19	0.0147841	0.2965168	0.0499	0.96	0.01737002	count	1
RMDN1	0.0129157	0.152737	0.0846	0.933	0.017456188	count	1

USP7	0.0130683	0.1393733	0.0938	0.925	0.017792634	count	1
DYRK3	0.0171383	0.2888874	0.0593	0.953	0.017967509	count	1
HIKESHI	0.0129747	0.0949151	0.1367	0.891	0.018112201	count	1
CCDC47	0.012803	0.0684077	0.1872	0.852	0.018164222	count	1
LINC00886	0.0421069	0.7986591	0.0527	0.958	0.018171872	count	1
AC092069.1	0.0156116	0.307909	0.0507	0.96	0.018226406	count	1
ZMYM2	0.0137453	0.1694035	0.0811	0.935	0.018289817	count	1
TWF2	0.0132026	0.106402	0.1241	0.901	0.018335712	count	1
NDUFS3	0.0130931	0.1036474	0.1263	0.899	0.018336725	count	1
SELENOH	0.0128807	0.0582339	0.2212	0.825	0.01836263	count	1
VCAN	0.0137588	0.2094278	0.0657	0.948	0.018448738	count	1
SEL1L	0.0136935	0.1491149	0.0918	0.927	0.018661312	count	1
AC104794.2	0.0171488	0.3103281	0.0553	0.956	0.018691329	count	1
RTN4	0.0132097	0.0599891	0.2202	0.826	0.018827297	count	1
NAA10	0.0132314	0.0636961	0.2077	0.835	0.01883407	count	1
CENPBD1	0.023398	0.4858201	0.0482	0.962	0.019016642	count	1
LSM8	0.0135315	0.082259	0.1645	0.869	0.019143954	count	1
TSG101	0.0136898	0.1094131	0.1251	0.9	0.019174684	count	1
STEAP1B	0.0766929	0.5908853	0.1298	0.897	0.019235898	count	1
ZNF566	0.0183742	0.4799148	0.0383	0.969	0.019260564	count	1
ATP1A1	0.0135504	0.067925	0.1995	0.842	0.019282216	count	1
AVIL	0.0322592	0.4905189	0.0658	0.948	0.019390363	count	1
TCF7L2	0.0142866	0.1495698	0.0955	0.924	0.019391542	count	1
JPX	0.0143635	0.1503613	0.0955	0.924	0.019400215	count	1
HACD3	0.0138335	0.1019091	0.1357	0.892	0.019419892	count	1
ZBTB12	0.0611456	1.1050162	0.0553	0.956	0.019423576	count	1
TRIL	0.0611456	1.1070819	0.0552	0.956	0.019423576	count	1
RCOR2	0.0611456	1.1070819	0.0552	0.956	0.019423576	count	1
AC025031.4	0.0611456	1.1070819	0.0552	0.956	0.019423576	count	1
GSDMB	0.0611456	1.1070819	0.0552	0.956	0.019423576	count	1
LINC00309	0.0611456	1.382313	0.0442	0.965	0.019423576	count	1
DNAJC14	0.0208216	0.3391933	0.0614	0.951	0.019556288	count	1
ARID4B	0.0138468	0.0759115	0.1824	0.855	0.019560408	count	1
CAPNS1	0.0137908	0.0591045	0.2333	0.8155	0.019655042	count	1
ZNF138	0.0154548	0.2217864	0.0697	0.944	0.019710613	count	1
SMARCAD1	0.0149678	0.2010304	0.0745	0.941	0.019795075	count	1
ISYNA1	0.0150574	0.23029	0.0654	0.948	0.019891563	count	1
MRPL21	0.0142727	0.0972162	0.1468	0.883	0.019967447	count	1
SVIL	0.014604	0.1228617	0.1189	0.905	0.020250731	count	1
ORMDL2	0.0147108	0.1130721	0.1301	0.896	0.020344797	count	1
CCDC127	0.0154728	0.1892739	0.0817	0.935	0.020356585	count	1
PPIL6	0.0231498	0.4613243	0.0502	0.96	0.020438239	count	1
PPP2R5D	0.0201811	0.3705749	0.0545	0.957	0.020471571	count	1
SSNA1	0.0145781	0.0918468	0.1587	0.874	0.020492209	count	1
WDR19	0.0202225	0.4687315	0.0431	0.966	0.020513465	count	1

KAT7	0.0162611	0.2074394	0.0784	0.938	0.020738158	count	1
B3GALNT2	0.0200222	0.2931786	0.0683	0.946	0.020825975	count	1
AL450998.2	0.0173613	0.2596054	0.0669	0.947	0.02084297	count	1
BHLHB9	0.021926	0.4637008	0.0473	0.962	0.020860043	count	1
KARS	0.0153132	0.1611435	0.095	0.924	0.020978802	count	1
TP53I3	0.015557	0.1411203	0.1102	0.912	0.021043709	count	1
FBXW11	0.0156506	0.1517757	0.1031	0.918	0.021075861	count	1
TPH1	0.1207569	0.7872883	0.1534	0.878	0.021100713	count	1
ZNF784	0.1207569	0.7846801	0.1539	0.878	0.021100713	count	1
PKN3	0.0162551	0.2358942	0.0689	0.945	0.021152282	count	1
MTHFD2L	0.0170143	0.2284227	0.0745	0.941	0.02116166	count	1
CHODL	0.0669149	0.5825311	0.1149	0.909	0.02121744	count	1
HSD17B8	0.0165929	0.2197468	0.0755	0.94	0.021250639	count	1
MDM1	0.0188701	0.2792347	0.0676	0.946	0.02147312	count	1
TCP1	0.0153852	0.0867437	0.1774	0.859	0.021558284	count	1
MROH6	0.0259277	0.4228113	0.0613	0.951	0.021558929	count	1
SLC20A2	0.0196067	0.2960969	0.0662	0.947	0.021599999	count	1
DOK1	0.0197264	0.2739547	0.072	0.943	0.021731618	count	1
DYNLRB1	0.0153787	0.0538195	0.2857	0.7751	0.021955123	count	1
C14orf119	0.0157488	0.098345	0.1601	0.873	0.021982217	count	1
TMLHE	0.0199487	0.30276	0.0659	0.947	0.022089571	count	1
CU638689.5	0.039239	0.9564982	0.041	0.967	0.022111879	count	1
GTF2H1	0.017302	0.1802321	0.096	0.924	0.022328083	count	1
ACTRT3	0.0321968	0.6615198	0.0487	0.961	0.022363706	count	1
ELAVL2	0.0321968	0.7003096	0.046	0.963	0.022363706	count	1
CCAR1	0.0165404	0.1655062	0.0999	0.92	0.022406055	count	1
CLASRP	0.0201126	0.2725511	0.0738	0.941	0.02248855	count	1
AC078802.1	0.0711061	0.9696316	0.0733	0.942	0.022516445	count	1
NHLRC1	0.0711061	0.9696316	0.0733	0.942	0.022516445	count	1
NEXMIF	0.0711061	1.106081	0.0643	0.949	0.022516445	count	1
PNPLA7	0.0711061	1.106081	0.0643	0.949	0.022516445	count	1
RPS12	0.0157174	0.0187349	0.8389	0.402	0.022666458	count	1
FAM216A	0.0262905	0.4060582	0.0647	0.948	0.022778343	count	1
PRH1	0.0183861	0.2322012	0.0792	0.937	0.022829465	count	1
UBXN2A	0.0176742	0.1809078	0.0977	0.922	0.022867863	count	1
AKT2	0.019503	0.2912844	0.067	0.947	0.022906157	count	1
TOMM70	0.0169659	0.1509539	0.1124	0.911	0.023014286	count	1
ZNF879	0.0383563	0.5877935	0.0653	0.948	0.023021899	count	1
KCTD12	0.0164787	0.1044373	0.1578	0.875	0.023262458	count	1
BUD31	0.0165367	0.0809065	0.2044	0.838	0.023279059	count	1
UBA2	0.0171521	0.1220759	0.1405	0.888	0.023474141	count	1
NFATC2	0.0184367	0.2219853	0.0831	0.934	0.023535876	count	1
AL450326.1	0.0223267	0.4198673	0.0532	0.958	0.023563189	count	1
TRAPPC13	0.0217842	0.3517006	0.0619	0.951	0.023595189	count	1
MAP3K13	0.0170443	0.111053	0.1535	0.878	0.023696097	count	1

FBXW7	0.0181961	0.1895324	0.096	0.924	0.023763854	count	1
MED8	0.0180041	0.1522069	0.1183	0.906	0.023907239	count	1
SLIRP	0.0172162	0.087617	0.1965	0.844	0.023973396	count	1
UMPS	0.0187921	0.215376	0.0873	0.93	0.023989173	count	1
SEC22B	0.0176686	0.1163093	0.1519	0.879	0.024303431	count	1
MRPS36	0.0174622	0.1009428	0.173	0.863	0.024382697	count	1
MED17	0.0257234	0.3304623	0.0778	0.938	0.024459962	count	1
AHSA1	0.0179816	0.1202786	0.1495	0.881	0.024552544	count	1
TAF1D	0.0173882	0.0786114	0.2212	0.825	0.024565481	count	1
CDK19	0.0236903	0.3121688	0.0759	0.94	0.024631074	count	1
MMS22L	0.0297133	0.4404781	0.0675	0.946	0.024690553	count	1
SYNE1	0.0181368	0.1282245	0.1414	0.888	0.024826223	count	1
PPHLN1	0.0181588	0.1272145	0.1427	0.887	0.024956721	count	1
YPEL3	0.0176767	0.0837682	0.211	0.833	0.025100043	count	1
RBX1	0.0176408	0.0609933	0.2892	0.772	0.025107006	count	1
PUDP	0.0235331	0.3575054	0.0658	0.948	0.025171127	count	1
AC124312.1	0.0363156	0.7222685	0.0503	0.96	0.025202696	count	1
RABGEF1	0.0256019	0.4349533	0.0589	0.953	0.025204	count	1
CWF19L1	0.0225773	0.290425	0.0777	0.938	0.025238719	count	1
ZNF513	0.0254366	0.349663	0.0727	0.942	0.025301023	count	1
FUNDC1	0.0195025	0.172894	0.1128	0.91	0.025315113	count	1
KLHDC10	0.019561	0.1888053	0.1036	0.917	0.025450797	count	1
PIK3R2	0.2935232	1.081219	0.2715	0.786	0.025565853	count	1
TMEM9B-AS1	0.2935232	1.102052	0.2663	0.79	0.025565853	count	1
PCDH18	0.2935232	1.159126	0.2532	0.8	0.025565853	count	1
AC091563.1	0.2935232	1.165386	0.2519	0.801	0.025565853	count	1
AL162586.1	0.2935232	1.425015	0.206	0.837	0.025565853	count	1
PLGLB1	0.2955192	1.109806	0.2663	0.79	0.025720168	count	1
AC078883.2	0.2955192	1.109806	0.2663	0.79	0.025720168	count	1
ATP2A1-AS1	0.2955192	1.109806	0.2663	0.79	0.025720168	count	1
POC5	0.0215695	0.2720091	0.0793	0.937	0.025823818	count	1
PARP15	0.2976949	1.193464	0.2494	0.803	0.025888095	count	1
EIF2S2	0.0182028	0.0574944	0.3166	0.7516	0.02599936	count	1
EFCAB13	0.0234343	0.2610478	0.0898	0.928	0.026069771	count	1
GPALPP1	0.019823	0.1734477	0.1143	0.909	0.026122831	count	1
FAM126A	0.020633	0.2195896	0.094	0.925	0.026126169	count	1
ARAP1	0.0252427	0.3462682	0.0729	0.942	0.026240492	count	1
ZNF83	0.0195675	0.1620973	0.1207	0.904	0.026417165	count	1
RNF135	0.0206459	0.2151604	0.096	0.924	0.026566329	count	1
PCID2	0.0197894	0.1492288	0.1326	0.895	0.026655451	count	1
HHEX	0.0191787	0.101838	0.1883	0.851	0.026827464	count	1
PXMP4	0.0232464	0.3043309	0.0764	0.939	0.026848828	count	1
ALG14	0.0213889	0.2291067	0.0934	0.926	0.026872437	count	1
SURF6	0.0210588	0.196335	0.1073	0.915	0.026991992	count	1
RPL28	0.0187386	0.0200064	0.9366	0.349	0.027015419	count	1

EIF3D	0.0192463	0.0679803	0.2831	0.777	0.027266832	count	1
CMPK1	0.0196681	0.0905766	0.2171	0.828	0.027544524	count	1
CDPF1	0.0260139	0.3333607	0.078	0.938	0.027633965	count	1
PCBD2	0.029142	0.3411755	0.0854	0.932	0.027697511	count	1
AL021453.1	0.0334248	0.4940737	0.0677	0.946	0.027756873	count	1
DCTN2	0.0198375	0.0889263	0.2231	0.823	0.027778171	count	1
WDR18	0.020749	0.1550016	0.1339	0.894	0.027858259	count	1
HYAL1	0.0237661	0.2242407	0.106	0.916	0.02790414	count	1
UBR2	0.0207339	0.1729006	0.1199	0.905	0.027954915	count	1
PPARD	0.028558	0.3809359	0.075	0.94	0.028103413	count	1
TMUB2	0.0217795	0.176681	0.1233	0.902	0.028150432	count	1
CIAO1	0.0209779	0.1522308	0.1378	0.89	0.028186094	count	1
TRIM68	0.0356752	0.5712601	0.0624	0.95	0.028205203	count	1
ALAD	0.0256261	0.3171479	0.0808	0.936	0.028215008	count	1
NOP10	0.0198404	0.0551059	0.36	0.7188	0.028321301	count	1
MIPEP	0.0310967	0.3731134	0.0833	0.934	0.028338032	count	1
MIS18A	0.024318	0.3011636	0.0807	0.936	0.02855094	count	1
CCNI	0.0199346	0.0393512	0.5066	0.612	0.028623444	count	1
RTL8A	0.0206507	0.1089263	0.1896	0.85	0.028645509	count	1
KIN	0.0207175	0.1288424	0.1608	0.872	0.02868491	count	1
ODC1	0.0204496	0.1256994	0.1627	0.871	0.028808001	count	1
ZFP28	0.0321379	0.4263878	0.0754	0.94	0.028820617	count	1
PYURF	0.0202733	0.0628453	0.3226	0.747	0.028839799	count	1
BRF1	0.0260825	0.2865455	0.091	0.927	0.02886488	count	1
CLIC1	0.0200934	0.0375863	0.5346	0.593	0.0288675	count	1
STK3	0.0219929	0.173366	0.1269	0.899	0.029013858	count	1
HSF2BP	0.0556731	0.8421513	0.0661	0.947	0.029028172	count	1
GALK2	0.0235468	0.2073977	0.1135	0.91	0.029082059	count	1
SLC44A1	0.021731	0.1651105	0.1316	0.895	0.029108962	count	1
KIF9-AS1	0.0486597	0.6777291	0.0718	0.943	0.029134648	count	1
UBE3C	0.0231667	0.2058697	0.1125	0.91	0.029366316	count	1
OSMR	0.0225406	0.2027153	0.1112	0.911	0.029368453	count	1
GRB2	0.0208553	0.0878747	0.2373	0.812	0.029373375	count	1
EIF5A	0.0206836	0.0628995	0.3288	0.742	0.029472411	count	1
NUDT16L1	0.022411	0.1770381	0.1266	0.899	0.029565001	count	1
TMEM223	0.0215594	0.1301922	0.1656	0.868	0.029638394	count	1
TMEM161B-AS1	0.0245363	0.2743186	0.0894	0.929	0.029650283	count	1
KIF9	0.0216547	0.1175928	0.1842	0.854	0.029817458	count	1
RPL21	0.0207036	0.0218052	0.9495	0.342	0.029828989	count	1
AC103736.1	0.1207569	0.6406867	0.1885	0.851	0.029842245	count	1
FURIN	0.0256295	0.2313966	0.1108	0.912	0.029994612	count	1
TRMT12	0.0270481	0.260471	0.1038	0.917	0.030079851	count	1
GLUD1	0.0216748	0.1119965	0.1935	0.847	0.030128674	count	1
KRCC1	0.0220456	0.1308444	0.1685	0.866	0.030387912	count	1
E4F1	0.0292709	0.3910755	0.0748	0.94	0.030413968	count	1

NT5DC3	0.0818304	0.8060173	0.1015	0.919	0.030606119	count	1
KCTD15	0.0315093	0.3597941	0.0876	0.93	0.030659538	count	1
ZUP1	0.0281787	0.3154166	0.0893	0.929	0.030679641	count	1
L3HYPDH	0.0243944	0.2333862	0.1045	0.917	0.030957532	count	1
BX890604.1	0.043059	0.5399079	0.0798	0.936	0.030990234	count	1
GAK	0.0274029	0.2285847	0.1199	0.905	0.03128511	count	1
SLC25A36	0.0224727	0.0990338	0.2269	0.821	0.03146922	count	1
SH3GL1	0.0244183	0.1824461	0.1338	0.894	0.031501968	count	1
PIAS4	0.0293691	0.3292744	0.0892	0.929	0.031593657	count	1
EMP2	0.0220663	0.0519793	0.4245	0.671	0.031611975	count	1
MYH9	0.022166	0.0659769	0.336	0.7369	0.031654447	count	1
ZNF85	0.025125	0.2445912	0.1027	0.918	0.031806962	count	1
AC104825.1	0.0611456	0.7814752	0.0782	0.938	0.031836186	count	1
GK	0.0568636	0.5143479	0.1106	0.912	0.031903699	count	1
ANO10	0.0256104	0.2126254	0.1204	0.904	0.031937002	count	1
HAS3	0.0378322	0.6332234	0.0597	0.952	0.03207425	count	1
TCEA2	0.0233169	0.1170046	0.1993	0.842	0.032221108	count	1
LRRC14	0.0315461	0.3063972	0.103	0.918	0.03223859	count	1
DPYD	0.0251174	0.2021458	0.1243	0.901	0.032251915	count	1
DDOST	0.0229697	0.0847406	0.2711	0.786	0.032338447	count	1
AF064858.1	0.0866033	0.7943942	0.109	0.913	0.032344637	count	1
FAHD1	0.0249402	0.1956404	0.1275	0.899	0.032366297	count	1
HAX1	0.022909	0.0808601	0.2833	0.777	0.032388259	count	1
USP24	0.0275941	0.2496636	0.1105	0.912	0.032582047	count	1
KIF21A	0.0301369	0.3339377	0.0902	0.928	0.032614589	count	1
TMEM206	0.0393555	0.4183439	0.0941	0.925	0.032648377	count	1
KLF8	0.0290232	0.2755144	0.1053	0.916	0.032720549	count	1
PHC1	0.0329828	0.4064273	0.0812	0.935	0.032775678	count	1
LSM12	0.0236692	0.1086873	0.2178	0.828	0.032793647	count	1
MIER1	0.0232072	0.0798295	0.2907	0.771	0.032819275	count	1
BORA	0.0693145	0.6916368	0.1002	0.92	0.032996685	count	1
RNMT	0.0237677	0.1124882	0.2113	0.833	0.033016852	count	1
TMED8	0.0363473	0.3889738	0.0934	0.926	0.033096733	count	1
VPS45	0.0293099	0.3131516	0.0936	0.925	0.033323058	count	1
NDUFA10	0.0237189	0.0861405	0.2754	0.783	0.033438882	count	1
B3GNTL1	0.0449528	0.6281024	0.0716	0.943	0.033458925	count	1
IKZF5	0.0322891	0.3619549	0.0892	0.929	0.033538485	count	1
THAP11	0.0248945	0.1644752	0.1514	0.88	0.03363573	count	1
KMT2B	0.0251875	0.1781868	0.1414	0.888	0.033638675	count	1
GCAT	0.0276634	0.285182	0.097	0.923	0.033710642	count	1
LSG1	0.0251824	0.182338	0.1381	0.89	0.033843567	count	1
EPDR1	0.0348195	0.4935763	0.0705	0.944	0.033865582	count	1
MMP1	0.4059749	0.629479	0.6449	0.519	0.033879784	count	1
RHPN1	0.0332011	0.300624	0.1104	0.912	0.033923242	count	1
PPP1R3G	0.1382599	1.12831	0.1225	0.902	0.033966337	count	1

GPC2	0.1382599	1.12831	0.1225	0.902	0.033966337	count	1
FAM71F2	0.1382599	1.12831	0.1225	0.902	0.033966337	count	1
SPIN2A	0.1382599	1.12831	0.1225	0.902	0.033966337	count	1
MRVI1	0.1382599	1.12831	0.1225	0.902	0.033966337	count	1
AP001062.1	0.1382599	1.12831	0.1225	0.902	0.033966337	count	1
SDC1	0.1382599	1.1346253	0.1219	0.903	0.033966337	count	1
CEP295NL	0.1382599	1.302298	0.1062	0.915	0.033966337	count	1
C1orf74	0.1382599	1.3181	0.1049	0.916	0.033966337	count	1
CDK6	0.0279265	0.229133	0.1219	0.903	0.034099483	count	1
DNAJB12	0.025156	0.1323056	0.1901	0.849	0.034358156	count	1
BBS12	0.0477861	0.5268264	0.0907	0.928	0.034359193	count	1
EIF1B	0.0241844	0.061981	0.3902	0.696	0.034434327	count	1
PACSIN3	0.0662787	0.768452	0.0862	0.931	0.034462658	count	1
PRDX3	0.0244724	0.0750533	0.3261	0.744	0.034591961	count	1
PEX19	0.0274217	0.2143875	0.1279	0.898	0.034710483	count	1
RPL19	0.0242662	0.0170048	1.427	0.154	0.034992565	count	1
ZNF347	0.0276528	0.2823226	0.0979	0.922	0.035002606	count	1
RABEP2	0.0289749	0.2411258	0.1202	0.904	0.035081861	count	1
MOB1A	0.0251532	0.097332	0.2584	0.796	0.035262708	count	1
STARD4-AS1	0.2083008	0.6465766	0.3222	0.747	0.035274498	count	1
LINC01126	0.1439601	1.1047751	0.1303	0.896	0.035298596	count	1
LINC00997	0.1439601	1.0982246	0.1311	0.896	0.035298596	count	1
LIN37	0.1439601	1.2729807	0.1131	0.91	0.035298596	count	1
ADCY7	0.1439601	1.6336445	0.0881	0.9298	0.035298596	count	1
SUPT5H	0.0271996	0.1696418	0.1603	0.873	0.035484382	count	1
TMEM108	0.0952922	0.7694125	0.1239	0.901	0.035496421	count	1
AC018362.2	0.2101562	0.7849648	0.2677	0.789	0.035564985	count	1
RTN4IP1	0.0453597	0.5590202	0.0811	0.935	0.035797887	count	1
CYTIP	0.4356511	0.7954889	0.5477	0.584	0.035948473	count	1
INTS5	0.0416682	0.3475865	0.1199	0.905	0.036011367	count	1
BRI3	0.0251643	0.0452645	0.5559	0.578	0.036075878	count	1
DCTN6	0.0260168	0.1055452	0.2465	0.805	0.036117341	count	1
NPIPA5	0.4396376	1.062097	0.4139	0.679	0.036222499	count	1
TGFBI	0.4396376	1.11474	0.3944	0.693	0.036222499	count	1
SLC25A39	0.0265733	0.1253344	0.212	0.832	0.036353372	count	1
KREMEN1	0.0977636	0.5726123	0.1707	0.864	0.036389795	count	1
RBM3	0.0255705	0.0542611	0.4712	0.638	0.036407167	count	1
ACAD10	0.0576936	0.7849258	0.0735	0.941	0.036408299	count	1
PKD2L2	0.1490635	1.0356163	0.1439	0.886	0.036486868	count	1
PYCR2	0.0273359	0.1451022	0.1884	0.851	0.036489871	count	1
TMPO	0.0267876	0.1328037	0.2017	0.84	0.036696923	count	1
SNX32	0.4466317	1.087529	0.4107	0.681	0.036701068	count	1
AC007038.1	0.4466317	1.090297	0.4096	0.682	0.036701068	count	1
LINC00242	0.4466317	1.090297	0.4096	0.682	0.036701068	count	1
PCLAF	0.4466317	1.242488	0.3595	0.719	0.036701068	count	1

AC136604.3	0.0861337	1.0919	0.0789	0.937	0.036707166	count	1
MTFR2	0.0861337	1.0919	0.0789	0.937	0.036707166	count	1
AC005225.2	0.0861337	1.0919	0.0789	0.937	0.036707166	count	1
AC106028.4	0.0861337	1.0919	0.0789	0.937	0.036707166	count	1
DPH1	0.0861337	1.0919	0.0789	0.937	0.036707166	count	1
ASB1	0.0861337	1.179391	0.073	0.942	0.036707166	count	1
PRR14L	0.0288452	0.2245831	0.1284	0.898	0.036805129	count	1
MINOS1	0.0261268	0.081706	0.3198	0.749	0.036816965	count	1
AC022784.3	0.2183519	0.9504848	0.2297	0.818	0.036843281	count	1
SARDH	0.2183519	0.9504848	0.2297	0.818	0.036843281	count	1
AL590428.1	0.2183519	1.0290359	0.2122	0.832	0.036843281	count	1
LRRN1	0.2183519	1.247814	0.175	0.861	0.036843281	count	1
AC004922.1	0.2183519	1.247814	0.175	0.861	0.036843281	count	1
LPCAT3	0.0298774	0.2073908	0.1441	0.885	0.036887106	count	1
POLR2J3	0.0992621	0.684842	0.1449	0.885	0.036930811	count	1
DUT	0.0258418	0.0602221	0.4291	0.668	0.036935179	count	1
UAP1	0.0283929	0.1588455	0.1787	0.858	0.037039425	count	1
MAPK13	0.2198601	1.1182927	0.1966	0.844	0.037077657	count	1
AC007448.3	0.2198601	1.1182927	0.1966	0.844	0.037077657	count	1
MAFIP	0.2198601	1.127178	0.1951	0.845	0.037077657	count	1
AL121672.2	0.2198601	1.14004	0.1929	0.847	0.037077657	count	1
FAM239A	0.2198601	1.375293	0.1599	0.873	0.037077657	count	1
WIPF2	0.0288112	0.2066821	0.1394	0.889	0.03709443	count	1
AC092053.3	0.4540692	1.099963	0.4128	0.68	0.037206925	count	1
SRSF12	0.4540692	1.099963	0.4128	0.68	0.037206925	count	1
AL513550.1	0.4540692	1.099963	0.4128	0.68	0.037206925	count	1
UFSP2	0.0273396	0.147828	0.1849	0.853	0.037272478	count	1
GNPNAT1	0.0306006	0.2193528	0.1395	0.889	0.037282974	count	1
EXOSC1	0.0274921	0.1370934	0.2005	0.841	0.037283532	count	1
ARHGEF16	0.2212237	1.0896208	0.203	0.839	0.037289334	count	1
CLEC4A	0.2212237	1.0896208	0.203	0.839	0.037289334	count	1
AC104590.1	0.2212237	1.0896208	0.203	0.839	0.037289334	count	1
ZGPAT	0.2212237	1.0896208	0.203	0.839	0.037289334	count	1
DMC1	0.2212237	1.0896208	0.203	0.839	0.037289334	count	1
AC133550.2	0.2212237	1.1031177	0.2005	0.841	0.037289334	count	1
ZSWIM5	0.2212237	1.1125946	0.1988	0.842	0.037289334	count	1
RNF26	0.0405899	0.3231759	0.1256	0.9	0.03749002	count	1
RPUSD1	0.0309483	0.2506515	0.1235	0.902	0.037547962	count	1
FBXW5	0.0271806	0.1116503	0.2434	0.808	0.037647651	count	1
ZNF514	0.0398263	0.3482574	0.1144	0.909	0.037795932	count	1
CHD1L	0.0338475	0.3231198	0.1048	0.917	0.037798283	count	1
CTSO	0.0287038	0.1728805	0.166	0.868	0.037858254	count	1
SPOP	0.0274572	0.1034229	0.2655	0.791	0.037998313	count	1
COPS9	0.0269039	0.0649432	0.4143	0.679	0.038120617	count	1
RPRD2	0.0297922	0.2002879	0.1487	0.882	0.03828354	count	1

NPC1	0.0344745	0.2944762	0.1171	0.907	0.038674608	count	1
TNFRSF14-AS1	0.0540616	0.4519896	0.1196	0.905	0.038821476	count	1
IQCC	0.1260407	0.8015755	0.1572	0.875	0.039219402	count	1
AP001269.4	0.0628357	0.7389298	0.085	0.932	0.039606469	count	1
CFAP57	0.4943126	1.20845	0.409	0.683	0.039890116	count	1
SPR	0.0302806	0.1965066	0.1541	0.878	0.039935733	count	1
POLE3	0.0298441	0.1513408	0.1972	0.844	0.040132039	count	1
TBCK	0.0338119	0.2840386	0.119	0.905	0.040238138	count	1
RALGPS2	0.0429972	0.3475338	0.1237	0.902	0.040256834	count	1
TMEM44	0.0316318	0.2139329	0.1479	0.882	0.040441239	count	1
INO80C	0.0296661	0.1289183	0.2301	0.818	0.040487619	count	1
GTF3C4	0.0347678	0.324045	0.1073	0.915	0.040530553	count	1
TXLNB	0.0455758	0.4647891	0.0981	0.922	0.040786632	count	1
TSC22D4	0.0320857	0.1959131	0.1638	0.87	0.040797417	count	1
ELP4	0.033121	0.2264811	0.1462	0.884	0.040812026	count	1
POP4	0.0298104	0.1251709	0.2382	0.812	0.040850949	count	1
C3orf58	0.0311229	0.1646762	0.189	0.85	0.040948398	count	1
GPATCH3	0.0356214	0.4993898	0.0713	0.943	0.040950988	count	1
ATP6V1E2	0.1684481	0.7998611	0.2106	0.833	0.040961756	count	1
RAET1E	0.1684481	0.9205483	0.183	0.855	0.040961756	count	1
TSC2	0.0337437	0.2172436	0.1553	0.877	0.041019105	count	1
AC087623.3	0.0735778	0.4796907	0.1534	0.878	0.041109394	count	1
ZNF589	0.0573891	0.4628667	0.124	0.901	0.041182811	count	1
MMS19	0.0389404	0.3453134	0.1128	0.91	0.041307914	count	1
VIM	0.0286426	0.0237916	1.2039	0.229	0.04131495	count	1
DYNLL2	0.0318282	0.1838118	0.1732	0.863	0.041661097	count	1
CARMN	0.0746551	0.7639967	0.0977	0.922	0.041700053	count	1
HEXIM1	0.0322541	0.1572452	0.2051	0.837	0.041776959	count	1
RPL7	0.029137	0.0252629	1.1533	0.249	0.041945906	count	1
ZBTB11-AS1	0.041858	0.3142957	0.1332	0.894	0.041957271	count	1
AC006333.2	0.0370834	0.3157444	0.1174	0.907	0.041957735	count	1
CRADD	0.0376125	0.3307735	0.1137	0.909	0.041988144	count	1
SCP2	0.0296394	0.0656643	0.4514	0.652	0.042152772	count	1
MORF4L2	0.0301905	0.086781	0.3479	0.728	0.042314586	count	1
NSF	0.0362389	0.24134	0.1502	0.881	0.042376253	count	1
PDCL	0.0305688	0.1095037	0.2792	0.78	0.04239673	count	1
TMEM184C	0.0366381	0.3545395	0.1033	0.918	0.042418701	count	1
ADH5	0.0301954	0.0884166	0.3415	0.733	0.042499092	count	1
CSNK2A1	0.0309338	0.1188742	0.2602	0.795	0.042651553	count	1
FAM149A	0.0517032	0.5551745	0.0931	0.926	0.042799822	count	1
EXOSC10	0.0338182	0.1962047	0.1724	0.863	0.042896839	count	1
NUDT5	0.0310437	0.1173295	0.2646	0.791	0.04307865	count	1
C5orf24	0.031015	0.1010622	0.3069	0.759	0.043183522	count	1
RNF139	0.033933	0.1894522	0.1791	0.858	0.043191259	count	1
PFDN5	0.0300909	0.0281831	1.0677	0.2858	0.04330317	count	1

PPFIA1	0.0319049	0.1614934	0.1976	0.843	0.043309968	count	1
SPPL3	0.0328605	0.174699	0.1881	0.851	0.043359161	count	1
IL1R1	0.0306418	0.0853513	0.359	0.72	0.043521727	count	1
ZP3	0.0568415	0.3663882	0.1551	0.877	0.043528609	count	1
CTNNBIP1	0.0331279	0.1598701	0.2072	0.836	0.043661194	count	1
LINC00685	0.0432597	0.3410893	0.1268	0.899	0.043759581	count	1
TRIM28	0.0324918	0.1413426	0.2299	0.818	0.043793968	count	1
SRSF9	0.0308401	0.0576067	0.5354	0.592	0.043935929	count	1
DPH3	0.0323783	0.129396	0.2502	0.802	0.043940397	count	1
CLEC11A	0.0789292	0.4088568	0.193	0.847	0.044040244	count	1
NOL7	0.0308843	0.0580277	0.5322	0.5946	0.044055782	count	1
PSME3	0.0358011	0.211878	0.169	0.866	0.044107418	count	1
TMEM123	0.0312401	0.0804924	0.3881	0.698	0.04427041	count	1
MFSD11	0.0338726	0.186771	0.1814	0.856	0.044421971	count	1
B3GALT4	0.0344717	0.2150021	0.1603	0.873	0.04445249	count	1
TMX2	0.0322766	0.1220151	0.2645	0.791	0.044521329	count	1
FUZ	0.0405559	0.2952187	0.1374	0.891	0.044588583	count	1
FUBP1	0.0336405	0.1362455	0.2469	0.805	0.044713211	count	1
RASAL3	0.1448686	0.9942973	0.1457	0.884	0.044806726	count	1
PACRGL	0.041	0.296279	0.1384	0.89	0.044832436	count	1
CR381653.1	0.094958	0.8019891	0.1184	0.906	0.044887555	count	1
AC105760.2	0.094958	0.9431545	0.1007	0.92	0.044887555	count	1
TRAPPC3	0.0322859	0.1015595	0.3179	0.751	0.04499566	count	1
CDIP1	0.0339367	0.1744113	0.1946	0.846	0.045062684	count	1
AC016773.1	0.5761798	1.139477	0.5057	0.613	0.045075474	count	1
POMGNT1	0.0352759	0.213476	0.1652	0.869	0.045185349	count	1
SLC22A18AS	0.0658707	0.7932671	0.083	0.934	0.045427723	count	1
PCNA	0.0336251	0.1398814	0.2404	0.81	0.045690784	count	1
LINC01704	0.5864397	1.080719	0.5426	0.587	0.045700263	count	1
UCKL1-AS1	0.5864397	1.138642	0.515	0.607	0.045700263	count	1
IRF5	0.5864397	1.183695	0.4954	0.62	0.045700263	count	1
PKP1	0.5864397	1.236804	0.4742	0.635	0.045700263	count	1
CCDC124	0.0326352	0.0893617	0.3652	0.715	0.045755952	count	1
SRI	0.0321935	0.0617618	0.5213	0.602	0.045789168	count	1
CRTC1	0.0442769	0.4760625	0.093	0.926	0.045927011	count	1
EXTL3	0.0499833	0.4296263	0.1163	0.907	0.046102243	count	1
UFC1	0.0323801	0.0531514	0.6092	0.5424	0.046233619	count	1
MYLPF	0.5971708	1.088783	0.5485	0.583	0.046347923	count	1
RALY	0.0330844	0.0839384	0.3942	0.694	0.04665411	count	1
KIAA1549	0.078495	0.8045834	0.0976	0.922	0.046663841	count	1
AC026401.3	0.126469	0.64329	0.1966	0.844	0.046666211	count	1
FUOM	0.0399642	0.2976732	0.1343	0.893	0.046718966	count	1
CTBP1-DT	0.0469037	0.3421489	0.1371	0.891	0.046985315	count	1
DNAJC24	0.0395584	0.2906776	0.1361	0.892	0.047057424	count	1
OSBPL8	0.0338403	0.106288	0.3184	0.75	0.047136414	count	1

USP8	0.0341174	0.117355	0.2907	0.771	0.047168301	count	1
BLOC1S5	0.0350655	0.160123	0.219	0.827	0.047212093	count	1
ADAMTS3	0.0472587	0.4318224	0.1094	0.913	0.047338829	count	1
HMGN3	0.0334225	0.05533	0.6041	0.5459	0.04782959	count	1
FBRSL1	0.0452632	0.3351163	0.1351	0.893	0.047982242	count	1
GPT2	0.1307031	1.236856	0.1057	0.916	0.048166428	count	1
PDS5A	0.0368574	0.1584045	0.2327	0.816	0.048168507	count	1
WAPL	0.0362883	0.1609605	0.2254	0.822	0.048181552	count	1
SLF2	0.0364226	0.2002479	0.1819	0.856	0.048211902	count	1
TNR	0.630739	0.9304949	0.6779	0.498	0.048335858	count	1
MPPE1	0.0400168	0.2209906	0.1811	0.856	0.048415831	count	1
FAM174A	0.0349889	0.1215458	0.2879	0.773	0.048490756	count	1
PBRM1	0.0357266	0.1276397	0.2799	0.78	0.048531443	count	1
NDUFB5	0.0341435	0.0681705	0.5009	0.617	0.04854997	count	1
SEC62	0.0338574	0.0432911	0.7821	0.434	0.048610793	count	1
VAMP1	0.1032859	0.7797271	0.1325	0.895	0.048712456	count	1
PCDHB7	0.1032859	0.8324744	0.1241	0.901	0.048712456	count	1
RAB11FIP1	0.0353377	0.1626147	0.2173	0.828	0.048841647	count	1
C9orf78	0.0345973	0.0867572	0.3988	0.69	0.0488861	count	1
GNL3	0.0362367	0.1301761	0.2784	0.781	0.048930883	count	1
TPT1	0.0339767	0.0190076	1.7875	0.074	0.049000474	count	1
FANCC	0.0711748	0.5604688	0.127	0.899	0.04903008	count	1
ZXDB	0.0608562	0.3593646	0.1693	0.866	0.049130469	count	1
KTN1	0.0342482	0.0452248	0.7573	0.449	0.049144618	count	1
ITPR1	0.036677	0.1547193	0.2371	0.813	0.049275708	count	1
IFT74	0.0373514	0.1956329	0.1909	0.849	0.049305058	count	1
AL591846.2	0.0597534	0.4794437	0.1246	0.901	0.049394308	count	1
GANC	0.0521837	0.3255603	0.1603	0.873	0.049437651	count	1
SVIP	0.0349911	0.0864616	0.4047	0.686	0.049446934	count	1
DGCR6L	0.035732	0.1330605	0.2685	0.788	0.049473345	count	1
TBC1D17	0.0417347	0.2691795	0.155	0.877	0.049638518	count	1
PRCC	0.0380583	0.2027342	0.1877	0.851	0.049665312	count	1
EFNB1	0.0460011	0.2686835	0.1712	0.864	0.049702293	count	1
SF1	0.035058	0.0748244	0.4685	0.639	0.049712302	count	1
GSPT1	0.0355932	0.0878645	0.4051	0.685	0.04994393	count	1
KLC1	0.0382226	0.1575693	0.2426	0.808	0.049950041	count	1
RUNX1	0.0842558	0.5651008	0.1491	0.881	0.050019099	count	1
CENPP	0.0842558	0.5663318	0.1488	0.882	0.050019099	count	1
AC245014.3	0.6600811	0.8135182	0.8114	0.417	0.050027158	count	1
MED7	0.0395221	0.2337308	0.1691	0.866	0.05005785	count	1
RPP38	0.0372969	0.1963183	0.19	0.849	0.05021338	count	1
SUDS3	0.0367488	0.1431919	0.2566	0.797	0.050273195	count	1
MICAL3	0.0481304	0.2614625	0.1841	0.854	0.05028432	count	1
GBP7	0.0431661	0.3272314	0.1319	0.895	0.050287646	count	1
STX7	0.0366542	0.1216778	0.3012	0.763	0.05032318	count	1

ARHGEF15	0.0378075	0.1939664	0.1949	0.845	0.050537613	count	1
EEA1	0.0360317	0.0965618	0.3731	0.709	0.050539244	count	1
ARFGEF1	0.0375202	0.1447597	0.2592	0.796	0.050581291	count	1
RMDN2	0.045249	0.3519925	0.1286	0.898	0.050711978	count	1
ECHDC1	0.0370745	0.1291371	0.2871	0.774	0.050728588	count	1
ZNF562	0.0425267	0.2881092	0.1476	0.883	0.050840241	count	1
NECAP2	0.0367332	0.1192236	0.3081	0.758	0.05090673	count	1
LY6G5B	0.6783599	1.097942	0.6178	0.537	0.051059308	count	1
TRIQK	0.0379961	0.1785624	0.2128	0.832	0.051100667	count	1
AC097634.1	0.0633509	0.4649136	0.1363	0.892	0.051121475	count	1
RTL8C	0.0366045	0.0911881	0.4014	0.688	0.051144581	count	1
RBFOX2	0.0377777	0.1398497	0.2701	0.787	0.05116212	count	1
UCHL5	0.0380512	0.1516115	0.251	0.802	0.051362446	count	1
AL158212.2	0.0866033	0.5611906	0.1543	0.877	0.051383617	count	1
ACOT13	0.0378797	0.1408701	0.2689	0.788	0.051399438	count	1
DCLK1	0.121912	0.5048425	0.2415	0.809	0.051420825	count	1
C1orf56	0.0380512	0.1455683	0.2614	0.794	0.051426108	count	1
PHF14	0.0366545	0.0937175	0.3911	0.696	0.051479075	count	1
SLC35D1	0.058643	0.5041942	0.1163	0.907	0.051482684	count	1
RNF216	0.0389065	0.175488	0.2217	0.825	0.051495468	count	1
FAM193A	0.0476732	0.275001	0.1734	0.862	0.051500068	count	1
CAPN1	0.0386185	0.1788517	0.2159	0.829	0.051532864	count	1
NSMCE2	0.0398073	0.1706449	0.2333	0.816	0.051710815	count	1
ZMYM4	0.0383207	0.1547491	0.2476	0.804	0.051758207	count	1
APH1B	0.0405956	0.2043217	0.1987	0.843	0.051824965	count	1
SARS	0.0371079	0.0918743	0.4039	0.686	0.05188106	count	1
IKZF2	0.07007	0.5281624	0.1327	0.894	0.051894722	count	1
ABCC5	0.04935	0.3867939	0.1276	0.898	0.051928451	count	1
SLC2A1	0.1010897	0.6573897	0.1538	0.878	0.052085762	count	1
UBE4B	0.0510945	0.3067879	0.1665	0.868	0.052094238	count	1
SRSF11	0.0365858	0.0638514	0.573	0.5667	0.052153654	count	1
ADAT2	0.0594965	0.4267837	0.1394	0.889	0.05222482	count	1
ZNF544	0.0468625	0.3229816	0.1451	0.885	0.052269392	count	1
ZBTB40	0.0559967	0.3759219	0.149	0.882	0.052329858	count	1
U2AF1	0.7020951	0.7553028	0.9296	0.353	0.052375413	count	1
RPS17	0.0382726	0.1254347	0.3051	0.76	0.052377042	count	1
ALG1L2	0.0649548	0.7123828	0.0912	0.927	0.052400571	count	1
SFMBT2	0.0764652	0.3558887	0.2149	0.83	0.052614823	count	1
EFNA1	0.037109	0.0879278	0.422	0.673	0.052676592	count	1
BRCC3	0.0424369	0.2235724	0.1898	0.849	0.052705409	count	1
TM4SF1	0.0365958	0.0506494	0.7225	0.47	0.052716036	count	1
FAM217B	0.0411938	0.2399108	0.1717	0.864	0.05275131	count	1
C1orf123	0.0374951	0.0793639	0.4724	0.637	0.052900547	count	1
WDR45B	0.0390868	0.1385287	0.2822	0.778	0.052902834	count	1
STYX	0.0409884	0.2068555	0.1982	0.843	0.052934902	count	1

HDAC2	0.0383314	0.1374841	0.2788	0.78	0.052941317	count	1
ING4	0.0406515	0.176993	0.2297	0.818	0.053119194	count	1
SNHG19	0.0596123	0.3446839	0.1729	0.863	0.053231715	count	1
RAB29	0.0403648	0.16956	0.2381	0.812	0.053277246	count	1
NCOR1	0.0378065	0.0895035	0.4224	0.673	0.053387006	count	1
BTG3	0.0386809	0.1213529	0.3187	0.75	0.053547199	count	1
SP100	0.0375762	0.0624141	0.602	0.5472	0.053564678	count	1
GALNT14	0.726381	0.9004072	0.8067	0.42	0.053694272	count	1
BCL2	0.0725705	0.471111	0.154	0.878	0.053719815	count	1
CD82	0.0480137	0.2930961	0.1638	0.87	0.053796851	count	1
UIMC1	0.0419937	0.1945194	0.2159	0.829	0.053880851	count	1
ZBTB47	0.0910809	0.552619	0.1648	0.869	0.053981898	count	1
ARL15	0.0425598	0.2049273	0.2077	0.835	0.054025337	count	1
MAN2C1	0.0437008	0.2915527	0.1499	0.881	0.054094061	count	1
PRKAR2A	0.0402617	0.1455143	0.2767	0.782	0.054105804	count	1
DDX5	0.0377579	0.0379787	0.9942	0.32	0.054199623	count	1
SEM1	0.0379407	0.0497418	0.7628	0.446	0.054212838	count	1
NDUFA3	0.0385062	0.0844172	0.4561	0.648	0.054261621	count	1
FAM53C	0.0462306	0.2688277	0.172	0.863	0.054351451	count	1
NHP2	0.038281	0.0756157	0.5063	0.613	0.054370287	count	1
LSM4	0.0387915	0.0904397	0.4289	0.668	0.054386654	count	1
CSKMT	0.0425239	0.2165662	0.1964	0.844	0.054395651	count	1
STRAP	0.038965	0.0844783	0.4612	0.645	0.054676131	count	1
RPS19BP1	0.0386859	0.0769368	0.5028	0.615	0.054856439	count	1
IDH3A	0.0441896	0.2104595	0.21	0.834	0.054876723	count	1
KANSL1-AS1	0.0444998	0.173671	0.2562	0.798	0.054892643	count	1
GSK3B	0.0411295	0.1488963	0.2762	0.782	0.055017873	count	1
HAUS8	0.0616675	0.4212517	0.1464	0.884	0.055049251	count	1
TRAF4	0.0433719	0.2119474	0.2046	0.838	0.055054013	count	1
ZNF471	0.0551884	0.4066936	0.1357	0.892	0.055227031	count	1
MACO1	0.0416397	0.1681595	0.2476	0.804	0.055510424	count	1
ATP5F1A	0.0394475	0.0640357	0.616	0.538	0.056000465	count	1
SRCAP	0.089406	0.6669666	0.134	0.893	0.056009331	count	1
CMTM4	0.109075	0.675269	0.1615	0.872	0.056081925	count	1
GGH	0.0525344	0.3688898	0.1424	0.887	0.056377125	count	1
CBWD6	0.079006	0.6477463	0.122	0.903	0.056443052	count	1
AC024257.3	0.778728	1.279618	0.6086	0.543	0.056443652	count	1
SET	0.0395376	0.0454105	0.8707	0.384	0.056618859	count	1
EID2B	0.0567844	0.3671707	0.1547	0.877	0.056812749	count	1
PFKP	0.0434958	0.160367	0.2712	0.786	0.056946398	count	1
IL18R1	0.0565492	0.3944042	0.1434	0.886	0.057109477	count	1
SPG7	0.0437686	0.1725779	0.2536	0.8	0.057185401	count	1
FZR1	0.0515939	0.2904883	0.1776	0.859	0.057244603	count	1
CEP164	0.0434489	0.1597206	0.272	0.786	0.057276235	count	1
METTL22	0.0654223	0.5549576	0.1179	0.906	0.057371697	count	1

SCAI	0.0518034	0.3729299	0.1389	0.89	0.057475906	count	1
PTGDR	0.0973059	0.5496305	0.177	0.859	0.0575846	count	1
CSF2RB	0.0423338	0.1957854	0.2162	0.829	0.057694748	count	1
KLHL23	0.0597313	0.411575	0.1451	0.885	0.057901614	count	1
CDK5	0.0463544	0.2373743	0.1953	0.845	0.057997094	count	1
CCDC82	0.0414507	0.1001575	0.4139	0.679	0.058004964	count	1
TYK2	0.0537344	0.3196067	0.1681	0.866	0.058011543	count	1
SETD2	0.0425879	0.1427459	0.2983	0.765	0.058169495	count	1
ARVCF	0.0615137	0.9192843	0.0669	0.947	0.058200089	count	1
IFNGR2	0.0418708	0.1128345	0.3711	0.711	0.058201472	count	1
KMT5C	0.0929861	0.5077461	0.1831	0.855	0.058203709	count	1
ZNF383	0.0543451	0.2996555	0.1814	0.856	0.058309167	count	1
SLU7	0.042073	0.1094096	0.3845	0.701	0.058470787	count	1
WDCP	0.0646032	0.488229	0.1323	0.895	0.058574478	count	1
STT3B	0.0451743	0.1692472	0.2669	0.79	0.0586234	count	1
DCLRE1C	0.0572382	0.3078915	0.1859	0.853	0.058808642	count	1
MED13	0.0436977	0.1363178	0.3206	0.749	0.058840547	count	1
TRIP12	0.0424014	0.1416317	0.2994	0.765	0.058926888	count	1
NOVA2	0.0449325	0.2250133	0.1997	0.842	0.058940237	count	1
AC073335.2	0.161711	0.928026	0.1743	0.862	0.059031565	count	1
CREBL2	0.0464147	0.223769	0.2074	0.836	0.059108763	count	1
GLRX	0.0429248	0.1188263	0.3612	0.718	0.05924814	count	1
ZNF708	0.0478927	0.2168156	0.2209	0.825	0.059366588	count	1
AL031280.1	0.3724766	0.9504006	0.3919	0.695	0.059449042	count	1
SAMD10	0.3724766	0.9504006	0.3919	0.695	0.059449042	count	1
ERMP1	0.0530988	0.3008385	0.1765	0.86	0.059466694	count	1
CAPN10	0.0595474	0.3264433	0.1824	0.855	0.059556408	count	1
GPR143	0.0907448	0.5637186	0.161	0.872	0.059642588	count	1
CHMP6	0.0460258	0.1705644	0.2698	0.787	0.059726305	count	1
MRPS10	0.0433835	0.1227473	0.3534	0.724	0.059776973	count	1
ATP5MF	0.0428433	0.0932905	0.4592	0.646	0.059999122	count	1
MT-CO2	0.0416287	0.0296132	1.4057	0.16	0.060015113	count	1
EP400	0.0483653	0.2348672	0.2059	0.837	0.060047962	count	1
CCDC173	0.3772349	1.0690324	0.3529	0.724	0.060104858	count	1
AL135905.1	0.3772349	1.0959641	0.3442	0.731	0.060104858	count	1
CLSTN3	0.0515913	0.2751915	0.1875	0.851	0.060256662	count	1
LZTS3	0.1439601	0.7778834	0.1851	0.853	0.06033275	count	1
GCNA	0.1439601	0.8414415	0.1711	0.864	0.06033275	count	1
AC005046.1	0.166021	1.0935173	0.1518	0.879	0.06052491	count	1
RASSF1-AS1	0.166021	1.0959745	0.1515	0.88	0.06052491	count	1
AC007620.2	0.166021	1.0959745	0.1515	0.88	0.06052491	count	1
LRRC4	0.166021	1.1963612	0.1388	0.89	0.06052491	count	1
AC069185.1	0.166021	1.1986076	0.1385	0.89	0.06052491	count	1
KLHL25	0.166021	1.1963612	0.1388	0.89	0.06052491	count	1
AL390198.1	0.166021	1.1986076	0.1385	0.89	0.06052491	count	1

ODF2L	0.0430646	0.0870835	0.4945	0.621	0.06057994	count	1
PAPOLA	0.0430955	0.0832307	0.5178	0.605	0.060779141	count	1
FST	0.3823346	1.2002587	0.3185	0.75	0.060805005	count	1
ZNF548	0.0579109	0.3143069	0.1842	0.854	0.060878661	count	1
OTOA	0.8696995	1.265138	0.6874	0.492	0.060930727	count	1
ADAMTS18	0.8703858	0.3179957	2.7371	0.00624	0.060963224	count	1
TPMT	0.0476632	0.1982414	0.2404	0.81	0.061198974	count	1
TRIR	0.0428289	0.0484921	0.8832	0.3772	0.061280901	count	1
UBTD2	0.0487487	0.2214201	0.2202	0.826	0.061484962	count	1
CETP	0.0861958	0.4036662	0.2135	0.831	0.061487788	count	1
MRPS33	0.0447355	0.1282478	0.3488	0.727	0.061512625	count	1
IL4I1	0.3878088	1.1088359	0.3497	0.727	0.061553452	count	1
LINC01191	0.3878088	1.357162	0.2857	0.775	0.061553452	count	1
SSH1	0.0476234	0.1797322	0.265	0.791	0.061644159	count	1
SUCLA2	0.0472396	0.1726265	0.2737	0.784	0.061712091	count	1
LINC02328	0.1115436	0.8281166	0.1347	0.893	0.061728771	count	1
USP39	0.0488589	0.2147821	0.2275	0.82	0.06177899	count	1
STAT5B	0.0475778	0.2216671	0.2146	0.83	0.061833506	count	1
ADPRHL1	0.1699031	0.9591927	0.1771	0.859	0.061866469	count	1
CLMN	0.1699031	0.9616227	0.1767	0.86	0.061866469	count	1
LINC00910	0.1699031	0.9616227	0.1767	0.86	0.061866469	count	1
EMID1	0.1323026	0.6469404	0.2045	0.838	0.061898895	count	1
MOB4	0.0447641	0.1174215	0.3812	0.703	0.061931564	count	1
LINS1	0.0521339	0.3038153	0.1716	0.864	0.062123998	count	1
ATP5MGL	0.895488	1.052035	0.8512	0.395	0.06213833	count	1
HPS1	0.0507869	0.2568967	0.1977	0.843	0.062400764	count	1
UBE2K	0.0451052	0.1173613	0.3843	0.701	0.062471371	count	1
UBR4	0.0487597	0.2138476	0.228	0.82	0.062480976	count	1
LINC00323	0.0846618	0.5634641	0.1503	0.881	0.062518853	count	1
HERPUD1	0.0440688	0.0676453	0.6515	0.515	0.062606617	count	1
ZBTB21	0.0533345	0.2526052	0.2111	0.833	0.062669335	count	1
PTAR1	0.0496337	0.2102778	0.236	0.813	0.062677958	count	1
LRP1B	0.9087614	1.049973	0.8655	0.387	0.062749182	count	1
SERPINB6	0.0441178	0.0616554	0.7156	0.474	0.062788285	count	1
CHKB-DT	0.1343699	0.644093	0.2086	0.835	0.062830039	count	1
HILPDA	0.0483372	0.2220317	0.2177	0.828	0.062866839	count	1
ELMOD3	0.0539498	0.3147153	0.1714	0.864	0.063389352	count	1
RAD23B	0.046369	0.1248442	0.3714	0.71	0.063446315	count	1
GBA2	0.0612989	0.3751631	0.1634	0.87	0.063458447	count	1
MCM7	0.0530119	0.2297966	0.2307	0.818	0.063486608	count	1
PKN1	0.0464012	0.1202474	0.3859	0.7	0.063540169	count	1
ADPGK	0.0528046	0.251303	0.2101	0.834	0.063690071	count	1
BCL6	0.0525824	0.238369	0.2206	0.825	0.063841678	count	1
KCMF1	0.0475699	0.1501937	0.3167	0.751	0.063843794	count	1
MT1F	0.2108671	0.8011686	0.2632	0.792	0.063845698	count	1

PTP4A1	0.0512684	0.1962941	0.2612	0.794	0.064034541	count	1
SLC17A5	0.054676	0.309377	0.1767	0.86	0.064044181	count	1
ZNF518B	0.0602127	0.3259277	0.1847	0.853	0.064153056	count	1
CYP26B1	0.9401578	1.5764863	0.5964	0.551	0.064165555	count	1
DCBLD2	0.0649777	0.3655965	0.1777	0.859	0.064305769	count	1
FANCM	0.0710152	0.3643613	0.1949	0.845	0.064325064	count	1
RSF1	0.0453887	0.0778424	0.5831	0.56	0.064413948	count	1
C8orf88	0.0702224	0.5695074	0.1233	0.902	0.06457566	count	1
CNN2	0.0528206	0.2287089	0.231	0.817	0.064648515	count	1
MUT	0.0534708	0.25769	0.2075	0.836	0.064916549	count	1
UBE2I	0.046016	0.0785786	0.5856	0.558	0.064968659	count	1
TCEAL8	0.0464331	0.0918237	0.5057	0.613	0.065015358	count	1
FOXP1	0.0455848	0.0682024	0.6684	0.504	0.065030157	count	1
ADARB1	0.0513987	0.2327918	0.2208	0.825	0.065061682	count	1
CCDC40	0.179855	0.7932727	0.2267	0.821	0.065290418	count	1
AC017100.1	0.179855	0.8905753	0.202	0.84	0.065290418	count	1
XAF1	0.0464657	0.1211901	0.3834	0.701	0.065457213	count	1
PCBD1	0.0466661	0.1004111	0.4648	0.642	0.065575599	count	1
AIMP2	0.05014	0.1810978	0.2769	0.782	0.065628646	count	1
VAMP5	0.0457748	0.0463805	0.9869	0.324	0.065664452	count	1
C8orf59	0.0461156	0.0634702	0.7266	0.4676	0.065704615	count	1
SLC9A8	0.0705908	0.4382325	0.1611	0.872	0.065828908	count	1
SCAF4	0.0519082	0.2595835	0.2	0.842	0.065861742	count	1
SPATA12	0.1815961	0.7852121	0.2313	0.817	0.065887191	count	1
PIP5KL1	0.1815961	0.7850543	0.2313	0.817	0.065887191	count	1
LINC01431	0.1815961	0.7852121	0.2313	0.817	0.065887191	count	1
CLN3	0.1815961	0.9917062	0.1831	0.855	0.065887196	count	1
MOB2	0.0468048	0.0839205	0.5577	0.577	0.066032049	count	1
CERK	0.0565881	0.2615296	0.2164	0.829	0.066066859	count	1
LNP1	0.2191255	0.9251614	0.2369	0.813	0.066168718	count	1
UXT	0.0464533	0.0573589	0.8099	0.418	0.066174494	count	1
CHTF8	0.0505072	0.1741174	0.2901	0.772	0.066196056	count	1
RPL41	0.0460447	0.0171323	2.6876	0.00724	0.066395361	count	1
TRIM39	0.0725269	0.3611368	0.2008	0.841	0.066671921	count	1
AL359711.2	0.2212237	0.7823925	0.2828	0.777	0.066756841	count	1
WTAP	0.0474848	0.0897886	0.5289	0.597	0.066827634	count	1
TMBIM4	0.0468316	0.0502336	0.9323	0.351	0.067014247	count	1
GGCT	0.0481247	0.1061805	0.4532	0.65	0.067054748	count	1
AC090630.1	0.0730414	0.5320083	0.1373	0.891	0.067139727	count	1
CHST12	0.0488707	0.1332472	0.3668	0.714	0.067236849	count	1
NABP2	0.0501681	0.1607785	0.312	0.755	0.067275343	count	1
TRMT61B	0.0559501	0.2299495	0.2433	0.808	0.067314065	count	1
PRPF6	0.0482059	0.0939383	0.5132	0.608	0.067383047	count	1
SSBP1	0.0473835	0.0586863	0.8074	0.42	0.067477995	count	1
TNFRSF19	1.0196217	1.6062464	0.6348	0.5256	0.067577215	count	1

PDCD2L	0.0659001	0.3310501	0.1991	0.842	0.067638743	count	1
STXBP5-AS1	0.1870214	0.6745995	0.2772	0.782	0.067742487	count	1
MTG2	0.0586188	0.282204	0.2077	0.835	0.067746239	count	1
PLIN2	0.0488722	0.1216388	0.4018	0.688	0.067946399	count	1
RINL	0.0547085	0.2523191	0.2168	0.828	0.068006624	count	1
PAFAH1B3	0.0500682	0.1411114	0.3548	0.723	0.068143094	count	1
SIAE	0.0611963	0.3091975	0.1979	0.843	0.068165661	count	1
RTRAF	0.0477561	0.0535109	0.8925	0.372	0.068168645	count	1
SHARPIN	0.0492451	0.1111678	0.443	0.658	0.068184293	count	1
XPO7	0.0599001	0.2596016	0.2307	0.818	0.068195572	count	1
SOCS7	0.079434	0.56762	0.1399	0.889	0.068223428	count	1
LETM2	0.2928941	0.6247556	0.4688	0.639	0.068260916	count	1
FBXO6	0.0600574	0.327535	0.1834	0.855	0.068373722	count	1
BTBD10	0.0513492	0.1714659	0.2995	0.765	0.068433499	count	1
MAX	0.0495758	0.1215974	0.4077	0.684	0.06847648	count	1
MFSD8	0.0635609	0.3752433	0.1694	0.866	0.068550366	count	1
OCIAD1	0.0480165	0.0532954	0.9009	0.3677	0.068644559	count	1
LARS2	0.1003099	0.565626	0.1773	0.859	0.068668569	count	1
AC015712.2	0.1170028	0.6344942	0.1844	0.854	0.068910674	count	1
AC025283.2	0.1170028	0.6674556	0.1753	0.861	0.068910674	count	1
DENND5A	0.0524378	0.2691035	0.1949	0.846	0.06893912	count	1
CCDC90B	0.0490643	0.0936889	0.5237	0.601	0.06903422	count	1
ZBTB16	0.0510633	0.1549206	0.3296	0.742	0.069209404	count	1
SNRPA1	0.0508945	0.1410228	0.3609	0.718	0.069299483	count	1
ZNF853	0.1015395	0.4443964	0.2285	0.819	0.069491824	count	1
UFM1	0.049606	0.0981796	0.5053	0.613	0.069548223	count	1
ALDOA	0.0558073	0.2386731	0.2338	0.815	0.069686162	count	1
EIF3B	0.0521629	0.1849047	0.2821	0.778	0.069810275	count	1
RNF227	0.091792	0.4997859	0.1837	0.854	0.069820114	count	1
CCDC120	0.091792	0.5163688	0.1778	0.859	0.069820114	count	1
LRRC75A	0.0507944	0.1176279	0.4318	0.666	0.06988093	count	1
ZNF345	0.0814399	0.3689756	0.2207	0.825	0.069922855	count	1
DNAJC21	0.0497715	0.0857528	0.5804	0.562	0.07001936	count	1
PRPF38B	0.0500076	0.0960634	0.5206	0.603	0.070170197	count	1
BLMH	0.0540408	0.152209	0.355	0.723	0.070214468	count	1
NFXL1	0.0669006	0.3591913	0.1863	0.852	0.070258423	count	1
WDR5	0.0638359	0.3131899	0.2038	0.839	0.07039019	count	1
KLF12	0.1952658	0.5161668	0.3783	0.705	0.070549425	count	1
TOM1	0.05944	0.2593586	0.2292	0.819	0.070606393	count	1
RSBN1L	0.050368	0.1119546	0.4499	0.653	0.070607701	count	1
L1TD1	1.095932	1.376548	0.7961	0.426	0.070631055	count	1
GRAP	0.062373	0.2765479	0.2255	0.822	0.070708745	count	1
ZBTB7B	0.0808434	0.3935809	0.2054	0.837	0.070718727	count	1
OGA	0.0515953	0.1506472	0.3425	0.732	0.070751478	count	1
ATP6V1A	0.0577492	0.2048951	0.2818	0.778	0.070793411	count	1

KMT2D	0.0636375	0.3290139	0.1934	0.847	0.07086863	count	1
MXRA5	0.4582498	0.7292065	0.6284	0.53	0.070900225	count	1
CXCL9	0.0543808	0.9115431	0.0597	0.9524	0.071021342	count	1
SOX12	0.0798263	0.3519403	0.2268	0.821	0.07105624	count	1
MTHFD2	0.0613916	0.2670254	0.2299	0.818	0.071179001	count	1
JKAMP	0.0529996	0.1428302	0.3711	0.711	0.071511798	count	1
FXYD5	0.0499097	0.0386614	1.2909	0.197	0.071770856	count	1
KTI12	0.0629377	0.2341126	0.2688	0.788	0.071916013	count	1
ICOSLG	0.3111045	1.1734445	0.2651	0.791	0.072052719	count	1
DNAJB9	0.0528504	0.1287844	0.4104	0.682	0.072059486	count	1
AC020915.1	0.2404646	1.1082836	0.217	0.828	0.072110751	count	1
AC145285.6	0.2404646	1.1136782	0.2159	0.829	0.072110751	count	1
DAGLA	0.2404646	1.2381161	0.1942	0.846	0.072110751	count	1
HFM1	0.2404646	1.242947	0.1935	0.847	0.072110751	count	1
STAM2	0.0560771	0.2008246	0.2792	0.78	0.072112047	count	1
WDTC1	0.0622408	0.2990982	0.2081	0.835	0.072158625	count	1
ATXN7	0.2408481	1.0846936	0.222	0.824	0.072216743	count	1
WWC2-AS2	0.2408481	1.0879188	0.2214	0.825	0.072216743	count	1
RLN2	0.2408481	1.0879188	0.2214	0.825	0.072216743	count	1
IPCEF1	0.2408481	1.1998428	0.2007	0.841	0.072216743	count	1
AF196972.1	0.2408481	1.1998428	0.2007	0.841	0.072216743	count	1
CCDC184	0.2411982	1.0258798	0.2351	0.814	0.072313478	count	1
CNN1	0.2411982	1.0995638	0.2194	0.826	0.072313478	count	1
PEX3	0.0557481	0.19636	0.2839	0.777	0.07248356	count	1
CRYBB2	0.3137937	1.1012745	0.2849	0.776	0.072608364	count	1
PLEKHM1	0.064063	0.3017218	0.2123	0.832	0.072613765	count	1
SMDT1	0.0512869	0.064614	0.7937	0.427	0.07298744	count	1
IFT27	0.0556616	0.165462	0.3364	0.737	0.073031699	count	1
ZNF414	0.0610927	0.2563075	0.2384	0.812	0.073122704	count	1
C12orf75	0.0598373	0.2783565	0.215	0.83	0.073204316	count	1
STT3A	0.05742	0.1992829	0.2881	0.773	0.073244735	count	1
LACTB	0.0535947	0.1266975	0.423	0.672	0.073321912	count	1
STIM1	0.0694363	0.2876201	0.2414	0.809	0.073413133	count	1
SEC24B-AS1	0.1032859	0.5689572	0.1815	0.856	0.073417065	count	1
SLC44A2	0.0534471	0.1279247	0.4178	0.676	0.073502972	count	1
MADD	0.0753656	0.3554557	0.212	0.832	0.073712214	count	1
KANSL2	0.0603917	0.2989074	0.202	0.84	0.073736272	count	1
SYP	0.0896922	0.4172687	0.215	0.83	0.073752961	count	1
C8orf48	0.1002362	0.6845203	0.1464	0.884	0.073788187	count	1
GATAD1	0.0533768	0.1257992	0.4243	0.671	0.073827293	count	1
DCAF11	0.060382	0.217174	0.278	0.781	0.073868174	count	1
WTIP	0.0613079	0.2122468	0.2889	0.773	0.07390379	count	1
POLR2F	0.0526907	0.0891299	0.5912	0.554	0.073904152	count	1
UCK2	0.0586405	0.2134824	0.2747	0.784	0.073923196	count	1
PSMD4	0.0522361	0.0703222	0.7428	0.458	0.073973849	count	1

ADAM19	0.1786348	0.5001582	0.3572	0.721	0.074109888	count	1
SHPRH	0.0560418	0.1738007	0.3224	0.747	0.074131962	count	1
SAV1	0.0537905	0.1230923	0.437	0.662	0.074370626	count	1
CNKSR3	0.055869	0.1687682	0.331	0.741	0.074479562	count	1
NAGPA	0.0590906	0.2354907	0.2509	0.802	0.074488854	count	1
YARS2	0.0608465	0.2187195	0.2782	0.781	0.074576001	count	1
NENF	0.0524641	0.0718729	0.73	0.465	0.074605983	count	1
C8orf76	0.0604939	0.2438395	0.2481	0.804	0.074807401	count	1
ABHD12	0.0619345	0.2056934	0.3011	0.763	0.074824536	count	1
KRIT1	0.0545359	0.1205527	0.4524	0.651	0.075058673	count	1
M6PR	0.0542405	0.1147606	0.4726	0.637	0.075165183	count	1
DERL2	0.0553073	0.1313918	0.4209	0.674	0.075171608	count	1
PSMD14	0.0555503	0.1290431	0.4305	0.667	0.075261556	count	1
TARDBP	0.0581574	0.1876403	0.3099	0.757	0.07530851	count	1
FAM3C	0.0536227	0.0906998	0.5912	0.554	0.07537837	count	1
PITPNA-AS1	0.0590001	0.2203654	0.2677	0.789	0.075491068	count	1
PINLYP	0.1029278	0.6576748	0.1565	0.876	0.07572841	count	1
BNIP2	0.0534237	0.0720966	0.741	0.459	0.07581282	count	1
RBL2	0.05574	0.1528843	0.3646	0.715	0.075852393	count	1
Z93930.2	0.0885024	0.4047642	0.2187	0.827	0.075897007	count	1
STRADA	0.0761273	0.3675849	0.2071	0.836	0.075979206	count	1
AC127024.6	1.244491	1.532698	0.812	0.417	0.076000175	count	1
BICD2	0.0615866	0.2403382	0.2562	0.798	0.076024411	count	1
COA4	0.0543655	0.0900204	0.6039	0.546	0.076414613	count	1
NADK2	0.0677274	0.3358934	0.2016	0.84	0.07642086	count	1
NOL3	0.0613133	0.2245489	0.2731	0.785	0.076538072	count	1
HIST1H2AG	0.1235043	0.781797	0.158	0.874	0.076757182	count	1
ANKRD42	0.0757114	0.2785575	0.2718	0.786	0.076963109	count	1
NAGA	0.0750717	0.3334086	0.2252	0.822	0.076968199	count	1
ICE1	0.0564855	0.1488932	0.3794	0.704	0.077044048	count	1
EPG5	0.0710903	0.3538338	0.2009	0.841	0.077064137	count	1
MRPL12	0.0558686	0.1230787	0.4539	0.65	0.077096354	count	1
UBE2V2	0.0555966	0.1114915	0.4987	0.618	0.077153635	count	1
RNF24	0.0725371	0.3810582	0.1904	0.849	0.077181236	count	1
SPART	0.0560948	0.1145612	0.4896	0.624	0.077502982	count	1
AC009831.1	0.1527033	0.7862891	0.1942	0.846	0.077609537	count	1
VSIG10L	0.1527033	0.8364236	0.1826	0.855	0.077609537	count	1
AC004865.2	0.5114354	1.105132	0.4628	0.644	0.077616612	count	1
FPGT-TNNI3K	0.5114354	1.1208902	0.4563	0.648	0.077616612	count	1
MTMR4	0.0763783	0.2825768	0.2703	0.787	0.077634722	count	1
CYSTM1	0.0547632	0.0623111	0.8789	0.38	0.078031546	count	1
AC004982.2	0.1421316	0.6403711	0.222	0.824	0.078046771	count	1
STK11IP	0.1421316	0.6680064	0.2128	0.832	0.078046771	count	1
DGKA	0.0722895	0.2773884	0.2606	0.794	0.078354462	count	1
AL133453.1	0.0659978	0.2602185	0.2536	0.8	0.078358655	count	1

SPRED2	0.0844373	0.3814206	0.2214	0.825	0.078582331	count	1
SLC12A5	0.0738978	0.3653266	0.2023	0.84	0.078617457	count	1
AC009283.1	0.5201394	1.1580766	0.4491	0.653	0.078688575	count	1
PEBP4	0.126742	0.8087607	0.1567	0.875	0.078709513	count	1
GATD3A	0.1551253	0.865671	0.1792	0.858	0.078789493	count	1
MPDU1	0.0617798	0.2028951	0.3045	0.761	0.078789648	count	1
AFF4	0.0568724	0.1058945	0.5371	0.591	0.078827023	count	1
LINC01268	1.336912	1.052506	1.2702	0.204	0.078988666	count	1
GPR4	0.0675137	0.2513281	0.2686	0.788	0.079002668	count	1
NIPSNAP2	0.0574169	0.116066	0.4947	0.621	0.079019516	count	1
RIF1	0.0581053	0.1471027	0.395	0.693	0.07902824	count	1
SMC3	0.0563054	0.0917839	0.6135	0.54	0.079072823	count	1
SMARCA5	0.0566701	0.0952088	0.5952	0.552	0.079354342	count	1
AIDA	0.0573743	0.1222978	0.4691	0.639	0.079380714	count	1
AKAP8L	0.0649068	0.1789756	0.3627	0.717	0.07938102	count	1
MAP7D3	0.0596674	0.1683485	0.3544	0.723	0.079389986	count	1
ORAI3	0.0589976	0.1661943	0.355	0.723	0.079408965	count	1
COLGALT1	0.0679449	0.2221276	0.3059	0.76	0.079504572	count	1
CNBP	0.0555905	0.0458882	1.2114	0.226	0.079580273	count	1
ACOT8	0.0653694	0.2654172	0.2463	0.805	0.079629454	count	1
ATRN	0.0754408	0.3176028	0.2375	0.812	0.079708703	count	1
SNX17	0.0572632	0.0986949	0.5802	0.562	0.079770717	count	1
ANKLE2	0.0596875	0.1738437	0.3433	0.731	0.079797887	count	1
HIST1H2BJ	0.1574207	0.7995351	0.1969	0.844	0.079906302	count	1
SDHC	0.0570669	0.084557	0.6749	0.5	0.080042705	count	1
NID1	0.0608506	0.222399	0.2736	0.784	0.08011918	count	1
ZNF202	0.270255	0.6755273	0.4001	0.689	0.080261202	count	1
SUCO	0.0596629	0.1632288	0.3655	0.715	0.080275492	count	1
C2orf76	0.0588685	0.1629792	0.3612	0.718	0.080325582	count	1
CENPE	0.2705779	0.7692817	0.3517	0.725	0.080348596	count	1
EEF1A1	0.0557568	0.0172843	3.2259	0.00127	0.080419347	count	1
WASHC2C	0.0689787	0.3319568	0.2078	0.835	0.08045434	count	1
LDB1	0.0742421	0.3140669	0.2364	0.813	0.080454744	count	1
THAP3	0.0673486	0.2687107	0.2506	0.802	0.080574991	count	1
IBTK	0.0614646	0.217142	0.2831	0.777	0.080576258	count	1
ABCD3	0.0770653	0.2799372	0.2753	0.783	0.080840881	count	1
SUMO4	0.3545907	0.6855013	0.5173	0.605	0.08090343	count	1
MBD5	0.0639304	0.1988568	0.3215	0.748	0.080971005	count	1
BCAP31	0.0567794	0.0578929	0.9808	0.327	0.080972723	count	1
PROB1	0.538956	0.9152697	0.5888	0.556	0.080980239	count	1
ZNF503-AS1	0.538956	1.1040778	0.4882	0.625	0.080980239	count	1
NFKBIE	0.0668022	0.2476257	0.2698	0.787	0.081030841	count	1
ADAM12	1.413403	0.8774818	1.6107	0.107	0.081275356	count	1
UBE2Q1	0.0602415	0.1397438	0.4311	0.666	0.081538314	count	1
TSEN15	0.065	0.2079373	0.3126	0.755	0.081697616	count	1

PRKRA	0.0603903	0.1324162	0.4561	0.648	0.081808996	count	1
FAM210B	0.0590984	0.125898	0.4694	0.639	0.081890657	count	1
AL590822.2	1.4393706	1.7865904	0.8057	0.4205	0.082015344	count	1
HNRNPA2B1	0.0571593	0.0385779	1.4817	0.139	0.082070937	count	1
CCNG1	0.0611158	0.1836928	0.3327	0.739	0.082082155	count	1
SCAF11	0.0579254	0.0766218	0.756	0.45	0.082099921	count	1
PDK3	0.0926551	0.4255662	0.2177	0.828	0.0823083	count	1
BRD8	0.0613409	0.1666949	0.368	0.713	0.082354012	count	1
FBXL19	0.0863957	0.7802539	0.1107	0.912	0.082473455	count	1
DCAF12	0.0729859	0.2675372	0.2728	0.785	0.082662266	count	1
DNM1L	0.0609055	0.1344207	0.4531	0.651	0.082705956	count	1
CALCOCO1	0.0652443	0.2020944	0.3228	0.747	0.082727752	count	1
PLEKHF2	0.0701147	0.215579	0.3252	0.745	0.082763057	count	1
CD1D	0.1271729	0.5649561	0.2251	0.822	0.082902876	count	1
CMC4	0.0988267	0.4571968	0.2162	0.829	0.082918773	count	1
NRDC	0.0595807	0.1091556	0.5458	0.585	0.082952543	count	1
RNF130	0.0594141	0.1037914	0.5724	0.567	0.083043991	count	1
SIVA1	0.0584524	0.0670175	0.8722	0.383	0.083179256	count	1
GH1	0.1221348	0.5719919	0.2135	0.831	0.083213749	count	1
PEX2	0.0609691	0.1387642	0.4394	0.66	0.083224181	count	1
CACNA1C	0.1222346	0.5277908	0.2316	0.817	0.083279933	count	1
EWSR1	0.0600124	0.1160663	0.5171	0.605	0.083284704	count	1
MRPL16	0.0595568	0.1000632	0.5952	0.552	0.083419627	count	1
CMTM3	0.0619403	0.1545187	0.4009	0.689	0.083555387	count	1
MIOS	0.0941105	0.3288515	0.2862	0.775	0.083581853	count	1
WNK3	0.1282826	0.4845749	0.2647	0.791	0.083605264	count	1
TRIM65	0.0809706	0.2847424	0.2844	0.776	0.083630819	count	1
STARD7	0.0625783	0.15734	0.3977	0.691	0.083723243	count	1
MX1	0.0594135	0.1122996	0.5291	0.597	0.083745775	count	1
RGL2	0.0603142	0.1207882	0.4993	0.618	0.083772834	count	1
ACTR3	0.0596284	0.0841356	0.7087	0.479	0.083839154	count	1
STK25	0.0610086	0.1074887	0.5676	0.57	0.083916775	count	1
POLR3E	0.0758934	0.2820477	0.2691	0.788	0.084010246	count	1
FNBP1	0.0633362	0.1720397	0.3681	0.713	0.084019298	count	1
POM121C	0.0649671	0.2090128	0.3108	0.756	0.084032022	count	1
TMEM9B	0.0599967	0.1026411	0.5845	0.559	0.084050803	count	1
RPA2	0.061978	0.1284732	0.4824	0.63	0.084224718	count	1
RER1	0.0600069	0.095473	0.6285	0.53	0.084276244	count	1
OTUD6B	0.0778255	0.3146084	0.2474	0.805	0.084306908	count	1
UBALD2	0.0604267	0.0993854	0.608	0.543	0.084403242	count	1
PATJ	0.083106	0.3920665	0.212	0.832	0.084403683	count	1
ZKSCAN7	1.5364507	2.0041915	0.7666	0.4434	0.08462775	count	1
CD37	0.1546678	0.5611269	0.2756	0.783	0.084658565	count	1
FAM208A	0.0621446	0.1545011	0.4022	0.688	0.084752015	count	1
MAP3K20	0.0659218	0.2088391	0.3157	0.752	0.085043396	count	1

CMSS1	0.0648665	0.1664333	0.3897	0.697	0.085240029	count	1
TIMM17B	0.063854	0.1722928	0.3706	0.711	0.085286648	count	1
TBC1D12	0.0656529	0.2053276	0.3197	0.749	0.085327217	count	1
MRPL24	0.0625554	0.1255998	0.4981	0.618	0.085423168	count	1
FAR1	0.0654873	0.1671966	0.3917	0.695	0.085489205	count	1
AP4M1	0.0699691	0.2199898	0.3181	0.75	0.085544925	count	1
KRR1	0.0611225	0.0923794	0.6616	0.508	0.085589725	count	1
RLIM	0.0658304	0.1855383	0.3548	0.723	0.08568698	count	1
AK6	0.0624107	0.1244946	0.5013	0.616	0.085910896	count	1
CFAP97	0.0608921	0.0838951	0.7258	0.468	0.086058272	count	1
STX10	0.0628052	0.1207182	0.5203	0.603	0.086103587	count	1
ZNF184	0.0795462	0.5105749	0.1558	0.876	0.086155626	count	1
PIGO	0.0841327	0.3598276	0.2338	0.815	0.086164584	count	1
ASCC2	0.0683274	0.2120835	0.3222	0.747	0.08620137	count	1
SGPL1	0.108031	0.4898862	0.2205	0.825	0.086469011	count	1
TGIF2	0.0770382	0.296729	0.2596	0.795	0.086475905	count	1
DHRS4-AS1	0.0684231	0.2220352	0.3082	0.758	0.086536783	count	1
RANGRF	0.0640471	0.1506949	0.425	0.671	0.086705693	count	1
SNX10	0.0933325	0.500129	0.1866	0.852	0.086747328	count	1
MRPL4	0.0622832	0.1086773	0.5731	0.567	0.086757644	count	1
CLEC16A	0.0962415	0.522674	0.1841	0.854	0.086836829	count	1
EXOC7	0.065557	0.1510394	0.434	0.664	0.086915397	count	1
AC046143.1	1.633515	1.05885	1.5427	0.123	0.087011873	count	1
SCNM1	0.0654605	0.1529867	0.4279	0.669	0.08735394	count	1
REPS2	0.2456476	0.6499389	0.378	0.705	0.087379456	count	1
PJVK	0.2456476	0.7231508	0.3397	0.734	0.087379456	count	1
AC097532.2	0.2457001	0.6425246	0.3824	0.702	0.087396703	count	1
AC093635.1	0.2457001	0.6438157	0.3816	0.703	0.087396703	count	1
RANBP3L	0.1898564	0.9518921	0.1995	0.842	0.087409056	count	1
LINC02104	0.1898564	0.9518921	0.1995	0.842	0.087409056	count	1
KDM4C	0.0782598	0.2895537	0.2703	0.787	0.08744113	count	1
ARMCX1	0.0661889	0.1639005	0.4038	0.686	0.08747688	count	1
POLR2E	0.0623555	0.0841891	0.7407	0.459	0.087585794	count	1
INPP5F	0.0745815	0.2747767	0.2714	0.786	0.087752441	count	1
CYP20A1	0.0642097	0.1369914	0.4687	0.639	0.087770716	count	1
AQP3	0.0659457	0.2365809	0.2787	0.78	0.087923816	count	1
MTDH	0.0616169	0.0556604	1.107	0.2684	0.088088943	count	1
MAPKAP1	0.0649792	0.160972	0.4037	0.686	0.088206084	count	1
GOLT1B	0.0686401	0.2042362	0.3361	0.737	0.088217497	count	1
SPIN2B	0.0754661	0.2517656	0.2997	0.764	0.088253443	count	1
PDCD1LG2	0.1014767	0.5190457	0.1955	0.845	0.088469952	count	1
LINC01209	0.1924013	0.7824249	0.2459	0.806	0.088517317	count	1
AC005498.2	0.1924013	0.7814691	0.2462	0.806	0.088517317	count	1
CLSPN	0.1924013	1.0627283	0.181	0.856	0.088517317	count	1
LSP1	0.1924013	1.1545768	0.1666	0.868	0.088517317	count	1

ZDHHC16	0.0817822	0.2963642	0.276	0.783	0.088556978	count	1
SLC43A2	0.0918221	0.4151092	0.2212	0.825	0.088622192	count	1
IDO1	0.3023984	0.8186508	0.3694	0.712	0.088867732	count	1
ANKRD17	0.0661206	0.1519494	0.4351	0.663	0.088978705	count	1
SYS1	0.0653204	0.1361222	0.4799	0.631	0.089323193	count	1
SH3PXD2A-AS1	0.3047506	0.7807381	0.3903	0.696	0.089489963	count	1
ZNF789	0.0898435	0.4115095	0.2183	0.827	0.089511872	count	1
ANKRD13C	0.0737213	0.2265644	0.3254	0.745	0.089570981	count	1
PSEN1	0.073464	0.2059922	0.3566	0.721	0.089622505	count	1
GDPGP1	0.1951832	0.6364819	0.3067	0.759	0.089726885	count	1
HGH1	0.0856372	0.4104287	0.2087	0.835	0.08974574	count	1
ACTR1A	0.065955	0.1260753	0.5231	0.601	0.08975334	count	1
LINC01465	0.3060024	0.786888	0.3889	0.697	0.089820675	count	1
EDA2R	0.3060024	0.9454096	0.3237	0.746	0.089820675	count	1
SLC45A4	0.2193746	0.7125479	0.3079	0.758	0.089926181	count	1
YBX1	0.0624968	0.0275547	2.2681	0.0234	0.089980187	count	1
MYO15B	0.0866102	0.7157649	0.121	0.904	0.090089134	count	1
SNTB2	0.0634717	0.0753874	0.8419	0.4	0.090124291	count	1
PVR	0.0823474	0.3487727	0.2361	0.813	0.090163411	count	1
MIR762HG	0.1130565	0.4624783	0.2445	0.807	0.090407667	count	1
S1PR4	0.0851811	0.4121209	0.2067	0.836	0.090510233	count	1
PSMD6	0.0652043	0.1083039	0.602	0.547	0.090520715	count	1
DOCK5	0.6209272	0.7147959	0.8687	0.385	0.090561977	count	1
SNX4	0.0683607	0.169034	0.4044	0.686	0.090578091	count	1
EIF6	0.064426	0.0884405	0.7285	0.466	0.090716258	count	1
ZNF615	0.1238986	0.4592302	0.2698	0.787	0.090770391	count	1
GNL1	0.0672036	0.1246011	0.5393	0.59	0.090971567	count	1
TOMM34	0.0755333	0.2508564	0.3011	0.763	0.091169995	count	1
ATP6V1F	0.0639766	0.0603987	1.0592	0.2896	0.09119112	count	1
RPL9	0.0633382	0.0230568	2.747	0.00605	0.091254727	count	1
EPHA4	0.0675362	0.1748507	0.3863	0.699	0.091314693	count	1
TMEM33	0.0702238	0.1825923	0.3846	0.701	0.091320507	count	1
ALDH5A1	0.0936279	0.6312994	0.1483	0.882	0.091350969	count	1
NCLN	0.0786771	0.279388	0.2816	0.778	0.091397941	count	1
ATXN7L1	0.091792	0.3672577	0.2499	0.803	0.091430339	count	1
ZSCAN16	0.0808324	0.3177016	0.2544	0.799	0.091485079	count	1
DIAPH1	0.0676033	0.1496461	0.4518	0.651	0.091485433	count	1
LINC02453	0.6292599	1.0407621	0.6046	0.545	0.091500269	count	1
AC026471.4	0.6292599	1.1369978	0.5534	0.58	0.091500269	count	1
SCN4A	0.6292955	1.149307	0.5475	0.584	0.091504255	count	1
ZC3HAV1L	0.1033864	0.4735889	0.2183	0.827	0.091684457	count	1
N4BP2L2	0.0644205	0.0668488	0.9637	0.3353	0.091880932	count	1
YBEY	0.068877	0.19324	0.3564	0.722	0.091903334	count	1
PEX1	0.0703818	0.2084705	0.3376	0.736	0.092051647	count	1
HARS2	0.083407	0.300767	0.2773	0.782	0.092260542	count	1

VIPAS39	0.0819896	0.3374354	0.243	0.808	0.092394659	count	1
FGFR1OP2	0.0658017	0.0915834	0.7185	0.473	0.092418233	count	1
CAPN2	0.0661004	0.0958053	0.6899	0.49	0.092513293	count	1
SUMO1	0.065124	0.0597247	1.0904	0.276	0.092696272	count	1
ZNF233	0.6400243	1.1323393	0.5652	0.572	0.092702806	count	1
DCP1B	0.0952035	0.3560551	0.2674	0.789	0.092868619	count	1
TMEM132A	0.1112838	0.4256044	0.2615	0.794	0.093169182	count	1
CCDC106	0.0741452	0.2221019	0.3338	0.739	0.09327084	count	1
ZCCHC10	0.0690145	0.1428227	0.4832	0.629	0.093282107	count	1
DDX39A	0.0700537	0.1521578	0.4604	0.645	0.093429634	count	1
ST6GAL1	0.0674283	0.1230588	0.5479	0.584	0.093555474	count	1
AC009506.1	0.0900835	0.368249	0.2446	0.807	0.093664308	count	1
ACBD3-AS1	0.1380295	0.8834793	0.1562	0.876	0.093716925	count	1
ITGA8	0.1382693	0.6059842	0.2282	0.82	0.093874801	count	1
CYB5R1	0.067694	0.1130024	0.599	0.549	0.093883013	count	1
PPIE	0.068232	0.1348729	0.5059	0.613	0.09389908	count	1
PACS2	0.0729458	0.1720009	0.4241	0.672	0.093906995	count	1
PSD	0.6512123	1.1619814	0.5604	0.575	0.093941334	count	1
EPC1	0.066947	0.0923445	0.725	0.469	0.093941849	count	1
LAPTM4A	0.065467	0.0418958	1.5626	0.118	0.093985348	count	1
NAGS	0.1148479	0.5056097	0.2271	0.82	0.094015619	count	1
MANEAL	0.1148479	0.6206732	0.185	0.853	0.094015619	count	1
CFAP20	0.0682453	0.1182161	0.5773	0.564	0.094087486	count	1
Z99289.3	0.2302831	0.7861675	0.2929	0.77	0.094093531	count	1
PRR29	0.2302831	0.7861675	0.2929	0.77	0.094093531	count	1
RELL2	0.2302831	0.8488473	0.2713	0.786	0.094093531	count	1
SLC50A1	0.071734	0.2042075	0.3513	0.725	0.094240569	count	1
CFAP36	0.0669311	0.0927325	0.7218	0.471	0.094276048	count	1
AC024060.1	0.2669377	0.8264679	0.323	0.747	0.094325535	count	1
TBC1D10B	0.0840973	0.263898	0.3187	0.75	0.09433848	count	1
PCSK6	0.2311419	1.1107653	0.2081	0.835	0.094420404	count	1
CCT8	0.0667763	0.0728788	0.9163	0.36	0.094479275	count	1
USP3	0.0718464	0.1840336	0.3904	0.696	0.09450276	count	1
AKIRIN1	0.069162	0.1243968	0.556	0.578	0.09451051	count	1
ELOA-AS1	0.0959234	0.4796145	0.2	0.841	0.094549602	count	1
AP003108.2	0.2315421	0.786798	0.2943	0.769	0.094572676	count	1
DTWD1	0.0691118	0.1353979	0.5104	0.61	0.094593958	count	1
AC015982.1	0.26818	0.7900044	0.3395	0.734	0.094727831	count	1
IRF3	0.0703841	0.133702	0.5264	0.599	0.09473707	count	1
BORCS7	0.0676725	0.085926	0.7876	0.431	0.094874161	count	1
ZNF215	0.2686326	0.7904218	0.3399	0.734	0.094874314	count	1
PTPRM	0.068836	0.1150116	0.5985	0.55	0.094955222	count	1
SAP18	0.0663647	0.0452707	1.466	0.1428	0.095012732	count	1
FAM198A	2.048468	1.290271	1.5876	0.112	0.095050971	count	1
ZC3H7A	0.0710882	0.1546524	0.4597	0.646	0.095160007	count	1

MIR4458HG	0.0800227	0.3216363	0.2488	0.804	0.095165138	count	1
CACNB1	0.6628334	1.120465	0.5916	0.554	0.095215654	count	1
TLE4	0.0698295	0.1292315	0.5403	0.589	0.095258292	count	1
RP9	0.0700135	0.1566093	0.4471	0.655	0.095267154	count	1
SPATA7	0.0773162	0.2285703	0.3383	0.735	0.095351648	count	1
INTS7	0.1405355	0.5783548	0.243	0.808	0.095365953	count	1
AC234582.1	0.140754	0.7836156	0.1796	0.857	0.095509643	count	1
SAMD4B	0.0739643	0.2035362	0.3634	0.716	0.095551272	count	1
NKAP	0.0688223	0.1071766	0.6421	0.521	0.095727406	count	1
EIF3K	0.066737	0.0369366	1.8068	0.0709	0.095859371	count	1
IRF7	0.070448	0.1478051	0.4766	0.634	0.095925841	count	1
MCRS1	0.074776	0.1961063	0.3813	0.703	0.096166679	count	1
NUDT22	0.0721361	0.1726282	0.4179	0.676	0.096242207	count	1
RPL22L1	0.0682017	0.0759803	0.8976	0.369	0.096439151	count	1
AURKA	0.1660209	0.7738397	0.2145	0.83	0.09661151	count	1
RBM48	0.0756086	0.2236028	0.3381	0.735	0.096666366	count	1
CHST2	0.1073838	0.3898764	0.2754	0.783	0.096722672	count	1
C1orf43	0.0682871	0.07218	0.9461	0.344	0.096727519	count	1
ZNF616	0.1373261	0.4833969	0.2841	0.776	0.096916518	count	1
H1F0	0.0697792	0.1555715	0.4485	0.654	0.096924671	count	1
ANKRD36	0.080224	0.2504588	0.3203	0.749	0.097015464	count	1
CWC27	0.0722646	0.1422167	0.5081	0.611	0.097024644	count	1
TATDN3	0.0810615	0.233006	0.3479	0.728	0.097124722	count	1
MYO1E	0.0727193	0.1744092	0.4169	0.677	0.097181916	count	1
IFFO1	0.0950913	0.4598765	0.2068	0.836	0.09725943	count	1
NDUFAF1	0.0860564	0.3429226	0.251	0.802	0.097352035	count	1
AMMECR1	0.1220327	0.5443878	0.2242	0.823	0.097423804	count	1
CWC25	0.0704185	0.1178801	0.5974	0.55	0.097524344	count	1
PRKACA	0.0758765	0.2147788	0.3533	0.724	0.097577153	count	1
AC008549.2	0.1680112	0.8390493	0.2002	0.841	0.097721608	count	1
GNG7	0.0899372	0.2767888	0.3249	0.745	0.097861459	count	1
PHYKPL	0.0747598	0.1643168	0.455	0.649	0.097956043	count	1
VPS18	0.1341635	0.4424275	0.3032	0.762	0.098084842	count	1
GHITM	0.0694437	0.0709888	0.9782	0.328	0.098245909	count	1
ENDOV	0.0761672	0.2144479	0.3552	0.722	0.098387695	count	1
POLR1D	0.0695617	0.0692221	1.0049	0.315	0.098399431	count	1
PTPN1	0.0727499	0.1459456	0.4985	0.618	0.098408566	count	1
EFCAB2	0.0808703	0.2991161	0.2704	0.787	0.09841317	count	1
TRMT10B	0.0817559	0.2555936	0.3199	0.749	0.098415381	count	1
TSC22D1	0.0690733	0.0732757	0.9427	0.346	0.098417882	count	1
CDC5L	0.0703199	0.0993257	0.708	0.479	0.098470136	count	1
TAF3	0.0749446	0.1644929	0.4556	0.649	0.098507141	count	1
PIH1D1	0.071267	0.1129654	0.6309	0.528	0.098578765	count	1
STX12	0.0723842	0.1162523	0.6226	0.534	0.098693695	count	1
HNRNPA0	0.0697498	0.0680932	1.0243	0.306	0.098710064	count	1

RIBC1	0.1699031	0.747524	0.2273	0.82	0.098775756	count	1
MUTYH	0.0974907	0.2961137	0.3292	0.742	0.09883809	count	1
NPM1	0.0688531	0.0334364	2.0592	0.0396	0.098955987	count	1
HNRNPM	0.0698908	0.0719852	0.9709	0.332	0.098957209	count	1
PLXNB2	0.0845301	0.2575781	0.3282	0.743	0.09908644	count	1
IMP3	0.0696875	0.0769117	0.9061	0.365	0.099091109	count	1
MUM1	0.0773926	0.2332635	0.3318	0.74	0.099138696	count	1
STX5	0.0750829	0.1576206	0.4764	0.634	0.099146444	count	1
FAM50A	0.0717132	0.1164598	0.6158	0.538	0.099195205	count	1
SEPHS1	0.0763005	0.178975	0.4263	0.67	0.099272983	count	1
PRDM15	0.1615798	0.5744885	0.2813	0.779	0.099521708	count	1
BANF1	0.0699936	0.0637012	1.0988	0.272	0.09957726	count	1
TUBA4A	0.0717069	0.106358	0.6742	0.5	0.099579056	count	1
ZNF850	0.1471501	0.5081905	0.2896	0.772	0.099709503	count	1
MED31	0.073306	0.1382417	0.5303	0.596	0.099880422	count	1
MICU2	0.0763473	0.1735042	0.44	0.66	0.099898048	count	1
CC2D1A	0.0947604	0.2869383	0.3302	0.741	0.099906464	count	1
PODXL2	0.4535928	1.0595536	0.4281	0.669	0.100006581	count	1
PCDHGA8	0.4535928	1.0921793	0.4153	0.678	0.100006581	count	1
AC127024.5	0.4535928	1.0909844	0.4158	0.678	0.100006581	count	1
HSPB6	0.4535928	1.0921793	0.4153	0.678	0.100006581	count	1
AL355312.3	0.4535928	1.1527405	0.3935	0.694	0.100006581	count	1
C3orf67	0.4535928	1.542747	0.294	0.769	0.100006581	count	1
ARPC1A	0.0704856	0.0669881	1.0522	0.293	0.100028907	count	1
PSMC6	0.0717931	0.0949614	0.756	0.45	0.100100724	count	1
ACTG1	0.0694814	0.0321389	2.1619	0.0307	0.100135128	count	1
LRRC42	0.0835166	0.2283902	0.3657	0.715	0.100288634	count	1
TAPBP	0.0707164	0.0812828	0.87	0.384	0.100309096	count	1
PSMA3-AS1	0.0745091	0.1368877	0.5443	0.586	0.100378377	count	1
BCL6B	0.0744382	0.1517381	0.4906	0.624	0.100444428	count	1
SRGAP2B	0.0918451	0.25202	0.3644	0.716	0.100466987	count	1
KMT5B	0.075516	0.1644976	0.4591	0.646	0.10047229	count	1
TAF2	0.0840187	0.2515662	0.334	0.738	0.100646964	count	1
FLYWCH1	0.0883466	0.2421272	0.3649	0.715	0.100728184	count	1
SMYD2	0.1073092	0.3635658	0.2952	0.768	0.100865883	count	1
AC005034.3	0.1737797	0.7006791	0.248	0.804	0.100932528	count	1
ARHGAP21	0.0734139	0.1630587	0.4502	0.653	0.101098355	count	1
PDCD2	0.0730805	0.1030365	0.7093	0.478	0.101276727	count	1
AC108866.1	0.460901	1.0843802	0.425	0.671	0.101360473	count	1
AC026250.1	0.460901	1.0843802	0.425	0.671	0.101360473	count	1
AC007952.7	0.460901	1.0843802	0.425	0.671	0.101360473	count	1
LINC00562	0.460901	1.3290762	0.3468	0.729	0.101360473	count	1
NUTF2	0.0725206	0.0975055	0.7438	0.457	0.101388682	count	1
SUMO3	0.0719226	0.0824542	0.8723	0.383	0.10141527	count	1
PDCD6IP	0.0728963	0.1063007	0.6858	0.493	0.101425689	count	1

RBBP7	0.0734548	0.1104991	0.6648	0.506	0.101430139	count	1
TPRN	0.1093994	0.4749464	0.2303	0.818	0.101439435	count	1
MAF1	0.0727458	0.0980024	0.7423	0.458	0.101484895	count	1
MDN1	0.0869815	0.2967207	0.2931	0.769	0.101627912	count	1
SHKBP1	0.0751606	0.1405544	0.5347	0.593	0.101750991	count	1
CASP3	0.0752402	0.1615256	0.4658	0.641	0.101800181	count	1
ZNF720	0.0791121	0.1839869	0.43	0.667	0.102000559	count	1
NIFK	0.0726167	0.0963351	0.7538	0.451	0.102038135	count	1
CLINT1	0.0728587	0.1006758	0.7237	0.469	0.102041128	count	1
ZNF17	0.1658512	0.5728455	0.2895	0.772	0.102048861	count	1
LUZP1	0.0720714	0.0736166	0.979	0.328	0.102077714	count	1
ZNF200	0.0903737	0.3188268	0.2835	0.777	0.102196428	count	1
SLC35A4	0.0845646	0.2363162	0.3578	0.72	0.102234742	count	1
TASP1	0.0893388	0.2530443	0.3531	0.724	0.102240552	count	1
UTRN	0.0724471	0.0898265	0.8065	0.42	0.102302305	count	1
LMAN1	0.0720757	0.0687709	1.0481	0.295	0.102395541	count	1
FOXN3-AS1	0.1226328	0.5759234	0.2129	0.831	0.102467837	count	1
HUS1	0.0820673	0.217803	0.3768	0.706	0.102626068	count	1
SLC38A10	0.0806368	0.1850554	0.4357	0.663	0.102633026	count	1
ANKRD49	0.0820808	0.2540772	0.3231	0.747	0.102788531	count	1
AC068620.1	0.4686546	1.0931605	0.4287	0.668	0.1027886	count	1
FGF13	0.4686546	1.0931605	0.4287	0.668	0.1027886	count	1
SLC16A1	0.0882911	0.2530397	0.3489	0.727	0.102821406	count	1
CCNT1	0.0833424	0.2139235	0.3896	0.697	0.102922337	count	1
ATXN7L3B	0.0738488	0.1081959	0.6825	0.495	0.102980808	count	1
CYP2R1	0.1232606	0.4228584	0.2915	0.771	0.102981104	count	1
ADO	0.0907008	0.2908279	0.3119	0.755	0.102983348	count	1
SNRPD1	0.0728413	0.0737443	0.9878	0.323	0.102986918	count	1
TMCC1	0.0897289	0.2955726	0.3036	0.761	0.103064067	count	1
MED14OS	0.1098851	0.4065289	0.2703	0.787	0.103248593	count	1
RHOC	0.0721126	0.0468605	1.5389	0.124	0.103553989	count	1
HSPA14	0.0862601	0.2317612	0.3722	0.71	0.103563284	count	1
OXR1	0.0749939	0.1212933	0.6183	0.536	0.103634884	count	1
QTRT1	0.0775595	0.1807891	0.429	0.668	0.10363498	count	1
CCT6A	0.0730138	0.0650882	1.1218	0.262	0.103725922	count	1
LACTB2	0.0786538	0.1792471	0.4388	0.661	0.103732965	count	1
HPS6	0.1216447	0.3603317	0.3376	0.736	0.103737517	count	1
TTC23	0.0934993	0.2635869	0.3547	0.723	0.10382911	count	1
ATXN7	0.7462608	1.1156939	0.6689	0.504	0.104008691	count	1
FAM91A1	0.0818851	0.200279	0.4089	0.683	0.104099756	count	1
MPPED2	0.7478608	0.8120451	0.921	0.357	0.104171369	count	1
SFT2D1	0.0767975	0.137831	0.5572	0.577	0.104191087	count	1
RBM26	0.0762088	0.1551229	0.4913	0.623	0.104209135	count	1
TRIM27	0.0801719	0.1745004	0.4594	0.646	0.104214695	count	1
ZNF701	0.0898331	0.4051739	0.2217	0.825	0.10426343	count	1

PSMC2	0.0760395	0.1204998	0.631	0.528	0.104440132	count	1
ORAOV1	0.0941863	0.3426439	0.2749	0.783	0.104585129	count	1
ZNF585B	0.1312528	0.5057084	0.2595	0.795	0.104605395	count	1
GRK3	0.2581662	0.7882263	0.3275	0.743	0.104616489	count	1
MAP3K6	0.0844845	0.2533619	0.3335	0.739	0.104672259	count	1
SMIM13	0.0948211	0.2763176	0.3432	0.732	0.104770148	count	1
IST1	0.0777812	0.1481523	0.525	0.6	0.10494592	count	1
RSRC1	0.0766164	0.1446333	0.5297	0.596	0.104988786	count	1
ADNP	0.0775183	0.132659	0.5843	0.559	0.104995605	count	1
ACKR2	0.2592157	1.0381929	0.2497	0.803	0.105008955	count	1
AP002761.3	0.2592157	1.0447896	0.2481	0.804	0.105008955	count	1
SMIM10L1	0.0753593	0.1180578	0.6383	0.523	0.10501543	count	1
TRAIP	0.2596637	1.0739662	0.2418	0.809	0.105176405	count	1
TINCR	0.2596637	1.0886744	0.2385	0.812	0.105176405	count	1
AC090198.1	0.2596637	1.2518561	0.2074	0.836	0.105176405	count	1
CCDC13	0.7585519	1.0609615	0.715	0.475	0.105252688	count	1
TUBGCP4	0.1031118	0.3122574	0.3302	0.741	0.105360458	count	1
ZRANB2	0.0746627	0.0978148	0.7633	0.445	0.105422052	count	1
AGTRAP	0.0751368	0.0962881	0.7803	0.435	0.105502284	count	1
CLUAP1	0.0798266	0.1794193	0.4449	0.656	0.105674826	count	1
KIAA1143	0.0767164	0.1279107	0.5998	0.549	0.105749762	count	1
DGAT1	0.0799115	0.2014764	0.3966	0.692	0.10578691	count	1
BEX5	0.0853717	0.1970555	0.4332	0.665	0.105935993	count	1
PLEKHG5	0.1076528	0.4999907	0.2153	0.83	0.105947138	count	1
OXSM	0.0912015	0.2854322	0.3195	0.749	0.106185989	count	1
EXOSC2	0.100852	0.3093623	0.326	0.744	0.10625636	count	1
R3HDM2	0.0767571	0.1321203	0.581	0.561	0.106266434	count	1
SPTBN1	0.0742154	0.0587319	1.2636	0.206	0.106334778	count	1
USF3	0.0834006	0.1961065	0.4253	0.671	0.106366443	count	1
DENND6A	0.0868055	0.2132838	0.407	0.684	0.10641911	count	1
VIM-AS1	0.1305599	0.4668612	0.2797	0.78	0.10657591	count	1
EPS15	0.0785427	0.1453408	0.5404	0.589	0.106637878	count	1
CSNK1G2	0.0837883	0.1838543	0.4557	0.649	0.106858902	count	1
EDEM3	0.0837894	0.2361368	0.3548	0.723	0.1069731	count	1
GAS1	0.184837	0.5188966	0.3562	0.722	0.10706047	count	1
ABHD14B	0.0774756	0.1154684	0.671	0.502	0.107073607	count	1
IL18BP	0.0804604	0.165331	0.4867	0.627	0.107179247	count	1
FRG1	0.0765877	0.0918042	0.8342	0.404	0.107367877	count	1
NDEL1	0.0834805	0.1887833	0.4422	0.658	0.107418142	count	1
ERBB3	0.374815	0.8190299	0.4576	0.647	0.107555634	count	1
RPL24	0.0746543	0.0210917	3.5395	0.000407	0.107582422	count	1
TMEM259	0.0784358	0.1191026	0.6586	0.51	0.107689494	count	1
MECR	0.0882478	0.2727229	0.3236	0.746	0.107767474	count	1
GMIP	0.376952	0.6502369	0.5797	0.562	0.108092548	count	1
AL132639.2	0.3772349	0.7643807	0.4935	0.622	0.108163558	count	1

NDUFA8	0.0768282	0.0843077	0.9113	0.362	0.108206092	count	1
RPL26	0.0751041	0.0230546	3.2577	0.00114	0.108233853	count	1
AC068338.3	0.1602601	0.6873742	0.2331	0.816	0.108279371	count	1
ERCC2	0.1153605	0.3973293	0.2903	0.772	0.108307211	count	1
ZNF740	0.1139128	0.4310433	0.2643	0.792	0.108320707	count	1
SRP9	0.0761185	0.0621378	1.225	0.221	0.108380947	count	1
BNC2	0.0951731	0.2956424	0.3219	0.748	0.108446753	count	1
HEXDC	0.08394	0.2338796	0.3589	0.72	0.108483918	count	1
ST3GAL2	0.0934384	0.2301523	0.406	0.685	0.108770865	count	1
DCAF5	0.0847191	0.171592	0.4937	0.622	0.108802967	count	1
ZNF711	0.1880493	0.4937417	0.3809	0.703	0.108834087	count	1
CXorf40B	0.0967087	0.2881538	0.3356	0.737	0.108835617	count	1
METTL6	0.09296	0.3156412	0.2945	0.768	0.108896307	count	1
ANKRD40	0.0821336	0.1681964	0.4883	0.625	0.108940732	count	1
TMEM183A	0.0812516	0.1422851	0.571	0.568	0.108941555	count	1
RAD21	0.0775979	0.0896166	0.8659	0.387	0.108971774	count	1
RPS13	0.0756525	0.0200919	3.7653	0.00017	0.109071258	count	1
RAB5C	0.0767796	0.0674061	1.1391	0.255	0.109103544	count	1
TADA2A	0.0889126	0.2613903	0.3402	0.734	0.109188122	count	1
KLC4	0.1616836	0.6254828	0.2585	0.796	0.109206783	count	1
HOMER1	0.2020612	0.4938738	0.4091	0.682	0.10925528	count	1
AC093157.1	0.085174	0.2122718	0.4012	0.688	0.109280554	count	1
AL035563.1	0.2022045	0.761962	0.2654	0.791	0.109328689	count	1
CGRRF1	0.0838902	0.2041744	0.4109	0.681	0.109354919	count	1
SNX9	0.0792377	0.1127193	0.703	0.482	0.109602742	count	1
CNPPD1	0.0831072	0.1642178	0.5061	0.613	0.109831043	count	1
LZTFL1	0.08839	0.2209268	0.4001	0.689	0.109834864	count	1
FAM98C	0.0849047	0.2042804	0.4156	0.678	0.109909359	count	1
ZNF304	0.1194802	0.3960161	0.3017	0.763	0.110620709	count	1
CNOT7	0.0812587	0.140683	0.5776	0.564	0.110644295	count	1
CRAT	0.0940801	0.2425831	0.3878	0.698	0.110850269	count	1
C1D	0.0802798	0.1205782	0.6658	0.506	0.11087072	count	1
SLC25A5	0.0776024	0.0525597	1.4765	0.1399	0.110930897	count	1
UCKL1	0.086554	0.1839275	0.4706	0.638	0.110936318	count	1
WDR46	0.0860031	0.1800447	0.4777	0.633	0.110949128	count	1
SMIM12	0.0816454	0.1387281	0.5885	0.556	0.111064044	count	1
SBSPON	15.939517	1799.33141	0.0089	0.993	0.111116848	count	1
SLC6A2	15.9395187	1799.330371	0.0089	0.993	0.111116848	count	1
CDC20B	15.9395188	1799.332264	0.0089	0.993	0.111116848	count	1
AP001269.2	15.939521	1799.332253	0.0089	0.993	0.111116848	count	1
AC026304.1	16.1706519	1801.538391	0.009	0.993	0.111116851	count	1
FCGR3B	16.2981114	2285.628495	0.0071	0.9943	0.111116852	count	1
AC016907.2	16.3808283	2571.920411	0.0064	0.995	0.111116853	count	1
MYO15A	16.3808306	2571.92186	0.0064	0.995	0.111116853	count	1
NTRK3	16.3808318	2571.923346	0.0064	0.995	0.111116853	count	1

SNCA-AS1	16.3808338	2571.923813	0.0064	0.995	0.111116853	count	1
AC105001.1	16.3816727	3007.477429	0.0054	0.996	0.111116853	count	1
AC136489.1	16.3816751	3007.476225	0.0054	0.996	0.111116853	count	1
LYG2	16.6184828	1881.080752	0.0088	0.993	0.111116855	count	1
AL391988.1	16.6700499	1739.419408	0.0096	0.9924	0.111116855	count	1
TBX15	16.6830307	5240.567881	0.0032	0.997	0.111116855	count	1
CCR5	16.6830309	5240.568023	0.0032	0.997	0.111116855	count	1
MSANTD1	16.6830309	5240.568023	0.0032	0.997	0.111116855	count	1
AC091849.2	16.6830308	5240.568058	0.0032	0.997	0.111116855	count	1
SAPCD1-AS1	16.6830309	5240.568023	0.0032	0.997	0.111116855	count	1
AL009178.3	16.6830308	5240.567846	0.0032	0.997	0.111116855	count	1
AL109910.2	16.6830309	5240.568235	0.0032	0.997	0.111116855	count	1
ABCB5	16.6830309	5240.568023	0.0032	0.997	0.111116855	count	1
AC099552.1	16.6830308	5240.567846	0.0032	0.997	0.111116855	count	1
VEGFD	16.6830309	5240.568023	0.0032	0.997	0.111116855	count	1
AL353616.2	16.6830309	5240.568023	0.0032	0.997	0.111116855	count	1
HTR3A	16.6830308	5240.568058	0.0032	0.997	0.111116855	count	1
PLEKHS1	16.6830309	5240.568023	0.0032	0.997	0.111116855	count	1
AC079315.1	16.6830309	5240.568023	0.0032	0.997	0.111116855	count	1
AL583810.3	16.6830309	5240.568023	0.0032	0.997	0.111116855	count	1
CDH8	16.6830309	5240.568023	0.0032	0.997	0.111116855	count	1
FBXW10	16.6830308	5240.567846	0.0032	0.997	0.111116855	count	1
LINC01901	16.6830309	5240.568023	0.0032	0.997	0.111116855	count	1
AC145212.1	16.6830309	5240.568023	0.0032	0.997	0.111116855	count	1
LINC01873	16.683031	5240.568199	0.0032	0.997	0.111116855	count	1
U91328.1	16.683031	5240.568199	0.0032	0.997	0.111116855	count	1
AC084757.2	16.683031	5240.568199	0.0032	0.997	0.111116855	count	1
AC011500.3	16.683031	5240.568199	0.0032	0.997	0.111116855	count	1
AC022154.1	16.683031	5240.568199	0.0032	0.997	0.111116855	count	1
FP236383.3	16.683031	5240.568199	0.0032	0.997	0.111116855	count	1
AC105271.1	16.6830311	5240.568164	0.0032	0.997	0.111116855	count	1
AC114808.2	16.6830311	5240.568164	0.0032	0.997	0.111116855	count	1
AC012065.4	16.6830311	5240.568164	0.0032	0.997	0.111116855	count	1
AL591503.1	16.6830311	5240.567952	0.0032	0.997	0.111116855	count	1
ZCCHC12	16.6830311	5240.567952	0.0032	0.997	0.111116855	count	1
PRDM12	16.6830311	5240.568164	0.0032	0.997	0.111116855	count	1
AL583810.1	16.6830311	5240.568164	0.0032	0.997	0.111116855	count	1
KRT24	16.6830311	5240.568164	0.0032	0.997	0.111116855	count	1
MMP11	16.6830311	5240.568164	0.0032	0.997	0.111116855	count	1
AC099566.1	16.6830313	5240.568306	0.0032	0.997	0.111116855	count	1
PROK2	16.6830312	5240.567917	0.0032	0.997	0.111116855	count	1
AC096586.1	16.6830313	5240.568306	0.0032	0.997	0.111116855	count	1
AC135457.1	16.6830312	5240.568129	0.0032	0.997	0.111116855	count	1
AL034374.1	16.6830312	5240.567917	0.0032	0.997	0.111116855	count	1
IFT74-AS1	16.6830312	5240.567917	0.0032	0.997	0.111116855	count	1

AL359182.2	16.6830312	5240.567917	0.0032	0.997	0.111116855	count	1
AC073389.3	16.6830312	5240.568129	0.0032	0.997	0.111116855	count	1
LINC02387	16.6830312	5240.568341	0.0032	0.997	0.111116855	count	1
AL160191.1	16.6830312	5240.568341	0.0032	0.997	0.111116855	count	1
C17orf64	16.6830312	5240.568129	0.0032	0.997	0.111116855	count	1
CIRBP-AS1	16.6830312	5240.567917	0.0032	0.997	0.111116855	count	1
AC011445.2	16.6830312	5240.568129	0.0032	0.997	0.111116855	count	1
AJ011932.1	16.6830312	5240.567917	0.0032	0.997	0.111116855	count	1
AL031283.1	16.6830314	5240.56827	0.0032	0.997	0.111116855	count	1
LINC00636	16.7009707	2553.480301	0.0065	0.9948	0.111116855	count	1
SYCP3	16.7009728	2553.481631	0.0065	0.9948	0.111116855	count	1
AL596202.1	16.7009731	2553.482536	0.0065	0.9948	0.111116855	count	1
AC004889.1	16.7009731	2553.482546	0.0065	0.9948	0.111116855	count	1
H3F3C	16.7009771	2553.484292	0.0065	0.9948	0.111116855	count	1
PLK4	15.7288872	1364.556518	0.0115	0.9908	0.111116855	count	1
AL354760.1	16.8947092	4282.004062	0.0039	0.997	0.111116856	count	1
AC079148.1	16.8947092	4282.004013	0.0039	0.997	0.111116856	count	1
CDCP2	16.8947093	4282.004086	0.0039	0.997	0.111116856	count	1
AC019171.1	16.8947093	4282.004111	0.0039	0.997	0.111116856	count	1
DLEC1	16.8947093	4282.004037	0.0039	0.997	0.111116856	count	1
LINC02067	16.8947093	4282.004111	0.0039	0.997	0.111116856	count	1
AC008875.1	16.8947094	4282.004135	0.0039	0.997	0.111116856	count	1
GRPR	16.8947093	4282.004086	0.0039	0.997	0.111116856	count	1
NANOG	16.8947093	4282.004111	0.0039	0.997	0.111116856	count	1
SYNDIG1L	16.8947093	4282.004062	0.0039	0.997	0.111116856	count	1
AC005291.1	16.8947093	4282.004111	0.0039	0.997	0.111116856	count	1
KCNJ6	16.8947093	4282.004111	0.0039	0.997	0.111116856	count	1
RAB42	16.8947129	4282.015126	0.0039	0.997	0.111116856	count	1
AL022326.1	16.8947196	4282.006338	0.0039	0.997	0.111116856	count	1
AP001267.1	16.8947228	4282.003425	0.0039	0.997	0.111116856	count	1
KHDRBS2	16.8947261	4282.008811	0.0039	0.997	0.111116856	count	1
EME1	16.8947261	4282.008811	0.0039	0.997	0.111116856	count	1
GHRHR	16.8947296	4282.011503	0.0039	0.997	0.111116856	count	1
SLC35G5	16.8947296	4282.011552	0.0039	0.997	0.111116856	count	1
CARMIL3	16.8947329	4282.016962	0.0039	0.997	0.111116856	count	1
AC011921.1	16.8947329	4282.016962	0.0039	0.997	0.111116856	count	1
SMO	16.894733	4282.016987	0.0039	0.997	0.111116856	count	1
AL021707.5	15.8887974	1671.56676	0.0095	0.992	0.111116858	count	1
AC009318.1	15.9395151	1799.330544	0.0089	0.993	0.111116859	count	1
AC093157.2	15.939517	1799.331404	0.0089	0.993	0.111116859	count	1
RNF128	15.939517	1799.331406	0.0089	0.993	0.111116859	count	1
KCNIP2-AS1	15.939517	1799.331407	0.0089	0.993	0.111116859	count	1
EIF2S3B	15.939517	1799.331407	0.0089	0.993	0.111116859	count	1
AC005261.2	15.9395176	1799.331404	0.0089	0.993	0.111116859	count	1
AP000446.1	15.939518	1799.33053	0.0089	0.993	0.111116859	count	1

AC002470.1	15.939518	1799.330533	0.0089	0.993	0.111116859	count	1
CCDC168	15.9395181	1799.332423	0.0089	0.993	0.111116859	count	1
AC023355.1	15.9395181	1799.332426	0.0089	0.993	0.111116859	count	1
CHST6	15.9395181	1799.332421	0.0089	0.993	0.111116859	count	1
IL1RL2	15.9395184	1799.331705	0.0089	0.993	0.111116859	count	1
CYP2E1	15.9395185	1799.331554	0.0089	0.993	0.111116859	count	1
AC124045.1	15.9395188	1799.330373	0.0089	0.993	0.111116859	count	1
KIF6	15.9395188	1799.332265	0.0089	0.993	0.111116859	count	1
TMEM71	15.9395191	1799.331549	0.0089	0.993	0.111116859	count	1
EPHB6	15.9395193	1799.331395	0.0089	0.993	0.111116859	count	1
AL451074.2	15.9395195	1799.331089	0.0089	0.993	0.111116859	count	1
COLQ	15.9395195	1799.331089	0.0089	0.993	0.111116859	count	1
CNTN2	15.9395206	1799.331234	0.0089	0.993	0.111116859	count	1
AL450306.1	15.9395206	1799.331236	0.0089	0.993	0.111116859	count	1
NCKAP1L	15.9395206	1799.331233	0.0089	0.993	0.111116859	count	1
AL121917.1	15.9395206	1799.332108	0.0089	0.993	0.111116859	count	1
AC005288.1	15.9395211	1799.332252	0.0089	0.993	0.111116859	count	1
TTTY10	15.9395211	1799.332253	0.0089	0.993	0.111116859	count	1
AC233992.3	15.9395212	1799.332105	0.0089	0.993	0.111116859	count	1
LINC02334	15.9395216	1799.33225	0.0089	0.993	0.111116859	count	1
CFAP47	15.9395224	1799.331075	0.0089	0.993	0.111116859	count	1
AC079174.2	15.9395232	1799.332807	0.0089	0.993	0.111116859	count	1
SLC2A1-AS1	17.5309873	3839.022159	0.0046	0.996	0.111116859	count	1
AC005523.1	17.5309881	3839.017634	0.0046	0.996	0.111116859	count	1
EFNB3	17.5309915	3839.022159	0.0046	0.996	0.111116859	count	1
ESRP2	17.5309928	3839.015285	0.0046	0.996	0.111116859	count	1
ADORA2B	17.5309934	3839.013044	0.0046	0.996	0.111116859	count	1
C17orf82	17.5309953	3839.022072	0.0046	0.996	0.111116859	count	1
AC044802.2	17.5309986	3839.024226	0.0046	0.996	0.111116859	count	1
SEC23A-AS1	17.5310042	3839.017352	0.0046	0.996	0.111116859	count	1
LPAR2	15.9765637	1371.779095	0.0116	0.9907	0.111116859	count	1
MMP25	16.0680819	1262.412484	0.0127	0.99	0.11111686	count	1
SP6	16.1377483	2415.69751	0.0067	0.995	0.111116861	count	1
GINS1	16.1706473	1801.537309	0.009	0.993	0.111116861	count	1
IL6R-AS1	16.1706479	1801.537308	0.009	0.993	0.111116861	count	1
AC096564.1	16.1706479	1801.537306	0.009	0.993	0.111116861	count	1
RERGL	16.1706479	1801.537306	0.009	0.993	0.111116861	count	1
ADPGK-AS1	16.1706485	1801.537855	0.009	0.993	0.111116861	count	1
AC007613.1	16.1706485	1801.537859	0.009	0.993	0.111116861	count	1
AL513314.2	16.1706497	1801.538404	0.009	0.993	0.111116861	count	1
PIPOX	16.1706508	1801.537846	0.009	0.993	0.111116861	count	1
TTC25	16.1706508	1801.537842	0.009	0.993	0.111116861	count	1
KIF14	16.1706509	1801.538245	0.009	0.993	0.111116861	count	1
INKA2	16.1706512	1801.537994	0.009	0.993	0.111116861	count	1
PTGFR	16.170652	1801.53769	0.009	0.993	0.111116861	count	1

AC005037.1	16.170652	1801.538391	0.009	0.993	0.111116861	count	1
AC020978.5	16.1706547	1801.537129	0.009	0.993	0.111116861	count	1
DLGAP3	16.1706549	1801.537678	0.009	0.993	0.111116861	count	1
OCA2	16.1706553	1801.537126	0.009	0.993	0.111116861	count	1
FOXF1	16.1706553	1801.537126	0.009	0.993	0.111116861	count	1
AC091057.6	16.1706559	1801.537675	0.009	0.993	0.111116861	count	1
RPS6KA6	16.170656	1801.537678	0.009	0.993	0.111116861	count	1
AC008555.2	16.1708534	2063.169049	0.0078	0.994	0.111116861	count	1
AC008758.4	16.255764	1545.249648	0.0105	0.9916	0.111116862	count	1
CELSR3	16.3475077	1793.193037	0.0091	0.993	0.111116863	count	1
AC087392.5	16.3478388	1970.89155	0.0083	0.993	0.111116863	count	1
AL136038.5	16.3478423	1970.891957	0.0083	0.993	0.111116863	count	1
HDAC2-AS2	16.3478429	1970.893982	0.0083	0.993	0.111116863	count	1
C4orf19	16.3753231	1763.010904	0.0093	0.9926	0.111116863	count	1
AC023509.3	16.3753246	1763.012068	0.0093	0.9926	0.111116863	count	1
AC044802.1	16.3753247	1763.012071	0.0093	0.9926	0.111116863	count	1
AC004223.2	16.3765432	1892.144756	0.0087	0.993	0.111116864	count	1
NEB	16.3808267	2571.919435	0.0064	0.995	0.111116864	count	1
TTLL6	16.3808268	2571.91944	0.0064	0.995	0.111116864	count	1
AC009720.1	16.380827	2571.920422	0.0064	0.995	0.111116864	count	1
AP005205.2	16.3808275	2571.919912	0.0064	0.995	0.111116864	count	1
AC024475.1	16.3808276	2571.919912	0.0064	0.995	0.111116864	count	1
AC107982.3	16.3808276	2571.919912	0.0064	0.995	0.111116864	count	1
ZBTB8B	16.3808277	2571.922396	0.0064	0.995	0.111116864	count	1
ARHGEF33	16.3808282	2571.920406	0.0064	0.995	0.111116864	count	1
AC004754.1	16.3808282	2571.920406	0.0064	0.995	0.111116864	count	1
AC103810.2	16.3808282	2571.92039	0.0064	0.995	0.111116864	count	1
AL512770.1	16.3808284	2571.920406	0.0064	0.995	0.111116864	count	1
AC090360.1	16.3808293	2571.919398	0.0064	0.995	0.111116864	count	1
ZNF709	16.3808295	2571.921871	0.0064	0.995	0.111116864	count	1
CNR2	16.3808298	2571.921377	0.0064	0.995	0.111116864	count	1
AC093525.7	16.3808301	2571.91988	0.0064	0.995	0.111116864	count	1
AL050403.2	16.3808301	2571.91988	0.0064	0.995	0.111116864	count	1
VIL1	16.3808302	2571.920857	0.0064	0.995	0.111116864	count	1
RNF150	16.3808302	2571.922364	0.0064	0.995	0.111116864	count	1
AC112250.2	16.3808303	2571.922369	0.0064	0.995	0.111116864	count	1
PON3	16.3808303	2571.920862	0.0064	0.995	0.111116864	count	1
SOX6	16.3808303	2571.920868	0.0064	0.995	0.111116864	count	1
AC092490.1	16.3808303	2571.920873	0.0064	0.995	0.111116864	count	1
AL121761.2	16.3808304	2571.920868	0.0064	0.995	0.111116864	count	1
SLITRK5	16.3808307	2571.921865	0.0064	0.995	0.111116864	count	1
AC005224.4	16.380831	2571.922842	0.0064	0.995	0.111116864	count	1
AL354732.1	16.3808313	2571.92384	0.0064	0.995	0.111116864	count	1
TTC39A-AS1	16.3808317	2571.923335	0.0064	0.995	0.111116864	count	1
ITGB1BP2	16.380832	2571.920342	0.0064	0.995	0.111116864	count	1

AL603839.3	16.3808321	2571.920342	0.0064	0.995	0.111116864	count	1
PSAT1	16.3808321	2571.920353	0.0064	0.995	0.111116864	count	1
CENPA	16.3808322	2571.920358	0.0064	0.995	0.111116864	count	1
FCER1A	16.3808326	2571.92134	0.0064	0.995	0.111116864	count	1
AC092718.3	16.3808326	2571.919833	0.0064	0.995	0.111116864	count	1
HDHD5-AS1	16.3808326	2571.919838	0.0064	0.995	0.111116864	count	1
IGHEP2	16.3808329	2571.92082	0.0064	0.995	0.111116864	count	1
AC073195.1	16.3808333	2571.921818	0.0064	0.995	0.111116864	count	1
AC024267.6	16.3808335	2571.922815	0.0064	0.995	0.111116864	count	1
LINC00449	16.380834	2571.922311	0.0064	0.995	0.111116864	count	1
AL136382.1	16.3808345	2571.921807	0.0064	0.995	0.111116864	count	1
AC020907.3	16.3808349	2571.922794	0.0064	0.995	0.111116864	count	1
AL031665.2	16.3808353	2571.922295	0.0064	0.995	0.111116864	count	1
CFAP58	16.3808355	2571.923282	0.0064	0.995	0.111116864	count	1
AL161756.1	16.3808355	2571.923282	0.0064	0.995	0.111116864	count	1
PCDHGA1	16.3808356	2571.923282	0.0064	0.995	0.111116864	count	1
PTPRD	16.381673	3007.475697	0.0054	0.996	0.111116864	count	1
KCNIP2	16.381673	3007.477442	0.0054	0.996	0.111116864	count	1
RAVER1	16.3816731	3007.475697	0.0054	0.996	0.111116864	count	1
AC099811.4	16.3816744	3007.475666	0.0054	0.996	0.111116864	count	1
AF287957.1	16.381676	3007.475057	0.0054	0.996	0.111116864	count	1
AC005014.2	16.3816763	3007.476212	0.0054	0.996	0.111116864	count	1
DGKI	16.3816785	3007.476765	0.0054	0.996	0.111116864	count	1
CXorf57	16.432348	1526.063632	0.0108	0.9914	0.111116864	count	1
BCAS4	16.4357094	2188.145925	0.0075	0.994	0.111116864	count	1
AC132192.1	16.4619503	1095.350522	0.015	0.988	0.111116864	count	1
GYPE	16.4688389	1276.07049	0.0129	0.9897	0.111116864	count	1
MAPT	16.4932752	1783.148506	0.0092	0.9926	0.111116864	count	1
AC012213.3	16.4932773	1783.150769	0.0092	0.9926	0.111116864	count	1
RORA-AS1	16.4936606	1917.383143	0.0086	0.9931	0.111116864	count	1
AC244100.2	16.4936607	1917.383149	0.0086	0.9931	0.111116864	count	1
GAS6-AS1	16.4936632	1917.382583	0.0086	0.9931	0.111116864	count	1
CNIH2	16.5426425	2038.323469	0.0081	0.994	0.111116865	count	1
AC103746.1	16.5426485	1779.934884	0.0093	0.9926	0.111116865	count	1
AC022098.4	16.5426492	1779.934468	0.0093	0.9926	0.111116865	count	1
AC022306.2	16.5426497	1779.934881	0.0093	0.9926	0.111116865	count	1
AC119674.1	16.5426504	1779.935528	0.0093	0.9926	0.111116865	count	1
TRIP13	16.5426519	1779.934468	0.0093	0.9926	0.111116865	count	1
AL031846.2	16.5426519	1779.935249	0.0093	0.9926	0.111116865	count	1
AC015468.3	16.5434964	1910.005895	0.0087	0.993	0.111116865	count	1
LINC02062	16.5532423	1522.677153	0.0109	0.9913	0.111116865	count	1
CRISPLD2	16.5545636	1720.824282	0.0096	0.9923	0.111116865	count	1
ZNF710-AS1	16.5684333	1243.825631	0.0133	0.9894	0.111116865	count	1
SH2D3A	16.6184828	1881.082317	0.0088	0.993	0.111116865	count	1
AC055876.5	16.6188419	1982.961697	0.0084	0.9933	0.111116865	count	1

AC097468.3	16.618992	2079.70339	0.008	0.9936	0.111116865	count	1
ANPEP	16.6194909	2346.334762	0.0071	0.994	0.111116865	count	1
NOX5	16.6646798	1451.741331	0.0115	0.9908	0.111116866	count	1
LINC02367	16.6712074	1825.661204	0.0091	0.9927	0.111116866	count	1
KCNIP3	16.6758669	1783.547096	0.0093	0.9925	0.111116866	count	1
LINC01730	16.6758707	1783.547087	0.0093	0.9925	0.111116866	count	1
AL109761.1	16.6830306	5240.567704	0.0032	0.997	0.111116866	count	1
REELD1	16.6830307	5240.568164	0.0032	0.997	0.111116866	count	1
AC012313.7	16.6830307	5240.567846	0.0032	0.997	0.111116866	count	1
AJAP1	16.6830309	5240.568023	0.0032	0.997	0.111116866	count	1
NPPA-AS1	16.6830309	5240.567987	0.0032	0.997	0.111116866	count	1
AL121992.2	16.6830309	5240.567881	0.0032	0.997	0.111116866	count	1
TMEM61	16.6830309	5240.568164	0.0032	0.997	0.111116866	count	1
CYP2J2	16.6830309	5240.567952	0.0032	0.997	0.111116866	count	1
AC099794.1	16.6830309	5240.567917	0.0032	0.997	0.111116866	count	1
GPR61	16.6830309	5240.567881	0.0032	0.997	0.111116866	count	1
PTPN22	16.6830309	5240.567846	0.0032	0.997	0.111116866	count	1
AL356356.1	16.6830309	5240.567846	0.0032	0.997	0.111116866	count	1
TNFAIP8L2	16.6830309	5240.567952	0.0032	0.997	0.111116866	count	1
LINC01641	16.6830309	5240.567917	0.0032	0.997	0.111116866	count	1
LINC00582	16.6830309	5240.567952	0.0032	0.997	0.111116866	count	1
AL357556.4	16.6830309	5240.567881	0.0032	0.997	0.111116866	count	1
CHRM3-AS2	16.6830308	5240.568164	0.0032	0.997	0.111116866	count	1
DNAJC5G	16.6830309	5240.567952	0.0032	0.997	0.111116866	count	1
AC012593.1	16.6830309	5240.568093	0.0032	0.997	0.111116866	count	1
ZEB2-AS1	16.6830309	5240.568023	0.0032	0.997	0.111116866	count	1
TUBA4B	16.6830309	5240.568058	0.0032	0.997	0.111116866	count	1
SNORC	16.6830309	5240.567846	0.0032	0.997	0.111116866	count	1
AC018809.2	16.6830309	5240.567987	0.0032	0.997	0.111116866	count	1
SAMMSON	16.6830309	5240.568023	0.0032	0.997	0.111116866	count	1
AC110491.3	16.6830309	5240.568129	0.0032	0.997	0.111116866	count	1
NPHP3-AS1	16.6830309	5240.568164	0.0032	0.997	0.111116866	count	1
LINC00881	16.6830309	5240.567881	0.0032	0.997	0.111116866	count	1
LINC02054	16.6830309	5240.567917	0.0032	0.997	0.111116866	count	1
FGFBP2	16.6830309	5240.568164	0.0032	0.997	0.111116866	count	1
AC106864.1	16.6830309	5240.568164	0.0032	0.997	0.111116866	count	1
AC108935.1	16.6830309	5240.567846	0.0032	0.997	0.111116866	count	1
FAM160A1	16.6830309	5240.567917	0.0032	0.997	0.111116866	count	1
C1QTNF3-AMACR	16.6830309	5240.568164	0.0032	0.997	0.111116866	count	1
LINC02122	16.6830308	5240.567704	0.0032	0.997	0.111116866	count	1
AC026427.1	16.6830309	5240.567917	0.0032	0.997	0.111116866	count	1
LINC00992	16.6830309	5240.567881	0.0032	0.997	0.111116866	count	1
REEP2	16.6830309	5240.567881	0.0032	0.997	0.111116866	count	1
PLAC8L1	16.6830309	5240.567952	0.0032	0.997	0.111116866	count	1
AL513548.1	16.6830309	5240.568023	0.0032	0.997	0.111116866	count	1

C6orf47-AS1	16.6830309	5240.567881	0.0032	0.997	0.111116866	count	1
AL356277.3	16.6830309	5240.567987	0.0032	0.997	0.111116866	count	1
AL589666.1	16.6830309	5240.568129	0.0032	0.997	0.111116866	count	1
KLHL32	16.6830309	5240.567846	0.0032	0.997	0.111116866	count	1
GRM1	16.6830309	5240.568129	0.0032	0.997	0.111116866	count	1
AL356417.3	16.6830309	5240.567987	0.0032	0.997	0.111116866	count	1
AC092171.2	16.6830309	5240.568023	0.0032	0.997	0.111116866	count	1
AC007255.1	16.6830308	5240.568164	0.0032	0.997	0.111116866	count	1
AC004988.1	16.6830309	5240.568058	0.0032	0.997	0.111116866	count	1
AC005537.1	16.6830309	5240.568023	0.0032	0.997	0.111116866	count	1
TAC1	16.6830308	5240.567846	0.0032	0.997	0.111116866	count	1
MAP7D2	16.6830309	5240.567881	0.0032	0.997	0.111116866	count	1
ERAS	16.6830308	5240.568058	0.0032	0.997	0.111116866	count	1
AL139398.1	16.6830309	5240.567952	0.0032	0.997	0.111116866	count	1
TSIX	16.6830309	5240.567987	0.0032	0.997	0.111116866	count	1
GPR174	16.6830309	5240.568129	0.0032	0.997	0.111116866	count	1
RHOXF1	16.6830309	5240.567775	0.0032	0.997	0.111116866	count	1
TPD52L3	16.6830309	5240.567881	0.0032	0.997	0.111116866	count	1
PIP5K1B	16.6830309	5240.567917	0.0032	0.997	0.111116866	count	1
NXNL2	16.6830309	5240.567917	0.0032	0.997	0.111116866	count	1
OR1L8	16.6830309	5240.567987	0.0032	0.997	0.111116866	count	1
CYSRT1	16.6830309	5240.568164	0.0032	0.997	0.111116866	count	1
AC091053.1	16.6830309	5240.568129	0.0032	0.997	0.111116866	count	1
SMTNL1	16.6830308	5240.568023	0.0032	0.997	0.111116866	count	1
AP003721.1	16.6830308	5240.56774	0.0032	0.997	0.111116866	count	1
AP002373.1	16.6830309	5240.567952	0.0032	0.997	0.111116866	count	1
AP000941.1	16.6830309	5240.567917	0.0032	0.997	0.111116866	count	1
ARMC3	16.6830308	5240.56774	0.0032	0.997	0.111116866	count	1
AL121749.1	16.6830309	5240.567917	0.0032	0.997	0.111116866	count	1
ANXA8	16.6830309	5240.567987	0.0032	0.997	0.111116866	count	1
AL121928.1	16.6830309	5240.567917	0.0032	0.997	0.111116866	count	1
AC018630.2	16.6830309	5240.567917	0.0032	0.997	0.111116866	count	1
TAS2R46	16.6830308	5240.56781	0.0032	0.997	0.111116866	count	1
SMCO3	16.6830309	5240.567987	0.0032	0.997	0.111116866	count	1
AC008083.3	16.6830309	5240.568129	0.0032	0.997	0.111116866	count	1
LINC01154	16.6830308	5240.568164	0.0032	0.997	0.111116866	count	1
AC020656.2	16.6830309	5240.568164	0.0032	0.997	0.111116866	count	1
PLA2G1B	16.6830309	5240.567846	0.0032	0.997	0.111116866	count	1
CCNA1	16.6830308	5240.568164	0.0032	0.997	0.111116866	count	1
AC004817.3	16.6830308	5240.568023	0.0032	0.997	0.111116866	count	1
AC005476.2	16.6830309	5240.567881	0.0032	0.997	0.111116866	count	1
AC123768.2	16.6830309	5240.567881	0.0032	0.997	0.111116866	count	1
AC010809.1	16.6830309	5240.567917	0.0032	0.997	0.111116866	count	1
BUB1B	16.6830309	5240.567881	0.0032	0.997	0.111116866	count	1
CAPN3	16.6830309	5240.567846	0.0032	0.997	0.111116866	count	1

AC027237.3	16.6830308	5240.56781	0.0032	0.997	0.111116866	count	1
AC118658.1	16.6830309	5240.568023	0.0032	0.997	0.111116866	count	1
SLC5A11	16.6830309	5240.567917	0.0032	0.997	0.111116866	count	1
AC007336.1	16.6830309	5240.568129	0.0032	0.997	0.111116866	count	1
CDH3	16.6830309	5240.568023	0.0032	0.997	0.111116866	count	1
AC092718.6	16.6830309	5240.567952	0.0032	0.997	0.111116866	count	1
AC093484.2	16.6830309	5240.567775	0.0032	0.997	0.111116866	count	1
GSDMA	16.6830309	5240.568341	0.0032	0.997	0.111116866	count	1
GALR2	16.6830309	5240.567987	0.0032	0.997	0.111116866	count	1
AC100788.1	16.6830309	5240.567917	0.0032	0.997	0.111116866	count	1
FSCN2	16.6830308	5240.567704	0.0032	0.997	0.111116866	count	1
AP005059.1	16.6830309	5240.567881	0.0032	0.997	0.111116866	count	1
AP005432.2	16.6830309	5240.567917	0.0032	0.997	0.111116866	count	1
AC023983.2	16.6830309	5240.568093	0.0032	0.997	0.111116866	count	1
AC016205.1	16.6830308	5240.568058	0.0032	0.997	0.111116866	count	1
SLC25A41	16.6830309	5240.567881	0.0032	0.997	0.111116866	count	1
CD70	16.6830309	5240.567987	0.0032	0.997	0.111116866	count	1
ANGPTL6	16.6830309	5240.568093	0.0032	0.997	0.111116866	count	1
AC018761.3	16.6830309	5240.567917	0.0032	0.997	0.111116866	count	1
AC024075.3	16.6830308	5240.568023	0.0032	0.997	0.111116866	count	1
AC024563.1	16.6830308	5240.56781	0.0032	0.997	0.111116866	count	1
CCDC8	16.6830308	5240.567704	0.0032	0.997	0.111116866	count	1
LILRB2	16.6830309	5240.568129	0.0032	0.997	0.111116866	count	1
LINC01660	16.6830309	5240.567917	0.0032	0.997	0.111116866	count	1
AC254562.2	16.6830309	5240.567775	0.0032	0.997	0.111116866	count	1
BACH1-IT2	16.6830309	5240.567917	0.0032	0.997	0.111116866	count	1
AP000275.2	16.6830309	5240.567952	0.0032	0.997	0.111116866	count	1
HCRTR1	16.683031	5240.568093	0.0032	0.997	0.111116866	count	1
AL451062.1	16.683031	5240.568164	0.0032	0.997	0.111116866	count	1
FYB2	16.683031	5240.568093	0.0032	0.997	0.111116866	count	1
AL583808.1	16.683031	5240.568093	0.0032	0.997	0.111116866	count	1
NBPF10	16.683031	5240.567987	0.0032	0.997	0.111116866	count	1
AL592295.3	16.683031	5240.568093	0.0032	0.997	0.111116866	count	1
TEX35	16.683031	5240.567987	0.0032	0.997	0.111116866	count	1
RGS18	16.683031	5240.567846	0.0032	0.997	0.111116866	count	1
AC012358.1	16.683031	5240.567952	0.0032	0.997	0.111116866	count	1
CNNM3-DT	16.683031	5240.567881	0.0032	0.997	0.111116866	count	1
AC019197.1	16.683031	5240.567917	0.0032	0.997	0.111116866	count	1
AC007879.3	16.683031	5240.567952	0.0032	0.997	0.111116866	count	1
BOK-AS1	16.683031	5240.567987	0.0032	0.997	0.111116866	count	1
CPB1	16.683031	5240.567952	0.0032	0.997	0.111116866	count	1
ERICH6	16.683031	5240.567846	0.0032	0.997	0.111116866	count	1
AC078795.2	16.683031	5240.567952	0.0032	0.997	0.111116866	count	1
DGKG	16.683031	5240.567987	0.0032	0.997	0.111116866	count	1
FGG	16.683031	5240.568058	0.0032	0.997	0.111116866	count	1

AC034229.1	16.683031	5240.568341	0.0032	0.997	0.111116866	count	1
AC079465.1	16.683031	5240.567881	0.0032	0.997	0.111116866	count	1
SPINK9	16.683031	5240.567881	0.0032	0.997	0.111116866	count	1
AC008443.4	16.683031	5240.567952	0.0032	0.997	0.111116866	count	1
AL138885.3	16.683031	5240.567881	0.0032	0.997	0.111116866	count	1
AL021807.1	16.683031	5240.568306	0.0032	0.997	0.111116866	count	1
CDSN	16.683031	5240.568023	0.0032	0.997	0.111116866	count	1
MSH5	16.683031	5240.567881	0.0032	0.997	0.111116866	count	1
CFAP206	16.683031	5240.568129	0.0032	0.997	0.111116866	count	1
GRIK2	16.683031	5240.567987	0.0032	0.997	0.111116866	count	1
FO393415.3	16.683031	5240.568093	0.0032	0.997	0.111116866	count	1
RAET1L	16.683031	5240.568093	0.0032	0.997	0.111116866	count	1
AL355297.2	16.683031	5240.567987	0.0032	0.997	0.111116866	count	1
AL009176.1	16.683031	5240.567846	0.0032	0.997	0.111116866	count	1
SLC29A4	16.683031	5240.567987	0.0032	0.997	0.111116866	count	1
AC005091.1	16.683031	5240.568058	0.0032	0.997	0.111116866	count	1
TRIM74	16.683031	5240.567881	0.0032	0.997	0.111116866	count	1
LMOD2	16.683031	5240.568058	0.0032	0.997	0.111116866	count	1
AC006372.2	16.683031	5240.567881	0.0032	0.997	0.111116866	count	1
AC078960.1	16.683031	5240.567881	0.0032	0.997	0.111116866	count	1
AL591501.1	16.683031	5240.567917	0.0032	0.997	0.111116866	count	1
IGSF1	16.683031	5240.568164	0.0032	0.997	0.111116866	count	1
PNMA3	16.683031	5240.567881	0.0032	0.997	0.111116866	count	1
AC022784.8	16.683031	5240.567952	0.0032	0.997	0.111116866	count	1
AC104964.1	16.683031	5240.567846	0.0032	0.997	0.111116866	count	1
FAM167A	16.683031	5240.567952	0.0032	0.997	0.111116866	count	1
EYA1	16.683031	5240.567952	0.0032	0.997	0.111116866	count	1
AP003465.1	16.683031	5240.56781	0.0032	0.997	0.111116866	count	1
SNX31	16.683031	5240.567987	0.0032	0.997	0.111116866	count	1
AC100803.1	16.683031	5240.567952	0.0032	0.997	0.111116866	count	1
C9orf24	16.683031	5240.568093	0.0032	0.997	0.111116866	count	1
KCNJ11	16.683031	5240.568093	0.0032	0.997	0.111116866	count	1
AP001453.1	16.683031	5240.567881	0.0032	0.997	0.111116866	count	1
AP001273.2	16.683031	5240.567952	0.0032	0.997	0.111116866	count	1
AP000757.1	16.683031	5240.56827	0.0032	0.997	0.111116866	count	1
AP003396.1	16.683031	5240.567846	0.0032	0.997	0.111116866	count	1
CACNA2D4	16.683031	5240.567987	0.0032	0.997	0.111116866	count	1
NRIP2	16.683031	5240.567987	0.0032	0.997	0.111116866	count	1
AC024896.1	16.683031	5240.567952	0.0032	0.997	0.111116866	count	1
AC008083.1	16.683031	5240.567881	0.0032	0.997	0.111116866	count	1
AC083809.1	16.683031	5240.567952	0.0032	0.997	0.111116866	count	1
HAL	16.683031	5240.567846	0.0032	0.997	0.111116866	count	1
AC083806.2	16.683031	5240.568129	0.0032	0.997	0.111116866	count	1
LINC00944	16.683031	5240.567881	0.0032	0.997	0.111116866	count	1
MMP17	16.683031	5240.567952	0.0032	0.997	0.111116866	count	1

AL445288.1	16.683031	5240.567917	0.0032	0.997	0.111116866	count	1
AL136526.1	16.683031	5240.568093	0.0032	0.997	0.111116866	count	1
TEX29	16.683031	5240.567881	0.0032	0.997	0.111116866	count	1
ATP4B	16.683031	5240.567987	0.0032	0.997	0.111116866	count	1
AC007262.2	16.683031	5240.568341	0.0032	0.997	0.111116866	count	1
SLC24A5	16.683031	5240.567917	0.0032	0.997	0.111116866	count	1
AC011939.3	16.683031	5240.56827	0.0032	0.997	0.111116866	count	1
AC108861.1	16.683031	5240.568164	0.0032	0.997	0.111116866	count	1
CHRNB4	16.683031	5240.567952	0.0032	0.997	0.111116866	count	1
CRTC3-AS1	16.683031	5240.567846	0.0032	0.997	0.111116866	count	1
CCDC78	16.683031	5240.568023	0.0032	0.997	0.111116866	count	1
AC093512.2	16.683031	5240.567952	0.0032	0.997	0.111116866	count	1
AC135050.1	16.683031	5240.567881	0.0032	0.997	0.111116866	count	1
SLC9A5	16.683031	5240.567952	0.0032	0.997	0.111116866	count	1
AC009120.4	16.683031	5240.568341	0.0032	0.997	0.111116866	count	1
AC009063.3	16.683031	5240.56827	0.0032	0.997	0.111116866	count	1
CDH15	16.683031	5240.568093	0.0032	0.997	0.111116866	count	1
VPS9D1-AS1	16.683031	5240.567881	0.0032	0.997	0.111116866	count	1
HASPIN	16.683031	5240.567881	0.0032	0.997	0.111116866	count	1
AC004771.4	16.683031	5240.568129	0.0032	0.997	0.111116866	count	1
AC068025.2	16.683031	5240.567952	0.0032	0.997	0.111116866	count	1
AC243585.2	16.683031	5240.567917	0.0032	0.997	0.111116866	count	1
MYCBPAP	16.683031	5240.568129	0.0032	0.997	0.111116866	count	1
AC064805.2	16.683031	5240.568093	0.0032	0.997	0.111116866	count	1
AC087289.4	16.683031	5240.568093	0.0032	0.997	0.111116866	count	1
AC110285.1	16.683031	5240.568164	0.0032	0.997	0.111116866	count	1
AC132938.3	16.683031	5240.567704	0.0032	0.997	0.111116866	count	1
ZNF750	16.683031	5240.568129	0.0032	0.997	0.111116866	count	1
AC015819.1	16.683031	5240.567846	0.0032	0.997	0.111116866	count	1
C18orf65	16.683031	5240.568412	0.0032	0.997	0.111116866	count	1
ZNF831	16.683031	5240.568023	0.0032	0.997	0.111116866	count	1
KF456478.1	16.683031	5240.56827	0.0032	0.997	0.111116866	count	1
CATSPERD	16.683031	5240.567987	0.0032	0.997	0.111116866	count	1
RTBDN	16.683031	5240.567952	0.0032	0.997	0.111116866	count	1
AC020913.1	16.683031	5240.567846	0.0032	0.997	0.111116866	count	1
DLL3	16.683031	5240.567952	0.0032	0.997	0.111116866	count	1
MIMT1	16.683031	5240.567952	0.0032	0.997	0.111116866	count	1
LINC00896	16.683031	5240.567846	0.0032	0.997	0.111116866	count	1
AP000553.3	16.683031	5240.568093	0.0032	0.997	0.111116866	count	1
U62317.2	16.683031	5240.568129	0.0032	0.997	0.111116866	count	1
AP001172.1	16.683031	5240.567917	0.0032	0.997	0.111116866	count	1
AL627309.3	16.6830311	5240.568093	0.0032	0.997	0.111116866	count	1
CCDC27	16.6830312	5240.568023	0.0032	0.997	0.111116866	count	1
AL121992.1	16.6830311	5240.568129	0.0032	0.997	0.111116866	count	1
AL109936.2	16.6830311	5240.56827	0.0032	0.997	0.111116866	count	1

E2F2	16.6830311	5240.568129	0.0032	0.997	0.111116866	count	1
AL391650.1	16.6830311	5240.56827	0.0032	0.997	0.111116866	count	1
AL929472.2	16.6830311	5240.56827	0.0032	0.997	0.111116866	count	1
TMCO2	16.6830311	5240.56827	0.0032	0.997	0.111116866	count	1
MPL	16.6830311	5240.568164	0.0032	0.997	0.111116866	count	1
AL513218.1	16.6830312	5240.568199	0.0032	0.997	0.111116866	count	1
MSH4	16.6830311	5240.56827	0.0032	0.997	0.111116866	count	1
LINC01780	16.6830311	5240.568129	0.0032	0.997	0.111116866	count	1
NBPF26	16.6830312	5240.568058	0.0032	0.997	0.111116866	count	1
BGLAP	16.6830311	5240.568341	0.0032	0.997	0.111116866	count	1
TSACC	16.6830312	5240.568093	0.0032	0.997	0.111116866	count	1
SLAMF1	16.6830312	5240.567987	0.0032	0.997	0.111116866	count	1
AL121985.1	16.6830312	5240.56827	0.0032	0.997	0.111116866	count	1
KIAA1614-AS1	16.6830312	5240.568058	0.0032	0.997	0.111116866	count	1
PDC	16.6830311	5240.568235	0.0032	0.997	0.111116866	count	1
CRB1	16.6830312	5240.568058	0.0032	0.997	0.111116866	count	1
INAVA	16.6830311	5240.568235	0.0032	0.997	0.111116866	count	1
LINC01136	16.6830312	5240.567952	0.0032	0.997	0.111116866	count	1
OBSCN-AS1	16.6830312	5240.568093	0.0032	0.997	0.111116866	count	1
AC080162.1	16.6830311	5240.56827	0.0032	0.997	0.111116866	count	1
AC012073.1	16.6830312	5240.568199	0.0032	0.997	0.111116866	count	1
SLC30A3	16.6830311	5240.568235	0.0032	0.997	0.111116866	count	1
AC007391.1	16.6830311	5240.56827	0.0032	0.997	0.111116866	count	1
KCNG3	16.6830311	5240.568058	0.0032	0.997	0.111116866	count	1
AC073283.1	16.6830311	5240.56827	0.0032	0.997	0.111116866	count	1
AC008280.3	16.6830312	5240.568093	0.0032	0.997	0.111116866	count	1
CD207	16.6830311	5240.56827	0.0032	0.997	0.111116866	count	1
AC006030.1	16.6830312	5240.568235	0.0032	0.997	0.111116866	count	1
PROM2	16.6830312	5240.567987	0.0032	0.997	0.111116866	count	1
AC010884.1	16.6830312	5240.567987	0.0032	0.997	0.111116866	count	1
AC112229.2	16.6830311	5240.56827	0.0032	0.997	0.111116866	count	1
AC104653.2	16.6830311	5240.568023	0.0032	0.997	0.111116866	count	1
AC104653.1	16.6830311	5240.56781	0.0032	0.997	0.111116866	count	1
AC009312.1	16.6830312	5240.568023	0.0032	0.997	0.111116866	count	1
AJ239322.2	16.6830311	5240.56827	0.0032	0.997	0.111116866	count	1
AC009480.1	16.6830311	5240.56827	0.0032	0.997	0.111116866	count	1
AC009336.1	16.6830311	5240.568235	0.0032	0.997	0.111116866	count	1
AC068196.1	16.6830311	5240.568341	0.0032	0.997	0.111116866	count	1
RUFY4	16.6830311	5240.567881	0.0032	0.997	0.111116866	count	1
IHH	16.6830311	5240.56827	0.0032	0.997	0.111116866	count	1
NHEJ1	16.6830311	5240.56827	0.0032	0.997	0.111116866	count	1
IRS1	16.6830312	5240.568058	0.0032	0.997	0.111116866	count	1
AC026191.1	16.6830311	5240.56827	0.0032	0.997	0.111116866	count	1
LINC00691	16.6830312	5240.568058	0.0032	0.997	0.111116866	count	1
AC126118.1	16.6830312	5240.568199	0.0032	0.997	0.111116866	count	1

AC098613.1	16.6830311	5240.56827	0.0032	0.997	0.111116866	count	1
GPR62	16.6830311	5240.568129	0.0032	0.997	0.111116866	count	1
FLNB-AS1	16.6830312	5240.568199	0.0032	0.997	0.111116866	count	1
GPR27	16.6830311	5240.56827	0.0032	0.997	0.111116866	count	1
ARHGAP31-AS1	16.6830312	5240.568199	0.0032	0.997	0.111116866	count	1
LINC02014	16.6830312	5240.568306	0.0032	0.997	0.111116866	count	1
AC072039.2	16.6830311	5240.568129	0.0032	0.997	0.111116866	count	1
AC080013.6	16.6830312	5240.568058	0.0032	0.997	0.111116866	count	1
GMNC	16.6830311	5240.56827	0.0032	0.997	0.111116866	count	1
TNK2-AS1	16.6830311	5240.568093	0.0032	0.997	0.111116866	count	1
AC068620.2	16.6830311	5240.56827	0.0032	0.997	0.111116866	count	1
AC069307.1	16.6830311	5240.568129	0.0032	0.997	0.111116866	count	1
AC020741.1	16.6830312	5240.567987	0.0032	0.997	0.111116866	count	1
PF4	16.6830311	5240.568129	0.0032	0.997	0.111116866	count	1
ODAPH	16.6830311	5240.568129	0.0032	0.997	0.111116866	count	1
PRKG2	16.6830312	5240.567987	0.0032	0.997	0.111116866	count	1
SNHG27	16.6830311	5240.56827	0.0032	0.997	0.111116866	count	1
AC096711.2	16.6830312	5240.568058	0.0032	0.997	0.111116866	count	1
TRIM60	16.6830312	5240.56781	0.0032	0.997	0.111116866	count	1
AC019163.1	16.6830312	5240.56781	0.0032	0.997	0.111116866	count	1
AC098864.1	16.6830311	5240.56827	0.0032	0.997	0.111116866	count	1
PLEKHG4B	16.6830311	5240.568199	0.0032	0.997	0.111116866	count	1
LINC02102	16.6830311	5240.56827	0.0032	0.997	0.111116866	count	1
AC034231.1	16.6830311	5240.567881	0.0032	0.997	0.111116866	count	1
C6	16.6830311	5240.56827	0.0032	0.997	0.111116866	count	1
DDX4	16.6830311	5240.56827	0.0032	0.997	0.111116866	count	1
AC104113.1	16.6830311	5240.56827	0.0032	0.997	0.111116866	count	1
AC018754.1	16.6830311	5240.56827	0.0032	0.997	0.111116866	count	1
AC010261.1	16.6830311	5240.567952	0.0032	0.997	0.111116866	count	1
AC106786.2	16.6830311	5240.56827	0.0032	0.997	0.111116866	count	1
AC010240.3	16.6830311	5240.568341	0.0032	0.997	0.111116866	count	1
AC005609.5	16.6830311	5240.568023	0.0032	0.997	0.111116866	count	1
PCDHB8	16.6830311	5240.56827	0.0032	0.997	0.111116866	count	1
SAP30L-AS1	16.6830311	5240.56827	0.0032	0.997	0.111116866	count	1
KIF4B	16.6830311	5240.56827	0.0032	0.997	0.111116866	count	1
AL357054.3	16.6830311	5240.568129	0.0032	0.997	0.111116866	count	1
U91328.3	16.6830311	5240.56827	0.0032	0.997	0.111116866	count	1
HIST1H3I	16.6830312	5240.568058	0.0032	0.997	0.111116866	count	1
HIST1H2BO	16.6830311	5240.56827	0.0032	0.997	0.111116866	count	1
AL662791.1	16.6830312	5240.568093	0.0032	0.997	0.111116866	count	1
SLC26A8	16.6830311	5240.56827	0.0032	0.997	0.111116866	count	1
ENPP5	16.6830312	5240.568058	0.0032	0.997	0.111116866	count	1
AL121974.1	16.6830312	5240.568023	0.0032	0.997	0.111116866	count	1
RIMS1	16.6830311	5240.56827	0.0032	0.997	0.111116866	count	1
AL603910.1	16.6830311	5240.56827	0.0032	0.997	0.111116866	count	1

AL080317.2	16.6830312	5240.568023	0.0032	0.997	0.111116866	count	1
AL356234.3	16.6830311	5240.56827	0.0032	0.997	0.111116866	count	1
LINC01277	16.6830311	5240.56827	0.0032	0.997	0.111116866	count	1
AL133260.2	16.6830311	5240.568129	0.0032	0.997	0.111116866	count	1
Z94721.1	16.6830311	5240.568129	0.0032	0.997	0.111116866	count	1
AC091729.1	16.6830312	5240.567987	0.0032	0.997	0.111116866	count	1
TRGC2	16.6830311	5240.567881	0.0032	0.997	0.111116866	count	1
AMPH	16.6830311	5240.56827	0.0032	0.997	0.111116866	count	1
AC004870.4	16.6830311	5240.56827	0.0032	0.997	0.111116866	count	1
C7orf57	16.6830311	5240.568023	0.0032	0.997	0.111116866	count	1
AC006012.1	16.6830311	5240.568199	0.0032	0.997	0.111116866	count	1
CLDN3	16.6830311	5240.568093	0.0032	0.997	0.111116866	count	1
CLDN4	16.6830312	5240.568199	0.0032	0.997	0.111116866	count	1
AC002454.1	16.6830312	5240.567987	0.0032	0.997	0.111116866	count	1
AC002429.2	16.6830311	5240.568023	0.0032	0.997	0.111116866	count	1
DLX6	16.6830311	5240.56827	0.0032	0.997	0.111116866	count	1
AC007938.2	16.6830311	5240.56827	0.0032	0.997	0.111116866	count	1
OR9A4	16.6830311	5240.568129	0.0032	0.997	0.111116866	count	1
CTAGE6	16.6830311	5240.568058	0.0032	0.997	0.111116866	count	1
AC093726.2	16.6830311	5240.568164	0.0032	0.997	0.111116866	count	1
PTCHD1	16.6830311	5240.56827	0.0032	0.997	0.111116866	count	1
AC115618.2	16.6830311	5240.56827	0.0032	0.997	0.111116866	count	1
AL035427.1	16.6830312	5240.568058	0.0032	0.997	0.111116866	count	1
INTS6L-AS1	16.6830311	5240.568023	0.0032	0.997	0.111116866	count	1
AL078639.1	16.6830312	5240.568058	0.0032	0.997	0.111116866	count	1
AC245187.2	16.6830311	5240.568129	0.0032	0.997	0.111116866	count	1
AC021242.3	16.6830312	5240.568058	0.0032	0.997	0.111116866	count	1
FAM66D	16.6830312	5240.568023	0.0032	0.997	0.111116866	count	1
ESCO2	16.6830312	5240.568235	0.0032	0.997	0.111116866	count	1
AC104051.2	16.6830312	5240.568058	0.0032	0.997	0.111116866	count	1
AC022182.1	16.6830311	5240.56827	0.0032	0.997	0.111116866	count	1
AC009812.3	16.6830312	5240.56781	0.0032	0.997	0.111116866	count	1
AC090568.2	16.6830311	5240.56827	0.0032	0.997	0.111116866	count	1
C8orf37-AS1	16.6830311	5240.568235	0.0032	0.997	0.111116866	count	1
FBXO43	16.6830311	5240.56827	0.0032	0.997	0.111116866	count	1
AC013546.1	16.6830311	5240.56827	0.0032	0.997	0.111116866	count	1
AC067930.2	16.6830312	5240.567987	0.0032	0.997	0.111116866	count	1
C9orf92	16.6830311	5240.56827	0.0032	0.997	0.111116866	count	1
CR786580.1	16.6830311	5240.56827	0.0032	0.997	0.111116866	count	1
TMEM252	16.6830311	5240.568093	0.0032	0.997	0.111116866	count	1
AL135924.2	16.6830311	5240.56827	0.0032	0.997	0.111116866	count	1
AL137847.1	16.6830312	5240.567987	0.0032	0.997	0.111116866	count	1
AL606807.1	16.6830312	5240.568199	0.0032	0.997	0.111116866	count	1
LINC01509	16.6830312	5240.567881	0.0032	0.997	0.111116866	count	1
TNFSF15	16.6830312	5240.567881	0.0032	0.997	0.111116866	count	1

AL161630.1	16.6830312	5240.56781	0.0032	0.997	0.111116866	count	1
TTLL11-IT1	16.6830311	5240.568129	0.0032	0.997	0.111116866	count	1
AL158151.3	16.6830311	5240.56827	0.0032	0.997	0.111116866	count	1
AL353803.1	16.6830312	5240.568199	0.0032	0.997	0.111116866	count	1
C9orf139	16.6830312	5240.568093	0.0032	0.997	0.111116866	count	1
ENTPD8	16.6830311	5240.56827	0.0032	0.997	0.111116866	count	1
AP006621.1	16.6830312	5240.568199	0.0032	0.997	0.111116866	count	1
OR51B5	16.6830312	5240.568199	0.0032	0.997	0.111116866	count	1
OR52N1	16.6830312	5240.568306	0.0032	0.997	0.111116866	count	1
AC087280.2	16.6830311	5240.568023	0.0032	0.997	0.111116866	count	1
AC018523.2	16.6830311	5240.56827	0.0032	0.997	0.111116866	count	1
OR9Q1	16.6830311	5240.568164	0.0032	0.997	0.111116866	count	1
AP000640.1	16.6830311	5240.568129	0.0032	0.997	0.111116866	count	1
MAJIN	16.6830312	5240.568023	0.0032	0.997	0.111116866	count	1
SHANK2	16.6830311	5240.56827	0.0032	0.997	0.111116866	count	1
ZNF705E	16.6830311	5240.567917	0.0032	0.997	0.111116866	count	1
CHRDL2	16.6830312	5240.568023	0.0032	0.997	0.111116866	count	1
MOGAT2	16.6830311	5240.56827	0.0032	0.997	0.111116866	count	1
AP002360.2	16.6830311	5240.56827	0.0032	0.997	0.111116866	count	1
AP001189.4	16.6830311	5240.56827	0.0032	0.997	0.111116866	count	1
CARD18	16.6830311	5240.568129	0.0032	0.997	0.111116866	count	1
CD3G	16.6830311	5240.568093	0.0032	0.997	0.111116866	count	1
TTC36	16.6830312	5240.568199	0.0032	0.997	0.111116866	count	1
AP003397.1	16.6830311	5240.568129	0.0032	0.997	0.111116866	count	1
AL157895.1	16.6830311	5240.56827	0.0032	0.997	0.111116866	count	1
AL391839.2	16.6830311	5240.56827	0.0032	0.997	0.111116866	count	1
AL731537.1	16.6830311	5240.568235	0.0032	0.997	0.111116866	count	1
STOX1	16.6830311	5240.568093	0.0032	0.997	0.111116866	count	1
SYNPO2L	16.6830311	5240.56827	0.0032	0.997	0.111116866	count	1
AL356608.1	16.6830311	5240.56827	0.0032	0.997	0.111116866	count	1
ATRNL1	16.6830311	5240.56827	0.0032	0.997	0.111116866	count	1
AL354950.1	16.6830312	5240.567917	0.0032	0.997	0.111116866	count	1
FGF23	16.6830312	5240.568023	0.0032	0.997	0.111116866	count	1
AC092745.1	16.6830312	5240.568023	0.0032	0.997	0.111116866	count	1
AC008115.4	16.6830312	5240.568058	0.0032	0.997	0.111116866	count	1
OVCH1	16.6830312	5240.568093	0.0032	0.997	0.111116866	count	1
AC004801.6	16.6830311	5240.56827	0.0032	0.997	0.111116866	count	1
DDN	16.6830312	5240.568058	0.0032	0.997	0.111116866	count	1
ANKRD33	16.6830312	5240.568058	0.0032	0.997	0.111116866	count	1
NPFF	16.6830311	5240.568235	0.0032	0.997	0.111116866	count	1
AC024884.2	16.6830311	5240.568023	0.0032	0.997	0.111116866	count	1
SLC26A10	16.6830311	5240.568341	0.0032	0.997	0.111116866	count	1
AC020656.1	16.6830312	5240.568023	0.0032	0.997	0.111116866	count	1
LINC02410	16.6830311	5240.568129	0.0032	0.997	0.111116866	count	1
AC131238.1	16.6830311	5240.56827	0.0032	0.997	0.111116866	count	1

SRRM4	16.6830312	5240.567952	0.0032	0.997	0.111116866	count	1
AL021546.1	16.6830311	5240.567775	0.0032	0.997	0.111116866	count	1
TMEM132C	16.6830311	5240.56827	0.0032	0.997	0.111116866	count	1
RIMBP2	16.6830311	5240.56827	0.0032	0.997	0.111116866	count	1
USP12-AS2	16.6830311	5240.56827	0.0032	0.997	0.111116866	count	1
FARP1-AS1	16.6830312	5240.567987	0.0032	0.997	0.111116866	count	1
AL391262.1	16.6830311	5240.56827	0.0032	0.997	0.111116866	count	1
AC004817.4	16.6830311	5240.568199	0.0032	0.997	0.111116866	count	1
AC005479.1	16.6830312	5240.568306	0.0032	0.997	0.111116866	count	1
AF111167.2	16.6830312	5240.567987	0.0032	0.997	0.111116866	count	1
AL118558.4	16.6830312	5240.56827	0.0032	0.997	0.111116866	count	1
ASPG	16.6830312	5240.567987	0.0032	0.997	0.111116866	count	1
AC026150.3	16.6830311	5240.568093	0.0032	0.997	0.111116866	count	1
ARHGAP11B	16.6830311	5240.56827	0.0032	0.997	0.111116866	count	1
AC020661.3	16.6830311	5240.568023	0.0032	0.997	0.111116866	count	1
EXD1	16.6830311	5240.568093	0.0032	0.997	0.111116866	count	1
FOXB1	16.6830311	5240.568093	0.0032	0.997	0.111116866	count	1
C15orf59	16.6830311	5240.56827	0.0032	0.997	0.111116866	count	1
AC025219.1	16.6830311	5240.56827	0.0032	0.997	0.111116866	count	1
AC021739.3	16.6830312	5240.568093	0.0032	0.997	0.111116866	count	1
PLIN1	16.6830311	5240.568235	0.0032	0.997	0.111116866	count	1
AC091167.5	16.6830312	5240.567987	0.0032	0.997	0.111116866	count	1
AC027013.1	16.6830311	5240.56827	0.0032	0.997	0.111116866	count	1
HBA1	16.6830311	5240.56827	0.0032	0.997	0.111116866	count	1
PDIA2	16.6830311	5240.568341	0.0032	0.997	0.111116866	count	1
TPSB2	16.6830311	5240.56827	0.0032	0.997	0.111116866	count	1
SYNGR3	16.6830311	5240.568199	0.0032	0.997	0.111116866	count	1
AC106820.5	16.6830311	5240.568093	0.0032	0.997	0.111116866	count	1
AC012173.1	16.6830311	5240.568199	0.0032	0.997	0.111116866	count	1
AC092375.2	16.6830312	5240.568306	0.0032	0.997	0.111116866	count	1
PDZD9	16.6830312	5240.568093	0.0032	0.997	0.111116866	count	1
VWA3A	16.6830311	5240.567881	0.0032	0.997	0.111116866	count	1
LINC02175	16.6830311	5240.568058	0.0032	0.997	0.111116866	count	1
AC007495.1	16.6830312	5240.567881	0.0032	0.997	0.111116866	count	1
AC092118.2	16.6830312	5240.568058	0.0032	0.997	0.111116866	count	1
AC092378.1	16.6830311	5240.56827	0.0032	0.997	0.111116866	count	1
AC027682.3	16.6830311	5240.568058	0.0032	0.997	0.111116866	count	1
AC004943.3	16.6830312	5240.568235	0.0032	0.997	0.111116866	count	1
AC009159.1	16.6830311	5240.568341	0.0032	0.997	0.111116866	count	1
AC009148.1	16.6830312	5240.567952	0.0032	0.997	0.111116866	count	1
AC134312.1	16.6830312	5240.568023	0.0032	0.997	0.111116866	count	1
CPNE7	16.6830311	5240.56827	0.0032	0.997	0.111116866	count	1
AC113189.1	16.6830311	5240.568164	0.0032	0.997	0.111116866	count	1
AC129492.1	16.6830312	5240.568058	0.0032	0.997	0.111116866	count	1
TEKT3	16.6830311	5240.568093	0.0032	0.997	0.111116866	count	1

SEZ6	16.6830311	5240.56827	0.0032	0.997	0.111116866	count	1
AC084809.1	16.6830311	5240.568093	0.0032	0.997	0.111116866	count	1
ARL5C	16.6830311	5240.56827	0.0032	0.997	0.111116866	count	1
STAC2	16.6830312	5240.567846	0.0032	0.997	0.111116866	count	1
AC138150.2	16.6830311	5240.56827	0.0032	0.997	0.111116866	count	1
AC091132.1	16.6830311	5240.568129	0.0032	0.997	0.111116866	count	1
MPO	16.6830311	5240.56827	0.0032	0.997	0.111116866	count	1
TMEM105	16.6830311	5240.568235	0.0032	0.997	0.111116866	count	1
AC139149.1	16.6830311	5240.56827	0.0032	0.997	0.111116866	count	1
AP005328.1	16.6830311	5240.56827	0.0032	0.997	0.111116866	count	1
AP005131.1	16.6830311	5240.56827	0.0032	0.997	0.111116866	count	1
AC027449.1	16.6830311	5240.568164	0.0032	0.997	0.111116866	count	1
AL133396.2	16.6830311	5240.56827	0.0032	0.997	0.111116866	count	1
AL158013.1	16.6830312	5240.567987	0.0032	0.997	0.111116866	count	1
AL035252.3	16.6830312	5240.568023	0.0032	0.997	0.111116866	count	1
AL034550.2	16.6830312	5240.568093	0.0032	0.997	0.111116866	count	1
AL121895.2	16.6830311	5240.56827	0.0032	0.997	0.111116866	count	1
AL359555.2	16.6830312	5240.56781	0.0032	0.997	0.111116866	count	1
AL391095.3	16.6830312	5240.567987	0.0032	0.997	0.111116866	count	1
UBE2C	16.6830311	5240.56827	0.0032	0.997	0.111116866	count	1
MIR1-1HG	16.6830311	5240.567917	0.0032	0.997	0.111116866	count	1
RTEL1	16.6830311	5240.56827	0.0032	0.997	0.111116866	count	1
AC005954.2	16.6830311	5240.56827	0.0032	0.997	0.111116866	count	1
DPP9-AS1	16.6830311	5240.568023	0.0032	0.997	0.111116866	count	1
ACER1	16.6830311	5240.56827	0.0032	0.997	0.111116866	count	1
ANGPTL8	16.6830312	5240.568058	0.0032	0.997	0.111116866	count	1
SYCE2	16.6830311	5240.568129	0.0032	0.997	0.111116866	count	1
C19orf67	16.6830312	5240.568058	0.0032	0.997	0.111116866	count	1
TMEM221	16.6830311	5240.56827	0.0032	0.997	0.111116866	count	1
AC011477.4	16.6830311	5240.568341	0.0032	0.997	0.111116866	count	1
ZNF729	16.6830311	5240.56827	0.0032	0.997	0.111116866	count	1
AC092295.1	16.6830311	5240.568341	0.0032	0.997	0.111116866	count	1
AC011479.1	16.6830311	5240.568093	0.0032	0.997	0.111116866	count	1
HIPK4	16.6830311	5240.568129	0.0032	0.997	0.111116866	count	1
LILRA5	16.6830311	5240.568093	0.0032	0.997	0.111116866	count	1
AC006115.1	16.6830312	5240.568058	0.0032	0.997	0.111116866	count	1
AC004076.2	16.6830312	5240.567987	0.0032	0.997	0.111116866	count	1
LINC00895	16.6830311	5240.568341	0.0032	0.997	0.111116866	count	1
IGLC6	16.6830311	5240.56827	0.0032	0.997	0.111116866	count	1
ADORA2A-AS1	16.6830312	5240.568058	0.0032	0.997	0.111116866	count	1
C22orf23	16.6830312	5240.568199	0.0032	0.997	0.111116866	count	1
PNPLA3	16.6830312	5240.568058	0.0032	0.997	0.111116866	count	1
AP003900.1	16.6830312	5240.568023	0.0032	0.997	0.111116866	count	1
LINC02246	16.6830311	5240.56827	0.0032	0.997	0.111116866	count	1
AJ009632.2	16.6830311	5240.56827	0.0032	0.997	0.111116866	count	1

AP001596.1	16.6830312	5240.568058	0.0032	0.997	0.111116866	count	1
AP000265.1	16.6830312	5240.567881	0.0032	0.997	0.111116866	count	1
RIPPLY3	16.6830312	5240.568199	0.0032	0.997	0.111116866	count	1
DSCR9	16.6830312	5240.568093	0.0032	0.997	0.111116866	count	1
LINC01423	16.6830312	5240.567952	0.0032	0.997	0.111116866	count	1
CBS	16.6830312	5240.567952	0.0032	0.997	0.111116866	count	1
AP001056.2	16.6830311	5240.56827	0.0032	0.997	0.111116866	count	1
LINC01786	16.6830313	5240.568129	0.0032	0.997	0.111116866	count	1
AL033528.2	16.6830313	5240.568376	0.0032	0.997	0.111116866	count	1
C1orf232	16.6830313	5240.568235	0.0032	0.997	0.111116866	count	1
HPCA	16.6830313	5240.568058	0.0032	0.997	0.111116866	count	1
COL9A2	16.6830313	5240.568376	0.0032	0.997	0.111116866	count	1
ARMH1	16.6830313	5240.568376	0.0032	0.997	0.111116866	count	1
LINC01144	16.6830313	5240.568376	0.0032	0.997	0.111116866	count	1
GPR88	16.6830313	5240.568376	0.0032	0.997	0.111116866	count	1
ALX3	16.6830313	5240.568376	0.0032	0.997	0.111116866	count	1
AL365361.1	16.6830313	5240.568376	0.0032	0.997	0.111116866	count	1
EDARADD	16.6830313	5240.568376	0.0032	0.997	0.111116866	count	1
TRIM54	16.6830313	5240.568376	0.0032	0.997	0.111116866	count	1
CLEC4F	16.6830313	5240.568199	0.0032	0.997	0.111116866	count	1
AC009237.15	16.6830313	5240.568376	0.0032	0.997	0.111116866	count	1
BUB1	16.6830313	5240.568376	0.0032	0.997	0.111116866	count	1
ACOXL	16.6830313	5240.567987	0.0032	0.997	0.111116866	count	1
AC009299.3	16.6830313	5240.568376	0.0032	0.997	0.111116866	count	1
DHRS9	16.6830313	5240.568376	0.0032	0.997	0.111116866	count	1
AC007966.1	16.6830313	5240.568376	0.0032	0.997	0.111116866	count	1
UNC80	16.6830313	5240.568376	0.0032	0.997	0.111116866	count	1
AC116096.1	16.6830313	5240.568235	0.0032	0.997	0.111116866	count	1
AC024933.1	16.6830313	5240.568376	0.0032	0.997	0.111116866	count	1
TPRG1	16.6830313	5240.568376	0.0032	0.997	0.111116866	count	1
AC005699.1	16.6830313	5240.568376	0.0032	0.997	0.111116866	count	1
BMPR1B-DT	16.6830313	5240.568376	0.0032	0.997	0.111116866	count	1
AP001961.1	16.6830313	5240.568376	0.0032	0.997	0.111116866	count	1
AC093879.2	16.6830313	5240.568376	0.0032	0.997	0.111116866	count	1
FABP2	16.6830313	5240.568376	0.0032	0.997	0.111116866	count	1
C4orf51	16.6830313	5240.568376	0.0032	0.997	0.111116866	count	1
SPATA9	16.6830313	5240.568376	0.0032	0.997	0.111116866	count	1
SLC27A6	16.6830313	5240.568164	0.0032	0.997	0.111116866	count	1
PCDHGB4	16.6830313	5240.568376	0.0032	0.997	0.111116866	count	1
UNC5A	16.6830313	5240.568376	0.0032	0.997	0.111116866	count	1
FAM153C	16.6830313	5240.568376	0.0032	0.997	0.111116866	count	1
ADAMTS2	16.6830313	5240.568376	0.0032	0.997	0.111116866	count	1
HIST1H3F	16.6830313	5240.568376	0.0032	0.997	0.111116866	count	1
HCG15	16.6830313	5240.568376	0.0032	0.997	0.111116866	count	1
AL662791.2	16.6830313	5240.568199	0.0032	0.997	0.111116866	count	1

COL19A1	16.6830313	5240.568376	0.0032	0.997	0.111116866	count	1
VNN2	16.6830313	5240.568376	0.0032	0.997	0.111116866	count	1
RAET1E-AS1	16.6830313	5240.568058	0.0032	0.997	0.111116866	count	1
LINC02544	16.6830313	5240.568376	0.0032	0.997	0.111116866	count	1
AC005154.6	16.6830313	5240.568058	0.0032	0.997	0.111116866	count	1
POU6F2-AS2	16.6830313	5240.568376	0.0032	0.997	0.111116866	count	1
NACAD	16.6830313	5240.567987	0.0032	0.997	0.111116866	count	1
CNTNAP2	16.6830313	5240.568058	0.0032	0.997	0.111116866	count	1
ASMT	16.6830313	5240.568376	0.0032	0.997	0.111116866	count	1
AC009630.2	16.6830313	5240.568482	0.0032	0.997	0.111116866	count	1
NKAIN3	16.6830313	5240.568376	0.0032	0.997	0.111116866	count	1
PAG1	16.6830313	5240.567987	0.0032	0.997	0.111116866	count	1
AC090572.3	16.6830313	5240.568376	0.0032	0.997	0.111116866	count	1
AC090579.1	16.6830313	5240.567952	0.0032	0.997	0.111116866	count	1
FOXD4L5	16.6830313	5240.568376	0.0032	0.997	0.111116866	count	1
C9orf147	16.6830313	5240.568376	0.0032	0.997	0.111116866	count	1
HMGA1P4	16.6830313	5240.568376	0.0032	0.997	0.111116866	count	1
AP000759.1	16.6830313	5240.568376	0.0032	0.997	0.111116866	count	1
AP001790.1	16.6830313	5240.568058	0.0032	0.997	0.111116866	count	1
CFAP300	16.6830313	5240.568447	0.0032	0.997	0.111116866	count	1
AP001527.1	16.6830313	5240.568376	0.0032	0.997	0.111116866	count	1
CASP5	16.6830313	5240.568376	0.0032	0.997	0.111116866	count	1
FXYD6-FXYD2	16.6830313	5240.568376	0.0032	0.997	0.111116866	count	1
AP003390.1	16.6830313	5240.568376	0.0032	0.997	0.111116866	count	1
MALRD1	16.6830313	5240.568376	0.0032	0.997	0.111116866	count	1
PCAT5	16.6830313	5240.568376	0.0032	0.997	0.111116866	count	1
MAT1A	16.6830313	5240.568376	0.0032	0.997	0.111116866	count	1
PRLHR	16.6830313	5240.568199	0.0032	0.997	0.111116866	count	1
LINC02449	16.6830313	5240.568058	0.0032	0.997	0.111116866	count	1
AC022509.3	16.6830313	5240.568376	0.0032	0.997	0.111116866	count	1
TROAP	16.6830313	5240.568376	0.0032	0.997	0.111116866	count	1
AC025165.2	16.6830313	5240.568376	0.0032	0.997	0.111116866	count	1
LINC02384	16.6830313	5240.568376	0.0032	0.997	0.111116866	count	1
AC126178.1	16.6830313	5240.568376	0.0032	0.997	0.111116866	count	1
AC126175.1	16.6830313	5240.568376	0.0032	0.997	0.111116866	count	1
AC069209.1	16.6830313	5240.568376	0.0032	0.997	0.111116866	count	1
UBE2L5	16.6830313	5240.568058	0.0032	0.997	0.111116866	count	1
GRTP1	16.6830313	5240.568376	0.0032	0.997	0.111116866	count	1
AC022613.1	16.6830313	5240.568058	0.0032	0.997	0.111116866	count	1
OIP5	16.6830313	5240.568376	0.0032	0.997	0.111116866	count	1
AC090510.2	16.6830313	5240.568376	0.0032	0.997	0.111116866	count	1
AL031600.3	16.6830313	5240.567987	0.0032	0.997	0.111116866	count	1
AC009021.1	16.6830313	5240.568376	0.0032	0.997	0.111116866	count	1
AC136944.2	16.6830313	5240.568376	0.0032	0.997	0.111116866	count	1
NOD2	16.6830313	5240.568376	0.0032	0.997	0.111116866	count	1

CMTM1	16.6830313	5240.568376	0.0032	0.997	0.111116866	count	1
SMPD3	16.6830313	5240.568376	0.0032	0.997	0.111116866	count	1
AC009097.3	16.6830313	5240.568376	0.0032	0.997	0.111116866	count	1
AC015853.2	16.6830313	5240.568376	0.0032	0.997	0.111116866	count	1
AC024267.3	16.6830313	5240.568376	0.0032	0.997	0.111116866	count	1
AC018521.7	16.6830313	5240.568376	0.0032	0.997	0.111116866	count	1
AC091180.4	16.6830313	5240.568376	0.0032	0.997	0.111116866	count	1
AC005920.1	16.6830313	5240.568376	0.0032	0.997	0.111116866	count	1
TBX2	16.6830313	5240.568376	0.0032	0.997	0.111116866	count	1
AC021504.1	16.6830313	5240.568376	0.0032	0.997	0.111116866	count	1
AL136531.2	16.6830313	5240.568376	0.0032	0.997	0.111116866	count	1
AL121760.1	16.6830313	5240.568376	0.0032	0.997	0.111116866	count	1
GNRH2	16.6830313	5240.568376	0.0032	0.997	0.111116866	count	1
AC005220.1	16.6830313	5240.568447	0.0032	0.997	0.111116866	count	1
LINC01775	16.6830313	5240.568376	0.0032	0.997	0.111116866	count	1
TEX45	16.6830313	5240.568341	0.0032	0.997	0.111116866	count	1
AC010323.1	16.6830313	5240.568376	0.0032	0.997	0.111116866	count	1
AC010422.6	16.6830313	5240.568376	0.0032	0.997	0.111116866	count	1
PTGER1	16.6830313	5240.568376	0.0032	0.997	0.111116866	count	1
FFAR2	16.6830313	5240.568058	0.0032	0.997	0.111116866	count	1
AC005515.2	16.6830313	5240.568376	0.0032	0.997	0.111116866	count	1
AC010616.1	16.6830313	5240.568376	0.0032	0.997	0.111116866	count	1
AC008403.3	16.6830313	5240.568023	0.0032	0.997	0.111116866	count	1
LHB	16.6830313	5240.568058	0.0032	0.997	0.111116866	count	1
AC008687.4	16.6830313	5240.568058	0.0032	0.997	0.111116866	count	1
AC020909.3	16.6830313	5240.568376	0.0032	0.997	0.111116866	count	1
AP000357.2	16.6830313	5240.568023	0.0032	0.997	0.111116866	count	1
CYTH4	16.6830313	5240.568376	0.0032	0.997	0.111116866	count	1
AL031590.1	16.6830313	5240.568376	0.0032	0.997	0.111116866	count	1
CHODL-AS1	16.6830313	5240.568376	0.0032	0.997	0.111116866	count	1
HUNK	16.6830313	5240.567987	0.0032	0.997	0.111116866	count	1
AL359881.1	16.6830314	5240.56827	0.0032	0.997	0.111116866	count	1
C1orf189	16.6830314	5240.568341	0.0032	0.997	0.111116866	count	1
AL512343.2	16.6830314	5240.568306	0.0032	0.997	0.111116866	count	1
ZMYND10	16.6830314	5240.56827	0.0032	0.997	0.111116866	count	1
PRG2	16.6830314	5240.568306	0.0032	0.997	0.111116866	count	1
AC024941.2	16.6830314	5240.568164	0.0032	0.997	0.111116866	count	1
AC060809.1	16.6830314	5240.568341	0.0032	0.997	0.111116866	count	1
AC022167.1	16.6830314	5240.568341	0.0032	0.997	0.111116866	count	1
AC027130.1	16.6830314	5240.568306	0.0032	0.997	0.111116866	count	1
AC087294.1	16.6830315	5240.568306	0.0032	0.997	0.111116866	count	1
ELL3	16.7009714	2553.480748	0.0065	0.9948	0.111116866	count	1
ENPP3	16.700973	2553.482536	0.0065	0.9948	0.111116866	count	1
SLC5A2	16.7009732	2553.48344	0.0065	0.9948	0.111116866	count	1
RASGRF2-AS1	16.7009736	2553.482078	0.0065	0.9948	0.111116866	count	1

TMEM254-AS1	16.7009737	2553.482983	0.0065	0.9948	0.111116866	count	1
TMSB15B	16.7009742	2553.482515	0.0065	0.9948	0.111116866	count	1
AL121894.2	16.7009742	2553.481631	0.0065	0.9948	0.111116866	count	1
AC004943.2	16.7009747	2553.484334	0.0065	0.9948	0.111116866	count	1
AP000911.1	16.7009751	2553.482972	0.0065	0.9948	0.111116866	count	1
TAPT1-AS1	16.7009763	2553.483845	0.0065	0.9948	0.111116866	count	1
DNAH2	16.7009765	2553.483866	0.0065	0.9948	0.111116866	count	1
NCF2	16.7009767	2553.4816	0.0065	0.9948	0.111116866	count	1
AC020917.3	16.7009766	2553.483866	0.0065	0.9948	0.111116866	count	1
COL11A2	16.7009774	2553.482037	0.0065	0.9948	0.111116866	count	1
LINC02158	16.7009777	2553.480686	0.0065	0.9948	0.111116866	count	1
SCGN	16.7009789	2553.482931	0.0065	0.9948	0.111116866	count	1
AC128687.2	16.700979	2553.483824	0.0065	0.9948	0.111116866	count	1
ASB15	16.7009792	2553.483835	0.0065	0.9948	0.111116866	count	1
CCDC116	16.7009792	2553.483835	0.0065	0.9948	0.111116866	count	1
KIF4A	16.7018313	2849.558405	0.0059	0.995	0.111116866	count	1
FHL5	16.7018341	2849.559392	0.0059	0.995	0.111116866	count	1
TRPV1	16.7018342	2849.56039	0.0059	0.995	0.111116866	count	1
AC133555.3	16.7018347	2849.558881	0.0059	0.995	0.111116866	count	1
AC010809.3	16.7018358	2849.55786	0.0059	0.995	0.111116866	count	1
DIO3	16.7030109	3364.747092	0.005	0.996	0.111116866	count	1
ROPN1B	16.727533	1764.409498	0.0095	0.9924	0.111116866	count	1
ITGA11	16.7275355	1764.409722	0.0095	0.9924	0.111116866	count	1
RNF31	16.7888099	1782.188735	0.0094	0.9925	0.111116867	count	1
Z99774.1	16.7888114	1782.187085	0.0094	0.9925	0.111116867	count	1
AL031666.2	16.7888135	1782.187956	0.0094	0.9925	0.111116867	count	1
AC007881.3	16.7892311	1912.1232	0.0088	0.993	0.111116867	count	1
SNTB1	16.8038218	1760.443694	0.0095	0.9924	0.111116867	count	1
PRKCZ-AS1	16.8947094	4282.004086	0.0039	0.997	0.111116867	count	1
AL390728.5	16.8947094	4282.004135	0.0039	0.997	0.111116867	count	1
CACNA1D	16.8947094	4282.004037	0.0039	0.997	0.111116867	count	1
OSTN-AS1	16.8947094	4282.004086	0.0039	0.997	0.111116867	count	1
KCTD8	16.8947094	4282.004111	0.0039	0.997	0.111116867	count	1
STK32A	16.8947093	4282.004111	0.0039	0.997	0.111116867	count	1
AC005082.1	16.8947094	4282.004086	0.0039	0.997	0.111116867	count	1
AC074183.1	16.8947094	4282.004086	0.0039	0.997	0.111116867	count	1
ASB11	16.8947093	4282.004062	0.0039	0.997	0.111116867	count	1
AL583839.1	16.8947094	4282.004135	0.0039	0.997	0.111116867	count	1
KC877392.1	16.8947094	4282.004135	0.0039	0.997	0.111116867	count	1
GAS2	16.8947094	4282.004062	0.0039	0.997	0.111116867	count	1
SPART-AS1	16.8947094	4282.004086	0.0039	0.997	0.111116867	count	1
AC092119.3	16.8947094	4282.004037	0.0039	0.997	0.111116867	count	1
AC106782.6	16.8947094	4282.004086	0.0039	0.997	0.111116867	count	1
AC145207.2	16.8947094	4282.004111	0.0039	0.997	0.111116867	count	1
MIA-RAB4B	16.8947094	4282.004086	0.0039	0.997	0.111116867	count	1

AL022322.2	16.8947094	4282.004086	0.0039	0.997	0.111116867	count	1
AP000295.1	16.8947094	4282.004062	0.0039	0.997	0.111116867	count	1
FOXD2	16.8947096	4282.004135	0.0039	0.997	0.111116867	count	1
DEPDC1	16.8947095	4282.00416	0.0039	0.997	0.111116867	count	1
RXFP4	16.8947095	4282.004062	0.0039	0.997	0.111116867	count	1
HAPLN2	16.8947096	4282.00416	0.0039	0.997	0.111116867	count	1
ADCY10	16.8947095	4282.004086	0.0039	0.997	0.111116867	count	1
AL121983.2	16.8947095	4282.004086	0.0039	0.997	0.111116867	count	1
AL078644.2	16.8947095	4282.004086	0.0039	0.997	0.111116867	count	1
AC026316.5	16.8947096	4282.004135	0.0039	0.997	0.111116867	count	1
CTBP1-AS	16.8947096	4282.004135	0.0039	0.997	0.111116867	count	1
AC141928.1	16.8947095	4282.004135	0.0039	0.997	0.111116867	count	1
CPEB2-DT	16.8947095	4282.004135	0.0039	0.997	0.111116867	count	1
AC069200.1	16.8947096	4282.004111	0.0039	0.997	0.111116867	count	1
AC092354.2	16.8947096	4282.004086	0.0039	0.997	0.111116867	count	1
AC008906.2	16.8947095	4282.004086	0.0039	0.997	0.111116867	count	1
CCNI2	16.8947095	4282.004111	0.0039	0.997	0.111116867	count	1
AL662796.1	16.8947096	4282.004086	0.0039	0.997	0.111116867	count	1
AL021368.1	16.8947095	4282.004062	0.0039	0.997	0.111116867	count	1
AC073073.2	16.8947095	4282.004062	0.0039	0.997	0.111116867	count	1
AC009275.1	16.8947096	4282.004086	0.0039	0.997	0.111116867	count	1
AC084116.1	16.8947096	4282.004086	0.0039	0.997	0.111116867	count	1
BX005266.2	16.8947095	4282.004062	0.0039	0.997	0.111116867	count	1
C9orf106	16.8947095	4282.004086	0.0039	0.997	0.111116867	count	1
OR2AG2	16.8947095	4282.004037	0.0039	0.997	0.111116867	count	1
AKR1C4	16.8947095	4282.004111	0.0039	0.997	0.111116867	count	1
ASAH2	16.8947096	4282.004135	0.0039	0.997	0.111116867	count	1
AC068987.2	16.8947095	4282.004111	0.0039	0.997	0.111116867	count	1
CCDC62	16.8947095	4282.004086	0.0039	0.997	0.111116867	count	1
AC027290.1	16.8947095	4282.004037	0.0039	0.997	0.111116867	count	1
LINC00284	16.8947096	4282.004111	0.0039	0.997	0.111116867	count	1
AL359513.1	16.8947096	4282.004111	0.0039	0.997	0.111116867	count	1
AL627171.2	16.8947096	4282.004135	0.0039	0.997	0.111116867	count	1
AC013553.4	16.8947095	4282.004062	0.0039	0.997	0.111116867	count	1
AL022341.2	16.8947095	4282.004111	0.0039	0.997	0.111116867	count	1
CCDC154	16.8947096	4282.004135	0.0039	0.997	0.111116867	count	1
AC007014.1	16.8947095	4282.004062	0.0039	0.997	0.111116867	count	1
TMED6	16.8947096	4282.004086	0.0039	0.997	0.111116867	count	1
AC010531.3	16.8947095	4282.004086	0.0039	0.997	0.111116867	count	1
AIPL1	16.8947095	4282.004062	0.0039	0.997	0.111116867	count	1
AC068234.3	16.8947095	4282.004111	0.0039	0.997	0.111116867	count	1
AC100778.3	16.8947095	4282.004135	0.0039	0.997	0.111116867	count	1
FAM129C	16.8947095	4282.004111	0.0039	0.997	0.111116867	count	1
ADM2	16.8947095	4282.004135	0.0039	0.997	0.111116867	count	1
UGT8	16.8947097	4282.004111	0.0039	0.997	0.111116867	count	1

MAGEE2	16.8947097	4282.00416	0.0039	0.997	0.111116867	count	1
C9orf153	16.8947097	4282.00416	0.0039	0.997	0.111116867	count	1
AP000350.5	16.8947097	4282.004135	0.0039	0.997	0.111116867	count	1
CYP2D6	16.8947097	4282.004135	0.0039	0.997	0.111116867	count	1
AL512604.3	16.8947098	4282.004086	0.0039	0.997	0.111116867	count	1
LINC01291	16.8947126	4282.009521	0.0039	0.997	0.111116867	count	1
HPDL	16.8947128	4282.009496	0.0039	0.997	0.111116867	count	1
DES	16.8947128	4282.009496	0.0039	0.997	0.111116867	count	1
FAM166B	16.8947127	4282.009569	0.0039	0.997	0.111116867	count	1
AC136475.9	16.8947128	4282.009545	0.0039	0.997	0.111116867	count	1
DNAH9	16.8947128	4282.015151	0.0039	0.997	0.111116867	count	1
AC016590.1	16.8947127	4282.009447	0.0039	0.997	0.111116867	count	1
LINC02580	16.894713	4282.009521	0.0039	0.997	0.111116867	count	1
TEKT4	16.8947129	4282.009472	0.0039	0.997	0.111116867	count	1
KBTBD12	16.8947129	4282.012238	0.0039	0.997	0.111116867	count	1
AC083829.2	16.8947129	4282.009472	0.0039	0.997	0.111116867	count	1
SNX15	16.8947129	4282.012238	0.0039	0.997	0.111116867	count	1
AL049871.1	16.8947129	4282.009496	0.0039	0.997	0.111116867	count	1
SLX1B	16.8947129	4282.012238	0.0039	0.997	0.111116867	count	1
AC012588.1	16.894713	4282.012262	0.0039	0.997	0.111116867	count	1
AP000253.1	16.8947129	4282.012238	0.0039	0.997	0.111116867	count	1
HAND2	16.8947131	4282.009472	0.0039	0.997	0.111116867	count	1
AL031432.1	16.894716	4282.006534	0.0039	0.997	0.111116867	count	1
IDI2-AS1	16.894716	4282.006559	0.0039	0.997	0.111116867	count	1
SLCO1B3	16.8947159	4282.006583	0.0039	0.997	0.111116867	count	1
GALNT4	16.894716	4282.006534	0.0039	0.997	0.111116867	count	1
AP000845.1	16.894716	4282.00651	0.0039	0.997	0.111116867	count	1
CCDC114	16.894716	4282.006607	0.0039	0.997	0.111116867	count	1
AC011476.3	16.894716	4282.006607	0.0039	0.997	0.111116867	count	1
PIK3IP1-AS1	16.894716	4282.006559	0.0039	0.997	0.111116867	count	1
AL391069.4	16.8947161	4282.006607	0.0039	0.997	0.111116867	count	1
AC244197.2	16.8947161	4282.006583	0.0039	0.997	0.111116867	count	1
IL23A	16.8947161	4282.006583	0.0039	0.997	0.111116867	count	1
LATS2-AS1	16.8947161	4282.006583	0.0039	0.997	0.111116867	count	1
SLC10A1	16.8947161	4282.006607	0.0039	0.997	0.111116867	count	1
TEX46	16.8947162	4282.006583	0.0039	0.997	0.111116867	count	1
AL356441.1	16.8947162	4282.006583	0.0039	0.997	0.111116867	count	1
TRAPPC12-AS1	16.8947162	4282.006559	0.0039	0.997	0.111116867	count	1
AC008277.1	16.8947162	4282.006559	0.0039	0.997	0.111116867	count	1
ZNF385B	16.8947162	4282.006534	0.0039	0.997	0.111116867	count	1
UNC5C	16.8947162	4282.006607	0.0039	0.997	0.111116867	count	1
LINC00243	16.8947163	4282.012164	0.0039	0.997	0.111116867	count	1
FGD2	16.8947162	4282.006559	0.0039	0.997	0.111116867	count	1
EYS	16.8947162	4282.006583	0.0039	0.997	0.111116867	count	1
ATP5MF-PTCD1	16.8947163	4282.006607	0.0039	0.997	0.111116867	count	1

GAL3ST4	16.8947162	4282.006607	0.0039	0.997	0.111116867	count	1
AC027702.1	16.8947162	4282.006534	0.0039	0.997	0.111116867	count	1
SAPCD2	16.8947162	4282.006559	0.0039	0.997	0.111116867	count	1
PARD3-AS1	16.8947163	4282.006583	0.0039	0.997	0.111116867	count	1
GLIPR1L1	16.8947162	4282.006607	0.0039	0.997	0.111116867	count	1
ALDH1L2	16.8947162	4282.006607	0.0039	0.997	0.111116867	count	1
AC107241.1	16.8947163	4282.006607	0.0039	0.997	0.111116867	count	1
AC010761.2	16.8947163	4282.006632	0.0039	0.997	0.111116867	count	1
LRRC46	16.8947163	4282.006607	0.0039	0.997	0.111116867	count	1
AP005203.1	16.8947162	4282.006559	0.0039	0.997	0.111116867	count	1
AC005614.1	16.8947162	4282.006607	0.0039	0.997	0.111116867	count	1
PRR34	16.8947163	4282.006559	0.0039	0.997	0.111116867	count	1
IQCJ-SCHIP1-AS1	16.8947164	4282.006583	0.0039	0.997	0.111116867	count	1
AC006435.1	16.8947164	4282.006559	0.0039	0.997	0.111116867	count	1
LINC02148	16.8947196	4282.006338	0.0039	0.997	0.111116867	count	1
PPT2-EGFL8	16.8947195	4282.017648	0.0039	0.997	0.111116867	count	1
AC026979.3	16.8947196	4282.009104	0.0039	0.997	0.111116867	count	1
AP000769.2	16.8947196	4282.017599	0.0039	0.997	0.111116867	count	1
COLCA1	16.8947196	4282.009104	0.0039	0.997	0.111116867	count	1
AL392046.1	16.8947196	4282.017623	0.0039	0.997	0.111116867	count	1
AL122035.1	16.8947195	4282.017623	0.0039	0.997	0.111116867	count	1
AC092718.5	16.8947196	4282.009104	0.0039	0.997	0.111116867	count	1
PHOSPHO1	16.8947195	4282.006363	0.0039	0.997	0.111116867	count	1
AL021392.1	16.8947195	4282.017648	0.0039	0.997	0.111116867	count	1
AL391152.1	16.8947197	4282.012042	0.0039	0.997	0.111116867	count	1
LINC00677	16.8947198	4282.006338	0.0039	0.997	0.111116867	count	1
NHLH1	16.8947227	4282.009055	0.0039	0.997	0.111116867	count	1
AC139099.3	16.8947227	4282.009055	0.0039	0.997	0.111116867	count	1
LNCSRLR	16.8947228	4282.009055	0.0039	0.997	0.111116867	count	1
DCT	16.8947229	4282.00908	0.0039	0.997	0.111116867	count	1
REM2	16.8947228	4282.003401	0.0039	0.997	0.111116867	count	1
SLC25A47	16.8947228	4282.009031	0.0039	0.997	0.111116867	count	1
SLC28A2	16.8947229	4282.00908	0.0039	0.997	0.111116867	count	1
AC106886.3	16.8947229	4282.009006	0.0039	0.997	0.111116867	count	1
CNTD1	16.8947229	4282.00908	0.0039	0.997	0.111116867	count	1
CU634019.6	16.8947229	4282.009031	0.0039	0.997	0.111116867	count	1
AL139424.3	16.8947231	4282.009104	0.0039	0.997	0.111116867	count	1
CNKSR1	16.894723	4282.003425	0.0039	0.997	0.111116867	count	1
CD38	16.894723	4282.00345	0.0039	0.997	0.111116867	count	1
AL353596.1	16.8947231	4282.00908	0.0039	0.997	0.111116867	count	1
CEL	16.8947231	4282.009031	0.0039	0.997	0.111116867	count	1
AC012313.5	16.8947231	4282.00908	0.0039	0.997	0.111116867	count	1
AC002116.2	16.8947232	4282.009031	0.0039	0.997	0.111116867	count	1
LINC02033	16.8947261	4282.014416	0.0039	0.997	0.111116867	count	1
STEAP3-AS1	16.8947262	4282.008835	0.0039	0.997	0.111116867	count	1

FOXD1	16.8947263	4282.008835	0.0039	0.997	0.111116867	count	1
SLC36A1	16.8947263	4282.014514	0.0039	0.997	0.111116867	count	1
HIST1H4F	16.8947263	4282.011601	0.0039	0.997	0.111116867	count	1
AC006960.3	16.8947263	4282.008835	0.0039	0.997	0.111116867	count	1
NLRP2B	16.8947262	4282.008835	0.0039	0.997	0.111116867	count	1
CCKBR	16.8947263	4282.008811	0.0039	0.997	0.111116867	count	1
AP002761.2	16.8947263	4282.008811	0.0039	0.997	0.111116867	count	1
ABCC2	16.8947262	4282.008835	0.0039	0.997	0.111116867	count	1
NOXRED1	16.8947263	4282.011577	0.0039	0.997	0.111116867	count	1
LINC01483	16.8947262	4282.011577	0.0039	0.997	0.111116867	count	1
SMIM11A	16.8947262	4282.008835	0.0039	0.997	0.111116867	count	1
FO393415.1	16.8947265	4282.014441	0.0039	0.997	0.111116867	count	1
AP001610.1	16.8947264	4282.011577	0.0039	0.997	0.111116867	count	1
BX005214.2	16.8947296	4282.011552	0.0039	0.997	0.111116867	count	1
AP001107.4	16.8947296	4282.011528	0.0039	0.997	0.111116867	count	1
CYYR1-AS1	16.8947296	4282.011552	0.0039	0.997	0.111116867	count	1
C1orf61	16.8947299	4282.011552	0.0039	0.997	0.111116867	count	1
AL845472.1	16.8947298	4282.011552	0.0039	0.997	0.111116867	count	1
AL157700.1	16.894733	4282.016987	0.0039	0.997	0.111116867	count	1
AC053513.1	16.8947332	4282.019729	0.0039	0.997	0.111116867	count	1
AC239868.3	16.9410811	2540.297844	0.0067	0.995	0.111116867	count	1
GSN-AS1	16.9410812	2540.297844	0.0067	0.995	0.111116867	count	1
AL139383.1	16.941082	2540.298284	0.0067	0.995	0.111116867	count	1
AL022322.1	16.9410824	2540.297844	0.0067	0.995	0.111116867	count	1
LINC01140	16.9410831	2540.298253	0.0067	0.995	0.111116867	count	1
AC010245.2	16.9410857	2540.297393	0.0067	0.995	0.111116867	count	1
NEO1	16.9410858	2540.296544	0.0067	0.995	0.111116867	count	1
LGI4	16.941909	2764.466513	0.0061	0.995	0.111116867	count	1
SV2C	16.9419124	2764.466045	0.0061	0.995	0.111116867	count	1
AL139011.1	16.9443018	3686.98782	0.0046	0.996	0.111116867	count	1
FCMR	17.0755785	1710.725459	0.01	0.992	0.111116868	count	1
AL132657.1	17.1067929	2539.795151	0.0067	0.9946	0.111116868	count	1
JPH1	17.1067942	2539.797572	0.0067	0.9946	0.111116868	count	1
AC068446.2	17.1335717	2530.381069	0.0068	0.9946	0.111116868	count	1
AC072022.1	17.1335763	2530.381464	0.0068	0.9946	0.111116868	count	1
AL117379.1	17.1335766	2530.381029	0.0068	0.9946	0.111116868	count	1
HSF4	17.1343427	2710.695345	0.0063	0.995	0.111116868	count	1
AL031055.1	17.1350811	2879.945103	0.0059	0.9953	0.111116868	count	1
AL137145.1	17.1365608	3474.934776	0.0049	0.9961	0.111116868	count	1
PRPH2	17.2274763	1700.68505	0.0101	0.9919	0.111116869	count	1
AL139022.2	17.2277672	2252.421055	0.0076	0.994	0.111116869	count	1
CITED1	17.2354197	3136.261146	0.0055	0.9956	0.111116869	count	1
LRRN4CL	17.2950334	2673.319207	0.0065	0.9948	0.111116869	count	1
AL137077.2	17.2950366	2673.317908	0.0065	0.9948	0.111116869	count	1
ZG16B	17.4655034	2445.821948	0.0071	0.9943	0.111116869	count	1

AC010359.1	17.4655035	2445.821948	0.0071	0.9943	0.111116869	count	1
RGPD2	17.4655098	2445.824603	0.0071	0.9943	0.111116869	count	1
ZNF385C	17.5309813	3839.01324	0.0046	0.996	0.11111687	count	1
SSC4D	17.5309816	3839.010934	0.0046	0.996	0.11111687	count	1
HRK	17.5309816	3839.010977	0.0046	0.996	0.11111687	count	1
AC079354.3	17.5309851	3839.01089	0.0046	0.996	0.11111687	count	1
FAXC	17.5309855	3839.010912	0.0046	0.996	0.11111687	count	1
MIR646HG	17.5309856	3839.010912	0.0046	0.996	0.11111687	count	1
C2orf50	17.5309859	3839.008584	0.0046	0.996	0.11111687	count	1
IGSF9B	17.530986	3839.008649	0.0046	0.996	0.11111687	count	1
CD28	17.5309866	3839.024422	0.0046	0.996	0.11111687	count	1
AL603832.1	17.5309876	3839.019853	0.0046	0.996	0.11111687	count	1
ASIP	17.5309884	3839.01535	0.0046	0.996	0.11111687	count	1
LINC01030	17.5309899	3839.008541	0.0046	0.996	0.11111687	count	1
APOBEC3H	17.53099	3839.008584	0.0046	0.996	0.11111687	count	1
RRH	17.5309911	3839.022116	0.0046	0.996	0.11111687	count	1
AC026471.3	17.5309911	3839.022116	0.0046	0.996	0.11111687	count	1
AC004832.6	17.5309922	3839.017613	0.0046	0.996	0.11111687	count	1
PTGER3	17.5309928	3839.015263	0.0046	0.996	0.11111687	count	1
ART5	17.5309927	3839.015285	0.0046	0.996	0.11111687	count	1
FO704657.1	17.5309929	3839.012979	0.0046	0.996	0.11111687	count	1
AC005753.1	17.530993	3839.013044	0.0046	0.996	0.11111687	count	1
AC103974.1	17.5309929	3839.015285	0.0046	0.996	0.11111687	count	1
CHRFAM7A	17.5309937	3839.010781	0.0046	0.996	0.11111687	count	1
PLS3-AS1	17.5309946	3839.024248	0.0046	0.996	0.11111687	count	1
AL133215.2	17.5309946	3839.02427	0.0046	0.996	0.11111687	count	1
AL035420.3	17.5309946	3839.00395	0.0046	0.996	0.11111687	count	1
S100Z	17.5309949	3839.02427	0.0046	0.996	0.11111687	count	1
FBXO24	17.5309957	3839.019766	0.0046	0.996	0.11111687	count	1
AC009121.2	17.5309955	3839.019723	0.0046	0.996	0.11111687	count	1
RPL34-AS1	17.5309959	3839.019766	0.0046	0.996	0.11111687	count	1
Z95114.1	17.5309962	3839.017526	0.0046	0.996	0.11111687	count	1
Z97200.1	17.5309968	3839.015263	0.0046	0.996	0.11111687	count	1
AC023024.1	17.5309977	3839.00841	0.0046	0.996	0.11111687	count	1
AC090948.3	17.5309986	3839.024183	0.0046	0.996	0.11111687	count	1
AC012087.1	17.5309986	3839.024226	0.0046	0.996	0.11111687	count	1
LINC01574	17.5309987	3839.024226	0.0046	0.996	0.11111687	count	1
AP003559.1	17.5309996	3839.019701	0.0046	0.996	0.11111687	count	1
CFAP58-DT	17.5309994	3839.021964	0.0046	0.996	0.11111687	count	1
AC011495.3	17.5309996	3839.019658	0.0046	0.996	0.11111687	count	1
ITGA7	17.5309998	3839.019723	0.0046	0.996	0.11111687	count	1
SHISA2	17.5310002	3839.017373	0.0046	0.996	0.11111687	count	1
TLE6	17.5310006	3839.017417	0.0046	0.996	0.11111687	count	1
AC034187.1	17.5310029	3839.024139	0.0046	0.996	0.11111687	count	1
AC055811.3	17.5310029	3839.024139	0.0046	0.996	0.11111687	count	1

ACHE	17.531003	3839.024161	0.0046	0.996	0.11111687	count	1
FLG-AS1	17.5310032	3839.02192	0.0046	0.996	0.11111687	count	1
CSTA	17.5310036	3839.021898	0.0046	0.996	0.11111687	count	1
AC096711.1	17.5310032	3839.021855	0.0046	0.996	0.11111687	count	1
AL157373.2	17.5310035	3839.019614	0.0046	0.996	0.11111687	count	1
AK8	17.5310032	3839.021877	0.0046	0.996	0.11111687	count	1
AC025034.1	17.5310033	3839.02192	0.0046	0.996	0.11111687	count	1
AC027307.1	17.5310032	3839.02192	0.0046	0.996	0.11111687	count	1
OLFM2	17.5310036	3839.019636	0.0046	0.996	0.11111687	count	1
WNT2	17.5310039	3839.019614	0.0046	0.996	0.11111687	count	1
AC021028.1	17.5310037	3839.021877	0.0046	0.996	0.11111687	count	1
TSPOAP1-AS1	17.531007	3839.024096	0.0046	0.996	0.11111687	count	1
XCL2	17.7262022	2273.181344	0.0078	0.9938	0.11111687	count	1
C16orf54	18.1047706	3272.19187	0.0055	0.9956	0.111116871	count	1
E2F8	18.1050301	3790.348531	0.0048	0.9962	0.111116871	count	1
GDF9	18.1050362	3790.344613	0.0048	0.9962	0.111116871	count	1
HIST1H2AL	18.1050371	3790.352449	0.0048	0.9962	0.111116871	count	1
WFDC3	18.1050401	3790.344529	0.0048	0.9962	0.111116871	count	1
HMGCS1	0.0910223	0.2760198	0.3298	0.742	0.111135943	count	1
AC138150.1	0.2760974	0.6501312	0.4247	0.671	0.11128595	count	1
ZHX1	0.0826712	0.1629102	0.5075	0.612	0.111349254	count	1
RNF141	0.0886546	0.2206285	0.4018	0.688	0.1114366	count	1
PFKM	0.0925056	0.2521347	0.3669	0.714	0.111526922	count	1
EIF3I	0.0783343	0.0573059	1.367	0.172	0.111566361	count	1
EFCAB11	0.0959724	0.2778926	0.3454	0.73	0.111697895	count	1
NOL9	0.0857378	0.2240498	0.3827	0.702	0.111755055	count	1
SMIM26	0.0793537	0.0805033	0.9857	0.324	0.111813619	count	1
SLC35B3	0.0902588	0.2229608	0.4048	0.686	0.111968391	count	1
BSCL2	0.3925648	1.0666544	0.368	0.713	0.111990107	count	1
AC079305.1	0.3925648	1.0788754	0.3639	0.716	0.111990107	count	1
AC004771.3	0.3925648	1.1669171	0.3364	0.737	0.111990107	count	1
GZF1	0.106378	0.3058748	0.3478	0.728	0.112008903	count	1
PICALM	0.0818435	0.114829	0.7127	0.476	0.112054606	count	1
SLC25A1	0.0863562	0.1793012	0.4816	0.63	0.112310027	count	1
PCNX4	0.0890198	0.17935	0.4963	0.62	0.112327282	count	1
SNRPB2	0.0795369	0.0826281	0.9626	0.336	0.112407824	count	1
TNS2	0.0806208	0.119375	0.6754	0.5	0.112440871	count	1
SMARCC1	0.0861786	0.1797507	0.4794	0.632	0.112565559	count	1
SMC2	0.0839464	0.1713811	0.4898	0.624	0.112587912	count	1
LRRC59	0.0860445	0.1611756	0.5339	0.593	0.112693455	count	1
KIAA1671	0.0874758	0.1806056	0.4843	0.628	0.112742222	count	1
PAX8	0.324968	1.0184978	0.3191	0.75	0.112764128	count	1
BABAM2	0.0841131	0.15287	0.5502	0.582	0.112810936	count	1
BCL2L13	0.0943258	0.2434144	0.3875	0.698	0.112911615	count	1
UHRF1BP1	0.1188835	0.4282761	0.2776	0.781	0.112967605	count	1

VWCE	0.1258619	0.5211827	0.2415	0.809	0.113038793	count	1
CBL	0.0900932	0.1964002	0.4587	0.646	0.113084434	count	1
OGFOD3	0.0849476	0.1704338	0.4984	0.618	0.1131403	count	1
ZNF205	0.0905876	0.2307377	0.3926	0.695	0.113227208	count	1
PIK3CG	0.2275296	0.7161634	0.3177	0.751	0.113329644	count	1
CSNK2B	0.0795691	0.0610905	1.3025	0.193	0.113353955	count	1
LINC01336	0.3981081	1.0885411	0.3657	0.715	0.113363367	count	1
AC009549.1	0.3981081	1.0885411	0.3657	0.715	0.113363367	count	1
AL512791.2	0.3981081	1.0885411	0.3657	0.715	0.113363367	count	1
AC138207.2	0.3981081	1.0986417	0.3624	0.717	0.113363367	count	1
HCG25	0.3981081	1.1058592	0.36	0.719	0.113363367	count	1
AC091948.1	0.3981081	1.2239933	0.3253	0.745	0.113363367	count	1
AL449266.1	0.3981081	1.2304758	0.3235	0.746	0.113363367	count	1
MRPS18C	0.0801514	0.081482	0.9837	0.325	0.113406129	count	1
DNAJB14	0.0815702	0.10649	0.766	0.444	0.113479687	count	1
POLR3GL	0.0804421	0.0820182	0.9808	0.327	0.113519825	count	1
TCF12	0.0864345	0.1558496	0.5546	0.579	0.113630102	count	1
ALYREF	0.0820102	0.103517	0.7922	0.428	0.113698932	count	1
IDH1-AS1	0.3280378	1.0895669	0.3011	0.763	0.113719549	count	1
AC008946.1	0.3280378	1.0895669	0.3011	0.763	0.113719549	count	1
SMAD2	0.0832159	0.1298428	0.6409	0.522	0.113796309	count	1
IL17RC	0.0969047	0.2798196	0.3463	0.729	0.113822279	count	1
NIPSNAP3B	0.1766615	0.7984132	0.2213	0.825	0.113869845	count	1
MRPS23	0.0826861	0.123772	0.6681	0.504	0.113999105	count	1
NRG2	0.252287	0.6332872	0.3984	0.69	0.114116402	count	1
ERCC5	0.147426	0.5200394	0.2835	0.777	0.114126392	count	1
OSBPL2	0.0926549	0.2825688	0.3279	0.743	0.11416233	count	1
GAS6-DT	0.1292934	0.5014571	0.2578	0.797	0.114182993	count	1
DDT	0.0805116	0.0752232	1.0703	0.285	0.114186171	count	1
NSA2	0.0802204	0.0637538	1.2583	0.208	0.114223016	count	1
MGRN1	0.1000494	0.3738477	0.2676	0.789	0.114392228	count	1
ZNF730	0.8545897	1.0536518	0.8111	0.417	0.114537185	count	1
TM4SF19-AS1	0.8545897	1.1133287	0.7676	0.443	0.114537185	count	1
AC093495.1	0.8545897	1.1174656	0.7648	0.444	0.114537185	count	1
RPS27	0.0795777	0.0217855	3.6528	0.000264	0.11465175	count	1
POLDIP3	0.1097066	0.2309653	0.475	0.635	0.114654582	count	1
DOK3	0.3313496	1.0961602	0.3023	0.762	0.114748055	count	1
LINC01801	0.3313496	1.1965484	0.2769	0.782	0.114748055	count	1
AC009133.2	0.3313496	1.6161832	0.205	0.8376	0.114748055	count	1
GTF2E1	0.1278047	0.3808425	0.3356	0.737	0.114748572	count	1
KCND3	0.1783644	0.788067	0.2263	0.821	0.114922389	count	1
NPTXR	0.1260632	0.4249845	0.2966	0.767	0.114951202	count	1
NOA1	0.0882091	0.1665858	0.5295	0.596	0.11504716	count	1
LARP7	0.0816358	0.0888423	0.9189	0.358	0.115142763	count	1
LINC01473	0.1415098	0.4123843	0.3432	0.732	0.115285618	count	1

EXOC1	0.0850506	0.154164	0.5517	0.581	0.115302543	count	1
UBE4A	0.0882745	0.183343	0.4815	0.63	0.115372825	count	1
ATP2B1-AS1	0.0856359	0.1575071	0.5437	0.587	0.115408505	count	1
DNALI1	0.0977374	0.4339266	0.2252	0.822	0.115453244	count	1
SQSTM1	0.0806468	0.0507638	1.5887	0.112	0.115467608	count	1
COA3	0.08218	0.0905174	0.9079	0.364	0.115645238	count	1
GALNT6	0.867729	1.0948134	0.7926	0.428	0.115749056	count	1
TNFRSF10B	0.0852629	0.1485311	0.574	0.566	0.115831664	count	1
ZNF547	0.1799852	1.3241574	0.1359	0.8919	0.115923382	count	1
RRBP1	0.0816185	0.0725802	1.1245	0.261	0.116012735	count	1
PHTF2	0.1012156	0.3192352	0.3171	0.751	0.116144363	count	1
TANK	0.0870446	0.1424745	0.6109	0.541	0.116322868	count	1
PLPP2	0.0914799	0.2001841	0.457	0.648	0.116499435	count	1
ZCCHC7	0.0860763	0.1426091	0.6036	0.546	0.116528831	count	1
GNG12	0.0833707	0.0967854	0.8614	0.389	0.116597504	count	1
LENG1	0.084934	0.1338607	0.6345	0.526	0.116635946	count	1
FEZ2	0.0823062	0.0798373	1.0309	0.303	0.116688484	count	1
RBM41	0.1020798	0.2832984	0.3603	0.719	0.11669319	count	1
KDM5A	0.0860511	0.1903062	0.4522	0.651	0.11674947	count	1
STRN3	0.0851698	0.1284389	0.6631	0.507	0.116837384	count	1
CPT1C	0.54777	0.8008113	0.684	0.494	0.116880334	count	1
SBF2	0.0927339	0.1976039	0.4693	0.639	0.117136673	count	1
SLC15A3	0.0952853	0.2585039	0.3686	0.712	0.117178268	count	1
CBWD2	0.1194747	0.3656875	0.3267	0.744	0.11739754	count	1
LIN52	0.1381992	0.5113361	0.2703	0.787	0.117522737	count	1
DDB1	0.0896592	0.1717552	0.522	0.602	0.117711053	count	1
MPLKIP	0.0842209	0.1095537	0.7688	0.442	0.117855552	count	1
RPL22	0.0818238	0.0229406	3.5668	0.000368	0.117868845	count	1
HDX	0.1010601	0.2797302	0.3613	0.718	0.117945278	count	1
RSAD2	0.0964578	0.2864156	0.3368	0.736	0.117958514	count	1
TTC8	0.0988376	0.2873915	0.3439	0.731	0.117982902	count	1
PALLD	0.0931496	0.2073556	0.4492	0.653	0.118083171	count	1
EPN2	0.087317	0.1520223	0.5744	0.566	0.118400235	count	1
BRPF1	0.1145208	0.3939043	0.2907	0.771	0.118734167	count	1
ZNF625	0.1845438	0.679997	0.2714	0.786	0.118734516	count	1
MAEA	0.0925027	0.1760667	0.5254	0.599	0.118756894	count	1
CTHRC1	0.0842237	0.1117756	0.7535	0.451	0.11891186	count	1
MFF	0.0869433	0.1204942	0.7216	0.471	0.118931216	count	1
TIAL1	0.0852427	0.0986589	0.864	0.388	0.119163737	count	1
TMEM250	0.0951172	0.2275312	0.418	0.676	0.11919463	count	1
MGME1	0.0999862	0.2535758	0.3943	0.693	0.119344111	count	1
TMEM67	0.101351	0.3928017	0.258	0.796	0.119350801	count	1
NUDT2	0.0888585	0.1396644	0.6362	0.525	0.119375641	count	1
AKR1C3	0.0836703	0.0684107	1.2231	0.221	0.119458972	count	1
AP000866.1	0.2408481	0.8061492	0.2988	0.765	0.119528814	count	1

SEMA6A-AS1	0.2408481	0.8155731	0.2953	0.768	0.119528814	count	1
NPIPB4	0.2408481	0.8514695	0.2829	0.777	0.119528814	count	1
LINC00619	0.4234574	0.6386683	0.663	0.507	0.119573175	count	1
ZNF7	0.0988325	0.2352107	0.4202	0.674	0.120130703	count	1
C6orf136	0.1040536	0.2509638	0.4146	0.678	0.120220939	count	1
ANXA1	0.0836589	0.0441296	1.8958	0.0581	0.120260675	count	1
ZNF587B	0.1606929	0.5591565	0.2874	0.774	0.120576841	count	1
GSR	0.0990253	0.2480112	0.3993	0.69	0.120607018	count	1
UPRT	0.1104751	0.3136188	0.3523	0.725	0.120618701	count	1
MRS2	0.1010641	0.29685	0.3405	0.734	0.120621325	count	1
SMPD2	0.1147295	0.3479403	0.3297	0.742	0.120688698	count	1
LINC00271	0.350726	0.8563162	0.4096	0.682	0.120719152	count	1
TMEM68	0.1064759	0.3112096	0.3421	0.732	0.120725125	count	1
HIGD2A	0.0848928	0.0671205	1.2648	0.206	0.120760004	count	1
FOXJ3	0.0888951	0.1311383	0.6779	0.498	0.120785819	count	1
SLC39A3	0.0901471	0.1705685	0.5285	0.597	0.120791071	count	1
KDM6B	0.0923837	0.1759487	0.5251	0.6	0.120886663	count	1
SHMT1	0.1126888	0.3513309	0.3207	0.748	0.12090654	count	1
RPGRIP1L	0.1397156	0.5237682	0.2668	0.79	0.121040199	count	1
ARV1	0.0912995	0.1735644	0.526	0.599	0.121120604	count	1
SINHCAF	0.0883018	0.1226125	0.7202	0.471	0.121168852	count	1
HPS3	0.1131953	0.3246037	0.3487	0.727	0.121443391	count	1
SH3PXD2A	0.1034965	0.2982017	0.3471	0.729	0.121857267	count	1
AP003469.4	0.5778316	1.092312	0.529	0.597	0.122010202	count	1
PDE8A	0.0980127	0.250219	0.3917	0.695	0.122097698	count	1
FNIP1	0.091906	0.1843762	0.4985	0.618	0.122213664	count	1
TOP1	0.0864332	0.0778586	1.1101	0.267	0.122335825	count	1
TSPAN17	0.1069291	0.2717971	0.3934	0.694	0.122640644	count	1
PDZD11	0.0902236	0.1330788	0.678	0.498	0.122994458	count	1
MRPL2	0.09073	0.1305783	0.6948	0.487	0.123115573	count	1
CCDC186	0.089401	0.1373183	0.651	0.515	0.123169489	count	1
SBDS	0.0861053	0.0523228	1.6457	0.0999	0.123170211	count	1
CYREN	0.1027674	0.2418294	0.425	0.671	0.123239037	count	1
SRRM2	0.086126	0.0557652	1.5444	0.123	0.123316381	count	1
CCT5	0.0873652	0.0737758	1.1842	0.236	0.123379538	count	1
ADAMTS10	0.1920987	0.5465236	0.3515	0.725	0.123379641	count	1
AFAP1	0.0930292	0.1726532	0.5388	0.59	0.123408095	count	1
SNRNP200	0.0920127	0.1775746	0.5182	0.604	0.123422906	count	1
USP6	0.5877755	1.0703813	0.5491	0.583	0.12368043	count	1
HMBOX1	0.0937277	0.1767198	0.5304	0.596	0.123685065	count	1
FBXL7	0.1168396	0.3171548	0.3684	0.713	0.123717121	count	1
PIN1	0.0878687	0.0842043	1.0435	0.297	0.123853777	count	1
BNIP1	0.1062288	0.2914682	0.3645	0.716	0.1239264	count	1
QRICH1	0.1033607	0.280825	0.3681	0.713	0.123945334	count	1
INTS1	0.1064829	0.2728902	0.3902	0.696	0.124220307	count	1

GOLM1	0.0936038	0.1877238	0.4986	0.618	0.124288502	count	1
RNF169	0.094634	0.1832553	0.5164	0.606	0.12437162	count	1
LACC1	0.116021	0.3071879	0.3777	0.706	0.124437305	count	1
AC022182.2	0.2771285	0.7749603	0.3576	0.721	0.124466085	count	1
AC025181.2	0.1201545	0.3293625	0.3648	0.715	0.124492626	count	1
IDI1	0.0939773	0.1478319	0.6357	0.525	0.124600263	count	1
LZTS2	0.0933052	0.1679685	0.5555	0.579	0.124613785	count	1
RPL13	0.086448	0.0172463	5.0126	5.71E-07	0.12466745	count	0.013366539
CPLANE1	0.1053867	0.3502586	0.3009	0.764	0.125095286	count	1
C11orf95	0.1239583	0.3216353	0.3854	0.7	0.125258641	count	1
RYK	0.0937063	0.1416037	0.6618	0.508	0.125401018	count	1
AC124242.1	0.5981651	1.324897	0.4515	0.652	0.125411503	count	1
GMPPB	0.1055111	0.2671859	0.3949	0.693	0.125569035	count	1
AL135818.2	0.3152229	0.7835856	0.4023	0.688	0.125574758	count	1
RELL1	0.0962736	0.2032562	0.4737	0.636	0.125613579	count	1
AC005523.2	0.1628269	0.5469859	0.2977	0.766	0.125673273	count	1
HMGN5	0.0958401	0.1917197	0.4999	0.617	0.125717767	count	1
AC008079.2	0.9822514	1.2197586	0.8053	0.421	0.12574747	count	1
TRADD	0.0933268	0.1445319	0.6457	0.519	0.125787345	count	1
PXK	0.0969186	0.1998405	0.485	0.628	0.125799095	count	1
ZNF410	0.2065214	0.5681417	0.3635	0.716	0.12584155	count	1
MECP2	0.0944357	0.150669	0.6268	0.531	0.125959679	count	1
HLCS	0.4501909	0.8059317	0.5586	0.576	0.125998505	count	1
ID3	0.0875221	0.0532983	1.6421	0.101	0.12606123	count	1
CDKN1B	0.0915654	0.1221192	0.7498	0.453	0.126164925	count	1
DDX20	0.1072545	0.2974707	0.3606	0.718	0.126245454	count	1
TMEM94	0.1178097	0.3528635	0.3339	0.739	0.126331507	count	1
MAGEF1	0.098286	0.2015731	0.4876	0.626	0.126725869	count	1
RAD51C	0.0967104	0.194993	0.496	0.62	0.126774696	count	1
PUF60	0.0906536	0.0937107	0.9674	0.333	0.126796732	count	1
RTCA	0.095169	0.1690564	0.5629	0.574	0.126880094	count	1
MRPS5	0.0921698	0.1174179	0.785	0.433	0.126996265	count	1
AHI1	0.0914383	0.1239863	0.7375	0.461	0.127043239	count	1
GIPC3	0.135738	0.3733501	0.3636	0.716	0.127060002	count	1
ZNF569	0.132431	0.3709073	0.357	0.721	0.127098986	count	1
C18orf54	0.1606059	0.5451041	0.2946	0.768	0.127297823	count	1
NKAPD1	0.096945	0.1670587	0.5803	0.562	0.127550422	count	1
AP5Z1	0.1222837	0.3164744	0.3864	0.699	0.127613725	count	1
UBE2E1	0.0945015	0.1385713	0.682	0.495	0.127645116	count	1
TMEM39B	0.0949149	0.1703176	0.5573	0.577	0.127667646	count	1
PRELID3A	0.2097421	0.6294566	0.3332	0.739	0.127704833	count	1
USP22	0.092397	0.1229214	0.7517	0.452	0.127727583	count	1
GCLC	0.1184353	0.3572128	0.3316	0.74	0.127756528	count	1
PSD3	0.6140222	0.7860095	0.7812	0.435	0.128026154	count	1
ZFYVE16	0.0957231	0.1703262	0.562	0.574	0.12809214	count	1

RILP	0.0951779	0.1663755	0.5721	0.567	0.128149875	count	1
LINC00847	0.108604	0.2926624	0.3711	0.711	0.128183825	count	1
TMEM116	0.1369841	0.5059467	0.2707	0.787	0.128202962	count	1
HSF2	0.108631	0.245824	0.4419	0.659	0.128215428	count	1
C14orf132	0.107985	0.2955613	0.3654	0.715	0.128489931	count	1
C5orf22	0.1132421	0.366637	0.3089	0.757	0.128830813	count	1
ICA1L	0.1218179	0.4519889	0.2695	0.788	0.128916981	count	1
TRAPPC12	0.1021634	0.189824	0.5382	0.59	0.129450427	count	1
AKAP8	0.1046555	0.2200562	0.4756	0.634	0.129508569	count	1
SSH2	0.1066524	0.2196336	0.4856	0.627	0.129568946	count	1
PIKFYVE	0.1130946	0.2615018	0.4325	0.665	0.129643441	count	1
AC245140.2	0.2628251	0.638837	0.4114	0.681	0.129653594	count	1
AC124283.1	0.2629826	0.5492893	0.4788	0.632	0.129725683	count	1
BORCS8	0.0999989	0.167081	0.5985	0.55	0.129780492	count	1
ETNK1	0.0970442	0.1507536	0.6437	0.52	0.129854767	count	1
DMAP1	0.0981875	0.1621862	0.6054	0.545	0.129898717	count	1
BLOC1S3	0.1428575	0.469547	0.3042	0.761	0.129928649	count	1
AP5M1	0.1079698	0.2754089	0.392	0.695	0.130032459	count	1
NBR1	0.0968313	0.1515642	0.6389	0.523	0.130056697	count	1
YTHDC2	0.1000888	0.1636829	0.6115	0.541	0.130096122	count	1
AL049597.2	0.2140834	0.8006196	0.2674	0.789	0.130211558	count	1
TNRC18	0.1072214	0.2508748	0.4274	0.669	0.130255304	count	1
AC020916.1	0.0961757	0.1660984	0.579	0.563	0.130276724	count	1
CMPK2	0.1170692	0.2903094	0.4033	0.687	0.130324852	count	1
CNTRL	0.1035031	0.2125028	0.4871	0.626	0.130337438	count	1
SRSF1	0.0969759	0.1257255	0.7713	0.441	0.130476625	count	1
FIP1L1	0.0970217	0.1431971	0.6775	0.498	0.130493657	count	1
ATP5MC2	0.090865	0.0356725	2.5472	0.0109	0.13060966	count	1
GTF3C6	0.0933362	0.0895625	1.0421	0.297	0.130821541	count	1
FOPNL	0.099267	0.1844842	0.5381	0.591	0.130821767	count	1
ORC4	0.1011065	0.1970513	0.5131	0.608	0.130898764	count	1
DPH2	0.1349661	0.3739509	0.3609	0.718	0.130947777	count	1
CARMIL1	0.1001077	0.1772286	0.5649	0.572	0.131211048	count	1
FLT1	0.0925135	0.0864473	1.0702	0.285	0.131612721	count	1
DACT1	1.055745	1.1425529	0.924	0.356	0.131656043	count	1
OSER1	0.0978203	0.1490548	0.6563	0.512	0.131784663	count	1
GALT	0.102746	0.1958671	0.5246	0.6	0.131845122	count	1
ZNF84	0.1094899	0.2145819	0.5102	0.61	0.131849307	count	1
TSPAN18	0.0946115	0.1033205	0.9157	0.36	0.131895652	count	1
ZNF595	0.1430818	0.3679301	0.3889	0.697	0.132003925	count	1
EIF5B	0.092668	0.0694154	1.335	0.182	0.132054426	count	1
MFAP3L	1.0615352	1.474305	0.72	0.472	0.132105588	count	1
TOGARAM1	0.1048474	0.1876208	0.5588	0.576	0.132188947	count	1
PNN	0.0935463	0.0778002	1.2024	0.229	0.132389297	count	1
AUH	0.1111276	0.2667728	0.4166	0.677	0.132864598	count	1

SCN3B	0.1357475	0.4402169	0.3084	0.758	0.133097714	count	1
LINC00960	0.3362734	0.539315	0.6235	0.533	0.133113738	count	1
HNRNPA3	0.0933743	0.0574552	1.6252	0.104	0.133153419	count	1
UBALD1	0.1146297	0.2759757	0.4154	0.678	0.133210771	count	1
AC006213.2	0.1731422	0.6951914	0.2491	0.803	0.133366772	count	1
LDLRAD3	0.1193384	0.2846158	0.4193	0.675	0.133431905	count	1
CCHCR1	0.114501	0.3742321	0.306	0.76	0.133488135	count	1
NRP1	0.094176	0.0885916	1.063	0.288	0.133510937	count	1
INPP4B	0.1447817	0.4778828	0.303	0.762	0.133537935	count	1
DEDD2	0.1114499	0.2448146	0.4552	0.649	0.133569052	count	1
NHLRC3	0.10413	0.2362845	0.4407	0.659	0.133612522	count	1
GSTCD	0.1647706	0.4898732	0.3364	0.737	0.133667691	count	1
ALG1	0.1232676	0.3685332	0.3345	0.738	0.133668691	count	1
STAT2	0.1005174	0.164892	0.6096	0.542	0.133688817	count	1
AC142472.1	0.2339429	0.7250509	0.3227	0.747	0.133847629	count	1
CARD19	0.0960808	0.1228748	0.7819	0.434	0.133930199	count	1
TMEM141	0.0956573	0.0942079	1.0154	0.31	0.13405546	count	1
FDXACB1	0.1924013	0.55203	0.3485	0.727	0.134194483	count	1
PPP1R12C	0.1105045	0.221537	0.4988	0.618	0.134487129	count	1
ZNF433	0.1699926	0.4977439	0.3415	0.733	0.134499366	count	1
GGPS1	0.1010415	0.1729896	0.5841	0.559	0.134684186	count	1
RCOR3	0.1045218	0.1748067	0.5979	0.55	0.134846341	count	1
GOPC	0.0963458	0.1026596	0.9385	0.348	0.13487138	count	1
ITGAE	0.0981295	0.1208461	0.812	0.417	0.134922035	count	1
NTN4	0.107374	0.2083712	0.5153	0.606	0.135184008	count	1
POC1B-AS1	0.170892	0.6710741	0.2547	0.799	0.135187976	count	1
GART	0.1045383	0.1947063	0.5369	0.591	0.135431352	count	1
TIMM23	0.1088179	0.2121193	0.513	0.608	0.135474252	count	1
PRRC2B	0.0997172	0.1595529	0.625	0.532	0.135488494	count	1
B3GNT5	0.1382643	0.4041996	0.3421	0.732	0.13551958	count	1
PHB2	0.0954036	0.0641327	1.4876	0.137	0.135534506	count	1
AL354836.1	1.107682	1.0945755	1.012	0.312	0.135606398	count	1
POLR2G	0.0970975	0.0883849	1.0986	0.272	0.135816721	count	1
KDM3A	0.1158688	0.2303529	0.503	0.615	0.135896066	count	1
AL512329.2	0.2765753	0.9451612	0.2926	0.77	0.135922203	count	1
SMG1	0.0992271	0.15764	0.6295	0.529	0.135983559	count	1
HDAC6	0.1277844	0.3074335	0.4156	0.678	0.136026509	count	1
LINC00346	0.1720009	0.4623584	0.372	0.71	0.136036655	count	1
CCP110	0.1128384	0.2380239	0.4741	0.635	0.136154534	count	1
PLEKHB2	0.1050574	0.1666383	0.6305	0.528	0.136209403	count	1
ZNF836	0.1178307	0.2897424	0.4067	0.684	0.136444688	count	1
AP000487.1	0.277743	1.082413	0.2566	0.798	0.136452244	count	1
AC010320.1	0.277743	1.406289	0.1975	0.843	0.136452244	count	1
FAM174B	0.1773052	0.5695838	0.3113	0.756	0.136462462	count	1
SRGAP1	0.1124729	0.2742184	0.4102	0.682	0.136587429	count	1

SLTM	0.0965331	0.0849151	1.1368	0.256	0.136704772	count	1
GTF2F1	0.0982711	0.1118282	0.8788	0.38	0.137067606	count	1
DNAJC19	0.097476	0.0919853	1.0597	0.289	0.137112118	count	1
APLF	0.1782011	0.5676229	0.3139	0.754	0.137127976	count	1
CYHR1	0.100249	0.1354888	0.7399	0.459	0.137252731	count	1
EMC8	0.1061489	0.1803703	0.5885	0.556	0.137284238	count	1
ARPP19	0.0996103	0.1196604	0.8324	0.405	0.137500935	count	1
AL592148.3	1.1343851	1.0324418	1.0987	0.272	0.137567443	count	1
TTC39A	1.1343851	1.1577762	0.9798	0.327	0.137567443	count	1
PHF7	0.1790737	0.5480477	0.3267	0.744	0.137775949	count	1
ZNF671	0.2586814	0.6329875	0.4087	0.683	0.137813156	count	1
ATF6B	0.1039897	0.1571542	0.6617	0.508	0.137828336	count	1
USP45	0.1518675	0.3474915	0.437	0.662	0.13793049	count	1
SEMA6C	0.1703545	0.405111	0.4205	0.674	0.138056051	count	1
ZNF165	0.6769326	0.9679097	0.6994	0.484	0.138078439	count	1
CINP	0.1022819	0.1470428	0.6956	0.487	0.138526308	count	1
SLC39A13	0.1100009	0.2046511	0.5375	0.591	0.138648525	count	1
AP001160.1	0.122974	0.3405237	0.3611	0.718	0.138650674	count	1
AL445686.2	0.282656	0.6686299	0.4227	0.673	0.138678143	count	1
PPP4R3B	0.1015028	0.1373231	0.7392	0.46	0.13891231	count	1
AQP7	0.155489	0.3233555	0.4809	0.631	0.138993618	count	1
LYPLAL1	0.1032276	0.1508101	0.6845	0.494	0.139002911	count	1
BMS1	0.1042498	0.1576497	0.6613	0.508	0.13900663	count	1
MYSM1	0.1102871	0.2058746	0.5357	0.592	0.13900715	count	1
PSIP1	0.0975611	0.0673683	1.4482	0.1477	0.13906962	count	1
C16orf87	0.1070497	0.1832881	0.5841	0.559	0.13910368	count	1
FAM228B	0.1067599	0.1987771	0.5371	0.591	0.139138091	count	1
FAM69B	0.0989837	0.0943192	1.0495	0.294	0.139303666	count	1
FAF2	0.1033396	0.1566021	0.6599	0.509	0.139333558	count	1
VAMP7	0.1068847	0.18451	0.5793	0.562	0.139496819	count	1
AL356275.1	0.1813938	0.5254105	0.3452	0.73	0.139497659	count	1
PIGBOS1	0.104316	0.1693373	0.616	0.538	0.139663438	count	1
SLC16A3	0.1141223	0.2270958	0.5025	0.615	0.139667173	count	1
MCM2	0.6880114	1.0352543	0.6646	0.506	0.139796535	count	1
ADGRE5	0.2191255	0.6046172	0.3624	0.717	0.139856785	count	1
MTM1	0.1142936	0.2377011	0.4808	0.631	0.139875289	count	1
EHMT2	0.1080896	0.1969772	0.5487	0.583	0.139897421	count	1
RPL36AL	0.0974519	0.0350405	2.7811	0.00545	0.139967455	count	1
NR2F6	0.1026595	0.1525951	0.6728	0.501	0.140000418	count	1
EXOSC5	0.1056632	0.2004568	0.5271	0.598	0.140038397	count	1
NR1D1	0.1128942	0.2569785	0.4393	0.66	0.140084091	count	1
INSR	0.1018891	0.1501621	0.6785	0.497	0.140125701	count	1
ZNF107	0.1262198	0.3235981	0.3901	0.697	0.140388335	count	1
C12orf65	0.1027484	0.1637553	0.6275	0.53	0.140446573	count	1
WDR34	0.1136605	0.2375054	0.4786	0.632	0.140576385	count	1

PIGU	0.1252793	0.3082408	0.4064	0.684	0.140617239	count	1
GPR108	0.1006573	0.1080814	0.9313	0.352	0.140644428	count	1
PITPNB	0.1002779	0.0990306	1.0126	0.311	0.140658769	count	1
MED4	0.1006163	0.0929198	1.0828	0.279	0.140732367	count	1
RPL36	0.0978375	0.0253851	3.8541	0.000119	0.140853597	count	1
THRAP3	0.0991295	0.0704632	1.4068	0.16	0.140971946	count	1
SP1	0.1119261	0.1991513	0.562	0.574	0.141060696	count	1
MTERF1	0.1326293	0.3928962	0.3376	0.736	0.141108638	count	1
MT-ND4	0.0979476	0.0305068	3.2107	0.00134	0.141173722	count	1
SHPK	0.4193417	0.7925292	0.5291	0.597	0.141233469	count	1
MTOR	0.1228648	0.3277962	0.3748	0.708	0.141235197	count	1
Z68871.1	0.5158501	0.7028268	0.734	0.463	0.141250449	count	1
ITGA2	0.1044417	0.1636194	0.6383	0.523	0.141284001	count	1
NAA16	0.1176931	0.2589453	0.4545	0.649	0.141321673	count	1
MAFG	0.1056468	0.1463011	0.7221	0.47	0.141439487	count	1
AHCYL2	0.1583823	0.4519238	0.3505	0.726	0.141514614	count	1
AC007950.2	0.6994707	1.0668252	0.6557	0.512	0.141557499	count	1
STAM-AS1	0.6994707	1.1267009	0.6208	0.535	0.141557499	count	1
ARHGAP5	0.1072777	0.1866164	0.5749	0.565	0.141575513	count	1
MAPK3	0.1010286	0.0884148	1.1427	0.253	0.14170273	count	1
MATR3	0.1294088	0.3710577	0.3488	0.727	0.141771752	count	1
EPRS	0.1015049	0.0999757	1.0153	0.31	0.141786456	count	1
AFAP1L1	0.1066607	0.1627399	0.6554	0.512	0.141895816	count	1
RAMP2-AS1	0.1270982	0.3040936	0.418	0.676	0.142003933	count	1
OXA1L	0.1030237	0.1203151	0.8563	0.392	0.142005206	count	1
ACIN1	0.1030533	0.1186655	0.8684	0.385	0.142025369	count	1
NPRL2	0.1231241	0.2816978	0.4371	0.662	0.142027703	count	1
ZNF431	0.1183649	0.2439442	0.4852	0.628	0.142121619	count	1
CFI	0.1006678	0.0819496	1.2284	0.219	0.142149051	count	1
AC008467.1	0.4225397	0.8430659	0.5012	0.616	0.142165853	count	1
BATF	0.5201394	0.7631699	0.6816	0.496	0.142221031	count	1
AL135999.1	0.5201394	0.8404645	0.6189	0.536	0.142221031	count	1
KLHL26	0.2130259	0.5563601	0.3829	0.702	0.142246172	count	1
TAF12	0.1031498	0.1233089	0.8365	0.403	0.142299528	count	1
SLC25A11	0.1044487	0.1321752	0.7902	0.429	0.142307309	count	1
CD200	0.1029883	0.1390003	0.7409	0.459	0.142414095	count	1
PPM1G	0.1009283	0.0820617	1.2299	0.219	0.142432283	count	1
HBEGF	0.1017322	0.1302355	0.7811	0.435	0.142446667	count	1
TATDN1	0.1031617	0.1235486	0.835	0.404	0.142451467	count	1
CLCN5	0.223563	0.502835	0.4446	0.657	0.142541172	count	1
ZNF888	0.2503522	0.8048107	0.3111	0.756	0.142643364	count	1
FTO	0.118555	0.3286497	0.3607	0.718	0.142675765	count	1
SNAPIN	0.1030887	0.1123888	0.9173	0.359	0.142691618	count	1
NEDD9	0.1021914	0.0873352	1.1701	0.242	0.142712864	count	1
KLHL5	0.1066336	0.1824161	0.5846	0.559	0.142756348	count	1

ZNF891	0.133394	0.4508669	0.2959	0.767	0.142802872	count	1
SLC37A3	0.1599621	0.3521858	0.4542	0.65	0.142890086	count	1
HINT3	0.1170028	0.2359883	0.4958	0.62	0.142894942	count	1
PDSS1	0.1691163	0.4792825	0.3529	0.724	0.143049628	count	1
PDCL3	0.1047385	0.1404035	0.746	0.456	0.143102219	count	1
SLC12A9-AS1	0.4258427	0.7943038	0.5361	0.592	0.143126651	count	1
NDUFA9	0.1044885	0.1354721	0.7713	0.441	0.143256225	count	1
CAP1	0.1010915	0.073059	1.3837	0.167	0.143273507	count	1
VPS37C	0.1222874	0.2884028	0.424	0.672	0.143351748	count	1
ABCB4	0.7113159	1.0638588	0.6686	0.504	0.143360618	count	1
MPP5	0.1140284	0.2250232	0.5067	0.612	0.143694176	count	1
IRF9	0.1103743	0.1660592	0.6647	0.506	0.144030999	count	1
MRPL11	0.1024609	0.0928961	1.103	0.27	0.144044274	count	1
IPO8	0.1120669	0.2379554	0.471	0.638	0.144146629	count	1
LRIG3	0.10628	0.1779601	0.5972	0.55	0.144197843	count	1
MAP3K21	0.5293048	0.7883733	0.6714	0.502	0.144284504	count	1
SERPINF1	0.5293048	0.8873005	0.5965	0.551	0.144284504	count	1
MOSPD2	0.114459	0.2070399	0.5528	0.58	0.144414414	count	1
TAPBPL	0.111956	0.1790243	0.6254	0.532	0.144514249	count	1
ARHGAP29	0.1010486	0.0608752	1.6599	0.097	0.144537567	count	1
CAPZA1	0.1037919	0.1100008	0.9436	0.345	0.144602723	count	1
MMADHC	0.1033251	0.0964972	1.0708	0.284	0.144619696	count	1
CCL5	0.1344247	0.8390153	0.1602	0.873	0.144758654	count	1
EIF3J	0.102857	0.0885561	1.1615	0.246	0.144833722	count	1
ADAMTS5	0.2171479	0.3401704	0.6384	0.523	0.144864034	count	1
FBXO21	0.1057257	0.1571557	0.6727	0.501	0.144922518	count	1
OXCT1	0.1207638	0.2480236	0.4869	0.626	0.144977471	count	1
ENTPD2	0.5327748	1.2052945	0.442	0.659	0.145062011	count	1
SLC25A23	0.1355686	0.4584533	0.2957	0.767	0.145096644	count	1
AL078459.1	1.244491	0.9693516	1.2838	0.199	0.145176427	count	1
DAZAP2	0.1033535	0.0876822	1.1787	0.239	0.145256805	count	1
DCPS	0.1099893	0.193408	0.5687	0.57	0.14529787	count	1
SEC11A	0.1014591	0.0431227	2.3528	0.0187	0.145504156	count	1
RSAD1	0.1297035	0.2879171	0.4505	0.652	0.145522996	count	1
EIF4A2	0.1023418	0.0610482	1.6764	0.0938	0.145528941	count	1
NHSL2	0.1079447	0.1529655	0.7057	0.48	0.145570297	count	1
AGO2	0.1120581	0.2184492	0.513	0.608	0.145581295	count	1
C12orf49	0.1136125	0.2232311	0.5089	0.611	0.14585388	count	1
CORO1A	0.4361316	0.7254019	0.6012	0.548	0.146105286	count	1
PRKAR1A	0.1050951	0.0987794	1.0639	0.287	0.146128215	count	1
SIKE1	0.1099554	0.162807	0.6754	0.499	0.146329458	count	1
SLC25A14	0.1732262	0.3887114	0.4456	0.656	0.146421434	count	1
RNF5	0.1071277	0.1179538	0.9082	0.364	0.146447986	count	1
DDX46	0.1034796	0.0776073	1.3334	0.183	0.146544672	count	1
MRPL36	0.1055831	0.1081614	0.9762	0.329	0.146619533	count	1

MTHFR	0.1187901	0.3147997	0.3774	0.706	0.14663175	count	1
HDGFL3	0.102914	0.0660431	1.5583	0.1193	0.146712114	count	1
AC005838.2	0.2305056	0.5756241	0.4004	0.689	0.146729076	count	1
VEGFB	0.1044873	0.1048545	0.9965	0.319	0.146740975	count	1
IBA57	0.1340353	0.3207223	0.4179	0.676	0.146771513	count	1
CHP1	0.1060998	0.1080759	0.9817	0.326	0.146781159	count	1
TRDMT1	0.1315331	0.3279702	0.4011	0.688	0.146897139	count	1
TECR	0.1043172	0.089155	1.1701	0.242	0.146905596	count	1
PPIF	0.14863	0.4358887	0.341	0.733	0.146952284	count	1
TMEM131	0.113639	0.1918264	0.5924	0.554	0.147044533	count	1
PPFIA3	0.1079726	0.1433758	0.7531	0.451	0.147062792	count	1
OTUD3	0.1537551	0.362488	0.4242	0.671	0.147122225	count	1
TMEM191B	0.5421358	1.1724927	0.4624	0.644	0.147149381	count	1
SERINC2	0.1368427	0.291035	0.4702	0.638	0.147324551	count	1
ZC3H13	0.1050088	0.0926955	1.1328	0.257	0.147326751	count	1
HTATSF1	0.1087729	0.1510433	0.7201	0.471	0.147373944	count	1
PCDHB11	0.5436322	0.653268	0.8322	0.405	0.14748169	count	1
FECH	0.1463694	0.4122498	0.3551	0.723	0.147487731	count	1
TTLL1	0.1315343	0.3990263	0.3296	0.742	0.147551829	count	1
RNF115	0.1051809	0.0990089	1.0623	0.288	0.147736041	count	1
MAPKAPK5-AS1	0.1120375	0.1660661	0.6747	0.5	0.147749898	count	1
ACTL6A	0.1119762	0.1851197	0.6049	0.545	0.147751268	count	1
BRD4	0.1067719	0.1059611	1.0077	0.314	0.14809805	count	1
EPB41L4A	0.109867	0.1534995	0.7157	0.474	0.148107877	count	1
MAP1LC3B2	0.1836064	0.5652476	0.3248	0.745	0.148432691	count	1
ZC3H4	0.1234321	0.2702911	0.4567	0.648	0.148494596	count	1
NUCB1	0.1044827	0.0636828	1.6407	0.101	0.148734785	count	1
ERF	0.111085	0.1312027	0.8467	0.397	0.148753017	count	1
ZNF665	0.1642354	0.3600737	0.4561	0.648	0.148876237	count	1
HSD17B4	0.1126953	0.1546406	0.7288	0.466	0.148937824	count	1
PDHB	0.1072003	0.1077088	0.9953	0.32	0.149078583	count	1
CUL2	0.1133035	0.160758	0.7048	0.481	0.149157913	count	1
LMAN2L	0.1559603	0.3315859	0.4703	0.638	0.149185693	count	1
NUDC	0.1052904	0.0693587	1.5181	0.129	0.149245808	count	1
TDP1	0.1675987	0.3975371	0.4216	0.673	0.14952868	count	1
TATDN2	0.1705539	0.4514147	0.3778	0.706	0.149610485	count	1
EIF1AD	0.1241529	0.2939835	0.4223	0.673	0.149690268	count	1
POLR1B	0.1531839	0.3763597	0.407	0.684	0.149841104	count	1
ARHGAP19	0.1629082	0.4474592	0.3641	0.716	0.149843999	count	1
DNAJC30	0.1165323	0.2023327	0.5759	0.565	0.149859719	count	1
RNF13	0.1062717	0.079367	1.339	0.181	0.150092607	count	1
RASGRP2	1.323205	1.0198439	1.2975	0.195	0.150170973	count	1
AC106869.1	0.3850555	0.6026172	0.639	0.523	0.150188238	count	1
ELOC	0.1055068	0.0614762	1.7162	0.0862	0.150204714	count	1
JRK	0.1859408	0.7443288	0.2498	0.803	0.150255056	count	1

VPS39	0.1233269	0.2723066	0.4529	0.651	0.150264492	count	1
POLD2	0.111729	0.1588121	0.7035	0.482	0.150314247	count	1
NAA25	0.1323846	0.2796011	0.4735	0.636	0.150351988	count	1
SPA17	0.1300006	0.3043358	0.4272	0.669	0.150384943	count	1
DGKQ	0.2493875	0.5724197	0.4357	0.663	0.150389567	count	1
AGBL3	0.1381778	0.3576447	0.3864	0.699	0.150437595	count	1
CORO7	0.2090604	0.3933489	0.5315	0.595	0.150483193	count	1
DNAJC7	0.1078361	0.0929912	1.1596	0.246	0.150494145	count	1
SH3BGRL	0.1055344	0.0589839	1.7892	0.0737	0.150569105	count	1
DAXX	0.1157627	0.1696287	0.6824	0.495	0.150706948	count	1
MBD4	0.108603	0.1242393	0.8741	0.382	0.150728771	count	1
FAM135A	0.133289	0.3206151	0.4157	0.678	0.150761581	count	1
MEX3B	0.2853961	0.5766346	0.4949	0.621	0.150975214	count	1
CDC14A	0.1342646	0.2471033	0.5434	0.587	0.151223426	count	1
DIDO1	0.1177394	0.1991602	0.5912	0.554	0.151264394	count	1
SMIM15	0.1120604	0.1536943	0.7291	0.466	0.151290913	count	1
RNF152	0.2102724	0.4777968	0.4401	0.66	0.151316991	count	1
MED30	0.1094794	0.1219852	0.8975	0.37	0.151334876	count	1
HAUS2	0.1159382	0.2000305	0.5796	0.562	0.151362606	count	1
USP35	0.2863183	0.7744607	0.3697	0.712	0.151425993	count	1
POLR2A	0.1137338	0.1491039	0.7628	0.446	0.151474316	count	1
PDHA1	0.1119482	0.1423149	0.7866	0.432	0.151537803	count	1
P4HA3	0.1335228	0.3559844	0.3751	0.708	0.151629201	count	1
WNT9B	0.5626931	0.7679536	0.7327	0.464	0.151681772	count	1
AC005229.4	0.1481344	0.377161	0.3928	0.695	0.151775901	count	1
CLK3	0.1156524	0.1765502	0.6551	0.512	0.151781686	count	1
UEVLD	0.1419984	0.3139694	0.4523	0.651	0.15187217	count	1
U47924.2	0.39029	0.8319335	0.4691	0.639	0.151987739	count	1
AC002553.1	0.39029	0.8319335	0.4691	0.639	0.151987739	count	1
AC106782.2	0.39029	0.8386633	0.4654	0.642	0.151987739	count	1
COX11	0.1164338	0.1718355	0.6776	0.498	0.152006618	count	1
ZNF358	0.1101029	0.1255018	0.8773	0.38	0.152156356	count	1
STXBP4	0.1841178	0.3932827	0.4682	0.64	0.152175326	count	1
KDM2A	0.1129532	0.158569	0.7123	0.476	0.152206533	count	1
CHMP5	0.1067766	0.0590436	1.8084	0.0706	0.152233514	count	1
RAMMET	0.1144182	0.1613925	0.7089	0.478	0.152246382	count	1
HECTD3	0.2118092	0.4522586	0.4683	0.64	0.152373578	count	1
IFT172	0.2118092	0.4888393	0.4333	0.665	0.152373578	count	1
MASP1	0.2122529	0.5636519	0.3766	0.707	0.152678491	count	1
ZNF552	0.2405964	0.6856045	0.3509	0.726	0.152790162	count	1
DDO	0.4599685	0.9987227	0.4606	0.645	0.152923313	count	1
ZNF564	0.2409683	0.6801166	0.3543	0.723	0.153012966	count	1
TCTEX1D2	0.1315181	0.2506283	0.5248	0.6	0.153116245	count	1
PPP1R11	0.1111592	0.109739	1.0129	0.311	0.153195547	count	1
AL731661.1	0.3482487	0.9907115	0.3515	0.725	0.15323156	count	1

PXT1	0.3482487	1.349242	0.2581	0.796	0.15323156	count	1
CCDC7	0.1898068	0.5896653	0.3219	0.748	0.153269401	count	1
CCDC174	0.1114483	0.1357501	0.821	0.412	0.153316034	count	1
POLR2C	0.1114909	0.1224415	0.9106	0.363	0.15337451	count	1
KATNAL2	0.1753352	0.4079541	0.4298	0.667	0.15368339	count	1
ERG28	0.1119387	0.1327267	0.8434	0.399	0.153740277	count	1
TMSB4X	0.1066534	0.0263884	4.0417	5.45E-05	0.153763148	count	1
BBIP1	0.1151651	0.1616302	0.7125	0.476	0.15376752	count	1
PLEKHN1	0.3959995	0.7802663	0.5075	0.612	0.153943353	count	1
RAB17	0.1591994	0.4997491	0.3186	0.75	0.153944023	count	1
ARHGAP1	0.1223911	0.2431014	0.5035	0.615	0.153966408	count	1
SERAC1	0.2319555	0.4410543	0.5259	0.599	0.154225602	count	1
TPM4	0.1076745	0.0485689	2.2169	0.0267	0.154447095	count	1
OSGIN1	0.7870227	1.1025608	0.7138	0.475	0.154481567	count	1
CDK12	0.113343	0.1451239	0.781	0.435	0.15463296	count	1
IRAK2	0.1522737	0.4842429	0.3145	0.753	0.154656423	count	1
BIN1	0.1916668	0.4513615	0.4246	0.671	0.154718028	count	1
GLCE	0.1178136	0.1982766	0.5942	0.552	0.154887177	count	1
ENO1-AS1	0.3527794	0.8346821	0.4227	0.673	0.155020891	count	1
CHCHD3	0.1142651	0.1311775	0.8711	0.384	0.155041174	count	1
RFC3	0.1340892	0.2530535	0.5299	0.596	0.155061683	count	1
SOD3	0.5787015	0.9212548	0.6282	0.53	0.155162498	count	1
AL133338.1	0.3196487	0.7652212	0.4177	0.676	0.155230812	count	1
TIGD4	0.3196487	0.8096796	0.3948	0.693	0.155230812	count	1
ZNRD1	0.1119642	0.1283002	0.8727	0.383	0.155253397	count	1
WASL	0.1108299	0.0897154	1.2354	0.217	0.155499972	count	1
ZNF335	0.1692208	0.440335	0.3843	0.701	0.155500442	count	1
CCNK	0.1175134	0.1706081	0.6888	0.491	0.155834848	count	1
GDPD3	0.3548589	0.8208832	0.4323	0.666	0.155840431	count	1
UROS	0.1183508	0.1698596	0.6968	0.486	0.155863293	count	1
METTL2B	0.1287966	0.2337361	0.551	0.582	0.155914221	count	1
IFITM3	0.1081558	0.024761	4.368	1.30E-05	0.15593238	count	0.301249
PRPF19	0.1214662	0.3073044	0.3953	0.693	0.156025511	count	1
BCLAF1	0.1107351	0.090451	1.2243	0.221	0.156050097	count	1
PIP4K2B	0.1261635	0.2392313	0.5274	0.598	0.156177217	count	1
GGACT	0.159827	0.5231142	0.3055	0.76	0.156198388	count	1
GTF2H3	0.1190353	0.1768449	0.6731	0.501	0.15620126	count	1
FAM71A	0.7993316	1.0627721	0.7521	0.452	0.156225108	count	1
C6orf62	0.1149531	0.1257557	0.9141	0.361	0.156247805	count	1
GAMT	0.1429182	0.3086172	0.4631	0.643	0.156357305	count	1
ZNF549	0.2176235	0.5881488	0.37	0.711	0.156364458	count	1
ANKRD10	0.1117697	0.1095667	1.0201	0.308	0.156422615	count	1
USO1	0.1154371	0.1404238	0.8221	0.411	0.156461234	count	1
SUSD6	0.12058	0.2186735	0.5514	0.581	0.156476455	count	1
NKIRAS1	0.1430318	0.3330255	0.4295	0.668	0.156479773	count	1

RPGR	0.1123286	0.1097411	1.0236	0.306	0.156693503	count	1
MINCR	0.2359774	0.4573975	0.5159	0.606	0.156756766	count	1
FGD5	0.114544	0.1528319	0.7495	0.454	0.156835629	count	1
C6orf226	0.1213328	0.193841	0.6259	0.531	0.156947492	count	1
CNIH4	0.1117428	0.0913358	1.2234	0.221	0.157077484	count	1
AC005696.1	0.4747411	1.0788407	0.44	0.66	0.157091059	count	1
AL390728.6	0.1363413	0.3056376	0.4461	0.656	0.157098543	count	1
MTPAP	0.139189	0.2628209	0.5296	0.596	0.157350414	count	1
PGRMC1	0.1111551	0.0814307	1.365	0.172	0.15738995	count	1
FMO5	0.1610764	0.4133378	0.3897	0.697	0.157392684	count	1
EVL	0.1170041	0.2375172	0.4926	0.622	0.157492876	count	1
RAP2B	0.1191834	0.1644583	0.7247	0.469	0.157559671	count	1
ISG20L2	0.1251285	0.2090836	0.5985	0.55	0.157588688	count	1
NDUFAF4	0.118804	0.1689845	0.703	0.482	0.15769207	count	1
GGNBP2	0.1123524	0.0894745	1.2557	0.209	0.157756796	count	1
ZBTB44	0.1238343	0.2015552	0.6144	0.539	0.157790802	count	1
SPATA5	0.1554372	0.3169768	0.4904	0.624	0.157807114	count	1
AC007920.2	0.4074079	1.0676622	0.3816	0.703	0.157828585	count	1
AC010491.1	0.4074079	1.0676622	0.3816	0.703	0.157828585	count	1
AIFM3	0.4074079	1.0858939	0.3752	0.708	0.157828585	count	1
FAM50B	0.132222	0.2385676	0.5542	0.579	0.157844332	count	1
CAPN12	0.8119562	1.0555195	0.7692	0.442	0.157995011	count	1
TMEM62	0.1655268	0.3756679	0.4406	0.66	0.158121612	count	1
C2orf69	0.1318993	0.2869124	0.4597	0.646	0.158220843	count	1
OCEL1	0.1217351	0.1991768	0.6112	0.541	0.158326734	count	1
TCF3	0.1306415	0.2612781	0.5	0.617	0.158459052	count	1
STK38	0.1333858	0.2498489	0.5339	0.593	0.158822956	count	1
EXT2	0.1258601	0.1981012	0.6353	0.525	0.158899726	count	1
ZSWIM4	0.3019838	0.535772	0.5636	0.573	0.159047288	count	1
PCDHB3	0.2647518	0.6529098	0.4055	0.685	0.159055768	count	1
CMC1	0.1151731	0.1145321	1.0056	0.315	0.15908699	count	1
DMWD	0.1272455	0.2157935	0.5897	0.555	0.159171738	count	1
ITPRIP	0.1306154	0.2116893	0.617	0.537	0.159379346	count	1
TAF6	0.1341176	0.2229529	0.6016	0.548	0.1596857	count	1
OPTN	0.1136513	0.0960207	1.1836	0.237	0.159764413	count	1
MRPL50	0.1167724	0.1324581	0.8816	0.378	0.159787616	count	1
NBPF11	0.2226318	0.6694606	0.3326	0.739	0.159793751	count	1
DISP2	0.4133539	1.082262	0.3819	0.703	0.159841748	count	1
EIF4EBP3	0.4133539	1.16747	0.3541	0.723	0.159841748	count	1
MRPL22	0.1146075	0.1009163	1.1357	0.256	0.15984223	count	1
AL022323.4	0.6007041	0.5706782	1.0526	0.293	0.159877629	count	1
SLC30A9	0.1203459	0.1789996	0.6723	0.501	0.159956203	count	1
ENAH	0.1181115	0.1365936	0.8647	0.387	0.159988197	count	1
GPR137B	0.1576594	0.3547154	0.4445	0.657	0.160018745	count	1
MIER3	0.1638478	0.4193465	0.3907	0.696	0.16004033	count	1

RNF220	0.1356932	0.2417872	0.5612	0.575	0.160269715	count	1
PARPBP	0.2234253	0.6427188	0.3476	0.728	0.160336376	count	1
PNMA8B	0.2234253	0.7515657	0.2973	0.766	0.160336376	count	1
ANKRD52	0.2040354	0.6751489	0.3022	0.763	0.160391261	count	1
LINC00921	0.2040354	0.7697597	0.2651	0.791	0.160391261	count	1
VTI1B	0.1151839	0.1084714	1.0619	0.288	0.160580599	count	1
GLS	0.1131753	0.0867443	1.3047	0.192	0.160595033	count	1
NFYA	0.1868578	0.6999058	0.267	0.79	0.160599579	count	1
C15orf61	0.1192153	0.1489329	0.8005	0.424	0.160616807	count	1
HINT1	0.111719	0.0343088	3.2563	0.00114	0.160652543	count	1
FITM2	0.1907256	0.5366639	0.3554	0.722	0.160721137	count	1
ZNF275	0.2045584	0.7929366	0.258	0.796	0.160786266	count	1
ZNF444	0.145777	0.295221	0.4938	0.621	0.161061265	count	1
DENND4A	0.1279475	0.2785748	0.4593	0.646	0.161317702	count	1
EIF3L	0.1136287	0.0600237	1.8931	0.0585	0.16147225	count	1
ACP1	0.1153346	0.0958695	1.203	0.229	0.161525248	count	1
PREP	0.1579018	0.3679918	0.4291	0.668	0.161590231	count	1
HIST1H2BE	0.490941	0.7778977	0.6311	0.528	0.161611147	count	1
AL662884.4	0.2437409	0.5693705	0.4281	0.669	0.161628732	count	1
FXR1	0.1142925	0.077042	1.4835	0.138	0.161667384	count	1
RWDD2B	0.1265352	0.2750043	0.4601	0.645	0.161712667	count	1
RPS9	0.1122932	0.0221855	5.0616	4.43E-07	0.161808004	count	0.010377718
AUP1	0.1155487	0.094366	1.2245	0.221	0.1618926	count	1
PPP1R13B	0.1230568	0.1458814	0.8435	0.399	0.161939205	count	1
MYH10	0.1219706	0.2111184	0.5777	0.563	0.161955053	count	1
TIMM44	0.1267797	0.2213865	0.5727	0.567	0.16202323	count	1
POLR2M	0.1301189	0.2302391	0.5651	0.572	0.162036768	count	1
PUM2	0.1254238	0.2329751	0.5384	0.59	0.162076084	count	1
STRIP1	0.1853379	0.3015144	0.6147	0.539	0.16218209	count	1
MED14	0.1437027	0.2734743	0.5255	0.599	0.162386068	count	1
CALM2	0.1132689	0.0429035	2.6401	0.00834	0.162465277	count	1
DDX1	0.1189385	0.1340568	0.8872	0.375	0.162680658	count	1
AFTPH	0.1248686	0.2303176	0.5422	0.588	0.162734927	count	1
TEK	0.1176509	0.124053	0.9484	0.343	0.162807429	count	1
UBE2D2	0.114358	0.0613675	1.8635	0.0625	0.162829207	count	1
LYRM2	0.1168683	0.1205411	0.9695	0.332	0.162924957	count	1
DYNC1I2	0.1152133	0.086909	1.3257	0.185	0.162937658	count	1
APH1A	0.1159137	0.0851341	1.3615	0.173	0.162993195	count	1
SLC10A3	0.1263991	0.2066997	0.6115	0.541	0.16305279	count	1
AC009133.1	0.2583076	0.5642806	0.4578	0.647	0.163354306	count	1
CXorf56	0.1458473	0.3505743	0.416	0.677	0.163387888	count	1
PHTF1	0.1314478	0.2359974	0.557	0.578	0.163433259	count	1
BANP	0.1531521	0.3122221	0.4905	0.624	0.163601961	count	1
PNPT1	0.1237442	0.1881028	0.6579	0.511	0.163648358	count	1
RBM6	0.1201264	0.1628475	0.7377	0.461	0.163718083	count	1

PAXIP1-AS2	0.1392384	0.2882051	0.4831	0.629	0.16395457	count	1
UBE2Q2	0.1183411	0.123505	0.9582	0.338	0.163969755	count	1
EIF3H	0.114695	0.0504734	2.2724	0.0231	0.164125462	count	1
FAM78B	0.2596636	0.8795811	0.2952	0.768	0.164159209	count	1
AFDN	0.1162228	0.0850137	1.3671	0.172	0.164222982	count	1
AL118516.1	0.1216578	0.1996556	0.6093	0.542	0.164498498	count	1
MRPL45	0.1216958	0.1385813	0.8782	0.38	0.16478265	count	1
AC027307.3	0.6240331	0.7929027	0.787	0.431	0.16479072	count	1
ZMYND19	0.1729113	0.3506646	0.4931	0.622	0.165001822	count	1
DBT	0.1546255	0.3525842	0.4385	0.661	0.165149213	count	1
CHMP2B	0.1181445	0.0970562	1.2173	0.224	0.165211772	count	1
IQSEC2	0.1396007	0.3227739	0.4325	0.665	0.165283356	count	1
RPLP0	0.1148872	0.0266888	4.3047	1.73E-05	0.16556869	count	0.4004777
PES1	0.1317275	0.2107098	0.6252	0.532	0.165627751	count	1
DNAJC1	0.1175464	0.0919417	1.2785	0.201	0.165681453	count	1
RPS25	0.1150372	0.0203416	5.6553	1.72E-08	0.165783376	count	0.000406092
NUP214	0.129708	0.1963781	0.6605	0.509	0.165908448	count	1
MDM4	0.1219669	0.1464357	0.8329	0.405	0.165913147	count	1
ZFP64	0.1719984	0.4157361	0.4137	0.679	0.166024239	count	1
TOB1	0.1199417	0.1180637	1.0159	0.31	0.166088223	count	1
GRHPR	0.1190511	0.0906206	1.3137	0.189	0.166219916	count	1
MANEA	0.1459938	0.2897963	0.5038	0.614	0.166251312	count	1
FCF1	0.1434187	0.2720042	0.5273	0.598	0.16627215	count	1
PSMD10	0.1310716	0.2103608	0.6231	0.533	0.166406679	count	1
PTER	0.1655772	0.3650473	0.4536	0.65	0.166434342	count	1
GALNT1	0.1224784	0.1166057	1.0504	0.294	0.166486533	count	1
ESRRA	0.1379762	0.2645038	0.5216	0.602	0.166563796	count	1
UFL1	0.1196231	0.1106602	1.081	0.28	0.166648643	count	1
ZNF43	0.1331414	0.2105652	0.6323	0.527	0.166950273	count	1
SUZ12	0.1206567	0.1242702	0.9709	0.332	0.166958245	count	1
AP000766.1	0.6345898	0.7826162	0.8109	0.418	0.166985058	count	1
WBP4	0.1201318	0.1187153	1.0119	0.312	0.167091006	count	1
ERLIN1	0.1255781	0.1846709	0.68	0.497	0.167258327	count	1
TFE3	0.1623976	0.3494343	0.4647	0.642	0.167415679	count	1
VPS13A	0.124985	0.1725061	0.7245	0.469	0.16761086	count	1
SRSF7	0.1179809	0.0621816	1.8974	0.0579	0.167633638	count	1
FERMT2	0.1187228	0.0751993	1.5788	0.115	0.167941427	count	1
MYOF	0.120266	0.1139248	1.0557	0.291	0.168022938	count	1
ZNF830	0.1275096	0.1775738	0.7181	0.473	0.168152984	count	1
ARPC4	0.1195975	0.08112	1.4743	0.141	0.168181802	count	1
KLC2	0.254245	0.5491207	0.463	0.643	0.168191276	count	1
LITAF	0.1221254	0.1489161	0.8201	0.412	0.168322458	count	1
MED10	0.1201153	0.0911933	1.3172	0.188	0.168407669	count	1
CHMP4A	0.1203348	0.0940462	1.2795	0.201	0.168434057	count	1
FRY-AS1	0.8896917	1.1810395	0.7533	0.451	0.168492304	count	1

TCP11L1	0.1746363	0.4334431	0.4029	0.687	0.168508341	count	1
MRPL3	0.1228519	0.1365914	0.8994	0.369	0.168547319	count	1
ALG8	0.1333087	0.2509755	0.5312	0.595	0.168643536	count	1
WDR73	0.1406996	0.2488769	0.5653	0.572	0.168671445	count	1
CUL5	0.1226671	0.1318425	0.9304	0.352	0.168764853	count	1
QRSL1	0.1411248	0.2449385	0.5762	0.565	0.168774217	count	1
TMEM87A	0.122818	0.1147879	1.07	0.285	0.168892584	count	1
MAD2L1BP	0.1355848	0.222279	0.61	0.542	0.16903917	count	1
ZNF75A	0.149178	0.2558938	0.583	0.56	0.169168648	count	1
UBE2N	0.1209418	0.0906842	1.3337	0.182	0.169239373	count	1
SPATA17	0.6455648	0.7858923	0.8214	0.411	0.169247417	count	1
ZNF32	0.1227448	0.1309299	0.9375	0.349	0.169317309	count	1
B3GAT2	0.3523024	0.515581	0.6833	0.494	0.169538388	count	1
PHC2	0.1246516	0.1293159	0.9639	0.335	0.169713208	count	1
EML2	0.1299518	0.2220668	0.5852	0.558	0.169794154	count	1
SCYL1	0.1362337	0.2142112	0.636	0.525	0.17008763	count	1
GTPBP1	0.1472753	0.4679291	0.3147	0.753	0.170121681	count	1
PLEKHJ1	0.133081	0.2017117	0.6598	0.509	0.170195269	count	1
AP000894.4	0.6504821	1.0646175	0.611	0.541	0.170254811	count	1
NTHL1	0.1234457	0.1239468	0.996	0.319	0.170281932	count	1
ATP9B	0.1424046	0.3595195	0.3961	0.692	0.17028898	count	1
METTL16	0.1341643	0.2176451	0.6164	0.538	0.170306373	count	1
TP53BP2	0.1369345	0.214318	0.6389	0.523	0.170708651	count	1
ZKSCAN5	0.2074566	0.5633198	0.3683	0.713	0.170744147	count	1
SMG7	0.1323217	0.1933242	0.6845	0.494	0.170793653	count	1
CCDC43	0.1256225	0.1433347	0.8764	0.381	0.170829932	count	1
SAC3D1	0.1474185	0.2951839	0.4994	0.618	0.170852497	count	1
MMP28	0.1220516	0.1138558	1.072	0.284	0.171000193	count	1
NBEAL2	0.1673623	0.4259417	0.3929	0.694	0.171072579	count	1
RCE1	0.1366469	0.2240538	0.6099	0.542	0.171079235	count	1
ENTPD4	0.1647707	0.3210157	0.5133	0.608	0.171098995	count	1
CRTC3	0.1390778	0.2226873	0.6245	0.532	0.17116871	count	1
WDR7	0.1704839	0.52492	0.3248	0.745	0.171258608	count	1
PRPF31	0.1236061	0.1119851	1.1038	0.27	0.171268908	count	1
USP10	0.1327078	0.190135	0.698	0.485	0.171289062	count	1
TMEM128	0.1334322	0.2190853	0.609	0.543	0.171300856	count	1
NCOA5	0.196259	0.4232139	0.4637	0.643	0.171427022	count	1
DHX40	0.1312335	0.178383	0.7357	0.462	0.171460034	count	1
LAMTOR3	0.1315195	0.1724665	0.7626	0.446	0.17171514	count	1
ACAD11	0.9159349	1.0474518	0.8744	0.382	0.171884418	count	1
VPS37D	0.2727275	0.583482	0.4674	0.64	0.171885425	count	1
PAK2	0.1208242	0.0663188	1.8219	0.0686	0.172050372	count	1
DYNC1LI1	0.1249681	0.1130121	1.1058	0.269	0.172079901	count	1
VBP1	0.1260113	0.1191359	1.0577	0.29	0.172084667	count	1
TERF2	0.1317064	0.1863459	0.7068	0.48	0.17241606	count	1

AC105942.1	0.1750059	0.4738047	0.3694	0.712	0.172423578	count	1
DUSP1	0.1198075	0.0470196	2.548	0.0109	0.172430962	count	1
CDK20	0.6617442	1.0672329	0.6201	0.535	0.172547599	count	1
PGK1	0.1211103	0.0565196	2.1428	0.0322	0.172662056	count	1
LMBRD2	0.1689583	0.3979419	0.4246	0.671	0.172669966	count	1
ZNF440	0.2151586	0.4542655	0.4736	0.636	0.172922917	count	1
TMEM218	0.132822	0.1774422	0.7485	0.454	0.173043825	count	1
ARID3B	0.9254604	0.830274	1.1146	0.265	0.173097162	count	1
TTTY15	0.1568869	0.2831208	0.5541	0.58	0.173145148	count	1
TMEM53	0.1533812	0.2751725	0.5574	0.577	0.173169014	count	1
DARS2	0.1819048	0.4994065	0.3642	0.716	0.173360415	count	1
NCBP3	0.1280238	0.1335792	0.9584	0.338	0.173417911	count	1
FBXO11	0.1314033	0.1513659	0.8681	0.385	0.173546731	count	1
RPL38	0.1208976	0.0330268	3.6606	0.000256	0.17363972	count	1
SLC27A4	0.252287	0.5266416	0.479	0.632	0.173676557	count	1
C16orf70	0.1896511	0.4446801	0.4265	0.67	0.173728022	count	1
LRSAM1	0.163863	0.4019453	0.4077	0.684	0.173734617	count	1
TUT4	0.1242697	0.1070992	1.1603	0.246	0.173751615	count	1
AC234772.3	0.5360749	0.6972042	0.7689	0.442	0.173929495	count	1
PFKL	0.1297942	0.1459483	0.8893	0.374	0.173970946	count	1
TLE2	0.1402597	0.2006957	0.6989	0.485	0.174031398	count	1
CXorf38	0.1337967	0.2277759	0.5874	0.557	0.174054787	count	1
IK	0.1226984	0.073641	1.6662	0.0958	0.174119707	count	1
TARS2	0.1787139	0.3175447	0.5628	0.574	0.17420651	count	1
ZNHIT3	0.125913	0.1194466	1.0541	0.292	0.174257376	count	1
EIF1AX	0.1231835	0.0711011	1.7325	0.0833	0.174304534	count	1
PEX6	0.1469827	0.299929	0.4901	0.624	0.174386837	count	1
HTR2B	0.1906174	0.4876704	0.3909	0.696	0.174587132	count	1
TRIM23	0.1377617	0.228744	0.6023	0.547	0.174642693	count	1
ARMC8	0.1362517	0.2013763	0.6766	0.499	0.174732895	count	1
SPAST	0.138199	0.2204936	0.6268	0.531	0.174785456	count	1
P4HA1	0.1322402	0.1797677	0.7356	0.462	0.174922441	count	1
SEC14L1	0.1235987	0.0931344	1.3271	0.185	0.175075349	count	1
JUN	0.1215209	0.0509231	2.3864	0.0171	0.175084971	count	1
PIGV	0.1838267	0.4100054	0.4484	0.654	0.175143677	count	1
CGGBP1	0.1256282	0.1062045	1.1829	0.237	0.175400113	count	1
HIST1H4C	0.1233092	0.080714	1.5277	0.127	0.175446422	count	1
APPBP2	0.1338752	0.2090027	0.6405	0.522	0.175464587	count	1
PTCD3	0.1411333	0.2203981	0.6404	0.522	0.175640729	count	1
AC004241.1	0.460901	0.7745338	0.5951	0.552	0.175651234	count	1
ARID3A	0.460901	0.7745338	0.5951	0.552	0.175651234	count	1
TTC21A	0.460901	0.7970103	0.5783	0.563	0.175651234	count	1
BAG5	0.1268707	0.1039757	1.2202	0.222	0.175662362	count	1
UBR7	0.1473636	0.2375293	0.6204	0.535	0.175725408	count	1
EIF3M	0.1238248	0.0654555	1.8917	0.0586	0.17580304	count	1

ACAA2	0.129809	0.1404756	0.9241	0.356	0.175827972	count	1
RPS15	0.1221353	0.0205453	5.9447	3.12E-09	0.176030905	count	7.39E-05
SLC25A25-AS1	0.5452533	0.7592324	0.7182	0.473	0.17638554	count	1
DIRC2	0.1657097	0.3429932	0.4831	0.629	0.176772043	count	1
CREBBP	0.1347321	0.1880139	0.7166	0.474	0.176802016	count	1
TUBGCP6	0.2476722	0.5306825	0.4667	0.641	0.176823202	count	1
ATP1B1	0.2476837	0.4995643	0.4958	0.62	0.17683098	count	1
HMBS	0.1691978	0.3217527	0.5259	0.599	0.176875871	count	1
ZSWIM6	0.1491192	0.2551969	0.5843	0.559	0.176893215	count	1
RBCK1	0.1260293	0.0989961	1.2731	0.203	0.1770822	count	1
TCOF1	0.1524201	0.2496196	0.6106	0.542	0.177147468	count	1
NUMA1	0.1296419	0.14112	0.9187	0.358	0.177172851	count	1
ATG4A	0.1519629	0.2379322	0.6387	0.523	0.177178042	count	1
NUP37	0.1380441	0.200675	0.6879	0.492	0.177183464	count	1
FXN	0.1421857	0.2256398	0.6301	0.529	0.177201404	count	1
PTMS	0.1234272	0.041585	2.9681	0.00302	0.177270059	count	1
BIRC6	0.1310402	0.1518736	0.8628	0.388	0.177292872	count	1
RARS	0.1328879	0.178026	0.7465	0.455	0.177405017	count	1
DSEL	0.1461342	0.2257468	0.6473	0.517	0.177429442	count	1
ALS2	0.1676094	0.3449238	0.4859	0.627	0.177631932	count	1
CC2D2A	0.1452557	0.2737491	0.5306	0.596	0.177745994	count	1
YWHAE	0.1241621	0.0472976	2.6251	0.00871	0.177761098	count	1
CUTC	0.1331664	0.1554995	0.8564	0.392	0.177775246	count	1
BDP1	0.1264232	0.1059787	1.1929	0.233	0.177854145	count	1
TMEM234	0.1497209	0.2664095	0.562	0.574	0.178057604	count	1
RPS6KA3	0.1306173	0.1282905	1.0181	0.309	0.178392993	count	1
PRKRIP1	0.1377033	0.1731899	0.7951	0.427	0.178426981	count	1
GPAM	0.1762523	0.4117878	0.428	0.669	0.178472321	count	1
UBTD1	0.1325417	0.1547826	0.8563	0.392	0.1786192	count	1
WDR55	0.1421681	0.2154899	0.6597	0.509	0.178653563	count	1
ARID4A	0.1277915	0.1054533	1.2118	0.226	0.178762518	count	1
POT1	0.1710394	0.3516802	0.4863	0.627	0.178762609	count	1
UBE2E3	0.1272655	0.0835354	1.5235	0.128	0.178958155	count	1
STARD3NL	0.1310559	0.1268576	1.0331	0.302	0.179063415	count	1
ZGRF1	0.3744372	0.8449427	0.4432	0.658	0.1790759	count	1
PLAA	0.1587541	0.2558234	0.6206	0.535	0.1791463	count	1
CDS2	0.1338556	0.1575541	0.8496	0.396	0.17918962	count	1
KLHL36	0.144609	0.2382444	0.607	0.544	0.179381591	count	1
LRRC23	0.1432061	0.2357067	0.6076	0.544	0.179470409	count	1
REV3L	0.1314844	0.1448011	0.908	0.364	0.179998841	count	1
MICU3	0.1518426	0.2715534	0.5592	0.576	0.180086872	count	1
RSPRY1	0.1422736	0.1955273	0.7276	0.467	0.180112258	count	1
PSTPIP2	0.5594828	0.8553303	0.6541	0.513	0.180160768	count	1
COG3	0.1527532	0.2742406	0.557	0.578	0.180182911	count	1
RPS3	0.1250193	0.0216631	5.7711	8.75E-09	0.180239698	count	0.000206728

ZNF667-AS1	0.1301437	0.1175602	1.107	0.268	0.180327829	count	1
TMEM14C	0.1260972	0.0537454	2.3462	0.019	0.180401404	count	1
WDR70	0.1403414	0.184306	0.7615	0.446	0.180444773	count	1
LILRB3	0.9853701	1.0456905	0.9423	0.346	0.180504061	count	1
SCML2	0.9853701	1.0945184	0.9003	0.368	0.180504061	count	1
NKAIN2	0.9853701	1.182762	0.8331	0.405	0.180504061	count	1
TRIM62	0.9853701	1.1993312	0.8216	0.411	0.180504061	count	1
ARL6IP1	0.1271982	0.0818522	1.554	0.12	0.180556149	count	1
PPP1R7	0.1301812	0.1063271	1.2243	0.221	0.180725707	count	1
GAPVD1	0.1421621	0.1994853	0.7126	0.476	0.180784707	count	1
DHRS12	0.1474935	0.3235023	0.4559	0.648	0.180789187	count	1
SOCS5	0.1453385	0.2595824	0.5599	0.576	0.18082977	count	1
SUPT20H	0.1398886	0.1873535	0.7467	0.455	0.180950948	count	1
LNPK	0.1625723	0.3106625	0.5233	0.601	0.181022343	count	1
PANK2	0.1426985	0.1901522	0.7504	0.453	0.181060758	count	1
SLC25A30	0.2009399	0.3948077	0.509	0.611	0.181097262	count	1
LIG1	0.1927144	0.3807056	0.5062	0.613	0.181175374	count	1
TRIM44	0.1297363	0.1111109	1.1676	0.243	0.181204844	count	1
RAI14	0.1300478	0.1137686	1.1431	0.253	0.181450424	count	1
MT-ND5	0.1261159	0.0377749	3.3386	0.000853	0.181482759	count	1
SLC25A38	0.1459611	0.2139596	0.6822	0.495	0.181597805	count	1
HDAC7	0.132664	0.1282329	1.0346	0.301	0.181609036	count	1
ZNF248	0.1780792	0.3344507	0.5325	0.594	0.181786005	count	1
CAPRIN1	0.1334627	0.1422094	0.9385	0.348	0.181877515	count	1
NUMBL	0.2020119	0.3713866	0.5439	0.587	0.182032358	count	1
CLPX	0.1433446	0.2048867	0.6996	0.484	0.182077723	count	1
TWF1	0.1350374	0.1457614	0.9264	0.354	0.182084714	count	1
IFT52	0.1366983	0.1497037	0.9131	0.361	0.182162112	count	1
RNF20	0.1386111	0.2025013	0.6845	0.494	0.182195069	count	1
UTP18	0.138199	0.1804309	0.7659	0.444	0.182279325	count	1
ZNF445	0.1721244	0.3587644	0.4798	0.631	0.182324191	count	1
HNRNPH3	0.1297389	0.0817921	1.5862	0.113	0.182372967	count	1
AP000254.1	0.3264742	0.960306	0.34	0.734	0.182428168	count	1
NBN	0.134324	0.149532	0.8983	0.369	0.182535323	count	1
OGDH	0.150395	0.2475607	0.6075	0.544	0.182550393	count	1
TACC2	0.1594431	0.2404058	0.6632	0.507	0.182704754	count	1
SLC39A6	0.160724	0.3274119	0.4909	0.624	0.182784418	count	1
PASK	0.5695479	0.7661472	0.7434	0.457	0.182807436	count	1
ERI3	0.1358989	0.145582	0.9335	0.351	0.182827789	count	1
HOMER2	0.2282103	0.5625473	0.4057	0.685	0.182963872	count	1
RSRP1	0.1290191	0.0709055	1.8196	0.0689	0.183050037	count	1
SPTLC1	0.1353437	0.1298498	1.0423	0.297	0.183197356	count	1
ADCK1	0.3280378	0.8010969	0.4095	0.682	0.183227844	count	1
TSPAN11	0.2785839	0.5427844	0.5132	0.608	0.183267648	count	1
KDM3B	0.1509981	0.2601195	0.5805	0.562	0.183274993	count	1

CDC37L1	0.1384575	0.2093724	0.6613	0.508	0.183566948	count	1
SF3B1	0.1302528	0.0742807	1.7535	0.0796	0.184223874	count	1
ST13	0.12869	0.0513171	2.5077	0.0122	0.184246115	count	1
WRB	0.1343746	0.1438919	0.9339	0.35	0.18424686	count	1
STARD10	0.1445547	0.2461045	0.5874	0.557	0.184391073	count	1
SHROOM2	0.2589328	0.5449781	0.4751	0.635	0.18441798	count	1
ZNF816	0.2590456	0.6421026	0.4034	0.687	0.184493863	count	1
FARSA	0.1427043	0.1876697	0.7604	0.447	0.184570327	count	1
AC098487.1	0.2431599	0.5231939	0.4648	0.642	0.184723481	count	1
USP33	0.1379887	0.1571313	0.8782	0.38	0.184839619	count	1
TMEM237	0.1607962	0.2885281	0.5573	0.577	0.184889362	count	1
IFNAR2	0.1372394	0.183907	0.7462	0.456	0.184925708	count	1
FAM96A	0.1359568	0.1436143	0.9467	0.344	0.185012145	count	1
CEP112	0.1343786	0.1273007	1.0556	0.291	0.185032007	count	1
SSB	0.1298085	0.0570836	2.274	0.023	0.185093629	count	1
DNAJC12	0.2372041	0.5168475	0.4589	0.646	0.185275111	count	1
SLC35E3	0.1404213	0.2479047	0.5664	0.571	0.185296752	count	1
GPR82	0.5793753	0.9361844	0.6189	0.536	0.18537277	count	1
RAB9A	0.138662	0.16045	0.8642	0.388	0.185452476	count	1
SRP72	0.1308899	0.0738526	1.7723	0.0765	0.185505036	count	1
TMSB10	0.1289236	0.0249812	5.1608	2.63E-07	0.185872862	count	0.006169454
UTP14A	0.1445038	0.2033312	0.7107	0.477	0.185928241	count	1
ZRSR2	0.1535948	0.2225907	0.69	0.49	0.186010512	count	1
AC007114.2	0.3586305	0.7676371	0.4672	0.64	0.1860347	count	1
NR2C1	0.1476497	0.2130727	0.693	0.488	0.18642159	count	1
MOCS3	0.5834607	0.6597215	0.8844	0.377	0.186433746	count	1
MXD3	0.3601635	1.109316	0.3247	0.745	0.186752633	count	1
COMMD10	0.1369077	0.1413829	0.9683	0.333	0.186800929	count	1
EAPP	0.1329394	0.091187	1.4579	0.145	0.186950489	count	1
PCDHGA10	0.1696192	0.3580798	0.4737	0.636	0.186959004	count	1
SLC18B1	0.214789	0.3872878	0.5546	0.579	0.187031198	count	1
EIF3G	0.1308704	0.0516546	2.5336	0.0113	0.187053208	count	1
MS4A6A	0.2018096	0.5440898	0.3709	0.711	0.187053269	count	1
FAM193B	0.2151171	0.3647393	0.5898	0.555	0.187306557	count	1
TUSC1	0.136659	0.1282221	1.0658	0.287	0.187336742	count	1
BCL7C	0.1324312	0.0782409	1.6926	0.0906	0.187414343	count	1
PSKH1	0.1649186	0.4005365	0.4117	0.681	0.187483981	count	1
RAD17	0.1504121	0.1897447	0.7927	0.428	0.187636993	count	1
ACSM1	0.5890233	1.0462768	0.563	0.574	0.187873231	count	1
AP002433.1	0.5890233	1.0624537	0.5544	0.579	0.187873231	count	1
MCF2L-AS1	0.5890233	1.0624537	0.5544	0.579	0.187873231	count	1
SLC16A8	0.5890233	1.14478	0.5145	0.607	0.187873231	count	1
EIF4A1	0.1327783	0.073355	1.8101	0.0704	0.187928729	count	1
RHBDL2	0.3952899	0.8109777	0.4874	0.626	0.18794246	count	1
AL356056.2	0.3952899	0.8557956	0.4619	0.644	0.18794246	count	1

TBC1D4	0.1596258	0.2701252	0.5909	0.555	0.188189302	count	1
ZFX	0.1459904	0.1726869	0.8454	0.398	0.188321103	count	1
MDFI	0.148318	0.2008627	0.7384	0.46	0.188347352	count	1
PARS2	0.7431726	1.0307432	0.721	0.471	0.188536368	count	1
NOMO2	0.7431726	1.0307432	0.721	0.471	0.188536368	count	1
ERP29	0.1322503	0.0590445	2.2398	0.0252	0.18864025	count	1
RAD54L2	0.1760041	0.3071528	0.573	0.567	0.188686352	count	1
CHEK2	0.3647517	0.5569252	0.6549	0.513	0.188897518	count	1
SAMD12	0.163209	0.2920739	0.5588	0.576	0.188903435	count	1
AL365203.2	0.3187285	0.6588366	0.4838	0.629	0.188946798	count	1
SNX5	0.1358267	0.1082489	1.2548	0.21	0.188962128	count	1
SLBP	0.1387933	0.139827	0.9926	0.321	0.189201774	count	1
CAMK2G	0.1477959	0.2311961	0.6393	0.523	0.189252101	count	1
ORC2	0.1563355	0.2355455	0.6637	0.507	0.189294197	count	1
CNTROB	0.2765187	0.5702823	0.4849	0.628	0.189338571	count	1
TMEM147-AS1	1.061359	1.033803	1.0267	0.305	0.189369249	count	1
PPAN	0.1621223	0.2671196	0.6069	0.544	0.189445284	count	1
AC018647.2	0.2771285	0.5472781	0.5064	0.613	0.189730376	count	1
KRTCAP2	0.1335615	0.0702742	1.9006	0.0575	0.189803504	count	1
LGALSL	0.1436652	0.1829472	0.7853	0.432	0.189859138	count	1
AGPAT3	0.153135	0.2568016	0.5963	0.551	0.189861185	count	1
IER5	0.136123	0.1140025	1.194	0.233	0.1899829	count	1
SNAP25	0.59751	0.6787438	0.8803	0.379	0.190058016	count	1
SMC5	0.1429976	0.1423126	1.0048	0.315	0.190094726	count	1
RPS6KA4	0.1977021	0.2949755	0.6702	0.503	0.190146306	count	1
MORN2	0.1440962	0.1880325	0.7663	0.444	0.19022357	count	1
EGLN2	0.3676243	0.778425	0.4723	0.637	0.190237414	count	1
SLC7A6OS	0.1490345	0.211377	0.7051	0.481	0.190258773	count	1
PPT1	0.1385543	0.1259705	1.0999	0.271	0.190349665	count	1
KLHDC2	0.139019	0.1381341	1.0064	0.314	0.190389169	count	1
AC092053.2	0.5990937	1.1692519	0.5124	0.608	0.190464185	count	1
DNAAF3	0.5990937	1.1683556	0.5128	0.608	0.190464185	count	1
MMP24	0.5990937	1.1747996	0.51	0.61	0.190464185	count	1
ARF6	0.1375505	0.1035966	1.3278	0.184	0.19052399	count	1
INTS6	0.1379862	0.1042151	1.3241	0.186	0.190715849	count	1
AC072061.1	0.3428573	0.7316211	0.4686	0.639	0.190771986	count	1
NYAP1	0.7551317	1.0631043	0.7103	0.478	0.190799121	count	1
AL445248.1	0.7551317	1.122288	0.6729	0.501	0.190799121	count	1
POLD1	0.4466783	0.617329	0.7236	0.469	0.190955501	count	1
ZNFX1	0.1416402	0.1679383	0.8434	0.399	0.191012462	count	1
TSPAN9	0.1394478	0.1314099	1.0612	0.289	0.191148419	count	1
ZNF846	0.1795833	0.3594043	0.4997	0.617	0.191277441	count	1
SAP30BP	0.1391437	0.1146447	1.2137	0.225	0.191310876	count	1
MCC	0.1705465	0.2857399	0.5969	0.551	0.191438496	count	1
AC004918.1	0.2455485	0.953933	0.2574	0.797	0.19148155	count	1

DHRS7B	0.1417785	0.1603552	0.8842	0.377	0.191488746	count	1
MAPK12	0.1473009	0.1805462	0.8159	0.415	0.191514007	count	1
PIAS1	0.1447971	0.2002277	0.7232	0.47	0.191643548	count	1
TXNDC12	0.1359696	0.084039	1.6179	0.106	0.191664253	count	1
AC092279.1	0.603849	0.96827	0.6236	0.533	0.191680942	count	1
ZSCAN25	0.23406	0.4564257	0.5128	0.608	0.191706869	count	1
LETM1	0.1511782	0.2111556	0.716	0.474	0.191736669	count	1
EPM2AIP1	0.152359	0.2419804	0.6296	0.529	0.191844414	count	1
C18orf32	0.1695725	0.2431732	0.6973	0.486	0.19193929	count	1
SNAP23	0.1372196	0.097515	1.4072	0.159	0.192002303	count	1
YME1L1	0.1371012	0.0935113	1.4661	0.143	0.192061254	count	1
VWA8	0.2401048	0.4940209	0.486	0.627	0.19206869	count	1
MTCH1	0.1357012	0.0710223	1.9107	0.0561	0.192118097	count	1
RABL2B	0.2295697	0.4189883	0.5479	0.584	0.19213045	count	1
AL158835.1	0.1901991	0.7162723	0.2655	0.791	0.192254488	count	1
CREM	0.1413411	0.1453403	0.9725	0.331	0.192403485	count	1
SOD2	0.134771	0.0888934	1.5161	0.13	0.192455465	count	1
SNRPC	0.1370239	0.0890678	1.5384	0.124	0.192534724	count	1
SNRPD2	0.1347411	0.0522364	2.5794	0.01	0.192575729	count	1
BCKDK	0.1463694	0.1705764	0.8581	0.391	0.192673176	count	1
SOS2	0.1469277	0.2090635	0.7028	0.482	0.192715151	count	1
BCAR3	0.1637147	0.2233052	0.7331	0.464	0.192948471	count	1
SNX6	0.1356029	0.0679714	1.995	0.0461	0.19307906	count	1
LINC01695	0.7674375	1.1800149	0.6504	0.516	0.193105092	count	1
AC114271.1	0.6096015	1.071166	0.5691	0.569	0.193147119	count	1
NUP210L	0.6096015	1.0907615	0.5589	0.576	0.193147119	count	1
TRIM24	0.1538184	0.1910261	0.8052	0.421	0.193170109	count	1
FPGS	0.1599523	0.2463067	0.6494	0.516	0.193216547	count	1
RBBP8	0.1578167	0.2190793	0.7204	0.471	0.19331495	count	1
XRCC4	0.1715215	0.2611016	0.6569	0.511	0.193324697	count	1
TRRAP	0.1755495	0.3858277	0.455	0.649	0.193380337	count	1
NKX3-1	0.2118945	0.3849205	0.5505	0.582	0.193434736	count	1
TRMT10A	0.1748198	0.2823743	0.6191	0.536	0.193523862	count	1
NEK8	0.3098382	0.6209426	0.499	0.618	0.193552269	count	1
SDHAF2	0.1408753	0.1340967	1.0506	0.294	0.193560745	count	1
CTIF	0.179568	0.3389625	0.5298	0.596	0.19356297	count	1
EXOC8	0.1777796	0.2734298	0.6502	0.516	0.193800183	count	1
NOSIP	0.1376775	0.0919649	1.4971	0.134	0.193811758	count	1
ADAT3	1.1014591	1.194945	0.9218	0.357	0.193818462	count	1
TMEM241	0.2959335	0.5508837	0.5372	0.591	0.193903888	count	1
PFAS	0.3280076	0.5640024	0.5816	0.561	0.193998411	count	1
ARRDC3	0.1390855	0.1399318	0.994	0.32	0.194020831	count	1
FAF1	0.1565932	0.2105659	0.7437	0.457	0.194108558	count	1
SAMD9	0.1436147	0.1475818	0.9731	0.331	0.194350431	count	1
UNKL	0.2130259	0.3877191	0.5494	0.583	0.194433225	count	1

SERTAD4-AS1	0.1405085	0.1090957	1.2879	0.198	0.194522056	count	1
VEZF1	0.1423797	0.1318702	1.0797	0.28	0.194756109	count	1
ZNF446	0.1985533	0.375289	0.5291	0.597	0.195002776	count	1
PDIK1L	0.1986278	0.4081894	0.4866	0.627	0.195073971	count	1
ANAPC7	0.1622689	0.2235904	0.7257	0.468	0.195124955	count	1
RPS26	0.1354617	0.0239625	5.6531	1.74E-08	0.195143031	count	0.000410797
PPP1R10	0.1378924	0.0763538	1.806	0.071	0.195216869	count	1
EGFL8	0.5220406	1.1097655	0.4704	0.638	0.195235381	count	1
AASS	0.1811597	0.2630418	0.6887	0.491	0.195245275	count	1
FAM219A	0.1592144	0.2570261	0.6194	0.536	0.195360648	count	1
ZNF441	0.2057743	0.4753386	0.4329	0.665	0.195433668	count	1
CPNE2	0.1403428	0.107176	1.3095	0.19	0.195437562	count	1
CENPO	0.78009	1.0594841	0.7363	0.462	0.195452545	count	1
NHS	0.78009	1.1106836	0.7024	0.483	0.195452545	count	1
NDST2	0.78009	1.1836432	0.6591	0.51	0.195452545	count	1
DET1	0.2142545	0.4547621	0.4711	0.638	0.195517069	count	1
ZNF623	0.2058987	0.4696733	0.4384	0.661	0.19554828	count	1
AL160006.1	0.6195796	0.9789903	0.6329	0.527	0.195675474	count	1
EBLN3P	0.1454168	0.1545762	0.9407	0.347	0.195772951	count	1
PARP4	0.148533	0.1776873	0.8359	0.403	0.195835493	count	1
GLMN	0.185154	0.3406916	0.5435	0.587	0.19583691	count	1
PINX1	0.160277	0.2523728	0.6351	0.525	0.195936703	count	1
CCDC36	0.2761542	0.6301783	0.4382	0.661	0.195956857	count	1
MCMBP	0.1547998	0.1842641	0.8401	0.401	0.196069379	count	1
EIF4A3	0.1415113	0.1022551	1.3839	0.166	0.19633817	count	1
ZNF532	0.159753	0.2611675	0.6117	0.541	0.196360111	count	1
CHAF1A	0.622855	0.6314215	0.9864	0.324	0.19650126	count	1
RPS5	0.1364129	0.0238132	5.7285	1.12E-08	0.196552897	count	0.000264555
C1GALT1C1	0.1441513	0.140333	1.0272	0.304	0.196609253	count	1
GDI2	0.1389496	0.0670735	2.0716	0.0384	0.196628648	count	1
AC011468.5	0.5264999	0.7718655	0.6821	0.495	0.196631425	count	1
ATG10	0.1763794	0.2851445	0.6186	0.536	0.197018098	count	1
RPN1	0.1417904	0.1096801	1.2928	0.196	0.197150491	count	1
RNF6	0.1540443	0.1928995	0.7986	0.425	0.197194095	count	1
MTFR1	0.2194631	0.3790852	0.5789	0.563	0.197207019	count	1
CRKL	0.1719572	0.3006884	0.5719	0.567	0.197531877	count	1
DAP3	0.1487248	0.1404239	1.0591	0.29	0.197579325	count	1
MRPL37	0.1441535	0.1323497	1.0892	0.276	0.197718611	count	1
LEPROT	0.1379762	0.0468434	2.9455	0.00325	0.197917673	count	1
AIP	0.141475	0.0937497	1.5091	0.131	0.198154087	count	1
SENP5	0.1512564	0.1791382	0.8444	0.399	0.198359549	count	1
C2CD2L	0.2799082	0.4991008	0.5608	0.575	0.19845991	count	1
STX4	0.1482236	0.1495572	0.9911	0.322	0.198709317	count	1
DIP2C	0.221267	0.4018925	0.5506	0.582	0.198770436	count	1
TMEM138	0.1612688	0.257879	0.6254	0.532	0.19888123	count	1

PCYT2	0.1816414	0.2804961	0.6476	0.517	0.198965741	count	1
MRPL38	0.4217984	1.0518498	0.401	0.688	0.199048604	count	1
EEF1AKMT1	0.1561388	0.2177056	0.7172	0.473	0.199053557	count	1
RHNO1	0.1801409	0.3460346	0.5206	0.603	0.199309062	count	1
VHL	0.1717952	0.2810532	0.6113	0.541	0.199345349	count	1
FXR2	0.185129	0.3626721	0.5105	0.61	0.199437842	count	1
RNF19A	0.1434321	0.114374	1.2541	0.21	0.199501522	count	1
RNF148	0.535989	1.0533278	0.5089	0.611	0.199587525	count	1
CARNS1	0.535989	1.1359852	0.4718	0.637	0.199587525	count	1
RPS6	0.138503	0.0219543	6.3087	3.27E-10	0.199625305	count	7.76E-06
NIPA2	0.1556336	0.1829379	0.8507	0.395	0.199781171	count	1
NCL	0.1393921	0.0487863	2.8572	0.00431	0.199928276	count	1
TBC1D10A	0.1652853	0.1974607	0.8371	0.403	0.200008052	count	1
CCDC115	0.1452703	0.1249981	1.1622	0.245	0.200046375	count	1
XPNPEP3	0.1731309	0.402621	0.43	0.667	0.200219787	count	1
BTRC	0.1801902	0.296448	0.6078	0.543	0.20029597	count	1
AP001816.1	0.1471252	0.1493962	0.9848	0.325	0.200341073	count	1
ABCF2	0.174456	0.2625774	0.6644	0.506	0.200358816	count	1
RANBP9	0.1550675	0.1983089	0.7819	0.434	0.200451114	count	1
BBS10	0.2138897	0.4150279	0.5154	0.606	0.20045179	count	1
MTA3	0.1672425	0.2567149	0.6515	0.515	0.200576425	count	1
INCA1	0.4728841	1.0195224	0.4638	0.643	0.200597078	count	1
7-Sep	0.1400682	0.0452666	3.0943	0.00199	0.200910642	count	1
FGD5-AS1	0.1450099	0.1104146	1.3133	0.189	0.200965454	count	1
IFRD1	0.145176	0.1082457	1.3412	0.18	0.201128143	count	1
GCC2	0.1425419	0.0894582	1.5934	0.111	0.201141672	count	1
RPS21	0.1399658	0.0294577	4.7514	2.12E-06	0.201265788	count	0.04947444
CELF1	0.1506845	0.2171081	0.6941	0.488	0.201296072	count	1
PNRC1	0.1399656	0.038249	3.6593	0.000258	0.201308086	count	1
PEX13	0.1592393	0.2016148	0.7898	0.43	0.201411344	count	1
NAB2	0.1727018	0.2933631	0.5887	0.556	0.20163476	count	1
STK35	0.1884473	0.3489727	0.54	0.589	0.201750928	count	1
ANAPC2	0.2469693	0.4566171	0.5409	0.589	0.201800394	count	1
CREB3L4	0.1686818	0.2880568	0.5856	0.558	0.201808718	count	1
CWF19L2	0.1459376	0.1456376	1.0021	0.316	0.201827013	count	1
CCDC22	0.1691278	0.2633912	0.6421	0.521	0.201850451	count	1
RTCB	0.1473069	0.1156418	1.2738	0.203	0.201851199	count	1
ZFP2	0.428871	0.694304	0.6177	0.537	0.201980637	count	1
FOXO3	0.1535134	0.1675168	0.9164	0.36	0.202023212	count	1
PHF12	0.1854808	0.3277222	0.566	0.571	0.202033144	count	1
CBX4	0.1670143	0.2586062	0.6458	0.518	0.202076241	count	1
VPS33B	0.393266	0.5444362	0.7223	0.47	0.202096518	count	1
ZBTB20-AS2	0.3250004	0.5077868	0.64	0.522	0.202284888	count	1
FBXL18	0.5450785	1.071377	0.5088	0.611	0.202400782	count	1
MYZAP	0.5450785	1.071377	0.5088	0.611	0.202400782	count	1

CBY1	0.1504752	0.1390604	1.0821	0.279	0.202487328	count	1
OSBPL10	0.1597995	0.2147077	0.7443	0.457	0.202580762	count	1
GPKOW	0.1658306	0.2615481	0.634	0.526	0.202652584	count	1
H6PD	0.1557012	0.2070443	0.752	0.452	0.202659341	count	1
NOC4L	0.1752734	0.2734631	0.6409	0.522	0.202660757	count	1
RPL39	0.1407593	0.0232534	6.0533	1.61E-09	0.202837984	count	3.81E-05
EPM2A	0.4310422	0.6196842	0.6956	0.487	0.202878101	count	1
HMG20A	0.1663628	0.2710765	0.6137	0.539	0.2029128	count	1
RFC1	0.1435209	0.0799806	1.7944	0.0729	0.203095122	count	1
SNHG15	0.1509984	0.1368067	1.1037	0.27	0.20312101	count	1
SHOC2	0.1480719	0.1286488	1.151	0.25	0.203250467	count	1
AC118553.1	0.4803236	1.0528615	0.4562	0.648	0.203303752	count	1
POLE2	0.4803236	1.1335337	0.4237	0.672	0.203303752	count	1
GATAD2B	0.1530627	0.1736774	0.8813	0.378	0.203312364	count	1
SMAD9	0.1503968	0.1423488	1.0565	0.291	0.203377061	count	1
MFSD5	0.1766072	0.294986	0.5987	0.549	0.203495075	count	1
POMGNT2	0.25525	0.5105619	0.4999	0.617	0.20359824	count	1
SAT2	0.1448178	0.0910771	1.5901	0.112	0.203601193	count	1
SUGT1	0.1440128	0.0807109	1.7843	0.0745	0.203785194	count	1
CX3CL1	0.150005	0.2161845	0.6939	0.488	0.204011526	count	1
TMEM19	0.1636652	0.2432704	0.6728	0.501	0.204013754	count	1
BX088645.1	0.8273562	1.0180743	0.8127	0.416	0.204014013	count	1
CAMTA2	0.1875997	0.266733	0.7033	0.482	0.20429588	count	1
RPS15A	0.1417536	0.0219076	6.4705	1.15E-10	0.204341616	count	2.73E-06
TMEM219	0.1430382	0.0518784	2.7572	0.00587	0.204596417	count	1
PCCA	0.1764069	0.2627385	0.6714	0.502	0.204617767	count	1
ESCO1	0.1560115	0.1739696	0.8968	0.37	0.204808221	count	1
ZNF384	0.1880817	0.2822197	0.6664	0.505	0.204810451	count	1
BUD23	0.1462849	0.0975685	1.4993	0.134	0.205181909	count	1
SETD1B	0.213829	0.3998553	0.5348	0.593	0.205185816	count	1
NUTM2A-AS1	0.1613471	0.1985818	0.8125	0.417	0.205205301	count	1
URB1	0.197117	0.3322466	0.5933	0.553	0.205386649	count	1
PRPF40A	0.1457648	0.0852223	1.7104	0.0873	0.205451951	count	1
AGAP3	0.1720007	0.2328276	0.7387	0.46	0.20572962	count	1
BDNF-AS	0.2371665	0.5519766	0.4297	0.667	0.205737176	count	1
AQR	0.1626738	0.2008386	0.81	0.418	0.205958	count	1
SUPT7L	0.1682911	0.2669011	0.6305	0.528	0.206006207	count	1
MAP3K11	0.1489395	0.1002135	1.4862	0.137	0.206066919	count	1
WASF2	0.1438928	0.0522828	2.7522	0.00596	0.206077867	count	1
PPP1R15A	0.1438032	0.0542258	2.6519	0.00805	0.206145183	count	1
AP001043.1	0.4881806	1.0736808	0.4547	0.649	0.206147781	count	1
GRB7	0.4881806	1.1438859	0.4268	0.67	0.206147781	count	1
MIS18BP1	0.1475417	0.1122503	1.3144	0.189	0.206184329	count	1
LINC00663	1.222943	1.0569253	1.1571	0.247	0.206385792	count	1
FBXO8	0.1569866	0.2034857	0.7715	0.44	0.206567008	count	1

SUPT16H	0.1490121	0.1191159	1.251	0.211	0.206632445	count	1
ZNF839	0.2534051	0.4644375	0.5456	0.585	0.206813137	count	1
SDHD	0.1456678	0.0714373	2.0391	0.0415	0.206852934	count	1
SIK3	0.1622025	0.226171	0.7172	0.473	0.206935036	count	1
TWSG1	0.1665653	0.2448625	0.6802	0.496	0.206981536	count	1
WASHC2A	0.167392	0.2632139	0.636	0.525	0.207030946	count	1
EDEM1	0.1763533	0.3058791	0.5765	0.564	0.207052945	count	1
TRA2B	0.1476854	0.0864289	1.7088	0.0876	0.207220213	count	1
SF3B4	0.1525921	0.1484901	1.0276	0.304	0.207266133	count	1
PRUNE1	0.2349623	0.5735301	0.4097	0.682	0.20734025	count	1
RPL31	0.144309	0.0336833	4.2843	1.90E-05	0.207354476	count	0.439736
MAGOH	0.1470849	0.0805934	1.825	0.0681	0.207755784	count	1
AC093249.6	0.849402	1.0398457	0.8169	0.414	0.207896872	count	1
LINC01765	0.849402	1.0603892	0.801	0.423	0.207896872	count	1
SUPT6H	0.1568426	0.1866924	0.8401	0.401	0.207911075	count	1
PPP1R12A	0.1477954	0.0944993	1.564	0.118	0.208050273	count	1
GRAMD1C	0.1902777	0.3408364	0.5583	0.577	0.208240719	count	1
PURA	0.1480765	0.1012077	1.4631	0.144	0.20824076	count	1
EEF1AKMT3	0.24972	0.46575	0.5362	0.592	0.208241907	count	1
LRRC47	0.168728	0.2117322	0.7969	0.426	0.208328389	count	1
SCCPDH	0.1540327	0.1446043	1.0652	0.287	0.208449486	count	1
EIF2S1	0.1508479	0.1222424	1.234	0.217	0.208522667	count	1
RNF14	0.1762901	0.3170934	0.556	0.578	0.208693353	count	1
SPEF2	0.2176235	0.4000526	0.544	0.586	0.20871373	count	1
COPS7B	0.1854347	0.3107102	0.5968	0.551	0.20873483	count	1
AC073896.2	0.2954528	0.4480473	0.6594	0.51	0.208777796	count	1
POM121	0.1722741	0.2647945	0.6506	0.515	0.208797223	count	1
EPHX4	0.1827874	0.3330139	0.5489	0.583	0.209025207	count	1
RBM28	0.1633891	0.2147043	0.761	0.447	0.209062328	count	1
TRIAP1	0.1536871	0.1456426	1.0552	0.291	0.209195611	count	1
F11R	0.161512	0.1898926	0.8505	0.395	0.209225142	count	1
DTYMK	0.1568572	0.1787636	0.8775	0.38	0.209243716	count	1
COG4	0.1727063	0.2244909	0.7693	0.442	0.209314867	count	1
THAP9-AS1	0.1574918	0.1599514	0.9846	0.325	0.209356326	count	1
CEBPZOS	0.1532475	0.1314738	1.1656	0.244	0.209363897	count	1
TIMM22	0.1664717	0.1903951	0.8743	0.382	0.209453678	count	1
COASY	0.1689372	0.2164615	0.7804	0.435	0.209579907	count	1
ANKRD13D	0.1664322	0.1958255	0.8499	0.395	0.209667238	count	1
UACA	0.1480799	0.0897181	1.6505	0.099	0.209746586	count	1
PLBD1	0.1674058	0.2344561	0.714	0.475	0.209796799	count	1
ARRDC4	0.2340526	0.5774225	0.4053	0.685	0.209823683	count	1
E2F4	0.154495	0.138563	1.115	0.265	0.210094945	count	1
PEX5	0.2343759	0.3605776	0.65	0.516	0.210102542	count	1
ARHGAP39	0.8622452	1.1717845	0.7358	0.462	0.210127083	count	1
TMCO3	0.156393	0.1776554	0.8803	0.379	0.21020329	count	1

FRA10AC1	0.1514852	0.1189565	1.2735	0.203	0.210418085	count	1
PPP2R2A	0.1493638	0.0911114	1.6394	0.101	0.210446058	count	1
TICAM1	0.1752132	0.2661327	0.6584	0.51	0.210496087	count	1
ARFRP1	0.1537326	0.1337834	1.1491	0.251	0.210588856	count	1
AC116913.1	0.3234723	0.6256069	0.5171	0.605	0.210597193	count	1
CDC73	0.151105	0.1182587	1.2778	0.201	0.21060151	count	1
STARD8	0.182995	0.2632121	0.6952	0.487	0.210739497	count	1
AC007032.1	0.2199559	0.4744547	0.4636	0.643	0.210880234	count	1
ACSL5	0.1706822	0.202913	0.8412	0.4	0.211057887	count	1
AC100786.1	0.5018498	0.7982508	0.6287	0.53	0.211060124	count	1
SLC45A3	0.5018498	0.8366869	0.5998	0.549	0.211060124	count	1
IPPK	0.3836772	0.5543093	0.6922	0.489	0.211225393	count	1
FAM129B	0.1609719	0.1534386	1.0491	0.294	0.211406874	count	1
RBMS1	0.1508936	0.0874804	1.7249	0.0847	0.211529642	count	1
MLKL	0.1631804	0.1577466	1.0344	0.301	0.211535929	count	1
SHC1	0.1532744	0.1144679	1.339	0.181	0.211540627	count	1
STEAP3	0.1787892	0.2871176	0.6227	0.534	0.211611218	count	1
STX8	0.1571836	0.1497622	1.0496	0.294	0.211612209	count	1
KIAA1257	0.4144333	0.6270699	0.6609	0.509	0.211749397	count	1
ALKBH4	0.1816099	0.2722705	0.667	0.505	0.211881216	count	1
STAT3	0.1487198	0.0648582	2.293	0.0219	0.21189468	count	1
WDR47	0.1891968	0.3116243	0.6071	0.544	0.211997775	count	1
C1orf109	0.197086	0.2879388	0.6845	0.494	0.212044276	count	1
ENTPD5	0.2239137	0.396445	0.5648	0.572	0.212099119	count	1
PRRG3	0.6863451	1.0423614	0.6585	0.51	0.212114678	count	1
CCDC57	0.2407866	0.4773757	0.5044	0.614	0.21227482	count	1
NR5A2	0.1624741	0.2159124	0.7525	0.452	0.212294422	count	1
PLEKHF1	0.1695571	0.2463705	0.6882	0.491	0.21246717	count	1
USP37	0.1832956	0.2705396	0.6775	0.498	0.212485351	count	1
TMEM131L	0.233588	0.4144902	0.5636	0.573	0.212513867	count	1
UBOX5	0.3433152	0.7033791	0.4881	0.626	0.212740595	count	1
INAFM1	0.1775632	0.2823154	0.629	0.529	0.212795604	count	1
UBA7	0.2033367	0.3117611	0.6522	0.514	0.213193235	count	1
PDCD10	0.1515707	0.0904065	1.6765	0.0937	0.213276216	count	1
NSMCE1	0.1540225	0.1090477	1.4124	0.158	0.213279634	count	1
MBIP	0.1623234	0.1802426	0.9006	0.368	0.213297483	count	1
ZNF337	0.186932	0.3194258	0.5852	0.558	0.213686162	count	1
KRAS	0.1521767	0.0847364	1.7959	0.0726	0.213727918	count	1
TMEM222	0.1602382	0.1672169	0.9583	0.338	0.213730572	count	1
PIK3C2A	0.1523333	0.094228	1.6166	0.106	0.213752566	count	1
ATG2B	0.177683	0.2936678	0.605	0.545	0.213902819	count	1
ATOH8	0.1786087	0.2030444	0.8797	0.379	0.214032343	count	1
ZNF780A	0.1737876	0.2632784	0.6601	0.509	0.214153054	count	1
MVD	0.1938862	0.2589119	0.7488	0.454	0.214223064	count	1
FOCAD-AS1	15.8064403	1591.705555	0.0099	0.992	0.214284072	count	1

AC136469.1	15.9579307	1169.134637	0.0136	0.9891	0.214284077	count	1
AL034417.2	16.0812974	2250.649461	0.0071	0.994	0.21428408	count	1
AC104248.1	16.0812974	2250.649463	0.0071	0.994	0.21428408	count	1
MREG	16.081359	1813.694207	0.0089	0.993	0.21428408	count	1
AC018553.1	16.0813636	1813.693826	0.0089	0.993	0.21428408	count	1
AC069503.2	16.0813644	1813.694512	0.0089	0.993	0.21428408	count	1
AL160272.1	16.0813658	1813.694134	0.0089	0.993	0.21428408	count	1
BEGAIN	16.0813658	1813.694136	0.0089	0.993	0.21428408	count	1
AC005264.1	16.0813658	1813.694133	0.0089	0.993	0.21428408	count	1
AC124016.2	16.1173956	1131.021599	0.0143	0.9886	0.214284081	count	1
IGSF10	16.1954455	2604.288729	0.0062	0.995	0.214284083	count	1
AC090844.3	16.1954455	2604.288706	0.0062	0.995	0.214284083	count	1
AC022382.1	16.1954456	2604.288712	0.0062	0.995	0.214284083	count	1
CPNE4	16.1954456	2604.288729	0.0062	0.995	0.214284083	count	1
AL391807.1	16.1954456	2604.288734	0.0062	0.995	0.214284083	count	1
ADAP1	16.1954456	2604.288706	0.0062	0.995	0.214284083	count	1
PRRT4	16.1954456	2604.28874	0.0062	0.995	0.214284083	count	1
AC079949.2	16.1954456	2604.288706	0.0062	0.995	0.214284083	count	1
RAPGEFL1	16.1954456	2604.288706	0.0062	0.995	0.214284083	count	1
HIST1H3E	16.1954481	2604.288695	0.0062	0.995	0.214284083	count	1
AC009318.4	16.1954501	2604.290123	0.0062	0.995	0.214284083	count	1
TMEM225B	16.1954502	2604.288431	0.0062	0.995	0.214284083	count	1
CXCR2	16.1954503	2604.288459	0.0062	0.995	0.214284083	count	1
AL080317.1	16.1954503	2604.288475	0.0062	0.995	0.214284083	count	1
AP006287.2	16.1954503	2604.288459	0.0062	0.995	0.214284083	count	1
CCDC38	16.1954503	2604.290139	0.0062	0.995	0.214284083	count	1
AL049780.2	16.1954503	2604.290161	0.0062	0.995	0.214284083	count	1
FOXJ1	16.1954503	2604.288475	0.0062	0.995	0.214284083	count	1
VPS33B-DT	16.1954504	2604.28847	0.0062	0.995	0.214284083	count	1
AC119403.1	16.1954504	2604.288459	0.0062	0.995	0.214284083	count	1
CRB3	16.1954504	2604.288475	0.0062	0.995	0.214284083	count	1
TMEM236	16.1954573	2604.289682	0.0062	0.995	0.214284083	count	1
AC005775.1	16.1957269	3330.79116	0.0049	0.996	0.214284083	count	1
MAP4K1	16.1957291	3330.791118	0.0049	0.996	0.214284083	count	1
HIST1H2AI	16.1957292	3330.791097	0.0049	0.996	0.214284083	count	1
AL512306.3	16.1957339	3330.792901	0.0049	0.996	0.214284083	count	1
AC080013.1	16.1957339	3330.792901	0.0049	0.996	0.214284083	count	1
FAM72B	16.2907275	1818.278145	0.009	0.993	0.214284084	count	1
AL596094.1	16.2907285	1818.278486	0.009	0.993	0.214284084	count	1
FRG1-DT	16.2907314	1818.280323	0.009	0.993	0.214284084	count	1
SSTR2	16.290732	1818.279079	0.009	0.993	0.214284084	count	1
RAD21-AS1	16.2907324	1818.279691	0.009	0.993	0.214284084	count	1
DENND2A	16.3775717	3179.446288	0.0052	0.996	0.214284086	count	1
TMEM35B	16.3775718	3179.44632	0.0052	0.996	0.214284086	count	1
TFR2	16.3775718	3179.44632	0.0052	0.996	0.214284086	count	1

EXTL3-AS1	16.3775718	3179.446383	0.0052	0.996	0.214284086	count	1
TNFSF14	16.3775718	3179.446367	0.0052	0.996	0.214284086	count	1
ITIH4	16.3775719	3179.446478	0.0052	0.996	0.214284086	count	1
LINC01102	16.377572	3179.446494	0.0052	0.996	0.214284086	count	1
LINC02427	16.377572	3179.446256	0.0052	0.996	0.214284086	count	1
UGT3A2	16.377572	3179.44632	0.0052	0.996	0.214284086	count	1
AC005618.1	16.377572	3179.446399	0.0052	0.996	0.214284086	count	1
AP000842.2	16.377572	3179.446367	0.0052	0.996	0.214284086	count	1
STK24-AS1	16.377572	3179.446383	0.0052	0.996	0.214284086	count	1
AC012174.1	16.377572	3179.446415	0.0052	0.996	0.214284086	count	1
LINC01909	16.377572	3179.446478	0.0052	0.996	0.214284086	count	1
AC024575.1	16.377572	3179.446573	0.0052	0.996	0.214284086	count	1
AC007773.1	16.377572	3179.446367	0.0052	0.996	0.214284086	count	1
Z73429.1	16.377572	3179.446241	0.0052	0.996	0.214284086	count	1
AC069148.1	16.3775721	3179.44643	0.0052	0.996	0.214284086	count	1
UPK1B	16.3775721	3179.446399	0.0052	0.996	0.214284086	count	1
AL354977.2	16.3775721	3179.446446	0.0052	0.996	0.214284086	count	1
CUZD1	16.3775721	3179.446494	0.0052	0.996	0.214284086	count	1
AC092338.2	16.3775721	3179.446446	0.0052	0.996	0.214284086	count	1
AC004069.1	16.3775722	3179.446494	0.0052	0.996	0.214284086	count	1
CDH12	16.3775722	3179.446367	0.0052	0.996	0.214284086	count	1
AC006042.2	16.3775722	3179.446383	0.0052	0.996	0.214284086	count	1
KCND2	16.3775722	3179.446557	0.0052	0.996	0.214284086	count	1
AC084357.2	16.3775722	3179.446509	0.0052	0.996	0.214284086	count	1
SRCIN1	16.3775722	3179.446541	0.0052	0.996	0.214284086	count	1
AC011451.1	16.3775722	3179.44643	0.0052	0.996	0.214284086	count	1
AC097059.1	16.3775723	3179.446415	0.0052	0.996	0.214284086	count	1
NRIP3	16.3775723	3179.446462	0.0052	0.996	0.214284086	count	1
AP000238.1	16.3775723	3179.44643	0.0052	0.996	0.214284086	count	1
LEFTY1	16.3783084	4499.87515	0.0036	0.997	0.214284086	count	1
AC005697.2	16.3783084	4499.875284	0.0036	0.997	0.214284086	count	1
CABP7	16.3783084	4499.87515	0.0036	0.997	0.214284086	count	1
GNGT1	16.3783085	4499.875351	0.0036	0.997	0.214284086	count	1
AC073529.1	16.3783085	4499.875418	0.0036	0.997	0.214284086	count	1
AC140125.2	16.3783086	4499.875239	0.0036	0.997	0.214284086	count	1
AC104316.2	16.3783086	4499.875597	0.0036	0.997	0.214284086	count	1
AC124254.1	16.3783086	4499.875597	0.0036	0.997	0.214284086	count	1
LINC01134	16.3783087	4499.875396	0.0036	0.997	0.214284086	count	1
AL355574.1	16.3783088	4499.875262	0.0036	0.997	0.214284086	count	1
AC135178.5	16.3783088	4499.875463	0.0036	0.997	0.214284086	count	1
RPS6KL1	16.396801	1549.260734	0.0106	0.9916	0.214284086	count	1
MAP9	16.4526101	1811.174522	0.0091	0.993	0.214284087	count	1
RFPL1S	16.4528294	1990.627381	0.0083	0.993	0.214284087	count	1
ARMC4	16.4772632	1782.25535	0.0092	0.993	0.214284088	count	1
C11orf65	16.5248147	1624.252484	0.0102	0.9919	0.214284089	count	1

AC012645.1	16.5871477	1801.58329	0.0092	0.993	0.214284089	count	1
AL359265.3	16.5891278	2597.48559	0.0064	0.995	0.214284089	count	1
TMPPE	16.5891288	2597.488007	0.0064	0.995	0.214284089	count	1
AL133346.1	16.5891289	2597.488018	0.0064	0.995	0.214284089	count	1
TAS2R30	16.589129	2597.488018	0.0064	0.995	0.214284089	count	1
AP000442.2	16.5891291	2597.488018	0.0064	0.995	0.214284089	count	1
OSBPL10-AS1	16.5891294	2597.486793	0.0064	0.995	0.214284089	count	1
AC109454.2	16.5891309	2597.489233	0.0064	0.995	0.214284089	count	1
NPTX1	16.5891357	2597.489047	0.0064	0.995	0.214284089	count	1
AC092587.1	16.5891393	2597.491432	0.0064	0.995	0.214284089	count	1
TRPM2-AS	16.5891415	2597.487646	0.0064	0.995	0.214284089	count	1
FCGR3A	16.5891418	2597.48641	0.0064	0.995	0.214284089	count	1
DCDC1	16.5891419	2597.48641	0.0064	0.995	0.214284089	count	1
PLEKHG6	16.589143	2597.488839	0.0064	0.995	0.214284089	count	1
AC104260.1	16.589143	2597.488839	0.0064	0.995	0.214284089	count	1
AP003392.5	16.5891434	2597.48885	0.0064	0.995	0.214284089	count	1
NRG3	16.5897408	3037.222323	0.0055	0.996	0.21428409	count	1
AL353622.1	16.5897451	3037.217818	0.0055	0.996	0.21428409	count	1
AC092802.1	16.7190272	2354.295751	0.0071	0.994	0.214284091	count	1
AL928970.1	16.7190286	2354.298201	0.0071	0.994	0.214284091	count	1
SLAMF8	16.7190345	2354.298025	0.0071	0.994	0.214284091	count	1
FGF22	16.7190345	2354.298025	0.0071	0.994	0.214284091	count	1
AC007383.3	16.7190432	2354.29785	0.0071	0.994	0.214284091	count	1
GLIS3-AS1	16.7190433	2354.297845	0.0071	0.994	0.214284091	count	1
F2RL1	16.7190452	2354.29906	0.0071	0.994	0.214284091	count	1
AC125603.3	16.7191308	3144.322756	0.0053	0.996	0.214284091	count	1
ENPP1	16.7582501	1802.23862	0.0093	0.993	0.214284092	count	1
AC009501.1	15.8053302	1342.174171	0.0118	0.9906	0.214284092	count	1
AC008537.4	16.8637875	2584.55139	0.0065	0.995	0.214284093	count	1
GOLGA8M	16.8637908	2584.553481	0.0065	0.995	0.214284093	count	1
EML5	16.8637917	2584.555638	0.0065	0.995	0.214284093	count	1
QRICH2	16.8637933	2584.55346	0.0065	0.995	0.214284093	count	1
DCST2	16.8637942	2584.552349	0.0065	0.995	0.214284093	count	1
AL121987.2	16.8778933	1779.058572	0.0095	0.9924	0.214284093	count	1
H2AFY2	15.8645632	1174.218025	0.0135	0.989	0.214284094	count	1
SLC29A2	15.8648797	1248.61995	0.0127	0.99	0.214284094	count	1
GLB1L2	17.0762026	2572.805774	0.0066	0.9947	0.214284095	count	1
AC068790.8	17.2265851	3111.987735	0.0055	0.996	0.214284096	count	1
AL080317.3	17.2280929	2573.750923	0.0067	0.995	0.214284096	count	1
AC034111.1	16.0705818	1167.397035	0.0138	0.989	0.2142841	count	1
GABBR2	16.0812875	2250.647534	0.0071	0.994	0.2142841	count	1
AC021086.1	16.081295	2250.649502	0.0071	0.994	0.2142841	count	1
AC007879.2	16.0812961	2250.650735	0.0071	0.994	0.2142841	count	1
AC005332.8	16.0813561	1813.692218	0.0089	0.993	0.2142841	count	1
ZSCAN23	16.0813591	1813.694227	0.0089	0.993	0.2142841	count	1

LINC01123	16.0813617	1813.692812	0.0089	0.993	0.2142841	count	1
CDK5R1	16.0813617	1813.692807	0.0089	0.993	0.2142841	count	1
TRPV4	16.0813633	1813.695192	0.0089	0.993	0.2142841	count	1
AQP10	16.0813634	1813.695196	0.0089	0.993	0.2142841	count	1
POU5F1B	16.0813634	1813.695196	0.0089	0.993	0.2142841	count	1
AC016722.3	16.0813645	1813.694514	0.0089	0.993	0.2142841	count	1
MSX2	16.0813645	1813.694515	0.0089	0.993	0.2142841	count	1
AP001029.2	16.0813646	1813.694145	0.0089	0.993	0.2142841	count	1
PARD6B	16.0813646	1813.695825	0.0089	0.993	0.2142841	count	1
CBARP	16.0813646	1813.694139	0.0089	0.993	0.2142841	count	1
CD226	16.0813647	1813.694153	0.0089	0.993	0.2142841	count	1
AL022157.1	16.0813648	1813.693781	0.0089	0.993	0.2142841	count	1
LINC00235	16.0813659	1813.694136	0.0089	0.993	0.2142841	count	1
EFCAB5	16.0813659	1813.694136	0.0089	0.993	0.2142841	count	1
AC009309.1	16.1264785	1062.688384	0.0152	0.9879	0.214284101	count	1
AC121338.2	16.1954455	2604.288718	0.0062	0.995	0.214284102	count	1
AL590434.1	16.1954456	2604.288695	0.0062	0.995	0.214284102	count	1
AP000442.1	16.1954456	2604.288706	0.0062	0.995	0.214284102	count	1
LINC02551	16.1954456	2604.288723	0.0062	0.995	0.214284102	count	1
AL137026.1	16.1954456	2604.288701	0.0062	0.995	0.214284102	count	1
ZGLP1	16.1954456	2604.288706	0.0062	0.995	0.214284102	count	1
PRKCG	16.1954456	2604.288701	0.0062	0.995	0.214284102	count	1
AL451042.2	16.1954457	2604.288723	0.0062	0.995	0.214284102	count	1
EFCAB12	16.1954457	2604.288712	0.0062	0.995	0.214284102	count	1
AC026412.3	16.1954457	2604.288734	0.0062	0.995	0.214284102	count	1
LY86	16.1954457	2604.288712	0.0062	0.995	0.214284102	count	1
AC004982.1	16.1954457	2604.288723	0.0062	0.995	0.214284102	count	1
DLX5	16.1954457	2604.288712	0.0062	0.995	0.214284102	count	1
LRFN5	16.1954457	2604.288723	0.0062	0.995	0.214284102	count	1
MAP3K9	16.1954457	2604.288723	0.0062	0.995	0.214284102	count	1
AC123768.3	16.195448	2604.28869	0.0062	0.995	0.214284102	count	1
AC009133.4	16.195448	2604.28869	0.0062	0.995	0.214284102	count	1
ZNF843	16.195448	2604.28869	0.0062	0.995	0.214284102	count	1
AC005329.2	16.195448	2604.288673	0.0062	0.995	0.214284102	count	1
FAM131C	16.1954502	2604.290123	0.0062	0.995	0.214284102	count	1
AC010173.1	16.1954502	2604.288442	0.0062	0.995	0.214284102	count	1
GRIN3B	16.1954502	2604.288453	0.0062	0.995	0.214284102	count	1
AC027319.1	16.1954502	2604.290134	0.0062	0.995	0.214284102	count	1
AC104667.2	16.1954503	2604.290145	0.0062	0.995	0.214284102	count	1
SMAD1-AS1	16.1954503	2604.290145	0.0062	0.995	0.214284102	count	1
AC005520.1	16.1954503	2604.288442	0.0062	0.995	0.214284102	count	1
AL137786.1	16.1954503	2604.288442	0.0062	0.995	0.214284102	count	1
AC009119.2	16.1954503	2604.290128	0.0062	0.995	0.214284102	count	1
ONECUT2	16.1954503	2604.288464	0.0062	0.995	0.214284102	count	1
AL109955.1	16.1954503	2604.288459	0.0062	0.995	0.214284102	count	1

AC008770.1	16.1954503	2604.288425	0.0062	0.995	0.214284102	count	1
AL360091.3	16.1954504	2604.290139	0.0062	0.995	0.214284102	count	1
KLB	16.1954504	2604.288481	0.0062	0.995	0.214284102	count	1
AC093799.1	16.1954504	2604.288464	0.0062	0.995	0.214284102	count	1
CCL27	16.1954504	2604.288475	0.0062	0.995	0.214284102	count	1
AP000808.1	16.1954504	2604.288464	0.0062	0.995	0.214284102	count	1
AC013565.3	16.1954504	2604.288459	0.0062	0.995	0.214284102	count	1
AC019254.1	16.1954504	2604.288486	0.0062	0.995	0.214284102	count	1
AC092115.2	16.1954504	2604.288475	0.0062	0.995	0.214284102	count	1
RNF112	16.1954504	2604.288459	0.0062	0.995	0.214284102	count	1
AC068234.2	16.1954504	2604.288459	0.0062	0.995	0.214284102	count	1
AC007663.2	16.1954504	2604.288459	0.0062	0.995	0.214284102	count	1
DRAXIN	16.1954526	2604.290112	0.0062	0.995	0.214284102	count	1
FCRL6	16.1954527	2604.290101	0.0062	0.995	0.214284102	count	1
AL590708.1	16.1954527	2604.290112	0.0062	0.995	0.214284102	count	1
EML6	16.1954528	2604.290117	0.0062	0.995	0.214284102	count	1
ADAM11	16.1954528	2604.290117	0.0062	0.995	0.214284102	count	1
PSD4	16.1954529	2604.290128	0.0062	0.995	0.214284102	count	1
AP002992.1	16.1957175	3330.789743	0.0049	0.996	0.214284102	count	1
TMEM200A	16.1957222	3330.791626	0.0049	0.996	0.214284102	count	1
HSD11B1	16.1957223	3330.791682	0.0049	0.996	0.214284102	count	1
E2F7	16.1957292	3330.797737	0.0049	0.996	0.214284102	count	1
AC079447.1	16.1957293	3330.795545	0.0049	0.996	0.214284102	count	1
TRIM34	16.1957293	3330.797744	0.0049	0.996	0.214284102	count	1
C16orf89	16.1957293	3330.79109	0.0049	0.996	0.214284102	count	1
LINC00482	16.1957294	3330.791125	0.0049	0.996	0.214284102	count	1
AC080013.5	16.1957315	3330.790787	0.0049	0.996	0.214284102	count	1
CYP21A2	16.1957315	3330.790773	0.0049	0.996	0.214284102	count	1
LINC01229	16.1957315	3330.79522	0.0049	0.996	0.214284102	count	1
MPP4	16.1957339	3330.792944	0.0049	0.996	0.214284102	count	1
STKLD1	16.2288102	1092.711526	0.0149	0.9882	0.214284103	count	1
TMEM31	16.2513469	1866.975096	0.0087	0.9931	0.214284104	count	1
FREM3	16.2905945	2256.435867	0.0072	0.994	0.214284104	count	1
TMEM40	16.2907277	1818.279407	0.009	0.993	0.214284104	count	1
DENND6A-DT	16.2907277	1818.279404	0.009	0.993	0.214284104	count	1
HIST1H2AK	16.29073	1818.27936	0.009	0.993	0.214284104	count	1
C11orf91	16.290732	1818.279079	0.009	0.993	0.214284104	count	1
AL353719.1	16.3069825	1068.064703	0.0153	0.9878	0.214284105	count	1
MYMX	16.3619029	1116.468059	0.0147	0.9883	0.214284106	count	1
AL606760.3	16.3635463	1080.227539	0.0151	0.9879	0.214284106	count	1
AL031432.3	16.3775718	3179.446446	0.0052	0.996	0.214284106	count	1
EXTL1	16.3775718	3179.446351	0.0052	0.996	0.214284106	count	1
AL136115.1	16.3775718	3179.446351	0.0052	0.996	0.214284106	count	1
EFR3B	16.3775718	3179.446383	0.0052	0.996	0.214284106	count	1
HCG14	16.3775718	3179.446415	0.0052	0.996	0.214284106	count	1

SPDYE1	16.3775718	3179.446462	0.0052	0.996	0.214284106	count	1
MIRLET7DHG	16.3775718	3179.446193	0.0052	0.996	0.214284106	count	1
UPK2	16.3775718	3179.446351	0.0052	0.996	0.214284106	count	1
KIF11	16.3775718	3179.446462	0.0052	0.996	0.214284106	count	1
AC022087.1	16.3775718	3179.446288	0.0052	0.996	0.214284106	count	1
AC022167.2	16.3775718	3179.446209	0.0052	0.996	0.214284106	count	1
AC012123.1	16.3775718	3179.446256	0.0052	0.996	0.214284106	count	1
LINC01311	16.3775718	3179.446209	0.0052	0.996	0.214284106	count	1
AL451007.2	16.3775719	3179.446509	0.0052	0.996	0.214284106	count	1
AC093459.1	16.3775719	3179.446272	0.0052	0.996	0.214284106	count	1
LINC01600	16.3775719	3179.446383	0.0052	0.996	0.214284106	count	1
LINC00265	16.3775719	3179.446383	0.0052	0.996	0.214284106	count	1
AC003991.1	16.3775719	3179.446383	0.0052	0.996	0.214284106	count	1
FAM86B2	16.3775719	3179.446383	0.0052	0.996	0.214284106	count	1
KCNC1	16.3775719	3179.446383	0.0052	0.996	0.214284106	count	1
AC079906.1	16.3775719	3179.446383	0.0052	0.996	0.214284106	count	1
EHD4-AS1	16.3775719	3179.446209	0.0052	0.996	0.214284106	count	1
AC005899.7	16.3775719	3179.446351	0.0052	0.996	0.214284106	count	1
GDF5	16.3775719	3179.446383	0.0052	0.996	0.214284106	count	1
JSRP1	16.3775719	3179.446383	0.0052	0.996	0.214284106	count	1
UPK3A	16.3775719	3179.446336	0.0052	0.996	0.214284106	count	1
MYT1L	16.377572	3179.446367	0.0052	0.996	0.214284106	count	1
FAM228A	16.377572	3179.446494	0.0052	0.996	0.214284106	count	1
AC013403.2	16.377572	3179.446367	0.0052	0.996	0.214284106	count	1
LYG1	16.377572	3179.446415	0.0052	0.996	0.214284106	count	1
AC097634.3	16.377572	3179.446351	0.0052	0.996	0.214284106	count	1
UBE2QL1	16.377572	3179.446494	0.0052	0.996	0.214284106	count	1
AL451064.1	16.377572	3179.446399	0.0052	0.996	0.214284106	count	1
AL136088.1	16.377572	3179.446446	0.0052	0.996	0.214284106	count	1
CCDC73	16.377572	3179.446351	0.0052	0.996	0.214284106	count	1
TIGD3	16.377572	3179.446494	0.0052	0.996	0.214284106	count	1
AP000911.2	16.377572	3179.446494	0.0052	0.996	0.214284106	count	1
DCUN1D2-AS	16.377572	3179.446462	0.0052	0.996	0.214284106	count	1
AC123768.4	16.377572	3179.446494	0.0052	0.996	0.214284106	count	1
AC023908.3	16.377572	3179.446288	0.0052	0.996	0.214284106	count	1
ACAN	16.377572	3179.446367	0.0052	0.996	0.214284106	count	1
SLC12A5-AS1	16.377572	3179.446272	0.0052	0.996	0.214284106	count	1
AC024592.3	16.377572	3179.446336	0.0052	0.996	0.214284106	count	1
ZNF560	16.377572	3179.446509	0.0052	0.996	0.214284106	count	1
AC010487.2	16.377572	3179.446383	0.0052	0.996	0.214284106	count	1
AC098484.2	16.3775721	3179.446383	0.0052	0.996	0.214284106	count	1
IL12RB2	16.3775721	3179.446336	0.0052	0.996	0.214284106	count	1
HIST2H4A	16.3775721	3179.446351	0.0052	0.996	0.214284106	count	1
SLC8A1-AS1	16.3775721	3179.446351	0.0052	0.996	0.214284106	count	1
CD86	16.3775721	3179.446478	0.0052	0.996	0.214284106	count	1

SERPINI2	16.3775721	3179.446336	0.0052	0.996	0.214284106	count	1
TMEM171	16.3775721	3179.446573	0.0052	0.996	0.214284106	count	1
AL023583.1	16.3775721	3179.446288	0.0052	0.996	0.214284106	count	1
HIST1H3C	16.3775721	3179.446415	0.0052	0.996	0.214284106	count	1
ZNF451-AS1	16.3775721	3179.44643	0.0052	0.996	0.214284106	count	1
CXorf58	16.3775721	3179.446446	0.0052	0.996	0.214284106	count	1
BRINP1	16.3775721	3179.446478	0.0052	0.996	0.214284106	count	1
AC136475.7	16.3775721	3179.446478	0.0052	0.996	0.214284106	count	1
AP000842.3	16.3775721	3179.446509	0.0052	0.996	0.214284106	count	1
AC005342.1	16.3775721	3179.446478	0.0052	0.996	0.214284106	count	1
LINC02415	16.3775721	3179.446478	0.0052	0.996	0.214284106	count	1
AL512506.3	16.3775721	3179.446478	0.0052	0.996	0.214284106	count	1
AC092757.3	16.3775721	3179.446478	0.0052	0.996	0.214284106	count	1
BTBD17	16.3775721	3179.446351	0.0052	0.996	0.214284106	count	1
NPHS1	16.3775721	3179.446288	0.0052	0.996	0.214284106	count	1
RHCE	16.3775722	3179.446446	0.0052	0.996	0.214284106	count	1
PCDHGA4	16.3775722	3179.446383	0.0052	0.996	0.214284106	count	1
TUBB2B	16.3775722	3179.446256	0.0052	0.996	0.214284106	count	1
TRGV9	16.3775722	3179.446383	0.0052	0.996	0.214284106	count	1
POU6F2	16.3775722	3179.446383	0.0052	0.996	0.214284106	count	1
CASC9	16.3775722	3179.446336	0.0052	0.996	0.214284106	count	1
AL359736.1	16.3775722	3179.446462	0.0052	0.996	0.214284106	count	1
LINC00637	16.3775722	3179.446367	0.0052	0.996	0.214284106	count	1
AC027237.2	16.3775722	3179.446415	0.0052	0.996	0.214284106	count	1
CCDC33	16.3775722	3179.446383	0.0052	0.996	0.214284106	count	1
ZNF132	16.3775722	3179.446288	0.0052	0.996	0.214284106	count	1
AL020996.1	16.3775723	3179.446478	0.0052	0.996	0.214284106	count	1
AC069277.1	16.3775723	3179.446494	0.0052	0.996	0.214284106	count	1
SPRY4-AS1	16.3775723	3179.446478	0.0052	0.996	0.214284106	count	1
AC104964.4	16.3775723	3179.446462	0.0052	0.996	0.214284106	count	1
MAMDC2-AS1	16.3775723	3179.446494	0.0052	0.996	0.214284106	count	1
AL136084.2	16.3775723	3179.446446	0.0052	0.996	0.214284106	count	1
AC022075.1	16.3775723	3179.446336	0.0052	0.996	0.214284106	count	1
LCTL	16.3775723	3179.44643	0.0052	0.996	0.214284106	count	1
AC022167.3	16.3775723	3179.446383	0.0052	0.996	0.214284106	count	1
CABYR	16.3775723	3179.446494	0.0052	0.996	0.214284106	count	1
TP53TG5	16.3775723	3179.446525	0.0052	0.996	0.214284106	count	1
EBI3	16.3775723	3179.446446	0.0052	0.996	0.214284106	count	1
CARD14	16.3775724	3179.44643	0.0052	0.996	0.214284106	count	1
AC011511.2	16.3775724	3179.44643	0.0052	0.996	0.214284106	count	1
AL360014.1	16.3783085	4499.87506	0.0036	0.997	0.214284106	count	1
ITGB7	16.3783085	4499.875239	0.0036	0.997	0.214284106	count	1
IL21R-AS1	16.3783085	4499.875508	0.0036	0.997	0.214284106	count	1
AL354824.1	16.3783085	4499.875441	0.0036	0.997	0.214284106	count	1
LINC00337	16.3783086	4499.875329	0.0036	0.997	0.214284106	count	1

MATN1	16.3783086	4499.875396	0.0036	0.997	0.214284106	count	1
ATP6V1G2	16.3783086	4499.87553	0.0036	0.997	0.214284106	count	1
AL138889.1	16.3783086	4499.875284	0.0036	0.997	0.214284106	count	1
TSPAN33	16.3783086	4499.87562	0.0036	0.997	0.214284106	count	1
AC011933.4	16.3783086	4499.875463	0.0036	0.997	0.214284106	count	1
AC010327.4	16.3783086	4499.875127	0.0036	0.997	0.214284106	count	1
SERPINC1	16.3783087	4499.875262	0.0036	0.997	0.214284106	count	1
PRSS40A	16.3783087	4499.875396	0.0036	0.997	0.214284106	count	1
COL4A3	16.3783087	4499.875262	0.0036	0.997	0.214284106	count	1
CLSTN2	16.3783087	4499.875508	0.0036	0.997	0.214284106	count	1
SPSB4	16.3783087	4499.875508	0.0036	0.997	0.214284106	count	1
EFCAB9	16.3783087	4499.875441	0.0036	0.997	0.214284106	count	1
AC013470.2	16.3783087	4499.875508	0.0036	0.997	0.214284106	count	1
AC092647.5	16.3783087	4499.875351	0.0036	0.997	0.214284106	count	1
AC021237.1	16.3783087	4499.875441	0.0036	0.997	0.214284106	count	1
AC022400.6	16.3783087	4499.875396	0.0036	0.997	0.214284106	count	1
LINC00621	16.3783087	4499.875217	0.0036	0.997	0.214284106	count	1
AC105020.2	16.3783087	4499.875441	0.0036	0.997	0.214284106	count	1
MATN4	16.3783087	4499.875351	0.0036	0.997	0.214284106	count	1
AC074135.1	16.3783087	4499.875239	0.0036	0.997	0.214284106	count	1
AL591848.3	16.3783088	4499.875418	0.0036	0.997	0.214284106	count	1
LINC01143	16.3783088	4499.875239	0.0036	0.997	0.214284106	count	1
TMEM156	16.3783088	4499.875239	0.0036	0.997	0.214284106	count	1
AL136309.2	16.3783088	4499.875239	0.0036	0.997	0.214284106	count	1
ALDH8A1	16.3783088	4499.875239	0.0036	0.997	0.214284106	count	1
AC013714.1	16.3783088	4499.875441	0.0036	0.997	0.214284106	count	1
CYP17A1	16.3783088	4499.875239	0.0036	0.997	0.214284106	count	1
AC023310.4	16.3783088	4499.875239	0.0036	0.997	0.214284106	count	1
AC009139.1	16.3783088	4499.875239	0.0036	0.997	0.214284106	count	1
AC005899.8	16.3783088	4499.875485	0.0036	0.997	0.214284106	count	1
AC087289.2	16.3783088	4499.875239	0.0036	0.997	0.214284106	count	1
AANAT	16.3783088	4499.875239	0.0036	0.997	0.214284106	count	1
FHOD3	16.3783088	4499.875239	0.0036	0.997	0.214284106	count	1
SLC13A3	16.3783088	4499.875239	0.0036	0.997	0.214284106	count	1
ZNF280B	16.3783088	4499.875485	0.0036	0.997	0.214284106	count	1
GRIK1	16.3783088	4499.875239	0.0036	0.997	0.214284106	count	1
AP000322.1	16.3783088	4499.875418	0.0036	0.997	0.214284106	count	1
NCR3LG1	16.3968016	1549.261134	0.0106	0.9916	0.214284106	count	1
ANKS1B	16.3974041	1641.824088	0.01	0.992	0.214284106	count	1
NPB	16.4526023	1811.174576	0.0091	0.993	0.214284107	count	1
GPR3	16.4526064	1811.175714	0.0091	0.993	0.214284107	count	1
PATL2	16.4526068	1811.174545	0.0091	0.993	0.214284107	count	1
AC092687.3	16.452607	1811.175147	0.0091	0.993	0.214284107	count	1
BX005040.1	16.4526104	1811.176878	0.0091	0.993	0.214284107	count	1
MAMDC4	16.4772628	1782.254749	0.0092	0.993	0.214284108	count	1

NLRP12	16.4772677	1782.257359	0.0092	0.993	0.214284108	count	1
AP005530.1	16.4782475	1912.620132	0.0086	0.993	0.214284108	count	1
PODN	16.5248122	1624.252697	0.0102	0.9919	0.214284108	count	1
CDC20	16.5248132	1624.253319	0.0102	0.9919	0.214284108	count	1
MTRNR2L6	16.5871418	1801.582743	0.0092	0.993	0.214284109	count	1
AC090971.1	16.5871419	1801.582756	0.0092	0.993	0.214284109	count	1
AP001318.2	16.5871429	1801.584153	0.0092	0.993	0.214284109	count	1
TRAPPC5	16.5871478	1801.583296	0.0092	0.993	0.214284109	count	1
MIA	16.5872858	2346.752824	0.0071	0.994	0.214284109	count	1
AC117382.2	16.587289	2346.752796	0.0071	0.994	0.214284109	count	1
FAM111B	16.589129	2597.488018	0.0064	0.995	0.214284109	count	1
AC099518.4	16.589129	2597.488029	0.0064	0.995	0.214284109	count	1
AMT	16.5891291	2597.48804	0.0064	0.995	0.214284109	count	1
CSF2RA	16.5891296	2597.486804	0.0064	0.995	0.214284109	count	1
PPP1R27	16.5891298	2597.486804	0.0064	0.995	0.214284109	count	1
AC127070.2	16.5891396	2597.491464	0.0064	0.995	0.214284109	count	1
SYT6	16.5891397	2597.486432	0.0064	0.995	0.214284109	count	1
AC141930.3	16.5891413	2597.492657	0.0064	0.995	0.214284109	count	1
SMCO2	16.5891413	2597.487614	0.0064	0.995	0.214284109	count	1
AP001636.3	16.5891414	2597.492679	0.0064	0.995	0.214284109	count	1
AC107871.1	16.5891415	2597.487646	0.0064	0.995	0.214284109	count	1
AL513217.1	16.5891416	2597.487657	0.0064	0.995	0.214284109	count	1
AC017071.1	16.5891421	2597.48641	0.0064	0.995	0.214284109	count	1
AC137630.2	16.5891431	2597.48885	0.0064	0.995	0.214284109	count	1
AC114488.3	16.5891432	2597.488839	0.0064	0.995	0.214284109	count	1
LRRC7	16.5891432	2597.488839	0.0064	0.995	0.214284109	count	1
ACTN3	16.5891432	2597.488861	0.0064	0.995	0.214284109	count	1
SPC24	16.5891433	2597.48885	0.0064	0.995	0.214284109	count	1
AP000962.1	16.5891433	2597.488861	0.0064	0.995	0.214284109	count	1
NSG1	16.5892266	3322.125778	0.005	0.996	0.214284109	count	1
AP003392.6	16.589234	3322.125484	0.005	0.996	0.214284109	count	1
PYHIN1	16.5892345	3322.123903	0.005	0.996	0.214284109	count	1
SCAANT1	16.6334315	1798.857262	0.0092	0.993	0.21428411	count	1
AL162411.1	16.6334391	1798.860392	0.0092	0.993	0.21428411	count	1
AC130371.2	16.6334392	1798.858643	0.0092	0.993	0.21428411	count	1
NDUFB2-AS1	16.6334408	1798.860074	0.0092	0.993	0.21428411	count	1
STIL	16.7031462	1791.849366	0.0093	0.9926	0.214284111	count	1
AC027682.4	16.7190249	2354.294521	0.0071	0.994	0.214284111	count	1
AC010864.1	16.7190264	2354.296966	0.0071	0.994	0.214284111	count	1
FOXA3	16.7190264	2354.296971	0.0071	0.994	0.214284111	count	1
KCNC3	16.7190264	2354.296961	0.0071	0.994	0.214284111	count	1
OTUB2	16.7190266	2354.296986	0.0071	0.994	0.214284111	count	1
AD000090.1	16.7190271	2354.295751	0.0071	0.994	0.214284111	count	1
LINC02587	16.719033	2354.29556	0.0071	0.994	0.214284111	count	1
HBA2	16.7190343	2354.298025	0.0071	0.994	0.214284111	count	1

AC021851.1	16.7190344	2354.298015	0.0071	0.994	0.214284111	count	1
AL158211.3	16.7190431	2354.297835	0.0071	0.994	0.214284111	count	1
PAGR1	16.7190431	2354.297845	0.0071	0.994	0.214284111	count	1
CYP51A1-AS1	16.7190432	2354.297845	0.0071	0.994	0.214284111	count	1
C9orf41-AS1	16.7190452	2354.299055	0.0071	0.994	0.214284111	count	1
TMEM253	16.7190452	2354.29905	0.0071	0.994	0.214284111	count	1
HIST2H4B	16.7190453	2354.29906	0.0071	0.994	0.214284111	count	1
TNNC1	16.7190453	2354.29906	0.0071	0.994	0.214284111	count	1
AC092634.5	16.7191281	3144.322762	0.0053	0.996	0.214284111	count	1
SYTL1	16.7191289	3144.321133	0.0053	0.996	0.214284111	count	1
AC007750.1	16.7191366	3144.315902	0.0053	0.996	0.214284111	count	1
HIST1H3B	16.7191369	3144.322501	0.0053	0.996	0.214284111	count	1
AC110285.5	16.751054	1758.722472	0.0095	0.9924	0.214284112	count	1
ZNF732	16.751056	1758.725988	0.0095	0.9924	0.214284112	count	1
AC010175.1	16.7520306	1845.788725	0.0091	0.9928	0.214284112	count	1
AP001922.5	16.7582456	1802.238645	0.0093	0.993	0.214284112	count	1
BRICD5	16.7586856	1933.646721	0.0087	0.993	0.214284112	count	1
TTC23L	16.8637868	2584.552489	0.0065	0.995	0.214284113	count	1
AL035530.2	16.8637868	2584.552489	0.0065	0.995	0.214284113	count	1
AC007216.2	16.8637874	2584.551379	0.0065	0.995	0.214284113	count	1
AL031708.1	16.8637876	2584.551379	0.0065	0.995	0.214284113	count	1
AL357673.1	16.8637883	2584.553557	0.0065	0.995	0.214284113	count	1
C17orf113	16.8637884	2584.553568	0.0065	0.995	0.214284113	count	1
AC073591.1	16.8637933	2584.553438	0.0065	0.995	0.214284113	count	1
AC055713.1	16.8637942	2584.555595	0.0065	0.995	0.214284113	count	1
AC012409.3	16.8637951	2584.554506	0.0065	0.995	0.214284113	count	1
AL033384.2	16.863796	3305.763433	0.0051	0.996	0.214284113	count	1
AC104184.1	16.8638001	2584.554398	0.0065	0.995	0.214284113	count	1
POU5F1	16.8638001	2584.554398	0.0065	0.995	0.214284113	count	1
AL049840.2	16.8638002	2584.554387	0.0065	0.995	0.214284113	count	1
AC139795.2	16.8638065	3305.765874	0.0051	0.996	0.214284113	count	1
ABCC9	16.8638076	3305.768701	0.0051	0.996	0.214284113	count	1
LINC00852	16.8638099	3305.764453	0.0051	0.996	0.214284113	count	1
AC079610.2	16.8644504	1800.565225	0.0094	0.993	0.214284113	count	1
AL031123.2	16.864455	1800.565207	0.0094	0.993	0.214284113	count	1
BANK1	16.8778905	1779.056117	0.0095	0.9924	0.214284113	count	1
AL355802.2	16.9105905	3309.931418	0.0051	0.996	0.214284113	count	1
CSF3R	16.9746327	1362.734159	0.0125	0.9901	0.214284114	count	1
CHSY3	17.0698818	1762.728024	0.0097	0.9923	0.214284115	count	1
CYP4B1	17.0762003	2572.805816	0.0066	0.9947	0.214284115	count	1
UBAP1L	17.0762003	2572.805806	0.0066	0.9947	0.214284115	count	1
MFSD14A	17.076202	2572.811754	0.0066	0.9947	0.214284115	count	1
C5orf17	17.0762038	2572.804763	0.0066	0.9947	0.214284115	count	1
CLTRN	17.0762101	2572.808668	0.0066	0.9947	0.214284115	count	1
PXYLP1	17.0768759	2799.747269	0.0061	0.995	0.214284115	count	1

AL162231.2	17.0768835	2799.747165	0.0061	0.995	0.214284115	count	1
AL691403.2	17.0768845	2799.751453	0.0061	0.995	0.214284115	count	1
TNNI3K	17.0768874	2799.751453	0.0061	0.995	0.214284115	count	1
SUSD1	17.2266178	2329.925139	0.0074	0.9941	0.214284116	count	1
10-Mar	17.22662	2329.923084	0.0074	0.9941	0.214284116	count	1
C4orf36	17.2266229	2329.926113	0.0074	0.9941	0.214284116	count	1
PIFO	17.2498309	2562.825227	0.0067	0.9946	0.214284116	count	1
AC015727.1	17.2498316	2562.822418	0.0067	0.9946	0.214284116	count	1
FOXI2	17.2498349	2562.826132	0.0067	0.9946	0.214284116	count	1
LINC01770	17.5561049	2312.755572	0.0076	0.9939	0.214284118	count	1
ATP8A2	17.8012539	2300.354198	0.0077	0.9938	0.21428412	count	1
RGS8	17.8023806	2424.233654	0.0073	0.9941	0.21428412	count	1
LOH12CR2	0.2323348	0.5325496	0.4363	0.663	0.21434396	count	1
AL645568.1	0.3663584	0.8397133	0.4363	0.663	0.214606579	count	1
CMTR2	0.1768592	0.2423527	0.7298	0.466	0.214722316	count	1
PELP1	0.1900902	0.3099293	0.6133	0.54	0.214756563	count	1
LINC01521	0.6974775	1.0930382	0.6381	0.523	0.214775908	count	1
USP3-AS1	0.6974775	1.2023554	0.5801	0.562	0.214775908	count	1
HIBCH	0.1599945	0.1483441	1.0785	0.281	0.215094174	count	1
TNRC6B	0.1524901	0.0879778	1.7333	0.0832	0.21510846	count	1
KDM7A	0.1752866	0.2544881	0.6888	0.491	0.215241151	count	1
AC009118.3	0.5877755	0.8963271	0.6558	0.512	0.215375806	count	1
EIF4H	0.1540764	0.0867414	1.7763	0.0758	0.215414698	count	1
MT-ND1	0.1496332	0.0376611	3.9731	7.27E-05	0.215543288	count	1
NCAPD3	0.2103111	0.3288799	0.6395	0.523	0.215543644	count	1
MINDY2	0.1572814	0.1429609	1.1002	0.271	0.215588787	count	1
AP005329.3	0.2590817	0.5315795	0.4874	0.626	0.215684552	count	1
EEF1B2	0.149798	0.0291348	5.1415	2.92E-07	0.215694053	count	0.006847692
CCDC59	0.1541595	0.0976569	1.5786	0.115	0.215699424	count	1
TYW5	0.2227916	0.649052	0.3433	0.731	0.21584761	count	1
PRMT9	0.201999	0.4829473	0.4183	0.676	0.215936077	count	1
SASS6	0.172414	0.3517418	0.4902	0.624	0.216012331	count	1
SDHAF3	0.1870316	0.2517286	0.743	0.458	0.21604018	count	1
UMAD1	0.1891696	0.3071658	0.6159	0.538	0.216201005	count	1
HARS	0.1590278	0.1360681	1.1687	0.243	0.216234909	count	1
SMAD5	0.1554942	0.1270789	1.2236	0.221	0.216317137	count	1
DRC3	0.2379533	0.4475826	0.5316	0.595	0.216336222	count	1
MMAB	0.1631839	0.1651542	0.9881	0.323	0.216477842	count	1
CXorf40A	0.2381659	0.446587	0.5333	0.594	0.21652223	count	1
POU2F2	0.3946589	0.8135409	0.4851	0.628	0.216646261	count	1
SRP19	0.1559023	0.1132405	1.3767	0.169	0.216683999	count	1
CIP2A	0.3339047	0.5646139	0.5914	0.554	0.216860245	count	1
ZSCAN32	0.3339047	0.6152517	0.5427	0.587	0.216860245	count	1
UGDH-AS1	0.5929136	0.7728726	0.7672	0.443	0.216910748	count	1
ANAPC5	0.1552271	0.1087183	1.4278	0.153	0.216916997	count	1

CCDC86	0.1884614	0.2923494	0.6446	0.519	0.216932207	count	1
MCPH1-AS1	0.3709142	1.0759818	0.3447	0.73	0.217025772	count	1
AL080250.1	0.7089825	1.0601761	0.6687	0.504	0.2175026	count	1
SLFN12L	0.7089825	1.1577281	0.6124	0.54	0.2175026	count	1
RALGPS1	0.7089825	1.160933	0.6107	0.541	0.2175026	count	1
TMEM109	0.153306	0.071666	2.1392	0.0325	0.217524243	count	1
AF001548.2	0.4274608	0.7854392	0.5442	0.586	0.217628995	count	1
TADA1	0.4274608	0.8129574	0.5258	0.599	0.217628995	count	1
RAB24	0.227287	0.5677306	0.4003	0.689	0.217679762	count	1
DUSP15	0.3520676	0.4845478	0.7266	0.468	0.217701431	count	1
ZBTB17	0.1981422	0.3035635	0.6527	0.514	0.217768928	count	1
RUBCN	0.256661	0.4737767	0.5417	0.588	0.217944788	count	1
DSN1	0.1839614	0.3152974	0.5835	0.56	0.218215118	count	1
SIRT3	0.19859	0.2822554	0.7036	0.482	0.218251115	count	1
JDP2	0.1642368	0.1519386	1.0809	0.28	0.218272957	count	1
USP16	0.1575627	0.1232566	1.2783	0.201	0.21833984	count	1
BPHL	0.2083953	0.3971617	0.5247	0.6	0.218367805	count	1
PER3	0.1590785	0.1489323	1.0681	0.286	0.21845455	count	1
SNRNP25	0.1647444	0.1762144	0.9349	0.35	0.218536219	count	1
CDK17	0.1646418	0.1597961	1.0303	0.303	0.218708018	count	1
SHLD3	0.2529513	0.4939277	0.5121	0.609	0.218840567	count	1
INTS12	0.169546	0.1798964	0.9425	0.346	0.218844015	count	1
TMEM74B	0.1888954	0.3973042	0.4754	0.635	0.218873732	count	1
ZNF451	0.1623813	0.1934968	0.8392	0.401	0.218992221	count	1
CAPN11	0.2138522	0.3864786	0.5533	0.58	0.21907666	count	1
HNRNPD	0.1544019	0.0658237	2.3457	0.0191	0.219204754	count	1
URB1-AS1	0.1874173	0.3015873	0.6214	0.534	0.219208875	count	1
NIPBL	0.158808	0.1224744	1.2967	0.195	0.21929962	count	1
ZNF334	0.3550112	0.5889668	0.6028	0.547	0.219364668	count	1
EIF4E	0.1615521	0.1310126	1.2331	0.218	0.219387198	count	1
ZNF24	0.1580338	0.1137715	1.389	0.165	0.219404568	count	1
CEP83	0.1927671	0.3051251	0.6318	0.528	0.219427188	count	1
BMT2	0.2142162	0.4019309	0.533	0.594	0.219439639	count	1
IARS2	0.1589099	0.1336051	1.1894	0.234	0.219524616	count	1
QTRT2	0.1690109	0.1858022	0.9096	0.363	0.219538291	count	1
LAMTOR2	0.1560758	0.0891976	1.7498	0.0803	0.219623495	count	1
TMEM242	0.1603287	0.1348732	1.1887	0.235	0.219751455	count	1
PNPLA6	0.2070257	0.4589305	0.4511	0.652	0.219829212	count	1
CFDP1	0.1564751	0.0955939	1.6369	0.102	0.219849646	count	1
NT5C2	0.2088	0.2914511	0.7164	0.474	0.22025086	count	1
SMIM10L2A	0.7208648	1.2105288	0.5955	0.552	0.220293653	count	1
FAU	0.152932	0.0203274	7.5234	7.17E-14	0.220421209	count	1.71E-09
AC005332.6	0.433911	0.8386547	0.5174	0.605	0.220522854	count	1
TRIOBP	0.1584236	0.0989746	1.6006	0.11	0.220575849	count	1
URI1	0.1599989	0.1419139	1.1274	0.26	0.220582692	count	1

PHKA2	0.2361665	0.4617789	0.5114	0.609	0.220590627	count	1
SRGAP2	0.1725165	0.1673008	1.0312	0.303	0.220643468	count	1
MDP1	0.2121719	0.3637636	0.5833	0.56	0.220677454	count	1
MSRB3	0.1576705	0.0968927	1.6273	0.104	0.220753155	count	1
BFAR	0.1597699	0.1172638	1.3625	0.173	0.220820512	count	1
LRRC37B	0.285439	0.4814979	0.5928	0.553	0.220851212	count	1
FUNDC2	0.1563789	0.0754847	2.0717	0.0384	0.220983599	count	1
RDH11	0.1625686	0.1392721	1.1673	0.243	0.221082522	count	1
UBR5	0.1728913	0.1904191	0.908	0.364	0.221118793	count	1
HSPA2	0.1649397	0.1515555	1.0883	0.277	0.221168363	count	1
CSTF2	0.1993485	0.3410594	0.5845	0.559	0.22117466	count	1
NR4A1	0.1553307	0.1079259	1.4392	0.1502	0.22119084	count	1
AC020911.2	0.2400918	0.4011446	0.5985	0.55	0.221235386	count	1
PPP1CC	0.1588293	0.1008859	1.5743	0.116	0.221395873	count	1
BEX4	0.1592742	0.1049666	1.5174	0.129	0.221441622	count	1
DDA1	0.1756097	0.2103162	0.835	0.404	0.221660623	count	1
CPT2	0.2131988	0.3698362	0.5765	0.564	0.221718305	count	1
ADAT1	0.2028584	0.3248479	0.6245	0.532	0.221720264	count	1
MSRB2	0.1593216	0.1177473	1.3531	0.176	0.221746476	count	1
KIF27	0.2184702	0.3609227	0.6053	0.545	0.221891143	count	1
BLOC1S4	0.1635625	0.1640454	0.9971	0.319	0.222050789	count	1
MITF	0.2731352	0.4998221	0.5465	0.585	0.222100335	count	1
PIK3C3	0.175918	0.1944059	0.9049	0.366	0.222310893	count	1
CREBZF	0.171693	0.1780663	0.9642	0.335	0.222314595	count	1
TRIP4	0.1847817	0.249033	0.742	0.458	0.22233646	count	1
TYW3	0.1602288	0.1161277	1.3798	0.168	0.222354164	count	1
MYLIP	0.1690137	0.176793	0.956	0.339	0.222419236	count	1
SNHG12	0.200502	0.2213702	0.9057	0.365	0.222429055	count	1
C10orf143	0.2352863	0.4454748	0.5282	0.597	0.222499255	count	1
KDR	0.1638215	0.181037	0.9049	0.366	0.222511247	count	1
PRR14	0.1872276	0.2457103	0.762	0.446	0.222599978	count	1
VARS2	0.305692	0.8615555	0.3548	0.723	0.222633922	count	1
ZNF397	0.1794413	0.20924	0.8576	0.391	0.22281107	count	1
ZNF718	0.2062555	0.4324714	0.4769	0.633	0.22295063	count	1
ETV3	0.1853152	0.2743029	0.6756	0.499	0.222969921	count	1
RNF213	0.15912	0.1068729	1.4889	0.137	0.22297614	count	1
BBS9	0.2196522	0.3867597	0.5679	0.57	0.223058216	count	1
SLC25A33	0.1793754	0.194986	0.9199	0.358	0.223064178	count	1
KXD1	0.1596829	0.1011071	1.5793	0.114	0.223114761	count	1
RELB	0.1662945	0.1519463	1.0944	0.274	0.223135769	count	1
ZDHHC24	0.1637849	0.133293	1.2288	0.219	0.223138078	count	1
AC078881.1	0.7331277	0.8900991	0.8236	0.41	0.223147568	count	1
EDEM2	0.1714889	0.1789263	0.9584	0.338	0.223220876	count	1
MOSPD1	0.1836664	0.2370647	0.7748	0.439	0.223326963	count	1
LINC01003	0.1782024	0.2047534	0.8703	0.384	0.223489078	count	1

SBNO1	0.1696777	0.2034041	0.8342	0.404	0.223539189	count	1
OTUB1	0.1622064	0.1319034	1.2297	0.219	0.223709545	count	1
IRF2BP1	0.1920164	0.3073136	0.6248	0.532	0.223831043	count	1
PIGX	0.1860567	0.3177535	0.5855	0.558	0.223850266	count	1
ANP32E	0.1618965	0.1152948	1.4042	0.16	0.22399599	count	1
TIMM50	0.1695795	0.1752022	0.9679	0.333	0.224014535	count	1
RBM12	0.1875413	0.2476665	0.7572	0.449	0.224062118	count	1
TPBG	0.165994	0.166398	0.9976	0.319	0.22424472	count	1
HIP1	0.163467	0.1287264	1.2699	0.204	0.224386082	count	1
MRPL44	0.1749718	0.2062852	0.8482	0.396	0.224399407	count	1
SNHG7	0.156287	0.0470012	3.3252	0.000895	0.224442837	count	1
AC025175.1	0.9482844	1.454747	0.6519	0.515	0.224478255	count	1
MID1	0.1803032	0.2357446	0.7648	0.444	0.224535348	count	1
ZADH2	0.1818079	0.236213	0.7697	0.442	0.224662063	count	1
MAU2	0.1939979	0.2808999	0.6906	0.49	0.224689175	count	1
RRP1	0.1821769	0.2113279	0.8621	0.389	0.22474659	count	1
RAB12	0.1671966	0.1664839	1.0043	0.315	0.224954146	count	1
AC023043.1	0.4443658	0.6508513	0.6827	0.495	0.225189126	count	1
MAIP1	0.1835189	0.2425736	0.7565	0.449	0.225231398	count	1
UBQLN4	0.2384255	0.3737016	0.638	0.524	0.225363418	count	1
COIL	0.1682801	0.1716358	0.9804	0.327	0.225375927	count	1
MAPKAPK2	0.1764661	0.1815432	0.972	0.331	0.225428722	count	1
RECQL	0.1630233	0.1109263	1.4697	0.142	0.225630039	count	1
ZNF507	0.1909191	0.2817487	0.6776	0.498	0.225756687	count	1
EMSY	0.1958548	0.2726684	0.7183	0.473	0.226061139	count	1
THBD	0.1584736	0.0694157	2.283	0.0225	0.226139399	count	1
NSMAF	0.1960351	0.4392729	0.4463	0.655	0.22626575	count	1
AL035071.1	0.2249317	0.3733156	0.6025	0.547	0.226350176	count	1
ELOF1	0.1693231	0.1669137	1.0144	0.31	0.226420148	count	1
METTL23	0.1640137	0.1251314	1.3107	0.19	0.226436997	count	1
FBXL4	0.2097048	0.2669779	0.7855	0.432	0.226595044	count	1
CBWD1	0.1761317	0.2416525	0.7289	0.466	0.226693283	count	1
PLGRKT	0.1719693	0.1751457	0.9819	0.326	0.226788287	count	1
KIAA0355	0.1635907	0.1229754	1.3303	0.184	0.226793154	count	1
RPL35	0.1575825	0.0283734	5.5539	3.06E-08	0.226795054	count	0.000721579
NELFCD	0.1785348	0.1926466	0.9267	0.354	0.226866541	count	1
PCBP1	0.1589672	0.0551838	2.8807	0.004	0.227046348	count	1
ZC3H14	0.1696352	0.1557435	1.0892	0.276	0.227096069	count	1
ZSWIM8	0.1938029	0.4006895	0.4837	0.629	0.227222901	count	1
ZNF528	0.2202921	0.3536757	0.6229	0.533	0.227228931	count	1
ZFAS1	0.159626	0.0594012	2.6873	0.00725	0.227432573	count	1
NRIP1	0.1797983	0.2108637	0.8527	0.394	0.227434016	count	1
AP1B1	0.1864512	0.230559	0.8087	0.419	0.227543363	count	1
RPL18	0.1578765	0.019792	7.9768	2.18E-15	0.227594633	count	5.22E-11
EXOC3L2	0.1804159	0.1990262	0.9065	0.365	0.227668374	count	1

MED23	0.1889528	0.2447595	0.772	0.44	0.227785719	count	1
FLII	0.1672511	0.1869854	0.8945	0.371	0.227891889	count	1
ASNS	0.2595481	0.7946257	0.3266	0.744	0.228100373	count	1
FKBP14	0.1929663	0.2529342	0.7629	0.446	0.228141275	count	1
COQ4	0.1699879	0.1568098	1.084	0.278	0.22814698	count	1
ACBD4	0.2166098	0.424985	0.5097	0.61	0.228283186	count	1
SCARF1	0.1682742	0.1488426	1.1306	0.258	0.228304742	count	1
AC010542.2	0.2882506	0.6947226	0.4149	0.678	0.228472608	count	1
C15orf65	0.2070732	0.3514025	0.5893	0.556	0.228490126	count	1
BEND5	0.1839295	0.2483592	0.7406	0.459	0.229002438	count	1
PPA2	0.1704829	0.1393678	1.2233	0.221	0.229046238	count	1
CLDN23	0.3393591	0.4444533	0.7635	0.445	0.229108054	count	1
XPO5	0.2425353	0.405529	0.5981	0.55	0.229108809	count	1
TMC6	0.2058257	0.3000209	0.686	0.493	0.229254222	count	1
KDM5C	0.2015646	0.3202993	0.6293	0.529	0.22925856	count	1
NUTM2B-AS1	0.170889	0.1387177	1.2319	0.218	0.229350188	count	1
MDFIC	0.1678337	0.1333753	1.2584	0.208	0.229443376	count	1
ZBED5	0.1764394	0.1982039	0.8902	0.373	0.229451786	count	1
PABPC1	0.160281	0.0482968	3.3187	0.000916	0.229494135	count	1
DBNL	0.1675406	0.1247639	1.3429	0.179	0.229518323	count	1
ISY1	0.1720902	0.1720919	1	0.317	0.229641124	count	1
C16orf72	0.176675	0.1729167	1.0217	0.307	0.229755882	count	1
SLC39A10	0.1660587	0.1251731	1.3266	0.185	0.229898704	count	1
PAK4	0.197975	0.2739784	0.7226	0.47	0.229950247	count	1
DHFR	0.3278985	0.5545583	0.5913	0.554	0.230070715	count	1
PNPO	0.1981033	0.2872451	0.6897	0.49	0.230096802	count	1
SLC35E1	0.1820098	0.2427284	0.7498	0.453	0.23047	count	1
THAP10	0.2472565	0.439016	0.5632	0.573	0.230562209	count	1
GATC	0.1917657	0.2658517	0.7213	0.471	0.230624953	count	1
PNMA1	0.1957052	0.2881267	0.6792	0.497	0.23070844	count	1
TMUB1	0.1712489	0.1563808	1.0951	0.274	0.230750124	count	1
TGDS	0.1857531	0.2431032	0.7641	0.445	0.230907835	count	1
UBXN8	0.1779115	0.2073777	0.8579	0.391	0.2310117	count	1
ANXA6	0.1798581	0.2023716	0.8888	0.374	0.231036974	count	1
STK17A	0.1692856	0.1385877	1.2215	0.222	0.231047931	count	1
GPIHBP1	0.1829165	0.2864185	0.6386	0.523	0.231068757	count	1
ABCF1	0.1671712	0.1348973	1.2392	0.215	0.231104957	count	1
TRMT2A	0.2117502	0.2853932	0.742	0.458	0.231224635	count	1
RALB	0.1656486	0.1143054	1.4492	0.147	0.231233249	count	1
RBMS2	0.1698896	0.14988	1.1335	0.257	0.231265101	count	1
PSMG4	0.1677962	0.1253864	1.3382	0.181	0.231426023	count	1
NUP54	0.1720472	0.1499494	1.1474	0.251	0.231523713	count	1
RACK1	0.1607652	0.0225024	7.1443	1.15E-12	0.231689723	count	2.74E-08
EPAS1	0.1621219	0.0688631	2.3543	0.0186	0.231737312	count	1
PHF2	0.192786	0.2303776	0.8368	0.403	0.231835088	count	1

ECI1	0.1679571	0.1236826	1.358	0.175	0.231924725	count	1
ZNF449	0.2267588	0.3443173	0.6586	0.51	0.231925562	count	1
PALD1	0.1838886	0.22641	0.8122	0.417	0.232007187	count	1
AC096733.2	0.5619732	0.5476953	1.0261	0.305	0.232135736	count	1
HIST2H2BE	0.6452817	0.7792442	0.8281	0.408	0.232235251	count	1
SENP7	0.1737876	0.1543394	1.126	0.26	0.232266516	count	1
AC002310.1	0.460901	0.6574645	0.701	0.483	0.232508192	count	1
PDGFRA	0.2357348	0.4681681	0.5035	0.615	0.232614623	count	1
ZMIZ1	0.1729101	0.1720441	1.005	0.315	0.232754723	count	1
AMER1	1.0010591	1.3200832	0.7583	0.448	0.232789359	count	1
AMDHD2	0.2243573	0.3240181	0.6924	0.489	0.233010678	count	1
C1orf50	0.2361566	0.4463942	0.529	0.597	0.233017505	count	1
C4orf33	0.1912733	0.2402827	0.796	0.426	0.233353626	count	1
SH3BP5-AS1	0.6497938	1.0416441	0.6238	0.533	0.233528618	count	1
PTPN13	0.6497938	1.0416441	0.6238	0.533	0.233528618	count	1
ATP7B	0.6497938	1.0523087	0.6175	0.537	0.233528618	count	1
C2orf92	0.6497938	1.1335482	0.5732	0.567	0.233528618	count	1
AP4S1	0.2189013	0.3668517	0.5967	0.551	0.233564597	count	1
IPO9-AS1	0.4295037	1.0410467	0.4126	0.68	0.233618072	count	1
HDHD2	0.2218058	0.2991019	0.7416	0.458	0.233618615	count	1
APOM	0.4634346	0.6289924	0.7368	0.461	0.233623062	count	1
PPIG	0.1640508	0.069933	2.3458	0.0191	0.233748648	count	1
FAM32A	0.1679069	0.1096723	1.531	0.126	0.233961592	count	1
MPHOSPH6	0.1814793	0.2054263	0.8834	0.377	0.234120433	count	1
FTSJ1	0.1919478	0.2438369	0.7872	0.431	0.234166034	count	1
TNKS2	0.1745427	0.1491136	1.1705	0.242	0.234472469	count	1
PILRB	0.1922236	0.3138451	0.6125	0.54	0.234498197	count	1
THSD1	0.1842597	0.1921569	0.9589	0.338	0.234572785	count	1
ZNF131	0.1763543	0.140487	1.2553	0.209	0.234700832	count	1
RAP1A	0.1662501	0.0762595	2.1801	0.0293	0.234705222	count	1
EBLN2	1.013993	1.0231568	0.991	0.322	0.234771075	count	1
SMAD3	0.1972161	0.2494576	0.7906	0.429	0.234882122	count	1
TCTN3	0.1800742	0.1792202	1.0048	0.315	0.234965023	count	1
MRRF	0.188249	0.2004471	0.9391	0.348	0.234992314	count	1
METTL5	0.1662292	0.0822432	2.0212	0.0434	0.23509925	count	1
PLXND1	0.1681679	0.1190605	1.4125	0.158	0.23518109	count	1
GMPR2	0.172589	0.1330568	1.2971	0.195	0.235238233	count	1
RPL29	0.1631876	0.0207373	7.8693	5.08E-15	0.235267223	count	1.22E-10
PSMC1	0.1671626	0.0822339	2.0328	0.0422	0.235285085	count	1
CTCF	0.1722434	0.1319929	1.3049	0.192	0.235308554	count	1
NFX1	0.1896234	0.2794714	0.6785	0.498	0.235310397	count	1
ZBTB7A	0.1686212	0.1027952	1.6404	0.101	0.235355692	count	1
CASP4	0.1660177	0.0793369	2.0926	0.0365	0.235401302	count	1
PPME1	0.1942273	0.2554664	0.7603	0.447	0.235529492	count	1
AKAP11	0.1807047	0.1885541	0.9584	0.338	0.23562076	count	1

MFAP1	0.1717549	0.1352529	1.2699	0.204	0.235761236	count	1
DAB2	0.1677521	0.0964001	1.7402	0.0819	0.235774563	count	1
STAU1	0.1663861	0.0740255	2.2477	0.0247	0.235815193	count	1
MAPK1IP1L	0.1689667	0.0973929	1.7349	0.0829	0.235888637	count	1
ZNF655	0.2139856	0.317999	0.6729	0.501	0.235952447	count	1
TSR1	0.1904625	0.2314964	0.8227	0.411	0.23633973	count	1
GLMP	0.1814205	0.1826485	0.9933	0.321	0.236383463	count	1
RPL36A	0.1667437	0.0678261	2.4584	0.014	0.236443762	count	1
GRSF1	0.173619	0.1324266	1.3111	0.19	0.236480136	count	1
SOGA1	0.2051696	0.2534848	0.8094	0.418	0.236622828	count	1
AL356056.1	0.435767	1.6497272	0.2641	0.7917	0.236631951	count	1
C17orf107	0.6607173	1.064504	0.6207	0.535	0.236642188	count	1
HSD17B1	0.6607173	1.0648625	0.6205	0.535	0.236642188	count	1
AC001226.2	0.6607173	1.963742	0.3365	0.7366	0.236642188	count	1
KBTBD4	0.1987291	0.2552833	0.7785	0.436	0.236657206	count	1
USP30-AS1	1.027097	1.0433647	0.9844	0.325	0.236757031	count	1
AC025171.4	0.5153981	0.6738645	0.7648	0.444	0.236799148	count	1
GFM2	0.2336183	0.3346125	0.6982	0.485	0.236819434	count	1
DEGS2	0.7951829	1.138282	0.6986	0.485	0.237182351	count	1
TOP2A	0.7951829	1.1430503	0.6957	0.487	0.237182351	count	1
FAM215B	0.2704182	0.3318373	0.8149	0.415	0.237220389	count	1
LINC01355	0.3684062	0.7496303	0.4915	0.623	0.237334999	count	1
KLF6	0.165614	0.0603061	2.7462	0.00607	0.237430421	count	1
VASH1-AS1	0.3689867	0.5522654	0.6681	0.504	0.237676362	count	1
FBN1	0.172245	0.1487428	1.158	0.247	0.23773351	count	1
SMPDL3A	0.2041862	0.3645382	0.5601	0.575	0.237777904	count	1
YES1	0.170511	0.1042831	1.6351	0.102	0.237807498	count	1
UTP20	0.2031009	0.3684631	0.5512	0.582	0.237945793	count	1
OSMR-AS1	0.5184911	0.6643902	0.7804	0.435	0.238008044	count	1
UPF3B	0.1769167	0.1590498	1.1123	0.266	0.238043017	count	1
UBE2T	0.2415136	0.4410071	0.5476	0.584	0.23812993	count	1
TMEM256	0.1699487	0.0927391	1.8325	0.067	0.238186555	count	1
PLXDC1	0.3889237	0.6429517	0.6049	0.545	0.238337411	count	1
ZNF555	0.3701546	0.5930588	0.6241	0.533	0.238362843	count	1
TP53	0.1871192	0.2006142	0.9327	0.351	0.23842689	count	1
CFAP44	0.5195811	0.9149684	0.5679	0.57	0.238433476	count	1
BTF3	0.1657383	0.0276668	5.9905	2.36E-09	0.238627152	count	5.59E-05
CACHD1	0.1994469	0.2397818	0.8318	0.406	0.238640263	count	1
ZBTB26	0.3548589	0.6718842	0.5282	0.597	0.238728756	count	1
AC108488.1	0.2503522	0.4743035	0.5278	0.598	0.238971876	count	1
ZFP91	0.1929986	0.2244097	0.86	0.39	0.23908237	count	1
UQCRC2	0.1691117	0.0774513	2.1835	0.0291	0.23913801	count	1
DDX59	0.1805457	0.1765967	1.0224	0.307	0.239250194	count	1
PPCS	0.1693701	0.0815761	2.0762	0.038	0.239272051	count	1
CHURC1	0.1687273	0.071498	2.3599	0.0183	0.239502022	count	1

PARD3B	0.2325865	0.3409876	0.6821	0.495	0.239549023	count	1
GPN1	0.1836362	0.2060768	0.8911	0.373	0.239577661	count	1
NMNAT3	0.2164188	0.3356246	0.6448	0.519	0.23970663	count	1
CHFR	0.2961338	0.3764953	0.7866	0.432	0.239766525	count	1
ANKRD26	0.1833149	0.2306081	0.7949	0.427	0.239794464	count	1
EIF2AK4	0.1717537	0.1193891	1.4386	0.15	0.239804956	count	1
AL451085.2	0.2896958	0.3853609	0.7518	0.452	0.239833053	count	1
AC010136.1	0.6720465	1.6216012	0.4144	0.6786	0.239845211	count	1
ZIK1	0.8074954	1.0721101	0.7532	0.451	0.239887397	count	1
PRDM10	0.8074954	1.106473	0.7298	0.466	0.239887397	count	1
TIMM9	0.1769598	0.1276968	1.3858	0.166	0.240096383	count	1
NAP1L2	0.311977	0.5248046	0.5945	0.552	0.24011328	count	1
XPC	0.1781385	0.1611395	1.1055	0.269	0.240210421	count	1
CFL2	0.1761378	0.1370118	1.2856	0.199	0.240260996	count	1
EARS2	0.4152955	0.6393878	0.6495	0.516	0.240272283	count	1
RTN4RL2	0.5243893	0.6210735	0.8443	0.399	0.240306548	count	1
ZFP69	0.5243893	0.7065267	0.7422	0.458	0.240306548	count	1
AL161421.1	0.2463365	0.4039123	0.6099	0.542	0.24034737	count	1
TSPAN10	0.2791069	0.4623671	0.6036	0.546	0.240384983	count	1
HNRNPK	0.1685142	0.05453	3.0903	0.00202	0.2404321	count	1
SARNP	0.204649	0.2620654	0.7809	0.435	0.241081948	count	1
VPS4B	0.1736122	0.1236427	1.4041	0.16	0.241091308	count	1
TBRG4	0.2147971	0.3790324	0.5667	0.571	0.241115189	count	1
B3GLCT	0.3750388	0.5721402	0.6555	0.512	0.241229159	count	1
ZC3H15	0.1697037	0.066934	2.5354	0.0113	0.241320854	count	1
SCNN1A	0.4173545	0.6748834	0.6184	0.536	0.24133667	count	1
TRIM59	0.3943713	0.6501039	0.6066	0.544	0.241352755	count	1
ACBD3	0.17046	0.0938156	1.817	0.0693	0.241448564	count	1
RMDN3	0.1893406	0.2004783	0.9444	0.345	0.241477945	count	1
NACA	0.1676665	0.0218609	7.6697	2.37E-14	0.241678345	count	5.67E-10
LINC02018	0.5901918	1.063755	0.5548	0.579	0.241732323	count	1
CADM2	0.5901918	1.0841315	0.5444	0.586	0.241732323	count	1
NT5DC1	0.1781932	0.1564143	1.1392	0.255	0.241762185	count	1
MORN3	0.3763219	0.7938956	0.474	0.636	0.241980931	count	1
PDP1	0.1974927	0.3099536	0.6372	0.524	0.242163399	count	1
RAB8A	0.1758782	0.1188229	1.4802	0.139	0.242192877	count	1
FEN1	0.3466058	0.488008	0.7102	0.478	0.242197761	count	1
ACSL1	0.3962187	1.0101715	0.3922	0.695	0.242373286	count	1
SNRNP35	0.1813004	0.1552554	1.1678	0.243	0.242532242	count	1
BTF3L4	0.1738618	0.100866	1.7237	0.0849	0.24259833	count	1
APBB1IP	0.8201165	1.2263686	0.6687	0.504	0.242633338	count	1
RHOT1	0.1933886	0.2413869	0.8012	0.423	0.242645877	count	1
ZNF280C	0.2723641	0.5699968	0.4778	0.633	0.242649885	count	1
FLRT2	0.2006003	0.2215519	0.9054	0.365	0.242654171	count	1
IL6ST	0.1692263	0.0621656	2.7222	0.00653	0.242724548	count	1

SLC30A4	0.2252018	0.3053833	0.7374	0.461	0.242932718	count	1
TSN	0.181408	0.1552608	1.1684	0.243	0.24303988	count	1
HTATIP2	0.17694	0.1288771	1.3729	0.17	0.243283276	count	1
ZRANB1	0.1931619	0.270227	0.7148	0.475	0.243287465	count	1
ZBTB11	0.1937361	0.1847722	1.0485	0.294	0.243391082	count	1
CARS2	0.2044842	0.2210486	0.9251	0.355	0.243405248	count	1
KBTBD11	0.5327748	0.8066467	0.6605	0.509	0.243559088	count	1
WISP2	0.5327748	0.8511304	0.626	0.531	0.243559088	count	1
AL163051.1	0.5327748	0.9586365	0.5558	0.578	0.243559088	count	1
ACAD9	0.2189882	0.2518421	0.8695	0.385	0.243599756	count	1
NRAV	0.3088955	0.5625673	0.5491	0.583	0.243859983	count	1
ATP6V1H	0.1972956	0.1978167	0.9974	0.319	0.243957795	count	1
FAM177A1	0.1722272	0.082352	2.0914	0.0366	0.244013895	count	1
ZNF33A	0.1824668	0.1661282	1.0983	0.272	0.244176675	count	1
CHRAC1	0.179587	0.1399998	1.2828	0.2	0.244295575	count	1
LRRC28	0.2016157	0.2290193	0.8803	0.379	0.24436484	count	1
DGCR6	0.6884227	0.6443589	1.0684	0.285	0.244428311	count	1
PEX11B	0.2065669	0.2973268	0.6947	0.487	0.244605282	count	1
GID8	0.1790057	0.1347651	1.3283	0.184	0.244606412	count	1
FBXL20	0.2179761	0.3701271	0.5889	0.556	0.244609313	count	1
RELA	0.1812978	0.1416617	1.2798	0.201	0.244743845	count	1
MRPS2	0.179352	0.133655	1.3419	0.18	0.244830829	count	1
PCIF1	0.1935078	0.2113856	0.9154	0.36	0.244880711	count	1
MORF4L2-AS1	0.3812907	0.8933306	0.4268	0.67	0.244887406	count	1
FRYL	0.1763722	0.1398828	1.2609	0.207	0.244937102	count	1
TDRKH-AS1	0.5999629	1.0471146	0.573	0.567	0.245011805	count	1
AC021188.1	0.5999629	1.1194748	0.5359	0.592	0.245011805	count	1
ACTL10	0.5999629	1.1547046	0.5196	0.603	0.245011805	count	1
SCML1	0.1838782	0.1567969	1.1727	0.241	0.245176056	count	1
ELOVL1	0.1753914	0.1041011	1.6848	0.0921	0.24527568	count	1
TM9SF1	0.2333658	0.3850276	0.6061	0.544	0.245463968	count	1
DPEP2	0.2606183	0.4003405	0.651	0.515	0.245529927	count	1
ZFYVE26	0.4914657	0.6621461	0.7422	0.458	0.245841006	count	1
ARFIP1	0.1825669	0.1525952	1.1964	0.232	0.245843271	count	1
FAM184B	0.2812005	0.6196035	0.4538	0.65	0.246231012	count	1
BCL7B	0.178534	0.1266096	1.4101	0.159	0.246351927	count	1
AMD1	0.1773467	0.0971892	1.8248	0.0681	0.246381815	count	1
TIPRL	0.1762694	0.1005351	1.7533	0.0797	0.246564486	count	1
MUC20	0.4931484	1.9218288	0.2566	0.7975	0.246567667	count	1
BBS4	0.2049048	0.3487384	0.5876	0.557	0.246736675	count	1
TRMT44	0.3053288	0.5196617	0.5876	0.557	0.246783146	count	1
CSK	0.2190782	0.3003744	0.7294	0.466	0.246835313	count	1
SF3B2	0.17412	0.0744771	2.3379	0.0195	0.246849736	count	1
PA2G4	0.1738893	0.0650885	2.6716	0.00759	0.247079672	count	1
CDC42	0.1722561	0.045446	3.7904	0.000154	0.247103137	count	1

CRYBG1	0.2031389	0.2786811	0.7289	0.466	0.247160962	count	1
LINC02315	0.3853148	1.072814	0.3592	0.72	0.247235725	count	1
CARM1	0.2306559	0.3053032	0.7555	0.45	0.24724972	count	1
STON2	1.0998431	1.0414184	1.0561	0.291	0.247392254	count	1
SRP54	0.1817728	0.1555085	1.1689	0.243	0.247425976	count	1
UNC50	0.178283	0.1183743	1.5061	0.132	0.247435741	count	1
DENND1B	0.282656	0.4021088	0.7029	0.482	0.24744462	count	1
AC004951.1	0.4585244	0.5184445	0.8844	0.377	0.247488716	count	1
AC040977.1	0.4958684	0.7962084	0.6228	0.533	0.247740641	count	1
VPS26A	0.17936	0.1100066	1.6304	0.103	0.247745584	count	1
RARB	0.2249316	0.379045	0.5934	0.553	0.247746261	count	1
CENPH	0.2569886	0.3780451	0.6798	0.497	0.247775926	count	1
HSPA9	0.1788912	0.1087683	1.6447	0.1	0.248073001	count	1
DTNBP1	0.1845606	0.1591125	1.1599	0.246	0.248108386	count	1
EPSTI1	0.1814246	0.1828445	0.9922	0.321	0.248186521	count	1
PSMG3	0.1836833	0.159308	1.153	0.249	0.248236792	count	1
NR4A2	0.180655	0.1313033	1.3759	0.169	0.248294648	count	1
DDX60	0.1882139	0.1981208	0.95	0.342	0.248329906	count	1
CLASP1	0.2077183	0.2884443	0.7201	0.472	0.248385698	count	1
PARD6G-AS1	0.6101494	1.0656121	0.5726	0.567	0.248407076	count	1
ZSWIM1	0.6101494	1.094702	0.5574	0.577	0.248407076	count	1
AC114291.1	0.6101494	1.1367817	0.5367	0.591	0.248407076	count	1
THRA	0.18394	0.1611559	1.1414	0.254	0.248438458	count	1
SLC35F5	0.1954927	0.2101719	0.9302	0.352	0.248466822	count	1
SPOUT1	0.2094263	0.2252532	0.9297	0.353	0.248573095	count	1
TSPAN4	0.1743143	0.0640341	2.7222	0.00653	0.248673497	count	1
MRPL23	0.1860974	0.1438072	1.2941	0.196	0.248719008	count	1
KEAP1	0.1893909	0.1641703	1.1536	0.249	0.248752879	count	1
RPS6KC1	0.2282854	0.3558094	0.6416	0.521	0.248848545	count	1
AKAP7	0.2125848	0.2688685	0.7907	0.429	0.248865254	count	1
PARP12	0.1962672	0.2608886	0.7523	0.452	0.248897707	count	1
VSIG10	0.2005473	0.238678	0.8402	0.401	0.249078042	count	1
ZNF232	0.2403195	0.3370388	0.713	0.476	0.249107647	count	1
A2M	0.1736179	0.0668036	2.5989	0.0094	0.249260811	count	1
DPAGT1	0.1947741	0.2086364	0.9336	0.351	0.249322025	count	1
RNF144A	0.224294	0.3526386	0.636	0.525	0.249371127	count	1
HNRNPDL	0.1748357	0.0519576	3.365	0.000776	0.249841109	count	1
PHF10	0.1889346	0.1705012	1.1081	0.268	0.250046586	count	1
CBX8	0.4103329	0.8193567	0.5008	0.617	0.2501362	count	1
CCDC6	0.1873357	0.1638072	1.1436	0.253	0.25046171	count	1
RPTOR	0.3179524	0.5139476	0.6186	0.536	0.250567991	count	1
TXN2	0.1778163	0.0859649	2.0685	0.0387	0.250594305	count	1
TAF15	0.1809332	0.1167392	1.5499	0.121	0.250789399	count	1
UBE3B	0.2258485	0.2917655	0.7741	0.439	0.251060793	count	1
RANBP10	0.2316365	0.3088482	0.75	0.453	0.251077826	count	1

GEMIN6	0.1931322	0.2057963	0.9385	0.348	0.251163289	count	1
RBM33	0.189026	0.2141482	0.8827	0.377	0.251234437	count	1
SDCBP2-AS1	0.4671462	0.7603465	0.6144	0.539	0.251563438	count	1
SLC35E2A	0.4671462	0.8780197	0.532	0.595	0.251563438	count	1
SLC11A1	0.4376707	0.9538075	0.4589	0.646	0.251772113	count	1
MIGA2	0.2747398	0.444881	0.6176	0.537	0.251799025	count	1
AHCY	0.1917627	0.1695549	1.131	0.258	0.251844953	count	1
DCP1A	0.1903005	0.1754667	1.0845	0.278	0.252090123	count	1
RNF2	0.2038463	0.189411	1.0762	0.282	0.25235296	count	1
BCL10	0.1805608	0.1195887	1.5098	0.131	0.252353201	count	1
CCT2	0.1789385	0.0778716	2.2979	0.0216	0.252498427	count	1
AC100830.1	0.3503771	1.4601076	0.24	0.8104	0.25276148	count	1
C3orf38	0.2024099	0.2319617	0.8726	0.383	0.252814007	count	1
CDC23	0.2188294	0.3459672	0.6325	0.527	0.252913224	count	1
FRMD8	0.2286441	0.2773998	0.8242	0.41	0.252937045	count	1
CCDC50	0.1781419	0.0791413	2.2509	0.0245	0.25314264	count	1
NDUFAF2	0.1827441	0.1286719	1.4202	0.156	0.253236902	count	1
EXOC2	0.2363991	0.2866768	0.8246	0.41	0.25324468	count	1
TJP2	0.4410279	0.8014887	0.5503	0.582	0.253484859	count	1
AC062029.1	0.4410279	0.9422508	0.4681	0.64	0.253484859	count	1
MAN2A2	0.2767817	0.3983522	0.6948	0.487	0.253589032	count	1
SCO1	0.2062229	0.2468868	0.8353	0.404	0.253602451	count	1
PHF6	0.186633	0.1466646	1.2725	0.203	0.253776894	count	1
GCA	0.1853349	0.1494565	1.2401	0.215	0.253796605	count	1
HHLA3	0.2065433	0.2553717	0.8088	0.419	0.253991269	count	1
FBXO30	0.2401282	0.3335708	0.7199	0.472	0.2540178	count	1
KDSR	0.1829849	0.11416	1.6029	0.109	0.25419181	count	1
CECR2	0.2322693	0.3511713	0.6614	0.508	0.25438081	count	1
ATXN2L	0.2168236	0.2288772	0.9473	0.344	0.254467465	count	1
PRICKLE3	0.3320264	0.5009517	0.6628	0.508	0.254518212	count	1
ARHGAP20	0.1988657	0.2558077	0.7774	0.437	0.254988422	count	1
FLT3LG	0.2000427	0.2490913	0.8031	0.422	0.25499004	count	1
SNX1	0.1915759	0.154051	1.2436	0.214	0.25505052	count	1
MRPL49	0.1997196	0.1761764	1.1336	0.257	0.255094312	count	1
COL4A5	0.6305582	0.807973	0.7804	0.435	0.255137221	count	1
CHN2	0.6305582	0.841414	0.7494	0.454	0.255137221	count	1
WDR4	0.30295	0.4274743	0.7087	0.479	0.255146793	count	1
APPL2	0.223947	0.2545897	0.8796	0.379	0.255148986	count	1
MEGF9	0.2569176	0.4136573	0.6211	0.535	0.255169527	count	1
TLE3	0.2083423	0.211162	0.9866	0.324	0.255287222	count	1
RPL7A	0.1771961	0.0202188	8.7639	3.23E-18	0.255496974	count	7.76E-14
DNAJB6	0.1816379	0.0799375	2.2723	0.0231	0.255534617	count	1
RPL30	0.1772979	0.0192865	9.1928	7.32E-20	0.255644935	count	1.76E-15
IRF2	0.1834267	0.1103277	1.6626	0.0965	0.255753192	count	1
WDR13	0.185848	0.1127431	1.6484	0.0994	0.255758086	count	1

IAH1	0.1894492	0.1458919	1.2986	0.194	0.255765527	count	1
PIBF1	0.1908126	0.1364868	1.398	0.162	0.255844401	count	1
AL441992.1	0.3172564	0.4690382	0.6764	0.499	0.255845298	count	1
ZBTB24	0.1989951	0.2436164	0.8168	0.414	0.255846699	count	1
ENPP4	0.1960613	0.1877276	1.0444	0.296	0.256000463	count	1
MCOLN1	0.2293998	0.2709703	0.8466	0.397	0.25604919	count	1
RAB27A	0.190013	0.142499	1.3334	0.182	0.256057262	count	1
AL035448.1	0.8840871	1.1388829	0.7763	0.438	0.256138787	count	1
ASXL2	0.2065465	0.2249936	0.918	0.359	0.256436394	count	1
GYPC	0.180578	0.0935526	1.9302	0.0537	0.256456838	count	1
SPACA6	0.4776414	0.6214419	0.7686	0.442	0.256495068	count	1
THOC7	0.1816593	0.0800614	2.269	0.0233	0.256657985	count	1
AL135960.1	0.3113193	0.5634155	0.5526	0.581	0.256713307	count	1
MYCBP	0.2149797	0.2592665	0.8292	0.407	0.256930592	count	1
C12orf10	0.1834383	0.0985873	1.8607	0.0629	0.256930941	count	1
WBP2	0.1847296	0.1065602	1.7336	0.0831	0.256987232	count	1
AC020910.4	0.3567866	0.6413814	0.5563	0.578	0.257031189	count	1
EIF2AK2	0.1832498	0.1060638	1.7277	0.0841	0.257134168	count	1
CCDC110	0.3119663	0.5942271	0.525	0.6	0.257216122	count	1
ABTB1	0.1886602	0.160685	1.1741	0.24	0.257320555	count	1
ZNF610	0.4030182	0.5455583	0.7387	0.46	0.257507679	count	1
ZNF800	0.1905357	0.1322614	1.4406	0.15	0.257595585	count	1
TAF8	0.2091649	0.2322983	0.9004	0.368	0.257604408	count	1
ZBTB6	0.2706779	0.4044193	0.6693	0.503	0.257607412	count	1
SS18L2	0.1877618	0.1224485	1.5334	0.125	0.257732497	count	1
BX537318.1	0.3581111	0.731629	0.4895	0.625	0.257911889	count	1
SHE	0.188353	0.121212	1.5539	0.12	0.257959308	count	1
RFX2	0.2395673	0.2825454	0.8479	0.397	0.258024635	count	1
UBAP1	0.2167072	0.2484826	0.8721	0.383	0.258346249	count	1
TTC5	0.2083132	0.2225107	0.9362	0.349	0.258602259	count	1
PRPSAP2	0.208703	0.2168289	0.9625	0.336	0.258687873	count	1
MCEE	0.2094562	0.2422701	0.8646	0.387	0.258799685	count	1
GTDC1	0.26331	0.3335403	0.7894	0.43	0.258847977	count	1
MDH1	0.1865568	0.112991	1.6511	0.0988	0.259088766	count	1
IWS1	0.1928829	0.1491586	1.2931	0.196	0.259145156	count	1
PAK1	0.8988705	0.8871042	1.0133	0.311	0.259163548	count	1
ARGLU1	0.1812658	0.0560803	3.2323	0.00124	0.25927546	count	1
NNT-AS1	0.1958852	0.2027778	0.966	0.334	0.259436815	count	1
TSGA10	0.3612514	0.445536	0.8108	0.418	0.259997779	count	1
HSD17B6	1.1933769	1.1742315	1.0163	0.31	0.260134629	count	1
RPS24	0.1804467	0.019349	9.3259	2.19E-20	0.260178026	count	5.27E-16
CCNH	0.1905888	0.1466085	1.3	0.194	0.260208393	count	1
SH3GLB2	0.1998647	0.1839259	1.0867	0.277	0.260213974	count	1
C12orf45	0.1981908	0.1740548	1.1387	0.255	0.260375046	count	1
METTL21A	0.1997199	0.2034729	0.9816	0.326	0.260387239	count	1

PSMB2	0.1838554	0.0746076	2.4643	0.0138	0.260406874	count	1
GGA2	0.2334101	0.287793	0.811	0.417	0.260423093	count	1
ACVR2A	0.2600722	0.3270154	0.7953	0.427	0.260511549	count	1
NAPRT	0.1892526	0.1327369	1.4258	0.154	0.260692306	count	1
TCERG1	0.193559	0.1682179	1.1506	0.25	0.260809351	count	1
NR4A3	0.3909277	0.5920377	0.6603	0.509	0.260827067	count	1
AC012306.2	0.2122452	0.2470887	0.859	0.39	0.260907915	count	1
APPL1	0.1843149	0.0800007	2.3039	0.0213	0.260996677	count	1
NUFIP1	0.2044096	0.2106984	0.9702	0.332	0.261023858	count	1
SNURF	0.9097917	1.047429	0.8686	0.385	0.261375236	count	1
SPATA41	0.9097917	1.047429	0.8686	0.385	0.261375236	count	1
AC099850.1	0.9097917	1.047429	0.8686	0.385	0.261375236	count	1
ZNF197	0.2442132	0.3217418	0.759	0.448	0.261388034	count	1
MPI	0.2163527	0.2939304	0.7361	0.462	0.261420581	count	1
CENPS	0.3108784	0.4250041	0.7315	0.465	0.261451835	count	1
LINC01089	0.4567361	0.5277868	0.8654	0.387	0.26145475	count	1
ORMDL1	0.1881843	0.1054446	1.7847	0.0744	0.261467454	count	1
ESAM	0.1831897	0.060029	3.0517	0.0023	0.261578139	count	1
TTC19	0.1920963	0.1234667	1.5559	0.12	0.261646711	count	1
WBP1	0.7518667	1.0320141	0.7285	0.466	0.261668241	count	1
MYL2	1.2064745	1.185584	1.0176	0.309	0.261838694	count	1
RCAN1	0.1861026	0.1558259	1.1943	0.232	0.261880227	count	1
SERBP1	0.1826063	0.0444337	4.1096	4.08E-05	0.261921231	count	0.9408888
OXNAD1	0.2694028	0.4137357	0.6511	0.515	0.262005544	count	1
ARMT1	0.2036675	0.2247306	0.9063	0.365	0.262026591	count	1
KIF20B	0.2319651	0.3132298	0.7406	0.459	0.262083337	count	1
ZNF599	0.2617519	0.4165847	0.6283	0.53	0.262135911	count	1
LINC00667	0.1934971	0.1694359	1.142	0.254	0.262218605	count	1
KAT8	0.1993973	0.1626433	1.226	0.22	0.262250344	count	1
INTS11	0.1900895	0.1368194	1.3893	0.165	0.262711619	count	1
SOX7	0.1895334	0.1437483	1.3185	0.187	0.26281143	count	1
GNAS	0.1826671	0.0336278	5.432	6.06E-08	0.262990542	count	0.001426585
SAMD9L	0.1919993	0.1371983	1.3994	0.162	0.263212367	count	1
RPS3A	0.1825712	0.021319	8.5638	1.79E-17	0.26323178	count	4.30E-13
MANSC1	0.1902191	0.1307618	1.4547	0.146	0.263266341	count	1
ITGB1BP1	0.1867341	0.0834289	2.2382	0.0253	0.263271916	count	1
NIN	0.20773	0.2523301	0.8232	0.41	0.263567061	count	1
EXO5	0.3956339	0.6086638	0.65	0.516	0.263680533	count	1
CDKAL1	0.2466099	0.2803104	0.8798	0.379	0.263882638	count	1
ZNF628	0.3280378	0.5623251	0.5834	0.56	0.263997945	count	1
HYLS1	0.3280378	0.5641498	0.5815	0.561	0.263997945	count	1
ADAP2	0.9230063	1.6448005	0.5612	0.5747	0.264025632	count	1
FH	0.2048758	0.1835428	1.1162	0.264	0.264241882	count	1
NEPRO	0.2078311	0.2039255	1.0192	0.308	0.264262567	count	1
FAAP24	0.3366787	0.6530254	0.5156	0.606	0.264355274	count	1

LRRC37A2	0.5351788	0.600088	0.8918	0.373	0.26446962	count	1
EFR3A	0.2011321	0.180037	1.1172	0.264	0.26466437	count	1
MTMR12	0.2099206	0.2321398	0.9043	0.366	0.264795442	count	1
MYLK-AS1	0.7638457	1.0603712	0.7204	0.471	0.264832648	count	1
MCM3AP-AS1	0.7638457	1.1412654	0.6693	0.503	0.264832648	count	1
KLRD1	0.7638457	1.2106035	0.631	0.528	0.264832648	count	1
FAM118A	0.1903586	0.1266297	1.5033	0.133	0.264888146	count	1
GALNT12	1.2305311	1.0179668	1.2088	0.227	0.264918824	count	1
AF117829.1	0.2461486	0.4329443	0.5685	0.57	0.264921612	count	1
TAF6L	0.2434311	0.3245005	0.7502	0.453	0.264933386	count	1
ELF2	0.1883794	0.0881551	2.1369	0.0327	0.264976871	count	1
MARK2	0.2280587	0.313507	0.7274	0.467	0.265048307	count	1
YTHDC1	0.1895087	0.1042402	1.818	0.0692	0.265221238	count	1
PIK3CB	0.2119077	0.2485035	0.8527	0.394	0.265252311	count	1
INF2	0.2066394	0.2155428	0.9587	0.338	0.26534178	count	1
PTGES3	0.1859016	0.0522563	3.5575	0.000381	0.265607493	count	1
SRRM1	0.1861981	0.059096	3.1508	0.00165	0.265636116	count	1
SYNJ2BP	0.1932788	0.1544328	1.2515	0.211	0.265701235	count	1
CCDC113	0.2867152	0.389372	0.7364	0.462	0.265746851	count	1
DOCK7	0.2867152	0.3940489	0.7276	0.467	0.265746851	count	1
SIRT2	0.2006933	0.169401	1.1847	0.236	0.265759379	count	1
CSNK1E	0.204948	0.1991177	1.0293	0.303	0.265805583	count	1
LINC00665	0.2417714	0.3849031	0.6281	0.53	0.265834882	count	1
EGLN1	0.1920643	0.1320336	1.4547	0.146	0.265932742	count	1
TMEM267	0.2237382	0.2775581	0.8061	0.42	0.265935322	count	1
TUBB2A	0.2095282	0.1713822	1.2226	0.222	0.266112688	count	1
DVL2	0.2613744	0.3084657	0.8473	0.397	0.266165468	count	1
C5orf15	0.2052359	0.2049031	1.0016	0.317	0.266175615	count	1
HVCN1	0.2252521	0.2725728	0.8264	0.409	0.266344206	count	1
SLC25A43	0.2615919	0.309867	0.8442	0.399	0.26637959	count	1
PRPF8	0.1963649	0.1489985	1.3179	0.188	0.266426225	count	1
HERC3	0.2230768	0.3150875	0.708	0.479	0.266447201	count	1
TANC1	0.2067831	0.1966199	1.0517	0.293	0.266454213	count	1
GPBP1	0.1884293	0.0803379	2.3455	0.0191	0.266503232	count	1
FP565260.1	1.243226	1.0293796	1.2077	0.227	0.266518511	count	1
STRN	0.2000623	0.1841523	1.0864	0.277	0.266613322	count	1
RPS8	0.184944	0.0196553	9.4094	1.02E-20	0.266709676	count	2.46E-16
NUAK1	0.1903648	0.1053395	1.8072	0.0708	0.267163838	count	1
GOSR1	0.1926263	0.1148901	1.6766	0.0937	0.267169795	count	1
CRYZL1	0.1987524	0.1646055	1.2074	0.227	0.267252379	count	1
NCOA7	0.1872341	0.0782459	2.3929	0.0168	0.267272029	count	1
THYN1	0.1910594	0.0986905	1.9359	0.053	0.267532033	count	1
CPSF3	0.2263462	0.2786905	0.8122	0.417	0.267615	count	1
EIF2B3	0.2147315	0.2199706	0.9762	0.329	0.267635663	count	1
RNF167	0.196621	0.1343942	1.463	0.144	0.267780226	count	1

RIC8A	0.200285	0.1527549	1.3112	0.19	0.267870781	count	1
RBBP5	0.2381925	0.2580549	0.923	0.356	0.2678846	count	1
NAE1	0.2027329	0.1647828	1.2303	0.219	0.267903763	count	1
AP000787.1	0.2976942	0.6629531	0.449	0.653	0.268071816	count	1
ZNF322	0.1979589	0.1519767	1.3026	0.193	0.268162953	count	1
CAMKK1	0.2790936	0.4313301	0.6471	0.518	0.268235616	count	1
ESS2	0.2540096	0.3292984	0.7714	0.441	0.268271343	count	1
KLF2	0.1862538	0.0412135	4.5192	6.47E-06	0.268299931	count	0.15040162
PDCD5	0.1888243	0.0665487	2.8374	0.00458	0.268336732	count	1
ZNF790	0.257626	0.3915922	0.6579	0.511	0.268381017	count	1
SHISA5	0.1906122	0.0861444	2.2127	0.027	0.268572857	count	1
ATAD2B	0.2401077	0.2544711	0.9436	0.345	0.268870992	count	1
BUB3	0.1939948	0.1032497	1.8789	0.0604	0.268920552	count	1
HIPK1	0.2072306	0.1954122	1.0605	0.289	0.268940462	count	1
GSDMD	0.1908087	0.0864598	2.2069	0.0274	0.268996204	count	1
RUFY1	0.2079024	0.2004114	1.0374	0.3	0.269602334	count	1
TBCC	0.2096022	0.1914976	1.0945	0.274	0.269820731	count	1
PELI1	0.2056068	0.1617334	1.2713	0.204	0.269881051	count	1
RNF144B	0.2610854	0.2782643	0.9383	0.348	0.269948	count	1
RBAK	0.3052085	0.4925495	0.6197	0.536	0.270436498	count	1
PAXX	0.1936631	0.1297308	1.4928	0.136	0.270467162	count	1
ALG10B	0.2704131	0.3742338	0.7226	0.47	0.270499271	count	1
CASTOR1	0.2816558	0.3314259	0.8498	0.395	0.270597952	count	1
DUSP6	0.1941542	0.0915676	2.1203	0.0341	0.270604252	count	1
CNOT3	0.2369598	0.2921558	0.8111	0.417	0.270639388	count	1
NBPF12	1.2773664	1.3247034	0.9643	0.335	0.270734257	count	1
EIF2B5	0.2215536	0.2070097	1.0703	0.285	0.270753069	count	1
KIF16B	0.2418397	0.3159676	0.7654	0.444	0.270764953	count	1
NT5C3A	0.1990193	0.1382045	1.44	0.15	0.270786907	count	1
WNT2B	0.2887668	0.9134731	0.3161	0.752	0.270900207	count	1
IFT46	0.245329	0.3051756	0.8039	0.422	0.270936928	count	1
EZH2	0.6054847	0.6260269	0.9672	0.334	0.271015263	count	1
9-Sep	0.2000658	0.133523	1.4984	0.134	0.271073267	count	1
NOP14-AS1	0.3112766	0.5710309	0.5451	0.586	0.271176034	count	1
CA5B	0.2128958	0.2024169	1.0518	0.293	0.271197214	count	1
CDC40	0.1952211	0.1177343	1.6581	0.0974	0.271239603	count	1
AFF1	0.1964186	0.1386762	1.4164	0.157	0.27139367	count	1
CDC26	0.1931835	0.0925528	2.0873	0.037	0.271503435	count	1
EXOC5	0.2034403	0.1497935	1.3581	0.175	0.271530582	count	1
FAM41C	0.4500175	0.6483105	0.6941	0.488	0.271641102	count	1
ZNF580	0.1995963	0.133832	1.4914	0.136	0.272049988	count	1
PCAT1	0.312422	0.4261393	0.7331	0.464	0.272120501	count	1
ZNF646	0.3474282	0.5701775	0.6093	0.542	0.272219344	count	1
GOLPH3	0.2066891	0.1564604	1.321	0.187	0.272371591	count	1
SFTPD	0.9656522	1.1415966	0.8459	0.398	0.272389224	count	1

AKT1S1	0.2106141	0.2234638	0.9425	0.346	0.272454711	count	1
SND1	0.2099148	0.2093166	1.0029	0.316	0.272593539	count	1
KCTD18	0.2508307	0.3406476	0.7363	0.462	0.272771442	count	1
USP25	0.2066889	0.1889694	1.0938	0.274	0.27280862	count	1
DLG2	0.2948351	0.3673465	0.8026	0.422	0.272929656	count	1
CCDC9	0.2641717	0.3691351	0.7157	0.474	0.273035552	count	1
TET3	0.6868359	0.8146432	0.8431	0.399	0.273201369	count	1
NSMCE4A	0.1982474	0.121644	1.6297	0.103	0.273265214	count	1
AL355001.2	0.2463549	0.3468898	0.7102	0.478	0.273297471	count	1
BAP1	0.23948	0.2939421	0.8147	0.415	0.273455469	count	1
ATPAF2	0.2529362	0.3224379	0.7844	0.433	0.273535574	count	1
CD2BP2	0.1989416	0.1217079	1.6346	0.102	0.2735389	count	1
CCDC14	0.2095178	0.2132647	0.9824	0.326	0.273779264	count	1
TJP1	0.1933741	0.0822169	2.352	0.0187	0.274060595	count	1
TRPC4AP	0.224797	0.2616167	0.8593	0.39	0.274152074	count	1
IL15	0.2067885	0.1753913	1.179	0.238	0.274167002	count	1
IFT43	0.197529	0.1196676	1.6506	0.0989	0.274197154	count	1
TMEM41B	0.2271661	0.240028	0.9464	0.344	0.274277069	count	1
PSMG3-AS1	0.3502514	0.4959737	0.7062	0.48	0.274278619	count	1
PTPRK	0.2077786	0.188791	1.1006	0.271	0.274379155	count	1
ZNF41	0.3600167	0.9487114	0.3795	0.704	0.274415055	count	1
KHDRBS1	0.1932151	0.067585	2.8588	0.00428	0.274589437	count	1
AC008543.1	0.274705	0.4016985	0.6839	0.494	0.274635823	count	1
DGKZ	0.2143821	0.1953887	1.0972	0.273	0.274677552	count	1
LINC00957	0.9785498	1.0282505	0.9517	0.341	0.274862388	count	1
LRFN1	0.9785498	1.0583541	0.9246	0.355	0.274862388	count	1
AC008443.5	0.9785498	1.0750999	0.9102	0.363	0.274862388	count	1
CTDSP2	0.2021852	0.1599966	1.2637	0.206	0.275135593	count	1
PCNT	0.2728172	0.3362084	0.8115	0.417	0.27515934	count	1
RERE	0.2002611	0.1456734	1.3747	0.169	0.275217367	count	1
COMMD1	0.1979398	0.1040788	1.9018	0.0573	0.275233959	count	1
FLOT1	0.1934447	0.0617406	3.1332	0.00175	0.275376602	count	1
MAT2B	0.2075432	0.1929571	1.0756	0.282	0.275418217	count	1
APBB2	0.2074867	0.1647026	1.2598	0.208	0.275470661	count	1
HSP90AB1	0.1912073	0.0280245	6.8228	1.09E-11	0.275473772	count	2.60E-07
AL139393.2	0.561748	0.7940796	0.7074	0.479	0.275541278	count	1
AC110769.2	0.8056046	0.7463874	1.0793	0.281	0.275642763	count	1
SNX12	0.2359175	0.2425984	0.9725	0.331	0.275652329	count	1
ISG20	0.2042709	0.1861037	1.0976	0.272	0.27574268	count	1
IFI30	0.2670795	0.4431391	0.6027	0.547	0.275942198	count	1
ZNF517	0.5197609	0.6384836	0.8141	0.416	0.275973585	count	1
ESF1	0.1970357	0.1033034	1.9073	0.0566	0.276024916	count	1
KLF13	0.1959006	0.0927457	2.1122	0.0348	0.27605041	count	1
PIP4P2	0.2121633	0.2219701	0.9558	0.339	0.276082027	count	1
CATSPER2	0.6195202	0.5423558	1.1423	0.253	0.276162626	count	1

CIRBP	0.1927695	0.0451332	4.2711	2.01E-05	0.276209919	count	0.4650135
HCCS	0.2152908	0.1965027	1.0956	0.273	0.276584513	count	1
SCOC	0.1977271	0.1039207	1.9027	0.0572	0.276744521	count	1
EMILIN2	0.2992226	0.4396867	0.6805	0.496	0.276802577	count	1
TCAF2	0.4176759	0.8117414	0.5145	0.607	0.276953068	count	1
CLDN12	0.5226581	0.6274319	0.833	0.405	0.277295048	count	1
NAA20	0.1961866	0.0762614	2.5726	0.0101	0.277328765	count	1
GLCCI1	0.3876537	0.5323662	0.7282	0.467	0.277411293	count	1
MALINC1	0.5665696	0.8005761	0.7077	0.479	0.277530275	count	1
CPNE3	0.1959057	0.0759831	2.5783	0.00998	0.277547418	count	1
THAP5	0.2037888	0.1630021	1.2502	0.211	0.277555012	count	1
RAVER2	0.3088942	0.3550843	0.8699	0.384	0.277650585	count	1
ZNF678	0.2672647	0.4333354	0.6168	0.537	0.278098482	count	1
KIFAP3	0.2023877	0.1495307	1.3535	0.176	0.278127732	count	1
SMU1	0.2033991	0.1268673	1.6032	0.109	0.278162537	count	1
AKNA	0.2588958	0.3526645	0.7341	0.463	0.278249405	count	1
IARS	0.2252243	0.2319509	0.971	0.332	0.278450143	count	1
FBXL2	0.4627965	0.6341476	0.7298	0.466	0.278465024	count	1
LYRM4	0.2113347	0.2030503	1.0408	0.298	0.278595743	count	1
AZIN2	0.5256045	0.6372319	0.8248	0.41	0.278636526	count	1
LENG8-AS1	0.525631	0.7455085	0.7051	0.481	0.278648586	count	1
VDAC3	0.1986197	0.0968905	2.0499	0.0405	0.278678836	count	1
MAD2L1	0.4403332	0.5511202	0.799	0.424	0.278843233	count	1
COPS2	0.1985349	0.0935213	2.1229	0.0339	0.279033073	count	1
NCOA6	0.2419238	0.2720331	0.8893	0.374	0.279046614	count	1
PFDN1	0.1986323	0.0905558	2.1935	0.0284	0.279194986	count	1
ADAM10	0.2025853	0.1221909	1.6579	0.0974	0.279374105	count	1
SMARCC2	0.2015944	0.124941	1.6135	0.107	0.279389226	count	1
AL512353.1	0.7067665	1.0437839	0.6771	0.498	0.279426964	count	1
FAM210A	0.2233387	0.2128276	1.0494	0.294	0.279748594	count	1
FBXO5	0.316403	0.4248266	0.7448	0.456	0.279831433	count	1
OGFRL1	0.2384848	0.2902848	0.8216	0.411	0.280183231	count	1
RPL14	0.1944366	0.0225919	8.6065	1.24E-17	0.280207638	count	2.98E-13
MSL1	0.2165084	0.1934402	1.1193	0.263	0.280225247	count	1
ETFRF1	0.2012719	0.1078096	1.8669	0.062	0.280313052	count	1
SPIN1	0.2164798	0.208346	1.039	0.299	0.280405381	count	1
GTF2IRD1	0.4234958	0.6373238	0.6645	0.506	0.280432227	count	1
PNRC2	0.1976591	0.0729283	2.7103	0.00676	0.280507578	count	1
TFAM	0.2014307	0.1110917	1.8132	0.0699	0.280602655	count	1
MLF2	0.200091	0.0923383	2.1669	0.0303	0.280635437	count	1
FBXO28	0.2340975	0.3061603	0.7646	0.445	0.280672297	count	1
CYCS	0.1991962	0.0808363	2.4642	0.0138	0.280695344	count	1
TSSC4	0.2026065	0.1181427	1.7149	0.0865	0.280695917	count	1
EEF1AKMT2	0.2094523	0.1694113	1.2364	0.216	0.280776659	count	1
WDR41	0.2317421	0.2950911	0.7853	0.432	0.280857697	count	1

ZCCHC9	0.2120401	0.1767583	1.1996	0.23	0.280943563	count	1
TXNDC9	0.203796	0.1150119	1.772	0.0765	0.281224667	count	1
NUDT7	0.2138342	0.2045123	1.0456	0.296	0.281241913	count	1
PPP1R12B	0.3131052	0.4923399	0.636	0.525	0.2812421	count	1
AC012640.2	0.4446019	0.8011473	0.555	0.579	0.281256667	count	1
PARP1	0.2011311	0.1290895	1.5581	0.119	0.281266298	count	1
COX16	0.2369098	0.2438152	0.9717	0.331	0.281306957	count	1
MAP3K10	0.408137	0.556717	0.7331	0.464	0.281309501	count	1
U2SURP	0.1983002	0.0760134	2.6088	0.00914	0.281426242	count	1
KPNB1	0.2028392	0.1098923	1.8458	0.065	0.281430942	count	1
BCCIP	0.2081302	0.1410251	1.4758	0.14	0.281714385	count	1
BRD7	0.2007646	0.1007438	1.9928	0.0464	0.281809915	count	1
UBE2G1	0.2155026	0.1752623	1.2296	0.219	0.28189274	count	1
MT-ND3	0.1959003	0.0360986	5.4268	6.23E-08	0.282093827	count	0.001466417
UBE2D3	0.1977838	0.0579344	3.4139	0.00065	0.282193349	count	1
EVA1C	0.2026781	0.109245	1.8553	0.0637	0.282218566	count	1
SWAP70	0.2007463	0.0860614	2.3326	0.0197	0.282260454	count	1
LINC00672	0.7160112	0.7956495	0.8999	0.368	0.282284512	count	1
AC092910.3	0.8321253	0.9591037	0.8676	0.386	0.282332107	count	1
SCAF1	0.2614577	0.3037825	0.8607	0.389	0.282488894	count	1
IDE	0.2601167	0.3189516	0.8155	0.415	0.282588637	count	1
PNPLA4	0.2445362	0.269807	0.9063	0.365	0.282910604	count	1
ZNF835	0.7183127	1.0704835	0.671	0.502	0.282992947	count	1
AK2	0.2018132	0.0966621	2.0878	0.0369	0.283014833	count	1
DDX3X	0.1988481	0.0649858	3.0599	0.00224	0.283042736	count	1
SMARCA1	0.2240756	0.2090867	1.0717	0.284	0.283099992	count	1
USP9X	0.2207933	0.18883	1.1693	0.242	0.283324371	count	1
LMBRD1	0.2060181	0.1319117	1.5618	0.118	0.283357792	count	1
GEM	0.2638787	0.6282041	0.4201	0.674	0.283448151	count	1
CEP89	0.2269489	0.2157302	1.052	0.293	0.283448779	count	1
GSPT2	0.2407168	0.2835176	0.849	0.396	0.283528671	count	1
PPP1R3B	0.2227034	0.2783528	0.8001	0.424	0.2835488	count	1
HSDL2	0.2163728	0.1690136	1.2802	0.201	0.283722362	count	1
ZNF100	0.2233305	0.2560576	0.8722	0.383	0.283742993	count	1
MFHAS1	0.240266	0.2398809	1.0016	0.317	0.283762182	count	1
CUL4A	0.2212284	0.1999418	1.1065	0.269	0.283876752	count	1
FRS3	0.5022253	0.7487969	0.6707	0.502	0.284126943	count	1
CASP2	0.2541121	0.274374	0.9262	0.354	0.284165125	count	1
MPDZ	0.2068039	0.1308365	1.5806	0.114	0.284215245	count	1
ZNF57	0.4737804	0.8430803	0.562	0.574	0.284291137	count	1
PLA2G16	0.2006232	0.0716477	2.8001	0.00514	0.28433658	count	1
ATP6V1C1	0.2239344	0.2002727	1.1181	0.264	0.284501321	count	1
EHD4	0.204043	0.1047356	1.9482	0.0515	0.284588681	count	1
CIAPIN1	0.2173555	0.1627933	1.3352	0.182	0.284826829	count	1
PPP2R3A	0.2527127	0.2625275	0.9626	0.336	0.284965953	count	1

DPYSL3	0.2051416	0.1432714	1.4318	0.152	0.285163599	count	1
SNRPF	0.2017954	0.0683946	2.9505	0.0032	0.285211462	count	1
TMEM159	0.2147449	0.1776865	1.2086	0.227	0.285294319	count	1
AC004520.1	0.6450071	0.7940032	0.8123	0.417	0.285384935	count	1
PER1	0.2231589	0.1712515	1.3031	0.193	0.285531164	count	1
ETS1	0.2031064	0.0943372	2.153	0.0314	0.285755472	count	1
CLEC7A	0.5056415	1.121137	0.451	0.652	0.285805205	count	1
LIAS	0.2309794	0.2482775	0.9303	0.352	0.285908169	count	1
TRAK1	0.273259	0.3946169	0.6925	0.489	0.286075597	count	1
GRAMD2B	0.2347328	0.2431756	0.9653	0.334	0.286077693	count	1
LINC01415	0.6472263	0.651763	0.993	0.321	0.286180362	count	1
RPUSD4	0.2388325	0.245084	0.9745	0.33	0.286249584	count	1
GSTA4	0.2269087	0.1945602	1.1663	0.244	0.286299537	count	1
SETX	0.209914	0.1480517	1.4178	0.156	0.286344996	count	1
CDC42BPB	0.2096236	0.1272409	1.6475	0.0996	0.286356628	count	1
USP11	0.2432332	0.2679659	0.9077	0.364	0.286434988	count	1
AL133415.1	1.0410032	1.0080027	1.0327	0.302	0.286477241	count	1
FGFBP3	0.3024459	0.4525059	0.6684	0.504	0.286491467	count	1
WIZ	0.2486532	0.3897796	0.6379	0.524	0.286640171	count	1
ZBED6	0.7302278	1.066334	0.6848	0.494	0.286641798	count	1
UFSP1	0.7302278	1.1285809	0.647	0.518	0.286641798	count	1
AREL1	0.2928741	0.578206	0.5065	0.613	0.286733833	count	1
LINC02035	0.543635	0.9717656	0.5594	0.576	0.28679272	count	1
PLEKHA8	0.3028167	0.4817122	0.6286	0.53	0.286826852	count	1
TNC	0.6491438	1.0068292	0.6447	0.519	0.286866665	count	1
LSM14A	0.2076267	0.1169158	1.7759	0.0759	0.286891965	count	1
LRRC8C-DT	0.2577797	0.3297651	0.7817	0.434	0.28692185	count	1
USHBP1	0.2271842	0.2203019	1.0312	0.303	0.286979111	count	1
RNF4	0.2292628	0.229195	1.0003	0.317	0.28707128	count	1
SSRP1	0.2145319	0.1555014	1.3796	0.168	0.287120895	count	1
COQ8A	0.2299393	0.2598739	0.8848	0.376	0.287133394	count	1
SLCO3A1	0.2138315	0.1510878	1.4153	0.157	0.287207461	count	1
RNF146	0.2143216	0.1714109	1.2503	0.211	0.287260887	count	1
LINC00115	0.6504821	0.7957176	0.8175	0.414	0.287345138	count	1
CAST	0.2003415	0.0451029	4.4419	9.27E-06	0.28756617	count	0.21520305
MFSD13A	0.2787642	0.413442	0.6743	0.5	0.287599339	count	1
JMJD1C	0.2029711	0.0758685	2.6753	0.00751	0.287603901	count	1
ZNF581	0.2202776	0.1864154	1.1816	0.237	0.287711905	count	1
HMCES	0.2245808	0.1785397	1.2579	0.209	0.28786865	count	1
APIP	0.213204	0.1497261	1.424	0.155	0.28814438	count	1
ARL2	0.2016518	0.0525888	3.8345	0.000129	0.288306353	count	1
RBM26-AS1	0.4044086	0.6759715	0.5983	0.55	0.288347193	count	1
LINC00941	0.4815092	0.8447663	0.57	0.569	0.288368942	count	1
AC006299.1	0.4815092	0.8753775	0.5501	0.582	0.288368942	count	1
NDUFS2	0.2102805	0.1227517	1.7131	0.0868	0.288408076	count	1

HIST1H3H	0.8569575	1.0582013	0.8098	0.418	0.288473586	count	1
AC084036.1	0.8569575	1.1251463	0.7616	0.446	0.288473586	count	1
CD24	0.8569575	1.1380347	0.753	0.452	0.288473586	count	1
AC105277.1	0.8569575	1.1798528	0.7263	0.468	0.288473586	count	1
AC016957.2	0.8569575	1.214359	0.7057	0.48	0.288473586	count	1
UBXN2B	0.3267823	0.5174017	0.6316	0.528	0.288507611	count	1
CNTNAP3	0.2450539	0.2792318	0.8776	0.38	0.288537019	count	1
SYF2	0.2027144	0.0620325	3.2679	0.0011	0.288690276	count	1
AC018362.1	1.0539079	1.1842623	0.8899	0.374	0.288803987	count	1
NAA80	0.263257	0.4553251	0.5782	0.563	0.288815102	count	1
C2orf73	0.8584322	0.7618037	1.1268	0.26	0.288834601	count	1
C18orf21	0.2210724	0.1904856	1.1606	0.246	0.288927865	count	1
RASSF7	0.2442246	0.2530282	0.9652	0.335	0.28909676	count	1
MORC2-AS1	0.3534642	0.4399043	0.8035	0.422	0.28919001	count	1
CXCL16	0.2420181	0.2692552	0.8988	0.369	0.289337467	count	1
OR2A1-AS1	0.7398378	0.777378	0.9517	0.341	0.289561895	count	1
SIDT2	0.2749554	0.4073171	0.675	0.5	0.289684186	count	1
KIF3C	0.2931585	0.4374662	0.6701	0.503	0.289735646	count	1
OXLD1	0.2179714	0.1802235	1.2095	0.227	0.289806495	count	1
AC118553.2	0.5503568	1.0960292	0.5021	0.616	0.28981008	count	1
ZYG11B	0.2321522	0.229848	1.01	0.313	0.289859114	count	1
OSER1-DT	0.3402654	0.3984058	0.8541	0.393	0.289933808	count	1
RPL11	0.2011484	0.0178047	11.2975	5.77E-29	0.290061124	count	1.40E-24
GSKIP	0.2282618	0.2265957	1.0074	0.314	0.29024079	count	1
PHLDA2	0.2331545	0.2431456	0.9589	0.338	0.29027984	count	1
ING1	0.2153939	0.1382222	1.5583	0.119	0.290406064	count	1
PYM1	0.2187912	0.1977752	1.1063	0.269	0.290497775	count	1
ELK1	0.2557666	0.2747097	0.931	0.352	0.290552595	count	1
RIT1	0.2131213	0.1411477	1.5099	0.131	0.290637194	count	1
PRR13	0.2058041	0.0811795	2.5352	0.0113	0.290637986	count	1
SLC1A3	0.6597477	1.1070394	0.596	0.551	0.290645745	count	1
AP000879.1	0.6597477	1.1070394	0.596	0.551	0.290645745	count	1
PRR29-AS1	0.6597477	1.1583474	0.5696	0.569	0.290645745	count	1
AC008264.2	0.4407367	0.7422322	0.5938	0.553	0.290676871	count	1
CRYBG3	0.2093283	0.1446514	1.4471	0.148	0.29109598	count	1
CYFIP1	0.2161096	0.1426263	1.5152	0.13	0.291096653	count	1
AP001505.1	1.0669602	1.0897499	0.9791	0.328	0.291132341	count	1
SYNPO2	1.0669602	1.1341388	0.9408	0.347	0.291132341	count	1
ZMYND15	1.459881	1.0631522	1.3732	0.17	0.291249738	count	1
FANCD2	0.6617442	0.8090307	0.8179	0.413	0.291354193	count	1
SETDB2	0.2979912	0.3765495	0.7914	0.429	0.291535028	count	1
SLC25A40	0.3491898	0.3939877	0.8863	0.376	0.291640518	count	1
DNTTIP2	0.207517	0.0903793	2.2961	0.0217	0.291706658	count	1
LCMT2	0.488157	0.8857253	0.5511	0.582	0.291862077	count	1
KATNA1	0.2236479	0.1758946	1.2715	0.204	0.291881238	count	1

STRBP	0.273622	0.2735796	1.0002	0.317	0.291897403	count	1
CKMT2-AS1	0.3164391	0.4091612	0.7734	0.439	0.291943837	count	1
DIS3	0.2148596	0.1303741	1.648	0.0995	0.292020306	count	1
AC008014.1	0.5183683	0.6900161	0.7512	0.453	0.292027528	count	1
FABP4	0.2033355	0.1235117	1.6463	0.0998	0.292233432	count	1
ZSCAN2	0.3657706	0.6134101	0.5963	0.551	0.292240171	count	1
GPANK1	0.223067	0.1678183	1.3292	0.184	0.292244464	count	1
PSENEN	0.2079808	0.0916942	2.2682	0.0234	0.292396159	count	1
AP003068.2	1.47146	1.1892128	1.2373	0.216	0.292442838	count	1
LDAH	0.4108749	0.5471683	0.7509	0.453	0.292543874	count	1
SNAP47	0.2492465	0.2708251	0.9203	0.357	0.292571471	count	1
PDLIM2	0.208506	0.0982583	2.122	0.0339	0.292620166	count	1
HLA-DQA2	1.4737498	0.2442288	6.0343	1.81E-09	0.292677445	count	4.29E-05
VRK3	0.2276126	0.2302483	0.9886	0.323	0.292757328	count	1
TMEM127	0.2290709	0.2143312	1.0688	0.285	0.293009985	count	1
CENPB	0.2253346	0.1812714	1.2431	0.214	0.293056141	count	1
ASB6	0.2782763	0.3499385	0.7952	0.427	0.293068623	count	1
ERO1B	0.2537764	0.2684334	0.9454	0.345	0.293366525	count	1
ILVBL	0.2245676	0.1872031	1.1996	0.23	0.293453725	count	1
LINC01106	1.0801527	1.1314039	0.9547	0.34	0.293460314	count	1
NTAN1	0.2087627	0.0903833	2.3097	0.021	0.29355734	count	1
WHAMM	0.2194303	0.1662481	1.3199	0.187	0.293631275	count	1
HSPBAP1	0.2946318	0.3862722	0.7628	0.446	0.293773614	count	1
DNMT1	0.2158247	0.135277	1.5954	0.111	0.29379581	count	1
TRAPPC8	0.2427628	0.2904293	0.8359	0.403	0.293987823	count	1
TULP3	0.2391748	0.2301299	1.0393	0.299	0.293999498	count	1
DENR	0.2148799	0.1217374	1.7651	0.0777	0.294116254	count	1
ZNF436-AS1	0.5601366	0.6720027	0.8335	0.405	0.294177612	count	1
AP003059.1	0.4927721	0.8798773	0.56	0.575	0.294279287	count	1
MOK	0.2474468	0.2825643	0.8757	0.381	0.294305148	count	1
ZCCHC3	0.2420753	0.2943195	0.8225	0.411	0.294321164	count	1
JAK1	0.2063601	0.0595199	3.4671	5.00E-04	0.294518604	count	1
HLA-G	0.6707406	1.12307	0.5972	0.55	0.294534415	count	1
DAGLB	0.3341366	0.4580322	0.7295	0.466	0.294634939	count	1
VPS35	0.2100249	0.09815	2.1398	0.0325	0.294761353	count	1
RCAN3	0.2882134	0.3422074	0.8422	0.4	0.294785959	count	1
PHF20	0.2156927	0.1448053	1.4895	0.136	0.294948483	count	1
ZNF764	0.3788421	0.5145137	0.7363	0.462	0.294989842	count	1
BTBD9	0.3893765	0.505977	0.7696	0.442	0.295017035	count	1
ATG4B	0.2432924	0.227569	1.0691	0.285	0.295203243	count	1
EEF1AKMT4	0.3019952	0.3761103	0.8029	0.422	0.295286564	count	1
NPDC1	0.2053423	0.0401068	5.1199	3.27E-07	0.295416364	count	0.007666515
AC044839.1	0.6106633	1.3507409	0.4521	0.651	0.295433458	count	1
CDC42SE1	0.2160395	0.1305744	1.6545	0.0981	0.295856575	count	1
ERLIN2	0.2361644	0.2497562	0.9456	0.344	0.296364502	count	1

ITGA9	0.2361681	0.2372351	0.9955	0.32	0.296369084	count	1
MOCS1	0.8896917	0.7539755	1.18	0.238	0.296391639	count	1
WDR43	0.2162332	0.1265292	1.709	0.0876	0.296584582	count	1
TAF10	0.2090876	0.0734506	2.8466	0.00445	0.296620111	count	1
SERTAD3	0.2126313	0.1065366	1.9959	0.046	0.296794137	count	1
TRNAU1AP	0.2239973	0.1667837	1.343	0.179	0.296801999	count	1
SCAMP3	0.219631	0.1584098	1.3865	0.166	0.296944236	count	1
FBXO32	0.3427256	0.6367372	0.5383	0.59	0.296959457	count	1
LEMD2	0.2587115	0.2658376	0.9732	0.331	0.296974702	count	1
POLM	0.2723971	0.2970407	0.917	0.359	0.297071679	count	1
JADE3	0.4729441	0.5729866	0.8254	0.409	0.297138902	count	1
RARA	0.2215361	0.162717	1.3615	0.173	0.297179786	count	1
G6PC3	0.2207053	0.1618479	1.3637	0.173	0.297339697	count	1
CBWD3	0.4340366	0.4661444	0.9311	0.352	0.297415589	count	1
BOD1L1	0.2094202	0.0930013	2.2518	0.0244	0.297519087	count	1
ARL13B	0.2267263	0.1620077	1.3995	0.162	0.297531627	count	1
TSC22D3	0.2092343	0.0654609	3.1963	0.00141	0.297607952	count	1
IP6K1	0.2407187	0.2369368	1.016	0.31	0.297783501	count	1
RASIP1	0.212179	0.0875862	2.4225	0.0155	0.297821697	count	1
SAMD5	0.3828354	0.5251722	0.729	0.466	0.297861811	count	1
WARS	0.21258	0.1201197	1.7697	0.0769	0.297960987	count	1
PCED1A	0.3081929	0.372761	0.8268	0.408	0.298082372	count	1
GTF2A1	0.2332258	0.210789	1.1064	0.269	0.298263194	count	1
SHLD2	0.2435781	0.2368639	1.0283	0.304	0.298299394	count	1
USP14	0.2179342	0.1455141	1.4977	0.134	0.298438728	count	1
WDR25	0.2684163	0.292766	0.9168	0.359	0.298444111	count	1
KDELC1	0.3445635	0.4457398	0.773	0.44	0.298456691	count	1
KMT2E	0.2102244	0.0741834	2.8338	0.00463	0.298469963	count	1
GPATCH1	0.2968814	0.2977682	0.997	0.319	0.298485845	count	1
BORCS5	0.2641138	0.3009844	0.8775	0.38	0.298666737	count	1
PHLDA3	0.2234614	0.1559549	1.4329	0.152	0.298761483	count	1
WDR83	0.2467763	0.2546025	0.9693	0.332	0.298764059	count	1
ZNF681	0.2771382	0.4176703	0.6635	0.507	0.298916503	count	1
PDS5B	0.2242341	0.1531669	1.464	0.143	0.298958159	count	1
ALDH1B1	0.3202432	0.3649661	0.8775	0.38	0.298990945	count	1
KIAA0895	0.6198079	0.758178	0.8175	0.414	0.299082135	count	1
ARSB	0.6839422	0.6667488	1.0258	0.305	0.299165507	count	1
UCK1	0.2557632	0.2559487	0.9993	0.318	0.2992155	count	1
WDR91	0.3247465	0.493507	0.658	0.511	0.29921781	count	1
GEMIN7	0.2494643	0.2574744	0.9689	0.333	0.299426051	count	1
ENTR1	0.2361469	0.2275068	1.038	0.299	0.29950218	count	1
EBP	0.235145	0.1995007	1.1787	0.239	0.299505569	count	1
YTHDF1	0.2457238	0.2161147	1.137	0.256	0.299805509	count	1
RPL35A	0.2080694	0.0211381	9.8433	1.71E-22	0.299908627	count	4.12E-18
FLVCR2	1.552245	1.4895899	1.0421	0.297	0.300431526	count	1

MITD1	0.2220094	0.1409953	1.5746	0.115	0.300549435	count	1
HMGN3-AS1	0.3471543	0.4936511	0.7032	0.482	0.300565485	count	1
EPS8L1	0.3018898	0.3721807	0.8111	0.417	0.300716381	count	1
LONRF3	0.3976967	0.7943573	0.5007	0.617	0.300805232	count	1
BOLA1	0.2335883	0.2160135	1.0814	0.28	0.300873072	count	1
UBC	0.2089011	0.0296223	7.0522	2.22E-12	0.300887738	count	5.30E-08
RYR3	0.2386531	0.3189553	0.7482	0.454	0.300923913	count	1
RPS18	0.2088066	0.0211653	9.8655	1.38E-22	0.301087685	count	3.33E-18
SLC39A8	0.3478224	0.471012	0.7385	0.46	0.30110894	count	1
ATF4	0.2127194	0.075747	2.8083	0.00502	0.301127829	count	1
AP004609.3	0.4588232	0.5950563	0.7711	0.441	0.301324314	count	1
RPS27A	0.2090124	0.0183895	11.3659	2.74E-29	0.301381375	count	6.63E-25
NUDT21	0.2207304	0.1469915	1.5017	0.133	0.301802556	count	1
PPP2R5E	0.2195543	0.1286076	1.7072	0.0879	0.301845911	count	1
MANBA	0.315841	0.4058262	0.7783	0.436	0.30193254	count	1
C2orf68	0.2755992	0.3461632	0.7962	0.426	0.301965245	count	1
ARID1B	0.2204225	0.167597	1.3152	0.189	0.30220041	count	1
VPS50	0.2524756	0.297842	0.8477	0.397	0.302295901	count	1
ECH1	0.2132758	0.0729866	2.9221	0.0035	0.302625175	count	1
TSR3	0.219181	0.1251083	1.7519	0.0799	0.30275493	count	1
ELP2	0.23322	0.1895462	1.2304	0.219	0.30277154	count	1
C1orf131	0.2208953	0.1425485	1.5496	0.121	0.30289723	count	1
SERTAD2	0.2302078	0.1742576	1.3211	0.187	0.30292615	count	1
KMT2E-AS1	0.2990093	0.3131864	0.9547	0.34	0.303020874	count	1
MGAT2	0.2406649	0.1899132	1.2672	0.205	0.303063251	count	1
APAF1	0.2486252	0.2504142	0.9929	0.321	0.303286092	count	1
INTS2	0.3341157	0.3575018	0.9346	0.35	0.30333511	count	1
PEX10	0.2527981	0.2388174	1.0585	0.29	0.30335321	count	1
OGG1	0.2528396	0.257031	0.9837	0.325	0.303402084	count	1
LURAP1	0.2848173	0.3839628	0.7418	0.458	0.303453533	count	1
FANCB	0.4625139	0.653979	0.7072	0.479	0.303484473	count	1
LIG3	0.2996833	0.3231613	0.9273	0.354	0.303677343	count	1
C2orf74	0.2203519	0.1395096	1.5795	0.114	0.303687355	count	1
AC079922.2	0.3575771	0.4599876	0.7774	0.437	0.303746409	count	1
C18orf25	0.2549676	0.301179	0.8466	0.397	0.303807241	count	1
FAM86B1	0.4148156	0.6249239	0.6638	0.507	0.304132076	count	1
AC245595.1	0.2498576	0.2610182	0.9572	0.339	0.304198071	count	1
ABHD10	0.2560196	0.279122	0.9172	0.359	0.304300894	count	1
TTC27	0.2857368	0.295634	0.9665	0.334	0.304401237	count	1
MIR22HG	0.2177674	0.1062718	2.0492	0.0405	0.304422957	count	1
PCOLCE	0.4866368	0.4959426	0.9812	0.327	0.304723646	count	1
NUP43	0.2897964	0.3479558	0.8329	0.405	0.304786638	count	1
AC048341.2	0.7912029	0.9264784	0.854	0.393	0.304826991	count	1
RAP2A	0.2565339	0.2548779	1.0065	0.314	0.304900099	count	1
DOCK9	0.219437	0.1307388	1.6784	0.0934	0.305159155	count	1

FBXO46	0.3745121	0.4134243	0.9059	0.365	0.305198345	count	1
XRRA1	0.2527105	0.305145	0.8282	0.408	0.305198404	count	1
GTPBP3	0.2815882	0.2766456	1.0179	0.309	0.305206531	count	1
GULP1	0.2304068	0.1849288	1.2459	0.213	0.305224842	count	1
SRD5A3	0.2292006	0.1585947	1.4452	0.149	0.305275714	count	1
CCDC112	0.2322817	0.1797151	1.2925	0.196	0.305286555	count	1
KIAA0825	0.3318366	0.4491078	0.7389	0.46	0.305409311	count	1
RAPGEF4	0.2370836	0.2012693	1.1779	0.239	0.305581235	count	1
TTC17	0.2269037	0.1944774	1.1667	0.243	0.305635402	count	1
NT5DC2	0.4882902	0.6316214	0.7731	0.44	0.305635619	count	1
PCF11-AS1	0.9290361	1.181872	0.7861	0.432	0.305645821	count	1
ATP7A	0.2542404	0.2766185	0.9191	0.358	0.305719376	count	1
PDHX	0.267539	0.2891276	0.9253	0.355	0.305804187	count	1
KLHL9	0.2543287	0.2880492	0.8829	0.377	0.305823586	count	1
CNBD2	0.3045907	0.3840824	0.793	0.428	0.305924545	count	1
ZFYVE28	0.3020706	0.3368323	0.8968	0.37	0.306001533	count	1
NT5C	0.2336138	0.2043629	1.1431	0.253	0.306119099	count	1
PATZ1	0.3204813	0.4131474	0.7757	0.438	0.306159365	count	1
KCNS3	0.7042498	0.8130093	0.8662	0.386	0.306206893	count	1
COL5A1	0.291199	0.3701651	0.7867	0.432	0.306210948	count	1
IL15RA	0.2246495	0.1515873	1.482	0.138	0.306465173	count	1
HEATR1	0.2454142	0.2784978	0.8812	0.378	0.306596854	count	1
RSBN1	0.2246395	0.1411183	1.5919	0.112	0.306639039	count	1
INO80E	0.2458311	0.2063937	1.1911	0.234	0.30669085	count	1
TMCO4	0.2623128	0.3105866	0.8446	0.398	0.306711562	count	1
MFSD3	0.3685484	0.4487104	0.8214	0.412	0.306718254	count	1
SPTLC3	0.3550214	0.3624298	0.9796	0.327	0.306955997	count	1
C16orf58	0.2740237	0.3185053	0.8603	0.39	0.307118098	count	1
EPB41L4A-DT	0.2921626	0.3960697	0.7377	0.461	0.30718916	count	1
H1FX-AS1	0.4336452	0.6089897	0.7121	0.476	0.307216113	count	1
TIGAR	0.2903534	0.3015712	0.9628	0.336	0.307286644	count	1
RAB9B	1.1628937	1.0283809	1.1308	0.258	0.307492805	count	1
AC016588.2	1.1628937	1.1251189	1.0336	0.301	0.307492805	count	1
PDSS2	0.2541371	0.3059075	0.8308	0.406	0.30751609	count	1
PSMA6	0.2443106	0.2376616	1.028	0.304	0.307591739	count	1
STXBP6	1.163873	0.9544909	1.2194	0.223	0.307653212	count	1
ARID2	0.2443668	0.2591934	0.9428	0.346	0.30766153	count	1
KANTR	0.3866457	0.5630839	0.6867	0.492	0.307669635	count	1
SRSF8	0.2231501	0.1160769	1.9224	0.0547	0.307694882	count	1
ZNF837	0.9383727	0.737322	1.2727	0.203	0.307800368	count	1
MBLAC1	0.4509484	0.548346	0.8224	0.411	0.307818182	count	1
MSRA	0.2255216	0.1293893	1.743	0.0815	0.307836722	count	1
IRF2BP2	0.2192972	0.1007979	2.1756	0.0297	0.307863308	count	1
LLGL2	0.2827993	0.3000589	0.9425	0.346	0.308073853	count	1
TMEM86B	0.4514336	1.7307145	0.2608	0.7942	0.308115298	count	1

TRAM2-AS1	0.2557601	0.3225227	0.793	0.428	0.308170434	count	1
OAS2	0.244521	0.2238461	1.0924	0.275	0.30822305	count	1
LINC00342	0.8032137	1.0422208	0.7707	0.441	0.308314074	count	1
AC233723.1	0.8032137	1.0422208	0.7707	0.441	0.308314074	count	1
AC084018.2	0.8032137	1.3963856	0.5752	0.565	0.308314074	count	1
IRAK4	0.2351362	0.1719932	1.3671	0.172	0.308465081	count	1
RNF187	0.224663	0.1235877	1.8178	0.0692	0.308644764	count	1
CEP128	0.7113835	0.6678349	1.0652	0.287	0.308656828	count	1
GIT1	0.2308334	0.1950893	1.1832	0.237	0.308662057	count	1
AC092040.1	0.4093474	0.6197444	0.6605	0.509	0.308873085	count	1
LEMD3	0.2634402	0.2764002	0.9531	0.341	0.308874313	count	1
MICALL1	0.4942905	0.534199	0.9253	0.355	0.308938193	count	1
PBX1	0.2293854	0.1516656	1.5124	0.131	0.309051343	count	1
NUP93	0.2714731	0.2764348	0.9821	0.326	0.3090827	count	1
TMX1	0.2241127	0.1209258	1.8533	0.0639	0.309181246	count	1
SMYD3	0.2408006	0.2378205	1.0125	0.311	0.309253856	count	1
PAIP1	0.2206931	0.097644	2.2602	0.0239	0.309502149	count	1
DHX37	0.4730209	0.4913211	0.9628	0.336	0.309610946	count	1
AL356512.1	1.176424	0.9991428	1.1774	0.239	0.30969642	count	1
PDRG1	0.2401275	0.2007636	1.1961	0.232	0.309716663	count	1
TACC1	0.2164975	0.0569796	3.7996	0.000148	0.309727375	count	1
MON2	0.2446771	0.2592617	0.9437	0.345	0.310180664	count	1
AL391834.2	0.411329	0.6374304	0.6453	0.519	0.310240955	count	1
AC091729.3	0.411329	0.672803	0.6114	0.541	0.310240955	count	1
SMPD4	0.2782335	0.3494373	0.7962	0.426	0.310400867	count	1
KIF2A	0.2231689	0.1233241	1.8096	0.0705	0.310494341	count	1
ARL6IP6	0.2309329	0.1613878	1.4309	0.153	0.310532656	count	1
CCNT2-AS1	15.6650279	1097.93939	0.0143	0.989	0.310563591	count	1
AC099568.2	15.6652551	1180.563565	0.0133	0.989	0.310563591	count	1
AC092718.1	15.6655052	1330.471684	0.0118	0.991	0.310563591	count	1
GOLGA6L9	15.7741604	1274.45979	0.0124	0.99	0.310563596	count	1
MEG3	15.7749112	1490.53614	0.0106	0.992	0.310563596	count	1
PRMT5-AS1	15.8269311	1281.889948	0.0123	0.99	0.310563599	count	1
ZNF311	15.8712932	1142.629118	0.0139	0.9889	0.310563601	count	1
SYTL2	16.1247305	1274.149076	0.0127	0.99	0.310563611	count	1
AL035587.1	16.1694426	1169.455765	0.0138	0.989	0.310563612	count	1
AQP9	16.189037	2640.513854	0.0061	0.995	0.310563613	count	1
FAT1	16.1953108	1815.526468	0.0089	0.993	0.310563613	count	1
LINC01684	16.1953108	1815.526465	0.0089	0.993	0.310563613	count	1
OSGEPL1-AS1	16.1953176	1815.52516	0.0089	0.993	0.310563613	count	1
HOOK1	16.1953178	1815.527436	0.0089	0.993	0.310563613	count	1
RAD9B	16.1953225	1815.529633	0.0089	0.993	0.310563613	count	1
COL25A1	16.2093524	1082.184429	0.015	0.988	0.310563614	count	1
C12orf60	16.2981106	1083.499671	0.015	0.988	0.310563616	count	1
ZSCAN22	16.3468109	1565.112431	0.0104	0.9917	0.310563618	count	1

PFN4	15.6650304	1097.939501	0.0143	0.989	0.310563619	count	1
SLC25A15	16.3883475	2126.373796	0.0077	0.994	0.310563619	count	1
AC087289.5	16.3883956	1823.185861	0.009	0.993	0.310563619	count	1
PLD6	16.4182117	2597.664106	0.0063	0.995	0.31056362	count	1
ASF1B	16.4182117	2597.664062	0.0063	0.995	0.31056362	count	1
LOXL3	16.4182118	2597.664106	0.0063	0.995	0.31056362	count	1
AL138820.1	16.4182163	2597.66393	0.0063	0.995	0.31056362	count	1
AC106881.1	16.4182165	2597.663952	0.0063	0.995	0.31056362	count	1
AC010761.1	16.4182256	2597.664948	0.0063	0.995	0.31056362	count	1
AC091891.2	16.4182257	2597.664948	0.0063	0.995	0.31056362	count	1
DNAH12	16.4185759	3100.105661	0.0053	0.996	0.31056362	count	1
AL157786.1	16.418576	3100.105648	0.0053	0.996	0.31056362	count	1
AC037487.2	16.4190648	3916.500606	0.0042	0.997	0.31056362	count	1
AL391834.1	16.4830535	1560.380156	0.0106	0.9916	0.310563621	count	1
PSMD6-AS1	16.4856667	2330.305771	0.0071	0.994	0.310563621	count	1
AC021752.1	15.7741588	1274.460025	0.0124	0.99	0.310563624	count	1
FAM74A7	16.708808	1808.07639	0.0092	0.993	0.310563626	count	1
RAB27B	16.7088203	1808.080053	0.0092	0.993	0.310563626	count	1
C9orf43	15.826932	1281.890652	0.0123	0.99	0.310563627	count	1
FMO3	16.7529692	2604.1717	0.0064	0.995	0.310563627	count	1
TTLL9	16.7529692	2604.1717	0.0064	0.995	0.310563627	count	1
NUF2	16.7529693	2604.171711	0.0064	0.995	0.310563627	count	1
AMY2B	16.7529779	2604.175924	0.0064	0.995	0.310563627	count	1
TOM1L1	16.7842261	3180.08239	0.0053	0.996	0.310563627	count	1
AC036214.1	16.7842262	3180.08258	0.0053	0.996	0.310563627	count	1
TUBB3	16.7842262	3180.08258	0.0053	0.996	0.310563627	count	1
TRPM2	16.7842264	3180.082643	0.0053	0.996	0.310563627	count	1
KLHL7-DT	16.7842265	3180.08239	0.0053	0.996	0.310563627	count	1
LINC02099	16.7842265	3180.082517	0.0053	0.996	0.310563627	count	1
AC090186.1	16.7842266	3180.082438	0.0053	0.996	0.310563627	count	1
LVRN	16.7849288	4108.888571	0.0041	0.997	0.310563627	count	1
C15orf38-AP3S2	16.7849289	4108.888408	0.0041	0.997	0.310563627	count	1
AC099811.3	16.7849293	4108.888653	0.0041	0.997	0.310563627	count	1
BDNF	16.7859246	5517.166162	0.003	0.998	0.310563627	count	1
UBE2F-SCLY	16.7859248	5517.166602	0.003	0.998	0.310563627	count	1
TFAP2B	16.7859248	5517.166327	0.003	0.998	0.310563627	count	1
UCP3	16.7859248	5517.166602	0.003	0.998	0.310563627	count	1
AC074050.4	16.8199204	1857.115591	0.0091	0.993	0.310563628	count	1
RAB39A	16.8679405	2361.860369	0.0071	0.994	0.310563629	count	1
AC004803.1	16.8679405	2361.860349	0.0071	0.994	0.310563629	count	1
HCK	16.8679509	2361.862844	0.0071	0.994	0.310563629	count	1
AL445483.1	16.8679511	2361.862844	0.0071	0.994	0.310563629	count	1
AC104041.1	16.8679567	2361.862692	0.0071	0.994	0.310563629	count	1
ACSM5	16.8683851	3785.791643	0.0045	0.996	0.310563629	count	1
PITRM1-AS1	16.9958466	3916.153958	0.0043	0.997	0.310563631	count	1

NCAM2	15.9279696	1098.24999	0.0145	0.988	0.310563631	count	1
LINC01119	15.9281412	1178.242024	0.0135	0.989	0.310563631	count	1
HIST1H4B	15.9407648	1312.474306	0.0121	0.99	0.310563632	count	1
CDC6	16.0168782	1096.059236	0.0146	0.988	0.310563635	count	1
PFKFB2	16.01698	1162.521421	0.0138	0.989	0.310563635	count	1
LINC00638	17.328201	2591.174587	0.0067	0.995	0.310563635	count	1
AC027601.4	17.3282659	3097.824574	0.0056	0.996	0.310563635	count	1
AC090510.1	16.0372886	1077.643678	0.0149	0.9881	0.310563636	count	1
HNRNPA1L2	16.1248336	1092.258303	0.0148	0.9882	0.310563639	count	1
TCAP	16.177106	1069.120732	0.0151	0.9879	0.310563641	count	1
AC003101.2	16.1875699	1570.236863	0.0103	0.992	0.310563641	count	1
CKMT2	16.1875704	1570.236594	0.0103	0.992	0.310563641	count	1
AC013468.1	16.1875706	1570.235585	0.0103	0.992	0.310563641	count	1
AOX1	16.187573	1570.237069	0.0103	0.992	0.310563641	count	1
AC006449.5	16.187614	1874.085588	0.0086	0.993	0.310563641	count	1
TMEM182	16.1876141	1874.085594	0.0086	0.993	0.310563641	count	1
AL357078.1	16.1896743	2278.380674	0.0071	0.994	0.310563641	count	1
AL355297.4	16.1953108	1815.526458	0.0089	0.993	0.310563641	count	1
AL672277.1	16.1953108	1815.526455	0.0089	0.993	0.310563641	count	1
MRPS24	16.1953178	1815.527427	0.0089	0.993	0.310563641	count	1
AL590705.1	16.1953179	1815.527427	0.0089	0.993	0.310563641	count	1
NECAB1	16.1953198	1815.527779	0.0089	0.993	0.310563641	count	1
EFCAB6	16.1953244	1815.529611	0.0089	0.993	0.310563641	count	1
AC097358.2	16.1953246	1815.529624	0.0089	0.993	0.310563641	count	1
SNRK-AS1	16.1953481	2117.403849	0.0076	0.994	0.310563641	count	1
AC016405.3	16.1953483	2117.403849	0.0076	0.994	0.310563641	count	1
AC068768.1	16.1953504	2117.400173	0.0076	0.994	0.310563641	count	1
NTF3	16.1953508	2117.402428	0.0076	0.994	0.310563641	count	1
CDCA5	16.2701408	1144.877873	0.0142	0.9887	0.310563643	count	1
AC233309.1	16.3468126	1565.112412	0.0104	0.9917	0.310563646	count	1
HOXD10	16.3468146	1565.112383	0.0104	0.9917	0.310563646	count	1
AL136985.3	16.3883549	2126.374877	0.0077	0.994	0.310563647	count	1
AP001363.2	16.3883949	1823.184845	0.009	0.993	0.310563647	count	1
SPAG5	16.3883985	1823.187219	0.009	0.993	0.310563647	count	1
LINC01220	16.388399	1823.188624	0.009	0.993	0.310563647	count	1
AC093627.5	16.3883991	1823.188631	0.009	0.993	0.310563647	count	1
AL121672.3	16.3884007	1823.18755	0.009	0.993	0.310563647	count	1
AL606760.1	16.3884074	1823.187116	0.009	0.993	0.310563647	count	1
RFX3-AS1	16.4182114	2597.663985	0.0063	0.995	0.310563648	count	1
TMEM200B	16.4182115	2597.663985	0.0063	0.995	0.310563648	count	1
AC005550.2	16.4182115	2597.663996	0.0063	0.995	0.310563648	count	1
AF131216.4	16.4182115	2597.66404	0.0063	0.995	0.310563648	count	1
COLCA2	16.4182115	2597.663974	0.0063	0.995	0.310563648	count	1
AKAP3	16.4182115	2597.663974	0.0063	0.995	0.310563648	count	1
PRC1	16.4182115	2597.664095	0.0063	0.995	0.310563648	count	1

AL360219.1	16.4182116	2597.664051	0.0063	0.995	0.310563648	count	1
LGALS8-AS1	16.4182116	2597.664095	0.0063	0.995	0.310563648	count	1
AC097662.1	16.4182116	2597.663996	0.0063	0.995	0.310563648	count	1
DDIT4L	16.4182116	2597.664084	0.0063	0.995	0.310563648	count	1
HIST1H2BF	16.4182116	2597.664084	0.0063	0.995	0.310563648	count	1
ATP6V1FNB	16.4182116	2597.664051	0.0063	0.995	0.310563648	count	1
LRRC24	16.4182116	2597.664051	0.0063	0.995	0.310563648	count	1
AC007216.4	16.4182116	2597.664051	0.0063	0.995	0.310563648	count	1
AC015726.1	16.4182116	2597.664084	0.0063	0.995	0.310563648	count	1
AC016708.1	16.4182117	2597.664084	0.0063	0.995	0.310563648	count	1
LINC01970	16.4182117	2597.664106	0.0063	0.995	0.310563648	count	1
ZIM2-AS1	16.4182117	2597.664073	0.0063	0.995	0.310563648	count	1
Z97192.1	16.4182118	2597.664127	0.0063	0.995	0.310563648	count	1
SDK1	16.4182163	2597.663876	0.0063	0.995	0.310563648	count	1
SEPT7-AS1	16.4182163	2597.66392	0.0063	0.995	0.310563648	count	1
AC137630.1	16.4182164	2597.663898	0.0063	0.995	0.310563648	count	1
AC012603.1	16.4182164	2597.663909	0.0063	0.995	0.310563648	count	1
CMYA5	16.4182165	2597.66393	0.0063	0.995	0.310563648	count	1
AL158152.2	16.4182165	2597.663963	0.0063	0.995	0.310563648	count	1
AL139339.1	16.4182165	2597.663974	0.0063	0.995	0.310563648	count	1
NEURL1	16.4182165	2597.663909	0.0063	0.995	0.310563648	count	1
AC012150.1	16.4182165	2597.66393	0.0063	0.995	0.310563648	count	1
RNASEH2B-AS1	16.4182165	2597.663952	0.0063	0.995	0.310563648	count	1
UNC79	16.4182165	2597.66392	0.0063	0.995	0.310563648	count	1
CACNA1F	16.4182167	2597.663963	0.0063	0.995	0.310563648	count	1
AL049629.1	16.4182258	2597.664948	0.0063	0.995	0.310563648	count	1
MMP2-AS1	16.4185759	3100.105596	0.0053	0.996	0.310563648	count	1
PAPPA	16.418576	3100.105596	0.0053	0.996	0.310563648	count	1
AC141424.1	16.418576	3100.105609	0.0053	0.996	0.310563648	count	1
NLGN3	16.4185761	3100.105648	0.0053	0.996	0.310563648	count	1
AC007292.2	16.4185761	3100.105661	0.0053	0.996	0.310563648	count	1
ANKRD34A	16.4185769	3100.11069	0.0053	0.996	0.310563648	count	1
WNT11	16.4185769	3100.11069	0.0053	0.996	0.310563648	count	1
CCR7	16.4190648	3916.500655	0.0042	0.997	0.310563648	count	1
GHET1	16.4443195	1700.512816	0.0097	0.9923	0.310563648	count	1
AC135050.3	16.4775501	1514.253725	0.0109	0.9913	0.310563649	count	1
AC104458.1	16.4830516	1560.38016	0.0106	0.9916	0.310563649	count	1
ABCA10	16.4847342	2160.913691	0.0076	0.994	0.310563649	count	1
FAM227A	16.5390162	1818.339988	0.0091	0.993	0.31056365	count	1
AL590068.1	16.5390164	1818.339991	0.0091	0.993	0.31056365	count	1
HIPK1-AS1	16.561463	1791.0746	0.0092	0.993	0.310563651	count	1
AC124319.2	16.561463	1791.074594	0.0092	0.993	0.310563651	count	1
GLDC	16.5614729	1791.07349	0.0092	0.993	0.310563651	count	1
PLAC8	16.582114	1166.87153	0.0142	0.9887	0.310563651	count	1
KIFC2	16.6021599	1556.047012	0.0107	0.9915	0.310563652	count	1

AC123595.1	16.708808	1808.076387	0.0092	0.993	0.310563654	count	1
AC026471.2	16.7088168	1808.077545	0.0092	0.993	0.310563654	count	1
CCDC188	16.7088168	1808.077545	0.0092	0.993	0.310563654	count	1
DGAT2	16.708821	1808.079058	0.0092	0.993	0.310563654	count	1
AC097381.1	16.7529652	2604.169053	0.0064	0.995	0.310563655	count	1
AC106739.1	16.7529652	2604.169031	0.0064	0.995	0.310563655	count	1
E2F1	16.7529652	2604.169053	0.0064	0.995	0.310563655	count	1
NTN5	16.7529691	2604.1717	0.0064	0.995	0.310563655	count	1
AC138356.1	16.7529692	2604.1717	0.0064	0.995	0.310563655	count	1
AP001469.3	16.7529692	2604.171733	0.0064	0.995	0.310563655	count	1
FBXO15	16.7529693	2604.171733	0.0064	0.995	0.310563655	count	1
AC092123.1	16.7529694	2604.171788	0.0064	0.995	0.310563655	count	1
FAAH2	16.7529695	2604.171755	0.0064	0.995	0.310563655	count	1
OR4D1	16.7529695	2604.171766	0.0064	0.995	0.310563655	count	1
SERF1B	16.7529745	2604.1717	0.0064	0.995	0.310563655	count	1
SLC25A30-AS1	16.7529785	2604.172902	0.0064	0.995	0.310563655	count	1
AP002807.1	16.7529786	2604.172946	0.0064	0.995	0.310563655	count	1
MEIG1	16.7529787	2604.172913	0.0064	0.995	0.310563655	count	1
AC008622.2	16.7531575	3107.84932	0.0054	0.996	0.310563655	count	1
LMO3	16.7531629	3107.845463	0.0054	0.996	0.310563655	count	1
AL592301.1	16.7533948	3926.049183	0.0043	0.997	0.310563655	count	1
LINC02210-CRHR1	16.7534037	3926.053159	0.0043	0.997	0.310563655	count	1
COL4A4	16.7534228	3044.994244	0.0055	0.996	0.310563655	count	1
TEX22	16.7536227	3485.812245	0.0048	0.996	0.310563655	count	1
AC245297.2	16.784226	3180.08239	0.0053	0.996	0.310563655	count	1
POT1-AS1	16.784226	3180.082343	0.0053	0.996	0.310563655	count	1
AC024243.1	16.7842262	3180.082327	0.0053	0.996	0.310563655	count	1
AP003721.4	16.7842262	3180.082548	0.0053	0.996	0.310563655	count	1
AL162591.2	16.7842263	3180.082374	0.0053	0.996	0.310563655	count	1
AC104187.1	16.7842263	3180.082628	0.0053	0.996	0.310563655	count	1
FGF9	16.7842263	3180.082533	0.0053	0.996	0.310563655	count	1
AL139220.2	16.7842264	3180.082517	0.0053	0.996	0.310563655	count	1
SYCP1	16.7842264	3180.082327	0.0053	0.996	0.310563655	count	1
EPHA6	16.7842264	3180.082548	0.0053	0.996	0.310563655	count	1
GABRB1	16.7842264	3180.082548	0.0053	0.996	0.310563655	count	1
AL513122.2	16.7842264	3180.082564	0.0053	0.996	0.310563655	count	1
AL137025.1	16.7842264	3180.082596	0.0053	0.996	0.310563655	count	1
NPIPB11	16.7842264	3180.082628	0.0053	0.996	0.310563655	count	1
AC027682.1	16.7842264	3180.082501	0.0053	0.996	0.310563655	count	1
RAP1GAP2	16.7842264	3180.082406	0.0053	0.996	0.310563655	count	1
ATP1B2	16.7842264	3180.082469	0.0053	0.996	0.310563655	count	1
AP000897.2	16.7842265	3180.082533	0.0053	0.996	0.310563655	count	1
MAST1	16.7842265	3180.082485	0.0053	0.996	0.310563655	count	1
AC006483.2	16.7842267	3180.082517	0.0053	0.996	0.310563655	count	1
AC084082.1	16.7842267	3180.08258	0.0053	0.996	0.310563655	count	1

AL392048.1	16.7842267	3180.082485	0.0053	0.996	0.310563655	count	1
AP001033.1	16.7842267	3180.082485	0.0053	0.996	0.310563655	count	1
ZNF697	16.7842269	3180.082754	0.0053	0.996	0.310563655	count	1
AC034102.6	16.7842269	3180.082533	0.0053	0.996	0.310563655	count	1
GRIN2D	16.7849288	4108.888428	0.0041	0.997	0.310563655	count	1
AL022328.3	16.7849288	4108.888326	0.0041	0.997	0.310563655	count	1
AC113349.2	16.7849289	4108.888551	0.0041	0.997	0.310563655	count	1
OR6A2	16.784929	4108.888612	0.0041	0.997	0.310563655	count	1
ZSCAN4	16.7849291	4108.88851	0.0041	0.997	0.310563655	count	1
CX3CR1	16.7849292	4108.888796	0.0041	0.997	0.310563655	count	1
MND1	16.7849292	4108.888694	0.0041	0.997	0.310563655	count	1
AL356417.1	16.7849292	4108.888756	0.0041	0.997	0.310563655	count	1
AL354710.2	16.7849292	4108.888633	0.0041	0.997	0.310563655	count	1
LINC01666	16.7849292	4108.888592	0.0041	0.997	0.310563655	count	1
KLHL31	16.7849293	4108.888776	0.0041	0.997	0.310563655	count	1
AC138627.1	16.7859245	5517.166355	0.003	0.998	0.310563655	count	1
AC080188.1	16.7859246	5517.166382	0.003	0.998	0.310563655	count	1
AL354956.1	16.7859247	5517.166162	0.003	0.998	0.310563655	count	1
AC096586.2	16.785925	5517.16685	0.003	0.998	0.310563655	count	1
SLC36A2	16.7859251	5517.166575	0.003	0.998	0.310563655	count	1
LINC01238	16.7859252	5517.16674	0.003	0.998	0.310563655	count	1
TNIP3	16.8190267	2063.985485	0.0081	0.993	0.310563656	count	1
GVQW2	16.8191048	1769.618905	0.0095	0.9924	0.310563656	count	1
VIP	16.8191083	1769.618275	0.0095	0.9924	0.310563656	count	1
AC017083.2	16.8679404	2361.860349	0.0071	0.994	0.310563657	count	1
AC087203.3	16.8679404	2361.860369	0.0071	0.994	0.310563657	count	1
ELFN1-AS1	16.8679405	2361.860359	0.0071	0.994	0.310563657	count	1
CRYGS	16.8679406	2361.860349	0.0071	0.994	0.310563657	count	1
GPC4	16.8679406	2361.860369	0.0071	0.994	0.310563657	count	1
TBX6	16.8679445	2361.865969	0.0071	0.994	0.310563657	count	1
AC011815.1	16.8679511	2361.862844	0.0071	0.994	0.310563657	count	1
AC108734.4	16.8679512	2361.862854	0.0071	0.994	0.310563657	count	1
PLSCR3	16.8681363	2914.762318	0.0058	0.995	0.310563657	count	1
PKN2-AS1	16.8681408	2914.76744	0.0058	0.995	0.310563657	count	1
CALN1	16.8681408	2914.76744	0.0058	0.995	0.310563657	count	1
CD79A	16.8681408	2914.767452	0.0058	0.995	0.310563657	count	1
MKI67	16.8683852	3785.791676	0.0045	0.996	0.310563657	count	1
AL162171.1	16.9402562	1789.73509	0.0095	0.9924	0.310563658	count	1
IQCD	16.9956375	2597.526611	0.0065	0.995	0.310563659	count	1
ERICH4	16.9956427	2597.525165	0.0065	0.995	0.310563659	count	1
AC005899.5	16.9956432	2597.525198	0.0065	0.995	0.310563659	count	1
ACRBP	16.995651	2597.528812	0.0065	0.995	0.310563659	count	1
AL136418.1	16.995651	2597.528834	0.0065	0.995	0.310563659	count	1
AL355816.2	16.9956515	2597.527432	0.0065	0.995	0.310563659	count	1
DUOX1	16.9956515	2597.52741	0.0065	0.995	0.310563659	count	1

TMEM74	16.9956518	2597.527432	0.0065	0.995	0.310563659	count	1
LINC00900	16.99574	3100.006348	0.0055	0.996	0.310563659	count	1
Z95115.1	16.9968941	3422.172279	0.005	0.996	0.310563659	count	1
AP001596.2	17.0197819	1752.836588	0.0097	0.992	0.310563659	count	1
TH	17.3269727	2345.852205	0.0074	0.994	0.310563663	count	1
VLDLR	17.3269851	2345.850912	0.0074	0.994	0.310563663	count	1
NRXN3	17.327016	2895.094589	0.006	0.995	0.310563663	count	1
GUK1	0.2158365	0.0345322	6.2503	4.73E-10	0.310571525	count	1.12E-05
DDX23	0.2486606	0.211674	1.1747	0.24	0.31059335	count	1
TPD52L2	0.2323317	0.1444897	1.6079	0.108	0.31076781	count	1
GCFC2	0.2466341	0.2667388	0.9246	0.355	0.310850292	count	1
ZNF35	0.2920339	0.3020775	0.9668	0.334	0.310885581	count	1
VGLL4	0.2202703	0.0917659	2.4003	0.0164	0.3110065	count	1
ZFAND5	0.2211937	0.0825975	2.678	0.00745	0.311075468	count	1
CRBN	0.2196241	0.0792079	2.7728	0.0056	0.31123331	count	1
NPEPL1	0.2506752	0.2228483	1.1249	0.261	0.311313096	count	1
TMEM243	0.2256569	0.1326835	1.7007	0.0891	0.311464657	count	1
RNF25	0.2707516	0.2861277	0.9463	0.344	0.311507649	count	1
ZNF626	0.2505172	0.2418986	1.0356	0.3	0.311571282	count	1
NDST1-AS1	0.9549024	1.1252401	0.8486	0.396	0.311576245	count	1
SERPING1	0.2180663	0.0700781	3.1118	0.00188	0.311582858	count	1
RPS14	0.2161702	0.0195025	11.0842	5.77E-28	0.311694691	count	1.39E-23
C6orf106	0.244122	0.1928332	1.266	0.206	0.31172737	count	1
MLX	0.230297	0.1526201	1.509	0.131	0.311782142	count	1
LINC02452	0.815528	1.1235891	0.7258	0.468	0.311857226	count	1
ZNF296	0.4998538	0.6076266	0.8226	0.411	0.311990401	count	1
CNNM3	0.4579259	0.5042072	0.9082	0.364	0.312083786	count	1
ABT1	0.2310857	0.1434552	1.6109	0.107	0.312156459	count	1
ZNF211	0.4144811	0.438506	0.9452	0.345	0.31241422	count	1
DDHD2	0.2606774	0.2554856	1.0203	0.308	0.312626517	count	1
CMAS	0.2317772	0.1466475	1.5805	0.114	0.313084505	count	1
NAPG	0.2298738	0.1420138	1.6187	0.106	0.313290214	count	1
INTS14	0.2534669	0.2400657	1.0558	0.291	0.313303951	count	1
AL583785.1	0.2445404	0.1870474	1.3074	0.191	0.31343542	count	1
UNC45A	0.2476033	0.2427346	1.0201	0.308	0.313495521	count	1
FIZ1	0.3004359	0.4085964	0.7353	0.462	0.31350175	count	1
FSIP1	0.604292	0.8032681	0.7523	0.452	0.313565755	count	1
THNSL1	0.3947375	0.4931871	0.8004	0.424	0.313613005	count	1
HSPA14	0.3287742	0.3509384	0.9368	0.349	0.313697427	count	1
FMR1	0.2340432	0.1709368	1.3692	0.171	0.313725851	count	1
EIF2A	0.2226312	0.0752854	2.9572	0.00313	0.313803394	count	1
ARMC1	0.2361826	0.1607342	1.4694	0.142	0.313961035	count	1
TPCN1	0.2748689	0.3224729	0.8524	0.394	0.313972914	count	1
PARP14	0.2223512	0.1063463	2.0908	0.0366	0.313976759	count	1
CLN6	0.72731	0.6708678	1.0841	0.278	0.314082389	count	1

TOR1AIP1	0.2343984	0.1935319	1.2112	0.226	0.314086683	count	1
FBXW9	0.3131009	0.3414852	0.9169	0.359	0.314116591	count	1
PCMT1	0.2239898	0.0889348	2.5186	0.0118	0.314129376	count	1
HMG20B	0.2285657	0.1221369	1.8714	0.0614	0.314176379	count	1
CD109	0.2293286	0.1423528	1.611	0.107	0.314185845	count	1
NDST1	0.2555326	0.2242607	1.1394	0.255	0.314298871	count	1
HIST1H3A	0.9681515	1.0408217	0.9302	0.352	0.314567408	count	1
NBAS	0.2529673	0.2208489	1.1454	0.252	0.314571866	count	1
HIST1H4H	0.3870177	0.5516536	0.7016	0.483	0.314642708	count	1
MTX2	0.2282122	0.1280047	1.7828	0.0747	0.314770367	count	1
RNF214	0.2366487	0.1867482	1.2672	0.205	0.315116797	count	1
TBL1X	0.2749825	0.3965371	0.6935	0.488	0.315192284	count	1
EZH1	0.2371462	0.1834146	1.293	0.196	0.31536918	count	1
LSM5	0.2238862	0.0874967	2.5588	0.0106	0.315407358	count	1
SGTA	0.254055	0.1995387	1.2732	0.203	0.315445349	count	1
PRMT6	0.3883375	0.622867	0.6235	0.533	0.315636521	count	1
ZNF687	0.3883431	0.8296392	0.4681	0.64	0.315640726	count	1
TOMM22	0.2241696	0.0862804	2.5982	0.00942	0.315661841	count	1
F2R	0.2277738	0.1220513	1.8662	0.0621	0.315697049	count	1
MTERF2	0.2628258	0.2674647	0.9827	0.326	0.315845017	count	1
CENPJ	0.2887491	0.3640822	0.7931	0.428	0.315934903	count	1
PABPC4L	0.398108	0.5634121	0.7066	0.48	0.316082391	count	1
6-Mar	0.2314069	0.1256799	1.8412	0.0657	0.316201267	count	1
GAB3	0.4085511	0.4930724	0.8286	0.407	0.316233857	count	1
AC144652.1	0.4085511	0.5323633	0.7674	0.443	0.316233857	count	1
SMIM19	0.2274519	0.1176247	1.9337	0.0533	0.316404512	count	1
GADD45A	0.2240799	0.1139046	1.9673	0.0493	0.316553751	count	1
SLX4IP	0.3036275	0.3598671	0.8437	0.399	0.316709674	count	1
GPHN	0.2842914	0.3315282	0.8575	0.391	0.31696826	count	1
F8A1	0.265041	0.2591421	1.0228	0.307	0.317043287	count	1
KLHL15	0.322133	0.4247126	0.7585	0.448	0.317102022	count	1
SCAP	0.2691455	0.3013664	0.8931	0.372	0.31713953	count	1
CHD9	0.2238651	0.0768823	2.9118	0.00362	0.317482993	count	1
CARD8	0.2289809	0.1231763	1.859	0.0631	0.317496242	count	1
TK2	0.2529251	0.20604	1.2276	0.22	0.31749719	count	1
PSMD12	0.2283771	0.102934	2.2187	0.0266	0.317518181	count	1
C17orf58	0.2410701	0.1892637	1.2737	0.203	0.317779043	count	1
AC020915.3	0.3228893	0.4028397	0.8015	0.423	0.317813104	count	1
NIPSNAP1	0.2572419	0.2381249	1.0803	0.28	0.317894677	count	1
YTHDF2	0.2282248	0.1054499	2.1643	0.0305	0.317928725	count	1
MOSMO	0.2356901	0.1615515	1.4589	0.145	0.318059796	count	1
CCZ1B	0.3567589	0.3634935	0.9815	0.326	0.318151796	count	1
ARL5B	0.2385054	0.1613197	1.4785	0.139	0.318221304	count	1
CHAMP1	0.2654499	0.2581187	1.0284	0.304	0.31823762	count	1
SNRPE	0.2252269	0.0744393	3.0256	0.0025	0.318253095	count	1

SUMF1	0.268039	0.2321023	1.1548	0.248	0.31829103	count	1
MIF	0.224558	0.0696829	3.2226	0.00129	0.31848852	count	1
GCH1	0.2710615	0.2479611	1.0932	0.274	0.318489908	count	1
MRPL19	0.232912	0.1323189	1.7602	0.0785	0.318549744	count	1
INVS	0.5738484	0.5423426	1.0581	0.29	0.318603511	count	1
ANKAR	0.2683363	0.2414055	1.1116	0.266	0.318636715	count	1
TOR1B	0.2826625	0.2837848	0.996	0.319	0.319080087	count	1
AC008035.1	0.8410586	0.8803614	0.9554	0.339	0.319100475	count	1
CACFD1	0.6712833	0.6265954	1.0713	0.284	0.319212889	count	1
MISP3	0.3703162	0.5532419	0.6694	0.503	0.319324335	count	1
LRFN4	0.3705404	0.3950186	0.938	0.348	0.319505094	count	1
KIAA0753	0.3025898	0.4157874	0.7278	0.467	0.319779305	count	1
EIF2D	0.2357674	0.1550745	1.5203	0.129	0.319956886	count	1
STX17-AS1	0.3712052	0.4541802	0.8173	0.414	0.32004097	count	1
ZBTB48	0.3719154	0.3806801	0.977	0.329	0.320613291	count	1
CENPV	0.4721614	0.779396	0.6058	0.545	0.320738771	count	1
NRF1	0.3867999	0.4458963	0.8675	0.386	0.32082447	count	1
FBXO36	0.3721992	0.5710315	0.6518	0.515	0.320841947	count	1
MAD2L2	0.2375639	0.1376499	1.7259	0.0845	0.321120055	count	1
C1orf52	0.2342076	0.1252346	1.8702	0.0616	0.3211674	count	1
PEF1	0.2305411	0.1015973	2.2692	0.0233	0.321170032	count	1
NHSL1	0.3152833	0.3656185	0.8623	0.389	0.321360824	count	1
AC083798.2	0.7491089	0.7776159	0.9633	0.335	0.32141048	count	1
CRCP	0.2377195	0.169871	1.3994	0.162	0.321506021	count	1
UNC93B1	0.2690832	0.2724097	0.9878	0.323	0.32178096	count	1
UGP2	0.2280427	0.0956977	2.3829	0.0172	0.321805466	count	1
NEIL1	0.2570325	0.2093416	1.2278	0.22	0.322166912	count	1
VEGFA	0.2978169	0.3305584	0.901	0.368	0.322225123	count	1
EXOSC6	0.2357496	0.1390926	1.6949	0.0902	0.322226806	count	1
UBE2J2	0.239823	0.1439351	1.6662	0.0958	0.322295773	count	1
ACTR10	0.2298521	0.0912238	2.5197	0.0118	0.322378475	count	1
WDR5B	0.3386181	0.588794	0.5751	0.565	0.322618746	count	1
ANKS3	0.3054266	0.3446195	0.8863	0.376	0.322669895	count	1
RPL5	0.2238303	0.0219744	10.186	6.04E-24	0.322684878	count	1.46E-19
TTC1	0.2298138	0.0910356	2.5244	0.0116	0.322789191	count	1
CYB5R4	0.2735586	0.316597	0.8641	0.388	0.323071431	count	1
RGS19	0.2429504	0.1718771	1.4135	0.158	0.323301824	count	1
RRP7A	0.2318495	0.1236124	1.8756	0.0608	0.323435185	count	1
RMND1	0.2437224	0.1973876	1.2347	0.217	0.323464856	count	1
NOL11	0.2468481	0.1807656	1.3656	0.172	0.3236781	count	1
OLMALINC	0.4773702	0.6454012	0.7396	0.46	0.323889656	count	1
HNRNPL	0.2396072	0.1399786	1.7117	0.0871	0.323953309	count	1
TOX4	0.2305898	0.0906109	2.5448	0.011	0.324040933	count	1
TMEM140	0.2349038	0.1451343	1.6185	0.106	0.324043826	count	1
ZNF575	0.757394	0.7498201	1.0101	0.313	0.324166105	count	1

SGMS1	0.2546445	0.2228008	1.1429	0.253	0.324346153	count	1
SLC39A1	0.2298194	0.0830056	2.7687	0.00567	0.324509627	count	1
AMMECR1L	0.3185233	0.3574286	0.8912	0.373	0.32452797	count	1
TFB2M	0.2690487	0.258754	1.0398	0.299	0.324549967	count	1
LRRC4C	0.6852517	0.4790977	1.4303	0.153	0.324557004	count	1
NCALD	0.4994955	0.5366689	0.9307	0.352	0.32489527	count	1
AL161729.1	0.7604131	1.0963236	0.6936	0.488	0.325166243	count	1
MYO9A	0.2408583	0.1600431	1.505	0.132	0.325175192	count	1
BRAP	0.2839344	0.2786331	1.019	0.308	0.325190237	count	1
NGDN	0.2387209	0.1317574	1.8118	0.0701	0.325277627	count	1
HNRNPH1	0.2286777	0.0713288	3.206	0.00136	0.325401184	count	1
RABL2A	0.4473531	0.5465002	0.8186	0.413	0.325709333	count	1
TTC30A	0.4473531	0.5518122	0.8107	0.418	0.325709333	count	1
ZBTB8A	0.2817537	0.3677967	0.7661	0.444	0.325950163	count	1
UBLCP1	0.2530921	0.2064788	1.2258	0.22	0.325969886	count	1
RUVBL1	0.2409241	0.1446801	1.6652	0.096	0.326075362	count	1
PRDM11	0.4023803	0.5106569	0.788	0.431	0.3261761	count	1
WAC-AS1	0.2391084	0.1472131	1.6242	0.104	0.326277492	count	1
ZNF317	0.3861074	0.5464698	0.7065	0.48	0.326303041	count	1
ZMAT2	0.2360487	0.1133149	2.0831	0.0373	0.326502932	count	1
RNF114	0.2372344	0.1257113	1.8871	0.0592	0.32652135	count	1
LRRC29	0.4640916	0.5537324	0.8381	0.402	0.326576416	count	1
EMP1	0.2288839	0.0627131	3.6497	0.000267	0.326675207	count	1
MKRN1	0.2618456	0.1964756	1.3327	0.183	0.326807629	count	1
TMEM101	0.2395524	0.1333766	1.7961	0.0726	0.326879894	count	1
RAI2	0.265067	0.2076814	1.2763	0.202	0.326886718	count	1
IRX5	0.8697416	0.8112145	1.0721	0.284	0.327074754	count	1
RB1	0.2386735	0.1409267	1.6936	0.0905	0.327148133	count	1
SNX25	0.3165385	0.4030245	0.7854	0.432	0.327386258	count	1
KDM1A	0.2473353	0.1897865	1.3032	0.193	0.327450611	count	1
FEM1A	0.3624377	0.3869738	0.9366	0.349	0.32753765	count	1
CNOT1	0.2528071	0.2002048	1.2627	0.207	0.327673421	count	1
AUTS2	0.5287614	0.4662165	1.1342	0.257	0.327697546	count	1
2-Mar	0.2605199	0.2002844	1.3007	0.193	0.327701629	count	1
VPS4A	0.2445536	0.149681	1.6338	0.102	0.328052002	count	1
UBN1	0.2357632	0.1215478	1.9397	0.0525	0.328203747	count	1
DCTN4	0.2675615	0.2577314	1.0381	0.299	0.328285145	count	1
MTRNR2L12	0.2288552	0.0544043	4.2066	2.68E-05	0.328285945	count	0.6191872
CDK1	0.3280244	0.3697947	0.887	0.375	0.328432641	count	1
ZCCHC8	0.2582779	0.2124928	1.2155	0.224	0.328577534	count	1
MRPL30	0.2598205	0.2263354	1.1479	0.251	0.3287474	count	1
IQUB	1.0329812	1.0416814	0.9916	0.321	0.328758276	count	1
POLE	1.3004679	1.0940855	1.1886	0.235	0.328764759	count	1
FYB1	0.6399247	0.6983865	0.9163	0.36	0.328819203	count	1
KDM5B	0.2449624	0.1700466	1.4406	0.15	0.328821361	count	1

DARS	0.2382598	0.1268625	1.8781	0.0605	0.328823282	count	1
NDOR1	0.8761731	0.8813602	0.9941	0.32	0.328839307	count	1
RPS7	0.2281739	0.0219524	10.394	7.53E-25	0.328925024	count	1.82E-20
AC010969.2	0.3895247	0.5739357	0.6787	0.497	0.328987456	count	1
ACTB	0.2283074	0.0311518	7.3289	3.03E-13	0.329044244	count	7.24E-09
SH2B2	0.3316757	0.3427234	0.9678	0.333	0.32905143	count	1
PDCD11	0.2855151	0.4793243	0.5957	0.551	0.329141297	count	1
SNHG8	0.2315069	0.0613172	3.7756	0.000163	0.329307781	count	1
ABHD11	0.3100829	0.3187702	0.9727	0.331	0.329414105	count	1
DDX10	0.269152	0.2284321	1.1783	0.239	0.329635545	count	1
BRD3	0.2524392	0.184778	1.3662	0.172	0.329706738	count	1
AHDC1	0.3240314	0.2912719	1.1125	0.266	0.329905507	count	1
TRIM47	0.236318	0.1082307	2.1835	0.0291	0.329910247	count	1
TRIO	0.2588781	0.3023374	0.8563	0.392	0.329997284	count	1
PLCG1	0.2804236	0.3483891	0.8049	0.421	0.330128327	count	1
RPS28	0.2291325	0.0214567	10.6788	4.10E-26	0.330296137	count	9.90E-22
CNIH3	0.3196487	0.3544507	0.9018	0.367	0.330475264	count	1
POLR2J2	0.4546853	0.7331246	0.6202	0.535	0.33052481	count	1
PIK3IP1	0.2486906	0.1649408	1.5078	0.132	0.330589889	count	1
SLC25A24	0.2479889	0.1566454	1.5831	0.114	0.330770504	count	1
S1PR1	0.2366952	0.1147931	2.0619	0.0393	0.330774981	count	1
TFDP1	0.2617848	0.201609	1.2985	0.194	0.33082429	count	1
ARHGAP5-AS1	0.534758	0.5701471	0.9379	0.348	0.330923852	count	1
B4GALT6	0.3922071	0.5801109	0.6761	0.499	0.331091973	count	1
SYNE3	0.2643836	0.2254458	1.1727	0.241	0.331240086	count	1
RXRB	0.2856059	0.264527	1.0797	0.28	0.331321696	count	1
AP1S1	0.2441962	0.1368279	1.7847	0.0744	0.331564108	count	1
IDNK	0.2713028	0.2607674	1.0404	0.298	0.33163948	count	1
PIK3CA	0.2509321	0.1817576	1.3806	0.168	0.331680939	count	1
NFKB1	0.2443733	0.1634192	1.4954	0.135	0.33188244	count	1
TMEM229B	0.7046596	1.0453734	0.6741	0.5	0.331898943	count	1
AL390957.1	0.7046596	2.3657216	0.2979	0.7658	0.331898943	count	1
LYRM7	0.2701168	0.2455665	1.1	0.271	0.331922443	count	1
APOPT1	0.2426904	0.1275169	1.9032	0.0571	0.332034087	count	1
GAR1	0.2474904	0.1570643	1.5757	0.115	0.332075708	count	1
PPCDC	0.2830198	0.3248602	0.8712	0.384	0.332215814	count	1
GLRX2	0.2460419	0.1860977	1.3221	0.186	0.332220058	count	1
HIF3A	0.2767414	0.1990414	1.3904	0.165	0.332227457	count	1
C1orf35	0.2449376	0.1417848	1.7275	0.0842	0.33223978	count	1
ZSWIM3	0.5671721	0.63631	0.8913	0.373	0.332368458	count	1
RIMKLA	0.2839755	0.2842091	0.9992	0.318	0.332387578	count	1
C8orf58	0.3000102	0.2763385	1.0857	0.278	0.332509884	count	1
GFER	0.2482818	0.1712038	1.4502	0.147	0.332544966	count	1
MBD6	0.3015073	0.2862778	1.0532	0.292	0.332607956	count	1
MT-ND2	0.2309693	0.0374729	6.1636	8.15E-10	0.332710783	count	1.93E-05

SAFB	0.2476678	0.1734142	1.4282	0.153	0.332759459	count	1
EIF4B	0.2334606	0.0545445	4.2802	1.93E-05	0.332805727	count	0.4465827
RGMB-AS1	0.891133	1.0214473	0.8724	0.383	0.332910673	count	1
ACSM6	0.891133	1.0419837	0.8552	0.392	0.332910673	count	1
SALL2	0.891133	1.223211	0.7285	0.466	0.332910673	count	1
FASTKD3	0.3327075	0.358688	0.9276	0.354	0.332912067	count	1
NUDT16	0.2417752	0.1212217	1.9945	0.0462	0.33297863	count	1
NAB1	0.2524505	0.2340298	1.0787	0.281	0.332988491	count	1
TMEM168	0.26451	0.2358738	1.1214	0.262	0.333048616	count	1
LTN1	0.2484324	0.1558363	1.5942	0.111	0.333099248	count	1
CASP8	0.2840383	0.2923001	0.9717	0.331	0.333383503	count	1
SCUBE3	0.6055864	0.9707494	0.6238	0.533	0.333407268	count	1
TRUB1	0.3121543	0.375393	0.8315	0.406	0.33348717	count	1
RTKN	0.3141395	0.2749089	1.1427	0.253	0.333566752	count	1
ANKMY2	0.3550485	0.4940518	0.7186	0.472	0.333658832	count	1
CASKIN2	0.2529038	0.1653329	1.5297	0.126	0.333925489	count	1
CCT7	0.2371883	0.0817437	2.9016	0.00374	0.33399964	count	1
ABCG1	0.2509716	0.1642349	1.5281	0.127	0.334026256	count	1
RCCD1	0.4607035	0.5073062	0.9081	0.364	0.334464398	count	1
RABGAP1L	0.2718317	0.221003	1.23	0.219	0.334543323	count	1
C17orf80	0.2737526	0.2540534	1.0775	0.281	0.334578958	count	1
TTN	1.9862977	2.6156418	0.7594	0.4477	0.334605803	count	1
RPS4X	0.2322225	0.021995	10.558	1.42E-25	0.334796479	count	3.43E-21
TAPT1	0.279016	0.2673249	1.0437	0.297	0.334901771	count	1
TUBA1B	0.233259	0.0435144	5.3605	8.98E-08	0.334961093	count	0.002112006
GALC	0.3101575	0.3450649	0.8988	0.369	0.33512178	count	1
EIF3J-DT	0.2533588	0.2001752	1.2657	0.206	0.335351818	count	1
ACSF2	0.543102	0.6400361	0.8485	0.396	0.335394846	count	1
COG8	0.5736699	0.8408117	0.6823	0.495	0.335616694	count	1
COQ10A	0.3161925	0.2870239	1.1016	0.271	0.33566671	count	1
DDX19B	0.2824054	0.2491603	1.1334	0.257	0.335792637	count	1
DENND4B	0.3532521	0.3915826	0.9021	0.367	0.335827698	count	1
CD72	0.7158857	1.1492905	0.6229	0.533	0.336101762	count	1
CLP1	0.2800417	0.2510649	1.1154	0.265	0.33610739	count	1
AC068338.2	0.4367624	0.6693004	0.6526	0.514	0.336144022	count	1
NOP2	0.9038435	1.124994	0.8034	0.422	0.336333815	count	1
PRKAG1	0.2561812	0.185516	1.3809	0.167	0.336381394	count	1
ZNF558	0.4637024	0.5277609	0.8786	0.38	0.336423186	count	1
KLHL18	0.2950939	0.3957231	0.7457	0.456	0.336450108	count	1
ADD3	0.2539614	0.1872632	1.3562	0.175	0.336463044	count	1
MAP3K4	0.2577317	0.2215488	1.1633	0.245	0.336544752	count	1
AC133919.1	0.6588308	1.5561638	0.4234	0.6721	0.336771228	count	1
CCAR2	0.3367847	0.3015408	1.1169	0.264	0.336806828	count	1
ZDHHC13	0.3013533	0.3163287	0.9527	0.341	0.3368417	count	1
CUL7	0.3368593	0.5284796	0.6374	0.524	0.336878038	count	1

DNPEP	0.2469288	0.1351164	1.8275	0.0677	0.337089337	count	1
BACE1-AS	0.7972356	0.7302281	1.0918	0.275	0.337192363	count	1
TTF1	0.2501015	0.1544491	1.6193	0.105	0.33726935	count	1
MT-ND4L	0.2360756	0.0593913	3.9749	7.22E-05	0.337281198	count	1
LARP6	0.249248	0.1554284	1.6036	0.109	0.337354382	count	1
MALL	0.2392246	0.0853489	2.8029	0.0051	0.337482622	count	1
XYLT2	0.3346785	0.4690961	0.7135	0.476	0.337587542	count	1
AC009961.1	0.6148065	0.823811	0.7463	0.456	0.337653716	count	1
NUDT9	0.2509318	0.1875322	1.3381	0.181	0.337656642	count	1
RUNDC3B	0.3319983	0.2902333	1.1439	0.253	0.337668545	count	1
TTPAL	0.4659196	0.4867339	0.9572	0.339	0.3378695	count	1
TRUB2	0.2594603	0.1822032	1.424	0.155	0.337880402	count	1
WBP11	0.2454837	0.1232693	1.9914	0.0465	0.337916528	count	1
PARP9	0.2453594	0.1320297	1.8584	0.0632	0.337938146	count	1
ZNF274	0.2906016	0.2825914	1.0283	0.304	0.337991072	count	1
ARHGEF26	1.077677	0.7722557	1.3955	0.163	0.338121497	count	1
TRNT1	0.2525265	0.1779299	1.4192	0.156	0.338184862	count	1
POLR1E	0.2711578	0.2437288	1.1125	0.266	0.338242594	count	1
FKRP	0.5229639	0.5361661	0.9754	0.329	0.338261463	count	1
COQ6	0.2746203	0.2530596	1.0852	0.278	0.338460869	count	1
SFXN2	1.3692294	1.0149767	1.349	0.177	0.338494644	count	1
PEX16	0.2521551	0.1504062	1.6765	0.0938	0.338516195	count	1
ST7L	0.2886633	0.2816711	1.0248	0.306	0.338683233	count	1
TSPAN31	0.2499142	0.1499406	1.6668	0.0957	0.338689879	count	1
LINC00893	0.9127407	0.8809086	1.0361	0.3	0.338710336	count	1
ELMO2	0.5237698	0.4767515	1.0986	0.272	0.338717404	count	1
ANKRA2	0.2994409	0.3179716	0.9417	0.346	0.338775737	count	1
RPS23	0.2349579	0.0189504	12.3986	2.15E-34	0.33878262	count	5.21E-30
AC062017.1	0.4405378	0.561044	0.7852	0.432	0.338789026	count	1
IQCB1	0.2691543	0.2423459	1.1106	0.267	0.338807963	count	1
GGA1	0.2837011	0.2804994	1.0114	0.312	0.338887516	count	1
ANP32A	0.241484	0.0926067	2.6076	0.00917	0.339069883	count	1
NELFB	0.2687292	0.1899258	1.4149	0.157	0.339080731	count	1
SPATA5L1	0.3881411	0.5031836	0.7714	0.441	0.339111608	count	1
ARNT	0.2890464	0.280949	1.0288	0.304	0.339122024	count	1
NAA60	0.2691688	0.2021204	1.3317	0.183	0.339229815	count	1
ABCF3	0.2815753	0.2572831	1.0944	0.274	0.339353165	count	1
RBM8A	0.2376927	0.0534325	4.4485	8.99E-06	0.339418009	count	0.20872982
AC009812.1	0.4683349	0.5559113	0.8425	0.4	0.339443277	count	1
TMEM100	0.5252347	0.6823093	0.7698	0.441	0.339545679	count	1
GTF2IRD2B	0.550894	0.5540427	0.9943	0.32	0.339550889	count	1
C20orf194	0.2948158	0.2916034	1.011	0.312	0.339583218	count	1
LETMD1	0.2479127	0.1333728	1.8588	0.0632	0.339625274	count	1
URGCP	0.306649	0.3080414	0.9955	0.32	0.339637569	count	1
COX7A2L	0.2388254	0.0646188	3.6959	0.000223	0.339639273	count	1

CDK18	0.9167941	1.0452361	0.8771	0.38	0.339787731	count	1
AC009005.1	0.9167941	1.1957325	0.7667	0.443	0.339787731	count	1
ERMARD	0.3042444	0.4208674	0.7229	0.47	0.339975729	count	1
RABGAP1	0.26256	0.1712892	1.5328	0.125	0.340418921	count	1
MED29	0.2533384	0.1608374	1.5751	0.115	0.340543013	count	1
TCAIM	0.2673715	0.2460219	1.0868	0.277	0.340675191	count	1
AC079630.1	0.4867787	0.8710392	0.5588	0.576	0.340810463	count	1
PRKAR1B	0.2574222	0.1991484	1.2926	0.196	0.340843514	count	1
MAP6	0.553338	0.6555901	0.844	0.399	0.340850546	count	1
PROS1	0.2446928	0.1272498	1.9229	0.0546	0.340947756	count	1
TENT4B	0.2916391	0.2786272	1.0467	0.295	0.341140008	count	1
SART3	0.2717922	0.215195	1.263	0.207	0.341240923	count	1
ITGA1	0.2676008	0.2286794	1.1702	0.242	0.341301024	count	1
MEF2C-AS1	0.3358099	0.3683278	0.9117	0.362	0.341376325	count	1
HSPA4	0.254961	0.1510339	1.6881	0.0915	0.341418815	count	1
FZD6	0.2472566	0.1250803	1.9768	0.0482	0.341567769	count	1
NME5	0.2868025	0.3618861	0.7925	0.428	0.3417171	count	1
RPL10A	0.2374973	0.0250608	9.4768	5.44E-21	0.342191361	count	1.31E-16
DFFB	0.7323108	0.7838037	0.9343	0.35	0.342193179	count	1
RBM4B	0.3135768	0.3214726	0.9754	0.329	0.342193616	count	1
ALKBH1	0.3745569	0.4095704	0.9145	0.361	0.342389626	count	1
CSPP1	0.2735063	0.2083792	1.3125	0.189	0.342488079	count	1
DHRS11	0.2821167	0.2679895	1.0527	0.293	0.342709381	count	1
ATL1	0.2898369	0.2571935	1.1269	0.26	0.342730044	count	1
PPP1R13L	0.3497489	0.3737356	0.9358	0.349	0.342959238	count	1
GON7	0.2662428	0.2043351	1.303	0.193	0.34298011	count	1
MTMR6	0.2593261	0.2401734	1.0797	0.28	0.343008702	count	1
PPP1R37	0.316055	0.2886698	1.0949	0.274	0.343066442	count	1
TGFB3	0.5094689	0.6856774	0.743	0.458	0.343117477	count	1
AP3S2	0.4000141	0.5028584	0.7955	0.426	0.343128458	count	1
EML1	0.2942904	0.2496543	1.1788	0.239	0.343181501	count	1
RNLS	0.4740931	0.6153039	0.7705	0.441	0.343187625	count	1
DNAJA4	0.4907148	0.8224088	0.5967	0.551	0.343263269	count	1
FBXL15	0.248126	0.128741	1.9273	0.054	0.343522686	count	1
RAB6A	0.2496478	0.1245876	2.0038	0.0452	0.343569907	count	1
CYBC1	0.2523483	0.1480068	1.705	0.0883	0.343656955	count	1
CFAP45	1.1051306	1.037468	1.0652	0.287	0.34370713	count	1
AC107375.1	1.1051306	1.2630355	0.875	0.382	0.34370713	count	1
GAS8	0.3411146	0.365857	0.9324	0.351	0.34378648	count	1
AL133467.1	0.9323904	0.7622392	1.2232	0.221	0.343902202	count	1
TRIM45	1.410076	1.2886917	1.0942	0.274	0.344006199	count	1
SECISBP2	0.2496373	0.1283567	1.9449	0.0519	0.344085509	count	1
KIF13A	0.2544716	0.1671548	1.5224	0.128	0.344097236	count	1
CEP85	0.5338731	0.7110544	0.7508	0.453	0.344416318	count	1
CTSA	0.2475728	0.1099144	2.2524	0.0244	0.344493248	count	1

CEP97	0.3113224	0.3840127	0.8107	0.418	0.344648699	count	1
LIN7B	0.3036884	0.3033501	1.0011	0.317	0.344725191	count	1
NEURL2	0.6304469	0.8161404	0.7725	0.44	0.344801571	count	1
MICB	0.4766544	0.6362237	0.7492	0.454	0.344849741	count	1
FAM234B	0.3592045	0.434332	0.827	0.408	0.344855014	count	1
DIAPH2	0.2516208	0.1584093	1.5884	0.112	0.344855545	count	1
BAZ1B	0.2531814	0.1610116	1.5724	0.116	0.345078558	count	1
EXD3	0.2830741	0.3128365	0.9049	0.366	0.345131698	count	1
ZKSCAN3	0.822481	0.7782461	1.0568	0.291	0.345255435	count	1
ZNF44	0.3199799	0.295029	1.0846	0.278	0.345359025	count	1
JUND	0.2398978	0.0405831	5.9113	3.81E-09	0.345364549	count	9.02E-05
TEX10	0.3895202	0.4032188	0.966	0.334	0.34546244	count	1
ASNSD1	0.2546496	0.1557949	1.6345	0.102	0.345495317	count	1
MED13L	0.2517338	0.14427	1.7449	0.0811	0.345526188	count	1
MRAP2	0.3491537	0.4435152	0.7872	0.431	0.345559331	count	1
MROH8	1.4221014	1.5489943	0.9181	0.359	0.345591791	count	1
CTSW	0.439041	1.2714402	0.3453	0.7299	0.345779903	count	1
TIMM10	0.2528424	0.102357	2.4702	0.0136	0.345906759	count	1
PCDHB5	0.5945238	0.8296408	0.7166	0.474	0.345956966	count	1
ZNF543	0.4508241	0.583747	0.7723	0.44	0.345972117	count	1
RBM18	0.2643667	0.177278	1.4913	0.136	0.345984192	count	1
ALDH4A1	1.4252894	1.042332	1.3674	0.172	0.346009363	count	1
MORN4	0.9410006	0.9222383	1.0203	0.308	0.346152702	count	1
IGBP1	0.2458613	0.0840113	2.9265	0.00346	0.346233876	count	1
HAUS6	0.2906978	0.2618885	1.11	0.267	0.346254496	count	1
PIGF	0.2539894	0.1665735	1.5248	0.127	0.346388898	count	1
REXO4	0.2743044	0.2284588	1.2007	0.23	0.346407629	count	1
SRSF5	0.2430207	0.0585251	4.1524	3.39E-05	0.346419779	count	0.7823103
ZNF674	0.5151349	0.6189768	0.8322	0.405	0.346477738	count	1
ZNF793	0.4206847	0.5063744	0.8308	0.406	0.346730591	count	1
TBC1D13	0.3360712	0.3883577	0.8654	0.387	0.346741855	count	1
PNO1	0.2621898	0.1620524	1.6179	0.106	0.346759958	count	1
THAP1	0.2576855	0.1721499	1.4969	0.135	0.346784085	count	1
GPATCH2	0.2558216	0.1419643	1.802	0.0717	0.346816391	count	1
CD99L2	0.2996256	0.2745051	1.0915	0.275	0.347163991	count	1
SLC25A51	0.4054555	0.5500611	0.7371	0.461	0.347459567	count	1
UBE2A	0.2510185	0.1110738	2.2599	0.0239	0.347470737	count	1
TBL2	0.3154561	0.2891801	1.0909	0.275	0.347491654	count	1
RTN3	0.2469896	0.0967556	2.5527	0.0107	0.347545141	count	1
MUC20-OT1	0.2716757	0.2082207	1.3047	0.192	0.347749163	count	1
TTC28	0.2700556	0.1970872	1.3702	0.171	0.347829036	count	1
AC005837.1	0.5400012	0.6456234	0.8364	0.403	0.347857491	count	1
SPSB1	0.2995319	0.2580427	1.1608	0.246	0.34811138	count	1
SEMA3B	0.8317314	0.7723828	1.0768	0.282	0.348173189	count	1
IQCE	0.3118569	0.2917154	1.069	0.285	0.348218342	count	1

ZNF75D	0.4987884	0.5605468	0.8898	0.374	0.348278928	count	1
PCBP2	0.2436388	0.0506488	4.8104	1.59E-06	0.348325199	count	0.03712809
AC125807.2	0.5995354	0.9652115	0.6211	0.535	0.34842275	count	1
RBL1	0.4543946	0.6856116	0.6628	0.508	0.348457437	count	1
MRPL46	0.2602021	0.1651866	1.5752	0.115	0.348628676	count	1
TMEM179B	0.245183	0.0694963	3.528	0.000426	0.348736332	count	1
TNFRSF11A	0.7506099	0.8006689	0.9375	0.349	0.348899144	count	1
HAUS4	0.3313527	0.391956	0.8454	0.398	0.348988658	count	1
MT-CO3	0.2420675	0.0337293	7.1768	9.13E-13	0.348994617	count	2.18E-08
CCDC157	1.4487014	1.0182299	1.4228	0.155	0.349040534	count	1
YJEFN3	1.4487014	1.1241443	1.2887	0.198	0.349040534	count	1
MARCKS	0.2459979	0.0796935	3.0868	0.00204	0.349141681	count	1
MED28	0.2536974	0.1234986	2.0543	0.04	0.349314434	count	1
ILKAP	0.2803019	0.2127415	1.3176	0.188	0.349457546	count	1
ZFYVE9	0.2949448	0.2765571	1.0665	0.286	0.349504343	count	1
GLOD4	0.2545786	0.1231583	2.0671	0.0388	0.349577333	count	1
RPL12	0.2424553	0.0241709	10.0309	2.78E-23	0.349586378	count	6.71E-19
SPTY2D1	0.2678538	0.1810262	1.4796	0.139	0.349613444	count	1
LANCL3	0.6410827	0.7208189	0.8894	0.374	0.349622483	count	1
DLG1	0.2664441	0.1921482	1.3867	0.166	0.34971438	count	1
TRAF2	0.4013064	0.3302005	1.2153	0.224	0.349814927	count	1
TFPI2	0.3071804	0.4968744	0.6182	0.536	0.349838446	count	1
BEND7	0.2627736	0.2053255	1.2798	0.201	0.349886665	count	1
TCF7L1	0.2839668	0.1929333	1.4718	0.141	0.350325725	count	1
TARBP2	0.3044484	0.3575255	0.8515	0.395	0.350377688	count	1
AC037459.3	0.8395854	0.9912536	0.847	0.397	0.350635129	count	1
PARN	0.305829	0.2594011	1.179	0.239	0.350760873	count	1
H2AFX	0.2582629	0.1369456	1.8859	0.0594	0.350786082	count	1
AL157938.3	0.8400797	0.6163871	1.3629	0.173	0.350789597	count	1
TBC1D23	0.2704697	0.2030729	1.3319	0.183	0.351314417	count	1
AKIP1	0.2653075	0.1658167	1.6	0.11	0.351350749	count	1
UBXN1	0.2459291	0.0547842	4.4891	7.45E-06	0.351354945	count	0.17308585
DNAJA2	0.2529239	0.1122468	2.2533	0.0243	0.351443104	count	1
MALSU1	0.2571704	0.1428844	1.7998	0.072	0.35146423	count	1
MMP15	0.3297057	0.3593488	0.9175	0.359	0.351530299	count	1
ARRDC1-AS1	0.3409468	0.3993257	0.8538	0.393	0.351556873	count	1
C10orf25	0.4871259	0.4878932	0.9984	0.318	0.351623052	count	1
ZNF598	0.3243631	0.2670937	1.2144	0.225	0.351764893	count	1
ASCC3	0.2676431	0.1610025	1.6624	0.0966	0.352065428	count	1
PRIMPOL	0.3116351	0.3669566	0.8492	0.396	0.352161889	count	1
ZWILCH	0.3759674	0.4395547	0.8553	0.392	0.352184187	count	1
LRBA	0.3155294	0.2882824	1.0945	0.274	0.352189868	count	1
MBNL3	0.4882484	0.6232746	0.7834	0.433	0.352347029	count	1
TMEM43	0.2552621	0.1274838	2.0023	0.0453	0.352398089	count	1
TMEM187	0.2908417	0.2681175	1.0848	0.278	0.352412348	count	1

TRPT1	0.2724144	0.2153933	1.2647	0.206	0.352508502	count	1
ALPK1	0.3045669	0.2923673	1.0417	0.298	0.352737527	count	1
BYSL	0.4051229	0.4109889	0.9857	0.324	0.352907443	count	1
SIMC1	0.4490867	0.4499796	0.998	0.318	0.352985468	count	1
SLC48A1	0.2598069	0.1442374	1.8012	0.0718	0.353197349	count	1
ARMC6	0.3240352	0.3313521	0.9779	0.328	0.353208657	count	1
FDX2	0.3771802	0.4020719	0.9381	0.348	0.353254129	count	1
FBXL12	0.2816355	0.2251717	1.2508	0.211	0.35339947	count	1
RPIA	0.2686254	0.1739519	1.5443	0.123	0.353537703	count	1
SNAPC2	0.2699566	0.1797219	1.5021	0.133	0.353648638	count	1
TCF4	0.2470075	0.0601832	4.1043	4.17E-05	0.353755331	count	0.9614769
TCTA	0.2864151	0.2303655	1.2433	0.214	0.353841954	count	1
TMEM216	0.2728155	0.2131221	1.2801	0.201	0.354070708	count	1
TMEM99	0.2766869	0.2201443	1.2568	0.209	0.354074945	count	1
LINC00662	0.2833603	0.2489557	1.1382	0.255	0.35415858	count	1
FANCA	0.8518583	1.0349968	0.8231	0.411	0.354454134	count	1
CORO1B	0.2542657	0.1060789	2.3969	0.0166	0.354482669	count	1
AGFG2	0.3269848	0.3434016	0.9522	0.341	0.354506116	count	1
HYI	0.25664	0.127721	2.0094	0.0446	0.354640392	count	1
HEATR3	0.4226399	0.7488873	0.5644	0.573	0.354807337	count	1
CDC37L1-DT	0.6128586	0.6319873	0.9697	0.332	0.354941874	count	1
NT5C3B	0.2615015	0.1457136	1.7946	0.0728	0.354985964	count	1
PDPK1	0.2924996	0.2218801	1.3183	0.188	0.355064314	count	1
BBS5	0.3842976	0.4202191	0.9145	0.361	0.355230302	count	1
STAT6	0.2701344	0.187125	1.4436	0.149	0.355307948	count	1
ITCH	0.2839719	0.2153129	1.3189	0.187	0.355375604	count	1
AC016727.1	0.855789	0.796098	1.075	0.282	0.355670041	count	1
MPHOSPH8	0.249255	0.0710096	3.5102	0.000455	0.355718201	count	1
WDHD1	0.4242012	0.6083995	0.6972	0.486	0.356015986	count	1
RBM10	0.2984536	0.2949336	1.0119	0.312	0.35610925	count	1
FBXO44	0.3542098	0.4029704	0.879	0.379	0.356362746	count	1
SBF2-AS1	0.6163924	0.6616223	0.9316	0.352	0.356662192	count	1
COPS3	0.2649663	0.1455276	1.8207	0.0688	0.356727476	count	1
TRMT1L	0.3185329	0.3247423	0.9809	0.327	0.356903497	count	1
MED19	0.2596346	0.1288693	2.0147	0.044	0.357083632	count	1
CAMSAP2	0.2634156	0.1911401	1.3781	0.168	0.357128615	count	1
BTG2	0.2487087	0.0770607	3.2274	0.00126	0.357165584	count	1
SPNS1	0.284697	0.2349619	1.2117	0.226	0.357177972	count	1
MRPL15	0.2630527	0.1411012	1.8643	0.0624	0.357247402	count	1
ADSSL1	0.3689125	0.4802194	0.7682	0.442	0.35729214	count	1
WDR17	0.9847776	1.0324158	0.9539	0.34	0.357367145	count	1
KIAA0040	0.2594208	0.1805411	1.4369	0.151	0.357693668	count	1
LARP4	0.2679342	0.1582623	1.693	0.0906	0.357701888	count	1
WDR76	0.3925648	0.4215294	0.9313	0.352	0.357809175	count	1
TMEM201	0.7754421	0.6665789	1.1633	0.245	0.357864653	count	1

PANX1	0.3695798	0.4920766	0.7511	0.453	0.357903814	count	1
STXBP2	0.9870235	0.883683	1.1169	0.264	0.357932303	count	1
ST6GALNAC1	0.2817886	0.2573091	1.0951	0.274	0.358045797	count	1
PLOD3	0.2688759	0.1556759	1.7272	0.0843	0.358085951	count	1
USP51	0.3738535	0.3880475	0.9634	0.335	0.358143666	count	1
FAM98A	0.2661513	0.1526318	1.7437	0.0813	0.358310271	count	1
STK36	0.8644019	1.11634	0.7743	0.439	0.358322095	count	1
SPATA6	0.2820574	0.2076369	1.3584	0.174	0.358382251	count	1
CARHSP1	0.2527869	0.0811296	3.1158	0.00185	0.35853153	count	1
CEP19	0.3346583	0.4124306	0.8114	0.417	0.358641396	count	1
SRFBP1	0.2597717	0.1373535	1.8913	0.0587	0.358815963	count	1
PRDX4	0.2518201	0.0750431	3.3557	0.000802	0.35882431	count	1
RIN2	0.2592232	0.1335321	1.9413	0.0523	0.358894337	count	1
IGF2R	0.284031	0.2163399	1.3129	0.189	0.358906118	count	1
PPP2R3C	0.2660259	0.1617267	1.6449	0.1	0.359204709	count	1
PKNOX1	0.3049389	0.263193	1.1586	0.247	0.35922343	count	1
PRELID3B	0.2731003	0.1756576	1.5547	0.12	0.359364504	count	1
NECAP1	0.2939554	0.2177156	1.3502	0.177	0.359414715	count	1
DSTYK	0.3096763	0.2630084	1.1774	0.239	0.359586755	count	1
ZNF141	0.318535	0.2808918	1.134	0.257	0.359720923	count	1
LCORL	0.290493	0.2539895	1.1437	0.253	0.359875975	count	1
AC087190.1	0.3469415	0.3630475	0.9556	0.339	0.359972478	count	1
SLC41A3	0.2578941	0.1092244	2.3611	0.0183	0.359996416	count	1
FBXW8	0.3612931	0.4198502	0.8605	0.39	0.360118005	count	1
ZNF567	0.2730204	0.2051727	1.3307	0.183	0.360213781	count	1
AC090114.2	0.3585236	0.374616	0.957	0.339	0.360494863	count	1
C11orf68	0.2971646	0.2782537	1.068	0.286	0.360608875	count	1
ABHD16A	0.9978332	1.1112029	0.898	0.369	0.360638741	count	1
OVGP1	0.9978332	1.2357706	0.8075	0.419	0.360638741	count	1
RTL6	0.3766293	0.4045963	0.9309	0.352	0.36065444	count	1
PBDC1	0.2626577	0.1430895	1.8356	0.0665	0.360835442	count	1
RNPS1	0.2569695	0.0918413	2.798	0.00518	0.360951739	count	1
MRPL39	0.2721422	0.167483	1.6249	0.104	0.360983296	count	1
TMEM161A	0.2822391	0.2018339	1.3984	0.162	0.361079562	count	1
AKAP10	0.316249	0.435866	0.7256	0.468	0.361131785	count	1
BCAS2	0.2607115	0.1151825	2.2635	0.0237	0.36114115	count	1
MRPL9	0.2608274	0.1169773	2.2297	0.0258	0.361183978	count	1
CCDC102B	0.3625524	0.4902356	0.7395	0.46	0.361311103	count	1
YEATS4	0.2799865	0.2067172	1.3544	0.176	0.361333097	count	1
ARRDC2	0.2814349	0.168343	1.6718	0.0947	0.36136296	count	1
CARS	0.2860723	0.2125607	1.3458	0.178	0.361444912	count	1
AAED1	0.2572902	0.0965761	2.6641	0.00776	0.361613867	count	1
DTD1	0.2636614	0.125549	2.1001	0.0358	0.361626885	count	1
AP1AR	0.2916772	0.2358796	1.2366	0.216	0.361848583	count	1
RPL37	0.251175	0.022439	11.1937	1.78E-28	0.361935949	count	4.30E-24

SDE2	0.2842226	0.1990431	1.4279	0.153	0.362190577	count	1
INSL3	0.8772123	1.0462192	0.8385	0.402	0.362235727	count	1
AC092171.3	0.8772123	1.4266673	0.6149	0.539	0.362235727	count	1
RORA	0.2643919	0.135179	1.9559	0.0506	0.362441259	count	1
PRRG2	0.3018372	0.2656944	1.136	0.256	0.362464678	count	1
PALB2	0.3384002	0.3856218	0.8775	0.38	0.362497092	count	1
SATB1	0.3404226	0.310796	1.0953	0.273	0.362507677	count	1
TLK1	0.2609215	0.1153813	2.2614	0.0238	0.362541714	count	1
ETFDH	0.3033331	0.2704236	1.1217	0.262	0.362621803	count	1
PHF1	0.273396	0.1667601	1.6395	0.101	0.362630135	count	1
CDKN2AIP	0.2714993	0.1569376	1.73	0.0837	0.3628356	count	1
KYAT1	0.4892575	0.7856965	0.6227	0.534	0.363031061	count	1
TRAF3IP2-AS1	0.3555006	0.4292361	0.8282	0.408	0.363227212	count	1
COG1	0.32722	0.2902514	1.1274	0.26	0.363255876	count	1
KCNJ12	0.3412175	0.344538	0.9904	0.322	0.363320693	count	1
NOP58	0.2634794	0.1248375	2.1106	0.0349	0.363499165	count	1
KRT222	0.2888231	0.1995699	1.4472	0.148	0.363598345	count	1
AFG3L2	0.2838248	0.2016635	1.4074	0.159	0.363743547	count	1
TBK1	0.2729481	0.2717083	1.0046	0.315	0.363756575	count	1
CEP250	0.5447356	0.52834	1.031	0.303	0.363867849	count	1
SIK1B	1.0110695	1.1338511	0.8917	0.373	0.363921852	count	1
GRHL1	0.4056084	0.4073927	0.9956	0.32	0.363949614	count	1
FAM160B2	0.3309531	0.2944058	1.1241	0.261	0.36397103	count	1
UPF1	0.2988969	0.2564325	1.1656	0.244	0.364027063	count	1
DIMT1	0.2718223	0.1489099	1.8254	0.068	0.364063797	count	1
AC243964.2	0.6735119	0.4627861	1.4553	0.146	0.364123608	count	1
FAM104A	0.27156	0.1710898	1.5872	0.113	0.364224374	count	1
AL357054.4	0.3179514	0.3453641	0.9206	0.357	0.364262697	count	1
ZNF438	0.3283667	0.3330075	0.9861	0.324	0.364486191	count	1
MED15	0.3043121	0.232258	1.3102	0.19	0.364575914	count	1
SLC45A1	0.5987303	0.5693303	1.0516	0.293	0.36466054	count	1
AC083843.3	0.7951935	0.6748954	1.1782	0.239	0.36488596	count	1
USP47	0.2728786	0.1861385	1.466	0.143	0.364935433	count	1
ZNF785	0.5467977	0.656141	0.8334	0.405	0.365068959	count	1
PSME2	0.2555916	0.0651613	3.9224	8.98E-05	0.365303232	count	1
DAZAP1	0.2705591	0.1297209	2.0857	0.0371	0.365686654	count	1
MTMR10	0.3457037	0.3480496	0.9933	0.321	0.365729678	count	1
PSMG2	0.2586813	0.0760639	3.4008	0.000681	0.365745884	count	1
SPATS2L	0.2623774	0.0991102	2.6473	0.00816	0.365747127	count	1
ZNF862	0.601293	0.5759884	1.0439	0.297	0.365986115	count	1
PABPC4	0.2700552	0.1449137	1.8636	0.0625	0.366065208	count	1
AC103591.3	0.4463665	0.5001906	0.8924	0.372	0.366118941	count	1
CHML	0.3711176	0.4193451	0.885	0.376	0.366178277	count	1
NEURL4	0.6785415	0.6754626	1.0046	0.315	0.366346033	count	1
AGTPBP1	0.3045165	0.2485832	1.225	0.221	0.36638665	count	1

RPS4Y1	0.2547671	0.0438182	5.8142	6.79E-09	0.366448231	count	0.000160522
TRAPPC4	0.2630888	0.0994161	2.6463	0.00818	0.366535667	count	1
LIMD1	0.4951049	0.5573801	0.8883	0.374	0.366913053	count	1
CNDP2	0.2748062	0.1589887	1.7285	0.084	0.366934841	count	1
N6AMT1	0.3152738	0.294474	1.0706	0.284	0.366994971	count	1
AGO4	0.3409325	0.374146	0.9112	0.362	0.367113618	count	1
RBMX	0.2606	0.0886897	2.9385	0.00333	0.367461379	count	1
C1orf174	0.2800216	0.1948289	1.4373	0.151	0.367547301	count	1
MRPL58	0.2862099	0.2283904	1.2532	0.21	0.367727205	count	1
GNL2	0.2822203	0.2030796	1.3897	0.165	0.367899669	count	1
IFITM2	0.2555253	0.0307507	8.3096	1.49E-16	0.367972917	count	3.58E-12
NAP1L5	0.3316359	0.3256502	1.0184	0.309	0.367992014	count	1
WDR27	0.4315677	0.5538649	0.7792	0.436	0.368111971	count	1
RPL10	0.2552963	0.0203142	12.5674	2.89E-35	0.368226784	count	7.00E-31
HBS1L	0.2762914	0.1729323	1.5977	0.11	0.368466447	count	1
LBR	0.2694834	0.1382846	1.9488	0.0514	0.368531631	count	1
ZNF468	0.4835464	0.5633698	0.8583	0.391	0.368594588	count	1
PPIP5K1	0.5136208	0.6945377	0.7395	0.46	0.368603147	count	1
HIRA	0.4978992	0.5559387	0.8956	0.371	0.368764232	count	1
CDKN2AIPNL	0.2835015	0.1908081	1.4858	0.137	0.368803575	count	1
PSME1	0.2570476	0.0457747	5.6155	2.16E-08	0.368808043	count	0.000509738
COA1	0.283524	0.1869806	1.5163	0.13	0.368832483	count	1
CEMIP2	0.2632363	0.1222644	2.153	0.0314	0.368969666	count	1
CAPN7	0.2932159	0.2213557	1.3246	0.185	0.369036639	count	1
ATF7IP	0.2708954	0.1851469	1.4631	0.144	0.369139156	count	1
FOXO4	0.5327748	0.5839876	0.9123	0.362	0.369164361	count	1
KBTBD2	0.2677493	0.120265	2.2263	0.0261	0.369165657	count	1
DYNC2LI1	0.2881628	0.2215994	1.3004	0.194	0.369223518	count	1
SLC35F6	0.2799054	0.1706703	1.64	0.101	0.369390481	count	1
RBM17	0.2616282	0.0883973	2.9597	0.00311	0.369512343	count	1
LRCH4	0.4849909	0.5235831	0.9263	0.354	0.369585222	count	1
BRAF	0.2926359	0.1946301	1.5035	0.133	0.369603875	count	1
RSU1	0.2650052	0.1018047	2.6031	0.00929	0.369617741	count	1
TMEM260	0.334787	0.2951075	1.1345	0.257	0.369728218	count	1
CCNT2	0.2859124	0.2190569	1.3052	0.192	0.369746268	count	1
COMMD7	0.2648689	0.1060668	2.4972	0.0126	0.369747423	count	1
HEATR6	0.3229922	0.3277023	0.9856	0.324	0.369867353	count	1
SH3RF3	0.4191493	0.466103	0.8993	0.369	0.369871107	count	1
TTLL5	0.3827374	0.4119695	0.929	0.353	0.369937331	count	1
FAM76A	0.285148	0.2054309	1.388	0.165	0.370402335	count	1
F2RL3	0.8112096	0.6262498	1.2953	0.195	0.370508502	count	1
GALNT11	0.2812057	0.1890078	1.4878	0.137	0.370510351	count	1
TMEM102	0.3577101	0.3962277	0.9028	0.367	0.370648669	count	1
AL359643.3	1.2475657	1.1520286	1.0829	0.279	0.370751954	count	1
TBC1D19	0.4435073	0.5113515	0.8673	0.386	0.37089665	count	1

ZNF490	0.9062537	0.9492425	0.9547	0.34	0.370972265	count	1
SLC16A14	0.4212275	0.4986962	0.8447	0.398	0.371572711	count	1
NASP	0.2615016	0.0674947	3.8744	0.000109	0.371751753	count	1
TDRD3	0.2866585	0.1945172	1.4737	0.141	0.37180663	count	1
TCF20	0.3590954	0.3963788	0.9059	0.365	0.372019739	count	1
ACADSB	0.3707452	0.4023104	0.9215	0.357	0.372172728	count	1
YBX3	0.2588712	0.0382636	6.7655	1.62E-11	0.3722375	count	3.86E-07
KLHL12	0.4155341	0.4077448	1.0191	0.308	0.37223873	count	1
AC008637.1	0.5594375	0.8083616	0.6921	0.489	0.37240223	count	1
PREB	0.3343051	0.2847997	1.1738	0.241	0.372443627	count	1
VMA21	0.2766244	0.1530389	1.8075	0.0708	0.372748563	count	1
GRPEL1	0.2721705	0.1259601	2.1608	0.0308	0.373040171	count	1
HIRIP3	0.2872218	0.1851638	1.5512	0.121	0.373061875	count	1
BAZ2A	0.278909	0.186089	1.4988	0.134	0.373064961	count	1
HNRNPA1	0.2606378	0.0459589	5.6711	1.57E-08	0.373327498	count	0.00037074
CPD	0.270903	0.1470122	1.8427	0.0655	0.373340565	count	1
STK16	0.3016991	0.233736	1.2908	0.197	0.373495439	count	1
UGGT2	0.2965411	0.2277949	1.3018	0.193	0.373592919	count	1
SPRTN	0.3066489	0.2349036	1.3054	0.192	0.373946516	count	1
RMC1	0.322863	0.2896694	1.1146	0.265	0.374470222	count	1
ZNF18	0.7521925	0.6194095	1.2144	0.225	0.374623947	count	1
CEP85L	0.2853348	0.1672548	1.706	0.0881	0.374655552	count	1
PEAR1	0.3179058	0.2470485	1.2868	0.198	0.375075353	count	1
AC027644.3	0.2992652	0.2393806	1.2502	0.211	0.375137735	count	1
RPRD1B	0.3188663	0.2681239	1.1893	0.234	0.375203605	count	1
NSMCE3	0.2748983	0.1573671	1.7469	0.0808	0.375243895	count	1
AC007383.2	0.6552053	0.7501149	0.8735	0.382	0.375315454	count	1
RAB23	0.6552053	0.7849871	0.8347	0.404	0.375315454	count	1
STYXL1	0.3014328	0.2634184	1.1443	0.253	0.375321779	count	1
MFSD10	0.2701175	0.1093884	2.4693	0.0136	0.375632099	count	1
AL451085.1	1.0594878	1.0577119	1.0017	0.317	0.375645892	count	1
HNRNPF	0.2646635	0.0669268	3.9545	7.86E-05	0.375699459	count	1
CIPC	0.5657049	0.5135481	1.1016	0.271	0.376019739	count	1
RWDD4	0.2760689	0.1425931	1.9361	0.053	0.376219685	count	1
SCRN2	0.2879399	0.159496	1.8053	0.0711	0.376232551	count	1
MVP	0.273223	0.1121137	2.437	0.0149	0.376571207	count	1
FUS	0.2644802	0.0637254	4.1503	3.42E-05	0.376573965	count	0.789165
TRPM7	0.3033413	0.2454357	1.2359	0.217	0.376591029	count	1
WHRN	0.6579435	0.6647171	0.9898	0.322	0.376614737	count	1
MPRIP	0.2826259	0.1663885	1.6986	0.0895	0.376833804	count	1
CTDSPL2	0.2828298	0.1929901	1.4655	0.143	0.376951939	count	1
AL592183.1	0.3524737	0.4227413	0.8338	0.404	0.376965732	count	1
CHAC2	0.4428564	0.4478469	0.9889	0.323	0.376972573	count	1
ACO2	0.2895709	0.1951217	1.4841	0.138	0.377111312	count	1
PQBP1	0.2703134	0.1078262	2.5069	0.0122	0.377264829	count	1

ZBTB33	0.3594105	0.3719409	0.9663	0.334	0.377268354	count	1
SDAD1	0.2732998	0.1240788	2.2026	0.0277	0.377405012	count	1
NVL	0.3700883	0.3391527	1.0912	0.275	0.377418712	count	1
USP12	0.2834682	0.1823226	1.5548	0.12	0.377946114	count	1
FMO4	0.4844546	0.5268979	0.9194	0.358	0.378093389	count	1
SCN11A	0.5950263	1.1952121	0.4978	0.619	0.378233826	count	1
SLC25A16	0.3354788	0.332489	1.009	0.313	0.378235936	count	1
AC068888.1	0.2982574	0.2199671	1.3559	0.175	0.378241758	count	1
DICER1	0.2786531	0.1651183	1.6876	0.0916	0.378279617	count	1
B4GALT3	0.2967854	0.1933217	1.5352	0.125	0.378325224	count	1
RPS2	0.2624423	0.0229276	11.4466	1.13E-29	0.3783862	count	2.73E-25
ARHGAP12	0.3021056	0.2358715	1.2808	0.2	0.378635469	count	1
HIST1H1C	0.2831177	0.1729445	1.637	0.102	0.378642703	count	1
PRR3	0.3541759	0.3305994	1.0713	0.284	0.378712183	count	1
MIEF1	0.3609907	0.4422468	0.8163	0.414	0.378854702	count	1
CFAP46	0.8360437	1.2005543	0.6964	0.486	0.379102025	count	1
MIATNB	1.0742627	0.8158585	1.3167	0.188	0.379135414	count	1
MAN1B1	0.2997102	0.2032806	1.4744	0.14	0.379235382	count	1
DNAJC2	0.2752293	0.1302714	2.1127	0.0347	0.37942278	count	1
APOL3	0.2789626	0.1431084	1.9493	0.0514	0.379481018	count	1
SUV39H1	0.9356252	1.1447804	0.8173	0.414	0.379618512	count	1
RUFY2	0.2891094	0.1721564	1.6793	0.0932	0.379769217	count	1
RPF2	0.2746485	0.1301978	2.1095	0.035	0.380060511	count	1
GNG10	0.362348	0.3686216	0.983	0.326	0.380216742	count	1
SLC15A4	0.3556649	0.4183337	0.8502	0.395	0.380239272	count	1
ZC3H6	0.2837991	0.1789732	1.5857	0.113	0.380493668	count	1
CEP57	0.2739767	0.1170835	2.34	0.0194	0.380493775	count	1
TACO1	0.2994764	0.2410391	1.2424	0.214	0.380556193	count	1
ARL16	0.2903881	0.1511905	1.9207	0.0549	0.380774155	count	1
TIMM29	0.3274695	0.3036816	1.0783	0.281	0.380791964	count	1
TUBB	0.2660666	0.0498996	5.332	1.05E-07	0.380893703	count	0.00246834
RNF208	0.6303938	0.7464885	0.8445	0.398	0.380899282	count	1
LDLR	0.29053	0.1744176	1.6657	0.0959	0.380958025	count	1
CDK11B	0.2866836	0.1815175	1.5794	0.114	0.381085291	count	1
LIX1L	0.2819204	0.1481394	1.9031	0.0571	0.381231882	count	1
ZNF823	0.5527074	0.5099677	1.0838	0.279	0.381241763	count	1
FOXO1	0.2741029	0.1180159	2.3226	0.0203	0.381314303	count	1
MOB3B	0.3567807	0.377219	0.9458	0.344	0.381383224	count	1
SFTA2	0.6313925	1.15088	0.5486	0.583	0.381406551	count	1
RFT1	0.414926	0.3202016	1.2958	0.195	0.381676203	count	1
DBF4	0.3137377	0.2698113	1.1628	0.245	0.381708157	count	1
GUCD1	0.3046736	0.2220793	1.3719	0.17	0.381796648	count	1
DHPS	0.2780796	0.1265989	2.1965	0.0281	0.381955608	count	1
CDC16	0.2960232	0.1859542	1.5919	0.112	0.382346233	count	1
C16orf91	0.2898806	0.1795212	1.6147	0.106	0.382409593	count	1

AC145124.1	0.3519372	0.3452013	1.0195	0.308	0.382460086	count	1
ATP8B1	0.2758184	0.1172743	2.3519	0.0187	0.382559223	count	1
CCDC144A	0.3541745	0.4505638	0.7861	0.432	0.38283148	count	1
DNAAF2	0.2960515	0.1906109	1.5532	0.12	0.38297169	count	1
NCOA2	0.3472677	0.3501584	0.9917	0.321	0.38301252	count	1
AC010654.1	1.318594	1.475757	0.8935	0.372	0.383087179	count	1
ZNF573	0.3893268	0.5469562	0.7118	0.477	0.383165189	count	1
AIFM1	0.3188289	0.2382561	1.3382	0.181	0.383200961	count	1
SCLT1	0.3106785	0.2353853	1.3199	0.187	0.383220934	count	1
MINDY1	0.3607596	0.4307768	0.8375	0.402	0.383255299	count	1
PHRF1	0.3527231	0.3353851	1.0517	0.293	0.383281121	count	1
ELP5	0.2940214	0.2300544	1.2781	0.201	0.383341392	count	1
PTPN23	0.3683884	0.376135	0.9794	0.327	0.383781403	count	1
FNTB	0.6362982	0.4860703	1.3091	0.191	0.383893878	count	1
MSC	0.4025121	0.4978137	0.8086	0.419	0.383953926	count	1
TBC1D25	0.4236622	0.4858829	0.8719	0.383	0.384198612	count	1
POP5	0.2831577	0.1465022	1.9328	0.0534	0.38434911	count	1
FLVCR1	0.637464	0.5467914	1.1658	0.244	0.384483959	count	1
5-Mar	0.2884459	0.1962144	1.4701	0.142	0.384972547	count	1
LEF1	0.3624667	0.2914969	1.2435	0.214	0.384991852	count	1
NRBP1	0.2808604	0.1250249	2.2464	0.0248	0.385005979	count	1
ADSL	0.4189198	0.3834916	1.0924	0.275	0.385103119	count	1
CDK4	0.2791959	0.120574	2.3156	0.0207	0.385598404	count	1
PPP1R18	0.2802735	0.1165956	2.4038	0.0163	0.385642378	count	1
MCMDC2	0.780828	1.0552231	0.74	0.459	0.385768646	count	1
NEIL2	0.3515627	0.4490426	0.7829	0.434	0.385794344	count	1
ZNF146	0.3041859	0.2039447	1.4915	0.136	0.386043408	count	1
ZNF22	0.2750616	0.1080937	2.5447	0.011	0.386209682	count	1
TXLNG	0.2966629	0.2327229	1.2747	0.203	0.386486456	count	1
ACD	0.303636	0.2038494	1.4895	0.136	0.386538161	count	1
CDK14	1.788776	1.030723	1.7355	0.0828	0.386658004	count	1
THOP1	0.4266836	0.4205352	1.0146	0.31	0.386746412	count	1
C16orf86	0.3478246	0.346082	1.005	0.315	0.38697445	count	1
GLIDR	0.9614359	1.1017502	0.8726	0.383	0.387061276	count	1
FLYWCH2	0.277528	0.1000233	2.7746	0.00556	0.387182154	count	1
AAR2	0.3331384	0.2716208	1.2265	0.22	0.387194185	count	1
MTHFSD	0.5434246	0.5044734	1.0772	0.281	0.387433551	count	1
ZNF281	0.30641	0.233188	1.314	0.189	0.387570412	count	1
ASB8	0.2890467	0.1566217	1.8455	0.0651	0.38773026	count	1
ERO1A	0.3049629	0.2277941	1.3388	0.181	0.387810619	count	1
CHAF1B	0.613006	0.6641631	0.923	0.356	0.387956112	count	1
RP2	0.3986075	0.3924592	1.0157	0.31	0.388157269	count	1
AC012368.1	0.4868197	0.6308147	0.7717	0.44	0.388208064	count	1
TBCCD1	0.5276796	0.4541007	1.162	0.245	0.388335946	count	1
NUDT17	0.6828575	0.6081502	1.1228	0.262	0.388335956	count	1

LENG9	0.3199927	0.2524936	1.2673	0.205	0.388422702	count	1
HSBP1L1	0.2981956	0.2018993	1.477	0.14	0.388457442	count	1
TMEM150A	0.3018424	0.198329	1.5219	0.128	0.388502382	count	1
OSTF1	0.276972	0.0969033	2.8582	0.00429	0.388560345	count	1
MRNIP	0.3011585	0.1989081	1.5141	0.13	0.388885625	count	1
PRPF38A	0.2844896	0.1219095	2.3336	0.0197	0.389202602	count	1
CETN3	0.3048732	0.2056711	1.4823	0.138	0.38922249	count	1
DLAT	0.3793634	0.3813811	0.9947	0.32	0.389264451	count	1
SRSF3	0.2736237	0.0601729	4.5473	5.67E-06	0.389270402	count	0.13191255
URM1	0.280097	0.1011916	2.768	0.00568	0.389309451	count	1
ARID5B	0.2718805	0.0696428	3.9039	9.69E-05	0.389556532	count	1
PPP2R2D	0.3069021	0.2058097	1.4912	0.136	0.389838189	count	1
TMEM120B	0.5019029	0.9736111	0.5155	0.606	0.390330059	count	1
HEXIM2	0.3525415	0.3729365	0.9453	0.345	0.390348683	count	1
FAHD2A	0.3250175	0.2944991	1.1036	0.27	0.390459064	count	1
GBP3	0.2864259	0.1813436	1.5795	0.114	0.390476908	count	1
HYPK	0.5022761	0.5440186	0.9233	0.356	0.390590721	count	1
RNF40	0.3455458	0.3225191	1.0714	0.284	0.390678871	count	1
ZNF213	0.5313493	0.5625447	0.9445	0.345	0.390727775	count	1
ACSS1	0.5313493	0.5724308	0.9282	0.353	0.390727775	count	1
DDX49	0.3363264	0.256058	1.3135	0.189	0.390791496	count	1
SAMM50	0.2943329	0.1472855	1.9984	0.0458	0.390807913	count	1
IVD	0.2980214	0.2011434	1.4816	0.139	0.390889342	count	1
TRIT1	0.3904795	0.4169547	0.9365	0.349	0.390937961	count	1
PSMA3	0.2751984	0.0651899	4.2215	2.51E-05	0.391204642	count	0.5800861
HEATR5B	0.3533839	0.367347	0.962	0.336	0.391247299	count	1
RBMX2	0.2862146	0.1363618	2.0989	0.0359	0.391332251	count	1
STK32B	0.7364591	1.099852	0.6696	0.503	0.391427022	count	1
MAP1LC3B	0.2739315	0.0594502	4.6077	4.26E-06	0.391534273	count	0.09918132
TCF4-AS2	1.1288768	1.059389	1.0656	0.287	0.391684574	count	1
SUPT3H	0.371419	0.3492856	1.0634	0.288	0.391735804	count	1
AL391069.3	0.6200483	0.6522935	0.9506	0.342	0.391736915	count	1
USP15	0.2835123	0.1249339	2.2693	0.0233	0.391994419	count	1
AC005520.2	0.3716986	0.4802339	0.774	0.439	0.392017581	count	1
NSUN2	0.357572	0.315124	1.1347	0.257	0.392137427	count	1
HAGLR	0.3576112	0.3724581	0.9601	0.337	0.392178772	count	1
KBTBD6	0.4030889	0.7484883	0.5385	0.59	0.392267375	count	1
PIEZO1	0.2964456	0.248524	1.1928	0.233	0.392511167	count	1
ZBTB25	0.3389141	0.3174465	1.0676	0.286	0.392535272	count	1
HNRNPH2	0.2904143	0.1314394	2.2095	0.0272	0.39272991	count	1
CDK13	0.2915573	0.1582244	1.8427	0.0655	0.392815251	count	1
RNPEP	0.3125733	0.2040624	1.5318	0.126	0.39296525	count	1
TAGLN2	0.2731869	0.0343349	7.9565	2.56E-15	0.393210632	count	6.13E-11
RPAP2	0.2957899	0.1900734	1.5562	0.12	0.39339971	count	1
SDCCAG8	0.2876902	0.1756175	1.6382	0.102	0.393480622	count	1

ZNF783	0.8013005	0.7561513	1.0597	0.289	0.393604509	count	1
LINC00294	0.8015824	0.6865274	1.1676	0.243	0.393711629	count	1
TMEM173	0.2775899	0.0793273	3.4993	0.000474	0.393998881	count	1
CENPL	0.7426729	0.594827	1.2486	0.212	0.394062347	count	1
FKTN	0.4239662	0.5028231	0.8432	0.399	0.394183853	count	1
AC010226.1	0.3577938	0.3510277	1.0193	0.308	0.394186199	count	1
TGS1	0.2875566	0.1519606	1.8923	0.0586	0.394388133	count	1
RPA3	0.2883507	0.1390455	2.0738	0.0382	0.394449615	count	1
ZNF737	0.4424486	0.4870542	0.9084	0.364	0.394558693	count	1
ADHFE1	0.6965192	0.7853616	0.8869	0.375	0.394686634	count	1
MRPS30	0.2930641	0.1593935	1.8386	0.0661	0.394709107	count	1
LIMS1	0.2765092	0.0676262	4.0888	4.46E-05	0.394905243	count	1
HELZ	0.303647	0.3211965	0.9454	0.345	0.394932252	count	1
ADA	0.3421557	0.3349648	1.0215	0.307	0.394960309	count	1
TPM3	0.2751858	0.0414853	6.6333	3.93E-11	0.3951684	count	9.35E-07
PGAP3	0.3951489	0.3478699	1.1359	0.256	0.395361531	count	1
C19orf48	0.3240918	0.2358582	1.3741	0.17	0.395448091	count	1
PRMT5	0.3323334	0.2856244	1.1635	0.245	0.39549611	count	1
OSCP1	0.4068845	0.4419752	0.9206	0.357	0.395743771	count	1
SLC2A8	0.3476881	0.3170563	1.0966	0.273	0.395871657	count	1
SNAPC5	0.2972264	0.1512732	1.9648	0.0495	0.396104736	count	1
PLPBP	0.2924764	0.1382591	2.1154	0.0345	0.396316828	count	1
NSRP1	0.2801026	0.0823028	3.4033	0.000675	0.396535063	count	1
PPP1R3F	0.4449905	0.4939197	0.9009	0.368	0.396654771	count	1
PLVAP	0.2761866	0.0518266	5.3291	1.07E-07	0.396767196	count	0.002515249
SCAF8	0.3249411	0.2427201	1.3387	0.181	0.39715673	count	1
TEX2	0.3937583	0.3589148	1.0971	0.273	0.397238656	count	1
AL357033.1	0.7021437	0.7507886	0.9352	0.35	0.397285445	count	1
DDRGK1	0.2827599	0.0949052	2.9794	0.00291	0.397364413	count	1
DYRK1A	0.3139591	0.1941624	1.617	0.106	0.397388192	count	1
PARP3	0.4224704	0.5134756	0.8228	0.411	0.397426832	count	1
AC106047.1	1.1547378	1.0304309	1.1206	0.263	0.397438463	count	1
GLA	0.3100075	0.2108255	1.4704	0.142	0.397490204	count	1
BRWD3	0.3684689	0.3219212	1.1446	0.252	0.397646194	count	1
FBXL3	0.3000361	0.161286	1.8603	0.063	0.39776449	count	1
RPL8	0.2760137	0.0204935	13.4684	4.30E-40	0.397948029	count	1.04E-35
NSFL1C	0.2920032	0.1253002	2.3304	0.0199	0.39802555	count	1
SMG9	0.3509585	0.2946256	1.1912	0.234	0.398050975	count	1
SFXN4	0.3669829	0.2861978	1.2823	0.2	0.398150674	count	1
ZNF561	0.3567714	0.3768809	0.9466	0.344	0.398224812	count	1
MCM8	0.8137675	0.7523136	1.0817	0.279	0.398322732	count	1
PDK1	0.6049606	0.495466	1.221	0.222	0.398396271	count	1
FNDC11	1.4135728	1.0184859	1.3879	0.165	0.398464705	count	1
WDR48	0.3058604	0.1935335	1.5804	0.114	0.398833963	count	1
CCT4	0.2811911	0.0729452	3.8548	0.000118	0.398844211	count	1

ANGEL2	0.3054782	0.1868109	1.6352	0.102	0.39885307	count	1
SACM1L	0.3316192	0.2667603	1.2431	0.214	0.399028817	count	1
ESD	0.2812549	0.0715816	3.9291	8.73E-05	0.399142001	count	1
TFCP2	0.3896302	0.3210516	1.2136	0.225	0.39927153	count	1
FLNC	0.3300791	0.2344979	1.4076	0.159	0.399613732	count	1
CCL19	1.4210457	1.031976	1.377	0.169	0.399622487	count	1
NABP1	0.3486395	0.3746788	0.9305	0.352	0.399633278	count	1
SLC9A9	0.4635692	0.4997303	0.9276	0.354	0.399685396	count	1
RHOG	0.2898301	0.1244868	2.3282	0.02	0.39980172	count	1
ZFY	0.3368682	0.307561	1.0953	0.273	0.399804111	count	1
PPP2R1A	0.2883137	0.1075027	2.6819	0.00736	0.39984366	count	1
VSIR	0.2865198	0.1146845	2.4983	0.0125	0.399901809	count	1
AC124016.1	0.7078604	0.7842493	0.9026	0.367	0.399917496	count	1
RPF1	0.3077576	0.1827536	1.684	0.0923	0.399930417	count	1
MORC2	0.4001249	0.3937891	1.0161	0.31	0.400068523	count	1
HIST1H2AC	0.3016631	0.1940155	1.5548	0.12	0.400079053	count	1
NAPA-AS1	0.6689177	0.5365567	1.2467	0.213	0.400248712	count	1
TUBB4B	0.2806921	0.0627609	4.4724	8.05E-06	0.400258032	count	0.1869693
ADAM22	0.7574512	0.6543403	1.1576	0.247	0.400287092	count	1
AL359258.2	1.4254123	1.1248169	1.2672	0.205	0.400295571	count	1
SPATA33	0.317503	0.2359156	1.3458	0.178	0.400455352	count	1
TTLL10	0.4730541	1.0748297	0.4401	0.66	0.400472884	count	1
STK10	0.4041716	0.3330785	1.2134	0.225	0.400482251	count	1
LRR1	0.3546021	0.3299282	1.0748	0.283	0.4005674	count	1
CIR1	0.2838275	0.0924422	3.0703	0.00216	0.400661705	count	1
STX18-AS1	15.7332099	1100.222497	0.0143	0.989	0.400817836	count	1
ADAM32	15.7332106	1100.221906	0.0143	0.989	0.400817836	count	1
AF129075.2	15.7332115	1100.222177	0.0143	0.989	0.400817836	count	1
OPRL1	15.7333813	1183.020422	0.0133	0.989	0.400817836	count	1
AL359198.1	15.7748343	1099.287547	0.0144	0.989	0.400817839	count	1
AC016355.1	15.8368302	1095.569191	0.0145	0.988	0.400817843	count	1
GPBAR1	15.8369983	1294.334945	0.0122	0.99	0.400817843	count	1
AC008972.2	15.9954197	1089.410252	0.0147	0.988	0.400817852	count	1
CAPN10-DT	16.0121211	1224.472169	0.0131	0.9896	0.400817853	count	1
SEMA5B	16.0691778	1239.816683	0.013	0.99	0.400817855	count	1
PHKA1	16.0883883	1202.040937	0.0134	0.9893	0.400817856	count	1
AC025431.1	16.1084134	1578.692076	0.0102	0.992	0.400817857	count	1
ITPKB-AS1	16.1084173	1578.693237	0.0102	0.992	0.400817857	count	1
TMEM169	16.1088555	1761.63123	0.0091	0.993	0.400817857	count	1
ANKDD1B	16.1578032	1214.837152	0.0133	0.9894	0.40081786	count	1
SLC7A5	16.1712689	1080.284627	0.015	0.9881	0.40081786	count	1
LINC01301	15.6141866	1244.538003	0.0125	0.99	0.400817863	count	1
PDZD7	15.6142029	1104.158321	0.0141	0.989	0.400817863	count	1
CHRNE	15.6142037	1104.158643	0.0141	0.989	0.400817863	count	1
AL359541.1	16.2826689	1099.729244	0.0148	0.9882	0.400817865	count	1

AC019077.1	16.2843702	2203.097634	0.0074	0.994	0.400817865	count	1
AL136531.1	16.2928561	1812.632484	0.009	0.993	0.400817865	count	1
PLXNC1	16.2928633	1812.635714	0.009	0.993	0.400817865	count	1
AL139384.2	16.2928633	1812.635714	0.009	0.993	0.400817865	count	1
AC138696.2	16.2928675	1812.634715	0.009	0.993	0.400817865	count	1
CDT1	16.2928698	1812.6347	0.009	0.993	0.400817865	count	1
PLSCR2	16.2928748	1812.63713	0.009	0.993	0.400817865	count	1
ENKUR	16.2930771	2439.375982	0.0067	0.995	0.400817865	count	1
FAM83D	16.3269033	1089.817564	0.015	0.988	0.400817866	count	1
AC007009.1	16.4317438	1571.362037	0.0105	0.992	0.40081787	count	1
FAM170B	16.4317439	1571.362047	0.0105	0.992	0.40081787	count	1
AC011511.1	16.4318006	1804.469323	0.0091	0.993	0.40081787	count	1
WDR31	15.733169	1240.143982	0.0127	0.99	0.400817871	count	1
AC127070.1	16.4727463	1823.002482	0.009	0.993	0.400817872	count	1
WBP2NL	16.4727486	1823.003248	0.009	0.993	0.400817872	count	1
IGF1	16.4727779	2054.72221	0.008	0.994	0.400817872	count	1
AL021154.1	15.7747865	1239.030523	0.0127	0.99	0.400817874	count	1
KLLN	15.7748364	1099.288758	0.0144	0.989	0.400817874	count	1
CFAP43	15.775209	1179.441277	0.0134	0.989	0.400817874	count	1
LCN2	16.596937	2588.721606	0.0064	0.995	0.400817875	count	1
MTCP1	16.5969496	2588.730815	0.0064	0.995	0.400817875	count	1
RIBC2	16.5969541	2588.73049	0.0064	0.995	0.400817875	count	1
AC087241.3	16.5969602	2588.735171	0.0064	0.995	0.400817875	count	1
AC084757.3	16.5969602	2588.735084	0.0064	0.995	0.400817875	count	1
AC116036.2	16.5969687	2588.733957	0.0064	0.995	0.400817875	count	1
CD19	16.5969687	2588.733968	0.0064	0.995	0.400817875	count	1
FOXRED2	16.5969688	2588.733957	0.0064	0.995	0.400817875	count	1
AC025580.3	16.5969689	2588.734011	0.0064	0.995	0.400817875	count	1
AL021707.6	16.5973552	2972.526466	0.0056	0.996	0.400817875	count	1
AC053503.5	16.5973555	2972.526665	0.0056	0.996	0.400817875	count	1
AC087392.1	16.5973579	2972.520118	0.0056	0.996	0.400817875	count	1
LINC02288	16.5978771	3620.283015	0.0046	0.996	0.400817875	count	1
LINC01266	16.5979067	3620.292904	0.0046	0.996	0.400817875	count	1
AC115522.1	16.6349284	2022.978373	0.0082	0.993	0.400817876	count	1
AC091982.3	16.6349359	1794.860977	0.0093	0.993	0.400817876	count	1
AC073896.3	15.836785	1234.951907	0.0128	0.99	0.400817878	count	1
TSPAN19	15.8368303	1095.569485	0.0145	0.988	0.400817878	count	1
KCNRG	15.8875376	1102.118596	0.0144	0.988	0.400817881	count	1
AP001437.1	16.8791756	1776.058148	0.0095	0.992	0.400817882	count	1
AC102953.2	16.8900171	2604.153109	0.0065	0.995	0.400817883	count	1
FBXO39	16.8900209	2604.163096	0.0065	0.995	0.400817883	count	1
LINC01273	16.8900211	2604.163096	0.0065	0.995	0.400817883	count	1
RSPH14	16.8900211	2604.163096	0.0065	0.995	0.400817883	count	1
MMP23B	16.8900214	2604.163129	0.0065	0.995	0.400817883	count	1
AL133351.1	16.8902454	2990.184823	0.0056	0.995	0.400817883	count	1

AC124066.1	16.8902503	2990.180362	0.0056	0.995	0.400817883	count	1
AL451050.2	16.89058	3381.272813	0.005	0.996	0.400817883	count	1
ATXN7L2	15.9291985	1146.287147	0.0139	0.989	0.400817883	count	1
SNHG22	16.9934353	2362.612863	0.0072	0.994	0.400817885	count	1
SHISAL2A	16.9936728	2788.095698	0.0061	0.995	0.400817885	count	1
AC098617.1	15.9839573	1241.884483	0.0129	0.99	0.400817886	count	1
SSC5D	15.9840301	1101.734407	0.0145	0.988	0.400817886	count	1
AL354696.2	15.9840302	1101.734406	0.0145	0.988	0.400817886	count	1
FEV	17.0730428	3180.591032	0.0054	0.996	0.400817886	count	1
PIWIL2	17.073043	3180.591127	0.0054	0.996	0.400817886	count	1
AC015813.2	17.0737139	3898.806008	0.0044	0.997	0.400817886	count	1
TMC8	17.0737139	3898.805891	0.0044	0.997	0.400817886	count	1
SNAP25-AS1	17.0737139	3898.805736	0.0044	0.997	0.400817886	count	1
ACBD7	17.0737143	3898.806319	0.0044	0.997	0.400817886	count	1
TFAP2C	17.074662	5037.876852	0.0034	0.997	0.400817886	count	1
PRR22	17.0860905	3229.285707	0.0053	0.996	0.400817886	count	1
RAB39B	17.4142497	2354.518192	0.0074	0.994	0.400817891	count	1
AP001020.1	16.1084159	1578.691366	0.0102	0.992	0.400817892	count	1
TRG-AS1	16.1084181	1578.692598	0.0102	0.992	0.400817892	count	1
AL096855.1	16.1085636	1812.742124	0.0089	0.993	0.400817892	count	1
GSTM5	16.1085676	1812.73981	0.0089	0.993	0.400817892	count	1
KCTD14	17.7021373	2341.556917	0.0076	0.994	0.400817895	count	1
PYGM	16.2848768	2304.776544	0.0071	0.994	0.4008179	count	1
AP001605.1	16.2928543	1812.633438	0.009	0.993	0.4008179	count	1
SH3RF3-AS1	16.2928634	1812.635714	0.009	0.993	0.4008179	count	1
HSPA1L	16.2928699	1812.6347	0.009	0.993	0.4008179	count	1
AL691447.2	16.2929716	2043.020607	0.008	0.994	0.4008179	count	1
ATAD3C	16.4317455	1571.363043	0.0105	0.992	0.400817905	count	1
HIST1H2AE	16.4317998	1804.46876	0.0091	0.993	0.400817905	count	1
AC009542.1	16.4323734	2279.351113	0.0072	0.994	0.400817905	count	1
EPS8L2	16.4571191	1164.788975	0.0141	0.9887	0.400817906	count	1
SYTL3	16.4726994	2087.813231	0.0079	0.994	0.400817907	count	1
ACER2	16.4727463	1823.002482	0.009	0.993	0.400817907	count	1
ERBB4	16.4727487	1823.003254	0.009	0.993	0.400817907	count	1
CD81-AS1	16.4727494	1823.00393	0.009	0.993	0.400817907	count	1
AP002449.1	16.4727503	1823.005448	0.009	0.993	0.400817907	count	1
AC093227.1	16.4727631	2054.719132	0.008	0.994	0.400817907	count	1
TIAF1	16.4727693	2054.722431	0.008	0.994	0.400817907	count	1
FBXO41	16.4727779	2054.722203	0.008	0.994	0.400817907	count	1
SH3TC2	16.5063017	1131.508556	0.0146	0.9884	0.400817908	count	1
ADGRG2	16.5591308	1567.331583	0.0106	0.9916	0.400817909	count	1
YY2	16.5969371	2588.72153	0.0064	0.995	0.40081791	count	1
TMC3-AS1	16.5969371	2588.721617	0.0064	0.995	0.40081791	count	1
ZNF285	16.5969374	2588.721617	0.0064	0.995	0.40081791	count	1
AC008429.1	16.5969494	2588.730837	0.0064	0.995	0.40081791	count	1

LINC02068	16.5969539	2588.730436	0.0064	0.995	0.40081791	count	1
ACSS3	16.5969604	2588.73516	0.0064	0.995	0.40081791	count	1
PEG10	16.5973439	2972.516609	0.0056	0.996	0.40081791	count	1
AC104109.2	16.5973555	2972.526677	0.0056	0.996	0.40081791	count	1
CRACR2A	16.5973556	2972.526702	0.0056	0.996	0.40081791	count	1
ODF3L2	16.5973581	2972.519994	0.0056	0.996	0.40081791	count	1
AL031283.2	16.5978713	3620.276631	0.0046	0.996	0.40081791	count	1
AC025754.2	16.6349358	1794.861008	0.0093	0.993	0.400817911	count	1
AL031595.2	16.6349359	1794.860971	0.0093	0.993	0.400817911	count	1
ACTR3B	16.7713922	1559.695863	0.0108	0.9914	0.400817915	count	1
STK31	16.890017	2604.153087	0.0065	0.995	0.400817918	count	1
AC011825.4	16.890017	2604.15312	0.0065	0.995	0.400817918	count	1
BAALC-AS2	16.8900212	2604.159686	0.0065	0.995	0.400817918	count	1
EDC4	16.8900216	2604.159786	0.0065	0.995	0.400817918	count	1
ZNF286B	16.8900354	2604.162191	0.0065	0.995	0.400817918	count	1
LINC02021	16.8902453	2990.188676	0.0056	0.995	0.400817918	count	1
PCDHGA2	16.8902595	2990.187713	0.0056	0.995	0.400817918	count	1
AC053527.1	16.8903421	3045.005414	0.0055	0.996	0.400817918	count	1
LINC01001	16.8905665	3641.614681	0.0046	0.996	0.400817918	count	1
AL358115.1	16.9934294	2362.61646	0.0072	0.994	0.40081792	count	1
CPS1	16.9936677	2788.092328	0.0061	0.995	0.40081792	count	1
AC016575.1	16.9939059	3156.892522	0.0054	0.996	0.40081792	count	1
LIN7A	17.0730427	3180.590969	0.0054	0.996	0.400817921	count	1
GNAO1	17.0730427	3180.590969	0.0054	0.996	0.400817921	count	1
ALOX12	17.0730428	3180.591064	0.0054	0.996	0.400817921	count	1
BCKDHA	17.0730428	3180.591064	0.0054	0.996	0.400817921	count	1
SLC13A5	17.0730432	3180.59119	0.0054	0.996	0.400817921	count	1
XCL1	17.0737138	3898.805775	0.0044	0.997	0.400817921	count	1
AP006333.1	17.0737139	3898.806047	0.0044	0.997	0.400817921	count	1
MAB21L1	17.073714	3898.806125	0.0044	0.997	0.400817921	count	1
AC138894.1	17.073714	3898.806008	0.0044	0.997	0.400817921	count	1
B3GNT3	17.4142513	2354.525578	0.0074	0.994	0.400817926	count	1
C16orf46	17.4142514	2354.525598	0.0074	0.994	0.400817926	count	1
C2orf70	17.4143254	2778.567256	0.0063	0.995	0.400817926	count	1
KCNK13	17.9222083	2749.804898	0.0065	0.9948	0.400817931	count	1
PAAF1	0.3332729	0.2426818	1.3733	0.17	0.400969467	count	1
HOXA-AS2	0.34575	0.3451809	1.0016	0.317	0.401411971	count	1
ZNF302	0.2986953	0.1646976	1.8136	0.0698	0.4016042	count	1
JAK3	0.3516966	0.353353	0.9953	0.32	0.401672249	count	1
CKS2	0.3319676	0.2223589	1.4929	0.136	0.401845467	count	1
PCDHGA5	0.4846366	0.659151	0.7352	0.462	0.402195879	count	1
SMIM8	0.3149058	0.2135794	1.4744	0.14	0.402203538	count	1
TBC1D8-AS1	0.5491061	0.8879044	0.6184	0.536	0.402239431	count	1
DNAJC3-DT	0.4099152	0.4164396	0.9843	0.325	0.402253403	count	1
AC116366.3	0.7136698	0.8076868	0.8836	0.377	0.402582509	count	1

GLUL	0.2855176	0.1069949	2.6685	0.00766	0.402763119	count	1
BRMS1	0.2938148	0.1428339	2.057	0.0398	0.402849141	count	1
UBE2L6	0.2862004	0.0886749	3.2275	0.00126	0.403657799	count	1
C9orf64	0.4686308	0.5272254	0.8889	0.374	0.40368484	count	1
SLC35D2	0.3092756	0.1740029	1.7774	0.0756	0.403745946	count	1
SLC5A6	0.8283547	0.7325874	1.1307	0.258	0.403792283	count	1
TAF11	0.2950684	0.1395512	2.1144	0.0346	0.404007257	count	1
GRIPAP1	0.3066065	0.2230855	1.3744	0.169	0.404421999	count	1
C5AR2	0.4358029	0.6686921	0.6517	0.515	0.40443148	count	1
COG7	0.3661132	0.3190436	1.1475	0.251	0.404804488	count	1
RRP15	0.2953849	0.1360074	2.1718	0.03	0.404852859	count	1
SFPQ	0.2851456	0.0690219	4.1312	3.72E-05	0.404902891	count	0.8580924
AC007365.1	1.0257686	1.0564187	0.971	0.332	0.404992158	count	1
CNEP1R1	0.357367	0.3555436	1.0051	0.315	0.405071147	count	1
RIPOR2	0.3310985	0.3574959	0.9262	0.354	0.405208644	count	1
CEP41	0.4094481	0.3513934	1.1652	0.244	0.40541205	count	1
RTL8B	0.3146214	0.2003011	1.5707	0.116	0.405691315	count	1
SYNGR2	0.28616	0.0795347	3.5979	0.000326	0.405846678	count	1
MYL12A	0.2818861	0.0307065	9.18	8.22E-20	0.405902801	count	1.98E-15
DZANK1	0.7215151	0.6650667	1.0849	0.278	0.40616604	count	1
RAB3A	0.5119909	0.5519075	0.9277	0.354	0.406250942	count	1
NPAT	0.3092377	0.2244132	1.378	0.168	0.40633629	count	1
MEAF6	0.2902597	0.1013654	2.8635	0.00422	0.406365117	count	1
THSD7A	0.3017628	0.1604162	1.8811	0.0601	0.406684101	count	1
PNPLA8	0.2962016	0.1311802	2.258	0.024	0.406751691	count	1
ACAP2	0.2890037	0.1008509	2.8657	0.00419	0.406816266	count	1
TMA16	0.2982662	0.157359	1.8955	0.0581	0.40730982	count	1
HOOK2	0.36372	0.2931284	1.2408	0.215	0.407342556	count	1
TRIM16L	0.6494642	0.7555141	0.8596	0.39	0.407364106	count	1
KLRF1	0.6494642	0.8136069	0.7983	0.425	0.407364106	count	1
LYSMD1	0.6494642	0.826638	0.7857	0.432	0.407364106	count	1
TEX30	0.3586361	0.2857691	1.255	0.21	0.407915402	count	1
TRMO	0.4017047	0.3404588	1.1799	0.238	0.40796874	count	1
SYPL1	0.2871113	0.0662948	4.3308	1.54E-05	0.408465255	count	0.356664
AC095055.1	1.0387998	1.1004815	0.944	0.345	0.408518293	count	1
MON1B	0.3369545	0.2530473	1.3316	0.183	0.408518702	count	1
RBM14	0.3961805	0.3508193	1.1293	0.259	0.408525445	count	1
SEC23B	0.371461	0.341822	1.0867	0.277	0.40865271	count	1
DPH6	0.3307847	0.2326035	1.4221	0.155	0.408744765	count	1
MRPS25	0.3177172	0.1741272	1.8246	0.0682	0.408959335	count	1
RNASEK	0.3033167	0.1539649	1.97	0.0489	0.40899895	count	1
RNF215	0.3610306	0.2774152	1.3014	0.193	0.409080081	count	1
MKNK2	0.2911565	0.1043491	2.7902	0.0053	0.409285204	count	1
CTNNBL1	0.3037451	0.1517085	2.0022	0.0454	0.409331647	count	1
NXT1	0.2924459	0.0942945	3.1014	0.00195	0.409530968	count	1

ENY2	0.2869344	0.0555182	5.1683	2.53E-07	0.409733995	count	0.005936139
EFCAB7	0.5052139	0.4352505	1.1607	0.246	0.409738214	count	1
B4GALT4	0.4429995	0.3803138	1.1648	0.244	0.410640557	count	1
AC008771.1	0.4151331	0.3978612	1.0434	0.297	0.410714266	count	1
DNAL4	0.3300015	0.2250084	1.4666	0.143	0.410740348	count	1
ZNF669	0.3500029	0.3413545	1.0253	0.305	0.410782096	count	1
SLCO2A1	0.2955132	0.1059048	2.7904	0.0053	0.4108864	count	1
MFNG	0.2942734	0.1163025	2.5302	0.0115	0.410960254	count	1
CAMK2D	0.3045804	0.1579823	1.9279	0.054	0.411041159	count	1
MYD88	0.3312227	0.2273522	1.4569	0.145	0.411078486	count	1
PLAGL1	0.3860661	0.2990782	1.2909	0.197	0.411289178	count	1
EEF2K	0.3283398	0.247859	1.3247	0.185	0.411398499	count	1
DHX16	0.3357343	0.2455723	1.3672	0.172	0.411446495	count	1
YJU2	0.3275991	0.2197207	1.491	0.136	0.411498569	count	1
ERI2	0.4970068	0.4952313	1.0036	0.316	0.411501945	count	1
G0S2	0.3311064	0.5522366	0.5996	0.549	0.411517343	count	1
BOLA3-AS1	0.5196513	0.5072218	1.0245	0.306	0.411700824	count	1
DAPK3	0.3353969	0.2011143	1.6677	0.0955	0.411722571	count	1
GAREM2	1.0519873	1.1161143	0.9425	0.346	0.412050965	count	1
THAP4	0.3294171	0.2162387	1.5234	0.128	0.41220117	count	1
FARSB	0.3428622	0.2615508	1.3109	0.19	0.412212181	count	1
ACAT1	0.2957284	0.1061946	2.7848	0.00539	0.412248688	count	1
PGBD1	0.3805828	0.54705	0.6957	0.487	0.412283028	count	1
CLUH	0.4571646	0.5146916	0.8882	0.374	0.412288248	count	1
SPSB3	0.2972969	0.1122443	2.6487	0.00813	0.412360352	count	1
DROSHA	0.3828943	0.3403283	1.1251	0.261	0.412542446	count	1
SLC3A2	0.2897625	0.064199	4.5135	6.64E-06	0.412642478	count	0.15432688
TCHP	0.3262617	0.2219289	1.4701	0.142	0.412680282	count	1
TRA2A	0.3001221	0.1276868	2.3505	0.0188	0.4127825	count	1
FBXL19-AS1	1.5101721	1.0270096	1.4705	0.142	0.412870994	count	1
L2HGDH	0.6602256	0.8409886	0.7851	0.432	0.413014562	count	1
PPP1R3E	0.4581599	0.5277058	0.8682	0.385	0.413117282	count	1
CRTC2	0.3776953	0.3130778	1.2064	0.228	0.413311694	count	1
FGD1	0.4996294	0.7838138	0.6374	0.524	0.413468487	count	1
EXOSC7	0.3044018	0.1577639	1.9295	0.0538	0.413722915	count	1
AC118549.1	0.3271183	0.2223606	1.4711	0.141	0.413744173	count	1
POLR3G	0.5000226	0.4872795	1.0262	0.305	0.413763141	count	1
CEBPG	0.3036403	0.1276438	2.3788	0.0174	0.413768416	count	1
GADD45B	0.2873883	0.0774324	3.7115	0.00021	0.41378006	count	1
ZNF354B	0.3590578	0.305307	1.1761	0.24	0.413843583	count	1
INTS9	0.5226718	0.571523	0.9145	0.361	0.413844439	count	1
MED24	0.3529629	0.3047253	1.1583	0.247	0.414153638	count	1
BBS7	0.3399439	0.2417205	1.4064	0.16	0.414335375	count	1
DCAF4	0.4533674	0.419877	1.0798	0.28	0.414453639	count	1
TBC1D9B	0.3229043	0.2006499	1.6093	0.108	0.414483228	count	1

MAGIX	0.5870808	0.5041167	1.1646	0.244	0.414498899	count	1
TTC39C	0.3769993	0.4306583	0.8754	0.381	0.414501479	count	1
ASH1L-AS1	0.4475816	0.4953317	0.9036	0.366	0.414585452	count	1
AKTIP	0.3206703	0.1972671	1.6256	0.104	0.414659873	count	1
PBX2	0.3481456	0.2347635	1.483	0.138	0.414759074	count	1
POLRMT	0.3969978	0.311537	1.2743	0.203	0.41481651	count	1
TBC1D32	0.7925962	0.533237	1.4864	0.137	0.414849019	count	1
CD302	0.3123364	0.1772688	1.7619	0.0782	0.414987792	count	1
SGPP1	0.6097355	0.6274288	0.9718	0.331	0.415095084	count	1
C6orf120	0.3301785	0.2400658	1.3754	0.169	0.415173654	count	1
ITFG2-AS1	0.8592071	1.1521228	0.7458	0.456	0.415180155	count	1
RFX1	0.3639867	0.2817249	1.292	0.196	0.415231874	count	1
C19orf66	0.3018458	0.1176749	2.5651	0.0104	0.415435642	count	1
METAP2	0.2942048	0.0803051	3.6636	0.000253	0.415586809	count	1
RBFA	0.3142774	0.1620224	1.9397	0.0525	0.41564935	count	1
SF3B3	0.3244234	0.2392442	1.356	0.175	0.415674465	count	1
IMP4	0.3029912	0.1207698	2.5088	0.0122	0.415685181	count	1
TAF13	0.3327034	0.2463314	1.3506	0.177	0.415696329	count	1
ILF3-DT	0.306978	0.1619406	1.8956	0.0581	0.415802182	count	1
SLC25A17	0.3802518	0.3417598	1.1126	0.266	0.41599432	count	1
HAPLN3	0.2953863	0.0972933	3.036	0.00242	0.416189557	count	1
NAA30	0.3624231	0.2983726	1.2147	0.225	0.416267729	count	1
PCED1B	0.4252176	0.665592	0.6389	0.523	0.416358892	count	1
C14orf93	0.4690879	0.5014567	0.9355	0.35	0.416423401	count	1
IDH3G	0.3036615	0.1224766	2.4793	0.0132	0.416463818	count	1
ZBTB42	0.7019604	0.6511795	1.078	0.281	0.416490567	count	1
MRPL32	0.2961104	0.0912522	3.245	0.00119	0.416505304	count	1
MINK1	0.3707102	0.3084236	1.202	0.229	0.416516002	count	1
SPG11	0.3431834	0.2538959	1.3517	0.177	0.416668265	count	1
AC009403.1	0.4941714	0.5660227	0.8731	0.383	0.416731012	count	1
ANAPC10	0.3106141	0.1642492	1.8911	0.0587	0.416936749	count	1
ISCA1	0.3067948	0.1275573	2.4052	0.0162	0.417043376	count	1
SRP68	0.3309469	0.2157044	1.5343	0.125	0.417094623	count	1
AC087672.2	0.7983775	0.7759131	1.029	0.304	0.417212059	count	1
OTUD4	0.3368206	0.2433323	1.3842	0.166	0.417277408	count	1
FBLN2	0.2950311	0.0948854	3.1093	0.00189	0.417284942	count	1
DNMT3A	0.327593	0.2731452	1.1993	0.231	0.417333101	count	1
MT1M	0.5048387	0.5207202	0.9695	0.332	0.417368008	count	1
ELOVL6	1.544083	1.0155081	1.5205	0.128	0.417648797	count	1
EIF4ENIF1	0.3490402	0.2678459	1.3031	0.193	0.417659578	count	1
AP1G1	0.3254966	0.2359261	1.3797	0.168	0.418467105	count	1
MRPL48	0.3217903	0.1966706	1.6362	0.102	0.418479724	count	1
AC004825.2	0.6409631	0.7475856	0.8574	0.391	0.418492404	count	1
AL023806.1	0.6409631	0.8491502	0.7548	0.45	0.418492404	count	1
MRPS18B	0.3002412	0.1164366	2.5786	0.00997	0.418510889	count	1

TMEM42	0.3399037	0.2989374	1.137	0.256	0.419124772	count	1
RASA1	0.3411543	0.2326643	1.4663	0.143	0.419309014	count	1
DNTTIP1	0.3149859	0.1733051	1.8175	0.0692	0.419516368	count	1
TSR2	0.3043642	0.120201	2.5321	0.0114	0.419891559	count	1
KPTN	0.5960199	0.6121015	0.9737	0.33	0.419965008	count	1
DCTPP1	0.3031466	0.1112351	2.7253	0.00647	0.420263361	count	1
TTYH3	0.4338642	0.3551368	1.2217	0.222	0.420329515	count	1
GOLPH3L	0.3287293	0.2031445	1.6182	0.106	0.42034104	count	1
GATD1	0.3974504	0.3338599	1.1905	0.234	0.420406105	count	1
NARF	0.3116257	0.1500267	2.0771	0.0379	0.420462896	count	1
LINC00467	0.3865043	0.3738664	1.0338	0.301	0.420515809	count	1
PLA2G6	1.0846177	1.0952367	0.9903	0.322	0.420638896	count	1
PSPN	0.8068833	0.976205	0.8266	0.409	0.420672196	count	1
MAGI2	0.371645	0.3893779	0.9545	0.34	0.420677039	count	1
AC089984.1	1.266879	1.0889057	1.1634	0.245	0.421040698	count	1
DDX3Y	0.3045262	0.1085691	2.8049	0.00507	0.421593072	count	1
SMN1	0.423066	0.3972205	1.0651	0.287	0.42167557	count	1
FAM173B	0.365148	0.3032907	1.204	0.229	0.421937743	count	1
RNF217-AS1	0.6776679	1.8777123	0.3609	0.7182	0.422097469	count	1
ZNF622	0.3211027	0.1800571	1.7833	0.0746	0.422866804	count	1
ACP2	0.3369245	0.2436719	1.3827	0.167	0.422982668	count	1
C5orf63	0.4174408	0.8702851	0.4797	0.632	0.423067091	count	1
MTREX	0.3352441	0.1980616	1.6926	0.0906	0.423363598	count	1
ATP13A1	0.4148702	0.3893929	1.0654	0.287	0.423745509	count	1
SUGP1	0.3680669	0.3476336	1.0588	0.29	0.42388232	count	1
UTP23	0.3051428	0.1279564	2.3847	0.0172	0.423907169	count	1
DCAF10	0.3456047	0.2427039	1.424	0.155	0.423962996	count	1
ANXA4	0.3024809	0.0970797	3.1158	0.00185	0.423991799	count	1
ZNF747	0.4646939	0.3725334	1.2474	0.212	0.424022491	count	1
MRPS17	0.3684502	0.3601303	1.0231	0.306	0.424309018	count	1
PRR12	0.8850414	0.6160184	1.4367	0.151	0.424529062	count	1
RLF	0.3284443	0.2221175	1.4787	0.139	0.424558337	count	1
XIAP	0.3136656	0.1387267	2.261	0.0238	0.424674298	count	1
UBAP2L	0.3356154	0.2209605	1.5189	0.129	0.424751793	count	1
PPIP5K2	0.3192465	0.1502881	2.1242	0.0337	0.424780611	count	1
RPL34	0.2947536	0.0199133	14.8019	9.18E-48	0.424990181	count	2.23E-43
LRRTM2	0.7638457	0.828875	0.9215	0.357	0.425196241	count	1
ARPIN	0.324055	0.1901366	1.7043	0.0884	0.42531412	count	1
USP54	0.6839048	0.5529293	1.2369	0.216	0.425322613	count	1
ZNF551	0.5852072	0.6814161	0.8588	0.391	0.425327631	count	1
NCK2	0.332805	0.177998	1.8697	0.0616	0.425463823	count	1
SLC29A1	0.3069595	0.1161894	2.6419	0.00829	0.425563652	count	1
CDC34	0.3160583	0.1384046	2.2836	0.0225	0.425642351	count	1
TEDC1	0.4027033	0.3388001	1.1886	0.235	0.425694962	count	1
NF2	0.4080346	0.3919648	1.041	0.298	0.425767636	count	1

SEC23IP	0.3460049	0.2911589	1.1884	0.235	0.425811815	count	1
TAZ	0.3350443	0.2400439	1.3958	0.163	0.425812206	count	1
ZNF487	0.4813581	0.5006155	0.9615	0.336	0.426417947	count	1
AL117339.5	0.7226668	0.6583252	1.0977	0.272	0.426502674	count	1
STXBP5	0.4322612	0.4236454	1.0203	0.308	0.426631025	count	1
CC2D1B	0.540898	0.5170255	1.0462	0.296	0.426715743	count	1
RAB2B	0.3485116	0.2945728	1.1831	0.237	0.426735621	count	1
PAQR8	0.5073498	0.4725869	1.0736	0.283	0.426803415	count	1
LSM6	0.3048129	0.103836	2.9355	0.00336	0.426827804	count	1
NXN	0.3174421	0.172994	1.835	0.0666	0.426833915	count	1
AL592295.4	1.1103661	1.0985178	1.0108	0.312	0.42726365	count	1
ANKRD65	0.3350452	0.2168204	1.5453	0.122	0.427480603	count	1
AC073332.1	0.4418068	0.447304	0.9877	0.323	0.427525342	count	1
UBXN11	0.3974611	0.3537142	1.1237	0.261	0.427529012	count	1
DCAF17	0.4688603	0.4469118	1.0491	0.294	0.427532125	count	1
ACYP2	0.3470589	0.2168308	1.6006	0.11	0.427747415	count	1
CHUK	0.4830778	0.4252981	1.1359	0.256	0.42781486	count	1
NAT10	0.3769359	0.3198881	1.1783	0.239	0.427984016	count	1
TNFAIP1	0.3084532	0.1266606	2.4353	0.0149	0.428043184	count	1
BCL3	0.3067018	0.1238751	2.4759	0.0133	0.428210809	count	1
GPER1	0.4301971	0.3762603	1.1433	0.253	0.428360385	count	1
RNF149	0.3207106	0.188659	1.6999	0.0893	0.428388339	count	1
ZNF256	0.8263081	0.7774084	1.0629	0.288	0.428500502	count	1
EIF3E	0.2992262	0.0447791	6.6823	2.83E-11	0.428861566	count	6.74E-07
SNW1	0.3047854	0.093272	3.2677	0.0011	0.428906762	count	1
MSH6	0.3240383	0.1741234	1.861	0.0629	0.429403849	count	1
HMGCL	0.3254646	0.1787044	1.8212	0.0687	0.42941946	count	1
SPRYD3	0.3273918	0.1859073	1.761	0.0783	0.429635253	count	1
SCOC-AS1	0.7295477	0.8204781	0.8892	0.374	0.429801616	count	1
LRTOMT	0.4399924	0.4449965	0.9888	0.323	0.42991132	count	1
EAF2	0.5329877	0.5556176	0.9593	0.338	0.429931854	count	1
CAPZB	0.3015827	0.0600061	5.0259	5.33E-07	0.429958265	count	0.012480728
MBLAC2	0.3831487	0.3356693	1.1414	0.254	0.429959399	count	1
LRPPRC	0.3239182	0.1713647	1.8902	0.0588	0.430011779	count	1
TOP3B	0.613405	0.5351357	1.1463	0.252	0.430521909	count	1
AL121658.1	1.315378	0.736398	1.7862	0.0742	0.430594967	count	1
LAYN	0.3310667	0.1945341	1.7018	0.0889	0.430657575	count	1
BRD9	0.3346805	0.2138368	1.5651	0.118	0.430792879	count	1
ARF5	0.3051339	0.0850215	3.5889	0.000338	0.430868543	count	1
TAF7	0.3084383	0.1011603	3.049	0.00232	0.43088393	count	1
NPM3	0.3768876	0.2627766	1.4343	0.152	0.430885732	count	1
ASTE1	0.376956	0.2901157	1.2993	0.194	0.430961177	count	1
ZNF878	0.777085	1.1276942	0.6891	0.491	0.431042915	count	1
RCHY1	0.346201	0.2256828	1.534	0.125	0.431060438	count	1
TNFRSF10D	0.3149712	0.1428486	2.2049	0.0275	0.431060654	count	1

MAPKBP1	0.5611524	0.4438146	1.2644	0.206	0.431139289	count	1
GTF3C3	0.3582931	0.2189782	1.6362	0.102	0.431156598	count	1
EPOP	0.8330226	0.6170076	1.3501	0.177	0.431182761	count	1
APC	0.3265804	0.1577033	2.0709	0.0385	0.431301618	count	1
VASP	0.3123934	0.1128426	2.7684	0.00567	0.431330318	count	1
OMA1	0.3815182	0.3093109	1.2334	0.218	0.431440103	count	1
SQLE	0.3601403	0.2711084	1.3284	0.184	0.431508908	count	1
EIF1	0.2997937	0.0254881	11.7621	3.35E-31	0.431973755	count	8.11E-27
G3BP1	0.3092263	0.1057038	2.9254	0.00347	0.432008227	count	1
ENSA	0.3038422	0.0671958	4.5217	6.39E-06	0.432134104	count	0.14856111
MAST3	0.8364251	0.5395937	1.5501	0.121	0.432537336	count	1
CFAP69	0.7353917	0.7675519	0.9581	0.338	0.432592399	count	1
IL17RA	0.4279112	0.3185034	1.3435	0.179	0.433073387	count	1
LRRC45	0.5374547	0.5670032	0.9479	0.343	0.433156081	count	1
TENT4A	0.4315999	0.352994	1.2227	0.222	0.433195511	count	1
SELENOW	0.3010276	0.0351125	8.5732	1.65E-17	0.433235997	count	3.96E-13
C12orf76	0.3240442	0.16955	1.9112	0.0561	0.433739368	count	1
ITM2B	0.3008804	0.0258407	11.6437	1.27E-30	0.433792566	count	3.07E-26
DCUN1D4	0.3526879	0.2382554	1.4803	0.139	0.433844175	count	1
PHF5A	0.31305	0.1073258	2.9168	0.00356	0.434004462	count	1
HDAC9	0.5075844	0.42354	1.1984	0.231	0.434186422	count	1
SLC4A1AP	0.3230736	0.1575522	2.0506	0.0404	0.434194031	count	1
APOL2	0.3444993	0.1873969	1.8383	0.0661	0.434336152	count	1
FES	0.3377751	0.2169988	1.5566	0.12	0.434353216	count	1
RWDD2A	0.6699986	0.631456	1.061	0.289	0.434403606	count	1
ZNF92	0.3597136	0.2382261	1.51	0.131	0.434555545	count	1
LAMC1	0.3173315	0.1421346	2.2326	0.0257	0.434569014	count	1
PRPF3	0.345141	0.194094	1.7782	0.0755	0.434631499	count	1
LMNB2	0.3909391	0.308071	1.269	0.205	0.434845302	count	1
GSS	0.3393834	0.1928547	1.7598	0.0786	0.435653204	count	1
LAP3	0.304949	0.0674008	4.5244	6.31E-06	0.435793889	count	0.14672643
UBAC1	0.3186323	0.1341545	2.3751	0.0176	0.435801372	count	1
CCDC58	0.3379746	0.2122603	1.5923	0.111	0.436008182	count	1
RPL39L	0.4561078	0.5202529	0.8767	0.381	0.436080551	count	1
AC027031.2	0.4277048	0.3482678	1.2281	0.22	0.436120521	count	1
CIC	0.354675	0.2328931	1.5229	0.128	0.436230939	count	1
MARF1	0.3486103	0.2261233	1.5417	0.123	0.436271364	count	1
AL590617.2	0.5852968	0.7862424	0.7444	0.457	0.436711672	count	1
STYK1	1.6910656	1.0689596	1.582	0.114	0.436790174	count	1
ALS2CL	0.47991	0.4417034	1.0865	0.277	0.436813296	count	1
PAPOLG	0.3928977	0.3233124	1.2152	0.224	0.436935738	count	1
AP001267.3	0.7067664	0.8436951	0.8377	0.402	0.43704291	count	1
TM7SF2	0.457289	0.475719	0.9613	0.337	0.437131937	count	1
SUPT4H1	0.3118826	0.0954598	3.2672	0.0011	0.437671712	count	1
ATE1	0.3397972	0.2156699	1.5755	0.115	0.437975558	count	1

BCS1L	0.3892642	0.2583093	1.507	0.132	0.438233769	count	1
CFAP54	1.020234	0.6577336	1.5511	0.121	0.438254144	count	1
STC2	0.4017256	0.3390029	1.185	0.236	0.438460989	count	1
CTSL	0.3105807	0.091224	3.4046	0.000672	0.438523957	count	1
S100PBP	0.3598949	0.2934018	1.2266	0.22	0.438818406	count	1
IPO5	0.3337431	0.183404	1.8197	0.0689	0.438825684	count	1
CELSR1	0.4760494	0.4488563	1.0606	0.289	0.438946532	count	1
USP46-AS1	1.0227968	0.7854569	1.3022	0.193	0.439021795	count	1
GLE1	0.5727977	0.6519081	0.8786	0.38	0.439024839	count	1
GALNT15	0.3320368	0.2103174	1.5787	0.115	0.43905389	count	1
C6orf163	1.360628	1.4449473	0.9416	0.346	0.439169486	count	1
CPEB2	0.404486	0.3671868	1.1016	0.271	0.439205129	count	1
PRKAA1	0.3328878	0.1729425	1.9248	0.0544	0.43930783	count	1
ZNF677	0.3325629	0.1716115	1.9379	0.0527	0.439317226	count	1
ZCCHC4	0.4650267	0.4069182	1.1428	0.253	0.439384174	count	1
LRRCC1	0.3415707	0.2017508	1.693	0.0906	0.439514304	count	1
3-Mar	0.3574855	0.2219019	1.611	0.107	0.439605503	count	1
SLFN5	0.3117536	0.109143	2.8564	0.00432	0.440200871	count	1
MRPS22	0.3191873	0.1262034	2.5291	0.0115	0.440211802	count	1
ORAI1	0.3163465	0.1073356	2.9473	0.00323	0.440369157	count	1
LRRFIP1	0.3066101	0.0459083	6.6787	2.90E-11	0.440387427	count	6.90E-07
CNOT10	0.3722862	0.2860761	1.3014	0.193	0.440587208	count	1
DHX8	0.3583545	0.228451	1.5686	0.117	0.44064862	count	1
USP27X-AS1	1.1641963	1.0599386	1.0984	0.272	0.440689348	count	1
TIGD5	0.4177682	0.3592364	1.1629	0.245	0.440820982	count	1
PLA2G12A	0.3965675	0.2830064	1.4013	0.161	0.440849972	count	1
SPOPL	0.3725156	0.3182011	1.1707	0.242	0.440850505	count	1
ZNF282	0.4206847	0.3828549	1.0988	0.272	0.441054949	count	1
RBM22	0.3166909	0.1019663	3.1058	0.00192	0.441091376	count	1
PEX14	0.4297253	0.3595439	1.1952	0.232	0.441211862	count	1
TCEAL1	0.3220332	0.1464694	2.1986	0.028	0.441259319	count	1
TBC1D31	0.6106633	0.8204145	0.7443	0.457	0.441353091	count	1
SMNDC1	0.3215225	0.1398715	2.2987	0.0216	0.441885851	count	1
LMO4	0.3187584	0.1190095	2.6784	0.00744	0.442006211	count	1
SIRT7	0.3556953	0.2170749	1.6386	0.101	0.44201507	count	1
CUL1	0.3436224	0.1915846	1.7936	0.073	0.442110009	count	1
VPS51	0.3201737	0.1179077	2.7155	0.00666	0.442166916	count	1
PIGN	0.3995684	0.4176515	0.9567	0.339	0.442171913	count	1
NR2F1-AS1	0.4688354	0.4049097	1.1579	0.247	0.442722183	count	1
TSFM	0.3337758	0.1598536	2.088	0.0369	0.442745489	count	1
ME2	0.365241	0.240914	1.5161	0.13	0.442749374	count	1
RCN1	0.3127336	0.0809232	3.8646	0.000114	0.442837962	count	1
ADCK2	0.3591079	0.2541924	1.4127	0.158	0.44292848	count	1
GOLGA8N	1.0362368	0.9091013	1.1398	0.254	0.443024155	count	1
RPP30	0.3309137	0.1535163	2.1556	0.0312	0.443146315	count	1

DDX11	0.4229118	0.3629252	1.1653	0.244	0.4432686	count	1
ZNF343	0.8053327	0.8168282	0.9859	0.324	0.443351165	count	1
SLC22A18	0.5647891	0.6219321	0.9081	0.364	0.443422236	count	1
RAP1B	0.3110933	0.0627343	4.9589	7.52E-07	0.443502687	count	0.017593792
CPSF1	0.4088515	0.345484	1.1834	0.237	0.443729642	count	1
RCBTB2	0.3543419	0.1940171	1.8263	0.0679	0.443847677	count	1
PAICS	0.3376006	0.1764934	1.9128	0.0559	0.444067819	count	1
CENPK	0.5412823	0.4813557	1.1245	0.261	0.444399817	count	1
FAM171A1	0.3572047	0.2165647	1.6494	0.0992	0.444458508	count	1
CDC42EP4	0.3379046	0.143271	2.3585	0.0184	0.444462294	count	1
AC099063.4	0.9421325	1.1048946	0.8527	0.394	0.444594186	count	1
TBC1D22B	0.661053	0.6958714	0.95	0.342	0.444672311	count	1
MED20	0.7218307	0.5836177	1.2368	0.216	0.444678728	count	1
OCLN	0.6891013	0.7657843	0.8999	0.368	0.444728032	count	1
MAPK9	0.3750091	0.2998197	1.2508	0.211	0.444776582	count	1
DDX21	0.3130692	0.0803712	3.8953	1.00E-04	0.445064149	count	1
SHQ1	0.4196617	0.3533477	1.1877	0.235	0.445312289	count	1
BID	0.3355037	0.1688839	1.9866	0.0471	0.445399495	count	1
RBM4	0.3629327	0.235938	1.5383	0.124	0.445426625	count	1
FAM189B	0.3765904	0.2647644	1.4224	0.155	0.445525543	count	1
SBF1	0.9450495	0.8852806	1.0675	0.286	0.445597645	count	1
PSMD3	0.3279179	0.1312939	2.4976	0.0126	0.445711262	count	1
BCHE	0.6180353	0.4348806	1.4212	0.155	0.445954624	count	1
ZNF586	0.5229152	0.5135403	1.0183	0.309	0.446055318	count	1
PIGW	0.5842291	0.58691	0.9954	0.32	0.446722175	count	1
CLBA1	0.6406533	0.7107849	0.9013	0.367	0.446872112	count	1
HIST1H4A	0.665195	0.7504352	0.8864	0.375	0.447023047	count	1
IL33	0.3130207	0.0714215	4.3827	1.22E-05	0.447125972	count	0.2828082
ZNF524	0.3209331	0.1106258	2.9011	0.00375	0.44751165	count	1
ZBTB46	0.3837531	0.2941491	1.3046	0.192	0.447889421	count	1
TOP3A	0.4694981	0.4306463	1.0902	0.276	0.44797404	count	1
C3orf80	0.4806806	0.403329	1.1918	0.233	0.448086001	count	1
ADCY4	0.3338789	0.1534939	2.1752	0.0297	0.448277543	count	1
C11orf74	0.3309651	0.1552875	2.1313	0.0332	0.448333568	count	1
RPL32	0.3110796	0.0202127	15.3903	2.42E-51	0.448574677	count	5.87E-47
TMEM18	0.3190246	0.1046781	3.0477	0.00233	0.448600759	count	1
BLVRA	0.3282434	0.1360376	2.4129	0.0159	0.448763816	count	1
SS18	0.3491954	0.2076994	1.6813	0.0928	0.448786635	count	1
NUB1	0.3200595	0.0956496	3.3462	0.00083	0.448810591	count	1
TNFAIP3	0.3223712	0.194856	1.6544	0.0982	0.448856709	count	1
ZNF672	0.4117237	0.2883706	1.4278	0.153	0.448880558	count	1
CIZ1	0.3752657	0.2514645	1.4923	0.136	0.449110865	count	1
NUP155	0.4413305	0.3351286	1.3169	0.188	0.449206173	count	1
REC8	0.6974775	0.6829041	1.0213	0.307	0.449219193	count	1
RRS1	0.3994907	0.271583	1.471	0.141	0.44929117	count	1

NDUFV3	0.3276914	0.138482	2.3663	0.018	0.449325252	count	1
LANCL2	0.6693201	0.5719202	1.1703	0.242	0.449358781	count	1
PQLC3	0.3291198	0.1601543	2.055	0.04	0.449366662	count	1
TMEM255B	0.3208499	0.1147739	2.7955	0.00522	0.449463768	count	1
DPYSL2	0.3178205	0.0878964	3.6159	0.000305	0.449494201	count	1
ZNF419	0.5736053	0.5397933	1.0626	0.288	0.449539729	count	1
COMMD6	0.3134612	0.042577	7.3622	2.37E-13	0.449619316	count	5.66E-09
DYM	0.3561475	0.2500226	1.4245	0.154	0.449730733	count	1
AC100810.1	0.3574532	0.219088	1.6316	0.103	0.449817286	count	1
SERTAD1	0.3154923	0.0695393	4.5369	5.95E-06	0.45036015	count	0.138397
BCLAF3	0.3870763	0.2997323	1.2914	0.197	0.450374372	count	1
ARIH1	0.3361715	0.1821858	1.8452	0.0651	0.450421696	count	1
FARS2	0.3741887	0.2516619	1.4869	0.137	0.450663849	count	1
RNF217	0.3462261	0.2120286	1.6329	0.103	0.450675185	count	1
SWT1	0.4899669	0.4284145	1.1437	0.253	0.450763101	count	1
HDGFL2	0.3379553	0.2060551	1.6401	0.101	0.450818005	count	1
PMM2	0.3540356	0.2108948	1.6787	0.0933	0.450827478	count	1
EIPR1	0.4139166	0.3688769	1.1221	0.262	0.451162405	count	1
INPP5K	0.350825	0.1816178	1.9317	0.0535	0.451217731	count	1
GALNT18	0.3573494	0.2330974	1.533	0.125	0.451217846	count	1
VPS9D1	0.7012709	0.6400596	1.0956	0.273	0.451245978	count	1
AC015813.1	1.4286232	1.041655	1.3715	0.17	0.451457425	count	1
CROT	0.3870193	0.4106535	0.9424	0.346	0.451574234	count	1
GPATCH4	0.3365332	0.1599057	2.1046	0.0354	0.451949468	count	1
PYROXD1	0.3753273	0.2674477	1.4034	0.161	0.451996723	count	1
PTBP3	0.3298068	0.1307769	2.5219	0.0117	0.45242289	count	1
ZNF283	0.6095668	0.544902	1.1187	0.263	0.45245936	count	1
NSL1	0.32594	0.1158628	2.8132	0.00494	0.452598819	count	1
AMBRA1	0.9656521	0.8617015	1.1206	0.263	0.452625447	count	1
RAB3GAP1	0.3473246	0.1804036	1.9253	0.0543	0.452962353	count	1
SCG3	1.442167	0.9571778	1.5067	0.132	0.453822135	count	1
RNF34	0.3654242	0.2352641	1.5533	0.12	0.453827704	count	1
SLC38A5	0.4763278	0.6254547	0.7616	0.446	0.454018904	count	1
AL162377.1	0.6121777	0.5754593	1.0638	0.288	0.454141958	count	1
NOL10	0.3798783	0.2280325	1.6659	0.0958	0.454469501	count	1
LIPE-AS1	0.4945756	0.4733238	1.0449	0.296	0.454662527	count	1
PIP4P1	0.345912	0.1593217	2.1712	0.03	0.455091603	count	1
LINC02356	1.077986	1.038472	1.0381	0.299	0.455201428	count	1
DDX18	0.3215952	0.0888869	3.618	0.000302	0.455382412	count	1
MIIP	0.3670914	0.2361925	1.5542	0.12	0.455850335	count	1
MRFAP1L1	0.3487951	0.2128829	1.6384	0.101	0.455980757	count	1
FKBP4	0.3503862	0.2164403	1.6189	0.106	0.4563058	count	1
ZNF253	0.4280514	0.3966265	1.0792	0.281	0.456328279	count	1
ZSCAN21	0.5109765	0.4139657	1.2343	0.217	0.456657422	count	1
ZNF600	0.6571654	0.5529398	1.1885	0.235	0.456663774	count	1

ABO	0.4033275	0.4308763	0.9361	0.349	0.456786576	count	1
CAT	0.3307971	0.1214565	2.7236	0.0065	0.45685371	count	1
ZNF354A	0.369714	0.2448432	1.51	0.131	0.457063292	count	1
MKNK1	0.3981236	0.3228441	1.2332	0.218	0.457239811	count	1
SLC8B1	0.3840445	0.2903254	1.3228	0.186	0.457278182	count	1
ZNF391	0.8382294	0.7806212	1.0738	0.283	0.457401575	count	1
STPG1	0.5046951	0.3591769	1.4051	0.16	0.457489781	count	1
CNRIP1	0.3341251	0.1269263	2.6324	0.00852	0.457624442	count	1
ZDHHC23	0.6010205	0.4778498	1.2578	0.209	0.457950689	count	1
ZNF234	0.6374764	0.5236983	1.2173	0.224	0.458004805	count	1
MTMR1	0.5859437	0.7552845	0.7758	0.438	0.458058191	count	1
IRF8	0.6599108	0.5484237	1.2033	0.229	0.458283254	count	1
GNAI3	0.3293028	0.0930278	3.5398	0.000407	0.458384215	count	1
ZNF333	0.5296949	0.4186644	1.2652	0.206	0.458460127	count	1
ZNF217	0.3501505	0.1900679	1.8422	0.0655	0.458543108	count	1
FGR	0.3943554	0.2510169	1.571	0.116	0.45855152	count	1
MLLT10	0.3437206	0.1701165	2.0205	0.0434	0.458593999	count	1
NFKBIL1	0.3408903	0.1622181	2.1014	0.0357	0.458600097	count	1
ELAC1	0.4115803	0.3271756	1.258	0.209	0.458712213	count	1
NDE1	0.3711772	0.2493225	1.4887	0.137	0.458828677	count	1
THAP6	0.4039076	0.2787661	1.4489	0.147	0.45903222	count	1
ZNF746	0.5219058	0.5261995	0.9918	0.321	0.459100211	count	1
NOP53	0.3207387	0.0500844	6.404	1.77E-10	0.459277075	count	4.20E-06
ADK	0.3834103	0.3043935	1.2596	0.208	0.459550292	count	1
RABGGTA	0.366717	0.2343005	1.5652	0.118	0.459577132	count	1
DTX4	0.5883959	0.4953475	1.1878	0.235	0.4597452	count	1
SLC25A21-AS1	0.456013	0.369704	1.2335	0.218	0.459771136	count	1
RRP8	0.363563	0.293568	1.2384	0.216	0.459901568	count	1
CHCHD6	0.3837131	0.3247538	1.1816	0.237	0.459902558	count	1
TFEB	0.3922984	0.2173865	1.8046	0.0712	0.460002904	count	1
TBL3	0.4184146	0.2969565	1.409	0.159	0.460078884	count	1
SPON1	0.987925	0.5532911	1.7855	0.0743	0.460106443	count	1
UBE2E2	0.3677292	0.2627236	1.3997	0.162	0.460248133	count	1
CEBPD	0.319807	0.0666666	4.7971	1.70E-06	0.460279841	count	0.0396865
PRIM2	0.4608181	0.3188681	1.4452	0.149	0.460670492	count	1
E2F5	0.7190224	0.528234	1.3612	0.174	0.460670885	count	1
FAM161B	0.6057123	0.4626107	1.3093	0.191	0.461071554	count	1
TRAPPC2B	0.3348679	0.1355572	2.4703	0.0136	0.461206228	count	1
CXXC4	0.9916392	0.8178323	1.2125	0.225	0.461342261	count	1
HERC6	0.426197	0.3961898	1.0757	0.282	0.461657284	count	1
ATF2	0.3552943	0.1779841	1.9962	0.046	0.46167306	count	1
MSH2	0.3815223	0.2329339	1.6379	0.102	0.461940531	count	1
GNPAT	0.3890741	0.2543151	1.5299	0.126	0.462020577	count	1
CCDC93	0.3609512	0.2215843	1.629	0.103	0.462030469	count	1
C2orf49	0.3419889	0.158861	2.1528	0.0314	0.462044867	count	1

NARFL	0.3964891	0.325116	1.2195	0.223	0.462244358	count	1
RENBP	0.5345668	0.559463	0.9555	0.339	0.462273252	count	1
PCDHB4	0.4913674	0.3788472	1.297	0.195	0.462376872	count	1
DOPEY2	0.5658819	0.4868714	1.1623	0.245	0.462399451	count	1
COL4A3BP	0.3322304	0.1189614	2.7928	0.00526	0.462511886	count	1
FIGNL1	0.5108791	0.4066749	1.2562	0.209	0.462618001	count	1
TMEM134	0.3371542	0.1343174	2.5101	0.0121	0.462645945	count	1
ARFIP2	0.3743755	0.2401132	1.5592	0.119	0.462686165	count	1
NR2F1	0.3928499	0.2445756	1.6063	0.108	0.462998975	count	1
SOAT1	0.3833005	0.273714	1.4004	0.162	0.46314797	count	1
POLL	0.409204	0.3143697	1.3017	0.193	0.463177168	count	1
YOD1	0.4922945	0.5650259	0.8713	0.384	0.463182175	count	1
DHRS4L2	0.3385917	0.129767	2.6092	0.00912	0.463294609	count	1
FOS	0.3214041	0.0428892	7.4938	8.95E-14	0.463350694	count	2.14E-09
CFAP298	0.3360373	0.1173813	2.8628	0.00423	0.463413037	count	1
FBXL5	0.3445765	0.1509899	2.2821	0.0226	0.463787795	count	1
DUSP4	0.3663124	0.2408892	1.5207	0.128	0.463800769	count	1
ZNF189	0.5197645	0.4343435	1.1967	0.232	0.463814869	count	1
LMBR1	0.3771004	0.2802681	1.3455	0.179	0.463850079	count	1
LINC02202	0.8025412	0.7424072	1.081	0.28	0.463937457	count	1
AC061992.1	0.9176153	1.3292513	0.6903	0.49	0.463944431	count	1
ZNRF2	0.4133409	0.2847293	1.4517	0.147	0.464225867	count	1
COX15	0.3827679	0.2626953	1.4571	0.145	0.464273437	count	1
LYPLA2	0.3433508	0.1456471	2.3574	0.0185	0.464358247	count	1
ATG14	0.3562148	0.2039306	1.7467	0.0808	0.464376307	count	1
RANBP3	0.405957	0.2828006	1.4355	0.151	0.464395062	count	1
RNASEH2B	0.3653577	0.2449823	1.4914	0.136	0.46450852	count	1
CNIH1	0.3289506	0.0879971	3.7382	0.000189	0.464633403	count	1
AP001160.3	0.7621101	0.7707638	0.9888	0.323	0.464757121	count	1
H3F3B	0.3225092	0.0289517	11.1396	3.19E-28	0.464765969	count	7.71E-24
CRTAP	0.329134	0.0806056	4.0833	4.57E-05	0.465305597	count	1
RBPMS	0.3297063	0.088417	3.729	0.000196	0.465496768	count	1
PEAK1	0.3846671	0.2849341	1.35	0.177	0.46564145	count	1
DSP	0.3708598	0.2914829	1.2723	0.203	0.465777046	count	1
ALG3	0.3554031	0.1804016	1.9701	0.0489	0.465857276	count	1
RNF123	0.6732971	0.6608901	1.0188	0.308	0.466145084	count	1
PWP1	0.3370121	0.1179027	2.8584	0.00429	0.466389899	count	1
TAMM41	0.4153939	0.3119974	1.3314	0.183	0.466435611	count	1
MFSD14B	0.3929376	0.2578645	1.5238	0.128	0.466463899	count	1
VAT1	0.3359881	0.1265796	2.6544	0.00799	0.466557232	count	1
LAMB2	0.3381663	0.1548195	2.1843	0.029	0.466564458	count	1
DLD	0.3506364	0.1610721	2.1769	0.0296	0.466605796	count	1
C22orf34	0.766222	0.6727112	1.139	0.255	0.46677921	count	1
WDR6	0.4110776	0.2718568	1.5121	0.131	0.466858918	count	1
COA7	0.4290905	0.4393073	0.9767	0.329	0.466917255	count	1

MEMO1	0.4336975	0.7816375	0.5549	0.579	0.467010896	count	1
RNPC3	0.3456388	0.2031274	1.7016	0.0889	0.467052133	count	1
ABCB7	0.3774429	0.2680381	1.4082	0.159	0.467067077	count	1
CROCC	0.486207	0.408244	1.191	0.234	0.467395236	count	1
C5orf34	0.5614836	0.7066961	0.7945	0.427	0.467581559	count	1
NMRK1	0.3355369	0.1118022	3.0012	0.00271	0.467632385	count	1
FAM49B	0.3671574	0.2230112	1.6464	0.0998	0.467661308	count	1
RBM42	0.3370034	0.1101861	3.0585	0.00225	0.467786196	count	1
JARID2	0.3881326	0.3028362	1.2817	0.2	0.467903688	count	1
RBM27	0.450987	0.3451242	1.3067	0.191	0.468061064	count	1
TPST1	0.3875029	0.2626888	1.4751	0.14	0.468080793	count	1
KIAA1586	0.3513915	0.1834514	1.9154	0.0555	0.46815975	count	1
KDM6A	0.3926019	0.4579804	0.8572	0.391	0.468200523	count	1
PRTG	0.9291746	0.5685532	1.6343	0.102	0.46827465	count	1
ZNF420	0.4735425	0.34671	1.3658	0.172	0.468667025	count	1
PSPC1	0.3795287	0.2241067	1.6935	0.0905	0.468897629	count	1
TSNAX	0.3409919	0.1275317	2.6738	0.00754	0.46899469	count	1
CEP44	0.3740754	0.2386112	1.5677	0.117	0.469164492	count	1
FOXK1	0.4026512	0.2741598	1.4687	0.142	0.469176784	count	1
BVES	0.8664531	2.0660066	0.4194	0.675	0.469215029	count	1
ZNF766	0.377425	0.2433852	1.5507	0.121	0.469662039	count	1
MTIF3	0.33214	0.0905877	3.6665	0.000251	0.469662315	count	1
NFKBIB	0.392367	0.2208591	1.7765	0.0758	0.469962266	count	1
UBIAD1	0.4843162	0.3384681	1.4309	0.153	0.470170584	count	1
NEDD1	0.4061489	0.2626432	1.5464	0.122	0.470409667	count	1
ZNF565	0.5547179	0.5659727	0.9801	0.327	0.470431163	count	1
C19orf54	0.6377854	0.5449375	1.1704	0.242	0.470526424	count	1
ANXA11	0.3325949	0.0784459	4.2398	2.31E-05	0.470734164	count	0.5340951
CCDC17	1.1338756	1.1479191	0.9878	0.323	0.470909662	count	1
THAP2	0.4094224	0.3130793	1.3077	0.191	0.471206417	count	1
ASNA1	0.3456831	0.127534	2.7105	0.00676	0.471699626	count	1
LINC02001	0.3781693	0.2130039	1.7754	0.0759	0.471812201	count	1
WDR60	0.3436701	0.1275862	2.6936	0.00711	0.47188439	count	1
C19orf25	0.3580577	0.1821039	1.9662	0.0494	0.472062314	count	1
TDP2	0.3689564	0.2005105	1.8401	0.0659	0.472083551	count	1
OARD1	0.3470465	0.1451567	2.3908	0.0169	0.472197773	count	1
INTS3	0.3923249	0.2392341	1.6399	0.101	0.472809616	count	1
LSMEM1	0.7429616	0.6287785	1.1816	0.237	0.473226228	count	1
ZSCAN5A	0.7429616	0.7155611	1.0383	0.299	0.473226228	count	1
SERINC5	0.4533209	0.3150029	1.4391	0.15	0.473355064	count	1
VPS41	0.3666126	0.1917612	1.9118	0.056	0.47340089	count	1
FBXO33	0.3981056	0.2561558	1.5542	0.12	0.473491953	count	1
TXNDC11	0.4832627	0.3469589	1.3929	0.164	0.473503594	count	1
ELF4	0.5104545	0.3279224	1.5566	0.12	0.473586162	count	1
ARFGAP2	0.3680642	0.2030132	1.813	0.0699	0.473758893	count	1

TMEM150C	0.36161	0.1856738	1.9476	0.0516	0.473870734	count	1
ZNF10	0.5245447	0.6955925	0.7541	0.451	0.473906834	count	1
HMGA1	0.381568	0.2210314	1.7263	0.0844	0.474049309	count	1
SLC39A4	0.3656604	0.2164434	1.6894	0.0913	0.474263827	count	1
SNAP29	0.3460595	0.1477288	2.3425	0.0192	0.474314258	count	1
RMI2	1.3101277	1.11573	1.1742	0.24	0.4743202	count	1
IQCH	1.0315594	1.0861999	0.9497	0.342	0.474415625	count	1
EPHA1-AS1	2.052575	1.1846763	1.7326	0.0833	0.474421042	count	1
ZNF79	1.1467159	1.0414591	1.1011	0.271	0.47442428	count	1
HLA-E	0.3293028	0.0285531	11.533	4.35E-30	0.474489001	count	1.05E-25
AC011416.3	0.6644482	0.6423908	1.0343	0.301	0.474519178	count	1
FAM120B	0.3946928	0.2711478	1.4556	0.146	0.474626857	count	1
AMOTL1	0.3734513	0.2039598	1.831	0.0672	0.475058108	count	1
DHX9	0.3505692	0.148627	2.3587	0.0184	0.475100164	count	1
PPP5C	0.381562	0.2215656	1.7221	0.0852	0.475321766	count	1
TMEM126A	0.3459169	0.1343577	2.5746	0.0101	0.475512837	count	1
MNDA	0.7476012	0.9313889	0.8027	0.422	0.475639017	count	1
CNOT4	0.3452099	0.1217602	2.8352	0.00461	0.475665901	count	1
TM4SF1-AS1	0.6459997	0.6307512	1.0242	0.306	0.475736605	count	1
PLEKHG2	0.5196479	0.4196439	1.2383	0.216	0.475757584	count	1
WDSUB1	0.4149727	0.3462029	1.1986	0.231	0.475847704	count	1
UTP3	0.3594674	0.1810137	1.9859	0.0471	0.475944491	count	1
HSPA12B	0.3575225	0.152647	2.3422	0.0192	0.476037197	count	1
ZNF286A	1.0366512	0.9539164	1.0867	0.277	0.476055784	count	1
HS6ST1	0.4199027	0.336103	1.2493	0.212	0.476475327	count	1
FAM208B	0.3616614	0.1792241	2.0179	0.0437	0.476504335	count	1
TNFRSF1B	0.3501503	0.1735143	2.018	0.0437	0.476585011	count	1
NAAA	0.3638768	0.1990128	1.8284	0.0676	0.477067764	count	1
USP31	0.4150178	0.393756	1.054	0.292	0.477400874	count	1
HDDC2	0.3719706	0.1851693	2.0088	0.0447	0.477515786	count	1
GALE	0.3682412	0.1849778	1.9907	0.0466	0.477554454	count	1
SELENOP	0.3355602	0.0737718	4.5486	5.63E-06	0.477700654	count	0.13098758
C4orf47	1.5889641	1.0157348	1.5643	0.118	0.47777114	count	1
TRIM52	0.3852916	0.3032114	1.2707	0.204	0.477899241	count	1
CENPT	0.3894882	0.2679284	1.4537	0.146	0.477929699	count	1
API5	0.3743664	0.2129303	1.7582	0.0788	0.478006265	count	1
LTBP1	0.4229236	0.3613002	1.1706	0.242	0.478064396	count	1
FYN	0.3531375	0.1543886	2.2873	0.0223	0.47819306	count	1
S100A10	0.3318533	0.038309	8.6625	7.71E-18	0.47825853	count	1.85E-13
ARNTL2	0.410907	0.3679514	1.1167	0.264	0.478451332	count	1
IPMK	0.4283803	0.3345227	1.2806	0.2	0.478524711	count	1
HARBI1	1.0445546	1.1334353	0.9216	0.357	0.478589511	count	1
YTHDF3-AS1	0.5886902	0.5442964	1.0816	0.28	0.478909838	count	1
KIAA0586	0.3789621	0.1905079	1.9892	0.0468	0.478967041	count	1
THAP12	0.3501806	0.1402854	2.4962	0.0126	0.479021003	count	1

ASL	0.3642794	0.1894137	1.9232	0.0546	0.479408778	count	1
NUP153	0.4258114	0.2731377	1.559	0.119	0.47943576	count	1
UBL7-AS1	0.4032785	0.2886697	1.397	0.163	0.47944639	count	1
CLEC2D	1.047437	0.768768	1.3625	0.173	0.479509972	count	1
ZNF8	0.557043	0.5435422	1.0248	0.306	0.479764264	count	1
SHLD1	0.459962	0.3598329	1.2783	0.201	0.479890968	count	1
SMC1B	0.8388856	1.194028	0.7026	0.482	0.480353574	count	1
CLDND1	0.3456793	0.1056475	3.272	0.00108	0.480736203	count	1
ASB13	0.5589211	0.4830037	1.1572	0.247	0.48121832	count	1
FAIM	0.3859144	0.231043	1.6703	0.095	0.481245556	count	1
ZNF559	0.4678897	0.3795307	1.2328	0.218	0.481339144	count	1
FO681492.1	0.5135883	0.5429486	0.9459	0.344	0.481603867	count	1
SPIDR	0.3795291	0.2250059	1.6868	0.0918	0.481672871	count	1
ATP8B3	1.347388	1.0600354	1.2711	0.204	0.482289117	count	1
PPIH	0.3569776	0.1464394	2.4377	0.0148	0.482472629	count	1
RFC2	0.3849508	0.21715	1.7727	0.0764	0.482491825	count	1
PPFIBP1	0.3421848	0.087545	3.9087	9.50E-05	0.48256389	count	1
CCNL1	0.3387451	0.0735059	4.6084	4.24E-06	0.482576029	count	0.09872416
FANCF	0.4339973	0.3288499	1.3197	0.187	0.482576611	count	1
NCAPH2	0.459883	0.3339716	1.377	0.169	0.48277277	count	1
ABHD3	0.5715772	0.4840899	1.1807	0.238	0.483218436	count	1
SPAG7	0.3481459	0.1051919	3.3096	0.000946	0.483339007	count	1
NPHP4	1.3529633	1.0737863	1.26	0.208	0.483461034	count	1
SNAPC1	0.3591599	0.154336	2.3271	0.02	0.483490427	count	1
SETD1A	0.515839	0.4375598	1.1789	0.239	0.483542606	count	1
RBM43	0.3541186	0.169596	2.088	0.0369	0.483750532	count	1
FAM76B	0.3646524	0.1812222	2.0122	0.0443	0.483792602	count	1
TIPIN	0.3970292	0.2863436	1.3865	0.166	0.484516083	count	1
TBCE	0.6810882	0.4613274	1.4764	0.14	0.484589574	count	1
LY96	0.3460204	0.0994449	3.4795	0.00051	0.484720827	count	1
ITGB4	0.3429469	0.1054808	3.2513	0.00116	0.484771758	count	1
ST8SIA4	0.3727511	0.2540491	1.4672	0.142	0.484949087	count	1
EXOC4	0.370441	0.2065488	1.7935	0.073	0.484983566	count	1
AC021016.3	15.5478498	1104.094005	0.0141	0.989	0.485756709	count	1
TMC1	15.5478498	1104.094004	0.0141	0.989	0.485756709	count	1
FBXO10	15.5479137	1217.736267	0.0128	0.99	0.485756709	count	1
STAMBPL1	15.7008653	1089.24976	0.0144	0.989	0.485756724	count	1
PACRG	15.7008721	1089.250003	0.0144	0.989	0.485756724	count	1
AC073263.2	15.7009049	1201.346701	0.0131	0.99	0.485756724	count	1
AC005696.4	15.7945594	1214.200892	0.013	0.99	0.485756732	count	1
AC005670.2	15.8342762	1100.224789	0.0144	0.989	0.485756735	count	1
AC127164.1	15.8933547	1209.916621	0.0131	0.99	0.485756739	count	1
AC245041.2	15.8935078	1461.45474	0.0109	0.991	0.485756739	count	1
AP000438.1	15.9421732	1103.536876	0.0144	0.988	0.485756742	count	1
DIABLO	15.9421745	1103.536258	0.0144	0.988	0.485756742	count	1

AC092295.2	16.0084837	1577.790645	0.0101	0.992	0.485756747	count	1
AC097359.2	16.0084948	1577.792735	0.0101	0.992	0.485756747	count	1
AL731569.1	16.009012	2009.721695	0.008	0.994	0.485756747	count	1
AC026254.2	16.0098107	2686.24849	0.006	0.995	0.485756747	count	1
NEFH	16.0347863	1264.174692	0.0127	0.99	0.485756749	count	1
AC108471.2	16.0348223	1186.617286	0.0135	0.989	0.485756749	count	1
AC145285.2	16.0458516	1145.491261	0.014	0.989	0.485756749	count	1
AC107027.1	15.5478538	1104.095039	0.0141	0.989	0.48575675	count	1
AC233280.1	16.118317	1110.726107	0.0145	0.9884	0.485756754	count	1
KCP	16.1907935	1099.742223	0.0147	0.988	0.485756758	count	1
SCUBE1	16.1907935	1099.742225	0.0147	0.988	0.485756758	count	1
TMEM220-AS1	16.207647	1579.56393	0.0103	0.992	0.485756759	count	1
ADAMTS12	16.2169611	1212.277712	0.0134	0.989	0.485756759	count	1
BHLHE40-AS1	16.2582868	1313.530746	0.0124	0.99	0.485756761	count	1
AC145343.1	16.2954234	1089.636116	0.015	0.9881	0.485756763	count	1
CYSLTR2	16.2971794	1929.33315	0.0084	0.993	0.485756763	count	1
FAM181B	16.2971795	1929.333206	0.0084	0.993	0.485756763	count	1
AL121821.1	16.2971796	1929.333228	0.0084	0.993	0.485756763	count	1
TDRD1	16.2978182	2284.857887	0.0071	0.994	0.485756763	count	1
SHCBP1L	16.2978183	2284.857937	0.0071	0.994	0.485756763	count	1
PSMA8	16.2978184	2284.857837	0.0071	0.994	0.485756763	count	1
GOLGA8Q	16.2984456	2592.423614	0.0063	0.995	0.485756763	count	1
SLC6A15	16.2997749	3567.184013	0.0046	0.996	0.485756763	count	1
LINC01915	16.3701595	1577.91325	0.0104	0.992	0.485756766	count	1
TLL2	16.3701604	1577.912616	0.0104	0.992	0.485756766	count	1
SYN3	16.3701633	1577.914376	0.0104	0.992	0.485756766	count	1
TMEM178B	16.3701642	1577.914951	0.0104	0.992	0.485756766	count	1
AC068305.2	16.370169	1577.915468	0.0104	0.992	0.485756766	count	1
GRM3	16.3706995	1893.064235	0.0086	0.993	0.485756766	count	1
LINC01659	16.3790737	1807.771676	0.0091	0.993	0.485756767	count	1
AC011481.2	16.3790761	1807.77166	0.0091	0.993	0.485756767	count	1
HIST1H2AH	16.3790835	1807.775221	0.0091	0.993	0.485756767	count	1
AC080112.1	16.3790835	1807.77523	0.0091	0.993	0.485756767	count	1
TGFA	16.3794054	2321.675019	0.0071	0.994	0.485756767	count	1
TMEM56	16.3990345	1398.094463	0.0117	0.9906	0.485756768	count	1
HBB	16.4017672	1398.413051	0.0117	0.9906	0.485756768	count	1
MIF-AS1	16.4923571	1086.600564	0.0152	0.988	0.485756772	count	1
MDH1B	16.5080677	1764.592658	0.0094	0.993	0.485756772	count	1
DPF3	16.5496356	1399.16045	0.0118	0.9906	0.485756774	count	1
WDR90	16.5670633	1203.642318	0.0138	0.989	0.485756774	count	1
APBA1	16.6116814	1552.733337	0.0107	0.9915	0.485756776	count	1
AC008040.5	16.6278325	1571.795647	0.0106	0.992	0.485756777	count	1
ALDH1L1	16.6278996	1761.005292	0.0094	0.992	0.485756777	count	1
RELT	16.6462657	1092.523001	0.0152	0.9878	0.485756777	count	1
RGMA	16.7340063	1757.299702	0.0095	0.992	0.48575678	count	1

TMEM79	16.7343758	1829.741076	0.0091	0.993	0.48575678	count	1
TESMIN	16.7467609	2579.884454	0.0065	0.995	0.485756781	count	1
MOCOS	16.7467609	2579.884497	0.0065	0.995	0.485756781	count	1
AC008440.1	16.7467609	2579.884475	0.0065	0.995	0.485756781	count	1
YPEL4	16.7467655	2579.884421	0.0065	0.995	0.485756781	count	1
OR2I1P	16.7467745	2579.895258	0.0065	0.995	0.485756781	count	1
MC1R	16.7471797	2890.186734	0.0058	0.995	0.485756781	count	1
AMIGO1	16.7471798	2890.186698	0.0058	0.995	0.485756781	count	1
FGF16	16.74718	2890.186806	0.0058	0.995	0.485756781	count	1
HSD17B13	16.7471837	2890.185796	0.0058	0.995	0.485756781	count	1
AL353708.3	16.7471885	2890.185712	0.0058	0.995	0.485756781	count	1
SLC5A9	16.747191	2890.192109	0.0058	0.995	0.485756781	count	1
PLEK	16.8238307	1833.450538	0.0092	0.9927	0.485756783	count	1
SDR42E1	16.8305789	2170.261952	0.0078	0.994	0.485756783	count	1
SLC4A5	17.0636132	3360.871327	0.0051	0.996	0.485756789	count	1
AC002480.2	17.1026791	2360.416068	0.0072	0.994	0.48575679	count	1
ALG1L	17.1026957	2360.418806	0.0072	0.994	0.48575679	count	1
SYT12	17.1037047	4010.678513	0.0043	0.997	0.48575679	count	1
NUP210	17.1037104	4010.68398	0.0043	0.997	0.48575679	count	1
AC008914.1	17.1346842	1552.18642	0.011	0.9912	0.48575679	count	1
PIK3C2G	17.193303	2914.72263	0.0059	0.995	0.485756792	count	1
TPBGL	17.4922195	2359.10182	0.0074	0.994	0.485756797	count	1
TLR6	17.4923462	2704.609299	0.0065	0.995	0.485756797	count	1
AL645608.8	17.5191705	1908.570623	0.0092	0.9927	0.485756797	count	1
S100A14	17.9748749	2464.078996	0.0073	0.9942	0.485756802	count	1
AC025171.5	16.3701643	1577.914956	0.0104	0.992	0.485756808	count	1
ARHGAP42	16.3790761	1807.771666	0.0091	0.993	0.485756808	count	1
RIOK1	0.3663176	0.157935	2.3194	0.0204	0.485759973	count	1
ALDH16A1	0.3833724	0.2019468	1.8984	0.0577	0.48644794	count	1
XRN2	0.3431775	0.0801249	4.283	1.91E-05	0.486514703	count	0.4420122
TRMT10C	0.3535564	0.1182146	2.9908	0.00281	0.486623597	count	1
MMAA	0.5329184	0.4824792	1.1045	0.269	0.486841448	count	1
QRFPR	0.9800452	0.9096875	1.0773	0.281	0.486919901	count	1
ADAMTS9-AS2	0.4706541	0.381698	1.2331	0.218	0.487251363	count	1
GAS2L1	0.359999	0.1377011	2.6144	0.00899	0.487512014	count	1
NFYC	0.372683	0.1683217	2.2141	0.0269	0.487587086	count	1
TBC1D15	0.3507099	0.1132547	3.0966	0.00198	0.488014404	count	1
CRAMP1	0.4622767	0.4807787	0.9615	0.336	0.488024007	count	1
PPP3R1	0.3809989	0.1897551	2.0078	0.0448	0.48804803	count	1
AC025287.2	1.1982225	1.0717937	1.118	0.264	0.488176341	count	1
UBE2R2	0.3484735	0.1014109	3.4363	0.000599	0.488246157	count	1
AC092140.1	1.3771384	1.1893065	1.1579	0.247	0.488479731	count	1
CHERP	0.4396188	0.3531251	1.2449	0.213	0.488541118	count	1
SSH3	0.4396188	0.4643412	0.9468	0.344	0.488541118	count	1
DNM2	0.379095	0.1822736	2.0798	0.0376	0.488850379	count	1

SAFB2	0.3674262	0.1697347	2.1647	0.0305	0.488856262	count	1
ERAP1	0.4299509	0.3211467	1.3388	0.181	0.489087268	count	1
C5orf51	0.3876405	0.2273567	1.705	0.0883	0.489162195	count	1
IKBIP	0.346902	0.0907581	3.8223	0.000135	0.489208354	count	1
UHRF2	0.3709728	0.1841781	2.0142	0.0441	0.489330452	count	1
TMEM238	0.3921048	0.2436127	1.6095	0.108	0.489410691	count	1
CADPS2	0.3595208	0.1515769	2.3719	0.0178	0.489426252	count	1
DLL4	0.3711079	0.1957341	1.896	0.0581	0.489506065	count	1
ZBTB41	0.4020095	0.3099794	1.2969	0.195	0.489574253	count	1
PLK2	0.3504338	0.1261065	2.7789	0.00549	0.489636168	count	1
AC064836.3	0.4536757	0.4761852	0.9527	0.341	0.489894186	count	1
FIGNL2	1.6726284	1.3318967	1.2558	0.209	0.490121966	count	1
HADHA	0.3445024	0.0655136	5.2585	1.56E-07	0.490343286	count	0.003663816
RFC5	0.5527124	0.4730428	1.1684	0.243	0.490426504	count	1
MT-CYB	0.3401125	0.0303151	11.2192	1.35E-28	0.490439746	count	3.26E-24
MAK	0.8618392	0.7345566	1.1733	0.241	0.490524684	count	1
MEF2C	0.3446675	0.0762332	4.5212	6.41E-06	0.490644108	count	0.14901968
ALDH9A1	0.3600053	0.1157571	3.11	0.00189	0.490876093	count	1
MINDY3	0.3978727	0.2298546	1.731	0.0836	0.490970131	count	1
WDR36	0.3965623	0.2308066	1.7182	0.0859	0.491524141	count	1
ZNF160	0.3806162	0.2019482	1.8847	0.0596	0.491526306	count	1
CCDC142	0.8174832	0.6020073	1.3579	0.175	0.491561041	count	1
GRWD1	0.4084604	0.2309867	1.7683	0.0771	0.491658928	count	1
TENT2	0.397264	0.2167375	1.8329	0.0669	0.491672968	count	1
CHTOP	0.3644757	0.1492153	2.4426	0.0146	0.491969527	count	1
ALG10	0.7798783	0.6416014	1.2155	0.224	0.492240599	count	1
ZNF480	0.3859556	0.236308	1.6333	0.103	0.492503883	count	1
REPS1	0.4728428	0.327888	1.4421	0.149	0.492531957	count	1
ETV6	0.3667519	0.1658772	2.211	0.0271	0.492535004	count	1
MIEF2	0.6368515	0.6008782	1.0599	0.289	0.492673022	count	1
FANK1	0.6368515	0.6307427	1.0097	0.313	0.492673022	count	1
EPB41L4A-AS1	0.3565794	0.1210371	2.946	0.00325	0.492678632	count	1
MET	0.3575291	0.1633351	2.1889	0.0287	0.492963301	count	1
AC064801.1	0.9970154	2.0978068	0.4753	0.6346	0.49299231	count	1
MTAP	0.3762774	0.1826119	2.0605	0.0394	0.493070592	count	1
SH2B1	0.445869	0.2742561	1.6257	0.104	0.493105305	count	1
AL356599.1	0.4914743	0.5112286	0.9614	0.336	0.493127573	count	1
AARSD1	0.584856	0.4248061	1.3768	0.169	0.493223919	count	1
ZNF627	0.8680626	0.7735429	1.1222	0.262	0.493256304	count	1
EFTUD2	0.4059299	0.2462499	1.6484	0.0994	0.493337206	count	1
ENTPD3-AS1	0.5056055	0.4094159	1.2349	0.217	0.493788361	count	1
AC060780.1	0.4446803	0.3445769	1.2905	0.197	0.493904574	count	1
LCOR	0.3953016	0.1970054	2.0066	0.0449	0.493931897	count	1
TTC9C	0.382588	0.2388591	1.6017	0.109	0.494025994	count	1
ZNF808	0.423826	0.2887942	1.4676	0.142	0.494299246	count	1

DUSP2	0.382637	0.2966772	1.2897	0.197	0.494458388	count	1
LINC01535	1.223588	1.0272211	1.1912	0.234	0.494748333	count	1
DNAJC17	0.3755395	0.1778909	2.1111	0.0349	0.494769015	count	1
FAN1	0.4267351	0.3238517	1.3177	0.188	0.494781124	count	1
EBAG9	0.3593496	0.1222275	2.94	0.00331	0.494806624	count	1
ABCE1	0.3572356	0.1176268	3.037	0.00241	0.494870134	count	1
DNAJC9	0.3852081	0.2002778	1.9234	0.0545	0.495003242	count	1
ZBTB22	0.4131199	0.2581288	1.6004	0.11	0.49506773	count	1
PARP11	0.4860612	0.3522733	1.3798	0.168	0.495376773	count	1
UGCG	0.3524736	0.1274485	2.7656	0.00572	0.496097041	count	1
MVB12B	0.5034867	0.3821016	1.3177	0.188	0.496181231	count	1
PSEN2	0.4222801	0.3124197	1.3516	0.177	0.496460902	count	1
VCAM1	0.3472957	0.1281439	2.7102	0.00677	0.49650727	count	1
TUBB6	0.3511126	0.0791432	4.4364	9.50E-06	0.496566258	count	0.2205235
CCDC148	1.1026526	1.05669	1.0435	0.297	0.496766186	count	1
SNRNP40	0.3605834	0.1175482	3.0675	0.00218	0.496887696	count	1
DPM2	0.3622586	0.1294263	2.799	0.00516	0.497049278	count	1
STX17	0.3715769	0.1666959	2.2291	0.0259	0.497110138	count	1
WDR77	0.435835	0.2618644	1.6644	0.0962	0.49716809	count	1
DUSP5	0.3525785	0.1008371	3.4965	0.000479	0.497343059	count	1
MRPS26	0.358337	0.1145968	3.1269	0.00178	0.497445764	count	1
C1orf112	0.8300874	0.6463848	1.2842	0.199	0.497534095	count	1
AC005070.3	0.7569293	0.6484293	1.1673	0.243	0.497693316	count	1
TGFBRAP1	0.4723828	0.3438719	1.3737	0.17	0.498078832	count	1
SIRPA	0.3835798	0.2015044	1.9036	0.0571	0.49812643	count	1
MALT1	0.3745216	0.1690773	2.2151	0.0268	0.498170704	count	1
SIRT5	0.4052388	0.2331755	1.7379	0.0823	0.498296946	count	1
TNFRSF1A	0.3507378	0.067695	5.1811	2.36E-07	0.498467412	count	0.005538212
AL359220.1	0.4790643	0.3462541	1.3836	0.167	0.498620633	count	1
AC006942.1	0.8807044	0.8112256	1.0856	0.278	0.498770995	count	1
GPR68	0.453334	0.390126	1.162	0.245	0.498808313	count	1
ADIPOR2	0.3808457	0.1732996	2.1976	0.0281	0.499523748	count	1
AC008608.2	0.8343548	0.6267691	1.3312	0.183	0.499545655	count	1
AARS2	0.6841398	0.6667703	1.0261	0.305	0.499638414	count	1
AC009779.2	0.5286835	0.4469008	1.183	0.237	0.49987404	count	1
ANKRD54	0.4134126	0.2875695	1.4376	0.151	0.50032488	count	1
EIF2B4	0.4097123	0.2279917	1.797	0.0724	0.500397518	count	1
SMCHD1	0.3586573	0.1305352	2.7476	0.00604	0.50043376	count	1
SCYL2	0.4163264	0.2819536	1.4766	0.14	0.500828903	count	1
LIPE	0.7968444	0.5030499	1.584	0.113	0.500838505	count	1
FAM168B	0.3942173	0.2632381	1.4976	0.134	0.50091025	count	1
CATSPERG	0.7340937	0.6681042	1.0988	0.272	0.501126978	count	1
TRPC1	0.6867319	0.5324575	1.2897	0.197	0.50124554	count	1
PPP2CA	0.3684966	0.1272014	2.897	0.0038	0.501633571	count	1
LINC02482	0.4501493	0.4427146	1.0168	0.309	0.501715959	count	1

NME6	0.4017629	0.2429423	1.6537	0.0983	0.501806694	count	1
MED26	0.8396219	0.5352583	1.5686	0.117	0.502021082	count	1
NDC1	0.7104249	0.5400436	1.3155	0.188	0.50212495	count	1
TESK2	0.9482844	0.7094	1.3367	0.181	0.502226902	count	1
TBC1D8	0.597045	0.509593	1.1716	0.241	0.50235691	count	1
TDRP	0.4223136	0.3105596	1.3598	0.174	0.502447749	count	1
ZNF736	0.5057819	0.4007875	1.262	0.207	0.502453322	count	1
HDAC1	0.3654622	0.1138607	3.2097	0.00134	0.502531238	count	1
EIF2B1	0.3777147	0.1530456	2.468	0.0136	0.502563909	count	1
LINC01534	0.7663697	0.7830829	0.9787	0.328	0.502757184	count	1
BHLHE40	0.356309	0.0956209	3.7263	0.000198	0.502757772	count	1
ZNF688	0.3833736	0.1964551	1.9515	0.0511	0.502786487	count	1
SETD9	0.4096698	0.2384887	1.7178	0.0859	0.502804845	count	1
NUDT6	0.4216585	0.3113113	1.3545	0.176	0.502807912	count	1
CBX2	0.552248	0.441957	1.2496	0.212	0.50288455	count	1
ALMS1	0.4979582	0.6156403	0.8088	0.419	0.503006815	count	1
ZDHHC11B	1.0258038	0.9078987	1.1299	0.259	0.50312696	count	1
RBM5	0.3788848	0.1688486	2.2439	0.0249	0.503272026	count	1
GRK4	0.7380008	0.621762	1.187	0.235	0.503334583	count	1
ZNF98	1.1244871	0.5930502	1.8961	0.0581	0.503396031	count	1
HERC4	0.4049061	0.2300414	1.7601	0.0785	0.503666227	count	1
CHST11	0.4360478	0.2768373	1.5751	0.115	0.503679594	count	1
KAT6A	0.3778586	0.1548414	2.4403	0.0147	0.503740957	count	1
CEP135	0.4118421	0.222629	1.8499	0.0644	0.503762921	count	1
DDX41	0.3919222	0.199188	1.9676	0.0492	0.503873398	count	1
MPP7	1.4547507	1.0915652	1.3327	0.183	0.503929496	count	1
KLHL28	0.3947735	0.2332282	1.6926	0.0906	0.503984211	count	1
TRAF3IP1	0.4011333	0.2349104	1.7076	0.0878	0.504082724	count	1
ZNF33B	0.3971062	0.2304288	1.7233	0.0849	0.504501443	count	1
ZNF45	1.1285647	1.0410318	1.0841	0.278	0.50462215	count	1
QARS	0.3792987	0.1530508	2.4783	0.0133	0.504642998	count	1
ECT2	0.4890102	0.353636	1.3828	0.167	0.50506193	count	1
PTCD1	1.1302771	0.7855315	1.4389	0.15	0.505135906	count	1
GMEB1	0.4175163	0.2579485	1.6186	0.106	0.505138569	count	1
GSN	0.3510104	0.0490693	7.1534	1.08E-12	0.505321743	count	2.58E-08
TRIM32	0.7158857	0.7958831	0.8995	0.368	0.505358174	count	1
LTA4H	0.3747454	0.1444554	2.5942	0.00953	0.505538467	count	1
CNTNAP3B	0.3609371	0.132114	2.732	0.00633	0.505706249	count	1
ASPH	0.3564467	0.084245	4.2311	2.40E-05	0.505715592	count	0.55476
WIPF1	0.4129808	0.3393684	1.2169	0.224	0.505942333	count	1
DHTKD1	0.5057588	0.490462	1.0312	0.303	0.506457997	count	1
RAB28	0.4135918	0.2277028	1.8164	0.0694	0.50666951	count	1
UBQLN2	0.4653819	0.2953891	1.5755	0.115	0.506767329	count	1
LINC00513	0.7182865	0.4293833	1.6728	0.0945	0.506776574	count	1
HS3ST1	0.3802257	0.1979614	1.9207	0.0549	0.506854863	count	1

SNAI1	0.3785776	0.1624747	2.3301	0.0199	0.50688518	count	1
GALM	0.4464548	0.2971917	1.5022	0.133	0.507051896	count	1
FAM161A	0.4592078	0.3704786	1.2395	0.215	0.507136305	count	1
RICTOR	0.4113818	0.1947367	2.1125	0.0347	0.507186039	count	1
B3GNT2	0.3810164	0.1794422	2.1233	0.0338	0.507301977	count	1
RTN4R	0.961309	1.0633427	0.904	0.366	0.50731339	count	1
CATSPERE	0.961309	1.1778588	0.8161	0.414	0.50731339	count	1
STAT5A	0.4085154	0.2332373	1.7515	0.08	0.507354968	count	1
CCDC134	0.9615013	0.779438	1.2336	0.217	0.507388141	count	1
IFNGR1	0.3574912	0.0810398	4.4113	1.07E-05	0.507523467	count	0.2481972
GPS2	0.3920726	0.210381	1.8636	0.0625	0.507536349	count	1
ZNF654	0.4288205	0.3344129	1.2823	0.2	0.507580765	count	1
PI4KB	0.4486847	0.2788017	1.6093	0.108	0.507706778	count	1
RAB40B	0.4077187	0.2315155	1.7611	0.0783	0.507747242	count	1
BLZF1	0.4022678	0.2297173	1.7511	0.08	0.507749763	count	1
AC013394.1	0.4214835	0.2856593	1.4755	0.14	0.507838587	count	1
OXSR1	0.4085258	0.2563095	1.5939	0.111	0.508052774	count	1
B2M	0.3523262	0.0197722	17.8193	2.55E-67	0.508213018	count	6.19E-63
PPP1R3D	0.4135708	0.2939607	1.4069	0.16	0.508255861	count	1
HEXA	0.3617842	0.1018418	3.5524	0.000388	0.508277672	count	1
AC078846.1	1.4777803	1.0167798	1.4534	0.146	0.508324711	count	1
IQCH-AS1	1.4777803	1.0688416	1.3826	0.167	0.508324711	count	1
PLSCR1	0.3558041	0.0605686	5.8744	4.75E-09	0.50836578	count	0.000112338
NOL8	0.3770879	0.1761364	2.1409	0.0324	0.508803658	count	1
ZNF717	0.5274902	0.4275438	1.2338	0.217	0.508804646	count	1
LINC02185	0.3631159	0.1583102	2.2937	0.0219	0.508904462	count	1
RAD51D	0.4609581	0.3697682	1.2466	0.213	0.508974018	count	1
POMZP3	0.4932483	0.5317648	0.9276	0.354	0.509160199	count	1
MAT2A	0.3853098	0.1722724	2.2366	0.0254	0.509187128	count	1
TBC1D22A	0.3898687	0.2402537	1.6227	0.105	0.509392608	count	1
KATNBL1	0.3680125	0.117831	3.1232	0.00181	0.50954033	count	1
CALHM2	0.3706688	0.1300537	2.8501	0.0044	0.509705999	count	1
DDX27	0.379115	0.1873781	2.0233	0.0431	0.509732051	count	1
MKKS	0.4558733	0.2884827	1.5802	0.114	0.509791179	count	1
BRF2	0.4389488	0.2842395	1.5443	0.123	0.509838338	count	1
PYROXD2	0.6197126	0.4982831	1.2437	0.214	0.51036494	count	1
HAT1	0.374009	0.1334457	2.8027	0.0051	0.510482232	count	1
EMG1	0.3880651	0.1730932	2.2419	0.025	0.51048966	count	1
GPD1L	0.455065	0.3688796	1.2336	0.217	0.510870288	count	1
MED18	0.5064237	0.3901369	1.2981	0.194	0.510968043	count	1
RBP7	0.3575507	0.0871638	4.1021	4.21E-05	0.511098313	count	0.9705734
APBB3	0.6209825	0.4306411	1.442	0.149	0.511285456	count	1
NPIPA1	1.291145	0.8860807	1.4571	0.145	0.511623721	count	1
LPIN3	0.8606933	0.809983	1.0626	0.288	0.511841623	count	1
SMIM1	0.9742595	1.0707682	0.9099	0.363	0.512325875	count	1

SRSF4	0.365583	0.0909298	4.0205	5.96E-05	0.512451241	count	1
APOOL	0.3978683	0.2198947	1.8094	0.0705	0.512589437	count	1
ZC3HAV1	0.3756164	0.127198	2.953	0.00317	0.51265574	count	1
MTMR2	0.4179468	0.2272269	1.8393	0.066	0.51267321	count	1
SETDB1	0.4443566	0.3669525	1.2109	0.226	0.512888451	count	1
TRAPPC9	0.6002292	0.4253203	1.4112	0.158	0.512905261	count	1
FICD	0.4791068	0.3557812	1.3466	0.178	0.51319807	count	1
PPM1F	0.4022223	0.2009107	2.002	0.0454	0.513291684	count	1
PRMT3	0.5577183	0.4535542	1.2297	0.219	0.513485441	count	1
DPP8	0.4006013	0.2025173	1.9781	0.048	0.513525255	count	1
MAGI1	0.3910536	0.2069663	1.8895	0.0589	0.513540124	count	1
LAS1L	0.4310816	0.2513906	1.7148	0.0865	0.513659896	count	1
VAC14	0.5654509	0.4109002	1.3761	0.169	0.513773149	count	1
THOC5	0.4636682	0.3226071	1.4373	0.151	0.51396672	count	1
CSRNP2	0.5657948	0.4009916	1.411	0.158	0.514056009	count	1
LIPT2	1.0576751	1.0153483	1.0417	0.298	0.514104981	count	1
TRAFD1	0.4368136	0.292275	1.4945	0.135	0.514205431	count	1
OLA1	0.3698719	0.1066808	3.4671	0.000534	0.514390112	count	1
ENPP2	0.380667	0.2467786	1.5425	0.123	0.514940054	count	1
ZPR1	0.3937243	0.1773061	2.2206	0.0265	0.515553477	count	1
CHD1	0.3671248	0.1029262	3.5669	0.000367	0.515634083	count	1
ZBTB39	0.6403216	0.4998964	1.2809	0.2	0.515647144	count	1
RBM23	0.3936283	0.186539	2.1102	0.0349	0.515723612	count	1
ACVR2B	0.5535415	0.4939663	1.1206	0.263	0.51577989	count	1
THAP7-AS1	0.594329	0.5698903	1.0429	0.297	0.516141574	count	1
NANS	0.3687172	0.1073712	3.434	0.000603	0.51615912	count	1
ZNF426	0.4364058	0.3250559	1.3426	0.18	0.516237124	count	1
FAM110D	0.3639391	0.0953962	3.815	0.000139	0.516396161	count	1
MLLT6	0.3822143	0.1748715	2.1857	0.0289	0.516610858	count	1
HIST2H2BF	1.1698209	0.7752333	1.509	0.131	0.516817054	count	1
NELFE	0.3769894	0.1211447	3.1119	0.00188	0.516844047	count	1
FAM118B	0.4340224	0.2928794	1.4819	0.138	0.517042793	count	1
KDM4A	0.4886288	0.3593952	1.3596	0.174	0.517148121	count	1
CAMKK2	0.3896812	0.211484	1.8426	0.0655	0.517190181	count	1
LINC02147	0.5696165	0.4156289	1.3705	0.171	0.517196842	count	1
PICK1	0.4887994	0.3228971	1.5138	0.13	0.517317965	count	1
FAAP100	0.5428729	0.3832431	1.4165	0.157	0.517390782	count	1
AC005726.1	0.924366	2.1611163	0.4277	0.6689	0.517467869	count	1
DOHH	0.4281755	0.2554682	1.676	0.0938	0.517626258	count	1
EEF2KMT	0.4760378	0.3305192	1.4403	0.15	0.517752441	count	1
CRNKL1	0.4064162	0.2070172	1.9632	0.0497	0.51804978	count	1
POLG2	0.4925833	0.3508321	1.404	0.16	0.518090517	count	1
ZNF12	0.4350078	0.3286114	1.3238	0.186	0.518175913	count	1
XPO4	0.5708577	0.3892563	1.4665	0.143	0.51821589	count	1
ELMO1	0.3840577	0.1521165	2.5248	0.0116	0.518384271	count	1

PUS7L	0.3874312	0.1740266	2.2263	0.0261	0.518408532	count	1
SIK1	1.0704753	1.0811255	0.9901	0.322	0.518443157	count	1
ZCCHC2	0.4091772	0.2178308	1.8784	0.0604	0.51844895	count	1
DNAJA1	0.3615429	0.0513859	7.0358	2.49E-12	0.518558567	count	5.94E-08
TAF1B	0.4032483	0.1943924	2.0744	0.0381	0.518560629	count	1
SNAPC3	0.4432101	0.2840416	1.5604	0.119	0.518801777	count	1
SP3	0.3887331	0.1966123	1.9772	0.0481	0.518824486	count	1
GNB1L	0.5720529	0.4085895	1.4001	0.162	0.519196696	count	1
WRAP73	0.4082208	0.2220036	1.8388	0.0661	0.519317121	count	1
RASSF3	0.402656	0.2012825	2.0005	0.0455	0.519435747	count	1
AC132872.1	0.9294971	0.8914004	1.0427	0.297	0.519629782	count	1
ZNF121	0.4671328	0.3051458	1.5309	0.126	0.519725429	count	1
BRIX1	0.3761036	0.1095018	3.4347	0.000602	0.520163037	count	1
FAM220A	1.1816753	1.09257	1.0816	0.28	0.520250948	count	1
TENM2	1.3276737	1.1236761	1.1815	0.237	0.520377252	count	1
RAB33A	1.0766794	0.9645509	1.1162	0.264	0.520531342	count	1
ZNF493	0.4174912	0.3119131	1.3385	0.181	0.520945785	count	1
CHKA	0.5259308	0.2861976	1.8376	0.0662	0.520978183	count	1
NME7	0.4076505	0.2039922	1.9984	0.0458	0.521009663	count	1
DAB2IP	0.4237059	0.3264298	1.298	0.194	0.52115852	count	1
YRDC	0.3909694	0.1471081	2.6577	0.00791	0.521180542	count	1
NIPAL2	0.5599998	0.483362	1.1586	0.247	0.521256446	count	1
FADD	0.4281356	0.234254	1.8277	0.0677	0.521326661	count	1
DNAAF5	0.4848644	0.2992186	1.6204	0.105	0.521696367	count	1
CCDC117	0.4752918	0.3366029	1.412	0.158	0.521722767	count	1
EGR4	1.920752	1.348822	1.424	0.155	0.521822872	count	1
C7orf31	0.6995268	0.6541993	1.0693	0.285	0.521950592	count	1
RCL1	0.4851548	0.2901112	1.6723	0.0946	0.521991223	count	1
UBAP2	0.415798	0.2288069	1.8172	0.0693	0.522057011	count	1
PHF8	0.5679856	0.4003186	1.4188	0.156	0.522057953	count	1
TBC1D24	0.720711	0.5527382	1.3039	0.192	0.522109325	count	1
EXOSC3	0.4169205	0.2347371	1.7761	0.0758	0.522189981	count	1
LDHA	0.3638459	0.0449258	8.0988	8.24E-16	0.522325667	count	1.98E-11
ALKBH3	0.4016807	0.2048598	1.9608	0.05	0.522590109	count	1
PCDHGA6	1.0834145	1.0810018	1.0022	0.316	0.522787553	count	1
ASCC1	0.4548225	0.3187693	1.4268	0.154	0.52287934	count	1
HSCB	0.3933874	0.1721489	2.2852	0.0224	0.522910408	count	1
TRIM7	1.339871	1.0490367	1.2772	0.202	0.523243648	count	1
RBM19	0.5045132	0.3795101	1.3294	0.184	0.523410241	count	1
AL627171.1	0.7745277	0.6357964	1.2182	0.223	0.523737329	count	1
PCDHGB6	0.7475033	0.6016555	1.2424	0.214	0.523888423	count	1
LMCD1	0.3659548	0.0676671	5.4082	6.91E-08	0.523941867	count	0.00162613
KLHL8	0.4777959	0.3316407	1.4407	0.15	0.524327897	count	1
ZNF670	0.5640003	0.5284367	1.0673	0.286	0.524642083	count	1
BAZ2B	0.3769178	0.1154155	3.2657	0.00111	0.525082249	count	1

PCTP	0.525924	0.3922217	1.3409	0.18	0.525171477	count	1
UNC13B	0.531133	0.6366846	0.8342	0.404	0.525740932	count	1
RYBP	0.3914957	0.158604	2.4684	0.0136	0.526011976	count	1
ADAMTSL4	1.0103904	0.615588	1.6413	0.101	0.526074297	count	1
AGAP9	1.958848	1.4010642	1.3981	0.162	0.526097193	count	1
ZFYVE19	0.4366109	0.287915	1.5165	0.13	0.526487766	count	1
ZNF792	0.5733816	0.4495116	1.2756	0.202	0.526549589	count	1
ABHD14A-ACY1	1.0118465	1.0482984	0.9652	0.335	0.526621128	count	1
C12orf29	0.4057709	0.1996245	2.0327	0.0422	0.527118674	count	1
TENT5C	0.5989432	0.4590646	1.3047	0.192	0.527168676	count	1
SCUBE2	1.2062277	1.2313376	0.9796	0.327	0.527264757	count	1
ELF1	0.372446	0.0732391	5.0853	3.92E-07	0.527378302	count	0.009185736
FAM122C	0.5378802	0.4538093	1.1853	0.236	0.527445329	count	1
TMEM246	0.5244336	0.4189382	1.2518	0.211	0.527833599	count	1
EPHB4	0.385571	0.1266823	3.0436	0.00236	0.528162028	count	1
SNX16	0.4398796	0.2357391	1.866	0.0622	0.528211132	count	1
PITPNM3	0.6738938	0.5493036	1.2268	0.22	0.528457646	count	1
RNASEH1	0.4156525	0.2177887	1.9085	0.0564	0.528557572	count	1
NOC3L	0.3850763	0.1754489	2.1948	0.0283	0.52889117	count	1
PRELID2	0.4337995	0.2743807	1.581	0.114	0.528915744	count	1
SLC49A3	0.4285618	0.240744	1.7802	0.0752	0.529322734	count	1
L3MBTL2	0.4437894	0.2827246	1.5697	0.117	0.529419731	count	1
CLYBL	0.7849959	0.4285386	1.8318	0.0671	0.529506108	count	1
SUFU	0.5698741	0.3773134	1.5103	0.131	0.529603802	count	1
UPP1	0.3775092	0.1087608	3.471	0.000526	0.529612429	count	1
MAPK14	0.4157412	0.2200235	1.8895	0.0589	0.529671209	count	1
PACS1	0.4295598	0.2365004	1.8163	0.0694	0.529743742	count	1
TAF4	0.7119172	0.5519435	1.2898	0.197	0.529773964	count	1
ANKRD55	0.9549937	0.5828605	1.6385	0.101	0.530262742	count	1
AC108673.3	0.8564417	0.6063661	1.4124	0.158	0.530343332	count	1
PCGF6	0.4622049	0.2788444	1.6576	0.0975	0.53100088	count	1
HDAC10	0.7598151	0.6639491	1.1444	0.253	0.531016525	count	1
AL109741.1	0.401761	0.2355646	1.7055	0.0882	0.531087664	count	1
DHRS13	0.6138216	0.515277	1.1912	0.234	0.531199346	count	1
AATF	0.4074379	0.1655211	2.4615	0.0139	0.531290357	count	1
NUP133	0.5325642	0.3374013	1.5784	0.115	0.53130662	count	1
ARL17B	0.5424825	0.7324067	0.7407	0.459	0.531601178	count	1
PTPRN2	0.3982538	0.1919315	2.075	0.0381	0.531941347	count	1
AC026979.2	0.5659759	0.4799767	1.1792	0.238	0.532008085	count	1
ZNF606	0.6497938	0.7101706	0.915	0.36	0.532025512	count	1
AC023157.3	0.4206173	0.2627878	1.6006	0.11	0.532051668	count	1
PTTG1	0.6151888	0.6046292	1.0175	0.309	0.532250755	count	1
FAHD2B	0.4620714	0.2995477	1.5426	0.123	0.532468182	count	1
AC015912.3	0.6645235	0.4922057	1.3501	0.177	0.532562045	count	1
MTFMT	0.4555643	0.3187643	1.4292	0.153	0.53269234	count	1

SCLY	1.3811996	1.0769639	1.2825	0.2	0.532747895	count	1
CHST10	0.7910266	0.699627	1.1306	0.258	0.532813651	count	1
SASH1	0.3787694	0.1130582	3.3502	0.000818	0.53282538	count	1
R3HDM4	0.4054525	0.200336	2.0239	0.0431	0.53298505	count	1
ZNF251	0.5078031	0.3834765	1.3242	0.186	0.533091461	count	1
IFT88	0.4142091	0.1992389	2.079	0.0377	0.533211818	count	1
ZNF611	0.5743802	0.4266356	1.3463	0.178	0.533402642	count	1
LRIF1	0.3991943	0.1919141	2.0801	0.0376	0.533562592	count	1
HOXD-AS2	0.4589569	0.3068511	1.4957	0.135	0.533615919	count	1
DCAF13	0.3928204	0.1365632	2.8765	0.00405	0.53371776	count	1
TRIM4	0.4142535	0.2295759	1.8044	0.0713	0.534094431	count	1
NEK6	0.5356314	0.342008	1.5661	0.117	0.53413599	count	1
C3orf33	0.9102891	0.7901935	1.152	0.249	0.534440506	count	1
LAPTM4B	0.3826605	0.1036538	3.6917	0.000227	0.534481776	count	1
PLEKHO2	0.4214558	0.2551341	1.6519	0.0987	0.534718506	count	1
TMEM65	0.4299934	0.2395378	1.7951	0.0727	0.534735021	count	1
UTP6	0.4025893	0.1784022	2.2566	0.0241	0.534742535	count	1
GPATCH11	0.3978931	0.1649893	2.4116	0.0159	0.534827705	count	1
SMYD4	0.4929053	0.436977	1.128	0.259	0.535078593	count	1
TGIF1	0.3950517	0.1441093	2.7413	0.00616	0.535109283	count	1
RRM1	0.4630726	0.3342391	1.3855	0.166	0.535152506	count	1
ZBTB37	0.6541896	0.4964862	1.3176	0.188	0.5351655	count	1
CDK11A	0.4218463	0.2215653	1.9039	0.057	0.535202343	count	1
AMZ2	0.419816	0.2225129	1.8867	0.0593	0.535241958	count	1
CD55	0.375762	0.0804666	4.6698	3.16E-06	0.535405369	count	0.073628
LINC00987	0.4453909	0.3321563	1.3409	0.18	0.535670933	count	1
SHB	0.434699	0.2304825	1.886	0.0594	0.535897395	count	1
CYP51A1	0.6203818	0.5485383	1.131	0.258	0.536238726	count	1
PPP3CC	0.4600086	0.2437345	1.8873	0.0592	0.536251156	count	1
SVBP	0.3884939	0.126689	3.0665	0.00219	0.536811078	count	1
ACTR8	0.4813196	0.3379996	1.424	0.155	0.536833807	count	1
FAM13A	0.4464121	0.292639	1.5255	0.127	0.536857181	count	1
SORBS3	0.3971489	0.1389274	2.8587	0.00429	0.537522329	count	1
KCNJ14	1.2435671	1.1297106	1.1008	0.271	0.537680496	count	1
AOC2	1.2435671	1.2117725	1.0262	0.305	0.537680496	count	1
GSK3A	0.4299867	0.200382	2.1458	0.032	0.538127671	count	1
DNAJC18	0.4734822	0.2988308	1.5844	0.113	0.538157385	count	1
FBXO22	0.431713	0.2104086	2.0518	0.0403	0.538897726	count	1
MBNL2	0.3895916	0.119089	3.2714	0.00108	0.538971676	count	1
SWSAP1	0.4896472	0.358397	1.3662	0.172	0.538981574	count	1
DDX60L	0.4293777	0.2209231	1.9436	0.052	0.539296143	count	1
PPP2R3B	0.5814428	0.4992792	1.1646	0.244	0.539343554	count	1
CEP152	0.5814428	0.5089179	1.1425	0.253	0.539343554	count	1
ALG6	0.4731324	0.3050072	1.5512	0.121	0.539561168	count	1
SMG5	0.5327848	0.4476381	1.1902	0.234	0.539591376	count	1

ARSD	0.4561041	0.2809934	1.6232	0.105	0.539927092	count	1
NUDT15	0.4099659	0.2001552	2.0482	0.0406	0.540198339	count	1
RPP25L	0.4157918	0.1961202	2.1201	0.0341	0.540602544	count	1
SLC25A35	0.8060487	0.6604942	1.2204	0.222	0.541002333	count	1
ZNF195	0.4214635	0.2581148	1.6329	0.103	0.541047985	count	1
MPND	0.4817534	0.3033731	1.588	0.112	0.541375272	count	1
NOB1	0.3888592	0.1203309	3.2316	0.00125	0.541614793	count	1
EAF1	0.4719827	0.29784	1.5847	0.113	0.541737652	count	1
USP21	1.14179	0.8618591	1.3248	0.185	0.54188269	count	1
TM2D3	0.390113	0.1156565	3.373	0.000754	0.541892326	count	1
ZNF484	0.4949532	0.3890075	1.2723	0.203	0.542132644	count	1
LYPD5	0.6390922	0.4573099	1.3975	0.162	0.542200559	count	1
SEPHS2	0.3877176	0.1069243	3.6261	0.000293	0.542646133	count	1
NAF1	0.4811574	0.3089141	1.5576	0.119	0.54269816	count	1
FAM234A	0.4266289	0.1996889	2.1365	0.0327	0.542710086	count	1
MCCC2	0.4699246	0.3122559	1.5049	0.132	0.542730623	count	1
PHYH	0.404153	0.1490874	2.7108	0.00675	0.5427999	count	1
ELAC2	0.4611337	0.3233868	1.426	0.154	0.543057163	count	1
GPN3	0.4010472	0.1500951	2.672	0.00759	0.543269777	count	1
AL451165.2	0.5504983	0.3891685	1.4146	0.157	0.543397205	count	1
PTDSS1	0.469151	0.2581353	1.8175	0.0693	0.543441587	count	1
RING1	0.4169682	0.1781113	2.3411	0.0193	0.543487454	count	1
ZNF117	0.5668931	0.3622419	1.565	0.118	0.543555983	count	1
MRPL10	0.4062526	0.1711451	2.3737	0.0177	0.543625883	count	1
IMPDH2	0.39151	0.1047766	3.7366	0.00019	0.543627286	count	1
HIBADH	0.4325482	0.2124397	2.0361	0.0418	0.54379794	count	1
CENPC	0.3898401	0.1124866	3.4657	0.000537	0.543947688	count	1
POLR3C	0.459841	0.2944922	1.5615	0.119	0.544182696	count	1
LHFPL6	0.3856873	0.0890665	4.3303	1.54E-05	0.544247181	count	0.356664
PHOSPHO2	0.5948469	0.5452693	1.0909	0.275	0.544323755	count	1
NOLC1	0.4014922	0.159562	2.5162	0.0119	0.544382676	count	1
PQLC1	0.397951	0.127346	3.125	0.0018	0.544411709	count	1
WDR20	0.4558802	0.3027231	1.5059	0.132	0.54448537	count	1
GRAPL	1.1516307	1.1156211	1.0323	0.302	0.545021149	count	1
DMAC2	0.4157281	0.1825477	2.2774	0.0228	0.545084633	count	1
HENMT1	0.6830613	0.6216477	1.0988	0.272	0.54538602	count	1
STARD4	0.5528583	0.4268092	1.2953	0.195	0.545541026	count	1
GNG11	0.3794694	0.0472982	8.0229	1.51E-15	0.545944499	count	3.62E-11
AC108134.3	0.7010001	0.4811833	1.4568	0.145	0.546654647	count	1
MAP4K5	0.4076459	0.1523756	2.6753	0.00751	0.546736678	count	1
KDM1B	0.5767283	0.4368475	1.3202	0.187	0.546777842	count	1
ZFP37	0.7617595	0.5183478	1.4696	0.142	0.54680985	count	1
PWWP2B	0.4816489	0.2978794	1.6169	0.106	0.547003358	count	1
TCFL5	0.5071834	0.3153846	1.6081	0.108	0.547027125	count	1
AC027097.1	0.450022	0.2476246	1.8174	0.0693	0.547113787	count	1

RSPH3	0.4894062	0.4148016	1.1799	0.238	0.54749736	count	1
HINFP	0.5260372	0.4564759	1.1524	0.249	0.547656842	count	1
NSUN6	0.4094564	0.1981942	2.0659	0.0389	0.548041595	count	1
CDC37	0.3839025	0.0600523	6.3928	1.91E-10	0.54820312	count	4.54E-06
TBC1D30	0.5424285	0.3370697	1.6092	0.108	0.548635929	count	1
CNOT9	0.4258224	0.19373	2.198	0.028	0.548697853	count	1
GTPBP2	0.7046116	0.4639853	1.5186	0.129	0.549060622	count	1
NAT1	1.1645312	1.2767005	0.9121	0.362	0.549100909	count	1
OTUD1	0.4453772	0.249405	1.7858	0.0742	0.549476984	count	1
EHBP1L1	0.418189	0.173626	2.4086	0.0161	0.54972833	count	1
AL139353.1	0.8971942	0.7828105	1.1461	0.252	0.549898918	count	1
AC004812.2	0.6016501	0.5190668	1.1591	0.247	0.549925818	count	1
HSF1	0.3991844	0.1286806	3.1021	0.00194	0.550067988	count	1
POLR1C	0.4321524	0.2087618	2.0701	0.0385	0.550091269	count	1
NDUFV2-AS1	0.5398308	0.4128646	1.3075	0.191	0.550094794	count	1
TOMM40	0.4147061	0.1578684	2.6269	0.00866	0.550360891	count	1
LTV1	0.4166001	0.1794309	2.3218	0.0203	0.550642658	count	1
ASAP1	0.3913363	0.090551	4.3217	1.60E-05	0.550699622	count	0.37048
CNP	0.4210494	0.1955673	2.153	0.0314	0.551006453	count	1
TLK2	0.4273712	0.1985715	2.1522	0.0315	0.551071643	count	1
DPM1	0.4131158	0.1630159	2.5342	0.0113	0.5513089	count	1
GIGYF1	0.4325152	0.2597868	1.6649	0.096	0.551562965	count	1
ZC3H3	1.173157	0.6639924	1.7668	0.0774	0.551807151	count	1
DENND1A	0.5237581	0.4120492	1.2711	0.204	0.551946665	count	1
SNX2	0.3951037	0.1051543	3.7574	0.000175	0.552006274	count	1
CTR9	0.4154081	0.1729046	2.4025	0.0163	0.552189659	count	1
AK9	0.4625953	0.295582	1.565	0.118	0.552211025	count	1
NOP16	0.4402854	0.2128184	2.0688	0.0387	0.552635274	count	1
SLC1A1	1.0829104	0.8013939	1.3513	0.177	0.552636993	count	1
GFOD2	0.4110844	0.1541744	2.6664	0.00771	0.552663694	count	1
NDRG1	0.3940057	0.0947438	4.1586	3.30E-05	0.552771396	count	0.76164
AC016831.1	0.7719614	0.4593256	1.6806	0.0929	0.552863309	count	1
RAB35	0.4432253	0.2349512	1.8865	0.0593	0.552871507	count	1
REEP6	0.7289769	0.6216481	1.1727	0.241	0.553130031	count	1
CPLX1	0.4047312	0.1180918	3.4273	0.000619	0.553308271	count	1
SPOCK2	0.6540531	0.5726383	1.1422	0.253	0.55334411	count	1
NPHP1	0.4727286	0.3900513	1.212	0.226	0.553375328	count	1
SP110	0.3928395	0.1008032	3.8971	9.97E-05	0.553576101	count	1
ANXA2R	0.4069223	0.1549453	2.6262	0.00868	0.55373212	count	1
TUT1	0.5289849	0.3300698	1.6026	0.109	0.553858105	count	1
MPRIP-AS1	0.8000368	0.6594872	1.2131	0.225	0.553962357	count	1
AURKC	0.9059973	1.005376	0.9012	0.368	0.55405764	count	1
USP4	0.4472988	0.2276166	1.9651	0.0495	0.554134486	count	1
EFHD1	1.4801696	1.0676009	1.3864	0.166	0.554247528	count	1
DEK	0.388289	0.0600138	6.47	1.16E-10	0.554480347	count	2.76E-06

ZC3H18	0.4402624	0.210768	2.0888	0.0368	0.554483929	count	1
TIMELESS	0.831678	0.8253778	1.0076	0.314	0.554808006	count	1
UBE2D1	0.4037415	0.1244614	3.2439	0.00119	0.554943274	count	1
LINC00472	0.8672841	0.6586083	1.3168	0.188	0.555148938	count	1
PLRG1	0.4088491	0.1458803	2.8026	0.0051	0.555255052	count	1
AP002360.1	0.6456639	0.5351898	1.2064	0.228	0.555525935	count	1
ADCK5	0.6698724	0.5358815	1.25	0.211	0.555895544	count	1
ZFP36L1	0.3884988	0.0768473	5.0555	4.57E-07	0.556029467	count	0.010705225
ZNF791	0.4176718	0.1467781	2.8456	0.00447	0.556036035	count	1
AKIRIN2	0.408966	0.1409716	2.9011	0.00375	0.556133596	count	1
MRPS31	0.4078917	0.1411636	2.8895	0.00389	0.556148647	count	1
SCGB3A1	0.5218452	0.3737482	1.3962	0.163	0.556149305	count	1
CNTLN	0.4194064	0.1780726	2.3553	0.0186	0.556265552	count	1
FAM122A	0.4389249	0.225659	1.9451	0.0519	0.556339188	count	1
C6orf47	0.472885	0.2863113	1.6516	0.0987	0.556341348	count	1
AAAS	0.4766891	0.3342919	1.426	0.154	0.556363885	count	1
PLP2	0.3947709	0.102057	3.8681	0.000112	0.556434687	count	1
NR1H2	0.4046367	0.1228913	3.2926	0.00101	0.556932399	count	1
AP4B1	0.6028746	0.3820764	1.5779	0.115	0.557272665	count	1
PCNX3	0.5475252	0.5050226	1.0842	0.278	0.557357595	count	1
PPP3CA	0.3943064	0.0876401	4.4992	7.10E-06	0.557785388	count	0.1649614
COQ3	0.5524019	0.4375729	1.2624	0.207	0.557960139	count	1
CSE1L	0.4628005	0.2459919	1.8814	0.06	0.558032813	count	1
JAGN1	0.4064384	0.1221704	3.3268	0.00089	0.558048654	count	1
CCDC146	0.6200845	0.4753856	1.3044	0.192	0.558226164	count	1
MEX3C	0.4221037	0.1846886	2.2855	0.0224	0.558309869	count	1
CHCHD7	0.4243644	0.2090448	2.03	0.0425	0.558565945	count	1
MAP2K1	0.5411191	0.3180082	1.7016	0.0889	0.558737341	count	1
CTDP1	0.6124692	0.4247793	1.4419	0.149	0.558803555	count	1
TBC1D20	0.4496774	0.2402782	1.8715	0.0614	0.559250008	count	1
ARRDC1	0.4193231	0.1633487	2.567	0.0103	0.55967686	count	1
RPAP3	0.4207082	0.1841207	2.285	0.0224	0.559797889	count	1
GNB5	0.4569728	0.3551691	1.2866	0.198	0.559921713	count	1
DNAJC27-AS1	0.8772123	0.7816801	1.1222	0.262	0.560132696	count	1
FN3KRP	0.4451744	0.2034262	2.1884	0.0287	0.561122881	count	1
VWA5A	0.4797303	0.2840211	1.6891	0.0913	0.561229094	count	1
TLCD1	1.1078256	1.0196407	1.0865	0.277	0.561450497	count	1
CEP70	0.4899918	0.2866446	1.7094	0.0875	0.561452848	count	1
TARS	0.4303957	0.1926011	2.2346	0.0255	0.561685912	count	1
POLD3	0.4310328	0.2114562	2.0384	0.0416	0.561815018	count	1
DLG4	0.7070949	0.4789863	1.4762	0.14	0.561840779	count	1
SMIM27	0.4780516	0.2503185	1.9098	0.0563	0.562172108	count	1
LSM2	0.3964568	0.0739578	5.3606	8.98E-08	0.562529832	count	0.002112006
TMEM186	0.5530689	0.418136	1.3227	0.186	0.562579317	count	1
CARD8-AS1	0.4239207	0.1809586	2.3426	0.0192	0.56286798	count	1

FOXN3	0.421217	0.1664184	2.5311	0.0114	0.563171114	count	1
RTP4	0.4380438	0.2617876	1.6733	0.0944	0.56322244	count	1
PXN-AS1	0.7896576	0.6640047	1.1892	0.234	0.563283087	count	1
TTTY14	0.5960198	0.5075052	1.1744	0.24	0.56335962	count	1
FKBP11	0.4058653	0.1188604	3.4146	0.000648	0.563914137	count	1
HPCAL1	0.4108288	0.1174831	3.4969	0.000478	0.564021184	count	1
FAM200A	0.5506507	0.4262906	1.2917	0.197	0.564149308	count	1
CCDC77	0.5846772	0.379088	1.5423	0.123	0.5643331	count	1
TYW1B	1.215353	1.0728344	1.1328	0.257	0.56479482	count	1
FKBP1B	0.4348648	0.204453	2.127	0.0335	0.564906191	count	1
CDK10	0.4441197	0.2058141	2.1579	0.031	0.564955331	count	1
AAMP	0.4184782	0.1337072	3.1298	0.00177	0.565023404	count	1
MFSD6	0.4358543	0.2263482	1.9256	0.0543	0.565026423	count	1
LSM14B	0.5307878	0.3920797	1.3538	0.176	0.565071664	count	1
QSER1	0.4369739	0.203426	2.1481	0.0318	0.565262974	count	1
NXT2	0.585835	0.4673425	1.2535	0.21	0.565351458	count	1
GVQW3	1.2184993	0.8609266	1.4153	0.157	0.565746787	count	1
TINF2	0.4249333	0.1741545	2.44	0.0148	0.565775761	count	1
HIF1A-AS2	15.4567872	1093.041497	0.0141	0.989	0.565971586	count	1
IGSF22	15.4569597	1187.601703	0.013	0.99	0.565971586	count	1
LINC00471	15.6160709	1197.028218	0.013	0.99	0.565971606	count	1
AC007249.2	15.6161843	1367.855675	0.0114	0.991	0.565971606	count	1
AL117335.1	15.7429041	1102.524559	0.0143	0.989	0.565971619	count	1
AC010524.1	15.7429045	1102.523657	0.0143	0.989	0.565971619	count	1
TMCC2	15.8507451	1195.694712	0.0133	0.989	0.56597163	count	1
AC009812.4	15.8775776	2353.673345	0.0067	0.995	0.565971632	count	1
AC109322.1	15.8887204	1365.837476	0.0116	0.991	0.565971633	count	1
LINC02186	15.9453082	1192.230571	0.0134	0.989	0.565971638	count	1
AC006064.4	15.9453129	1192.231024	0.0134	0.989	0.565971638	count	1
AL359397.2	15.9923501	1103.892137	0.0145	0.988	0.565971642	count	1
AC010809.2	16.081475	1371.282313	0.0117	0.991	0.565971648	count	1
AC090825.1	16.1131174	1575.340788	0.0102	0.992	0.56597165	count	1
DKK4	16.1134018	1734.771546	0.0093	0.993	0.56597165	count	1
AC015987.1	16.11422	2376.80474	0.0068	0.995	0.565971651	count	1
LINC01703	15.6159753	1101.721273	0.0142	0.989	0.565971652	count	1
AL049775.3	15.6162818	1590.051802	0.0098	0.992	0.565971652	count	1
COL1A1	16.1606954	1376.050776	0.0117	0.991	0.565971654	count	1
LYPD6	16.242089	1409.154224	0.0115	0.991	0.565971659	count	1
FOXP2	16.2423333	1275.901731	0.0127	0.9898	0.565971659	count	1
C10orf95	16.2965783	1579.159907	0.0103	0.992	0.565971662	count	1
KIF25	15.7429069	1102.523981	0.0143	0.989	0.565971666	count	1
AC226118.1	15.7429069	1102.523979	0.0143	0.989	0.565971666	count	1
AC022211.2	15.7611336	1183.038361	0.0133	0.989	0.565971668	count	1
AL353770.4	16.4020775	1095.052845	0.015	0.988	0.565971668	count	1
KCNE1	16.4804183	1929.442365	0.0085	0.993	0.565971672	count	1

Z97192.2	16.4810248	2229.948086	0.0074	0.994	0.565971672	count	1
AC000403.1	16.5777224	2018.714665	0.0082	0.993	0.565971676	count	1
GJC3	15.8507177	1100.460103	0.0144	0.989	0.565971676	count	1
KIF18A	15.8507196	1100.459405	0.0144	0.989	0.565971676	count	1
APOBEC3D	15.8508138	1183.301274	0.0134	0.989	0.565971676	count	1
AC105285.1	15.850814	1183.301817	0.0134	0.989	0.565971676	count	1
AC010997.5	15.8763637	1717.712164	0.0092	0.993	0.565971679	count	1
TAF1A-AS1	15.8763638	1717.712169	0.0092	0.993	0.565971679	count	1
BAIAP3	15.8763638	1717.71218	0.0092	0.993	0.565971679	count	1
KCNAB3	15.8767526	1862.364763	0.0085	0.993	0.565971679	count	1
AC009318.3	15.888711	1100.118553	0.0144	0.988	0.56597168	count	1
DHX34	15.9452977	1362.464407	0.0117	0.991	0.565971685	count	1
AC015908.2	16.8759628	2571.715953	0.0066	0.995	0.565971688	count	1
ANKHD1-EIF4EBP3	15.9836479	1174.182712	0.0136	0.989	0.565971688	count	1
TENM3	16.9652938	1724.65075	0.0098	0.9922	0.56597169	count	1
DOC2A	16.0815367	1199.974918	0.0134	0.989	0.565971695	count	1
AL138963.3	16.0815614	1104.390441	0.0146	0.988	0.565971695	count	1
CALHM6	16.0815665	1104.390229	0.0146	0.988	0.565971695	count	1
SPON2	16.0915623	1187.464474	0.0136	0.989	0.565971696	count	1
AC090092.1	16.1140014	2231.121225	0.0072	0.994	0.565971697	count	1
FCN2	16.2970839	1906.830368	0.0085	0.993	0.565971709	count	1
PAN3-AS1	16.2976743	1098.750927	0.0148	0.988	0.565971709	count	1
LINC02352	16.38947	1100.523392	0.0149	0.988	0.565971714	count	1
CCDC141	16.4346649	1828.268871	0.009	0.9928	0.565971716	count	1
TDGF1	16.447988	1578.975834	0.0104	0.992	0.565971717	count	1
AC109347.2	16.4485097	2382.281657	0.0069	0.994	0.565971717	count	1
LINC00511	16.4804184	1929.442443	0.0085	0.993	0.565971719	count	1
C2CD4A	16.4810248	2229.94802	0.0074	0.994	0.565971719	count	1
DCST1-AS1	16.6907929	1574.64461	0.0106	0.992	0.565971728	count	1
AC097478.1	16.8759627	2571.7159	0.0066	0.995	0.565971734	count	1
AC006059.1	16.875963	2571.715985	0.0066	0.995	0.565971734	count	1
CASC1	16.875963	2571.715942	0.0066	0.995	0.565971734	count	1
WNT9A	17.1997601	2356.842196	0.0073	0.994	0.565971743	count	1
AL139280.1	17.1997605	2356.842276	0.0073	0.994	0.565971743	count	1
ACTN1-AS1	17.2000478	2647.857372	0.0065	0.995	0.565971743	count	1
ZFAND1	0.4053302	0.107421	3.7733	0.000164	0.56599198	count	1
POPDC2	0.7707863	0.7660566	1.0062	0.314	0.5662523	count	1
CDC42SE2	0.4228431	0.1599619	2.6434	0.00825	0.566278397	count	1
ZDHHC8	0.5186312	0.3085995	1.6806	0.093	0.566574731	count	1
RNF113A	0.4482082	0.2207813	2.0301	0.0424	0.566649992	count	1
RDH13	0.5194815	0.2834861	1.8325	0.067	0.567449662	count	1
CNST	0.4297283	0.1946337	2.2079	0.0273	0.567704413	count	1
SLC35C2	0.4484739	0.2645941	1.695	0.0902	0.568136798	count	1
SLC35C1	0.4891105	0.2759979	1.7722	0.0765	0.568702752	count	1
SUCLG2	0.4210407	0.1367868	3.0781	0.0021	0.569033276	count	1

TONSL	0.5900966	0.4259917	1.3852	0.166	0.569096178	count	1
IDH3B	0.4222686	0.1453885	2.9044	0.00371	0.56914818	count	1
MSTO1	0.6028074	0.4582449	1.3155	0.188	0.569165415	count	1
RNF38	0.4585176	0.2310482	1.9845	0.0473	0.569165747	count	1
STK32C	0.4725591	0.2770432	1.7057	0.0882	0.569376616	count	1
MED1	0.4534685	0.2557827	1.7729	0.0764	0.569387116	count	1
PIM1	0.4367362	0.2119026	2.061	0.0394	0.56945388	count	1
ARHGEF1	0.4349537	0.187974	2.3139	0.0207	0.569508859	count	1
AL354920.1	1.2312274	0.7738962	1.5909	0.112	0.569574768	count	1
RSL24D1	0.4020742	0.0695386	5.782	8.21E-09	0.570021526	count	0.000194011
ASMTL	0.435635	0.1900649	2.292	0.022	0.570061342	count	1
N4BP2L1	0.4282599	0.1905111	2.248	0.0247	0.57013696	count	1
PRKAB2	0.6542755	0.5108256	1.2808	0.2	0.570217242	count	1
USP28	0.5494131	0.3306557	1.6616	0.0967	0.570281285	count	1
TK1	0.6900528	0.6428976	1.0733	0.283	0.570417456	count	1
CADM4	1.3686811	1.0724118	1.2763	0.202	0.570447314	count	1
VAMP3	0.4026305	0.0780799	5.1566	2.69E-07	0.570580273	count	0.006309126
CCDC138	0.7046081	0.4846707	1.4538	0.146	0.570718023	count	1
FOSB	0.3997092	0.0711691	5.6163	2.14E-08	0.570775859	count	0.00050504
ALOX12-AS1	0.8619703	0.6075308	1.4188	0.156	0.570857942	count	1
CCDC137	0.4334958	0.2026463	2.1392	0.0325	0.570946245	count	1
PACSIN2	0.4428036	0.1864896	2.3744	0.0176	0.570968255	count	1
SNX33	0.7049905	0.547322	1.2881	0.198	0.570984437	count	1
TIMM23B	0.6455696	0.4721106	1.3674	0.172	0.571228588	count	1
GTPBP6	0.4226467	0.1367795	3.09	0.00202	0.571322997	count	1
GSTK1	0.4004624	0.0654044	6.1229	1.05E-09	0.571421985	count	2.49E-05
AC017083.1	0.5470074	0.3850043	1.4208	0.155	0.571425183	count	1
MARCKSL1	0.3990061	0.0642709	6.2082	6.17E-10	0.571474888	count	1.46E-05
NUP50-DT	0.587229	0.4988448	1.1772	0.239	0.571662915	count	1
LRP2BP	1.0595365	0.8029467	1.3196	0.187	0.571983335	count	1
PPP1R35	0.423228	0.157644	2.6847	0.0073	0.572099034	count	1
ZBTB18	0.5148001	0.3506468	1.4681	0.142	0.572160734	count	1
AC011498.1	1.8790351	1.3302966	1.4125	0.158	0.572507568	count	1
FCHO2	0.4137886	0.1213712	3.4093	0.000661	0.572846598	count	1
CASP8AP2	0.4290876	0.1886281	2.2748	0.023	0.572930574	count	1
CCDC61	0.4623854	0.2412103	1.9169	0.0553	0.573047037	count	1
SLC25A42	0.947756	0.615623	1.5395	0.124	0.573470053	count	1
SFMBT1	0.8679007	0.5977213	1.452	0.147	0.573966272	count	1
NFYC-AS1	0.8085459	1.5987974	0.5057	0.6131	0.574292176	count	1
KLHL2	0.495926	0.2843059	1.7443	0.0812	0.574682235	count	1
DUS1L	0.4498641	0.1854452	2.4259	0.0153	0.574697917	count	1
LARS	0.4103154	0.1062592	3.8615	0.000115	0.574722296	count	1
CDK5RAP2	0.4662199	0.2483019	1.8776	0.0605	0.575223427	count	1
ALDH6A1	0.4436507	0.203072	2.1847	0.029	0.575314283	count	1
REL	0.4140078	0.1208805	3.4249	0.000624	0.575488323	count	1

TAF1	0.4597922	0.2783875	1.6516	0.0987	0.575761539	count	1
LDB2	0.4040544	0.0660558	6.1169	1.09E-09	0.575812985	count	2.58E-05
DDIT3	0.4280658	0.1750529	2.4454	0.0145	0.575870104	count	1
LEO1	0.427732	0.1511581	2.8297	0.00469	0.575927226	count	1
LYRM1	0.4171343	0.1472144	2.8335	0.00464	0.576060291	count	1
MSMO1	0.4498738	0.2044431	2.2005	0.0279	0.576195807	count	1
KCTD13	0.7458942	0.5598355	1.3323	0.183	0.576253762	count	1
MTA2	0.5090988	0.2854418	1.7835	0.0746	0.576615158	count	1
DTX3L	0.4297056	0.2108128	2.0383	0.0416	0.576643632	count	1
AL441883.1	1.1522612	0.8576114	1.3436	0.179	0.576780436	count	1
RHBDD1	0.5988673	0.4923849	1.2163	0.224	0.576784893	count	1
ISCA2	0.4264895	0.1597803	2.6692	0.00765	0.577456316	count	1
ACO1	0.4681693	0.2095915	2.2337	0.0256	0.577552926	count	1
SRP14-AS1	0.5346574	0.3568881	1.4981	0.134	0.577632496	count	1
AP1S2	0.4112287	0.0985274	4.1737	3.09E-05	0.577818674	count	0.713481
BTAF1	0.4809519	0.2430035	1.9792	0.0479	0.577995442	count	1
NIPA1	0.6543376	0.5808768	1.1265	0.26	0.578091929	count	1
DDX56	0.4543553	0.1947252	2.3333	0.0197	0.57819457	count	1
SULT1C2	0.5700054	0.3427765	1.6629	0.0964	0.578474719	count	1
CCDC149	0.4652077	0.2314638	2.0099	0.0445	0.578744943	count	1
CBLN3	1.262769	1.1372253	1.1104	0.267	0.578902646	count	1
MOV10	0.4851626	0.3297926	1.4711	0.141	0.579330192	count	1
WDR78	0.608134	0.4420517	1.3757	0.169	0.579401529	count	1
ATG16L1	0.9163909	0.6066912	1.5105	0.131	0.579504102	count	1
PSMG1	0.4322196	0.1571992	2.7495	0.00601	0.579601889	count	1
COPG1	0.5290274	0.2777285	1.9048	0.0569	0.579816432	count	1
SHISAL1	1.016386	0.7571187	1.3424	0.18	0.580392364	count	1
AACS	0.7720918	0.7078788	1.0907	0.275	0.580456112	count	1
7-Mar	0.4206473	0.1232044	3.4142	0.000649	0.580736272	count	1
GTF3C5	0.4788369	0.2658511	1.8011	0.0718	0.58091933	count	1
TMEM177	0.7048005	0.5591006	1.2606	0.208	0.580943526	count	1
CYB5B	0.4209898	0.1194621	3.524	0.000432	0.581000168	count	1
ECSIT	0.4398537	0.1725545	2.5491	0.0109	0.581119772	count	1
C9orf72	0.498984	0.3075364	1.6225	0.105	0.581235287	count	1
MYNN	0.4481819	0.2105928	2.1282	0.0334	0.581458479	count	1
TDRD10	0.6043376	0.4028033	1.5003	0.134	0.581567999	count	1
RBMXL1	0.4704341	0.2945678	1.597	0.11	0.581909587	count	1
RNF185	0.7207415	0.4799327	1.5018	0.133	0.581914475	count	1
SCN9A	0.8505215	0.4595508	1.8508	0.0643	0.582026537	count	1
AC129507.4	2.645151	1.124274	2.3528	0.0187	0.582191658	count	1
NFE2L1	0.4176336	0.116455	3.5862	0.000341	0.582439252	count	1
AC091271.1	0.4868364	0.2748952	1.771	0.0767	0.582457756	count	1
AC024940.1	1.2751477	1.1244739	1.134	0.257	0.582502387	count	1
B3GALT6	0.4627163	0.2164209	2.138	0.0326	0.582632278	count	1
SCN1B	0.4232247	0.1213355	3.4881	0.000494	0.582901172	count	1

JMJD1C-AS1	0.6821249	0.5269171	1.2946	0.196	0.582965878	count	1
AFDN-DT	1.0228492	0.7674105	1.3329	0.183	0.583087998	count	1
ZXDA	0.7385693	0.5606022	1.3175	0.188	0.583097434	count	1
SLC44A4	1.0887879	1.1437833	0.9519	0.341	0.583126564	count	1
HOTAIRM1	0.4457059	0.1732658	2.5724	0.0102	0.583317069	count	1
INTS4	0.6344749	0.4178847	1.5183	0.129	0.583435681	count	1
HMGXB4	0.4269887	0.1388741	3.0746	0.00213	0.583928004	count	1
NMI	0.4221718	0.1222459	3.4535	0.000562	0.583930507	count	1
AC108477.1	1.1736542	1.1341178	1.0349	0.301	0.583985492	count	1
CRIP1	0.4176352	0.1387069	3.0109	0.00263	0.584139884	count	1
BZW2	0.4256507	0.1383526	3.0766	0.00211	0.584434409	count	1
POLI	0.4828948	0.2867503	1.684	0.0923	0.584622732	count	1
FBXL6	0.5204619	0.3635325	1.4317	0.152	0.584696775	count	1
AL391121.1	0.5815164	0.4542382	1.2802	0.201	0.585006601	count	1
FGGY	0.5448307	0.3451717	1.5784	0.115	0.585050801	count	1
TTC30B	0.6853783	0.4944748	1.3861	0.166	0.585392726	count	1
OSBP	0.4417454	0.1932348	2.2861	0.0223	0.585401352	count	1
SNAPC4	0.7801279	0.6323602	1.2337	0.217	0.585481139	count	1
ZNF880	0.4484168	0.2183567	2.0536	0.0401	0.586124464	count	1
MKRN2	0.4809452	0.2500666	1.9233	0.0545	0.586390351	count	1
MIR222HG	0.4483656	0.2084927	2.1505	0.0316	0.586396497	count	1
FAM13A-AS1	1.4349969	1.9323812	0.7426	0.4578	0.586539198	count	1
DENND6B	0.7820689	0.5464969	1.4311	0.153	0.586691662	count	1
SCAPER	0.4502835	0.1800062	2.5015	0.0124	0.586761147	count	1
ARRB1	0.4428642	0.1856723	2.3852	0.0171	0.586859056	count	1
CD79B	0.4268189	0.118103	3.614	0.000307	0.586861153	count	1
ELK4	0.4249963	0.1370853	3.1002	0.00195	0.58729196	count	1
SART1	0.4922696	0.2830461	1.7392	0.0821	0.587482272	count	1
DISC1	0.594395	0.455783	1.3041	0.192	0.587784446	count	1
TEAD4	0.4720752	0.2027301	2.3286	0.02	0.58778693	count	1
POLG	0.6055706	0.6068158	0.9979	0.318	0.587902423	count	1
SPIN3	0.8949013	0.6721027	1.3315	0.183	0.587978827	count	1
FAM72A	0.7842	0.608474	1.2888	0.198	0.58801929	count	1
RALGAPA2	0.4300158	0.1396056	3.0802	0.00209	0.588125239	count	1
HTT	0.4850531	0.2835534	1.7106	0.0873	0.588190243	count	1
UPF2	0.4232421	0.1136808	3.7231	0.000201	0.588475541	count	1
ZFYVE21	0.4155075	0.0827141	5.0234	5.40E-07	0.589012767	count	0.0126441
CHN1	0.458353	0.2037065	2.2501	0.0245	0.590112936	count	1
P2RY1	0.938461	0.5800788	1.6178	0.106	0.590209892	count	1
AKAP1	0.5017092	0.3042696	1.6489	0.0993	0.590238025	count	1
CRYL1	0.4284037	0.122051	3.51	0.000455	0.590495202	count	1
ERVK3-1	0.6030002	0.3461051	1.7422	0.0816	0.590680133	count	1
DOLPP1	0.6811202	0.5525368	1.2327	0.218	0.590735477	count	1
ZNF721	0.4502766	0.1999322	2.2521	0.0244	0.590806102	count	1
LMTK2	0.750444	0.5269309	1.4242	0.155	0.591030903	count	1

RPL17	0.4245475	0.1025788	4.1387	3.60E-05	0.591249676	count	0.830484
PRCP	0.4128656	0.0576151	7.1659	9.87E-13	0.591396324	count	2.36E-08
ZWINT	1.0429553	1.0862032	0.9602	0.337	0.591398715	count	1
ZNF492	1.0429553	1.1156752	0.9348	0.35	0.591398715	count	1
AC006449.6	0.5933132	0.4094421	1.4491	0.147	0.591427259	count	1
AL357060.1	0.6720088	0.6234445	1.0779	0.281	0.591846743	count	1
AC093462.1	1.4579475	1.0408199	1.4008	0.161	0.591910878	count	1
AC006504.5	0.721731	0.5856306	1.2324	0.218	0.592937483	count	1
ATG4C	0.4735172	0.2365591	2.0017	0.0454	0.593149129	count	1
TTLL11	1.6832075	1.0723471	1.5696	0.117	0.593180346	count	1
ZNF813	1.6832075	1.2004704	1.4021	0.161	0.593180346	count	1
NIPSNAP3A	0.4576661	0.2083432	2.1967	0.0281	0.593498257	count	1
CSRNP1	0.4253269	0.1022942	4.1579	3.31E-05	0.593774805	count	0.7638818
NRROS	0.5821864	0.5224048	1.1144	0.265	0.593941853	count	1
INTS13	0.5293338	0.3477868	1.522	0.128	0.594122225	count	1
TFIP11	0.5301804	0.3134547	1.6914	0.0909	0.59502058	count	1
KHNYN	0.5549764	0.4184734	1.3262	0.185	0.59523015	count	1
RFTN1	0.5063031	0.2251826	2.2484	0.0246	0.595407832	count	1
CDC42EP3	0.4219305	0.0908152	4.646	3.54E-06	0.595452756	count	0.0824643
TRAPPC6A	0.430937	0.1275891	3.3775	0.000742	0.595824749	count	1
KCTD7	1.209798	0.9570578	1.2641	0.206	0.595903724	count	1
BX284668.6	0.6682541	0.5526475	1.2092	0.227	0.596605088	count	1
RUFY3	0.5063662	0.2569408	1.9708	0.0489	0.596907388	count	1
GEMIN8	0.5160726	0.2762465	1.8682	0.0618	0.59692616	count	1
KPNA3	0.436827	0.1439657	3.0342	0.00243	0.5970317	count	1
GABPB1	0.5029106	0.2814908	1.7866	0.0741	0.59708576	count	1
PLCD1	0.6431521	0.486819	1.3211	0.187	0.597089307	count	1
CLK4	0.4666813	0.2227348	2.0952	0.0362	0.597196243	count	1
CYTL1	0.4231616	0.1384602	3.0562	0.00226	0.597252081	count	1
PIGA	0.9535849	0.6005793	1.5878	0.112	0.597460775	count	1
L3MBTL4	1.4824959	1.035445	1.4317	0.152	0.597546807	count	1
RBKS	0.6229847	0.405205	1.5375	0.124	0.597800713	count	1
ZNF341-AS1	0.9549047	0.8048146	1.1865	0.236	0.598090247	count	1
TSTD2	0.6171473	0.4153276	1.4859	0.137	0.59809744	count	1
PARP6	0.6447076	0.4278728	1.5068	0.132	0.59838113	count	1
HES1	0.4192056	0.1151068	3.6419	0.000276	0.598474317	count	1
HADHB	0.426671	0.0971617	4.3914	1.17E-05	0.598546237	count	0.2712879
ATP2B1	0.4242263	0.0961139	4.4138	1.05E-05	0.598696167	count	0.2436
NCBP2	0.4400498	0.1390254	3.1652	0.00157	0.598779276	count	1
RNF126	0.4440791	0.1427204	3.1115	0.00188	0.598804993	count	1
TFAP4	0.6534287	0.425708	1.5349	0.125	0.598971296	count	1
AL356488.3	0.8818438	0.6432385	1.3709	0.171	0.59902844	count	1
TIPARP-AS1	0.8253527	0.6395252	1.2906	0.197	0.599045123	count	1
IER2	0.416545	0.0539894	7.7153	1.67E-14	0.599051154	count	4.00E-10
AC067852.2	0.6381013	0.570458	1.1186	0.263	0.599114297	count	1

DHRS7	0.4233051	0.0827642	5.1146	3.36E-07	0.599436587	count	0.007876176
POLK	0.4391785	0.1568053	2.8008	0.00513	0.59945928	count	1
NET1	0.433255	0.1102055	3.9313	8.65E-05	0.599574555	count	1
VTA1	0.4739769	0.2133061	2.2221	0.0264	0.600177158	count	1
TBPL1	0.4439539	0.1488478	2.9826	0.00288	0.600356019	count	1
CMIP	0.4275389	0.0987026	4.3316	1.53E-05	0.600383591	count	0.3543633
ATG5	0.4479565	0.1796343	2.4937	0.0127	0.600392881	count	1
SH3PXD2B	0.6637962	0.3802932	1.7455	0.081	0.600396752	count	1
ZFP62	0.5544562	0.3444112	1.6099	0.108	0.600494565	count	1
THOC1	0.472631	0.2357913	2.0044	0.0451	0.600845808	count	1
NAA15	0.4603497	0.1946594	2.3649	0.0181	0.601054168	count	1
GOLGA1	0.4646109	0.2813391	1.6514	0.0988	0.601064311	count	1
INPP1	0.427494	0.0961534	4.446	9.10E-06	0.601490598	count	0.2112747
FN3K	0.8295194	0.7116031	1.1657	0.244	0.601508995	count	1
NEK11	0.6756374	0.4583097	1.4742	0.141	0.602419697	count	1
F3	1.5049192	1.0429256	1.443	0.149	0.602597104	count	1
ABRAXAS2	0.4727321	0.2232011	2.118	0.0343	0.60263305	count	1
DHX32	0.5439248	0.2875054	1.8919	0.0586	0.602652973	count	1
CCDC122	0.4751109	0.2581024	1.8408	0.0658	0.603915318	count	1
NUS1	0.5055311	0.2841443	1.7791	0.0753	0.603924504	count	1
DTWD2	0.8337186	0.6222228	1.3399	0.18	0.603986322	count	1
TNFRSF25	0.8337186	1.0359332	0.8048	0.421	0.603986322	count	1
ZNF350	0.5476703	0.3730083	1.4683	0.142	0.604106829	count	1
PTPN12	0.4274	0.1117841	3.8231	0.000135	0.604358238	count	1
CCNB1	0.8113896	0.6117706	1.3263	0.185	0.604825541	count	1
ZBTB45	0.7713304	0.6160515	1.2521	0.211	0.604870316	count	1
GRK2	0.4825799	0.2359101	2.0456	0.0409	0.604870624	count	1
FAM53B	0.603166	0.4455904	1.3536	0.176	0.604950038	count	1
ZNF136	0.5301189	0.2971009	1.7843	0.0745	0.605097014	count	1
MTX1	0.4417678	0.1476588	2.9918	0.0028	0.605128772	count	1
BTBD3	0.4607099	0.2223933	2.0716	0.0384	0.605499554	count	1
MXI1	0.440031	0.1277528	3.4444	0.000581	0.605910336	count	1
ZNF696	1.1506628	0.7701872	1.494	0.135	0.605952401	count	1
DERA	0.443146	0.1395111	3.1764	0.00151	0.605992943	count	1
SMN2	1.2412306	0.7430881	1.6704	0.095	0.606011614	count	1
SIK2	0.5024241	0.2781	1.8066	0.0709	0.606261008	count	1
CYB5D1	0.5796107	0.3629484	1.597	0.11	0.606543029	count	1
DCAF15	0.5219558	0.2996409	1.7419	0.0816	0.606740503	count	1
PLCXD1	1.7642153	1.0592859	1.6655	0.0959	0.606933304	count	1
ACER3	0.5318878	0.2758756	1.928	0.054	0.607011803	count	1
AC242426.2	1.082417	0.6261014	1.7288	0.084	0.6073822	count	1
LINC00909	0.5044661	0.2909849	1.7337	0.0831	0.607490244	count	1
DCAF16	0.5342835	0.2586783	2.0654	0.039	0.60765647	count	1
PRKN	0.8980845	0.7417434	1.2108	0.226	0.60772098	count	1
AC092164.1	1.247845	1.0242221	1.2183	0.223	0.608108546	count	1

AC105020.6	1.247845	1.0642476	1.1725	0.241	0.608108546	count	1
ERAL1	0.4865519	0.2642867	1.841	0.0657	0.608263643	count	1
FNTA	0.4370048	0.1046729	4.175	3.07E-05	0.608336656	count	0.7088937
PPM1D	0.4747439	0.1690711	2.808	0.00502	0.609268023	count	1
MAD1L1	0.5721794	0.3091405	1.8509	0.0643	0.609295073	count	1
LMBR1L	0.5440984	0.2593999	2.0975	0.036	0.609762927	count	1
CLK2	0.7184034	0.4160251	1.7268	0.0843	0.609826189	count	1
VPS26C	0.4872607	0.2501226	1.9481	0.0515	0.609848097	count	1
KRI1	0.5610379	0.3637739	1.5423	0.123	0.609969182	count	1
ACKR4	0.5268341	0.3570059	1.4757	0.14	0.610473536	count	1
RAB8B	0.4472593	0.1477142	3.0279	0.00249	0.610587386	count	1
TSPAN14	0.4428256	0.1204081	3.6777	0.00024	0.610789865	count	1
HLA-A	0.4238408	0.0312829	13.5487	1.55E-40	0.610858249	count	3.76E-36
PDXDC1	0.4759057	0.2115601	2.2495	0.0246	0.611210855	count	1
PCMTD2	0.4568496	0.1786056	2.5579	0.0106	0.6113252	count	1
ANKDD1A	0.6533159	0.5313555	1.2295	0.219	0.611899971	count	1
KLHDC8B	0.4676659	0.1943247	2.4066	0.0162	0.612162099	count	1
TEF	0.5558763	0.370669	1.4997	0.134	0.612607865	count	1
MARS2	1.3847332	1.0312563	1.3428	0.179	0.612909589	count	1
MAFG-DT	1.3847332	1.0621034	1.3038	0.192	0.612909589	count	1
SP2-AS1	1.036308	0.7605287	1.3626	0.173	0.612935311	count	1
HELZ2	0.5480303	0.2817002	1.9454	0.0518	0.613918584	count	1
CCND2	0.4586833	0.1965923	2.3332	0.0197	0.61394565	count	1
PLCB3	0.5217652	0.306779	1.7008	0.0891	0.614251295	count	1
AL139289.2	1.1002448	1.2227678	0.8998	0.368	0.614462046	count	1
ANKRD46	0.5032655	0.238976	2.1059	0.0353	0.614628918	count	1
RAPGEF1	0.4791037	0.2065999	2.319	0.0205	0.614724558	count	1
ZNF724	1.5606795	1.073225	1.4542	0.146	0.614759998	count	1
LENG8	0.5964377	0.3393125	1.7578	0.0789	0.61530535	count	1
TOR3A	0.4562932	0.1785586	2.5554	0.0107	0.615868979	count	1
RAD9A	0.5272972	0.2533104	2.0816	0.0375	0.615881742	count	1
SH2D3C	0.4676232	0.1700424	2.75	0.006	0.615950646	count	1
USP49	1.3966185	1.1638006	1.2	0.23	0.616053534	count	1
IFT81	0.5568508	0.3516805	1.5834	0.113	0.616113747	count	1
CHRNB1	0.5337704	0.2976582	1.7932	0.073	0.616386237	count	1
ZNF781	0.6109952	0.3888451	1.5713	0.116	0.616583657	count	1
SRSF6	0.4539186	0.1419104	3.1986	0.0014	0.61664479	count	1
SNRNP48	0.478184	0.2060343	2.3209	0.0204	0.616884426	count	1
DRG1	0.4728939	0.1815064	2.6054	0.00923	0.617810066	count	1
RFC4	0.5995702	0.383691	1.5626	0.118	0.618281139	count	1
ADGRL4	0.4324041	0.0626507	6.9018	6.34E-12	0.618333598	count	1.51E-07
SUN1	0.4462763	0.1310743	3.4048	0.000672	0.618668037	count	1
MORC3	0.4683987	0.1821176	2.572	0.0102	0.618705778	count	1
SPRED1	0.5220942	0.2595229	2.0117	0.0443	0.618877746	count	1
RSL1D1	0.4353358	0.0684324	6.3615	2.33E-10	0.61943783	count	5.53E-06

MAF	0.7194898	0.3818038	1.8844	0.0596	0.619643424	count	1
FOXK2	0.5514006	0.2682343	2.0557	0.0399	0.619704657	count	1
AC097376.2	0.5970901	0.3841505	1.5543	0.12	0.61974221	count	1
LINC01011	1.0523716	0.6106756	1.7233	0.0849	0.619850327	count	1
TRMT6	0.4752614	0.2186969	2.1732	0.0299	0.620110878	count	1
AGPAT2	0.4412448	0.1060325	4.1614	3.26E-05	0.62012848	count	0.7524406
TMSB4Y	0.7952308	0.6500693	1.2233	0.221	0.620527197	count	1
HGSNAT	0.481453	0.2076709	2.3183	0.0205	0.620541959	count	1
TIFA	0.4506781	0.1629668	2.7655	0.00572	0.620670062	count	1
LLPH	0.4551216	0.1590086	2.8622	0.00424	0.620739259	count	1
ATP11C	0.4608551	0.1620675	2.8436	0.00449	0.621105664	count	1
GTPBP4	0.4634405	0.1469135	3.1545	0.00162	0.621224481	count	1
AL161785.1	1.5934061	1.0990748	1.4498	0.147	0.621641813	count	1
CPTP	0.4660166	0.1695444	2.7486	0.00602	0.622105274	count	1
ERCC3	0.5181931	0.2679032	1.9343	0.0532	0.622159546	count	1
AC093827.4	0.6387275	0.4790511	1.3333	0.183	0.622360439	count	1
PSMB8	0.4355625	0.0603152	7.2214	6.62E-13	0.622543266	count	1.58E-08
CHMP4B	0.4545942	0.1356366	3.3516	0.000814	0.62270778	count	1
GDF3	0.8189055	0.7614358	1.0755	0.282	0.622953807	count	1
TMOD3	0.4357821	0.0657451	6.6284	4.07E-11	0.62316121	count	9.68E-07
UTP25	0.5288975	0.287485	1.8397	0.0659	0.623736306	count	1
CTNS	0.8972458	0.6383474	1.4056	0.16	0.624442557	count	1
TCF15	0.569974	0.457445	1.246	0.213	0.624536562	count	1
AL353194.1	0.6610285	0.602033	1.098	0.272	0.624576198	count	1
MCM4	0.6767863	0.7107186	0.9523	0.341	0.624844967	count	1
WFS1	0.5175674	0.2511387	2.0609	0.0394	0.624961874	count	1
GGT5	0.46407	0.174121	2.6652	0.00774	0.625030353	count	1
DNASE1L3	0.4402817	0.1673776	2.6305	0.00857	0.625229369	count	1
COX19	0.5013259	0.2371182	2.1142	0.0346	0.625392184	count	1
POP1	0.7398966	0.4639954	1.5946	0.111	0.625530592	count	1
BCDIN3D	0.5711382	0.4009319	1.4245	0.154	0.625730631	count	1
ZKSCAN4	1.3056884	1.0737288	1.216	0.224	0.626008101	count	1
ZNF692	0.9336519	0.571673	1.6332	0.103	0.626465686	count	1
SF3A3	0.4575214	0.1279964	3.5745	0.000357	0.626573638	count	1
ZNF143	0.4983184	0.2327967	2.1406	0.0324	0.626815681	count	1
RIN1	0.6224385	0.4481287	1.389	0.165	0.627130579	count	1
REEP1	2.3929193	1.3893091	1.7224	0.0851	0.627430724	count	1
PRPF39	0.5941127	0.3059204	1.9421	0.0522	0.627599519	count	1
SIAH1	0.4774211	0.1653523	2.8873	0.00392	0.62766576	count	1
CCDC28A	0.4795255	0.2046589	2.343	0.0192	0.627711178	count	1
AHNAK	0.4388629	0.0813984	5.3915	7.58E-08	0.62788028	count	0.001783271
RAB11A	0.4396808	0.0570912	7.7014	1.86E-14	0.627888941	count	4.45E-10
EED	0.5222695	0.258561	2.0199	0.0435	0.628062465	count	1
AZIN1-AS1	1.0193138	0.7198695	1.416	0.157	0.628173028	count	1
SURF2	0.4785707	0.2147792	2.2282	0.0259	0.628826683	count	1

SPIN4	0.9764117	0.7569731	1.2899	0.197	0.628903637	count	1
RXRA	0.4886933	0.2106395	2.32	0.0204	0.629172597	count	1
COTL1	0.4451708	0.096998	4.5895	4.64E-06	0.629335642	count	0.10800528
TWISTNB	0.4562578	0.1368158	3.3348	0.000865	0.629368443	count	1
AC025159.1	0.6159261	0.428995	1.4357	0.151	0.629693452	count	1
SGF29	0.5024195	0.2304469	2.1802	0.0293	0.629696915	count	1
ATG16L2	0.6116546	0.3161289	1.9348	0.0531	0.629733288	count	1
EFHD2	0.4542145	0.1161128	3.9118	9.38E-05	0.630107507	count	1
ST7-AS1	1.141756	0.7725408	1.4779	0.14	0.630611037	count	1
BOLA3	0.4684641	0.1441574	3.2497	0.00117	0.630861796	count	1
CAMK1	0.4734534	0.1702798	2.7804	0.00547	0.63095087	count	1
RCC1	0.8800983	0.5448204	1.6154	0.106	0.630963709	count	1
LGALS8	0.4796973	0.2047056	2.3434	0.0192	0.631542196	count	1
ZC3HC1	0.5981593	0.4066239	1.471	0.141	0.631555762	count	1
STX11	0.5345889	0.2972299	1.7986	0.0722	0.631599375	count	1
GDAP1	0.556817	0.3690912	1.5086	0.132	0.631872698	count	1
KCNJ15	1.4584527	1.0874871	1.3411	0.18	0.631940676	count	1
GON4L	0.4614128	0.1795808	2.5694	0.0102	0.632045971	count	1
ECD	0.5004278	0.2036531	2.4573	0.0141	0.632062416	count	1
LINC01354	0.6383389	0.5317365	1.2005	0.23	0.632204871	count	1
RARS2	0.4728891	0.2059638	2.296	0.0218	0.632227166	count	1
TRMT5	0.5157745	0.3033559	1.7002	0.0892	0.633274409	count	1
IFIT5	0.4994603	0.2311785	2.1605	0.0308	0.633393499	count	1
CDYL	0.4981184	0.2078834	2.3961	0.0166	0.633531449	count	1
FAM47E	0.6158429	0.5200788	1.1841	0.236	0.633692156	count	1
MKS1	0.672016	0.4924678	1.3646	0.172	0.633846187	count	1
HAUS5	0.7662075	0.4070706	1.8822	0.0599	0.63449041	count	1
LRRC8C	0.4817454	0.1792582	2.6874	0.00724	0.634494902	count	1
UBXN10-AS1	1.2324422	1.0247819	1.2026	0.229	0.634604946	count	1
MT1A	1.470066	1.1754603	1.2506	0.211	0.634838094	count	1
ZC4H2	0.7992142	0.5470792	1.4609	0.144	0.635125482	count	1
NSMF	0.6416597	0.390613	1.6427	0.101	0.635187174	count	1
KIZ	0.5562511	0.3179922	1.7493	0.0804	0.635295608	count	1
YPEL2	0.450687	0.1018565	4.4247	1.00E-05	0.635559163	count	0.23207
HSPBP1	0.4993005	0.2214873	2.2543	0.0243	0.635575611	count	1
ZBTB10	0.4879283	0.1967677	2.4797	0.0132	0.635893992	count	1
PRICKLE2	0.6609129	0.401988	1.6441	0.1	0.636252767	count	1
VIRMA	0.4759933	0.1758081	2.7075	0.00682	0.636309082	count	1
ZNF780B	0.5899834	0.3900562	1.5126	0.131	0.63633143	count	1
AC009948.1	0.6105949	0.3576909	1.707	0.0879	0.636436235	count	1
RINT1	0.6378901	0.4404241	1.4484	0.148	0.636634378	count	1
FKBP9	0.4595258	0.1173044	3.9174	9.17E-05	0.636758016	count	1
MYOM2	0.6282865	0.5537467	1.1346	0.257	0.636951018	count	1
AL138762.1	0.9203673	0.7754848	1.1868	0.235	0.637097979	count	1
NTNG2	0.9549155	1.4076473	0.6784	0.4976	0.637481248	count	1

C1QTNF2	0.5752875	0.3379378	1.7023	0.0888	0.637759432	count	1
NELFA	0.5945331	0.3367777	1.7654	0.0776	0.637811704	count	1
METTL2A	0.5250999	0.2686151	1.9548	0.0507	0.638166777	count	1
C12orf66	0.6451109	0.4656839	1.3853	0.166	0.63828289	count	1
ZNF578	0.8040538	0.643443	1.2496	0.212	0.638339224	count	1
ANKZF1	0.6252904	0.3621823	1.7265	0.0844	0.638456623	count	1
GMNN	0.5286981	0.2395011	2.2075	0.0274	0.639017501	count	1
BCAR1	0.4735141	0.1472714	3.2152	0.00132	0.639443847	count	1
RBM15B	0.5406009	0.2475743	2.1836	0.0291	0.639822847	count	1
THOC6	0.5729921	0.3941771	1.4536	0.146	0.640206464	count	1
GMPS	0.4816826	0.15035	3.2037	0.00137	0.640259254	count	1
HDAC11	0.7910644	0.4793929	1.6501	0.099	0.641046907	count	1
KIF17	0.6119827	0.3107638	1.9693	0.049	0.641456205	count	1
IMPACT	0.4852994	0.1930535	2.5138	0.012	0.641472189	count	1
AL358472.4	0.6332423	0.4150131	1.5258	0.127	0.641535748	count	1
HCN3	15.527758	1089.463283	0.0143	0.989	0.641960448	count	1
HIST1H2BH	15.527758	1089.463285	0.0143	0.989	0.641960448	count	1
AMDHD1	15.5279382	1170.769964	0.0133	0.989	0.641960448	count	1
LINC01481	15.6785643	1098.968003	0.0143	0.989	0.641960468	count	1
AL445250.1	15.6785709	1098.968724	0.0143	0.989	0.641960468	count	1
CAMK2N2	15.6785712	1098.969314	0.0143	0.989	0.641960468	count	1
AC146944.4	15.678685	1180.977621	0.0133	0.989	0.641960468	count	1
ANGPT1	15.6786853	1180.978276	0.0133	0.989	0.641960468	count	1
AL138828.1	15.9027198	1099.449368	0.0145	0.988	0.641960493	count	1
AMACR	15.9391607	1099.350034	0.0145	0.988	0.641960497	count	1
PLAG1	15.9936342	1163.486809	0.0137	0.989	0.641960502	count	1
NCF4	16.2069619	1572.586039	0.0103	0.992	0.641960519	count	1
AL139089.1	16.2207774	1153.161309	0.0141	0.989	0.641960521	count	1
MAN2B1	16.2801225	1083.484732	0.015	0.988	0.641960525	count	1
ATP6V0C	16.2928842	1314.880206	0.0124	0.99	0.641960526	count	1
CCDC65	16.3773161	1578.026167	0.0104	0.992	0.641960531	count	1
PCDHGB5	16.3773257	1578.027456	0.0104	0.992	0.641960531	count	1
ITGB8	16.499082	1091.81246	0.0151	0.9879	0.641960538	count	1
LYPD3	16.5192599	1579.094627	0.0105	0.992	0.641960539	count	1
CLGN	15.8908795	821.4775498	0.0193	0.9846	0.641960544	count	1
SLC9B1	16.636541	2422.574614	0.0069	0.995	0.641960545	count	1
GABRB3	16.6414891	1578.214184	0.0105	0.992	0.641960546	count	1
SGK494	15.953653	1004.705362	0.0159	0.9873	0.64196055	count	1
CLTCL1	16.7490814	1576.396	0.0106	0.992	0.64196055	count	1
BX255925.3	16.7491868	1714.263302	0.0098	0.992	0.64196055	count	1
BMPR1B	15.9896248	1555.323157	0.0103	0.992	0.641960553	count	1
AL354822.1	17.274168	1694.272918	0.0102	0.9919	0.641960568	count	1
IL1R2	17.2742091	1734.522876	0.01	0.992	0.641960568	count	1
MTRNR2L1	17.2872782	2352.638857	0.0073	0.994	0.641960568	count	1
AL583856.2	16.2079036	2068.360172	0.0078	0.994	0.641960571	count	1

NPIPB2	17.6288013	2608.536775	0.0068	0.995	0.641960576	count	1
CDHR3	16.6359767	2189.811748	0.0076	0.994	0.641960597	count	1
OXGR1	16.6359769	2189.811764	0.0076	0.994	0.641960597	count	1
ITGA2B	16.636541	2422.574721	0.0069	0.995	0.641960597	count	1
GLT1D1	16.6409012	5131.302278	0.0032	0.997	0.641960597	count	1
NMNAT2	16.6610407	1228.496182	0.0136	0.989	0.641960598	count	1
DNMT3B	16.9323391	1709.094802	0.0099	0.992	0.641960609	count	1
AC022364.1	16.9354571	1457.480881	0.0116	0.9907	0.64196061	count	1
KBTBD8	1.254607	1.3284144	0.9444	0.345	0.642080648	count	1
FBXO45	0.584662	0.3160691	1.8498	0.0645	0.642284241	count	1
FGF14-AS2	0.6341037	0.4185779	1.5149	0.13	0.642331881	count	1
DRG2	0.4893596	0.1763256	2.7753	0.00555	0.642378698	count	1
FTSJ3	0.5328905	0.2450342	2.1748	0.0297	0.642709367	count	1
RANBP6	0.5229496	0.2625451	1.9918	0.0465	0.642718942	count	1
MXRA7	0.4618955	0.1144208	4.0368	5.57E-05	0.642867849	count	1
RRAGC	0.4921974	0.1947489	2.5273	0.0115	0.643217124	count	1
SFI1	1.006333	0.6255049	1.6088	0.108	0.643412703	count	1
POU5F2	1.5059134	1.0155407	1.4829	0.138	0.643613065	count	1
EPB41L5	0.7183654	0.4656282	1.5428	0.123	0.643659902	count	1
COQ10B	0.4665323	0.1230064	3.7927	0.000152	0.64385896	count	1
JRKL	0.611223	0.3912255	1.5623	0.118	0.644295064	count	1
CLASP2	0.5544993	0.3193191	1.7365	0.0826	0.644387686	count	1
ZNF799	0.7671997	0.38784	1.9781	0.048	0.645255133	count	1
AL138724.1	0.6653497	0.464125	1.4336	0.152	0.645702667	count	1
GNB4	0.4647959	0.1239824	3.7489	0.000181	0.64621434	count	1
DRAM2	0.4792933	0.1573762	3.0455	0.00234	0.646459175	count	1
PELO	0.4820106	0.1574971	3.0604	0.00223	0.646531613	count	1
CC2D2B	1.2680162	0.7673915	1.6524	0.0986	0.646543957	count	1
ZNF34	0.570959	0.2941455	1.9411	0.0524	0.647004847	count	1
CCM2L	0.4891206	0.1525287	3.2067	0.00136	0.647281893	count	1
THOC3	0.5676211	0.2871301	1.9769	0.0482	0.647561589	count	1
MAP2K7	0.6147103	0.3280262	1.874	0.061	0.647687309	count	1
DHRS1	0.4911102	0.2686348	1.8282	0.0676	0.647816069	count	1
NGLY1	0.490325	0.1769079	2.7716	0.00561	0.647986213	count	1
PPP5D1	1.119712	0.7683364	1.4573	0.145	0.648037866	count	1
XAB2	0.5806835	0.3022117	1.9214	0.0548	0.648273322	count	1
CHIC2	0.4677411	0.1139241	4.1057	4.15E-05	0.649179257	count	0.956907
ULK3	0.6282837	0.3313098	1.8964	0.058	0.649458831	count	1
DCUN1D2	1.193663	0.765986	1.5583	0.119	0.650151923	count	1
ARHGAP10	0.548565	0.317312	1.7288	0.084	0.650253682	count	1
RFXANK	0.488797	0.1725111	2.8334	0.00464	0.650301245	count	1
METTL13	0.6429747	0.3362701	1.9121	0.056	0.650517321	count	1
LY6E	0.4533244	0.0683809	6.6294	4.04E-11	0.650564125	count	9.61E-07
DBR1	0.6920241	0.4557959	1.5183	0.129	0.650625246	count	1
AL031777.3	0.9146888	0.5648054	1.6195	0.105	0.650626415	count	1

TUT7	0.526696	0.2423029	2.1737	0.0298	0.650857055	count	1
STRADB	0.727881	0.4884635	1.4901	0.136	0.651102177	count	1
PXN	0.4951437	0.2095106	2.3633	0.0182	0.651138599	count	1
IFIT1	0.4659079	0.1586313	2.937	0.00334	0.651468336	count	1
EHMT1	0.4927199	0.1763969	2.7932	0.00525	0.651668132	count	1
TDRKH	1.5400934	1.0171889	1.5141	0.13	0.651746528	count	1
COG2	0.5499479	0.3590152	1.5318	0.126	0.651817406	count	1
AC084033.3	0.5064488	0.2065714	2.4517	0.0143	0.651937054	count	1
ST3GAL5	0.9844898	0.7592645	1.2966	0.195	0.652566232	count	1
MAP3K3	0.4941516	0.1675169	2.9499	0.00321	0.652649393	count	1
AC058791.1	0.494632	0.1617591	3.0578	0.00225	0.652973405	count	1
AL365205.1	1.1326778	0.7746141	1.4622	0.144	0.653318254	count	1
PPP1R16A	0.540185	0.2558784	2.1111	0.0349	0.653495182	count	1
NPHP3	0.6285312	0.3034579	2.0712	0.0384	0.653610444	count	1
WRAP53	0.7121344	0.4418765	1.6116	0.107	0.653612679	count	1
FUT4	1.4002915	1.0220261	1.3701	0.171	0.653631953	count	1
STXBP1	0.4987564	0.1701817	2.9307	0.00341	0.653761147	count	1
HS2ST1	0.572013	0.3119465	1.8337	0.0668	0.654298434	count	1
PAF1	0.511798	0.2116186	2.4185	0.0156	0.654465168	count	1
PHLPP1	0.6430509	0.4174049	1.5406	0.124	0.65500326	count	1
OSGEPL1	0.5896762	0.3700478	1.5935	0.111	0.655219787	count	1
NECTIN2	0.462883	0.081698	5.6658	1.61E-08	0.655282513	count	0.000380153
MRPS9	0.4934569	0.1793659	2.7511	0.00598	0.655622387	count	1
FAM114A2	0.5025721	0.2170073	2.3159	0.0206	0.65571198	count	1
IRF2BPL	0.497537	0.1556693	3.1961	0.00141	0.655813586	count	1
SF3A1	0.4961431	0.191604	2.5894	0.00966	0.656394788	count	1
LYRM9	0.5183428	0.2244019	2.3099	0.021	0.656630159	count	1
HSPD1	0.4596415	0.0620786	7.4042	1.74E-13	0.656740246	count	4.16E-09
IFI16	0.4591932	0.0594139	7.7287	1.51E-14	0.656792758	count	3.61E-10
HDAC4	0.6781919	0.4402489	1.5405	0.124	0.656881299	count	1
PCYOX1L	0.7989092	0.4910177	1.627	0.104	0.65735176	count	1
DHODH	0.666762	0.41586	1.6033	0.109	0.657617967	count	1
ANP32B	0.4603781	0.0592956	7.7641	1.15E-14	0.657656824	count	2.75E-10
OLFML2A	0.4992435	0.2474824	2.0173	0.0438	0.65770362	count	1
NLN	0.6931064	0.4028256	1.7206	0.0854	0.65784001	count	1
HIST4H4	1.7827204	0.9837849	1.8121	0.0701	0.657945597	count	1
RIOK2	0.5166515	0.2545633	2.0296	0.0425	0.658216227	count	1
AL136295.5	0.854107	0.4345999	1.9653	0.0495	0.658278807	count	1
HACE1	0.6520183	0.3988954	1.6346	0.102	0.658836922	count	1
ZBTB34	1.5709409	0.9670447	1.6245	0.104	0.658895435	count	1
ELL	0.6069309	0.3116441	1.9475	0.0516	0.65935456	count	1
ECI2	0.4744949	0.1092207	4.3444	1.45E-05	0.65958883	count	0.3358925
PAXIP1-AS1	0.688369	0.473591	1.4535	0.146	0.659874283	count	1
LCA5	0.5294682	0.2446996	2.1637	0.0306	0.660122582	count	1
RREB1	0.5837229	0.2733793	2.1352	0.0328	0.660618335	count	1

SPATC1L	0.514721	0.2508545	2.0519	0.0403	0.660778922	count	1
USF1	0.580301	0.3790876	1.5308	0.126	0.661202161	count	1
EXOSC9	0.4970821	0.1756595	2.8298	0.00469	0.661374753	count	1
C8orf44	0.8394675	0.4872397	1.7229	0.085	0.661615571	count	1
NFASC	0.7773567	0.6193204	1.2552	0.21	0.662302551	count	1
RABL3	0.5549237	0.2638098	2.1035	0.0355	0.662998707	count	1
POU6F1	1.0974295	0.8244743	1.3311	0.183	0.663005454	count	1
ZNF330	0.5056026	0.1870008	2.7037	0.0069	0.663240575	count	1
ESYT1	0.5144249	0.2186244	2.353	0.0187	0.663405825	count	1
DTNB	0.7249585	0.5541679	1.3082	0.191	0.663946871	count	1
LINC01290	1.4386455	0.7360379	1.9546	0.0507	0.664266766	count	1
LRIG2	0.7008278	0.4132637	1.6958	0.09	0.664353408	count	1
SCNN1B	0.9115167	0.547986	1.6634	0.0963	0.664606955	count	1
FAM221A	0.6251185	0.3440879	1.8167	0.0694	0.664802151	count	1
ZBTB2	0.6226901	0.3244607	1.9192	0.0551	0.665759153	count	1
PYCR3	1.1038826	0.7282736	1.5158	0.13	0.66580311	count	1
MARK1	1.2370876	0.7595894	1.6286	0.104	0.665952985	count	1
RUSC2	0.7826846	0.5554904	1.409	0.159	0.666173268	count	1
BST1	0.5554323	0.2765008	2.0088	0.0447	0.666202171	count	1
SCAMP1-AS1	0.5869516	0.3019979	1.9436	0.052	0.666223233	count	1
NPFFR2	1.6040998	1.1571998	1.3862	0.166	0.666381406	count	1
FAM213A	0.4674526	0.0787732	5.9342	3.32E-09	0.666471569	count	7.86E-05
ASXL1	0.4836018	0.1328022	3.6415	0.000276	0.667413596	count	1
AC011978.2	1.1077307	0.6668015	1.6613	0.0968	0.667465715	count	1
RRN3	0.5681327	0.2996693	1.8959	0.0581	0.667742656	count	1
NRARP	0.4781741	0.1161574	4.1166	3.96E-05	0.667861464	count	0.9132948
CWC22	0.4982784	0.1596825	3.1204	0.00182	0.667989337	count	1
FRAT1	0.8310883	0.4863642	1.7088	0.0876	0.668079618	count	1
SP140L	0.494742	0.1649264	2.9998	0.00273	0.668361024	count	1
AKT3	0.4793189	0.1287981	3.7215	0.000202	0.669282711	count	1
UBN2	0.5346682	0.2484924	2.1516	0.0315	0.669544295	count	1
PYGO2	0.8166575	0.6266305	1.3033	0.193	0.669607673	count	1
ACAD8	0.6118707	0.3416872	1.7907	0.0734	0.670125975	count	1
OAS3	0.5516991	0.3098811	1.7804	0.0751	0.670297195	count	1
PAK1IP1	0.4968772	0.1450469	3.4256	0.000622	0.67086233	count	1
TMEM202-AS1	1.6251827	1.050141	1.5476	0.122	0.671035997	count	1
CNNM4	1.0651636	1.006696	1.0581	0.29	0.671146306	count	1
DUBR	0.9236965	0.7527275	1.2271	0.22	0.671653551	count	1
MBTD1	0.6826787	0.4459775	1.5307	0.126	0.671736967	count	1
DENND2C	0.7347498	0.4219805	1.7412	0.0818	0.671800192	count	1
TAB3	1.0232764	0.5078879	2.0148	0.044	0.671936562	count	1
IGSF8	0.5639779	0.2911446	1.9371	0.0528	0.671946994	count	1
PDPR	0.652394	0.325372	2.0051	0.0451	0.672226164	count	1
LYST	0.4775258	0.1119501	4.2655	2.06E-05	0.672625151	count	0.4765398
PSMD9	0.5372687	0.2532105	2.1218	0.0339	0.672691557	count	1

PML	0.4979198	0.1527051	3.2607	0.00113	0.672925221	count	1
PARP10	0.6674223	0.3983823	1.6753	0.094	0.672949116	count	1
SLC25A29	0.5041278	0.1613448	3.1245	0.0018	0.673024268	count	1
PTPN2	0.4938485	0.1432589	3.4472	0.000575	0.673998905	count	1
MRI1	0.6744906	0.3750721	1.7983	0.0722	0.674591227	count	1
AP2A1	0.5389145	0.270839	1.9898	0.0467	0.674682767	count	1
MLF1	0.4983545	0.1461771	3.4093	0.000661	0.675113179	count	1
JPT2	0.5668203	0.2649585	2.1393	0.0325	0.675175785	count	1
MT-ATP8	0.5049051	0.158871	3.1781	0.0015	0.675416647	count	1
COQ2	0.7955302	0.4125587	1.9283	0.0539	0.675466032	count	1
CIT	1.6461886	1.0901788	1.51	0.131	0.675593643	count	1
HADH	0.5400037	0.2544209	2.1225	0.0339	0.676000282	count	1
TRIM41	0.6480263	0.6887429	0.9409	0.347	0.67609873	count	1
ZNF597	0.9315783	0.6044986	1.5411	0.123	0.676187581	count	1
TSEN2	0.9057902	0.5355943	1.6912	0.0909	0.676211613	count	1
SPTLC2	0.4862753	0.1206671	4.0299	5.73E-05	0.67636506	count	1
NFATC3	0.5431199	0.2551463	2.1287	0.0334	0.677372129	count	1
PAQR3	0.7019216	0.4240376	1.6553	0.098	0.677396838	count	1
NUP85	0.6684377	0.3726143	1.7939	0.0729	0.678486053	count	1
MYC	0.4858776	0.1323509	3.6711	0.000246	0.678583288	count	1
NIP7	0.5077669	0.1680075	3.0223	0.00253	0.678952132	count	1
AC104695.3	0.9112508	0.9775059	0.9322	0.351	0.679474952	count	1
RRAGA	0.4854869	0.0986661	4.9205	9.14E-07	0.679593782	count	0.021375718
TNFRSF14	0.4850837	0.1100438	4.4081	1.08E-05	0.679797698	count	0.250506
FAM189A2	0.5919662	0.3957776	1.4957	0.135	0.679837852	count	1
CUEDC1	0.60872	0.2880739	2.1131	0.0347	0.680037552	count	1
CCT6B	1.1377987	0.900006	1.2642	0.206	0.680309455	count	1
RRNAD1	0.5872291	0.3264895	1.7986	0.0722	0.68037157	count	1
ADGRL2	0.5029777	0.16162	3.1121	0.00188	0.681441147	count	1
EHD2	0.4935543	0.1300148	3.7961	0.00015	0.681473792	count	1
MED25	0.6579044	0.3855484	1.7064	0.088	0.68152657	count	1
RAB4B	1.6753568	1.0976683	1.5263	0.127	0.681791969	count	1
PPP2R5C	0.4984776	0.149959	3.3241	0.000899	0.681873993	count	1
BAALC-AS1	0.8921369	0.7657591	1.165	0.244	0.682045861	count	1
METTL14	0.557548	0.240078	2.3224	0.0203	0.682543891	count	1
UBR5-AS1	0.8528704	0.7188703	1.1864	0.236	0.68256379	count	1
MHENCR	0.8359505	0.4798466	1.7421	0.0816	0.682809036	count	1
TEC	1.285245	1.1064842	1.1616	0.246	0.682905431	count	1
KLHDC1	0.6401453	0.3991646	1.6037	0.109	0.682969346	count	1
KCTD6	0.5820667	0.307503	1.8929	0.0585	0.683284982	count	1
CENPQ	0.5550925	0.2650944	2.0939	0.0364	0.68366191	count	1
GPR155	0.7405016	0.706698	1.0478	0.295	0.683699838	count	1
KPNA5	0.6013161	0.3185838	1.8875	0.0592	0.683719194	count	1
KLHDC4	0.6560986	0.4272707	1.5356	0.125	0.683803888	count	1
GASAL1	0.6148792	0.322475	1.9067	0.0567	0.683947049	count	1

LINC01686	0.6609252	0.5069384	1.3038	0.192	0.684382704	count	1
NADK	0.5374897	0.2094146	2.5666	0.0103	0.684581869	count	1
PCAT6	1.3882453	1.0380825	1.3373	0.181	0.684625026	count	1
ZNF3	0.5988894	0.3623637	1.6527	0.0985	0.685309221	count	1
GNS	0.4971369	0.1530913	3.2473	0.00118	0.685417453	count	1
EXOSC8	0.4970542	0.1209774	4.1087	4.10E-05	0.685788112	count	0.94546
AC093673.1	0.6193081	0.3465938	1.7868	0.0741	0.685937101	count	1
AEN	0.9495734	0.6392192	1.4855	0.138	0.686463029	count	1
RBM15	0.8788932	0.5690189	1.5446	0.123	0.687031629	count	1
IL16	0.8789724	0.770358	1.141	0.254	0.687082173	count	1
TNFSF10	0.478164	0.0626558	7.6316	3.17E-14	0.68712771	count	7.58E-10
CALCOCO2	0.4826795	0.0720326	6.7008	2.50E-11	0.687155317	count	5.95E-07
RDH14	0.5197154	0.1855412	2.8011	0.00513	0.687241326	count	1
ZNF485	1.1008309	0.8346985	1.3188	0.187	0.687453637	count	1
NDFIP2	0.5609127	0.2459083	2.281	0.0226	0.68753644	count	1
WDR75	0.5522863	0.2409136	2.2925	0.022	0.687558322	count	1
EGR3	0.5230789	0.1823537	2.8685	0.00416	0.68775177	count	1
KRBOX4	0.545036	0.2372668	2.2971	0.0217	0.688693328	count	1
CR1L	1.157986	1.1310568	1.0238	0.306	0.688787111	count	1
TNK1	1.0585742	0.7422644	1.4261	0.154	0.689159933	count	1
UBE2V1	0.5818139	0.2542081	2.2887	0.0222	0.689241586	count	1
MIS12	0.6073455	0.3419499	1.7761	0.0758	0.690158566	count	1
POLR3D	0.5499601	0.2075578	2.6497	0.0081	0.691110896	count	1
KLF7	0.5142639	0.1530298	3.3605	0.000788	0.691331388	count	1
HPF1	0.4991757	0.1241936	4.0193	5.99E-05	0.691515808	count	1
DALRD3	0.5246214	0.1892964	2.7714	0.00562	0.69172134	count	1
GAS7	1.4119638	1.2217356	1.1557	0.248	0.691736081	count	1
PRKCH	0.5171583	0.1633029	3.1669	0.00156	0.691742498	count	1
MLLT3	0.5351015	0.2212647	2.4184	0.0157	0.691796827	count	1
MYO18A	0.5612521	0.2935507	1.9119	0.056	0.691925404	count	1
PTPDC1	0.9593015	0.5268897	1.8207	0.0688	0.691973746	count	1
CPNE8	0.4927502	0.1008939	4.8838	1.10E-06	0.692187662	count	0.0257147
1-Mar	0.7710968	0.4552407	1.6938	0.0904	0.692751996	count	1
WDR3	0.5527311	0.2303698	2.3993	0.0165	0.692950372	count	1
SENCR	0.5246477	0.1920379	2.732	0.00634	0.693015526	count	1
MRPL1	0.5158805	0.1676804	3.0766	0.00211	0.693267982	count	1
ZNF230	0.9104525	0.6431705	1.4156	0.157	0.693319633	count	1
MT-CO1	0.4810567	0.0314574	15.2923	9.73E-51	0.69379387	count	2.36E-46
TNIP2	0.5047724	0.1319768	3.8247	0.000134	0.693887953	count	1
FASTKD2	0.5607356	0.2333836	2.4026	0.0163	0.69413486	count	1
TRPS1	1.1158316	0.772868	1.4438	0.149	0.694198918	count	1
GUCY1A1	0.5262189	0.2297155	2.2907	0.0221	0.69442082	count	1
SLCO4A1	0.5323447	0.2083846	2.5546	0.0107	0.694427377	count	1
POLB	0.5293531	0.1989677	2.6605	0.00785	0.695119972	count	1
ZNF114	1.0714808	0.7721743	1.3876	0.165	0.695362034	count	1

NKILA	1.3218459	1.0198438	1.2961	0.195	0.695395866	count	1
ANKRD16	0.6217526	0.3173653	1.9591	0.0502	0.696124751	count	1
USP9Y	0.8107239	0.7036267	1.1522	0.249	0.69617645	count	1
ELK3	0.4902057	0.0712819	6.877	7.53E-12	0.696202944	count	1.80E-07
PUM3	0.5074263	0.1277948	3.9706	7.35E-05	0.696425322	count	1
TEAD2	0.5257814	0.1731637	3.0363	0.00242	0.696867252	count	1
MAFF	0.4968222	0.1096717	4.5301	6.15E-06	0.696931371	count	0.14304285
CCL16	2.0285666	1.2957169	1.5656	0.118	0.697080017	count	1
TNPO3	0.5934536	0.305091	1.9452	0.0519	0.697597462	count	1
CHKB	1.035579	0.7507258	1.3794	0.168	0.697615615	count	1
RPP40	0.6210048	0.3220782	1.9281	0.0539	0.697769953	count	1
NXPE3	0.5381267	0.206603	2.6046	0.00925	0.697925467	count	1
DNM3	0.5510834	0.2295349	2.4009	0.0164	0.698133269	count	1
GPAT3	0.6089058	0.3293512	1.8488	0.0646	0.698148992	count	1
NCK1-DT	0.6632971	0.4484785	1.479	0.139	0.698376593	count	1
N4BP1	0.5184845	0.1818662	2.8509	0.00439	0.698839891	count	1
HELQ	0.5669949	0.2587758	2.1911	0.0285	0.699696355	count	1
ETS2	0.4880359	0.0591303	8.2536	2.35E-16	0.699884023	count	5.64E-12
A2M-AS1	1.1287222	0.809802	1.3938	0.163	0.699942282	count	1
AC027277.1	0.8299824	0.4776791	1.7375	0.0824	0.700112578	count	1
MFAP3	0.6445702	0.3400807	1.8953	0.0582	0.700285867	count	1
PPM1K	0.5616812	0.2937827	1.9119	0.056	0.700527203	count	1
SLC9A1	0.9004862	0.5449042	1.6526	0.0985	0.700729756	count	1
GPAT4	0.6266334	0.316605	1.9792	0.0479	0.701227247	count	1
SEMA4D	2.0583702	1.2396256	1.6605	0.0969	0.70127567	count	1
AKAP17A	0.5421104	0.1880138	2.8834	0.00397	0.701568377	count	1
LMNB1	0.8641939	0.4791305	1.8037	0.0714	0.701906954	count	1
RORB	1.5852427	1.1241421	1.4102	0.159	0.7020671	count	1
ANGEL1	0.8652063	0.5794849	1.4931	0.136	0.702586512	count	1
CEBPZ	0.5025701	0.115848	4.3382	1.49E-05	0.702795581	count	0.3451287
C1QTNF1	0.5130132	0.1712855	2.9951	0.00277	0.702848923	count	1
AC008074.3	1.4500869	1.0955887	1.3236	0.186	0.702897179	count	1
ARHGAP31	0.531126	0.182407	2.9118	0.00362	0.703512925	count	1
EEFSEC	0.603844	0.3241015	1.8631	0.0625	0.704019516	count	1
LIX1L-AS1	1.0484172	0.788712	1.3293	0.184	0.704099394	count	1
CEACAM1	1.5952604	1.106796	1.4413	0.15	0.704491508	count	1
AL355353.1	1.1962781	0.7900563	1.5142	0.13	0.7045501	count	1
FBXO4	0.5587173	0.2389182	2.3385	0.0194	0.704695925	count	1
CCDC32	0.5278455	0.1737488	3.038	0.0024	0.704825221	count	1
FGF7	1.351116	0.8401647	1.6082	0.108	0.705144064	count	1
MATN1-AS1	1.3519759	1.1760739	1.1496	0.25	0.705427265	count	1
C1QTNF1-AS1	1.1427799	0.7867998	1.4524	0.146	0.706149829	count	1
LINC02256	0.6988561	0.4852685	1.4401	0.15	0.706358893	count	1
TSC1	0.6803147	0.3379958	2.0128	0.0442	0.706800287	count	1
DOCK4	0.5236728	0.1600493	3.2719	0.00108	0.706820909	count	1

HOXA3	0.6943288	0.4164658	1.6672	0.0956	0.706886675	count	1
GTF2B	0.5130659	0.1477278	3.473	0.000522	0.707393097	count	1
MCPH1	0.5773778	0.2447636	2.3589	0.0184	0.707942057	count	1
DKC1	0.5405117	0.1909385	2.8308	0.00468	0.707959109	count	1
ABCD4	0.5123504	0.1469007	3.4877	0.000495	0.708233578	count	1
MTRR	0.6959547	0.495931	1.4033	0.161	0.708382816	count	1
HCG11	0.6146919	0.3070008	2.0022	0.0454	0.708414752	count	1
ICA1	0.5076891	0.1078263	4.7084	2.62E-06	0.708586284	count	0.06109578
RAE1	0.5745799	0.2750823	2.0888	0.0368	0.7086864	count	1
MAVS	0.5763802	0.2729863	2.1114	0.0348	0.708827257	count	1
NIF3L1	0.5831759	0.3027356	1.9264	0.0542	0.709217996	count	1
DENND4C	0.5679181	0.2562141	2.2166	0.0267	0.709692462	count	1
MBD1	0.5837088	0.22979	2.5402	0.0111	0.709837832	count	1
ATN1	0.5599811	0.2057522	2.7216	0.00654	0.710382219	count	1
PRRG1	0.5431782	0.1800267	3.0172	0.00257	0.710603285	count	1
WASHC1	0.5651003	0.2538637	2.226	0.0261	0.710996467	count	1
ZNF727	1.283296	1.0664941	1.2033	0.229	0.711279547	count	1
ACSS2	0.61558	0.3053374	2.0161	0.0439	0.711329345	count	1
MAN1A1	0.5159944	0.1317783	3.9156	9.24E-05	0.711802914	count	1
PDE6D	0.5480987	0.2196359	2.4955	0.0126	0.711876359	count	1
SLC9B2	0.6858791	0.3989245	1.7193	0.0857	0.712059253	count	1
AC007952.4	0.7059695	0.3172381	2.2254	0.0261	0.712835158	count	1
NAV3	0.6575008	0.2993467	2.1965	0.0281	0.713256878	count	1
NUPL2	0.5459497	0.252125	2.1654	0.0304	0.713354781	count	1
TGFBR2	0.4966184	0.0567188	8.7558	3.46E-18	0.713622631	count	8.32E-14
ACTR1B	0.5559305	0.1921546	2.8931	0.00384	0.713806555	count	1
JPH3	15.593645	1287.908012	0.0121	0.99	0.714146334	count	1
MEFV	15.8524924	1364.282297	0.0116	0.991	0.714146369	count	1
LRRC3	15.8688349	1165.749642	0.0136	0.989	0.714146371	count	1
AC130466.1	15.9512787	1098.036767	0.0145	0.988	0.71414638	count	1
AC010894.2	15.951524	1541.125461	0.0104	0.992	0.71414638	count	1
JARID2-AS1	15.5933974	1157.164819	0.0135	0.989	0.71414639	count	1
NR6A1	16.0738958	1152.058279	0.014	0.989	0.714146393	count	1
HIST1H2BK	16.0826168	1243.231117	0.0129	0.99	0.714146394	count	1
AC130650.2	16.088212	883.6064679	0.0182	0.9855	0.714146394	count	1
AC106782.1	16.0919507	1782.013591	0.009	0.993	0.714146395	count	1
AC008555.1	16.0919508	1782.013602	0.009	0.993	0.714146395	count	1
LMNTD2	16.1748829	1093.353286	0.0148	0.988	0.714146402	count	1
PDZRN3	16.1748992	1232.661479	0.0131	0.99	0.714146402	count	1
HS3ST3B1	16.1891063	1207.051533	0.0134	0.989	0.714146404	count	1
LINC02019	15.7367365	1167.96829	0.0135	0.989	0.714146411	count	1
CCDC150	15.7368449	1235.722479	0.0127	0.99	0.714146411	count	1
ZMYND12	16.3313681	1156.110932	0.0141	0.989	0.714146415	count	1
GPR85	16.3966527	1175.230514	0.014	0.989	0.71414642	count	1
NUDT10	16.4515642	1697.702971	0.0097	0.992	0.714146423	count	1

AC024337.2	16.451567	1697.704774	0.0097	0.992	0.714146423	count	1
ZNF860	16.4679912	1169.48155	0.0141	0.989	0.714146425	count	1
SLC19A2	16.5267808	1171.470773	0.0141	0.989	0.714146428	count	1
DCLRE1B	16.5295539	1164.881924	0.0142	0.9887	0.714146428	count	1
MESP1	16.7696688	1929.401942	0.0087	0.993	0.714146441	count	1
KRBOX1	16.7702134	2159.115261	0.0078	0.994	0.714146441	count	1
AC091132.5	16.824163	1304.89744	0.0129	0.9897	0.714146444	count	1
PRRT3-AS1	16.89138	1094.355368	0.0154	0.9877	0.714146447	count	1
USP20	16.9009789	1312.017866	0.0129	0.9897	0.714146447	count	1
AL359644.1	16.5290831	1281.836742	0.0129	0.99	0.714146485	count	1
SPACA6P-AS	16.5852447	1700.005524	0.0098	0.992	0.714146488	count	1
AC005730.2	16.7702133	2159.115149	0.0078	0.994	0.714146498	count	1
TDRD12	16.7707466	2366.837874	0.0071	0.994	0.714146498	count	1
CLEC3B	0.5560235	0.2034849	2.7325	0.00633	0.714466967	count	1
TDRD7	0.7531982	0.4092334	1.8405	0.0658	0.714672795	count	1
QDPR	0.5922078	0.2791761	2.1213	0.034	0.71483624	count	1
METTL18	0.6156052	0.3005283	2.0484	0.0406	0.71513398	count	1
IQCN	1.2948854	0.9339634	1.3864	0.166	0.71548754	count	1
AL031848.2	1.295651	1.0230076	1.2665	0.205	0.715764587	count	1
PCF11	0.5289185	0.1257408	4.2064	2.68E-05	0.717174575	count	0.6191872
FANCL	0.5527713	0.1821663	3.0344	0.00243	0.717785243	count	1
MYCT1	0.5068767	0.0807165	6.2797	3.93E-10	0.71791214	count	9.32E-06
NFIL3	0.537686	0.1607362	3.3451	0.000833	0.717959036	count	1
PPP1R9B	0.5973172	0.2761768	2.1628	0.0306	0.718116235	count	1
CCDC71	0.7424608	0.4616399	1.6083	0.108	0.718133286	count	1
FOXRED1	0.8715995	0.5699995	1.5291	0.126	0.718344561	count	1
C20orf204	0.5639818	0.2256981	2.4988	0.0125	0.718524925	count	1
DVL1	0.6006768	0.2477333	2.4247	0.0154	0.719246108	count	1
RIPK3	0.657488	0.3714851	1.7699	0.0769	0.719357743	count	1
MMGT1	0.5663677	0.2236707	2.5322	0.0114	0.719545351	count	1
TMEM209	0.5987248	0.2777245	2.1558	0.0312	0.719728139	count	1
MACROD1	0.5561802	0.2032506	2.7364	0.00625	0.720238292	count	1
RASA4B	1.6653216	1.0253007	1.6242	0.104	0.720905736	count	1
ZNF174	0.7205533	0.4302092	1.6749	0.0941	0.721052377	count	1
FANCG	0.9563843	0.6196656	1.5434	0.123	0.721099012	count	1
AC087645.2	1.4008297	0.7086576	1.9767	0.0482	0.72122002	count	1
FANCI	1.6673118	1.1544479	1.4443	0.149	0.721358373	count	1
MCUR1	0.5190574	0.1147017	4.5253	6.29E-06	0.721380664	count	0.14627395
ZNF394	0.5634581	0.2130972	2.6441	0.00824	0.721483582	count	1
ORC5	0.654115	0.4028516	1.6237	0.105	0.721712145	count	1
EPHX2	0.5794659	0.2184634	2.6525	0.00804	0.721876289	count	1
MVK	0.7106762	0.4610506	1.5414	0.123	0.721891842	count	1
KLF4	0.5049962	0.0746257	6.7671	1.60E-11	0.72224859	count	3.81E-07
BORCS6	0.556664	0.218533	2.5473	0.0109	0.722248943	count	1
TMEM63B	1.0146563	0.6269464	1.6184	0.106	0.722743533	count	1

SIRT6	0.6014824	0.2809031	2.1412	0.0323	0.722884709	count	1
CRLF3	0.8220994	0.4097931	2.0061	0.0449	0.723144164	count	1
AC079834.2	1.2438988	1.0647295	1.1683	0.243	0.723579942	count	1
KIAA1551	0.5191357	0.1336757	3.8835	0.000105	0.723769488	count	1
ESYT2	0.5364074	0.2308893	2.3232	0.0202	0.724091662	count	1
AC139887.2	1.0508136	0.640617	1.6403	0.101	0.724247598	count	1
FNBP1L	0.5152744	0.1129868	4.5605	5.33E-06	0.724544119	count	0.12401311
CCNG2	0.535248	0.1679191	3.1875	0.00145	0.724643473	count	1
UNG	0.5844685	0.2189477	2.6694	0.00764	0.725174742	count	1
SDCBP	0.5054677	0.0495972	10.1915	5.72E-24	0.725219492	count	1.38E-19
RAP1GDS1	0.5980133	0.2738691	2.1836	0.0291	0.725252311	count	1
ABHD6	0.5560299	0.2171127	2.561	0.0105	0.725373868	count	1
ZNF583	0.6251257	0.3151827	1.9834	0.0474	0.725553364	count	1
THG1L	0.6252363	0.2908975	2.1493	0.0317	0.725674281	count	1
POLA1	1.0943696	0.6120637	1.788	0.0739	0.726885448	count	1
ZNF467	0.5402637	0.1544241	3.4986	0.000475	0.726900827	count	1
TVP23B	0.5973946	0.280411	2.1304	0.0332	0.726914796	count	1
NUP107	0.6017307	0.281588	2.1369	0.0327	0.727068973	count	1
AGMO	1.0233785	0.5979514	1.7115	0.0871	0.727501145	count	1
ZNF48	0.8282308	0.4814794	1.7202	0.0855	0.72772812	count	1
DDX50	0.5378686	0.1551935	3.4658	0.000537	0.727966445	count	1
KLF10	0.5197834	0.0998648	5.2049	2.08E-07	0.728400067	count	0.004882176
ZNF761	0.7965887	0.4498984	1.7706	0.0767	0.728552239	count	1
DDTL	0.9240218	0.7255852	1.2735	0.203	0.728757579	count	1
CANT1	0.666879	0.3675731	1.8143	0.0697	0.728848216	count	1
AEBP2	0.6033424	0.2880263	2.0947	0.0363	0.72892535	count	1
LYSMD2	0.5228334	0.1079922	4.8414	1.36E-06	0.729027524	count	0.0317764
PTPN18	0.5487558	0.1962587	2.7961	0.00521	0.72927726	count	1
MAPK11	0.5427704	0.1718508	3.1584	0.0016	0.729391822	count	1
DNAJA3	0.70455	0.3527606	1.9972	0.0459	0.72963597	count	1
ZNF765	0.8434497	0.5321826	1.5849	0.113	0.729636392	count	1
AC036176.1	0.9728964	0.7433578	1.3088	0.191	0.730913644	count	1
HIST1H4E	0.908544	0.5758995	1.5776	0.115	0.731349266	count	1
BCL2L12	0.6167109	0.2492966	2.4738	0.0134	0.731550457	count	1
TPRA1	0.5745674	0.2566324	2.2389	0.0252	0.731577326	count	1
DDX47	1.5539303	1.0370105	1.4985	0.134	0.731668952	count	1
NUDT8	0.5839389	0.2553185	2.2871	0.0223	0.732269498	count	1
RNF138	0.5937017	0.2202536	2.6955	0.00707	0.732306315	count	1
INTS6L	0.8923474	0.5545151	1.6092	0.108	0.732428276	count	1
TAF4B	0.7673131	0.4699505	1.6328	0.103	0.732980508	count	1
METAP1D	0.6304969	0.3472756	1.8156	0.0695	0.733302319	count	1
RAB3D	0.6782019	0.3503203	1.9359	0.053	0.733922877	count	1
AQP1	0.5111971	0.0623997	8.1923	3.87E-16	0.734572775	count	9.28E-12
TYSND1	0.7151212	0.4824847	1.4822	0.138	0.735149134	count	1
NAXD	0.686513	0.3555947	1.9306	0.0536	0.735409407	count	1

ATXN3	0.5350708	0.1600437	3.3433	0.000839	0.735527262	count	1
BRI3BP	0.7707082	0.493372	1.5621	0.118	0.735827952	count	1
TCEANC	1.733081	1.0769466	1.6093	0.108	0.73590656	count	1
PEX11G	0.7432159	0.4372696	1.6997	0.0893	0.735942816	count	1
ABHD17B	0.7634267	0.3613374	2.1128	0.0347	0.736017883	count	1
RRP9	0.6566161	0.3020474	2.1739	0.0298	0.737546706	count	1
ZNF574	0.900734	0.5636789	1.598	0.11	0.73807952	count	1
CCDC51	0.650582	0.3487403	1.8655	0.0622	0.738387199	count	1
AC136475.5	0.9857753	0.7601014	1.2969	0.195	0.738505785	count	1
KLHL7	0.6530382	0.3096155	2.1092	0.035	0.738650492	count	1
TMEM107	0.5559911	0.1892353	2.9381	0.00333	0.739251246	count	1
TMTC1	0.5375473	0.1620048	3.3181	0.000918	0.73937582	count	1
ABHD18	0.8442565	0.4600889	1.835	0.0666	0.739648793	count	1
CARNMT1	0.6497368	0.3036959	2.1394	0.0325	0.739764076	count	1
ITFG2	0.7160254	0.3609577	1.9837	0.0474	0.740385652	count	1
PSMB10	0.5454139	0.1584213	3.4428	0.000584	0.740463353	count	1
PCDHB13	0.942997	0.7791237	1.2103	0.226	0.740787676	count	1
LHFPL2	0.6487203	0.3234404	2.0057	0.045	0.740899681	count	1
BRMS1L	0.6348346	0.2777095	2.286	0.0223	0.741725265	count	1
NIT1	0.5803108	0.2123632	2.7326	0.00632	0.741783707	count	1
LTBR	0.5388036	0.1233161	4.3693	1.29E-05	0.742110618	count	0.2989446
LRRC40	0.6261713	0.2617082	2.3926	0.0168	0.74217595	count	1
ARL14EP	0.5498189	0.1624759	3.384	0.000724	0.742445191	count	1
FABP5	0.5175969	0.1161095	4.4578	8.61E-06	0.742891534	count	0.19994142
AC018653.3	1.0526371	0.6592284	1.5968	0.11	0.743280874	count	1
PAOX	0.8625506	0.5550951	1.5539	0.12	0.743511009	count	1
SLC2A11	1.0236671	0.5408067	1.8929	0.0585	0.744467536	count	1
MYADM	0.5276739	0.0884747	5.9641	2.77E-09	0.744485756	count	6.56E-05
SEC24C	0.6932332	0.5043365	1.3745	0.169	0.745471461	count	1
RFX5	0.6620508	0.2733294	2.4222	0.0155	0.745716948	count	1
PAN3	0.5783368	0.2160983	2.6763	0.00749	0.746075097	count	1
RASSF1	0.5555964	0.1707802	3.2533	0.00115	0.746311568	count	1
FOCAD	0.9994356	0.624599	1.6001	0.11	0.746498384	count	1
ZNF676	0.6426703	0.3680944	1.7459	0.0809	0.746618859	count	1
SNTA1	1.0280017	0.7383539	1.3923	0.164	0.746890306	count	1
TRIM21	0.6788961	0.3110333	2.1827	0.0291	0.746977434	count	1
HLX	0.5793688	0.2242752	2.5833	0.00984	0.747367205	count	1
APOBEC3G	0.6150506	0.2549642	2.4123	0.0159	0.747407648	count	1
KBTBD3	0.6086598	0.2579356	2.3597	0.0184	0.747884765	count	1
STIM2	0.5470442	0.1451525	3.7688	0.000167	0.748015811	count	1
RBM45	0.7342594	0.4540861	1.617	0.106	0.74819721	count	1
GPCPD1	0.5438454	0.1558336	3.4899	0.000491	0.748222192	count	1
KLF9	0.5338098	0.0874633	6.1032	1.18E-09	0.748618238	count	2.80E-05
ABI3	0.5440257	0.1231072	4.4191	1.03E-05	0.748657776	count	0.2389909
TUBD1	0.6232839	0.2827685	2.2042	0.0276	0.749161413	count	1

GPR146	0.5338116	0.114186	4.6749	3.08E-06	0.749316722	count	0.07177016
TRIB2	0.5690649	0.1967621	2.8921	0.00386	0.749637346	count	1
SPATA24	1.1893138	0.6514037	1.8258	0.068	0.74965284	count	1
DEDD	0.6019395	0.2258784	2.6649	0.00775	0.749671433	count	1
ZNF525	0.9585192	0.7263816	1.3196	0.187	0.75053816	count	1
PLEKHG1	0.5752219	0.1941797	2.9623	0.00308	0.750602534	count	1
PCDHGA9	1.6277346	1.0750607	1.5141	0.13	0.750725953	count	1
CYP7B1	1.3163182	0.7631612	1.7248	0.0847	0.751326263	count	1
CTC1	1.1452192	0.8543709	1.3404	0.18	0.751338365	count	1
DONSON	1.5004153	0.7276947	2.0619	0.0393	0.751655508	count	1
SRPK1	0.5511864	0.1386439	3.9756	7.20E-05	0.751670027	count	1
FAM167B	0.5361555	0.1137784	4.7123	2.57E-06	0.751849574	count	0.0599324
ZDHHC17	0.58515	0.216375	2.7043	0.00689	0.75187741	count	1
RIPK1	0.5818634	0.2684913	2.1672	0.0303	0.75203839	count	1
ZNF845	0.9849232	0.5931694	1.6604	0.0969	0.752789954	count	1
SDR42E2	0.7836266	0.7252038	1.0806	0.28	0.753112258	count	1
DCK	0.6629646	0.3177531	2.0864	0.037	0.753820457	count	1
EID3	0.7404213	0.4043119	1.8313	0.0672	0.753830841	count	1
DUSP22	0.5594982	0.1581834	3.537	0.000411	0.75413705	count	1
PLD2	0.6449257	0.2623458	2.4583	0.014	0.754638368	count	1
LRWD1	0.7266912	0.3277289	2.2174	0.0267	0.754680911	count	1
WASF3	0.6404203	0.2956299	2.1663	0.0304	0.754853481	count	1
HDDC3	0.5777575	0.2078821	2.7793	0.00549	0.75507972	count	1
PPAT	0.8413374	0.44114	1.9072	0.0566	0.755205702	count	1
TFB1M	0.6352329	0.3766186	1.6867	0.0918	0.755465021	count	1
SMIM33	1.646979	1.3114712	1.2558	0.209	0.755512194	count	1
ARHGAP27	0.7113275	0.3397165	2.0939	0.0364	0.756002057	count	1
ZNF346	1.0166365	0.6348384	1.6014	0.109	0.756474639	count	1
DMTF1	0.6855055	0.3189356	2.1494	0.0317	0.756617327	count	1
AC116614.1	2.1078644	1.1351777	1.8569	0.0634	0.757741366	count	1
GIMAP1	0.5318461	0.069681	7.6326	3.15E-14	0.758058927	count	7.54E-10
C20orf27	0.5777048	0.1863667	3.0998	0.00196	0.75855446	count	1
PGAP2	0.6087727	0.2426729	2.5086	0.0122	0.75874283	count	1
LPAR6	0.5336445	0.0875575	6.0948	1.25E-09	0.759034081	count	2.96E-05
MCM3	0.581657	0.2034121	2.8595	0.00427	0.759201557	count	1
ZNF415	0.6752853	0.3812539	1.7712	0.0766	0.759570826	count	1
C1GALT1	0.5916545	0.2630979	2.2488	0.0246	0.759974855	count	1
BET1	0.5842329	0.1947658	2.9997	0.00273	0.76117247	count	1
ATP11A	0.6496993	0.268367	2.4209	0.0155	0.761688716	count	1
NAA40	0.8288461	0.643357	1.2883	0.198	0.761808528	count	1
BASP1	0.9368562	0.87172	1.0747	0.283	0.762145966	count	1
PRIM1	0.6732971	0.3126846	2.1533	0.0314	0.762398703	count	1
SYNGAP1	0.8205341	0.6755783	1.2146	0.225	0.762807848	count	1
DYNLRB2	1.6777548	1.1135923	1.5066	0.132	0.763013081	count	1
C11orf80	1.12804	1.0462281	1.0782	0.281	0.763388561	count	1

KIFC3	0.5777584	0.1770525	3.2632	0.00112	0.763538978	count	1
BTN2A2	0.6248108	0.2404259	2.5988	0.00941	0.764616878	count	1
KHK	0.9073893	0.4838413	1.8754	0.0608	0.765018221	count	1
CENPF	1.0613905	0.8158793	1.3009	0.193	0.765347158	count	1
EHHADH	0.7591131	0.3987314	1.9038	0.057	0.765857267	count	1
ADH1B	1.8831964	1.7424152	1.0808	0.2799	0.766264516	count	1
ADAM15	0.5389101	0.0749075	7.1943	8.05E-13	0.766424947	count	1.92E-08
ZNF788P	1.2873263	2.0439749	0.6298	0.5289	0.766666474	count	1
NRM	0.6515383	0.2564402	2.5407	0.0111	0.767209085	count	1
TTC31	1.009337	0.6576233	1.5348	0.125	0.767397011	count	1
ZNF222	0.8960334	0.5481267	1.6347	0.102	0.767531418	count	1
DELE1	0.5820517	0.1819094	3.1997	0.00139	0.767729191	count	1
KIAA1324L	0.7872212	0.4733132	1.6632	0.0964	0.768532537	count	1
CASP6	0.603134	0.2410969	2.5016	0.0124	0.768701601	count	1
HIPK3	0.54625	0.0971149	5.6248	2.04E-08	0.769310927	count	0.000481542
C11orf71	0.6756778	0.2875212	2.35	0.0188	0.769612481	count	1
GFM1	0.5989505	0.2219303	2.6988	0.007	0.76962386	count	1
PPP1R21	0.6498047	0.2640971	2.4605	0.0139	0.770252258	count	1
CEP78	0.8123328	0.3917887	2.0734	0.0382	0.770427305	count	1
TRAM1L1	1.5662321	1.1552777	1.3557	0.175	0.770524047	count	1
HERPUD2	0.5866434	0.1808666	3.2435	0.00119	0.770750501	count	1
SENP1	0.659736	0.4377365	1.5072	0.132	0.770932344	count	1
PNPLA2	0.5516177	0.1112268	4.9594	7.50E-07	0.771211574	count	0.0175485
EYA3	0.641923	0.2390462	2.6854	0.00729	0.771815304	count	1
ERICH1	0.5515542	0.1131351	4.8752	1.15E-06	0.773245295	count	0.0268801
TCTEX1D1	0.7803093	0.3674256	2.1237	0.0338	0.774078382	count	1
CASC10	0.7092019	0.4160192	1.7047	0.0884	0.774514024	count	1
SLCO2B1	0.9216524	0.5258824	1.7526	0.0798	0.774905414	count	1
PSMD5	0.6209987	0.3314131	1.8738	0.0611	0.775165933	count	1
RGS14	0.6611353	0.3006345	2.1991	0.028	0.776092585	count	1
TMEM170A	0.5753508	0.1590182	3.6181	0.000302	0.776305853	count	1
USP36	0.6424359	0.297113	2.1623	0.0307	0.776467215	count	1
ZNF300	1.9398103	0.9986076	1.9425	0.0522	0.776746821	count	1
ZBTB7C	1.1571336	0.5251846	2.2033	0.0277	0.777649435	count	1
AKNAD1	1.2027501	0.8068574	1.4907	0.136	0.778052788	count	1
TBC1D2	0.8240496	0.4371103	1.8852	0.0595	0.780062332	count	1
BAZ1A	0.5659076	0.1264781	4.4744	7.97E-06	0.780369667	count	0.18511919
BOLA2B	1.7523018	1.072727	1.6335	0.102	0.78042005	count	1
ZNF112	0.7941967	1.4656785	0.5419	0.588	0.780471308	count	1
ZNF16	1.2648141	0.8249077	1.5333	0.125	0.782274154	count	1
NEK3	0.5807434	0.1713347	3.3895	0.00071	0.782650529	count	1
CAVIN2	0.5469165	0.0715598	7.6428	2.91E-14	0.782724558	count	6.96E-10
METTL1	0.7113396	0.2971477	2.3939	0.0167	0.782851838	count	1
ZNF99	15.91738	1265.853655	0.0126	0.99	0.782891838	count	1
CORT	15.6540149	1082.419191	0.0145	0.9885	0.78289186	count	1

AC018413.1	15.6542119	1145.822177	0.0137	0.989	0.78289186	count	1
RIPPLY2	16.1833766	1653.874805	0.0098	0.9922	0.782891868	count	1
CHGB	16.2132312	1207.948617	0.0134	0.989	0.782891871	count	1
RAG1	16.2942249	1163.315074	0.014	0.989	0.782891878	count	1
AC231981.1	15.7910445	1217.742239	0.013	0.99	0.782891881	count	1
RASGEF1A	15.9172703	1148.368241	0.0139	0.989	0.782891899	count	1
AC104596.1	15.9969117	1096.365292	0.0146	0.988	0.782891908	count	1
HIST3H2A	16.0689951	660.2327815	0.0243	0.981	0.782891916	count	1
ZNF772	16.9695812	1551.350505	0.0109	0.991	0.782891921	count	1
LRCOL1	17.0967533	1572.625947	0.0109	0.991	0.782891926	count	1
EIF3CL	16.1863737	3090.78725	0.0052	0.996	0.782891929	count	1
AC002480.3	16.2042228	1799.721792	0.009	0.993	0.78289193	count	1
PRSS57	16.2044579	1103.243824	0.0147	0.988	0.78289193	count	1
ZNF404	16.2130244	1275.312679	0.0127	0.99	0.782891931	count	1
ZBED8	16.2266949	1272.334142	0.0128	0.99	0.782891932	count	1
ZNF749	16.2754833	1075.301282	0.0151	0.9879	0.782891937	count	1
AC067930.4	17.4404797	2343.661935	0.0074	0.994	0.782891938	count	1
DDN-AS1	16.3499129	1291.27282	0.0127	0.99	0.782891943	count	1
FZD3	16.365151	1227.566002	0.0133	0.989	0.782891944	count	1
FAM19A2	16.5202543	1880.505582	0.0088	0.993	0.782891956	count	1
CRYBB1	16.5281827	1126.791613	0.0147	0.9883	0.782891957	count	1
SYN2	16.5583954	1173.582709	0.0141	0.989	0.782891959	count	1
HIST1H2BG	16.5584631	1625.438521	0.0102	0.992	0.782891959	count	1
C19orf73	16.5585367	1276.716634	0.013	0.99	0.782891959	count	1
MAB21L2	16.6462853	1577.66621	0.0106	0.992	0.782891964	count	1
AC132192.2	16.6815168	1097.639352	0.0152	0.988	0.782891966	count	1
FTCDNL1	16.6890645	1085.96422	0.0154	0.9877	0.782891967	count	1
AP003031.2	16.7453942	2049.976204	0.0082	0.993	0.78289197	count	1
RGS20	16.7566694	1686.788088	0.0099	0.992	0.782891971	count	1
TEPP	16.8891129	2322.433318	0.0073	0.994	0.782891978	count	1
KLK10	16.8891129	2322.433318	0.0073	0.994	0.782891978	count	1
AC008105.3	16.8920025	4035.114874	0.0042	0.997	0.782891978	count	1
SCRG1	1.0112395	0.6520445	1.5509	0.121	0.783048516	count	1
PIGZ	0.6827343	0.3720817	1.8349	0.0666	0.783853487	count	1
ZNF787	0.5843897	0.1655446	3.5301	0.000422	0.78400189	count	1
SSPN	0.7326742	0.3995504	1.8337	0.0668	0.784106076	count	1
CORO1C	0.5933894	0.1871944	3.1699	0.00154	0.784368923	count	1
ETV5	0.7686469	0.3984469	1.9291	0.0538	0.784391568	count	1
TPK1	0.6835458	0.3169575	2.1566	0.0311	0.784722837	count	1
ZNF714	0.8812388	0.6701086	1.3151	0.189	0.785437599	count	1
TRAF3IP2	0.6552663	0.2707192	2.4205	0.0156	0.785576181	count	1
MRPS35	0.5779173	0.147742	3.9117	9.39E-05	0.786540408	count	1
TANGO6	1.0698604	0.5640012	1.8969	0.0579	0.786724599	count	1
DDX31	0.9088017	0.8821252	1.0302	0.303	0.786771972	count	1
UBE2D4	0.5956974	0.1922496	3.0986	0.00196	0.787345952	count	1

CHD7	0.6360096	0.2079066	3.0591	0.00224	0.787378015	count	1
PHF11	0.5808466	0.1502437	3.866	0.000113	0.787596754	count	1
MBNL1-AS1	0.6613676	0.3175313	2.0828	0.0374	0.787969463	count	1
SMURF1	0.9128136	0.5785442	1.5778	0.115	0.789653846	count	1
PHYHIP	0.9612475	0.7626584	1.2604	0.208	0.790307789	count	1
SPINK5	0.8368998	1.6559942	0.5054	0.6133	0.790576524	count	1
SERPINB8	0.722088	0.4011884	1.7999	0.072	0.790598447	count	1
GK5	0.8205982	0.4200722	1.9535	0.0509	0.790644208	count	1
NUP50	0.5983273	0.1859284	3.2181	0.00131	0.790737367	count	1
CDK5RAP1	0.6956468	0.3518321	1.9772	0.0481	0.790758598	count	1
DNM3OS	0.7291341	0.3018157	2.4158	0.0158	0.791124426	count	1
SDHAF4	0.7881652	0.3894863	2.0236	0.0431	0.791868823	count	1
CENPU	1.2336792	0.9058414	1.3619	0.173	0.79200277	count	1
PUS3	0.6290857	0.247059	2.5463	0.0109	0.792474636	count	1
ALKBH2	0.6199192	0.2037429	3.0427	0.00237	0.79261238	count	1
GTPBP10	0.6895231	0.3266238	2.1111	0.0349	0.793301685	count	1
PER2	0.607772	0.2239425	2.714	0.00669	0.793652194	count	1
SLC12A9	0.7011053	0.2760944	2.5394	0.0112	0.794091403	count	1
EBPL	0.5618034	0.0852662	6.5888	5.29E-11	0.794362457	count	1.26E-06
CASP1	0.577832	0.1383166	4.1776	3.04E-05	0.794369634	count	0.7019968
USP32	0.774534	0.4513099	1.7162	0.0862	0.794557566	count	1
ZNF267	0.6046925	0.1773013	3.4105	0.000658	0.794613462	count	1
DHX15	0.600864	0.2021847	2.9719	0.00299	0.794677478	count	1
KDM4D	1.2400116	0.8777323	1.4127	0.158	0.79482366	count	1
ARHGAP26	0.5870898	0.2127793	2.7591	0.00583	0.795046595	count	1
PRKD2	0.6104716	0.1870679	3.2634	0.00111	0.795295483	count	1
NEBL	1.117326	0.828819	1.3481	0.178	0.795456008	count	1
TMEM106A	0.8617814	0.9009118	0.9566	0.339	0.795620006	count	1
TMEM185B	0.6835637	0.3176271	2.1521	0.0315	0.79703375	count	1
CCDC97	0.7185687	0.3589734	2.0017	0.0454	0.798999078	count	1
TMEM268	1.3052218	1.1032296	1.1831	0.237	0.79905599	count	1
EP300	0.6262278	0.1941866	3.2249	0.00127	0.799754917	count	1
DLC1	0.577689	0.1251842	4.6147	4.12E-06	0.799979661	count	0.09594244
SLC31A2	0.7915834	0.3813511	2.0757	0.038	0.800141585	count	1
FBL	0.5825062	0.1138212	5.1177	3.30E-07	0.800181215	count	0.00773652
TTC7A	0.8406579	0.461931	1.8199	0.0689	0.800644933	count	1
WDYHV1	0.6988491	0.3238643	2.1578	0.031	0.80108476	count	1
MTG1	1.3791861	0.8575635	1.6083	0.108	0.802081864	count	1
LRRC8B	0.8539454	0.5864115	1.4562	0.145	0.804437778	count	1
P2RY8	1.0234019	0.9309102	1.0994	0.272	0.804495103	count	1
SRPK2	0.5850787	0.1326847	4.4095	1.08E-05	0.804715578	count	0.250506
RFFL	1.0476959	0.6557554	1.5977	0.11	0.804982778	count	1
KIAA0319L	0.6377157	0.2414496	2.6412	0.00831	0.805295051	count	1
VPS72	0.6054919	0.1722526	3.5151	0.000447	0.80564023	count	1
CFP	0.7227085	0.3572829	2.0228	0.0432	0.806062467	count	1

MTMR14	0.6596472	0.2673692	2.4672	0.0137	0.806379595	count	1
ZC3H10	1.573507	0.8272701	1.902	0.0573	0.806504781	count	1
ZNF689	0.7729558	0.4783982	1.6157	0.106	0.806681178	count	1
UXS1	0.6441373	0.222095	2.9003	0.00376	0.807318295	count	1
JUNB	0.5605524	0.0452399	12.3907	2.36E-34	0.807725944	count	5.71E-30
ZNF19	1.5782091	1.0722495	1.4719	0.141	0.807881572	count	1
KIAA1614	1.7081762	1.1375411	1.5016	0.133	0.808027388	count	1
AC007686.3	1.4804452	1.0007652	1.4793	0.139	0.809085328	count	1
EDA	1.8862285	1.1265573	1.6743	0.0942	0.809117316	count	1
APTR	0.8277061	0.4815304	1.7189	0.0857	0.809276868	count	1
CD300LG	1.5847138	1.1056463	1.4333	0.152	0.809777837	count	1
TAF9B	0.6478738	0.278784	2.3239	0.0202	0.81004051	count	1
CCND3	0.58114	0.112297	5.175	2.44E-07	0.810148334	count	0.005725704
HS3ST2	1.276193	0.7207803	1.7706	0.0767	0.810714134	count	1
PET117	0.7148102	0.3531763	2.0239	0.0431	0.810951863	count	1
CSF1	0.6087834	0.2186445	2.7844	0.0054	0.811069544	count	1
RIC8B	0.8627046	0.3813266	2.2624	0.0238	0.81152253	count	1
AC087477.2	1.185998	0.7329948	1.618	0.106	0.811739446	count	1
TMEM161B	0.8929908	0.4598258	1.942	0.0522	0.811763036	count	1
REEP4	0.7883188	0.3656037	2.1562	0.0312	0.811924221	count	1
AC073072.1	2.5649738	1.4967118	1.7137	0.0867	0.811950918	count	1
GOLGA5	0.6466335	0.2369424	2.7291	0.00639	0.812032327	count	1
ARHGEF3	0.5985621	0.1499111	3.9928	6.70E-05	0.812892274	count	1
CCDC153	1.4110539	0.7392966	1.9086	0.0564	0.813834785	count	1
JAM2	0.5692457	0.064834	8.7801	2.81E-18	0.814070021	count	6.75E-14
CST3	0.5658482	0.0449588	12.5859	2.31E-35	0.815263225	count	5.59E-31
ZDHHC18	0.934629	0.5181878	1.8036	0.0714	0.815318604	count	1
UTP15	0.729036	0.3439458	2.1196	0.0341	0.815347172	count	1
OAS1	0.6010205	0.1846022	3.2558	0.00114	0.81584139	count	1
SLC25A26	0.6835918	0.2539943	2.6914	0.00716	0.816092035	count	1
ZNF227	0.7933806	0.3892814	2.0381	0.0416	0.816572984	count	1
HIF1AN	0.8994011	0.5503931	1.6341	0.102	0.816678145	count	1
TBP	0.8102276	0.3439675	2.3555	0.0186	0.816809747	count	1
NDRG2	0.6266578	0.1926498	3.2528	0.00116	0.817146049	count	1
PPM1N	1.6103814	1.3579836	1.1859	0.236	0.81716734	count	1
PLSCR4	0.5899761	0.1334614	4.4206	1.02E-05	0.81776967	count	0.2366808
GIMAP2	0.6050474	0.164562	3.6767	0.000241	0.817988865	count	1
FAM207A	0.6208984	0.201259	3.0851	0.00206	0.81800574	count	1
AP002748.3	1.0249144	0.6081456	1.6853	0.092	0.818951914	count	1
AC012358.3	1.0728928	0.7104778	1.5101	0.131	0.819882372	count	1
CD40	0.579416	0.0877894	6.6001	4.91E-11	0.820133194	count	1.17E-06
BOC	0.6820409	0.370934	1.8387	0.0661	0.820331872	count	1
METTL3	0.7837187	0.3203244	2.4466	0.0145	0.821090289	count	1
SEC24B	0.6778468	0.2769244	2.4478	0.0144	0.821098454	count	1
IL3RA	0.5787874	0.0895593	6.4626	1.21E-10	0.821286838	count	2.87E-06

STARD13	0.821909	0.3518679	2.3358	0.0196	0.821739475	count	1
RBM7	0.6115561	0.1570639	3.8937	0.000101	0.822030923	count	1
ZNF512B	0.9064145	0.5149555	1.7602	0.0785	0.822039598	count	1
BHLHE41	1.078171	0.6735967	1.6006	0.11	0.822976615	count	1
AC106897.1	0.9929677	0.6203054	1.6008	0.11	0.823295248	count	1
MAFB	0.5875812	0.1739301	3.3783	0.00074	0.823457057	count	1
WDR12	0.7005361	0.2508862	2.7922	0.00527	0.824962957	count	1
TIMP4	2.71246	1.1463351	2.3662	0.018	0.82512246	count	1
WDR74	0.6406131	0.241699	2.6505	0.00808	0.826058779	count	1
IFIH1	0.6356531	0.1988605	3.1965	0.00141	0.826631457	count	1
KIF21B	1.5360637	1.1286654	1.361	0.174	0.827115768	count	1
ZNF223	1.0855819	1.171558	0.9266	0.354	0.827305427	count	1
TMEM69	0.7236844	0.2883436	2.5098	0.0121	0.827507574	count	1
ACYP1	0.6401976	0.2300814	2.7825	0.00543	0.827744873	count	1
TRIM16	0.6718129	0.2704535	2.484	0.013	0.828564483	count	1
FRMD4A	0.672787	0.2416576	2.7841	0.00541	0.828610383	count	1
KLHDC3	0.6907351	0.2846863	2.4263	0.0153	0.828727177	count	1
CYP46A1	1.0631199	1.8772896	0.5663	0.5712	0.828749913	count	1
KIF22	0.6313661	0.1915501	3.2961	0.000993	0.829100743	count	1
GJC2	0.6987824	0.2707956	2.5805	0.00992	0.829912086	count	1
OGFR	0.6350503	0.2029279	3.1294	0.00177	0.83045752	count	1
ZNF775	0.9297675	0.7499206	1.2398	0.215	0.830786814	count	1
FZD5	0.87815	0.4626828	1.898	0.0578	0.83129962	count	1
RIOX1	1.0673594	0.585804	1.822	0.0686	0.831307452	count	1
SLC25A28	0.638644	0.1759284	3.6301	0.000288	0.831364248	count	1
SOCS3	0.579363	0.0580996	9.9719	4.94E-23	0.83227613	count	1.19E-18
ADAM23	1.3897168	0.6506837	2.1358	0.0328	0.832659323	count	1
HIC2	0.9899198	1.2764199	0.7755	0.4381	0.832834393	count	1
THRB	0.8631824	0.4772329	1.8087	0.0706	0.832837425	count	1
SLC9A3R1	0.8996646	0.4622665	1.9462	0.0517	0.8333262	count	1
AL135791.1	1.3931187	0.8205462	1.6978	0.0897	0.833970729	count	1
A4GALT	0.6067384	0.1303979	4.653	3.43E-06	0.834305027	count	0.07990528
SRF	0.8183411	0.3445796	2.3749	0.0176	0.834411158	count	1
HLA-B	0.5790345	0.030147	19.207	2.84E-77	0.834898279	count	6.90E-73
RPP21	0.7737975	0.4910795	1.5757	0.115	0.835107049	count	1
SOCS6	0.716919	0.3423225	2.0943	0.0363	0.835349218	count	1
FANCE	1.6779825	1.0137627	1.6552	0.098	0.835930958	count	1
GLB1L	0.8753702	0.6394504	1.3689	0.171	0.836124135	count	1
RSPO3	0.6576454	0.2013551	3.2661	0.0011	0.837001281	count	1
ZNF529	0.8688045	0.4893342	1.7755	0.0759	0.837520499	count	1
COL9A3	1.0785544	0.4966129	2.1718	0.03	0.838031866	count	1
FUT1	0.9165308	0.687618	1.3329	0.183	0.838280396	count	1
PDE9A	0.6687016	0.3265053	2.0481	0.0406	0.838573486	count	1
PKD1L1	1.839098	1.0688822	1.7206	0.0854	0.839004831	count	1
ARHGAP25	0.8078383	0.4461512	1.8107	0.0703	0.8391831	count	1

PARP2	0.7399014	0.3139534	2.3567	0.0185	0.839773702	count	1
XRCC1	0.6955754	0.2807068	2.4779	0.0133	0.840016881	count	1
NAIP	0.7685363	0.5273765	1.4573	0.145	0.840256482	count	1
LGALS3	0.5845175	0.0411861	14.1921	3.53E-44	0.841329172	count	8.56E-40
LINC02447	2.0580464	1.1649055	1.7667	0.0774	0.841482305	count	1
SLC2A3	0.5946452	0.0871442	6.8237	1.09E-11	0.842371022	count	2.60E-07
AC020928.1	0.9597103	0.4955373	1.9367	0.0529	0.843301845	count	1
SORBS1	1.1140274	0.6949525	1.603	0.109	0.843750857	count	1
GPM6B	0.9902652	0.5860667	1.6897	0.0912	0.844205761	count	1
PARP16	0.9902652	0.6023215	1.6441	0.1	0.844205761	count	1
HIC1	0.7443915	0.2966636	2.5092	0.0122	0.844478616	count	1
GINS3	1.0073985	0.4982807	2.0217	0.0433	0.844657342	count	1
BARD1	0.6716327	0.2650114	2.5344	0.0113	0.845537189	count	1
AL139274.2	0.8869151	0.4862567	1.824	0.0683	0.845581984	count	1
GEMIN4	1.8713864	1.1474459	1.6309	0.103	0.846144597	count	1
TMEM70	0.6039412	0.1310428	4.6087	4.24E-06	0.846465601	count	0.09872416
FBF1	15.8902962	749.9137325	0.0212	0.9831	0.848509986	count	1
SYNGAP1-AS1	15.711039	1136.141458	0.0138	0.989	0.848510022	count	1
MYO1F	16.0400163	1094.527635	0.0147	0.988	0.84851007	count	1
TRIM6	16.8089715	1690.270794	0.0099	0.992	0.848510073	count	1
AC009237.14	16.1214952	1093.603626	0.0147	0.988	0.84851008	count	1
LINC01506	16.2410062	1319.979122	0.0123	0.99	0.848510093	count	1
AC005363.2	16.2410285	1269.051154	0.0128	0.99	0.848510093	count	1
LINC01376	16.2410318	1215.983616	0.0134	0.989	0.848510093	count	1
C6orf52	16.3747144	1159.976895	0.0141	0.989	0.848510106	count	1
MEGF11	16.3823948	1141.981514	0.0143	0.989	0.848510106	count	1
AL031963.3	16.5163487	1161.383257	0.0142	0.989	0.848510118	count	1
AC007743.1	16.5799347	1073.654832	0.0154	0.9877	0.848510122	count	1
FRMPD4	16.6342975	1466.907037	0.0113	0.991	0.848510126	count	1
ALKAL2	16.7435522	1315.099495	0.0127	0.9898	0.848510133	count	1
AL035681.1	17.0002054	5560.556669	0.0031	0.998	0.848510147	count	1
TES	0.629332	0.1749119	3.598	0.000326	0.848535986	count	1
AC087239.1	1.4342735	0.6759492	2.1219	0.0339	0.849586074	count	1
FAM241A	0.602673	0.1087256	5.5431	3.25E-08	0.849603153	count	0.000766253
TMEM60	0.6605163	0.2365151	2.7927	0.00526	0.849655784	count	1
MGAT5B	1.6111323	1.0837079	1.4867	0.137	0.850272037	count	1
G2E3	0.6615545	0.2389627	2.7684	0.00567	0.85034004	count	1
LAMA5	0.6524057	0.2246308	2.9043	0.00371	0.850635338	count	1
FLT4	0.7679918	0.4385291	1.7513	0.08	0.852305841	count	1
NCKIPSD	1.0026487	0.5137018	1.9518	0.0511	0.852736445	count	1
TPPP	1.0582896	0.776846	1.3623	0.173	0.853201777	count	1
VTI1A	0.8091229	0.2993504	2.7029	0.00692	0.853515419	count	1
TGFBR3	0.6035004	0.1031891	5.8485	5.54E-09	0.853943402	count	0.000130999
DHRS4	0.7511659	0.2941816	2.5534	0.0107	0.854088079	count	1
AC083973.1	0.9755413	1.0748332	0.9076	0.364	0.854718667	count	1

PMS2	0.7683197	0.4583776	1.6762	0.0938	0.855604905	count	1
THUMPD3-AS1	0.6332291	0.1552064	4.0799	4.63E-05	0.855741425	count	1
PDZD4	1.9184728	0.8951346	2.1432	0.0322	0.85621786	count	1
CERS4	0.8919143	0.4763121	1.8725	0.0612	0.856659063	count	1
SSBP2	0.6432903	0.1941857	3.3128	0.000936	0.85682581	count	1
NRBF2	0.6228402	0.1405315	4.432	9.70E-06	0.857503901	count	0.2251564
ZMYM3	0.9206184	0.4849878	1.8982	0.0578	0.857711268	count	1
MTRNR2L8	0.6083731	0.0968664	6.2805	3.91E-10	0.857833868	count	9.28E-06
RNF19B	0.83366	0.3563382	2.3395	0.0194	0.85820501	count	1
ANAPC4	0.7316545	0.3618762	2.0218	0.0433	0.859208701	count	1
TMEM41A	0.679932	0.2358312	2.8831	0.00397	0.859657077	count	1
AC073508.3	1.7705559	1.0583543	1.6729	0.0945	0.860041481	count	1
LIFR	0.6061339	0.1127404	5.3764	8.24E-08	0.860123158	count	0.001938295
B4GALT5	0.6799609	0.2341039	2.9045	0.00371	0.860482798	count	1
TXNRD3	1.1736542	0.7762682	1.5119	0.131	0.860563294	count	1
KSR1	0.9348818	0.4841901	1.9308	0.0536	0.860772761	count	1
HCG18	0.707764	0.3232629	2.1894	0.0286	0.860780485	count	1
SKIDA1	0.7798276	0.4081024	1.9109	0.0561	0.861206053	count	1
TKFC	0.7613639	0.3401963	2.238	0.0253	0.862213442	count	1
DTX2	1.1769645	0.6518827	1.8055	0.0711	0.862348982	count	1
EMCN	0.6030159	0.0796173	7.5739	4.91E-14	0.862564484	count	1.17E-09
NDUFAF6	0.7319068	0.3371658	2.1708	0.03	0.86319533	count	1
PLCG2	0.6047021	0.1110713	5.4443	5.66E-08	0.863248706	count	0.001332704
HES4	0.6700566	0.2472129	2.7104	0.00676	0.863291197	count	1
HLA-F	0.605381	0.0727249	8.3243	1.32E-16	0.863332383	count	3.17E-12
MLH1	0.7997626	0.3373692	2.3706	0.0178	0.864091466	count	1
PARG	0.78336	0.3459688	2.2643	0.0236	0.864760419	count	1
ZNF571	1.1818761	0.6156759	1.9196	0.055	0.864991865	count	1
ZNF429	0.7908839	0.3465792	2.282	0.0226	0.865804658	count	1
LRRC70	1.3003278	0.6170796	2.1072	0.0352	0.865999317	count	1
MT1X	0.6503804	0.1866223	3.485	5.00E-04	0.866037419	count	1
SRBD1	0.7878978	0.3127867	2.519	0.0118	0.866117396	count	1
APOL6	0.6567839	0.2003813	3.2777	0.00106	0.866264351	count	1
WNK1	0.6179158	0.123898	4.9873	6.50E-07	0.866995713	count	0.0152126
NANP	1.3045304	0.7896767	1.652	0.0987	0.867917683	count	1
CD320	0.6077193	0.0916805	6.6287	4.06E-11	0.868440563	count	9.66E-07
SLC16A13	1.5701806	1.0847161	1.4475	0.148	0.868578943	count	1
DDX54	0.6805759	0.2354734	2.8902	0.00388	0.868611654	count	1
ITGB3BP	0.66951	0.2161843	3.0969	0.00197	0.869228982	count	1
TTC12	1.982611	1.1161273	1.7763	0.0758	0.869313085	count	1
GIPC2	0.6346536	0.1359717	4.6675	3.19E-06	0.86951115	count	0.07432062
DDX55	0.7256376	0.3653334	1.9862	0.0471	0.869671636	count	1
P3H2	0.8412034	0.3012233	2.7926	0.00526	0.869913236	count	1
GABBR1	0.8413619	0.4081488	2.0614	0.0394	0.870058387	count	1
HOXD9	0.729383	0.3313915	2.201	0.0278	0.87070125	count	1

AK7	1.1347596	0.6898338	1.645	0.1	0.871156361	count	1
TTI2	0.7636747	0.334457	2.2833	0.0225	0.872172377	count	1
EPB41L3	0.6334871	0.1520031	4.1676	3.17E-05	0.872264267	count	0.7318579
ITPKB	0.6909885	0.3091824	2.2349	0.0255	0.873856756	count	1
USPL1	0.831498	0.3744446	2.2206	0.0265	0.874573257	count	1
DMTN	1.0525185	0.5567234	1.8906	0.0588	0.874691832	count	1
IGF2	0.7434335	0.4488756	1.6562	0.0978	0.875889754	count	1
AC106739.2	1.0056546	0.6230697	1.614	0.107	0.87619677	count	1
FAM155A	0.9785498	0.4301425	2.2749	0.023	0.876204186	count	1
PLA1A	0.7192422	0.2890217	2.4885	0.0129	0.876613592	count	1
LIPT1	0.7194617	0.2614008	2.7523	0.00596	0.876866494	count	1
NCK1	0.6328962	0.1218157	5.1955	2.19E-07	0.877229984	count	0.005140149
C17orf49	0.9455374	0.4140449	2.2837	0.0225	0.87723373	count	1
AC147067.1	0.7387883	0.3111881	2.3741	0.0177	0.877872353	count	1
AC011511.5	2.0266475	0.993083	2.0408	0.0414	0.877900689	count	1
RIPK2	0.6945782	0.2705989	2.5668	0.0103	0.878205448	count	1
RAPH1	0.6575549	0.199538	3.2954	0.000995	0.878826235	count	1
TPST2	0.6324787	0.1260251	5.0187	5.53E-07	0.879211728	count	0.012946836
SKP2	1.1780436	0.7562633	1.5577	0.119	0.879779346	count	1
C22orf46	1.0289065	0.5006666	2.0551	0.04	0.881810362	count	1
TRAP1	0.6926743	0.2042313	3.3916	0.000705	0.882732895	count	1
SERPINI1	0.7028724	0.3694788	1.9023	0.0572	0.883132852	count	1
DUSP23	0.6176673	0.0701769	8.8016	2.33E-18	0.883391402	count	5.60E-14
S100A9	1.107244	0.7932173	1.3959	0.163	0.883710745	count	1
HERC5	0.6501213	0.1829984	3.5526	0.000388	0.884029324	count	1
ZSCAN9	0.8802123	0.4751835	1.8524	0.0641	0.88409256	count	1
AASDH	0.7085693	0.2326739	3.0453	0.00235	0.885334373	count	1
ASGR1	0.6763703	0.2394547	2.8246	0.00477	0.885544963	count	1
GPD2	0.8532699	0.3698021	2.3074	0.0211	0.885725686	count	1
MFSD4A	0.7926419	0.4804318	1.6499	0.0991	0.886258125	count	1
RUSC1	0.7303564	0.257641	2.8348	0.00462	0.888070242	count	1
BMP2K	0.7623049	0.2773036	2.749	0.00602	0.888653605	count	1
SHANK3	0.6684625	0.2656115	2.5167	0.0119	0.888876448	count	1
TYW1	0.7929737	0.3247751	2.4416	0.0147	0.889471761	count	1
NLRP1	0.6969619	0.2512562	2.7739	0.00558	0.890107426	count	1
PGAM5	0.933075	0.4262421	2.1891	0.0287	0.890300762	count	1
PPP1R36	1.0264269	0.6006056	1.709	0.0876	0.890829829	count	1
Z93241.1	0.8701139	0.5092239	1.7087	0.0876	0.891221559	count	1
ZNF236	0.8705028	0.4058207	2.145	0.032	0.891571485	count	1
ENTPD1	0.6413384	0.120723	5.3125	1.17E-07	0.89184784	count	0.002749617
NARS2	0.9650708	0.5221104	1.8484	0.0647	0.892388205	count	1
CYYR1	0.6272358	0.0737999	8.4991	3.08E-17	0.892501355	count	7.39E-13
ZCWPW1	0.9358629	0.5394953	1.7347	0.0829	0.892558629	count	1
ATP8A1	0.7303577	0.2667915	2.7376	0.00623	0.893290809	count	1
C12orf43	0.7067332	0.2373827	2.9772	0.00293	0.893731787	count	1

PMM1	0.9788154	0.5354707	1.828	0.0677	0.89441647	count	1
BUD13	0.7901672	0.3292687	2.3998	0.0165	0.894808406	count	1
BIN3	0.7691686	0.2711908	2.8363	0.0046	0.896077614	count	1
MED6	0.6752999	0.1763712	3.8289	0.000132	0.896098294	count	1
ZNF768	0.8370111	0.3588425	2.3325	0.0197	0.896338127	count	1
LONRF1	0.8056648	0.3499678	2.3021	0.0214	0.896536994	count	1
LINC00526	1.1548699	0.7542377	1.5312	0.126	0.897978094	count	1
KLHL22	1.4277801	0.7402247	1.9288	0.0539	0.898040069	count	1
HS1BP3	1.0704864	0.5181103	2.0661	0.0389	0.89853014	count	1
HACL1	0.7114411	0.2362491	3.0114	0.00262	0.898602041	count	1
GRB10	0.6656281	0.1920637	3.4657	0.000537	0.898609173	count	1
HOXA4	0.7914901	0.3250008	2.4353	0.0149	0.898808769	count	1
AC025171.3	0.9443198	0.4469892	2.1126	0.0347	0.89939158	count	1
ABCA5	0.7140442	0.3309111	2.1578	0.031	0.900054048	count	1
RARRES3	0.6390736	0.110899	5.7627	9.20E-09	0.900598015	count	0.00021735
STAP2	0.7569329	0.3454651	2.1911	0.0285	0.901480206	count	1
PPOX	0.8351255	0.3560156	2.3458	0.0191	0.902191844	count	1
ARL4A	0.6320171	0.0760629	8.3091	1.49E-16	0.903280973	count	3.58E-12
GEMIN2	0.8490644	0.3616823	2.3475	0.019	0.903818395	count	1
TJAP1	0.9244734	0.4080537	2.2656	0.0236	0.903818838	count	1
C11orf1	0.6675075	0.1534407	4.3503	1.41E-05	0.90469539	count	0.3266547
VEGFC	0.6664281	0.1842263	3.6174	0.000303	0.905259949	count	1
CDK7	0.7784189	0.3076461	2.5302	0.0115	0.906063867	count	1
CDC7	1.9731515	0.810722	2.4338	0.015	0.906868951	count	1
C15orf41	1.145713	0.6898583	1.6608	0.0969	0.90711392	count	1
EGOT	1.4510245	0.8353665	1.737	0.0825	0.907381948	count	1
UTP4	0.7779228	0.2860726	2.7193	0.00658	0.907624799	count	1
LINC01137	0.9371817	0.4829684	1.9405	0.0524	0.907718137	count	1
SPATA2	1.124316	0.557887	2.0153	0.044	0.907858644	count	1
POLR3B	1.2648354	0.9313287	1.3581	0.175	0.908452146	count	1
ELP3	0.8007735	0.3301493	2.4255	0.0154	0.908473461	count	1
ELMSAN1	0.670305	0.1519273	4.412	1.06E-05	0.909707921	count	0.2458988
PSMC3IP	2.2075777	1.0795488	2.0449	0.041	0.909962196	count	1
TLR4	0.6735969	0.2196586	3.0666	0.00219	0.910193096	count	1
CLIC2	0.6538722	0.1171683	5.5806	2.63E-08	0.91096842	count	0.000620443
ZNF541	15.8113748	670.1419827	0.0236	0.9812	0.911273021	count	1
AL451074.6	16.0962118	3570.134184	0.0045	0.996	0.911273064	count	1
MGARP	16.159811	1144.77831	0.0141	0.989	0.911273072	count	1
ECHDC3	15.7642203	1075.846827	0.0147	0.9883	0.911273081	count	1
MEST	16.3606372	1249.645859	0.0131	0.99	0.911273094	count	1
AC114490.3	15.8688885	709.0895625	0.0224	0.982	0.911273099	count	1
RBPMS-AS1	15.8896831	1087.166496	0.0146	0.988	0.911273102	count	1
ZBTB20-AS4	16.0810328	1243.94099	0.0129	0.99	0.91127313	count	1
IL17RE	16.0810343	1243.939467	0.0129	0.99	0.91127313	count	1
AC040169.1	16.1598572	1243.431165	0.013	0.99	0.91127314	count	1

CA13	16.2312278	1193.6583	0.0136	0.989	0.911273148	count	1
ASCL2	16.5099705	1963.958332	0.0084	0.993	0.911273176	count	1
STK26	16.5457055	1134.6548	0.0146	0.988	0.911273179	count	1
RADIL	16.56226	1226.366038	0.0135	0.989	0.91127318	count	1
LINC00630	16.5957854	1201.801893	0.0138	0.989	0.911273183	count	1
RASAL2-AS1	16.6100624	1430.985049	0.0116	0.991	0.911273184	count	1
C1QTNF9B	16.698314	1169.315986	0.0143	0.989	0.91127319	count	1
AL359636.1	17.09162	2797.876791	0.0061	0.995	0.911273214	count	1
LRRC2	17.2232772	1600.46755	0.0108	0.991	0.91127322	count	1
PITHD1	0.6665034	0.1344328	4.9579	7.56E-07	0.912417261	count	0.01768662
PECR	0.927342	0.5169084	1.794	0.0729	0.912666174	count	1
ADRA2C	0.9275809	0.4842133	1.9156	0.0555	0.912868028	count	1
AC008555.5	0.9277183	0.469905	1.9743	0.0485	0.912984062	count	1
PAIP2B	1.4085632	0.7709601	1.827	0.0678	0.913722618	count	1
LARGE1	1.4085632	1.0508806	1.3404	0.18	0.913722618	count	1
MDC1	1.112974	0.4976281	2.2366	0.0254	0.91383806	count	1
PTAFR	0.9287346	0.5856537	1.5858	0.113	0.913842466	count	1
SLC25A19	1.1339978	0.5694928	1.9912	0.0466	0.913858563	count	1
AC009113.1	1.850186	1.038841	1.781	0.075	0.915566132	count	1
ZNF596	1.3186644	0.8246747	1.599	0.11	0.915948392	count	1
LIFR-AS1	0.8261001	0.365372	2.261	0.0238	0.917180463	count	1
AP5B1	1.7302014	1.0624793	1.6285	0.104	0.917371695	count	1
FCHO1	1.366403	1.1054012	1.2361	0.217	0.917483057	count	1
GNGT2	0.8885687	0.3580339	2.4818	0.0131	0.917943893	count	1
UBE3D	0.9875321	0.4441158	2.2236	0.0263	0.918008392	count	1
SMIM10	0.6863956	0.1717895	3.9956	6.62E-05	0.918110453	count	1
CDK9	0.7027689	0.1811072	3.8804	0.000107	0.918523559	count	1
AC016831.7	0.7481735	0.3012388	2.4837	0.0131	0.918906958	count	1
SNRPA	0.6783608	0.1604497	4.2279	2.44E-05	0.919296459	count	0.5639572
LIPA	0.6702106	0.1416811	4.7304	2.35E-06	0.919600877	count	0.05481845
AL121944.1	1.1671078	0.6788517	1.7192	0.0857	0.919912954	count	1
STOX2	0.7742181	0.286104	2.7061	0.00685	0.920698305	count	1
BATF3	0.818065	0.3001897	2.7252	0.00647	0.920886015	count	1
PGM5	0.6655355	0.1270291	5.2392	1.73E-07	0.922202587	count	0.004061867
ODF2	0.819672	0.3890328	2.1069	0.0352	0.92253582	count	1
IL27RA	0.7609583	0.3147156	2.4179	0.0157	0.923083512	count	1
GGA3	1.0312425	0.5696714	1.8102	0.0704	0.924327992	count	1
CEPT1	0.8192499	0.3556206	2.3037	0.0213	0.924900065	count	1
RAB11FIP5	0.7199606	0.2218449	3.2453	0.00119	0.92512352	count	1
RBP5	0.7739036	0.2674052	2.8941	0.00383	0.925448038	count	1
DNAH6	1.1535377	0.5572772	2.07	0.0385	0.925870328	count	1
AOC3	0.7068497	0.2341723	3.0185	0.00256	0.926443066	count	1
ASB16-AS1	0.9995367	0.5855094	1.7071	0.0879	0.927275203	count	1
DGKH	0.7424501	0.3239267	2.292	0.022	0.927588244	count	1
TRIB1	0.6844579	0.1724913	3.9681	7.43E-05	0.92777266	count	1

PGBD2	0.917667	0.4200387	2.1847	0.029	0.928142814	count	1
GMDS-DT	0.8280849	0.3560248	2.3259	0.0201	0.928264503	count	1
PPWD1	0.7079126	0.1932012	3.6641	0.000253	0.929116089	count	1
CD3EAP	0.9190401	0.389792	2.3578	0.0185	0.929347379	count	1
ZNF691	1.0673579	0.5482628	1.9468	0.0517	0.930011002	count	1
MAN1B1-DT	1.2437341	0.9278452	1.3405	0.18	0.932382028	count	1
EIF2S3	0.6668977	0.101083	6.5975	4.99E-11	0.933224741	count	1.19E-06
SLC35G1	1.1473408	0.6439673	1.7817	0.0749	0.935532505	count	1
PUS7	0.8383748	0.3650126	2.2968	0.0217	0.935737996	count	1
GRINA	0.7082993	0.1757248	4.0307	5.71E-05	0.93608764	count	1
HOXB4	0.7073988	0.2020143	3.5017	0.00047	0.936410458	count	1
GNAL	0.7996148	0.308594	2.5912	0.00962	0.937288058	count	1
FGD6	0.719907	0.20296	3.547	0.000396	0.937680842	count	1
CDC25B	0.7136833	0.2280621	3.1293	0.00177	0.937748475	count	1
PPID	0.7032848	0.2043527	3.4415	0.000587	0.937755853	count	1
MMP16	1.3246925	0.6480155	2.0442	0.041	0.938442045	count	1
ECHDC2	0.7146687	0.2777746	2.5728	0.0101	0.93857175	count	1
MIF4GD	0.7048216	0.244952	2.8774	0.00404	0.939750501	count	1
TRAPPC11	0.8871811	0.3914476	2.2664	0.0235	0.939794185	count	1
IQCJ-SCHIP1	1.1787322	0.6204782	1.8997	0.0576	0.941166622	count	1
KIAA1841	0.9707151	0.4372368	2.2201	0.0265	0.942211491	count	1
ZNF782	1.8255091	1.0662614	1.7121	0.087	0.943661751	count	1
AC097534.2	0.7221077	0.2186843	3.3021	0.000972	0.943908624	count	1
PVT1	1.3785561	0.7264304	1.8977	0.0578	0.944136313	count	1
AC016831.5	0.7995125	0.2629457	3.0406	0.00238	0.944991274	count	1
SCYL3	1.1661879	0.5961285	1.9563	0.0505	0.947258453	count	1
CDKN3	0.7715884	0.3341191	2.3093	0.021	0.947292015	count	1
JAK2	0.9105764	0.3759567	2.422	0.0155	0.947735884	count	1
MIER2	0.80097	0.2875385	2.7856	0.00538	0.948461265	count	1
MAP3K14	0.9967792	0.5556888	1.7938	0.073	0.948897238	count	1
MTMR3	1.0072754	0.5250449	1.9185	0.0552	0.949490893	count	1
RNASEL	0.9732527	0.452323	2.1517	0.0315	0.951102898	count	1
TRIM25	0.720234	0.2076597	3.4683	0.000532	0.95138451	count	1
OFD1	0.7074757	0.1587884	4.4555	8.70E-06	0.951930796	count	0.202014
ZNF512	0.8913055	0.4457873	1.9994	0.0457	0.952143258	count	1
ZNF66	1.8582163	1.1872904	1.5651	0.118	0.952232739	count	1
PSMB9	0.6663512	0.067392	9.8877	1.12E-22	0.953120283	count	2.70E-18
NME2	0.8977161	0.39219	2.289	0.0222	0.953988987	count	1
NFYB	0.7065905	0.1607123	4.3966	1.14E-05	0.954213226	count	0.2643546
DYRK2	1.0142417	0.4064024	2.4957	0.0126	0.954951117	count	1
BCKDHB	0.8333083	0.3110571	2.679	0.00743	0.955280668	count	1
PDLIM3	0.6800523	0.1113364	6.1081	1.15E-09	0.956626565	count	2.72E-05
NOP56	0.7059387	0.1446708	4.8796	1.12E-06	0.956857478	count	0.02618112
PREX2	0.6894164	0.1233716	5.5881	2.52E-08	0.957431153	count	0.000594569
KITLG	0.7771918	0.2340239	3.321	0.000909	0.958684039	count	1

TRIM11	1.0191371	0.5086239	2.0037	0.0452	0.958778169	count	1
10-Sep	0.682195	0.1038822	6.567	6.11E-11	0.95901495	count	1.45E-06
CASP7	0.7643385	0.2152063	3.5517	0.000389	0.959454758	count	1
BX322562.1	0.9759824	0.4936086	1.9772	0.0481	0.95999221	count	1
CCDC69	0.6855323	0.1227209	5.5861	2.55E-08	0.96170264	count	0.000601596
NFKBIZ	0.6788747	0.1090823	6.2235	5.60E-10	0.962896087	count	1.33E-05
NIFK-AS1	0.9075152	0.3837791	2.3647	0.0181	0.963187599	count	1
AC025171.2	1.0266922	0.4782312	2.1469	0.0319	0.964668288	count	1
NLE1	0.9094542	0.3494687	2.6024	0.00931	0.965004228	count	1
BCAT2	0.7494609	0.2420759	3.096	0.00198	0.966054093	count	1
SLC43A1	1.2751788	0.6056158	2.1056	0.0353	0.966085654	count	1
WSCD1	0.9123274	0.3725913	2.4486	0.0144	0.967693895	count	1
NMNAT1	0.8839567	0.3711833	2.3815	0.0173	0.96803581	count	1
GMFG	0.6809195	0.0841327	8.0934	8.60E-16	0.968177928	count	2.06E-11
PIM2	1.6960899	0.8488473	1.9981	0.0458	0.968384814	count	1
MCAT	0.7835186	0.2895504	2.706	0.00685	0.96841934	count	1
AC022706.1	0.848662	0.4882788	1.7381	0.0823	0.96879374	count	1
SLC6A16	1.347852	0.7487763	1.8001	0.072	0.968864724	count	1
C6orf141	0.7574083	0.2721399	2.7832	0.00542	0.969145328	count	1
EGR1	0.6765531	0.0781114	8.6614	7.79E-18	0.969886273	count	1.87E-13
CRACR2B	0.7026293	0.1476428	4.759	2.05E-06	0.9708945	count	0.04784495
ZNF398	1.392424	0.8853087	1.5728	0.116	0.971018813	count	1
FALEC	16.1827135	3152.718183	0.0051	0.996	0.971419139	count	1
CAPN14	16.1830455	3225.835394	0.005	0.996	0.971419139	count	1
HHAT	15.8535339	802.3222269	0.0198	0.984	0.971419159	count	1
ARHGAP8	16.5737508	4421.851965	0.0037	0.997	0.971419181	count	1
AC104211.2	17.329391	1653.525609	0.0105	0.992	0.971419228	count	1
NUP62CL	16.3881543	1134.177706	0.0144	0.988	0.971419234	count	1
SEC16B	16.4944698	1175.700955	0.014	0.989	0.971419244	count	1
AC068473.5	16.6995872	2073.448024	0.0081	0.994	0.971419262	count	1
SLC6A4	17.1782243	2366.679698	0.0073	0.994	0.971419292	count	1
EDNRB	0.7477648	0.3002497	2.4905	0.0128	0.972106496	count	1
TRMT1	0.8930142	0.3224412	2.7695	0.00565	0.973275901	count	1
NFRKB	0.88945	0.4994817	1.7807	0.0751	0.973419426	count	1
NUBPL	1.1153208	0.5481296	2.0348	0.042	0.973965954	count	1
PGAP1	1.3999768	0.6380842	2.194	0.0283	0.974563238	count	1
STXBP3	0.7218354	0.1889303	3.8206	0.000136	0.97520873	count	1
SMTN	0.9072627	0.3651169	2.4849	0.013	0.975470856	count	1
HMGCR	0.8797736	0.298922	2.9432	0.00328	0.977409098	count	1
IL6R	0.9492372	0.6405859	1.4818	0.139	0.977648001	count	1
SPRY2	0.8083112	0.2598845	3.1103	0.00189	0.979850791	count	1
NUP35	0.8647643	0.31163	2.775	0.00556	0.980103144	count	1
ZNF436	0.9259905	0.5165397	1.7927	0.0731	0.980448622	count	1
SPNS2	0.7137566	0.1226817	5.818	6.64E-09	0.98047804	count	0.000156983
CCDC96	1.1116652	0.4815244	2.3086	0.021	0.981939611	count	1

MTRNR2L10	1.3743734	0.8138661	1.6887	0.0914	0.981944006	count	1
AC092683.1	1.201796	0.4624476	2.5988	0.00941	0.982669754	count	1
KRT7	0.9548927	0.4099067	2.3295	0.0199	0.982697903	count	1
ITM2A	0.6860235	0.0696977	9.8428	1.72E-22	0.982863617	count	4.15E-18
SYCP2	1.132027	1.0299081	1.0992	0.272	0.985462021	count	1
GBP5	0.8783031	0.4761261	1.8447	0.0652	0.988248039	count	1
SMAD1	0.710226	0.131058	5.4192	6.50E-08	0.988773405	count	0.00152984
RND1	0.7219203	0.2320019	3.1117	0.00188	0.989418683	count	1
OASL	0.9358223	0.4258066	2.1978	0.028	0.989590227	count	1
ENOX2	0.8538026	0.3312627	2.5774	0.01	0.991264122	count	1
ATP23	0.8211468	0.3186123	2.5773	0.01	0.991325402	count	1
GIMAP7	0.6900315	0.0548182	12.5876	2.27E-35	0.992555147	count	5.50E-31
LRRC57	0.8057826	0.2450959	3.2876	0.00102	0.99313036	count	1
TRIM14	1.1437348	0.5220895	2.1907	0.0286	0.993461376	count	1
TCTN2	1.8886416	1.1367836	1.6614	0.0967	0.993566173	count	1
KIAA1522	0.8197028	0.2786365	2.9418	0.00329	0.994253896	count	1
SETD4	1.0682191	0.4434392	2.4089	0.0161	0.996691755	count	1
ZNF30	1.4496782	1.1715763	1.2374	0.216	0.99745117	count	1
GCNT2	1.0052633	0.3891156	2.5835	0.00983	0.997453023	count	1
INPP4A	1.0484293	0.4307684	2.4339	0.015	0.997472875	count	1
CPED1	1.4067141	0.5788873	2.43	0.0152	0.997593871	count	1
PSMB8-AS1	0.7642091	0.1990146	3.84	0.000126	1.00042917	count	1
LINC00649	1.3741002	0.7469277	1.8397	0.0659	1.000517539	count	1
HSD3B7	0.7841521	0.2243113	3.4958	0.00048	1.001091295	count	1
MTHFD1	0.9173262	0.361812	2.5354	0.0113	1.004263736	count	1
INIP	0.8222929	0.2694811	3.0514	0.0023	1.004351503	count	1
VMO1	1.4668532	0.7615415	1.9262	0.0542	1.00518576	count	1
AC004839.1	1.7222892	1.1195853	1.5383	0.124	1.005570305	count	1
ARMC5	0.9245062	0.3317754	2.7865	0.00536	1.007560916	count	1
SSBP3	0.7774305	0.1959925	3.9666	7.47E-05	1.008028489	count	1
SERF1A	1.7419213	1.0879862	1.6011	0.109	1.011938341	count	1
FLI1	0.7438079	0.1504867	4.9427	8.17E-07	1.012036364	count	0.01910963
ZNRF3	1.3984798	0.6983488	2.0026	0.0453	1.012653484	count	1
EIF3C	1.535251	1.1901727	1.2899	0.197	1.012760528	count	1
CBFA2T3	1.1011391	0.4499822	2.4471	0.0145	1.012823723	count	1
CPSF7	0.899947	0.3195796	2.816	0.0049	1.013217981	count	1
MTO1	0.9194165	0.3610351	2.5466	0.0109	1.013407007	count	1
AL031058.1	1.7486453	1.0333996	1.6921	0.0907	1.014097575	count	1
CEP68	0.7488702	0.1698321	4.4095	1.08E-05	1.017112078	count	0.250506
IPP	0.9476094	0.405118	2.3391	0.0194	1.017877908	count	1
POLE4	0.716743	0.0747629	9.5869	1.95E-21	1.01856119	count	4.70E-17
MKL2	0.7231769	0.1074883	6.728	2.08E-11	1.019920124	count	4.95E-07
FAM124A	1.1523528	0.4938781	2.3333	0.0197	1.020782135	count	1
ING3	0.7801787	0.2105828	3.7049	0.000216	1.021551093	count	1
TLR3	0.8488002	0.3427757	2.4763	0.0133	1.022394978	count	1

PRKAG2	0.7816371	0.2141428	3.6501	0.000267	1.022410572	count	1
MAK16	0.8506781	0.2676512	3.1783	0.0015	1.022855108	count	1
FBXO31	0.8408011	0.3831366	2.1945	0.0283	1.024068282	count	1
SLC40A1	0.7513259	0.1760135	4.2686	2.03E-05	1.024807343	count	0.4696202
SYT7	1.1301664	0.6450563	1.752	0.0799	1.024889416	count	1
MPV17L2	0.8750539	0.2851107	3.0692	0.00217	1.025073384	count	1
U2AF2	0.8419516	0.2377683	3.5411	0.000405	1.025377628	count	1
PPIL3	0.8414534	0.3177039	2.6485	0.00813	1.026240299	count	1
NPAS3	1.630442	0.7639265	2.1343	0.0329	1.026498	count	1
HDHD3	0.929203	0.5716558	1.6255	0.104	1.026504727	count	1
AC093297.2	1.8916812	1.1504952	1.6442	0.1	1.026571175	count	1
ST6GALNAC3	0.8381302	0.2688365	3.1176	0.00184	1.027930587	count	1
CSTF1	0.8683078	0.2979168	2.9146	0.00359	1.02797241	count	1
AC018742.1	1.707475	1.0290034	1.6593	0.0972	1.028214954	count	1
AC073352.2	16.4037855	1160.551843	0.0141	0.989	1.029157749	count	1
MXRA8	16.074135	1331.524663	0.0121	0.99	1.029157778	count	1
IL10RB	16.3481819	1089.085347	0.015	0.988	1.029157816	count	1
AC010931.2	16.6178068	1217.927023	0.0136	0.989	1.029157844	count	1
NUDT13	16.776817	1144.049629	0.0147	0.988	1.029157857	count	1
FAM110B	17.424286	1488.703966	0.0117	0.991	1.029157894	count	1
SLC38A9	1.1240599	0.4814963	2.3345	0.0196	1.029778928	count	1
ISM1	2.0406482	1.0991402	1.8566	0.0635	1.031352564	count	1
TMEM135	0.9101884	0.3793209	2.3995	0.0165	1.032374417	count	1
CCNE2	2.0485466	1.0417179	1.9665	0.0493	1.033190255	count	1
DHCR24	1.2862198	0.6430926	2.0001	0.0456	1.033648815	count	1
SYDE1	1.222003	0.5488211	2.2266	0.0261	1.03370711	count	1
RTN2	0.8316156	0.2745602	3.0289	0.00248	1.033838934	count	1
TNFRSF10A	0.8158861	0.217695	3.7478	0.000182	1.033889996	count	1
PRMT7	1.117753	0.64781	1.7254	0.0846	1.034108245	count	1
CDCA4	1.0277468	0.3915688	2.6247	0.00872	1.035094939	count	1
TIAM1	1.0767666	0.5121539	2.1024	0.0356	1.035140298	count	1
ISLR2	1.4078005	0.7738631	1.8192	0.069	1.03564989	count	1
CNOT11	0.9287869	0.3016667	3.0789	0.0021	1.036082494	count	1
SLF1	0.8424968	0.2661113	3.166	0.00156	1.03814921	count	1
NOV	1.0558115	0.73584	1.4348	0.151	1.039541755	count	1
VRK1	0.8757171	0.269965	3.2438	0.00119	1.039918694	count	1
CADM1	1.4164347	0.5778479	2.4512	0.0143	1.039982051	count	1
CISH	1.667144	1.0324388	1.6148	0.106	1.04010063	count	1
PCAT19	0.763482	0.1398163	5.4606	5.17E-08	1.041372488	count	0.001217638
LINC00324	0.9168919	0.3510538	2.6118	0.00905	1.042068243	count	1
TCIRG1	0.8126697	0.2055083	3.9544	7.86E-05	1.043245068	count	1
AGK	0.9273544	0.3219216	2.8807	0.004	1.044003008	count	1
CDKN1A	0.7351528	0.0974432	7.5444	6.13E-14	1.044257704	count	1.47E-09
ABLIM3	0.8366408	0.2839538	2.9464	0.00324	1.045215399	count	1
PUS1	1.0476959	0.6457615	1.6224	0.105	1.045842043	count	1

ZNF546	1.0646848	0.5352118	1.9893	0.0468	1.04684048	count	1
FAM20A	1.5132346	0.7786314	1.9435	0.0521	1.046852332	count	1
TAF1A	1.9688205	1.0036215	1.9617	0.0499	1.047159452	count	1
PRPS2	0.914103	0.3275967	2.7903	0.0053	1.047335996	count	1
CHST15	0.9008673	0.3219252	2.7984	0.00517	1.048217436	count	1
PCGF1	0.9257949	0.3898556	2.3747	0.0176	1.048331915	count	1
DDX43	1.6902865	0.6483657	2.607	0.00918	1.048492081	count	1
NFKBIA	0.727795	0.0772866	9.4168	9.48E-21	1.048557349	count	2.28E-16
SPATA1	1.8630934	1.0903023	1.7088	0.0876	1.049185421	count	1
GKAP1	0.8279361	0.3106488	2.6652	0.00774	1.049257093	count	1
ZNF70	1.7705728	1.0712791	1.6528	0.0985	1.049344223	count	1
FAM84A	1.1511869	0.7213208	1.5959	0.111	1.049609057	count	1
ANKEF1	1.6299475	1.0511638	1.5506	0.121	1.050767646	count	1
PKP4	0.7781502	0.174243	4.4659	8.29E-06	1.050787685	count	0.19251867
ZFP36L2	0.7320622	0.0644144	11.3649	2.77E-29	1.052054426	count	6.70E-25
NYNRIN	0.9358036	0.3299119	2.8365	0.00459	1.052542704	count	1
C1orf167	1.295258	0.7930236	1.6333	0.103	1.053099092	count	1
TIMP3	0.7364075	0.1010809	7.2853	4.17E-13	1.053391689	count	9.96E-09
NRN1	0.7375122	0.0712052	10.3576	1.09E-24	1.05380652	count	2.63E-20
NR2C2AP	0.8439396	0.23576	3.5797	0.00035	1.054881206	count	1
GIMAP8	0.7527559	0.1270554	5.9246	3.52E-09	1.055312584	count	8.33E-05
RRP12	1.450656	0.751882	1.9294	0.0538	1.056919095	count	1
NINJ2	0.8686585	0.30926	2.8088	0.00501	1.057174801	count	1
TFF3	0.7844873	0.2511362	3.1238	0.0018	1.05841596	count	1
C9orf85	0.8096657	0.2198825	3.6823	0.000236	1.05869214	count	1
LINC01116	0.7852792	0.1629274	4.8198	1.51E-06	1.061775742	count	0.03526605
GIMAP6	0.7669166	0.1241291	6.1784	7.43E-10	1.061799734	count	1.76E-05
LRCH2	1.05925	0.4855788	2.1814	0.0292	1.061853334	count	1
HYKK	1.1829087	0.474863	2.4911	0.0128	1.062618857	count	1
MAML1	1.0677547	0.4336884	2.462	0.0139	1.062645126	count	1
PRPSAP1	0.7657671	0.1219022	6.2818	3.87E-10	1.063368543	count	9.18E-06
HSD17B7	0.9198513	0.348858	2.6368	0.00842	1.063512447	count	1
TAP1	0.7700651	0.1374688	5.6017	2.33E-08	1.063973777	count	0.00054981
EHBP1	0.8790309	0.2437701	3.606	0.000316	1.064318808	count	1
MAP3K8	0.7583192	0.1100543	6.8904	6.86E-12	1.065152722	count	1.64E-07
ATP5S	0.8007043	0.1870039	4.2818	1.92E-05	1.06538218	count	0.4443072
ZNF140	1.1614137	0.4338396	2.6771	0.00747	1.066380121	count	1
TTI1	1.4337411	0.7335003	1.9547	0.0507	1.066624762	count	1
KRT27	1.0146853	0.5262649	1.9281	0.0539	1.066682606	count	1
ZNF180	1.1078715	0.7640175	1.4501	0.147	1.067322522	count	1
ALDH1A2	0.7879515	0.1740631	4.5268	6.24E-06	1.067906375	count	0.14512368
ZNF142	1.829439	1.048665	1.7445	0.0812	1.068164102	count	1
PIM3	0.7462201	0.0749938	9.9504	6.08E-23	1.068762334	count	1.47E-18
DNAJC11	1.1539453	0.838946	1.3755	0.169	1.069921368	count	1
IRS2	2.2201883	1.2589959	1.7635	0.0779	1.070270881	count	1

SHC2	0.8634702	0.2585785	3.3393	0.000851	1.071045734	count	1
BTBD8	1.2813236	0.6790473	1.8869	0.0593	1.071287519	count	1
ZNF658	1.184668	0.4793916	2.4712	0.0135	1.073729881	count	1
PRRG4	0.9145926	0.3752601	2.4372	0.0149	1.07401306	count	1
PITPNC1	0.790256	0.1749647	4.5167	6.55E-06	1.075103849	count	0.1522482
BCAM	0.752397	0.0694813	10.8288	8.61E-27	1.075526774	count	2.08E-22
RHBDF2	1.4203487	0.6169016	2.3024	0.0214	1.077037992	count	1
POSTN	0.7632169	0.1109647	6.878	7.48E-12	1.077839515	count	1.78E-07
TMOD2	0.8321878	0.2345746	3.5476	0.000395	1.077849324	count	1
RASL10A	0.8782497	0.2783275	3.1555	0.00162	1.079346613	count	1
KCTD3	0.9237487	0.2836764	3.2563	0.00114	1.079406421	count	1
ISOC1	1.0803789	0.4355885	2.4803	0.0132	1.079612084	count	1
NSDHL	1.105522	0.4030785	2.7427	0.00613	1.080083717	count	1
GLI4	0.9674591	0.3901697	2.4796	0.0132	1.081135819	count	1
GPRC5C	0.8409233	0.2277124	3.6929	0.000226	1.081398509	count	1
ZNF883	2.1110403	1.024906	2.0597	0.0395	1.081896413	count	1
TMEM97	1.1838498	0.522892	2.264	0.0236	1.082704947	count	1
SPATA6L	1.1998218	0.4876181	2.4606	0.0139	1.084518238	count	1
LINC01513	16.0194751	1119.933302	0.0143	0.9886	1.084674173	count	1
MAST4-AS1	16.2646307	1316.307469	0.0124	0.99	1.084674212	count	1
UBD	16.3302189	1086.716507	0.015	0.988	1.084674221	count	1
ZNF426-DT	16.3907137	1320.160307	0.0124	0.99	1.084674305	count	1
GRAMD2A	16.4347898	1180.040952	0.0139	0.989	1.08467431	count	1
TCHH	17.0718414	1575.076516	0.0108	0.991	1.084674366	count	1
PHLDB2	0.8364362	0.2916409	2.868	0.00416	1.085543865	count	1
FZD4	0.784155	0.1577528	4.9708	7.08E-07	1.087603054	count	0.016567908
LFNG	0.790566	0.1687992	4.6835	2.96E-06	1.087863149	count	0.06898872
NR2F2	0.7646585	0.0792973	9.6429	1.15E-21	1.09110854	count	2.77E-17
LRCH1	0.8581117	0.2470308	3.4737	0.000521	1.09158504	count	1
C5orf30	1.1965378	0.47697	2.5086	0.0122	1.091859009	count	1
FAM13C	0.8240391	0.2145907	3.8401	0.000126	1.09280937	count	1
SPECC1	1.0842569	0.4482481	2.4189	0.0156	1.095323689	count	1
NRP2	0.7924248	0.1455319	5.445	5.64E-08	1.096439318	count	0.001328051
NFKB2	0.9131579	0.264519	3.4521	0.000565	1.097500754	count	1
ABRAXAS1	0.84243	0.1997067	4.2183	2.54E-05	1.097617608	count	0.586994
CSTF2T	0.9031575	0.3015585	2.995	0.00277	1.09769721	count	1
RFESD	1.8376724	1.0556108	1.7409	0.0818	1.098670973	count	1
RN7SL832P	1.2503873	0.5713299	2.1886	0.0287	1.098678119	count	1
ELP1	1.4998009	0.6208198	2.4158	0.0158	1.099394673	count	1
AP001528.2	0.8213017	0.1951475	4.2086	2.65E-05	1.100101575	count	0.612309
CD93	0.7771743	0.1099028	7.0715	1.93E-12	1.100594547	count	4.61E-08
KANK3	0.7774648	0.0911498	8.5295	2.38E-17	1.100711296	count	5.71E-13
GNAI1	0.8522481	0.2381023	3.5793	0.00035	1.10073927	count	1
TRIM26	0.9420379	0.3839754	2.4534	0.0142	1.101143031	count	1
IGFBP4	0.7649264	0.0455146	16.8062	2.00E-60	1.101440279	count	4.86E-56

TRPM4	0.976981	0.4004589	2.4397	0.0148	1.103239133	count	1
FAM227B	1.2128959	0.6324855	1.9177	0.0553	1.10357778	count	1
LINC00920	1.8545426	0.7663862	2.4199	0.0156	1.104065853	count	1
DHX58	1.0323759	0.4049523	2.5494	0.0108	1.110241135	count	1
ACADS	0.8831454	0.2603678	3.3919	0.000704	1.110508077	count	1
KAT2A	1.0014597	0.410259	2.441	0.0147	1.111404611	count	1
AREG	0.9004703	0.6952499	1.2952	0.195	1.1127302	count	1
DNLZ	2.2574354	1.0373892	2.1761	0.0296	1.11361773	count	1
TMCO6	1.1780233	0.5294538	2.225	0.0262	1.11377486	count	1
MRM3	0.972151	0.3485135	2.7894	0.00532	1.115425436	count	1
SNN	1.0017524	0.3773491	2.6547	0.00798	1.118477358	count	1
SGO2	1.0723173	0.4199092	2.5537	0.0107	1.118506333	count	1
EPS8	0.7983728	0.1089103	7.3306	3.00E-13	1.118989768	count	7.17E-09
VRK2	0.9158997	0.2610039	3.5091	0.000457	1.119563631	count	1
TSPAN12	0.8178954	0.1613591	5.0688	4.27E-07	1.122749907	count	0.010004183
PMP22	0.7935944	0.0929904	8.5342	2.29E-17	1.124303601	count	5.50E-13
S100A8	1.7618166	1.2228904	1.4407	0.15	1.124612318	count	1
FGD4	0.8694943	0.2458165	3.5372	0.000411	1.12461361	count	1
TSHZ1	0.9103258	0.2719117	3.3479	0.000825	1.125376063	count	1
SKAP2	0.8270056	0.1532159	5.3976	7.33E-08	1.125803578	count	0.001724602
HOXD1	0.9637592	0.3108734	3.1002	0.00195	1.126504638	count	1
P2RX7	1.4837459	0.744824	1.9921	0.0465	1.126826511	count	1
PTGER4	0.9230764	0.3415539	2.7026	0.00692	1.130592929	count	1
CIITA	0.9550868	0.4605132	2.074	0.0382	1.132271506	count	1
OPN3	1.495018	0.7532033	1.9849	0.0473	1.132588012	count	1
PAN2	2.054661	0.9348647	2.1978	0.028	1.132694295	count	1
GBP4	0.7990103	0.1247385	6.4055	1.76E-10	1.134040489	count	4.18E-06
PROX1	1.3873396	0.6193318	2.2401	0.0252	1.135439174	count	1
AL136084.3	1.1051105	0.5784109	1.9106	0.0562	1.136558771	count	1
AL662844.4	2.209593	1.1078096	1.9946	0.0462	1.137859577	count	1
TRAF6	1.151002	0.5543828	2.0762	0.038	1.137866729	count	1
ARAP2	0.9224768	0.2807324	3.286	0.00103	1.138088003	count	1
LRRC3B	16.0152922	717.980888	0.0223	0.9822	1.138133166	count	1
SEMA4F	15.7500594	708.5564013	0.0222	0.982	1.138133187	count	1
CD274	15.7783881	728.7539913	0.0217	0.983	1.138133194	count	1
AC073349.1	15.9507541	1174.381225	0.0136	0.9892	1.138133232	count	1
CALML4	15.9705505	694.7218907	0.023	0.982	1.138133236	count	1
NT5M	16.2470601	691.2977267	0.0235	0.9813	1.138133285	count	1
LRRIQ3	16.4604295	1218.392614	0.0135	0.9892	1.138133314	count	1
SLC27A2	16.6041646	2598.491179	0.0064	0.995	1.13813333	count	1
CCDC183	16.6203842	1134.817852	0.0146	0.988	1.138133332	count	1
S100P	17.683279	2840.473366	0.0062	0.995	1.138133401	count	1
C1orf21	0.8321651	0.1493522	5.5718	2.76E-08	1.140342661	count	0.000651056
AP000692.2	1.2802983	0.7965286	1.6073	0.108	1.140465117	count	1
AGPAT4	1.0610851	0.5468044	1.9405	0.0524	1.141324601	count	1

DUS4L	1.227505	0.5656007	2.1703	0.0301	1.142069456	count	1
PLCXD3	0.8719695	0.198953	4.3828	1.22E-05	1.144108238	count	0.2828082
MAP2K6	1.2863509	0.4910541	2.6196	0.00885	1.144581195	count	1
ARMH4	0.9726657	0.4015874	2.4221	0.0155	1.144927072	count	1
PLCE1	1.1514999	0.4073819	2.8266	0.00474	1.145045536	count	1
LRP5	0.8739745	0.2199856	3.9729	7.28E-05	1.145629479	count	1
FHIT	1.4313668	0.7321221	1.9551	0.0507	1.146364477	count	1
TAP2	0.8837446	0.1932563	4.5729	5.02E-06	1.147870569	count	0.11682544
ZNF454	2.0053975	1.116181	1.7967	0.0725	1.149294869	count	1
SNAI2	1.1933468	0.5048345	2.3638	0.0182	1.149629249	count	1
GATM	1.2388127	0.5725031	2.1639	0.0306	1.150342943	count	1
ZC3H12A	0.8843156	0.2590594	3.4136	0.00065	1.15211191	count	1
UBXN10	1.5016829	0.7703527	1.9493	0.0514	1.152893007	count	1
KLHL3	0.8893355	0.3037728	2.9276	0.00344	1.153568991	count	1
ANKHD1	1.4757757	0.5677583	2.5993	0.00939	1.155445184	count	1
SOWAHC	1.1820352	0.4619313	2.5589	0.0106	1.155753469	count	1
HID1-AS1	1.012496	0.4294355	2.3577	0.0185	1.156858347	count	1
GPAT2	1.184794	0.7981364	1.4845	0.138	1.157945419	count	1
GIMAP5	1.2509901	0.4640542	2.6958	0.00706	1.159202638	count	1
C1orf115	0.8451461	0.1489026	5.6758	1.52E-08	1.159361277	count	0.000358963
FILIP1L	0.8141116	0.1063571	7.6545	2.66E-14	1.160595775	count	6.36E-10
BIRC3	0.8518377	0.1842598	4.623	3.96E-06	1.161024401	count	0.09223236
6-Sep	0.8909038	0.1949262	4.5705	5.08E-06	1.163139654	count	0.11820652
GTF3C2	1.2449039	0.5009702	2.485	0.013	1.163754277	count	1
INTS6-AS1	1.5987848	0.7449225	2.1462	0.0319	1.16515394	count	1
MT2A	0.8193791	0.1499398	5.4647	5.05E-08	1.166490381	count	0.001189528
TFPI	0.8153835	0.0740624	11.0094	1.28E-27	1.169119635	count	3.09E-23
NOP14	0.921202	0.2322249	3.9669	7.47E-05	1.169145566	count	1
CARD10	0.8876734	0.2105885	4.2152	2.58E-05	1.170556468	count	0.5962122
ADD2	1.8963685	0.8004433	2.3691	0.0179	1.171177839	count	1
ZNF501	1.2974017	0.6319488	2.053	0.0402	1.172873162	count	1
GPRASP2	1.9027964	1.035118	1.8382	0.0661	1.173286478	count	1
FJX1	1.1552533	0.4341034	2.6612	0.00783	1.173628212	count	1
BEX2	1.0597879	0.4150646	2.5533	0.0107	1.175423652	count	1
SLC16A1-AS1	1.2883484	0.5801421	2.2207	0.0264	1.176465003	count	1
AC083862.2	1.7189497	0.9834514	1.7479	0.0806	1.17719845	count	1
SLC12A7	1.0011129	0.389947	2.5673	0.0103	1.177503394	count	1
ISLR	1.019399	0.3577969	2.8491	0.00442	1.177515712	count	1
GPRASP1	1.8412535	0.9178736	2.006	0.045	1.177656702	count	1
MRTO4	0.8882395	0.1834548	4.8417	1.36E-06	1.178050683	count	0.0317764
HOXB3	1.2418178	0.4818236	2.5773	0.01	1.178636934	count	1
SEMA3A	1.490206	0.5612343	2.6552	0.00797	1.178850845	count	1
MEPCE	1.0613687	0.3106893	3.4162	0.000644	1.18049623	count	1
CBLB	0.9356478	0.2797624	3.3444	0.000836	1.18168454	count	1
ENTPD6	1.5628155	0.7616652	2.0518	0.0403	1.1839473	count	1

EML4	0.8784505	0.1602219	5.4827	4.57E-08	1.184627062	count	0.001076783
APEX2	1.2782039	0.3935299	3.248	0.00118	1.188054484	count	1
ARHGAP28	1.278503	0.4889338	2.6149	0.00897	1.188270977	count	1
AC040162.1	15.6890875	663.4221824	0.0236	0.981	1.189681851	count	1
MACROD2	15.8185108	732.4447027	0.0216	0.983	1.189681886	count	1
GPR135	15.9083133	776.1545949	0.0205	0.984	1.189681907	count	1
FAAH	16.1435769	2390.682636	0.0068	0.995	1.189681955	count	1
SNAI3-AS1	16.5636722	1706.996048	0.0097	0.992	1.189682017	count	1
MAMDC2	16.6575295	1702.296089	0.0098	0.9922	1.189682027	count	1
KCTD1	2.1663807	1.1016289	1.9665	0.0493	1.191943787	count	1
SLC9A3	1.5147611	0.7559885	2.0037	0.0452	1.192068147	count	1
SFRP2	1.2722929	1.5485261	0.8216	0.4114	1.192706186	count	1
NSD2	1.2020293	0.5064333	2.3735	0.0177	1.192948861	count	1
C16orf45	0.8750247	0.1668438	5.2446	1.68E-07	1.193130415	count	0.00394464
TBKBP1	1.2115673	0.5225438	2.3186	0.0205	1.193644605	count	1
SAP30	0.85757	0.1263481	6.7874	1.39E-11	1.195293876	count	3.31E-07
PSTK	1.2237398	0.5674289	2.1566	0.0311	1.196071416	count	1
PRKAB1	1.1489534	0.4478878	2.5653	0.0104	1.196651488	count	1
IRX3	1.7738137	0.6497389	2.73	0.00637	1.199173109	count	1
PRCD	1.294047	0.8714988	1.4849	0.138	1.199478933	count	1
CYP4F12	2.3421696	1.0640088	2.2013	0.0278	1.199672724	count	1
CCRL2	1.0974332	0.3427196	3.2021	0.00138	1.200290242	count	1
STARD3	0.8871774	0.1715818	5.1706	2.50E-07	1.200740442	count	0.005866
NLRC5	1.0374183	0.433251	2.3945	0.0167	1.201370175	count	1
HLA-DRB5	0.8352933	0.0482742	17.3031	9.11E-64	1.201598717	count	2.21E-59
SPRY1	0.8420026	0.0825903	10.1949	5.52E-24	1.204374057	count	1.33E-19
SH3BP5	0.8450627	0.0748045	11.2969	5.81E-29	1.204417886	count	1.40E-24
EPHX1	0.8554078	0.1039456	8.2294	2.87E-16	1.205849512	count	6.88E-12
RAMP3	0.838838	0.0633159	13.2485	6.90E-39	1.205939272	count	1.67E-34
RMI1	1.3810336	0.4830037	2.8593	0.00428	1.207302577	count	1
AC098818.2	1.6930441	0.8153061	2.0766	0.0379	1.20769229	count	1
MYBBP1A	1.1193538	0.3118462	3.5894	0.000337	1.208419501	count	1
PLCB4	0.9089918	0.2167492	4.1937	2.83E-05	1.211790185	count	0.65373
INTU	1.0428964	0.3251546	3.2074	0.00135	1.212133546	count	1
NDC80	1.7041368	1.0119906	1.6839	0.0923	1.212521538	count	1
C11orf96	0.8564586	0.1598495	5.3579	9.11E-08	1.213223178	count	0.002142217
HELB	1.2659658	0.6360799	1.9903	0.0467	1.21328991	count	1
AKAP2	1.1057767	0.3580224	3.0886	0.00203	1.216018682	count	1
ETV7	1.5952279	0.7041673	2.2654	0.0236	1.217240447	count	1
PPP2R5A	0.9003562	0.1646756	5.4675	4.97E-08	1.219920914	count	0.001170783
AC016596.1	1.9690247	1.0264956	1.9182	0.0552	1.22071619	count	1
NDNF	1.8958324	1.1577491	1.6375	0.102	1.221221994	count	1
HSD17B11	0.8637566	0.0970307	8.9019	9.73E-19	1.221971041	count	2.34E-14
MPP1	0.9609246	0.2099681	4.5765	4.94E-06	1.22641327	count	0.1149785
INHBB	1.0275294	0.2627095	3.9113	9.40E-05	1.226713613	count	1

SLC16A11	2.0790089	1.0755476	1.933	0.0533	1.227197088	count	1
SPAG1	1.0837532	0.3079815	3.5189	0.00044	1.22888578	count	1
LCN6	0.9490056	0.2514176	3.7746	0.000164	1.231058947	count	1
SHROOM1	1.3868627	0.5075518	2.7325	0.00633	1.233421469	count	1
LINC00842	2.3515791	1.1395675	2.0636	0.0392	1.234526084	count	1
ITPKC	0.9107292	0.1888232	4.8232	1.49E-06	1.234707493	count	0.0348064
WDR89	1.2735036	0.542788	2.3462	0.019	1.23483285	count	1
PMAIP1	1.0269397	0.3083557	3.3304	0.000879	1.2354723	count	1
KIF19	1.4881268	0.6648014	2.2385	0.0253	1.23556671	count	1
DNAH14	1.2334522	0.5614879	2.1968	0.0281	1.238587091	count	1
IL2RG	16.5265963	1235.513174	0.0134	0.989	1.239452187	count	1
FAM74A1	16.5299198	1158.095475	0.0143	0.9886	1.239452187	count	1
MTMR8	16.8333539	1912.152155	0.0088	0.993	1.239452221	count	1
ZNF563	17.1070215	1735.685071	0.0099	0.992	1.239452244	count	1
TNMD	16.567221	1004.998334	0.0165	0.987	1.239452275	count	1
AC092803.2	1.4966158	0.6775491	2.2089	0.0273	1.240493471	count	1
GAB1	0.9816275	0.3200618	3.067	0.00218	1.242086039	count	1
ZNF516	2.3967732	1.1015713	2.1758	0.0297	1.243951477	count	1
MYO5C	0.9475478	0.2075981	4.5643	5.23E-06	1.245636993	count	0.12169164
METTL7A	0.9130122	0.1573145	5.8037	7.22E-09	1.246710195	count	0.000170652
RNF122	1.1055256	0.3392684	3.2586	0.00113	1.247404667	count	1
LY6E-DT	1.6965283	1.064072	1.5944	0.111	1.247734703	count	1
SNTG2	1.1953803	0.441482	2.7077	0.00682	1.247890117	count	1
SYNE2	0.8764402	0.1046472	8.3752	8.65E-17	1.248228145	count	2.08E-12
ADIRF	0.8721127	0.0471779	18.4856	5.01E-72	1.255368354	count	1.22E-67
STON1	1.1170867	0.4564609	2.4473	0.0145	1.255538219	count	1
AC104506.1	1.0316889	0.2859249	3.6083	0.000314	1.25605667	count	1
RNF125	0.9300026	0.1902255	4.8889	1.07E-06	1.257439031	count	0.02501553
AC108134.2	2.1927131	1.2290267	1.7841	0.0745	1.258208497	count	1
KCNB1	1.4280945	0.6636571	2.1519	0.0315	1.260383814	count	1
KATNB1	1.6882158	0.7527333	2.2428	0.025	1.262176978	count	1
LAMA3	1.3000798	0.5137988	2.5303	0.0115	1.262975075	count	1
SPATA13	1.2024037	0.4030898	2.983	0.00288	1.264536056	count	1
GNA14	1.1238276	0.4083698	2.752	0.00596	1.265475286	count	1
KLF11	0.9644619	0.193671	4.9799	6.75E-07	1.26584594	count	0.01579635
STK33	2.1226864	1.2751112	1.6647	0.0961	1.267199008	count	1
CXorf36	0.9049092	0.1039898	8.7019	5.51E-18	1.268703173	count	1.32E-13
AMIGO2	1.0034481	0.2585151	3.8816	0.000106	1.268915811	count	1
CLEC2B	0.8922893	0.0933259	9.561	2.48E-21	1.269837721	count	5.97E-17
TRANK1	1.3325002	0.4827277	2.7604	0.00581	1.271432761	count	1
TYMP	0.9157392	0.1388577	6.5948	5.08E-11	1.271713943	count	1.21E-06
MGLL	0.8898329	0.1022486	8.7026	5.47E-18	1.271761586	count	1.31E-13
COP1	1.223063	0.3613857	3.3844	0.000723	1.271877162	count	1
ZNF366	1.2642432	0.3646784	3.4667	0.000535	1.27675198	count	1
C3orf70	1.1994458	0.436005	2.751	0.00598	1.276846176	count	1

CCSER1	1.1967252	0.4616985	2.592	0.00959	1.279155091	count	1
IRF1	0.8952511	0.0991196	9.032	3.09E-19	1.280916111	count	7.43E-15
TSPAN7	0.8952236	0.06681	13.3996	1.03E-39	1.281661529	count	2.50E-35
FAM117A	1.0846536	0.3387483	3.2019	0.00138	1.281848515	count	1
CGAS	1.0083858	0.253939	3.971	7.34E-05	1.285079713	count	1
TPD52L1	0.9124699	0.1031192	8.8487	1.55E-18	1.287294963	count	3.73E-14
ASAP3	1.3321274	0.4588266	2.9033	0.00372	1.287401614	count	1
P2RY11	15.5984163	1123.064003	0.0139	0.989	1.287562283	count	1
CAHM	16.1840452	719.318984	0.0225	0.982	1.287562523	count	1
HLA-DQB2	16.339882	1090.546254	0.015	0.988	1.287562551	count	1
CNN3	0.9022953	0.0687379	13.1266	3.15E-38	1.28832451	count	7.63E-34
C14orf28	1.8908191	0.8005142	2.362	0.0182	1.288412023	count	1
PARD6G	1.4944506	0.6468278	2.3104	0.0209	1.290601421	count	1
SYT1	1.7159029	0.8777939	1.9548	0.0507	1.292965326	count	1
SLC16A7	1.0654161	0.3083808	3.4549	0.000559	1.295228552	count	1
MPV17L	2.1304209	1.0681916	1.9944	0.0462	1.296282509	count	1
CD36	0.9488341	0.2851472	3.3275	0.000888	1.296488872	count	1
CXXC1	1.143004	0.3338139	3.4241	0.000626	1.297259529	count	1
DPP4	1.2537835	0.3562938	3.519	0.00044	1.298193143	count	1
HOXD3	1.3941428	0.6352021	2.1948	0.0283	1.298509489	count	1
EEPD1	1.1808824	0.4759398	2.4812	0.0132	1.298899891	count	1
CTTNBP2	1.4389452	0.4498521	3.1987	0.0014	1.299868452	count	1
FAM184A	1.5787245	0.6669533	2.3671	0.018	1.301076032	count	1
ENDOD1	0.9410369	0.1447912	6.4993	9.54E-11	1.303026579	count	2.27E-06
ICAM1	0.9182086	0.1186634	7.7379	1.41E-14	1.304840942	count	3.38E-10
DICER1-AS1	2.2675943	1.1428374	1.9842	0.0473	1.30613509	count	1
C2CD4C	2.5497417	1.0799123	2.3611	0.0183	1.306540596	count	1
FRY	0.9754848	0.159681	6.109	1.14E-09	1.309503418	count	2.70E-05
ZNF608	0.9912147	0.2069177	4.7904	1.75E-06	1.309714736	count	0.040852
CCDC85A	1.5491018	0.478272	3.239	0.00121	1.310961257	count	1
CNFN	2.2952857	1.1186001	2.0519	0.0403	1.313068884	count	1
BDKRB2	1.1486315	0.357924	3.2091	0.00135	1.314988046	count	1
PMS1	1.0707179	0.2658941	4.0269	5.80E-05	1.318104711	count	1
LRMP	1.297582	0.7476171	1.7356	0.0827	1.323066935	count	1
ZNF395	1.1957854	0.431454	2.7715	0.00562	1.324544615	count	1
AC090204.1	1.7115462	0.7548025	2.2675	0.0234	1.325057573	count	1
C9orf40	1.0254091	0.2486324	4.1242	3.83E-05	1.326147208	count	0.8833895
GBP2	0.9459044	0.1212806	7.7993	8.75E-15	1.326719922	count	2.10E-10
BTN3A2	0.9507454	0.1414812	6.7199	2.20E-11	1.329700858	count	5.24E-07
GBP1	0.9430748	0.1611451	5.8523	5.42E-09	1.330733388	count	0.000128167
RAPGEF3	0.9888015	0.1862427	5.3092	1.19E-07	1.330954182	count	0.0027965
RPH3AL	1.0351353	0.2431346	4.2575	2.14E-05	1.331333937	count	0.4950034
CCDC121	1.142763	0.3778836	3.0241	0.00252	1.333403529	count	1
ZFP36	0.9257878	0.0540254	17.1361	1.23E-62	1.333982329	count	2.99E-58
RIOX2	1.0675337	0.25454	4.194	2.83E-05	1.33410249	count	0.65373

GPR157	16.3757522	713.526538	0.023	0.9817	1.334120051	count	1
CLEC12A	16.3047974	768.2608393	0.0212	0.983	1.334120125	count	1
HLA-DOB	16.4757155	985.1700095	0.0167	0.987	1.334120154	count	1
KCNMB3	1.5087439	0.570028	2.6468	0.00817	1.33451062	count	1
TRPM6	1.4777909	0.6114787	2.4167	0.0157	1.336073186	count	1
CLEC1A	1.0599389	0.2445394	4.3344	1.51E-05	1.342606813	count	0.3497462
RALGDS	0.9963727	0.2228225	4.4716	8.08E-06	1.343895147	count	0.187658
NR2F2-AS1	1.5301425	0.4928337	3.1048	0.00192	1.34801777	count	1
IFIT2	0.9970504	0.2214762	4.5018	7.02E-06	1.348209097	count	0.16311672
CLDN11	1.3734363	0.7121163	1.9287	0.0539	1.349584841	count	1
AC092384.1	1.441249	0.5253991	2.7432	0.00612	1.350086169	count	1
CD14	0.9867193	0.1870881	5.2741	1.44E-07	1.350542567	count	0.003382848
CDKL1	2.3328911	1.079128	2.1618	0.0307	1.350811716	count	1
ZDHHC12	1.0716335	0.2559192	4.1874	2.91E-05	1.353338571	count	0.6720645
HMGB2	0.9583709	0.1030714	9.2981	2.82E-20	1.354524802	count	6.79E-16
ACACB	1.0704072	0.2795754	3.8287	0.000132	1.354955129	count	1
POU3F1	1.7113862	0.7574644	2.2594	0.0239	1.357100997	count	1
ITGA6	0.9560241	0.1080133	8.851	1.52E-18	1.357570604	count	3.65E-14
JHY	1.4460082	0.4729536	3.0574	0.00225	1.362465742	count	1
RASAL2	0.9960143	0.1518449	6.5594	6.43E-11	1.363765017	count	1.53E-06
GNG4	1.5610128	0.8822353	1.7694	0.0769	1.367222874	count	1
CCDC68	0.9988783	0.1394885	7.161	1.02E-12	1.371857355	count	2.43E-08
PLAC9	0.9767937	0.1329649	7.3463	2.67E-13	1.373115803	count	6.38E-09
PDE1A	15.7324571	749.9606789	0.021	0.983	1.379221959	count	1
WDR49	16.0899999	683.3986733	0.0235	0.981	1.379222062	count	1
ANKRD29	0.9908923	0.1334408	7.4257	1.49E-13	1.379351601	count	3.56E-09
CADM3	1.2424931	0.4818736	2.5785	0.00998	1.380139339	count	1
DDX58	1.0622482	0.2241589	4.7388	2.26E-06	1.38136857	count	0.05273032
MME	1.7974336	0.81508	2.2052	0.0275	1.383008847	count	1
ANGPTL1	2.110843	1.1222046	1.881	0.0601	1.389023088	count	1
GIMAP4	0.9743244	0.0724706	13.4444	5.83E-40	1.393359187	count	1.41E-35
RASA2	1.1501039	0.3293449	3.4921	0.000487	1.395465228	count	1
MBP	2.2071346	1.0235628	2.1563	0.0311	1.395475899	count	1
RASL11A	1.4247653	0.5110876	2.7877	0.00534	1.396234902	count	1
BAIAP2	1.0769004	0.2544003	4.2331	2.38E-05	1.399345346	count	0.5501846
BTN3A3	1.0908515	0.2470011	4.4164	1.04E-05	1.400930928	count	0.2412904
TTC32	1.2261408	0.2954748	4.1497	3.43E-05	1.401356037	count	0.7914382
SEMA3D	2.7217335	1.1162618	2.4383	0.0148	1.401796127	count	1
ACP5	1.185865	0.5746044	2.0638	0.0391	1.404405358	count	1
CYP39A1	1.5224531	0.5519626	2.7583	0.00585	1.406093266	count	1
HLA-DMB	1.0322589	0.1778393	5.8044	7.19E-09	1.40629545	count	0.00016995
MCUB	1.0177273	0.138328	7.3574	2.46E-13	1.40884087	count	5.88E-09
TTC21B	1.341269	0.4194227	3.1979	0.0014	1.411346111	count	1
HLA-DRB1	0.9819013	0.0455967	21.5345	3.28E-95	1.415099375	count	7.97E-91
BDKRB1	1.6690497	0.7887146	2.1162	0.0344	1.419170871	count	1

AC021054.1	2.2103915	1.3275023	1.6651	0.096	1.420721287	count	1
PTGES	16.2626116	1308.535679	0.0124	0.9901	1.422956664	count	1
PRR5	1.0948873	0.226634	4.8311	1.43E-06	1.425950203	count	0.03340766
SPARCL1	0.9927242	0.0423732	23.4281	7.23E-111	1.431229734	count	1.76E-106
PALM	1.1287806	0.2518637	4.4817	7.71E-06	1.432670709	count	0.17909559
CYTH1	1.0822392	0.2094741	5.1665	2.56E-07	1.433240126	count	0.006006016
FAM160A2	1.3310761	0.3267669	4.0735	4.76E-05	1.436882528	count	1
IGF2BP2	1.266634	0.3894165	3.2526	0.00116	1.441279456	count	1
OSBPL1A	1.0675795	0.1938611	5.5069	3.99E-08	1.442037553	count	0.000940323
AC116366.1	1.354426	0.5493036	2.4657	0.0137	1.443230188	count	1
GSAP	1.670613	0.9387701	1.7796	0.0753	1.445094331	count	1
ETV1	1.1918101	0.3059031	3.896	1.00E-04	1.448100672	count	1
PLPP6	1.7771448	0.7545635	2.3552	0.0186	1.450877214	count	1
SAMHD1	1.049704	0.1516663	6.9211	5.55E-12	1.452638144	count	1.32E-07
CPSF4	1.3711748	0.4017367	3.4131	0.000651	1.452718246	count	1
AC068870.2	1.7593421	0.7692113	2.2872	0.0223	1.455680576	count	1
MBOAT2	1.8850862	0.6939604	2.7164	0.00664	1.457281064	count	1
AC007681.1	1.7371336	0.6789445	2.5586	0.0106	1.457308635	count	1
SLC38A1	1.4409372	0.3887722	3.7064	0.000214	1.458142593	count	1
SV2B	1.8219577	0.7754179	2.3496	0.0189	1.458833565	count	1
NUDCD1	1.1004415	0.1964116	5.6027	2.32E-08	1.464401358	count	0.000547474
TUBAL3	15.683563	706.6257327	0.0222	0.9823	1.465404312	count	1
NR3C2	1.3601149	0.4518618	3.01	0.00264	1.467359703	count	1
WARS2	1.4029287	0.4820547	2.9103	0.00364	1.469154416	count	1
RAB3IL1	1.2780567	0.3439734	3.7156	0.000207	1.476525995	count	1
HOXB2	1.1621008	0.2474508	4.6963	2.78E-06	1.477775674	count	0.06481292
FAM84B	1.1609389	0.248804	4.6661	3.22E-06	1.480197751	count	0.07501634
EBF3	1.2048901	0.3339861	3.6076	0.000315	1.480457794	count	1
SYBU	1.2672436	0.2805411	4.5171	6.53E-06	1.486706662	count	0.15178985
LRRC32	1.1060443	0.1685175	6.5634	6.26E-11	1.486915027	count	1.49E-06
NFKBID	1.178975	0.2989126	3.9442	8.21E-05	1.487237927	count	1
MTERF3	1.2874745	0.395866	3.2523	0.00116	1.488774089	count	1
TNFAIP8L3	1.285465	0.345983	3.7154	0.000207	1.489541458	count	1
ADAMTS9	1.0876262	0.173238	6.2782	3.96E-10	1.491242034	count	9.39E-06
CDH26	1.5320703	0.48576	3.154	0.00163	1.491529374	count	1
TCP11L2	1.1091832	0.2218039	5.0007	6.07E-07	1.49158869	count	0.014207442
AIM2	1.4837617	0.4902322	3.0267	0.0025	1.498478327	count	1
TSPAN1	1.1792109	0.3548266	3.3233	0.000901	1.498884729	count	1
SLC10A6	1.392824	0.54125	2.5733	0.0101	1.50096003	count	1
BTN3A1	1.2101479	0.2696711	4.4875	7.50E-06	1.506249575	count	0.17424
ABCF2	16.2238434	754.8850647	0.0215	0.983	1.506638947	count	1
HOXD8	1.1098998	0.1860471	5.9657	2.75E-09	1.507091214	count	6.51E-05
AC067735.1	2.5339806	1.0637958	2.382	0.0173	1.508068623	count	1
ADAMTS4	1.1425337	0.2331548	4.9003	1.01E-06	1.510934931	count	0.02361481
PPP1R16B	1.224164	0.3299692	3.7099	0.000211	1.51093833	count	1

LINC02197	1.7502589	1.3888782	1.2602	0.2077	1.515681358	count	1
BAIAP2L1	2.0789021	0.7441562	2.7936	0.00525	1.52255455	count	1
EIF4E3	1.3320632	0.424489	3.138	0.00172	1.524106385	count	1
ODF3B	1.0968456	0.149191	7.352	2.56E-13	1.524683921	count	6.12E-09
TSPYL5	1.5945905	0.6259498	2.5475	0.0109	1.528180915	count	1
ATAD5	1.9022996	1.0158059	1.8727	0.0612	1.528588058	count	1
OSBPL5	1.4267083	0.3730275	3.8247	0.000134	1.535424206	count	1
CRHBP	1.1483728	0.2144506	5.355	9.26E-08	1.535442932	count	0.002177396
SLC25A25	1.1539741	0.1998112	5.7753	8.54E-09	1.536368833	count	0.000201783
AP1G2	1.6468014	0.6766143	2.4339	0.015	1.537161511	count	1
MYPOP	1.6791971	0.6256651	2.6839	0.00732	1.538885991	count	1
TXNIP	1.0702922	0.0653379	16.3809	1.25E-57	1.540726404	count	3.03E-53
SERPINF2	1.7944937	0.7658257	2.3432	0.0192	1.540756152	count	1
IFIT3	1.0945276	0.1947802	5.6193	2.11E-08	1.541627933	count	0.000497981
KALRN	1.4921745	0.5718486	2.6094	0.00912	1.542909736	count	1
EGR2	1.2210358	0.3690222	3.3088	0.000949	1.544732629	count	1
ASB9	1.1958764	0.2400385	4.982	6.68E-07	1.545377674	count	0.015633204
EPHA7	16.0069823	672.7528064	0.0238	0.981	1.546727345	count	1
WNT4	16.0786464	690.8709493	0.0233	0.981	1.546727366	count	1
CXCL14	16.3001408	3413.458153	0.0048	0.996	1.546727519	count	1
C1QL2	17.0890467	1027.83943	0.0166	0.9867	1.54672755	count	1
CSRP2	1.1073306	0.1685376	6.5702	5.98E-11	1.547870302	count	1.42E-06
RAB3IP	2.1451709	0.7396671	2.9002	0.00376	1.548133144	count	1
KCNK5	2.2745856	1.0830732	2.1001	0.0358	1.553451327	count	1
GPSM2	1.4668072	0.7348253	1.9961	0.046	1.56025802	count	1
PIP4K2A	1.2049052	0.2097875	5.7435	1.03E-08	1.56770453	count	0.000243317
MEOX1	1.1903835	0.2175754	5.4711	4.87E-08	1.569752744	count	0.001147323
TCEA3	1.2340929	0.2604232	4.7388	2.26E-06	1.571139065	count	0.05273032
BAALC	1.2156654	0.2845248	4.2726	2.00E-05	1.573719768	count	0.46274
LINC01117	1.5731778	0.5730097	2.7455	0.00608	1.581060121	count	1
TPD52	1.2276171	0.2330355	5.2679	1.49E-07	1.581146195	count	0.003499861
MAP3K1	1.1941369	0.254326	4.6953	2.79E-06	1.584945658	count	0.06504327
LINC01725	15.7404464	1042.312013	0.0151	0.988	1.585731913	count	1
PCLO	2.0334897	0.8031893	2.5318	0.0114	1.589753677	count	1
ROPN1L	1.6070071	0.5262387	3.0538	0.00228	1.599513141	count	1
TACSTD2	1.2805659	0.3526599	3.6312	0.000287	1.612418388	count	1
FRMD3	1.6503565	0.4861421	3.3948	0.000696	1.616296921	count	1
CA4	1.5272453	0.9702471	1.5741	0.116	1.61645799	count	1
CD74	1.1327057	0.0469639	24.1187	8.52E-117	1.633151048	count	2.07E-112
RASSF9	1.1605755	0.1351695	8.5861	1.48E-17	1.633747253	count	3.55E-13
HOPX	1.7453992	0.5980289	2.9186	0.00354	1.639398303	count	1
MCF2L	1.41502	0.4034448	3.5073	0.00046	1.640968572	count	1
TLN2	1.4690723	0.407121	3.6084	0.000314	1.655283622	count	1
RNF165	16.2749763	724.2590639	0.0225	0.982	1.660713668	count	1
AL121603.2	1.3984112	0.4635808	3.0165	0.00258	1.661890472	count	1

PDE10A	1.9263033	0.612624	3.1443	0.00168	1.662744528	count	1
DAPK1	1.5736772	0.4237548	3.7137	0.000208	1.665633842	count	1
ERI1	1.8024119	0.5668127	3.1799	0.00149	1.666568331	count	1
LINC01985	1.3152294	0.2673631	4.9193	9.20E-07	1.666685288	count	0.02151512
MYRIP	1.3076571	0.208008	6.2866	3.76E-10	1.685540969	count	8.92E-06
GMFB	1.9711865	0.7298058	2.701	0.00696	1.686754346	count	1
MAOB	1.6082109	0.3911427	4.1116	4.04E-05	1.688581085	count	0.9317048
SELE	1.1934186	0.2062988	5.7849	8.07E-09	1.695030561	count	0.00019071
TMEM273	1.2246458	0.1484025	8.2522	2.38E-16	1.696271908	count	5.71E-12
PARM1	16.00952	935.4906331	0.0171	0.986	1.696791806	count	1
PAK3	16.0800325	703.6077151	0.0229	0.9818	1.69679183	count	1
RBM11	16.2878991	717.5366734	0.0227	0.982	1.696791893	count	1
HSPB8	1.3487788	0.3398763	3.9684	7.42E-05	1.700892695	count	1
CCDC178	2.0052902	0.7650035	2.6213	0.00881	1.704550919	count	1
TSHZ2	1.2173684	0.130297	9.343	1.87E-20	1.705486811	count	4.50E-16
RAB3C	1.2858164	0.2014431	6.383	2.03E-10	1.70627728	count	4.82E-06
CSF3	1.2987223	0.4191182	3.0987	0.00196	1.715125371	count	1
RAB38	1.8924905	0.5007858	3.779	0.000161	1.721829422	count	1
SLC1A5	1.4681334	0.2917352	5.0324	5.15E-07	1.723180389	count	0.012060785
MYLK	1.2964007	0.2856421	4.5385	5.91E-06	1.725288457	count	0.13747251
ICAM4	1.4715247	0.3662712	4.0176	6.04E-05	1.73652594	count	1
SMAGP	1.2596583	0.1405789	8.9605	5.81E-19	1.739845277	count	1.40E-14
MGST1	1.6883535	0.4575359	3.6901	0.000228	1.740610727	count	1
PTK2B	1.8459146	0.5787138	3.1897	0.00144	1.749916745	count	1
RHOU	1.3075482	0.1894117	6.9032	6.28E-12	1.751590477	count	1.50E-07
LINC01197	1.3437374	0.2389112	5.6244	2.05E-08	1.751620457	count	0.000483862
SNCA	1.4144331	0.2768412	5.1092	3.46E-07	1.755762696	count	0.008109548
HLA-DMA	1.2310296	0.0886355	13.8887	1.92E-42	1.75754789	count	4.66E-38
ACCS	2.6207152	1.0618032	2.4682	0.0136	1.762730136	count	1
OLFML3	15.8406358	679.8270635	0.0233	0.9814	1.766349337	count	1
RBP4	15.6857905	783.5656845	0.02	0.984	1.766349362	count	1
FAM117B	16.1525912	718.6712986	0.0225	0.982	1.766349459	count	1
HIST1H1D	1.9937118	0.7373201	2.704	0.00689	1.769596184	count	1
ZNF385D	1.2635947	0.1175672	10.7479	2.00E-26	1.772329657	count	4.83E-22
SNCG	1.2438642	0.0731603	17.0019	9.86E-62	1.782258433	count	2.39E-57
AP002387.2	1.9821456	0.7677687	2.5817	0.00988	1.784721738	count	1
APOLD1	1.3087806	0.1360874	9.6172	1.47E-21	1.786378494	count	3.54E-17
FAR2	1.5119961	0.3801932	3.9769	7.16E-05	1.794066539	count	1
HID1	1.5815934	0.4304737	3.6741	0.000243	1.794684288	count	1
FBLN1	16.2844183	2047.319535	0.008	0.994	1.799909934	count	1
RASSF10	16.7276754	911.9144112	0.0183	0.9854	1.799910043	count	1
NBL1	1.28804	0.1222107	10.5395	1.72E-25	1.804471766	count	4.15E-21
ATF3	1.2660379	0.1143691	11.0698	6.73E-28	1.806160282	count	1.63E-23
NOCT	1.6991941	0.5871179	2.8941	0.00383	1.815457822	count	1
OTULINL	1.4161821	0.3600239	3.9336	8.58E-05	1.821458066	count	1

CXCL11	1.4515836	0.7808379	1.859	0.0631	1.822255753	count	1
CHST7	1.4153562	0.2376666	5.9552	2.93E-09	1.827901229	count	6.94E-05
GJB2	16.4736621	660.3707678	0.0249	0.9801	1.83270737	count	1
HLF	16.7920734	725.0610168	0.0232	0.9815	1.832707441	count	1
F8	1.4101881	0.238594	5.9104	3.83E-09	1.834431101	count	9.06E-05
MMRN1	1.299934	0.132007	9.8475	1.64E-22	1.834437017	count	3.95E-18
NEURL1B	1.4027394	0.2386478	5.8779	4.65E-09	1.841442362	count	0.000109986
EGLN3	1.3606723	0.2335759	5.8254	6.36E-09	1.847679796	count	0.000150369
OLFM1	1.3012537	0.1169745	11.1243	3.75E-28	1.849222287	count	9.06E-24
CA8	1.8758315	0.4732476	3.9637	7.57E-05	1.850934334	count	1
PDK4	1.3371855	0.1432183	9.3367	1.98E-20	1.855125528	count	4.77E-16
ZNF462	1.4679023	0.2885685	5.0868	3.88E-07	1.862440657	count	0.009092392
LAG3	2.7758092	1.1286404	2.4594	0.014	1.86540881	count	1
GCNT4	2.587673	1.0304028	2.5113	0.0121	1.86817396	count	1
CTSH	1.3168033	0.114222	11.5285	4.57E-30	1.868694353	count	1.11E-25
SLC8A1	1.4540714	0.3431814	4.237	2.34E-05	1.869376856	count	0.541008
HES5	1.6566484	0.5261665	3.1485	0.00166	1.870667124	count	1
MYCN	2.1367842	1.0348588	2.0648	0.039	1.88058416	count	1
MINDY4	1.6272474	0.3430081	4.7441	2.20E-06	1.881537114	count	0.0513348
PDE7B	2.6377438	1.0663711	2.4736	0.0134	1.883763496	count	1
C21orf91	1.3592965	0.1532053	8.8724	1.26E-18	1.896144599	count	3.03E-14
AL035701.1	15.6370249	1056.170883	0.0148	0.9882	1.896146298	count	1
ADGRA2	1.4528493	0.2798707	5.1911	2.24E-07	1.92955047	count	0.00525728
CASZ1	2.1646197	0.9249791	2.3402	0.0193	1.949348752	count	1
MYO7A	16.0929055	700.6501268	0.023	0.9817	1.95691332	count	1
LNX1	1.9021021	0.7029758	2.7058	0.00686	1.963175604	count	1
LPCAT4	1.4360663	0.1863832	7.7049	1.81E-14	1.967413366	count	4.33E-10
CD83	1.6595828	0.4515259	3.6755	0.000242	1.967842079	count	1
DAAM1	1.3958976	0.1307081	10.6795	4.07E-26	1.978780037	count	9.83E-22
STARD9	1.6712649	0.4448203	3.7572	0.000175	1.979427631	count	1
L1CAM	1.9441295	0.6429434	3.0238	0.00252	1.981530203	count	1
RGMB	2.7541707	1.3482374	2.0428	0.0412	1.989516305	count	1
TMEM176B	1.7038587	0.4893814	3.4817	0.000506	2.001569964	count	1
SYT15	1.6932944	0.3366501	5.0298	5.22E-07	2.021848123	count	0.012224196
TLL1	1.6808447	0.3253313	5.1666	2.55E-07	2.023976248	count	0.00598281
IFITM1	1.4195799	0.0970228	14.6314	9.50E-47	2.024072168	count	2.30E-42
DLL1	1.731442	0.4041251	4.2844	1.89E-05	2.030828572	count	0.4374594
HLA-DRA	1.4188088	0.0616974	22.9962	3.23E-107	2.043032488	count	7.85E-103
LRRIQ1	16.9905043	1119.88325	0.0152	0.988	2.043518566	count	1
ICAM5	16.9365393	700.0885495	0.0242	0.9807	2.043518664	count	1
HLA-DPB1	1.4392892	0.0769077	18.7145	1.13E-73	2.064478797	count	2.75E-69
DENND2D	3.0532719	1.2078409	2.5279	0.0115	2.068504193	count	1
ADGRG6	1.6984727	0.3249428	5.227	1.85E-07	2.07220629	count	0.00434306
MAOA	1.6026788	0.2386931	6.7144	2.28E-11	2.0723191	count	5.43E-07
GUCY1B1	2.2391504	0.7681972	2.9148	0.00359	2.087206888	count	1

AC007906.2	16.124792	760.7726837	0.0212	0.9831	2.098495304	count	1
AL033384.1	16.5256466	757.6035902	0.0218	0.983	2.098495349	count	1
C2CD4B	1.4685602	0.1817163	8.0816	9.46E-16	2.106678081	count	2.27E-11
GRAMD4	2.6477579	1.5464659	1.7121	0.087	2.108382878	count	1
ADM5	1.5094078	0.1931006	7.8167	7.65E-15	2.116537856	count	1.83E-10
C7	1.6369173	0.1911425	8.5639	1.78E-17	2.123217238	count	4.27E-13
PTPN5	1.9963188	0.5934542	3.3639	0.000779	2.146705419	count	1
SEMA6A	1.5727569	0.1955663	8.0421	1.30E-15	2.148087571	count	3.12E-11
PDIA5	1.5851506	0.1759922	9.0069	3.86E-19	2.169229981	count	9.29E-15
ANKS1A	1.8426044	0.5204146	3.5406	0.000406	2.172902241	count	1
DOC2B	1.6394385	0.2205314	7.434	1.40E-13	2.174592878	count	3.35E-09
NPR3	2.826138	1.0449104	2.7047	0.00688	2.184659132	count	1
SECTM1	2.0750239	0.5722567	3.626	0.000293	2.192120843	count	1
SESN3	1.6611986	0.2822274	5.886	4.43E-09	2.199723096	count	0.000104792
AK4	1.8316647	0.3418404	5.3582	9.10E-08	2.204334845	count	0.002140047
KL	2.8970559	1.1577947	2.5022	0.0124	2.207268142	count	1
HLA-DQB1	1.5579265	0.1237547	12.5888	2.23E-35	2.211818885	count	5.40E-31
SPHK1	1.6076125	0.1901838	8.4529	4.53E-17	2.21811582	count	1.09E-12
PDE2A	1.702302	0.2602915	6.54	7.30E-11	2.226041598	count	1.74E-06
PNMT	2.5878129	0.7454459	3.4715	0.000526	2.243171281	count	1
CBR3	1.6667939	0.2521862	6.6094	4.61E-11	2.249139082	count	1.10E-06
C2orf40	1.766749	0.3575758	4.9409	8.24E-07	2.24966669	count	0.019272536
TACR1	1.8057663	0.3395265	5.3185	1.13E-07	2.262102225	count	0.002656065
CORO2A	3.1625068	1.1653261	2.7138	0.00669	2.265743337	count	1
PLAT	1.5898673	0.1315185	12.0885	8.06E-33	2.269761586	count	1.95E-28
LRG1	1.6793807	0.3596656	4.6693	3.17E-06	2.273840202	count	0.07385783
DSG2	1.9145409	0.4511869	4.2433	2.27E-05	2.274983541	count	0.5248921
LRRC1	1.831381	0.2807451	6.5233	8.15E-11	2.279027731	count	1.94E-06
TMEM88	1.6455238	0.1749511	9.4056	1.05E-20	2.282846359	count	2.53E-16
IL17RD	16.87881	1091.133214	0.0155	0.988	2.299578079	count	1
CTSC	1.6110203	0.1142656	14.0989	1.21E-43	2.307248985	count	2.93E-39
CYSLTR1	1.7066808	0.2362678	7.2235	6.52E-13	2.307342008	count	1.56E-08
HLA-DPA1	1.6054366	0.0707931	22.6779	1.48E-104	2.310302444	count	3.60E-100
RGS16	1.6221448	0.159564	10.1661	7.35E-24	2.328321333	count	1.77E-19
LHX6	2.1348862	0.4915679	4.343	1.46E-05	2.339739223	count	0.3381944
CCL14	1.6664746	0.1130707	14.7383	2.20E-47	2.352897965	count	5.34E-43
EBF1	2.007401	0.3742101	5.3644	8.80E-08	2.415791928	count	0.00206976
TESC	1.7241786	0.1622322	10.6278	6.94E-26	2.416887475	count	1.68E-21
GPM6A	1.9000011	0.3075754	6.1774	7.48E-10	2.422260874	count	1.77E-05
CPVL	1.8610719	0.2864202	6.4977	9.64E-11	2.440063055	count	2.29E-06
TPO	2.5822368	0.642531	4.0189	6.00E-05	2.451789096	count	1
ABCG2	1.9022802	0.2786025	6.8279	1.06E-11	2.453415749	count	2.53E-07
CCL23	1.7951564	0.4164085	4.311	1.68E-05	2.476231589	count	0.3889536
PPL	2.4788174	0.6213477	3.9894	6.80E-05	2.483715846	count	1
NOSTRIN	1.7658941	0.1437854	12.2815	8.53E-34	2.48463701	count	2.07E-29

DSC2	16.30026	480.0425002	0.034	0.973	2.49664457	count	1
PLEKHA7	2.7010069	0.8541923	3.1621	0.00158	2.504568086	count	1
AKAP12	1.8784725	0.3477755	5.4014	7.18E-08	2.548212777	count	0.001689526
AVPR2	1.9344862	0.2688849	7.1945	8.04E-13	2.549173642	count	1.92E-08
CFD	2.3757898	2.1947817	1.0825	0.2791	2.590785696	count	1
PGM5-AS1	2.5181421	0.8411521	2.9937	0.00278	2.652194526	count	1
TAGAP	3.2905107	1.1023115	2.9851	0.00286	2.668288991	count	1
C1QTNF9	16.2951576	415.1515496	0.0393	0.9687	2.669999241	count	1
PERP	1.932561	0.1741924	11.0944	5.17E-28	2.714510764	count	1.25E-23
AKR1C2	2.1556176	0.4469758	4.8227	1.49E-06	2.726063998	count	0.0348064
RASA4	1.979035	0.22316	8.8682	1.31E-18	2.736208216	count	3.15E-14
TNFSF9	2.10402	0.4865239	4.3246	1.58E-05	2.753295738	count	0.3658648
AKR1C1	2.0674796	0.2605432	7.9353	3.02E-15	2.827942563	count	7.23E-11
PCDH19	2.6606202	0.7337784	3.6259	0.000293	2.834850225	count	1
FGF11	3.1366339	1.2217471	2.5673	0.0103	2.877467808	count	1
MEOX2	2.1434058	0.2461009	8.7095	5.16E-18	2.907229787	count	1.24E-13
SH3BGRL2	2.204275	0.276819	7.9629	2.43E-15	2.927982249	count	5.82E-11
TMEM176A	2.8061656	0.8719231	3.2184	0.0013	2.946988654	count	1
CYGB	2.4953529	0.4532871	5.505	4.03E-08	3.023906274	count	0.00094971
HLA-DOA	2.6218398	0.5293939	4.9525	7.77E-07	3.072435549	count	0.018176361
PRKAG2-AS1	16.5383351	412.5679922	0.0401	0.968	3.221359956	count	1
SCARB1	2.6976102	0.515471	5.2333	1.79E-07	3.272129886	count	0.004202383
STC1	2.3771056	0.225871	10.5242	2.01E-25	3.375508163	count	4.85E-21
DOCK11	4.0659912	1.1290709	3.6012	0.000322	3.429395336	count	1
ACKR1	2.455559	0.1223453	20.0707	9.40E-84	3.533927825	count	2.28E-79
GABRA2	16.7974686	504.5740619	0.0333	0.973	3.637975553	count	1
ELOVL7	3.5644515	0.7709427	4.6235	3.95E-06	3.655693569	count	0.0920034
CXCL10	2.8210981	1.9493698	1.4472	0.148	3.688322094	count	1
HGF	17.0638014	480.5248833	0.0355	0.9717	3.692598786	count	1
HLA-DQA1	2.6402895	0.2339352	11.2864	6.51E-29	3.722708	count	1.57E-24
SEMA3E	4.2609449	1.0458166	4.0743	4.75E-05	3.748341768	count	1
NTS	16.8195852	946.4827049	0.0178	0.9858	3.884696162	count	1
MLPH	3.6311874	0.6642205	5.4668	4.99E-08	3.937819304	count	0.001175444
IL6	3.0676273	0.5282481	5.8072	7.08E-09	4.117609721	count	0.000167357
CADM3-AS1	4.4317353	1.2175843	3.6398	0.000278	4.207177102	count	1
