# Supplementary material for: Decoding the transcriptome of calcified atherosclerotic plaque at single-cell resolution
Source: Commun Biol. 2022 Oct 12;5:1084. doi: 10.1038/s42003-022-04056-7 (PMC9556750; doi:10.1038/s42003-022-04056-7)
Supplement: Supplementary file 11 — Supplementary Data 9 [file 42003_2022_4056_MOESM11_ESM.pdf]

							Contigency Table Values Used In Overlap Analysis (Fisher Exact Test)<br>Coordinate 1 - Network; Non-DE Genes = 1, DE Genes = 2<br>Coordinate 2 - Module; Non-DE Genes = 1, DE Genes = 2			
module_id	no_genes	eigen_pct_expl	cond_cor	cond_cor_pval	de_overlap_est	de_overlap_pval	cont_mat_11	cont_mat_12	cont_mat_21	cont_mat_22
1	318	10.94	-0.5437	6.00E-213	10.3617	2.38E-69	6522	175	514	143
2	14	59.96	0.3501	7.30E-81	134.9992	2.66E-13	6696	1	644	13
35	89	11.33	-0.3275	1.90E-70	5.3817	5.00E-11	6638	59	627	30
36	63	13.75	-0.0261	0.085	4.8567	2.67E-07	6654	43	637	20
6	123	6.28	-0.2089	5.80E-29	2.946	8.10E-06	6601	96	630	27
3	19	39.38	-0.0953	2.60E-07	7.4882	0.0001205	6686	11	649	8
18	117	5.54	-0.3021	9.00E-60	1.7522	0.03017	6597	100	640	17
29	221	4.97	-0.2374	5.00E-37	1.312	0.1288	6501	196	632	25
32	175	4.94	-0.2901	4.50E-55	1.2486	0.2167	6541	156	638	19
13	111	4.97	-0.2304	5.90E-35	1.2399	0.2868	6598	99	645	12
24	177	4.53	-0.2302	6.90E-35	1.0133	0.5194	6536	161	641	16
16	470	4.14	-0.1889	6.20E-24	0.9725	0.591	6268	429	616	41
10	190	4.5	-0.1889	6.20E-24	0.8068	0.8131	6521	176	643	14
39	108	4.86	-0.1875	1.30E-23	0.7033	0.8602	6596	101	650	7
38	400	4.5	-0.2336	7.00E-36	0.8182	0.871	6327	370	627	30
25	204	4.85	-0.2394	1.20E-37	0.7457	0.8834	6507	190	643	14
12	193	4.97	-0.1885	7.60E-24	0.7309	0.8912	6517	180	644	13
19	444	4.16	-0.2305	5.60E-35	0.7811	0.9229	6285	412	625	32
28	123	5.12	-0.1662	7.10E-19	0.611	0.9319	6581	116	650	7
17	156	4.85	-0.196	1.20E-25	0.6189	0.9462	6550	147	648	9
34	160	4.88	-0.2308	4.70E-35	0.6021	0.9556	6546	151	648	9
11	179	5.09	-0.2391	1.40E-37	0.5971	0.9648	6528	169	647	10
23	245	3.69	-0.1856	3.70E-23	0.6095	0.9777	6466	231	643	14
31	98	4.57	-0.2371	6.20E-37	0.4303	0.9798	6603	94	653	4
15	299	4.09	-0.2027	2.40E-27	0.6043	0.987	6415	282	640	17
20	233	4.29	-0.2059	3.60E-28	0.5452	0.9898	6476	221	645	12
9	126	5.04	-0.1968	7.50E-26	0.4168	0.9904	6576	121	652	5
22	128	4.67	-0.1832	1.30E-22	0.4099	0.9916	6574	123	652	5
37	311	4.48	-0.1744	1.20E-20	0.5785	0.9924	6403	294	640	17
4	255	4.49	-0.1893	5.00E-24	0.5385	0.993	6455	242	644	13
27	135	4.83	-0.2037	1.30E-27	0.3874	0.9947	6567	130	652	5
26	99	5.37	-0.2204	4.40E-32	0.3154	0.9948	6601	96	654	3
8	176	4.53	-0.2179	2.20E-31	0.416	0.9967	6528	169	650	7
5	245	4.3	-0.208	1.00E-28	0.4703	0.9976	6463	234	646	11
33	129	4.43	-0.1704	9.00E-20	0.3221	0.9977	6572	125	653	4
7	200	4.7	-0.2081	9.70E-29	0.309	0.9998	6503	194	651	6
14	143	4.43	-0.2062	3.10E-28	0.2149	0.9998	6557	140	654	3
30	255	4.19	-0.2514	2.00E-41	0.3643	0.9998	6451	246	648	9
21	223	4.26	-0.2548	1.50E-42	0.2753	1	6480	217	651	6
