# Supplementary material for: Decoding the transcriptome of calcified atherosclerotic plaque at single-cell resolution
Source: Commun Biol. 2022 Oct 12;5:1084. doi: 10.1038/s42003-022-04056-7 (PMC9556750; doi:10.1038/s42003-022-04056-7)
Supplement: Supplementary file 12 — Supplementary Data 10 [file 42003_2022_4056_MOESM12_ESM.pdf]

STable14.ECNet.Module.Genes.Rev2

gene_short_nar	gene_card	module_i	gene_description	biotype	de_norm_e	de_pval	de_qval	coeff_va	strength
B3GALT6	B3GALT6	1	beta-1,3-galactosyltransferase 6	protein_codin	0.5820	0.0320	1	7.482	0.126
FAM213B	FAM213B	1	peroxiredoxin like 2B	protein_codin	-0.02736	0.885	1	3.186	0.07952
ACOT7	ACOT7	1	acyl-CoA thioesterase 7	protein_codin	-1.228	2.94E-09	0.000692	52.52	0.06236
ERRFI1	ERRFI1	1	ERBB receptor feedback inhibitor 1	protein_codin	-0.7920	0.00155	1	47.11	0.1822
DHRS3	DHRS3	1	dehydrogenase/reductase 3	protein_codin	-1.529	1.29E-17	3.1E-13	52.5	0.08236
DNAJC16	DNAJC16	1	DnaJ heat shock protein family (Hsp40) member C16	protein_codin	-0.4318	0.147	1	2.946	0.126
MFAP2	MFAP2	1	microfibril associated protein 2	protein_codin	-3.062	1.35E-20	3.25E-16	31.44	0.1754
PLA2G2A	PLA2G2A	1	phospholipase A2 group IIA	protein_codin	-2.915	0.0030	1	Inf	0.5992
CDA	CDA	1	cytidine deaminase	protein_codin	-1.792	7.41E-33	1.79E-28	43.58	0.05444
NIPAL3	NIPAL3	1	NIPA like domain containing 3	protein_codin	-0.8988	0.000482	1	52.58	0.3412
SH3BGRL3	SH3BGRL3	1	SH3 domain binding glutamate rich protein like 3	protein_codin	-0.6564	1.75E-41	4.24E-37	52.58	0.06242
TMEM222	TMEM222	1	transmembrane protein 222	protein_codin	0.2137	0.338	1	51.97	0.1509
WASF2	WASF2	1	WASP family member 2	protein_codin	0.2061	0.00596	1	51.5	0.02689
FGR	FGR	1	FGR proto-oncogene, Src family tyrosine kinase	protein_codin	0.4586	0.116	1	28.8	0.177
EPB41	EPB41	1	erythrocyte membrane protein band 4.1	protein_codin	-0.08875	0.754	1	7.835	0.1442
SNRNP40	SNRNP40	1	small nuclear ribonucleoprotein U5 subunit 40	protein_codin	0.4969	0.00218	1	6.414	0.08134
NFIA	NFIA	1	nuclear factor I A	protein_codin	-0.5147	7.27E-07	0.01701	43.2	0.02688
LEPR	LEPR	1	leptin receptor	protein_codin	-2.046	2.65E-33	6.42E-29	47.92	0.02771
CYR61	CYR61	1	cellular communication network factor 1	protein_codin	-2.026	3.28E-32	7.94E-28	44.26	0.02812
EVI5	EVI5	1	ecotropic viral integration site 5	protein_codin	-1.548	4.42E-21	1.07E-16	47.92	0.02664
BCAS2	BCAS2	1	BCAS2 pre-mRNA processing factor	protein_codin	0.3611	0.0237	1	13.35	0.118
TSPAN2	TSPAN2	1	tetraspanin 2	protein_codin	-0.8459	2.41E-06	0.05622	47.85	0.06339
CD58	CD58	1	CD58 molecule	protein_codin	-0.5859	0.000575	1	51.96	0.05472
POLR3GL	POLR3GL	1	RNA polymerase III subunit G like	protein_codin	0.1125	0.327	1	29.22	0.1409
GJA5	GJA5	1	gap junction protein alpha 5	protein_codin	-3.558	2.23E-24	5.38E-20	48.05	0.08781
CTSK	CTSK	1	cathepsin K	protein_codin	-3.385	3.77E-27	9.11E-23	40.83	0.1268
S100A10	S100A10	1	S100 calcium binding protein A10	protein_codin	0.4782	7.71E-18	1.85E-13	Inf	0.1334
S100A6	S100A6	1	S100 calcium binding protein A6	protein_codin	-1.257	2.25E-145	5.47E-141	Inf	0.03195
S100A4	S100A4	1	S100 calcium binding protein A4	protein_codin	-4.57	7.58E-69	1.84E-64	Inf	0.3945
PMVK	PMVK	1	phosphomevalonate kinase	protein_codin	-0.07575	0.696	1	40.49	0.08882
NAXE	NAXE	1	NAD(P)HX epimerase	protein_codin	-0.805	1.59E-08	0.000375	52.58	0.06842
ITLN1	ITLN1	1	intelectin 1	protein_codin	-6.03	3.21E-56	7.79E-52	NA	0.5614
TSTD1	TSTD1	1	thiosulfate sulfurtransferase like domain containing 1	protein_codin	-4.614	1.1E-15	2.64E-11	27.86	0.1172
SFT2D2	SFT2D2	1	SFT2 domain containing 2	protein_codin	-0.6111	0.00224	1	7.987	0.2256
F5	F5	1	coagulation factor V	protein_codin	-5.268	2.17E-19	5.22E-15	20.57	0.1781
SELP	SELP	1	selectin P	protein_codin	-2.127	1.39E-72	3.38E-68	52.07	0.03327
SELL	SELL	1	selectin L	protein_codin	-4.128	3.27E-55	7.94E-51	29.18	0.1679
FMO2	FMO2	1	flavin containing dimethylaniline monoxygenase 2	protein_codin	-1.946	1.23E-10	2.92E-06	52.54	0.2672
HMCN1	HMCN1	1	hemicentin 1	protein_codin	-4.574	7.04E-42	1.71E-37	52.58	0.1186
C1orf27	C1orf27	1	odr-4 GPCR localization factor homolog	protein_codin	NA	NA	NA	8.168	0.2592
BTG2	BTG2	1	BTG anti-proliferation factor 2	protein_codin	0.3572	0.00126	1	52.58	0.09580
FMOD	FMOD	1	fibromodulin	protein_codin	-2.752	7.1E-22	1.71E-17	43.83	0.05407
PRELP	PRELP	1	proline and arginine rich end leucine rich repeat protein	protein_codin	-3.702	2.08E-28	5.03E-24	51.6	0.3205
SOX13	SOX13	1	SRY-box transcription factor 13	protein_codin	-0.1259	0.682	1	9.488	0.2059
PIK3C2B	PIK3C2B	1	phosphatidylinositol-4-phosphate 3-kinase catalytic subunit ty	protein_codin	-0.1422	0.506	1	32.47	0.1370
CD34	CD34	1	CD34 molecule	protein_codin	-0.6082	3.01E-10	7.15E-06	43.79	0.05984
PLXNA2	PLXNA2	1	plexin A2	protein_codin	-2.145	1.45E-22	3.5E-19	52.57	0.1141
AHCTF1	AHCTF1	1	AT-hook containing transcription factor 1	protein_codin	-0.3532	0.0886	1	18.85	0.05329
SMC6	SMC6	1	structural maintenance of chromosomes 6	protein_codin	-0.7738	0.00012	1	52.36	0.02772
GDF7	GDF7	1	growth differentiation factor 7	protein_codin	-3.248	2.04E-27	4.93E-23	40.53	0.3117
ITSN2	ITSN2	1	intersectin 2	protein_codin	-1.068	4.78E-12	1.14E-07	47.09	0.06491
SLC4A1AP	SLC4A1AP	1	solute carrier family 4 member 1 adaptor protein	protein_codin	0.4342	0.0404	1	46.68	0.1146
CRIM1	CRIM1	1	cysteine rich transmembrane BMP regulator 1	protein_codin	-1.582	4.64E-64	1.13E-59	52.07	0.1849
EFEMP1	EFEMP1	1	EGF containing fibulin extracellular matrix protein 1	protein_codin	-3.636	1.31E-76	3.18E-72	48.05	0.1587
RAB1A	RAB1A	1	RAB1A, member RAS oncogene family	protein_codin	-0.004756	0.968	1	28.41	0.1326
GMCL1	GMCL1	1	germ cell-less 1, spermatogenesis associated	protein_codin	-0.1042	0.722	1	9.549	0.2058
IL1RL1	IL1RL1	1	interleukin 1 receptor like 1	protein_codin	-1.638	1E-04	1	52.58	0.3522
FHL2	FHL2	1	four and a half LIM domains 2	protein_codin	-1.044	4.44E-05	1	43.46	0.1474
C2orf40	C2orf40	1	ECRG4 augurin precursor	protein_codin	2.25	8.24E-07	0.01927	47.8	0.2926
MALL	MALL	1	mal, T cell differentiation protein like	protein_codin	0.3375	0.0051	1	52.58	0.05507
FMNL2	FMNL2	1	formin like 2	protein_codin	-0.1172	0.688	1	8.375	0.232
HOXD-AS2	HOXD-AS2	1	HOXD cluster antisense RNA 2	antisense_RN	0.5336	0.135	1	9.586	0.1741
ITGAV	ITGAV	1	integrin subunit alpha V	protein_codin	-1.627	1.75E-14	4.19E-10	52.58	0.1187
COL3A1	COL3A1	1	collagen type III alpha 1 chain	protein_codin	-2.913	1.55E-30	3.75E-26	52.58	0.2168
FN1	FN1	1	fibronectin 1	protein_codin	-4.963	9.04E-54	2.19E-49	Inf	0.3485
SERPINE2	SERPINE2	1	serpin family E member 2	protein_codin	-3.092	1.23E-16	2.95E-12	52.54	0.3262
MTERF4	MTERF4	1	mitochondrial transcription termination factor 4	protein_codin	-0.2385	0.272	1	7.312	0.0587
ARPC4	ARPC4	1	actin related protein 2/3 complex subunit 4	protein_codin	0.1682	0.142	1	32.03	0.02682
FBLN2	FBLN2	1	fibulin 2	protein_codin	0.4172	0.00189	1	52.07	0.08442
SLC6A6	SLC6A6	1	solute carrier family 6 member 6	protein_codin	-1.845	4.51E-12	1.08E-07	36.63	0.1954
MYD88	MYD88	1	MYD88 innate immune signal transduction adaptor	protein_codin	0.4111	0.145	1	3.292	0.141
CCDC51	CCDC51	1	coiled-coil domain containing 51	protein_codin	0.7384	0.0622	1	2.662	0.2264
SEMA3F	SEMA3F	1	semaphorin 3F	protein_codin	-1.466	1.79E-14	4.28E-10	46.34	0.02922
KCTD6	KCTD6	1	potassium channel tetramerization domain containing 6	protein_codin	0.6832	0.0585	1	2.607	0.1347
CRYBG3	CRYBG3	1	crystallin beta-gamma domain containing 3	protein_codin	0.2911	0.148	1	24	0.1112
COL8A1	COL8A1	1	collagen type VIII alpha 1 chain	protein_codin	-3.563	5.8E-73	1.41E-68	52.58	0.09534
ABI3BP	ABI3BP	1	ABI family member 3 binding protein	protein_codin	-1.833	1.73E-16	4.15E-12	52.58	0.1152
BBX	BBX	1	BBX high mobility group box domain containing	protein_codin	-0.08945	0.512	1	37.33	0.1147
FSTL1	FSTL1	1	follistatin like 1	protein_codin	-1.049	4.14E-09	9.79E-05	52.58	0.105
HEG1	HEG1	1	heart development protein with EGF like domains 1	protein_codin	-1.055	2.94E-22	7.09E-19	33.78	0.02822
CP	CP	1	ceruloplasmin	protein_codin	-4.464	4.72E-41	1.14E-36	52.58	0.2795
LXN	LXN	1	latexin	protein_codin	-0.4389	0.000346	1	52.58	0.09968
ATP13A3	ATP13A3	1	ATPase 13A3	protein_codin	-2.737	3.93E-22	9.48E-19	25.67	0.1917
APOD	APOD	1	apolipoprotein D	protein_codin	-1.095	0.0056	1	52.58	0.4452
AFAP1	AFAP1	1	actin filament associated protein 1	protein_codin	0.1234	0.59	1	9.202	0.2672
ANXA3	ANXA3	1	annexin A3	protein_codin	-2.263	1.88E-27	4.54E-23	35.24	0.2712
RASGEF1B	RASGEF1B	1	RasGEF domain family member 1B	protein_codin	-1.77	7.6E-13	1.82E-08	25.92	0.05657
TSPAN5	TSPAN5	1	tetraspanin 5	protein_codin	-0.6351	0.01	1	35.96	0.2904
DKK2.00	DKK2.00	1	dickkopf WNT signaling pathway inhibitor 2	protein_codin	-5.523	8.45E-18	2.03E-13	52.06	0.192
FAT4	FAT4	1	FAT atypical cadherin 4	protein_codin	-0.1149	0.674	1	22.62	0.2602
JADE1	JADE1	1	jade family PHD finger 1	protein_codin	-0.5616	0.0099	1	26.33	0.1719
PCDH10	PCDH10	1	protocadherin 10	protein_codin	-2.663	1.4E-23	3.38E-19	47.92	0.05506
HHIP	HHIP	1	hedgehog interacting protein	protein_codin	-4.185	1.97E-13	4.71E-09	48	0.2184
PLRG1	PLRG1	1	pleiotropic regulator 1	protein_codin	0.5552	0.0051	1	6.947	0.2317
GUCY1A3	GUCY1A3	1	guanylate cyclase 1 soluble subunit alpha 1	protein_codin	NA	NA	NA	52.58	0.2632
FNIP2	FNIP2	1	folliculin interacting protein 2	protein_codin	-0.5368	0.000772	1	52.58	0.176
PALLD	PALLD	1	palladin, cytoskeletal associated protein	protein_codin	0.1181	0.652	1	47.88	0.08412
SORBS2	SORBS2	1	sorbin and SH3 domain containing 2	protein_codin	-0.7118	5.8E-05	1	52.58	0.129
ZFR	ZFR	1	zinc finger RNA binding protein	protein_codin	-0.1144	0.432	1	26.4	0.106
PLK2	PLK2	1	polo like kinase 2	protein_codin	0.4890	0.00549	1	33.74	0.234
MAP1B	MAP1B	1	microtubule associated protein 1B	protein_codin	-2.154	9.07E-27	2.19E-22	52.58	0.1161
SERINC5	SERINC5	1	serine incorporator 5	protein_codin	0.4734	0.15	1	3.312	0.08307
VCAN	VCAN	1	versican	protein_codin	0.01845	0.948	1	52.07	0.1522
MCTP1	MCTP1	1	multiple C2 and transmembrane domain containing 1	protein_codin	-0.04798	0.692	1	52.58	0.09528
LNPEP	LNPEP	1	leucyl and cystinyl aminopeptidase	protein_codin	-0.1911	0.354	1	7.637	0.2846
SRFBP1	SRFBP1	1	serum response factor binding protein 1	protein_codin	0.3589	0.0587	1	31.21	0.196
C5orf24	C5orf24	1	chromosome 5 open reading frame 24	protein_codin	0.04318	0.758	1	13.93	0.05679
FGF18	FGF18	1	fibroblast growth factor 18	protein_codin	-3.029	3.14E-10	7.45E-06	47.57	0.09154
FBXW11	FBXW11	1	F-box and WD repeat domain containing 11	protein_codin	0.02108	0.918	1	7.888	0.2009
RPL26L1	RPL26L1	1	ribosomal protein L26 like 1	protein_codin	-0.1452	0.292	1	26.68	0.08471
NSD1	NSD1	1	nuclear receptor binding SET domain protein 1	protein_codin	-0.03507	0.854	1	17.07	0.1104
SSR1	SSR1	1	signal sequence receptor subunit 1	protein_codin	-0.09835	0.522	1	25.65	0.1711
BMP6	BMP6	1	bone morphogenetic protein 6	protein_codin	-4.047	6.69E-37	1.62E-32	34.64	0.1127
TMEM14B	TMEM14B	1	transmembrane protein 14B	protein_codin	0.01460	0.878	1	26.1	0.1134
EDN1	EDN1	1	endothelin 1	protein_codin	-3.245	5.5E-92	1.34E-86	52.58	0.1779
RNF144B	RNF144B	1	ring finger protein 144B	protein_codin	0.2890	0.348	1	29.02	0.1712
ID4	ID4	1	inhibitor of DNA binding 4, HLH protein	protein_codin	-1.412	0.000110	1	43.85	0.4630
SLC39A7	SLC39A7	1	solute carrier family 39 member 7	protein_codin	-0.9881	7.69E-10	1.82E-05	17.08	0.1021
PFDN6	PFDN6	1	prefoldin subunit 6	protein_codin	-0.1929	0.328	1	6.476	0.1152
SAYSD1	SAYSD1	1	SAYSVFN motif domain containing 1	protein_codin	-0.0417	0.884	1	8.98	0.1744
DST	DST	1	dystonin	protein_codin	-0.7892	4.35E-07	0.01019	52.58	0.2211
FAM46A	FAM46A	1	terminal nucleotidyltransferase 5A	protein_codin	NA	NA	NA	52.46	0.02676
RARS2	RARS2	1	arginyl-tRNA synthetase 2, mitochondrial	protein_codin	0.6322	0.0218	1	23.38	0.1202
QRSL1	QRSL1	1	glutaminyl-tRNA amidotransferase subunit QRSL1	protein_codin	0.1688	0.565	1	8.976	0.279
HEY2	HEY2	1	hes related family bHLH transcription factor with YRPW motif	protein_codin	-2.828	4.72E-09	0.000112	24.68	0.3059
CTGF	CTGF	1	cellular communication network factor 2	protein_codin	-1.998	9.83E-44	2.38E-39	52.58	0.1441
ABRACL	ABRACL	1	ABRA C-terminal like	protein_codin	-0.271	0.0526	1	24.6	0.02679
ZBTB2	ZBTB2	1	zinc finger and BTB domain containing 2	protein_codin	0.6658	0.0551	1	2.95	0.1162
ACTB	ACTB	1	actin beta	protein_codin	0.329	3.03E-13	7.24E-09	52.58	0.1182
AC004540.2	AC004540.2	1	novel transcri	antisense_RN	-4.313	2.37E-31	5.74E-27	27.54	0.2112
CREB5	CREB5	1	cAMP responsive element binding protein 5	protein_codin	-2.16	1.53E-15	3.67E-11	11.56	0.09388
INMT	INMT	1	indolethylamine N-methyltransferase	protein_codin	-1.919	6.81E-27	1.65E-22	52.58	0.2271
AEBP1	AEBP1	1	AE binding protein 1	protein_codin	-0.9442	0.00115	1	52.58	0.4567
IGFBP3	IGFBP3	1	insulin like growth factor binding protein 3	protein_codin	-2.823	3.11E-25	7.51E-21	52.58	0.3035
SEC61G	SEC61G	1	SEC61 translocon gamma subunit	protein_codin	-0.4232	1.2E-07	0.00282	51.93	0.05676
ELN	ELN	1	elastin	protein_codin	-3.058	6.64E-82	1.61E-77	52.58	0.2489
TMEM120A	TMEM120A	1	transmembrane protein 120A	protein_codin	-2.342	1.05E-62	2.55E-58	42.66	0.06026
FGL2	FGL2	1	fibrinogen like 2	protein_codin	-2.118	2.83E-19	6.81E-15	52.07	0.2452
GNG11	GNG11	1	G protein subunit gamma 11	protein_codin	0.5459	1.51E-15	3.62E-11	47.62	0.02744
SGCE	SGCE	1	sarcoglycan epsilon	protein_codin	-2.443	1.01E-22	2.44E-19	49.74	0.2577
SERPINE1	SERPINE1	1	serpin family E member 1	protein_codin	-3.568	2.25E-56	5.46E-52	52.58	0.2041
CAV2	CAV2	1	caveolin 2	protein_codin	0.005065	0.95	1	52.58	0.02579
CAV1	CAV1	1	caveolin 1	protein_codin	-0.0005124	0.992	1	52.58	0.09263
PODXL	PODXL	1	podocalyxin like	protein_codin	-1.2	2.1E-20	5.06E-16	52.58	0.1269
BPGM	BPGM	1	bisphosphoglycerate mutase	protein_codin	-1.218	7.39E-09	0.000175	28.33	0.1129
CALD1	CALD1	1	caldesmon 1	protein_codin	-1.445	2.46E-62	5.97E-58	29.29	0.05930
HIPK2	HIPK2	1	homeodomain interacting protein kinase 2	protein_codin	-1.185	3.56E-08	0.000839	52.58	0.05490
MRPS33	MRPS33	1	mitochondrial ribosomal protein S33	protein_codin	0.06151	0.727	1	6.196	0.08357
RARRES2	RARRES2	1	retinoic acid receptor responder 2	protein_codin	-2.418	1.17E-13	2.8E-09	52.58	0.3008
BMX	BMX	1	BMX non-receptor tyrosine kinase	protein_codin	-2.798	2.45E-93	5.96E-89	52.07	0.09292
WDR13	WDR13	1	WD repeat domain 13	protein_codin	0.2558	0.0994	1	5.758	0.08614
GRIPAP1	GRIPAP1	1	GRIP1 associated protein 1	protein_codin	0.4044	0.168	1	8.667	0.2051
CCDC22	CCDC22	1	coiled-coil domain containing 22	protein_codin	0.2019	0.522	1	1.812	0.1080
ARMCX3	ARMCX3	1	armadillo repeat containing X-linked 3	protein_codin	-0.2650	0.0512	1	41.11	0.02832
TCEAL2	TCEAL2	1	transcription elongation factor A like 2	protein_codin	-1.505	6.63E-08	0.00156	41.7	0.2542
FHL1	FHL1	1	four and a half LIM domains 1	protein_codin	-0.7988	3.02E-06	0.07028	49.07	0.1832
RBMX	RBMX	1	RNA binding motif protein X-linked	protein_codin	0.3675	0.00333	1	49.57	0.1096
BGN	BGN	1	biglycan	protein_codin	-4.834	4.32E-85	1.05E-80	52.58	0.03067
FLNA	FLNA	1	filamin A	protein_codin	-0.4102	0.0032	1	52.58	0.1132
MCPH1	MCPH1	1	microcephalin 1	protein_codin	0.7079	0.0184	1	7.945	0.2541
CTSB	CTSB	1	cathepsin B	protein_codin	-0.8186	1.49E-11	3.55E-07	52.58	0.05644
SLC7A2	SLC7A2	1	solute carrier family 7 member 2	protein_codin	-3.39	3.49E-15	8.36E-11	52.58	0.1647
NRG1	NRG1	1	neuregulin 1	protein_codin	-3.711	1.91E-18	4.59E-14	47.94	0.291
SFRP1	SFRP1	1	secreted frizzled related protein 1	protein_codin	-2.83	1.45E-19	3.49E-15	51.32	0.2615
SULF1	SULF1	1	sulfatase 1	protein_codin	-4.237	4.36E-53	1.06E-48	52.58	0.03431
WWP1	WWP1	1	WW domain containing E3 ubiquitin protein ligase 1	protein_codin	-0.06093	0.702	1	36.87	0.1369
OSGIN2	OSGIN2	1	oxidative stress induced growth inhibitor family member 2	protein_codin	-1.917	9.1E-11	2.16E-06	10.9	0.137
EXT1	EXT1	1	exostosin glycosyltransferase 1	protein_codin	-2.268	8.23E-22	1.98E-17	47.98	0.0955
SCX	SCX	1	scleraxis bHLH transcription factor	protein_codin	-4.402	2.83E-28	6.84E-24	52.58	0.8958
CBWD1	CBWD1	1	COBW domain containing 1	protein_codin	0.2267	0.466	1	8.32	0.1149
JAK2	JAK2	1	Janus kinase 2	protein_codin	0.9477	0.0155	1	2.427	0.172
LINC01235	LINC01235	1	long intergenic non-protein coding RNA 1235	lincRNA	-1.658	1.25E-07	0.002937	32.26	0.3527
AL583785.1	AL583785.1	1	novel transcri	lincRNA	0.3134	0.192	1	28.75	0.1632
DDX58	DDX58	1	DExD/H-box helicase 58	protein_codin	1.381	2.26E-06	0.05272	31.54	0.1134
GALT	GALT	1	galactose-1-phosphate uridylyltransferase	protein_codin	0.1310	0.6	1	28.88	0.02830
IL11RA	IL11RA	1	interleukin 11 receptor subunit alpha	protein_codin	-2.392	1.51E-18	3.63E-14	51.26	0.2391
CCL19	CCL19	1	C-C motif chemokine ligand 19	protein_codin	0.3996	0.168	1	Inf	0.7062
TPM2	TPM2	1	tropomyosin 2	protein_codin	-3.195	3.04E-34	7.36E-30	50.52	0.3387
OGN	OGN	1	osteoglycin	protein_codin	-4.895	1.01E-26	2.44E-22	37	0.276
OMD	OMD	1	osteomodulin	protein_codin	-4.834	5.01E-46	1.22E-41	52.58	0.3099
PTGDS	PTGDS	1	prostaglandin D2 synthase	protein_codin	-2.244	2.06E-23	4.97E-19	52.58	0.3392
CLIC3	CLIC3	1	chloride intracellular channel 3	protein_codin	-2.082	3.3E-17	7.92E-13	48.05	0.1387
DENND5A	DENND5A	1	DENN domain containing 5A	protein_codin	0.06894	0.848	1	3.315	0.3052
HSD17B12	HSD17B12	1	hydroxysteroid 17-beta dehydrogenase 12	protein_codin	-0.7004	1.03E-11	2.46E-07	51.97	0.05231
C11orf96	C11orf96	1	chromosome 11 open reading frame 96	protein_codin	1.213	9.11E-08	0.002142	30.35	0.1685
PTPRJ	PTPRJ	1	protein tyrosine phosphatase receptor type J	protein_codin	-3.949	7.78E-18	1.87E-13	49.84	0.1944
APLNR	APLNR	1	apelin receptor	protein_codin	0.002676	0.992	1	51.36	0.1098
FTH1	FTH1	1	ferritin heavy chain 1	protein_codin	-0.8701	1.08E-88	2.63E-84	NA	0.1062
EFEMP2	EFEMP2	1	EGF containing fibulin extracellular matrix protein 2	protein_codin	-1.798	1.21E-12	2.89E-08	45.3	0.02890
BUD13	BUD13	1	BUD13 homolog	protein_codin	0.8949	0.0160	1	2.121	0.3502
TAGLN	TAGLN	1	transgelin	protein_codin	-1.73	2.02E-11	4.81E-07	48.05	0.412
MPZL2	MPZL2	1	myelin protein zero like 2	protein_codin	-2.644	6.62E-109	1.61E-104	52.58	0.02792
SC5D	SC5D	1	sterol-C5-desaturase	protein_codin	-1.486	3.05E-08	0.000719	52.03	0.1509
ROBO3	ROBO3	1	roundabout guidance receptor 3	protein_codin	-1.084	6.33E-06	0.1472	7.902	0.2246
ANKRD16	ANKRD16	1	ankyrin repeat domain 16	protein_codin	0.6961	0.0502	1	3.387	0.2502
CELF2	CELF2	1	CUGBP Elav-like family member 2	protein_codin	-0.7667	0.00152	1	52.48	0.1672
CCDC3	CCDC3	1	coiled-coil domain containing 3	protein_codin	-0.5091	0.00769	1	52.58	0.03026
FAM107B	FAM107B	1	family with sequence similarity 107 member B	protein_codin	-2.594	2.06E-64	5E-60	51.34	0.05696
CISD1	CISD1	1	CDGSH iron sulfur domain 1	protein_codin	-0.1299	0.315	1	51.14	0.08059
CDH23	CDH23	1	cadherin related 23	protein_codin	-0.2782	0.207	1	48.63	0.1289
PAPSS2	PAPSS2	1	3'-phosphoadenosine 5'-phosphosulfate synthase 2	protein_codin	-3.248	3.52E-20	8.47E-16	27.49	0.1519
ACTA2	ACTA2	1	actin alpha 2, smooth muscle	protein_codin	-0.9124	0.0312	1	52.54	0.7301
PDLIM1	PDLIM1	1	PDZ and LIM domain 1	protein_codin	-0.1040	0.0877	1	52.58	0.02725
CRTAC1	CRTAC1	1	cartilage acidic protein 1	protein_codin	-2.182	4.14E-108	1.01E-103	52.58	0.13
EMG1	EMG1	1	EMG1 N1-specific pseudouridine methyltransferase	protein_codin	0.5105	0.025	1	5.957	0.1426
H2AFJ	H2AFJ	1	H2A histone family member J	protein_codin	-1.258	8.55E-22	2.06E-17	47.3	0.0598
SSPN	SSPN	1	sarcospan	protein_codin	0.7841	0.0666	1	26.47	0.1392
STK38L	STK38L	1	serine/threonine kinase 38 like	protein_codin	-0.7835	3.58E-05	0.8259	35.06	0.0855
PTHLH	PTHLH	1	parathyroid hormone like hormone	protein_codin	-0.29	0.382	1	52.58	0.1976
CCDC91	CCDC91	1	coiled-coil domain containing 91	protein_codin	-0.8716	4.36E-05	1	9.115	0.1989
NR4A1	NR4A1	1	nuclear receptor subfamily 4 group A member 1	protein_codin	0.2212	0.1502	1	52.02	0.1988
MYL6B	MYL6B	1	myosin light chain 6B	protein_codin	-0.6619	1.66E-06	0.03879	34.25	0.06819
MYL6	MYL6	1	myosin light chain 6	protein_codin	-0.1172	0.00327	1	52.53	0.02842
KCNMB4	KCNMB4	1	potassium calcium-activated channel subfamily M regulatory	protein_codin	-2.957	1.16E-24	2.8E-20	41.8	0.1631
PHLDA1	PHLDA1	1	pleckstrin homology like domain family A member 1	protein_codin	-1.428	3.7E-06	0.08619	52.07	0.18
PAWR	PAWR	1	pro-apoptotic WT1 regulator	protein_codin	-1.698	6.68E-20	1.61E-15	30.78	0.1155
NDUFA12	NDUFA12	1	NADH ubiquinone oxidoreductase subunit A12	protein_codin	-0.09434	0.2607	1	25.93	0.02811
TMPO	TMPO	1	thymopoietin	protein_codin	0.0367	0.86	1	18.49	0.08222
HCFC2	HCFC2	1	host cell factor C2	protein_codin	-0.3719	0.147	1	3.022	0.2356
C12orf75	C12orf75	1	chromosome 12 open reading frame 75	protein_codin	0.0732	0.83	1	51.6	0.1557
UNG	UNG	1	uracil DNA glycosylase	protein_codin	0.7252	0.00764	1	10.57	0.1642
RNF34	RNF34	1	ring finger protein 34	protein_codin	0.4538	0.12	1	9.805	0.1226
KMT5A	KMT5A	1	lysine methyltransferase 5A	protein_codin	-0.1896	0.282	1	17.26	0.109
FLT1	FLT1	1	fms related tyrosine kinase 1	protein_codin	0.1316	0.285	1	41.03	0.0845
PCDH17	PCDH17	1	protocadherin 17	protein_codin	-1.025	1.5E-09	3.55E-05	52.53	0.0987
RNF219	RNF219	1	ORC ubiquitin ligase 1	protein_codin	-0.08434	0.726	1	9.56	0.3171
GAS6	GAS6	1	growth arrest specific 6	protein_codin	-0.9512	1E-24	2.41E-20	43.84	0.05319
CDC16	CDC16	1	cell division cycle 16	protein_codin	0.3827	0.112	1	8.436	0.05297
ANG	ANG	1	angiogenin	protein_codin	-0.1694	0.585	1	3.152	0.205
GEMIN2	GEMIN2	1	gem nuclear organelle associated protein 2	protein_codin	0.9038	0.018	1	3.176	0.2968
BMP4	BMP4	1	bone morphogenetic protein 4	protein_codin	-2.048	9.13E-34	2.21E-29	52.58	0.1424
FBXO34	FBXO34	1	F-box protein 34	protein_codin	-0.3436	0.0594	1	19.8	0.1922
RHOJ	RHOJ	1	ras homolog family member J	protein_codin	-0.03234	0.842	1	28.24	0.08267
RBM25	RBM25	1	RNA binding motif protein 25	protein_codin	-0.005427	0.952	1	43.93	0.03222
LTBP2	LTBP2	1	latent transforming growth factor beta binding protein 2	protein_codin	-4.133	4.06E-38	9.84E-34	46.73	0.2204
ITPK1	ITPK1	1	inositol-tetrakisphosphate 1-kinase	protein_codin	-0.6979	0.00772	1	11.83	0.0347
NUDT14	NUDT14	1	nudix hydrolase 14	protein_codin	-0.5831	5.16E-05	1	11.58	0.0531
MEIS2	MEIS2	1	Meis homeobox 2	protein_codin	-3.144	1.08E-41	2.62E-37	28.03	0.0956
THBS1	THBS1	1	thrombospondin 1	protein_codin	-3.418	4.34E-43	1.05E-38	48.04	0.08385
TPM1	TPM1	1	tropomyosin 1	protein_codin	-0.5369	3.75E-06	0.08725	52.58	0.04454
RAB11A	RAB11A	1	RAB11A, member RAS oncogene family	protein_codin	0.6279	1.86E-14	4.45E-10	37.18	0.05937
TSPAN3	TSPAN3	1	tetraspanin 3	protein_codin	-0.8692	4.59E-13	1.1E-08	44.98	0.05447
WDR61	WDR61	1	WD repeat domain 61	protein_codin	-0.07992	0.622	1	13.55	0.0811
C15orf40	C15orf40	1	chromosome 15 open reading frame 40	protein_codin	0.0007782	0.997	1	19.62	0.1672
MFGE8	MFGE8	1	milk fat globule-EGF factor 8 protein	protein_codin	-0.8896	7.79E-09	0.000184	26.98	0.09294
ALDH1A3	ALDH1A3	1	aldehyde dehydrogenase 1 family member A3	protein_codin	-0.5794	0.0125	1	52.58	0.05541
MCRIP2	MCRIP2	1	MAPK regulated corepressor interacting protein 2	protein_codin	-0.4761	0.0954	1	3.267	0.201
EMP2	EMP2	1	epithelial membrane protein 2	protein_codin	0.03161	0.672	1	24.29	0.08037
NDE1	NDE1	1	nudE neurodevelopment protein 1	protein_codin	0.4588	0.137	1	7.871	0.2002
C16orf62	C16orf62	1	VPS35 endosomal protein sorting factor like	protein_codin	NA	NA	NA	3.618	0.0557
KAT8	KAT8	1	lysine acetyltransferase 8	protein_codin	0.2622	0.22	1	7.126	0.1112
SIAH1	SIAH1	1	siah E3 ubiquitin protein ligase 1	protein_codin	0.6277	0.00392	1	40.68	0.2282
FTO	FTO	1	FTO alpha-ketoglutarate dependent dioxygenase	protein_codin	0.1427	0.718	1	3.475	0.2495
MMP2	MMP2	1	matrix metallopeptidase 2	protein_codin	-4.187	1.01E-64	2.45E-60	51.45	0.156
PLLP	PLLP	1	plasmolipin	protein_codin	-1.486	2.23E-10	5.29E-06	50.74	0.09621
NQO1	NQO1	1	NAD(P)H quinone dehydrogenase 1	protein_codin	-1.125	2.14E-11	5.09E-07	47.5	0.115
COG4	COG4	1	component of oligomeric golgi complex 4	protein_codin	0.2092	0.442	1	2.826	0.08779
TERF2IP	TERF2IP	1	TERF2 interacting protein	protein_codin	-0.1796	0.0586	1	41.23	0.05737
CDH13	CDH13	1	cadherin 13	protein_codin	-1.473	1.18E-16	2.83E-12	47.96	0.062
FOXC2	FOXC2	1	forkhead box C2	protein_codin	-2.733	8.56E-20	2.06E-15	28.02	0.3869
ZCCHC14	ZCCHC14	1	zinc finger CCHC-type containing 14	protein_codin	-0.05046	0.863	1	3.255	0.3741
METTL16	METTL16	1	methyltransferase like 16	protein_codin	0.1702	0.538	1	9.886	0.1972
RNASEK	RNASEK	1	ribonuclease K	protein_codin	0.409	0.0489	1	40.85	0.2507
MYH10	MYH10	1	myosin heavy chain 10	protein_codin	0.162	0.562	1	32.45	0.3277
RASD1	RASD1	1	ras related dexamethasone induced 1	protein_codin	-0.921	0.00145	1	35.45	0.3208
ALDH3A2	ALDH3A2	1	aldehyde dehydrogenase 3 family member A2	protein_codin	-1.414	5.02E-13	1.2E-08	18.86	0.09600
FLOT2	FLOT2	1	flotillin 2	protein_codin	-0.4326	0.0611	1	8.866	0.1945
ZNF207	ZNF207	1	zinc finger protein 207	protein_codin	-0.006483	0.967	1	26.69	0.02667
TMEM98	TMEM98	1	transmembrane protein 98	protein_codin	-3.228	5.19E-33	1.26E-28	50.21	0.1485
NPEPPS	NPEPPS	1	aminopeptidase puromycin sensitive	protein_codin	-1.113	1.29E-08	0.000305	19.53	0.09495
MED13	MED13	1	mediator complex subunit 13	protein_codin	0.05884	0.748	1	17.66	0.08036
WBP2	WBP2	1	WW domain binding protein 2	protein_codin	0.257	0.0831	1	29.54	0.05282
ACTG1	ACTG1	1	actin gamma 1	protein_codin	0.1001	0.0307	1	52.58	0.06032
NPLOC4	NPLOC4	1	NPL4 homolog, ubiquitin recognition factor	protein_codin	-0.7372	0.00214	1	29.82	0.1096
METRNL	METRNL	1	meteorin like, glial cell differentiation regulator	protein_codin	-0.827	1.03E-16	2.47E-12	48	0.03149
SPIRE1	SPIRE1	1	spire type actin nucleation factor 1	protein_codin	-0.2952	0.268	1	3.042	0.1857
RPRD1A	RPRD1A	1	regulation of nuclear pre-mRNA domain containing 1A	protein_codin	-0.1112	0.664	1	8.423	0.1242
NEDD4L	NEDD4L	1	NEDD4 like E3 ubiquitin protein ligase	protein_codin	-0.4064	0.172	1	11.75	0.1672
DSEL	DSEL	1	dermatan sulfate epimerase like	protein_codin	0.1774	0.517	1	26.04	0.3090
BMP2	BMP2	1	bone morphogenetic protein 2	protein_codin	-2.046	1.48E-14	3.54E-10	52.58	0.2002
JAG1	JAG1	1	jagged canonical Notch ligand 1	protein_codin	-0.906	6.32E-07	0.01479	37.18	0.09272
MYL9	MYL9	1	myosin light chain 9	protein_codin	-0.5881	3.19E-05	0.7364	52.58	0.3430
KCNK15	KCNK15	1	potassium two pore domain channel subfamily K member 15	protein_codin	-2.915	2.49E-22	6.01E-19	30.22	0.272
STK4	STK4	1	serine/threonine kinase 4	protein_codin	-0.5182	0.00662	1	24.14	0.1461
SLPI	SLPI	1	secretory leukocyte peptidase inhibitor	protein_codin	-2.843	3.88E-58	9.42E-54	52.58	0.3571
PMEPA1	PMEPA1	1	prostate transmembrane protein, androgen induced 1	protein_codin	-1.903	4.51E-30	1.09E-25	46.63	0.03106
NELFCD	NELFCD	1	negative elongation factor complex member C/D	protein_codin	0.2269	0.354	1	7.28	0.1714
PPDPF	PPDPF	1	pancreatic progenitor cell differentiation and proliferation facto	protein_codin	-0.02211	0.762	1	52.54	0.02850
TPD52L2	TPD52L2	1	TPD52 like 2	protein_codin	0.3109	0.108	1	21.15	0.2295
NDUFS7	NDUFS7	1	NADH ubiquinone oxidoreductase core subunit S7	protein_codin	-0.09341	0.414	1	47.29	0.05502
ANGPTL4	ANGPTL4	1	angiopoietin like 4	protein_codin	-1.127	3.04E-06	0.07085	52.58	0.09950
IL27RA	IL27RA	1	interleukin 27 receptor subunit alpha	protein_codin	0.9231	0.0157	1	9.752	0.2523
CPAMD8	CPAMD8	1	C3 and PZP like alpha-2-macroglobulin domain containing 8	protein_codin	-1.688	1.3E-12	3.1E-08	24.52	0.1138
C19orf60	C19orf60	1	required for excision 1-B domain containing	protein_codin	NA	NA	NA	26.35	0.05292
HOMER3	HOMER3	1	homer scaffold protein 3	protein_codin	-2.136	1.08E-34	2.62E-30	51.86	0.1312
LSR	LSR	1	lipolysis stimulated lipoprotein receptor	protein_codin	-0.7395	0.000254	1	50.93	0.1483
SPINT2	SPINT2	1	serine peptidase inhibitor, Kunitz type 2	protein_codin	-3.245	2.2E-40	5.33E-36	31.07	0.111
PPP1R14A	PPP1R14A	1	protein phosphatase 1 regulatory inhibitor subunit 14A	protein_codin	-1.512	3.96E-09	9.37E-05	52.48	0.2394
C19orf33	C19orf33	1	chromosome 19 open reading frame 33	protein_codin	-3.074	6.59E-23	1.59E-18	51.71	0.09362
LTBP4	LTBP4	1	latent transforming growth factor beta binding protein 4	protein_codin	-2.053	1.29E-50	3.13E-46	52.02	0.03362
TGFB1	TGFB1	1	transforming growth factor beta 1	protein_codin	-0.7076	2.54E-08	0.000599	52.58	0.02865
RABAC1	RABAC1	1	Rab acceptor 1	protein_codin	-0.2472	4.99E-05	1	28.83	0.0286
SNRPD2	SNRPD2	1	small nuclear ribonucleoprotein D2 polypeptide	protein_codin	0.1926	0.01	1	45.86	0.02684
TMEM160	TMEM160	1	transmembrane protein 160	protein_codin	-0.3432	0.0109	1	45.26	0.05476
EMP3	EMP3	1	epithelial membrane protein 3	protein_codin	-2.348	1.08E-51	2.62E-47	52.58	0.1970
FTL	FTL	1	ferritin light chain	protein_codin	-0.885	6.53E-111	1.59E-106	37.18	0.06861
BCL2L13	BCL2L13	1	BCL2 like 13	protein_codin	0.1129	0.698	1	3.15	0.1660
TBX1	TBX1	1	T-box transcription factor 1	protein_codin	-3.617	2.69E-28	6.5E-24	31.82	0.08416
TXNRD2	TXNRD2	1	thioredoxin reductase 2	protein_codin	-1.294	9.73E-07	0.02275	30.38	0.2287
CHCHD10	CHCHD10	1	coiled-coil-helix-coiled-coil-helix domain containing 10	protein_codin	-1.307	1.82E-14	4.36E-10	48.48	0.05547
HPS4	HPS4	1	HPS4 biogenesis of lysosomal organelles complex 3 subunit	protein_codin	-0.06860	0.83	1	3.429	0.2176
SF3A1	SF3A1	1	splicing factor 3a subunit 1	protein_codin	0.6564	0.00966	1	7.322	0.1069
MFNG	MFNG	1	MFNG O-fucosylpeptide 3-beta-N-acetylglucosaminyltransfer	protein_codin	0.411	0.0115	1	25.58	0.02796
ALG12	ALG12	1	ALG12 alpha-1,6-mannosyltransferase	protein_codin	-0.228	0.452	1	3.292	0.141
MAPK12	MAPK12	1	mitogen-activated protein kinase 12	protein_codin	0.1915	0.415	1	8.1	0.171

MRPS6	MRPS6	1	mitochondrial ribosomal protein S6	protein_codin	-0.2986	0.0599	1	21.29	0.1124
COL18A1	COL18A1	1	collagen type XVIII alpha 1 chain	protein_codin	-0.5239	4.68E-05	1	46.25	0.02662
COL6A2	COL6A2	1	collagen type VI alpha 2 chain	protein_codin	-1.226	8.36E-09	0.000198	45.9	0.3177
CD74	CD74	2	CD74 molecule	protein_codin	1.633	8.52E-117	2.07E-112	Inf	0.4868
HLA-A	HLA-A	2	major histocompatibility complex, class I, A	protein_codin	0.6109	1.55E-40	3.76E-36	52.58	0.1469
HLA-E	HLA-E	2	major histocompatibility complex, class I, E	protein_codin	0.4745	4.35E-30	1.05E-25	37.18	0.02736
HLA-C	HLA-C	2	major histocompatibility complex, class I, C	protein_codin	0.006719	0.8749	1	52.58	0.1574
HLA-B	HLA-B	2	major histocompatibility complex, class I, B	protein_codin	0.8349	2.84E-77	6.9E-73	52.58	0.2602
HLA-DRA	HLA-DRA	2	major histocompatibility complex, class II, DR alpha	protein_codin	2.042	3.23E-107	7.85E-102	52.58	0.4375
HLA-DRB5	HLA-DRB5	2	major histocompatibility complex, class II, DR beta 5	protein_codin	1.202	9.11E-64	2.21E-59	48	0.3382
HLA-DRB1	HLA-DRB1	2	major histocompatibility complex, class II, DR beta 1	protein_codin	1.415	3.28E-95	7.97E-91	52.58	0.4532
HLA-DQA1	HLA-DQA1	2	major histocompatibility complex, class II, DQ alpha 1	protein_codin	2.722	6.51E-29	1.57E-24	48.05	0.3668
HLA-DQB1	HLA-DQB1	2	major histocompatibility complex, class II, DQ beta 1	protein_codin	2.212	2.23E-35	5.4E-31	52.34	0.3052
HLA-DMA	HLA-DMA	2	major histocompatibility complex, class II, DM alpha	protein_codin	1.758	1.92E-42	4.66E-38	52.58	0.2648
HLA-DPA1	HLA-DPA1	2	major histocompatibility complex, class II, DP alpha 1	protein_codin	2.39	1.48E-104	3.6E-100	52.58	0.4362
HLA-DPB1	HLA-DPB1	2	major histocompatibility complex, class II, DP beta 1	protein_codin	2.064	1.13E-72	2.75E-68	52.58	0.327
B2M	B2M	2	beta-2-microglobulin	protein_codin	0.5082	2.55E-67	6.19E-63	NA	0.1552
MTRNR2L12	MTRNR2L1	3	MT-RNR2 like 12	protein_codin	0.3282	2.68E-05	0.6192	50.96	0.309
MTRNR2L8	MTRNR2L8	3	MT-RNR2 like 8	protein_codin	0.8579	3.91E-10	9.28E-06	46.4	0.2717
NEAT1	NEAT1	3	nuclear paraspeckle assembly transcript 1	lincRNA	-0.5891	5.88E-11	1.4E-06	52.58	0.09228
MALAT1	MALAT1	3	metastasis associated lung adenocarcinoma transcript 1	lincRNA	-0.218	2.38E-08	0.000562	NA	0.1025
NOC4L	NOC4L	3	nucleolar complex associated 4 homolog	protein_codin	0.2027	0.522	1	2.852	0.1152
CDC42BPB	CDC42BPB	3	CDC42 binding protein kinase beta	protein_codin	0.2864	0.0996	1	32.7	0.05472
CBX4	CBX4	3	chromobox 4	protein_codin	0.2021	0.518	1	25.63	0.1379
MT-ND1	MT-ND1	3	mitochondrially encoded NADH:ubiquinone oxidoreductase c	protein_codin	0.2155	7.27E-05	1	30.32	0.429
MT-ND2	MT-ND2	3	mitochondrially encoded NADH:ubiquinone oxidoreductase c	protein_codin	0.3327	8.15E-10	1.93E-05	52.58	0.4548
MT-CO1	MT-CO1	3	mitochondrially encoded cytochrome c oxidase I	protein_codin	0.6838	9.73E-51	2.36E-46	Inf	0.5635
MT-CO2	MT-CO2	3	mitochondrially encoded cytochrome c oxidase II	protein_codin	0.06002	0.16	1	52.58	0.6296
MT-ATP8	MT-ATP8	3	mitochondrially encoded ATP synthase membrane subunit 8	protein_codin	0.6754	0.0015	1	19.12	0.3015
MT-ATP6	MT-ATP6	3	mitochondrially encoded ATP synthase membrane subunit 6	protein_codin	-0.1118	0.0121	1	52.58	0.6027
MT-CO3	MT-CO3	3	mitochondrially encoded cytochrome c oxidase III	protein_codin	0.349	9.13E-13	2.18E-08	52.58	0.6246
MT-ND3	MT-ND3	3	mitochondrially encoded NADH:ubiquinone oxidoreductase c	protein_codin	0.2821	6.23E-08	0.001460	52.58	0.53
MT-ND4L	MT-ND4L	3	mitochondrially encoded NADH:ubiquinone oxidoreductase c	protein_codin	0.3372	7.22E-05	1	35.78	0.3417
MT-ND4	MT-ND4	3	mitochondrially encoded NADH:ubiquinone oxidoreductase c	protein_codin	0.1412	0.00134	1	48.05	0.6167
MT-ND5	MT-ND5	3	mitochondrially encoded NADH:ubiquinone oxidoreductase c	protein_codin	0.1815	0.000853	1	52.58	0.4082
MT-CYB	MT-CYB	3	mitochondrially encoded cytochrome b	protein_codin	0.4904	1.35E-29	3.26E-24	Inf	0.6557
PER3	PER3	4	period circadian regulator 3	protein_codin	0.2185	0.286	1	16.16	0.1097
SPEN	SPEN	4	spen family transcriptional repressor	protein_codin	-0.4599	0.0156	1	8.505	0.1761
SZRD1	SZRD1	4	SUZ RNA binding domain containing 1	protein_codin	-0.1898	0.308	1	17.52	0.1424
TMEM57	TMEM57	4	macoilin 1	protein_codin	NA	NA	NA	7.125	0.1469
ATPIF1	ATPIF1	4	ATP synthase inhibitory factor subunit 1	protein_codin	NA	NA	NA	51.48	0.0569
RBBP4	RBBP4	4	RB binding protein 4, chromatin remodeling factor	protein_codin	-0.04881	0.775	1	19.03	0.08786
PPIE	PPIE	4	peptidylprolyl isomerase E	protein_codin	0.0939	0.613	1	7.107	0.2264
EIF2B3	EIF2B3	4	eukaryotic translation initiation factor 2B subunit gamma	protein_codin	0.2676	0.329	1	7.058	0.2628
BEND5	BEND5	4	BEN domain containing 5	protein_codin	0.229	0.458	1	7.718	0.2970
MYSM1	MYSM1	4	Myb like, SWIRM and MPN domains 1	protein_codin	0.139	0.592	1	9.079	0.1828
ADGRL4	ADGRL4	4	adhesion G protein-coupled receptor L4	protein_codin	0.6182	6.34E-12	1.51E-07	52.42	0.02670
AC093157.1	AC093157.:	4	novel transcri	antisense_RN	0.1093	0.688	1	3.354	0.1756
TAF13	TAF13	4	TATA-box binding protein associated factor 13	protein_codin	0.4157	0.177	1	6.988	0.1162
WDR77	WDR77	4	WD repeat domain 77	protein_codin	0.4972	0.0962	1	3.444	0.205
APH1A	APH1A	4	aph-1 homolog A, gamma-secretase subunit	protein_codin	0.162	0.172	1	8.633	0.1385
RFX5	RFX5	4	regulatory factor X5	protein_codin	0.7457	0.0155	1	2.655	0.08619
CKS1B	CKS1B	4	CDC28 protein kinase regulatory subunit 1B	protein_codin	-0.03662	0.865	1	22.85	0.05559
ACBD6	ACBD6	4	acyl-CoA binding domain containing 6	protein_codin	-0.0554	0.732	1	16.85	0.1114
ATP2B4	ATP2B4	4	ATPase plasma membrane Ca2+ transporting 4	protein_codin	-0.1226	0.446	1	52.52	0.1103
PPP1R15B	PPP1R15B	4	protein phosphatase 1 regulatory subunit 15B	protein_codin	-0.01267	0.942	1	16.06	0.1642
RBBP5	RBBP5	4	RB binding protein 5, histone lysine methyltransferase comple	protein_codin	0.2679	0.356	1	3.193	0.2906
GPATCH2	GPATCH2	4	G-patch domain containing 2	protein_codin	0.3468	0.0717	1	18.9	0.1197
LGALS8	LGALS8	4	galectin 8	protein_codin	0.6315	0.0192	1	8.815	0.1992
TMEM18	TMEM18	4	transmembrane protein 18	protein_codin	0.4486	0.00237	1	9.594	0.07966
IAH1	IAH1	4	isoamyl acetate hydrolyzing esterase 1 (putative)	protein_codin	0.2558	0.194	1	18.08	0.2324
ADAM17	ADAM17	4	ADAM metallopeptidase domain 17	protein_codin	-0.2871	0.137	1	7.95	0.1519
NOL10	NOL10	4	nucleolar protein 10	protein_codin	0.4545	0.0958	1	3.243	0.2018
LCLAT1	LCLAT1	4	lysocardiolipin acyltransferase 1	protein_codin	-0.4037	0.155	1	9.94	0.1625
EPAS1	EPAS1	4	endothelial PAS domain protein 1	protein_codin	0.2317	0.0186	1	52.58	0.02990
CCDC88A	CCDC88A	4	coiled-coil domain containing 88A	protein_codin	-0.2298	0.266	1	32.15	0.2057
PELI1	PELI1	4	pellino E3 ubiquitin protein ligase 1	protein_codin	0.2699	0.206	1	51.96	0.2654
FAM136A	FAM136A	4	family with sequence similarity 136 member A	protein_codin	-0.4775	0.0338	1	3.534	0.199
MPHOSPH10	MPHOSPH	4	M-phase phosphoprotein 10	protein_codin	-0.01398	0.926	1	13.08	0.1425
WDR54	WDR54	4	WD repeat domain 54	protein_codin	-0.269	0.219	1	8.202	0.08607
TCF7L1	TCF7L1	4	transcription factor 7 like 1	protein_codin	0.3503	0.143	1	6.9	0.1101
ARID5A	ARID5A	4	AT-rich interaction domain 5A	protein_codin	-0.3408	0.0161	1	50.62	0.02612
ANKRD39	ANKRD39	4	ankyrin repeat domain 39	protein_codin	-0.193	0.485	1	3.114	0.1970
RANBP2	RANBP2	4	RAN binding protein 2	protein_codin	-0.2536	0.206	1	7.88	0.1168
ZC3H8	ZC3H8	4	zinc finger CCCH-type containing 8	protein_codin	-0.06482	0.786	1	5.984	0.1365
SLC35F5	SLC35F5	4	solute carrier family 35 member F5	protein_codin	0.2485	0.352	1	7.837	0.1098
ATF2	ATF2	4	activating transcription factor 2	protein_codin	0.4617	0.046	1	7.758	0.08934
NFE2L2	NFE2L2	4	nuclear factor, erythroid 2 like 2	protein_codin	-0.4088	8.12E-06	0.1886	52.58	0.02777
SESTD1	SESTD1	4	SEC14 and spectrin domain containing 1	protein_codin	-0.7816	0.00129	1	9.062	0.1984
GLS	GLS	4	glutaminase	protein_codin	0.1606	0.192	1	36.63	0.05685
PNKD	PNKD	4	PNKD metallo-beta-lactamase domain containing	protein_codin	-0.1009	0.396	1	8.858	0.1387
ZFAND2B	ZFAND2B	4	zinc finger AN1-type containing 2B	protein_codin	-0.253	0.151	1	6.784	0.1439
SP100	SP100	4	SP100 nuclear antigen	protein_codin	0.05356	0.5472	1	52.57	0.02612
LMCD1	LMCD1	4	LIM and cysteine rich domains 1	protein_codin	0.5239	6.91E-08	0.001626	52.58	0.06005
TAMM41	TAMM41	4	TAM41 mitochondrial translocator assembly and maintenance	protein_codin	0.4664	0.183	1	3.042	0.3879
SATB1	SATB1	4	SATB homeobox 1	protein_codin	0.3625	0.273	1	3.046	0.285
CRTAP	CRTAP	4	cartilage associated protein	protein_codin	0.4651	4.57E-05	1	22.53	0.0920
TMEM115	TMEM115	4	transmembrane protein 115	protein_codin	-0.3459	0.0792	1	8.132	0.1117
MANF	MANF	4	mesencephalic astrocyte derived neurotrophic factor	protein_codin	-0.3717	0.00937	1	41.82	0.08342
RBM15B	RBM15B	4	RNA binding motif protein 15B	protein_codin	0.6398	0.0291	1	8.56	0.2023
DUSP7	DUSP7	4	dual specificity phosphatase 7	protein_codin	-0.03273	0.903	1	30.85	0.2381
RYBP	RYBP	4	RING1 and YY1 binding protein	protein_codin	0.526	0.0126	1	7.266	0.05594
QTRT2	QTRT2	4	queuine tRNA-ribosyltransferase accessory subunit 2	protein_codin	0.2195	0.363	1	3.51	0.1142
MYLK	MYLK	4	myosin light chain kinase	protein_codin	1.725	5.91E-06	0.1375	32.47	0.1912
SRPRB	SRPRB	4	SRP receptor subunit beta	protein_codin	-0.49	0.00590	1	7.988	0.1121
ZBTB38	ZBTB38	4	zinc finger and BTB domain containing 38	protein_codin	-0.3168	0.0119	1	16.41	0.1385
PHC3	PHC3	4	polyhomeotic homolog 3	protein_codin	-0.1956	0.526	1	3.386	0.2799
DCUN1D1	DCUN1D1	4	defective in cullin neddylation 1 domain containing 1	protein_codin	-0.1632	0.483	1	8.829	0.08996
IGF2BP2	IGF2BP2	4	insulin like growth factor 2 mRNA binding protein 2	protein_codin	1.441	0.00116	1	9.092	0.2569
CTBP1	CTBP1	4	C-terminal binding protein 1	protein_codin	-0.08552	0.686	1	8.006	0.171
FBXL5	FBXL5	4	F-box and leucine rich repeat protein 5	protein_codin	0.4636	0.0220	1	6.933	0.1191
UBE2K	UBE2K	4	ubiquitin conjugating enzyme E2 K	protein_codin	0.06247	0.702	1	35.18	0.1769
GABRA2	GABRA2	4	gamma-aminobutyric acid type A receptor alpha2 subunit	protein_codin	3.638	0.973	1	46.41	0.2138
USP46	USP46	4	ubiquitin specific peptidase 46	protein_codin	-0.4193	0.124	1	9.722	0.1459
CLOCK	CLOCK	4	clock circadian regulator	protein_codin	-0.03837	0.874	1	26.69	0.08742
AASDH	AASDH	4	aminoadipate-semialdehyde dehydrogenase	protein_codin	0.8857	0.00235	1	7.531	0.14
SRP72	SRP72	4	signal recognition particle 72	protein_codin	0.1855	0.0765	1	23.72	0.02973
USO1	USO1	4	USO1 vesicle transport factor	protein_codin	0.1565	0.411	1	7.308	0.05392
NUDT9	NUDT9	4	nudix hydrolase 9	protein_codin	0.3377	0.189	1	23.23	0.34
PPM1K	PPM1K	4	protein phosphatase, Mg2+/Mn2+ dependent 1K	protein_codin	0.7005	0.056	1	8.532	0.191
ADH5	ADH5	4	alcohol dehydrogenase 5 (class III), chi polypeptide	protein_codin	0.0425	0.732	1	9.278	0.06187
TIFA	TIFA	4	TRAF interacting protein with forkhead associated domain	protein_codin	0.6207	0.00572	1	48.03	0.1314
INTU	INTU	4	inturned planar cell polarity protein	protein_codin	1.212	0.00135	1	10.56	0.2074
RAB33B	RAB33B	4	RAB33B, member RAS oncogene family	protein_codin	-0.1145	0.652	1	3.504	0.0829
SCOC	SCOC	4	short coiled-coil protein	protein_codin	0.2767	0.0572	1	10.85	0.1396
ANAPC10	ANAPC10	4	anaphase promoting complex subunit 10	protein_codin	0.4169	0.0587	1	6.867	0.1982
LRBA	LRBA	4	LPS responsive beige-like anchor protein	protein_codin	0.3522	0.274	1	2.808	0.21
HMGB2	HMGB2	4	high mobility group box 2	protein_codin	1.359	2.82E-20	6.79E-16	34.63	0.05978
PDLIM3	PDLIM3	4	PDZ and LIM domain 3	protein_codin	0.9566	1.15E-09	2.72E-05	29.72	0.1127
CMBL	CMBL	4	carboxymethylenebutenolidase homolog	protein_codin	-0.2918	0.188	1	9.207	0.1439
FAM105A	FAM105A	4	OTU deubiquitinase with linear linkage specificity like	protein_codin	NA	NA	NA	22.95	0.2594
ARL15.00	ARL15.00	4	ADP ribosylation factor like GTPase 15	protein_codin	0.05400	0.835	1	30.93	0.09438
IL6ST	IL6ST	4	interleukin 6 signal transducer	protein_codin	0.2427	0.00652	1	52.58	0.02652
DIMT1	DIMT1	4	DIMT1 rRNA methyltransferase and ribosome maturation factor	protein_codin	0.3641	0.068	1	7.392	0.05521
CDK7	CDK7	4	cyclin dependent kinase 7	protein_codin	0.9061	0.0115	1	3.187	0.2972
SCAMP1-AS1	SCAMP1-A	4	SCAMP1 antisense RNA 1	lincRNA	0.6662	0.052	1	3.662	0.236
SSBP2	SSBP2	4	single stranded DNA binding protein 2	protein_codin	0.8569	0.000936	1	52	0.1984
PPIP5K2	PPIP5K2	4	diphosphoinositol pentakisphosphate kinase 2	protein_codin	0.4246	0.0337	1	20.3	0.1632
FBXL17	FBXL17	4	F-box and leucine rich repeat protein 17	protein_codin	-0.8581	0.00346	1	3.352	0.08671
EPB41L4A-AS1	EPB41L4A-	4	EPB41L4A antisense RNA 1	lincRNA	0.4927	0.00325	1	50.12	0.1754
ATG12	ATG12	4	autophagy related 12	protein_codin	0.01112	0.942	1	14.1	0.1735
CDC42SE2	CDC42SE2	4	CDC42 small effector 2	protein_codin	0.5662	0.00825	1	21.05	0.05516
AFF4	AFF4	4	AF4/FMR2 family member 4	protein_codin	0.07882	0.592	1	24.53	0.08697
ECSCR	ECSCR	4	endothelial cell surface expressed chemotaxis and apoptosis	protein_codin	-0.05026	0.358	1	39.72	0.02702
NDUFA2	NDUFA2	4	NADH:ubiquinone oxidoreductase subunit A2	protein_codin	0.002885	0.979	1	14.67	0.1345
HMGXB3	HMGXB3	4	HMG-box containing 3	protein_codin	-0.1612	0.476	1	23.29	0.2115
FAM50B	FAM50B	4	family with sequence similarity 50 member B	protein_codin	0.1576	0.579	1	10.68	0.08394
C6orf141	C6orf141	4	chromosome 6 open reading frame 141	protein_codin	0.9691	0.00542	1	9.532	0.2812
GINM1	GINM1	4	glycoprotein integral membrane 1	protein_codin	-0.05167	0.583	1	17.22	0.1382
SYNJ2	SYNJ2	4	synaptojanin 2	protein_codin	-0.3628	0.26	1	9.853	0.08661
ACAT2	ACAT2	4	acetyl-CoA acetyltransferase 2	protein_codin	-0.05369	0.83	1	7.704	0.1099
ARL4A	ARL4A	4	ADP ribosylation factor like GTPase 4A	protein_codin	0.9032	1.49E-16	3.58E-12	52.58	0.05796
MALSU1	MALSU1	4	mitochondrial assembly of ribosomal large subunit 1	protein_codin	0.3515	0.072	1	17.06	0.05932
RALA	RALA	4	RAS like proto-oncogene A	protein_codin	-0.4982	6.63E-05	1	50.88	0.05786
PURB	PURB	4	purine rich element binding protein B	protein_codin	-0.08272	0.638	1	7.6	0.2552
GRB10	GRB10	4	growth factor receptor bound protein 10	protein_codin	0.8986	0.000537	1	40.29	0.1681
ABCB1	ABCB1	4	ATP binding cassette subfamily B member 1	protein_codin	-0.3827	0.124	1	29.76	0.02612
DLD	DLD	4	dihydrolipoamide dehydrogenase	protein_codin	0.4666	0.0296	1	6.879	0.08829
FLNC	FLNC	4	filamin C	protein_codin	0.3996	0.159	1	8.892	0.3397
ZC3HAV1	ZC3HAV1	4	zinc finger CCCH-type containing, antiviral 1	protein_codin	0.5127	0.00317	1	52.58	0.06442
GSTK1	GSTK1	4	glutathione S-transferase kappa 1	protein_codin	0.5714	1.05E-09	2.49E-05	43.23	0.05288
GIMAP8	GIMAP8	4	GTPase, IMAP family member 8	protein_codin	1.059	3.52E-09	8.33E-05	32.63	0.1381
DNAJB6	DNAJB6	4	DnaJ heat shock protein family (Hsp40) member B6	protein_codin	0.2555	0.0231	1	29.35	0.1176
PTPRN2	PTPRN2	4	protein tyrosine phosphatase receptor type N2	protein_codin	0.5319	0.0381	1	7.862	0.08812
PRKX	PRKX	4	protein kinase X-linked	protein_codin	-0.4982	0.0897	1	2.872	0.08358
MED14	MED14	4	mediator complex subunit 14	protein_codin	0.1624	0.599	1	3.292	0.1369
TAF1	TAF1	4	TATA-box binding protein associated factor 1	protein_codin	0.5758	0.0987	1	7.409	0.1116
BEX3	BEX3	4	brain expressed X-linked 3	protein_codin	-0.6586	2.4E-12	5.73E-08	45.36	0.02725
ALG13	ALG13	4	ALG13 UDP-N-acetylglucosaminyltransferase subunit	protein_codin	-0.1788	0.258	1	16.47	0.1646
IL13RA1	IL13RA1	4	interleukin 13 receptor subunit alpha 1	protein_codin	-0.2095	0.167	1	28.38	0.08396
RNF113A	RNF113A	4	ring finger protein 113A	protein_codin	0.5666	0.0424	1	9.012	0.267
CUL4B	CUL4B	4	cullin 4B	protein_codin	-0.03041	0.911	1	32.92	0.1141
SLC10A3	SLC10A3	4	solute carrier family 10 member 3	protein_codin	0.1631	0.542	1	9.077	0.1662
SORBS3	SORBS3	4	sorbin and SH3 domain containing 3	protein_codin	0.5375	0.00429	1	6.693	0.1412
KCTD9	KCTD9	4	potassium channel tetramerization domain containing 9	protein_codin	-0.2626	0.309	1	8.555	0.08308
SARAF	SARAF	4	store-operated calcium entry associated regulatory factor	protein_codin	-0.3344	5.17E-05	1	29.14	0.05618
COPS5	COPS5	4	COP9 signalosome subunit 5	protein_codin	-0.03774	0.772	1	12	0.02698
CPNE3	CPNE3	4	copine 3	protein_codin	0.2775	0.00996	1	38.52	0.1126
RIPK2	RIPK2	4	receptor interacting serine/threonine kinase 2	protein_codin	0.8782	0.0102	1	52.58	0.2174
PDP1	PDP1	4	pyruvate dehyrogenase phosphatase catalytic subunit 1	protein_codin	0.2422	0.524	1	31.44	0.2001
RIDA	RIDA	4	reactive intermediate imine deaminase A homolog	protein_codin	-0.1786	0.51	1	10.96	0.2572
KLF10	KLF10	4	Kruppel like factor 10	protein_codin	0.7284	2.08E-07	0.004882	47.92	0.0604
AGO2	AGO2	4	argonaute RISC catalytic component 2	protein_codin	0.1456	0.608	1	30.27	0.2195
PPP1R16A	PPP1R16A	4	protein phosphatase 1 regulatory subunit 16A	protein_codin	0.6535	0.0349	1	3.123	0.2232
SMIM27	SMIM27	4	small integral membrane protein 27	protein_codin	0.5622	0.0562	1	3.22	0.0594
POLR1E	POLR1E	4	RNA polymerase I subunit E	protein_codin	0.3382	0.266	1	3.524	0.1762
EXOSC3	EXOSC3	4	exosome component 3	protein_codin	0.5222	0.0758	1	7.934	0.3910
C9orf85	C9orf85	4	chromosome 9 open reading frame 85	protein_codin	1.059	0.000236	1	7.988	0.2006
SPOUT1	SPOUT1	4	SPOUT domain containing methyltransferase 1	protein_codin	0.2486	0.353	1	2.618	0.2002
HPS5	HPS5	4	HPS5 biogenesis of lysosomal organelles complex 2 subunit	protein_codin	-0.8352	0.00432	1	3.408	0.1364
SVIP	SVIP	4	small VCP interacting protein	protein_codin	0.04945	0.686	1	51.29	0.0271
SELENOH	SELENOH	4	selenoprotein H	protein_codin	0.01836	0.825	1	51.51	0.02942
EML3	EML3	4	EMAP like 3	protein_codin	-0.2511	0.376	1	2.913	0.1952
ANKRD13D	ANKRD13D	4	ankyrin repeat domain 13D	protein_codin	0.2097	0.395	1	7.007	0.3104
CTSC	CTSC	4	cathepsin C	protein_codin	2.307	1.21E-42	2.93E-38	48.05	0.1149
MRE11	MRE11	4	MRE11 homolog, double strand break repair nuclease	protein_codin	-0.06565	0.782	1	6.871	0.1754
REXO2	REXO2	4	RNA exonuclease 2	protein_codin	-0.2777	0.00428	1	52.58	0.02632
ATP5C1	ATP5C1	4	ATP synthase F1 subunit gamma	protein_codin	NA	NA	NA	31.21	0.029
RPP38	RPP38	4	ribonuclease P/MRP subunit p38	protein_codin	0.05023	0.845	1	21.38	0.1388
KIF5B	KIF5B	4	kinesin family member 5B	protein_codin	-0.3731	9.84E-05	1	25.25	0.05377
8-Mar	42522	4	membrane associated ring-CH-type finger 8	protein_codin	-0.4931	0.0522	1	3.503	0.08578
KIF1BP	KIF1BP	4	KIF1 binding protein	protein_codin	0.003536	0.988	1	3.532	0.115
ADK	ADK	4	adenosine kinase	protein_codin	0.4596	0.208	1	11.03	0.1149
GLUD1	GLUD1	4	glutamate dehydrogenase 1	protein_codin	0.03013	0.847	1	45.08	0.07970
FAM45A	FAM45A	4	DENN domain containing 10	protein_codin	-0.08866	0.667	1	20.63	0.1372
TIAL1	TIAL1	4	TIA1 cytotoxic granule associated RNA binding protein like 1	protein_codin	0.1192	0.388	1	11.92	0.08880
FKBP4	FKBP4	4	FKBP prolyl isomerase 4	protein_codin	0.4562	0.106	1	7.253	0.08657
LTBR	LTBR	4	lymphotoxin beta receptor	protein_codin	0.7421	1.29E-05	0.2989	13.52	0.1672
LDHB	LDHB	4	lactate dehydrogenase B	protein_codin	-0.3022	2E-04	1	52.58	0.02657
ANO6	ANO6	4	anoctamin 6	protein_codin	-0.4396	0.0202	1	7.788	0.05879
CALCOCO1	CALCOCO:	4	calcium binding and coiled-coil domain 1	protein_codin	0.08273	0.747	1	6.914	0.1962
NAB2	NAB2	4	NGFI-A binding protein 2	protein_codin	0.2016	0.556	1	3.181	0.3889
AGAP2-AS1	AGAP2-AS	4	AGAP2 antisense RNA 1	antisense_RN	-0.1228	0.626	1	3.642	0.1168
ZDHHC17	ZDHHC17	4	zinc finger DHHC-type containing 17	protein_codin	0.7519	0.00689	1	7.842	0.1194
EEA1	EEA1	4	early endosome antigen 1	protein_codin	0.05054	0.709	1	35.79	0.05422
LTA4H	LTA4H	4	leukotriene A4 hydrolase	protein_codin	0.5055	0.00952	1	6.87	0.1336
TXNRD1	TXNRD1	4	thioredoxin reductase 1	protein_codin	-0.6051	0.00139	1	52.58	0.05368
C12orf76	C12orf76	4	chromosome 12 open reading frame 76	protein_codin	0.4337	0.0561	1	41.9	0.1948
ATXN2	ATXN2	4	ataxin 2	protein_codin	-0.3997	0.0357	1	32.62	0.05746
CLIP1	CLIP1	4	CAP-Gly domain containing linker protein 1	protein_codin	-0.07236	0.657	1	14.68	0.1287
GTF2H3	GTF2H3	4	general transcription factor IIH subunit 3	protein_codin	0.1562	0.502	1	6.604	0.1167
N4BP2L1	N4BP2L1	4	NEDD4 binding protein 2 like 1	protein_codin	0.5701	0.0247	1	7.672	0.08426
NUDT15	NUDT15	4	nudix hydrolase 15	protein_codin	0.5402	0.0406	1	9.072	0.2081
MYCBP2	MYCBP2	4	MYC binding protein 2	protein_codin	-0.07382	0.525	1	29.95	0.02856
DAD1	DAD1	4	defender against cell death 1	protein_codin	-0.1754	0.00351	1	52.57	0.02706
LRP10	LRP10	4	LDL receptor related protein 10	protein_codin	-0.2052	0.139	1	46.34	0.1102
DCAF11	DCAF11	4	DDB1 and CUL4 associated factor 11	protein_codin	0.07387	0.783	1	3.388	0.2581
GMPR2	GMPR2	4	guanosine monophosphate reductase 2	protein_codin	0.2352	0.195	1	17.75	0.05456
MGAT2	MGAT2	4	mannosyl (alpha-1,6-)-glycoprotein beta-1,2-N-acetylglucosar	protein_codin	0.3031	0.205	1	29.84	0.08594
GNPNAT1	GNPNAT1	4	glucosamine-phosphate N-acetyltransferase 1	protein_codin	0.02728	0.888	1	8.488	0.1108
KIAA0586	KIAA0586	4	KIAA0586	protein_codin	0.479	0.0468	1	3.206	0.1667
MNAT1	MNAT1	4	MNAT1 component of CDK activating kinase	protein_codin	-0.1676	0.329	1	6.811	0.174
TRMT5	TRMT5	4	tRNA methyltransferase 5	protein_codin	0.6332	0.0892	1	8.993	0.2619
PPP2R5E	PPP2R5E	4	protein phosphatase 2 regulatory subunit B'epsilon	protein_codin	0.3016	0.0879	1	13.4	0.08198
MPP5	MPP5	4	membrane palmitoylated protein 5	protein_codin	0.1437	0.612	1	3.152	0.1899
PNMA1	PNMA1	4	PNMA family member 1	protein_codin	0.2307	0.497	1	8.912	0.2445
FCF1	FCF1	4	FCF1 rRNA-processing protein	protein_codin	0.1662	0.598	1	2.373	0.199
SEL1L	SEL1L	4	SEL1L adaptor subunit of ERAD E3 ubiquitin ligase	protein_codin	0.01866	0.927	1	20.16	0.1347
RPS6KA5	RPS6KA5	4	ribosomal protein S6 kinase A5	protein_codin	0.009229	0.975	1	8.672	0.3101
IFI27L1	IFI27L1	4	interferon alpha inducible protein 27 like 1	protein_codin	0.0172	0.943	1	28.03	0.1680
KIF26A	KIF26A	4	kinesin family member 26A	protein_codin	-0.8205	0.000587	1	52.21	0.08842
TTBK2	TTBK2	4	tau tubulin kinase 2	protein_codin	-0.1915	0.466	1	3.667	0.2358
UBR1	UBR1	4	ubiquitin protein ligase E3 component n-recognin 1	protein_codin	-0.01382	0.955	1	52.49	0.2592
TRPM7	TRPM7	4	transient receptor potential cation channel subfamily M member 7	protein_codin	0.3766	0.217	1	49.43	0.2541
FAM96A	FAM96A	4	cytosolic iron-sulfur assembly component 2A	protein_codin	0.185	0.344	1	7.287	0.2541
MTFMT	MTFMT	4	mitochondrial methionyl-tRNA formyltransferase	protein_codin	0.5327	0.153	1	2.967	0.226
SCAMP2	SCAMP2	4	secretory carrier membrane protein 2	protein_codin	-0.1101	0.499	1	33.03	0.1177
CTSH	CTSH	4	cathepsin H	protein_codin	1.869	4.57E-30	1.11E-25	51.96	0.1408
LINC01197	LINC01197	4	long intergenic non-protein coding RNA 1197	lincRNA	1.752	2.05E-08	0.000484	19.68	0.08366
PKD1	PKD1	4	polycystin 1, transient receptor potential channel interacting	protein_codin	-0.9729	3.77E-05	0.8696	3.193	0.2579
GSPT1	GSPT1	4	G1 to S phase transition 1	protein_codin	0.04994	0.685	1	43.8	0.05652
NTAN1	NTAN1	4	N-terminal asparagine amidase	protein_codin	0.2936	0.023	1	8.967	0.02877
STX4	STX4	4	syntaxin 4	protein_codin	0.1987	0.322	1	19.63	0.05432
FAM192A	FAM192A	4	family with sequence similarity 192 member A	protein_codin	-0.2392	0.187	1	7.433	0.1122
NFAT5	NFAT5	4	nuclear factor of activated T cells 5	protein_codin	-0.06181	0.732	1	45.58	0.08256
GAN	GAN	4	gigaxonin	protein_codin	-0.006282	0.983	1	9.375	0.2347
KLHL36	KLHL36	4	kelch like family member 36	protein_codin	0.1794	0.544	1	10.7	0.05558
MYO1C	MYO1C	4	myosin IC	protein_codin	-0.4287	0.00916	1	51.98	0.08297
BLMH	BLMH	4	bleomycin hydrolase	protein_codin	0.07021	0.723	1	6.078	0.03349
CWC25	CWC25	4	CWC25 spliceosome associated protein homolog	protein_codin	0.09752	0.55	1	15.49	0.1431
CAVIN1	CAVIN1	4	caveolae associated protein 1	protein_codin	-0.05934	0.407	1	52.58	0.02776
NAGLU	NAGLU	4	N-acetyl-alpha-glucosaminidase	protein_codin	-0.8919	0.00114	1	2.828	0.1719
VAT1	VAT1	4	vesicle amine transport 1	protein_codin	0.4666	0.00790	1	14.05	0.1438
HDAC5	HDAC5	4	histone deacetylase 5	protein_codin	-0.04805	0.848	1	3.525	0.254
HEXIM1	HEXIM1	4	HEXIM P-TEFb complex subunit 1	protein_codin	0.04178	0.837	1	17.94	0.1859
COPZ2	COPZ2	4	coatomer protein complex subunit zeta 2	protein_codin	-0.3657	0.109	1	49.82	0.1080
TRIM25	TRIM25	4	tripartite motif containing 25	protein_codin	0.9514	0.000532	1	8.216	0.1411
WDR45B	WDR45B	4	WD repeat domain 45B	protein_codin	0.0529	0.779	1	7.402	0.1642
NDUFV2	NDUFV2	4	NADH:ubiquinone oxidoreductase core subunit V2	protein_codin	-0.03	0.724	1	20.17	0.02907
ZCCHC3	ZCCHC3	4	zinc finger CCHC-type containing 3	protein_codin	0.2942	0.413	1	3.275	0.1167
SOGA1	SOGA1	4	suppressor of glucose, autophagy associated 1	protein_codin	0.2366	0.418	1	2.938	0.2225
SNX21	SNX21	4	sorting nexin family member 21	protein_codin	-0.06725	0.822	1	2.63	0.03062
RAE1	RAE1	4	ribonucleic acid export 1	protein_codin	0.7087	0.0368	1	3.246	0.2002
SBNO2	SBNO2	4	strawberry notch homolog 2	protein_codin	-0.2472	0.347	1	6.628	0.1967
YIPF2	YIPF2	4	Yip1 domain family member 2	protein_codin	-0.2505	0.207	1	21.4	0.05356
DHPS	DHPS	4	deoxyhypusine synthase	protein_codin	0.382	0.0281	1	6.554	0.2092
USHBP1	USHBP1	4	USH1 protein network component harmonin binding protein 1	protein_codin	0.287	0.303	1	3.218	0.2542
NR2C2AP	NR2C2AP	4	nuclear receptor 2C2 associated protein	protein_codin	1.059	0.00035	1	3.425	0.196
ZNF254	ZNF254	4	zinc finger protein 254	protein_codin	-0.3061	0.302	1	8.617	0.2575
CEBPG	CEBPG	4	CCAAT enhancer binding protein gamma	protein_codin	0.4138	0.0174	1	15.58	0.282
DMKN	DMKN	4	dermokine	protein_codin	-0.6397	0.0204	1	10.6	0.2277
ZNF146	ZNF146	4	zinc finger protein 146	protein_codin	0.386	0.136	1	9.685	0.2796
YIF1B	YIF1B	4	Yip1 interacting factor homolog B, membrane trafficking prote	protein_codin	-0.07571	0.732	1	8.713	0.06341
FBL	FBL	4	fibrillarin	protein_codin	0.8002	3.3E-07	0.007737	33.59	0.1125
B9D2	B9D2	4	B9 domain containing 2	protein_codin	-0.1045	0.719	1	8.967	0.1747
STRN4	STRN4	4	striatin 4	protein_codin	-0.235	0.375	1	10.63	0.1710
CARD8-AS1	CARD8-AS	4	CARD8 antisense RNA 1	antisense_RN	0.5629	0.0192	1	7.013	0.2277
BCL2L12	BCL2L12	4	BCL2 like 12	protein_codin	0.7316	0.0134	1	3.575	0.1764
ZNF480	ZNF480	4	zinc finger protein 480	protein_codin	0.4925	0.103	1	30.83	0.6961
ZNF808	ZNF808	4	zinc finger protein 808	protein_codin	0.4942	0.142	1	2.844	0.2357
TSEN34	TSEN34	4	tRNA splicing endonuclease subunit 34	protein_codin	-0.2722	0.0744	1	18.23	0.14
LENG8	LENG8	4	leukocyte receptor cluster member 8	protein_codin	0.6157	0.0789	1	2.864	0.2832
SLC27A5	SLC27A5	4	solute carrier family 27 member 5	protein_codin	-0.639	0.00711	1	29.46	0.05556
PPM1F	PPM1F	4	protein phosphatase, Mg2+/Mn2+ dependent 1F	protein_codin	0.5132	0.0454	1	27.63	0.2479
LIMK2	LIMK2	4	LIM domain kinase 2	protein_codin	-0.2731	0.427	1	10.02	0.1622
PARVB	PARVB	4	parvin beta	protein_codin	-0.01106	0.93	1	35.54	0.05632
URB1-AS1	URB1-AS1	4	URB1 antisense RNA 1 (head to head)	lincRNA	0.2192	0.534	1	9.436	0.1388
WRB	WRB	4	guided entry of tail-anchored proteins factor 1	protein_codin	0.1842	0.35	1	7.23	0.1384
ABCG1	ABCG1	4	ATP binding cassette subfamily G member 1	protein_codin	0.334	0.127	1	7.907	0.1093
RRP1B	RRP1B	4	ribosomal RNA processing 1B	protein_codin	-0.1205	0.57	1	3.356	0.08136
PFKL	PFKL	4	phosphofructokinase, liver type	protein_codin	0.174	0.374	1	21.5	0.09108
RER1	RER1	5	retention in endoplasmic reticulum sorting receptor 1	protein_codin	0.08428	0.53	1	44.93	0.2029
C1orf174	C1orf174	5	chromosome 1 open reading frame 174	protein_codin	0.3675	0.153	1	8.333	0.1116
AKR7A2	AKR7A2	5	aldo-keto reductase family 7 member A2	protein_codin	-0.1296	0.346	1	12.66	0.1417
MAP3K6	MAP3K6	5	mitogen-activated protein kinase kinase kinase 6	protein_codin	0.1047	0.728	1	10.42	0.2652
PHACTR4	PHACTR4	5	phosphatase and actin regulator 4	protein_codin	-0.1786	0.288	1	13.28	0.1396
RNF19B	RNF19B	5	ring finger protein 19B	protein_codin	0.8582	0.0194	1	3.019	0.1882
NFYC	NFYC	5	nuclear transcription factor Y subunit gamma	protein_codin	0.4876	0.0269	1	7.618	0.1432
MED8	MED8	5	mediator complex subunit 8	protein_codin	0.02391	0.906	1	8.398	0.1198
B4GALT2	B4GALT2	5	beta-1,4-galactosyltransferase 2	protein_codin	-0.2624	0.318	1	8.432	0.1751
HSPB11	HSPB11	5	heat shock protein family B (small) member 11	protein_codin	-0.1594	0.347	1	6.935	0.129
FUBP1	FUBP1	5	far upstream element binding protein 1	protein_codin	0.04471	0.805	1	21.48	0.1394
CTBS	CTBS	5	chitobiase	protein_codin	-0.5337	0.000626	1	41.55	0.1136
AC104506.1	AC104506.:	5	novel transcript, antisense to EXT	antisense_RN	1.256	0.000314	1	6.803	0.2249
BOLA1	BOLA1	5	bolA family member 1	protein_codin	0.3009	0.28	1	7.218	0.2984
PI4KB	PI4KB	5	phosphatidylinositol 4-kinase beta	protein_codin	0.5077	0.108	1	3.118	0.2182
KIFAP3	KIFAP3	5	kinesin associated protein 3	protein_codin	0.2781	0.176	1	6.963	0.179
CAPN2	CAPN2	5	calpain 2	protein_codin	0.09251	0.49	1	50.43	0.03218
PARP1	PARP1	5	poly(ADP-ribose) polymerase 1	protein_codin	0.2817	0.119	1	25.76	0.1169
TSNAX	TSNAX	5	translin associated factor X	protein_codin	0.469	0.00754	1	38.82	0.1064
NTPCR	NTPCR	5	nucleoside-triphosphatase, cancer-related	protein_codin	-0.3982	0.0144	1	14.16	0.1390
MTR	MTR	5	5-methyltetrahydrofolate-homocysteine methyltransferase	protein_codin	-0.1236	0.737	1	8.113	0.2604
FH	FH	5	fumarate hydratase	protein_codin	0.2642	0.264	1	6.714	0.02686
CEP170	CEP170	5	centrosomal protein 170	protein_codin	-0.04962	0.797	1	6.74	0.1960
SH3YL1	SH3YL1	5	SH3 and SYLF domain containing 1	protein_codin	-0.1392	0.603	1	9.095	0.1434
PREB	PREB	5	prolactin regulatory element binding	protein_codin	0.3724	0.241	1	2.993	0.3414
SNX17	SNX17	5	sorting nexin 17	protein_codin	0.07977	0.563	1	35.2	0.05609

**TRMT61B**	TRMT61B	5	tRNA methyltransferase 61B	protein_codin	0.0673	0.808	1	2.854	0.0569
**VRK2**	VRK2	5	VRK serine/threonine kinase 2	protein_codin	1.12	0.000457	1	7.372	0.1657
**REL**	REL	5	REL proto-oncogene, NF-kB subunit	protein_codin	0.5755	0.000624	1	42.37	0.07966
**PNO1**	PNO1	5	partner of NOB1 homolog	protein_codin	0.3469	0.106	1	8.448	0.09056
**NAGK**	NAGK	5	N-acetylglucosamine kinase	protein_codin	-0.05127	0.786	1	7.432	0.1347
**DUSP11**	DUSP11	5	dual specificity phosphatase 11	protein_codin	-0.04711	0.8	1	22.79	0.119
**ACTR1B**	ACTR1B	5	actin related protein 1B	protein_codin	0.7138	0.00384	1	7.218	0.1652
**CNOT11**	CNOT11	5	CCR4-NOT transcription complex subunit 11	protein_codin	1.036	0.0021	1	8.729	0.1419
**NIFK**	NIFK	5	nucleolar protein interacting with the FHA domain of MKI67	protein_codin	0.102	0.452	1	43.94	0.166
**DARS**	DARS	5	aspartyl-tRNA synthetase	protein_codin	0.2289	0.0605	1	37.92	0.05563
**HAT1**	HAT1	5	histone acetyltransferase 1	protein_codin	0.5105	0.0051	1	7.014	0.1379
**COQ10B**	COQ10B	5	coenzyme Q10B	protein_codin	0.6429	0.000152	1	10.42	0.05268
**ACSL3**	ACSL3	5	acyl-CoA synthetase long chain family member 3	protein_codin	-0.01452	0.938	1	7.377	0.1091
**TRIP12**	TRIP12	5	thyroid hormone receptor interactor 12	protein_codin	0.05880	0.765	1	13.19	0.1449
**UBE2F**	UBE2F	5	ubiquitin conjugating enzyme E2 F (putative)	protein_codin	-0.2542	0.288	1	7.832	0.1935
**CRELD1**	CRELD1	5	cysteine rich with EGF like domains 1	protein_codin	-0.3791	0.0906	1	3.086	0.1665
**IQSEC1**	IQSEC1	5	IQ motif and Sec7 domain 1	protein_codin	-0.5426	0.0381	1	3.364	0.1396
**CAPN7**	CAPN7	5	calpain 7	protein_codin	0.368	0.185	1	7.944	0.2302
**EAF1**	EAF1	5	ELL associated factor 1	protein_codin	0.5417	0.113	1	3.093	0.3279
**HACL1**	HACL1	5	2-hydroxyacyl-CoA lyase 1	protein_codin	0.8988	0.00262	1	3.438	0.1677
**TMF1**	TMF1	5	TATA element modulatory factor 1	protein_codin	-0.2194	0.129	1	25.67	0.05352
**RABL3**	RABL3	5	RAB, member of RAS oncogene family like 3	protein_codin	0.662	0.0350	1	8.375	0.2888
**PIK3CB**	PIK3CB	5	phosphatidylinositol-4,5-bisphosphate 3-kinase catalytic subu	protein_codin	0.2652	0.394	1	3.279	0.2429
**TTC14**	TTC14	5	tetratricopeptide repeat domain 14	protein_codin	-0.3022	0.188	1	8.126	0.2817
**KLHL24**	KLHL24	5	kelch like family member 24	protein_codin	-0.5334	0.00922	1	6.528	0.0930
**CCDC50**	CCDC50	5	coiled-coil domain containing 50	protein_codin	0.2531	0.0245	1	32.86	0.03004
**LSG1**	LSG1	5	large 60S subunit nuclear export GTPase 1	protein_codin	0.02384	0.89	1	5.702	0.1997
**ATP5I**	ATP5I	5	ATP synthase membrane subunit e	protein_codin	NA	NA	NA	19.98	0.1381
**ADD1**	ADD1	5	adducin 1	protein_codin	-0.4277	0.00229	1	22.16	0.1117
**HTT**	HTT	5	huntingtin	protein_codin	0.5882	0.0872	1	2.882	0.2756
**CC2D2A**	CC2D2A	5	coiled-coil and C2 domain containing 2A	protein_codin	0.1777	0.596	1	35.62	0.3487
**QDPR**	QDPR	5	quinoid dihydropteridine reductase	protein_codin	0.7149	0.034	1	9.052	0.1161
**RFC1**	RFC1	5	replication factor C subunit 1	protein_codin	0.2031	0.0729	1	44.15	0.05218
**SGCB**	SGCB	5	sarcoglycan beta	protein_codin	-0.8097	1E-04	1	44.6	0.1074
**MRPL1**	MRPL1	5	mitochondrial ribosomal protein L1	protein_codin	0.6932	0.00211	1	40.78	0.2265
**USP53**	USP53	5	ubiquitin specific peptidase 53	protein_codin	-0.6387	0.000303	1	39.25	0.02750
**ABCE1**	ABCE1	5	ATP binding cassette subfamily E member 1	protein_codin	0.4949	0.00241	1	12.79	0.1372
**RWDD4**	RWDD4	5	RWD domain containing 4	protein_codin	0.3762	0.052	1	15.22	0.1109
**XRCC4**	XRCC4	5	X-ray repair cross complementing 4	protein_codin	0.1932	0.511	1	10.72	0.1838
**REEP5**	REEP5	5	receptor accessory protein 5	protein_codin	-0.5097	7.43E-10	1.76E-05	46.58	0.02671
**CCDC112**	CCDC112	5	coiled-coil domain containing 112	protein_codin	0.3052	0.196	1	20.72	0.2341
**FAM53C**	FAM53C	5	family with sequence similarity 53 member C	protein_codin	0.05435	0.862	1	3.062	0.1647
**NR3C1**	NR3C1	5	nuclear receptor subfamily 3 group C member 1	protein_codin	-0.2952	0.0117	1	18.74	0.2437
**RBM22**	RBM22	5	RNA binding motif protein 22	protein_codin	0.4411	0.00192	1	44.36	0.05726
**TTC1**	TTC1	5	tetratricopeptide repeat domain 1	protein_codin	0.3229	0.0116	1	22.09	0.221
**PWWP2A**	PWWP2A	5	PWWP domain containing 2A	protein_codin	-0.4459	0.0606	1	3.568	0.1712
**ZNF354A**	ZNF354A	5	zinc finger protein 354A	protein_codin	0.4571	0.133	1	8.423	0.1089
**RNF130**	RNF130	5	ring finger protein 130	protein_codin	0.08304	0.567	1	11.58	0.08582
**MGAT1**	MGAT1	5	mannosyl (alpha-1,3-)-glycoprotein beta-1,2-N-acetylglucosar	protein_codin	0.01427	0.925	1	22.63	0.05268
**PPP1R11**	PPP1R11	5	protein phosphatase 1 regulatory inhibitor subunit 11	protein_codin	0.1532	0.311	1	41.52	0.1058
**MRPS18B**	MRPS18B	5	mitochondrial ribosomal protein S18B	protein_codin	0.4185	0.00997	1	12.37	0.1696
**BAG6**	BAG6	5	BCL2 associated athanogene 6	protein_codin	-0.02412	0.913	1	6.063	0.1965
**RING1**	RING1	5	ring finger protein 1	protein_codin	0.5435	0.0192	1	8.632	0.1364
**MRPL14**	MRPL14	5	mitochondrial ribosomal protein L14	protein_codin	-0.05809	0.685	1	28.67	0.1429
**MANEA**	MANEA	5	mannosidase endo-alpha	protein_codin	0.1662	0.614	1	10.02	0.1942
**PEX3**	PEX3	5	peroxisomal biogenesis factor 3	protein_codin	0.07248	0.777	1	8.446	0.08493
**PCMT1**	PCMT1	5	protein-L-isoaspartate (D-aspartate) O-methyltransferase	protein_codin	0.3141	0.0118	1	20.56	0.1088
**TFB1M**	TFB1M	5	transcription factor B1, mitochondrial	protein_codin	0.7555	0.0918	1	2.856	0.2294
**WIPI2**	WIPI2	5	WD repeat domain, phosphoinositide interacting 2	protein_codin	-0.1156	0.517	1	4.888	0.1597
**NUPL2**	NUPL2	5	nucleoporin 42	protein_codin	0.7134	0.0304	1	6.33	0.1419
**CCDC126**	CCDC126	5	coiled-coil domain containing 126	protein_codin	-0.2249	0.466	1	3.214	0.1476
**STARD3NL**	STARD3NL	5	STARD3 N-terminal like	protein_codin	0.1791	0.302	1	17.88	0.1192
**RUNDC3B**	RUNDC3B	5	RUN domain containing 3B	protein_codin	0.3377	0.252	1	9.93	0.3682
**COPS6**	COPS6	5	COP9 signalosome subunit 6	protein_codin	-0.04232	0.711	1	31.41	0.05302
**SRPK2**	SRPK2	5	SRSF protein kinase 2	protein_codin	0.8047	1.08E-05	0.2505	11.62	0.1646
**DOCK4**	DOCK4	5	dedicator of cytokinesis 4	protein_codin	0.7066	0.00106	1	50.85	0.1131
**MET**	MET	5	MET proto-oncogene, receptor tyrosine kinase	protein_codin	0.492	0.0287	1	12.33	0.1490
**SYAP1**	SYAP1	5	synapse associated protein 1	protein_codin	-0.08338	0.592	1	31.72	0.05527
**CDK16**	CDK16	5	cyclin dependent kinase 16	protein_codin	-0.2294	0.538	1	2.59	0.3009
**PQBP1**	PQBP1	5	polyglutamine binding protein 1	protein_codin	0.3772	0.0122	1	12.77	0.1155
**SMC1A**	SMC1A	5	structural maintenance of chromosomes 1A	protein_codin	-0.1754	0.384	1	8.003	0.1089
**YIPF6**	YIPF6	5	Yip1 domain family member 6	protein_codin	-0.1675	0.332	1	7.437	0.08865
**ABCB7**	ABCB7	5	ATP binding cassette subfamily B member 7	protein_codin	0.4671	0.158	1	3.575	0.3762
**PSMD10**	PSMD10	5	proteasome 26S subunit, non-ATPase 10	protein_codin	0.1664	0.533	1	7.935	0.145
**IRAK1**	IRAK1	5	interleukin 1 receptor associated kinase 1	protein_codin	-0.7374	0.00213	1	9.768	0.1488
**ASH2L**	ASH2L	5	ASH2 like, histone lysine methyltransferase complex subunit	protein_codin	-0.5081	0.0591	1	2.892	0.08259
**TACC1**	TACC1	5	transforming acidic coiled-coil containing protein 1	protein_codin	0.3097	0.000148	1	50.54	0.02612
**UBE2V2**	UBE2V2	5	ubiquitin conjugating enzyme E2 V2	protein_codin	0.07715	0.618	1	14.96	0.1955
**ARMC1**	ARMC1	5	armadillo repeat containing 1	protein_codin	0.314	0.142	1	5.548	0.08382
**NUDCD1**	NUDCD1	5	NudC domain containing 1	protein_codin	1.464	2.32E-08	0.000548	19.68	0.2767
**PARP10**	PARP10	5	poly(ADP-ribose) polymerase family member 10	protein_codin	0.6729	0.094	1	9.911	0.2062
**GLIS3**	GLIS3	5	GLIS family zinc finger 3	protein_codin	-0.5238	0.0288	1	22.14	0.0849
**FXN**	FXN	5	frataxin	protein_codin	0.1772	0.529	1	8.058	0.1749
**TMEM2**	TMEM2	5	cell migration inducing hyaluronidase 2	protein_codin	NA	NA	NA	51.22	0.0593
**SLC35D2**	SLC35D2	5	solute carrier family 35 member D2	protein_codin	0.4037	0.0756	1	3.268	0.1436
**RNF20**	RNF20	5	ring finger protein 20	protein_codin	0.1822	0.494	1	8.686	0.1407
**SMC2**	SMC2	5	structural maintenance of chromosomes 2	protein_codin	0.1126	0.624	1	18.19	0.08128
**CDK9**	CDK9	5	cyclin dependent kinase 9	protein_codin	0.9185	0.000107	1	19.82	0.254
**FBXW5**	FBXW5	5	F-box and WD repeat domain containing 5	protein_codin	0.03766	0.808	1	13.79	0.08234
**RNH1**	RNH1	5	ribonuclease/angiogenin inhibitor 1	protein_codin	-0.1697	0.0331	1	38.14	0.05486
**PGAP2**	PGAP2	5	post-GPI attachment to proteins 2	protein_codin	0.7587	0.0122	1	7.875	0.1770
**TMEM9B**	TMEM9B	5	TMEM9 domain family member B	protein_codin	0.08405	0.558	1	11.97	0.05399
**SWAP70**	SWAP70	5	switching B cell complex subunit SWAP70	protein_codin	0.2822	0.0197	1	33.19	0.08284
**PARVA**	PARVA	5	parvin alpha	protein_codin	-0.5232	0.00207	1	18.13	0.1112
**LIN7C**	LIN7C	5	lin-7 homolog C, crumbs cell polarity complex component	protein_codin	-0.1668	0.466	1	7.026	0.08272
**TTC9C**	TTC9C	5	tetratricopeptide repeat domain 9C	protein_codin	0.494	0.109	1	10.33	0.1931
**COX8A**	COX8A	5	cytochrome c oxidase subunit 8A	protein_codin	-0.3317	1.06E-05	0.2459	50.96	0.08179
**STIP1**	STIP1	5	stress induced phosphoprotein 1	protein_codin	-0.07969	0.676	1	23.45	0.1386
**ARL2.00**	ARL2.00	5	ADP ribosylation factor like GTPase 2	protein_codin	0.2882	0.000129	1	29.58	0.05532
**RNASEH2C**	RNASEH2C	5	ribonuclease H2 subunit C	protein_codin	-0.5238	3.41E-07	0.007992	30.87	0.1101
**NUDT8**	NUDT8	5	nudix hydrolase 8	protein_codin	0.7322	0.0222	1	26.3	0.2288
**PCF11**	PCF11	5	PCF11 cleavage and polyadenylation factor subunit	protein_codin	0.7172	2.68E-05	0.6192	42.46	0.06205
**TAF1D**	TAF1D	5	TATA-box binding protein associated factor, RNA polymerase	protein_codin	0.02457	0.825	1	42.22	0.03019
**MAML2**	MAML2	5	mastermind like transcriptional coactivator 2	protein_codin	-0.2748	0.263	1	2.856	0.2222
**BIRC3**	BIRC3	5	baculoviral IAP repeat containing 3	protein_codin	1.161	3.96E-06	0.09222	43.68	0.118
**ACAT1**	ACAT1	5	acetyl-CoA acetyltransferase 1	protein_codin	0.4122	0.00539	1	20.69	0.02696
**SDHD**	SDHD	5	succinate dehydrogenase complex subunit D	protein_codin	0.2069	0.0415	1	24.22	0.05352
**SIK3**	SIK3	5	SIK family kinase 3	protein_codin	0.2069	0.472	1	7.279	0.02769
**VPS26B**	VPS26B	5	VPS26, retromer complex component B	protein_codin	-0.06026	0.838	1	2.877	0.2275
**PIP4K2A**	PIP4K2A	5	phosphatidylinositol-5-phosphate 4-kinase type 2 alpha	protein_codin	1.568	1.03E-08	0.000242	7.756	0.08172
**MSRB2**	MSRB2	5	methionine sulfoxide reductase B2	protein_codin	0.2217	0.176	1	14.19	0.1905
**GOT1**	GOT1	5	glutamic-oxaloacetic transaminase 1	protein_codin	-0.012	0.962	1	8.277	0.14
**DOCK1**	DOCK1	5	dedicator of cytokinesis 1	protein_codin	-0.1052	0.577	1	6.496	0.05641
**PWWP2B**	PWWP2B	5	PWWP domain containing 2B	protein_codin	0.547	0.106	1	2.423	0.2590
**ZNF511**	ZNF511	5	zinc finger protein 511	protein_codin	-0.0846	0.722	1	8.103	0.1111
**CLEC2B**	CLEC2B	5	C-type lectin domain family 2 member B	protein_codin	1.27	2.48E-21	5.98E-17	51.45	0.03077
**MAGOHB**	MAGOHB	5	mago homolog B, exon junction complex subunit	protein_codin	-0.352	0.0712	1	7.797	0.1135
**ETNK1**	ETNK1	5	ethanolamine kinase 1	protein_codin	0.1299	0.52	1	8.604	0.1701
**TM7SF3**	TM7SF3	5	transmembrane 7 superfamily member 3	protein_codin	-0.1342	0.472	1	7.72	0.1985
**FGD4**	FGD4	5	FYVE, RhoGEF and PH domain containing 4	protein_codin	1.129	0.000411	1	8.559	0.1209
**AC008124.1**	AC008124.1	5	novel transcri	lincRNA	-0.2629	0.374	1	3.428	0.167
**HDAC7**	HDAC7	5	histone deacetylase 7	protein_codin	0.1816	0.302	1	6.444	0.1502
**RND1**	RND1	5	Rho family GTPase 1	protein_codin	0.9894	0.00188	1	51.38	0.1101
**AC068888.1**	AC068888.1	5	novel transcript, antisense to TENC1 & EIF	antisense_RN	0.3782	0.175	1	10.03	0.1849
**METTL1**	METTL1	5	methyltransferase like 1	protein_codin	0.7829	0.0167	1	3.186	0.2542
**NTN4**	NTN4	5	netrin 4	protein_codin	0.1352	0.606	1	9.637	0.2584
**DRAM1**	DRAM1	5	DNA damage regulated autophagy modulator 1	protein_codin	-0.6752	0.00996	1	25.82	0.08705
**TMEM263**	TMEM263	5	transmembrane protein 263	protein_codin	0.007132	0.972	1	21.25	0.1131
**SH2B3**	SH2B3	5	SH2B adaptor protein 3	protein_codin	-0.3062	0.174	1	27.6	0.2170
**ANKLE2**	ANKLE2	5	ankyrin repeat and LEM domain containing 2	protein_codin	0.0799	0.723	1	7.604	0.06119
**ZC3H13**	ZC3H13	5	zinc finger CCCH-type containing 13	protein_codin	0.1472	0.257	1	40.32	0.0264
**ARHGEF7**	ARHGEF7	5	Rho guanine nucleotide exchange factor 7	protein_codin	-0.02749	0.916	1	9.173	0.2534
**UPF3A**	UPF3A	5	UPF3A regulator of nonsense mediated mRNA decay	protein_codin	-0.07115	0.597	1	33.46	0.05888
**ACIN1**	ACIN1	5	apoptotic chromatin condensation inducer 1	protein_codin	0.142	0.385	1	15.29	0.2224
**PSMA3-AS1**	PSMA3-AS	5	PSMA3 antisense RNA 1	antisense_RN	0.1004	0.586	1	38.98	0.07992
**TIMM9**	TIMM9	5	translocase of inner mitochondrial membrane 9	protein_codin	0.2401	0.166	1	6.923	0.1897
**CHURC1**	CHURC1	5	churchill domain containing 1	protein_codin	0.2395	0.0182	1	51.03	0.02632
**SIPA1L1**	SIPA1L1	5	signal induced proliferation associated 1 like 1	protein_codin	-0.7837	0.00312	1	3.418	0.2252
**SLIRP**	SLIRP	5	SRA stem-loop interacting RNA binding protein	protein_codin	0.02397	0.844	1	10.52	0.02682
**GTF2A1**	GTF2A1	5	general transcription factor IIA subunit 1	protein_codin	0.2982	0.269	1	3.638	0.172
**EFCAB11**	EFCAB11	5	EF-hand calcium binding domain 11	protein_codin	0.1117	0.73	1	3.195	0.2235
**MOAP1**	MOAP1	5	modulator of apoptosis 1	protein_codin	-0.3205	0.19	1	7.949	0.116
**UBE3A**	UBE3A	5	ubiquitin protein ligase E3A	protein_codin	-0.3046	0.0414	1	15.72	0.1356
**KLF13**	KLF13	5	Kruppel like factor 13	protein_codin	0.2761	0.0346	1	50.83	0.0570
**ZNF770**	ZNF770	5	zinc finger protein 770	protein_codin	-0.1741	0.434	1	8.418	0.1592
**CCNDBP1**	CCNDBP1	5	cyclin D1 binding protein 1	protein_codin	0.00238	0.987	1	12.28	0.1349
**GABPB1**	GABPB1	5	GA binding protein transcription factor subunit beta 1	protein_codin	0.5971	0.0741	1	3.503	0.2604
**GNB5**	GNB5	5	G protein subunit beta 5	protein_codin	0.5599	0.196	1	33.94	0.2249
**SLTM**	SLTM	5	SAFB like transcription modulator	protein_codin	0.1367	0.256	1	13.85	0.05262
**UBE2Q2**	UBE2Q2	5	ubiquitin conjugating enzyme E2 Q2	protein_codin	0.164	0.338	1	12.46	0.1126
**MRPS11**	MRPS11	5	mitochondrial ribosomal protein S11	protein_codin	-0.1888	0.297	1	7.426	0.08434
**ABHD2**	ABHD2	5	abhydrolase domain containing 2	protein_codin	-1.65	1.58E-20	3.81E-16	40.11	0.05292
**HCFC1R1**	HCFC1R1	5	host cell factor C1 regulator 1	protein_codin	-0.661	5.14E-08	0.001211	22.09	0.115
**CLUAP1**	CLUAP1	5	clusterin associated protein 1	protein_codin	0.1057	0.656	1	8.562	0.09284
**CDIP1**	CDIP1	5	cell death inducing p53 target 1	protein_codin	0.04506	0.848	1	6.605	0.1152
**LINC02185**	LINC02185	5	long intergenic non-protein coding RNA 2185	lincRNA	0.5089	0.0219	1	22.36	0.05861
**MKL2**	MKL2	5	myocardin related transcription factor B	protein_codin	1.02	2.08E-11	4.95E-07	30.64	0.199
**COQ7**	COQ7	5	coenzyme Q7, hydroxylase	protein_codin	-0.2451	0.414	1	10.33	0.2017
**SGF29**	SGF29	5	SAGA complex associated factor 29	protein_codin	0.6297	0.0292	1	8.148	0.1815
**YPEL3**	YPEL3	5	yippee like 3	protein_codin	0.0251	0.833	1	51.72	0.02856
**HSD3B7**	HSD3B7	5	hydroxy-delta-5-steroid dehydrogenase, 3 beta- and steroid d	protein_codin	1.001	0.00046	1	7.852	0.2220
**ITFG1**	ITFG1	5	integrin alpha FG-GAP repeat containing 1	protein_codin	-0.25	0.177	1	7.88	0.1377
**ENKD1**	ENKD1	5	enkurin domain containing 1	protein_codin	-0.08859	0.767	1	2.43	0.2297
**AP1G1**	AP1G1	5	adaptor related protein complex 1 subunit gamma 1	protein_codin	0.4185	0.168	1	7.495	0.2268
**VPS53**	VPS53	5	VPS53 subunit of GARP complex	protein_codin	-0.177	0.62	1	3.515	0.3071
**CRK**	CRK	5	CRK proto-oncogene, adaptor protein	protein_codin	-0.01165	0.937	1	12.56	0.1369
**SCARF1**	SCARF1	5	scavenger receptor class F member 1	protein_codin	0.2282	0.258	1	7.075	0.08805
**TRAPPC1**	TRAPPC1	5	trafficking protein particle complex 1	protein_codin	-0.1264	0.258	1	35.09	0.08266
**PER1**	PER1	5	period circadian regulator 1	protein_codin	0.2858	0.193	1	7.568	0.02612
**DRG2**	DRG2	5	developmentally regulated GTP binding protein 2	protein_codin	0.6424	0.00550	1	6.675	0.05756
**ALKBH5**	ALKBH5	5	alkB homolog 5, RNA demethylase	protein_codin	-0.3486	0.122	1	7.4	0.08609
**KIAA0100**	KIAA0100	5	KIAA0100	protein_codin	-0.2564	0.292	1	3.18	0.252
**GGNBP2**	GGNBP2	5	gametogenetin binding protein 2	protein_codin	0.1579	0.209	1	37.97	0.1109
**ARHGAP23**	ARHGAP22	5	Rho GTPase activating protein 23	protein_codin	-0.2271	0.346	1	25.23	0.1646
**MEOX1**	MEOX1	5	mesenchyme homeobox 1	protein_codin	1.57	4.87E-08	0.001147	44.96	0.05531
**GOSR2**	GOSR2	5	golgi SNAP receptor complex member 2	protein_codin	-0.2749	0.211	1	9.154	0.05922
**PNPO**	PNPO	5	pyridoxamine 5'-phosphate oxidase	protein_codin	0.2301	0.49	1	3.652	0.05347
**LRRC59**	LRRC59	5	leucine rich repeat containing 59	protein_codin	0.1127	0.592	1	7.896	0.05680
**LUC7L3**	LUC7L3	5	LUC7 like 3 pre-mRNA splicing factor	protein_codin	-0.3531	0.0102	1	35.16	0.1767
**HID1**	HID1	5	HID1 domain containing	protein_codin	1.795	0.000243	1	3.433	0.195
**ASPSCR1**	ASPSCR1	5	ASPSCR1 tether for SLC2A4, UBX domain containing	protein_codin	-0.1846	0.526	1	3.243	0.2
**DCXR**	DCXR	5	dicarbonyl and L-xylulose reductase	protein_codin	-0.1106	0.518	1	19.42	0.1639
**SMCHD1**	SMCHD1	5	structural maintenance of chromosomes flexible hinge domai	protein_codin	0.5004	0.00604	1	25.17	0.2021
**OSBPL1A**	OSBPL1A	5	oxysterol binding protein like 1A	protein_codin	1.442	3.99E-09	0.00094	48.33	0.23
**DSG2**	DSG2	5	desmoglein 2	protein_codin	2.275	2.27E-05	0.5249	8.914	0.352
**POLI**	POLI	5	DNA polymerase iota	protein_codin	0.5840	0.0922	1	3.33	0.1925
**TSHZ1**	TSHZ1	5	teashirt zinc finger homeobox 1	protein_codin	1.125	0.000825	1	44.38	0.1721
**NXT1**	NXT1	5	nuclear transport factor 2 like export factor 1	protein_codin	0.4095	0.00195	1	21.55	0.02681
**ABHD12**	ABHD12	5	abhydrolase domain containing 12	protein_codin	0.07482	0.763	1	7.928	0.2495
**BCL2L1**	BCL2L1	5	BCL2 like 1	protein_codin	-0.3231	0.124	1	52.48	0.2620
**NCOA6**	NCOA6	5	nuclear receptor coactivator 6	protein_codin	0.279	0.374	1	3.23	0.1989
**CSE1L**	CSE1L	5	chromosome segregation 1 like	protein_codin	0.558	0.06	1	3.479	0.1432
**NPEPL1**	NPEPL1	5	aminopeptidase like 1	protein_codin	0.3112	0.262	1	7.826	0.1641
**DAZAP1**	DAZAP1	5	DAZ associated protein 1	protein_codin	0.3657	0.0371	1	7.317	0.1082
**CSNK1G2**	CSNK1G2	5	casein kinase 1 gamma 2	protein_codin	0.1069	0.648	1	7.115	0.1147
**MFSD12**	MFSD12	5	major facilitator superfamily domain containing 12	protein_codin	-0.3601	0.232	1	2.93	0.1846
**PRDX2**	PRDX2	5	peroxiredoxin 2	protein_codin	-0.4942	7.54E-12	1.8E-07	30.4	0.08134
**CCDC130**	CCDC130	5	coiled-coil domain containing 130	protein_codin	-0.03497	0.868	1	24.07	0.2234
**PRKACA**	PRKACA	5	protein kinase cAMP-activated catalytic subunit alpha	protein_codin	0.09758	0.724	1	3.088	0.1756
**SSBP4**	SSBP4	5	single stranded DNA binding protein 4	protein_codin	-0.449	0.000251	1	25.96	0.03022
**COPE**	COPE	5	coatomer protein complex subunit epsilon	protein_codin	-0.3975	1.33E-05	0.3082	43.9	0.1238
**NDUFA13**	NDUFA13	5	NADH:ubiquinone oxidoreductase subunit A13	protein_codin	-0.1568	0.358	1	15.96	0.1907
**ZNF181**	ZNF181	5	zinc finger protein 181	protein_codin	-0.1864	0.548	1	8.699	0.1752
**GRAMD1A**	GRAMD1A	5	GRAM domain containing 1A	protein_codin	-0.3025	0.0429	1	50.06	0.1419
**ZC3H4**	ZC3H4	5	zinc finger CCCH-type containing 4	protein_codin	0.1485	0.648	1	3.274	0.2557
**CYTH2**	CYTH2	5	cytohesin 2	protein_codin	-0.08258	0.587	1	14.96	0.02960
**NUCB1**	NUCB1	5	nucleobindin 1	protein_codin	0.1487	0.102	1	19.07	0.0851
**ALDH16A1**	ALDH16A1	5	aldehyde dehydrogenase 16 family member A1	protein_codin	0.4864	0.0577	1	6.966	0.02612
**ZNF415**	ZNF415	5	zinc finger protein 415	protein_codin	0.7596	0.0766	1	9.682	0.3657
**TFPT**	TFPT	5	TCF3 fusion partner	protein_codin	-0.2758	0.206	1	21.2	0.1072
**ZNF787**	ZNF787	5	zinc finger protein 787	protein_codin	0.784	0.000422	1	5.875	0.1920
**ZNF274**	ZNF274	5	zinc finger protein 274	protein_codin	0.338	0.304	1	9.077	0.1712
**TFIP11**	TFIP11	5	tuftelin interacting protein 11	protein_codin	0.595	0.0909	1	2.168	0.1955
**DUSP18**	DUSP18	5	dual specificity phosphatase 18	protein_codin	-0.6079	0.0512	1	3.12	0.3325
**APOL3**	APOL3	5	apolipoprotein L3	protein_codin	0.3795	0.0514	1	28.99	0.05646
**TMEM184B**	TMEM184B	5	transmembrane protein 184B	protein_codin	-0.1682	0.476	1	9.618	0.05652
**MRPL39**	MRPL39	5	mitochondrial ribosomal protein L39	protein_codin	0.361	0.106	1	8.142	0.2564
**CCT8**	CCT8	5	chaperonin containing TCP1 subunit 8	protein_codin	0.09448	0.36	1	28.64	0.05249
**CRYZL1**	CRYZL1	5	crystallin zeta like 1	protein_codin	0.2672	0.227	1	45.67	0.136
**DVL1**	DVL1	6	dishevelled segment polarity protein 1	protein_codin	0.7192	0.0154	1	2.919	0.08174
**NMNAT1**	NMNAT1	6	nicotinamide nucleotide adenylyltransferase 1	protein_codin	0.968	0.0172	1	2.638	0.1944
**RBP7**	RBP7	6	retinol binding protein 7	protein_codin	0.5111	4.21E-05	0.9706	52.58	0.2691
**TMEM234**	TMEM234	6	transmembrane protein 234	protein_codin	0.1781	0.574	1	3.214	0.2881
**MRPS15**	MRPS15	6	mitochondrial ribosomal protein S15	protein_codin	-0.1125	0.398	1	31.8	0.1360
**PLEKHO1**	PLEKHO1	6	pleckstrin homology domain containing O1	protein_codin	-1.426	1.62E-11	3.86E-07	42.54	0.12
**PLA2G4A**	PLA2G4A	6	phospholipase A2 group IVA	protein_codin	-2.347	7.35E-20	1.77E-15	21.25	0.05463
**NAV1**	NAV1	6	neuron navigator 1	protein_codin	-2.28	4.4E-32	1.07E-28	52.56	0.02711
**AIDA**	AIDA	6	axin interactor, dorsalization associated	protein_codin	0.07938	0.638	1	12.76	0.05378
**PSEN2**	PSEN2	6	presenilin 2	protein_codin	0.4960	0.177	1	11.88	0.1932
**NID1**	NID1	6	nidogen 1	protein_codin	0.08012	0.786	1	33	0.3441
**IGKC**	IGKC	6	immunoglobulin kappa constant	IG_C_gene	-1.811	2.2E-38	5.33E-34	52.58	0.4582
**NRP2**	NRP2	6	neuropilin 2	protein_codin	1.096	5.64E-08	0.001320	47.62	0.4038
**PPARG**	PPARG	6	peroxisome proliferator activated receptor gamma	protein_codin	-0.01052	0.957	1	52.49	0.2351
**DNASE1L3**	DNASE1L3	6	deoxyribonuclease 1 like 3	protein_codin	0.6252	0.00857	1	52.58	0.2172
**ALCAM**	ALCAM	6	activated leukocyte cell adhesion molecule	protein_codin	-2.177	1.44E-15	3.45E-11	21.76	0.1389
**TM4SF18**	TM4SF18	6	transmembrane 4 L six family member 18	protein_codin	-0.2675	0.0742	1	52.58	0.08454
**TRA2B**	TRA2B	6	transformer 2 beta homolog	protein_codin	0.2072	0.0870	1	52.9	0.0542
**P3H2**	P3H2	6	prolyl 3-hydroxylase 2	protein_codin	0.8699	0.00520	1	52.29	0.3038
**LRCH3**	LRCH3	6	leucine rich repeats and calponin homology domain containin	protein_codin	-0.312	0.252	1	8.396	0.3721
**COMMD8**	COMMD8	6	COMM domain containing 8	protein_codin	-0.1629	0.362	1	6.642	0.1685
**POLR2B**	POLR2B	6	RNA polymerase II subunit B	protein_codin	-0.3072	0.0951	1	17.14	0.1674
**SNCA**	SNCA	6	synuclein alpha	protein_codin	1.756	3.46E-07	0.00811	51.27	0.06034
**MMRN1**	MMRN1	6	multimerin 1	protein_codin	1.834	1.64E-22	3.95E-18	52.58	0.07698
**CPE**	CPE	6	carboxypeptidase E	protein_codin	-1.652	5.92E-24	1.43E-19	52.58	0.166
**C5orf42**	C5orf42	6	ciliogenesis and planar polarity effector 1	protein_codin	NA	NA	NA	9.423	0.2797
**C7**	C7	6	complement C7	protein_codin	2.122	1.78E-17	4.28E-13	52.07	0.1792
**SELENOP**	SELENOP	6	selenoprotein P	protein_codin	0.4777	5.63E-06	0.131	52.58	0.1245
**CRHBP**	CRHBP	6	corticotropin releasing hormone binding protein	protein_codin	1.535	9.26E-08	0.002177	52.58	0.3256
**NR2F1**	NR2F1	6	nuclear receptor subfamily 2 group F member 1	protein_codin	0.462	0.108	1	33.79	0.3230
**LTC4S**	LTC4S	6	leukotriene C4 synthase	protein_codin	-1.187	7.09E-44	1.72E-39	48	0.1462
**TPMT**	TPMT	6	thiopurine S-methyltransferase	protein_codin	0.0612	0.81	1	7.972	0.02779
**EFHC1**	EFHC1	6	EF-hand domain containing 1	protein_codin	-0.0316	0.906	1	8.534	0.2357
**MARCKS**	MARCKS	6	myristoylated alanine rich protein kinase C substrate	protein_codin	0.3491	0.00204	1	48.03	0.08879
**FAM126A**	FAM126A	6	family with sequence similarity 126 member A	protein_codin	0.02612	0.925	1	9.486	0.1141
**NUDCD3**	NUDCD3	6	NudC domain containing 3	protein_codin	-0.09297	0.702	1	7.862	0.3792
**POR**	POR	6	cytochrome p450 oxidoreductase	protein_codin	-0.8490	0.000488	1	9.702	0.1329
**CD36**	CD36	6	CD36 molecule	protein_codin	1.296	0.000888	1	47.46	0.2819
**NAMPT**	NAMPT	6	nicotinamide phosphoribosyltransferase	protein_codin	-0.1571	0.111	1	52.52	0.02672
**TIMP1**	TIMP1	6	TIMP metallopeptidase inhibitor 1	protein_codin	-1.658	1.7E-68	4.13E-64	Inf	0.1492
**STARD8**	STARD8	6	StAR related lipid transfer domain containing 8	protein_codin	0.2107	0.487	1	9.652	0.2031
**MMGT1**	MMGT1	6	membrane magnesium transporter 1	protein_codin	0.7295	0.0114	1	6.762	0.2515
**DLC1**	DLC1	6	DLC1 Rho GTPase activating protein	protein_codin	0.8	4.12E-06	0.09594	42.09	0.02957
**CLU**	CLU	6	clusterin	protein_codin	-0.1050	0.0977	1	Inf	0.09517
**PLAT**	PLAT	6	plasminogen activator, tissue type	protein_codin	2.27	8.06E-32	1.95E-28	52.07	0.1695
**FABP5**	FABP5	6	fatty acid binding protein 5	protein_codin	0.7429	8.61E-06	0.1999	52.58	0.2618
**FABP4**	FABP4	6	fatty acid binding protein 4	protein_codin	0.2922	0.0996	1	Inf	0.564
**ZHX1**	ZHX1	6	zinc fingers and homeoboxes 1	protein_codin	0.1112	0.612	1	21.35	0.2171
**THEM6**	THEM6	6	thioesterase superfamily member 6	protein_codin	-0.6775	0.000543	1	23.34	0.1122
**PUM3**	PUM3	6	pumilio RNA binding family member 3	protein_codin	0.6864	7.35E-05	1	34.36	0.1214
**PLIN2**	PLIN2	6	perilipin 2	protein_codin	0.06795	0.688	1	52.07	0.4394
**FAM214B**	FAM214B	6	family with sequence similarity 214 member B	protein_codin	-0.314	0.376	1	9.452	0.1364
**CKS2**	CKS2	6	CDC28 protein kinase regulatory subunit 2	protein_codin	0.4019	0.136	1	51.63	0.2570
**TOR1B**	TOR1B	6	torsin family 1 member B	protein_codin	0.3391	0.319	1	11.96	0.1961
**ABL1**	ABL1	6	ABL proto-oncogene 1, non-receptor tyrosine kinase	protein_codin	-0.2618	0.36	1	3.533	0.1392
**PLEKHA7**	PLEKHA7	6	pleckstrin homology domain containing A7	protein_codin	2.505	0.00156	1	2.37	0.2885
**UBXN1**	UBXN1	6	UBX domain protein 1	protein_codin	0.3514	7.45E-06	0.1731	29.97	0.02681
**ZDHHC24**	ZDHHC24	6	zinc finger DHHC-type containing 24	protein_codin	0.2231	0.219	1	20	0.1791
**NUMA1**	NUMA1	6	nuclear mitotic apparatus protein 1	protein_codin	0.1772	0.358	1	7.518	0.2515
**PRCP**	PRCP	6	prolylcarboxypeptidase	protein_codin	0.5914	9.87E-13	2.36E-08	52.58	0.02886
**ENDOD1**	ENDOD1	6	endonuclease domain containing 1	protein_codin	1.302	9.54E-11	2.27E-06	22.67	0.2686
**SLC35F2**	SLC35F2	6	solute carrier family 35 member F2	protein_codin	-0.6952	0.0107	1	11.18	0.06345
**FXYD6**	FXYD6	6	FXYD domain containing ion transport regulator 6	protein_codin	-1.016	7.59E-12	1.81E-07	35.44	0.06132
**IDI1**	IDI1	6	isopentenyl-diphosphate delta isomerase 1	protein_codin	0.1246	0.525	1	21.96	0.05664
**MAPK8**	MAPK8	6	mitogen-activated protein kinase 8	protein_codin	-0.1495	0.568	1	10.86	0.3227
**JMJD1C**	JMJD1C	6	jumonji domain containing 1C	protein_codin	0.2876	0.00751	1	47.14	0.1422
**COMTD1**	COMTD1	6	catechol-O-methyltransferase domain containing 1	protein_codin	-0.04131	0.877	1	9.329	0.1922
**NUTM2A-AS1**	NUTM2A-A	6	NUTM2A antisense RNA 1	antisense_RN	0.2052	0.417	1	9.423	0.2484
**COPS7A**	COPS7A	6	COP9 signalosome subunit 7A	protein_codin	-0.6078	0.00155	1	7.614	0.216
**PTMS**	PTMS	6	parathymosin	protein_codin	0.1772	0.00302	1	52.58	0.02744
**APOLD1**	APOLD1	6	apolipoprotein L domain containing 1	protein_codin	1.786	1.47E-21	3.54E-17	27.93	0.08419
**PLBD1**	PLBD1	6	phospholipase B domain containing 1	protein_codin	0.2096	0.475	1	9.758	0.1655
**MGP**	MGP	6	matrix Gla protein	protein_codin	-3.033	2.12E-174	5.16E-170	NA	0.1392
**KLHL42**	KLHL42	6	kelch like family member 42	protein_codin	-0.6071	0.0312	1	10.29	0.2064
**ZCRB1**	ZCRB1	6	zinc finger CCHC-type and RNA binding motif containing 1	protein_codin	-0.45	0.000194	1	46.21	0.1108
**CTDSP2**	CTDSP2	6	CTD small phosphatase 2	protein_codin	0.2751	0.208	1	18.25	0.1161
**SLC16A7**	SLC16A7	6	solute carrier family 16 member 7	protein_codin	1.295	0.000550	1	8.482	0.1630
**DCN**	DCN	6	decorin	protein_codin	-4.68	3.17E-29	7.67E-25	52.58	0.2184
**UBE2N**	UBE2N	6	ubiquitin conjugating enzyme E2 N	protein_codin	0.1692	0.182	1	33.33	0.1104
**TMCC3**	TMCC3	6	transmembrane and coiled-coil domain family 3	protein_codin	-0.2137	0.428	1	8.865	0.05562
**MICU2**	MICU2	6	mitochondrial calcium uptake 2	protein_codin	0.0999	0.66	1	9.273	0.1131
**PDS5B**	PDS5B	6	PDS5 cohesin associated factor B	protein_codin	0.296	0.142	1	7.792	0.1107
**POSTN**	POSTN	6	periostin	protein_codin	1.079	7.48E-12	1.78E-07	52.58	0.187
**TPP2**	TPP2	6	tripeptidyl peptidase 2	protein_codin	-0.003658	0.988	1	8.398	0.2294
**RNASE1**	RNASE1	6	ribonuclease A family member 1, pancreatic	protein_codin	-0.5815	7.95E-29	1.92E-24	52.58	0.09011
**FLRT2**	FLRT2	6	fibronectin leucine rich transmembrane protein 2	protein_codin	0.2427	0.369	1	43.65	0.3372
**LGMN**	LGMN	6	legumain	protein_codin	-0.01172	0.954	1	48.03	0.1752
**IGHA1**	IGHA1	6	immunoglobulin heavy constant alpha 1	IG_C_gene	-0.1822	8.47E-08	0.001992	52.58	0.7424
**TMEM87A**	TMEM87A	6	transmembrane protein 87A	protein_codin	0.1689	0.285	1	15.15	0.2616
**HAGH**	HAGH	6	hydroxyacylglutathione hydrolase	protein_codin	-0.4382	0.00408	1	36.97	0.1514
**CREBBP**	CREBBP	6	CREB binding protein	protein_codin	0.1769	0.474	1	3.419	0.1429
**ALDOA**	ALDOA	6	aldolase, fructose-bisphosphate A	protein_codin	0.06966	0.815	1	9.088	0.2581
**ZFHX3**	ZFHX3	6	zinc finger homeobox 3	protein_codin	-0.3459	0.199	1	10.81	0.02608
**GCSH**	GCSH	6	glycine cleavage system protein H	protein_codin	-0.2059	0.305	1	21.98	0.05580
**COTL1**	COTL1	6	coactosin like F-actin binding protein 1	protein_codin	0.6293	4.64E-06	0.108	52	0.0552
**PITPNA**	PITPNA	6	phosphatidylinositol transfer protein alpha	protein_codin	-0.03505	0.85	1	21.32	0.08586
**USP22**	USP22	6	ubiquitin specific peptidase 22	protein_codin	0.1277	0.452	1	5.933	0.08232
**WIPF2**	WIPF2	6	WAS/WASL interacting protein family member 2	protein_codin	0.03700	0.888	1	8.943	0.1517

**RAMP2**	RAMP2	6	receptor activity modifying protein 2	protein_codin	-0.08719	0.0991	1	52.58	0.02900
**LSM12.00**	LSM12.00	6	LSM12 homolog	protein_codin	0.03279	0.828	1	32.7	0.128
**NMT1**	NMT1	6	N-myristoyltransferase 1	protein_codin	-0.2422	0.242	1	22.55	0.1731
**GALNT1**	GALNT1	6	polypeptide N-acetylgalactosaminyltransferase 1	protein_codin	0.1665	0.294	1	52.52	0.1221
**TCF4**	TCF4	6	transcription factor 4	protein_codin	0.3538	4.17E-05	0.9619	51.56	0.02721
**ESF1**	ESF1	6	ESF1 nucleolar pre-rRNA processing protein homolog	protein_codin	0.270	0.0566	1	29.21	0.2482
**SMIM26**	SMIM26	6	small integral membrane protein 26	protein_codin	0.1119	0.324	1	52.32	0.1398
**CST3**	CST3	6	cystatin C	protein_codin	0.8157	2.31E-35	5.6E-31	52.58	0.08482
**NOL4L**	NOL4L	6	nucleolar protein 4 like	protein_codin	-0.02522	0.92	1	10.28	0.1697
**PROCR**	PROCR	6	protein C receptor	protein_codin	-3.188	1.25E-138	3.04E-134	52.58	0.09972
**MAFB**	MAFB	6	MAF bZIP transcription factor B	protein_codin	0.8235	0.00074	1	48.05	0.2855
**PLTP**	PLTP	6	phospholipid transfer protein	protein_codin	-2.102	6.7E-18	1.61E-13	52.58	0.3151
**ZMYND8**	ZMYND8	6	zinc finger MYND-type containing 8	protein_codin	-0.8555	0.000681	1	8.855	0.255
**ZNF626**	ZNF626	6	zinc finger protein 626	protein_codin	0.3116	0.2	1	7.989	0.1096
**ZNF302**	ZNF302	6	zinc finger protein 302	protein_codin	0.4016	0.0696	1	6.896	0.1686
**EIF3K**	EIF3K	6	eukaryotic translation initiation factor 3 subunit K	protein_codin	0.09586	0.0709	1	52.48	0.02742
**PAK4**	PAK4	6	p21 (RAC1) activated kinase 4	protein_codin	0.22	0.47	1	9.532	0.3157
**NDUFA3**	NDUFA3	6	NADH ubiquinone oxidoreductase subunit A3	protein_codin	0.05426	0.648	1	35.55	0.08405
**EIF1AY**	EIF1AY	6	eukaryotic translation initiation factor 1A Y-linked	protein_codin	-0.1358	0.467	1	52.58	0.3462
**ZDHHC8**	ZDHHC8	6	zinc finger DHHC-type containing 8	protein_codin	0.5666	0.093	1	2.832	0.1129
**SDF2L1**	SDF2L1	6	stromal cell derived factor 2 like 1	protein_codin	-0.5392	0.000627	1	39.03	0.05476
**IGLC2**	IGLC2	6	immunoglobulin lambda constant 2	IG_C_gene	-1.366	5.68E-28	1.37E-23	52.07	0.4252
**IGLC3**	IGLC3	6	immunoglobulin lambda constant 3 (Kern-Oz+ marker)	IG_C_gene	-0.5352	0.0104	1	43.25	0.4644
**HMGXB4**	HMGXB4	6	HMG-box containing 4	protein_codin	0.5839	0.00213	1	7.16	0.02698
**ATF4**	ATF4	6	activating transcription factor 4	protein_codin	0.3011	0.00502	1	39.58	0.0856
**MRPL20**	MRPL20	7	mitochondrial ribosomal protein L20	protein_codin	-0.0611	0.528	1	51.99	0.08562
**NOL9**	NOL9	7	nucleolar protein 9	protein_codin	0.1110	0.702	1	10.17	0.1517
**LZIC**	LZIC	7	leucine zipper and CTNNBIP1 domain containing	protein_codin	-0.1412	0.496	1	42.19	0.1702
**GPN2**	GPN2	7	GPN-loop GTPase 2	protein_codin	0.016	0.955	1	3.408	0.05592
**EYA3**	EYA3	7	EYA transcriptional coactivator and phosphatase 3	protein_codin	0.7718	0.00720	1	3.376	0.1105
**SRSF4**	SRSF4	7	serine and arginine rich splicing factor 4	protein_codin	0.5125	5.96E-05	1	17.63	0.08225
**HDAC1**	HDAC1	7	histone deacetylase 1	protein_codin	0.5025	0.00134	1	26.46	0.08447
**TIE1**	TIE1	7	tyrosine kinase with immunoglobulin like and EGF like domains 1	protein_codin	-0.2771	0.0421	1	25.13	0.0875
**KCNN3**	KCNN3	7	potassium calcium-activated channel subfamily N member 3	protein_codin	-0.1724	0.314	1	22.5	0.1768
**ARHGEF2**	ARHGEF2	7	Rho/Rac guanine nucleotide exchange factor 2	protein_codin	-0.3744	0.112	1	7.092	0.05657
**ISG20L2**	ISG20L2	7	interferon stimulated exonuclease gene 20 like 2	protein_codin	0.1570	0.55	1	7.212	0.2574
**F11R**	F11R	7	F11 receptor	protein_codin	0.2092	0.395	1	9.179	0.1666
**TIPRL**	TIPRL	7	TOR signaling pathway regulator	protein_codin	0.2466	0.0797	1	11.4	0.1787
**PRRC2C**	PRRC2C	7	proline rich coiled-coil 2C	protein_codin	-0.1782	0.0264	1	34.38	0.05414
**RNF2**	RNF2	7	ring finger protein 2	protein_codin	0.2524	0.282	1	3.335	0.1414
**TATDN3**	TATDN3	7	TatD DNase domain containing 3	protein_codin	0.09712	0.728	1	9.692	0.1125
**MRPL55**	MRPL55	7	mitochondrial ribosomal protein L55	protein_codin	9.93E-05	1	1	18.08	0.1214
**CNST**	CNST	7	consortin, connexin sorting protein	protein_codin	0.5677	0.0272	1	23.25	0.119
**PUM2**	PUM2	7	pumilio RNA binding family member 2	protein_codin	0.1621	0.59	1	3.306	0.3231
**EIF2B4**	EIF2B4	7	eukaryotic translation initiation factor 2B subunit delta	protein_codin	0.5004	0.0724	1	32.32	0.1712
**SOS1**	SOS1	7	SOS Ras/Rac guanine nucleotide exchange factor 1	protein_codin	-0.258	0.19	1	52.54	0.1169
**ACTR2**	ACTR2	7	actin related protein 2	protein_codin	-0.02185	0.842	1	23.72	0.08535
**RTKN**	RTKN	7	rhotekin	protein_codin	0.3336	0.253	1	2.856	0.1679
**CAPG**	CAPG	7	capping actin protein, gelsolin like	protein_codin	-0.6772	0.00882	1	52.19	0.09588
**RNF103**	RNF103	7	ring finger protein 103	protein_codin	-0.08882	0.755	1	9.617	0.297
**CCDC115**	CCDC115	7	coiled-coil domain containing 115	protein_codin	0.2	0.245	1	16.56	0.08176
**STAT1**	STAT1	7	signal transducer and activator of transcription 1	protein_codin	-0.01136	0.944	1	52.21	0.1827
**GTF3C3**	GTF3C3	7	general transcription factor IIIC subunit 3	protein_codin	0.4312	0.102	1	3.38	0.1774
**RAPH1**	RAPH1	7	Ras association (RalGDS/AF-6) and pleckstrin homology don	protein_codin	0.8788	0.000995	1	41.27	0.1672
**DIS3L2**	DIS3L2	7	DIS3 like 3'-5' exoribonuclease 2	protein_codin	-0.1896	0.573	1	3.418	0.1795
**EIF4E2**	EIF4E2	7	eukaryotic translation initiation factor 4E family member 2	protein_codin	-0.1511	0.345	1	17.53	0.1415
**THUMPD3**	THUMPD3	7	THUMP domain containing 3	protein_codin	-0.1455	0.512	1	8.263	0.1958
**OGG1**	OGG1	7	8-oxoguanine DNA glycosylase	protein_codin	0.3034	0.325	1	8.992	0.1619
**TADA3**	TADA3	7	transcriptional adaptor 3	protein_codin	-0.3777	0.0722	1	8.203	0.05576
**CMC1**	CMC1	7	C-X9-C motif containing 1	protein_codin	0.1591	0.315	1	15.36	0.05349
**EXOSC7**	EXOSC7	7	exosome component 7	protein_codin	0.4137	0.0528	1	6.072	0.2538
**DALRD3**	DALRD3	7	DALR anticodon binding domain containing 3	protein_codin	0.6917	0.00562	1	7.272	0.1409
**FAM208A**	FAM208A	7	transcription activation suppressor	protein_codin	0.08475	0.688	1	17.58	0.2756
**FLNB**	FLNB	7	filamin B	protein_codin	-0.2854	0.0537	1	30.88	0.1984
**ZNF654**	ZNF654	7	zinc finger protein 654	protein_codin	0.5076	0.2	1	3.057	0.2879
**DTX3L**	DTX3L	7	deltex E3 ubiquitin ligase 3L	protein_codin	0.5766	0.0416	1	8.292	0.2825
**SIAH2**	SIAH2	7	siah E3 ubiquitin protein ligase 2	protein_codin	-0.03851	0.868	1	6.738	0.2864
**KPNA4**	KPNA4	7	karyopherin subunit alpha 4	protein_codin	-0.1925	0.163	1	33.05	0.05929
**FYTTD1**	FYTTD1	7	forty-two-three domain containing 1	protein_codin	-0.1917	0.175	1	42.82	0.1414
**TNIP2**	TNIP2	7	TNFAIP3 interacting protein 2	protein_codin	0.6839	0.000134	1	12.13	0.09256
**ARAP2**	ARAP2	7	ArfGAP with RhoGAP domain, ankyrin repeat and PH domain 2	protein_codin	1.136	0.00102	1	25.35	0.2126
**KLHL5**	KLHL5	7	kelch like family member 5	protein_codin	0.1426	0.558	1	24.11	0.2992
**LIAS**	LIAS	7	lipoic acid synthetase	protein_codin	0.2859	0.352	1	3.447	0.2527
**SMIM14**	SMIM14	7	small integral membrane protein 14	protein_codin	-0.4786	0.000809	1	28.62	0.05758
**OCIAD1**	OCIAD1	7	OCIA domain containing 1	protein_codin	0.06864	0.3677	1	31.1	0.05459
**DCK**	DCK	7	deoxycytidine kinase	protein_codin	0.7538	0.037	1	3.156	0.1729
**PYURF**	PYURF	7	PIGY upstream reading frame	protein_codin	0.02884	0.747	1	14.28	0.02745
**ANKRD50**	ANKRD50	7	ankyrin repeat domain 50	protein_codin	-0.178	0.492	1	8.333	0.1627
**PGRMC2**	PGRMC2	7	progesterone receptor membrane component 2	protein_codin	-0.8206	2.01E-10	4.77E-06	51.44	0.02739
**ARHGAP10**	ARHGAP10	7	Rho GTPase activating protein 10	protein_codin	0.6502	0.086	1	3.505	0.2014
**TMA16**	TMA16	7	translation machinery associated 16 homolog	protein_codin	0.4072	0.0581	1	7.276	0.05822
**PAPD4**	PAPD4	7	terminal nucleotidyltransferase 2	protein_codin	NA	NA	NA	7.596	0.2246
**HSPA4**	HSPA4	7	heat shock protein family A (Hsp70) member 4	protein_codin	0.3414	0.0915	1	7.42	0.05882
**DIAPH1**	DIAPH1	7	diaphanous related formin 1	protein_codin	0.09146	0.652	1	19.14	0.2846
**RGS14**	RGS14	7	regulator of G protein signaling 14	protein_codin	0.7761	0.028	1	10.58	0.2292
**PRPF4B**	PRPF4B	7	pre-mRNA processing factor 4B	protein_codin	-7.47E-05	0.999	1	24.03	0.1412
**NUP153**	NUP153	7	nucleoporin 153	protein_codin	0.4794	0.119	1	10.33	0.08212
**HIST1H1C**	HIST1H1C	7	histone cluster 1 H1 family member c	protein_codin	0.3786	0.102	1	8.157	0.1901
**SRPK1**	SRPK1	7	SRSF protein kinase 1	protein_codin	0.7517	7.2E-05	1	14.26	0.08215
**MAD2L1BP**	MAD2L1BP	7	MAD2L1 binding protein	protein_codin	0.169	0.542	1	8.387	0.1382
**FBXL4**	FBXL4	7	F-box and leucine rich repeat protein 4	protein_codin	0.2266	0.432	1	3.106	0.3806
**CD164**	CD164	7	CD164 molecule	protein_codin	-0.5072	5.52E-05	1	12.67	0.08603
**PRKAR1B**	PRKAR1B	7	protein kinase cAMP-dependent type I regulatory subunit beta	protein_codin	0.3406	0.196	1	3.376	0.3474
**EIF3B**	EIF3B	7	eukaryotic translation initiation factor 3 subunit B	protein_codin	0.06981	0.778	1	20.99	0.0922
**VPS41**	VPS41	7	VPS41 subunit of HOPS complex	protein_codin	0.4734	0.058	1	6.099	0.08888
**UBE2D4**	UBE2D4	7	ubiquitin conjugating enzyme E2 D4 (putative)	protein_codin	0.7872	0.00196	1	7.82	0.1409
**TMEM243**	TMEM243	7	transmembrane protein 243	protein_codin	0.3115	0.0891	1	39.47	0.1674
**BUD31**	BUD31	7	BUD31 homolog	protein_codin	0.02326	0.838	1	21.15	0.05416
**COG5**	COG5	7	component of oligomeric golgi complex 5	protein_codin	-0.255	0.525	1	9.083	0.2557
**MTPN**	MTPN	7	myotrophin	protein_codin	-0.1127	0.252	1	49.36	0.08258
**TCAF1**	TCAF1	7	TRPM8 channel associated factor 1	protein_codin	-0.02197	0.922	1	7.205	0.06784
**PDIA4**	PDIA4	7	protein disulfide isomerase family A member 4	protein_codin	-0.3932	0.0146	1	13.01	0.05608
**PRPS2**	PRPS2	7	phosphoribosyl pyrophosphate synthetase 2	protein_codin	1.047	0.0052	1	3.292	0.1501
**MAOA**	MAOA	7	monoamine oxidase A	protein_codin	2.072	2.28E-11	5.43E-07	48.81	0.05432
**OTUD5**	OTUD5	7	OTU deubiquitinase 5	protein_codin	-0.08414	0.742	1	7.976	0.2072
**HMGN5**	HMGN5	7	high mobility group nucleosome binding domain 5	protein_codin	0.1257	0.617	1	22.03	0.1468
**FAM199X**	FAM199X	7	family with sequence similarity 199, X-linked	protein_codin	-0.4486	0.0670	1	9.638	0.2366
**XIAP**	XIAP	7	X-linked inhibitor of apoptosis	protein_codin	0.4247	0.0228	1	17.37	0.05957
**SOX7**	SOX7	7	SRY-box transcription factor 7	protein_codin	0.2626	0.187	1	35.14	0.3016
**POLR3D**	POLR3D	7	RNA polymerase III subunit D	protein_codin	0.6911	0.0081	1	23.4	0.2508
**SLC39A14**	SLC39A14	7	solute carrier family 39 member 14	protein_codin	-0.6832	0.013	1	3.778	0.1717
**RBPMS**	RBPMS	7	RNA binding protein, mRNA processing factor	protein_codin	0.4655	0.000196	1	52.23	0.05284
**FGFR1**	FGFR1	7	fibroblast growth factor receptor 1	protein_codin	-0.2309	0.265	1	5.836	0.2352
**CSPP1**	CSPP1	7	centrosome and spindle pole associated protein 1	protein_codin	0.3425	0.188	1	9.108	0.2639
**LACTB2**	LACTB2	7	lactamase beta 2	protein_codin	0.1037	0.662	1	6.717	0.171
**GLI4**	GLI4	7	GLI family zinc finger 4	protein_codin	1.081	0.0132	1	3.106	0.1732
**AK3**	AK3	7	adenylate kinase 3	protein_codin	-0.3342	0.0411	1	19.58	0.1522
**DMAC1**	DMAC1	7	distal membrane arm assembly complex 1	protein_codin	-0.1071	0.462	1	41.85	0.1407
**DNAJA1**	DNAJA1	7	DnaJ heat shock protein family (Hsp40) member A1	protein_codin	0.5186	2.49E-12	5.94E-08	52.58	0.02786
**UBAP1**	UBAP1	7	ubiquitin associated protein 1	protein_codin	0.2582	0.383	1	8.703	0.3332
**SHB**	SHB	7	SH2 domain containing adaptor protein B	protein_codin	0.5359	0.0594	1	3.384	0.1437
**POLE3**	POLE3	7	DNA polymerase epsilon 3, accessory subunit	protein_codin	0.04013	0.844	1	19.2	0.05800
**RAB14**	RAB14	7	RAB14, member RAS oncogene family	protein_codin	-0.05642	0.582	1	29.36	0.02949
**SURF2**	SURF2	7	surfeit 2	protein_codin	0.6288	0.0259	1	6.641	0.1635
**PTDSS2**	PTDSS2	7	phosphatidylserine synthase 2	protein_codin	-0.2769	0.33	1	3.053	0.08058
**SBF2**	SBF2	7	SET binding factor 2	protein_codin	0.1171	0.628	1	27.48	0.0665
**ALKBH3**	ALKBH3	7	alkB homolog 3, alpha-ketoglutaratedependent dioxygenase	protein_codin	0.5226	0.05	1	3.094	0.1234
**MARK2**	MARK2	7	microtubule affinity regulating kinase 2	protein_codin	0.265	0.467	1	3.428	0.1409
**TRMT112**	TRMT112	7	tRNA methyltransferase subunit 11-2	protein_codin	0.01091	0.872	1	24.93	0.02784
**AP001528.2**	AP001528.2	7	novel transcri	antisense_RN	1.1	2.65E-05	0.6123	18.46	0.2787
**CHORDC1**	CHORDC1	7	cysteine and histidine rich domain containing 1	protein_codin	-0.04756	0.786	1	15.85	0.2215
**C11orf57**	C11orf57	7	NKAP domain containing 1	protein_codin	NA	NA	NA	5.55	0.05644
**SENCR**	SENCR	7	smooth muscle and endothelial cell enriched migration/differe	antisense_RN	0.692	0.00634	1	6.628	0.3342
**LARP4B**	LARP4B	7	La ribonucleoprotein domain family member 4B	protein_codin	-0.3304	0.248	1	3.542	0.08688
**PFKP**	PFKP	7	phosphofructokinase, platelet	protein_codin	0.05695	0.798	1	21.32	0.1363
**IL15RA**	IL15RA	7	interleukin 15 receptor subunit alpha	protein_codin	0.3065	0.138	1	18.13	0.211
**KIN**	KIN	7	Kin17 DNA and RNA binding protein	protein_codin	0.02866	0.872	1	33.25	0.1772
**CDC123**	CDC123	7	cell division cycle 123	protein_codin	-0.09355	0.508	1	12.7	0.1065
**STAM**	STAM	7	signal transducing adaptor molecule	protein_codin	-0.5862	0.00966	1	7.563	0.1501
**BMI1**	BMI1	7	BMI1 proto-oncogene, polycomb ring finger	protein_codin	-0.2317	0.392	1	3.352	0.09123
**MAP3K8**	MAP3K8	7	mitogen-activated protein kinase kinase kinase 8	protein_codin	1.095	6.86E-12	1.64E-07	51.55	0.02931
**UBE2D1**	UBE2D1	7	ubiquitin conjugating enzyme E2 D1	protein_codin	0.5549	0.00119	1	15.15	0.0911
**TFAM**	TFAM	7	transcription factor A, mitochondrial	protein_codin	0.2806	0.0696	1	11.8	0.1456
**VPS26A**	VPS26A	7	VPS26, retromer complex component A	protein_codin	0.2477	0.103	1	14.29	0.1109
**USMG5**	USMG5	7	ATP synthase membrane subunit DAPIT	protein_codin	NA	NA	NA	37.42	0.05568
**SLC38A2**	SLC38A2	7	solute carrier family 38 member 2	protein_codin	0.007826	0.944	1	47.19	0.08498
**COX14**	COX14	7	cytochrome c oxidase assembly factor COX14	protein_codin	-0.3262	0.00688	1	50.1	0.05507
**SPRYD3**	SPRYD3	7	SPRY domain containing 3	protein_codin	0.4296	0.0782	1	8.058	0.1459
**PA2G4**	PA2G4	7	proliferation-associated 2G4	protein_codin	0.2471	0.00759	1	26.13	0.05401
**LLPH**	LLPH	7	LLP homolog, long-term synaptic facilitation factor	protein_codin	0.6207	0.00424	1	12.99	0.172
**TMBIM4**	TMBIM4	7	transmembrane BAX inhibitor motif containing 4	protein_codin	0.06701	0.352	1	36.12	0.05582
**VEZT**	VEZT	7	vezatin, adherens junctions transmembrane protein	protein_codin	-0.1077	0.548	1	19.75	0.1105
**GNPTAB**	GNPTAB	7	N-acetylglucosamine-1-phosphate transferase subunits alpha	protein_codin	0.01147	0.962	1	8.283	0.1379
**MAPKAPK5-AS1**	MAPKAPK5	7	MAPKAPK5 antisense RNA 1	lincRNA	0.1477	0.5	1	7.92	0.08471
**MED13L**	MED13L	7	mediator complex subunit 13L	protein_codin	0.3455	0.0811	1	13.8	0.1455
**PEBP1**	PEBP1	7	phosphatidylethanolamine binding protein 1	protein_codin	-0.3781	5.3E-09	0.000127	36.9	0.0266
**CAMKK2**	CAMKK2	7	calcium/calmodulin dependent protein kinase kinase 2	protein_codin	0.5172	0.0655	1	7.982	0.147
**USP12**	USP12	7	ubiquitin specific peptidase 12	protein_codin	0.3779	0.12	1	7.785	0.1352
**UFM1**	UFM1	7	ubiquitin fold modifier 1	protein_codin	0.06955	0.613	1	21.47	0.2192
**FOXO1**	FOXO1	7	forkhead box O1	protein_codin	0.3813	0.0202	1	11.38	0.1421
**UCHL3**	UCHL3	7	ubiquitin C-terminal hydrolase L3	protein_codin	-0.106	0.585	1	8.268	0.1675
**CHMP4A**	CHMP4A	7	charged multivesicular body protein 4A	protein_codin	0.1684	0.202	1	22.8	0.1086
**PPP2R3C**	PPP2R3C	7	protein phosphatase 2 regulatory subunit B''gamma	protein_codin	0.3592	0.1	1	7.008	0.08641
**AP5M1**	AP5M1	7	adaptor related protein complex 5 subunit mu 1	protein_codin	0.13	0.695	1	3.265	0.2238
**GSTZ1**	GSTZ1	7	glutathione S-transferase zeta 1	protein_codin	-0.02347	0.938	1	3.592	0.1618
**GOLGA5**	GOLGA5	7	golgin A5	protein_codin	0.812	0.00639	1	7.066	0.08116
**EVL**	EVL	7	Enah/Vasp-like	protein_codin	0.1575	0.622	1	18.21	0.2532
**CYFIP1**	CYFIP1	7	cytoplasmic FMR1 interacting protein 1	protein_codin	0.2911	0.13	1	7.217	0.1389
**STARD9**	STARD9	7	StAR related lipid transfer domain containing 9	protein_codin	1.976	0.000175	1	9.086	0.234
**LYSMD2**	LYSMD2	7	LysM domain containing 2	protein_codin	0.729	1.36E-06	0.03179	22.82	0.2452
**RORA**	RORA	7	RAR related orphan receptor A	protein_codin	0.3624	0.0506	1	12	0.169
**HERC1**	HERC1	7	HECT and RLD domain containing E3 ubiquitin protein ligase	protein_codin	-0.2457	0.312	1	7.368	0.2249
**ZNF609**	ZNF609	7	zinc finger protein 609	protein_codin	-0.3022	0.294	1	9.572	0.1412
**TBC1D2B**	TBC1D2B	7	TBC1 domain family member 2B	protein_codin	-0.4482	0.0938	1	3.515	0.2247
**STUB1**	STUB1	7	STIP1 homology and U-box containing protein 1	protein_codin	-0.172	0.134	1	8.272	0.1135
**JMJD8**	JMJD8	7	jumonji domain containing 8	protein_codin	-0.2687	0.158	1	8.033	0.08703
**GLYR1**	GLYR1	7	glyoxylate reductase 1 homolog	protein_codin	-0.137	0.562	1	7.016	0.112
**MARF1**	MARF1	7	meiosis regulator and mRNA stability factor 1	protein_codin	0.4362	0.123	1	10.44	0.08559
**FBRS**	FBRS	7	fibrosin	protein_codin	-0.2128	0.404	1	3.252	0.2304
**CIAPIN1**	CIAPIN1	7	cytokine induced apoptosis inhibitor 1	protein_codin	0.2846	0.182	1	8.487	0.1086
**SLC7A6OS**	SLC7A6OS	7	solute carrier family 7 member 6 opposite strand	protein_codin	0.1902	0.482	1	3.417	0.3181
**ST3GAL2**	ST3GAL2	7	ST3 beta-galactoside alpha-2,3-sialyltransferase 2	protein_codin	0.1086	0.685	1	3.414	0.1412
**KARS**	KARS	7	lysyl-tRNA synthetase	protein_codin	0.02096	0.924	1	23.17	0.1204
**UBE2G1**	UBE2G1	7	ubiquitin conjugating enzyme E2 G1	protein_codin	0.2819	0.218	1	6.432	0.2877
**RABEP1**	RABEP1	7	rabaptin, RAB GTPase binding effector protein 1	protein_codin	-0.1334	0.508	1	7.258	0.1129
**ZBTB4**	ZBTB4	7	zinc finger and BTB domain containing 4	protein_codin	-0.7247	0.000410	1	7.116	0.1659
**POLR2A**	POLR2A	7	RNA polymerase II subunit A	protein_codin	0.1515	0.446	1	21.38	0.08048
**ZSWIM7**	ZSWIM7	7	zinc finger SWIM-type containing 7	protein_codin	-0.1716	0.336	1	17.57	0.1144
**C17orf75**	C17orf75	7	chromosome 17 open reading frame 75	protein_codin	-0.08199	0.748	1	3.278	0.2047
**LINC02001**	LINC02001	7	small nucleolar RNA host gene 30	lincRNA	0.4716	0.0759	1	7.357	0.17
**CCL23**	CCL23	7	C-C motif chemokine ligand 23	protein_codin	2.476	1.68E-05	0.389	52.58	0.7394
**NT5C3B**	NT5C3B	7	5'-nucleotidase, cytosolic IIIB	protein_codin	0.359	0.0729	1	7.648	0.1792
**UBE2Z**	UBE2Z	7	ubiquitin conjugating enzyme E2 Z	protein_codin	-0.08189	0.626	1	50.27	0.08119
**CD79B**	CD79B	7	CD79b molecule	protein_codin	0.5869	0.000307	1	52.53	0.05337
**GRB2**	GRB2	7	growth factor receptor bound protein 2	protein_codin	0.02937	0.812	1	19.16	0.1671
**ITGB4**	ITGB4	7	integrin subunit beta 4	protein_codin	0.4849	0.00116	1	45.22	0.1371
**SLC38A10**	SLC38A10	7	solute carrier family 38 member 10	protein_codin	0.1026	0.663	1	8.628	0.2403
**GPS1**	GPS1	7	G protein pathway suppressor 1	protein_codin	-0.169	0.425	1	8.157	0.1672
**LPIN2**	LPIN2	7	lipin 2	protein_codin	0.003082	0.992	1	3.009	0.3226
**LINC00667**	LINC00667	7	long intergenic non-protein coding RNA 667	lincRNA	0.2622	0.254	1	5.12	0.02642
**PMAIP1**	PMAIP1	7	phorbol-12-myristate-13-acetate-induced protein 1	protein_codin	1.235	0.000870	1	9.865	0.106
**ZNF407**	ZNF407	7	zinc finger protein 407	protein_codin	-0.4762	0.0661	1	9.038	0.1676
**XRN2**	XRN2	7	5'-3' exoribonuclease 2	protein_codin	0.4865	1.91E-05	0.442	40.13	0.1096
**CHD6**	CHD6	7	chromodomain helicase DNA binding protein 6	protein_codin	0.01326	0.942	1	20.62	0.1894
**RNF114**	RNF114	7	ring finger protein 114	protein_codin	0.3265	0.0592	1	33.48	0.2235
**TPGS1**	TPGS1	7	tubulin polyglutamylase complex subunit 1	protein_codin	-0.07026	0.638	1	14.47	0.1392
**WDR18**	WDR18	7	WD repeat domain 18	protein_codin	0.02786	0.894	1	28.46	0.1095
**LSM7.00**	LSM7.00	7	LSM7 homolog, U6 small nuclear RNA and mRNA degradatio	protein_codin	-0.2106	0.0342	1	40.66	0.1391
**SPPL2B**	SPPL2B	7	signal peptide peptidase like 2B	protein_codin	-0.8077	0.00304	1	3.34	0.2931
**ZNF414**	ZNF414	7	zinc finger protein 414	protein_codin	0.07312	0.812	1	3.205	0.2277
**WDR83**	WDR83	7	WD repeat domain 83	protein_codin	0.2988	0.332	1	2.885	0.1948
**ASNA1**	ASNA1	7	guided entry of tail-anchored proteins factor 3, ATPase	protein_codin	0.4717	0.00670	1	6.696	0.0882
**MYO9B**	MYO9B	7	myosin IXB	protein_codin	-1.073	7.08E-06	0.1645	33.68	0.06292
**TSHZ3**	TSHZ3	7	teashirt zinc finger homeobox 3	protein_codin	-0.615	0.0407	1	3.21	0.1502
**PPP5C**	PPP5C	7	protein phosphatase 5 catalytic subunit	protein_codin	0.4753	0.0852	1	9.853	0.1369
**AP2S1**	AP2S1	7	adaptor related protein complex 2 subunit sigma 1	protein_codin	-0.2226	0.0111	1	40.13	0.02849
**SAE1**	SAE1	7	SUMO1 activating enzyme subunit 1	protein_codin	0.004914	0.982	1	3.337	0.1072
**BBC3**	BBC3	7	BCL2 binding component 3	protein_codin	-0.3131	0.193	1	42.38	0.09722
**VRK3**	VRK3	7	VRK serine/threonine kinase 3	protein_codin	0.2926	0.322	1	3.106	0.229
**ZNF677**	ZNF677	7	zinc finger protein 677	protein_codin	0.4392	0.0527	1	23.9	0.202
**SNRPD3**	SNRPD3	7	small nuclear ribonucleoprotein D3 polypeptide	protein_codin	-0.3319	0.00027	1	39.83	0.02708
**SRRD**	SRRD	7	SRR1 domain containing	protein_codin	-0.07451	0.77	1	8.265	0.1779
**RBFOX2**	RBFOX2	7	RNA binding fox-1 homolog 2	protein_codin	0.05116	0.787	1	18.52	0.05741
**APOBEC3G**	APOBEC3G	7	apolipoprotein B mRNA editing enzyme catalytic subunit 3G	protein_codin	0.7474	0.0159	1	3.513	0.1426
**SON**	SON	7	SON DNA binding protein	protein_codin	-0.08319	0.273	1	52.58	0.1082
**NOC2L**	NOC2L	8	NOC2 like nucleolar associated transcriptional repressor	protein_codin	-0.2255	0.288	1	8.712	0.2234
**KIAA2013**	KIAA2013	8	KIAA2013	protein_codin	-0.2092	0.341	1	7.882	0.1366
**ARID1A**	ARID1A	8	AT-rich interaction domain 1A	protein_codin	-0.3294	0.126	1	6.362	0.1089
**ZCCHC17**	ZCCHC17	8	zinc finger CCHC-type containing 17	protein_codin	-0.2357	0.108	1	34.67	0.05422
**TRAPPC3**	TRAPPC3	8	trafficking protein particle complex 3	protein_codin	0.045	0.752	1	45.78	0.08477
**MACF1**	MACF1	8	microtubule actin crosslinking factor 1	protein_codin	-0.5147	2.23E-06	0.05202	52.58	0.08629
**ELOVL1**	ELOVL1	8	ELOVL fatty acid elongase 1	protein_codin	0.2452	0.0921	1	11.19	0.1702
**GPBP1L1**	GPBP1L1	8	GC-rich promoter binding protein 1 like 1	protein_codin	0.016	0.935	1	24.18	0.1656
**UQCRH**	UQCRH	8	ubiquinol-cytochrome c reductase hinge protein	protein_codin	-0.03185	0.596	1	28.74	0.03135
**RNF11**	RNF11	8	ring finger protein 11	protein_codin	-0.6762	4.01E-06	0.09326	40.35	0.05382
**C1orf52**	C1orf52	8	chromosome 1 open reading frame 52	protein_codin	0.3212	0.0616	1	45.56	0.1636
**CAPZA1**	CAPZA1	8	capping actin protein of muscle Z-line subunit alpha 1	protein_codin	0.1446	0.345	1	10.12	0.202
**WARS2**	WARS2	8	tryptophanyl tRNA synthetase 2, mitochondrial	protein_codin	1.499	0.00364	1	2.864	0.1479
**SHE**	SHE	8	Src homology 2 domain containing E	protein_codin	0.258	0.12	1	36.84	0.0593
**DAP3**	DAP3	8	death associated protein 3	protein_codin	0.1976	0.29	1	6.077	0.1728
**RIT1**	RIT1	8	Ras like without CAAX 1	protein_codin	0.2906	0.133	1	52.58	0.2266
**COPA**	COPA	8	coatomer protein complex subunit alpha	protein_codin	-0.5826	0.000346	1	19.85	0.1691
**POU2F1**	POU2F1	8	POU class 2 homeobox 1	protein_codin	-0.7155	0.0142	1	3.162	0.1469
**NEK7**	NEK7	8	NIMA related kinase 7	protein_codin	-0.02276	0.928	1	3.526	0.1684
**SNRPE**	SNRPE	8	small nuclear ribonucleoprotein polypeptide E	protein_codin	0.3182	0.0025	1	22.94	0.02701
**DEGS1**	DEGS1	8	delta 4-desaturase, sphingolipid 1	protein_codin	-0.3834	0.0117	1	52.46	0.1947
**RNF187**	RNF187	8	ring finger protein 187	protein_codin	0.3086	0.0692	1	6.892	0.1089
**GNPAT**	GNPAT	8	glyceronephosphate O-acyltransferase	protein_codin	0.462	0.126	1	3.108	0.2726
**ERO1B**	ERO1B	8	endoplasmic reticulum oxidoreductase 1 beta	protein_codin	0.2934	0.345	1	9.552	0.1952
**PQLC3**	PQLC3	8	solute carrier family 66 member 3	protein_codin	0.4494	0.06	1	6.76	0.2198
**MORN2**	MORN2	8	MORN repeat containing 2	protein_codin	0.1902	0.444	1	9.079	0.1137
**STAMBP**	STAMBP	8	STAM binding protein	protein_codin	-0.04163	0.848	1	7.267	0.2209
**KIAA1211L**	KIAA1211L	8	KIAA1211 like	protein_codin	-1.011	4.98E-05	1	19.19	0.02768
**PDCL3**	PDCL3	8	phosducin like 3	protein_codin	0.1431	0.456	1	7.726	0.08141
**NIFK-AS1**	NIFK-AS1	8	NIFK antisense RNA 1	antisense_RN	0.9632	0.0181	1	3.267	0.2262
**FKBP7**	FKBP7	8	FKBP prolyl isomerase 7	protein_codin	-0.1309	0.587	1	8.918	0.2006
**LRRFIP1**	LRRFIP1	8	LRR binding FLII interacting protein 1	protein_codin	0.4404	2.9E-11	6.9E-07	52.58	0.02916
**ILKAP**	ILKAP	8	ILK associated serine/threonine phosphatase	protein_codin	0.3495	0.188	1	3.287	0.145
**CTDSPL**	CTDSPL	8	CTD small phosphatase like	protein_codin	-0.5289	0.0245	1	49.05	0.05846
**DHX30**	DHX30	8	DExH-box helicase 30	protein_codin	-0.136	0.538	1	9.568	0.1095
**SHISA5**	SHISA5	8	shisa family member 5	protein_codin	0.2686	0.027	1	22.25	0.1107
**QARS**	QARS	8	glutaminyl-tRNA synthetase	protein_codin	0.5046	0.0132	1	19.8	0.05406
**USP4**	USP4	8	ubiquitin specific peptidase 4	protein_codin	0.5541	0.0495	1	7.607	0.2215
**RAB7A**	RAB7A	8	RAB7A, member RAS oncogene family	protein_codin	-0.2242	0.0164	1	21.74	0.08426
**ANAPC13**	ANAPC13	8	anaphase promoting complex subunit 13	protein_codin	-0.09802	0.503	1	14.1	0.05564
**RASA2**	RASA2	8	RAS p21 protein activator 2	protein_codin	1.395	0.000487	1	10.66	0.2425
**RNF13**	RNF13	8	ring finger protein 13	protein_codin	0.1501	0.182	1	17.73	0.1687
**EIF2B5**	EIF2B5	8	eukaryotic translation initiation factor 2B subunit epsilon	protein_codin	0.2706	0.285	1	3.429	0.2254
**SLC30A9**	SLC30A9	8	solute carrier family 30 member 9	protein_codin	0.16	0.501	1	7.619	0.1964
**PAICS**	PAICS	8	phosphoribosylaminoimidazole carboxylase and phosphoribo	protein_codin	0.4441	0.0559	1	20.58	0.05605
**NDUFS4**	NDUFS4	8	NADH ubiquinone oxidoreductase subunit S4	protein_codin	-0.1492	0.222	1	11.29	0.1127
**SCAMP1**	SCAMP1	8	secretory carrier membrane protein 1	protein_codin	-0.1676	0.37	1	7.218	0.2317
**ZNF608**	ZNF608	8	zinc finger protein 608	protein_codin	1.33	1.75E-06	0.04085	17.35	0.1989
**SFXN1**	SFXN1	8	sideroflexin 1	protein_codin	-0.8787	0.00119	1	3.287	0.2087
**B4GALT7**	B4GALT7	8	beta-1,4-galactosyltransferase 7	protein_codin	-0.03314	0.892	1	2.949	0.1429
**BLOC1S5**	BLOC1S5	8	biogenesis of lysosomal organelles complex 1 subunit 5	protein_codin	0.04723	0.827	1	22.33	0.1141
**MYLIP**	MYLIP	8	myosin regulatory light chain interacting protein	protein_codin	0.2224	0.336	1	21.78	0.112
**MCM3**	MCM3	8	minichromosome maintenance complex component 3	protein_codin	0.7592	0.00427	1	24.03	0.242
**RWDD1**	RWDD1	8	RWD domain containing 1	protein_codin	-0.2485	0.0021	1	35.92	0.02962
**PHACTR2**	PHACTR2	8	phosphatase and actin regulator 2	protein_codin	-0.2762	0.0051	1	44.65	0.1677
**SCAF8**	SCAF8	8	SR-related CTD associated factor 8	protein_codin	0.3972	0.183	1	8.333	0.1685
**LFNG**	LFNG	8	LFNG O-fucosylpeptide 3-beta-N-acetylglucosaminyltransferase	protein_codin	1.086	2.96E-06	0.06899	51.92	0.08980
**MEOX2**	MEOX2	8	mesenchyme homeobox 2	protein_codin	2.907	5.16E-18	1.24E-13	16.44	0.09275
**RHBDD2**	RHBDD2	8	rhomboid domain containing 2	protein_codin	-0.3236	0.0715	1	21.52	0.1211
**SEMA3E**	SEMA3E	8	semaphorin 3E	protein_codin	3.748	4.75E-05	1	46.82	0.2097
**PRKRIP1**	PRKRIP1	8	PRKR interacting protein 1	protein_codin	0.1784	0.427	1	9.002	0.2474
**CAPZA2**	CAPZA2	8	capping actin protein of muscle Z-line subunit alpha 2	protein_codin	-0.07977	0.312	1	48	0.02829
**TSPAN12**	TSPAN12	8	tetraspanin 12	protein_codin	1.123	4.27E-07	0.01	11.19	0.1406
**FMC1**	FMC1	8	formation of mitochondrial complex V assembly factor 1 homolog	protein_codin	-0.4161	0.0807	1	7.696	0.06075
**PRKAG2**	PRKAG2	8	protein kinase AMP-activated non-catalytic subunit gamma 2	protein_codin	1.022	0.000267	1	43.22	0.2268
**CASK**	CASK	8	calcium/calmodulin dependent serine protein kinase	protein_codin	-0.01238	0.962	1	8.17	0.2002
**ARHGEF9**	ARHGEF9	8	Cdc42 guanine nucleotide exchange factor 9	protein_codin	-0.448	0.108	1	3.294	0.1934
**TSPAN6**	TSPAN6	8	tetraspanin 6	protein_codin	-0.5332	0.00127	1	30.62	0.164
**ENOX2**	ENOX2	8	ecto-NOX disulfide-thiol exchanger 2	protein_codin	0.9912	0.01	1	8.464	0.1998
**HCFC1**	HCFC1	8	host cell factor C1	protein_codin	-0.1098	0.69	1	2.704	0.2320
**G6PD**	G6PD	8	glucose-6-phosphate dehydrogenase	protein_codin	-0.2545	0.319	1	8.266	0.2192
**DKC1**	DKC1	8	dyskerin pseudouridine synthase 1	protein_codin	0.709	0.00466	1	24.02	0.3314
**HOOK3**	HOOK3	8	hook microtubule tethering protein 3	protein_codin	0.01151	0.942	1	13.27	0.1122
**YTHDF3**	YTHDF3	8	YTH N6-methyladenosine RNA binding protein 3	protein_codin	-0.01256	0.962	1	7.972	0.2219
**TRAM1**	TRAM1	8	translocation associated membrane protein 1	protein_codin	-0.08997	0.352	1	52.32	0.0852
**AZIN1**	AZIN1	8	antizyme inhibitor 1	protein_codin	-0.2854	0.166	1	23.96	0.136
**WDYHV1**	WDYHV1	8	WDYHV motif containing 1	protein_codin	0.8011	0.032	1	2.587	0.1129
**AUH**	AUH	8	AU RNA binding methylglutaconyl-CoA hydratase	protein_codin	0.1329	0.677	1	2.642	0.05409
**XPA**	XPA	8	XPA, DNA damage recognition and repair factor	protein_codin	-0.1859	0.258	1	6.942	0.05582
**FAM129B**	FAM129B	8	niban apoptosis regulator 2	protein_codin	0.2114	0.294	1	21.19	0.1214
**DOLK**	DOLK	8	dolichol kinase	protein_codin	-0.3671	0.193	1	1.375	0.1736
**UCK1**	UCK1	8	uridine-cytidine kinase 1	protein_codin	0.2992	0.318	1	2.344	0.3034
**SURF6**	SURF6	8	surfeit 6	protein_codin	0.02699	0.915	1	3.458	0.1391
**USP47**	USP47	8	ubiquitin specific peptidase 47	protein_codin	0.3649	0.143	1	8.218	0.08950
**ZDHHC5**	ZDHHC5	8	zinc finger DHHC-type containing 5	protein_codin	-0.2744	0.193	1	8.856	0.177
**MTA2**	MTA2	8	metastasis associated 1 family member 2	protein_codin	0.5766	0.0746	1	2.882	0.2048
**KAT5**	KAT5	8	lysine acetyltransferase 5	protein_codin	-0.2376	0.358	1	2.964	0.2255
**PACS1**	PACS1	8	phosphofurin acidic cluster sorting protein 1	protein_codin	0.5297	0.0694	1	3.438	0.2932
**ANAPC15**	ANAPC15	8	anaphase promoting complex subunit 15	protein_codin	-0.8365	5.06E-05	1	7.903	0.1726
**CLNS1A**	CLNS1A	8	chloride nucleotide-sensitive channel 1A	protein_codin	0.006308	0.963	1	26.71	0.1429
**HYOU1**	HYOU1	8	hypoxia up-regulated 1	protein_codin	-0.802	0.00147	1	2.627	0.1747
**VWA5A**	VWA5A	8	von Willebrand factor A domain containing 5A	protein_codin	0.5612	0.0912	1	2.997	0.1989

**TMEM218**	TMEM218	8	transmembrane protein 218	protein_codin	0.172	0.454	1	2.9	0.1642
**RPUSD4**	RPUSD4	8	RNA pseudouridine synthase D4	protein_codin	0.2862	0.33	1	3.292	0.2012
**PCBD1**	PCBD1	8	pterin-4 alpha-carbinolamine dehydratase 1	protein_codin	0.06558	0.642	1	27.52	0.1103
**RPP30**	RPP30	8	ribonuclease P/MRP subunit p30	protein_codin	0.4431	0.0312	1	7.985	0.2238
**NPM3**	NPM3	8	nucleophosmin/nucleoplasmin 3	protein_codin	0.4309	0.152	1	2.758	0.1197
**MGEA5**	MGEA5	8	O-GlcNAcase	protein_codin	NA	NA	NA	7.05	0.173
**SH3PXD2A**	SH3PXD2A	8	SH3 and PX domains 2A	protein_codin	0.1219	0.729	1	9.577	0.06812
**SFR1**	SFR1	8	SWI5 dependent homologous recombination repair protein 1	protein_codin	-0.1219	0.578	1	6.553	0.1952
**ADD3**	ADD3	8	adducin 3	protein_codin	0.3365	0.175	1	22.86	0.1702
**VTI1A**	VTI1A	8	vesicle transport through interaction with t-SNAREs 1A	protein_codin	0.8535	0.00692	1	3.168	0.1997
**WDR11**	WDR11	8	WD repeat domain 11	protein_codin	0.017	0.95	1	7.826	0.2571
**HTRA1**	HTRA1	8	HtrA serine peptidase 1	protein_codin	-1.229	5.2E-37	1.26E-32	52.54	0.02946
**STK32C**	STK32C	8	serine/threonine kinase 32C	protein_codin	0.5694	0.0882	1	3.362	0.2002
**GOLT1B**	GOLT1B	8	golgi transport 1B	protein_codin	0.08822	0.737	1	3.279	0.2532
**PRKAG1**	PRKAG1	8	protein kinase AMP-activated non-catalytic subunit gamma 1	protein_codin	0.3364	0.167	1	6.864	0.08869
**SPATS2**	SPATS2	8	spermatogenesis associated serine rich 2	protein_codin	-0.03375	0.862	1	19.78	0.08541
**CBX5**	CBX5	8	chromobox 5	protein_codin	-0.2056	0.202	1	6.606	0.19
**BLOC1S1**	BLOC1S1	8	biogenesis of lysosomal organelles complex 1 subunit 1	protein_codin	-0.02625	0.819	1	33.62	0.08159
**DDIT3**	DDIT3	8	DNA damage inducible transcript 3	protein_codin	0.5759	0.0145	1	39.84	0.03062
**OS9**	OS9	8	OS9 endoplasmic reticulum lectin	protein_codin	-0.4741	5.05E-06	0.1175	40.98	0.05762
**9-Mar**	43533	8	membrane associated ring-CH-type finger 9	protein_codin	-0.3308	0.213	1	9.429	0.0815
**BRAP**	BRAP	8	BRCA1 associated protein	protein_codin	0.3252	0.308	1	3.243	0.08482
**SPPL3**	SPPL3	8	signal peptide peptidase like 3	protein_codin	0.04336	0.852	1	6.588	0.1637
**DENR**	DENR	8	density regulated re-initiation and release factor	protein_codin	0.2941	0.0777	1	15.93	0.1739
**MRPL57**	MRPL57	8	mitochondrial ribosomal protein L57	protein_codin	-0.3318	0.000858	1	31.72	0.08321
**NUFIP1**	NUFIP1	8	nuclear FMR1 interacting protein 1	protein_codin	0.261	0.332	1	8.075	0.3211
**ANKRD10**	ANKRD10	8	ankyrin repeat domain 10	protein_codin	0.1564	0.308	1	47.46	0.08677
**MTHFD1**	MTHFD1	8	methylenetetrahydrofolate dehydrogenase, cyclohydrolase an	protein_codin	1.006	0.0112	1	3.232	0.1742
**HSPA2**	HSPA2	8	heat shock protein family A (Hsp70) member 2	protein_codin	0.2212	0.277	1	52.58	0.3855
**ISCA2**	ISCA2	8	iron-sulfur cluster assembly 2	protein_codin	0.5775	0.00765	1	3.247	0.1401
**PPP4R3A**	PPP4R3A	8	protein phosphatase 4 regulatory subunit 3A	protein_codin	-0.3241	0.113	1	8.746	0.1381
**GSKIP**	GSKIP	8	GSK3B interacting protein	protein_codin	0.2902	0.314	1	52.14	0.282
**EIF2AK4**	EIF2AK4	8	eukaryotic translation initiation factor 2 alpha kinase 4	protein_codin	0.2398	0.15	1	12.63	0.1431
**TNFAIP8L3**	TNFAIP8L3	8	TNF alpha induced protein 8 like 3	protein_codin	1.49	0.000207	1	29.56	0.142
**ARPP19**	ARPP19	8	cAMP regulated phosphoprotein 19	protein_codin	0.1375	0.405	1	46.72	0.08297
**APH1B**	APH1B	8	aph-1 homolog B, gamma-secretase subunit	protein_codin	0.05182	0.843	1	7.946	0.1982
**PLEKHO2**	PLEKHO2	8	pleckstrin homology domain containing O2	protein_codin	0.5347	0.0987	1	9.548	0.2474
**UBL7-AS1**	UBL7-AS1	8	UBL7 antisense RNA 1 (head to head)	antisense_RN	0.4794	0.163	1	9.222	0.2842
**CSK**	CSK	8	C-terminal Src kinase	protein_codin	0.2468	0.466	1	9.255	0.1068
**FLYWCH2**	FLYWCH2	8	FLYWCH family member 2	protein_codin	0.3872	0.00556	1	27.54	0.1424
**ROGDI**	ROGDI	8	rogdi atypical leucine zipper	protein_codin	-0.2402	0.458	1	9.687	0.1071
**RRN3**	RRN3	8	RRN3 homolog, RNA polymerase I transcription factor	protein_codin	0.6677	0.0581	1	9.015	0.3437
**C16orf45**	C16orf45	8	bMERB domain containing 1	protein_codin	1.192	1.68E-07	0.003945	13.87	0.1605
**IL4R**	IL4R	8	interleukin 4 receptor	protein_codin	0.01287	0.962	1	9.454	0.146
**NFATC2IP**	NFATC2IP	8	nuclear factor of activated T cells 2 interacting protein	protein_codin	-0.09872	0.748	1	9.324	0.2308
**FUS**	FUS	8	FUS RNA binding protein	protein_codin	0.3766	3.42E-05	0.7892	46.54	0.1606
**NIP7**	NIP7	8	nucleolar pre-rRNA processing protein NIP7	protein_codin	0.679	0.00253	1	8.606	0.08085
**YWHAE**	YWHAE	8	tyrosine 3-monooxygenase/tryptophan 5-monooxygenase act	protein_codin	0.1779	0.00871	1	50.2	0.02645
**INPP5K**	INPP5K	8	inositol polyphosphate-5-phosphatase K	protein_codin	0.4512	0.0535	1	9.348	0.05706
**TAX1BP3**	TAX1BP3	8	Tax1 binding protein 3	protein_codin	-0.2908	0.076	1	18.29	0.09017
**EMC6**	EMC6	8	ER membrane protein complex subunit 6	protein_codin	-0.2368	0.0862	1	13.42	0.1469
**FLII**	FLII	8	FLII actin remodeling protein	protein_codin	0.2279	0.371	1	7.785	0.0274
**CBX1**	CBX1	8	chromobox 1	protein_codin	-0.3479	0.0669	1	39.36	0.07984
**PPP1R9B**	PPP1R9B	8	protein phosphatase 1 regulatory subunit 9B	protein_codin	0.7181	0.0306	1	3.584	0.2551
**NAT9**	NAT9	8	N-acetyltransferase 9 (putative)	protein_codin	-0.2094	0.458	1	3.434	0.145
**PRPSAP1**	PRPSAP1	8	phosphoribosyl pyrophosphate synthetase associated protein	protein_codin	1.062	3.87E-10	9.18E-06	24.41	0.1149
**ST6GALNAC1**	ST6GALNA	8	ST6 N-acetylgalactosaminide alpha-2,6-sialyltransferase 1	protein_codin	0.358	0.274	1	10.43	0.2272
**CANT1**	CANT1	8	calcium activated nucleotidase 1	protein_codin	0.7288	0.0697	1	2.137	0.1394
**SLC16A3**	SLC16A3	8	solute carrier family 16 member 3	protein_codin	0.1397	0.615	1	10.17	0.1799
**TGIF1**	TGIF1	8	TGFB induced factor homeobox 1	protein_codin	0.5351	0.00610	1	52.48	0.1452
**RBBP8**	RBBP8	8	RB binding protein 8, endonuclease	protein_codin	0.1932	0.471	1	7.95	0.1765
**RNF138**	RNF138	8	ring finger protein 138	protein_codin	0.7322	0.00707	1	6.914	0.1397
**DYM**	DYM	8	dymeclin	protein_codin	0.4497	0.154	1	8.882	0.1447
**TMX4**	TMX4	8	thioredoxin related transmembrane protein 4	protein_codin	-0.6868	2.32E-06	0.05412	43.81	0.08642
**TASP1**	TASP1	8	taspase 1	protein_codin	0.1022	0.724	1	2.967	0.1952
**PHF20**	PHF20	8	PHD finger protein 20	protein_codin	0.2949	0.136	1	14.26	0.1407
**SYS1**	SYS1	8	SYS1 golgi trafficking protein	protein_codin	0.08932	0.633	1	17.23	0.08462
**DAPK3**	DAPK3	8	death associated protein kinase 3	protein_codin	0.4117	0.0950	1	8.329	0.1409
**KHSRP**	KHSRP	8	KH-type splicing regulatory protein	protein_codin	-0.1372	0.576	1	8.266	0.1449
**C19orf66**	C19orf66	8	shiftless antiviral inhibitor of ribosomal frameshifting	protein_codin	0.4154	0.0104	1	36.54	0.1977
**ECSIT**	ECSIT	8	ECSIT signalling integrator	protein_codin	0.5811	0.0109	1	26.92	0.2522
**ZNF507**	ZNF507	8	zinc finger protein 507	protein_codin	0.2258	0.498	1	10.2	0.2213
**FXYD5**	FXYD5	8	FXYD domain containing ion transport regulator 5	protein_codin	0.07177	0.197	1	52.58	0.02998
**ECH1**	ECH1	8	enoyl-CoA hydratase 1	protein_codin	0.3026	0.0035	1	21.32	0.08058
**ETHE1**	ETHE1	8	ETHE1 persulfide dioxygenase	protein_codin	-0.1801	0.136	1	22	0.1116
**XRCC1**	XRCC1	8	X-ray repair cross complementing 1	protein_codin	0.84	0.0132	1	3.249	0.2304
**GEMIN7**	GEMIN7	8	gem nuclear organelle associated protein 7	protein_codin	0.2994	0.332	1	2.796	0.225
**ARHGAP35**	ARHGAP35	8	Rho GTPase activating protein 35	protein_codin	-0.09922	0.733	1	9.15	0.3222
**PPP1R12C**	PPP1R12C	8	protein phosphatase 1 regulatory subunit 12C	protein_codin	0.1345	0.618	1	3.075	0.1084
**EPN1**	EPN1	8	epsin 1	protein_codin	-0.02718	0.871	1	7.318	0.08532
**THAP7**	THAP7	8	THAP domain containing 7	protein_codin	-0.04556	0.877	1	6.893	0.1096
**BCR**	BCR	8	BCR activator of RhoGEF and GTPase	protein_codin	-0.03149	0.873	1	37.5	0.135
**PIK3IP1**	PIK3IP1	8	phosphoinositide-3-kinase interacting protein 1	protein_codin	0.3306	0.132	1	19.07	0.1632
**N6AMT1**	N6AMT1	8	N-6 adenine-specific DNA methyltransferase 1	protein_codin	0.367	0.284	1	2.86	0.202
**MIB2**	MIB2	9	mindbomb E3 ubiquitin protein ligase 2	protein_codin	-0.2567	0.414	1	3.024	0.3092
**PLOD1**	PLOD1	9	procollagen-lysine,2-oxoglutarate 5-dioxygenase 1	protein_codin	-0.7598	0.0022	1	28.5	0.1686
**FUCA1**	FUCA1	9	alpha-L-fucosidase 1	protein_codin	-0.2712	0.254	1	6.937	0.1962
**DNAJB4**	DNAJB4	9	DnaJ heat shock protein family (Hsp40) member B4	protein_codin	-0.05047	0.681	1	52.02	0.1442
**CEPT1**	CEPT1	9	choline/ethanolamine phosphotransferase 1	protein_codin	0.9249	0.0212	1	2.77	0.1186
**MPZL1**	MPZL1	9	myelin protein zero like 1	protein_codin	-0.4582	0.0145	1	8.503	0.02832
**UCHL5**	UCHL5	9	ubiquitin C-terminal hydrolase L5	protein_codin	0.05136	0.802	1	7.636	0.1486
**ADIPOR1**	ADIPOR1	9	adiponectin receptor 1	protein_codin	-0.158	0.396	1	6.396	0.106
**EGLN1**	EGLN1	9	egl-9 family hypoxia inducible factor 1	protein_codin	0.2659	0.146	1	30.72	0.1559
**LYST**	LYST	9	lysosomal trafficking regulator	protein_codin	0.6726	2.06E-05	0.4769	44.47	0.11
**HNRNPU**	HNRNPU	9	heterogeneous nuclear ribonucleoprotein U	protein_codin	-0.0266	0.769	1	27.87	0.02658
**ZNF669**	ZNF669	9	zinc finger protein 669	protein_codin	0.4106	0.305	1	9.687	0.1971
**DDX1**	DDX1	9	DEAD-box helicase 1	protein_codin	0.1627	0.375	1	19.08	0.1162
**CDC42EP3**	CDC42EP3	9	CDC42 effector protein 3	protein_codin	0.5855	3.54E-06	0.08246	52.58	0.02682
**MCFD2**	MCFD2	9	multiple coagulation factor deficiency 2	protein_codin	-0.09436	0.452	1	20	0.08481
**PCYOX1**	PCYOX1	9	prenylcysteine oxidase 1	protein_codin	-0.4022	0.0421	1	20.03	0.2222
**ATOH8**	ATOH8	9	atonal bHLH transcription factor 8	protein_codin	0.214	0.379	1	21.58	0.2205
**DNAJC10**	DNAJC10	9	DnaJ heat shock protein family (Hsp40) member C10	protein_codin	-0.4718	0.00492	1	15	0.1234
**ANKRD28**	ANKRD28	9	ankyrin repeat domain 28	protein_codin	-0.2319	0.142	1	52.53	0.1106
**GOLGB1**	GOLGB1	9	golgin B1	protein_codin	-0.4469	3.17E-05	0.7319	52.58	0.05348
**CHCHD6**	CHCHD6	9	coiled-coil-helix-coiled-coil-helix domain containing 6	protein_codin	0.4599	0.237	1	35.08	0.8036
**CDV3**	CDV3	9	CDV3 homolog	protein_codin	-0.01486	0.876	1	24.57	0.1091
**FAM193A**	FAM193A	9	family with sequence similarity 193 member A	protein_codin	0.0515	0.862	1	9.836	0.3129
**CENPC**	CENPC	9	centromere protein C	protein_codin	0.5436	0.000537	1	10.86	0.1462
**NOCT**	NOCT	9	nocturnin	protein_codin	1.815	0.00382	1	9.214	0.09376
**MAML3**	MAML3	9	mastermind like transcriptional coactivator 3	protein_codin	-0.03828	0.924	1	9.764	0.1986
**SMARCA5**	SMARCA5	9	SWI/SNF related, matrix associated, actin dependent regulato	protein_codin	0.07935	0.552	1	29.52	0.1459
**WWC2**	WWC2	9	WW and C2 domain containing 2	protein_codin	-0.5706	0.0249	1	8.486	0.2555
**ANKRD37**	ANKRD37	9	ankyrin repeat domain 37	protein_codin	-0.4372	0.0455	1	51.72	0.3199
**LIFR**	LIFR	9	LIF receptor subunit alpha	protein_codin	0.8601	8.24E-08	0.001936	29.07	0.03185
**BRD8**	BRD8	9	bromodomain containing 8	protein_codin	0.08235	0.713	1	7.882	0.2856
**RARS**	RARS	9	arginyl-tRNA synthetase	protein_codin	0.1774	0.455	1	8.007	0.1462
**HIVEP1**	HIVEP1	9	human immunodeficiency virus type I enhancer binding protein	protein_codin	-0.3969	0.0542	1	22.03	0.08012
**FLOT1**	FLOT1	9	flotillin 1	protein_codin	0.2754	0.00175	1	29.38	0.02803
**PBX2**	PBX2	9	PBX homeobox 2	protein_codin	0.4146	0.138	1	3.186	0.2037
**RXRB**	RXRB	9	retinoid X receptor beta	protein_codin	0.3313	0.28	1	32.83	0.1909
**OGFRL1**	OGFRL1	9	opioid growth factor receptor like 1	protein_codin	0.2802	0.411	1	9.445	0.3342
**STX7**	STX7	9	syntaxin 7	protein_codin	0.05032	0.763	1	33.06	0.1136
**ARID1B**	ARID1B	9	AT-rich interaction domain 1B	protein_codin	0.3022	0.188	1	15.93	0.1802
**IGF2R**	IGF2R	9	insulin like growth factor 2 receptor	protein_codin	0.3589	0.189	1	9.823	0.1972
**RPS6KA2**	RPS6KA2	9	ribosomal protein S6 kinase A2	protein_codin	-0.2768	0.164	1	20.27	0.2
**7-Sep**	43715	9	septin 7	protein_codin	0.2009	0.00199	1	34.92	0.08584
**YWHAG**	YWHAG	9	tyrosine 3-monooxygenase/tryptophan 5-monooxygenase act	protein_codin	-0.08388	0.588	1	28.78	0.1107
**LAMTOR4**	LAMTOR4	9	late endosomal/lysosomal adaptor, MAPK and MTOR activato	protein_codin	-0.128	0.0867	1	27.25	0.08428
**MEPCE**	MEPCE	9	methylphosphate capping enzyme	protein_codin	1.18	0.000644	1	10.22	0.1916
**TRIM24**	TRIM24	9	tripartite motif containing 24	protein_codin	0.1932	0.421	1	3.675	0.1762
**TMUB1**	TMUB1	9	transmembrane and ubiquitin like domain containing 1	protein_codin	0.2306	0.274	1	5.675	0.1103
**HTATSF1**	HTATSF1	9	HIV-1 Tat specific factor 1	protein_codin	0.1474	0.471	1	7.653	0.2221
**TMEM185A**	TMEM185A	9	transmembrane protein 185A	protein_codin	-0.03461	0.924	1	3.042	0.177
**ASAH1**	ASAH1	9	N-acylsphingosine amidohydrolase 1	protein_codin	-0.08105	0.597	1	13.7	0.11
**PPP3CC**	PPP3CC	9	protein phosphatase 3 catalytic subunit gamma	protein_codin	0.5362	0.0592	1	3.47	0.115
**WRN**	WRN	9	WRN RecQ like helicase	protein_codin	-0.09619	0.78	1	3.524	0.1395
**SOX17**	SOX17	9	SRY-box transcription factor 17	protein_codin	-0.8296	2.22E-05	0.5134	52.58	0.1206
**CHCHD7**	CHCHD7	9	coiled-coil-helix-coiled-coil-helix domain containing 7	protein_codin	0.5586	0.0425	1	6.626	0.2005
**PLEKHF2**	PLEKHF2	9	pleckstrin homology and FYVE domain containing 2	protein_codin	0.08276	0.745	1	3.452	0.2396
**SLC25A32**	SLC25A32	9	solute carrier family 25 member 32	protein_codin	-0.2264	0.302	1	9.24	0.1075
**EBLN3P**	EBLN3P	9	endogenous Bornavirus like nucleoprotein 3, pseudogene	lincRNA	0.1958	0.347	1	7.911	0.1121
**PTAR1**	PTAR1	9	protein prenyltransferase alpha subunit repeat containing 1	protein_codin	0.06268	0.813	1	7.527	0.1967
**ERP44**	ERP44	9	endoplasmic reticulum protein 44	protein_codin	-0.0889	0.512	1	38.36	0.05505
**PSMB7**	PSMB7	9	proteasome subunit beta 7	protein_codin	-0.1527	0.221	1	43.97	0.1369
**ODF2**	ODF2	9	outer dense fiber of sperm tails 2	protein_codin	0.9227	0.0352	1	8.678	0.226
**FAM69B**	FAM69B	9	divergent protein kinase domain 1B	protein_codin	0.1392	0.294	1	42.82	0.1734
**AP2A2**	AP2A2	9	adaptor related protein complex 2 subunit alpha 2	protein_codin	0.004931	0.981	1	25.16	0.1446
**HIPK3**	HIPK3	9	homeodomain interacting protein kinase 3	protein_codin	0.7692	2.04E-08	0.000482	52.02	0.08622
**TSPAN18**	TSPAN18	9	tetraspanin 18	protein_codin	0.1319	0.36	1	39.64	0.1141
**UQCC3**	UQCC3	9	ubiquinol-cytochrome c reductase complex assembly factor 3	protein_codin	-0.03286	0.845	1	14.38	0.1076
**SLC3A2**	SLC3A2	9	solute carrier family 3 member 2	protein_codin	0.4126	6.64E-06	0.1542	38.84	0.05227
**SART1**	SART1	9	spliceosome associated factor 1, recruiter of U4/U6.U5 tri-snR	protein_codin	0.5875	0.0821	1	3.252	0.1999
**TMEM126B**	TMEM126B	9	transmembrane protein 126B	protein_codin	-0.3002	0.068	1	14.58	0.09294
**KMT2A**	KMT2A	9	lysine methyltransferase 2A	protein_codin	0.01694	0.892	1	15.07	0.1074
**TBRG1**	TBRG1	9	transforming growth factor beta regulator 1	protein_codin	-0.1126	0.532	1	7.287	0.06407
**ROBO4**	ROBO4	9	roundabout guidance receptor 4	protein_codin	-0.007913	0.967	1	41.27	0.1474
**RBM17**	RBM17	9	RNA binding motif protein 17	protein_codin	0.3695	0.00311	1	33.68	0.1742
**CREM**	CREM	9	cAMP responsive element modulator	protein_codin	0.1924	0.333	1	38.14	0.08652
**GHITM**	GHITM	9	growth hormone inducible transmembrane protein	protein_codin	0.09825	0.328	1	15.52	0.02857
**HHEX**	HHEX	9	hematopoietically expressed homeobox	protein_codin	0.02683	0.852	1	27.48	0.05571
**WNK1**	WNK1	9	WNK lysine deficient protein kinase 1	protein_codin	0.867	6.5E-07	0.01521	50.64	0.1446
**TULP3**	TULP3	9	TUB like protein 3	protein_codin	0.294	0.296	1	9.882	0.129
**C12orf4**	C12orf4	9	chromosome 12 open reading frame 4	protein_codin	-0.1442	0.636	1	3.134	0.2232
**FAR2**	FAR2	9	fatty acyl-CoA reductase 2	protein_codin	1.794	7.16E-05	1	26.19	0.2254
**ITGA5**	ITGA5	9	integrin subunit alpha 5	protein_codin	-0.2031	0.153	1	44.09	0.0859
**SLC35E3**	SLC35E3	9	solute carrier family 35 member E3	protein_codin	0.1852	0.571	1	8.288	0.1388
**TCHP**	TCHP	9	trichoplein keratin filament binding	protein_codin	0.4127	0.142	1	9.108	0.3209
**INTS6**	INTS6	9	integrator complex subunit 6	protein_codin	0.1907	0.186	1	49.95	0.02929
**TDRD3**	TDRD3	9	tudor domain containing 3	protein_codin	0.3718	0.143	1	8.955	0.1748
**RAP2A**	RAP2A	9	RAP2A, member of RAS oncogene family	protein_codin	0.3569	0.314	1	3.206	0.1076
**TOGARAM1**	TOGARAM	9	TOG array regulator of axonemal microtubules 1	protein_codin	0.1322	0.576	1	3.504	0.09202
**C14orf37**	C14orf37	9	armadillo like helical domain containing 4	protein_codin	NA	NA	NA	8.771	0.2944
**MED6**	MED6	9	mediator complex subunit 6	protein_codin	0.8961	0.000132	1	7.733	0.1624
**NDN**	NDN	9	necdin, MAGE family member	protein_codin	-0.2105	0.268	1	14.46	0.109
**SNAPC5**	SNAPC5	9	small nuclear RNA activating complex polypeptide 5	protein_codin	0.3961	0.0495	1	5.379	0.05276
**NEIL1**	NEIL1	9	nei like DNA glycosylase 1	protein_codin	0.3222	0.22	1	8.715	0.3368
**ZC3H7A**	ZC3H7A	9	zinc finger CCCH-type containing 7A	protein_codin	0.09516	0.646	1	7.537	0.1745
**NSMCE1**	NSMCE1	9	NSE1 homolog, SMC5-SMC6 complex component	protein_codin	0.2132	0.158	1	33.2	0.02657
**OGFOD1**	OGFOD1	9	2-oxoglutarate and iron dependent oxygenase domain contai	protein_codin	-0.175	0.553	1	3.625	0.3446
**NXN**	NXN	9	nucleoredoxin	protein_codin	0.4268	0.0666	1	8.238	0.07958
**STX8**	STX8	9	syntaxin 8	protein_codin	0.2116	0.294	1	8.133	0.05617
**SCO1**	SCO1	9	SCO cytochrome c oxidase assembly protein 1	protein_codin	0.2536	0.404	1	2.977	0.1635
**PEMT**	PEMT	9	phosphatidylethanolamine N-methyltransferase	protein_codin	-0.6881	0.000411	1	8.41	0.1426
**PRPSAP2**	PRPSAP2	9	phosphoribosyl pyrophosphate synthetase associated protein	protein_codin	0.2587	0.236	1	9.552	0.155
**UTP6**	UTP6	9	UTP6 small subunit processome component	protein_codin	0.5347	0.0241	1	23.33	0.1116
**MIEN1**	MIEN1	9	migration and invasion enhancer 1	protein_codin	-0.425	0.00157	1	12.92	0.1162
**KAT2A**	KAT2A	9	lysine acetyltransferase 2A	protein_codin	1.111	0.0147	1	10.8	0.2303
**DDX42**	DDX42	9	DEAD-box helicase 42	protein_codin	-0.0009179	0.996	1	13.47	0.1102
**GNA13**	GNA13	9	G protein subunit alpha 13	protein_codin	-0.1052	0.703	1	31.22	0.176
**EXOC7**	EXOC7	9	exocyst complex component 7	protein_codin	0.08692	0.664	1	7.553	0.1369
**C18orf25**	C18orf25	9	chromosome 18 open reading frame 25	protein_codin	0.3038	0.397	1	3.214	0.2524
**SMAD2**	SMAD2	9	SMAD family member 2	protein_codin	0.1138	0.522	1	14.82	0.112
**CYB5A**	CYB5A	9	cytochrome b5 type A	protein_codin	-0.1946	0.06	1	19.07	0.02712
**NSFL1C**	NSFL1C	9	NSFL1 cofactor	protein_codin	0.398	0.0199	1	50.37	0.1931
**NFS1**	NFS1	9	NFS1 cysteine desulfurase	protein_codin	-0.1852	0.52	1	3.037	0.1966
**R3HDM4**	R3HDM4	9	R3H domain containing 4	protein_codin	0.532	0.0431	1	8.732	0.2522
**UQCR11**	UQCR11	9	ubiquinol-cytochrome c reductase, complex III subunit XI	protein_codin	-0.5305	6.68E-11	1.59E-06	49.22	0.08158
**MAP2K7**	MAP2K7	9	mitogen-activated protein kinase kinase 7	protein_codin	0.6477	0.062	1	2.777	0.1917
**ZNF91**	ZNF91	9	zinc finger protein 91	protein_codin	-0.2184	0.39	1	6.038	0.3758
**SDHAF1**	SDHAF1	9	succinate dehydrogenase complex assembly factor 1	protein_codin	-0.2932	0.26	1	9.122	0.1166
**ZNF576**	ZNF576	9	zinc finger protein 576	protein_codin	-0.1297	0.6	1	2.53	0.1682
**NECTIN2**	NECTIN2	9	nectin cell adhesion molecule 2	protein_codin	0.6552	1.61E-06	0.00038	51.7	0.02742
**PPFIA3**	PPFIA3	9	PTPRF interacting protein alpha 3	protein_codin	0.1471	0.452	1	17.32	0.1142
**ZNF83**	ZNF83	9	zinc finger protein 83	protein_codin	0.02642	0.904	1	21.53	0.2548
**BID**	BID	9	BH3 interacting domain death agonist	protein_codin	0.4454	0.0471	1	8.17	0.1674
**C22orf39**	C22orf39	9	chromosome 22 open reading frame 39	protein_codin	-0.01299	0.945	1	8.282	0.1062
**SMARCB1**	SMARCB1	9	SWI/SNF related, matrix associated, actin dependent regulato	protein_codin	-0.04552	0.79	1	6.524	0.1405
**RBX1**	RBX1	9	ring-box 1	protein_codin	0.02511	0.772	1	28.43	0.03154
**PKNOX1**	PKNOX1	9	PBX/knotted 1 homeobox 1	protein_codin	0.3592	0.247	1	10.15	0.2218
**RRP1**	RRP1	9	ribosomal RNA processing 1	protein_codin	0.2247	0.388	1	8.077	0.06082
**EFHD2**	EFHD2	10	EF-hand domain family member D2	protein_codin	0.6301	9.38E-05	1	34.53	0.02826
**CDC42**	CDC42	10	cell division cycle 42	protein_codin	0.2471	0.000154	1	39.6	0.02682
**PITHD1**	PITHD1	10	PITH domain containing 1	protein_codin	0.9124	7.56E-07	0.01769	12.64	0.115
**SNHG12**	SNHG12	10	small nucleolar RNA host gene 12	antisense_RN	0.2226	0.365	1	32.25	0.08985
**MEAF6**	MEAF6	10	MYST/Esa1 associated factor 6	protein_codin	0.4064	0.00422	1	10.48	0.1079
**EBNA1BP2**	EBNA1BP2	10	EBNA1 binding protein 2	protein_codin	-0.006372	0.968	1	14.86	0.03026
**TACSTD2**	TACSTD2	10	tumor associated calcium signal transducer 2	protein_codin	1.612	0.000287	1	34.74	0.277
**JAK1**	JAK1	10	Janus kinase 1	protein_codin	0.2945	5E-04	1	24.6	0.05279
**AK4**	AK4	10	adenylate kinase 4	protein_codin	2.206	9.1E-08	0.00214	8.609	0.1152
**SRSF11**	SRSF11	10	serine and arginine rich splicing factor 11	protein_codin	0.05215	0.5667	1	27.6	0.1068
**ZNF644**	ZNF644	10	zinc finger protein 644	protein_codin	0.01182	0.939	1	48.95	0.05847
**LIX1L**	LIX1L	10	limb and CNS expressed 1 like	protein_codin	0.3812	0.0571	1	19.52	0.1097
**C1orf56**	C1orf56	10	chromosome 1 open reading frame 56	protein_codin	0.05141	0.794	1	19.68	0.1482
**SLC39A1**	SLC39A1	10	solute carrier family 39 member 1	protein_codin	0.3245	0.00567	1	42.03	0.08068
**SSR2**	SSR2	10	signal sequence receptor subunit 2	protein_codin	-0.01319	0.8625	1	51.49	0.07996
**PEAR1**	PEAR1	10	platelet endothelial aggregation receptor 1	protein_codin	0.3751	0.198	1	3.403	0.05636
**SDHC**	SDHC	10	succinate dehydrogenase complex subunit C	protein_codin	0.08006	0.5	1	25.65	0.08229
**IER5**	IER5	10	immediate early response 5	protein_codin	0.18	0.233	1	47.43	0.0262
**SRGAP2**	SRGAP2	10	SLIT-ROBO Rho GTPase activating protein 2	protein_codin	0.2206	0.303	1	7.798	0.08377
**HADHA**	HADHA	10	hydroxyacyl-CoA dehydrogenase trifunctional multienzyme co	protein_codin	0.4902	1.56E-07	0.003664	52.58	0.1104
**DPY30**	DPY30	10	dpy-30 histone methyltransferase complex regulatory subunit	protein_codin	-0.04574	0.736	1	12.9	0.1088
**PREPL**	PREPL	10	prolyl endopeptidase like	protein_codin	-0.5649	0.0121	1	3.525	0.169
**USP34**	USP34	10	ubiquitin specific peptidase 34	protein_codin	-0.1527	0.311	1	12.78	0.1618
**MCEE**	MCEE	10	methylmalonyl-CoA epimerase	protein_codin	0.2586	0.387	1	3.046	0.4272
**INHBB**	INHBB	10	inhibin subunit beta B	protein_codin	1.227	9.4E-05	1	23.62	0.08048
**TLK1**	TLK1	10	tousled like kinase 1	protein_codin	0.3625	0.0228	1	28.88	0.08702
**TFPI**	TFPI	10	tissue factor pathway inhibitor	protein_codin	1.169	1.28E-27	3.09E-23	52.58	0.09046
**MOB4**	MOB4	10	MOB family member 4, phocein	protein_codin	0.06193	0.703	1	30.93	0.1117
**C2orf69**	C2orf69	10	chromosome 2 open reading frame 69	protein_codin	0.1582	0.646	1	3.2	0.1436
**CASP10**	CASP10	10	caspase 10	protein_codin	-0.01792	0.944	1	8.158	0.2451
**SUMO1**	SUMO1	10	small ubiquitin like modifier 1	protein_codin	0.0927	0.276	1	49.66	0.02771
**NOP58**	NOP58	10	NOP58 ribonucleoprotein	protein_codin	0.3635	0.0349	1	13.68	0.1617
**RNPEPL1**	RNPEPL1	10	arginyl aminopeptidase like 1	protein_codin	0.008566	0.968	1	22.75	0.2232
**TGFBR2**	TGFBR2	10	transforming growth factor beta receptor 2	protein_codin	0.7136	3.46E-19	8.32E-14	44.21	0.02831
**CCDC12**	CCDC12	10	coiled-coil domain containing 12	protein_codin	-0.1131	0.395	1	52.12	0.1411
**CHMP2B**	CHMP2B	10	charged multivesicular body protein 2B	protein_codin	0.1652	0.224	1	10.73	0.11
**ARL13B**	ARL13B	10	ADP ribosylation factor like GTPase 13B	protein_codin	0.2975	0.162	1	7.872	0.1647
**CLDND1**	CLDND1	10	claudin domain containing 1	protein_codin	0.4807	0.00109	1	31.2	0.03119
**ABHD10**	ABHD10	10	abhydrolase domain containing 10	protein_codin	0.3042	0.358	1	3.196	0.252
**UMPS**	UMPS	10	uridine monophosphate synthetase	protein_codin	0.02399	0.93	1	8.466	0.2932
**H1FX**	H1FX	10	H1 histone family member X	protein_codin	-0.0397	0.7097	1	50.61	0.1114
**U2SURP**	U2SURP	10	U2 snRNP associated SURP domain containing	protein_codin	0.2814	0.00914	1	18.9	0.05298
**TSC22D2**	TSC22D2	10	TSC22 domain family member 2	protein_codin	-0.1421	0.422	1	24.29	0.05396
**MFN1**	MFN1	10	mitofusin 1	protein_codin	-0.09918	0.665	1	3.333	0.2566
**EIF4G1**	EIF4G1	10	eukaryotic translation initiation factor 4 gamma 1	protein_codin	-0.2225	0.218	1	6.563	0.1432
**C3orf70**	C3orf70	10	chromosome 3 open reading frame 70	protein_codin	1.277	0.00598	1	3.176	0.2961
**OPA1**	OPA1	10	OPA1 mitochondrial dynamin like GTPase	protein_codin	-0.04085	0.904	1	3.183	0.1926
**GAK**	GAK	10	cyclin G associated kinase	protein_codin	0.03129	0.905	1	9.293	0.1998
**LNX1**	LNX1	10	ligand of numb-protein X 1	protein_codin	1.962	0.00686	1	34.58	0.2642
**THAP6**	THAP6	10	THAP domain containing 6	protein_codin	0.459	0.147	1	8.907	0.3216
**WDFY3**	WDFY3	10	WD repeat and FYVE domain containing 3	protein_codin	-0.6672	0.0249	1	8.407	0.2026
**TMEM267**	TMEM267	10	transmembrane protein 267	protein_codin	0.2659	0.42	1	2.917	0.3491
**IPO11**	IPO11	10	importin 11	protein_codin	-0.04706	0.727	1	36.15	0.1076
**LYSMD3**	LYSMD3	10	LysM domain containing 3	protein_codin	-0.2977	0.188	1	7.922	0.1126
**3-Mar**	43527	10	membrane associated ring-CH-type finger 3	protein_codin	0.4396	0.107	1	10.17	0.08522
**CDKN2AIPNL**	CDKN2AIP	10	CDKN2A interacting protein N-terminal like	protein_codin	0.3688	0.137	1	6.962	0.08569
**SAR1B**	SAR1B	10	secretion associated Ras related GTPase 1B	protein_codin	-0.6735	4.31E-05	0.9935	19.11	0.1131
**G3BP1**	G3BP1	10	G3BP stress granule assembly factor 1	protein_codin	0.432	0.00347	1	23.32	0.1641
**CLTB**	CLTB	10	clathrin light chain B	protein_codin	-0.5533	4.15E-11	9.87E-07	43.26	0.06088
**GMDS**	GMDS	10	GDP-mannose 4,6-dehydratase	protein_codin	-0.7912	3.17E-09	7.5E-05	47.48	0.08592
**SERPINB9**	SERPINB9	10	serpin family B member 9	protein_codin	-1.179	1.26E-08	0.000298	30.45	0.1456
**NQO2**	NQO2	10	N-ribosyldihydronicotinamide:quinone reductase 2	protein_codin	-0.1899	0.336	1	24.83	0.1362
**SLC35B3**	SLC35B3	10	solute carrier family 35 member B3	protein_codin	0.112	0.686	1	3.212	0.1372
**ACOT13**	ACOT13	10	acyl-CoA thioesterase 13	protein_codin	0.0514	0.788	1	39.29	0.08058
**HIST1H1E**	HIST1H1E	10	histone cluster 1 H1 family member e	protein_codin	-0.1645	0.449	1	8.623	0.3135
**BTN2A1**	BTN2A1	10	butyrophilin subfamily 2 member A1	protein_codin	-0.08642	0.747	1	3.08	0.1071
**HSD17B8**	HSD17B8	10	hydroxysteroid 17-beta dehydrogenase 8	protein_codin	0.02125	0.94	1	8.573	0.1408
**ZFAND3**	ZFAND3	10	zinc finger AN1-type containing 3	protein_codin	-0.07276	0.744	1	6.585	0.08532
**ATG5**	ATG5	10	autophagy related 5	protein_codin	0.6004	0.0127	1	6.104	0.02682
**PTPRK**	PTPRK	10	protein tyrosine phosphatase receptor type K	protein_codin	0.2744	0.271	1	9.128	0.2465
**SASH1**	SASH1	10	SAM and SH3 domain containing 1	protein_codin	0.5326	0.000816	1	33.93	0.08417
**RMND1**	RMND1	10	required for meiotic nuclear division 1 homolog	protein_codin	0.3235	0.217	1	9.173	0.145
**FAM120B**	FAM120B	10	family with sequence similarity 120B	protein_codin	0.4746	0.146	1	3.169	0.1736
**DPY19L1**	DPY19L1	10	dpy-19 like C-mannosyltransferase 1	protein_codin	-0.6112	0.0162	1	8.965	0.225
**TAF6**	TAF6	10	TATA-box binding protein associated factor 6	protein_codin	0.1597	0.548	1	3.447	0.232
**NA**	NA	10	RNA polymerase II subunit J3	NA	NA	NA	NA	15.22	0.1954
**PNPLA8**	PNPLA8	10	patatin like phospholipase domain containing 8	protein_codin	0.4066	0.026	1	52.58	0.03233
**TMEM176B**	TMEM176B	10	transmembrane protein 176B	protein_codin	2.002	0.000506	1	38.76	0.2588
**TMEM176A**	TMEM176A	10	transmembrane protein 176A	protein_codin	2.947	0.0012	1	27.78	0.3222
**RBM33**	RBM33	10	RNA binding motif protein 33	protein_codin	0.2512	0.377	1	6.369	0.1976
**OFD1**	OFD1	10	OFD1 centriole and centriolar satellite protein	protein_codin	0.9519	8.7E-06	0.202	6.863	0.3044
**APEX2**	APEX2	10	apurinic/apyrimidinic endodeoxyribonuclease 2	protein_codin	1.188	0.00118	1	3.002	0.2798
**OPHN1**	OPHN1	10	oligophrenin 1	protein_codin	-0.67	0.0206	1	9.785	0.2465
**COX7B**	COX7B	10	cytochrome c oxidase subunit 7B	protein_codin	-0.3292	6.12E-05	1	17.43	0.02679
**CYSLTR1**	CYSLTR1	10	cysteinyl leukotriene receptor 1	protein_codin	2.307	6.52E-13	1.56E-08	52.58	0.1975
**CHM**	CHM	10	CHM Rab escort protein	protein_codin	-0.06108	0.742	1	7.309	0.141
**TCEAL9**	TCEAL9	10	transcription elongation factor A like 9	protein_codin	-0.1292	0.254	1	52.54	0.03116
**DOCK11**	DOCK11	10	dedicator of cytokinesis 11	protein_codin	3.429	0.000322	1	26.34	0.1736
**MCTS1**	MCTS1	10	MCTS1 re-initiation and release factor	protein_codin	-0.07309	0.642	1	12.85	0.1896
**BCAP31**	BCAP31	10	B cell receptor associated protein 31	protein_codin	0.08097	0.327	1	24.95	0.05671
**ELP3**	ELP3	10	elongator acetyltransferase complex subunit 3	protein_codin	0.9085	0.0154	1	2.387	0.2574
**GTF2E2**	GTF2E2	10	general transcription factor IIE subunit 2	protein_codin	-0.2658	0.235	1	9.263	0.2036
**MAK16**	MAK16	10	MAK16 homolog	protein_codin	1.022	0.0015	1	7.804	0.3407
**RNF122**	RNF122	10	ring finger protein 122	protein_codin	1.247	0.00112	1	3.527	0.2322
**PLPP5**	PLPP5	10	phospholipid phosphatase 5	protein_codin	-0.126	0.468	1	16.66	0.1665
**GPAT4**	GPAT4	10	glycerol-3-phosphate acyltransferase 4	protein_codin	0.7012	0.0479	1	2.412	0.2166
**COX6C**	COX6C	10	cytochrome c oxidase subunit 6C	protein_codin	-0.2988	6.06E-07	0.01418	49.32	0.02652
**PSIP1**	PSIP1	10	PC4 and SFRS1 interacting protein 1	protein_codin	0.1391	0.1477	1	52.56	0.1076
**PGM5-AS1**	PGM5-AS1	10	PGM5 antisense RNA 1	antisense_RN	2.652	0.00276	1	37.14	0.4226
**AGTPBP1**	AGTPBP1	10	ATP/GTP binding protein 1	protein_codin	0.3664	0.222	1	3.572	0.196
**SPTLC1**	SPTLC1	10	serine palmitoyltransferase long chain base subunit 1	protein_codin	0.1832	0.297	1	6.662	0.112
**PRPF4**	PRPF4	10	pre-mRNA processing factor 4	protein_codin	-0.02996	0.914	1	3.464	0.1631
**SH3GLB2**	SH3GLB2	10	SH3 domain containing GRB2 like, endophilin B2	protein_codin	0.2602	0.277	1	28.48	0.1185
**TTF1**	TTF1	10	transcription termination factor 1	protein_codin	0.3372	0.105	1	39.1	0.2205
**SLC39A13**	SLC39A13	10	solute carrier family 39 member 13	protein_codin	0.1386	0.596	1	8.086	0.02947
**OSBP**	OSBP	10	oxysterol binding protein	protein_codin	0.5854	0.0222	1	6.219	0.1686
**MACROD1**	MACROD1	10	mono-ADP ribosylhydrolase 1	protein_codin	0.7202	0.00625	1	6.724	0.1969

**CORO1B**	CORO1B	10	coronin 1B	protein_codin	0.3545	0.0166	1	32.02	0.1922
**CCND1**	CCND1	10	cyclin D1	protein_codin	-0.5767	2.6E-05	0.6008	52.58	0.02612
**NCOA4**	NCOA4	10	nuclear receptor coactivator 4	protein_codin	-0.2879	0.0545	1	15.57	0.122
**LIPA**	LIPA	10	lipase A, lysosomal acid type	protein_codin	0.9196	2.35E-06	0.05482	14.88	0.05658
**CALHM2**	CALHM2	10	calcium homeostasis modulator family member 2	protein_codin	0.5097	0.0044	1	30.38	0.02798
**SEC23IP**	SEC23IP	10	SEC23 interacting protein	protein_codin	0.4258	0.225	1	9.108	0.2732
**TACC2**	TACC2	10	transforming acidic coiled-coil containing protein 2	protein_codin	0.1827	0.507	1	10.78	0.1632
**BUB3**	BUB3	10	BUB3 mitotic checkpoint protein	protein_codin	0.2689	0.0604	1	27.67	0.1409
**PTPRE**	PTPRE	10	protein tyrosine phosphatase receptor type E	protein_codin	-0.1966	0.248	1	33.69	0.1006
**LINC00987**	LINC00987	10	long intergenic non-protein coding RNA 987	lincRNA	0.5357	0.18	1	9.127	0.2025
**IRAK4**	IRAK4	10	interleukin 1 receptor associated kinase 4	protein_codin	0.3085	0.172	1	21.42	0.1128
**RPAP3**	RPAP3	10	RNA polymerase II associated protein 3	protein_codin	0.5598	0.0224	1	7.738	0.1437
**IRAK3**	IRAK3	10	interleukin 1 receptor associated kinase 3	protein_codin	-0.06848	0.69	1	18.82	0.08476
**NTS**	NTS	10	neurotensin	protein_codin	3.885	0.9856	1	52.58	0.5711
**SNRPF**	SNRPF	10	small nuclear ribonucleoprotein polypeptide F	protein_codin	0.2852	0.0032	1	11.99	0.03035
**SCYL2**	SCYL2	10	SCY1 like pseudokinase 2	protein_codin	0.5006	0.14	1	8.715	0.229
**TPCN1**	TPCN1	10	two pore segment channel 1	protein_codin	0.314	0.394	1	2.158	0.2115
**TMEM233**	TMEM233	10	transmembrane protein 233	protein_codin	-0.39	0.322	1	44.09	0.1392
**HSPH1**	HSPH1	10	heat shock protein family H (Hsp110) member 1	protein_codin	-0.4692	0.00167	1	47.89	0.06112
**RNASEH2B**	RNASEH2B	10	ribonuclease H2 subunit B	protein_codin	0.4643	0.138	1	7.29	0.1408
**DHRS12**	DHRS12	10	dehydrogenase/reductase 12	protein_codin	0.1808	0.648	1	9.054	0.1716
**MZT1**	MZT1	10	mitotic spindle organizing protein 1	protein_codin	-0.3088	0.132	1	3.419	0.1782
**RBM26**	RBM26	10	RNA binding motif protein 26	protein_codin	0.1042	0.622	1	6.74	0.08226
**TFDP1**	TFDP1	10	transcription factor Dp-1	protein_codin	0.3308	0.194	1	3.15	0.08857
**C14orf119**	C14orf119	10	chromosome 14 open reading frame 119	protein_codin	0.02198	0.872	1	12.78	0.02946
**MBIP**	MBIP	10	MAP3K12 binding inhibitory protein 1	protein_codin	0.2132	0.368	1	9.105	0.1082
**PSMC6**	PSMC6	10	proteasome 26S subunit, ATPase 6	protein_codin	0.1001	0.45	1	11.86	0.1458
**NDUFB1**	NDUFB1	10	NADH ubiquinone oxidoreductase subunit B1	protein_codin	-0.1867	0.0682	1	43.06	0.1112
**PACS2**	PACS2	10	phosphofurin acidic cluster sorting protein 2	protein_codin	0.09391	0.672	1	6.857	0.1426
**RYR3**	RYR3	10	ryanodine receptor 3	protein_codin	0.3009	0.454	1	9.268	0.3476
**GCHFR**	GCHFR	10	GTP cyclohydrolase I feedback regulator	protein_codin	-0.225	0.178	1	39.98	0.1347
**CASC4**	CASC4	10	cancer susceptibility 4	protein_codin	-0.3025	0.0525	1	7.096	0.08198
**TRIM69**	TRIM69	10	tripartite motif containing 69	protein_codin	-0.02021	0.922	1	23.12	0.162
**SCAPER**	SCAPER	10	S-phase cyclin A associated protein in the ER	protein_codin	0.5868	0.0124	1	8.726	0.1417
**SNHG9**	SNHG9	10	small nucleolar RNA host gene 9	lincRNA	-0.4869	0.029	1	8.417	0.2218
**TNFRSF12A**	TNFRSF12A	10	TNF receptor superfamily member 12A	protein_codin	-1.258	4.82E-11	1.15E-06	52.06	0.1164
**UBN1**	UBN1	10	ubinuclein 1	protein_codin	0.3282	0.0525	1	25.55	0.08688
**FOPNL**	FOPNL	10	FGFR1OP N-terminal like	protein_codin	0.1308	0.592	1	8.486	0.2246
**GPRC5B**	GPRC5B	10	G protein-coupled receptor class C group 5 member B	protein_codin	-0.4299	0.0246	1	20.03	0.1964
**THUMPD1**	THUMPD1	10	THUMP domain containing 1	protein_codin	-0.1159	0.405	1	18.82	0.08368
**PPP4C**	PPP4C	10	protein phosphatase 4 catalytic subunit	protein_codin	0.01096	0.928	1	26.48	0.08418
**ZNF771**	ZNF771	10	zinc finger protein 771	protein_codin	-1.542	8.4E-10	1.99E-05	3.418	0.05725
**ARMC5**	ARMC5	10	armadillo repeat containing 5	protein_codin	1.008	0.00536	1	3.34	0.308
**AKTIP**	AKTIP	10	AKT interacting protein	protein_codin	0.4147	0.106	1	9.262	0.1429
**CHTF8**	CHTF8	10	chromosome transmission fidelity factor 8	protein_codin	0.0662	0.772	1	9.169	0.1632
**MLKL**	MLKL	10	mixed lineage kinase domain like pseudokinase	protein_codin	0.2115	0.301	1	20.35	0.02716
**ZDHHC7**	ZDHHC7	10	zinc finger DHHC-type containing 7	protein_codin	-0.01108	0.968	1	2.796	0.1954
**SPATA33**	SPATA33	10	spermatogenesis associated 33	protein_codin	0.4005	0.178	1	3.686	0.3862
**CDK10**	CDK10	10	cyclin dependent kinase 10	protein_codin	0.568	0.031	1	7.062	0.08362
**ITGAE**	ITGAE	10	integrin subunit alpha E	protein_codin	0.1349	0.417	1	15.68	0.1632
**CYB5D2**	CYB5D2	10	cytochrome b5 domain containing 2	protein_codin	-0.4454	0.00895	1	7.737	0.1542
**RPAIN**	RPAIN	10	RPA interacting protein	protein_codin	-0.1789	0.186	1	12.34	0.1719
**MED31**	MED31	10	mediator complex subunit 31	protein_codin	0.09988	0.596	1	20.45	0.3414
**ASGR1**	ASGR1	10	asialoglycoprotein receptor 1	protein_codin	0.8853	0.00477	1	6.888	0.1359
**VAMP2**	VAMP2	10	vesicle associated membrane protein 2	protein_codin	-0.1294	0.156	1	39.88	0.1652
**AC007952.4**	AC007952.4	10	novel transcri	lincRNA	0.7128	0.0261	1	52.28	0.2852
**LYRM9**	LYRM9	10	LYR motif containing 9	protein_codin	0.6566	0.021	1	7.396	0.3031
**UNC119**	UNC119	10	unc-119 lipid binding chaperone	protein_codin	-0.3632	0.181	1	2.707	0.2359
**COASY**	COASY	10	Coenzyme A synthase	protein_codin	0.2096	0.435	1	3.106	0.2003
**KANSL1**	KANSL1	10	KAT8 regulatory NSL complex subunit 1	protein_codin	-0.2279	0.184	1	16.45	0.0839
**NSF**	NSF	10	N-ethylmaleimide sensitive factor, vesicle fusing ATPase	protein_codin	0.04238	0.882	1	2.917	0.1172
**CEP95**	CEP95	10	centrosomal protein 95	protein_codin	0.0127	0.968	1	8.828	0.1736
**ARMC7**	ARMC7	10	armadillo repeat containing 7	protein_codin	-0.7372	0.00698	1	52.28	0.1709
**CHMP1B**	CHMP1B	10	charged multivesicular body protein 1B	protein_codin	-0.426	0.00642	1	52.04	0.1151
**GATA6**	GATA6	10	GATA binding protein 6	protein_codin	-1.052	3.98E-09	9.42E-05	30.55	0.08368
**INO80C**	INO80C	10	INO80 complex subunit C	protein_codin	0.04049	0.818	1	20.06	0.05856
**ACSS2**	ACSS2	10	acyl-CoA synthetase short chain family member 2	protein_codin	0.7112	0.0429	1	3.26	0.3174
**GSS**	GSS	10	glutathione synthetase	protein_codin	0.4357	0.0786	1	3.487	0.05359
**IFT52**	IFT52	10	intraflagellar transport 52	protein_codin	0.1822	0.361	1	5.598	0.05992
**CD40**	CD40	10	CD40 molecule	protein_codin	0.8201	4.91E-11	1.17E-06	50.32	0.08485
**DOK5**	DOK5	10	docking protein 5	protein_codin	-0.9628	0.0017	1	27.19	0.1022
**OSBPL2**	OSBPL2	10	oxysterol binding protein like 2	protein_codin	0.1142	0.743	1	3.502	0.2239
**SLCO4A1**	SLCO4A1	10	solute carrier organic anion transporter family member 4A1	protein_codin	0.6944	0.0107	1	8.94	0.1751
**SGTA**	SGTA	10	small glutamine rich tetratricopeptide repeat containing alpha	protein_codin	0.3154	0.203	1	3.602	0.136
**ALKBH7**	ALKBH7	10	alkB homolog 7	protein_codin	-0.2125	0.0255	1	45.53	0.05486
**TNFSF9**	TNFSF9	10	TNF superfamily member 9	protein_codin	2.752	1.58E-05	0.3659	52.58	0.2121
**QTRT1**	QTRT1	10	queuine tRNA-ribosyltransferase catalytic subunit 1	protein_codin	0.1036	0.668	1	8.567	0.2309
**AP1M1**	AP1M1	10	adaptor related protein complex 1 subunit mu 1	protein_codin	0.001958	0.994	1	3.133	0.1545
**PLAUR**	PLAUR	10	plasminogen activator, urokinase receptor	protein_codin	-0.7256	0.000324	1	52.58	0.2476
**RELB**	RELB	10	RELB proto-oncogene, NF-kB subunit	protein_codin	0.2231	0.274	1	18.28	0.08317
**RASL10A**	RASL10A	10	RAS like family 10 member A	protein_codin	1.079	0.00162	1	26.8	0.2589
**PRR34-AS1**	PRR34-AS1	10	PRR34 antisense RNA 1	antisense_RN	-0.09796	0.717	1	8.397	0.142
**RWDD2B**	RWDD2B	10	RWD domain containing 2B	protein_codin	0.1617	0.645	1	9.955	0.1918
**AGRN**	AGRN	11	agrin	protein_codin	-0.159	0.506	1	21.63	0.1102
**NADK**	NADK	11	NAD kinase	protein_codin	0.6846	0.0102	1	6.677	0.2614
**FAAP20**	FAAP20	11	FA core complex associated protein 20	protein_codin	-0.07163	0.625	1	24.67	0.0547
**AGTRAP**	AGTRAP	11	angiotensin II receptor associated protein	protein_codin	0.1055	0.435	1	40.44	0.05552
**MUL1**	MUL1	11	mitochondrial E3 ubiquitin protein ligase 1	protein_codin	-0.1382	0.625	1	1.817	0.2615
**PTP4A2**	PTP4A2	11	protein tyrosine phosphatase 4A2	protein_codin	-0.6097	5.5E-07	0.01288	42.68	0.06159
**KHDRBS1**	KHDRBS1	11	KH RNA binding domain containing, signal transduction asso	protein_codin	0.2746	0.00428	1	38.94	0.05342
**ZMYM4**	ZMYM4	11	zinc finger MYM-type containing 4	protein_codin	0.05176	0.806	1	6.882	0.1784
**C1orf109**	C1orf109	11	chromosome 1 open reading frame 109	protein_codin	0.212	0.494	1	2.872	0.1366
**RNF220**	RNF220	11	ring finger protein 220	protein_codin	0.1602	0.575	1	2.554	0.2005
**BTF3L4**	BTF3L4	11	basic transcription factor 3 like 4	protein_codin	0.2426	0.0849	1	29.18	0.1652
**USP24**	USP24	11	ubiquitin specific peptidase 24	protein_codin	0.03258	0.912	1	9.269	0.3152
**CACHD1**	CACHD1	11	cache domain containing 1	protein_codin	0.2386	0.406	1	9.006	0.1832
**PIGK**	PIGK	11	phosphatidylinositol glycan anchor biosynthesis class K	protein_codin	-0.2646	0.19	1	7.226	0.05675
**ATP1A1**	ATP1A1	11	ATPase Na+/K+ transporting subunit alpha 1	protein_codin	0.01928	0.842	1	52.58	0.05282
**MEF2D**	MEF2D	11	myocyte enhancer factor 2D	protein_codin	-0.2974	0.284	1	9.088	0.1152
**HSD17B7**	HSD17B7	11	hydroxysteroid 17-beta dehydrogenase 7	protein_codin	1.064	0.00842	1	9.339	0.3496
**ABL2**	ABL2	11	ABL proto-oncogene 2, non-receptor tyrosine kinase	protein_codin	-1.148	2.2E-10	5.22E-06	51.18	0.1426
**TOR1AIP2**	TOR1AIP2	11	torsin 1A interacting protein 2	protein_codin	-0.6399	1.03E-05	0.239	51.22	0.128
**ELK4**	ELK4	11	ETS transcription factor ELK4	protein_codin	0.5872	0.00195	1	27.29	0.2494
**SLC35F6**	SLC35F6	11	solute carrier family 35 member F6	protein_codin	0.3694	0.101	1	21.99	0.1134
**BABAM2**	BABAM2	11	BRISC and BRCA1 A complex member 2	protein_codin	0.1128	0.582	1	6.46	0.1074
**RASGRP3**	RASGRP3	11	RAS guanyl releasing protein 3	protein_codin	-0.2869	0.195	1	22.48	0.08159
**UGP2**	UGP2	11	UDP-glucose pyrophosphorylase 2	protein_codin	0.3218	0.0172	1	52.52	0.1744
**MTHFD2**	MTHFD2	11	methylenetetrahydrofolate dehydrogenase (NADP+ depender	protein_codin	0.07118	0.818	1	3.412	0.2196
**VAMP5**	VAMP5	11	vesicle associated membrane protein 5	protein_codin	0.06566	0.324	1	45.49	0.02646
**CHMP3**	CHMP3	11	charged multivesicular body protein 3	protein_codin	-0.2269	0.0336	1	29.63	0.08076
**MRPS9**	MRPS9	11	mitochondrial ribosomal protein S9	protein_codin	0.6556	0.00596	1	8.06	0.1712
**DBI**	DBI	11	diazepam binding inhibitor, acyl-CoA binding protein	protein_codin	-0.2892	0.000886	1	52.58	0.08336
**7-Mar**	43531	11	membrane associated ring-CH-type finger 7	protein_codin	0.5807	0.000646	1	12.5	0.0827
**COBLL1**	COBLL1	11	cordon-bleu WH2 repeat protein like 1	protein_codin	-0.05096	0.802	1	7.568	0.1101
**SSB**	SSB	11	small RNA binding exonuclease protection factor La	protein_codin	0.1851	0.022	1	41.54	0.05479
**AGPS**	AGPS	11	alkylglycerone phosphate synthase	protein_codin	-0.8947	0.000893	1	8.489	0.3127
**CASP8**	CASP8	11	caspase 8	protein_codin	0.3334	0.333	1	12.21	0.3248
**AAMP**	AAMP	11	angio associated migratory cell protein	protein_codin	0.568	0.00177	1	15.66	0.2342
**CUL3**	CUL3	11	cullin 3	protein_codin	-0.05337	0.804	1	7.946	0.146
**MFF**	MFF	11	mitochondrial fission factor	protein_codin	0.1189	0.471	1	6.992	0.1903
**LZTFL1**	LZTFL1	11	leucine zipper transcription factor like 1	protein_codin	0.1096	0.688	1	3.216	0.1726
**STAB1**	STAB1	11	stabilin 1	protein_codin	-1.208	4.94E-10	1.17E-05	43.19	0.05387
**FILIP1L**	FILIP1L	11	filamin A interacting protein 1 like	protein_codin	1.161	2.66E-14	6.36E-10	52.53	0.02967
**SLC35A5**	SLC35A5	11	solute carrier family 35 member A5	protein_codin	-0.1793	0.499	1	2.969	0.1802
**PARP14**	PARP14	11	poly(ADP-ribose) polymerase family member 14	protein_codin	0.314	0.0366	1	50.58	0.1142
**TIPARP**	TIPARP	11	TCDD inducible poly(ADP-ribose) polymerase	protein_codin	-0.007303	0.96	1	52.58	0.05836
**AP2M1**	AP2M1	11	adaptor related protein complex 2 subunit mu 1	protein_codin	-0.09872	0.263	1	49.55	0.1647
**MAEA**	MAEA	11	macrophage erythroblast attacher	protein_codin	0.1188	0.599	1	7.546	0.05552
**SEC31A**	SEC31A	11	SEC31 homolog A, COPII coat complex component	protein_codin	-0.07086	0.62	1	21.29	0.05644
**C4orf3**	C4orf3	11	chromosome 4 open reading frame 3	protein_codin	-0.001443	0.9849	1	35.22	0.02788
**FBXW7**	FBXW7	11	F-box and WD repeat domain containing 7	protein_codin	0.02376	0.924	1	23.87	0.1379
**HPF1**	HPF1	11	histone PARylation factor 1	protein_codin	0.6915	5.99E-05	1	24.79	0.1371
**UFSP2**	UFSP2	11	UFM1 specific peptidase 2	protein_codin	0.03727	0.852	1	19.34	0.1399
**HMGCS1**	HMGCS1	11	3-hydroxy-3-methylglutaryl-CoA synthase 1	protein_codin	0.1111	0.742	1	30.66	0.08566
**KIF2A**	KIF2A	11	kinesin family member 2A	protein_codin	0.3105	0.0705	1	26.68	0.1372
**ZNF366**	ZNF366	11	zinc finger protein 366	protein_codin	1.277	0.000535	1	3.479	0.1702
**EPB41L4A**	EPB41L4A	11	erythrocyte membrane protein band 4.1 like 4A	protein_codin	0.1481	0.474	1	19.08	0.2512
**GRAMD2B**	GRAMD2B	11	GRAM domain containing 2B	protein_codin	0.2861	0.334	1	9.199	0.08142
**KLHL3**	KLHL3	11	kelch like family member 3	protein_codin	1.154	0.00344	1	9.51	0.3294
**LMAN2**	LMAN2	11	lectin, mannose binding 2	protein_codin	-0.2236	0.0237	1	26.63	0.0525
**C6orf136**	C6orf136	11	chromosome 6 open reading frame 136	protein_codin	0.1202	0.678	1	9.652	0.1106
**NFKBIL1**	NFKBIL1	11	NFKB inhibitor like 1	protein_codin	0.4586	0.0357	1	47.25	0.08555
**HLA-DMB**	HLA-DMB	11	major histocompatibility complex, class II, DM beta	protein_codin	1.406	7.19E-09	0.00017	49.4	0.1959
**C6orf89**	C6orf89	11	chromosome 6 open reading frame 89	protein_codin	0.005894	0.974	1	19.43	0.1677
**MTCH1**	MTCH1	11	mitochondrial carrier 1	protein_codin	0.1921	0.0561	1	24.81	0.08848
**GLO1**	GLO1	11	glyoxalase I	protein_codin	-0.006376	0.966	1	14.03	0.05276
**MRPS18A**	MRPS18A	11	mitochondrial ribosomal protein S18A	protein_codin	-0.1042	0.597	1	7.085	0.06019
**AKIRIN2**	AKIRIN2	11	akirin 2	protein_codin	0.5561	0.00375	1	14.86	0.1406
**CASP8AP2**	CASP8AP2	11	caspase 8 associated protein 2	protein_codin	0.5729	0.022	1	8.149	0.2275
**ETV1**	ETV1	11	ETS variant 1	protein_codin	1.448	1E-04	1	9.967	0.1884
**GGCT**	GGCT	11	gamma-glutamylcyclotransferase	protein_codin	0.06705	0.65	1	11.18	0.09026
**CCM2**	CCM2	11	CCM2 scaffold protein	protein_codin	0.002646	0.99	1	51.67	0.1142
**PON2**	PON2	11	paraoxonase 2	protein_codin	-0.5612	3.04E-06	0.07085	35.48	0.05772
**DNAJC2**	DNAJC2	11	DnaJ heat shock protein family (Hsp40) member C2	protein_codin	0.3794	0.0347	1	12.94	0.1139
**HILPDA**	HILPDA	11	hypoxia inducible lipid droplet associated	protein_codin	0.06287	0.828	1	52.06	0.3325
**TMEM140**	TMEM140	11	transmembrane protein 140	protein_codin	0.326	0.106	1	15.48	0.08358
**BRAF**	BRAF	11	B-Raf proto-oncogene, serine/threonine kinase	protein_codin	0.3696	0.133	1	3.578	0.2527
**WDR60**	WDR60	11	WD repeat domain 60	protein_codin	0.4719	0.00711	1	11.62	0.08515
**GEMIN8**	GEMIN8	11	gem nuclear organelle associated protein 8	protein_codin	0.5969	0.0618	1	2.873	0.1668
**DYNLT3**	DYNLT3	11	dynein light chain Tctex-type 3	protein_codin	-0.3572	0.0264	1	27.7	0.02768
**NKAP**	NKAP	11	NFKB activating protein	protein_codin	0.09573	0.521	1	26.54	0.142
**MOSPD1**	MOSPD1	11	motile sperm domain containing 1	protein_codin	0.2232	0.438	1	3.383	0.262
**MECP2**	MECP2	11	methyl-CpG binding protein 2	protein_codin	0.126	0.531	1	7.792	0.1977
**SLC25A37**	SLC25A37	11	solute carrier family 25 member 37	protein_codin	-0.02796	0.836	1	52.58	0.1122
**DPYSL2**	DPYSL2	11	dihydropyrimidinase like 2	protein_codin	0.4495	0.000305	1	32.04	0.05367
**IKBKB**	IKBKB	11	inhibitor of nuclear factor kappa B kinase subunit beta	protein_codin	-0.07109	0.808	1	7.322	0.272
**DPY19L4**	DPY19L4	11	dpy-19 like 4	protein_codin	-0.01096	0.967	1	7.966	0.2332
**NDRG1**	NDRG1	11	N-myc downstream regulated 1	protein_codin	0.5528	3.3E-05	0.7616	11.05	0.08164
**TSTA3**	TSTA3	11	tissue specific transplantation antigen P35B	protein_codin	-0.2548	0.146	1	38.62	0.128
**PUF60**	PUF60	11	poly(U) binding splicing factor 60	protein_codin	0.1268	0.333	1	13.6	0.0644
**SLC39A4**	SLC39A4	11	solute carrier family 39 member 4	protein_codin	0.4742	0.0912	1	10.11	0.1672
**DENND4C**	DENND4C	11	DENN domain containing 4C	protein_codin	0.7097	0.0267	1	7.578	0.3077
**SMU1**	SMU1	11	SMU1 DNA replication regulator and spliceosomal factor	protein_codin	0.2782	0.109	1	6.467	0.1701
**ISCA1**	ISCA1	11	iron-sulfur cluster assembly 1	protein_codin	0.417	0.0162	1	43.12	0.02615
**TMEM245**	TMEM245	11	transmembrane protein 245	protein_codin	-0.536	0.0164	1	21.11	0.2312
**POLR2L**	POLR2L	11	RNA polymerase II subunit L	protein_codin	-0.2811	4.58E-05	1	52.58	0.05326
**RRP8**	RRP8	11	ribosomal RNA processing 8	protein_codin	0.4599	0.216	1	3.29	0.2984
**QSER1**	QSER1	11	glutamine and serine rich 1	protein_codin	0.5651	0.0318	1	8.912	0.1399
**TMX2**	TMX2	11	thioredoxin related transmembrane protein 2	protein_codin	0.04452	0.792	1	20.4	0.2017
**TMEM179B**	TMEM179B	11	transmembrane protein 179B	protein_codin	0.3487	0.000426	1	38.27	0.0282
**CARD16**	CARD16	11	caspase recruitment domain family member 16	protein_codin	-0.08777	0.46	1	25.79	0.02626
**NPAT**	NPAT	11	nuclear protein, coactivator of histone transcription	protein_codin	0.4062	0.168	1	9.334	0.1609
**ZMYND11**	ZMYND11	11	zinc finger MYND-type containing 11	protein_codin	-0.2026	0.254	1	17.45	0.08782
**WAC-AS1**	WAC-AS1	11	WAC antisense RNA 1 (head to head)	antisense_RN	0.3262	0.104	1	22.2	0.1467
**JCAD**	JCAD	11	junctional cadherin 5 associated	protein_codin	-0.3712	0.0466	1	16.74	0.0532
**AP3M1**	AP3M1	11	adaptor related protein complex 3 subunit mu 1	protein_codin	-0.1092	0.699	1	3.352	0.05254
**SAMD8**	SAMD8	11	sterile alpha motif domain containing 8	protein_codin	-0.7435	0.00226	1	8.189	0.09316
**FRA10AC1**	FRA10AC1	11	FRA10A associated CGG repeat 1	protein_codin	0.2106	0.203	1	32	0.2786
**PCGF6**	PCGF6	11	polycomb group ring finger 6	protein_codin	0.531	0.0979	1	8.907	0.1172
**STN1.00**	STN1.00	11	STN1 subunit of CST complex	protein_codin	-0.2417	0.242	1	26.27	0.1417
**GRK5**	GRK5	11	G protein-coupled receptor kinase 5	protein_codin	-0.009547	0.97	1	3.423	0.1541
**UROS**	UROS	11	uroporphyrinogen III synthase	protein_codin	0.1559	0.486	1	3.198	0.2239
**PPP2R2D**	PPP2R2D	11	protein phosphatase 2 regulatory subunit Bdelta	protein_codin	0.3898	0.136	1	9.662	0.1384
**ADIPOR2**	ADIPOR2	11	adiponectin receptor 2	protein_codin	0.4995	0.0281	1	8.187	0.08492
**ETV6**	ETV6	11	ETS variant 6	protein_codin	0.4923	0.0271	1	7.277	0.05576
**ETFRF1**	ETFRF1	11	electron transfer flavoprotein regulatory factor 1	protein_codin	0.2802	0.062	1	10.76	0.1936
**SCAF11**	SCAF11	11	SR-related CTD associated factor 11	protein_codin	0.0821	0.45	1	29.06	0.08516
**PTGES3**	PTGES3	11	prostaglandin E synthase 3	protein_codin	0.2656	0.000381	1	25.05	0.02668
**MON2**	MON2	11	MON2 homolog, regulator of endosome-to-Golgi trafficking	protein_codin	0.3102	0.345	1	10.88	0.1469
**ARL1.00**	ARL1.00	11	ADP ribosylation factor like GTPase 1	protein_codin	-0.1477	0.271	1	12.21	0.08797
**WASHC4**	WASHC4	11	WASH complex subunit 4	protein_codin	-0.4471	0.0391	1	8.602	0.1688
**MAPKAPK5**	MAPKAPK5	11	MAPK activated protein kinase 5	protein_codin	-0.1282	0.656	1	2.98	0.3701
**PSPC1**	PSPC1	11	paraspeckle component 1	protein_codin	0.4689	0.0905	1	29.46	0.197
**ABCC4**	ABCC4	11	ATP binding cassette subfamily C member 4	protein_codin	-0.3087	0.27	1	3.566	0.1426
**OSGEP**	OSGEP	11	O-sialoglycoprotein endopeptidase	protein_codin	-0.125	0.53	1	6.35	0.1089
**TRAPPC6B**	TRAPPC6B	11	trafficking protein particle complex 6B	protein_codin	-0.1634	0.534	1	31.86	0.3142
**FBXO33**	FBXO33	11	F-box protein 33	protein_codin	0.4735	0.12	1	3.262	0.2007
**AL358220.1**	AL358220.1	11	novel transcript, antisense to PRK	antisense_RN	0.4986	0.167	1	29.64	0.1961
**SLC25A29**	SLC25A29	11	solute carrier family 25 member 29	protein_codin	0.672	0.0018	1	38.63	0.1418
**LPCAT4**	LPCAT4	11	lysophosphatidylcholine acyltransferase 4	protein_codin	1.967	1.81E-14	4.33E-10	30.3	0.08815
**GABPB1-AS1**	GABPB1-AS	11	GABPB1 antisense RNA 1	antisense_RN	-0.09512	0.68	1	20.18	0.1922
**PIGBOS1**	PIGBOS1	11	PIGB opposite strand 1	protein_codin	0.1397	0.538	1	47.87	0.3084
**MYO9A**	MYO9A	11	myosin IXA	protein_codin	0.3252	0.132	1	7.282	0.1379
**BBS4**	BBS4	11	Bardet-Biedl syndrome 4	protein_codin	0.2467	0.557	1	3.646	0.2886
**SNRNP25**	SNRNP25	11	small nuclear ribonucleoprotein U11/U12 subunit 25	protein_codin	0.2185	0.35	1	25.15	0.1166
**JPT2**	JPT2	11	Jupiter microtubule associated homolog 2	protein_codin	0.6752	0.0325	1	9.262	0.1682
**TRAP1**	TRAP1	11	TNF receptor associated protein 1	protein_codin	0.8827	0.000705	1	7.353	0.1632
**SNX29**	SNX29	11	sorting nexin 29	protein_codin	-0.6827	0.0119	1	10.52	0.2846
**IQCK**	IQCK	11	IQ motif containing K	protein_codin	-0.6268	0.000785	1	37.13	0.1367
**DCUN1D3**	DCUN1D3	11	defective in cullin neddylation 1 domain containing 3	protein_codin	-0.3352	0.177	1	9.812	0.1128
**BCKDK**	BCKDK	11	branched chain keto acid dehydrogenase kinase	protein_codin	0.1927	0.391	1	24.15	0.02688
**BRD7**	BRD7	11	bromodomain containing 7	protein_codin	0.2818	0.0464	1	27.04	0.3557
**RIPOR1**	RIPOR1	11	RHO family interacting cell polarization regulator 1	protein_codin	-0.06606	0.726	1	19.19	0.1421
**UTP4**	UTP4	11	UTP4 small subunit processome component	protein_codin	0.9076	0.00658	1	2.948	0.08732
**PSMD7**	PSMD7	11	proteasome 26S subunit, non-ATPase 7	protein_codin	-0.2078	0.078	1	17.32	0.09067
**GPS2**	GPS2	11	G protein pathway suppressor 2	protein_codin	0.5075	0.0625	1	2.997	0.1932
**TMEM107**	TMEM107	11	transmembrane protein 107	protein_codin	0.7392	0.00333	1	44.08	0.1819
**TTC19**	TTC19	11	tetratricopeptide repeat domain 19	protein_codin	0.2616	0.12	1	6.55	0.05642
**RARA-AS1**	RARA-AS1	11	RARA antisense RNA 1	antisense_RN	-0.1847	0.467	1	9.942	0.164
**JUP**	JUP	11	junction plakoglobin	protein_codin	-0.5335	0.0219	1	9.877	0.2037
**NBR1**	NBR1	11	NBR1 autophagy cargo receptor	protein_codin	0.1301	0.522	1	41.35	0.1119
**NT5C**	NT5C	11	5', 3'-nucleotidase, cytosolic	protein_codin	0.3061	0.252	1	8.692	0.2294
**AFMID**	AFMID	11	arylformamidase	protein_codin	-0.107	0.667	1	9.73	0.2434
**ARL16.00**	ARL16.00	11	ADP ribosylation factor like GTPase 16	protein_codin	0.3806	0.0549	1	7.762	0.0816
**P4HB**	P4HB	11	prolyl 4-hydroxylase subunit beta	protein_codin	-1.099	4.02E-32	9.73E-28	34.14	0.029
**MAFG**	MAFG	11	MAF bZIP transcription factor G	protein_codin	0.1414	0.47	1	7.588	0.027
**SEH1L**	SEH1L	11	SEH1 like nucleoporin	protein_codin	-0.08159	0.8	1	2.912	0.311
**C18orf8**	C18orf8	11	regulator of MON1-CCZ1	protein_codin	NA	NA	NA	10.29	0.1509
**ATP5A1**	ATP5A1	11	ATP synthase F1 subunit alpha	protein_codin	NA	NA	NA	30.52	0.137
**POLR3F**	POLR3F	11	RNA polymerase III subunit F	protein_codin	0.004718	0.987	1	1.809	0.1948
**APMAP**	APMAP	11	adipocyte plasma membrane associated protein	protein_codin	-0.05549	0.778	1	7.779	0.1158
**ROMO1**	ROMO1	11	reactive oxygen species modulator 1	protein_codin	-0.03381	0.769	1	16.38	0.146
**MRGBP**	MRGBP	11	MRG domain binding protein	protein_codin	-0.2802	0.252	1	8.222	0.1226
**DIDO1**	DIDO1	11	death inducer-obliterator 1	protein_codin	0.1517	0.554	1	3.187	0.1135
**PTBP1**	PTBP1	11	polypyrimidine tract binding protein 1	protein_codin	-0.1842	0.318	1	17.29	0.05896
**MEX3D**	MEX3D	11	mex-3 RNA binding family member D	protein_codin	-0.7652	0.00142	1	3.439	0.02615
**MRPL54**	MRPL54	11	mitochondrial ribosomal protein L54	protein_codin	-0.1176	0.328	1	10.02	0.1072
**TMED1**	TMED1	11	transmembrane p24 trafficking protein 1	protein_codin	-0.1371	0.485	1	7.798	0.08237
**URI1**	URI1	11	URI1 prefoldin like chaperone	protein_codin	0.2206	0.26	1	15.06	0.177
**PSENEN**	PSENEN	11	presenilin enhancer, gamma-secretase subunit	protein_codin	0.2924	0.0234	1	22.63	0.1737
**TIMM50**	TIMM50	11	translocase of inner mitochondrial membrane 50	protein_codin	0.224	0.333	1	8.442	0.2258
**KCNN4**	KCNN4	11	potassium calcium-activated channel subfamily N member 4	protein_codin	-1.688	2.98E-12	7.11E-08	31.54	0.0852
**RCN3**	RCN3	11	reticulocalbin 3	protein_codin	-3.254	7.5E-25	1.81E-20	24.53	0.08449
**NR1H2**	NR1H2	11	nuclear receptor subfamily 1 group H member 2	protein_codin	0.5569	0.00101	1	32	0.05467
**PRPF31**	PRPF31	11	pre-mRNA processing factor 31	protein_codin	0.1712	0.27	1	6.006	0.1551
**UBE2L3**	UBE2L3	11	ubiquitin conjugating enzyme E2 L3	protein_codin	-0.06282	0.546	1	26.96	0.1698
**YDJC**	YDJC	11	YdjC chitooligosaccharide deacetylase homolog	protein_codin	-0.1887	0.478	1	3.105	0.2796
**RPS19BP1**	RPS19BP1	11	ribosomal protein S19 binding protein 1	protein_codin	0.05486	0.615	1	28.44	0.1152
**XRCC6**	XRCC6	11	X-ray repair cross complementing 6	protein_codin	-0.06514	0.537	1	16.84	0.05482
**PLXNB2**	PLXNB2	11	plexin B2	protein_codin	0.09906	0.742	1	2.976	0.252
**PPP6R2**	PPP6R2	11	protein phosphatase 6 regulatory subunit 2	protein_codin	-0.04677	0.899	1	3.279	0.3034
**PTTG1IP**	PTTG1IP	11	PTTG1 interacting protein	protein_codin	-0.5627	1.4E-14	3.35E-10	28.25	0.08236
**ANKRD65**	ANKRD65	12	ankyrin repeat domain 65	protein_codin	0.4275	0.122	1	9.854	0.1704
**LRRC47**	LRRC47	12	leucine rich repeat containing 47	protein_codin	0.2082	0.426	1	7.924	0.171
**KAZN**	KAZN	12	kazrin, periplakin interacting protein	protein_codin	-0.1844	0.498	1	3.747	0.1562
**CAPZB**	CAPZB	12	capping actin protein of muscle Z-line subunit beta	protein_codin	0.43	5.33E-07	0.01248	42.62	0.082
**NBL1**	NBL1	12	NBL1, DAN family BMP antagonist	protein_codin	1.804	1.72E-25	4.15E-21	48.49	0.06385
**ECE1**	ECE1	12	endothelin converting enzyme 1	protein_codin	-0.788	9.79E-12	2.33E-07	24.83	0.02806
**AK2**	AK2	12	adenylate kinase 2	protein_codin	0.282	0.0369	1	11.8	0.171
**ERI3**	ERI3	12	ERI1 exoribonuclease family member 3	protein_codin	0.1829	0.352	1	7.247	0.1377
**LEPROT**	LEPROT	12	leptin receptor overlapping transcript	protein_codin	0.1979	0.00325	1	26.97	0.05409
**SELENOF**	SELENOF	12	selenoprotein F	protein_codin	-0.08992	0.322	1	36.98	0.02712
**DR1**	DR1	12	down-regulator of transcription 1	protein_codin	-0.1445	0.455	1	7.198	0.1902
**AGL**	AGL	12	amylo-alpha-1, 6-glucosidase, 4-alpha-glucanotransferase	protein_codin	-0.5885	0.0111	1	7.552	0.08396
**RHOC**	RHOC	12	ras homolog family member C	protein_codin	0.1036	0.126	1	52.58	0.02747
**PSMB4**	PSMB4	12	proteasome subunit beta 4	protein_codin	-0.3744	4.11E-05	0.9477	43.28	0.08476
**PBXIP1**	PBXIP1	12	PBX homeobox interacting protein 1	protein_codin	-0.2738	0.26	1	8.768	0.1129
**SHC1**	SHC1	12	SHC adaptor protein 1	protein_codin	0.2115	0.181	1	29.33	0.05556
**DEDD**	DEDD	12	death effector domain containing	protein_codin	0.7497	0.00775	1	3.505	0.2224
**UFC1**	UFC1	12	ubiquitin-fold modifier conjugating enzyme 1	protein_codin	0.04622	0.5424	1	29.84	0.08983
**RC3H1**	RC3H1	12	ring finger and CCCH-type domains 1	protein_codin	-0.06158	0.826	1	9.796	0.1781
**STX6**	STX6	12	syntaxin 6	protein_codin	-0.352	0.101	1	9.352	0.1106
**RRP15**	RRP15	12	ribosomal RNA processing 15 homolog	protein_codin	0.4069	0.03	1	42.38	0.1132
**GUK1**	GUK1	12	guanylate kinase 1	protein_codin	0.3106	4.73E-10	1.12E-05	35.48	0.02622
**RAB4A**	RAB4A	12	RAB4A, member RAS oncogene family	protein_codin	-0.167	0.284	1	25.87	0.02686
**CCDC121**	CCDC121	12	coiled-coil domain containing 121	protein_codin	1.332	0.00252	1	8.255	0.2995
**HTRA2**	HTRA2	12	HtrA serine peptidase 2	protein_codin	-0.006726	0.966	1	6.868	0.1615
**LINC00116**	LINC00116	12	mitoregulin	protein_codin	NA	NA	NA	24.26	0.02616
**ANAPC1**	ANAPC1	12	anaphase promoting complex subunit 1	protein_codin	-0.03178	0.92	1	9.417	0.2026
**MAP3K2**	MAP3K2	12	mitogen-activated protein kinase kinase kinase 2	protein_codin	-0.6042	0.0041	1	23.4	0.1965
**WDR33**	WDR33	12	WD repeat domain 33	protein_codin	-0.08312	0.59	1	6.026	0.1718
**PLEKHB2**	PLEKHB2	12	pleckstrin homology domain containing B2	protein_codin	0.1362	0.528	1	43.04	0.05772
**ORMDL1**	ORMDL1	12	ORMDL sphingolipid biosynthesis regulator 1	protein_codin	0.2615	0.0744	1	28.69	0.1341
**PPIL3**	PPIL3	12	peptidylprolyl isomerase like 3	protein_codin	1.026	0.00812	1	8.352	0.1239
**COPS8**	COPS8	12	COP9 signalosome subunit 8	protein_codin	-0.1786	0.198	1	14.37	0.1472
**ZNF385D**	ZNF385D	12	zinc finger protein 385D	protein_codin	1.772	2E-26	4.83E-22	18.56	0.05396
**EIF1B**	EIF1B	12	eukaryotic translation initiation factor 1B	protein_codin	0.03443	0.696	1	46.32	0.05604
**IP6K2**	IP6K2	12	inositol hexakisphosphate kinase 2	protein_codin	-0.04876	0.779	1	18.14	0.1412
**MAGI1**	MAGI1	12	membrane associated guanylate kinase, WW and PDZ doma	protein_codin	0.5135	0.0589	1	9.002	0.224
**EIF4E3**	EIF4E3	12	eukaryotic translation initiation factor 4E family member 3	protein_codin	1.526	0.00172	1	3.293	0.3067
**ASTE1**	ASTE1	12	asteroid homolog 1	protein_codin	0.433	0.194	1	3.028	0.2246
**RYK**	RYK	12	receptor like tyrosine kinase	protein_codin	0.1254	0.508	1	7.689	0.1847
**GYG1**	GYG1	12	glycogenin 1	protein_codin	-0.5532	0.0013	1	7.911	0.1092
**MBNL1-AS1**	MBNL1-AS	12	MBNL1 antisense RNA 1	antisense_RN	0.788	0.0374	1	48.08	0.2252
**RAP2B**	RAP2B	12	RAP2B, member of RAS oncogene family	protein_codin	0.1576	0.469	1	8.168	0.02776
**POLR2H**	POLR2H	12	RNA polymerase II subunit H	protein_codin	-0.03633	0.812	1	32.09	0.08952
**UVSSA**	UVSSA	12	UV stimulated scaffold protein A	protein_codin	0.004624	0.992	1	11.58	0.4527
**NELFA**	NELFA	12	negative elongation factor complex member A	protein_codin	0.6376	0.0776	1	2.161	0.2251
**MFSD10**	MFSD10	12	major facilitator superfamily domain containing 10	protein_codin	0.3756	0.0126	1	32.47	0.05459
**KIAA0232**	KIAA0232	12	KIAA0232	protein_codin	-0.1272	0.576	1	8.261	0.1727
**CHIC2**	CHIC2	12	cysteine rich hydrophobic domain 2	protein_codin	0.6492	4.15E-05	0.9568	32.14	0.1392
**UBA6**	UBA6	12	ubiquitin like modifier activating enzyme 6	protein_codin	-0.1686	0.51	1	8.604	0.1144
**MRPS18C**	MRPS18C	12	mitochondrial ribosomal protein S18C	protein_codin	0.1136	0.325	1	41.22	0.1089
**HSD17B11**	HSD17B11	12	hydroxysteroid 17-beta dehydrogenase 11	protein_codin	1.222	9.73E-19	2.34E-14	23.7	0.1372
**CFI**	CFI	12	complement factor I	protein_codin	0.1421	0.219	1	49.28	0.2585
**GAB1**	GAB1	12	GRB2 associated binding protein 1	protein_codin	1.242	0.00218	1	7.086	0.1685
**EXOC3**	EXOC3	12	exocyst complex component 3	protein_codin	0.01521	0.958	1	8.943	0.1142

**TRIO**	TRIO	12	trio Rho guanine nucleotide exchange factor	protein_codin	0.32	0.392	1	3.422	0.4972
**CARD6**	CARD6	12	caspase recruitment domain family member 6	protein_codin	-0.3269	0.107	1	43.7	0.134
**HEXB**	HEXB	12	hexosaminidase subunit beta	protein_codin	-0.2894	0.0482	1	27.62	0.1471
**MEF2C**	MEF2C	12	myocyte enhancer factor 2C	protein_codin	0.4906	6.41E-06	0.149	24.58	0.0568
**WDR36**	WDR36	12	WD repeat domain 36	protein_codin	0.4915	0.0859	1	3.542	0.1679
**SLC35A4**	SLC35A4	12	solute carrier family 35 member A4	protein_codin	0.1022	0.72	1	3.114	0.2868
**WDR55**	WDR55	12	WD repeat domain 55	protein_codin	0.1787	0.509	1	3.368	0.1897
**RNF14**	RNF14	12	ring finger protein 14	protein_codin	0.2087	0.578	1	9.439	0.3730
**NDST1**	NDST1	12	N-deacetylase and N-sulfotransferase 1	protein_codin	0.3142	0.255	1	8.512	0.1677
**BNIP1**	BNIP1	12	BCL2 interacting protein 1	protein_codin	0.1229	0.716	1	3.272	0.1231
**PAK1IP1**	PAK1IP1	12	PAK1 interacting protein 1	protein_codin	0.6709	0.000622	1	15.14	0.1340
**MCUR1**	MCUR1	12	mitochondrial calcium uniporter regulator 1	protein_codin	0.7214	6.29E-06	0.1463	23.19	0.0870
**ZNRD1**	ZNRD1	12	zinc ribbon domain containing 1	protein_codin	0.1552	0.383	1	13.96	0.05641
**HLA-DOA**	HLA-DOA	12	major histocompatibility complex, class II, DO alpha	protein_codin	3.072	7.77E-07	0.01818	8.152	0.2307
**RNF8**	RNF8	12	ring finger protein 8	protein_codin	-0.2411	0.192	1	7.308	0.05650
**LCA5**	LCA5	12	lebercilin LCA5	protein_codin	0.6601	0.0300	1	9.438	0.2062
**HDAC2**	HDAC2	12	histone deacetylase 2	protein_codin	0.0529	0.78	1	14.17	0.02812
**DCBLD1**	DCBLD1	12	discoidin, CUB and LCCL domain containing 1	protein_codin	-0.02479	0.938	1	3.603	0.129
**GNA12**	GNA12	12	G protein subunit alpha 12	protein_codin	-0.2606	0.375	1	9.902	0.1187
**KDELR2**	KDELR2	12	KDEL endoplasmic reticulum protein retention receptor 2	protein_codin	-0.3693	0.000285	1	27.44	0.02841
**HIBADH**	HIBADH	12	3-hydroxyisobutyrate dehydrogenase	protein_codin	0.5438	0.0418	1	7.226	0.1731
**ZNF138**	ZNF138	12	zinc finger protein 138	protein_codin	0.01971	0.944	1	9.825	0.167
**SDHAF3**	SDHAF3	12	succinate dehydrogenase complex assembly factor 3	protein_codin	0.216	0.458	1	2.942	0.1345
**TRIM56**	TRIM56	12	tripartite motif containing 56	protein_codin	-0.1247	0.488	1	32.87	0.1684
**KMT2E**	KMT2E	12	lysine methyltransferase 2E	protein_codin	0.2985	0.00462	1	40.79	0.142
**ATP6V1F**	ATP6V1F	12	ATPase H+ transporting V1 subunit F	protein_codin	0.09110	0.2890	1	32.93	0.146
**LUC7L2**	LUC7L2	12	LUC7 like 2, pre-mRNA splicing factor	protein_codin	-0.1922	0.318	1	8.111	0.05830
**SSBP1**	SSBP1	12	single stranded DNA binding protein 1	protein_codin	0.06748	0.42	1	31.95	0.03081
**GIMAP6**	GIMAP6	12	GTPase, IMAP family member 6	protein_codin	1.062	7.43E-10	1.76E-05	29.88	0.1111
**SLC4A2**	SLC4A2	12	solute carrier family 4 member 2	protein_codin	-0.1996	0.491	1	3.024	0.1541
**MAOB**	MAOB	12	monoamine oxidase B	protein_codin	1.680	4.04E-05	0.9317	7.286	0.152
**PDZD11**	PDZD11	12	PDZ domain containing 11	protein_codin	0.122	0.498	1	18.27	0.1352
**BEX5**	BEX5	12	brain expressed X-linked 5	protein_codin	0.1059	0.665	1	26.14	0.1094
**THOC2**	THOC2	12	THO complex 2	protein_codin	-0.001982	0.991	1	50.17	0.117
**RBMX2**	RBMX2	12	RNA binding motif protein X-linked 2	protein_codin	0.3917	0.0359	1	19.28	0.2348
**TAZ**	TAZ	12	tafazzin	protein_codin	0.4259	0.162	1	7.514	0.1695
**ERI1**	ERI1	12	exoribonuclease 1	protein_codin	1.667	0.00149	1	49.7	0.2814
**PPP2CB**	PPP2CB	12	protein phosphatase 2 catalytic subunit beta	protein_codin	-0.09170	0.556	1	29.88	0.08382
**SPIDR**	SPIDR	12	scaffold protein involved in DNA repair	protein_codin	0.4817	0.0918	1	7.697	0.1919
**MAF1.00**	MAF1.00	12	MAF1 homolog, negative regulator of RNA polymerase III	protein_codin	0.1015	0.458	1	9.987	0.08426
**CYHR1**	CYHR1	12	cysteine and histidine rich 1	protein_codin	0.1372	0.459	1	6.765	0.327
**RCL1**	RCL1	12	RNA terminal phosphate cyclase like 1	protein_codin	0.522	0.0940	1	3.195	0.1892
**ZDHHC21**	ZDHHC21	12	zinc finger DHHC-type containing 21	protein_codin	-0.1935	0.488	1	8.764	0.1759
**CREB3**	CREB3	12	cAMP responsive element binding protein 3	protein_codin	-0.1497	0.44	1	8.543	0.1145
**CTSL**	CTSL	12	cathepsin L	protein_codin	0.4383	0.000672	1	43.28	0.08114
**NANS**	NANS	12	N-acetylneuraminate synthase	protein_codin	0.5162	0.000603	1	27.53	0.08128
**STOM**	STOM	12	stomatin	protein_codin	-0.07886	0.297	1	52.06	0.05551
**PPP6C**	PPP6C	12	protein phosphatase 6 catalytic subunit	protein_codin	-0.307	0.0712	1	6.186	0.03091
**TRUB2**	TRUB2	12	TruB pseudouridine synthase family member 2	protein_codin	0.3379	0.155	1	8.486	0.1732
**FNBP1**	FNBP1	12	formin binding protein 1	protein_codin	0.08402	0.713	1	25.04	0.1335
**TALDO1**	TALDO1	12	transaldolase 1	protein_codin	-0.3335	0.000276	1	52.58	0.1089
**NUP160**	NUP160	12	nucleoporin 160	protein_codin	-0.6721	0.00924	1	31.06	0.1898
**CLP1**	CLP1	12	cleavage and polyadenylation factor I subunit 1	protein_codin	0.3361	0.265	1	10.42	0.08282
**DDB1**	DDB1	12	damage specific DNA binding protein 1	protein_codin	0.1177	0.602	1	25.48	0.1992
**EIF1AD**	EIF1AD	12	eukaryotic translation initiation factor 1A domain containing	protein_codin	0.1497	0.673	1	2.852	0.2279
**YIF1A**	YIF1A	12	Yip1 interacting factor homolog A, membrane trafficking prote	protein_codin	-0.1342	0.352	1	29.88	0.1402
**CTSF**	CTSF	12	cathepsin F	protein_codin	-0.6750	2.92E-06	0.06800	36.45	0.112
**MRPL48**	MRPL48	12	mitochondrial ribosomal protein L48	protein_codin	0.4185	0.102	1	9.167	0.1701
**NDUFC2**	NDUFC2	12	NADH ubiquinone oxidoreductase subunit C2	protein_codin	-0.5979	2.2E-18	5.29E-14	52.58	0.05512
**SRSF8**	SRSF8	12	serine and arginine rich splicing factor 8	protein_codin	0.3077	0.0547	1	11.53	0.1377
**PCSK7**	PCSK7	12	proprotein convertase subtilisin/kexin type 7	protein_codin	-0.5768	0.0127	1	27.71	0.0808
**CCDC84**	CCDC84	12	coiled-coil domain containing 84	protein_codin	-0.1949	0.537	1	3.302	0.2361
**FLI1**	FLI1	12	Fli-1 proto-oncogene, ETS transcription factor	protein_codin	1.012	8.17E-07	0.01911	6.53	0.1122
**BEND7**	BEND7	12	BEN domain containing 7	protein_codin	0.3499	0.201	1	8.515	0.1395
**KIAA1217**	KIAA1217	12	KIAA1217	protein_codin	-0.0211	0.927	1	7.458	0.168
**HK1**	HK1	12	hexokinase 1	protein_codin	-0.1727	0.407	1	9.093	0.08257
**ANXA7**	ANXA7	12	annexin A7	protein_codin	-0.2962	0.00382	1	33.46	0.08227
**PTEN**	PTEN	12	phosphatase and tensin homolog	protein_codin	-0.5082	0.000207	1	11.29	0.05476
**CCND2**	CCND2	12	cyclin D2	protein_codin	0.6139	0.0197	1	43.03	0.1695
**CLSTN3**	CLSTN3	12	calsyntenin 3	protein_codin	0.06026	0.852	1	3.167	0.2532
**RECQL**	RECQL	12	RecQ like helicase	protein_codin	0.2256	0.142	1	28.84	0.08134
**KANSL2**	KANSL2	12	KAT8 regulatory NSL complex subunit 2	protein_codin	0.07374	0.84	1	2.934	0.1967
**STAT6**	STAT6	12	signal transducer and activator of transcription 6	protein_codin	0.3552	0.149	1	8.79	0.2281
**MSRB3**	MSRB3	12	methionine sulfoxide reductase B3	protein_codin	0.2209	0.104	1	20.42	0.232
**NUP37**	NUP37	12	nucleoporin 37	protein_codin	0.1772	0.492	1	3.267	0.1402
**MMAB**	MMAB	12	metabolism of cobalamin associated B	protein_codin	0.2165	0.323	1	47.32	0.1165
**GPN3**	GPN3	12	GPN-loop GTPase 3	protein_codin	0.5432	0.00759	1	5.733	0.08672
**EEF1AKMT1**	EEF1AKMT	12	EEF1A lysine methyltransferase 1	protein_codin	0.1991	0.473	1	7.956	0.2065
**PAN3**	PAN3	12	poly(A) specific ribonuclease subunit PAN3	protein_codin	0.7461	0.00749	1	7.125	0.3479
**NHLRC3**	NHLRC3	12	NHL repeat containing 3	protein_codin	0.1336	0.658	1	3.303	0.2891
**CDADC1**	CDADC1	12	cytidine and dCMP deaminase domain containing 1	protein_codin	-0.01212	0.968	1	3.449	0.2836
**THSD1**	THSD1	12	thrombospondin type 1 domain containing 1	protein_codin	0.2340	0.328	1	3.513	0.1488
**OXA1L**	OXA1L	12	OXA1L mitochondrial inner membrane protein	protein_codin	0.142	0.392	1	15.86	0.179
**IRF9**	IRF9	12	interferon regulatory factor 9	protein_codin	0.144	0.506	1	20.27	0.1149
**SYNJ2BP**	SYNJ2BP	12	synaptojanin 2 binding protein	protein_codin	0.2657	0.211	1	16.97	0.1384
**SQOR**	SQOR	12	sulfide quinone oxidoreductase	protein_codin	-0.06252	0.699	1	16.74	0.1657
**SPPL2A**	SPPL2A	12	signal peptide peptidase like 2A	protein_codin	-0.112	0.407	1	22.88	0.02779
**MYO5A**	MYO5A	12	myosin VA	protein_codin	-0.4319	0.116	1	7.724	0.1765
**ICE2**	ICE2	12	interactor of little elongation complex ELL subunit 2	protein_codin	-0.0001953	0.999	1	19.92	0.1109
**ADPGK**	ADPGK	12	ADP dependent glucokinase	protein_codin	0.06360	0.834	1	2.676	0.1732
**SNRPA1**	SNRPA1	12	small nuclear ribonucleoprotein polypeptide A'	protein_codin	0.0692	0.718	1	22.09	0.08127
**GFER**	GFER	12	growth factor, augmenter of liver regeneration	protein_codin	0.3325	0.147	1	8.575	0.1981
**NAGPA**	NAGPA	12	N-acetylglucosamine-1-phosphodiester alpha-N-acetylglucos	protein_codin	0.07449	0.802	1	8.996	0.2166
**KNOP1**	KNOP1	12	lysine rich nucleolar protein 1	protein_codin	-0.1254	0.543	1	32.65	0.1617
**LONP2**	LONP2	12	lon peptidase 2, peroxisomal	protein_codin	-0.08402	0.673	1	6.538	0.1335
**ARL2BP**	ARL2BP	12	ADP ribosylation factor like GTPase 2 binding protein	protein_codin	-0.3657	0.00115	1	36.58	0.05552
**E2F4**	E2F4	12	E2F transcription factor 4	protein_codin	0.2101	0.265	1	21.62	0.1118
**ACD**	ACD	12	ACD shelterin complex subunit and telomerase recruitment factor	protein_codin	0.3865	0.136	1	3.053	0.1952
**GLG1**	GLG1	12	golgi glycoprotein 1	protein_codin	-0.8059	5.93E-08	0.001390	13.86	0.1107
**TMEM170A**	TMEM170A	12	transmembrane protein 170A	protein_codin	0.7762	0.000302	1	18.68	0.1707
**MBTPS1**	MBTPS1	12	membrane bound transcription factor peptidase, site 1	protein_codin	-0.07257	0.695	1	7.795	0.2006
**PITPNA-AS1**	PITPNA-AS	12	PITPNA antisense RNA 1	antisense_RN	0.07540	0.786	1	3.375	0.2205
**ZZEF1**	ZZEF1	12	zinc finger ZZ-type and EF-hand domain containing 1	protein_codin	-0.2196	0.496	1	3.111	0.3735
**TMEM88**	TMEM88	12	transmembrane protein 88	protein_codin	2.282	1.05E-20	2.53E-16	46.18	0.08516
**TP53I13**	TP53I13	12	tumor protein p53 inducible protein 13	protein_codin	-0.3492	0.0890	1	8.95	0.0307
**MRPL45**	MRPL45	12	mitochondrial ribosomal protein L45	protein_codin	0.1649	0.38	1	20.97	0.2017
**CASC3**	CASC3	12	CASC3 exon junction complex subunit	protein_codin	-0.07685	0.758	1	3.571	0.1442
**GHDC**	GHDC	12	GH3 domain containing	protein_codin	-0.1292	0.593	1	8.856	0.166
**SLC25A39**	SLC25A39	12	solute carrier family 25 member 39	protein_codin	0.03635	0.832	1	7.438	0.1098
**HOXB2**	HOXB2	12	homeobox B2	protein_codin	1.479	2.78E-06	0.06481	45.29	0.2082
**HOXB4**	HOXB4	12	homeobox B4	protein_codin	0.9364	0.00047	1	7.303	0.2372
**MRPS7**	MRPS7	12	mitochondrial ribosomal protein S7	protein_codin	-0.05151	0.713	1	46.86	0.2192
**SAP30BP**	SAP30BP	12	SAP30 binding protein	protein_codin	0.1917	0.225	1	17.08	0.1948
**UBALD2**	UBALD2	12	UBA like domain containing 2	protein_codin	0.0844	0.543	1	51.9	0.1402
**RFNG**	RFNG	12	RFNG O-fucosylpeptide 3-beta-N-acetylglucosaminyltransferase	protein_codin	-0.03490	0.877	1	3.315	0.2219
**ENOSF1**	ENOSF1	12	enolase superfamily member 1	protein_codin	-0.5932	0.013	1	9.31	0.05641
**RBCK1**	RBCK1	12	RANBP2-type and C3HC4-type zinc finger containing 1	protein_codin	0.1771	0.203	1	10.33	0.2042
**NOP56**	NOP56	12	NOP56 ribonucleoprotein	protein_codin	0.9569	1.12E-06	0.02618	30.5	0.2881
**COMMD7**	COMMD7	12	COMM domain containing 7	protein_codin	0.3697	0.0120	1	9.445	0.02757
**STAU1**	STAU1	12	staufen double-stranded RNA binding protein 1	protein_codin	0.2359	0.0247	1	51.82	0.08761
**LAMA5**	LAMA5	12	laminin subunit alpha 5	protein_codin	0.8506	0.00371	1	6.312	0.3097
**PLPP2**	PLPP2	12	phospholipid phosphatase 2	protein_codin	0.1165	0.648	1	47.1	0.08124
**PALM**	PALM	12	paralemmin	protein_codin	1.432	7.71E-06	0.1792	47.03	0.08342
**SCAMP4**	SCAMP4	12	secretory carrier membrane protein 4	protein_codin	-0.394	0.132	1	8.386	0.3254
**DNASE2**	DNASE2	12	deoxyribonuclease 2, lysosomal	protein_codin	-0.3102	0.108	1	8.652	0.2886
**TRMT1**	TRMT1	12	tRNA methyltransferase 1	protein_codin	0.9732	0.00565	1	8.967	0.1554
**MPV17L2**	MPV17L2	12	MPV17 mitochondrial inner membrane protein like 2	protein_codin	1.025	0.00217	1	11.13	0.08004
**ZNF85**	ZNF85	12	zinc finger protein 85	protein_codin	0.03181	0.918	1	9.02	0.1689
**CEP89**	CEP89	12	centrosomal protein 89	protein_codin	0.2834	0.293	1	3.516	0.1789
**KMT2B**	KMT2B	12	lysine methyltransferase 2B	protein_codin	0.03364	0.888	1	3.24	0.1181
**PSMD8**	PSMD8	12	proteasome 26S subunit, non-ATPase 8	protein_codin	-0.3132	2E-04	1	52.42	0.055
**AKT1S1**	AKT1S1	12	AKT1 substrate 1	protein_codin	0.2725	0.346	1	3.619	0.2237
**ZNF160**	ZNF160	12	zinc finger protein 160	protein_codin	0.4915	0.0596	1	9.123	0.2499
**MYH9**	MYH9	12	myosin heavy chain 9	protein_codin	0.03165	0.7369	1	45.92	0.02832
**POLDIP3**	POLDIP3	12	DNA polymerase delta interacting protein 3	protein_codin	0.1147	0.635	1	3.066	0.1620
**BRWD1**	BRWD1	12	bromodomain and WD repeat domain containing 1	protein_codin	-0.09156	0.672	1	23.35	0.1119
**NDUFV3**	NDUFV3	12	NADH ubiquinone oxidoreductase subunit V3	protein_codin	0.4492	0.018	1	16.72	0.1371
**SUMO3**	SUMO3	12	small ubiquitin like modifier 3	protein_codin	0.1014	0.383	1	17.95	0.1104
**TMCO4**	TMCO4	13	transmembrane and coiled-coil domains 4	protein_codin	0.3067	0.398	1	3.819	0.2258
**ZMYM1**	ZMYM1	13	zinc finger MYM-type containing 1	protein_codin	-0.008168	0.979	1	10.68	0.2752
**HYI**	HYI	13	hydroxypyruvate isomerase (putative)	protein_codin	0.3540	0.0440	1	34.82	0.1467
**PIK3R3**	PIK3R3	13	phosphoinositide-3-kinase regulatory subunit 3	protein_codin	-0.3199	0.0579	1	52.58	0.05575
**LRRC41**	LRRC41	13	leucine rich repeat containing 41	protein_codin	-0.2236	0.352	1	3.177	0.1652
**ZCCHC11**	ZCCHC11	13	terminal uridylyl transferase 4	protein_codin	NA	NA	NA	47.82	0.08649
**GPX7**	GPX7	13	glutathione peroxidase 7	protein_codin	-0.5798	0.0228	1	10.63	0.1652
**SCP2**	SCP2	13	sterol carrier protein 2	protein_codin	0.04215	0.652	1	45.29	0.0888
**SIKE1**	SIKE1	13	suppressor of IKBKE 1	protein_codin	0.1462	0.499	1	7.384	0.2572
**FAM189B**	FAM189B	13	family with sequence similarity 189 member B	protein_codin	0.4455	0.150	1	2.944	0.1435
**KLHL20**	KLHL20	13	kelch like family member 20	protein_codin	-0.103	0.682	1	7.228	0.1431
**PPM1G**	PPM1G	13	protein phosphatase, Mg2+/Mn2+ dependent 1G	protein_codin	0.1424	0.219	1	34.19	0.05236
**MOGS**	MOGS	13	mannosyl-oligosaccharide glucosidase	protein_codin	-0.3479	0.185	1	3.157	0.1532
**RBMS1**	RBMS1	13	RNA binding motif single stranded interacting protein 1	protein_codin	0.2115	0.0847	1	32.62	0.1119
**DPH3**	DPH3	13	diphthamide biosynthesis 3	protein_codin	0.04384	0.802	1	45.29	0.1642
**EPM2AIP1**	EPM2AIP1	13	EPM2A interacting protein 1	protein_codin	0.1919	0.529	1	3.433	0.2609
**CSRNP1**	CSRNP1	13	cysteine and serine rich nuclear protein 1	protein_codin	0.5938	3.31E-05	0.7639	19.38	0.05442
**SEC22C**	SEC22C	13	SEC22 homolog C, vesicle trafficking protein	protein_codin	-0.0954	0.635	1	26.29	0.05645
**DCP1A**	DCP1A	13	decapping mRNA 1A	protein_codin	0.2521	0.278	1	7.968	0.1952
**USF3**	USF3	13	upstream transcription factor family member 3	protein_codin	0.1064	0.671	1	8.339	0.2284
**ISY1**	ISY1	13	ISY1 splicing factor homolog	protein_codin	0.2296	0.317	1	8.743	0.1646
**DNAJC13**	DNAJC13	13	DnaJ heat shock protein family (Hsp40) member C13	protein_codin	-0.2614	0.329	1	3.516	0.06179
**RPL22L1**	RPL22L1	13	ribosomal protein L22 like 1	protein_codin	0.09644	0.369	1	47.13	0.02714
**ZNF639**	ZNF639	13	zinc finger protein 639	protein_codin	-0.05244	0.808	1	3.447	0.1988
**SLBP**	SLBP	13	stem-loop binding protein	protein_codin	0.1892	0.321	1	19.52	0.2588
**WFS1**	WFS1	13	wolframin ER transmembrane glycoprotein	protein_codin	0.625	0.0394	1	3.53	0.1432
**NUP54**	NUP54	13	nucleoporin 54	protein_codin	0.2315	0.252	1	22.88	0.05540
**BDH2**	BDH2	13	3-hydroxybutyrate dehydrogenase 2	protein_codin	-0.2414	0.0901	1	12.82	0.1488
**CFAP97**	CFAP97	13	cilia and flagella associated protein 97	protein_codin	0.08606	0.468	1	52.44	0.08270
**PLCXD3**	PLCXD3	13	phosphatidylinositol specific phospholipase C X domain conta	protein_codin	1.146	1.22E-05	0.2828	44.43	0.05632
**CCNH**	CCNH	13	cyclin H	protein_codin	0.2602	0.194	1	6.844	0.1719
**SNX2**	SNX2	13	sorting nexin 2	protein_codin	0.552	0.000175	1	35.22	0.09602
**PRRC1**	PRRC1	13	proline rich coiled-coil 1	protein_codin	-0.1859	0.432	1	6.056	0.168
**DDX46**	DDX46	13	DEAD-box helicase 46	protein_codin	0.1465	0.182	1	47.38	0.1141
**UIMC1**	UIMC1	13	ubiquitin interaction motif containing 1	protein_codin	0.05388	0.829	1	7.425	0.1685
**SERPINB6**	SERPINB6	13	serpin family B member 6	protein_codin	0.06279	0.474	1	43.7	0.0552
**C6orf48**	C6orf48	13	small nucleolar RNA host gene 32	protein_codin	-0.9536	3.23E-14	7.73E-10	52.57	0.08272
**AGPAT1**	AGPAT1	13	1-acylglycerol-3-phosphate O-acyltransferase 1	protein_codin	-0.6947	0.000615	1	9.439	0.1122
**TAPBP**	TAPBP	13	TAP binding protein	protein_codin	0.1002	0.384	1	52.48	0.1126
**CD2AP**	CD2AP	13	CD2 associated protein	protein_codin	-0.2826	0.0589	1	13.05	0.08084
**SH3BGRL2**	SH3BGRL2	13	SH3 domain binding glutamate rich protein like 2	protein_codin	2.926	2.43E-15	5.82E-11	30.15	0.1117
**NT5E**	NT5E	13	5'-nucleotidase ecto	protein_codin	-0.575	0.00586	1	21.76	0.1211
**TNRC18**	TNRC18	13	trinucleotide repeat containing 18	protein_codin	0.1302	0.669	1	3.413	0.2851
**CPVL**	CPVL	13	carboxypeptidase vitellogenic like	protein_codin	2.44	9.64E-11	2.29E-06	32.88	0.05602
**ELMO1**	ELMO1	13	engulfment and cell motility 1	protein_codin	0.5184	0.0110	1	19.63	0.08588
**BCL7B**	BCL7B	13	BAF chromatin remodeling complex subunit BCL7B	protein_codin	0.2464	0.158	1	40.81	0.02638
**ZNF394**	ZNF394	13	zinc finger protein 394	protein_codin	0.7215	0.00824	1	8.062	0.1126
**ZNF800**	ZNF800	13	zinc finger protein 800	protein_codin	0.2570	0.15	1	17.5	0.116
**RBM28**	RBM28	13	RNA binding motif protein 28	protein_codin	0.2091	0.447	1	8.247	0.2568
**ADCK2**	ADCK2	13	aarF domain containing kinase 2	protein_codin	0.4429	0.158	1	2.648	0.1944
**TMEM47**	TMEM47	13	transmembrane protein 47	protein_codin	-0.8901	4.84E-07	0.01134	16.66	0.05407
**CXorf38**	CXorf38	13	chromosome X open reading frame 38	protein_codin	0.1741	0.557	1	9.356	0.2265
**RPL36A**	RPL36A	13	ribosomal protein L36a	protein_codin	0.2364	0.014	1	25.18	0.08052
**ARMCX2**	ARMCX2	13	armadillo repeat containing X-linked 2	protein_codin	-1.112	0.000581	1	28.63	0.02608
**CD99L2**	CD99L2	13	CD99 molecule like 2	protein_codin	0.3472	0.275	1	3.229	0.2515
**EGR3**	EGR3	13	early growth response 3	protein_codin	0.6879	0.00416	1	31.66	0.1122
**PTDSS1**	PTDSS1	13	phosphatidylserine synthase 1	protein_codin	0.5434	0.0692	1	2.479	0.1389
**RRAGA**	RRAGA	13	Ras related GTP binding A	protein_codin	0.6796	9.14E-07	0.02138	52.48	0.08157
**BAG1**	BAG1	13	BCL2 associated athanogene 1	protein_codin	-0.2194	0.0334	1	27.44	0.02824
**AKAP2**	AKAP2	13	NA	protein_codin	1.210	0.00203	1	3.484	0.3944
**ANGPTL2**	ANGPTL2	13	angiopoietin like 2	protein_codin	-1.332	4.68E-09	0.000111	47.56	0.0274
**SSNA1**	SSNA1	13	SS nuclear autoantigen 1	protein_codin	0.02040	0.874	1	11.86	0.1361
**METTL15**	METTL15	13	methyltransferase like 15	protein_codin	-0.2329	0.422	1	10.18	0.2304
**TP53I11**	TP53I11	13	tumor protein p53 inducible protein 11	protein_codin	-0.5582	0.0489	1	48.04	0.108
**FAM111A**	FAM111A	13	family with sequence similarity 111 member A	protein_codin	-0.06280	0.749	1	17.5	0.1105
**OTUB1**	OTUB1	13	OTU deubiquitinase, ubiquitin aldehyde binding 1	protein_codin	0.2237	0.219	1	6.662	0.1919
**SIPA1**	SIPA1	13	signal-induced proliferation-associated 1	protein_codin	-0.3160	0.262	1	11.63	0.2301
**PDE2A**	PDE2A	13	phosphodiesterase 2A	protein_codin	2.226	7.3E-11	1.74E-06	14.73	0.2171
**MSANTD2**	MSANTD2	13	Myb/SANT DNA binding domain containing 2	protein_codin	-0.4042	0.242	1	9.346	0.1101
**DDX50**	DDX50	13	DExD-box helicase 50	protein_codin	0.728	0.000537	1	17.56	0.2204
**ABLIM1**	ABLIM1	13	actin binding LIM protein 1	protein_codin	-0.09285	0.508	1	31.47	0.02812
**TNFRSF1A**	TNFRSF1A	13	TNF receptor superfamily member 1A	protein_codin	0.4985	2.36E-07	0.005538	52.42	0.02779
**MRPL51**	MRPL51	13	mitochondrial ribosomal protein L51	protein_codin	-0.2464	0.00392	1	51.53	0.1352
**NECAP1**	NECAP1	13	NECAP endocytosis associated 1	protein_codin	0.3594	0.177	1	3.262	0.1362
**CDK17**	CDK17	13	cyclin dependent kinase 17	protein_codin	0.2187	0.303	1	41.53	0.1916
**RITA1**	RITA1	13	RBPJ interacting and tubulin associated 1	protein_codin	-0.09637	0.744	1	2.597	0.195
**USPL1**	USPL1	13	ubiquitin specific peptidase like 1	protein_codin	0.8740	0.0265	1	3.167	0.3342
**MAX**	MAX	13	MYC associated factor X	protein_codin	0.06848	0.684	1	14.6	0.1104
**EIF2S1**	EIF2S1	13	eukaryotic translation initiation factor 2 subunit alpha	protein_codin	0.2085	0.217	1	14.63	0.1721
**FBLN5**	FBLN5	13	fibulin 5	protein_codin	-1.904	3.36E-07	0.007870	51.78	0.2390
**WDR20**	WDR20	13	WD repeat domain 20	protein_codin	0.5445	0.132	1	3.456	0.2749
**KLC1**	KLC1	13	kinesin light chain 1	protein_codin	0.04995	0.808	1	24.43	0.02733
**ZFYVE21**	ZFYVE21	13	zinc finger FYVE-type containing 21	protein_codin	0.580	5.4E-07	0.01264	19.58	0.0554
**TMOD2**	TMOD2	13	tropomodulin 2	protein_codin	1.079	0.000395	1	9.142	0.2392
**POLR2M**	POLR2M	13	RNA polymerase II subunit M	protein_codin	0.162	0.572	1	8.717	0.08585
**CSNK1G1**	CSNK1G1	13	casein kinase 1 gamma 1	protein_codin	-0.4454	0.212	1	2.435	0.3642
**PDCD7**	PDCD7	13	programmed cell death 7	protein_codin	-0.08303	0.608	1	15.19	0.1479
**TLE3**	TLE3	13	TLE family member 3, transcriptional corepressor	protein_codin	0.2552	0.324	1	7.675	0.1964
**HDGFL3**	HDGFL3	13	HDGF like 3	protein_codin	0.1467	0.1192	1	50.83	0.08632
**GDE1**	GDE1	13	glycerophosphodiester phosphodiesterase 1	protein_codin	-0.7777	0.000793	1	9.915	0.1136
**LPCAT2**	LPCAT2	13	lysophosphatidylcholine acyltransferase 2	protein_codin	-1.319	1.78E-16	4.27E-12	48.32	0.1882
**ATMIN**	ATMIN	13	ATM interactor	protein_codin	-0.1937	0.448	1	7.608	0.2538
**CYBA**	CYBA	13	cytochrome b-245 alpha chain	protein_codin	-1.394	1.13E-29	2.73E-24	30.23	0.05387
**APRT**	APRT	13	adenine phosphoribosyltransferase	protein_codin	-0.2318	0.00586	1	24.58	0.0522
**FGF11**	FGF11	13	fibroblast growth factor 11	protein_codin	2.877	0.0102	1	8.93	0.1477
**AATF**	AATF	13	apoptosis antagonizing transcription factor	protein_codin	0.5317	0.0139	1	7.447	0.08216
**PCGF2**	PCGF2	13	polycomb group ring finger 2	protein_codin	-0.08062	0.565	1	13.23	0.3179
**MSL1**	MSL1	13	MSL complex subunit 1	protein_codin	0.2802	0.263	1	3.405	0.2905
**COG1**	COG1	13	component of oligomeric golgi complex 1	protein_codin	0.3632	0.26	1	3.238	0.2452
**RRBP1**	RRBP1	13	ribosome binding protein 1	protein_codin	0.116	0.262	1	21.25	0.054
**PDRG1**	PDRG1	13	p53 and DNA damage regulated 1	protein_codin	0.3097	0.232	1	8.343	0.08422
**PCIF1**	PCIF1	13	PDX1 C-terminal inhibiting factor 1	protein_codin	0.2449	0.36	1	3.306	0.2169
**GATAD2A**	GATAD2A	13	GATA zinc finger domain containing 2A	protein_codin	-0.09611	0.751	1	8.623	0.02828
**ZNF43**	ZNF43	13	zinc finger protein 43	protein_codin	0.167	0.527	1	6.952	0.1516
**UQCRFS1**	UQCRFS1	13	ubiquinol-cytochrome c reductase, Rieske iron-sulfur polypep	protein_codin	-0.05241	0.688	1	27.63	0.05992
**POP4**	POP4	13	POP4 homolog, ribonuclease P/MRP subunit	protein_codin	0.04085	0.812	1	7.046	0.1162
**COQ8B**	COQ8B	13	coenzyme Q8B	protein_codin	-0.0588	0.838	1	9.76	0.1657
**CCDC61**	CCDC61	13	coiled-coil domain containing 61	protein_codin	0.572	0.0552	1	2.957	0.1372
**MICAL3**	MICAL3	13	microtubule associated monooxygenase, calponin and LIM do	protein_codin	0.05020	0.854	1	3.53	0.08808
**ARFGAP3**	ARFGAP3	13	ADP ribosylation factor GTPase activating protein 3	protein_codin	-0.2635	0.0741	1	12.78	0.1091
**BRD1**	BRD1	13	bromodomain containing 1	protein_codin	-0.04917	0.822	1	7.98	0.1089
**MIIP**	MIIP	14	migration and invasion inhibitory protein	protein_codin	0.4559	0.12	1	3.058	0.3222
**UBR4**	UBR4	14	ubiquitin protein ligase E3 component n-recognin 4	protein_codin	0.06248	0.82	1	8.638	0.2549
**KPNA6**	KPNA6	14	karyopherin subunit alpha 6	protein_codin	-0.2904	0.202	1	3.427	0.1176
**BSDC1**	BSDC1	14	BSD domain containing 1	protein_codin	-0.3071	0.252	1	8.275	0.1721
**TAL1**	TAL1	14	TAL bHLH transcription factor 1, erythroid differentiation facto	protein_codin	-0.1397	0.643	1	2.766	0.3307
**ZZZ3**	ZZZ3	14	zinc finger ZZ-type containing 3	protein_codin	NA	NA	NA	7.106	0.149
**CTTNBP2NL**	CTTNBP2N	14	CTTNBP2 N-terminal like	protein_codin	-0.4371	0.000602	1	50.79	0.1136
**CDC42SE1**	CDC42SE1	14	CDC42 small effector 1	protein_codin	0.2959	0.0981	1	16.2	0.1182
**CREB3L4**	CREB3L4	14	cAMP responsive element binding protein 3 like 4	protein_codin	0.2016	0.558	1	10.24	0.1896
**PRCC**	PRCC	14	proline rich mitotic checkpoint control factor	protein_codin	0.04967	0.852	1	9.507	0.1458
**NCSTN**	NCSTN	14	nicastrin	protein_codin	-0.1822	0.391	1	8.541	0.0982
**RFWD2**	RFWD2	14	COP1 E3 ubiquitin ligase	protein_codin	NA	NA	NA	2.672	0.1689
**TOR1AIP1**	TOR1AIP1	14	torsin 1A interacting protein 1	protein_codin	0.3141	0.229	1	7.886	0.1104
**NUCKS1**	NUCKS1	14	nuclear casein kinase and cyclin dependent kinase substrate 1	protein_codin	-0.2375	0.000431	1	34.8	0.02874
**COQ8A**	COQ8A	14	coenzyme Q8A	protein_codin	0.2871	0.376	1	3.405	0.2490
**CDC42BPA**	CDC42BPA	14	CDC42 binding protein kinase alpha	protein_codin	-0.2822	0.106	1	31.7	0.1679
**ADI1**	ADI1	14	acireductone dioxygenase 1	protein_codin	-0.4916	4.41E-05	1	49.82	0.05285
**E2F6**	E2F6	14	E2F transcription factor 6	protein_codin	0.01484	0.958	1	10.87	0.3101
**TIA1**	TIA1	14	TIA1 cytotoxic granule associated RNA binding protein	protein_codin	-0.2854	0.205	1	26.24	0.2285
**TPRKB**	TPRKB	14	TP53RK binding protein	protein_codin	-0.004405	0.974	1	28.64	0.1204
**10-Sep**	43718	14	septin 10	protein_codin	0.959	6.11E-11	1.45E-06	36.89	0.1634
**CREB1**	CREB1	14	cAMP responsive element binding protein 1	protein_codin	-0.006075	0.974	1	18.36	0.1147
**CAMK1**	CAMK1	14	calcium/calmodulin dependent protein kinase I	protein_codin	0.631	0.00547	1	22.63	0.167
**MKRN2**	MKRN2	14	makorin ring finger protein 2	protein_codin	0.5864	0.0545	1	8.536	0.1149
**GLB1**	GLB1	14	galactosidase beta 1	protein_codin	-0.04129	0.891	1	8.738	0.3237
**NME6**	NME6	14	NME/NM23 nucleoside diphosphate kinase 6	protein_codin	0.5019	0.0982	1	10.12	0.1082
**KLHDC8B**	KLHDC8B	14	kelch domain containing 8B	protein_codin	0.6122	0.0162	1	9.166	0.2309
**GLT8D1**	GLT8D1	14	glycosyltransferase 8 domain containing 1	protein_codin	-0.4935	0.00571	1	20.94	0.1655
**SPCS1**	SPCS1	14	signal peptidase complex subunit 1	protein_codin	-0.218	0.000904	1	20.56	0.02622
**PXK**	PXK	14	PX domain containing serine/threonine kinase like	protein_codin	0.1258	0.628	1	6.513	0.1369
**TBC1D23**	TBC1D23	14	TBC1 domain family member 23	protein_codin	0.3512	0.183	1	27.25	0.1755
**TIMMDC1**	TIMMDC1	14	translocase of inner mitochondrial membrane domain contain	protein_codin	-0.2797	0.0232	1	18.99	0.1642
**PPP2R3A**	PPP2R3A	14	protein phosphatase 2 regulatory subunit B''alpha	protein_codin	0.285	0.336	1	3.452	0.1062
**COMMD2**	COMMD2	14	COMM domain containing 2	protein_codin	-0.07521	0.664	1	7.493	0.08266
**ACAP2**	ACAP2	14	ArfGAP with coiled-coil, ankyrin repeat and PH domains 2	protein_codin	0.4068	0.00419	1	49.67	0.1411
**RNF4**	RNF4	14	ring finger protein 4	protein_codin	0.2871	0.317	1	2.977	0.3265
**STX18**	STX18	14	syntaxin 18	protein_codin	-0.03787	0.889	1	3.319	0.2454
**ATP10D**	ATP10D	14	ATPase phospholipid transporting 10D (putative)	protein_codin	-0.7349	0.000746	1	20.08	0.189
**MED10**	MED10	14	mediator complex subunit 10	protein_codin	0.1684	0.188	1	9.726	0.112
**DAP**	DAP	14	death associated protein	protein_codin	-0.312	0.0156	1	22.28	0.05269
**TARS**	TARS	14	threonyl-tRNA synthetase	protein_codin	0.5617	0.0255	1	8.298	0.1752
**MRNIP**	MRNIP	14	MRN complex interacting protein	protein_codin	0.3889	0.13	1	27.29	0.2198
**WRNIP1**	WRNIP1	14	WRN helicase interacting protein 1	protein_codin	-0.4509	0.0470	1	9.776	0.125
**RIPK1**	RIPK1	14	receptor interacting serine/threonine kinase 1	protein_codin	0.752	0.0302	1	3.534	0.1082
**NOL7**	NOL7	14	nucleolar protein 7	protein_codin	0.06406	0.5946	1	25.94	0.08112
**MAPK14**	MAPK14	14	mitogen-activated protein kinase 14	protein_codin	0.5297	0.0589	1	8.838	0.2529
**SRSF3**	SRSF3	14	serine and arginine rich splicing factor 3	protein_codin	0.3892	5.67E-06	0.1319	34.45	0.08802
**PHIP**	PHIP	14	pleckstrin homology domain interacting protein	protein_codin	-0.4536	0.00602	1	35.37	0.08329
**CRYBG1**	CRYBG1	14	crystallin beta-gamma domain containing 1	protein_codin	0.2472	0.466	1	10.8	0.3195
**HBS1L**	HBS1L	14	HBS1 like translational GTPase	protein_codin	0.3685	0.11	1	6.912	0.1491
**LRP11**	LRP11	14	LDL receptor related protein 11	protein_codin	-0.006626	0.982	1	8.762	0.05256
**MYCT1**	MYCT1	14	MYC target 1	protein_codin	0.7179	3.93E-10	9.32E-06	21.13	0.05832
**DNAAF5**	DNAAF5	14	dynein axonemal assembly factor 5	protein_codin	0.5217	0.105	1	2.828	0.1121
**MRM2**	MRM2	14	mitochondrial rRNA methyltransferase 2	protein_codin	-0.1198	0.673	1	8.298	0.2821
**ICA1**	ICA1	14	islet cell autoantigen 1	protein_codin	0.7080	2.62E-06	0.0611	38.06	0.1974
**YKT6**	YKT6	14	YKT6 v-SNARE homolog	protein_codin	-0.1772	0.538	1	2.824	0.4081
**ZNF680**	ZNF680	14	zinc finger protein 680	protein_codin	-0.4188	0.13	1	8.7	0.1111
**TRIM4**	TRIM4	14	tripartite motif containing 4	protein_codin	0.5341	0.0712	1	7.052	0.2465
**LINC01003**	LINC01003	14	long intergenic non-protein coding RNA 1003	lincRNA	0.2220	0.384	1	7.624	0.1075
**DHRSX**	DHRSX	14	dehydrogenase/reductase X-linked	protein_codin	-0.2257	0.418	1	3.49	0.2332
**MIR222HG**	MIR222HG	14	miR222/221 cluster host gene	lincRNA	0.5864	0.0316	1	51.32	0.1154
**HMGB3**	HMGB3	14	high mobility group box 3	protein_codin	-0.4899	0.0602	1	9.214	0.08089
**DUSP4**	DUSP4	14	dual specificity phosphatase 4	protein_codin	0.4638	0.128	1	52.58	0.1468
**CEBPD**	CEBPD	14	CCAAT enhancer binding protein delta	protein_codin	0.4602	1.7E-06	0.03969	52.58	0.02612
**RB1CC1**	RB1CC1	14	RB1 inducible coiled-coil 1	protein_codin	-0.5532	0.000252	1	26.08	0.2571
**IMPAD1**	IMPAD1	14	inositol monophosphatase domain containing 1	protein_codin	-0.07232	0.747	1	7.638	0.09252
**RAB2A**	RAB2A	14	RAB2A, member RAS oncogene family	protein_codin	-0.2832	8E-04	1	14.13	0.03432
**LAPTM4B**	LAPTM4B	14	lysosomal protein transmembrane 4 beta	protein_codin	0.5345	0.000227	1	31.1	0.1619

**ANKRD46**	ANKRD46	14	ankyrin repeat domain 46	protein_codin	0.6146	0.0352	1	7.908	0.1070
**FAM49B**	FAM49B	14	family with sequence similarity 49 member B	protein_codin	0.4677	0.0996	1	3.028	0.1219
**PHF20L1**	PHF20L1	14	PHD finger protein 20 like 1	protein_codin	-0.168	0.262	1	12.72	0.1469
**DCAF12**	DCAF12	14	DDB1 and CUL4 associated factor 12	protein_codin	0.08266	0.785	1	3.238	0.05771
**HRCT1**	HRCT1	14	histidine rich carboxyl terminus 1	protein_codin	-0.65	9.88E-05	1	47.59	0.08132
**TOMM5**	TOMM5	14	translocase of outer mitochondrial membrane 5	protein_codin	-0.1896	0.307	1	44.71	0.054
**FAM189A2**	FAM189A2	14	family with sequence similarity 189 member A2	protein_codin	0.6796	0.135	1	3.238	0.3020
**TLE1**	TLE1	14	TLE family member 1, transcriptional corepressor	protein_codin	-0.4847	0.021	1	43.38	0.1472
**DAPK1**	DAPK1	14	death associated protein kinase 1	protein_codin	1.666	0.000206	1	3.209	0.3389
**TMOD1**	TMOD1	14	tropomodulin 1	protein_codin	-0.2352	0.224	1	29.25	0.1162
**STXBP1**	STXBP1	14	syntaxin binding protein 1	protein_codin	0.6536	0.00341	1	7.464	0.1107
**RRM1**	RRM1	14	ribonucleotide reductase catalytic subunit M1	protein_codin	0.5352	0.166	1	9.038	0.2195
**GALNT18**	GALNT18	14	polypeptide N-acetylgalactosaminyltransferase 18	protein_codin	0.4512	0.125	1	48.58	0.3395
**TSG101**	TSG101	14	tumor susceptibility 101	protein_codin	0.01917	0.9	1	11.65	0.08026
**PEX16**	PEX16	14	peroxisomal biogenesis factor 16	protein_codin	0.3385	0.0928	1	3.209	0.2004
**WDR74**	WDR74	14	WD repeat domain 74	protein_codin	0.8261	0.00806	1	9.176	0.1172
**EHD1**	EHD1	14	EH domain containing 1	protein_codin	-0.8769	0.000835	1	11.74	0.3472
**ZNF32**	ZNF32	14	zinc finger protein 32	protein_codin	0.1692	0.349	1	15.58	0.1751
**SGMS1**	SGMS1	14	sphingomyelin synthase 1	protein_codin	0.3242	0.252	1	9.282	0.1729
**ATAD1**	ATAD1	14	ATPase family AAA domain containing 1	protein_codin	-0.7588	0.000234	1	3.418	0.1392
**CH25H**	CH25H	14	cholesterol 25-hydroxylase	protein_codin	-0.306	0.498	1	48.05	0.6872
**SHOC2**	SHOC2	14	SHOC2 leucine rich repeat scaffold protein	protein_codin	0.2032	0.25	1	14.83	0.1370
**PDZD8**	PDZD8	14	PDZ domain containing 8	protein_codin	-0.6636	0.00387	1	31.78	0.1696
**ERGIC2**	ERGIC2	14	ERGIC and golgi 2	protein_codin	-0.007769	0.955	1	13.29	0.0822
**SENP1**	SENP1	14	SUMO specific peptidase 1	protein_codin	0.7709	0.132	1	3.317	0.2202
**ASB8**	ASB8	14	ankyrin repeat and SOCS box containing 8	protein_codin	0.3877	0.0651	1	6.672	0.1689
**NABP2**	NABP2	14	nucleic acid binding protein 2	protein_codin	0.06726	0.755	1	7.898	0.1682
**BAZ2A**	BAZ2A	14	bromodomain adjacent to zinc finger domain 2A	protein_codin	0.3731	0.134	1	7.808	0.1946
**SOCS2**	SOCS2	14	suppressor of cytokine signaling 2	protein_codin	-0.04302	0.704	1	48	0.1089
**WSB2**	WSB2	14	WD repeat and SOCS box containing 2	protein_codin	-0.3645	0.0979	1	8.793	0.1741
**VPS37B**	VPS37B	14	VPS37B subunit of ESCRT-I	protein_codin	-0.3325	0.184	1	6.863	0.142
**SPATA13**	SPATA13	14	spermatogenesis associated 13	protein_codin	1.265	0.00288	1	9.015	0.2161
**SPART**	SPART	14	spartin	protein_codin	0.0775	0.624	1	29.2	0.1146
**SPRY2**	SPRY2	14	sprouty RTK signaling antagonist 2	protein_codin	0.9799	0.00189	1	44.72	0.1671
**UGGT2**	UGGT2	14	UDP-glucose glycoprotein glucosyltransferase 2	protein_codin	0.3736	0.193	1	7.462	0.2979
**CFL2**	CFL2	14	cofilin 2	protein_codin	0.2402	0.196	1	18.7	0.1426
**ERO1A**	ERO1A	14	endoplasmic reticulum oxidoreductase 1 alpha	protein_codin	0.3878	0.183	1	8.587	0.279
**AL133453.1**	AL133453.1	14	novel transcript, antisense to ERO	antisense_RN	0.07836	0.8	1	3.327	0.1919
**NAA30**	NAA30	14	N(alpha)-acetyltransferase 30, NatC catalytic subunit	protein_codin	0.4162	0.225	1	3.403	0.2257
**VTI1B**	VTI1B	14	vesicle transport through interaction with t-SNAREs 1B	protein_codin	0.1606	0.288	1	48.36	0.08297
**ALDH6A1**	ALDH6A1	14	aldehyde dehydrogenase 6 family member A1	protein_codin	0.5752	0.029	1	22.78	0.1742
**BTBD7**	BTBD7	14	BTB domain containing 7	protein_codin	-0.08934	0.592	1	45.74	0.1922
**MAN2C1**	MAN2C1	14	mannosidase alpha class 2C member 1	protein_codin	0.05409	0.882	1	2.518	0.1415
**UBE2I**	UBE2I	14	ubiquitin conjugating enzyme E2 I	protein_codin	0.06497	0.558	1	14.34	0.08575
**SMG1**	SMG1	14	SMG1 nonsense mediated mRNA decay associated PI3K rel	protein_codin	0.138	0.529	1	17.68	0.1965
**N4BP1**	N4BP1	14	NEDD4 binding protein 1	protein_codin	0.6988	0.00439	1	6.853	0.08429
**PDPR**	PDPR	14	pyruvate dehydrogenase phosphatase regulatory subunit	protein_codin	0.6722	0.0451	1	11.82	0.2531
**ANKFY1**	ANKFY1	14	ankyrin repeat and FYVE domain containing 1	protein_codin	-0.4175	0.154	1	3.176	0.08244
**PHF23**	PHF23	14	PHD finger protein 23	protein_codin	-0.264	0.268	1	39.88	0.05284
**TP53**	TP53	14	tumor protein p53	protein_codin	0.2384	0.352	1	3.529	0.1719
**TMEM199**	TMEM199	14	transmembrane protein 199	protein_codin	0.000726	0.998	1	3.284	0.2282
**MYO18A**	MYO18A	14	myosin XVIIIA	protein_codin	0.6919	0.056	1	3.442	0.3112
**GOSR1**	GOSR1	14	golgi SNAP receptor complex member 1	protein_codin	0.2672	0.0937	1	14.54	0.1679
**PSMD11**	PSMD11	14	proteasome 26S subunit, non-ATPase 11	protein_codin	-0.05319	0.703	1	13.67	0.08238
**MMP28**	MMP28	14	matrix metallopeptidase 28	protein_codin	0.171	0.284	1	43.22	0.1375
**UBTF**	UBTF	14	upstream binding transcription factor	protein_codin	-0.03229	0.852	1	16.52	0.1167
**DYNLL2**	DYNLL2	14	dynein light chain LC8-type 2	protein_codin	0.04166	0.862	1	8.906	0.1142
**RNF213**	RNF213	14	ring finger protein 213	protein_codin	0.222	0.137	1	10.53	0.06351
**IMPACT**	IMPACT	14	impact RWD domain protein	protein_codin	0.6415	0.012	1	21.45	0.0552
**VPS4B**	VPS4B	14	vacuolar protein sorting 4 homolog B	protein_codin	0.2411	0.16	1	13.53	0.1977
**SRSF6**	SRSF6	14	serine and arginine rich splicing factor 6	protein_codin	0.6166	0.0014	1	15.76	0.1462
**EIF3G**	EIF3G	14	eukaryotic translation initiation factor 3 subunit G	protein_codin	0.1871	0.0112	1	45.82	0.1114
**MRPL4**	MRPL4	14	mitochondrial ribosomal protein L4	protein_codin	0.08676	0.567	1	6.175	0.1401
**MVB12A**	MVB12A	14	multivesicular body subunit 12A	protein_codin	-0.1474	0.323	1	12.17	0.05875
**RINL**	RINL	14	Ras and Rab interactor like	protein_codin	0.06806	0.828	1	3.349	0.1102
**PLD3**	PLD3	14	phospholipase D family member 3	protein_codin	-0.1089	0.516	1	15.4	0.1115
**TBC1D17**	TBC1D17	14	TBC1 domain family member 17	protein_codin	0.04964	0.877	1	2.844	0.2252
**ZNF766**	ZNF766	14	zinc finger protein 766	protein_codin	0.4697	0.129	1	7.873	0.1994
**UBE2M**	UBE2M	14	ubiquitin conjugating enzyme E2 M	protein_codin	-0.1036	0.318	1	16.55	0.05867
**HDHD5**	HDHD5	14	haloacid dehalogenase like hydrolase domain containing 5	protein_codin	-0.1481	0.579	1	9.792	0.157
**YWHAH**	YWHAH	14	tyrosine 3-monooxygenase/tryptophan 5-monooxygenase act	protein_codin	-0.2746	0.00578	1	46.58	0.02632
**ACO2**	ACO2	14	aconitase 2	protein_codin	0.3771	0.138	1	7.323	0.2586
**BTG3**	BTG3	14	BTG anti-proliferation factor 3	protein_codin	0.05359	0.75	1	28.26	0.05277
**GABPA**	GABPA	14	GA binding protein transcription factor subunit alpha	protein_codin	0.00505	0.979	1	21.28	0.1645
**ADARB1**	ADARB1	14	adenosine deaminase RNA specific B1	protein_codin	0.06506	0.825	1	3.185	0.1699
**GNB1**	GNB1	15	G protein subunit beta 1	protein_codin	-0.1554	0.215	1	20.42	0.02660
**HES5**	HES5	15	hes family bHLH transcription factor 5	protein_codin	1.871	0.00166	1	52.58	0.4790
**PNRC2**	PNRC2	15	proline rich nuclear receptor coactivator 2	protein_codin	0.2805	0.00670	1	34.18	0.1071
**MTFR1L**	MTFR1L	15	mitochondrial fission regulator 1 like	protein_codin	-0.437	0.0712	1	9.714	0.1385
**TRNAU1AP**	TRNAU1AP	15	tRNA selenocysteine 1 associated protein 1	protein_codin	0.2968	0.179	1	7.594	0.1336
**MTF1**	MTF1	15	metal regulatory transcription factor 1	protein_codin	-0.5936	0.0336	1	9.697	0.2510
**SVBP**	SVBP	15	small vasohibin binding protein	protein_codin	0.5366	0.00219	1	15.96	0.1472
**TMEM69**	TMEM69	15	transmembrane protein 69	protein_codin	0.8275	0.0121	1	2.834	0.2492
**USP1**	USP1	15	ubiquitin specific peptidase 1	protein_codin	-0.1842	0.282	1	50.08	0.1902
**GNG12**	GNG12	15	G protein subunit gamma 12	protein_codin	0.1166	0.388	1	21.98	0.1647
**WLS**	WLS	15	Wnt ligand secretion mediator	protein_codin	-0.6966	1.18E-05	0.2738	34.96	0.1397
**ST6GALNAC3**	ST6GALNA	15	ST6 N-acetylgalactosaminide alpha-2,6-sialyltransferase 3	protein_codin	1.026	0.00184	1	9.129	0.08172
**ODF2L**	ODF2L	15	outer dense fiber of sperm tails 2 like	protein_codin	0.06058	0.621	1	31.17	0.1354
**RNPC3**	RNPC3	15	RNA binding region (RNP1, RRM) containing 3	protein_codin	0.4671	0.0889	1	8.054	0.2171
**RAP1A**	RAP1A	15	RAP1A, member of RAS oncogene family	protein_codin	0.2347	0.0292	1	37.05	0.2229
**ST7L**	ST7L	15	suppression of tumorigenicity 7 like	protein_codin	0.3387	0.306	1	3.317	0.1922
**PHTF1**	PHTF1	15	putative homeodomain transcription factor 1	protein_codin	0.1634	0.578	1	3.229	0.1111
**HIPK1**	HIPK1	15	homeodomain interacting protein kinase 1	protein_codin	0.2689	0.289	1	6.895	0.1525
**ENSA**	ENSA	15	endosulfine alpha	protein_codin	0.4321	6.39E-06	0.1486	25.94	0.02712
**C1orf43**	C1orf43	15	chromosome 1 open reading frame 43	protein_codin	0.09672	0.344	1	26.68	0.02768
**RUSC1**	RUSC1	15	RUN and SH3 domain containing 1	protein_codin	0.8881	0.00462	1	7.616	0.14
**ARPC5**	ARPC5	15	actin related protein 2/3 complex subunit 5	protein_codin	-0.4571	8.92E-10	2.11E-05	52.02	0.02642
**GLRX2**	GLRX2	15	glutaredoxin 2	protein_codin	0.3322	0.186	1	7.127	0.1398
**NR5A2**	NR5A2	15	nuclear receptor subfamily 5 group A member 2	protein_codin	0.2122	0.452	1	9.628	0.149
**ARL8A**	ARL8A	15	ADP ribosylation factor like GTPase 8A	protein_codin	-0.2387	0.152	1	7.282	0.08069
**LBR**	LBR	15	lamin B receptor	protein_codin	0.3685	0.0514	1	18.03	0.1939
**ARID4B**	ARID4B	15	AT-rich interaction domain 4B	protein_codin	0.01956	0.855	1	41.73	0.02735
**SF3B6**	SF3B6	15	splicing factor 3b subunit 6	protein_codin	-0.08741	0.2612	1	52.32	0.05781
**NCOA1**	NCOA1	15	nuclear receptor coactivator 1	protein_codin	-0.2422	0.295	1	6.037	0.2009
**OST4**	OST4	15	oligosaccharyltransferase complex subunit 4, non-catalytic	protein_codin	-0.4128	1.27E-11	3.03E-07	34.85	0.02786
**GPATCH11**	GPATCH11	15	G-patch domain containing 11	protein_codin	0.5348	0.0159	1	7.268	0.08596
**EHBP1**	EHBP1	15	EH domain binding protein 1	protein_codin	1.064	0.000316	1	7.567	0.1824
**C1D**	C1D	15	C1D nuclear receptor corepressor	protein_codin	0.1109	0.506	1	13.67	0.1442
**ANXA4**	ANXA4	15	annexin A4	protein_codin	0.424	0.00185	1	37.05	0.1388
**PCGF1**	PCGF1	15	polycomb group ring finger 1	protein_codin	1.046	0.0176	1	2.632	0.3507
**CIAO1**	CIAO1	15	cytosolic iron-sulfur assembly component 1	protein_codin	0.02810	0.89	1	5.488	0.1120
**FAHD2B**	FAHD2B	15	fumarylacetoacetate hydrolase domain containing 2B	protein_codin	0.5325	0.122	1	3.142	0.1691
**NCK2**	NCK2	15	NCK adaptor protein 2	protein_codin	0.4255	0.0616	1	26.74	0.195
**C2orf76**	C2orf76	15	chromosome 2 open reading frame 76	protein_codin	0.08033	0.718	1	6.954	0.2056
**GYPC**	GYPC	15	glycophorin C (Gerbich blood group)	protein_codin	0.2565	0.0537	1	36.02	0.03871
**LIMS2**	LIMS2	15	LIM zinc finger domain containing 2	protein_codin	-0.02228	0.932	1	24.95	0.08514
**SPOPL**	SPOPL	15	speckle type BTB/POZ protein like	protein_codin	0.4409	0.242	1	3.272	0.1456
**ORC4**	ORC4	15	origin recognition complex subunit 4	protein_codin	0.1309	0.608	1	3.014	0.2141
**NMI**	NMI	15	N-myc and STAT interactor	protein_codin	0.5839	0.000562	1	37.66	0.1130
**STAM2**	STAM2	15	signal transducing adaptor molecule 2	protein_codin	0.07211	0.78	1	8.75	0.2802
**GCA**	GCA	15	grancalcin	protein_codin	0.2538	0.215	1	7.357	0.1416
**PPIG**	PPIG	15	peptidylprolyl isomerase G	protein_codin	0.2337	0.0191	1	42.2	0.1460
**ANKAR**	ANKAR	15	ankyrin and armadillo repeat containing	protein_codin	0.3186	0.266	1	3.352	0.2590
**HECW2**	HECW2	15	HECT, C2 and WW domain containing E3 ubiquitin protein lig	protein_codin	-0.3918	0.26	1	46.58	0.1552
**RFTN2**	RFTN2	15	raftlin family member 2	protein_codin	-0.1332	0.596	1	7.222	0.1642
**IGFBP5**	IGFBP5	15	insulin like growth factor binding protein 5	protein_codin	-0.6611	0.0726	1	52.58	0.2248
**CNOT9**	CNOT9	15	CCR4-NOT transcription complex subunit 9	protein_codin	0.5487	0.028	1	7.592	0.2074
**ARIH2**	ARIH2	15	ariadne RBR E3 ubiquitin protein ligase 2	protein_codin	-0.1381	0.528	1	8.686	0.2764
**RBM5**	RBM5	15	RNA binding motif protein 5	protein_codin	0.5032	0.0249	1	8.2	0.2767
**ABHD14A**	ABHD14A	15	abhydrolase domain containing 14A	protein_codin	-0.113	0.638	1	7.132	0.1122
**ARHGEF3**	ARHGEF3	15	Rho guanine nucleotide exchange factor 3	protein_codin	0.8129	6.7E-05	1	16.22	0.2285
**ARL6IP5**	ARL6IP5	15	ADP ribosylation factor like GTPase 6 interacting protein 5	protein_codin	-0.4022	2.23E-10	5.29E-06	51.82	0.0275
**FOXP1**	FOXP1	15	forkhead box P1	protein_codin	0.06503	0.504	1	51.25	0.05332
**CD47**	CD47	15	CD47 molecule	protein_codin	-0.08817	0.482	1	19.78	0.05768
**TPRA1**	TPRA1	15	transmembrane protein adipocyte associated 1	protein_codin	0.7316	0.0252	1	7.126	0.02677
**SLC35G2**	SLC35G2	15	solute carrier family 35 member G2	protein_codin	-0.003648	0.988	1	7.668	0.1769
**SSR3**	SSR3	15	signal sequence receptor subunit 3	protein_codin	-0.618	1.88E-12	4.49E-08	52.58	0.0261
**DNAJB11**	DNAJB11	15	DnaJ heat shock protein family (Hsp40) member B11	protein_codin	-0.04992	0.774	1	7.924	0.1115
**HS2ST1**	HS2ST1	15	heparan sulfate-glucosamine 3-sulfotransferase 1	protein_codin	0.5069	0.0549	1	42.52	0.2587
**FAM114A1**	FAM114A1	15	family with sequence similarity 114 member A1	protein_codin	-0.5981	3.39E-05	0.7823	43.37	0.1114
**FIP1L1**	FIP1L1	15	factor interacting with PAPOLA and CPSF1	protein_codin	0.1305	0.498	1	7.282	0.2027
**HNRNPDL**	HNRNPDL	15	heterogeneous nuclear ribonucleoprotein D like	protein_codin	0.2498	0.000776	1	51.2	0.05655
**COPS4**	COPS4	15	COP9 signalosome subunit 4	protein_codin	0.005471	0.976	1	7.122	0.1451
**ABRAXAS1**	ABRAXAS1	15	abraxas 1, BRCA1 A complex subunit	protein_codin	1.098	2.54E-05	0.587	7.385	0.1504
**NFKB1**	NFKB1	15	nuclear factor kappa B subunit 1	protein_codin	0.3319	0.135	1	7.692	0.08187
**PLA2G12A**	PLA2G12A	15	phospholipase A2 group XIIA	protein_codin	0.4408	0.162	1	2.655	0.171
**EXOSC9**	EXOSC9	15	exosome component 9	protein_codin	0.6614	0.00469	1	8.032	0.2220
**OTUD4**	OTUD4	15	OTU deubiquitinase 4	protein_codin	0.4173	0.166	1	8.455	0.2204
**GPM6A**	GPM6A	15	glycoprotein M6A	protein_codin	2.422	7.48E-10	1.77E-05	29.62	0.176
**CDKN2AIP**	CDKN2AIP	15	CDKN2A interacting protein	protein_codin	0.3626	0.0837	1	32.62	0.05576
**ANKH**	ANKH	15	ANKH inorganic pyrophosphate transport regulator	protein_codin	-0.3598	0.14	1	11.03	0.1098
**DAB2**	DAB2	15	DAB adaptor protein 2	protein_codin	0.2358	0.0819	1	46.45	0.08417
**MRPS30**	MRPS30	15	mitochondrial ribosomal protein S30	protein_codin	0.3947	0.0661	1	20.85	0.1359
**POC5**	POC5	15	POC5 centriolar protein	protein_codin	0.02582	0.937	1	3.724	0.4486
**WDR41**	WDR41	15	WD repeat domain 41	protein_codin	0.2809	0.432	1	3.496	0.2697
**RASA1**	RASA1	15	RAS p21 protein activator 1	protein_codin	0.4192	0.143	1	9.476	0.09172
**RIOK2**	RIOK2	15	RIO kinase 2	protein_codin	0.6582	0.0425	1	9.92	0.2332
**AC116366.1**	AC116366.1	15	novel transcri	antisense_RN	1.442	0.0137	1	9.346	0.4716
**TXNDC15**	TXNDC15	15	thioredoxin domain containing 15	protein_codin	-0.01422	0.93	1	6.462	0.1419
**DNAJC18**	DNAJC18	15	DnaJ heat shock protein family (Hsp40) member C18	protein_codin	0.5382	0.113	1	9.16	0.25
**CD14**	CD14	15	CD14 molecule	protein_codin	1.351	1.44E-07	0.003382	52.54	0.3467
**ARAP3**	ARAP3	15	ArfGAP with RhoGAP domain, ankyrin repeat and PH domain 3	protein_codin	0.007020	0.979	1	3.352	0.1682
**GNPDA1**	GNPDA1	15	glucosamine-6-phosphate deaminase 1	protein_codin	-0.1152	0.606	1	8.424	0.1181
**FAF2**	FAF2	15	Fas associated factor family member 2	protein_codin	0.1392	0.508	1	7.485	0.1376
**PDLIM7**	PDLIM7	15	PDZ and LIM domain 7	protein_codin	-0.3447	0.0256	1	45.15	0.05482
**PHYKPL**	PHYKPL	15	5-phosphohydroxy-L-lysine phospho-lyase	protein_codin	0.09796	0.648	1	7.964	0.07992
**SQSTM1**	SQSTM1	15	sequestosome 1	protein_codin	0.1155	0.112	1	52.02	0.142
**TUBB2A**	TUBB2A	15	tubulin beta 2A class IIa	protein_codin	0.2661	0.222	1	47.44	0.122
**SNRNP48**	SNRNP48	15	small nuclear ribonucleoprotein U11/U12 subunit 48	protein_codin	0.6169	0.0204	1	8.978	0.1472
**GFOD1**	GFOD1	15	glucose-fructose oxidoreductase domain containing 1	protein_codin	-0.208	0.297	1	15.32	0.1098
**GMNN**	GMNN	15	geminin DNA replication inhibitor	protein_codin	0.636	0.0274	1	24.64	0.1692
**BTN3A3**	BTN3A3	15	butyrophilin subfamily 3 member A3	protein_codin	1.401	1.04E-05	0.2412	8.012	0.1801
**ATF6B**	ATF6B	15	activating transcription factor 6 beta	protein_codin	0.1379	0.508	1	7.262	0.1089
**FKBPL**	FKBPL	15	FKBP prolyl isomerase like	protein_codin	-0.09406	0.687	1	22.62	0.2005
**PSMB9**	PSMB9	15	proteasome subunit beta 9	protein_codin	0.9531	1.12E-22	2.7E-18	36.98	0.06117
**ZBTB22**	ZBTB22	15	zinc finger and BTB domain containing 22	protein_codin	0.4951	0.11	1	2.804	0.08792
**UQCC2**	UQCC2	15	ubiquinol-cytochrome c reductase complex assembly factor 2	protein_codin	-1.117	1.03E-11	2.46E-07	17.58	0.08150
**PIM1**	PIM1	15	Pim-1 proto-oncogene, serine/threonine kinase	protein_codin	0.5695	0.0394	1	52.43	0.2947
**CCND3**	CCND3	15	cyclin D3	protein_codin	0.8101	2.44E-07	0.005726	51.05	0.08803
**C6orf226**	C6orf226	15	chromosome 6 open reading frame 226	protein_codin	0.1569	0.532	1	3.378	0.1704
**KLHDC3**	KLHDC3	15	kelch domain containing 3	protein_codin	0.8287	0.0152	1	30.7	0.1441
**MRPL2**	MRPL2	15	mitochondrial ribosomal protein L2	protein_codin	0.1231	0.487	1	21.2	0.1205
**CENPQ**	CENPQ	15	centromere protein Q	protein_codin	0.6837	0.0364	1	3.628	0.2886
**PTP4A1**	PTP4A1	15	protein tyrosine phosphatase 4A1	protein_codin	0.06403	0.796	1	7.244	0.08029
**SENP6**	SENP6	15	SUMO specific peptidase 6	protein_codin	-0.1081	0.49	1	28.92	0.1326
**MYO6**	MYO6	15	myosin VI	protein_codin	-0.1724	0.4	1	15.96	0.08479
**BCKDHB**	BCKDHB	15	branched chain keto acid dehydrogenase E1 subunit beta	protein_codin	0.9551	0.00742	1	3.307	0.1986
**NDUFAF4**	NDUFAF4	15	NADH ubiquinone oxidoreductase complex assembly factor 4	protein_codin	0.1577	0.482	1	8.555	0.1682
**PDSS2**	PDSS2	15	decaprenyl diphosphate synthase subunit 2	protein_codin	0.3075	0.406	1	3.066	0.4007
**AK9**	AK9	15	adenylate kinase 9	protein_codin	0.5522	0.118	1	3.605	0.2212
**TUBE1**	TUBE1	15	tubulin epsilon 1	protein_codin	-0.07258	0.78	1	7.334	0.02819
**TSPYL1**	TSPYL1	15	TSPY like 1	protein_codin	-0.2937	0.0722	1	15.92	0.05862
**HSF2**	HSF2	15	heat shock transcription factor 2	protein_codin	0.1282	0.658	1	3.48	0.08030
**AHI1**	AHI1	15	Abelson helper integration site 1	protein_codin	0.127	0.462	1	44.48	0.2061
**ADGRG6**	ADGRG6	15	adhesion G protein-coupled receptor G6	protein_codin	2.072	1.85E-07	0.004342	35.82	0.1702
**UTRN**	UTRN	15	utrophin	protein_codin	0.1022	0.42	1	37.18	0.027
**AKAP12**	AKAP12	15	A-kinase anchoring protein 12	protein_codin	2.548	7.18E-08	0.00169	44.26	0.3882
**PHF14**	PHF14	15	PHD finger protein 14	protein_codin	0.05148	0.696	1	21.43	0.1119
**LSM5.00**	LSM5.00	15	LSM5 homolog, U6 small nuclear RNA and mRNA degradatio	protein_codin	0.3154	0.0106	1	19.54	0.05412
**H2AFV**	H2AFV	15	H2A histone family member V	protein_codin	-0.2852	0.0135	1	18.03	0.06241
**GUSB**	GUSB	15	glucuronidase beta	protein_codin	-0.392	0.0102	1	6.892	0.2487
**TYW1**	TYW1	15	tRNA-yW synthesizing protein 1 homolog	protein_codin	0.8895	0.0147	1	3.302	0.2372
**KRIT1**	KRIT1	15	KRIT1 ankyrin repeat containing	protein_codin	0.07506	0.652	1	15.06	0.1101
**VPS50**	VPS50	15	VPS50 subunit of EARP/GARPII complex	protein_codin	0.3022	0.397	1	2.658	0.3192
**TMEM209**	TMEM209	15	transmembrane protein 209	protein_codin	0.7197	0.0312	1	3.288	0.2975
**ZNF467**	ZNF467	15	zinc finger protein 467	protein_codin	0.7269	0.000475	1	18.64	0.05441
**PNPLA4**	PNPLA4	15	patatin like phospholipase domain containing 4	protein_codin	0.2829	0.365	1	3.214	0.222
**SCML1**	SCML1	15	Scm polycomb group protein like 1	protein_codin	0.2452	0.241	1	20.53	0.08388
**GNL3L**	GNL3L	15	G protein nucleolar 3 like	protein_codin	-0.2679	0.378	1	3.302	0.2815
**MAGEH1**	MAGEH1	15	MAGE family member H1	protein_codin	-0.1171	0.593	1	7.555	0.1482
**EFNB1**	EFNB1	15	ephrin B1	protein_codin	0.0497	0.864	1	31.1	0.3495
**ACSL4**	ACSL4	15	acyl-CoA synthetase long chain family member 4	protein_codin	-0.2372	0.258	1	25.17	0.1754
**WDR44**	WDR44	15	WD repeat domain 44	protein_codin	-0.01354	0.965	1	2.976	0.3452
**6-Sep**	43714	15	septin 6	protein_codin	1.162	5.08E-06	0.1182	41.03	0.1476
**ATP11C**	ATP11C	15	ATPase phospholipid transporting 11C	protein_codin	0.6211	0.00449	1	52.32	0.027
**AVPR2**	AVPR2	15	arginine vasopressin receptor 2	protein_codin	2.549	8.04E-12	1.92E-08	44.11	0.0841
**GDI1**	GDI1	15	GDP dissociation inhibitor 1	protein_codin	-0.2886	0.232	1	6.97	0.1206
**MSRA**	MSRA	15	methionine sulfoxide reductase A	protein_codin	0.3076	0.0815	1	14.49	0.08209
**XPO7**	XPO7	15	exportin 7	protein_codin	0.0682	0.818	1	3.402	0.2862
**FAM91A1**	FAM91A1	15	family with sequence similarity 91 member A1	protein_codin	0.1041	0.683	1	7.71	0.2575
**FAM84B**	FAM84B	15	LRAT domain containing 2	protein_codin	1.48	3.22E-06	0.07502	52.48	0.2002
**EXOSC4**	EXOSC4	15	exosome component 4	protein_codin	-0.4154	0.0537	1	6.818	0.08555
**DGAT1**	DGAT1	15	diacylglycerol O-acyltransferase 1	protein_codin	0.1056	0.692	1	3.424	0.2618
**MLLT3**	MLLT3	15	MLLT3 super elongation complex subunit	protein_codin	0.6918	0.0157	1	8.096	0.1994
**TUSC1**	TUSC1	15	tumor suppressor candidate 1	protein_codin	0.1872	0.287	1	41.83	0.07935
**APTX**	APTX	15	aprataxin	protein_codin	-0.1879	0.476	1	10.22	0.2587
**UBE2R2**	UBE2R2	15	ubiquitin conjugating enzyme E2 R2	protein_codin	0.4882	0.000590	1	9.22	0.08389
**GOLM1**	GOLM1	15	golgi membrane protein 1	protein_codin	0.1242	0.618	1	18.28	0.02725
**COL15A1**	COL15A1	15	collagen type XV alpha 1 chain	protein_codin	-0.2676	0.0426	1	37.18	0.09256
**ST6GALNAC4**	ST6GALNA	15	ST6 N-acetylgalactosaminide alpha-2,6-sialyltransferase 4	protein_codin	-0.3736	0.0649	1	8.996	0.0866
**PRRC2B**	PRRC2B	15	proline rich coiled-coil 2B	protein_codin	0.1355	0.532	1	17.67	0.107
**RXRA**	RXRA	15	retinoid X receptor alpha	protein_codin	0.6292	0.0204	1	25.03	0.1472
**MRPS2**	MRPS2	15	mitochondrial ribosomal protein S2	protein_codin	0.2448	0.18	1	6.83	0.1362
**DPH7**	DPH7	15	diphthamide biosynthesis 7	protein_codin	-0.1497	0.552	1	2.878	0.1474
**RIC8A**	RIC8A	15	RIC8 guanine nucleotide exchange factor A	protein_codin	0.2679	0.19	1	8.288	0.05632
**TOLLIP**	TOLLIP	15	toll interacting protein	protein_codin	-0.4618	0.0396	1	3.468	0.2398
**IGF2**	IGF2	15	insulin like growth factor 2	protein_codin	0.8759	0.0979	1	52.54	0.8169
**CARS**	CARS	15	cysteinyl-tRNA synthetase	protein_codin	0.2614	0.178	1	3.472	0.2097
**TRIM21**	TRIM21	15	tripartite motif containing 21	protein_codin	0.747	0.0291	1	2.942	0.1792
**ARFIP2**	ARFIP2	15	ADP ribosylation factor interacting protein 2	protein_codin	0.4627	0.119	1	8.128	0.1150
**WEE1**	WEE1	15	WEE1 G2 checkpoint kinase	protein_codin	-0.1455	0.642	1	25.79	0.1457
**GTF2H1**	GTF2H1	15	general transcription factor IIH subunit 1	protein_codin	0.02233	0.924	1	7.025	0.138
**CSTF3**	CSTF3	15	cleavage stimulation factor subunit 3	protein_codin	-0.2625	0.372	1	2.59	0.2262
**APIP**	APIP	15	APAF1 interacting protein	protein_codin	0.2881	0.155	1	20.45	0.1289
**CREB3L1**	CREB3L1	15	cAMP responsive element binding protein 3 like 1	protein_codin	-0.2292	0.393	1	11.13	0.1555
**SLC43A3**	SLC43A3	15	solute carrier family 43 member 3	protein_codin	-0.2961	0.228	1	8.758	0.05334
**UBE2L6**	UBE2L6	15	ubiquitin conjugating enzyme E2 L6	protein_codin	0.4037	0.00126	1	43.32	0.06552
**CTNND1**	CTNND1	15	catenin delta 1	protein_codin	-0.1842	0.323	1	19.19	0.3101
**ATL3**	ATL3	15	atlastin GTPase 3	protein_codin	0.01364	0.933	1	26.95	0.08647
**RELA**	RELA	15	RELA proto-oncogene, NF-kB subunit	protein_codin	0.2447	0.203	1	22.05	0.1908
**RAB6A**	RAB6A	15	RAB6A, member RAS oncogene family	protein_codin	0.3436	0.0452	1	12.06	0.1932
**UVRAG**	UVRAG	15	UV radiation resistance associated	protein_codin	-0.09305	0.55	1	52.15	0.1222
**AAMDC**	AAMDC	15	adipogenesis associated Mth938 domain containing	protein_codin	-0.9596	7.86E-18	1.89E-13	36.55	0.05279
**CASP1**	CASP1	15	caspase 1	protein_codin	0.7944	3.04E-05	0.702	6.692	0.1204
**CEP164**	CEP164	15	centrosomal protein 164	protein_codin	0.05728	0.786	1	7.306	0.1362
**BCL9L**	BCL9L	15	BCL9 like	protein_codin	-0.1808	0.579	1	3.297	0.238
**DCPS**	DCPS	15	decapping enzyme, scavenger	protein_codin	0.1452	0.57	1	3.224	0.08198
**FRMD4A**	FRMD4A	15	FERM domain containing 4A	protein_codin	0.8286	0.00541	1	9.68	0.05490
**WAC**	WAC	15	WW domain containing adaptor with coiled-coil	protein_codin	0.01514	0.91	1	35.13	0.0265
**WASHC2A**	WASHC2A	15	WASH complex subunit 2A	protein_codin	0.207	0.525	1	3.032	0.2622
**FAM13C**	FAM13C	15	family with sequence similarity 13 member C	protein_codin	1.092	0.000126	1	45.11	0.2642
**ZCCHC24**	ZCCHC24	15	zinc finger CCHC-type containing 24	protein_codin	-0.405	0.0561	1	47.3	0.1882
**WAPL**	WAPL	15	WAPL cohesin release factor	protein_codin	0.04818	0.822	1	7.468	0.1479
**TNKS2**	TNKS2	15	tankyrase 2	protein_codin	0.2345	0.242	1	24.17	0.1661
**MYOF**	MYOF	15	myoferlin	protein_codin	0.168	0.292	1	32.95	0.05512
**TCTN3**	TCTN3	15	tectonic family member 3	protein_codin	0.235	0.315	1	9.202	0.1399
**BLOC1S2**	BLOC1S2	15	biogenesis of lysosomal organelles complex 1 subunit 2	protein_codin	-0.2102	0.247	1	6.932	0.1181
**DPCD**	DPCD	15	deleted in primary ciliary dyskinesia homolog (mouse)	protein_codin	-0.01955	0.928	1	32.84	0.05687
**POLL**	POLL	15	DNA polymerase lambda	protein_codin	0.4632	0.193	1	2.872	0.2896
**FBXL15**	FBXL15	15	F-box and leucine rich repeat protein 15	protein_codin	0.3433	0.054	1	41.08	0.1435
**ERC1**	ERC1	15	ELKS/RAB6-interacting/CAST family member 1	protein_codin	-0.07064	0.748	1	8.494	0.09297
**A2M**	A2M	15	alpha-2-macroglobulin	protein_codin	0.2492	0.0094	1	48.05	0.1252
**CREBL2**	CREBL2	15	cAMP responsive element binding protein like 2	protein_codin	0.05911	0.836	1	2.866	0.2265
**MGST1**	MGST1	15	microsomal glutathione S-transferase 1	protein_codin	1.741	0.000228	1	29.22	0.1767
**PYROXD1**	PYROXD1	15	pyridine nucleotide-disulphide oxidoreductase domain 1	protein_codin	0.452	0.162	1	3.805	0.1989
**MRPS35**	MRPS35	15	mitochondrial ribosomal protein S35	protein_codin	0.7865	9.39E-05	1	38.87	0.2181
**YAF2**	YAF2	15	YY1 associated factor 2	protein_codin	-0.4395	0.0452	1	8.938	0.1424
**LMBR1L**	LMBR1L	15	limb development membrane protein 1 like	protein_codin	0.6098	0.036	1	9.992	0.2826
**RNF41**	RNF41	15	ring finger protein 41	protein_codin	-0.5002	0.0411	1	7.833	0.05941
**XPOT**	XPOT	15	exportin for tRNA	protein_codin	-0.2458	0.377	1	10.93	0.17
**GNS**	GNS	15	glucosamine (N-acetyl)-6-sulfatase	protein_codin	0.6854	0.00118	1	14.79	0.1649
**TMTC3**	TMTC3	15	transmembrane O-mannosyltransferase targeting cadherins 3	protein_codin	-0.1142	0.68	1	3.658	0.195
**MRPL42**	MRPL42	15	mitochondrial ribosomal protein L42	protein_codin	-0.1232	0.367	1	13.27	0.1772
**CKAP4**	CKAP4	15	cytoskeleton associated protein 4	protein_codin	-0.8048	8.29E-05	1	25.68	0.0835
**VPS29**	VPS29	15	VPS29 retromer complex component	protein_codin	-0.07386	0.433	1	20.82	0.03175
**TRAFD1**	TRAFD1	15	TRAF-type zinc finger domain containing 1	protein_codin	0.5142	0.135	1	2.782	0.1774
**EP400**	EP400	15	E1A binding protein p400	protein_codin	0.06005	0.837	1	3.152	0.2172
**LATS2**	LATS2	15	large tumor suppressor kinase 2	protein_codin	-0.3538	0.153	1	32.48	0.1115
**COG3**	COG3	15	component of oligomeric golgi complex 3	protein_codin	0.1802	0.578	1	3.64	0.1782
**LRCH1**	LRCH1	15	leucine rich repeats and calponin homology domain containing 1	protein_codin	1.092	0.000521	1	30.85	0.1954
**LPAR6**	LPAR6	15	lysophosphatidic acid receptor 6	protein_codin	0.759	1.25E-09	2.96E-05	52.58	0.1684
**RAB2B**	RAB2B	15	RAB2B, member RAS oncogene family	protein_codin	0.4267	0.237	1	7.958	0.1991
**RALGAPA1**	RALGAPA1	15	Ral GTPase activating protein catalytic alpha subunit 1	protein_codin	-0.08297	0.752	1	6.21	0.1685
**KLHL28**	KLHL28	15	kelch like family member 28	protein_codin	0.504	0.0906	1	7.298	0.1319
**ERG28**	ERG28	15	ergosterol biosynthesis 28 homolog	protein_codin	0.1537	0.398	1	19.07	0.08052
**PPP2R5C**	PPP2R5C	15	protein phosphatase 2 regulatory subunit B'gamma	protein_codin	0.6819	0.000889	1	14.27	0.1434
**BAG5**	BAG5	15	BCL2 associated athanogene 5	protein_codin	0.1757	0.222	1	13.17	0.05645
**SIVA1**	SIVA1	15	SIVA1 apoptosis inducing factor	protein_codin	0.08318	0.383	1	42.2	0.05284
**DPH6**	DPH6	15	diphthamine biosynthesis 6	protein_codin	0.4087	0.155	1	2.892	0.2048
**LRRC57**	LRRC57	15	leucine rich repeat containing 57	protein_codin	0.9931	0.00102	1	7.368	0.2252
**DUT**	DUT	15	deoxyuridine triphosphatase	protein_codin	0.03694	0.668	1	30.8	0.114
**BNIP2**	BNIP2	15	BCL2 interacting protein 2	protein_codin	0.07581	0.458	1	29.13	0.08245
**OAZ2**	OAZ2	15	ornithine decarboxylase antizyme 2	protein_codin	-0.128	0.452	1	40	0.06126
**NPTN**	NPTN	15	neuroplastin	protein_codin	-0.4274	0.00679	1	46.11	0.08318
**SNUPN**	SNUPN	15	snurportin 1	protein_codin	0.01394	0.96	1	9.72	0.2327
**FBXO22**	FBXO22	15	F-box protein 22	protein_codin	0.5389	0.0402	1	30.13	0.1182
**RPS17**	RPS17	15	ribosomal protein S17	protein_codin	0.05238	0.76	1	41.06	0.02796
**WHAMM**	WHAMM	15	WASP homolog associated with actin, golgi membranes and	protein_codin	0.2936	0.187	1	8.454	0.2722
**TMEM8A**	TMEM8A	15	transmembrane protein 8A	protein_codin	-0.1716	0.577	1	3.246	0.08679
**TMEM204**	TMEM204	15	transmembrane protein 204	protein_codin	-0.2767	0.028	1	50.8	0.1674
**DEXI**	DEXI	15	Dexi homolog	protein_codin	-0.488	0.00652	1	7.925	0.06256
**PHKB**	PHKB	15	phosphorylase kinase regulatory subunit beta	protein_codin	-0.02842	0.877	1	18.37	0.2554
**RSPRY1**	RSPRY1	15	ring finger and SPRY domain containing 1	protein_codin	0.1801	0.467	1	3.302	0.1129
**NOB1**	NOB1	15	NIN1 (RPN12) binding protein 1 homolog	protein_codin	0.5416	0.00125	1	27.24	0.2394
**SF3B3**	SF3B3	15	splicing factor 3b subunit 3	protein_codin	0.4157	0.175	1	3.195	0.222
**ZC3H18**	ZC3H18	15	zinc finger CCCH-type containing 18	protein_codin	0.5545	0.0368	1	8.528	0.3527
**RILP**	RILP	15	Rab interacting lysosomal protein	protein_codin	0.1281	0.567	1	6.41	0.08387

**HIC1**	HIC1	15	HIC ZBTB transcriptional repressor 1	protein_codin	0.8445	0.0122	1	10.37	0.08607
**TMEM256**	TMEM256	15	transmembrane protein 256	protein_codin	0.2382	0.067	1	9.75	0.1089
**TNFSF12**	TNFSF12	15	TNF superfamily member 12	protein_codin	-0.3185	0.0151	1	11.02	0.1392
**BORCS6**	BORCS6	15	BLOC-1 related complex subunit 6	protein_codin	0.7222	0.0109	1	6.706	0.2321
**NDEL1**	NDEL1	15	nudE neurodevelopment protein 1 like 1	protein_codin	0.1074	0.658	1	8.429	0.1102
**TVP23B**	TVP23B	15	trans-golgi network vesicle protein 23 homolog B	protein_codin	0.7269	0.0332	1	2.498	0.2282
**DHRS7B**	DHRS7B	15	dehydrogenase/reductase 7B	protein_codin	0.1915	0.377	1	18.65	0.1325
**RHOT1**	RHOT1	15	ras homolog family member T1	protein_codin	0.2426	0.422	1	3.529	0.1638
**PSMB3**	PSMB3	15	proteasome subunit beta 3	protein_codin	-0.2238	0.0018	1	33.72	0.08037
**STARD3**	STARD3	15	StAR related lipid transfer domain containing 3	protein_codin	1.201	2.5E-07	0.005866	49.9	0.1139
**MRPL10**	MRPL10	15	mitochondrial ribosomal protein L10	protein_codin	0.5436	0.0177	1	25.72	0.2292
**SRSF1**	SRSF1	15	serine and arginine rich splicing factor 1	protein_codin	0.1305	0.442	1	19.58	0.05511
**CA4**	CA4	15	carbonic anhydrase 4	protein_codin	1.610	0.116	1	52.58	0.9640
**GPRC5C**	GPRC5C	15	G protein-coupled receptor class C group 5 member C	protein_codin	1.081	0.000226	1	35.55	0.1410
**JPT1**	JPT1	15	Jupiter microtubule associated homolog 1	protein_codin	-0.6012	6.93E-06	0.161	33.32	0.09287
**SRP68**	SRP68	15	signal recognition particle 68	protein_codin	0.4171	0.125	1	6.862	0.1438
**JMJD6**	JMJD6	15	jumonji domain containing 6, arginine demethylase and lysine	protein_codin	-0.212	0.358	1	26.17	0.2265
**TNRC6C**	TNRC6C	15	trinucleotide repeat containing adaptor 6C	protein_codin	-0.1427	0.582	1	9.587	0.08670
**YES1**	YES1	15	YES proto-oncogene 1, Src family tyrosine kinase	protein_codin	0.2378	0.102	1	10.82	0.05542
**PPP4R1**	PPP4R1	15	protein phosphatase 4 regulatory subunit 1	protein_codin	-0.05556	0.807	1	9.305	0.1072
**SEC11C**	SEC11C	15	SEC11 homolog C, signal peptidase complex subunit	protein_codin	-0.402	0.000195	1	46.84	0.03067
**PQLC1**	PQLC1	15	solute carrier family 66 member 2	protein_codin	0.5444	0.0018	1	6.487	0.129
**TCF15**	TCF15	15	transcription factor 15	protein_codin	0.6245	0.212	1	35.44	0.6451
**CDC25B**	CDC25B	15	cell division cycle 25B	protein_codin	0.9377	0.00177	1	38.43	0.2441
**KIZ**	KIZ	15	kizuna centrosomal protein	protein_codin	0.6352	0.0804	1	9.272	0.3682
**MANBAL**	MANBAL	15	mannosidase beta like	protein_codin	-0.2346	0.286	1	22.49	0.1472
**ZHX3**	ZHX3	15	zinc fingers and homeoboxes 3	protein_codin	-0.4941	0.0941	1	11.88	0.1981
**ACOT8**	ACOT8	15	acyl-CoA thioesterase 8	protein_codin	0.07963	0.805	1	3.312	0.1814
**ZNFX1**	ZNFX1	15	zinc finger NFX1-type containing 1	protein_codin	0.191	0.396	1	8.086	0.08771
**PCMTD2**	PCMTD2	15	protein-L-isoaspartate (D-aspartate) O-methyltransferase dom	protein_codin	0.6112	0.0106	1	21.1	0.2115
**AP3D1**	AP3D1	15	adaptor related protein complex 3 subunit delta 1	protein_codin	-0.5931	0.00744	1	9.96	0.02779
**TLE2**	TLE2	15	TLE family member 2, transcriptional corepressor	protein_codin	0.174	0.485	1	8.547	0.158
**ELAVL1**	ELAVL1	15	ELAV like RNA binding protein 1	protein_codin	-0.1936	0.294	1	22.27	0.08897
**CD320**	CD320	15	CD320 molecule	protein_codin	0.8684	4.06E-11	9.66E-07	52.58	0.1582
**ICAM4**	ICAM4	15	intercellular adhesion molecule 4 (Landsteiner-Wiener blood g	protein_codin	1.737	6.04E-05	1	23.21	0.1492
**ZNF136**	ZNF136	15	zinc finger protein 136	protein_codin	0.6051	0.0745	1	3.327	0.2180
**AKAP8L**	AKAP8L	15	A-kinase anchoring protein 8 like	protein_codin	0.07938	0.717	1	28.42	0.1379
**MAU2**	MAU2	15	MAU2 sister chromatid cohesion factor	protein_codin	0.2247	0.49	1	2.782	0.2294
**PDCD5**	PDCD5	15	programmed cell death 5	protein_codin	0.2682	0.00458	1	52.58	0.05414
**ACTN4**	ACTN4	15	actinin alpha 4	protein_codin	-0.1387	0.188	1	30.67	0.02828
**PPP1R37**	PPP1R37	15	protein phosphatase 1 regulatory subunit 37	protein_codin	0.3431	0.274	1	2.652	0.2287
**TRAPPC6A**	TRAPPC6A	15	trafficking protein particle complex 6A	protein_codin	0.5956	0.000742	1	14.6	0.221
**SNRNP70**	SNRNP70	15	small nuclear ribonucleoprotein U1 subunit 70	protein_codin	-0.009507	0.952	1	14.88	0.08271
**PTOV1**	PTOV1	15	PTOV1 extended AT-hook containing adaptor protein	protein_codin	-0.04995	0.802	1	7.468	0.1172
**C19orf48**	C19orf48	15	chromosome 19 open reading frame 48	protein_codin	0.3954	0.17	1	2.748	0.2224
**LENG9**	LENG9	15	leukocyte receptor cluster member 9	protein_codin	0.3884	0.205	1	8.748	0.1655
**UBE2S**	UBE2S	15	ubiquitin conjugating enzyme E2 S	protein_codin	-0.3737	0.00667	1	46.6	0.02678
**ZNF582-AS1**	ZNF582-AS	15	ZNF582 antisense RNA 1 (head to head)	lincRNA	-0.157	0.616	1	9.748	0.2080
**ZNF667-AS1**	ZNF667-AS	15	ZNF667 antisense RNA 1 (head to head)	lincRNA	0.1802	0.268	1	15.62	0.2070
**AC005261.2**	AC005261.2	15	novel transcri	lincRNA	0.1111	0.992	1	9.874	0.1154
**CHMP2A**	CHMP2A	15	charged multivesicular body protein 2A	protein_codin	-0.2286	0.00265	1	32.42	0.05550
**XBP1**	XBP1	15	X-box binding protein 1	protein_codin	-0.1472	0.297	1	52.02	0.112
**AP1B1**	AP1B1	15	adaptor related protein complex 1 subunit beta 1	protein_codin	0.2275	0.419	1	3.308	0.05968
**SELENOM**	SELENOM	15	selenoprotein M	protein_codin	-0.7068	1.47E-26	3.55E-22	52.58	0.03051
**CERK**	CERK	15	ceramide kinase	protein_codin	0.06607	0.829	1	9.103	0.1104
**DSCR3**	DSCR3	15	VPS26 endosomal protein sorting factor C	protein_codin	NA	NA	NA	7.813	0.307
**TFF3**	TFF3	15	trefoil factor 3	protein_codin	1.058	0.0018	1	52.58	0.3614
**TARDBP**	TARDBP	16	TAR DNA binding protein	protein_codin	0.07531	0.757	1	7.257	0.2584
**PLEKHM2**	PLEKHM2	16	pleckstrin homology and RUN domain containing M2	protein_codin	-0.04294	0.828	1	8.911	0.05771
**SDHB**	SDHB	16	succinate dehydrogenase complex iron sulfur subunit B	protein_codin	-0.009165	0.952	1	14.36	0.1207
**PINK1**	PINK1	16	PTEN induced kinase 1	protein_codin	-0.7709	8.37E-09	0.000198	20.7	0.02686
**HP1BP3**	HP1BP3	16	heterochromatin protein 1 binding protein 3	protein_codin	-0.04982	0.65	1	50.24	0.1700
**USP48**	USP48	16	ubiquitin specific peptidase 48	protein_codin	-0.3697	0.0331	1	19.64	0.05466
**MAN1C1**	MAN1C1	16	mannosidase alpha class 1C member 1	protein_codin	-0.5041	0.0757	1	10.37	0.419
**LAPTM5**	LAPTM5	16	lysosomal protein transmembrane 5	protein_codin	-1.66	1.14E-10	2.71E-06	34.65	0.1748
**ZBTB8OS**	ZBTB8OS	16	zinc finger and BTB domain containing 8 opposite strand	protein_codin	-0.01309	0.943	1	18.38	0.121
**YARS**	YARS	16	tyrosyl-tRNA synthetase	protein_codin	-0.1378	0.617	1	3.256	0.09552
**PHC2**	PHC2	16	polyhomeotic homolog 2	protein_codin	0.1697	0.335	1	4.419	0.2345
**MYCBP**	MYCBP	16	MYC binding protein	protein_codin	0.2569	0.407	1	3.119	0.3202
**CAP1**	CAP1	16	cyclase associated actin cytoskeleton regulatory protein 1	protein_codin	0.1432	0.167	1	21.77	0.05468
**PPIH**	PPIH	16	peptidylprolyl isomerase H	protein_codin	0.4825	0.0148	1	42.06	0.08788
**PLK3**	PLK3	16	polo like kinase 3	protein_codin	-0.2302	0.349	1	21.33	0.02782
**EPS15**	EPS15	16	epidermal growth factor receptor pathway substrate 15	protein_codin	0.1060	0.588	1	19.02	0.1382
**ITGB3BP**	ITGB3BP	16	integrin subunit beta 3 binding protein	protein_codin	0.8692	0.00197	1	22.26	0.0581
**PRKACB**	PRKACB	16	protein kinase cAMP-activated catalytic subunit beta	protein_codin	-1.568	7.9E-11	1.88E-06	44.5	0.08106
**SH3GLB1**	SH3GLB1	16	SH3 domain containing GRB2 like, endophilin B1	protein_codin	-0.3279	1E-04	1	22.03	0.1085
**GBP1**	GBP1	16	guanylate binding protein 1	protein_codin	1.331	5.42E-09	0.000129	52.58	0.3857
**GBP2**	GBP2	16	guanylate binding protein 2	protein_codin	1.327	8.75E-15	2.1E-10	33.83	0.1254
**GBP4**	GBP4	16	guanylate binding protein 4	protein_codin	1.134	1.76E-10	4.18E-06	52.58	0.3252
**BCAR3**	BCAR3	16	BCAR3 adaptor protein, NSP family member	protein_codin	0.1929	0.464	1	3.48	0.3204
**VCAM1**	VCAM1	16	vascular cell adhesion molecule 1	protein_codin	0.4965	0.00677	1	52.58	0.116
**CLCC1**	CLCC1	16	chloride channel CLIC like 1	protein_codin	-0.2122	0.426	1	11.52	0.1665
**TMEM167B**	TMEM167B	16	transmembrane protein 167B	protein_codin	-0.2985	0.222	1	8.672	0.1889
**SRGAP2B**	SRGAP2B	16	SLIT-ROBO Rho GTPase activating protein 2B	protein_codin	0.1005	0.716	1	3.587	0.3386
**HIST2H2AC**	HIST2H2AC	16	histone cluster 2 H2A family member c	protein_codin	-0.5856	0.0162	1	30.68	0.02636
**SF3B4**	SF3B4	16	splicing factor 3b subunit 4	protein_codin	0.2072	0.306	1	7.264	0.1197
**GOLPH3L**	GOLPH3L	16	golgi phosphoprotein 3 like	protein_codin	0.4202	0.106	1	3.062	0.1419
**CTSS**	CTSS	16	cathepsin S	protein_codin	-0.3267	0.0272	1	52.58	0.1535
**PIP5K1A**	PIP5K1A	16	phosphatidylinositol-4-phosphate 5-kinase type 1 alpha	protein_codin	-0.2245	0.416	1	1.823	0.2824
**MRPL9**	MRPL9	16	mitochondrial ribosomal protein L9	protein_codin	0.3612	0.0256	1	52.14	0.05864
**ADAR**	ADAR	16	adenosine deaminase RNA specific	protein_codin	-0.06474	0.67	1	12.82	0.2532
**IFI16**	IFI16	16	interferon gamma inducible protein 16	protein_codin	0.6569	1.51E-14	3.61E-10	34.76	0.02887
**NIT1**	NIT1	16	nitrilase 1	protein_codin	0.7419	0.00632	1	8.519	0.1615
**ADAMTS4**	ADAMTS4	16	ADAM metallopeptidase with thrombospondin type 1 motif 4	protein_codin	1.511	1.01E-06	0.02361	52.54	0.1715
**SELE**	SELE	16	selectin E	protein_codin	1.690	8.07E-09	0.000193	51.58	0.1767
**MRPS14**	MRPS14	16	mitochondrial ribosomal protein S14	protein_codin	-0.05605	0.772	1	7.647	0.1972
**KIAA0040**	KIAA0040	16	KIAA0040	protein_codin	0.3577	0.152	1	52.58	0.1201
**ZNF281**	ZNF281	16	zinc finger protein 281	protein_codin	0.3876	0.185	1	9.298	0.2885
**DSTYK**	DSTYK	16	dual serine/threonine and tyrosine protein kinase	protein_codin	0.3596	0.228	1	3.5	0.2208
**LPGAT1**	LPGAT1	16	lysophosphatidylglycerol acyltransferase 1	protein_codin	-0.09322	0.572	1	41.86	0.05537
**JMJD4**	JMJD4	16	jumonji domain containing 4	protein_codin	-0.432	0.158	1	3.028	0.2417
**C1orf131**	C1orf131	16	chromosome 1 open reading frame 131	protein_codin	0.3029	0.123	1	28.71	0.1102
**LPIN1**	LPIN1	16	lipin 1	protein_codin	-0.1982	0.47	1	9.325	0.1115
**TRIB2**	TRIB2	16	tribbles pseudokinase 2	protein_codin	0.7496	0.00386	1	7.755	0.2270
**RDH14**	RDH14	16	retinol dehydrogenase 14	protein_codin	0.6872	0.00517	1	6.943	0.03905
**UBXN2A**	UBXN2A	16	UBX domain protein 2A	protein_codin	0.02287	0.922	1	26.03	0.2166
**ASXL2**	ASXL2	16	ASXL transcriptional regulator 2	protein_codin	0.2564	0.358	1	3.352	0.3048
**NRBP1**	NRBP1	16	nuclear receptor binding protein 1	protein_codin	0.385	0.0248	1	14.03	0.1422
**SUPT7L**	SUPT7L	16	SPT7 like, STAGA complex gamma subunit	protein_codin	0.206	0.528	1	8.854	0.3372
**FOSL2**	FOSL2	16	FOS like 2, AP-1 transcription factor subunit	protein_codin	-0.03481	0.82	1	46.32	0.08369
**CEBPZOS**	CEBPZOS	16	CEBPZ opposite strand	protein_codin	0.2094	0.244	1	17.86	0.1372
**CRIPT**	CRIPT	16	CXXC repeat containing interactor of PDZ3 domain	protein_codin	0.01586	0.942	1	26.44	0.0866
**PPP4R3B**	PPP4R3B	16	protein phosphatase 4 regulatory subunit 3B	protein_codin	0.1389	0.46	1	17.62	0.175
**FANCL**	FANCL	16	FA complementation group L	protein_codin	0.7179	0.00247	1	8.452	0.1125
**PPP3R1**	PPP3R1	16	protein phosphatase 3 regulatory subunit B, alpha	protein_codin	0.488	0.0448	1	7.3	0.1185
**GGCX**	GGCX	16	gamma-glutamyl carboxylase	protein_codin	-0.3392	0.138	1	10.32	0.3155
**FAHD2A**	FAHD2A	16	fumarylacetoacetate hydrolase domain containing 2A	protein_codin	0.3905	0.27	1	3.01	0.165
**EIF5B**	EIF5B	16	eukaryotic translation initiation factor 5B	protein_codin	0.1321	0.182	1	46.12	0.02746
**RAB3GAP1**	RAB3GAP1	16	RAB3 GTPase activating protein catalytic subunit 1	protein_codin	0.452	0.0542	1	25.15	0.1962
**RIF1**	RIF1	16	replication timing regulatory factor 1	protein_codin	0.07903	0.692	1	19.5	0.1684
**PSMD14**	PSMD14	16	proteasome 26S subunit, non-ATPase 14	protein_codin	0.07526	0.667	1	17.27	0.05426
**DPP4**	DPP4	16	dipeptidyl peptidase 4	protein_codin	1.298	0.00044	1	3.782	0.1699
**SPATS2L**	SPATS2L	16	spermatogenesis associated serine rich 2 like	protein_codin	0.3657	0.00816	1	10.38	0.1151
**SP110**	SP110	16	SP110 nuclear body protein	protein_codin	0.5536	9.97E-05	1	38.65	0.1135
**HDLBP**	HDLBP	16	high density lipoprotein binding protein	protein_codin	-0.5451	6.97E-06	0.162	21.32	0.1092
**TRNT1**	TRNT1	16	tRNA nucleotidyl transferase 1	protein_codin	0.3382	0.156	1	8.176	0.1427
**VHL**	VHL	16	von Hippel-Lindau tumor suppressor	protein_codin	0.1992	0.541	1	2.617	0.1109
**SEC13**	SEC13	16	SEC13 homolog, nuclear pore and COPII coat complex comp	protein_codin	-0.2335	0.163	1	7.477	0.1952
**CMTM8**	CMTM8	16	CKLF like MARVEL transmembrane domain containing 8	protein_codin	-0.2692	0.0884	1	22.96	0.1674
**CMTM6**	CMTM6	16	CKLF like MARVEL transmembrane domain containing 6	protein_codin	-0.1316	0.475	1	50.21	0.08212
**LRRFIP2**	LRRFIP2	16	LRR binding FLII interacting protein 2	protein_codin	0.008349	0.952	1	19.85	0.1369
**SLC25A38**	SLC25A38	16	solute carrier family 25 member 38	protein_codin	0.1816	0.495	1	9.884	0.227
**PRKAR2A**	PRKAR2A	16	protein kinase cAMP-dependent type II regulatory subunit alpha	protein_codin	0.05411	0.792	1	17.8	0.1391
**P4HTM**	P4HTM	16	prolyl 4-hydroxylase, transmembrane	protein_codin	-0.282	0.105	1	7.006	0.1369
**TCTA**	TCTA	16	T cell leukemia translocation altered	protein_codin	0.3536	0.214	1	8.128	0.1462
**DAG1**	DAG1	16	dystroglycan 1	protein_codin	-0.9575	3.17E-05	0.7319	9.613	0.1055
**HYAL2**	HYAL2	16	hyaluronidase 2	protein_codin	-0.5471	6.48E-11	1.54E-06	52.58	0.03298
**NPRL2**	NPRL2	16	NPR2 like, GATOR1 complex subunit	protein_codin	0.142	0.662	1	3.181	0.1958
**ABHD14B**	ABHD14B	16	abhydrolase domain containing 14B	protein_codin	0.1071	0.502	1	6.517	0.1365
**DENND6A**	DENND6A	16	DENN domain containing 6A	protein_codin	0.1064	0.686	1	3.407	0.1791
**PROS1**	PROS1	16	protein S	protein_codin	0.3409	0.0546	1	30.29	0.2719
**ST3GAL6**	ST3GAL6	16	ST3 beta-galactoside alpha-2,3-sialyltransferase 6	protein_codin	-0.06312	0.822	1	10.52	0.2
**SENP7**	SENP7	16	SUMO specific peptidase 7	protein_codin	0.2322	0.26	1	7.866	0.2221
**NFKBIZ**	NFKBIZ	16	NFKB inhibitor zeta	protein_codin	0.9629	5.6E-10	1.33E-05	50.88	0.08292
**ATG3**	ATG3	16	autophagy related 3	protein_codin	-0.008807	0.948	1	24.87	0.1405
**PLA1A**	PLA1A	16	phospholipase A1 member A	protein_codin	0.8766	0.0129	1	52.52	0.1718
**COX17**	COX17	16	cytochrome c oxidase copper chaperone COX17	protein_codin	-0.2097	0.0526	1	52.58	0.02726
**KPNA1**	KPNA1	16	karyopherin subunit alpha 1	protein_codin	-0.3146	0.225	1	3.025	0.03328
**PDIA5**	PDIA5	16	protein disulfide isomerase family A member 5	protein_codin	2.166	3.86E-19	9.29E-15	41.2	0.1349
**KALRN**	KALRN	16	kalirin RhoGEF kinase	protein_codin	1.542	0.00912	1	3.019	0.204
**SNX4**	SNX4	16	sorting nexin 4	protein_codin	0.09056	0.686	1	8.8	0.08211
**RPN1**	RPN1	16	ribophorin I	protein_codin	0.1972	0.196	1	13.6	0.08177
**COPG1**	COPG1	16	coatomer protein complex subunit gamma 1	protein_codin	0.5798	0.0566	1	2.988	0.1431
**HMCES**	HMCES	16	5-hydroxymethylcytosine binding, ES cell specific	protein_codin	0.2879	0.208	1	6.842	0.112
**NPHP3**	NPHP3	16	nephrocystin 3	protein_codin	0.6536	0.0384	1	31.7	0.1342
**CCNL1**	CCNL1	16	cyclin L1	protein_codin	0.4826	4.24E-06	0.09872	36.98	0.02776
**SKIL**	SKIL	16	SKI like proto-oncogene	protein_codin	-1.224	1.43E-32	3.46E-28	34.95	0.02627
**TNFSF10**	TNFSF10	16	TNF superfamily member 10	protein_codin	0.6871	3.17E-14	7.58E-10	52.58	0.1478
**PIK3CA**	PIK3CA	16	phosphatidylinositol-4,5-bisphosphate 3-kinase catalytic subu	protein_codin	0.3317	0.168	1	8.672	0.1389
**TCTEX1D2**	TCTEX1D2	16	Tctex1 domain containing 2	protein_codin	0.1531	0.6	1	8.719	0.1704
**PAK2**	PAK2	16	p21 (RAC1) activated kinase 2	protein_codin	0.1721	0.0686	1	44.22	0.08542
**MFSD7**	MFSD7	16	solute carrier family 49 member 3	protein_codin	NA	NA	NA	10.07	0.1825
**CPLX1**	CPLX1	16	complexin 1	protein_codin	0.5532	0.000610	1	45.27	0.1105
**TMEM129**	TMEM129	16	transmembrane protein 129	protein_codin	-0.1624	0.577	1	3.048	0.1979
**HAUS3**	HAUS3	16	HAUS augmin like complex subunit 3	protein_codin	-0.05381	0.858	1	3.367	0.2526
**LYAR**	LYAR	16	Ly1 antibody reactive	protein_codin	0.004349	0.98	1	13.8	0.1391
**CYTL1**	CYTL1	16	cytokine like 1	protein_codin	0.5972	0.00226	1	52.58	0.2149
**RAB28**	RAB28	16	RAB28, member RAS oncogene family	protein_codin	0.5067	0.0694	1	10.02	0.1414
**FAM200B**	FAM200B	16	family with sequence similarity 200 member B	protein_codin	-0.1207	0.464	1	50.69	0.1175
**LAP3**	LAP3	16	leucine aminopeptidase 3	protein_codin	0.4358	6.31E-06	0.1467	52.07	0.06579
**TMEM33**	TMEM33	16	transmembrane protein 33	protein_codin	0.09132	0.709	1	6.724	0.2029
**REST**	REST	16	RE1 silencing transcription factor	protein_codin	-0.329	0.026	1	23.68	0.09128
**CXCL8**	CXCL8	16	C-X-C motif chemokine ligand 8	protein_codin	-0.4015	0.406	1	47.6	0.5096
**CXCL1**	CXCL1	16	C-X-C motif chemokine ligand 1	protein_codin	-1.668	0.0022	1	52.58	0.421
**CXCL3**	CXCL3	16	C-X-C motif chemokine ligand 3	protein_codin	-0.1690	0.618	1	52.58	0.2488
**CXCL2**	CXCL2	16	C-X-C motif chemokine ligand 2	protein_codin	-0.9601	5.1E-08	0.001201	52.58	0.2502
**CXCL9**	CXCL9	16	C-X-C motif chemokine ligand 9	protein_codin	0.07102	0.9524	1	Inf	0.2972
**CXCL10**	CXCL10	16	C-X-C motif chemokine ligand 10	protein_codin	3.688	0.148	1	52.58	1.852
**CXCL11**	CXCL11	16	C-X-C motif chemokine ligand 11	protein_codin	1.822	0.0631	1	52.58	0.6482
**PDLIM5**	PDLIM5	16	PDZ and LIM domain 5	protein_codin	-0.05712	0.614	1	28.77	0.08525
**CASP6**	CASP6	16	caspase 6	protein_codin	0.7687	0.0124	1	8.672	0.1679
**CAMK2D**	CAMK2D	16	calcium/calmodulin dependent protein kinase II delta	protein_codin	0.411	0.054	1	42.73	0.1651
**SNHG8**	SNHG8	16	small nucleolar RNA host gene 8	lincRNA	0.3293	0.000163	1	47.5	0.02622
**ELF2**	ELF2	16	E74 like ETS transcription factor 2	protein_codin	0.265	0.0327	1	36.62	0.1077
**LSM6.00**	LSM6.00	16	LSM6 homolog, U6 small nuclear RNA and mRNA degradatio	protein_codin	0.4269	0.00336	1	11.5	0.02838
**CBR4**	CBR4	16	carbonyl reductase 4	protein_codin	-0.5879	0.0442	1	8.822	0.2518
**CLCN3**	CLCN3	16	chloride voltage-gated channel 3	protein_codin	-0.2912	0.0912	1	18.57	0.1434
**SAP30**	SAP30	16	Sin3A associated protein 30	protein_codin	1.195	1.39E-11	3.31E-07	52.58	0.1067
**DCTD**	DCTD	16	dCMP deaminase	protein_codin	-0.3707	0.0306	1	8.173	0.08162
**ING2**	ING2	16	inhibitor of growth family member 2	protein_codin	-0.2252	0.224	1	47.05	0.111
**WDR70**	WDR70	16	WD repeat domain 70	protein_codin	0.1804	0.446	1	3.588	0.1816
**PPWD1**	PPWD1	16	peptidylprolyl isomerase domain and WD repeat containing 1	protein_codin	0.9291	0.000253	1	6.617	0.306
**SREK1**	SREK1	16	splicing regulatory glutamic acid and lysine rich protein 1	protein_codin	-0.07444	0.632	1	51.62	0.2479
**CCDC125**	CCDC125	16	coiled-coil domain containing 125	protein_codin	0.0005678	0.996	1	3.86	0.08621
**AK6**	AK6	16	adenylate kinase 6	protein_codin	0.08581	0.616	1	34.68	0.2259
**TAF9**	TAF9	16	TATA-box binding protein associated factor 9	protein_codin	-0.009816	0.95	1	52.54	0.1705
**ZBED3**	ZBED3	16	zinc finger BED-type containing 3	protein_codin	-0.1139	0.654	1	3.566	0.2316
**ZFYVE16**	ZFYVE16	16	zinc finger FYVE-type containing 16	protein_codin	0.1281	0.574	1	19.92	0.2829
**FNIP1**	FNIP1	16	folliculin interacting protein 1	protein_codin	0.1222	0.618	1	7.422	0.08742
**PPP2CA**	PPP2CA	16	protein phosphatase 2 catalytic subunit alpha	protein_codin	0.5016	0.0036	1	14.28	0.1115
**SRA1**	SRA1	16	steroid receptor RNA activator 1	protein_codin	-0.03537	0.812	1	14.16	0.1419
**ZMAT2**	ZMAT2	16	zinc finger matrin-type 2	protein_codin	0.3265	0.0372	1	13.98	0.1654
**NDFIP1**	NDFIP1	16	Nedd4 family interacting protein 1	protein_codin	-0.3136	0.00255	1	15.82	0.08344
**LARS**	LARS	16	leucyl-tRNA synthetase	protein_codin	0.5747	0.000115	1	20.57	0.1084
**TCERG1**	TCERG1	16	transcription elongation regulator 1	protein_codin	0.2606	0.25	1	17.47	0.1687
**FAM114A2**	FAM114A2	16	family with sequence similarity 114 member A2	protein_codin	0.6557	0.0206	1	25.38	0.166
**UBTD2**	UBTD2	16	ubiquitin domain containing 2	protein_codin	0.06148	0.826	1	3.654	0.1642
**DDX41**	DDX41	16	DEAD-box helicase 41	protein_codin	0.5026	0.0492	1	3.174	0.2272
**ZNF354B**	ZNF354B	16	zinc finger protein 354B	protein_codin	0.4138	0.26	1	36.02	0.404
**RUFY1**	RUFY1	16	RUN and FYVE domain containing 1	protein_codin	0.2696	0.3	1	8.832	0.1630
**MGAT4B**	MGAT4B	16	alpha-1,3-mannosyl-glycoprotein 4-beta-N-acetylglucosaminy	protein_codin	-0.7586	1.39E-06	0.03247	17.6	0.1361
**TRIM52**	TRIM52	16	tripartite motif containing 52	protein_codin	0.4779	0.206	1	10.32	0.1632
**RREB1**	RREB1	16	ras responsive element binding protein 1	protein_codin	0.6606	0.0329	1	8.465	0.2377
**NEDD9**	NEDD9	16	neural precursor cell expressed, developmentally down-regul	protein_codin	0.1427	0.242	1	43.7	0.02644
**CD83**	CD83	16	CD83 molecule	protein_codin	1.968	0.000242	1	25.02	0.2345
**SOX4**	SOX4	16	SRY-box transcription factor 4	protein_codin	-0.6615	1.63E-07	0.003829	51.46	0.1972
**BTN3A2**	BTN3A2	16	butyrophilin subfamily 3 member A2	protein_codin	1.32	2.2E-11	5.24E-07	14.92	0.08853
**DHX16**	DHX16	16	DEAH-box helicase 16	protein_codin	0.4114	0.172	1	8.406	0.08714
**PPP1R18**	PPP1R18	16	protein phosphatase 1 regulatory subunit 18	protein_codin	0.3856	0.0162	1	14.32	0.1714
**IER3**	IER3	16	immediate early response 3	protein_codin	-0.2952	0.00629	1	52.58	0.1285
**EHMT2**	EHMT2	16	euchromatic histone lysine methyltransferase 2	protein_codin	0.1399	0.582	1	3.084	0.227
**TAP2**	TAP2	16	transporter 2, ATP binding cassette subfamily B member	protein_codin	1.148	5.02E-06	0.1168	29	0.1174
**BRD2**	BRD2	16	bromodomain containing 2	protein_codin	-0.02332	0.852	1	29.08	0.116
**MDFI**	MDFI	16	MyoD family inhibitor	protein_codin	0.1882	0.46	1	23.26	0.09490
**TFEB**	TFEB	16	transcription factor EB	protein_codin	0.46	0.0712	1	9.727	0.1749
**LRRC1**	LRRC1	16	leucine rich repeat containing 1	protein_codin	2.279	8.15E-11	1.94E-06	6.884	0.1427
**LMBRD1**	LMBRD1	16	LMBR1 domain containing 1	protein_codin	0.2834	0.118	1	5.226	0.284
**MB21D1**	MB21D1	16	cyclic GMP-AMP synthase	protein_codin	NA	NA	NA	9.206	0.1398
**TPBG**	TPBG	16	trophoblast glycoprotein	protein_codin	0.2242	0.319	1	8.118	0.0829
**PGM3**	PGM3	16	phosphoglucomutase 3	protein_codin	-0.4577	0.0519	1	10.47	0.1455
**CDC40**	CDC40	16	cell division cycle 40	protein_codin	0.2712	0.0974	1	13.26	0.08971
**AMD1.00**	AMD1.00	16	adenosylmethionine decarboxylase 1	protein_codin	0.2464	0.0681	1	42.37	0.08316
**TNFAIP3**	TNFAIP3	16	TNF alpha induced protein 3	protein_codin	0.4489	0.0982	1	29.93	0.2304
**SOD2**	SOD2	16	superoxide dismutase 2	protein_codin	0.1925	0.12	1	48	0.02647
**PHF10**	PHF10	16	PHD finger protein 10	protein_codin	0.25	0.268	1	5.934	0.2519
**PDCD2**	PDCD2	16	programmed cell death 2	protein_codin	0.1012	0.478	1	11.42	0.1435
**ZFAND2A**	ZFAND2A	16	zinc finger AN1-type containing 2A	protein_codin	-0.1942	0.406	1	52.44	0.2374
**NUDT1**	NUDT1	16	nudix hydrolase 1	protein_codin	-0.0422	0.884	1	3.408	0.1219
**BRAT1**	BRAT1	16	BRCA1 associated ATM activator 1	protein_codin	-0.06696	0.798	1	3.228	0.1084
**FOXK1**	FOXK1	16	forkhead box K1	protein_codin	0.4692	0.142	1	8.826	0.2442
**THSD7A**	THSD7A	16	thrombospondin type 1 domain containing 7A	protein_codin	0.4067	0.0601	1	52.03	0.08726
**TMEM106B**	TMEM106B	16	transmembrane protein 106B	protein_codin	-0.1544	0.365	1	6.552	0.1684
**IL6**	IL6	16	interleukin 6	protein_codin	4.119	7.08E-09	0.000167	52.54	0.1685
**KLHL7**	KLHL7	16	kelch like family member 7	protein_codin	0.7387	0.035	1	3.075	0.3037
**GARS**	GARS	16	glycyl-tRNA synthetase	protein_codin	-0.1578	0.462	1	6.7	0.1479
**HERPUD2**	HERPUD2	16	HERPUD family member 2	protein_codin	0.7706	0.00116	1	5.643	0.2195
**MRPL32**	MRPL32	16	mitochondrial ribosomal protein L32	protein_codin	0.4165	0.00119	1	44.15	0.08075
**BLVRA**	BLVRA	16	biliverdin reductase A	protein_codin	0.4486	0.0156	1	18.62	0.1119
**VKORC1L1**	VKORC1L1	16	vitamin K epoxide reductase complex subunit 1 like 1	protein_codin	-0.2646	0.262	1	3.433	0.1445
**POM121**	POM121	16	POM121 transmembrane nucleoporin	protein_codin	0.2088	0.515	1	3.268	0.2260
**TBL2**	TBL2	16	transducin beta like 2	protein_codin	0.3475	0.275	1	2.828	0.02802
**GNAI1**	GNAI1	16	G protein subunit alpha i1	protein_codin	1.101	0.00035	1	24	0.2
**CDK6**	CDK6	16	cyclin dependent kinase 6	protein_codin	0.0341	0.903	1	7.772	0.08114
**GIGYF1**	GIGYF1	16	GRB10 interacting GYF protein 1	protein_codin	0.5516	0.096	1	3.287	0.08614
**WASL**	WASL	16	WASP like actin nucleation promoting factor	protein_codin	0.1555	0.217	1	10.9	0.07971
**SND1**	SND1	16	staphylococcal nuclease and tudor domain containing 1	protein_codin	0.2726	0.316	1	6.8	0.1388
**PRKAG2-AS1**	PRKAG2-A	16	PRKAG2 antisense RNA 1	antisense_RN	3.221	0.968	1	52.32	0.1624
**AP1S2**	AP1S2	16	adaptor related protein complex 1 subunit sigma 2	protein_codin	0.5779	3.09E-05	0.7135	45.26	0.02636
**RBBP7**	RBBP7	16	RB binding protein 7, chromatin remodeling factor	protein_codin	0.1014	0.506	1	16.52	0.08814
**RPGR**	RPGR	16	retinitis pigmentosa GTPase regulator	protein_codin	0.1567	0.306	1	40.82	0.05434
**USP11**	USP11	16	ubiquitin specific peptidase 11	protein_codin	0.2864	0.364	1	3.428	0.2321
**FTSJ1**	FTSJ1	16	FtsJ RNA 2'-O-methyltransferase 1	protein_codin	0.2342	0.432	1	2.885	0.1076
**TIMM17B**	TIMM17B	16	translocase of inner mitochondrial membrane 17B	protein_codin	0.08520	0.711	1	7.562	0.3411
**TSPYL2**	TSPYL2	16	TSPY like 2	protein_codin	-0.09944	0.64	1	50.65	0.1092
**SPIN2B**	SPIN2B	16	spindlin family member 2B	protein_codin	0.08825	0.764	1	2.798	0.05375
**HDAC8**	HDAC8	16	histone deacetylase 8	protein_codin	-0.1027	0.709	1	35.12	0.1944
**PGK1.00**	PGK1.00	16	phosphoglycerate kinase 1	protein_codin	0.1727	0.0322	1	46.08	0.02779
**ARMCX6**	ARMCX6	16	armadillo repeat containing X-linked 6	protein_codin	-0.5952	0.000947	1	18.85	0.05738
**TCEAL4**	TCEAL4	16	transcription elongation factor A like 4	protein_codin	-0.1925	0.0202	1	27.12	0.02711
**NDUFA1**	NDUFA1	16	NADH ubiquinone oxidoreductase subunit A1	protein_codin	-0.2637	0.0011	1	52.32	0.05438
**UBL4A**	UBL4A	16	ubiquitin like 4A	protein_codin	-0.2397	0.337	1	3.462	0.08656
**FUNDC2**	FUNDC2	16	FUN14 domain containing 2	protein_codin	0.221	0.0384	1	52.27	0.1438
**FDFT1**	FDFT1	16	farnesyl-diphosphate farnesyltransferase 1	protein_codin	-0.0342	0.869	1	52.06	0.1959
**LONRF1**	LONRF1	16	LON peptidase N-terminal domain and ring finger 1	protein_codin	0.8965	0.0214	1	3.525	0.2301
**TUSC3**	TUSC3	16	tumor suppressor candidate 3	protein_codin	-0.1915	0.426	1	21.48	0.1412
**DCTN6**	DCTN6	16	dynactin subunit 6	protein_codin	0.03612	0.805	1	15.64	0.05477
**ERLIN2**	ERLIN2	16	ER lipid raft associated 2	protein_codin	0.2964	0.344	1	3.109	0.1799
**RAB11FIP1**	RAB11FIP1	16	RAB11 family interacting protein 1	protein_codin	0.04884	0.828	1	28.3	0.1158
**AP3M2**	AP3M2	16	adaptor related protein complex 3 subunit mu 2	protein_codin	-0.1605	0.558	1	8.078	0.2559
**HGSNAT**	HGSNAT	16	heparan-alpha-glucosaminide N-acetyltransferase	protein_codin	0.6205	0.0205	1	6.875	0.1622
**MATN2**	MATN2	16	matrilin 2	protein_codin	-0.647	0.0129	1	20.22	0.02821
**RNF19A**	RNF19A	16	ring finger protein 19A, RBR E3 ubiquitin protein ligase	protein_codin	0.1995	0.21	1	41.94	0.05605
**C8orf76**	C8orf76	16	chromosome 8 open reading frame 76	protein_codin	0.07481	0.804	1	9.552	0.2812
**CHRAC1**	CHRAC1	16	chromatin accessibility complex subunit 1	protein_codin	0.2447	0.2	1	16.82	0.1382
**GPAA1**	GPAA1	16	glycosylphosphatidylinositol anchor attachment 1	protein_codin	-0.2848	0.0596	1	6.896	0.1415
**C8orf82**	C8orf82	16	chromosome 8 open reading frame 82	protein_codin	-0.1089	0.656	1	30.29	0.165
**ZNF7**	ZNF7	16	zinc finger protein 7	protein_codin	0.1201	0.674	1	3.357	0.2026
**UHRF2**	UHRF2	16	ubiquitin like with PHD and ring finger domains 2	protein_codin	0.4892	0.0441	1	52.4	0.1849
**CAAP1**	CAAP1	16	caspase activity and apoptosis inhibitor 1	protein_codin	-0.1358	0.558	1	8.711	0.2274
**KLF9**	KLF9	16	Kruppel like factor 9	protein_codin	0.7486	1.18E-09	2.8E-05	35.53	0.02612
**ZFAND5**	ZFAND5	16	zinc finger AN1-type containing 5	protein_codin	0.3111	0.00747	1	52	0.02666
**NAA35**	NAA35	16	N(alpha)-acetyltransferase 35, NatC auxiliary subunit	protein_codin	-0.08292	0.762	1	3.293	0.1747
**GADD45G**	GADD45G	16	growth arrest and DNA damage inducible gamma	protein_codin	-0.1368	0.622	1	37.18	0.3579
**RAD23B**	RAD23B	16	RAD23 homolog B, nucleotide excision repair protein	protein_codin	0.06345	0.71	1	16.02	0.15
**FKBP15**	FKBP15	16	FKBP prolyl isomerase 15	protein_codin	-0.2420	0.413	1	3.287	0.1655
**FBXW2**	FBXW2	16	F-box and WD repeat domain containing 2	protein_codin	-0.2815	0.214	1	7.282	0.1682
**FPGS**	FPGS	16	folylpolyglutamate synthase	protein_codin	0.1932	0.516	1	8.996	0.2881
**AK1**	AK1	16	adenylate kinase 1	protein_codin	-0.6349	0.0832	1	33.56	0.1160
**SLC25A25**	SLC25A25	16	solute carrier family 25 member 25	protein_codin	1.536	8.54E-09	0.000202	52.58	0.2074
**NTMT1**	NTMT1	16	N-terminal Xaa-Pro-Lys N-methyltransferase 1	protein_codin	-0.2541	0.242	1	7.929	0.08287
**AIF1L**	AIF1L	16	allograft inflammatory factor 1 like	protein_codin	-0.8795	6.6E-06	0.1534	50.82	0.0532
**LCN6**	LCN6	16	lipocalin 6	protein_codin	1.231	0.000164	1	28.11	0.08305
**TMEM203**	TMEM203	16	transmembrane protein 203	protein_codin	-0.1327	0.545	1	8.467	0.1155
**NAP1L4**	NAP1L4	16	nucleosome assembly protein 1 like 4	protein_codin	-0.03217	0.821	1	13.22	0.1956
**TRIM5**	TRIM5	16	tripartite motif containing 5	protein_codin	-0.102	0.713	1	8.434	0.2940
**ADM**	ADM	16	adrenomedullin	protein_codin	-0.6419	0.00498	1	52.58	0.3827
**CTR9**	CTR9	16	CTR9 homolog, Paf1/RNA polymerase II complex component	protein_codin	0.5522	0.0167	1	42.6	0.1147
**DKK3.00**	DKK3.00	16	dickkopf WNT signaling pathway inhibitor 3	protein_codin	-1.027	1.25E-06	0.02921	46.97	0.2343
**SERGEF**	SERGEF	16	secretion regulating guanine nucleotide exchange factor	protein_codin	-0.3346	0.144	1	3.495	0.1376
**SPTY2D1**	SPTY2D1	16	SPT2 chromatin protein domain containing 1	protein_codin	0.3496	0.138	1	9.005	0.08249
**CAPRIN1**	CAPRIN1	16	cell cycle associated protein 1	protein_codin	0.1819	0.348	1	42.18	0.1942
**FJX1**	FJX1	16	four-jointed box kinase 1	protein_codin	1.174	0.00782	1	50.96	0.6652
**TTC17**	TTC17	16	tetratricopeptide repeat domain 17	protein_codin	0.3050	0.242	1	26.05	0.1609
**MED19**	MED19	16	mediator complex subunit 19	protein_codin	0.3571	0.044	1	28.28	0.05619
**SLC15A3**	SLC15A3	16	solute carrier family 15 member 3	protein_codin	0.1172	0.712	1	10.68	0.1942
**B3GAT3**	B3GAT3	16	beta-1,3-glucuronyltransferase 3	protein_codin	-0.01828	0.929	1	5.98	0.1664
**TMEM223**	TMEM223	16	transmembrane protein 223	protein_codin	0.02964	0.868	1	38.52	0.1396

RARRES3	RARRES3	16	phospholipase A and acyltransferase 4	protein_codin	0.9006	9.2E-09	0.000217	47.62	0.1706
TRPT1	TRPT1	16	tRNA phosphotransferase 1	protein_codin	0.3525	0.206	1	7.188	0.2822
EHBP1L1	EHBP1L1	16	EH domain binding protein 1 like 1	protein_codin	0.5497	0.0161	1	20.92	0.2204
BRMS1	BRMS1	16	BRMS1 transcriptional repressor and anoikis regulator	protein_codin	0.4028	0.0398	1	18.52	0.1536
GRK2	GRK2	16	G protein-coupled receptor kinase 2	protein_codin	0.6049	0.0409	1	32.71	0.2267
UNC93B1	UNC93B1	16	unc-93 homolog B1, TLR signaling regulator	protein_codin	0.3219	0.322	1	3.566	0.2175
XRRA1	XRRA1	16	X-ray radiation resistance associated 1	protein_codin	0.3052	0.408	1	2.98	0.2504
ACER3	ACER3	16	alkaline ceramidase 3	protein_codin	0.607	0.054	1	10.12	0.2227
RAB38	RAB38	16	RAB38, member RAS oncogene family	protein_codin	1.722	0.000161	1	10.27	0.1985
ANKRD49	ANKRD49	16	ankyrin repeat domain 49	protein_codin	0.1028	0.747	1	8.876	0.08228
CWC15	CWC15	16	CWC15 spliceosome associated protein homolog	protein_codin	-0.004087	0.978	1	10.71	0.1711
SESN3	SESN3	16	sestrin 3	protein_codin	2.2	4.43E-09	0.000105	42	0.1684
LAYN	LAYN	16	layilin	protein_codin	0.4307	0.0889	1	22.96	0.2601
PTS	PTS	16	6-pyruvoyltetrahydropterin synthase	protein_codin	-0.2224	0.208	1	15.68	0.08634
H2AFX	H2AFX	16	H2A histone family member X	protein_codin	0.3508	0.0594	1	42	0.1397
OAF	OAF	16	out at first homolog	protein_codin	-0.0008554	0.997	1	24.02	0.2192
ZBTB44	ZBTB44	16	zinc finger and BTB domain containing 44	protein_codin	0.1579	0.538	1	30.16	0.2144
AKR1C1	AKR1C1	16	aldo-keto reductase family 1 member C1	protein_codin	2.828	3.02E-15	7.23E-11	28.24	0.1362
AKR1C2	AKR1C2	16	aldo-keto reductase family 1 member C2	protein_codin	2.726	1.49E-06	0.03481	51.52	0.2996
USP6NL	USP6NL	16	USP6 N-terminal like	protein_codin	-0.09409	0.728	1	3.383	0.2638
PHYH	PHYH	16	phytanoyl-CoA 2-hydroxylase	protein_codin	0.5428	0.00675	1	41.38	0.1116
SVIL	SVIL	16	supervillin	protein_codin	0.02025	0.905	1	42.08	0.08342
CSGALNACT2	CSGALNAC	16	chondroitin sulfate N-acetylgalactosaminyltransferase 2	protein_codin	-0.1336	0.652	1	3.422	0.3381
CCDC6	CCDC6	16	coiled-coil domain containing 6	protein_codin	0.2505	0.252	1	39.71	0.1379
EGR2	EGR2	16	early growth response 2	protein_codin	1.545	0.000946	1	52.06	0.1231
SIRT1	SIRT1	16	sirtuin 1	protein_codin	-0.01162	0.967	1	8.69	0.2286
RUFY2	RUFY2	16	RUN and FYVE domain containing 2	protein_codin	0.3796	0.0932	1	25.54	0.2264
PSAP	PSAP	16	prosaposin	protein_codin	-0.6403	2.01E-18	4.83E-14	52.57	0.02759
ECD	ECD	16	ecdysoneless cell cycle regulator	protein_codin	0.6321	0.0141	1	3.327	0.08500
PPP3CB	PPP3CB	16	protein phosphatase 3 catalytic subunit beta	protein_codin	-0.006742	0.98	1	3.343	0.1447
VCL	VCL	16	vinculin	protein_codin	-0.6862	0.000102	1	18.88	0.1151
ZNF503	ZNF503	16	zinc finger protein 503	protein_codin	-0.499	0.0439	1	42.37	0.1132
TRIM8	TRIM8	16	tripartite motif containing 8	protein_codin	-0.2109	0.207	1	52.58	0.1629
MXI1	MXI1	16	MAX interactor 1, dimerization protein	protein_codin	0.6059	0.000581	1	22.68	0.2225
PDCD4	PDCD4	16	programmed cell death 4	protein_codin	-0.1017	0.44	1	47.36	0.05859
NSMCE4A	NSMCE4A	16	NSE4 homolog A, SMC5-SMC6 complex component	protein_codin	0.2732	0.103	1	13.86	0.2208
EEF1AKMT2	EEF1AKMT	16	EEF1A lysine methyltransferase 2	protein_codin	0.2808	0.216	1	5.972	0.2261
GLRX3	GLRX3	16	glutaredoxin 3	protein_codin	-0.05232	0.736	1	15.06	0.1369
KDM5A	KDM5A	16	lysine demethylase 5A	protein_codin	0.1167	0.652	1	35.84	0.2322
TEAD4	TEAD4	16	TEA domain transcription factor 4	protein_codin	0.5879	0.02	1	25.97	0.08268
MANSC1	MANSC1	16	MANSC domain containing 1	protein_codin	0.2632	0.146	1	17.43	0.2467
AEBP2	AEBP2	16	AE binding protein 2	protein_codin	0.7289	0.0362	1	9.02	0.1436
KRAS	KRAS	16	KRAS proto-oncogene, GTPase	protein_codin	0.2137	0.0726	1	25.36	0.02680
PUS7L	PUS7L	16	pseudouridine synthase 7 like	protein_codin	0.5184	0.0261	1	19.68	0.1771
CCNT1	CCNT1	16	cyclin T1	protein_codin	0.1029	0.697	1	9.292	0.2001
LETMD1	LETMD1	16	LETM1 domain containing 1	protein_codin	0.3396	0.0632	1	16.72	0.2586
IGFBP6	IGFBP6	16	insulin like growth factor binding protein 6	protein_codin	-0.6979	0.0162	1	52.53	0.6424
ESYT1	ESYT1	16	extended synaptotagmin 1	protein_codin	0.6634	0.0187	1	25.14	0.151
R3HDM2	R3HDM2	16	R3H domain containing 2	protein_codin	0.1062	0.562	1	14.54	0.1725
ATXN7L3B	ATXN7L3B	16	ataxin 7 like 3B	protein_codin	0.102	0.495	1	39.79	0.08256
OSBPL8	OSBPL8	16	oxysterol binding protein like 8	protein_codin	0.04714	0.75	1	38.6	0.05586
NUDT4	NUDT4	16	nudix hydrolase 4	protein_codin	-1.014	6.16E-10	1.46E-05	52.58	0.02826
PWP1	PWP1	16	PWP1 homolog, endonuclein	protein_codin	0.4664	0.00429	1	15.75	0.08387
KCTD10	KCTD10	16	potassium channel tetramerization domain containing 10	protein_codin	-0.1506	0.579	1	7.546	0.1888
GLTP	GLTP	16	glycolipid transfer protein	protein_codin	-0.2212	0.168	1	44.41	0.08657
DDX54	DDX54	16	DEAD-box helicase 54	protein_codin	0.8686	0.00388	1	7.747	0.3651
SLC8B1	SLC8B1	16	solute carrier family 8 member B1	protein_codin	0.4572	0.186	1	3.048	0.3674
COQ5	COQ5	16	coenzyme Q5, methyltransferase	protein_codin	-0.01864	0.942	1	3.156	0.1402
RNF10	RNF10	16	ring finger protein 10	protein_codin	-0.8668	1.63E-07	0.003829	14.68	0.02779
TMED2	TMED2	16	transmembrane p24 trafficking protein 2	protein_codin	-0.3392	0.00285	1	9.99	0.08376
MTMR6	MTMR6	16	myotubularin related protein 6	protein_codin	0.342	0.28	1	8.604	0.08192
UBL3	UBL3	16	ubiquitin like 3	protein_codin	-0.2337	0.212	1	7.974	0.2021
SMAD9	SMAD9	16	SMAD family member 9	protein_codin	0.2034	0.292	1	7.07	0.1744
MRPS31	MRPS31	16	mitochondrial ribosomal protein S31	protein_codin	0.5561	0.00389	1	13.71	0.02862
ESD	ESD	16	esterase D	protein_codin	0.3991	8.73E-05	1	21.62	0.08448
MED4	MED4	16	mediator complex subunit 4	protein_codin	0.1407	0.279	1	19.22	0.2294
UBAC2	UBAC2	16	UBA domain containing 2	protein_codin	-0.01076	0.956	1	7.598	0.2196
TEX30	TEX30	16	testis expressed 30	protein_codin	0.4079	0.21	1	3.418	0.2799
CUL4A	CUL4A	16	cullin 4A	protein_codin	0.2839	0.269	1	7.157	0.1642
NDRG2	NDRG2	16	NDRG family member 2	protein_codin	0.8171	0.00110	1	22.68	0.1108
PSME1	PSME1	16	proteasome activator subunit 1	protein_codin	0.3688	2.16E-08	0.00051	52.02	0.02636
PSME2	PSME2	16	proteasome activator subunit 2	protein_codin	0.3657	8.98E-05	1	52.58	0.1421
RABGGTA	RABGGTA	16	Rab geranylgeranyltransferase subunit alpha	protein_codin	0.4596	0.118	1	10.41	0.142
ARHGAP5	ARHGAP5	16	Rho GTPase activating protein 5	protein_codin	0.1416	0.565	1	24.07	0.2011
EAPP	EAPP	16	E2F associated phosphoprotein	protein_codin	0.187	0.145	1	39.03	0.166
SRP54	SRP54	16	signal recognition particle 54	protein_codin	0.2474	0.243	1	17.9	0.3391
NFKBIA	NFKBIA	16	NFKB inhibitor alpha	protein_codin	1.049	9.48E-21	2.28E-16	52.58	0.2944
KLHDC2	KLHDC2	16	kelch domain containing 2	protein_codin	0.1904	0.314	1	18.87	0.1392
DAAM1	DAAM1	16	dishevelled associated activator of morphogenesis 1	protein_codin	1.979	4.07E-26	9.83E-22	31.22	0.1152
SNAPC1	SNAPC1	16	small nuclear RNA activating complex polypeptide 1	protein_codin	0.4835	0.02	1	45.76	0.1791
PIGH	PIGH	16	phosphatidylinositol glycan anchor biosynthesis class H	protein_codin	-0.07426	0.754	1	7.288	0.2499
ZFP36L1	ZFP36L1	16	ZFP36 ring finger protein like 1	protein_codin	0.556	4.57E-07	0.01071	52.58	0.03586
DCAF5	DCAF5	16	DDB1 and CUL4 associated factor 5	protein_codin	0.1088	0.622	1	8.177	0.1511
YLPM1	YLPM1	16	YLP motif containing 1	protein_codin	-0.1872	0.437	1	3.717	0.1132
SPTLC2	SPTLC2	16	serine palmitoyltransferase long chain base subunit 2	protein_codin	0.6764	5.73E-05	1	51.82	0.1626
BDKRB2	BDKRB2	16	bradykinin receptor B2	protein_codin	1.315	0.00135	1	9.028	0.2832
WARS	WARS	16	tryptophanyl-tRNA synthetase	protein_codin	0.296	0.0769	1	52.58	0.2971
TNFAIP2	TNFAIP2	16	TNF alpha induced protein 2	protein_codin	-0.06817	0.772	1	20.86	0.1626
INF2	INF2	16	inverted formin, FH2 and WH2 domain containing	protein_codin	0.2652	0.338	1	3.133	0.1405
MTA1	MTA1	16	metastasis associated 1	protein_codin	-0.2324	0.277	1	8.316	0.2261
RTF1	RTF1	16	RTF1 homolog, Paf1/RNA polymerase II complex component	protein_codin	-0.02322	0.834	1	52.49	0.1199
SNAP23	SNAP23	16	synaptosome associated protein 23	protein_codin	0.192	0.158	1	20.82	0.1065
CTDSPL2	CTDSPL2	16	CTD small phosphatase like 2	protein_codin	0.377	0.142	1	8.416	0.1729
BLOC1S6	BLOC1S6	16	biogenesis of lysosomal organelles complex 1 subunit 6	protein_codin	-0.4535	0.0149	1	17.32	0.1159
TMOD3	TMOD3	16	tropomodulin 3	protein_codin	0.6232	4.07E-11	9.68E-07	33.02	0.09238
RAB27A	RAB27A	16	RAB27A, member RAS oncogene family	protein_codin	0.2561	0.182	1	17.42	0.1116
C2CD4B	C2CD4B	16	C2 calcium dependent domain containing 4B	protein_codin	2.107	9.46E-16	2.27E-11	52.58	0.2685
TLN2	TLN2	16	talin 2	protein_codin	1.659	0.000314	1	3.613	0.2250
LACTB	LACTB	16	lactamase beta	protein_codin	0.07332	0.672	1	14.95	0.05519
USP3	USP3	16	ubiquitin specific peptidase 3	protein_codin	0.0945	0.696	1	9.015	0.1816
ANP32A	ANP32A	16	acidic nuclear phosphoprotein 32 family member A	protein_codin	0.3391	0.00917	1	20.87	0.05296
LARP6	LARP6	16	La ribonucleoprotein domain family member 6	protein_codin	0.3374	0.109	1	17.78	0.181
COMMD4	COMMD4	16	COMM domain containing 4	protein_codin	-0.03636	0.814	1	16.98	0.1117
HMG20A	HMG20A	16	high mobility group 20A	protein_codin	0.2029	0.538	1	11.43	0.2566
FAM103A1	FAM103A1	16	RNA guanine-7 methyltransferase activating subunit	protein_codin	NA	NA	NA	7.116	0.1812
MEF2A	MEF2A	16	myocyte enhancer factor 2A	protein_codin	-0.2904	0.0142	1	43.69	0.05367
GNPTG	GNPTG	16	N-acetylglucosamine-1-phosphate transferase subunit gamma	protein_codin	-0.5068	0.000226	1	14.23	0.2317
TBL3	TBL3	16	transducin beta like 3	protein_codin	0.4601	0.158	1	2.86	0.3128
NTHL1	NTHL1	16	nth like DNA glycosylase 1	protein_codin	0.1702	0.319	1	48.05	0.1665
RNPS1	RNPS1	16	RNA binding protein with serine rich domain 1	protein_codin	0.361	0.00516	1	40.17	0.08344
IL32	IL32	16	interleukin 32	protein_codin	-0.8456	2.86E-05	0.6606	52.58	0.296
NMRAL1	NMRAL1	16	NmrA like redox sensor 1	protein_codin	-0.7817	0.000296	1	7.068	0.08167
NUBP1	NUBP1	16	nucleotide binding protein 1	protein_codin	-0.3522	0.0920	1	25.25	0.1458
CCP110	CCP110	16	centriolar coiled-coil protein 110	protein_codin	0.1362	0.635	1	8.723	0.1442
METTL9	METTL9	16	methyltransferase like 9	protein_codin	-0.1632	0.158	1	34.85	0.05626
SPNS1	SPNS1	16	sphingolipid transporter 1 (putative)	protein_codin	0.3572	0.226	1	8.242	0.1191
PHKG2	PHKG2	16	phosphorylase kinase catalytic subunit gamma 2	protein_codin	0.01176	0.971	1	10.16	0.1652
ZNF267	ZNF267	16	zinc finger protein 267	protein_codin	0.7946	0.000658	1	33.99	0.1396
CYLD	CYLD	16	CYLD lysine 63 deubiquitinase	protein_codin	-0.2649	0.265	1	8.819	0.2234
AMFR	AMFR	16	autocrine motility factor receptor	protein_codin	-0.6910	0.00127	1	7.829	0.08732
CX3CL1	CX3CL1	16	C-X3-C motif chemokine ligand 1	protein_codin	0.206	0.488	1	51.83	0.1365
POLR2C	POLR2C	16	RNA polymerase II subunit C	protein_codin	0.1534	0.362	1	6.796	0.1176
KIFC3	KIFC3	16	kinesin family member C3	protein_codin	0.7635	0.00112	1	20.48	0.03615
NAE1	NAE1	16	NEDD8 activating enzyme E1 subunit 1	protein_codin	0.2679	0.219	1	9.059	0.3132
TRADD	TRADD	16	TNFRSF1A associated via death domain	protein_codin	0.1258	0.519	1	21.53	0.1384
THAP11	THAP11	16	THAP domain containing 11	protein_codin	0.03364	0.88	1	5.323	0.1416
PSMB10	PSMB10	16	proteasome subunit beta 10	protein_codin	0.7405	0.000584	1	16.52	0.2025
NFATC3	NFATC3	16	nuclear factor of activated T cells 3	protein_codin	0.6774	0.0334	1	26.96	0.09266
VPS4A	VPS4A	16	vacuolar protein sorting 4 homolog A	protein_codin	0.3281	0.102	1	18.37	0.2
HSBP1	HSBP1	16	heat shock factor binding protein 1	protein_codin	-0.1136	0.1512	1	20.07	0.146
ANKRD11	ANKRD11	16	ankyrin repeat domain 11	protein_codin	-0.2951	0.0214	1	30.71	0.08658
DEF8	DEF8	16	differentially expressed in FDCP 8 homolog	protein_codin	-0.5511	0.0112	1	9.417	0.1747
CAMTA2	CAMTA2	16	calmodulin binding transcription activator 2	protein_codin	0.2042	0.482	1	2.872	0.1932
TXNDC17	TXNDC17	16	thioredoxin domain containing 17	protein_codin	-0.1218	0.252	1	38.49	0.05257
TRIM16	TRIM16	16	tripartite motif containing 16	protein_codin	0.8286	0.012	1	9.457	0.1677
SUPT6H	SUPT6H	16	SPT6 homolog, histone chaperone and transcription elongatio	protein_codin	0.2079	0.402	1	7.368	0.2531
CCL2	CCL2	16	C-C motif chemokine ligand 2	protein_codin	-0.1094	0.642	1	Inf	0.4501
SLFN12	SLFN12	16	schlafen family member 12	protein_codin	-0.9077	4.18E-05	0.9637	7.818	0.1411
DHRS11	DHRS11	16	dehydrogenase/reductase 11	protein_codin	0.3427	0.299	1	2.93	0.262
FKBP10	FKBP10	16	FKBP prolyl isomerase 10	protein_codin	-0.07858	0.702	1	21.69	0.09003
TUBG1	TUBG1	16	tubulin gamma 1	protein_codin	-0.2042	0.377	1	7.118	0.08361
ARL4D	ARL4D	16	ADP ribosylation factor like GTPase 4D	protein_codin	-1.512	2.28E-15	5.46E-11	29.68	0.164
DHX8	DHX8	16	DEAH-box helicase 8	protein_codin	0.4406	0.117	1	7.938	0.2356
G6PC3	G6PC3	16	glucose-6-phosphatase catalytic subunit 3	protein_codin	0.2972	0.172	1	8.153	0.1145
GRN	GRN	16	granulin precursor	protein_codin	-0.4093	7.85E-06	0.1822	51.27	0.02641
ATP5G1	ATP5G1	16	ATP synthase membrane subunit c locus 1	protein_codin	NA	NA	NA	25.62	0.08012
SPOP	SPOP	16	speckle type BTB/POZ protein	protein_codin	0.038	0.799	1	14.07	0.2068
KAT7	KAT7	16	lysine acetyltransferase 7	protein_codin	0.02074	0.938	1	8.946	0.142
PDK2	PDK2	16	pyruvate dehydrogenase kinase 2	protein_codin	-0.1559	0.542	1	7.74	0.164
ANKRD40	ANKRD40	16	ankyrin repeat domain 40	protein_codin	0.1089	0.625	1	8.644	0.1915
AC005921.2	AC005921.2	16	novel transcript, antisense to ANKRD	antisense_RN	-0.04638	0.82	1	7.852	0.1617
CLTC	CLTC	16	clathrin heavy chain	protein_codin	-0.4089	0.00487	1	14.69	0.1428
DCAF7	DCAF7	16	DDB1 and CUL4 associated factor 7	protein_codin	-0.2392	0.265	1	25.79	0.08888
FTSJ3	FTSJ3	16	FtsJ RNA 2'-O-methyltransferase 3	protein_codin	0.6427	0.0297	1	29	0.167
SMARCD2	SMARCD2	16	SWI/SNF related, matrix associated, actin dependent regulato	protein_codin	-0.4761	0.0327	1	3.552	0.1464
GALK1	GALK1	16	galactokinase 1	protein_codin	-0.2337	0.338	1	25.51	0.2052
9-Sep	43717	16	septin 9	protein_codin	0.2711	0.134	1	19.16	0.217
USP36	USP36	16	ubiquitin specific peptidase 36	protein_codin	0.7765	0.0307	1	33.12	0.2575
ALYREF	ALYREF	16	Aly/REF export factor	protein_codin	0.1137	0.428	1	29.41	0.1112
SECTM1	SECTM1	16	secreted and transmembrane 1	protein_codin	2.192	0.000293	1	29.42	0.2797
FN3KRP	FN3KRP	16	fructosamine 3 kinase related protein	protein_codin	0.5611	0.0287	1	3.317	0.2374
ZNF521	ZNF521	16	zinc finger protein 521	protein_codin	-0.2686	0.114	1	50.71	0.1398
ZNF397	ZNF397	16	zinc finger protein 397	protein_codin	0.2228	0.392	1	24.96	0.1082
IER3IP1	IER3IP1	16	immediate early response 3 interacting protein 1	protein_codin	-0.03689	0.797	1	12.88	0.1199
CSNK2A1	CSNK2A1	16	casein kinase 2 alpha 1	protein_codin	0.04265	0.795	1	15.29	0.1395
SNRPB	SNRPB	16	small nuclear ribonucleoprotein polypeptides B and B1	protein_codin	0.01349	0.88	1	43.99	0.02852
PTPRA	PTPRA	16	protein tyrosine phosphatase receptor type A	protein_codin	0.0142	0.916	1	42.73	0.08608
CENPB	CENPB	16	centromere protein B	protein_codin	0.2931	0.214	1	28.83	0.1429
PANK2	PANK2	16	pantothenate kinase 2	protein_codin	0.1811	0.452	1	6.962	0.1906
GPCPD1	GPCPD1	16	glycerophosphocholine phosphodiesterase 1	protein_codin	0.7482	0.000491	1	16.07	0.146
NAA20	NAA20	16	N(alpha)-acetyltransferase 20, NatB catalytic subunit	protein_codin	0.2772	0.0101	1	30.02	0.05749
HM13	HM13	16	histocompatibility minor 13	protein_codin	-0.1839	0.146	1	50.38	0.08272
EIF6	EIF6	16	eukaryotic translation initiation factor 6	protein_codin	0.09072	0.466	1	22.01	0.1452
AAR2	AAR2	16	AAR2 splicing factor	protein_codin	0.3872	0.22	1	2.766	0.1385
PLCG1	PLCG1	16	phospholipase C gamma 1	protein_codin	0.3301	0.422	1	2.77	0.3384
DPM1	DPM1	16	dolichyl-phosphate mannosyltransferase subunit 1, catalytic	protein_codin	0.5512	0.0112	1	21.9	0.03515
GID8	GID8	16	GID complex subunit 8 homolog	protein_codin	0.2446	0.186	1	17.38	0.129
CNN2	CNN2	16	calponin 2	protein_codin	0.06465	0.817	1	3.4	0.226
SF3A2	SF3A2	16	splicing factor 3a subunit 2	protein_codin	-0.0258	0.936	1	9.222	0.2381
PLIN3	PLIN3	16	perilipin 3	protein_codin	-0.499	0.00817	1	46.62	0.08406
RANBP3	RANBP3	16	RAN binding protein 3	protein_codin	0.4644	0.153	1	2.58	0.1162
ICAM1	ICAM1	16	intercellular adhesion molecule 1	protein_codin	1.305	1.41E-14	3.38E-10	52.47	0.2331
ELOF1	ELOF1	16	elongation factor 1 homolog	protein_codin	0.2264	0.31	1	7.635	0.2036
PKN1	PKN1	16	protein kinase N1	protein_codin	0.06254	0.7	1	14.53	0.1415
USE1	USE1	16	unconventional SNARE in the ER 1	protein_codin	-0.06314	0.732	1	18.47	0.2255
FKBP8	FKBP8	16	FKBP prolyl isomerase 8	protein_codin	-0.01902	0.868	1	17.2	0.05412
DDX49	DDX49	16	DEAD-box helicase 49	protein_codin	0.3908	0.189	1	8.641	0.0842
C19orf12	C19orf12	16	chromosome 19 open reading frame 12	protein_codin	-0.1347	0.627	1	2.632	0.1425
POLR2I	POLR2I	16	RNA polymerase II subunit I	protein_codin	-0.06105	0.599	1	27.99	0.1424
PAF1	PAF1	16	PAF1 homolog, Paf1/RNA polymerase II complex component	protein_codin	0.6545	0.0156	1	7.628	0.1409
ARHGEF1	ARHGEF1	16	Rho guanine nucleotide exchange factor 1	protein_codin	0.5695	0.0207	1	6.568	0.1938
BCL3	BCL3	16	BCL3 transcription coactivator	protein_codin	0.4282	0.0132	1	45.21	0.1137
CALM3	CALM3	16	calmodulin 3	protein_codin	-0.03436	0.768	1	31.66	0.1082
ZNF331	ZNF331	16	zinc finger protein 331	protein_codin	-0.3785	0.241	1	48.63	0.08177
CDC42EP5	CDC42EP5	16	CDC42 effector protein 5	protein_codin	-0.2032	0.0949	1	37.9	0.05463
DDX3Y	DDX3Y	16	DEAD-box helicase 3 Y-linked	protein_codin	0.4216	0.00507	1	24.64	0.1707
COMT	COMT	16	catechol-O-methyltransferase	protein_codin	-0.3612	0.000523	1	48.57	0.08185
GUCD1	GUCD1	16	guanylyl cyclase domain containing 1	protein_codin	0.3818	0.17	1	3.566	0.2276
TCN2	TCN2	16	transcobalamin 2	protein_codin	-0.1598	0.207	1	52.38	0.1516
APOL1	APOL1	16	apolipoprotein L1	protein_codin	-0.03177	0.835	1	21.43	0.1768
LGALS1	LGALS1	16	galectin 1	protein_codin	-1.958	1.99E-48	4.83E-44	52.58	0.1425
CBX7	CBX7	16	chromobox 7	protein_codin	-0.1761	0.515	1	10.79	0.2824
PRR5	PRR5	16	proline rich 5	protein_codin	1.426	1.43E-06	0.03341	7.916	0.1442
CRELD2	CRELD2	16	cysteine rich with EGF like domains 2	protein_codin	-0.288	0.0768	1	28.96	0.08733
HSPA13	HSPA13	16	heat shock protein family A (Hsp70) member 13	protein_codin	-0.3735	0.216	1	2.868	0.2057
SCAF4	SCAF4	16	SR-related CTD associated factor 4	protein_codin	0.06586	0.842	1	2.753	0.2272
MORC3	MORC3	16	MORC family CW-type zinc finger 3	protein_codin	0.6187	0.0102	1	8.038	0.05611
AGPAT3	AGPAT3	16	1-acylglycerol-3-phosphate O-acyltransferase 3	protein_codin	0.1899	0.552	1	8.453	0.2861
DIP2A	DIP2A	16	disco interacting protein 2 homolog A	protein_codin	-0.01096	0.971	1	10.22	0.1846
MT-ND6	MT-ND6	16	mitochondrially encoded NADH:ubiquinone oxidoreductase c	protein_codin	-0.7092	0.000236	1	25.82	0.2546
PARK7	PARK7	17	Parkinsonism associated deglycase	protein_codin	-0.3556	6.12E-08	0.001441	51.93	0.02702
EXOSC10	EXOSC10	17	exosome component 10	protein_codin	0.0429	0.862	1	3.168	0.129
MRTO4	MRTO4	17	MRT4 homolog, ribosome maturation factor	protein_codin	1.176	1.36E-06	0.03179	7.342	0.0275
GALE	GALE	17	UDP-galactose-4-epimerase	protein_codin	0.4776	0.0466	1	3.472	0.12
SRRM1	SRRM1	17	serine and arginine repetitive matrix 1	protein_codin	0.2656	0.00165	1	28.73	0.08548
ZNF362	ZNF362	17	zinc finger protein 362	protein_codin	-0.248	0.374	1	3.2	0.192
GNL2	GNL2	17	G protein nucleolar 2	protein_codin	0.3679	0.165	1	8.303	0.1389
SF3A3	SF3A3	17	splicing factor 3a subunit 3	protein_codin	0.6266	0.000257	1	15.43	0.1099
MIER1	MIER1	17	MIER1 transcriptional regulator	protein_codin	0.03282	0.771	1	29.16	0.1602
TGFBR3	TGFBR3	17	transforming growth factor beta receptor 3	protein_codin	0.8537	5.54E-09	0.000133	18.15	0.2471
ALG14	ALG14	17	ALG14 UDP-N-acetylglucosaminyltransferase subunit	protein_codin	0.02687	0.926	1	31.22	0.1169
SLC35A3	SLC35A3	17	solute carrier family 35 member A3	protein_codin	-0.06962	0.782	1	7.842	0.08486
TRIM33	TRIM33	17	tripartite motif containing 33	protein_codin	-0.4896	0.0402	1	8.808	0.1729
RPRD2	RPRD2	17	regulation of nuclear pre-mRNA domain containing 2	protein_codin	0.03828	0.882	1	3.332	0.08325
NPR1.00	NPR1.00	17	natriuretic peptide receptor 1	protein_codin	-0.1896	0.342	1	20.82	0.1471
HAX1	HAX1	17	HCLS1 associated protein X-1	protein_codin	0.02236	0.777	1	18.18	0.1826
DPM3	DPM3	17	dolichyl-phosphate mannosyltransferase subunit 3, regulatory	protein_codin	0.01515	0.914	1	33.14	0.1344
NENF	NENF	17	neudesin neurotrophic factor	protein_codin	0.07461	0.465	1	19.74	0.1372
ANGEL2	ANGEL2	17	angel homolog 2	protein_codin	0.3989	0.102	1	6.443	0.1666
SNAP47	SNAP47	17	synaptosome associated protein 47	protein_codin	0.2926	0.357	1	3.584	0.176
TOMM20	TOMM20	17	translocase of outer mitochondrial membrane 20	protein_codin	-0.09002	0.212	1	36.23	0.05445
ROCK2	ROCK2	17	Rho associated coiled-coil containing protein kinase 2	protein_codin	-0.08518	0.532	1	19.57	0.05275
HNRNPLL	HNRNPLL	17	heterogeneous nuclear ribonucleoprotein L like	protein_codin	-0.3119	0.197	1	3.38	0.06037
SNRPG	SNRPG	17	small nuclear ribonucleoprotein polypeptide G	protein_codin	-0.02098	0.832	1	20.22	0.08214
STARD7	STARD7	17	StAR related lipid transfer domain containing 7	protein_codin	0.08372	0.692	1	7.678	0.08776
RNF149	RNF149	17	ring finger protein 149	protein_codin	0.4284	0.0892	1	3.382	0.1492
UBR3	UBR3	17	ubiquitin protein ligase E3 component n-recognin 3	protein_codin	-0.6295	0.0174	1	3.385	0.1132
NIF3L1	NIF3L1	17	NGG1 interacting factor 3 like 1	protein_codin	0.7092	0.0542	1	2.885	0.2484
TMBIM1	TMBIM1	17	transmembrane BAX inhibitor motif containing 1	protein_codin	-0.4211	0.00114	1	42.48	0.1099
GMPPA	GMPPA	17	GDP-mannose pyrophosphorylase A	protein_codin	-0.2008	0.479	1	2.977	0.2532
MRPL44	MRPL44	17	mitochondrial ribosomal protein L44	protein_codin	0.2244	0.396	1	9.245	0.1168
ARL8B	ARL8B	17	ADP ribosylation factor like GTPase 8B	protein_codin	-0.2795	0.132	1	6.904	0.1977
KAT2B	KAT2B	17	lysine acetyltransferase 2B	protein_codin	-0.5057	0.0516	1	11.05	0.1702
TMA7	TMA7	17	translation machinery associated 7 homolog	protein_codin	-0.02354	0.692	1	46.07	0.02686
SLC25A26	SLC25A26	17	solute carrier family 25 member 26	protein_codin	0.8161	0.00716	1	3.313	0.1126
SUCLG2	SUCLG2	17	succinate-CoA ligase GDP-forming beta subunit	protein_codin	0.569	0.0021	1	6.76	0.1512
CD200	CD200	17	CD200 molecule	protein_codin	0.1424	0.458	1	35.35	0.2361
HACD2	HACD2	17	3-hydroxyacyl-CoA dehydratase 2	protein_codin	-0.1579	0.535	1	10.2	0.2356
ST6GAL1	ST6GAL1	17	ST6 beta-galactoside alpha-2,6-sialyltransferase 1	protein_codin	0.09256	0.586	1	14.54	0.1704
RTP4	RTP4	17	receptor transporter protein 4	protein_codin	0.5632	0.0944	1	8.696	0.0827
RNF168	RNF168	17	ring finger protein 168	protein_codin	-0.2647	0.147	1	16.65	0.206
GNPDA2	GNPDA2	17	glucosamine-6-phosphate deaminase 2	protein_codin	-0.03926	0.879	1	9.252	0.0816
TMEM150C	TMEM150C	17	transmembrane protein 150C	protein_codin	0.4739	0.0516	1	24.42	0.08362
MCUB	MCUB	17	mitochondrial calcium uniporter dominant negative beta subunit	protein_codin	1.409	2.46E-13	5.88E-09	23.33	0.02819
ZNF330	ZNF330	17	zinc finger protein 330	protein_codin	0.6632	0.0069	1	45.72	0.2276
CTSO	CTSO	17	cathepsin O	protein_codin	0.03786	0.868	1	8.058	0.08846
DDX60L	DDX60L	17	DExD/H-box 60 like	protein_codin	0.5392	0.052	1	9.809	0.1422
RNF180	RNF180	17	ring finger protein 180	protein_codin	-0.3619	0.208	1	9.08	0.3132
SREK1IP1	SREK1IP1	17	SREK1 interacting protein 1	protein_codin	-0.04852	0.725	1	21.68	0.1432
POLK	POLK	17	DNA polymerase kappa	protein_codin	0.5995	0.00512	1	16.94	0.1706
RASGRF2	RASGRF2	17	Ras protein specific guanine nucleotide releasing factor 2	protein_codin	-0.6438	0.0148	1	3.853	0.126
ZCCHC9	ZCCHC9	17	zinc finger CCHC-type containing 9	protein_codin	0.2809	0.23	1	8.435	0.07934
TMEM161B-AS1	TMEM161B	17	TMEM161B antisense RNA 1	antisense_RN	0.02965	0.929	1	3.042	0.1415
ST8SIA4	ST8SIA4	17	ST8 alpha-N-acetyl-neuraminide alpha-2,8-sialyltransferase 4	protein_codin	0.4849	0.142	1	7.445	0.1698
SLC25A46	SLC25A46	17	solute carrier family 25 member 46	protein_codin	-0.1327	0.622	1	3.442	0.08107
TMEM173	TMEM173	17	transmembrane protein 173	protein_codin	0.394	0.000474	1	43.54	0.05506
SLU7	SLU7	17	SLU7 homolog, splicing factor	protein_codin	0.05847	0.702	1	11.68	0.1965
KIAA1191	KIAA1191	17	KIAA1191	protein_codin	-0.2112	0.342	1	7.986	0.2631
LYRM4	LYRM4	17	LYR motif containing 4	protein_codin	0.2786	0.298	1	3.375	0.05535
ABT1	ABT1	17	activator of basal transcription 1	protein_codin	0.3122	0.107	1	6.608	0.1972
LSM2.00	LSM2.00	17	LSM2 homolog, U6 small nuclear RNA and mRNA degradatio	protein_codin	0.5625	8.98E-08	0.002112	19.64	0.02846
RNF5	RNF5	17	ring finger protein 5	protein_codin	0.1464	0.364	1	15.72	0.1925
B3GALT4	B3GALT4	17	beta-1,3-galactosyltransferase 4	protein_codin	0.04445	0.873	1	3.21	0.2326
SNRPC	SNRPC	17	small nuclear ribonucleoprotein polypeptide C	protein_codin	0.1925	0.126	1	22.53	0.1917
TAF8	TAF8	17	TATA-box binding protein associated factor 8	protein_codin	0.2576	0.368	1	3.592	0.1812
REV3L	REV3L	17	REV3 like, DNA directed polymerase zeta catalytic subunit	protein_codin	0.18	0.364	1	49.22	0.02624
HINT3	HINT3	17	histidine triad nucleotide binding protein 3	protein_codin	0.1429	0.62	1	8.255	0.08122
CCDC28A	CCDC28A	17	coiled-coil domain containing 28A	protein_codin	0.6277	0.0192	1	6.693	0.2275
CITED2	CITED2	17	Cbp/p300 interacting transactivator with Glu/Asp rich carboxy	protein_codin	-0.7051	1.34E-05	0.3105	51.82	0.08836
DLL1	DLL1	17	delta like canonical Notch ligand 1	protein_codin	2.031	1.89E-05	0.4375	44.38	0.1404
RPA3	RPA3	17	replication protein A3	protein_codin	0.3944	0.0382	1	7.089	0.1111
AQP1	AQP1	17	aquaporin 1 (Colton blood group)	protein_codin	0.7346	3.87E-16	9.28E-12	51.92	0.05739
MPLKIP	MPLKIP	17	M-phase specific PLK1 interacting protein	protein_codin	0.1179	0.442	1	12.49	0.1401
RCC1L	RCC1L	17	RCC1 like	protein_codin	-0.1806	0.462	1	7.944	0.1119
ANKIB1	ANKIB1	17	ankyrin repeat and IBR domain containing 1	protein_codin	-0.2101	0.375	1	3.335	0.08347
FAM133B	FAM133B	17	family with sequence similarity 133 member B	protein_codin	-0.1017	0.387	1	25.79	0.08352
SYPL1	SYPL1	17	synaptophysin like 1	protein_codin	0.4085	1.54E-05	0.3567	24.46	0.1432
IMMP2L	IMMP2L	17	inner mitochondrial membrane peptidase subunit 2	protein_codin	-0.3686	0.0655	1	21.33	0.2346
IFRD1	IFRD1	17	interferon related developmental regulator 1	protein_codin	0.2011	0.18	1	51.89	0.02838
AASS	AASS	17	aminoadipate-semialdehyde synthase	protein_codin	0.1952	0.492	1	2.92	0.1196
NA	NA	17	novel transcri	NA	NA	NA	NA	8.707	0.1874
AC016831.5	AC016831.5	17	novel transcri	lincRNA	0.945	0.00236	1	42.83	0.1202
AC058791.1	AC058791.1	17	novel transcri	lincRNA	0.652	0.00225	1	19.33	0.1839
AKR1B1	AKR1B1	17	aldo-keto reductase family 1 member B	protein_codin	-0.3154	0.0801	1	31.9	0.07884
C7orf73	C7orf73	17	short transmembrane mitochondrial protein 1	protein_codin	NA	NA	NA	52.14	0.02871
TRAPPC2	TRAPPC2	17	trafficking protein particle complex 2	protein_codin	-0.2702	0.356	1	3.418	0.1089
CHIC1	CHIC1	17	cysteine rich hydrophobic domain 1	protein_codin	-0.01577	0.962	1	11.48	0.2444
JPX	JPX	17	JPX transcript, XIST activator	lincRNA	0.0194	0.924	1	7.657	0.1067
RTL8A	RTL8A	17	retrotransposon Gag like 8A	protein_codin	0.02865	0.85	1	49.68	0.08312
DDHD2	DDHD2	17	DDHD domain containing 2	protein_codin	0.3126	0.308	1	3.127	0.1729
NSD3	NSD3	17	nuclear receptor binding SET domain protein 3	protein_codin	-0.4486	5.77E-05	1	19.03	0.2079
TCEA1	TCEA1	17	transcription elongation factor A1	protein_codin	-0.08476	0.41	1	33.53	0.1166
RNF139	RNF139	17	ring finger protein 139	protein_codin	0.04319	0.858	1	7.268	0.1417
ST3GAL1	ST3GAL1	17	ST3 beta-galactoside alpha-2,3-sialyltransferase 1	protein_codin	-0.7345	0.000148	1	19.7	0.172
AQP3	AQP3	17	aquaporin 3 (Gill blood group)	protein_codin	0.08792	0.79	1	37.18	0.7501
ZCCHC7	ZCCHC7	17	zinc finger CCHC-type containing 7	protein_codin	0.1165	0.546	1	7.126	0.2248
RFK	RFK	17	riboflavin kinase	protein_codin	-0.7630	1.84E-08	0.000434	34.53	0.08479
CIZ1	CIZ1	17	CDKN1A interacting zinc finger protein 1	protein_codin	0.4491	0.136	1	2.806	0.2452
REXO4	REXO4	17	REX4 homolog, 3'-5' exonuclease	protein_codin	0.3464	0.23	1	3.607	0.2326
OSBPL5	OSBPL5	17	oxysterol binding protein like 5	protein_codin	1.535	0.000134	1	8.255	0.1704
ZNF143	ZNF143	17	zinc finger protein 143	protein_codin	0.6268	0.0324	1	10.02	0.1761
TEAD1	TEAD1	17	TEA domain transcription factor 1	protein_codin	-0.3697	0.0879	1	8.416	0.2881
PDHX	PDHX	17	pyruvate dehydrogenase complex component X	protein_codin	0.3059	0.255	1	2.876	0.142
PSMC3	PSMC3	17	proteasome 26S subunit, ATPase 3	protein_codin	-0.001286	0.992	1	46.19	0.1072
PLCB3	PLCB3	17	phospholipase C beta 3	protein_codin	0.6142	0.0891	1	2.893	0.1242
FIBP	FIBP	17	FGF1 intracellular binding protein	protein_codin	-0.5292	5.45E-05	1	13.48	0.05682
EED	EED	17	embryonic ectoderm development	protein_codin	0.6281	0.0426	1	8.375	0.1397

**BIRC2**	BIRC2	17	baculoviral IAP repeat containing 2	protein_codin	-0.2144	0.232	1	20.12	0.1589
**RSU1**	RSU1	17	Ras suppressor protein 1	protein_codin	0.3696	0.00929	1	52.15	0.1089
**CAMK2G**	CAMK2G	17	calcium/calmodulin dependent protein kinase II gamma	protein_codin	0.1892	0.522	1	10.62	0.2982
**KAT6B**	KAT6B	17	lysine acetyltransferase 6B	protein_codin	-0.1508	0.488	1	8.868	0.1475
**BAG3**	BAG3	17	BCL2 associated athanogene 3	protein_codin	-0.1729	0.248	1	52.58	0.1186
**TWF1**	TWF1	17	twinfilin actin binding protein 1	protein_codin	0.1821	0.354	1	19.68	0.05267
**TMEM106C**	TMEM106C	17	transmembrane protein 106C	protein_codin	-1.114	1.75E-07	0.004109	23.88	0.1126
**ACVR1B**	ACVR1B	17	activin A receptor type 1B	protein_codin	-0.7057	0.013	1	2.848	0.1691
**THAP2**	THAP2	17	THAP domain containing 2	protein_codin	0.4712	0.193	1	3.923	0.2199
**HIP1R**	HIP1R	17	huntingtin interacting protein 1 related	protein_codin	-0.9074	0.000327	1	3.707	0.08093
**TMEM255B**	TMEM255B	17	transmembrane protein 255B	protein_codin	0.4495	0.00522	1	27.72	0.1481
**SLC22A17**	SLC22A17	17	solute carrier family 22 member 17	protein_codin	-0.7785	0.00222	1	3.422	0.1754
**DNAAF2**	DNAAF2	17	dynein axonemal assembly factor 2	protein_codin	0.382	0.12	1	9.296	0.1471
**MAP4K5**	MAP4K5	17	mitogen-activated protein kinase kinase kinase kinase 5	protein_codin	0.5467	0.00751	1	19.41	0.1937
**KTN1**	KTN1	17	kinectin 1	protein_codin	0.04914	0.449	1	52.48	0.02610
**ACTN1**	ACTN1	17	actinin alpha 1	protein_codin	-0.04065	0.6679	1	24.94	0.02845
**CRIP2**	CRIP2	17	cysteine rich protein 2	protein_codin	-0.07382	0.172	1	52.58	0.03486
**CRIP1**	CRIP1	17	cysteine rich protein 1	protein_codin	0.5841	0.00263	1	47.57	0.1475
**CLPX**	CLPX	17	caseinolytic mitochondrial matrix peptidase chaperone subuni	protein_codin	0.1821	0.494	1	3.605	0.1153
**PML**	PML	17	promyelocytic leukemia	protein_codin	0.6729	0.00112	1	6.862	0.1070
**CLK3**	CLK3	17	CDC like kinase 3	protein_codin	0.1519	0.512	1	6.32	0.2882
**ULK3**	ULK3	17	unc-51 like kinase 3	protein_codin	0.6495	0.058	1	2.856	0.1484
**EEF2K**	EEF2K	17	eukaryotic elongation factor 2 kinase	protein_codin	0.4114	0.185	1	8.506	0.2277
**CD2BP2**	CD2BP2	17	CD2 cytoplasmic tail binding protein 2	protein_codin	0.2735	0.102	1	16.79	0.08125
**TERF2**	TERF2	17	telomeric repeat binding factor 2	protein_codin	0.1724	0.48	1	9.418	0.1189
**SPG7**	SPG7	17	SPG7 matrix AAA peptidase subunit, paraplegin	protein_codin	0.05719	0.8	1	3.474	0.3729
**CHMP1A**	CHMP1A	17	charged multivesicular body protein 1A	protein_codin	-0.2322	0.222	1	3.325	0.0538
**TAOK1**	TAOK1	17	TAO kinase 1	protein_codin	-0.1129	0.555	1	19	0.02639
**CISD3**	CISD3	17	CDGSH iron sulfur domain 3	protein_codin	-0.1121	0.483	1	6.715	0.1445
**NOL11**	NOL11	17	nucleolar protein 11	protein_codin	0.3237	0.172	1	48.04	0.2957
**KPNA2**	KPNA2	17	karyopherin subunit alpha 2	protein_codin	-0.1646	0.483	1	22.38	0.1242
**PTPRM**	PTPRM	17	protein tyrosine phosphatase receptor type M	protein_codin	0.09496	0.55	1	14.53	0.2158
**AC027097.1**	AC027097.1	17	uncharacterized LOC100505549	antisense_RN	0.5471	0.0692	1	44.58	0.2341
**RIN2**	RIN2	17	Ras and Rab interactor 2	protein_codin	0.3589	0.0522	1	33.62	0.2642
**CHMP4B**	CHMP4B	17	charged multivesicular body protein 4B	protein_codin	0.6227	0.000814	1	14.68	0.08329
**RPN2**	RPN2	17	ribophorin II	protein_codin	-0.4594	4.06E-06	0.09455	15.53	0.1387
**PIGT**	PIGT	17	phosphatidylinositol glycan anchor biosynthesis class T	protein_codin	-0.2226	0.137	1	49.83	0.1125
**CTSA**	CTSA	17	cathepsin A	protein_codin	0.3445	0.0244	1	27.53	0.1117
**KLF16**	KLF16	17	Kruppel like factor 16	protein_codin	-0.04526	0.848	1	3.493	0.09486
**SAFB2**	SAFB2	17	scaffold attachment factor B2	protein_codin	0.4889	0.0305	1	7.87	0.164
**EID2**	EID2	17	EP300 interacting inhibitor of differentiation 2	protein_codin	-0.1004	0.643	1	8.575	0.1899
**NUP62**	NUP62	17	nucleoporin 62	protein_codin	-0.118	0.666	1	8.478	0.2379
**RNF215**	RNF215	17	ring finger protein 215	protein_codin	0.4091	0.193	1	2.958	0.2851
**FBXO7**	FBXO7	17	F-box protein 7	protein_codin	-0.06196	0.673	1	15.49	0.1412
**TXN2**	TXN2	17	thioredoxin 2	protein_codin	0.2509	0.0387	1	16.69	0.06356
**POLR3H**	POLR3H	17	RNA polymerase III subunit H	protein_codin	-0.5702	0.0722	1	3.292	0.1866
**NUP50**	NUP50	17	nucleoporin 50	protein_codin	0.7907	0.00131	1	7.755	0.1736
**DYRK1A**	DYRK1A	17	dual specificity tyrosine phosphorylation regulated kinase 1A	protein_codin	0.3974	0.106	1	6.728	0.2715
**SPATC1L**	SPATC1L	17	spermatogenesis and centriole associated 1 like	protein_codin	0.6609	0.0407	1	10.24	0.2212
**EIF4G3**	EIF4G3	18	eukaryotic translation initiation factor 4 gamma 3	protein_codin	-0.2507	0.158	1	39.42	0.1987
**MECR**	MECR	18	mitochondrial trans-2-enoyl-CoA reductase	protein_codin	0.1079	0.748	1	2.963	0.2806
**C1orf122**	C1orf122	18	chromosome 1 open reading frame 122	protein_codin	-0.8017	4.76E-11	1.13E-06	52.45	0.03026
**MKNK1**	MKNK1	18	MAPK interacting serine/threonine kinase 1	protein_codin	0.4572	0.218	1	3.63	0.29
**OSBPL9**	OSBPL9	18	oxysterol binding protein like 9	protein_codin	-0.2042	0.263	1	7.689	0.2001
**NRDC**	NRDC	18	nardilysin convertase	protein_codin	0.08295	0.585	1	31.23	0.1107
**GIPC2**	GIPC2	18	GIPC PDZ domain containing family member 2	protein_codin	0.8695	3.19E-06	0.07432	35.2	0.126
**VANGL1**	VANGL1	18	VANGL planar cell polarity protein 1	protein_codin	-0.2032	0.443	1	10.76	0.02961
**MRPS21**	MRPS21	18	mitochondrial ribosomal protein S21	protein_codin	-0.08609	0.333	1	41.48	0.08212
**GLMP**	GLMP	18	glycosylated lysosomal membrane protein	protein_codin	0.2364	0.323	1	3.269	0.05807
**CADM3**	CADM3	18	cell adhesion molecule 3	protein_codin	1.38	0.00996	1	52.53	0.2497
**NDUFS2**	NDUFS2	18	NADH ubiquinone oxidoreductase core subunit S2	protein_codin	0.2884	0.0866	1	16.65	0.1082
**LAMC1**	LAMC1	18	laminin subunit gamma 1	protein_codin	0.4346	0.0257	1	42.93	0.0526
**PTGS2**	PTGS2	18	prostaglandin-endoperoxide synthase 2	protein_codin	-1.744	5.26E-35	1.27E-30	52.58	0.02738
**TRAPPC12**	TRAPPC12	18	trafficking protein particle complex 12	protein_codin	0.1295	0.59	1	9.609	0.3692
**PDIA6**	PDIA6	18	protein disulfide isomerase family A member 6	protein_codin	-0.4769	8.35E-11	1.98E-06	52.58	0.03249
**GPN1**	GPN1	18	GPN-loop GTPase 1	protein_codin	0.2396	0.373	1	10.25	0.1757
**CYP1B1**	CYP1B1	18	cytochrome P450 family 1 subfamily B member 1	protein_codin	-1.453	6.62E-26	1.6E-21	52.58	0.02809
**ATL2**	ATL2	18	atlastin GTPase 2	protein_codin	-0.4347	0.107	1	22	0.2717
**MSH6**	MSH6	18	mutS homolog 6	protein_codin	0.4294	0.0629	1	8.1	0.1971
**TMEM131**	TMEM131	18	transmembrane protein 131	protein_codin	0.147	0.554	1	6.792	0.1719
**DDX18**	DDX18	18	DEAD-box helicase 18	protein_codin	0.4554	0.000302	1	27.68	0.1613
**MZT2B**	MZT2B	18	mitotic spindle organizing protein 2B	protein_codin	-0.1395	0.0546	1	22.66	0.05706
**METTL21A**	METTL21A	18	methyltransferase like 21A	protein_codin	0.2604	0.326	1	51.43	0.1627
**PIKFYVE**	PIKFYVE	18	phosphoinositide kinase, FYVE-type zinc finger containing	protein_codin	0.1296	0.665	1	3.113	0.1424
**LANCL1**	LANCL1	18	LanC like 1	protein_codin	-0.1418	0.637	1	3.656	0.3188
**FARSB**	FARSB	18	phenylalanyl-tRNA synthetase subunit beta	protein_codin	0.4122	0.19	1	3.023	0.2768
**GALNT15**	GALNT15	18	polypeptide N-acetylgalactosaminyltransferase 15	protein_codin	0.4391	0.115	1	20.2	0.1937
**PPP4R2**	PPP4R2	18	protein phosphatase 4 regulatory subunit 2	protein_codin	-0.0870	0.557	1	38.03	0.1099
**ATP11B**	ATP11B	18	ATPase phospholipid transporting 11B (putative)	protein_codin	-0.0711	0.793	1	10.25	0.2707
**PDS5A**	PDS5A	18	PDS5 cohesin associated factor A	protein_codin	0.04817	0.816	1	7.633	0.1996
**ENOPH1**	ENOPH1	18	enolase-phosphatase 1	protein_codin	-0.06471	0.768	1	3.375	0.1732
**FAM13A**	FAM13A	18	family with sequence similarity 13 member A	protein_codin	0.5369	0.127	1	3.693	0.3665
**METAP1**	METAP1	18	methionyl aminopeptidase 1	protein_codin	-0.1268	0.62	1	10.12	0.3776
**C4orf32**	C4orf32	18	family with sequence similarity 241 member A	protein_codin	NA	NA	NA	51.97	0.1111
**UBLCP1**	UBLCP1	18	ubiquitin like domain containing CTD phosphatase 1	protein_codin	0.326	0.22	1	6.986	0.1126
**YIPF3**	YIPF3	18	Yip1 domain family member 3	protein_codin	-0.1152	0.268	1	37.41	0.1449
**TECPR1**	TECPR1	18	tectonin beta-propeller repeat containing 1	protein_codin	-0.2272	0.39	1	3.594	0.2104
**CHCHD3**	CHCHD3	18	coiled-coil-helix-coiled-coil-helix domain containing 3	protein_codin	0.155	0.384	1	45.47	0.2026
**MKRN1**	MKRN1	18	makorin ring finger protein 1	protein_codin	0.3266	0.183	1	9.394	0.05397
**GALNT11**	GALNT11	18	polypeptide N-acetylgalactosaminyltransferase 11	protein_codin	0.3705	0.127	1	24.78	0.0547
**MAGED1**	MAGED1	18	MAGE family member D1	protein_codin	-0.4546	0.0448	1	7.594	0.1457
**GLA**	GLA	18	galactosidase alpha	protein_codin	0.3975	0.142	1	7.642	0.08475
**LOXL2**	LOXL2	18	lysyl oxidase like 2	protein_codin	0.01158	0.968	1	44.66	0.1705
**HMBOX1**	HMBOX1	18	homeobox containing 1	protein_codin	0.1237	0.596	1	23.35	0.1824
**TPD52**	TPD52	18	tumor protein D52	protein_codin	1.581	1.49E-07	0.0035	45.33	0.1613
**CPQ**	CPQ	18	carboxypeptidase Q	protein_codin	-0.7639	1.31E-06	0.03061	16.28	0.1403
**HAS2**	HAS2	18	hyaluronan synthase 2	protein_codin	-0.7728	0.00197	1	48.05	0.22
**DERL1**	DERL1	18	derlin 1	protein_codin	-0.1247	0.503	1	18.69	0.08246
**NFIB**	NFIB	18	nuclear factor I B	protein_codin	-0.1679	0.0446	1	52.58	0.1475
**NUDT2**	NUDT2	18	nudix hydrolase 2	protein_codin	0.1194	0.525	1	19.34	0.1505
**BICD2**	BICD2	18	BICD cargo adaptor 2	protein_codin	0.07602	0.798	1	8.109	0.3465
**ERCC6L2**	ERCC6L2	18	ERCC excision repair 6 like 2	protein_codin	-0.8572	0.00201	1	29.58	0.3048
**HSPA5**	HSPA5	18	heat shock protein family A (Hsp70) member 5	protein_codin	-0.4645	4.23E-07	0.009911	52.54	0.1179
**RALGDS**	RALGDS	18	ral guanine nucleotide dissociation stimulator	protein_codin	1.344	8.08E-06	0.1877	30.48	0.05442
**MRPL41**	MRPL41	18	mitochondrial ribosomal protein L41	protein_codin	-0.2093	0.0506	1	52.47	0.02686
**CDKN1C**	CDKN1C	18	cyclin dependent kinase inhibitor 1C	protein_codin	-0.1184	0.413	1	52.58	0.1665
**TAF10**	TAF10	18	TATA-box binding protein associated factor 10	protein_codin	0.2966	0.00445	1	19.18	0.08032
**TMEM41B**	TMEM41B	18	transmembrane protein 41B	protein_codin	0.2742	0.344	1	3.054	0.2029
**SSRP1**	SSRP1	18	structure specific recognition protein 1	protein_codin	0.2871	0.168	1	7.936	0.1409
**PLA2G16**	PLA2G16	18	phospholipase A and acyltransferase 3	protein_codin	0.2842	0.00514	1	25.28	0.08282
**LTBP3**	LTBP3	18	latent transforming growth factor beta binding protein 3	protein_codin	-0.9527	4.43E-09	0.000105	46.09	0.1071
**SF3B2**	SF3B2	18	splicing factor 3b subunit 2	protein_codin	0.2468	0.0195	1	24.4	0.02957
**RBM4**	RBM4	18	RNA binding motif protein 4	protein_codin	0.4454	0.124	1	3.298	0.2269
**CPT1A**	CPT1A	18	carnitine palmitoyltransferase 1A	protein_codin	-0.09207	0.657	1	8.03	0.2464
**SEPHS1**	SEPHS1	18	selenophosphate synthetase 1	protein_codin	0.09927	0.67	1	27.66	0.08048
**HACD1**	HACD1	18	3-hydroxyacyl-CoA dehydratase 1	protein_codin	-1.062	8.55E-05	1	11.14	0.2222
**TIMM23**	TIMM23	18	translocase of inner mitochondrial membrane 23	protein_codin	0.1355	0.608	1	9.538	0.1096
**FAM204A**	FAM204A	18	family with sequence similarity 204 member A	protein_codin	-0.02756	0.806	1	44.58	0.1428
**HSP90B1**	HSP90B1	18	heat shock protein 90 beta family member 1	protein_codin	-0.7070	5.23E-32	1.27E-27	52.58	0.2206
**ACACB**	ACACB	18	acetyl-CoA carboxylase beta	protein_codin	1.352	0.000132	1	24.3	0.1988
**GIT2**	GIT2	18	GIT ArfGAP 2	protein_codin	-0.1851	0.487	1	2.993	0.1363
**MPHOSPH8**	MPHOSPH	18	M-phase phosphoprotein 8	protein_codin	0.3557	0.000455	1	52.52	0.1676
**AKAP11**	AKAP11	18	A-kinase anchoring protein 11	protein_codin	0.2356	0.328	1	29.82	0.08686
**CCNB1IP1**	CCNB1IP1	18	cyclin B1 interacting protein 1	protein_codin	-0.2035	0.246	1	6.75	0.266
**JKAMP**	JKAMP	18	JNK1/MAPK8 associated membrane protein	protein_codin	0.07151	0.711	1	7.367	0.08916
**IRF2BPL**	IRF2BPL	18	interferon regulatory factor 2 binding protein like	protein_codin	0.6559	0.00141	1	35.63	0.08396
**GON7**	GON7	18	GON7 subunit of KEOPS complex	protein_codin	0.342	0.193	1	3.136	0.1782
**PDIA3**	PDIA3	18	protein disulfide isomerase family A member 3	protein_codin	-0.5265	1.29E-15	3.09E-11	50.94	0.06462
**PPIB**	PPIB	18	peptidylprolyl isomerase B	protein_codin	-0.5152	2.79E-22	6.73E-18	46.26	0.0291
**SMAD3**	SMAD3	18	SMAD family member 3	protein_codin	0.2349	0.429	1	9.006	0.1748
**SLCO3A1**	SLCO3A1	18	solute carrier organic anion transporter family member 3A1	protein_codin	0.2872	0.157	1	18.7	0.1489
**C16orf91**	C16orf91	18	chromosome 16 open reading frame 91	protein_codin	0.3824	0.106	1	8.352	0.2244
**THOC6**	THOC6	18	THO complex 6	protein_codin	0.6402	0.146	1	2.906	0.222
**LYRM1**	LYRM1	18	LYR motif containing 1	protein_codin	0.5761	0.00464	1	32.98	0.1484
**SH2B1**	SH2B1	18	SH2B adaptor protein 1	protein_codin	0.4931	0.104	1	2.758	0.2049
**HERPUD1**	HERPUD1	18	homocysteine inducible ER protein with ubiquitin like domain	protein_codin	0.06261	0.515	1	37.18	0.0532
**CPNE2**	CPNE2	18	copine 2	protein_codin	0.1954	0.19	1	23.25	0.1115
**RPA1**	RPA1	18	replication protein A1	protein_codin	-0.006508	0.982	1	8.298	0.3553
**MYBBP1A**	MYBBP1A	18	MYB binding protein 1a	protein_codin	1.208	0.000337	1	3.493	0.1981
**PLD2**	PLD2	18	phospholipase D2	protein_codin	0.7546	0.014	1	3.296	0.1242
**CHD3**	CHD3	18	chromodomain helicase DNA binding protein 3	protein_codin	-0.9519	3.22E-05	0.7433	7.266	0.1113
**POLDIP2**	POLDIP2	18	DNA polymerase delta interacting protein 2	protein_codin	0.005857	0.979	1	8.393	0.2583
**FAM104A**	FAM104A	18	family with sequence similarity 104 member A	protein_codin	0.3642	0.113	1	7.234	0.2318
**CDC42EP4**	CDC42EP4	18	CDC42 effector protein 4	protein_codin	0.4445	0.0184	1	38.47	0.1137
**CASKIN2**	CASKIN2	18	CASK interacting protein 2	protein_codin	0.3229	0.128	1	52.9	0.056
**RPL17**	RPL17	18	ribosomal protein L17	protein_codin	0.5912	3.6E-05	0.8305	21.38	0.05747
**LINC00909**	LINC00909	18	long intergenic non-protein coding RNA 909	lincRNA	0.6075	0.0821	1	9.512	0.3368
**ZADH2**	ZADH2	18	zinc binding alcohol dehydrogenase domain containing 2	protein_codin	0.2247	0.442	1	8.088	0.2661
**C20orf27**	C20orf27	18	chromosome 20 open reading frame 27	protein_codin	0.7586	0.00196	1	7.283	0.2257
**PCNA**	PCNA	18	proliferating cell nuclear antigen	protein_codin	0.04566	0.81	1	7.864	0.2162
**MED16**	MED16	18	mediator complex subunit 16	protein_codin	-0.282	0.292	1	2.757	0.1679
**BTBD2**	BTBD2	18	BTB domain containing 2	protein_codin	-0.3535	0.204	1	8.173	0.1972
**MLLT1**	MLLT1	18	MLLT1 super elongation complex subunit	protein_codin	-0.8262	0.000301	1	7.282	0.1782
**RAB3D**	RAB3D	18	RAB3D, member RAS oncogene family	protein_codin	0.7329	0.053	1	9.803	0.2296
**CALR**	CALR	18	calreticulin	protein_codin	-0.7954	2E-31	4.84E-27	48.04	0.07223
**NFIX**	NFIX	18	nuclear factor I X	protein_codin	-1.344	3.88E-09	9.18E-05	47.83	0.05798
**ITPKC**	ITPKC	18	inositol-trisphosphate 3-kinase C	protein_codin	1.235	1.49E-06	0.03481	36.92	0.1675
**OPA3**	OPA3	18	outer mitochondrial membrane lipid metabolism regulator OPA3	protein_codin	-0.2352	0.423	1	10.55	0.05763
**SLC1A5**	SLC1A5	18	solute carrier family 1 member 5	protein_codin	1.723	5.15E-07	0.01206	19.56	0.1915
**GRWD1**	GRWD1	18	glutamate rich WD repeat containing 1	protein_codin	0.4917	0.0771	1	33.93	0.0835
**BCAT2**	BCAT2	18	branched chain amino acid transaminase 2	protein_codin	0.9661	0.00198	1	3.379	0.1421
**GGA1**	GGA1	18	golgi associated, gamma adaptin ear containing, ARF binding	protein_codin	0.3389	0.312	1	2.834	0.1666
**H1F0**	H1F0	18	H1 histone family member 0	protein_codin	0.09692	0.654	1	51.46	0.2645
**ZC3H7B**	ZC3H7B	18	zinc finger CCCH-type containing 7B	protein_codin	-0.2786	0.304	1	2.968	0.3389
**C21orf91**	C21orf91	18	chromosome 21 open reading frame 91	protein_codin	1.896	1.26E-18	3.03E-14	19.68	0.1136
**TTC3**	TTC3	18	tetratricopeptide repeat domain 3	protein_codin	-0.5400	1.75E-05	0.4051	29.06	0.1388
**CDK11B**	CDK11B	19	cyclin dependent kinase 11B	protein_codin	0.3811	0.114	1	8.274	0.1149
**TNFRSF14**	TNFRSF14	19	TNF receptor superfamily member 14	protein_codin	0.6796	1.08E-05	0.2505	9.76	0.0852
**ICMT**	ICMT	19	isoprenylcysteine carboxyl methyltransferase	protein_codin	-0.09259	0.729	1	7.312	0.2217
**RERE**	RERE	19	arginine-glutamic acid dipeptide repeats	protein_codin	0.2752	0.168	1	6.898	0.1141
**MAD2L2**	MAD2L2	19	mitotic arrest deficient 2 like 2	protein_codin	0.3211	0.0845	1	19.43	0.1972
**LUZP1**	LUZP1	19	leucine zipper protein 1	protein_codin	0.1021	0.329	1	50.79	0.08062
**RSRP1**	RSRP1	19	arginine and serine rich protein 1	protein_codin	0.1831	0.0689	1	41.33	0.02879
**DHDDS**	DHDDS	19	dehydrodolichyl diphosphate synthase subunit	protein_codin	-0.1948	0.493	1	9.133	0.2774
**GMEB1**	GMEB1	19	glucocorticoid modulatory element binding protein 1	protein_codin	0.5051	0.106	1	8.288	0.1409
**SYNC**	SYNC	19	syncoilin, intermediate filament protein	protein_codin	-0.4774	0.0615	1	3.553	0.1704
**SFPQ**	SFPQ	19	splicing factor proline and glutamine rich	protein_codin	0.4049	3.72E-05	0.8581	31.64	0.02699
**THRAP3**	THRAP3	19	thyroid hormone receptor associated protein 3	protein_codin	0.141	0.16	1	17.36	0.05472
**PABPC4**	PABPC4	19	poly(A) binding protein cytoplasmic 4	protein_codin	0.3661	0.0625	1	7.138	0.1212
**AKR1A1**	AKR1A1	19	aldo-keto reductase family 1 member A1	protein_codin	-0.02115	0.857	1	16.69	0.1141
**NASP**	NASP	19	nuclear autoantigenic sperm protein	protein_codin	0.3719	0.000109	1	46.49	0.05527
**ANKRD13C**	ANKRD13C	19	ankyrin repeat domain 13C	protein_codin	0.08957	0.745	1	3.315	0.2637
**USP33**	USP33	19	ubiquitin specific peptidase 33	protein_codin	0.1848	0.38	1	6.645	0.08175
**HS2ST1**	HS2ST1	19	heparan sulfate 2-O-sulfotransferase 1	protein_codin	0.6542	0.0666	1	2.573	0.3765
**FNBP1L**	FNBP1L	19	formin binding protein 1 like	protein_codin	0.7265	5.33E-06	0.124	49.28	0.1794
**SNX7**	SNX7	19	sorting nexin 7	protein_codin	-0.3436	0.0748	1	25.38	0.109
**PRPF38B**	PRPF38B	19	pre-mRNA processing factor 38B	protein_codin	0.07017	0.603	1	36.14	0.1392
**SARS**	SARS	19	seryl-tRNA synthetase	protein_codin	0.05186	0.686	1	26.68	0.02662
**PSMA5**	PSMA5	19	proteasome subunit alpha 5	protein_codin	-0.2172	0.0745	1	21.85	0.1435
**NRAS**	NRAS	19	NRAS proto-oncogene, GTPase	protein_codin	-0.05479	0.814	1	6.842	0.1918
**AC245595.1**	AC245595.1	19	long intergenic non-protein coding RNA 2802	lincRNA	0.3042	0.338	1	32.27	0.06286
**PIAS3**	PIAS3	19	protein inhibitor of activated STAT 3	protein_codin	-0.3312	0.213	1	2.676	0.1387
**ARNT**	ARNT	19	aryl hydrocarbon receptor nuclear translocator	protein_codin	0.3391	0.306	1	2.624	0.27
**PSMD4**	PSMD4	19	proteasome 26S subunit, non-ATPase 4	protein_codin	0.07397	0.458	1	15.54	0.08637
**SLC50A1**	SLC50A1	19	solute carrier family 50 member 1	protein_codin	0.09424	0.729	1	42.68	0.1138
**GBA**	GBA	19	glucosylceramidase beta	protein_codin	-0.5636	0.0919	1	3.148	0.2485
**FDPS**	FDPS	19	farnesyl diphosphate synthase	protein_codin	-0.5148	4.58E-07	0.01072	34.63	0.0302
**ATF6**	ATF6	19	activating transcription factor 6	protein_codin	-0.4019	0.0221	1	19.7	0.1071
**PBX1**	PBX1	19	PBX homeobox 1	protein_codin	0.3091	0.133	1	19.6	0.1958
**CREG1**	CREG1	19	cellular repressor of E1A stimulated genes 1	protein_codin	-0.2015	0.188	1	14.48	0.1442
**SUCO**	SUCO	19	SUN domain containing ossification factor	protein_codin	0.08028	0.715	1	8.187	0.1451
**TOR3A**	TOR3A	19	torsin family 3 member A	protein_codin	0.6159	0.0107	1	33.48	0.1087
**SOAT1**	SOAT1	19	sterol O-acyltransferase 1	protein_codin	0.4631	0.162	1	3.023	0.3426
**DHX9**	DHX9	19	DExH-box helicase 9	protein_codin	0.4751	0.0184	1	18.23	0.1125
**SMG7**	SMG7	19	SMG7 nonsense mediated mRNA decay factor	protein_codin	0.1709	0.494	1	7.457	0.2622
**C1orf21**	C1orf21	19	chromosome 1 open reading frame 21	protein_codin	1.14	2.76E-08	0.000651	49.87	0.1741
**TRMT1L**	TRMT1L	19	tRNA methyltransferase 1 like	protein_codin	0.3569	0.327	1	9.203	0.2035
**CSRP1**	CSRP1	19	cysteine and glycine rich protein 1	protein_codin	-0.3406	0.0212	1	52.56	0.02694
**IPO9**	IPO9	19	importin 9	protein_codin	-0.0202	0.945	1	2.58	0.2099
**TMEM183A**	TMEM183A	19	transmembrane protein 183A	protein_codin	0.1089	0.568	1	7.06	0.08505
**EIF2D**	EIF2D	19	eukaryotic translation initiation factor 2D	protein_codin	0.32	0.129	1	20.73	0.1977
**SERTAD4**	SERTAD4	19	SERTA domain containing 4	protein_codin	-0.397	0.152	1	9.247	0.1072
**PPP2R5A**	PPP2R5A	19	protein phosphatase 2 regulatory subunit B'alpha	protein_codin	1.22	4.97E-08	0.001171	15.68	0.1909
**CNIH4**	CNIH4	19	cornichon family AMPA receptor auxiliary protein 4	protein_codin	0.1571	0.229	1	23.18	0.1422
**ENAH**	ENAH	19	ENAH actin regulator	protein_codin	0.16	0.387	1	16.78	0.1954
**SDE2**	SDE2	19	SDE2 telomere maintenance homolog	protein_codin	0.3622	0.153	1	28.5	0.06176
**ITPKB**	ITPKB	19	inositol-trisphosphate 3-kinase B	protein_codin	0.8729	0.0255	1	9.599	0.4807
**ARF1**	ARF1	19	ADP ribosylation factor 1	protein_codin	-0.1575	0.0212	1	32.1	0.02614
**ID2**	ID2	19	inhibitor of DNA binding 2	protein_codin	-0.3415	0.0117	1	52.58	0.3288
**PPP1CB**	PPP1CB	19	protein phosphatase 1 catalytic subunit beta	protein_codin	-0.72	4.27E-14	1.02E-09	50.15	0.0296
**FEZ2**	FEZ2	19	fasciculation and elongation protein zeta 2	protein_codin	0.1167	0.303	1	37.13	0.08172
**SLC8A1**	SLC8A1	19	solute carrier family 8 member A1	protein_codin	1.869	2.34E-05	0.541	18.65	0.1153
**MTA3**	MTA3	19	metastasis associated 1 family member 3	protein_codin	0.2006	0.515	1	2.755	0.1531
**CNRIP1**	CNRIP1	19	cannabinoid receptor interacting protein 1	protein_codin	0.4576	0.00852	1	14.83	0.1092
**GFPT1**	GFPT1	19	glutamine--fructose-6-phosphate transaminase 1	protein_codin	-1.068	0.000164	1	2.826	0.2697
**DCTN1**	DCTN1	19	dynactin subunit 1	protein_codin	-0.2495	0.344	1	3.663	0.02644
**TMEM150A**	TMEM150A	19	transmembrane protein 150A	protein_codin	0.3885	0.128	1	6.762	0.1439
**MRPL35**	MRPL35	19	mitochondrial ribosomal protein L35	protein_codin	-0.08711	0.697	1	22.95	0.08247
**RPIA**	RPIA	19	ribose 5-phosphate isomerase A	protein_codin	0.3535	0.122	1	8.323	0.2035
**SEMA4C**	SEMA4C	19	semaphorin 4C	protein_codin	-0.06321	0.79	1	7.637	0.1206
**IL1R1**	IL1R1	19	interleukin 1 receptor type 1	protein_codin	0.04252	0.72	1	46.55	0.0322
**PTPN4**	PTPN4	19	protein tyrosine phosphatase non-receptor type 4	protein_codin	-0.06886	0.803	1	2.113	0.1775
**CLASP1**	CLASP1	19	cytoplasmic linker associated protein 1	protein_codin	0.2484	0.472	1	3.07	0.2891
**AC012306.2**	AC012306.2	19	novel transcri	lincRNA	0.2609	0.39	1	8.7	0.2466
**IMP4**	IMP4	19	IMP U3 small nucleolar ribonucleoprotein 4	protein_codin	0.4157	0.0122	1	13.93	0.252
**RND3**	RND3	19	Rho family GTPase 3	protein_codin	-1.282	1.45E-07	0.003406	47.09	0.08049
**NR4A2**	NR4A2	19	nuclear receptor subfamily 4 group A member 2	protein_codin	0.2482	0.168	1	43.84	0.1206
**WDSUB1**	WDSUB1	19	WD repeat, sterile alpha motif and U-box domain containing 1	protein_codin	0.4758	0.233	1	9.697	0.297
**SLC25A12**	SLC25A12	19	solute carrier family 25 member 12	protein_codin	0.005661	0.985	1	3.015	0.05678
**PRKRA**	PRKRA	19	protein activator of interferon induced protein kinase EIF2AK2	protein_codin	0.08181	0.648	1	7.945	0.08208
**SLC40A1**	SLC40A1	19	solute carrier family 40 member 1	protein_codin	1.025	2.03E-05	0.4696	28.12	0.09486
**MFSD6**	MFSD6	19	major facilitator superfamily domain containing 6	protein_codin	0.565	0.0542	1	7.107	0.3442
**NABP1**	NABP1	19	nucleic acid binding protein 1	protein_codin	0.3996	0.353	1	30.89	0.1742
**SLC39A10**	SLC39A10	19	solute carrier family 39 member 10	protein_codin	0.2299	0.185	1	30.65	0.1124
**MAIP1**	MAIP1	19	matrix AAA peptidase interacting protein 1	protein_codin	0.2252	0.448	1	3.475	0.1125
**KLF7**	KLF7	19	Kruppel like factor 7	protein_codin	0.6917	0.000788	1	14.58	0.08152
**SPAG16**	SPAG16	19	sperm associated antigen 16	protein_codin	-0.0951	0.67	1	8.508	0.1672
**CNPPD1**	CNPPD1	19	cyclin Pas1/PHO80 domain containing 1	protein_codin	0.1096	0.613	1	3.146	0.1163
**DNAJB2**	DNAJB2	19	DnaJ heat shock protein family (Hsp40) member B2	protein_codin	-0.3942	0.109	1	8.364	0.2253
**OBSL1**	OBSL1	19	obscurin like 1	protein_codin	-0.05779	0.868	1	3.272	0.2419
**EPHA4**	EPHA4	19	EPH receptor A4	protein_codin	0.09133	0.696	1	7.772	0.1363
**PSMD1**	PSMD1	19	proteasome 26S subunit, non-ATPase 1	protein_codin	-0.01085	0.947	1	27.35	0.05696
**SUMF1**	SUMF1	19	sulfatase modifying factor 1	protein_codin	0.3182	0.248	1	7.988	0.08459
**TBC1D5**	TBC1D5	19	TBC1 domain family member 5	protein_codin	-0.4397	0.0104	1	19.74	0.2599
**OSBPL10**	OSBPL10	19	oxysterol binding protein like 10	protein_codin	0.2026	0.457	1	7.687	0.2209
**PDCD6IP**	PDCD6IP	19	programmed cell death 6 interacting protein	protein_codin	0.1014	0.493	1	30.43	0.1462
**GOLGA4**	GOLGA4	19	golgin A4	protein_codin	-0.4075	0.0016	1	45.98	0.05341
**SS18L2**	SS18L2	19	SS18 like 2	protein_codin	0.2577	0.125	1	14.33	0.05568
**ANO10**	ANO10	19	anoctamin 10	protein_codin	0.03194	0.904	1	30.88	0.3592
**TMEM42**	TMEM42	19	transmembrane protein 42	protein_codin	0.4191	0.256	1	9.033	0.1142
**SACM1L**	SACM1L	19	SAC1 like phosphatidylinositide phosphatase	protein_codin	0.396	0.214	1	3.196	0.2207
**WDR6**	WDR6	19	WD repeat domain 6	protein_codin	0.4669	0.133	1	2.547	0.2857
**QRICH1**	QRICH1	19	glutamine rich 1	protein_codin	0.1229	0.713	1	2.522	0.1466
**RBM6**	RBM6	19	RNA binding motif protein 6	protein_codin	0.1637	0.463	1	17.36	0.1569
**RASSF1**	RASSF1	19	Ras association domain family member 1	protein_codin	0.7463	0.00115	1	42.03	0.201
**NISCH**	NISCH	19	nischarin	protein_codin	-0.5422	0.0517	1	3.177	0.2006
**EOGT**	EOGT	19	EGF domain specific O-linked N-acetylglucosamine transferase	protein_codin	-0.5795	0.0296	1	3.214	0.08495
**FRMD4B**	FRMD4B	19	FERM domain containing 4B	protein_codin	-0.6394	7.12E-10	1.69E-05	46.68	0.08777
**C3orf38**	C3orf38	19	chromosome 3 open reading frame 38	protein_codin	0.2526	0.383	1	3.622	0.1551
**TOMM70**	TOMM70	19	translocase of outer mitochondrial membrane 70	protein_codin	0.02301	0.913	1	8.488	0.3026
**ZBTB11**	ZBTB11	19	zinc finger and BTB domain containing 11	protein_codin	0.2434	0.296	1	23.03	0.02746
**PHLDB2**	PHLDB2	19	pleckstrin homology like domain family B member 2	protein_codin	1.086	0.00416	1	46.02	0.3158
**CCDC80**	CCDC80	19	coiled-coil domain containing 80	protein_codin	-1.508	1.34E-16	3.22E-12	52.58	0.4231
**ATP6V1A**	ATP6V1A	19	ATPase H+ transporting V1 subunit A	protein_codin	0.07079	0.779	1	3.127	0.1452
**GSK3B**	GSK3B	19	glycogen synthase kinase 3 beta	protein_codin	0.05502	0.782	1	7.523	0.1687
**ZNF148**	ZNF148	19	zinc finger protein 148	protein_codin	-0.1124	0.53	1	6.35	0.2626
**ABTB1**	ABTB1	19	ankyrin repeat and BTB domain containing 1	protein_codin	0.2572	0.26	1	7.893	0.1429
**EEFSEC**	EEFSEC	19	eukaryotic elongation factor, selenocysteine-tRNA specific	protein_codin	0.704	0.0625	1	2.573	0.2035
**CEP70**	CEP70	19	centrosomal protein 70	protein_codin	0.5615	0.0875	1	2.785	0.2528
**PLOD2**	PLOD2	19	procollagen-lysine,2-oxoglutarate 5-dioxygenase 2	protein_codin	-0.03786	0.873	1	20.7	0.0365
**WWTR1**	WWTR1	19	WW domain containing transcription regulator 1	protein_codin	-1.106	1.88E-41	4.56E-37	34.57	0.02639
**MBNL1**	MBNL1	19	muscleblind like splicing regulator 1	protein_codin	-0.09426	0.35	1	28.38	0.1331
**SEC62**	SEC62	19	SEC62 homolog, preprotein translocation factor	protein_codin	0.04861	0.434	1	46.63	0.05433
**PIGX**	PIGX	19	phosphatidylinositol glycan anchor biosynthesis class X	protein_codin	0.2239	0.558	1	3.525	0.1981
**LETM1**	LETM1	19	leucine zipper and EF-hand containing transmembrane protein 1	protein_codin	0.1917	0.474	1	3.206	0.1936
**SH3BP2**	SH3BP2	19	SH3 domain binding protein 2	protein_codin	-0.3115	0.21	1	8.024	0.3752
**BOD1L1**	BOD1L1	19	biorientation of chromosomes in cell division 1 like 1	protein_codin	0.2975	0.0244	1	18.4	0.167
**MED28**	MED28	19	mediator complex subunit 28	protein_codin	0.3493	0.06	1	14.78	0.0822
**STIM2**	STIM2	19	stromal interaction molecule 2	protein_codin	0.748	0.000167	1	14.19	0.08898
**DCUN1D4**	DCUN1D4	19	defective in cullin neddylation 1 domain containing 4	protein_codin	0.4338	0.138	1	3.534	0.1948
**RCHY1**	RCHY1	19	ring finger and CHY zinc finger domain containing 1	protein_codin	0.4311	0.125	1	3.433	0.1951
**INTS12**	INTS12	19	integrator complex subunit 12	protein_codin	0.2186	0.348	1	3.462	0.3642
**SH3D19**	SH3D19	19	SH3 domain containing 19	protein_codin	-0.2481	0.242	1	25.56	0.1492
**ARFIP1**	ARFIP1	19	ADP ribosylation factor interacting protein 1	protein_codin	0.2458	0.232	1	22.4	0.1112
**LPCAT1**	LPCAT1	19	lysophosphatidylcholine acyltransferase 1	protein_codin	-0.5492	0.0677	1	8.723	0.2822
**BRIX1**	BRIX1	19	biogenesis of ribosomes BRX1	protein_codin	0.5202	0.000602	1	13.96	0.08577
**TTC33**	TTC33	19	tetratricopeptide repeat domain 33	protein_codin	-0.1172	0.658	1	8.727	0.1824
**ANXA2R**	ANXA2R	19	annexin A2 receptor	protein_codin	0.5537	0.00868	1	52.07	0.2229
**NNT-AS1**	NNT-AS1	19	NNT antisense RNA 1	antisense_RN	0.2594	0.334	1	22.78	0.1545
**ERBIN**	ERBIN	19	erbb2 interacting protein	protein_codin	-0.0872	0.613	1	19.75	0.05952
**TNPO1**	TNPO1	19	transportin 1	protein_codin	-0.4257	0.0119	1	6.993	0.2163
**F2R**	F2R	19	coagulation factor II thrombin receptor	protein_codin	0.3157	0.0623	1	38.29	0.06413
**FAM172A**	FAM172A	19	family with sequence similarity 172 member A	protein_codin	-0.2172	0.288	1	7.893	0.1433
**ERAP2**	ERAP2	19	endoplasmic reticulum aminopeptidase 2	protein_codin	-0.1296	0.563	1	20.47	0.1956
**DCP2**	DCP2	19	decapping mRNA 2	protein_codin	-0.1855	0.312	1	20.06	0.1786
**SNX24**	SNX24	19	sorting nexin 24	protein_codin	-0.9409	0.000322	1	11.16	0.1435
**PHAX**	PHAX	19	phosphorylated adaptor for RNA export	protein_codin	-0.1186	0.46	1	14.82	0.114
**C5orf15**	C5orf15	19	chromosome 5 open reading frame 15	protein_codin	0.2662	0.317	1	6.932	0.1115
**FAM13B**	FAM13B	19	family with sequence similarity 13 member B	protein_codin	-0.01322	0.958	1	9.68	0.08003
**ETF1**	ETF1	19	eukaryotic translation termination factor 1	protein_codin	-0.254	0.122	1	17.72	0.02642
**PURA**	PURA	19	purine rich element binding protein A	protein_codin	0.2082	0.144	1	32.74	0.1429
**HBEGF**	HBEGF	19	heparin binding EGF like growth factor	protein_codin	0.1424	0.435	1	50.27	0.02828
**AFAP1L1**	AFAP1L1	19	actin filament associated protein 1 like 1	protein_codin	0.1419	0.512	1	19.54	0.179
**GPX3**	GPX3	19	glutathione peroxidase 3	protein_codin	-0.9597	1.5E-06	0.03504	43.43	0.1727
**THOC3**	THOC3	19	THO complex 3	protein_codin	0.6476	0.0482	1	3.088	0.1452
**MAPK9**	MAPK9	19	mitogen-activated protein kinase 9	protein_codin	0.4449	0.213	1	3.643	0.2986
**RANBP9**	RANBP9	19	RAN binding protein 9	protein_codin	0.2005	0.434	1	8.523	0.1669

**RIPOR2**	RIPOR2	19	RHO family interacting cell polarization regulator 2	protein_codin	0.4052	0.354	1	3.807	0.3912
**BTN3A1**	BTN3A1	19	butyrophilin subfamily 3 member A1	protein_codin	1.506	7.5E-06	0.1742	8.186	0.3076
**ZSCAN16-AS1**	ZSCAN16-A	19	ZSCAN16 antisense RNA 1	antisense_RN	-0.4188	0.0892	1	8.364	0.05911
**HLA-F**	HLA-F	19	major histocompatibility complex, class I, F	protein_codin	0.8632	1.32E-10	3.17E-12	28.7	0.02679
**SMIM29**	SMIM29	19	small integral membrane protein 29	protein_codin	-0.2019	0.284	1	8.709	0.05412
**FKBP5**	FKBP5	19	FKBP prolyl isomerase 5	protein_codin	-1.128	7.96E-17	1.91E-12	52.37	0.05292
**PEX6**	PEX6	19	peroxisomal biogenesis factor 6	protein_codin	0.1744	0.624	1	3.418	0.1761
**RRP36**	RRP36	19	ribosomal RNA processing 36	protein_codin	-0.003498	0.984	1	20.34	0.1652
**GSTA4**	GSTA4	19	glutathione S-transferase alpha 4	protein_codin	0.2863	0.244	1	3.32	0.09972
**MTO1**	MTO1	19	mitochondrial tRNA translation optimization 1	protein_codin	1.012	0.0109	1	9.414	0.2842
**CEP162**	CEP162	19	centrosomal protein 162	protein_codin	-0.04009	0.885	1	3.664	0.2495
**OSTM1**	OSTM1	19	osteoclastogenesis associated transmembrane protein 1	protein_codin	-0.0662	0.77	1	25.79	0.2462
**NT5DC1**	NT5DC1	19	5'-nucleotidase domain containing 1	protein_codin	0.2418	0.259	1	16.74	0.1382
**RNF217**	RNF217	19	ring finger protein 217	protein_codin	0.4507	0.102	1	8.168	0.1654
**KATNA1**	KATNA1	19	katanin catalytic subunit A1	protein_codin	0.2919	0.204	1	9.003	0.1988
**LATS1**	LATS1	19	large tumor suppressor kinase 1	protein_codin	-0.07276	0.825	1	11.04	0.3394
**SYNE1**	SYNE1	19	spectrin repeat containing nuclear envelope protein 1	protein_codin	0.02482	0.888	1	44.41	0.09685
**TMEM181**	TMEM181	19	transmembrane protein 181	protein_codin	-0.5715	0.0381	1	3.693	0.1719
**DYNLT1**	DYNLT1	19	dynein light chain Tctex-type 1	protein_codin	0.01466	0.89	1	46.88	0.0271
**EZR**	EZR	19	ezrin	protein_codin	-0.2222	0.328	1	21.73	0.1112
**WTAP**	WTAP	19	WT1 associated protein	protein_codin	0.06683	0.597	1	16.38	0.05352
**AIMP2**	AIMP2	19	aminoacyl tRNA synthetase complex interacting multifunction	protein_codin	0.06563	0.782	1	9.534	0.1509
**EIF2AK1**	EIF2AK1	19	eukaryotic translation initiation factor 2 alpha kinase 1	protein_codin	-0.08198	0.706	1	21.05	0.05312
**TWISTNB**	TWISTNB	19	TWIST neighbor	protein_codin	0.6294	0.000865	1	33.85	0.05529
**FKBP14**	FKBP14	19	FKBP prolyl isomerase 14	protein_codin	0.2281	0.446	1	10.06	0.3194
**RP9**	RP9	19	RP9 pre-mRNA splicing factor	protein_codin	0.09527	0.659	1	16.97	0.1966
**PSMA2**	PSMA2	19	proteasome subunit alpha 2	protein_codin	-0.1961	0.158	1	13	0.08565
**OGDH**	OGDH	19	oxoglutarate dehydrogenase	protein_codin	0.1826	0.544	1	3.067	0.1176
**GTF2I**	GTF2I	19	general transcription factor IIi	protein_codin	-0.6996	6.43E-08	0.001512	27.86	0.03567
**STYXL1**	STYXL1	19	serine/threonine/tyrosine interacting like 1	protein_codin	0.3752	0.252	1	3.456	0.1689
**DBF4**	DBF4	19	DBF4 zinc finger	protein_codin	0.3817	0.245	1	8.462	0.1462
**PDK4**	PDK4	19	pyruvate dehydrogenase kinase 4	protein_codin	1.855	1.98E-20	4.77E-16	52.58	0.06178
**MCM7**	MCM7	19	minichromosome maintenance complex component 7	protein_codin	0.06349	0.818	1	9.242	0.1944
**DNAJB9**	DNAJB9	19	DnaJ heat shock protein family (Hsp40) member B9	protein_codin	0.07206	0.682	1	45.58	0.1175
**ING3**	ING3	19	inhibitor of growth family member 3	protein_codin	1.022	0.000216	1	7.815	0.1936
**KLHDC10**	KLHDC10	19	kelch domain containing 10	protein_codin	0.02545	0.917	1	7.014	0.1649
**NUB1**	NUB1	19	negative regulator of ubiquitin like proteins 1	protein_codin	0.4488	0.00082	1	50.53	0.0894
**IL3RA**	IL3RA	19	interleukin 3 receptor subunit alpha	protein_codin	0.8217	1.21E-10	2.87E-06	52.52	0.06482
**SLC25A6**	SLC25A6	19	solute carrier family 25 member 6	protein_codin	-0.03852	0.516	1	44.26	0.04179
**CD99**	CD99	19	CD99 molecule (Xg blood group)	protein_codin	-0.3232	6.3E-10	1.49E-05	41.02	0.03709
**WWC3**	WWC3	19	WWC family member 3	protein_codin	-0.3382	0.227	1	10.28	0.1172
**MOSPD2**	MOSPD2	19	motile sperm domain containing 2	protein_codin	0.1444	0.58	1	3.295	0.3112
**CA5B**	CA5B	19	carbonic anhydrase 5B	protein_codin	0.2712	0.293	1	10.01	0.08542
**TXLNG**	TXLNG	19	taxilin gamma	protein_codin	0.3865	0.203	1	9.011	0.1542
**ACOT9**	ACOT9	19	acyl-CoA thioesterase 9	protein_codin	-0.01903	0.918	1	7.388	0.1151
**MID1IP1**	MID1IP1	19	MID1 interacting protein 1	protein_codin	-0.02265	0.919	1	52.58	0.4171
**RBM10**	RBM10	19	RNA binding motif protein 10	protein_codin	0.3561	0.312	1	3.484	0.3104
**FAM104B**	FAM104B	19	family with sequence similarity 104 member B	protein_codin	-0.7037	0.00101	1	7.609	0.06212
**LAS1L**	LAS1L	19	LAS1 like, ribosome biogenesis factor	protein_codin	0.5127	0.0865	1	8.512	0.1172
**NHSL2**	NHSL2	19	NHS like 2	protein_codin	0.1456	0.48	1	7.783	0.1962
**PHF6**	PHF6	19	PHD finger protein 6	protein_codin	0.2538	0.203	1	19.14	0.1812
**VBP1**	VBP1	19	VHL binding protein 1	protein_codin	0.1721	0.29	1	6.568	0.08408
**FBXO25**	FBXO25	19	F-box protein 25	protein_codin	0.01504	0.949	1	3.435	0.2228
**CLN8**	CLN8	19	CLN8 transmembrane ER and ERGIC protein	protein_codin	0.008928	0.978	1	9.283	0.229
**C8orf58**	C8orf58	19	chromosome 8 open reading frame 58	protein_codin	0.3325	0.278	1	34.18	0.2334
**ADGRA2**	ADGRA2	19	adhesion G protein-coupled receptor A2	protein_codin	1.93	2.24E-07	0.005257	21.02	0.1335
**MTDH**	MTDH	19	metadherin	protein_codin	0.08806	0.2684	1	51.88	0.1076
**RRM2B**	RRM2B	19	ribonucleotide reductase regulatory TP53 inducible subunit M2B	protein_codin	-0.7026	0.018	1	8.182	0.2513
**OXR1**	OXR1	19	oxidation resistance 1	protein_codin	0.1036	0.536	1	14.96	0.08050
**NSMCE2**	NSMCE2	19	NSE2 (MMS21) homolog, SMC5-SMC6 complex SUMO ligase	protein_codin	0.05171	0.816	1	7.86	0.1171
**EFR3A**	EFR3A	19	EFR3 homolog A	protein_codin	0.2647	0.264	1	23.2	0.2469
**PTK2**	PTK2	19	protein tyrosine kinase 2	protein_codin	-0.366	0.0132	1	33.34	0.1121
**FAM219A**	FAM219A	19	family with sequence similarity 219 member A	protein_codin	0.1954	0.536	1	2.893	0.2746
**GRHPR**	GRHPR	19	glyoxylate and hydroxypyruvate reductase	protein_codin	0.1662	0.186	1	23.56	0.1076
**NMRK1**	NMRK1	19	nicotinamide riboside kinase 1	protein_codin	0.4676	0.00271	1	26.24	0.08034
**GNAQ**	GNAQ	19	G protein subunit alpha q	protein_codin	-0.07015	0.636	1	23.65	0.141
**NINJ1**	NINJ1	19	ninjurin 1	protein_codin	-0.2282	0.233	1	48.92	0.1722
**FAM120AOS**	FAM120AO	19	family with sequence similarity 120A opposite strand	protein_codin	-0.4009	0.0171	1	16.85	0.08404
**FAM120A**	FAM120A	19	family with sequence similarity 120A	protein_codin	-0.3432	0.105	1	18.73	0.202
**MFSD14B**	MFSD14B	19	major facilitator superfamily domain containing 14B	protein_codin	0.4665	0.128	1	3.637	0.1101
**NIPSNAP3A**	NIPSNAP3	19	nipsnap homolog 3A	protein_codin	0.5935	0.0281	1	3.537	0.0899
**HSDL2**	HSDL2	19	hydroxysteroid dehydrogenase like 2	protein_codin	0.2837	0.202	1	22.58	0.2546
**INIP**	INIP	19	INTS3 and NABP interacting protein	protein_codin	1.004	0.0027	1	27.86	0.0809
**TLR4**	TLR4	19	toll like receptor 4	protein_codin	0.9102	0.00219	1	18.67	0.2769
**NDUFA8**	NDUFA8	19	NADH ubiquinone oxidoreductase subunit A8	protein_codin	0.1082	0.362	1	47.89	0.02839
**GOLGA1**	GOLGA1	19	golgin A1	protein_codin	0.6011	0.0988	1	3.343	0.2074
**SWI5**	SWI5	19	SWI5 homologous recombination repair protein	protein_codin	-0.1136	0.534	1	6.369	0.1724
**FUBP3**	FUBP3	19	far upstream element binding protein 3	protein_codin	-0.1374	0.629	1	10.09	0.1952
**SDCCAG3**	SDCCAG3	19	endosome associated trafficking regulator 1	protein_codin	NA	NA	NA	3.161	0.2379
**SIGIRR**	SIGIRR	19	single Ig and TIR domain containing	protein_codin	-0.169	0.107	1	41.96	0.05966
**HRAS**	HRAS	19	HRas proto-oncogene, GTPase	protein_codin	-0.1332	0.419	1	17.12	0.1402
**TSPAN4**	TSPAN4	19	tetraspanin 4	protein_codin	0.2497	0.00652	1	44.85	0.03012
**FAM160A2**	FAM160A2	19	family with sequence similarity 160 member A2	protein_codin	1.437	4.76E-05	1	3.295	0.1111
**TIMM10B**	TIMM10B	19	translocase of inner mitochondrial membrane 10B	protein_codin	-0.1929	0.428	1	9.068	0.2472
**AKIP1**	AKIP1	19	A-kinase interacting protein 1	protein_codin	0.3514	0.11	1	6.792	0.1397
**IPO7**	IPO7	19	importin 7	protein_codin	-0.1649	0.374	1	16.87	0.1427
**ZBED5**	ZBED5	19	zinc finger BED-type containing 5	protein_codin	0.2295	0.373	1	9.378	0.08647
**PSMA1**	PSMA1	19	proteasome subunit alpha 1	protein_codin	-0.1511	0.0819	1	52.05	0.03036
**PIK3C2A**	PIK3C2A	19	phosphatidylinositol-4-phosphate 3-kinase catalytic subunit ty	protein_codin	0.2138	0.106	1	22.93	0.09439
**CAT**	CAT	19	catalase	protein_codin	0.4569	0.0060	1	24.99	0.0870
**DNAJC4**	DNAJC4	19	DnaJ heat shock protein family (Hsp40) member C4	protein_codin	-0.01209	0.933	1	10.05	0.08468
**GPR137**	GPR137	19	G protein-coupled receptor 137	protein_codin	-0.3236	0.22	1	9.955	0.2537
**RHOD**	RHOD	19	ras homolog family member D	protein_codin	-0.5501	0.0129	1	8.838	0.05636
**CTTN**	CTTN	19	cortactin	protein_codin	-0.1979	0.116	1	43.39	0.1066
**POLD3**	POLD3	19	DNA polymerase delta 3, accessory subunit	protein_codin	0.5618	0.0416	1	8.903	0.1425
**SERPINH1**	SERPINH1	19	serpin family H member 1	protein_codin	-0.2215	0.0722	1	52.58	0.1949
**LRRC32**	LRRC32	19	leucine rich repeat containing 32	protein_codin	1.487	6.26E-11	1.49E-06	47.83	0.08339
**FZD4**	FZD4	19	frizzled class receptor 4	protein_codin	1.088	7.08E-07	0.01657	30.15	0.08367
**DCUN1D5**	DCUN1D5	19	defective in cullin neddylation 1 domain containing 5	protein_codin	-0.1454	0.443	1	7.08	0.2016
**DYNC2H1**	DYNC2H1	19	dynein cytoplasmic 2 heavy chain 1	protein_codin	-0.01986	0.953	1	3.8	0.3447
**PPP2R1B**	PPP2R1B	19	protein phosphatase 2 scaffold subunit Abeta	protein_codin	-0.07709	0.8	1	11.93	0.2705
**TIMM8B**	TIMM8B	19	translocase of inner mitochondrial membrane 8 homolog B	protein_codin	-0.4996	0.000167	1	29.9	0.08694
**FBXO18**	FBXO18	19	F-box DNA helicase 1	protein_codin	NA	NA	NA	3.538	0.3469
**TAF3**	TAF3	19	TATA-box binding protein associated factor 3	protein_codin	0.09851	0.649	1	8.08	0.2296
**COMMD3**	COMMD3	19	COMM domain containing 3	protein_codin	-0.1621	0.321	1	7.103	0.1129
**ZEB1**	ZEB1	19	zinc finger E-box binding homeobox 1	protein_codin	-0.1394	0.238	1	25.7	0.02835
**BMS1**	BMS1	19	BMS1 ribosome biogenesis factor	protein_codin	0.139	0.508	1	8.578	0.1122
**SYT15**	SYT15	19	synaptotagmin 15	protein_codin	2.022	5.22E-07	0.01222	3.543	0.1332
**HERC4**	HERC4	19	HECT and RLD domain containing E3 ubiquitin protein ligase 4	protein_codin	0.5037	0.0785	1	3.412	0.1122
**DDX21**	DDX21	19	DExD-box helicase 21	protein_codin	0.4451	1E-04	1	30.3	0.05617
**EIF4EBP2**	EIF4EBP2	19	eukaryotic translation initiation factor 4E binding protein 2	protein_codin	-0.06249	0.788	1	7.585	0.08508
**FUT11**	FUT11	19	fucosyltransferase 11	protein_codin	-0.1648	0.607	1	31.43	0.3516
**VDAC2**	VDAC2	19	voltage dependent anion channel 2	protein_codin	-0.246	0.0016	1	22.87	0.02998
**PLAC9**	PLAC9	19	placenta associated 9	protein_codin	1.372	2.67E-12	6.38E-09	27.98	0.1393
**FAM213A**	FAM213A	19	peroxiredoxin like 2A	protein_codin	0.6665	3.32E-09	7.86E-05	46.75	0.08261
**NOC3L**	NOC3L	19	NOC3 like DNA replication regulator	protein_codin	0.5289	0.0282	1	41.37	0.222
**HPS1**	HPS1	19	HPS1 biogenesis of lysosomal organelles complex 3 subunit	protein_codin	0.0624	0.843	1	8.618	0.2619
**NDUFB8**	NDUFB8	19	NADH ubiquinone oxidoreductase subunit B8	protein_codin	-0.277	0.0012	1	30.29	0.08825
**ACTR1A**	ACTR1A	19	actin related protein 1A	protein_codin	0.08975	0.601	1	7.027	0.1422
**CASP7**	CASP7	19	caspase 7	protein_codin	0.9598	0.000389	1	6.227	0.1555
**CCDC186**	CCDC186	19	coiled-coil domain containing 186	protein_codin	0.1232	0.515	1	25.13	0.1979
**ATE1**	ATE1	19	arginyltransferase 1	protein_codin	0.438	0.115	1	3.443	0.2321
**BCCIP**	BCCIP	19	BRCA2 and CDKN1A interacting protein	protein_codin	0.2817	0.16	1	6.059	0.2015
**ECHS1**	ECHS1	19	enoyl-CoA hydratase, short chain 1	protein_codin	-0.06512	0.576	1	16.58	0.1666
**ING4**	ING4	19	inhibitor of growth family member 4	protein_codin	0.05312	0.818	1	8.29	0.3460
**C1S**	C1S	19	complement C1s	protein_codin	-3.175	3.31E-55	8.03E-51	52.58	0.1411
**C1R**	C1R	19	complement C1r	protein_codin	-2.495	7.85E-97	1.91E-92	47.62	0.06968
**GABARAPL1**	GABARAPL	19	GABA type A receptor associated protein like 1	protein_codin	-0.2308	0.129	1	51.86	0.08518
**CDKN1B**	CDKN1B	19	cyclin dependent kinase inhibitor 1B	protein_codin	0.1262	0.453	1	13.76	0.02872
**GPRC5A**	GPRC5A	19	G protein-coupled receptor class C group 5 member A	protein_codin	-1.01	1.55E-05	0.3589	29.81	0.1438
**PPFIBP1**	PPFIBP1	19	PPFIA binding protein 1	protein_codin	0.4826	9.5E-05	1	36.65	0.09169
**KIAA1551**	KIAA1551	19	retroelement silencing factor 1	protein_codin	0.7238	0.000105	1	17.93	0.1694
**PPHLN1**	PPHLN1	19	periphilin 1	protein_codin	0.02496	0.887	1	16.26	0.2417
**MCRS1**	MCRS1	19	microspherule protein 1	protein_codin	0.09617	0.703	1	3.093	0.2209
**CERS5**	CERS5	19	ceramide synthase 5	protein_codin	-0.01163	0.96	1	7.036	0.08658
**ORMDL2**	ORMDL2	19	ORMDL sphingolipid biosynthesis regulator 2	protein_codin	0.02034	0.896	1	15.55	0.05419
**CDK4**	CDK4	19	cyclin dependent kinase 4	protein_codin	0.3856	0.0207	1	34.96	0.1099
**AC084033.3**	AC084033.3	19	GIHCG inhibitor of miR-200b/200a/429 expression	lincRNA	0.6519	0.0142	1	3.38	0.1685
**TMEM5**	TMEM5	19	ribitol xylosyltransferase 1	protein_codin	NA	NA	NA	9.088	0.1504
**MDM1**	MDM1	19	Mdm1 nuclear protein	protein_codin	0.02147	0.946	1	10.06	0.4591
**NUP107**	NUP107	19	nucleoporin 107	protein_codin	0.7271	0.0327	1	8.421	0.2255
**CHPT1**	CHPT1	19	choline phosphotransferase 1	protein_codin	-0.06496	0.78	1	3.442	0.06016
**CHST11**	CHST11	19	carbohydrate sulfotransferase 11	protein_codin	0.5037	0.115	1	8.992	0.1712
**C12orf49**	C12orf49	19	chromosome 12 open reading frame 49	protein_codin	0.1459	0.611	1	3.14	0.14
**TESC**	TESC	19	tescalcin	protein_codin	2.417	6.94E-20	1.68E-21	26.88	0.02823
**FBXO21**	FBXO21	19	F-box protein 21	protein_codin	0.1449	0.501	1	7.416	0.1365
**PXN**	PXN	19	paxillin	protein_codin	0.6511	0.0182	1	8.18	0.2571
**C12orf43**	C12orf43	19	chromosome 12 open reading frame 43	protein_codin	0.8937	0.00293	1	22.81	0.1347
**CCDC92**	CCDC92	19	coiled-coil domain containing 92	protein_codin	-0.1852	0.468	1	8.535	0.05422
**GOLGA3**	GOLGA3	19	golgin A3	protein_codin	-0.4403	0.145	1	9.507	0.2696
**ZMYM2**	ZMYM2	19	zinc finger MYM-type containing 2	protein_codin	0.01829	0.935	1	5.938	0.222
**IFT88**	IFT88	19	intraflagellar transport 88	protein_codin	0.5332	0.0377	1	8.894	0.2592
**SACS**	SACS	19	sacsin molecular chaperone	protein_codin	-0.5194	0.206	1	11.53	0.5177
**RFXAP**	RFXAP	19	regulatory factor X associated protein	protein_codin	-0.2204	0.395	1	10.86	0.2089
**COG6**	COG6	19	component of oligomeric golgi complex 6	protein_codin	-0.7239	0.00967	1	3.129	0.2085
**TBC1D4**	TBC1D4	19	TBC1 domain family member 4	protein_codin	0.1882	0.555	1	3.332	0.2941
**ARGLU1**	ARGLU1	19	arginine and glutamate rich 1	protein_codin	0.2593	0.00124	1	44.16	0.05445
**TMCO3**	TMCO3	19	transmembrane and coiled-coil domains 3	protein_codin	0.2102	0.379	1	22.32	0.1747
**PNP**	PNP	19	purine nucleoside phosphorylase	protein_codin	-0.1784	0.122	1	36.97	0.02811
**HNRNPC**	HNRNPC	19	heterogeneous nuclear ribonucleoprotein C (C1/C2)	protein_codin	-0.02506	0.796	1	49.55	0.1096
**ADCY4**	ADCY4	19	adenylate cyclase 4	protein_codin	0.4482	0.0297	1	7.312	0.1148
**G2E3**	G2E3	19	G2/M-phase specific E3 ubiquitin protein ligase	protein_codin	0.8503	0.00567	1	8.198	0.1711
**AL121603.2**	AL121603.2	19	novel transcript, antisense to BAZ	antisense_RN	1.662	0.00258	1	30.35	0.3597
**SAV1**	SAV1	19	salvador family WW domain containing protein 1	protein_codin	0.07437	0.662	1	24.92	0.1375
**CGRRF1**	CGRRF1	19	cell growth regulator with ring finger domain 1	protein_codin	0.1094	0.688	1	6.849	0.276
**ACTR10**	ACTR10	19	actin related protein 10	protein_codin	0.3224	0.0118	1	47.92	0.05564
**SRSF5**	SRSF5	19	serine and arginine rich splicing factor 5	protein_codin	0.3464	3.39E-05	0.7823	48	0.03345
**SNW1**	SNW1	19	SNW domain containing 1	protein_codin	0.4289	0.0011	1	18.76	0.1139
**NRDE2**	NRDE2	19	NRDE-2, necessary for RNA interference, domain containing	protein_codin	-0.3106	0.323	1	3.408	0.1134
**ATXN3**	ATXN3	19	ataxin 3	protein_codin	0.7359	0.000839	1	15.25	0.05760
**ATG2B**	ATG2B	19	autophagy related 2B	protein_codin	0.2139	0.545	1	9.517	0.1268
**MOK**	MOK	19	MOK protein kinase	protein_codin	0.2943	0.383	1	9.468	0.3164
**CINP**	CINP	19	cyclin dependent kinase 2 interacting protein	protein_codin	0.1385	0.487	1	22.2	0.1426
**PPP1R13B**	PPP1R13B	19	protein phosphatase 1 regulatory subunit 13B	protein_codin	0.1619	0.396	1	6.942	0.145
**BTBD6**	BTBD6	19	BTB domain containing 6	protein_codin	-0.3114	0.0704	1	7.183	0.1146
**GOLGA8A**	GOLGA8A	19	golgin A8 family member A	protein_codin	-0.1602	0.568	1	9.217	0.1962
**DLL4**	DLL4	19	delta like canonical Notch ligand 4	protein_codin	0.4895	0.0581	1	45.5	0.05782
**COPS2**	COPS2	19	COP9 signalosome subunit 2	protein_codin	0.279	0.0326	1	22.82	0.1092
**FAM214A**	FAM214A	19	family with sequence similarity 214 member A	protein_codin	-0.628	0.00289	1	7.542	0.1715
**DNAAF4**	DNAAF4	19	dynein axonemal assembly factor 4	protein_codin	-0.06859	0.758	1	10.14	0.2655
**ADAM10**	ADAM10	19	ADAM metallopeptidase domain 10	protein_codin	0.2794	0.0974	1	34	0.1952
**RNF111**	RNF111	19	ring finger protein 111	protein_codin	0.01225	0.963	1	8.036	0.1226
**TRIP4**	TRIP4	19	thyroid hormone receptor interactor 4	protein_codin	0.2222	0.458	1	3.39	0.1445
**RPL4**	RPL4	19	ribosomal protein L4	protein_codin	0.004724	0.927	1	52.52	0.02668
**AAGAB**	AAGAB	19	alpha and gamma adaptin binding protein	protein_codin	-0.2576	0.329	1	3.41	0.342
**C15orf61**	C15orf61	19	chromosome 15 open reading frame 61	protein_codin	0.1606	0.424	1	8.14	0.111
**UBL7**	UBL7	19	ubiquitin like 7	protein_codin	-0.1772	0.426	1	23.65	0.1401
**MPI**	MPI	19	mannose phosphate isomerase	protein_codin	0.2614	0.462	1	2.638	0.2685
**BTBD1**	BTBD1	19	BTB domain containing 1	protein_codin	0.005864	0.982	1	3.49	0.1365
**CHD2**	CHD2	19	chromodomain helicase DNA binding protein 2	protein_codin	-0.07798	0.697	1	21.1	0.2174
**FAM234A**	FAM234A	19	family with sequence similarity 234 member A	protein_codin	0.5427	0.0327	1	9.482	0.1826
**FAM173A**	FAM173A	19	adenine nucleotide translocase lysine methyltransferase	protein_codin	-0.3436	0.0786	1	20.94	0.1969
**PGP**	PGP	19	phosphoglycolate phosphatase	protein_codin	-0.05211	0.752	1	18.2	0.08384
**FLYWCH1**	FLYWCH1	19	FLYWCH-type zinc finger 1	protein_codin	0.1007	0.715	1	9.533	0.3974
**PPL**	PPL	19	periplakin	protein_codin	2.484	6.8E-05	1	7.383	0.3147
**USP7**	USP7	19	ubiquitin specific peptidase 7	protein_codin	0.01779	0.925	1	18.96	0.2214
**CIITA**	CIITA	19	class II major histocompatibility complex transactivator	protein_codin	1.132	0.0382	1	8.298	0.2496
**ITPRIPL2**	ITPRIPL2	19	ITPRIP like 2	protein_codin	-0.7059	7.92E-05	1	15.93	0.08438
**TMEM159**	TMEM159	19	transmembrane protein 159	protein_codin	0.2852	0.227	1	46.25	0.09927
**DCTN5**	DCTN5	19	dynactin subunit 5	protein_codin	-0.1268	0.714	1	11.29	0.1694
**TNRC6A**	TNRC6A	19	trinucleotide repeat containing adaptor 6A	protein_codin	-0.3351	0.117	1	8.437	0.1372
**LCMT1**	LCMT1	19	leucine carboxyl methyltransferase 1	protein_codin	-0.02377	0.913	1	8.282	0.1099
**TUFM**	TUFM	19	Tu translation elongation factor, mitochondrial	protein_codin	-0.1488	0.0981	1	50.47	0.0812
**RABEP2**	RABEP2	19	rabaptin, RAB GTPase binding effector protein 2	protein_codin	0.02508	0.904	1	2.92	0.2882
**VKORC1**	VKORC1	19	vitamin K epoxide reductase complex subunit 1	protein_codin	-0.1747	0.0836	1	14.38	0.1619
**RBL2**	RBL2	19	RB transcriptional corepressor like 2	protein_codin	0.07585	0.715	1	21.05	0.2202
**CFAP20**	CFAP20	19	cilia and flagella associated protein 20	protein_codin	0.09409	0.564	1	45.2	0.119
**NDRG4**	NDRG4	19	NDRG family member 4	protein_codin	-1.232	1.6E-06	0.03726	33.83	0.1405
**IST1**	IST1	19	IST1 factor associated with ESCRT-III	protein_codin	0.1069	0.6	1	7.33	0.2532
**BCAR1**	BCAR1	19	BCAR1 scaffold protein, Cas family member	protein_codin	0.6394	0.00132	1	18.59	0.08767
**PLCG2**	PLCG2	19	phospholipase C gamma 2	protein_codin	0.8632	5.66E-08	0.001332	52.58	0.2111
**DOC2B**	DOC2B	19	double C2 domain beta	protein_codin	2.175	1.4E-13	3.35E-09	32.38	0.1376
**KIF1C**	KIF1C	19	kinesin family member 1C	protein_codin	-0.433	0.0727	1	8.334	0.1069
**CTDNEP1**	CTDNEP1	19	CTD nuclear envelope phosphatase 1	protein_codin	-0.2592	0.0726	1	27.29	0.08395
**SAT2**	SAT2	19	spermidine/spermine N1-acetyltransferase family member 2	protein_codin	0.2036	0.112	1	9.697	0.1079
**NUFIP2**	NUFIP2	19	nuclear FMR1 interacting protein 2	protein_codin	-0.05842	0.737	1	16.5	0.1786
**CCL4**	CCL4	19	C-C motif chemokine ligand 4	protein_codin	-0.9409	0.000775	1	3.405	0.3095
**ZNHIT3**	ZNHIT3	19	zinc finger HIT-type containing 3	protein_codin	0.1742	0.292	1	16.43	0.1172
**NR1D1**	NR1D1	19	nuclear receptor subfamily 1 group D member 1	protein_codin	0.1401	0.66	1	8.452	0.1147
**ACLY**	ACLY	19	ATP citrate lyase	protein_codin	-0.06608	0.783	1	3.366	0.08782
**STAT5A**	STAT5A	19	signal transducer and activator of transcription 5A	protein_codin	0.5074	0.08	1	29.33	0.256
**ATP6V0A1**	ATP6V0A1	19	ATPase H+ transporting V0 subunit a1	protein_codin	-0.188	0.565	1	8.397	0.2174
**BECN1**	BECN1	19	beclin 1	protein_codin	0.004539	0.979	1	18.85	0.08429
**AOC3**	AOC3	19	amine oxidase copper containing 3	protein_codin	0.9264	0.00256	1	51.45	0.2029
**DUSP3**	DUSP3	19	dual specificity phosphatase 3	protein_codin	-0.6225	0.0336	1	8.948	0.1934
**KPNB1**	KPNB1	19	karyopherin subunit beta 1	protein_codin	0.2814	0.065	1	10.22	0.115
**SCRN2**	SCRN2	19	secernin 2	protein_codin	0.3762	0.0711	1	5.996	0.1679
**SPATA20**	SPATA20	19	spermatogenesis associated 20	protein_codin	-0.6097	0.0115	1	3.249	0.1915
**YPEL2**	YPEL2	19	yippee like 2	protein_codin	0.6356	1E-05	0.2321	48	0.125
**METTL2A**	METTL2A	19	methyltransferase like 2A	protein_codin	0.6382	0.0507	1	3.419	0.2031
**C17orf58**	C17orf58	19	chromosome 17 open reading frame 58	protein_codin	0.3179	0.203	1	8.397	0.08441
**PCYT2**	PCYT2	19	phosphate cytidylyltransferase 2, ethanolamine	protein_codin	0.196	0.517	1	2.872	0.1551
**RIOK3**	RIOK3	19	RIO kinase 3	protein_codin	-0.1208	0.38	1	12.21	0.1094
**ANKRD29**	ANKRD29	19	ankyrin repeat domain 29	protein_codin	1.379	1.49E-13	3.56E-09	17.39	0.1619
**ZNF24**	ZNF24	19	zinc finger protein 24	protein_codin	0.2194	0.169	1	13.26	0.1432
**PIAS2**	PIAS2	19	protein inhibitor of activated STAT 2	protein_codin	-0.5079	0.0514	1	3.606	0.222
**ACAA2**	ACAA2	19	acetyl-CoA acyltransferase 2	protein_codin	0.1759	0.356	1	7.725	0.1129
**CCDC68**	CCDC68	19	coiled-coil domain containing 68	protein_codin	1.372	1.02E-12	2.44E-08	15.67	0.1952
**TIMM21**	TIMM21	19	translocase of inner mitochondrial membrane 21	protein_codin	-0.02495	0.932	1	2.913	0.1976
**CNDP2**	CNDP2	19	carnosine dipeptidase 2	protein_codin	0.3669	0.086	1	17.48	0.05759
**IDH3B**	IDH3B	19	isocitrate dehydrogenase (NAD(+)) 3 beta	protein_codin	0.5691	0.00371	1	19.44	0.09092
**ASXL1**	ASXL1	19	ASXL transcriptional regulator 1	protein_codin	0.6674	0.000276	1	26.75	0.1977
**RBM12**	RBM12	19	RNA binding motif protein 12	protein_codin	0.2241	0.449	1	3.419	0.08579
**RBM39**	RBM39	19	RNA binding motif protein 39	protein_codin	-0.001706	0.993	1	46	0.02719
**BLCAP**	BLCAP	19	BLCAP apoptosis inducing factor	protein_codin	-0.1248	0.539	1	7.725	0.1388
**PKIG**	PKIG	19	cAMP-dependent protein kinase inhibitor gamma	protein_codin	-0.8988	1.25E-15	3E-11	28.16	0.05472
**UBE2V1**	UBE2V1	19	ubiquitin conjugating enzyme E2 V1	protein_codin	0.6892	0.0222	1	3.553	0.1938
**DNAJC5**	DNAJC5	19	DnaJ heat shock protein family (Hsp40) member C5	protein_codin	-0.5992	0.0307	1	3.15	0.1495
**TCEA2**	TCEA2	19	transcription elongation factor A2	protein_codin	0.03222	0.842	1	15.37	0.1364
**CDC34**	CDC34	19	cell division cycle 34	protein_codin	0.4256	0.0225	1	18.23	0.1431
**CFD**	CFD	19	complement factor D	protein_codin	2.598	0.2791	1	52.02	4.484
**MPND**	MPND	19	MPN domain containing	protein_codin	0.5414	0.112	1	3.011	0.2604
**UBXN6**	UBXN6	19	UBX domain protein 6	protein_codin	-0.3722	0.0592	1	7.249	0.08139
**SEMA6B**	SEMA6B	19	semaphorin 6B	protein_codin	-0.2029	0.295	1	43.48	0.2889
**GPR108**	GPR108	19	G protein-coupled receptor 108	protein_codin	0.1406	0.352	1	12.88	0.02615
**RAB11B**	RAB11B	19	RAB11B, member RAS oncogene family	protein_codin	-0.2576	0.0748	1	36.17	0.1442
**HNRNPM**	HNRNPM	19	heterogeneous nuclear ribonucleoprotein M	protein_codin	0.09886	0.332	1	21.48	0.1122
**DOCK6**	DOCK6	19	dedicator of cytokinesis 6	protein_codin	-0.4848	0.0359	1	9.757	0.1432
**STX10**	STX10	19	syntaxin 10	protein_codin	0.0861	0.603	1	18.17	0.1455
**ILVBL**	ILVBL	19	ilvB acetolactate synthase like	protein_codin	0.2935	0.23	1	3.033	0.2201
**EPS15L1**	EPS15L1	19	epidermal growth factor receptor pathway substrate 15 like 1	protein_codin	-0.1282	0.662	1	3.167	0.1614
**DDA1**	DDA1	19	DET1 and DDB1 associated 1	protein_codin	0.2217	0.404	1	9.205	0.1484
**COLGALT1**	COLGALT1	19	collagen beta(1-O)galactosyltransferase 1	protein_codin	0.0795	0.76	1	9.255	0.2304
**RFXANK**	RFXANK	19	regulatory factor X associated ankyrin containing protein	protein_codin	0.6503	0.00464	1	7.544	0.2414
**NFKBID**	NFKBID	19	NFKB inhibitor delta	protein_codin	1.487	8.21E-05	1	43.06	0.2074
**ZNF567**	ZNF567	19	zinc finger protein 567	protein_codin	0.3602	0.183	1	8.344	0.2358
**PSMC4**	PSMC4	19	proteasome 26S subunit, ATPase 4	protein_codin	-0.1391	0.288	1	13.54	0.0541
**PCAT19**	PCAT19	19	prostate cancer associated transcript 19	lincRNA	1.041	5.17E-08	0.001218	12.17	0.07927
**PAFAH1B3**	PAFAH1B3	19	platelet activating factor acetylhydrolase 1b catalytic subunit 3	protein_codin	0.06814	0.723	1	50.92	0.1950
**ZNF224**	ZNF224	19	zinc finger protein 224	protein_codin	-0.5179	0.0242	1	3.408	0.1757
**DMWD**	DMWD	19	DM1 locus, WD repeat containing	protein_codin	0.1592	0.555	1	8.137	0.1885
**NAPA**	NAPA	19	NSF attachment protein alpha	protein_codin	-0.4384	0.00337	1	13.11	0.1684
**EHD2**	EHD2	19	EH domain containing 2	protein_codin	0.6815	0.00015	1	29.96	0.1958
**TRPM4**	TRPM4	19	transient receptor potential cation channel subfamily M member 4	protein_codin	1.102	0.0148	1	2.607	0.1456
**TEAD2**	TEAD2	19	TEA domain transcription factor 2	protein_codin	0.6969	0.00242	1	7.965	0.2492
**PIH1D1**	PIH1D1	19	PIH1 domain containing 1	protein_codin	0.09858	0.528	1	16.96	0.1122
**PPP2R1A**	PPP2R1A	19	protein phosphatase 2 scaffold subunit Aalpha	protein_codin	0.3998	0.00736	1	37.12	0.2198
**HSPBP1**	HSPBP1	19	HSPA (Hsp70) binding protein 1	protein_codin	0.6356	0.0242	1	7.448	0.1452
**TRIM28**	TRIM28	19	tripartite motif containing 28	protein_codin	0.04379	0.818	1	7.479	0.2036
**RPS4Y1**	RPS4Y1	19	ribosomal protein S4 Y-linked 1	protein_codin	0.3664	6.79E-09	0.000161	52.58	0.1436
**ZFY**	ZFY	19	zinc finger protein Y-linked	protein_codin	0.3998	0.273	1	2.925	0.1742
**UTY**	UTY	19	ubiquitously transcribed tetratricopeptide repeat containing, Y	protein_codin	-0.08715	0.817	1	3.558	0.2672
**GAS2L1**	GAS2L1	19	growth arrest specific 2 like 1	protein_codin	0.4875	0.00899	1	39.54	0.0808
**ASCC2**	ASCC2	19	activating signal cointegrator 1 complex subunit 2	protein_codin	0.0862	0.747	1	3.114	0.1139
**CARD10**	CARD10	19	caspase recruitment domain family member 10	protein_codin	1.171	2.58E-05	0.5962	6.953	0.221
**CBY1**	CBY1	19	chibby family member 1, beta catenin antagonist	protein_codin	0.2025	0.279	1	7.676	0.06208
**SNU13**	SNU13	19	small nuclear ribonucleoprotein 13	protein_codin	-0.1035	0.1417	1	51.93	0.02701
**FAM118A**	FAM118A	19	family with sequence similarity 118 member A	protein_codin	0.2649	0.133	1	34.73	0.2452
**LMF2**	LMF2	19	lipase maturation factor 2	protein_codin	-0.02367	0.901	1	5.262	0.2588
**LTN1**	LTN1	19	listerin E3 ubiquitin protein ligase 1	protein_codin	0.3331	0.111	1	20.05	0.08786
**CBR1**	CBR1	19	carbonyl reductase 1	protein_codin	-0.05835	0.808	1	9.08	0.1362
**TRAPPC10**	TRAPPC10	19	trafficking protein particle complex 10	protein_codin	-0.3152	0.163	1	8.874	0.1686
**C21orf2**	C21orf2	19	cilia and flagella associated protein 410	protein_codin	-1.01	0.000175	1	9.333	0.2669
**SDF4**	SDF4	20	stromal cell derived factor 4	protein_codin	-0.6351	2.58E-09	6.11E-05	29.95	0.02639
**VAMP3**	VAMP3	20	vesicle associated membrane protein 3	protein_codin	0.5706	2.69E-07	0.006309	33.42	0.1925
**ZNF593**	ZNF593	20	zinc finger protein 593	protein_codin	-0.1329	0.401	1	6.25	0.1079
**LSM10.00**	LSM10.00	20	LSM10, U7 small nuclear RNA associated	protein_codin	-0.4565	0.0102	1	7.688	0.0296
**AKIRIN1**	AKIRIN1	20	akirin 1	protein_codin	0.09451	0.578	1	38.77	0.02789
**TSPAN1**	TSPAN1	20	tetraspanin 1	protein_codin	1.499	0.000901	1	52.48	0.1742
**ATG4C**	ATG4C	20	autophagy related 4C cysteine peptidase	protein_codin	0.5931	0.0454	1	3.293	0.1642
**LRRC40**	LRRC40	20	leucine rich repeat containing 40	protein_codin	0.7422	0.0166	1	3.262	0.2317
**TMED5**	TMED5	20	transmembrane p24 trafficking protein 5	protein_codin	-0.2245	0.275	1	22.24	0.1409
**AL109741.1**	AL109741.1	20	novel transcri	antisense_RN	0.5311	0.0882	1	9.102	0.1479
**S1PR1**	S1PR1	20	sphingosine-1-phosphate receptor 1	protein_codin	0.3309	0.0392	1	20.49	0.1111
**SNX27**	SNX27	20	sorting nexin 27	protein_codin	-0.06040	0.814	1	7.423	0.1184
**ACKR1**	ACKR1	20	atypical chemokine receptor 1 (Duffy blood group)	protein_codin	3.534	9.4E-84	2.28E-79	52.58	0.02811
**DUSP12**	DUSP12	20	dual specificity phosphatase 12	protein_codin	-0.2679	0.252	1	8.947	0.1770
**RASAL2**	RASAL2	20	RAS protein activator like 2	protein_codin	1.364	6.43E-11	1.53E-06	24.33	0.0818
**MAPKAPK2**	MAPKAPK2	20	MAPK activated protein kinase 2	protein_codin	0.2254	0.333	1	8.746	0.05415
**HLX**	HLX	20	H2.0 like homeobox	protein_codin	0.7474	0.00984	1	52.58	0.3186
**ACBD3**	ACBD3	20	acyl-CoA binding domain containing 3	protein_codin	0.2414	0.0692	1	28.27	0.05862
**SPRTN**	SPRTN	20	SprT-like N-terminal domain	protein_codin	0.3729	0.192	1	3.168	0.1382
**SMYD3**	SMYD3	20	SET and MYND domain containing 3	protein_codin	0.3092	0.311	1	7.932	0.3335
**PXDN**	PXDN	20	peroxidasin	protein_codin	-0.8036	0.00472	1	50.84	0.1712
**TAF1B**	TAF1B	20	TATA-box binding protein associated factor, RNA polymerase	protein_codin	0.5186	0.0381	1	21.24	0.1682
**PTRHD1**	PTRHD1	20	peptidyl-tRNA hydrolase domain containing 1	protein_codin	-0.2787	0.0764	1	15.16	0.1105
**CEBPZ**	CEBPZ	20	CCAAT enhancer binding protein zeta	protein_codin	0.7029	1.49E-05	0.3451	21.35	0.1404

**STON1**	STON1	20	stonin 1	protein_codin	1.25	0.0145	1	3.578	0.3157
**PSME4**	PSME4	20	proteasome activator subunit 4	protein_codin	-0.508	0.128	1	9.532	0.1941
**RAB11FIP5**	RAB11FIP5	20	RAB11 family interacting protein 5	protein_codin	0.9251	0.00119	1	27.22	0.1616
**KRCC1**	KRCC1	20	lysine rich coiled-coil 1	protein_codin	0.02039	0.866	1	15.82	0.1395
**ANKRD36C**	ANKRD36C	20	ankyrin repeat domain 36C	protein_codin	-0.09166	0.787	1	3.302	0.388
**CHCHD5**	CHCHD5	20	coiled-coil-helix-coiled-coil-helix domain containing 5	protein_codin	-0.004552	0.977	1	14.95	0.08279
**TSN**	TSN	20	translin	protein_codin	0.242	0.243	1	7.538	0.2516
**ARL6IP6**	ARL6IP6	20	ADP ribosylation factor like GTPase 6 interacting protein 6	protein_codin	0.3105	0.153	1	5.598	0.1985
**HIBCH**	HIBCH	20	3-hydroxyisobutyryl-CoA hydrolase	protein_codin	0.2151	0.282	1	8.196	0.0811
**SF3B1**	SF3B1	20	splicing factor 3b subunit 1	protein_codin	0.1842	0.0796	1	22.11	0.05571
**STK16**	STK16	20	serine/threonine kinase 16	protein_codin	0.3735	0.197	1	3.228	0.1592
**NDUFA10**	NDUFA10	20	NADH ubiquinone oxidoreductase subunit A10	protein_codin	0.02344	0.782	1	17.85	0.05832
**NGLY1**	NGLY1	20	N-glycanase 1	protein_codin	0.648	0.00561	1	8.268	0.05506
**ITGA9**	ITGA9	20	integrin subunit alpha 9	protein_codin	0.2964	0.32	1	10.58	0.08188
**ACAA1**	ACAA1	20	acetyl-CoA acyltransferase 1	protein_codin	-0.4444	0.00841	1	43.76	0.08868
**ABHD5**	ABHD5	20	abhydrolase domain containing 5	protein_codin	-0.3306	0.0679	1	46.02	0.02688
**CCRL2**	CCRL2	20	C-C motif chemokine receptor like 2	protein_codin	1.2	0.00138	1	8.533	0.1656
**KIF9**	KIF9	20	kinesin family member 9	protein_codin	0.02982	0.854	1	15.66	0.1132
**NAA50**	NAA50	20	N(alpha)-acetyltransferase 50, NatE catalytic subunit	protein_codin	-0.03974	0.846	1	6.173	0.2292
**CCDC191**	CCDC191	20	coiled-coil domain containing 191	protein_codin	-0.2902	0.328	1	9.08	0.3265
**ZBTB20**	ZBTB20	20	zinc finger and BTB domain containing 20	protein_codin	-0.2464	0.0314	1	26	0.08956
**RUVBL1**	RUVBL1	20	RuvB like AAA ATPase 1	protein_codin	0.3261	0.096	1	18.94	0.3064
**ACKR4**	ACKR4	20	atypical chemokine receptor 4	protein_codin	0.6105	0.14	1	28.32	0.3769
**STAG1**	STAG1	20	stromal antigen 1	protein_codin	-0.09198	0.682	1	3.465	0.194
**MRAS**	MRAS	20	muscle RAS oncogene homolog	protein_codin	-0.3035	0.155	1	52.58	0.08845
**FAIM**	FAIM	20	Fas apoptotic inhibitory molecule	protein_codin	0.4812	0.095	1	7.696	0.2242
**PLSCR4**	PLSCR4	20	phospholipid scramblase 4	protein_codin	0.8179	1.02E-05	0.2367	20.61	0.112
**NMD3**	NMD3	20	NMD3 ribosome export adaptor	protein_codin	-0.4564	0.0315	1	8.413	0.1092
**TBL1XR1**	TBL1XR1	20	transducin beta like 1 X-linked receptor 1	protein_codin	-0.1404	0.368	1	12.62	0.1177
**MRPL47**	MRPL47	20	mitochondrial ribosomal protein L47	protein_codin	-0.03719	0.834	1	15.52	0.2532
**USP13**	USP13	20	ubiquitin specific peptidase 13	protein_codin	0.001569	0.996	1	49.22	0.2699
**PARL**	PARL	20	presenilin associated rhomboid like	protein_codin	-0.08929	0.666	1	6.443	0.08196
**TMEM128**	TMEM128	20	transmembrane protein 128	protein_codin	0.1712	0.543	1	3.283	0.1535
**CCDC149**	CCDC149	20	coiled-coil domain containing 149	protein_codin	0.5787	0.0445	1	7.965	0.137
**ATP8A1**	ATP8A1	20	ATPase phospholipid transporting 8A1	protein_codin	0.8932	0.00622	1	7.862	0.1936
**SEC24B**	SEC24B	20	SEC24 homolog B, COPII coat complex component	protein_codin	0.8211	0.0144	1	3.433	0.314
**ALPK1**	ALPK1	20	alpha kinase 1	protein_codin	0.3527	0.298	1	3.557	0.329
**IL15**	IL15	20	interleukin 15	protein_codin	0.2742	0.238	1	23.14	0.05621
**MYO10**	MYO10	20	myosin X	protein_codin	-0.2255	0.357	1	21.11	0.05531
**DNAJC21**	DNAJC21	20	DnaJ heat shock protein family (Hsp40) member C21	protein_codin	0.07002	0.562	1	18.5	0.05356
**ELOVL7**	ELOVL7	20	ELOVL fatty acid elongase 7	protein_codin	3.656	3.95E-06	0.092	8.479	0.1461
**TTC37**	TTC37	20	tetratricopeptide repeat domain 37	protein_codin	-0.1993	0.278	1	18.19	0.08566
**PGGT1B**	PGGT1B	20	protein geranylgeranyltransferase type I subunit beta	protein_codin	-0.1252	0.615	1	8.003	0.1674
**COMMD10**	COMMD10	20	COMM domain containing 10	protein_codin	0.1868	0.333	1	7.535	0.1126
**YIPF5**	YIPF5	20	Yip1 domain family member 5	protein_codin	-0.09748	0.464	1	27.17	0.05325
**MED7**	MED7	20	mediator complex subunit 7	protein_codin	0.05006	0.866	1	3.432	0.3438
**BOD1**	BOD1	20	biorientation of chromosomes in cell division 1	protein_codin	-0.04107	0.782	1	34.96	0.02902
**HIGD2A**	HIGD2A	20	HIG1 hypoxia inducible domain family member 2A	protein_codin	0.1209	0.206	1	28.14	0.1128
**BTN2A2**	BTN2A2	20	butyrophilin subfamily 2 member A2	protein_codin	0.7646	0.00941	1	3.283	0.05756
**HCG11**	HCG11	20	HLA complex group 11	lincRNA	0.7084	0.0454	1	8.783	0.222
**PRRC2A**	PRRC2A	20	proline rich coiled-coil 2A	protein_codin	-0.3642	0.113	1	3.392	0.2332
**CSNK2B**	CSNK2B	20	casein kinase 2 beta	protein_codin	0.1134	0.192	1	24.06	0.02768
**NELFE**	NELFE	20	negative elongation factor complex member E	protein_codin	0.5168	0.00188	1	15.4	0.1441
**DNPH1**	DNPH1	20	2'-deoxynucleoside 5'-phosphate N-hydrolase 1	protein_codin	-0.3622	0.0617	1	5.434	0.1896
**TMEM30A**	TMEM30A	20	transmembrane protein 30A	protein_codin	-0.5591	4.46E-05	1	11.56	0.05447
**SYNCRIP**	SYNCRIP	20	synaptotagmin binding cytoplasmic RNA interacting protein	protein_codin	-0.2475	0.0228	1	41.37	0.03168
**MAP3K7**	MAP3K7	20	mitogen-activated protein kinase kinase kinase 7	protein_codin	-0.5355	0.0532	1	9.886	0.1091
**TCP1**	TCP1	20	t-complex 1	protein_codin	0.02156	0.858	1	17.37	0.08717
**PMS2**	PMS2	20	PMS1 homolog 2, mismatch repair system component	protein_codin	0.8556	0.0928	1	2.655	0.4495
**HOTAIRM1**	HOTAIRM1	20	HOXA transcript antisense RNA, myeloid-specific 1	antisense_RN	0.5832	0.0102	1	44.58	0.117
**GATAD1**	GATAD1	20	GATA zinc finger domain containing 1	protein_codin	0.07383	0.672	1	14.08	0.1394
**SLC12A9**	SLC12A9	20	solute carrier family 12 member 9	protein_codin	0.7941	0.0112	1	3.126	0.2227
**CCDC71L**	CCDC71L	20	coiled-coil domain containing 71 like	protein_codin	-0.1696	0.388	1	52.58	0.1862
**ARF5**	ARF5	20	ADP ribosylation factor 5	protein_codin	0.4309	0.000338	1	16.29	0.05346
**FASTK**	FASTK	20	Fas activated serine/threonine kinase	protein_codin	-0.5893	0.00701	1	7.292	0.1905
**ASMTL**	ASMTL	20	acetylserotonin O-methyltransferase like	protein_codin	0.5701	0.022	1	8.279	0.1404
**ZBED1**	ZBED1	20	zinc finger BED-type containing 1	protein_codin	-0.1119	0.624	1	8.23	0.1686
**MID1**	MID1	20	midline 1	protein_codin	0.2245	0.444	1	31.15	0.1095
**WDR45**	WDR45	20	WD repeat domain 45	protein_codin	-0.006027	0.974	1	20.1	0.1479
**GSPT2**	GSPT2	20	G1 to S phase transition 2	protein_codin	0.2833	0.398	1	3.148	0.3271
**HUWE1**	HUWE1	20	HECT, UBA and WWE domain containing E3 ubiquitin protein	protein_codin	-0.002420	0.99	1	19.87	0.0318
**ATP7A**	ATP7A	20	ATPase copper transporting alpha	protein_codin	0.3057	0.358	1	2.925	0.3122
**TCEAL1**	TCEAL1	20	transcription elongation factor A like 1	protein_codin	0.4412	0.028	1	16.28	0.1382
**NAA10**	NAA10	20	N(alpha)-acetyltransferase 10, NatA catalytic subunit	protein_codin	0.01883	0.835	1	42.86	0.1097
**ZDHHC2**	ZDHHC2	20	zinc finger DHHC-type containing 2	protein_codin	-1.003	9.56E-08	0.002246	23.58	0.1126
**NUDT18**	NUDT18	20	nudix hydrolase 18	protein_codin	-0.004855	0.987	1	2.893	0.1106
**PLPBP**	PLPBP	20	pyridoxal phosphate binding protein	protein_codin	0.3962	0.0345	1	17.17	0.2606
**POLB**	POLB	20	DNA polymerase beta	protein_codin	0.6951	0.00785	1	24.92	0.3429
**PRKDC**	PRKDC	20	protein kinase, DNA-activated, catalytic subunit	protein_codin	-0.09952	0.452	1	17.84	0.08606
**SDCBP**	SDCBP	20	syndecan binding protein	protein_codin	0.7252	5.72E-24	1.38E-19	52.02	0.05276
**ZC2HC1A**	ZC2HC1A	20	zinc finger C2HC-type containing 1A	protein_codin	-0.1058	0.588	1	8.296	0.116
**ZNF706**	ZNF706	20	zinc finger protein 706	protein_codin	-0.1282	0.147	1	41.88	0.0317
**C8orf33**	C8orf33	20	chromosome 8 open reading frame 33	protein_codin	-0.3082	0.0566	1	14.81	0.02908
**CNTLN**	CNTLN	20	centlein	protein_codin	0.5562	0.0186	1	15.05	0.2031
**PLAA**	PLAA	20	phospholipase A2 activating protein	protein_codin	0.1791	0.535	1	2.826	0.2255
**RPP25L**	RPP25L	20	ribonuclease P/MRP subunit p25 like	protein_codin	0.5406	0.0341	1	3.353	0.2246
**SIGMAR1**	SIGMAR1	20	sigma non-opioid intracellular receptor 1	protein_codin	-0.2167	0.393	1	2.732	0.1344
**SPIN1**	SPIN1	20	spindlin 1	protein_codin	0.2804	0.298	1	8.192	0.2216
**MRPL50**	MRPL50	20	mitochondrial ribosomal protein L50	protein_codin	0.1596	0.378	1	7.378	0.1445
**PSMD5**	PSMD5	20	proteasome 26S subunit, non-ATPase 5	protein_codin	0.7752	0.0611	1	2.652	0.2622
**CNTRL**	CNTRL	20	centriolin	protein_codin	0.1303	0.626	1	9.192	0.2312
**PTRH1**	PTRH1	20	peptidyl-tRNA hydrolase 1 homolog	protein_codin	-0.1641	0.492	1	3.438	0.2872
**SPTAN1**	SPTAN1	20	spectrin alpha, non-erythrocytic 1	protein_codin	-0.1429	0.241	1	52.58	0.05988
**ZER1**	ZER1	20	zyg-11 related cell cycle regulator	protein_codin	-0.3589	0.222	1	3.032	0.1994
**SETX**	SETX	20	senataxin	protein_codin	0.2882	0.158	1	17.2	0.2992
**MRPL23**	MRPL23	20	mitochondrial ribosomal protein L23	protein_codin	0.2487	0.196	1	6.836	0.1775
**RCN1**	RCN1	20	reticulocalbin 1	protein_codin	0.4428	0.000114	1	45.82	0.1195
**DGKZ**	DGKZ	20	diacylglycerol kinase zeta	antisense_RN	0.2747	0.273	1	9.214	0.03465
**PTPMT1**	PTPMT1	20	protein tyrosine phosphatase mitochondrial 1	protein_codin	0.01502	0.954	1	3.525	0.09339
**MRPL16**	MRPL16	20	mitochondrial ribosomal protein L16	protein_codin	0.08342	0.552	1	27.02	0.08372
**CYB561A3**	CYB561A3	20	cytochrome b561 family member A3	protein_codin	-0.1492	0.597	1	3.032	0.1694
**TMEM258**	TMEM258	20	transmembrane protein 258	protein_codin	-0.2646	0.0011	1	36.23	0.08893
**HNRNPUL2**	HNRNPUL2	20	heterogeneous nuclear ribonucleoprotein U like 2	protein_codin	-0.6809	0.00496	1	3.238	0.1968
**NXF1**	NXF1	20	nuclear RNA export factor 1	protein_codin	-0.2029	0.508	1	2.788	0.2247
**BAD**	BAD	20	BCL2 associated agonist of cell death	protein_codin	-0.3565	0.00222	1	25.06	0.08806
**ESRRA**	ESRRA	20	estrogen related receptor alpha	protein_codin	0.1666	0.602	1	3.117	0.112
**SF1**	SF1	20	splicing factor 1	protein_codin	0.04971	0.638	1	51.04	0.1106
**ZFPL1**	ZFPL1	20	zinc finger protein like 1	protein_codin	-0.2111	0.305	1	6.379	0.1072
**TMEM134**	TMEM134	20	transmembrane protein 134	protein_codin	0.4626	0.0121	1	6.703	0.1682
**NDUFV1**	NDUFV1	20	NADH ubiquinone oxidoreductase core subunit V1	protein_codin	-0.006428	0.965	1	49.45	0.05646
**TCIRG1**	TCIRG1	20	T cell immune regulator 1, ATPase H+ transporting V0 subun	protein_codin	1.042	7.86E-05	1	29.84	0.2185
**RSF1**	RSF1	20	remodeling and spacing factor 1	protein_codin	0.06441	0.56	1	32.46	0.02997
**YAP1**	YAP1	20	Yes associated protein 1	protein_codin	-0.1564	0.503	1	8.105	0.1107
**TMEM123**	TMEM123	20	transmembrane protein 123	protein_codin	0.04427	0.698	1	19.62	0.08136
**CWF19L2**	CWF19L2	20	CWF19 like cell cycle control factor 2	protein_codin	0.2018	0.316	1	27.9	0.1702
**FDX1**	FDX1	20	ferredoxin 1	protein_codin	-0.1257	0.438	1	51.88	0.08875
**C11orf1**	C11orf1	20	chromosome 11 open reading frame 1	protein_codin	0.9047	1.41E-05	0.3267	15.02	0.2374
**PAFAH1B2**	PAFAH1B2	20	platelet activating factor acetylhydrolase 1b catalytic subunit 2	protein_codin	-0.01039	0.952	1	17.51	0.2842
**THY1**	THY1	20	Thy-1 cell surface antigen	protein_codin	-1.463	5.9E-07	0.01381	52.58	0.174
**NUDT5**	NUDT5	20	nudix hydrolase 5	protein_codin	0.04308	0.792	1	35.65	0.1185
**PRPF18**	PRPF18	20	pre-mRNA processing factor 18	protein_codin	-0.165	0.466	1	9.215	0.1914
**MLLT10**	MLLT10	20	MLLT10 histone lysine methyltransferase DOT1L cofactor	protein_codin	0.4586	0.0434	1	7.386	0.1629
**EPC1**	EPC1	20	enhancer of polycomb homolog 1	protein_codin	0.09394	0.468	1	19.86	0.09002
**C10orf10**	C10orf10	20	DEPP1 autophagy regulator	protein_codin	NA	NA	NA	52.58	0.06789
**PARG**	PARG	20	poly(ADP-ribose) glycohydrolase	protein_codin	0.8649	0.0226	1	10.23	0.3899
**PALD1**	PALD1	20	phosphatase domain containing paladin 1	protein_codin	0.232	0.417	1	8.489	0.1354
**VSIR**	VSIR	20	V-set immunoregulatory receptor	protein_codin	0.3999	0.0125	1	45.27	0.08627
**AL135925.1**	AL135925.1	20	novel transcri	lincRNA	-0.1777	0.492	1	8.223	0.05651
**BTAF1**	BTAF1	20	B-TFIID TATA-box binding protein associated factor 1	protein_codin	0.578	0.0479	1	45.3	0.4347
**EXOC6**	EXOC6	20	exocyst complex component 6	protein_codin	-0.01627	0.942	1	30.35	0.142
**PLCE1**	PLCE1	20	phospholipase C epsilon 1	protein_codin	1.145	0.00474	1	2.68	0.1409
**MARVELD1**	MARVELD1	20	MARVEL domain containing 1	protein_codin	-0.8382	0.000921	1	27.38	0.0861
**ARL3.00**	ARL3.00	20	ADP ribosylation factor like GTPase 3	protein_codin	-0.1497	0.288	1	33.3	0.109
**ACSL5**	ACSL5	20	acyl-CoA synthetase long chain family member 5	protein_codin	0.2111	0.4	1	7.252	0.1251
**OAT**	OAT	20	ornithine aminotransferase	protein_codin	-0.158	0.5	1	9.911	0.0882
**ABRAXAS2**	ABRAXAS2	20	abraxas 2, BRISC complex subunit	protein_codin	0.6026	0.0342	1	7.527	0.1432
**M6PR**	M6PR	20	mannose-6-phosphate receptor, cation dependent	protein_codin	0.07517	0.637	1	15.73	0.2549
**LRP6**	LRP6	20	LDL receptor related protein 6	protein_codin	-0.5772	0.00594	1	8.127	0.08275
**FAM60A**	FAM60A	20	SIN3-HDAC complex associated factor	protein_codin	NA	NA	NA	29.6	0.02847
**ATF1**	ATF1	20	activating transcription factor 1	protein_codin	0.007615	0.968	1	6.603	0.3072
**C12orf10**	C12orf10	20	chromosome 12 open reading frame 10	protein_codin	0.2569	0.0629	1	24.12	0.1132
**COPZ1**	COPZ1	20	coatomer protein complex subunit zeta 1	protein_codin	-0.3007	0.018	1	24.1	0.08336
**CS**	CS	20	citrate synthase	protein_codin	-0.5536	0.0712	1	2.828	0.3451
**TBK1**	TBK1	20	TANK binding kinase 1	protein_codin	0.3638	0.315	1	8.572	0.2304
**PPP1R12A**	PPP1R12A	20	protein phosphatase 1 regulatory subunit 12A	protein_codin	0.2081	0.118	1	17.98	0.2106
**CCDC59**	CCDC59	20	coiled-coil domain containing 59	protein_codin	0.2157	0.115	1	9.635	0.1381
**NEDD1**	NEDD1	20	NEDD1 gamma-tubulin ring complex targeting factor	protein_codin	0.4704	0.122	1	8.856	0.1669
**NUAK1**	NUAK1	20	NUAK family kinase 1	protein_codin	0.2672	0.0706	1	48.15	0.1446
**IFT81**	IFT81	20	intraflagellar transport 81	protein_codin	0.6161	0.113	1	9.586	0.2025
**TCTN1**	TCTN1	20	tectonic family member 1	protein_codin	-0.8024	0.000806	1	3.247	0.2251
**PPP1CC**	PPP1CC	20	protein phosphatase 1 catalytic subunit gamma	protein_codin	0.2214	0.116	1	28.68	0.2617
**C1QTNF9**	C1QTNF9	20	C1q and TNF related 9	protein_codin	2.67	0.9687	1	3.983	0.2959
**POMP**	POMP	20	proteasome maturation protein	protein_codin	-0.256	0.000215	1	51.99	0.02606
**MRPL52**	MRPL52	20	mitochondrial ribosomal protein L52	protein_codin	-0.1016	0.373	1	27.36	0.05527
**EMC9**	EMC9	20	ER membrane protein complex subunit 9	protein_codin	-0.1724	0.526	1	2.379	0.2304
**SPTSSA**	SPTSSA	20	serine palmitoyltransferase small subunit A	protein_codin	-0.08626	0.553	1	16.36	0.1129
**ATP5S**	ATP5S	20	distal membrane arm assembly complex 2 like	protein_codin	1.065	1.92E-05	0.4443	16.05	0.08427
**RDH11**	RDH11	20	retinol dehydrogenase 11	protein_codin	0.2211	0.243	1	6.917	0.1492
**VASH1**	VASH1	20	vasohibin 1	protein_codin	-1.066	1.1E-05	0.2551	10.54	0.2006
**PSMC1**	PSMC1	20	proteasome 26S subunit, ATPase 1	protein_codin	0.2352	0.0422	1	51.88	0.1372
**RCOR1**	RCOR1	20	REST corepressor 1	protein_codin	0.01732	0.953	1	30.12	0.23
**MFAP1**	MFAP1	20	microfibril associated protein 1	protein_codin	0.2359	0.204	1	48.88	0.1165
**SPG11**	SPG11	20	SPG11 vesicle trafficking associated, spatacsin	protein_codin	0.4167	0.177	1	8.32	0.2516
**GALK2**	GALK2	20	galactokinase 2	protein_codin	0.02908	0.91	1	34.17	0.3562
**ARIH1**	ARIH1	20	ariadne RBR E3 ubiquitin protein ligase 1	protein_codin	0.4504	0.0651	1	18.88	0.1342
**PSMA4**	PSMA4	20	proteasome subunit alpha 4	protein_codin	-0.1715	0.082	1	24.25	0.1068
**ECI1**	ECI1	20	enoyl-CoA delta isomerase 1	protein_codin	0.2319	0.175	1	14.79	0.218
**SOCS1**	SOCS1	20	suppressor of cytokine signaling 1	protein_codin	-0.2287	0.463	1	35.62	0.3236
**GTF3C1**	GTF3C1	20	general transcription factor IIIC subunit 1	protein_codin	-0.001179	0.997	1	2.772	0.1396
**MAPK3**	MAPK3	20	mitogen-activated protein kinase 3	protein_codin	0.1417	0.253	1	21.62	0.08366
**CHD9**	CHD9	20	chromodomain helicase DNA binding protein 9	protein_codin	0.3175	0.00362	1	39.05	0.05896
**NUDT21**	NUDT21	20	nudix hydrolase 21	protein_codin	0.3016	0.133	1	19.19	0.1872
**MNT**	MNT	20	MAX network transcriptional repressor	protein_codin	-0.08444	0.772	1	3.566	0.222
**LINC00324**	LINC00324	20	long intergenic non-protein coding RNA 324	lincRNA	1.042	0.00905	1	48.36	0.2215
**EPN2**	EPN2	20	epsin 2	protein_codin	0.1184	0.566	1	17.74	0.1992
**SDF2**	SDF2	20	stromal cell derived factor 2	protein_codin	-0.2109	0.117	1	32.44	0.09082
**PLEKHH3**	PLEKHH3	20	pleckstrin homology, MyTH4 and FERM domain containing H	protein_codin	-0.4212	0.136	1	31.05	0.115
**TMEM101**	TMEM101	20	transmembrane protein 101	protein_codin	0.3269	0.0726	1	6.336	0.1371
**EFTUD2**	EFTUD2	20	elongation factor Tu GTP binding domain containing 2	protein_codin	0.4932	0.0994	1	3.282	0.2414
**PPM1D**	PPM1D	20	protein phosphatase, Mg2+/Mn2+ dependent 1D	protein_codin	0.6093	0.00502	1	43.04	0.08242
**SMURF2**	SMURF2	20	SMAD specific E3 ubiquitin protein ligase 2	protein_codin	-0.209	0.31	1	7.997	0.2551
**PITPNC1**	PITPNC1	20	phosphatidylinositol transfer protein cytoplasmic 1	protein_codin	1.075	6.55E-06	0.1522	14.32	0.1164
**CYGB**	CYGB	20	cytoglobin	protein_codin	2.024	4.03E-08	0.00095	28.58	0.2017
**C17orf62**	C17orf62	20	cytochrome b-245 chaperone 1	protein_codin	NA	NA	NA	18.92	0.2358
**MPPE1**	MPPE1	20	metallophosphoesterase 1	protein_codin	0.04842	0.856	1	3.353	0.2429
**TRAPPC8**	TRAPPC8	20	trafficking protein particle complex 8	protein_codin	0.294	0.403	1	3.115	0.2629
**SETBP1**	SETBP1	20	SET binding protein 1	protein_codin	-1.402	4.78E-07	0.0112	10.85	0.1415
**EIF2S2**	EIF2S2	20	eukaryotic translation initiation factor 2 subunit beta	protein_codin	0.026	0.7516	1	39.06	0.05666
**TP53RK**	TP53RK	20	TP53 regulating kinase	protein_codin	-0.1182	0.678	1	7.938	0.1412
**PFDN4**	PFDN4	20	prefoldin subunit 4	protein_codin	-0.1042	0.492	1	16.41	0.08168
**VAPB**	VAPB	20	VAMP associated protein B and C	protein_codin	-0.1748	0.447	1	8.258	0.1909
**STAP2**	STAP2	20	signal transducing adaptor family member 2	protein_codin	0.9015	0.0285	1	9.567	0.4037
**ICAM3**	ICAM3	20	intercellular adhesion molecule 3	protein_codin	-0.01152	0.958	1	8.306	0.1399
**HOOK2**	HOOK2	20	hook microtubule tethering protein 2	protein_codin	0.4072	0.215	1	9.062	0.1441
**LYL1**	LYL1	20	LYL1 basic helix-loop-helix family member	protein_codin	-0.4232	0.000641	1	38.7	0.1055
**SLC35E1**	SLC35E1	20	solute carrier family 35 member E1	protein_codin	0.2305	0.453	1	3.248	0.1394
**MAP1S**	MAP1S	20	microtubule associated protein 1S	protein_codin	-0.03246	0.92	1	3.228	0.2288
**ZNF506**	ZNF506	20	zinc finger protein 506	protein_codin	0.003479	0.988	1	7.688	0.2262
**ZNF100**	ZNF100	20	zinc finger protein 100	protein_codin	0.2837	0.383	1	3.376	0.2135
**PEPD**	PEPD	20	peptidase D	protein_codin	-0.5987	2.94E-07	0.006894	24.9	0.027
**FAM98C**	FAM98C	20	family with sequence similarity 98 member C	protein_codin	0.1099	0.678	1	3.623	0.1964
**SAMD4B**	SAMD4B	20	sterile alpha motif domain containing 4B	protein_codin	0.09555	0.716	1	3.355	0.1354
**SYMPK**	SYMPK	20	symplekin	protein_codin	-0.2675	0.352	1	10.12	0.2905
**LIN7B**	LIN7B	20	lin-7 homolog B, crumbs cell polarity complex component	protein_codin	0.3447	0.317	1	3.312	0.2622
**FCGRT**	FCGRT	20	Fc fragment of IgG receptor and transporter	protein_codin	-0.2039	0.00155	1	37.16	0.03008
**ADM5**	ADM5	20	adrenomedullin 5 (putative)	protein_codin	2.117	7.65E-15	1.83E-10	33.75	0.2092
**GGT5**	GGT5	20	gamma-glutamyltransferase 5	protein_codin	0.625	0.00774	1	20.12	0.2281
**NIPSNAP1**	NIPSNAP1	20	nipsnap homolog 1	protein_codin	0.3179	0.28	1	2.826	0.1669
**ANKRD54**	ANKRD54	20	ankyrin repeat domain 54	protein_codin	0.5002	0.158	1	3.34	0.279
**EPHA2**	EPHA2	21	EPH receptor A2	protein_codin	-0.05209	0.857	1	3.106	0.223
**TCEA3**	TCEA3	21	transcription elongation factor A3	protein_codin	1.571	2.26E-06	0.05272	8.736	0.2787
**ADPRHL2**	ADPRHL2	21	ADP-ribosylhydrolase like 2	protein_codin	-0.3577	0.15	1	3.434	0.1734
**MAP7D1**	MAP7D1	21	MAP7 domain containing 1	protein_codin	-0.1506	0.407	1	20.15	0.08449
**UROD**	UROD	21	uroporphyrinogen decarboxylase	protein_codin	-0.3639	0.0229	1	33.19	0.1097
**CMPK1**	CMPK1	21	cytidine/uridine monophosphate kinase 1	protein_codin	0.02754	0.828	1	42.23	0.08218
**TXNDC12**	TXNDC12	21	thioredoxin domain containing 12	protein_codin	0.1917	0.106	1	9.235	0.112
**C1orf123**	C1orf123	21	CXXC motif containing zinc binding protein	protein_codin	0.0529	0.637	1	18.42	0.1768
**IFI44**	IFI44	21	interferon induced protein 44	protein_codin	-0.3684	0.0365	1	38.08	0.07992
**ADGRL2**	ADGRL2	21	adhesion G protein-coupled receptor L2	protein_codin	0.6814	0.00188	1	34.35	0.2817
**DPH5**	DPH5	21	diphthamide biosynthesis 5	protein_codin	0.01127	0.968	1	2.827	0.1722
**GSTM3**	GSTM3	21	glutathione S-transferase mu 3	protein_codin	-0.2096	0.358	1	9.227	0.02751
**CSF1**	CSF1	21	colony stimulating factor 1	protein_codin	0.8111	0.0054	1	52.58	0.2956
**SLC16A1**	SLC16A1	21	solute carrier family 16 member 1	protein_codin	0.1026	0.727	1	3.308	0.2261
**METTL13**	METTL13	21	eEF1A lysine and N-terminal methyltransferase	protein_codin	0.6505	0.056	1	2.673	0.1439
**XPR1**	XPR1	21	xenotropic and polytropic retrovirus receptor 1	protein_codin	-0.2232	0.455	1	2.972	0.309
**TMEM9**	TMEM9	21	transmembrane protein 9	protein_codin	-0.1754	0.334	1	7.568	0.1381
**SLC41A1**	SLC41A1	21	solute carrier family 41 member 1	protein_codin	-0.2062	0.512	1	9.332	0.3352
**CD46**	CD46	21	CD46 molecule	protein_codin	-0.2855	0.0126	1	33.63	0.0264
**FAM89A**	FAM89A	21	family with sequence similarity 89 member A	protein_codin	-0.09569	0.627	1	33.07	0.08114
**HEATR1**	HEATR1	21	HEAT repeat containing 1	protein_codin	0.3066	0.378	1	8.433	0.2849
**SNTG2**	SNTG2	21	syntrophin gamma 2	protein_codin	1.249	0.00682	1	3.582	0.1842
**RAB10**	RAB10	21	RAB10, member RAS oncogene family	protein_codin	-0.02063	0.875	1	41.37	0.1157
**PPM1B**	PPM1B	21	protein phosphatase, Mg2+/Mn2+ dependent 1B	protein_codin	-0.2181	0.413	1	9.454	0.107
**ACYP2**	ACYP2	21	acylphosphatase 2	protein_codin	0.4277	0.11	1	3.315	0.0269
**MTIF2**	MTIF2	21	mitochondrial translational initiation factor 2	protein_codin	-0.2574	0.269	1	26.73	0.08696
**SNRNP27**	SNRNP27	21	small nuclear ribonucleoprotein U4/U6.U5 subunit 27	protein_codin	-0.0248	0.892	1	7.282	0.1116
**BOLA3**	BOLA3	21	bolA family member 3	protein_codin	0.6309	0.00117	1	7.119	0.02679
**SUCLG1**	SUCLG1	21	succinate-CoA ligase alpha subunit	protein_codin	-0.01068	0.941	1	34.73	0.1687
**SULT1C4**	SULT1C4	21	sulfotransferase family 1C member 4	protein_codin	-0.2072	0.414	1	8.512	0.1972
**LIMS1**	LIMS1	21	LIM zinc finger domain containing 1	protein_codin	0.3949	4.46E-05	1	51.83	0.05726
**ACTR3**	ACTR3	21	actin related protein 3	protein_codin	0.08384	0.479	1	14.41	0.02621
**ERCC3**	ERCC3	21	ERCC excision repair 3, TFIIH core complex helicase subunit	protein_codin	0.6222	0.0532	1	3.344	0.197
**MBD5**	MBD5	21	methyl-CpG binding domain protein 5	protein_codin	0.08097	0.748	1	7.023	0.1652
**RBM43**	RBM43	21	RNA binding motif protein 43	protein_codin	0.4839	0.0369	1	7.244	0.1711
**DYNC1I2**	DYNC1I2	21	dynein cytoplasmic 1 intermediate chain 2	protein_codin	0.1629	0.185	1	52.5	0.05479
**SCRN3**	SCRN3	21	secernin 3	protein_codin	-0.06509	0.828	1	3.49	0.1682
**NBEAL1**	NBEAL1	21	neurobeachin like 1	protein_codin	-0.3042	0.086	1	7.337	0.02734
**FASTKD2**	FASTKD2	21	FAST kinase domains 2	protein_codin	0.6941	0.0162	1	3.404	0.1396
**TNS1**	TNS1	21	tensin 1	protein_codin	-0.03709	0.879	1	8.486	0.1729
**SH3BP4**	SH3BP4	21	SH3 domain binding protein 4	protein_codin	-0.4452	0.0352	1	23.55	0.2236
**ZDHHC3**	ZDHHC3	21	zinc finger DHHC-type containing 3	protein_codin	-0.1594	0.373	1	7.08	0.05397
**NDUFAF3**	NDUFAF3	21	NADH ubiquinone oxidoreductase complex assembly factor 3	protein_codin	-0.1302	0.152	1	40.66	0.114
**ABHD6**	ABHD6	21	abhydrolase domain containing 6	protein_codin	0.7254	0.0105	1	8.238	0.2822
**TRMT10C**	TRMT10C	21	tRNA methyltransferase 10C, mitochondrial RNase P subunit	protein_codin	0.4866	0.00281	1	6.258	0.1081
**ACAD9**	ACAD9	21	acyl-CoA dehydrogenase family member 9	protein_codin	0.2436	0.385	1	2.774	0.2575
**RBP1**	RBP1	21	retinol binding protein 1	protein_codin	-1.52	1.92E-17	4.61E-13	34.14	0.08377
**RNF7**	RNF7	21	ring finger protein 7	protein_codin	-0.295	0.000586	1	50.36	0.02762
**RAPGEF2**	RAPGEF2	21	Rap guanine nucleotide exchange factor 2	protein_codin	-0.06879	0.794	1	31.2	0.4586
**TLL1**	TLL1	21	tolloid like 1	protein_codin	2.024	2.55E-07	0.005882	22.62	0.167
**DDX60**	DDX60	21	DExD/H-box helicase 60	protein_codin	0.2482	0.342	1	20.72	0.1697
**SLC25A4**	SLC25A4	21	solute carrier family 25 member 4	protein_codin	-0.7536	6.67E-06	0.155	15.75	0.1681
**MTMR12**	MTMR12	21	myotubularin related protein 12	protein_codin	0.2649	0.366	1	7.312	0.3892
**PTGER4**	PTGER4	21	prostaglandin E receptor 4	protein_codin	1.131	0.00692	1	24.98	0.2279
**SMIM15**	SMIM15	21	small integral membrane protein 15	protein_codin	0.1517	0.466	1	8.255	0.2262
**ZSWIM6**	ZSWIM6	21	zinc finger SWIM-type containing 6	protein_codin	0.1769	0.558	1	49.09	0.2039
**HMGCR**	HMGCR	21	3-hydroxy-3-methylglutaryl-CoA reductase	protein_codin	0.9774	0.00328	1	35.08	0.2348
**COL4A3BP**	COL4A3BP	21	ceramide transporter 1	protein_codin	0.4625	0.00526	1	21.13	0.1368
**UBE2D2**	UBE2D2	21	ubiquitin conjugating enzyme E2 D2	protein_codin	0.1626	0.0625	1	17.28	0.05488
**PCDH1**	PCDH1	21	protocadherin 1	protein_codin	-0.2429	0.26	1	22.55	0.05526
**PRELID2**	PRELID2	21	PRELI domain containing 2	protein_codin	0.5289	0.114	1	3.422	0.217
**RNF145**	RNF145	21	ring finger protein 145	protein_codin	-0.2036	0.403	1	51.32	0.09388
**PSMG4**	PSMG4	21	proteasome assembly chaperone 4	protein_codin	0.2314	0.182	1	14.48	0.1392
**CDYL**	CDYL	21	chromodomain Y like	protein_codin	0.6335	0.0166	1	8.438	0.1406
**TRIM38**	TRIM38	21	tripartite motif containing 38	protein_codin	-0.001452	0.994	1	33.65	0.2479
**TRIM27**	TRIM27	21	tripartite motif containing 27	protein_codin	0.1042	0.646	1	6.508	0.1686
**STK38**	STK38	21	serine/threonine kinase 38	protein_codin	0.1588	0.593	1	2.925	0.266
**SLC29A1**	SLC29A1	21	solute carrier family 29 member 1 (Augustine blood group)	protein_codin	0.4256	0.00829	1	49.62	0.1372
**SNX14**	SNX14	21	sorting nexin 14	protein_codin	-0.1992	0.409	1	7.623	0.1126
**RPF2**	RPF2	21	ribosome production factor 2 homolog	protein_codin	0.3801	0.035	1	33.25	0.08361
**TRMT11**	TRMT11	21	tRNA methyltransferase 11 homolog	protein_codin	-0.09615	0.695	1	7.207	0.1948
**AIG1**	AIG1	21	androgen induced 1	protein_codin	-0.1622	0.218	1	40.97	0.08702
**STX11**	STX11	21	syntaxin 11	protein_codin	0.6316	0.0722	1	10.52	0.02707
**MPC1**	MPC1	21	mitochondrial pyruvate carrier 1	protein_codin	-0.3596	0.017	1	15.07	0.1952
**C6orf120**	C6orf120	21	chromosome 6 open reading frame 120	protein_codin	0.4152	0.169	1	8.15	0.2036
**NT5C3A**	NT5C3A	21	5'-nucleotidase, cytosolic IIIA	protein_codin	0.2706	0.15	1	43.77	0.1435
**BAZ1B**	BAZ1B	21	bromodomain adjacent to zinc finger domain 1B	protein_codin	0.3451	0.116	1	16.62	0.2358
**DNAJC30**	DNAJC30	21	DnaJ heat shock protein family (Hsp40) member C30	protein_codin	0.1499	0.565	1	7.932	0.1632
**AKAP9**	AKAP9	21	A-kinase anchoring protein 9	protein_codin	-0.03692	0.756	1	51.92	0.1404
**FIS1**	FIS1	21	fission, mitochondrial 1	protein_codin	-0.1046	0.0949	1	50.73	0.05418
**TMEM168**	TMEM168	21	transmembrane protein 168	protein_codin	0.332	0.262	1	9.812	0.2303
**SMIM30**	SMIM30	21	small integral membrane protein 30	protein_codin	-0.3696	0.0319	1	7.878	0.194
**AKAP17A**	AKAP17A	21	A-kinase anchoring protein 17A	protein_codin	0.7016	0.00397	1	7.53	0.1395
**UBA1**	UBA1	21	ubiquitin like modifier activating enzyme 1	protein_codin	-0.1364	0.672	1	3.479	0.3232
**UXT**	UXT	21	ubiquitously expressed prefoldin like chaperone	protein_codin	0.06617	0.418	1	47.64	0.06005
**NONO**	NONO	21	non-POU domain containing octamer binding	protein_codin	-0.2019	0.158	1	13.28	0.229
**ATG4A**	ATG4A	21	autophagy related 4A cysteine peptidase	protein_codin	0.1772	0.522	1	9.629	0.08811
**LAMP2**	LAMP2	21	lysosomal associated membrane protein 2	protein_codin	-0.5502	7.37E-05	1	13.71	0.02692
**LDOC1**	LDOC1	21	LDOC1 regulator of NFKB signaling	protein_codin	-0.3471	0.121	1	8.385	0.1452
**TDRP**	TDRP	21	testis development related protein	protein_codin	0.5026	0.174	1	2.936	0.3802
**VPS37A**	VPS37A	21	VPS37A subunit of ESCRT-I	protein_codin	-0.2101	0.379	1	25.55	0.1482
**TNFRSF10A**	TNFRSF10A	21	TNF receptor superfamily member 10a	protein_codin	1.034	0.000182	1	9.088	0.1994
**LEPROTL1**	LEPROTL1	21	leptin receptor overlapping transcript like 1	protein_codin	-0.08599	0.617	1	7.332	0.1686
**KAT6A**	KAT6A	21	lysine acetyltransferase 6A	protein_codin	0.5037	0.0147	1	18.35	0.166
**LY96**	LY96	21	lymphocyte antigen 96	protein_codin	0.4847	0.00051	1	34.07	0.126
**ZNF704**	ZNF704	21	zinc finger protein 704	protein_codin	-0.03057	0.913	1	7.962	0.2237
**OTUD6B-AS1**	OTUD6B-A	21	OTUD6B antisense RNA 1 (head to head)	antisense_RN	0.006796	0.962	1	42.96	0.02846
**FAM92A**	FAM92A	21	family with sequence similarity 92 member A	protein_codin	-0.5202	0.0169	1	9.249	0.05246
**STK3**	STK3	21	serine/threonine kinase 3	protein_codin	0.02901	0.896	1	8.33	0.2249
**SPAG1**	SPAG1	21	sperm associated antigen 1	protein_codin	1.229	0.00044	1	10.14	0.2822
**RAD21**	RAD21	21	RAD21 cohesin complex component	protein_codin	0.109	0.387	1	37.17	0.1467
**CYC1**	CYC1	21	cytochrome c1	protein_codin	-0.07966	0.488	1	19.62	0.07988
**CDC37L1**	CDC37L1	21	cell division cycle 37 like 1	protein_codin	0.1836	0.508	1	8.794	0.2367
**NDUFB6**	NDUFB6	21	NADH ubiquinone oxidoreductase subunit B6	protein_codin	-0.02562	0.826	1	20.42	0.1706
**B4GALT1**	B4GALT1	21	beta-1,4-galactosyltransferase 1	protein_codin	-0.2981	0.0481	1	43.09	0.03127
**FAM122A**	FAM122A	21	family with sequence similarity 122A	protein_codin	0.5562	0.0519	1	7.971	0.1112
**CARNMT1**	CARNMT1	21	carnosine N-methyltransferase 1	protein_codin	0.7396	0.0325	1	10.82	0.1335
**TLE4**	TLE4	21	TLE family member 4, transcriptional corepressor	protein_codin	0.09526	0.588	1	46.9	0.2352

**PDCL**	PDCL	21	phosducin like	protein_codin	0.0424	0.78	1	14.49	0.05468
**RC3H2**	RC3H2	21	ring finger and CCCH-type domains 2	protein_codin	-0.00303	0.992	1	3.474	0.1969
**RABGAP1**	RABGAP1	21	RAB GTPase activating protein 1	protein_codin	0.3404	0.125	1	24.3	0.05851
**TSC1**	TSC1	21	TSC complex subunit 1	protein_codin	0.7068	0.0442	1	11.38	0.1495
**MAN1B1**	MAN1B1	21	mannosidase alpha class 1B member 1	protein_codin	0.3792	0.14	1	3.402	0.1975
**DPP7**	DPP7	21	dipeptidyl peptidase 7	protein_codin	-0.8417	1.22E-17	2.93E-13	51.88	0.1922
**PSMD13**	PSMD13	21	proteasome 26S subunit, non-ATPase 13	protein_codin	-0.1214	0.357	1	41.72	0.05706
**DCHS1**	DCHS1	21	dachsous cadherin-related 1	protein_codin	-0.7379	0.00107	1	9.012	0.2516
**EIF4G2**	EIF4G2	21	eukaryotic translation initiation factor 4 gamma 2	protein_codin	-0.2269	0.0081	1	42.92	0.03087
**ARL14EP**	ARL14EP	21	ADP ribosylation factor like GTPase 14 effector protein	protein_codin	0.7424	0.000724	1	16.78	0.218
**SLC35C1**	SLC35C1	21	solute carrier family 35 member C1	protein_codin	0.5687	0.0765	1	9.006	0.2609
**METTL12**	METTL12	21	citrate synthase lysine methyltransferase	protein_codin	NA	NA	NA	9.124	0.2691
**SCYL1**	SCYL1	21	SCY1 like pseudokinase 1	protein_codin	0.1701	0.525	1	3.396	0.3372
**NADSYN1**	NADSYN1	21	NAD synthetase 1	protein_codin	-0.385	0.17	1	3.042	0.12
**RNF169**	RNF169	21	ring finger protein 169	protein_codin	0.1244	0.606	1	3.29	0.3138
**UBE4A**	UBE4A	21	ubiquitination factor E4A	protein_codin	0.1154	0.63	1	8.76	0.2539
**AKR1C3**	AKR1C3	21	aldo-keto reductase family 1 member C3	protein_codin	0.1195	0.222	1	51.57	0.05497
**FAM171A1**	FAM171A1	21	family with sequence similarity 171 member A1	protein_codin	0.4445	0.0992	1	8.802	0.1848
**OTUD1**	OTUD1	21	OTU deubiquitinase 1	protein_codin	0.5495	0.0742	1	52.58	0.6004
**NRBF2**	NRBF2	21	nuclear receptor binding factor 2	protein_codin	0.8575	9.7E-06	0.2252	14.14	0.08222
**MICU1**	MICU1	21	mitochondrial calcium uptake 1	protein_codin	-0.00895	0.968	1	3.348	0.1098
**LRMDA**	LRMDA	21	leucine rich melanocyte differentiation associated	protein_codin	-0.1698	0.277	1	12.45	0.08044
**MCMBP**	MCMBP	21	minichromosome maintenance complex binding protein	protein_codin	0.1961	0.402	1	3.37	0.08472
**ZNF384**	ZNF384	21	zinc finger protein 384	protein_codin	0.2048	0.505	1	2.676	0.2679
**C12orf57**	C12orf57	21	chromosome 12 open reading frame 57	protein_codin	-0.8403	8.46E-22	2.04E-17	46.01	0.0606
**SLC48A1**	SLC48A1	21	solute carrier family 48 member 1	protein_codin	0.3532	0.0718	1	7.264	0.09115
**DDX23**	DDX23	21	DEAD-box helicase 23	protein_codin	0.3106	0.28	1	3.608	0.2446
**TMBIM6**	TMBIM6	21	transmembrane BAX inhibitor motif containing 6	protein_codin	-0.002678	0.967	1	47.92	0.08007
**RARG**	RARG	21	retinoic acid receptor gamma	protein_codin	-0.5791	0.0445	1	12.28	0.2936
**RBMS2**	RBMS2	21	RNA binding motif single stranded interacting protein 2	protein_codin	0.2312	0.257	1	6.648	0.2329
**SRGAP1**	SRGAP1	21	SLIT-ROBO Rho GTPase activating protein 1	protein_codin	0.1366	0.682	1	7.756	0.1391
**MDM2**	MDM2	21	MDM2 proto-oncogene	protein_codin	-0.1522	0.522	1	19.06	0.1638
**ACTR6**	ACTR6	21	actin related protein 6	protein_codin	-0.1909	0.29	1	39.65	0.07970
**HECTD4**	HECTD4	21	HECT domain E3 ubiquitin protein ligase 4	protein_codin	-0.3687	0.324	1	2.443	0.3817
**C12orf65**	C12orf65	21	chromosome 12 open reading frame 65	protein_codin	0.1404	0.53	1	7.52	0.1934
**ZNF84**	ZNF84	21	zinc finger protein 84	protein_codin	0.1318	0.61	1	8.454	0.1616
**NUP58**	NUP58	21	nucleoporin 58	protein_codin	-0.1305	0.625	1	3.125	0.2982
**GTF2F2**	GTF2F2	21	general transcription factor IIF subunit 2	protein_codin	-0.06234	0.772	1	19.02	0.05601
**TGDS**	TGDS	21	TDP-glucose 4,6-dehydratase	protein_codin	0.2309	0.445	1	3.412	0.2332
**MCF2L**	MCF2L	21	MCF.2 cell line derived transforming sequence like	protein_codin	1.641	0.00046	1	32.18	0.3844
**DHRS4-AS1**	DHRS4-AS	21	DHRS4 antisense RNA 1	antisense_RN	0.08654	0.758	1	2.977	0.2231
**CTAGE5**	CTAGE5	21	MIA SH3 domain ER export factor 2	protein_codin	NA	NA	NA	23.02	0.1125
**NEMF**	NEMF	21	nuclear export mediator factor	protein_codin	-0.001936	0.989	1	25.14	0.1099
**PPM1A**	PPM1A	21	protein phosphatase, Mg2+/Mn2+ dependent 1A	protein_codin	-0.3079	0.21	1	8.375	0.1412
**PCNX1**	PCNX1	21	pecanex 1	protein_codin	-0.3157	0.23	1	9.452	0.2182
**PSEN1**	PSEN1	21	presenilin 1	protein_codin	0.08962	0.723	1	3.232	0.05971
**ABCD4**	ABCD4	21	ATP binding cassette subfamily D member 4	protein_codin	0.7082	0.000496	1	35.96	0.2132
**EMC7**	EMC7	21	ER membrane protein complex subunit 7	protein_codin	-0.2574	0.0292	1	30.15	0.03052
**NOP10**	NOP10	21	NOP10 ribonucleoprotein	protein_codin	0.02832	0.7188	1	26.03	0.02966
**AQR**	AQR	21	aquarius intron-binding spliceosomal factor	protein_codin	0.206	0.418	1	8.947	0.08170
**SPRED1**	SPRED1	21	sprouty related EVH1 domain containing 1	protein_codin	0.6189	0.0442	1	10.43	0.2016
**RMDN3**	RMDN3	21	regulator of microtubule dynamics 3	protein_codin	0.2415	0.345	1	7.394	0.1089
**ZNF280D**	ZNF280D	21	zinc finger protein 280D	protein_codin	-0.1196	0.642	1	9.412	0.1632
**TIPIN**	TIPIN	21	TIMELESS interacting protein	protein_codin	0.4845	0.166	1	3.255	0.2042
**TLNRD1**	TLNRD1	21	talin rod domain containing 1	protein_codin	-0.05016	0.787	1	30.2	0.05290
**CPPED1**	CPPED1	21	calcineurin like phosphoesterase domain containing 1	protein_codin	-0.2944	0.235	1	48.38	0.08287
**PARN**	PARN	21	poly(A)-specific ribonuclease	protein_codin	0.3508	0.228	1	3.288	0.1925
**KIF22**	KIF22	21	kinesin family member 22	protein_codin	0.8291	0.000993	1	20.74	0.1181
**MAZ**	MAZ	21	MYC associated zinc finger protein	protein_codin	-0.00667	0.967	1	16.54	0.1695
**CYB5B**	CYB5B	21	cytochrome b5 type B	protein_codin	0.581	0.000432	1	15.38	0.1409
**DDX19B**	DDX19B	21	DEAD-box helicase 19B	protein_codin	0.3358	0.257	1	8.927	0.2036
**EMC8**	EMC8	21	ER membrane protein complex subunit 8	protein_codin	0.1372	0.556	1	6.8	0.1135
**TLCD2**	TLCD2	21	TLC domain containing 2	protein_codin	-0.38	0.144	1	9.708	0.05564
**LRRC75A**	LRRC75A	21	leucine rich repeat containing 75A	protein_codin	0.06988	0.666	1	27.44	0.05956
**MAP2K3**	MAP2K3	21	mitogen-activated protein kinase kinase 3	protein_codin	-0.0992	0.652	1	8.179	0.1988
**NF1**	NF1	21	neurofibromin 1	protein_codin	-0.664	0.00256	1	8.69	0.05536
**NLE1**	NLE1	21	notchless homolog 1	protein_codin	0.968	0.00931	1	2.424	0.1931
**SPAG9**	SPAG9	21	sperm associated antigen 9	protein_codin	-0.09336	0.312	1	45.88	0.1107
**TUBD1**	TUBD1	21	tubulin delta 1	protein_codin	0.7492	0.0270	1	3.173	0.405
**PRKAR1A**	PRKAR1A	21	protein kinase cAMP-dependent type I regulatory subunit alpha	protein_codin	0.1461	0.287	1	11.85	0.02882
**MXRA7**	MXRA7	21	matrix remodeling associated 7	protein_codin	0.6429	5.57E-05	1	22.96	0.1099
**CHMP6**	CHMP6	21	charged multivesicular body protein 6	protein_codin	0.05971	0.787	1	6.68	0.2222
**DUS1L**	DUS1L	21	dihydrouridine synthase 1 like	protein_codin	0.5747	0.0152	1	6.345	0.1122
**CSNK1D**	CSNK1D	21	casein kinase 1 delta	protein_codin	-0.1345	0.462	1	19.11	0.02996
**VAPA**	VAPA	21	VAMP associated protein A	protein_codin	-0.1239	0.106	1	38.37	0.0262
**FAM210A**	FAM210A	21	family with sequence similarity 210 member A	protein_codin	0.2797	0.294	1	7.347	0.08467
**SMAD4**	SMAD4	21	SMAD family member 4	protein_codin	-0.08842	0.74	1	9.035	0.3516
**ZNF532**	ZNF532	21	zinc finger protein 532	protein_codin	0.1964	0.542	1	2.952	0.4862
**TBC1D20**	TBC1D20	21	TBC1 domain family member 20	protein_codin	0.5592	0.0614	1	3.566	0.2229
**C20orf194**	C20orf194	21	chromosome 20 open reading frame 194	protein_codin	0.3396	0.312	1	3.152	0.2272
**TM9SF4**	TM9SF4	21	transmembrane 9 superfamily member 4	protein_codin	0.002999	0.992	1	8.409	0.1982
**AHCY**	AHCY	21	adenosylhomocysteinase	protein_codin	0.2518	0.258	1	46.4	0.1129
**STK11**	STK11	21	serine/threonine kinase 11	protein_codin	-0.1872	0.486	1	3.416	0.1916
**ABHD17A**	ABHD17A	21	abhydrolase domain containing 17A	protein_codin	-0.2147	0.0516	1	47.22	0.0575
**AES**	AES	21	TLE family member 5, transcriptional modulator	protein_codin	-0.6162	4.24E-14	1.01E-09	52.48	0.05432
**GNA11**	GNA11	21	G protein subunit alpha 11	protein_codin	-0.2052	0.357	1	8.114	0.1387
**HMG20B**	HMG20B	21	high mobility group 20B	protein_codin	0.3142	0.0614	1	12.19	0.1122
**PIP5K1C**	PIP5K1C	21	phosphatidylinositol-4-phosphate 5-kinase type 1 gamma	protein_codin	-0.2108	0.358	1	7.56	0.08898
**CCDC94**	CCDC94	21	YJU2 splicing factor homolog	protein_codin	NA	NA	NA	3.115	0.1357
**LRG1**	LRG1	21	leucine rich alpha-2-glycoprotein 1	protein_codin	2.274	3.17E-06	0.07380	52.58	0.2042
**NDUFA11**	NDUFA11	21	NADH ubiquinone oxidoreductase subunit A11	protein_codin	-0.2524	0.0046	1	30.54	0.08279
**MCOLN1**	MCOLN1	21	mucolipin 1	protein_codin	0.256	0.397	1	3.307	0.3392
**PPAN**	PPAN	21	peter pan homolog	protein_codin	0.1894	0.544	1	2.632	0.2875
**TECR**	TECR	21	trans-2,3-enoyl-CoA reductase	protein_codin	0.1469	0.242	1	10.87	0.1391
**ZNF708**	ZNF708	21	zinc finger protein 708	protein_codin	0.05937	0.825	1	31.88	0.1732
**MED29**	MED29	21	mediator complex subunit 29	protein_codin	0.3405	0.115	1	20.73	0.2232
**SMG9**	SMG9	21	SMG9 nonsense mediated mRNA decay factor	protein_codin	0.3981	0.234	1	2.82	0.3421
**ZNF226**	ZNF226	21	zinc finger protein 226	protein_codin	-0.2484	0.314	1	9.003	0.3407
**TOMM40**	TOMM40	21	translocase of outer mitochondrial membrane 40	protein_codin	0.5504	0.00866	1	3.215	0.05634
**ERCC1**	ERCC1	21	ERCC excision repair 1, endonuclease non-catalytic subunit	protein_codin	-0.3442	0.0597	1	18.49	0.08525
**NOP53**	NOP53	21	NOP53 ribosome biogenesis factor	protein_codin	0.4592	1.77E-10	4.2E-06	52.58	0.02820
**PLA2G4C**	PLA2G4C	21	phospholipase A2 group IVC	protein_codin	-0.5754	0.0214	1	8.914	0.119
**PNKP**	PNKP	21	polynucleotide kinase 3'-phosphatase	protein_codin	-0.423	0.0442	1	28.27	0.1104
**CTU1**	CTU1	21	cytosolic thiouridylase subunit 1	protein_codin	-0.04512	0.89	1	2.848	0.3906
**ZNF880**	ZNF880	21	zinc finger protein 880	protein_codin	0.5861	0.0401	1	7.329	0.3667
**CNOT3**	CNOT3	21	CCR4-NOT transcription complex subunit 3	protein_codin	0.2706	0.417	1	3.317	0.1419
**LENG1**	LENG1	21	leukocyte receptor cluster member 1	protein_codin	0.1166	0.526	1	7.128	0.1712
**ZNF581**	ZNF581	21	zinc finger protein 581	protein_codin	0.2877	0.237	1	7.615	0.1418
**U2AF2**	U2AF2	21	U2 small nuclear RNA auxiliary factor 2	protein_codin	1.025	0.000405	1	3.409	0.08841
**AC003002.1**	AC003002.1	21	trafficking protein particle complex 2B	protein_codin	NA	NA	NA	16.32	0.1109
**ATP6V1E1**	ATP6V1E1	21	ATPase H+ transporting V1 subunit E1	protein_codin	-0.1127	0.427	1	25.65	0.02714
**PITPNB**	PITPNB	21	phosphatidylinositol transfer protein beta	protein_codin	0.1407	0.311	1	28.23	0.08129
**IFT27**	IFT27	21	intraflagellar transport 27	protein_codin	0.07303	0.737	1	6.464	0.2321
**SAMM50**	SAMM50	21	SAMM50 sorting and assembly machinery component	protein_codin	0.3908	0.0456	1	7.976	0.242
**ZBTB21**	ZBTB21	21	zinc finger and BTB domain containing 21	protein_codin	0.06267	0.832	1	9.077	0.1354
**VPS13D**	VPS13D	22	vacuolar protein sorting 13 homolog D	protein_codin	-0.2577	0.368	1	9.132	0.2457
**PRDM2**	PRDM2	22	PR/SET domain 2	protein_codin	-0.04818	0.842	1	8.288	0.2780
**MINOS1**	MINOS1	22	mitochondrial contact site and cristae organizing system subu	protein_codin	0.03682	0.749	1	51.97	0.1395
**TMEM54**	TMEM54	22	transmembrane protein 54	protein_codin	-0.3416	0.0536	1	52.58	0.1446
**TM2D1**	TM2D1	22	TM2 domain containing 1	protein_codin	-0.3408	0.00871	1	26.45	0.0541
**GADD45A**	GADD45A	22	growth arrest and DNA damage inducible alpha	protein_codin	0.3166	0.0492	1	52.58	0.05582
**ZRANB2**	ZRANB2	22	zinc finger RANBP2-type containing 2	protein_codin	0.1054	0.445	1	38.14	0.1682
**ILF2**	ILF2	22	interleukin enhancer binding factor 2	protein_codin	-0.005306	0.954	1	22.02	0.05621
**INTS3**	INTS3	22	integrator complex subunit 3	protein_codin	0.4728	0.102	1	7.542	0.1621
**JTB**	JTB	22	jumping translocation breakpoint	protein_codin	-0.3122	0.000996	1	50.82	0.1702
**TSEN15**	TSEN15	22	tRNA splicing endonuclease subunit 15	protein_codin	0.0817	0.755	1	7.823	0.1987
**SRP9**	SRP9	22	signal recognition particle 9	protein_codin	0.1084	0.223	1	17.93	0.142
**RHOU**	RHOU	22	ras homolog family member U	protein_codin	1.752	6.28E-12	1.5E-07	26.74	0.05325
**COX7A2L**	COX7A2L	22	cytochrome c oxidase subunit 7A2 like	protein_codin	0.3396	0.000222	1	32.75	0.02702
**C2orf49**	C2orf49	22	chromosome 2 open reading frame 49	protein_codin	0.462	0.0314	1	7.342	0.1672
**SP3**	SP3	22	Sp3 transcription factor	protein_codin	0.5188	0.0481	1	21.29	0.1712
**CIR1**	CIR1	22	corepressor interacting with RBPJ, 1	protein_codin	0.4007	0.00216	1	26.83	0.2424
**HSPE1**	HSPE1	22	heat shock protein family E (Hsp10) member 1	protein_codin	-0.06147	0.472	1	52.02	0.05681
**INO80D**	INO80D	22	INO80 complex subunit D	protein_codin	-0.2086	0.38	1	6.112	0.2009
**DNPEP**	DNPEP	22	aspartyl aminopeptidase	protein_codin	0.3371	0.0677	1	6.714	0.1451
**RPUSD3**	RPUSD3	22	RNA pseudouridine synthase D3	protein_codin	-0.04689	0.862	1	2.984	0.1094
**ATG7**	ATG7	22	autophagy related 7	protein_codin	-0.2229	0.436	1	9.983	0.2264
**SMARCC1**	SMARCC1	22	SWI/SNF related, matrix associated, actin dependent regulato	protein_codin	0.1126	0.632	1	8.837	0.05684
**PSMD6**	PSMD6	22	proteasome 26S subunit, non-ATPase 6	protein_codin	0.09052	0.547	1	34.11	0.1442
**SMC4**	SMC4	22	structural maintenance of chromosomes 4	protein_codin	-0.4071	0.0192	1	14.16	0.05438
**LINC00969**	LINC00969	22	MUC20 overlapping transcript	lincRNA	NA	NA	NA	6.81	0.2087
**SENP5**	SENP5	22	SUMO specific peptidase 5	protein_codin	0.1984	0.399	1	6.547	0.1966
**NAAA**	NAAA	22	N-acylethanolamine acid amidase	protein_codin	0.4771	0.0670	1	8.211	0.1987
**SCARB2**	SCARB2	22	scavenger receptor class B member 2	protein_codin	-0.1757	0.157	1	23.88	0.02981
**SCLT1**	SCLT1	22	sodium channel and clathrin linker 1	protein_codin	0.3832	0.187	1	3.552	0.0839
**CCDC127**	CCDC127	22	coiled-coil domain containing 127	protein_codin	0.02036	0.935	1	9.449	0.2248
**SLC30A5**	SLC30A5	22	solute carrier family 30 member 5	protein_codin	-0.423	0.0919	1	3.074	0.21
**ZCCHC10**	ZCCHC10	22	zinc finger CCHC-type containing 10	protein_codin	0.09328	0.628	1	7.41	0.05568
**FLT4**	FLT4	22	fms related tyrosine kinase 4	protein_codin	0.8522	0.08	1	2.876	0.3452
**TMEM14C**	TMEM14C	22	transmembrane protein 14C	protein_codin	0.1804	0.018	1	50.06	0.1357
**HMGN3**	HMGN3	22	high mobility group nucleosomal binding domain 3	protein_codin	0.04783	0.5459	1	52.07	0.05631
**ZBTB24**	ZBTB24	22	zinc finger and BTB domain containing 24	protein_codin	0.2558	0.414	1	8.212	0.2321
**NUS1**	NUS1	22	NUS1 dehydrodolichyl diphosphate synthase subunit	protein_codin	0.6039	0.0752	1	8.863	0.2004
**SHPRH**	SHPRH	22	SNF2 histone linker PHD RING helicase	protein_codin	0.07412	0.747	1	22.9	0.1402
**SNX9**	SNX9	22	sorting nexin 9	protein_codin	0.1096	0.482	1	34.02	0.1427
**RNF216**	RNF216	22	ring finger protein 216	protein_codin	0.0515	0.825	1	3.306	0.2571
**RAPGEF5**	RAPGEF5	22	Rap guanine nucleotide exchange factor 5	protein_codin	-0.04056	0.776	1	51.33	0.05331
**CYCS**	CYCS	22	cytochrome c, somatic	protein_codin	0.2807	0.0138	1	51.85	0.0838
**FKBP9**	FKBP9	22	FKBP prolyl isomerase 9	protein_codin	0.6368	9.17E-05	1	49.02	0.1075
**COA1**	COA1	22	cytochrome c oxidase assembly factor 1 homolog	protein_codin	0.3688	0.12	1	26.86	0.1382
**ZNF92**	ZNF92	22	zinc finger protein 92	protein_codin	0.4346	0.132	1	10.95	0.2212
**RFC2**	RFC2	22	replication factor C subunit 2	protein_codin	0.4825	0.0764	1	3.343	0.1114
**HGF**	HGF	22	hepatocyte growth factor	protein_codin	3.692	0.9717	1	48.06	0.2021
**IFT22**	IFT22	22	intraflagellar transport 22	protein_codin	-0.2051	0.279	1	26.28	0.05702
**ARMC10**	ARMC10	22	armadillo repeat containing 10	protein_codin	-0.04478	0.808	1	7.194	0.05952
**AR**	AR	22	androgen receptor	protein_codin	-1.548	9.18E-10	2.18E-05	51.05	0.222
**C1GALT1C1**	C1GALT1C	22	C1GALT1 specific chaperone 1	protein_codin	0.1966	0.304	1	7.582	0.08075
**SMIM10**	SMIM10	22	small integral membrane protein 10	protein_codin	0.9181	6.62E-05	1	15.52	0.1729
**F8**	F8	22	coagulation factor VIII	protein_codin	1.834	3.83E-09	9.06E-05	30.58	0.08266
**MFHAS1**	MFHAS1	22	malignant fibrous histiocytoma amplified sequence 1	protein_codin	0.2838	0.317	1	8.606	0.1829
**LSM1.00**	LSM1.00	22	LSM1 homolog, mRNA degradation associated	protein_codin	-0.3122	0.0457	1	17.41	0.05582
**KIAA2026**	KIAA2026	22	KIAA2026	protein_codin	-0.005518	0.982	1	17.23	0.3397
**IL33**	IL33	22	interleukin 33	protein_codin	0.4471	1.22E-05	0.2828	51.56	0.05406
**C9orf72**	C9orf72	22	C9orf72-SMCR8 complex subunit	protein_codin	0.5812	0.105	1	2.617	0.1696
**SMC5**	SMC5	22	structural maintenance of chromosomes 5	protein_codin	0.1901	0.315	1	20.46	0.1335
**SLC44A1**	SLC44A1	22	solute carrier family 44 member 1	protein_codin	0.02911	0.899	1	39.37	0.1362
**CRAT**	CRAT	22	carnitine O-acetyltransferase	protein_codin	0.1109	0.698	1	3.163	0.1612
**GPR107**	GPR107	22	G protein-coupled receptor 107	protein_codin	-0.4542	0.11	1	3.388	0.2308
**ACP2**	ACP2	22	acid phosphatase 2, lysosomal	protein_codin	0.422	0.167	1	7.69	0.2031
**TIMM10**	TIMM10	22	translocase of inner mitochondrial membrane 10	protein_codin	0.3459	0.0130	1	20.82	0.1350
**RAD9A**	RAD9A	22	RAD9 checkpoint clamp component A	protein_codin	0.6159	0.0379	1	2.888	0.2561
**FAM76B**	FAM76B	22	family with sequence similarity 76 member B	protein_codin	0.4838	0.0442	1	23.7	0.2021
**STT3A**	STT3A	22	STT3 oligosaccharyltransferase complex catalytic subunit A	protein_codin	0.07324	0.772	1	3.546	0.1126
**FAM118B**	FAM118B	22	family with sequence similarity 118 member B	protein_codin	0.517	0.138	1	3.182	0.289
**IFIT5**	IFIT5	22	interferon induced protein with tetratricopeptide repeats 5	protein_codin	0.6334	0.0309	1	3.282	0.1386
**PCGF5**	PCGF5	22	polycomb group ring finger 5	protein_codin	-0.5319	0.0029	1	21.17	0.1154
**TM9SF3**	TM9SF3	22	transmembrane 9 superfamily member 3	protein_codin	-0.3928	0.000533	1	43.02	0.05238
**SMC3**	SMC3	22	structural maintenance of chromosomes 3	protein_codin	0.07907	0.54	1	24.22	0.1762
**INPP5F**	INPP5F	22	inositol polyphosphate-5-phosphatase F	protein_codin	0.08775	0.786	1	3.822	0.1126
**CHST15**	CHST15	22	carbohydrate sulfotransferase 15	protein_codin	1.048	0.00517	1	31.33	0.3195
**NINJ2**	NINJ2	22	ninjurin 2	protein_codin	1.057	0.00501	1	26.43	0.2879
**RBP5**	RBP5	22	retinol binding protein 5	protein_codin	0.9254	0.00382	1	18.02	0.2582
**YARS2**	YARS2	22	tyrosyl-tRNA synthetase 2	protein_codin	0.07458	0.782	1	9.026	0.1975
**LARP4**	LARP4	22	La ribonucleoprotein domain family member 4	protein_codin	0.3577	0.0906	1	20.9	0.2242
**DDX55**	DDX55	22	DEAD-box helicase 55	protein_codin	0.8697	0.0471	1	3.522	0.1462
**EIF2B1**	EIF2B1	22	eukaryotic translation initiation factor 2B subunit alpha	protein_codin	0.5026	0.0136	1	5.172	0.1772
**STARD13**	STARD13	22	StAR related lipid transfer domain containing 13	protein_codin	0.8217	0.0196	1	3.015	0.2770
**LHFPL6**	LHFPL6	22	LHFPL tetraspan subfamily member 6	protein_codin	0.5442	1.54E-05	0.3567	24.65	0.1377
**TMEM55B**	TMEM55B	22	phosphatidylinositol-4,5-bisphosphate 4-phosphatase 1	protein_codin	NA	NA	NA	7.532	0.02859
**IFT43**	IFT43	22	intraflagellar transport 43	protein_codin	0.2742	0.0989	1	34.06	0.1341
**AHSA1**	AHSA1	22	activator of HSP90 ATPase activity 1	protein_codin	0.02455	0.882	1	7.037	0.1372
**ZC3H14**	ZC3H14	22	zinc finger CCCH-type containing 14	protein_codin	0.2271	0.276	1	7.478	0.1711
**SYNE3**	SYNE3	22	spectrin repeat containing nuclear envelope family member 3	protein_codin	0.3312	0.241	1	8.264	0.1177
**C14orf132**	C14orf132	22	chromosome 14 open reading frame 132	protein_codin	0.1285	0.715	1	9.47	0.2824
**CCNK**	CCNK	22	cyclin K	protein_codin	0.1558	0.499	1	7.102	0.1774
**ZNF106**	ZNF106	22	zinc finger protein 106	protein_codin	-0.08734	0.518	1	22.09	0.08259
**PIAS1**	PIAS1	22	protein inhibitor of activated STAT 1	protein_codin	0.1916	0.47	1	6.416	0.2254
**ISLR**	ISLR	22	immunoglobulin superfamily containing leucine rich repeat	protein_codin	1.179	0.00442	1	28.28	0.3719
**CRTC3**	CRTC3	22	CREB regulated transcription coactivator 3	protein_codin	0.1712	0.532	1	7.862	0.2929
**CHSY1**	CHSY1	22	chondroitin sulfate synthase 1	protein_codin	-0.63	0.000124	1	42.55	0.1396
**MRPL28**	MRPL28	22	mitochondrial ribosomal protein L28	protein_codin	-0.163	0.272	1	15.47	0.1397
**NME3**	NME3	22	NME/NM23 nucleoside diphosphate kinase 3	protein_codin	-0.01762	0.852	1	18.02	0.0568
**NUPR1**	NUPR1	22	nuclear protein 1, transcriptional regulator	protein_codin	-0.4170	0.00011	1	52.47	0.05671
**ZNF688**	ZNF688	22	zinc finger protein 688	protein_codin	0.5026	0.0511	1	6.733	0.2348
**DERL2**	DERL2	22	derlin 2	protein_codin	0.07517	0.674	1	6.638	0.08942
**TRAF4**	TRAF4	22	TNF receptor associated factor 4	protein_codin	0.05505	0.838	1	10.78	0.1141
**LASP1**	LASP1	22	LIM and SH3 protein 1	protein_codin	-0.4516	0.0405	1	23.5	0.118
**COIL**	COIL	22	coilin	protein_codin	0.2254	0.327	1	8.409	0.2592
**AC091271.1**	AC091271.1	22	novel transcri	lincRNA	0.5825	0.0767	1	3.722	0.1995
**ACOX1**	ACOX1	22	acyl-CoA oxidase 1	protein_codin	-0.2060	0.508	1	8.148	0.1117
**TEN1**	TEN1	22	TEN1 subunit of CST complex	protein_codin	-0.7642	0.000563	1	7.581	0.02671
**TIMP2**	TIMP2	22	TIMP metallopeptidase inhibitor 2	protein_codin	-0.8397	3.89E-16	9.33E-12	46.79	0.02650
**RALBP1**	RALBP1	22	ralA binding protein 1	protein_codin	-0.05297	0.628	1	30.16	0.1489
**SIRPA**	SIRPA	22	signal regulatory protein alpha	protein_codin	0.4981	0.0571	1	3.317	0.2225
**TOMM34**	TOMM34	22	translocase of outer mitochondrial membrane 34	protein_codin	0.09117	0.762	1	3.196	0.1176
**SNAI1**	SNAI1	22	snail family transcriptional repressor 1	protein_codin	0.5069	0.0199	1	40.4	0.06144
**FAM217B**	FAM217B	22	family with sequence similarity 217 member B	protein_codin	0.05275	0.864	1	7.506	0.09242
**ARFRP1**	ARFRP1	22	ADP ribosylation factor related protein 1	protein_codin	0.2106	0.252	1	7.084	0.1547
**SLC39A3**	SLC39A3	22	solute carrier family 39 member 3	protein_codin	0.1208	0.597	1	3.186	0.1401
**TNFAIP8L1**	TNFAIP8L1	22	TNF alpha induced protein 8 like 1	protein_codin	-0.05854	0.75	1	31.19	0.1747
**SLC44A2**	SLC44A2	22	solute carrier family 44 member 2	protein_codin	0.0735	0.676	1	15.52	0.08849
**SAMD1**	SAMD1	22	sterile alpha motif domain containing 1	protein_codin	-0.1664	0.492	1	6.707	0.1989
**ZNF431**	ZNF431	22	zinc finger protein 431	protein_codin	0.1421	0.628	1	3.357	0.3122
**PLEKHF1**	PLEKHF1	22	pleckstrin homology and FYVE domain containing 1	protein_codin	0.2125	0.492	1	10.86	0.109
**RBM42**	RBM42	22	RNA binding motif protein 42	protein_codin	0.4679	0.00225	1	14.83	0.02767
**DEDD2**	DEDD2	22	death effector domain containing 2	protein_codin	0.1336	0.648	1	2.877	0.362
**CIC**	CIC	22	capicua transcriptional repressor	protein_codin	0.4362	0.128	1	3.33	0.1391
**RDH13**	RDH13	22	retinol dehydrogenase 13	protein_codin	0.5674	0.067	1	9.557	0.3909
**SLC35E4**	SLC35E4	22	solute carrier family 35 member E4	protein_codin	-0.3896	0.137	1	3.398	0.1082
**EIF4ENIF1**	EIF4ENIF1	22	eukaryotic translation initiation factor 4E nuclear import factor 1	protein_codin	0.4177	0.193	1	2.862	0.1751
**APOL2**	APOL2	22	apolipoprotein L2	protein_codin	0.4347	0.0661	1	37.2	0.1722
**TOB2**	TOB2	22	transducer of ERBB2, 2	protein_codin	-0.1872	0.382	1	7.743	0.02801
**TYMP**	TYMP	22	thymidine phosphorylase	protein_codin	1.272	5.08E-11	1.21E-06	43.72	0.09128
**MTHFR**	MTHFR	23	methylenetetrahydrofolate reductase	protein_codin	0.1466	0.706	1	8.555	0.129
**KDM1A**	KDM1A	23	lysine demethylase 1A	protein_codin	0.3275	0.193	1	6.164	0.149
**LYPLA2**	LYPLA2	23	lysophospholipase 2	protein_codin	0.4644	0.0185	1	21.76	0.0867
**PPT1**	PPT1	23	palmitoyl-protein thioesterase 1	protein_codin	0.1902	0.272	1	17.82	0.245
**MRPL37**	MRPL37	23	mitochondrial ribosomal protein L37	protein_codin	0.1977	0.276	1	15.96	0.1182
**KYAT3**	KYAT3	23	kynurenine aminotransferase 3	protein_codin	-0.05844	0.86	1	3.084	0.2791
**DNTTIP2**	DNTTIP2	23	deoxynucleotidyltransferase terminal interacting protein 2	protein_codin	0.2917	0.0217	1	9.042	0.1144
**FLAD1**	FLAD1	23	flavin adenine dinucleotide synthetase 1	protein_codin	0.01246	0.97	1	2.729	0.2361
**POGK**	POGK	23	pogo transposable element derived with KRAB domain	protein_codin	-0.729	0.0188	1	3.167	0.2476
**FAM20B**	FAM20B	23	FAM20B glycosaminoglycan xylosylkinase	protein_codin	-0.2411	0.38	1	8.967	0.2290
**IVNS1ABP**	IVNS1ABP	23	influenza virus NS1A binding protein	protein_codin	-0.2577	0.202	1	20.15	0.1365
**SERTAD4-AS1**	SERTAD4-A	23	SERTAD4 antisense RNA 1	antisense_RN	0.1945	0.198	1	10.96	0.1985
**AL390728.6**	AL390728.6	23	novel transcri	lincRNA	0.1571	0.656	1	3.308	0.2892
**TPO**	TPO	23	thyroid peroxidase	protein_codin	2.452	6E-05	1	9.937	0.2799
**CPSF3**	CPSF3	23	cleavage and polyadenylation specific factor 3	protein_codin	0.2676	0.417	1	3.222	0.1172
**HADHB**	HADHB	23	hydroxyacyl-CoA dehydrogenase trifunctional multienzyme co	protein_codin	0.5985	1.17E-05	0.2712	19.66	0.08875
**SOCS5**	SOCS5	23	suppressor of cytokine signaling 5	protein_codin	0.1808	0.576	1	9.622	0.2764
**TEX261**	TEX261	23	testis expressed 261	protein_codin	-0.2956	0.296	1	3.088	0.02814
**PTCD3**	PTCD3	23	pentatricopeptide repeat domain 3	protein_codin	0.1756	0.522	1	3.312	0.3955
**MRPS5**	MRPS5	23	mitochondrial ribosomal protein S5	protein_codin	0.127	0.432	1	14.19	0.1772
**LIPT1**	LIPT1	23	lipoyltransferase 1	protein_codin	0.8769	0.00596	1	3.568	0.1498
**MRPL30**	MRPL30	23	mitochondrial ribosomal protein L30	protein_codin	0.3287	0.252	1	2.708	0.2552
**PTPN18**	PTPN18	23	protein tyrosine phosphatase non-receptor type 18	protein_codin	0.7292	0.00521	1	36.46	0.2447
**ARL5A**	ARL5A	23	ADP ribosylation factor like GTPase 5A	protein_codin	-1.202	2.08E-05	0.4811	2.747	0.1741
**CD302**	CD302	23	CD302 molecule	protein_codin	0.415	0.0782	1	7.892	0.08266
**MAP3K20**	MAP3K20	23	mitogen-activated protein kinase kinase kinase 20	protein_codin	0.08504	0.752	1	9.492	0.1682
**OLA1**	OLA1	23	Obg like ATPase 1	protein_codin	0.5144	0.000534	1	10.9	0.08758
**MTX2**	MTX2	23	metaxin 2	protein_codin	0.3148	0.0747	1	7.022	0.05537
**GULP1**	GULP1	23	GULP PTB domain containing engulfment adaptor 1	protein_codin	0.3052	0.213	1	7.005	0.1675
**NAB1**	NAB1	23	NGFI-A binding protein 1	protein_codin	0.332	0.282	1	6.667	0.1981
**ABI2**	ABI2	23	abl interactor 2	protein_codin	-0.1092	0.582	1	8.666	0.1364
**RPE**	RPE	23	ribulose-5-phosphate-3-epimerase	protein_codin	-0.4195	0.0868	1	8.276	0.1477
**WDFY1**	WDFY1	23	WD repeat and FYVE domain containing 1	protein_codin	-0.05002	0.829	1	6.582	0.1976
**INPP5D**	INPP5D	23	inositol polyphosphate-5-phosphatase D	protein_codin	-0.6101	0.111	1	3.202	0.2756
**BOK**	BOK	23	BCL2 family apoptosis regulator BOK	protein_codin	-0.0565	0.834	1	3.48	0.2342
**UBP1**	UBP1	23	upstream binding protein 1	protein_codin	0.0057	0.984	1	3.357	0.1112
**SETD2**	SETD2	23	SET domain containing 2, histone lysine methyltransferase	protein_codin	0.05817	0.765	1	7.234	0.08432
**LAMB2**	LAMB2	23	laminin subunit beta 2	protein_codin	0.4666	0.029	1	14.74	0.2512
**ALAS1**	ALAS1	23	5'-aminolevulinate synthase 1	protein_codin	-0.0564	0.828	1	7.39	0.08358
**SMIM4**	SMIM4	23	small integral membrane protein 4	protein_codin	-0.1067	0.575	1	19.42	0.1352
**TKT**	TKT	23	transketolase	protein_codin	-0.5138	3.06E-06	0.07131	52.58	0.1395
**CGGBP1**	CGGBP1	23	CGG triplet repeat binding protein 1	protein_codin	0.1754	0.237	1	13.11	0.2431
**CCDC14**	CCDC14	23	coiled-coil domain containing 14	protein_codin	0.2738	0.326	1	6.468	0.162
**YEATS2**	YEATS2	23	YEATS domain containing 2	protein_codin	-0.1396	0.736	1	3.002	0.258
**ALG3**	ALG3	23	ALG3 alpha-1,3- mannosyltransferase	protein_codin	0.4659	0.0489	1	7.747	0.1407
**MRFAP1L1**	MRFAP1L1	23	Morf4 family associated protein 1 like 1	protein_codin	0.456	0.102	1	3.499	0.07898
**ANAPC4**	ANAPC4	23	anaphase promoting complex subunit 4	protein_codin	0.8592	0.0432	1	3.029	0.1629
**NOA1**	NOA1	23	nitric oxide associated 1	protein_codin	0.115	0.596	1	27.76	0.08606
**RUFY3**	RUFY3	23	RUN and FYVE domain containing 3	protein_codin	0.5969	0.0489	1	7.967	0.2535
**AREG**	AREG	23	amphiregulin	protein_codin	1.112	0.195	1	52.58	0.9742
**CCNG2**	CCNG2	23	cyclin G2	protein_codin	0.7248	0.00145	1	16.54	0.1199
**THAP9-AS1**	THAP9-AS1	23	THAP9 antisense RNA 1	antisense_RN	0.2094	0.325	1	18.47	0.1526
**SLC10A6**	SLC10A6	23	solute carrier family 10 member 6	protein_codin	1.501	0.0101	1	3.413	0.2056
**USP38**	USP38	23	ubiquitin specific peptidase 38	protein_codin	-0.1252	0.673	1	2.955	0.08178
**ETFDH**	ETFDH	23	electron transfer flavoprotein dehydrogenase	protein_codin	0.3626	0.262	1	8.847	0.1457
**PPID**	PPID	23	peptidylprolyl isomerase D	protein_codin	0.9379	0.000587	1	20.01	0.2928
**KLHL2**	KLHL2	23	kelch like family member 2	protein_codin	0.5747	0.0812	1	2.4	0.1464
**GALNT7**	GALNT7	23	polypeptide N-acetylgalactosaminyltransferase 7	protein_codin	-0.06821	0.805	1	8.309	0.05731
**CLPTM1L**	CLPTM1L	23	CLPTM1 like	protein_codin	-0.2295	0.256	1	7.307	0.1367
**PRKAA1**	PRKAA1	23	protein kinase AMP-activated catalytic subunit alpha 1	protein_codin	0.4392	0.0564	1	24.62	0.1925
**MAP3K1**	MAP3K1	23	mitogen-activated protein kinase kinase kinase 1	protein_codin	1.585	2.79E-06	0.06504	7.782	0.2827
**MRPS27**	MRPS27	23	mitochondrial ribosomal protein S27	protein_codin	-0.03772	0.862	1	8.035	0.1375
**LHFPL2**	LHFPL2	23	LHFPL tetraspan subfamily member 2	protein_codin	0.7409	0.045	1	11.92	0.2016
**ARRDC3**	ARRDC3	23	arrestin domain containing 3	protein_codin	0.194	0.32	1	48	0.1246
**CHD1**	CHD1	23	chromodomain helicase DNA binding protein 1	protein_codin	0.5156	0.000367	1	48.2	0.1098
**YTHDC2**	YTHDC2	23	YTH domain containing 2	protein_codin	0.1301	0.542	1	6.558	0.1816
**CDO1**	CDO1	23	cysteine dioxygenase type 1	protein_codin	-0.5192	0.0397	1	31.66	0.1674
**DPYSL3**	DPYSL3	23	dihydropyrimidinase like 3	protein_codin	0.2852	0.152	1	52.58	0.02752
**TNIP1**	TNIP1	23	TNFAIP3 interacting protein 1	protein_codin	-0.05764	0.795	1	8.434	0.1156
**ATOX1**	ATOX1	23	antioxidant 1 copper chaperone	protein_codin	-0.272	0.00341	1	50.74	0.08505
**RPP40**	RPP40	23	ribonuclease P/MRP subunit p40	protein_codin	0.6879	0.0529	1	8.927	0.2769
**SIRT5**	SIRT5	23	sirtuin 5	protein_codin	0.4982	0.0822	1	7.966	0.1141
**CDKAL1**	CDKAL1	23	CDK5 regulatory subunit associated protein 1 like 1	protein_codin	0.2639	0.379	1	3.042	0.1671
**HIST1H4C**	HIST1H4C	23	histone cluster 1 H4 family member c	protein_codin	0.1754	0.127	1	21.52	0.1128

**ABCF1**	ABCF1	23	ATP binding cassette subfamily F member 1	protein_codin	0.2311	0.219	1	13.43	0.08118
**DAXX**	DAXX	23	death domain associated protein	protein_codin	0.1507	0.499	1	8.592	0.2034
**KIAA1586**	KIAA1586	23	KIAA1586	protein_codin	0.4682	0.0555	1	3.44	0.1721
**ZNF451**	ZNF451	23	zinc finger protein 451	protein_codin	0.219	0.403	1	7.942	0.2214
**SMIM8**	SMIM8	23	small integral membrane protein 8	protein_codin	0.4022	0.14	1	8.188	0.2147
**UBE2J1**	UBE2J1	23	ubiquitin conjugating enzyme E2 J1	protein_codin	-0.04505	0.757	1	41.62	0.1674
**CCNC**	CCNC	23	cyclin C	protein_codin	-0.04336	0.808	1	19.22	0.1398
**CEP57L1**	CEP57L1	23	centrosomal protein 57 like 1	protein_codin	-0.06011	0.811	1	3.764	0.2572
**FAM229B**	FAM229B	23	family with sequence similarity 229 member B	protein_codin	-0.5967	0.00219	1	25.75	0.1154
**KPNA5**	KPNA5	23	karyopherin subunit alpha 5	protein_codin	0.6837	0.0592	1	8.943	0.275
**AKAP7**	AKAP7	23	A-kinase anchoring protein 7	protein_codin	0.2489	0.429	1	8.767	0.141
**LTV1**	LTV1	23	LTV1 ribosome biogenesis factor	protein_codin	0.5506	0.0202	1	6.995	0.1462
**PPIL4**	PPIL4	23	peptidylprolyl isomerase like 4	protein_codin	-0.08521	0.544	1	11.25	0.0832
**MRPL18**	MRPL18	23	mitochondrial ribosomal protein L18	protein_codin	-0.03525	0.787	1	49.38	0.1099
**ZNRF2**	ZNRF2	23	zinc and ring finger 2	protein_codin	0.4642	0.147	1	2.826	0.1701
**CCT6A**	CCT6A	23	chaperonin containing TCP1 subunit 6A	protein_codin	0.1037	0.262	1	46.88	0.02874
**GTPBP10**	GTPBP10	23	GTP binding protein 10	protein_codin	0.7932	0.0349	1	2.925	0.1674
**PEX1**	PEX1	23	peroxisomal biogenesis factor 1	protein_codin	0.09205	0.736	1	7.173	0.2594
**PDAP1**	PDAP1	23	PDGFA associated protein 1	protein_codin	-0.148	0.148	1	14.3	0.08598
**UBN2**	UBN2	23	ubinuclein 2	protein_codin	0.6695	0.0315	1	7.419	0.1962
**PDHA1**	PDHA1	23	pyruvate dehydrogenase E1 alpha 1 subunit	protein_codin	0.1515	0.432	1	23.72	0.1442
**SH3KBP1**	SH3KBP1	23	SH3 domain containing kinase binding protein 1	protein_codin	-0.5896	0.00996	1	9.503	0.1729
**ZFX**	ZFX	23	zinc finger protein X-linked	protein_codin	0.1882	0.398	1	6.49	0.1611
**MAGT1**	MAGT1	23	magnesium transporter 1	protein_codin	-0.3049	0.0841	1	14.6	0.05497
**SMARCA1**	SMARCA1	23	SWI/SNF related, matrix associated, actin dependent regulato	protein_codin	0.2831	0.286	1	43.78	0.08002
**RTL8C**	RTL8C	23	retrotransposon Gag like 8C	protein_codin	0.05114	0.688	1	11.54	0.1990
**MAP7D3**	MAP7D3	23	MAP7 domain containing 3	protein_codin	0.07938	0.723	1	40.68	0.2489
**PINX1**	PINX1	23	PIN2 (TERF1) interacting telomerase inhibitor 1	protein_codin	0.1959	0.525	1	3.429	0.3454
**PPP2R2A**	PPP2R2A	23	protein phosphatase 2 regulatory subunit Balpha	protein_codin	0.2104	0.103	1	33.58	0.1731
**BNIP3L**	BNIP3L	23	BCL2 interacting protein 3 like	protein_codin	-0.9595	1.26E-18	3.03E-14	26.11	0.05632
**SCARA3**	SCARA3	23	scavenger receptor class A member 3	protein_codin	-0.1042	0.65	1	9.022	0.1635
**LYPLA1**	LYPLA1	23	lysophospholipase 1	protein_codin	-0.07518	0.574	1	12.66	0.08817
**ASPH**	ASPH	23	aspartate beta-hydroxylase	protein_codin	0.5057	2.4E-05	0.5548	46.06	0.1124
**CTHRC1**	CTHRC1	23	collagen triple helix repeat containing 1	protein_codin	0.1189	0.452	1	34.27	0.05708
**EIF3H**	EIF3H	23	eukaryotic translation initiation factor 3 subunit H	protein_codin	0.1641	0.0231	1	38.82	0.03112
**MED30**	MED30	23	mediator complex subunit 30	protein_codin	0.1513	0.37	1	6.596	0.09719
**MRPL13**	MRPL13	23	mitochondrial ribosomal protein L13	protein_codin	-0.0003482	0.998	1	11.57	0.1635
**COMMD5**	COMMD5	23	COMM domain containing 5	protein_codin	-0.04134	0.852	1	8.379	0.0570
**HAUS6**	HAUS6	23	HAUS augmin like complex subunit 6	protein_codin	0.3462	0.267	1	8.508	0.1789
**TESK1**	TESK1	23	testis associated actin remodelling kinase 1	protein_codin	-0.01504	0.958	1	2.627	0.1622
**C9orf40**	C9orf40	23	chromosome 9 open reading frame 40	protein_codin	1.326	3.83E-05	0.8834	24	0.1732
**HABP4**	HABP4	23	hyaluronan binding protein 4	protein_codin	-0.03549	0.858	1	22.35	0.05411
**DAB2IP**	DAB2IP	23	DAB2 interacting protein	protein_codin	0.5212	0.194	1	8.234	0.2265
**RBM18**	RBM18	23	RNA binding motif protein 18	protein_codin	0.346	0.138	1	50.98	0.1640
**SH2D3C**	SH2D3C	23	SH2 domain containing 3C	protein_codin	0.616	0.006	1	20.68	0.09179
**SEC16A**	SEC16A	23	SEC16 homolog A, endoplasmic reticulum export factor	protein_codin	-0.02636	0.93	1	2.86	0.1384
**PNPLA2**	PNPLA2	23	patatin like phospholipase domain containing 2	protein_codin	0.7712	7.5E-07	0.01750	42.86	0.2239
**KCNQ1OT1**	KCNQ1OT1	23	KCNQ1 opposite strand/antisense transcript 1	antisense_RN	-0.5046	0.265	1	36.72	0.3675
**KBTBD4**	KBTBD4	23	kelch repeat and BTB domain containing 4	protein_codin	0.2367	0.436	1	2.56	0.1952
**MTCH2**	MTCH2	23	mitochondrial carrier 2	protein_codin	-0.09195	0.653	1	22.32	0.08376
**CAPN1**	CAPN1	23	calpain 1	protein_codin	0.05153	0.828	1	25.48	0.1442
**LRP5**	LRP5	23	LDL receptor related protein 5	protein_codin	1.146	7.28E-05	1	23.62	0.1662
**PPP6R3**	PPP6R3	23	protein phosphatase 6 regulatory subunit 3	protein_codin	-0.166	0.452	1	7.596	0.08308
**FCHSD2**	FCHSD2	23	FCH and double SH3 domains 2	protein_codin	-0.09368	0.655	1	19.53	0.1709
**ALG8**	ALG8	23	ALG8 alpha-1,3-glucosyltransferase	protein_codin	0.1686	0.595	1	3.394	0.282
**DPAGT1**	DPAGT1	23	dolichyl-phosphate N-acetylglucosaminephosphotransferase	protein_codin	0.2493	0.352	1	2.945	0.1072
**MINDY3**	MINDY3	23	MINDY lysine 48 deubiquitinase 3	protein_codin	0.491	0.0836	1	3.094	0.1987
**NSUN6**	NSUN6	23	NOP2/Sun RNA methyltransferase 6	protein_codin	0.548	0.0389	1	52.58	0.1632
**ZNF33A**	ZNF33A	23	zinc finger protein 33A	protein_codin	0.2442	0.272	1	51.88	0.1745
**C10orf128**	C10orf128	23	transmembrane protein 273	protein_codin	NA	NA	NA	32.1	0.03078
**TBC1D12**	TBC1D12	23	TBC1 domain family member 12	protein_codin	0.08532	0.748	1	7.064	0.119
**ENTPD1**	ENTPD1	23	ectonucleoside triphosphate diphosphohydrolase 1	protein_codin	0.8918	1.17E-07	0.00279	41.98	0.08324
**SLC25A28**	SLC25A28	23	solute carrier family 25 member 28	protein_codin	0.8314	0.000288	1	17.19	0.2874
**COX15**	COX15	23	cytochrome c oxidase assembly homolog COX15	protein_codin	0.4642	0.145	1	8.375	0.1979
**BTRC**	BTRC	23	beta-transducin repeat containing E3 ubiquitin protein ligase	protein_codin	0.2002	0.543	1	3.492	0.08177
**BBIP1**	BBIP1	23	BBSome interacting protein 1	protein_codin	0.1538	0.476	1	8.685	0.2545
**TCF7L2**	TCF7L2	23	transcription factor 7 like 2	protein_codin	0.01939	0.924	1	19.44	0.1368
**CACUL1**	CACUL1	23	CDK2 associated cullin domain 1	protein_codin	-0.2793	0.192	1	7.758	0.109
**MGMT**	MGMT	23	O-6-methylguanine-DNA methyltransferase	protein_codin	-0.2067	0.0477	1	51.62	0.05468
**BNIP3**	BNIP3	23	BCL2 interacting protein 3	protein_codin	-0.03548	0.804	1	28.19	0.08152
**NDUFA9**	NDUFA9	23	NADH ubiquinone oxidoreductase subunit A9	protein_codin	0.1432	0.441	1	6.924	0.09228
**LRRC23**	LRRC23	23	leucine rich repeat containing 23	protein_codin	0.1795	0.544	1	10.46	0.3609
**WBP11**	WBP11	23	WW domain binding protein 11	protein_codin	0.3379	0.0465	1	14.02	0.1176
**ITPR2**	ITPR2	23	inositol 1,4,5-trisphosphate receptor type 2	protein_codin	-1.019	9.5E-15	2.27E-10	47.99	0.1388
**RAPGEF3**	RAPGEF3	23	Rap guanine nucleotide exchange factor 3	protein_codin	1.331	1.19E-07	0.002796	16.15	0.1585
**PFKM**	PFKM	23	phosphofructokinase, muscle	protein_codin	0.1115	0.714	1	3.168	0.2266
**TUBA1C**	TUBA1C	23	tubulin alpha 1c	protein_codin	-0.9881	9.05E-15	2.17E-10	52.58	0.03145
**TNS2**	TNS2	23	tensin 2	protein_codin	0.1124	0.5	1	12.26	0.05364
**AAAS**	AAAS	23	aladin WD repeat nucleoporin	protein_codin	0.5564	0.154	1	9.606	0.179
**SP1**	SP1	23	Sp1 transcription factor	protein_codin	0.1411	0.574	1	7.464	0.1948
**PCBP2**	PCBP2	23	poly(rC) binding protein 2	protein_codin	0.3482	1.59E-06	0.03712	32.22	0.08089
**STAT2**	STAT2	23	signal transducer and activator of transcription 2	protein_codin	0.1337	0.542	1	22.53	0.1922
**METTL25**	METTL25	23	methyltransferase like 25	protein_codin	-1.082	9.94E-06	0.2307	52.58	0.08761
**GLT8D2**	GLT8D2	23	glycosyltransferase 8 domain containing 2	protein_codin	-1.699	4.26E-13	1.02E-08	28.4	0.1182
**PTPN11**	PTPN11	23	protein tyrosine phosphatase non-receptor type 11	protein_codin	0.01001	0.949	1	14.6	0.1151
**ACADS**	ACADS	23	acyl-CoA dehydrogenase short chain	protein_codin	1.111	0.000706	1	3.287	0.2352
**CCDC122**	CCDC122	23	coiled-coil domain containing 122	protein_codin	0.6039	0.0656	1	9.39	0.1808
**GPALPP1**	GPALPP1	23	GPALPP motifs containing 1	protein_codin	0.02612	0.909	1	9.062	0.1947
**DIS3**	DIS3	23	DIS3 homolog, exosome endoribonuclease and 3'-5' exoribon	protein_codin	0.292	0.0995	1	52.54	0.1719
**FBXL3**	FBXL3	23	F-box and leucine rich repeat protein 3	protein_codin	0.3979	0.062	1	7.906	0.08452
**CHAMP1**	CHAMP1	23	chromosome alignment maintaining phosphoprotein 1	protein_codin	0.3182	0.304	1	9.046	0.3101
**EGLN3**	EGLN3	23	egl-9 family hypoxia inducible factor 3	protein_codin	1.848	6.36E-09	0.00015	52.58	0.1415
**EXOC5**	EXOC5	23	exocyst complex component 5	protein_codin	0.2715	0.175	1	7.342	0.1919
**DHRS7**	DHRS7	23	dehydrogenase/reductase 7	protein_codin	0.5994	3.36E-07	0.007870	21.34	0.1072
**ZBTB1**	ZBTB1	23	zinc finger and BTB domain containing 1	protein_codin	-0.02179	0.919	1	8.519	0.3049
**SETD3**	SETD3	23	SET domain containing 3, actin histidine methyltransferase	protein_codin	-0.08232	0.675	1	5.962	0.2016
**CHST14**	CHST14	23	carbohydrate sulfotransferase 14	protein_codin	-0.1081	0.699	1	2.682	0.3414
**MINDY2**	MINDY2	23	MINDY lysine 48 deubiquitinase 2	protein_codin	0.2156	0.271	1	15.06	0.0879
**HACD3**	HACD3	23	3-hydroxyacyl-CoA dehydratase 3	protein_codin	0.01942	0.892	1	26.32	0.08111
**DENND4A**	DENND4A	23	DENN domain containing 4A	protein_codin	0.1612	0.646	1	8.386	0.2604
**TM6SF1**	TM6SF1	23	transmembrane 6 superfamily member 1	protein_codin	-0.8535	0.000174	1	41.92	0.02656
**AC013394.1**	AC013394.1	23	novel prote	protein_codin	0.5079	0.14	1	2.948	0.2017
**NME4**	NME4	23	NME/NM23 nucleoside diphosphate kinase 4	protein_codin	-0.3715	0.009	1	15.45	0.1112
**PIGQ**	PIGQ	23	phosphatidylinositol glycan anchor biosynthesis class Q	protein_codin	-0.1176	0.692	1	3.377	0.2981
**PRR14**	PRR14	23	proline rich 14	protein_codin	0.2226	0.446	1	2.944	0.1087
**DNAJA2**	DNAJA2	23	DnaJ heat shock protein family (Hsp40) member A2	protein_codin	0.3514	0.0242	1	26.29	0.2294
**CRNDE**	CRNDE	23	colorectal neoplasia differentially expressed	lincRNA	-0.03562	0.866	1	17.86	0.1429
**NLRC5**	NLRC5	23	NLR family CARD domain containing 5	protein_codin	1.201	0.0167	1	9.332	0.3145
**CTCF**	CTCF	23	CCCTC-binding factor	protein_codin	0.2352	0.192	1	17.63	0.1521
**USP10**	USP10	23	ubiquitin specific peptidase 10	protein_codin	0.1717	0.485	1	6.835	0.2017
**NCBP3**	NCBP3	23	nuclear cap binding subunit 3	protein_codin	0.1734	0.338	1	19.49	0.08722
**EIF4A1**	EIF4A1	23	eukaryotic translation initiation factor 4A1	protein_codin	0.1879	0.0704	1	26.93	0.02638
**MPDU1**	MPDU1	23	mannose-P-dolichol utilization defect 1	protein_codin	0.07879	0.762	1	7.192	0.1077
**ARHGEF15**	ARHGEF15	23	Rho guanine nucleotide exchange factor 15	protein_codin	0.05054	0.845	1	24.58	0.1650
**TAF15**	TAF15	23	TATA-box binding protein associated factor 15	protein_codin	0.2508	0.123	1	12.33	0.0274
**DDX52**	DDX52	23	DExD-box helicase 52	protein_codin	0.0082	0.966	1	18.7	0.1371
**CSF3**	CSF3	23	colony stimulating factor 3	protein_codin	1.715	0.00190	1	52.58	0.2174
**RARA**	RARA	23	retinoic acid receptor alpha	protein_codin	0.2972	0.173	1	7.719	0.08551
**TMUB2**	TMUB2	23	transmembrane and ubiquitin like domain containing 2	protein_codin	0.02815	0.902	1	3.519	0.1687
**EFCAB13**	EFCAB13	23	EF-hand calcium binding domain 13	protein_codin	0.02607	0.928	1	3.19	0.3195
**ABI3**	ABI3	23	ABI family member 3	protein_codin	0.7487	1.03E-05	0.239	50.16	0.05426
**C17orf67**	C17orf67	23	chromosome 17 open reading frame 67	protein_codin	-0.1704	0.548	1	3.412	0.2493
**DGKE**	DGKE	23	diacylglycerol kinase epsilon	protein_codin	-0.6776	0.00161	1	9.093	0.258
**SKA2**	SKA2	23	spindle and kinetochore associated complex subunit 2	protein_codin	-0.1011	0.676	1	6.924	0.1692
**TLK2**	TLK2	23	tousled like kinase 2	protein_codin	0.5511	0.0315	1	7.922	0.1142
**ABCA5**	ABCA5	23	ATP binding cassette subfamily A member 5	protein_codin	0.9001	0.032	1	8.277	0.4570
**SPHK1**	SPHK1	23	sphingosine kinase 1	protein_codin	2.218	4.53E-17	1.09E-12	52.48	0.02941
**ENDOV**	ENDOV	23	endonuclease V	protein_codin	0.09838	0.722	1	3.43	0.1915
**HGS**	HGS	23	hepatocyte growth factor-regulated tyrosine kinase substrate	protein_codin	-0.124	0.674	1	2.952	0.2049
**OGFOD3**	OGFOD3	23	2-oxoglutarate and iron dependent oxygenase domain contai	protein_codin	0.1131	0.618	1	24.48	0.08372
**FOXK2**	FOXK2	23	forkhead box K2	protein_codin	0.6197	0.0399	1	3.345	0.162
**CXXC1**	CXXC1	23	CXXC finger protein 1	protein_codin	1.297	0.000626	1	7.762	0.08881
**KDSR**	KDSR	23	3-ketodihydrosphingosine reductase	protein_codin	0.2542	0.108	1	27.86	0.08244
**SDCBP2**	SDCBP2	23	syndecan binding protein 2	protein_codin	-0.2114	0.435	1	27.07	0.08225
**TMEM230**	TMEM230	23	transmembrane protein 230	protein_codin	-0.2371	0.00792	1	50.47	0.02606
**CRLS1**	CRLS1	23	cardiolipin synthase 1	protein_codin	-0.04579	0.834	1	3.368	0.1495
**MKKS**	MKKS	23	McKusick-Kaufman syndrome	protein_codin	0.5098	0.114	1	3.12	0.2082
**RPRD1B**	RPRD1B	23	regulation of nuclear pre-mRNA domain containing 1B	protein_codin	0.3752	0.234	1	9.042	0.1965
**ZNF217**	ZNF217	23	zinc finger protein 217	protein_codin	0.4585	0.0655	1	9.336	0.2740
**TCF3**	TCF3	23	transcription factor 3	protein_codin	0.1585	0.617	1	3.429	0.146
**TICAM1**	TICAM1	23	toll like receptor adaptor molecule 1	protein_codin	0.2105	0.51	1	2.796	0.1380
**SAFB**	SAFB	23	scaffold attachment factor B	protein_codin	0.3328	0.153	1	8.275	0.1931
**SNAPC2**	SNAPC2	23	small nuclear RNA activating complex polypeptide 2	protein_codin	0.3536	0.132	1	27.77	0.1982
**RAB11B-AS1**	RAB11B-AS	23	RAB11B antisense RNA 1	antisense_RN	-0.5826	0.0326	1	3.6	0.1941
**ZNF121**	ZNF121	23	zinc finger protein 121	protein_codin	0.5197	0.128	1	3.418	0.111
**UBL5**	UBL5	23	ubiquitin like 5	protein_codin	-0.4538	1.2E-11	2.86E-07	43.18	0.02928
**DDX39A**	DDX39A	23	DExD-box helicase 39A	protein_codin	0.09343	0.645	1	7.447	0.1421
**RAB8A**	RAB8A	23	RAB8A, member RAS oncogene family	protein_codin	0.2422	0.138	1	15.55	0.1072
**BABAM1**	BABAM1	23	BRISC and BRCA1 A complex member 1	protein_codin	-0.1586	0.297	1	15.92	0.1142
**ZNF430**	ZNF430	23	zinc finger protein 430	protein_codin	-0.4936	0.0606	1	8.397	0.3569
**ZNF493**	ZNF493	23	zinc finger protein 493	protein_codin	0.5209	0.182	1	8.63	0.2770
**LSM14A**	LSM14A	23	LSM14A mRNA processing body assembly factor	protein_codin	0.2869	0.0756	1	13.44	0.1065
**KIAA0355**	KIAA0355	23	KIAA0355	protein_codin	0.2268	0.186	1	10.25	0.2221
**GMFG**	GMFG	23	glia maturation factor gamma	protein_codin	0.9682	8.6E-16	2.06E-11	35.94	0.02608
**SERTAD3**	SERTAD3	23	SERTA domain containing 3	protein_codin	0.2968	0.046	1	25.84	0.02636
**SHKBP1**	SHKBP1	23	SH3KBP1 binding protein 1	protein_codin	0.1018	0.593	1	20.38	0.1452
**SNRPA**	SNRPA	23	small nuclear ribonucleoprotein polypeptide A	protein_codin	0.9192	2.44E-05	0.564	16.15	0.1935
**CLPTM1**	CLPTM1	23	CLPTM1 regulator of GABA type A receptor forward traffickin	protein_codin	-0.1605	0.444	1	25.82	0.08326
**PRKD2**	PRKD2	23	protein kinase D2	protein_codin	0.7952	0.00111	1	8.642	0.2472
**PRMT1**	PRMT1	23	protein arginine methyltransferase 1	protein_codin	0.006802	0.944	1	14.04	0.1792
**ZNF583**	ZNF583	23	zinc finger protein 583	protein_codin	0.7256	0.0474	1	10.42	0.08382
**DGCR2**	DGCR2	23	DiGeorge syndrome critical region gene 2	protein_codin	-0.09914	0.774	1	3.446	0.2801
**TBC1D10A**	TBC1D10A	23	TBC1 domain family member 10A	protein_codin	0.2	0.403	1	3.614	0.09198
**MPST**	MPST	23	mercaptopyruvate sulfurtransferase	protein_codin	-0.1874	0.226	1	14.53	0.08126
**TRIOBP**	TRIOBP	23	TRIO and F-actin binding protein	protein_codin	0.2206	0.11	1	50.97	0.08085
**GCAT**	GCAT	23	glycine C-acetyltransferase	protein_codin	0.02371	0.922	1	9.392	0.1957
**KDELR3**	KDELR3	23	KDEL endoplasmic reticulum protein retention receptor 3	protein_codin	-0.7787	0.000364	1	9.213	0.1727
**TOMM22**	TOMM22	23	translocase of outer mitochondrial membrane 22	protein_codin	0.3157	0.00942	1	21.06	0.08342
**TNRC6B**	TNRC6B	23	trinucleotide repeat containing adaptor 6B	protein_codin	0.2151	0.0832	1	27.52	0.1401
**A4GALT**	A4GALT	23	alpha 1,4-galactosyltransferase (P blood group)	protein_codin	0.8342	3.43E-06	0.07991	14.37	0.1656
**FAM207A**	FAM207A	23	family with sequence similarity 207 member A	protein_codin	0.818	0.00200	1	7.25	0.1479
**SLC25A33**	SLC25A33	24	solute carrier family 25 member 33	protein_codin	0.2231	0.358	1	10.12	0.1105
**FAM110D**	FAM110D	24	family with sequence similarity 110 member D	protein_codin	0.5164	0.000126	1	52.54	0.02912
**UTP11**	UTP11	24	UTP11 small subunit processome component	protein_codin	-0.143	0.343	1	12.54	0.1952
**PDE4B**	PDE4B	24	phosphodiesterase 4B	protein_codin	-1.9	1.13E-11	2.69E-07	25.24	0.1508
**ACADM**	ACADM	24	acyl-CoA dehydrogenase medium chain	protein_codin	-0.0229	0.892	1	35.32	0.2005
**MTF2**	MTF2	24	metal response element binding transcription factor 2	protein_codin	-0.04717	0.796	1	7.686	0.05567
**LRIF1**	LRIF1	24	ligand dependent nuclear receptor interacting factor 1	protein_codin	0.5336	0.0376	1	21.34	0.1642
**WDR3**	WDR3	24	WD repeat domain 3	protein_codin	0.692	0.0165	1	3.327	0.2657
**SRGAP2C**	SRGAP2C	24	SLIT-ROBO Rho GTPase activating protein 2C	protein_codin	-0.07622	0.787	1	11.15	0.1939
**PRPF3**	PRPF3	24	pre-mRNA processing factor 3	protein_codin	0.4346	0.0755	1	8.446	0.1399
**MTX1**	MTX1	24	metaxin 1	protein_codin	0.6051	0.0029	1	7.096	0.1968
**PEA15**	PEA15	24	proliferation and apoptosis adaptor protein 15	protein_codin	-0.02101	0.856	1	40.64	0.02626
**PEX19**	PEX19	24	peroxisomal biogenesis factor 19	protein_codin	0.03471	0.898	1	3.047	0.1534
**UAP1**	UAP1	24	UDP-N-acetylglucosamine pyrophosphorylase 1	protein_codin	0.03704	0.858	1	52.58	0.1103
**TMCO1**	TMCO1	24	transmembrane and coiled-coil domains 1	protein_codin	-0.2519	0.0112	1	51.94	0.1366
**DNM3OS**	DNM3OS	24	DNM3 opposite strand/antisense RNA	antisense_RN	0.7911	0.0156	1	10.62	0.4052
**2-Mar**	43526	24	mitochondrial amidoxime reducing component 2	protein_codin	0.3277	0.193	1	7.354	0.2445
**KIDINS220**	KIDINS220	24	kinase D interacting substrate 220	protein_codin	-0.01698	0.932	1	6.913	0.3392
**YWHAQ**	YWHAQ	24	tyrosine 3-monooxygenase/tryptophan 5-monooxygenase act	protein_codin	-0.1222	0.244	1	52.48	0.05697
**TMEM87B**	TMEM87B	24	transmembrane protein 87B	protein_codin	-1.064	5.68E-07	0.0132	9.696	0.1968
**BAZ2B**	BAZ2B	24	bromodomain adjacent to zinc finger domain 2B	protein_codin	0.5251	0.00111	1	17.88	0.1108
**NDUFB3**	NDUFB3	24	NADH ubiquinone oxidoreductase subunit B3	protein_codin	-0.1642	0.163	1	10.34	0.1711
**USP37**	USP37	24	ubiquitin specific peptidase 37	protein_codin	0.2125	0.498	1	3.205	0.2297
**GIGYF2**	GIGYF2	24	GRB10 interacting GYF protein 2	protein_codin	-0.2638	0.203	1	23.35	0.1424
**TRAF3IP1**	TRAF3IP1	24	TRAF3 interacting protein 1	protein_codin	0.5041	0.0879	1	10.78	0.2068
**CRBN**	CRBN	24	cereblon	protein_codin	0.3112	0.0056	1	51.98	0.1387
**MTMR14**	MTMR14	24	myotubularin related protein 14	protein_codin	0.8064	0.0137	1	7.945	0.1094
**XPC**	XPC	24	XPC complex subunit, DNA damage recognition and repair fa	protein_codin	0.2402	0.268	1	44.12	0.1134
**RBSN**	RBSN	24	rabenosyn, RAB effector	protein_codin	-0.1005	0.728	1	3.002	0.1420
**RAB5A**	RAB5A	24	RAB5A, member RAS oncogene family	protein_codin	-0.1087	0.477	1	25.77	0.08292
**NR1D2**	NR1D2	24	nuclear receptor subfamily 1 group D member 2	protein_codin	-0.2254	0.233	1	50.17	0.1962
**AZI2**	AZI2	24	5-azacytidine induced 2	protein_codin	-0.2292	0.254	1	23.66	0.08018
**IFRD2**	IFRD2	24	interferon related developmental regulator 2	protein_codin	-0.03049	0.864	1	7.233	0.2592
**PDHB**	PDHB	24	pyruvate dehydrogenase E1 beta subunit	protein_codin	0.1491	0.32	1	12.93	0.05621
**FAM107A**	FAM107A	24	family with sequence similarity 107 member A	protein_codin	-0.5155	0.000573	1	52.02	0.05248
**THOC7**	THOC7	24	THO complex 7	protein_codin	0.2567	0.0222	1	45.8	0.05520
**SEC22A**	SEC22A	24	SEC22 homolog A, vesicle trafficking protein	protein_codin	-0.04292	0.892	1	2.876	0.1072
**ARMC8**	ARMC8	24	armadillo repeat containing 8	protein_codin	0.1747	0.499	1	28.25	0.1366
**SERP1**	SERP1	24	stress associated endoplasmic reticulum protein 1	protein_codin	-0.04337	0.558	1	52.58	0.0582
**LPP**	LPP	24	LIM domain containing preferred translocation partner in lipom	protein_codin	-0.2059	0.118	1	44.47	0.05590
**UBXN7**	UBXN7	24	UBX domain protein 7	protein_codin	-0.0812	0.753	1	8.138	0.2309
**DLG1**	DLG1	24	discs large MAGUK scaffold protein 1	protein_codin	0.3497	0.166	1	28.52	0.2214
**ZNF721**	ZNF721	24	zinc finger protein 721	protein_codin	0.5908	0.0244	1	8.676	0.1232
**BST1**	BST1	24	bone marrow stromal cell antigen 1	protein_codin	0.6662	0.0447	1	2.819	0.1938
**DCAF16**	DCAF16	24	DDB1 and CUL4 associated factor 16	protein_codin	0.6077	0.038	1	2.985	0.1196
**LARP7**	LARP7	24	La ribonucleoprotein domain family member 7	protein_codin	0.1151	0.358	1	14.04	0.1239
**ANXA5**	ANXA5	24	annexin A5	protein_codin	-0.3368	3.84E-07	0.008999	45.35	0.02974
**NAA15**	NAA15	24	N(alpha)-acetyltransferase 15, NatA auxiliary subunit	protein_codin	0.6011	0.0181	1	24.87	0.1724
**BRD9**	BRD9	24	bromodomain containing 9	protein_codin	0.4308	0.118	1	7.018	0.08038
**OXCT1**	OXCT1	24	3-oxoacid CoA-transferase 1	protein_codin	0.145	0.626	1	10.09	0.2038
**MRPS36**	MRPS36	24	mitochondrial ribosomal protein S36	protein_codin	0.02438	0.862	1	27.27	0.02668
**PJA2**	PJA2	24	praja ring finger ubiquitin ligase 2	protein_codin	-0.1022	0.402	1	17.48	0.05414
**HCG18**	HCG18	24	HLA complex group 18	antisense_RN	0.8608	0.0286	1	8.037	0.05928
**LEMD2**	LEMD2	24	LEM domain containing 2	protein_codin	0.297	0.333	1	2.917	0.05582
**HMGA1**	HMGA1	24	high mobility group AT-hook 1	protein_codin	0.474	0.0844	1	7.503	0.1796
**MEA1**	MEA1	24	male-enhanced antigen 1	protein_codin	0.008881	0.948	1	32.45	0.1365
**ICK**	ICK	24	intestinal cell kinase	protein_codin	-0.1920	0.457	1	2.57	0.1370
**IRAK1BP1**	IRAK1BP1	24	interleukin 1 receptor associated kinase 1 binding protein 1	protein_codin	-0.1038	0.725	1	10.08	0.1102
**SEC63**	SEC63	24	SEC63 homolog, protein translocation regulator	protein_codin	-0.3928	0.0101	1	13.72	0.02902
**GTF3C6**	GTF3C6	24	general transcription factor IIIC subunit 6	protein_codin	0.1308	0.297	1	25.79	0.03003
**TPD52L1**	TPD52L1	24	TPD52 like 1	protein_codin	1.287	1.55E-18	3.73E-14	43.35	0.1182
**RNF146**	RNF146	24	ring finger protein 146	protein_codin	0.2872	0.211	1	8.443	0.1365
**EPB41L2**	EPB41L2	24	erythrocyte membrane protein band 4.1 like 2	protein_codin	-0.8738	4.52E-06	0.1052	42.82	0.1462
**MTHFD1L**	MTHFD1L	24	methylenetetrahydrofolate dehydrogenase (NADP+ depender	protein_codin	-0.8428	0.00149	1	3.683	0.1652
**ZNF3**	ZNF3	24	zinc finger protein 3	protein_codin	0.6852	0.0985	1	3.219	0.3072
**PPP1R35**	PPP1R35	24	protein phosphatase 1 regulatory subunit 35	protein_codin	0.5721	0.0072	1	6.828	0.1163
**AP1S1**	AP1S1	24	adaptor related protein complex 1 subunit sigma 1	protein_codin	0.3316	0.0744	1	7.386	0.1402
**EXOC4**	EXOC4	24	exocyst complex component 4	protein_codin	0.485	0.072	1	6.68	0.2317
**SLC35B4**	SLC35B4	24	solute carrier family 35 member B4	protein_codin	-0.2952	0.35	1	9.602	0.1762
**ZYX**	ZYX	24	zyxin	protein_codin	-0.2597	0.278	1	7.129	0.1976
**GIMAP1**	GIMAP1	24	GTPase, IMAP family member 1	protein_codin	0.7581	3.15E-14	7.54E-10	37.05	0.02885
**CDK5**	CDK5	24	cyclin dependent kinase 5	protein_codin	0.058	0.845	1	3.408	0.1267
**RBM3**	RBM3	24	RNA binding motif protein 3	protein_codin	0.03641	0.638	1	35.23	0.05782
**RBM41**	RBM41	24	RNA binding motif protein 41	protein_codin	0.1187	0.719	1	3.584	0.1789
**PGRMC1**	PGRMC1	24	progesterone receptor membrane component 1	protein_codin	0.1574	0.172	1	40.6	0.1696
**UTP14A**	UTP14A	24	UTP14A small subunit processome component	protein_codin	0.1859	0.477	1	3.606	0.172
**AGPAT5**	AGPAT5	24	1-acylglycerol-3-phosphate O-acyltransferase 5	protein_codin	-0.3265	0.153	1	20.28	0.2012
**CSGALNACT1**	CSGALNAC	24	chondroitin sulfate N-acetylgalactosaminyltransferase 1	protein_codin	-1.045	4.85E-12	1.16E-07	47.5	0.05594
**PDLIM2**	PDLIM2	24	PDZ and LIM domain 2	protein_codin	0.2926	0.0326	1	31.68	0.05426
**ATP6V1H**	ATP6V1H	24	ATPase H+ transporting V1 subunit H	protein_codin	0.244	0.318	1	8.902	0.2466
**LYN**	LYN	24	LYN proto-oncogene, Src family tyrosine kinase	protein_codin	-0.02969	0.894	1	22.04	0.1382
**SGK3**	SGK3	24	serum/glucocorticoid regulated kinase family member 3	protein_codin	-0.03868	0.888	1	8.029	0.1179
**PREX2**	PREX2	24	phosphatidylinositol-3,4,5-trisphosphate dependent Rac exch	protein_codin	0.9574	2.52E-08	0.000595	44.87	0.02926
**SNX16**	SNX16	24	sorting nexin 16	protein_codin	0.5282	0.0622	1	3.519	0.148
**RMDN1**	RMDN1	24	regulator of microtubule dynamics 1	protein_codin	0.01746	0.932	1	3.404	0.1684
**UBR5**	UBR5	24	ubiquitin protein ligase E3 component n-recognin 5	protein_codin	0.2211	0.364	1	25.84	0.186
**TAF2**	TAF2	24	TATA-box binding protein associated factor 2	protein_codin	0.1006	0.738	1	3.178	0.1917
**TATDN1**	TATDN1	24	TatD DNase domain containing 1	protein_codin	0.1425	0.404	1	6.204	0.08336
**SNAPC3**	SNAPC3	24	small nuclear RNA activating complex polypeptide 3	protein_codin	0.5188	0.119	1	3.246	0.2517
**UBAP2**	UBAP2	24	ubiquitin associated protein 2	protein_codin	0.5221	0.0692	1	10.02	0.3854
**RECK**	RECK	24	reversion inducing cysteine rich protein with kazal motifs	protein_codin	-0.4601	0.105	1	2.652	0.1949
**LHX6**	LHX6	24	LIM homeobox 6	protein_codin	2.34	1.46E-05	0.3382	25.36	0.08335
**AGPAT2**	AGPAT2	24	1-acylglycerol-3-phosphate O-acyltransferase 2	protein_codin	0.6201	3.26E-05	0.7524	50.22	0.1115
**EHMT1**	EHMT1	24	euchromatic histone lysine methyltransferase 1	protein_codin	0.6517	0.00525	1	8.024	0.3296
**DEAF1**	DEAF1	24	DEAF1 transcription factor	protein_codin	-0.5402	0.00872	1	3.27	0.0832
**NUP98**	NUP98	24	nucleoporin 98	protein_codin	-0.03317	0.906	1	10.08	0.2261
**HTATIP2**	HTATIP2	24	HIV-1 Tat interactive protein 2	protein_codin	0.2432	0.17	1	16.36	0.1389
**ARHGAP1**	ARHGAP1	24	Rho GTPase activating protein 1	protein_codin	0.154	0.619	1	3.367	0.1979
**FNBP4**	FNBP4	24	formin binding protein 4	protein_codin	-0.5438	0.000980	1	44.02	0.1356
**CREB3F**	CREB3F	24	CREB/ATF bZIP transcription factor	protein_codin	0.2223	0.335	1	9.032	0.1126
**HIKESHI**	HIKESHI	24	heat shock protein nuclear import factor hikeshi	protein_codin	0.01811	0.893	1	12.11	0.05812
**TMEM135**	TMEM135	24	transmembrane protein 135	protein_codin	1.032	0.0165	1	33.79	0.208
**AASDHPPT**	AASDHPPT	24	aminoadipate-semialdehyde dehydrogenase-phosphopanteth	protein_codin	-0.1412	0.41	1	17.25	0.1412
**CSTF2T**	CSTF2T	24	cleavage stimulation factor subunit 2 tau variant	protein_codin	1.096	0.00277	1	3.088	0.2765
**MCU**	MCU	24	mitochondrial calcium uniporter	protein_codin	-0.1742	0.595	1	2.864	0.3021
**DNAJC9**	DNAJC9	24	DnaJ heat shock protein family (Hsp40) member C9	protein_codin	0.495	0.0545	1	6.721	0.2839
**ZNF518A**	ZNF518A	24	zinc finger protein 518A	protein_codin	-0.362	0.116	1	3.621	0.08159
**MRPL43**	MRPL43	24	mitochondrial ribosomal protein L43	protein_codin	-0.1796	0.0979	1	37.77	0.1094
**AFAP1L2**	AFAP1L2	24	actin filament associated protein 1 like 2	protein_codin	-0.05836	0.835	1	8.788	0.1495
**PRDX3**	PRDX3	24	peroxiredoxin 3	protein_codin	0.03450	0.744	1	8.025	0.02617
**VWF**	VWF	24	von Willebrand factor	protein_codin	-0.7893	1.16E-22	2.8E-19	52.58	0.027
**C1RL**	C1RL	24	complement C1r subcomponent like	protein_codin	-0.2858	0.257	1	28.85	0.2272
**FGFR1OP2**	FGFR1OP2	24	FGFR1 oncogene partner 2	protein_codin	0.09242	0.473	1	19.96	0.0602
**CPNE8**	CPNE8	24	copine 8	protein_codin	0.6922	1.1E-06	0.02571	15.96	0.1346
**ARF3**	ARF3	24	ADP ribosylation factor 3	protein_codin	-0.1327	0.5	1	8.403	0.1705
**KRT7**	KRT7	24	keratin 7	protein_codin	0.9827	0.0199	1	52.58	0.7206
**PRR13**	PRR13	24	proline rich 13	protein_codin	0.2906	0.0112	1	15.16	0.1116
**CNPY2**	CNPY2	24	canopy FGF signaling regulator 2	protein_codin	-0.6549	5.54E-08	0.001305	23.15	0.05292
**CSRP2**	CSRP2	24	cysteine and glycine rich protein 2	protein_codin	1.548	5.98E-11	1.42E-06	52.58	0.06062
**NR2C1**	NR2C1	24	nuclear receptor subfamily 2 group C member 1	protein_codin	0.1864	0.488	1	11.02	0.2882
**ISCU**	ISCU	24	iron-sulfur cluster assembly enzyme	protein_codin	-0.1216	0.125	1	44.93	0.05522
**ALKBH2**	ALKBH2	24	alkB homolog 2, alpha-ketoglutarate dependent dioxygenase	protein_codin	0.7926	0.00237	1	40.62	0.1951
**SUDS3**	SUDS3	24	SDS3 homolog, SIN3A corepressor complex component	protein_codin	0.05027	0.797	1	7.41	0.1103
**RSRC2**	RSRC2	24	arginine and serine rich coiled-coil 2	protein_codin	-0.07305	0.652	1	25.02	0.1701
**SFSWAP**	SFSWAP	24	splicing factor SWAP	protein_codin	-0.3024	0.133	1	19.74	0.09679
**MTIF3**	MTIF3	24	mitochondrial translational initiation factor 3	protein_codin	0.4697	0.000251	1	21.85	0.0292
**DNAJC15**	DNAJC15	24	DnaJ heat shock protein family (Hsp40) member C15	protein_codin	-0.225	0.0221	1	52.57	0.08026
**RCBTB2**	RCBTB2	24	RCC1 and BTB domain containing protein 2	protein_codin	0.4438	0.0679	1	6.622	0.1918
**CLDN10**	CLDN10	24	claudin 10	protein_codin	-0.7916	0.0956	1	52.58	0.5737
**ATP11A**	ATP11A	24	ATPase phospholipid transporting 11A	protein_codin	0.7617	0.0155	1	33.88	0.1684
**TOX4**	TOX4	24	TOX high mobility group box family member 4	protein_codin	0.324	0.011	1	24.32	0.1394
**METTL3**	METTL3	24	methyltransferase like 3	protein_codin	0.8211	0.0145	1	2.61	0.05626
**NYNRIN**	NYNRIN	24	NYN domain and retroviral integrase containing	protein_codin	1.052	0.00459	1	3.227	0.09376
**BAZ1A**	BAZ1A	24	bromodomain adjacent to zinc finger domain 1A	protein_codin	0.7804	7.97E-06	0.1851	50.88	0.08485
**SYNE2**	SYNE2	24	spectrin repeat containing nuclear envelope protein 2	protein_codin	1.248	8.65E-17	2.08E-12	52.49	0.08327
**UBR7**	UBR7	24	ubiquitin protein ligase E3 component n-recognin 7 (putative)	protein_codin	0.1757	0.535	1	3.023	0.115
**NEDD4**	NEDD4	24	NEDD4 E3 ubiquitin protein ligase	protein_codin	0.01724	0.922	1	15.86	0.1518
**GLCE**	GLCE	24	glucuronic acid epimerase	protein_codin	0.1549	0.552	1	3.582	0.1931
**LRRC49**	LRRC49	24	leucine rich repeat containing 49	protein_codin	-0.6819	0.0196	1	3.322	0.2774
**MESD**	MESD	24	mesoderm development LRP chaperone	protein_codin	-0.2306	0.0429	1	12.47	0.1675
**MRPL46**	MRPL46	24	mitochondrial ribosomal protein L46	protein_codin	0.3486	0.115	1	3.326	0.1326
**IDH2**	IDH2	24	isocitrate dehydrogenase (NADP(+)) 2, mitochondrial	protein_codin	-0.425	0.0912	1	9.545	0.08044
**UNC45A**	UNC45A	24	unc-45 myosin chaperone A	protein_codin	0.3135	0.308	1	3.227	0.2287
**UBALD1**	UBALD1	24	UBA like domain containing 1	protein_codin	0.1332	0.678	1	2.438	0.2612
**PDXDC1**	PDXDC1	24	pyridoxal dependent decarboxylase domain containing 1	protein_codin	0.6112	0.0246	1	8.586	0.1686
**GGA2**	GGA2	24	golgi associated, gamma adaptin ear containing, ARF binding	protein_codin	0.2604	0.417	1	2.662	0.1452

**MON1B**	MON1B	24	MON1 homolog B, secretory trafficking associated	protein_codin	0.4085	0.182	1	30.9	0.2829
**TRAPPC2L**	TRAPPC2L	24	trafficking protein particle complex 2 like	protein_codin	-0.5672	1.43E-06	0.03341	31.26	0.1408
**RPH3AL**	RPH3AL	24	rabphilin 3A like (without C2 domains)	protein_codin	1.331	2.14E-05	0.495	24.71	0.1438
**MIS12**	MIS12	24	MIS12 kinetochore complex component	protein_codin	0.6902	0.0758	1	3.317	0.2284
**TNFAIP1**	TNFAIP1	24	TNF alpha induced protein 1	protein_codin	0.429	0.0149	1	27.66	0.1371
**PIP4K2B**	PIP4K2B	24	phosphatidylinositol-5-phosphate 4-kinase type 2 beta	protein_codin	0.1562	0.598	1	10.56	0.1991
**MSI2**	MSI2	24	musashi RNA binding protein 2	protein_codin	-1.288	3.68E-10	8.73E-06	10.53	0.2311
**CUEDC1**	CUEDC1	24	CUE domain containing 1	protein_codin	0.68	0.0347	1	8.673	0.3388
**PTRH2**	PTRH2	24	peptidyl-tRNA hydrolase 2	protein_codin	-0.1752	0.383	1	8.323	0.2231
**C17orf80**	C17orf80	24	chromosome 17 open reading frame 80	protein_codin	0.3346	0.282	1	8.396	0.2609
**HID1-AS1**	HID1-AS1	24	HID1 antisense RNA 1	antisense_RN	1.157	0.0185	1	3.55	0.422
**GAA**	GAA	24	glucosidase alpha, acid	protein_codin	-0.6025	0.00649	1	9.087	0.1686
**ARHGDIA**	ARHGDIA	24	Rho GDP dissociation inhibitor alpha	protein_codin	-0.009315	0.936	1	19.05	0.03059
**HAUS1**	HAUS1	24	HAUS augmin like complex subunit 1	protein_codin	-0.168	0.343	1	7.657	0.2215
**NORAD**	NORAD	24	non-coding RNA activated by DNA damage	lincRNA	-0.665	1.76E-11	4.19E-07	52.57	0.0279
**PPP1R16B**	PPP1R16B	24	protein phosphatase 1 regulatory subunit 16B	protein_codin	1.511	0.000211	1	9.73	0.1677
**CSTF1**	CSTF1	24	cleavage stimulation factor subunit 1	protein_codin	1.028	0.00359	1	8.255	0.1106
**MIER2**	MIER2	24	MIER family member 2	protein_codin	0.9485	0.00538	1	10.46	0.2449
**MIDN**	MIDN	24	midnolin	protein_codin	-0.2152	0.124	1	47.78	0.1704
**PLEKHJ1**	PLEKHJ1	24	pleckstrin homology domain containing J1	protein_codin	0.1702	0.509	1	7.644	0.22
**DPP9**	DPP9	24	dipeptidyl peptidase 9	protein_codin	-0.5745	0.1	1	3.192	0.4272
**TIMM44**	TIMM44	24	translocase of inner mitochondrial membrane 44	protein_codin	0.162	0.567	1	3.422	0.08851
**CDKN2D**	CDKN2D	24	cyclin dependent kinase inhibitor 2D	protein_codin	-0.187	0.258	1	24.49	0.02614
**GIPC1**	GIPC1	24	GIPC PDZ domain containing family member 1	protein_codin	-0.1617	0.349	1	7.088	0.1098
**WTIP**	WTIP	24	WT1 interacting protein	protein_codin	0.0739	0.773	1	3.248	0.2727
**COX7A1**	COX7A1	24	cytochrome c oxidase subunit 7A1	protein_codin	-0.5672	3.88E-08	0.000915	47.88	0.1132
**EMC10**	EMC10	24	ER membrane protein complex subunit 10	protein_codin	-0.1505	0.395	1	7.512	0.08196
**MRPL40**	MRPL40	24	mitochondrial ribosomal protein L40	protein_codin	-0.1341	0.308	1	12.08	0.1166
**TRMT2A**	TRMT2A	24	tRNA methyltransferase 2 homolog A	protein_codin	0.2312	0.458	1	2.644	0.1388
**USP25**	USP25	24	ubiquitin specific peptidase 25	protein_codin	0.2729	0.274	1	7.698	0.1228
**PRMT2**	PRMT2	24	protein arginine methyltransferase 2	protein_codin	-0.4132	0.000776	1	20.06	0.08584
**UBE2J2**	UBE2J2	25	ubiquitin conjugating enzyme E2 J2	protein_codin	0.3222	0.0958	1	20.92	0.1702
**SKI**	SKI	25	SKI proto-oncogene	protein_codin	-0.8091	5.6E-05	1	8.849	0.02960
**SYF2**	SYF2	25	SYF2 pre-mRNA splicing factor	protein_codin	0.2887	0.0011	1	29.52	0.08290
**SPATA6**	SPATA6	25	spermatogenesis associated 6	protein_codin	0.3584	0.174	1	9.138	0.1458
**FAF1**	FAF1	25	Fas associated factor 1	protein_codin	0.1941	0.457	1	7.878	0.199
**MAGOH**	MAGOH	25	mago homolog, exon junction complex subunit	protein_codin	0.2079	0.0681	1	15.98	0.1437
**SSBP3**	SSBP3	25	single stranded DNA binding protein 3	protein_codin	1.008	7.47E-05	1	5.779	0.2249
**FPGT**	FPGT	25	fucose-1-phosphate guanylyltransferase	protein_codin	0.00688	0.982	1	2.415	0.1632
**GTF2B**	GTF2B	25	general transcription factor IIB	protein_codin	0.7074	0.000522	1	33.27	0.1144
**SLC30A7**	SLC30A7	25	solute carrier family 30 member 7	protein_codin	-0.06810	0.797	1	22.81	0.2015
**RSBN1**	RSBN1	25	round spermatid basic protein 1	protein_codin	0.3066	0.112	1	6.893	0.2596
**SEC22B**	SEC22B	25	SEC22 homolog B, vesicle trafficking protein (gene/pseudogene)	protein_codin	0.0242	0.879	1	15.4	0.1166
**EFNA1**	EFNA1	25	ephrin A1	protein_codin	0.05268	0.673	1	52.58	0.2359
**CADM3-AS1**	CADM3-AS	25	CADM3 antisense RNA 1	antisense_RN	4.207	0.000278	1	16.68	0.1721
**IGSF8**	IGSF8	25	immunoglobulin superfamily member 8	protein_codin	0.6719	0.0529	1	3.16	0.3296
**MPC2**	MPC2	25	mitochondrial pyruvate carrier 2	protein_codin	-0.4372	0.00011	1	19.92	0.1132
**DCAF6**	DCAF6	25	DDB1 and CUL4 associated factor 6	protein_codin	-0.1019	0.706	1	8.63	0.196
**METTL18**	METTL18	25	methyltransferase like 18	protein_codin	0.7151	0.0406	1	3.515	0.08571
**ZBTB41**	ZBTB41	25	zinc finger and BTB domain containing 41	protein_codin	0.4896	0.195	1	9.493	0.4151
**C1orf35**	C1orf35	25	chromosome 1 open reading frame 35	protein_codin	0.3322	0.0842	1	16.32	0.1632
**DESI2**	DESI2	25	desumoylating isopeptidase 2	protein_codin	-0.05712	0.732	1	7.23	0.05788
**CFAP36**	CFAP36	25	cilia and flagella associated protein 36	protein_codin	0.09428	0.471	1	52.58	0.02703
**SERTAD2**	SERTAD2	25	SERTA domain containing 2	protein_codin	0.3029	0.187	1	7.404	0.2825
**WDR92**	WDR92	25	WD repeat domain 92	protein_codin	-0.505	0.152	1	2.872	0.2658
**DGUOK**	DGUOK	25	deoxyguanosine kinase	protein_codin	-0.2765	0.0467	1	45.44	0.1154
**MGAT4A**	MGAT4A	25	alpha-1,3-mannosyl-glycoprotein 4-beta-N-acetylglucosaminy	protein_codin	-0.7217	2.19E-06	0.0511	41.32	0.1674
**NPHP1**	NPHP1	25	nephrocystin 1	protein_codin	0.5534	0.226	1	9.067	0.1812
**BCL2L11**	BCL2L11	25	BCL2 like 11	protein_codin	-0.5202	0.045	1	9.452	0.099
**MZT2A**	MZT2A	25	mitotic spindle organizing protein 2A	protein_codin	-0.06792	0.61	1	37.58	0.02717
**CCNT2**	CCNT2	25	cyclin T2	protein_codin	0.3697	0.192	1	9.803	0.1756
**CYBRD1**	CYBRD1	25	cytochrome b reductase 1	protein_codin	-0.7841	1.1E-05	0.2551	7.675	0.1341
**HOXD1**	HOXD1	25	homeobox D1	protein_codin	1.127	0.00195	1	52.07	0.2106
**HNRNPA3**	HNRNPA3	25	heterogeneous nuclear ribonucleoprotein A3	protein_codin	0.1332	0.106	1	52	0.02622
**NUP35**	NUP35	25	nucleoporin 35	protein_codin	0.9801	0.00556	1	2.885	0.166
**FAM171B**	FAM171B	25	family with sequence similarity 171 member B	protein_codin	-1.121	9.6E-08	0.002257	43.25	0.1481
**CFLAR**	CFLAR	25	CASP8 and FADD like apoptosis regulator	protein_codin	-0.01379	0.908	1	38.19	0.08529
**IDH1**	IDH1	25	isocitrate dehydrogenase (NADP(+)) 1, cytosolic	protein_codin	-0.1325	0.587	1	9.22	0.2488
**CYP27A1**	CYP27A1	25	cytochrome P450 family 27 subfamily A member 1	protein_codin	-0.7564	0.00107	1	3.641	0.2015
**EDEM1**	EDEM1	25	ER degradation enhancing alpha-mannosidase like protein 1	protein_codin	0.2071	0.564	1	9.713	0.1902
**RFTN1**	RFTN1	25	raftlin, lipid raft linker 1	protein_codin	0.5954	0.0246	1	25.5	0.2888
**CMTM7**	CMTM7	25	CKLF like MARVEL transmembrane domain containing 7	protein_codin	-0.7636	0.00492	1	9.403	0.1955
**MLH1**	MLH1	25	mutL homolog 1	protein_codin	0.8641	0.0179	1	3.454	0.1202
**HIGD1A**	HIGD1A	25	HIG1 hypoxia inducible domain family member 1A	protein_codin	-0.1539	0.368	1	7.175	0.02708
**RRP9**	RRP9	25	ribosomal RNA processing 9, U3 small nucleolar RNA binding	protein_codin	0.7375	0.0296	1	3.154	0.1982
**PDE12**	PDE12	25	phosphodiesterase 12	protein_codin	-0.2647	0.316	1	3.173	0.03369
**UBA3**	UBA3	25	ubiquitin like modifier activating enzyme 3	protein_codin	-0.05990	0.75	1	7.433	0.08157
**GTPBP8**	GTPBP8	25	GTP binding protein 8 (putative)	protein_codin	-0.09503	0.692	1	6.813	0.08252
**MBD4**	MBD4	25	methyl-CpG binding domain 4, DNA glycosylase	protein_codin	0.1507	0.382	1	13.53	0.1439
**TOPBP1**	TOPBP1	25	DNA topoisomerase II binding protein 1	protein_codin	-0.2348	0.373	1	31.17	0.3206
**HLTF**	HLTF	25	helicase like transcription factor	protein_codin	-0.2751	0.134	1	18.87	0.1946
**B3GALNT1**	B3GALNT1	25	beta-1,3-N-acetylgalactosaminyltransferase 1 (globoside bloo	protein_codin	-1.578	2.04E-08	0.000482	8.635	0.164
**MYNN**	MYNN	25	myoneurin	protein_codin	0.5815	0.0334	1	3.398	0.1924
**FXR1**	FXR1	25	FMR1 autosomal homolog 1	protein_codin	0.1617	0.138	1	24.88	0.1192
**TMEM41A**	TMEM41A	25	transmembrane protein 41A	protein_codin	0.8597	0.00397	1	7.352	0.2794
**HES1**	HES1	25	hes family bHLH transcription factor 1	protein_codin	0.5985	0.000276	1	52.58	0.1942
**FAM43A**	FAM43A	25	family with sequence similarity 43 member A	protein_codin	-0.07839	0.745	1	52.57	0.1574
**NCBP2-AS2**	NCBP2-AS2	25	NCBP2 antisense 2 (head to head)	protein_codin	-0.4295	0.00862	1	6.048	0.1089
**PIGG**	PIGG	25	phosphatidylinositol glycan anchor biosynthesis class G	protein_codin	-0.933	0.00117	1	2.824	0.3298
**GRPEL1**	GRPEL1	25	GrpE like 1, mitochondrial	protein_codin	0.372	0.0309	1	14.07	0.0567
**WDR1**	WDR1	25	WD repeat domain 1	protein_codin	-0.1244	0.377	1	34.28	0.1122
**PGM2**	PGM2	25	phosphoglucomutase 2	protein_codin	-0.3162	0.0867	1	8.323	0.05756
**CNOT6L**	CNOT6L	25	CCR4-NOT transcription complex subunit 6 like	protein_codin	-0.08514	0.592	1	13.57	0.1822
**ABCG2**	ABCG2	25	ATP binding cassette subfamily G member 2 (Junior blood gr	protein_codin	2.452	1.06E-11	2.53E-07	36.47	0.1209
**CISD2**	CISD2	25	CDGSH iron sulfur domain 2	protein_codin	-0.4	0.0498	1	8.752	0.05735
**NR3C2**	NR3C2	25	nuclear receptor subfamily 3 group C member 2	protein_codin	1.467	0.00264	1	3.033	0.2541
**NEK1**	NEK1	25	NIMA related kinase 1	protein_codin	-0.2002	0.402	1	9.537	0.08216
**FRG1**	FRG1	25	FSHD region gene 1	protein_codin	0.1074	0.406	1	10.06	0.08090
**SDHA**	SDHA	25	succinate dehydrogenase complex flavoprotein subunit A	protein_codin	-0.1429	0.465	1	7.062	0.222
**MRPL36**	MRPL36	25	mitochondrial ribosomal protein L36	protein_codin	0.1466	0.329	1	35.87	0.05369
**RICTOR**	RICTOR	25	RPTOR independent companion of MTOR complex 2	protein_codin	0.5072	0.0347	1	25.2	0.06258
**PELO**	PELO	25	pelota mRNA surveillance and ribosome rescue factor	protein_codin	0.6465	0.00222	1	32.06	0.1111
**PDE4D**	PDE4D	25	phosphodiesterase 4D	protein_codin	-0.333	0.201	1	7.21	0.1816
**FCHO2**	FCHO2	25	FCH domain only 2	protein_codin	0.5728	0.000661	1	46.32	0.2384
**EDIL3**	EDIL3	25	EGF like repeats and discoidin domains 3	protein_codin	-0.935	9.66E-05	1	37.93	0.1482
**SNCAIP**	SNCAIP	25	synuclein alpha interacting protein	protein_codin	-2.037	2.43E-13	5.81E-09	30.2	0.1803
**UBE2B**	UBE2B	25	ubiquitin conjugating enzyme E2 B	protein_codin	-0.1152	0.1546	1	26.76	0.05716
**CAMLG**	CAMLG	25	calcium modulating ligand	protein_codin	-0.04810	0.666	1	27.15	0.1311
**CTNNA1**	CTNNA1	25	catenin alpha 1	protein_codin	-0.08407	0.466	1	33.6	0.1122
**PAIP2**	PAIP2	25	poly(A) binding protein interacting protein 2	protein_codin	-0.01531	0.892	1	25.78	0.02647
**SPRY4**	SPRY4	25	sprouty RTK signaling antagonist 4	protein_codin	-0.1245	0.646	1	10.8	0.05606
**ARHGAP26**	ARHGAP26	25	Rho GTPase activating protein 26	protein_codin	0.795	0.00582	1	15.88	0.145
**ANXA6**	ANXA6	25	annexin A6	protein_codin	0.231	0.374	1	8.637	0.2175
**MRPL22**	MRPL22	25	mitochondrial ribosomal protein L22	protein_codin	0.1596	0.256	1	11.78	0.07915
**NUDCD2**	NUDCD2	25	NudC domain containing 2	protein_codin	-0.1586	0.243	1	13.87	0.1364
**MAT2B**	MAT2B	25	methionine adenosyltransferase 2B	protein_codin	0.2754	0.282	1	7.747	0.05335
**NOP16**	NOP16	25	NOP16 nucleolar protein	protein_codin	0.5526	0.0387	1	8.475	0.02641
**RNF44**	RNF44	25	ring finger protein 44	protein_codin	-0.3429	0.188	1	2.349	0.1626
**CANX**	CANX	25	calnexin	protein_codin	-0.3274	2E-04	1	52.05	0.02881
**DUSP22**	DUSP22	25	dual specificity phosphatase 22	protein_codin	0.7541	0.000411	1	7.432	0.2035
**PXDC1**	PXDC1	25	PX domain containing 1	protein_codin	-1.317	2.87E-20	6.91E-16	47.13	0.082
**ZSCAN26**	ZSCAN26	25	zinc finger and SCAN domain containing 26	protein_codin	-0.1661	0.572	1	2.921	0.1132
**TRIM26**	TRIM26	25	tripartite motif containing 26	protein_codin	1.101	0.0142	1	3.152	0.362
**NOTCH4**	NOTCH4	25	notch receptor 4	protein_codin	-0.8289	7.01E-05	1	30.01	0.09125
**TAF11**	TAF11	25	TATA-box binding protein associated factor 11	protein_codin	0.404	0.0346	1	16.32	0.1965
**LYRM2**	LYRM2	25	LYR motif containing 2	protein_codin	0.1629	0.332	1	24.62	0.05560
**ASF1A**	ASF1A	25	anti-silencing function 1A histone chaperone	protein_codin	-0.09074	0.62	1	47.14	0.1411
**SGK1**	SGK1	25	serum/glucocorticoid regulated kinase 1	protein_codin	-0.3877	0.00612	1	47.11	0.1728
**BCLAF1**	BCLAF1	25	BCL2 associated transcription factor 1	protein_codin	0.1561	0.229	1	37.57	0.08482
**VTA1**	VTA1	25	vesicle trafficking 1	protein_codin	0.6002	0.0264	1	8.026	0.09037
**TULP4**	TULP4	25	TUB like protein 4	protein_codin	-0.4232	0.0226	1	7.91	0.1096
**GPR146**	GPR146	25	G protein-coupled receptor 146	protein_codin	0.7493	3.08E-06	0.07177	52.49	0.192
**IQCE**	IQCE	25	IQ motif containing E	protein_codin	0.3482	0.285	1	3.485	0.2639
**CRCP**	CRCP	25	CGRP receptor component	protein_codin	0.3215	0.162	1	5.531	0.2015
**TP53TG1**	TP53TG1	25	TP53 target 1	lincRNA	-0.01447	0.938	1	6.96	0.054
**PILRB**	PILRB	25	paired immunoglobin like type 2 receptor beta	protein_codin	0.2345	0.54	1	2.593	0.3008
**NDUFA5**	NDUFA5	25	NADH:ubiquinone oxidoreductase subunit A5	protein_codin	-0.08986	0.395	1	21.21	0.1362
**AGAP3**	AGAP3	25	ArfGAP with GTPase domain, ankyrin repeat and PH domain 3	protein_codin	0.2057	0.46	1	49.46	0.2564
**NOM1**	NOM1	25	nucleolar protein with MIF4G domain 1	protein_codin	-0.2132	0.487	1	9.153	0.3970
**SRPX**	SRPX	25	sushi repeat containing protein X-linked	protein_codin	-0.37	0.0384	1	51.79	0.1661
**DDX3X**	DDX3X	25	DEAD-box helicase 3 X-linked	protein_codin	0.282	0.00224	1	47.52	0.08652
**ELK1**	ELK1	25	ETS transcription factor ELK1	protein_codin	0.2906	0.252	1	11.22	0.2679
**PRAF2**	PRAF2	25	PRA1 domain family member 2	protein_codin	-0.01732	0.913	1	39.72	0.08522
**HNRNPH2**	HNRNPH2	25	heterogeneous nuclear ribonucleoprotein H2	protein_codin	0.3927	0.0272	1	7.197	0.174
**BAG4**	BAG4	25	BCL2 associated athanogene 4	protein_codin	-0.1898	0.452	1	3.502	0.1127
**C8orf4**	C8orf4	25	transcriptional and immune response regulator	protein_codin	NA	NA	NA	48	0.1944
**LRRCC1**	LRRCC1	25	leucine rich repeat and coiled-coil centrosomal protein 1	protein_codin	0.4395	0.0906	1	21.58	0.2226
**VIRMA**	VIRMA	25	vir like m6A methyltransferase associated	protein_codin	0.6367	0.00682	1	7.648	0.1716
**MTERF3**	MTERF3	25	mitochondrial transcription termination factor 3	protein_codin	1.489	0.00116	1	3.334	0.2497
**TRIB1**	TRIB1	25	tribbles pseudokinase 1	protein_codin	0.9279	7.43E-05	1	23.46	0.1118
**DENND3**	DENND3	25	DENN domain containing 3	protein_codin	-0.5801	0.0426	1	9.342	0.1656
**TLN1**	TLN1	25	talin 1	protein_codin	-0.1875	0.0646	1	42.47	0.1414
**GKAP1**	GKAP1	25	G kinase anchoring protein 1	protein_codin	1.049	0.00774	1	9.363	0.1856
**ZCCHC6**	ZCCHC6	25	terminal uridylyl transferase 7	protein_codin	NA	NA	NA	8.293	0.2664
**IARS**	IARS	25	isoleucyl-tRNA synthetase	protein_codin	0.2785	0.332	1	3.745	0.1737
**ALG2**	ALG2	25	ALG2 alpha-1,3/1,6-mannosyltransferase	protein_codin	-0.2364	0.287	1	6.903	0.1702
**TXN**	TXN	25	thioredoxin	protein_codin	0.006905	0.919	1	52.58	0.02682
**OLFML2A**	OLFML2A	25	olfactomedin like 2A	protein_codin	0.6577	0.0428	1	23.88	0.2022
**IER5L**	IER5L	25	immediate early response 5 like	protein_codin	-0.3526	0.0146	1	52.01	0.03382
**TMEM141**	TMEM141	25	transmembrane protein 141	protein_codin	0.1341	0.31	1	11.47	0.1142
**NRARP**	NRARP	25	NOTCH regulated ankyrin repeat protein	protein_codin	0.6679	3.96E-05	0.9133	39.86	0.1659
**RASSF7**	RASSF7	25	Ras association domain family member 7	protein_codin	0.2891	0.335	1	3.227	0.1986
**SDHAF2**	SDHAF2	25	succinate dehydrogenase complex assembly factor 2	protein_codin	0.1936	0.294	1	6.26	0.08492
**RDX**	RDX	25	radixin	protein_codin	-0.4542	9.76E-08	0.002295	37.18	0.09954
**SRGN**	SRGN	25	serglycin	protein_codin	-1.435	1.05E-42	2.55E-38	52.58	0.05552
**SUPV3L1**	SUPV3L1	25	Suv3 like RNA helicase	protein_codin	-0.1271	0.646	1	9.818	0.1415
**DDIT4**	DDIT4	25	DNA damage inducible transcript 4	protein_codin	-0.6771	2.96E-07	0.006941	52.58	0.09618
**SLF2**	SLF2	25	SMC5-SMC6 complex localization factor 2	protein_codin	0.04821	0.856	1	47.32	0.2075
**DUSP5**	DUSP5	25	dual specificity phosphatase 5	protein_codin	0.4972	0.000479	1	44.26	0.1868
**PLEKHA1**	PLEKHA1	25	pleckstrin homology domain containing A1	protein_codin	-0.1072	0.508	1	12.42	0.1726
**DHX32**	DHX32	25	DEAH-box helicase 32 (putative)	protein_codin	0.6027	0.0586	1	3.196	0.3354
**ATF7IP**	ATF7IP	25	activating transcription factor 7 interacting protein	protein_codin	0.3691	0.144	1	15.73	0.2052
**EIF4B**	EIF4B	25	eukaryotic translation initiation factor 4B	protein_codin	0.3326	1.93E-05	0.4466	42.33	0.02666
**LEMD3**	LEMD3	25	LEM domain containing 3	protein_codin	0.3089	0.341	1	3.265	0.2127
**MTERF2**	MTERF2	25	mitochondrial transcription termination factor 2	protein_codin	0.3158	0.326	1	3.284	0.09612
**ANKRD13A**	ANKRD13A	25	ankyrin repeat domain 13A	protein_codin	-0.1574	0.572	1	2.83	0.1915
**NAA25**	NAA25	25	N(alpha)-acetyltransferase 25, NatB auxiliary subunit	protein_codin	0.1504	0.638	1	3.102	0.1457
**STX2**	STX2	25	syntaxin 2	protein_codin	-0.2320	0.296	1	21.86	0.1477
**POLR1D**	POLR1D	25	RNA polymerase I and III subunit D	protein_codin	0.0984	0.319	1	27.5	0.1669
**DNAJC3**	DNAJC3	25	DnaJ heat shock protein family (Hsp40) member C3	protein_codin	-0.6948	3.55E-11	8.45E-07	18.97	0.05732
**SDR39U1**	SDR39U1	25	short chain dehydrogenase/reductase family 39U member 1	protein_codin	-0.133	0.551	1	3.526	0.3396
**MIPOL1**	MIPOL1	25	mirror-image polydactyly 1	protein_codin	-0.2301	0.412	1	11.45	0.2352
**SOCS4**	SOCS4	25	suppressor of cytokine signaling 4	protein_codin	-0.1895	0.363	1	22.54	0.1099
**ERH**	ERH	25	ERH mRNA splicing and mitosis factor	protein_codin	-0.02361	0.763	1	24.41	0.08446
**EIF5**	EIF5	25	eukaryotic translation initiation factor 5	protein_codin	-0.3501	6.53E-05	1	24.21	0.02612
**TRMT61A**	TRMT61A	25	tRNA methyltransferase 61A	protein_codin	0.0134	0.967	1	9.996	0.2599
**HERC2**	HERC2	25	HECT and RLD domain containing E3 ubiquitin protein ligase 2	protein_codin	-0.3105	0.0684	1	27.64	0.02882
**MYO1E**	MYO1E	25	myosin IE	protein_codin	0.09716	0.677	1	20.68	0.0279
**GTF2A2**	GTF2A2	25	general transcription factor IIA subunit 2	protein_codin	-0.06095	0.506	1	33.08	0.1088
**SIN3A**	SIN3A	25	SIN3 transcription regulator family member A	protein_codin	-0.149	0.526	1	24.36	0.2676
**IMP3**	IMP3	25	IMP U3 small nucleolar ribonucleoprotein 3	protein_codin	0.09909	0.369	1	52.52	0.05518
**SEC11A**	SEC11A	25	SEC11 homolog A, signal peptidase complex subunit	protein_codin	0.1457	0.0187	1	52.51	0.05432
**METTL26**	METTL26	25	methyltransferase like 26	protein_codin	-0.3398	0.00167	1	20.43	0.05668
**RHOT2**	RHOT2	25	ras homolog family member T2	protein_codin	-0.1043	0.682	1	7.667	0.1906
**NAA60**	NAA60	25	N(alpha)-acetyltransferase 60, NatF catalytic subunit	protein_codin	0.3392	0.183	1	3.606	0.2698
**NPIPB5**	NPIPB5	25	nuclear pore complex interacting protein family member B5	protein_codin	-0.2656	0.303	1	3.233	0.3352
**INO80E**	INO80E	25	INO80 complex subunit E	protein_codin	0.3067	0.234	1	7.637	0.1148
**MT2A**	MT2A	25	metallothionein 2A	protein_codin	1.166	5.05E-08	0.00119	52.58	0.3665
**MT1E**	MT1E	25	metallothionein 1E	protein_codin	-0.6948	0.0276	1	48	0.5254
**MT1M**	MT1M	25	metallothionein 1M	protein_codin	0.4174	0.332	1	52.58	0.7581
**MT1X**	MT1X	25	metallothionein 1X	protein_codin	0.866	5E-04	1	52.07	0.4014
**NUP93**	NUP93	25	nucleoporin 93	protein_codin	0.3091	0.326	1	9.421	0.3890
**NCOR1**	NCOR1	25	nuclear receptor corepressor 1	protein_codin	0.05330	0.673	1	13.88	0.08512
**ERAL1**	ERAL1	25	Era like 12S mitochondrial rRNA chaperone 1	protein_codin	0.6082	0.0657	1	3.123	0.2580
**SSH2**	SSH2	25	slingshot protein phosphatase 2	protein_codin	0.1296	0.627	1	8.42	0.08290
**CPD**	CPD	25	carboxypeptidase D	protein_codin	0.3737	0.0656	1	50.14	0.05875
**COPRS**	COPRS	25	coordinator of PRMT5 and differentiation stimulator	protein_codin	-0.3475	0.00409	1	21.72	0.08687
**NFE2L1**	NFE2L1	25	nuclear factor, erythroid 2 like 1	protein_codin	0.5824	0.000341	1	23.8	0.2871
**4-Sep**	43712	25	septin 4	protein_codin	-0.6594	0.0122	1	9.396	0.117
**ERN1.00**	ERN1.00	25	endoplasmic reticulum to nucleus signaling 1	protein_codin	-0.7412	0.00821	1	52.58	1.489
**BPTF**	BPTF	25	bromodomain PHD finger transcription factor	protein_codin	-0.01819	0.886	1	42.62	0.08168
**KCNJ2**	KCNJ2	25	potassium voltage-gated channel subfamily J member 2	protein_codin	-1.279	0.00662	1	47.09	0.1877
**CYTH1**	CYTH1	25	cytohesin 1	protein_codin	1.432	2.56E-07	0.006006	15.79	0.1384
**RAB40B**	RAB40B	25	RAB40B, member RAS oncogene family	protein_codin	0.5077	0.0782	1	2.934	0.1747
**SS18**	SS18	25	SS18 subunit of BAF chromatin remodeling complex	protein_codin	0.4488	0.0926	1	6.863	0.4763
**ELP2**	ELP2	25	elongator acetyltransferase complex subunit 2	protein_codin	0.3026	0.219	1	6.575	0.224
**MBD2**	MBD2	25	methyl-CpG binding domain protein 2	protein_codin	-0.1422	0.258	1	19.46	0.06054
**PRNP**	PRNP	25	prion protein	protein_codin	-0.3439	0.0685	1	38.31	0.061
**SAMHD1**	SAMHD1	25	SAM and HD domain containing deoxynucleoside triphosphat	protein_codin	1.452	5.55E-12	1.32E-07	46.02	0.1085
**ADNP**	ADNP	25	activity dependent neuroprotector homeobox	protein_codin	0.105	0.558	1	7.135	0.1647
**STMN3**	STMN3	25	stathmin 3	protein_codin	-1.224	4.42E-09	0.000105	23.95	0.1515
**DOHH**	DOHH	25	deoxyhypusine hydroxylase	protein_codin	0.5176	0.0938	1	3.22	0.1704
**OCEL1**	OCEL1	25	occludin/ELL domain containing 1	protein_codin	0.1582	0.541	1	3.193	0.195
**ISYNA1**	ISYNA1	25	inositol-3-phosphate synthase 1	protein_codin	0.01989	0.948	1	21.76	0.2201
**HNRNPL**	HNRNPL	25	heterogeneous nuclear ribonucleoprotein L	protein_codin	0.324	0.0871	1	7.063	0.112
**MRPS12**	MRPS12	25	mitochondrial ribosomal protein S12	protein_codin	-0.04927	0.737	1	15.18	0.02628
**HNRNPUL1**	HNRNPUL1	25	heterogeneous nuclear ribonucleoprotein U like 1	protein_codin	0.0101	0.952	1	7.453	0.1403
**ERF**	ERF	25	ETS2 repressor factor	protein_codin	0.1488	0.397	1	34.17	0.1382
**HIF3A**	HIF3A	25	hypoxia inducible factor 3 subunit alpha	protein_codin	0.3322	0.165	1	9.153	0.2788
**RANBP1**	RANBP1	25	RAN binding protein 1	protein_codin	-0.03512	0.73	1	13.76	0.02859
**TTC28**	TTC28	25	tetratricopeptide repeat domain 28	protein_codin	0.3479	0.175	1	9.526	0.2673
**HMOX1**	HMOX1	25	heme oxygenase 1	protein_codin	-0.6499	0.0226	1	48.05	0.7502
**POLR2F**	POLR2F	25	RNA polymerase II subunit F	protein_codin	0.0739	0.554	1	19.9	0.1397
**ODF3B**	ODF3B	25	outer dense fiber of sperm tails 3B	protein_codin	1.525	2.56E-13	6.12E-09	50.34	0.1442
**COL6A1**	COL6A1	25	collagen type VI alpha 1 chain	protein_codin	-0.5507	0.0272	1	3.08	0.1782
**DFFA**	DFFA	26	DNA fragmentation factor subunit alpha	protein_codin	-0.1136	0.552	1	24.5	0.09022
**FAM76A**	FAM76A	26	family with sequence similarity 76 member A	protein_codin	0.3704	0.165	1	3.453	0.3085
**LMO4**	LMO4	26	LIM domain only 4	protein_codin	0.442	0.00744	1	44.94	0.05269
**HDGF**	HDGF	26	heparin binding growth factor	protein_codin	-0.01266	0.925	1	11.5	0.08044
**RGS2**	RGS2	26	regulator of G protein signaling 2	protein_codin	-0.2791	0.317	1	47.94	0.2561
**COA6**	COA6	26	cytochrome c oxidase assembly factor 6	protein_codin	-0.0409	0.829	1	52.58	0.2079
**ATRAID**	ATRAID	26	all-trans retinoic acid induced differentiation factor	protein_codin	-0.1124	0.17	1	44.36	0.0282
**WDR43**	WDR43	26	WD repeat domain 43	protein_codin	0.2966	0.0876	1	26.3	0.08564
**PRKD3**	PRKD3	26	protein kinase D3	protein_codin	-0.3682	0.0455	1	7.289	0.1945
**GALM**	GALM	26	galactose mutarotase	protein_codin	0.5071	0.133	1	3.143	0.2254
**PPP1R21**	PPP1R21	26	protein phosphatase 1 regulatory subunit 21	protein_codin	0.7702	0.0129	1	3.401	0.1397
**SPTBN1**	SPTBN1	26	spectrin beta, non-erythrocytic 1	protein_codin	0.1062	0.206	1	52.58	0.03592
**CCT4**	CCT4	26	chaperonin containing TCP1 subunit 4	protein_codin	0.3988	0.000118	1	50.29	0.05794
**AUP1**	AUP1	26	AUP1 lipid droplet regulating VLDL assembly factor	protein_codin	0.1619	0.222	1	44.1	0.1067
**KDM3A**	KDM3A	26	lysine demethylase 3A	protein_codin	0.1359	0.615	1	8.89	0.1239
**EPC2**	EPC2	26	enhancer of polycomb homolog 2	protein_codin	-0.3539	0.0855	1	6.605	0.2571
**ACVR1**	ACVR1	26	activin A receptor type 1	protein_codin	-1.224	3.97E-08	0.000936	52.3	0.0268
**TMEM237**	TMEM237	26	transmembrane protein 237	protein_codin	0.1849	0.577	1	2.173	0.1076
**KANSL1L**	KANSL1L	26	KAT8 regulatory NSL complex subunit 1 like	protein_codin	-0.3436	0.202	1	3.21	0.1096
**PPP1R7**	PPP1R7	26	protein phosphatase 1 regulatory subunit 7	protein_codin	0.1807	0.223	1	11.58	0.05592
**DTYMK**	DTYMK	26	deoxythymidylate kinase	protein_codin	0.2092	0.38	1	8.138	0.2321
**TOP2B**	TOP2B	26	DNA topoisomerase II beta	protein_codin	-0.7872	0.000195	1	28.67	0.2295
**WDR48**	WDR48	26	WD repeat domain 48	protein_codin	0.3988	0.114	1	8.211	0.1627
**MRFAP1**	MRFAP1	26	Morf4 family associated protein 1	protein_codin	-0.2075	0.0107	1	52.58	0.07948
**RBPJ**	RBPJ	26	recombination signal binding protein for immunoglobulin kapp	protein_codin	-0.1601	0.336	1	49.84	0.05355
**BMP2K**	BMP2K	26	BMP2 inducible kinase	protein_codin	0.8887	0.00602	1	8.298	0.08722
**NDUFAF2**	NDUFAF2	26	NADH:ubiquinone oxidoreductase complex assembly factor 2	protein_codin	0.2532	0.156	1	27.58	0.05546
**TNFAIP8**	TNFAIP8	26	TNF alpha induced protein 8	protein_codin	-0.2702	0.352	1	9.045	0.142
**LYRM7**	LYRM7	26	LYR motif containing 7	protein_codin	0.3319	0.272	1	8.375	0.1892
**PDLIM4**	PDLIM4	26	PDZ and LIM domain 4	protein_codin	-0.6832	2.03E-07	0.004765	51.99	0.02729
**HDAC3**	HDAC3	26	histone deacetylase 3	protein_codin	-0.02124	0.922	1	7.497	0.08312
**GPSM3**	GPSM3	26	G protein signaling modulator 3	protein_codin	-0.4930	0.0954	1	32.52	0.1675
**UBR2**	UBR2	26	ubiquitin protein ligase E3 component n-recognin 2	protein_codin	0.02795	0.905	1	7.646	0.08846
**TBPL1**	TBPL1	26	TATA-box binding protein like 1	protein_codin	0.6004	0.00288	1	19.45	0.1429
**HEBP2**	HEBP2	26	heme binding protein 2	protein_codin	0.01044	0.9187	1	51.68	0.09547
**FUCA2**	FUCA2	26	alpha-L-fucosidase 2	protein_codin	-0.5834	0.00294	1	7.358	0.06197
**AHR**	AHR	26	aryl hydrocarbon receptor	protein_codin	-0.5548	0.000247	1	52.51	0.0849
**VOPP1**	VOPP1	26	VOPP1 WW domain binding protein	protein_codin	-0.3281	0.0808	1	24.35	0.1482
**TMEM248**	TMEM248	26	transmembrane protein 248	protein_codin	-0.1060	0.514	1	16.51	0.1681
**CPSF4**	CPSF4	26	cleavage and polyadenylation specific factor 4	protein_codin	1.452	0.000651	1	2.554	0.1481
**BCAP29**	BCAP29	26	B cell receptor associated protein 29	protein_codin	-0.475	1.07E-07	0.002515	47.96	0.05364
**AGK**	AGK	26	acylglycerol kinase	protein_codin	1.046	0.006	1	2.905	0.2841
**EBP**	EBP	26	EBP cholestenol delta-isomerase	protein_codin	0.2995	0.238	1	3.264	0.1162
**SHROOM4**	SHROOM4	26	shroom family member 4	protein_codin	-0.1756	0.358	1	46.82	0.1125
**PRPS1**	PRPS1	26	phosphoribosyl pyrophosphate synthetase 1	protein_codin	-0.1974	0.396	1	6.958	0.1772
**SMIM19**	SMIM19	26	small integral membrane protein 19	protein_codin	0.3164	0.0532	1	28	0.1393
**PCMTD1**	PCMTD1	26	protein-L-isoaspartate (D-aspartate) O-methyltransferase dom	protein_codin	-0.448	5.06E-06	0.1177	30.58	0.02713
**TGS1**	TGS1	26	trimethylguanosine synthase 1	protein_codin	0.3944	0.0586	1	14.01	0.1989
**VCPIP1**	VCPIP1	26	valosin containing protein interacting protein 1	protein_codin	-0.2896	0.354	1	3.179	0.1105
**DCAF13**	DCAF13	26	DDB1 and CUL4 associated factor 13	protein_codin	0.5337	0.00405	1	16.15	0.08208
**NAPRT**	NAPRT	26	nicotinate phosphoribosyltransferase	protein_codin	0.2607	0.154	1	6.806	0.2529
**HSF1**	HSF1	26	heat shock transcription factor 1	protein_codin	0.5501	0.00194	1	6.332	0.366
**TEK**	TEK	26	TEK receptor tyrosine kinase	protein_codin	0.1626	0.343	1	12.71	0.1492
**RNF38**	RNF38	26	ring finger protein 38	protein_codin	0.5692	0.0472	1	3.1	0.2741
**PKN3**	PKN3	26	protein kinase N3	protein_codin	0.02115	0.945	1	27.09	0.1142
**SPACA9**	SPACA9	26	sperm acrosome associated 9	protein_codin	-0.06491	0.844	1	3.48	0.266
**UBAC1**	UBAC1	26	UBA domain containing 1	protein_codin	0.4359	0.0176	1	6.737	0.1893
**ASRGL1**	ASRGL1	26	asparaginase and isoaspartyl peptidase 1	protein_codin	-0.1166	0.637	1	46.3	0.08646
**C11orf68**	C11orf68	26	chromosome 11 open reading frame 68	protein_codin	0.3606	0.298	1	9.102	0.1975
**PICALM**	PICALM	26	phosphatidylinositol binding clathrin assembly protein	protein_codin	0.1121	0.476	1	14.63	0.1681
**CEP295**	CEP295	26	centrosomal protein 295	protein_codin	-0.2081	0.498	1	10.11	0.1786
**SRPRA**	SRPRA	26	SRP receptor subunit alpha	protein_codin	-0.1334	0.273	1	48.25	0.08195
**ST3GAL4**	ST3GAL4	26	ST3 beta-galactoside alpha-2,3-sialyltransferase 4	protein_codin	-0.6371	0.0227	1	10.28	0.2189
**ZNF33B**	ZNF33B	26	zinc finger protein 33B	protein_codin	0.5045	0.0849	1	3.564	0.1974
**NOLC1**	NOLC1	26	nucleolar and coiled-body phosphoprotein 1	protein_codin	0.5444	0.0119	1	14.16	0.1788
**ATN1**	ATN1	26	atrophin 1	protein_codin	0.7104	0.00654	1	3.36	0.2295
**CAND1**	CAND1	26	cullin associated and neddylation dissociated 1	protein_codin	-0.01515	0.937	1	8.487	0.08694
**ZFC3H1**	ZFC3H1	26	zinc finger C3H1-type containing	protein_codin	-0.3011	0.0834	1	16.9	0.08586
**N4BP2L2**	N4BP2L2	26	NEDD4 binding protein 2 like 2	protein_codin	0.09188	0.3352	1	27.72	0.06329
**KPNA3**	KPNA3	26	karyopherin subunit alpha 3	protein_codin	0.597	0.00243	1	35.96	0.1732
**CKAP2**	CKAP2	26	cytoskeleton associated protein 2	protein_codin	-0.402	0.167	1	3.778	0.1395
**STK24**	STK24	26	serine/threonine kinase 24	protein_codin	-0.2239	0.248	1	47.54	0.1111
**BRMS1L**	BRMS1L	26	BRMS1 like transcriptional repressor	protein_codin	0.7417	0.0222	1	8.363	0.1952
**ARID4A**	ARID4A	26	AT-rich interaction domain 4A	protein_codin	0.1788	0.226	1	10.21	0.1104
**COX16**	COX16	26	cytochrome c oxidase assembly factor COX16	protein_codin	0.2817	0.338	1	2.952	0.08252
**FEM1B**	FEM1B	26	fem-1 homolog B	protein_codin	-0.01440	0.958	1	3.456	0.2177
**ZFAND6**	ZFAND6	26	zinc finger AN1-type containing 6	protein_codin	0.01711	0.902	1	27.88	0.08421
**SRRM2**	SRRM2	26	serine/arginine repetitive matrix 2	protein_codin	0.1232	0.123	1	32.93	0.106
**ZNF75A**	ZNF75A	26	zinc finger protein 75a	protein_codin	0.1692	0.56	1	3.129	0.2214
**USB1**	USB1	26	U6 snRNA biogenesis phosphodiesterase 1	protein_codin	-0.5866	0.0228	1	7.863	0.1071
**DYNC1LI2**	DYNC1LI2	26	dynein cytoplasmic 1 light intermediate chain 2	protein_codin	-0.02416	0.83	1	14.45	0.1124
**MED11**	MED11	26	mediator complex subunit 11	protein_codin	-0.1159	0.637	1	7.134	0.08862

**MRPS23**	MRPS23	26	mitochondrial ribosomal protein S23	protein_codin	0.114	0.506	1	7.146	0.0221
**RAD51C**	RAD51C	26	RAD51 paralog C	protein_codin	0.1269	0.62	1	3.511	0.0948
**RAB12**	RAB12	26	RAB12, member RAS oncogene family	protein_codin	0.225	0.315	1	24.62	0.1654
**TXNL1**	TXNL1	26	thioredoxin like 1	protein_codin	0.005897	0.957	1	18.18	0.1918
**TRPC4AP**	TRPC4AP	26	transient receptor potential cation channel subfamily C memb	protein_codin	0.2742	0.39	1	2.982	0.2447
**CPNE1**	CPNE1	26	copine 1	protein_codin	-0.3174	0.0441	1	7.607	0.1141
**PTPN1**	PTPN1	26	protein tyrosine phosphatase non-receptor type 1	protein_codin	0.09841	0.618	1	18.04	0.08806
**SLC2A4RG**	SLC2A4RG	26	SLC2A4 regulator	protein_codin	-0.1354	0.318	1	11.58	0.08677
**HDGFL2**	HDGFL2	26	HDGF like 2	protein_codin	0.4509	0.101	1	6.946	0.1459
**PIN1**	PIN1	26	peptidylprolyl cis/trans isomerase, NIMA-interacting 1	protein_codin	0.1229	0.297	1	28.27	0.08326
**KXD1**	KXD1	26	KxDL motif containing 1	protein_codin	0.2231	0.114	1	12.87	0.02685
**EXOSC5**	EXOSC5	26	exosome component 5	protein_codin	0.14	0.598	1	9.328	0.2768
**SLC25A1**	SLC25A1	26	solute carrier family 25 member 1	protein_codin	0.1122	0.63	1	6.793	0.1082
**DDX17**	DDX17	26	DEAD-box helicase 17	protein_codin	-0.2105	0.0596	1	32.02	0.08132
**DNAL4**	DNAL4	26	dynein axonemal light chain 4	protein_codin	0.4107	0.143	1	8.212	0.1932
**MAPK11**	MAPK11	26	mitogen-activated protein kinase 11	protein_codin	0.7294	0.0016	1	7.585	0.1745
**PAXBP1**	PAXBP1	26	PAX3 and PAX7 binding protein 1	protein_codin	-0.002282	0.992	1	7.172	0.3561
**SSU72**	SSU72	27	SSU72 homolog, RNA polymerase II CTD phosphatase	protein_codin	-0.1131	0.328	1	19.99	0.1394
**CDK11A**	CDK11A	27	cyclin dependent kinase 11A	protein_codin	0.5352	0.057	1	8.956	0.1389
**SPSB1**	SPSB1	27	splA/ryanodine receptor domain and SOCS box containing 1	protein_codin	0.3481	0.248	1	10.98	0.09438
**NECAP2**	NECAP2	27	NECAP endocytosis associated 2	protein_codin	0.05091	0.758	1	15.13	0.1364
**RABGGTB**	RABGGTB	27	Rab geranylgeranyltransferase subunit beta	protein_codin	-0.3382	0.0620	1	45.88	0.1107
**LRRC8C**	LRRC8C	27	leucine rich repeat containing 8 VRAC subunit C	protein_codin	0.6345	0.00724	1	22.46	0.1939
**SLC25A24**	SLC25A24	27	solute carrier family 25 member 24	protein_codin	0.3309	0.114	1	8.484	0.1387
**GATAD2B**	GATAD2B	27	GATA zinc finger domain containing 2B	protein_codin	0.2032	0.378	1	7.592	0.1721
**KIAA0907**	KIAA0907	27	KH domain containing 4, pre-mRNA splicing factor	protein_codin	NA	NA	NA	3.274	0.05839
**PFDN2**	PFDN2	27	prefoldin subunit 2	protein_codin	-0.3694	1.66E-05	0.3843	28.88	0.02651
**TIMM17A**	TIMM17A	27	translocase of inner mitochondrial membrane 17A	protein_codin	-0.05326	0.817	1	6.797	0.1364
**EPRS**	EPRS	27	glutamyl-prolyl-tRNA synthetase	protein_codin	0.1419	0.31	1	20.14	0.02618
**ZNF672**	ZNF672	27	zinc finger protein 672	protein_codin	0.4489	0.152	1	3.093	0.1481
**VPS54**	VPS54	27	VPS54 subunit of GARP complex	protein_codin	0.01081	0.972	1	3.189	0.1671
**GCFC2**	GCFC2	27	GC-rich sequence DNA-binding factor 2	protein_codin	0.3109	0.255	1	8.32	0.2379
**TXNDC9**	TXNDC9	27	thioredoxin domain containing 9	protein_codin	0.2812	0.0769	1	13.8	0.1129
**RALB**	RALB	27	RAS like proto-oncogene B	protein_codin	0.2312	0.147	1	52.34	0.08003
**UGGT1**	UGGT1	27	UDP-glucose glycoprotein glucosyltransferase 1	protein_codin	-0.3685	0.192	1	3.354	0.3085
**TANK**	TANK	27	TRAF family member associated NFKB activator	protein_codin	0.1162	0.541	1	5.417	0.06829
**SSFA2**	SSFA2	27	ITPR interacting domain containing 2	protein_codin	-0.7639	9.37E-12	2.22E-07	47.53	0.1131
**CYP20A1**	CYP20A1	27	cytochrome P450 family 20 subfamily A member 1	protein_codin	0.08777	0.639	1	17.49	0.3319
**CTDSP1**	CTDSP1	27	CTD small phosphatase 1	protein_codin	-0.2482	0.094	1	15.2	0.05338
**JAGN1**	JAGN1	27	jagunal homolog 1	protein_codin	0.558	0.00089	1	12.9	0.1419
**LSM3.00**	LSM3.00	27	LSM3 homolog, U6 small nuclear RNA and mRNA degradatio	protein_codin	-0.1482	0.152	1	34.12	0.1342
**CNOT10**	CNOT10	27	CCR4-NOT transcription complex subunit 10	protein_codin	0.4406	0.192	1	10.33	0.2349
**C3orf14**	C3orf14	27	chromosome 3 open reading frame 14	protein_codin	-0.52	0.0501	1	7.657	0.08659
**NECTIN3**	NECTIN3	27	nectin cell adhesion molecule 3	protein_codin	-0.3885	0.139	1	9.259	0.4185
**C3orf58**	C3orf58	27	divergent protein kinase domain 2A	protein_codin	0.04096	0.85	1	19.77	0.127
**LRPAP1**	LRPAP1	27	LDL receptor related protein associated protein 1	protein_codin	-0.425	7.66E-06	0.1779	41.94	0.05459
**HNRNPD**	HNRNPD	27	heterogeneous nuclear ribonucleoprotein D	protein_codin	0.2192	0.0191	1	34.35	0.05437
**AFF1**	AFF1	27	AF4/FMR2 family member 1	protein_codin	0.2714	0.157	1	30.41	0.3735
**EIF4E**	EIF4E	27	eukaryotic translation initiation factor 4E	protein_codin	0.2194	0.218	1	7.448	0.02626
**UBE2D3**	UBE2D3	27	ubiquitin conjugating enzyme E2 D3	protein_codin	0.2822	0.00069	1	52.52	0.09071
**PIK3R1**	PIK3R1	27	phosphoinositide-3-kinase regulatory subunit 1	protein_codin	-0.6632	0.0014	1	47.2	0.08562
**MSH3**	MSH3	27	mutS homolog 3	protein_codin	-0.2027	0.488	1	11.22	0.08208
**FAM174A**	FAM174A	27	family with sequence similarity 174 member A	protein_codin	0.04840	0.772	1	16.68	0.05444
**FER**	FER	27	FER tyrosine kinase	protein_codin	-0.1462	0.58	1	9.262	0.1974
**HNRNPA0**	HNRNPA0	27	heterogeneous nuclear ribonucleoprotein A0	protein_codin	0.09871	0.306	1	46.05	0.0542
**CLINT1**	CLINT1	27	clathrin interactor 1	protein_codin	0.102	0.469	1	24.38	0.1101
**RIOK1**	RIOK1	27	RIO kinase 1	protein_codin	0.4858	0.0204	1	20.52	0.2867
**ATXN1**	ATXN1	27	ataxin 1	protein_codin	-0.8539	0.000929	1	10.33	0.08179
**ENPP4**	ENPP4	27	ectonucleotide pyrophosphatase/phosphodiesterase 4	protein_codin	0.256	0.296	1	7.878	0.1970
**ASCC3**	ASCC3	27	activating signal cointegrator 1 complex subunit 3	protein_codin	0.3521	0.0966	1	8.311	0.172
**HDDC2**	HDDC2	27	HD domain containing 2	protein_codin	0.4775	0.0447	1	23.84	0.1125
**SF3B5**	SF3B5	27	splicing factor 3b subunit 5	protein_codin	-0.2325	0.00897	1	28.06	0.02967
**PSMG3**	PSMG3	27	proteasome assembly chaperone 3	protein_codin	0.2482	0.249	1	5.484	0.1080
**CHST12**	CHST12	27	carbohydrate sulfotransferase 12	protein_codin	0.06724	0.714	1	43.82	0.2435
**UPP1**	UPP1	27	uridine phosphorylase 1	protein_codin	0.5296	0.000526	1	52.06	0.1684
**SAMD9**	SAMD9	27	sterile alpha motif domain containing 9	protein_codin	0.1944	0.333	1	50.67	0.0801
**SRRT**	SRRT	27	serrate, RNA effector molecule	protein_codin	-0.08312	0.726	1	8.235	0.1432
**ORAI2**	ORAI2	27	ORAI calcium release-activated calcium modulator 2	protein_codin	-0.2934	0.215	1	27.27	0.1144
**PUS7**	PUS7	27	pseudouridine synthase 7	protein_codin	0.9357	0.0217	1	34.01	0.1411
**GIMAP2**	GIMAP2	27	GTPase, IMAP family member 2	protein_codin	0.818	0.000241	1	7.182	0.1074
**CHST7**	CHST7	27	carbohydrate sulfotransferase 7	protein_codin	1.828	2.93E-09	6.94E-05	18.85	0.1744
**DIAPH2**	DIAPH2	27	diaphanous related formin 2	protein_codin	0.3449	0.112	1	51.42	0.1369
**TCEAL8**	TCEAL8	27	transcription elongation factor A like 8	protein_codin	0.06502	0.613	1	49.8	0.1212
**GSR**	GSR	27	glutathione-disulfide reductase	protein_codin	0.1200	0.69	1	2.725	0.1688
**CHD7**	CHD7	27	chromodomain helicase DNA binding protein 7	protein_codin	0.7874	0.00224	1	3.627	0.1667
**ENY2**	ENY2	27	ENY2 transcription and export complex 2 subunit	protein_codin	0.4097	2.53E-07	0.005936	43.37	0.02758
**MYC**	MYC	27	MYC proto-oncogene, bHLH transcription factor	protein_codin	0.6786	0.000246	1	52.58	0.05619
**VCP**	VCP	27	valosin containing protein	protein_codin	-0.4341	0.00208	1	45.12	0.05326
**OSTF1**	OSTF1	27	osteoclast stimulating factor 1	protein_codin	0.3886	0.00429	1	24.98	0.08414
**C9orf78**	C9orf78	27	chromosome 9 open reading frame 78	protein_codin	0.04880	0.69	1	19.98	0.05398
**NELFB**	NELFB	27	negative elongation factor complex member B	protein_codin	0.3391	0.157	1	6.902	0.1942
**COMMD9**	COMMD9	27	COMM domain containing 9	protein_codin	0.00659	0.976	1	6.049	0.1137
**CPSF7**	CPSF7	27	cleavage and polyadenylation specific factor 7	protein_codin	1.012	0.0049	1	2.827	0.1951
**CDC42EP2**	CDC42EP2	27	CDC42 effector protein 2	protein_codin	-0.3359	0.0669	1	17.76	0.08080
**MAP3K11**	MAP3K11	27	mitogen-activated protein kinase kinase kinase 11	protein_codin	0.2061	0.137	1	51.89	0.08182
**PPFIA1**	PPFIA1	27	PTPRF interacting protein alpha 1	protein_codin	0.04231	0.843	1	22.45	0.1386
**IL18BP**	IL18BP	27	interleukin 18 binding protein	protein_codin	0.1072	0.627	1	17.94	0.1869
**C11orf54**	C11orf54	27	chromosome 11 open reading frame 54	protein_codin	-0.1075	0.628	1	6.29	0.1414
**AMOTL1**	AMOTL1	27	angiomotin like 1	protein_codin	0.4751	0.0672	1	8.407	0.1562
**CBL**	CBL	27	Cbl proto-oncogene	protein_codin	0.1131	0.646	1	8.488	0.276
**ARHGAP21**	ARHGAP21	27	Rho GTPase activating protein 21	protein_codin	0.1011	0.653	1	17.72	0.2624
**P4HA1**	P4HA1	27	prolyl 4-hydroxylase subunit alpha 1	protein_codin	0.1749	0.462	1	23.64	0.167
**TSPAN14**	TSPAN14	27	tetraspanin 14	protein_codin	0.6109	0.00024	1	28.67	0.1962
**DYRK4**	DYRK4	27	dual specificity tyrosine phosphorylation regulated kinase 4	protein_codin	-1.438	1.2E-13	2.87E-09	8.896	0.1384
**LPCAT3**	LPCAT3	27	lysophosphatidylcholine acyltransferase 3	protein_codin	0.03689	0.885	1	3.24	0.1422
**HEBP1**	HEBP1	27	heme binding protein 1	protein_codin	-0.1919	0.0520	1	46.68	0.05986
**PLEKHA5**	PLEKHA5	27	pleckstrin homology domain containing A5	protein_codin	-0.5264	0.0567	1	8.109	0.2279
**SMARCD1**	SMARCD1	27	SWI/SNF related, matrix associated, actin dependent regulato	protein_codin	-0.3815	0.0485	1	7.29	0.2262
**KRT8**	KRT8	27	keratin 8	protein_codin	-0.02444	0.922	1	22.28	0.1702
**MAP3K12**	MAP3K12	27	mitogen-activated protein kinase kinase kinase 12	protein_codin	-0.4558	0.0319	1	6.51	0.1969
**CPSF6**	CPSF6	27	cleavage and polyadenylation specific factor 6	protein_codin	-0.06977	0.742	1	8.779	0.1392
**CNOT2**	CNOT2	27	CCR4-NOT transcription complex subunit 2	protein_codin	-0.09565	0.52	1	11.99	0.1108
**KITLG**	KITLG	27	KIT ligand	protein_codin	0.9587	0.000909	1	6.788	0.2169
**SSH1**	SSH1	27	slingshot protein phosphatase 1	protein_codin	0.06164	0.792	1	9.77	0.1682
**RILPL2**	RILPL2	27	Rab interacting lysosomal protein like 2	protein_codin	-0.07732	0.477	1	40.19	0.14
**PCCA**	PCCA	27	propionyl-CoA carboxylase subunit alpha	protein_codin	0.2066	0.502	1	3.252	0.08026
**DHRS1**	DHRS1	27	dehydrogenase/reductase 1	protein_codin	0.6479	0.0670	1	3.317	0.06172
**HECTD1**	HECTD1	27	HECT domain E3 ubiquitin protein ligase 1	protein_codin	-0.2355	0.137	1	34.42	0.1657
**FAM177A1**	FAM177A1	27	family with sequence similarity 177 member A1	protein_codin	0.244	0.0366	1	33.79	0.02827
**PNN**	PNN	27	pinin, desmosome associated protein	protein_codin	0.1324	0.229	1	16.2	0.1092
**ARF6**	ARF6	27	ADP ribosylation factor 6	protein_codin	0.1905	0.186	1	12.83	0.1637
**NIN**	NIN	27	ninein	protein_codin	0.2636	0.41	1	29.17	0.2781
**DDHD1**	DDHD1	27	DDHD domain containing 1	protein_codin	-0.6086	0.109	1	11.53	0.1958
**MAPK1IP1L**	MAPK1IP1L	27	mitogen-activated protein kinase 1 interacting protein 1 like	protein_codin	0.2359	0.0829	1	11.28	0.08088
**ATP6V1D**	ATP6V1D	27	ATPase H+ transporting V1 subunit D	protein_codin	-0.3525	0.0479	1	16.47	0.1102
**ACYP1**	ACYP1	27	acylphosphatase 1	protein_codin	0.8277	0.00542	1	8.124	0.1692
**NEK9**	NEK9	27	NIMA related kinase 9	protein_codin	0.01328	0.965	1	2.972	0.2232
**SECISBP2L**	SECISBP2L	27	SECIS binding protein 2 like	protein_codin	-0.1217	0.488	1	42.6	0.2549
**GOT2**	GOT2	27	glutamic-oxaloacetic transaminase 2	protein_codin	0.01156	0.97	1	9.082	0.2831
**PARD6A**	PARD6A	27	par-6 family cell polarity regulator alpha	protein_codin	-0.3474	0.232	1	3.584	0.192
**WWP2**	WWP2	27	WW domain containing E3 ubiquitin protein ligase 2	protein_codin	-0.3322	0.232	1	3.229	0.08617
**DDX19A**	DDX19A	27	DEAD-box helicase 19A	protein_codin	-0.2812	0.379	1	2.952	0.2584
**ABR**	ABR	27	ABR activator of RhoGEF and GTPase	protein_codin	-0.5125	0.0996	1	3.169	0.1409
**TMEM220**	TMEM220	27	transmembrane protein 220	protein_codin	-0.2506	0.338	1	49.63	0.1397
**THRA**	THRA	27	thyroid hormone receptor alpha	protein_codin	0.2484	0.254	1	8.434	0.3441
**RAB5C**	RAB5C	27	RAB5C, member RAS oncogene family	protein_codin	0.1091	0.255	1	33.05	0.02905
**TOB1**	TOB1	27	transducer of ERBB2, 1	protein_codin	0.1661	0.31	1	20.58	0.1892
**RPS6KB1**	RPS6KB1	27	ribosomal protein S6 kinase B1	protein_codin	-0.3366	0.207	1	9.068	0.2016
**TACO1**	TACO1	27	translational activator of cytochrome c oxidase I	protein_codin	0.3806	0.214	1	31.75	0.1771
**METTL23**	METTL23	27	methyltransferase like 23	protein_codin	0.2264	0.19	1	6.678	0.02637
**ESCO1**	ESCO1	27	establishment of sister chromatid cohesion N-acetyltransfera	protein_codin	0.2049	0.37	1	8.185	0.1388
**SNRPD1**	SNRPD1	27	small nuclear ribonucleoprotein D1 polypeptide	protein_codin	0.102	0.322	1	45.58	0.08106
**MALT1**	MALT1	27	MALT1 paracaspase	protein_codin	0.4982	0.0268	1	6.988	0.1751
**LMAN1**	LMAN1	27	lectin, mannose binding 1	protein_codin	0.1024	0.296	1	27.88	0.08574
**CCM2L**	CCM2L	27	CCM2 like scaffold protein	protein_codin	0.6472	0.00136	1	7.392	0.1669
**ITCH**	ITCH	27	itchy E3 ubiquitin protein ligase	protein_codin	0.3554	0.187	1	8.022	0.2555
**ERGIC3**	ERGIC3	27	ERGIC and golgi 3	protein_codin	-0.4224	0.00109	1	48.83	0.1636
**SLC35C2**	SLC35C2	27	solute carrier family 35 member C2	protein_codin	0.5681	0.0902	1	3.42	0.262
**B4GALT5**	B4GALT5	27	beta-1,4-galactosyltransferase 5	protein_codin	0.8605	0.00371	1	9.137	0.1489
**GTF2F1**	GTF2F1	27	general transcription factor IIF subunit 1	protein_codin	0.1371	0.38	1	52.2	0.2257
**TRIP10**	TRIP10	27	thyroid hormone receptor interactor 10	protein_codin	-0.0008512	0.997	1	6.338	0.1101
**XAB2**	XAB2	27	XPA binding protein 2	protein_codin	0.6482	0.0549	1	2.59	0.056
**KANK3**	KANK3	27	KN motif and ankyrin repeat domains 3	protein_codin	1.101	2.38E-17	5.72E-13	47.48	0.0329
**ZNF426**	ZNF426	27	zinc finger protein 426	protein_codin	0.5162	0.18	1	8.266	0.196
**DNM2**	DNM2	27	dynamin 2	protein_codin	0.4880	0.0370	1	9.328	0.2910
**ACP5**	ACP5	27	acid phosphatase 5, tartrate resistant	protein_codin	1.404	0.0391	1	52.58	0.1092
**ZNF44**	ZNF44	27	zinc finger protein 44	protein_codin	0.3454	0.278	1	3.418	0.2081
**SUGP2**	SUGP2	27	SURP and G-patch domain containing 2	protein_codin	-0.6812	0.0126	1	9.796	0.2512
**TMEM161A**	TMEM161A	27	transmembrane protein 161A	protein_codin	0.3611	0.162	1	2.716	0.1431
**ZNF428**	ZNF428	27	zinc finger protein 428	protein_codin	-0.5091	3.88E-05	0.8949	29.48	0.02916
**JOSD2**	JOSD2	27	Josephin domain containing 2	protein_codin	-0.1231	0.440	1	15.45	0.1845
**ZNF264**	ZNF264	27	zinc finger protein 264	protein_codin	-0.1164	0.672	1	9.84	0.2059
**CCNL2**	CCNL2	28	cyclin L2	protein_codin	-0.2449	0.194	1	7.797	0.05296
**PSMB2**	PSMB2	28	proteasome subunit beta 2	protein_codin	0.2604	0.0128	1	52.58	0.0575
**YRDC**	YRDC	28	yrdC N6-threonylcarbamoyltransferase domain containing	protein_codin	0.5212	0.00791	1	16.68	0.06163
**ZFYVE9**	ZFYVE9	28	zinc finger FYVE-type containing 9	protein_codin	0.3495	0.286	1	3.257	0.268
**PGM1**	PGM1	28	phosphoglucomutase 1	protein_codin	-0.5347	0.0394	1	3.037	0.1792
**DDX20**	DDX20	28	DEAD-box helicase 20	protein_codin	0.1262	0.718	1	11.8	0.312
**OTUD7B**	OTUD7B	28	OTU deubiquitinase 7B	protein_codin	-0.5652	0.0447	1	3.102	0.1899
**KRTCAP2**	KRTCAP2	28	keratinocyte associated protein 2	protein_codin	0.1898	0.0575	1	21.22	0.1088
**BATF3**	BATF3	28	basic leucine zipper ATF-like transcription factor 3	protein_codin	0.9209	0.00647	1	8.34	0.2262
**UNC50**	UNC50	28	unc-50 inner nuclear membrane RNA binding protein	protein_codin	0.2474	0.132	1	15.47	0.1966
**TANC1**	TANC1	28	tetratricopeptide repeat, ankyrin repeat and coiled-coil contair	protein_codin	0.2665	0.292	1	6.709	0.2254
**NOSTRIN**	NOSTRIN	28	nitric oxide synthase trafficking	protein_codin	2.485	8.53E-34	2.07E-29	27.1	0.1104
**PMS1**	PMS1	28	PMS1 homolog 1, mismatch repair system component	protein_codin	1.318	5.8E-05	1	8.317	0.0795
**INPP1**	INPP1	28	inositol polyphosphate-1-phosphatase	protein_codin	0.6015	9.1E-06	0.2113	27.85	0.09686
**ITPR1**	ITPR1	28	inositol 1,4,5-trisphosphate receptor type 1	protein_codin	0.04926	0.813	1	16.1	0.05679
**BHLHE40**	BHLHE40	28	basic helix-loop-helix family member e40	protein_codin	0.5029	0.000199	1	37.56	0.05395
**HRH1**	HRH1	28	histamine receptor H1	protein_codin	-0.06042	0.772	1	19.68	0.1121
**MYRIP**	MYRIP	28	myosin VIIA and Rab interacting protein	protein_codin	1.686	3.76E-10	8.92E-06	30.1	0.2332
**KIAA1143**	KIAA1143	28	KIAA1143	protein_codin	0.1057	0.549	1	38.24	0.3043
**PBRM1**	PBRM1	28	polybromo 1	protein_codin	0.04853	0.78	1	15.72	0.1636
**CCDC66**	CCDC66	28	coiled-coil domain containing 66	protein_codin	-0.02447	0.88	1	13.87	0.2569
**LRIG1**	LRIG1	28	leucine rich repeats and immunoglobulin like domains 1	protein_codin	-1.082	5.53E-07	0.01295	51.92	0.06586
**SLC25A36**	SLC25A36	28	solute carrier family 25 member 36	protein_codin	0.03147	0.822	1	12.52	0.08197
**GNB4**	GNB4	28	G protein subunit beta 4	protein_codin	0.6462	0.000181	1	23.03	0.1219
**NDUFB5**	NDUFB5	28	NADH:ubiquinone oxidoreductase subunit B5	protein_codin	0.04855	0.617	1	50.62	0.199
**YTHDC1**	YTHDC1	28	YTH domain containing 1	protein_codin	0.2652	0.0692	1	18.4	0.07941
**GRSF1**	GRSF1	28	G-rich RNA sequence binding factor 1	protein_codin	0.2365	0.19	1	6.695	0.2206
**NAF1**	NAF1	28	nuclear assembly factor 1 ribonucleoprotein	protein_codin	0.5427	0.119	1	2.989	0.09007
**RAD1**	RAD1	28	RAD1 checkpoint DNA exonuclease	protein_codin	-0.008052	0.98	1	8.298	0.1795
**SRP19**	SRP19	28	signal recognition particle 19	protein_codin	0.2167	0.169	1	50.18	0.05867
**LINC01184**	LINC01184	28	long intergenic non-protein coding RNA 1184	lincRNA	0.003625	0.987	1	8.948	0.165
**PCDH12**	PCDH12	28	protocadherin 12	protein_codin	-0.1317	0.552	1	46.75	0.1156
**TUBB**	TUBB	28	tubulin beta class I	protein_codin	0.3809	1.05E-07	0.002469	32.2	0.02645
**CDC5L**	CDC5L	28	cell division cycle 5 like	protein_codin	0.09847	0.479	1	10.58	0.1142
**TMEM14A**	TMEM14A	28	transmembrane protein 14A	protein_codin	-0.158	0.397	1	7.652	0.2214
**SMAP1**	SMAP1	28	small ArfGAP 1	protein_codin	-0.02031	0.903	1	14.02	0.1132
**C6orf203**	C6orf203	28	mitochondrial transcription rescue factor 1	protein_codin	-0.02506	0.924	1	3.05	0.1099
**RAB32**	RAB32	28	RAB32, member RAS oncogene family	protein_codin	-0.602	9.34E-05	1	15.89	0.05446
**KBTBD2**	KBTBD2	28	kelch repeat and BTB domain containing 2	protein_codin	0.3692	0.0261	1	12.86	0.1671
**SUMF2**	SUMF2	28	sulfatase modifying factor 2	protein_codin	-0.1902	0.171	1	30.37	0.08815
**SBDS**	SBDS	28	SBDS ribosome maturation factor	protein_codin	0.1232	0.0999	1	30.84	0.02688
**BUD23**	BUD23	28	BUD23 rRNA methyltransferase and ribosome maturation fac	protein_codin	0.2052	0.134	1	10.2	0.1691
**AP4M1**	AP4M1	28	adaptor related protein complex 4 subunit mu 1	protein_codin	0.08554	0.75	1	3.009	0.1685
**MOSPD3**	MOSPD3	28	motile sperm domain containing 3	protein_codin	0.01551	0.946	1	6.566	0.1541
**ABCF2**	ABCF2	28	ATP binding cassette subfamily F member 2	protein_codin	1.507	0.983	1	9.657	0.1442
**PIR**	PIR	28	pirin	protein_codin	-0.1336	0.461	1	52.58	0.1672
**ZRSR2**	ZRSR2	28	zinc finger CCCH-type, RNA binding motif and serine/arginine	protein_codin	0.186	0.49	1	10.45	0.2509
**PRRG1**	PRRG1	28	proline rich and Gla domain 1	protein_codin	0.7106	0.00257	1	41.55	0.1401
**TSR2**	TSR2	28	TSR2 ribosome maturation factor	protein_codin	0.4199	0.0114	1	14.37	0.2336
**APOOL**	APOOL	28	apolipoprotein O like	protein_codin	0.5126	0.0705	1	6.707	0.1449
**CNOT7**	CNOT7	28	CCR4-NOT transcription complex subunit 7	protein_codin	0.1106	0.564	1	17.22	0.1372
**ELOC**	ELOC	28	elongin C	protein_codin	0.1502	0.0862	1	46.52	0.056
**TMEM70**	TMEM70	28	transmembrane protein 70	protein_codin	0.8465	4.24E-06	0.09872	52.58	0.1742
**NBN**	NBN	28	nibrin	protein_codin	0.1825	0.369	1	18.22	0.1074
**RUNX1T1**	RUNX1T1	28	RUNX1 partner transcriptional co-repressor 1	protein_codin	-0.6875	6.35E-06	0.1476	51.9	0.1131
**NDUFB9**	NDUFB9	28	NADH:ubiquinone oxidoreductase subunit B9	protein_codin	-0.02097	0.811	1	27.22	0.05237
**GRINA**	GRINA	28	glutamate ionotropic receptor NMDA type subunit associated	protein_codin	0.9361	5.71E-05	1	20.8	0.1116
**CCDC107**	CCDC107	28	coiled-coil domain containing 107	protein_codin	-0.2748	0.0724	1	6.605	0.1627
**CRACR2B**	CRACR2B	28	calcium release activated channel regulator 2B	protein_codin	0.9709	2.05E-06	0.04784	26.86	0.1066
**CCDC34**	CCDC34	28	coiled-coil domain containing 34	protein_codin	-0.6449	0.0199	1	10.59	0.2720
**PRPF19**	PRPF19	28	pre-mRNA processing factor 19	protein_codin	0.156	0.693	1	3.32	0.1924
**VPS51**	VPS51	28	VPS51 subunit of GARP complex	protein_codin	0.4422	0.00666	1	26.8	0.09248
**RCE1**	RCE1	28	Ras converting CAAX endopeptidase 1	protein_codin	0.1711	0.542	1	3.335	0.2272
**FADD**	FADD	28	Fas associated via death domain	protein_codin	0.5212	0.0677	1	9.777	0.2012

**SEC61A1**	SEC61A1		29	SEC61 translocon alpha 1 subunit	protein_codin	-0.6571	0.00164	1	8.652	0.1112
**GATA2-AS1**	GATA2-AS1		29	GATA2 antisense RNA 1	antisense_RN	-0.5637	0.00265	1	8.074	0.1372
**EIF2A**	EIF2A		29	eukaryotic translation initiation factor 2A	protein_codin	0.3128	0.00312	1	32.42	0.05666
**PCYT1A**	PCYT1A		29	phosphate cytidylyltransferase 1, choline, alpha	protein_codin	-0.2251	0.428	1	3.255	0.3792
**NCBP2**	NCBP2		29	nuclear cap binding protein subunit 2	protein_codin	0.5888	0.00157	1	19.21	0.166
**CEP135**	CEP135		29	centrosomal protein 135	protein_codin	0.5038	0.0644	1	25.48	0.3079
**UTP3**	UTP3		29	UTP3 small subunit processome component	protein_codin	0.4759	0.0471	1	21.92	0.1114
**MTHFD2L**	MTHFD2L		29	methylenetetrahydrofolate dehydrogenase (NADP+ dependen	protein_codin	0.02116	0.942	1	8.288	0.2188
**CCT5**	CCT5		29	chaperonin containing TCP1 subunit 5	protein_codin	0.1234	0.238	1	24.72	0.08622
**OSMR**	OSMR		29	oncostatin M receptor	protein_codin	0.02937	0.911	1	6.744	0.2022
**MOCS2**	MOCS2		29	molybdenum cofactor synthesis 2	protein_codin	-0.1075	0.485	1	13.18	0.1079
**MAN2A1**	MAN2A1		29	mannosidase alpha class 2A member 1	protein_codin	-0.3582	0.202	1	3.366	0.2027
**NREP**	NREP		29	neuronal regeneration related protein	protein_codin	-0.03402	0.894	1	8.792	0.2187
**VDAC1**	VDAC1		29	voltage dependent anion channel 1	protein_codin	-0.1467	0.19	1	49.66	0.05377
**H2AFY**	H2AFY		29	H2A histone family member Y	protein_codin	-0.05007	0.722	1	43.58	0.08175
**SIL1**	SIL1		29	SIL1 nucleotide exchange factor	protein_codin	-0.4956	0.022	1	9.076	0.2
**TCOF1**	TCOF1		29	treacle ribosome biogenesis factor 1	protein_codin	0.1771	0.542	1	11.48	0.2244
**CCDC69**	CCDC69		29	coiled-coil domain containing 69	protein_codin	0.9617	2.55E-08	0.000602	9.74	0.1122
**FAXDC2**	FAXDC2		29	fatty acid hydroxylase domain containing 2	protein_codin	-0.3406	0.222	1	9.222	0.2055
**TAP1**	TAP1		29	transporter 1, ATP binding cassette subfamily B member	protein_codin	1.064	2.23E-08	0.00050	45.19	0.128
**OARD1**	OARD1		29	O-acyl-ADP-ribose deacylase 1	protein_codin	0.4722	0.0169	1	7.334	0.128
**SLC35A1**	SLC35A1		29	solute carrier family 35 member A1	protein_codin	-0.1067	0.682	1	3.143	0.1792
**ORC3**	ORC3		29	origin recognition complex subunit 3	protein_codin	-0.07096	0.796	1	9.518	0.2508
**MDN1**	MDN1		29	midasin AAA ATPase 1	protein_codin	0.1016	0.768	1	3.402	0.08334
**ECHDC1**	ECHDC1		29	ethylmalonyl-CoA decarboxylase 1	protein_codin	0.05073	0.774	1	39.87	0.2065
**PERP**	PERP		29	p53 apoptosis effector related to PMP22	protein_codin	2.715	5.17E-28	1.25E-23	47.56	0.08209
**TAGAP**	TAGAP		29	T cell activation RhoGTPase activating protein	protein_codin	2.668	0.00286	1	44.16	0.1687
**COX19**	COX19		29	cytochrome c oxidase assembly factor COX19	protein_codin	0.6254	0.0346	1	7.114	0.09042
**ZNF12**	ZNF12		29	zinc finger protein 12	protein_codin	0.5182	0.188	1	3.317	0.3127
**BZW2**	BZW2		29	basic leucine zipper and W2 domains 2	protein_codin	0.5844	0.00211	1	14.22	0.1089
**MINDY4**	MINDY4		29	MINDY lysine 48 deubiquitinase 4	protein_codin	1.882	2.2E-06	0.05122	21.77	0.08076
**TMED4**	TMED4		29	transmembrane p24 trafficking protein 4	protein_codin	-0.2932	0.0695	1	16.4	0.326
**TPST1**	TPST1		29	tyrosylprotein sulfotransferase 1	protein_codin	0.4681	0.14	1	8.442	0.1870
**AC027644.3**	AC027644.3		29	novel transcript, antisense to RABGE	antisense_RN	0.3751	0.211	1	8.489	0.1675
**RSBN1L**	RSBN1L		29	round spermatid basic protein 1 like	protein_codin	0.07061	0.653	1	25.11	0.1748
**TMEM60**	TMEM60		29	transmembrane protein 60	protein_codin	0.8497	0.00526	1	6.638	0.2538
**CACNA2D1**	CACNA2D1		29	calcium voltage-gated channel auxiliary subunit alpha2delta 1	protein_codin	-0.4132	0.119	1	10.62	0.1412
**TRIP6**	TRIP6		29	thyroid hormone receptor interactor 6	protein_codin	-1.444	4.34E-15	1.04E-10	8.155	0.02785
**PLOD3**	PLOD3		29	procollagen-lysine,2-oxoglutarate 5-dioxygenase 3	protein_codin	0.3581	0.0842	1	7.828	0.1402
**HBP1**	HBP1		29	HMG-box transcription factor 1	protein_codin	-0.0192	0.906	1	6.312	0.2466
**METTL2B**	METTL2B		29	methyltransferase like 2B	protein_codin	0.1559	0.582	1	9.7	0.1670
**NOS3**	NOS3		29	nitric oxide synthase 3	protein_codin	-0.9002	8.09E-08	0.001902	47.94	0.02937
**KMT2C**	KMT2C		29	lysine methyltransferase 2C	protein_codin	-0.155	0.418	1	16.79	0.1101
**ESYT2**	ESYT2		29	extended synaptotagmin 2	protein_codin	0.7241	0.0202	1	17.52	0.174
**OTC**	OTC		29	ornithine carbamoyltransferase	protein_codin	-2.117	9.53E-17	2.29E-12	51.28	0.1228
**NDUFB11**	NDUFB11		29	NADH ubiquinone oxidoreductase subunit B11	protein_codin	-0.3167	2.85E-05	0.6583	25.4	0.05358
**HSD17B10**	HSD17B10		29	hydroxysteroid 17-beta dehydrogenase 10	protein_codin	-0.1922	0.226	1	6.476	0.08255
**PBDC1**	PBDC1		29	polysaccharide biosynthesis domain containing 1	protein_codin	0.3606	0.0665	1	29.58	0.1729
**ITM2A**	ITM2A		29	integral membrane protein 2A	protein_codin	0.9829	1.72E-22	4.15E-18	52.58	0.02716
**PCDH19**	PCDH19		29	protocadherin 19	protein_codin	2.835	0.000293	1	9.849	0.3467
**UBE2A**	UBE2A		29	ubiquitin conjugating enzyme E2 A	protein_codin	0.3475	0.0226	1	14.44	0.1134
**IDS**	IDS		29	iduronate 2-sulfatase	protein_codin	-1.015	1.99E-11	4.74E-07	30.2	0.06209
**IDH3G**	IDH3G		29	isocitrate dehydrogenase (NAD(+)) 3 gamma	protein_codin	0.4165	0.0132	1	6.665	0.08114
**F8A1**	F8A1		29	coagulation factor VIII associated 1	protein_codin	0.317	0.307	1	2.325	0.2492
**ERICH1**	ERICH1		29	glutamate rich 1	protein_codin	0.7732	1.15E-06	0.02688	9.17	0.2872
**MTUS1**	MTUS1		29	microtubule associated scaffold protein 1	protein_codin	-0.9048	1.41E-39	3.42E-35	51.57	0.02918
**BIN3**	BIN3		29	bridging integrator 3	protein_codin	0.8961	0.0046	1	8.277	0.1711
**R3HCC1**	R3HCC1		29	R3H domain and coiled-coil containing 1	protein_codin	0.01432	0.948	1	9.526	0.1422
**UBXN8**	UBXN8		29	UBX domain protein 8	protein_codin	0.231	0.392	1	8.622	0.1962
**TM2D2**	TM2D2		29	TM2 domain containing 2	protein_codin	-0.2335	0.15	1	14.54	0.1912
**ZBTB10**	ZBTB10		29	zinc finger and BTB domain containing 10	protein_codin	0.6359	0.0132	1	8.344	0.2305
**NDUFAF6**	NDUFAF6		29	NADH ubiquinone oxidoreductase complex assembly factor 6	protein_codin	0.8632	0.03	1	3.338	0.2272
**PKHD1L1**	PKHD1L1		29	PKHD1 like 1	protein_codin	-2.437	3.67E-15	8.79E-11	32.37	0.3551
**EBAG9**	EBAG9		29	estrogen receptor binding site associated antigen 9	protein_codin	0.4946	0.00331	1	34.43	0.08809
**CHMP5**	CHMP5		29	charged multivesicular body protein 5	protein_codin	0.1522	0.0706	1	22.95	0.06417
**ALDH1A1**	ALDH1A1		29	aldehyde dehydrogenase 1 family member A1	protein_codin	-3.807	1.83E-47	4.44E-43	44.94	0.08159
**GNA14**	GNA14		29	G protein subunit alpha 14	protein_codin	1.265	0.00596	1	9.378	0.1967
**SEC61B**	SEC61B		29	SEC61 translocon beta subunit	protein_codin	-0.4576	9.72E-14	2.32E-09	47.6	0.02617
**ZNF462**	ZNF462		29	zinc finger protein 462	protein_codin	1.862	3.88E-07	0.009092	7.816	0.05684
**MRRF**	MRRF		29	mitochondrial ribosome recycling factor	protein_codin	0.235	0.348	1	9.078	0.2465
**ZBTB43**	ZBTB43		29	zinc finger and BTB domain containing 43	protein_codin	-0.2992	0.308	1	10.29	0.3531
**PAXX**	PAXX		29	PAXX non-homologous end joining factor	protein_codin	0.2705	0.136	1	32.11	0.1097
**SMPD1**	SMPD1		29	sphingomyelin phosphodiesterase 1	protein_codin	-0.112	0.677	1	3.262	0.167
**ILK**	ILK		29	integrin linked kinase	protein_codin	-0.0711	0.626	1	12.58	0.161
**TNKS1BP1**	TNKS1BP1		29	tankyrase 1 binding protein 1	protein_codin	-0.2714	0.272	1	7.86	0.1125
**PPP1CA**	PPP1CA		29	protein phosphatase 1 catalytic subunit alpha	protein_codin	-0.07457	0.542	1	23.79	0.05779
**TMEM126A**	TMEM126A		29	transmembrane protein 126A	protein_codin	0.4755	0.0101	1	6.645	0.1457
**SMCO4**	SMCO4		29	single-pass membrane protein with coiled-coil domains 4	protein_codin	-0.2872	0.208	1	9.158	0.1670
**CCDC82**	CCDC82		29	coiled-coil domain containing 82	protein_codin	0.058	0.679	1	10.06	0.206
**KBTBD3**	KBTBD3		29	kelch repeat and BTB domain containing 3	protein_codin	0.7479	0.0184	1	8.036	0.2467
**NNMT**	NNMT		29	nicotinamide N-methyltransferase	protein_codin	-0.1858	0.0129	1	52.58	0.02787
**ZPR1**	ZPR1		29	ZPR1 zinc finger	protein_codin	0.5156	0.0265	1	27.25	0.1129
**TRAPPC4**	TRAPPC4		29	trafficking protein particle complex 4	protein_codin	0.3665	0.00816	1	26.75	0.166
**PUS3**	PUS3		29	pseudouridine synthase 3	protein_codin	0.7925	0.0109	1	3.58	0.1724
**BAMBI**	BAMBI		29	BMP and activin membrane bound inhibitor	protein_codin	-2.228	1.77E-09	4.19E-05	52.58	0.6542
**NRP1**	NRP1		29	neuropilin 1	protein_codin	0.1335	0.288	1	52.58	0.06178
**ARID5B**	ARID5B		29	AT-rich interaction domain 5B	protein_codin	0.3896	9.69E-05	1	52.42	0.08201
**GBF1**	GBF1		29	golgi brefeldin A resistant guanine nucleotide exchange factor	protein_codin	-0.6288	0.0422	1	2.348	0.2212
**FAM160B1**	FAM160B1		29	family with sequence similarity 160 member B1	protein_codin	0.008139	0.976	1	3.828	0.4309
**CTBP2**	CTBP2		29	C-terminal binding protein 2	protein_codin	-0.02365	0.898	1	6.822	0.1479
**TUBGCP2**	TUBGCP2		29	tubulin gamma complex associated protein 2	protein_codin	-0.009210	0.962	1	8.664	0.2454
**CHD4**	CHD4		29	chromodomain helicase DNA binding protein 4	protein_codin	-0.3907	0.00669	1	12.86	0.111
**MLF2.00**	MLF2.00		29	myeloid leukemia factor 2	protein_codin	0.2806	0.0302	1	42.12	0.1609
**TMTC1**	TMTC1		29	transmembrane O-mannosyltransferase targeting cadherins 1	protein_codin	0.7394	0.000918	1	42.05	0.08612
**ARID2**	ARID2		29	AT-rich interaction domain 2	protein_codin	0.3077	0.348	1	8.192	0.2296
**AMIGO2**	AMIGO2		29	adhesion molecule with Ig like domain 2	protein_codin	1.266	0.000106	1	9.238	0.2238
**SMAGP**	SMAGP		29	small cell adhesion glycoprotein	protein_codin	1.74	5.81E-19	1.4E-14	28.65	0.09282
**PRIM1**	PRIM1		29	DNA primase subunit 1	protein_codin	0.7626	0.0314	1	4.006	0.1717
**ATP23**	ATP23		29	ATP23 metallopeptidase and ATP synthase assembly factor h	protein_codin	0.9912	0.01	1	7.744	0.1741
**USP15**	USP15		29	ubiquitin specific peptidase 15	protein_codin	0.392	0.0232	1	11	0.2291
**RASSF3**	RASSF3		29	Ras association domain family member 3	protein_codin	0.5194	0.0455	1	27.78	0.1706
**YEATS4**	YEATS4		29	YEATS domain containing 4	protein_codin	0.3617	0.176	1	8.935	0.1452
**RASSF9**	RASSF9		29	Ras association domain family member 9	protein_codin	1.634	1.48E-17	3.56E-13	21.55	0.1168
**IKBIP**	IKBIP		29	IKBKB interacting protein	protein_codin	0.4892	0.000135	1	30.59	0.1364
**TCP11L2**	TCP11L2		29	t-complex 11 like 2	protein_codin	1.492	6.07E-07	0.01421	19.72	0.1945
**MLXIP**	MLXIP		29	MLX interacting protein	protein_codin	-0.3246	0.106	1	25.25	0.1652
**PXMP2**	PXMP2		29	peroxisomal membrane protein 2	protein_codin	-0.3919	0.0926	1	8.254	0.1659
**ZNF605**	ZNF605		29	zinc finger protein 605	protein_codin	-0.0316	0.928	1	3.032	0.2059
**RFC3**	RFC3		29	replication factor C subunit 3	protein_codin	0.1551	0.596	1	3.408	0.1954
**EXOSC8**	EXOSC8		29	exosome component 8	protein_codin	0.6858	4.1E-05	0.9455	12.72	0.1625
**WBP4**	WBP4		29	WW domain binding protein 4	protein_codin	0.1671	0.312	1	33.22	0.2895
**TRIM13**	TRIM13		29	tripartite motif containing 13	protein_codin	-0.4658	0.0154	1	8.58	0.08522
**TUBGCP3**	TUBGCP3		29	tubulin gamma complex associated protein 3	protein_codin	-0.1326	0.668	1	3.088	0.1674
**NGDN**	NGDN		29	neuroguidin	protein_codin	0.3252	0.0701	1	5.178	0.02634
**SCFD1**	SCFD1		29	sec1 family domain containing 1	protein_codin	-0.02503	0.892	1	20.46	0.1442
**FKBP3**	FKBP3		29	FKBP prolyl isomerase 3	protein_codin	-0.1996	0.212	1	26.55	0.1371
**MIS18BP1**	MIS18BP1		29	MIS18 binding protein 1	protein_codin	0.2062	0.188	1	43.79	0.1159
**TMX1**	TMX1		29	thioredoxin related transmembrane protein 1	protein_codin	0.3092	0.0629	1	15.84	0.1402
**CDKN3**	CDKN3		29	cyclin dependent kinase inhibitor 3	protein_codin	0.9472	0.022	1	47.38	0.3088
**ELMSAN1**	ELMSAN1		29	ELM2 and Myb/SANT domain containing 1	protein_codin	0.9097	1.06E-05	0.2459	52.57	0.02972
**TRIP11**	TRIP11		29	thyroid hormone receptor interactor 11	protein_codin	-0.1414	0.362	1	17.42	0.262
**CPSF2**	CPSF2		29	cleavage and polyadenylation specific factor 2	protein_codin	-0.1142	0.668	1	8.222	0.3022
**NIPA2**	NIPA2		29	NIPA magnesium transporter 2	protein_codin	0.1996	0.395	1	3.292	0.169
**EMC4**	EMC4		29	ER membrane protein complex subunit 4	protein_codin	-0.1302	0.196	1	29.96	0.1126
**IVD**	IVD		29	isovaleryl-CoA dehydrogenase	protein_codin	0.3909	0.128	1	8.965	0.1427
**INO80**	INO80		29	INO80 complex ATPase subunit	protein_codin	-0.07797	0.722	1	6.092	0.06032
**VPS13C**	VPS13C		29	vacuolar protein sorting 13 homolog C	protein_codin	-0.1252	0.394	1	35.07	0.08162
**HEXA**	HEXA		29	hexosaminidase subunit alpha	protein_codin	0.5082	0.000389	1	19.1	0.02924
**TARSL2**	TARSL2		29	threonyl-tRNA synthetase like 2	protein_codin	-0.002687	0.988	1	9.741	0.1657
**BFAR**	BFAR		29	bifunctional apoptosis regulator	protein_codin	0.2206	0.172	1	52.38	0.05984
**ARHGAP17**	ARHGAP17		29	Rho GTPase activating protein 17	protein_codin	-0.1367	0.546	1	27.98	0.227
**CSNK2A2**	CSNK2A2		29	casein kinase 2 alpha 2	protein_codin	-0.4076	0.00776	1	45.06	0.05476
**CDH11**	CDH11		29	cadherin 11	protein_codin	-2.928	1.98E-37	4.8E-33	49.35	0.06015
**FBXO31**	FBXO31		29	F-box protein 31	protein_codin	1.026	0.0282	1	9.829	0.3299
**PAFAH1B1**	PAFAH1B1		29	platelet activating factor acetylhydrolase 1b regulatory subunit 1	protein_codin	0.0162	0.908	1	44.79	0.07985
**SPNS2**	SPNS2		29	sphingolipid transporter 2	protein_codin	0.9805	6.64E-09	0.000157	37.74	0.1694
**RNF167**	RNF167		29	ring finger protein 167	protein_codin	0.2676	0.144	1	5.607	0.1682
**CHRNB1**	CHRNB1		29	cholinergic receptor nicotinic beta 1 subunit	protein_codin	0.6164	0.072	1	2.346	0.3836
**KDM6B**	KDM6B		29	lysine demethylase 6B	protein_codin	0.1209	0.6	1	18.25	0.09032
**PIGS**	PIGS		29	phosphatidylinositol glycan anchor biosynthesis class S	protein_codin	-0.001525	0.996	1	3.127	0.1961
**TEFM**	TEFM		29	transcription elongation factor, mitochondrial	protein_codin	-0.2575	0.414	1	3.129	0.3317
**CDK5RAP3**	CDK5RAP3		29	CDK5 regulatory subunit associated protein 3	protein_codin	-0.1255	0.496	1	23.17	0.0822
**SUPT4H1**	SUPT4H1		29	SPT4 homolog, DSIF elongation factor subunit	protein_codin	0.4377	0.0011	1	22.34	0.05596
**HELZ**	HELZ		29	helicase with zinc finger	protein_codin	0.3949	0.345	1	6.748	0.2011
**C1QTNF1**	C1QTNF1		29	C1q and TNF related 1	protein_codin	0.7028	0.00277	1	18.13	0.1835
**OXLD1**	OXLD1		29	oxidoreductase like domain containing 1	protein_codin	0.2898	0.227	1	45.73	0.05727
**PSMG2**	PSMG2		29	proteasome assembly chaperone 2	protein_codin	0.3657	0.00681	1	14.09	0.1109
**ATP9B**	ATP9B		29	ATPase phospholipid transporting 9B (putative)	protein_codin	0.1702	0.692	1	2.852	0.09132
**HSBP1L1**	HSBP1L1		29	heat shock factor binding protein 1 like 1	protein_codin	0.3885	0.14	1	8.57	0.2338
**DDRGK1**	DDRGK1		29	DDRGK domain containing 1	protein_codin	0.3974	0.00291	1	21.62	0.1235
**RALGAPA2**	RALGAPA2		29	Ral GTPase activating protein catalytic alpha subunit 2	protein_codin	0.5881	0.00209	1	13.49	0.08854
**DLGAP4**	DLGAP4		29	DLG associated protein 4	protein_codin	-0.2217	0.252	1	19.52	0.08406
**PTGIS**	PTGIS		29	prostaglandin I2 synthase	protein_codin	-2.884	5.47E-78	1.33E-73	51.97	0.05572
**STX16**	STX16		29	syntaxin 16	protein_codin	-0.2226	0.325	1	8.797	0.2375
**PRELID3B**	PRELID3B		29	PRELI domain containing 3B	protein_codin	0.3594	0.12	1	24.29	0.08328
**RGS19**	RGS19		29	regulator of G protein signaling 19	protein_codin	0.3232	0.158	1	7.335	0.09392
**MKNK2**	MKNK2		29	MAPK interacting serine/threonine kinase 2	protein_codin	0.4092	0.0052	1	52.26	0.1452
**TIMM13**	TIMM13		29	translocase of inner mitochondrial membrane 13	protein_codin	-0.1366	0.267	1	39.32	0.05561
**WDR83OS**	WDR83OS		29	WD repeat domain 83 opposite strand	protein_codin	-0.1698	0.0101	1	22.76	0.1129
**RAD23A**	RAD23A		29	RAD23 homolog A, nucleotide excision repair protein	protein_codin	-0.02355	0.744	1	27.45	0.2735
**ARRDC2**	ARRDC2		29	arrestin domain containing 2	protein_codin	0.3614	0.0947	1	8.232	0.1424
**LSM4.00**	LSM4.00		29	LSM4 homolog, U6 small nuclear RNA and mRNA degradatio	protein_codin	0.05430	0.668	1	20.96	0.1115
**COX6B1**	COX6B1		29	cytochrome c oxidase subunit 6B1	protein_codin	-0.3131	1.34E-06	0.03121	29.82	0.0535
**RASIP1**	RASIP1		29	Ras interacting protein 1	protein_codin	0.2976	0.0155	1	34.75	0.08347
**ISOC2**	ISOC2		29	isochorismatase domain containing 2	protein_codin	-0.2324	0.227	1	21.99	0.1437
**CCDC106**	CCDC106		29	coiled-coil domain containing 106	protein_codin	0.09327	0.728	1	2.962	0.165
**MED15**	MED15		29	mediator complex subunit 15	protein_codin	0.3646	0.19	1	9.332	0.3109
**MIF**	MIF		29	macrophage migration inhibitory factor	protein_codin	0.3185	0.00129	1	49.98	0.03067
**HSCB**	HSCB		29	HscB mitochondrial iron-sulfur cluster cochaperone	protein_codin	0.5229	0.0224	1	22.74	0.1161
**PES1.00**	PES1.00		29	pescadillo ribosomal biogenesis factor 1	protein_codin	0.1659	0.528	1	9.532	0.1958
**RTCB**	RTCB		29	RNA 2',3'-cyclic phosphate and 5'-OH ligase	protein_codin	0.2019	0.203	1	16.02	0.2265
**CSF2RB**	CSF2RB		29	colony stimulating factor 2 receptor beta common subunit	protein_codin	0.05766	0.829	1	51.87	0.1102
**CBX6**	CBX6		29	chromobox 6	protein_codin	-0.3358	0.0505	1	44.65	0.07872
**ATXN10**	ATXN10		29	ataxin 10	protein_codin	-0.009592	0.952	1	36.29	0.1379
**ARSA**	ARSA		29	arylsulfatase A	protein_codin	-0.8814	0.000472	1	8.748	0.09206
**MIR99AHG**	MIR99AHG		29	mir-99a-let-7c cluster host gene	lincRNA	-0.8182	9.71E-06	0.2254	34.36	0.1231
**USP16**	USP16		29	ubiquitin specific peptidase 16	protein_codin	0.2182	0.205	1	13.52	0.0947
**IFNAR2**	IFNAR2		29	interferon alpha and beta receptor subunit 2	protein_codin	0.1849	0.456	1	7.272	0.1201
**CSTB**	CSTB		29	cystatin B	protein_codin	-0.4385	1.82E-09	4.31E-05	46.57	0.08663
**WRAP73**	WRAP73		30	WD repeat containing, antisense to TP73	protein_codin	0.5192	0.0661	1	10.37	0.2209
**PPP1R8**	PPP1R8		30	protein phosphatase 1 regulatory subunit 8	protein_codin	-0.2496	0.377	1	9.546	0.3199
**TXLNA**	TXLNA		30	taxilin alpha	protein_codin	-0.2241	0.33	1	7.067	0.1938
**ZBTB8A**	ZBTB8A		30	zinc finger and BTB domain containing 8A	protein_codin	0.326	0.444	1	9.028	0.2101
**P3H1**	P3H1		30	prolyl 3-hydroxylase 1	protein_codin	-0.3532	0.207	1	3.492	0.0572
**DMAP1**	DMAP1		30	DNA methyltransferase 1 associated protein 1	protein_codin	0.1299	0.545	1	7.64	0.1398
**KTI12**	KTI12		30	KTI12 chromatin associated homolog	protein_codin	0.07192	0.798	1	3.177	0.08660
**ECHDC2**	ECHDC2		30	enoyl-CoA hydratase domain containing 2	protein_codin	0.9386	0.0101	1	8.95	0.2434
**LRRC42**	LRRC42		30	leucine rich repeat containing 42	protein_codin	0.1002	0.715	1	3.182	0.08341
**ALG6**	ALG6		30	ALG6 alpha-1,3-glucosyltransferase	protein_codin	0.5396	0.122	1	2.832	0.1998
**PKN2**	PKN2		30	protein kinase N2	protein_codin	-0.03012	0.836	1	48.25	0.1907
**DRAM2**	DRAM2		30	DNA damage regulated autophagy modulator 2	protein_codin	0.6465	0.00234	1	19.09	0.0851
**MOV10**	MOV10		30	Mov10 RISC complex RNA helicase	protein_codin	0.5792	0.142	1	10.95	0.2669
**POGZ**	POGZ		30	pogo transposable element derived with ZNF domain	protein_codin	-0.08086	0.724	1	7.766	0.137
**THEM4**	THEM4		30	thioesterase superfamily member 4	protein_codin	-0.4275	0.0841	1	10.68	0.2275
**LAMTOR2**	LAMTOR2		30	late endosomal/lysosomal adaptor, MAPK and MTOR activator 2	protein_codin	0.2196	0.0802	1	50.92	0.05714
**UCK2**	UCK2		30	uridine-cytidine kinase 2	protein_codin	0.07392	0.786	1	9.92	0.1089
**VAMP4**	VAMP4		30	vesicle associated membrane protein 4	protein_codin	-0.2842	0.262	1	6.674	0.05541
**CACYBP**	CACYBP		30	calcyclin binding protein	protein_codin	-0.1332	0.252	1	50.9	0.05334
**RABIF**	RABIF		30	RAB interacting factor	protein_codin	-0.1207	0.644	1	3.362	0.1964
**CD55**	CD55		30	CD55 molecule (Cromer blood group)	protein_codin	0.5354	3.16E-06	0.07362	51.45	0.02627
**TRAF5**	TRAF5		30	TNF receptor associated factor 5	protein_codin	-0.01598	0.96	1	9.379	0.2064
**KCTD3**	KCTD3		30	potassium channel tetramerization domain containing 3	protein_codin	1.079	0.00114	1	7.888	0.2581
**RAB3GAP2**	RAB3GAP2		30	RAB3 GTPase activating non-catalytic protein subunit 2	protein_codin	-0.5132	0.0556	1	10.16	0.2226
**SDCCAG8**	SDCCAG8		30	serologically defined colon cancer antigen 8	protein_codin	0.3935	0.102	1	15.03	0.3471
**AKT3**	AKT3		30	AKT serine/threonine kinase 3	protein_codin	0.6692	0.000202	1	22.68	0.1132
**ASAP2**	ASAP2		30	ArfGAP with SH3 domain, ankyrin repeat and PH domain 2	protein_codin	-0.6072	0.0032	1	17.17	0.1105
**ITGB1BP1**	ITGB1BP1		30	integrin subunit beta 1 binding protein 1	protein_codin	0.2632	0.0252	1	17.82	0.02698
**TP53I3**	TP53I3		30	tumor protein p53 inducible protein 3	protein_codin	0.02104	0.912	1	15.73	0.05671
**MRPL33**	MRPL33		30	mitochondrial ribosomal protein L33	protein_codin	-0.4859	4.26E-07	0.009981	52.58	0.0269
**MAP4K3**	MAP4K3		30	mitogen-activated protein kinase kinase kinase kinase 3	protein_codin	-0.211	0.41	1	8.245	0.2246
**ERLEC1**	ERLEC1		30	endoplasmic reticulum lectin 1	protein_codin	-0.5245	4.33E-06	0.1008	18.62	0.08562
**MAP4K4**	MAP4K4		30	mitogen-activated protein kinase kinase kinase kinase 4	protein_codin	-0.3946	0.0111	1	43.03	0.02897
**CCDC93**	CCDC93		30	coiled-coil domain containing 93	protein_codin	0.462	0.103	1	3.428	0.2536
**STEAP3**	STEAP3		30	STEAP3 metalloreductase	protein_codin	0.2116	0.536	1	11.95	0.1124
**FAM168B**	FAM168B		30	family with sequence similarity 168 member B	protein_codin	0.5009	0.134	1	7.66	0.1725
**R3HDM1**	R3HDM1		30	R3H domain containing 1	protein_codin	-0.4445	0.062	1	9.235	0.1077
**HOXD8**	HOXD8		30	homeobox D8	protein_codin	1.507	2.75E-09	6.51E-05	30.93	0.1077
**ASNSD1**	ASNSD1		30	asparagine synthetase domain containing 1	protein_codin	0.3455	0.102	1	7.669	0.1402
**ORC2**	ORC2		30	origin recognition complex subunit 2	protein_codin	0.1892	0.507	1	3.422	0.2562
**PDE6D**	PDE6D		30	phosphodiesterase 6D	protein_codin	0.7119	0.0126	1	7.156	0.1956
**FGD5-AS1**	FGD5-AS1		30	FGD5 antisense RNA 1	antisense_RN	0.201	0.188	1	27.19	0.0894
**TEX264**	TEX264		30	testis expressed 264, ER-phagy receptor	protein_codin	-0.008712	0.942	1	20.22	0.06012
**TWF2**	TWF2		30	twinfilin actin binding protein 2	protein_codin	0.01834	0.902	1	34.36	0.1209
**APPL1**	APPL1		30	adaptor protein, phosphotyrosine interacting with PH domain	protein_codin	0.261	0.0212	1	12.62	0.03216
**RIOX2**	RIOX2		30	ribosomal oxygenase 2	protein_codin	1.334	2.83E-05	0.6537	7.827	0.2518
**CMSS1**	CMSS1		30	cms1 ribosomal small subunit homolog	protein_codin	0.08524	0.697	1	19.68	0.1375
**NXPE3**	NXPE3		30	neurexophilin and PC-esterase domain family member 3	protein_codin	0.6979	0.00925	1	3.605	0.2750
**GATA2**	GATA2		30	GATA binding protein 2	protein_codin	-0.5638	0.000778	1	30.14	0.1375
**PLXND1**	PLXND1		30	plexin D1	protein_codin	0.2352	0.158	1	39.38	0.1622
**DHX36**	DHX36		30	DEAH-box helicase 36	protein_codin	-0.007197	0.954	1	8.448	0.1452
**RSRC1**	RSRC1		30	arginine and serine rich coiled-coil 1	protein_codin	0.105	0.596	1	6.906	0.1428
**MLF1.00**	MLF1.00		30	myeloid leukemia factor 1	protein_codin	0.6751	0.000661	1	14.4	0.08168
**ACTL6A**	ACTL6A		30	actin like 6A	protein_codin	0.1476	0.545	1	7.764	0.1119
**ABCF3**	ABCF3		30	ATP binding cassette subfamily F member 3	protein_codin	0.3394	0.274	1	9.385	0.1990
**BCL6**	BCL6		30	BCL6 transcription repressor	protein_codin	0.06384	0.825	1	29.06	0.07922
**TAPT1**	TAPT1		30	transmembrane anterior posterior transformation 1	protein_codin	0.3349	0.297	1	2.118	0.1757
**SLAIN2**	SLAIN2		30	SLAIN motif family member 2	protein_codin	-0.2231	0.196	1	17.55	0.1387
**SRD5A3**	SRD5A3		30	steroid 5 alpha-reductase 3	protein_codin	0.3052	0.149	1	8.806	0.2240
**EXOC1**	EXOC1		30	exocyst complex component 1	protein_codin	0.1152	0.582	1	19.24	0.1676
**SDAD1**	SDAD1		30	SDA1 domain containing 1	protein_codin	0.3774	0.0277	1	15.5	0.1672
**RAP1GDS1**	RAP1GDS1		30	Rap1 GTPase-GDP dissociation stimulator 1	protein_codin	0.7252	0.0291	1	3.428	0.08235
**LAMTOR3**	LAMTOR3		30	late endosomal/lysosomal adaptor, MAPK and MTOR activator 3	protein_codin	0.1717	0.446	1	7.873	0.1056
**FLJ20021**	FLJ20021		30	novel transcri	lincRNA	NA	NA	NA	18.56	0.1654
**HADH**	HADH		30	hydroxyacyl-CoA dehydrogenase	protein_codin	0.670	0.0329	1	3.438	0.1484
**MGST2**	MGST2		30	microsomal glutathione S-transferase 2	protein_codin	-0.02234	0.732	1	46.73	0.05670
**SMAD1**	SMAD1		30	SMAD family member 1	protein_codin	0.9889	6.5E-08	0.00152	18.57	0.1159
**STOX2**	STOX2		30	storkhead box 2	protein_codin	0.9207	0.00685	1	8.455	0.330
**LIFR-AS1**	LIFR-AS1		30	LIFR antisense RNA 1	antisense_RN	0.9172	0.0228	1	3.718	0.2009
**GPBP1**	GPBP1		30	GC-rich promoter binding protein 1	protein_codin	0.2665	0.0191	1	30.22	0.05815
**RAB3C**	RAB3C		30	RAB3C, member RAS oncogene family	protein_codin	1.706	2.03E-10	4.82E-06	34.07	0.08040
**CWC27**	CWC27		30	CWC27 spliceosome associated cyclophilin	protein_codin	0.09702	0.611	1	22	0.1172
**RAD17**	RAD17		30	RAD17 checkpoint clamp loader component	protein_codin	0.1876	0.428	1	7.485	0.1992
**TMED7**	TMED7		30	transmembrane p24 trafficking protein 7	protein_codin	-0.4238	0.0182	1	5.812	0.1397
**HSD17B4**	HSD17B4		30	hydroxysteroid 17-beta dehydrogenase 4	protein_codin	0.1489	0.466	1	5.79	0.112
**JADE2**	JADE2		30	jade family PHD finger 2	protein_codin	-0.01228	0.964	1	2.905	0.1497
**HSPA9**	HSPA9		30	heat shock protein family A (Hsp70) member 9	protein_codin	0.2481	0.1	1	11.79	0.05657
**HARS**	HARS		30	histidyl-tRNA synthetase	protein_codin	0.2162	0.242	1	6.238	0.07965
**ABLIM3**	ABLIM3		30	actin binding LIM protein family member 3	protein_codin	1.045	0.00324	1	9.446	0.1079
**CNOT8**	CNOT8		30	CCR4-NOT transcription complex subunit 8	protein_codin	-0.4527	0.0504	1	6.637	0.1972
**PRR7**	PRR7		30	proline rich 7, synaptic	protein_codin	-0.03175	0.896	1	3.547	0.1557
**DBN1**	DBN1		30	drebrin 1	protein_codin	-0.3868	0.0246	1	17.73	0.05449
**TMED9**	TMED9		30	transmembrane p24 trafficking protein 9	protein_codin	-0.4049	1.83E-05	0.4236	22.22	0.05822
**TBC1D9B**	TBC1D9B		30	TBC1 domain family member 9B	protein_codin	0.4145	0.108	1	7.6	0.1941
**DSP**	DSP		30	desmoplakin	protein_codin	0.4658	0.202	1	3.552	0.42
**DTNBP1**	DTNBP1		30	dystrobrevin binding protein 1	protein_codin	0.2481	0.246	1	7.944	0.2457
**KIF13A**	KIF13A		30	kinesin family member 13A	protein_codin	0.3441	0.128	1	19.13	0.1106
**C6orf62**	C6orf62		30	chromosome 6 open reading frame 62	protein_codin	0.1562	0.362	1	12.74	0.1398
**CARMIL1**	CARMIL1		30	capping protein regulator and myosin 1 linker 1	protein_codin	0.1312	0.572	1	24.07	0.08062
**GPANK1**	GPANK1		30	G-patch domain and ankyrin repeats 1	protein_codin	0.2922	0.184	1	5.9	0.1112
**PSMB8-AS1**	PSMB8-AS		30	PSMB8 antisense RNA 1 (head to head)	lincRNA	1	0.000129	1	7.548	0.1718
**RGL2**	RGL2		30	ral guanine nucleotide dissociation stimulator like 2	protein_codin	0.08377	0.618	1	14.62	0.112
**C6orf106**	C6orf106		30	inflammation and lipid regulator with UBA-like and NBR1-like	protein_codin	0.3117	0.206	1	9.757	0.1341
**TBCC**	TBCC		30	tubulin folding cofactor C	protein_codin	0.2696	0.274	1	2.992	0.2622
**RPL7L1**	RPL7L1		30	ribosomal protein L7 like 1	protein_codin	-0.172	0.176	1	23.96	0.02622
**CNPY3**	CNPY3		30	canopy FGF signaling regulator 3	protein_codin	-0.01495	0.932	1	20.87	0.05436
**MAN1A1**	MAN1A1		30	mannosidase alpha class 1A member 1	protein_codin	0.7118	9.24E-05	1	29.88	0.05792
**CNKSR3**	CNKSR3		30	CNKSR family member 3	protein_codin	0.07446	0.742	1	52.07	0.1426
**ZDHHC14**	ZDHHC14		30	zinc finger DHHC-type containing 14	protein_codin	-0.3966	0.154	1	11	0.08568
**GTF2H5**	GTF2H5		30	general transcription factor IIH subunit 5	protein_codin	-0.01496	0.911	1	31.82	0.1085
**TBP**	TBP		30	TATA-box binding protein	protein_codin	0.8169	0.0186	1	2.868	0.2564
**C1GALT1**	C1GALT1		30	core 1 synthase, glycoprotein-N-acetylgalactosamine 3-beta-(	protein_codin	0.76	0.0246	1	7.322	0.2446
**YAE1D1**	YAE1D1		30	YAE1 maturation factor of ABCE1	protein_codin	-0.1209	0.703	1	2.98	0.2515
**DBNL**	DBNL		30	drebrin like	protein_codin	0.2295	0.179	1	6.857	0.1756
**HUS1**	HUS1		30	HUS1 checkpoint clamp component	protein_codin	0.1026	0.706	1	10.13	0.2071
**MDH2**	MDH2		30	malate dehydrogenase 2	protein_codin	-0.01012	0.917	1	20.58	0.02838
**BET1**	BET1		30	Bet1 golgi vesicular membrane trafficking protein	protein_codin	0.7612	0.00272	1	7.954	0.364
**CASD1**	CASD1		30	CAS1 domain containing 1	protein_codin	-0.182	0.477	1	9.326	0.1449
**SLC25A13**	SLC25A13		30	solute carrier family 25 member 13	protein_codin	-0.2478	0.32	1	7.507	0.1402
**POLR2J**	POLR2J		30	RNA polymerase II subunit J	protein_codin	-0.6452	2.69E-07	0.006309	47.52	0.03028
**PMPCB**	PMPCB		30	peptidase, mitochondrial processing beta subunit	protein_codin	-0.05516	0.736	1	37.17	0.1391
**MKLN1**	MKLN1		30	muskelin 1	protein_codin	-0.4806	0.0326	1	19.42	0.17
**C7orf49**	C7orf49		30	cell cycle regulator of NHEJ	protein_codin	NA	NA	NA	8.408	0.211
**CUL1**	CUL1		30	cullin 1	protein_codin	0.4421	0.072	1	9.04	0.1647
**INSIG1**	INSIG1		30	insulin induced gene 1	protein_codin	-0.2014	0.276	1	52.58	0.152
**BCLAF3**	BCLAF3		30	BCLAF1 and THRAP3 family member 3	protein_codin	0.4504	0.197	1	2.8	0.2220
**EIF1AX**	EIF1AX		30	eukaryotic translation initiation factor 1A X-linked	protein_codin	0.1742	0.0832	1	42.39	0.08471
**SMS**	SMS		30	spermine synthase	protein_codin	-0.1196	0.611	1	47.33	0.03239
**KRBOX4**	KRBOX4		30	KRAB box domain containing 4	protein_codin	0.6887	0.0217	1	3.512	0.1778
**NBDY**	NBDY		30	negative regulator of P-body association	protein_codin	-0.0496	0.615	1	43.13	0.1347
**UPF3B**	UPF3B		30	UPF3B regulator of nonsense mediated mRNA decay	protein_codin	0.238	0.266	1	17.11	0.1427
**RAP2C**	RAP2C		30	RAP2C, member of RAS oncogene family	protein_codin	-0.119	0.716	1	10.24	0.2409
**MTM1**	MTM1		30	myotubularin 1	protein_codin	0.1399	0.638	1	2.854	0.1538
**DNASE1L1**	DNASE1L1		30	deoxyribonuclease 1 like 1	protein_codin	-0.05988	0.752	1	18.54	0.02605
**ATP6AP1**	ATP6AP1		30	ATPase H+ transporting accessory protein 1	protein_codin	-0.3158	0.0476	1	19.25	0.08307
**VAMP7**	VAMP7		30	vesicle associated membrane protein 7	protein_codin	0.1395	0.562	1	3.345	0.26
**BMP1**	BMP1		30	bone morphogenetic protein 1	protein_codin	-0.3188	0.287	1	3.088	0.2726
**EPHX2**	EPHX2		30	epoxide hydrolase 2	protein_codin	0.7219	0.00804	1	9.845	0.05529
**KIF13B**	KIF13B		30	kinesin family member 13B	protein_codin	-0.8022	5.01E-05	1	23.38	0.1381
**MRPL15**	MRPL15		30	mitochondrial ribosomal protein L15	protein_codin	0.3572	0.0624	1	18.98	0.1079
**PEX2**	PEX2		30	peroxisomal biogenesis factor 2	protein_codin	0.08322	0.66	1	6.827	0.1754
**ATP6V1C1**	ATP6V1C1		30	ATPase H+ transporting V1 subunit C1	protein_codin	0.2845	0.264	1	3.405	0.1451
**BAALC**	BAALC		30	BAALC binder of MAP3K1 and KLF4	protein_codin	1.574	2E-05	0.4627	50.21	0.1467
**PTP4A3**	PTP4A3		30	protein tyrosine phosphatase 4A3	protein_codin	-0.3021	0.129	1	24.38	0.1731
**PLEC**	PLEC		30	plectin	protein_codin	0.009486	0.942	1	34.24	0.09292
**DCAF10**	DCAF10		30	DDB1 and CUL4 associated factor 10	protein_codin	0.424	0.155	1	3.482	0.2061
**CNTNAP3B**	CNTNAP3B		30	contactin associated protein like 3B	protein_codin	0.5057	0.00632	1	34.11	0.1167
**STX17**	STX17		30	syntaxin 17	protein_codin	0.4971	0.0256	1	8.137	0.1119

**KIAA0368**	KIAA0368	20	Ecm29 proteasome adaptor and scaffold	protein_codin	NA	NA	NA	29.19	0.1769
**RRAS2**	RRAS2	20	RAS related 2	protein_codin	-0.5381	0.0206	1	10.24	0.05692
**COPB1**	COPB1	20	coatomer protein complex subunit beta 1	protein_codin	0.01136	0.949	1	28.17	0.0549
**C11orf58**	C11orf58	20	chromosome 11 open reading frame 58	protein_codin	-0.2069	0.00219	1	17.59	0.1424
**SERPING1**	SERPING1	20	serpin family G member 1	protein_codin	0.3116	0.00189	1	27.64	0.1615
**STX3**	STX3	20	syntaxin 3	protein_codin	-0.6196	0.019	1	3.303	0.09275
**TMEM109**	TMEM109	20	transmembrane protein 109	protein_codin	0.2175	0.0325	1	26.11	0.08876
**ROM1**	ROM1	20	retinal outer segment membrane protein 1	protein_codin	-0.9532	1.56E-06	0.03642	47.07	0.05782
**FRMD8**	FRMD8	20	FERM domain containing 8	protein_codin	0.2529	0.41	1	2.812	0.1142
**DRAP1**	DRAP1	20	DR1 associated protein 1	protein_codin	-0.4651	0.000127	1	31.06	0.05618
**MRPL21**	MRPL21	20	mitochondrial ribosomal protein L21	protein_codin	0.01997	0.882	1	23.96	0.08252
**ATG16L2**	ATG16L2	20	autophagy related 16 like 2	protein_codin	0.6297	0.0531	1	2.876	0.1186
**RAB30-AS1**	RAB30-AS1	20	RAB30 divergent transcript	lincRNA	-0.2112	0.42	1	9.66	0.2794
**ARHGAP20**	ARHGAP20	20	Rho GTPase activating protein 20	protein_codin	0.259	0.437	1	33.42	0.1752
**RAB18**	RAB18	20	RAB18, member RAS oncogene family	protein_codin	-0.1670	0.285	1	18.22	0.05719
**MTPAP**	MTPAP	20	mitochondrial poly(A) polymerase	protein_codin	0.1574	0.596	1	3.015	0.1119
**CCNY**	CCNY	20	cyclin Y	protein_codin	-0.03274	0.834	1	16.95	0.08147
**FZD8**	FZD8	20	frizzled class receptor 8	protein_codin	-1.062	2.26E-05	0.5226	45.38	0.3082
**ZNF37A**	ZNF37A	20	zinc finger protein 37A	protein_codin	-0.07027	0.792	1	9.764	0.1655
**HNRNPH3**	HNRNPH3	20	heterogeneous nuclear ribonucleoprotein H3	protein_codin	0.1824	0.113	1	37.1	0.08715
**MMRN2**	MMRN2	20	multimerin 2	protein_codin	-0.156	0.08	1	33.72	0.08407
**FAM35A**	FAM35A	20	shieldin complex subunit 2	protein_codin	NA	NA	NA	8.119	0.196
**LZTS2**	LZTS2	20	leucine zipper tumor suppressor 2	protein_codin	0.1246	0.579	1	7.614	0.1357
**SFXN3**	SFXN3	20	sideroflexin 3	protein_codin	-0.6037	0.00419	1	10.35	0.05461
**RAB11FIP2**	RAB11FIP2	20	RAB11 family interacting protein 2	protein_codin	-0.0198	0.942	1	3.271	0.1649
**TAPBPL**	TAPBPL	20	TAP binding protein like	protein_codin	0.1445	0.532	1	7.948	0.1481
**GRASP**	GRASP	20	general receptor for phosphoinositides 1 associated scaffold p	protein_codin	-0.2735	0.0026	1	47.98	0.0814
**ATF7**	ATF7	20	activating transcription factor 7	protein_codin	-0.04566	0.845	1	8.201	0.0866
**TBC1D15**	TBC1D15	20	TBC1 domain family member 15	protein_codin	0.488	0.00196	1	19.4	0.1405
**KRR1**	KRR1	20	KRR1 small subunit processome component homolog	protein_codin	0.08558	0.508	1	10.59	0.1112
**APAF1**	APAF1	20	apoptotic peptidase activating factor 1	protein_codin	0.3032	0.321	1	3.436	0.195
**UTP20**	UTP20	20	UTP20 small subunit processome component	protein_codin	0.2379	0.582	1	9.241	0.2062
**TDG**	TDG	20	thymine DNA glycosylase	protein_codin	-0.2229	0.206	1	44.8	0.1715
**ANAPC7**	ANAPC7	20	anaphase promoting complex subunit 7	protein_codin	0.1951	0.468	1	2.628	0.1427
**ORAI1**	ORAI1	20	ORAI calcium release-activated calcium modulator 1	protein_codin	0.4404	0.00322	1	24.26	0.1392
**RNF6**	RNF6	20	ring finger protein 6	protein_codin	0.1972	0.425	1	8.938	0.1186
**DGKH**	DGKH	20	diacylglycerol kinase eta	protein_codin	0.9270	0.022	1	8.278	0.2734
**CARS2**	CARS2	20	cysteinyl-tRNA synthetase 2, mitochondrial	protein_codin	0.2434	0.355	1	3.177	0.2470
**PCID2**	PCID2	20	PCI domain containing 2	protein_codin	0.02666	0.895	1	7.645	0.1138
**TTC5**	TTC5	20	tetratricopeptide repeat domain 5	protein_codin	0.2586	0.349	1	3.323	0.09487
**SOS2**	SOS2	20	SOS Ras/Rho guanine nucleotide exchange factor 2	protein_codin	0.1927	0.482	1	25.53	0.1467
**FERMT2**	FERMT2	20	fermitin family member 2	protein_codin	0.1679	0.115	1	35.67	0.02962
**DNAL1**	DNAL1	20	dynein axonemal light chain 1	protein_codin	-0.5327	0.0769	1	9.542	0.4014
**DLST**	DLST	20	dihydrolipoamide S-succinyltransferase	protein_codin	0.004218	0.988	1	8.706	0.08147
**MLH3**	MLH3	20	mutL homolog 3	protein_codin	-0.1238	0.603	1	9.829	0.2024
**GPATCH2L**	GPATCH2L	20	G-patch domain containing 2 like	protein_codin	-0.398	0.149	1	52.58	0.3086
**APOPT1**	APOPT1	20	cytochrome c oxidase assembly factor 8	protein_codin	0.332	0.0571	1	13.93	0.1442
**AKT1**	AKT1	20	AKT serine/threonine kinase 1	protein_codin	-0.4738	0.00757	1	36.92	0.02866
**MAPK6**	MAPK6	20	mitogen-activated protein kinase 6	protein_codin	-0.3455	0.176	1	3.403	0.2282
**MYO5C**	MYO5C	20	myosin VC	protein_codin	1.246	5.23E-06	0.1217	8.002	0.2834
**TCF12**	TCF12	20	transcription factor 12	protein_codin	0.1136	0.579	1	5.919	0.1431
**UACA**	UACA	20	uveal autoantigen with coiled-coil domains and ankyrin repeats	protein_codin	0.2097	0.099	1	33.77	0.0579
**RCN2**	RCN2	20	reticulocalbin 2	protein_codin	-0.72	4.16E-12	9.92E-08	32.24	0.02659
**PEAK1**	PEAK1	20	pseudopodium enriched atypical kinase 1	protein_codin	0.4656	0.177	1	9.677	0.3502
**IQGAP1**	IQGAP1	20	IQ motif containing GTPase activating protein 1	protein_codin	-0.2062	0.0519	1	37.17	0.1136
**FURIN**	FURIN	20	furin, paired basic amino acid cleaving enzyme	protein_codin	0.02996	0.912	1	9.142	0.1194
**SELENOS**	SELENOS	20	selenoprotein S	protein_codin	-0.1766	0.0697	1	17.42	0.08259
**NPRL3**	NPRL3	20	NPR3 like, GATOR1 complex subunit	protein_codin	-0.3682	0.153	1	9.088	0.167
**RPUSD1**	RPUSD1	20	RNA pseudouridine synthase domain containing 1	protein_codin	0.03755	0.902	1	2.785	0.259
**LITAF**	LITAF	20	lipopolysaccharide induced TNF factor	protein_codin	0.1682	0.412	1	52.58	0.02642
**C16orf52**	C16orf52	20	modulator of smoothened	protein_codin	NA	NA	NA	7.894	0.3162
**TBC1D10B**	TBC1D10B	20	TBC1 domain family member 10B	protein_codin	0.09434	0.75	1	2.412	0.3154
**CKLF**	CKLF	20	chemokine like factor	protein_codin	-0.05509	0.748	1	36.53	0.1779
**PIEZO1**	PIEZO1	20	piezo type mechanosensitive ion channel component 1	protein_codin	0.3925	0.233	1	8.035	0.2291
**SMG6**	SMG6	20	SMG6 nonsense mediated mRNA decay factor	protein_codin	-0.4470	0.0344	1	6.1	0.2546
**C1QBP**	C1QBP	20	complement C1q binding protein	protein_codin	-0.04426	0.644	1	23.26	0.05881
**TMEM11**	TMEM11	20	transmembrane protein 11	protein_codin	-0.1097	0.586	1	7.982	0.1094
**IFT20**	IFT20	20	intraflagellar transport 20	protein_codin	-0.2314	0.132	1	14.11	0.1172
**SLFN11**	SLFN11	20	schlafen family member 11	protein_codin	-0.02146	0.934	1	8.72	0.2219
**TMEM99**	TMEM99	20	transmembrane protein 99 (putative)	protein_codin	0.3541	0.209	1	9.262	0.227
**NKIRAS2**	NKIRAS2	20	NFKB inhibitor interacting Ras like 2	protein_codin	-0.1552	0.464	1	7.722	0.1076
**DHX58**	DHX58	20	DExH-box helicase 58	protein_codin	1.11	0.0106	1	3.382	0.1429
**MLX**	MLX	20	MAX dimerization protein MLX	protein_codin	0.3119	0.131	1	45.95	0.2382
**EZH1**	EZH1	20	enhancer of zeste 1 polycomb repressive complex 2 subunit	protein_codin	0.3154	0.196	1	24.07	0.2279
**ATXN7L3**	ATXN7L3	20	ataxin 7 like 3	protein_codin	-0.2476	0.38	1	2.93	0.3752
**CDC27**	CDC27	20	cell division cycle 27	protein_codin	-0.2972	0.0221	1	37.63	0.2305
**FAM117A**	FAM117A	20	family with sequence similarity 117 member A	protein_codin	1.282	0.00138	1	7.942	0.2284
**ITGA3**	ITGA3	20	integrin subunit alpha 3	protein_codin	-0.4049	0.122	1	9.676	0.1431
**COX11**	COX11	20	cytochrome c oxidase copper chaperone COX11	protein_codin	0.152	0.498	1	8.045	0.05239
**TRIM47**	TRIM47	20	tripartite motif containing 47	protein_codin	0.3299	0.0291	1	42.56	0.0842
**MRPL12**	MRPL12	20	mitochondrial ribosomal protein L12	protein_codin	0.0771	0.65	1	16.38	0.1624
**ANAPC11**	ANAPC11	20	anaphase promoting complex subunit 11	protein_codin	-0.4505	2.89E-07	0.006779	44.22	0.08282
**CENPX**	CENPX	20	centromere protein X	protein_codin	-0.1605	0.202	1	51.48	0.05297
**THOC1**	THOC1	20	THO complex 1	protein_codin	0.6009	0.0451	1	9.186	0.2795
**ANKRD12**	ANKRD12	20	ankyrin repeat domain 12	protein_codin	-0.02308	0.8155	1	24.62	0.05285
**PTPN2**	PTPN2	20	protein tyrosine phosphatase non-receptor type 2	protein_codin	0.674	0.000575	1	15.48	0.1152
**LDLRAD4**	LDLRAD4	20	low density lipoprotein receptor class A domain containing 4	protein_codin	-0.2191	0.382	1	8.234	0.1781
**MAPRE2**	MAPRE2	20	microtubule associated protein RP/EB family member 2	protein_codin	-0.4701	0.0344	1	9.11	0.1137
**PIK3C3**	PIK3C3	20	phosphatidylinositol 3-kinase catalytic subunit type 3	protein_codin	0.2222	0.366	1	3.424	0.1072
**PSMF1**	PSMF1	20	proteasome inhibitor subunit 1	protein_codin	-0.06019	0.65	1	47.25	0.1419
**DYNLRB1**	DYNLRB1	20	dynein light chain roadblock-type 1	protein_codin	0.02196	0.7751	1	46.11	0.02805
**TMEM189**	TMEM189	20	transmembrane protein 189	protein_codin	-0.6281	0.0341	1	3.59	0.1727
**NFATC2**	NFATC2	20	nuclear factor of activated T cells 2	protein_codin	0.02354	0.934	1	22.22	0.216
**TSHZ2**	TSHZ2	20	teashirt zinc finger homeobox 2	protein_codin	1.705	1.87E-20	4.5E-16	45.62	0.1122
**RAB22A**	RAB22A	20	RAB22A, member RAS oncogene family	protein_codin	-0.08376	0.644	1	21.09	0.1076
**PRPF6**	PRPF6	20	pre-mRNA processing factor 6	protein_codin	0.06738	0.608	1	23.56	0.08217
**SHC2**	SHC2	20	SHC adaptor protein 2	protein_codin	1.072	0.000853	1	7.15	0.191
**RNF126**	RNF126	20	ring finger protein 126	protein_codin	0.5988	0.00189	1	6.36	0.166
**TMEM259**	TMEM259	20	transmembrane protein 259	protein_codin	0.1077	0.51	1	6.092	0.1142
**C19orf25**	C19orf25	20	chromosome 19 open reading frame 25	protein_codin	0.4721	0.0494	1	3.207	0.1389
**C19orf70**	C19orf70	20	mitochondrial contact site and cristae organizing system subu	protein_codin	-0.2281	0.0176	1	31.02	0.05426
**PET100**	PET100	20	PET100 cytochrome c oxidase chaperone	protein_codin	-0.3679	0.0124	1	15.43	0.05571
**TMEM205**	TMEM205	20	transmembrane protein 205	protein_codin	-0.37	0.00109	1	31.41	0.03045
**TMEM147**	TMEM147	20	transmembrane protein 147	protein_codin	-0.2359	0.029	1	40.22	0.08547
**NFKBIB**	NFKBIB	20	NFKB inhibitor beta	protein_codin	0.47	0.0758	1	7.762	0.1101
**TMEM91**	TMEM91	20	transmembrane protein 91	protein_codin	-0.6135	0.00514	1	23.19	0.05668
**GPR4**	GPR4	20	G protein-coupled receptor 4	protein_codin	0.079	0.788	1	9.677	0.11
**EML2**	EML2	20	EMAP like 2	protein_codin	0.1696	0.558	1	3.549	0.2192
**FLT3LG**	FLT3LG	20	fms related tyrosine kinase 3 ligand	protein_codin	0.259	0.422	1	8.202	0.3091
**NOSIP**	NOSIP	20	nitric oxide synthase interacting protein	protein_codin	0.1938	0.134	1	42.77	0.0852
**AC010642.2**	AC010642.2	20	novel transcript, antisense to ERVK3	antisense_RN	-0.2435	0.219	1	24.44	0.1175
**ZMAT5**	ZMAT5	20	zinc finger matrin-type 5	protein_codin	-0.01945	0.934	1	6.13	0.02702
**CSNK1E**	CSNK1E	20	casein kinase 1 epsilon	protein_codin	0.2659	0.303	1	8.506	0.1412
**SHANK3**	SHANK3	20	SH3 and multiple ankyrin repeat domains 3	protein_codin	0.8889	0.0119	1	22.72	0.3729
**BACH1**	BACH1	20	BTB domain and CNC homolog 1	protein_codin	-0.07014	0.764	1	27.13	0.2209
**TMEM50B**	TMEM50B	20	transmembrane protein 50B	protein_codin	-0.09461	0.511	1	12.85	0.08345
**ITSN1**	ITSN1	20	intersectin 1	protein_codin	-0.07262	0.8	1	26.74	0.09731
**ID3**	ID3	21	inhibitor of DNA binding 3, HLH protein	protein_codin	0.1261	0.109	1	52.58	0.15
**TAF12**	TAF12	21	TATA-box binding protein associated factor 12	protein_codin	0.1422	0.403	1	6.272	0.3089
**KIAA1522**	KIAA1522	21	KIAA1522	protein_codin	0.9942	0.00329	1	3.147	0.3457
**S100PBP**	S100PBP	21	S100P binding protein	protein_codin	0.4388	0.22	1	2.686	0.2317
**RIMKLA**	RIMKLA	21	ribosomal modification protein rimK like family member A	protein_codin	0.3324	0.318	1	35.94	0.1714
**SNAPIN**	SNAPIN	21	SNAP associated protein	protein_codin	0.1427	0.359	1	16.78	0.08037
**QSOX1**	QSOX1	21	quiescin sulfhydryl oxidase 1	protein_codin	-0.1748	0.642	1	8.98	0.1418
**RCOR3**	RCOR3	21	REST corepressor 3	protein_codin	0.1349	0.55	1	8.256	0.1379
**COG2**	COG2	21	component of oligomeric golgi complex 2	protein_codin	0.6519	0.128	1	2.766	0.3067
**MPV17**	MPV17	21	mitochondrial inner membrane protein MPV17	protein_codin	-0.01703	0.925	1	17.82	0.111
**BIRC6**	BIRC6	21	baculoviral IAP repeat containing 6	protein_codin	0.1772	0.388	1	7.317	0.1696
**STRN**	STRN	21	striatin	protein_codin	0.2666	0.277	1	7.296	0.1096
**C2orf74**	C2orf74	21	chromosome 2 open reading frame 74	protein_codin	0.3037	0.114	1	50.02	0.1137
**TACR1**	TACR1	21	tachykinin receptor 1	protein_codin	2.262	1.13E-07	0.002656	31.53	0.02629
**SLC20A1**	SLC20A1	21	solute carrier family 20 member 1	protein_codin	-0.6197	0.000238	1	33.94	0.05552
**BTD**	BTD	21	biotinidase	protein_codin	-0.01166	0.96	1	3.529	0.1066
**CLEC3B**	CLEC3B	21	C-type lectin domain family 3 member B	protein_codin	0.7145	0.00632	1	22.25	0.2299
**TFG**	TFG	21	trafficking from ER to golgi regulator	protein_codin	-0.1001	0.399	1	22.16	0.02682
**NCK1**	NCK1	21	NCK adaptor protein 1	protein_codin	0.8772	2.19E-07	0.00514	21.78	0.1379
**XRN1**	XRN1	21	5'-3' exoribonuclease 1	protein_codin	-0.3292	0.0425	1	50.27	0.08810
**FNDC3B**	FNDC3B	21	fibronectin type III domain containing 3B	protein_codin	-0.5674	0.00545	1	8.352	0.1104
**DHX15**	DHX15	21	DEAH-box helicase 15	protein_codin	0.7947	0.00299	1	6.737	0.02626
**AGA**	AGA	21	aspartylglucosaminidase	protein_codin	-0.7118	0.00277	1	3.692	0.1734
**6-Mar**	43530	21	membrane associated ring-CH-type finger 6	protein_codin	0.3162	0.0657	1	35.85	0.1984
**DHX29**	DHX29	21	DExH-box helicase 29	protein_codin	-0.1136	0.492	1	43.27	0.2015
**ADRB2**	ADRB2	21	adrenoceptor beta 2	protein_codin	-0.6194	0.00259	1	29.28	0.02951
**GCNT2**	GCNT2	21	glucosaminyl (N-acetyl) transferase 2 (I blood group)	protein_codin	0.9975	0.00982	1	48.41	0.2382
**GJA1**	GJA1	21	gap junction protein alpha 1	protein_codin	-1.203	7.83E-22	1.89E-18	52.58	0.05372
**MED23**	MED23	21	mediator complex subunit 23	protein_codin	0.2279	0.44	1	3.282	0.1631
**PLAGL1**	PLAGL1	21	PLAG1 like zinc finger 1	protein_codin	0.4112	0.197	1	2.988	0.2355
**SFT2D1**	SFT2D1	21	SFT2 domain containing 1	protein_codin	0.1042	0.577	1	24.48	0.1399
**LMBR1**	LMBR1	21	limb development membrane protein 1	protein_codin	0.4629	0.179	1	3.257	0.2798
**THAP1**	THAP1	21	THAP domain containing 1	protein_codin	0.3468	0.135	1	8.726	0.3412
**MRPS28**	MRPS28	21	mitochondrial ribosomal protein S28	protein_codin	-0.3264	0.134	1	7.472	0.08322
**IMPA1**	IMPA1	21	inositol monophosphatase 1	protein_codin	-0.2308	0.214	1	3.5	0.2963
**WASHC1**	WASHC1	21	WASH complex subunit 1	protein_codin	0.711	0.0261	1	3.315	0.2588
**MTAP**	MTAP	21	methylthioadenosine phosphorylase	protein_codin	0.4931	0.0394	1	7.335	0.2214
**HINT2**	HINT2	21	histidine triad nucleotide binding protein 2	protein_codin	-0.1462	0.322	1	49.62	0.07908
**CNTNAP3**	CNTNAP3	21	contactin associated protein like 3	protein_codin	0.2885	0.38	1	3.562	0.3953
**IDNK**	IDNK	21	IDNK gluconokinase	protein_codin	0.3316	0.298	1	3.57	0.1469
**MFSD14C**	MFSD14C	21	major facilitator superfamily domain containing 14C	protein_codin	-0.2098	0.494	1	8.972	0.2619
**PTBP3**	PTBP3	21	polypyrimidine tract binding protein 3	protein_codin	0.4524	0.0117	1	44.66	0.2585
**CDK5RAP2**	CDK5RAP2	21	CDK5 regulatory subunit associated protein 2	protein_codin	0.5752	0.0605	1	9.53	0.1447
**STRBP**	STRBP	21	spermatid perinuclear RNA binding protein	protein_codin	0.2919	0.317	1	3.646	0.3145
**ENDOG**	ENDOG	21	endonuclease G	protein_codin	0.01329	0.956	1	9.062	0.1985
**LRRC8A**	LRRC8A	21	leucine rich repeat containing 8 VRAC subunit A	protein_codin	-0.5336	0.00689	1	30.07	0.1402
**IMMP1L**	IMMP1L	21	inner mitochondrial membrane peptidase subunit 1	protein_codin	-0.1059	0.667	1	8.698	0.1085
**C11orf49**	C11orf49	21	chromosome 11 open reading frame 49	protein_codin	-0.4527	0.122	1	2.823	0.1486
**TMEM128**	TMEM128	21	transmembrane protein 128	protein_codin	0.1989	0.532	1	2.993	0.165
**LAMTOR1**	LAMTOR1	21	late endosomal/lysosomal adaptor, MAPK and MTOR activato	protein_codin	-0.1244	0.1267	1	34.32	0.0546
**MTMR2**	MTMR2	21	myotubularin related protein 2	protein_codin	0.5127	0.066	1	7.946	0.111
**RBM7**	RBM7	21	RNA binding motif protein 7	protein_codin	0.822	0.000101	1	16.02	0.1445
**ABI1**	ABI1	21	abl interactor 1	protein_codin	-0.1690	0.464	1	8.57	0.08096
**ANKRD26**	ANKRD26	21	ankyrin repeat domain 26	protein_codin	0.2398	0.427	1	9.384	0.1360
**ARHGAP12**	ARHGAP12	21	Rho GTPase activating protein 12	protein_codin	0.3786	0.2	1	26.96	0.1681
**WASHC2C**	WASHC2C	21	WASH complex subunit 2C	protein_codin	0.08045	0.835	1	3.224	0.2302
**NFKB2**	NFKB2	21	nuclear factor kappa B subunit 2	protein_codin	1.098	0.000565	1	3.558	0.09007
**CUEDC2**	CUEDC2	21	CUE domain containing 2	protein_codin	-0.3492	0.00481	1	11.75	0.1384
**TSPAN9**	TSPAN9	21	tetraspanin 9	protein_codin	0.1911	0.288	1	40.1	0.1772
**ARNTL2**	ARNTL2	21	aryl hydrocarbon receptor nuclear translocator like 2	protein_codin	0.4785	0.264	1	11.58	0.05686
**CAPRIN2**	CAPRIN2	21	caprin family member 2	protein_codin	-0.6472	0.074	1	31.07	0.1877
**SARNP**	SARNP	21	SAP domain containing ribonucleoprotein	protein_codin	0.2411	0.435	1	2.627	0.2312
**MBD6**	MBD6	21	methyl-CpG binding domain protein 6	protein_codin	0.3326	0.292	1	2.145	0.1370
**SAP18**	SAP18	21	Sin3A associated protein 18	protein_codin	0.09560	0.1429	1	46.68	0.0272
**ZDHHC20**	ZDHHC20	21	zinc finger DHHC-type containing 20	protein_codin	-0.4954	0.0341	1	3.703	0.1442
**ALG5**	ALG5	21	ALG5 dolichyl-phosphate beta-glucosyltransferase	protein_codin	-0.06918	0.662	1	12.38	0.05262
**PABPN1**	PABPN1	21	poly(A) binding protein nuclear 1	protein_codin	-0.1557	0.405	1	19.36	0.08729
**PRPF39**	PRPF39	21	pre-mRNA processing factor 39	protein_codin	0.6276	0.0522	1	3.219	0.2267
**VPS39**	VPS39	21	VPS39 subunit of HOPS complex	protein_codin	0.1503	0.652	1	3.006	0.3744
**SNN**	SNN	21	stannin	protein_codin	1.118	0.00798	1	9.133	0.2501
**MVP**	MVP	21	major vault protein	protein_codin	0.3766	0.0149	1	12.96	0.1472
**DCTPP1**	DCTPP1	21	dCTP pyrophosphatase 1	protein_codin	0.4203	0.00647	1	14.07	0.05845
**ATP6V0D1**	ATP6V0D1	21	ATPase H+ transporting V0 subunit d1	protein_codin	-0.1897	0.26	1	21.73	0.1394
**CXCL16**	CXCL16	21	C-X-C motif chemokine ligand 16	protein_codin	0.2893	0.368	1	8.882	0.2167
**SPAG7**	SPAG7	21	sperm associated antigen 7	protein_codin	0.4823	0.000946	1	12.17	0.2058
**ACADVL**	ACADVL	21	acyl-CoA dehydrogenase very long chain	protein_codin	-0.4311	0.00352	1	13.18	0.05432
**COPS3**	COPS3	21	COP9 signalosome subunit 3	protein_codin	0.3567	0.0688	1	19.17	0.1399
**CDK12**	CDK12	21	cyclin dependent kinase 12	protein_codin	0.1546	0.435	1	18.18	0.09415
**NARF**	NARF	21	nuclear prelamin A recognition factor	protein_codin	0.4205	0.0379	1	44.34	0.1164
**DLGAP1-AS1**	DLGAP1-AS	21	DLGAP1 antisense RNA 1	antisense_RN	-0.08403	0.775	1	3.396	0.3451
**TWSG1**	TWSG1	21	twisted gastrulation BMP signaling modulator 1	protein_codin	0.207	0.496	1	8.617	0.1692
**MBD1**	MBD1	21	methyl-CpG binding domain protein 1	protein_codin	0.7096	0.0111	1	2.628	0.1117
**ME2**	ME2	21	malic enzyme 2	protein_codin	0.4427	0.13	1	8.364	0.05826
**ITPA**	ITPA	21	inosine triphosphatase	protein_codin	-0.2879	0.188	1	43.64	0.1079
**ID1**	ID1	21	inhibitor of DNA binding 1, HLH protein	protein_codin	-0.5409	1.11E-10	2.64E-06	52.58	0.2051
**RALGAPB**	RALGAPB	21	Ral GTPase activating protein non-catalytic beta subunit	protein_codin	-0.4709	0.0842	1	11.34	0.1906
**SOX18**	SOX18	21	SRY-box transcription factor 18	protein_codin	-0.1775	0.0499	1	52.58	0.1553
**C19orf24**	C19orf24	21	family with sequence similarity 174 member C	protein_codin	0.003526	0.98	1	12	0.1332
**ZNF358**	ZNF358	21	zinc finger protein 358	protein_codin	0.1522	0.38	1	16.72	0.1689
**SMARCA4**	SMARCA4	21	SWI/SNF related, matrix associated, actin dependent regulato	protein_codin	-0.1606	0.43	1	7.738	0.2359
**ZNF791**	ZNF791	21	zinc finger protein 791	protein_codin	0.556	0.00447	1	51.82	0.05472
**DBP**	DBP	21	D-box binding PAR bZIP transcription factor	protein_codin	-0.06665	0.81	1	29.43	0.2775
**EWSR1**	EWSR1	21	EWS RNA binding protein 1	protein_codin	0.08328	0.605	1	13.2	0.1651
**EIF3D**	EIF3D	21	eukaryotic translation initiation factor 3 subunit D	protein_codin	0.02727	0.777	1	36.42	0.06284
**TST**	TST	21	thiosulfate sulfurtransferase	protein_codin	-0.2758	0.212	1	9.348	0.05432
**JOSD1**	JOSD1	21	Josephin domain containing 1	protein_codin	-0.2805	0.147	1	21.12	0.1426
**TRABD**	TRABD	21	TraB domain containing	protein_codin	-0.1789	0.468	1	7.807	0.2595
**GART**	GART	21	phosphoribosylglycinamide formyltransferase, phosphoribosy	protein_codin	0.1354	0.599	1	8.722	0.2495
**THAP3**	THAP3	22	THAP domain containing 3	protein_codin	0.08057	0.802	1	2.613	0.1954
**STX12**	STX12	22	syntaxin 12	protein_codin	0.09866	0.534	1	14.17	0.05726
**CCDC28B**	CCDC28B	22	coiled-coil domain containing 28B	protein_codin	-0.1215	0.688	1	3.825	0.1694
**GON4L**	GON4L	22	gon-4 like	protein_codin	0.632	0.0102	1	42.15	0.1451
**ALDH9A1**	ALDH9A1	22	aldehyde dehydrogenase 9 family member A1	protein_codin	0.4909	0.00189	1	14.23	0.08218
**CEP350**	CEP350	22	centrosomal protein 350	protein_codin	-0.1907	0.304	1	7.2	0.1931
**CFH**	CFH	22	complement factor H	protein_codin	-1.144	6.41E-39	1.55E-34	33.95	0.03587
**PHLDA3**	PHLDA3	22	pleckstrin homology like domain family A member 3	protein_codin	0.2988	0.152	1	42.96	0.1479
**ACP1**	ACP1	22	acid phosphatase 1	protein_codin	0.1615	0.229	1	29.03	0.08132
**FNDC4**	FNDC4	22	fibronectin type III domain containing 4	protein_codin	-0.662	0.0246	1	30.17	0.1312
**SRSF7**	SRSF7	22	serine and arginine rich splicing factor 7	protein_codin	0.1676	0.0579	1	51.45	0.08258
**RHOQ**	RHOQ	22	ras homolog family member Q	protein_codin	-1.264	1.12E-06	0.02618	45.75	0.05752
**XPO1**	XPO1	22	exportin 1	protein_codin	-0.2389	0.172	1	19.62	0.0849
**PRADC1**	PRADC1	22	protease associated domain containing 1	protein_codin	-0.8379	9.06E-05	1	9.942	0.1087
**TGOLN2**	TGOLN2	22	trans-golgi network protein 2	protein_codin	-0.517	1.98E-06	0.04622	46.36	0.1351
**TMEM127**	TMEM127	22	transmembrane protein 127	protein_codin	0.292	0.285	1	7.554	0.2521
**TMEM185B**	TMEM185B	22	transmembrane protein 185B	protein_codin	0.797	0.0315	1	2.603	0.1812
**PRPF40A**	PRPF40A	22	pre-mRNA processing factor 40 homolog A	protein_codin	0.2055	0.0872	1	31.41	0.111
**TTC21B**	TTC21B	22	tetratricopeptide repeat domain 21B	protein_codin	1.411	0.0014	1	2.627	0.3169
**RAPGEF4**	RAPGEF4	22	Rap guanine nucleotide exchange factor 4	protein_codin	0.3056	0.228	1	6.654	0.1121
**NCKAP1**	NCKAP1	22	NCK associated protein 1	protein_codin	-0.377	0.00969	1	49.29	0.02862
**BCS1L**	BCS1L	22	BCS1 homolog, ubiquinol-cytochrome c reductase complex c	protein_codin	0.4382	0.132	1	3.269	0.1731
**ATG4B**	ATG4B	22	autophagy related 4B cysteine peptidase	protein_codin	0.2952	0.285	1	8.333	0.1301
**SETD5**	SETD5	22	SET domain containing 5	protein_codin	-0.198	0.37	1	7.86	0.0951
**CCDC174**	CCDC174	22	coiled-coil domain containing 174	protein_codin	0.1532	0.412	1	13.88	0.1237
**SCAP**	SCAP	22	SREBF chaperone	protein_codin	0.3171	0.372	1	10.09	0.2668
**SLMAP**	SLMAP	22	sarcolemma associated protein	protein_codin	-0.2496	0.368	1	3.575	0.05674
**HCLS1**	HCLS1	22	hematopoietic cell-specific Lyn substrate 1	protein_codin	-0.6017	0.0446	1	12.09	0.319
**ZMAT3**	ZMAT3	22	zinc finger matrin-type 3	protein_codin	-1.263	2.74E-06	0.06389	44.12	0.1442
**RGS12**	RGS12	22	regulator of G protein signaling 12	protein_codin	-0.3256	0.211	1	10.11	0.1445
**TBC1D1**	TBC1D1	22	TBC1 domain family member 1	protein_codin	-0.6776	0.000119	1	28.74	0.09917
**SPP1**	SPP1	22	secreted phosphoprotein 1	protein_codin	-4.428	3.52E-10	8.35E-06	Inf	0.9351
**SEC24D**	SEC24D	22	SEC24 homolog D, COPII coat complex component	protein_codin	-0.0859	0.792	1	3.495	0.1687
**KIAA1109**	KIAA1109	22	KIAA1109	protein_codin	-0.1979	0.249	1	16.48	0.1127
**OTULIN**	OTULIN	22	OTU deubiquitinase with linear linkage specificity	protein_codin	-0.2542	0.33	1	3.405	0.2022
**C5orf51**	C5orf51	22	chromosome 5 open reading frame 51	protein_codin	0.4892	0.0882	1	7.876	0.1377
**GPX8**	GPX8	22	glutathione peroxidase 8 (putative)	protein_codin	-0.386	0.0955	1	46.11	0.1672
**SKIV2L2**	SKIV2L2	22	Mtr4 exosome RNA helicase	protein_codin	NA	NA	NA	3.51	0.1381
**BDP1**	BDP1	22	B double prime 1, subunit of RNA polymerase III transcription	protein_codin	0.1779	0.233	1	17.69	0.1107
**NSA2**	NSA2	22	NSA2 ribosome biogenesis factor	protein_codin	0.1142	0.208	1	45.99	0.05664
**P4HA2**	P4HA2	22	prolyl 4-hydroxylase subunit alpha 2	protein_codin	-1.495	6.72E-08	0.001581	9.224	0.1494
**KIF3A**	KIF3A	22	kinesin family member 3A	protein_codin	-0.08402	0.72	1	8.576	0.1175
**SMAD5**	SMAD5	22	SMAD family member 5	protein_codin	0.2162	0.221	1	32.35	0.1346
**KDM3B**	KDM3B	22	lysine demethylase 3B	protein_codin	0.1822	0.562	1	3.63	0.3354
**CYSTM1**	CYSTM1	22	cysteine rich transmembrane module containing 1	protein_codin	0.07860	0.38	1	52.06	0.0274
**ERGIC1**	ERGIC1	22	endoplasmic reticulum-golgi intermediate compartment 1	protein_codin	-0.7516	0.000171	1	7.962	0.126
**CREBRF**	CREBRF	22	CREB3 regulatory factor	protein_codin	-0.2265	0.133	1	33.38	0.02926
**CPEB4**	CPEB4	22	cytoplasmic polyadenylation element binding protein 4	protein_codin	-1.078	1.17E-10	2.78E-06	34.75	0.08648
**ECI2**	ECI2	22	enoyl-CoA delta isomerase 2	protein_codin	0.6596	1.45E-05	0.3359	10.85	0.05762
**TDP2**	TDP2	22	tyrosyl-DNA phosphodiesterase 2	protein_codin	0.4721	0.0659	1	7.173	0.1455
**DDX39B**	DDX39B	22	DExD-box helicase 39B	protein_codin	-0.1303	0.592	1	7.066	0.1762
**FBXO9**	FBXO9	22	F-box protein 9	protein_codin	-0.4056	0.0152	1	14.63	0.08015
**IBTK**	IBTK	22	inhibitor of Bruton tyrosine kinase	protein_codin	0.08058	0.777	1	9.076	0.2169
**MAP3K4**	MAP3K4	22	mitogen-activated protein kinase kinase kinase 4	protein_codin	0.3365	0.245	1	8.423	0.2571
**QKI**	QKI	22	QKI, KH domain containing RNA binding	protein_codin	-0.1097	0.298	1	36.38	0.02654
**MAD1L1**	MAD1L1	22	mitotic arrest deficient 1 like 1	protein_codin	0.6092	0.0642	1	3.217	0.1926
**FSCN1**	FSCN1	22	fascin actin-bundling protein 1	protein_codin	-0.01047	0.948	1	52.58	0.02632
**HOXA4**	HOXA4	22	homeobox A4	protein_codin	0.8988	0.0149	1	8.792	0.1665
**JAZF1**	JAZF1	22	JAZF zinc finger 1	protein_codin	-0.6034	0.0526	1	10.29	0.3381
**STK17A**	STK17A	22	serine/threonine kinase 17a	protein_codin	0.231	0.222	1	51.83	0.08532
**DDX56**	DDX56	22	DEAD-box helicase 56	protein_codin	0.5782	0.0197	1	3.432	0.1932
**EIF4H**	EIF4H	22	eukaryotic translation initiation factor 4H	protein_codin	0.2154	0.0756	1	29.55	0.1409
**EPHB4**	EPHB4	22	EPH receptor B4	protein_codin	0.5282	0.00236	1	25.21	0.05832
**CLDN15**	CLDN15	22	claudin 15	protein_codin	-1.686	4.02E-18	9.66E-14	45.58	0.2237
**ALKBH4**	ALKBH4	22	alkB homolog 4, lysine demethylase	protein_codin	0.2119	0.505	1	3.176	0.3162
**CNOT4**	CNOT4	22	CCR4-NOT transcription complex subunit 4	protein_codin	0.4757	0.00461	1	15.04	0.08268
**GIMAP4**	GIMAP4	22	GTPase, IMAP family member 4	protein_codin	1.392	5.83E-40	1.41E-35	46.95	0.05486
**UBE3C**	UBE3C	22	ubiquitin protein ligase E3C	protein_codin	0.02937	0.91	1	8.138	0.1491
**ARSD**	ARSD	22	arylsulfatase D	protein_codin	0.5399	0.105	1	11.27	0.05502
**HCCS**	HCCS	22	holocytochrome c synthase	protein_codin	0.2766	0.273	1	28.33	0.08871
**GPKOW**	GPKOW	22	G-patch domain and KOW motifs	protein_codin	0.2027	0.526	1	2.554	0.1751
**MAGED2**	MAGED2	22	MAGE family member D2	protein_codin	-0.2250	0.0424	1	47.1	0.0538
**RLIM**	RLIM	22	ring finger protein, LIM domain interacting	protein_codin	0.08568	0.722	1	8.862	0.1724
**ATRX**	ATRX	22	ATRX chromatin remodeler	protein_codin	-0.2261	0.0244	1	32.13	0.05757
**SH3BGRL**	SH3BGRL	22	SH3 domain binding glutamate rich protein like	protein_codin	0.1506	0.0737	1	42.75	0.2082
**BEX4**	BEX4	22	brain expressed X-linked 4	protein_codin	0.2214	0.129	1	45.71	0.086
**AIFM1**	AIFM1	22	apoptosis inducing factor mitochondria associated 1	protein_codin	0.3832	0.185	1	8.266	0.2094
**FMR1**	FMR1	22	fragile X mental retardation 1	protein_codin	0.3137	0.171	1	7.112	0.2896
**CETN2**	CETN2	22	centrin 2	protein_codin	-0.05127	0.794	1	38.89	0.05607
**AC108010.1**	AC108010.1	22	novel transcri	antisense_RN	0.4469	0.103	1	3.223	0.1702
**ZNF395**	ZNF395	22	zinc finger protein 395	protein_codin	1.325	0.00562	1	3.037	0.2506
**BRF2**	BRF2	22	BRF2 RNA polymerase III transcription initiation factor subuni	protein_codin	0.5096	0.123	1	3.298	0.05846
**ARFGEF1**	ARFGEF1	22	ADP ribosylation factor guanine nucleotide exchange factor 1	protein_codin	0.05058	0.796	1	38.48	0.1674
**UBE2W**	UBE2W	22	ubiquitin conjugating enzyme E2 W	protein_codin	-0.08506	0.678	1	52.52	0.1724
**GDAP1**	GDAP1	22	ganglioside induced differentiation associated protein 1	protein_codin	0.6319	0.132	1	36.77	0.1724
**RBM12B**	RBM12B	22	RNA binding motif protein 12B	protein_codin	-0.07798	0.822	1	3.62	0.3899
**TMEM65**	TMEM65	22	transmembrane protein 65	protein_codin	0.5347	0.0727	1	3.058	0.2552
**PLGRKT**	PLGRKT	22	plasminogen receptor with a C-terminal lysine	protein_codin	0.2269	0.326	1	7.288	0.1349
**TOPORS**	TOPORS	22	TOP1 binding arginine/serine rich protein	protein_codin	-0.01163	0.938	1	43.79	0.05875
**VPS13A**	VPS13A	22	vacuolar protein sorting 13 homolog A	protein_codin	0.1676	0.468	1	20.79	0.1202
**CARD19**	CARD19	22	caspase recruitment domain family member 19	protein_codin	0.1339	0.434	1	12.79	0.142
**MAPKAP1**	MAPKAP1	22	MAPK associated protein 1	protein_codin	0.08821	0.686	1	7.406	0.08668
**SET**	SET	22	SET nuclear proto-oncogene	protein_codin	0.05662	0.384	1	51.38	0.02684
**ZDHHC12**	ZDHHC12	22	zinc finger DHHC-type containing 12	protein_codin	1.352	2.91E-05	0.6721	9.644	0.1457
**TOR1A**	TOR1A	22	torsin family 1 member A	protein_codin	-0.07671	0.727	1	8.926	0.08289
**GTF3C5**	GTF3C5	22	general transcription factor IIIC subunit 5	protein_codin	0.5809	0.0718	1	3.136	0.266
**PHPT1**	PHPT1	22	phosphohistidine phosphatase 1	protein_codin	-0.4436	1.24E-06	0.02896	47.32	0.02874
**BTBD10**	BTBD10	22	BTB domain containing 10	protein_codin	0.06843	0.765	1	7.784	0.171
**ELP4**	ELP4	22	elongator acetyltransferase complex subunit 4	protein_codin	0.04081	0.884	1	3.128	0.1421
**ARFGAP2**	ARFGAP2	22	ADP ribosylation factor GTPase activating protein 2	protein_codin	0.4738	0.0699	1	9.765	0.2317
**DDB2**	DDB2	22	damage specific DNA binding protein 2	protein_codin	-0.1824	0.418	1	45.43	0.206
**CELF1**	CELF1	22	CUGBP Elav-like family member 1	protein_codin	0.2017	0.488	1	6.232	0.1634

**ZFP91**	ZFP91	32	ZFP91 zinc finger protein	protein_codin	0.2391	0.39	1	3.606	0.1667
**POLR2G**	POLR2G	32	RNA polymerase II subunit G	protein_codin	0.1358	0.272	1	25.17	0.02732
**NDUFS8**	NDUFS8	32	NADH ubiquinone oxidoreductase core subunit S8	protein_codin	-0.372	0.00075	1	19.83	0.02748
**STARD10**	STARD10	32	StAR related lipid transfer domain containing 10	protein_codin	0.1844	0.557	1	8.342	0.3344
**COA4**	COA4	32	cytochrome c oxidase assembly factor 4 homolog	protein_codin	0.07641	0.546	1	25.5	0.164
**CCDC90B**	CCDC90B	32	coiled-coil domain containing 90B	protein_codin	0.06903	0.601	1	52.45	0.114
**C11orf71**	C11orf71	32	chromosome 11 open reading frame 71	protein_codin	0.7696	0.0188	1	10.52	0.1905
**EI24**	EI24	32	EI24 autophagy associated transmembrane protein	protein_codin	-0.02666	0.86	1	51.68	0.1185
**ARL5B**	ARL5B	32	ADP ribosylation factor like GTPase 5B	protein_codin	0.3182	0.138	1	16.88	0.2326
**ASCC1**	ASCC1	32	activating signal cointegrator 1 complex subunit 1	protein_codin	0.5229	0.154	1	3.288	0.1158
**NUTM2B-AS1**	NUTM2B-A	32	NUTM2B antisense RNA 1	antisense_RN	0.2294	0.218	1	7.619	0.136
**UBTD1**	UBTD1	32	ubiquitin domain containing 1	protein_codin	0.1786	0.392	1	16.6	0.1614
**CUTC**	CUTC	32	cutC copper transporter	protein_codin	0.1778	0.392	1	23.14	0.2597
**BORCS7**	BORCS7	32	BLOC-1 related complex subunit 7	protein_codin	0.09487	0.432	1	41.6	0.1408
**RIMKLB**	RIMKLB	32	ribosomal modification protein rimK like family member B	protein_codin	-0.02984	0.919	1	7.866	0.1397
**PYM1**	PYM1	32	PYM homolog 1, exon junction complex associated factor	protein_codin	0.2905	0.268	1	3.334	0.1632
**NAV3**	NAV3	32	neuron navigator 3	protein_codin	0.7132	0.0281	1	10.55	0.3962
**HSPB8**	HSPB8	32	heat shock protein family B (small) member 8	protein_codin	1.701	7.42E-05	1	51.94	0.1432
**TRIAP1**	TRIAP1	32	TP53 regulated inhibitor of apoptosis 1	protein_codin	0.2092	0.292	1	52.52	0.2472
**ANAPC5**	ANAPC5	32	anaphase promoting complex subunit 5	protein_codin	0.2169	0.153	1	30.38	0.2249
**PSMD9**	PSMD9	32	proteasome 26S subunit, non-ATPase 9	protein_codin	0.6727	0.0326	1	3.002	0.2865
**RAN**	RAN	32	RAN, member RAS oncogene family	protein_codin	-0.07767	0.2537	1	39.88	0.0286
**FRY**	FRY	32	FRY microtubule binding protein	protein_codin	1.32	1.14E-09	2.7E-05	28.94	0.108
**IPO5**	IPO5	32	importin 5	protein_codin	0.4388	0.0689	1	6.914	0.2882
**PARP2**	PARP2	32	poly(ADP-ribose) polymerase 2	protein_codin	0.8398	0.0185	1	9.083	0.1692
**STRN3**	STRN3	32	striatin 3	protein_codin	0.1168	0.507	1	15.48	0.1548
**CNIH1**	CNIH1	32	cornichon family AMPA receptor auxiliary protein 1	protein_codin	0.4640	0.000189	1	16.26	0.05865
**PCNX4**	PCNX4	32	pecanex 4	protein_codin	0.1122	0.62	1	21.43	0.1116
**COQ6**	COQ6	32	coenzyme Q6, monooxygenase	protein_codin	0.3385	0.278	1	2.985	0.1946
**SPATA7**	SPATA7	32	spermatogenesis associated 7	protein_codin	0.09535	0.735	1	8.752	0.2537
**FOXN3**	FOXN3	32	forkhead box N3	protein_codin	0.5632	0.0114	1	7.91	0.1472
**TMEM251**	TMEM251	32	transmembrane protein 251	protein_codin	-0.2092	0.468	1	3.265	0.1952
**ANKRD9**	ANKRD9	32	ankyrin repeat domain 9	protein_codin	-0.4502	0.068	1	8.477	0.1666
**DNAJC17**	DNAJC17	32	DnaJ heat shock protein family (Hsp40) member C17	protein_codin	0.4969	0.0349	1	8.019	0.1419
**RPS27L**	RPS27L	32	ribosomal protein S27 like	protein_codin	-0.5592	4.9E-16	1.18E-11	52.02	0.1652
**SPG21**	SPG21	32	SPG21 abhydrolase domain containing, maspardin	protein_codin	-0.0668	0.746	1	7.994	0.1076
**LRRC28**	LRRC28	32	leucine rich repeat containing 28	protein_codin	0.2444	0.379	1	3.282	0.17
**POLR3K**	POLR3K	32	RNA polymerase III subunit K	protein_codin	-0.01428	0.942	1	6.858	0.1404
**DECR2**	DECR2	32	2,4-dienoyl-CoA reductase 2	protein_codin	-0.3506	0.214	1	2.762	0.2605
**TSR3**	TSR3	32	TSR3 ribosome maturation factor	protein_codin	0.3028	0.0796	1	42.25	0.167
**SPSB3**	SPSB3	32	splA/ryanodine receptor domain and SOCS box containing 3	protein_codin	0.4124	0.00812	1	14.8	0.03179
**NDUFB10**	NDUFB10	32	NADH ubiquinone oxidoreductase subunit B10	protein_codin	-0.1946	0.00727	1	38.37	0.03033
**NUDT16L1**	NUDT16L1	32	nudix hydrolase 16 like 1	protein_codin	0.02957	0.896	1	49.18	0.1756
**PMM2**	PMM2	32	phosphomannomutase 2	protein_codin	0.4508	0.0922	1	3.416	0.3294
**UQCRC2**	UQCRC2	32	ubiquinol-cytochrome c reductase core protein 2	protein_codin	0.2391	0.0291	1	13.4	0.1405
**TK2**	TK2	32	thymidine kinase 2	protein_codin	0.3175	0.22	1	7.688	0.1744
**CBFB**	CBFB	32	core-binding factor subunit beta	protein_codin	-0.03051	0.909	1	8.94	0.1118
**EXOSC6**	EXOSC6	32	exosome component 6	protein_codin	0.3222	0.0902	1	6.896	0.05467
**NAA38**	NAA38	32	N(alpha)-acetyltransferase 38, NatC auxiliary subunit	protein_codin	-0.4962	7.61E-07	0.0178	52.54	0.05545
**MAP2K4**	MAP2K4	32	mitogen-activated protein kinase kinase 4	protein_codin	-0.01727	0.958	1	2.707	0.2791
**PMP22**	PMP22	32	peripheral myelin protein 22	protein_codin	1.124	2.29E-17	5.5E-13	39.55	0.03052
**RAB34**	RAB34	32	RAB34, member RAS oncogene family	protein_codin	-3.296	4.42E-46	1.07E-41	21.36	0.06536
**SMARCE1**	SMARCE1	32	SWI/SNF related, matrix associated, actin dependent regulato	protein_codin	0.003184	0.984	1	17.5	0.08032
**NME1**	NME1	32	NME/NM23 nucleoside diphosphate kinase 1	protein_codin	-0.2689	0.0414	1	46.68	0.06445
**CCDC137**	CCDC137	32	coiled-coil domain containing 137	protein_codin	0.5709	0.0325	1	8.927	0.3099
**SIRT7**	SIRT7	32	sirtuin 7	protein_codin	0.442	0.101	1	3.27	0.1995
**RNF125**	RNF125	32	ring finger protein 125	protein_codin	1.257	1.07E-06	0.02502	27.67	0.02956
**PLCB1**	PLCB1	32	phospholipase C beta 1	protein_codin	-0.1206	0.476	1	52.58	0.1566
**RTFDC1**	RTFDC1	32	replication termination factor 2	protein_codin	NA	NA	NA	14.55	0.05621
**ZBTB46**	ZBTB46	32	zinc finger and BTB domain containing 46	protein_codin	0.4479	0.192	1	3.388	0.1486
**DNMT1**	DNMT1	32	DNA methyltransferase 1	protein_codin	0.2938	0.111	1	7.196	0.1687
**ILF3**	ILF3	32	interleukin enhancer binding factor 3	protein_codin	-0.3698	0.0122	1	28.57	0.08149
**GADD45GIP1**	GADD45GI	32	GADD45G interacting protein 1	protein_codin	-0.6624	8.5E-15	2.04E-10	31.36	0.02838
**CCDC124**	CCDC124	32	coiled-coil domain containing 124	protein_codin	0.04576	0.715	1	11.67	0.08936
**GDF15**	GDF15	32	growth differentiation factor 15	protein_codin	-3.854	2.4E-14	5.74E-10	52.58	0.2519
**UBA2**	UBA2	32	ubiquitin like modifier activating enzyme 2	protein_codin	0.02347	0.888	1	14.29	0.08357
**AKT2**	AKT2	32	AKT serine/threonine kinase 2	protein_codin	0.02291	0.947	1	1.816	0.1942
**BAX**	BAX	32	BCL2 associated X, apoptosis regulator	protein_codin	-0.6666	2.9E-09	6.87E-05	37.14	0.105
**RUVBL2**	RUVBL2	32	RuvB like AAA ATPase 2	protein_codin	0.01348	0.95	1	3.282	0.1392
**GSTT2B**	GSTT2B	32	glutathione S-transferase theta 2B (gene/pseudogene)	protein_codin	-1.076	1.86E-07	0.004366	8.965	0.142
**EP300**	EP300	32	E1A binding protein p300	protein_codin	0.7998	0.00127	1	6.092	0.1114
**DESI1**	DESI1	32	desumoylating isopeptidase 1	protein_codin	-0.0279	0.901	1	8.452	0.1982
**PACSIN2**	PACSIN2	32	protein kinase C and casein kinase substrate in neurons 2	protein_codin	0.571	0.0176	1	26.88	0.2771
**CPTP**	CPTP	33	ceramide-1-phosphate transfer protein	protein_codin	0.6221	0.00602	1	6.717	0.196
**CEP104**	CEP104	33	centrosomal protein 104	protein_codin	-0.08015	0.768	1	2.972	0.2229
**H6PD**	H6PD	33	hexose-6-phosphate dehydrogenase/glucose 1-dehydrogena	protein_codin	0.2027	0.452	1	9.155	0.3864
**TNFRSF1B**	TNFRSF1B	33	TNF receptor superfamily member 1B	protein_codin	0.4766	0.0427	1	6.937	0.05368
**DDOST**	DDOST	33	dolichyl-diphosphooligosaccharide--protein glycosyltransfera	protein_codin	0.02234	0.798	1	19.56	0.02747
**PUM1**	PUM1	33	pumilio RNA binding family member 1	protein_codin	-0.04191	0.858	1	16.56	0.08608
**ZC3H12A**	ZC3H12A	33	zinc finger CCCH-type containing 12A	protein_codin	1.152	0.00065	1	30.2	0.02635
**SERBP1**	SERBP1	33	SERPINE1 mRNA binding protein 1	protein_codin	0.2619	4.08E-05	0.9409	44.47	0.02708
**TYW3**	TYW3	33	tRNA-yW synthesizing protein 3 homolog	protein_codin	0.2224	0.168	1	12.89	0.05682
**ZNHIT6**	ZNHIT6	33	zinc finger HIT-type containing 6	protein_codin	0.01228	0.962	1	8.842	0.2665
**SASS6**	SASS6	33	SAS-6 centriolar assembly protein	protein_codin	0.216	0.624	1	27.92	0.2212
**SYT11**	SYT11	33	synaptotagmin 11	protein_codin	-0.7182	0.00487	1	9.277	0.142
**LMNA**	LMNA	33	lamin A/C	protein_codin	-0.03362	0.622	1	52.48	0.02726
**B4GALT3**	B4GALT3	33	beta-1,4-galactosyltransferase 3	protein_codin	0.3782	0.125	1	9.387	0.1394
**CDC73**	CDC73	33	cell division cycle 73	protein_codin	0.2106	0.202	1	49.26	0.08106
**KDM5B**	KDM5B	33	lysine demethylase 5B	protein_codin	0.3288	0.15	1	40.32	0.1991
**GALNT2**	GALNT2	33	polypeptide N-acetylgalactosaminyltransferase 2	protein_codin	-0.9159	1.2E-05	0.2782	9.322	0.116
**ODC1**	ODC1	33	ornithine decarboxylase 1	protein_codin	0.02881	0.871	1	52.52	0.2432
**ATAD2B**	ATAD2B	33	ATPase family AAA domain containing 2B	protein_codin	0.2689	0.345	1	3.044	0.08219
**DNMT3A**	DNMT3A	33	DNA methyltransferase 3 alpha	protein_codin	0.4172	0.233	1	3.542	0.2534
**SPAST**	SPAST	33	spastin	protein_codin	0.1748	0.532	1	3.032	0.1519
**THUMPD2**	THUMPD2	33	THUMP domain containing 2	protein_codin	-0.1708	0.502	1	3.687	0.2226
**DYNC2LI1**	DYNC2LI1	33	dynein cytoplasmic 2 light intermediate chain 1	protein_codin	0.3692	0.194	1	7.178	0.1462
**CEP68**	CEP68	33	centrosomal protein 68	protein_codin	1.017	1.08E-05	0.2505	14.32	0.2338
**ZNF638**	ZNF638	33	zinc finger protein 638	protein_codin	-0.2065	0.152	1	14.09	0.1216
**EXOC6B**	EXOC6B	33	exocyst complex component 6B	protein_codin	-0.2274	0.422	1	2.827	0.0312
**CCT7**	CCT7	33	chaperonin containing TCP1 subunit 7	protein_codin	0.334	0.00374	1	17.57	0.0274
**TRAK2**	TRAK2	33	trafficking kinesin protein 2	protein_codin	-0.05903	0.824	1	7.518	0.1942
**ARL4C**	ARL4C	33	ADP ribosylation factor like GTPase 4C	protein_codin	-0.05179	0.83	1	52.47	0.08682
**ING5**	ING5	33	inhibitor of growth family member 5	protein_codin	0.01312	0.958	1	3.736	0.195
**THUMPD3-AS1**	THUMPD3-	33	THUMPD3 antisense RNA 1	antisense_RN	0.8557	4.63E-05	1	26.12	0.1152
**RAF1**	RAF1	33	Raf-1 proto-oncogene, serine/threonine kinase	protein_codin	0.009234	0.97	1	9.354	0.1672
**MRPS25**	MRPS25	33	mitochondrial ribosomal protein S25	protein_codin	0.409	0.0682	1	46.71	0.2012
**DYNC1LI1**	DYNC1LI1	33	dynein cytoplasmic 1 light intermediate chain 1	protein_codin	0.1721	0.268	1	14.63	0.1642
**CLASP2**	CLASP2	33	cytoplasmic linker associated protein 2	protein_codin	0.6444	0.0826	1	2.8	0.3884
**IP6K1**	IP6K1	33	inositol hexakisphosphate kinase 1	protein_codin	0.2979	0.31	1	8.686	0.1747
**GBE1**	GBE1	33	1,4-alpha-glucan branching enzyme 1	protein_codin	-0.04168	0.884	1	11.48	0.1361
**FAM162A**	FAM162A	33	family with sequence similarity 162 member A	protein_codin	-0.01425	0.912	1	21.72	0.14
**SLC41A3**	SLC41A3	33	solute carrier family 41 member 3	protein_codin	0.38	0.0182	1	9.307	0.1115
**PCGF3**	PCGF3	33	polycomb group ring finger 3	protein_codin	-0.3034	0.255	1	34.48	0.2901
**ANKRD17**	ANKRD17	33	ankyrin repeat domain 17	protein_codin	0.08896	0.663	1	7.625	0.2002
**PKD2**	PKD2	33	polycystin 2, transient receptor potential cation channel	protein_codin	-0.2532	0.455	1	49.58	0.1601
**LARP1B**	LARP1B	33	La ribonucleoprotein domain family member 1B	protein_codin	-0.8101	0.00585	1	35.32	0.2777
**TMEM192**	TMEM192	33	transmembrane protein 192	protein_codin	-0.03856	0.873	1	7.587	0.1739
**TRIM23**	TRIM23	33	tripartite motif containing 23	protein_codin	0.1746	0.547	1	33.55	0.09134
**MAST4**	MAST4	33	microtubule associated serine/threonine kinase family membe	protein_codin	-1.18	4.52E-11	1.08E-06	47.55	0.1931
**PAM**	PAM	33	peptidylglycine alpha-amidating monooxygenase	protein_codin	-0.5531	0.0047	1	8.111	0.09548
**APC**	APC	33	APC regulator of WNT signaling pathway	protein_codin	0.4317	0.0385	1	19.84	0.2811
**CEP120**	CEP120	33	centrosomal protein 120	protein_codin	-0.08456	0.784	1	9.982	0.2121
**FBXO38**	FBXO38	33	F-box protein 38	protein_codin	-1.096	0.00052	1	3.028	0.2116
**NHP2**	NHP2	33	NHP2 ribonucleoprotein	protein_codin	0.05437	0.613	1	23.34	0.03018
**FAM8A1**	FAM8A1	33	family with sequence similarity 8 member A1	protein_codin	-0.2219	0.405	1	7.779	0.1967
**MRS2**	MRS2	33	magnesium transporter MRS2	protein_codin	0.1206	0.734	1	11.72	0.2172
**HIST1H2AC**	HIST1H2AC	33	histone cluster 1 H2A family member c	protein_codin	0.4001	0.12	1	51.76	0.1402
**GNL1**	GNL1	33	G protein nucleolar 1 (putative)	protein_codin	0.09097	0.59	1	6.737	0.1119
**NRM**	NRM	33	nurim	protein_codin	0.7672	0.0111	1	3.432	0.3276
**STK19**	STK19	33	serine/threonine kinase 19	protein_codin	-0.1132	0.572	1	6.041	0.1966
**IFNGR1**	IFNGR1	33	interferon gamma receptor 1	protein_codin	0.5075	1.07E-05	0.2482	51.43	0.05802
**INTS1**	INTS1	33	integrator complex subunit 1	protein_codin	0.1242	0.696	1	3.152	0.2034
**THAP5**	THAP5	33	THAP domain containing 5	protein_codin	0.2776	0.211	1	7.776	0.1914
**ATP6AP2**	ATP6AP2	33	ATPase H+ transporting accessory protein 2	protein_codin	-0.1474	0.132	1	22.1	0.05448
**TAF9B**	TAF9B	33	TATA-box binding protein associated factor 9b	protein_codin	0.81	0.0202	1	2.968	0.2627
**MORF4L2**	MORF4L2	33	mortality factor 4 like 2	protein_codin	0.04231	0.728	1	45.94	0.05803
**PPP1R3B**	PPP1R3B	33	protein phosphatase 1 regulatory subunit 3B	protein_codin	0.2835	0.424	1	10.86	0.1914
**FAM160B2**	FAM160B2	33	family with sequence similarity 160 member B2	protein_codin	0.364	0.262	1	2.655	0.2854
**CA8**	CA8	33	carbonic anhydrase 8	protein_codin	1.851	7.57E-05	1	7.857	0.2282
**SHARPIN**	SHARPIN	33	SHANK associated RH domain interactor	protein_codin	0.06818	0.658	1	14.83	0.08214
**SLC52A2**	SLC52A2	33	solute carrier family 52 member 2	protein_codin	-0.1472	0.49	1	6.692	0.2318
**IFT74**	IFT74	33	intraflagellar transport 74	protein_codin	0.04931	0.849	1	20.31	0.05507
**AAED1**	AAED1	33	peroxiredoxin like 2C	protein_codin	0.3616	0.00776	1	38.5	0.02899
**COQ4**	COQ4	33	coenzyme Q4	protein_codin	0.2281	0.278	1	6.548	0.1659
**MOB2**	MOB2	33	MOB kinase activator 2	protein_codin	0.06603	0.577	1	50.68	0.1362
**DNAJC24**	DNAJC24	33	DnaJ heat shock protein family (Hsp40) member C24	protein_codin	0.04706	0.892	1	9.338	0.1697
**API5**	API5	33	apoptosis inhibitor 5	protein_codin	0.479	0.0788	1	9.81	0.1832
**AHNAK**	AHNAK	33	AHNAK nucleoprotein	protein_codin	0.6279	7.58E-08	0.001782	47.08	0.1114
**PPP2R5B**	PPP2R5B	33	protein phosphatase 2 regulatory subunit B'beta	protein_codin	-0.8596	0.00179	1	10.68	0.1484
**RPS6KB2**	RPS6KB2	33	ribosomal protein S6 kinase B2	protein_codin	-0.1249	0.651	1	3.396	0.3076
**ATM**	ATM	33	ATM serine/threonine kinase	protein_codin	-0.314	0.179	1	7.162	0.09648
**OPTN**	OPTN	33	optineurin	protein_codin	0.1598	0.237	1	47.45	0.05447
**WBP1L**	WBP1L	33	WW domain binding protein 1 like	protein_codin	-0.6879	0.00124	1	7.186	0.2317
**SMIM10L1**	SMIM10L1	33	small integral membrane protein 10 like 1	protein_codin	0.105	0.522	1	31.92	0.188
**METTL7A**	METTL7A	33	methyltransferase like 7A	protein_codin	1.247	7.22E-09	0.000171	52.54	0.311
**SMARCC2**	SMARCC2	33	SWI/SNF related, matrix associated, actin dependent regulato	protein_codin	0.2794	0.107	1	28.52	0.05464
**CCT2**	CCT2	33	chaperonin containing TCP1 subunit 2	protein_codin	0.2525	0.0216	1	13.54	0.02776
**ATP2B1**	ATP2B1	33	ATPase plasma membrane Ca2+ transporting 1	protein_codin	0.5987	1.05E-05	0.2436	45.54	0.08106
**WASHC3**	WASHC3	33	WASH complex subunit 3	protein_codin	-0.1571	0.292	1	6.135	0.08362
**SART3**	SART3	33	spliceosome associated factor 3, U4/U6 recycling protein	protein_codin	0.3412	0.207	1	3.505	0.1757
**FNDC3A**	FNDC3A	33	fibronectin type III domain containing 3A	protein_codin	-0.06692	0.68	1	14.25	0.1389
**VPS36**	VPS36	33	vacuolar protein sorting 36 homolog	protein_codin	-0.01996	0.887	1	12.32	0.09642
**NDFIP2**	NDFIP2	33	Nedd4 family interacting protein 2	protein_codin	0.6875	0.0226	1	8.232	0.222
**RBM23**	RBM23	33	RNA binding motif protein 23	protein_codin	0.5157	0.0349	1	7.366	0.2008
**STYX**	STYX	33	serine/threonine/tyrosine interacting protein	protein_codin	0.05293	0.843	1	9.292	0.2726
**ALDH1A2**	ALDH1A2	33	aldehyde dehydrogenase 1 family member A2	protein_codin	1.068	6.24E-06	0.1451	51.96	0.2345
**LUC7L**	LUC7L	33	LUC7 like	protein_codin	-0.1001	0.636	1	7.763	0.03398
**TSC2**	TSC2	33	TSC complex subunit 2	protein_codin	0.04102	0.877	1	3.092	0.1649
**HIRIP3**	HIRIP3	33	HIRA interacting protein 3	protein_codin	0.3731	0.121	1	23.52	0.2318
**CMTR2**	CMTR2	33	cap methyltransferase 2	protein_codin	0.2147	0.468	1	8.726	0.2289
**NLRP1**	NLRP1	33	NLR family pyrin domain containing 1	protein_codin	0.8901	0.00559	1	8.243	0.1392
**SYNRG**	SYNRG	33	synergin gamma	protein_codin	-0.07487	0.732	1	8.594	0.1389
**MLLT6**	MLLT6	33	MLLT6, PHD finger containing	protein_codin	0.5166	0.0289	1	7.523	0.2509
**PSMD3**	PSMD3	33	proteasome 26S subunit, non-ATPase 3	protein_codin	0.4457	0.0126	1	17.72	0.05845
**KRT222**	KRT222	33	keratin 222	protein_codin	0.3636	0.148	1	43.19	0.2512
**GPATCH8**	GPATCH8	33	G-patch domain containing 8	protein_codin	-0.3596	0.0657	1	7.972	0.1428
**KANSL1-AS1**	KANSL1-AS	33	KANSL1 antisense RNA 1	antisense_RN	0.05489	0.798	1	25.75	0.08859
**TRIM37**	TRIM37	33	tripartite motif containing 37	protein_codin	-0.3719	0.192	1	9.631	0.2011
**CCDC47**	CCDC47	33	coiled-coil domain containing 47	protein_codin	0.01816	0.852	1	34.62	0.05399
**CEP112**	CEP112	33	centrosomal protein 112	protein_codin	0.185	0.292	1	16.31	0.2292
**PSMD12**	PSMD12	33	proteasome 26S subunit, non-ATPase 12	protein_codin	0.3175	0.0266	1	22.44	0.1129
**MRPL58**	MRPL58	33	mitochondrial ribosomal protein L58	protein_codin	0.3677	0.21	1	7.612	0.1386
**ATP5H**	ATP5H	33	ATP synthase peripheral stalk subunit d	protein_codin	NA	NA	NA	27.48	0.1126
**AFG3L2**	AFG3L2	33	AFG3 like matrix AAA peptidase subunit 2	protein_codin	0.3637	0.158	1	6.878	0.1412
**HDHD2**	HDHD2	33	haloacid dehalogenase like hydrolase domain containing 2	protein_codin	0.2336	0.458	1	3.042	0.388
**VPS16**	VPS16	33	VPS16 core subunit of CORVET and HOPS complexes	protein_codin	0.0094	0.976	1	2.852	0.1392
**MRPS26**	MRPS26	33	mitochondrial ribosomal protein S26	protein_codin	0.4974	0.00179	1	14.18	0.05437
**CRNKL1**	CRNKL1	33	crooked neck pre-mRNA splicing factor 1	protein_codin	0.518	0.0497	1	3.625	0.2545
**ARFGEF2**	ARFGEF2	33	ADP ribosylation factor guanine nucleotide exchange factor 2	protein_codin	-0.7757	0.0022	1	32.12	0.1692
**ZFAS1**	ZFAS1	33	ZNFX1 antisense RNA 1	antisense_RN	0.2274	0.00725	1	28.96	0.02684
**BRD4**	BRD4	33	bromodomain containing 4	protein_codin	0.1481	0.314	1	11.96	0.08819
**ELL**	ELL	33	elongation factor for RNA polymerase II	protein_codin	0.6594	0.0516	1	2.82	0.1441
**ZNF675**	ZNF675	33	zinc finger protein 675	protein_codin	-0.04211	0.894	1	10.02	0.2354
**ZNF570**	ZNF570	33	zinc finger protein 570	protein_codin	-0.1285	0.684	1	3.496	0.3172
**DMAC2**	DMAC2	33	distal membrane arm assembly complex 2	protein_codin	0.5451	0.0229	1	7.341	0.2529
**HSD17B14**	HSD17B14	33	hydroxysteroid 17-beta dehydrogenase 14	protein_codin	-0.1774	0.543	1	9.042	0.1996
**MYADM**	MYADM	33	myeloid associated differentiation marker	protein_codin	0.7445	2.77E-09	6.56E-05	51.21	0.1125
**TMEM238**	TMEM238	33	transmembrane protein 238	protein_codin	0.4894	0.108	1	7.94	0.1066
**CABIN1**	CABIN1	33	calcineurin binding protein 1	protein_codin	-0.5162	0.129	1	3.01	0.1742
**APOL6**	APOL6	33	apolipoprotein L6	protein_codin	0.8662	0.00106	1	19.34	0.08416
**NDUFA6**	NDUFA6	33	NADH ubiquinone oxidoreductase subunit A6	protein_codin	-0.2162	0.0722	1	19.65	0.06422
**AL118516.1**	AL118516.1	33	novel transcript, antisense to TBC1D2	antisense_RN	0.1645	0.542	1	52.54	0.5089
**KIF1B**	KIF1B	34	kinesin family member 1B	protein_codin	-0.2377	0.318	1	8.736	0.05882
**KIF17**	KIF17	34	kinesin family member 17	protein_codin	0.6415	0.049	1	10.2	0.1156
**KIAA0319L**	KIAA0319L	34	KIAA0319 like	protein_codin	0.8052	0.00831	1	8.282	0.1368
**DPYD**	DPYD	34	dihydropyrimidine dehydrogenase	protein_codin	0.03225	0.902	1	3.346	0.1959
**SCAMP3**	SCAMP3	34	secretory carrier membrane protein 3	protein_codin	0.2969	0.166	1	6.59	0.1469
**ASH1L**	ASH1L	34	ASH1 like histone lysine methyltransferase	protein_codin	-0.1497	0.303	1	27.62	0.1161
**AIM2**	AIM2	34	absent in melanoma 2	protein_codin	1.498	0.0025	1	31.1	0.1695
**BLZF1**	BLZF1	34	basic leucine zipper nuclear factor 1	protein_codin	0.5077	0.08	1	28.5	0.2374
**PIGC**	PIGC	34	phosphatidylinositol glycan anchor biosynthesis class C	protein_codin	-0.02215	0.92	1	8.77	0.08312
**CYB5R1**	CYB5R1	34	cytochrome b5 reductase 1	protein_codin	0.09388	0.549	1	15.29	0.08349
**G0S2**	G0S2	34	G0/G1 switch 2	protein_codin	0.4115	0.549	1	48.05	0.8571
**WDR26**	WDR26	34	WD repeat domain 26	protein_codin	-0.6326	0.0127	1	3.543	0.2572
**SCCPDH**	SCCPDH	34	saccharopine dehydrogenase (putative)	protein_codin	0.2084	0.287	1	7.266	0.05316
**TTC32**	TTC32	34	tetratricopeptide repeat domain 32	protein_codin	1.401	3.43E-05	0.7914	47.83	0.1254
**YPEL5**	YPEL5	34	yippee like 5	protein_codin	-0.4006	0.000889	1	52.28	0.1184
**PNPT1**	PNPT1	34	polyribonucleotide nucleotidyltransferase 1	protein_codin	0.1636	0.511	1	21.52	0.2347
**COMMD1**	COMMD1	34	copper metabolism domain containing 1	protein_codin	0.2752	0.0572	1	33.82	0.05298
**SPR**	SPR	34	sepiapterin reductase	protein_codin	0.02994	0.878	1	8.488	0.1148
**MAT2A**	MAT2A	34	methionine adenosyltransferase 2A	protein_codin	0.5092	0.0254	1	8.042	0.115
**RNF181**	RNF181	34	ring finger protein 181	protein_codin	-0.145	0.197	1	25.35	0.08079
**REV1**	REV1	34	REV1 DNA directed polymerase	protein_codin	-0.1862	0.364	1	7.233	0.08698
**ZC3H6**	ZC3H6	34	zinc finger CCCH-type containing 6	protein_codin	0.3805	0.113	1	17.54	0.08186
**INSIG2**	INSIG2	34	insulin induced gene 2	protein_codin	-1.088	7.29E-08	0.001715	8.906	0.1776
**UBXN4**	UBXN4	34	UBX domain protein 4	protein_codin	-0.09792	0.2376	1	33.14	0.02663
**MMADHC**	MMADHC	34	metabolism of cobalamin associated D	protein_codin	0.1446	0.284	1	11.82	0.08109
**HOXD9**	HOXD9	34	homeobox D9	protein_codin	0.8707	0.0279	1	31.29	0.3107
**HAGLROS**	HAGLROS	34	HAGLR opposite strand lncRNA	lincRNA	-0.2547	0.35	1	9.366	0.3122
**ZC3H15**	ZC3H15	34	zinc finger CCCH-type containing 15	protein_codin	0.2412	0.0112	1	42.97	0.08314
**WDR12**	WDR12	34	WD repeat domain 12	protein_codin	0.825	0.00527	1	8.513	0.1079
**CHCHD4**	CHCHD4	34	coiled-coil-helix-coiled-coil-helix domain containing 4	protein_codin	-0.03615	0.904	1	3.484	0.1687
**IQCB1**	IQCB1	34	IQ motif containing B1	protein_codin	0.3386	0.267	1	9.938	0.2008
**CEP63**	CEP63	34	centrosomal protein 63	protein_codin	-0.3135	0.136	1	6.923	0.02954
**MRPS22**	MRPS22	34	mitochondrial ribosomal protein S22	protein_codin	0.4402	0.0115	1	16.93	0.06664
**TFDP2**	TFDP2	34	transcription factor Dp-2	protein_codin	-0.3291	0.0616	1	7.006	0.1699
**SENP2**	SENP2	34	SUMO specific peptidase 2	protein_codin	-0.07261	0.832	1	3.509	0.2782
**AC147067.1**	AC147067.1	34	novel transcri	lincRNA	0.8779	0.0177	1	36.16	0.3632
**11-Sep**	43719	34	septin 11	protein_codin	-0.6974	4.91E-08	0.001157	28.92	0.05482
**PPP3CA**	PPP3CA	34	protein phosphatase 3 catalytic subunit alpha	protein_codin	0.5579	7.1E-06	0.165	36.04	0.08156
**BBS7**	BBS7	34	Bardet-Biedl syndrome 7	protein_codin	0.4142	0.16	1	10.13	0.1726
**FGF2**	FGF2	34	fibroblast growth factor 2	protein_codin	-0.801	9.3E-05	1	16.88	0.1182
**TLR3**	TLR3	34	toll like receptor 3	protein_codin	1.022	0.0132	1	8.161	0.3119
**ZNF622**	ZNF622	34	zinc finger protein 622	protein_codin	0.4229	0.0746	1	8.295	0.1176
**MCCC2**	MCCC2	34	methylcrotonoyl-CoA carboxylase 2	protein_codin	0.5427	0.132	1	3.562	0.2114
**JMY**	JMY	34	junction mediating and regulatory protein, p53 cofactor	protein_codin	-0.3691	0.15	1	9.654	0.2855
**SLF1**	SLF1	34	SMC5-SMC6 complex localization factor 1	protein_codin	1.038	0.00156	1	26.81	0.2796
**ERAP1**	ERAP1	34	endoplasmic reticulum aminopeptidase 1	protein_codin	0.4891	0.183	1	3.272	0.2739
**AP3S1**	AP3S1	34	adaptor related protein complex 3 subunit sigma 1	protein_codin	-0.07185	0.61	1	11.33	0.05572
**ALDH7A1**	ALDH7A1	34	aldehyde dehydrogenase 7 family member A1	protein_codin	-0.2256	0.352	1	8.412	0.2249
**8-Sep**	43716	34	septin 8	protein_codin	-0.3811	0.192	1	11.61	0.2729
**SLC26A2**	SLC26A2	34	solute carrier family 26 member 2	protein_codin	-0.4989	0.0916	1	8.497	0.1414
**DCTN4**	DCTN4	34	dynactin subunit 4	protein_codin	0.3282	0.296	1	2.944	0.3252
**GALNT10**	GALNT10	34	polypeptide N-acetylgalactosaminyltransferase 10	protein_codin	-0.2696	0.286	1	8.398	0.2258
**ZNF322**	ZNF322	34	zinc finger protein 322	protein_codin	0.2682	0.193	1	6.454	0.1686
**NEU1**	NEU1	34	neuraminidase 1	protein_codin	-0.1509	0.422	1	20.09	0.1891
**CCDC167**	CCDC167	34	coiled-coil domain containing 167	protein_codin	-0.5738	0.0122	1	6.997	0.05325
**NFKBIE**	NFKBIE	34	NFKB inhibitor epsilon	protein_codin	0.08103	0.787	1	8.808	0.1446
**MUT**	MUT	34	methylmalonyl-CoA mutase	protein_codin	0.06492	0.838	1	3.237	0.117
**CD109**	CD109	34	CD109 molecule	protein_codin	0.3142	0.107	1	13.67	0.1791
**PNISR**	PNISR	34	PNN interacting serine and arginine rich protein	protein_codin	0.0159	0.88	1	39.34	0.08338
**SERINC1**	SERINC1	34	serine incorporator 1	protein_codin	-0.1337	0.252	1	48.96	0.1102
**AFDN**	AFDN	34	afadin, adherens junction formation factor	protein_codin	0.1642	0.172	1	15.25	0.1127
**SNX13**	SNX13	34	sorting nexin 13	protein_codin	-0.08095	0.75	1	6.944	0.1881
**PHKG1**	PHKG1	34	phosphorylase kinase catalytic subunit gamma 1	protein_codin	-0.1192	0.693	1	3.603	0.117
**CHCHD2**	CHCHD2	34	coiled-coil-helix-coiled-coil-helix domain containing 2	protein_codin	-0.07428	0.152	1	51.97	0.02607
**PTPN12**	PTPN12	34	protein tyrosine phosphatase non-receptor type 12	protein_codin	0.6064	0.000135	1	49.8	0.08102
**TSC22D4**	TSC22D4	34	TSC22 domain family member 4	protein_codin	0.0406	0.87	1	3.416	0.1376
**GNB2**	GNB2	34	G protein subunit beta 2	protein_codin	-0.07874	0.414	1	21.25	0.05991
**POP7**	POP7	34	POP7 homolog, ribonuclease P/MRP subunit	protein_codin	-0.02566	0.88	1	6.972	0.128
**TES**	TES	34	testin LIM domain protein	protein_codin	0.8485	0.000326	1	16.16	0.2047
**CADPS2**	CADPS2	34	calcium dependent secretion activator 2	protein_codin	0.4894	0.0179	1	14.8	0.1735
**REPIN1**	REPIN1	34	replication initiator 1	protein_codin	-0.143	0.563	1	3.493	0.1126
**GTPBP6**	GTPBP6	34	GTP binding protein 6 (putative)	protein_codin	0.5712	0.00202	1	7.412	0.196
**UBQLN2**	UBQLN2	34	ubiquilin 2	protein_codin	0.5068	0.115	1	2.95	0.2302
**ZDHHC9**	ZDHHC9	34	zinc finger DHHC-type containing 9	protein_codin	-0.4706	0.105	1	2.86	0.1794
**RTL8B**	RTL8B	34	retrotransposon Gag like 8B	protein_codin	0.4057	0.116	1	6.922	0.1752
**LAGE3**	LAGE3	34	L antigen family member 3	protein_codin	-0.046	0.767	1	36.76	0.1121
**BRCC3**	BRCC3	34	BRCA1/BRCA2-containing complex subunit 3	protein_codin	0.05271	0.849	1	10.98	0.1942
**INTS10**	INTS10	34	integrator complex subunit 10	protein_codin	-0.09706	0.61	1	7.438	0.1725
**ATP6V1B2**	ATP6V1B2	34	ATPase H+ transporting V1 subunit B2	protein_codin	-0.08273	0.773	1	2.57	0.2458
**TNFRSF10D**	TNFRSF10	34	TNF receptor superfamily member 10d	protein_codin	0.4311	0.0279	1	25.55	0.05386
**CCDC25**	CCDC25	34	coiled-coil domain containing 25	protein_codin	-0.1487	0.235	1	18.66	0.05621
**TTI2**	TTI2	34	TELO2 interacting protein 2	protein_codin	0.8722	0.0229	1	11.28	0.2494
**ZFAND1**	ZFAND1	34	zinc finger AN1-type containing 1	protein_codin	0.566	0.000164	1	23.16	0.1906
**FZD6**	FZD6	34	frizzled class receptor 6	protein_codin	0.3416	0.0482	1	49.46	0.07974
**PGM5**	PGM5	34	phosphoglucomutase 5	protein_codin	0.9222	1.73E-07	0.004062	27.52	0.06158
**PTGR1**	PTGR1	34	prostaglandin reductase 1	protein_codin	-0.2111	0.284	1	21.63	0.05407
**CDC26**	CDC26	34	cell division cycle 26	protein_codin	0.2715	0.037	1	18.68	0.05491
**RABEPK**	RABEPK	34	Rab9 effector protein with kelch motifs	protein_codin	-0.1632	0.53	1	2.826	0.08067
**C9orf16**	C9orf16	34	chromosome 9 open reading frame 16	protein_codin	-0.2056	0.00517	1	46.5	0.03061
**URM1**	URM1	34	ubiquitin related modifier 1	protein_codin	0.3892	0.00568	1	13.22	0.05326
**RABL6**	RABL6	34	RAB, member RAS oncogene family like 6	protein_codin	0.003236	0.988	1	21.5	0.08415
**EXD3**	EXD3	34	exonuclease 3'-5' domain containing 3	protein_codin	0.3451	0.366	1	2.861	0.1181
**TMEM80**	TMEM80	34	transmembrane protein 80	protein_codin	-0.2298	0.374	1	7.442	0.1411
**CHID1**	CHID1	34	chitinase domain containing 1	protein_codin	-0.641	3.46E-05	0.7983	7.31	0.222
**CAVIN3**	CAVIN3	34	caveolae associated protein 3	protein_codin	-0.3766	0.000116	1	52.54	0.05237
**LDLRAD3**	LDLRAD3	34	low density lipoprotein receptor class A domain containing 3	protein_codin	0.1334	0.675	1	10.97	0.1138
**CKAP5**	CKAP5	34	cytoskeleton associated protein 5	protein_codin	-0.3668	0.143	1	8.419	0.08197
**STX5**	STX5	34	syntaxin 5	protein_codin	0.09915	0.634	1	7.506	0.1459
**ARRB1**	ARRB1	34	arrestin beta 1	protein_codin	0.5869	0.0171	1	6.752	0.1116
**ZBTB16**	ZBTB16	34	zinc finger and BTB domain containing 16	protein_codin	0.06921	0.742	1	37.83	0.08156
**ADO**	ADO	34	2-aminoethanethiol dioxygenase	protein_codin	0.102	0.755	1	3.026	0.3105
**CHCHD1**	CHCHD1	34	coiled-coil-helix-coiled-coil-helix domain containing 1	protein_codin	-0.2176	0.176	1	7.172	0.08083
**ZMIZ1**	ZMIZ1	34	zinc finger MIZ-type containing 1	protein_codin	0.2326	0.315	1	40.46	0.1402
**FMNL3**	FMNL3	34	formin like 3	protein_codin	-0.1831	0.552	1	8.643	0.1421
**ATG101**	ATG101	34	autophagy related 101	protein_codin	-0.03687	0.824	1	18.45	0.2706
**CSAD**	CSAD	34	cysteine sulfinic acid decarboxylase	protein_codin	-0.208	0.395	1	8.837	0.2362
**ATP2B1-AS1**	ATP2B1-AS	34	ATP2B1 antisense RNA 1	lincRNA	0.1154	0.587	1	51.34	0.2317
**ZMYM5**	ZMYM5	34	zinc finger MYM-type containing 5	protein_codin	-0.2376	0.31	1	9.187	0.1708
**PARP4**	PARP4	34	poly(ADP-ribose) polymerase family member 4	protein_codin	0.1858	0.402	1	9.078	0.1176
**ELF1**	ELF1	34	E74 like ETS transcription factor 1	protein_codin	0.5274	3.92E-07	0.009186	24.15	0.02627
**EBPL**	EBPL	34	EBP like	protein_codin	0.7944	5.29E-11	1.26E-06	51.26	0.08216
**CLN5**	CLN5	34	CLN5 intracellular trafficking protein	protein_codin	-0.2202	0.242	1	7.718	0.1105
**ING1**	ING1	34	inhibitor of growth family member 1	protein_codin	0.2904	0.119	1	35.96	0.1792
**METTL17**	METTL17	34	methyltransferase like 17	protein_codin	-0.1827	0.54	1	2.444	0.2317
**TINF2**	TINF2	34	TERF1 interacting nuclear factor 2	protein_codin	0.5658	0.0148	1	7.272	0.2072

ATG14	ATG14	24	autophagy related 14	protein_codin	0.4644	0.0809	1	8.168	0.2147
L3HYPDH	L3HYPDH	24	trans-L-3-hydroxyproline dehydratase	protein_codin	0.02096	0.917	1	2.82	0.3025
DDX24	DDX24	24	DEAD-box helicase 24	protein_codin	-0.4516	3.39E-10	8.04E-06	52.5	0.05357
TJP1	TJP1	24	tight junction protein 1	protein_codin	0.2741	0.0187	1	30.06	0.1146
CCDC32	CCDC32	24	coiled-coil domain containing 32	protein_codin	0.7048	0.0024	1	8.242	0.2222
EIF3J-AS1	EIF3J-AS1	24	EIF3J divergent transcript	lincRNA	NA	NA	NA	7.478	0.3997
EID1	EID1	24	EP300 interacting inhibitor of differentiation 1	protein_codin	-0.1357	0.00962	1	52.58	0.02667
DPP8	DPP8	24	dipeptidyl peptidase 8	protein_codin	0.5135	0.048	1	9.265	0.1682
WDR73	WDR73	24	WD repeat domain 73	protein_codin	0.1687	0.572	1	2.797	0.2564
HAPLN3	HAPLN3	24	hyaluronan and proteoglycan link protein 3	protein_codin	0.4162	0.00242	1	47.02	0.08106
MSRB1	MSRB1	24	methionine sulfoxide reductase B1	protein_codin	-0.6457	0.00738	1	8.244	0.2042
ZNF205	ZNF205	24	zinc finger protein 205	protein_codin	0.1132	0.695	1	3.042	0.1342
ERCC4	ERCC4	24	ERCC excision repair 4, endonuclease catalytic subunit	protein_codin	-0.4024	0.0785	1	6.857	0.08086
PAPD5	PAPD5	24	terminal nucleotidyltransferase 4B	protein_codin	NA	NA	NA	3.284	0.1964
ADGRG1	ADGRG1	24	adhesion G protein-coupled receptor G1	protein_codin	-0.1395	0.48	1	31.02	0.2308
NUTF2	NUTF2	24	nuclear transport factor 2	protein_codin	0.1014	0.457	1	28.74	0.08462
ZFP90	ZFP90	24	ZFP90 zinc finger protein	protein_codin	-0.02715	0.922	1	8.673	0.2214
MPHOSPH6	MPHOSPH	24	M-phase phosphoprotein 6	protein_codin	0.2341	0.377	1	3.107	0.08666
GLOD4	GLOD4	24	glyoxalase domain containing 4	protein_codin	0.3496	0.0388	1	15.08	0.08452
MRM3	MRM3	24	mitochondrial rRNA methyltransferase 3	protein_codin	1.115	0.00532	1	3.038	0.2228
ELAC2	ELAC2	24	elaC ribonuclease Z 2	protein_codin	0.5431	0.154	1	2.706	0.06246
MYO1D	MYO1D	24	myosin ID	protein_codin	-1.519	7.48E-11	1.78E-06	8.232	0.05676
DUSP14	DUSP14	24	dual specificity phosphatase 14	protein_codin	-0.8386	0.000295	1	25.75	0.05772
STAT3	STAT3	24	signal transducer and activator of transcription 3	protein_codin	0.2115	0.0215	1	30.52	0.08678
CCDC43	CCDC43	24	coiled-coil domain containing 43	protein_codin	0.1708	0.382	1	6.982	0.1856
CALCOCO2	CALCOCO2	24	calcium binding and coiled-coil domain 2	protein_codin	0.6872	2.5E-11	5.95E-07	35.04	0.06506
VEZF1	VEZF1	24	vascular endothelial zinc finger 1	protein_codin	0.1968	0.28	1	18.54	0.1441
PSMC5	PSMC5	24	proteasome 26S subunit, ATPase 5	protein_codin	-0.2276	0.00975	1	46.46	0.02658
SRSF2	SRSF2	24	serine and arginine rich splicing factor 2	protein_codin	-0.1421	0.184	1	20.72	0.05686
MCRIP1	MCRIP1	24	MAPK regulated corepressor interacting protein 1	protein_codin	-0.2832	0.0214	1	12.12	0.1132
CEP192	CEP192	24	centrosomal protein 192	protein_codin	-0.2092	0.457	1	9.074	0.1622
TXNL4A	TXNL4A	24	thioredoxin like 4A	protein_codin	-0.2592	0.0222	1	36.18	0.02671
OGFR	OGFR	24	opioid growth factor receptor	protein_codin	0.8305	0.00177	1	5.938	0.1702
MAP2K2	MAP2K2	24	mitogen-activated protein kinase kinase 2	protein_codin	-0.05829	0.586	1	34.5	0.05768
ZFP14	ZFP14	24	ZFP14 zinc finger protein	protein_codin	-0.1089	0.729	1	3.267	0.3754
SIRT2	SIRT2	24	sirtuin 2	protein_codin	0.2658	0.236	1	3.41	0.2946
GSK3A	GSK3A	24	glycogen synthase kinase 3 alpha	protein_codin	0.5381	0.032	1	47.82	0.1174
UFD1	UFD1	24	ubiquitin recognition factor in ER associated degradation 1	protein_codin	-0.5974	3.55E-08	0.000837	18.5	0.119
DDT	DDT	24	D-dopachrome tautomerase	protein_codin	0.1142	0.285	1	40.67	0.05505
KIAA1671	KIAA1671	24	KIAA1671	protein_codin	0.1127	0.628	1	7.022	0.1152
PHF5A	PHF5A	24	PHD finger protein 5A	protein_codin	0.434	0.00356	1	12.07	0.1961
TBC1D22A	TBC1D22A	24	TBC1 domain family member 22A	protein_codin	0.5094	0.105	1	3.282	0.2634
IFNGR2	IFNGR2	24	interferon gamma receptor 2	protein_codin	0.0582	0.711	1	15.25	0.05622
PSMG1	PSMG1	24	proteasome assembly chaperone 1	protein_codin	0.5796	0.00601	1	20.2	0.0800
MCM3AP	MCM3AP	24	minichromosome maintenance complex component 3 associa	protein_codin	-0.1076	0.798	1	2.856	0.1419
HES4	HES4	25	hes family bHLH transcription factor 4	protein_codin	0.8632	0.00676	1	52.54	0.3292
TNFRSF4	TNFRSF4	25	TNF receptor superfamily member 4	protein_codin	-1.197	0.000193	1	51.97	0.2961
VWA1	VWA1	25	von Willebrand factor A domain containing 1	protein_codin	-0.2507	0.0306	1	48.05	0.09971
TPRG1L	TPRG1L	25	tumor protein p63 regulated 1 like	protein_codin	-0.5365	0.00664	1	8.417	0.1107
CLIC4	CLIC4	25	chloride intracellular channel 4	protein_codin	-0.2319	0.0451	1	52.52	0.05650
FAM167B	FAM167B	25	family with sequence similarity 167 member B	protein_codin	0.7519	2.57E-06	0.05992	52.07	0.03342
CITED4	CITED4	25	Cbp/p300 interacting transactivator with Glu/Asp rich carboxy	protein_codin	-1.442	1.44E-07	0.003382	48.03	0.1322
PLPP3	PLPP3	25	phospholipid phosphatase 3	protein_codin	-0.3825	0.0405	1	34.36	0.177
RPF1	RPF1	25	ribosome production factor 1 homolog	protein_codin	0.3999	0.0922	1	6.571	0.1092
NES	NES	25	nestin	protein_codin	-0.8108	1.93E-10	4.58E-06	52.54	0.09095
FAM129A	FAM129A	25	niban apoptosis regulator 1	protein_codin	-0.8056	0.0033	1	47.2	0.03836
PTPN14	PTPN14	25	protein tyrosine phosphatase non-receptor type 14	protein_codin	-0.6336	0.000208	1	35.72	0.1131
LBH	LBH	25	LBH regulator of WNT signaling pathway	protein_codin	-0.3844	0.0567	1	50.96	0.144
MSH2	MSH2	25	mutS homolog 2	protein_codin	0.4619	0.102	1	2.915	0.129
CYTOR	CYTOR	25	cytoskeleton regulator RNA	lincRNA	-0.7382	0.000447	1	52.58	0.2402
MIR4435-2HG	MIR4435-2	25	MIR4435-2 host gene	lincRNA	-1.415	9.91E-06	0.23	52.58	0.4806
ITGA6	ITGA6	25	integrin subunit alpha 6	protein_codin	1.358	1.52E-19	3.66E-14	46.55	0.09705
MYO1B	MYO1B	25	myosin IB	protein_codin	-0.3716	0.228	1	41.22	0.2572
ARPC2	ARPC2	25	actin related protein 2/3 complex subunit 2	protein_codin	-0.4286	1.9E-10	4.51E-06	52.58	0.05986
AGFG1	AGFG1	25	ArfGAP with FG repeats 1	protein_codin	-0.2493	0.222	1	22.47	0.0856
ACKR3	ACKR3	25	atypical chemokine receptor 3	protein_codin	-1.846	1.27E-17	3.05E-13	44.26	0.1086
PER2	PER2	25	period circadian regulator 2	protein_codin	0.7937	0.00669	1	21.58	0.1407
SLC4A7	SLC4A7	25	solute carrier family 4 member 7	protein_codin	-0.7397	0.0107	1	24.9	0.05278
CTNNB1	CTNNB1	25	catenin beta 1	protein_codin	-0.1086	0.379	1	52.58	0.2649
POGLUT1	POGLUT1	25	protein O-glucosyltransferase 1	protein_codin	-0.1771	0.394	1	8.332	0.1524
MGLL	MGLL	25	monoglyceride lipase	protein_codin	1.272	5.47E-18	1.31E-13	36.79	0.2082
GMPS	GMPS	25	guanine monophosphate synthase	protein_codin	0.6402	0.00137	1	18.98	0.1656
SERPINI1	SERPINI1	25	serpin family I member 1	protein_codin	0.8831	0.0572	1	47.27	0.379
MXD4	MXD4	25	MAX dimerization protein 4	protein_codin	-0.9368	3.02E-10	7.17E-06	49.75	0.03326
MSX1	MSX1	25	msh homeobox 1	protein_codin	-0.2041	0.382	1	43.68	0.1204
KDR	KDR	25	kinase insert domain receptor	protein_codin	0.2225	0.366	1	36.96	0.2777
IGFBP7	IGFBP7	25	insulin like growth factor binding protein 7	protein_codin	-0.4893	3.97E-20	9.56E-16	Inf	0.1152
EMCN	EMCN	25	endomucin	protein_codin	0.8626	4.91E-14	1.17E-09	52.58	0.2249
ELMOD2	ELMOD2	25	ELMO domain containing 2	protein_codin	-0.6314	0.00672	1	9.66	0.169
TBC1D9	TBC1D9	25	TBC1 domain family member 9	protein_codin	-0.1405	0.573	1	7.887	0.1152
ITGA1	ITGA1	25	integrin subunit alpha 1	protein_codin	0.3417	0.242	1	10.42	0.1416
ESM1	ESM1	25	endothelial cell specific molecule 1	protein_codin	-4.08	1.16E-07	0.002720	52.52	0.4611
PLPP1	PLPP1	25	phospholipid phosphatase 1	protein_codin	-0.599	0.000484	1	52.02	0.4048
SLC12A2	SLC12A2	25	solute carrier family 12 member 2	protein_codin	-0.628	0.00869	1	30.05	0.08256
IK	IK	25	IK cytokine	protein_codin	0.1741	0.0956	1	26.21	0.05657
HNRNPH1	HNRNPH1	25	heterogeneous nuclear ribonucleoprotein H1	protein_codin	0.3254	0.00136	1	46.29	0.1564
TBC1D7	TBC1D7	25	TBC1 domain family member 7	protein_codin	-0.3271	0.219	1	3.171	0.1115
ADGRF5	ADGRF5	25	adhesion G protein-coupled receptor F5	protein_codin	-0.197	0.273	1	51.6	0.1722
FILIP1	FILIP1	25	filamin A interacting protein 1	protein_codin	-1.108	2.97E-12	7.09E-08	52.53	0.1185
ARHGAP18	ARHGAP18	25	Rho GTPase activating protein 18	protein_codin	-1.526	7.24E-27	1.75E-22	52.07	0.1056
SNHG15	SNHG15	25	small nucleolar RNA host gene 15	lincRNA	0.2031	0.27	1	52.37	0.1187
LAMB1	LAMB1	25	laminin subunit beta 1	protein_codin	-1.242	3.71E-12	8.85E-08	51.57	0.08414
MDFIC	MDFIC	25	MyoD family inhibitor domain containing	protein_codin	0.2294	0.208	1	29.93	0.09461
PLXNA4	PLXNA4	25	plexin A4	protein_codin	-2.211	1.56E-15	3.74E-11	25.57	0.1255
SAT1	SAT1	25	spermidine/spermine N1-acetyltransferase 1	protein_codin	-0.3152	0.0037	1	52.58	0.05774
ANGPT2	ANGPT2	25	angiopoietin 2	protein_codin	-1.984	1.61E-16	3.86E-12	52.58	0.4058
EIF4EBP1	EIF4EBP1	25	eukaryotic translation initiation factor 4E binding protein 1	protein_codin	-0.8246	2.98E-07	0.006987	52.58	0.141
ENPP2	ENPP2	25	ectonucleotide pyrophosphatase/phosphodiesterase 2	protein_codin	0.5145	0.122	1	47.53	0.3077
LY6H	LY6H	25	lymphocyte antigen 6 family member H	protein_codin	-4.809	1.93E-05	0.4466	52.58	0.4192
GPIHBP1	GPIHBP1	25	glycosylphosphatidylinositol anchored high density lipoprotein	protein_codin	0.2311	0.522	1	30.64	0.2320
RGS3	RGS3	25	regulator of G protein signaling 3	protein_codin	-1.608	5.11E-20	1.23E-15	52.58	0.2044
GSN	GSN	25	gelsolin	protein_codin	0.5052	1.08E-12	2.58E-08	52.58	0.02806
OLFM1	OLFM1	25	olfactomedin 1	protein_codin	1.849	3.75E-29	9.06E-24	52.58	0.14
PDGFD	PDGFD	25	platelet derived growth factor D	protein_codin	-2.282	3.84E-21	9.25E-17	42.94	0.3466
FEZ1	FEZ1	25	fasciculation and elongation protein zeta 1	protein_codin	-3.733	2.72E-28	6.58E-24	26.36	0.1737
TSPAN15	TSPAN15	25	tetraspanin 15	protein_codin	-1.03	2.22E-05	0.5134	37.96	0.1902
KRT18	KRT18	25	keratin 18	protein_codin	-0.06298	0.752	1	52.02	0.06295
ELK3	ELK3	25	ETS transcription factor ELK3	protein_codin	0.6962	7.53E-12	1.8E-07	50.88	0.05677
MEDAG	MEDAG	25	mesenteric estrogen dependent adipogenesis	protein_codin	-2.37	3.93E-25	9.49E-21	20.79	0.0934
RGCC	RGCC	25	regulator of cell cycle	protein_codin	-0.7222	0.00916	1	52.58	0.3602
EDNRB	EDNRB	25	endothelin receptor type B	protein_codin	0.9721	0.0129	1	52.02	0.8591
TM9SF2	TM9SF2	25	transmembrane 9 superfamily member 2	protein_codin	-0.5194	0.00011	1	52.57	0.1366
COL4A1	COL4A1	25	collagen type IV alpha 1 chain	protein_codin	-1.836	7.52E-27	1.82E-22	52.58	0.154
COL4A2	COL4A2	25	collagen type IV alpha 2 chain	protein_codin	-1.592	6.07E-24	1.47E-19	52.58	0.18
PGF	PGF	25	placental growth factor	protein_codin	-2.18	6.33E-19	1.52E-14	37.16	0.2989
EIF2B2	EIF2B2	25	eukaryotic translation initiation factor 2B subunit beta	protein_codin	-0.1617	0.448	1	47.17	0.11
EML1	EML1	25	EMAP like 1	protein_codin	0.3432	0.238	1	11.46	0.1927
TMED3	TMED3	25	transmembrane p24 trafficking protein 3	protein_codin	-0.9671	2.25E-07	0.005281	35.63	0.02771
PDE8A	PDE8A	25	phosphodiesterase 8A	protein_codin	0.1221	0.695	1	3.544	0.1926
RFLNB	RFLNB	25	refilin B	protein_codin	-1.092	7.29E-07	0.01706	52.52	0.122
ICAM2	ICAM2	25	intercellular adhesion molecule 2	protein_codin	-0.2771	0.0108	1	51.92	0.0607
SEC14L1	SEC14L1	25	SEC14 like lipid binding 1	protein_codin	0.1751	0.185	1	47.81	0.08002
PGS1	PGS1	25	phosphatidylglycerophosphate synthase 1	protein_codin	-0.1948	0.447	1	2.842	0.1075
EIF4A3	EIF4A3	25	eukaryotic translation initiation factor 4A3	protein_codin	0.1962	0.166	1	31.02	0.1644
HEXDC	HEXDC	25	hexosaminidase D	protein_codin	0.1085	0.72	1	3.334	0.0828
BTBD3	BTBD3	25	BTB domain containing 3	protein_codin	0.6055	0.0384	1	46.34	0.1512
MAP1LC3A	MAP1LC3A	25	microtubule associated protein 1 light chain 3 alpha	protein_codin	-0.01730	0.906	1	29.22	0.08216
NCOA3	NCOA3	25	nuclear receptor coactivator 3	protein_codin	-0.5202	0.0082	1	47.52	0.2088
INSR	INSR	25	insulin receptor	protein_codin	0.1401	0.497	1	47.53	0.1084
PLVAP	PLVAP	25	plasmalemma vesicle associated protein	protein_codin	0.3968	1.07E-07	0.002515	52.58	0.06307
EXOC3L2	EXOC3L2	25	exocyst complex component 3 like 2	protein_codin	0.2277	0.365	1	50.92	0.142
CLDN5	CLDN5	25	claudin 5	protein_codin	-0.307	0.0167	1	52.58	0.1712
TIMP3	TIMP3	25	TIMP metallopeptidase inhibitor 3	protein_codin	1.052	4.17E-13	9.96E-09	35.3	0.1270
APP	APP	25	amyloid beta precursor protein	protein_codin	-0.0382	0.671	1	37.18	0.1232
JUN	JUN	26	Jun proto-oncogene, AP-1 transcription factor subunit	protein_codin	0.1751	0.0171	1	52.58	0.3562
MIGA1	MIGA1	26	mitoguardin 1	protein_codin	-0.1562	0.494	1	22.32	0.1697
MCL1	MCL1	26	MCL1 apoptosis regulator, BCL2 family member	protein_codin	-0.2928	0.00120	1	32.1	0.1062
ETV3	ETV3	26	ETS variant 3	protein_codin	0.222	0.498	1	34.85	0.2526
RGS16	RGS16	26	regulator of G protein signaling 16	protein_codin	2.326	7.35E-24	1.77E-19	37.16	0.06394
ATF3	ATF3	26	activating transcription factor 3	protein_codin	1.806	6.73E-29	1.63E-24	47.48	0.1071
RHOB	RHOB	26	ras homolog family member B	protein_codin	-1.579	6.81E-55	1.65E-50	52.58	0.08514
NDUFAF7	NDUFAF7	26	NADH ubiquinone oxidoreductase complex assembly factor 7	protein_codin	0.01513	0.962	1	3.439	0.1131
EML4	EML4	26	EMAP like 4	protein_codin	1.185	4.57E-08	0.001077	46.82	0.1686
IMMT	IMMT	26	inner membrane mitochondrial protein	protein_codin	-0.2404	0.2	1	6.314	0.08422
ADAMTS9	ADAMTS9	26	ADAM metallopeptidase with thrombospondin type 1 motif 9	protein_codin	1.493	3.96E-10	9.39E-06	52.58	0.08166
AP3B1	AP3B1	26	adaptor related protein complex 3 subunit beta 1	protein_codin	-0.0448	0.81	1	18.77	0.1745
EGR1	EGR1	26	early growth response 1	protein_codin	0.9696	7.79E-18	1.87E-13	52.58	0.1319
DUSP1	DUSP1	26	dual specificity phosphatase 1	protein_codin	0.1724	0.0109	1	52.58	0.2216
HSPA1A	HSPA1A	26	heat shock protein family A (Hsp70) member 1A	protein_codin	-1.763	1.05E-64	2.55E-60	Inf	0.2782
HSPA1B	HSPA1B	26	heat shock protein family A (Hsp70) member 1B	protein_codin	-1.374	1.01E-37	2.45E-33	52.58	0.2682
CDKN1A	CDKN1A	26	cyclin dependent kinase inhibitor 1A	protein_codin	1.044	6.13E-14	1.47E-09	41.02	0.02622
PPIL1	PPIL1	26	peptidylprolyl isomerase like 1	protein_codin	-0.0412	0.888	1	2.415	0.1456
POLR1C	POLR1C	26	RNA polymerase I and III subunit C	protein_codin	0.5501	0.0385	1	3.489	0.2808
HSPB1	HSPB1	26	heat shock protein family B (small) member 1	protein_codin	-0.183	0.000154	1	52.54	0.02952
ZKSCAN1	ZKSCAN1	26	zinc finger with KRAB and SCAN domains 1	protein_codin	-0.2642	0.259	1	40.56	0.1382
STC1	STC1	26	stanniocalcin 1	protein_codin	3.376	2.01E-25	4.85E-21	52.58	0.087
TMEM55A	TMEM55A	26	phosphatidylinositol-4,5-bisphosphate 4-phosphatase 2	protein_codin	NA	NA	NA	29.54	0.2585
POLR2K	POLR2K	26	RNA polymerase II subunit K	protein_codin	-0.04136	0.672	1	15.17	0.08158
NFX1	NFX1	26	nuclear transcription factor, X-box binding 1	protein_codin	0.2352	0.498	1	9.199	0.3019
STOML2	STOML2	26	stomatin like 2	protein_codin	-0.03217	0.812	1	32.93	0.1119
C9orf3	C9orf3	26	aminopeptidase O (putative)	protein_codin	-0.644	4.48E-08	0.001056	52.07	0.02776
KLF4	KLF4	26	Kruppel like factor 4	protein_codin	0.7222	1.6E-11	3.81E-07	52.07	0.08282
GAPVD1	GAPVD1	26	GTPase activating protein and VPS9 domains 1	protein_codin	0.1806	0.476	1	26.21	0.08762
B4GAT1	B4GAT1	26	beta-1,4-glucuronyltransferase 1	protein_codin	-0.3144	0.175	1	7.186	0.1329
CHKA	CHKA	26	choline kinase alpha	protein_codin	0.521	0.0662	1	2.848	0.3101
TMEM25	TMEM25	26	transmembrane protein 25	protein_codin	-0.2535	0.35	1	8.754	0.1134
KLF6	KLF6	26	Kruppel like factor 6	protein_codin	0.2374	0.00607	1	52.58	0.08766
ANXA11	ANXA11	26	annexin A11	protein_codin	0.4707	2.31E-05	0.5341	12.22	0.02906
XPNPEP1	XPNPEP1	26	X-prolyl aminopeptidase 1	protein_codin	-0.1775	0.487	1	8.342	0.2751
EMP1	EMP1	26	epithelial membrane protein 1	protein_codin	0.3267	0.000267	1	52.58	0.03185
TSPAN31	TSPAN31	26	tetraspanin 31	protein_codin	0.3387	0.0957	1	7.52	0.1685
DUSP6	DUSP6	26	dual specificity phosphatase 6	protein_codin	0.2706	0.0341	1	51.35	0.1752
FOS	FOS	26	Fos proto-oncogene, AP-1 transcription factor subunit	protein_codin	0.4634	8.95E-14	2.14E-09	Inf	0.3936
C14orf159	C14orf159	26	D-glutamate cyclase	protein_codin	NA	NA	NA	7.068	0.08272
DYNC1H1	DYNC1H1	26	dynein cytoplasmic 1 heavy chain 1	protein_codin	-0.2476	0.0886	1	32.92	0.05768
HSP90AA1	HSP90AA1	26	heat shock protein 90 alpha family class A member 1	protein_codin	-0.4593	7.15E-18	1.72E-13	52.54	0.1101
FAM98B	FAM98B	26	family with sequence similarity 98 member B	protein_codin	-0.4964	0.0656	1	3.439	0.1656
USP8	USP8	26	ubiquitin specific peptidase 8	protein_codin	0.04717	0.771	1	27.63	0.1161
ARPIN	ARPIN	26	actin related protein 2/3 complex inhibitor	protein_codin	0.4252	0.0884	1	8.129	0.1686
ZNF720	ZNF720	26	zinc finger protein 720	protein_codin	0.102	0.667	1	8.375	0.05292
THBD	THBD	26	thrombomodulin	protein_codin	0.2261	0.0225	1	52.58	0.1376
CIRBP	CIRBP	26	cold inducible RNA binding protein	protein_codin	0.2762	2.01E-05	0.465	52.42	0.02609
GADD45B	GADD45B	26	growth arrest and DNA damage inducible beta	protein_codin	0.4138	0.00021	1	52.58	0.2134
JUNB	JUNB	26	JunB proto-oncogene, AP-1 transcription factor subunit	protein_codin	0.8077	2.36E-34	5.72E-30	52.58	0.2455
IER2	IER2	26	immediate early response 2	protein_codin	0.5991	1.67E-14	4E-10	52.58	0.3654
DNAJB1	DNAJB1	26	DnaJ heat shock protein family (Hsp40) member B1	protein_codin	-1.246	1.17E-33	2.83E-29	52.07	0.2712
KLF2	KLF2	26	Kruppel like factor 2	protein_codin	0.2682	6.47E-06	0.1504	52.58	0.1732
JUND	JUND	26	JunD proto-oncogene, AP-1 transcription factor subunit	protein_codin	0.3454	3.81E-09	9.02E-05	52.58	0.1226
UPF1	UPF1	26	UPF1 RNA helicase and ATPase	protein_codin	0.364	0.244	1	8.536	0.2785
BORCS8	BORCS8	26	BLOC-1 related complex subunit 8	protein_codin	0.1298	0.55	1	3.399	0.02857
ZFP36	ZFP36	26	ZFP36 ring finger protein	protein_codin	1.334	1.23E-62	2.99E-58	48.04	0.2962
SERTAD1	SERTAD1	26	SERTA domain containing 1	protein_codin	0.4504	5.95E-06	0.1384	42.9	0.02717
FOSB	FOSB	26	FosB proto-oncogene, AP-1 transcription factor subunit	protein_codin	0.5706	2.14E-08	0.000505	48	0.2672
NOVA2	NOVA2	26	NOVA alternative splicing regulator 2	protein_codin	0.05894	0.842	1	8.092	0.1652
IRF3	IRF3	26	interferon regulatory factor 3	protein_codin	0.09474	0.599	1	6.324	0.1368
ZNF524	ZNF524	26	zinc finger protein 524	protein_codin	0.4475	0.00375	1	32.55	0.1474
DGCR6L	DGCR6L	26	DiGeorge syndrome critical region gene 6 like	protein_codin	0.04947	0.788	1	19.48	0.1395
ISG15	ISG15	27	ISG15 ubiquitin like modifier	protein_codin	-0.9177	2.81E-11	6.69E-07	52.58	0.6777
MEGF6	MEGF6	27	multiple EGF like domains 6	protein_codin	-1.065	4.52E-05	1	3.685	0.2008
PGD	PGD	27	phosphogluconate dehydrogenase	protein_codin	-0.06340	0.85	1	9.986	0.1125
IFI6	IFI6	27	interferon alpha inducible protein 6	protein_codin	-1.044	7.65E-19	1.84E-14	52.58	0.3512
RPA2	RPA2	27	replication protein A2	protein_codin	0.08422	0.63	1	18.42	0.05697
EIF3I	EIF3I	27	eukaryotic translation initiation factor 3 subunit I	protein_codin	0.1116	0.172	1	29.87	0.05889
FHL3	FHL3	27	four and a half LIM domains 3	protein_codin	-0.4844	0.0366	1	9.512	0.08275
RLF	RLF	27	rearranged L-myc fusion	protein_codin	0.4246	0.135	1	6.996	0.1944
POMGNT1	POMGNT1	27	protein O-linked mannose N-acetylglucosaminyltransferase 1	protein_codin	0.04519	0.869	1	3.157	0.1154
YIPF1	YIPF1	27	Yip1 domain family member 1	protein_codin	-0.2795	0.318	1	3.053	0.3314
IFI44L	IFI44L	27	interferon induced protein 44 like	protein_codin	-0.1976	0.158	1	36.75	0.5068
BCL10	BCL10	27	BCL10 immune signaling adaptor	protein_codin	0.2524	0.132	1	24.61	0.08492
CNN3	CNN3	27	calponin 3	protein_codin	1.288	3.15E-38	7.63E-34	51.45	0.02825
PTBP2	PTBP2	27	polypyrimidine tract binding protein 2	protein_codin	-0.7928	0.000452	1	31.8	0.1739
CDC14A	CDC14A	27	cell division cycle 14A	protein_codin	0.1512	0.587	1	3.048	0.3372
LAMTOR5	LAMTOR5	27	late endosomal/lysosomal adaptor, MAPK and MTOR activato	protein_codin	-0.4776	2.89E-08	0.000682	34.72	0.02756
MAN1A2	MAN1A2	27	mannosidase alpha class 1A member 2	protein_codin	-0.05806	0.713	1	14.44	0.1089
POLR3C	POLR3C	27	RNA polymerase III subunit C	protein_codin	0.5442	0.119	1	9.576	0.3889
SELENBP1	SELENBP1	27	selenium binding protein 1	protein_codin	-1.058	9.44E-06	0.2193	3.497	0.05611
RRNAD1	RRNAD1	27	ribosomal RNA adenine dimethylase domain containing 1	protein_codin	0.6804	0.0722	1	3.238	0.06119
NME7	NME7	27	NME/NM23 family member 7	protein_codin	0.521	0.0458	1	8.542	0.126
RABGAP1L	RABGAP1L	27	RAB GTPase activating protein 1 like	protein_codin	0.3345	0.219	1	7.564	0.1171
TROVE2	TROVE2	27	Ro60, Y RNA binding protein	protein_codin	-0.048	0.718	1	19.52	0.1401
RNPEP	RNPEP	27	arginyl aminopeptidase	protein_codin	0.392	0.126	1	10.04	0.169
DIEXF	DIEXF	27	UTP25 small subunit processor component	protein_codin	NA	NA	NA	2.755	0.2886
NSL1	NSL1	27	NSL1 component of MIS12 kinetochore complex	protein_codin	0.4526	0.00494	1	5.643	0.2302
MIA3	MIA3	27	MIA SH3 domain ER export factor 3	protein_codin	-0.09138	0.501	1	50.38	0.0562
BROX	BROX	27	BRO1 domain and CAAX motif containing	protein_codin	0.01022	0.956	1	18.31	0.1714
FBXO28	FBXO28	27	F-box protein 28	protein_codin	0.2807	0.445	1	9.918	0.3696
PYCR2	PYCR2	27	pyrroline-5-carboxylate reductase 2	protein_codin	0.03649	0.852	1	7.305	0.05317
EXOC8	EXOC8	27	exocyst complex component 8	protein_codin	0.1938	0.516	1	2.666	0.1615
CMPK2	CMPK2	27	cytidine/uridine monophosphate kinase 2	protein_codin	0.1302	0.687	1	35.46	0.2261
RSAD2	RSAD2	27	radical S-adenosyl methionine domain containing 2	protein_codin	0.118	0.726	1	52.52	0.5134
FAM228B	FAM228B	27	family with sequence similarity 228 member B	protein_codin	0.1391	0.592	1	9.215	0.1365
YIPF4	YIPF4	27	Yip1 domain family member 4	protein_codin	-0.2167	0.221	1	51.58	0.08084
FAM98A	FAM98A	27	family with sequence similarity 98 member A	protein_codin	0.3582	0.0812	1	18.35	0.1199
EIF2AK2	EIF2AK2	27	eukaryotic translation initiation factor 2 alpha kinase 2	protein_codin	0.2571	0.0841	1	50.83	0.1838
GEMIN6	GEMIN6	27	gem nuclear organelle associated protein 6	protein_codin	0.2512	0.348	1	7.621	0.1659
SRBD1	SRBD1	27	S1 RNA binding domain 1	protein_codin	0.8661	0.0118	1	3.63	0.1708
PIGF	PIGF	27	phosphatidylinositol glycan anchor biosynthesis class F	protein_codin	0.3464	0.127	1	7.566	0.1372
FBXO11	FBXO11	27	F-box protein 11	protein_codin	0.1735	0.385	1	23.46	0.2077
FOXN2	FOXN2	27	forkhead box N2	protein_codin	-0.07734	0.715	1	8.37	0.1459
MDH1	MDH1	27	malate dehydrogenase 1	protein_codin	0.2591	0.0988	1	32.33	0.08162
LGALSL	LGALSL	27	galectin like	protein_codin	0.1899	0.432	1	40.79	0.0851
MRPL19	MRPL19	27	mitochondrial ribosomal protein L19	protein_codin	0.3185	0.0785	1	6.632	0.1711
KCMF1	KCMF1	27	potassium channel modulatory factor 1	protein_codin	0.06384	0.752	1	6.221	0.1657
RMND5A	RMND5A	27	required for meiotic nuclear division 5 homolog A	protein_codin	-0.0222	0.935	1	7.63	0.2297
DUSP2	DUSP2	27	dual specificity phosphatase 2	protein_codin	0.4945	0.197	1	52.58	0.33
COA5	COA5	27	cytochrome c oxidase assembly factor 5	protein_codin	-0.01226	0.943	1	6.486	0.1932
IFIH1	IFIH1	27	interferon induced with helicase C domain 1	protein_codin	0.8266	0.00141	1	17.84	0.1632
WIPF1	WIPF1	27	WAS/WASL interacting protein family member 1	protein_codin	0.5059	0.224	1	3.156	0.3135
PLEKHA3	PLEKHA3	27	pleckstrin homology domain containing A3	protein_codin	-0.4505	0.00047	1	13.34	0.02726
UBE2E3	UBE2E3	27	ubiquitin conjugating enzyme E2 E3	protein_codin	0.179	0.128	1	35.43	0.02632
RETREG2	RETREG2	27	reticulophagy regulator family member 2	protein_codin	-0.355	0.061	1	6.328	0.1397
SP140L	SP140L	27	SP140 nuclear body protein like	protein_codin	0.6684	0.00272	1	16.33	0.1964
SNED1	SNED1	27	sushi, nidogen and EGF like domains 1	protein_codin	-0.009332	0.973	1	8.992	0.2446
2-Sep	43710	27	septin 2	protein_codin	-0.352	6.01E-05	1	45.62	0.1176
VGLL4	VGLL4	27	vestigial like family member 4	protein_codin	0.311	0.0164	1	36.9	0.06151
FGD5	FGD5	27	FYVE, RhoGEF and PH domain containing 5	protein_codin	0.1569	0.454	1	51.37	0.2268
UBE2E2	UBE2E2	27	ubiquitin conjugating enzyme E2 E2	protein_codin	0.4602	0.162	1	10.05	0.0547
LINC01985	LINC01985	27	long intergenic non-protein coding RNA 1985	lincRNA	1.667	9.2E-07	0.02152	22.78	0.1707
OXSR1	OXSR1	27	oxidative stress responsive kinase 1	protein_codin	0.5081	0.111	1	3.244	0.2017
NKTR	NKTR	27	natural killer cell triggering receptor	protein_codin	-0.09522	0.467	1	52.06	0.2227
NIT2	NIT2	27	nitrilase family member 2	protein_codin	-0.07075	0.777	1	6.792	0.1406
IFT57	IFT57	27	intraflagellar transport 57	protein_codin	-0.1282	0.382	1	14.88	0.1097
DZIP3	DZIP3	27	DAZ interacting zinc finger protein 3	protein_codin	-0.08715	0.814	1	3.727	0.1692
CCDC58	CCDC58	27	coiled-coil domain containing 58	protein_codin	0.436	0.111	1	3.152	0.1916
PFN2	PFN2	27	profilin 2	protein_codin	-1.102	0.000108	1	8.673	0.2522
MFSD1	MFSD1	27	major facilitator superfamily domain containing 1	protein_codin	-0.0446	0.848	1	24.67	0.2252
MAP3K13	MAP3K13	27	mitogen-activated protein kinase kinase kinase 13	protein_codin	0.0237	0.878	1	26.26	0.1866
TMEM175	TMEM175	27	transmembrane protein 175	protein_codin	-0.09126	0.728	1	2.916	0.1179
NOP14	NOP14	27	NOP14 nucleolar protein	protein_codin	1.169	7.47E-05	1	6.393	0.2224
BLOC1S4	BLOC1S4	27	biogenesis of lysosomal organelles complex 1 subunit 4	protein_codin	0.2221	0.319	1	8.072	0.1637
RELL1	RELL1	27	RELT like 1	protein_codin	0.1256	0.626	1	23.64	0.1089
FRYL	FRYL	27	FRY like transcription coactivator	protein_codin	0.2449	0.207	1	48.75	0.2482
GPAT3	GPAT3	27	glycerol-3-phosphate acyltransferase 3	protein_codin	0.6981	0.0646	1	3.332	0.1817
HERC5	HERC5	27	HECT and RLD domain containing E3 ubiquitin protein ligase 5	protein_codin	0.884	0.000388	1	51.3	0.1482
AIMP1	AIMP1	27	aminoacyl tRNA synthetase complex interacting multifunction	protein_codin	-0.0932	0.53	1	10.68	0.111
GAR1	GAR1	27	GAR1 ribonucleoprotein	protein_codin	0.3321	0.115	1	20.47	0.287
AP1AR	AP1AR	27	adaptor related protein complex 1 associated regulatory prote	protein_codin	0.3618	0.216	1	10.57	0.2042
SETD7	SETD7	27	SET domain containing 7, histone lysine methyltransferase	protein_codin	-0.1345	0.583	1	6.67	0.2201
CEP44	CEP44	27	centrosomal protein 44	protein_codin	0.4692	0.117	1	7.783	0.1979
CASP3	CASP3	27	caspase 3	protein_codin	0.1016	0.643	1	15.86	0.1424
SLC12A7	SLC12A7	27	solute carrier family 12 member 7	protein_codin	1.176	0.0102	1	31.97	0.2109
NDUFS6	NDUFS6	27	NADH ubiquinone oxidoreductase subunit S6	protein_codin	-0.3505	0.000211	1	28.57	0.1479
RAI14	RAI14	27	retinoic acid induced 14	protein_codin	0.1815	0.252	1	42.21	0.1159
ZNF131	ZNF131	27	zinc finger protein 131	protein_codin	0.2347	0.209	1	43.76	0.08289
PAIP1	PAIP1	27	poly(A) binding protein interacting protein 1	protein_codin	0.3095	0.0229	1	44.24	0.09479
ITGA2	ITGA2	27	integrin subunit alpha 2	protein_codin	0.1412	0.522	1	42.41	0.08422
SETD9	SETD9	27	SET domain containing 9	protein_codin	0.5029	0.0856	1	3.172	0.1942
TBCA	TBCA	27	tubulin folding cofactor A	protein_codin	-0.05895	0.4479	1	52.4	0.08209
TMEM167A	TMEM167A	27	transmembrane protein 167A	protein_codin	-0.03962	0.787	1	14.57	0.1156
CETN3	CETN3	27	centrin 3	protein_codin	0.3892	0.138	1	7.494	0.2565
SEMA6A	SEMA6A	27	semaphorin 6A	protein_codin	2.148	1.3E-15	3.12E-11	45.48	0.1114
DMXL1	DMXL1	27	Dmx like 1	protein_codin	-0.3191	0.216	1	8.928	0.1721
TAF7	TAF7	27	TATA-box binding protein associated factor 7	protein_codin	0.4309	0.00232	1	24.32	0.05578
KIAA0141	KIAA0141	27	DAP3 binding cell death enhancer 1	protein_codin	NA	NA	NA	7.193	0.1732
EBF1	EBF1	27	EBF transcription factor 1	protein_codin	2.416	8.8E-08	0.00207	8.13	0.1386
CCNG1	CCNG1	27	cyclin G1	protein_codin	0.08206	0.736	1	20.16	0.08938
CLK4	CLK4	27	CDC like kinase 4	protein_codin	0.5972	0.0362	1	3.438	0.1986
PPP1R10	PPP1R10	27	protein phosphatase 1 regulatory subunit 10	protein_codin	0.1952	0.071	1	52.58	0.08217
TNXB	TNXB	27	tenascin XB	protein_codin	-0.2122	0.279	1	52.58	0.1137
ANKS1A	ANKS1A	27	ankyrin repeat and sterile alpha motif domain containing 1A	protein_codin	2.172	0.000406	1	9.839	0.3352
KCTD20	KCTD20	27	potassium channel tetramerization domain containing 20	protein_codin	-0.1896	0.322	1	22.69	0.1176
UFL1	UFL1	27	UFM1 specific ligase 1	protein_codin	0.1666	0.28	1	29.12	0.2511
FOXO3	FOXO3	27	forkhead box O3	protein_codin	0.202	0.36	1	29.68	0.05552
TSPYL4	TSPYL4	27	TSPY like 4	protein_codin	-0.4868	0.106	1	2.628	0.2211
CEP85L	CEP85L	27	centrosomal protein 85 like	protein_codin	0.3747	0.0881	1	8.578	0.08034
HECA	HECA	27	hdc homolog, cell cycle regulator	protein_codin	-0.3034	0.195	1	7.326	0.1675
RNASET2	RNASET2	27	ribonuclease T2	protein_codin	-0.9382	7.34E-05	1	7.592	0.1192
SUN1	SUN1	27	Sad1 and UNC84 domain containing 1	protein_codin	0.6187	0.000672	1	14.11	0.1384
MAFK	MAFK	27	MAF bZIP transcription factor K	protein_codin	-0.6076	0.0288	1	2.832	0.1469
TRA2A	TRA2A	27	transformer 2 alpha homolog	protein_codin	0.4126	0.0188	1	13.48	0.1154
TAX1BP1	TAX1BP1	27	Tax1 binding protein 1	protein_codin	-0.143	0.1316	1	50.56	0.08162
CDK13	CDK13	27	cyclin dependent kinase 13	protein_codin	0.3926	0.0650	1	21.55	0.1902
POLD2	POLD2	27	DNA polymerase delta 2, accessory subunit	protein_codin	0.1502	0.482	1	23.38	0.1991
NIPSNAP2	NIPSNAP2	27	nipsnap homolog 2	protein_codin	0.07902	0.621	1	27.62	0.05372
HIP1	HIP1	27	huntingtin interacting protein 1	protein_codin	0.2244	0.204	1	45.13	0.1084
RBM48	RBM48	27	RNA binding motif protein 48	protein_codin	0.09667	0.735	1	2.942	0.3370
SAMD9L	SAMD9L	27	sterile alpha motif domain containing 9 like	protein_codin	0.2632	0.162	1	23.09	0.144

**SEM1**	SEM1	27	SEM1 26S proteasome complex subunit	protein_codin	0.05421	0.446	1	43.8	0.05377
**ARPC1A**	ARPC1A	27	actin related protein 2/3 complex subunit 1A	protein_codin	0.1	0.292	1	10.77	0.05558
**CUX1**	CUX1	27	cut like homeobox 1	protein_codin	-0.2147	0.314	1	27.68	0.09596
**CBLL1**	CBLL1	27	Cbl proto-oncogene like 1	protein_codin	-0.219	0.396	1	3.357	0.1688
**ZNF277**	ZNF277	27	zinc finger protein 277	protein_codin	-0.2285	0.297	1	48.62	0.1722
**LSM8.00**	LSM8.00	27	LSM8 homolog, U6 small nuclear RNA associated	protein_codin	0.01914	0.869	1	15.66	0.06089
**UBE2H**	UBE2H	27	ubiquitin conjugating enzyme E2 H	protein_codin	-0.2496	0.122	1	46.38	0.1446
**KDM7A**	KDM7A	27	lysine demethylase 7A	protein_codin	0.2152	0.492	1	8.726	0.1379
**TPK1**	TPK1	27	thiamin pyrophosphokinase 1	protein_codin	0.7847	0.0311	1	11.95	0.2242
**PAXIP1-AS2**	PAXIP1-AS	27	PAXIP1 antisense RNA 2	antisense_RN	0.164	0.629	1	3.176	0.2658
**APOO**	APOO	27	apolipoprotein O	protein_codin	-0.0368	0.902	1	2.744	0.1145
**FUNDC1**	FUNDC1	27	FUN14 domain containing 1	protein_codin	0.02532	0.91	1	8.406	0.2222
**SLC35A2**	SLC35A2	27	solute carrier family 35 member A2	protein_codin	-0.003366	0.989	1	7.558	0.166
**RRAGB**	RRAGB	27	Ras related GTP binding B	protein_codin	-0.09619	0.715	1	3.616	0.3220
**PIN4**	PIN4	27	peptidylprolyl cis/trans isomerase, NIMA-interacting 4	protein_codin	-0.1355	0.334	1	13.28	0.1730
**FTX**	FTX	27	FTX transcript, XIST regulator	lincRNA	-0.05476	0.782	1	39.1	0.0812
**ARMCX1**	ARMCX1	27	armadillo repeat containing X-linked 1	protein_codin	0.08748	0.686	1	7.449	0.08212
**FAM58A**	FAM58A	27	cyclin Q	protein_codin	NA	NA	NA	3.152	0.08139
**MPP1**	MPP1	27	membrane palmitoylated protein 1	protein_codin	1.226	4.94E-06	0.115	8.383	0.08294
**TNKS**	TNKS	27	tankyrase	protein_codin	-0.0884	0.738	1	2.892	0.2782
**VDAC3**	VDAC3	27	voltage dependent anion channel 3	protein_codin	0.2787	0.0405	1	24.74	0.164
**STAU2**	STAU2	27	staufen double-stranded RNA binding protein 2	protein_codin	-0.2596	0.259	1	8.147	0.1396
**TRIQK**	TRIQK	27	triple QxxK/R motif containing	protein_codin	0.0511	0.832	1	7.528	0.1701
**LY6E**	LY6E	27	lymphocyte antigen 6 family member E	protein_codin	0.6506	4.04E-11	9.61E-07	52.58	0.282
**GSDMD**	GSDMD	27	gasdermin D	protein_codin	0.269	0.0274	1	18.55	0.1742
**ZNF34**	ZNF34	27	zinc finger protein 34	protein_codin	0.647	0.0524	1	9.232	0.1892
**RANBP6**	RANBP6	27	RAN binding protein 6	protein_codin	0.6427	0.0465	1	28.38	0.1371
**TRMT10B**	TRMT10B	27	tRNA methyltransferase 10B	protein_codin	0.09842	0.749	1	10.54	0.3105
**WDR34**	WDR34	27	WD repeat domain 34	protein_codin	0.1406	0.632	1	3.339	0.2049
**PTPA**	PTPA	27	protein phosphatase 2 phosphatase activator	protein_codin	-0.4496	0.042	1	6.914	0.1126
**NUP214**	NUP214	27	nucleoporin 214	protein_codin	0.1659	0.509	1	8.802	0.2477
**RAPGEF1**	RAPGEF1	27	Rap guanine nucleotide exchange factor 1	protein_codin	0.6147	0.0205	1	7.895	0.08686
**SURF1**	SURF1	27	SURF1 cytochrome c oxidase assembly factor	protein_codin	-0.006994	0.971	1	7.552	0.1496
**HSPC324**	HSPC324	27	novel transcri	antisense_RN	NA	NA	NA	19.42	0.2082
**TUBB4B**	TUBB4B	27	tubulin beta 4B class IVb	protein_codin	0.4002	8.05E-06	0.187	35.18	0.02611
**ARRDC1**	ARRDC1	27	arrestin domain containing 1	protein_codin	0.5597	0.0102	1	23.2	0.02622
**IFITM2**	IFITM2	27	interferon induced transmembrane protein 2	protein_codin	0.368	1.49E-16	3.58E-12	52.58	0.04344
**IFITM1**	IFITM1	27	interferon induced transmembrane protein 1	protein_codin	2.024	9.5E-47	2.3E-42	51.97	0.0908
**IFITM3**	IFITM3	27	interferon induced transmembrane protein 3	protein_codin	0.1559	1.3E-05	0.3012	52.58	0.1405
**RHOG**	RHOG	27	ras homolog family member G	protein_codin	0.3998	0.02	1	14.06	0.1707
**TRIM22**	TRIM22	27	tripartite motif containing 22	protein_codin	-0.1911	0.24	1	34.13	0.1218
**PTPN5**	PTPN5	27	protein tyrosine phosphatase non-receptor type 5	protein_codin	2.147	0.000770	1	8.11	0.3688
**PRRG4**	PRRG4	27	proline rich and Gla domain 4	protein_codin	1.074	0.0149	1	10.4	0.1391
**FBXO3**	FBXO3	27	F-box protein 3	protein_codin	-0.3096	0.0865	1	5.685	0.1096
**TRIM44**	TRIM44	27	tripartite motif containing 44	protein_codin	0.1812	0.242	1	11.32	0.2228
**PHF21A**	PHF21A	27	PHD finger protein 21A	protein_codin	-0.274	0.34	1	35.96	0.3957
**TKFC**	TKFC	27	triokinase and FMN cyclase	protein_codin	0.8622	0.0252	1	2.824	0.1646
**GANAB**	GANAB	27	glucosidase II alpha subunit	protein_codin	-0.5646	0.0156	1	22.82	0.1717
**RTN3**	RTN3	27	reticulon 3	protein_codin	0.3475	0.0107	1	50.55	0.1392
**NUDT22**	NUDT22	27	nudix hydrolase 22	protein_codin	0.09624	0.676	1	7.38	0.1552
**MRPL49**	MRPL49	27	mitochondrial ribosomal protein L49	protein_codin	0.2551	0.257	1	8.349	0.1112
**DPF2**	DPF2	27	double PHD fingers 2	protein_codin	-0.5477	0.0172	1	3.278	0.272
**SSSCA1**	SSSCA1	27	zinc ribbon domain containing 2	protein_codin	-0.03918	0.81	1	15.68	0.1967
**MUS81**	MUS81	27	MUS81 structure-specific endonuclease subunit	protein_codin	0.01115	0.968	1	2.317	0.3368
**FOSL1**	FOSL1	27	FOS like 1, AP-1 transcription factor subunit	protein_codin	-0.06775	0.8	1	10.6	0.07996
**RAB1B**	RAB1B	27	RAB1B, member RAS oncogene family	protein_codin	0.01243	0.946	1	44.33	0.08174
**MRPL11**	MRPL11	27	mitochondrial ribosomal protein L11	protein_codin	0.144	0.27	1	22.7	0.1482
**AIP**	AIP	27	aryl hydrocarbon receptor interacting protein	protein_codin	0.1982	0.132	1	45.55	0.05259
**KMT5B**	KMT5B	27	lysine methyltransferase 5B	protein_codin	0.1005	0.646	1	7.818	0.112
**KDELC2**	KDELC2	27	protein O-glucosyltransferase 3	protein_codin	-0.06809	0.804	1	3.172	0.115
**DIXDC1**	DIXDC1	27	DIX domain containing 1	protein_codin	-0.8376	2.78E-08	0.000656	32.34	0.0542
**ARHGEF12**	ARHGEF12	27	Rho guanine nucleotide exchange factor 12	protein_codin	-0.316	0.069	1	17.68	0.1387
**NRGN**	NRGN	27	neurogranin	protein_codin	-0.6394	7.92E-05	1	19.18	0.05756
**ETS1**	ETS1	27	ETS proto-oncogene 1, transcription factor	protein_codin	0.2858	0.0314	1	34.6	0.02898
**UPF2**	UPF2	27	UPF2 regulator of nonsense mediated mRNA decay	protein_codin	0.5885	0.000201	1	10.8	0.2457
**DNAJC1**	DNAJC1	27	DnaJ heat shock protein family (Hsp40) member C1	protein_codin	0.1657	0.202	1	25.86	0.189
**YME1L1**	YME1L1	27	YME1 like 1 ATPase	protein_codin	0.1921	0.143	1	35.83	0.09152
**CUL2**	CUL2	27	cullin 2	protein_codin	0.1492	0.482	1	6.066	0.276
**ERCC6**	ERCC6	27	ERCC excision repair 6, chromatin remodeling factor	protein_codin	-1.161	1.5E-06	0.03504	9.142	0.1408
**CCAR1**	CCAR1	27	cell division cycle and apoptosis regulator 1	protein_codin	0.02241	0.92	1	14.8	0.1498
**ANAPC16**	ANAPC16	27	anaphase promoting complex subunit 16	protein_codin	-0.004498	0.9532	1	29.24	0.02627
**IFIT2**	IFIT2	27	interferon induced protein with tetratricopeptide repeats 2	protein_codin	1.348	7.02E-06	0.1633	52.54	0.2579
**IFIT3**	IFIT3	27	interferon induced protein with tetratricopeptide repeats 3	protein_codin	1.542	2.11E-08	0.000498	52.58	0.2235
**IFIT1**	IFIT1	27	interferon induced protein with tetratricopeptide repeats 1	protein_codin	0.6515	0.00334	1	52.07	0.4583
**HECTD2**	HECTD2	27	HECT domain E3 ubiquitin protein ligase 2	protein_codin	-0.1772	0.594	1	9.378	0.1411
**5-Mar**	43529	27	membrane associated ring-CH-type finger 5	protein_codin	0.385	0.142	1	6.685	0.1114
**FRAT2**	FRAT2	27	FRAT regulator of WNT signaling pathway 2	protein_codin	-0.3908	0.122	1	3.504	0.1157
**R3HCC1L**	R3HCC1L	27	R3H domain and coiled-coil containing 1 like	protein_codin	-0.1734	0.517	1	3.132	0.2586
**ERLIN1**	ERLIN1	27	ER lipid raft associated 1	protein_codin	0.1672	0.497	1	3.599	0.08328
**ITPRIP**	ITPRIP	27	inositol 1,4,5-trisphosphate receptor interacting protein	protein_codin	0.1594	0.537	1	26.75	0.3322
**ZDHHC6**	ZDHHC6	27	zinc finger DHHC-type containing 6	protein_codin	-0.5169	0.0221	1	3.262	0.3559
**FUOM**	FUOM	27	fucose mutarotase	protein_codin	0.04672	0.892	1	9.262	0.2794
**CLEC1A**	CLEC1A	27	C-type lectin domain family 1 member A	protein_codin	1.342	1.51E-05	0.3497	8.615	0.3088
**PRH1**	PRH1	27	proline rich protein HaeIII subfamily 1	protein_codin	0.02283	0.937	1	2.938	0.3418
**DERA**	DERA	27	deoxyribose-phosphate aldolase	protein_codin	0.606	0.00151	1	6.798	0.1145
**CMAS**	CMAS	27	cytidine monophosphate N-acetylneuraminic acid synthetase	protein_codin	0.3131	0.114	1	19.79	0.1674
**MED21**	MED21	27	mediator complex subunit 21	protein_codin	-0.1517	0.404	1	7.596	0.109
**IPO8**	IPO8	27	importin 8	protein_codin	0.1441	0.638	1	9.486	0.1392
**BICD1**	BICD1	27	BICD cargo adaptor 1	protein_codin	-0.08532	0.754	1	28.33	0.252
**DNM1L**	DNM1L	27	dynamin 1 like	protein_codin	0.08271	0.652	1	7.355	0.08076
**FKBP11**	FKBP11	27	FKBP prolyl isomerase 11	protein_codin	0.5639	0.000648	1	36.4	0.02646
**SHMT2**	SHMT2	27	serine hydroxymethyltransferase 2	protein_codin	0.007422	0.978	1	3.01	0.1652
**TSFM**	TSFM	27	Ts translation elongation factor, mitochondrial	protein_codin	0.4427	0.0369	1	24.65	0.1401
**RAB21**	RAB21	27	RAB21, member RAS oncogene family	protein_codin	-0.1779	0.205	1	49.29	0.1197
**C12orf29**	C12orf29	27	chromosome 12 open reading frame 29	protein_codin	0.5271	0.0422	1	8.739	0.2047
**C12orf45**	C12orf45	27	chromosome 12 open reading frame 45	protein_codin	0.2604	0.259	1	22.7	0.1641
**OAS1**	OAS1	27	2'-5'-oligoadenylate synthetase 1	protein_codin	0.8158	0.00114	1	43.02	0.4505
**OAS3**	OAS3	27	2'-5'-oligoadenylate synthetase 3	protein_codin	0.6702	0.0751	1	3.722	0.2411
**OAS2**	OAS2	27	2'-5'-oligoadenylate synthetase 2	protein_codin	0.3082	0.275	1	10.43	0.184
**GATC**	GATC	27	glutamyl-tRNA amidotransferase subunit C	protein_codin	0.2306	0.471	1	32.33	0.272
**POP5**	POP5	27	POP5 homolog, ribonuclease P/MRP subunit	protein_codin	0.3842	0.0534	1	37.9	0.1184
**OASL**	OASL	27	2'-5'-oligoadenylate synthetase like	protein_codin	0.9896	0.028	1	23.82	0.32
**ZCCHC8**	ZCCHC8	27	zinc finger CCHC-type containing 8	protein_codin	0.3286	0.224	1	10.43	0.1692
**SCARB1**	SCARB1	27	scavenger receptor class B member 1	protein_codin	3.272	1.79E-07	0.004202	49.85	0.2842
**GTF3A**	GTF3A	27	general transcription factor IIIA	protein_codin	-0.0136	0.902	1	40.97	0.1104
**EPSTI1**	EPSTI1	27	epithelial stromal interaction 1	protein_codin	0.2482	0.321	1	32.96	0.3311
**RB1**	RB1	27	RB transcriptional corepressor 1	protein_codin	0.3271	0.0905	1	34.49	0.2817
**DLEU2**	DLEU2	27	deleted in lymphocytic leukemia 2	antisense_RN	-0.2542	0.382	1	3.496	0.1667
**NEK3**	NEK3	27	NIMA related kinase 3	protein_codin	0.7827	0.00071	1	7.149	0.2760
**SUGT1**	SUGT1	27	SGT1 homolog, MIS12 kinetochore complex assembly cocha	protein_codin	0.2036	0.0746	1	24.4	0.2238
**MBNL2**	MBNL2	27	muscleblind like splicing regulator 2	protein_codin	0.539	0.00106	1	31.85	0.1892
**THTPA**	THTPA	27	thiamine triphosphatase	protein_codin	-0.6347	0.0249	1	3.037	0.2102
**PSMA6**	PSMA6	27	proteasome subunit alpha 6	protein_codin	0.3076	0.304	1	3.497	0.1437
**PRKCH**	PRKCH	27	protein kinase C eta	protein_codin	0.6917	0.00156	1	7.384	0.08061
**IFI27**	IFI27	27	interferon alpha inducible protein 27	protein_codin	-0.2322	6.18E-05	1	52.53	0.3440
**DICER1**	DICER1	27	dicer 1, ribonuclease III	protein_codin	0.3782	0.0916	1	16.77	0.1394
**TMEM121**	TMEM121	27	transmembrane protein 121	protein_codin	-0.3922	0.103	1	23.39	0.1095
**NSMCE3**	NSMCE3	27	NSE3 homolog, SMC5-SMC6 complex component	protein_codin	0.3752	0.0806	1	7.371	0.116
**AVEN**	AVEN	27	apoptosis and caspase activation inhibitor	protein_codin	-0.1421	0.561	1	3.368	0.08425
**KATNBL1**	KATNBL1	27	katanin regulatory subunit B1 like 1	protein_codin	0.5095	0.00181	1	23.44	0.1415
**DTWD1**	DTWD1	27	DTW domain containing 1	protein_codin	0.09459	0.61	1	7.043	0.2645
**IDH3A**	IDH3A	27	isocitrate dehydrogenase (NAD(+)) 3 alpha	protein_codin	0.05488	0.834	1	2.822	0.1706
**IREB2**	IREB2	27	iron responsive element binding protein 2	protein_codin	-0.7291	0.00289	1	10.52	0.1070
**AKAP13**	AKAP13	27	A-kinase anchoring protein 13	protein_codin	-0.1404	0.249	1	52.58	0.05718
**ISG20**	ISG20	27	interferon stimulated exonuclease gene 20	protein_codin	0.2757	0.272	1	47.97	0.2189
**FES**	FES	27	FES proto-oncogene, tyrosine kinase	protein_codin	0.4344	0.12	1	9.322	0.3365
**TM2D3**	TM2D3	27	TM2 domain containing 3	protein_codin	0.5419	0.000754	1	14	0.121
**METRN**	METRN	27	meteorin, glial cell differentiation regulator	protein_codin	-0.02527	0.879	1	16.32	0.1387
**MRPS34**	MRPS34	27	mitochondrial ribosomal protein S34	protein_codin	-0.2219	0.0646	1	18.11	0.0267
**TRAF7**	TRAF7	27	TNF receptor associated factor 7	protein_codin	-0.3391	0.112	1	9.472	0.06096
**C16orf72**	C16orf72	27	chromosome 16 open reading frame 72	protein_codin	0.2298	0.307	1	24.16	0.2311
**SEPHS2**	SEPHS2	27	selenophosphate synthetase 2	protein_codin	0.5426	0.000293	1	34.94	0.08256
**PYCARD**	PYCARD	27	PYD and CARD domain containing	protein_codin	-0.1159	0.429	1	11.25	0.1192
**VPS35**	VPS35	27	VPS35 retromer complex component	protein_codin	0.2948	0.0325	1	23.12	0.1777
**CNOT1**	CNOT1	27	CCR4-NOT transcription complex subunit 1	protein_codin	0.3277	0.207	1	8.282	0.2205
**TMEM208**	TMEM208	27	transmembrane protein 208	protein_codin	-0.2226	0.108	1	13.67	0.1158
**SNTB2**	SNTB2	27	syntrophin beta 2	protein_codin	0.09012	0.4	1	52.48	0.02650
**MVD**	MVD	27	mevalonate diphosphate decarboxylase	protein_codin	0.2142	0.454	1	2.644	0.1348
**TCF25**	TCF25	27	transcription factor 25	protein_codin	-0.07839	0.471	1	46.67	0.1147
**TIMM22**	TIMM22	27	translocase of inner mitochondrial membrane 22	protein_codin	0.2095	0.382	1	3.338	0.06026
**TSR1**	TSR1	27	TSR1 ribosome maturation factor	protein_codin	0.2362	0.413	1	9.825	0.09019
**XAF1**	XAF1	27	XIAP associated factor 1	protein_codin	0.06546	0.702	1	52.28	0.09025
**LGALS9**	LGALS9	27	galectin 9	protein_codin	-0.5091	0.000890	1	23.68	0.02678
**AP2B1**	AP2B1	27	adaptor related protein complex 2 subunit beta 1	protein_codin	-0.1454	0.379	1	15.08	0.08787
**CNP**	CNP	27	2',3'-cyclic nucleotide 3' phosphodiesterase	protein_codin	0.551	0.0314	1	7.15	0.1170
**IFI35**	IFI35	27	interferon induced protein 35	protein_codin	-0.004575	0.975	1	43.04	0.05817
**SLC35B1**	SLC35B1	27	solute carrier family 35 member B1	protein_codin	-0.1622	0.388	1	19.82	0.1398
**MRPL27**	MRPL27	27	mitochondrial ribosomal protein L27	protein_codin	-0.2522	0.0844	1	12.38	0.1396
**UTP18**	UTP18	27	UTP18 small subunit processome component	protein_codin	0.1822	0.444	1	7.197	0.2808
**DDX5**	DDX5	27	DEAD-box helicase 5	protein_codin	0.0542	0.32	1	52.58	0.02748
**MIF4GD**	MIF4GD	27	MIF4G domain containing	protein_codin	0.9398	0.00404	1	8.453	0.0932
**TSEN54**	TSEN54	27	tRNA splicing endonuclease subunit 54	protein_codin	-0.2039	0.504	1	3.102	0.1134
**MFSD11**	MFSD11	27	major facilitator superfamily domain containing 11	protein_codin	0.04442	0.856	1	3.458	0.1369
**SMAD7**	SMAD7	27	SMAD family member 7	protein_codin	-1.19	9.1E-08	0.00214	10.54	0.1468
**C18orf32**	C18orf32	27	chromosome 18 open reading frame 32	protein_codin	0.1919	0.486	1	2.133	0.202
**ATP8B1**	ATP8B1	27	ATPase phospholipid transporting 8B1	protein_codin	0.3826	0.0187	1	26.92	0.1109
**SOCS6**	SOCS6	27	suppressor of cytokine signaling 6	protein_codin	0.8352	0.0362	1	2.697	0.2162
**PLCB4**	PLCB4	27	phospholipase C beta 4	protein_codin	1.212	2.83E-05	0.6537	29.92	0.1726
**SNX5**	SNX5	27	sorting nexin 5	protein_codin	0.186	0.21	1	24.32	0.08558
**MGME1**	MGME1	27	mitochondrial genome maintenance exonuclease 1	protein_codin	0.1192	0.693	1	9.166	0.171
**KIF3B**	KIF3B	27	kinesin family member 3B	protein_codin	-0.3222	0.207	1	48.95	0.1371
**MAPRE1**	MAPRE1	27	microtubule associated protein RP/EB family member 1	protein_codin	0.006715	0.968	1	41.93	0.08012
**CDK5RAP1**	CDK5RAP1	27	CDK5 regulatory subunit associated protein 1	protein_codin	0.7906	0.0481	1	2.906	0.05467
**EDEM2**	EDEM2	27	ER degradation enhancing alpha-mannosidase like protein 2	protein_codin	0.2232	0.338	1	7.513	0.08482
**NDRG3**	NDRG3	27	NDRG family member 3	protein_codin	-0.4402	0.13	1	2.942	0.2830
**CTNNBL1**	CTNNBL1	27	catenin beta like 1	protein_codin	0.4092	0.0454	1	19.94	0.1082
**SERINC3**	SERINC3	27	serine incorporator 3	protein_codin	-0.2305	0.0394	1	18.93	0.1091
**DNTTIP1**	DNTTIP1	27	deoxynucleotidyltransferase terminal interacting protein 1	protein_codin	0.4195	0.0692	1	3.217	0.1122
**HELZ2**	HELZ2	27	helicase with zinc finger 2	protein_codin	0.6139	0.0518	1	3.85	0.2321
**ZBTB7A**	ZBTB7A	27	zinc finger and BTB domain containing 7A	protein_codin	0.2354	0.103	1	51.3	0.1142
**2-Mar**	43526	27	membrane associated ring-CH-type finger 2	protein_codin	-0.1402	0.226	1	36.96	0.1654
**AC020916.1**	AC020916.1	27	novel transcri	lincRNA	0.1302	0.563	1	45.82	0.2214
**RFX1**	RFX1	27	regulatory factor X1	protein_codin	0.4152	0.198	1	2.934	0.3058
**NDUFB7**	NDUFB7	27	NADH:ubiquinone oxidoreductase subunit B7	protein_codin	-0.267	0.0021	1	40.29	0.08109
**SMIM7**	SMIM7	27	small integral membrane protein 7	protein_codin	-0.4039	0.000717	1	11.52	0.08757
**BST2**	BST2	27	bone marrow stromal cell antigen 2	protein_codin	-0.8867	2.55E-30	6.17E-26	44.26	0.2364
**ZNF429**	ZNF429	27	zinc finger protein 429	protein_codin	0.8656	0.0226	1	10.02	0.2232
**CAPNS1**	CAPNS1	27	calpain small subunit 1	protein_codin	0.01966	0.8150	1	25.44	0.03026
**ZNF780A**	ZNF780A	27	zinc finger protein 780A	protein_codin	0.2142	0.508	1	3.605	0.264
**CARD8**	CARD8	27	caspase recruitment domain family member 8	protein_codin	0.3175	0.0631	1	31.43	0.05552
**KDELR1**	KDELR1	27	KDEL endoplasmic reticulum protein retention receptor 1	protein_codin	-0.04547	0.542	1	33.24	0.05438
**ATF5**	ATF5	27	activating transcription factor 5	protein_codin	-0.2802	0.232	1	21.32	0.07962
**ZNF347**	ZNF347	27	zinc finger protein 347	protein_codin	0.035	0.922	1	8.51	0.2492
**TPST2**	TPST2	27	tyrosylprotein sulfotransferase 2	protein_codin	0.8792	5.53E-07	0.01296	27.32	0.08230
**KCTD17**	KCTD17	27	potassium channel tetramerization domain containing 17	protein_codin	-0.4362	0.133	1	3.126	0.1981
**SMDT1**	SMDT1	27	single-pass membrane protein with aspartate rich tail 1	protein_codin	0.07296	0.427	1	38.04	0.07896
**RRP7A**	RRP7A	27	ribosomal RNA processing 7 homolog A	protein_codin	0.3234	0.0606	1	15.76	0.174
**PIGP**	PIGP	27	phosphatidylinositol glycan anchor biosynthesis class P	protein_codin	-0.2641	0.0921	1	15.3	0.1705
**ERG**	ERG	27	ETS transcription factor ERG	protein_codin	-0.02534	0.822	1	31.79	0.1349
**MX1**	MX1	27	MX dynamin like GTPase 1	protein_codin	0.08375	0.597	1	52.07	0.5269
**PDE9A**	PDE9A	27	phosphodiesterase 9A	protein_codin	0.8386	0.0406	1	3.58	0.3455
**INTS11**	INTS11	28	integrator complex subunit 11	protein_codin	0.2627	0.165	1	6.701	0.2056
**FLJ37453**	FLJ37453	28	novel transcri	antisense_RN	NA	NA	NA	9.286	0.2771
**BX284668.5**	BX284668.5	28	novel transcri	lincRNA	-0.05342	0.846	1	2.535	0.07952
**STMN1**	STMN1	28	stathmin 1	protein_codin	-0.08704	0.509	1	52.07	0.08168
**HMGN2**	HMGN2	28	high mobility group nucleosomal binding domain 2	protein_codin	-0.04438	0.592	1	52.58	0.08470
**PEF1**	PEF1	28	penta-EF-hand domain containing 1	protein_codin	0.3212	0.0222	1	11.32	0.1715
**TMEM39B**	TMEM39B	28	transmembrane protein 39B	protein_codin	0.1277	0.577	1	25.88	0.1112
**GJA4**	GJA4	28	gap junction protein alpha 4	protein_codin	-1.522	2.87E-12	6.85E-08	52.58	0.1358
**AGO3**	AGO3	28	argonaute RISC catalytic component 3	protein_codin	-0.7405	0.00189	1	24.96	0.2251
**SNIP1**	SNIP1	28	Smad nuclear interacting protein 1	protein_codin	-0.5576	0.0284	1	9.75	0.1357
**ZMPSTE24**	ZMPSTE24	28	zinc metallopeptidase STE24	protein_codin	-0.05718	0.792	1	8.286	0.08158
**FOXJ3**	FOXJ3	28	forkhead box J3	protein_codin	0.1206	0.498	1	6.956	0.2201
**PPCS**	PPCS	28	phosphopantothenoylcysteine synthetase	protein_codin	0.2392	0.038	1	17.87	0.1340
**ATPAF1**	ATPAF1	28	ATP synthase mitochondrial F1 complex assembly factor 1	protein_codin	-0.4562	0.0827	1	3.119	0.219
**ZYG11B**	ZYG11B	28	zyg-11 family member B, cell cycle regulator	protein_codin	0.2899	0.313	1	9.442	0.1419
**HHLA3**	HHLA3	28	HERV-H LTR-associating 3	protein_codin	0.254	0.419	1	3.584	0.2260
**RBMXL1**	RBMXL1	28	RBMX like 1	protein_codin	0.5819	0.11	1	10.22	0.2282
**RPAP2**	RPAP2	28	RNA polymerase II associated protein 2	protein_codin	0.3934	0.12	1	8.124	0.2620
**ARHGAP29**	ARHGAP29	28	Rho GTPase activating protein 29	protein_codin	0.1445	0.097	1	52.41	0.02712
**TRMT13**	TRMT13	28	tRNA methyltransferase 13 homolog	protein_codin	-0.07984	0.75	1	3.547	0.172
**RTCA**	RTCA	28	RNA 3'-terminal phosphate cyclase	protein_codin	0.1269	0.574	1	7.806	0.1387
**STXBP3**	STXBP3	28	syntaxin binding protein 3	protein_codin	0.9752	0.000136	1	16.36	0.1642
**C1orf54**	C1orf54	28	chromosome 1 open reading frame 54	protein_codin	-1.207	4.24E-16	1.02E-11	42.96	0.02870
**SCNM1**	SCNM1	28	sodium channel modifier 1	protein_codin	0.08735	0.668	1	22.87	0.05226
**VPS72**	VPS72	28	vacuolar protein sorting 72 homolog	protein_codin	0.8056	0.000447	1	7.127	0.149
**CHTOP**	CHTOP	28	chromatin target of PRMT1	protein_codin	0.492	0.0146	1	21.8	0.1392
**SLC27A3**	SLC27A3	28	solute carrier family 27 member 3	protein_codin	-0.3511	0.115	1	9.224	0.05355
**YY1AP1**	YY1AP1	28	YY1 associated protein 1	protein_codin	-0.03881	0.892	1	9.734	0.05368
**SEMA4A**	SEMA4A	28	semaphorin 4A	protein_codin	-0.4026	0.1	1	27.73	0.05717
**DUSP23**	DUSP23	28	dual specificity phosphatase 23	protein_codin	0.8834	2.33E-18	5.6E-14	37.16	0.03237
**DCAF8**	DCAF8	28	DDB1 and CUL4 associated factor 8	protein_codin	-0.01436	0.958	1	8.629	0.1744
**DNM3**	DNM3	28	dynamin 3	protein_codin	0.6981	0.0164	1	9.348	0.167
**GLUL**	GLUL	28	glutamate-ammonia ligase	protein_codin	0.4026	0.00766	1	52.54	0.3186
**TPR**	TPR	28	translocated promoter region, nuclear basket protein	protein_codin	-0.3591	6.37E-05	1	43.49	0.02634
**DDX59**	DDX59	28	DEAD-box helicase 59	protein_codin	0.2392	0.307	1	8.522	0.02600
**MDM4**	MDM4	28	MDM4 regulator of p53	protein_codin	0.1659	0.405	1	17.45	0.2185
**RAB29**	RAB29	28	RAB29, member RAS oncogene family	protein_codin	0.05328	0.812	1	8.842	0.1365
**TP53BP2**	TP53BP2	28	tumor protein p53 binding protein 2	protein_codin	0.1707	0.522	1	27.48	0.2524
**GJC2**	GJC2	28	gap junction protein gamma 2	protein_codin	0.8299	0.00992	1	26.95	0.2883
**ARV1**	ARV1	28	ARV1 homolog, fatty acid homeostasis modulator	protein_codin	0.1211	0.598	1	9.38	0.1677
**IRF2BP2**	IRF2BP2	28	interferon regulatory factor 2 binding protein 2	protein_codin	0.3079	0.0297	1	27.41	0.1095
**ADSS**	ADSS	28	adenylosuccinate synthase	protein_codin	-0.1996	0.212	1	33.38	0.1441
**COX20**	COX20	28	cytochrome c oxidase assembly factor COX20	protein_codin	-0.03902	0.782	1	21.34	0.1084
**RNASEH1**	RNASEH1	28	ribonuclease H1	protein_codin	0.5286	0.0564	1	7.662	0.1646
**KLF11**	KLF11	28	Kruppel like factor 11	protein_codin	1.266	6.75E-07	0.0158	18.52	0.167
**HPCAL1**	HPCAL1	28	hippocalcin like 1	protein_codin	0.564	0.000478	1	20.68	0.08945
**TMEM214**	TMEM214	28	transmembrane protein 214	protein_codin	-0.06866	0.83	1	3.014	0.202
**PEX13**	PEX13	28	peroxisomal biogenesis factor 13	protein_codin	0.2014	0.43	1	3.374	0.2352
**B3GNT2**	B3GNT2	28	UDP-GlcNAc:betaGal beta-1,3-N-acetylglucosaminyltransfera	protein_codin	0.5072	0.0326	1	17.22	0.1917
**AFTPH**	AFTPH	28	aftiphilin	protein_codin	0.1627	0.588	1	8.352	0.2971
**ETAA1**	ETAA1	28	ETAA1 activator of ATR kinase	protein_codin	-0.3649	0.226	1	11.1	0.086
**MOB1A**	MOB1A	28	MOB kinase activator 1A	protein_codin	0.03526	0.796	1	25.02	0.0802
**POLE4**	POLE4	28	DNA polymerase epsilon 4, accessory subunit	protein_codin	1.019	1.95E-21	4.7E-17	46.52	0.05741
**SNRNP200**	SNRNP200	28	small nuclear ribonucleoprotein U5 subunit 200	protein_codin	0.1234	0.606	1	7.717	0.2781
**MITD1**	MITD1	28	microtubule interacting and trafficking domain containing 1	protein_codin	0.3005	0.115	1	7.426	0.136
**UXS1**	UXS1	28	UDP-glucuronate decarboxylase 1	protein_codin	0.8072	0.00376	1	30.26	0.1792
**POLR2D**	POLR2D	28	RNA polymerase II subunit D	protein_codin	-0.01382	0.962	1	8.75	0.2192
**CHN1**	CHN1	28	chimerin 1	protein_codin	0.5901	0.0245	1	3.411	0.1665
**LINC01116**	LINC01116	28	long intergenic non-protein coding RNA 1116	lincRNA	1.062	1.51E-06	0.03527	7.204	0.115
**CWC22**	CWC22	28	CWC22 spliceosome associated protein homolog	protein_codin	0.668	0.00182	1	16.85	0.3515
**WDR75**	WDR75	28	WD repeat domain 75	protein_codin	0.6876	0.022	1	3.11	0.1619
**FAM126B**	FAM126B	28	family with sequence similarity 126 member B	protein_codin	-0.4389	0.0945	1	8.773	0.106
**CCNYL1**	CCNYL1	28	cyclin Y like 1	protein_codin	-0.1467	0.553	1	7.236	0.05339
**BARD1**	BARD1	28	BRCA1 associated RING domain 1	protein_codin	0.8455	0.0112	1	9.438	0.1938
**TUBA4A**	TUBA4A	28	tubulin alpha 4a	protein_codin	0.09958	0.5	1	11.87	0.142
**CAB39**	CAB39	28	calcium binding protein 39	protein_codin	-0.1222	0.613	1	3.484	0.2475
**IL17RC**	IL17RC	28	interleukin 17 receptor C	protein_codin	0.1136	0.729	1	3.702	0.3134
**EMC3**	EMC3	28	ER membrane protein complex subunit 3	protein_codin	-0.03162	0.816	1	28.38	0.1377
**TMEM43**	TMEM43	28	transmembrane protein 43	protein_codin	0.3524	0.0452	1	11.86	0.1109
**NR2C2**	NR2C2	28	nuclear receptor subfamily 2 group C member 2	protein_codin	-0.109	0.743	1	9.557	0.3079
**MAP4**	MAP4	28	microtubule associated protein 4	protein_codin	0.01334	0.898	1	44.55	0.027
**UQCRC1**	UQCRC1	28	ubiquinol-cytochrome c reductase core protein 1	protein_codin	-0.4066	0.0226	1	20.79	0.1694
**HYAL1**	HYAL1	28	hyaluronidase 1	protein_codin	0.0279	0.916	1	9.878	0.2537
**WDR82**	WDR82	28	WD repeat domain 82	protein_codin	-0.02952	0.892	1	8.074	0.1112
**GNL3**	GNL3	28	G protein nucleolar 3	protein_codin	0.04893	0.791	1	7.422	0.169
**ARF4**	ARF4	28	ADP ribosylation factor 4	protein_codin	-0.1862	0.0566	1	26.82	0.1122
**PCNP**	PCNP	28	PEST proteolytic signal containing nuclear protein	protein_codin	-0.09757	0.419	1	18.98	0.08290
**ARHGAP31**	ARHGAP31	28	Rho GTPase activating protein 31	protein_codin	0.7035	0.00362	1	7.162	0.2268
**LRRC58**	LRRC58	28	leucine rich repeat containing 58	protein_codin	-0.3182	0.315	1	3.62	0.2979
**ATP2C1**	ATP2C1	28	ATPase secretory pathway Ca2+ transporting 1	protein_codin	-0.2437	0.279	1	6.803	0.1934
**AMOTL2**	AMOTL2	28	angiomotin like 2	protein_codin	-0.0921	0.736	1	31.28	0.1126
**COPB2**	COPB2	28	coatomer protein complex subunit beta 2	protein_codin	-0.03893	0.785	1	32.4	0.1379
**SELENOT**	SELENOT	28	selenoprotein T	protein_codin	-0.4629	8.41E-05	1	44.82	0.08128
**PDCD10**	PDCD10	28	programmed cell death 10	protein_codin	0.2132	0.0937	1	9.19	0.05847
**GOLIM4**	GOLIM4	28	golgi integral membrane protein 4	protein_codin	-0.5782	4.44E-06	0.1034	17.09	0.2051
**PRKCI**	PRKCI	28	protein kinase C iota	protein_codin	-0.03702	0.884	1	48.86	0.1682
**DNAJC19**	DNAJC19	28	DnaJ heat shock protein family (Hsp40) member C19	protein_codin	0.1371	0.288	1	20.7	0.05249
**PSMD2**	PSMD2	28	proteasome 26S subunit, non-ATPase 2	protein_codin	0.01216	0.947	1	6.437	0.0852
**MAGEF1**	MAGEF1	28	MAGE family member F1	protein_codin	0.1267	0.626	1	7.782	0.2232
**TMEM44**	TMEM44	28	transmembrane protein 44	protein_codin	0.04044	0.882	1	6.654	0.1670
**PPP1R2**	PPP1R2	28	protein phosphatase 1 regulatory inhibitor subunit 2	protein_codin	-0.1459	0.232	1	30.7	0.0302
**ZNF141**	ZNF141	28	zinc finger protein 141	protein_codin	0.3597	0.257	1	3.106	0.2379
**LCORL**	LCORL	28	ligand dependent nuclear receptor corepressor like	protein_codin	0.3599	0.253	1	8.432	0.2640
**SMIM20**	SMIM20	28	small integral membrane protein 20	protein_codin	-0.4329	0.0394	1	7.655	0.05558
**HELQ**	HELQ	28	helicase, POLQ like	protein_codin	0.6997	0.0285	1	8.171	0.02754
**SMARCAD1**	SMARCAD1	28	SWI/SNF-related, matrix-associated actin-dependent regulato	protein_codin	0.0196	0.941	1	6.79	0.1992
**TRMT10A**	TRMT10A	28	tRNA methyltransferase 10A	protein_codin	0.1935	0.536	1	3.307	0.19
**DNAJB14**	DNAJB14	28	DnaJ heat shock protein family (Hsp40) member B14	protein_codin	0.1135	0.444	1	31.33	0.2535
**TET2**	TET2	28	tet methylcytosine dioxygenase 2	protein_codin	-0.2021	0.452	1	3.662	0.166
**PPA2**	PPA2	28	pyrophosphatase (inorganic) 2	protein_codin	0.229	0.221	1	7.12	0.1985
**PAPSS1**	PAPSS1	28	3'-phosphoadenosine 5'-phosphosulfate synthase 1	protein_codin	-1.669	1.52E-26	3.67E-22	32.93	0.05375
**METTL14**	METTL14	28	methyltransferase like 14	protein_codin	0.6825	0.0202	1	7.127	0.3451
**NUDT6**	NUDT6	28	nudix hydrolase 6	protein_codin	0.5026	0.176	1	9.252	0.3412
**C4orf33**	C4orf33	28	chromosome 4 open reading frame 33	protein_codin	0.2234	0.428	1	8.663	0.1457
**FAM198B**	FAM198B	28	golgi associated kinase 1B	protein_codin	-0.9569	4.07E-16	9.76E-12	51.69	0.07994
**MSMO1**	MSMO1	28	methylsterol monooxygenase 1	protein_codin	0.5762	0.0279	1	23.82	0.1807
**AC097534.2**	AC097534.2	28	novel transcript, antisense to SAP	antisense_RN	0.9439	0.000972	1	24.34	0.2034
**FBXO8**	FBXO8	28	F-box protein 8	protein_codin	0.2066	0.44	1	7.204	0.1408
**SPCS3**	SPCS3	28	signal peptidase complex subunit 3	protein_codin	-0.000110	0.999	1	24.01	0.08226
**VEGFC**	VEGFC	28	vascular endothelial growth factor C	protein_codin	0.9052	0.000303	1	34.84	0.1377
**IRF2**	IRF2	28	interferon regulatory factor 2	protein_codin	0.2559	0.0965	1	10.42	0.1705
**FBXO4**	FBXO4	28	F-box protein 4	protein_codin	0.7047	0.0194	1	7.384	0.1971
**ADAMTS6**	ADAMTS6	28	ADAM metallopeptidase with thrombospondin type 1 motif 6	protein_codin	-0.8996	0.0106	1	27.66	0.816
**SGTB**	SGTB	28	small glutamine rich tetratricopeptide repeat containing beta	protein_codin	-0.1945	0.49	1	8.812	0.3335
**GLRX**	GLRX	28	glutaredoxin	protein_codin	0.05925	0.718	1	42.1	0.08476
**SKP1**	SKP1	28	S-phase kinase associated protein 1	protein_codin	-0.2534	1.17E-06	0.02725	32.37	0.05541
**CXXC5**	CXXC5	28	CXXC finger protein 5	protein_codin	-0.2586	0.15	1	16.64	0.1431
**SAP30L**	SAP30L	28	SAP30 like	protein_codin	-0.2196	0.367	1	9.236	0.1115
**LARP1**	LARP1	28	La ribonucleoprotein domain family member 1	protein_codin	-0.0735	0.696	1	6.819	0.06121
**THG1L**	THG1L	28	tRNA-histidine guanylyltransferase 1 like	protein_codin	0.7257	0.0317	1	10.02	0.1756
**PANK3**	PANK3	28	pantothenate kinase 3	protein_codin	-0.4798	0.0805	1	8.453	0.1716
**EEF1E1**	EEF1E1	28	eukaryotic translation elongation factor 1 epsilon 1	protein_codin	-0.07652	0.661	1	18.17	0.08977
**JARID2**	JARID2	28	jumonji and AT-rich interaction domain containing 2	protein_codin	0.4679	0.2	1	3.271	0.1436
**MICA**	MICA	28	MHC class I polypeptide-related sequence A	protein_codin	-0.4065	0.0674	1	52.58	0.2992
**C6orf47**	C6orf47	28	chromosome 6 open reading frame 47	protein_codin	0.5562	0.0987	1	3.237	0.1347
**DDAH2**	DDAH2	28	dimethylarginine dimethylaminohydrolase 2	protein_codin	-0.2011	0.0487	1	43.18	0.08532

**HLA-DQA2**	HLA-DQA2	28	major histocompatibility complex, class II, DQ alpha 2	protein_codin	0.2927	1.81E-09	4.29E-05	20.93	0.1994
**PHF1**	PHF1	28	PHD finger protein 1	protein_codin	0.3626	0.101	1	7.979	0.1405
**MRPS10**	MRPS10	28	mitochondrial ribosomal protein S10	protein_codin	0.05978	0.724	1	14.49	0.08603
**ELOVL5**	ELOVL5	28	ELOVL fatty acid elongase 5	protein_codin	-0.258	0.193	1	47.52	0.02957
**ZNF292**	ZNF292	28	zinc finger protein 292	protein_codin	-0.01610	0.945	1	18.47	0.2519
**SNX3**	SNX3	28	sorting nexin 3	protein_codin	-0.02886	0.662	1	52.58	0.1254
**FYN**	FYN	28	FYN proto-oncogene, Src family tyrosine kinase	protein_codin	0.4782	0.0222	1	17.79	0.1422
**GOPC**	GOPC	28	golgi associated PDZ and coiled-coil motif containing	protein_codin	0.1349	0.348	1	11.97	0.1626
**RSPO3**	RSPO3	28	R-spondin 3	protein_codin	0.837	0.0011	1	52.58	0.111
**ARFGEF3**	ARFGEF3	28	ARFGEF family member 3	protein_codin	-0.6382	0.0258	1	3.544	0.2674
**PLEKHG1**	PLEKHG1	28	pleckstrin homology and RhoGEF domain containing G1	protein_codin	0.7500	0.00308	1	6.678	0.1961
**ARMT1**	ARMT1	28	acidic residue methyltransferase 1	protein_codin	0.262	0.365	1	3.555	0.1442
**MTRF1L**	MTRF1L	28	mitochondrial translational release factor 1 like	protein_codin	-0.7952	2.08E-06	0.04854	40.1	0.08250
**TMEM242**	TMEM242	28	transmembrane protein 242	protein_codin	0.2198	0.235	1	6.683	0.1962
**C7orf50**	C7orf50	28	chromosome 7 open reading frame 50	protein_codin	-0.5842	1.6E-06	0.03730	27.53	0.08821
**CCZ1**	CCZ1	28	CCZ1 homolog, vacuolar protein trafficking and biogenesis as	protein_codin	-0.01160	0.959	1	7.946	0.1334
**TSPAN13**	TSPAN13	28	tetraspanin 13	protein_codin	-0.01148	0.953	1	50.96	0.0828
**SKAP2**	SKAP2	28	src kinase associated phosphoprotein 2	protein_codin	1.120	7.33E-08	0.001725	5.886	0.02620
**MTURN**	MTURN	28	maturin, neural progenitor differentiation regulator homolog	protein_codin	-0.295	0.0860	1	17.22	0.2314
**ASL**	ASL	28	argininosuccinate lyase	protein_codin	0.4794	0.0540	1	3.32	0.1174
**POM121C**	POM121C	28	POM121 transmembrane nucleoporin C	protein_codin	0.08403	0.756	1	3.096	0.1692
**SRI**	SRI	28	sorcin	protein_codin	0.04579	0.602	1	50.02	0.05632
**RASA4**	RASA4	28	RAS p21 protein activator 4	protein_codin	2.730	1.31E-18	3.15E-14	52.04	0.05552
**PSMC2**	PSMC2	28	proteasome 26S subunit, ATPase 2	protein_codin	0.1044	0.528	1	16.35	0.1631
**FAM3C**	FAM3C	28	family with sequence similarity 3 member C	protein_codin	0.07536	0.554	1	26.85	0.1954
**TNPO3**	TNPO3	28	transportin 3	protein_codin	0.6970	0.0519	1	3.485	0.1361
**PARP12**	PARP12	28	poly(ADP-ribose) polymerase family member 12	protein_codin	0.2489	0.452	1	3.629	0.2334
**CASP2**	CASP2	28	caspase 2	protein_codin	0.2842	0.354	1	3.269	0.2570
**RHEB**	RHEB	28	Ras homolog, mTORC1 binding	protein_codin	-0.1962	0.0429	1	51.85	0.05259
**RAB9A**	RAB9A	28	RAB9A, member RAS oncogene family	protein_codin	0.1855	0.388	1	7.327	0.07906
**ASB9**	ASB9	28	ankyrin repeat and SOCS box containing 9	protein_codin	1.545	6.68E-07	0.01563	8.052	0.1416
**RAI2**	RAI2	28	retinoic acid induced 2	protein_codin	0.3269	0.202	1	7.875	0.1436
**RPS6KA3**	RPS6KA3	28	ribosomal protein S6 kinase A3	protein_codin	0.1784	0.309	1	17.17	0.2421
**USP9X**	USP9X	28	ubiquitin specific peptidase 9 X-linked	protein_codin	0.2837	0.242	1	8.928	0.1391
**CXorf36**	CXorf36	28	divergent protein kinase domain 2B	protein_codin	1.269	5.51E-18	1.32E-13	37.22	0.0279
**ARAF**	ARAF	28	A-Raf proto-oncogene, serine/threonine kinase	protein_codin	-0.2842	0.264	1	3.325	0.2279
**KLF8**	KLF8	28	Kruppel like factor 8	protein_codin	0.03272	0.916	1	9.785	0.1982
**MSN**	MSN	28	moesin	protein_codin	-0.0235	0.816	1	35.7	0.05552
**SNX12**	SNX12	28	sorting nexin 12	protein_codin	0.2757	0.333	1	3.071	0.1472
**OGT**	OGT	28	O-linked N-acetylglucosamine (GlcNAc) transferase	protein_codin	-0.049	0.864	1	6.7	0.2392
**HDX**	HDX	28	highly divergent homeobox	protein_codin	0.1179	0.718	1	2.864	0.2237
**ZMAT1**	ZMAT1	28	zinc finger matrin-type 1	protein_codin	-0.3482	0.144	1	8.785	0.1082
**BEX2**	BEX2	28	brain expressed X-linked 2	protein_codin	1.175	0.0107	1	11.53	0.196
**PLS3**	PLS3	28	plastin 3	protein_codin	-0.5639	1.81E-06	0.04225	37.16	0.128
**STAG2**	STAG2	28	stromal antigen 2	protein_codin	-0.07626	0.612	1	11.79	0.2
**HPRT1**	HPRT1	28	hypoxanthine phosphoribosyltransferase 1	protein_codin	-0.02632	0.87	1	50.76	0.1097
**TMEM187**	TMEM187	28	transmembrane protein 187	protein_codin	0.3524	0.278	1	3.145	0.2876
**EMD**	EMD	28	emerin	protein_codin	-0.09412	0.542	1	16.27	0.08496
**FAM50A**	FAM50A	28	family with sequence similarity 50 member A	protein_codin	0.0992	0.528	1	29.13	0.1195
**FAM3A**	FAM3A	28	family with sequence similarity 3 member A	protein_codin	-0.1894	0.393	1	6.9	0.05688
**IKBKG**	IKBKG	28	inhibitor of nuclear factor kappa B kinase regulatory subunit g	protein_codin	-0.205	0.466	1	8.332	0.1105
**CLIC2**	CLIC2	28	chloride intracellular channel 2	protein_codin	0.911	2.63E-09	0.00062	9.717	0.2456
**MTMR9**	MTMR9	28	myotubularin related protein 9	protein_codin	-0.1448	0.606	1	3.748	0.1665
**PCM1**	PCM1	28	pericentriolar material 1	protein_codin	-0.2270	0.0505	1	30.94	0.05472
**TNFRSF10B**	TNFRSF10	28	TNF receptor superfamily member 10b	protein_codin	0.1159	0.566	1	16.45	0.06071
**RNF170**	RNF170	28	ring finger protein 170	protein_codin	-0.6540	0.0218	1	3.440	0.1978
**RRS1**	RRS1	28	ribosome biogenesis regulator 1 homolog	protein_codin	0.4492	0.141	1	2.917	0.2044
**TERF1**	TERF1	28	telomeric repeat binding factor 1	protein_codin	-0.4760	0.0142	1	6.751	0.08904
**HEY1**	HEY1	28	hes related family bHLH transcription factor with YRPW motif	protein_codin	-0.658	0.0132	1	52.49	0.1809
**C8orf59**	C8orf59	28	ribosomal biogenesis factor	protein_codin	0.0657	0.4670	1	15.78	0.08336
**SYBU**	SYBU	28	syntabulin	protein_codin	1.487	6.53E-06	0.1518	45.96	0.1932
**UTP23**	UTP23	28	UTP23 small subunit processome component	protein_codin	0.4239	0.0172	1	39.34	0.08387
**TRMT12**	TRMT12	28	tRNA methyltransferase 12 homolog	protein_codin	0.03006	0.917	1	3.037	0.1449
**MTSS1**	MTSS1	28	MTSS I-BAR domain containing 1	protein_codin	-0.2262	0.316	1	23.67	0.2605
**SQLE**	SQLE	28	squalene epoxidase	protein_codin	0.4315	0.186	1	52.58	0.1482
**SMARCA2**	SMARCA2	28	SWI/SNF related, matrix associated, actin dependent regulato	protein_codin	-0.2587	0.114	1	13.72	0.05244
**MPDZ**	MPDZ	28	multiple PDZ domain crumbs cell polarity complex componen	protein_codin	0.2842	0.114	1	13.27	0.0862
**KLHL9**	KLHL9	28	kelch like family member 9	protein_codin	0.3058	0.377	1	2.95	0.2831
**DCTN3**	DCTN3	28	dynactin subunit 3	protein_codin	-0.2761	0.00434	1	41.58	0.1092
**UBQLN1**	UBQLN1	28	ubiquilin 1	protein_codin	-0.1108	0.605	1	6.828	0.2214
**SECISBP2**	SECISBP2	28	SECIS binding protein 2	protein_codin	0.3441	0.0519	1	14.07	0.1454
**NOL8**	NOL8	28	nucleolar protein 8	protein_codin	0.5089	0.0324	1	18.44	0.1091
**PHF2**	PHF2	28	PHD finger protein 2	protein_codin	0.2319	0.403	1	3.252	0.1744
**MSANTD3**	MSANTD3	28	Myb/SANT DNA binding domain containing 3	protein_codin	-0.2712	0.296	1	27.53	0.0829
**PTGES2**	PTGES2	28	prostaglandin E synthase 2	protein_codin	-0.1062	0.642	1	8.997	0.1645
**GOLGA2**	GOLGA2	28	golgin A2	protein_codin	-0.142	0.375	1	16.2	0.08888
**MED27**	MED27	28	mediator complex subunit 27	protein_codin	0.00794	0.974	1	3.505	0.05462
**SURF4**	SURF4	28	surfeit 4	protein_codin	-0.3970	0.0204	1	18.73	0.1135
**BRD3**	BRD3	28	bromodomain containing 3	protein_codin	0.3297	0.172	1	8.287	0.1340
**PMPCA**	PMPCA	28	peptidase, mitochondrial processing alpha subunit	protein_codin	-0.6250	0.0134	1	3.275	0.09050
**IRF7**	IRF7	28	interferon regulatory factor 7	protein_codin	0.09580	0.634	1	19.25	0.1182
**TSSC4**	TSSC4	28	tumor suppressing subtransferable candidate 4	protein_codin	0.2807	0.0865	1	14.93	0.05486
**PHLDA2**	PHLDA2	28	pleckstrin homology like domain family A member 2	protein_codin	0.2903	0.338	1	22.4	0.1991
**ZNF195**	ZNF195	28	zinc finger protein 195	protein_codin	0.541	0.103	1	9.833	0.2237
**TPP1**	TPP1	28	tripeptidyl peptidase 1	protein_codin	-0.303	0.177	1	24.06	0.08633
**RNF141**	RNF141	28	ring finger protein 141	protein_codin	0.1114	0.688	1	9.863	0.05712
**FAR1**	FAR1	28	fatty acyl-CoA reductase 1	protein_codin	0.08549	0.695	1	7.708	0.1685
**EXT2**	EXT2	28	exostosin glycosyltransferase 2	protein_codin	0.1589	0.525	1	30.77	0.02698
**NDUFS3**	NDUFS3	28	NADH:ubiquinone oxidoreductase core subunit S3	protein_codin	0.01834	0.896	1	27.44	0.1668
**PRDX5**	PRDX5	28	peroxiredoxin 5	protein_codin	-0.3900	7.51E-07	0.01757	39.87	0.05442
**CCS**	CCS	28	copper chaperone for superoxide dismutase	protein_codin	-0.1344	0.414	1	19.95	0.2302
**KDM2A**	KDM2A	28	lysine demethylase 2A	protein_codin	0.1522	0.476	1	7.526	0.2922
**PGM2L1**	PGM2L1	28	phosphoglucomutase 2 like 1	protein_codin	-0.7425	1.89E-05	0.4375	26.43	0.09776
**THAP12**	THAP12	28	THAP domain containing 12	protein_codin	0.479	0.0120	1	18.83	0.1366
**PRSS23**	PRSS23	28	serine protease 23	protein_codin	-0.1110	0.388	1	52.07	0.05388
**CEP57**	CEP57	28	centrosomal protein 57	protein_codin	0.3805	0.0194	1	13.13	0.2078
**DDX10**	DDX10	28	DEAD-box helicase 10	protein_codin	0.3296	0.238	1	3.696	0.2632
**DDX6**	DDX6	28	DEAD-box helicase 6	protein_codin	-0.06944	0.593	1	37.36	0.02801
**JAM3**	JAM3	28	junctional adhesion molecule 3	protein_codin	-0.3570	0.112	1	3.538	0.2189
**THYN1**	THYN1	28	thymocyte nuclear protein 1	protein_codin	0.2675	0.053	1	10.74	0.1419
**FAM208B**	FAM208B	28	transcription activation suppressor family member 2	protein_codin	0.4765	0.0437	1	7.403	0.262
**CXCL12**	CXCL12	28	C-X-C motif chemokine ligand 12	protein_codin	-0.9275	7.29E-08	0.001715	47.57	0.4406
**REEP3**	REEP3	28	receptor accessory protein 3	protein_codin	-0.33	0.0258	1	47.66	0.1442
**PPA1**	PPA1	28	pyrophosphatase (inorganic) 1	protein_codin	0.006385	0.95	1	47.96	0.06897
**MRPS16**	MRPS16	28	mitochondrial ribosomal protein S16	protein_codin	-0.1284	0.397	1	15.33	0.08258
**LCOR**	LCOR	28	ligand dependent nuclear receptor corepressor	protein_codin	0.4939	0.0449	1	27.58	0.08493
**EXOSC1**	EXOSC1	28	exosome component 1	protein_codin	0.03728	0.841	1	20.79	0.1079
**SLK**	SLK	28	STE20 like kinase	protein_codin	-0.2252	0.052	1	26.2	0.05309
**SMNDC1**	SMNDC1	28	survival motor neuron domain containing 1	protein_codin	0.4419	0.0216	1	13.86	0.1645
**EIF3A**	EIF3A	28	eukaryotic translation initiation factor 3 subunit A	protein_codin	-0.3359	0.00932	1	51.58	0.02729
**ZRANB1**	ZRANB1	28	zinc finger RANBP2-type containing 1	protein_codin	0.2432	0.475	1	3.233	0.3871
**EBF3**	EBF3	28	EBF transcription factor 3	protein_codin	1.48	0.000315	1	7.657	0.1699
**FOXJ2**	FOXJ2	28	forkhead box J2	protein_codin	-0.2058	0.478	1	2.673	0.2905
**EPS8**	EPS8	28	epidermal growth factor receptor pathway substrate 8	protein_codin	1.119	3E-13	7.17E-09	13.11	0.1129
**STRAP**	STRAP	28	serine/threonine kinase receptor associated protein	protein_codin	0.05468	0.645	1	21.44	0.05741
**AMN1**	AMN1	28	antagonist of mitotic exit network 1 homolog	protein_codin	-0.2268	0.429	1	9.949	0.1961
**GXYLT1**	GXYLT1	28	glucoside xylosyltransferase 1	protein_codin	-0.128	0.629	1	8.856	0.1147
**SMUG1**	SMUG1	28	single-strand-selective monofunctional uracil-DNA glycosylase	protein_codin	-0.07142	0.827	1	3.297	0.1729
**RAB5B**	RAB5B	28	RAB5B, member RAS oncogene family	protein_codin	-0.154	0.617	1	3.196	0.2572
**DCTN2**	DCTN2	28	dynactin subunit 2	protein_codin	0.02778	0.823	1	22.13	0.08718
**RAP1B**	RAP1B	28	RAP1B, member of RAS oncogene family	protein_codin	0.4435	7.52E-07	0.01759	31.84	0.0295
**CEP290**	CEP290	28	centrosomal protein 290	protein_codin	-0.009718	0.962	1	26.18	0.2347
**CEP83**	CEP83	28	centrosomal protein 83	protein_codin	0.2194	0.528	1	9.918	0.1688
**METAP2**	METAP2	28	methionyl aminopeptidase 2	protein_codin	0.4156	0.000253	1	26.97	0.1668
**NFYB**	NFYB	28	nuclear transcription factor Y subunit beta	protein_codin	0.9542	1.14E-05	0.2644	41.16	0.2295
**APPL2**	APPL2	28	adaptor protein, phosphotyrosine interacting with PH domain	protein_codin	0.2551	0.379	1	2.766	0.1722
**CORO1C**	CORO1C	28	coronin 1C	protein_codin	0.7864	0.00154	1	7.822	0.1971
**HVCN1**	HVCN1	28	hydrogen voltage gated channel 1	protein_codin	0.2662	0.409	1	9.272	0.2558
**TAOK3**	TAOK3	28	TAO kinase 3	protein_codin	-0.3825	0.0518	1	50.75	0.1075
**RAB35**	RAB35	28	RAB35, member RAS oncogene family	protein_codin	0.5529	0.0593	1	7.855	0.1647
**SBNO1**	SBNO1	28	strawberry notch homolog 1	protein_codin	0.2235	0.404	1	21.75	0.2266
**NCOR2**	NCOR2	28	nuclear receptor corepressor 2	protein_codin	-0.2790	0.2	1	6.83	0.08329
**WASF3**	WASF3	28	WASP family member 3	protein_codin	0.7549	0.0304	1	8.94	0.4001
**SUPT20H**	SUPT20H	28	SPT20 homolog, SAGA complex component	protein_codin	0.181	0.455	1	8.336	0.2189
**SPRYD7**	SPRYD7	28	SPRY domain containing 7	protein_codin	-0.1702	0.429	1	7.522	0.1921
**KCTD12**	KCTD12	28	potassium channel tetramerization domain containing 12	protein_codin	0.02326	0.875	1	36.96	0.08798
**DOCK9**	DOCK9	28	dedicator of cytokinesis 9	protein_codin	0.3052	0.0934	1	24.21	0.05571
**EFNB2**	EFNB2	28	ephrin B2	protein_codin	-0.1362	0.284	1	48.03	0.05817
**ABHD13**	ABHD13	28	abhydrolase domain containing 13	protein_codin	-0.08132	0.758	1	3.094	0.2172
**APEX1**	APEX1	28	apurinic/apyrimidinic endodeoxyribonuclease 1	protein_codin	-0.2522	0.0154	1	48.18	0.1100
**SUPT16H**	SUPT16H	28	SPT16 homolog, facilitates chromatin remodeling subunit	protein_codin	0.2066	0.211	1	42.53	0.05258
**CHD8**	CHD8	28	chromodomain helicase DNA binding protein 8	protein_codin	-0.1320	0.566	1	27.22	0.1356
**MMP14**	MMP14	28	matrix metallopeptidase 14	protein_codin	-0.3105	0.249	1	3.543	0.1513
**PRMT5**	PRMT5	28	protein arginine methyltransferase 5	protein_codin	0.3957	0.245	1	2.909	0.2591
**DHRS4**	DHRS4	28	dehydrogenase/reductase 4	protein_codin	0.8541	0.0107	1	2.96	0.2345
**DHRS4L2**	DHRS4L2	28	dehydrogenase/reductase 4 like 2	protein_codin	0.4632	0.00912	1	17.15	0.08626
**SNX6**	SNX6	28	sorting nexin 6	protein_codin	0.1931	0.0461	1	39.05	0.08326
**ATL1**	ATL1	28	atlastin GTPase 1	protein_codin	0.3427	0.26	1	3.667	0.1409
**C14orf166**	C14orf166	28	RNA transcription, translation and transport factor	protein_codin	NA	NA	NA	43.8	0.05322
**GCH1**	GCH1	28	GTP cyclohydrolase 1	protein_codin	0.3185	0.274	1	3.409	0.1977
**FUT8**	FUT8	28	fucosyltransferase 8	protein_codin	-1.5	3.13E-11	7.45E-07	20.25	0.1216
**NUMB**	NUMB	28	NUMB endocytic adaptor protein	protein_codin	-0.347	0.068	1	23.93	0.05429
**JDP2**	JDP2	28	Jun dimerization protein 2	protein_codin	0.2182	0.28	1	23.67	0.02971
**PAPOLA**	PAPOLA	28	poly(A) polymerase alpha	protein_codin	0.06078	0.605	1	17.98	0.02751
**MARK3**	MARK3	28	microtubule affinity regulating kinase 3	protein_codin	-0.245	0.238	1	8.68	0.1404
**JAG2**	JAG2	28	jagged canonical Notch ligand 2	protein_codin	-0.2708	0.255	1	24.43	0.1414
**SLC12A6**	SLC12A6	28	solute carrier family 12 member 6	protein_codin	-0.05448	0.845	1	7.882	0.2398
**SRP14**	SRP14	28	signal recognition particle 14	protein_codin	-0.07371	0.148	1	52.58	0.109
**EHD4**	EHD4	28	EH domain containing 4	protein_codin	0.2860	0.0515	1	40.96	0.1196
**HAUS2**	HAUS2	28	HAUS augmin like complex subunit 2	protein_codin	0.1514	0.562	1	8.109	0.1402
**EIF3J**	EIF3J	28	eukaryotic translation initiation factor 3 subunit J	protein_codin	0.1449	0.246	1	41.29	0.1891
**FBN1**	FBN1	28	fibrillin 1	protein_codin	0.2377	0.247	1	52.38	0.1675
**LEO1**	LEO1	28	LEO1 homolog, Paf1/RNA polymerase II complex component	protein_codin	0.5759	0.00469	1	18.58	0.05398
**RSL24D1**	RSL24D1	28	ribosomal L24 domain containing 1	protein_codin	0.57	8.21E-09	0.000194	33.26	0.1441
**CCPG1**	CCPG1	28	cell cycle progression 1	protein_codin	-0.496	1.29E-07	0.003031	33.95	0.1968
**SNX1**	SNX1	28	sorting nexin 1	protein_codin	0.2551	0.214	1	7.986	0.1372
**INTS14**	INTS14	28	integrator complex subunit 14	protein_codin	0.3133	0.293	1	2.849	0.3541
**FAM219B**	FAM219B	28	family with sequence similarity 219 member B	protein_codin	-0.2772	0.334	1	2.61	0.1379
**COX5A**	COX5A	28	cytochrome c oxidase subunit 5A	protein_codin	-0.3302	2.34E-05	0.541	36.48	0.02772
**ETFA**	ETFA	28	electron transfer flavoprotein subunit alpha	protein_codin	-0.06027	0.693	1	14.82	0.0568
**NMB**	NMB	28	neuromedin B	protein_codin	-0.1442	0.614	1	49.83	0.3079
**IGF1R**	IGF1R	28	insulin like growth factor 1 receptor	protein_codin	-0.2067	0.393	1	20.76	0.1942
**NUBP2**	NUBP2	28	nucleotide binding protein 2	protein_codin	-0.08758	0.628	1	6.796	0.1692
**ZNF598**	ZNF598	28	zinc finger protein 598	protein_codin	0.3519	0.225	1	3.661	0.14
**SLC9A3R2**	SLC9A3R2	28	SLC9A3 regulator 2	protein_codin	-0.4608	2.95E-07	0.006917	52.58	0.0267
**PDPK1**	PDPK1	28	3-phosphoinositide dependent protein kinase 1	protein_codin	0.3551	0.188	1	3.197	0.05775
**ARL6IP1**	ARL6IP1	28	ADP ribosylation factor like GTPase 6 interacting protein 1	protein_codin	0.1800	0.12	1	27.33	0.08526
**ATXN2L**	ATXN2L	28	ataxin 2 like	protein_codin	0.2545	0.344	1	7.979	0.2105
**TAOK2**	TAOK2	28	TAO kinase 2	protein_codin	-0.402	0.138	1	7.365	0.2541
**RNF40**	RNF40	28	ring finger protein 40	protein_codin	0.3907	0.284	1	9.642	0.2272
**ORAI3**	ORAI3	28	ORAI calcium release-activated calcium modulator 3	protein_codin	0.07941	0.723	1	8.304	0.2272
**TGFB1I1**	TGFB1I1	28	transforming growth factor beta 1 induced transcript 1	protein_codin	-0.2519	0.161	1	50.88	0.03044
**C16orf87**	C16orf87	28	chromosome 16 open reading frame 87	protein_codin	0.1391	0.558	1	6.505	0.1679
**COQ9**	COQ9	28	coenzyme Q9	protein_codin	-0.08503	0.722	1	7.039	0.08268
**CCDC102A**	CCDC102A	28	coiled-coil domain containing 102A	protein_codin	-0.02311	0.926	1	33.27	0.1672
**CDH5**	CDH5	28	cadherin 5	protein_codin	-0.4712	1.23E-05	0.2852	52.05	0.05252
**GFOD2**	GFOD2	28	glucose-fructose oxidoreductase domain containing 2	protein_codin	0.5527	0.00771	1	44.63	0.164
**CENPT**	CENPT	28	centromere protein T	protein_codin	0.4779	0.146	1	3.156	0.1671
**DHX38**	DHX38	28	DEAH-box helicase 38	protein_codin	0.01706	0.947	1	8.117	0.1382
**CFDP1**	CFDP1	28	craniofacial development protein 1	protein_codin	0.2198	0.102	1	50.29	0.2524
**CMIP**	CMIP	28	c-Maf inducing protein	protein_codin	0.6004	1.53E-05	0.3544	51.2	0.02758
**ZFPM1**	ZFPM1	28	zinc finger protein, FOG family member 1	protein_codin	-0.2834	0.307	1	3.311	0.2629
**ZNF276**	ZNF276	28	zinc finger protein 276	protein_codin	-0.3347	0.187	1	10.95	0.1947
**SLC25A11**	SLC25A11	28	solute carrier family 25 member 11	protein_codin	0.1427	0.429	1	16.86	0.08315
**ELP5**	ELP5	28	elongator acetyltransferase complex subunit 5	protein_codin	0.3832	0.203	1	29.85	0.1600
**EIF5A**	EIF5A	28	eukaryotic translation initiation factor 5A	protein_codin	0.02947	0.742	1	34.22	0.02631
**CD68**	CD68	28	CD68 molecule	protein_codin	-1.476	2.86E-08	0.000675	10.38	0.1426
**RANGRF**	RANGRF	28	RAN guanine nucleotide release factor	protein_codin	0.08671	0.671	1	8.517	0.1372
**MPRIP**	MPRIP	28	myosin phosphatase Rho interacting protein	protein_codin	0.3768	0.0895	1	20.09	0.1392
**SUZ12**	SUZ12	28	SUZ12 polycomb repressive complex 2 subunit	protein_codin	0.167	0.332	1	12.79	0.1645
**TADA2A**	TADA2A	28	transcriptional adaptor 2A	protein_codin	0.1092	0.734	1	2.776	0.3312
**PNMT**	PNMT	28	phenylethanolamine N-methyltransferase	protein_codin	2.242	0.000526	1	11.93	0.3344
**P3H4**	P3H4	28	prolyl 3-hydroxylase family member 4 (non-enzymatic)	protein_codin	-0.5187	0.0819	1	2.997	0.2667
**STAT5B**	STAT5B	28	signal transducer and activator of transcription 5B	protein_codin	0.06183	0.83	1	3.333	0.259
**VPS25**	VPS25	28	vacuolar protein sorting 25 homolog	protein_codin	-0.03303	0.849	1	5.747	0.1390
**PSME3**	PSME3	28	proteasome activator subunit 3	protein_codin	0.04413	0.866	1	3.266	0.08364
**ZNF652**	ZNF652	28	zinc finger protein 652	protein_codin	-0.285	0.155	1	7.729	0.141
**PHB**	PHB	28	prohibitin	protein_codin	-0.06437	0.533	1	41.93	0.08835
**RSAD1**	RSAD1	28	radical S-adenosyl methionine domain containing 1	protein_codin	0.1455	0.652	1	2.158	0.2225
**SCPEP1**	SCPEP1	28	serine carboxypeptidase 1	protein_codin	-0.2934	0.0999	1	46.7	0.08315
**AKAP1**	AKAP1	28	A-kinase anchoring protein 1	protein_codin	0.5902	0.0992	1	8.822	0.1242
**DHX40**	DHX40	28	DEAH-box helicase 40	protein_codin	0.1715	0.462	1	8.922	0.07888
**APPBP2**	APPBP2	28	amyloid beta precursor protein binding protein 2	protein_codin	0.1755	0.522	1	3.369	0.1695
**ACE**	ACE	28	angiotensin I converting enzyme	protein_codin	-0.5110	0.0566	1	47.47	0.251
**LIMD2**	LIMD2	28	LIM domain containing 2	protein_codin	-0.2048	0.38	1	8.727	0.2252
**UNK**	UNK	28	unk zinc finger	protein_codin	-0.1392	0.631	1	3.019	0.1071
**USP14**	USP14	28	ubiquitin specific peptidase 14	protein_codin	0.2984	0.134	1	6.717	0.0541
**METTL4**	METTL4	28	methyltransferase like 4	protein_codin	-0.3879	0.168	1	8.842	0.1538
**NAPG**	NAPG	28	NSF attachment protein gamma	protein_codin	0.3133	0.106	1	7.069	0.1469
**RNMT**	RNMT	28	RNA guanine-7 methyltransferase	protein_codin	0.02302	0.833	1	12.04	0.07968
**ROCK1**	ROCK1	28	Rho associated coiled-coil containing protein kinase 1	protein_codin	-0.04641	0.741	1	15.33	0.02608
**MEX3C**	MEX3C	28	mex-3 RNA binding family member C	protein_codin	0.5582	0.0224	1	7.768	0.2047
**NARS**	NARS	28	asparaginyl-tRNA synthetase	protein_codin	-0.09275	0.517	1	14.4	0.02694
**NFATC1**	NFATC1	28	nuclear factor of activated T cells 1	protein_codin	-0.4962	0.0605	1	47.72	0.202
**RBFA**	RBFA	28	ribosome binding factor A	protein_codin	0.4156	0.0525	1	7.768	0.2002
**FKBP1A**	FKBP1A	28	FKBP prolyl isomerase 1A	protein_codin	-0.1069	0.0279	1	52.58	0.02660
**HSPA12B**	HSPA12B	28	heat shock protein family A (Hsp70) member 12B	protein_codin	0.470	0.0192	1	31.93	0.05726
**TRMT6**	TRMT6	28	tRNA methyltransferase 6	protein_codin	0.6201	0.0299	1	8.99	0.1388
**SNRPB2**	SNRPB2	28	small nuclear ribonucleoprotein polypeptide B2	protein_codin	0.1124	0.336	1	17.95	0.1691
**DTD1**	DTD1	28	D-aminoacyl-tRNA deacylase 1	protein_codin	0.3610	0.0358	1	13.74	0.1942
**CD93**	CD93	28	CD93 molecule	protein_codin	1.101	1.93E-12	4.61E-08	52.58	0.03066
**RALY**	RALY	28	RALY heterogeneous nuclear ribonucleoprotein	protein_codin	0.04665	0.694	1	41.28	0.05645
**MMP24-AS1**	MMP24-AS	28	MMP24 opposite strand	protein_codin	NA	NA	NA	23.33	0.05702
**TOP1.00**	TOP1.00	28	DNA topoisomerase I	protein_codin	0.1227	0.267	1	42.64	0.05306
**DDX27**	DDX27	28	DEAD-box helicase 27	protein_codin	0.5097	0.0431	1	41.7	0.3016
**PPP1R3D**	PPP1R3D	28	protein phosphatase 1 regulatory subunit 3D	protein_codin	0.5087	0.16	1	3.494	0.256
**MBD3**	MBD3	28	methyl-CpG binding domain protein 3	protein_codin	-0.4892	0.0562	1	8.817	0.1370
**REXO1**	REXO1	28	RNA exonuclease 1 homolog	protein_codin	-0.1181	0.628	1	3.007	0.1130
**SIRT6**	SIRT6	28	sirtuin 6	protein_codin	0.7229	0.0322	1	9.243	0.1768
**ZNF562**	ZNF562	28	zinc finger protein 562	protein_codin	0.05084	0.883	1	9.396	0.431
**CDC37**	CDC37	28	cell division cycle 37	protein_codin	0.5482	1.91E-10	4.54E-06	46.24	0.02706
**KEAP1**	KEAP1	28	kelch like ECH associated protein 1	protein_codin	0.2489	0.249	1	7.408	0.1727
**ILF3-AS1**	ILF3-AS1	28	ILF3 divergent transcript	lincRNA	NA	NA	NA	18.93	0.2619
**LDLR**	LDLR	28	low density lipoprotein receptor	protein_codin	0.381	0.0959	1	28.93	0.1721
**FARSA**	FARSA	28	phenylalanyl-tRNA synthetase subunit alpha	protein_codin	0.1860	0.447	1	9.523	0.1748
**DCAF15**	DCAF15	28	DDB1 and CUL4 associated factor 15	protein_codin	0.6067	0.0810	1	9.657	0.2756
**AKAP8**	AKAP8	28	A-kinase anchoring protein 8	protein_codin	0.1295	0.634	1	9.617	0.2519
**FAM32A**	FAM32A	28	family with sequence similarity 32 member A	protein_codin	0.234	0.128	1	12.53	0.1662
**SIN3B**	SIN3B	28	SIN3 transcription regulator family member B	protein_codin	-0.4952	0.0262	1	6.963	0.2064
**GTPBP3**	GTPBP3	28	GTP binding protein 3, mitochondrial	protein_codin	0.3052	0.309	1	2.793	0.3071
**SCN1B**	SCN1B	28	sodium voltage-gated channel beta subunit 1	protein_codin	0.5829	0.000494	1	28.7	0.02709
**SUPT5H**	SUPT5H	28	SPT5 homolog, DSIF elongation factor subunit	protein_codin	0.03548	0.873	1	8.655	0.05245
**RTN2**	RTN2	28	reticulon 2	protein_codin	1.034	0.00248	1	9.757	0.1769
**PRRG2**	PRRG2	28	proline rich and Gla domain 2	protein_codin	0.3625	0.256	1	28.07	0.3492
**AP2A1**	AP2A1	28	adaptor related protein complex 2 subunit alpha 1	protein_codin	0.6747	0.0467	1	7.688	0.202
**MBOAT7**	MBOAT7	28	membrane bound O-acyltransferase domain containing 7	protein_codin	-0.1348	0.634	1	3.573	0.1148
**ZNF580**	ZNF580	28	zinc finger protein 580	protein_codin	0.272	0.136	1	7.349	0.1912
**ZSCAN18**	ZSCAN18	28	zinc finger and SCAN domain containing 18	protein_codin	-0.1131	0.518	1	7.542	0.056
**SNAP29**	SNAP29	28	synaptosome associated protein 29	protein_codin	0.4747	0.0192	1	19.88	0.1995
**MAPK1**	MAPK1	28	mitogen-activated protein kinase 1	protein_codin	-0.4698	0.0115	1	6.95	0.2875
**UQCR10**	UQCR10	28	ubiquinol-cytochrome c reductase, complex III subunit X	protein_codin	-0.2770	0.00805	1	30.07	0.02682
**SMTN**	SMTN	28	smoothelin	protein_codin	0.9755	0.013	1	52.58	0.2599
**DRG1**	DRG1	28	developmentally regulated GTP binding protein 1	protein_codin	0.6179	0.00923	1	8.099	0.2016
**PRR14L**	PRR14L	28	proline rich 14 like	protein_codin	0.03681	0.898	1	7.049	0.1672
**TOM1**	TOM1	28	target of myb1 membrane trafficking protein	protein_codin	0.07061	0.819	1	10.3	0.2932
**CDC42EP1**	CDC42EP1	28	CDC42 effector protein 1	protein_codin	-0.1118	0.541	1	19.53	0.07952
**SUN2**	SUN2	28	Sad1 and UNC84 domain containing 2	protein_codin	-0.1195	0.555	1	7.654	0.1999
**L3MBTL2**	L3MBTL2	28	L3MBTL histone methyl-lysine binding protein 2	protein_codin	0.5294	0.117	1	8.822	0.1092
**MCAT**	MCAT	28	malonyl-CoA-acyl carrier protein transacylase	protein_codin	0.9684	0.00685	1	8.443	0.08779
**C21orf59**	C21orf59	28	cilia and flagella associated protein 298	protein_codin	NA	NA	NA	26.48	0.2206
**CBR3**	CBR3	28	carbonyl reductase 3	protein_codin	2.249	4.61E-11	1.1E-06	52.58	0.09617
**PDXK**	PDXK	28	pyridoxal kinase	protein_codin	-0.2229	0.372	1	8.398	0.08432
**UBE2G2**	UBE2G2	28	ubiquitin conjugating enzyme E2 G2	protein_codin	-0.1110	0.447	1	6.093	0.1751
**YBEY**	YBEY	28	ybeY metalloendoribonuclease	protein_codin	0.0919	0.722	1	8.052	0.1968
**PEX10**	PEX10	28	peroxisomal biogenesis factor 10	protein_codin	0.3034	0.29	1	8.582	0.1731
**PHF13**	PHF13	29	PHD finger protein 13	protein_codin	-0.04727	0.863	1	8.096	0.08668
**CAMTA1**	CAMTA1	29	calmodulin binding transcription activator 1	protein_codin	-0.1128	0.228	1	18.45	0.1134
**CTNNBIP1**	CTNNBIP1	29	catenin beta interacting protein 1	protein_codin	0.04366	0.836	1	39.93	0.08532
**HMGCL**	HMGCL	29	3-hydroxy-3-methylglutaryl-CoA lyase	protein_codin	0.4294	0.0687	1	6.178	0.0824
**SELENON**	SELENON	29	selenoprotein N	protein_codin	-0.1380	0.433	1	15.13	0.1142
**DNAJC8**	DNAJC8	29	DnaJ heat shock protein family (Hsp40) member C8	protein_codin	-0.0790	0.398	1	29.15	0.1096
**SMIM12**	SMIM12	29	small integral membrane protein 12	protein_codin	0.1111	0.556	1	7.482	0.1666
**GCLM**	GCLM	29	glutamate-cysteine ligase modifier subunit	protein_codin	-0.454	0.0502	1	9.995	0.1404
**CERS2**	CERS2	29	ceramide synthase 2	protein_codin	-0.02680	0.856	1	14.49	0.05625
**MRPL24**	MRPL24	29	mitochondrial ribosomal protein L24	protein_codin	0.08542	0.618	1	43.93	0.1129
**EDEM3**	EDEM3	29	ER degradation enhancing alpha-mannosidase like protein 3	protein_codin	0.107	0.723	1	8.947	0.2809
**SLC30A1**	SLC30A1	29	solute carrier family 30 member 1	protein_codin	-0.6781	0.0156	1	9.204	0.3716
**LYPLAL1**	LYPLAL1	29	lysophospholipase like 1	protein_codin	0.139	0.494	1	21.85	0.1942
**EFCAB2**	EFCAB2	29	EF-hand calcium binding domain 2	protein_codin	0.09841	0.787	1	3.307	0.1351
**NFU1**	NFU1	29	NFU1 iron-sulfur cluster scaffold	protein_codin	-0.05152	0.767	1	5.082	0.05496
**GCC2**	GCC2	29	GRIP and coiled-coil domain containing 2	protein_codin	0.2011	0.111	1	14.18	0.02726
**METTL5**	METTL5	29	methyltransferase like 5	protein_codin	0.2351	0.0434	1	35.32	0.08151
**GORASP2**	GORASP2	29	golgi reassembly stacking protein 2	protein_codin	-0.09972	0.578	1	7.113	0.1484
**CAVIN2**	CAVIN2	29	caveolae associated protein 2	protein_codin	0.7827	2.91E-14	6.96E-10	52.58	0.09826
**MLPH**	MLPH	29	melanophilin	protein_codin	2.938	4.99E-08	0.001179	46.83	0.02862
**STT3B**	STT3B	29	STT3 oligosaccharyltransferase complex catalytic subunit B	protein_codin	0.05862	0.79	1	7.767	0.1664
**ELP6**	ELP6	29	elongator acetyltransferase complex subunit 6	protein_codin	-0.2272	0.233	1	5.553	0.1695
**GMPPB**	GMPPB	29	GDP-mannose pyrophosphorylase B	protein_codin	0.1256	0.693	1	3.097	0.2521
**PARP9**	PARP9	29	poly(ADP-ribose) polymerase family member 9	protein_codin	0.3379	0.0632	1	14.84	0.02788
**NUDT16**	NUDT16	29	nudix hydrolase 16	protein_codin	0.332	0.0462	1	14.13	0.1828
**UBA5**	UBA5	29	ubiquitin like modifier activating enzyme 5	protein_codin	-0.3096	0.129	1	6.384	0.1254
**GFM1**	GFM1	29	G elongation factor mitochondrial 1	protein_codin	0.7896	0.007	1	9.136	0.2812
**ICE1**	ICE1	29	interactor of little elongation complex ELL subunit 1	protein_codin	0.07704	0.704	1	19.2	0.2382
**GOLPH3**	GOLPH3	29	golgi phosphoprotein 3	protein_codin	0.2724	0.187	1	7.862	0.08235
**NIPBL**	NIPBL	29	NIPBL cohesin loading factor	protein_codin	0.2193	0.195	1	11.55	0.1107
**UTP15**	UTP15	29	UTP15 small subunit processome component	protein_codin	0.8153	0.0341	1	2.68	0.2823
**CSNK1G3**	CSNK1G3	29	casein kinase 1 gamma 3	protein_codin	-0.08364	0.635	1	7.222	0.08637
**PFDN1**	PFDN1	29	prefoldin subunit 1	protein_codin	0.2792	0.0284	1	46.68	0.03398
**NEURL1B**	NEURL1B	29	neuralized E3 ubiquitin protein ligase 1B	protein_codin	1.841	4.65E-09	0.00011	17.57	0.08376
**GMDS-AS1**	GMDS-AS1	29	GMDS divergent transcript	lincRNA	NA	NA	NA	3.4	0.1904
**FARS2**	FARS2	29	phenylalanyl-tRNA synthetase 2, mitochondrial	protein_codin	0.4507	0.137	1	2.659	0.2470
**GMPR**	GMPR	29	guanosine monophosphate reductase	protein_codin	-0.5317	0.0342	1	9.308	0.1157
**CUTA**	CUTA	29	cutA divalent cation tolerance homolog	protein_codin	-0.169	0.0940	1	37.17	0.05629
**NUDT3**	NUDT3	29	nudix hydrolase 3	protein_codin	-0.4662	0.0129	1	7.1	0.142
**PHF3**	PHF3	29	PHD finger protein 3	protein_codin	-0.2272	0.0787	1	10.29	0.1665
**TRAF3IP2**	TRAF3IP2	29	TRAF3 interacting protein 2	protein_codin	0.7856	0.0156	1	7.509	0.1101
**TAB2**	TAB2	29	TGF-beta activated kinase 1 (MAP3K7) binding protein 2	protein_codin	-0.659	0.0209	1	3.297	0.1979

**DMTF1**	DMTF1		29	cyclin D binding myb like transcription factor 1	protein_codin	0.7566	0.0317	1	3.238	0.2975
**CNPY4**	CNPY4		29	canopy FGF signaling regulator 4	protein_codin	-0.3846	0.132	1	3.552	0.2212
**CREB3L2**	CREB3L2		29	cAMP responsive element binding protein 3 like 2	protein_codin	-1.657	1.17E-15	2.8E-11	47.12	0.08595
**IGBP1**	IGBP1		29	immunoglobulin binding protein 1	protein_codin	0.3462	0.00346	1	33.49	0.07934
**VMA21**	VMA21		29	vacuolar ATPase assembly factor VMA21	protein_codin	0.3727	0.0706	1	6.62	0.05822
**GOLGA7**	GOLGA7		29	golgin A7	protein_codin	-0.17	0.162	1	10.96	0.1792
**FNTA**	FNTA		29	farnesyltransferase, CAAX box, alpha	protein_codin	0.6082	3.07E-05	0.7089	27.87	0.1402
**EMC2**	EMC2		29	ER membrane protein complex subunit 2	protein_codin	-0.02625	0.843	1	12.15	0.08882
**ACO1**	ACO1		29	aconitase 1	protein_codin	0.5776	0.0256	1	7.222	0.171
**DPM2**	DPM2		29	dolichyl-phosphate mannosyltransferase subunit 2, regulatory	protein_codin	0.497	0.00516	1	6.186	0.02687
**NACC2**	NACC2		29	NACC family member 2	protein_codin	-0.2166	0.515	1	30.82	0.175
**TMEM250**	TMEM250		29	transmembrane protein 250	protein_codin	0.1192	0.676	1	3.086	0.2329
**NOTCH1**	NOTCH1		29	notch receptor 1	protein_codin	-0.08129	0.688	1	19.58	0.1908
**C11orf74**	C11orf74		29	chromosome 11 open reading frame 74	protein_codin	0.4482	0.0322	1	20.85	0.1516
**TMEM216**	TMEM216		29	transmembrane protein 216	protein_codin	0.3541	0.202	1	7.671	0.1347
**FADS3**	FADS3		29	fatty acid desaturase 3	protein_codin	-0.9775	4.99E-07	0.01166	31	0.06279
**RAB3IL1**	RAB3IL1		29	RAB3A interacting protein like 1	protein_codin	1.477	0.000207	1	10.02	0.1739
**NAALADL1**	NAALADL1		29	N-acetylated alpha-linked acidic dipeptidase like 1	protein_codin	-0.4884	0.111	1	3.097	0.2052
**PAAF1**	PAAF1		29	proteasomal ATPase associated factor 1	protein_codin	0.401	0.17	1	8.932	0.07962
**RNF214**	RNF214		29	ring finger protein 214	protein_codin	0.3151	0.205	1	26.37	0.1651
**ARCN1**	ARCN1		29	archain 1	protein_codin	-0.2446	0.127	1	40.57	0.05348
**HSPA14**	HSPA14		29	heat shock protein family A (Hsp70) member 14	protein_codin	0.3137	0.349	1	8.687	0.1392
**ST8SIA6**	ST8SIA6		29	ST8 alpha-N-acetyl-neuraminide alpha-2,8-sialyltransferase 6	protein_codin	-1.158	2.86E-08	0.000675	16.47	0.1652
**ACBD5**	ACBD5		29	acyl-CoA binding domain containing 5	protein_codin	-0.182	0.396	1	9.152	0.1416
**ZNF22**	ZNF22		29	zinc finger protein 22	protein_codin	0.3862	0.011	1	42.5	0.142
**PGAM1**	PGAM1		29	phosphoglycerate mutase 1	protein_codin	-0.3658	0.000122	1	42.88	0.02797
**PHB2**	PHB2		29	prohibitin 2	protein_codin	0.1355	0.137	1	36.97	0.1119
**AC023157.3**	AC023157.3		29	novel transcript, antisense to AM	antisense_RN	0.5321	0.11	1	52.58	0.6001
**ATP5B**	ATP5B		29	ATP synthase F1 subunit beta	protein_codin	NA	NA	NA	40.42	0.08908
**LRIG3**	LRIG3		29	leucine rich repeats and immunoglobulin like domains 3	protein_codin	0.1442	0.55	1	52.58	0.1937
**FGD6**	FGD6		29	FYVE, RhoGEF and PH domain containing 6	protein_codin	0.9377	0.000386	1	23.12	0.1396
**VSIG10**	VSIG10		29	V-set and immunoglobulin domain containing 10	protein_codin	0.2491	0.402	1	3.142	0.1474
**RILPL1**	RILPL1		29	Rab interacting lysosomal protein like 1	protein_codin	-0.092	0.676	1	9.028	0.08836
**CRYL1**	CRYL1		29	crystallin lambda 1	protein_codin	0.5805	0.000455	1	15.42	0.02652
**SUCLA2**	SUCLA2		29	succinate-CoA ligase ADP-forming beta subunit	protein_codin	0.06171	0.786	1	8.374	0.08186
**PIBF1**	PIBF1		29	progesterone immunomodulatory binding factor 1	protein_codin	0.2559	0.162	1	19.62	0.1404
**LAMP1**	LAMP1		29	lysosomal associated membrane protein 1	protein_codin	-0.3414	0.00129	1	41.24	0.1122
**NOVA1**	NOVA1		29	NOVA alternative splicing regulator 1	protein_codin	-0.3842	0.125	1	10.06	0.2627
**SEC23A**	SEC23A		29	Sec23 homolog A, coat complex II component	protein_codin	-0.2212	0.285	1	8.902	0.2011
**VRK1**	VRK1		29	VRK serine/threonine kinase 1	protein_codin	1.04	0.00119	1	7.677	0.1174
**ZFYVE19**	ZFYVE19		29	zinc finger FYVE-type containing 19	protein_codin	0.5265	0.13	1	2.552	0.1709
**CHP1**	CHP1		29	calcineurin like EF-hand protein 1	protein_codin	0.1468	0.326	1	29.77	0.1337
**RAB8B**	RAB8B		29	RAB8B, member RAS oncogene family	protein_codin	0.6106	0.00249	1	16.68	0.1656
**HDDC3**	HDDC3		29	HD domain containing 3	protein_codin	0.7551	0.00549	1	7.687	0.1407
**FAHD1**	FAHD1		29	fumarylacetoacetate hydrolase domain containing 1	protein_codin	0.02237	0.898	1	8.255	0.151
**MLST8**	MLST8		29	MTOR associated protein, LST8 homolog	protein_codin	-0.05536	0.818	1	3.262	0.1947
**NOL3**	NOL3		29	nucleolar protein 3	protein_codin	0.07654	0.785	1	9.935	0.2187
**AARS**	AARS		29	alanyl-tRNA synthetase	protein_codin	0.007934	0.978	1	3.308	0.1442
**GSE1**	GSE1		29	Gse1 coiled-coil protein	protein_codin	-0.09022	0.737	1	48.25	0.114
**NSRP1**	NSRP1		29	nuclear speckle splicing regulatory protein 1	protein_codin	0.3965	0.000675	1	44.58	0.07897
**RNF135**	RNF135		29	ring finger protein 135	protein_codin	0.02657	0.924	1	8.696	0.3702
**ZNF830**	ZNF830		29	zinc finger protein 830	protein_codin	0.1682	0.473	1	27.42	0.195
**DNAJC7**	DNAJC7		29	DnaJ heat shock protein family (Hsp40) member C7	protein_codin	0.1505	0.248	1	37.48	0.1682
**BAIAP2**	BAIAP2		29	BAI1 associated protein 2	protein_codin	1.396	2.38E-05	0.5502	19.94	0.2568
**TUBB6**	TUBB6		29	tubulin beta 6 class V	protein_codin	0.4966	9.5E-06	0.2205	29.62	0.02722
**ZCCHC2**	ZCCHC2		29	zinc finger CCHC-type containing 2	protein_codin	0.5184	0.0604	1	8.29	0.1405
**TMX3**	TMX3		29	thioredoxin related transmembrane protein 3	protein_codin	-0.2859	0.112	1	18.92	0.02641
**FBXL12**	FBXL12		29	F-box and leucine rich repeat protein 12	protein_codin	0.3534	0.211	1	9.784	0.1075
**CCDC159**	CCDC159		29	coiled-coil domain containing 159	protein_codin	-0.0872	0.756	1	2.75	0.05688
**PRKCSH**	PRKCSH		29	protein kinase C substrate 80K-H	protein_codin	-0.3392	0.0406	1	43.96	0.1371
**USF2**	USF2		29	upstream transcription factor 2, c-fos interacting	protein_codin	-0.1388	0.367	1	42.96	0.08159
**BCAM**	BCAM		29	basal cell adhesion molecule (Lutheran blood group)	protein_codin	1.076	8.61E-27	2.08E-22	50.97	0.0281
**ATP5O**	ATP5O		29	ATP synthase peripheral stalk subunit OSCP	protein_codin	NA	NA	NA	32.28	0.08887
**SLC5A3**	SLC5A3		29	solute carrier family 5 member 3	protein_codin	-0.456	0.228	1	3.79	0.2232
**RCAN1**	RCAN1		29	regulator of calcineurin 1	protein_codin	0.2619	0.232	1	52.58	0.02745
