# Supplementary material for: Decoding the transcriptome of calcified atherosclerotic plaque at single-cell resolution
Source: Commun Biol. 2022 Oct 12;5:1084. doi: 10.1038/s42003-022-04056-7 (PMC9556750; doi:10.1038/s42003-022-04056-7)
Supplement: Supplementary file 13 — Supplementary Data 11 [file 42003_2022_4056_MOESM13_ESM.pdf]

							Contigency Table Values Used In Overlap Analysis (Fisher Exact Test)<br>Coordinate 1 - Network; Non-DE Genes = 1, DE Genes = 2<br>Coordinate 2 - Module; Non-DE Genes = 1, DE Genes = 2			
module_id	no_genes	eigen_pct_expl	cond_cor	cond_cor_pval	de_overlap_est	de_overlap_pval	cont_mat_11	cont_mat_12	cont_mat_21	cont_mat_22
30	142	11.62	0.2973	4.20E-76	3.8317	6.09E-11	6663	99	755	43
31	284	9.35	0.1772	1.40E-27	2.4812	1.22E-08	6540	222	736	62
24	177	8.42	0.2783	1.30E-66	2.3819	1.31E-05	6623	139	760	38
13	401	6.96	-0.0285	0.042	1.7982	4.01E-05	6429	333	730	68
11	250	6.38	0.0739	3.70E-06	1.9663	0.0001054	6558	204	752	46
28	15	46.45	0.0641	5.00E-05	7.4671	0.0004272	6754	8	791	7
14	23	35.35	-0.0641	5.10E-05	3.73	0.00766	6746	16	791	7
2	166	8.98	-0.0402	0.0074	1.7453	0.008006	6624	138	770	28
9	348	6.43	0.02	0.11	1.3785	0.03053	6462	300	750	48
18	422	5.07	0.0705	9.60E-06	1.1761	0.1654	6391	371	747	51
26	160	5.99	0.0743	3.30E-06	1.2159	0.243	6622	140	778	20
4	323	5.7	-0.0244	0.069	1.1009	0.3217	6476	286	761	37
12	160	5.76	7.00E-04	0.48	1.1453	0.3279	6621	141	779	19
6	245	5.99	0.0612	1.00E-04	1.0968	0.356	6545	217	770	28
29	108	6.5	0.0926	9.60E-09	0.9604	0.5971	6665	97	787	11
15	287	5.56	0.0721	6.20E-06	0.9507	0.6294	6504	258	769	29
1	167	7.53	0.0095	0.28	0.8958	0.698	6611	151	782	16
21	214	6.16	0.0291	0.039	0.8214	0.821	6567	195	779	19
20	308	5.66	0.0545	0.00048	0.7405	0.9396	6479	283	773	25
17	213	6.19	0.0342	0.019	0.6819	0.9485	6565	197	782	16
3	183	5.74	0.0228	0.084	0.6422	0.9579	6592	170	785	13
25	298	5.64	0.0603	0.00013	0.6328	0.9863	6485	277	777	21
19	206	5.7	0.0419	0.0056	0.5169	0.9942	6568	194	786	12
7	86	6.94	0.0406	0.007	0.3037	0.9959	6679	83	795	3
10	522	5.13	0.0519	0.00083	0.6101	0.9988	6276	486	762	36
5	209	5.82	0.0325	0.025	0.4186	0.9992	6563	199	788	10
27	276	5.76	0.0412	0.0062	0.4772	0.9992	6501	261	783	15
23	257	5.67	0.0416	0.0058	0.4424	0.9995	6518	244	785	13
16	226	6.24	0.0359	0.015	0.3846	0.9998	6546	216	788	10
8	407	5.46	0.0444	0.0036	0.4235	1	6375	387	778	20
22	477	5.96	0.0367	0.013	0.4912	1	6312	450	771	27
