# Supplementary material for: Decoding the transcriptome of calcified atherosclerotic plaque at single-cell resolution
Source: Commun Biol. 2022 Oct 12;5:1084. doi: 10.1038/s42003-022-04056-7 (PMC9556750; doi:10.1038/s42003-022-04056-7)
Supplement: Supplementary file 14 — Supplementary Data 12 [file 42003_2022_4056_MOESM14_ESM.pdf]

STable16.VSMCNet.Module.Genes.Rev2

gene_short_nar	gene_cards	module_i	gene_description	biotype	de_norm_e	de_pval	de_qval	coeff_va	strength
CCNL2	CCNL2	1	cyclin L2	protein_codin	0.5082	0.0036	1	7.632	0.3716
SKI	SKI	1	SKI proto-oncogene	protein_codin	0.2656	0.1	1	41.52	0.0654
LRRC47	LRRC47	1	leucine rich repeat containing 47	protein_codin	-0.04727	0.776	1	3.222	0.06656
FBXO2	FBXO2	1	F-box protein 2	protein_codin	0.6732	0.0156	1	60.59	0.2817
FOXJ3	FOXJ3	1	forkhead box J3	protein_codin	-0.02003	0.918	1	6.365	0.4985
CYP4B1	CYP4B1	1	cytochrome P450 family 4 subfamily B member 1	protein_codin	3.751	4.55E-07	0.01061	60.33	0.1317
C1orf123	C1orf123	1	CXXC motif containing zinc binding protein	protein_codin	0.0578	0.479	1	24.12	0.1388
STXBP3	STXBP3	1	syntaxin binding protein 3	protein_codin	0.2182	0.171	1	14.33	0.276
APH1A	APH1A	1	aph-1 homolog A, gamma-secretase subunit	protein_codin	-0.04911	0.66	1	11.78	0.03535
CHTOP	CHTOP	1	chromatin target of PRMT1	protein_codin	0.3385	0.0278	1	42.22	0.1412
SSR2	SSR2	1	signal sequence receptor subunit 2	protein_codin	0.06931	0.202	1	59.5	0.06847
TIPRL	TIPRL	1	TOR signaling pathway regulator	protein_codin	0.009184	0.934	1	35.38	0.02381
NME7	NME7	1	NME/NM23 family member 7	protein_codin	0.07662	0.642	1	25.86	0.07254
CACYBP	CACYBP	1	calcyclin binding protein	protein_codin	0.05162	0.531	1	25.42	0.03372
PTGS2	PTGS2	1	prostaglandin-endoperoxide synthase 2	protein_codin	0.01062	0.971	1	60.6	0.2918
PHLDA3	PHLDA3	1	pleckstrin homology like domain family A member 3	protein_codin	0.5243	7.1E-07	0.01655	42.8	0.1042
RNPEP	RNPEP	1	arginyl aminopeptidase	protein_codin	0.9011	8.78E-06	0.2026	21.06	0.1337
ADIPOR1	ADIPOR1	1	adiponectin receptor 1	protein_codin	-0.02152	0.881	1	20	0.1345
RAB29	RAB29	1	RAB29, member RAS oncogene family	protein_codin	-0.6415	0.00165	1	8.947	0.3207
MAPKAPK2	MAPKAPK2	1	MAPK activated protein kinase 2	protein_codin	-0.3629	0.0482	1	3.535	0.2777
CD46	CD46	1	CD46 molecule	protein_codin	0.2582	0.00263	1	20.79	0.1354
NTPCR	NTPCR	1	nucleoside-triphosphatase, cancer-related	protein_codin	-0.2301	0.101	1	47.93	0.06876
FH	FH	1	fumarate hydratase	protein_codin	0.389	0.0198	1	5.188	0.1661
TRAPPC12	TRAPPC12	1	trafficking protein particle complex 12	protein_codin	-0.1627	0.367	1	7.471	0.2021
ACTR2	ACTR2	1	actin related protein 2	protein_codin	-0.277	0.00158	1	27.22	0.02602
DGUOK	DGUOK	1	deoxyguanosine kinase	protein_codin	-0.2056	0.0235	1	35.58	0.03672
ATOH8	ATOH8	1	atonal bHLH transcription factor 8	protein_codin	-0.04152	0.832	1	19.27	0.2129
KDM3A	KDM3A	1	lysine demethylase 3A	protein_codin	0.3936	0.142	1	3.367	0.2384
RAB3GAP1	RAB3GAP1	1	RAB3 GTPase activating protein catalytic subunit 1	protein_codin	0.08694	0.522	1	16.1	0.06964
TTC21B	TTC21B	1	tetratricopeptide repeat domain 21B	protein_codin	0.3418	0.12	1	9.204	0.3351
AGAP1	AGAP1	1	ArfGAP with GTPase domain, ankyrin repeat and PH	protein_codin	0.4898	0.0585	1	10.29	0.4142
MKRN2	MKRN2	1	makorin ring finger protein 2	protein_codin	0.0919	0.609	1	8.573	0.06454
KAT2B	KAT2B	1	lysine acetyltransferase 2B	protein_codin	-0.3211	0.164	1	8.51	0.1916
GLB1	GLB1	1	galactosidase beta 1	protein_codin	0.7309	0.00015	1	51.93	0.168
EIF1B	EIF1B	1	eukaryotic translation initiation factor 1B	protein_codin	0.1814	0.00255	1	60.6	0.06553
CCDC51	CCDC51	1	coiled-coil domain containing 51	protein_codin	0.3494	0.0887	1	8.818	0.0379
ALAS1	ALAS1	1	5'-aminolevulinate synthase 1	protein_codin	0.009109	0.976	1	8.645	0.4966
PPM1M	PPM1M	1	protein phosphatase, Mg2+/Mn2+ dependent 1M	protein_codin	0.3369	0.195	1	9.006	0.2745
RYBP	RYBP	1	RING1 and YY1 binding protein	protein_codin	0.1346	0.532	1	8.055	0.1794
LNP1	LNP1	1	leukemia NUP98 fusion partner 1	protein_codin	-0.8559	9.07E-06	0.2092	60.6	0.2556
SMC4	SMC4	1	structural maintenance of chromosomes 4	protein_codin	-1.002	7.29E-10	1.72E-05	53.26	0.2102
ZMAT3	ZMAT3	1	zinc finger matrin-type 3	protein_codin	-0.3596	0.0177	1	40.94	0.1452
ATP11B	ATP11B	1	ATPase phospholipid transporting 11B (putative)	protein_codin	-0.1394	0.576	1	9.68	0.4502
NOA1	NOA1	1	nitric oxide associated 1	protein_codin	-0.05657	0.745	1	26.33	0.0331
SCARB2	SCARB2	1	scavenger receptor class B member 2	protein_codin	0.2558	0.0199	1	43.6	0.1029
THAP9-AS1	THAP9-AS1	1	THAP9 antisense RNA 1	antisense_RN	-0.4524	0.0218	1	9.206	0.2680
HSPA4L	HSPA4L	1	heat shock protein family A (Hsp70) member 4 like	protein_codin	0.3866	0.081	1	27.12	0.1397
AC097376.2	AC097376.2	1	novel transcript, antisense to RAB3	antisense_RN	0.308	0.217	1	10.26	0.2021
TMEM131L	TMEM131L	1	transmembrane 131 like	protein_codin	0.3588	0.109	1	3.518	0.2186
RNF180	RNF180	1	ring finger protein 180	protein_codin	-0.3918	0.0332	1	21.67	0.2648
TMEM161B-AS1	TMEM161B-AS	1	TMEM161B antisense RNA 1	antisense_RN	0.0258	0.916	1	3.122	0.109
YTHDC2	YTHDC2	1	YTH domain containing 2	protein_codin	0.2784	0.132	1	9.181	0.2184
PHAX	PHAX	1	phosphorylated adaptor for RNA export	protein_codin	0.09429	0.387	1	12.33	0.1376
UBE2B	UBE2B	1	ubiquitin conjugating enzyme E2 B	protein_codin	0.1622	0.0086	1	36.83	0.06535
SRA1	SRA1	1	steroid receptor RNA activator 1	protein_codin	-0.114	0.336	1	25.07	0.1001
CSNK1A1	CSNK1A1	1	casein kinase 1 alpha 1	protein_codin	-0.0759	0.311	1	41.66	0.06554
C1QTNF2	C1QTNF2	1	C1q and TNF related 2	protein_codin	-0.07654	0.512	1	30.02	0.09941
CCNG1	CCNG1	1	cyclin G1	protein_codin	-0.1179	0.401	1	29.66	0.2122
HCG18	HCG18	1	HLA complex group 18	antisense_RN	0.2071	0.425	1	10.46	0.4048
BRD2	BRD2	1	bromodomain containing 2	protein_codin	0.02415	0.867	1	27.44	0.1072
FBXO9	FBXO9	1	F-box protein 9	protein_codin	-0.1102	0.34	1	23.72	0.03222
FAM46A	FAM46A	1	terminal nucleotidyltransferase 5A	protein_codin	NA	NA	NA	60.6	0.1196
NUS1	NUS1	1	NUS1 dehydrodolichyl diphosphate synthase subunit	protein_codin	-0.04184	0.85	1	3.335	0.2492
PEX7	PEX7	1	peroxisomal biogenesis factor 7	protein_codin	0.004897	0.985	1	3.254	0.2849
CCDC28A	CCDC28A	1	coiled-coil domain containing 28A	protein_codin	0.4126	0.0716	1	6.592	0.1454
MRM2	MRM2	1	mitochondrial rRNA methyltransferase 2	protein_codin	-0.09382	0.612	1	29.03	0.1322
CDK13	CDK13	1	cyclin dependent kinase 13	protein_codin	0.586	0.001	1	8.13	0.03653
POR	POR	1	cytochrome p450 oxidoreductase	protein_codin	0.4534	0.00782	1	8.444	0.1649
PHTF2	PHTF2	1	putative homeodomain transcription factor 2	protein_codin	-0.0965	0.66	1	8.002	0.1447
AC058791.1	AC058791.1	1	novel transcri	lincRNA	0.5648	0.0435	1	10.52	0.5772
AKAP17A	AKAP17A	1	A-kinase anchoring protein 17A	protein_codin	0.6288	0.00418	1	8.836	0.3158
MID1IP1	MID1IP1	1	MID1 interacting protein 1	protein_codin	0.3842	0.0126	1	42.6	0.1046
CDK16	CDK16	1	cyclin dependent kinase 16	protein_codin	0.1812	0.455	1	8.829	0.3188
ARAF	ARAF	1	A-Raf proto-oncogene, serine/threonine kinase	protein_codin	0.2776	0.171	1	2.916	0.1692
WDR45	WDR45	1	WD repeat domain 45	protein_codin	-0.01302	0.895	1	41.33	0.02745
VCPIP1	VCPIP1	1	valosin containing protein interacting protein 1	protein_codin	0.08694	0.72	1	7.768	0.4014
TMEM55A	TMEM55A	1	phosphatidylinositol-4,5-bisphosphate 4-phosphatase 2	protein_codin	NA	NA	NA	10.86	0.0980
ATP6V1C1	ATP6V1C1	1	ATPase H+ transporting V1 subunit C1	protein_codin	0.1192	0.539	1	8.582	0.4186
MAF1.00	MAF1.00	1	MAF1 homolog, negative regulator of RNA polymeras	protein_codin	-0.3004	0.00018	1	33.58	0.1002
DNAJA1	DNAJA1	1	DnaJ heat shock protein family (Hsp40) member A1	protein_codin	0.2221	0.0036	1	60.6	0.0695
TESK1	TESK1	1	testis associated actin remodelling kinase 1	protein_codin	-0.2712	0.122	1	7.411	0.1712
MAMDC2	MAMDC2	1	MAM domain containing 2	protein_codin	0.8536	0.00016	1	49.94	0.1014
HSDL2	HSDL2	1	hydroxysteroid dehydrogenase like 2	protein_codin	0.3246	0.012	1	53.92	0.2334
CDC26	CDC26	1	cell division cycle 26	protein_codin	0.01362	0.906	1	25.84	0.07431
PHPT1	PHPT1	1	phosphohistidine phosphatase 1	protein_codin	-0.2711	6.93E-07	0.01615	33.64	0.07435
EPS8L2	EPS8L2	1	EPS8 like 2	protein_codin	0.7842	0.00229	1	8.557	0.1702
CAPRIN1	CAPRIN1	1	cell cycle associated protein 1	protein_codin	0.2923	0.0177	1	33.01	0.1692
EXT2	EXT2	1	exostosin glycosyltransferase 2	protein_codin	0.09616	0.572	1	23.06	0.06852
MDK	MDK	1	midkine	protein_codin	-0.03944	0.802	1	42.84	0.1354
DDB2	DDB2	1	damage specific DNA binding protein 2	protein_codin	-0.08555	0.612	1	31.02	0.3869
SSRP1	SSRP1	1	structure specific recognition protein 1	protein_codin	0.09032	0.686	1	7.568	0.2415
FAM111A	FAM111A	1	family with sequence similarity 111 member A	protein_codin	0.2552	0.209	1	59.26	0.1766
RAB3IL1	RAB3IL1	1	RAB3A interacting protein like 1	protein_codin	0.7948	3.24E-06	0.07502	8.482	0.1375
DNAJC4	DNAJC4	1	DnaJ heat shock protein family (Hsp40) member C4	protein_codin	-0.1182	0.391	1	37.74	0.1759
SYVN1	SYVN1	1	synoviolin 1	protein_codin	0.1484	0.505	1	2.917	0.2476
CDC42EP2	CDC42EP2	1	CDC42 effector protein 2	protein_codin	2.383	5.18E-14	1.24E-09	37.95	0.03304
SIPA1	SIPA1	1	signal-induced proliferation-associated 1	protein_codin	0.1229	0.614	1	3.262	0.148
NOX4	NOX4	1	NADPH oxidase 4	protein_codin	2.211	4.17E-11	9.89E-07	36.45	0.1407
PGR	PGR	1	progesterone receptor	protein_codin	-0.6272	0.00078	1	9.279	0.1779
FEZ1	FEZ1	1	fasciculation and elongation protein zeta 1	protein_codin	-0.004582	0.958	1	31.25	0.03418
GTPBP4	GTPBP4	1	GTP binding protein 4	protein_codin	0.09374	0.557	1	18.49	0.06853
GDI2	GDI2	1	GDP dissociation inhibitor 2	protein_codin	0.1298	0.0869	1	32.07	0.03297
SIRT1	SIRT1	1	sirtuin 1	protein_codin	0.7467	0.00279	1	9.029	0.2048
FBXL15	FBXL15	1	F-box and leucine rich repeat protein 15	protein_codin	-0.1918	0.177	1	14.87	0.1419
UROS	UROS	1	uroporphyrinogen III synthase	protein_codin	-0.1298	0.396	1	21.45	0.136
LTBR	LTBR	1	lymphotoxin beta receptor	protein_codin	0.4702	0.00326	1	40.17	0.1026
CBX5	CBX5	1	chromobox 5	protein_codin	0.1425	0.21	1	20.41	0.03495
ITGA5	ITGA5	1	integrin subunit alpha 5	protein_codin	0.8482	0.00400	1	41.44	0.1995
LEMD3	LEMD3	1	LEM domain containing 3	protein_codin	0.2192	0.32	1	7.636	0.1854
MDM2	MDM2	1	MDM2 proto-oncogene	protein_codin	-0.1724	0.299	1	38.31	0.2136
CPSF6	CPSF6	1	cleavage and polyadenylation specific factor 6	protein_codin	-0.09427	0.591	1	7.77	0.2475
KITLG	KITLG	1	KIT ligand	protein_codin	0.3002	0.0832	1	28.96	0.03412
BTG1	BTG1	1	BTG anti-proliferation factor 1	protein_codin	0.1203	0.12	1	60.6	0.03196
UBE2N	UBE2N	1	ubiquitin conjugating enzyme E2 N	protein_codin	-0.1546	0.101	1	22.8	0.1002
ALDH2	ALDH2	1	aldehyde dehydrogenase 2 family member	protein_codin	0.8228	1.09E-31	2.63E-27	40.4	0.03249
SPART	SPART	1	spartin	protein_codin	0.07987	0.402	1	19.85	0.1057
AKAP11	AKAP11	1	A-kinase anchoring protein 11	protein_codin	0.09994	0.572	1	18.06	0.07117
DIS3	DIS3	1	DIS3 homolog, exosome endoribonuclease and 3'-5' e	protein_codin	-0.2718	0.0866	1	19.2	0.3674
RAB2B	RAB2B	1	RAB2B, member RAS oncogene family	protein_codin	0.2842	0.16	1	7.284	0.1694
BAG5	BAG5	1	BCL2 associated athanogene 5	protein_codin	0.4417	0.0006	1	31.93	0.03282
AVEN	AVEN	1	apoptosis and caspase activation inhibitor	protein_codin	-0.3772	0.0302	1	52.96	0.1387
RSL24D1	RSL24D1	1	ribosomal L24 domain containing 1	protein_codin	-0.3453	8.83E-08	0.00207	57.01	0.1062
PIGBOS1	PIGBOS1	1	PIGB opposite strand 1	protein_codin	0.1352	0.381	1	8.098	0.1050
RPS27L	RPS27L	1	ribosomal protein S27 like	protein_codin	-0.4509	2.75E-24	6.62E-20	50.5	0.2332
ANP32A	ANP32A	1	acidic nuclear phosphoprotein 32 family member A	protein_codin	-0.1766	0.104	1	24.87	0.06701
HDGFL3	HDGFL3	1	HDGF like 3	protein_codin	-0.3414	0.00038	1	48.28	0.1024
POLR3K	POLR3K	1	RNA polymerase III subunit K	protein_codin	-0.2164	0.222	1	3.535	0.2438
DNAJA3	DNAJA3	1	DnaJ heat shock protein family (Hsp40) member A3	protein_codin	-0.04952	0.881	1	3.12	0.219
USP7	USP7	1	ubiquitin specific peptidase 7	protein_codin	0.1238	0.359	1	16.37	0.1032
LCMT1	LCMT1	1	leucine carboxyl methyltransferase 1	protein_codin	-0.3162	0.04	1	18.69	0.06630
NSMCE1	NSMCE1	1	NSE1 homolog, SMC5-SMC6 complex component	protein_codin	-0.02012	0.85	1	22.38	0.03427
KAT8	KAT8	1	lysine acetyltransferase 8	protein_codin	0.2499	0.101	1	7.992	0.2056
ZNF267	ZNF267	1	zinc finger protein 267	protein_codin	0.4562	0.0934	1	11.53	0.4109
SIAH1	SIAH1	1	siah E3 ubiquitin protein ligase 1	protein_codin	0.1648	0.252	1	21.84	0.1672
INPP5K	INPP5K	1	inositol polyphosphate-5-phosphatase K	protein_codin	0.1142	0.629	1	8.333	0.06986
TSR1	TSR1	1	TSR1 ribosome maturation factor	protein_codin	0.05629	0.81	1	9.679	0.03380
COPRS	COPRS	1	coordinator of PRMT5 and differentiation stimulator	protein_codin	-0.7276	1.4E-22	3.37E-18	60.47	0.1359
ITGA3	ITGA3	1	integrin subunit alpha 3	protein_codin	-1.095	8.68E-10	2.05E-05	24.26	0.2230
MTMR4	MTMR4	1	myotubularin related protein 4	protein_codin	0.2162	0.414	1	2.694	0.309
HELZ	HELZ	1	helicase with zinc finger	protein_codin	0.7759	0.00112	1	9.282	0.3092
PSMD12	PSMD12	1	proteasome 26S subunit, non-ATPase 12	protein_codin	0.1854	0.0671	1	10.62	0.06448
NOL11	NOL11	1	nucleolar protein 11	protein_codin	0.1367	0.481	1	3.403	0.2
INO80C	INO80C	1	INO80 complex subunit C	protein_codin	-0.02255	0.911	1	3.624	0.175
RPRD1B	RPRD1B	1	regulation of nuclear pre-mRNA domain containing 1B	protein_codin	0.1288	0.602	1	3.002	0.3156
TOP1.00	TOP1.00	1	DNA topoisomerase I	protein_codin	-0.1066	0.295	1	15.52	0.3021
STAU1	STAU1	1	staufen double-stranded RNA binding protein 1	protein_codin	-0.00346	0.966	1	48.03	0.0646
POLR2E	POLR2E	1	RNA polymerase II subunit E	protein_codin	-0.04807	0.544	1	30.28	0.1011
SLC25A23	SLC25A23	1	solute carrier family 25 member 23	protein_codin	-0.1831	0.325	1	6.916	0.2852
KANK2	KANK2	1	KN motif and ankyrin repeat domains 2	protein_codin	-0.3468	0.00035	1	44.74	0.2109
GADD45GIP1	GADD45GIP1	1	GADD45G interacting protein 1	protein_codin	-0.3898	5.91E-09	0.00014	59.52	0.03294
FXYD1	FXYD1	1	FXYD domain containing ion transport regulator 1	protein_codin	-0.4753	2.59E-19	6.22E-15	60.6	0.03411
EXOSC5	EXOSC5	1	exosome component 5	protein_codin	-0.4081	0.0229	1	6.619	0.1030
RPS19	RPS19	1	ribosomal protein S19	protein_codin	-0.6259	2.81E-62	6.82E-58	60.6	0.1134
SMG9	SMG9	1	SMG9 nonsense mediated mRNA decay factor	protein_codin	0.6366	0.00432	1	3.132	0.1406
TOMM40	TOMM40	1	translocase of outer mitochondrial membrane 40	protein_codin	0.193	0.226	1	8.082	0.06946
DACT3	DACT3	1	dishevelled binding antagonist of beta catenin 3	protein_codin	-0.8175	5.22E-09	0.00012	31.41	0.2416
ARHGAP35	ARHGAP35	1	Rho GTPase activating protein 35	protein_codin	0.1271	0.536	1	9.532	0.2030
HSD17B14	HSD17B14	1	hydroxysteroid 17-beta dehydrogenase 14	protein_codin	0.3626	0.187	1	11.92	0.1719
BAX	BAX	1	BCL2 associated X, apoptosis regulator	protein_codin	-0.5187	8.63E-08	0.00202	60.45	0.114
LENG9	LENG9	1	leukocyte receptor cluster member 9	protein_codin	0.354	0.177	1	50.02	0.1011
U2AF2	U2AF2	1	U2 small nuclear RNA auxiliary factor 2	protein_codin	0.2299	0.238	1	6.834	0.1687
ZNF444	ZNF444	1	zinc finger protein 444	protein_codin	0.07321	0.752	1	2.519	0.2697
UFD1	UFD1	1	ubiquitin recognition factor in ER associated degradat	protein_codin	-0.2739	0.00449	1	18.03	0.1374
RANBP1	RANBP1	1	RAN binding protein 1	protein_codin	-0.05682	0.518	1	42.58	0.03300
MPST	MPST	1	mercaptopyruvate sulfurtransferase	protein_codin	0.4348	0.00235	1	15.53	0.1032
SMDT1	SMDT1	1	single-pass membrane protein with aspartate rich tail 1	protein_codin	-0.3117	1.08E-05	0.2488	27.68	0.102
BTG3	BTG3	1	BTG anti-proliferation factor 3	protein_codin	0.418	0.00040	1	52.76	0.1722
FAAP20	FAAP20	2	FA core complex associated protein 20	protein_codin	-0.1841	0.101	1	40	0.1326
UBR4	UBR4	2	ubiquitin protein ligase E3 component n-recognin 4	protein_codin	0.1796	0.322	1	26.88	0.2140
MINOS1	MINOS1	2	mitochondrial contact site and cristae organizing syst	protein_codin	-0.3356	4.08E-06	0.09426	42.74	0.06574
GPBP1L1	GPBP1L1	2	GC-rich promoter binding protein 1 like 1	protein_codin	-0.1552	0.381	1	6.598	0.372
NEXN	NEXN	2	nexilin F-actin binding protein	protein_codin	-0.7031	5.21E-19	1.25E-14	60.6	0.06961
HIST2H2AC	HIST2H2AC	2	histone cluster 2 H2A family member c	protein_codin	0.3765	0.117	1	9.918	0.2885
ARNT	ARNT	2	aryl hydrocarbon receptor nuclear translocator	protein_codin	0.4001	0.0998	1	3.245	0.1372
EDEM3	EDEM3	2	ER degradation enhancing alpha-mannosidase like p	protein_codin	0.5259	0.00798	1	8.985	0.2115
CSRP1	CSRP1	2	cysteine and glycine rich protein 1	protein_codin	-0.5897	2.79E-20	6.7E-16	60	0.1591
COA6	COA6	2	cytochrome c oxidase assembly factor 6	protein_codin	0.165	0.251	1	44.42	0.1374
NOL10	NOL10	2	nucleolar protein 10	protein_codin	-0.07796	0.712	1	7.744	0.2127
OSR1	OSR1	2	odd-skipped related transcription factor 1	protein_codin	1.129	1.5E-12	3.57E-08	60.52	0.101
PREB	PREB	2	prolactin regulatory element binding	protein_codin	0.2136	0.349	1	3.474	0.1697
MPV17	MPV17	2	mitochondrial inner membrane protein MPV17	protein_codin	0.005721	0.966	1	15.85	0.1002
THUMPD2	THUMPD2	2	THUMP domain containing 2	protein_codin	-0.232	0.129	1	58.7	0.2272
EFEMP1	EFEMP1	2	EGF containing fibulin extracellular matrix protein 1	protein_codin	0.3602	5.49E-08	0.00129	60.6	0.02544
ACTG2	ACTG2	2	actin gamma 2, smooth muscle	protein_codin	-0.1666	0.279	1	60.6	0.352
NMI	NMI	2	N-myc and STAT interactor	protein_codin	-0.172	0.371	1	8.882	0.1792
TANC1	TANC1	2	tetratricopeptide repeat, ankyrin repeat and coiled-coi	protein_codin	-0.4239	0.0629	1	32.74	0.4540
SSFA2	SSFA2	2	ITPR interacting domain containing 2	protein_codin	-0.5358	0.0010	1	20.63	0.03258
TRAF3IP1	TRAF3IP1	2	TRAF3 interacting protein 1	protein_codin	-0.2645	0.126	1	23.96	0.2399
LMCD1	LMCD1	2	LIM and cysteine rich domains 1	protein_codin	-0.4112	3.57E-06	0.08262	60	0.07342
ARPC4	ARPC4	2	actin related protein 2/3 complex subunit 4	protein_codin	-0.3622	5.16E-05	1	51.09	0.02527
FYCO1	FYCO1	2	FYVE and coiled-coil domain containing 1	protein_codin	-0.2395	0.2	1	9.436	0.2711
TEX264	TEX264	2	testis expressed 264, ER-phagy receptor	protein_codin	0.149	0.226	1	15.3	0.137
SELENOK	SELENOK	2	selenoprotein K	protein_codin	0.02346	0.709	1	38.44	0.1040
ZNF148	ZNF148	2	zinc finger protein 148	protein_codin	-0.01522	0.9	1	59.68	0.2504
ACKR4	ACKR4	2	atypical chemokine receptor 4	protein_codin	1.355	7.26E-12	1.73E-08	22.55	0.1711
ARHGEF26	ARHGEF26	2	Rho guanine nucleotide exchange factor 26	protein_codin	-0.4097	0.0485	1	9.937	0.2416
PTX3	PTX3	2	pentraxin 3	protein_codin	0.0934	0.781	1	60.6	0.4495
EIF5A2	EIF5A2	2	eukaryotic translation initiation factor 5A2	protein_codin	-0.185	0.372	1	27.42	0.2636
DNAJB11	DNAJB11	2	DnaJ heat shock protein family (Hsp40) member B11	protein_codin	-0.08542	0.629	1	39.96	0.1049
CCDC50	CCDC50	2	coiled-coil domain containing 50	protein_codin	0.2238	0.0534	1	10.76	0.2030
PPP1R2	PPP1R2	2	protein phosphatase 1 regulatory inhibitor subunit 2	protein_codin	0.2721	0.00697	1	53.27	0.1322
OCIAD2	OCIAD2	2	OCIA domain containing 2	protein_codin	-1.52	6.4E-19	1.54E-14	52.58	0.2814
MTHFD2L	MTHFD2L	2	methylenetetrahydrofolate dehydrogenase (NADP+ d	protein_codin	-0.04844	0.825	1	3.129	0.2935
MRPS18C	MRPS18C	2	mitochondrial ribosomal protein S18C	protein_codin	-0.4718	6.84E-08	0.0016	19.29	0.0676
CASP6	CASP6	2	caspase 6	protein_codin	0.1497	0.532	1	8.155	0.068
MYOZ2	MYOZ2	2	myozenin 2	protein_codin	-0.6893	0.00537	1	60.6	0.2936
MGARP	MGARP	2	mitochondria localized glutamic acid rich protein	protein_codin	0.6826	0.0332	1	48.03	0.0785
CCDC127	CCDC127	2	coiled-coil domain containing 127	protein_codin	0.322	0.0432	1	8.168	0.1002
CMBL	CMBL	2	carboxymethylenebutenolidase homolog	protein_codin	0.328	0.0164	1	58.64	0.1061
NPR3.00	NPR3.00	2	natriuretic peptide receptor 3	protein_codin	1.269	7.24E-05	1	60.6	0.1922
NIPBL	NIPBL	2	NIPBL cohesin loading factor	protein_codin	0.1938	0.154	1	12.68	0.3102
WDR70	WDR70	2	WD repeat domain 70	protein_codin	0.05125	0.826	1	8.636	0.1387
XRCC4	XRCC4	2	X-ray repair cross complementing 4	protein_codin	-0.4426	0.111	1	59.41	0.2592
ETF1	ETF1	2	eukaryotic translation termination factor 1	protein_codin	0.07349	0.615	1	16.82	0.14
CYFIP2	CYFIP2	2	cytoplasmic FMR1 interacting protein 2	protein_codin	-1.084	1.85E-06	0.04292	59.68	0.06592
SLU7	SLU7	2	SLU7 homolog, splicing factor	protein_codin	-0.143	0.186	1	27.58	0.1676
RARS	RARS	2	arginyl-tRNA synthetase	protein_codin	-0.2109	0.3	1	5.982	0.1762
CPEB4	CPEB4	2	cytoplasmic polyadenylation element binding protein 4	protein_codin	-0.3762	0.0300	1	52.76	0.2388
KIAA1191	KIAA1191	2	KIAA1191	protein_codin	0.03289	0.857	1	7.687	0.06959
PSMG4	PSMG4	2	proteasome assembly chaperone 4	protein_codin	-0.1637	0.212	1	17.87	0.2114
NRN1	NRN1	2	neuritin 1	protein_codin	-1.592	1.54E-05	0.3543	59.76	0.5091
MCUR1	MCUR1	2	mitochondrial calcium uniporter regulator 1	protein_codin	-0.2766	0.022	1	28.88	0.1367
SCUBE3	SCUBE3	2	signal peptide, CUB domain and EGF like domain co	protein_codin	-0.2651	0.212	1	49.92	0.2059
ELOVL5	ELOVL5	2	ELOVL fatty acid elongase 5	protein_codin	0.3968	0.03	1	23.83	0.2734
COL21A1	COL21A1	2	collagen type XXI alpha 1 chain	protein_codin	-0.8812	8.67E-15	2.07E-10	60.58	0.06496
LRP11	LRP11	2	LDL receptor related protein 11	protein_codin	0.1264	0.638	1	8.164	0.2412
RNASET2	RNASET2	2	ribonuclease T2	protein_codin	0.03029	0.85	1	60.58	0.6301
HDAC9	HDAC9	2	histone deacetylase 9	protein_codin	-1.077	5.84E-08	0.00137	7.785	0.2149
SUGCT	SUGCT	2	succinyl-CoA:glutarate-CoA transferase	protein_codin	-1.813	7.07E-39	1.71E-34	59.34	0.04232
MRPS33	MRPS33	2	mitochondrial ribosomal protein S33	protein_codin	-0.3062	0.0217	1	30.5	0.143
XG	XG	2	Xg glycoprotein (Xg blood group)	protein_codin	1.378	5.25E-09	0.00012	39.86	0.1446
PIR	PIR	2	pirin	protein_codin	0.2718	0.092	1	60.6	0.317
DLG3	DLG3	2	discs large MAGUK scaffold protein 3	protein_codin	-0.4852	0.00837	1	8.776	0.2719
RTL8B	RTL8B	2	retrotransposon Gag like 8B	protein_codin	-0.2001	0.242	1	8.118	0.2092
ASAH1	ASAH1	2	N-acylsphingosine amidohydrolase 1	protein_codin	0.2298	0.00590	1	60.3	0.1358
ZFAND1	ZFAND1	2	zinc finger AN1-type containing 1	protein_codin	-0.1919	0.0585	1	19.08	0.1344
GEM	GEM	2	GTP binding protein overexpressed in skeletal muscle	protein_codin	-0.3855	3.5E-06	0.08102	59.94	0.06578
NOV	NOV	2	cellular communication network factor 3	protein_codin	-0.06696	0.529	1	60.54	0.214
HAUS6	HAUS6	2	HAUS augmin like complex subunit 6	protein_codin	-0.1196	0.552	1	8.136	0.1714
TOPORS	TOPORS	2	TOP1 binding arginine/serine rich protein	protein_codin	0.1763	0.255	1	15.77	0.1742
TPM2	TPM2	2	tropomyosin 2	protein_codin	-0.7445	5.61E-37	1.36E-32	Inf	0.1837
NDUFA8	NDUFA8	2	NADH:ubiquinone oxidoreductase subunit A8	protein_codin	-0.2298	0.0264	1	30.16	0.06452
SLC2A8	SLC2A8	2	solute carrier family 2 member 8	protein_codin	0.3288	0.2	1	9.053	0.2992
C9orf78	C9orf78	2	chromosome 9 open reading frame 78	protein_codin	-0.05802	0.531	1	31.97	0.09872
ASS1	ASS1	2	argininosuccinate synthase 1	protein_codin	-1.135	3.39E-10	8.02E-06	40.56	0.1105
AIF1L	AIF1L	2	allograft inflammatory factor 1 like	protein_codin	0.7931	0.0020	1	11.09	0.1097
RABL6	RABL6	2	RAB, member RAS oncogene family like 6	protein_codin	-0.2997	0.0384	1	51.92	0.1969
CHID1	CHID1	2	chitinase domain containing 1	protein_codin	0.1277	0.24	1	35.07	0.06634
MRPL23	MRPL23	2	mitochondrial ribosomal protein L23	protein_codin	-0.4663	4.3E-05	0.9837	14.56	0.09788
SERGEF	SERGEF	2	secretion regulating guanine nucleotide exchange factor	protein_codin	-0.4381	0.0150	1	20.03	0.1006
BDNF	BDNF	2	brain derived neurotrophic factor	protein_codin	0.5214	0.212	1	42.58	0.5132
NDUFS3	NDUFS3	2	NADH:ubiquinone oxidoreductase core subunit S3	protein_codin	-0.01109	0.912	1	45.3	0.1337
UBXN1	UBXN1	2	UBX domain protein 1	protein_codin	-0.3964	8.24E-10	1.95E-05	54.7	0.03541
DPF2	DPF2	2	double PHD fingers 2	protein_codin	0.06101	0.719	1	27.54	0.1384
CCDC85B	CCDC85B	2	coiled-coil domain containing 85B	protein_codin	-0.3303	4.27E-08	0.001	60.6	0.0698
NADSYN1	NADSYN1	2	NAD synthetase 1	protein_codin	0.07474	0.79	1	2.621	0.2426
ANAPC15	ANAPC15	2	anaphase promoting complex subunit 15	protein_codin	-0.2293	0.158	1	7.379	0.2432
TAGLN	TAGLN	2	transgelin	protein_codin	-0.6685	1.43E-36	3.46E-32	NA	0.149
HYOU1	HYOU1	2	hypoxia up-regulated 1	protein_codin	-0.02796	0.912	1	9.922	0.2851
BEND7	BEND7	2	BEN domain containing 7	protein_codin	0.1362	0.58	1	10.16	0.1837
FAM107B	FAM107B	2	family with sequence similarity 107 member B	protein_codin	-0.1125	0.517	1	54.75	0.06852
HSPA14	HSPA14	2	heat shock protein family A (Hsp70) member 14	protein_codin	0.3382	0.259	1	3.689	0.1681
BICC1	BICC1	2	BicC family RNA binding protein 1	protein_codin	1.3	2.67E-22	6.42E-18	35.74	0.07112
ACTA2	ACTA2	2	actin alpha 2, smooth muscle	protein_codin	-0.7314	3.72E-30	8.98E-26	NA	0.1509
FAS	FAS	2	Fas cell surface death receptor	protein_codin	-0.09294	0.52	1	54.82	0.2516
PCGF5	PCGF5	2	polycomb group ring finger 5	protein_codin	-0.4102	0.00036	1	60.6	0.1426
KAZALD1	KAZALD1	2	Kazal type serine peptidase inhibitor domain 1	protein_codin	0.08395	0.659	1	20.54	0.07026
SH3PXD2A	SH3PXD2A	2	SH3 and PX domains 2A	protein_codin	0.3028	0.22	1	7.279	0.1325
FAM160B1	FAM160B1	2	family with sequence similarity 160 member B1	protein_codin	0.2721	0.212	1	9.895	0.3380
ZNF511	ZNF511	2	zinc finger protein 511	protein_codin	-0.1537	0.25	1	18.16	0.1357
CLEC2B	CLEC2B	2	C-type lectin domain family 2 member B	protein_codin	0.4501	0.0852	1	60.45	0.2264
H2AFJ	H2AFJ	2	H2A histone family member J	protein_codin	0.03329	0.562	1	59.19	0.03247
BCDIN3D	BCDIN3D	2	BCDIN3 domain containing RNA methyltransferase	protein_codin	0.5887	0.0232	1	3.196	0.2520
KRT7	KRT7	2	keratin 7	protein_codin	0.2227	0.42	1	54.86	0.3549
KRT18	KRT18	2	keratin 18	protein_codin	-0.2148	0.489	1	60.6	0.4092
ITGA7	ITGA7	2	integrin subunit alpha 7	protein_codin	-0.3153	0.0434	1	30.48	0.2879
MYL6	MYL6	2	myosin light chain 6	protein_codin	-0.4004	1.2E-24	2.89E-20	60.6	0.03563
RBMS2	RBMS2	2	RNA binding motif single stranded interacting protein 2	protein_codin	0.4294	0.0937	1	10.15	0.1350
DDIT3	DDIT3	2	DNA damage inducible transcript 3	protein_codin	0.4066	0.00188	1	37.62	0.17
TSFM	TSFM	2	Ts translation elongation factor, mitochondrial	protein_codin	-0.277	0.104	1	8.14	0.1004
ARL1.00	ARL1.00	2	ADP ribosylation factor like GTPase 1	protein_codin	0.1926	0.0218	1	60.02	0.0331
C12orf49	C12orf49	2	chromosome 12 open reading frame 49	protein_codin	0.6852	0.0025	1	10.53	0.1695
ZNF664	ZNF664	2	zinc finger protein 664	protein_codin	0.2982	0.189	1	3.312	0.1345
NUP58	NUP58	2	nucleoporin 58	protein_codin	0.2086	0.404	1	10.26	0.2808
KPNA3	KPNA3	2	karyopherin subunit alpha 3	protein_codin	0.2671	0.102	1	20.53	0.07585
IPO5	IPO5	2	importin 5	protein_codin	0.419	0.00552	1	19.96	0.2112
ITGBL1	ITGBL1	2	integrin subunit beta like 1	protein_codin	0.3376	0.00017	1	55.36	0.02515
TEX30	TEX30	2	testis expressed 30	protein_codin	0.5087	0.0352	1	3.096	0.2055
NAXD	NAXD	2	NAD(P)HX dehydratase	protein_codin	-0.02582	0.904	1	3.245	0.1375
THTPA	THTPA	2	thiamine triphosphatase	protein_codin	-0.1383	0.441	1	3.227	0.1017
MBIP	MBIP	2	MAP3K12 binding inhibitory protein 1	protein_codin	0.09017	0.542	1	7.521	0.2392
FBXO33	FBXO33	2	F-box protein 33	protein_codin	-0.01016	0.966	1	9.309	0.3192
PSMC1	PSMC1	2	proteasome 26S subunit, ATPase 1	protein_codin	0.02972	0.751	1	58.29	0.06786
TC2N	TC2N	2	tandem C2 domains, nuclear	protein_codin	-0.585	0.0157	1	60.53	0.4186
PPP2R5C	PPP2R5C	2	protein phosphatase 2 regulatory subunit B'gamma	protein_codin	0.1369	0.335	1	15.46	0.2382
ZNF770	ZNF770	2	zinc finger protein 770	protein_codin	0.4966	0.00536	1	7.567	0.305
EIF2AK4	EIF2AK4	2	eukaryotic translation initiation factor 2 alpha kinase 4	protein_codin	0.0868	0.544	1	7.334	0.208
TPM1	TPM1	2	tropomyosin 1	protein_codin	-0.392	9.84E-15	2.35E-10	60.6	0.03959
PHKB	PHKB	2	phosphorylase kinase regulatory subunit beta	protein_codin	0.3289	0.0414	1	8.056	0.07062
LONP2	LONP2	2	lon peptidase 2, peroxisomal	protein_codin	0.2090	0.142	1	6.75	0.1682
WFDC1	WFDC1	2	WAP four-disulfide core domain 1	protein_codin	-0.5531	0.0005	1	40.68	0.08352
SMG6	SMG6	2	SMG6 nonsense mediated mRNA decay factor	protein_codin	0.1746	0.327	1	20.45	0.2367
EMC6	EMC6	2	ER membrane protein complex subunit 6	protein_codin	-0.1387	0.235	1	15.27	0.02334
UBE2G1	UBE2G1	2	ubiquitin conjugating enzyme E2 G1	protein_codin	-0.154	0.332	1	24.01	0.07082
NLRP1	NLRP1	2	NLR family pyrin domain containing 1	protein_codin	0.09405	0.629	1	60.58	0.2381
TNFAIP1	TNFAIP1	2	TNF alpha induced protein 1	protein_codin	-0.1313	0.562	1	7.908	0.1626
PIGS	PIGS	2	phosphatidylinositol glycan anchor biosynthesis class S	protein_codin	-0.07264	0.811	1	3.386	0.2889
NUFIP2	NUFIP2	2	nuclear FMR1 interacting protein 2	protein_codin	0.3282	0.0250	1	30.16	0.1426
WIPF2	WIPF2	2	WAS/WASL interacting protein family member 2	protein_codin	0.115	0.647	1	7.921	0.213
KRT17	KRT17	2	keratin 17	protein_codin	-1.934	4.57E-14	1.09E-09	60.6	0.2702
KANSL1-AS1	KANSL1-AS1	2	KANSL1 antisense RNA 1	antisense_RN	-0.1295	0.352	1	19.42	0.1037
ERN1.00	ERN1.00	2	endoplasmic reticulum to nucleus signaling 1	protein_codin	0.4232	0.118	1	10.15	0.1792
FDXR	FDXR	2	ferredoxin reductase	protein_codin	-0.3345	0.104	1	8.79	0.2984
MYL12A	MYL12A	2	myosin light chain 12A	protein_codin	-0.349	2.22E-14	5.29E-10	60.6	0.08648
MYL12B	MYL12B	2	myosin light chain 12B	protein_codin	-0.1363	0.00040	1	60.6	0.05255

RNF24	RNF24	2	ring finger protein 24	protein_codin	-0.5794	0.00082	1	48.8	0.1075
MKKS	MKKS	2	McKusick-Kaufman syndrome	protein_codin	-0.05617	0.768	1	8.686	0.1774
KIZ	KIZ	2	kizuna centrosomal protein	protein_codin	0.1096	0.521	1	5.97	0.09805
MYL9	MYL9	2	myosin light chain 9	protein_codin	-0.5454	2.01E-22	4.84E-19	Inf	0.07686
SLPI	SLPI	2	secretory leukocyte peptidase inhibitor	protein_codin	2.348	1.18E-14	2.82E-10	Inf	0.1681
B4GALT5	B4GALT5	2	beta-1,4-galactosyltransferase 5	protein_codin	0.5165	0.0569	1	8.468	0.06636
UCKL1	UCKL1	2	uridine-cytidine kinase 1 like 1	protein_codin	0.2132	0.289	1	3.02	0.2002
GAMT	GAMT	2	guanidinoacetate N-methyltransferase	protein_codin	-0.1339	0.249	1	37.93	0.03235
CNN1	CNN1	2	calponin 1	protein_codin	-0.3246	0.00175	1	60.6	0.1934
CRLF1	CRLF1	2	cytokine receptor like factor 1	protein_codin	-0.9836	8.12E-05	1	60.58	0.2005
ZNF181	ZNF181	2	zinc finger protein 181	protein_codin	0.0009882	0.997	1	9.316	0.1682
PPP1R14A	PPP1R14A	2	protein phosphatase 1 regulatory inhibitor subunit 14A	protein_codin	-0.8257	1.35E-32	3.26E-29	60.54	0.06802
FBXO17	FBXO17	2	F-box protein 17	protein_codin	0.5001	0.00131	1	39.96	0.03731
PLEKHA4	PLEKHA4	2	pleckstrin homology domain containing A4	protein_codin	0.06896	0.624	1	13.92	0.1724
TOM1	TOM1	2	target of myb1 membrane trafficking protein	protein_codin	0.1892	0.371	1	3.452	0.1675
MRPS6	MRPS6	2	mitochondrial ribosomal protein S6	protein_codin	-0.4332	1.87E-06	0.04326	40.88	0.07156
SLC5A3	SLC5A3	2	solute carrier family 5 member 3	protein_codin	-0.5833	0.0232	1	9.025	0.4629
SH3BGR	SH3BGR	2	SH3 domain binding glutamate rich protein	protein_codin	-1.074	1.43E-17	3.42E-13	59.65	0.06818
SAMD11	SAMD11	3	sterile alpha motif domain containing 11	protein_codin	0.4611	0.049	1	26.86	0.2762
SDF4	SDF4	3	stromal cell derived factor 4	protein_codin	-0.09214	0.449	1	37.8	0.0235
TPRG1L	TPRG1L	3	tumor protein p63 regulated 1 like	protein_codin	0.1603	0.29	1	23.18	0.1017
S100PBP	S100PBP	3	S100P binding protein	protein_codin	0.2004	0.438	1	3.36	0.3689
FNDC5	FNDC5	3	fibronectin type III domain containing 5	protein_codin	0.01605	0.952	1	11.62	0.2124
MIGA1	MIGA1	3	mitoguardin 1	protein_codin	0.4067	0.00702	1	7.399	0.3104
DPYD	DPYD	3	dihydropyrimidine dehydrogenase	protein_codin	0.7432	0.00048	1	8.232	0.2884
PALMD	PALMD	3	palmdelphin	protein_codin	0.418	0.141	1	29.65	0.2692
SLC25A24	SLC25A24	3	solute carrier family 25 member 24	protein_codin	0.1892	0.237	1	44.79	0.1695
SARS	SARS	3	seryl-tRNA synthetase	protein_codin	-0.004058	0.969	1	35.03	0.06798
CTTNBP2NL	CTTNBP2NL	3	CTTNBP2 N-terminal like	protein_codin	-0.03028	0.856	1	23.95	0.2166
THEM4	THEM4	3	thioesterase superfamily member 4	protein_codin	0.3002	0.27	1	7.971	0.2438
ILF2	ILF2	3	interleukin enhancer binding factor 2	protein_codin	-0.1626	0.0677	1	28.11	0.06806
HAX1	HAX1	3	HCLS1 associated protein X-1	protein_codin	-0.2804	0.00041	1	28.95	0.1011
FDPS	FDPS	3	farnesyl diphosphate synthase	protein_codin	-0.08543	0.41	1	55.22	0.1135
UFC1	UFC1	3	ubiquitin-fold modifier conjugating enzyme 1	protein_codin	-0.4668	4.64E-10	1.1E-05	36.88	0.07302
ANGPTL1	ANGPTL1	3	angiopoietin like 1	protein_codin	0.8697	7.88E-10	1.86E-05	59.45	0.07027
SERTAD4-AS1	SERTAD4-AS1	3	SERTAD4 antisense RNA 1	antisense_RN	-0.7319	9.88E-09	0.00022	30.29	0.2105
TLR5	TLR5	3	toll like receptor 5	protein_codin	2.043	1.66E-08	0.00036	11.14	0.2396
TSNAX	TSNAX	3	translin associated factor X	protein_codin	0.202	0.109	1	18.11	0.1015
IAH1	IAH1	3	isoamyl acetate hydrolyzing esterase 1 (putative)	protein_codin	0.1156	0.212	1	45.38	0.09875
KCNS3	KCNS3	3	potassium voltage-gated channel modifier subfamily S	protein_codin	-0.7449	0.00015	1	11.01	0.3268
YPEL5	YPEL5	3	yippee like 5	protein_codin	-0.2949	0.00277	1	58.61	0.1042
SOS1	SOS1	3	SOS Ras/Rac guanine nucleotide exchange factor 1	protein_codin	-0.722	2.87E-07	0.0067	15.57	0.1741
CCDC88A	CCDC88A	3	coiled-coil domain containing 88A	protein_codin	-0.4278	0.00928	1	33.42	0.2424
SERTAD2	SERTAD2	3	SERTA domain containing 2	protein_codin	-0.1027	0.629	1	7.953	0.1019
TEX41	TEX41	3	testis expressed 41	lincRNA	-0.4909	0.00446	1	22.15	0.2131
MMADHC	MMADHC	3	metabolism of cobalamin associated D	protein_codin	-0.3105	0.00175	1	22.35	0.03326
TANK	TANK	3	TRAF family member associated NFKB activator	protein_codin	-0.1944	0.146	1	16.06	0.1437
STK39	STK39	3	serine/threonine kinase 39	protein_codin	0.3109	0.309	1	35.06	0.2435
SLC25A12	SLC25A12	3	solute carrier family 25 member 12	protein_codin	0.1068	0.577	1	3.454	0.1992
CWC22	CWC22	3	CWC22 spliceosome associated protein homolog	protein_codin	0.1752	0.16	1	60.19	0.201
SLC40A1	SLC40A1	3	solute carrier family 40 member 1	protein_codin	-0.2487	0.0249	1	60.59	0.1017
TMEM237	TMEM237	3	transmembrane protein 237	protein_codin	0.6098	0.00010	1	17.54	0.07112
NBEAL1	NBEAL1	3	neurobeachin like 1	protein_codin	0.00959	0.936	1	23.8	0.07251
FASTKD2	FASTKD2	3	FAST kinase domains 2	protein_codin	0.2422	0.227	1	6.774	0.1365
KCNE4	KCNE4	3	potassium voltage-gated channel subfamily E regulat	protein_codin	0.3369	0.0119	1	40.64	0.06604
NCL	NCL	3	nucleolin	protein_codin	-0.06358	0.209	1	52.94	0.03299
EXOG	EXOG	3	exo/endonuclease G	protein_codin	0.5408	0.0226	1	3.147	0.2058
DAG1	DAG1	3	dystroglycan 1	protein_codin	0.0873	0.64	1	7.394	0.1397
PXK	PXK	3	PX domain containing serine/threonine kinase like	protein_codin	0.5687	0.00011	1	34.12	0.03209
TMEM45A	TMEM45A	3	transmembrane protein 45A	protein_codin	0.1484	0.245	1	60.6	0.03209
CD200	CD200	3	CD200 molecule	protein_codin	-0.4555	0.00013	1	60.58	0.0709
CCDC58	CCDC58	3	coiled-coil domain containing 58	protein_codin	0.09354	0.812	1	3.432	0.2217
SEC61A1	SEC61A1	3	SEC61 translocon alpha 1 subunit	protein_codin	0.2601	0.122	1	20.56	0.07111
ATP2C1	ATP2C1	3	ATPase secretory pathway Ca2+ transporting 1	protein_codin	0.07254	0.739	1	7.576	0.2435
PCCB	PCCB	3	propionyl-CoA carboxylase subunit beta	protein_codin	0.1289	0.519	1	3.417	0.1389
AGTR1	AGTR1	3	angiotensin II receptor type 1	protein_codin	1.891	9.88E-10	2.33E-05	27.93	0.2522
EIF2B5	EIF2B5	3	eukaryotic translation initiation factor 2B subunit epsilon	protein_codin	0.6448	0.00250	1	8.323	0.1067
MAGEF1	MAGEF1	3	MAGE family member F1	protein_codin	-0.0543	0.687	1	6.388	0.2071
C4orf48	C4orf48	3	chromosome 4 open reading frame 48	protein_codin	-0.9259	3.73E-05	0.8539	34.42	0.06537
C1QTNF7	C1QTNF7	3	C1q and TNF related 7	protein_codin	0.2308	0.211	1	30.79	0.1076
SRP72	SRP72	3	signal recognition particle 72	protein_codin	0.01262	0.882	1	24	0.03234
ABRAXAS1	ABRAXAS1	3	abraxas 1, BRCA1 A complex subunit	protein_codin	0.2393	0.136	1	8.383	0.3061
HPF1	HPF1	3	histone PARylation factor 1	protein_codin	-0.1206	0.241	1	11.57	0.1882
BDP1	BDP1	3	B double prime 1, subunit of RNA polymerase III tran	protein_codin	-0.003262	0.98	1	59.76	0.2238
MRPS27	MRPS27	3	mitochondrial ribosomal protein S27	protein_codin	-0.1484	0.367	1	8.97	0.2779
SLC25A46	SLC25A46	3	solute carrier family 25 member 46	protein_codin	0.4185	0.0453	1	9.705	0.1339
CDC42SE2	CDC42SE2	3	CDC42 small effector 2	protein_codin	-0.1172	0.559	1	3.573	0.1862
HNRNPA0	HNRNPA0	3	heterogeneous nuclear ribonucleoprotein A0	protein_codin	0.1987	0.0111	1	39.27	0.06952
HARS	HARS	3	histidyl-tRNA synthetase	protein_codin	-0.2306	0.0971	1	49.43	0.1359
CCDC69	CCDC69	3	coiled-coil domain containing 69	protein_codin	1.389	3.84E-09	9.05E-05	24.2	0.2086
TBC1D9B	TBC1D9B	3	TBC1 domain family member 9B	protein_codin	0.3497	0.122	1	7.443	0.1386
SSR1	SSR1	3	signal sequence receptor subunit 1	protein_codin	0.1064	0.335	1	49.34	0.1003
SLC35B3	SLC35B3	3	solute carrier family 35 member B3	protein_codin	0.2617	0.27	1	3.448	0.09892
HIVEP1	HIVEP1	3	human immunodeficiency virus type I enhancer bindin	protein_codin	0.4283	0.0214	1	19.32	0.06621
EDN1	EDN1	3	endothelin 1	protein_codin	-0.9169	0.00687	1	60.6	0.7135
ZNF322	ZNF322	3	zinc finger protein 322	protein_codin	0.1274	0.336	1	14.63	0.1684
BEND6	BEND6	3	BEN domain containing 6	protein_codin	-0.3692	0.174	1	29.58	0.1505
HECA	HECA	3	hdc homolog, cell cycle regulator	protein_codin	-0.3073	0.152	1	3.177	0.06636
TCP1	TCP1	3	t-complex 1	protein_codin	0.0882	0.379	1	29.58	0.2077
PDGFA	PDGFA	3	platelet derived growth factor subunit A	protein_codin	-0.5436	2.72E-09	6.41E-05	54.8	0.03474
TRA2A	TRA2A	3	transformer 2 alpha homolog	protein_codin	0.2078	0.105	1	13.05	0.1688
RCC1L	RCC1L	3	RCC1 like	protein_codin	-0.02555	0.875	1	8.43	0.1035
CBLL1	CBLL1	3	Cbl proto-oncogene like 1	protein_codin	-0.0739	0.757	1	3.134	0.3177
EXOC4	EXOC4	3	exocyst complex component 4	protein_codin	0.3427	0.0733	1	27.88	0.09972
PAXIP1-AS1	PAXIP1-AS1	3	PAXIP1 antisense RNA 1 (head to head)	lincRNA	-0.01550	0.952	1	8.412	0.2508
AIFM1	AIFM1	3	apoptosis inducing factor mitochondria associated 1	protein_codin	0.5055	0.0802	1	8.482	0.2068
MTUS1	MTUS1	3	microtubule associated scaffold protein 1	protein_codin	0.1106	0.298	1	60.57	0.2909
CHMP7	CHMP7	3	charged multivesicular body protein 7	protein_codin	-0.1521	0.429	1	2.543	0.1368
PPP2CB	PPP2CB	3	protein phosphatase 2 catalytic subunit beta	protein_codin	0.03124	0.752	1	23.17	0.07755
SLC20A2	SLC20A2	3	solute carrier family 20 member 2	protein_codin	-0.1179	0.547	1	27.44	0.1885
ASPH	ASPH	3	aspartate beta-hydroxylase	protein_codin	0.4682	5.52E-09	0.00012	32.96	0.0352
LRRCC1	LRRCC1	3	leucine rich repeat and coiled-coil centrosomal protein 1	protein_codin	-0.1269	0.482	1	41.02	0.1667
AZIN1	AZIN1	3	antizyme inhibitor 1	protein_codin	0.2798	0.0986	1	8.712	0.1025
PLAA	PLAA	3	phospholipase A2 activating protein	protein_codin	0.4983	0.0376	1	3.113	0.2048
NDUFB6	NDUFB6	3	NADH:ubiquinone oxidoreductase subunit B6	protein_codin	-0.09183	0.22	1	26.59	0.2081
RNF20	RNF20	3	ring finger protein 20	protein_codin	-0.1159	0.432	1	20.33	0.0682
NIPSNAP3A	NIPSNAP3A	3	nipsnap homolog 3A	protein_codin	-0.1602	0.336	1	52.39	0.1866
PRPF4	PRPF4	3	pre-mRNA processing factor 4	protein_codin	0.08882	0.667	1	3.311	0.2079
AK1	AK1	3	adenylate kinase 1	protein_codin	0.03407	0.864	1	25.29	0.1994
COQ4	COQ4	3	coenzyme Q4	protein_codin	0.3521	0.0177	1	7.217	0.1359
FANCF	FANCF	3	FA complementation group F	protein_codin	-0.1175	0.632	1	2.963	0.1398
PDHX	PDHX	3	pyruvate dehydrogenase complex component X	protein_codin	0.05232	0.828	1	3.214	0.1422
CKAP5	CKAP5	3	cytoskeleton associated protein 5	protein_codin	-0.004799	0.979	1	7.348	0.2582
MED19	MED19	3	mediator complex subunit 19	protein_codin	0.1621	0.289	1	15.84	0.1344
CAPN1	CAPN1	3	calpain 1	protein_codin	0.206	0.139	1	7.405	0.1384
UNC93B1	UNC93B1	3	unc-93 homolog B1, TLR signaling regulator	protein_codin	0.3615	0.151	1	8.078	0.2109
MRPL21	MRPL21	3	mitochondrial ribosomal protein L21	protein_codin	-0.2868	0.00290	1	16.09	0.09848
PGM2L1	PGM2L1	3	phosphoglucomutase 2 like 1	protein_codin	-1.102	9.36E-09	0.00022	58.93	0.1232
MTMR2	MTMR2	3	myotubularin related protein 2	protein_codin	-0.2419	0.202	1	6.888	0.3824
SNX19	SNX19	3	sorting nexin 19	protein_codin	0.2102	0.498	1	3.209	0.1901
FBXO18	FBXO18	3	F-box DNA helicase 1	protein_codin	NA	NA	NA	7.806	0.1368
APBB1IP	APBB1IP	3	amyloid beta precursor protein binding family B memb	protein_codin	2.971	2.44E-07	0.0057	39.29	0.06725
HK1	HK1	3	hexokinase 1	protein_codin	-0.5693	0.00103	1	21.09	0.09729
ANXA7	ANXA7	3	annexin A7	protein_codin	-0.08403	0.246	1	59.29	0.0720
TSPAN14	TSPAN14	3	tetraspanin 14	protein_codin	0.3506	0.19	1	2.923	0.6514
COX15	COX15	3	cytochrome c oxidase assembly homolog COX15	protein_codin	0.3372	0.193	1	2.761	0.3086
NFKB2	NFKB2	3	nuclear factor kappa B subunit 2	protein_codin	-0.4021	0.0806	1	10.56	0.2482
ARL3.00	ARL3.00	3	ADP ribosylation factor like GTPase 3	protein_codin	-0.1071	0.192	1	57.4	0.03242
WBP1L	WBP1L	3	WW domain binding protein 1 like	protein_codin	0.1674	0.371	1	8.437	0.0668
ATE1	ATE1	3	arginyltransferase 1	protein_codin	0.3115	0.126	1	6.625	0.1787
CACNA1C	CACNA1C	3	calcium voltage-gated channel subunit alpha1 C	protein_codin	-0.5203	0.144	1	3.55	0.7664
FAM60A	FAM60A	3	SIN3-HDAC complex associated factor	protein_codin	NA	NA	NA	34.52	0.2890
BICD1	BICD1	3	BICD cargo adaptor 1	protein_codin	0.06661	0.79	1	54.39	0.3525
PRKAG1	PRKAG1	3	protein kinase AMP-activated non-catalytic subunit g	protein_codin	0.005404	0.972	1	7.311	0.17
COX14	COX14	3	cytochrome c oxidase assembly factor COX14	protein_codin	-0.2602	0.0009	1	24.95	0.03942
ACVRL1	ACVRL1	3	activin A receptor like type 1	protein_codin	1.668	4.81E-12	1.14E-07	43.54	0.06702
UBE3B	UBE3B	3	ubiquitin protein ligase E3B	protein_codin	-0.02955	0.895	1	2.966	0.322
HECTD4	HECTD4	3	HECT domain E3 ubiquitin protein ligase 4	protein_codin	-0.01290	0.962	1	3.301	0.4288
ZNF605	ZNF605	3	zinc finger protein 605	protein_codin	-0.04882	0.832	1	8.036	0.4495
ZNF84	ZNF84	3	zinc finger protein 84	protein_codin	0.1429	0.533	1	3.349	0.2988
COG6	COG6	3	component of oligomeric golgi complex 6	protein_codin	0.1384	0.516	1	9.377	0.1370
WBP4	WBP4	3	WW domain binding protein 4	protein_codin	0.3292	0.0094	1	19.88	0.1335
DCAF11	DCAF11	3	DDB1 and CUL4 associated factor 11	protein_codin	-0.1195	0.572	1	7.996	0.1764
SEC23A	SEC23A	3	Sec23 homolog A, coat complex II component	protein_codin	0.3475	0.0224	1	33.34	0.06625
MAP4K5	MAP4K5	3	mitogen-activated protein kinase kinase kinase kinase 5	protein_codin	0.1272	0.359	1	15.16	0.3572
ATL1	ATL1	3	atlastin GTPase 1	protein_codin	0.2323	0.136	1	49.53	0.1339
ATG14	ATG14	3	autophagy related 14	protein_codin	0.2849	0.169	1	8.349	0.2145
PSMA3-AS1	PSMA3-AS1	3	PSMA3 antisense RNA 1	antisense_RN	0.0321	0.869	1	20.33	0.282
VTI1B	VTI1B	3	vesicle transport through interaction with t-SNAREs 1B	protein_codin	0.2284	0.0229	1	22.53	0.1012
PGF	PGF	3	placental growth factor	protein_codin	-0.2839	0.0217	1	60.56	0.07076
DICER1	DICER1	3	dicer 1, ribonuclease III	protein_codin	0.169	0.341	1	20.66	0.2407
EHD4	EHD4	3	EH domain containing 4	protein_codin	-0.5905	0.011	1	26.34	0.1345
TMOD2	TMOD2	3	tropomodulin 2	protein_codin	1.623	1.05E-09	2.48E-05	8.303	0.3238
ZNF280D	ZNF280D	3	zinc finger protein 280D	protein_codin	0.1971	0.213	1	23.59	0.1762
POLR2M	POLR2M	3	RNA polymerase II subunit M	protein_codin	0.149	0.417	1	9.868	0.171
CRTC3	CRTC3	3	CREB regulated transcription coactivator 3	protein_codin	0.1298	0.472	1	6.054	0.3081
UBE2I	UBE2I	3	ubiquitin conjugating enzyme E2 I	protein_codin	0.03462	0.699	1	35.96	0.07141
SNHG19	SNHG19	3	small nucleolar RNA host gene 19	lincRNA	0.132	0.504	1	8.365	0.2394
ZNF205	ZNF205	3	zinc finger protein 205	protein_codin	0.389	0.137	1	8.143	0.4035
GSPT1	GSPT1	3	G1 to S phase transition 1	protein_codin	-0.01682	0.871	1	11.57	0.03264
SNX29	SNX29	3	sorting nexin 29	protein_codin	0.2045	0.324	1	6.558	0.3492
RRN3	RRN3	3	RRN3 homolog, RNA polymerase I transcription facto	protein_codin	0.1901	0.452	1	3.078	0.3874
C16orf62	C16orf62	3	VPS35 endosomal protein sorting factor like	protein_codin	NA	NA	NA	3.187	0.5913
MAZ	MAZ	3	MYC associated zinc finger protein	protein_codin	-0.1118	0.359	1	17	0.13
PPP4C	PPP4C	3	protein phosphatase 4 catalytic subunit	protein_codin	0.02462	0.849	1	14.3	0.06532
AKTIP	AKTIP	3	AKT interacting protein	protein_codin	0.1597	0.262	1	7.081	0.1350
CIAPIN1	CIAPIN1	3	cytokine induced apoptosis inhibitor 1	protein_codin	0.2208	0.251	1	8.652	0.1721
GLG1	GLG1	3	golgi glycoprotein 1	protein_codin	0.3737	0.00016	1	32.37	0.06607
DEF8	DEF8	3	differentially expressed in FDCP 8 homolog	protein_codin	-0.1888	0.194	1	17.98	0.2452
SSH2	SSH2	3	slingshot protein phosphatase 2	protein_codin	0.2074	0.462	1	3.283	0.3267
AP2B1	AP2B1	3	adaptor related protein complex 2 subunit beta 1	protein_codin	0.4216	0.0149	1	8.088	0.1387
PCGF2	PCGF2	3	polycomb group ring finger 2	protein_codin	0.1214	0.497	1	8.32	0.06937
CASC3	CASC3	3	CASC3 exon junction complex subunit	protein_codin	0.01582	0.926	1	8.195	0.3179
STAT5A	STAT5A	3	signal transducer and activator of transcription 5A	protein_codin	0.1083	0.652	1	2.916	0.2742
COASY	COASY	3	Coenzyme A synthase	protein_codin	0.3785	0.0562	1	7.862	0.1779
COA3	COA3	3	cytochrome c oxidase assembly factor 3	protein_codin	-0.114	0.197	1	20.03	0.03519
DUSP3	DUSP3	3	dual specificity phosphatase 3	protein_codin	-0.3703	0.0262	1	24.84	0.1684
APPBP2	APPBP2	3	amyloid beta precursor protein binding protein 2	protein_codin	0.574	0.00985	1	22.26	0.1082
ICAM2	ICAM2	3	intercellular adhesion molecule 2	protein_codin	-0.2075	0.482	1	28.68	0.1382
UNK	UNK	3	unk zinc finger	protein_codin	-0.287	0.175	1	2.79	0.2944
TWSG1	TWSG1	3	twisted gastrulation BMP signaling modulator 1	protein_codin	-0.2977	0.009	1	26.53	0.2229
GNAL	GNAL	3	G protein subunit alpha L	protein_codin	-0.006302	0.975	1	8.611	0.06937
CENPB	CENPB	3	centromere protein B	protein_codin	-0.4462	0.0115	1	23.5	0.2532
UBE2V1	UBE2V1	3	ubiquitin conjugating enzyme E2 V1	protein_codin	-0.1157	0.615	1	2.758	0.2177
NPEPL1	NPEPL1	3	aminopeptidase like 1	protein_codin	0.3532	0.101	1	9.608	0.2054
SF3A2	SF3A2	3	splicing factor 3a subunit 2	protein_codin	-0.07026	0.739	1	2.811	0.2426
AES	AES	3	TLE family member 5, transcriptional modulator	protein_codin	-0.1207	0.098	1	29.53	0.1680
KHSRP	KHSRP	3	KH-type splicing regulatory protein	protein_codin	-0.02034	0.92	1	8.043	0.2796
PET100	PET100	3	PET100 cytochrome c oxidase chaperone	protein_codin	-0.1816	0.0995	1	13.85	0.128
TIMM44	TIMM44	3	translocase of inner mitochondrial membrane 44	protein_codin	0.1627	0.347	1	7.196	0.1680
MAP1S	MAP1S	3	microtubule associated protein 1S	protein_codin	0.1842	0.557	1	2.373	0.2786
CCDC8	CCDC8	3	coiled-coil domain containing 8	protein_codin	-0.04844	0.827	1	3.212	0.2869
ZNF480	ZNF480	3	zinc finger protein 480	protein_codin	0.265	0.364	1	8.63	0.1775
A1BG	A1BG	3	alpha-1-B glycoprotein	protein_codin	-0.5563	0.00089	1	19.69	0.2421
TRIM28	TRIM28	3	tripartite motif containing 28	protein_codin	-0.09419	0.472	1	17.43	0.07093
TTC28	TTC28	3	tetratricopeptide repeat domain 28	protein_codin	0.2245	0.365	1	7.797	0.4132
PIK3IP1	PIK3IP1	3	phosphoinositide-3-kinase interacting protein 1	protein_codin	0.1158	0.413	1	17.46	0.1380
HMOX1	HMOX1	3	heme oxygenase 1	protein_codin	-0.4051	0.142	1	59.96	0.04213
NDUFA6	NDUFA6	3	NADH:ubiquinone oxidoreductase subunit A6	protein_codin	-0.1876	0.0262	1	16.48	0.1108
UBE2G2	UBE2G2	3	ubiquitin conjugating enzyme E2 G2	protein_codin	-0.09895	0.466	1	15.35	0.1782
PRMT2	PRMT2	3	protein arginine methyltransferase 2	protein_codin	-0.3973	5.99E-06	0.1383	45.69	0.1080
AURKAIP1	AURKAIP1	4	aurora kinase A interacting protein 1	protein_codin	-0.1913	0.0186	1	18.13	0.03527
TARDBP	TARDBP	4	TAR DNA binding protein	protein_codin	0.02221	0.887	1	3.292	0.1412
CDC42	CDC42	4	cell division cycle 42	protein_codin	0.0103	0.846	1	48.96	0.03459
TMEM234	TMEM234	4	transmembrane protein 234	protein_codin	0.423	0.0801	1	10.3	0.2400
SYNC	SYNC	4	syncoilin, intermediate filament protein	protein_codin	-0.2395	0.156	1	7.325	0.2707
SFPQ	SFPQ	4	splicing factor proline and glutamine rich	protein_codin	0.4216	3.07E-06	0.07112	25.7	0.09825
C1orf109	C1orf109	4	chromosome 1 open reading frame 109	protein_codin	-0.03349	0.866	1	3	0.1726
SMAP2	SMAP2	4	small ArfGAP2	protein_codin	0.02436	0.913	1	59.98	0.1972
CITED4	CITED4	4	Cbp/p300 interacting transactivator with Glu/Asp rich	protein_codin	0.4696	0.00216	1	35.7	0.1368
LURAP1	LURAP1	4	leucine rich adaptor protein 1	protein_codin	-0.01435	0.952	1	8.814	0.167
PATJ	PATJ	4	PATJ crumbs cell polarity complex component	protein_codin	-0.7486	0.00376	1	3.393	0.317
PGM1	PGM1	4	phosphoglucomutase 1	protein_codin	-0.4794	0.00459	1	45.24	0.1370
CRYZ	CRYZ	4	crystallin zeta	protein_codin	0.3668	0.0851	1	3.379	0.1660
PKN2	PKN2	4	protein kinase N2	protein_codin	0.3553	0.00132	1	26.57	0.3060
ATP1A1	ATP1A1	4	ATPase Na+/K+ transporting subunit alpha 1	protein_codin	0.397	0.00229	1	27.71	0.1009
ITGA10	ITGA10	4	integrin subunit alpha 10	protein_codin	-1.207	2.47E-14	5.89E-10	40.07	0.07262
PEX11B	PEX11B	4	peroxisomal biogenesis factor 11 beta	protein_codin	0.5182	0.101	1	3.345	0.3814
POLR3GL	POLR3GL	4	RNA polymerase III subunit G like	protein_codin	-0.154	0.162	1	11.4	0.1462
NBPF14	NBPF14	4	NBPF member 14	protein_codin	0.2499	0.304	1	8.527	0.2542
SELENBP1	SELENBP1	4	selenium binding protein 1	protein_codin	0.3547	0.00045	1	17.1	0.1062
ADAM15	ADAM15	4	ADAM metallopeptidase domain 15	protein_codin	1.023	4.35E-06	0.1006	53.4	0.1295
ARHGEF2	ARHGEF2	4	Rho/Rac guanine nucleotide exchange factor 2	protein_codin	0.1137	0.684	1	6.756	0.3902
NAXE	NAXE	4	NAD(P)HX epimerase	protein_codin	0.2538	0.014	1	51.92	0.1791
HDGF	HDGF	4	heparin binding growth factor	protein_codin	-0.1874	0.0962	1	53.07	0.2421
SFT2D2	SFT2D2	4	SFT2 domain containing 2	protein_codin	0.4255	0.0182	1	20.3	0.1699
GORAB	GORAB	4	golgin, RAB6 interacting	protein_codin	0.1409	0.46	1	8.599	0.178
LMOD1	LMOD1	4	leiomodin 1	protein_codin	-0.9279	2.25E-41	5.45E-37	60.6	0.04435
PPP1R12B	PPP1R12B	4	protein phosphatase 1 regulatory subunit 12B	protein_codin	-0.7735	7.64E-11	1.81E-06	58.71	0.1130
SLC41A1	SLC41A1	4	solute carrier family 41 member 1	protein_codin	-0.5951	0.00341	1	3.167	0.308
FBXO28	FBXO28	4	F-box protein 28	protein_codin	-0.6181	0.0184	1	2.901	0.669
LBR	LBR	4	lamin B receptor	protein_codin	0.5752	0.00212	1	8.222	0.2138
ARF1	ARF1	4	ADP ribosylation factor 1	protein_codin	-0.1534	0.00232	1	59.2	0.09762
AGT	AGT	4	angiotensinogen	protein_codin	-0.2239	0.079	1	60.6	0.1806
COLEC11	COLEC11	4	collectin subfamily member 11	protein_codin	-1.327	2.92E-07	0.00682	54.3	0.2415
YWHAQ	YWHAQ	4	tyrosine 3-monooxygenase/tryptophan 5-monooxyge	protein_codin	0.02425	0.702	1	40.1	0.03246
NBAS	NBAS	4	neuroblastoma amplified sequence	protein_codin	0.1545	0.362	1	6.332	0.1231
EMILIN1	EMILIN1	4	elastin microfibril interfacer 1	protein_codin	-0.7754	9.71E-13	2.31E-08	59.92	0.1102
ZNF512	ZNF512	4	zinc finger protein 512	protein_codin	-0.6696	0.0007	1	3.43	0.1982
FOSL2	FOSL2	4	FOS like 2, AP-1 transcription factor subunit	protein_codin	0.7407	1.82E-05	0.4183	21.7	0.1035
GPATCH11	GPATCH11	4	G-patch domain containing 11	protein_codin	0.1142	0.459	1	20.06	0.06817
FOXN2	FOXN2	4	forkhead box N2	protein_codin	-0.008619	0.961	1	9.132	0.3479
SPTBN1	SPTBN1	4	spectrin beta, non-erythrocytic 1	protein_codin	0.5582	1.64E-12	3.9E-08	44.97	0.03400
VRK2	VRK2	4	VRK serine/threonine kinase 2	protein_codin	-0.05439	0.796	1	3.452	0.1752
FANCL	FANCL	4	FA complementation group L	protein_codin	-0.07612	0.699	1	3.458	0.2019
CEP68	CEP68	4	centrosomal protein 68	protein_codin	-0.2572	0.211	1	8.056	0.1639
PDCL3	PDCL3	4	phosducin like 3	protein_codin	-0.2642	0.0486	1	17.53	0.1362
ST6GAL2	ST6GAL2	4	ST6 beta-galactoside alpha-2,6-sialyltransferase 2	protein_codin	-1.569	2.03E-11	4.82E-07	10.95	0.2796
DARS	DARS	4	aspartyl-tRNA synthetase	protein_codin	-0.03836	0.756	1	12.54	0.1351
KLHL23	KLHL23	4	kelch like family member 23	protein_codin	0.8879	2.35E-05	0.5284	24.63	0.1802
SSB	SSB	4	small RNA binding exonuclease protection factor La	protein_codin	-0.09837	0.1045	1	60.14	0.1231
METAP1D	METAP1D	4	methionyl aminopeptidase type 1D, mitochondrial	protein_codin	1.275	0.00036	1	8.511	0.5736
SLC39A10	SLC39A10	4	solute carrier family 39 member 10	protein_codin	0.4907	0.0105	1	21.4	0.2736
CYP20A1	CYP20A1	4	cytochrome P450 family 20 subfamily A member 1	protein_codin	0.1006	0.354	1	29.03	0.1327
SPEG	SPEG	4	striated muscle enriched protein kinase	protein_codin	-0.8638	5.62E-05	1	36.07	0.3701
COL4A4	COL4A4	4	collagen type IV alpha 4 chain	protein_codin	0.4493	0.0817	1	41.09	0.2766
GIGYF2	GIGYF2	4	GRB10 interacting GYF protein 2	protein_codin	-0.09121	0.639	1	19.83	0.2574
SETMAR	SETMAR	4	SET domain and mariner transposase fusion gene	protein_codin	0.1893	0.47	1	10.82	0.1672
TTLL3	TTLL3	4	tubulin tyrosine ligase like 3	protein_codin	0.4749	0.0871	1	10.59	0.06936
UBP1	UBP1	4	upstream binding protein 1	protein_codin	0.03329	0.884	1	2.207	0.2452
PDCD6IP	PDCD6IP	4	programmed cell death 6 interacting protein	protein_codin	0.4837	0.00034	1	12.86	0.06441
LRRFIP2	LRRFIP2	4	LRR binding FLII interacting protein 2	protein_codin	-0.4969	4.18E-07	0.00975	51.73	0.1402
P4HTM	P4HTM	4	prolyl 4-hydroxylase, transmembrane	protein_codin	0.02267	0.865	1	40.58	0.128
TWF2	TWF2	4	twinfilin actin binding protein 2	protein_codin	-0.1653	0.221	1	37.63	0.07259
CLDND1	CLDND1	4	claudin domain containing 1	protein_codin	0.3344	0.00064	1	21.03	0.0684
MYLK	MYLK	4	myosin light chain kinase	protein_codin	-0.9447	1.86E-28	4.49E-24	60.53	0.06932
KALRN	KALRN	4	kalirin RhoGEF kinase	protein_codin	-1.118	4.97E-05	1	25.48	0.3096
ABTB1	ABTB1	4	ankyrin repeat and BTB domain containing 1	protein_codin	0.1216	0.401	1	37.29	0.1064
NPHP3	NPHP3	4	nephrocystin 3	protein_codin	0.3277	0.197	1	3.183	0.2907
SRPRB	SRPRB	4	SRP receptor subunit beta	protein_codin	0.16	0.369	1	51.79	0.03852
MRPS22	MRPS22	4	mitochondrial ribosomal protein S22	protein_codin	0.01882	0.887	1	47.88	0.09865
KCNAB1	KCNAB1	4	potassium voltage-gated channel subfamily A membe	protein_codin	0.3706	0.0126	1	59.6	0.06769
AP2M1	AP2M1	4	adaptor related protein complex 2 subunit mu 1	protein_codin	-0.208	0.004	1	59.1	0.06869
EIF4G1	EIF4G1	4	eukaryotic translation initiation factor 4 gamma 1	protein_codin	0.183	0.285	1	16.86	0.203
MAP3K13	MAP3K13	4	mitogen-activated protein kinase kinase kinase 13	protein_codin	-0.1616	0.238	1	55.67	0.1385
TMEM41A	TMEM41A	4	transmembrane protein 41A	protein_codin	0.4253	0.0252	1	7.823	0.1650
SENP2	SENP2	4	SUMO specific peptidase 2	protein_codin	0.3758	0.0717	1	33.77	0.1651
LPP	LPP	4	LIM domain containing preferred translocation partne	protein_codin	-0.6815	8.67E-20	2.08E-15	59.88	0.1097
SENP5	SENP5	4	SUMO specific peptidase 5	protein_codin	-0.0086	0.965	1	26.08	0.2096
ADRA2C	ADRA2C	4	adrenoceptor alpha 2C	protein_codin	-1.606	1.47E-14	3.51E-10	22.02	0.488
WFS1	WFS1	4	wolframin ER transmembrane glycoprotein	protein_codin	-0.2584	0.212	1	9.423	0.2350
PCDH7	PCDH7	4	protocadherin 7	protein_codin	-1.391	1.82E-37	4.41E-33	47.92	0.1079
KLHL5	KLHL5	4	kelch like family member 5	protein_codin	-0.4542	0.0493	1	8.209	0.1757
FRYL	FRYL	4	FRY like transcription coactivator	protein_codin	-0.01542	0.942	1	22.83	0.3468
MRPL1	MRPL1	4	mitochondrial ribosomal protein L1	protein_codin	-0.07426	0.633	1	6.859	0.2059
SPARCL1	SPARCL1	4	SPARC like 1	protein_codin	-0.5983	3.31E-22	7.96E-18	NA	0.09068
CISD2	CISD2	4	CDGSH iron sulfur domain 2	protein_codin	0.1488	0.299	1	43.76	0.2376
NPNT	NPNT	4	nephronectin	protein_codin	-0.8497	4.23E-06	0.09784	18.5	0.435
ANKRD50	ANKRD50	4	ankyrin repeat domain 50	protein_codin	-0.0004347	0.998	1	10.14	0.2147
TRIM2	TRIM2	4	tripartite motif containing 2	protein_codin	0.6268	0.00300	1	7.182	0.3045
GUCY1A3	GUCY1A3	4	guanylate cyclase 1 soluble subunit alpha 1	protein_codin	NA	NA	NA	60.6	0.06645
PDGFC	PDGFC	4	platelet derived growth factor C	protein_codin	-1.003	3.65E-07	0.00852	26.44	0.1905
GRIA2	GRIA2	4	glutamate ionotropic receptor AMPA type subunit 2	protein_codin	-2.478	6.9E-11	1.64E-06	50.68	0.7982
DCTD	DCTD	4	dCMP deaminase	protein_codin	0.001200	0.993	1	46.79	0.03205
RWDD4	RWDD4	4	RWD domain containing 4	protein_codin	0.4579	0.00028	1	22.87	0.03512
CFAP97	CFAP97	4	cilia and flagella associated protein 97	protein_codin	-0.1897	0.0137	1	19.12	0.03429
PDCD6	PDCD6	4	programmed cell death 6	protein_codin	0.1453	0.0367	1	52.57	0.07912
ZFR	ZFR	4	zinc finger RNA binding protein	protein_codin	-0.1224	0.217	1	41.71	0.2464
ZNF131	ZNF131	4	zinc finger protein 131	protein_codin	0.2548	0.149	1	9.228	0.2388
SKIV2L2	SKIV2L2	4	Mtr4 exosome RNA helicase	protein_codin	NA	NA	NA	7.729	0.06571
RASA1	RASA1	4	RAS p21 protein activator 1	protein_codin	0.1362	0.538	1	3.349	0.1980
FAM172A	FAM172A	4	family with sequence similarity 172 member A	protein_codin	0.3474	0.0432	1	17.66	0.3177
CDKN2AIPNL	CDKN2AIPNL	4	CDKN2A interacting protein N-terminal like	protein_codin	-0.2677	0.133	1	3.567	0.2545
CDC23	CDC23	4	cell division cycle 23	protein_codin	0.2296	0.303	1	2.796	0.3622
WDR55	WDR55	4	WD repeat domain 55	protein_codin	0.2773	0.196	1	3.554	0.2462
CARMN	CARMN	4	cardiac mesoderm enhancer-associated non-coding R	lincRNA	-1.075	1.46E-05	0.3359	54.58	0.7980
TCOF1	TCOF1	4	treacle ribosome biogenesis factor 1	protein_codin	0.3492	0.296	1	9.19	0.1717
KIF13A	KIF13A	4	kinesin family member 13A	protein_codin	-0.1533	0.354	1	13.06	0.03321
HIST1H1C	HIST1H1C	4	histone cluster 1 H1 family member c	protein_codin	-0.08982	0.555	1	50.81	0.3522
GABBR1	GABBR1	4	gamma-aminobutyric acid type B receptor subunit 1	protein_codin	-0.6549	0.0311	1	3.058	0.6341
C6orf48	C6orf48	4	small nucleolar RNA host gene 32	protein_codin	-0.5273	3.52E-10	8.33E-06	53.73	0.1082
TEAD3	TEAD3	4	TEA domain transcription factor 3	protein_codin	-0.122	0.544	1	8.127	0.2358
CCDC167	CCDC167	4	coiled-coil domain containing 167	protein_codin	-0.7636	1.25E-05	0.2878	24.64	0.03302
BICRAL	BICRAL	4	BRD4 interacting chromatin remodeling complex asso	protein_codin	0.1467	0.559	1	2.929	0.4856
SMAP1	SMAP1	4	small ArfGAP 1	protein_codin	-0.149	0.209	1	13.35	0.1795
MTO1	MTO1	4	mitochondrial tRNA translation optimization 1	protein_codin	-0.1746	0.469	1	2.979	0.2442
MDN1	MDN1	4	midasin AAA ATPase 1	protein_codin	0.5719	0.062	1	11.22	0.2808

**FHL5**	FHL5	4	four and a half LIM domains 5	protein_codin	-0.211	0.132	1	54.2	0.1442
**ASCC3**	ASCC3	4	activating signal cointegrator 1 complex subunit 3	protein_codin	-0.1798	0.219	1	57.52	0.1310
**RPF2**	RPF2	4	ribosome production factor 2 homolog	protein_codin	0.1249	0.335	1	25.2	0.06800
**FRK**	FRK	4	fyn related Src family tyrosine kinase	protein_codin	-0.634	0.0215	1	8.833	0.3476
**KPNA5**	KPNA5	4	karyopherin subunit alpha 5	protein_codin	0.3837	0.104	1	3.083	0.2114
**AHI1**	AHI1	4	Abelson helper integration site 1	protein_codin	0.7026	1.67E-07	0.00391	54.27	0.1687
**HEBP2**	HEBP2	4	heme binding protein 2	protein_codin	-0.2335	0.00180	1	37.77	0.03294
**FUCA2**	FUCA2	4	alpha-L-fucosidase 2	protein_codin	0.5109	7.21E-05	1	42.8	0.06982
**GINM1**	GINM1	4	glycoprotein integral membrane 1	protein_codin	0.1662	0.016	1	47.41	0.09821
**PDCD2**	PDCD2	4	programmed cell death 2	protein_codin	-0.1693	0.140	1	12.95	0.07010
**AIMP2**	AIMP2	4	aminoacyl tRNA synthetase complex interacting multi	protein_codin	-0.1118	0.512	1	7.432	0.06726
**MEOX2**	MEOX2	4	mesenchyme homeobox 2	protein_codin	0.971	6.64E-07	0.01548	46.8	0.1365
**RAPGEF5**	RAPGEF5	4	Rap guanine nucleotide exchange factor 5	protein_codin	-0.5335	0.00245	1	44.45	0.3765
**FKBP9**	FKBP9	4	FKBP prolyl isomerase 9	protein_codin	0.6542	4.18E-08	0.00099	33.92	0.0694
**TRIM73**	TRIM73	4	tripartite motif containing 73	protein_codin	0.8274	0.00363	1	3.158	0.4475
**TMEM120A**	TMEM120A	4	transmembrane protein 120A	protein_codin	-0.139	0.279	1	6.565	0.2672
**DMTF1**	DMTF1	4	cyclin D binding myb like transcription factor 1	protein_codin	0.1168	0.752	1	7.947	0.4067
**AP4M1**	AP4M1	4	adaptor related protein complex 4 subunit mu 1	protein_codin	0.2321	0.357	1	8.794	0.1352
**NA**	NA	4	RNA polymerase II subunit J3	NA	NA	NA	NA	15.72	0.1005
**NAPEPLD**	NAPEPLD	4	N-acyl phosphatidylethanolamine phospholipase D	protein_codin	0.06395	0.832	1	3.164	0.3268
**KMT2E**	KMT2E	4	lysine methyltransferase 2E	protein_codin	-0.1475	0.0632	1	26.4	0.0694
**SYPL1**	SYPL1	4	synaptophysin like 1	protein_codin	0.1778	0.0265	1	54.5	0.1654
**AC006333.2**	AC006333.2	4	novel transcript, antisense to WA	antisense_RN	-0.4792	0.0698	1	7.896	0.1489
**TMEM209**	TMEM209	4	transmembrane protein 209	protein_codin	0.1521	0.574	1	37.94	0.09962
**HIPK2**	HIPK2	4	homeodomain interacting protein kinase 2	protein_codin	-0.1797	0.456	1	9.821	0.2391
**SMARCD3**	SMARCD3	4	SWI/SNF related, matrix associated, actin dependent	protein_codin	0.1299	0.432	1	16.04	0.1682
**HCCS**	HCCS	4	holocytochrome c synthase	protein_codin	-0.4901	0.0626	1	7.836	0.282
**GEMIN8**	GEMIN8	4	gem nuclear organelle associated protein 8	protein_codin	0.08901	0.742	1	3.278	0.1038
**PDHA1**	PDHA1	4	pyruvate dehydrogenase E1 alpha 1 subunit	protein_codin	-0.1958	0.126	1	17.83	0.03496
**DMD**	DMD	4	dystrophin	protein_codin	-0.7399	1.94E-06	0.04501	15.35	0.2412
**RBM3**	RBM3	4	RNA binding motif protein 3	protein_codin	-0.003402	0.957	1	50.26	0.02386
**HDAC8**	HDAC8	4	histone deacetylase 8	protein_codin	0.1672	0.499	1	3.353	0.3566
**ATRX**	ATRX	4	ATRX chromatin remodeler	protein_codin	-0.1661	0.0548	1	53.14	0.2362
**ARMCX5**	ARMCX5	4	armadillo repeat containing X-linked 5	protein_codin	-0.2606	0.219	1	8.772	0.2005
**BEX4**	BEX4	4	brain expressed X-linked 4	protein_codin	0.3984	4.99E-05	1	21.48	0.1371
**LRCH2**	LRCH2	4	leucine rich repeats and calponin homology domain c	protein_codin	-0.1014	0.66	1	9.058	0.279
**SLC25A43**	SLC25A43	4	solute carrier family 25 member 43	protein_codin	0.001859	0.992	1	3.356	0.1922
**LDOC1**	LDOC1	4	LDOC1 regulator of NFKB signaling	protein_codin	-0.7333	6.88E-12	1.64E-08	37.85	0.07338
**CD99L2**	CD99L2	4	CD99 molecule like 2	protein_codin	0.2458	0.187	1	9.305	0.1326
**FLNA**	FLNA	4	filamin A	protein_codin	-0.7339	2.29E-20	5.5E-16	60.6	0.1432
**MPP1**	MPP1	4	membrane palmitoylated protein 1	protein_codin	-0.06892	0.735	1	3.227	0.09959
**LONRF1**	LONRF1	4	LON peptidase N-terminal domain and ring finger 1	protein_codin	0.3592	0.0507	1	10.26	0.06985
**BIN3**	BIN3	4	bridging integrator 3	protein_codin	-0.3041	0.281	1	9.287	0.4427
**HMBOX1**	HMBOX1	4	homeobox containing 1	protein_codin	0.4273	0.0956	1	3.47	0.2972
**WRN**	WRN	4	WRN RecQ like helicase	protein_codin	0.521	0.0490	1	7.658	0.2098
**NSD3**	NSD3	4	nuclear receptor binding SET domain protein 3	protein_codin	0.05812	0.565	1	14.38	0.09912
**SFRP1**	SFRP1	4	secreted frizzled related protein 1	protein_codin	4.274	2.62E-32	6.33E-29	55.36	0.07006
**UBE2V2**	UBE2V2	4	ubiquitin conjugating enzyme E2 V2	protein_codin	-0.0158	0.86	1	9.976	0.09721
**TMEM68**	TMEM68	4	transmembrane protein 68	protein_codin	0.2937	0.212	1	10.8	0.1022
**ELOC**	ELOC	4	elongin C	protein_codin	0.0331	0.667	1	21.41	0.1032
**PLEKHF2**	PLEKHF2	4	pleckstrin homology and FYVE domain containing 2	protein_codin	-0.1054	0.612	1	3.096	0.246
**AC027031.2**	AC027031.2	4	novel transcri	lincRNA	-0.459	0.0207	1	24.16	0.2441
**LYNX1**	LYNX1	4	Ly6/neurotoxin 1	protein_codin	-0.6433	0.00340	1	10.55	0.4179
**CYC1**	CYC1	4	cytochrome c1	protein_codin	-0.02612	0.79	1	14.93	0.1676
**SHARPIN**	SHARPIN	4	SHANK associated RH domain interactor	protein_codin	-0.05063	0.687	1	36.43	0.1232
**CCDC171**	CCDC171	4	coiled-coil domain containing 171	protein_codin	0.1214	0.604	1	11.2	0.3192
**NMRK1**	NMRK1	4	nicotinamide riboside kinase 1	protein_codin	-0.7431	6.52E-07	0.0152	7.501	0.1786
**VPS13A**	VPS13A	4	vacuolar protein sorting 13 homolog A	protein_codin	0.6255	0.0542	1	19.8	0.3882
**BICD2**	BICD2	4	BICD cargo adaptor 2	protein_codin	-0.1752	0.379	1	8.546	0.2026
**SLC35D2**	SLC35D2	4	solute carrier family 35 member D2	protein_codin	0.2784	0.2	1	3.552	0.1735
**CDC14B**	CDC14B	4	cell division cycle 14B	protein_codin	0.2578	0.236	1	8.298	0.1805
**TGFBR1**	TGFBR1	4	transforming growth factor beta receptor 1	protein_codin	0.1832	0.349	1	34.25	0.3174
**TMEM246**	TMEM246	4	transmembrane protein 246	protein_codin	0.6476	0.00832	1	10.06	0.1405
**RC3H2**	RC3H2	4	ring finger and CCCH-type domains 2	protein_codin	0.4205	0.102	1	3.26	0.3358
**NCS1**	NCS1	4	neuronal calcium sensor 1	protein_codin	-0.4727	0.00017	1	15.52	0.1854
**SURF1**	SURF1	4	SURF1 cytochrome c oxidase assembly factor	protein_codin	-0.2371	0.0380	1	47.83	0.1642
**NACC2**	NACC2	4	NACC family member 2	protein_codin	-0.5167	0.0318	1	3.109	0.5638
**SDCCAG3**	SDCCAG3	4	endosome associated trafficking regulator 1	protein_codin	NA	NA	NA	3.482	0.1492
**AGPAT2**	AGPAT2	4	1-acylglycerol-3-phosphate O-acyltransferase 2	protein_codin	-0.418	0.00265	1	20.65	0.2095
**PSMA1**	PSMA1	4	proteasome subunit alpha 1	protein_codin	0.04629	0.52	1	47.83	0.03265
**GTF2H1**	GTF2H1	4	general transcription factor IIH subunit 1	protein_codin	0.2329	0.217	1	3.426	0.2075
**PEX16**	PEX16	4	peroxisomal biogenesis factor 16	protein_codin	-0.03389	0.854	1	5.833	0.2086
**C11orf49**	C11orf49	4	chromosome 11 open reading frame 49	protein_codin	0.275	0.157	1	8.277	0.1395
**ZDHHC5**	ZDHHC5	4	zinc finger DHHC-type containing 5	protein_codin	0.2069	0.349	1	8.128	0.277
**OSBP**	OSBP	4	oxysterol binding protein	protein_codin	-0.08583	0.552	1	6.783	0.2691
**TMEM216**	TMEM216	4	transmembrane protein 216	protein_codin	-0.05803	0.768	1	8.907	0.2405
**METTL12**	METTL12	4	citrate synthase lysine methyltransferase	protein_codin	NA	NA	NA	7.25	0.1709
**HNRNPUL2**	HNRNPUL2	4	heterogeneous nuclear ribonucleoprotein U like 2	protein_codin	-0.2388	0.292	1	10.42	0.3027
**MALAT1**	MALAT1	4	metastasis associated lung adenocarcinoma transcript 1	lincRNA	0.025	0.5285	1	NA	0.06475
**KAT5**	KAT5	4	lysine acetyltransferase 5	protein_codin	0.08311	0.67	1	6.225	0.1681
**ZDHHC24**	ZDHHC24	4	zinc finger DHHC-type containing 24	protein_codin	0.2733	0.117	1	9.193	0.1016
**CDK2AP2**	CDK2AP2	4	cyclin dependent kinase 2 associated protein 2	protein_codin	-0.05062	0.656	1	59.1	0.1782
**NUDT8**	NUDT8	4	nudix hydrolase 8	protein_codin	0.1474	0.472	1	49.64	0.06568
**TCIRG1**	TCIRG1	4	T cell immune regulator 1, ATPase H+ transporting V	protein_codin	0.1792	0.28	1	30.84	0.2111
**RNF121**	RNF121	4	ring finger protein 121	protein_codin	-0.2562	0.252	1	8.594	0.1357
**RAB30**	RAB30	4	RAB30, member RAS oncogene family	protein_codin	0.9748	5.69E-07	0.01327	8.462	0.1804
**MRE11**	MRE11	4	MRE11 homolog, double strand break repair nuclease	protein_codin	0.1992	0.304	1	7.504	0.306
**DCUN1D5**	DCUN1D5	4	defective in cullin neddylation 1 domain containing 5	protein_codin	-0.1019	0.465	1	7.258	0.1356
**KBTBD3**	KBTBD3	4	kelch repeat and BTB domain containing 3	protein_codin	0.2293	0.292	1	9.52	0.1046
**DDX6**	DDX6	4	DEAD-box helicase 6	protein_codin	0.2052	0.0596	1	38.54	0.1426
**H2AFX**	H2AFX	4	H2A histone family member X	protein_codin	0.5019	0.00783	1	34.68	0.1704
**ZBTB44**	ZBTB44	4	zinc finger and BTB domain containing 44	protein_codin	0.3252	0.118	1	9.376	0.2418
**THYN1**	THYN1	4	thymocyte nuclear protein 1	protein_codin	0.1332	0.171	1	52.1	0.03365
**KIN**	KIN	4	Kin17 DNA and RNA binding protein	protein_codin	0.001171	0.994	1	6.505	0.1012
**CDC123**	CDC123	4	cell division cycle 123	protein_codin	0.15	0.177	1	25.97	0.06625
**ITGA8**	ITGA8	4	integrin subunit alpha 8	protein_codin	-1.337	3.2E-50	7.76E-46	60.48	0.2384
**BMI1**	BMI1	4	BMI1 proto-oncogene, polycomb ring finger	protein_codin	0.083	0.679	1	9.253	0.3455
**ZNF33B**	ZNF33B	4	zinc finger protein 33B	protein_codin	-0.5444	0.00705	1	3.518	0.3397
**PRKG1**	PRKG1	4	protein kinase cGMP-dependent 1	protein_codin	-0.9453	9.78E-16	2.34E-12	53.26	0.1841
**CSTF2T**	CSTF2T	4	cleavage stimulation factor subunit 2 tau variant	protein_codin	0.2778	0.221	1	7.646	0.1325
**KCNMA1**	KCNMA1	4	potassium calcium-activated channel subfamily M alp	protein_codin	-1.416	4.18E-26	1.01E-21	42.16	0.03905
**GHITM**	GHITM	4	growth hormone inducible transmembrane protein	protein_codin	-0.01036	0.894	1	58.62	0.03258
**LDB3**	LDB3	4	LIM domain binding 3	protein_codin	-0.6961	0.00765	1	26.15	0.302
**PAPSS2**	PAPSS2	4	3'-phosphoadenosine 5'-phosphosulfate synthase 2	protein_codin	0.8117	0.00085	1	7.848	0.06678
**GOT1**	GOT1	4	glutamic-oxaloacetic transaminase 1	protein_codin	0.1021	0.614	1	3.173	0.1321
**BLOC1S2**	BLOC1S2	4	biogenesis of lysosomal organelles complex 1 subunit 2	protein_codin	0.2926	0.0521	1	42.7	0.1765
**TCF7L2**	TCF7L2	4	transcription factor 7 like 2	protein_codin	0.4335	0.00130	1	49.93	0.07042
**PRDX3**	PRDX3	4	peroxiredoxin 3	protein_codin	0.1765	0.0377	1	21.08	0.1026
**ZRANB1**	ZRANB1	4	zinc finger RANBP2-type containing 1	protein_codin	0.09871	0.662	1	54.17	0.2385
**ECHS1**	ECHS1	4	enoyl-CoA hydratase, short chain 1	protein_codin	-0.2025	0.0145	1	49.11	0.1414
**NRIP2**	NRIP2	4	nuclear receptor interacting protein 2	protein_codin	0.6763	0.00147	1	29.72	0.3806
**MRPS35**	MRPS35	4	mitochondrial ribosomal protein S35	protein_codin	-0.06323	0.595	1	13.25	0.1715
**ERGIC2**	ERGIC2	4	ERGIC and golgi 2	protein_codin	-0.09748	0.294	1	33.01	0.06841
**FGD4**	FGD4	4	FYVE, RhoGEF and PH domain containing 4	protein_codin	0.7535	2.21E-05	0.5074	43.37	0.2246
**ATF7**	ATF7	4	activating transcription factor 7	protein_codin	-0.1649	0.3	1	23.6	0.2454
**ATP23**	ATP23	4	ATP23 metallopeptidase and ATP synthase assembly	protein_codin	0.1088	0.612	1	8.363	0.1759
**LRIG3**	LRIG3	4	leucine rich repeats and immunoglobulin like domains 3	protein_codin	0.5347	0.0079	1	8.779	0.1677
**DYRK2**	DYRK2	4	dual specificity tyrosine phosphorylation regulated kin	protein_codin	0.4288	0.0657	1	9.03	0.1761
**APPL2**	APPL2	4	adaptor protein, phosphotyrosine interacting with PH	protein_codin	-0.3355	0.0797	1	6.235	0.3172
**SSH1**	SSH1	4	slingshot protein phosphatase 1	protein_codin	-0.4934	0.0886	1	3.398	0.496
**SUCLA2**	SUCLA2	4	succinate-CoA ligase ADP-forming beta subunit	protein_codin	0.1497	0.212	1	56.85	0.2186
**FNDC3A**	FNDC3A	4	fibronectin type III domain containing 3A	protein_codin	-0.08409	0.455	1	41.92	0.03359
**NGDN**	NGDN	4	neuroguidin	protein_codin	-0.1495	0.295	1	5.076	0.2762
**DHRS4**	DHRS4	4	dehydrogenase/reductase 4	protein_codin	0.06841	0.729	1	7.279	0.1988
**PRPF39**	PRPF39	4	pre-mRNA processing factor 39	protein_codin	0.14	0.576	1	3.335	0.168
**SAV1**	SAV1	4	salvador family WW domain containing protein 1	protein_codin	0.4349	0.00040	1	32.7	0.2427
**HSPA2**	HSPA2	4	heat shock protein family A (Hsp70) member 2	protein_codin	-1.017	8.89E-12	2.11E-07	51.45	0.1055
**CHURC1**	CHURC1	4	churchill domain containing 1	protein_codin	-0.3091	5.5E-06	0.127	16.83	0.03222
**DIO2**	DIO2	4	iodothyronine deiodinase 2	protein_codin	-0.1609	0.434	1	60.6	0.1601
**MEIS2**	MEIS2	4	Meis homeobox 2	protein_codin	0.1001	0.509	1	16.21	0.1347
**CHST14**	CHST14	4	carbohydrate sulfotransferase 14	protein_codin	0.3288	0.17	1	3.337	0.201
**INO80**	INO80	4	INO80 complex ATPase subunit	protein_codin	0.2103	0.245	1	17.96	0.4192
**SHF**	SHF	4	Src homology 2 domain containing F	protein_codin	0.09421	0.659	1	8.928	0.1465
**FEM1B**	FEM1B	4	fem-1 homolog B	protein_codin	-0.115	0.525	1	7.295	0.09954
**HMG20A**	HMG20A	4	high mobility group 20A	protein_codin	-0.2678	0.37	1	2.708	0.3768
**ABHD2**	ABHD2	4	abhydrolase domain containing 2	protein_codin	-0.04205	0.794	1	53.43	0.106
**UNC45A**	UNC45A	4	unc-45 myosin chaperone A	protein_codin	-0.1263	0.409	1	22.85	0.2418
**C16orf91**	C16orf91	4	chromosome 16 open reading frame 91	protein_codin	-0.3054	0.0651	1	7.96	0.2168
**TMEM204**	TMEM204	4	transmembrane protein 204	protein_codin	-0.4709	0.00356	1	50.05	0.1008
**NAA60**	NAA60	4	N(alpha)-acetyltransferase 60, NatF catalytic subunit	protein_codin	0.3319	0.354	1	3.157	0.3368
**CARHSP1**	CARHSP1	4	calcium regulated heat stable protein 1	protein_codin	0.4392	2.11E-05	0.4845	55.36	0.09751
**MIR193BHG**	MIR193BHG	4	MIR193B host gene	lincRNA	-0.5992	0.0566	1	10.45	0.3496
**MARF1**	MARF1	4	meiosis regulator and mRNA stability factor 1	protein_codin	-0.1629	0.454	1	7.113	0.2431
**MYH11**	MYH11	4	myosin heavy chain 11	protein_codin	-0.9913	1.11E-39	2.69E-35	60.6	0.1242
**DCUN1D3**	DCUN1D3	4	defective in cullin neddylation 1 domain containing 3	protein_codin	0.6391	0.0164	1	3.333	0.3795
**ARHGAP17**	ARHGAP17	4	Rho GTPase activating protein 17	protein_codin	0.2489	0.271	1	7.345	0.3527
**ZNF688**	ZNF688	4	zinc finger protein 688	protein_codin	0.1888	0.19	1	21.52	0.06567
**CTF1**	CTF1	4	cardiotrophin 1	protein_codin	-0.5391	0.0106	1	9.763	0.3062
**VPS4A**	VPS4A	4	vacuolar protein sorting 4 homolog A	protein_codin	-0.1684	0.231	1	20.93	0.107
**ZNRF1**	ZNRF1	4	zinc and ring finger 1	protein_codin	0.2114	0.388	1	8.205	0.3622
**BCAR1**	BCAR1	4	BCAR1 scaffold protein, Cas family member	protein_codin	-0.6063	0.00378	1	9.33	0.196
**MVD**	MVD	4	mevalonate diphosphate decarboxylase	protein_codin	0.1787	0.425	1	2.699	0.06796
**SPATA33**	SPATA33	4	spermatogenesis associated 33	protein_codin	0.09281	0.701	1	10.85	0.3915
**HIC1**	HIC1	4	HIC ZBTB transcriptional repressor 1	protein_codin	1.695	2.55E-12	6.06E-08	60.02	0.07294
**KIF1C**	KIF1C	4	kinesin family member 1C	protein_codin	-0.4751	0.00427	1	7.883	0.068
**RNASEK**	RNASEK	4	ribonuclease K	protein_codin	0.1792	0.412	1	8.819	0.1419
**CHD3**	CHD3	4	chromodomain helicase DNA binding protein 3	protein_codin	0.1288	0.389	1	39.38	0.166
**SREBF1**	SREBF1	4	sterol regulatory element binding transcription factor 1	protein_codin	1.616	4.54E-09	0.00011	23.79	0.1054
**ERAL1**	ERAL1	4	Era like 12S mitochondrial rRNA chaperone 1	protein_codin	0.2326	0.275	1	7.7	0.2167
**FLOT2**	FLOT2	4	flotillin 2	protein_codin	-0.1947	0.367	1	3.51	0.2387
**TP53I13**	TP53I13	4	tumor protein p53 inducible protein 13	protein_codin	0.4105	0.00471	1	16.21	0.1364
**NSRP1**	NSRP1	4	nuclear speckle splicing regulatory protein 1	protein_codin	-0.1199	0.185	1	38.32	0.1357
**SLFN5**	SLFN5	4	schlafen family member 5	protein_codin	0.2301	0.206	1	44.82	0.2196
**TMEM101**	TMEM101	4	transmembrane protein 101	protein_codin	-0.2173	0.164	1	3.288	0.06459
**AKAP1**	AKAP1	4	A-kinase anchoring protein 1	protein_codin	-0.7789	2.86E-06	0.06627	57.28	0.19
**SOX9**	SOX9	4	SRY-box transcription factor 9	protein_codin	0.6668	0.022	1	58.94	0.1718
**TNRC6C**	TNRC6C	4	trinucleotide repeat containing adaptor 6C	protein_codin	-0.06564	0.732	1	19.95	0.3552
**GAA**	GAA	4	glucosidase alpha, acid	protein_codin	0.4793	0.00097	1	17.73	0.1788
**USP14**	USP14	4	ubiquitin specific peptidase 14	protein_codin	0.2229	0.0892	1	25.93	0.1051
**TGIF1**	TGIF1	4	TGFB induced factor homeobox 1	protein_codin	0.1537	0.404	1	8.022	0.1284
**ZNF397**	ZNF397	4	zinc finger protein 397	protein_codin	0.1128	0.619	1	9.18	0.25
**GALNT1**	GALNT1	4	polypeptide N-acetylgalactosaminyltransferase 1	protein_codin	-0.4032	0.00353	1	60.6	0.07185
**RPL17**	RPL17	4	ribosomal protein L17	protein_codin	0.0725	0.554	1	32.98	0.1086
**PYGB**	PYGB	4	glycogen phosphorylase B	protein_codin	-0.08321	0.697	1	7.023	0.3542
**ASXL1**	ASXL1	4	ASXL transcriptional regulator 1	protein_codin	0.5735	0.00020	1	16.23	0.1056
**GSS**	GSS	4	glutathione synthetase	protein_codin	-0.05699	0.801	1	8.329	0.1312
**ROMO1**	ROMO1	4	reactive oxygen species modulator 1	protein_codin	-0.2722	0.00065	1	14.65	0.1032
**ZMYND8**	ZMYND8	4	zinc finger MYND-type containing 8	protein_codin	-0.2532	0.244	1	27.35	0.2209
**ZFAS1**	ZFAS1	4	ZNFX1 antisense RNA 1	antisense_RN	-0.4396	1.76E-08	0.00041	36.08	0.06878
**ZNF217**	ZNF217	4	zinc finger protein 217	protein_codin	0.5797	0.00567	1	7.357	0.2889
**C19orf70**	C19orf70	4	mitochondrial contact site and cristae organizing syst	protein_codin	-0.3122	7.23E-06	0.1668	60.6	0.06576
**NDUFA11**	NDUFA11	4	NADH:ubiquinone oxidoreductase subunit A11	protein_codin	-0.1433	0.0215	1	26.3	0.06876
**RGL3**	RGL3	4	ral guanine nucleotide dissociation stimulator like 3	protein_codin	0.2238	0.459	1	8.95	0.3565
**ECSIT**	ECSIT	4	ECSIT signalling integrator	protein_codin	-0.1937	0.212	1	22.47	0.2413
**PRKACA**	PRKACA	4	protein kinase cAMP-activated catalytic subunit alpha	protein_codin	-0.2441	0.159	1	7.952	0.1672
**ZNF708**	ZNF708	4	zinc finger protein 708	protein_codin	0.2787	0.151	1	8.87	0.1482
**URI1**	URI1	4	URI1 prefoldin like chaperone	protein_codin	-0.1771	0.109	1	48.2	0.03387
**PEPD**	PEPD	4	peptidase D	protein_codin	-0.03644	0.696	1	33.9	0.1049
**KIAA0355**	KIAA0355	4	KIAA0355	protein_codin	-0.4436	0.0528	1	9.096	0.1679
**U2AF1L4**	U2AF1L4	4	U2 small nuclear RNA auxiliary factor 1 like 4	protein_codin	-0.03848	0.87	1	10.33	0.242
**ZNF566**	ZNF566	4	zinc finger protein 566	protein_codin	0.3132	0.182	1	2.998	0.2796
**ECH1**	ECH1	4	enoyl-CoA hydratase 1	protein_codin	0.2599	0.0005	1	26.93	0.03295
**SAMD4B**	SAMD4B	4	sterile alpha motif domain containing 4B	protein_codin	0.2895	0.236	1	5.896	0.2875
**FBL**	FBL	4	fibrillarin	protein_codin	-0.3082	0.0293	1	42.02	0.1059
**PSMC4**	PSMC4	4	proteasome 26S subunit, ATPase 4	protein_codin	-0.2229	0.0237	1	27.32	0.1982
**SNRPA**	SNRPA	4	small nuclear ribonucleoprotein polypeptide A	protein_codin	0.1495	0.257	1	14.4	0.1685
**APOE**	APOE	4	apolipoprotein E	protein_codin	-2.259	2.17E-27	5.23E-23	NA	0.479
**APOC1**	APOC1	4	apolipoprotein C1	protein_codin	-2.503	1.35E-15	3.23E-11	60.6	0.8251
**ZNF766**	ZNF766	4	zinc finger protein 766	protein_codin	-0.576	0.00126	1	9.371	0.1719
**DGCR2**	DGCR2	4	DiGeorge syndrome critical region gene 2	protein_codin	0.0455	0.826	1	3.393	0.3087
**LZTR1**	LZTR1	4	leucine zipper like transcription regulator 1	protein_codin	0.3322	0.229	1	2.615	0.3476
**GGT5**	GGT5	4	gamma-glutamyltransferase 5	protein_codin	-0.4083	0.00356	1	28.13	0.2132
**THOC5**	THOC5	4	THO complex 5	protein_codin	0.03332	0.876	1	3.277	0.4432
**L3MBTL2**	L3MBTL2	4	L3MBTL histone methyl-lysine binding protein 2	protein_codin	-0.01033	0.964	1	2.836	0.2114
**MIR99AHG**	MIR99AHG	4	mir-99a-let-7c cluster host gene	lincRNA	1.706	4.13E-13	9.83E-09	21.04	0.1374
**MRPL39**	MRPL39	4	mitochondrial ribosomal protein L39	protein_codin	0.03837	0.814	1	7.153	0.1679
**PDXK**	PDXK	4	pyridoxal kinase	protein_codin	-0.2549	0.0998	1	37.53	0.06761
**H6PD**	H6PD	5	hexose-6-phosphate dehydrogenase/glucose 1-dehyd	protein_codin	0.7965	0.00023	1	8.024	0.1656
**PLA2G5**	PLA2G5	5	phospholipase A2 group V	protein_codin	-0.5698	0.00315	1	60.52	0.1187
**RBBP4**	RBBP4	5	RB binding protein 4, chromatin remodeling factor	protein_codin	0.1592	0.287	1	16.98	0.2161
**DNALI1**	DNALI1	5	dynein axonemal light intermediate chain 1	protein_codin	0.4053	0.152	1	3.299	0.0688
**NFYC**	NFYC	5	nuclear transcription factor Y subunit gamma	protein_codin	-0.09442	0.582	1	7.607	0.2501
**RNF220**	RNF220	5	ring finger protein 220	protein_codin	0.06891	0.771	1	3.016	0.1664
**BTF3L4**	BTF3L4	5	basic transcription factor 3 like 4	protein_codin	0.08602	0.409	1	11.64	0.2092
**ZYG11B**	ZYG11B	5	zyg-11 family member B, cell cycle regulator	protein_codin	0.4454	0.0262	1	8.543	0.1724
**OMA1**	OMA1	5	OMA1 zinc metallopeptidase	protein_codin	0.5409	0.00960	1	9.445	0.2512
**ATG4C**	ATG4C	5	autophagy related 4C cysteine peptidase	protein_codin	0.09545	0.662	1	3.434	0.1446
**ITGB3BP**	ITGB3BP	5	integrin subunit beta 3 binding protein	protein_codin	0.2685	0.199	1	7.03	0.2745
**LRRC40**	LRRC40	5	leucine rich repeat containing 40	protein_codin	0.2063	0.347	1	3.211	0.3455
**PIGK**	PIGK	5	phosphatidylinositol glycan anchor biosynthesis class K	protein_codin	0.0005236	0.997	1	35.76	0.1372
**ZNHIT6**	ZNHIT6	5	zinc finger HIT-type containing 6	protein_codin	-0.1796	0.267	1	18.45	0.1714
**RAP1A**	RAP1A	5	RAP1A, member of RAS oncogene family	protein_codin	-0.01311	0.879	1	49.63	0.1499
**MAN1A2**	MAN1A2	5	mannosidase alpha class 1A member 2	protein_codin	0.04392	0.706	1	24.58	0.06775
**PRKAB2**	PRKAB2	5	protein kinase AMP-activated non-catalytic subunit beta 2	protein_codin	0.4449	0.0441	1	9.602	0.1082
**MTMR11**	MTMR11	5	myotubularin related protein 11	protein_codin	-0.5058	0.00990	1	7.486	0.2805
**VPS72**	VPS72	5	vacuolar protein sorting 72 homolog	protein_codin	-0.1072	0.492	1	20.49	0.1649
**SYT11**	SYT11	5	synaptotagmin 11	protein_codin	-0.2804	0.147	1	8.481	0.3056
**HSPA6**	HSPA6	5	heat shock protein family A (Hsp70) member 6	protein_codin	1.116	0.0981	1	55.36	1.145
**FAM129A**	FAM129A	5	niban apoptosis regulator 1	protein_codin	-0.6188	2.09E-09	4.93E-05	53.75	0.03621
**IPO9**	IPO9	5	importin 9	protein_codin	-0.02522	0.916	1	3.452	0.2795
**SHISA4**	SHISA4	5	shisa family member 4	protein_codin	-0.6382	4.13E-08	0.00097	28.32	0.1771
**PYCR2**	PYCR2	5	pyrroline-5-carboxylate reductase 2	protein_codin	0.1944	0.191	1	7.334	0.06732
**IRF2BP2**	IRF2BP2	5	interferon regulatory factor 2 binding protein 2	protein_codin	0.4677	1.86E-05	0.4275	29.94	0.2346
**HEATR1**	HEATR1	5	HEAT repeat containing 1	protein_codin	0.3368	0.132	1	3.705	0.1842
**KLF11**	KLF11	5	Kruppel like factor 11	protein_codin	1.071	0.00048	1	11.47	0.2211
**PUM2**	PUM2	5	pumilio RNA binding family member 2	protein_codin	0.05609	0.789	1	31.81	0.356
**YIPF4**	YIPF4	5	Yip1 domain family member 4	protein_codin	0.3212	0.0252	1	13.3	0.06645
**MAP4K3**	MAP4K3	5	mitogen-activated protein kinase kinase kinase kinase 3	protein_codin	-0.1242	0.512	1	7.042	0.2206
**USP34**	USP34	5	ubiquitin specific peptidase 34	protein_codin	0.2532	0.0802	1	11.56	0.1868
**GTDC1**	GTDC1	5	glycosyltransferase like domain containing 1	protein_codin	-0.1728	0.474	1	3.346	0.2962
**ACVR1**	ACVR1	5	activin A receptor type 1	protein_codin	-0.1784	0.436	1	3.438	0.1794
**HAT1**	HAT1	5	histone acetyltransferase 1	protein_codin	-0.1753	0.165	1	15.24	0.2339
**HNRNPA3**	HNRNPA3	5	heterogeneous nuclear ribonucleoprotein A3	protein_codin	-0.2008	0.0029	1	25.32	0.06726
**GULP1**	GULP1	5	GULP PTB domain containing engulfment adaptor 1	protein_codin	-0.3029	0.00718	1	24.88	0.1675
**HSPD1**	HSPD1	5	heat shock protein family D (Hsp60) member 1	protein_codin	0.04934	0.482	1	37.9	0.02351
**SPATS2L**	SPATS2L	5	spermatogenesis associated serine rich 2 like	protein_codin	-0.278	0.00128	1	60.6	0.06531
**MFF**	MFF	5	mitochondrial fission factor	protein_codin	0.05979	0.607	1	29.27	0.03190
**SETD2**	SETD2	5	SET domain containing 2, histone lysine methyltransf	protein_codin	0.2409	0.187	1	18	0.3396
**SMIM4**	SMIM4	5	small integral membrane protein 4	protein_codin	-0.4897	0.00116	1	21.3	0.1017
**LRRC58**	LRRC58	5	leucine rich repeat containing 58	protein_codin	0.5004	0.00496	1	21.33	0.101
**KPNA1**	KPNA1	5	karyopherin subunit alpha 1	protein_codin	0.8688	0.00072	1	8.369	0.4101
**SNX4**	SNX4	5	sorting nexin 4	protein_codin	0.2907	0.0706	1	8.265	0.1701
**PLXNA1**	PLXNA1	5	plexin A1	protein_codin	-0.1659	0.549	1	10.82	0.6937
**TPRA1**	TPRA1	5	transmembrane protein adipocyte associated 1	protein_codin	0.1995	0.234	1	29.95	0.09874
**TNIP2**	TNIP2	5	TNFAIP3 interacting protein 2	protein_codin	-0.368	0.0182	1	21.24	0.1847
**AFAP1**	AFAP1	5	actin filament associated protein 1	protein_codin	0.03399	0.852	1	8.354	0.204
**LGI2**	LGI2	5	leucine rich repeat LGI family member 2	protein_codin	-0.3236	0.0959	1	44.74	0.3362
**KLF3**	KLF3	5	Kruppel like factor 3	protein_codin	0.9539	1.11E-17	2.66E-13	52.35	0.0321
**CNOT6L**	CNOT6L	5	CCR4-NOT transcription complex subunit 6 like	protein_codin	0.01502	0.937	1	8.249	0.1696
**LINC01088**	LINC01088	5	long intergenic non-protein coding RNA 1088	antisense_RN	-1.471	1.81E-14	4.32E-10	25.45	0.3637
**WDFY3**	WDFY3	5	WD repeat and FYVE domain containing 3	protein_codin	0.2302	0.324	1	30.05	0.2496
**MANBA**	MANBA	5	mannosidase beta	protein_codin	0.9238	0.00032	1	8.616	0.2067
**PLA2G12A**	PLA2G12A	5	phospholipase A2 group XIIA	protein_codin	-0.02172	0.926	1	7.263	0.2764
**LARP7**	LARP7	5	La ribonucleoprotein domain family member 7	protein_codin	-0.02182	0.809	1	27	0.0667
**SMARCA5**	SMARCA5	5	SWI/SNF related, matrix associated, actin dependent	protein_codin	-0.03722	0.708	1	15.14	0.1062
**ZNF622**	ZNF622	5	zinc finger protein 622	protein_codin	0.1233	0.525	1	6.795	0.1345
**PAIP1**	PAIP1	5	poly(A) binding protein interacting protein 1	protein_codin	0.1309	0.156	1	53.89	0.03418
**SMIM15**	SMIM15	5	small integral membrane protein 15	protein_codin	0.2142	0.0660	1	15.88	0.06479
**SREK1IP1**	SREK1IP1	5	SREK1 interacting protein 1	protein_codin	0.02787	0.836	1	21.02	0.1712
**MAST4**	MAST4	5	microtubule associated serine/threonine kinase family	protein_codin	-0.4538	0.0158	1	8.712	0.2377
**CHD1**	CHD1	5	chromodomain helicase DNA binding protein 1	protein_codin	0.4356	0.002	1	14.96	0.1329
**KDM3B**	KDM3B	5	lysine demethylase 3B	protein_codin	0.011	0.962	1	3.414	0.6177
**HMGXB3**	HMGXB3	5	HMG-box containing 3	protein_codin	-0.1418	0.406	1	6.052	0.1714
**NOP16**	NOP16	5	NOP16 nucleolar protein	protein_codin	-0.1368	0.494	1	10.7	0.2556
**BTN2A1**	BTN2A1	5	butyrophilin subfamily 2 member A1	protein_codin	-0.125	0.51	1	3.412	0.2129
**NFKBIL1**	NFKBIL1	5	NFKB inhibitor like 1	protein_codin	0.08404	0.586	1	6.393	0.1006
**CENPQ**	CENPQ	5	centromere protein Q	protein_codin	0.1605	0.468	1	3.606	0.3217
**TRAM2**	TRAM2	5	translocation associated membrane protein 2	protein_codin	-0.06526	0.742	1	31.73	0.3972
**LMBRD1**	LMBRD1	5	LMBR1 domain containing 1	protein_codin	0.5542	1.36E-05	0.3133	32.6	0.0332
**SLC35A1**	SLC35A1	5	solute carrier family 35 member A1	protein_codin	0.2658	0.179	1	7.173	0.171
**PM20D2**	PM20D2	5	peptidase M20 domain containing 2	protein_codin	-0.3004	0.168	1	9.27	0.1451
**UBE2J1**	UBE2J1	5	ubiquitin conjugating enzyme E2 J1	protein_codin	0.3946	0.0162	1	17.11	0.0977
**MRPL18**	MRPL18	5	mitochondrial ribosomal protein L18	protein_codin	0.1486	0.0938	1	54.4	0.2004
**EIF3B**	EIF3B	5	eukaryotic translation initiation factor 3 subunit B	protein_codin	-0.1044	0.541	1	7.37	0.2419
**FSCN1**	FSCN1	5	fascin actin-bundling protein 1	protein_codin	0.1027	0.722	1	9.826	0.2094
**CCZ1B**	CCZ1B	5	CCZ1 homolog B, vacuolar protein trafficking and bio	protein_codin	0.1616	0.45	1	3.015	0.2508
**TAX1BP1**	TAX1BP1	5	Tax1 binding protein 1	protein_codin	-0.09314	0.1609	1	52.67	0.03302
**TMED4**	TMED4	5	transmembrane p24 trafficking protein 4	protein_codin	0.09397	0.217	1	48.43	0.03434
**CCM2**	CCM2	5	CCM2 scaffold protein	protein_codin	-0.1324	0.419	1	20.08	0.03382
**RFC2**	RFC2	5	replication factor C subunit 2	protein_codin	0.2229	0.236	1	7.17	0.07459
**RSBN1L**	RSBN1L	5	round spermatid basic protein 1 like	protein_codin	-0.07752	0.445	1	52.6	0.2056
**GTPBP10**	GTPBP10	5	GTP binding protein 10	protein_codin	0.7066	0.00616	1	8.118	0.3162
**TAF6**	TAF6	5	TATA-box binding protein associated factor 6	protein_codin	0.2702	0.155	1	7.193	0.0665
**PNPLA8**	PNPLA8	5	patatin like phospholipase domain containing 8	protein_codin	0.2982	0.022	1	15.82	0.06472
**BRAF**	BRAF	5	B-Raf proto-oncogene, serine/threonine kinase	protein_codin	-0.1788	0.417	1	6.648	0.4775
**WDR44**	WDR44	5	WD repeat domain 44	protein_codin	-0.2792	0.195	1	34.38	0.1422
**XIAP**	XIAP	5	X-linked inhibitor of apoptosis	protein_codin	0.002992	0.985	1	7.432	0.1746
**SMARCA1**	SMARCA1	5	SWI/SNF related, matrix associated, actin dependent	protein_codin	0.008589	0.948	1	59.07	0.2849
**G6PD**	G6PD	5	glucose-6-phosphate dehydrogenase	protein_codin	-0.05444	0.717	1	20.73	0.03537
**FBXO25**	FBXO25	5	F-box protein 25	protein_codin	-0.2141	0.345	1	6.952	0.182
**ZDHHC2**	ZDHHC2	5	zinc finger DHHC-type containing 2	protein_codin	-0.08096	0.721	1	9.98	0.1392
**NUDT18**	NUDT18	5	nudix hydrolase 18	protein_codin	0.09124	0.66	1	8.775	0.1234
**PNMA2**	PNMA2	5	PNMA family member 2	protein_codin	1.026	0.00028	1	11.5	0.1001
**DPYSL2**	DPYSL2	5	dihydropyrimidinase like 2	protein_codin	0.7574	5.82E-12	1.38E-07	49.57	0.06797
**EPHX2**	EPHX2	5	epoxide hydrolase 2	protein_codin	0.4762	0.0214	1	3.386	0.1254
**ATP6V1H**	ATP6V1H	5	ATPase H+ transporting V1 subunit H	protein_codin	0.2982	0.152	1	9.132	0.03252
**LACTB2**	LACTB2	5	lactamase beta 2	protein_codin	0.1567	0.415	1	22.96	0.2524
**SBSPON**	SBSPON	5	somatomedin B and thrombospondin type 1 domain c	protein_codin	-0.5249	0.00080	1	27.83	0.1581
**MED30**	MED30	5	mediator complex subunit 30	protein_codin	0.2642	0.0386	1	6.534	0.2456
**CYHR1**	CYHR1	5	cysteine and histidine rich 1	protein_codin	-0.4204	4.55E-05	1	32.37	0.06427
**ZDHHC21**	ZDHHC21	5	zinc finger DHHC-type containing 21	protein_codin	-0.03932	0.855	1	31.76	0.1552
**DCAF10**	DCAF10	5	DDB1 and CUL4 associated factor 10	protein_codin	0.1232	0.547	1	9.458	0.2737
**INIP**	INIP	5	INTS3 and NABP interacting protein	protein_codin	0.1322	0.436	1	7.824	0.1752
**DAB2IP**	DAB2IP	5	DAB2 interacting protein	protein_codin	-0.05532	0.824	1	9.028	0.4728
**NUP98**	NUP98	5	nucleoporin 98	protein_codin	0.04229	0.855	1	3.3	0.2808
**ARFIP2**	ARFIP2	5	ADP ribosylation factor interacting protein 2	protein_codin	-0.07958	0.665	1	3.363	0.2488
**EIF3M**	EIF3M	5	eukaryotic translation initiation factor 3 subunit M	protein_codin	-0.1471	0.0646	1	33.13	0.102

**DDB1**	DDB1	5	damage specific DNA binding protein 1	protein_codin	0.6127	0.00060	1	24.67	0.3162
**GANAB**	GANAB	5	glucosidase II alpha subunit	protein_codin	0.1118	0.469	1	22.34	0.102
**TRPT1**	TRPT1	5	tRNA phosphotransferase 1	protein_codin	0.04679	0.769	1	23.16	0.2159
**ESRRA**	ESRRA	5	estrogen related receptor alpha	protein_codin	0.5452	0.0204	1	9.302	0.1731
**MRPL49**	MRPL49	5	mitochondrial ribosomal protein L49	protein_codin	-0.6352	2.85E-05	0.6532	22.32	0.2542
**FIBP**	FIBP	5	FGF1 intracellular binding protein	protein_codin	-0.2953	0.0102	1	20.75	0.07258
**SSH3**	SSH3	5	slingshot protein phosphatase 3	protein_codin	0.006725	0.981	1	9.045	0.5559
**PPP1CA**	PPP1CA	5	protein phosphatase 1 catalytic subunit alpha	protein_codin	-0.1694	0.152	1	28.48	0.02199
**AAMDC**	AAMDC	5	adipogenesis associated Mth938 domain containing	protein_codin	0.2185	0.0100	1	29.1	0.2147
**CREB2F**	CREB2F	5	CREB/ATF bZIP transcription factor	protein_codin	0.5102	0.00196	1	18.93	0.1694
**CWC15**	CWC15	5	CWC15 spliceosome associated protein homolog	protein_codin	0.02210	0.825	1	21.12	0.03288
**CRYAB**	CRYAB	5	crystallin alpha B	protein_codin	-0.05658	0.381	1	Inf	0.1836
**MCAM**	MCAM	5	melanoma cell adhesion molecule	protein_codin	-0.4753	7.1E-06	0.1639	55.32	0.03240
**EI24**	EI24	5	EI24 autophagy associated transmembrane protein	protein_codin	-0.1889	0.0705	1	27.58	0.03879
**MAPK8**	MAPK8	5	mitogen-activated protein kinase 8	protein_codin	-0.0836	0.711	1	2.873	0.3588
**VPS26A**	VPS26A	5	VPS26, retromer complex component A	protein_codin	0.06521	0.566	1	31.56	0.1426
**HIF1AN**	HIF1AN	5	hypoxia inducible factor 1 subunit alpha inhibitor	protein_codin	-0.09784	0.7	1	3.172	0.1467
**MGEA5**	MGEA5	5	O-GlcNAcase	protein_codin	NA	NA	NA	16.62	0.1746
**PCGF6**	PCGF6	5	polycomb group ring finger 6	protein_codin	0.2229	0.32	1	10.7	0.2072
**SHOC2**	SHOC2	5	SHOC2 leucine rich repeat scaffold protein	protein_codin	0.2129	0.12	1	16.74	0.1018
**TSPAN9**	TSPAN9	5	tetraspanin 9	protein_codin	-0.5848	0.00056	1	9.976	0.2432
**NDUFA9**	NDUFA9	5	NADH:ubiquinone oxidoreductase subunit A9	protein_codin	-0.02478	0.837	1	13.52	0.07928
**NTF3**	NTF3	5	neurotrophin 3	protein_codin	0.3617	0.0752	1	22.07	0.3551
**ING4**	ING4	5	inhibitor of growth family member 4	protein_codin	-0.1062	0.5	1	6.778	0.172
**ATF7IP**	ATF7IP	5	activating transcription factor 7 interacting protein	protein_codin	0.5648	0.00446	1	17.24	0.2710
**PYROXD1**	PYROXD1	5	pyridine nucleotide-disulphide oxidoreductase domain	protein_codin	-0.101	0.654	1	9.304	0.1012
**GOLT1B**	GOLT1B	5	golgi transport 1B	protein_codin	0.1403	0.389	1	7.978	0.03776
**IPO8**	IPO8	5	importin 8	protein_codin	-0.1249	0.521	1	3.468	0.2094
**RAP1B**	RAP1B	5	RAP1B, member of RAS oncogene family	protein_codin	0.1934	0.005	1	53.14	0.03257
**NTN4**	NTN4	5	netrin 4	protein_codin	0.1505	0.247	1	31.42	0.1392
**NUP37**	NUP37	5	nucleoporin 37	protein_codin	0.581	0.00802	1	6.945	0.318
**TMEM263**	TMEM263	5	transmembrane protein 263	protein_codin	0.1948	0.105	1	26	0.1310
**RFXAP**	RFXAP	5	regulatory factor X associated protein	protein_codin	0.2553	0.24	1	3.527	0.1752
**CLN5**	CLN5	5	CLN5 intracellular trafficking protein	protein_codin	0.4093	0.00172	1	15.86	0.1019
**FBXL3**	FBXL3	5	F-box and leucine rich repeat protein 3	protein_codin	0.04732	0.689	1	39.32	0.2376
**KLHDC1**	KLHDC1	5	kelch domain containing 1	protein_codin	0.1406	0.561	1	2.938	0.2787
**TMEM30B**	TMEM30B	5	transmembrane protein 30B	protein_codin	0.8579	6.79E-05	1	26.07	0.1708
**RDH11**	RDH11	5	retinol dehydrogenase 11	protein_codin	0.1025	0.432	1	15.22	0.06510
**SEL1L**	SEL1L	5	SEL1L adaptor subunit of ERAD E3 ubiquitin ligase	protein_codin	0.2076	0.124	1	39.62	0.2716
**DDX24**	DDX24	5	DEAD-box helicase 24	protein_codin	0.1166	0.0602	1	53.47	0.06918
**WDR25**	WDR25	5	WD repeat domain 25	protein_codin	-0.05621	0.806	1	2.911	0.2392
**NDN**	NDN	5	necdin, MAGE family member	protein_codin	0.5057	2.12E-10	5.02E-06	38.23	0.06624
**INAFM2**	INAFM2	5	InaF motif containing 2	protein_codin	-0.4313	0.132	1	10.18	0.2402
**COPS2**	COPS2	5	COP9 signalosome subunit 2	protein_codin	-0.08528	0.345	1	17.4	0.03701
**ETFA**	ETFA	5	electron transfer flavoprotein subunit alpha	protein_codin	-0.06873	0.527	1	12.58	0.1134
**TMED3**	TMED3	5	transmembrane p24 trafficking protein 3	protein_codin	0.2847	0.00258	1	57.78	0.03578
**C15orf40**	C15orf40	5	chromosome 15 open reading frame 40	protein_codin	0.1295	0.39	1	15.3	0.178
**BTBD1**	BTBD1	5	BTB domain containing 1	protein_codin	0.1123	0.657	1	7.587	0.1344
**TRAP1**	TRAP1	5	TNF receptor associated protein 1	protein_codin	0.2472	0.187	1	7.222	0.1382
**ERCC4**	ERCC4	5	ERCC excision repair 4, endonuclease catalytic subuni	protein_codin	0.2755	0.124	1	8.137	0.268
**PRRT2**	PRRT2	5	proline rich transmembrane protein 2	protein_codin	0.6826	0.00282	1	7.176	0.3532
**COQ9**	COQ9	5	coenzyme Q9	protein_codin	0.08049	0.692	1	8.912	0.32
**POLR2C**	POLR2C	5	RNA polymerase II subunit C	protein_codin	-0.5133	1.68E-06	0.039	13.02	0.2501
**GOT2**	GOT2	5	glutamic-oxaloacetic transaminase 2	protein_codin	0.2164	0.399	1	8.826	0.2359
**TK2**	TK2	5	thymidine kinase 2	protein_codin	0.3536	0.0385	1	51.26	0.329
**CBFB**	CBFB	5	core-binding factor subunit beta	protein_codin	0.35	0.146	1	3.468	0.3925
**NFATC3**	NFATC3	5	nuclear factor of activated T cells 3	protein_codin	0.1471	0.605	1	2.932	0.27
**CHTF8**	CHTF8	5	chromosome transmission fidelity factor 8	protein_codin	0.186	0.352	1	6.552	0.2145
**IL34**	IL34	5	interleukin 34	protein_codin	-0.769	3.56E-08	0.00084	23.45	0.03227
**KLHL36**	KLHL36	5	kelch like family member 36	protein_codin	0.6694	0.00536	1	6.932	0.2338
**CDK10**	CDK10	5	cyclin dependent kinase 10	protein_codin	0.02221	0.927	1	2.652	0.1352
**CRK**	CRK	5	CRK proto-oncogene, adaptor protein	protein_codin	-0.3216	0.0164	1	21.11	0.1416
**SLC25A11**	SLC25A11	5	solute carrier family 25 member 11	protein_codin	-0.07912	0.485	1	39.57	0.1066
**MED31**	MED31	5	mediator complex subunit 31	protein_codin	-0.01272	0.941	1	8.658	0.1010
**AC016876.1**	AC016876.1	5	novel transcript, antisense to MPDU1 and CD	antisense_RN	0.1796	0.415	1	2.922	0.07807
**SCO1**	SCO1	5	SCO cytochrome c oxidase assembly protein 1	protein_codin	0.219	0.287	1	3.547	0.06882
**ACLY**	ACLY	5	ATP citrate lyase	protein_codin	0.7543	0.00025	1	9.825	0.1032
**HOXB2**	HOXB2	5	homeobox B2	protein_codin	-0.2957	0.0245	1	14.68	0.1386
**MSI2**	MSI2	5	musashi RNA binding protein 2	protein_codin	-0.1102	0.632	1	9.212	0.4996
**DDX42**	DDX42	5	DEAD-box helicase 42	protein_codin	0.002294	0.988	1	31.63	0.1298
**SLC38A10**	SLC38A10	5	solute carrier family 38 member 10	protein_codin	0.535	0.00526	1	20.45	0.03381
**HGS**	HGS	5	hepatocyte growth factor-regulated tyrosine kinase su	protein_codin	-0.1624	0.464	1	8.642	0.3634
**MAFG**	MAFG	5	MAF bZIP transcription factor G	protein_codin	0.3484	0.039	1	18.76	0.3896
**VAPA**	VAPA	5	VAMP associated protein A	protein_codin	0.03857	0.5301	1	50.63	0.1379
**PTPN2**	PTPN2	5	protein tyrosine phosphatase non-receptor type 2	protein_codin	-0.113	0.437	1	17.22	0.06601
**ACAA2**	ACAA2	5	acetyl-CoA acyltransferase 2	protein_codin	0.4325	0.00215	1	33.23	0.03336
**JAG1**	JAG1	5	jagged canonical Notch ligand 1	protein_codin	-0.2042	0.0538	1	53.63	0.1708
**PXMP4**	PXMP4	5	peroxisomal membrane protein 4	protein_codin	0.5066	0.0432	1	10.27	0.2508
**CPNE1**	CPNE1	5	copine 1	protein_codin	-0.04738	0.715	1	11.49	0.09952
**REXO1**	REXO1	5	RNA exonuclease 1 homolog	protein_codin	0.4805	0.0311	1	2.585	0.4599
**PIP5K1C**	PIP5K1C	5	phosphatidylinositol-4-phosphate 5-kinase type 1 gam	protein_codin	0.1047	0.607	1	3.452	0.3092
**PLIN3**	PLIN3	5	perilipin 3	protein_codin	-0.3246	0.00212	1	56.62	0.06431
**ICAM3**	ICAM3	5	intercellular adhesion molecule 3	protein_codin	-0.6311	0.00090	1	3.537	0.1044
**KEAP1**	KEAP1	5	kelch like ECH associated protein 1	protein_codin	-0.0276	0.868	1	6.238	0.2479
**ZNF791**	ZNF791	5	zinc finger protein 791	protein_codin	0.1532	0.414	1	49.96	0.06838
**HOOK2**	HOOK2	5	hook microtubule tethering protein 2	protein_codin	-0.08787	0.695	1	9.287	0.1972
**LSM4.00**	LSM4.00	5	LSM4 homolog, U6 small nuclear RNA and mRNA de	protein_codin	-0.2288	0.0259	1	38.9	0.1375
**ZNF506**	ZNF506	5	zinc finger protein 506	protein_codin	0.4658	0.0421	1	8.606	0.1374
**ZNF429**	ZNF429	5	zinc finger protein 429	protein_codin	0.1863	0.396	1	8.594	0.3176
**GPI**	GPI	5	glucose-6-phosphate isomerase	protein_codin	0.06974	0.621	1	18.07	0.1319
**LIN7B**	LIN7B	5	lin-7 homolog B, crumbs cell polarity complex compon	protein_codin	-0.2415	0.341	1	8.818	0.2032
**ZNF667-AS1**	ZNF667-AS1	5	ZNF667 antisense RNA 1 (head to head)	lincRNA	-0.1173	0.362	1	33.77	0.1042
**YWHAH**	YWHAH	5	tyrosine 3-monooxygenase/tryptophan 5-monooxyger	protein_codin	0.3796	1.07E-06	0.02489	40.53	0.03277
**EIF3D**	EIF3D	5	eukaryotic translation initiation factor 3 subunit D	protein_codin	-0.3496	5.91E-05	1	44.09	0.07051
**FAM118A**	FAM118A	5	family with sequence similarity 118 member A	protein_codin	0.6492	0.00022	1	54.24	0.3488
**C21orf2**	C21orf2	5	cilia and flagella associated protein 410	protein_codin	-0.1506	0.335	1	59.26	0.07406
**ADARB1**	ADARB1	5	adenosine deaminase RNA specific B1	protein_codin	-0.7114	0.00110	1	7.955	0.06481
**COL18A1**	COL18A1	5	collagen type XVIII alpha 1 chain	protein_codin	-0.9312	4.68E-21	1.13E-16	60.6	0.2231
**MT-ATP8**	MT-ATP8	5	mitochondrially encoded ATP synthase membrane su	protein_codin	-0.4679	5.66E-05	1	39.34	0.3852
**PUSL1**	PUSL1	6	pseudouridine synthase like 1	protein_codin	0.145	0.569	1	10.78	0.2038
**DVL1**	DVL1	6	dishevelled segment polarity protein 1	protein_codin	0.1486	0.514	1	3.206	0.09948
**MXRA8**	MXRA8	6	matrix remodeling associated 8	protein_codin	0.5908	1.32E-14	3.15E-10	39.06	0.03604
**SSU72**	SSU72	6	SSU72 homolog, RNA polymerase II CTD phosphatas	protein_codin	0.02497	0.802	1	29.64	0.1016
**TNFRSF14**	TNFRSF14	6	TNF receptor superfamily member 14	protein_codin	0.4872	0.0188	1	22.32	0.2872
**CLSTN1**	CLSTN1	6	calsyntenin 1	protein_codin	0.3447	0.0121	1	6.356	0.07214
**LZIC**	LZIC	6	leucine zipper and CTNNBIP1 domain containing	protein_codin	-0.05388	0.639	1	13.96	0.1682
**PLOD1**	PLOD1	6	procollagen-lysine,2-oxoglutarate 5-dioxygenase 1	protein_codin	-0.9094	1.99E-05	0.4573	28.38	0.2062
**VPS13D**	VPS13D	6	vacuolar protein sorting 13 homolog D	protein_codin	0.8664	0.00204	1	25.11	0.5149
**HSPB7**	HSPB7	6	heat shock protein family B (small) member 7	protein_codin	-0.6342	1.67E-05	0.384	50.42	0.1736
**USP48**	USP48	6	ubiquitin specific peptidase 48	protein_codin	0.3364	0.027	1	14.54	0.2806
**SELENON**	SELENON	6	selenoprotein N	protein_codin	0.5079	0.0424	1	2.74	0.3601
**ATPIF1**	ATPIF1	6	ATP synthase inhibitory factor subunit 1	protein_codin	NA	NA	NA	54.6	0.0364
**BSDC1**	BSDC1	6	BSD domain containing 1	protein_codin	0.4287	0.0349	1	7.647	0.1098
**ATP6V0B**	ATP6V0B	6	ATPase H+ transporting V0 subunit b	protein_codin	-0.3361	1.93E-06	0.04479	60.6	0.03298
**TMEM53**	TMEM53	6	transmembrane protein 53	protein_codin	0.3902	0.103	1	10.79	0.1387
**SLC35D1**	SLC35D1	6	solute carrier family 35 member D1	protein_codin	0.6314	0.038	1	3.392	0.1017
**DBT**	DBT	6	dihydrolipoamide branched chain transacylase E2	protein_codin	0.5296	0.0500	1	8.441	0.1387
**GSTM5**	GSTM5	6	glutathione S-transferase mu 5	protein_codin	2.861	4.99E-28	1.2E-23	59.53	0.07202
**NRAS**	NRAS	6	NRAS proto-oncogene, GTPase	protein_codin	0.5158	0.0947	1	2.876	0.1352
**CSDE1**	CSDE1	6	cold shock domain containing E1	protein_codin	-0.3193	1.61E-06	0.03739	54.74	0.06460
**SNAPIN**	SNAPIN	6	SNAP associated protein	protein_codin	0.09189	0.437	1	13.86	0.244
**C1orf43**	C1orf43	6	chromosome 1 open reading frame 43	protein_codin	-0.09299	0.249	1	29.32	0.07257
**SLC50A1**	SLC50A1	6	solute carrier family 50 member 1	protein_codin	0.3335	0.0879	1	7.96	0.1388
**PIGM**	PIGM	6	phosphatidylinositol glycan anchor biosynthesis class	protein_codin	-0.04636	0.842	1	8.018	0.2781
**NIT1**	NIT1	6	nitrilase 1	protein_codin	0.1754	0.298	1	24.48	0.1474
**RFWD2**	RFWD2	6	COP1 E3 ubiquitin ligase	protein_codin	NA	NA	NA	8.26	0.1132
**DHX9**	DHX9	6	DExH-box helicase 9	protein_codin	0.2615	0.0625	1	34.6	0.2692
**RGL1**	RGL1	6	ral guanine nucleotide dissociation stimulator like 1	protein_codin	1.303	6.5E-10	1.54E-05	7.586	0.1751
**FAM89A**	FAM89A	6	family with sequence similarity 89 member A	protein_codin	-0.8043	0.00010	1	3.586	0.1736
**TMEM214**	TMEM214	6	transmembrane protein 214	protein_codin	0.4884	0.0201	1	9.182	0.1435
**VIT**	VIT	6	vitrin	protein_codin	4.306	0.947	1	50.42	0.03317
**DYNC2LI1**	DYNC2LI1	6	dynein cytoplasmic 2 light intermediate chain 1	protein_codin	0.3796	0.0340	1	7.745	0.1399
**PPP4R3B**	PPP4R3B	6	protein phosphatase 4 regulatory subunit 3B	protein_codin	-0.1527	0.277	1	40.12	0.3959
**UGP2**	UGP2	6	UDP-glucose pyrophosphorylase 2	protein_codin	0.1215	0.166	1	60.6	0.06752
**MPHOSPH10**	MPHOSPH10	6	M-phase phosphoprotein 10	protein_codin	-0.08667	0.466	1	29.03	0.1734
**TMEM150A**	TMEM150A	6	transmembrane protein 150A	protein_codin	-0.1375	0.491	1	11.23	0.1415
**FBLN7**	FBLN7	6	fibulin 7	protein_codin	-0.3024	0.12	1	3.55	0.2479
**PLA2R1**	PLA2R1	6	phospholipase A2 receptor 1	protein_codin	-0.07072	0.786	1	7.368	0.2376
**INO80D**	INO80D	6	INO80 complex subunit D	protein_codin	0.4635	0.0319	1	23.67	0.1782
**DNAJB2**	DNAJB2	6	DnaJ heat shock protein family (Hsp40) member B2	protein_codin	0.01934	0.911	1	8.184	0.09861
**TRIP12**	TRIP12	6	thyroid hormone receptor interactor 12	protein_codin	-0.119	0.411	1	34.67	0.2005
**PSMD1**	PSMD1	6	proteasome 26S subunit, non-ATPase 1	protein_codin	-0.008634	0.941	1	12.37	0.1407
**SNED1**	SNED1	6	sushi, nidogen and EGF like domains 1	protein_codin	0.4338	0.0592	1	26.92	0.2426
**THAP4**	THAP4	6	THAP domain containing 4	protein_codin	0.0872	0.649	1	3.026	0.1788
**UBE2E2**	UBE2E2	6	ubiquitin conjugating enzyme E2 E2	protein_codin	0.3722	0.0107	1	15.53	0.102
**MLH1**	MLH1	6	mutL homolog 1	protein_codin	0.3366	0.103	1	3.325	0.2092
**PRICKLE2**	PRICKLE2	6	prickle planar cell polarity protein 2	protein_codin	-0.2601	0.3	1	3.532	0.3212
**UMPS**	UMPS	6	uridine monophosphate synthetase	protein_codin	0.1772	0.429	1	7.918	0.1048
**COPB2**	COPB2	6	coatomer protein complex subunit beta 2	protein_codin	0.3293	0.00092	1	29.32	0.0659
**GK5**	GK5	6	glycerol kinase 5	protein_codin	0.4485	0.0929	1	7.73	0.4491
**PLSCR4**	PLSCR4	6	phospholipid scramblase 4	protein_codin	0.683	9.5E-09	0.00022	55.18	0.1052
**DHX36**	DHX36	6	DEAH-box helicase 36	protein_codin	0.02765	0.774	1	15.55	0.1721
**TRA2B**	TRA2B	6	transformer 2 beta homolog	protein_codin	0.4168	0.00033	1	32.94	0.1019
**CTBP1**	CTBP1	6	C-terminal binding protein 1	protein_codin	0.5765	0.00362	1	8.727	0.4245
**SLBP**	SLBP	6	stem-loop binding protein	protein_codin	0.2303	0.0806	1	42.78	0.1714
**TMEM129**	TMEM129	6	transmembrane protein 129	protein_codin	-0.2309	0.149	1	3.449	0.1722
**ADD1**	ADD1	6	adducin 1	protein_codin	0.4066	4.4E-05	1	15.23	0.03198
**MRFAP1**	MRFAP1	6	Morf4 family associated protein 1	protein_codin	-0.3578	1.63E-11	3.87E-07	60.48	0.06557
**OCIAD1**	OCIAD1	6	OCIA domain containing 1	protein_codin	0.01149	0.859	1	59.38	0.06864
**CLOCK**	CLOCK	6	clock circadian regulator	protein_codin	0.000585	0.996	1	25.12	0.2710
**HNRNPDL**	HNRNPDL	6	heterogeneous nuclear ribonucleoprotein D like	protein_codin	0.06762	0.214	1	59.92	0.06563
**ENOPH1**	ENOPH1	6	enolase-phosphatase 1	protein_codin	0.07066	0.691	1	3.422	0.06801
**UBE2D3**	UBE2D3	6	ubiquitin conjugating enzyme E2 D3	protein_codin	0.1526	0.0322	1	12.23	0.1012
**EXOSC9**	EXOSC9	6	exosome component 9	protein_codin	0.109	0.566	1	8.457	0.206
**ETFDH**	ETFDH	6	electron transfer flavoprotein dehydrogenase	protein_codin	0.6486	0.00513	1	7.222	0.1725
**PPID**	PPID	6	peptidylprolyl isomerase D	protein_codin	0.04355	0.744	1	17.98	0.09914
**PRIMPOL**	PRIMPOL	6	primase and DNA directed polymerase	protein_codin	0.1098	0.672	1	3.598	0.2118
**BRD9**	BRD9	6	bromodomain containing 9	protein_codin	0.1573	0.369	1	8.324	0.2622
**CLPTM1L**	CLPTM1L	6	CLPTM1 like	protein_codin	0.2022	0.189	1	7.812	0.1481
**SNHG18**	SNHG18	6	small nucleolar RNA host gene 18	lincRNA	-0.5785	2.49E-05	0.5712	16.44	0.2162
**PDE4D**	PDE4D	6	phosphodiesterase 4D	protein_codin	-1.464	3.14E-10	7.43E-06	27.77	0.3169
**SLC30A5**	SLC30A5	6	solute carrier family 30 member 5	protein_codin	0.09032	0.736	1	6.687	0.1731
**SRFBP1**	SRFBP1	6	serum response factor binding protein 1	protein_codin	0.1324	0.322	1	35.12	0.1042
**3-Mar**	43527	6	membrane associated ring-CH-type finger 3	protein_codin	1.525	2.5E-05	0.5735	10.29	0.1142
**JADE2**	JADE2	6	jade family PHD finger 2	protein_codin	0.2596	0.25	1	3.018	0.2011
**BRD8**	BRD8	6	bromodomain containing 8	protein_codin	-0.002637	0.985	1	8.166	0.2674
**NDUFA2**	NDUFA2	6	NADH:ubiquinone oxidoreductase subunit A2	protein_codin	-0.3136	9.35E-05	1	27.93	0.1442
**RPL26L1**	RPL26L1	6	ribosomal protein L26 like 1	protein_codin	-0.2916	0.0189	1	36.14	0.2124
**CREBRF**	CREBRF	6	CREB3 regulatory factor	protein_codin	0.177	0.155	1	48.43	0.2564
**PHYKPL**	PHYKPL	6	5-phosphohydroxy-L-lysine phospho-lyase	protein_codin	0.7394	3.21E-05	0.7352	59.44	0.2735
**NOL7**	NOL7	6	nucleolar protein 7	protein_codin	-0.1545	0.0216	1	57.99	0.03297
**NELFE**	NELFE	6	negative elongation factor complex member E	protein_codin	-0.1547	0.211	1	15.26	0.1069
**PHF1**	PHF1	6	PHD finger protein 1	protein_codin	-0.08846	0.55	1	7.082	0.176
**SRPK1**	SRPK1	6	SRSF protein kinase 1	protein_codin	-0.1603	0.305	1	6.066	0.08330
**GLO1**	GLO1	6	glyoxalase I	protein_codin	-0.009076	0.918	1	16.09	0.1369
**MRPL14**	MRPL14	6	mitochondrial ribosomal protein L14	protein_codin	-0.2485	0.0162	1	60.37	0.06558
**MANEA**	MANEA	6	mannosidase endo-alpha	protein_codin	-0.006562	0.971	1	3.556	0.2086
**HSF2**	HSF2	6	heat shock transcription factor 2	protein_codin	-0.1361	0.487	1	3.417	0.1402
**BCLAF1**	BCLAF1	6	BCL2 associated transcription factor 1	protein_codin	0.2032	0.0354	1	35.6	0.06803
**PSMG3**	PSMG3	6	proteasome assembly chaperone 3	protein_codin	0.03386	0.817	1	5.176	0.1686
**KLHL7**	KLHL7	6	kelch like family member 7	protein_codin	0.3232	0.206	1	7.408	0.2767
**MALSU1**	MALSU1	6	mitochondrial assembly of ribosomal large subunit 1	protein_codin	-0.2007	0.128	1	18.07	0.1672
**CREB5**	CREB5	6	cAMP responsive element binding protein 5	protein_codin	2.431	5.72E-12	1.36E-08	57.96	0.1759
**CPVL**	CPVL	6	carboxypeptidase vitellogenic like	protein_codin	3.569	2.1E-15	5.02E-11	55.35	0.1797
**GARS**	GARS	6	glycyl-tRNA synthetase	protein_codin	0.249	0.179	1	25.11	0.06776
**LSM5.00**	LSM5.00	6	LSM5 homolog, U6 small nuclear RNA and mRNA de	protein_codin	-0.2053	0.0185	1	60.48	0.034
**TNS3**	TNS3	6	tensin 3	protein_codin	-0.6513	0.00286	1	9.178	0.4111
**CCL26**	CCL26	6	C-C motif chemokine ligand 26	protein_codin	0.2987	0.505	1	60.6	1.337
**PEX1**	PEX1	6	peroxisomal biogenesis factor 1	protein_codin	0.2072	0.297	1	7.052	0.3914
**DYNC1I1**	DYNC1I1	6	dynein cytoplasmic 1 intermediate chain 1	protein_codin	0.2148	0.371	1	6.522	0.3059
**POLR2J**	POLR2J	6	RNA polymerase II subunit J	protein_codin	-0.2559	0.00046	1	26.72	0.1019
**CREB3L2**	CREB3L2	6	cAMP responsive element binding protein 3 like 2	protein_codin	0.3092	0.0642	1	38.46	0.09856
**TRAPPC2**	TRAPPC2	6	trafficking protein particle complex 2	protein_codin	0.5833	0.0112	1	8.798	0.3548
**MAOB**	MAOB	6	monoamine oxidase B	protein_codin	-1.37	2.84E-11	6.74E-07	8.674	0.4194
**TSR2**	TSR2	6	TSR2 ribosome maturation factor	protein_codin	-0.54	7.34E-08	0.00172	59.75	0.136
**YIPF6**	YIPF6	6	Yip1 domain family member 6	protein_codin	0.07366	0.537	1	33.55	0.2058
**CHM**	CHM	6	CHM Rab escort protein	protein_codin	0.5116	0.00065	1	44.23	0.1714
**FAM199X**	FAM199X	6	family with sequence similarity 199, X-linked	protein_codin	-0.02814	0.889	1	3.275	0.1788
**STAG2**	STAG2	6	stromal antigen 2	protein_codin	0.2633	0.0287	1	21.95	0.1402
**RAP2C**	RAP2C	6	RAP2C, member of RAS oncogene family	protein_codin	0.4766	0.126	1	8.906	0.388
**CDR1**	CDR1	6	NA	antisense_RN	0.161	0.506	1	22.05	0.2735
**CXorf40B**	CXorf40B	6	chromosome X open reading frame 40B	protein_codin	0.3298	0.309	1	2.248	0.3341
**VMA21**	VMA21	6	vacuolar ATPase assembly factor VMA21	protein_codin	0.05802	0.656	1	7.752	0.06706
**IDH3G**	IDH3G	6	isocitrate dehydrogenase (NAD(+)) 3 gamma	protein_codin	-0.2753	0.00216	1	22.33	0.2024
**DNASE1L1**	DNASE1L1	6	deoxyribonuclease 1 like 1	protein_codin	-0.9278	1.82E-05	0.4183	30.1	0.1808
**VAMP7**	VAMP7	6	vesicle associated membrane protein 7	protein_codin	-0.01884	0.92	1	3.523	0.1397
**AC145124.1**	AC145124.1	6	novel transcri	antisense_RN	-0.02753	0.897	1	3.465	0.2736
**PHYHIP**	PHYHIP	6	phytanoyl-CoA 2-hydroxylase interacting protein	protein_codin	2.679	5.98E-06	1	11.65	0.2161
**SLC39A14**	SLC39A14	6	solute carrier family 39 member 14	protein_codin	-0.0777	0.654	1	60.58	0.06526
**TNFRSF10B**	TNFRSF10B	6	TNF receptor superfamily member 10b	protein_codin	0.2826	0.228	1	9.828	0.07920
**GOLGA7**	GOLGA7	6	golgin A7	protein_codin	-0.02124	0.837	1	23.65	0.0671
**RNF170**	RNF170	6	ring finger protein 170	protein_codin	0.3505	0.179	1	7.297	0.1732
**MMP16**	MMP16	6	matrix metallopeptidase 16	protein_codin	0.2128	0.486	1	11.27	0.222
**C8orf88**	C8orf88	6	chromosome 8 open reading frame 88	protein_codin	-0.1007	0.542	1	9.402	0.06522
**OTUD6B-AS1**	OTUD6B-AS1	6	OTUD6B antisense RNA 1 (head to head)	antisense_RN	0.2834	0.00414	1	37.83	0.1348
**ENY2**	ENY2	6	ENY2 transcription and export complex 2 subunit	protein_codin	-0.05349	0.502	1	19.23	0.03597
**PTPRD**	PTPRD	6	protein tyrosine phosphatase receptor type D	protein_codin	0.1688	0.458	1	33.05	0.392
**RNF38**	RNF38	6	ring finger protein 38	protein_codin	-0.0976	0.679	1	34.1	0.3142
**TOMM5**	TOMM5	6	translocase of outer mitochondrial membrane 5	protein_codin	0.2658	0.225	1	8.73	0.2748
**CARD19**	CARD19	6	caspase recruitment domain family member 19	protein_codin	-0.3503	0.00587	1	42.18	0.2
**ALG2**	ALG2	6	ALG2 alpha-1,3/1,6-mannosyltransferase	protein_codin	0.6208	0.00027	1	51.62	0.1016
**HSPA5**	HSPA5	6	heat shock protein family A (Hsp70) member 5	protein_codin	0.3584	1.99E-06	0.04616	60.6	0.06570
**STXBP1**	STXBP1	6	syntaxin binding protein 1	protein_codin	1.905	9.9E-11	2.35E-06	7.014	0.221
**ABL1**	ABL1	6	ABL proto-oncogene 1, non-receptor tyrosine kinase	protein_codin	0.9702	3.87E-06	0.08954	31.72	0.2774
**RIC3**	RIC3	6	RIC3 acetylcholine receptor chaperone	protein_codin	0.492	0.0486	1	3.629	0.3206
**ZBED5**	ZBED5	6	zinc finger BED-type containing 5	protein_codin	0.2653	0.117	1	19.64	0.2791
**SPTY2D1**	SPTY2D1	6	SPT2 chromatin protein domain containing 1	protein_codin	0.4205	0.0128	1	7.508	0.1396
**HTATIP2**	HTATIP2	6	HIV-1 Tat interactive protein 2	protein_codin	-0.1548	0.229	1	16.92	0.1007
**LGR4**	LGR4	6	leucine rich repeat containing G protein-coupled rece	protein_codin	0.412	0.211	1	9.048	0.4411
**TRIM44**	TRIM44	6	tripartite motif containing 44	protein_codin	0.08206	0.575	1	27.95	0.1082
**PHF21A**	PHF21A	6	PHD finger protein 21A	protein_codin	0.2314	0.386	1	7.772	0.2788
**FAM180B**	FAM180B	6	family with sequence similarity 180 member B	protein_codin	4.299	0.958	1	33.76	0.1052
**C1QTNF4**	C1QTNF4	6	C1q and TNF related 4	protein_codin	2.689	3.44E-08	0.00081	59.96	0.3894
**EML3**	EML3	6	EMAP like 3	protein_codin	0.2888	0.222	1	9.094	0.3032
**CEP295**	CEP295	6	centrosomal protein 295	protein_codin	0.3472	0.22	1	10.15	0.3987
**DIXDC1**	DIXDC1	6	DIX domain containing 1	protein_codin	-0.222	0.0297	1	29.54	0.1345
**PTS**	PTS	6	6-pyruvoyltetrahydropterin synthase	protein_codin	0.05409	0.679	1	39.39	0.2104
**IFT46**	IFT46	6	intraflagellar transport 46	protein_codin	-0.4393	0.0515	1	8.477	0.2392
**VWA5A**	VWA5A	6	von Willebrand factor A domain containing 5A	protein_codin	0.6902	0.00852	1	9.334	0.2556
**VPS26B**	VPS26B	6	VPS26, retromer complex component B	protein_codin	0.07854	0.692	1	10.42	0.1321
**ACAD8**	ACAD8	6	acyl-CoA dehydrogenase family member 8	protein_codin	0.06529	0.764	1	2.808	0.1058
**SVIL**	SVIL	6	supervillin	protein_codin	-1.083	2.35E-51	5.7E-47	60.58	0.09994
**TFAM**	TFAM	6	transcription factor A, mitochondrial	protein_codin	-0.122	0.354	1	16.63	0.2408
**ADK**	ADK	6	adenosine kinase	protein_codin	0.784	9.93E-07	0.0231	55.24	0.09540
**SFR1**	SFR1	6	SWI5 dependent homologous recombination repair pr	protein_codin	0.4203	0.0299	1	7.045	0.0652
**SFXN4**	SFXN4	6	sideroflexin 4	protein_codin	-0.0009182	0.996	1	8.155	0.1017
**SPATS2**	SPATS2	6	spermatogenesis associated serine rich 2	protein_codin	0.2699	0.189	1	6.349	0.3010
**ATG101**	ATG101	6	autophagy related 101	protein_codin	0.2123	0.138	1	36.86	0.07050
**EIF4B**	EIF4B	6	eukaryotic translation initiation factor 4B	protein_codin	-0.2676	0.00017	1	55.25	0.06499
**SMARCC2**	SMARCC2	6	SWI/SNF related, matrix associated, actin dependent	protein_codin	0.2518	0.0697	1	31.46	0.06803
**MON2**	MON2	6	MON2 homolog, regulator of endosome-to-Golgi traffi	protein_codin	0.7673	0.00047	1	29.79	0.4098
**SLC35E3**	SLC35E3	6	solute carrier family 35 member E3	protein_codin	0.7383	0.00013	1	20.7	0.1092
**EID3**	EID3	6	EP300 interacting inhibitor of differentiation 3	protein_codin	-0.3825	0.176	1	8.905	0.4219
**C12orf76**	C12orf76	6	chromosome 12 open reading frame 76	protein_codin	-0.3146	0.0346	1	40.39	0.0681
**ALG5**	ALG5	6	ALG5 dolichyl-phosphate beta-glucosyltransferase	protein_codin	0.09329	0.469	1	40.52	0.06562
**DGKH**	DGKH	6	diacylglycerol kinase eta	protein_codin	-0.7453	0.00046	1	10.42	0.3854
**MBNL2**	MBNL2	6	muscleblind like splicing regulator 2	protein_codin	0.4863	9.33E-06	0.215	59.66	0.03742
**SUPT16H**	SUPT16H	6	SPT16 homolog, facilitates chromatin remodeling sub	protein_codin	0.1232	0.229	1	21.73	0.04247
**TRAC**	TRAC	6	T cell receptor alpha constant	TR_C_gene	1.434	8.38E-06	0.1932	54.29	0.1371
**NUBPL**	NUBPL	6	nucleotide binding protein like	protein_codin	0.1166	0.619	1	3.424	0.1824
**MIS18BP1**	MIS18BP1	6	MIS18 binding protein 1	protein_codin	0.07282	0.68	1	47.9	0.3141
**ALDH6A1**	ALDH6A1	6	aldehyde dehydrogenase 6 family member A1	protein_codin	0.1199	0.427	1	21.44	0.2321
**EIF2B2**	EIF2B2	6	eukaryotic translation initiation factor 2B subunit beta	protein_codin	0.257	0.118	1	48	0.1692
**GSTZ1**	GSTZ1	6	glutathione S-transferase zeta 1	protein_codin	-0.2067	0.379	1	3.314	0.2212
**CPSF2**	CPSF2	6	cleavage and polyadenylation specific factor 2	protein_codin	-0.1645	0.397	1	7.068	0.1365
**MOAP1**	MOAP1	6	modulator of apoptosis 1	protein_codin	-0.2309	0.127	1	25.97	0.1036
**TECPR2**	TECPR2	6	tectonin beta-propeller repeat containing 2	protein_codin	0.07447	0.762	1	10.35	0.215
**LYSMD2**	LYSMD2	6	LysM domain containing 2	protein_codin	-0.06116	0.645	1	17.49	0.06762
**CLK3**	CLK3	6	CDC like kinase 3	protein_codin	-0.0692	0.738	1	8.635	0.2455
**MAN2C1**	MAN2C1	6	mannosidase alpha class 2C member 1	protein_codin	0.302	0.332	1	8.643	0.3495
**PTPN9**	PTPN9	6	protein tyrosine phosphatase non-receptor type 9	protein_codin	0.4246	0.0875	1	2.772	0.1029
**CHSY1**	CHSY1	6	chondroitin sulfate synthase 1	protein_codin	-0.2956	0.127	1	39.47	0.1785
**SNRNP25**	SNRNP25	6	small nuclear ribonucleoprotein U11/U12 subunit 25	protein_codin	-0.2767	0.0549	1	18.66	0.1701
**LMF1**	LMF1	6	lipase maturation factor 1	protein_codin	0.8836	0.00277	1	8.547	0.1832
**GFER**	GFER	6	growth factor, augmenter of liver regeneration	protein_codin	-0.1156	0.589	1	3.466	0.2084
**VASN**	VASN	6	vasorin	protein_codin	0.675	1.46E-11	3.47E-07	60.58	0.03264
**SPNS1**	SPNS1	6	sphingolipid transporter 1 (putative)	protein_codin	0.2108	0.299	1	9.622	0.1052
**TBC1D10B**	TBC1D10B	6	TBC1 domain family member 10B	protein_codin	-0.1829	0.538	1	3.236	0.1468
**1-Sep**	43709	6	septin 1	protein_codin	-0.143	0.38	1	8.312	0.1639
**DCTPP1**	DCTPP1	6	dCTP pyrophosphatase 1	protein_codin	-0.1653	0.265	1	21.38	0.1308
**ORAI3**	ORAI3	6	ORAI calcium release-activated calcium modulator 3	protein_codin	0.9142	6.58E-07	0.01524	42.33	0.0688
**HSD3B7**	HSD3B7	6	hydroxy-delta-5-steroid dehydrogenase, 3 beta- and s	protein_codin	1.05	1.36E-06	0.0316	60	0.03735
**ITFG1**	ITFG1	6	integrin alpha FG-GAP repeat containing 1	protein_codin	0.1745	0.179	1	15.27	0.03754
**FTO**	FTO	6	FTO alpha-ketoglutarate dependent dioxygenase	protein_codin	0.2568	0.252	1	7.85	0.2912
**MBTPS1**	MBTPS1	6	membrane bound transcription factor peptidase, site 1	protein_codin	0.05018	0.695	1	28.26	0.06728
**ZC3H18**	ZC3H18	6	zinc finger CCCH-type containing 18	protein_codin	-0.3375	0.139	1	7.212	0.3250
**ACSF3**	ACSF3	6	acyl-CoA synthetase family member 3	protein_codin	0.03741	0.849	1	2.673	0.07712
**XAF1**	XAF1	6	XIAP associated factor 1	protein_codin	-0.1135	0.585	1	30.25	0.23
**DVL2**	DVL2	6	dishevelled segment polarity protein 2	protein_codin	0.2115	0.292	1	3.171	0.1385
**CD68**	CD68	6	CD68 molecule	protein_codin	0.8498	7.36E-07	0.01715	57.86	0.05175
**FXR2**	FXR2	6	FMR1 autosomal homolog 2	protein_codin	-0.1112	0.615	1	2.846	0.2462
**TOM1L2**	TOM1L2	6	target of myb1 like 2 membrane trafficking protein	protein_codin	-0.0445	0.881	1	8.584	0.5916
**DRG2**	DRG2	6	developmentally regulated GTP binding protein 2	protein_codin	0.3338	0.0628	1	7.2	0.2857
**SUPT6H**	SUPT6H	6	SPT6 homolog, histone chaperone and transcription e	protein_codin	0.3346	0.0617	1	49.83	0.1669
**STARD3**	STARD3	6	StAR related lipid transfer domain containing 3	protein_codin	-0.05342	0.828	1	3.124	0.1470
**RARA-AS1**	RARA-AS1	6	RARA antisense RNA 1	antisense_RN	0.3473	0.132	1	33.21	0.1148
**RAMP2**	RAMP2	6	receptor activity modifying protein 2	protein_codin	3.358	1.24E-17	2.97E-13	60	0.152
**DHX8**	DHX8	6	DEAH-box helicase 8	protein_codin	0.3245	0.209	1	7.524	0.2009
**SCRN2**	SCRN2	6	secernin 2	protein_codin	0.2972	0.121	1	29	0.1092
**XYLT2**	XYLT2	6	xylosyltransferase 2	protein_codin	-0.03282	0.908	1	3.015	0.1929
**VEZF1**	VEZF1	6	vascular endothelial zinc finger 1	protein_codin	0.298	0.024	1	14.72	0.1702
**SUPT4H1**	SUPT4H1	6	SPT4 homolog, DSIF elongation factor subunit	protein_codin	-0.3319	0.00073	1	25.06	0.1404
**CYB561**	CYB561	6	cytochrome b561	protein_codin	0.6609	0.00638	1	9.17	0.1079
**TACO1**	TACO1	6	translational activator of cytochrome c oxidase I	protein_codin	0.1018	0.602	1	8.959	0.2075
**HAUS1**	HAUS1	6	HAUS augmin like complex subunit 1	protein_codin	-0.06506	0.752	1	9.837	0.1349
**C20orf27**	C20orf27	6	chromosome 20 open reading frame 27	protein_codin	0.05807	0.714	1	46.99	0.2318
**COMMD7**	COMMD7	6	COMM domain containing 7	protein_codin	-0.1905	0.154	1	6.686	0.1358

**PLTP**	PLTP	6	phospholipid transfer protein	protein_codin	1.753	1.91E-39	4.63E-35	60.58	0.03309
**PMEPA1**	PMEPA1	6	prostate transmembrane protein, androgen induced 1	protein_codin	-0.6358	2.79E-12	6.64E-09	59.41	0.03486
**SHC2**	SHC2	6	SHC adaptor protein 2	protein_codin	1.771	7.23E-07	0.01685	7.99	0.258
**NDUFS7**	NDUFS7	6	NADH:ubiquinone oxidoreductase core subunit S7	protein_codin	-0.01008	0.895	1	53.37	0.2464
**SH3GL1**	SH3GL1	6	SH3 domain containing GRB2 like 1, endophilin A2	protein_codin	0.2469	0.281	1	7.535	0.06907
**HDGFL2**	HDGFL2	6	HDGF like 2	protein_codin	0.09786	0.482	1	20.8	0.1072
**SAFB**	SAFB	6	scaffold attachment factor B	protein_codin	0.1429	0.396	1	20.71	0.211
**MRPL4**	MRPL4	6	mitochondrial ribosomal protein L4	protein_codin	-0.005409	0.964	1	12.19	0.1319
**TMED1**	TMED1	6	transmembrane p24 trafficking protein 1	protein_codin	0.05859	0.675	1	16.96	0.2047
**WDR83**	WDR83	6	WD repeat domain 83	protein_codin	0.243	0.235	1	3.378	0.288
**PKN1**	PKN1	6	protein kinase N1	protein_codin	0.004861	0.976	1	18.42	0.158
**DDA1**	DDA1	6	DET1 and DDB1 associated 1	protein_codin	-0.1316	0.39	1	18.2	0.2107
**FKBP8**	FKBP8	6	FKBP prolyl isomerase 8	protein_codin	-0.05097	0.459	1	46.18	0.03263
**POP4**	POP4	6	POP4 homolog, ribonuclease P/MRP subunit	protein_codin	-0.2682	0.0593	1	38.38	0.07079
**LGI4**	LGI4	6	leucine rich repeat LGI family member 4	protein_codin	1.062	3.23E-07	0.00754	9.188	0.09439
**ZFP14**	ZFP14	6	ZFP14 zinc finger protein	protein_codin	0.3738	0.0851	1	7.92	0.2819
**EIF3K**	EIF3K	6	eukaryotic translation initiation factor 3 subunit K	protein_codin	-0.3149	1.84E-12	4.37E-08	54.7	0.04032
**TMEM91**	TMEM91	6	transmembrane protein 91	protein_codin	0.2179	0.202	1	8.815	0.2709
**CCDC61**	CCDC61	6	coiled-coil domain containing 61	protein_codin	0.5677	0.0166	1	3.185	0.2334
**DBP**	DBP	6	D-box binding PAR bZIP transcription factor	protein_codin	0.3038	0.0425	1	47.61	0.1692
**BCL2L12**	BCL2L12	6	BCL2 like 12	protein_codin	0.3208	0.161	1	3.052	0.103
**DDX3Y**	DDX3Y	6	DEAD-box helicase 3 Y-linked	protein_codin	0.3168	0.0332	1	30.27	0.1362
**PPIL2**	PPIL2	6	peptidylprolyl isomerase like 2	protein_codin	0.3469	0.12	1	2.806	0.2171
**GAS2L1**	GAS2L1	6	growth arrest specific 2 like 1	protein_codin	0.5233	0.0272	1	10.17	0.3569
**APOL3**	APOL3	6	apolipoprotein L3	protein_codin	0.6623	5.9E-05	1	46.62	0.07123
**TST**	TST	6	thiosulfate sulfurtransferase	protein_codin	0.1106	0.652	1	7.503	0.2167
**SYNGR1**	SYNGR1	6	synaptogyrin 1	protein_codin	0.7203	6.03E-06	0.1392	33.37	0.152
**NADK**	NADK	7	NAD kinase	protein_codin	0.5983	0.0123	1	10.38	0.2368
**PER3**	PER3	7	period circadian regulator 3	protein_codin	0.4019	0.0105	1	49.61	0.174
**SDHB**	SDHB	7	succinate dehydrogenase complex iron sulfur subunit B	protein_codin	-0.04471	0.634	1	26.54	0.1332
**ID3**	ID3	7	inhibitor of DNA binding 3, HLH protein	protein_codin	0.3019	2.59E-06	0.06004	60.6	0.1714
**LYPLA2**	LYPLA2	7	lysophospholipase 2	protein_codin	-0.2753	0.12	1	6.97	0.1312
**ADPRHL2**	ADPRHL2	7	ADP-ribosylhydrolase like 2	protein_codin	-0.1679	0.331	1	8.692	0.2737
**CEPT1**	CEPT1	7	choline/ethanolamine phosphotransferase 1	protein_codin	0.4219	0.0945	1	9.789	0.4345
**RSBN1**	RSBN1	7	round spermatid basic protein 1	protein_codin	0.06672	0.622	1	44.74	0.03332
**KIFAP3**	KIFAP3	7	kinesin associated protein 3	protein_codin	0.4628	0.00017	1	12.36	0.09988
**RABIF**	RABIF	7	RAB interacting factor	protein_codin	-0.1162	0.564	1	3.263	0.1037
**RAB4A**	RAB4A	7	RAB4A, member RAS oncogene family	protein_codin	-0.2962	0.00399	1	28.22	0.0685
**NRBP1**	NRBP1	7	nuclear receptor binding protein 1	protein_codin	-0.0624	0.619	1	28.73	0.03405
**CYBRD1**	CYBRD1	7	cytochrome b reductase 1	protein_codin	1.378	2.02E-36	4.89E-32	33.78	0.03511
**SETD5**	SETD5	7	SET domain containing 5	protein_codin	-0.1418	0.381	1	7.226	0.2469
**WDR48**	WDR48	7	WD repeat domain 48	protein_codin	-0.009802	0.961	1	26.34	0.179
**NT5DC2**	NT5DC2	7	5'-nucleotidase domain containing 2	protein_codin	0.1402	0.302	1	54.46	0.142
**RNF13**	RNF13	7	ring finger protein 13	protein_codin	0.589	3.36E-10	7.95E-06	48.52	0.06572
**UBXN7**	UBXN7	7	UBX domain protein 7	protein_codin	0.369	0.112	1	55.37	0.3538
**ANKRD17**	ANKRD17	7	ankyrin repeat domain 17	protein_codin	0.426	0.0211	1	48.35	0.4994
**TIFA**	TIFA	7	TRAF interacting protein with forkhead associated do	protein_codin	-0.1727	0.355	1	25.19	0.1011
**BRIX1**	BRIX1	7	biogenesis of ribosomes BRX1	protein_codin	0.1342	0.311	1	13.82	0.1326
**OXCT1**	OXCT1	7	3-oxoacid CoA-transferase 1	protein_codin	-0.1762	0.285	1	7.176	0.2034
**FBXO4**	FBXO4	7	F-box protein 4	protein_codin	-0.3927	0.0745	1	7.73	0.2142
**ISOC1**	ISOC1	7	isochorismatase domain containing 1	protein_codin	0.04971	0.81	1	10.09	0.2088
**ZMAT2**	ZMAT2	7	zinc finger matrin-type 2	protein_codin	-0.2372	0.0303	1	58.76	0.2069
**BNIP1**	BNIP1	7	BCL2 interacting protein 1	protein_codin	0.113	0.627	1	3.182	0.1068
**UIMC1**	UIMC1	7	ubiquitin interaction motif containing 1	protein_codin	-0.1237	0.476	1	7.376	0.2748
**SERPINB6**	SERPINB6	7	serpin family B member 6	protein_codin	-0.08752	0.259	1	33.36	0.1004
**GNL1**	GNL1	7	G protein nucleolar 1 (putative)	protein_codin	0.04829	0.739	1	18.54	0.06626
**CDC5L**	CDC5L	7	cell division cycle 5 like	protein_codin	0.2799	0.00996	1	33.98	0.07325
**TMEM30A**	TMEM30A	7	transmembrane protein 30A	protein_codin	-0.005422	0.952	1	60.58	0.1319
**FILIP1**	FILIP1	7	filamin A interacting protein 1	protein_codin	-0.345	0.0168	1	27.62	0.06576
**PHIP**	PHIP	7	pleckstrin homology domain interacting protein	protein_codin	0.1096	0.436	1	22.38	0.1785
**STX7**	STX7	7	syntaxin 7	protein_codin	0.2405	0.0742	1	30.86	0.2432
**GGCT**	GGCT	7	gamma-glutamylcyclotransferase	protein_codin	-0.4758	0.00126	1	35.03	0.2158
**POM121C**	POM121C	7	POM121 transmembrane nucleoporin C	protein_codin	0.2617	0.27	1	9.66	0.1042
**MAGI2**	MAGI2	7	membrane associated guanylate kinase, WW and PD	protein_codin	-0.1993	0.3	1	26.04	0.3746
**ANKIB1**	ANKIB1	7	ankyrin repeat and IBR domain containing 1	protein_codin	0.1073	0.542	1	8.52	0.1836
**BPGM**	BPGM	7	bisphosphoglycerate mutase	protein_codin	0.3644	0.047	1	8.814	0.1339
**SAT1**	SAT1	7	spermidine/spermine N1-acetyltransferase 1	protein_codin	0.02752	0.788	1	60	0.03696
**UBE2A**	UBE2A	7	ubiquitin conjugating enzyme E2 A	protein_codin	0.1885	0.122	1	11.77	0.1442
**MCTS1**	MCTS1	7	MCTS1 re-initiation and release factor	protein_codin	-0.3985	0.00207	1	32.51	0.1406
**ARHGEF10**	ARHGEF10	7	Rho guanine nucleotide exchange factor 10	protein_codin	0.9442	0.00228	1	11.38	0.7558
**POLR3D**	POLR3D	7	RNA polymerase III subunit D	protein_codin	0.09143	0.694	1	54.45	0.2332
**IMPA1**	IMPA1	7	inositol monophosphatase 1	protein_codin	-0.1228	0.482	1	29.12	0.1709
**SNX16**	SNX16	7	sorting nexin 16	protein_codin	0.3596	0.104	1	9.364	0.1719
**JAK2**	JAK2	7	Janus kinase 2	protein_codin	-0.7134	0.00038	1	10.08	0.1717
**SLC44A1**	SLC44A1	7	solute carrier family 44 member 1	protein_codin	0.1045	0.505	1	19.21	0.103
**ALKBH3**	ALKBH3	7	alkB homolog 3, alpha-ketoglutaratedependent dioxy	protein_codin	0.01615	0.926	1	8.064	0.2237
**C11orf1**	C11orf1	7	chromosome 11 open reading frame 1	protein_codin	-0.5354	0.00503	1	24.81	0.2542
**CDON**	CDON	7	cell adhesion associated, oncogene regulated	protein_codin	2.301	5.37E-09	0.00012	9.423	0.3271
**ZNF37A**	ZNF37A	7	zinc finger protein 37A	protein_codin	0.06426	0.734	1	24.26	0.2452
**MYOF**	MYOF	7	myoferlin	protein_codin	0.5333	0.00012	1	29.41	0.2045
**PHB2**	PHB2	7	prohibitin 2	protein_codin	-0.2045	0.00453	1	55.85	0.03246
**ITPR2**	ITPR2	7	inositol 1,4,5-trisphosphate receptor type 2	protein_codin	0.02682	0.896	1	36.82	0.1061
**LRRK2**	LRRK2	7	leucine rich repeat kinase 2	protein_codin	0.5118	0.00538	1	51.82	0.199
**SCAF11**	SCAF11	7	SR-related CTD associated factor 11	protein_codin	0.3033	0.00342	1	24.78	0.1372
**THAP2**	THAP2	7	THAP domain containing 2	protein_codin	0.1346	0.616	1	9.635	0.3888
**ATXN7L3B**	ATXN7L3B	7	ataxin 7 like 3B	protein_codin	0.1878	0.109	1	46.9	0.1036
**TDG**	TDG	7	thymine DNA glycosylase	protein_codin	0.4893	0.00408	1	8.298	0.2445
**GIT2**	GIT2	7	GIT ArfGAP 2	protein_codin	0.3434	0.137	1	8.447	0.2747
**MAPKAPK5-AS**	MAPKAPK5-AS	7	MAPKAPK5 antisense RNA 1	lincRNA	-0.3171	0.0626	1	22.45	0.3369
**SNRNP35**	SNRNP35	7	small nuclear ribonucleoprotein U11/U12 subunit 35	protein_codin	-0.1288	0.366	1	7.057	0.2056
**ZMYM5**	ZMYM5	7	zinc finger MYM-type containing 5	protein_codin	0.123	0.539	1	22.41	0.1359
**MICU2**	MICU2	7	mitochondrial calcium uptake 2	protein_codin	0.483	0.0054	1	20.51	0.1062
**CGRRF1**	CGRRF1	7	cell growth regulator with ring finger domain 1	protein_codin	-0.1246	0.4	1	8.407	0.1321
**SIPA1L1**	SIPA1L1	7	signal induced proliferation associated 1 like 1	protein_codin	0.2352	0.287	1	21.36	0.2484
**MINDY2**	MINDY2	7	MINDY lysine 48 deubiquitinase 2	protein_codin	-0.03218	0.779	1	20.29	0.03228
**RSL1D1**	RSL1D1	7	ribosomal L1 domain containing 1	protein_codin	0.03405	0.615	1	53.48	0.09961
**BCKDK**	BCKDK	7	branched chain keto acid dehydrogenase kinase	protein_codin	0.3384	0.039	1	21.88	0.03221
**USP10**	USP10	7	ubiquitin specific peptidase 10	protein_codin	0.3962	0.0305	1	8.87	0.1381
**METTL16**	METTL16	7	methyltransferase like 16	protein_codin	0.05233	0.796	1	28.97	0.2914
**TMEM256**	TMEM256	7	transmembrane protein 256	protein_codin	-0.1882	0.0685	1	48.32	0.167
**CDK12**	CDK12	7	cyclin dependent kinase 12	protein_codin	0.3926	0.0312	1	7.458	0.2804
**G6PC3**	G6PC3	7	glucose-6-phosphatase catalytic subunit 3	protein_codin	-0.04438	0.819	1	27.4	0.1391
**CHMP1B**	CHMP1B	7	charged multivesicular body protein 1B	protein_codin	0.2903	0.088	1	50.99	0.4777
**ID1**	ID1	7	inhibitor of DNA binding 1, HLH protein	protein_codin	0.09447	0.451	1	24.94	0.1118
**EIF6**	EIF6	7	eukaryotic translation initiation factor 6	protein_codin	-0.2879	0.00188	1	59	0.06435
**C20orf24**	C20orf24	7	RAB5 interacting factor	protein_codin	NA	NA	NA	19.51	0.2111
**STX16**	STX16	7	syntaxin 16	protein_codin	0.1779	0.46	1	8.226	0.4192
**MRPL54**	MRPL54	7	mitochondrial ribosomal protein L54	protein_codin	-0.1709	0.071	1	33	0.1771
**RAB8A**	RAB8A	7	RAB8A, member RAS oncogene family	protein_codin	-0.113	0.376	1	14.73	0.2031
**USE1**	USE1	7	unconventional SNARE in the ER 1	protein_codin	-0.2499	0.0587	1	56.76	0.1309
**COPE**	COPE	7	coatomer protein complex subunit epsilon	protein_codin	-0.1557	0.0207	1	35.33	0.03419
**SUN2**	SUN2	7	Sad1 and UNC84 domain containing 2	protein_codin	-0.3397	0.00107	1	10.84	0.07
**NAGA**	NAGA	7	alpha-N-acetylgalactosaminidase	protein_codin	0.2224	0.345	1	3.245	0.2094
**MEGF6**	MEGF6	8	multiple EGF like domains 6	protein_codin	0.7292	0.00354	1	58.79	0.1435
**MTHFR**	MTHFR	8	methylenetetrahydrofolate reductase	protein_codin	0.6506	0.015	1	3.785	0.2809
**KDM1A**	KDM1A	8	lysine demethylase 1A	protein_codin	-0.007328	0.965	1	26.93	0.1785
**PIGV**	PIGV	8	phosphatidylinositol glycan anchor biosynthesis class	protein_codin	0.08032	0.689	1	8.964	0.1199
**SRSF4**	SRSF4	8	serine and arginine rich splicing factor 4	protein_codin	-0.1893	0.0675	1	48.25	0.1356
**PEF1**	PEF1	8	penta-EF-hand domain containing 1	protein_codin	-0.2563	0.0261	1	12.58	0.2443
**AGO3**	AGO3	8	argonaute RISC catalytic component 3	protein_codin	0.3167	0.186	1	7.307	0.3182
**FHL3**	FHL3	8	four and a half LIM domains 3	protein_codin	-0.5233	0.00058	1	38.51	0.06521
**ECHDC2**	ECHDC2	8	enoyl-CoA hydratase domain containing 2	protein_codin	1.041	2.14E-10	5.07E-06	32.88	0.2809
**SCP2**	SCP2	8	sterol carrier protein 2	protein_codin	0.4365	1.82E-10	4.31E-06	59.75	0.06679
**JAK1**	JAK1	8	Janus kinase 1	protein_codin	0.3266	4.88E-05	1	26.82	0.06647
**GNG12**	GNG12	8	G protein subunit gamma 12	protein_codin	-0.03323	0.693	1	19.27	0.07209
**ODF2L**	ODF2L	8	outer dense fiber of sperm tails 2 like	protein_codin	0.2583	0.0996	1	47.87	0.1076
**DR1**	DR1	8	down-regulator of transcription 1	protein_codin	-0.115	0.43	1	7.408	0.2459
**AGL**	AGL	8	amylo-alpha-1, 6-glucosidase, 4-alpha-glucanotransfe	protein_codin	0.01593	0.926	1	41.88	0.1581
**WDR47**	WDR47	8	WD repeat domain 47	protein_codin	0.2287	0.252	1	8.372	0.1369
**SRGAP2B**	SRGAP2B	8	SLIT-ROBO Rho GTPase activating protein 2B	protein_codin	0.2812	0.262	1	3.727	0.4059
**PDE4DIP**	PDE4DIP	8	phosphodiesterase 4D interacting protein	protein_codin	-0.2156	0.12	1	14.56	0.0695
**OTUD7B**	OTUD7B	8	OTU deubiquitinase 7B	protein_codin	-0.209	0.459	1	8.487	0.3171
**PRPF3**	PRPF3	8	pre-mRNA processing factor 3	protein_codin	-0.07788	0.734	1	3.285	0.2496
**MCL1**	MCL1	8	MCL1 apoptosis regulator, BCL2 family member	protein_codin	0.7188	4.49E-11	1.07E-06	42.5	0.1025
**C1orf56**	C1orf56	8	chromosome 1 open reading frame 56	protein_codin	-0.04934	0.762	1	20.23	0.3452
**NPR1.00**	NPR1.00	8	natriuretic peptide receptor 1	protein_codin	0.8696	7.1E-07	0.01655	54.5	0.1025
**FLAD1**	FLAD1	8	flavin adenine dinucleotide synthetase 1	protein_codin	0.2593	0.293	1	2.861	0.1974
**ISG20L2**	ISG20L2	8	interferon stimulated exonuclease gene 20 like 2	protein_codin	0.3389	0.165	1	8.348	0.2054
**IFI16**	IFI16	8	interferon gamma inducible protein 16	protein_codin	0.0628	0.436	1	59.92	0.0969
**COPA**	COPA	8	coatomer protein complex subunit alpha	protein_codin	0.04854	0.726	1	16.16	0.1742
**B4GALT3**	B4GALT3	8	beta-1,4-galactosyltransferase 3	protein_codin	-0.0739	0.734	1	2.862	0.1883
**TMCO1**	TMCO1	8	transmembrane and coiled-coil domains 1	protein_codin	0.1879	0.0211	1	60.31	0.06902
**SOAT1**	SOAT1	8	sterol O-acyltransferase 1	protein_codin	0.7526	0.00065	1	25.8	0.2095
**TOR1AIP2**	TOR1AIP2	8	torsin 1A interacting protein 2	protein_codin	0.02409	0.849	1	14.44	0.03357
**ZNF281**	ZNF281	8	zinc finger protein 281	protein_codin	0.5264	0.0798	1	7.753	0.32
**CYB5R1**	CYB5R1	8	cytochrome b5 reductase 1	protein_codin	-0.1953	0.0975	1	12.5	0.1352
**PPP1R15B**	PPP1R15B	8	protein phosphatase 1 regulatory subunit 15B	protein_codin	0.893	0.00203	1	58.96	0.4292
**MDM4**	MDM4	8	MDM4 regulator of p53	protein_codin	0.371	0.0416	1	49.87	0.3699
**C1orf132**	C1orf132	8	MIR29B2 and MIR29C host gene	lincRNA	NA	NA	NA	10.35	1.48
**TATDN3**	TATDN3	8	TatD DNase domain containing 3	protein_codin	-0.04436	0.871	1	3.338	0.145
**ANGEL2**	ANGEL2	8	angel homolog 2	protein_codin	0.1766	0.359	1	3.298	0.2092
**TGFB2**	TGFB2	8	transforming growth factor beta 2	protein_codin	0.5703	0.0769	1	38.63	0.06481
**AIDA**	AIDA	8	axin interactor, dorsalization associated	protein_codin	0.004973	0.969	1	30.88	0.2148
**SDE2**	SDE2	8	SDE2 telomere maintenance homolog	protein_codin	0.101	0.626	1	2.883	0.1558
**ARID4B**	ARID4B	8	AT-rich interaction domain 4B	protein_codin	-0.1893	0.0358	1	49.34	0.1045
**EFCAB2**	EFCAB2	8	EF-hand calcium binding domain 2	protein_codin	0.5103	0.0876	1	35.53	0.2796
**CNST**	CNST	8	consortin, connexin sorting protein	protein_codin	-0.3718	0.0932	1	3.703	0.2158
**DNMT3A**	DNMT3A	8	DNA methyltransferase 3 alpha	protein_codin	0.1284	0.619	1	3.369	0.2826
**STRN**	STRN	8	striatin	protein_codin	0.7604	0.00197	1	8.154	0.3217
**CEBPZ**	CEBPZ	8	CCAAT enhancer binding protein zeta	protein_codin	0.03323	0.794	1	10.85	0.2096
**EHBP1**	EHBP1	8	EH domain binding protein 1	protein_codin	1.321	3.39E-12	8.07E-09	18.36	0.2854
**MEIS1**	MEIS1	8	Meis homeobox 1	protein_codin	0.4488	0.0244	1	6.539	0.3384
**C1D**	C1D	8	C1D nuclear receptor corepressor	protein_codin	-0.1219	0.187	1	12.41	0.135
**MTHFD2**	MTHFD2	8	methylenetetrahydrofolate dehydrogenase (NADP+ d	protein_codin	-0.4478	2.35E-05	0.5394	50	0.03762
**IMMT**	IMMT	8	inner membrane mitochondrial protein	protein_codin	-0.008219	0.954	1	36.25	0.06842
**MRPL35**	MRPL35	8	mitochondrial ribosomal protein L35	protein_codin	0.3206	0.0647	1	21.63	0.06932
**CYTOR**	CYTOR	8	cytoskeleton regulator RNA	lincRNA	-0.2383	0.0822	1	59.19	0.195
**EIF5B**	EIF5B	8	eukaryotic translation initiation factor 5B	protein_codin	-0.3458	3.64E-06	0.08422	42.17	0.06608
**REV1**	REV1	8	REV1 DNA directed polymerase	protein_codin	0.2669	0.126	1	7.695	0.2042
**LINC00116**	LINC00116	8	mitoregulin	protein_codin	NA	NA	NA	21.43	0.1701
**MIR4435-2HG**	MIR4435-2HG	8	MIR4435-2 host gene	lincRNA	-0.05214	0.757	1	29.52	0.1919
**CCDC115**	CCDC115	8	coiled-coil domain containing 115	protein_codin	-0.3915	0.00016	1	32.93	0.2026
**ZEB2**	ZEB2	8	zinc finger E-box binding homeobox 2	protein_codin	0.1554	0.105	1	47.9	0.2105
**EPC2**	EPC2	8	enhancer of polycomb homolog 2	protein_codin	0.06854	0.685	1	7.505	0.3495
**RIF1**	RIF1	8	replication timing regulatory factor 1	protein_codin	-0.04035	0.81	1	18.97	0.1232
**METTL8**	METTL8	8	methyltransferase like 8	protein_codin	0.4327	0.0712	1	10.24	0.1411
**MTX2**	MTX2	8	metaxin 2	protein_codin	-0.1293	0.402	1	6.723	0.1309
**UBE2E3**	UBE2E3	8	ubiquitin conjugating enzyme E2 E3	protein_codin	-0.1389	0.0982	1	49.3	0.1739
**DNAJC10**	DNAJC10	8	DnaJ heat shock protein family (Hsp40) member C10	protein_codin	0.3818	0.00186	1	48.45	0.1374
**ITGAV**	ITGAV	8	integrin subunit alpha V	protein_codin	-0.09039	0.479	1	60.58	0.0669
**CALCRL**	CALCRL	8	calcitonin receptor like receptor	protein_codin	-0.3459	0.02	1	60.58	0.209
**SF3B1**	SF3B1	8	splicing factor 3b subunit 1	protein_codin	0.1888	0.0276	1	16.99	0.2001
**CFLAR**	CFLAR	8	CASP8 and FADD like apoptosis regulator	protein_codin	0.3003	0.0352	1	20.73	0.243
**FZD7**	FZD7	8	frizzled class receptor 7	protein_codin	0.8199	0.0009	1	31.72	0.3566
**RAPH1**	RAPH1	8	Ras association (RalGDS/AF-6) and pleckstrin homol	protein_codin	-0.353	0.0587	1	9.897	0.1764
**CUL3**	CUL3	8	cullin 3	protein_codin	0.04145	0.79	1	6.814	0.2379
**JAGN1**	JAGN1	8	jagunal homolog 1	protein_codin	-0.02437	0.84	1	15.62	0.06914
**NR2C2**	NR2C2	8	nuclear receptor subfamily 2 group C member 2	protein_codin	0.1043	0.72	1	9.103	0.2854
**GOLGA4**	GOLGA4	8	golgin A4	protein_codin	0.0522	0.626	1	27.7	0.06783
**SEC22C**	SEC22C	8	SEC22 homolog C, vesicle trafficking protein	protein_codin	0.5206	0.0008	1	7.546	0.1372
**NKTR**	NKTR	8	natural killer cell triggering receptor	protein_codin	0.378	0.00988	1	31.24	0.332
**SCAP**	SCAP	8	SREBF chaperone	protein_codin	-0.07585	0.751	1	2.006	0.3132
**WDR6**	WDR6	8	WD repeat domain 6	protein_codin	0.1754	0.48	1	10.03	0.2952
**RBM15B**	RBM15B	8	RNA binding motif protein 15B	protein_codin	0.628	0.00202	1	7.719	0.1415
**CCDC66**	CCDC66	8	coiled-coil domain containing 66	protein_codin	0.2479	0.0649	1	30.73	0.2305
**PTPRG**	PTPRG	8	protein tyrosine phosphatase receptor type G	protein_codin	-0.08053	0.654	1	18.17	0.2528
**SUCLG2**	SUCLG2	8	succinate-CoA ligase GDP-forming beta subunit	protein_codin	-0.135	0.253	1	11.93	0.0354
**CMSS1**	CMSS1	8	cms1 ribosomal small subunit homolog	protein_codin	-0.4415	0.00213	1	55.83	0.235
**BBX**	BBX	8	BBX high mobility group box domain containing	protein_codin	-0.1233	0.145	1	19.87	0.06629
**FAM162A**	FAM162A	8	family with sequence similarity 162 member A	protein_codin	-0.2025	0.017	1	54.66	0.2084
**SEC22A**	SEC22A	8	SEC22 homolog A, vesicle trafficking protein	protein_codin	0.3197	0.156	1	9.187	0.2367
**PODXL2**	PODXL2	8	podocalyxin like 2	protein_codin	0.9743	1.25E-05	0.2879	10.2	0.2818
**HMCES**	HMCES	8	5-hydroxymethylcytosine binding, ES cell specific	protein_codin	0.09361	0.586	1	6.457	0.202
**ANAPC13**	ANAPC13	8	anaphase promoting complex subunit 13	protein_codin	0.3632	0.0128	1	38.41	0.07712
**SLC25A36**	SLC25A36	8	solute carrier family 25 member 36	protein_codin	0.4763	7.58E-05	1	45.45	0.2065
**RASA2**	RASA2	8	RAS p21 protein activator 2	protein_codin	0.1932	0.37	1	6.913	0.1341
**U2SURP**	U2SURP	8	U2 snRNP associated SURP domain containing	protein_codin	0.2604	0.00779	1	27.79	0.2294
**HPS3**	HPS3	8	HPS3 biogenesis of lysosomal organelles complex 2	protein_codin	0.3757	0.129	1	3.507	0.1472
**GMPS**	GMPS	8	guanine monophosphate synthase	protein_codin	-0.1068	0.432	1	6.657	0.2377
**SHOX2**	SHOX2	8	short stature homeobox 2	protein_codin	-0.1817	0.472	1	26.08	0.1795
**MYNN**	MYNN	8	myoneurin	protein_codin	0.07	0.712	1	9.845	0.2752
**MAEA**	MAEA	8	macrophage erythroblast attacher	protein_codin	-0.02579	0.872	1	6.523	0.2499
**CC2D2A**	CC2D2A	8	coiled-coil and C2 domain containing 2A	protein_codin	0.6563	8.04E-05	1	20.4	0.1015
**LDB2**	LDB2	8	LIM domain binding 2	protein_codin	0.9438	0.00012	1	21.61	0.1848
**ANAPC4**	ANAPC4	8	anaphase promoting complex subunit 4	protein_codin	0.3566	0.129	1	8.758	0.1403
**CEP135**	CEP135	8	centrosomal protein 135	protein_codin	0.2183	0.374	1	36.19	0.3294
**POLR2B**	POLR2B	8	RNA polymerase II subunit B	protein_codin	0.242	0.11	1	15.58	0.3088
**RCHY1**	RCHY1	8	ring finger and CHY zinc finger domain containing 1	protein_codin	-0.3232	0.0817	1	3.474	0.2024
**PKD2**	PKD2	8	polycystin 2, transient receptor potential cation chann	protein_codin	0.1644	0.232	1	53.53	0.1198
**KIAA1109**	KIAA1109	8	KIAA1109	protein_codin	0.2633	0.066	1	56.56	0.2294
**IL15**	IL15	8	interleukin 15	protein_codin	-0.2203	0.322	1	8.653	0.1806
**GAB1**	GAB1	8	GRB2 associated binding protein 1	protein_codin	-0.1057	0.592	1	10.17	0.06896
**TMEM184C**	TMEM184C	8	transmembrane protein 184C	protein_codin	0.08096	0.717	1	2.25	0.1322
**PRMT9**	PRMT9	8	protein arginine methyltransferase 9	protein_codin	0.9299	0.0005	1	9.167	0.1872
**ARHGAP10**	ARHGAP10	8	Rho GTPase activating protein 10	protein_codin	0.05952	0.549	1	28.14	0.1372
**CTSO**	CTSO	8	cathepsin O	protein_codin	0.1786	0.224	1	45.84	0.3165
**NEK1**	NEK1	8	NIMA related kinase 1	protein_codin	0.5046	0.00523	1	8.887	0.06752
**WWC2**	WWC2	8	WW and C2 domain containing 2	protein_codin	0.2238	0.311	1	8.796	0.06871
**CDKN2AIP**	CDKN2AIP	8	CDKN2A interacting protein	protein_codin	0.6973	9.37E-05	1	17.36	0.3737
**MRPL36**	MRPL36	8	mitochondrial ribosomal protein L36	protein_codin	-0.442	1.52E-05	0.3497	34.39	0.2461
**OTULIN**	OTULIN	8	OTU deubiquitinase with linear linkage specificity	protein_codin	0.2658	0.216	1	7.392	0.2789
**DNAJC21**	DNAJC21	8	DnaJ heat shock protein family (Hsp40) member C21	protein_codin	-0.1525	0.105	1	45.98	0.06812
**NDUFS4**	NDUFS4	8	NADH:ubiquinone oxidoreductase subunit S4	protein_codin	-0.0835	0.295	1	59.9	0.1006
**IL6ST**	IL6ST	8	interleukin 6 signal transducer	protein_codin	0.3004	0.00011	1	42.71	0.03266
**NDUFAF2**	NDUFAF2	8	NADH:ubiquinone oxidoreductase complex assembly	protein_codin	-0.2467	0.014	1	26.29	0.1059
**CWC27**	CWC27	8	CWC27 spliceosome associated cyclophilin	protein_codin	-0.1323	0.352	1	7.154	0.174
**MCCC2**	MCCC2	8	methylcrotonoyl-CoA carboxylase 2	protein_codin	-0.2686	0.214	1	3.253	0.2421
**ANKRA2**	ANKRA2	8	ankyrin repeat family A member 2	protein_codin	0.1523	0.44	1	7.068	0.2762
**ZBED3**	ZBED3	8	zinc finger BED-type containing 3	protein_codin	-0.1098	0.622	1	3.607	0.2724
**ZFYVE16**	ZFYVE16	8	zinc finger FYVE-type containing 16	protein_codin	0.2165	0.165	1	17.35	0.3143
**ERAP2**	ERAP2	8	endoplasmic reticulum aminopeptidase 2	protein_codin	0.6365	0.00436	1	59.68	0.1051
**SRP19**	SRP19	8	signal recognition particle 19	protein_codin	0.07601	0.548	1	13.54	0.07166
**CCDC112**	CCDC112	8	coiled-coil domain containing 112	protein_codin	-0.7203	2.46E-05	0.5644	38.74	0.2474
**TMED7**	TMED7	8	transmembrane p24 trafficking protein 7	protein_codin	-0.08307	0.512	1	57.52	0.207
**LOX**	LOX	8	lysyl oxidase	protein_codin	1.347	7.28E-12	1.73E-08	55.04	0.03723
**GRAMD2B**	GRAMD2B	8	GRAM domain containing 2B	protein_codin	0.1737	0.386	1	60.58	0.376
**KIF3A**	KIF3A	8	kinesin family member 3A	protein_codin	0.4538	0.00926	1	22.13	0.06763
**AFF4**	AFF4	8	AF4/FMR2 family member 4	protein_codin	0.3747	0.00427	1	29.17	0.1851
**DNAJC18**	DNAJC18	8	DnaJ heat shock protein family (Hsp40) member C18	protein_codin	0.006257	0.975	1	8.862	0.2052
**PANK3**	PANK3	8	pantothenate kinase 3	protein_codin	-0.08984	0.687	1	3.016	0.211
**SFXN1**	SFXN1	8	sideroflexin 1	protein_codin	0.5256	0.00412	1	8.663	0.1288
**CLK4**	CLK4	8	CDC like kinase 4	protein_codin	0.155	0.367	1	7.944	0.3068
**MAPK9**	MAPK9	8	mitogen-activated protein kinase 9	protein_codin	0.00571	0.981	1	7.09	0.2062
**CDYL**	CDYL	8	chromodomain Y like	protein_codin	0.1234	0.549	1	6.907	0.1384
**SNRNP48**	SNRNP48	8	small nuclear ribonucleoprotein U11/U12 subunit 48	protein_codin	0.1929	0.387	1	8.301	0.2397
**EEF1E1**	EEF1E1	8	eukaryotic translation elongation factor 1 epsilon 1	protein_codin	-0.04664	0.729	1	15.23	0.1345
**MYLIP**	MYLIP	8	myosin regulatory light chain interacting protein	protein_codin	-0.48	0.00047	1	57.35	0.1731
**ZSCAN16-AS1**	ZSCAN16-AS1	8	ZSCAN16 antisense RNA 1	antisense_RN	-0.1498	0.307	1	20	0.1648
**MRPS18B**	MRPS18B	8	mitochondrial ribosomal protein S18B	protein_codin	0.01438	0.9	1	23.15	0.06614
**RGL2**	RGL2	8	ral guanine nucleotide dissociation stimulator like 2	protein_codin	-0.3096	0.172	1	28.66	0.1292
**MAPK14**	MAPK14	8	mitogen-activated protein kinase 14	protein_codin	0.6413	0.00739	1	8.464	0.06592
**KCTD20**	KCTD20	8	potassium channel tetramerization domain containing 20	protein_codin	0.3164	0.0437	1	58.62	0.1393
**SAYSD1**	SAYSD1	8	SAYSVFN motif domain containing 1	protein_codin	0.1853	0.382	1	3.673	0.03202
**MRPS10**	MRPS10	8	mitochondrial ribosomal protein S10	protein_codin	0.1113	0.379	1	25.97	0.07379
**RPL7L1**	RPL7L1	8	ribosomal protein L7 like 1	protein_codin	0.1432	0.191	1	20.58	0.06949
**GSTA4**	GSTA4	8	glutathione S-transferase alpha 4	protein_codin	0.04716	0.762	1	7.242	0.167
**IBTK**	IBTK	8	inhibitor of Bruton tyrosine kinase	protein_codin	0.212	0.234	1	25.34	0.2701
**ORC3**	ORC3	8	origin recognition complex subunit 3	protein_codin	0.2097	0.254	1	6.303	0.206
**LYRM2**	LYRM2	8	LYR motif containing 2	protein_codin	0.1545	0.222	1	51.27	0.1052
**MAP3K7**	MAP3K7	8	mitogen-activated protein kinase kinase kinase 7	protein_codin	-0.1058	0.609	1	3.312	0.1671
**ATG5**	ATG5	8	autophagy related 5	protein_codin	0.006051	0.967	1	23.87	0.2042
**TNFAIP3**	TNFAIP3	8	TNF alpha induced protein 3	protein_codin	-0.4203	0.182	1	28.4	0.353
**FBXO30**	FBXO30	8	F-box protein 30	protein_codin	-0.05706	0.819	1	10.43	0.2768
**COX19**	COX19	8	cytochrome c oxidase assembly factor COX19	protein_codin	0.2128	0.272	1	26.9	0.1076
**MPP6**	MPP6	8	membrane palmitoylated protein 6	protein_codin	-0.4123	0.0321	1	9.937	0.2262
**YAE1D1**	YAE1D1	8	YAE1 maturation factor of ABCE1	protein_codin	0.2732	0.177	1	6.78	0.2361
**URGCP**	URGCP	8	upregulator of cell proliferation	protein_codin	-0.1741	0.452	1	2.758	0.3939
**SUMF2**	SUMF2	8	sulfatase modifying factor 2	protein_codin	0.2555	0.00807	1	45.83	0.06686
**PHKG1**	PHKG1	8	phosphorylase kinase catalytic subunit gamma 1	protein_codin	-0.4504	0.012	1	8.847	0.2784
**EIF4H**	EIF4H	8	eukaryotic translation initiation factor 4H	protein_codin	-0.273	0.0015	1	28.61	0.032
**RHBDD2**	RHBDD2	8	rhomboid domain containing 2	protein_codin	0.4977	0.00379	1	18.25	0.1767
**MDH2**	MDH2	8	malate dehydrogenase 2	protein_codin	-0.0519	0.436	1	28.03	0.06666
**CDK14**	CDK14	8	cyclin dependent kinase 14	protein_codin	0.616	0.0125	1	7.896	0.2445
**SLC25A13**	SLC25A13	8	solute carrier family 25 member 13	protein_codin	0.9099	0.00092	1	8.312	0.2217
**SDHAF3**	SDHAF3	8	succinate dehydrogenase complex assembly factor 3	protein_codin	0.09312	0.58	1	7.749	0.2345
**CPSF4**	CPSF4	8	cleavage and polyadenylation specific factor 4	protein_codin	0.2283	0.324	1	3.183	0.2879
**ATP5J2**	ATP5J2	8	ATP synthase membrane subunit f	protein_codin	NA	NA	NA	39.32	0.03246
**TNPO3**	TNPO3	8	transportin 3	protein_codin	0.3355	0.194	1	2.852	0.1234
**KMT2C**	KMT2C	8	lysine methyltransferase 2C	protein_codin	0.2505	0.12	1	7.623	0.1797
**DNAJB6**	DNAJB6	8	DnaJ heat shock protein family (Hsp40) member B6	protein_codin	-0.0949	0.291	1	33.32	0.03326
**MSL3**	MSL3	8	MSL complex subunit 3	protein_codin	0.06801	0.72	1	53.41	0.1462
**ZRSR2**	ZRSR2	8	zinc finger CCCH-type, RNA binding motif and serine	protein_codin	-0.2564	0.319	1	7.605	0.1746
**RBBP7**	RBBP7	8	RB binding protein 7, chromatin remodeling factor	protein_codin	-0.09458	0.284	1	30.38	0.1398
**NHS**	NHS	8	NHS actin remodeling regulator	protein_codin	-0.1923	0.556	1	10.57	0.4670
**SMS**	SMS	8	spermine synthase	protein_codin	0.3787	0.00106	1	60.6	0.09933
**CCDC22**	CCDC22	8	coiled-coil domain containing 22	protein_codin	0.3633	0.105	1	2.813	0.1033
**USP51**	USP51	8	ubiquitin specific peptidase 51	protein_codin	0.2142	0.329	1	3.828	0.3464
**PBDC1**	PBDC1	8	polysaccharide biosynthesis domain containing 1	protein_codin	0.08426	0.472	1	29.96	0.06597
**TCEAL8**	TCEAL8	8	transcription elongation factor A like 8	protein_codin	0.07544	0.392	1	27.58	0.07148
**MORC4**	MORC4	8	MORC family CW-type zinc finger 4	protein_codin	0.4073	0.0217	1	41.93	0.1440
**DOCK11**	DOCK11	8	dedicator of cytokinesis 11	protein_codin	0.1898	0.288	1	28.66	0.137
**FGF13**	FGF13	8	fibroblast growth factor 13	protein_codin	-0.4796	0.0249	1	3.976	0.3777
**F8A1**	F8A1	8	coagulation factor VIII associated 1	protein_codin	0.8234	2.09E-05	0.48	10.06	0.1658
**POLB**	POLB	8	DNA polymerase beta	protein_codin	0.565	0.023	1	8.433	0.1460
**FNTA**	FNTA	8	farnesyltransferase, CAAX box, alpha	protein_codin	0.2094	0.0901	1	13.42	0.2074
**HGSNAT**	HGSNAT	8	heparan-alpha-glucosaminide N-acetyltransferase	protein_codin	0.2066	0.377	1	8.536	0.4865
**FAM110B**	FAM110B	8	family with sequence similarity 110 member B	protein_codin	0.5758	0.0271	1	9.847	0.2414
**WWP1**	WWP1	8	WW domain containing E3 ubiquitin protein ligase 1	protein_codin	0.6339	0.0129	1	7.316	0.3390
**VPS13B**	VPS13B	8	vacuolar protein sorting 13 homolog B	protein_codin	0.5727	0.011	1	56.32	0.2777
**EMC2**	EMC2	8	ER membrane protein complex subunit 2	protein_codin	0.117	0.29	1	60.23	0.1001
**AGO2**	AGO2	8	argonaute RISC catalytic component 2	protein_codin	0.05087	0.787	1	21.58	0.1707
**EXOSC4**	EXOSC4	8	exosome component 4	protein_codin	-0.2078	0.279	1	8.438	0.06747
**WASHC1**	WASHC1	8	WASH complex subunit 1	protein_codin	0.2552	0.185	1	6.903	0.03305
**GLIS3**	GLIS3	8	GLIS family zinc finger 3	protein_codin	-0.6524	0.00525	1	54.78	0.106
**RCL1**	RCL1	8	RNA terminal phosphate cyclase like 1	protein_codin	-0.2823	0.135	1	3.418	0.2472
**MPDZ**	MPDZ	8	multiple PDZ domain crumbs cell polarity complex co	protein_codin	-0.01584	0.932	1	7.26	0.282
**MLLT3**	MLLT3	8	MLLT3 super elongation complex subunit	protein_codin	0.651	0.0172	1	9.303	0.4765

**CHMP5**	CHMP5	8	charged multivesicular body protein 5	protein_codin	-0.08419	0.211	1	58.44	0.06862
**SIGMAR1**	SIGMAR1	8	sigma non-opioid intracellular receptor 1	protein_codin	0.2916	0.071	1	8.464	0.2372
**CREB3**	CREB3	8	cAMP responsive element binding protein 3	protein_codin	-0.2178	0.0739	1	16.65	0.03463
**EBLN3P**	EBLN3P	8	endogenous Bornavirus like nucleoprotein 3, pseudog	lincRNA	0.2397	0.0846	1	36.95	0.1659
**FAM122A**	FAM122A	8	family with sequence similarity 122A	protein_codin	0.2752	0.059	1	8.08	0.1742
**IARS**	IARS	8	isoleucyl-tRNA synthetase	protein_codin	0.198	0.332	1	9.106	0.1075
**FAM102A**	FAM102A	8	family with sequence similarity 102 member A	protein_codin	0.5062	0.0602	1	8.412	0.3179
**DNM1**	DNM1	8	dynamin 1	protein_codin	2.664	5.5E-09	0.00012	23.82	0.3635
**SPTAN1**	SPTAN1	8	spectrin alpha, non-erythrocytic 1	protein_codin	0.3917	0.00354	1	14.72	0.06652
**PAXX**	PAXX	8	PAXX non-homologous end joining factor	protein_codin	-0.00919	0.942	1	28.54	0.1024
**FAM160A2**	FAM160A2	8	family with sequence similarity 160 member A2	protein_codin	0.09107	0.739	1	3.158	0.3112
**RRP8**	RRP8	8	ribosomal RNA processing 8	protein_codin	0.02591	0.896	1	9.924	0.2037
**TPP1**	TPP1	8	tripeptidyl peptidase 1	protein_codin	0.5772	0.00149	1	19.62	0.1990
**ZNF143**	ZNF143	8	zinc finger protein 143	protein_codin	-0.158	0.455	1	7.502	0.2479
**CTR9**	CTR9	8	CTR9 homolog, Paf1/RNA polymerase II complex co	protein_codin	0.2105	0.222	1	7.54	0.1816
**ARL14EP**	ARL14EP	8	ADP ribosylation factor like GTPase 14 effector prote	protein_codin	0.1207	0.371	1	16.87	0.1088
**CAT**	CAT	8	catalase	protein_codin	0.8798	3.04E-15	7.26E-11	16.2	0.0360
**SLC39A13**	SLC39A13	8	solute carrier family 39 member 13	protein_codin	0.002326	0.988	1	8.137	0.07536
**TIMM10**	TIMM10	8	translocase of inner mitochondrial membrane 10	protein_codin	-0.2199	0.0596	1	59.22	0.1732
**CTNND1**	CTNND1	8	catenin delta 1	protein_codin	0.3782	0.0768	1	6.958	0.3008
**OTUB1**	OTUB1	8	OTU deubiquitinase, ubiquitin aldehyde binding 1	protein_codin	-0.09757	0.567	1	16.04	0.2752
**VPS51**	VPS51	8	VPS51 subunit of GARP complex	protein_codin	-0.3011	0.00485	1	13.65	0.06744
**NEAT1**	NEAT1	8	nuclear paraspeckle assembly transcript 1	lincRNA	0.7092	1.61E-06	0.03729	60.6	0.6115
**SCYL1**	SCYL1	8	SCY1 like pseudokinase 1	protein_codin	0.01114	0.946	1	7.897	0.1026
**EFEMP2**	EFEMP2	8	EGF containing fibulin extracellular matrix protein 2	protein_codin	0.0362	0.62	1	60.58	0.03501
**RCE1**	RCE1	8	Ras converting CAAX endopeptidase 1	protein_codin	0.1528	0.569	1	8.719	0.2467
**RPS6KB2**	RPS6KB2	8	ribosomal protein S6 kinase B2	protein_codin	0.05214	0.802	1	7.162	0.2682
**CPT1A**	CPT1A	8	carnitine palmitoyltransferase 1A	protein_codin	0.8288	0.00014	1	7.732	0.2451
**LAMTOR1**	LAMTOR1	8	late endosomal/lysosomal adaptor, MAPK and MTOR	protein_codin	-0.2122	0.00285	1	24.78	0.0357
**PAK1**	PAK1	8	p21 (RAC1) activated kinase 1	protein_codin	-0.1452	0.517	1	8.685	0.126
**ALG8**	ALG8	8	ALG8 alpha-1,3-glucosyltransferase	protein_codin	0.5429	0.0116	1	7.742	0.2031
**EED**	EED	8	embryonic ectoderm development	protein_codin	0.3274	0.206	1	7.603	0.2074
**TMEM135**	TMEM135	8	transmembrane protein 135	protein_codin	0.06187	0.796	1	56	0.3442
**CEP57**	CEP57	8	centrosomal protein 57	protein_codin	-0.1984	0.131	1	15.11	0.132
**DYNC2H1**	DYNC2H1	8	dynein cytoplasmic 2 heavy chain 1	protein_codin	0.4516	0.0537	1	52.82	0.4122
**RDX**	RDX	8	radixin	protein_codin	-0.2658	0.00380	1	21	0.1679
**SIDT2**	SIDT2	8	SID1 transmembrane family member 2	protein_codin	0.3262	0.162	1	2.87	0.434
**BCL9L**	BCL9L	8	BCL9 like	protein_codin	0.2816	0.167	1	8.606	0.1385
**RNF26**	RNF26	8	ring finger protein 26	protein_codin	-0.02072	0.926	1	3.27	0.2552
**PRPF18**	PRPF18	8	pre-mRNA processing factor 18	protein_codin	0.148	0.468	1	31.2	0.3717
**COMMD3**	COMMD3	8	COMM domain containing 3	protein_codin	-0.1353	0.21	1	11.94	0.1011
**MTPAP**	MTPAP	8	mitochondrial poly(A) polymerase	protein_codin	0.3172	0.202	1	2.212	0.06790
**CUL2**	CUL2	8	cullin 2	protein_codin	0.3619	0.0319	1	8.587	0.1389
**WASHC2A**	WASHC2A	8	WASH complex subunit 2A	protein_codin	0.3276	0.174	1	7.738	0.3232
**SGMS1**	SGMS1	8	sphingomyelin synthase 1	protein_codin	0.2474	0.204	1	9.02	0.1130
**ECD**	ECD	8	ecdysoneless cell cycle regulator	protein_codin	0.1085	0.64	1	3.518	0.1670
**SAMD8**	SAMD8	8	sterile alpha motif domain containing 8	protein_codin	0.2652	0.239	1	7.356	0.2875
**ZNF503**	ZNF503	8	zinc finger protein 503	protein_codin	0.9242	3.41E-17	8.16E-13	55.04	0.1008
**BTAF1**	BTAF1	8	B-TFIID TATA-box binding protein associated factor 1	protein_codin	-0.02042	0.931	1	7.102	0.1440
**HPS1**	HPS1	8	HPS1 biogenesis of lysosomal organelles complex 3	protein_codin	0.2952	0.112	1	8.095	0.3242
**CUTC**	CUTC	8	cutC copper transporter	protein_codin	0.408	0.0133	1	19.36	0.08431
**NDUFB8**	NDUFB8	8	NADH:ubiquinone oxidoreductase subunit B8	protein_codin	-0.2382	2.47E-05	0.5667	53.34	0.06652
**ACTR1A**	ACTR1A	8	actin related protein 1A	protein_codin	-0.06907	0.618	1	14.62	0.09882
**SLK**	SLK	8	STE20 like kinase	protein_codin	0.08799	0.446	1	12.5	0.09998
**ZDHHC6**	ZDHHC6	8	zinc finger DHHC-type containing 6	protein_codin	0.1976	0.322	1	7.054	0.0995
**SEC23IP**	SEC23IP	8	SEC23 interacting protein	protein_codin	0.3297	0.109	1	7.928	0.2803
**MGMT**	MGMT	8	O-6-methylguanine-DNA methyltransferase	protein_codin	0.3847	5.87E-05	1	56.45	0.07584
**CHD4**	CHD4	8	chromodomain helicase DNA binding protein 4	protein_codin	-0.1865	0.139	1	14.53	0.2111
**P3H3**	P3H3	8	prolyl 3-hydroxylase 3	protein_codin	0.2425	0.117	1	21.86	0.2317
**PDE3A**	PDE3A	8	phosphodiesterase 3A	protein_codin	-0.8457	9.8E-10	2.31E-05	39.62	0.06787
**CPNE8**	CPNE8	8	copine 8	protein_codin	0.173	0.219	1	16.33	0.1061
**KIF21A**	KIF21A	8	kinesin family member 21A	protein_codin	1.392	2.73E-06	0.06327	9.758	0.3172
**STAT6**	STAT6	8	signal transducer and activator of transcription 6	protein_codin	0.337	0.0900	1	7.492	0.3184
**TSPAN31**	TSPAN31	8	tetraspanin 31	protein_codin	-0.07915	0.531	1	14.72	0.1726
**TMEM5**	TMEM5	8	ribitol xylosyltransferase 1	protein_codin	NA	NA	NA	8.056	0.246
**NDUFA12**	NDUFA12	8	NADH:ubiquinone oxidoreductase subunit A12	protein_codin	-0.4203	6.42E-10	1.52E-05	51.03	0.03245
**LTA4H**	LTA4H	8	leukotriene A4 hydrolase	protein_codin	-0.4882	0.00100	1	7.032	0.1682
**CHST11**	CHST11	8	carbohydrate sulfotransferase 11	protein_codin	-0.5598	0.0218	1	3.427	0.3122
**IFT81**	IFT81	8	intraflagellar transport 81	protein_codin	0.1305	0.439	1	22.86	0.269
**FAM216A**	FAM216A	8	family with sequence similarity 216 member A	protein_codin	0.1606	0.45	1	8.424	0.2492
**MED13L**	MED13L	8	mediator complex subunit 13L	protein_codin	-0.0625	0.66	1	56.95	0.1718
**FBXO21**	FBXO21	8	F-box protein 21	protein_codin	0.01512	0.917	1	16.6	0.171
**WSB2**	WSB2	8	WD repeat and SOCS box containing 2	protein_codin	0.2738	0.125	1	21.42	0.06558
**EP400**	EP400	8	E1A binding protein p400	protein_codin	-0.06428	0.804	1	3.192	0.2180
**CENPJ**	CENPJ	8	centromere protein J	protein_codin	0.05626	0.816	1	11.85	0.3875
**POLR1D**	POLR1D	8	RNA polymerase I and III subunit D	protein_codin	-0.2043	0.0197	1	27.45	0.06852
**PDS5B**	PDS5B	8	PDS5 cohesin associated factor B	protein_codin	0.01745	0.91	1	45.72	0.1135
**GTF2F2**	GTF2F2	8	general transcription factor IIF subunit 2	protein_codin	-0.2786	0.0967	1	19.52	0.2088
**WDFY2**	WDFY2	8	WD repeat and FYVE domain containing 2	protein_codin	0.4238	0.109	1	3.605	0.2412
**DHRS12**	DHRS12	8	dehydrogenase/reductase 12	protein_codin	0.01122	0.966	1	7.452	0.199
**PCDH9**	PCDH9	8	protocadherin 9	protein_codin	1.324	1.5E-07	0.00351	36.8	0.2525
**PIBF1**	PIBF1	8	progesterone immunomodulatory binding factor 1	protein_codin	0.116	0.479	1	19.33	0.06626
**ARGLU1**	ARGLU1	8	arginine and glutamate rich 1	protein_codin	0.2742	0.001	1	31.38	0.1414
**ARHGEF7**	ARHGEF7	8	Rho guanine nucleotide exchange factor 7	protein_codin	-0.002680	0.989	1	52.4	0.1377
**TMEM55B**	TMEM55B	8	phosphatidylinositol-4,5-bisphosphate 4-phosphatase	protein_codin	NA	NA	NA	24.05	0.03541
**OXA1L**	OXA1L	8	OXA1L mitochondrial inner membrane protein	protein_codin	0.04104	0.788	1	19.57	0.1770
**CHMP4A**	CHMP4A	8	charged multivesicular body protein 4A	protein_codin	-0.4175	6.09E-05	1	25.4	0.176
**DHRS1**	DHRS1	8	dehydrogenase/reductase 1	protein_codin	-0.3785	0.0289	1	6.968	0.1022
**AKAP6**	AKAP6	8	A-kinase anchoring protein 6	protein_codin	-0.9057	0.00032	1	28.32	0.1860
**AP5M1**	AP5M1	8	adaptor related protein complex 5 subunit mu 1	protein_codin	0.247	0.262	1	3.75	0.2084
**L3HYPDH**	L3HYPDH	8	trans-L-3-hydroxyproline dehydratase	protein_codin	-0.3058	0.0982	1	9.505	0.03252
**PCNX4**	PCNX4	8	pecanex 4	protein_codin	0.3102	0.142	1	24.58	0.3045
**DCAF5**	DCAF5	8	DDB1 and CUL4 associated factor 5	protein_codin	0.5515	0.0116	1	7.548	0.41
**ELMSAN1**	ELMSAN1	8	ELM2 and Myb/SANT domain containing 1	protein_codin	0.7276	0.00012	1	21.67	0.2358
**TGFB3**	TGFB3	8	transforming growth factor beta 3	protein_codin	0.6166	0.0157	1	25.18	0.2018
**IRF2BPL**	IRF2BPL	8	interferon regulatory factor 2 binding protein like	protein_codin	0.6177	7.58E-05	1	55	0.1707
**TRIP11**	TRIP11	8	thyroid hormone receptor interactor 11	protein_codin	0.1668	0.102	1	12.97	0.24
**MOK**	MOK	8	MOK protein kinase	protein_codin	1.222	2.54E-07	0.00592	3.462	0.1362
**BTBD6**	BTBD6	8	BTB domain containing 6	protein_codin	0.2538	0.077	1	7.292	0.2015
**NIPA2**	NIPA2	8	NIPA magnesium transporter 2	protein_codin	0.3258	0.104	1	8.722	0.236
**RMDN3**	RMDN3	8	regulator of microtubule dynamics 3	protein_codin	-0.3139	0.139	1	7.424	0.2164
**DNAJC17**	DNAJC17	8	DnaJ heat shock protein family (Hsp40) member C17	protein_codin	0.3668	0.135	1	7.308	0.06528
**LRRC57**	LRRC57	8	leucine rich repeat containing 57	protein_codin	0.556	0.0186	1	7.642	0.2651
**GALK2**	GALK2	8	galactokinase 2	protein_codin	0.1213	0.595	1	11.5	0.1102
**FAM214A**	FAM214A	8	family with sequence similarity 214 member A	protein_codin	0.06484	0.803	1	10.19	0.665
**DNAAF4**	DNAAF4	8	dynein axonemal assembly factor 4	protein_codin	-0.228	0.326	1	3.76	0.4324
**INTS14**	INTS14	8	integrator complex subunit 14	protein_codin	0.01254	0.961	1	3.221	0.2069
**C15orf61**	C15orf61	8	chromosome 15 open reading frame 61	protein_codin	0.5186	0.00026	1	14.14	0.06798
**PML**	PML	8	promyelocytic leukemia	protein_codin	0.03997	0.834	1	6.768	0.2075
**WDR61**	WDR61	8	WD repeat domain 61	protein_codin	-0.07376	0.568	1	12.54	0.03977
**PSMA4**	PSMA4	8	proteasome subunit alpha 4	protein_codin	0.06472	0.441	1	48.93	0.03225
**FAH**	FAH	8	fumarylacetoacetate hydrolase	protein_codin	-0.7247	0.00336	1	35.71	0.1368
**AC013394.1**	AC013394.1	8	novel prote	protein_codin	0.3992	0.116	1	3.113	0.2805
**LUC7L**	LUC7L	8	LUC7 like	protein_codin	0.4624	0.00556	1	8.022	0.06825
**MRPL28**	MRPL28	8	mitochondrial ribosomal protein L28	protein_codin	-0.2078	0.082	1	14.71	0.2391
**METRN**	METRN	8	meteorin, glial cell differentiation regulator	protein_codin	-0.2157	0.0542	1	41.76	0.06497
**MSRB1**	MSRB1	8	methionine sulfoxide reductase B1	protein_codin	-0.4595	0.00832	1	9.596	0.2742
**PKD1**	PKD1	8	polycystin 1, transient receptor potential channel inter	protein_codin	0.2522	0.227	1	8.21	0.5266
**CREBBP**	CREBBP	8	CREB binding protein	protein_codin	0.1568	0.388	1	7.676	0.1305
**ROGDI**	ROGDI	8	rogdi atypical leucine zipper	protein_codin	-0.3248	0.0992	1	3.177	0.3492
**ITPRIPL2**	ITPRIPL2	8	ITPRIP like 2	protein_codin	-0.02298	0.84	1	43.72	0.296
**AC099518.5**	AC099518.5	8	novel transcript, antisense to ITPRIP	antisense_RN	0.007246	0.972	1	55.88	0.2348
**DCTN5**	DCTN5	8	dynactin subunit 5	protein_codin	0.2888	0.271	1	3.142	0.1676
**TUFM**	TUFM	8	Tu translation elongation factor, mitochondrial	protein_codin	-0.08302	0.288	1	59.25	0.03582
**ALDOA**	ALDOA	8	aldolase, fructose-bisphosphate A	protein_codin	0.3558	0.109	1	7.379	0.1796
**ZNF771**	ZNF771	8	zinc finger protein 771	protein_codin	-0.4582	0.00899	1	6.587	0.1395
**NAE1**	NAE1	8	NEDD8 activating enzyme E1 subunit 1	protein_codin	0.4312	0.00498	1	19.88	0.1376
**CES2**	CES2	8	carboxylesterase 2	protein_codin	0.08701	0.706	1	7.161	0.202
**NUTF2**	NUTF2	8	nuclear transport factor 2	protein_codin	-0.3163	0.00160	1	20.58	0.2227
**ZFPM1**	ZFPM1	8	zinc finger protein, FOG family member 1	protein_codin	-0.0608	0.692	1	7.076	0.1772
**RPAIN**	RPAIN	8	RPA interacting protein	protein_codin	-0.07996	0.455	1	42.72	0.3015
**POLR2A**	POLR2A	8	RNA polymerase II subunit A	protein_codin	-0.008291	0.958	1	8.502	0.2088
**KDM6B**	KDM6B	8	lysine demethylase 6B	protein_codin	0.6652	0.00873	1	30.9	0.3125
**VAMP2**	VAMP2	8	vesicle associated membrane protein 2	protein_codin	0.1098	0.0849	1	40.79	0.171
**CENPV**	CENPV	8	centromere protein V	protein_codin	-0.1781	0.505	1	7.817	0.2172
**ALKBH5**	ALKBH5	8	alkB homolog 5, RNA demethylase	protein_codin	0.1542	0.322	1	59.33	0.07512
**WSB1**	WSB1	8	WD repeat and SOCS box containing 1	protein_codin	0.5868	2.58E-07	0.00602	48.79	0.2554
**LYRM9**	LYRM9	8	LYR motif containing 9	protein_codin	-0.4101	0.0127	1	8.692	0.1791
**TAOK1**	TAOK1	8	TAO kinase 1	protein_codin	0.2607	0.0808	1	18.9	0.2112
**BLMH**	BLMH	8	bleomycin hydrolase	protein_codin	-0.165	0.413	1	3.432	0.2729
**SYNRG**	SYNRG	8	synergin gamma	protein_codin	0.6018	0.001	1	6.132	0.3145
**CDC27**	CDC27	8	cell division cycle 27	protein_codin	0.2015	0.0916	1	16.52	0.1316
**STXBP4**	STXBP4	8	syntaxin binding protein 4	protein_codin	0.3122	0.195	1	11.26	0.1992
**RPS6KB1**	RPS6KB1	8	ribosomal protein S6 kinase B1	protein_codin	0.2947	0.26	1	7.398	0.2672
**TLK2**	TLK2	8	tousled like kinase 2	protein_codin	0.4608	0.0322	1	7.828	0.1436
**MAP3K3**	MAP3K3	8	mitogen-activated protein kinase kinase kinase 3	protein_codin	0.1985	0.471	1	9.842	0.2115
**GRB2**	GRB2	8	growth factor receptor bound protein 2	protein_codin	-0.1077	0.321	1	11.68	0.1722
**PGS1**	PGS1	8	phosphatidylglycerophosphate synthase 1	protein_codin	-0.3432	0.125	1	2.628	0.2047
**USP36**	USP36	8	ubiquitin specific peptidase 36	protein_codin	0.3166	0.275	1	3.296	0.33
**C1QTNF1**	C1QTNF1	8	C1q and TNF related 1	protein_codin	-0.7028	1.62E-12	3.86E-08	59.92	0.03541
**TBC1D16**	TBC1D16	8	TBC1 domain family member 16	protein_codin	1.071	0.00159	1	10.55	0.1907
**ENDOV**	ENDOV	8	endonuclease V	protein_codin	0.5462	0.0352	1	8.002	0.1692
**OXLD1**	OXLD1	8	oxidoreductase like domain containing 1	protein_codin	-0.4262	0.0148	1	52.92	0.1778
**MCRIP1**	MCRIP1	8	MAPK regulated corepressor interacting protein 1	protein_codin	-0.4447	1.75E-06	0.04062	25.82	0.1
**RFNG**	RFNG	8	RFNG O-fucosylpeptide 3-beta-N-acetylglucosaminyl	protein_codin	0.05179	0.697	1	15.2	0.142
**DUS1L**	DUS1L	8	dihydrouridine synthase 1 like	protein_codin	0.4282	0.0164	1	7.812	0.348
**CSNK1D**	CSNK1D	8	casein kinase 1 delta	protein_codin	0.2858	0.102	1	7.364	0.2296
**LINC00667**	LINC00667	8	long intergenic non-protein coding RNA 667	lincRNA	0.1579	0.246	1	16.91	0.06632
**MAPRE2**	MAPRE2	8	microtubule associated protein RP/EB family member	protein_codin	0.03312	0.836	1	40.14	0.3096
**ZNF24**	ZNF24	8	zinc finger protein 24	protein_codin	0.2411	0.0396	1	34.36	0.2168
**ELP2**	ELP2	8	elongator acetyltransferase complex subunit 2	protein_codin	-0.009752	0.96	1	6.084	0.1727
**SMAD2**	SMAD2	8	SMAD family member 2	protein_codin	0.1072	0.481	1	21.32	0.03828
**ZCCHC2**	ZCCHC2	8	zinc finger CCHC-type containing 2	protein_codin	-0.005212	0.982	1	7.702	0.2127
**TSHZ1**	TSHZ1	8	teashirt zinc finger homeobox 1	protein_codin	-0.04042	0.86	1	9.89	0.2290
**RBCK1**	RBCK1	8	RANBP2-type and C3HC4-type zinc finger containing	protein_codin	-0.07175	0.599	1	15.58	0.1826
**NSFL1C**	NSFL1C	8	NSFL1 cofactor	protein_codin	-0.05013	0.667	1	14.39	0.1030
**DDRGK1**	DDRGK1	8	DDRGK domain containing 1	protein_codin	0.1647	0.154	1	11.79	0.2419
**CRLS1**	CRLS1	8	cardiolipin synthase 1	protein_codin	0.04866	0.759	1	23.25	0.1444
**BCL2L1**	BCL2L1	8	BCL2 like 1	protein_codin	-0.2622	0.152	1	8.562	0.1734
**SOGA1**	SOGA1	8	suppressor of glucose, autophagy associated 1	protein_codin	0.7927	0.00282	1	2.622	0.4095
**CTNNBL1**	CTNNBL1	8	catenin beta like 1	protein_codin	-0.1734	0.248	1	6.252	0.1796
**BCAS4**	BCAS4	8	breast carcinoma amplified sequence 4	protein_codin	-0.8503	0.00043	1	3.028	0.2657
**NELFCD**	NELFCD	8	negative elongation factor complex member C/D	protein_codin	0.2997	0.0798	1	23.64	0.09949
**GNA11**	GNA11	8	G protein subunit alpha 11	protein_codin	0.3858	0.0307	1	51.58	0.2385
**ZNF561**	ZNF561	8	zinc finger protein 561	protein_codin	-0.5263	0.0307	1	2.738	0.1828
**ASNA1**	ASNA1	8	guided entry of tail-anchored proteins factor 3, ATPas	protein_codin	-0.1209	0.345	1	15.42	0.1421
**STX10**	STX10	8	syntaxin 10	protein_codin	-0.3257	0.0162	1	59.19	0.2345
**NDUFB7**	NDUFB7	8	NADH:ubiquinone oxidoreductase subunit B7	protein_codin	-0.3008	4.11E-08	0.00096	54.67	0.03378
**KXD1**	KXD1	8	KxDL motif containing 1	protein_codin	-0.3857	0.0002	1	20.47	0.03321
**LRP3**	LRP3	8	LDL receptor related protein 3	protein_codin	1.113	1.04E-07	0.00244	9.197	0.1662
**LSM14A**	LSM14A	8	LSM14A mRNA processing body assembly factor	protein_codin	-0.09474	0.349	1	26.87	0.2068
**GRAMD1A**	GRAMD1A	8	GRAM domain containing 1A	protein_codin	-0.6474	0.00163	1	55.72	0.1732
**RBM42**	RBM42	8	RNA binding motif protein 42	protein_codin	0.007432	0.945	1	5.942	0.1358
**ACTN4**	ACTN4	8	actinin alpha 4	protein_codin	-0.4427	2.37E-09	5.58E-05	42.76	0.03403
**TIMM50**	TIMM50	8	translocase of inner mitochondrial membrane 50	protein_codin	-0.03376	0.85	1	5.476	0.1031
**ZNF428**	ZNF428	8	zinc finger protein 428	protein_codin	0.001076	0.988	1	40.74	0.03916
**PRMT1**	PRMT1	8	protein arginine methyltransferase 1	protein_codin	-0.09387	0.249	1	53.8	0.1419
**MED25**	MED25	8	mediator complex subunit 25	protein_codin	0.3594	0.147	1	2.61	0.3768
**C22orf39**	C22orf39	8	chromosome 22 open reading frame 39	protein_codin	-0.1516	0.266	1	16.92	0.1991
**SF3A1**	SF3A1	8	splicing factor 3a subunit 1	protein_codin	-0.01141	0.945	1	3.195	0.172
**CDC42EP1**	CDC42EP1	8	CDC42 effector protein 1	protein_codin	-0.04505	0.721	1	28.87	0.09785
**TOMM22**	TOMM22	8	translocase of outer mitochondrial membrane 22	protein_codin	-0.1568	0.0905	1	26.55	0.03246
**KIAA0930**	KIAA0930	8	KIAA0930	protein_codin	0.6366	0.00072	1	8.5	0.09981
**CERK**	CERK	8	ceramide kinase	protein_codin	-0.1723	0.392	1	9.993	0.2150
**BRD1**	BRD1	8	bromodomain containing 1	protein_codin	0.4262	0.0106	1	22.72	0.1278
**LMF2**	LMF2	8	lipase maturation factor 2	protein_codin	0.3872	0.0156	1	7.057	0.167
**GABPA**	GABPA	8	GA binding protein transcription factor subunit alpha	protein_codin	0.411	0.0196	1	8.645	0.1032
**N6AMT1**	N6AMT1	8	N-6 adenine-specific DNA methyltransferase 1	protein_codin	0.3348	0.192	1	9.348	0.4204
**GART**	GART	8	phosphoribosylglycinamide formyltransferase, phosp	protein_codin	-0.3466	0.11	1	9.118	0.4818
**PIGP**	PIGP	8	phosphatidylinositol glycan anchor biosynthesis class	protein_codin	0.06895	0.542	1	42.78	0.07242
**PFKL**	PFKL	8	phosphofructokinase, liver type	protein_codin	-0.12	0.456	1	20.82	0.1760
**POFUT2**	POFUT2	8	protein O-fucosyltransferase 2	protein_codin	0.5407	0.0196	1	3.39	0.3905
**MT-ND6**	MT-ND6	8	mitochondrially encoded NADH:ubiquinone oxidoredu	protein_codin	-0.3508	0.0276	1	24.34	0.09922
**ISG15**	ISG15	9	ISG15 ubiquitin like modifier	protein_codin	-1.122	1.25E-11	2.97E-07	60.6	0.8872
**TMEM57**	TMEM57	9	macoilin 1	protein_codin	NA	NA	NA	8.017	0.1724
**LDLRAP1**	LDLRAP1	9	low density lipoprotein receptor adaptor protein 1	protein_codin	-0.06653	0.781	1	3.181	0.2316
**IFI6**	IFI6	9	interferon alpha inducible protein 6	protein_codin	-1.062	1.18E-19	2.83E-15	60.58	0.4626
**PHACTR4**	PHACTR4	9	phosphatase and actin regulator 4	protein_codin	-0.1583	0.476	1	8.472	0.3111
**AK2**	AK2	9	adenylate kinase 2	protein_codin	-0.274	0.0238	1	16.6	0.1068
**ZMYM4**	ZMYM4	9	zinc finger MYM-type containing 4	protein_codin	0.2771	0.084	1	16.52	0.1015
**PSMB2**	PSMB2	9	proteasome subunit beta 2	protein_codin	-0.07246	0.354	1	45.37	0.03816
**BEND5**	BEND5	9	BEN domain containing 5	protein_codin	-0.432	0.0237	1	59.86	0.1537
**PRPF38A**	PRPF38A	9	pre-mRNA processing factor 38A	protein_codin	-0.1441	0.376	1	7.55	0.2751
**SRSF11**	SRSF11	9	serine and arginine rich splicing factor 11	protein_codin	0.236	0.00222	1	60.2	0.1054
**ZRANB2**	ZRANB2	9	zinc finger RANBP2-type containing 2	protein_codin	0.1105	0.232	1	31.85	0.06612
**IFI44L**	IFI44L	9	interferon induced protein 44 like	protein_codin	-0.868	8.47E-05	1	59.84	0.7159
**BCL10**	BCL10	9	BCL10 immune signaling adaptor	protein_codin	0.1208	0.442	1	38.34	0.1672
**SELENOF**	SELENOF	9	selenoprotein F	protein_codin	0.3732	6.18E-07	0.01441	49.06	0.04127
**GBP3**	GBP3	9	guanylate binding protein 3	protein_codin	-0.2884	0.211	1	60.45	0.3282
**GBP1**	GBP1	9	guanylate binding protein 1	protein_codin	0.1613	0.329	1	59.88	0.7830
**GBP2**	GBP2	9	guanylate binding protein 2	protein_codin	0.1114	0.297	1	60	0.3817
**GBP4**	GBP4	9	guanylate binding protein 4	protein_codin	0.5485	0.044	1	60.58	1.095
**VCAM1**	VCAM1	9	vascular cell adhesion molecule 1	protein_codin	-1.158	5.17E-18	1.24E-13	60.6	0.1401
**CHI3L2**	CHI3L2	9	chitinase 3 like 2	protein_codin	-0.4338	0.225	1	60.6	0.1989
**OLFML3**	OLFML3	9	olfactomedin like 3	protein_codin	1.906	6.23E-40	1.51E-35	52.68	0.03198
**CD58**	CD58	9	CD58 molecule	protein_codin	-0.07252	0.574	1	37.36	0.06887
**CTSS**	CTSS	9	cathepsin S	protein_codin	0.4889	0.0142	1	53.22	0.2071
**S100A3**	S100A3	9	S100 calcium binding protein A3	protein_codin	-1.175	7.23E-09	0.00017	50.05	0.06797
**SLC27A3**	SLC27A3	9	solute carrier family 27 member 3	protein_codin	0.6313	9.26E-06	0.212	37.8	0.138
**SEMA4A**	SEMA4A	9	semaphorin 4A	protein_codin	0.7167	0.0379	1	30.52	0.3558
**PMF1**	PMF1	9	polyamine modulated factor 1	protein_codin	0.1046	0.504	1	59.25	0.2124
**DCAF8**	DCAF8	9	DDB1 and CUL4 associated factor 8	protein_codin	0.1506	0.498	1	3.026	0.3745
**UAP1**	UAP1	9	UDP-N-acetylglucosamine pyrophosphorylase 1	protein_codin	2.583	1.31E-39	3.17E-35	54.81	0.03537
**RCSD1**	RCSD1	9	RCSD domain containing 1	protein_codin	-0.05835	0.807	1	48.26	0.1307
**SUCO**	SUCO	9	SUN domain containing ossification factor	protein_codin	0.4086	0.0386	1	27.93	0.2405
**STX6**	STX6	9	syntaxin 6	protein_codin	-0.3701	0.109	1	7.696	0.3098
**RGS2**	RGS2	9	regulator of G protein signaling 2	protein_codin	-0.9438	1.26E-06	0.02929	55.36	0.1502
**RCOR3**	RCOR3	9	REST corepressor 3	protein_codin	-0.2546	0.121	1	20.6	0.2135
**C1orf198**	C1orf198	9	chromosome 1 open reading frame 198	protein_codin	-0.3673	0.0114	1	37.45	0.06690
**PXDN**	PXDN	9	peroxidasin	protein_codin	0.385	0.0786	1	22.88	0.2105
**ITGB1BP1**	ITGB1BP1	9	integrin subunit beta 1 binding protein 1	protein_codin	0.09404	0.232	1	59.42	0.0367
**CYS1**	CYS1	9	cystin 1	protein_codin	-0.04196	0.792	1	18.22	0.03344
**ROCK2**	ROCK2	9	Rho associated coiled-coil containing protein kinase 2	protein_codin	-0.1553	0.062	1	25	0.06489
**MOB1A**	MOB1A	9	MOB kinase activator 1A	protein_codin	-0.05992	0.59	1	26.28	0.1434
**MAT2A**	MAT2A	9	methionine adenosyltransferase 2A	protein_codin	0.1282	0.422	1	18.69	0.1652
**VAMP5**	VAMP5	9	vesicle associated membrane protein 5	protein_codin	-0.2174	0.0062	1	54.38	0.03877
**ACTR1B**	ACTR1B	9	actin related protein 1B	protein_codin	-0.1802	0.236	1	6.768	0.109
**C2orf49**	C2orf49	9	chromosome 2 open reading frame 49	protein_codin	-0.07344	0.59	1	6.647	0.2444
**C2orf40**	C2orf40	9	ECRG4 augurin precursor	protein_codin	-0.2124	0.044	1	Inf	0.1896
**AC012306.2**	AC012306.2	9	novel transcri	lincRNA	0.3982	0.0929	1	3.564	0.2796
**IMP4**	IMP4	9	IMP U3 small nucleolar ribonucleoprotein 4	protein_codin	-0.3179	0.0157	1	40.2	0.03285
**RND3**	RND3	9	Rho family GTPase 3	protein_codin	0.4767	0.00012	1	60.52	0.04459
**TNFAIP6**	TNFAIP6	9	TNF alpha induced protein 6	protein_codin	-0.565	0.0867	1	60.54	0.522
**ARL5A**	ARL5A	9	ADP ribosylation factor like GTPase 5A	protein_codin	0.07227	0.765	1	9.098	0.2088
**IFIH1**	IFIH1	9	interferon induced with helicase C domain 1	protein_codin	-0.3649	0.115	1	30.58	0.2487
**CHN1**	CHN1	9	chimerin 1	protein_codin	-0.8163	0.00014	1	37.12	0.3892
**PLEKHA3**	PLEKHA3	9	pleckstrin homology domain containing A3	protein_codin	-0.1211	0.266	1	12.14	0.1702
**TFPI**	TFPI	9	tissue factor pathway inhibitor	protein_codin	0.5093	6.33E-11	1.5E-06	55.3	0.03409
**STAT1**	STAT1	9	signal transducer and activator of transcription 1	protein_codin	-0.2155	0.102	1	60.46	0.03306
**NABP1**	NABP1	9	nucleic acid binding protein 1	protein_codin	0.7215	0.00962	1	29.42	0.4501
**MOB4**	MOB4	9	MOB family member 4, phocein	protein_codin	-0.091	0.434	1	16.45	0.07616
**KLF7**	KLF7	9	Kruppel like factor 7	protein_codin	0.1933	0.29	1	8.25	0.192
**CTDSP1**	CTDSP1	9	CTD small phosphatase 1	protein_codin	-0.2529	0.0259	1	30.52	0.1034
**STK16**	STK16	9	serine/threonine kinase 16	protein_codin	0.1568	0.349	1	8.749	0.06608
**ASB1**	ASB1	9	ankyrin repeat and SOCS box containing 1	protein_codin	-0.4395	0.12	1	2.628	0.3495
**MTERF4**	MTERF4	9	mitochondrial transcription termination factor 4	protein_codin	0.05292	0.766	1	5.788	0.2012
**BHLHE40**	BHLHE40	9	basic helix-loop-helix family member e40	protein_codin	1.11	1.39E-08	0.00033	47.57	0.2648
**CRELD1**	CRELD1	9	cysteine rich with EGF like domains 1	protein_codin	-0.3536	0.0636	1	9.02	0.1642
**TIMP4**	TIMP4	9	TIMP metallopeptidase inhibitor 4	protein_codin	-0.6096	0.0151	1	60.6	1.434
**WDR82**	WDR82	9	WD repeat domain 82	protein_codin	0.4964	0.0117	1	8	0.174
**ARL13B**	ARL13B	9	ADP ribosylation factor like GTPase 13B	protein_codin	0.07689	0.642	1	22.24	0.1024
**ZBTB11**	ZBTB11	9	zinc finger and BTB domain containing 11	protein_codin	0.06696	0.758	1	31.88	0.1751
**NFKBIZ**	NFKBIZ	9	NFKB inhibitor zeta	protein_codin	1.722	3.24E-21	7.79E-17	55.22	0.2725
**PARP14**	PARP14	9	poly(ADP-ribose) polymerase family member 14	protein_codin	0.09437	0.558	1	60.58	0.3722
**RAB7A**	RAB7A	9	RAB7A, member RAS oncogene family	protein_codin	0.2475	0.00080	1	58.95	0.0327
**RBP1**	RBP1	9	retinol binding protein 1	protein_codin	-0.331	0.0294	1	34.95	0.03961
**PLSCR1**	PLSCR1	9	phospholipid scramblase 1	protein_codin	0.2107	0.0358	1	59.96	0.106
**CP**	CP	9	ceruloplasmin	protein_codin	-0.08306	0.512	1	60.6	0.07274
**FNDC3B**	FNDC3B	9	fibronectin type III domain containing 3B	protein_codin	0.2226	0.154	1	14.52	0.1412
**TNFSF10**	TNFSF10	9	TNF superfamily member 10	protein_codin	0.501	0.00060	1	60.6	0.2551
**PAK2**	PAK2	9	p21 (RAC1) activated kinase 2	protein_codin	-0.121	0.197	1	47.06	0.03392
**ZNF141**	ZNF141	9	zinc finger protein 141	protein_codin	0.32	0.149	1	10.42	0.1089
**NELFA**	NELFA	9	negative elongation factor complex member A	protein_codin	-4.64E-05	1	1	8.492	0.2311
**STX18**	STX18	9	syntaxin 18	protein_codin	0.1428	0.496	1	7.182	0.03229
**MRFAP1L1**	MRFAP1L1	9	Morf4 family associated protein 1 like 1	protein_codin	0.1016	0.514	1	22.82	0.1346
**LAP3**	LAP3	9	leucine aminopeptidase 3	protein_codin	-0.3776	0.00175	1	55.32	0.4042
**EXOC1**	EXOC1	9	exocyst complex component 1	protein_codin	0.1709	0.374	1	19.06	0.2770
**CXCL2**	CXCL2	9	C-X-C motif chemokine ligand 2	protein_codin	0.7432	0.0338	1	60.54	0.3551
**CXCL9**	CXCL9	9	C-X-C motif chemokine ligand 9	protein_codin	-0.2502	0.342	1	60.6	0.598
**CXCL10**	CXCL10	9	C-X-C motif chemokine ligand 10	protein_codin	0.9338	0.2532	1	NA	1.982
**11-Sep**	43719	9	septin 11	protein_codin	0.1803	0.0452	1	29.1	0.03281
**CCNG2**	CCNG2	9	cyclin G2	protein_codin	0.2207	0.145	1	20.04	0.1757
**PPM1K**	PPM1K	9	protein phosphatase, Mg2+/Mn2+ dependent 1K	protein_codin	0.1102	0.429	1	36.02	0.03482
**TSPAN5**	TSPAN5	9	tetraspanin 5	protein_codin	-0.703	0.00112	1	30.34	0.2207
**SLC39A8**	SLC39A8	9	solute carrier family 39 member 8	protein_codin	0.7415	0.00699	1	59.62	0.4441
**SCLT1**	SCLT1	9	sodium channel and clathrin linker 1	protein_codin	0.6258	0.0168	1	25.72	0.2298
**LRBA**	LRBA	9	LPS responsive beige-like anchor protein	protein_codin	0.2933	0.248	1	10.66	0.1837
**SPCS3**	SPCS3	9	signal peptidase complex subunit 3	protein_codin	0.3606	0.00064	1	30.96	0.07079
**ICE1**	ICE1	9	interactor of little elongation complex ELL subunit 1	protein_codin	0.05402	0.706	1	17.04	0.1024
**EGFLAM**	EGFLAM	9	EGF like, fibronectin type III and laminin G domains	protein_codin	0.09804	0.626	1	53.62	0.1172
**GHR**	GHR	9	growth hormone receptor	protein_codin	0.6919	0.00202	1	25.84	0.06945
**NNT**	NNT	9	nicotinamide nucleotide transhydrogenase	protein_codin	0.3256	0.161	1	3.282	0.4105
**SCAMP1**	SCAMP1	9	secretory carrier membrane protein 1	protein_codin	0.1873	0.142	1	37.19	0.03291
**GLRX**	GLRX	9	glutaredoxin	protein_codin	-0.01053	0.937	1	60	0.08694
**ELL2**	ELL2	9	elongation factor for RNA polymerase II 2	protein_codin	0.1622	0.404	1	60.46	0.3151
**FBXL17**	FBXL17	9	F-box and leucine rich repeat protein 17	protein_codin	0.001222	0.996	1	8.516	0.1875
**TNFAIP8**	TNFAIP8	9	TNF alpha induced protein 8	protein_codin	-0.5241	0.00116	1	38.64	0.1429
**IRF1**	IRF1	9	interferon regulatory factor 1	protein_codin	0.4948	0.00136	1	60.58	0.4818
**ARHGAP26**	ARHGAP26	9	Rho GTPase activating protein 26	protein_codin	0.2643	0.387	1	11.05	0.1884
**TNIP1**	TNIP1	9	TNFAIP3 interacting protein 1	protein_codin	-0.3488	0.0350	1	23.53	0.3347
**LTC4S**	LTC4S	9	leukotriene C4 synthase	protein_codin	-0.4658	0.00600	1	60.6	0.3514
**MGAT4B**	MGAT4B	9	alpha-1,3-mannosyl-glycoprotein 4-beta-N-acetylgluc	protein_codin	-0.4426	0.00676	1	60.27	0.07601
**FAM8A1**	FAM8A1	9	family with sequence similarity 8 member A1	protein_codin	0.3019	0.124	1	10.05	0.141
**GMNN**	GMNN	9	geminin DNA replication inhibitor	protein_codin	0.5497	0.0187	1	3.762	0.2779
**TRIM38**	TRIM38	9	tripartite motif containing 38	protein_codin	-0.2153	0.126	1	18.38	0.2115
**HIST1H2AC**	HIST1H2AC	9	histone cluster 1 H2A family member c	protein_codin	0.1044	0.665	1	30.72	0.2547
**IER3**	IER3	9	immediate early response 3	protein_codin	-0.2251	0.0976	1	60	0.1438

**EHMT2**	EHMT2	9	euchromatic histone lysine methyltransferase 2	protein_codin	0.2922	0.224	1	3.244	0.2427
**PSMB8**	PSMB8	9	proteasome subunit beta 8	protein_codin	-0.1025	0.259	1	42.29	0.1518
**PSMB9**	PSMB9	9	proteasome subunit beta 9	protein_codin	-0.2468	0.0311	1	60.58	0.2689
**TAP1**	TAP1	9	transporter 1, ATP binding cassette subfamily B mem	protein_codin	-0.4101	0.0485	1	26.22	0.2302
**CUTA**	CUTA	9	cutA divalent cation tolerance homolog	protein_codin	-0.2009	7E-04	1	39.17	0.03539
**PIM1**	PIM1	9	Pim-1 proto-oncogene, serine/threonine kinase	protein_codin	0.4604	0.0701	1	39.97	0.1896
**RRP36**	RRP36	9	ribosomal RNA processing 36	protein_codin	-0.1647	0.252	1	18.6	0.1964
**MRPS18A**	MRPS18A	9	mitochondrial ribosomal protein S18A	protein_codin	0.2445	0.0795	1	16.96	0.2151
**BCKDHB**	BCKDHB	9	branched chain keto acid dehydrogenase E1 subunit	protein_codin	0.7087	0.00102	1	8.829	0.2712
**RNGTT**	RNGTT	9	RNA guanylyltransferase and 5'-phosphatase	protein_codin	0.284	0.196	1	2.811	0.07477
**SOBP**	SOBP	9	sine oculis binding protein homolog	protein_codin	0.9645	0.00204	1	27.19	0.1188
**SESN1**	SESN1	9	sestrin 1	protein_codin	0.06924	0.699	1	9.242	0.03509
**AMD1.00**	AMD1.00	9	adenosylmethionine decarboxylase 1	protein_codin	0.3282	0.00741	1	59.98	0.168
**LAMA4**	LAMA4	9	laminin subunit alpha 4	protein_codin	0.6262	1.98E-06	0.04592	60.45	0.09992
**NCOA7**	NCOA7	9	nuclear receptor coactivator 7	protein_codin	0.683	8.26E-09	0.00019	60.52	0.0928
**ARHGAP18**	ARHGAP18	9	Rho GTPase activating protein 18	protein_codin	-0.423	0.0177	1	33.12	0.06696
**EPB41L2**	EPB41L2	9	erythrocyte membrane protein band 4.1 like 2	protein_codin	0.8413	1.43E-21	3.44E-17	56.68	0.03436
**SOD2**	SOD2	9	superoxide dismutase 2	protein_codin	0.3713	0.0072	1	40.71	0.2296
**WTAP**	WTAP	9	WT1 associated protein	protein_codin	-0.009049	0.932	1	60.42	0.1731
**ZFAND2A**	ZFAND2A	9	zinc finger AN1-type containing 2A	protein_codin	-0.2461	0.198	1	8.871	0.06529
**C1GALT1**	C1GALT1	9	core 1 synthase, glycoprotein-N-acetylgalactosamine	protein_codin	0.2029	0.182	1	35.06	0.06172
**RP9**	RP9	9	RP9 pre-mRNA splicing factor	protein_codin	-0.06365	0.672	1	18.23	0.1359
**DPY19L1**	DPY19L1	9	dpy-19 like C-mannosyltransferase 1	protein_codin	0.2935	0.305	1	37.05	0.2496
**HERPUD2**	HERPUD2	9	HERPUD family member 2	protein_codin	0.2232	0.29	1	8.996	0.1026
**PSMA2**	PSMA2	9	proteasome subunit alpha 2	protein_codin	-0.3027	0.0082	1	13.18	0.1038
**TBRG4**	TBRG4	9	transforming growth factor beta regulator 4	protein_codin	0.1164	0.609	1	3.528	0.2117
**RAMP3**	RAMP3	9	receptor activity modifying protein 3	protein_codin	-1.28	0.00132	1	59.96	0.606
**IGFBP3**	IGFBP3	9	insulin like growth factor binding protein 3	protein_codin	-1.838	3.58E-22	8.62E-18	60.6	0.1984
**SAMD9**	SAMD9	9	sterile alpha motif domain containing 9	protein_codin	0.0422	0.841	1	22.76	0.244
**SAMD9L**	SAMD9L	9	sterile alpha motif domain containing 9 like	protein_codin	0.126	0.529	1	58.14	0.2269
**TFPI2**	TFPI2	9	tissue factor pathway inhibitor 2	protein_codin	-1.955	1.53E-16	3.66E-12	60.6	0.1582
**GNG11**	GNG11	9	G protein subunit gamma 11	protein_codin	-0.1531	0.046	1	46.03	0.1706
**ZNF655**	ZNF655	9	zinc finger protein 655	protein_codin	0.4262	0.0596	1	7.296	0.2085
**ST7**	ST7	9	suppression of tumorigenicity 7	protein_codin	0.2488	0.265	1	3.114	0.2172
**CTTNBP2**	CTTNBP2	9	cortactin binding protein 2	protein_codin	0.572	0.0205	1	10.86	0.2508
**KDM7A**	KDM7A	9	lysine demethylase 7A	protein_codin	0.336	0.132	1	8.142	0.1654
**MKRN1**	MKRN1	9	makorin ring finger protein 1	protein_codin	0.1777	0.248	1	22.27	0.1069
**ZYX**	ZYX	9	zyxin	protein_codin	-0.06878	0.479	1	25.58	0.0764
**NUB1**	NUB1	9	negative regulator of ubiquitin like proteins 1	protein_codin	0.002874	0.977	1	30.4	0.1329
**MAOA**	MAOA	9	monoamine oxidase A	protein_codin	0.1892	0.132	1	30.15	0.2107
**PDZD11**	PDZD11	9	PDZ domain containing 11	protein_codin	-0.2408	0.0468	1	14.92	0.2069
**TAF1**	TAF1	9	TATA-box binding protein associated factor 1	protein_codin	0.02469	0.919	1	3.323	0.3612
**PABPC5**	PABPC5	9	poly(A) binding protein cytoplasmic 5	protein_codin	0.2496	0.242	1	2.886	0.208
**ARMCX1**	ARMCX1	9	armadillo repeat containing X-linked 1	protein_codin	0.08761	0.462	1	30.75	0.1417
**RTL8A**	RTL8A	9	retrotransposon Gag like 8A	protein_codin	-0.2605	0.0058	1	19.87	0.2052
**TMEM187**	TMEM187	9	transmembrane protein 187	protein_codin	-0.07422	0.736	1	3.743	0.1792
**EGR3**	EGR3	9	early growth response 3	protein_codin	0.4742	0.0829	1	57.45	0.4697
**RIPK2**	RIPK2	9	receptor interacting serine/threonine kinase 2	protein_codin	0.4606	0.0301	1	29.92	0.3229
**TRIQK**	TRIQK	9	triple QxxK/R motif containing	protein_codin	-0.001229	0.994	1	7.907	0.1581
**LAPTM4B**	LAPTM4B	9	lysosomal protein transmembrane 4 beta	protein_codin	0.4318	0.00026	1	34.95	0.1704
**RSPO2**	RSPO2	9	R-spondin 2	protein_codin	-0.2365	0.437	1	31.73	0.4379
**HAS2**	HAS2	9	hyaluronan synthase 2	protein_codin	1.028	0.00012	1	19.54	0.06832
**LY6E**	LY6E	9	lymphocyte antigen 6 family member E	protein_codin	0.1937	0.0229	1	55.38	0.2761
**HSF1**	HSF1	9	heat shock transcription factor 1	protein_codin	0.01042	0.944	1	6.709	0.2321
**HACD4**	HACD4	9	3-hydroxyacyl-CoA dehydratase 4	protein_codin	-0.7298	0.00018	1	9.575	0.2376
**DDX58**	DDX58	9	DExD/H-box helicase 58	protein_codin	-0.05892	0.768	1	52.98	0.2442
**CCL19**	CCL19	9	C-C motif chemokine ligand 19	protein_codin	0.3886	0.342	1	NA	0.9116
**CCL21**	CCL21	9	C-C motif chemokine ligand 21	protein_codin	1.549	0.0109	1	NA	1.198
**GKAP1**	GKAP1	9	G kinase anchoring protein 1	protein_codin	-0.7035	5.07E-05	1	9.665	0.3541
**NINJ1**	NINJ1	9	ninjurin 1	protein_codin	-0.5208	1.07E-06	0.02488	37.58	0.1832
**HDHD3**	HDHD3	9	haloacid dehalogenase like hydrolase domain contair	protein_codin	0.3352	0.194	1	2.779	0.3137
**ENG**	ENG	9	endoglin	protein_codin	-0.8695	5.88E-17	1.41E-12	55.3	0.07085
**SPOUT1**	SPOUT1	9	SPOUT domain containing methyltransferase 1	protein_codin	-0.7467	0.0125	1	3.162	0.2046
**UCK1**	UCK1	9	uridine-cytidine kinase 1	protein_codin	-0.2308	0.209	1	7.66	0.2482
**RXRA**	RXRA	9	retinoid X receptor alpha	protein_codin	0.04389	0.816	1	6.445	0.3552
**UBAC1**	UBAC1	9	UBA domain containing 1	protein_codin	0.1509	0.343	1	19.48	0.2539
**SEC16A**	SEC16A	9	SEC16 homolog A, endoplasmic reticulum export factor	protein_codin	0.037	0.872	1	2.767	0.4047
**FBXW5**	FBXW5	9	F-box and WD repeat domain containing 5	protein_codin	-0.31	0.00329	1	54.77	0.07032
**CLIC3**	CLIC3	9	chloride intracellular channel 3	protein_codin	-0.1153	0.549	1	59.87	0.1841
**DPH7**	DPH7	9	diphthamide biosynthesis 7	protein_codin	-0.002694	0.988	1	7.69	0.1699
**IFITM2**	IFITM2	9	interferon induced transmembrane protein 2	protein_codin	-0.1423	0.00146	1	60.6	0.06004
**IFITM1**	IFITM1	9	interferon induced transmembrane protein 1	protein_codin	1.531	9.65E-21	2.32E-16	45.58	0.1426
**IFITM3**	IFITM3	9	interferon induced transmembrane protein 3	protein_codin	-0.3003	1.74E-18	4.17E-14	60.58	0.2948
**IRF7**	IRF7	9	interferon regulatory factor 7	protein_codin	-0.3072	0.226	1	59.79	0.2567
**TMEM80**	TMEM80	9	transmembrane protein 80	protein_codin	0.1647	0.237	1	7.143	0.1362
**SVIP**	SVIP	9	small VCP interacting protein	protein_codin	-0.5303	2.47E-05	0.5667	13.63	0.1096
**CD82**	CD82	9	CD82 molecule	protein_codin	-0.8105	8.01E-07	0.01865	60.53	0.08829
**RARRES3**	RARRES3	9	phospholipase A and acyltransferase 4	protein_codin	-0.2822	0.00643	1	34.96	0.2237
**MACROD1**	MACROD1	9	mono-ADP ribosylhydrolase 1	protein_codin	0.128	0.371	1	7.737	0.2839
**GPR137**	GPR137	9	G protein-coupled receptor 137	protein_codin	0.01774	0.942	1	8.602	0.2761
**RPS6KA4**	RPS6KA4	9	ribosomal protein S6 kinase A4	protein_codin	0.2796	0.235	1	2.834	0.3105
**PPP2R5B**	PPP2R5B	9	protein phosphatase 2 regulatory subunit B'beta	protein_codin	-0.2569	0.328	1	10.45	0.2621
**LTBP3**	LTBP3	9	latent transforming growth factor beta binding protein 3	protein_codin	0.1836	0.0474	1	53.34	0.03327
**FAM168A**	FAM168A	9	family with sequence similarity 168 member A	protein_codin	0.3986	0.0874	1	3.242	0.4636
**RNF169**	RNF169	9	ring finger protein 169	protein_codin	-0.1975	0.215	1	6.722	0.208
**HIKESHI**	HIKESHI	9	heat shock protein nuclear import factor hikeshi	protein_codin	0.01232	0.93	1	49.15	0.1037
**BIRC3**	BIRC3	9	baculoviral IAP repeat containing 3	protein_codin	-0.6013	0.0116	1	53.75	0.3939
**NPAT**	NPAT	9	nuclear protein, coactivator of histone transcription	protein_codin	0.4828	0.0554	1	32.07	0.2497
**ST3GAL4**	ST3GAL4	9	ST3 beta-galactoside alpha-2,3-sialyltransferase 4	protein_codin	0.06589	0.656	1	35.63	0.06644
**AKR1C2**	AKR1C2	9	aldo-keto reductase family 1 member C2	protein_codin	-0.2759	0.0278	1	60.58	0.06702
**IL15RA**	IL15RA	9	interleukin 15 receptor subunit alpha	protein_codin	0.7452	0.00017	1	42.82	0.08254
**CELF2**	CELF2	9	CUGBP Elav-like family member 2	protein_codin	0.8402	1.37E-09	3.23E-05	20.82	0.2429
**NUDT5**	NUDT5	9	nudix hydrolase 5	protein_codin	0.2022	0.166	1	7.077	0.06948
**OPTN**	OPTN	9	optineurin	protein_codin	-0.005715	0.945	1	46.42	0.0321
**RAB18**	RAB18	9	RAB18, member RAS oncogene family	protein_codin	0.01624	0.888	1	13.58	0.0997
**MAP3K8**	MAP3K8	9	mitogen-activated protein kinase kinase kinase 8	protein_codin	0.3152	0.0375	1	54.95	0.1746
**CXCL12**	CXCL12	9	C-X-C motif chemokine ligand 12	protein_codin	0.1252	0.202	1	60	0.1162
**FAM13C**	FAM13C	9	family with sequence similarity 13 member C	protein_codin	-0.8953	1.83E-09	4.32E-05	53.52	0.1318
**KIF1BP**	KIF1BP	9	KIF1 binding protein	protein_codin	0.3067	0.0584	1	23.03	0.06587
**SRGN**	SRGN	9	serglycin	protein_codin	-1.396	6.6E-16	1.58E-11	47.26	0.1802
**UNC5B**	UNC5B	9	unc-5 netrin receptor B	protein_codin	-1.258	6.98E-10	1.65E-05	44.05	0.1752
**NUTM2A-AS1**	NUTM2A-AS1	9	NUTM2A antisense RNA 1	antisense_RN	-0.1716	0.465	1	8.288	0.364
**IFIT2**	IFIT2	9	interferon induced protein with tetratricopeptide repeats 2	protein_codin	-0.8021	0.00177	1	60.6	0.9339
**IFIT3**	IFIT3	9	interferon induced protein with tetratricopeptide repeats 3	protein_codin	-0.3724	0.0968	1	60.6	0.9424
**IFIT1**	IFIT1	9	interferon induced protein with tetratricopeptide repeats 1	protein_codin	0.4468	0.0508	1	60.6	0.7724
**TNKS2**	TNKS2	9	tankyrase 2	protein_codin	0.7583	2.03E-05	0.4664	7.478	0.2392
**IDE**	IDE	9	insulin degrading enzyme	protein_codin	0.03015	0.909	1	3.229	0.2869
**SFRP5**	SFRP5	9	secreted frizzled related protein 5	protein_codin	-4.359	4.15E-11	9.84E-07	60.6	0.5092
**CRTAC1**	CRTAC1	9	cartilage acidic protein 1	protein_codin	-3.217	1.62E-30	3.91E-26	60.6	0.3382
**BBIP1**	BBIP1	9	BBSome interacting protein 1	protein_codin	0.1487	0.32	1	20.07	0.04358
**PDZD8**	PDZD8	9	PDZ domain containing 8	protein_codin	0.4579	0.011	1	59.6	0.04531
**ABRAXAS2**	ABRAXAS2	9	abraxas 2, BRISC complex subunit	protein_codin	0.3723	0.0614	1	6.157	0.1684
**ADIPOR2**	ADIPOR2	9	adiponectin receptor 2	protein_codin	0.6602	0.00273	1	31.48	0.2054
**MGP**	MGP	9	matrix Gla protein	protein_codin	-0.4885	1.88E-12	4.48E-08	NA	0.03264
**KRAS**	KRAS	9	KRAS proto-oncogene, GTPase	protein_codin	0.1933	0.12	1	60.29	0.07051
**MAP3K12**	MAP3K12	9	mitogen-activated protein kinase kinase kinase 12	protein_codin	-0.0682	0.714	1	8.876	0.3934
**LINC02381**	LINC02381	9	long intergenic non-protein coding RNA 2381	lincRNA	2.357	1.19E-10	2.82E-06	8.3	0.2448
**MMP19**	MMP19	9	matrix metallopeptidase 19	protein_codin	0.4063	0.127	1	11.42	0.1759
**RNF41**	RNF41	9	ring finger protein 41	protein_codin	0.01116	0.949	1	27.16	0.1419
**LLPH**	LLPH	9	LLP homolog, long-term synaptic facilitation factor	protein_codin	0.02752	0.847	1	17.34	0.1234
**ATP2B1-AS1**	ATP2B1-AS1	9	ATP2B1 antisense RNA 1	lincRNA	-0.1349	0.565	1	11.09	0.2729
**TCP11L2**	TCP11L2	9	t-complex 11 like 2	protein_codin	0.5166	0.0177	1	7.128	0.2097
**OAS1**	OAS1	9	2'-5'-oligoadenylate synthetase 1	protein_codin	0.1532	0.514	1	54.93	0.5985
**PSMD9**	PSMD9	9	proteasome 26S subunit, non-ATPase 9	protein_codin	0.367	0.0876	1	6.275	0.2072
**SGCG**	SGCG	9	sarcoglycan gamma	protein_codin	-0.5599	0.0327	1	60.58	0.1838
**SERP2**	SERP2	9	stress associated endoplasmic reticulum protein famil	protein_codin	-0.4302	0.0326	1	3.708	0.2784
**MED4**	MED4	9	mediator complex subunit 4	protein_codin	-0.003836	0.969	1	27.67	0.03488
**RNASEH2B**	RNASEH2B	9	ribonuclease H2 subunit B	protein_codin	-0.4047	0.00264	1	58.19	0.1127
**GPR180**	GPR180	9	G protein-coupled receptor 180	protein_codin	0.6528	0.0203	1	2.808	0.2142
**TNFSF13B**	TNFSF13B	9	TNF superfamily member 13b	protein_codin	1.012	3.33E-07	0.00777	60.6	0.12
**EFS**	EFS	9	embryonal Fyn-associated substrate	protein_codin	0.7252	0.0018	1	28.28	0.08003
**PSME1**	PSME1	9	proteasome activator subunit 1	protein_codin	-0.03033	0.597	1	60.6	0.1156
**PSME2**	PSME2	9	proteasome activator subunit 2	protein_codin	-0.4182	1.04E-06	0.02419	60.6	0.3138
**HECTD1**	HECTD1	9	HECT domain E3 ubiquitin protein ligase 1	protein_codin	0.03320	0.813	1	16.58	0.2372
**NFKBIA**	NFKBIA	9	NFKB inhibitor alpha	protein_codin	0.4782	7.75E-09	0.00018	60.6	0.3619
**CTAGE5**	CTAGE5	9	MIA SH3 domain ER export factor 2	protein_codin	NA	NA	NA	8.775	0.2437
**FRMD6**	FRMD6	9	FERM domain containing 6	protein_codin	1.018	1.75E-07	0.00409	32.26	0.1002
**DNAL1**	DNAL1	9	dynein axonemal light chain 1	protein_codin	0.3624	0.146	1	3.805	0.2925
**COQ6**	COQ6	9	coenzyme Q6, monooxygenase	protein_codin	0.3482	0.111	1	9.278	0.1642
**ISCA2**	ISCA2	9	iron-sulfur cluster assembly 2	protein_codin	0.7245	0.00053	1	8.685	0.2808
**DLST**	DLST	9	dihydrolipoamide S-succinyltransferase	protein_codin	0.3722	0.0722	1	8.589	0.2068
**IFI27**	IFI27	9	interferon alpha inducible protein 27	protein_codin	-1.495	1.48E-14	3.53E-10	60.6	0.4087
**IFI27L2**	IFI27L2	9	interferon alpha inducible protein 27 like 2	protein_codin	-0.5022	4.27E-07	0.00996	57.28	0.1061
**PAPOLA**	PAPOLA	9	poly(A) polymerase alpha	protein_codin	-0.1237	0.231	1	39.96	0.1721
**WARS**	WARS	9	tryptophanyl-tRNA synthetase	protein_codin	0.04382	0.845	1	59.82	0.3822
**TNFAIP2**	TNFAIP2	9	TNF alpha induced protein 2	protein_codin	1.955	4.4E-19	1.06E-13	60.6	0.328
**CKB**	CKB	9	creatine kinase B	protein_codin	1.815	1.38E-32	3.33E-28	29.57	0.03317
**TRMT61A**	TRMT61A	9	tRNA methyltransferase 61A	protein_codin	-0.3069	0.109	1	3.428	0.1011
**PACS2**	PACS2	9	phosphofurin acidic cluster sorting protein 2	protein_codin	0.4805	0.0374	1	9.414	0.2439
**EMC7**	EMC7	9	ER membrane protein complex subunit 7	protein_codin	0.2058	0.0166	1	49.38	0.03246
**TMEM87A**	TMEM87A	9	transmembrane protein 87A	protein_codin	-0.03387	0.716	1	17.12	0.1795
**EIF3J**	EIF3J	9	eukaryotic translation initiation factor 3 subunit J	protein_codin	-0.3311	0.00461	1	18.69	0.2725
**RAB27A**	RAB27A	9	RAB27A, member RAS oncogene family	protein_codin	-0.1705	0.42	1	7.563	0.3186
**TLN2**	TLN2	9	talin 2	protein_codin	-0.2558	0.221	1	24.98	0.09932
**ANKDD1A**	ANKDD1A	9	ankyrin repeat and death domain containing 1A	protein_codin	0.6809	0.0277	1	3.511	0.2201
**TIPIN**	TIPIN	9	TIMELESS interacting protein	protein_codin	0.09388	0.698	1	10.71	0.3281
**UACA**	UACA	9	uveal autoantigen with coiled-coil domains and ankyri	protein_codin	-0.7126	2.45E-10	5.8E-06	54.8	0.07521
**NPTN**	NPTN	9	neuroplastin	protein_codin	-0.1048	0.196	1	59.33	0.06536
**RCN2**	RCN2	9	reticulocalbin 2	protein_codin	-0.3642	9E-06	0.2075	59.85	0.1049
**PEAK1**	PEAK1	9	pseudopodium enriched atypical kinase 1	protein_codin	-0.09592	0.666	1	26.83	0.3105
**CIB2**	CIB2	9	calcium and integrin binding family member 2	protein_codin	-0.7568	0.00026	1	9.297	0.2704
**ISG20**	ISG20	9	interferon stimulated exonuclease gene 20	protein_codin	-0.241	0.2	1	60.6	0.529
**LINC01197**	LINC01197	9	long intergenic non-protein coding RNA 1197	lincRNA	0.5106	0.00975	1	31.6	0.3694
**NR2F2**	NR2F2	9	nuclear receptor subfamily 2 group F member 2	protein_codin	0.03631	0.605	1	54.37	0.1436
**FAM234A**	FAM234A	9	family with sequence similarity 234 member A	protein_codin	0.1664	0.452	1	7.008	0.172
**TSR3**	TSR3	9	TSR3 ribosome maturation factor	protein_codin	-0.0206	0.86	1	30.36	0.06607
**SNHG9**	SNHG9	9	small nucleolar RNA host gene 9	lincRNA	0.6326	0.00152	1	20.1	0.09839
**SLC9A3R2**	SLC9A3R2	9	SLC9A3 regulator 2	protein_codin	-0.7223	1.53E-10	3.62E-06	60	0.09482
**ECI1**	ECI1	9	enoyl-CoA delta isomerase 1	protein_codin	-0.03446	0.742	1	48.18	0.1772
**IL32**	IL32	9	interleukin 32	protein_codin	0.5098	0.00058	1	60.58	0.4061
**CLUAP1**	CLUAP1	9	clusterin associated protein 1	protein_codin	-0.1247	0.372	1	7.329	0.2042
**SOCS1**	SOCS1	9	suppressor of cytokine signaling 1	protein_codin	1.205	1.51E-08	0.00036	54.6	0.1542
**NTAN1**	NTAN1	9	N-terminal asparagine amidase	protein_codin	0.1064	0.336	1	40.36	0.102
**C16orf45**	C16orf45	9	bMERB domain containing 1	protein_codin	0.364	0.00671	1	59.92	0.06465
**HIRIP3**	HIRIP3	9	HIRA interacting protein 3	protein_codin	-0.1626	0.311	1	21.36	0.1712
**PHKG2**	PHKG2	9	phosphorylase kinase catalytic subunit gamma 2	protein_codin	0.1652	0.487	1	3.057	0.1028
**OGFOD1**	OGFOD1	9	2-oxoglutarate and iron dependent oxygenase domai	protein_codin	-0.06752	0.796	1	9.086	0.1385
**FAM192A**	FAM192A	9	family with sequence similarity 192 member A	protein_codin	-0.2709	0.0958	1	8.394	0.06675
**CX3CL1**	CX3CL1	9	C-X3-C motif chemokine ligand 1	protein_codin	-0.66	0.00021	1	60.6	0.4501
**ENKD1**	ENKD1	9	enkurin domain containing 1	protein_codin	0.4796	0.054	1	8.776	0.1852
**PSMB10**	PSMB10	9	proteasome subunit beta 10	protein_codin	0.5144	0.0109	1	25.64	0.276
**PITPNA-AS1**	PITPNA-AS1	9	PITPNA antisense RNA 1	antisense_RN	-0.1168	0.501	1	8.014	0.1369
**RILP**	RILP	9	Rab interacting lysosomal protein	protein_codin	-0.376	0.0766	1	26.47	0.1381
**TAX1BP3**	TAX1BP3	9	Tax1 binding protein 3	protein_codin	0.01452	0.91	1	24.34	0.0667
**MFAP4**	MFAP4	9	microfibril associated protein 4	protein_codin	0.5263	1.48E-12	3.53E-08	60.6	0.1011
**SDF2**	SDF2	9	stromal cell derived factor 2	protein_codin	0.02034	0.847	1	26.37	0.07082
**CCL2**	CCL2	9	C-C motif chemokine ligand 2	protein_codin	-0.05382	0.791	1	Inf	0.6577
**CCL5**	CCL5	9	C-C motif chemokine ligand 5	protein_codin	-0.7276	0.00615	1	60.6	0.6049
**RARA**	RARA	9	retinoic acid receptor alpha	protein_codin	0.3944	0.0432	1	23.97	0.1359
**IFI35**	IFI35	9	interferon induced protein 35	protein_codin	0.01209	0.925	1	45.2	0.3139
**LSM12.00**	LSM12.00	9	LSM12 homolog	protein_codin	0.148	0.229	1	11.03	0.1912
**SGCA**	SGCA	9	sarcoglycan alpha	protein_codin	-0.8872	3.44E-18	8.25E-14	54.01	0.03203
**LUC7L3**	LUC7L3	9	LUC7 like 3 pre-mRNA splicing factor	protein_codin	-0.3232	0.00066	1	56.76	0.213
**RGS9**	RGS9	9	regulator of G protein signaling 9	protein_codin	0.4128	0.066	1	10.32	0.1774
**GALK1**	GALK1	9	galactokinase 1	protein_codin	-0.211	0.182	1	8.488	0.1317
**ANKRD29**	ANKRD29	9	ankyrin repeat domain 29	protein_codin	0.443	0.0321	1	20.79	0.1752
**CCDC102B**	CCDC102B	9	coiled-coil domain containing 102B	protein_codin	-0.909	4.15E-09	9.78E-05	60.54	0.4222
**SNRPB**	SNRPB	9	small nuclear ribonucleoprotein polypeptides B and B	protein_codin	0.001098	0.99	1	33.78	0.0328
**CDK5RAP1**	CDK5RAP1	9	CDK5 regulatory subunit associated protein 1	protein_codin	0.2449	0.297	1	8.712	0.3087
**PROCR**	PROCR	9	protein C receptor	protein_codin	0.4162	0.00187	1	60.53	0.1066
**WISP2**	WISP2	9	cellular communication network factor 5	protein_codin	1.816	7.7E-34	1.86E-29	60.6	0.1017
**CD40**	CD40	9	CD40 molecule	protein_codin	0.4629	0.00259	1	55.09	0.1411
**NCOA3**	NCOA3	9	nuclear receptor coactivator 3	protein_codin	-0.04012	0.812	1	7.195	0.2606
**DPM1**	DPM1	9	dolichyl-phosphate mannosyltransferase subunit 1, c	protein_codin	-0.1309	0.416	1	7.423	0.2816
**TSHZ2**	TSHZ2	9	teashirt zinc finger homeobox 2	protein_codin	4.517	1.82E-12	4.34E-08	31.23	0.03203
**YTHDF1**	YTHDF1	9	YTH N6-methyladenosine RNA binding protein 1	protein_codin	0.5856	0.00632	1	9.03	0.2487
**FSTL3**	FSTL3	9	follistatin like 3	protein_codin	1.226	1.44E-12	3.42E-08	59.94	0.03726
**CFD**	CFD	9	complement factor D	protein_codin	10.72	3.76E-05	0.8607	NA	0.03447
**MEX3D**	MEX3D	9	mex-3 RNA binding family member D	protein_codin	0.3472	0.121	1	10.95	0.1331
**MLLT1**	MLLT1	9	MLLT1 super elongation complex subunit	protein_codin	-0.01002	0.967	1	8.011	0.2519
**MAP2K7**	MAP2K7	9	mitogen-activated protein kinase kinase 7	protein_codin	0.1296	0.622	1	37.29	0.3112
**ICAM1**	ICAM1	9	intercellular adhesion molecule 1	protein_codin	0.6217	0.0672	1	60.54	0.6811
**CARM1**	CARM1	9	coactivator associated arginine methyltransferase 1	protein_codin	0.5628	0.0332	1	3.013	0.1662
**DDX39A**	DDX39A	9	DExD-box helicase 39A	protein_codin	0.1129	0.56	1	25.76	0.1772
**NOTCH3**	NOTCH3	9	notch receptor 3	protein_codin	-0.2443	0.0546	1	29.2	0.1376
**BST2**	BST2	9	bone marrow stromal cell antigen 2	protein_codin	-0.4875	2.3E-09	5.43E-05	55.35	0.2046
**COLGALT1**	COLGALT1	9	collagen beta(1-O)galactosyltransferase 1	protein_codin	0.1023	0.582	1	8.877	0.1836
**XRCC1**	XRCC1	9	X-ray repair cross complementing 1	protein_codin	0.1898	0.347	1	3.194	0.248
**BCL3**	BCL3	9	BCL3 transcription coactivator	protein_codin	0.5303	0.0124	1	53.38	0.1282
**TRMT2A**	TRMT2A	9	tRNA methyltransferase 2 homolog A	protein_codin	0.04401	0.846	1	2.682	0.2775
**GSTT2B**	GSTT2B	9	glutathione S-transferase theta 2B (gene/pseudogene)	protein_codin	-0.6711	3.68E-08	0.00088	39.03	0.1462
**GUCD1**	GUCD1	9	guanylyl cyclase domain containing 1	protein_codin	0.1507	0.406	1	7.047	0.1412
**HSCB**	HSCB	9	HscB mitochondrial iron-sulfur cluster cochaperone	protein_codin	-0.07617	0.669	1	8.296	0.2614
**PRR14L**	PRR14L	9	proline rich 14 like	protein_codin	0.2571	0.272	1	10.05	0.3142
**APOL6**	APOL6	9	apolipoprotein L6	protein_codin	0.6792	0.00025	1	59.03	0.2836
**APOL2**	APOL2	9	apolipoprotein L2	protein_codin	0.1186	0.506	1	57.6	0.2654
**APOL1**	APOL1	9	apolipoprotein L1	protein_codin	0.5906	0.00200	1	36.07	0.188
**CSNK1E**	CSNK1E	9	casein kinase 1 epsilon	protein_codin	0.4352	0.0316	1	7.788	0.2681
**APOBEC3G**	APOBEC3G	9	apolipoprotein B mRNA editing enzyme catalytic subu	protein_codin	-0.5231	0.0167	1	3.55	0.3271
**POLDIP3**	POLDIP3	9	DNA polymerase delta interacting protein 3	protein_codin	0.01405	0.942	1	7.142	0.2107
**PIM3**	PIM3	9	Pim-3 proto-oncogene, serine/threonine kinase	protein_codin	-0.4423	0.00476	1	60.6	0.1565
**TYMP**	TYMP	9	thymidine phosphorylase	protein_codin	-0.1606	0.215	1	59.96	0.2231
**JAM2**	JAM2	9	junctional adhesion molecule 2	protein_codin	0.431	0.0842	1	24.23	0.1748
**MX1**	MX1	9	MX dynamin like GTPase 1	protein_codin	-0.5115	0.00056	1	40.65	0.6156
**NOC2L**	NOC2L	10	NOC2 like nucleolar associated transcriptional repres	protein_codin	0.3535	0.0377	1	7.07	0.2706
**MIB2**	MIB2	10	mindbomb E3 ubiquitin protein ligase 2	protein_codin	0.1005	0.661	1	8.715	0.1766
**MMP23B**	MMP23B	10	matrix metallopeptidase 23B	protein_codin	1.288	1.36E-24	3.28E-20	55.36	0.03222
**GNB1**	GNB1	10	G protein subunit beta 1	protein_codin	-0.1839	0.0994	1	12.03	0.1711
**SPSB1**	SPSB1	10	splA/ryanodine receptor domain and SOCS box conta	protein_codin	0.411	0.0159	1	60.35	0.1332
**PGD**	PGD	10	phosphogluconate dehydrogenase	protein_codin	0.5229	0.0498	1	8.25	0.1695
**SZRD1**	SZRD1	10	SUZ RNA binding domain containing 1	protein_codin	-0.4957	3.17E-05	0.7262	15.13	0.21
**TMEM222**	TMEM222	10	transmembrane protein 222	protein_codin	-0.3011	0.0632	1	22.44	0.2862
**STX12**	STX12	10	syntaxin 12	protein_codin	0.1427	0.258	1	13.98	0.1187
**TXLNA**	TXLNA	10	taxilin alpha	protein_codin	0.2789	0.149	1	23.85	0.1727
**HDAC1**	HDAC1	10	histone deacetylase 1	protein_codin	-0.5583	0.00115	1	21.33	0.09803
**TMEM54**	TMEM54	10	transmembrane protein 54	protein_codin	0.1638	0.309	1	36.42	0.06452
**SF3A3**	SF3A3	10	splicing factor 3a subunit 3	protein_codin	-0.03902	0.742	1	12.53	0.03326
**RRAGC**	RRAGC	10	Ras related GTP binding C	protein_codin	0.2678	0.136	1	20.21	0.1062
**MED8**	MED8	10	mediator complex subunit 8	protein_codin	-0.08947	0.5	1	7.224	0.06693
**DMAP1**	DMAP1	10	DNA methyltransferase 1 associated protein 1	protein_codin	-0.01669	0.92	1	7.722	0.277
**ERI3**	ERI3	10	ERI1 exoribonuclease family member 3	protein_codin	0.09714	0.45	1	17	0.144
**UROD**	UROD	10	uroporphyrinogen decarboxylase	protein_codin	-0.05297	0.616	1	59.52	0.126
**MKNK1**	MKNK1	10	MAPK interacting serine/threonine kinase 1	protein_codin	0.59	0.00341	1	8.842	0.1768
**EFCAB14**	EFCAB14	10	EF-hand calcium binding domain 14	protein_codin	-0.2103	0.0967	1	33.17	0.2116
**MAGOH**	MAGOH	10	mago homolog, exon junction complex subunit	protein_codin	-0.1074	0.252	1	11.07	0.06827
**HSPB11**	HSPB11	10	heat shock protein family B (small) member 11	protein_codin	-0.04071	0.705	1	35.33	0.09682
**PTGER3**	PTGER3	10	prostaglandin E receptor 3	protein_codin	-2.018	3.28E-19	7.87E-15	19.64	0.5232
**RPF1**	RPF1	10	ribosome production factor 1 homolog	protein_codin	-0.05754	0.756	1	10.02	0.2481
**EVI5**	EVI5	10	ecotropic viral integration site 5	protein_codin	0.2501	0.0436	1	43.23	0.09935
**TRMT13**	TRMT13	10	tRNA methyltransferase 13 homolog	protein_codin	0.1548	0.495	1	31.26	0.1005
**SLC30A7**	SLC30A7	10	solute carrier family 30 member 7	protein_codin	0.2232	0.251	1	8.703	0.3112
**DPH5**	DPH5	10	diphthamide biosynthesis 5	protein_codin	-0.07284	0.728	1	3.022	0.07329
**GSTM4**	GSTM4	10	glutathione S-transferase mu 4	protein_codin	-0.002312	0.989	1	23.19	0.172
**SLC16A4**	SLC16A4	10	solute carrier family 16 member 4	protein_codin	0.4605	0.00208	1	32.37	0.1154
**DRAM2**	DRAM2	10	DNA damage regulated autophagy modulator 2	protein_codin	0.2042	0.197	1	21.63	0.06605
**NGF**	NGF	10	nerve growth factor	protein_codin	0.7154	0.00087	1	22.63	0.1691
**SCNM1**	SCNM1	10	sodium channel modifier 1	protein_codin	-0.2444	0.119	1	19.27	0.1031
**PI4KB**	PI4KB	10	phosphatidylinositol 4-kinase beta	protein_codin	0.2257	0.318	1	7.714	0.3335
**POGZ**	POGZ	10	pogo transposable element derived with ZNF domain	protein_codin	0.2704	0.12	1	24.99	0.2816
**SLC39A1**	SLC39A1	10	solute carrier family 39 member 1	protein_codin	-0.1754	0.0379	1	34.99	0.02228
**ASH1L**	ASH1L	10	ASH1 like histone lysine methyltransferase	protein_codin	0.2172	0.05	1	22.02	0.2079
**KIAA0907**	KIAA0907	10	KH domain containing 4, pre-mRNA splicing factor	protein_codin	NA	NA	NA	2.719	0.7611
**GLMP**	GLMP	10	glycosylated lysosomal membrane protein	protein_codin	0.3596	0.021	1	20.16	0.1092
**UHMK1**	UHMK1	10	U2AF homology motif kinase 1	protein_codin	0.2788	0.112	1	26.7	0.3646
**POU2F1**	POU2F1	10	POU class 2 homeobox 1	protein_codin	0.2868	0.329	1	7.998	0.8172
**DCAF6**	DCAF6	10	DDB1 and CUL4 associated factor 6	protein_codin	0.2278	0.265	1	6.97	0.4052
**METTL18**	METTL18	10	methyltransferase like 18	protein_codin	-0.1221	0.595	1	3.248	0.3325
**PRRC2C**	PRRC2C	10	proline rich coiled-coil 2C	protein_codin	-0.0419	0.576	1	28.45	0.0332
**CEP350**	CEP350	10	centrosomal protein 350	protein_codin	0.1492	0.29	1	30.82	0.309
**RNF2**	RNF2	10	ring finger protein 2	protein_codin	0.321	0.131	1	3.335	0.1708
**NEK7**	NEK7	10	NIMA related kinase 7	protein_codin	-0.005827	0.969	1	56.78	0.2142
**TRAF5**	TRAF5	10	TNF receptor associated factor 5	protein_codin	-0.0778	0.729	1	9.294	0.3771
**PPP2R5A**	PPP2R5A	10	protein phosphatase 2 regulatory subunit B'alpha	protein_codin	0.1378	0.368	1	20.57	0.2072
**PTPN14**	PTPN14	10	protein tyrosine phosphatase non-receptor type 14	protein_codin	-0.398	0.0795	1	3.54	0.3709
**RRP15**	RRP15	10	ribosomal RNA processing 15 homolog	protein_codin	0.3762	0.026	1	16.33	0.1689
**EPRS**	EPRS	10	glutamyl-prolyl-tRNA synthetase	protein_codin	0.3728	0.00074	1	40.77	0.03202
**RAB3GAP2**	RAB3GAP2	10	RAB3 GTPase activating non-catalytic protein subunit 2	protein_codin	0.002369	0.99	1	29.43	0.282
**GALNT2**	GALNT2	10	polypeptide N-acetylgalactosaminyltransferase 2	protein_codin	0.01212	0.937	1	60.58	0.1024
**GNPAT**	GNPAT	10	glyceronephosphate O-acyltransferase	protein_codin	-0.3386	0.075	1	6.786	0.1308
**NID1**	NID1	10	nidogen 1	protein_codin	1.426	3.07E-08	0.00072	9.4	0.0661
**MTR**	MTR	10	5-methyltetrahydrofolate-homocysteine methyltransfe	protein_codin	0.2762	0.168	1	8.524	0.02393
**AKT3**	AKT3	10	AKT serine/threonine kinase 3	protein_codin	-0.3127	0.0239	1	6.946	0.3098
**ZNF672**	ZNF672	10	zinc finger protein 672	protein_codin	-0.1075	0.639	1	9.126	0.2711
**CPSF3**	CPSF3	10	cleavage and polyadenylation specific factor 3	protein_codin	0.04711	0.887	1	3.042	0.2019
**ADAM17**	ADAM17	10	ADAM metallopeptidase domain 17	protein_codin	0.5998	0.00808	1	53.58	0.3024
**ODC1**	ODC1	10	ornithine decarboxylase 1	protein_codin	-0.1245	0.326	1	58.18	0.0679
**ATAD2B**	ATAD2B	10	ATPase family AAA domain containing 2B	protein_codin	0.5047	0.064	1	3.342	0.7154
**BIRC6**	BIRC6	10	baculoviral IAP repeat containing 6	protein_codin	0.2182	0.226	1	20.24	0.2765
**EIF2AK2**	EIF2AK2	10	eukaryotic translation initiation factor 2 alpha kinase 2	protein_codin	0.07297	0.517	1	55.67	0.1031
**GALM**	GALM	10	galactose mutarotase	protein_codin	-0.0006277	0.997	1	9.711	0.2836
**PPM1B**	PPM1B	10	protein phosphatase, Mg2+/Mn2+ dependent 1B	protein_codin	-0.393	0.0226	1	25.64	0.349
**FBXO11**	FBXO11	10	F-box protein 11	protein_codin	0.3746	0.0462	1	24.58	0.1725
**REL**	REL	10	REL proto-oncogene, NF-kB subunit	protein_codin	0.06819	0.746	1	9.172	0.3924
**PEX13**	PEX13	10	peroxisomal biogenesis factor 13	protein_codin	0.6024	0.0109	1	7.217	0.102
**AFTPH**	AFTPH	10	aftiphilin	protein_codin	-0.07017	0.714	1	7.158	0.3419
**PPP3R1**	PPP3R1	10	protein phosphatase 3 regulatory subunit B, alpha	protein_codin	0.2539	0.257	1	8.049	0.1772
**TIA1**	TIA1	10	TIA1 cytotoxic granule associated RNA binding protein	protein_codin	0.3048	0.0922	1	23.03	0.1742
**TPRKB**	TPRKB	10	TP53RK binding protein	protein_codin	-0.2446	0.0838	1	16.35	0.1381
**DOK1**	DOK1	10	docking protein 1	protein_codin	-0.3241	0.142	1	2.873	0.1756
**RNF181**	RNF181	10	ring finger protein 181	protein_codin	-0.2923	0.0002	1	44.25	0.03851
**USP39**	USP39	10	ubiquitin specific peptidase 39	protein_codin	0.1712	0.445	1	3.272	0.03417
**CHMP3**	CHMP3	10	charged multivesicular body protein 3	protein_codin	-0.3273	0.00069	1	39.9	0.178
**AC092683.1**	AC092683.1	10	novel transcri	lincRNA	0.581	0.0531	1	8.249	0.2506
**COX5B**	COX5B	10	cytochrome c oxidase subunit 5B	protein_codin	-0.07833	0.0893	1	60.45	0.02233
**LIMS1**	LIMS1	10	LIM zinc finger domain containing 1	protein_codin	-0.4141	2.85E-07	0.00666	43.82	0.03201
**TSN**	TSN	10	translin	protein_codin	-0.3017	0.0396	1	7.724	0.09848
**ERCC3**	ERCC3	10	ERCC excision repair 3, TFIIH core complex helicase	protein_codin	0.3608	0.0828	1	7.406	0.1389

**LIMS2**	LIMS2		10	LIM zinc finger domain containing 2	protein_codin	-0.2744	0.0412	1	12.3	0.0679
**CCNT2**	CCNT2		10	cyclin T2	protein_codin	0.4228	0.012	1	8.56	0.09866
**PRPF40A**	PRPF40A		10	pre-mRNA processing factor 40 homolog A	protein_codin	-0.0824	0.42	1	33.65	0.09887
**PDE1A**	PDE1A		10	phosphodiesterase 1A	protein_codin	-0.7816	1.01E-09	2.39E-05	42.92	0.2312
**ZC3H15**	ZC3H15		10	zinc finger CCCH-type containing 15	protein_codin	-0.05685	0.47	1	20.93	0.1632
**STK17B**	STK17B		10	serine/threonine kinase 17b	protein_codin	-0.2651	0.142	1	60.53	0.1038
**NDUFB3**	NDUFB3		10	NADH:ubiquinone oxidoreductase subunit B3	protein_codin	-0.08115	0.344	1	52.16	0.1005
**WDR12**	WDR12		10	WD repeat domain 12	protein_codin	0.4493	0.0461	1	7.746	0.1721
**METTL21A**	METTL21A		10	methyltransferase like 21A	protein_codin	0.4622	0.0719	1	8.688	0.2368
**RETREG2**	RETREG2		10	reticulophagy regulator family member 2	protein_codin	0.1844	0.279	1	7.835	0.06671
**SP110**	SP110		10	SP110 nuclear body protein	protein_codin	-0.3212	0.0194	1	12.1	0.2094
**TWIST2**	TWIST2		10	twist family bHLH transcription factor 2	protein_codin	1.897	4.47E-15	1.07E-10	38.39	0.03382
**DTYMK**	DTYMK		10	deoxythymidylate kinase	protein_codin	0.3269	0.0846	1	52.9	0.1452
**ING5**	ING5		10	inhibitor of growth family member 5	protein_codin	0.3766	0.0535	1	29.44	0.09768
**ITPR1**	ITPR1		10	inositol 1,4,5-trisphosphate receptor type 1	protein_codin	-0.7812	0.00467	1	3.399	0.358
**HRH1**	HRH1		10	histamine receptor H1	protein_codin	-0.05875	0.732	1	55.45	0.1039
**METTL6**	METTL6		10	methyltransferase like 6	protein_codin	0.2322	0.348	1	8.906	0.2545
**EAF1**	EAF1		10	ELL associated factor 1	protein_codin	0.2679	0.242	1	9.839	0.1787
**NKIRAS1**	NKIRAS1		10	NFKB inhibitor interacting Ras like 1	protein_codin	0.09145	0.672	1	32.4	0.357
**CMC1**	CMC1		10	C-X9-C motif containing 1	protein_codin	-0.1197	0.324	1	6.88	0.1709
**DYNC1LI1**	DYNC1LI1		10	dynein cytoplasmic 1 light intermediate chain 1	protein_codin	-0.1546	0.142	1	29.1	0.2369
**CNOT10**	CNOT10		10	CCR4-NOT transcription complex subunit 10	protein_codin	0.5122	0.0328	1	9.496	0.3318
**TCAIM**	TCAIM		10	T cell activation inhibitor, mitochondrial	protein_codin	0.4534	0.0333	1	3.245	0.3044
**SACM1L**	SACM1L		10	SAC1 like phosphatidylinositide phosphatase	protein_codin	0.08867	0.686	1	6.982	0.1768
**CCDC12**	CCDC12		10	coiled-coil domain containing 12	protein_codin	0.07612	0.52	1	15.24	0.1808
**IP6K1**	IP6K1		10	inositol hexakisphosphate kinase 1	protein_codin	0.2202	0.339	1	2.976	0.1796
**RBM6**	RBM6		10	RNA binding motif protein 6	protein_codin	0.4638	0.00203	1	14.69	0.344
**PDE12**	PDE12		10	phosphodiesterase 12	protein_codin	0.3365	0.167	1	8.237	0.3036
**KCTD6**	KCTD6		10	potassium channel tetramerization domain containing 6	protein_codin	0.4102	0.0651	1	7.847	0.2802
**C3orf14**	C3orf14		10	chromosome 3 open reading frame 14	protein_codin	-0.1496	0.396	1	60.45	0.06426
**CHMP2B**	CHMP2B		10	charged multivesicular body protein 2B	protein_codin	0.05556	0.554	1	35.46	0.1512
**CBLB**	CBLB		10	Cbl proto-oncogene B	protein_codin	1.244	2.55E-07	0.00596	21.56	0.2882
**DUBR**	DUBR		10	DPPA2 upstream binding RNA	lincRNA	0.04187	0.859	1	3.242	0.4856
**PHLDB2**	PHLDB2		10	pleckstrin homology like domain family B member 2	protein_codin	0.1121	0.402	1	52.06	0.06758
**RUVBL1**	RUVBL1		10	RuvB like AAA ATPase 1	protein_codin	0.1554	0.362	1	7.634	0.0975
**TMCC1**	TMCC1		10	transmembrane and coiled-coil domain family 1	protein_codin	-0.2861	0.202	1	3.134	0.1816
**MRPL3**	MRPL3		10	mitochondrial ribosomal protein L3	protein_codin	0.2308	0.0327	1	13.46	0.0976
**CEP63**	CEP63		10	centrosomal protein 63	protein_codin	-0.1284	0.366	1	23.64	0.1745
**TRPC1**	TRPC1		10	transient receptor potential cation channel subfamily (	protein_codin	-0.04495	0.82	1	34.17	0.2706
**GOLIM4**	GOLIM4		10	golgi integral membrane protein 4	protein_codin	0.1421	0.0606	1	54.17	0.0352
**SKIL**	SKIL		10	SKI like proto-oncogene	protein_codin	0.8815	2.82E-12	6.7E-08	34.45	0.1041
**ZNF639**	ZNF639		10	zinc finger protein 639	protein_codin	-0.03206	0.849	1	28.54	0.2781
**ACTL6A**	ACTL6A		10	actin like 6A	protein_codin	0.343	0.0242	1	6.486	0.1659
**CHRD**	CHRD		10	chordin	protein_codin	1.525	2.03E-08	0.00048	10.44	0.2102
**EIF4A2**	EIF4A2		10	eukaryotic translation initiation factor 4A2	protein_codin	-0.1768	0.0017	1	59.93	0.02234
**TMEM175**	TMEM175		10	transmembrane protein 175	protein_codin	0.6578	0.00086	1	22.81	0.03357
**DCAF16**	DCAF16		10	DDB1 and CUL4 associated factor 16	protein_codin	0.4521	0.0387	1	10.03	0.1412
**RFC1**	RFC1		10	replication factor C subunit 1	protein_codin	0.03159	0.759	1	36.96	0.179
**TMEM33**	TMEM33		10	transmembrane protein 33	protein_codin	0.5333	0.0021	1	8.743	0.2422
**ATP10D**	ATP10D		10	ATPase phospholipid transporting 10D (putative)	protein_codin	-0.3212	0.0367	1	17.53	0.1734
**DCK**	DCK		10	deoxycytidine kinase	protein_codin	-0.07923	0.702	1	10.97	0.1474
**LAMTOR3**	LAMTOR3		10	late endosomal/lysosomal adaptor, MAPK and MTOR	protein_codin	-0.06406	0.685	1	7.972	0.2352
**SGMS2**	SGMS2		10	sphingomyelin synthase 2	protein_codin	1.071	6.61E-07	0.01541	24.39	0.2825
**CYP2U1**	CYP2U1		10	cytochrome P450 family 2 subfamily U member 1	protein_codin	0.4374	0.0187	1	8.84	0.1819
**SEC24B**	SEC24B		10	SEC24 homolog B, COPII coat complex component	protein_codin	0.1814	0.438	1	2.793	0.1729
**GAR1**	GAR1		10	GAR1 ribonucleoprotein	protein_codin	-0.09714	0.562	1	40.19	0.03427
**ALPK1**	ALPK1		10	alpha kinase 1	protein_codin	0.3002	0.279	1	9.878	0.4058
**CAMK2D**	CAMK2D		10	calcium/calmodulin dependent protein kinase II delta	protein_codin	0.009712	0.949	1	16.09	0.03286
**METTL14**	METTL14		10	methyltransferase like 14	protein_codin	0.2166	0.337	1	3.443	0.2442
**MFSD8**	MFSD8		10	major facilitator superfamily domain containing 8	protein_codin	0.2771	0.188	1	3	0.4142
**SCOC**	SCOC		10	short coiled-coil protein	protein_codin	0.02041	0.819	1	35.95	0.0322
**ZNF330**	ZNF330		10	zinc finger protein 330	protein_codin	0.1278	0.415	1	8.008	0.06453
**MAP9**	MAP9		10	microtubule associated protein 9	protein_codin	0.0277	0.814	1	55.38	0.2047
**RAPGEF2**	RAPGEF2		10	Rap guanine nucleotide exchange factor 2	protein_codin	-0.2016	0.429	1	8.662	0.371
**SEMA5A**	SEMA5A		10	semaphorin 5A	protein_codin	0.02606	0.882	1	42.63	0.1736
**GOLPH3**	GOLPH3		10	golgi phosphoprotein 3	protein_codin	0.2528	0.0712	1	20.66	0.2429
**RAI14**	RAI14		10	retinoic acid induced 14	protein_codin	-0.5251	0.0046	1	49.26	0.2115
**SPEF2**	SPEF2		10	sperm flagellar 2	protein_codin	0.7262	0.00866	1	7.96	0.1754
**TTC33**	TTC33		10	tetratricopeptide repeat domain 33	protein_codin	-0.1996	0.29	1	3.305	0.1388
**MRPS30**	MRPS30		10	mitochondrial ribosomal protein S30	protein_codin	0.05891	0.732	1	5.505	0.1709
**PELO**	PELO		10	pelota mRNA surveillance and ribosome rescue facto	protein_codin	-0.3368	0.0276	1	30.12	0.1959
**PART1**	PART1		10	prostate androgen-regulated transcript 1	lincRNA	-1.855	9.35E-21	2.25E-16	34.98	0.5977
**CDK7**	CDK7		10	cyclin dependent kinase 7	protein_codin	0.2188	0.436	1	8.754	0.3474
**JMY**	JMY		10	junction mediating and regulatory protein, p53 cofacto	protein_codin	-0.1009	0.58	1	9.261	0.1741
**ATG10**	ATG10		10	autophagy related 10	protein_codin	0.08124	0.727	1	3.264	0.218
**ERAP1**	ERAP1		10	endoplasmic reticulum aminopeptidase 1	protein_codin	-0.1219	0.594	1	7.83	0.2529
**FAM174A**	FAM174A		10	family with sequence similarity 174 member A	protein_codin	0.006137	0.965	1	6.958	0.2435
**PPIP5K2**	PPIP5K2		10	diphosphoinositol pentakisphosphate kinase 2	protein_codin	0.04967	0.725	1	7.163	0.2816
**DCP2**	DCP2		10	decapping mRNA 2	protein_codin	-0.004577	0.98	1	8.496	0.1798
**LVRN**	LVRN		10	laeverin	protein_codin	2.599	0.967	1	11.11	0.4406
**DPYSL3**	DPYSL3		10	dihydropyrimidinase like 3	protein_codin	-0.295	0.0178	1	54.88	0.1851
**SLC26A2**	SLC26A2		10	solute carrier family 26 member 2	protein_codin	-0.01006	0.961	1	29.61	0.2091
**B4GALT7**	B4GALT7		10	beta-1,4-galactosyltransferase 7	protein_codin	-0.1539	0.365	1	8.572	0.07142
**RMND5B**	RMND5B		10	required for meiotic nuclear division 5 homolog B	protein_codin	0.1488	0.404	1	30.22	0.1684
**RUFY1**	RUFY1		10	RUN and FYVE domain containing 1	protein_codin	0.2377	0.166	1	9.292	0.318
**ECI2**	ECI2		10	enoyl-CoA delta isomerase 2	protein_codin	0.5099	1.68E-05	0.3863	10.96	0.1759
**PHACTR1**	PHACTR1		10	phosphatase and actin regulator 1	protein_codin	-0.6586	0.0127	1	8.902	0.3422
**HIST1H1A**	HIST1H1A		10	histone cluster 1 H1 family member a	protein_codin	-0.5728	0.0286	1	10.86	0.3434
**TRIM27**	TRIM27		10	tripartite motif containing 27	protein_codin	-0.02588	0.882	1	5.79	0.2471
**DHX16**	DHX16		10	DEAH-box helicase 16	protein_codin	0.2822	0.309	1	3.108	0.3934
**NEU1**	NEU1		10	neuraminidase 1	protein_codin	0.5338	0.00146	1	59.56	0.06717
**DAXX**	DAXX		10	death domain associated protein	protein_codin	0.3172	0.105	1	6.727	0.1335
**RNF8**	RNF8		10	ring finger protein 8	protein_codin	-0.1372	0.41	1	43.54	0.1021
**ZFAND3**	ZFAND3		10	zinc finger AN1-type containing 3	protein_codin	0.2242	0.184	1	7.237	0.1345
**CNPY3**	CNPY3		10	canopy FGF signaling regulator 3	protein_codin	0.03354	0.814	1	18.29	0.2372
**EFHC1**	EFHC1		10	EF-hand domain containing 1	protein_codin	0.7829	0.0001	1	5.925	0.0331
**TRAM2-AS1**	TRAM2-AS1		10	TRAM2 antisense RNA 1 (head to head)	lincRNA	-0.03775	0.862	1	32.5	0.1706
**AL391807.1**	AL391807.1		10	novel transcri	antisense_RN	0.4427	0.0642	1	27.77	0.2797
**ADGRB3**	ADGRB3		10	adhesion G protein-coupled receptor B3	protein_codin	0.7211	0.0266	1	31.68	0.736
**SLC17A5**	SLC17A5		10	solute carrier family 17 member 5	protein_codin	1.232	1.4E-07	0.00329	26.84	0.1721
**PGM3**	PGM3		10	phosphoglucomutase 3	protein_codin	0.5137	0.0225	1	7.955	0.1759
**ANKRD6**	ANKRD6		10	ankyrin repeat domain 6	protein_codin	0.4387	0.189	1	8.661	0.2314
**PNISR**	PNISR		10	PNN interacting serine and arginine rich protein	protein_codin	0.08992	0.241	1	43.78	0.1315
**TUBE1**	TUBE1		10	tubulin epsilon 1	protein_codin	0.5263	0.0342	1	3.536	0.4561
**TSPYL1**	TSPYL1		10	TSPY like 1	protein_codin	0.06003	0.541	1	36.7	0.102
**ASF1A**	ASF1A		10	anti-silencing function 1A histone chaperone	protein_codin	-0.06753	0.657	1	6.364	0.2464
**CITED2**	CITED2		10	Cbp/p300 interacting transactivator with Glu/Asp rich	protein_codin	0.06766	0.536	1	59.97	0.1654
**AIG1**	AIG1		10	androgen induced 1	protein_codin	-0.4589	8.6E-09	0.0002	32.27	0.1115
**MPC1**	MPC1		10	mitochondrial pyruvate carrier 1	protein_codin	0.2546	0.00279	1	30.94	0.137
**PMS2**	PMS2		10	PMS1 homolog 2, mismatch repair system componer	protein_codin	-0.08026	0.769	1	3.236	0.2411
**ANKMY2**	ANKMY2		10	ankyrin repeat and MYND domain containing 2	protein_codin	0.2686	0.156	1	7.992	0.1406
**SNX13**	SNX13		10	sorting nexin 13	protein_codin	0.5076	0.0122	1	8.572	0.1486
**POLD2**	POLD2		10	DNA polymerase delta 2, accessory subunit	protein_codin	-0.1812	0.134	1	15	0.2002
**YKT6**	YKT6		10	YKT6 v-SNARE homolog	protein_codin	0.05364	0.845	1	9.28	0.1750
**VOPP1**	VOPP1		10	VOPP1 WW domain binding protein	protein_codin	-0.7374	8.62E-07	0.02007	8.353	0.1745
**ASL**	ASL		10	argininosuccinate lyase	protein_codin	-0.5392	0.00912	1	7.669	0.3122
**CRCP**	CRCP		10	CGRP receptor component	protein_codin	0.2179	0.232	1	8.288	0.2049
**POM121**	POM121		10	POM121 transmembrane nucleoporin	protein_codin	0.1196	0.588	1	3.035	0.2052
**GTF2I**	GTF2I		10	general transcription factor IIi	protein_codin	0.02874	0.782	1	10.45	0.2088
**HIP1**	HIP1		10	huntingtin interacting protein 1	protein_codin	-0.1799	0.282	1	18.39	0.2460
**FGL2**	FGL2		10	fibrinogen like 2	protein_codin	0.7166	1.04E-10	2.46E-06	60.6	0.06900
**ZNF394**	ZNF394		10	zinc finger protein 394	protein_codin	0.05579	0.795	1	31.76	0.3164
**PMPCB**	PMPCB		10	peptidase, mitochondrial processing beta subunit	protein_codin	-0.256	0.0192	1	13.2	0.09642
**IFRD1**	IFRD1		10	interferon related developmental regulator 1	protein_codin	0.6619	2.72E-07	0.00626	59.87	0.1346
**SMIM30**	SMIM30		10	small integral membrane protein 30	protein_codin	-0.3558	0.0402	1	52.06	0.1399
**TSPAN12**	TSPAN12		10	tetraspanin 12	protein_codin	0.3272	0.129	1	9.957	0.1324
**IMPDH1**	IMPDH1		10	inosine monophosphate dehydrogenase 1	protein_codin	-0.26	0.254	1	8.818	0.254
**CNOT4**	CNOT4		10	CCR4-NOT transcription complex subunit 4	protein_codin	0.2697	0.0106	1	26.35	0.1986
**C7orf73**	C7orf73		10	short transmembrane mitochondrial protein 1	protein_codin	NA	NA	NA	60.6	0.03581
**ZC3HAV1**	ZC3HAV1		10	zinc finger CCCH-type containing, antiviral 1	protein_codin	0.3794	0.0436	1	60.58	0.2322
**LUC7L2**	LUC7L2		10	LUC7 like 2, pre-mRNA splicing factor	protein_codin	0.04712	0.729	1	19.42	0.3169
**PRKAG2**	PRKAG2		10	protein kinase AMP-activated non-catalytic subunit g	protein_codin	0.8806	8.34E-07	0.01962	23.02	0.1361
**SCML1**	SCML1		10	Scm polycomb group protein like 1	protein_codin	0.02718	0.9	1	8.569	0.2075
**CDKL5**	CDKL5		10	cyclin dependent kinase like 5	protein_codin	-0.07247	0.796	1	3.386	0.2687
**CXorf38**	CXorf38		10	chromosome X open reading frame 38	protein_codin	0.01232	0.962	1	10.52	0.3092
**FTSJ1**	FTSJ1		10	FtsJ RNA 2'-O-methyltransferase 1	protein_codin	-0.1513	0.476	1	9.172	0.1771
**WDR13**	WDR13		10	WD repeat domain 13	protein_codin	-0.1458	0.192	1	13.53	0.1382
**PJA1**	PJA1		10	praja ring finger ubiquitin ligase 1	protein_codin	0.04521	0.812	1	9.059	0.2036
**NHSL2**	NHSL2		10	NHS like 2	protein_codin	0.06726	0.839	1	8.657	0.2409
**SLC16A2**	SLC16A2		10	solute carrier family 16 member 2	protein_codin	0.502	0.0300	1	2.628	0.3518
**DIAPH2**	DIAPH2		10	diaphanous related formin 2	protein_codin	0.2683	0.0582	1	24.88	0.1354
**ZMAT1**	ZMAT1		10	zinc finger matrin-type 1	protein_codin	0.2747	0.357	1	8.514	0.5067
**RAB9B**	RAB9B		10	RAB9B, member RAS oncogene family	protein_codin	0.00806	0.977	1	34.17	0.3511
**PRPS1**	PRPS1		10	phosphoribosyl pyrophosphate synthetase 1	protein_codin	0.06767	0.691	1	3.185	0.07192
**CUL4B**	CUL4B		10	cullin 4B	protein_codin	-0.1444	0.532	1	32.58	0.2282
**SMIM10**	SMIM10		10	small integral membrane protein 10	protein_codin	-0.5844	9.38E-08	0.0022	38.81	0.2019
**CETN2**	CETN2		10	centrin 2	protein_codin	-0.2716	0.0249	1	34.37	0.09937
**FAM50A**	FAM50A		10	family with sequence similarity 50 member A	protein_codin	-0.04632	0.621	1	57.66	0.1342
**SLC10A3**	SLC10A3		10	solute carrier family 10 member 3	protein_codin	0.5428	0.00505	1	7.38	0.1380
**DLC1**	DLC1		10	DLC1 Rho GTPase activating protein	protein_codin	0.4672	0.00023	1	49.2	0.136
**PCM1**	PCM1		10	pericentriolar material 1	protein_codin	-0.2272	0.0117	1	21.33	0.1981
**XPO7**	XPO7		10	exportin 7	protein_codin	-0.03664	0.869	1	2.452	0.2495
**GTF2E2**	GTF2E2		10	general transcription factor IIE subunit 2	protein_codin	0.1762	0.394	1	6.523	0.1004
**FUT10**	FUT10		10	fucosyltransferase 10	protein_codin	0.09096	0.688	1	3.674	0.282
**ERLIN2**	ERLIN2		10	ER lipid raft associated 2	protein_codin	0.1032	0.556	1	25.7	0.2077
**ADGRA2**	ADGRA2		10	adhesion G protein-coupled receptor A2	protein_codin	1.153	1.18E-07	0.00276	9.158	0.1752
**PLPP5**	PLPP5		10	phospholipid phosphatase 5	protein_codin	-0.3097	0.0227	1	29.33	0.03223
**ADAM9**	ADAM9		10	ADAM metallopeptidase domain 9	protein_codin	0.8937	1.11E-07	0.0026	15.69	0.079
**PRKDC**	PRKDC		10	protein kinase, DNA-activated, catalytic subunit	protein_codin	0.4235	8.73E-05	1	60.01	0.06840
**LYPLA1**	LYPLA1		10	lysophospholipase 1	protein_codin	-0.316	0.0429	1	6.396	0.1
**IMPAD1**	IMPAD1		10	inositol monophosphatase domain containing 1	protein_codin	0.01166	0.92	1	16.24	0.09860
**PREX2**	PREX2		10	phosphatidylinositol-3,4,5-trisphosphate dependent R	protein_codin	0.3511	0.0894	1	9.681	0.4552
**TMEM70**	TMEM70		10	transmembrane protein 70	protein_codin	0.1165	0.438	1	20.15	0.205
**OSGIN2**	OSGIN2		10	oxidative stress induced growth inhibitor family member 2	protein_codin	-0.4768	0.0324	1	9.26	0.1707
**VIRMA**	VIRMA		10	vir like m6A methyltransferase associated	protein_codin	0.186	0.261	1	8.16	0.2809
**UTP23**	UTP23		10	UTP23 small subunit processome component	protein_codin	0.1045	0.432	1	27.48	0.2680
**C8orf76**	C8orf76		10	chromosome 8 open reading frame 76	protein_codin	0.5679	0.0202	1	8.368	0.2794
**FBXO32**	FBXO32		10	F-box protein 32	protein_codin	-1.413	2.86E-30	6.91E-26	60.59	0.09992
**FAM91A1**	FAM91A1		10	family with sequence similarity 91 member A1	protein_codin	0.3235	0.119	1	3.442	0.1802
**NSMCE2**	NSMCE2		10	NSE2 (MMS21) homolog, SMC5-SMC6 complex SUM	protein_codin	0.2448	0.187	1	5.973	0.3451
**VPS28**	VPS28		10	VPS28 subunit of ESCRT-I	protein_codin	-0.2453	5.84E-06	0.1349	40.86	0.03222
**ZNF7**	ZNF7		10	zinc finger protein 7	protein_codin	0.371	0.0772	1	3.342	0.1988
**CBWD1**	CBWD1		10	COBW domain containing 1	protein_codin	0.002434	0.99	1	29.77	0.2475
**ACO1**	ACO1		10	aconitase 1	protein_codin	0.317	0.0715	1	9.051	0.212
**SMU1**	SMU1		10	SMU1 DNA replication regulator and spliceosomal fa	protein_codin	0.3028	0.0198	1	6.308	0.144
**UBAP2**	UBAP2		10	ubiquitin associated protein 2	protein_codin	0.1665	0.527	1	3.562	0.1790
**ZCCHC7**	ZCCHC7		10	zinc finger CCHC-type containing 7	protein_codin	0.2871	0.0566	1	15.53	0.06854
**C9orf85**	C9orf85		10	chromosome 9 open reading frame 85	protein_codin	0.202	0.266	1	7.138	0.3062
**NAA35**	NAA35		10	N(alpha)-acetyltransferase 35, NatC auxiliary subunit	protein_codin	0.1102	0.571	1	10.27	0.2098
**ABCA1**	ABCA1		10	ATP binding cassette subfamily A member 1	protein_codin	0.5596	0.00374	1	18.2	0.2127
**RAD23B**	RAD23B		10	RAD23 homolog B, nucleotide excision repair protein	protein_codin	0.3121	0.0254	1	16.88	0.1019
**LPAR1**	LPAR1		10	lysophosphatidic acid receptor 1	protein_codin	0.1737	0.574	1	10.33	0.3067
**POLE3**	POLE3		10	DNA polymerase epsilon 3, accessory subunit	protein_codin	0.03445	0.822	1	17.69	0.1830
**PSMD5**	PSMD5		10	proteasome 26S subunit, non-ATPase 5	protein_codin	0.3435	0.171	1	3.368	0.1366
**RABGAP1**	RABGAP1		10	RAB GTPase activating protein 1	protein_codin	-0.6836	4.84E-07	0.01129	40.2	0.3852
**PBX3**	PBX3		10	PBX homeobox 3	protein_codin	1.118	1.01E-08	0.00024	41.71	0.166
**DOLK**	DOLK		10	dolichol kinase	protein_codin	0.6146	0.0476	1	8.938	0.2430
**IER5L**	IER5L		10	immediate early response 5 like	protein_codin	0.359	0.00106	1	60.47	0.1007
**NTMT1**	NTMT1		10	N-terminal Xaa-Pro-Lys N-methyltransferase 1	protein_codin	-0.01011	0.952	1	19.49	0.1069
**GPR107**	GPR107		10	G protein-coupled receptor 107	protein_codin	0.5145	0.0374	1	7.772	0.06866
**BRD3**	BRD3		10	bromodomain containing 3	protein_codin	0.06622	0.701	1	8.4	0.06448
**TUBB4B**	TUBB4B		10	tubulin beta 4B class IVb	protein_codin	0.4383	1.18E-07	0.00276	60.58	0.06529
**EHMT1**	EHMT1		10	euchromatic histone lysine methyltransferase 1	protein_codin	0.4728	0.0086	1	7.458	0.2352
**TALDO1**	TALDO1		10	transaldolase 1	protein_codin	-0.02552	0.712	1	59.85	0.09827
**RRM1**	RRM1		10	ribonucleotide reductase catalytic subunit M1	protein_codin	-0.1169	0.522	1	8.729	0.2016
**TMEM41B**	TMEM41B		10	transmembrane protein 41B	protein_codin	0.3746	0.0637	1	8.553	0.1450
**C11orf74**	C11orf74		10	chromosome 11 open reading frame 74	protein_codin	-0.0448	0.794	1	8.237	0.2019
**ARHGAP1**	ARHGAP1		10	Rho GTPase activating protein 1	protein_codin	-0.2326	0.114	1	20.57	0.4744
**PACSIN3**	PACSIN3		10	protein kinase C and casein kinase substrate in neuro	protein_codin	-0.308	0.104	1	7.872	0.2058
**ACP2**	ACP2		10	acid phosphatase 2, lysosomal	protein_codin	0.6657	0.00281	1	10.56	0.0679
**CELF1**	CELF1		10	CUGBP Elav-like family member 1	protein_codin	0.004745	0.982	1	27.71	0.5311
**TMEM109**	TMEM109		10	transmembrane protein 109	protein_codin	0.2069	0.00432	1	50.1	0.07272
**SDHAF2**	SDHAF2		10	succinate dehydrogenase complex assembly factor 2	protein_codin	0.2758	0.0546	1	6.872	0.2142
**UQCC3**	UQCC3		10	ubiquinol-cytochrome c reductase complex assembly	protein_codin	0.08341	0.542	1	6.568	0.1388
**STX5**	STX5		10	syntaxin 5	protein_codin	0.1657	0.357	1	8.092	0.1695
**RASGRP2**	RASGRP2		10	RAS guanyl releasing protein 2	protein_codin	0.01712	0.925	1	36.86	0.2082
**RNASEH2C**	RNASEH2C		10	ribonuclease H2 subunit C	protein_codin	-0.2387	0.00194	1	39.7	0.03603
**MUS81**	MUS81		10	MUS81 structure-specific endonuclease subunit	protein_codin	0.1412	0.507	1	7.08	0.2081
**CORO1B**	CORO1B		10	coronin 1B	protein_codin	0.283	0.0142	1	14.37	0.03292
**ARHGEF17**	ARHGEF17		10	Rho guanine nucleotide exchange factor 17	protein_codin	-0.5253	0.0032	1	19.2	0.2819
**THAP12**	THAP12		10	THAP domain containing 12	protein_codin	0.2036	0.191	1	19.5	0.2115
**CCDC90B**	CCDC90B		10	coiled-coil domain containing 90B	protein_codin	-0.3765	8.79E-05	1	40.75	0.1307
**FZD4**	FZD4		10	frizzled class receptor 4	protein_codin	0.9622	1.41E-05	0.3245	18.34	0.247
**CWF19L2**	CWF19L2		10	CWF19 like cell cycle control factor 2	protein_codin	0.1842	0.205	1	29.37	0.2117
**DDX10**	DDX10		10	DEAD-box helicase 10	protein_codin	0.03725	0.86	1	30.04	0.2437
**SDHD**	SDHD		10	succinate dehydrogenase complex subunit D	protein_codin	0.4054	3.21E-07	0.00749	21.61	0.035
**ZBTB16**	ZBTB16		10	zinc finger and BTB domain containing 16	protein_codin	-0.009522	0.931	1	35.47	0.09854
**ZPR1**	ZPR1		10	ZPR1 zinc finger	protein_codin	0.006379	0.974	1	9.906	0.08235
**CCDC84**	CCDC84		10	coiled-coil domain containing 84	protein_codin	0.078	0.746	1	31.91	0.2706
**DPAGT1**	DPAGT1		10	dolichyl-phosphate N-acetylglucosaminephosphotran	protein_codin	0.09079	0.659	1	3.154	0.2301
**MSANTD2**	MSANTD2		10	Myb/SANT DNA binding domain containing 2	protein_codin	0.2544	0.319	1	2.976	0.2147
**RPP38**	RPP38		10	ribonuclease P/MRP subunit p38	protein_codin	0.03067	0.879	1	7.881	0.1032
**RSU1**	RSU1		10	Ras suppressor protein 1	protein_codin	-0.3626	2E-05	0.4589	41.31	0.06649
**JCAD**	JCAD		10	junctional cadherin 5 associated	protein_codin	-0.4496	0.0345	1	7.258	0.1804
**ARHGAP12**	ARHGAP12		10	Rho GTPase activating protein 12	protein_codin	1.076	1.11E-09	2.62E-05	19.7	0.2094
**ZNF33A**	ZNF33A		10	zinc finger protein 33A	protein_codin	-0.06515	0.706	1	49.97	0.2919
**ZNF22**	ZNF22		10	zinc finger protein 22	protein_codin	-0.04462	0.685	1	11.69	0.03670
**JMJD1C**	JMJD1C		10	jumonji domain containing 1C	protein_codin	0.2152	0.0348	1	25.09	0.1344
**SLC25A16**	SLC25A16		10	solute carrier family 25 member 16	protein_codin	0.1278	0.618	1	9.123	0.1770
**DNAJB12**	DNAJB12		10	DnaJ heat shock protein family (Hsp40) member B12	protein_codin	0.4852	0.00341	1	16.21	0.1017
**DNAJC9**	DNAJC9		10	DnaJ heat shock protein family (Hsp40) member C9	protein_codin	-0.157	0.347	1	9.142	0.06795
**GLUD1**	GLUD1		10	glutamate dehydrogenase 1	protein_codin	0.3226	0.00326	1	52.4	0.03806
**ATAD1**	ATAD1		10	ATPase family AAA domain containing 1	protein_codin	-0.1406	0.277	1	6.827	0.2749
**HECTD2**	HECTD2		10	HECT domain E3 ubiquitin protein ligase 2	protein_codin	0.1569	0.471	1	27.85	0.2069
**PDLIM1**	PDLIM1		10	PDZ and LIM domain 1	protein_codin	-0.4675	2.32E-11	5.51E-07	60.54	0.03246
**MRPL43**	MRPL43		10	mitochondrial ribosomal protein L43	protein_codin	-0.2279	0.00207	1	24.79	0.1371
**BORCS7**	BORCS7		10	BLOC-1 related complex subunit 7	protein_codin	-0.1227	0.205	1	26.96	0.132
**CACUL1**	CACUL1		10	CDK2 associated cullin domain 1	protein_codin	0.4738	0.0247	1	8.757	0.144
**PLEKHA1**	PLEKHA1		10	pleckstrin homology domain containing A1	protein_codin	0.1707	0.299	1	16.16	0.2706
**DHX32**	DHX32		10	DEAH-box helicase 32 (putative)	protein_codin	0.1642	0.551	1	3.449	0.1692
**TUBGCP2**	TUBGCP2		10	tubulin gamma complex associated protein 2	protein_codin	0.4372	0.00958	1	16.08	0.2442
**ERC1**	ERC1		10	ELKS/RAB6-interacting/CAST family member 1	protein_codin	0.2119	0.205	1	17.5	0.1458
**TULP3**	TULP3		10	TUB like protein 3	protein_codin	0.5438	0.00203	1	47.22	0.1002
**KCNA5**	KCNA5		10	potassium voltage-gated channel subfamily A member 5	protein_codin	-0.2009	0.294	1	49.42	0.1640
**LPCAT3**	LPCAT3		10	lysophosphatidylcholine acyltransferase 3	protein_codin	-0.04162	0.841	1	2.55	0.1349
**LRP6**	LRP6		10	LDL receptor related protein 6	protein_codin	0.4355	0.0559	1	26.27	0.2072
**CREBL2**	CREBL2		10	cAMP responsive element binding protein like 2	protein_codin	0.3212	0.0732	1	8	0.03557
**LMO3**	LMO3		10	LIM domain only 3	protein_codin	0.4256	0.00199	1	26.01	0.1002
**AEBP2**	AEBP2		10	AE binding protein 2	protein_codin	0.2476	0.108	1	7.01	0.1732
**BCAT1**	BCAT1		10	branched chain amino acid transaminase 1	protein_codin	0.5828	0.00666	1	39.58	0.1345
**RASSF8-AS1**	RASSF8-AS1		10	RASSF8 antisense RNA 1	antisense_RN	0.4405	0.0203	1	19.39	0.03422
**CCDC91**	CCDC91		10	coiled-coil domain containing 91	protein_codin	-0.1755	0.182	1	14.79	0.128
**TMTC1**	TMTC1		10	transmembrane O-mannosyltransferase targeting cad	protein_codin	-0.2635	0.129	1	8.089	0.2380
**DNM1L**	DNM1L		10	dynamin 1 like	protein_codin	0.3399	0.0252	1	7.568	0.2074
**PFKM**	PFKM		10	phosphofructokinase, muscle	protein_codin	-0.0861	0.597	1	9.13	0.2065
**ASB8**	ASB8		10	ankyrin repeat and SOCS box containing 8	protein_codin	-0.1881	0.135	1	6.981	0.128
**LMBR1L**	LMBR1L		10	limb development membrane protein 1 like	protein_codin	0.4597	0.0852	1	2.828	0.3697
**MCRS1**	MCRS1		10	microspherule protein 1	protein_codin	0.05182	0.762	1	29.24	0.1412
**GDF11**	GDF11		10	growth differentiation factor 11	protein_codin	-0.2154	0.362	1	11.68	0.3432
**COQ10A**	COQ10A		10	coenzyme Q10A	protein_codin	0.4928	0.0842	1	2.947	0.2757
**MARS**	MARS		10	methionyl-tRNA synthetase	protein_codin	-0.07295	0.737	1	3.042	0.4045
**AGAP2-AS1**	AGAP2-AS1		10	AGAP2 antisense RNA 1	antisense_RN	-0.3909	0.114	1	33.43	0.05460
**TMEM19**	TMEM19		10	transmembrane protein 19	protein_codin	0.8647	0.0006	1	10.3	0.07661
**TMTC3**	TMTC3		10	transmembrane O-mannosyltransferase targeting cad	protein_codin	0.2763	0.131	1	25.26	0.1452
**EEA1**	EEA1		10	early endosome antigen 1	protein_codin	0.06941	0.361	1	52.35	0.06726
**TMPO**	TMPO		10	thymopoietin	protein_codin	0.2007	0.217	1	19.07	0.3349
**C12orf45**	C12orf45		10	chromosome 12 open reading frame 45	protein_codin	0.1495	0.382	1	44.45	0.1401
**ANKRD13A**	ANKRD13A		10	ankyrin repeat domain 13A	protein_codin	-0.1009	0.679	1	7.499	0.1941
**PTPN11**	PTPN11		10	protein tyrosine phosphatase non-receptor type 11	protein_codin	-0.1972	0.175	1	24.61	0.126
**SUDS3**	SUDS3		10	SDS3 homolog, SIN3A corepressor complex component	protein_codin	-0.1544	0.232	1	13.24	0.1232
**RAB35**	RAB35		10	RAB35, member RAS oncogene family	protein_codin	0.5122	0.0255	1	3.016	0.3507
**C12orf65**	C12orf65		10	chromosome 12 open reading frame 65	protein_codin	-0.1993	0.14	1	29.25	0.1725
**RILPL1**	RILPL1		10	Rab interacting lysosomal protein like 1	protein_codin	-0.3487	0.0359	1	21.53	0.1021
**DDX55**	DDX55		10	DEAD-box helicase 55	protein_codin	0.1134	0.587	1	7.343	0.1620
**CCDC92**	CCDC92		10	coiled-coil domain containing 92	protein_codin	-0.2273	0.192	1	8.326	0.2992
**IFT88**	IFT88		10	intraflagellar transport 88	protein_codin	0.7814	0.00026	1	23.67	0.3144
**SACS**	SACS		10	sacsin molecular chaperone	protein_codin	-0.1684	0.502	1	24.09	0.3162
**MTMR6**	MTMR6		10	myotubularin related protein 6	protein_codin	-0.1226	0.421	1	3.264	0.2487
**N4BP2L2**	N4BP2L2		10	NEDD4 binding protein 2 like 2	protein_codin	-0.006408	0.929	1	26.75	0.1115
**RCBTB2**	RCBTB2		10	RCC1 and BTB domain containing protein 2	protein_codin	-0.5267	0.00907	1	3.124	0.2140
**INTS6**	INTS6		10	integrator complex subunit 6	protein_codin	0.6413	0.00017	1	60.58	0.2728
**SUGT1**	SUGT1		10	SGT1 homolog, MIS12 kinetochore complex assembl	protein_codin	0.08982	0.261	1	42.18	0.03307
**KCTD12**	KCTD12		10	potassium channel tetramerization domain containing 12	protein_codin	0.09691	0.659	1	25.52	0.2101
**RAP2A**	RAP2A		10	RAP2A, member of RAS oncogene family	protein_codin	0.05985	0.762	1	60.58	0.4050
**TTC5**	TTC5		10	tetratricopeptide repeat domain 5	protein_codin	0.4972	0.0162	1	8.815	0.1376
**CHD8**	CHD8		10	chromodomain helicase DNA binding protein 8	protein_codin	0.326	0.0752	1	8.587	0.2852
**DHRS4L2**	DHRS4L2		10	dehydrogenase/reductase 4 like 2	protein_codin	-0.08297	0.491	1	13.44	0.07418
**SPTSSA**	SPTSSA		10	serine palmitoyltransferase small subunit A	protein_codin	0.3032	0.00476	1	60.59	0.06646
**MGAT2**	MGAT2		10	mannosyl (alpha-1,6-)-glycoprotein beta-1,2-N-acetylg	protein_codin	0.4492	0.0226	1	9.811	0.1642
**NEMF**	NEMF		10	nuclear export mediator factor	protein_codin	0.1547	0.17	1	42.22	0.07044
**ARF6**	ARF6		10	ADP ribosylation factor 6	protein_codin	0.3693	0.00886	1	34.52	0.108
**NIN**	NIN		10	ninein	protein_codin	-0.1174	0.547	1	27.38	0.1076
**GNPNAT1**	GNPNAT1		10	glucosamine-phosphate N-acetyltransferase 1	protein_codin	0.3571	0.169	1	3.526	0.1868
**MAPK1IP1L**	MAPK1IP1L		10	mitogen-activated protein kinase 1 interacting protein	protein_codin	0.303	0.022	1	12.87	0.2464
**ZBTB25**	ZBTB25		10	zinc finger and BTB domain containing 25	protein_codin	0.1763	0.527	1	8.67	0.2198
**ABCD4**	ABCD4		10	ATP binding cassette subfamily D member 4	protein_codin	0.249	0.294	1	6.806	0.2085
**KLC1**	KLC1		10	kinesin light chain 1	protein_codin	0.1445	0.382	1	8.089	0.2048
**DTWD1**	DTWD1		10	DTW domain containing 1	protein_codin	-0.04942	0.672	1	34.09	0.169
**GABPB1-AS1**	GABPB1-AS1		10	GABPB1 antisense RNA 1	antisense_RN	0.4116	0.0877	1	20.37	0.1916
**GTF2A2**	GTF2A2		10	general transcription factor IIA subunit 2	protein_codin	-0.04683	0.545	1	27.6	0.09942
**APH1B**	APH1B		10	aph-1 homolog B, gamma-secretase subunit	protein_codin	0.1879	0.314	1	26.14	0.1238
**DPP8**	DPP8		10	dipeptidyl peptidase 8	protein_codin	0.2012	0.274	1	29.18	0.2662
**TLE3**	TLE3		10	TLE family member 3, transcriptional corepressor	protein_codin	0.305	0.199	1	8.316	0.4231
**ADPGK**	ADPGK		10	ADP dependent glucokinase	protein_codin	0.3705	0.0995	1	9.017	0.1430
**SNUPN**	SNUPN		10	snurportin 1	protein_codin	0.2418	0.122	1	20.35	0.09968
**UBE2Q2**	UBE2Q2		10	ubiquitin conjugating enzyme E2 Q2	protein_codin	-0.2786	0.024	1	59.56	0.097
**NMB**	NMB		10	neuromedin B	protein_codin	-0.04909	0.809	1	42.58	0.2392
**PDE8A**	PDE8A		10	phosphodiesterase 8A	protein_codin	0.2138	0.432	1	8.962	0.1751
**FAM173A**	FAM173A		10	adenine nucleotide translocase lysine methyltransferase	protein_codin	-0.2315	0.0506	1	26.72	0.1105
**RPUSD1**	RPUSD1		10	RNA pseudouridine synthase domain containing 1	protein_codin	0.01596	0.944	1	3.43	0.03481
**TBL3**	TBL3		10	transducin beta like 3	protein_codin	0.07923	0.72	1	2.202	0.2752
**TSC2**	TSC2		10	TSC complex subunit 2	protein_codin	-0.06346	0.785	1	2.777	0.3175
**SRRM2**	SRRM2		10	serine/arginine repetitive matrix 2	protein_codin	0.2347	0.00014	1	57.51	0.0652
**MKL2**	MKL2		10	myocardin related transcription factor B	protein_codin	-0.2419	0.269	1	7.677	0.2726
**EEF2K**	EEF2K		10	eukaryotic elongation factor 2 kinase	protein_codin	1.137	1E-05	0.2304	7.996	0.2588
**SULT1A1**	SULT1A1		10	sulfotransferase family 1A member 1	protein_codin	1.474	5.09E-07	0.01187	52.87	0.06626
**CD2BP2**	CD2BP2		10	CD2 cytoplasmic tail binding protein 2	protein_codin	-0.3512	0.00143	1	11.38	0.1669

**SEPHS2**	SEPHS2	10	selenophosphate synthetase 2	protein_codin	0.04662	0.754	1	60.6	0.2098
**FUS**	FUS	10	FUS RNA binding protein	protein_codin	0.4534	5.97E-07	0.01392	32.19	0.1388
**DYNC1LI2**	DYNC1LI2	10	dynein cytoplasmic 1 light intermediate chain 2	protein_codin	-0.3092	0.00028	1	60.34	0.03587
**TRADD**	TRADD	10	TNFRSF1A associated via death domain	protein_codin	0.02641	0.854	1	19.96	0.103
**ACD**	ACD	10	ACD shelterin complex subunit and telomerase recrui	protein_codin	0.1764	0.376	1	8.911	0.1029
**CYB5B**	CYB5B	10	cytochrome b5 type B	protein_codin	0.1848	0.292	1	7.712	0.1227
**EXOSC6**	EXOSC6	10	exosome component 6	protein_codin	0.5168	0.00189	1	6.806	0.1988
**SF3B3**	SF3B3	10	splicing factor 3b subunit 3	protein_codin	0.1376	0.562	1	3.376	0.2188
**MAF**	MAF	10	MAF bZIP transcription factor	protein_codin	0.8138	2.04E-05	0.4687	51.88	0.06832
**ATMIN**	ATMIN	10	ATM interactor	protein_codin	-0.1189	0.525	1	8.476	0.1815
**COTL1**	COTL1	10	coactosin like F-actin binding protein 1	protein_codin	-0.01357	0.952	1	59.8	0.2928
**EMC8**	EMC8	10	ER membrane protein complex subunit 8	protein_codin	-0.2105	0.245	1	25.72	0.2019
**CTDNEP1**	CTDNEP1	10	CTD nuclear envelope phosphatase 1	protein_codin	0.09129	0.372	1	53.88	0.1028
**CHRNB1**	CHRNB1	10	cholinergic receptor nicotinic beta 1 subunit	protein_codin	-0.5859	0.00487	1	7.962	0.1325
**ELAC2**	ELAC2	10	elaC ribonuclease Z 2	protein_codin	0.3538	0.105	1	3.562	0.3015
**FLCN**	FLCN	10	folliculin	protein_codin	0.6663	0.0157	1	8.837	0.4248
**FLII**	FLII	10	FLII actin remodeling protein	protein_codin	0.171	0.266	1	20.92	0.1239
**TMEM11**	TMEM11	10	transmembrane protein 11	protein_codin	-0.07507	0.632	1	22.97	0.1434
**KIAA0100**	KIAA0100	10	KIAA0100	protein_codin	0.8599	4.25E-05	0.9723	7.907	0.1969
**CCL4**	CCL4	10	C-C motif chemokine ligand 4	protein_codin	-1.709	3.39E-07	0.00791	60.58	2.549
**AATF**	AATF	10	apoptosis antagonizing transcription factor	protein_codin	-0.1723	0.338	1	25.36	0.1731
**MLLT6**	MLLT6	10	MLLT6, PHD finger containing	protein_codin	0.3813	0.0657	1	7.807	0.2379
**TMEM99**	TMEM99	10	transmembrane protein 99 (putative)	protein_codin	0.7925	5.28E-05	1	7.122	0.1515
**CNP**	CNP	10	2',3'-cyclic nucleotide 3' phosphodiesterase	protein_codin	0.2006	0.185	1	8.105	0.09899
**RAB5C**	RAB5C	10	RAB5C, member RAS oncogene family	protein_codin	-0.1677	0.0345	1	30.83	0.0324
**ATP6V0A1**	ATP6V0A1	10	ATPase H+ transporting V0 subunit a1	protein_codin	0.00352	0.988	1	6.762	0.5389
**TUBG1**	TUBG1	10	tubulin gamma 1	protein_codin	-0.2515	0.257	1	33.14	0.2474
**AC100793.2**	AC100793.2	10	novel transcript, antisense to CCR	antisense_RN	0.1262	0.679	1	28.96	0.5884
**TMUB2**	TMUB2	10	transmembrane and ubiquitin like domain containing 2	protein_codin	0.06427	0.698	1	7.473	0.2348
**KANSL1**	KANSL1	10	KAT8 regulatory NSL complex subunit 1	protein_codin	0.1947	0.162	1	6.227	0.1050
**KAT7**	KAT7	10	lysine acetyltransferase 7	protein_codin	-0.2418	0.115	1	22.12	0.1350
**RAD51C**	RAD51C	10	RAD51 paralog C	protein_codin	-0.2818	0.0656	1	8.48	0.1315
**YPEL2**	YPEL2	10	yippee like 2	protein_codin	0.01066	0.941	1	55.03	0.127
**FTSJ3**	FTSJ3	10	FtsJ RNA 2'-O-methyltransferase 3	protein_codin	0.2341	0.235	1	3.132	0.1106
**CEP112**	CEP112	10	centrosomal protein 112	protein_codin	0.07332	0.779	1	8.776	0.311
**ARSG**	ARSG	10	arylsulfatase G	protein_codin	0.5082	0.0196	1	11.44	0.1761
**WIPI1**	WIPI1	10	WD repeat domain, phosphoinositide interacting 1	protein_codin	0.8367	3.02E-06	0.06996	21.14	0.1392
**MIF4GD**	MIF4GD	10	MIF4G domain containing	protein_codin	-0.4969	0.00025	1	20.35	0.1737
**SRP68**	SRP68	10	signal recognition particle 68	protein_codin	-0.4702	0.00432	1	7.486	0.2398
**MRPL12**	MRPL12	10	mitochondrial ribosomal protein L12	protein_codin	0.1765	0.22	1	58.62	0.0658
**RAB40B**	RAB40B	10	RAB40B, member RAS oncogene family	protein_codin	0.008547	0.962	1	7.633	0.2392
**METTL4**	METTL4	10	methyltransferase like 4	protein_codin	0.08329	0.788	1	3.182	0.4112
**PIK3C3**	PIK3C3	10	phosphatidylinositol 3-kinase catalytic subunit type 3	protein_codin	-0.05178	0.796	1	3.472	0.3268
**HDHD2**	HDHD2	10	haloacid dehalogenase like hydrolase domain contair	protein_codin	0.2107	0.322	1	3.233	0.17
**DYM**	DYM	10	dymeclin	protein_codin	0.05897	0.761	1	28.54	0.1461
**MBD1**	MBD1	10	methyl-CpG binding domain protein 1	protein_codin	0.3185	0.142	1	7.772	0.1692
**NRSN2**	NRSN2	10	neurensin 2	protein_codin	-0.1433	0.522	1	3.333	0.1451
**CSNK2A1**	CSNK2A1	10	casein kinase 2 alpha 1	protein_codin	0.2098	0.124	1	35.24	0.1426
**IDH3B**	IDH3B	10	isocitrate dehydrogenase (NAD(+)) 3 beta	protein_codin	0.03009	0.842	1	7.257	0.1769
**TMX4**	TMX4	10	thioredoxin related transmembrane protein 4	protein_codin	0.06461	0.496	1	58.74	0.2392
**KIF16B**	KIF16B	10	kinesin family member 16B	protein_codin	0.08169	0.727	1	8.456	0.203
**RIN2**	RIN2	10	Ras and Rab interactor 2	protein_codin	-0.1936	0.203	1	40.72	0.06518
**CHMP4B**	CHMP4B	10	charged multivesicular body protein 4B	protein_codin	-0.2958	0.00502	1	25.03	0.06905
**RBM39**	RBM39	10	RNA binding motif protein 39	protein_codin	0.4071	2.21E-07	0.00517	39.75	0.1401
**AAR2**	AAR2	10	AAR2 splicing factor	protein_codin	0.1011	0.65	1	8.888	0.2471
**ZHX3**	ZHX3	10	zinc fingers and homeoboxes 3	protein_codin	0.6896	0.00115	1	8.602	0.3407
**CHD6**	CHD6	10	chromodomain helicase DNA binding protein 6	protein_codin	0.0917	0.576	1	7.119	0.243
**PIGT**	PIGT	10	phosphatidylinositol glycan anchor biosynthesis class	protein_codin	0.4529	7.63E-07	0.01777	41.68	0.03219
**CTSA**	CTSA	10	cathepsin A	protein_codin	0.6909	7.02E-09	0.00017	35.1	0.06454
**PCIF1**	PCIF1	10	PDX1 C-terminal inhibiting factor 1	protein_codin	-0.1465	0.432	1	6.882	0.1729
**DDX27**	DDX27	10	DEAD-box helicase 27	protein_codin	0.2054	0.195	1	17.69	0.09952
**ZNFX1**	ZNFX1	10	zinc finger NFX1-type containing 1	protein_codin	0.612	0.00467	1	53.48	0.1961
**PTPN1**	PTPN1	10	protein tyrosine phosphatase non-receptor type 1	protein_codin	-0.4564	0.00074	1	45.98	0.06403
**FAM210B**	FAM210B	10	family with sequence similarity 210 member B	protein_codin	0.09591	0.472	1	33.75	0.0686
**RAB22A**	RAB22A	10	RAB22A, member RAS oncogene family	protein_codin	0.05248	0.69	1	16.82	0.07261
**VAPB**	VAPB	10	VAMP associated protein B and C	protein_codin	0.3199	0.0568	1	7.607	0.1736
**MRGBP**	MRGBP	10	MRG domain binding protein	protein_codin	-0.04071	0.846	1	9.658	0.3235
**PRPF6**	PRPF6	10	pre-mRNA processing factor 6	protein_codin	-0.07607	0.502	1	23.88	0.06952
**TCEA2**	TCEA2	10	transcription elongation factor A2	protein_codin	-0.3592	0.00129	1	28.82	0.09872
**DOHH**	DOHH	10	deoxyhypusine hydroxylase	protein_codin	-0.08854	0.648	1	7.742	0.3772
**HMG20B**	HMG20B	10	high mobility group 20B	protein_codin	-0.5193	1.78E-06	0.04131	51.38	0.1062
**TBXA2R**	TBXA2R	10	thromboxane A2 receptor	protein_codin	-1.464	7.68E-14	1.83E-09	23.45	0.2876
**KDM4B**	KDM4B	10	lysine demethylase 4B	protein_codin	-0.1179	0.677	1	3.203	0.4404
**HSD11B1L**	HSD11B1L	10	hydroxysteroid 11-beta dehydrogenase 1 like	protein_codin	-0.2895	0.199	1	8.84	0.1664
**CLPP**	CLPP	10	caseinolytic mitochondrial matrix peptidase proteolytic	protein_codin	-0.1618	0.152	1	14.37	0.09907
**GPR108**	GPR108	10	G protein-coupled receptor 108	protein_codin	0.07495	0.598	1	15.62	0.1377
**DNMT1**	DNMT1	10	DNA methyltransferase 1	protein_codin	0.3818	0.0236	1	47.8	0.02597
**CDKN2D**	CDKN2D	10	cyclin dependent kinase inhibitor 2D	protein_codin	-0.6663	1.68E-05	0.3863	60.14	0.0987
**DNM2**	DNM2	10	dynamin 2	protein_codin	-0.05912	0.786	1	8.175	0.2831
**ZNF136**	ZNF136	10	zinc finger protein 136	protein_codin	0.01318	0.95	1	3.26	0.2445
**MRI1**	MRI1	10	methylthioribose-1-phosphate isomerase 1	protein_codin	0.2519	0.157	1	3.412	0.2169
**ADGRE5**	ADGRE5	10	adhesion G protein-coupled receptor E5	protein_codin	-0.423	0.00448	1	16.32	0.2379
**BABAM1**	BABAM1	10	BRISC and BRCA1 A complex member 1	protein_codin	-0.214	0.0476	1	13.14	0.1731
**MAU2**	MAU2	10	MAU2 sister chromatid cohesion factor	protein_codin	0.7636	0.00700	1	3.23	0.3622
**ZNF254**	ZNF254	10	zinc finger protein 254	protein_codin	0.05329	0.811	1	3.063	0.2867
**PLEKHF1**	PLEKHF1	10	pleckstrin homology and FYVE domain containing 1	protein_codin	0.8223	3.53E-05	0.8085	8.902	0.07450
**ZNF507**	ZNF507	10	zinc finger protein 507	protein_codin	0.04028	0.849	1	7.98	0.209
**CEBPG**	CEBPG	10	CCAAT enhancer binding protein gamma	protein_codin	0.2743	0.152	1	23.94	0.1812
**TMEM147**	TMEM147	10	transmembrane protein 147	protein_codin	0.0319	0.734	1	31.85	0.144
**YIF1B**	YIF1B	10	Yip1 interacting factor homolog B, membrane trafficki	protein_codin	-0.05926	0.805	1	7.302	0.06411
**MED29**	MED29	10	mediator complex subunit 29	protein_codin	0.05766	0.736	1	43.75	0.2371
**NECTIN2**	NECTIN2	10	nectin cell adhesion molecule 2	protein_codin	0.259	0.12	1	21.9	0.1008
**ERCC1**	ERCC1	10	ERCC excision repair 1, endonuclease non-catalytic s	protein_codin	-0.05219	0.657	1	27.96	0.1408
**EML2**	EML2	10	EMAP like 2	protein_codin	-0.4409	0.0447	1	3.356	0.379
**DMPK**	DMPK	10	DM1 protein kinase	protein_codin	-0.1418	0.595	1	7.853	0.313
**PTGIR**	PTGIR	10	prostaglandin I2 receptor	protein_codin	-0.02555	0.862	1	25	0.1002
**NAPA**	NAPA	10	NSF attachment protein alpha	protein_codin	0.05856	0.633	1	34.78	0.09755
**PLA2G4C**	PLA2G4C	10	phospholipase A2 group IVC	protein_codin	0.2277	0.284	1	32	0.3441
**PTOV1**	PTOV1	10	PTOV1 extended AT-hook containing adaptor protein	protein_codin	0.1388	0.249	1	29.32	0.09827
**NR1H2**	NR1H2	10	nuclear receptor subfamily 1 group H member 2	protein_codin	-0.3637	0.00113	1	12.28	0.1396
**MBOAT7**	MBOAT7	10	membrane bound O-acyltransferase domain containing 7	protein_codin	-0.02148	0.936	1	2.892	0.2382
**LENG8**	LENG8	10	leukocyte receptor cluster member 8	protein_codin	0.6137	0.0106	1	7.636	0.2876
**ISOC2**	ISOC2	10	isochorismatase domain containing 2	protein_codin	0.01961	0.881	1	27.94	0.06881
**CCDC106**	CCDC106	10	coiled-coil domain containing 106	protein_codin	0.04171	0.841	1	8.009	0.1386
**ZNF787**	ZNF787	10	zinc finger protein 787	protein_codin	-0.4404	0.017	1	30.8	0.06786
**UTY**	UTY	10	ubiquitously transcribed tetratricopeptide repeat conta	protein_codin	0.3225	0.227	1	10.34	0.3097
**KDM5D**	KDM5D	10	lysine demethylase 5D	protein_codin	0.1817	0.569	1	3.612	0.4228
**HPS4**	HPS4	10	HPS4 biogenesis of lysosomal organelles complex 3 s	protein_codin	0.4748	0.0611	1	8.136	0.2858
**TXN2**	TXN2	10	thioredoxin 2	protein_codin	0.1018	0.252	1	20.09	0.2368
**JOSD1**	JOSD1	10	Josephin domain containing 1	protein_codin	0.5702	0.00349	1	24.83	0.1382
**CBX7**	CBX7	10	chromobox 7	protein_codin	-0.09364	0.595	1	6.666	0.2880
**SLC25A17**	SLC25A17	10	solute carrier family 25 member 17	protein_codin	0.3668	0.16	1	2.793	0.2035
**TEF**	TEF	10	TEF transcription factor, PAR bZIP family member	protein_codin	0.4959	0.0505	1	7.221	0.3418
**PHF5A**	PHF5A	10	PHD finger protein 5A	protein_codin	0.02601	0.85	1	17.83	0.07486
**SELENOO**	SELENOO	10	selenoprotein O	protein_codin	0.522	0.0327	1	3.336	0.1345
**PLXNB2**	PLXNB2	10	plexin B2	protein_codin	-0.3885	0.0412	1	8.948	0.3707
**USP25**	USP25	10	ubiquitin specific peptidase 25	protein_codin	0.3158	0.179	1	10.86	0.4202
**IFNAR1**	IFNAR1	10	interferon alpha and beta receptor subunit 1	protein_codin	0.3873	0.00076	1	58.25	0.07115
**ITSN1**	ITSN1	10	intersectin 1	protein_codin	0.07066	0.665	1	58.53	0.1729
**DSCR3**	DSCR3	10	VPS26 endosomal protein sorting factor C	protein_codin	NA	NA	NA	3.173	0.1959
**ERG**	ERG	10	ETS transcription factor ERG	protein_codin	-1.374	5.15E-14	1.23E-09	26.97	0.1379
**RRP1B**	RRP1B	10	ribosomal RNA processing 1B	protein_codin	0.4618	0.0293	1	7.558	0.2164
**KLHL21**	KLHL21	11	kelch like family member 21	protein_codin	-0.5387	0.0222	1	3.377	0.5097
**MAD2L2**	MAD2L2	11	mitotic arrest deficient 2 like 2	protein_codin	0.07741	0.681	1	54.78	0.06502
**C1orf167**	C1orf167	11	chromosome 1 open reading frame 167	protein_codin	1.149	8.08E-06	0.1863	24.62	0.1741
**NBL1**	NBL1	11	NBL1, DAN family BMP antagonist	protein_codin	0.3274	6.69E-06	0.1544	60.6	0.03725
**SH3BGRL3**	SH3BGRL3	11	SH3 domain binding glutamate rich protein like 3	protein_codin	-0.3614	7.27E-10	1.72E-05	60.6	0.07200
**ZMPSTE24**	ZMPSTE24	11	zinc metallopeptidase STE24	protein_codin	0.1107	0.441	1	49.43	0.2458
**NASP**	NASP	11	nuclear autoantigenic sperm protein	protein_codin	-0.264	0.04	1	59.76	0.07011
**LRRC41**	LRRC41	11	leucine rich repeat containing 41	protein_codin	-0.04177	0.812	1	7.303	0.2386
**CYP4X1**	CYP4X1	11	cytochrome P450 family 4 subfamily X member 1	protein_codin	0.8125	5.04E-05	1	50.33	0.1169
**CDKN2C**	CDKN2C	11	cyclin dependent kinase inhibitor 2C	protein_codin	-0.6824	3.09E-08	0.00072	22.54	0.236
**YIPF1**	YIPF1	11	Yip1 domain family member 1	protein_codin	0.6712	0.0185	1	2.934	0.2441
**MYSM1**	MYSM1	11	Myb like, SWIRM and MPN domains 1	protein_codin	-0.0462	0.827	1	6.792	0.1462
**DIRAS3**	DIRAS3	11	DIRAS family GTPase 3	protein_codin	-0.2029	0.374	1	9.866	0.3611
**PRPF38B**	PRPF38B	11	pre-mRNA processing factor 38B	protein_codin	0.1757	0.222	1	11.96	0.1350
**LRIG2**	LRIG2	11	leucine rich repeats and immunoglobulin like domains 2	protein_codin	0.429	0.0946	1	3.155	0.4771
**HIPK1**	HIPK1	11	homeodomain interacting protein kinase 1	protein_codin	0.2638	0.15	1	7.63	0.2721
**SIKE1**	SIKE1	11	suppressor of IKBKE 1	protein_codin	0.1094	0.501	1	8.59	0.277
**SRGAP2C**	SRGAP2C	11	SLIT-ROBO Rho GTPase activating protein 2C	protein_codin	-0.4101	0.0552	1	35.96	0.1752
**ECM1**	ECM1	11	extracellular matrix protein 1	protein_codin	1.584	5.68E-20	1.36E-15	59.2	0.03972
**GABPB2**	GABPB2	11	GA binding protein transcription factor subunit beta 2	protein_codin	0.5806	0.0227	1	8.912	0.2032
**S100A10**	S100A10	11	S100 calcium binding protein A10	protein_codin	0.5527	7.59E-15	1.81E-10	Inf	0.18
**PBXIP1**	PBXIP1	11	PBX homeobox interacting protein 1	protein_codin	0.04572	0.671	1	20.72	0.137
**CRABP2**	CRABP2	11	cellular retinoic acid binding protein 2	protein_codin	0.9006	4.76E-05	1	60.6	0.2420
**ATP1B1**	ATP1B1	11	ATPase Na+/K+ transporting subunit beta 1	protein_codin	0.1596	0.272	1	42.84	0.1896
**HMCN1**	HMCN1	11	hemicentin 1	protein_codin	-0.2604	0.132	1	59.96	0.3435
**TIMM17A**	TIMM17A	11	translocase of inner mitochondrial membrane 17A	protein_codin	-0.0237	0.868	1	7.724	0.1008
**NUCKS1**	NUCKS1	11	nuclear casein kinase and cyclin dependent kinase su	protein_codin	-0.2158	4.02E-07	0.00938	60.6	0.06745
**G0S2**	G0S2	11	G0/G1 switch 2	protein_codin	0.6581	0.00022	1	60.6	0.1096
**GREM2**	GREM2	11	gremlin 2, DAN family BMP antagonist	protein_codin	0.8286	0.0189	1	40.88	0.5919
**PPM1G**	PPM1G	11	protein phosphatase, Mg2+/Mn2+ dependent 1G	protein_codin	-0.144	0.15	1	52.84	0.06562
**SRBD1**	SRBD1	11	S1 RNA binding domain 1	protein_codin	0.2992	0.191	1	29.57	0.1917
**FAM161A**	FAM161A	11	FAM161 centrosomal protein A	protein_codin	-0.0955	0.659	1	9.624	0.298
**COMMD1**	COMMD1	11	copper metabolism domain containing 1	protein_codin	-0.1658	0.107	1	25.7	0.07612
**SNRPG**	SNRPG	11	small nuclear ribonucleoprotein polypeptide G	protein_codin	-0.2482	0.0019	1	24.9	0.06511
**ZNF638**	ZNF638	11	zinc finger protein 638	protein_codin	0.4085	0.00417	1	23.62	0.2511
**EXOC6B**	EXOC6B	11	exocyst complex component 6B	protein_codin	-0.1263	0.592	1	3.124	0.3602
**KRCC1**	KRCC1	11	lysine rich coiled-coil 1	protein_codin	0.2444	0.0645	1	13.67	0.2822
**ANKRD36B**	ANKRD36B	11	ankyrin repeat domain 36B	protein_codin	0.6082	0.0136	1	35.01	0.7106
**C2orf76**	C2orf76	11	chromosome 2 open reading frame 76	protein_codin	0.2468	0.251	1	8.642	0.2064
**MAP3K2**	MAP3K2	11	mitogen-activated protein kinase kinase kinase 2	protein_codin	0.2085	0.158	1	44.05	0.2482
**ATF2**	ATF2	11	activating transcription factor 2	protein_codin	-0.121	0.461	1	7.857	0.2612
**ZNF385B**	ZNF385B	11	zinc finger protein 385B	protein_codin	2.491	4.79E-11	1.14E-06	45.22	0.1045
**FRZB**	FRZB	11	frizzled related protein	protein_codin	-0.5386	8.14E-12	1.93E-07	60.6	0.1919
**NCKAP1**	NCKAP1	11	NCK associated protein 1	protein_codin	-0.01212	0.924	1	50.94	0.2425
**FAM171B**	FAM171B	11	family with sequence similarity 171 member B	protein_codin	0.7677	0.00045	1	7.73	0.2735
**INPP1**	INPP1	11	inositol polyphosphate-1-phosphatase	protein_codin	0.2465	0.157	1	26.49	0.06746
**CAVIN2**	CAVIN2	11	caveolae associated protein 2	protein_codin	1.353	9.15E-06	0.2109	60.58	0.3617
**HTR2B**	HTR2B	11	5-hydroxytryptamine receptor 2B	protein_codin	-1.514	9.15E-08	0.00214	21.98	0.1720
**RAMP1**	RAMP1	11	receptor activity modifying protein 1	protein_codin	-0.8107	1.46E-25	3.52E-21	42.84	0.03820
**ILKAP**	ILKAP	11	ILK associated serine/threonine phosphatase	protein_codin	0.2579	0.126	1	6.781	0.03236
**HACL1**	HACL1	11	2-hydroxyacyl-CoA lyase 1	protein_codin	-0.1462	0.481	1	9.818	0.1426
**ZNF385D**	ZNF385D	11	zinc finger protein 385D	protein_codin	-1.319	6.66E-14	1.59E-09	28.41	0.2558
**IMPDH2**	IMPDH2	11	inosine monophosphate dehydrogenase 2	protein_codin	-0.2111	0.0357	1	40.69	0.1015
**GMPPB**	GMPPB	11	GDP-mannose pyrophosphorylase B	protein_codin	0.4214	0.02	1	9.001	0.06739
**ABHD14A**	ABHD14A	11	abhydrolase domain containing 14A	protein_codin	1.245	2.13E-19	5.11E-15	59.67	0.07502
**FRMD4B**	FRMD4B	11	FERM domain containing 4B	protein_codin	-0.1549	0.565	1	32.96	0.1485
**GAP43**	GAP43	11	growth associated protein 43	protein_codin	-1.273	6.34E-11	1.5E-06	60.53	0.1087
**LSAMP**	LSAMP	11	limbic system associated membrane protein	protein_codin	1.422	0.00269	1	24.86	0.3902
**HEG1**	HEG1	11	heart development protein with EGF like domains 1	protein_codin	0.9454	1.64E-05	0.3772	59.96	0.07342
**XRN1**	XRN1	11	5'-3' exoribonuclease 1	protein_codin	0.4474	0.00168	1	15.16	0.134
**SIAH2**	SIAH2	11	siah E3 ubiquitin protein ligase 2	protein_codin	0.2195	0.286	1	8.728	0.1652
**IGSF10**	IGSF10	11	immunoglobulin superfamily member 10	protein_codin	2.509	3.96E-07	0.00924	59.58	0.5529
**SUCNR1**	SUCNR1	11	succinate receptor 1	protein_codin	0.04067	0.836	1	60.6	0.3479
**MBNL1-AS1**	MBNL1-AS1	11	MBNL1 antisense RNA 1	antisense_RN	-0.6426	1.19E-07	0.00279	40.66	0.03326
**NMD3**	NMD3	11	NMD3 ribosome export adaptor	protein_codin	-0.1991	0.152	1	40.9	0.07236
**MECOM**	MECOM	11	MDS1 and EVI1 complex locus	protein_codin	-0.7352	0.004	1	60.6	0.3477
**PIK3CA**	PIK3CA	11	phosphatidylinositol-4,5-bisphosphate 3-kinase cataly	protein_codin	-0.07826	0.637	1	45.14	0.3159
**RELL1**	RELL1	11	RELT like 1	protein_codin	0.9495	0.00018	1	3.693	0.2234
**RASGEF1B**	RASGEF1B	11	RasGEF domain family member 1B	protein_codin	0.006702	0.96	1	27.56	0.2205
**ARHGAP24**	ARHGAP24	11	Rho GTPase activating protein 24	protein_codin	0.08665	0.666	1	23.76	0.1074
**MCUB**	MCUB	11	mitochondrial calcium uniporter dominant negative be	protein_codin	1.092	6.8E-08	0.00159	32.01	0.1043
**PDE5A**	PDE5A	11	phosphodiesterase 5A	protein_codin	-0.5996	6.73E-11	1.6E-06	60.6	0.3256
**NDNF**	NDNF	11	neuron derived neurotrophic factor	protein_codin	2.012	5.78E-08	0.00138	42.48	0.2798
**INTU**	INTU	11	inturned planar cell polarity protein	protein_codin	0.4354	0.0386	1	8.372	0.1729
**RAB33B**	RAB33B	11	RAB33B, member RAS oncogene family	protein_codin	0.5353	0.00542	1	9.526	0.2432
**DCLK2**	DCLK2	11	doublecortin like kinase 2	protein_codin	0.1836	0.571	1	8.272	0.3316
**MAB21L2**	MAB21L2	11	mab-21 like 2	protein_codin	0.03587	0.891	1	26.07	0.1036
**PLK2**	PLK2	11	polo like kinase 2	protein_codin	-0.02984	0.822	1	37.42	0.1947
**RGS7BP**	RGS7BP	11	regulator of G protein signaling 7 binding protein	protein_codin	-0.6218	6.07E-05	1	53.77	0.1822
**MAP1B**	MAP1B	11	microtubule associated protein 1B	protein_codin	-0.4254	8.34E-12	1.98E-07	60.58	0.03273
**POLK**	POLK	11	DNA polymerase kappa	protein_codin	0.2052	0.175	1	15.19	0.07341
**F2R**	F2R	11	coagulation factor II thrombin receptor	protein_codin	-1.349	2.13E-21	5.12E-17	59.27	0.1763
**WDR41**	WDR41	11	WD repeat domain 41	protein_codin	0.1736	0.352	1	52.94	0.2405
**NUDT12**	NUDT12	11	nudix hydrolase 12	protein_codin	0.4476	0.0429	1	3.106	0.3156
**MAN2A1**	MAN2A1	11	mannosidase alpha class 2A member 1	protein_codin	0.1902	0.49	1	10.25	0.3648
**CDO1**	CDO1	11	cysteine dioxygenase type 1	protein_codin	0.4504	0.00017	1	60.6	0.2472
**AP3S1**	AP3S1	11	adaptor related protein complex 3 subunit sigma 1	protein_codin	0.2249	0.00494	1	54.53	0.1109
**CSNK1G3**	CSNK1G3	11	casein kinase 1 gamma 3	protein_codin	0.17	0.291	1	14.72	0.1096
**HSPA4**	HSPA4	11	heat shock protein family A (Hsp70) member 4	protein_codin	0.149	0.246	1	16.77	0.03246
**CXXC5**	CXXC5	11	CXXC finger protein 5	protein_codin	0.08354	0.514	1	58.52	0.1474
**G3BP1**	G3BP1	11	G3BP stress granule assembly factor 1	protein_codin	0.7121	2.8E-05	0.6419	60.58	0.2369
**NUDCD2**	NUDCD2	11	NudC domain containing 2	protein_codin	0.2988	0.0141	1	14.73	0.1347
**TMEM14C**	TMEM14C	11	transmembrane protein 14C	protein_codin	-0.01587	0.759	1	55.35	0.03289
**ID4**	ID4	11	inhibitor of DNA binding 4, HLH protein	protein_codin	-0.05694	0.461	1	60.6	0.06703
**SLC39A7**	SLC39A7	11	solute carrier family 39 member 7	protein_codin	0.4463	0.00013	1	44.67	0.1664
**TFEB**	TFEB	11	transcription factor EB	protein_codin	0.3385	0.145	1	2.61	0.2837
**CD2AP**	CD2AP	11	CD2 associated protein	protein_codin	-0.3948	0.0352	1	9.143	0.246
**PRSS35**	PRSS35	11	serine protease 35	protein_codin	-1.203	2.13E-10	5.04E-06	32.18	0.1893
**PDSS2**	PDSS2	11	decaprenyl diphosphate synthase subunit 2	protein_codin	0.09941	0.682	1	3.214	0.1320
**SMPDL3A**	SMPDL3A	11	sphingomyelin phosphodiesterase acid like 3A	protein_codin	1.732	1.09E-05	0.251	11.5	0.1452
**TPD52L1**	TPD52L1	11	TPD52 like 1	protein_codin	-0.5814	1.75E-05	0.4023	54.64	0.09782
**IFNGR1**	IFNGR1	11	interferon gamma receptor 1	protein_codin	0.4079	3.03E-05	0.6944	36.41	0.06750
**SFT2D1**	SFT2D1	11	SFT2 domain containing 1	protein_codin	0.6311	3.45E-07	0.00805	13.68	0.1306
**PHF14**	PHF14	11	PHD finger protein 14	protein_codin	0.01029	0.932	1	10.87	0.2743
**TMEM106B**	TMEM106B	11	transmembrane protein 106B	protein_codin	0.354	0.00068	1	26.6	0.1030
**BZW2**	BZW2	11	basic leucine zipper and W2 domains 2	protein_codin	0.362	0.0704	1	43.58	0.2132
**TSPAN13**	TSPAN13	11	tetraspanin 13	protein_codin	0.5549	0.0909	1	60.52	0.3795
**SFRP4**	SFRP4	11	secreted frizzled related protein 4	protein_codin	3.276	3.37E-20	8.09E-16	NA	0.2052
**RALA**	RALA	11	RAS like proto-oncogene A	protein_codin	0.007275	0.954	1	40.55	0.1392
**NSUN5**	NSUN5	11	NOP2/Sun RNA methyltransferase 5	protein_codin	0.2204	0.29	1	7.345	0.2872
**FZD1**	FZD1	11	frizzled class receptor 1	protein_codin	0.5049	0.0078	1	32.71	0.2028
**DLX6-AS1**	DLX6-AS1	11	DLX6 antisense RNA 1	antisense_RN	0.5242	0.015	1	59.17	0.4262
**DLX6**	DLX6	11	distal-less homeobox 6	protein_codin	0.7798	0.0006	1	36.41	0.3172
**DLX5**	DLX5	11	distal-less homeobox 5	protein_codin	0.7649	1.08E-05	0.2488	60.58	0.1294
**SRPK2**	SRPK2	11	SRSF protein kinase 2	protein_codin	0.325	0.00495	1	45.44	0.1042
**C7orf49**	C7orf49	11	cell cycle regulator of NHEJ	protein_codin	NA	NA	NA	2.552	0.3132
**FAM180A**	FAM180A	11	family with sequence similarity 180 member A	protein_codin	0.7288	0.00158	1	49.22	0.3559
**PTN**	PTN	11	pleiotrophin	protein_codin	-0.8052	1.86E-07	0.00435	60.6	0.6564
**TRIM24**	TRIM24	11	tripartite motif containing 24	protein_codin	-0.2969	0.202	1	9.036	0.2751
**ESYT2**	ESYT2	11	extended synaptotagmin 2	protein_codin	0.2162	0.129	1	31.6	0.134
**TMSB4X**	TMSB4X	11	thymosin beta 4 X-linked	protein_codin	-0.7213	3.3E-94	8.02E-90	60.6	0.035
**RPS6KA3**	RPS6KA3	11	ribosomal protein S6 kinase A3	protein_codin	0.1877	0.446	1	31.16	0.3570
**UXT**	UXT	11	ubiquitously expressed prefoldin like chaperone	protein_codin	-0.4268	4.55E-09	0.00011	47.45	0.1040
**HNRNPH2**	HNRNPH2	11	heterogeneous nuclear ribonucleoprotein H2	protein_codin	0.1796	0.199	1	16.02	0.1382
**TCEAL2**	TCEAL2	11	transcription elongation factor A like 2	protein_codin	-0.4033	0.00722	1	40.19	0.1541
**BEX2**	BEX2	11	brain expressed X-linked 2	protein_codin	-0.6545	0.0395	1	27.79	0.5532
**TSC22D3**	TSC22D3	11	TSC22 domain family member 3	protein_codin	0.6964	1.52E-22	3.66E-18	60.54	0.0425
**CLIC2**	CLIC2	11	chloride intracellular channel 2	protein_codin	1.203	1.46E-06	0.03392	59.06	0.2047
**R3HCC1**	R3HCC1	11	R3H domain and coiled-coil containing 1	protein_codin	-0.1126	0.438	1	18.48	0.2814
**DDHD2**	DDHD2	11	DDHD domain containing 2	protein_codin	0.186	0.345	1	3.556	0.3432
**MSC-AS1**	MSC-AS1	11	MSC antisense RNA 1	antisense_RN	-0.7452	1.57E-06	0.03646	20.79	0.1329
**ZNF704**	ZNF704	11	zinc finger protein 704	protein_codin	-0.1125	0.368	1	26.75	0.1031
**NCALD**	NCALD	11	neurocalcin delta	protein_codin	-0.6842	5.81E-05	1	59.92	0.1308
**ENPP2**	ENPP2	11	ectonucleotide pyrophosphatase/phosphodiesterase 2	protein_codin	-0.8145	3.79E-08	0.00089	54.82	0.2865
**PLEC**	PLEC	11	plectin	protein_codin	-0.21	0.157	1	13.63	0.1395
**GRINA**	GRINA	11	glutamate ionotropic receptor NMDA type subunit ass	protein_codin	0.2503	0.0926	1	45.65	0.03547
**CNTFR**	CNTFR	11	ciliary neurotrophic factor receptor	protein_codin	2.67	0.962	1	24.46	0.2086
**TMEM8B**	TMEM8B	11	transmembrane protein 8B	protein_codin	-0.069	0.748	1	8.789	0.1797
**UBQLN1**	UBQLN1	11	ubiquilin 1	protein_codin	-0.2387	0.146	1	7.835	0.3322
**AUH**	AUH	11	AU RNA binding methylglutaconyl-CoA hydratase	protein_codin	0.7068	0.00177	1	3.368	0.1716
**NANS**	NANS	11	N-acetylneuraminate synthase	protein_codin	0.1249	0.311	1	29.76	0.1670
**CTNNAL1**	CTNNAL1	11	catenin alpha like 1	protein_codin	-0.04374	0.754	1	42.07	0.06537
**ALAD**	ALAD	11	aminolevulinate dehydratase	protein_codin	-0.2517	0.248	1	7.388	0.4217
**MAN1B1**	MAN1B1	11	mannosidase alpha class 1B member 1	protein_codin	-0.1675	0.41	1	27.86	0.1397
**PGAP2**	PGAP2	11	post-GPI attachment to proteins 2	protein_codin	0.1854	0.504	1	2.783	0.2270
**GALNT18**	GALNT18	11	polypeptide N-acetylgalactosaminyltransferase 18	protein_codin	-0.4183	0.0646	1	8.426	0.1371
**SPON1**	SPON1	11	spondin 1	protein_codin	0.964	0.00290	1	42.13	0.1757
**TPH1**	TPH1	11	tryptophan hydroxylase 1	protein_codin	-0.7448	0.0013	1	47.89	0.3571
**MPPED2**	MPPED2	11	metallophosphoesterase domain containing 2	protein_codin	-0.7857	0.00127	1	11.94	0.3179
**LMO2**	LMO2	11	LIM domain only 2	protein_codin	-0.3123	0.0295	1	60.52	0.2184
**CD44**	CD44	11	CD44 molecule (Indian blood group)	protein_codin	-0.1797	0.0964	1	60.58	0.03242
**C11orf24**	C11orf24	11	chromosome 11 open reading frame 24	protein_codin	0.2095	0.304	1	9.782	0.1402
**CCND1**	CCND1	11	cyclin D1	protein_codin	0.1586	0.147	1	60.6	0.2450
**XRRA1**	XRRA1	11	X-ray radiation resistance associated 1	protein_codin	0.5257	0.161	1	3.216	0.3636
**WNT11**	WNT11	11	Wnt family member 11	protein_codin	3.167	0.00022	1	34.05	0.2942
**LRRC32**	LRRC32	11	leucine rich repeat containing 32	protein_codin	-0.3946	0.0132	1	54.58	0.03233
**FAM76B**	FAM76B	11	family with sequence similarity 76 member B	protein_codin	0.2907	0.276	1	8.752	0.2197
**CUL5**	CUL5	11	cullin 5	protein_codin	0.1185	0.403	1	15.85	0.03446
**IL18**	IL18	11	interleukin 18	protein_codin	1.643	9.82E-07	0.02285	56.25	0.1434
**NRGN**	NRGN	11	neurogranin	protein_codin	-0.4888	3.77E-05	0.8629	60.6	0.082
**STT3A**	STT3A	11	STT3 oligosaccharyltransferase complex catalytic sub	protein_codin	0.4688	0.0057	1	8.965	0.06562
**SEPHS1**	SEPHS1	11	selenophosphate synthetase 1	protein_codin	-0.2043	0.235	1	8.293	0.09990
**MINDY3**	MINDY3	11	MINDY lysine 48 deubiquitinase 3	protein_codin	0.1942	0.309	1	7.399	0.1994
**PRTFDC1**	PRTFDC1	11	phosphoribosyl transferase domain containing 1	protein_codin	-0.1167	0.526	1	25.58	0.2015
**ANKRD26**	ANKRD26	11	ankyrin repeat domain 26	protein_codin	0.3841	0.146	1	23.58	0.2187
**FZD8**	FZD8	11	frizzled class receptor 8	protein_codin	0.5492	0.0697	1	24.65	0.2532
**8-Mar**	43532	11	membrane associated ring-CH-type finger 8	protein_codin	0.4608	0.121	1	9.058	0.597
**DDIT4**	DDIT4	11	DNA damage inducible transcript 4	protein_codin	1.338	3.2E-17	7.66E-13	60.6	0.1676
**TBC1D12**	TBC1D12	11	TBC1 domain family member 12	protein_codin	1.166	1.66E-07	0.00386	21.79	0.1717
**TCTN3**	TCTN3	11	tectonic family member 3	protein_codin	0.5606	0.0117	1	26.33	0.2044
**ABLIM1**	ABLIM1	11	actin binding LIM protein 1	protein_codin	2.766	6.33E-15	1.51E-10	39.06	0.06952
**DYRK4**	DYRK4	11	dual specificity tyrosine phosphorylation regulated kin	protein_codin	-0.5012	0.00032	1	19.17	0.1420
**IFFO1**	IFFO1	11	intermediate filament family orphan 1	protein_codin	0.1728	0.352	1	3.282	0.3242
**NECAP1**	NECAP1	11	NECAP endocytosis associated 1	protein_codin	0.5029	0.00687	1	3.492	0.1332
**HEBP1**	HEBP1	11	heme binding protein 1	protein_codin	-0.0007275	0.992	1	29.17	0.06901
**BHLHE41**	BHLHE41	11	basic helix-loop-helix family member e41	protein_codin	0.8662	4.01E-07	0.00936	38.02	0.1707
**PTHLH**	PTHLH	11	parathyroid hormone like hormone	protein_codin	0.1468	0.612	1	60.6	0.467
**R3HDM2**	R3HDM2	11	R3H domain containing 2	protein_codin	0.3315	0.00490	1	27.61	0.1365
**ARHGEF25**	ARHGEF25	11	Rho guanine nucleotide exchange factor 25	protein_codin	-0.1758	0.376	1	22.93	0.3811
**TMEM119**	TMEM119	11	transmembrane protein 119	protein_codin	1.201	0.00763	1	9.504	0.3207
**ALKBH2**	ALKBH2	11	alkB homolog 2, alpha-ketoglutarate dependent dioxy	protein_codin	0.1598	0.364	1	9.046	0.3082
**TMEM223**	TMEM223	11	transmembrane protein 223	protein_codin	1.673	2.71E-05	0.6213	18.54	0.4024
**P2RX4**	P2RX4	11	purinergic receptor P2X 4	protein_codin	-0.3112	0.126	1	30.45	0.1714
**CLIP1**	CLIP1	11	CAP-Gly domain containing linker protein 1	protein_codin	0.2257	0.0744	1	21.71	0.1720
**IL17D**	IL17D	11	interleukin 17D	protein_codin	1.403	1.98E-07	0.00462	27.22	0.1341
**LMO7**	LMO7	11	LIM domain 7	protein_codin	-0.7633	3.49E-08	0.00082	60.6	0.1245
**PSMB5**	PSMB5	11	proteasome subunit beta 5	protein_codin	0.03256	0.62	1	60.46	0.03272
**TRAPPC6B**	TRAPPC6B	11	trafficking protein particle complex 6B	protein_codin	-0.1519	0.371	1	26.61	0.176
**DNAAF2**	DNAAF2	11	dynein axonemal assembly factor 2	protein_codin	0.1511	0.482	1	9.664	0.2385
**TXNDC16**	TXNDC16	11	thioredoxin domain containing 16	protein_codin	0.1534	0.51	1	8.87	0.3176
**PSEN1**	PSEN1	11	presenilin 1	protein_codin	0.4845	0.0548	1	8.134	0.3913
**NEK9**	NEK9	11	NIMA related kinase 9	protein_codin	-0.2043	0.396	1	3.329	0.2454
**JDP2**	JDP2	11	Jun dimerization protein 2	protein_codin	1.307	2.76E-18	6.62E-14	60.33	0.03582
**VIPAS39**	VIPAS39	11	VPS33B interacting protein, apical-basolateral polarity	protein_codin	-0.1221	0.585	1	2.75	0.1741
**DPH6**	DPH6	11	diphthamine biosynthesis 6	protein_codin	-0.1504	0.496	1	8.733	0.2108

**SPRED1**	SPRED1	11	sprouty related EVH1 domain containing 1	protein_codin	-0.2117	0.254	1	59.76	0.1724
**ANXA2**	ANXA2	11	annexin A2	protein_codin	0.4825	3.16E-22	7.61E-18	Inf	0.06558
**HACD3**	HACD3	11	3-hydroxyacyl-CoA dehydratase 3	protein_codin	0.6296	6.19E-07	0.01442	60.57	0.03587
**MAP2K1**	MAP2K1	11	mitogen-activated protein kinase kinase 1	protein_codin	0.5067	0.0222	1	7.862	0.1041
**LARP6**	LARP6	11	La ribonucleoprotein domain family member 6	protein_codin	0.1795	0.102	1	35.58	0.0642
**UBL7**	UBL7	11	ubiquitin like 7	protein_codin	0.03382	0.857	1	8.592	0.06782
**SEC11A**	SEC11A	11	SEC11 homolog A, signal peptidase complex subunit	protein_codin	-0.1971	0.00015	1	59.27	0.06887
**TXNDC11**	TXNDC11	11	thioredoxin domain containing 11	protein_codin	-0.02038	0.935	1	3.112	0.3832
**PDXDC1**	PDXDC1	11	pyridoxal dependent decarboxylase domain containing 1	protein_codin	0.01484	0.938	1	7.696	0.3005
**GGA2**	GGA2	11	golgi associated, gamma adaptin ear containing, ARF	protein_codin	0.05470	0.824	1	8.662	0.1704
**RBBP6**	RBBP6	11	RB binding protein 6, ubiquitin ligase	protein_codin	0.2145	0.122	1	50.02	0.1377
**AMFR**	AMFR	11	autocrine motility factor receptor	protein_codin	0.08666	0.621	1	7.789	0.1377
**NOL3**	NOL3	11	nucleolar protein 3	protein_codin	-0.1923	0.16	1	14.64	0.03324
**TPPP3**	TPPP3	11	tubulin polymerization promoting protein family member 3	protein_codin	0.935	1.03E-06	0.02390	60.6	0.2797
**MYH10**	MYH10	11	myosin heavy chain 10	protein_codin	-0.9977	1.74E-31	4.2E-27	60.58	0.0692
**ALDH3A2**	ALDH3A2	11	aldehyde dehydrogenase 3 family member A2	protein_codin	-0.2291	0.102	1	35.55	0.132
**TMEM199**	TMEM199	11	transmembrane protein 199	protein_codin	0.1487	0.531	1	3.328	0.3191
**RNF135**	RNF135	11	ring finger protein 135	protein_codin	0.181	0.409	1	9.158	0.1774
**ERBB2**	ERBB2	11	erb-b2 receptor tyrosine kinase 2	protein_codin	0.1419	0.449	1	7.205	0.2357
**NR1D1**	NR1D1	11	nuclear receptor subfamily 1 group D member 1	protein_codin	0.277	0.128	1	23.24	0.2081
**STAT5B**	STAT5B	11	signal transducer and activator of transcription 5B	protein_codin	0.2876	0.237	1	3.152	0.2977
**NAGLU**	NAGLU	11	N-acetyl-alpha-glucosaminidase	protein_codin	0.4138	0.00257	1	60.18	0.06552
**VAT1**	VAT1	11	vesicle amine transport 1	protein_codin	1.162	2.15E-16	5.14E-12	55.36	0.1726
**SOST**	SOST	11	sclerostin	protein_codin	0.1956	0.349	1	60.58	0.3052
**NT5C**	NT5C	11	5', 3'-nucleotidase, cytosolic	protein_codin	-0.1162	0.207	1	46.95	0.1015
**NARF**	NARF	11	nuclear prelamin A recognition factor	protein_codin	0.1402	0.412	1	7.964	0.06876
**TPGS2**	TPGS2	11	tubulin polyglutamylase complex subunit 2	protein_codin	0.03166	0.796	1	15.32	0.03588
**SLC14A1**	SLC14A1	11	solute carrier family 14 member 1 (Kidd blood group)	protein_codin	-0.8237	0.00097	1	53.75	0.4170
**IER3IP1**	IER3IP1	11	immediate early response 3 interacting protein 1	protein_codin	0.221	0.0527	1	39.36	0.1349
**ME2**	ME2	11	malic enzyme 2	protein_codin	-0.04389	0.831	1	8.958	0.4160
**CCDC68**	CCDC68	11	coiled-coil domain containing 68	protein_codin	-1.345	3.38E-10	8E-06	60.6	0.2368
**ZNF407**	ZNF407	11	zinc finger protein 407	protein_codin	0.418	0.164	1	9.088	0.4614
**RAE1**	RAE1	11	ribonucleic acid export 1	protein_codin	0.2486	0.182	1	9.649	0.1395
**STMN3**	STMN3	11	stathmin 3	protein_codin	0.3309	0.0617	1	22.33	0.1102
**MED16**	MED16	11	mediator complex subunit 16	protein_codin	0.1729	0.421	1	3.349	0.2370
**DNASE2**	DNASE2	11	deoxyribonuclease 2, lysosomal	protein_codin	0.2388	0.0509	1	16.07	0.03586
**NR2F6**	NR2F6	11	nuclear receptor subfamily 2 group F member 6	protein_codin	-0.04902	0.721	1	19.04	0.109
**C19orf60**	C19orf60	11	required for excision 1-B domain containing	protein_codin	NA	NA	NA	59.37	0.06907
**UPF1**	UPF1	11	UPF1 RNA helicase and ATPase	protein_codin	0.2875	0.182	1	7.802	0.2062
**ZNF91**	ZNF91	11	zinc finger protein 91	protein_codin	0.3819	0.0389	1	7.716	0.2047
**ZNF302**	ZNF302	11	zinc finger protein 302	protein_codin	0.5572	5E-04	1	17.66	0.167
**FXYD5**	FXYD5	11	FXYD domain containing ion transport regulator 5	protein_codin	-1.182	4.57E-38	1.11E-33	42.84	0.03263
**KCNK6**	KCNK6	11	potassium two pore domain channel subfamily K mem	protein_codin	-0.533	0.0652	1	21.03	0.247
**CLASRP**	CLASRP	11	CLK4 associating serine/arginine rich protein	protein_codin	0.2797	0.307	1	3.244	0.2142
**EMP3**	EMP3	11	epithelial membrane protein 3	protein_codin	0.3114	1.43E-06	0.03322	60.58	0.03465
**ATF5**	ATF5	11	activating transcription factor 5	protein_codin	1.202	1.5E-06	0.03484	60.58	0.1492
**ETFB**	ETFB	11	electron transfer flavoprotein subunit beta	protein_codin	0.008324	0.919	1	33.5	0.03409
**ZNF649**	ZNF649	11	zinc finger protein 649	protein_codin	0.7642	0.00992	1	8.892	0.1711
**CNOT3**	CNOT3	11	CCR4-NOT transcription complex subunit 3	protein_codin	-0.05324	0.805	1	9.272	0.3467
**MED15**	MED15	11	mediator complex subunit 15	protein_codin	0.02279	0.925	1	10.92	0.2517
**XBP1**	XBP1	11	X-box binding protein 1	protein_codin	0.3728	0.00017	1	41.12	0.03757
**ZMAT5**	ZMAT5	11	zinc finger matrin-type 5	protein_codin	-0.03686	0.812	1	7.647	0.1404
**EP300**	EP300	11	E1A binding protein p300	protein_codin	-0.0336	0.856	1	8.112	0.3101
**BRWD1**	BRWD1	11	bromodomain and WD repeat domain containing 1	protein_codin	0.2821	0.188	1	19.88	0.3854
**YBEY**	YBEY	11	ybeY metalloendoribonuclease	protein_codin	-0.0314	0.882	1	29.92	0.07735
**ELOA**	ELOA	12	elongin A	protein_codin	0.1532	0.384	1	8.054	0.07245
**TMEM39B**	TMEM39B	12	transmembrane protein 39B	protein_codin	0.06117	0.758	1	3.282	0.268
**LSM10.00**	LSM10.00	12	LSM10, U7 small nuclear RNA associated	protein_codin	-0.3214	0.00677	1	15.72	0.1387
**TRABD2B**	TRABD2B	12	TraB domain containing 2B	protein_codin	0.2985	0.24	1	9.132	0.1512
**BOLA1**	BOLA1	12	bolA family member 1	protein_codin	0.2158	0.216	1	6.014	0.24
**PEA15**	PEA15	12	proliferation and apoptosis adaptor protein 15	protein_codin	-0.197	0.0759	1	56.53	0.03360
**DEDD**	DEDD	12	death effector domain containing	protein_codin	-0.3588	0.0884	1	7.622	0.3252
**CAMSAP2**	CAMSAP2	12	calmodulin regulated spectrin associated protein fami	protein_codin	-0.09073	0.549	1	45.86	0.1352
**BROX**	BROX	12	BRO1 domain and CAAX motif containing	protein_codin	0.1879	0.179	1	14.94	0.167
**COQ8A**	COQ8A	12	coenzyme Q8A	protein_codin	0.2916	0.202	1	2.825	0.2441
**SMYD3**	SMYD3	12	SET and MYND domain containing 3	protein_codin	-0.02772	0.899	1	7.772	0.1414
**ACP1**	ACP1	12	acid phosphatase 1	protein_codin	-0.002269	0.981	1	24.53	0.1074
**CCDC121**	CCDC121	12	coiled-coil domain containing 121	protein_codin	0.4513	0.0896	1	36.79	0.3224
**ACYP2**	ACYP2	12	acylphosphatase 2	protein_codin	-0.03409	0.852	1	21.09	0.1734
**ELMOD3**	ELMOD3	12	ELMO domain containing 3	protein_codin	0.4429	0.0792	1	8.227	0.09880
**TMEM127**	TMEM127	12	transmembrane protein 127	protein_codin	0.3157	0.152	1	7.622	0.2407
**POLR2D**	POLR2D	12	RNA polymerase II subunit D	protein_codin	0.5762	0.00964	1	3.401	0.1372
**RPE**	RPE	12	ribulose-5-phosphate-3-epimerase	protein_codin	-0.06853	0.724	1	3.306	0.2049
**BARD1**	BARD1	12	BRCA1 associated RING domain 1	protein_codin	-0.6105	0.00066	1	58.58	0.06664
**GMPPA**	GMPPA	12	GDP-mannose pyrophosphorylase A	protein_codin	0.07789	0.699	1	6.718	0.1092
**FARSB**	FARSB	12	phenylalanyl-tRNA synthetase subunit beta	protein_codin	0.5806	0.00535	1	7.68	0.1365
**EFHD1**	EFHD1	12	EF-hand domain family member D1	protein_codin	-0.2833	0.00132	1	58.82	0.06792
**OXSR1**	OXSR1	12	oxidative stress responsive kinase 1	protein_codin	0.5081	0.0225	1	8.852	0.1324
**POMGNT2**	POMGNT2	12	protein O-linked mannose N-acetylglucosaminyltransf	protein_codin	0.8682	0.00011	1	2.224	0.1365
**LZTFL1**	LZTFL1	12	leucine zipper transcription factor like 1	protein_codin	-0.1388	0.497	1	10.46	0.3216
**EIF4E3**	EIF4E3	12	eukaryotic translation initiation factor 4E family member 3	protein_codin	0.5349	0.0662	1	3.23	0.3472
**TMEM39A**	TMEM39A	12	transmembrane protein 39A	protein_codin	0.6226	0.016	1	2.468	0.2432
**CCDC14**	CCDC14	12	coiled-coil domain containing 14	protein_codin	0.08582	0.675	1	24.08	0.3461
**NCK1**	NCK1	12	NCK adaptor protein 1	protein_codin	1.036	6.94E-10	1.64E-05	14.18	0.07036
**PARL**	PARL	12	presenilin associated rhomboid like	protein_codin	0.0301	0.82	1	18.82	0.1351
**PCYT1A**	PCYT1A	12	phosphate cytidylyltransferase 1, choline, alpha	protein_codin	0.4969	0.0215	1	31.05	0.1752
**HNRNPD**	HNRNPD	12	heterogeneous nuclear ribonucleoprotein D	protein_codin	-0.1155	0.116	1	43.97	0.06696
**MSMO1**	MSMO1	12	methylsterol monooxygenase 1	protein_codin	0.429	0.0176	1	40.11	0.3926
**DAP**	DAP	12	death associated protein	protein_codin	-0.4511	1.36E-05	0.3131	52.05	0.06592
**HMGCS1**	HMGCS1	12	3-hydroxy-3-methylglutaryl-CoA synthase 1	protein_codin	0.4711	0.108	1	60.53	0.5457
**FST**	FST	12	follistatin	protein_codin	0.06999	0.741	1	60.58	0.1827
**CETN3**	CETN3	12	centrin 3	protein_codin	0.2554	0.139	1	3.41	0.2358
**EPB41L4A-AS1**	EPB41L4A-AS1	12	EPB41L4A antisense RNA 1	lincRNA	-0.5218	7.65E-07	0.01782	12.38	0.1012
**PRRC1**	PRRC1	12	proline rich coiled-coil 1	protein_codin	0.4152	0.0174	1	20.79	0.07006
**PDLIM4**	PDLIM4	12	PDZ and LIM domain 4	protein_codin	-0.2488	0.0252	1	60.5	0.03242
**RAD50**	RAD50	12	RAD50 double strand break repair protein	protein_codin	0.1193	0.618	1	3.152	0.1748
**ZCCHC10**	ZCCHC10	12	zinc finger CCHC-type containing 10	protein_codin	-0.05682	0.752	1	8.195	0.142
**CTNNA1**	CTNNA1	12	catenin alpha 1	protein_codin	0.0217	0.812	1	15.22	0.104
**KIAA0141**	KIAA0141	12	DAP3 binding cell death enhancer 1	protein_codin	NA	NA	NA	7.966	0.2002
**GNPDA1**	GNPDA1	12	glucosamine-6-phosphate deaminase 1	protein_codin	0.2657	0.0786	1	45.52	0.1015
**NR3C1**	NR3C1	12	nuclear receptor subfamily 3 group C member 1	protein_codin	0.4172	1.65E-06	0.03821	25.61	0.1711
**FAXDC2**	FAXDC2	12	fatty acid hydroxylase domain containing 2	protein_codin	1.333	2.97E-15	7.09E-11	28.79	0.1624
**GMDS**	GMDS	12	GDP-mannose 4,6-dehydratase	protein_codin	-0.7074	2.05E-15	4.9E-11	42.6	0.1080
**WRNIP1**	WRNIP1	12	WRN helicase interacting protein 1	protein_codin	-0.1932	0.319	1	6.952	0.1422
**DEK**	DEK	12	DEK proto-oncogene	protein_codin	0.0346	0.5759	1	60.53	0.1051
**TAF11**	TAF11	12	TATA-box binding protein associated factor 11	protein_codin	-0.04422	0.779	1	6.595	0.1367
**SRF**	SRF	12	serum response factor	protein_codin	0.1833	0.372	1	7.485	0.2089
**PLN**	PLN	12	phospholamban	protein_codin	-1.469	1.56E-71	3.79E-67	54.98	0.02581
**SF3B5**	SF3B5	12	splicing factor 3b subunit 5	protein_codin	-0.2843	8.68E-05	1	32.33	0.07202
**SCAF8**	SCAF8	12	SR-related CTD associated factor 8	protein_codin	0.6211	0.0114	1	3.083	0.3492
**ACAT2**	ACAT2	12	acetyl-CoA acetyltransferase 2	protein_codin	-0.09498	0.604	1	24.65	0.2137
**VPS41**	VPS41	12	VPS41 subunit of HOPS complex	protein_codin	0.2997	0.101	1	7.954	0.1796
**STEAP1**	STEAP1	12	STEAP family member 1	protein_codin	-0.9436	4.55E-10	1.08E-05	60.58	0.1909
**FAM133B**	FAM133B	12	family with sequence similarity 133 member B	protein_codin	0.3379	0.00300	1	42.96	0.1746
**PON2**	PON2	12	paraoxonase 2	protein_codin	0.3974	0.0242	1	22.97	0.1085
**FAM200A**	FAM200A	12	family with sequence similarity 200 member A	protein_codin	0.3696	0.114	1	3.268	0.2470
**SERPINE1**	SERPINE1	12	serpin family E member 1	protein_codin	-2.035	1.61E-15	3.85E-11	60.6	0.4179
**KMT2E-AS1**	KMT2E-AS1	12	KMT2E antisense RNA 1	antisense_RN	0.3079	0.212	1	2.228	0.3797
**DNAJB9**	DNAJB9	12	DnaJ heat shock protein family (Hsp40) member B9	protein_codin	0.3892	0.00476	1	54.13	0.06945
**TES**	TES	12	testin LIM domain protein	protein_codin	-0.9889	4.28E-13	1.02E-08	39.29	0.2759
**GALNT11**	GALNT11	12	polypeptide N-acetylgalactosaminyltransferase 11	protein_codin	0.462	0.00498	1	18.76	0.1002
**INSIG1**	INSIG1	12	insulin induced gene 1	protein_codin	0.7017	0.0267	1	60.6	0.5582
**AP1S2**	AP1S2	12	adaptor related protein complex 1 subunit sigma 2	protein_codin	-0.4783	0.00052	1	14.55	0.1412
**PDK3**	PDK3	12	pyruvate dehydrogenase kinase 3	protein_codin	-0.06147	0.779	1	54.99	0.3457
**DDX3X**	DDX3X	12	DEAD-box helicase 3 X-linked	protein_codin	0.5657	4.02E-08	0.00094	29.41	0.06667
**FAM104B**	FAM104B	12	family with sequence similarity 104 member B	protein_codin	0.19	0.295	1	26.38	0.1736
**ABCB7**	ABCB7	12	ATP binding cassette subfamily B member 7	protein_codin	0.4835	0.0332	1	3.455	0.1666
**MAGT1**	MAGT1	12	magnesium transporter 1	protein_codin	0.178	0.170	1	40	0.09762
**UBL4A**	UBL4A	12	ubiquitin like 4A	protein_codin	-0.03245	0.844	1	52.02	0.1695
**BRCC3**	BRCC3	12	BRCA1/BRCA2-containing complex subunit 3	protein_codin	-0.03293	0.872	1	9.782	0.1466
**MCPH1**	MCPH1	12	microcephalin 1	protein_codin	-0.01505	0.934	1	9.172	0.2072
**FDFT1**	FDFT1	12	farnesyl-diphosphate farnesyltransferase 1	protein_codin	0.1547	0.356	1	19.67	0.104
**DUSP26**	DUSP26	12	dual specificity phosphatase 26	protein_codin	-1.096	1.09E-09	2.57E-05	21.76	0.3104
**RAB11FIP1**	RAB11FIP1	12	RAB11 family interacting protein 1	protein_codin	-0.3631	0.0442	1	17.4	0.226
**COPS5**	COPS5	12	COP9 signalosome subunit 5	protein_codin	0.1062	0.326	1	25.74	0.09994
**ZFHX4**	ZFHX4	12	zinc finger homeobox 4	protein_codin	1.969	1.6E-07	0.00374	59.96	0.388
**FAM92A**	FAM92A	12	family with sequence similarity 92 member A	protein_codin	0.1932	0.127	1	30.77	0.04214
**DCAF13**	DCAF13	12	DDB1 and CUL4 associated factor 13	protein_codin	-0.1329	0.382	1	48.62	0.07545
**ANGPT1**	ANGPT1	12	angiopoietin 1	protein_codin	-0.8717	4.86E-20	1.17E-15	40.53	0.1669
**MRPL13**	MRPL13	12	mitochondrial ribosomal protein L13	protein_codin	-0.1868	0.0628	1	12.55	0.1027
**TSTA3**	TSTA3	12	tissue specific transplantation antigen P35B	protein_codin	-0.1877	0.27	1	20.67	0.03201
**SCX**	SCX	12	scleraxis bHLH transcription factor	protein_codin	-1.865	1.63E-18	3.91E-14	52.88	0.3858
**SLC52A2**	SLC52A2	12	solute carrier family 52 member 2	protein_codin	0.179	0.312	1	23.59	0.2558
**PTPRD-AS1**	PTPRD-AS1	12	PTPRD antisense RNA 1	lincRNA	-0.3116	0.146	1	9.893	0.3512
**UBE2R2**	UBE2R2	12	ubiquitin conjugating enzyme E2 R2	protein_codin	0.121	0.309	1	31.94	0.2497
**ZCCHC6**	ZCCHC6	12	terminal uridylyl transferase 7	protein_codin	NA	NA	NA	8.808	0.217
**NFIL3**	NFIL3	12	nuclear factor, interleukin 3 regulated	protein_codin	1.311	5.51E-11	1.31E-06	59.89	0.09847
**RGS3**	RGS3	12	regulator of G protein signaling 3	protein_codin	-1.02	2.99E-16	7.15E-12	54.87	0.04284
**PMPCA**	PMPCA	12	peptidase, mitochondrial processing alpha subunit	protein_codin	0.03122	0.868	1	8.052	0.1028
**CHST1**	CHST1	12	carbohydrate sulfotransferase 1	protein_codin	-0.7662	0.00032	1	22.1	0.09952
**ARFGAP2**	ARFGAP2	12	ADP ribosylation factor GTPase activating protein 2	protein_codin	-0.04529	0.805	1	6.852	0.272
**NUP160**	NUP160	12	nucleoporin 160	protein_codin	0.3196	0.119	1	9.025	0.3118
**FADS1**	FADS1	12	fatty acid desaturase 1	protein_codin	-0.2254	0.191	1	9.292	0.2934
**CTSC**	CTSC	12	cathepsin C	protein_codin	-1.349	4.86E-22	1.17E-17	60.6	0.1181
**TMEM123**	TMEM123	12	transmembrane protein 123	protein_codin	0.05856	0.611	1	34.38	0.07462
**CASP4**	CASP4	12	caspase 4	protein_codin	0.2006	0.111	1	42.9	0.0685
**CISD1**	CISD1	12	CDGSH iron sulfur domain 1	protein_codin	-0.4523	2.08E-05	0.4778	24.87	0.04188
**DDX21**	DDX21	12	DExD-box helicase 21	protein_codin	0.0152	0.872	1	59.74	0.0737
**ANAPC16**	ANAPC16	12	anaphase promoting complex subunit 16	protein_codin	-0.07967	0.1916	1	48	0.06415
**PLAU**	PLAU	12	plasminogen activator, urokinase	protein_codin	0.2488	0.282	1	55.3	0.2422
**AP3M1**	AP3M1	12	adaptor related protein complex 3 subunit mu 1	protein_codin	-0.0127	0.949	1	7.875	0.3571
**IFIT5**	IFIT5	12	interferon induced protein with tetratricopeptide repeats 5	protein_codin	0.3622	0.0547	1	6.426	0.1345
**UBTD1**	UBTD1	12	ubiquitin domain containing 1	protein_codin	-0.2428	0.259	1	9.722	0.109
**TRUB1**	TRUB1	12	TruB pseudouridine synthase family member 1	protein_codin	0.9307	0.00090	1	3.342	0.3406
**RAB11FIP2**	RAB11FIP2	12	RAB11 family interacting protein 2	protein_codin	0.1979	0.222	1	8.088	0.2837
**SMIM10L1**	SMIM10L1	12	small integral membrane protein 10 like 1	protein_codin	-0.3086	0.0142	1	12.21	0.1132
**TWF1**	TWF1	12	twinfilin actin binding protein 1	protein_codin	0.3017	0.0302	1	16.64	0.06844
**LETMD1**	LETMD1	12	LETM1 domain containing 1	protein_codin	-0.04505	0.732	1	30.12	0.1664
**C12orf43**	C12orf43	12	chromosome 12 open reading frame 43	protein_codin	-0.03238	0.842	1	8.479	0.1765
**TMED2**	TMED2	12	transmembrane p24 trafficking protein 2	protein_codin	-9.25E-05	0.999	1	44.32	0.06602
**RASL11A**	RASL11A	12	RAS like family 11 member A	protein_codin	0.02937	0.813	1	55.35	0.267
**RGCC**	RGCC	12	regulator of cell cycle	protein_codin	0.1555	0.495	1	60.6	0.4086
**PARP2**	PARP2	12	poly(ADP-ribose) polymerase 2	protein_codin	0.2742	0.189	1	8.822	0.1686
**ARHGEF40**	ARHGEF40	12	Rho guanine nucleotide exchange factor 40	protein_codin	0.1452	0.547	1	7.799	0.3762
**GMPR2**	GMPR2	12	guanosine monophosphate reductase 2	protein_codin	-0.07889	0.589	1	18.17	0.1332
**MNAT1**	MNAT1	12	MNAT1 component of CDK activating kinase	protein_codin	-0.03187	0.809	1	13.93	0.1076
**UBR7**	UBR7	12	ubiquitin protein ligase E3 component n-recognin 7 (p	protein_codin	-0.1082	0.693	1	2.808	0.3752
**HERC2**	HERC2	12	HECT and RLD domain containing E3 ubiquitin prote	protein_codin	0.2997	0.0856	1	8.077	0.2166
**KLF13**	KLF13	12	Kruppel like factor 13	protein_codin	0.7862	3.73E-11	8.85E-07	29.75	0.06962
**ACTC1**	ACTC1	12	actin alpha cardiac muscle 1	protein_codin	-2.465	7.77E-19	1.86E-14	60.6	0.8421
**IDH3A**	IDH3A	12	isocitrate dehydrogenase (NAD(+)) 3 alpha	protein_codin	-0.3558	0.0209	1	7.08	0.111
**IREB2**	IREB2	12	iron responsive element binding protein 2	protein_codin	0.4815	0.0262	1	6.996	0.1635
**EMP2**	EMP2	12	epithelial membrane protein 2	protein_codin	-0.1426	0.0132	1	33.64	0.03337
**CPPED1**	CPPED1	12	calcineurin like phosphoesterase domain containing 1	protein_codin	-0.7181	4.7E-05	1	9.833	0.02731
**RRAD**	RRAD	12	RRAD, Ras related glycolysis inhibitor and calcium ch	protein_codin	-0.4807	0.0001	1	60.6	0.1052
**NIP7**	NIP7	12	nucleolar pre-rRNA processing protein NIP7	protein_codin	-0.07373	0.725	1	8.436	0.06686
**TERF2**	TERF2	12	telomeric repeat binding factor 2	protein_codin	-0.1718	0.377	1	30.66	0.2372
**NFAT5**	NFAT5	12	nuclear factor of activated T cells 5	protein_codin	0.1452	0.336	1	15.8	0.1445
**DHX38**	DHX38	12	DEAH-box helicase 38	protein_codin	0.3875	0.0705	1	7.805	0.1672
**ZFHX3**	ZFHX3	12	zinc finger homeobox 3	protein_codin	-0.2161	0.0563	1	59.87	0.07022
**ANKRD11**	ANKRD11	12	ankyrin repeat domain 11	protein_codin	0.2615	0.0307	1	46.85	0.09918
**TCF25**	TCF25	12	transcription factor 25	protein_codin	0.2471	0.00412	1	42.05	0.1057
**ALDOC**	ALDOC	12	aldolase, fructose-bisphosphate C	protein_codin	-0.4721	0.0349	1	7.86	0.4079
**ZNHIT3**	ZNHIT3	12	zinc finger HIT-type containing 3	protein_codin	-0.4114	0.00012	1	11.08	0.06581
**GPATCH8**	GPATCH8	12	G-patch domain containing 8	protein_codin	0.2229	0.135	1	34.14	0.204
**NFE2L1**	NFE2L1	12	nuclear factor, erythroid 2 like 1	protein_codin	0.03587	0.797	1	16.14	0.2169
**CUEDC1**	CUEDC1	12	CUE domain containing 1	protein_codin	0.6511	0.0132	1	8.297	0.3304
**9-Sep**	43717	12	septin 9	protein_codin	-0.3509	0.0664	1	30.09	0.2467
**LDLRAD4**	LDLRAD4	12	low density lipoprotein receptor class A domain conta	protein_codin	-0.2803	0.209	1	3.2	0.3889
**ESCO1**	ESCO1	12	establishment of sister chromatid cohesion N-acetyltr	protein_codin	0.3521	0.0257	1	20.44	0.3202
**ZCCHC3**	ZCCHC3	12	zinc finger CCHC-type containing 3	protein_codin	0.03511	0.874	1	3.126	0.271
**AHCY**	AHCY	12	adenosylhomocysteinase	protein_codin	-0.08129	0.656	1	48.84	0.182
**PHF20**	PHF20	12	PHD finger protein 20	protein_codin	0.08891	0.449	1	28.09	0.02378
**ACOT8**	ACOT8	12	acyl-CoA thioesterase 8	protein_codin	-0.04587	0.831	1	31.52	0.1676
**WDR18**	WDR18	12	WD repeat domain 18	protein_codin	0.06165	0.714	1	3.392	0.197
**MFSD12**	MFSD12	12	major facilitator superfamily domain containing 12	protein_codin	-0.5457	0.0172	1	10.26	0.1465
**SMARCA4**	SMARCA4	12	SWI/SNF related, matrix associated, actin dependent	protein_codin	0.3232	0.179	1	7.828	0.3268
**PGLS**	PGLS	12	6-phosphogluconolactonase	protein_codin	-0.11	0.174	1	39.84	0.1315
**ZNF146**	ZNF146	12	zinc finger protein 146	protein_codin	0.5025	0.0442	1	6.733	0.3595
**EID2B**	EID2B	12	EP300 interacting inhibitor of differentiation 2B	protein_codin	0.2011	0.516	1	10.41	0.2212
**ZC3H4**	ZC3H4	12	zinc finger CCCH-type containing 4	protein_codin	-0.1313	0.579	1	8.222	0.3566
**RTCB**	RTCB	12	RNA 2',3'-cyclic phosphate and 5'-OH ligase	protein_codin	-0.01257	0.926	1	16.16	0.03357
**TMEM184B**	TMEM184B	12	transmembrane protein 184B	protein_codin	0.975	0.00022	1	3.47	0.06832
**POLR3H**	POLR3H	12	RNA polymerase III subunit H	protein_codin	-0.1377	0.535	1	9.245	0.1674
**CRELD2**	CRELD2	12	cysteine rich with EGF like domains 2	protein_codin	0.145	0.232	1	42.05	0.06789
**PHF13**	PHF13	13	PHD finger protein 13	protein_codin	-0.1064	0.669	1	3.685	0.456
**EXOSC10**	EXOSC10	13	exosome component 10	protein_codin	0.2988	0.142	1	7.348	0.4179
**PDPN**	PDPN	13	podoplanin	protein_codin	0.4929	0.14	1	60.6	0.7235
**KAZN**	KAZN	13	kazrin, periplakin interacting protein	protein_codin	0.7825	0.00106	1	9.971	0.148
**FBXO42**	FBXO42	13	F-box protein 42	protein_codin	0.1554	0.512	1	7.838	0.4152
**CROCC**	CROCC	13	ciliary rootlet coiled-coil, rootletin	protein_codin	0.01101	0.964	1	3.263	0.06736
**MRTO4**	MRTO4	13	MRT4 homolog, ribosome maturation factor	protein_codin	0.188	0.320	1	7.57	0.1697
**ECE1**	ECE1	13	endothelin converting enzyme 1	protein_codin	-0.25	0.0327	1	55.05	0.3065
**SRRM1**	SRRM1	13	serine and arginine repetitive matrix 1	protein_codin	0.02418	0.707	1	30.28	0.03266
**GPN2**	GPN2	13	GPN-loop GTPase 2	protein_codin	-0.1981	0.469	1	9.193	0.3657
**PUM1**	PUM1	13	pumilio RNA binding family member 1	protein_codin	0.07522	0.61	1	17.87	0.2055
**COL16A1**	COL16A1	13	collagen type XVI alpha 1 chain	protein_codin	0.5028	0.0051	1	19	0.4062
**CMPK1**	CMPK1	13	cytidine/uridine monophosphate kinase 1	protein_codin	-0.1322	0.126	1	22.01	0.03302
**AC105942.1**	AC105942.1	13	novel transcri	antisense_RN	-0.03809	0.868	1	8.066	0.2752
**SNX7**	SNX7	13	sorting nexin 7	protein_codin	0.3449	0.00422	1	12.67	0.1046
**RNPC3**	RNPC3	13	RNA binding region (RNP1, RRM) containing 3	protein_codin	-0.1266	0.509	1	43.43	0.3268
**SORT1**	SORT1	13	sortilin 1	protein_codin	-0.5172	0.00027	1	41.01	0.2118
**CTSK**	CTSK	13	cathepsin K	protein_codin	1.228	2.75E-30	6.64E-26	60	0.07292
**SCAMP3**	SCAMP3	13	secretory carrier membrane protein 3	protein_codin	0.2462	0.225	1	54.07	0.06725
**RIT1**	RIT1	13	Ras like without CAAX 1	protein_codin	-0.0002291	0.999	1	9.006	0.1035
**MEF2D**	MEF2D	13	myocyte enhancer factor 2D	protein_codin	0.2062	0.291	1	9.079	0.2744
**RGS4**	RGS4	13	regulator of G protein signaling 4	protein_codin	-0.9281	1.71E-06	0.0397	36.56	0.2134
**DPT**	DPT	13	dermatopontin	protein_codin	0.5929	0.0002	1	55.36	0.1768
**TOR1AIP1**	TOR1AIP1	13	torsin 1A interacting protein 1	protein_codin	0.1964	0.207	1	15.34	0.2069
**MR1**	MR1	13	major histocompatibility complex, class I-related	protein_codin	0.3585	0.111	1	6.563	0.2862
**TPR**	TPR	13	translocated promoter region, nuclear basket protein	protein_codin	-0.1846	0.0321	1	34.48	0.0996
**KCNT2**	KCNT2	13	potassium sodium-activated channel subfamily T mer	protein_codin	-0.957	2.96E-06	0.06857	42.58	0.3272
**CFH**	CFH	13	complement factor H	protein_codin	-0.7903	4.46E-22	1.07E-17	60.6	0.1206
**PRELP**	PRELP	13	proline and arginine rich end leucine rich repeat prote	protein_codin	0.7785	2.33E-22	5.61E-18	60.6	0.1342
**SLC30A1**	SLC30A1	13	solute carrier family 30 member 1	protein_codin	0.618	0.0138	1	10.15	0.1366
**SNAP47**	SNAP47	13	synaptosome associated protein 47	protein_codin	-0.1856	0.336	1	3.202	0.1697
**COG2**	COG2	13	component of oligomeric golgi complex 2	protein_codin	0.1543	0.494	1	8.736	0.2212
**GPR137B**	GPR137B	13	G protein-coupled receptor 137B	protein_codin	0.5204	0.0292	1	29.79	0.1044
**RNASEH1-AS1**	RNASEH1-AS1	13	RNASEH1 antisense RNA 1	antisense_RN	-0.3886	0.0586	1	8.653	0.06826
**ID2**	ID2	13	inhibitor of DNA binding 2	protein_codin	0.9423	2.84E-18	6.81E-14	42.61	0.03259
**GDF7**	GDF7	13	growth differentiation factor 7	protein_codin	-0.3207	0.245	1	25.23	0.1772
**ASXL2**	ASXL2	13	ASXL transcriptional regulator 2	protein_codin	0.771	0.0105	1	8.592	0.1747
**CGREF1**	CGREF1	13	cell growth regulator with EF-hand domain 1	protein_codin	0.56	0.0175	1	25.38	0.1044
**SUPT7L**	SUPT7L	13	SPT7 like, STAGA complex gamma subunit	protein_codin	-0.2989	0.135	1	3.326	0.1314
**CRIM1**	CRIM1	13	cysteine rich transmembrane BMP regulator 1	protein_codin	0.003908	0.971	1	59.88	0.03307
**PRKD3**	PRKD3	13	protein kinase D3	protein_codin	0.4047	0.0175	1	7.582	0.1395
**CDC42EP3**	CDC42EP3	13	CDC42 effector protein 3	protein_codin	-1.117	2.85E-15	6.8E-11	39.04	0.169
**AC007288.1**	AC007288.1	13	MAP4K3 divergent transcript	antisense_RN	-0.0519	0.852	1	7.819	0.5158
**LGALSL**	LGALSL	13	galectin like	protein_codin	0.5382	0.00065	1	30.97	0.1298
**UNC50**	UNC50	13	unc-50 inner nuclear membrane RNA binding protein	protein_codin	0.3623	0.00046	1	29.33	0.102
**SLC20A1**	SLC20A1	13	solute carrier family 20 member 1	protein_codin	-0.4174	0.162	1	60.6	0.6685
**ACTR3**	ACTR3	13	actin related protein 3	protein_codin	-0.1108	0.199	1	59.72	0.06918
**DDX18**	DDX18	13	DEAD-box helicase 18	protein_codin	0.06767	0.46	1	60.28	0.206
**IWS1**	IWS1	13	interacts with SUPT6H, CTD assembly factor 1	protein_codin	0.5742	0.00366	1	16.09	0.1751
**CACNB4**	CACNB4	13	calcium voltage-gated channel auxiliary subunit beta	protein_codin	1.05	0.0005	1	4.141	0.2861
**DAPL1**	DAPL1	13	death associated protein like 1	protein_codin	0.128	0.66	1	60.53	0.3639
**FAP**	FAP	13	fibroblast activation protein alpha	protein_codin	-0.8417	9.37E-09	0.00022	60.58	0.1109
**GORASP2**	GORASP2	13	golgi reassembly stacking protein 2	protein_codin	-0.06227	0.575	1	32.64	0.07526
**DLX1**	DLX1	13	distal-less homeobox 1	protein_codin	1.505	1.21E-06	0.02812	10.53	0.2068
**COL3A1**	COL3A1	13	collagen type III alpha 1 chain	protein_codin	-0.6302	4.63E-05	1	Inf	0.7222
**COL5A2**	COL5A2	13	collagen type V alpha 2 chain	protein_codin	-0.6957	2.9E-06	0.06719	60.6	0.4138
**PLCL1**	PLCL1	13	phospholipase C like 1 (inactive)	protein_codin	-0.45	0.00905	1	18.44	0.0986
**TRAK2**	TRAK2	13	trafficking kinesin protein 2	protein_codin	-0.01856	0.924	1	7.111	0.138
**NRP2**	NRP2	13	neuropilin 2	protein_codin	-1.173	2.09E-08	0.00049	53.54	0.1726
**FN1**	FN1	13	fibronectin 1	protein_codin	-1.125	5.12E-25	1.23E-20	NA	0.4315
**SCG2**	SCG2	13	secretogranin II	protein_codin	-0.7665	0.0177	1	55.21	0.468
**SERPINE2**	SERPINE2	13	serpin family E member 2	protein_codin	-0.2978	0.270	1	60.6	0.2937
**ARL4C**	ARL4C	13	ADP ribosylation factor like GTPase 4C	protein_codin	-0.9646	0.00046	1	54.86	0.1349
**COPS8**	COPS8	13	COP9 signalosome subunit 8	protein_codin	-0.2683	0.00800	1	60.6	0.1777
**COL6A3**	COL6A3	13	collagen type VI alpha 3 chain	protein_codin	0.2571	0.0662	1	55.36	0.4185
**SUMF1**	SUMF1	13	sulfatase modifying factor 1	protein_codin	0.3927	0.0286	1	6.329	0.1819
**ATG7**	ATG7	13	autophagy related 7	protein_codin	-0.4883	0.0269	1	9.26	0.3249
**HDAC11**	HDAC11	13	histone deacetylase 11	protein_codin	-0.3411	0.0992	1	8.84	0.2158
**RBSN**	RBSN	13	rabenosyn, RAB effector	protein_codin	-0.2556	0.229	1	10.18	0.252
**GALNT15**	GALNT15	13	polypeptide N-acetylgalactosaminyltransferase 15	protein_codin	2.838	3.96E-17	9.48E-13	53.83	0.1105
**TGFBR2**	TGFBR2	13	transforming growth factor beta receptor 2	protein_codin	0.7016	2.72E-11	6.45E-07	59.93	0.03898
**CTNNB1**	CTNNB1	13	catenin beta 1	protein_codin	0.3638	0.00156	1	60.53	0.2611
**FAM198A**	FAM198A	13	golgi associated kinase 1A	protein_codin	1.675	1.76E-05	0.4046	7.694	0.2205
**MAP4**	MAP4	13	microtubule associated protein 4	protein_codin	-0.3149	5.17E-06	0.1195	60.46	0.03359
**NDUFAF3**	NDUFAF3	13	NADH:ubiquinone oxidoreductase complex assembly	protein_codin	-0.09107	0.156	1	58.94	0.06714
**CYB561D2**	CYB561D2	13	cytochrome b561 family member D2	protein_codin	0.9852	3.95E-05	0.904	3.832	0.1402
**GXYLT2**	GXYLT2	13	glucoside xylosyltransferase 2	protein_codin	1.295	3.3E-15	7.88E-11	42.48	0.1096
**PROS1**	PROS1	13	protein S	protein_codin	0.756	1.59E-10	3.77E-06	59.79	0.1426
**COL8A1**	COL8A1	13	collagen type VIII alpha 1 chain	protein_codin	-0.5294	0.00034	1	60.6	0.2874
**TBC1D23**	TBC1D23	13	TBC1 domain family member 23	protein_codin	-0.05907	0.719	1	5.604	0.1329
**TFG**	TFG	13	trafficking from ER to golgi regulator	protein_codin	0.1242	0.189	1	54.45	0.0776
**ABI3BP**	ABI3BP	13	ABI family member 3 binding protein	protein_codin	2.283	9.15E-52	2.22E-47	60.6	0.2126
**ATG3**	ATG3	13	autophagy related 3	protein_codin	-0.205	0.0672	1	28.41	0.03442
**PPP2R3A**	PPP2R3A	13	protein phosphatase 2 regulatory subunit B''alpha	protein_codin	0.1525	0.56	1	9.945	0.3081
**ATP1B3**	ATP1B3	13	ATPase Na+/K+ transporting subunit beta 3	protein_codin	-0.353	0.00058	1	60	0.1752
**PLOD2**	PLOD2	13	procollagen-lysine,2-oxoglutarate 5-dioxygenase 2	protein_codin	-0.2038	0.222	1	31.43	0.08162
**LXN**	LXN	13	latexin	protein_codin	0.5832	0.00300	1	60.48	0.362
**RARRES1**	RARRES1	13	retinoic acid receptor responder 1	protein_codin	2.976	1.48E-16	3.54E-12	60.6	0.4171
**MFSD1**	MFSD1	13	major facilitator superfamily domain containing 1	protein_codin	0.4764	0.00380	1	17.12	0.1471
**KPNA4**	KPNA4	13	karyopherin subunit alpha 4	protein_codin	-0.004022	0.975	1	13.54	0.09805
**RPL22L1**	RPL22L1	13	ribosomal protein L22 like 1	protein_codin	0.04817	0.656	1	60.6	0.03272
**HES1**	HES1	13	hes family bHLH transcription factor 1	protein_codin	0.2747	0.0259	1	59.93	0.07988
**ACAP2**	ACAP2	13	ArfGAP with coiled-coil, ankyrin repeat and PH domai	protein_codin	0.4496	0.00101	1	43.51	0.1698
**CYTL1**	CYTL1	13	cytokine like 1	protein_codin	0.1695	0.7668	1	Inf	0.8052
**DHX15**	DHX15	13	DEAH-box helicase 15	protein_codin	0.4196	0.019	1	24.14	0.2115
**SOD3**	SOD3	13	superoxide dismutase 3	protein_codin	-0.1212	0.00796	1	60.6	0.07132
**SLAIN2**	SLAIN2	13	SLAIN motif family member 2	protein_codin	-0.2175	0.0725	1	13.98	0.06557
**HOPX**	HOPX	13	HOP homeobox	protein_codin	-0.5856	0.0351	1	60.6	0.2946
**SDAD1**	SDAD1	13	SDA1 domain containing 1	protein_codin	0.01731	0.88	1	50.52	0.182
**HSD17B11**	HSD17B11	13	hydroxysteroid 17-beta dehydrogenase 11	protein_codin	0.547	2.72E-06	0.06304	42.05	0.06966
**IBSP**	IBSP	13	integrin binding sialoprotein	protein_codin	-3.917	1.5E-07	0.00353	Inf	0.6471
**SPP1**	SPP1	13	secreted phosphoprotein 1	protein_codin	-5.449	1.99E-09	4.7E-05	60.6	0.9288

**FAT4**	FAT4	12	FAT atypical cadherin 4	protein_codin	-0.1043	0.603	1	43.55	0.2462
**CPE**	CPE	12	carboxypeptidase E	protein_codin	0.4	1.55E-12	3.69E-08	59.99	0.03477
**SCRG1**	SCRG1	12	stimulator of chondrogenesis 1	protein_codin	-0.7313	1.37E-06	0.03183	60.53	0.1843
**ANKH**	ANKH	12	ANKH inorganic pyrophosphate transport regulator	protein_codin	-0.8525	2.32E-09	5.47E-05	37.53	0.1596
**RETREG1**	RETREG1	12	reticulophagy regulator 1	protein_codin	2.081	9.74E-12	2.31E-07	51.88	0.2181
**SELENOP**	SELENOP	12	selenoprotein P	protein_codin	1.478	2.26E-42	5.48E-38	54.85	0.1145
**ITGA1**	ITGA1	12	integrin subunit alpha 1	protein_codin	-0.6279	1.04E-05	0.2396	60.6	0.07397
**RAD17**	RAD17	12	RAD17 checkpoint clamp loader component	protein_codin	0.09128	0.637	1	8.33	0.06919
**VCAN**	VCAN	12	versican	protein_codin	-0.754	3.01E-18	7.22E-14	60.6	0.2994
**HAPLN1**	HAPLN1	12	hyaluronan and proteoglycan link protein 1	protein_codin	0.05919	0.782	1	59.93	0.1789
**EDIL3**	EDIL3	12	EGF like repeats and discoidin domains 3	protein_codin	-1.265	4.59E-56	1.11E-51	60.6	0.03919
**PGGT1B**	PGGT1B	12	protein geranylgeranyltransferase type I subunit beta	protein_codin	0.4079	0.046	1	9.212	0.06826
**AC010226.1**	AC010226.1	12	novel transcri	antisense_RN	0.1207	0.592	1	3.463	0.2382
**PRR16**	PRR16	12	proline rich 16	protein_codin	-0.2019	0.314	1	29.79	0.1766
**PPIC**	PPIC	12	peptidylprolyl isomerase C	protein_codin	0.2177	0.00241	1	42.63	0.03458
**H2AFY**	H2AFY	12	H2A histone family member Y	protein_codin	-0.03403	0.752	1	60.58	0.1302
**TGFBI**	TGFBI	12	transforming growth factor beta induced	protein_codin	-0.397	0.0237	1	42.19	0.3693
**SMAD5**	SMAD5	12	SMAD family member 5	protein_codin	0.1938	0.125	1	13.47	0.1696
**SIL1**	SIL1	12	SIL1 nucleotide exchange factor	protein_codin	-0.02703	0.834	1	13.17	0.04051
**UBE2D2**	UBE2D2	12	ubiquitin conjugating enzyme E2 D2	protein_codin	-0.04899	0.514	1	33.43	0.03254
**PCDHB4**	PCDHB4	12	protocadherin beta 4	protein_codin	0.5637	0.00673	1	52.35	0.1907
**GM2A**	GM2A	12	GM2 ganglioside activator	protein_codin	0.01394	0.956	1	3.262	0.4162
**SPARC**	SPARC	12	secreted protein acidic and cysteine rich	protein_codin	-0.3529	1.68E-08	0.00039	42.86	0.2447
**SGCD**	SGCD	12	sarcoglycan delta	protein_codin	0.09251	0.632	1	22.58	0.2016
**HRH2**	HRH2	12	histamine receptor H2	protein_codin	0.1707	0.419	1	60.6	0.1924
**HIGD2A**	HIGD2A	12	HIG1 hypoxia inducible domain family member 2A	protein_codin	0.3176	0.00041	1	47.55	0.1224
**HNRNPH1**	HNRNPH1	12	heterogeneous nuclear ribonucleoprotein H1	protein_codin	0.3513	3.08E-05	0.7057	55.64	0.1682
**FAM50B**	FAM50B	12	family with sequence similarity 50 member B	protein_codin	-0.239	0.264	1	32.25	0.2448
**GMPR**	GMPR	12	guanosine monophosphate reductase	protein_codin	-0.4069	0.0428	1	10.01	0.2007
**PPP1R11**	PPP1R11	12	protein phosphatase 1 regulatory inhibitor subunit 11	protein_codin	-0.1236	0.351	1	57.83	0.1322
**FLOT1**	FLOT1	12	flotillin 1	protein_codin	-0.07067	0.522	1	48.08	0.03258
**ANKS1A**	ANKS1A	12	ankyrin repeat and sterile alpha motif domain contain	protein_codin	0.7777	0.00158	1	3.402	0.4011
**PEX6**	PEX6	12	peroxisomal biogenesis factor 6	protein_codin	0.2665	0.241	1	2.843	0.1050
**VEGFA**	VEGFA	12	vascular endothelial growth factor A	protein_codin	0.9215	8.99E-06	0.2072	50.75	0.37
**SLC29A1**	SLC29A1	12	solute carrier family 29 member 1 (Augustine blood g	protein_codin	0.679	2.47E-08	0.00058	59.48	0.0492
**RAB23**	RAB23	12	RAB23, member RAS oncogene family	protein_codin	0.05958	0.756	1	60.58	0.1100
**CD109**	CD109	12	CD109 molecule	protein_codin	-0.5208	0.00342	1	60.53	0.0712
**CD164**	CD164	12	CD164 molecule	protein_codin	0.1614	0.0279	1	60.6	0.07465
**ENPP1**	ENPP1	12	ectonucleotide pyrophosphatase/phosphodiesterase :	protein_codin	-1.202	2.51E-13	5.98E-09	60.6	0.07402
**ABRACL**	ABRACL	12	ABRA C-terminal like	protein_codin	-0.9219	7.67E-08	0.0018	53.71	0.2157
**VTA1**	VTA1	12	vesicle trafficking 1	protein_codin	0.1665	0.329	1	8.305	0.1414
**PHACTR2**	PHACTR2	12	phosphatase and actin regulator 2	protein_codin	-0.3283	0.00234	1	36.93	0.06629
**UTRN**	UTRN	12	utrophin	protein_codin	0.5264	3.26E-05	0.7467	60.58	0.06734
**MTHFD1L**	MTHFD1L	12	methylenetetrahydrofolate dehydrogenase (NADP+ d	protein_codin	-0.1937	0.316	1	45.4	0.1019
**ZDHHC14**	ZDHHC14	12	zinc finger DHHC-type containing 14	protein_codin	-0.5644	0.0415	1	3.401	0.4392
**DYNLT1**	DYNLT1	12	dynein light chain Tctex-type 1	protein_codin	-0.229	0.00245	1	35.49	0.06604
**SMOC2**	SMOC2	12	SPARC related modular calcium binding 2	protein_codin	-0.7656	2.68E-19	6.43E-15	60.6	0.07414
**LINC01615**	LINC01615	12	long intergenic non-protein coding RNA 1615	lincRNA	-0.8315	5.83E-05	1	48.47	0.1488
**THBS2**	THBS2	12	thrombospondin 2	protein_codin	-0.3021	0.0102	1	55.36	0.1175
**NUDT1**	NUDT1	12	nudix hydrolase 1	protein_codin	-0.1471	0.312	1	60.43	0.09893
**GPNMB**	GPNMB	12	glycoprotein nmb	protein_codin	1.802	3.82E-61	9.28E-57	60.53	0.08391
**HOXA3**	HOXA3	12	homeobox A3	protein_codin	0.8547	0.00029	1	23.29	0.1411
**INHBA**	INHBA	12	inhibin subunit beta A	protein_codin	-0.7113	3.08E-07	0.00719	60.6	0.1090
**STK17A**	STK17A	12	serine/threonine kinase 17a	protein_codin	-0.1429	0.365	1	49.96	0.1358
**AEBP1**	AEBP1	12	AE binding protein 1	protein_codin	-0.3098	8.07E-06	0.1861	60.6	0.2687
**ELN**	ELN	12	elastin	protein_codin	0.9724	3.61E-10	8.54E-06	60	0.2699
**YWHAG**	YWHAG	12	tyrosine 3-monooxygenase/tryptophan 5-monooxyger	protein_codin	0.01096	0.937	1	41.7	0.03242
**CDK6**	CDK6	12	cyclin dependent kinase 6	protein_codin	-0.6275	0.0262	1	10.59	0.1852
**COL1A2**	COL1A2	12	collagen type I alpha 2 chain	protein_codin	0.3966	2.57E-06	0.05956	60.6	0.3690
**TMEM130**	TMEM130	12	transmembrane protein 130	protein_codin	-1.17	7.92E-06	0.1827	25.73	0.2757
**ZNF277**	ZNF277	12	zinc finger protein 277	protein_codin	-0.1732	0.324	1	8.036	0.09916
**AASS**	AASS	12	aminoadipate-semialdehyde synthase	protein_codin	0.159	0.492	1	8.853	0.2024
**SLC35B4**	SLC35B4	12	solute carrier family 35 member B4	protein_codin	0.5788	0.026	1	3.346	0.1732
**UBN2**	UBN2	12	ubinuclein 2	protein_codin	0.1688	0.398	1	7.538	0.2072
**PAXIP1-AS2**	PAXIP1-AS2	12	PAXIP1 antisense RNA 2	antisense_RN	0.2638	0.282	1	3.419	0.3027
**MXRA5**	MXRA5	12	matrix remodeling associated 5	protein_codin	-0.8423	0.001	1	39.87	0.2215
**SH3KBP1**	SH3KBP1	12	SH3 domain containing kinase binding protein 1	protein_codin	-0.09209	0.587	1	39.62	0.1344
**ZFX**	ZFX	12	zinc finger protein X-linked	protein_codin	0.1271	0.415	1	23.54	0.06612
**TIMP1**	TIMP1	12	TIMP metallopeptidase inhibitor 1	protein_codin	-0.8883	1.08E-28	2.61E-24	NA	0.3082
**MAGED1**	MAGED1	12	MAGE family member D1	protein_codin	0.2134	0.165	1	18.43	0.1605
**AR**	AR	12	androgen receptor	protein_codin	0.06866	0.556	1	20.63	0.03536
**PGK1.00**	PGK1.00	12	phosphoglycerate kinase 1	protein_codin	-0.03832	0.586	1	60.6	0.04787
**C1GALT1C1**	C1GALT1C1	12	C1GALT1 specific chaperone 1	protein_codin	0.534	9.29E-06	0.2141	36.85	0.07137
**ENOX2**	ENOX2	12	ecto-NOX disulfide-thiol exchanger 2	protein_codin	-0.0074	0.966	1	9.772	0.1666
**PHF6**	PHF6	12	PHD finger protein 6	protein_codin	0.5413	0.00385	1	8.57	0.1715
**RTL8C**	RTL8C	12	retrotransposon Gag like 8C	protein_codin	0.2298	0.0028	1	43.49	0.1345
**BGN**	BGN	12	biglycan	protein_codin	-0.9115	2.94E-60	7.14E-56	60	0.03318
**TAZ**	TAZ	12	tafazzin	protein_codin	0.4843	0.0901	1	2.665	0.2791
**LAGE3**	LAGE3	12	L antigen family member 3	protein_codin	0.08219	0.625	1	20.82	0.1679
**FAM3A**	FAM3A	12	family with sequence similarity 3 member A	protein_codin	-0.1138	0.449	1	6.138	0.207
**TNKS**	TNKS	12	tankyrase	protein_codin	0.3462	0.0637	1	3.542	0.1736
**NEIL2**	NEIL2	12	nei like DNA glycosylase 2	protein_codin	0.1788	0.391	1	3.109	0.1796
**CTSB**	CTSB	12	cathepsin B	protein_codin	0.1654	0.0889	1	60.58	0.2034
**CLU**	CLU	12	clusterin	protein_codin	0.1826	0.0258	1	NA	0.2359
**SCARA3**	SCARA3	12	scavenger receptor class A member 3	protein_codin	-0.2569	0.0559	1	47.38	0.1407
**SARAF**	SARAF	12	store-operated calcium entry associated regulatory fa	protein_codin	-0.1719	0.0021	1	39.5	0.03302
**BAG4**	BAG4	12	BCL2 associated athanogene 4	protein_codin	0.314	0.076	1	7.718	0.1065
**THAP1**	THAP1	12	THAP domain containing 1	protein_codin	0.2532	0.152	1	8.549	0.1185
**SULF1**	SULF1	12	sulfatase 1	protein_codin	-0.903	1.35E-17	3.23E-13	60.6	0.1826
**MSC**	MSC	12	musculin	protein_codin	-0.8035	1.62E-07	0.00379	60.2	0.06935
**STAU2**	STAU2	12	staufen double-stranded RNA binding protein 2	protein_codin	-0.2137	0.316	1	3.554	0.1455
**LY96**	LY96	12	lymphocyte antigen 96	protein_codin	0.2126	0.239	1	48.8	0.1807
**SDC2**	SDC2	12	syndecan 2	protein_codin	-0.1455	0.0886	1	60.58	0.04725
**SLC25A32**	SLC25A32	12	solute carrier family 25 member 32	protein_codin	0.3949	0.0852	1	7.497	0.131
**NUDCD1**	NUDCD1	12	NudC domain containing 1	protein_codin	-0.0459	0.842	1	3.28	0.2372
**TRPS1**	TRPS1	12	transcriptional repressor GATA binding 1	protein_codin	-0.3269	0.0686	1	40.64	0.3235
**TNFRSF11B**	TNFRSF11B	12	TNF receptor superfamily member 11b	protein_codin	-0.9843	3.09E-10	7.31E-06	60.6	0.2639
**ASAP1**	ASAP1	12	ArfGAP with SH3 domain, ankyrin repeat and PH dor	protein_codin	-0.7976	3.26E-06	0.07549	18.19	0.2265
**BOP1.00**	BOP1.00	12	BOP1 ribosomal biogenesis factor	protein_codin	-0.6616	0.00283	1	8.95	0.2472
**CDKN2A**	CDKN2A	12	cyclin dependent kinase inhibitor 2A	protein_codin	-0.8897	1.36E-05	0.3131	59.88	0.03664
**RECK**	RECK	12	reversion inducing cysteine rich protein with kazal mo	protein_codin	1.475	2.45E-15	5.85E-11	49.23	0.06884
**ERCC6L2**	ERCC6L2	12	ERCC excision repair 6 like 2	protein_codin	0.306	0.232	1	26.96	0.3401
**COL15A1**	COL15A1	12	collagen type XV alpha 1 chain	protein_codin	-1.185	9.8E-12	2.33E-07	60.6	0.3736
**ERP44**	ERP44	12	endoplasmic reticulum protein 44	protein_codin	0.4318	1.02E-05	0.235	56.65	0.03409
**SVEP1**	SVEP1	12	sushi, von Willebrand factor type A, EGF and pentrax	protein_codin	3.365	2.27E-13	5.41E-09	60.58	0.2381
**TNC**	TNC	12	tenascin C	protein_codin	-0.8472	0.0222	1	60.6	0.3444
**CDK5RAP2**	CDK5RAP2	12	CDK5 regulatory subunit associated protein 2	protein_codin	-0.1774	0.212	1	60.58	0.1350
**ANGPTL2**	ANGPTL2	12	angiopoietin like 2	protein_codin	-0.0764	0.686	1	57.98	0.1342
**FPGS**	FPGS	12	folylpolyglutamate synthase	protein_codin	0.2974	0.334	1	2.922	0.06781
**GOLGA2**	GOLGA2	12	golgin A2	protein_codin	0.3212	0.00502	1	51.34	0.1072
**CERCAM**	CERCAM	12	cerebral endothelial cell adhesion molecule	protein_codin	0.1776	0.12	1	54.77	0.06801
**PTPA**	PTPA	12	protein phosphatase 2 phosphatase activator	protein_codin	-0.6345	0.00025	1	58.84	0.1895
**PRRC2B**	PRRC2B	12	proline rich coiled-coil 2B	protein_codin	-0.06426	0.688	1	8.387	0.3515
**SURF4**	SURF4	12	surfeit 4	protein_codin	0.4529	0.00186	1	26.79	0.09996
**COL5A1**	COL5A1	12	collagen type V alpha 1 chain	protein_codin	-0.1645	0.422	1	60.6	0.3772
**PTDSS2**	PTDSS2	12	phosphatidylserine synthase 2	protein_codin	0.4145	0.165	1	8.074	0.175
**CDKN1C**	CDKN1C	12	cyclin dependent kinase inhibitor 1C	protein_codin	1.355	9.59E-39	2.32E-34	54.22	0.08021
**SAA1**	SAA1	12	serum amyloid A1	protein_codin	0.8984	0.016	1	60.6	1.137
**HPS5**	HPS5	12	HPS5 biogenesis of lysosomal organelles complex 2	protein_codin	-0.2344	0.325	1	10.41	0.2460
**FIBIN**	FIBIN	12	fin bud initiation factor homolog	protein_codin	-0.2223	0.0569	1	60.6	0.163
**CCDC34**	CCDC34	12	coiled-coil domain containing 34	protein_codin	0.01416	0.949	1	21.39	0.3090
**PAMR1**	PAMR1	12	peptidase domain containing associated with muscle	protein_codin	2.528	6.05E-07	0.01411	57.13	0.1769
**HSD17B12**	HSD17B12	12	hydroxysteroid 17-beta dehydrogenase 12	protein_codin	-0.02659	0.768	1	47.16	0.1962
**STIP1**	STIP1	12	stress induced phosphoprotein 1	protein_codin	-0.04595	0.773	1	7.992	0.1685
**SAC3D1**	SAC3D1	12	SAC3 domain containing 1	protein_codin	-0.07364	0.713	1	59.99	0.1180
**FAM89B**	FAM89B	12	family with sequence similarity 89 member B	protein_codin	-0.4139	6.98E-08	0.00164	53.79	0.06829
**C11orf68**	C11orf68	12	chromosome 11 open reading frame 68	protein_codin	-0.2133	0.192	1	8.203	0.269
**CST6**	CST6	12	cystatin E/M	protein_codin	-0.3847	0.0822	1	60.58	0.154
**RAB1B**	RAB1B	12	RAB1B, member RAS oncogene family	protein_codin	-0.002132	0.988	1	15.73	0.06746
**TMEM134**	TMEM134	12	transmembrane protein 134	protein_codin	-0.2771	0.0532	1	6.319	0.1396
**COA4**	COA4	12	cytochrome c oxidase assembly factor 4 homolog	protein_codin	-0.3256	0.00106	1	40.06	0.1062
**SYTL2**	SYTL2	12	synaptotagmin like 2	protein_codin	0.07692	0.715	1	35.63	0.139
**ANGPTL5**	ANGPTL5	12	angiopoietin like 5	protein_codin	2.022	7.11E-11	1.69E-06	45.23	0.2046
**CEP164**	CEP164	12	centrosomal protein 164	protein_codin	0.2711	0.162	1	6.833	0.1658
**TMEM25**	TMEM25	12	transmembrane protein 25	protein_codin	-0.6721	8.57E-05	1	9.39	0.1391
**TMEM218**	TMEM218	12	transmembrane protein 218	protein_codin	0.0638	0.707	1	8.693	0.06565
**RPUSD4**	RPUSD4	12	RNA pseudouridine synthase D4	protein_codin	-0.1829	0.364	1	3.141	0.169
**SRPRA**	SRPRA	12	SRP receptor subunit alpha	protein_codin	0.2977	0.00214	1	31.85	0.06577
**ETS1**	ETS1	12	ETS proto-oncogene 1, transcription factor	protein_codin	-0.18	0.31	1	34.2	0.1814
**PITRM1**	PITRM1	12	pitrilysin metallopeptidase 1	protein_codin	-0.1397	0.586	1	3.278	0.352
**ITIH5**	ITIH5	12	inter-alpha-trypsin inhibitor heavy chain 5	protein_codin	0.824	2.11E-06	0.04893	59.38	0.06862
**CCDC3**	CCDC3	12	coiled-coil domain containing 3	protein_codin	-0.1323	0.196	1	55.36	0.1712
**ACBD5**	ACBD5	12	acyl-CoA binding domain containing 5	protein_codin	0.5007	0.0316	1	3.652	0.1755
**EPC1**	EPC1	12	enhancer of polycomb homolog 1	protein_codin	0.3028	0.00388	1	22.2	0.1074
**ZNF22**	ZNF22	12	zinc finger protein 22	protein_codin	-0.003872	0.98	1	13.13	0.06686
**GDF10**	GDF10	12	growth differentiation factor 10	protein_codin	1.426	0.00013	1	60.6	0.2900
**ERCC6**	ERCC6	12	ERCC excision repair 6, chromatin remodeling factor	protein_codin	0.2706	0.232	1	8.807	0.2432
**CHST3**	CHST3	12	carbohydrate sulfotransferase 3	protein_codin	0.4402	0.154	1	7.952	0.2104
**ASCC1**	ASCC1	12	activating signal cointegrator 1 complex subunit 1	protein_codin	0.02387	0.895	1	7.642	0.2496
**FAM213A**	FAM213A	12	peroxiredoxin like 2A	protein_codin	0.02866	0.842	1	27.53	0.1344
**MINPP1**	MINPP1	12	multiple inositol-polyphosphate phosphatase 1	protein_codin	0.4587	0.0976	1	2.895	0.1474
**LIPA**	LIPA	12	lipase A, lysosomal acid type	protein_codin	0.4652	0.00458	1	8.272	0.2329
**R3HCC1L**	R3HCC1L	12	R3H domain and coiled-coil containing 1 like	protein_codin	0.05006	0.838	1	9.149	0.235
**LZTS2**	LZTS2	12	leucine zipper tumor suppressor 2	protein_codin	0.2994	0.0517	1	7.328	0.2046
**XPNPEP1**	XPNPEP1	12	X-prolyl aminopeptidase 1	protein_codin	0.4528	0.0619	1	55.95	0.2047
**EIF3A**	EIF3A	12	eukaryotic translation initiation factor 3 subunit A	protein_codin	-0.07297	0.528	1	59.23	0.1371
**NSMCE4A**	NSMCE4A	12	NSE4 homolog A, SMC5-SMC6 complex component	protein_codin	-0.08868	0.554	1	22.43	0.1386
**HTRA1**	HTRA1	12	HtrA serine peptidase 1	protein_codin	-0.4987	3.55E-17	8.5E-13	60.6	0.1944
**CD9**	CD9	12	CD9 molecule	protein_codin	-0.3909	1.05E-12	2.5E-08	60.6	0.06879
**TAPBPL**	TAPBPL	12	TAP binding protein like	protein_codin	0.6074	0.00025	1	46.33	0.06959
**TPI1**	TPI1	12	triosephosphate isomerase 1	protein_codin	-0.1875	0.00027	1	55.36	0.03776
**SPSB2**	SPSB2	12	splA/ryanodine receptor domain and SOCS box conta	protein_codin	-0.6277	0.00442	1	3.188	0.1766
**SLC2A3**	SLC2A3	12	solute carrier family 2 member 3	protein_codin	1.483	4.68E-13	1.11E-08	55.35	0.1600
**M6PR**	M6PR	12	mannose-6-phosphate receptor, cation dependent	protein_codin	0.09649	0.457	1	14.19	0.1666
**A2M**	A2M	12	alpha-2-macroglobulin	protein_codin	-0.9373	4.31E-36	1.04E-31	51	0.1841
**PRH1**	PRH1	12	proline rich protein HaeIII subfamily 1	protein_codin	0.3526	0.152	1	9.187	0.2880
**CDKN1B**	CDKN1B	12	cyclin dependent kinase inhibitor 1B	protein_codin	-0.03428	0.757	1	37.88	0.1802
**RECQL**	RECQL	12	RecQ like helicase	protein_codin	0.05087	0.67	1	28	0.1434
**ADCY6**	ADCY6	12	adenylate cyclase 6	protein_codin	0.1429	0.479	1	26.76	0.1751
**KRT8**	KRT8	12	keratin 8	protein_codin	-0.26	0.109	1	60.6	0.1496
**USP15**	USP15	12	ubiquitin specific peptidase 15	protein_codin	0.5211	2.43E-05	0.5576	46.68	0.2829
**YEATS4**	YEATS4	12	YEATS domain containing 4	protein_codin	0.2437	0.208	1	6.328	0.2859
**KCNMB4**	KCNMB4	12	potassium calcium-activated channel subfamily M reg	protein_codin	-0.6826	0.0118	1	10.66	0.4051
**ATP2B1**	ATP2B1	12	ATPase plasma membrane Ca2+ transporting 1	protein_codin	0.1319	0.396	1	50.26	0.2695
**LUM**	LUM	12	lumican	protein_codin	-0.2772	0.00703	1	NA	0.07141
**ELK3**	ELK3	12	ETS transcription factor ELK3	protein_codin	0.354	0.0982	1	8.972	0.1322
**NFYB**	NFYB	12	nuclear transcription factor Y subunit beta	protein_codin	0.2882	0.0227	1	59.29	0.1018
**NUAK1**	NUAK1	12	NUAK family kinase 1	protein_codin	-0.6879	0.0367	1	32.01	0.4138
**CORO1C**	CORO1C	12	coronin 1C	protein_codin	-0.2836	0.0901	1	34.34	0.06890
**DDX54**	DDX54	12	DEAD-box helicase 54	protein_codin	-0.07167	0.656	1	6.128	0.1359
**RITA1**	RITA1	12	RBPJ interacting and tubulin associated 1	protein_codin	-0.133	0.581	1	2.779	0.3394
**SPPL3**	SPPL3	12	signal peptide peptidase like 3	protein_codin	0.159	0.362	1	8.852	0.1982
**CAMKK2**	CAMKK2	12	calcium/calmodulin dependent protein kinase kinase 2	protein_codin	0.5878	0.00088	1	8.409	0.1775
**FRY**	FRY	12	FRY microtubule binding protein	protein_codin	-0.4063	0.0395	1	59.88	0.1430
**SMAD9**	SMAD9	12	SMAD family member 9	protein_codin	0.3672	0.00513	1	32.34	0.07015
**POSTN**	POSTN	12	periostin	protein_codin	-2.206	1.5E-20	3.6E-16	60.6	0.5227
**FOXO1**	FOXO1	12	forkhead box O1	protein_codin	0.7507	2.83E-05	0.6487	20.43	0.2002
**DZIP1**	DZIP1	12	DAZ interacting zinc finger protein 1	protein_codin	0.738	0.0125	1	3.902	0.3226
**UBAC2**	UBAC2	12	UBA domain containing 2	protein_codin	0.03296	0.81	1	19.05	0.1016
**COL4A1**	COL4A1	12	collagen type IV alpha 1 chain	protein_codin	-1.026	2.38E-15	5.68E-11	60.6	0.3850
**COL4A2**	COL4A2	12	collagen type IV alpha 2 chain	protein_codin	-0.9798	1.32E-19	3.17E-15	60.6	0.2197
**TFDP1**	TFDP1	12	transcription factor Dp-1	protein_codin	0.7819	0.00056	1	7.752	0.1075
**GAS6**	GAS6	12	growth arrest specific 6	protein_codin	-0.4804	2.62E-15	6.26E-11	60.6	0.08071
**MMP14**	MMP14	12	matrix metallopeptidase 14	protein_codin	-0.03215	0.85	1	36.53	0.1511
**NOVA1**	NOVA1	12	NOVA alternative splicing regulator 1	protein_codin	1.533	2.06E-13	4.91E-09	37.08	0.2129
**EGLN3**	EGLN3	12	egl-9 family hypoxia inducible factor 3	protein_codin	0.5237	0.0376	1	42.86	0.2594
**MAX**	MAX	12	MYC associated factor X	protein_codin	0.1606	0.279	1	18.53	0.1702
**SUSD6**	SUSD6	12	sushi domain containing 6	protein_codin	0.3132	0.206	1	9.958	0.1822
**SMOC1**	SMOC1	12	SPARC related modular calcium binding 1	protein_codin	-0.9929	3.1E-07	0.00724	46.74	0.4996
**LTBP2**	LTBP2	12	latent transforming growth factor beta binding protein 2	protein_codin	-0.5044	3.54E-05	0.8107	60.6	0.1538
**MLH3**	MLH3	12	mutL homolog 3	protein_codin	0.6834	0.00029	1	41.95	0.1449
**TMED10**	TMED10	12	transmembrane p24 trafficking protein 10	protein_codin	0.1177	0.0732	1	53.66	0.03222
**SPATA7**	SPATA7	12	spermatogenesis associated 7	protein_codin	0.8522	0.0375	1	7.302	0.6625
**GSKIP**	GSKIP	12	GSK3B interacting protein	protein_codin	0.1346	0.54	1	8.553	0.1080
**AHNAK2**	AHNAK2	12	AHNAK nucleoprotein 2	protein_codin	0.8447	3.36E-06	0.07779	60.47	0.1488
**LPCAT4**	LPCAT4	12	lysophosphatidylcholine acyltransferase 4	protein_codin	0.7276	0.00455	1	2.419	0.2094
**CCDC32**	CCDC32	12	coiled-coil domain containing 32	protein_codin	0.3103	0.0984	1	3.285	0.1662
**ZFYVE19**	ZFYVE19	12	zinc finger FYVE-type containing 19	protein_codin	-0.04509	0.847	1	9.997	0.1667
**HAUS2**	HAUS2	12	HAUS augmin like complex subunit 2	protein_codin	0.4292	0.0669	1	29.52	0.1381
**VPS13C**	VPS13C	12	vacuolar protein sorting 13 homolog C	protein_codin	0.6066	7.69E-07	0.01793	34.77	0.07282
**SMAD6**	SMAD6	12	SMAD family member 6	protein_codin	-0.271	0.257	1	10.89	0.2424
**ADAMTSL3**	ADAMTSL3	12	ADAMTS like 3	protein_codin	1.152	3.23E-11	7.66E-07	14.23	0.06862
**ACAN**	ACAN	12	aggrecan	protein_codin	-0.3459	0.182	1	59.93	0.4807
**HAPLN3**	HAPLN3	12	hyaluronan and proteoglycan link protein 3	protein_codin	-0.7133	0.00016	1	60.58	0.1076
**MFGE8**	MFGE8	12	milk fat globule-EGF factor 8 protein	protein_codin	-0.8413	5.12E-47	1.24E-42	60.6	0.03747
**ARPIN**	ARPIN	12	actin related protein 2/3 complex inhibitor	protein_codin	0.0692	0.689	1	30.53	0.1058
**FURIN**	FURIN	12	furin, paired basic amino acid cleaving enzyme	protein_codin	0.1398	0.564	1	2.914	0.2372
**SLCO3A1**	SLCO3A1	12	solute carrier organic anion transporter family membe	protein_codin	-0.2433	0.0764	1	14.76	0.1788
**LINS1**	LINS1	12	lines homolog 1	protein_codin	0.4236	0.103	1	3.13	0.2504
**SNRPA1**	SNRPA1	12	small nuclear ribonucleoprotein polypeptide A'	protein_codin	0.002574	0.984	1	52.13	0.2061
**NME3**	NME3	12	NME/NM23 nucleoside diphosphate kinase 3	protein_codin	-0.1572	0.0107	1	60.43	0.03264
**PMM2**	PMM2	12	phosphomannomutase 2	protein_codin	0.4642	0.0459	1	9.586	0.1494
**THUMPD1**	THUMPD1	12	THUMP domain containing 1	protein_codin	0.175	0.0996	1	32.03	0.1747
**NFATC2IP**	NFATC2IP	12	nuclear factor of activated T cells 2 interacting protein	protein_codin	0.0142	0.937	1	6.637	0.1349
**ZNF423**	ZNF423	12	zinc finger protein 423	protein_codin	1.856	2.41E-07	0.00562	32.67	0.1688
**CNEP1R1**	CNEP1R1	12	CTD nuclear envelope phosphatase 1 regulatory sub	protein_codin	0.4098	0.115	1	2.582	0.2043
**MMP2**	MMP2	12	matrix metallopeptidase 2	protein_codin	2.048	4.9E-46	1.19E-41	60.6	0.1242
**KIFC3**	KIFC3	12	kinesin family member C3	protein_codin	-0.4773	0.122	1	12.05	0.4932
**CFAP20**	CFAP20	12	cilia and flagella associated protein 20	protein_codin	0.2552	0.14	1	7.362	0.2162
**CSNK2A2**	CSNK2A2	12	casein kinase 2 alpha 2	protein_codin	-0.1414	0.324	1	19.7	0.06996
**COG4**	COG4	12	component of oligomeric golgi complex 4	protein_codin	-0.1216	0.631	1	3.402	0.2868
**AP1G1**	AP1G1	12	adaptor related protein complex 1 subunit gamma 1	protein_codin	0.3598	0.0658	1	28.03	0.2007
**MAP1LC3B**	MAP1LC3B	12	microtubule associated protein 1 light chain 3 beta	protein_codin	0.1665	0.0640	1	31.68	0.07051
**ITGAE**	ITGAE	12	integrin subunit alpha E	protein_codin	0.1455	0.313	1	14.46	0.1374
**SOX15**	SOX15	12	SRY-box transcription factor 15	protein_codin	0.4028	0.113	1	9.934	0.2867
**RANGRF**	RANGRF	12	RAN guanine nucleotide release factor	protein_codin	-0.1496	0.319	1	19.03	0.2019
**LRRC75A**	LRRC75A	12	leucine rich repeat containing 75A	protein_codin	-0.1738	0.252	1	35.93	0.345
**MYO1D**	MYO1D	12	myosin ID	protein_codin	-0.9419	6.9E-13	1.64E-08	44.98	0.1519
**SLFN12**	SLFN12	12	schlafen family member 12	protein_codin	0.1938	0.411	1	31.23	0.1516
**GGNBP2**	GGNBP2	12	gametogenetin binding protein 2	protein_codin	0.1615	0.142	1	21.08	0.0320
**CWC25**	CWC25	12	CWC25 spliceosome associated protein homolog	protein_codin	0.1193	0.416	1	15.06	0.2120
**TUBG2**	TUBG2	12	tubulin gamma 2	protein_codin	0.1517	0.496	1	3.062	0.3403
**BECN1**	BECN1	12	beclin 1	protein_codin	-0.192	0.320	1	24.7	0.2080
**ACBD4**	ACBD4	12	acyl-CoA binding domain containing 4	protein_codin	0.2886	0.222	1	3.423	0.3129
**HEXIM1**	HEXIM1	12	HEXIM P-TEFb complex subunit 1	protein_codin	0.07394	0.614	1	17.67	0.1354
**UBE2Z**	UBE2Z	12	ubiquitin conjugating enzyme E2 Z	protein_codin	-0.08976	0.548	1	41.8	0.1005
**SNF8**	SNF8	12	SNF8 subunit of ESCRT-II	protein_codin	-0.2369	0.00758	1	50.85	0.06571
**SPOP**	SPOP	12	speckle type BTB/POZ protein	protein_codin	-0.07615	0.543	1	20.23	0.2352
**COL1A1**	COL1A1	12	collagen type I alpha 1 chain	protein_codin	-0.3686	0.0162	1	Inf	0.5479
**SPATA20**	SPATA20	12	spermatogenesis associated 20	protein_codin	0.1613	0.363	1	9.984	0.172
**ANKRD40**	ANKRD40	12	ankyrin repeat domain 40	protein_codin	0.2785	0.0902	1	7.648	0.3516
**AC005921.2**	AC005921.2	12	novel transcript, antisense to ANKRD	antisense_RN	0.3478	0.0474	1	7.513	0.2377
**TUBD1**	TUBD1	12	tubulin delta 1	protein_codin	-0.317	0.127	1	3.599	0.3268
**BPTF**	BPTF	12	bromodomain PHD finger transcription factor	protein_codin	0.03126	0.796	1	34.54	0.1783
**METTL23**	METTL23	12	methyltransferase like 23	protein_codin	0.06246	0.613	1	37.17	0.06888
**CENPX**	CENPX	12	centromere protein X	protein_codin	-0.1818	0.0728	1	59.98	0.06598
**RALBP1**	RALBP1	12	ralA binding protein 1	protein_codin	-0.1446	0.0919	1	31.83	0.03479
**MPPE1**	MPPE1	12	metallophosphoesterase 1	protein_codin	0.5862	0.0146	1	3.503	0.3112
**SMAD4**	SMAD4	12	SMAD family member 4	protein_codin	0.007741	0.967	1	7.995	0.3130
**ATP8B1**	ATP8B1	12	ATPase phospholipid transporting 8B1	protein_codin	-0.9005	4.21E-06	0.09728	29.43	0.4025
**PQLC1**	PQLC1	12	solute carrier family 66 member 2	protein_codin	-0.388	0.0158	1	8.571	0.1427
**VPS16**	VPS16	12	VPS16 core subunit of CORVET and HOPS complexe	protein_codin	-0.1513	0.459	1	8.67	0.1442
**PET117**	PET117	12	PET117 cytochrome c oxidase chaperone	protein_codin	0.1617	0.481	1	3.133	0.1727
**POLR3F**	POLR3F	12	RNA polymerase III subunit F	protein_codin	0.03016	0.909	1	2.99	0.1407
**REM1**	REM1	12	RRAD and GEM like GTPase 1	protein_codin	-0.06926	0.722	1	24.7	0.167
**MAP1LC3A**	MAP1LC3A	12	microtubule associated protein 1 light chain 3 alpha	protein_codin	0.287	0.0141	1	58.02	0.1319
**MAFB**	MAFB	12	MAF bZIP transcription factor B	protein_codin	0.5693	7.56E-05	1	50.57	0.124
**STK11**	STK11	12	serine/threonine kinase 11	protein_codin	0.09892	0.619	1	9.158	0.1674
**MKNK2**	MKNK2	12	MAPK interacting serine/threonine kinase 2	protein_codin	0.3796	0.0895	1	34.76	0.252
**TLE2**	TLE2	12	TLE family member 2, transcriptional corepressor	protein_codin	-0.1319	0.568	1	7.812	0.1517
**MCOLN1**	MCOLN1	12	mucolipin 1	protein_codin	0.3726	0.105	1	8.982	0.1688
**ANGPTL4**	ANGPTL4	12	angiopoietin like 4	protein_codin	0.8663	2.8E-05	0.6419	60	0.3728
**HNRNPM**	HNRNPM	12	heterogeneous nuclear ribonucleoprotein M	protein_codin	-0.04152	0.615	1	23.2	0.1317
**AC092069.1**	AC092069.1	12	novel transcript, antisense to CA	antisense_RN	-0.1588	0.525	1	9.958	0.308
**PODNL1**	PODNL1	12	podocan like 1	protein_codin	-0.01882	0.951	1	27.53	0.2804
**AKAP8L**	AKAP8L	12	A-kinase anchoring protein 8 like	protein_codin	0.01862	0.916	1	7.923	0.1935
**COMP**	COMP	12	cartilage oligomeric matrix protein	protein_codin	1.158	0.0338	1	60.6	0.8988
**ZNF493**	ZNF493	12	zinc finger protein 493	protein_codin	0.1596	0.477	1	9.373	0.2436
**CEP89**	CEP89	12	centrosomal protein 89	protein_codin	0.3893	0.0488	1	9.103	0.1372
**USF2**	USF2	12	upstream transcription factor 2, c-fos interacting	protein_codin	-0.1615	0.182	1	42.15	0.1333
**DMKN**	DMKN	12	dermokine	protein_codin	-1.579	2.99E-17	7.16E-13	38.35	0.1458
**CIC**	CIC	12	capicua transcriptional repressor	protein_codin	-0.2213	0.346	1	10.58	0.2027
**PLAUR**	PLAUR	12	plasminogen activator, urokinase receptor	protein_codin	-0.1272	0.496	1	60.6	0.3084
**CALM3**	CALM3	12	calmodulin 3	protein_codin	-0.6442	3.44E-21	8.27E-17	39.02	0.03269
**TMEM160**	TMEM160	12	transmembrane protein 160	protein_codin	-0.4158	6.33E-05	1	40.04	0.06494
**JOSD2**	JOSD2	12	Josephin domain containing 2	protein_codin	-0.1399	0.245	1	20.8	0.1004
**LENG1**	LENG1	12	leukocyte receptor cluster member 1	protein_codin	0.1278	0.419	1	18.95	0.1826
**SSC5D**	SSC5D	12	scavenger receptor cysteine rich family member with	protein_codin	1.366	7.73E-07	0.018	10.13	0.148
**ZNF581**	ZNF581	12	zinc finger protein 581	protein_codin	-0.1997	0.275	1	6.81	0.06479
**MMP11**	MMP11	12	matrix metallopeptidase 11	protein_codin	-2.066	7.44E-11	1.76E-06	60.6	0.7709
**TPST2**	TPST2	12	tyrosylprotein sulfotransferase 2	protein_codin	-1.069	1.05E-06	0.02442	59.84	0.2266
**MN1**	MN1	12	MN1 proto-oncogene, transcriptional regulator	protein_codin	1.13	7.45E-06	0.1719	32.03	0.3349
**TIMP3**	TIMP3	12	TIMP metallopeptidase inhibitor 3	protein_codin	2.511	7.2E-73	1.75E-68	Inf	0.09484
**PACSIN2**	PACSIN2	12	protein kinase C and casein kinase substrate in neur	protein_codin	-0.3185	0.0102	1	28.09	0.1705
**SAMM50**	SAMM50	12	SAMM50 sorting and assembly machinery componen	protein_codin	-0.04944	0.762	1	24.93	0.1371
**TBC1D22A**	TBC1D22A	12	TBC1 domain family member 22A	protein_codin	-0.4261	0.0767	1	2.409	0.267
**ODF3B**	ODF3B	12	outer dense fiber of sperm tails 3B	protein_codin	-0.1277	0.407	1	58.83	0.06657
**COL6A1**	COL6A1	12	collagen type VI alpha 1 chain	protein_codin	-0.06445	0.604	1	60.6	0.2841
**COL6A2**	COL6A2	12	collagen type VI alpha 2 chain	protein_codin	0.07784	0.146	1	60.6	0.08671
**TXNIP**	TXNIP	14	thioredoxin interacting protein	protein_codin	2.356	######	######	60.6	0.03236
**STAM2**	STAM2	14	signal transducing adaptor molecule 2	protein_codin	0.2765	0.154	1	23.46	0.1344
**MTRNR2L12**	MTRNR2L12	14	MT-RNR2 like 12	protein_codin	0.1038	0.0961	1	60.6	0.2372
**FXR1**	FXR1	14	FMR1 autosomal homolog 1	protein_codin	0.07422	0.454	1	16.93	0.2680
**FBXO45**	FBXO45	14	F-box protein 45	protein_codin	0.086	0.704	1	11.22	0.1037
**MSX1**	MSX1	14	msh homeobox 1	protein_codin	1.979	3.12E-26	7.52E-22	60.6	0.1047
**SH2D4A**	SH2D4A	14	SH2 domain containing 4A	protein_codin	1.744	2.36E-10	5.59E-06	23.22	0.1069
**MTRNR2L8**	MTRNR2L8	14	MT-RNR2 like 8	protein_codin	-0.3966	7.87E-05	1	30.65	0.1531
**QSER1**	QSER1	14	glutamine and serine rich 1	protein_codin	0.6695	0.00212	1	24.98	0.1409
**WAPL**	WAPL	14	WAPL cohesin release factor	protein_codin	0.1987	0.265	1	5.543	0.2741
**CSK**	CSK	14	C-terminal Src kinase	protein_codin	-0.307	0.131	1	3.409	0.2126
**MCAT**	MCAT	14	malonyl-CoA-acyl carrier protein transacylase	protein_codin	0.05889	0.77	1	3.145	0.1052
**MT-ND1**	MT-ND1	14	mitochondrially encoded NADH:ubiquinone oxidoredu	protein_codin	-0.3186	9.62E-18	2.31E-13	60.6	0.59
**MT-ND2**	MT-ND2	14	mitochondrially encoded NADH:ubiquinone oxidoredu	protein_codin	-0.2626	1.55E-12	3.69E-08	Inf	0.5372
**MT-CO1**	MT-CO1	14	mitochondrially encoded cytochrome c oxidase I	protein_codin	0.2058	2.54E-10	6.01E-06	NA	0.5309
**MT-CO2**	MT-CO2	14	mitochondrially encoded cytochrome c oxidase II	protein_codin	0.001037	0.974	1	Inf	0.7577
**MT-ATP6**	MT-ATP6	14	mitochondrially encoded ATP synthase membrane su	protein_codin	-0.3836	2.69E-34	6.5E-30	60.6	0.7321
**MT-CO3**	MT-CO3	14	mitochondrially encoded cytochrome c oxidase III	protein_codin	-0.01213	0.706	1	Inf	0.6512

MT-ND3	MT-ND3	14	mitochondrially encoded NADH:ubiquinone oxidoredu	protein_codin	-0.1529	1.81E-05	0.416	59.94	0.6208
MT-ND4L	MT-ND4L	14	mitochondrially encoded NADH:ubiquinone oxidoredu	protein_codin	0.0583	0.304	1	54.69	0.3419
MT-ND4	MT-ND4	14	mitochondrially encoded NADH:ubiquinone oxidoredu	protein_codin	-0.1068	0.00048	1	Inf	0.6784
MT-ND5	MT-ND5	14	mitochondrially encoded NADH:ubiquinone oxidoredu	protein_codin	-0.1518	9.97E-05	1	60.6	0.5650
MT-CYB	MT-CYB	14	mitochondrially encoded cytochrome b	protein_codin	0.02262	0.442	1	Inf	0.7290
B3GALT6	B3GALT6	15	beta-1,3-galactosyltransferase 6	protein_codin	0.1778	0.397	1	8.329	0.2627
DFFA	DFFA	15	DNA fragmentation factor subunit alpha	protein_codin	-0.07907	0.641	1	7.514	0.09944
MFAP2	MFAP2	15	microfibril associated protein 2	protein_codin	0.6103	7.14E-06	0.1648	60.52	0.03294
EMC1	EMC1	15	ER membrane protein complex subunit 1	protein_codin	0.4614	0.0389	1	2.984	0.315
HP1BP3	HP1BP3	15	heterochromatin protein 1 binding protein 3	protein_codin	0.2164	0.00428	1	54.09	0.1379
PITHD1	PITHD1	15	PITH domain containing 1	protein_codin	-0.1615	0.152	1	13.44	0.09772
HMGCL	HMGCL	15	3-hydroxy-3-methylglutaryl-CoA lyase	protein_codin	-0.1701	0.289	1	7.442	0.1442
DNAJC8	DNAJC8	15	DnaJ heat shock protein family (Hsp40) member C8	protein_codin	-0.1482	0.0292	1	24.33	0.09916
GMEB1	GMEB1	15	glucocorticoid modulatory element binding protein 1	protein_codin	-0.08548	0.699	1	3.484	0.3520
ZCCHC17	ZCCHC17	15	zinc finger CCHC-type containing 17	protein_codin	-0.07443	0.538	1	55.02	0.1000
AGO1	AGO1	15	argonaute RISC component 1	protein_codin	-0.2717	0.298	1	2.972	0.3832
ZC3H12A	ZC3H12A	15	zinc finger CCCH-type containing 12A	protein_codin	1.071	0.00925	1	29.13	0.323
PABPC4	PABPC4	15	poly(A) binding protein cytoplasmic 4	protein_codin	-0.0259	0.867	1	57.58	0.0725
PPCS	PPCS	15	phosphopantothenoylcysteine synthetase	protein_codin	-0.2415	0.00616	1	56.49	0.1399
PPIH	PPIH	15	peptidylprolyl isomerase H	protein_codin	-0.06482	0.688	1	8.537	0.1358
PTPRF	PTPRF	15	protein tyrosine phosphatase receptor type F	protein_codin	0.7137	0.00477	1	9.106	0.4499
TMEM69	TMEM69	15	transmembrane protein 69	protein_codin	0.5054	0.0179	1	2.913	0.2015
HHLA3	HHLA3	15	HERV-H LTR-associating 3	protein_codin	0.8018	0.0106	1	8.282	0.3135
C1orf52	C1orf52	15	chromosome 1 open reading frame 52	protein_codin	0.7112	9.61E-06	0.2214	16.98	0.06758
MTF2	MTF2	15	metal response element binding transcription factor 2	protein_codin	0.2083	0.369	1	8.358	0.1667
CLCC1	CLCC1	15	chloride channel CLIC like 1	protein_codin	0.3752	0.0482	1	8.486	0.1112
NOTCH2	NOTCH2	15	notch receptor 2	protein_codin	0.3824	0.0637	1	21.05	0.3121
C1orf54	C1orf54	15	chromosome 1 open reading frame 54	protein_codin	-0.3916	0.00046	1	39.75	0.236
MLLT11	MLLT11	15	MLLT11 transcription factor 7 cofactor	protein_codin	-0.2812	0.227	1	10.34	0.03563
PRCC	PRCC	15	proline rich mitotic checkpoint control factor	protein_codin	-0.1177	0.506	1	6.634	0.2082
MPZL1	MPZL1	15	myelin protein zero like 1	protein_codin	0.8497	5.12E-10	1.21E-05	19.88	0.03465
FMO2	FMO2	15	flavin containing dimethylaniline monoxygenase 2	protein_codin	-0.3153	0.00365	1	55.3	0.1079
ABL2	ABL2	15	ABL proto-oncogene 2, non-receptor tyrosine kinase	protein_codin	-0.1392	0.494	1	3.235	0.4008
NAV1	NAV1	15	neuron navigator 1	protein_codin	0.5855	0.00607	1	60.59	0.1724
KCTD3	KCTD3	15	potassium channel tetramerization domain containing 3	protein_codin	-0.2899	0.144	1	7.94	0.2831
GPATCH2	GPATCH2	15	G-patch domain containing 2	protein_codin	0.03217	0.841	1	22.12	0.2027
IARS2	IARS2	15	isoleucyl-tRNA synthetase 2, mitochondrial	protein_codin	0.2234	0.0852	1	6.934	0.2087
CDC42BPA	CDC42BPA	15	CDC42 binding protein kinase alpha	protein_codin	-0.02511	0.859	1	16.6	0.1750
JMJD4	JMJD4	15	jumonji domain containing 4	protein_codin	0.3167	0.194	1	3.055	0.3520
HIST3H2A	HIST3H2A	15	histone cluster 3 H2A	protein_codin	1.502	7.71E-07	0.01790	31.93	0.1760
RHOU	RHOU	15	ras homolog family member U	protein_codin	0.2517	0.179	1	8.617	0.2490
ARV1	ARV1	15	ARV1 homolog, fatty acid homeostasis modulator	protein_codin	0.08832	0.606	1	8.13	0.03458
TMEM18	TMEM18	15	transmembrane protein 18	protein_codin	-0.05436	0.604	1	42.65	0.2338
ASAP2	ASAP2	15	ArfGAP with SH3 domain, ankyrin repeat and PH dom	protein_codin	0.3380	0.0136	1	47.98	0.07736
HPCAL1	HPCAL1	15	hippocalcin like 1	protein_codin	-0.3468	0.174	1	9.295	0.2710
TP53I3	TP53I3	15	tumor protein p53 inducible protein 3	protein_codin	1.348	2.58E-11	6.12E-07	17.4	0.1650
SLC4A1AP	SLC4A1AP	15	solute carrier family 4 member 1 adaptor protein	protein_codin	0.09344	0.52	1	7.668	0.1784
LRPPRC	LRPPRC	15	leucine rich pentatricopeptide repeat containing	protein_codin	0.3352	0.0762	1	8.671	0.2185
MCEE	MCEE	15	methylmalonyl-CoA epimerase	protein_codin	0.6943	0.0002	1	24.08	0.06787
RNF149	RNF149	15	ring finger protein 149	protein_codin	0.5903	0.00123	1	21.15	0.1788
WDSUB1	WDSUB1	15	WD repeat, sterile alpha motif and U-box domain con	protein_codin	0.2603	0.352	1	3.296	0.2197
FIGN	FIGN	15	fidgetin, microtubule severing factor	protein_codin	-0.3531	0.106	1	8.421	0.4931
PPIG	PPIG	15	peptidylprolyl isomerase G	protein_codin	0.05107	0.4635	1	60.21	0.1316
TLK1	TLK1	15	tousled like kinase 1	protein_codin	0.1611	0.208	1	13.19	0.1079
WIPF1	WIPF1	15	WAS/WASL interacting protein family member 1	protein_codin	-0.02649	0.874	1	8.163	0.0992
IDH1	IDH1	15	isocitrate dehydrogenase (NADP(+)) 1, cytosolic	protein_codin	0.2717	0.132	1	9.621	0.1717
ACADL	ACADL	15	acyl-CoA dehydrogenase long chain	protein_codin	0.2388	0.355	1	6.882	0.2885
DOCK10	DOCK10	15	dedicator of cytokinesis 10	protein_codin	-1.451	5.55E-09	0.00012	10.17	0.5797
UBE2F	UBE2F	15	ubiquitin conjugating enzyme E2 F (putative)	protein_codin	-0.2557	0.119	1	7.925	0.2025
MTMR14	MTMR14	15	myotubularin related protein 14	protein_codin	0.635	0.00975	1	2.956	0.2435
DPH3	DPH3	15	diphthamide biosynthesis 3	protein_codin	0.3233	0.0324	1	42.47	0.1434
THRB	THRB	15	thyroid hormone receptor beta	protein_codin	-0.4822	0.0211	1	9.068	0.2036
ELP6	ELP6	15	elongator acetyltransferase complex subunit 6	protein_codin	0.5248	0.00125	1	8.142	0.1752
QRICH1	QRICH1	15	glutamine rich 1	protein_codin	0.4228	0.112	1	10.12	0.4734
GNL3	GNL3	15	G protein nucleolar 3	protein_codin	0.5696	0.00022	1	46.79	0.1082
DCBLD2	DCBLD2	15	discoidin, CUB and LCCL domain containing 2	protein_codin	-0.231	0.161	1	17.32	0.1647
IFT57	IFT57	15	intraflagellar transport 57	protein_codin	0.1822	0.0605	1	26.1	0.1340
DZIP3	DZIP3	15	DAZ interacting zinc finger protein 3	protein_codin	0.3876	0.0708	1	8.077	0.3172
ATP6V1A	ATP6V1A	15	ATPase H+ transporting V1 subunit A	protein_codin	0.07141	0.736	1	8.119	0.2744
QTRT2	QTRT2	15	queuine tRNA-ribosyltransferase accessory subunit 2	protein_codin	0.4433	0.0698	1	10.45	0.228
PARP9	PARP9	15	poly(ADP-ribose) polymerase family member 9	protein_codin	0.04266	0.816	1	41.09	0.2419
SLC35G2	SLC35G2	15	solute carrier family 35 member G2	protein_codin	0.9446	0.00208	1	3.735	0.2472
TSC22D2	TSC22D2	15	TSC22 domain family member 2	protein_codin	0.5111	0.0152	1	21.57	0.1192
MBNL1	MBNL1	15	muscleblind like splicing regulator 1	protein_codin	-0.1362	0.0948	1	60.53	0.07052
NCBP2	NCBP2	15	nuclear cap binding protein subunit 2	protein_codin	-0.2279	0.09	1	7.245	0.1359
EVC	EVC	15	EvC ciliary complex subunit 1	protein_codin	0.6766	0.062	1	3.284	0.3059
FAM200B	FAM200B	15	family with sequence similarity 200 member B	protein_codin	-0.11	0.306	1	25.46	0.137
LIAS	LIAS	15	lipoic acid synthetase	protein_codin	-0.09587	0.652	1	8.103	0.1414
APBB2	APBB2	15	amyloid beta precursor protein binding family B mem	protein_codin	-0.2334	0.189	1	8.777	0.2162
UCHL1	UCHL1	15	ubiquitin C-terminal hydrolase L1	protein_codin	1.022	2.6E-08	0.00061	60	0.02747
YTHDC1	YTHDC1	15	YTH domain containing 1	protein_codin	-0.008249	0.944	1	49.26	0.2092
PTPN13	PTPN13	15	protein tyrosine phosphatase non-receptor type 13	protein_codin	0.479	0.0256	1	25.56	0.06768
PYURF	PYURF	15	PIGY upstream reading frame	protein_codin	0.1958	0.00568	1	27.54	0.1026
NAP1L5	NAP1L5	15	nucleosome assembly protein 1 like 5	protein_codin	0.2796	0.225	1	34.12	0.2460
SMARCAD1	SMARCAD1	15	SWI/SNF-related, matrix-associated actin-dependent	protein_codin	0.6694	0.0029	1	26.58	0.2037
SLC9B2	SLC9B2	15	solute carrier family 9 member B2	protein_codin	0.9748	0.00032	1	37.67	0.3139
PLRG1	PLRG1	15	pleiotropic regulator 1	protein_codin	0.2268	0.0949	1	6.511	0.2430
FNIP2	FNIP2	15	folliculin interacting protein 2	protein_codin	-0.1763	0.446	1	23.14	0.1750
SAP30	SAP30	15	Sin3A associated protein 30	protein_codin	0.004466	0.97	1	13.08	0.06532
C5orf42	C5orf42	15	ciliogenesis and planar polarity effector 1	protein_codin	NA	NA	NA	10.03	0.453
AK6	AK6	15	adenylate kinase 6	protein_codin	0.1654	0.172	1	29.89	0.1420
FOXD1	FOXD1	15	forkhead box D1	protein_codin	0.6883	0.00011	1	43.4	0.1893
BHMT2	BHMT2	15	betaine--homocysteine S-methyltransferase 2	protein_codin	0.7908	0.00065	1	9.215	0.09956
CKMT2	CKMT2	15	creatine kinase, mitochondrial 2	protein_codin	-0.6698	0.00027	1	25.08	0.177
TMEM167A	TMEM167A	15	transmembrane protein 167A	protein_codin	-0.1818	0.0454	1	60.58	0.06994
MEF2C	MEF2C	15	myocyte enhancer factor 2C	protein_codin	-1.001	6.41E-34	1.55E-29	60.52	0.03230
NR2F1	NR2F1	15	nuclear receptor subfamily 2 group F member 1	protein_codin	2.321	1.44E-08	0.00034	59.72	0.2250
REEP5	REEP5	15	receptor accessory protein 5	protein_codin	0.1193	0.0481	1	59.12	0.03261
SNX2	SNX2	15	sorting nexin 2	protein_codin	-0.03037	0.737	1	30.22	0.09851
CEP120	CEP120	15	centrosomal protein 120	protein_codin	0.4272	0.219	1	2.752	0.3336
P4HA2	P4HA2	15	prolyl 4-hydroxylase subunit alpha 2	protein_codin	0.1385	0.241	1	33.8	0.04102
MRPL22	MRPL22	15	mitochondrial ribosomal protein L22	protein_codin	-0.03188	0.761	1	12	0.1340
EBF1	EBF1	15	EBF transcription factor 1	protein_codin	0.7602	7.84E-11	1.86E-06	58.23	0.03389
MAT2B	MAT2B	15	methionine adenosyltransferase 2B	protein_codin	0.1176	0.515	1	9.192	0.1397
PRPF4B	PRPF4B	15	pre-mRNA processing factor 4B	protein_codin	0.2677	0.00376	1	31.64	0.1009
RANBP9	RANBP9	15	RAN binding protein 9	protein_codin	-0.3635	0.06	1	6.518	0.2068
E2F3	E2F3	15	E2F transcription factor 3	protein_codin	-1.099	2.82E-06	0.06534	8.6	0.06836
MICA	MICA	15	MHC class I polypeptide-related sequence A	protein_codin	0.4713	0.0652	1	26.45	0.2722
RXRB	RXRB	15	retinoid X receptor beta	protein_codin	-0.01893	0.918	1	7.134	0.2650
NUDT3	NUDT3	15	nudix hydrolase 3	protein_codin	-0.3411	0.139	1	8.476	0.2350
MEA1	MEA1	15	male-enhanced antigen 1	protein_codin	-0.2565	0.0232	1	39.15	0.2437
ENPP4	ENPP4	15	ectonucleotide pyrophosphatase/phosphodiesterase 4	protein_codin	0.0279	0.882	1	9.458	0.2732
CYB5R4	CYB5R4	15	cytochrome b5 reductase 4	protein_codin	0.2706	0.234	1	10.63	0.1997
NDUFAF4	NDUFAF4	15	NADH:ubiquinone oxidoreductase complex assembly	protein_codin	0.1809	0.313	1	22.2	0.1017
OSTM1	OSTM1	15	osteoclastogenesis associated transmembrane protein 1	protein_codin	0.264	0.0579	1	51.98	0.2026
FYN	FYN	15	FYN proto-oncogene, Src family tyrosine kinase	protein_codin	2.121	1.15E-16	2.75E-12	22.16	0.1077
FAM26E	FAM26E	15	calcium homeostasis modulator family member 5	protein_codin	NA	NA	NA	6.47	0.1985
MAN1A1	MAN1A1	15	mannosidase alpha class 1A member 1	protein_codin	0.7156	9.1E-07	0.02118	59.29	0.07186
GJA1	GJA1	15	gap junction protein alpha 1	protein_codin	-0.8393	2.9E-09	6.94E-05	19.19	0.1121
ECHDC1	ECHDC1	15	ethylmalonyl-CoA decarboxylase 1	protein_codin	0.922	2.91E-10	6.88E-06	30.41	0.03452
LAMA2	LAMA2	15	laminin subunit alpha 2	protein_codin	0.9218	8.13E-07	0.01892	58.54	0.2069
MAP3K5	MAP3K5	15	mitogen-activated protein kinase kinase kinase 5	protein_codin	-0.2344	0.204	1	24.68	0.2242
SYNE1	SYNE1	15	spectrin repeat containing nuclear envelope protein 1	protein_codin	0.3888	0.0012	1	45.25	0.03229
SNX9	SNX9	15	sorting nexin 9	protein_codin	0.2709	0.00225	1	30.78	0.03444
MAFK	MAFK	15	MAF bZIP transcription factor K	protein_codin	0.5454	0.0528	1	8.132	0.313
HIBADH	HIBADH	15	3-hydroxyisobutyrate dehydrogenase	protein_codin	0.2646	0.0768	1	18.72	0.03290
KBTBD2	KBTBD2	15	kelch repeat and BTB domain containing 2	protein_codin	0.6626	0.0262	1	30.43	0.3133
UPP1	UPP1	15	uridine phosphorylase 1	protein_codin	-0.8958	5.14E-06	0.1188	22.97	0.1392
RBM48	RBM48	15	RNA binding motif protein 48	protein_codin	0.1852	0.382	1	3.408	0.3479
POP7	POP7	15	POP7 homolog, ribonuclease P/MRP subunit	protein_codin	-0.221	0.0690	1	43.64	0.1724
TMEM168	TMEM168	15	transmembrane protein 168	protein_codin	0.2429	0.313	1	9.464	0.219
LSM8.00	LSM8.00	15	LSM8 homolog, U6 small nuclear RNA associated	protein_codin	-0.0982	0.347	1	22.17	0.06988
RBM33	RBM33	15	RNA binding motif protein 33	protein_codin	0.4165	0.0715	1	7.468	0.1537
NOM1	NOM1	15	nucleolar protein with MIF4G domain 1	protein_codin	0.4014	0.132	1	35.38	0.07026
PRPS2	PRPS2	15	phosphoribosyl pyrophosphate synthetase 2	protein_codin	-0.1747	0.414	1	9.62	0.1405
USP11	USP11	15	ubiquitin specific peptidase 11	protein_codin	-0.03806	0.792	1	8.296	0.1770
ALG13	ALG13	15	ALG13 UDP-N-acetylglucosaminyltransferase subuni	protein_codin	0.04149	0.776	1	53.26	0.2039
6-Sep	43714	15	septin 6	protein_codin	0.5058	0.0186	1	6.838	0.2691
ATP11C	ATP11C	15	ATPase phospholipid transporting 11C	protein_codin	-0.1752	0.497	1	7.898	0.3658
MICU3	MICU3	15	mitochondrial calcium uptake family member 3	protein_codin	0.2319	0.149	1	21.4	0.4198
UBXN8	UBXN8	15	UBX domain protein 8	protein_codin	0.398	0.119	1	8.48	0.1032
ASH2L	ASH2L	15	ASH2 like, histone lysine methyltransferase complex	protein_codin	-0.01555	0.959	1	10.08	0.2362
CSPP1	CSPP1	15	centrosome and spindle pole associated protein 1	protein_codin	-0.1906	0.302	1	22.75	0.2050
CRISPLD1	CRISPLD1	15	cysteine rich secretory protein LCCL domain containi	protein_codin	-0.7783	2.48E-09	5.85E-05	60.6	0.1095
MRPS28	MRPS28	15	mitochondrial ribosomal protein S28	protein_codin	-0.0569	0.745	1	2.9	0.00737
STK3	STK3	15	serine/threonine kinase 3	protein_codin	1.044	7.05E-09	0.00017	18.6	0.1724
ANKRD46	ANKRD46	15	ankyrin repeat domain 46	protein_codin	-0.02232	0.921	1	3.423	0.1329
ST3GAL1	ST3GAL1	15	ST3 beta-galactoside alpha-2,3-sialyltransferase 1	protein_codin	0.6136	0.0148	1	9.292	0.3295
APTX	APTX	15	aprataxin	protein_codin	-0.1276	0.532	1	8.890	0.1142
UBAP1	UBAP1	15	ubiquitin associated protein 1	protein_codin	0.3119	0.149	1	3.213	0.2770
ALDH1B1	ALDH1B1	15	aldehyde dehydrogenase 1 family member B1	protein_codin	-1.498	1.09E-19	2.62E-15	59.03	0.2062
SPIN1	SPIN1	15	spindlin 1	protein_codin	0.03529	0.789	1	17.82	0.3050
MRPL50	MRPL50	15	mitochondrial ribosomal protein L50	protein_codin	0.2179	0.15	1	7.865	0.126
FBXW2	FBXW2	15	F-box and WD repeat domain containing 2	protein_codin	-0.4415	0.0126	1	8.590	0.126
PSMB7	PSMB7	15	proteasome subunit beta 7	protein_codin	-0.0495	0.608	1	19.45	0.128
PTRH1	PTRH1	15	peptidyl-tRNA hydrolase 1 homolog	protein_codin	0.4260	0.0442	1	30.82	0.196
TRUB2	TRUB2	15	TruB pseudouridine synthase family member 2	protein_codin	0.08304	0.66	1	9.268	0.07260
TSPAN4	TSPAN4	15	tetraspanin 4	protein_codin	0.9667	1.42E-22	3.42E-18	54.67	0.03240
OSBPL5	OSBPL5	15	oxysterol binding protein like 5	protein_codin	-0.1224	0.616	1	8.868	0.2386
TRIM22	TRIM22	15	tripartite motif containing 22	protein_codin	0.1246	0.402	1	49.94	0.2092
AKIP1	AKIP1	15	A-kinase interacting protein 1	protein_codin	0.09058	0.559	1	8.147	0.2021
CRY2	CRY2	15	cryptochrome circadian regulator 2	protein_codin	0.469	0.0316	1	3.292	0.2952
SLC43A3	SLC43A3	15	solute carrier family 43 member 3	protein_codin	1.411	2.1E-09	4.95E-05	20.7	0.1882
SELENOH	SELENOH	15	selenoprotein H	protein_codin	-0.1473	0.082	1	38.38	0.03190
WDR74	WDR74	15	WD repeat domain 74	protein_codin	0.0892	0.614	1	6.148	0.1032
SF1	SF1	15	splicing factor 1	protein_codin	0.4789	8.99E-07	0.02092	50.22	0.2697
BRMS1	BRMS1	15	BRMS1 transcriptional repressor and anoikis regulato	protein_codin	-0.1393	0.424	1	3.234	0.1662
RAB30-AS1	RAB30-AS1	15	RAB30 divergent transcript	lincRNA	-0.0673	0.711	1	3.417	0.1771
AP001528.2	AP001528.2	15	novel transcri	antisense_RN	0.7606	2.34E-06	0.05420	13.8	0.1413
KDELC2	KDELC2	15	protein O-glucosyltransferase 3	protein_codin	0.598	1.68E-05	0.3863	15.02	0.179
TIMM8B	TIMM8B	15	translocase of inner mitochondrial membrane 8 homo	protein_codin	-0.4	0.00142	1	45.25	0.1004
ESAM	ESAM	15	endothelial cell adhesion molecule	protein_codin	-0.2697	0.0327	1	16.42	0.03249
ASB13	ASB13	15	ankyrin repeat and SOCS box containing 13	protein_codin	0.7619	0.106	1	2.876	0.478
STAM	STAM	15	signal transducing adaptor molecule	protein_codin	0.3473	0.0468	1	24.4	0.2385
VSTM4	VSTM4	15	V-set and transmembrane domain containing 4	protein_codin	0.009242	0.962	1	8.829	0.2106
CCDC6	CCDC6	15	coiled-coil domain containing 6	protein_codin	-0.1619	0.187	1	28.6	0.1331
ANK3	ANK3	15	ankyrin 3	protein_codin	-1.153	2.12E-07	0.00496	59.93	0.6462
SORBS1	SORBS1	15	sorbin and SH3 domain containing 1	protein_codin	-0.9327	7.94E-12	1.89E-07	35.2	0.03806
SLF2	SLF2	15	SMC5-SMC6 complex localization factor 2	protein_codin	0.1162	0.607	1	23.71	0.4848
SMNDC1	SMNDC1	15	survival motor neuron domain containing 1	protein_codin	-0.09736	0.41	1	13.63	0.1032
FAM45A	FAM45A	15	DENN domain containing 10	protein_codin	0.5506	0.00753	1	50.21	0.1122
TIAL1	TIAL1	15	TIA1 cytotoxic granule associated RNA binding protei	protein_codin	0.3874	0.00276	1	27.47	0.07003
WDR11	WDR11	15	WD repeat domain 11	protein_codin	-0.01588	0.944	1	3.557	0.26
MAGOHB	MAGOHB	15	mago homolog B, exon junction complex subunit	protein_codin	-0.1346	0.384	1	50.92	0.1382
WBP11	WBP11	15	WW domain binding protein 11	protein_codin	0.07779	0.562	1	14.68	0.1754
DERA	DERA	15	deoxyribose-phosphate aldolase	protein_codin	0.5511	0.00197	1	7.94	0.1371
ETNK1	ETNK1	15	ethanolamine kinase 1	protein_codin	0.0179	0.932	1	8.024	0.2862
TM7SF3	TM7SF3	15	transmembrane 7 superfamily member 3	protein_codin	0.2512	0.0982	1	20.03	0.06598
RPAP3	RPAP3	15	RNA polymerase II associated protein 3	protein_codin	0.4668	0.0196	1	9.189	0.1434
HDAC7	HDAC7	15	histone deacetylase 7	protein_codin	0.8388	7.16E-05	1	27.44	0.1021
NCKAP5L	NCKAP5L	15	NCK associated protein 5 like	protein_codin	0.5211	0.0369	1	10.96	0.3181
TNS2	TNS2	15	tensin 2	protein_codin	0.716	2.6E-06	0.06027	14.2	0.1352
SPRYD3	SPRYD3	15	SPRY domain containing 3	protein_codin	0.001804	0.99	1	18.03	0.1390
CSAD	CSAD	15	cysteine sulfinic acid decarboxylase	protein_codin	0.02932	0.879	1	19.52	0.3187
RAB5B	RAB5B	15	RAB5B, member RAS oncogene family	protein_codin	0.05757	0.738	1	7.238	0.2758
XPOT	XPOT	15	exportin for tRNA	protein_codin	0.3416	0.237	1	10.63	0.2169
MSRB3	MSRB3	15	methionine sulfoxide reductase B3	protein_codin	-0.654	8.29E-19	1.99E-14	34.9	0.03242
METAP2	METAP2	15	methionyl aminopeptidase 2	protein_codin	-0.09986	0.304	1	15.14	0.1488
CHPT1	CHPT1	15	choline phosphotransferase 1	protein_codin	1.08	9.46E-21	2.27E-16	36.27	0.03482
PWP1	PWP1	15	PWP1 homolog, endonuclein	protein_codin	-0.006819	0.951	1	33.25	0.2351
ATXN2	ATXN2	15	ataxin 2	protein_codin	0.3516	0.0175	1	13.88	0.1820
RNF10	RNF10	15	ring finger protein 10	protein_codin	0.2025	0.302	1	19.58	0.2357
CDK2AP1	CDK2AP1	15	cyclin dependent kinase 2 associated protein 1	protein_codin	0.002105	0.991	1	2.61	0.2489
GOLGA3	GOLGA3	15	golgin A3	protein_codin	0.4958	0.00320	1	17.26	0.2460
EEF1AKMT1	EEF1AKMT1	15	EEF1A lysine methyltransferase 1	protein_codin	0.1209	0.474	1	6.937	0.103
ELF1	ELF1	15	E74 like ETS transcription factor 1	protein_codin	0.3585	0.0105	1	28.94	0.1071
LPAR6	LPAR6	15	lysophosphatidic acid receptor 6	protein_codin	0.02613	0.889	1	20.03	0.1721
DLEU2	DLEU2	15	deleted in lymphocytic leukemia 2	antisense_RN	-0.5433	0.0619	1	3.157	0.4122
RNF219	RNF219	15	ORC ubiquitin ligase 1	protein_codin	0.121	0.517	1	3.657	0.2502
DOCK9	DOCK9	15	dedicator of cytokinesis 9	protein_codin	0.9658	0.00038	1	8.135	0.54
UPF3A	UPF3A	15	UPF3A regulator of nonsense mediated mRNA decay	protein_codin	0.04594	0.662	1	57.79	0.1085
STRN3	STRN3	15	striatin 3	protein_codin	0.4252	0.0032	1	14.32	0.06907
ARHGAP5	ARHGAP5	15	Rho GTPase activating protein 5	protein_codin	0.1234	0.365	1	55.13	0.1369
MIPOL1	MIPOL1	15	mirror-image polydactyly 1	protein_codin	0.3673	0.1	1	7.396	0.3744
SOS2	SOS2	15	SOS Ras/Rho guanine nucleotide exchange factor 2	protein_codin	0.2692	0.191	1	5.782	0.3748
BMP4	BMP4	15	bone morphogenetic protein 4	protein_codin	1.254	3.07E-08	0.00072	55.18	0.06682
GMFB	GMFB	15	glia maturation factor beta	protein_codin	0.8752	0.00016	1	8.972	0.2026
SNAPC1	SNAPC1	15	small nuclear RNA activating complex polypeptide 1	protein_codin	0.1785	0.242	1	16.07	0.292
PPP2R5E	PPP2R5E	15	protein phosphatase 2 regulatory subunit B'epsilon	protein_codin	0.04675	0.732	1	11.96	0.2368
YLPM1	YLPM1	15	YLP motif containing 1	protein_codin	0.1809	0.518	1	8.268	0.3802
GON7	GON7	15	GON7 subunit of KEOPS complex	protein_codin	-0.04852	0.807	1	7.555	0.1654
ZFYVE21	ZFYVE21	15	zinc finger FYVE-type containing 21	protein_codin	0.1888	0.0321	1	43.03	0.103
GOLGA8B	GOLGA8B	15	golgin A8 family member B	protein_codin	-0.3166	0.214	1	10.36	0.8857
RAB8B	RAB8B	15	RAB8B, member RAS oncogene family	protein_codin	0.9226	5.79E-10	1.37E-05	24.13	0.06650
NEO1	NEO1	15	neogenin 1	protein_codin	0.189	0.282	1	21.16	0.1750
SIN3A	SIN3A	15	SIN3 transcription regulator family member A	protein_codin	0.4332	0.132	1	8.977	0.4596
CIB1	CIB1	15	calcium and integrin binding 1	protein_codin	-0.2063	0.00563	1	59.93	0.03284
NGRN	NGRN	15	neugrin, neurite outgrowth associated	protein_codin	0.2257	0.314	1	8.857	0.132
IQGAP1	IQGAP1	15	IQ motif containing GTPase activating protein 1	protein_codin	0.103	0.202	1	14.47	0.102
TTC23	TTC23	15	tetratricopeptide repeat domain 23	protein_codin	-0.06169	0.789	1	2.622	0.1391
NPRL3	NPRL3	15	NPR3 like, GATOR1 complex subunit	protein_codin	0.4476	0.0616	1	9.006	0.2152
TMEM8A	TMEM8A	15	transmembrane protein 8A	protein_codin	-0.3523	0.0867	1	8.537	0.2450
MCRIP2	MCRIP2	15	MAPK regulated corepressor interacting protein 2	protein_codin	-0.3718	0.0907	1	7.622	0.1004
AC009041.2	AC009041.2	15	novel transcri	lincRNA	-0.006114	0.982	1	9.777	0.352
ARL6IP1	ARL6IP1	15	ADP ribosylation factor like GTPase 6 interacting prot	protein_codin	-0.02412	0.852	1	16.75	0.105
UBFD1	UBFD1	15	ubiquitin family domain containing 1	protein_codin	0.4147	0.115	1	26.02	0.1011
IL4R	IL4R	15	interleukin 4 receptor	protein_codin	0.3116	0.194	1	56.22	0.2432
SH2B1	SH2B1	15	SH2B adaptor protein 1	protein_codin	-0.2698	0.17	1	10.57	0.07126
BCL7C	BCL7C	15	BAF chromatin remodeling complex subunit BCL7C	protein_codin	-0.3874	1.54E-06	0.03577	51.38	0.03600
PAPD5	PAPD5	15	terminal nucleotidyltransferase 4B	protein_codin	NA	NA	NA	9.312	0.242
HERPUD1	HERPUD1	15	homocysteine inducible ER protein with ubiquitin like	protein_codin	0.4825	5.92E-10	1.4E-05	60.53	0.06490
B3GNT9	B3GNT9	15	UDP-GlcNAc:betaGal beta-1,3-N-acetylglucosaminylt	protein_codin	0.5554	0.0189	1	3.446	0.09821
TIMM22	TIMM22	15	translocase of inner mitochondrial membrane 22	protein_codin	-0.03284	0.86	1	8.132	0.1679
NCBP3	NCBP3	15	nuclear cap binding subunit 3	protein_codin	0.1314	0.352	1	7.686	0.1026
RNF167	RNF167	15	ring finger protein 167	protein_codin	0.08444	0.529	1	6.713	0.1082
ACADVL	ACADVL	15	acyl-CoA dehydrogenase very long chain	protein_codin	0.252	0.0292	1	60.58	0.06489
EIF4A1	EIF4A1	15	eukaryotic translation initiation factor 4A1	protein_codin	0.002061	0.985	1	50.52	0.06545
MPDU1	MPDU1	15	mannose-P-dolichol utilization defect 1	protein_codin	0.2755	0.254	1	2.993	0.3145
NDEL1	NDEL1	15	nudE neurodevelopment protein 1 like 1	protein_codin	-0.1326	0.502	1	26.5	0.1234
LINC02001	LINC02001	15	small nucleolar RNA host gene 30	lincRNA	-0.2302	0.17	1	6.774	0.2039
LASP1	LASP1	15	LIM and SH3 protein 1	protein_codin	0.2503	0.114	1	18.9	0.1434
THRA	THRA	15	thyroid hormone receptor alpha	protein_codin	-0.03197	0.775	1	22.03	0.2381
DHX40	DHX40	15	DEAH-box helicase 40	protein_codin	-0.007355	0.964	1	6.572	0.1747
AMZ2	AMZ2	15	archaelysin family metallopeptidase 2	protein_codin	0.2055	0.239	1	8.354	0.1052
FAM104A	FAM104A	15	family with sequence similarity 104 member A	protein_codin	-0.07668	0.685	1	6.548	0.3994
MFSD11	MFSD11	15	major facilitator superfamily domain containing 11	protein_codin	0.09725	0.632	1	8.362	0.3152
DCXR	DCXR	15	dicarbonyl and L-xylulose reductase	protein_codin	-0.1037	0.282	1	39.54	0.06445
C17orf62	C17orf62	15	cytochrome b-245 chaperone 1	protein_codin	NA	NA	NA	24.4	0.09727
KIAA1328	KIAA1328	15	KIAA1328	protein_codin	0.2572	0.259	1	10.63	0.07100
NARS	NARS	15	asparaginyl-tRNA synthetase	protein_codin	-0.04699	0.642	1	24.06	0.1341
TRMT6	TRMT6	15	tRNA methyltransferase 6	protein_codin	0.07467	0.728	1	3.632	0.1760
RRBP1	RRBP1	15	ribosome binding protein 1	protein_codin	0.5890	5.62E-11	1.33E-06	50.18	0.03492
SEC23B	SEC23B	15	SEC23 homolog B, coat complex II component	protein_codin	0.03587	0.887	1	9.048	0.1048
EDEM2	EDEM2	15	ER degradation enhancing alpha-mannosidase like p	protein_codin	-0.1256	0.637	1	50.96	0.1005
TP53RK	TP53RK	15	TP53 regulating kinase	protein_codin	-0.1587	0.361	1	51.79	0.2391
PRELID3B	PRELID3B	15	PRELI domain containing 3B	protein_codin	0.1102	0.56	1	6.727	0.1331
ARFRP1	ARFRP1	15	ADP ribosylation factor related protein 1	protein_codin	-0.1148	0.477	1	6.586	0.1694
TPGS1	TPGS1	15	tubulin polyglutamylase complex subunit 1	protein_codin	-0.4762	3.66E-06	0.08469	30.02	0.1009
TIMM13	TIMM13	15	translocase of inner mitochondrial membrane 13	protein_codin	-0.1668	0.0922	1	58.02	0.1011
CCDC130	CCDC130	15	coiled-coil domain containing 130	protein_codin	-0.01898	0.917	1	24.15	0.1372
TMEM161A	TMEM161A	15	transmembrane protein 161A	protein_codin	0.2871	0.219	1	2.729	0.2104
HNRNPUL1	HNRNPUL1	15	heterogeneous nuclear ribonucleoprotein U like 1	protein_codin	-0.2341	0.0772	1	7.158	0.06881
DEDD2	DEDD2	15	death effector domain containing 2	protein_codin	-0.3324	0.129	1	2.932	0.2441
RTN2	RTN2	15	reticulon 2	protein_codin	0.3342	0.152	1	3.614	0.1696
PPP5C	PPP5C	15	protein phosphatase 5 catalytic subunit	protein_codin	0.2626	0.224	1	3.032	0.2470
ALDH16A1	ALDH16A1	15	aldehyde dehydrogenase 16 family member A1	protein_codin	0.05247	0.805	1	8.398	0.2097
PNKP	PNKP	15	polynucleotide kinase 3'-phosphatase	protein_codin	0.547	0.082	1	7.082	0.2590
PPP2R1A	PPP2R1A	15	protein phosphatase 2 scaffold subunit Aalpha	protein_codin	0.01282	0.911	1	24.72	0.1042
PRPF31	PRPF31	15	pre-mRNA processing factor 31	protein_codin	-0.4316	0.00012	1	41.26	0.2415
EPN1	EPN1	15	epsin 1	protein_codin	0.003592	0.985	1	45.9	0.09801
ZNF583	ZNF583	15	zinc finger protein 583	protein_codin	-0.02135	0.924	1	3.042	0.2061
TTTY15	TTTY15	15	NA	lincRNA	-0.2782	0.265	1	7.536	0.4390
SCARF2	SCARF2	15	scavenger receptor class F member 2	protein_codin	0.1076	0.645	1	8.36	0.2469
UQCR10	UQCR10	15	ubiquinol-cytochrome c reductase, complex III subunit X	protein_codin	-0.1642	0.0129	1	15.3	0.07144
IFT27	IFT27	15	intraflagellar transport 27	protein_codin	0.4682	0.00566	1	7.256	0.1034
H1F0	H1F0	15	H1 histone family member 0	protein_codin	0.495	0.00019	1	57.75	0.1304
EIF3L	EIF3L	15	eukaryotic translation initiation factor 3 subunit L	protein_codin	-0.3664	5.19E-07	0.0121	18.48	0.0329
POLR2F	POLR2F	15	RNA polymerase II subunit F	protein_codin	-0.2603	0.00179	1	28.54	0.03651
TOB2	TOB2	15	transducer of ERBB2, 2	protein_codin	0.2854	0.109	1	7.547	0.236
PARVB	PARVB	15	parvin beta	protein_codin	1.167	0.00046	1	3.596	0.1035
PAXBP1	PAXBP1	15	PAX3 and PAX7 binding protein 1	protein_codin	0.3128	0.111	1	19.27	0.287
SPATC1L	SPATC1L	15	spermatogenesis and centriole associated 1 like	protein_codin	-0.2503	0.232	1	7.998	0.1682
SNRNP40	SNRNP40	16	small nuclear ribonucleoprotein U5 subunit 40	protein_codin	0.004771	0.971	1	6.842	0.1021
ZNF362	ZNF362	16	zinc finger protein 362	protein_codin	-0.2944	0.107	1	6.798	0.246
B4GALT2	B4GALT2	16	beta-1,4-galactosyltransferase 2	protein_codin	-0.01227	0.939	1	22.87	0.1764
EPS15	EPS15	16	epidermal growth factor receptor pathway substrate 15	protein_codin	0.2682	0.046	1	15.68	0.1339
TM2D1	TM2D1	16	TM2 domain containing 1	protein_codin	0.04362	0.62	1	17.28	0.09982
MIER1	MIER1	16	MIER1 transcriptional regulator	protein_codin	0.009964	0.903	1	31.13	0.2157
PRKACB	PRKACB	16	protein kinase cAMP-activated catalytic subunit beta	protein_codin	-0.4834	0.0188	1	9.303	0.1379
GPSM2	GPSM2	16	G protein signaling modulator 2	protein_codin	1.226	2.8E-06	0.06488	27.65	0.06790
PSMD4	PSMD4	16	proteasome 26S subunit, non-ATPase 4	protein_codin	-0.09178	0.241	1	20.96	0.1003
ATP8B2	ATP8B2	16	ATPase phospholipid transporting 8B2	protein_codin	0.4753	0.057	1	7.548	0.4679
SHC1	SHC1	16	SHC adaptor protein 1	protein_codin	-0.04822	0.825	1	54.11	0.06579
THBS3	THBS3	16	thrombospondin 3	protein_codin	0.6398	0.00166	1	22.41	0.1025
DUSP23	DUSP23	16	dual specificity phosphatase 23	protein_codin	-0.7503	1.57E-07	0.00367	17.95	0.03359
NCSTN	NCSTN	16	nicastrin	protein_codin	0.7115	0.00016	1	22.67	0.1694
FCGR2A	FCGR2A	16	Fc fragment of IgG receptor IIa	protein_codin	-0.1929	0.31	1	9.785	0.2871
PBX1	PBX1	16	PBX homeobox 1	protein_codin	0.4486	0.00112	1	12.8	0.06841
MRPS14	MRPS14	16	mitochondrial ribosomal protein S14	protein_codin	0.07220	0.581	1	15.72	0.03364
TRMT1L	TRMT1L	16	tRNA methyltransferase 1 like	protein_codin	0.09375	0.66	1	8.393	0.2049
DDX59	DDX59	16	DEAD-box helicase 59	protein_codin	0.1182	0.524	1	8.83	0.2246
KDM5B	KDM5B	16	lysine demethylase 5B	protein_codin	0.482	0.00463	1	20.82	0.03341
PQLC3	PQLC3	16	solute carrier family 66 member 3	protein_codin	0.5188	1.2E-06	0.02789	39.29	0.09926
MAPRE3	MAPRE3	16	microtubule associated protein RP/EB family member 3	protein_codin	-0.04205	0.851	1	3.356	0.1318
FEZ2	FEZ2	16	fasciculation and elongation protein zeta 2	protein_codin	-0.2719	0.0146	1	59.7	0.06783
SOCS5	SOCS5	16	suppressor of cytokine signaling 5	protein_codin	0.3323	0.111	1	24.24	0.3006
ETAA1	ETAA1	16	ETAA1 activator of ATR kinase	protein_codin	-0.1079	0.589	1	8.489	0.09964

AAK1	AAK1	16	AP2 associated kinase 1	protein_codin	-0.2448	0.146	1	6.942	0.1342
ANKRD36	ANKRD36	16	ankyrin repeat domain 36	protein_codin	0.1457	0.541	1	9.762	0.3447
CNOT11	CNOT11	16	CCR4-NOT transcription complex subunit 11	protein_codin	0.2972	0.197	1	2.785	0.221
GCC2	GCC2	16	GRIP and coiled-coil domain containing 2	protein_codin	0.1979	0.0506	1	37.42	0.1022
RBM43	RBM43	16	RNA binding motif protein 43	protein_codin	0.1896	0.161	1	51.38	0.1342
CD302	CD302	16	CD302 molecule	protein_codin	0.1056	0.275	1	59.32	0.06922
METTL5	METTL5	16	methyltransferase like 5	protein_codin	-0.3708	2.42E-05	0.5553	50.67	0.1695
CIR1	CIR1	16	corepressor interacting with RBPJ, 1	protein_codin	-0.07228	0.392	1	18.14	0.2025
HIBCH	HIBCH	16	3-hydroxyisobutyryl-CoA hydrolase	protein_codin	-0.08124	0.585	1	16.88	0.1988
NIF3L1	NIF3L1	16	NGG1 interacting factor 3 like 1	protein_codin	0.4527	0.0276	1	32.01	0.07247
SUMO1	SUMO1	16	small ubiquitin like modifier 1	protein_codin	-0.01738	0.798	1	40.14	0.0321
ABI2	ABI2	16	abl interactor 2	protein_codin	-0.1475	0.25	1	18	0.1762
MAP2	MAP2	16	microtubule associated protein 2	protein_codin	-0.2276	0.407	1	55.56	0.434
CNOT9	CNOT9	16	CCR4-NOT transcription complex subunit 9	protein_codin	0.08091	0.666	1	9.075	0.2117
CRBN	CRBN	16	cereblon	protein_codin	-0.07687	0.426	1	21.87	0.09712
VHL	VHL	16	von Hippel-Lindau tumor suppressor	protein_codin	0.4382	0.0586	1	3.39	0.2094
RAB5A	RAB5A	16	RAB5A, member RAS oncogene family	protein_codin	0.1419	0.329	1	35.14	0.1017
SLC4A7	SLC4A7	16	solute carrier family 4 member 7	protein_codin	0.3229	0.0561	1	34.34	0.07542
CMTM6	CMTM6	16	CKLF like MARVEL transmembrane domain containin	protein_codin	0.3252	0.0102	1	52.6	0.06604
KLHDC8B	KLHDC8B	16	kelch domain containing 8B	protein_codin	0.4254	0.024	1	24.57	0.07508
TCTA	TCTA	16	T cell leukemia translocation altered	protein_codin	-0.2711	0.0996	1	8.862	0.2702
APEH	APEH	16	acylaminoacyl-peptide hydrolase	protein_codin	-0.008327	0.965	1	6.752	0.2442
TUSC2	TUSC2	16	tumor suppressor 2, mitochondrial calcium regulator	protein_codin	-0.1219	0.385	1	7.094	0.2509
DCP1A	DCP1A	16	decapping mRNA 1A	protein_codin	0.2872	0.124	1	8.984	0.1712
VGLL3	VGLL3	16	vestigial like family member 3	protein_codin	0.9157	0.00022	1	38.77	0.2382
NSUN3	NSUN3	16	NOP2/Sun RNA methyltransferase 3	protein_codin	-0.06597	0.767	1	3.254	0.3195
NECTIN3	NECTIN3	16	nectin cell adhesion molecule 3	protein_codin	0.3807	0.0262	1	8.135	0.2297
USF3	USF3	16	upstream transcription factor family member 3	protein_codin	0.2052	0.345	1	23.22	0.3732
KLF15	KLF15	16	Kruppel like factor 15	protein_codin	-0.3164	0.229	1	8.048	0.3117
SEC62	SEC62	16	SEC62 homolog, preprotein translocation factor	protein_codin	0.2824	3.66E-08	0.00086	34.68	0.02324
MFN1	MFN1	16	mitofusin 1	protein_codin	0.1892	0.259	1	23.45	0.1680
KLHL24	KLHL24	16	kelch like family member 24	protein_codin	0.1697	0.385	1	7.675	0.2062
ATP13A3	ATP13A3	16	ATPase 13A3	protein_codin	-0.3371	0.0876	1	36.72	0.1345
ATP5I	ATP5I	16	ATP synthase membrane subunit e	protein_codin	NA	NA	NA	34.77	0.106
KIAA0232	KIAA0232	16	KIAA0232	protein_codin	-0.1696	0.174	1	45.01	0.2787
UBA6	UBA6	16	ubiquitin like modifier activating enzyme 6	protein_codin	0.4618	0.0359	1	29.22	0.4212
ANTXR2	ANTXR2	16	ANTXR cell adhesion molecule 2	protein_codin	-0.3189	0.0536	1	17.36	0.2096
LARP1B	LARP1B	16	La ribonucleoprotein domain family member 1B	protein_codin	-0.1001	0.652	1	25.29	0.2467
SETD7	SETD7	16	SET domain containing 7, histone lysine methyltransf	protein_codin	0.26	0.145	1	22.16	0.2112
HMGB2	HMGB2	16	high mobility group box 2	protein_codin	0.145	0.229	1	42.84	0.125
CASP3	CASP3	16	caspase 3	protein_codin	0.5298	0.0117	1	10.28	0.2692
CCT5	CCT5	16	chaperonin containing TCP1 subunit 5	protein_codin	-0.0215	0.796	1	42.82	0.03434
6-Mar	43530	16	membrane associated ring-CH-type finger 6	protein_codin	-0.08563	0.539	1	37.84	0.2452
C5orf51	C5orf51	16	chromosome 5 open reading frame 51	protein_codin	-0.09882	0.581	1	6.666	0.1342
TMEM267	TMEM267	16	transmembrane protein 267	protein_codin	0.4182	0.0576	1	7.602	0.1684
SREK1	SREK1	16	splicing regulatory glutamic acid and lysine rich prote	protein_codin	0.1688	0.285	1	27.44	0.2764
HSD17B4	HSD17B4	16	hydroxysteroid 17-beta dehydrogenase 4	protein_codin	0.1046	0.455	1	20.68	0.0742
YIPF5	YIPF5	16	Yip1 domain family member 5	protein_codin	0.07729	0.462	1	25	0.1089
TCERG1	TCERG1	16	transcription elongation regulator 1	protein_codin	0.1388	0.29	1	17.75	0.1021
PPP2R2B	PPP2R2B	16	protein phosphatase 2 regulatory subunit Bbeta	protein_codin	-0.796	9.94E-06	0.229	27.58	0.1750
DCTN4	DCTN4	16	dynactin subunit 4	protein_codin	-0.08759	0.642	1	8.916	0.1766
KCNMB1	KCNMB1	16	potassium calcium-activated channel subfamily M reg	protein_codin	-0.7511	1.04E-12	2.47E-08	21.45	0.03291
DDX41	DDX41	16	DEAD-box helicase 41	protein_codin	-0.4269	0.0908	1	3.09	0.2918
ZNF354A	ZNF354A	16	zinc finger protein 354A	protein_codin	-0.03227	0.884	1	7.943	0.1844
LYRM4	LYRM4	16	LYR motif containing 4	protein_codin	-0.05798	0.795	1	7.885	0.07012
PAK1IP1	PAK1IP1	16	PAK1 interacting protein 1	protein_codin	0.1847	0.187	1	39.28	0.03409
HCG11	HCG11	16	HLA complex group 11	lincRNA	0.6832	0.00022	1	20.01	0.09896
ICK	ICK	16	intestinal cell kinase	protein_codin	-0.3954	0.00354	1	17.65	0.1059
PHF3	PHF3	16	PHD finger protein 3	protein_codin	0.1845	0.0797	1	29.19	0.105
ZBTB24	ZBTB24	16	zinc finger and BTB domain containing 24	protein_codin	0.1392	0.501	1	7.232	0.3362
HDAC2	HDAC2	16	histone deacetylase 2	protein_codin	0.05364	0.677	1	13.67	0.1371
HDDC2	HDDC2	16	HD domain containing 2	protein_codin	-0.4037	2.41E-05	0.553	17.06	0.07154
PERP	PERP	16	p53 apoptosis effector related to PMP22	protein_codin	0.436	0.00168	1	35.26	0.03271
C6orf120	C6orf120	16	chromosome 6 open reading frame 120	protein_codin	0.5586	0.00165	1	28.49	0.0721
PHF10	PHF10	16	PHD finger protein 10	protein_codin	-0.2638	0.0831	1	6.252	0.1044
FKBP14	FKBP14	16	FKBP prolyl isomerase 14	protein_codin	0.4552	0.00373	1	22	0.104
BAZ1B	BAZ1B	16	bromodomain adjacent to zinc finger domain 1B	protein_codin	-0.1104	0.422	1	29.16	0.3545
AKAP9	AKAP9	16	A-kinase anchoring protein 9	protein_codin	-0.09488	0.351	1	24.45	0.1750
ZKSCAN1	ZKSCAN1	16	zinc finger with KRAB and SCAN domains 1	protein_codin	0.2763	0.0774	1	14.68	0.1800
LAMTOR4	LAMTOR4	16	late endosomal/lysosomal adaptor, MAPK and MTOR	protein_codin	-0.1135	0.0688	1	38.2	0.06534
ZNHIT1	ZNHIT1	16	zinc finger HIT-type containing 1	protein_codin	-0.1303	0.0805	1	46.55	0.03288
ORAI2	ORAI2	16	ORAI calcium release-activated calcium modulator 2	protein_codin	0.2424	0.31	1	32.76	0.07702
DLD	DLD	16	dihydrolipoamide dehydrogenase	protein_codin	0.09579	0.547	1	7.717	0.1015
FOXP2	FOXP2	16	forkhead box P2	protein_codin	1.376	0.00026	1	8.624	0.3236
ZNF800	ZNF800	16	zinc finger protein 800	protein_codin	0.6837	0.00016	1	24.05	0.2031
REPIN1	REPIN1	16	replication initiator 1	protein_codin	-0.1468	0.429	1	8.492	0.2421
RGN	RGN	16	regucalcin	protein_codin	-0.4417	0.0022	1	40.46	0.1996
TFE3	TFE3	16	transcription factor binding to IGHM enhancer 3	protein_codin	-0.1132	0.62	1	3.281	0.2106
RRAGB	RRAGB	16	Ras related GTP binding B	protein_codin	0.2595	0.235	1	9.396	0.4125
NKAP	NKAP	16	NFKB activating protein	protein_codin	0.2118	0.0714	1	12.1	0.1
FAM58A	FAM58A	16	cyclin Q	protein_codin	NA	NA	NA	23.97	0.1074
MTMR9	MTMR9	16	myotubularin related protein 9	protein_codin	0.5362	0.0315	1	3.277	0.3588
KAT6A	KAT6A	16	lysine acetyltransferase 6A	protein_codin	-0.05472	0.76	1	26.64	0.3061
ZBTB10	ZBTB10	16	zinc finger and BTB domain containing 10	protein_codin	-0.239	0.101	1	8.465	0.3106
PABPC1	PABPC1	16	poly(A) binding protein cytoplasmic 1	protein_codin	-0.03852	0.569	1	50.47	0.03336
TTC39B	TTC39B	16	tetratricopeptide repeat domain 39B	protein_codin	-0.4873	0.0096	1	7.695	0.2451
PSIP1	PSIP1	16	PC4 and SFRS1 interacting protein 1	protein_codin	0.02472	0.6882	1	56.62	0.06406
SECISBP2	SECISBP2	16	SECIS binding protein 2	protein_codin	0.3871	0.0112	1	7.309	0.3492
NAP1L4	NAP1L4	16	nucleosome assembly protein 1 like 4	protein_codin	0.0788	0.487	1	22.15	0.1426
ZNF195	ZNF195	16	zinc finger protein 195	protein_codin	0.4287	0.112	1	11.2	0.349
TMEM9B	TMEM9B	16	TMEM9 domain family member B	protein_codin	0.7096	1.79E-06	0.04154	16.57	0.06787
FAR1	FAR1	16	fatty acyl-CoA reductase 1	protein_codin	0.3902	0.12	1	6.527	0.1652
RRAS2	RRAS2	16	RAS related 2	protein_codin	0.1341	0.571	1	8.751	0.1121
API5	API5	16	apoptosis inhibitor 5	protein_codin	0.05819	0.724	1	6.081	0.1679
NR1H3	NR1H3	16	nuclear receptor subfamily 1 group H member 3	protein_codin	1.055	0.00052	1	3.435	0.4182
FADS3	FADS3	16	fatty acid desaturase 3	protein_codin	-0.3868	0.00038	1	28.69	0.03457
POLR2G	POLR2G	16	RNA polymerase II subunit G	protein_codin	-0.3711	0.00076	1	51.98	0.09842
TMEM223	TMEM223	16	transmembrane protein 223	protein_codin	0.259	0.136	1	7.638	0.1735
NXF1	NXF1	16	nuclear RNA export factor 1	protein_codin	0.1187	0.641	1	2.752	0.2091
EHD1	EHD1	16	EH domain containing 1	protein_codin	-0.2256	0.252	1	10.32	0.2374
SART1	SART1	16	spliceosome associated factor 1, recruiter of U4/U6.U	protein_codin	-0.1658	0.404	1	3.358	0.203
NDUFV1	NDUFV1	16	NADH:ubiquinone oxidoreductase core subunit V1	protein_codin	0.05609	0.626	1	8.046	0.1349
NDUFS8	NDUFS8	16	NADH:ubiquinone oxidoreductase core subunit S8	protein_codin	0.1447	0.0779	1	27.11	0.06705
NDUFC2	NDUFC2	16	NADH:ubiquinone oxidoreductase subunit C2	protein_codin	-0.2822	2.57E-07	0.006	60.6	0.03256
TAF1D	TAF1D	16	TATA-box binding protein associated factor, RNA pol	protein_codin	0.07790	0.445	1	35.22	0.09892
SESN3	SESN3	16	sestrin 3	protein_codin	-0.6705	7.98E-07	0.01858	40.22	0.09901
C11orf57	C11orf57	16	NKAP domain containing 1	protein_codin	NA	NA	NA	7.553	0.1111
RNF214	RNF214	16	ring finger protein 214	protein_codin	-0.1071	0.457	1	20.88	0.1725
TAF3	TAF3	16	TATA-box binding protein associated factor 3	protein_codin	-0.2255	0.155	1	7.768	0.3418
CAMK1D	CAMK1D	16	calcium/calmodulin dependent protein kinase ID	protein_codin	0.7106	0.0151	1	10.17	0.06552
YME1L1	YME1L1	16	YME1 like 1 ATPase	protein_codin	0.2949	0.0329	1	60.37	0.2402
UBE2D1	UBE2D1	16	ubiquitin conjugating enzyme E2 D1	protein_codin	0.1196	0.395	1	39.46	0.2062
BUB3	BUB3	16	BUB3 mitotic checkpoint protein	protein_codin	-0.1025	0.384	1	13.62	0.2232
BCCIP	BCCIP	16	BRCA2 and CDKN1A interacting protein	protein_codin	0.1464	0.308	1	7.595	0.1658
LRRC23	LRRC23	16	leucine rich repeat containing 23	protein_codin	-0.6161	0.0048	1	3.552	0.1475
YAF2	YAF2	16	YY1 associated factor 2	protein_codin	-0.08446	0.582	1	43.27	0.03512
AC008124.1	AC008124.1	16	novel transcri	lincRNA	-0.09484	0.652	1	7.745	0.1379
ARID2	ARID2	16	AT-rich interaction domain 2	protein_codin	0.1375	0.544	1	7.912	0.4417
ESYT1	ESYT1	16	extended synaptotagmin 1	protein_codin	0.1675	0.388	1	7.009	0.1654
IRAK3	IRAK3	16	interleukin 1 receptor associated kinase 3	protein_codin	0.7578	0.00048	1	22.67	0.227
ZFC3H1	ZFC3H1	16	zinc finger C3H1-type containing	protein_codin	0.2028	0.209	1	17.34	0.2764
RAB21	RAB21	16	RAB21, member RAS oncogene family	protein_codin	-0.05249	0.644	1	28.17	0.2357
NEDD1	NEDD1	16	NEDD1 gamma-tubulin ring complex targeting factor	protein_codin	0.4654	0.0382	1	9.807	0.3025
SCYL2	SCYL2	16	SCY1 like pseudokinase 2	protein_codin	-0.3566	0.212	1	8.088	0.486
HCFC2	HCFC2	16	host cell factor C2	protein_codin	0.6406	0.00030	1	9.253	0.1321
GPN3	GPN3	16	GPN-loop GTPase 3	protein_codin	-0.005202	0.964	1	38.39	0.06682
SBNO1	SBNO1	16	strawberry notch homolog 1	protein_codin	-0.009038	0.952	1	8.029	0.03625
LATS2	LATS2	16	large tumor suppressor kinase 2	protein_codin	0.6003	0.00512	1	8.575	0.2382
PARP4	PARP4	16	poly(ADP-ribose) polymerase family member 4	protein_codin	0.9242	0.00045	1	7.419	0.1215
USP12	USP12	16	ubiquitin specific peptidase 12	protein_codin	0.08752	0.586	1	22.42	0.1354
STARD13	STARD13	16	StAR related lipid transfer domain containing 13	protein_codin	-0.3742	0.124	1	2.631	0.3981
CDADC1	CDADC1	16	cytidine and dCMP deaminase domain containing 1	protein_codin	0.4968	0.0414	1	3.372	0.3535
NDFIP2	NDFIP2	16	Nedd4 family interacting protein 2	protein_codin	0.6697	0.00141	1	26.66	0.07396
LRP10	LRP10	16	LDL receptor related protein 10	protein_codin	-0.09106	0.244	1	29.93	0.1322
ACIN1	ACIN1	16	apoptotic chromatin condensation inducer 1	protein_codin	0.2892	0.0371	1	35.06	0.06632
ZBTB1	ZBTB1	16	zinc finger and BTB domain containing 1	protein_codin	0.213	0.259	1	26.07	0.03726
PCNX1	PCNX1	16	pecanex 1	protein_codin	0.08166	0.714	1	10.46	0.2021
GTF2A1	GTF2A1	16	general transcription factor IIA subunit 1	protein_codin	0.04779	0.762	1	23.3	0.1662
TMEM251	TMEM251	16	transmembrane protein 251	protein_codin	-0.2947	0.092	1	3.328	0.1115
EML1	EML1	16	EMAP like 1	protein_codin	0.7218	0.00030	1	34.93	0.1631
EIF5	EIF5	16	eukaryotic translation initiation factor 5	protein_codin	-0.05207	0.4036	1	49.95	0.03532
EIF3J-AS1	EIF3J-AS1	16	EIF3J divergent transcript	lincRNA	NA	NA	NA	7.406	0.1398
DUT	DUT	16	deoxyuridine triphosphatase	protein_codin	0.000395	0.995	1	55.38	0.03226
TNFAIP8L3	TNFAIP8L3	16	TNF alpha induced protein 8 like 3	protein_codin	-0.3603	0.0354	1	20.66	0.1074
TEX9	TEX9	16	testis expressed 9	protein_codin	-1.04	5.16E-05	1	60.6	0.1664
CLPX	CLPX	16	caseinolytic mitochondrial matrix peptidase chaperon	protein_codin	0.2002	0.245	1	7.273	0.207
ITGA11	ITGA11	16	integrin subunit alpha 11	protein_codin	0.2909	0.114	1	47.65	0.2302
WHAMM	WHAMM	16	WASP homolog associated with actin, golgi membran	protein_codin	0.3475	0.0406	1	8.255	0.3650
CHD2	CHD2	16	chromodomain helicase DNA binding protein 2	protein_codin	0.1115	0.552	1	25.48	0.09852
GLIS2	GLIS2	16	GLIS family zinc finger 2	protein_codin	0.2747	0.162	1	20.44	0.1042
NAGPA	NAGPA	16	N-acetylglucosamine-1-phosphodiester alpha-N-acety	protein_codin	0.4928	0.0671	1	8.991	0.1044
BRD7	BRD7	16	bromodomain containing 7	protein_codin	-0.2219	0.0207	1	30.2	0.1054
LPCAT2	LPCAT2	16	lysophosphatidylcholine acyltransferase 2	protein_codin	-0.6905	0.00488	1	8.411	0.1411
RSPRY1	RSPRY1	16	ring finger and SPRY domain containing 1	protein_codin	-0.2512	0.162	1	3.295	0.1401
E2F4	E2F4	16	E2F transcription factor 4	protein_codin	0.08416	0.611	1	8.029	0.1749
RIPOR1	RIPOR1	16	RHO family interacting cell polarization regulator 1	protein_codin	-0.1279	0.568	1	3.253	0.4751
SLC12A4	SLC12A4	16	solute carrier family 12 member 4	protein_codin	0.1485	0.441	1	8.457	0.1372
WWP2	WWP2	16	WW domain containing E3 ubiquitin protein ligase 2	protein_codin	-0.05722	0.832	1	28.58	0.3162
TERF2IP	TERF2IP	16	TERF2 interacting protein	protein_codin	-0.03002	0.676	1	60.52	0.1434
MED11	MED11	16	mediator complex subunit 11	protein_codin	0.2774	0.07	1	20.68	0.2096
PSMB6	PSMB6	16	proteasome subunit beta 6	protein_codin	-0.2368	3.76E-05	0.8607	60.6	0.03412
SAT2	SAT2	16	spermidine/spermine N1-acetyltransferase family mer	protein_codin	0.1825	0.0598	1	56.45	0.03276
TMEM220	TMEM220	16	transmembrane protein 220	protein_codin	-0.1952	0.184	1	7.994	0.06682
MAP2K4	MAP2K4	16	mitogen-activated protein kinase kinase 4	protein_codin	-0.1886	0.372	1	3.052	0.3039
KPNB1	KPNB1	16	karyopherin subunit beta 1	protein_codin	-0.01345	0.907	1	22.66	0.1714
TOB1	TOB1	16	transducer of ERBB2, 1	protein_codin	0.8384	2.36E-13	5.62E-09	31.36	0.1
TBX2	TBX2	16	T-box transcription factor 2	protein_codin	-0.09634	0.514	1	48.71	0.1781
CANT1	CANT1	16	calcium activated nucleotidase 1	protein_codin	0.1128	0.652	1	3.324	0.3882
PCYT2	PCYT2	16	phosphate cytidylyltransferase 2, ethanolamine	protein_codin	-0.07109	0.725	1	9.232	0.06788
DLGAP1-AS1	DLGAP1-AS1	16	DLGAP1 antisense RNA 1	antisense_RN	0.2052	0.382	1	8.934	0.3390
ANKRD12	ANKRD12	16	ankyrin repeat domain 12	protein_codin	-0.03833	0.651	1	55.06	0.123
PPP4R1	PPP4R1	16	protein phosphatase 4 regulatory subunit 1	protein_codin	-0.0116	0.957	1	7.889	0.3282
CEP192	CEP192	16	centrosomal protein 192	protein_codin	0.159	0.542	1	3.049	0.5222
MIB1	MIB1	16	mindbomb E3 ubiquitin protein ligase 1	protein_codin	-0.622	0.00073	1	8.869	0.1418
SETBP1	SETBP1	16	SET binding protein 1	protein_codin	0.2156	0.236	1	45.99	0.104
RBM12	RBM12	16	RNA binding motif protein 12	protein_codin	0.7782	0.00287	1	3.562	0.4540
SCAND1	SCAND1	16	SCAN domain containing 1	protein_codin	-0.1812	0.0117	1	30.97	0.07032
FAM217B	FAM217B	16	family with sequence similarity 217 member B	protein_codin	0.3053	0.104	1	22.35	0.3252
DIDO1	DIDO1	16	death inducer-obliterator 1	protein_codin	-0.1129	0.582	1	23.71	0.2431
TPD52L2	TPD52L2	16	TPD52 like 2	protein_codin	-0.2245	0.05	1	14.33	0.1039
CCDC94	CCDC94	16	YJU2 splicing factor homolog	protein_codin	NA	NA	NA	7.77	0.1432
EIF3G	EIF3G	16	eukaryotic translation initiation factor 3 subunit G	protein_codin	-0.4371	1.89E-15	4.51E-11	51.4	0.03479
DHPS	DHPS	16	deoxyhypusine synthase	protein_codin	-0.01158	0.922	1	29.36	0.06786
TECR	TECR	16	trans-2,3-enoyl-CoA reductase	protein_codin	0.2229	0.0152	1	26.63	0.0349
MVB12A	MVB12A	16	multivesicular body subunit 12A	protein_codin	0.05391	0.632	1	29.49	0.03439
PGPEP1	PGPEP1	16	pyroglutamyl-peptidase I	protein_codin	0.2035	0.418	1	33.27	0.2726
ZNF431	ZNF431	16	zinc finger protein 431	protein_codin	0.09521	0.685	1	7.952	0.4416
C19orf12	C19orf12	16	chromosome 19 open reading frame 12	protein_codin	0.2758	0.111	1	8.949	0.09952
ZNF576	ZNF576	16	zinc finger protein 576	protein_codin	0.08727	0.617	1	31.06	0.11
SIX5	SIX5	16	SIX homeobox 5	protein_codin	0.04031	0.851	1	3.085	0.2782
ZFY	ZFY	16	zinc finger protein Y-linked	protein_codin	-0.04776	0.822	1	8.952	0.128
MRPL40	MRPL40	16	mitochondrial ribosomal protein L40	protein_codin	-0.3014	0.00225	1	49.45	0.1452
DGCR6L	DGCR6L	16	DiGeorge syndrome critical region gene 6 like	protein_codin	-0.4082	0.0002	1	34.63	0.06736
THAP7	THAP7	16	THAP domain containing 7	protein_codin	-0.0695	0.626	1	7.642	0.06497
PES1.00	PES1.00	16	pescadillo ribosomal biogenesis factor 1	protein_codin	0.09027	0.65	1	8.566	0.2787
RBFOX2	RBFOX2	16	RNA binding fox-1 homolog 2	protein_codin	0.06036	0.602	1	49.75	0.1026
KCTD17	KCTD17	16	potassium channel tetramerization domain containing 1	protein_codin	0.1464	0.494	1	3.041	0.1390
RWDD2B	RWDD2B	16	RWD domain containing 2B	protein_codin	0.512	0.0666	1	3.671	0.2164
MIS18A	MIS18A	16	MIS18 kinetochore protein A	protein_codin	-0.3638	0.103	1	10.48	0.2379
RUNX1	RUNX1	16	RUNX family transcription factor 1	protein_codin	0.2937	0.222	1	24.46	0.07006
CBR3	CBR3	16	carbonyl reductase 3	protein_codin	1.158	6.49E-14	1.55E-09	60.58	0.03225
ACAP3	ACAP3	17	ArfGAP with coiled-coil, ankyrin repeat and PH doma	protein_codin	1.336	0.00026	1	56.79	0.1776
SLC25A33	SLC25A33	17	solute carrier family 25 member 33	protein_codin	0.6335	0.00311	1	8.292	0.1032
AGTRAP	AGTRAP	17	angiotensin II receptor associated protein	protein_codin	0.5072	0.0002	1	43.93	0.1334
MIIP	MIIP	17	migration and invasion inhibitory protein	protein_codin	-0.008614	0.962	1	9.43	0.2815
PLEKHM2	PLEKHM2	17	pleckstrin homology and RUN domain containing M2	protein_codin	0.2186	0.14	1	7.232	0.1305
AKR7A2	AKR7A2	17	aldo-keto reductase family 7 member A2	protein_codin	-0.05279	0.536	1	27.55	0.03279
FAM46B	FAM46B	17	terminal nucleotidyltransferase 5B	protein_codin	NA	NA	NA	8.362	0.2722
ZMYM1	ZMYM1	17	zinc finger MYM-type containing 1	protein_codin	0.2792	0.344	1	7.405	0.2922
AHCYL1	AHCYL1	17	adenosylhomocysteinase like 1	protein_codin	0.3409	0.00322	1	31.53	0.1351
PIAS3	PIAS3	17	protein inhibitor of activated STAT 3	protein_codin	-0.3267	0.141	1	8.255	0.3645
INTS3	INTS3	17	integrator complex subunit 3	protein_codin	-0.04344	0.867	1	7.575	0.5066
VAMP4	VAMP4	17	vesicle associated membrane protein 4	protein_codin	-0.01557	0.922	1	20.45	0.2342
DNM3OS	DNM3OS	17	DNM3 opposite strand/antisense RNA	antisense_RN	-0.0147	0.942	1	3.517	0.2122
RABGAP1L	RABGAP1L	17	RAB GTPase activating protein 1 like	protein_codin	0.7364	3.38E-05	0.7742	20.88	0.1677
DIEXF	DIEXF	17	UTP25 small subunit processor component	protein_codin	NA	NA	NA	3.631	0.1971
ENAH	ENAH	17	ENAH actin regulator	protein_codin	-0.1892	0.0672	1	60.53	0.03244
C1orf35	C1orf35	17	chromosome 1 open reading frame 35	protein_codin	-0.328	0.0198	1	18.72	0.09916
DESI2	DESI2	17	desumoylating isopeptidase 2	protein_codin	-0.02355	0.818	1	17.95	0.176
PDIA6	PDIA6	17	protein disulfide isomerase family A member 6	protein_codin	0.1518	0.0222	1	29.04	0.03452
HADHA	HADHA	17	hydroxyacyl-CoA dehydrogenase trifunctional multien	protein_codin	0.07129	0.316	1	30.34	0.09982
PLEKHH2	PLEKHH2	17	pleckstrin homology, MyTH4 and FERM domain cont	protein_codin	0.1974	0.297	1	37.37	0.0989
RHOQ	RHOQ	17	ras homolog family member Q	protein_codin	0.4748	6.48E-08	0.00152	60.54	0.06487
PCBP1	PCBP1	17	poly(rC) binding protein 1	protein_codin	0.06316	0.29	1	26.28	0.03450
TEX261	TEX261	17	testis expressed 261	protein_codin	0.02281	0.899	1	3.229	0.03488
NAGK	NAGK	17	N-acetylglucosamine kinase	protein_codin	-0.1588	0.204	1	33.93	0.1307
LIPT1	LIPT1	17	lipoyltransferase 1	protein_codin	0.3779	0.117	1	3.612	0.2045
RANBP2	RANBP2	17	RAN binding protein 2	protein_codin	0.1482	0.389	1	21.43	0.2785
SPOPL	SPOPL	17	speckle type BTB/POZ protein like	protein_codin	0.08374	0.656	1	3.34	0.3227
COBLL1	COBLL1	17	cordon-bleu WH2 repeat protein like 1	protein_codin	0.1238	0.36	1	35.61	0.1419
PPIL3	PPIL3	17	peptidylprolyl isomerase like 3	protein_codin	-0.2449	0.199	1	8.82	0.3004
NDUFS1	NDUFS1	17	NADH:ubiquinone oxidoreductase core subunit S1	protein_codin	0.03294	0.804	1	14.17	0.2879
TNS1	TNS1	17	tensin 1	protein_codin	-0.5869	5.3E-09	0.00012	33.2	0.2468
DNPEP	DNPEP	17	aspartyl aminopeptidase	protein_codin	-0.0629	0.659	1	7.196	0.2102
DES	DES	17	desmin	protein_codin	-0.2445	0.322	1	55.36	0.1968
CHPF	CHPF	17	chondroitin polymerizing factor	protein_codin	0.2995	0.0438	1	42.72	0.03861
PPP1R7	PPP1R7	17	protein phosphatase 1 regulatory subunit 7	protein_codin	-0.06216	0.471	1	41.93	0.0325
STT3B	STT3B	17	STT3 oligosaccharyltransferase complex catalytic sub	protein_codin	0.3356	0.014	1	29.5	0.1057
MANF	MANF	17	mesencephalic astrocyte derived neurotrophic factor	protein_codin	-0.237	0.0302	1	39.66	0.1422
NDUFB4	NDUFB4	17	NADH:ubiquinone oxidoreductase subunit B4	protein_codin	-0.188	0.00077	1	53.58	0.07112
COPG1	COPG1	17	coatomer protein complex subunit gamma 1	protein_codin	0.7547	0.00080	1	7.482	0.1345
H1FX	H1FX	17	H1 histone family member X	protein_codin	0.3757	1.09E-06	0.02525	60.6	0.1236
MLF1.00	MLF1.00	17	myeloid leukemia factor 1	protein_codin	0.4786	0.024	1	9.728	0.198
NDUFB5	NDUFB5	17	NADH:ubiquinone oxidoreductase subunit B5	protein_codin	-0.1422	0.0782	1	54.43	0.09962
DNAJC19	DNAJC19	17	DnaJ heat shock protein family (Hsp40) member C19	protein_codin	-0.09909	0.482	1	43.48	0.208
DCUN1D1	DCUN1D1	17	defective in cullin neddylation 1 domain containing 1	protein_codin	0.362	0.0488	1	51.29	0.1024
GRPEL1	GRPEL1	17	GrpE like 1, mitochondrial	protein_codin	-0.1272	0.392	1	7.755	0.2506
FIP1L1	FIP1L1	17	factor interacting with PAPOLA and CPSF1	protein_codin	0.1936	0.172	1	42.19	0.06500
RUFY3	RUFY3	17	RUN and FYVE domain containing 3	protein_codin	1.169	7.86E-07	0.01831	8.742	0.2789
TET2	TET2	17	tet methylcytosine dioxygenase 2	protein_codin	0.6971	0.0026	1	45.39	0.388
PAPSS1	PAPSS1	17	3'-phosphoadenosine 5'-phosphosulfate synthase 1	protein_codin	-0.01205	0.935	1	18.35	0.0334
AP1AR	AP1AR	17	adaptor related protein complex 1 associated regulato	protein_codin	-0.1387	0.552	1	8.225	0.1316
NAA15	NAA15	17	N(alpha)-acetyltransferase 15, NatA auxiliary subunit	protein_codin	0.1732	0.292	1	24.79	0.08041
FBXO8	FBXO8	17	F-box protein 8	protein_codin	0.2318	0.193	1	6.155	0.2411
C5orf38	C5orf38	17	chromosome 5 open reading frame 38	protein_codin	-1.535	3.35E-15	8E-11	10.63	0.1352
CDH6	CDH6	17	cadherin 6	protein_codin	1.209	1.51E-07	0.00352	40.36	0.3388
PRKAA1	PRKAA1	17	protein kinase AMP-activated catalytic subunit alpha	protein_codin	0.4725	0.0048	1	20.86	0.1379
KIF2A	KIF2A	17	kinesin family member 2A	protein_codin	0.186	0.182	1	13.43	0.108
PAPD4	PAPD4	17	terminal nucleotidyltransferase 2	protein_codin	NA	NA	NA	3.401	0.3200
LNPEP	LNPEP	17	leucyl and cystinyl aminopeptidase	protein_codin	-0.1819	0.217	1	21.18	0.03308
TMEM173	TMEM173	17	transmembrane protein 173	protein_codin	-0.04719	0.719	1	29.19	0.1006
LARS	LARS	17	leucyl-tRNA synthetase	protein_codin	0.08256	0.456	1	22.08	0.09882
FAM114A2	FAM114A2	17	family with sequence similarity 114 member A2	protein_codin	-0.02567	0.886	1	8.563	0.1724
LARP1	LARP1	17	La ribonucleoprotein domain family member 1	protein_codin	-0.03932	0.799	1	7.512	0.1679
UBTD2	UBTD2	17	ubiquitin domain containing 2	protein_codin	0.7725	0.00065	1	7.818	0.1371
HIST1H4C	HIST1H4C	17	histone cluster 1 H4 family member c	protein_codin	0.1578	0.0466	1	60.52	0.03296
ZNRD1	ZNRD1	17	zinc ribbon domain containing 1	protein_codin	0.127	0.422	1	27.88	0.3079
DDAH2	DDAH2	17	dimethylarginine dimethylaminohydrolase 2	protein_codin	0.3204	1.13E-07	0.00269	60.47	0.03265
C2	C2	17	complement C2	protein_codin	0.5836	0.0196	1	28.42	0.2098
RING1	RING1	17	ring finger protein 1	protein_codin	0.04357	0.775	1	6.813	0.3177
C6orf106	C6orf106	17	inflammation and lipid regulator with UBA-like and NB	protein_codin	-0.07271	0.677	1	9.071	0.1022
STK38	STK38	17	serine/threonine kinase 38	protein_codin	0.8107	0.00342	1	30.74	0.0709
OGFRL1	OGFRL1	17	opioid growth factor receptor like 1	protein_codin	0.5377	0.0394	1	9.649	0.292
TBX18	TBX18	17	T-box transcription factor 18	protein_codin	-0.5864	0.0136	1	3.296	0.3159
RRAGD	RRAGD	17	Ras related GTP binding D	protein_codin	0.3158	0.229	1	10.3	0.3072
CCNC	CCNC	17	cyclin C	protein_codin	0.02041	0.895	1	25.62	0.1836
GTF3C6	GTF3C6	17	general transcription factor IIIC subunit 6	protein_codin	-0.3219	0.00037	1	57.71	0.09737
TBPL1	TBPL1	17	TATA-box binding protein like 1	protein_codin	-0.09186	0.619	1	8.656	0.1698
SLC22A3	SLC22A3	17	solute carrier family 22 member 3	protein_codin	-1.388	8.84E-10	2.09E-05	9.996	0.4772
BRAT1	BRAT1	17	BRCA1 associated ATM activator 1	protein_codin	0.6944	0.00290	1	3.318	0.4109
RBAK-RBAKDN	RBAK-RBAKD	17	RBAK-RBAKDN readthrough	protein_codin	-0.1463	0.469	1	2.958	0.1351
CYTH3	CYTH3	17	cytohesin 3	protein_codin	0.1468	0.429	1	7.709	0.317
DNAJC30	DNAJC30	17	DnaJ heat shock protein family (Hsp40) member C30	protein_codin	-0.2249	0.262	1	3.352	0.2014
BUD23	BUD23	17	BUD23 rRNA methyltransferase and ribosome matur	protein_codin	-0.09768	0.472	1	32.72	0.1320
TRIM4	TRIM4	17	tripartite motif containing 4	protein_codin	0.02848	0.869	1	8.124	0.1725
TRIM56	TRIM56	17	tripartite motif containing 56	protein_codin	-0.06853	0.724	1	44.09	0.3912
AP1S1	AP1S1	17	adaptor related protein complex 1 subunit sigma 1	protein_codin	-0.2584	0.105	1	20.93	0.03342
ARMC10	ARMC10	17	armadillo repeat containing 10	protein_codin	-0.06872	0.592	1	42.42	0.09758
DNAJC2	DNAJC2	17	DnaJ heat shock protein family (Hsp40) member C2	protein_codin	-0.0302	0.852	1	15.49	0.06537
NAMPT	NAMPT	17	nicotinamide phosphoribosyltransferase	protein_codin	0.4797	0.0002	1	60.6	0.1048
SND1	SND1	17	staphylococcal nuclease and tudor domain containing 1	protein_codin	-0.2884	0.0781	1	8.116	0.2165
CUL1	CUL1	17	cullin 1	protein_codin	0.1911	0.216	1	21.37	0.07342
MOSPD2	MOSPD2	17	motile sperm domain containing 2	protein_codin	-0.01772	0.925	1	8.652	0.2706
TXLNG	TXLNG	17	taxilin gamma	protein_codin	0.4048	0.0305	1	9.582	0.1759
EIF1AX	EIF1AX	17	eukaryotic translation initiation factor 1A X-linked	protein_codin	-0.1548	0.0291	1	44.28	0.03462
RBM10	RBM10	17	RNA binding motif protein 10	protein_codin	-0.03914	0.84	1	3.488	0.1391
GSPT2	GSPT2	17	G1 to S phase transition 2	protein_codin	0.3494	0.0925	1	6.996	0.3782
SPIN2B	SPIN2B	17	spindlin family member 2B	protein_codin	0.1242	0.587	1	32.71	0.2841
HEPH	HEPH	17	hephaestin	protein_codin	-0.3745	0.0758	1	9.858	0.3826
JPX	JPX	17	JPX transcript, XIST activator	lincRNA	0.1565	0.311	1	18.79	0.3948
NAP1L3	NAP1L3	17	nucleosome assembly protein 1 like 3	protein_codin	-0.1584	0.438	1	60.02	0.1685
UTP14A	UTP14A	17	UTP14A small subunit processome component	protein_codin	0.2796	0.137	1	29.42	0.272
[illegible]	[illegible]	[illegible]	[illegible]	[illegible]	[illegible]	[illegible]	[illegible]	[illegible]	[illegible]

**OLFM1**	OLFM1	17	olfactomedin 1	protein_codin	1.392	6.67E-09	0.00016	59.82	0.2434
**NRARP**	NRARP	17	NOTCH regulated ankyrin repeat protein	protein_codin	0.9735	0.0028	1	58.32	0.3542
**MRPL41**	MRPL41	17	mitochondrial ribosomal protein L41	protein_codin	-0.2054	0.00902	1	27.32	0.1419
**TIMM10B**	TIMM10B	17	translocase of inner mitochondrial membrane 10B	protein_codin	-0.3012	0.0567	1	7.076	0.03229
**TMEM179B**	TMEM179B	17	transmembrane protein 179B	protein_codin	0.2217	0.0112	1	36.4	0.03285
**PAAF1**	PAAF1	17	proteasomal ATPase associated factor 1	protein_codin	-0.08144	0.67	1	3.486	0.03236
**ANKRD49**	ANKRD49	17	ankyrin repeat domain 49	protein_codin	0.286	0.15	1	8.556	0.1655
**FDX1**	FDX1	17	ferredoxin 1	protein_codin	-0.01446	0.919	1	19.93	0.03612
**RBM7**	RBM7	17	RNA binding motif protein 7	protein_codin	0.0487	0.712	1	6.455	0.2066
**AKR1C1**	AKR1C1	17	aldo-keto reductase family 1 member C1	protein_codin	0.8396	3.79E-09	8.93E-05	50.75	0.06946
**AKR1C3**	AKR1C3	17	aldo-keto reductase family 1 member C3	protein_codin	-0.2175	0.0248	1	33.53	0.03726
**MPP7**	MPP7	17	membrane palmitoylated protein 7	protein_codin	-0.8393	2.26E-05	0.5189	34.25	0.3452
**WAC-AS1**	WAC-AS1	17	WAC antisense RNA 1 (head to head)	antisense_RN	0.0004173	0.998	1	57.44	0.1051
**ZNF438**	ZNF438	17	zinc finger protein 438	protein_codin	0.04577	0.816	1	8.237	0.1347
**CCAR1**	CCAR1	17	cell division cycle and apoptosis regulator 1	protein_codin	-0.001744	0.992	1	18.64	0.2457
**MYOZ1**	MYOZ1	17	myozenin 1	protein_codin	0.01182	0.957	1	20.19	0.2231
**COMTD1**	COMTD1	17	catechol-O-methyltransferase domain containing 1	protein_codin	-0.4704	0.00283	1	7.802	0.3184
**LRMDA**	LRMDA	17	leucine rich melanocyte differentiation associated	protein_codin	-0.4274	0.0222	1	19.92	0.2481
**ADRA2A**	ADRA2A	17	adrenoceptor alpha 2A	protein_codin	1.347	2.59E-05	0.594	60.46	0.5362
**DOCK1**	DOCK1	17	dedicator of cytokinesis 1	protein_codin	0.3558	0.0762	1	52.37	0.3222
**PPP2R2D**	PPP2R2D	17	protein phosphatase 2 regulatory subunit Bdelta	protein_codin	0.06229	0.764	1	5.992	0.1652
**MED21**	MED21	17	mediator complex subunit 21	protein_codin	-0.03015	0.818	1	6.148	0.0324
**IRAK4**	IRAK4	17	interleukin 1 receptor associated kinase 4	protein_codin	0.1579	0.422	1	27.78	0.2
**SLC38A1**	SLC38A1	17	solute carrier family 38 member 1	protein_codin	0.07819	0.684	1	8.049	0.2851
**AC074032.1**	AC074032.1	17	novel transcript, antisense to CER	antisense_RN	0.2124	0.38	1	8.547	0.3236
**NABP2**	NABP2	17	nucleic acid binding protein 2	protein_codin	-0.4116	0.00427	1	7.242	0.1742
**OSBPL8**	OSBPL8	17	oxysterol binding protein like 8	protein_codin	-0.09768	0.269	1	47.45	0.03262
**CEP290**	CEP290	17	centrosomal protein 290	protein_codin	-0.04165	0.815	1	13.88	0.2027
**ACTR6**	ACTR6	17	actin related protein 6	protein_codin	0.09862	0.447	1	15.13	0.1312
**SART3**	SART3	17	spliceosome associated factor 3, U4/U6 recycling pro	protein_codin	0.1578	0.421	1	2.922	0.2584
**POP5**	POP5	17	POP5 homolog, ribonuclease P/MRP subunit	protein_codin	0.02502	0.848	1	35.5	0.07445
**ZCCHC8**	ZCCHC8	17	zinc finger CCHC-type containing 8	protein_codin	-0.2809	0.152	1	3.118	0.1237
**KMT5A**	KMT5A	17	lysine methyltransferase 5A	protein_codin	-0.3478	0.0158	1	39.46	0.2109
**RILPL2**	RILPL2	17	Rab interacting lysosomal protein like 2	protein_codin	-0.8623	4.4E-16	1.06E-11	42.82	0.06992
**PXMP2**	PXMP2	17	peroxisomal membrane protein 2	protein_codin	-0.8015	3.33E-06	0.0771	20.14	0.2494
**PSPC1**	PSPC1	17	paraspeckle component 1	protein_codin	-0.2068	0.282	1	7.382	0.1021
**UFM1**	UFM1	17	ubiquitin fold modifier 1	protein_codin	0.1604	0.181	1	22.14	0.1039
**NEK3**	NEK3	17	NIMA related kinase 3	protein_codin	-0.1175	0.676	1	10.54	0.2541
**PCID2**	PCID2	17	PCI domain containing 2	protein_codin	0.1835	0.16	1	13.4	0.196
**TMCO3**	TMCO3	17	transmembrane and coiled-coil domains 3	protein_codin	0.1497	0.297	1	41.88	0.17
**APEX1**	APEX1	17	apurinic/apyrimidinic endodeoxyribonuclease 1	protein_codin	-0.1798	0.0596	1	15.86	0.1002
**HNRNPC**	HNRNPC	17	heterogeneous nuclear ribonucleoprotein C (C1/C2)	protein_codin	-0.082	0.275	1	28.49	0.1417
**PCK2**	PCK2	17	phosphoenolpyruvate carboxykinase 2, mitochondrial	protein_codin	1.295	1.73E-08	0.00041	10.4	0.1696
**HIF1A**	HIF1A	17	hypoxia inducible factor 1 subunit alpha	protein_codin	-0.7533	2.95E-10	6.98E-06	60	0.142
**AHSA1**	AHSA1	17	activator of HSP90 ATPase activity 1	protein_codin	0.1023	0.371	1	13.42	0.1066
**INF2**	INF2	17	inverted formin, FH2 and WH2 domain containing	protein_codin	0.01579	0.94	1	28.73	0.2442
**TUBGCP4**	TUBGCP4	17	tubulin gamma complex associated protein 4	protein_codin	0.8764	0.00238	1	3.522	0.2086
**FBXL22**	FBXL22	17	F-box and leucine rich repeat protein 22	protein_codin	-0.9339	0.00018	1	26.16	0.1734
**GNPTG**	GNPTG	17	N-acetylglucosamine-1-phosphate transferase subuni	protein_codin	0.7373	1.81E-10	4.29E-06	53.61	0.1034
**JPT2**	JPT2	17	Jupiter microtubule associated homolog 2	protein_codin	-0.1579	0.534	1	9.094	0.1712
**NTHL1**	NTHL1	17	nth like DNA glycosylase 1	protein_codin	-0.3062	0.0531	1	8.658	0.2719
**PGP**	PGP	17	phosphoglycolate phosphatase	protein_codin	-0.06136	0.7	1	20.78	0.03565
**CCP110**	CCP110	17	centriolar coiled-coil protein 110	protein_codin	0.3366	0.157	1	7.405	0.5392
**ZNF720**	ZNF720	17	zinc finger protein 720	protein_codin	0.2138	0.296	1	7.079	0.3442
**C16orf87**	C16orf87	17	chromosome 16 open reading frame 87	protein_codin	-0.3124	0.129	1	8.305	0.355
**AARS**	AARS	17	alanyl-tRNA synthetase	protein_codin	0.2789	0.215	1	10.79	0.1996
**MRM3**	MRM3	17	mitochondrial rRNA methyltransferase 3	protein_codin	-0.1482	0.556	1	8.2	0.1786
**TVP23B**	TVP23B	17	trans-golgi network vesicle protein 23 homolog B	protein_codin	0.2464	0.293	1	3.355	0.1042
**PRPSAP2**	PRPSAP2	17	phosphoribosyl pyrophosphate synthetase associated	protein_codin	0.05337	0.793	1	7.52	0.03434
**CPD**	CPD	17	carboxypeptidase D	protein_codin	0.6877	0.00955	1	3.14	0.3925
**FBXL20**	FBXL20	17	F-box and leucine rich repeat protein 20	protein_codin	0.1868	0.414	1	2.979	0.4916
**RETREG3**	RETREG3	17	reticulophagy regulator family member 3	protein_codin	0.1468	0.561	1	8.736	0.3342
**GOSR2**	GOSR2	17	golgi SNAP receptor complex member 2	protein_codin	-0.2478	0.142	1	7.916	0.0670
**NXPH3**	NXPH3	17	neurexophilin 3	protein_codin	-0.4564	0.00326	1	59.03	0.103
**TMEM100**	TMEM100	17	transmembrane protein 100	protein_codin	2.181	2.06E-05	0.4732	60.6	0.5296
**SRSF1**	SRSF1	17	serine and arginine rich splicing factor 1	protein_codin	0.1782	0.244	1	7.207	0.1326
**UBALD2**	UBALD2	17	UBA like domain containing 2	protein_codin	-0.1219	0.369	1	37.93	0.06734
**SGSH**	SGSH	17	N-sulfoglucosamine sulfohydrolase	protein_codin	0.3145	0.239	1	9.19	0.3174
**ASPSCR1**	ASPSCR1	17	ASPSCR1 tether for SLC2A4, UBX domain containing	protein_codin	-0.2697	0.169	1	9.257	0.102
**RNMT**	RNMT	17	RNA guanine-7 methyltransferase	protein_codin	0.4988	4.09E-05	0.9259	35.72	0.112
**XRN2**	XRN2	17	5'-3' exoribonuclease 2	protein_codin	-0.3583	3.19E-05	0.7308	34.88	0.06606
**ABHD12**	ABHD12	17	abhydrolase domain containing 12	protein_codin	-0.08907	0.59	1	30.94	0.1070
**TM9SF4**	TM9SF4	17	transmembrane 9 superfamily member 4	protein_codin	0.3826	0.0484	1	29.63	0.2892
**NECAB3**	NECAB3	17	N-terminal EF-hand calcium binding protein 3	protein_codin	-0.01727	0.934	1	3.506	0.3591
**STK4**	STK4	17	serine/threonine kinase 4	protein_codin	0.416	0.0240	1	6.73	0.2357
**CSNK1G2**	CSNK1G2	17	casein kinase 1 gamma 2	protein_codin	0.04852	0.802	1	31.96	0.2062
**DAPK3**	DAPK3	17	death associated protein kinase 3	protein_codin	-0.2952	0.02	1	7.923	0.1767
**XAB2**	XAB2	17	XPA binding protein 2	protein_codin	-0.04275	0.842	1	3.154	0.1792
**2-Mar**	43526	17	membrane associated ring-CH-type finger 2	protein_codin	-0.1946	0.0524	1	60.53	0.1339
**PDE4A**	PDE4A	17	phosphodiesterase 4A	protein_codin	0.2869	0.245	1	10.41	0.2748
**PLPPR2**	PLPPR2	17	phospholipid phosphatase related 2	protein_codin	0.4947	0.0292	1	3.273	0.2692
**NFIX**	NFIX	17	nuclear factor I X	protein_codin	0.5156	1.11E-06	0.02581	40.38	0.1062
**SAMD1**	SAMD1	17	sterile alpha motif domain containing 1	protein_codin	-0.1335	0.487	1	9.806	0.2317
**ZNF570**	ZNF570	17	zinc finger protein 570	protein_codin	-0.3072	0.172	1	9.229	0.1366
**FAM98C**	FAM98C	17	family with sequence similarity 98 member C	protein_codin	-0.1479	0.388	1	7.652	0.2046
**NFKBIB**	NFKBIB	17	NFKB inhibitor beta	protein_codin	-0.3025	0.184	1	3.288	0.1018
**TRAPPC6A**	TRAPPC6A	17	trafficking protein particle complex 6A	protein_codin	0.02452	0.901	1	7.826	0.1729
**SAE1**	SAE1	17	SUMO1 activating enzyme subunit 1	protein_codin	-0.2283	0.187	1	8.423	0.203
**CABIN1**	CABIN1	17	calcineurin binding protein 1	protein_codin	0.171	0.254	1	22.32	0.1967
**SMTN**	SMTN	17	smoothelin	protein_codin	-0.6574	0.00038	1	28.63	0.1395
**DDX17**	DDX17	17	DEAD-box helicase 17	protein_codin	0.224	0.0110	1	26.14	0.03346
**APOBEC3C**	APOBEC3C	17	apolipoprotein B mRNA editing enzyme catalytic subu	protein_codin	0.1939	0.239	1	20.14	0.137
**ST13**	ST13	17	ST13 Hsp70 interacting protein	protein_codin	-0.1376	0.0168	1	33.13	0.0409
**RRP7A**	RRP7A	17	ribosomal RNA processing 7 homolog A	protein_codin	0.07582	0.581	1	6.83	0.06712
**ATXN10**	ATXN10	17	ataxin 10	protein_codin	0.1878	0.0897	1	11.67	0.03442
**IFNGR2**	IFNGR2	17	interferon gamma receptor 2	protein_codin	0.08561	0.51	1	25.46	0.03321
**CBR1**	CBR1	17	carbonyl reductase 1	protein_codin	0.1886	0.015	1	33.38	0.0332
**HES4**	HES4	18	hes family bHLH transcription factor 4	protein_codin	0.5593	2.51E-06	0.05819	59.41	0.07574
**PEX10**	PEX10	18	peroxisomal biogenesis factor 10	protein_codin	-0.2635	0.209	1	2.713	0.1714
**NOL9**	NOL9	18	nucleolar protein 9	protein_codin	0.0652	0.726	1	9.234	0.3449
**DHRS3**	DHRS3	18	dehydrogenase/reductase 3	protein_codin	2.703	2.63E-45	6.38E-41	59.94	0.08177
**EFHD2**	EFHD2	18	EF-hand domain family member D2	protein_codin	-0.1251	0.64	1	11.43	0.7779
**NBPF1**	NBPF1	18	NBPF member 1	protein_codin	-0.3351	0.151	1	8.265	0.1347
**CAMK2N1**	CAMK2N1	18	calcium/calmodulin dependent protein kinase II inhibitor 1	protein_codin	1.254	5.24E-15	1.25E-10	54.79	0.1007
**MTFR1L**	MTFR1L	18	mitochondrial fission regulator 1 like	protein_codin	0.1803	0.181	1	7.288	0.1139
**EPB41**	EPB41	18	erythrocyte membrane protein band 4.1	protein_codin	0.4446	0.0817	1	56.32	0.2076
**MARCKSL1**	MARCKSL1	18	MARCKS like 1	protein_codin	0.0522	0.837	1	55.17	0.3914
**C1orf216**	C1orf216	18	chromosome 1 open reading frame 216	protein_codin	-0.2129	0.346	1	8.776	0.1645
**TRAPPC3**	TRAPPC3	18	trafficking protein particle complex 3	protein_codin	-0.1645	0.117	1	13.44	0.1345
**PLK3**	PLK3	18	polo like kinase 3	protein_codin	0.0928	0.654	1	22.8	0.1681
**IPP**	IPP	18	intracisternal A particle-promoted polypeptide	protein_codin	0.3555	0.112	1	3.287	0.2404
**ATPAF1**	ATPAF1	18	ATP synthase mitochondrial F1 complex assembly fa	protein_codin	0.08555	0.566	1	19.88	0.1044
**LEPR**	LEPR	18	leptin receptor	protein_codin	4.543	2.46E-23	5.92E-19	60.54	0.18
**ADGRL2**	ADGRL2	18	adhesion G protein-coupled receptor L2	protein_codin	-0.2651	0.152	1	3.622	0.3462
**GNG5**	GNG5	18	G protein subunit gamma 5	protein_codin	-0.5083	8.54E-22	2.05E-17	54.85	0.03316
**RBMXL1**	RBMXL1	18	RBMX like 1	protein_codin	-0.5368	0.0357	1	3.366	0.2482
**FAM69A**	FAM69A	18	divergent protein kinase domain 1A	protein_codin	0.5777	0.00973	1	8.806	0.2508
**ALG14**	ALG14	18	ALG14 UDP-N-acetylglucosaminyltransferase subuni	protein_codin	0.232	0.225	1	8.503	0.1666
**SLC35A3**	SLC35A3	18	solute carrier family 35 member A3	protein_codin	-0.07688	0.7	1	7.604	0.1864
**FAM102B**	FAM102B	18	family with sequence similarity 102 member B	protein_codin	0.6795	0.0248	1	9.803	0.1774
**GSTM2**	GSTM2	18	glutathione S-transferase mu 2	protein_codin	0.2274	0.496	1	9.19	0.3972
**CASQ2**	CASQ2	18	calsequestrin 2	protein_codin	0.6093	0.00104	1	60.58	0.3032
**POLR3C**	POLR3C	18	RNA polymerase III subunit C	protein_codin	0.06259	0.774	1	3.046	0.2242
**GOLPH3L**	GOLPH3L	18	golgi phosphoprotein 3 like	protein_codin	0.04262	0.86	1	3.213	0.2802
**UBAP2L**	UBAP2L	18	ubiquitin associated protein 2 like	protein_codin	-0.1866	0.364	1	3.388	0.1455
**UBE2Q1**	UBE2Q1	18	ubiquitin conjugating enzyme E2 Q1	protein_codin	0.02527	0.888	1	9.09	0.1822
**ATP1A2**	ATP1A2	18	ATPase Na+/K+ transporting subunit alpha 2	protein_codin	-0.448	0.0269	1	18.96	0.1852
**TSTD1**	TSTD1	18	thiosulfate sulfurtransferase like domain containing 1	protein_codin	-0.8387	1.85E-06	0.04292	28.56	0.09786
**DUSP12**	DUSP12	18	dual specificity phosphatase 12	protein_codin	-0.1026	0.559	1	8.222	0.1667
**HSD17B7**	HSD17B7	18	hydroxysteroid 17-beta dehydrogenase 7	protein_codin	0.738	0.00056	1	8.702	0.06616
**RGS5**	RGS5	18	regulator of G protein signaling 5	protein_codin	-0.9749	7.58E-22	1.82E-17	60.6	0.4481
**BLZF1**	BLZF1	18	basic leucine zipper nuclear factor 1	protein_codin	0.1943	0.271	1	18.53	0.1707
**KIAA0040**	KIAA0040	18	KIAA0040	protein_codin	-0.2825	0.0542	1	41.19	0.1848
**RNASEL**	RNASEL	18	ribonuclease L	protein_codin	0.4992	0.0132	1	50.61	0.1406
**TSEN15**	TSEN15	18	tRNA splicing endonuclease subunit 15	protein_codin	0.104	0.555	1	23.88	0.2086
**RBBP5**	RBBP5	18	RB binding protein 5, histone lysine methyltransferas	protein_codin	0.3787	0.109	1	9.897	0.1386
**DSTYK**	DSTYK	18	dual serine/threonine and tyrosine protein kinase	protein_codin	0.128	0.581	1	10.04	0.33
**ELK4**	ELK4	18	ETS transcription factor ELK4	protein_codin	-0.001919	0.992	1	9.225	0.2362
**EIF2D**	EIF2D	18	eukaryotic translation initiation factor 2D	protein_codin	0.08099	0.637	1	7.146	0.1681
**HLX**	HLX	18	H2.0 like homeobox	protein_codin	1.975	2.76E-07	0.00645	24.68	0.1334
**MRPL55**	MRPL55	18	mitochondrial ribosomal protein L55	protein_codin	-0.2094	0.107	1	14.03	0.1548
**LYST**	LYST	18	lysosomal trafficking regulator	protein_codin	-0.03657	0.866	1	9.008	0.2567
**AHCTF1**	AHCTF1	18	AT-hook containing transcription factor 1	protein_codin	-0.06475	0.719	1	8.143	0.4115
**HADHB**	HADHB	18	hydroxyacyl-CoA dehydrogenase trifunctional multien	protein_codin	-0.02238	0.81	1	20.97	0.03359
**WDR43**	WDR43	18	WD repeat domain 43	protein_codin	0.3022	0.0391	1	41	0.2061
**LBH**	LBH	18	LBH regulator of WNT signaling pathway	protein_codin	0.2982	0.00490	1	60.6	0.03496
**ATL2**	ATL2	18	atlastin GTPase 2	protein_codin	-0.2587	0.171	1	7.918	0.2457
**GEMIN6**	GEMIN6	18	gem nuclear organelle associated protein 6	protein_codin	0.1682	0.424	1	8.145	0.1376
**THADA**	THADA	18	THADA armadillo repeat containing	protein_codin	0.1209	0.62	1	8.586	0.2032
**PIGF**	PIGF	18	phosphatidylinositol glycan anchor biosynthesis class F	protein_codin	0.4156	0.0381	1	8.316	0.2202
**XPO1**	XPO1	18	exportin 1	protein_codin	0.04064	0.768	1	17.45	0.165
**DUSP11**	DUSP11	18	dual specificity phosphatase 11	protein_codin	0.1896	0.234	1	21.73	0.2105
**AUP1**	AUP1	18	AUP1 lipid droplet regulating VLDL assembly factor	protein_codin	0.001757	0.988	1	26.86	0.2402
**PTCD3**	PTCD3	18	pentatricopeptide repeat domain 3	protein_codin	0.2352	0.267	1	8.918	0.319
**SNRNP200**	SNRNP200	18	small nuclear ribonucleoprotein U5 subunit 200	protein_codin	0.09642	0.539	1	7.806	0.3074
**ARID5A**	ARID5A	18	AT-rich interaction domain 5A	protein_codin	-0.2141	0.119	1	60.6	0.1145
**RALB**	RALB	18	RAS like proto-oncogene B	protein_codin	-0.1393	0.511	1	8.435	0.3132
**MZT2A**	MZT2A	18	mitotic spindle organizing protein 2A	protein_codin	-0.1907	0.0567	1	27.97	0.187
**UBR3**	UBR3	18	ubiquitin protein ligase E3 component n-recognin 3	protein_codin	-0.07152	0.752	1	3.563	0.1714
**MAP3K20**	MAP3K20	18	mitogen-activated protein kinase kinase kinase 20	protein_codin	0.006489	0.957	1	60.6	0.03961
**LNPK**	LNPK	18	lunapark, ER junction formation factor	protein_codin	0.2997	0.0376	1	19.6	0.06906
**FKBP7**	FKBP7	18	FKBP prolyl isomerase 7	protein_codin	-0.002535	0.982	1	23.4	0.103
**PMS1**	PMS1	18	PMS1 homolog 1, mismatch repair system componen	protein_codin	0.6574	0.00328	1	7.008	0.2032
**MYO1B**	MYO1B	18	myosin IB	protein_codin	-0.3068	0.0271	1	41.52	0.05228
**C2orf69**	C2orf69	18	chromosome 2 open reading frame 69	protein_codin	0.3167	0.225	1	8.672	0.2004
**KANSL1L**	KANSL1L	18	KAT8 regulatory NSL complex subunit 1 like	protein_codin	0.106	0.591	1	9.138	0.1984
**ATIC**	ATIC	18	5-aminoimidazole-4-carboxamide ribonucleotide form	protein_codin	-0.01987	0.912	1	8.805	0.1015
**CNPPD1**	CNPPD1	18	cyclin Pas1/PHO80 domain containing 1	protein_codin	0.2604	0.131	1	8.207	0.1364
**AGFG1**	AGFG1	18	ArfGAP with FG repeats 1	protein_codin	0.2745	0.209	1	8.295	0.1806
**CAB39**	CAB39	18	calcium binding protein 39	protein_codin	-0.2499	0.17	1	8.612	0.3116
**ITM2C**	ITM2C	18	integral membrane protein 2C	protein_codin	-0.5058	1.89E-06	0.04385	53.5	0.03832
**PER2**	PER2	18	period circadian regulator 2	protein_codin	0.5104	0.00268	1	57.42	0.1756
**CAMK1**	CAMK1	18	calcium/calmodulin dependent protein kinase I	protein_codin	-0.07399	0.56	1	52.34	0.1327
**SH3BP5**	SH3BP5	18	SH3 domain binding protein 5	protein_codin	1.174	2.19E-17	5.24E-13	47.25	0.03805
**BTD**	BTD	18	biotinidase	protein_codin	0.5865	0.00312	1	26.19	0.1085
**NR1D2**	NR1D2	18	nuclear receptor subfamily 1 group D member 2	protein_codin	0.199	0.142	1	27.64	0.3052
**CLASP2**	CLASP2	18	cytoplasmic linker associated protein 2	protein_codin	-0.1674	0.519	1	8.95	0.382
**KIAA1143**	KIAA1143	18	KIAA1143	protein_codin	0.3792	0.00661	1	16.46	0.2102
**HYAL2**	HYAL2	18	hyaluronidase 2	protein_codin	0.1672	0.415	1	60.53	0.1752
**PCBP4**	PCBP4	18	poly(rC) binding protein 4	protein_codin	-0.2663	0.157	1	25.74	0.0346
**TKT**	TKT	18	transketolase	protein_codin	-0.08247	0.334	1	59.87	0.07162
**MITF**	MITF	18	melanocyte inducing transcription factor	protein_codin	0.1629	0.344	1	7.656	0.1396
**FOXP1**	FOXP1	18	forkhead box P1	protein_codin	-0.2633	0.0026	1	54.43	0.1424
**PCNP**	PCNP	18	PEST proteolytic signal containing nuclear protein	protein_codin	-0.1984	0.00670	1	36.42	0.07352
**NAA50**	NAA50	18	N(alpha)-acetyltransferase 50, NatE catalytic subunit	protein_codin	0.2243	0.27	1	20.11	0.1722
**POGLUT1**	POGLUT1	18	protein O-glucosyltransferase 1	protein_codin	-0.2488	0.198	1	9.08	0.1017
**MGLL**	MGLL	18	monoglyceride lipase	protein_codin	0.7187	1.85E-10	4.38E-06	46.9	0.1771
**MBD4**	MBD4	18	methyl-CpG binding domain 4, DNA glycosylase	protein_codin	0.1092	0.41	1	16.73	0.1985
**DNAJC13**	DNAJC13	18	DnaJ heat shock protein family (Hsp40) member C13	protein_codin	0.2906	0.198	1	9.482	0.2518
**UBA5**	UBA5	18	ubiquitin like modifier activating enzyme 5	protein_codin	-0.07992	0.604	1	19.28	0.03432
**RNF7**	RNF7	18	ring finger protein 7	protein_codin	-0.3929	1.13E-08	0.00027	60.6	0.06586
**TFDP2**	TFDP2	18	transcription factor Dp-2	protein_codin	0.08757	0.441	1	59.99	0.09968
**SLC9A9**	SLC9A9	18	solute carrier family 9 member A9	protein_codin	1.266	4E-08	0.00094	25.28	0.3655
**TM4SF1**	TM4SF1	18	transmembrane 4 L six family member 1	protein_codin	-0.7939	1.32E-13	3.15E-09	60.6	0.068
**PHC3**	PHC3	18	polyhomeotic homolog 3	protein_codin	-0.02877	0.88	1	8.695	0.2752
**DVL3**	DVL3	18	dishevelled segment polarity protein 3	protein_codin	0.3652	0.112	1	7.346	0.2756
**POLR2H**	POLR2H	18	RNA polymerase II subunit H	protein_codin	-0.1558	0.259	1	60.56	0.09686
**LSG1**	LSG1	18	large 60S subunit nuclear export GTPase 1	protein_codin	0.3628	0.0262	1	7.672	0.03387
**PIGX**	PIGX	18	phosphatidylinositol glycan anchor biosynthesis class X	protein_codin	0.1382	0.482	1	8.232	0.1994
**GAK**	GAK	18	cyclin G associated kinase	protein_codin	-0.3149	0.176	1	9.096	0.3809
**SPON2**	SPON2	18	spondin 2	protein_codin	2.466	2.38E-05	0.5462	60.6	0.4125
**SHISA3**	SHISA3	18	shisa family member 3	protein_codin	2.371	3.98E-14	9.49E-10	60.43	0.1036
**DCUN1D4**	DCUN1D4	18	defective in cullin neddylation 1 domain containing 4	protein_codin	0.2946	0.115	1	3.203	0.203
**PAICS**	PAICS	18	phosphoribosylaminoimidazole carboxylase and phos	protein_codin	0.08188	0.66	1	9.746	0.1742
**UTP3**	UTP3	18	UTP3 small subunit processome component	protein_codin	0.2924	0.271	1	5.964	0.3254
**BTC**	BTC	18	betacellulin	protein_codin	-0.8198	4.55E-05	1	9.839	0.4934
**USO1**	USO1	18	USO1 vesicle transport factor	protein_codin	-0.1219	0.336	1	12.68	0.1045
**AFF1**	AFF1	18	AF4/FMR2 family member 1	protein_codin	0.2482	0.12	1	21.24	0.1681
**BMPR1B**	BMPR1B	18	bone morphogenetic protein receptor type 1B	protein_codin	0.9106	0.00058	1	48.07	0.1797
**RAP1GDS1**	RAP1GDS1	18	Rap1 GTPase-GDP dissociation stimulator 1	protein_codin	0.04038	0.84	1	30.6	0.03399
**PPP3CA**	PPP3CA	18	protein phosphatase 3 catalytic subunit alpha	protein_codin	0.2194	0.0801	1	20.87	0.1355
**FLJ20021**	FLJ20021	18	novel transcri	lincRNA	NA	NA	NA	32.97	0.1005
**CFI**	CFI	18	complement factor I	protein_codin	0.7835	5.72E-06	0.1321	26.72	0.06917
**SYNPO2**	SYNPO2	18	synaptopodin 2	protein_codin	-0.7573	8.1E-16	1.94E-11	55.35	0.03440
**USP53**	USP53	18	ubiquitin specific peptidase 53	protein_codin	1.799	2.77E-23	6.67E-19	60.6	0.236
**FGF2**	FGF2	18	fibroblast growth factor 2	protein_codin	0.473	0.00111	1	52.83	0.07556
**INPP4B**	INPP4B	18	inositol polyphosphate-4-phosphatase type II B	protein_codin	-0.03429	0.835	1	60.53	0.1767
**PDLIM3**	PDLIM3	18	PDZ and LIM domain 3	protein_codin	-0.6843	5.81E-25	1.4E-20	42.84	0.1009
**CYP4V2**	CYP4V2	18	cytochrome P450 family 4 subfamily V member 2	protein_codin	0.7352	0.00425	1	8.246	0.3082
**RANBP3L**	RANBP3L	18	RAN binding protein 3 like	protein_codin	-0.4642	0.0272	1	3.367	0.2032
**NNT-AS1**	NNT-AS1	18	NNT antisense RNA 1	antisense_RN	-0.04135	0.79	1	16.33	0.0682
**HEXB**	HEXB	18	hexosaminidase subunit beta	protein_codin	0.9055	3.23E-17	7.73E-13	22.96	0.03282
**NSA2**	NSA2	18	NSA2 ribosome biogenesis factor	protein_codin	-0.3302	2.64E-07	0.00617	42.79	0.1032
**SLF1**	SLF1	18	SMC5-SMC6 complex localization factor 1	protein_codin	0.6529	0.00678	1	3.452	0.2796
**LINC01184**	LINC01184	18	long intergenic non-protein coding RNA 1184	lincRNA	-0.1706	0.396	1	8.454	0.1019
**SLC12A2**	SLC12A2	18	solute carrier family 12 member 2	protein_codin	-0.08062	0.788	1	31.11	0.5692
**FNIP1**	FNIP1	18	folliculin interacting protein 1	protein_codin	0.7109	7.63E-06	0.176	15.38	0.06711
**SAR1B**	SAR1B	18	secretion associated Ras related GTPase 1B	protein_codin	-0.01759	0.884	1	12.66	0.1776
**PAIP2**	PAIP2	18	poly(A) binding protein interacting protein 2	protein_codin	-0.0122	0.882	1	41.85	0.1015
**HDAC3**	HDAC3	18	histone deacetylase 3	protein_codin	0.1906	0.252	1	6.386	0.1372
**RBM22**	RBM22	18	RNA binding motif protein 22	protein_codin	0.02709	0.811	1	59.65	0.1396
**GPX3**	GPX3	18	glutathione peroxidase 3	protein_codin	1.377	1.37E-31	3.31E-27	40.9	0.1012
**GALNT10**	GALNT10	18	polypeptide N-acetylgalactosaminyltransferase 10	protein_codin	0.4238	0.11	1	10.4	0.2859
**MED7**	MED7	18	mediator complex subunit 7	protein_codin	0.2903	0.109	1	8.588	0.2151
**NHP2**	NHP2	18	NHP2 ribonucleoprotein	protein_codin	-0.4202	3.99E-08	0.00094	53.9	0.2167
**HNRNPAB**	HNRNPAB	18	heterogeneous nuclear ribonucleoprotein A/B	protein_codin	0.1432	0.134	1	60.6	0.03366
**MRNIP**	MRNIP	18	MRN complex interacting protein	protein_codin	0.02032	0.92	1	18.68	0.2702
**FOXC1**	FOXC1	18	forkhead box C1	protein_codin	-0.354	3.06E-05	0.7011	60.58	0.1001
**NEDD9**	NEDD9	18	neural precursor cell expressed, developmentally dov	protein_codin	0.1645	0.445	1	22	0.3677
**ACOT13**	ACOT13	18	acyl-CoA thioesterase 13	protein_codin	-0.1587	0.301	1	16.76	0.1026
**PBX2**	PBX2	18	PBX homeobox 2	protein_codin	0.3007	0.308	1	3.025	0.419
**WDR46**	WDR46	18	WD repeat domain 46	protein_codin	-0.141	0.402	1	8.557	0.1027
**C6orf226**	C6orf226	18	chromosome 6 open reading frame 226	protein_codin	-0.2783	0.117	1	8.753	0.1041
**MRPL2**	MRPL2	18	mitochondrial ribosomal protein L2	protein_codin	-0.01947	0.88	1	17.42	0.1026
**SLC35B2**	SLC35B2	18	solute carrier family 35 member B2	protein_codin	0.3914	0.0312	1	24.09	0.1328
**ADGRF5**	ADGRF5	18	adhesion G protein-coupled receptor F5	protein_codin	-0.08687	0.769	1	59.85	0.442
**MCM3**	MCM3	18	minichromosome maintenance complex component 3	protein_codin	-0.07974	0.633	1	7.132	0.2179
**SENP6**	SENP6	18	SUMO specific peptidase 6	protein_codin	-0.02207	0.847	1	49.92	0.1365
**IRAK1BP1**	IRAK1BP1	18	interleukin 1 receptor associated kinase 1 binding pro	protein_codin	1.062	0.00028	1	9.958	0.108
**LCA5**	LCA5	18	lebercilin LCA5	protein_codin	0.1996	0.234	1	8.423	0.1356
**MRAP2**	MRAP2	18	melanocortin 2 receptor accessory protein 2	protein_codin	-1.419	3.11E-15	7.42E-11	17.06	0.04112
**RARS2**	RARS2	18	arginyl-tRNA synthetase 2, mitochondrial	protein_codin	0.2277	0.214	1	8.63	0.07057
**C6orf203**	C6orf203	18	mitochondrial transcription rescue factor 1	protein_codin	-0.03938	0.792	1	6.646	0.07182
**CEP57L1**	CEP57L1	18	centrosomal protein 57 like 1	protein_codin	0.4554	0.0174	1	22.73	0.1782
**MICAL1**	MICAL1	18	microtubule associated monooxygenase, calponin an	protein_codin	0.6207	0.072	1	8.857	0.3196
**REV3L**	REV3L	18	REV3 like, DNA directed polymerase zeta catalytic su	protein_codin	0.6862	0.00027	1	39.75	0.2746
**FAM162B**	FAM162B	18	family with sequence similarity 162 member B	protein_codin	0.8226	0.0006	1	59.42	0.4049
**L3MBTL3**	L3MBTL3	18	L3MBTL histone methyl-lysine binding protein 3	protein_codin	0.4419	0.0576	1	50.63	0.3674
**AKAP7**	AKAP7	18	A-kinase anchoring protein 7	protein_codin	0.3113	0.272	1	8.172	0.1439
**MED23**	MED23	18	mediator complex subunit 23	protein_codin	0.02209	0.927	1	3.36	0.3185
**UST**	UST	18	uronyl 2-sulfotransferase	protein_codin	1.388	1.51E-06	0.03507	10.36	0.1076
**IGF2R**	IGF2R	18	insulin like growth factor 2 receptor	protein_codin	0.5489	0.00246	1	20.72	0.3542
**SUN1**	SUN1	18	Sad1 and UNC84 domain containing 1	protein_codin	0.4096	0.00718	1	17.48	0.2724
**MAD1L1**	MAD1L1	18	mitotic arrest deficient 1 like 1	protein_codin	0.2906	0.246	1	2.778	0.2168
**UMAD1**	UMAD1	18	UBAP1-MVB12-associated (UMA) domain containing 1	protein_codin	0.1317	0.49	1	10.32	0.1022
**AHR**	AHR	18	aryl hydrocarbon receptor	protein_codin	0.2503	0.226	1	60.36	0.2106
**HOTAIRM1**	HOTAIRM1	18	HOXA transcript antisense RNA, myeloid-specific 1	antisense_RN	-0.2793	0.165	1	41.57	0.287
**COA1**	COA1	18	cytochrome c oxidase assembly factor 1 homolog	protein_codin	-0.295	0.0897	1	7.082	0.1469
**BCL7B**	BCL7B	18	BAF chromatin remodeling complex subunit BCL7B	protein_codin	-0.04509	0.706	1	16.03	0.07284
**CCDC146**	CCDC146	18	coiled-coil domain containing 146	protein_codin	1.391	2.18E-07	0.0053	45.15	0.3531
**CD36**	CD36	18	CD36 molecule	protein_codin	-0.1246	0.751	1	59.36	1.737
**SRI**	SRI	18	sorcin	protein_codin	-0.0397	0.5297	1	60.52	0.03298
**STEAP4**	STEAP4	18	STEAP4 metalloreductase	protein_codin	-0.1903	0.347	1	60.6	0.2897
**SEM1**	SEM1	18	SEM1 26S proteasome complex subunit	protein_codin	-0.07162	0.219	1	53.41	0.07137
**PLOD3**	PLOD3	18	procollagen-lysine,2-oxoglutarate 5-dioxygenase 3	protein_codin	0.5148	0.0136	1	3.615	0.1819
**PSMC2**	PSMC2	18	proteasome 26S subunit, ATPase 2	protein_codin	-0.03782	0.726	1	13.06	0.1707
**COG5**	COG5	18	component of oligomeric golgi complex 5	protein_codin	-0.1721	0.4	1	2.618	0.2412
**THAP5**	THAP5	18	THAP domain containing 5	protein_codin	0.04777	0.722	1	16.55	0.102
**ARF5**	ARF5	18	ADP ribosylation factor 5	protein_codin	0.2237	0.0239	1	41.54	0.2154
**KLHDC10**	KLHDC10	18	kelch domain containing 10	protein_codin	-0.08644	0.715	1	10.06	0.4056
**SLC37A3**	SLC37A3	18	solute carrier family 37 member 3	protein_codin	0.3736	0.129	1	8.956	0.2424
**ABCB8**	ABCB8	18	ATP binding cassette subfamily B member 8	protein_codin	-0.07594	0.799	1	11.16	0.1788
**RAB9A**	RAB9A	18	RAB9A, member RAS oncogene family	protein_codin	0.6058	1.25E-05	0.2878	7.615	0.1372
**OFD1**	OFD1	18	OFD1 centriole and centriolar satellite protein	protein_codin	0.09224	0.55	1	7.742	0.1319
**TMEM47**	TMEM47	18	transmembrane protein 47	protein_codin	-0.6321	1.12E-09	2.64E-05	42.86	0.1014
**TSPAN7**	TSPAN7	18	tetraspanin 7	protein_codin	1.125	7.32E-06	0.1689	57.06	0.1326
**CASK**	CASK	18	calcium/calmodulin dependent serine protein kinase	protein_codin	-0.7223	2.39E-05	0.5485	24.46	0.234
**PQBP1**	PQBP1	18	polyglutamine binding protein 1	protein_codin	-0.4433	3.78E-05	0.8651	12.34	0.09727
**OTUD5**	OTUD5	18	OTU deubiquitinase 5	protein_codin	0.3124	0.134	1	3.287	0.5337
**FTX**	FTX	18	FTX transcript, XIST regulator	lincRNA	0.1415	0.382	1	32.62	0.415
**BEX5**	BEX5	18	brain expressed X-linked 5	protein_codin	-0.8929	6.13E-06	0.1415	27.88	0.3716
**PSMD10**	PSMD10	18	proteasome 26S subunit, non-ATPase 10	protein_codin	0.1521	0.416	1	7.733	0.1835
**RBMX**	RBMX	18	RNA binding motif protein X-linked	protein_codin	-0.1763	0.0454	1	52.12	0.101
**TMEM185A**	TMEM185A	18	transmembrane protein 185A	protein_codin	0.7372	0.00236	1	7.83	0.2282
**BCAP31**	BCAP31	18	B cell receptor associated protein 31	protein_codin	-0.01567	0.835	1	36.48	0.03486
**IKBKG**	IKBKG	18	inhibitor of nuclear factor kappa B kinase regulatory s	protein_codin	-0.3121	0.094	1	3.071	0.2241
**DKC1**	DKC1	18	dyskerin pseudouridine synthase 1	protein_codin	-0.006832	0.97	1	7.66	0.203
**PDGFRL**	PDGFRL	18	platelet derived growth factor receptor like	protein_codin	2.149	1.54E-55	3.74E-51	60.6	0.03202
**CSGALNACT1**	CSGALNACT1	18	chondroitin sulfate N-acetylgalactosaminyltransferase 1	protein_codin	-1.09	2.3E-08	0.00054	60.46	0.3875
**SLC25A37**	SLC25A37	18	solute carrier family 25 member 37	protein_codin	1.298	8.96E-17	2.14E-12	49.82	0.1006
**GSR**	GSR	18	glutathione-disulfide reductase	protein_codin	0.46	0.0396	1	3.337	0.2082
**MAK16**	MAK16	18	MAK16 homolog	protein_codin	0.5748	0.0145	1	3.178	0.1332
**C8orf4**	C8orf4	18	transcriptional and immune response regulator	protein_codin	NA	NA	NA	55.31	0.3906
**CYP7B1**	CYP7B1	18	cytochrome P450 family 7 subfamily B member 1	protein_codin	0.7912	2.54E-05	0.5826	51.72	0.07959
**TERF1**	TERF1	18	telomeric repeat binding factor 1	protein_codin	0.01852	0.875	1	11.35	0.1727
**FABP5**	FABP5	18	fatty acid binding protein 5	protein_codin	0.0926	0.669	1	60.58	0.4377
**FABP4**	FABP4	18	fatty acid binding protein 4	protein_codin	1.227	0.002	1	60.6	1.248
**RUNX1T1**	RUNX1T1	18	RUNX1 partner transcriptional co-repressor 1	protein_codin	0.8538	3.41E-06	0.07894	36.71	0.192
**OSR2**	OSR2	18	odd-skipped related transciption factor 2	protein_codin	3.485	2.41E-20	5.79E-16	32.84	0.1497
**POLR2K**	POLR2K	18	RNA polymerase II subunit K	protein_codin	-0.2468	0.00066	1	22.15	0.1352
**RNF19A**	RNF19A	18	ring finger protein 19A, RBR E3 ubiquitin protein ligase	protein_codin	0.9064	1.63E-09	3.85E-05	37.45	0.2082
**CTHRC1**	CTHRC1	18	collagen triple helix repeat containing 1	protein_codin	1.029	1.19E-08	0.00028	60.6	0.03301
**ZFPM2**	ZFPM2	18	zinc finger protein, FOG family member 2	protein_codin	0.1919	0.359	1	26.58	0.4165
**ATAD2**	ATAD2	18	ATPase family AAA domain containing 2	protein_codin	-0.2157	0.337	1	60.46	0.1022
**RNF139**	RNF139	18	ring finger protein 139	protein_codin	0.7988	0.00046	1	8.083	0.3142
**TATDN1**	TATDN1	18	TatD DNase domain containing 1	protein_codin	0.08484	0.505	1	60.22	0.1116
**MTSS1**	MTSS1	18	MTSS I-BAR domain containing 1	protein_codin	1.728	5.83E-06	0.1346	23.1	0.2197

**EFR3A**	EFR3A	18	EFR3 homolog A	protein_codin	-0.3169	0.0129	1	17.68	0.1794
**PHF20L1**	PHF20L1	18	PHD finger protein 20 like 1	protein_codin	0.2248	0.0796	1	51.43	0.133
**CHRAC1**	CHRAC1	18	chromatin accessibility complex subunit 1	protein_codin	0.01772	0.889	1	38.63	0.1489
**PTP4A3**	PTP4A3	18	protein tyrosine phosphatase 4A3	protein_codin	0.1517	0.282	1	59	0.03224
**GPAA1**	GPAA1	18	glycosylphosphatidylinositol anchor attachment 1	protein_codin	-0.09563	0.338	1	41.72	0.09899
**KANK1**	KANK1	18	KN motif and ankyrin repeat domains 1	protein_codin	-0.09137	0.557	1	31.88	0.2162
**SMARCA2**	SMARCA2	18	SWI/SNF related, matrix associated, actin dependent	protein_codin	0.1968	0.116	1	27.1	0.1436
**CNTLN**	CNTLN	18	centlein	protein_codin	0.3329	0.0919	1	7.958	0.207
**CAAP1**	CAAP1	18	caspase activity and apoptosis inhibitor 1	protein_codin	0.2033	0.319	1	9.037	0.204
**TMEM245**	TMEM245	18	transmembrane protein 245	protein_codin	-0.0511	0.776	1	5.859	0.1355
**SLC31A1**	SLC31A1	18	solute carrier family 31 member 1	protein_codin	0.06309	0.836	1	3.164	0.2379
**TLR4**	TLR4	18	toll like receptor 4	protein_codin	0.5571	0.0654	1	3.848	0.2461
**OLFML2A**	OLFML2A	18	olfactomedin like 2A	protein_codin	1.18	4.75E-08	0.00111	7.432	0.1481
**GOLGA1**	GOLGA1	18	golgin A1	protein_codin	0.1844	0.388	1	7.072	0.2001
**GAPVD1**	GAPVD1	18	GTPase activating protein and VPS9 domains 1	protein_codin	0.3935	0.0526	1	8.903	0.2091
**SH3GLB2**	SH3GLB2	18	SH3 domain containing GRB2 like, endophilin B2	protein_codin	0.2505	0.0711	1	57.88	0.1326
**CRAT**	CRAT	18	carnitine O-acetyltransferase	protein_codin	0.42	0.0756	1	29.52	0.133
**SPACA9**	SPACA9	18	sperm acrosome associated 9	protein_codin	0.2009	0.435	1	31.94	0.3259
**TMEM250**	TMEM250	18	transmembrane protein 250	protein_codin	-0.008215	0.966	1	3.378	0.2719
**NSMF**	NSMF	18	NMDA receptor synaptonuclear signaling and neuron	protein_codin	0.06902	0.731	1	8.819	0.2642
**AP2A2**	AP2A2	18	adaptor related protein complex 2 subunit alpha 2	protein_codin	0.6282	0.016	1	3.328	0.2898
**TOLLIP**	TOLLIP	18	toll interacting protein	protein_codin	-0.00808	0.964	1	8.604	0.1141
**PHLDA2**	PHLDA2	18	pleckstrin homology like domain family A member 2	protein_codin	0.3823	0.00916	1	60.6	0.1930
**MRVI1**	MRVI1	18	murine retrovirus integration site 1 homolog	protein_codin	-0.498	0.00102	1	36.28	0.06870
**PARVA**	PARVA	18	parvin alpha	protein_codin	-0.07411	0.545	1	34.57	0.03211
**NAV2**	NAV2	18	neuron navigator 2	protein_codin	0.2919	0.27	1	24.03	0.2574
**METTL15**	METTL15	18	methyltransferase like 15	protein_codin	0.6936	0.00242	1	7.732	0.3156
**DNAJC24**	DNAJC24	18	DnaJ heat shock protein family (Hsp40) member C24	protein_codin	0.3	0.192	1	2.782	0.1458
**ATG13**	ATG13	18	autophagy related 13	protein_codin	0.2264	0.421	1	3.438	0.2446
**PTPMT1**	PTPMT1	18	protein tyrosine phosphatase mitochondrial 1	protein_codin	0.4636	0.0132	1	9.188	0.2341
**TTC9C**	TTC9C	18	tetratricopeptide repeat domain 9C	protein_codin	0.4413	0.0508	1	7.188	0.2452
**RTN3**	RTN3	18	reticulon 3	protein_codin	0.3937	1.18E-06	0.02742	52.33	0.03267
**B4GAT1**	B4GAT1	18	beta-1,4-glucuronyltransferase 1	protein_codin	0.8866	2.1E-08	0.00049	22.79	0.03364
**PELI3**	PELI3	18	pellino E3 ubiquitin protein ligase family member 3	protein_codin	-0.4412	0.0682	1	2.75	0.3788
**RBM4B**	RBM4B	18	RNA binding motif protein 4B	protein_codin	0.06264	0.762	1	10.34	0.2035
**GRK2**	GRK2	18	G protein-coupled receptor kinase 2	protein_codin	-0.4309	0.0925	1	2.603	0.4086
**ANKRD13D**	ANKRD13D	18	ankyrin repeat domain 13D	protein_codin	0.3126	0.127	1	8.093	0.1964
**MRGPRF**	MRGPRF	18	MAS related GPR family member F	protein_codin	0.02718	0.819	1	59.92	0.1772
**ANO1**	ANO1	18	anoctamin 1	protein_codin	-0.4777	0.00578	1	34.35	0.179
**PPFIA1**	PPFIA1	18	PTPRF interacting protein alpha 1	protein_codin	0.4495	0.00617	1	12.17	0.2031
**FCHSD2**	FCHSD2	18	FCH and double SH3 domains 2	protein_codin	-0.2089	0.181	1	42.04	0.2509
**TMEM126A**	TMEM126A	18	transmembrane protein 126A	protein_codin	-0.2281	0.0722	1	17.62	0.1371
**JRKL**	JRKL	18	JRK like	protein_codin	-0.2453	0.214	1	3.594	0.2059
**ARHGAP42**	ARHGAP42	18	Rho GTPase activating protein 42	protein_codin	0.1221	0.482	1	32.93	0.3175
**BIRC2**	BIRC2	18	baculoviral IAP repeat containing 2	protein_codin	0.5324	0.00168	1	48.08	0.1010
**CASP1**	CASP1	18	caspase 1	protein_codin	-0.4886	0.0336	1	10.76	0.1421
**FXYD6**	FXYD6	18	FXYD domain containing ion transport regulator 6	protein_codin	0.407	0.0492	1	48.9	0.1819
**CBL**	CBL	18	Cbl proto-oncogene	protein_codin	-0.5112	0.0399	1	3.75	0.3738
**THY1**	THY1	18	Thy-1 cell surface antigen	protein_codin	-0.7285	6.77E-06	0.1563	60.54	0.2988
**SC5D**	SC5D	18	sterol-C5-desaturase	protein_codin	0.129	0.539	1	9.675	0.1022
**CLMP**	CLMP	18	CXADR like membrane protein	protein_codin	0.5388	0.00679	1	57.9	0.1144
**PFKFB3**	PFKFB3	18	6-phosphofructo-2-kinase/fructose-2,6-biphosphatase 3	protein_codin	-0.6487	0.00463	1	11.47	0.4522
**DNAJC1**	DNAJC1	18	DnaJ heat shock protein family (Hsp40) member C1	protein_codin	0.3207	0.00246	1	23.26	0.06715
**PIP4K2A**	PIP4K2A	18	phosphatidylinositol-5-phosphate 4-kinase type 2 alpha	protein_codin	-0.08537	0.536	1	14.49	0.07050
**ZNF248**	ZNF248	18	zinc finger protein 248	protein_codin	-0.1821	0.422	1	7.212	0.2477
**WASHC2C**	WASHC2C	18	WASH complex subunit 2C	protein_codin	0.4107	0.109	1	2.888	0.3666
**ARID5B**	ARID5B	18	AT-rich interaction domain 5B	protein_codin	0.01	0.889	1	59.3	0.06540
**FAM35A**	FAM35A	18	shieldin complex subunit 2	protein_codin	NA	NA	NA	26.96	0.277
**5-Mar**	43529	18	membrane associated ring-CH-type finger 5	protein_codin	-0.08506	0.639	1	8.752	0.1052
**LCOR**	LCOR	18	ligand dependent nuclear receptor corepressor	protein_codin	0.1976	0.322	1	7.779	0.2676
**EXOSC1**	EXOSC1	18	exosome component 1	protein_codin	0.1462	0.352	1	22.48	0.1694
**SLC25A28**	SLC25A28	18	solute carrier family 25 member 28	protein_codin	0.1518	0.35	1	8.255	0.1986
**HSPA12A**	HSPA12A	18	heat shock protein family A (Hsp70) member 12A	protein_codin	1.256	9.72E-05	1	7.645	0.5663
**BAG3**	BAG3	18	BCL2 associated athanogene 3	protein_codin	0.2425	0.0866	1	28.78	0.1302
**EEF1AKMT2**	EEF1AKMT2	18	EEF1A lysine methyltransferase 2	protein_codin	0.1176	0.512	1	8.904	0.1369
**NINJ2**	NINJ2	18	ninjurin 2	protein_codin	1.482	8.03E-08	0.00186	9.958	0.102
**ETV6**	ETV6	18	ETS variant 6	protein_codin	-0.743	0.00048	1	27.9	0.1116
**ARHGDIB**	ARHGDIB	18	Rho GDP dissociation inhibitor beta	protein_codin	-1.172	4.27E-33	1.03E-28	54.86	0.1165
**RERG**	RERG	18	RAS like estrogen regulated growth inhibitor	protein_codin	-0.8837	3.11E-12	7.39E-08	43.38	0.1122
**KCNJ8**	KCNJ8	18	potassium voltage-gated channel subfamily J member 8	protein_codin	0.3992	0.0342	1	34.63	0.2752
**ABCC9**	ABCC9	18	ATP binding cassette subfamily C member 9	protein_codin	1.632	1.19E-20	2.86E-16	55.33	0.282
**PPHLN1**	PPHLN1	18	periphilin 1	protein_codin	-0.0935	0.466	1	15.12	0.1686
**ANO6**	ANO6	18	anoctamin 6	protein_codin	0.355	0.00142	1	35.45	0.1727
**SLC11A2**	SLC11A2	18	solute carrier family 11 member 2	protein_codin	0.6975	0.00263	1	10.42	0.379
**DAZAP2**	DAZAP2	18	DAZ associated protein 2	protein_codin	0.2676	0.00466	1	18.93	0.07132
**NDUFA4L2**	NDUFA4L2	18	NDUFA4 mitochondrial complex associated like 2	protein_codin	-0.5394	4.77E-07	0.01112	60.6	0.2151
**TSPAN8**	TSPAN8	18	tetraspanin 8	protein_codin	1.918	4.93E-10	1.17E-05	55.26	0.1037
**PHLDA1**	PHLDA1	18	pleckstrin homology like domain family A member 1	protein_codin	0.004395	0.977	1	50.86	0.1454
**MRPL42**	MRPL42	18	mitochondrial ribosomal protein L42	protein_codin	0.1255	0.287	1	5.944	0.1357
**SOCS2**	SOCS2	18	suppressor of cytokine signaling 2	protein_codin	-0.3555	0.0368	1	18.12	0.2347
**SNRPF**	SNRPF	18	small nuclear ribonucleoprotein polypeptide F	protein_codin	-0.3907	7.84E-06	0.1808	30.63	0.09942
**CMKLR1**	CMKLR1	18	chemerin chemokine-like receptor 1	protein_codin	-0.4517	0.0664	1	9.883	0.164
**VPS29**	VPS29	18	VPS29 retromer complex component	protein_codin	-0.1638	0.017	1	54.96	0.03318
**PLBD2**	PLBD2	18	phospholipase B domain containing 2	protein_codin	0.7026	0.0007	1	9.526	0.03257
**ANAPC5**	ANAPC5	18	anaphase promoting complex subunit 5	protein_codin	0.244	0.0407	1	23.06	0.03217
**RNF6**	RNF6	18	ring finger protein 6	protein_codin	-0.3247	0.186	1	7.998	0.4119
**PAN3**	PAN3	18	poly(A) specific ribonuclease subunit PAN3	protein_codin	0.2772	0.125	1	8.457	0.4684
**NUFIP1**	NUFIP1	18	nuclear FMR1 interacting protein 1	protein_codin	0.1593	0.434	1	10.26	0.2442
**ESD**	ESD	18	esterase D	protein_codin	0.01044	0.838	1	60.58	0.03406
**TRIM13**	TRIM13	18	tripartite motif containing 13	protein_codin	-0.3376	0.0298	1	21.92	0.2484
**CARS2**	CARS2	18	cysteinyl-tRNA synthetase 2, mitochondrial	protein_codin	0.04375	0.82	1	8.722	0.172
**PNP**	PNP	18	purine nucleoside phosphorylase	protein_codin	-0.91	0.00125	1	11.55	0.3693
**ANG**	ANG	18	angiogenin	protein_codin	0.6072	9.01E-07	0.02097	20.9	0.09978
**METTL3**	METTL3	18	methyltransferase like 3	protein_codin	-0.3781	0.065	1	8.482	0.07074
**BRMS1L**	BRMS1L	18	BRMS1 like transcriptional repressor	protein_codin	0.4352	0.029	1	8.543	0.2751
**PNN**	PNN	18	pinin, desmosome associated protein	protein_codin	-0.0619	0.502	1	23.76	0.1032
**C14orf28**	C14orf28	18	chromosome 14 open reading frame 28	protein_codin	-0.3508	0.07	1	8.078	0.2571
**KLHL28**	KLHL28	18	kelch like family member 28	protein_codin	0.2894	0.107	1	26.48	0.2144
**STYX**	STYX	18	serine/threonine/tyrosine interacting protein	protein_codin	-0.292	0.188	1	2.446	0.2085
**FBXO34**	FBXO34	18	F-box protein 34	protein_codin	0.3985	0.0781	1	7.1	0.1015
**JKAMP**	JKAMP	18	JNK1/MAPK8 associated membrane protein	protein_codin	0.3913	0.00373	1	6.223	0.1074
**RTN1**	RTN1	18	reticulon 1	protein_codin	-0.6802	0.0112	1	60.52	0.3881
**PIGH**	PIGH	18	phosphatidylinositol glycan anchor biosynthesis class H	protein_codin	0.3571	0.0337	1	7.489	0.1415
**NPC2**	NPC2	18	NPC intracellular cholesterol transporter 2	protein_codin	-0.2706	7.66E-06	0.1767	34.98	0.03671
**PTPN21**	PTPN21	18	protein tyrosine phosphatase non-receptor type 21	protein_codin	-0.5571	0.0238	1	8.373	0.4161
**ITPK1**	ITPK1	18	inositol-tetrakisphosphate 1-kinase	protein_codin	0.5592	0.044	1	8.278	0.1405
**ANKRD9**	ANKRD9	18	ankyrin repeat domain 9	protein_codin	0.6575	0.00132	1	26.14	0.1002
**CRIP2**	CRIP2	18	cysteine rich protein 2	protein_codin	-0.5039	9.45E-18	2.26E-13	59.34	0.0849
**CRIP1**	CRIP1	18	cysteine rich protein 1	protein_codin	0.5674	1.31E-09	3.09E-05	59.94	0.1621
**TUBGCP5**	TUBGCP5	18	tubulin gamma complex associated protein 5	protein_codin	0.02965	0.85	1	3.726	0.2382
**IVD**	IVD	18	isovaleryl-CoA dehydrogenase	protein_codin	0.05842	0.741	1	7.414	0.1421
**FGF7**	FGF7	18	fibroblast growth factor 7	protein_codin	2.137	2.23E-48	5.41E-44	60.6	0.03202
**NEDD4**	NEDD4	18	NEDD4 E3 ubiquitin protein ligase	protein_codin	-0.4534	0.0106	1	33.28	0.4463
**TCF12**	TCF12	18	transcription factor 12	protein_codin	0.6586	0.00017	1	17.24	0.2209
**LACTB**	LACTB	18	lactamase beta	protein_codin	0.1465	0.41	1	7.964	0.03772
**MYO9A**	MYO9A	18	myosin IXA	protein_codin	0.458	0.0141	1	17.16	0.4988
**CSPG4**	CSPG4	18	chondroitin sulfate proteoglycan 4	protein_codin	-0.8615	0.00046	1	9.535	0.2098
**RPS17**	RPS17	18	ribosomal protein S17	protein_codin	-0.1259	0.416	1	21.52	0.1432
**FES**	FES	18	FES proto-oncogene, tyrosine kinase	protein_codin	0.188	0.441	1	2.917	0.1072
**LRRC28**	LRRC28	18	leucine rich repeat containing 28	protein_codin	-0.4041	0.0979	1	8.307	0.2311
**MRPS34**	MRPS34	18	mitochondrial ribosomal protein S34	protein_codin	-0.09725	0.235	1	29.83	0.07
**SPSB3**	SPSB3	18	splA/ryanodine receptor domain and SOCS box conta	protein_codin	-0.004177	0.972	1	6.389	0.2142
**ZNF598**	ZNF598	18	zinc finger protein 598	protein_codin	0.2877	0.2	1	3.144	0.3021
**FLYWCH1**	FLYWCH1	18	FLYWCH-type zinc finger 1	protein_codin	0.3498	0.154	1	8.403	0.3140
**GLYR1**	GLYR1	18	glyoxylate reductase 1 homolog	protein_codin	0.3152	0.0882	1	26.17	0.168
**COQ7**	COQ7	18	coenzyme Q7, hydroxylase	protein_codin	0.1243	0.512	1	9.719	0.1694
**KNOP1**	KNOP1	18	lysine rich nucleolar protein 1	protein_codin	0.7258	0.00068	1	7.096	0.1504
**RBL2**	RBL2	18	RB transcriptional corepressor like 2	protein_codin	0.4399	0.0074	1	6.853	0.2059
**CENPT**	CENPT	18	centromere protein T	protein_codin	0.3132	0.246	1	7.65	0.1346
**SLC7A6OS**	SLC7A6OS	18	solute carrier family 7 member 6 opposite strand	protein_codin	0.2227	0.329	1	8.874	0.2105
**ZFP90**	ZFP90	18	ZFP90 zinc finger protein	protein_codin	0.08844	0.702	1	29.83	0.1367
**KARS**	KARS	18	lysyl-tRNA synthetase	protein_codin	-0.2045	0.279	1	8.464	0.3052
**SPAG7**	SPAG7	18	sperm associated antigen 7	protein_codin	0.03301	0.772	1	30.28	0.2376
**DERL2**	DERL2	18	derlin 2	protein_codin	0.1448	0.237	1	30.62	0.07206
**BORCS6**	BORCS6	18	BLOC-1 related complex subunit 6	protein_codin	0.1848	0.352	1	7.69	0.1027
**STX8**	STX8	18	syntaxin 8	protein_codin	0.226	0.0626	1	36.07	0.109
**RASD1**	RASD1	18	ras related dexamethasone induced 1	protein_codin	2.47	4.82E-09	0.00011	60.6	0.5286
**ULK2**	ULK2	18	unc-51 like autophagy activating kinase 2	protein_codin	0.01918	0.944	1	3.152	0.3186
**ZNF207**	ZNF207	18	zinc finger protein 207	protein_codin	0.2982	0.0317	1	14.79	0.252
**PIP4K2B**	PIP4K2B	18	phosphatidylinositol-5-phosphate 4-kinase type 2 beta	protein_codin	-0.2493	0.332	1	2.74	0.382
**PLXDC1**	PLXDC1	18	plexin domain containing 1	protein_codin	0.6279	0.0248	1	24.1	0.309
**ARL4D**	ARL4D	18	ADP ribosylation factor like GTPase 4D	protein_codin	1.832	4.86E-19	1.17E-14	50.48	0.0687
**NMT1**	NMT1	18	N-myristoyltransferase 1	protein_codin	0.1977	0.194	1	7.218	0.1461
**PLCD3**	PLCD3	18	phospholipase C delta 3	protein_codin	0.1478	0.552	1	45.7	0.1368
**SPAG9**	SPAG9	18	sperm associated antigen 9	protein_codin	0.2558	0.00532	1	48.58	0.1321
**4-Sep**	43712	18	septin 4	protein_codin	-0.6133	4.66E-06	0.00109	60.52	0.1116
**VMP1**	VMP1	18	vacuole membrane protein 1	protein_codin	0.4298	0.0002	1	60.58	0.2594
**MRC2**	MRC2	18	mannose receptor C type 2	protein_codin	0.687	1.31E-06	0.03044	32.24	0.172
**DCAF7**	DCAF7	18	DDB1 and CUL4 associated factor 7	protein_codin	0.1068	0.579	1	25.8	0.1762
**LIMD2**	LIMD2	18	LIM domain containing 2	protein_codin	-0.7894	0.00779	1	10.87	0.2606
**CCDC47**	CCDC47	18	coiled-coil domain containing 47	protein_codin	0.2777	0.00599	1	52.96	0.1719
**GPRC5C**	GPRC5C	18	G protein-coupled receptor class C group 5 member C	protein_codin	-0.1742	0.185	1	37.42	0.1668
**MRPL58**	MRPL58	18	mitochondrial ribosomal protein L58	protein_codin	-0.05129	0.776	1	7.702	0.1508
**TRIM47**	TRIM47	18	tripartite motif containing 47	protein_codin	0.01467	0.902	1	59.92	0.1039
**PRPSAP1**	PRPSAP1	18	phosphoribosyl pyrophosphate synthetase associated	protein_codin	-0.03886	0.781	1	19.7	0.2706
**CYGB**	CYGB	18	cytoglobin	protein_codin	1.962	1.12E-15	2.68E-11	59.92	0.2202
**SEC14L1**	SEC14L1	18	SEC14 like lipid binding 1	protein_codin	0.2718	0.0881	1	17.48	0.06436
**AFMID**	AFMID	18	arylformamidase	protein_codin	0.1142	0.627	1	7.402	0.2449
**OGFOD3**	OGFOD3	18	2-oxoglutarate and iron dependent oxygenase domai	protein_codin	-0.02553	0.856	1	37.3	0.1416
**NDUFV2**	NDUFV2	18	NADH:ubiquinone oxidoreductase core subunit V2	protein_codin	-0.09112	0.139	1	41.6	0.03298
**APCDD1**	APCDD1	18	APC down-regulated 1	protein_codin	-0.004757	0.96	1	59.98	0.2478
**PSMG2**	PSMG2	18	proteasome assembly chaperone 2	protein_codin	0.006206	0.935	1	54.54	0.07126
**GATA6**	GATA6	18	GATA binding protein 6	protein_codin	0.05366	0.691	1	41.29	0.03503
**TRAPPC8**	TRAPPC8	18	trafficking protein particle complex 8	protein_codin	-0.3108	0.217	1	2.873	0.2134
**DTNA**	DTNA	18	dystrobrevin alpha	protein_codin	-0.8863	2.98E-05	0.6829	3.587	0.4112
**MALT1**	MALT1	18	MALT1 paracaspase	protein_codin	0.4844	0.097	1	3.106	0.3557
**RNF152**	RNF152	18	ring finger protein 152	protein_codin	-0.3242	0.0872	1	44.14	0.2062
**VPS4B**	VPS4B	18	vacuolar protein sorting 4 homolog B	protein_codin	0.3855	0.00115	1	59.87	0.245
**HSBP1L1**	HSBP1L1	18	heat shock factor binding protein 1 like 1	protein_codin	-0.1781	0.52	1	56.53	0.07183
**PSMF1**	PSMF1	18	proteasome inhibitor subunit 1	protein_codin	-0.1525	0.0948	1	43.92	0.1368
**CRNKL1**	CRNKL1	18	crooked neck pre-mRNA splicing factor 1	protein_codin	0.006474	0.967	1	20.58	0.2111
**THBD**	THBD	18	thrombomodulin	protein_codin	-0.09988	0.562	1	59.99	0.2732
**COX4I2**	COX4I2	18	cytochrome c oxidase subunit 4I2	protein_codin	1.153	8.72E-07	0.0203	37.36	0.5293
**SLC35C2**	SLC35C2	18	solute carrier family 35 member C2	protein_codin	0.2347	0.246	1	7.508	0.1042
**SLC2A4RG**	SLC2A4RG	18	SLC2A4 regulator	protein_codin	-0.2103	0.0227	1	34.43	0.03254
**C19orf25**	C19orf25	18	chromosome 19 open reading frame 25	protein_codin	-0.2232	0.212	1	5.314	0.3742
**SPPL2B**	SPPL2B	18	signal peptide peptidase like 2B	protein_codin	0.3232	0.159	1	3.654	0.1359
**ELAVL1**	ELAVL1	18	ELAV like RNA binding protein 1	protein_codin	0.1872	0.22	1	18.13	0.1021
**SLC44A2**	SLC44A2	18	solute carrier family 44 member 2	protein_codin	-0.1046	0.606	1	23.42	0.1071
**QTRT1**	QTRT1	18	queuine tRNA-ribosyltransferase catalytic subunit 1	protein_codin	-0.05296	0.747	1	21.32	0.06795
**BRD4**	BRD4	18	bromodomain containing 4	protein_codin	0.07971	0.514	1	23.58	0.06616
**ISYNA1**	ISYNA1	18	inositol-3-phosphate synthase 1	protein_codin	0.2942	0.0057	1	54.18	0.06951
**RFXANK**	RFXANK	18	regulatory factor X associated ankyrin containing protein	protein_codin	-0.01193	0.941	1	7.866	0.1675
**ZNF599**	ZNF599	18	zinc finger protein 599	protein_codin	0.02736	0.921	1	11.03	0.1276
**EID2**	EID2	18	EP300 interacting inhibitor of differentiation 2	protein_codin	0.2422	0.196	1	6.078	0.1756
**GSK3A**	GSK3A	18	glycogen synthase kinase 3 alpha	protein_codin	-0.3427	0.04	1	6.488	0.1306
**ERF**	ERF	18	ETS2 repressor factor	protein_codin	0.458	0.0376	1	10.62	0.07095
**EHD2**	EHD2	18	EH domain containing 2	protein_codin	-0.1367	0.264	1	40.62	0.1691
**NOP53**	NOP53	18	NOP53 ribosome biogenesis factor	protein_codin	0.00522	0.932	1	49.38	0.03268
**KDELR1**	KDELR1	18	KDEL endoplasmic reticulum protein retention receptor 1	protein_codin	0.1328	0.0266	1	47.38	0.03322
**NOSIP**	NOSIP	18	nitric oxide synthase interacting protein	protein_codin	0.08282	0.450	1	11.78	0.1024
**AKT1S1**	AKT1S1	18	AKT1 substrate 1	protein_codin	0.03279	0.844	1	6.227	0.1677
**CTU1**	CTU1	18	cytosolic thiouridylase subunit 1	protein_codin	-0.1626	0.472	1	3.382	0.1657
**ZNF880**	ZNF880	18	zinc finger protein 880	protein_codin	0.2168	0.317	1	8.092	0.06668
**SNRPD3**	SNRPD3	18	small nuclear ribonucleoprotein D3 polypeptide	protein_codin	-0.05278	0.461	1	31.74	0.06642
**GGA1**	GGA1	18	golgi associated, gamma adaptin ear containing, ARF	protein_codin	0.1772	0.449	1	7.864	0.179
**XPNPEP3**	XPNPEP3	18	X-prolyl aminopeptidase 3	protein_codin	0.3313	0.197	1	3.435	0.2803
**CSDC2**	CSDC2	18	cold shock domain containing C2	protein_codin	-0.5815	0.00041	1	57.22	0.2386
**NCAPH2**	NCAPH2	18	non-SMC condensin II complex subunit H2	protein_codin	0.0978	0.669	1	10.49	0.173
**SETD4**	SETD4	18	SET domain containing 4	protein_codin	0.7756	0.0162	1	3.182	0.2581
**DYRK1A**	DYRK1A	18	dual specificity tyrosine phosphorylation regulated kin	protein_codin	0.3797	0.0771	1	34.64	0.1742
**PKNOX1**	PKNOX1	18	PBX/knotted 1 homeobox 1	protein_codin	0.1376	0.512	1	3.629	0.218
**THAP3**	THAP3	19	THAP domain containing 3	protein_codin	0.0922	0.632	1	6.679	0.1105
**VAMP3**	VAMP3	19	vesicle associated membrane protein 3	protein_codin	0.003637	0.966	1	58.82	0.06859
**SPEN**	SPEN	19	spen family transcriptional repressor	protein_codin	0.07082	0.679	1	18.56	0.3893
**FUCA1**	FUCA1	19	alpha-L-fucosidase 1	protein_codin	-0.02942	0.842	1	7.334	0.1026
**KHDRBS1**	KHDRBS1	19	KH RNA binding domain containing, signal transducti	protein_codin	-0.01625	0.839	1	48.34	0.1676
**STK40**	STK40	19	serine/threonine kinase 40	protein_codin	0.2385	0.35	1	2.92	0.3158
**GNL2**	GNL2	19	G protein nucleolar 2	protein_codin	0.2613	0.122	1	9.167	0.2105
**UTP11**	UTP11	19	UTP11 small subunit processome component	protein_codin	0.07312	0.579	1	7.436	0.1321
**SVBP**	SVBP	19	small vasohibin binding protein	protein_codin	0.3238	0.0106	1	51.93	0.09851
**POMGNT1**	POMGNT1	19	protein O-linked mannose N-acetylglucosaminyltransf	protein_codin	-0.4465	0.0602	1	3.459	0.1366
**ZFYVE9**	ZFYVE9	19	zinc finger FYVE-type containing 9	protein_codin	0.01775	0.926	1	3.404	0.168
**AC092807.3**	AC092807.3	19	novel transcri	lincRNA	-0.5371	0.0662	1	10.62	0.3134
**EXTL2**	EXTL2	19	exostosin like glycosyltransferase 2	protein_codin	0.1606	0.307	1	18.7	0.0997
**DDX20**	DDX20	19	DEAD-box helicase 20	protein_codin	-0.3021	0.159	1	2.852	0.1046
**RNF115**	RNF115	19	ring finger protein 115	protein_codin	-0.2925	0.0119	1	21.93	0.1426
**PIP5K1A**	PIP5K1A	19	phosphatidylinositol-4-phosphate 5-kinase type 1 alpha	protein_codin	0.06416	0.79	1	2.219	0.1657
**SNX27**	SNX27	19	sorting nexin 27	protein_codin	0.3382	0.0754	1	24.37	0.2450
**PFDN2**	PFDN2	19	prefoldin subunit 2	protein_codin	-0.05894	0.457	1	47.52	0.1332
**ALDH9A1**	ALDH9A1	19	aldehyde dehydrogenase 9 family member A1	protein_codin	0.4052	0.00056	1	13.22	0.068
**ACBD6**	ACBD6	19	acyl-CoA binding domain containing 6	protein_codin	0.04769	0.69	1	34.82	0.1077
**IER5**	IER5	19	immediate early response 5	protein_codin	-0.0703	0.659	1	60.6	0.4262
**GLUL**	GLUL	19	glutamate-ammonia ligase	protein_codin	1.026	8.26E-24	1.98E-19	54.7	0.09980
**LYPLAL1**	LYPLAL1	19	lysophospholipase like 1	protein_codin	0.01951	0.896	1	48.65	0.09739
**WDR26**	WDR26	19	WD repeat domain 26	protein_codin	0.1448	0.596	1	3.209	0.3731
**TRMT61B**	TRMT61B	19	tRNA methyltransferase 61B	protein_codin	0.5465	0.0146	1	3.632	0.2427
**SPAST**	SPAST	19	spastin	protein_codin	0.6882	0.00160	1	8.11	0.1076
**MTA3**	MTA3	19	metastasis associated 1 family member 3	protein_codin	0.1862	0.432	1	3.532	0.2712
**HAAO**	HAAO	19	3-hydroxyanthranilate 3,4-dioxygenase	protein_codin	-0.5588	0.0002	1	8.566	0.1802
**NFU1**	NFU1	19	NFU1 iron-sulfur cluster scaffold	protein_codin	-0.4295	0.0002	1	16.38	0.3885
**FMNL2**	FMNL2	19	formin like 2	protein_codin	0.6048	0.00212	1	22.67	0.1326
**ORMDL1**	ORMDL1	19	ORMDL sphingolipid biosynthesis regulator 1	protein_codin	0.02872	0.755	1	45.74	0.1646
**CLK1**	CLK1	19	CDC like kinase 1	protein_codin	0.2323	0.0848	1	27.11	0.1117
**RNF25**	RNF25	19	ring finger protein 25	protein_codin	-0.3725	0.0919	1	2.773	0.1671
**RPUSD3**	RPUSD3	19	RNA pseudouridine synthase D3	protein_codin	0.2214	0.256	1	7.546	0.1382
**VGLL4**	VGLL4	19	vestigial like family member 4	protein_codin	-0.5243	5.41E-06	0.00012	41.25	0.1402
**IQSEC1**	IQSEC1	19	IQ motif and Sec7 domain 1	protein_codin	-0.2698	0.125	1	8.228	0.2322
**LSM3.00**	LSM3.00	19	LSM3 homolog, U6 small nuclear RNA and mRNA de	protein_codin	-0.1107	0.185	1	37.22	0.1029
**RFTN1**	RFTN1	19	raftlin, lipid raft linker 1	protein_codin	0.5788	0.00207	1	15.69	0.2064
**NGLY1**	NGLY1	19	N-glycanase 1	protein_codin	0.03009	0.894	1	7.143	0.1776
**RASSF1**	RASSF1	19	Ras association domain family member 1	protein_codin	0.5209	0.00128	1	16.6	0.1412
**NPRL2**	NPRL2	19	NPR2 like, GATOR1 complex subunit	protein_codin	0.0206	0.916	1	3.178	0.2085
**SLC25A26**	SLC25A26	19	solute carrier family 25 member 26	protein_codin	-0.2404	0.172	1	3.172	0.2586
**C3orf38**	C3orf38	19	chromosome 3 open reading frame 38	protein_codin	0.03648	0.833	1	8.963	0.06892
**TRMT10C**	TRMT10C	19	tRNA methyltransferase 10C, mitochondrial RNase P	protein_codin	-0.00882	0.947	1	40.07	0.1001
**SLC35A5**	SLC35A5	19	solute carrier family 35 member A5	protein_codin	0.4224	0.0386	1	7.088	0.1406
**RABL3**	RABL3	19	RAB, member of RAS oncogene family like 3	protein_codin	0.01022	0.962	1	3.444	0.2421
**IQCB1**	IQCB1	19	IQ motif containing B1	protein_codin	0.04088	0.831	1	3.279	0.247
**STAG1**	STAG1	19	stromal antigen 1	protein_codin	0.6215	0.00203	1	7.03	0.1786
**ZBTB38**	ZBTB38	19	zinc finger and BTB domain containing 38	protein_codin	0.08094	0.345	1	60.6	0.1718
**NAALADL2**	NAALADL2	19	N-acetylated alpha-linked acidic dipeptidase like 2	protein_codin	0.4317	0.0456	1	29.55	0.2249
**ALG3**	ALG3	19	ALG3 alpha-1,3- mannosyltransferase	protein_codin	0.1222	0.462	1	6.822	0.06506
**ZNF721**	ZNF721	19	zinc finger protein 721	protein_codin	0.5151	0.0282	1	6.449	0.3757
**MED28**	MED28	19	mediator complex subunit 28	protein_codin	-0.1761	0.159	1	17.22	0.2318
**PDLIM5**	PDLIM5	19	PDZ and LIM domain 5	protein_codin	-0.4847	6.89E-09	0.00016	59.78	0.03292
**GUCY1B3**	GUCY1B3	19	guanylate cyclase 1 soluble subunit beta 1	protein_codin	NA	NA	NA	55.22	0.03358
**SLC25A4**	SLC25A4	19	solute carrier family 25 member 4	protein_codin	-0.4939	3.7E-12	8.79E-08	60.6	0.1041
**ANKRD37**	ANKRD37	19	ankyrin repeat domain 37	protein_codin	-0.2599	0.147	1	60.6	0.639
**FAM149A**	FAM149A	19	family with sequence similarity 149 member A	protein_codin	0.3626	0.142	1	3.428	0.137
**SUB1**	SUB1	19	SUB1 regulator of transcription	protein_codin	-0.276	5.36E-06	0.1238	36.7	0.03542
**FCHO2**	FCHO2	19	FCH domain only 2	protein_codin	0.2408	0.077	1	40.42	0.1336
**PJA2**	PJA2	19	praja ring finger ubiquitin ligase 2	protein_codin	0.04560	0.519	1	41.73	0.07092
**CAMLG**	CAMLG	19	calcium modulating ligand	protein_codin	-0.3353	2.56E-05	0.5871	60.35	0.03724
**TTC1**	TTC1	19	tetratricopeptide repeat domain 1	protein_codin	-0.2157	0.0237	1	59.58	0.1356
**SH3PXD2B**	SH3PXD2B	19	SH3 and PX domains 2B	protein_codin	0.8692	0.00123	1	9.576	0.2872
**BOD1**	BOD1	19	biorientation of chromosomes in cell division 1	protein_codin	-0.3075	0.00582	1	15.87	0.07557
**RNF130**	RNF130	19	ring finger protein 130	protein_codin	0.1573	0.111	1	20.13	0.1345
**DUSP22**	DUSP22	19	dual specificity phosphatase 22	protein_codin	0.0403	0.777	1	18.34	0.1384
**SERPINB1**	SERPINB1	19	serpin family B member 1	protein_codin	0.5314	1.63E-07	0.00381	34.94	0.1357
**PXDC1**	PXDC1	19	PX domain containing 1	protein_codin	0.2029	0.065	1	36.3	0.09814
**BAG6**	BAG6	19	BCL2 associated athanogene 6	protein_codin	0.08436	0.64	1	8.745	0.06468
**AGPAT1**	AGPAT1	19	1-acylglycerol-3-phosphate O-acyltransferase 1	protein_codin	-0.03982	0.839	1	51.97	0.1446
**FKBP5**	FKBP5	19	FKBP prolyl isomerase 5	protein_codin	0.5892	1E-05	0.2304	24.76	0.06599
**OARD1**	OARD1	19	O-acyl-ADP-ribose deacylase 1	protein_codin	-0.2322	0.135	1	7.462	0.2624
**CCND3**	CCND3	19	cyclin D3	protein_codin	-0.5682	0.00035	1	40.68	0.03752
**UBR2**	UBR2	19	ubiquitin protein ligase E3 component n-recognin 2	protein_codin	0.5413	0.00182	1	8.086	0.2721
**TBCC**	TBCC	19	tubulin folding cofactor C	protein_codin	0.4715	0.00716	1	52.57	0.2151
**TPBG**	TPBG	19	trophoblast glycoprotein	protein_codin	0.7192	2.96E-05	0.6784	27.82	0.1996
**FAM229B**	FAM229B	19	family with sequence similarity 229 member B	protein_codin	-0.1052	0.402	1	36.36	0.06998
**HIVEP2**	HIVEP2	19	human immunodeficiency virus type I enhancer bindin	protein_codin	-0.2598	0.302	1	9.965	0.1427
**TAB2**	TAB2	19	TGF-beta activated kinase 1 (MAP3K7) binding protein 2	protein_codin	0.1561	0.541	1	3.363	0.3312
**MAP3K4**	MAP3K4	19	mitogen-activated protein kinase kinase kinase 4	protein_codin	-0.1098	0.532	1	9.219	0.2086
**MPLKIP**	MPLKIP	19	M-phase specific PLK1 interacting protein	protein_codin	-0.0335	0.786	1	14.22	0.105
**NIPSNAP2**	NIPSNAP2	19	nipsnap homolog 2	protein_codin	-0.2455	0.0298	1	39.56	0.1043
**PDK4**	PDK4	19	pyruvate dehydrogenase kinase 4	protein_codin	0.6318	1.73E-07	0.00405	60.6	0.3079
**SRRT**	SRRT	19	serrate, RNA effector molecule	protein_codin	0.6032	0.00188	1	9.476	0.1367
**ATP6V0E2**	ATP6V0E2	19	ATPase H+ transporting V0 subunit e2	protein_codin	0.1882	0.312	1	8.188	0.2106
**TBL1X**	TBL1X	19	transducin beta like 1 X-linked	protein_codin	-0.451	0.0203	1	26.58	0.2482
**TSPYL2**	TSPYL2	19	TSPY like 2	protein_codin	0.06605	0.615	1	59.53	0.09971
**NBDY**	NBDY	19	negative regulator of P-body association	protein_codin	-0.2034	0.0182	1	49.33	0.1027
**TAF9B**	TAF9B	19	TATA-box binding protein associated factor 9b	protein_codin	0.167	0.381	1	7.303	0.2405
**HMGN5**	HMGN5	19	high mobility group nucleosome binding domain 5	protein_codin	-0.2293	0.207	1	20.68	0.2532
**APOOL**	APOOL	19	apolipoprotein O like	protein_codin	-0.4933	0.00062	1	45.93	0.1702
**PAK3**	PAK3	19	p21 (RAC1) activated kinase 3	protein_codin	0.9573	9.31E-05	1	59.48	0.07361
**IL13RA2**	IL13RA2	19	interleukin 13 receptor subunit alpha 2	protein_codin	-1.059	6.26E-05	1	25.7	0.1903
**RNF113A**	RNF113A	19	ring finger protein 113A	protein_codin	0.02936	0.899	1	9.424	0.07283
**NAA10**	NAA10	19	N(alpha)-acetyltransferase 10, NatA catalytic subunit	protein_codin	-0.3008	0.00232	1	54.05	0.06542
**TMEM67**	TMEM67	19	transmembrane protein 67	protein_codin	0.586	0.0228	1	29.48	0.2124
**MTERF3**	MTERF3	19	mitochondrial transcription termination factor 3	protein_codin	0.3542	0.182	1	10.52	0.06616
**NDRG1**	NDRG1	19	N-myc downstream regulated 1	protein_codin	0.9018	1.04E-06	0.02419	23.34	0.09912
**KIAA2026**	KIAA2026	19	KIAA2026	protein_codin	0.2614	0.111	1	6.974	0.07036
**SNAPC3**	SNAPC3	19	small nuclear RNA activating complex polypeptide 3	protein_codin	0.2809	0.179	1	9.508	0.2676
**DENND4C**	DENND4C	19	DENN domain containing 4C	protein_codin	0.3847	0.122	1	7.417	0.4734
**SMIM27**	SMIM27	19	small integral membrane protein 27	protein_codin	-0.3298	0.226	1	3.243	0.1367
**NUDT2**	NUDT2	19	nudix hydrolase 2	protein_codin	-0.2392	0.0914	1	8.058	0.1385
**GOLM1**	GOLM1	19	golgi membrane protein 1	protein_codin	0.1236	0.582	1	48.95	0.06936
**S1PR3**	S1PR3	19	sphingosine-1-phosphate receptor 3	protein_codin	-0.61	0.00119	1	10.57	0.3800
**PRRX2**	PRRX2	19	paired related homeobox 2	protein_codin	0.2628	0.0126	1	55.3	0.03214
**NUP214**	NUP214	19	nucleoporin 214	protein_codin	0.2057	0.32	1	30.66	0.1760
**SETX**	SETX	19	senataxin	protein_codin	0.2674	0.122	1	7.508	0.2726
**BET1L**	BET1L	19	Bet1 golgi vesicular membrane trafficking protein like	protein_codin	-0.3912	0.179	1	33.62	0.1139
**WEE1**	WEE1	19	WEE1 G2 checkpoint kinase	protein_codin	0.3143	0.119	1	47.02	0.108
**FNBP4**	FNBP4	19	formin binding protein 4	protein_codin	0.2496	0.089	1	32.19	0.06748
**TMX2**	TMX2	19	thioredoxin related transmembrane protein 2	protein_codin	0.09467	0.534	1	15.98	0.07102
**RBM4**	RBM4	19	RNA binding motif protein 4	protein_codin	0.05281	0.789	1	3.193	0.2365
**PPME1**	PPME1	19	protein phosphatase methylesterase 1	protein_codin	0.01040	0.959	1	9.49	0.1767

**AMOTL1**	AMOTL1	19	angiomotin like 1	protein_codin	-0.03894	0.834	1	7.766	0.1679
**HACD1**	HACD1	19	3-hydroxyacyl-CoA dehydratase 1	protein_codin	-0.2899	0.0419	1	44.28	0.1674
**CSGALNACT2**	CSGALNACT2	19	chondroitin sulfate N-acetylgalactosaminyltransferase 2	protein_codin	0.2629	0.129	1	8.443	0.1368
**C10orf10**	C10orf10	19	DEPP1 autophagy regulator	protein_codin	NA	NA	NA	55.36	0.1425
**NRBF2**	NRBF2	19	nuclear receptor binding factor 2	protein_codin	0.5206	6.86E-05	1	31.53	0.102
**DDX50**	DDX50	19	DExD-box helicase 50	protein_codin	0.07649	0.602	1	15.62	0.1044
**ZDHHC16**	ZDHHC16	19	zinc finger DHHC-type containing 16	protein_codin	0.3072	0.166	1	2.454	0.3108
**SFXN3**	SFXN3	19	sideroflexin 3	protein_codin	0.2146	0.261	1	3.527	0.2762
**FBXW4**	FBXW4	19	F-box and WD repeat domain containing 4	protein_codin	-0.1294	0.565	1	3.178	0.3106
**LDB1**	LDB1	19	LIM domain binding 1	protein_codin	0.7028	0.0286	1	3.282	0.2387
**CASP7**	CASP7	19	caspase 7	protein_codin	0.1247	0.494	1	3.613	0.1105
**YARS2**	YARS2	19	tyrosyl-tRNA synthetase 2	protein_codin	0.2433	0.315	1	3.14	0.1772
**PUS7L**	PUS7L	19	pseudouridine synthase 7 like	protein_codin	0.4262	0.0215	1	21.13	0.2111
**TUBA1C**	TUBA1C	19	tubulin alpha 1c	protein_codin	0.008446	0.92	1	60	0.1102
**SP1**	SP1	19	Sp1 transcription factor	protein_codin	0.08226	0.708	1	7.038	0.1786
**ORMDL2**	ORMDL2	19	ORMDL sphingolipid biosynthesis regulator 2	protein_codin	-0.2666	0.0589	1	42.08	0.02366
**SPRYD4**	SPRYD4	19	SPRY domain containing 4	protein_codin	-0.1605	0.468	1	3.202	0.1681
**BAZ2A**	BAZ2A	19	bromodomain adjacent to zinc finger domain 2A	protein_codin	0.4715	0.0432	1	9.187	0.2002
**CEP83**	CEP83	19	centrosomal protein 83	protein_codin	0.2127	0.4	1	10.12	0.3514
**ALDH1L2**	ALDH1L2	19	aldehyde dehydrogenase 1 family member L2	protein_codin	1.171	1.01E-06	0.0235	29.88	0.06644
**HSPB8**	HSPB8	19	heat shock protein family B (small) member 8	protein_codin	1.139	5.05E-12	1.2E-07	55.27	0.1539
**COQ5**	COQ5	19	coenzyme Q5, methyltransferase	protein_codin	-0.0387	0.829	1	2.656	0.09940
**VPS36**	VPS36	19	vacuolar protein sorting 36 homolog	protein_codin	0.5472	4.22E-05	0.9655	26.59	0.09895
**TGDS**	TGDS	19	TDP-glucose 4,6-dehydratase	protein_codin	-0.1018	0.65	1	3.618	0.1769
**METTL17**	METTL17	19	methyltransferase like 17	protein_codin	0.3186	0.0986	1	3.285	0.2377
**TOX4**	TOX4	19	TOX high mobility group box family member 4	protein_codin	-0.002502	0.979	1	28.47	0.2379
**RALGAPA1**	RALGAPA1	19	Ral GTPase activating protein catalytic alpha subunit 1	protein_codin	0.4282	0.0154	1	21.82	0.1022
**FKBP3**	FKBP3	19	FKBP prolyl isomerase 3	protein_codin	-0.003696	0.97	1	42.04	0.1352
**ACTR10**	ACTR10	19	actin related protein 10	protein_codin	0.05972	0.521	1	35.97	0.09917
**MTHFD1**	MTHFD1	19	methylenetetrahydrofolate dehydrogenase, cyclohydr	protein_codin	-0.369	0.0682	1	10.86	0.1032
**ATP6V1D**	ATP6V1D	19	ATPase H+ transporting V1 subunit D	protein_codin	-0.009726	0.937	1	55.43	0.106
**APOPT1**	APOPT1	19	cytochrome c oxidase assembly factor 8	protein_codin	-0.3681	0.00563	1	18.87	0.1850
**AQR**	AQR	19	aquarius intron-binding spliceosomal factor	protein_codin	0.3256	0.0902	1	7.106	0.348
**FAM98B**	FAM98B	19	family with sequence similarity 98 member B	protein_codin	0.3068	0.157	1	3.229	0.171
**GCHFR**	GCHFR	19	GTP cyclohydrolase I feedback regulator	protein_codin	0.1693	0.422	1	37.62	0.07466
**SECISBP2L**	SECISBP2L	19	SECIS binding protein 2 like	protein_codin	0.07892	0.52	1	14.49	0.07301
**LEO1**	LEO1	19	LEO1 homolog, Paf1/RNA polymerase II complex cor	protein_codin	0.01955	0.894	1	48.32	0.2712
**RORA**	RORA	19	RAR related orphan receptor A	protein_codin	0.3979	0.019	1	31.14	0.1701
**RPP25**	RPP25	19	ribonuclease P and MRP subunit p25	protein_codin	0.6868	0.0223	1	10.02	0.171
**SNX33**	SNX33	19	sorting nexin 33	protein_codin	1.152	6.45E-05	1	7.827	0.2419
**WDR73**	WDR73	19	WD repeat domain 73	protein_codin	-0.01888	0.924	1	6.598	0.3502
**MRPL46**	MRPL46	19	mitochondrial ribosomal protein L46	protein_codin	0.1712	0.272	1	7.472	0.1009
**NR2F2-AS1**	NR2F2-AS1	19	NR2F2 antisense RNA 1	antisense_RN	-0.08875	0.692	1	8.38	0.3565
**MLST8**	MLST8	19	MTOR associated protein, LST8 homolog	protein_codin	0.009479	0.965	1	7.199	0.03488
**PDPK1**	PDPK1	19	3-phosphoinositide dependent protein kinase 1	protein_codin	0.3542	0.0592	1	9.223	0.2445
**HMOX2**	HMOX2	19	heme oxygenase 2	protein_codin	0.2913	0.0552	1	36.26	0.0320
**TNRC6A**	TNRC6A	19	trinucleotide repeat containing adaptor 6A	protein_codin	0.1179	0.561	1	17.43	0.1814
**STX4**	STX4	19	syntaxin 4	protein_codin	-0.3882	0.00735	1	7.488	0.1477
**SNTB2**	SNTB2	19	syntrophin beta 2	protein_codin	0.5102	0.00026	1	29.69	0.07407
**MIS12**	MIS12	19	MIS12 kinetochore complex component	protein_codin	0.008649	0.969	1	9.214	0.1404
**PEMT**	PEMT	19	phosphatidylethanolamine N-methyltransferase	protein_codin	0.1088	0.587	1	9.343	0.06842
**EPN2**	EPN2	19	epsin 2	protein_codin	0.2122	0.122	1	39.3	0.2045
**POLDIP2**	POLDIP2	19	DNA polymerase delta interacting protein 2	protein_codin	0.04474	0.8	1	30.25	0.1002
**RAB34**	RAB34	19	RAB34, member RAS oncogene family	protein_codin	-0.232	2E-04	1	25.98	0.03304
**SLFN11**	SLFN11	19	schlafen family member 11	protein_codin	1.225	1.94E-08	0.00046	9.774	0.2355
**HDAC5**	HDAC5	19	histone deacetylase 5	protein_codin	-0.3067	0.131	1	3.323	0.2395
**AC091271.1**	AC091271.1	19	novel transcri	lincRNA	0.2026	0.472	1	3.726	0.2736
**RNF213**	RNF213	19	ring finger protein 213	protein_codin	0.3195	0.0553	1	18.66	0.3086
**CTIF**	CTIF	19	cap binding complex dependent translation initiation f	protein_codin	0.09677	0.709	1	2.968	0.392
**SLX4IP**	SLX4IP	19	SLX4 interacting protein	protein_codin	-0.6739	0.00298	1	3.392	0.3455
**SMIM26**	SMIM26	19	small integral membrane protein 26	protein_codin	-0.2725	0.00076	1	14.78	0.1017
**GGT7**	GGT7	19	gamma-glutamyltransferase 7	protein_codin	0.1017	0.686	1	2.207	0.1376
**SAMHD1**	SAMHD1	19	SAM and HD domain containing deoxynucleoside trip	protein_codin	1.196	6.69E-16	1.6E-11	58.37	0.1349
**ATP9A**	ATP9A	19	ATPase phospholipid transporting 9A (putative)	protein_codin	-0.1032	0.685	1	3.408	0.3479
**AP3D1**	AP3D1	19	adaptor related protein complex 3 subunit delta 1	protein_codin	-0.02345	0.91	1	6.373	0.2040
**RANBP3**	RANBP3	19	RAN binding protein 3	protein_codin	0.03215	0.871	1	7.394	0.2369
**GTF2F1**	GTF2F1	19	general transcription factor IIF subunit 1	protein_codin	0.08251	0.502	1	12.56	0.209
**ZNF358**	ZNF358	19	zinc finger protein 358	protein_codin	0.1414	0.24	1	34.87	0.0660
**CCDC124**	CCDC124	19	coiled-coil domain containing 124	protein_codin	-0.2854	0.00173	1	20.23	0.1679
**UQCRFS1**	UQCRFS1	19	ubiquinol-cytochrome c reductase, Rieske iron-sulfur	protein_codin	0.1476	0.122	1	17.79	0.2072
**POLR2I**	POLR2I	19	RNA polymerase II subunit I	protein_codin	-0.3567	4.83E-05	1	56.59	0.06745
**COQ8B**	COQ8B	19	coenzyme Q8B	protein_codin	-0.0002717	0.999	1	2.889	0.1685
**ZNF226**	ZNF226	19	zinc finger protein 226	protein_codin	-0.036	0.886	1	3.624	0.3345
**BCAM**	BCAM	19	basal cell adhesion molecule (Lutheran blood group)	protein_codin	1.032	1.46E-12	3.47E-08	55.36	0.03246
**BBC3**	BBC3	19	BCL2 binding component 3	protein_codin	-0.1736	0.431	1	24.58	0.03311
**FLT3LG**	FLT3LG	19	fms related tyrosine kinase 3 ligand	protein_codin	0.4607	0.0399	1	7.575	0.1057
**ZNF160**	ZNF160	19	zinc finger protein 160	protein_codin	0.3274	0.0991	1	3.453	0.1407
**ZNF677**	ZNF677	19	zinc finger protein 677	protein_codin	0.02851	0.872	1	24.92	0.1012
**NAT14**	NAT14	19	N-acetyltransferase 14 (putative)	protein_codin	0.1388	0.546	1	9.273	0.236
**ZNF264**	ZNF264	19	zinc finger protein 264	protein_codin	0.06956	0.745	1	10.54	0.1032
**MAPK1**	MAPK1	19	mitogen-activated protein kinase 1	protein_codin	0.4666	0.00243	1	7.199	0.06615
**DRG1**	DRG1	19	developmentally regulated GTP binding protein 1	protein_codin	0.2562	0.117	1	6.345	0.1367
**CBX6**	CBX6	19	chromobox 6	protein_codin	0.1615	0.0905	1	32.27	0.2066
**RBX1**	RBX1	19	ring-box 1	protein_codin	-0.2405	0.00029	1	42.73	0.07306
**ZC3H7B**	ZC3H7B	19	zinc finger CCCH-type containing 7B	protein_codin	0.3247	0.249	1	3.154	0.45
**PRR34-AS1**	PRR34-AS1	19	PRR34 antisense RNA 1	antisense_RN	-0.5504	0.00026	1	24.79	0.4156
**AL118516.1**	AL118516.1	19	novel transcript, antisense to TBC1D2	antisense_RN	0.2699	0.145	1	40.86	0.2776
**CCT8**	CCT8	19	chaperonin containing TCP1 subunit 8	protein_codin	-0.243	0.0036	1	60.6	0.1150
**MAP3K7CL**	MAP3K7CL	19	MAP3K7 C-terminal like	protein_codin	0.2299	0.209	1	60.6	0.692
**MORC3**	MORC3	19	MORC family CW-type zinc finger 3	protein_codin	0.2511	0.0971	1	40.47	0.1074
**TTC3**	TTC3	19	tetratricopeptide repeat domain 3	protein_codin	-0.1025	0.272	1	35.44	0.03379
**ANGPTL7**	ANGPTL7	20	angiopoietin like 7	protein_codin	3.421	0.0158	1	60.6	1.074
**FLJ37453**	FLJ37453	20	novel transcri	antisense_RN	NA	NA	NA	2.927	0.1752
**EIF4G3**	EIF4G3	20	eukaryotic translation initiation factor 4 gamma 3	protein_codin	0.3502	0.0228	1	22.18	0.03367
**NUDC**	NUDC	20	nuclear distribution C, dynein complex regulator	protein_codin	-0.2037	0.00302	1	23.5	0.02828
**RPA2**	RPA2	20	replication protein A2	protein_codin	0.2815	0.0295	1	14.28	0.0344
**TRNAU1AP**	TRNAU1AP	20	tRNA selenocysteine 1 associated protein 1	protein_codin	0.2838	0.11	1	7.823	0.1662
**CCDC28B**	CCDC28B	20	coiled-coil domain containing 28B	protein_codin	-0.09068	0.686	1	10.24	0.1412
**OSBPL9**	OSBPL9	20	oxysterol binding protein like 9	protein_codin	0.1308	0.341	1	28.27	0.1701
**SSBP3**	SSBP3	20	single stranded DNA binding protein 3	protein_codin	-0.0724	0.626	1	8.206	0.1012
**GCLM**	GCLM	20	glutamate-cysteine ligase modifier subunit	protein_codin	0.1225	0.51	1	50.49	0.1036
**TRIM33**	TRIM33	20	tripartite motif containing 33	protein_codin	0.0348	0.838	1	8.716	0.1048
**TSPAN2**	TSPAN2	20	tetraspanin 2	protein_codin	-1.332	4.23E-22	1.02E-18	46.38	0.1026
**GDAP2**	GDAP2	20	ganglioside induced differentiation associated protein 2	protein_codin	0.4752	0.126	1	8.855	0.2834
**SF3B4**	SF3B4	20	splicing factor 3b subunit 4	protein_codin	0.1858	0.129	1	13.79	0.04219
**CIART**	CIART	20	circadian associated repressor of transcription	protein_codin	-0.04548	0.846	1	11.05	0.1445
**RPRD2**	RPRD2	20	regulation of nuclear pre-mRNA domain containing 2	protein_codin	-0.04462	0.806	1	8.629	0.2572
**CERS2**	CERS2	20	ceramide synthase 2	protein_codin	0.2379	0.0509	1	35.38	0.03597
**ADAR**	ADAR	20	adenosine deaminase RNA specific	protein_codin	-0.05564	0.742	1	17.22	0.3114
**PEX19**	PEX19	20	peroxisomal biogenesis factor 19	protein_codin	-0.5138	0.00074	1	7.537	0.1348
**NDUFS2**	NDUFS2	20	NADH:ubiquinone oxidoreductase core subunit S2	protein_codin	0.1469	0.236	1	14.58	0.1656
**MYOC**	MYOC	20	myocilin	protein_codin	2.716	3.99E-05	0.913	60.6	0.7319
**FAM20B**	FAM20B	20	FAM20B glycosaminoglycan xylosylkinase	protein_codin	-0.000239	0.999	1	3.308	0.1429
**TROVE2**	TROVE2	20	Ro60, Y RNA binding protein	protein_codin	0.09314	0.345	1	44.49	0.03398
**NVL**	NVL	20	nuclear VCP like	protein_codin	0.2462	0.36	1	3.232	0.529
**RNF187**	RNF187	20	ring finger protein 187	protein_codin	-0.01576	0.889	1	40.9	0.1045
**C1orf131**	C1orf131	20	chromosome 1 open reading frame 131	protein_codin	0.2692	0.0915	1	37.49	0.2025
**GGPS1**	GGPS1	20	geranylgeranyl diphosphate synthase 1	protein_codin	-0.2036	0.171	1	8.077	0.1347
**LGALS8**	LGALS8	20	galectin 8	protein_codin	-0.02048	0.917	1	8.41	0.1814
**HNRNPU**	HNRNPU	20	heterogeneous nuclear ribonucleoprotein U	protein_codin	0.4137	4.67E-06	0.108	24.23	0.1346
**DDX1**	DDX1	20	DEAD-box helicase 1	protein_codin	0.2713	0.0258	1	12.08	0.09938
**GPN1**	GPN1	20	GPN-loop GTPase 1	protein_codin	0.1824	0.449	1	3.157	0.4966
**LTBP1**	LTBP1	20	latent transforming growth factor beta binding protein 1	protein_codin	-0.5993	1.4E-15	3.34E-11	60	0.06611
**CYP1B1**	CYP1B1	20	cytochrome P450 family 1 subfamily B member 1	protein_codin	0.4295	0.0258	1	60.6	0.2195
**SRSF7**	SRSF7	20	serine and arginine rich splicing factor 7	protein_codin	0.4136	1.01E-07	0.00237	34.47	0.2038
**MORN2**	MORN2	20	MORN repeat containing 2	protein_codin	0.03152	0.849	1	20.53	0.1992
**PKDCC**	PKDCC	20	protein kinase domain containing, cytoplasmic	protein_codin	1.113	9.32E-11	2.21E-06	47.61	0.1384
**MCFD2**	MCFD2	20	multiple coagulation factor deficiency 2	protein_codin	-0.00379	0.966	1	60.6	0.06877
**MSH6**	MSH6	20	mutS homolog 6	protein_codin	0.1579	0.324	1	7.893	0.1008
**PNPT1**	PNPT1	20	polyribonucleotide nucleotidyltransferase 1	protein_codin	0.342	0.091	1	26.62	0.2871
**KCMF1**	KCMF1	20	potassium channel modulatory factor 1	protein_codin	0.1539	0.31	1	8.083	0.176
**CAPG**	CAPG	20	capping actin protein, gelsolin like	protein_codin	-0.008346	0.96	1	36.85	0.06525
**FAHD2B**	FAHD2B	20	fumarylacetoacetate hydrolase domain containing 2B	protein_codin	0.02322	0.921	1	3.266	0.2692
**MITD1**	MITD1	20	microtubule interacting and trafficking domain contain	protein_codin	-0.129	0.402	1	7.62	0.1335
**IL1R1**	IL1R1	20	interleukin 1 receptor type 1	protein_codin	0.2779	0.0372	1	53.53	0.03452
**MRPS9**	MRPS9	20	mitochondrial ribosomal protein S9	protein_codin	-0.07823	0.612	1	6.904	0.3107
**SLC35F5**	SLC35F5	20	solute carrier family 35 member F5	protein_codin	0.4208	0.0427	1	7.45	0.06452
**CLASP1**	CLASP1	20	cytoplasmic linker associated protein 1	protein_codin	-0.1029	0.699	1	3.065	0.4612
**UGGT1**	UGGT1	20	UDP-glucose glycoprotein glucosyltransferase 1	protein_codin	0.4733	0.0347	1	10.05	0.2259
**SP3**	SP3	20	Sp3 transcription factor	protein_codin	0.1515	0.395	1	6.056	0.2381
**NAB1**	NAB1	20	NGFI-A binding protein 1	protein_codin	0.09182	0.641	1	8.095	0.1759
**USP37**	USP37	20	ubiquitin specific peptidase 37	protein_codin	0.2685	0.252	1	9.005	0.296
**OBSL1**	OBSL1	20	obscurin like 1	protein_codin	0.07862	0.707	1	27.74	0.2807
**PDE6D**	PDE6D	20	phosphodiesterase 6D	protein_codin	0.1702	0.417	1	3.497	0.2442
**NDUFA10**	NDUFA10	20	NADH:ubiquinone oxidoreductase subunit A10	protein_codin	-0.01877	0.847	1	52.63	0.1382
**HDLBP**	HDLBP	20	high density lipoprotein binding protein	protein_codin	0.2259	0.00644	1	36.56	0.03268
**THUMPD3**	THUMPD3	20	THUMP domain containing 3	protein_codin	0.03097	0.856	1	19.87	0.1084
**IL17RC**	IL17RC	20	interleukin 17 receptor C	protein_codin	0.497	0.0252	1	6.864	0.3126
**TBC1D5**	TBC1D5	20	TBC1 domain family member 5	protein_codin	0.06054	0.7	1	7.707	0.1067
**TOP2B**	TOP2B	20	DNA topoisomerase II beta	protein_codin	0.1218	0.437	1	21.11	0.2427
**EPM2AIP1**	EPM2AIP1	20	EPM2A interacting protein 1	protein_codin	-0.01555	0.924	1	8.144	0.1734
**ZDHHC3**	ZDHHC3	20	zinc finger DHHC-type containing 3	protein_codin	0.08502	0.588	1	7.985	0.06485
**KIF9**	KIF9	20	kinesin family member 9	protein_codin	-0.2248	0.334	1	55.63	0.1191
**UQCRC1**	UQCRC1	20	ubiquinol-cytochrome c reductase core protein 1	protein_codin	0.08513	0.435	1	12.53	0.06914
**TMF1**	TMF1	20	TATA element modulatory factor 1	protein_codin	-0.102	0.32	1	60.52	0.1027
**ROBO1**	ROBO1	20	roundabout guidance receptor 1	protein_codin	0.01574	0.937	1	32.46	0.06607
**CGGBP1**	CGGBP1	20	CGG triplet repeat binding protein 1	protein_codin	0.2153	0.057	1	25.58	0.2359
**CEP97**	CEP97	20	centrosomal protein 97	protein_codin	0.449	0.0432	1	3.568	0.2919
**ACAD9**	ACAD9	20	acyl-CoA dehydrogenase family member 9	protein_codin	0.313	0.159	1	7.858	0.09972
**PLXND1**	PLXND1	20	plexin D1	protein_codin	-0.1213	0.57	1	11.19	0.1371
**MRAS**	MRAS	20	muscle RAS oncogene homolog	protein_codin	0.256	0.174	1	60.54	0.3311
**BCL6**	BCL6	20	BCL6 transcription repressor	protein_codin	0.3855	0.14	1	9.328	0.244
**APOD**	APOD	20	apolipoprotein D	protein_codin	7.73	9.85E-07	0.02292	NA	0.6842
**UVSSA**	UVSSA	20	UV stimulated scaffold protein A	protein_codin	0.5173	0.113	1	3.511	0.5696
**HAUS3**	HAUS3	20	HAUS augmin like complex subunit 3	protein_codin	0.01309	0.968	1	3.243	0.1827
**NOP14**	NOP14	20	NOP14 nucleolar protein	protein_codin	0.3726	0.156	1	28.79	0.1721
**HTT**	HTT	20	huntingtin	protein_codin	0.1198	0.631	1	2.856	0.2482
**EVC2**	EVC2	20	EvC ciliary complex subunit 2	protein_codin	1.057	0.00069	1	9.803	0.2029
**FBXL5**	FBXL5	20	F-box and leucine rich repeat protein 5	protein_codin	0.2962	0.0414	1	46.7	0.2069
**FGFBP2**	FGFBP2	20	fibroblast growth factor binding protein 2	protein_codin	2.242	0.00441	1	60.6	0.7181
**TAPT1**	TAPT1	20	transmembrane anterior posterior transformation 1	protein_codin	0.1346	0.557	1	8.21	0.4938
**SLIT2**	SLIT2	20	slit guidance ligand 2	protein_codin	2.216	6.27E-27	1.51E-22	24.44	0.1035
**N4BP2**	N4BP2	20	NEDD4 binding protein 2	protein_codin	0.2176	0.289	1	11.46	0.4216
**PARM1**	PARM1	20	prostate androgen-regulated mucin-like protein 1	protein_codin	-0.2429	0.15	1	47.63	0.2105
**THAP6**	THAP6	20	THAP domain containing 6	protein_codin	-0.01977	0.91	1	7.682	0.136
**COPS4**	COPS4	20	COP9 signalosome subunit 4	protein_codin	0.2694	0.0344	1	14.73	0.1691
**PPA2**	PPA2	20	pyrophosphatase (inorganic) 2	protein_codin	0.2267	0.188	1	18.06	0.0664
**BBS7**	BBS7	20	Bardet-Biedl syndrome 7	protein_codin	0.1311	0.475	1	3.633	0.1742
**MGST2**	MGST2	20	microsomal glutathione S-transferase 2	protein_codin	-0.5293	6.04E-05	1	38.17	0.1379
**EDNRA**	EDNRA	20	endothelin receptor type A	protein_codin	0.2975	0.122	1	27.16	0.03206
**TMEM192**	TMEM192	20	transmembrane protein 192	protein_codin	0.4236	0.0574	1	9.446	0.202
**SNX18**	SNX18	20	sorting nexin 18	protein_codin	-0.3168	0.00598	1	26.87	0.06906
**PIK3R1**	PIK3R1	20	phosphoinositide-3-kinase regulatory subunit 1	protein_codin	1.302	7.76E-35	1.88E-30	59.64	0.03190
**SSBP2**	SSBP2	20	single stranded DNA binding protein 2	protein_codin	0.1703	0.102	1	10.79	0.2146
**RHOBTB3**	RHOBTB3	20	Rho related BTB domain containing 3	protein_codin	1.018	1.82E-12	4.33E-08	51.38	0.07867
**FEM1C**	FEM1C	20	fem-1 homolog C	protein_codin	0.4963	0.0482	1	7.96	0.1122
**PRDM6**	PRDM6	20	PR/SET domain 6	protein_codin	-0.08258	0.604	1	55.05	0.03735
**UQCRQ**	UQCRQ	20	ubiquinol-cytochrome c reductase complex III subunit VII	protein_codin	-0.2934	4.36E-06	0.1008	49.9	0.03274
**RNF14**	RNF14	20	ring finger protein 14	protein_codin	0.2017	0.302	1	6.643	0.03777
**PDGFRB**	PDGFRB	20	platelet derived growth factor receptor beta	protein_codin	0.02096	0.844	1	42.58	0.1066
**ERGIC1**	ERGIC1	20	endoplasmic reticulum-golgi intermediate compartme	protein_codin	0.2283	0.0828	1	14.54	0.1674
**SQSTM1**	SQSTM1	20	sequestosome 1	protein_codin	0.2402	1E-05	0.2304	60.6	0.03222
**C6orf47**	C6orf47	20	chromosome 6 open reading frame 47	protein_codin	0.04901	0.789	1	7.213	0.1062
**PFDN6**	PFDN6	20	prefoldin subunit 6	protein_codin	-0.0592	0.7	1	6.986	0.06634
**ZBTB22**	ZBTB22	20	zinc finger and BTB domain containing 22	protein_codin	-0.01043	0.962	1	9.817	0.2722
**KCNK17**	KCNK17	20	potassium two pore domain channel subfamily K mem	protein_codin	0.8995	1.87E-06	0.04339	55.14	0.148
**TAF8**	TAF8	20	TATA-box binding protein associated factor 8	protein_codin	-0.2063	0.385	1	8.372	0.4097
**MYO6**	MYO6	20	myosin VI	protein_codin	0.4005	0.0512	1	19.2	0.1748
**FOXO3**	FOXO3	20	forkhead box O3	protein_codin	0.3073	0.0749	1	28.2	0.3827
**ARMT1**	ARMT1	20	acidic residue methyltransferase 1	protein_codin	0.4258	0.0236	1	27.83	0.1325
**FAM20C**	FAM20C	20	FAM20C golgi associated secretory pathway kinase	protein_codin	0.2185	0.222	1	20.36	0.06522
**IQCE**	IQCE	20	IQ motif containing E	protein_codin	0.3712	0.119	1	8.173	0.2786
**CCZ1**	CCZ1	20	CCZ1 homolog, vacuolar protein trafficking and bioge	protein_codin	0.373	0.0416	1	8.039	0.2124
**EPDR1**	EPDR1	20	ependymin related 1	protein_codin	-0.2815	0.0347	1	54.86	0.1502
**BLVRA**	BLVRA	20	biliverdin reductase A	protein_codin	-0.3821	0.00402	1	15.3	0.1711
**CCT6A**	CCT6A	20	chaperonin containing TCP1 subunit 6A	protein_codin	-0.2045	0.0132	1	60.57	0.06576
**VKORC1L1**	VKORC1L1	20	vitamin K epoxide reductase complex subunit 1 like 1	protein_codin	0.2035	0.26	1	8.824	0.1835
**GUSB**	GUSB	20	glucuronidase beta	protein_codin	0.4927	0.00027	1	23.16	0.03212
**SBDS**	SBDS	20	SBDS ribosome maturation factor	protein_codin	-0.1074	0.0241	1	59.88	0.03352
**TYW1**	TYW1	20	tRNA-yW synthesizing protein 1 homolog	protein_codin	0.8968	6.46E-05	1	10.45	0.1725
**CNPY4**	CNPY4	20	canopy FGF signaling regulator 4	protein_codin	0.179	0.239	1	19.92	0.2491
**HBP1**	HBP1	20	HMG-box transcription factor 1	protein_codin	0.2985	0.022	1	29.7	0.1305
**WASL**	WASL	20	WASP like actin nucleation promoting factor	protein_codin	-0.1297	0.116	1	28.14	0.09890
**CHCHD3**	CHCHD3	20	coiled-coil-helix-coiled-coil-helix domain containing 3	protein_codin	0.06381	0.616	1	45.25	0.2484
**TCAF1**	TCAF1	20	TRPM8 channel associated factor 1	protein_codin	-0.2697	0.176	1	22.92	0.1376
**UBE3C**	UBE3C	20	ubiquitin protein ligase E3C	protein_codin	0.3665	0.108	1	3.495	0.321
**WDR60**	WDR60	20	WD repeat domain 60	protein_codin	0.09222	0.54	1	13.92	0.3258
**ASMTL**	ASMTL	20	acetylserotonin O-methyltransferase like	protein_codin	-0.0667	0.7	1	8.579	0.1660
**BCLAF3**	BCLAF3	20	BCLAF1 and THRAP3 family member 3	protein_codin	0.5753	0.0182	1	9.529	0.1369
**EIF2S3**	EIF2S3	20	eukaryotic translation initiation factor 2 subunit gamm	protein_codin	-0.03809	0.702	1	56.06	0.1006
**KRBOX4**	KRBOX4	20	KRAB box domain containing 4	protein_codin	-0.2002	0.386	1	8.335	0.4481
**CHST7**	CHST7	20	carbohydrate sulfotransferase 7	protein_codin	0.8937	0.00136	1	37.46	0.1694
**UBQLN2**	UBQLN2	20	ubiquilin 2	protein_codin	0.4875	0.035	1	7.677	0.2386
**RPL36A**	RPL36A	20	ribosomal protein L36a	protein_codin	-0.568	2.92E-09	6.88E-05	53.53	0.03306
**ACSL4**	ACSL4	20	acyl-CoA synthetase long chain family member 4	protein_codin	0.07526	0.664	1	9.028	0.2042
**GPC3**	GPC3	20	glypican 3	protein_codin	2.017	2.17E-10	5.14E-06	54.79	0.2818

**NDUFB1**	NDUFB1	20	NADH:ubiquinone oxidoreductase subunit B1	protein_codin	-0.1203	0.096	1	45.42	0.1395
**LGMN**	LGMN	20	legumain	protein_codin	0.3211	0.00478	1	40.48	0.1047
**IFI27L1**	IFI27L1	20	interferon alpha inducible protein 27 like 1	protein_codin	0.4206	0.0471	1	9.995	0.1902
**GLRX5**	GLRX5	20	glutaredoxin 5	protein_codin	-0.1237	0.149	1	27.8	0.1681
**EVL**	EVL	20	Enah/Vasp-like	protein_codin	-0.3083	0.0797	1	60.6	0.1031
**CDC42BPB**	CDC42BPB	20	CDC42 binding protein kinase beta	protein_codin	0.3734	0.0468	1	21.8	0.1016
**AKT1**	AKT1	20	AKT serine/threonine kinase 1	protein_codin	-0.1355	0.256	1	15.62	0.1702
**LINC02256**	LINC02256	20	long intergenic non-protein coding RNA 2256	lincRNA	0.4738	0.119	1	3.236	0.5138
**KATNBL1**	KATNBL1	20	katanin regulatory subunit B1 like 1	protein_codin	0.02562	0.874	1	17.68	0.1449
**BLOC1S6**	BLOC1S6	20	biogenesis of lysosomal organelles complex 1 subuni	protein_codin	-0.02003	0.87	1	15.64	0.2688
**USP8**	USP8	20	ubiquitin specific peptidase 8	protein_codin	0.269	0.0427	1	24.27	0.1345
**GLDN**	GLDN	20	gliomedin	protein_codin	-1.337	2.65E-16	6.34E-12	31.1	0.2074
**MAPK6**	MAPK6	20	mitogen-activated protein kinase 6	protein_codin	0.1638	0.461	1	8.297	0.175
**TRIP4**	TRIP4	20	thyroid hormone receptor interactor 4	protein_codin	0.1137	0.597	1	8.255	0.1748
**SPG21**	SPG21	20	SPG21 abhydrolase domain containing, maspardin	protein_codin	0.2287	0.185	1	20.87	0.1739
**SMAD3**	SMAD3	20	SMAD family member 3	protein_codin	0.8485	3.08E-05	0.7057	25.74	0.04222
**LRRC49**	LRRC49	20	leucine rich repeat containing 49	protein_codin	0.4275	0.075	1	8.364	0.1117
**FAM219B**	FAM219B	20	family with sequence similarity 219 member B	protein_codin	0.00601	0.972	1	3.285	0.3388
**CTSH**	CTSH	20	cathepsin H	protein_codin	2.563	2.58E-29	6.25E-25	60.58	0.02379
**MEF2A**	MEF2A	20	myocyte enhancer factor 2A	protein_codin	-0.1695	0.1	1	43.12	0.1647
**TARSL2**	TARSL2	20	threonyl-tRNA synthetase like 2	protein_codin	-0.1697	0.441	1	9.342	0.1757
**NARFL**	NARFL	20	cytosolic iron-sulfur assembly component 3	protein_codin	-0.1532	0.532	1	8.884	0.2812
**PYCARD**	PYCARD	20	PYD and CARD domain containing	protein_codin	0.1842	0.272	1	59.9	0.141
**DNAJA2**	DNAJA2	20	DnaJ heat shock protein family (Hsp40) member A2	protein_codin	-0.1101	0.292	1	11.07	0.1009
**USB1**	USB1	20	U6 snRNA biogenesis phosphodiesterase 1	protein_codin	0.2726	0.206	1	7.619	0.2358
**ZDHHC1**	ZDHHC1	20	zinc finger DHHC-type containing 1	protein_codin	0.3176	0.166	1	3.045	0.09952
**NQO1**	NQO1	20	NAD(P)H quinone dehydrogenase 1	protein_codin	-0.0009719	0.995	1	42.8	0.2296
**PITPNA**	PITPNA	20	phosphatidylinositol transfer protein alpha	protein_codin	0.186	0.299	1	24.55	0.2372
**CXCL16**	CXCL16	20	C-X-C motif chemokine ligand 16	protein_codin	0.3402	0.126	1	10.44	0.09815
**PER1**	PER1	20	period circadian regulator 1	protein_codin	0.7624	4.56E-05	1	20.07	0.3527
**DHRS7B**	DHRS7B	20	dehydrogenase/reductase 7B	protein_codin	0.4062	0.0138	1	20.5	0.1681
**GOSR1**	GOSR1	20	golgi SNAP receptor complex member 1	protein_codin	0.2222	0.0788	1	38.7	0.1471
**SUZ12**	SUZ12	20	SUZ12 polycomb repressive complex 2 subunit	protein_codin	0.1433	0.358	1	19.46	0.2704
**GHDC**	GHDC	20	GH3 domain containing	protein_codin	0.01002	0.967	1	3.18	0.1697
**STAT3**	STAT3	20	signal transducer and activator of transcription 3	protein_codin	0.5074	3.99E-08	0.00094	53.34	0.1041
**FZD2**	FZD2	20	frizzled class receptor 2	protein_codin	0.818	0.00086	1	58.96	0.4972
**CDK5RAP3**	CDK5RAP3	20	CDK5 regulatory subunit associated protein 3	protein_codin	0.2228	0.12	1	19.7	0.1362
**SLC35B1**	SLC35B1	20	solute carrier family 35 member B1	protein_codin	-0.05379	0.764	1	21.13	0.09842
**LRRC59**	LRRC59	20	leucine rich repeat containing 59	protein_codin	0.01752	0.9	1	17.26	0.1076
**RSAD1**	RSAD1	20	radical S-adenosyl methionine domain containing 1	protein_codin	-0.09096	0.649	1	2.996	0.09971
**RNFT1**	RNFT1	20	ring finger protein, transmembrane 1	protein_codin	0.525	0.0136	1	8.022	0.2929
**BCAS3**	BCAS3	20	BCAS3 microtubule associated cell migration factor	protein_codin	-0.3139	0.252	1	8.252	0.3032
**METTL2A**	METTL2A	20	methyltransferase like 2A	protein_codin	0.3657	0.0671	1	8.19	0.3535
**PSMC5**	PSMC5	20	proteasome 26S subunit, ATPase 5	protein_codin	-0.2824	2.92E-05	0.6683	49.18	0.02371
**RAB12**	RAB12	20	RAB12, member RAS oncogene family	protein_codin	0.004004	0.98	1	9.254	0.1376
**SPIRE1**	SPIRE1	20	spire type actin nucleation factor 1	protein_codin	-0.3405	0.0989	1	9.122	0.3067
**ROCK1**	ROCK1	20	Rho associated coiled-coil containing protein kinase 1	protein_codin	-0.1966	0.0122	1	49.78	0.1334
**SS18**	SS18	20	SS18 subunit of BAF chromatin remodeling complex	protein_codin	-0.3562	0.0226	1	8.055	0.2084
**DSEL**	DSEL	20	dermatan sulfate epimerase like	protein_codin	-0.04224	0.803	1	57.18	0.07274
**TIMM21**	TIMM21	20	translocase of inner mitochondrial membrane 21	protein_codin	0.1625	0.466	1	3.306	0.1391
**FOXS1**	FOXS1	20	forkhead box S1	protein_codin	-0.3902	0.0258	1	60.6	0.2111
**RALY**	RALY	20	RALY heterogeneous nuclear ribonucleoprotein	protein_codin	-0.1383	0.125	1	14.8	0.06711
**PREX1**	PREX1	20	phosphatidylinositol-3,4,5-trisphosphate dependent R	protein_codin	-0.6576	0.00346	1	34.77	0.3192
**OSBPL2**	OSBPL2	20	oxysterol binding protein like 2	protein_codin	-0.2308	0.244	1	2.963	0.1079
**BTBD2**	BTBD2	20	BTB domain containing 2	protein_codin	0.05394	0.835	1	2.512	0.1767
**UBXN6**	UBXN6	20	UBX domain protein 6	protein_codin	0.3393	0.0468	1	8.142	0.2454
**PTPRS**	PTPRS	20	protein tyrosine phosphatase receptor type S	protein_codin	1.518	2.7E-05	0.8471	8.024	0.3354
**INSR**	INSR	20	insulin receptor	protein_codin	0.4795	0.00343	1	60.6	0.1816
**ILF3-AS1**	ILF3-AS1	20	ILF3 divergent transcript	lincRNA	NA	NA	NA	28.68	0.1837
**TRMT1**	TRMT1	20	tRNA methyltransferase 1	protein_codin	-0.03675	0.909	1	9.17	0.3518
**SLC35E1**	SLC35E1	20	solute carrier family 35 member E1	protein_codin	0.06201	0.755	1	6.96	0.1969
**OCEL1**	OCEL1	20	occludin/ELL domain containing 1	protein_codin	0.2596	0.0927	1	8.196	0.1124
**SCN1B**	SCN1B	20	sodium voltage-gated channel beta subunit 1	protein_codin	0.8247	0.00046	1	50.3	0.1166
**HSPB6**	HSPB6	20	heat shock protein family B (small) member 6	protein_codin	0.2115	0.0456	1	50.47	0.03402
**CAPNS1**	CAPNS1	20	calpain small subunit 1	protein_codin	-0.2297	0.00199	1	59.19	0.03926
**SIRT2**	SIRT2	20	sirtuin 2	protein_codin	0.5159	0.00332	1	8.216	0.3706
**NUMBL**	NUMBL	20	NUMB like endocytic adaptor protein	protein_codin	0.9153	8.6E-06	0.1983	60.52	0.2859
**ARHGEF1**	ARHGEF1	20	Rho guanine nucleotide exchange factor 1	protein_codin	0.1409	0.614	1	3.3	0.3196
**VASP**	VASP	20	vasodilator stimulated phosphoprotein	protein_codin	-0.3054	0.0246	1	13.61	0.07187
**SLC1A5**	SLC1A5	20	solute carrier family 1 member 5	protein_codin	0.07429	0.731	1	34.7	0.1648
**FTL**	FTL	20	ferritin light chain	protein_codin	-0.2114	1.41E-09	3.33E-05	NA	0.1049
**AP2A1**	AP2A1	20	adaptor related protein complex 2 subunit alpha 1	protein_codin	0.589	0.00148	1	24.23	0.2522
**CHMP2A**	CHMP2A	20	charged multivesicular body protein 2A	protein_codin	-0.204	0.001	1	59.83	0.03235
**BCL2L13**	BCL2L13	20	BCL2 like 13	protein_codin	0.2617	0.29	1	3.624	0.182
**TXNRD2**	TXNRD2	20	thioredoxin reductase 2	protein_codin	0.1789	0.462	1	55.26	0.2201
**NIPSNAP1**	NIPSNAP1	20	nipsnap homolog 1	protein_codin	0.1071	0.61	1	7.779	0.09927
**FBXO7**	FBXO7	20	F-box protein 7	protein_codin	0.3077	0.0205	1	15.03	0.06981
**NUP50**	NUP50	20	nucleoporin 50	protein_codin	0.3817	0.0491	1	31.61	0.07021
**PPARA**	PPARA	20	peroxisome proliferator activated receptor alpha	protein_codin	0.3403	0.141	1	3.258	0.2881
**ALG12**	ALG12	20	ALG12 alpha-1,6-mannosyltransferase	protein_codin	-0.2729	0.272	1	3.109	0.3091
**NRIP1**	NRIP1	20	nuclear receptor interacting protein 1	protein_codin	0.2048	0.241	1	9.748	0.1022
**ZBTB21**	ZBTB21	20	zinc finger and BTB domain containing 21	protein_codin	0.5879	0.0144	1	8.326	0.2072
**ICMT**	ICMT	21	isoprenylcysteine carboxyl methyltransferase	protein_codin	0.5044	0.00506	1	8.669	0.2407
**PRDM2**	PRDM2	21	PR/SET domain 2	protein_codin	0.2294	0.216	1	20.25	0.1401
**LUZP1**	LUZP1	21	leucine zipper protein 1	protein_codin	-0.409	0.00413	1	60.35	0.06779
**PNRC2**	PNRC2	21	proline rich nuclear receptor coactivator 2	protein_codin	0.3599	3.96E-06	0.09162	29.74	0.1356
**EIF3I**	EIF3I	21	eukaryotic translation initiation factor 3 subunit I	protein_codin	-0.1962	0.00616	1	58.26	0.06752
**P3H1**	P3H1	21	prolyl 3-hydroxylase 1	protein_codin	0.01251	0.949	1	8.305	0.1049
**EBNA1BP2**	EBNA1BP2	21	EBNA1 binding protein 2	protein_codin	-0.104	0.351	1	12.83	0.1775
**FOXD2**	FOXD2	21	forkhead box D2	protein_codin	-0.3002	0.106	1	58.43	0.2069
**ABCD3**	ABCD3	21	ATP binding cassette subfamily D member 3	protein_codin	0.03004	0.9	1	9.711	0.103
**TMEM56**	TMEM56	21	TLC domain containing 4	protein_codin	-0.03785	0.862	1	9.083	0.2792
**WARS2**	WARS2	21	tryptophanyl tRNA synthetase 2, mitochondrial	protein_codin	0.02324	0.9	1	6.876	0.1685
**DPM3**	DPM3	21	dolichyl-phosphate mannosyltransferase subunit 3, re	protein_codin	0.1448	0.159	1	31.52	0.03706
**RUSC1**	RUSC1	21	RUN and SH3 domain containing 1	protein_codin	0.1259	0.567	1	8.818	0.102
**QSOX1**	QSOX1	21	quiescin sulfhydryl oxidase 1	protein_codin	-0.0386	0.81	1	23.98	0.07142
**TMEM183A**	TMEM183A	21	transmembrane protein 183A	protein_codin	-0.2949	0.0357	1	18.78	0.03289
**EGLN1**	EGLN1	21	egl-9 family hypoxia inducible factor 1	protein_codin	0.2668	0.16	1	22.92	0.2532
**SH3YL1**	SH3YL1	21	SH3 and SYLF domain containing 1	protein_codin	0.3143	0.161	1	9.669	0.3436
**TRIB2**	TRIB2	21	tribbles pseudokinase 2	protein_codin	-0.3219	0.172	1	45.97	0.2017
**NCOA1**	NCOA1	21	nuclear receptor coactivator 1	protein_codin	0.3022	0.0807	1	20.5	0.1387
**FNDC4**	FNDC4	21	fibronectin type III domain containing 4	protein_codin	-1.187	1.39E-10	3.29E-06	53.81	0.1481
**PRADC1**	PRADC1	21	protease associated domain containing 1	protein_codin	0.1585	0.306	1	21.62	0.07756
**POLE4**	POLE4	21	DNA polymerase epsilon 4, accessory subunit	protein_codin	-0.5448	2.01E-09	4.74E-05	45.93	0.03824
**NCK2**	NCK2	21	NCK adaptor protein 2	protein_codin	-0.4873	0.0025	1	8.866	0.2374
**ZC3H6**	ZC3H6	21	zinc finger CCCH-type containing 6	protein_codin	0.2538	0.0592	1	26.12	0.1639
**7-Mar**	43531	21	membrane associated ring-CH-type finger 7	protein_codin	0.226	0.0852	1	13.12	0.2886
**GCA**	GCA	21	grancalcin	protein_codin	-0.3858	0.0372	1	7.162	0.142
**DLX2**	DLX2	21	distal-less homeobox 2	protein_codin	1.276	4.15E-05	0.9496	28.66	0.1697
**OLA1**	OLA1	21	Obg like ATPase 1	protein_codin	-0.6025	1.28E-07	0.003	30.6	0.2435
**NOP58**	NOP58	21	NOP58 ribonucleoprotein	protein_codin	0.1488	0.282	1	14.96	0.1092
**PARD3B**	PARD3B	21	par-3 family cell polarity regulator beta	protein_codin	0.52	0.0165	1	8.532	0.3102
**OGG1**	OGG1	21	8-oxoguanine DNA glycosylase	protein_codin	0.3912	0.066	1	2.486	0.2567
**TMEM43**	TMEM43	21	transmembrane protein 43	protein_codin	0.5412	5.93E-07	0.01382	48.94	0.1007
**HIGD1A**	HIGD1A	21	HIG1 hypoxia inducible domain family member 1A	protein_codin	0.3026	0.00028	1	33.96	0.03295
**NME6**	NME6	21	NME/NM23 nucleoside diphosphate kinase 6	protein_codin	-0.04676	0.797	1	7.805	0.2009
**ARIH2**	ARIH2	21	ariadne RBR E3 ubiquitin protein ligase 2	protein_codin	-0.07707	0.652	1	50.79	0.2361
**APPL1**	APPL1	21	adaptor protein, phosphotyrosine interacting with PH	protein_codin	0.1954	0.0342	1	14	0.07112
**GBE1**	GBE1	21	1,4-alpha-glucan branching enzyme 1	protein_codin	-0.1733	0.337	1	50.64	0.1701
**CRYBG3**	CRYBG3	21	crystallin beta-gamma domain containing 3	protein_codin	1.069	7E-08	0.00164	16.72	0.1027
**CHCHD6**	CHCHD6	21	coiled-coil-helix-coiled-coil-helix domain containing 6	protein_codin	0.08854	0.626	1	8.414	0.06526
**TBL1XR1**	TBL1XR1	21	transducin beta like 1 X-linked receptor 1	protein_codin	0.1177	0.27	1	52.81	0.2042
**GNB4**	GNB4	21	G protein subunit beta 4	protein_codin	0.135	0.387	1	15.42	0.2548
**PSMD2**	PSMD2	21	proteasome 26S subunit, non-ATPase 2	protein_codin	-0.03579	0.734	1	25.65	0.1361
**IDUA**	IDUA	21	iduronidase alpha-L-	protein_codin	0.5008	0.0159	1	28.18	0.2096
**GNPDA2**	GNPDA2	21	glucosamine-6-phosphate deaminase 2	protein_codin	0.2476	0.127	1	7.85	0.07228
**REST**	REST	21	RE1 silencing transcription factor	protein_codin	0.008106	0.94	1	21.86	0.0662
**NUP54**	NUP54	21	nucleoporin 54	protein_codin	0.12	0.434	1	21.57	0.1322
**HELQ**	HELQ	21	helicase, POLQ like	protein_codin	-0.03746	0.859	1	3.425	0.1340
**DNAJB14**	DNAJB14	21	DnaJ heat shock protein family (Hsp40) member B14	protein_codin	0.1271	0.197	1	33.26	0.1012
**ELF2**	ELF2	21	E74 like ETS transcription factor 2	protein_codin	0.3452	0.00466	1	11.78	0.1762
**FBXW7**	FBXW7	21	F-box and WD repeat domain containing 7	protein_codin	0.08747	0.666	1	8.546	0.1395
**NAF1**	NAF1	21	nuclear assembly factor 1 ribonucleoprotein	protein_codin	0.1065	0.576	1	54.49	0.1027
**CEP44**	CEP44	21	centrosomal protein 44	protein_codin	0.239	0.164	1	9.046	0.4541
**MED10**	MED10	21	mediator complex subunit 10	protein_codin	0.02746	0.767	1	19.18	0.1362
**PTGER4**	PTGER4	21	prostaglandin E receptor 4	protein_codin	0.3144	0.17	1	9.403	0.4088
**PPP2CA**	PPP2CA	21	protein phosphatase 2 catalytic subunit alpha	protein_codin	0.1918	0.149	1	44.02	0.2234
**C5orf24**	C5orf24	21	chromosome 5 open reading frame 24	protein_codin	-0.1021	0.291	1	20.92	0.09912
**SPOCK1**	SPOCK1	21	SPARC (osteonectin), cwcv and kazal like domains p	protein_codin	1.07	5.41E-06	0.125	55.32	0.03214
**FAM13B**	FAM13B	21	family with sequence similarity 13 member B	protein_codin	-0.01268	0.951	1	24.1	0.471
**UBLCP1**	UBLCP1	21	ubiquitin like domain containing CTD phosphatase 1	protein_codin	-0.3589	0.0467	1	26.07	0.09959
**PDLIM7**	PDLIM7	21	PDZ and LIM domain 7	protein_codin	-0.4202	1.76E-06	0.04085	55.38	0.06892
**CANX**	CANX	21	calnexin	protein_codin	0.01771	0.792	1	60.6	0.03276
**CARMIL1**	CARMIL1	21	capping protein regulator and myosin 1 linker 1	protein_codin	0.1803	0.225	1	18.98	0.1492
**DDX39B**	DDX39B	21	DExD-box helicase 39B	protein_codin	0.3392	0.127	1	6.411	0.232
**KLHDC3**	KLHDC3	21	kelch domain containing 3	protein_codin	-0.1183	0.461	1	7.874	0.2092
**SMIM8**	SMIM8	21	small integral membrane protein 8	protein_codin	-0.1357	0.436	1	6.268	0.2442
**TSPYL4**	TSPYL4	21	TSPY like 4	protein_codin	0.05087	0.799	1	8.572	0.241
**TNRC18**	TNRC18	21	trinucleotide repeat containing 18	protein_codin	0.03402	0.867	1	6.882	0.3405
**SCRN1**	SCRN1	21	secernin 1	protein_codin	0.02799	0.872	1	8.447	0.172
**STARD3NL**	STARD3NL	21	STARD3 N-terminal like	protein_codin	-0.08168	0.632	1	6.016	0.1326
**HGF**	HGF	21	hepatocyte growth factor	protein_codin	-0.3856	0.0142	1	51	0.209
**MOSPD3**	MOSPD3	21	motile sperm domain containing 3	protein_codin	0.08985	0.595	1	8.211	0.1706
**FIS1**	FIS1	21	fission, mitochondrial 1	protein_codin	-0.261	8.48E-07	0.01974	50.84	0.06529
**CUX1**	CUX1	21	cut like homeobox 1	protein_codin	-0.1003	0.41	1	21.39	0.06807
**CPED1**	CPED1	21	cadherin like and PC-esterase domain containing 1	protein_codin	-0.03365	0.756	1	32.35	0.207
**RBM28**	RBM28	21	RNA binding motif protein 28	protein_codin	0.2934	0.14	1	25.78	0.2379
**EBP**	EBP	21	EBP cholestenol delta-isomerase	protein_codin	0.8195	0.00016	1	6.166	0.06869
**SLC35A2**	SLC35A2	21	solute carrier family 35 member A2	protein_codin	0.1475	0.485	1	7.428	0.1777
**GRIPAP1**	GRIPAP1	21	GRIP1 associated protein 1	protein_codin	0.3868	0.0882	1	8.852	0.1376
**ARHGEF9**	ARHGEF9	21	Cdc42 guanine nucleotide exchange factor 9	protein_codin	-0.4305	0.00996	1	33.21	0.1061
**LAS1L**	LAS1L	21	LAS1 like, ribosome biogenesis factor	protein_codin	-0.0596	0.797	1	3.297	0.1304
**IL13RA1**	IL13RA1	21	interleukin 13 receptor subunit alpha 1	protein_codin	-0.08357	0.476	1	48.35	0.1404
**RBMX2**	RBMX2	21	RNA binding motif protein X-linked 2	protein_codin	-0.02356	0.859	1	15.75	0.368
**AC100810.1**	AC100810.1	21	novel transcri	antisense_RN	-0.5185	0.00198	1	23.55	0.1765
**BRF2**	BRF2	21	BRF2 RNA polymerase III transcription initiation facto	protein_codin	-0.04048	0.82	1	7.607	0.1367
**SNAI2**	SNAI2	21	snail family transcriptional repressor 2	protein_codin	0.4659	0.0121	1	38.53	0.1681
**CHCHD7**	CHCHD7	21	coiled-coil-helix-coiled-coil-helix domain containing 7	protein_codin	-0.1465	0.416	1	9.183	0.3267
**NCOA2**	NCOA2	21	nuclear receptor coactivator 2	protein_codin	0.5043	0.0188	1	9.5	0.138
**COMMD5**	COMMD5	21	COMM domain containing 5	protein_codin	0.05016	0.736	1	8.372	0.1640
**CDC37L1**	CDC37L1	21	cell division cycle 37 like 1	protein_codin	-0.2444	0.0606	1	17.72	0.1132
**AK3**	AK3	21	adenylate kinase 3	protein_codin	0.576	1.05E-05	0.2419	60.53	0.1448
**GLIPR2**	GLIPR2	21	GLI pathogenesis related 2	protein_codin	0.1575	0.171	1	54.47	0.03201
**GNAQ**	GNAQ	21	G protein subunit alpha q	protein_codin	-0.3504	0.0382	1	18.99	0.2701
**HNRNPK**	HNRNPK	21	heterogeneous nuclear ribonucleoprotein K	protein_codin	0.03276	0.574	1	45.73	0.09718
**FAM120AOS**	FAM120AOS	21	family with sequence similarity 120A opposite strand	protein_codin	-0.01482	0.892	1	27.71	0.106
**KIAA0368**	KIAA0368	21	Ecm29 proteasome adaptor and scaffold	protein_codin	NA	NA	NA	30.04	0.2339
**PTBP3**	PTBP3	21	polypyrimidine tract binding protein 3	protein_codin	0.3112	0.145	1	26.46	0.1805
**PHF19**	PHF19	21	PHD finger protein 19	protein_codin	0.1692	0.432	1	6.401	0.2511
**MAPKAP1**	MAPKAP1	21	MAPK associated protein 1	protein_codin	0.1312	0.382	1	19.57	0.2082
**CIZ1**	CIZ1	21	CDKN1A interacting zinc finger protein 1	protein_codin	0.3222	0.136	1	6.963	0.06484
**PHYHD1**	PHYHD1	21	phytanoyl-CoA dioxygenase domain containing 1	protein_codin	0.3977	0.0232	1	21.49	0.03279
**PSMD13**	PSMD13	21	proteasome 26S subunit, non-ATPase 13	protein_codin	-0.08529	0.409	1	11.28	0.03399
**APBB1**	APBB1	21	amyloid beta precursor protein binding family B meml	protein_codin	-0.3573	0.0954	1	7.938	0.1768
**MRPL17**	MRPL17	21	mitochondrial ribosomal protein L17	protein_codin	0.03276	0.795	1	30.68	0.1237
**HIPK3**	HIPK3	21	homeodomain interacting protein kinase 3	protein_codin	0.3257	0.011	1	6.333	0.1379
**APIP**	APIP	21	APAF1 interacting protein	protein_codin	0.1987	0.155	1	7.668	0.2182
**COMMD9**	COMMD9	21	COMM domain containing 9	protein_codin	-0.006247	0.977	1	3.264	0.2094
**KMT5B**	KMT5B	21	lysine methyltransferase 5B	protein_codin	0.1402	0.411	1	21.42	0.1765
**ENDOD1**	ENDOD1	21	endonuclease domain containing 1	protein_codin	-0.167	0.179	1	23.5	0.1047
**CEP126**	CEP126	21	centrosomal protein 126	protein_codin	0.3129	0.0492	1	28.74	0.2414
**ARHGAP20**	ARHGAP20	21	Rho GTPase activating protein 20	protein_codin	0.7026	0.0094	1	11.77	0.2702
**TMEM136**	TMEM136	21	TLC domain containing 5	protein_codin	-0.4678	0.00598	1	9.336	0.3639
**DCPS**	DCPS	21	decapping enzyme, scavenger	protein_codin	0.4426	0.017	1	8.194	0.1959
**DCLRE1C**	DCLRE1C	21	DNA cross-link repair 1C	protein_codin	0.2626	0.368	1	10.78	0.207
**ARHGAP21**	ARHGAP21	21	Rho GTPase activating protein 21	protein_codin	1.123	5.86E-15	1.4E-10	38.22	0.1009
**ABI1**	ABI1	21	abl interactor 1	protein_codin	0.6177	0.00103	1	5.996	0.238
**PARD3**	PARD3	21	par-3 family cell polarity regulator	protein_codin	0.2562	0.209	1	30.57	0.3071
**NCOA4**	NCOA4	21	nuclear receptor coactivator 4	protein_codin	-0.1819	0.154	1	15.01	0.1759
**REEP3**	REEP3	21	receptor accessory protein 3	protein_codin	-0.1901	0.0344	1	53.75	0.06562
**EIF4EBP2**	EIF4EBP2	21	eukaryotic translation initiation factor 4E binding prote	protein_codin	0.1496	0.334	1	7.611	0.3972
**FRA10AC1**	FRA10AC1	21	FRA10A associated CGG repeat 1	protein_codin	-0.02026	0.874	1	25.28	0.1305
**TM9SF3**	TM9SF3	21	transmembrane 9 superfamily member 3	protein_codin	0.1248	0.152	1	54.08	0.06840
**AVPI1**	AVPI1	21	arginine vasopressin induced 1	protein_codin	0.2154	0.216	1	58.48	0.1442
**GRK5**	GRK5	21	G protein-coupled receptor kinase 5	protein_codin	0.9095	0.0001	1	46.07	0.06662
**GLRX3**	GLRX3	21	glutaredoxin 3	protein_codin	0.1193	0.406	1	42.46	0.09998
**FUOM**	FUOM	21	fucose mutarotase	protein_codin	-0.001397	0.995	1	8.528	0.1482
**EMG1**	EMG1	21	EMG1 N1-specific pseudouridine methyltransferase	protein_codin	-0.1432	0.337	1	22.57	0.2362
**KIAA1551**	KIAA1551	21	retroelement silencing factor 1	protein_codin	-0.3276	0.0245	1	60.58	0.1384
**ZCRB1**	ZCRB1	21	zinc finger CCHC-type and RNA binding motif contain	protein_codin	-0.3864	1.44E-06	0.03346	55.53	0.03232
**SLC38A2**	SLC38A2	21	solute carrier family 38 member 2	protein_codin	0.436	1.15E-05	0.2648	54.75	0.1479
**AMIGO2**	AMIGO2	21	adhesion molecule with Ig like domain 2	protein_codin	-0.03263	0.886	1	10.06	0.1711
**AC068888.1**	AC068888.1	21	novel transcript, antisense to TENC1 & EIF	antisense_RN	-0.01476	0.916	1	15.61	0.09939
**PCBP2**	PCBP2	21	poly(rC) binding protein 2	protein_codin	-0.01882	0.786	1	58.83	0.03331
**PA2G4**	PA2G4	21	proliferation-associated 2G4	protein_codin	-0.2251	0.00203	1	54.18	0.03221
**CDK4**	CDK4	21	cyclin dependent kinase 4	protein_codin	0.2264	0.0244	1	47.79	0.0716
**FRS2**	FRS2	21	fibroblast growth factor receptor substrate 2	protein_codin	0.2962	0.181	1	8.541	0.1726
**GLIPR1**	GLIPR1	21	GLI pathogenesis related 1	protein_codin	-0.5226	7.13E-11	1.69E-06	59.34	0.1006
**PLXNC1**	PLXNC1	21	plexin C1	protein_codin	2.448	2.19E-09	5.17E-05	47.29	0.2825
**IKBIP**	IKBIP	21	IKBKB interacting protein	protein_codin	-0.2237	0.0622	1	40.12	0.1410
**KCTD10**	KCTD10	21	potassium channel tetramerization domain containing 10	protein_codin	-0.18	0.315	1	8.683	0.2352
**ORAI1**	ORAI1	21	ORAI calcium release-activated calcium modulator 1	protein_codin	0.6312	0.0112	1	56.23	0.1056
**ZMYM2**	ZMYM2	21	zinc finger MYM-type containing 2	protein_codin	0.5067	0.0249	1	7.733	0.3490
**SPRYD7**	SPRYD7	21	SPRY domain containing 7	protein_codin	-0.208	0.185	1	22.42	0.1122
**MYCBP2**	MYCBP2	21	MYC binding protein 2	protein_codin	0.8906	7.57E-13	1.8E-08	47.25	0.1042
**SPRY2**	SPRY2	21	sprouty RTK signaling antagonist 2	protein_codin	1.509	1.49E-13	3.55E-09	54.47	0.2325
**DHRS4-AS1**	DHRS4-AS1	21	DHRS4 antisense RNA 1	antisense_RN	-0.1294	0.431	1	7.933	0.1065
**EMC9**	EMC9	21	ER membrane protein complex subunit 9	protein_codin	-0.258	0.172	1	9.012	0.2422
**RABGGTA**	RABGGTA	21	Rab geranylgeranyltransferase subunit alpha	protein_codin	0.4418	0.0544	1	3.365	0.09866
**PYGL**	PYGL	21	glycogen phosphorylase L	protein_codin	0.632	0.0013	1	8.856	0.1772
**PSMC6**	PSMC6	21	proteasome 26S subunit, ATPase 6	protein_codin	0.0854	0.377	1	20.04	0.1042
**PSMA3**	PSMA3	21	proteasome subunit alpha 3	protein_codin	0.07146	0.38	1	59.03	0.1445
**KIAA0586**	KIAA0586	21	KIAA0586	protein_codin	0.02916	0.876	1	23.96	0.1654
**PPM1A**	PPM1A	21	protein phosphatase, Mg2+/Mn2+ dependent 1A	protein_codin	0.4268	0.0095	1	7.986	0.1626
**TRMT5**	TRMT5	21	tRNA methyltransferase 5	protein_codin	0.04262	0.826	1	3.204	0.1034
**CCNK**	CCNK	21	cyclin K	protein_codin	0.3132	0.119	1	8.975	0.1727
**MEG3**	MEG3	21	maternally expressed 3	lincRNA	1.867	7.04E-11	1.67E-06	60.6	0.1431
**RCOR1**	RCOR1	21	REST corepressor 1	protein_codin	0.006552	0.975	1	9.568	0.274
**SNRPN**	SNRPN	21	small nuclear ribonucleoprotein polypeptide N	protein_codin	-0.6162	6.59E-16	1.58E-11	42.67	0.03687
**UBE3A**	UBE3A	21	ubiquitin protein ligase E3A	protein_codin	-0.06186	0.635	1	35.77	0.09777
**TRIM69**	TRIM69	21	tripartite motif containing 69	protein_codin	-0.1765	0.266	1	21.49	0.1756
**TRPM7**	TRPM7	21	transient receptor potential cation channel subfamily	protein_codin	0.165	0.415	1	52.46	0.1752
**ALDH1A2**	ALDH1A2	21	aldehyde dehydrogenase 1 family member A2	protein_codin	-1.12	1.25E-06	0.02905	40.61	0.2856
**ICE2**	ICE2	21	interactor of little elongation complex ELL subunit 2	protein_codin	-0.04783	0.825	1	9.293	0.2600
**USP3**	USP3	21	ubiquitin specific peptidase 3	protein_codin	-0.006348	0.972	1	24.22	0.1302
**MTFMT**	MTFMT	21	mitochondrial methionyl-tRNA formyltransferase	protein_codin	0.2443	0.205	1	9.356	0.108
**CD276**	CD276	21	CD276 molecule	protein_codin	0.7844	0.00014	1	41.86	0.1044
**SCAPER**	SCAPER	21	S-phase cyclin A associated protein in the ER	protein_codin	-0.2371	0.244	1	8.266	0.2068
**STUB1**	STUB1	21	STIP1 homology and U-box containing protein 1	protein_codin	-0.1737	0.0211	1	17.79	0.03884
**TELO2**	TELO2	21	telomere maintenance 2	protein_codin	0.1247	0.641	1	2.606	0.3707
**THOC6**	THOC6	21	THO complex 6	protein_codin	0.1166	0.611	1	3.456	0.2765
**NUDT16L1**	NUDT16L1	21	nudix hydrolase 16 like 1	protein_codin	0.1018	0.525	1	8.535	0.09942
**C16orf72**	C16orf72	21	chromosome 16 open reading frame 72	protein_codin	0.03222	0.869	1	6.882	0.07036
**ZC3H7A**	ZC3H7A	21	zinc finger CCCH-type containing 7A	protein_codin	0.07792	0.572	1	15.56	0.1360
**GTF3C1**	GTF3C1	21	general transcription factor IIIC subunit 1	protein_codin	0.8422	0.00067	1	7.536	0.1781
**NUDT21**	NUDT21	21	nudix hydrolase 21	protein_codin	0.1912	0.202	1	7.562	0.03511
**DDX19B**	DDX19B	21	DEAD-box helicase 19B	protein_codin	-0.01127	0.959	1	3.363	0.356
**TMEM170A**	TMEM170A	21	transmembrane protein 170A	protein_codin	0.000717	0.997	1	8.913	0.03366
**WWOX**	WWOX	21	WW domain containing oxidoreductase	protein_codin	-0.0759	0.786	1	7.958	0.3052
**MLYCD**	MLYCD	21	malonyl-CoA decarboxylase	protein_codin	0.3143	0.199	1	9.05	0.1354
**RHOT1**	RHOT1	21	ras homolog family member T1	protein_codin	0.076	0.676	1	7.492	0.2637
**TAF15**	TAF15	21	TATA-box binding protein associated factor 15	protein_codin	-0.1409	0.326	1	19.96	0.1069
**TADA2A**	TADA2A	21	transcriptional adaptor 2A	protein_codin	0.1725	0.51	1	3.148	0.2841
**DDX52**	DDX52	21	DExD-box helicase 52	protein_codin	0.1433	0.405	1	8.195	0.1949
**JUP**	JUP	21	junction plakoglobin	protein_codin	-0.1634	0.485	1	8.647	0.1682
**DNAJC7**	DNAJC7	21	DnaJ heat shock protein family (Hsp40) member C7	protein_codin	-0.195	0.132	1	12.21	0.1051
**GRN**	GRN	21	granulin precursor	protein_codin	0.6837	1.11E-12	2.64E-08	53.77	0.06572
**ZNF652**	ZNF652	21	zinc finger protein 652	protein_codin	0.1926	0.169	1	15.77	0.3050
**NME1**	NME1	21	NME/NM23 nucleoside diphosphate kinase 1	protein_codin	-0.3414	0.00182	1	60.6	0.03406
**KPNA2**	KPNA2	21	karyopherin subunit alpha 2	protein_codin	0.4375	0.12	1	59.52	0.2182
**NDUFAF8**	NDUFAF8	21	NADH:ubiquinone oxidoreductase complex assembly	protein_codin	-0.268	0.0175	1	11.25	0.132
**ARL16.00**	ARL16.00	21	ADP ribosylation factor like GTPase 16	protein_codin	-0.2552	0.0581	1	43.72	0.2062
**LPIN2**	LPIN2	21	lipin 2	protein_codin	0.2786	0.061	1	20.57	0.2085
**IMPACT**	IMPACT	21	impact RWD domain protein	protein_codin	-0.2451	0.122	1	7.796	0.2148
**CDH2**	CDH2	21	cadherin 2	protein_codin	-1.33	4.39E-09	0.0001	32.06	0.0681
**KDSR**	KDSR	21	3-ketodihydrosphingosine reductase	protein_codin	0.6633	1.41E-08	0.00033	19.16	0.0350
**ESF1**	ESF1	21	ESF1 nucleolar pre-rRNA processing protein homolog	protein_codin	-0.1849	0.171	1	40.38	0.2786
**KIF3B**	KIF3B	21	kinesin family member 3B	protein_codin	0.1468	0.476	1	8.18	0.1306
**NORAD**	NORAD	21	non-coding RNA activated by DNA damage	lincRNA	0.1439	0.0451	1	27.58	0.1092
**NDRG3**	NDRG3	21	NDRG family member 3	protein_codin	-0.1694	0.372	1	7.407	0.1852
**SDC4**	SDC4	21	syndecan 4	protein_codin	-0.4984	0.00154	1	27.39	0.03582
**ARFGEF2**	ARFGEF2	21	ADP ribosylation factor guanine nucleotide exchange	protein_codin	0.1903	0.41	1	2.969	0.3106
**ILF3**	ILF3	21	interleukin enhancer binding factor 3	protein_codin	0.08914	0.439	1	12.56	0.143
**CCDC159**	CCDC159	21	coiled-coil domain containing 159	protein_codin	-0.243	0.251	1	9.238	0.3062
**ZNF567**	ZNF567	21	zinc finger protein 567	protein_codin	0.3034	0.179	1	3.521	0.2369
**CLPTM1**	CLPTM1	21	CLPTM1 regulator of GABA type A receptor forward t	protein_codin	-0.09862	0.545	1	6.429	0.2699
**NUCB1**	NUCB1	21	nucleobindin 1	protein_codin	0.03904	0.614	1	57.71	0.03226
**IRF3**	IRF3	21	interferon regulatory factor 3	protein_codin	0.05572	0.726	1	19.34	0.2061
**PPP1R12C**	PPP1R12C	21	protein phosphatase 1 regulatory subunit 12C	protein_codin	0.6322	0.00240	1	51.68	0.1719
**ARVCF**	ARVCF	21	ARVCF delta catenin family member	protein_codin	-0.3401	0.18	1	56.32	0.1819
**NF2**	NF2	21	neurofibromin 2	protein_codin	-0.2597	0.298	1	3.318	0.2072
**HMGXB4**	HMGXB4	21	HMG-box containing 4	protein_codin	0.3127	0.0374	1	16.06	0.07237
**TRMU**	TRMU	21	tRNA 5-methylaminomethyl-2-thiouridylate methyltrar	protein_codin	-0.04122	0.88	1	8.508	0.1439
**ARSA**	ARSA	21	arylsulfatase A	protein_codin	0.6999	0.00078	1	8.445	0.06618
**IFNAR2**	IFNAR2	21	interferon alpha and beta receptor subunit 2	protein_codin	-0.7438	0.00822	1	3.462	0.1976
**ATP5O**	ATP5O	21	ATP synthase peripheral stalk subunit OSCP	protein_codin	NA	NA	NA	41.61	0.1004
**CPTP**	CPTP	22	ceramide-1-phosphate transfer protein	protein_codin	-0.2978	0.189	1	2.536	0.3071
**CDK11B**	CDK11B	22	cyclin dependent kinase 11B	protein_codin	0.2088	0.246	1	23.43	0.287
**ERRFI1**	ERRFI1	22	ERBB receptor feedback inhibitor 1	protein_codin	0.3964	0.00547	1	23.25	0.1315
**RERE**	RERE	22	arginine-glutamic acid dipeptide repeats	protein_codin	-0.118	0.451	1	6.471	0.3076
**SRM**	SRM	22	spermidine synthase	protein_codin	-0.1668	0.0457	1	59.92	0.03329
**DHDDS**	DHDDS	22	dehydrodolichyl diphosphate synthase subunit	protein_codin	-0.155	0.479	1	7.915	0.1694
**YTHDF2**	YTHDF2	22	YTH N6-methyladenosine RNA binding protein 2	protein_codin	0.2242	0.102	1	16.1	0.1456
**KPNA6**	KPNA6	22	karyopherin subunit alpha 6	protein_codin	0.1194	0.536	1	52.16	0.2442
**SMIM12**	SMIM12	22	small integral membrane protein 12	protein_codin	-0.1876	0.192	1	18.37	0.1756
**MEAF6**	MEAF6	22	MYST/Esa1 associated factor 6	protein_codin	0.114	0.189	1	15.63	0.1096
**SNIP1**	SNIP1	22	Smad nuclear interacting protein 1	protein_codin	0.325	0.111	1	32.9	0.1695
**SCMH1**	SCMH1	22	Scm polycomb group protein homolog 1	protein_codin	-0.5508	0.0118	1	8.719	0.2622
**HYI**	HYI	22	hydroxypyruvate isomerase (putative)	protein_codin	-0.01242	0.896	1	15.34	0.03247
**AKR1A1**	AKR1A1	22	aldo-keto reductase family 1 member A1	protein_codin	0.178	0.0298	1	32.48	0.03372
**DOCK7**	DOCK7	22	dedicator of cytokinesis 7	protein_codin	0.3096	0.0841	1	7.536	0.3192
**ROR1**	ROR1	22	receptor tyrosine kinase like orphan receptor 1	protein_codin	-0.2176	0.379	1	10.79	0.4459
**AK4**	AK4	22	adenylate kinase 4	protein_codin	-0.3666	0.0772	1	8.024	0.2059
**SGIP1**	SGIP1	22	SH3 domain GRB2 like endophilin interacting protein 1	protein_codin	-1.512	3.88E-13	9.24E-09	9.096	0.1738
**ACADM**	ACADM	22	acyl-CoA dehydrogenase medium chain	protein_codin	-0.07617	0.525	1	14.72	0.06504
**USP33**	USP33	22	ubiquitin specific peptidase 33	protein_codin	0.3077	0.0479	1	6.745	0.1779
**FUBP1**	FUBP1	22	far upstream element binding protein 1	protein_codin	-0.01995	0.892	1	16.61	0.2456

**TTLL7**	TTLL7	22	tubulin tyrosine ligase like 7	protein_codin	-1.129	1.58E-2	3.81E-20	34.8	0.06838
**KYAT3**	KYAT3	22	kynurenine aminotransferase 3	protein_codin	0.02549	0.898	1	8.838	0.1050
**ZNF326**	ZNF326	22	zinc finger protein 326	protein_codin	0.09751	0.55	1	39.64	0.1972
**RPAP2**	RPAP2	22	RNA polymerase II associated protein 2	protein_codin	0.1988	0.29	1	21.83	0.2006
**DNTTIP2**	DNTTIP2	22	deoxynucleotidyltransferase terminal interacting prote	protein_codin	0.2393	0.0633	1	26.07	0.1412
**TAF13**	TAF13	22	TATA-box binding protein associated factor 13	protein_codin	0.305	0.127	1	33.72	0.1960
**GSTM3**	GSTM3	22	glutathione S-transferase mu 3	protein_codin	0.7263	2.61E-12	6.2E-08	46.27	0.133
**LRIF1**	LRIF1	22	ligand dependent nuclear receptor interacting factor 1	protein_codin	0.329	0.0716	1	23.29	0.1762
**WDR77**	WDR77	22	WD repeat domain 77	protein_codin	-0.07682	0.729	1	26	0.1752
**SLC16A1**	SLC16A1	22	solute carrier family 16 member 1	protein_codin	0.4318	0.0793	1	10.07	0.1507
**SEC22B**	SEC22B	22	SEC22 homolog B, vesicle trafficking protein (gene/p	protein_codin	0.2938	0.0147	1	41.93	0.06677
**AC245297.3**	AC245297.3	22	novel transcri	lincRNA	-0.4505	0.00163	1	20.03	0.2080
**VPS45**	VPS45	22	vacuolar protein sorting 45 homolog	protein_codin	0.2985	0.186	1	3.158	0.2169
**KRTCAP2**	KRTCAP2	22	keratinocyte associated protein 2	protein_codin	0.3846	3.14E-07	0.00723	25.43	0.1640
**MTX1**	MTX1	22	metaxin 1	protein_codin	0.2388	0.11	1	7.023	0.1704
**FAM189B**	FAM189B	22	family with sequence similarity 189 member B	protein_codin	-0.3504	0.144	1	3.155	0.3112
**PPOX**	PPOX	22	protoporphyrinogen oxidase	protein_codin	0.08421	0.758	1	3.656	0.3075
**ATF6**	ATF6	22	activating transcription factor 6	protein_codin	0.6074	0.00103	1	7.546	0.1347
**SRGAP2**	SRGAP2	22	SLIT-ROBO Rho GTPase activating protein 2	protein_codin	0.8107	0.00187	1	9.073	0.2461
**2-Mar**	43526	22	mitochondrial amidoxime reducing component 2	protein_codin	0.7809	0.00038	1	8.723	0.1788
**SPRTN**	SPRTN	22	SprT-like N-terminal domain	protein_codin	0.1812	0.399	1	3.2	0.03852
**RNASEH1**	RNASEH1	22	ribonuclease H1	protein_codin	-0.144	0.392	1	21.85	0.1601
**TAF1B**	TAF1B	22	TATA-box binding protein associated factor, RNA pol	protein_codin	0.5258	0.00327	1	22.06	0.1686
**LPIN1**	LPIN1	22	lipin 1	protein_codin	0.3666	0.0865	1	8.323	0.5227
**HS1BP3**	HS1BP3	22	HCLS1 binding protein 3	protein_codin	-0.1458	0.482	1	2.742	0.3581
**FAM228B**	FAM228B	22	family with sequence similarity 228 member B	protein_codin	-0.3164	0.108	1	8.675	0.1732
**EIF2B4**	EIF2B4	22	eukaryotic translation initiation factor 2B subunit delta	protein_codin	-0.1005	0.591	1	7.772	0.2332
**TTC27**	TTC27	22	tetratricopeptide repeat domain 27	protein_codin	0.5895	0.0124	1	8.297	0.1369
**FAM98A**	FAM98A	22	family with sequence similarity 98 member A	protein_codin	0.1002	0.485	1	16.09	0.0654
**AC009414.2**	AC009414.2	22	CRIM1 divergent transcript	lincRNA	-0.3447	0.106	1	29.18	0.1336
**NDUFAF7**	NDUFAF7	22	NADH:ubiquinone oxidoreductase complex assembly	protein_codin	0.4482	0.135	1	3.007	0.2967
**CRIPT**	CRIPT	22	CXXC repeat containing interactor of PDZ3 domain	protein_codin	-0.03971	0.761	1	47.03	0.2050
**PPP1R21**	PPP1R21	22	protein phosphatase 1 regulatory subunit 21	protein_codin	-0.1690	0.425	1	9.505	0.348
**CCT4**	CCT4	22	chaperonin containing TCP1 subunit 4	protein_codin	0.12	0.156	1	42.68	0.03205
**WDR54**	WDR54	22	WD repeat domain 54	protein_codin	-0.08082	0.661	1	8.344	0.309
**MOGS**	MOGS	22	mannosyl-oligosaccharide glucosidase	protein_codin	0.0419	0.847	1	9.25	0.2409
**PCGF1**	PCGF1	22	polycomb group ring finger 1	protein_codin	-0.1536	0.545	1	2.805	0.1314
**MRPL19**	MRPL19	22	mitochondrial ribosomal protein L19	protein_codin	0.3863	0.00257	1	12.66	0.1011
**TCF7L1**	TCF7L1	22	transcription factor 7 like 1	protein_codin	0.243	0.21	1	8.508	0.1677
**TGOLN2**	TGOLN2	22	trans-golgi network protein 2	protein_codin	0.1982	0.0102	1	54.34	0.03479
**RNF103**	RNF103	22	ring finger protein 103	protein_codin	0.3479	0.187	1	2.403	0.4085
**RMND5A**	RMND5A	22	required for meiotic nuclear division 5 homolog A	protein_codin	0.1756	0.455	1	8.887	0.2399
**IGKC**	IGKC	22	immunoglobulin kappa constant	IG_C_gene	-3.079	2.72E-08	0.00064	Inf	3.541
**STARD7**	STARD7	22	StAR related lipid transfer domain containing 7	protein_codin	0.03455	0.858	1	7.107	0.1702
**TMEM131**	TMEM131	22	transmembrane protein 131	protein_codin	0.1627	0.346	1	23.68	0.1794
**MRPL30**	MRPL30	22	mitochondrial ribosomal protein L30	protein_codin	0.1403	0.524	1	34.63	0.2367
**TTL**	TTL	22	tubulin tyrosine ligase	protein_codin	-0.05858	0.862	1	3.447	0.2059
**INSIG2**	INSIG2	22	insulin induced gene 2	protein_codin	-0.5352	0.00017	1	36.88	0.1440
**FAM168B**	FAM168B	22	family with sequence similarity 168 member B	protein_codin	-0.04729	0.808	1	9.038	0.2769
**ARL6IP6**	ARL6IP6	22	ADP ribosylation factor like GTPase 6 interacting prot	protein_codin	0.2803	0.124	1	7.284	0.2519
**RBMS1**	RBMS1	22	RNA binding motif single stranded interacting protein	protein_codin	0.5063	3.96E-07	0.00924	31.8	0.07198
**NUP35**	NUP35	22	nucleoporin 35	protein_codin	0.8105	0.00048	1	32.63	0.2090
**ANKRD44**	ANKRD44	22	ankyrin repeat domain 44	protein_codin	-0.8116	0.00187	1	3.262	0.3592
**COQ10B**	COQ10B	22	coenzyme Q10B	protein_codin	0.394	0.00179	1	30.44	0.11
**WDFY1**	WDFY1	22	WD repeat and FYVE domain containing 1	protein_codin	0.0162	0.934	1	26.08	0.07157
**SP140L**	SP140L	22	SP140 nuclear body protein like	protein_codin	0.208	0.355	1	8.027	0.07037
**2-Sep**	43710	22	septin 2	protein_codin	-0.1503	0.0308	1	42.53	0.03356
**ARL8B**	ARL8B	22	ADP ribosylation factor like GTPase 8B	protein_codin	0.2833	0.02	1	6.346	0.1417
**TAMM41**	TAMM41	22	TAM41 mitochondrial translocator assembly and mai	protein_codin	0.2845	0.208	1	3.196	0.1405
**AZI2**	AZI2	22	5-azacytidine induced 2	protein_codin	0.1533	0.255	1	30.63	0.1380
**SUSD5**	SUSD5	22	sushi domain containing 5	protein_codin	-1.115	4.96E-09	0.00012	41.45	0.1672
**MYD88**	MYD88	22	MYD88 innate immune signal transduction adaptor	protein_codin	0.1447	0.652	1	8.884	0.2469
**SMARCC1**	SMARCC1	22	SWI/SNF related, matrix associated, actin dependent	protein_codin	0.1276	0.475	1	25.6	0.2125
**DHX30**	DHX30	22	DExH-box helicase 30	protein_codin	0.1933	0.322	1	7.754	0.2005
**FAM208A**	FAM208A	22	transcription activation suppressor	protein_codin	0.06929	0.641	1	18.8	0.244
**THOC7**	THOC7	22	THO complex 7	protein_codin	0.1208	0.195	1	26.4	0.03354
**UBA3**	UBA3	22	ubiquitin like modifier activating enzyme 3	protein_codin	0.151	0.346	1	6.405	0.06685
**SENP7**	SENP7	22	SUMO specific peptidase 7	protein_codin	0.4419	0.0112	1	20.49	0.3779
**ALCAM**	ALCAM	22	activated leukocyte cell adhesion molecule	protein_codin	-0.8268	3.31E-11	7.85E-07	42.6	0.03998
**ITGB5**	ITGB5	22	integrin subunit beta 5	protein_codin	-0.02598	0.772	1	60.6	0.06773
**CDV3**	CDV3	22	CDV3 homolog	protein_codin	0.2501	0.00782	1	60.46	0.1145
**TOPBP1**	TOPBP1	22	DNA topoisomerase II binding protein 1	protein_codin	0.3705	0.156	1	7.884	0.1374
**CEP70**	CEP70	22	centrosomal protein 70	protein_codin	0.02212	0.922	1	3.462	0.2628
**HLTF**	HLTF	22	helicase like transcription factor	protein_codin	0.2165	0.0930	1	24.38	0.2059
**WWTR1**	WWTR1	22	WW domain containing transcription regulator 1	protein_codin	-0.8619	3.33E-1	7.96E-12	54.86	0.06528
**EIF2A**	EIF2A	22	eukaryotic translation initiation factor 2A	protein_codin	-0.1666	0.0608	1	59.44	0.1352
**RAP2B**	RAP2B	22	RAP2B, member of RAS oncogene family	protein_codin	0.303	0.099	1	53.06	0.3142
**ABCF3**	ABCF3	22	ATP binding cassette subfamily F member 3	protein_codin	0.06101	0.776	1	2.626	0.2421
**RPL39L**	RPL39L	22	ribosomal protein L39 like	protein_codin	-0.4546	0.0117	1	60.42	0.1679
**OPA1**	OPA1	22	OPA1 mitochondrial dynamin like GTPase	protein_codin	0.09695	0.635	1	8.619	0.4624
**TCTEX1D2**	TCTEX1D2	22	Tctex1 domain containing 2	protein_codin	0.2244	0.297	1	10.1	0.2182
**NCBP2-AS2**	NCBP2-AS2	22	NCBP2 antisense 2 (head to head)	protein_codin	-0.1306	0.299	1	19.65	0.1062
**LETM1**	LETM1	22	leucine zipper and EF-hand containing transmembra	protein_codin	0.4654	0.0403	1	8.641	0.07226
**RNF4**	RNF4	22	ring finger protein 4	protein_codin	0.1202	0.562	1	9.702	0.3071
**MFSD10**	MFSD10	22	major facilitator superfamily domain containing 10	protein_codin	0.225	0.0557	1	25.82	0.1117
**MAN2B2**	MAN2B2	22	mannosidase alpha class 2B member 2	protein_codin	-0.07982	0.692	1	6.958	0.274
**BLOC1S4**	BLOC1S4	22	biogenesis of lysosomal organelles complex 1 subuni	protein_codin	-0.06114	0.667	1	18.98	0.1315
**WDR1**	WDR1	22	WD repeat domain 1	protein_codin	-0.4614	1.77E-06	0.04109	45.22	0.0702
**BOD1L1**	BOD1L1	22	biorientation of chromosomes in cell division 1 like 1	protein_codin	0.08806	0.272	1	42	0.1032
**TBC1D1**	TBC1D1	22	TBC1 domain family member 1	protein_codin	-0.3686	0.00387	1	59.95	0.218
**SGCB**	SGCB	22	sarcoglycan beta	protein_codin	-0.102	0.269	1	31.87	0.1637
**USP46**	USP46	22	ubiquitin specific peptidase 46	protein_codin	0.2119	0.261	1	7.243	0.1369
**TMEM165**	TMEM165	22	transmembrane protein 165	protein_codin	0.126	0.144	1	38.23	0.0332
**CENPC**	CENPC	22	centromere protein C	protein_codin	0.326	0.00660	1	20.34	0.4026
**SEC31A**	SEC31A	22	SEC31 homolog A, COPII coat complex component	protein_codin	0.2174	0.0125	1	34.33	0.07041
**BDH2**	BDH2	22	3-hydroxybutyrate dehydrogenase 2	protein_codin	0.4849	7.61E-07	0.01772	60.53	0.1652
**SNHG8**	SNHG8	22	small nucleolar RNA host gene 8	lincRNA	-0.2588	0.00197	1	59.96	0.09852
**PCDH10**	PCDH10	22	protocadherin 10	protein_codin	-0.6275	0.00011	1	35.63	0.116
**FAM198B**	FAM198B	22	golgi associated kinase 1B	protein_codin	-1.077	7.04E-08	0.00165	51.03	0.1734
**CBR4**	CBR4	22	carbonyl reductase 4	protein_codin	0.02779	0.9	1	3.452	0.1702
**TRAPPC11**	TRAPPC11	22	trafficking protein particle complex 11	protein_codin	-0.1264	0.619	1	3.148	0.1351
**EXOC3**	EXOC3	22	exocyst complex component 3	protein_codin	0.1252	0.524	1	3.486	0.2062
**MTMR12**	MTMR12	22	myotubularin related protein 12	protein_codin	0.03565	0.896	1	8.873	0.2571
**CCDC152**	CCDC152	22	coiled-coil domain containing 152	protein_codin	-0.5303	0.0359	1	32.32	0.2092
**MOCS2**	MOCS2	22	molybdenum cofactor synthesis 2	protein_codin	-0.09739	0.316	1	51.49	0.06603
**TRIM23**	TRIM23	22	tripartite motif containing 23	protein_codin	0.05603	0.734	1	6.387	0.1706
**MRPS36**	MRPS36	22	mitochondrial ribosomal protein S36	protein_codin	-0.2423	0.00796	1	18.75	0.1015
**ARSK**	ARSK	22	arylsulfatase family member K	protein_codin	0.2287	0.226	1	6.698	0.1950
**WDR36**	WDR36	22	WD repeat domain 36	protein_codin	0.5615	0.0209	1	3.405	0.3752
**COMMD10**	COMMD10	22	COMM domain containing 10	protein_codin	0.1187	0.548	1	23.14	0.1072
**SNX24**	SNX24	22	sorting nexin 24	protein_codin	0.5233	0.0308	1	3.532	0.372
**SEC24A**	SEC24A	22	SEC24 homolog A, COPII coat complex component	protein_codin	0.9968	0.00062	1	3.108	0.2301
**HSPA9**	HSPA9	22	heat shock protein family A (Hsp70) member 9	protein_codin	0.005409	0.961	1	48.67	0.1674
**PFDN1**	PFDN1	22	prefoldin subunit 1	protein_codin	-0.1047	0.254	1	18.23	0.06443
**SLC35A4**	SLC35A4	22	solute carrier family 35 member A4	protein_codin	0.257	0.267	1	2.767	0.318
**SYNPO**	SYNPO	22	synaptopodin	protein_codin	-1.018	2.75E-15	6.57E-11	44.22	0.02362
**THG1L**	THG1L	22	tRNA-histidine guanylyltransferase 1 like	protein_codin	-0.2193	0.412	1	7.234	0.3012
**FBXW11**	FBXW11	22	F-box and WD repeat domain containing 11	protein_codin	-0.1593	0.342	1	8	0.3196
**TRIM7**	TRIM7	22	tripartite motif containing 7	protein_codin	0.6724	0.0068	1	29.33	0.1048
**RIPK1**	RIPK1	22	receptor interacting serine/threonine kinase 1	protein_codin	0.4138	0.0602	1	9.128	0.25
**BPHL**	BPHL	22	biphenyl hydrolase like	protein_codin	0.5209	0.0179	1	8.302	0.2768
**TUBB2A**	TUBB2A	22	tubulin beta 2A class IIa	protein_codin	-0.07992	0.574	1	60.58	0.06548
**FARS2**	FARS2	22	phenylalanyl-tRNA synthetase 2, mitochondrial	protein_codin	0.08910	0.687	1	3.096	0.2404
**BLOC1S5**	BLOC1S5	22	biogenesis of lysosomal organelles complex 1 subuni	protein_codin	0.5164	0.017	1	8.552	0.1384
**ATXN1**	ATXN1	22	ataxin 1	protein_codin	0.05687	0.769	1	7.167	0.06884
**BTN3A2**	BTN3A2	22	butyrophilin subfamily 3 member A2	protein_codin	-0.0665	0.696	1	17.53	0.1079
**PPP1R10**	PPP1R10	22	protein phosphatase 1 regulatory subunit 10	protein_codin	0.8785	2.94E-11	6.98E-07	25.58	0.1764
**NRM**	NRM	22	nurim	protein_codin	-0.7187	0.00058	1	3.036	0.2697
**GPANK1**	GPANK1	22	G-patch domain and ankyrin repeats 1	protein_codin	-0.1962	0.24	1	60.49	0.1119
**STK19**	STK19	22	serine/threonine kinase 19	protein_codin	-0.24	0.132	1	7.262	0.07405
**SMIM29**	SMIM29	22	small integral membrane protein 29	protein_codin	-0.4693	0.00493	1	23.28	0.1684
**DAAM2**	DAAM2	22	dishevelled associated activator of morphogenesis 2	protein_codin	-0.2398	0.24	1	9.158	0.1780
**SYNCRIP**	SYNCRIP	22	synaptotagmin binding cytoplasmic RNA interacting p	protein_codin	-0.266	0.0126	1	55.08	0.1768
**QRSL1**	QRSL1	22	glutaminyl-tRNA amidotransferase subunit QRSL1	protein_codin	0.6767	0.00871	1	2.963	0.1029
**CDC40**	CDC40	22	cell division cycle 40	protein_codin	0.1595	0.148	1	26.83	0.1295
**RWDD1**	RWDD1	22	RWD domain containing 1	protein_codin	-0.2062	0.00063	1	34.26	0.1336
**HINT3**	HINT3	22	histidine triad nucleotide binding protein 3	protein_codin	-0.3894	0.0388	1	7.217	0.06976
**TFB1M**	TFB1M	22	transcription factor B1, mitochondrial	protein_codin	-0.3467	0.0812	1	3.419	0.168
**ARID1B**	ARID1B	22	AT-rich interaction domain 1B	protein_codin	0.506	0.00457	1	15.73	0.2161
**TMEM242**	TMEM242	22	transmembrane protein 242	protein_codin	-0.1532	0.227	1	6.508	0.03262
**TULP4**	TULP4	22	TUB like protein 4	protein_codin	0.2558	0.124	1	8.603	0.3054
**LINC02544**	LINC02544	22	long intergenic non-protein coding RNA 2544	lincRNA	-0.8425	0.00022	1	28.57	0.4190
**C7orf50**	C7orf50	22	chromosome 7 open reading frame 50	protein_codin	-0.2678	0.0051	1	22.92	0.09729
**GNA12**	GNA12	22	G protein subunit alpha 12	protein_codin	0.1342	0.538	1	2.905	0.1037
**EIF2AK1**	EIF2AK1	22	eukaryotic translation initiation factor 2 alpha kinase 1	protein_codin	0.3899	0.0093	1	7.476	0.1674
**ZNF12**	ZNF12	22	zinc finger protein 12	protein_codin	0.32	0.159	1	8.06	0.1444
**NUPL2**	NUPL2	22	nucleoporin 42	protein_codin	0.1354	0.489	1	7.904	0.2491
**NT5C3A**	NT5C3A	22	5'-nucleotidase, cytosolic IIIA	protein_codin	0.3555	0.0604	1	8.524	0.2447
**MRPL32**	MRPL32	22	mitochondrial ribosomal protein L32	protein_codin	0.03085	0.729	1	27.67	0.108
**NUDCD3**	NUDCD3	22	NudC domain containing 3	protein_codin	0.3205	0.0805	1	6.186	0.07787
**AC027644.3**	AC027644.3	22	novel transcript, antisense to RABGE	antisense_RN	-0.3118	0.0322	1	8.045	0.4067
**TMEM60**	TMEM60	22	transmembrane protein 60	protein_codin	0.3354	0.0502	1	7.82	0.0711
**KRIT1**	KRIT1	22	KRIT1 ankyrin repeat containing	protein_codin	0.1813	0.244	1	15.57	0.114
**BET1**	BET1	22	Bet1 golgi vesicular membrane trafficking protein	protein_codin	-0.0334	0.868	1	7.477	0.2019
**ASNS**	ASNS	22	asparagine synthetase (glutamine-hydrolyzing)	protein_codin	-0.4422	0.0845	1	8.958	0.3217
**BUD31**	BUD31	22	BUD31 homolog	protein_codin	-0.3557	0.00029	1	22.3	0.1325
**PPP1R35**	PPP1R35	22	protein phosphatase 1 regulatory subunit 35	protein_codin	-0.06519	0.682	1	24.33	0.2380
**GIGYF1**	GIGYF1	22	GRB10 interacting GYF protein 1	protein_codin	0.3242	0.149	1	3.527	0.3248
**PRKAR2B**	PRKAR2B	22	protein kinase cAMP-dependent type II regulatory sub	protein_codin	0.7681	0.00014	1	24.05	0.2639
**ING3**	ING3	22	inhibitor of growth family member 3	protein_codin	0.5081	0.00703	1	24.63	0.1801
**UBE2H**	UBE2H	22	ubiquitin conjugating enzyme E2 H	protein_codin	-0.06707	0.474	1	47.88	0.212
**EPHB6**	EPHB6	22	EPH receptor B6	protein_codin	0.652	0.00399	1	59.13	0.2382
**PDIA4**	PDIA4	22	protein disulfide isomerase family A member 4	protein_codin	0.2729	0.0375	1	37.8	0.07354
**RARRES2**	RARRES2	22	retinoic acid receptor responder 2	protein_codin	0.5628	2.32E-15	5.54E-11	55.38	0.0321
**UBA1**	UBA1	22	ubiquitin like modifier activating enzyme 1	protein_codin	0.1845	0.275	1	8.782	0.2047
**OPHN1**	OPHN1	22	oligophrenin 1	protein_codin	0.1834	0.444	1	3.13	0.5416
**NONO**	NONO	22	non-POU domain containing octamer binding	protein_codin	-0.1007	0.369	1	13.97	0.1031
**OGT**	OGT	22	O-linked N-acetylglucosamine (GlcNAc) transferase	protein_codin	0.6029	0.00141	1	7.177	0.3519
**HDX**	HDX	22	highly divergent homeobox	protein_codin	-0.3648	0.0548	1	10.23	0.3704
**ARMCX6**	ARMCX6	22	armadillo repeat containing X-linked 6	protein_codin	-0.2897	0.0786	1	26.97	0.1381
**TBC1D8B**	TBC1D8B	22	TBC1 domain family member 8B	protein_codin	0.5715	0.00765	1	10.06	0.2342
**UPF3B**	UPF3B	22	UPF3B regulator of nonsense mediated mRNA decay	protein_codin	-0.1173	0.486	1	48.68	0.2672
**MMGT1**	MMGT1	22	membrane magnesium transporter 1	protein_codin	-0.02563	0.862	1	8.505	0.2031
**MECP2**	MECP2	22	methyl-CpG binding protein 2	protein_codin	-0.1788	0.24	1	44.54	0.1117
**EMD**	EMD	22	emerin	protein_codin	-0.01622	0.882	1	45.77	0.06438
**GDI1**	GDI1	22	GDP dissociation inhibitor 1	protein_codin	0.02929	0.858	1	7.948	0.2414
**INTS10**	INTS10	22	integrator complex subunit 10	protein_codin	0.2511	0.0706	1	18.22	0.212
**DCTN6**	DCTN6	22	dynactin subunit 6	protein_codin	-0.1376	0.148	1	26.75	0.06660
**TM2D2**	TM2D2	22	TM2 domain containing 2	protein_codin	0.2381	0.0929	1	7.225	0.1448
**PENK**	PENK	22	proenkephalin	protein_codin	3.04	0.9897	1	60.6	0.72
**ARFGEF1**	ARFGEF1	22	ADP ribosylation factor guanine nucleotide exchange	protein_codin	-0.03255	0.837	1	7.95	0.2396
**PEX2**	PEX2	22	peroxisomal biogenesis factor 2	protein_codin	0.1002	0.51	1	17.2	0.1338
**CPNE3**	CPNE3	22	copine 3	protein_codin	0.1699	0.0240	1	28.33	0.06466
**PDP1**	PDP1	22	pyruvate dehyrogenase phosphatase catalytic subuni	protein_codin	0.302	0.0962	1	18.03	0.1020
**RAD21**	RAD21	22	RAD21 cohesin complex component	protein_codin	0.1053	0.305	1	34.1	0.1388
**DERL1**	DERL1	22	derlin 1	protein_codin	-0.1067	0.517	1	18.29	0.1820
**WDYHV1**	WDYHV1	22	WDYHV motif containing 1	protein_codin	-0.06146	0.771	1	8.783	0.218
**SQLE**	SQLE	22	squalene epoxidase	protein_codin	0.1229	0.575	1	26.63	0.1462
**WASHC5**	WASHC5	22	WASH complex subunit 5	protein_codin	0.4163	0.0609	1	3.425	0.3179
**GSDMD**	GSDMD	22	gasdermin D	protein_codin	-0.1682	0.192	1	60.35	0.0352
**SLC39A4**	SLC39A4	22	solute carrier family 39 member 4	protein_codin	-0.2875	0.306	1	3.427	0.2152
**PPP1R16A**	PPP1R16A	22	protein phosphatase 1 regulatory subunit 16A	protein_codin	-1.04	4.04E-06	0.09347	28.88	0.2149
**C8orf82**	C8orf82	22	chromosome 8 open reading frame 82	protein_codin	0.1227	0.42	1	8.126	0.3124
**C8orf33**	C8orf33	22	chromosome 8 open reading frame 33	protein_codin	-0.296	0.0308	1	15.47	0.1481
**UHRF2**	UHRF2	22	ubiquitin like with PHD and ring finger domains 2	protein_codin	0.2750	0.289	1	9.628	0.3081
**RRAGA**	RRAGA	22	Ras related GTP binding A	protein_codin	0.03535	0.640	1	39.74	0.06874
**MTAP**	MTAP	22	methylthioadenosine phosphorylase	protein_codin	0.0822	0.582	1	7.606	0.2782
**TRMT10B**	TRMT10B	22	tRNA methyltransferase 10B	protein_codin	0.1588	0.601	1	3.419	0.4047
**FXN**	FXN	22	frataxin	protein_codin	-0.1829	0.533	1	3.36	0.5162
**STX17**	STX17	22	syntaxin 17	protein_codin	-0.1389	0.346	1	21.24	0.1699
**ZNF618**	ZNF618	22	zinc finger protein 618	protein_codin	0.5034	0.0681	1	9.193	0.2648
**ATP6V1G1**	ATP6V1G1	22	ATPase H+ transporting V1 subunit G1	protein_codin	-0.1006	0.0493	1	35.66	0.1002
**CNTRL**	CNTRL	22	centriolin	protein_codin	-0.197	0.34	1	9.596	0.3077
**RBM18**	RBM18	22	RNA binding motif protein 18	protein_codin	0.2772	0.168	1	30.14	0.2787
**ZDHHC12**	ZDHHC12	22	zinc finger DHHC-type containing 12	protein_codin	0.08539	0.708	1	6.474	0.2462
**TTF1**	TTF1	22	transcription termination factor 1	protein_codin	0.2723	0.09	1	17.12	0.3768
**REXO4**	REXO4	22	REX4 homolog, 3'-5' exonuclease	protein_codin	-0.1562	0.495	1	3.486	0.2072
**TMEM203**	TMEM203	22	transmembrane protein 203	protein_codin	-0.3903	0.00304	1	21.03	0.3112
**NELFB**	NELFB	22	negative elongation factor complex member B	protein_codin	0.1366	0.447	1	3.31	0.2125
**ARRDC1**	ARRDC1	22	arrestin domain containing 1	protein_codin	0.2017	0.432	1	3.248	0.1636
**RNF141**	RNF141	22	ring finger protein 141	protein_codin	-0.05206	0.795	1	8.636	0.1829
**EIF4G2**	EIF4G2	22	eukaryotic translation initiation factor 4 gamma 2	protein_codin	-0.03768	0.632	1	24.43	0.0708
**TEAD1**	TEAD1	22	TEA domain transcription factor 1	protein_codin	0.8842	1.78E-05	0.4092	18.82	0.1442
**BTBD10**	BTBD10	22	BTB domain containing 10	protein_codin	-0.239	0.129	1	7.678	0.1131
**PIK3C2A**	PIK3C2A	22	phosphatidylinositol-4-phosphate 3-kinase catalytic s	protein_codin	-0.04938	0.752	1	20.73	0.2059
**TSG101**	TSG101	22	tumor susceptibility 101	protein_codin	0.1062	0.294	1	43.84	0.1788
**DGKZ**	DGKZ	22	diacylglycerol kinase zeta	protein_codin	0.0352	0.870	1	3.259	0.3434
**KBTBD4**	KBTBD4	22	kelch repeat and BTB domain containing 4	protein_codin	-0.06887	0.779	1	2.733	0.1715
**TNKS1BP1**	TNKS1BP1	22	tankyrase 1 binding protein 1	protein_codin	0.7395	0.00544	1	8.6	0.1842
**PRPF19**	PRPF19	22	pre-mRNA processing factor 19	protein_codin	0.325	0.0593	1	31.19	0.1314
**TMEM138**	TMEM138	22	transmembrane protein 138	protein_codin	0.2327	0.266	1	6.738	0.2754
**TMEM258**	TMEM258	22	transmembrane protein 258	protein_codin	-0.0454	0.484	1	33.73	0.1006
**ROM1**	ROM1	22	retinal outer segment membrane protein 1	protein_codin	-0.8803	3.23E-06	0.0748	10.2	0.0771
**NAALADL1**	NAALADL1	22	N-acetylated alpha-linked acidic dipeptidase like 1	protein_codin	-0.1517	0.379	1	28.15	0.1006
**EIF1AD**	EIF1AD	22	eukaryotic translation initiation factor 1A domain cont	protein_codin	-0.2599	0.260	1	8.517	0.3069
**MRPL11**	MRPL11	22	mitochondrial ribosomal protein L11	protein_codin	-0.07456	0.471	1	54	0.1041
**CCS**	CCS	22	copper chaperone for superoxide dismutase	protein_codin	0.3903	0.00118	1	12.38	0.1064
**RHOD**	RHOD	22	ras homolog family member D	protein_codin	-0.7812	1.36E-09	3.21E-05	38.85	0.1379
**RAD9A**	RAD9A	22	RAD9 checkpoint clamp component A	protein_codin	0.02329	0.931	1	3.436	0.3850
**RAB6A**	RAB6A	22	RAB6A, member RAS oncogene family	protein_codin	0.02803	0.840	1	6.919	0.1697
**EMSY**	EMSY	22	EMSY transcriptional repressor, BRCA2 interacting	protein_codin	0.1872	0.552	1	3.719	0.2026
**RSF1**	RSF1	22	remodeling and spacing factor 1	protein_codin	-0.1579	0.0518	1	37.64	0.138
**PRCP**	PRCP	22	prolylcarboxypeptidase	protein_codin	1.793	8.13E-28	1.96E-22	53.93	0.1019
**SLC36A4**	SLC36A4	22	solute carrier family 36 member 4	protein_codin	0.09805	0.716	1	3.195	0.3277
**SMCO4**	SMCO4	22	single-pass membrane protein with coiled-coil domain	protein_codin	-0.5674	0.00295	1	10.03	0.06686
**HSPB2**	HSPB2	22	heat shock protein family B (small) member 2	protein_codin	0.1609	0.272	1	6.726	0.2127
**KMT2A**	KMT2A	22	lysine methyltransferase 2A	protein_codin	-0.007767	0.92	1	50.37	0.4006
**PHLDB1**	PHLDB1	22	pleckstrin homology like domain family B member 1	protein_codin	0.02229	0.909	1	19.6	0.3485
**PFKP**	PFKP	22	phosphofructokinase, platelet	protein_codin	-0.5658	0.00327	1	48.72	0.1080
**LINC00702**	LINC00702	22	long intergenic non-protein coding RNA 702	lincRNA	-1.088	1.83E-07	0.00429	9.007	0.09952
**NSUN6**	NSUN6	22	NOP2/Sun RNA methyltransferase 6	protein_codin	-0.4426	0.0761	1	7.592	0.5610
**BMS1**	BMS1	22	BMS1 ribosome biogenesis factor	protein_codin	0.2219	0.197	1	8.588	0.1031
**PARG**	PARG	22	poly(ADP-ribose) glycohydrolase	protein_codin	-0.1133	0.6	1	3.363	0.2793
**MRPS16**	MRPS16	22	mitochondrial ribosomal protein S16	protein_codin	0.1443	0.242	1	14.48	0.07848
**AL135925.1**	AL135925.1	22	novel transcri	lincRNA	0.2744	0.27	1	27.4	0.3127
**RPP30**	RPP30	22	ribonuclease P/MRP subunit p30	protein_codin	0.1117	0.431	1	18.58	0.03221
**PLCE1**	PLCE1	22	phospholipase C epsilon 1	protein_codin	0.9538	2.93E-05	0.6716	55.48	0.2950
**POLL**	POLL	22	DNA polymerase lambda	protein_codin	0.0006143	0.997	1	7.322	0.172
**GBF1**	GBF1	22	golgi brefeldin A resistant guanine nucleotide exchan	protein_codin	0.08369	0.679	1	3.422	0.1854
**USMG5**	USMG5	22	ATP synthase membrane subunit DAPIT	protein_codin	NA	NA	NA	58.55	0.03241
**STN1.00**	STN1.00	22	STN1 subunit of CST complex	protein_codin	-0.7072	0.00037	1	3.597	0.1996
**PDCD4**	PDCD4	22	programmed cell death 4	protein_codin	0.4957	0.00038	1	45.06	0.1332
**VTI1A**	VTI1A	22	vesicle transport through interaction with t-SNAREs 1	protein_codin	0.466	0.056	1	3.188	0.2482
**CCDC186**	CCDC186	22	coiled-coil domain containing 186	protein_codin	0.2096	0.0782	1	49.43	0.1001
**FAM204A**	FAM204A	22	family with sequence similarity 204 member A	protein_codin	2.44E-05	1	1	23.33	0.1726
**CPXM2**	CPXM2	22	carboxypeptidase X, M14 family member 2	protein_codin	0.9454	2.29E-12	5.44E-08	42.25	0.03595
**INPP5A**	INPP5A	22	inositol polyphosphate-5-phosphatase A	protein_codin	0.3378	0.112	1	10.17	0.1376
**FKBP4**	FKBP4	22	FKBP prolyl isomerase 4	protein_codin	0.003545	0.982	1	17.96	0.1412
**CCND2**	CCND2	22	cyclin D2	protein_codin	0.2676	0.0082	1	39.86	0.06806
**ATN1**	ATN1	22	atrophin 1	protein_codin	0.4405	0.0406	1	3.516	0.3091
**STRAP**	STRAP	22	serine/threonine kinase receptor associated protein	protein_codin	-0.009072	0.926	1	52.98	0.07349
**CMAS**	CMAS	22	cytidine monophosphate N-acetylneuraminic acid syn	protein_codin	-0.122	0.415	1	17.18	0.1964
**FGFR1OP2**	FGFR1OP2	22	FGFR1 oncogene partner 2	protein_codin	0.03577	0.74	1	20.54	0.1726
**AMN1**	AMN1	22	antagonist of mitotic exit network 1 homolog	protein_codin	-0.6418	0.0011	1	10.4	0.2379
**FKBP11**	FKBP11	22	FKBP prolyl isomerase 11	protein_codin	0.001396	0.992	1	60.6	0.05044
**SMARCD1**	SMARCD1	22	SWI/SNF related, matrix associated, actin dependent	protein_codin	0.2367	0.212	1	8.736	0.1748
**CERS5**	CERS5	22	ceramide synthase 5	protein_codin	0.1463	0.539	1	26.97	0.2511
**GRASP**	GRASP	22	general receptor for phosphoinositides 1 associated s	protein_codin	-0.1193	0.637	1	42.43	0.08747
**PRR13**	PRR13	22	proline rich 13	protein_codin	0.2312	0.0141	1	40.56	0.03269
**SARNP**	SARNP	22	SAP domain containing ribonucleoprotein	protein_codin	-0.3818	0.0838	1	3.328	0.2125
**MYL6B**	MYL6B	22	myosin light chain 6B	protein_codin	-0.5003	1.7E-06	0.03947	57.47	0.103
**NAB2**	NAB2	22	NGFI-A binding protein 2	protein_codin	0.01271	0.953	1	3.443	0.2769
**CTDSP2**	CTDSP2	22	CTD small phosphatase 2	protein_codin	0.1679	0.307	1	19.03	0.249
**CNOT2**	CNOT2	22	CCR4-NOT transcription complex subunit 2	protein_codin	0.1007	0.448	1	16.33	0.2815
**PAWR**	PAWR	22	pro-apoptotic WT1 regulator	protein_codin	-0.4313	3.18E-06	0.07365	60.46	0.1008
**RASSF9**	RASSF9	22	Ras association domain family member 9	protein_codin	0.4037	0.0823	1	8.678	0.1427
**C12orf29**	C12orf29	22	chromosome 12 open reading frame 29	protein_codin	0.2914	0.0490	1	19.79	0.4464
**NUDT4**	NUDT4	22	nudix hydrolase 4	protein_codin	-0.1602	0.215	1	59.97	0.1861
**DRAM1**	DRAM1	22	DNA damage regulated autophagy modulator 1	protein_codin	0.7638	3.66E-05	0.838	45.66	0.1814
**WASHC3**	WASHC3	22	WASH complex subunit 3	protein_codin	0.09357	0.341	1	60.16	0.1077
**HSP90B1**	HSP90B1	22	heat shock protein 90 beta family member 1	protein_codin	0.05929	0.203	1	42.84	0.1565
**SLC41A2**	SLC41A2	22	solute carrier family 41 member 2	protein_codin	0.3997	0.0233	1	23.88	0.1336
**ERP29**	ERP29	22	endoplasmic reticulum protein 29	protein_codin	-0.1651	0.015	1	48.96	0.04183
**TPCN1**	TPCN1	22	two pore segment channel 1	protein_codin	0.5783	0.00555	1	27.46	0.2772
**GATC**	GATC	22	glutamyl-tRNA amidotransferase subunit C	protein_codin	-0.04764	0.859	1	8.558	0.2736
**UBC**	UBC	22	ubiquitin C	protein_codin	0.1043	0.00141	1	60.58	0.03305
**ZDHHC20**	ZDHHC20	22	zinc finger DHHC-type containing 20	protein_codin	-0.1535	0.504	1	7.633	0.2752
**GTF3A**	GTF3A	22	general transcription factor IIIA	protein_codin	-0.006267	0.944	1	53.9	0.1011
**NHLRC3**	NHLRC3	22	NHL repeat containing 3	protein_codin	0.5752	0.00548	1	8.919	0.2068
**DNAJC15**	DNAJC15	22	DnaJ heat shock protein family (Hsp40) member C15	protein_codin	-0.07122	0.442	1	20.89	0.03317
**COG3**	COG3	22	component of oligomeric golgi complex 3	protein_codin	0.3407	0.242	1	9.143	0.2452
**UCHL3**	UCHL3	22	ubiquitin C-terminal hydrolase L3	protein_codin	0.06679	0.644	1	19.68	0.1058
**RBM26**	RBM26	22	RNA binding motif protein 26	protein_codin	0.1466	0.392	1	20.33	0.3156
**UGGT2**	UGGT2	22	UDP-glucose glycoprotein glucosyltransferase 2	protein_codin	0.5213	0.00582	1	19.94	0.203
**RASA3**	RASA3	22	RAS p21 protein activator 3	protein_codin	0.3228	0.212	1	10.33	0.3049
**RBM23**	RBM23	22	RNA binding motif protein 23	protein_codin	-0.1005	0.566	1	6.082	0.3520
**PRMT5**	PRMT5	22	protein arginine methyltransferase 5	protein_codin	-0.1495	0.49	1	2.968	0.06490
**PPP2R3C**	PPP2R3C	22	protein phosphatase 2 regulatory subunit B"gamma	protein_codin	0.0637	0.702	1	7.73	0.1006
**C14orf166**	C14orf166	22	RNA transcription, translation and transport factor	protein_codin	NA	NA	NA	60.6	0.03288
**SAMD4A**	SAMD4A	22	sterile alpha motif domain containing 4A	protein_codin	0.1711	0.509	1	3.476	0.2827
**SOCS4**	SOCS4	22	suppressor of cytokine signaling 4	protein_codin	-0.1139	0.606	1	7.693	0.1402
**DACT1**	DACT1	22	dishevelled binding antagonist of beta catenin 1	protein_codin	-0.3877	0.125	1	25.96	0.1171
**RHOJ**	RHOJ	22	ras homolog family member J	protein_codin	-0.03262	0.892	1	26.99	0.1112
**SYNE2**	SYNE2	22	spectrin repeat containing nuclear envelope protein 2	protein_codin	-0.02652	0.839	1	40.53	0.3032
**FCF1**	FCF1	22	FCF1 rRNA-processing protein	protein_codin	-0.1377	0.470	1	7.619	0.1371
**SPTLC2**	SPTLC2	22	serine palmitoyltransferase long chain base subunit 2	protein_codin	0.1887	0.419	1	7.762	0.2012
**CINP**	CINP	22	cyclin dependent kinase 2 interacting protein	protein_codin	-0.1595	0.270	1	18	0.1670
**MARK3**	MARK3	22	microtubule affinity regulating kinase 3	protein_codin	0.661	0.00263	1	8.117	0.4021
**IGHA1**	IGHA1	22	immunoglobulin heavy constant alpha 1	IG_C_gene	-1.557	3.88E-09	9.1E-05	60.58	0.4820
**CYFIP1**	CYFIP1	22	cytoplasmic FMR1 interacting protein 1	protein_codin	0.02146	0.899	1	17.77	0.2134
**GANC**	GANC	22	glucosidase alpha, neutral C	protein_codin	0.2543	0.294	1	2.252	0.3219
**SNAP23**	SNAP23	22	synaptosome associated protein 23	protein_codin	-0.02205	0.856	1	25.76	0.07300
**CCNDBP1**	CCNDBP1	22	cyclin D1 binding protein 1	protein_codin	0.4583	2.61E-06	0.06049	16.86	0.1662
**PDIA3**	PDIA3	22	protein disulfide isomerase family A member 3	protein_codin	0.2871	1.16E-08	0.00027	40.9	0.04252
**MYEF2**	MYEF2	22	myelin expression factor 2	protein_codin	-0.7763	0.0017	1	10.19	0.1860
**SPPL2A**	SPPL2A	22	signal peptide peptidase like 2A	protein_codin	0.3202	0.00132	1	36.12	0.1014
**TMOD3**	TMOD3	22	tropomodulin 3	protein_codin	-0.09238	0.312	1	40.63	0.03315

**GNB5**	GNB5	22	G protein subunit beta 5	protein_codin	-0.457	0.0052	1	7.786	0.168
**ARPP19**	ARPP19	22	cAMP regulated phosphoprotein 19	protein_codin	-0.1472	0.22	1	16.67	0.09909
**MNS1**	MNS1	22	meiosis specific nuclear structural 1	protein_codin	0.8379	0.00043	1	22.27	0.243
**CGNL1**	CGNL1	22	cingulin like 1	protein_codin	0.7143	0.0007	1	37.42	0.1022
**RNF111**	RNF111	22	ring finger protein 111	protein_codin	0.03905	0.839	1	3.379	0.1756
**SLTM**	SLTM	22	SAFB like transcription modulator	protein_codin	-0.03248	0.771	1	13.82	0.07522
**BNIP2**	BNIP2	22	BCL2 interacting protein 2	protein_codin	0.3852	0.00049	1	12.27	0.1091
**SNX1**	SNX1	22	sorting nexin 1	protein_codin	0.3827	0.0121	1	19.4	0.2368
**PPIB**	PPIB	22	peptidylprolyl isomerase B	protein_codin	-0.1429	0.00063	1	60.6	0.02369
**OAZ2**	OAZ2	22	ornithine decarboxylase antizyme 2	protein_codin	0.06734	0.595	1	25.75	0.1672
**SNAPC5**	SNAPC5	22	small nuclear RNA activating complex polypeptide 5	protein_codin	-0.1766	0.205	1	6.343	0.18
**BBS4**	BBS4	22	Bardet-Biedl syndrome 4	protein_codin	0.4063	0.0299	1	26.58	0.2502
**FBXO22**	FBXO22	22	F-box protein 22	protein_codin	-0.2813	0.206	1	5.894	0.2161
**FAM103A1**	FAM103A1	22	RNA guanine-7 methyltransferase activating subunit	protein_codin	NA	NA	NA	6.797	0.1814
**MRPS11**	MRPS11	22	mitochondrial ribosomal protein S11	protein_codin	-0.04741	0.777	1	7.542	0.1719
**IGF1R**	IGF1R	22	insulin like growth factor 1 receptor	protein_codin	0.3511	0.0667	1	22.13	0.2127
**SELENOS**	SELENOS	22	selenoprotein S	protein_codin	0.4052	2.59E-06	0.00061	60.54	0.03350
**TM2D3**	TM2D3	22	TM2 domain containing 3	protein_codin	-0.0003581	0.998	1	37.97	0.09790
**PIGQ**	PIGQ	22	phosphatidylinositol glycan anchor biosynthesis class Q	protein_codin	0.1928	0.452	1	8.71	0.2087
**HAGH**	HAGH	22	hydroxyacylglutathione hydrolase	protein_codin	-0.2129	0.0642	1	12.39	0.0932
**FLYWCH2**	FLYWCH2	22	FLYWCH family member 2	protein_codin	0.2224	0.0741	1	16.17	0.03268
**CDIP1**	CDIP1	22	cell death inducing p53 target 1	protein_codin	0.4338	0.138	1	10.56	0.2392
**TMEM186**	TMEM186	22	transmembrane protein 186	protein_codin	0.1209	0.624	1	2.608	0.1852
**BFAR**	BFAR	22	bifunctional apoptosis regulator	protein_codin	0.2525	0.0887	1	7.423	0.0379
**GDE1**	GDE1	22	glycerophosphodiester phosphodiesterase 1	protein_codin	0.3492	0.032	1	24.77	0.2019
**C16orf52**	C16orf52	22	modulator of smoothened	protein_codin	NA	NA	NA	50.88	0.2369
**SGF29**	SGF29	22	SAGA complex associated factor 29	protein_codin	0.2805	0.0634	1	7.565	0.135
**CYLD**	CYLD	22	CYLD lysine 63 deubiquitinase	protein_codin	-0.5953	6E-04	1	7.135	0.2845
**CCDC102A**	CCDC102A	22	coiled-coil domain containing 102A	protein_codin	-0.05199	0.791	1	23.94	0.1701
**CNOT1**	CNOT1	22	CCR4-NOT transcription complex subunit 1	protein_codin	0.2932	0.212	1	2.862	0.3971
**MON1B**	MON1B	22	MON1 homolog B, secretory trafficking associated	protein_codin	-0.289	0.255	1	8.836	0.2641
**SPG7**	SPG7	22	SPG7 matrix AAA peptidase subunit, paraplegin	protein_codin	0.09456	0.701	1	8.492	0.352
**ABR**	ABR	22	ABR activator of RhoGEF and GTPase	protein_codin	0.1866	0.391	1	7.61	0.3942
**MYO1C**	MYO1C	22	myosin IC	protein_codin	0.1272	0.406	1	13.9	0.2442
**TLCD2**	TLCD2	22	TLC domain containing 2	protein_codin	0.3085	0.29	1	9.701	0.2382
**ZZEF1**	ZZEF1	22	zinc finger ZZ-type and EF-hand domain containing 1	protein_codin	-0.07623	0.764	1	3.243	0.3231
**PELP1**	PELP1	22	proline, glutamate and leucine rich protein 1	protein_codin	0.291	0.234	1	3.147	0.2596
**COPS3**	COPS3	22	COP9 signalosome subunit 3	protein_codin	0.02179	0.871	1	15.45	0.1469
**MAP2K3**	MAP2K3	22	mitogen-activated protein kinase kinase 3	protein_codin	0.181	0.444	1	9.582	0.1361
**UNC119**	UNC119	22	unc-119 lipid binding chaperone	protein_codin	-0.4412	0.0254	1	3.332	0.1339
**MRPL45**	MRPL45	22	mitochondrial ribosomal protein L45	protein_codin	0.04462	0.792	1	6.775	0.1427
**MED1**	MED1	22	mediator complex subunit 1	protein_codin	-0.1288	0.556	1	3.07	0.1824
**NT5C3B**	NT5C3B	22	5'-nucleotidase, cytosolic IIIB	protein_codin	-0.3981	0.00032	1	14.53	0.06431
**VPS25**	VPS25	22	vacuolar protein sorting 25 homolog	protein_codin	0.05109	0.684	1	15.68	0.1675
**AOC3**	AOC3	22	amine oxidase copper containing 3	protein_codin	-0.6021	1.62E-05	0.3726	60.58	0.02583
**NBR1**	NBR1	22	NBR1 autophagy cargo receptor	protein_codin	0.2058	0.256	1	7.979	0.2819
**SLC25A39**	SLC25A39	22	solute carrier family 25 member 39	protein_codin	-0.09962	0.691	1	8.14	0.06770
**DCAKD**	DCAKD	22	dephospho-CoA kinase domain containing	protein_codin	0.08726	0.699	1	10.49	0.1067
**MRPL10**	MRPL10	22	mitochondrial ribosomal protein L10	protein_codin	-0.08465	0.549	1	8.007	0.0991
**CBX1**	CBX1	22	chromobox 1	protein_codin	0.08111	0.486	1	24.99	0.1042
**ATP5G1**	ATP5G1	22	ATP synthase membrane subunit c locus 1	protein_codin	NA	NA	NA	59.99	0.03721
**PHB**	PHB	22	prohibitin	protein_codin	-0.3331	0.00025	1	30.01	0.06650
**SKA2**	SKA2	22	spindle and kinetochore associated complex subunit 2	protein_codin	-0.1859	0.269	1	17.82	0.176
**HEATR6**	HEATR6	22	HEAT repeat containing 6	protein_codin	0.2721	0.212	1	9.63	0.1712
**ABCA5**	ABCA5	22	ATP binding cassette subfamily A member 5	protein_codin	0.6196	0.00322	1	9.788	0.3597
**JPT1**	JPT1	22	Jupiter microtubule associated homolog 1	protein_codin	0.03834	0.835	1	21.9	0.0320
**ACOX1**	ACOX1	22	acyl-CoA oxidase 1	protein_codin	0.8234	5.25E-05	1	7.583	0.3040
**TEN1**	TEN1	22	TEN1 subunit of CST complex	protein_codin	-0.07094	0.752	1	10.62	0.1469
**CHMP6**	CHMP6	22	charged multivesicular body protein 6	protein_codin	-0.4669	0.0445	1	8.566	0.101
**NPLOC4**	NPLOC4	22	NPL4 homolog, ubiquitin recognition factor	protein_codin	-0.008405	0.977	1	3.388	0.2802
**FOXK2**	FOXK2	22	forkhead box K2	protein_codin	-0.2044	0.313	1	3.228	0.1715
**SMCHD1**	SMCHD1	22	structural maintenance of chromosomes flexible hinge	protein_codin	0.2725	0.0520	1	27.33	0.172
**PTPRM**	PTPRM	22	protein tyrosine phosphatase receptor type M	protein_codin	-0.214	0.392	1	3.546	0.476
**IMPA2**	IMPA2	22	inositol monophosphatase 2	protein_codin	1.042	1.5E-05	0.3451	8.422	0.2030
**AFG3L2**	AFG3L2	22	AFG3 like matrix AAA peptidase subunit 2	protein_codin	0.5855	0.00315	1	20.97	0.2685
**FAM210A**	FAM210A	22	family with sequence similarity 210 member A	protein_codin	0.2505	0.295	1	9.096	0.2584
**TTC39C**	TTC39C	22	tetratricopeptide repeat domain 39C	protein_codin	0.6965	0.0116	1	8.399	0.06949
**OSBPL1A**	OSBPL1A	22	oxysterol binding protein like 1A	protein_codin	0.4623	7.86E-06	0.1813	28.81	0.06635
**SMAD7**	SMAD7	22	SMAD family member 7	protein_codin	-0.1304	0.615	1	7.62	0.4187
**C18orf32**	C18orf32	22	chromosome 18 open reading frame 32	protein_codin	0.1438	0.471	1	3.372	0.2690
**CXXC1**	CXXC1	22	CXXC finger protein 1	protein_codin	0.1499	0.552	1	2.877	0.3221
**SEC11C**	SEC11C	22	SEC11 homolog C, signal peptidase complex subunit	protein_codin	-0.2925	0.00357	1	57.29	0.1232
**RBFA**	RBFA	22	ribosome binding factor A	protein_codin	0.06251	0.682	1	7.285	0.2474
**ADNP2**	ADNP2	22	ADNP homeobox 2	protein_codin	0.329	0.151	1	3.364	0.2702
**PTPRA**	PTPRA	22	protein tyrosine phosphatase receptor type A	protein_codin	0.4514	4.76E-06	0.1101	40.39	0.09928
**GPCPD1**	GPCPD1	22	glycerophosphocholine phosphodiesterase 1	protein_codin	0.3522	0.0318	1	14.57	0.06554
**DYNLRB1**	DYNLRB1	22	dynein light chain roadblock-type 1	protein_codin	-0.3074	4.23E-08	0.00099	27.53	0.03296
**TRPC4AP**	TRPC4AP	22	transient receptor potential cation channel subfamily C	protein_codin	0.2982	0.169	1	3.573	0.2854
**DLGAP4**	DLGAP4	22	DLG associated protein 4	protein_codin	0.3492	0.0194	1	56.53	0.2471
**SERINC3**	SERINC3	22	serine incorporator 3	protein_codin	0.1884	0.0696	1	17.67	0.06832
**DNTTIP1**	DNTTIP1	22	deoxynucleotidyltransferase terminal interacting prote	protein_codin	-0.2512	0.129	1	7.727	0.2152
**SULF2**	SULF2	22	sulfatase 2	protein_codin	0.5353	0.0245	1	22.58	0.2772
**CSE1L**	CSE1L	22	chromosome segregation 1 like	protein_codin	0.1086	0.682	1	3.263	0.1691
**RNF114**	RNF114	22	ring finger protein 114	protein_codin	-0.02723	0.845	1	16.34	0.3462
**TMEM189**	TMEM189	22	transmembrane protein 189	protein_codin	-0.1068	0.637	1	2.802	0.245
**PFDN4**	PFDN4	22	prefoldin subunit 4	protein_codin	-0.05803	0.644	1	33.99	0.06570
**CSTF1**	CSTF1	22	cleavage stimulation factor subunit 1	protein_codin	0.04796	0.837	1	2.54	0.1002
**GID8**	GID8	22	GID complex subunit 8 homolog	protein_codin	0.05294	0.702	1	20.62	0.1082
**CDC34**	CDC34	22	cell division cycle 34	protein_codin	0.1425	0.41	1	8.086	0.3027
**RNF126**	RNF126	22	ring finger protein 126	protein_codin	-0.3216	0.0519	1	7.133	0.1392
**R3HDM4**	R3HDM4	22	R3H domain containing 4	protein_codin	0.1257	0.552	1	7.814	0.1452
**MUM1**	MUM1	22	PWWP domain containing 2A, DNA repair factor	protein_codin	0.4996	0.00165	1	16.62	0.2784
**DAZAP1**	DAZAP1	22	DAZ associated protein 1	protein_codin	0.337	0.0482	1	19.84	0.2032
**CERS4**	CERS4	22	ceramide synthase 4	protein_codin	0.1678	0.464	1	9.988	0.06740
**CD320**	CD320	22	CD320 molecule	protein_codin	-0.02277	0.846	1	55.04	0.06762
**ZNF426**	ZNF426	22	zinc finger protein 426	protein_codin	-0.01494	0.952	1	7.584	0.3042
**C19orf66**	C19orf66	22	shiftless antiviral inhibitor of ribosomal frameshifting	protein_codin	-0.1218	0.419	1	20.52	0.2309
**CCDC151**	CCDC151	22	coiled-coil domain containing 151	protein_codin	-0.7888	2.01E-05	0.4618	20.7	0.11
**ELOF1**	ELOF1	22	elongation factor 1 homolog	protein_codin	-0.1637	0.222	1	19.56	0.1380
**CALR**	CALR	22	calreticulin	protein_codin	-0.1229	0.0211	1	59.93	0.03912
**SYDE1**	SYDE1	22	synapse defective Rho GTPase homolog 1	protein_codin	0.09956	0.619	1	26.39	0.147
**SMIM7**	SMIM7	22	small integral membrane protein 7	protein_codin	-0.06084	0.452	1	14.48	0.1358
**SIN3B**	SIN3B	22	SIN3 transcription regulator family member B	protein_codin	0.6344	0.00507	1	6.577	0.1694
**MYO9B**	MYO9B	22	myosin IXB	protein_codin	-0.3914	0.0339	1	8.567	0.1412
**GATAD2A**	GATAD2A	22	GATA zinc finger domain containing 2A	protein_codin	0.03894	0.86	1	7.805	0.3216
**ZNF626**	ZNF626	22	zinc finger protein 626	protein_codin	0.4312	0.021	1	3.567	0.1669
**ZNF43**	ZNF43	22	zinc finger protein 43	protein_codin	0.3284	0.0919	1	7.481	0.2381
**LINC00662**	LINC00662	22	long intergenic non-protein coding RNA 662	lincRNA	-0.2413	0.236	1	3.44	0.4180
**HNRNPL**	HNRNPL	22	heterogeneous nuclear ribonucleoprotein L	protein_codin	0.3424	0.0244	1	45.62	0.3067
**TGFB1**	TGFB1	22	transforming growth factor beta 1	protein_codin	-1.125	9.33E-16	2.24E-12	43.91	0.1067
**PPP1R13L**	PPP1R13L	22	protein phosphatase 1 regulatory subunit 13 like	protein_codin	-0.591	0.0206	1	3.805	0.3045
**INAFM1**	INAFM1	22	InaF motif containing 1	protein_codin	-0.4975	3.14E-06	0.07272	35.64	0.2089
**SNRNP70**	SNRNP70	22	small nuclear ribonucleoprotein U1 subunit 70	protein_codin	-0.09	0.467	1	12.05	0.2034
**FUZ**	FUZ	22	fuzzy planar cell polarity protein	protein_codin	0.343	0.086	1	7.261	0.1345
**EMC10**	EMC10	22	ER membrane protein complex subunit 10	protein_codin	-0.4124	0.00027	1	30.33	0.06474
**CLEC11A**	CLEC11A	22	C-type lectin domain containing 11A	protein_codin	0.2436	0.119	1	48.56	0.03298
**NDUFA3**	NDUFA3	22	NADH:ubiquinone oxidoreductase subunit A3	protein_codin	0.1005	0.287	1	16.68	0.07352
**UBE2S**	UBE2S	22	ubiquitin conjugating enzyme E2 S	protein_codin	0.3276	0.00875	1	59.01	0.1389
**AC005261.2**	AC005261.2	22	novel transcri	lincRNA	-0.9164	0.189	1	7.701	0.2026
**AC003002.1**	AC003002.1	22	trafficking protein particle complex 2B	protein_codin	NA	NA	NA	7.618	0.2092
**YDJC**	YDJC	22	YdjC chitooligosaccharide deacetylase homolog	protein_codin	-0.3086	0.22	1	3.298	0.2002
**SDF2L1**	SDF2L1	22	stromal cell derived factor 2 like 1	protein_codin	-0.2812	0.0231	1	60.6	0.1379
**IGLC2**	IGLC2	22	immunoglobulin lambda constant 2	IG_C_gene	-1.468	1.2E-20	2.88E-16	60.6	0.8441
**IGLC3**	IGLC3	22	immunoglobulin lambda constant 3 (Kern-Oz+ marke	IG_C_gene	-1.074	1.73E-07	0.00405	55.36	0.7022
**LRRC75B**	LRRC75B	22	leucine rich repeat containing 75B	protein_codin	1.206	2.95E-05	0.6761	3.654	0.1384
**KIAA1671**	KIAA1671	22	KIAA1671	protein_codin	-0.1444	0.572	1	7.212	0.286
**SRRD**	SRRD	22	SRR1 domain containing	protein_codin	0.7382	0.00324	1	2.889	0.1750
**CCDC117**	CCDC117	22	coiled-coil domain containing 117	protein_codin	-0.1791	0.436	1	8.699	0.2042
**GCAT**	GCAT	22	glycine C-acetyltransferase	protein_codin	-0.3529	0.0935	1	2.927	0.1362
**DNAL4**	DNAL4	22	dynein axonemal light chain 4	protein_codin	0.5719	0.0424	1	7.95	0.169
**PMM1**	PMM1	22	phosphomannomutase 1	protein_codin	-0.09788	0.465	1	56	0.0322
**TRABD**	TRABD	22	TraB domain containing	protein_codin	0.1846	0.32	1	6.768	0.06436
**RABL2B**	RABL2B	22	RAB, member of RAS oncogene family like 2B	protein_codin	0.4292	0.156	1	3.223	0.2126
**USP16**	USP16	22	ubiquitin specific peptidase 16	protein_codin	0.09254	0.429	1	37.94	0.1342
**SCAF4**	SCAF4	22	SR-related CTD associated factor 4	protein_codin	0.7271	0.0118	1	2.694	0.1832
**TMEM50B**	TMEM50B	22	transmembrane protein 50B	protein_codin	0.2433	0.024	1	11.53	0.06571
**BACE2**	BACE2	22	beta-secretase 2	protein_codin	0.2423	0.0292	1	60.6	0.03766
**NDUFV3**	NDUFV3	22	NADH:ubiquinone oxidoreductase subunit V3	protein_codin	0.08726	0.432	1	34.19	0.1341
**RRP1**	RRP1	22	ribosomal RNA processing 1	protein_codin	0.1216	0.646	1	2.636	0.3538
**RER1**	RER1	22	retention in endoplasmic reticulum sorting receptor 1	protein_codin	0.1232	0.137	1	60.58	0.06882
**LINC00982**	LINC00982	22	PRDM16 divergent transcript	antisense_RN	-0.7426	0.00025	1	21.78	0.276
**CTNNBIP1**	CTNNBIP1	22	catenin beta interacting protein 1	protein_codin	-0.5178	0.0198	1	3.665	0.1752
**NECAP2**	NECAP2	22	NECAP endocytosis associated 2	protein_codin	-0.4117	0.0172	1	22.03	0.1766
**PINK1**	PINK1	22	PTEN induced kinase 1	protein_codin	-0.03962	0.757	1	52.83	0.06681
**HSPG2**	HSPG2	22	heparan sulfate proteoglycan 2	protein_codin	0.5849	2.09E-05	0.48	36.81	0.2510
**SRSF10**	SRSF10	22	serine and arginine rich splicing factor 10	protein_codin	-0.006581	0.944	1	20.61	0.1987
**AHDC1**	AHDC1	22	AT-hook DNA binding motif containing 1	protein_codin	0.7926	0.00166	1	3.349	0.3322
**PPP1R8**	PPP1R8	22	protein phosphatase 1 regulatory subunit 8	protein_codin	0.07184	0.742	1	3.182	0.3079
**ZBTB8OS**	ZBTB8OS	22	zinc finger and BTB domain containing 8 opposite str	protein_codin	-0.206	0.118	1	6.702	0.1161
**PHC2**	PHC2	22	polyhomeotic homolog 2	protein_codin	-0.3052	0.0124	1	43	0.0424
**COL8A2**	COL8A2	22	collagen type VIII alpha 2 chain	protein_codin	0.6993	0.00162	1	23.06	0.06852
**MYCBP**	MYCBP	22	MYC binding protein	protein_codin	-0.1417	0.562	1	3.415	0.1358
**GPX7**	GPX7	22	glutathione peroxidase 7	protein_codin	0.01689	0.922	1	44.17	0.1387
**PRKAA2**	PRKAA2	22	protein kinase AMP-activated catalytic subunit alpha 2	protein_codin	-0.252	0.32	1	7.742	0.1479
**ANKRD13C**	ANKRD13C	22	ankyrin repeat domain 13C	protein_codin	0.2213	0.299	1	9.254	0.1738
**SH3GLB1**	SH3GLB1	22	SH3 domain containing GRB2 like, endophilin B1	protein_codin	-0.2599	0.00252	1	60.6	0.07552
**BCAR3**	BCAR3	22	BCAR3 adaptor protein, NSP family member	protein_codin	0.07571	0.702	1	29.92	0.1695
**SASS6**	SASS6	22	SAS-6 centriolar assembly protein	protein_codin	0.3546	0.174	1	7.5	0.2315
**AMPD2**	AMPD2	22	adenosine monophosphate deaminase 2	protein_codin	-0.5038	0.0294	1	8.5	0.2059
**RBM8A**	RBM8A	22	RNA binding motif protein 8A	protein_codin	-0.0762	0.35	1	40.57	0.1072
**CDC42SE1**	CDC42SE1	22	CDC42 small effector 1	protein_codin	0.2196	0.216	1	51.19	0.1052
**GPATCH4**	GPATCH4	22	G-patch domain containing 4	protein_codin	0.1913	0.369	1	17.08	0.1045
**KIRREL1**	KIRREL1	22	kirre like nephrin family adhesion molecule 1	protein_codin	0.4409	0.0942	1	9.212	0.1764
**PIGC**	PIGC	22	phosphatidylinositol glycan anchor biosynthesis class C	protein_codin	-0.003322	0.982	1	19.83	0.1392
**SMG7**	SMG7	22	SMG7 nonsense mediated mRNA decay factor	protein_codin	0.1115	0.652	1	7.805	0.2502
**C1orf27**	C1orf27	22	odr-4 GPCR localization factor homolog	protein_codin	NA	NA	NA	25.92	0.2002
**ZBTB41**	ZBTB41	22	zinc finger and BTB domain containing 41	protein_codin	0.1286	0.547	1	7.67	0.2696
**TMEM9**	TMEM9	22	transmembrane protein 9	protein_codin	0.1542	0.169	1	10.96	0.03576
**ARL8A**	ARL8A	22	ADP ribosylation factor like GTPase 8A	protein_codin	-0.02262	0.873	1	7.158	0.1701
**PARP1**	PARP1	22	poly(ADP-ribose) polymerase 1	protein_codin	-0.05138	0.724	1	59.79	0.1320
**B3GALNT2**	B3GALNT2	22	beta-1,3-N-acetylgalactosaminyltransferase 2	protein_codin	-0.008529	0.974	1	27.19	0.3627
**FKBP1B**	FKBP1B	22	FKBP prolyl isomerase 1B	protein_codin	0.05651	0.823	1	3.764	0.1064
**ITSN2**	ITSN2	22	intersectin 2	protein_codin	0.3322	0.0160	1	29.61	0.2352
**PTRHD1**	PTRHD1	22	peptidyl-tRNA hydrolase domain containing 1	protein_codin	0.04342	0.755	1	15.52	0.2112
**RAB10**	RAB10	22	RAB10, member RAS oncogene family	protein_codin	0.1996	0.052	1	52.95	0.07081
**RMDN2**	RMDN2	22	regulator of microtubule dynamics 2	protein_codin	0.1385	0.572	1	9.635	0.1671
**SNRNP27**	SNRNP27	22	small nuclear ribonucleoprotein U4/U6.U5 subunit 27	protein_codin	0.06111	0.655	1	31.22	0.2371
**SUCLG1**	SUCLG1	22	succinate-CoA ligase alpha subunit	protein_codin	0.3344	0.00098	1	20.65	0.06585
**ST3GAL5**	ST3GAL5	22	ST3 beta-galactoside alpha-2,3-sialyltransferase 5	protein_codin	1.455	4.17E-06	0.09646	8.409	0.1787
**CIAO1**	CIAO1	22	cytosolic iron-sulfur assembly component 1	protein_codin	0.006474	0.968	1	8.626	0.1748
**COA5**	COA5	22	cytochrome c oxidase assembly factor 5	protein_codin	-0.1551	0.226	1	37.14	0.06567
**CHCHD5**	CHCHD5	22	coiled-coil-helix-coiled-coil-helix domain containing 5	protein_codin	-0.4118	0.00075	1	52.14	0.1714
**MBD5**	MBD5	22	methyl-CpG binding domain protein 5	protein_codin	0.4122	0.0918	1	7.028	0.3292
**CERS6**	CERS6	22	ceramide synthase 6	protein_codin	0.5366	0.064	1	3.474	0.3187
**DYNC1I2**	DYNC1I2	22	dynein cytoplasmic 1 intermediate chain 2	protein_codin	0.03579	0.627	1	57.72	0.06537
**XRCC5**	XRCC5	22	X-ray repair cross complementing 5	protein_codin	0.1038	0.144	1	50.33	0.06565
**ACSL3**	ACSL3	22	acyl-CoA synthetase long chain family member 3	protein_codin	0.1696	0.142	1	24.61	0.1059
**EIF4E2**	EIF4E2	22	eukaryotic translation initiation factor 4E family member 2	protein_codin	-0.1731	0.172	1	38.2	0.04036
**RNPEPL1**	RNPEPL1	22	arginyl aminopeptidase like 1	protein_codin	0.4	0.075	1	8.921	0.1006
**TRNT1**	TRNT1	22	tRNA nucleotidyl transferase 1	protein_codin	-0.08938	0.653	1	27.85	0.215
**TADA3**	TADA3	22	transcriptional adaptor 3	protein_codin	-0.1873	0.0672	1	26.75	0.0322
**EMC3**	EMC3	22	ER membrane protein complex subunit 3	protein_codin	0.05422	0.645	1	26.01	0.1726
**CAPN7**	CAPN7	22	calpain 7	protein_codin	0.3952	0.0272	1	8.061	0.2842
**ANKRD28**	ANKRD28	22	ankyrin repeat domain 28	protein_codin	-0.5317	0.00942	1	24.02	0.1066
**SLC25A38**	SLC25A38	22	solute carrier family 25 member 38	protein_codin	-0.3677	0.0444	1	7.568	0.2698
**DALRD3**	DALRD3	22	DALR anticodon binding domain containing 3	protein_codin	-0.1151	0.494	1	51.09	0.1677
**QARS**	QARS	22	glutaminyl-tRNA synthetase	protein_codin	0.231	0.227	1	8.406	0.06992
**NISCH**	NISCH	22	nischarin	protein_codin	0.5122	0.0279	1	8.088	0.2784
**PDZRN3**	PDZRN3	22	PDZ domain containing ring finger 3	protein_codin	0.02015	0.918	1	3.537	0.2094
**NXPE3**	NXPE3	22	neurexophilin and PC-esterase domain family member 3	protein_codin	0.03271	0.91	1	8.576	0.4600
**B4GALT4**	B4GALT4	22	beta-1,4-galactosyltransferase 4	protein_codin	0.4749	0.0118	1	23.5	0.1372
**PDIA5**	PDIA5	22	protein disulfide isomerase family A member 5	protein_codin	-0.02105	0.92	1	8.132	0.2832
**EEFSEC**	EEFSEC	22	eukaryotic elongation factor, selenocysteine-tRNA sp	protein_codin	0.294	0.182	1	8.122	0.1322
**NUDT16**	NUDT16	22	nudix hydrolase 16	protein_codin	-0.2958	0.0209	1	18.01	0.09842
**RYK**	RYK	22	receptor like tyrosine kinase	protein_codin	0.01499	0.92	1	15.34	0.1715
**MSL2**	MSL2	22	MSL complex subunit 2	protein_codin	0.3675	0.225	1	3.148	0.3869
**COMMD2**	COMMD2	22	COMM domain containing 2	protein_codin	0.1577	0.207	1	14.25	0.1041
**B3GALNT1**	B3GALNT1	22	beta-1,3-N-acetylgalactosaminyltransferase 1 (globos	protein_codin	-0.4185	0.0326	1	9.377	0.2125
**LRCH3**	LRCH3	22	leucine rich repeats and calponin homology domain c	protein_codin	0.1338	0.584	1	6.926	0.4949
**PGM2**	PGM2	22	phosphoglucomutase 2	protein_codin	-0.2363	0.232	1	3.329	0.2387
**METAP1**	METAP1	22	methionyl aminopeptidase 1	protein_codin	-0.6334	0.00022	1	31.15	0.06617
**HADH**	HADH	22	hydroxyacyl-CoA dehydrogenase	protein_codin	-0.1991	0.159	1	37.62	0.1317
**PCDH18**	PCDH18	22	protocadherin 18	protein_codin	0.8852	6.98E-06	0.1611	44.69	0.1705
**CLCN3**	CLCN3	22	chloride voltage-gated channel 3	protein_codin	0.7303	5.69E-05	1	40.47	0.1858
**AC097534.2**	AC097534.2	22	novel transcript, antisense to SAP	antisense_RN	-0.09927	0.654	1	32.4	0.3457
**FRG1**	FRG1	22	FSHD region gene 1	protein_codin	-0.2716	0.0041	1	20.25	0.1324
**RAD1**	RAD1	22	RAD1 checkpoint DNA exonuclease	protein_codin	0.05584	0.811	1	9.811	0.07254
**MSH3**	MSH3	22	mutS homolog 3	protein_codin	-0.7767	6.95E-05	1	9.424	0.4379
**LYSMD3**	LYSMD3	22	LysM domain containing 3	protein_codin	0.4555	0.0251	1	8.158	0.3206
**FER**	FER	22	FER tyrosine kinase	protein_codin	0.4274	0.0150	1	23.88	0.246
**NREP**	NREP	22	neuronal regeneration related protein	protein_codin	-0.7503	0.00010	1	29.9	0.205
**ATG12**	ATG12	22	autophagy related 12	protein_codin	0.231	0.0652	1	12.83	0.1791
**8-Sep**	43716	22	septin 8	protein_codin	0.3859	0.0204	1	21.58	0.1705
**ANXA6**	ANXA6	22	annexin A6	protein_codin	-0.01847	0.855	1	31.02	0.1714
**MSX2**	MSX2	22	msh homeobox 2	protein_codin	-0.2572	0.291	1	48.66	0.4547
**ZNF354B**	ZNF354B	22	zinc finger protein 354B	protein_codin	0.2186	0.297	1	3.408	0.2721
**ADAMTS2**	ADAMTS2	22	ADAM metallopeptidase with thrombospondin type 1	protein_codin	1.544	5.51E-08	0.00129	10.53	0.09907
**NUP153**	NUP153	22	nucleoporin 153	protein_codin	0.4399	0.054	1	3.196	0.6012
**MRS2**	MRS2	22	magnesium transporter MRS2	protein_codin	0.5945	0.0471	1	3.698	0.328
**ABT1**	ABT1	22	activator of basal transcription 1	protein_codin	0.1082	0.639	1	27.81	0.1982
**NKAPL**	NKAPL	22	NFKB activating protein like	protein_codin	-0.1596	0.601	1	10.98	0.5654
**ZSCAN26**	ZSCAN26	22	zinc finger and SCAN domain containing 26	protein_codin	0.1936	0.367	1	3.297	0.1039
**B3GALT4**	B3GALT4	22	beta-1,3-galactosyltransferase 4	protein_codin	-0.09	0.64	1	8.653	0.2788
**PPIL1**	PPIL1	22	peptidylprolyl isomerase like 1	protein_codin	-0.2588	0.232	1	10.98	0.3161
**DST**	DST	22	dystonin	protein_codin	0.2709	0.0186	1	46.07	0.1732
**ME1**	ME1	22	malic enzyme 1	protein_codin	-0.6158	0.00296	1	11.48	0.1792
**CEP162**	CEP162	22	centrosomal protein 162	protein_codin	0.3766	0.117	1	8.944	0.203
**ZNF292**	ZNF292	22	zinc finger protein 292	protein_codin	-0.1144	0.425	1	32.32	0.2449
**FBXL4**	FBXL4	22	F-box and leucine rich repeat protein 4	protein_codin	0.1339	0.541	1	8.448	0.1349
**RNF146**	RNF146	22	ring finger protein 146	protein_codin	0.6712	1.09E-06	0.02520	14.05	0.09779
**LATS1**	LATS1	22	large tumor suppressor kinase 1	protein_codin	0.128	0.606	1	10.58	0.2072
**PCMT1**	PCMT1	22	protein-L-isoaspartate (D-aspartate) O-methyltransfer	protein_codin	0.2433	0.00454	1	30.86	0.03289
**RMND1**	RMND1	22	required for meiotic nuclear division 1 homolog	protein_codin	-0.2894	0.15	1	28.54	0.2326
**INTS1**	INTS1	22	integrator complex subunit 1	protein_codin	-0.01062	0.969	1	3.036	0.264
**FAM126A**	FAM126A	22	family with sequence similarity 126 member A	protein_codin	0.4079	0.074	1	8.532	0.1350
**OGDH**	OGDH	22	oxoglutarate dehydrogenase	protein_codin	0.2748	0.149	1	10.02	0.2967
**PURB**	PURB	22	purine rich element binding protein B	protein_codin	0.2714	0.209	1	9.607	0.4309
**HUS1**	HUS1	22	HUS1 checkpoint clamp component	protein_codin	0.1985	0.405	1	11.39	0.1705
**VPS50**	VPS50	22	VPS50 subunit of EARP/GARPII complex	protein_codin	0.5565	0.0162	1	2.985	0.2117
**NPTX2**	NPTX2	22	neuronal pentraxin 2	protein_codin	-1.376	2.34E-10	5.54E-06	52.78	0.2280
**CADPS2**	CADPS2	22	calcium dependent secretion activator 2	protein_codin	0.4638	0.0175	1	25.38	0.1779
**AKR1B1**	AKR1B1	22	aldo-keto reductase family 1 member B	protein_codin	0.5106	0.00010	1	13.63	0.1054
**SLC25A6**	SLC25A6	22	solute carrier family 25 member 6	protein_codin	-0.1994	3.53E-05	0.8085	60.58	0.05860
**CD99**	CD99	22	CD99 molecule (Xg blood group)	protein_codin	0.2678	1.54E-09	3.63E-05	60.58	0.04502
**NDUFB11**	NDUFB11	22	NADH:ubiquinone oxidoreductase subunit B11	protein_codin	-0.1756	0.00266	1	57.81	0.06457
**TCEAL9**	TCEAL9	22	transcription elongation factor A like 9	protein_codin	0.05307	0.514	1	60.53	0.09935
**THOC2**	THOC2	22	THO complex 2	protein_codin	-0.04724	0.697	1	25.93	0.172
**IRAK1**	IRAK1	22	interleukin 1 receptor associated kinase 1	protein_codin	0.1864	0.372	1	9.829	0.1395
**VBP1**	VBP1	22	VHL binding protein 1	protein_codin	0.03135	0.796	1	13.94	0.1104
**MSRA**	MSRA	22	methionine sulfoxide reductase A	protein_codin	-0.5414	0.00096	1	28.46	0.1364
**CNOT7**	CNOT7	22	CCR4-NOT transcription complex subunit 7	protein_codin	-0.123	0.455	1	7.536	0.1758
**DOCK5**	DOCK5	22	dedicator of cytokinesis 5	protein_codin	-0.6402	0.0057	1	11.34	0.2749
**SPIDR**	SPIDR	22	scaffold protein involved in DNA repair	protein_codin	0.1435	0.541	1	7.811	0.1792
**PCMTD1**	PCMTD1	22	protein-L-isoaspartate (D-aspartate) O-methyltransfer	protein_codin	0.1126	0.192	1	21.57	0.1367
**MRPL15**	MRPL15	22	mitochondrial ribosomal protein L15	protein_codin	0.09046	0.695	1	6.841	0.1422
**MATN2**	MATN2	22	matrilin 2	protein_codin	1.528	1.26E-08	0.0003	55.41	0.3154
**KLHL9**	KLHL9	22	kelch like family member 9	protein_codin	0.4071	0.107	1	33.1	0.2796
**RUSC2**	RUSC2	22	RUN and SH3 domain containing 2	protein_codin	0.1619	0.56	1	8.466	0.268
**PTAR1**	PTAR1	22	protein prenyltransferase alpha subunit repeat contai	protein_codin	0.4696	0.0468	1	7.183	0.2289
**OSTF1**	OSTF1	22	osteoclast stimulating factor 1	protein_codin	0.5163	0.00026	1	6.839	0.06551
**TLE4**	TLE4	22	TLE family member 4, transcriptional corepressor	protein_codin	-0.04549	0.833	1	18.76	0.4865
**MFSD14C**	MFSD14C	22	major facilitator superfamily domain containing 14C	protein_codin	-0.09803	0.704	1	32.38	0.1764
**XPA**	XPA	22	XPA, DNA damage recognition and repair factor	protein_codin	0.006613	0.954	1	29.62	0.1041
**SLC31A2**	SLC31A2	22	solute carrier family 31 member 2	protein_codin	1.238	8.99E-06	0.2072	10.14	0.2696
**ARPC5L**	ARPC5L	22	actin related protein 2/3 complex subunit 5 like	protein_codin	-0.5415	9.42E-05	1	48.02	0.1370
**ZBTB43**	ZBTB43	22	zinc finger and BTB domain containing 43	protein_codin	0.328	0.162	1	7.698	0.1048
**FUBP3**	FUBP3	22	far upstream element binding protein 3	protein_codin	0.01229	0.942	1	9.216	0.3816
**GTF3C4**	GTF3C4	22	general transcription factor IIIC subunit 4	protein_codin	0.6244	0.0444	1	2.972	0.4698
**POLR2L**	POLR2L	22	RNA polymerase II subunit L	protein_codin	0.1679	0.006	1	31.28	0.03258
**SBF2**	SBF2	22	SET binding factor 2	protein_codin	0.5422	0.0115	1	28.77	0.5184
**IMMP1L**	IMMP1L	22	inner mitochondrial membrane peptidase subunit 1	protein_codin	-0.1877	0.294	1	9.222	0.1002
**MTCH2**	MTCH2	22	mitochondrial carrier 2	protein_codin	0.02704	0.868	1	19.26	0.06822
**CYB561A3**	CYB561A3	22	cytochrome b561 family member A3	protein_codin	-0.2604	0.197	1	9.774	0.09667
**CPSF7**	CPSF7	22	cleavage and polyadenylation specific factor 7	protein_codin	0.363	0.0746	1	2.811	0.1107
**B3GAT3**	B3GAT3	22	beta-1,3-glucuronyltransferase 3	protein_codin	-0.3089	0.0135	1	57.09	0.1045
**KDM2A**	KDM2A	22	lysine demethylase 2A	protein_codin	0.3756	0.112	1	8.523	0.3722
**FADD**	FADD	22	Fas associated via death domain	protein_codin	0.2549	0.191	1	7.816	0.1762
**PICALM**	PICALM	22	phosphatidylinositol binding clathrin assembly protein	protein_codin	0.1355	0.368	1	27.86	0.2205
**CHORDC1**	CHORDC1	22	cysteine and histidine rich domain containing 1	protein_codin	0.1893	0.222	1	14.76	0.2884
**PCSK7**	PCSK7	22	proprotein convertase subtilisin/kexin type 7	protein_codin	-0.1544	0.44	1	24.47	0.2726
**TRAPPC4**	TRAPPC4	22	trafficking protein particle complex 4	protein_codin	-0.02264	0.82	1	60.52	0.09847
**ARHGEF12**	ARHGEF12	22	Rho guanine nucleotide exchange factor 12	protein_codin	-0.007769	0.96	1	7.634	0.2512
**PHYH**	PHYH	22	phytanoyl-CoA 2-hydroxylase	protein_codin	0.299	0.00823	1	53.1	0.1635
**NMT2**	NMT2	22	N-myristoyltransferase 2	protein_codin	0.154	0.477	1	2.993	0.2111
**MLLT10**	MLLT10	22	MLLT10 histone lysine methyltransferase DOT1L cof	protein_codin	0.2848	0.151	1	3.513	0.2388
**HERC4**	HERC4	22	HECT and RLD domain containing E3 ubiquitin prote	protein_codin	0.4122	0.0566	1	8.29	0.2572
**KDM5A**	KDM5A	22	lysine demethylase 5A	protein_codin	0.2997	0.085	1	17.21	0.1371
**WNK1**	WNK1	22	WNK lysine deficient protein kinase 1	protein_codin	-0.4133	0.00406	1	40.54	0.2422
**ENO2**	ENO2	22	enolase 2	protein_codin	-0.1937	0.311	1	8.85	0.3832
**PLEKHA5**	PLEKHA5	22	pleckstrin homology domain containing A5	protein_codin	0.7726	3.13E-09	7.38E-05	16.27	0.1088
**RASSF8**	RASSF8	22	Ras association domain family member 8	protein_codin	0.4293	0.00011	1	38.11	0.1014
**AC023157.3**	AC023157.3	22	novel transcript, antisense to AM	antisense_RN	-0.06092	0.799	1	35.23	0.2807
**PDZRN4**	PDZRN4	22	PDZ domain containing ring finger 4	protein_codin	-0.4256	0.145	1	10.65	0.4248
**CCNT1**	CCNT1	22	cyclin T1	protein_codin	0.4186	0.0814	1	3.206	0.1389
**AAAS**	AAAS	22	aladin WD repeat nucleoporin	protein_codin	-0.01764	0.938	1	2.923	0.1798
**COPZ1**	COPZ1	22	coatomer protein complex subunit zeta 1	protein_codin	0.05474	0.621	1	11.21	0.03379
**DTX3**	DTX3	22	deltex E3 ubiquitin ligase 3	protein_codin	0.1827	0.382	1	3.19	0.3029
**CAND1**	CAND1	22	cullin associated and neddylation dissociated 1	protein_codin	0.283	0.0722	1	6.726	0.09940
**NUP107**	NUP107	22	nucleoporin 107	protein_codin	0.2306	0.22	1	3.211	0.1022
**METTL25**	METTL25	22	methyltransferase like 25	protein_codin	0.1952	0.279	1	32.63	0.1325
**UNG**	UNG	22	uracil DNA glycosylase	protein_codin	0.9753	3.62E-05	0.8288	3.363	0.06974
**TRIAP1**	TRIAP1	22	TP53 regulated inhibitor of apoptosis 1	protein_codin	-0.1416	0.332	1	21.29	0.2039
**RNF34**	RNF34	22	ring finger protein 34	protein_codin	0.07725	0.675	1	8.02	0.1674
**RSRC2**	RSRC2	22	arginine and serine rich coiled-coil 2	protein_codin	0.01042	0.896	1	42.63	0.09964
**NOC4L**	NOC4L	22	nucleolar complex associated 4 homolog	protein_codin	-0.3795	0.076	1	8.873	0.1712
**FBRSL1**	FBRSL1	22	fibrosin like 1	protein_codin	1.272	6.03E-06	0.1382	9.398	0.2702
**KBTBD7**	KBTBD7	22	kelch repeat and BTB domain containing 7	protein_codin	0.05002	0.822	1	8.168	0.1687

**NAA16**	NAA16	22	N(alpha)-acetyltransferase 16, NatA auxiliary subunit	protein_codin	-0.1631	0.464	1	3.729	0.3659
**PHF11**	PHF11	22	PHD finger protein 11	protein_codin	-0.07452	0.596	1	39.1	0.1830
**OSGEP**	OSGEP	22	O-sialoglycoprotein endopeptidase	protein_codin	-0.4513	0.0059	1	8.367	0.2081
**SCFD1**	SCFD1	22	sec1 family domain containing 1	protein_codin	0.2174	0.109	1	6.65	0.2769
**BAZ1A**	BAZ1A	22	bromodomain adjacent to zinc finger domain 1A	protein_codin	0.3194	0.102	1	23.54	0.07229
**SRP54**	SRP54	22	signal recognition particle 54	protein_codin	0.2182	0.0688	1	15.33	0.1329
**ERG28**	ERG28	22	ergosterol biosynthesis 28 homolog	protein_codin	0.2234	0.0874	1	7.279	0.133
**ZC3H14**	ZC3H14	22	zinc finger CCCH-type containing 14	protein_codin	-0.089	0.607	1	18.88	0.2050
**SETD3**	SETD3	22	SET domain containing 3, actin histidine methyltransf	protein_codin	-0.03972	0.811	1	7.71	0.2419
**DYNC1H1**	DYNC1H1	22	dynein cytoplasmic 1 heavy chain 1	protein_codin	0.4988	2.77E-05	0.6351	56.22	0.1422
**WDR20**	WDR20	22	WD repeat domain 20	protein_codin	0.1105	0.595	1	3.49	0.06812
**MTA1**	MTA1	22	metastasis associated 1	protein_codin	-0.0809	0.669	1	3.06	0.1807
**COX5A**	COX5A	22	cytochrome c oxidase subunit 5A	protein_codin	-0.1015	0.184	1	60.47	0.03226
**ZFAND6**	ZFAND6	22	zinc finger AN1-type containing 6	protein_codin	-0.06887	0.542	1	54.99	0.06504
**RHBDF1**	RHBDF1	22	rhomboid 5 homolog 1	protein_codin	0.8939	4.31E-06	0.09966	9.111	0.176
**JMJD8**	JMJD8	22	jumonji domain containing 8	protein_codin	-0.2216	0.0635	1	55.52	0.2132
**RNPS1**	RNPS1	22	RNA binding protein with serine rich domain 1	protein_codin	-0.07588	0.471	1	10.34	0.1696
**ZNF75A**	ZNF75A	22	zinc finger protein 75a	protein_codin	0.06566	0.77	1	10.26	0.2952
**NMRAL1**	NMRAL1	22	NmrA like redox sensor 1	protein_codin	0.1188	0.507	1	6.28	0.2395
**LYRM1**	LYRM1	22	LYR motif containing 1	protein_codin	0.5259	0.00072	1	16.38	0.1712
**INO80E**	INO80E	22	INO80 complex subunit E	protein_codin	0.1423	0.52	1	7.035	0.1826
**CKLF**	CKLF	22	chemokine like factor	protein_codin	0.2579	0.0758	1	28.01	0.2071
**CTCF**	CTCF	22	CCCTC-binding factor	protein_codin	-0.07922	0.552	1	7.449	0.1054
**NUDT7**	NUDT7	22	nudix hydrolase 7	protein_codin	0.1816	0.422	1	7.664	0.03612
**ZDHHC7**	ZDHHC7	22	zinc finger DHHC-type containing 7	protein_codin	0.5313	0.0211	1	10.58	0.3470
**CTU2**	CTU2	22	cytosolic thiouridylase subunit 2	protein_codin	0.03806	0.865	1	3.664	0.169
**VPS53**	VPS53	22	VPS53 subunit of GARP complex	protein_codin	0.2044	0.365	1	7.462	0.2085
**GLOD4**	GLOD4	22	glyoxalase domain containing 4	protein_codin	-0.04368	0.709	1	14.97	0.1869
**ELP5**	ELP5	22	elongator acetyltransferase complex subunit 5	protein_codin	0.5446	0.0151	1	3.322	0.1365
**ADPRM**	ADPRM	22	ADP-ribose/CDP-alcohol diphosphatase, manganese	protein_codin	0.22	0.342	1	2.77	0.2058
**ZSWIM7**	ZSWIM7	22	zinc finger SWIM-type containing 7	protein_codin	-0.2672	0.114	1	18.73	0.2406
**NCOR1**	NCOR1	22	nuclear receptor corepressor 1	protein_codin	0.07762	0.419	1	22.32	0.0666
**TEFM**	TEFM	22	transcription elongation factor, mitochondrial	protein_codin	0.07307	0.716	1	36.66	0.2261
**MSL1**	MSL1	22	MSL complex subunit 1	protein_codin	0.3436	0.0746	1	8.906	0.1329
**P3H4**	P3H4	22	prolyl 3-hydroxylase family member 4 (non-enzymatic	protein_codin	-0.1766	0.372	1	27.07	0.1798
**NKIRAS2**	NKIRAS2	22	NFKB inhibitor interacting Ras like 2	protein_codin	-0.3183	0.05	1	8.725	0.1675
**COIL**	COIL	22	coilin	protein_codin	-0.08153	0.612	1	7.185	0.3137
**EXOC7**	EXOC7	22	exocyst complex component 7	protein_codin	-0.07836	0.679	1	6.245	0.2912
**CCDC137**	CCDC137	22	coiled-coil domain containing 137	protein_codin	0.1807	0.382	1	9.471	0.1404
**ENOSF1**	ENOSF1	22	enolase superfamily member 1	protein_codin	0.3787	0.0805	1	27.35	0.354
**YES1**	YES1	22	YES proto-oncogene 1, Src family tyrosine kinase	protein_codin	0.06202	0.737	1	8.316	0.1015
**C18orf21**	C18orf21	22	chromosome 18 open reading frame 21	protein_codin	0.3582	0.109	1	3.462	0.1342
**MAVS**	MAVS	22	mitochondrial antiviral signaling protein	protein_codin	0.6236	0.012	1	3.517	0.3165
**APMAP**	APMAP	22	adipocyte plasma membrane associated protein	protein_codin	0.06729	0.654	1	6.14	0.06979
**MAPRE1**	MAPRE1	22	microtubule associated protein RP/EB family member 1	protein_codin	0.158	0.393	1	7.009	0.1345
**MMP24-AS1**	MMP24-AS1	22	MMP24 opposite strand	protein_codin	NA	NA	NA	32.07	0.06451
**SRSF6**	SRSF6	22	serine and arginine rich splicing factor 6	protein_codin	0.9153	6.49E-10	1.53E-05	55.49	0.138
**IFT52**	IFT52	22	intraflagellar transport 52	protein_codin	0.1035	0.496	1	7.13	0.07249
**CEBPB**	CEBPB	22	CCAAT enhancer binding protein beta	protein_codin	0.5333	3.55E-10	8.4E-06	60.58	0.03832
**RTFDC1**	RTFDC1	22	replication termination factor 2	protein_codin	NA	NA	NA	13.46	0.03444
**SCAMP4**	SCAMP4	22	secretory carrier membrane protein 4	protein_codin	0.3736	0.101	1	3.556	0.1335
**PLEKHJ1**	PLEKHJ1	22	pleckstrin homology domain containing J1	protein_codin	-0.1307	0.368	1	16.28	0.0724
**MPND**	MPND	22	MPN domain containing	protein_codin	-0.5519	0.0196	1	2.613	0.2831
**TRIP10**	TRIP10	22	thyroid hormone receptor interactor 10	protein_codin	1.045	2.6E-08	0.00061	52.11	0.2004
**C19orf53**	C19orf53	22	chromosome 19 open reading frame 53	protein_codin	-0.3852	4.4E-12	1.05E-07	53.8	0.03542
**EPS15L1**	EPS15L1	22	epidermal growth factor receptor pathway substrate 1	protein_codin	0.5457	0.0258	1	2.883	0.4156
**DDX49**	DDX49	22	DEAD-box helicase 49	protein_codin	-0.5025	0.00250	1	8.315	0.1318
**HOMER3**	HOMER3	22	homer scaffold protein 3	protein_codin	-0.03086	0.852	1	52.92	0.03514
**SUGP2**	SUGP2	22	SURP and G-patch domain containing 2	protein_codin	0.4305	0.0838	1	3.335	0.7232
**PSENEN**	PSENEN	22	presenilin enhancer, gamma-secretase subunit	protein_codin	0.2892	0.00680	1	22.53	0.03295
**AKT2**	AKT2	22	AKT serine/threonine kinase 2	protein_codin	-0.02267	0.89	1	3.398	0.1032
**SHKBP1**	SHKBP1	22	SH3KBP1 binding protein 1	protein_codin	0.1522	0.422	1	6.558	0.2357
**TBC1D17**	TBC1D17	22	TBC1 domain family member 17	protein_codin	0.4437	0.0307	1	7.738	0.2372
**NUP62**	NUP62	22	nucleoporin 62	protein_codin	0.1783	0.447	1	7.909	0.1412
**C19orf48**	C19orf48	22	chromosome 19 open reading frame 48	protein_codin	0.00724	0.972	1	11.24	0.1495
**CDC42EP5**	CDC42EP5	22	CDC42 effector protein 5	protein_codin	-0.8498	3.8E-10	8.99E-06	49.83	0.03250
**ZNF524**	ZNF524	22	zinc finger protein 524	protein_codin	-0.6505	5.7E-06	0.1316	47	0.1752
**ZNF580**	ZNF580	22	zinc finger protein 580	protein_codin	-0.2579	0.0179	1	59.29	0.138
**ZSCAN18**	ZSCAN18	22	zinc finger and SCAN domain containing 18	protein_codin	0.1709	0.235	1	16.8	0.2072
**RPS4Y1**	RPS4Y1	22	ribosomal protein S4 Y-linked 1	protein_codin	-0.3807	1.33E-10	3.15E-06	60.6	0.1447
**EIF1AY**	EIF1AY	22	eukaryotic translation initiation factor 1A Y-linked	protein_codin	-0.3466	0.0050	1	15.37	0.1744
**HDHD5**	HDHD5	22	haloacid dehalogenase like hydrolase domain contair	protein_codin	-0.3089	0.17	1	8.523	0.1706
**ASCC2**	ASCC2	22	activating signal cointegrator 1 complex subunit 2	protein_codin	0.6032	0.00512	1	7.214	0.1370
**EIF4ENIF1**	EIF4ENIF1	22	eukaryotic translation initiation factor 4E nuclear impo	protein_codin	-0.1573	0.445	1	2.972	0.06670
**A4GALT**	A4GALT	22	alpha 1,4-galactosyltransferase (P blood group)	protein_codin	0.6717	2.18E-07	0.0051	57.98	0.09975
**PSMG1**	PSMG1	22	proteasome assembly chaperone 1	protein_codin	-0.1369	0.347	1	18.38	0.1024
**C2CD2**	C2CD2	22	C2 calcium dependent domain containing 2	protein_codin	-0.0321	0.922	1	3.23	0.6349
**FAM207A**	FAM207A	22	family with sequence similarity 207 member A	protein_codin	-0.5498	0.00048	1	59.68	0.2414
**DIP2A**	DIP2A	22	disco interacting protein 2 homolog A	protein_codin	0.4817	0.151	1	2.57	0.7289
**KIAA2013**	KIAA2013	24	KIAA2013	protein_codin	-0.031	0.846	1	7.499	0.253
**PLA2G2A**	PLA2G2A	24	phospholipase A2 group IIA	protein_codin	7.253	1.98E-06	0.04593	Inf	0.2658
**FAF1**	FAF1	24	Fas associated factor 1	protein_codin	-0.2948	0.132	1	3.342	0.1129
**WNT2B**	WNT2B	24	Wnt family member 2B	protein_codin	1.978	3.32E-06	0.07687	58.4	0.1085
**CHD1L**	CHD1L	24	chromodomain helicase DNA binding protein 1 like	protein_codin	-0.0487	0.854	1	8.333	0.1348
**RRNAD1**	RRNAD1	24	ribosomal RNA adenine dimethylase domain containi	protein_codin	0.3233	0.231	1	8.695	0.1786
**LAMC1**	LAMC1	24	laminin subunit gamma 1	protein_codin	0.4013	0.00110	1	60.52	0.1758
**ATP2B4**	ATP2B4	24	ATPase plasma membrane Ca2+ transporting 4	protein_codin	0.252	0.044	1	60.02	0.1465
**MIA3**	MIA3	24	MIA SH3 domain ER export factor 3	protein_codin	0.2046	0.0502	1	42.98	0.09788
**SMC6**	SMC6	24	structural maintenance of chromosomes 6	protein_codin	0.05917	0.765	1	8.984	0.2056
**ADCY3**	ADCY3	24	adenylate cyclase 3	protein_codin	1.801	3.65E-18	8.75E-14	18.02	0.1044
**CLIP4**	CLIP4	24	CAP-Gly domain containing linker protein family mem	protein_codin	0.8229	0.00010	1	9.616	0.06961
**HNRNPLL**	HNRNPLL	24	heterogeneous nuclear ribonucleoprotein L like	protein_codin	0.3287	0.111	1	7.806	0.1747
**RAB1A**	RAB1A	24	RAB1A, member RAS oncogene family	protein_codin	0.1197	0.171	1	38.4	0.06853
**GCFC2**	GCFC2	24	GC-rich sequence DNA-binding factor 2	protein_codin	0.006446	0.976	1	7.842	0.2849
**ANKRD36C**	ANKRD36C	24	ankyrin repeat domain 36C	protein_codin	0.09439	0.641	1	52.08	0.4436
**NIFK**	NIFK	24	nucleolar protein interacting with the FHA domain of	protein_codin	-0.05906	0.622	1	51.92	0.06624
**SCRN3**	SCRN3	24	secernin 3	protein_codin	-0.1466	0.542	1	3.015	0.182
**DUSP19**	DUSP19	24	dual specificity phosphatase 19	protein_codin	-0.4064	0.0842	1	3.254	0.2182
**MAIP1**	MAIP1	24	matrix AAA peptidase interacting protein 1	protein_codin	-0.1495	0.462	1	10.17	0.2162
**AOX1**	AOX1	24	aldehyde oxidase 1	protein_codin	3.192	6.3E-15	1.5E-10	56.95	0.06769
**THUMPD3-AS1**	THUMPD3-AS	24	THUMPD3 antisense RNA 1	antisense_RN	0.4736	0.00214	1	60.56	0.5002
**SS18L2**	SS18L2	24	SS18 like 2	protein_codin	0.2719	0.0871	1	46.83	0.103
**PRKAR2A**	PRKAR2A	24	protein kinase cAMP-dependent type II regulatory sub	protein_codin	-0.001817	0.989	1	18.36	0.07497
**ABHD14B**	ABHD14B	24	abhydrolase domain containing 14B	protein_codin	0.06872	0.572	1	60.07	0.127
**GLT8D1**	GLT8D1	24	glycosyltransferase 8 domain containing 1	protein_codin	0.8288	3.43E-10	8.11E-06	45	0.0540
**GTPBP8**	GTPBP8	24	GTP binding protein 8 (putative)	protein_codin	-0.02047	0.927	1	60.58	0.2552
**ZBTB20**	ZBTB20	24	zinc finger and BTB domain containing 20	protein_codin	0.3892	2.31E-05	0.5303	45.25	0.06482
**SLC41A3**	SLC41A3	24	solute carrier family 41 member 3	protein_codin	-0.5319	6.4E-05	1	16.12	0.2061
**RPN1**	RPN1	24	ribophorin I	protein_codin	0.8298	4.8E-10	1.14E-05	22	0.06846
**ISY1**	ISY1	24	ISY1 splicing factor homolog	protein_codin	-0.2317	0.254	1	3.292	0.1083
**TIPARP**	TIPARP	24	TCDD inducible poly(ADP-ribose) polymerase	protein_codin	1.261	4.96E-11	1.18E-06	60.52	0.2295
**PDCD10**	PDCD10	24	programmed cell death 10	protein_codin	0.1346	0.146	1	21.11	0.03421
**RNF168**	RNF168	24	ring finger protein 168	protein_codin	0.3171	0.0620	1	7.948	0.1422
**SMIM20**	SMIM20	24	small integral membrane protein 20	protein_codin	-0.04979	0.682	1	28.52	0.128
**UBE2K**	UBE2K	24	ubiquitin conjugating enzyme E2 K	protein_codin	0.1958	0.125	1	35.42	0.1022
**CHIC2**	CHIC2	24	cysteine rich hydrophobic domain 2	protein_codin	0.1865	0.249	1	18.58	0.143
**SHROOM3**	SHROOM3	24	shroom family member 3	protein_codin	-0.5789	0.0118	1	28.17	0.135
**ABCE1**	ABCE1	24	ATP binding cassette subfamily E member 1	protein_codin	0.3286	0.0253	1	33.97	0.06896
**ENPP6**	ENPP6	24	ectonucleotide pyrophosphatase/phosphodiesterase 6	protein_codin	2.782	0.971	1	59.3	0.4005
**LIFR**	LIFR	24	LIF receptor subunit alpha	protein_codin	0.3225	0.118	1	58.33	0.1844
**DAB2**	DAB2	24	DAB adaptor protein 2	protein_codin	0.8883	2.89E-12	6.87E-08	48.36	0.07605
**C7**	C7	24	complement C7	protein_codin	0.897	0.00010	1	60.6	0.363
**CCL28**	CCL28	24	C-C motif chemokine ligand 28	protein_codin	1.394	1.87E-06	0.04339	28.19	0.09989
**TAF9**	TAF9	24	TATA-box binding protein associated factor 9	protein_codin	0.02845	0.82	1	39.16	0.143
**TNPO1**	TNPO1	24	transportin 1	protein_codin	0.6292	7.54E-06	0.174	14.43	0.1732
**COL4A3BP**	COL4A3BP	24	ceramide transporter 1	protein_codin	0.1633	0.248	1	14.13	0.1016
**AP3B1**	AP3B1	24	adaptor related protein complex 3 subunit beta 1	protein_codin	0.2673	0.0792	1	18.71	0.225
**PAM**	PAM	24	peptidylglycine alpha-amidating monooxygenase	protein_codin	0.3764	0.00040	1	47.6	0.03962
**APC**	APC	24	APC regulator of WNT signaling pathway	protein_codin	0.03666	0.812	1	19.83	0.3518
**MCC**	MCC	24	MCC regulator of WNT signaling pathway	protein_codin	1.049	0.0039	1	53.01	0.3240
**CXCL14**	CXCL14	24	C-X-C motif chemokine ligand 14	protein_codin	7.6	0.00422	1	NA	0.5396
**CD14**	CD14	24	CD14 molecule	protein_codin	0.4562	0.032	1	58.16	0.173
**IK**	IK	24	IK cytokine	protein_codin	-0.2617	0.0003	1	47.3	0.06665
**CNOT8**	CNOT8	24	CCR4-NOT transcription complex subunit 8	protein_codin	1.06	1.02E-07	0.00239	9.51	0.09849
**RNF145**	RNF145	24	ring finger protein 145	protein_codin	0.1533	0.436	1	8.902	0.1027
**FGF18**	FGF18	24	fibroblast growth factor 18	protein_codin	3.268	7E-07	0.01633	60.6	0.1468
**GFPT2**	GFPT2	24	glutamine-fructose-6-phosphate transaminase 2	protein_codin	1.896	6.28E-12	1.49E-07	22.04	0.1452
**ABCF1**	ABCF1	24	ATP binding cassette subfamily F member 1	protein_codin	0.02189	0.857	1	26.53	0.1348
**YIPF3**	YIPF3	24	Yip1 domain family member 3	protein_codin	0.2194	0.0162	1	59.18	0.03257
**MAD2L1BP**	MAD2L1BP	24	MAD2L1 binding protein	protein_codin	0.4352	0.0672	1	2.919	0.1759
**SNX14**	SNX14	24	sorting nexin 14	protein_codin	0.2884	0.127	1	7.786	0.3067
**RSPO3**	RSPO3	24	R-spondin 3	protein_codin	2.968	8.09E-13	1.93E-08	60.6	0.1475
**KATNA1**	KATNA1	24	katanin catalytic subunit A1	protein_codin	-0.08004	0.617	1	7.47	0.1332
**AFDN**	AFDN	24	afadin, adherens junction formation factor	protein_codin	0.7234	0.00038	1	40.02	0.2070
**RNF216**	RNF216	24	ring finger protein 216	protein_codin	0.2662	0.122	1	6.652	0.222
**RPA3**	RPA3	24	replication protein A3	protein_codin	-0.1903	0.167	1	60.3	0.2127
**ETV1**	ETV1	24	ETS variant 1	protein_codin	-0.2508	0.301	1	29.09	0.2564
**TWIST1**	TWIST1	24	twist family bHLH transcription factor 1	protein_codin	-0.09443	0.354	1	40.87	0.07152
**ZNRF2**	ZNRF2	24	zinc and ring finger 2	protein_codin	0.07687	0.722	1	8.775	0.2334
**GNAI1**	GNAI1	24	G protein subunit alpha i1	protein_codin	0.1527	0.371	1	25.76	0.203
**TP53TG1**	TP53TG1	24	TP53 target 1	lincRNA	-0.2204	0.106	1	18.22	0.1047
**LRRC17**	LRRC17	24	leucine rich repeat containing 17	protein_codin	0.3346	0.0019	1	50.08	0.1052
**LAMB1**	LAMB1	24	laminin subunit beta 1	protein_codin	-0.6619	0.00413	1	54.86	0.1803
**TMEM176B**	TMEM176B	24	transmembrane protein 176B	protein_codin	3.997	6.09E-22	1.47E-18	60.54	0.1544
**TMEM176A**	TMEM176A	24	transmembrane protein 176A	protein_codin	4.097	4.2E-17	1.01E-12	60.6	0.249
**SRPX**	SRPX	24	sushi repeat containing protein X-linked	protein_codin	2.597	1.06E-33	2.56E-29	60.6	0.2628
**PRAF2**	PRAF2	24	PRA1 domain family member 2	protein_codin	-0.3938	0.00122	1	38.19	0.06850
**SMC1A**	SMC1A	24	structural maintenance of chromosomes 1A	protein_codin	0.1868	0.193	1	14.12	0.2762
**ITM2A**	ITM2A	24	integral membrane protein 2A	protein_codin	3.761	3.23E-61	7.84E-57	42.84	0.03632
**SRPX2**	SRPX2	24	sushi repeat containing protein X-linked 2	protein_codin	1.133	2.35E-05	0.5394	23.09	0.1099
**HPRT1**	HPRT1	24	hypoxanthine phosphoribosyltransferase 1	protein_codin	-0.1234	0.419	1	20.77	0.1996
**ERICH1**	ERICH1	24	glutamate rich 1	protein_codin	0.6297	8.2E-05	1	46.96	0.035
**PPP3CC**	PPP3CC	24	protein phosphatase 3 catalytic subunit gamma	protein_codin	-0.1251	0.565	1	10.01	0.2112
**SORBS3**	SORBS3	24	sorbin and SH3 domain containing 3	protein_codin	0.3033	0.00947	1	20.23	0.06716
**TACC1**	TACC1	24	transforming acidic coiled-coil containing protein 1	protein_codin	0.5968	2.74E-11	6.5E-07	44.03	0.06537
**RDH10**	RDH10	24	retinol dehydrogenase 10	protein_codin	2.302	2.23E-11	5.29E-07	26.06	0.157
**KHDRBS3**	KHDRBS3	24	KH RNA binding domain containing, signal transducti	protein_codin	0.2437	0.12	1	48.42	0.1368
**PLGRKT**	PLGRKT	24	plasminogen receptor with a C-terminal lysine	protein_codin	0.004785	0.977	1	8.592	0.07456
**RANBP6**	RANBP6	24	RAN binding protein 6	protein_codin	0.04267	0.869	1	8.03	0.3089
**NTRK2**	NTRK2	24	neurotrophic receptor tyrosine kinase 2	protein_codin	1.533	4.71E-28	1.14E-23	60.48	0.02865
**PPP6C**	PPP6C	24	protein phosphatase 6 catalytic subunit	protein_codin	-0.05444	0.739	1	21.3	0.1771
**RABEPK**	RABEPK	24	Rab9 effector protein with kelch motifs	protein_codin	0.3153	0.127	1	25.92	0.04771
**LRRC8A**	LRRC8A	24	leucine rich repeat containing 8 VRAC subunit A	protein_codin	-0.5736	0.00546	1	10.58	0.4171
**SURF2**	SURF2	24	surfeit 2	protein_codin	-0.4093	0.00229	1	7.977	0.03861
**RHOG**	RHOG	24	ras homolog family member G	protein_codin	-0.1241	0.394	1	16.56	0.2034
**CSTF3**	CSTF3	24	cleavage stimulation factor subunit 3	protein_codin	0.1386	0.551	1	8.069	0.03206
**DRAP1**	DRAP1	24	DR1 associated protein 1	protein_codin	-0.08286	0.282	1	30.96	0.1008
**POLD3**	POLD3	24	DNA polymerase delta 3, accessory subunit	protein_codin	0.5502	0.00468	1	3.826	0.213
**ATM**	ATM	24	ATM serine/threonine kinase	protein_codin	0.09736	0.545	1	21.62	0.2032
**PUS3**	PUS3	24	pseudouridine synthase 3	protein_codin	-0.1803	0.377	1	10.22	0.3852
**ZEB1**	ZEB1	24	zinc finger E-box binding homeobox 1	protein_codin	0.1207	0.309	1	57.52	0.1419
**NRP1**	NRP1	24	neuropilin 1	protein_codin	1.469	1.38E-25	3.33E-21	54.81	0.1694
**VSIR**	VSIR	24	V-set immunoregulatory receptor	protein_codin	1.273	4.66E-22	1.12E-17	43.86	0.06751
**MICU1**	MICU1	24	mitochondrial calcium uptake 1	protein_codin	0.4092	0.00672	1	47.32	0.09914
**P4HA1**	P4HA1	24	prolyl 4-hydroxylase subunit alpha 1	protein_codin	0.4493	0.00038	1	54.23	0.06496
**VCL**	VCL	24	vinculin	protein_codin	-0.5	3.81E-08	0.00089	55.35	0.06569
**SHTN1**	SHTN1	24	shootin 1	protein_codin	-0.3868	0.187	1	8.796	0.178
**CTBP2**	CTBP2	24	C-terminal binding protein 2	protein_codin	-0.05634	0.697	1	15.73	0.1069
**C1S**	C1S	24	complement C1s	protein_codin	1.847	7.06E-97	1.72E-92	60.6	0.08132
**C1R**	C1R	24	complement C1r	protein_codin	1.355	2.2E-86	5.35E-82	55.36	0.03622
**MGST1**	MGST1	24	microsomal glutathione S-transferase 1	protein_codin	4.208	5.81E-51	1.41E-46	40.63	0.03621
**MFSD5**	MFSD5	24	major facilitator superfamily domain containing 5	protein_codin	0.6486	0.0182	1	3.029	0.1487
**PPP1R1A**	PPP1R1A	24	protein phosphatase 1 regulatory inhibitor subunit 1A	protein_codin	0.07320	0.758	1	26.08	0.1992
**LRP1**	LRP1	24	LDL receptor related protein 1	protein_codin	1.342	9.33E-38	2.26E-33	42.63	0.1051
**9-Mar**	43522	24	membrane associated ring-CH-type finger 9	protein_codin	-0.1433	0.497	1	8.048	0.3212
**METTL1**	METTL1	24	methyltransferase like 1	protein_codin	0.06027	0.795	1	8.236	0.06856
**UHRF1BP1L**	UHRF1BP1L	24	UHRF1 binding protein 1 like	protein_codin	0.3649	0.0802	1	40.1	0.2106
**IGF1**	IGF1	24	insulin like growth factor 1	protein_codin	5.385	9E-19	2.16E-14	54.83	0.108
**WASHC4**	WASHC4	24	WASH complex subunit 4	protein_codin	0.1506	0.347	1	7.759	0.1208
**MTERF2**	MTERF2	24	mitochondrial transcription termination factor 2	protein_codin	0.5428	0.0188	1	3.307	0.06422
**MAPKAPK5**	MAPKAPK5	24	MAPK activated protein kinase 5	protein_codin	0.1826	0.411	1	2.83	0.176
**TAOK3**	TAOK3	24	TAO kinase 3	protein_codin	0.01656	0.905	1	13.43	0.06598
**PXN**	PXN	24	paxillin	protein_codin	1.374	5.87E-08	0.00128	33.14	0.1552
**SFSWAP**	SFSWAP	24	splicing factor SWAP	protein_codin	0.2396	0.141	1	7.11	0.247
**MRPL57**	MRPL57	24	mitochondrial ribosomal protein L57	protein_codin	-0.124	0.122	1	54.29	0.2742
**EBPL**	EBPL	24	EBP like	protein_codin	-0.168	0.112	1	22.42	0.1064
**GPC6**	GPC6	24	glypican 6	protein_codin	-0.1264	0.327	1	17.95	0.06787
**SLC22A17**	SLC22A17	24	solute carrier family 22 member 17	protein_codin	-0.05222	0.757	1	25.03	0.1718
**DDHD1**	DDHD1	24	DDHD domain containing 1	protein_codin	0.8123	0.00286	1	11.04	0.3817
**GALNT16**	GALNT16	24	polypeptide N-acetylgalactosaminyltransferase 16	protein_codin	0.6696	0.00100	1	3.632	0.06844
**SRSF5**	SRSF5	24	serine and arginine rich splicing factor 5	protein_codin	0.2358	0.00040	1	50.22	0.1017
**ATXN3**	ATXN3	24	ataxin 3	protein_codin	0.3623	0.0277	1	7.523	0.06842
**SYNE3**	SYNE3	24	spectrin repeat containing nuclear envelope family m	protein_codin	0.5348	0.0119	1	3.616	0.2485
**C14orf132**	C14orf132	24	chromosome 14 open reading frame 132	protein_codin	1.185	0.00116	1	29.83	0.3652
**NOP10**	NOP10	24	NOP10 ribonucleoprotein	protein_codin	-0.1672	0.0247	1	49.8	0.06879
**SPG11**	SPG11	24	SPG11 vesicle trafficking associated, spatacsin	protein_codin	0.731	0.00240	1	9.836	0.483
**DENND4A**	DENND4A	24	DENN domain containing 4A	protein_codin	0.3008	0.271	1	3.345	0.5852
**TBC1D2B**	TBC1D2B	24	TBC1 domain family member 2B	protein_codin	0.7033	0.00014	1	18.85	0.21
**IDH2**	IDH2	24	isocitrate dehydrogenase (NADP(+)) 2, mitochondrial	protein_codin	0.1169	0.548	1	9.418	0.06926
**HDDC3**	HDDC3	24	HD domain containing 3	protein_codin	0.08281	0.691	1	3.458	0.1396
**SYNM**	SYNM	24	synemin	protein_codin	-0.4212	0.00016	1	53.24	0.07068
**ANKS3**	ANKS3	24	ankyrin repeat and sterile alpha motif domain containi	protein_codin	0.1802	0.394	1	7.645	0.3132
**C16orf89**	C16orf89	24	chromosome 16 open reading frame 89	protein_codin	3.986	2.68E-17	6.42E-13	42.52	0.1381
**KIF22**	KIF22	24	kinesin family member 22	protein_codin	0.1152	0.446	1	7.866	0.1047
**CHD9**	CHD9	24	chromodomain helicase DNA binding protein 9	protein_codin	0.3933	5E-05	1	54.83	0.03487
**BBS2**	BBS2	24	Bardet-Biedl syndrome 2	protein_codin	0.4026	0.081	1	7.373	0.2082
**IST1**	IST1	24	IST1 factor associated with ESCRT-III	protein_codin	-0.006007	0.97	1	7.796	0.1412
**HP**	HP	24	haptoglobin	protein_codin	2.782	0.9886	1	Inf	1.207
**B9D1**	B9D1	24	B9 domain containing 1	protein_codin	0.09442	0.734	1	35.48	0.2172
**ZNF830**	ZNF830	24	zinc finger protein 830	protein_codin	0.2127	0.209	1	8.448	0.1826
**DUSP14**	DUSP14	24	dual specificity phosphatase 14	protein_codin	1.047	3E-07	0.00701	59.92	0.06816
**IGFBP4**	IGFBP4	24	insulin like growth factor binding protein 4	protein_codin	1.036	1.29E-26	3.09E-22	60.6	0.04604
**FKBP10**	FKBP10	24	FKBP prolyl isomerase 10	protein_codin	-0.3233	0.00192	1	59.33	0.06896
**EZH1**	EZH1	24	enhancer of zeste 1 polycomb repressive complex 2 s	protein_codin	0.5808	0.00055	1	17.47	0.2412
**CALCOCO2**	CALCOCO2	24	calcium binding and coiled-coil domain 2	protein_codin	0.1212	0.132	1	43.14	0.1005
**MRPL27**	MRPL27	24	mitochondrial ribosomal protein L27	protein_codin	-0.05648	0.579	1	47.15	0.0372
**DYNLL2**	DYNLL2	24	dynein light chain LC8-type 2	protein_codin	-0.07648	0.631	1	7.906	0.3819
**COLEC12**	COLEC12	24	collectin subfamily member 12	protein_codin	1.647	2.92E-18	7E-14	60.6	0.06811
**CNDP2**	CNDP2	24	carnosine dipeptidase 2	protein_codin	0.5382	0.0138	1	7.025	0.09975
**MBP**	MBP	24	myelin basic protein	protein_codin	0.9403	1.38E-08	0.00032	59.25	0.2095
**TBC1D20**	TBC1D20	24	TBC1 domain family member 20	protein_codin	0.3645	0.0544	1	8.736	0.1405
**ATRN**	ATRN	24	attractin	protein_codin	0.8242	0.00348	1	9.163	0.568
**RASSF2**	RASSF2	24	Ras association domain family member 2	protein_codin	2.037	4.99E-06	0.1153	31.37	0.2501
**ISM1**	ISM1	24	isthmin 1	protein_codin	2.233	6.03E-13	1.44E-08	51.06	0.2267
**PIGU**	PIGU	24	phosphatidylinositol glycan anchor biosynthesis class	protein_codin	0.2069	0.49	1	2.767	0.2049
**KCNK15**	KCNK15	24	potassium two pore domain channel subfamily K mem	protein_codin	0.4369	0.00376	1	59.94	0.06631
**C3**	C3	24	complement C3	protein_codin	6.478	7.31E-12	1.74E-07	42.84	0.3301
**RAB11B-AS1**	RAB11B-AS1	24	RAB11B antisense RNA 1	antisense_RN	0.5117	0.0477	1	9.286	0.06645
**ZNF675**	ZNF675	24	zinc finger protein 675	protein_codin	0.3553	0.174	1	3.484	0.4961
**SUPT5H**	SUPT5H	24	SPT5 homolog, DSIF elongation factor subunit	protein_codin	-0.1556	0.475	1	7.818	0.4368
**AXL**	AXL	24	AXL receptor tyrosine kinase	protein_codin	0.9254	3.72E-20	8.93E-16	60.58	0.06794
**FCGRT**	FCGRT	24	Fc fragment of IgG receptor and transporter	protein_codin	1.079	2.12E-37	5.13E-33	55.33	0.03280
**TCN2**	TCN2	24	transcobalamin 2	protein_codin	0.863	0.0008	1	26.9	0.1722
**PICK1**	PICK1	24	protein interacting with PRKCA 1	protein_codin	-0.5342	0.0179	1	2.867	0.2911
**FBLN1**	FBLN1	24	fibulin 1	protein_codin	4.361	#######	1.6E-95	42.86	0.03290
**EVA1C**	EVA1C	24	eva-1 homolog C	protein_codin	-0.4045	0.0634	1	8.843	0.249
**UBE2J2**	UBE2J2	25	ubiquitin conjugating enzyme E2 J2	protein_codin	0.1658	0.241	1	7.51	0.1364
**INTS11**	INTS11	25	integrator complex subunit 11	protein_codin	0.2054	0.248	1	6.229	0.1824
**PRDM16**	PRDM16	25	PR/SET domain 16	protein_codin	-0.4092	0.275	1	3.476	0.4320
**CEP104**	CEP104	25	centrosomal protein 104	protein_codin	0.12	0.558	1	9.244	0.1815
**KIF1B**	KIF1B	25	kinesin family member 1B	protein_codin	0.1203	0.529	1	7.713	0.3119
**MUL1**	MUL1	25	mitochondrial E3 ubiquitin protein ligase 1	protein_codin	0.2596	0.182	1	2.633	0.1385
**ZNF593**	ZNF593	25	zinc finger protein 593	protein_codin	0.06671	0.631	1	34.49	0.0640
**TRNP1**	TRNP1	25	TMF1 regulated nuclear protein 1	protein_codin	-0.4512	0.00450	1	50.28	0.3196
**MAP3K6**	MAP3K6	25	mitogen-activated protein kinase kinase kinase 6	protein_codin	0.2487	0.293	1	2.822	0.172
**EYA3**	EYA3	25	EYA transcriptional coactivator and phosphatase 3	protein_codin	0.1066	0.714	1	2.963	0.172
**YARS**	YARS	25	tyrosyl-tRNA synthetase	protein_codin	-0.2898	0.168	1	3.533	0.1677
**SPATA6**	SPATA6	25	spermatogenesis associated 6	protein_codin	0.9919	5.92E-09	0.00014	15.68	0.04187
**RNF11**	RNF11	25	ring finger protein 11	protein_codin	-0.2805	0.0109	1	26.56	0.1476
**GADD45A**	GADD45A	25	growth arrest and DNA damage inducible alpha	protein_codin	-1.227	1.89E-45	4.58E-41	54.28	0.09952
**RABGGTB**	RABGGTB	25	Rab geranylgeranyltransferase subunit beta	protein_codin	0.262	0.122	1	5.692	0.03477
**ZNF644**	ZNF644	25	zinc finger protein 644	protein_codin	0.1513	0.198	1	30.7	0.06482
**MAGI3**	MAGI3	25	membrane associated guanylate kinase, WW and PD	protein_codin	0.826	0.00116	1	8.115	0.4332
**PMVK**	PMVK	25	phosphomevalonate kinase	protein_codin	-0.2657	0.0103	1	11.06	0.1429
**CCT3**	CCT3	25	chaperonin containing TCP1 subunit 3	protein_codin	-0.3258	1.48E-05	0.3409	22.63	0.03274
**OLFML2B**	OLFML2B	25	olfactomedin like 2B	protein_codin	0.8449	1.02E-12	2.43E-08	60.6	0.1377
**MPC2**	MPC2	25	mitochondrial pyruvate carrier 2	protein_codin	-0.2639	0.00090	1	29.49	0.07022
**PRRX1**	PRRX1	25	paired related homeobox 1	protein_codin	0.7009	5.88E-22	1.41E-17	60.6	0.06834
**IVNS1ABP**	IVNS1ABP	25	influenza virus NS1A binding protein	protein_codin	-0.0607	0.8	1	3.403	0.2692
**GLRX2**	GLRX2	25	glutaredoxin 2	protein_codin	0.05193	0.739	1	8.292	0.1381
**CDC73**	CDC73	25	cell division cycle 73	protein_codin	0.003595	0.975	1	11.74	0.1312
**SERTAD4**	SERTAD4	25	SERTA domain containing 4	protein_codin	-0.7397	0.00030	1	47.07	0.2425
**BATF3**	BATF3	25	basic leucine zipper ATF-like transcription factor 3	protein_codin	-0.9344	5.8E-05	1	31.03	0.3116
**RPS6KC1**	RPS6KC1	25	ribosomal protein S6 kinase C1	protein_codin	-0.1782	0.49	1	3.155	0.1662
**TP53BP2**	TP53BP2	25	tumor protein p53 binding protein 2	protein_codin	-0.02955	0.855	1	8.766	0.108
**CNIH4**	CNIH4	25	cornichon family AMPA receptor auxiliary protein 4	protein_codin	0.127	0.169	1	57.66	0.1679
**ADSS**	ADSS	25	adenylosuccinate synthase	protein_codin	-0.1506	0.354	1	19.03	0.09958
**DPY30**	DPY30	25	dpy-30 histone methyltransferase complex regulatory	protein_codin	-0.1183	0.219	1	24.96	0.1037
**EML4**	EML4	25	EMAP like 4	protein_codin	-0.2802	0.0766	1	15.58	0.2704
**MDH1**	MDH1	25	malate dehydrogenase 1	protein_codin	0.1612	0.0262	1	58.34	0.03336
**GMCL1**	GMCL1	25	germ cell-less 1, spermatogenesis associated	protein_codin	-0.1562	0.338	1	9.034	0.2399
**STAMBP**	STAMBP	25	STAM binding protein	protein_codin	-0.06379	0.676	1	16.25	0.03268
**HTRA2**	HTRA2	25	HtrA serine peptidase 2	protein_codin	-0.3479	0.007	1	19.54	0.1309
**MRPS5**	MRPS5	25	mitochondrial ribosomal protein S5	protein_codin	0.004069	0.975	1	13.88	0.2076
**SEMA4C**	SEMA4C	25	semaphorin 4C	protein_codin	0.42	0.0841	1	8.254	0.1497
**NPHP1**	NPHP1	25	nephrocystin 1	protein_codin	0.6416	0.0133	1	31.63	0.1019
**ZC3H8**	ZC3H8	25	zinc finger CCCH-type containing 8	protein_codin	-0.5993	0.00097	1	24.25	0.1842
**NIFK-AS1**	NIFK-AS1	25	NIFK antisense RNA 1	antisense_RN	-0.2169	0.29	1	7.628	0.06793
**SMPD4**	SMPD4	25	sphingomyelin phosphodiesterase 4	protein_codin	0.04111	0.889	1	3.116	0.294
**PTPN18**	PTPN18	25	protein tyrosine phosphatase non-receptor type 18	protein_codin	0.1457	0.454	1	27.06	0.353
**R3HDM1**	R3HDM1	25	R3H domain containing 1	protein_codin	-0.09988	0.62	1	10.33	0.2766
**PSMD14**	PSMD14	25	proteasome 26S subunit, non-ATPase 14	protein_codin	0.2237	0.12	1	18.09	0.09945
**AGPS**	AGPS	25	alkylglycerone phosphate synthase	protein_codin	0.4997	0.0107	1	7.845	0.3136
**WDR75**	WDR75	25	WD repeat domain 75	protein_codin	0.08652	0.689	1	9.474	0.03388
**ASNSD1**	ASNSD1	25	asparagine synthetase domain containing 1	protein_codin	0.5944	0.00087	1	19.94	0.1368
**ANKAR**	ANKAR	25	ankyrin and armadillo repeat containing	protein_codin	0.3645	0.0996	1	3.467	0.4168
**RFTN2**	RFTN2	25	raftlin family member 2	protein_codin	0.1376	0.459	1	9	0.1766
**BMPR2**	BMPR2	25	bone morphogenetic protein receptor type 2	protein_codin	-0.3093	0.029	1	34.83	0.1567
**LANCL1**	LANCL1	25	LanC like 1	protein_codin	-0.1129	0.724	1	3.428	0.4293
**SPAG16**	SPAG16	25	sperm associated antigen 16	protein_codin	0.01029	0.948	1	15.65	0.1703
**PNKD**	PNKD	25	PNKD metallo-beta-lactamase domain containing	protein_codin	-0.0306	0.86	1	18.76	0.06479
**COPS7B**	COPS7B	25	COP9 signalosome subunit 7B	protein_codin	-0.3686	0.0958	1	2	0.3559
**ATG4B**	ATG4B	25	autophagy related 4B cysteine peptidase	protein_codin	0.3892	0.149	1	3.003	0.5231
**XPC**	XPC	25	XPC complex subunit, DNA damage recognition and	protein_codin	0.1589	0.27	1	16.96	0.2738
**RBMS3**	RBMS3	25	RNA binding motif single stranded interacting protein 3	protein_codin	0.3763	0.00078	1	60.47	0.1718
**EXOSC7**	EXOSC7	25	exosome component 7	protein_codin	0.07661	0.564	1	17.49	0.06697
**SHISA5**	SHISA5	25	shisa family member 5	protein_codin	0.2036	0.039	1	35.48	0.1082

**LAMB2**	LAMB2	25	laminin subunit beta 2	protein_codin	0.8114	2.16E-10	5.11E-06	38.48	0.1404
**RBM5**	RBM5	25	RNA binding motif protein 5	protein_codin	0.5088	0.00563	1	7.261	0.2441
**DENND6A**	DENND6A	25	DENN domain containing 6A	protein_codin	0.04007	0.85	1	33.04	0.2972
**PPP4R2**	PPP4R2	25	protein phosphatase 4 regulatory subunit 2	protein_codin	0.3547	0.00598	1	56.37	0.2397
**ABHD10**	ABHD10	25	abhydrolase domain containing 10	protein_codin	0.27	0.209	1	9.043	0.06518
**GSK3B**	GSK3B	25	glycogen synthase kinase 3 beta	protein_codin	0.2458	0.175	1	8.098	0.1713
**GOLGB1**	GOLGB1	25	golgin B1	protein_codin	-0.1947	0.0487	1	42.88	0.1681
**RSRC1**	RSRC1	25	arginine and serine rich coiled-coil 1	protein_codin	0.1178	0.312	1	22.9	0.17
**GFM1**	GFM1	25	G elongation factor mitochondrial 1	protein_codin	0.2662	0.149	1	8.368	0.1413
**PRKCI**	PRKCI	25	protein kinase C iota	protein_codin	0.3417	0.0660	1	7.692	0.1749
**MCCC1**	MCCC1	25	methylcrotonoyl-CoA carboxylase 1	protein_codin	0.2463	0.247	1	2.872	0.1312
**LINC00969**	LINC00969	25	MUC20 overlapping transcript	lincRNA	NA	NA	NA	22.38	0.4292
**LYAR**	LYAR	25	Ly1 antibody reactive	protein_codin	0.1443	0.286	1	44.17	0.1987
**AC093323.1**	AC093323.1	25	Putative MORF4 family-associated protein 1-like prot	lincRNA	0.222	0.407	1	11.35	0.3114
**NUDT9**	NUDT9	25	nudix hydrolase 9	protein_codin	0.06352	0.625	1	15.68	0.09917
**HERC5**	HERC5	25	HECT and RLD domain containing E3 ubiquitin prote	protein_codin	-0.2872	0.0953	1	9.083	0.2055
**EIF4E**	EIF4E	25	eukaryotic translation initiation factor 4E	protein_codin	-0.1688	0.175	1	16.6	0.1348
**SEC24D**	SEC24D	25	SEC24 homolog D, COPII coat complex component	protein_codin	0.2038	0.26	1	8.258	0.2144
**ELMOD2**	ELMOD2	25	ELMO domain containing 2	protein_codin	0.119	0.568	1	8.567	0.2458
**ING2**	ING2	25	inhibitor of growth family member 2	protein_codin	1.278	2.51E-14	5.98E-10	33.42	0.06915
**DMXL1**	DMXL1	25	Dmx like 1	protein_codin	0.1671	0.409	1	29.74	0.1387
**LYRM7**	LYRM7	25	LYR motif containing 7	protein_codin	-0.1528	0.453	1	7.557	0.3796
**TAF7**	TAF7	25	TATA-box binding protein associated factor 7	protein_codin	0.2733	0.00074	1	30.25	0.06568
**TPMT**	TPMT	25	thiopurine S-methyltransferase	protein_codin	-0.2181	0.293	1	32.22	0.2314
**HIST1H1E**	HIST1H1E	25	histone cluster 1 H1 family member e	protein_codin	0.2421	0.179	1	36.28	0.1413
**PPP1R18**	PPP1R18	25	protein phosphatase 1 regulatory subunit 18	protein_codin	-0.367	0.0182	1	8.152	0.06638
**TAP2**	TAP2	25	transporter 2, ATP binding cassette subfamily B memb	protein_codin	-0.2233	0.314	1	11.65	0.2133
**HSD17B8**	HSD17B8	25	hydroxysteroid 17-beta dehydrogenase 8	protein_codin	0.1798	0.458	1	8.422	0.2041
**TMEM14A**	TMEM14A	25	transmembrane protein 14A	protein_codin	0.04572	0.668	1	23.7	0.06804
**KIAA1586**	KIAA1586	25	KIAA1586	protein_codin	0.3306	0.0736	1	22.97	0.07153
**ZNF451**	ZNF451	25	zinc finger protein 451	protein_codin	0.5076	0.0214	1	7.673	0.2836
**COL12A1**	COL12A1	25	collagen type XII alpha 1 chain	protein_codin	0.2212	0.218	1	50.32	0.1058
**CASP8AP2**	CASP8AP2	25	caspase 8 associated protein 2	protein_codin	0.2792	0.128	1	23.19	0.1308
**SERINC1**	SERINC1	25	serine incorporator 1	protein_codin	0.1635	0.0367	1	58.92	0.1084
**RNF217**	RNF217	25	ring finger protein 217	protein_codin	-0.06371	0.752	1	26.38	0.1371
**PTPRK**	PTPRK	25	protein tyrosine phosphatase receptor type K	protein_codin	0.2774	0.241	1	3.127	0.402
**SGK1**	SGK1	25	serum/glucocorticoid regulated kinase 1	protein_codin	0.2193	0.375	1	50.45	0.2072
**SHPRH**	SHPRH	25	SNF2 histone linker PHD RING helicase	protein_codin	0.2146	0.153	1	18.26	0.1758
**MTRF1L**	MTRF1L	25	mitochondrial translational release factor 1 like	protein_codin	-0.3983	0.00813	1	28.04	0.2496
**RGS17**	RGS17	25	regulator of G protein signaling 17	protein_codin	-0.00294	0.989	1	45.88	0.1662
**SNHG15**	SNHG15	25	small nucleolar RNA host gene 15	lincRNA	0.2005	0.198	1	18.38	0.2418
**EGFR**	EGFR	25	epidermal growth factor receptor	protein_codin	0.6573	0.00016	1	18.52	0.1458
**ZNF680**	ZNF680	25	zinc finger protein 680	protein_codin	0.5747	0.00754	1	8.96	0.2181
**AUTS2**	AUTS2	25	activator of transcription and developmental regulator	protein_codin	0.8096	0.0032	1	9.576	0.1922
**TMEM243**	TMEM243	25	transmembrane protein 243	protein_codin	0.5364	7.31E-05	1	14.68	0.1237
**GATAD1**	GATAD1	25	GATA zinc finger domain containing 1	protein_codin	0.459	0.00269	1	36.96	0.1397
**CASD1**	CASD1	25	CAS1 domain containing 1	protein_codin	0.5428	0.0144	1	9.742	0.2441
**SGCE**	SGCE	25	sarcoglycan epsilon	protein_codin	0.598	9.32E-08	0.00022	44.92	0.1432
**TAC1**	TAC1	25	tachykinin precursor 1	protein_codin	0.9384	0.0662	1	60.6	0.7768
**MEPCE**	MEPCE	25	methylphosphate capping enzyme	protein_codin	1.153	0.00023	1	37.94	0.2433
**ALKBH4**	ALKBH4	25	alkB homolog 4, lysine demethylase	protein_codin	0.09598	0.632	1	3.36	0.2103
**MDFIC**	MDFIC	25	MyoD family inhibitor domain containing	protein_codin	-0.3735	0.016	1	58.94	0.1447
**MTPN**	MTPN	25	myotrophin	protein_codin	-0.164	0.0218	1	25.35	0.06668
**ZBED1**	ZBED1	25	zinc finger BED-type containing 1	protein_codin	0.1568	0.418	1	9.31	0.1722
**PNPLA4**	PNPLA4	25	patatin like phospholipase domain containing 4	protein_codin	-0.1653	0.432	1	3.55	0.1433
**CA5B**	CA5B	25	carbonic anhydrase 5B	protein_codin	0.1502	0.437	1	9.577	0.09969
**SYAP1**	SYAP1	25	synapse associated protein 1	protein_codin	0.2798	0.0198	1	13.24	0.2092
**MSN**	MSN	25	moesin	protein_codin	-0.2046	0.0203	1	44.32	0.03472
**GLA**	GLA	25	galactosidase alpha	protein_codin	0.5585	0.00825	1	7.343	0.2451
**ARMCX2**	ARMCX2	25	armadillo repeat containing X-linked 2	protein_codin	-0.1773	0.257	1	26.42	0.1100
**TCEAL4**	TCEAL4	25	transcription elongation factor A like 4	protein_codin	-0.7098	4.5E-34	1.09E-29	59.93	0.1481
**TCEAL3**	TCEAL3	25	transcription elongation factor A like 3	protein_codin	-0.6503	5.34E-16	1.28E-12	55.36	0.1421
**TCEAL1**	TCEAL1	25	transcription elongation factor A like 1	protein_codin	-0.3357	0.00128	1	53.13	0.1427
**MORF4L2**	MORF4L2	25	mortality factor 4 like 2	protein_codin	-0.1464	0.0277	1	33.66	0.09854
**COL4A5**	COL4A5	25	collagen type IV alpha 5 chain	protein_codin	-0.1948	0.44	1	8.208	0.3095
**ARHGEF6**	ARHGEF6	25	Rac/Cdc42 guanine nucleotide exchange factor 6	protein_codin	0.2377	0.415	1	3.503	0.1467
**FUNDC2**	FUNDC2	25	FUN14 domain containing 2	protein_codin	-0.1053	0.17	1	24	0.02355
**PINX1**	PINX1	25	PIN2 (TERF1) interacting telomerase inhibitor 1	protein_codin	-0.2218	0.402	1	3.198	0.2729
**ATP6V1B2**	ATP6V1B2	25	ATPase H+ transporting V1 subunit B2	protein_codin	0.7268	8.28E-05	1	7.248	0.1282
**BMP1**	BMP1	25	bone morphogenetic protein 1	protein_codin	0.9006	0.00128	1	9.883	0.2027
**RBPMS**	RBPMS	25	RNA binding protein, mRNA processing factor	protein_codin	-0.4577	2.51E-09	5.92E-05	60.5	0.1009
**YTHDF3**	YTHDF3	25	YTH N6-methyladenosine RNA binding protein 3	protein_codin	0.2387	0.26	1	9.768	0.1784
**UBR5**	UBR5	25	ubiquitin protein ligase E3 component n-recognin 5	protein_codin	0.2312	0.256	1	20.79	0.2752
**OXR1**	OXR1	25	oxidation resistance 1	protein_codin	0.08829	0.447	1	48.65	0.2057
**EBAG9**	EBAG9	25	estrogen receptor binding site associated antigen 9	protein_codin	-0.03547	0.794	1	32.1	0.03494
**EXT1**	EXT1	25	exostosin glycosyltransferase 1	protein_codin	-0.2994	0.0878	1	19.65	0.1907
**TMEM65**	TMEM65	25	transmembrane protein 65	protein_codin	0.07866	0.724	1	8.292	0.1398
**NDUFB9**	NDUFB9	25	NADH:ubiquinone oxidoreductase subunit B9	protein_codin	0.1267	0.0465	1	45.45	0.06691
**PUF60**	PUF60	25	poly(U) binding splicing factor 60	protein_codin	0.03384	0.728	1	25.78	0.1642
**LURAP1L**	LURAP1L	25	leucine rich adaptor protein 1 like	protein_codin	0.1896	0.271	1	8.319	0.0340
**VCP**	VCP	25	valosin containing protein	protein_codin	0.2707	0.00727	1	57.62	0.03259
**TMOD1**	TMOD1	25	tropomodulin 1	protein_codin	-0.2052	0.142	1	56.82	0.06477
**GALNT12**	GALNT12	25	polypeptide N-acetylgalactosaminyltransferase 12	protein_codin	1.814	2.5E-08	0.00059	27.98	0.1659
**STOM**	STOM	25	stomatin	protein_codin	0.7068	8.27E-12	1.97E-08	54.66	0.03208
**URM1**	URM1	25	ubiquitin related modifier 1	protein_codin	-0.3825	0.00423	1	40.27	0.1099
**MED27**	MED27	25	mediator complex subunit 27	protein_codin	-0.2746	0.06	1	6.813	0.1662
**TMEM141**	TMEM141	25	transmembrane protein 141	protein_codin	-0.2917	0.00776	1	12.52	0.1697
**RIC8A**	RIC8A	25	RIC8 guanine nucleotide exchange factor A	protein_codin	-0.05196	0.711	1	7.352	0.167
**SIGIRR**	SIGIRR	25	single Ig and TIR domain containing	protein_codin	0.505	0.00015	1	13.1	0.03252
**MOB2**	MOB2	25	MOB kinase activator 2	protein_codin	-0.1918	0.108	1	44.68	0.1680
**KCNQ1OT1**	KCNQ1OT1	25	KCNQ1 opposite strand/antisense transcript 1	antisense_RN	1.543	1.86E-05	0.4275	49.54	0.7314
**OLFML1**	OLFML1	25	olfactomedin like 1	protein_codin	1.213	2.28E-16	5.45E-12	23.8	0.1730
**LIN7C**	LIN7C	25	lin-7 homolog C, crumbs cell polarity complex compo	protein_codin	0.2236	0.155	1	52.14	0.3105
**FBXO3**	FBXO3	25	F-box protein 3	protein_codin	0.3827	0.0218	1	5.362	0.137
**MARK2**	MARK2	25	microtubule affinity regulating kinase 2	protein_codin	0.09814	0.721	1	2.222	0.3451
**EHBP1L1**	EHBP1L1	25	EH domain binding protein 1 like 1	protein_codin	-0.5258	0.0006	1	20.12	0.211
**AIP**	AIP	25	aryl hydrocarbon receptor interacting protein	protein_codin	-0.326	0.00017	1	20.08	0.1338
**AP002387.1**	AP002387.1	25	novel transcri	antisense_RN	NA	NA	NA	11.53	0.3945
**NUMA1**	NUMA1	25	nuclear mitotic apparatus protein 1	protein_codin	0.1746	0.215	1	36.77	0.3096
**UVRAG**	UVRAG	25	UV radiation resistance associated	protein_codin	-0.1448	0.316	1	19.86	0.1522
**PCF11**	PCF11	25	PCF11 cleavage and polyadenylation factor subunit	protein_codin	0.5624	0.00134	1	17.06	0.315
**TMEM126B**	TMEM126B	25	transmembrane protein 126B	protein_codin	-0.2014	0.108	1	29.28	0.2757
**C11orf54**	C11orf54	25	chromosome 11 open reading frame 54	protein_codin	-0.09845	0.605	1	8.241	0.03228
**SRSF8**	SRSF8	25	serine and arginine rich splicing factor 8	protein_codin	-0.1859	0.122	1	28.16	0.1022
**MAML2**	MAML2	25	mastermind like transcriptional coactivator 2	protein_codin	-0.4919	0.00190	1	23.15	0.1001
**CCDC82**	CCDC82	25	coiled-coil domain containing 82	protein_codin	0.1443	0.22	1	25.6	0.1685
**YAP1**	YAP1	25	Yes associated protein 1	protein_codin	-0.2108	0.118	1	16.77	0.2479
**C11orf71**	C11orf71	25	chromosome 11 open reading frame 71	protein_codin	0.3892	0.0568	1	55.24	0.2544
**NFRKB**	NFRKB	25	nuclear factor related to kappaB binding protein	protein_codin	0.1769	0.488	1	2.55	0.2092
**JAM3**	JAM3	25	junctional adhesion molecule 3	protein_codin	0.1252	0.299	1	13.54	0.09719
**ZMYND11**	ZMYND11	25	zinc finger MYND-type containing 11	protein_codin	-0.06833	0.571	1	15.44	0.03826
**DIP2C**	DIP2C	25	disco interacting protein 2 homolog C	protein_codin	0.1427	0.626	1	2.592	0.4485
**IDI1**	IDI1	25	isopentenyl-diphosphate delta isomerase 1	protein_codin	-0.1937	0.222	1	19.62	0.1047
**FAM208B**	FAM208B	25	transcription activation suppressor family member 2	protein_codin	0.003242	0.985	1	17.22	0.24
**WAC**	WAC	25	WW domain containing adaptor with coiled-coil	protein_codin	0.1403	0.25	1	49.82	0.03432
**ZNF25**	ZNF25	25	zinc finger protein 25	protein_codin	-0.2832	0.308	1	8.565	0.211
**SAR1A**	SAR1A	25	secretion associated Ras related GTPase 1A	protein_codin	0.2788	0.00236	1	30.56	0.1774
**PPP3CB**	PPP3CB	25	protein phosphatase 3 catalytic subunit beta	protein_codin	0.135	0.376	1	20.68	0.2172
**FUT11**	FUT11	25	fucosyltransferase 11	protein_codin	0.5735	0.0195	1	8.962	0.2387
**KAT6B**	KAT6B	25	lysine acetyltransferase 6B	protein_codin	-0.04618	0.732	1	15.18	0.1394
**ZFYVE27**	ZFYVE27	25	zinc finger FYVE-type containing 27	protein_codin	-0.1434	0.535	1	9.102	0.2771
**SMC3**	SMC3	25	structural maintenance of chromosomes 3	protein_codin	0.03951	0.665	1	52.28	0.2040
**TACC2**	TACC2	25	transforming acidic coiled-coil containing protein 2	protein_codin	0.2965	0.174	1	8.358	0.1738
**ART4**	ART4	25	ADP-ribosyltransferase 4 (Dombrock blood group)	protein_codin	-0.8719	0.00011	1	37.8	0.2001
**CAPRIN2**	CAPRIN2	25	caprin family member 2	protein_codin	-0.3039	0.206	1	27.62	0.3450
**DDX23**	DDX23	25	DEAD-box helicase 23	protein_codin	0.2386	0.320	1	7.252	0.2114
**ARF3**	ARF3	25	ADP ribosylation factor 3	protein_codin	-0.06388	0.657	1	17.1	0.1681
**METTL7A**	METTL7A	25	methyltransferase like 7A	protein_codin	0.8089	1.8E-14	4.29E-10	39.6	0.06471
**SHMT2**	SHMT2	25	serine hydroxymethyltransferase 2	protein_codin	0.1267	0.522	1	26.52	0.03335
**AC084033.3**	AC084033.3	25	GIHCG inhibitor of miR-200b/200a/429 expression	lincRNA	0.1108	0.615	1	6.415	0.2172
**TBC1D15**	TBC1D15	25	TBC1 domain family member 15	protein_codin	-0.01524	0.915	1	60.27	0.2348
**CCDC59**	CCDC59	25	coiled-coil domain containing 59	protein_codin	-0.1442	0.207	1	22.99	0.09871
**GLTP**	GLTP	25	glycolipid transfer protein	protein_codin	-0.01483	0.917	1	15.86	0.06603
**ANAPC7**	ANAPC7	25	anaphase promoting complex subunit 7	protein_codin	0.008854	0.964	1	6.81	0.06449
**PPP1CC**	PPP1CC	25	protein phosphatase 1 catalytic subunit gamma	protein_codin	0.08529	0.37	1	38.53	0.1985
**NAA25**	NAA25	25	N(alpha)-acetyltransferase 25, NatB auxiliary subunit	protein_codin	0.4043	0.0655	1	8.84	0.1029
**RBM19**	RBM19	25	RNA binding motif protein 19	protein_codin	0.2702	0.258	1	3.31	0.07277
**ACADS**	ACADS	25	acyl-CoA dehydrogenase short chain	protein_codin	0.7305	0.00235	1	7.027	0.1056
**MLXIP**	MLXIP	25	MLX interacting protein	protein_codin	0.1355	0.539	1	30.94	0.2072
**STX2**	STX2	25	syntaxin 2	protein_codin	0.3206	0.0276	1	41.28	0.107
**MPHOSPH8**	MPHOSPH8	25	M-phase phosphoprotein 8	protein_codin	0.01673	0.817	1	55.48	0.105
**N4BP2L1**	N4BP2L1	25	NEDD4 binding protein 2 like 1	protein_codin	0.4317	0.0225	1	60.49	0.06862
**SUPT20H**	SUPT20H	25	SPT20 homolog, SAGA complex component	protein_codin	0.3866	0.0421	1	7.63	0.07214
**UTP14C**	UTP14C	25	UTP14C small subunit processome component	protein_codin	-0.09138	0.737	1	3.722	0.32
**IRS2**	IRS2	25	insulin receptor substrate 2	protein_codin	0.1029	0.647	1	53.89	0.2096
**IRF9**	IRF9	25	interferon regulatory factor 9	protein_codin	-0.1804	0.424	1	8.597	0.2097
**SDR39U1**	SDR39U1	25	short chain dehydrogenase/reductase family 39U me	protein_codin	-0.03988	0.818	1	24.5	0.248
**FERMT2**	FERMT2	25	fermitin family member 2	protein_codin	-0.115	0.099	1	20.06	0.07079
**ARID4A**	ARID4A	25	AT-rich interaction domain 4A	protein_codin	-0.2703	0.0311	1	43.62	0.2214
**DAAM1**	DAAM1	25	dishevelled associated activator of morphogenesis 1	protein_codin	-0.3797	0.00065	1	60.58	0.1019
**DHRS7**	DHRS7	25	dehydrogenase/reductase 7	protein_codin	0.2982	2.69E-05	0.6168	13.33	0.06598
**EIF2S1**	EIF2S1	25	eukaryotic translation initiation factor 2 subunit alpha	protein_codin	0.04067	0.734	1	38.35	0.1374
**GPATCH2L**	GPATCH2L	25	G-patch domain containing 2 like	protein_codin	0.2977	0.146	1	26.54	0.3134
**PPP4R3A**	PPP4R3A	25	protein phosphatase 4 regulatory subunit 3A	protein_codin	-0.00208	0.991	1	5.592	0.268
**BTBD7**	BTBD7	25	BTB domain containing 7	protein_codin	0.2567	0.172	1	19.53	0.2877
**SLC25A29**	SLC25A29	25	solute carrier family 25 member 29	protein_codin	-0.2442	0.368	1	10.62	0.4413
**NSMCE3**	NSMCE3	25	NSE3 homolog, SMC5-SMC6 complex component	protein_codin	0.4418	0.00103	1	13.88	0.1382
**CTDSPL2**	CTDSPL2	25	CTD small phosphatase like 2	protein_codin	0.327	0.0812	1	21.15	0.1637
**THSD4**	THSD4	25	thrombospondin type 1 domain containing 4	protein_codin	0.5519	0.0145	1	59.67	0.03515
**ARIH1**	ARIH1	25	ariadne RBR E3 ubiquitin protein ligase 1	protein_codin	0.1238	0.532	1	7.795	0.1802
**STOML1**	STOML1	25	stomatin like 1	protein_codin	-0.05642	0.792	1	8.784	0.1319
**IMP3**	IMP3	25	IMP U3 small nucleolar ribonucleoprotein 3	protein_codin	0.2475	0.00746	1	53.86	0.1022
**TLNRD1**	TLNRD1	25	talin rod domain containing 1	protein_codin	1.329	1.66E-08	0.00039	23.18	0.1730
**RHOT2**	RHOT2	25	ras homolog family member T2	protein_codin	-0.1499	0.518	1	7.919	0.2845
**NUBP1**	NUBP1	25	nucleotide binding protein 1	protein_codin	0.06169	0.764	1	5.983	0.14
**NDE1**	NDE1	25	nudE neurodevelopment protein 1	protein_codin	0.04389	0.858	1	7.385	0.2880
**MVP**	MVP	25	major vault protein	protein_codin	0.3694	0.00172	1	24.45	0.03959
**MAPK3**	MAPK3	25	mitogen-activated protein kinase 3	protein_codin	0.2668	0.0333	1	13.41	0.1701
**FBRS**	FBRS	25	fibrosin	protein_codin	-0.2636	0.29	1	3.173	0.1694
**ARL2BP**	ARL2BP	25	ADP ribosylation factor like GTPase 2 binding protein	protein_codin	0.5068	1.16E-06	0.02697	38.12	0.1024
**CMC2**	CMC2	25	C-X9-C motif containing 2	protein_codin	-0.37	0.0189	1	24.93	0.1819
**ZCCHC14**	ZCCHC14	25	zinc finger CCHC-type containing 14	protein_codin	-0.05033	0.829	1	3.562	0.3912
**TRAPPC2L**	TRAPPC2L	25	trafficking protein particle complex 2 like	protein_codin	-0.1593	0.127	1	48.18	0.1068
**ANKFY1**	ANKFY1	25	ankyrin repeat and FYVE domain containing 1	protein_codin	0.3138	0.232	1	11.25	0.5472
**ZBTB4**	ZBTB4	25	zinc finger and BTB domain containing 4	protein_codin	0.5376	0.00336	1	7.796	0.1668
**TNFSF12**	TNFSF12	25	TNF superfamily member 12	protein_codin	-0.1693	0.0127	1	46.06	0.06976
**TP53**	TP53	25	tumor protein p53	protein_codin	-0.2219	0.419	1	8.158	0.1719
**CCDC144NL-AS**	CCDC144NL-A	25	CCDC144NL antisense RNA 1	antisense_RN	-1.12	3.95E-05	0.904	26.68	0.1741
**IFT20**	IFT20	25	intraflagellar transport 20	protein_codin	-0.2244	0.0603	1	13.36	0.1364
**C17orf75**	C17orf75	25	chromosome 17 open reading frame 75	protein_codin	-0.155	0.474	1	30.16	0.211
**LINC00672**	LINC00672	25	long intergenic non-protein coding RNA 672	protein_codin	-0.6779	0.00327	1	8.335	0.2459
**PSMD3**	PSMD3	25	proteasome 26S subunit, non-ATPase 3	protein_codin	0.1582	0.166	1	14.92	0.09935
**UTP18**	UTP18	25	UTP18 small subunit processome component	protein_codin	0.4762	0.0431	1	3.453	0.1395
**COX11**	COX11	25	cytochrome c oxidase copper chaperone COX11	protein_codin	0.03347	0.802	1	15.57	0.1146
**TRIM25**	TRIM25	25	tripartite motif containing 25	protein_codin	1.122	6.58E-05	1	9.493	0.3096
**MED13**	MED13	25	mediator complex subunit 13	protein_codin	-0.01469	0.932	1	18.55	0.2332
**SMARCD2**	SMARCD2	25	SWI/SNF related, matrix associated, actin dependent	protein_codin	0.5093	0.0181	1	7.953	0.1336
**ABCA8**	ABCA8	25	ATP binding cassette subfamily A member 8	protein_codin	2.937	1.53E-38	3.7E-34	46.08	0.2246
**ABCA9**	ABCA9	25	ATP binding cassette subfamily A member 9	protein_codin	3.416	1.17E-20	2.81E-16	60	0.212
**ABCA6**	ABCA6	25	ATP binding cassette subfamily A member 6	protein_codin	2.483	1.38E-25	3.33E-21	60.6	0.1579
**ABCA10**	ABCA10	25	ATP binding cassette subfamily A member 10	protein_codin	3.851	1.31E-05	0.3016	54.18	0.4977
**COG1**	COG1	25	component of oligomeric golgi complex 1	protein_codin	-0.264	0.2	1	8.814	0.405
**SYNGR2**	SYNGR2	25	synaptogyrin 2	protein_codin	-0.7807	5.75E-08	0.00125	39.04	0.2087
**ALYREF**	ALYREF	25	Aly/REF export factor	protein_codin	0.3277	0.0209	1	55.36	0.1362
**GPS1**	GPS1	25	G protein pathway suppressor 1	protein_codin	0.4198	0.0558	1	7.12	0.1472
**WDR45B**	WDR45B	25	WD repeat domain 45B	protein_codin	-0.229	0.109	1	8.003	0.09982
**EPB41L3**	EPB41L3	25	erythrocyte membrane protein band 4.1 like 3	protein_codin	3.178	0.975	1	22.13	0.1695
**SNRPD1**	SNRPD1	25	small nuclear ribonucleoprotein D1 polypeptide	protein_codin	-0.1842	0.0409	1	60.5	0.06696
**RIOK3**	RIOK3	25	RIO kinase 3	protein_codin	0.2686	0.0292	1	22.8	0.06817
**TMX3**	TMX3	25	thioredoxin related transmembrane protein 3	protein_codin	0.1143	0.436	1	18.87	0.09902
**LINC00909**	LINC00909	25	long intergenic non-protein coding RNA 909	lincRNA	0.114	0.605	1	3.579	0.4290
**ZADH2**	ZADH2	25	zinc binding alcohol dehydrogenase domain containing 2	protein_codin	0.3588	0.121	1	9.508	0.1682
**MRPS26**	MRPS26	25	mitochondrial ribosomal protein S26	protein_codin	-0.1523	0.119	1	9.713	0.2041
**PRNP**	PRNP	25	prion protein	protein_codin	0.2682	0.00167	1	60.58	0.0659
**TASP1**	TASP1	25	taspase 1	protein_codin	-0.1033	0.579	1	7.216	0.2508
**SNRPB2**	SNRPB2	25	small nuclear ribonucleoprotein polypeptide B2	protein_codin	0.07937	0.398	1	25.62	0.2052
**DTD1**	DTD1	25	D-aminoacyl-tRNA deacylase 1	protein_codin	-0.1068	0.499	1	7.707	0.1361
**ERGIC3**	ERGIC3	25	ERGIC and golgi 3	protein_codin	-0.01505	0.881	1	20.9	0.2034
**DOK5**	DOK5	25	docking protein 5	protein_codin	-0.1599	0.429	1	53.74	0.2762
**ADRM1**	ADRM1	25	adhesion regulating molecule 1	protein_codin	-0.1312	0.16	1	16.25	0.03244
**CNN2**	CNN2	25	calponin 2	protein_codin	-0.4012	0.00238	1	29.28	0.2469
**ZNF846**	ZNF846	25	zinc finger protein 846	protein_codin	-0.07498	0.729	1	3.048	0.2474
**TIMM29**	TIMM29	25	translocase of inner mitochondrial membrane 29	protein_codin	0.06456	0.724	1	3.187	0.211
**FARSA**	FARSA	25	phenylalanyl-tRNA synthetase subunit alpha	protein_codin	-0.04725	0.812	1	3.502	0.1644
**ILVBL**	ILVBL	25	ilvB acetolactate synthase like	protein_codin	0.06836	0.711	1	8.426	0.2870
**AP1M1**	AP1M1	25	adaptor related protein complex 1 subunit mu 1	protein_codin	0.5153	0.0132	1	8.693	0.1412
**ABHD8**	ABHD8	25	abhydrolase domain containing 8	protein_codin	0.1953	0.446	1	2.923	0.1655
**ZNF430**	ZNF430	25	zinc finger protein 430	protein_codin	0.2974	0.213	1	3.057	0.3201
**GPATCH1**	GPATCH1	25	G-patch domain containing 1	protein_codin	0.2264	0.41	1	3.075	0.3117
**ZNF568**	ZNF568	25	zinc finger protein 568	protein_codin	-0.4094	0.0838	1	3.026	0.2774
**DMAC2**	DMAC2	25	distal membrane arm assembly complex 2	protein_codin	0.03147	0.927	1	7.352	0.5320
**DMWD**	DMWD	25	DM1 locus, WD repeat containing	protein_codin	-0.1623	0.482	1	30.2	0.6361
**IRF2BP1**	IRF2BP1	25	interferon regulatory factor 2 binding protein 1	protein_codin	0.02706	0.895	1	8.61	0.06736
**BCAT2**	BCAT2	25	branched chain amino acid transaminase 2	protein_codin	-0.08546	0.66	1	8.557	0.2332
**RUVBL2**	RUVBL2	25	RuvB like AAA ATPase 2	protein_codin	0.4033	0.0251	1	23.61	0.1302
**ZNF83**	ZNF83	25	zinc finger protein 83	protein_codin	0.4282	0.0252	1	19.23	0.1154
**AC010642.2**	AC010642.2	25	novel transcript, antisense to ERVK3	antisense_RN	-0.1933	0.319	1	8.403	0.2179
**PEX26**	PEX26	25	peroxisomal biogenesis factor 26	protein_codin	-0.1123	0.633	1	2.585	0.4184
**COMT**	COMT	25	catechol-O-methyltransferase	protein_codin	0.3529	2.33E-06	0.05402	35.18	0.0362
**CRKL**	CRKL	25	CRK like proto-oncogene, adaptor protein	protein_codin	0.1332	0.586	1	1.723	0.4184
**SMARCB1**	SMARCB1	25	SWI/SNF related, matrix associated, actin dependent	protein_codin	0.1438	0.228	1	27.08	0.1689
**DDT**	DDT	25	D-dopachrome tautomerase	protein_codin	-0.2497	0.00210	1	57.2	0.02544
**TRIOBP**	TRIOBP	25	TRIO and F-actin binding protein	protein_codin	0.8608	6.13E-08	0.00144	60.6	0.138
**ANKRD54**	ANKRD54	25	ankyrin repeat domain 54	protein_codin	-0.1036	0.65	1	3.293	0.221
**FAM109B**	FAM109B	25	PH domain containing endocytic trafficking adaptor 2	protein_codin	NA	NA	NA	32.82	0.1306
**PPP6R2**	PPP6R2	25	protein phosphatase 6 regulatory subunit 2	protein_codin	0.178	0.388	1	3.114	0.5187
**C21orf59**	C21orf59	25	cilia and flagella associated protein 298	protein_codin	NA	NA	NA	6.812	0.1069
**CDK11A**	CDK11A	26	cyclin dependent kinase 11A	protein_codin	0.2563	0.195	1	9.07	0.2772
**CLIC4**	CLIC4	26	chloride intracellular channel 4	protein_codin	-0.3915	6.17E-06	0.1424	39.58	0.09741
**SNHG12**	SNHG12	26	small nucleolar RNA host gene 12	antisense_RN	0.1287	0.631	1	31.37	0.2875
**MRPS15**	MRPS15	26	mitochondrial ribosomal protein S15	protein_codin	-0.2316	0.0349	1	12.08	0.1036
**YRDC**	YRDC	26	yrdC N6-threonylcarbamoyltransferase domain conta	protein_codin	-0.1805	0.448	1	7.7	0.2800
**KTI12**	KTI12	26	KTI12 chromatin associated homolog	protein_codin	0.3942	0.070	1	8.172	0.2769
**NEGR1**	NEGR1	26	neuronal growth regulator 1	protein_codin	0.6294	0.00166	1	57.32	0.1049
**F3**	F3	26	coagulation factor III, tissue factor	protein_codin	3.08	2.74E-30	6.62E-26	50.53	0.07356
**CAPZA1**	CAPZA1	26	capping actin protein of muscle Z-line subunit alpha 1	protein_codin	0.3047	0.0185	1	24.98	0.0341
**BCAS2**	BCAS2	26	BCAS2 pre-mRNA processing factor	protein_codin	0.01514	0.895	1	35.33	0.1031
**WDR3**	WDR3	26	WD repeat domain 3	protein_codin	0.2474	0.262	1	7.978	0.2104
**MRPL9**	MRPL9	26	mitochondrial ribosomal protein L9	protein_codin	0.1746	0.181	1	17.73	0.03297
**IGSF8**	IGSF8	26	immunoglobulin superfamily member 8	protein_codin	0.3166	0.0296	1	7.322	0.06754
**DDR2**	DDR2	26	discoidin domain receptor tyrosine kinase 2	protein_codin	0.9579	1.28E-08	0.0003	60	0.1464
**TOR3A**	TOR3A	26	torsin family 3 member A	protein_codin	0.2593	0.124	1	6.284	0.1151
**LPGAT1**	LPGAT1	26	lysophosphatidylglycerol acyltransferase 1	protein_codin	-0.1712	0.284	1	46.54	0.2026
**SDCCAG8**	SDCCAG8	26	serologically defined colon cancer antigen 8	protein_codin	0.5196	0.00078	1	30.26	0.21
**LCLAT1**	LCLAT1	26	lysocardiolipin acyltransferase 1	protein_codin	0.09870	0.693	1	3.503	0.1732
**FAM136A**	FAM136A	26	family with sequence similarity 136 member A	protein_codin	-0.419	0.0136	1	8.273	0.1022
**MAP4K4**	MAP4K4	26	mitogen-activated protein kinase kinase kinase kinase 4	protein_codin	-0.5277	0.00030	1	60.37	0.1022
**10-Sep**	43718	26	septin 10	protein_codin	0.07607	0.388	1	15.8	0.1007
**CCDC93**	CCDC93	26	coiled-coil domain containing 93	protein_codin	0.0265	0.888	1	3.463	0.1052
**WDR33**	WDR33	26	WD repeat domain 33	protein_codin	0.08366	0.561	1	15.82	0.06713
**ERBB4**	ERBB4	26	erb-b2 receptor tyrosine kinase 4	protein_codin	-0.06615	0.769	1	8.728	0.6504
**CYP27A1**	CYP27A1	26	cytochrome P450 family 27 subfamily A member 1	protein_codin	0.5306	0.0004	1	46.27	0.0688
**TUBA4A**	TUBA4A	26	tubulin alpha 4a	protein_codin	-0.7493	6.21E-07	0.01449	55.4	0.03263
**MRPL44**	MRPL44	26	mitochondrial ribosomal protein L44	protein_codin	-0.07897	0.636	1	3.383	0.217
**COPS9**	COPS9	26	COP9 signalosome subunit 9	protein_codin	-0.4197	1.21E-10	2.87E-06	46.15	0.03292
**CCDC174**	CCDC174	26	coiled-coil domain containing 174	protein_codin	0.2487	0.0765	1	39.46	0.1738
**ABHD5**	ABHD5	26	abhydrolase domain containing 5	protein_codin	1.006	5.18E-06	0.1197	31.77	0.1109
**ZNF445**	ZNF445	26	zinc finger protein 445	protein_codin	0.2547	0.374	1	3.166	0.358
**IP6K2**	IP6K2	26	inositol hexakisphosphate kinase 2	protein_codin	-0.04453	0.77	1	17.72	0.0661
**C3orf58**	C3orf58	26	divergent protein kinase domain 2A	protein_codin	-0.04223	0.768	1	18.68	0.2103
**DLG1**	DLG1	26	discs large MAGUK scaffold protein 1	protein_codin	0.6153	0.00325	1	25.68	0.2157
**PDS5A**	PDS5A	26	PDS5 cohesin associated factor A	protein_codin	0.3974	0.0772	1	7.629	0.2409
**LIMCH1**	LIMCH1	26	LIM and calponin homology domains 1	protein_codin	-1.019	9.25E-31	2.23E-26	59.33	0.03403
**COMMD8**	COMMD8	26	COMM domain containing 8	protein_codin	0.4456	0.00476	1	16.82	0.06886
**FAM13A**	FAM13A	26	family with sequence similarity 13 member A	protein_codin	0.817	5.35E-06	0.1236	25.35	0.1022
**INTS12**	INTS12	26	integrator complex subunit 12	protein_codin	0.01829	0.920	1	10.23	0.3106
**ANK2**	ANK2	26	ankyrin 2	protein_codin	1.945	3.77E-09	8.89E-05	47.72	0.1082
**IRF2**	IRF2	26	interferon regulatory factor 2	protein_codin	-0.09844	0.397	1	10.22	0.1035
**UFSP2**	UFSP2	26	UFM1 specific peptidase 2	protein_codin	0.001721	0.989	1	15.64	0.03621
**TRIO**	TRIO	26	trio Rho guanine nucleotide exchange factor	protein_codin	1.53	3.16E-05	0.724	53.9	0.2534
**OSMR**	OSMR	26	oncostatin M receptor	protein_codin	0.7076	1.26E-05	0.2901	17.49	0.07471
**RICTOR**	RICTOR	26	RPTOR independent companion of MTOR complex 2	protein_codin	0.389	0.0436	1	28.86	0.2768
**PPWD1**	PPWD1	26	peptidylprolyl isomerase domain and WD repeat cont	protein_codin	0.3869	0.0817	1	6.922	0.1706
**ATP6AP1L**	ATP6AP1L	26	ATPase H+ transporting accessory protein 1 like	protein_codin	-0.9713	6.08E-05	1	11.26	0.07434
**C5orf15**	C5orf15	26	chromosome 5 open reading frame 15	protein_codin	0.4977	0.00037	1	31.98	0.06449
**PCDHGA10**	PCDHGA10	26	protocadherin gamma subfamily A, 10	protein_codin	0.6177	0.0282	1	27.49	0.2081
**PWWP2A**	PWWP2A	26	PWWP domain containing 2A	protein_codin	0.2279	0.195	1	20.33	0.2066
**C6orf62**	C6orf62	26	chromosome 6 open reading frame 62	protein_codin	0.5476	0.00287	1	20.38	0.133
**C6orf89**	C6orf89	26	chromosome 6 open reading frame 89	protein_codin	0.1837	0.161	1	15.5	0.242
**DSE**	DSE	26	dermatan sulfate epimerase	protein_codin	1.355	3.93E-13	9.36E-09	30.91	0.1017
**CEP85L**	CEP85L	26	centrosomal protein 85 like	protein_codin	0.4802	0.0308	1	23.58	0.3048
**WIPI2**	WIPI2	26	WD repeat domain, phosphoinositide interacting 2	protein_codin	-0.0768	0.62	1	49.9	0.1014
**ARL4A**	ARL4A	26	ADP ribosylation factor like GTPase 4A	protein_codin	0.738	0.00420	1	33.61	0.3957
**TWISTNB**	TWISTNB	26	TWIST neighbor	protein_codin	0.05779	0.66	1	42.42	0.1392
**CCDC126**	CCDC126	26	coiled-coil domain containing 126	protein_codin	0.1518	0.5	1	3.405	0.2412
**CBX3**	CBX3	26	chromobox 3	protein_codin	-0.04313	0.644	1	26.22	0.177
**DDX56**	DDX56	26	DEAD-box helicase 56	protein_codin	0.09129	0.647	1	7.18	0.06892
**TMEM248**	TMEM248	26	transmembrane protein 248	protein_codin	0.2444	0.0247	1	31.85	0.1829
**PRKRIP1**	PRKRIP1	26	PRKR interacting protein 1	protein_codin	0.1589	0.361	1	8.205	0.3797
**CAPZA2**	CAPZA2	26	capping actin protein of muscle Z-line subunit alpha 2	protein_codin	-0.07525	0.316	1	53.82	0.03322
**NDUFB2**	NDUFB2	26	NADH:ubiquinone oxidoreductase subunit B2	protein_codin	-0.3658	7.69E-10	1.82E-05	60.58	0.03345
**ATP7A**	ATP7A	26	ATPase copper transporting alpha	protein_codin	0.5038	0.057	1	8.556	0.452
**HCFC1**	HCFC1	26	host cell factor C1	protein_codin	0.2458	0.280	1	3.302	0.1808
**MTFR1**	MTFR1	26	mitochondrial fission regulator 1	protein_codin	0.3471	0.104	1	7.698	0.0999
**RBM12B**	RBM12B	26	RNA binding motif protein 12B	protein_codin	-0.142	0.516	1	7.933	0.3571
**ZNF706**	ZNF706	26	zinc finger protein 706	protein_codin	0.08451	0.255	1	50.16	0.1662
**NRBP2**	NRBP2	26	nuclear receptor binding protein 2	protein_codin	0.5933	0.0406	1	7.971	0.3445
**BNC2**	BNC2	26	basonuclin 2	protein_codin	1.693	9.13E-07	0.02125	10.82	0.4858
**POLR1E**	POLR1E	26	RNA polymerase I subunit E	protein_codin	-0.1399	0.4	1	9.174	0.1072
**PCSK5**	PCSK5	26	proprotein convertase subtilisin/kexin type 5	protein_codin	1.229	2.4E-05	0.5508	10.24	0.3881
**ISCA1**	ISCA1	26	iron-sulfur cluster assembly 1	protein_codin	0.04556	0.67	1	38.8	0.1403
**PTGR1**	PTGR1	26	prostaglandin reductase 1	protein_codin	0.1394	0.220	1	49.64	0.1005
**PDCL**	PDCL	26	phosducin like	protein_codin	0.2598	0.0733	1	15.89	0.2392
**FAM129B**	FAM129B	26	niban apoptosis regulator 2	protein_codin	-0.8323	1.4E-05	0.3222	8.805	0.03213
**ST6GALNAC4**	ST6GALNAC4	26	ST6 N-acetylgalactosaminide alpha-2,6-sialyltransfer	protein_codin	-0.4077	0.0292	1	48.83	0.03229
**WDR34**	WDR34	26	WD repeat domain 34	protein_codin	0.09712	0.592	1	9.037	0.1003
**PNPLA2**	PNPLA2	26	patatin like phospholipase domain containing 2	protein_codin	-0.2743	0.0152	1	54.57	0.1048

[illegible]

**ITPA**	ITPA	27	inosine triphosphatase	protein_codin	-0.1607	0.255	1	7.202	0.09900
**PANK2**	PANK2	27	pantothenate kinase 2	protein_codin	0.09977	0.655	1	7.462	0.2016
**CDS2**	CDS2	27	CDP-diacylglycerol synthase 2	protein_codin	-0.008391	0.952	1	17.68	0.1022
**SNTA1**	SNTA1	27	syntrophin alpha 1	protein_codin	-0.4182	9.07E-05	1	50.95	0.06867
**ITCH**	ITCH	27	itchy E3 ubiquitin protein ligase	protein_codin	0.6152	0.00162	1	29.74	0.1702
**OSER1**	OSER1	27	oxidative stress responsive serine rich 1	protein_codin	0.01582	0.919	1	45.62	0.07
**SYS1**	SYS1	27	SYS1 golgi trafficking protein	protein_codin	0.5722	0.00038	1	17.78	0.03484
**SLC2A10**	SLC2A10	27	solute carrier family 2 member 10	protein_codin	1.046	9.98E-05	1	3.185	0.2047
**ADNP**	ADNP	27	activity dependent neuroprotector homeobox	protein_codin	0.2506	0.0696	1	37.98	0.1682
**LSM14B**	LSM14B	27	LSM family member 14B	protein_codin	0.143	0.518	1	3.405	0.1061
**LAMA5**	LAMA5	27	laminin subunit alpha 5	protein_codin	-0.6971	0.00148	1	10.43	0.4234
**LSM7.00**	LSM7.00	27	LSM7 homolog, U6 small nuclear RNA and mRNA de	protein_codin	-0.389	1.17E-06	0.0272	52.64	0.2381
**SLC39A3**	SLC39A3	27	solute carrier family 39 member 3	protein_codin	-0.1732	0.262	1	21.79	0.06758
**SGTA**	SGTA	27	small glutamine rich tetratricopeptide repeat containin	protein_codin	-0.2316	0.197	1	9.675	0.1682
**FBXL12**	FBXL12	27	F-box and leucine rich repeat protein 12	protein_codin	-0.06553	0.681	1	7.795	0.3531
**PPAN**	PPAN	27	peter pan homolog	protein_codin	-0.1082	0.572	1	3.254	0.1408
**ZNF441**	ZNF441	27	zinc finger protein 441	protein_codin	-0.2036	0.41	1	2.608	0.2112
**GCDH**	GCDH	27	glutaryl-CoA dehydrogenase	protein_codin	-0.4494	0.0727	1	3.392	0.1215
**AC020916.1**	AC020916.1	27	novel transcri	lincRNA	1.076	0.0001	1	60.6	0.4787
**ARRDC2**	ARRDC2	27	arrestin domain containing 2	protein_codin	-0.3368	0.164	1	8.8	0.1699
**NR2C2AP**	NR2C2AP	27	nuclear receptor 2C2 associated protein	protein_codin	-0.3908	0.0526	1	28.86	0.1032
**NDUFA13**	NDUFA13	27	NADH:ubiquinone oxidoreductase subunit A13	protein_codin	-0.252	0.007	1	22.03	0.1295
**PDCD5**	PDCD5	27	programmed cell death 5	protein_codin	-0.1759	0.0147	1	53.67	0.06703
**KMT2B**	KMT2B	27	lysine methyltransferase 2B	protein_codin	0.2312	0.232	1	6.772	0.1431
**CLIP3**	CLIP3	27	CAP-Gly domain containing linker protein 3	protein_codin	-0.2667	0.377	1	8.88	0.2834
**ZNF224**	ZNF224	27	zinc finger protein 224	protein_codin	0.3209	0.167	1	7.684	0.4638
**RELB**	RELB	27	RELB proto-oncogene, NF-kB subunit	protein_codin	-0.6656	0.0265	1	25.63	0.138
**AP2S1**	AP2S1	27	adaptor related protein complex 2 subunit sigma 1	protein_codin	-0.4221	9.24E-08	0.00216	58.98	0.03361
**TSEN34**	TSEN34	27	tRNA splicing endonuclease subunit 34	protein_codin	-0.2323	0.0339	1	13.85	0.1002
**HSPBP1**	HSPBP1	27	HSPA (Hsp70) binding protein 1	protein_codin	-0.1246	0.52	1	9.309	0.3216
**ZNF579**	ZNF579	27	zinc finger protein 579	protein_codin	-0.4757	0.0382	1	2.454	0.1881
**ATP6V1E1**	ATP6V1E1	27	ATPase H+ transporting V1 subunit E1	protein_codin	0.2017	0.0507	1	21.85	0.2709
**SNAP29**	SNAP29	27	synaptosome associated protein 29	protein_codin	-0.04254	0.771	1	7.556	0.2452
**UBE2L3**	UBE2L3	27	ubiquitin conjugating enzyme E2 L3	protein_codin	0.09705	0.219	1	41.32	0.03395
**PITPNB**	PITPNB	27	phosphatidylinositol transfer protein beta	protein_codin	-0.2745	0.00407	1	18.65	0.1215
**CBY1**	CBY1	27	chibby family member 1, beta catenin antagonist	protein_codin	-0.06457	0.636	1	14.94	0.1356
**ACO2**	ACO2	27	aconitase 2	protein_codin	-0.01688	0.914	1	21.34	0.06826
**C21orf91**	C21orf91	27	chromosome 21 open reading frame 91	protein_codin	0.5554	0.0152	1	7.552	0.3507
**TRAPPC10**	TRAPPC10	27	trafficking protein particle complex 10	protein_codin	0.2668	0.142	1	7.508	0.1351
**CD74**	CD74	28	CD74 molecule	protein_codin	0.7625	1.61E-06	0.03729	42.86	0.7086
**HLA-F**	HLA-F	28	major histocompatibility complex, class I, F	protein_codin	-0.1764	0.131	1	48.81	0.2411
**HLA-A**	HLA-A	28	major histocompatibility complex, class I, A	protein_codin	-0.1043	0.017	1	40.88	0.2841
**HLA-E**	HLA-E	28	major histocompatibility complex, class I, E	protein_codin	0.3465	8.03E-09	0.00019	60	0.1174
**HLA-C**	HLA-C	28	major histocompatibility complex, class I, C	protein_codin	-0.3713	1.36E-17	3.26E-13	60.58	0.282
**HLA-B**	HLA-B	28	major histocompatibility complex, class I, B	protein_codin	0.07474	0.159	1	55.32	0.328
**HLA-DRA**	HLA-DRA	28	major histocompatibility complex, class II, DR alpha	protein_codin	-0.057	0.722	1	60	0.7356
**HLA-DRB5**	HLA-DRB5	28	major histocompatibility complex, class II, DR beta 5	protein_codin	1.095	1.13E-07	0.00265	53.42	0.5926
**HLA-DRB1**	HLA-DRB1	28	major histocompatibility complex, class II, DR beta 1	protein_codin	1.179	6.22E-12	1.48E-07	60.6	0.6452
**HLA-DQA1**	HLA-DQA1	28	major histocompatibility complex, class II, DQ alpha 1	protein_codin	-0.5689	0.0245	1	60.56	0.7366
**HLA-DMA**	HLA-DMA	28	major histocompatibility complex, class II, DM alpha	protein_codin	0.2845	0.185	1	49.75	0.5412
**HLA-DPA1**	HLA-DPA1	28	major histocompatibility complex, class II, DP alpha 1	protein_codin	0.9573	3.31E-08	0.00079	60.6	0.5752
**HLA-DPB1**	HLA-DPB1	28	major histocompatibility complex, class II, DP beta 1	protein_codin	0.4434	0.0022	1	60.6	0.5917
**B2M**	B2M	28	beta-2-microglobulin	protein_codin	-0.05917	0.0718	1	NA	0.3974
**RAB31**	RAB31	28	RAB31, member RAS oncogene family	protein_codin	-0.8884	8.64E-14	2.06E-09	56.68	0.1345
**MRPL20**	MRPL20	29	mitochondrial ribosomal protein L20	protein_codin	-0.2677	6.17E-05	1	30.92	0.07564
**TCEA3**	TCEA3	29	transcription elongation factor A3	protein_codin	0.06004	0.574	1	37.99	0.03536
**MAN1C1**	MAN1C1	29	mannosidase alpha class 1C member 1	protein_codin	2.088	2.04E-15	4.87E-11	8.214	0.06652
**MACF1**	MACF1	29	microtubule actin crosslinking factor 1	protein_codin	-0.1884	0.0782	1	30.58	0.03525
**NRDC**	NRDC	29	nardilysin convertase	protein_codin	0.2297	0.0775	1	27.25	0.2815
**LRRC42**	LRRC42	29	leucine rich repeat containing 42	protein_codin	0.5948	0.00218	1	7.833	0.06527
**PTGFRN**	PTGFRN	29	prostaglandin F2 receptor inhibitor	protein_codin	1.497	4.64E-05	1	8.028	0.1730
**ACBD3**	ACBD3	29	acyl-CoA binding domain containing 3	protein_codin	0.1799	0.109	1	50.65	0.09958
**RDH14**	RDH14	29	retinol dehydrogenase 14	protein_codin	0.4255	0.00048	1	29.52	0.1102
**UBXN2A**	UBXN2A	29	UBX domain protein 2A	protein_codin	0.8973	3.29E-08	0.00077	37.47	0.1371
**B3GNT2**	B3GNT2	29	UDP-GlcNAc:betaGal beta-1,3-N-acetylglucosaminylt	protein_codin	-0.003142	0.985	1	20.28	0.1032
**ANTXR1**	ANTXR1	29	ANTXR cell adhesion molecule 1	protein_codin	-0.621	5.65E-10	1.34E-05	57.21	0.1694
**GFPT1**	GFPT1	29	glutamine--fructose-6-phosphate transaminase 1	protein_codin	0.3517	0.0308	1	42.86	0.0355
**GGCX**	GGCX	29	gamma-glutamyl carboxylase	protein_codin	1.06	0.00018	1	7.262	0.2756
**FAHD2A**	FAHD2A	29	fumarylacetoacetate hydrolase domain containing 2A	protein_codin	0.125	0.506	1	8.132	0.1368
**CCDC74A**	CCDC74A	29	coiled-coil domain containing 74A	protein_codin	0.5248	0.0515	1	9.238	0.2778
**MGAT5**	MGAT5	29	alpha-1,6-mannosylglycoprotein 6-beta-N-acetylgluc	protein_codin	-0.0551	0.794	1	9.975	0.3015
**HNMT**	HNMT	29	histamine N-methyltransferase	protein_codin	0.05489	0.64	1	59.17	0.06706
**PDHB**	PDHB	29	pyruvate dehydrogenase E1 beta subunit	protein_codin	-0.1498	0.135	1	44.86	0.06903
**NIT2**	NIT2	29	nitrilase family member 2	protein_codin	0.1806	0.188	1	39.07	0.06497
**TOMM70**	TOMM70	29	translocase of outer mitochondrial membrane 70	protein_codin	-0.2563	0.0767	1	54.15	0.1742
**FAIM**	FAIM	29	Fas apoptotic inhibitory molecule	protein_codin	-0.1887	0.208	1	20.97	0.2112
**CLDN11**	CLDN11	29	claudin 11	protein_codin	2.257	2.3E-06	0.05332	59.86	0.1897
**C3orf70**	C3orf70	29	chromosome 3 open reading frame 70	protein_codin	-0.09808	0.626	1	28.75	0.2905
**MXD4**	MXD4	29	MAX dimerization protein 4	protein_codin	-0.1671	0.121	1	28.78	0.1027
**NAAA**	NAAA	29	N-acylethanolamine acid amidase	protein_codin	0.3233	0.0352	1	37.82	0.2335
**MAPK10**	MAPK10	29	mitogen-activated protein kinase 10	protein_codin	1.129	1.3E-07	0.00304	7.289	0.1695
**NFKB1**	NFKB1	29	nuclear factor kappa B subunit 1	protein_codin	0.9217	3.87E-05	0.8857	7.995	0.209
**SPRY1**	SPRY1	29	sprouty RTK signaling antagonist 1	protein_codin	-0.4234	0.0002	1	59.88	0.07242
**SH3RF1**	SH3RF1	29	SH3 domain containing ring finger 1	protein_codin	0.3757	0.114	1	3.595	0.1744
**HAND2**	HAND2	29	heart and neural crest derivatives expressed 2	protein_codin	0.579	0.0018	1	22.56	0.06850
**AGA**	AGA	29	aspartylglucosaminidase	protein_codin	-0.03554	0.849	1	24.9	0.2022
**SDHA**	SDHA	29	succinate dehydrogenase complex flavoprotein subunit A	protein_codin	0.4236	0.0011	1	7.305	0.1352
**SGTB**	SGTB	29	small glutamine rich tetratricopeptide repeat containin	protein_codin	-0.08517	0.626	1	27.74	0.1736
**TTC37**	TTC37	29	tetratricopeptide repeat domain 37	protein_codin	0.3638	0.00579	1	30.35	0.2034
**PURA**	PURA	29	purine rich element binding protein A	protein_codin	-0.1164	0.202	1	27.22	0.1358
**PCDHGC3**	PCDHGC3	29	protocadherin gamma subfamily C, 3	protein_codin	0.52	0.0274	1	8.084	0.1359
**CLINT1**	CLINT1	29	clathrin interactor 1	protein_codin	0.4552	5.18E-05	1	52.62	0.1028
**NSD1**	NSD1	29	nuclear receptor binding SET domain protein 1	protein_codin	0.2084	0.142	1	41.98	0.3502
**CAP2**	CAP2	29	cyclase associated actin cytoskeleton regulatory prot	protein_codin	-0.1554	0.262	1	44.36	0.1304
**TDP2**	TDP2	29	tyrosyl-DNA phosphodiesterase 2	protein_codin	-0.04672	0.794	1	7.732	0.1748
**LEMD2**	LEMD2	29	LEM domain containing 2	protein_codin	0.4806	0.0651	1	8.11	0.3921
**EZR**	EZR	29	ezrin	protein_codin	-0.5075	0.0009	1	60.6	0.03397
**TBL2**	TBL2	29	transducin beta like 2	protein_codin	-0.1365	0.484	1	25.76	0.197
**CACNA2D1**	CACNA2D1	29	calcium voltage-gated channel auxiliary subunit alpha	protein_codin	-0.07219	0.675	1	7.646	0.217
**TMEM140**	TMEM140	29	transmembrane protein 140	protein_codin	0.1932	0.247	1	22.85	0.236
**FMC1**	FMC1	29	formation of mitochondrial complex V assembly facto	protein_codin	-0.1437	0.369	1	21.71	0.1484
**PIN4**	PIN4	29	peptidylprolyl cis/trans isomerase, NIMA-interacting 4	protein_codin	-0.48	1.8E-05	0.4136	12.52	0.07192
**IDS**	IDS	29	iduronate 2-sulfatase	protein_codin	0.07403	0.457	1	25.43	0.03706
**VPS37A**	VPS37A	29	VPS37A subunit of ESCRT-I	protein_codin	-0.06566	0.66	1	7.332	0.09902
**KIF13B**	KIF13B	29	kinesin family member 13B	protein_codin	-0.5663	0.00196	1	28.88	0.1357
**VDAC3**	VDAC3	29	voltage dependent anion channel 3	protein_codin	0.2542	0.00624	1	27.8	0.04142
**ZHX1**	ZHX1	29	zinc fingers and homeoboxes 1	protein_codin	-0.01689	0.904	1	47.76	0.09920
**BAG1**	BAG1	29	BCL2 associated athanogene 1	protein_codin	-0.1221	0.157	1	27.11	0.0645
**GALT**	GALT	29	galactose-1-phosphate uridylyltransferase	protein_codin	-0.03048	0.869	1	8.349	0.2730
**ALDH1A1**	ALDH1A1	29	aldehyde dehydrogenase 1 family member A1	protein_codin	2.106	1.34E-33	3.24E-29	60.6	0.112
**MFSD14B**	MFSD14B	29	major facilitator superfamily domain containing 14B	protein_codin	0.8823	0.00080	1	9.222	0.3092
**HABP4**	HABP4	29	hyaluronan binding protein 4	protein_codin	-0.5488	3.63E-06	0.084	34.86	0.1007
**SMC2**	SMC2	29	structural maintenance of chromosomes 2	protein_codin	-0.11	0.492	1	53.1	0.1322
**FKBP15**	FKBP15	29	FKBP prolyl isomerase 15	protein_codin	0.004167	0.985	1	3.55	0.1734
**GTF3C5**	GTF3C5	29	general transcription factor IIIC subunit 5	protein_codin	-0.05296	0.819	1	2.865	0.3176
**GATD1**	GATD1	29	glutamine amidotransferase like class 1 domain conta	protein_codin	-0.009814	0.962	1	3.415	0.1998
**ZFP91**	ZFP91	29	ZFP91 zinc finger protein	protein_codin	-0.4118	0.0646	1	8.132	0.2004
**SLC3A2**	SLC3A2	29	solute carrier family 3 member 2	protein_codin	0.1845	0.0792	1	42.02	0.1625
**ATL3**	ATL3	29	atlastin GTPase 3	protein_codin	0.1096	0.312	1	54.29	0.2102
**PRDX5**	PRDX5	29	peroxiredoxin 5	protein_codin	-0.1079	0.055	1	60.6	0.02262
**BAMBI**	BAMBI	29	BMP and activin membrane bound inhibitor	protein_codin	-0.07062	0.76	1	59.85	0.2785
**TET1**	TET1	29	tet methylcytosine dioxygenase 1	protein_codin	1.375	8.9E-07	0.02072	10.06	0.3125
**RBP4**	RBP4	29	retinol binding protein 4	protein_codin	3.624	7.98E-10	1.89E-05	59.94	0.1067
**ENTPD1**	ENTPD1	29	ectonucleoside triphosphate diphosphohydrolase 1	protein_codin	-0.02958	0.917	1	29.92	0.284
**NT5C2**	NT5C2	29	5'-nucleotidase, cytosolic II	protein_codin	-0.1851	0.452	1	8.994	0.3504
**CALHM2**	CALHM2	29	calcium homeostasis modulator family member 2	protein_codin	0.5326	2.35E-05	0.5394	30.89	0.06471
**GFRA1**	GFRA1	29	GDNF family receptor alpha 1	protein_codin	2.923	2.46E-09	5.8E-05	48.52	0.2079
**MCMBP**	MCMBP	29	minichromosome maintenance complex binding protein	protein_codin	0.4696	0.0542	1	3.131	0.2461
**BORCS5**	BORCS5	29	BLOC-1 related complex subunit 5	protein_codin	0.1234	0.627	1	3.299	0.1850
**PPFIBP1**	PPFIBP1	29	PPFIA binding protein 1	protein_codin	-0.1546	0.102	1	46.27	0.1357
**RASSF3**	RASSF3	29	Ras association domain family member 3	protein_codin	-0.5748	0.00018	1	60.6	0.03232
**TMBIM4**	TMBIM4	29	transmembrane BAX inhibitor motif containing 4	protein_codin	0.301	7.18E-08	0.00166	49.42	0.07172
**ZDHHC17**	ZDHHC17	29	zinc finger DHHC-type containing 17	protein_codin	0.1579	0.402	1	54.15	0.1096
**ACACB**	ACACB	29	acetyl-CoA carboxylase beta	protein_codin	1.009	6.84E-05	1	3.341	0.3612
**MMAB**	MMAB	29	metabolism of cobalamin associated B	protein_codin	0.2536	0.136	1	6.432	0.1032
**DENR**	DENR	29	density regulated re-initiation and release factor	protein_codin	-0.1032	0.41	1	32.53	0.1339
**CCDC122**	CCDC122	29	coiled-coil domain containing 122	protein_codin	0.1436	0.632	1	9.521	0.2444
**CUL4A**	CUL4A	29	cullin 4A	protein_codin	0.05831	0.764	1	7.537	0.1037
**CCNB1IP1**	CCNB1IP1	29	cyclin B1 interacting protein 1	protein_codin	0.1671	0.29	1	17.02	0.3152
**TINF2**	TINF2	29	TERF1 interacting nuclear factor 2	protein_codin	-0.06908	0.621	1	48.38	0.1694
**ERO1A**	ERO1A	29	endoplasmic reticulum oxidoreductase 1 alpha	protein_codin	-0.4337	0.0136	1	26.67	0.2062
**ACYP1**	ACYP1	29	acylphosphatase 1	protein_codin	0.8596	1.77E-06	0.04109	51.74	0.1377
**ZNF106**	ZNF106	29	zinc finger protein 106	protein_codin	0.2958	0.00407	1	48.26	0.1302
**ADAM10**	ADAM10	29	ADAM metallopeptidase domain 10	protein_codin	-0.05085	0.79	1	3.392	0.1716
**CRABP1**	CRABP1	29	cellular retinoic acid binding protein 1	protein_codin	1.969	0.00011	1	32.78	0.4541
**SMARCE1**	SMARCE1	29	SWI/SNF related, matrix associated, actin dependent	protein_codin	0.2741	0.039	1	7.392	0.1812
**SMURF2**	SMURF2	29	SMAD specific E3 ubiquitin protein ligase 2	protein_codin	0.3362	0.0675	1	24.37	0.2036
**GNA13**	GNA13	29	G protein subunit alpha 13	protein_codin	0.04916	0.814	1	8.518	0.1396
**JMJD6**	JMJD6	29	jumonji domain containing 6, arginine demethylase a	protein_codin	0.1418	0.42	1	9.884	0.2024
**EMILIN2**	EMILIN2	29	elastin microfibril interfacer 2	protein_codin	3.257	1.38E-07	0.00322	49.66	0.07134
**NAPG**	NAPG	29	NSF attachment protein gamma	protein_codin	0.1542	0.249	1	31.58	0.1066
**ZNF532**	ZNF532	29	zinc finger protein 532	protein_codin	0.07726	0.664	1	19.64	0.2862
**PCNA**	PCNA	29	proliferating cell nuclear antigen	protein_codin	0.09804	0.469	1	55.16	0.03848
**C19orf24**	C19orf24	29	family with sequence similarity 174 member C	protein_codin	-0.4268	0.00162	1	17.17	0.03869
**RAB11B**	RAB11B	29	RAB11B, member RAS oncogene family	protein_codin	0.0745	0.485	1	25.37	0.1677
**ZNF414**	ZNF414	29	zinc finger protein 414	protein_codin	0.2036	0.352	1	3.363	0.1019
**AKAP8**	AKAP8	29	A-kinase anchoring protein 8	protein_codin	0.3372	0.0982	1	3.216	0.1763
**GTPBP3**	GTPBP3	29	GTP binding protein 3, mitochondrial	protein_codin	0.04284	0.872	1	2.383	0.2815
**PAF1**	PAF1	29	PAF1 homolog, Paf1/RNA polymerase II complex co	protein_codin	0.2949	0.0882	1	22.13	0.2770
**TEAD2**	TEAD2	29	TEA domain transcription factor 2	protein_codin	-0.004232	0.982	1	9.306	0.1797
**TNRC6B**	TNRC6B	29	trinucleotide repeat containing adaptor 6B	protein_codin	0.4038	0.00014	1	53.53	0.1540
**ARFGAP3**	ARFGAP3	29	ADP ribosylation factor GTPase activating protein 3	protein_codin	-0.04643	0.622	1	23.61	0.1406
**FBXO44**	FBXO44	30	F-box protein 44	protein_codin	0.4716	0.046	1	7.955	0.06946
**FABP3**	FABP3	30	fatty acid binding protein 3	protein_codin	0.1238	0.499	1	60.6	0.2590
**CTPS1**	CTPS1	30	CTP synthase 1	protein_codin	-0.5145	0.00526	1	7.932	0.1787
**PLPP3**	PLPP3	30	phospholipid phosphatase 3	protein_codin	1.881	1.47E-37	3.56E-33	60.6	0.07268
**ZZZ3**	ZZZ3	30	zinc finger ZZ-type containing 3	protein_codin	NA	NA	NA	6.876	0.02396
**TGFBR3**	TGFBR3	30	transforming growth factor beta receptor 3	protein_codin	2.241	4.71E-20	1.13E-15	60.49	0.1637
**RTCA**	RTCA	30	RNA 3'-terminal phosphate cyclase	protein_codin	0.006076	0.977	1	3.497	0.2840
**CSF1**	CSF1	30	colony stimulating factor 1	protein_codin	1.439	2.55E-14	6.08E-10	33.71	0.1076
**LAMTOR5**	LAMTOR5	30	late endosomal/lysosomal adaptor, MAPK and MTOR	protein_codin	-0.06828	0.339	1	44.53	0.06853
**ST7L**	ST7L	30	suppression of tumorigenicity 7 like	protein_codin	0.1886	0.304	1	3.288	0.2179
**ADAMTSL4**	ADAMTSL4	30	ADAMTS like 4	protein_codin	2.295	5.4E-12	1.28E-07	26.93	0.07719
**CKS1B**	CKS1B	30	CDC28 protein kinase regulatory subunit 1B	protein_codin	-0.1558	0.259	1	50.45	0.03302
**GBA**	GBA	30	glucosylceramidase beta	protein_codin	0.4127	0.0664	1	3.688	0.1731
**CADM3**	CADM3	30	cell adhesion molecule 3	protein_codin	3.314	5.18E-07	0.01206	47.12	0.1517
**LINC01133**	LINC01133	30	long intergenic non-protein coding RNA 1133	lincRNA	2.945	7.79E-15	1.86E-10	33.21	0.1681
**SDHC**	SDHC	30	succinate dehydrogenase complex subunit C	protein_codin	0.08382	0.34	1	31.54	0.0706
**PRG4**	PRG4	30	proteoglycan 4	protein_codin	5.665	0.0781	1	60.6	0.4234
**UCHL5**	UCHL5	30	ubiquitin C-terminal hydrolase L5	protein_codin	0.05641	0.714	1	23.98	0.1682
**CD55**	CD55	30	CD55 molecule (Cromer blood group)	protein_codin	1.486	5.09E-16	1.22E-11	60.6	0.2808
**HSD11B1**	HSD11B1	30	hydroxysteroid 11-beta dehydrogenase 1	protein_codin	3.01	3.67E-20	8.81E-16	60.58	0.03298
**NSL1**	NSL1	30	NSL1 component of MIS12 kinetochore complex	protein_codin	0.1728	0.169	1	14.41	0.2392
**ERO1B**	ERO1B	30	endoplasmic reticulum oxidoreductase 1 beta	protein_codin	0.3809	0.101	1	2.963	0.1004
**COX20**	COX20	30	cytochrome c oxidase assembly factor COX20	protein_codin	-0.4028	5.09E-05	1	39.12	0.1352
**KIDINS220**	KIDINS220	30	kinase D interacting substrate 220	protein_codin	0.3275	0.00663	1	27.43	0.1707
**SLC35F6**	SLC35F6	30	solute carrier family 35 member F6	protein_codin	0.8895	2.51E-05	0.5757	8.698	0.1006
**CEBPZOS**	CEBPZOS	30	CEBPZ opposite strand	protein_codin	0.08026	0.526	1	16.2	0.2415
**SLC8A1**	SLC8A1	30	solute carrier family 8 member A1	protein_codin	-0.4512	0.0276	1	8.186	0.4497
**UXS1**	UXS1	30	UDP-glucuronate decarboxylase 1	protein_codin	0.4217	0.0227	1	24.21	0.1008
**GPC1**	GPC1	30	glypican 1	protein_codin	-0.9344	3.18E-08	0.00075	32.97	0.2049
**FBLN2**	FBLN2	30	fibulin 2	protein_codin	3.218	1.8E-82	4.37E-78	60.6	0.0347
**MRPS25**	MRPS25	30	mitochondrial ribosomal protein S25	protein_codin	0.5294	0.00377	1	7.663	0.06752
**ACAA1**	ACAA1	30	acetyl-CoA acyltransferase 1	protein_codin	-0.2548	0.0488	1	16.16	0.07038
**SNRK**	SNRK	30	SNF related kinase	protein_codin	0.1753	0.512	1	53.8	0.3469
**SEMA3B**	SEMA3B	30	semaphorin 3B	protein_codin	2.464	2.86E-19	6.86E-14	60.42	0.1845
**FLNB**	FLNB	30	filamin B	protein_codin	1.187	2.3E-06	0.05322	22.6	0.1367
**DNASE1L3**	DNASE1L3	30	deoxyribonuclease 1 like 3	protein_codin	2.961	5.4E-05	1	59.93	0.2176
**FSTL1**	FSTL1	30	follistatin like 1	protein_codin	1.617	2E-47	4.85E-43	60.6	0.07810
**DTX3L**	DTX3L	30	deltex E3 ubiquitin ligase 3L	protein_codin	0.593	0.017	1	9.996	0.1432
**AMOTL2**	AMOTL2	30	angiomotin like 2	protein_codin	1.512	4.35E-08	0.00102	48.47	0.1792
**PCOLCE2**	PCOLCE2	30	procollagen C-endopeptidase enhancer 2	protein_codin	2.055	6.31E-40	1.53E-35	60.6	0.2687
**HTRA3**	HTRA3	30	HtrA serine peptidase 3	protein_codin	3.953	1.07E-09	2.53E-05	60	0.3171
**RAB28**	RAB28	30	RAB28, member RAS oncogene family	protein_codin	0.6048	0.00136	1	6.465	0.03806
**UGDH**	UGDH	30	UDP-glucose 6-dehydrogenase	protein_codin	1.482	2.97E-20	7.13E-16	41.77	0.03895
**TLR3**	TLR3	30	toll like receptor 3	protein_codin	0.7422	0.00227	1	7.419	0.1996
**LPCAT1**	LPCAT1	30	lysophosphatidylcholine acyltransferase 1	protein_codin	0.7499	0.0112	1	8.344	0.2247
**C1QTNF3**	C1QTNF3	30	C1q and TNF related 3	protein_codin	3.575	5.16E-09	0.00012	60.6	0.3030
**SETD9**	SETD9	30	SET domain containing 9	protein_codin	-0.2642	0.189	1	7.294	0.2130
**LHFPL2**	LHFPL2	30	LHFPL tetraspan subfamily member 2	protein_codin	-0.1729	0.503	1	56.01	0.2137
**DIAPH1**	DIAPH1	30	diaphanous related formin 1	protein_codin	0.2179	0.219	1	8.228	0.1144
**SLIT3**	SLIT3	30	slit guidance ligand 3	protein_codin	2.006	5.13E-37	1.24E-32	47.67	0.06461
**TNXB**	TNXB	30	tenascin XB	protein_codin	3.308	1.91E-44	4.63E-40	59.86	0.1541
**SNRPC**	SNRPC	30	small nuclear ribonucleoprotein polypeptide C	protein_codin	-0.09927	0.302	1	25.53	0.03468
**PI16**	PI16	30	peptidase inhibitor 16	protein_codin	6.018	4.76E-18	1.14E-13	60.6	0.2222
**NT5E**	NT5E	30	5'-nucleotidase ecto	protein_codin	1.073	1.22E-13	2.91E-09	41.82	0.07296
**MARCKS**	MARCKS	30	myristoylated alanine rich protein kinase C substrate	protein_codin	-0.07745	0.375	1	55.36	0.07540
**LTV1**	LTV1	30	LTV1 ribosome biogenesis factor	protein_codin	0.2629	0.132	1	8.355	0.109
**RAB32**	RAB32	30	RAB32, member RAS oncogene family	protein_codin	1.008	1.35E-17	3.23E-13	55.18	0.06922
**AKAP12**	AKAP12	30	A-kinase anchoring protein 12	protein_codin	1.27	1.26E-26	3.04E-22	60.6	0.0387
**GTF2H5**	GTF2H5	30	general transcription factor IIH subunit 5	protein_codin	-0.002341	0.981	1	51.63	0.2035
**UBE2D4**	UBE2D4	30	ubiquitin conjugating enzyme E2 D4 (putative)	protein_codin	-0.0435	0.786	1	7.938	0.06921
**SEMA3C**	SEMA3C	30	semaphorin 3C	protein_codin	3.647	5.18E-25	1.25E-20	32.57	0.3087
**TRIP6**	TRIP6	30	thyroid hormone receptor interactor 6	protein_codin	0.1088	0.264	1	21.29	0.2717
**GPM6B**	GPM6B	30	glycoprotein M6B	protein_codin	-0.5624	0.00044	1	33.58	0.02538
**EBF2**	EBF2	30	EBF transcription factor 2	protein_codin	1.51	4.18E-08	0.00099	11.5	0.2117
**PPP2R2A**	PPP2R2A	30	protein phosphatase 2 regulatory subunit Balpha	protein_codin	0.7564	1.38E-06	0.00032	39.15	0.1016
**FGFR1**	FGFR1	30	fibroblast growth factor receptor 1	protein_codin	0.7963	8.52E-15	2.03E-10	53.96	0.03303
**PLEKHA2**	PLEKHA2	30	pleckstrin homology domain containing A2	protein_codin	1.21	6.28E-05	1	3.342	0.4387
**PLAT**	PLAT	30	plasminogen activator, tissue type	protein_codin	3.753	1.36E-37	3.29E-33	60.6	0.1127
**HOOK3**	HOOK3	30	hook microtubule tethering protein 3	protein_codin	0.4302	7.68E-05	1	34.07	0.0389
**SGK3**	SGK3	30	serum/glucocorticoid regulated kinase family member 3	protein_codin	-0.4111	0.0826	1	7.571	0.1676
**DPY19L4**	DPY19L4	30	dpy-19 like 4	protein_codin	0.6189	0.00382	1	26.53	0.1427
**ZNF34**	ZNF34	30	zinc finger protein 34	protein_codin	0.1563	0.484	1	7.595	0.2687
**SMC5**	SMC5	30	structural maintenance of chromosomes 5	protein_codin	0.256	0.0744	1	13.81	0.2075
**TSTD2**	TSTD2	30	thiosulfate sulfurtransferase like domain containing 2	protein_codin	0.3258	0.129	1	7.979	0.2832
**DPM2**	DPM2	30	dolichyl-phosphate mannosyltransferase subunit 2, re	protein_codin	-0.08053	0.572	1	7.462	0.2769
**PTGES**	PTGES	30	prostaglandin E synthase	protein_codin	2.523	4.87E-06	0.1126	27.42	0.2361
**MRPS2**	MRPS2	30	mitochondrial ribosomal protein S2	protein_codin	-0.06808	0.64	1	20.87	0.03212
**DEAF1**	DEAF1	30	DEAF1 transcription factor	protein_codin	0.09449	0.609	1	9.558	0.1005
**CD248**	CD248	30	CD248 molecule	protein_codin	3.522	7.77E-30	1.88E-25	50.93	0.1088
**MRPL48**	MRPL48	30	mitochondrial ribosomal protein L48	protein_codin	0.1528	0.425	1	5.803	0.1346
**TSKU**	TSKU	30	tsukushi, small leucine rich proteoglycan	protein_codin	2.46	9.56E-20	2.3E-15	58.7	0.1416
**BACE1**	BACE1	30	beta-secretase 1	protein_codin	0.4617	0.0196	1	8.905	0.1422
**NTM**	NTM	30	neurotrimin	protein_codin	2.874	3.29E-07	0.00768	59.84	0.3074
**CCNY**	CCNY	30	cyclin Y	protein_codin	0.005134	0.972	1	18.17	0.1896
**NOC3L**	NOC3L	30	NOC3 like DNA replication regulator	protein_codin	0.009292	0.952	1	13.32	0.0686
**NHLRC2**	NHLRC2	30	NHL repeat containing 2	protein_codin	0.01579	0.935	1	6.662	0.2
**ITFG2**	ITFG2	30	integrin alpha FG-GAP repeat containing 2	protein_codin	0.8011	0.00073	1	6.726	0.213
**COPS7A**	COPS7A	30	COP9 signalosome subunit 7A	protein_codin	0.1967	0.144	1	37.84	0.2441
**MFAP5**	MFAP5	30	microfibril associated protein 5	protein_codin	6.056	3.61E-24	8.7E-20	60.6	0.1092
**PLBD1**	PLBD1	30	phospholipase B domain containing 1	protein_codin	3.099	1.26E-25	3.04E-21	39.7	0.07060
**SLC16A7**	SLC16A7	30	solute carrier family 16 member 7	protein_codin	1.546	5.61E-14	1.34E-09	59.28	0.1006
**NCOR2**	NCOR2	30	nuclear receptor corepressor 2	protein_codin	0.6408	0.00303	1	21.98	0.09968
**DCLK1**	DCLK1	30	doublecortin like kinase 1	protein_codin	2.16	2.09E-16	5E-12	59.46	0.07040
**EXOSC8**	EXOSC8	30	exosome component 8	protein_codin	0.3769	0.0032	1	14.11	0.1680
**TM9SF2**	TM9SF2	30	transmembrane 9 superfamily member 2	protein_codin	0.2987	0.00833	1	41.24	0.04015
**ING1**	ING1	30	inhibitor of growth family member 1	protein_codin	0.1469	0.358	1	17.58	0.1082
**F10**	F10	30	coagulation factor X	protein_codin	3.032	2.95E-58	7.16E-54	34.86	0.07720
**CHAMP1**	CHAMP1	30	chromosome alignment maintaining phosphoprotein 1	protein_codin	-0.000537	0.998	1	7.275	0.107
**FAM177A1**	FAM177A1	30	family with sequence similarity 177 member A1	protein_codin	-0.5069	1.3E-09	3.07E-05	41.2	0.09928
**TIMM9**	TIMM9	30	translocase of inner mitochondrial membrane 9	protein_codin	-0.171	0.254	1	6.531	0.2389
**NUMB**	NUMB	30	NUMB endocytic adaptor protein	protein_codin	0.2069	0.284	1	5.796	0.1342
**FLRT2**	FLRT2	30	fibronectin leucine rich transmembrane protein 2	protein_codin	2.593	2.92E-19	7.01E-15	27.63	0.1754
**FOXN3**	FOXN3	30	forkhead box N3	protein_codin	-0.008640	0.951	1	32.02	0.1721
**CHP1**	CHP1	30	calcineurin like EF-hand protein 1	protein_codin	0.4188	0.00359	1	32.58	0.1066
**FBN1**	FBN1	30	fibrillin 1	protein_codin	2.766	2.86E-45	6.93E-41	60.6	0.4022
**FAM96A**	FAM96A	30	cytosolic iron-sulfur assembly component 2A	protein_codin	0.4558	0.015	1	8.871	0.06797
**LOXL1**	LOXL1	30	lysyl oxidase like 1	protein_codin	0.2978	0.0212	1	60.6	0.07205
**COMMD4**	COMMD4	30	COMM domain containing 4	protein_codin	-0.02242	0.854	1	55.58	0.1691
**FAHD1**	FAHD1	30	fumarylacetoacetate hydrolase domain containing 1	protein_codin	0.1205	0.494	1	8.73	0.1972
**PPL**	PPL	30	periplakin	protein_codin	1.374	2.46E-06	0.05702	51.59	0.1372
**FOPNL**	FOPNL	30	FGFR1OP N-terminal like	protein_codin	-0.09926	0.66	1	8.419	0.1649
**IQCK**	IQCK	30	IQ motif containing K	protein_codin	0.9294	4.53E-05	1	24.62	0.1987
**GPRC5B**	GPRC5B	30	G protein-coupled receptor class C group 5 member B	protein_codin	2.423	3.41E-06	0.07894	49.94	0.222
**TMEM159**	TMEM159	30	transmembrane protein 159	protein_codin	-0.4465	3.22E-06	0.07456	17.52	0.1384
**CMTM3**	CMTM3	30	CKLF like MARVEL transmembrane domain containin	protein_codin	0.7211	4.44E-07	0.01030	47.62	0.1060
**GFOD2**	GFOD2	30	glucose-fructose oxidoreductase domain containing 2	protein_codin	-0.1163	0.647	1	2.755	0.3020
**THAP11**	THAP11	30	THAP domain containing 11	protein_codin	-0.07372	0.593	1	7.869	0.07112
**DDX19A**	DDX19A	30	DEAD-box helicase 19A	protein_codin	0.2776	0.231	1	8.132	0.4582
**PHF23**	PHF23	30	PHD finger protein 23	protein_codin	-0.2247	0.332	1	31.73	0.06435
**TMEM107**	TMEM107	30	transmembrane protein 107	protein_codin	0.481	0.00496	1	7.878	0.1037
**USP22**	USP22	30	ubiquitin specific peptidase 22	protein_codin	-0.0969	0.519	1	7.127	0.1372
**C17orf51**	C17orf51	30	long intergenic non-protein coding RNA 2693	protein_codin	-0.03347	0.872	1	8.708	0.3126
**NSF**	NSF	30	N-ethylmaleimide sensitive factor, vesicle fusing ATPase	protein_codin	-0.166	0.536	1	8.685	0.3531
**C17orf58**	C17orf58	30	chromosome 17 open reading frame 58	protein_codin	2.835	1.69E-23	4.07E-19	60.6	0.08190
**C17orf80**	C17orf80	30	chromosome 17 open reading frame 80	protein_codin	0.2226	0.372	1	8.664	0.2079
**METRNL**	METRNL	30	meteorin like, glial cell differentiation regulator	protein_codin	1.261	1.66E-20	3.99E-16	54.12	0.04644
**ATP9B**	ATP9B	30	ATPase phospholipid transporting 9B (putative)	protein_codin	-0.203	0.479	1	2.979	0.3868
**NAA20**	NAA20	30	N(alpha)-acetyltransferase 20, NatB catalytic subunit	protein_codin	-0.1583	0.0919	1	49.22	0.1366
**PDRG1**	PDRG1	30	p53 and DNA damage regulated 1	protein_codin	0.2178	0.199	1	7.708	0.1725
**LPIN3**	LPIN3	30	lipin 3	protein_codin	0.8577	0.0007	1	8.01	0.1394
**MATN4**	MATN4	30	matrilin 4	protein_codin	1.596	0.00800	1	48.21	0.195
**ARFGAP1**	ARFGAP1	30	ADP ribosylation factor GTPase activating protein 1	protein_codin	0.5513	0.0359	1	2.006	0.2179
**ZNF557**	ZNF557	30	zinc finger protein 557	protein_codin	-0.1348	0.647	1	3.419	0.3579
**TMEM205**	TMEM205	30	transmembrane protein 205	protein_codin	0.05124	0.632	1	53.01	0.07245
**PRKCSH**	PRKCSH	30	protein kinase C substrate 80K-H	protein_codin	0.1309	0.389	1	16.67	0.1412
**GIPC1**	GIPC1	30	GIPC PDZ domain containing family member 1	protein_codin	-0.02941	0.82	1	17.01	0.06754
**LTBP4**	LTBP4	30	latent transforming growth factor beta binding protein 4	protein_codin	1.568	1.41E-70	3.43E-66	60.6	0.04006
**CYTH2**	CYTH2	30	cytohesin 2	protein_codin	-0.195	0.111	1	37.28	0.06605
**VRK3**	VRK3	30	VRK serine/threonine kinase 3	protein_codin	0.6553	0.00538	1	8.177	0.3411
**DESI1**	DESI1	30	desumoylating isopeptidase 1	protein_codin	-0.4617	0.0292	1	9.03	0.1017
**ADAMTS5**	ADAMTS5	30	ADAM metallopeptidase with thrombospondin type 1	protein_codin	2.508	2.33E-05	0.5349	59.05	1.158
**PCNT**	PCNT	30	pericentrin	protein_codin	0.1447	0.498	1	3.401	0.4553
**ACOT7**	ACOT7	31	acyl-CoA thioesterase 7	protein_codin	-0.4372	0.0722	1	60.56	0.2395
**CAMTA1**	CAMTA1	31	calmodulin binding transcription activator 1	protein_codin	-0.3297	1.57E-06	0.03646	42.48	0.09982
**FBLIM1**	FBLIM1	31	filamin binding LIM protein 1	protein_codin	-1.154	4.61E-42	1.12E-37	42.63	0.03202
**STMN1**	STMN1	31	stathmin 1	protein_codin	-0.644	0.00096	1	60.6	0.2668
**ARID1A**	ARID1A	31	AT-rich interaction domain 1A	protein_codin	0.05506	0.777	1	27.96	0.3539
**GJA4**	GJA4	31	gap junction protein alpha 4	protein_codin	0.1772	0.272	1	38.68	0.2947
**HEYL**	HEYL	31	hes related family bHLH transcription factor with YRP	protein_codin	-0.1578	0.577	1	7.93	0.2831
**PPIE**	PPIE	31	peptidylprolyl isomerase E	protein_codin	-0.22	0.0622	1	15.68	0.1026
**RLF**	RLF	31	rearranged L-myc fusion	protein_codin	0.122	0.502	1	28.13	0.1052
**MRPL37**	MRPL37	31	mitochondrial ribosomal protein L37	protein_codin	-0.1143	0.459	1	6.854	0.1324
**JUN**	JUN	31	Jun proto-oncogene, AP-1 transcription factor subunit	protein_codin	1.458	4.5E-89	1.09E-84	55.36	0.4916
**DNAJB4**	DNAJB4	31	DnaJ heat shock protein family (Hsp40) member B4	protein_codin	0.1932	0.0926	1	60.6	0.2685
**CYR61**	CYR61	31	cellular communication network factor 1	protein_codin	0.3154	1.37E-05	0.3153	60.6	0.1579

LMO4	LMO4	31	LIM domain only 4	protein_codin	0.2399	0.0165	1	60.6	0.07309
GTF2B	GTF2B	31	general transcription factor IIB	protein_codin	0.3525	0.00912	1	59.96	0.102
TMED5	TMED5	31	transmembrane p24 trafficking protein 5	protein_codin	0.5806	0.00042	1	47.35	0.03967
FNBP1L	FNBP1L	31	formin binding protein 1 like	protein_codin	0.3347	0.0992	1	9.72	0.2747
ATP5F1	ATP5F1	31	ATP synthase peripheral stalk-membrane subunit b	protein_codin	NA	NA	NA	18.02	0.03679
LINC00623	LINC00623	31	long intergenic non-protein coding RNA 623	lincRNA	-1.004	2.31E-07	0.0054	9.115	0.1319
PLEKHO1	PLEKHO1	31	pleckstrin homology domain containing O1	protein_codin	-0.3442	0.016	1	38.79	0.1012
ENSA	ENSA	31	endosulfine alpha	protein_codin	-0.04383	0.593	1	12.37	0.1011
ADAMTS4	ADAMTS4	31	ADAM metallopeptidase with thrombospondin type 1	protein_codin	0.8694	0.00208	1	60.53	0.6162
POGK	POGK	31	pogo transposable element derived with KRAB domai	protein_codin	-0.09782	0.654	1	2.99	0.2161
RASAL2	RASAL2	31	RAS protein activator like 2	protein_codin	-0.0947	0.608	1	8.829	0.1397
RGS16	RGS16	31	regulator of G protein signaling 16	protein_codin	0.06419	0.709	1	55.38	0.2629
BTG2	BTG2	31	BTG anti-proliferation factor 2	protein_codin	2.2	1.31E-72	3.18E-68	60	0.2201
PLXNA2	PLXNA2	31	plexin A2	protein_codin	-0.6633	0.00726	1	9.924	0.1019
ATF3	ATF3	31	activating transcription factor 3	protein_codin	0.9658	6.56E-17	1.57E-12	60.6	0.1108
SRP9	SRP9	31	signal recognition particle 9	protein_codin	-0.02044	0.752	1	56.07	0.02365
RHOB	RHOB	31	ras homolog family member B	protein_codin	1.285	8.15E-46	1.98E-42	60.6	0.07176
BABAM2	BABAM2	31	BRISC and BRCA1 A complex member 2	protein_codin	0.1614	0.372	1	8.738	0.1412
PREPL	PREPL	31	prolyl endopeptidase like	protein_codin	-0.07722	0.58	1	15.34	0.1018
MTIF2	MTIF2	31	mitochondrial translational initiation factor 2	protein_codin	0.3019	0.137	1	3.527	0.02239
PAPOLG	PAPOLG	31	poly(A) polymerase gamma	protein_codin	0.5693	0.0151	1	10.1	0.1434
PNO1	PNO1	31	partner of NOB1 homolog	protein_codin	0.07726	0.648	1	8.996	0.1752
CNRIP1	CNRIP1	31	cannabinoid receptor interacting protein 1	protein_codin	0.09507	0.49	1	58.53	0.1739
SPR	SPR	31	sepiapterin reductase	protein_codin	0.3632	0.0889	1	10.01	0.1812
ANKRD39	ANKRD39	31	ankyrin repeat domain 39	protein_codin	0.1306	0.522	1	6.554	0.266
TXNDC9	TXNDC9	31	thioredoxin domain containing 9	protein_codin	0.1523	0.272	1	57.58	0.1026
FHL2	FHL2	31	four and a half LIM domains 2	protein_codin	-0.537	9.44E-06	0.2175	42.86	0.04649
CBWD2	CBWD2	31	COBW domain containing 2	protein_codin	0.1436	0.428	1	3.048	0.06771
MZT2B	MZT2B	31	mitotic spindle organizing protein 2B	protein_codin	-0.4519	2.78E-19	6.67E-15	54.57	0.02354
NR4A2	NR4A2	31	nuclear receptor subfamily 4 group A member 2	protein_codin	1.251	1.92E-08	0.00045	60.32	0.1185
PRKRA	PRKRA	31	protein activator of interferon induced protein kinase	protein_codin	-0.1153	0.332	1	56.62	0.1022
KCTD18	KCTD18	31	potassium channel tetramerization domain containing 18	protein_codin	0.2243	0.424	1	3.18	0.4094
CREB1	CREB1	31	cAMP responsive element binding protein 1	protein_codin	-0.2685	0.0492	1	18.75	0.2706
AAMP	AAMP	31	angio associated migratory cell protein	protein_codin	0.01501	0.892	1	14.79	0.09977
BCS1L	BCS1L	31	BCS1 homolog, ubiquinol-cytochrome c reductase co	protein_codin	-0.177	0.389	1	3.551	0.189
RAF1	RAF1	31	Raf-1 proto-oncogene, serine/threonine kinase	protein_codin	0.2383	0.238	1	7.671	0.176
UBE2E1	UBE2E1	31	ubiquitin conjugating enzyme E2 E1	protein_codin	0.08539	0.577	1	20.47	0.2197
CSRNP1	CSRNP1	31	cysteine and serine rich nuclear protein 1	protein_codin	1.427	6.64E-17	1.58E-12	20.83	0.1087
USP4	USP4	31	ubiquitin specific peptidase 4	protein_codin	-0.109	0.614	1	3.635	0.2081
GNAI2	GNAI2	31	G protein subunit alpha i2	protein_codin	-0.3635	1.63E-07	0.00381	60.6	0.03190
SLMAP	SLMAP	31	sarcolemma associated protein	protein_codin	-0.7453	5.6E-09	0.00013	58.61	0.2208
LRIG1	LRIG1	31	leucine rich repeats and immunoglobulin like domains 1	protein_codin	-0.6995	0.00036	1	9.852	0.2112
EOGT	EOGT	31	EGF domain specific O-linked N-acetylglucosamine tr	protein_codin	-0.9093	5.57E-06	0.1287	7.898	0.2122
ZNF654	ZNF654	31	zinc finger protein 654	protein_codin	-0.1119	0.646	1	8.86	0.2002
EPHA3	EPHA3	31	EPH receptor A3	protein_codin	0.1504	0.351	1	42.17	0.1088
RIOX2	RIOX2	31	ribosomal oxygenase 2	protein_codin	0.3413	0.161	1	9.174	0.2341
FILIP1L	FILIP1L	31	filamin A interacting protein 1 like	protein_codin	-0.1852	0.0179	1	60.6	0.2238
NEPRO	NEPRO	31	nucleolus and neural progenitor protein	protein_codin	0.2503	0.195	1	8.206	0.2525
BOC	BOC	31	BOC cell adhesion associated, oncogene regulated	protein_codin	2.903	1.16E-10	2.75E-06	51.79	0.2404
HACD2	HACD2	31	3-hydroxyacyl-CoA dehydratase 2	protein_codin	-0.1108	0.544	1	9.233	0.1316
CCNL1	CCNL1	31	cyclin L1	protein_codin	1.368	9.49E-22	2.28E-17	27	0.2330
PPM1L	PPM1L	31	protein phosphatase, Mg2+/Mn2+ dependent 1L	protein_codin	-0.6737	0.0009	1	10.35	0.2075
SERPINI1	SERPINI1	31	serpin family I member 1	protein_codin	0.4525	0.012	1	60.38	0.1815
MRPL47	MRPL47	31	mitochondrial ribosomal protein L47	protein_codin	-0.3682	0.00164	1	17.06	0.1712
BST1	BST1	31	bone marrow stromal cell antigen 1	protein_codin	2.091	1.98E-22	4.77E-18	27.18	0.1349
PDGFRA	PDGFRA	31	platelet derived growth factor receptor alpha	protein_codin	0.7005	2.63E-09	6.2E-05	60.19	0.0641
GRSF1	GRSF1	31	G-rich RNA sequence binding factor 1	protein_codin	-0.2798	0.052	1	7.766	0.1402
H2AFZ	H2AFZ	31	H2A histone family member Z	protein_codin	-0.3617	5.72E-09	0.00013	60.6	0.04829
C4orf32	C4orf32	31	family with sequence similarity 241 member A	protein_codin	NA	NA	NA	60.6	0.3591
SH3D19	SH3D19	31	SH3 domain containing 19	protein_codin	0.9147	1.75E-09	4.13E-05	52.88	0.0650
SORBS2	SORBS2	31	sorbin and SH3 domain containing 2	protein_codin	0.03592	0.766	1	60.46	0.02388
MIR4458HG	MIR4458HG	31	MIR4458 host gene	lincRNA	0.07605	0.771	1	7.908	0.2090
BASP1	BASP1	31	brain abundant membrane attached signal protein 1	protein_codin	0.2464	0.0418	1	60	0.03259
NADK2	NADK2	31	NAD kinase 2, mitochondrial	protein_codin	-0.1615	0.509	1	8.204	0.2414
DIMT1	DIMT1	31	DIMT1 rRNA methyltransferase and ribosome matura	protein_codin	0.1768	0.242	1	20.4	0.1720
ERBIN	ERBIN	31	erbb2 interacting protein	protein_codin	-0.02262	0.906	1	8.435	0.2330
CCNH	CCNH	31	cyclin H	protein_codin	0.7577	8.23E-07	0.01916	32.58	0.3187
RIOK2	RIOK2	31	RIO kinase 2	protein_codin	0.2568	0.222	1	8.321	0.09942
EGR1	EGR1	31	early growth response 1	protein_codin	1.829	4.55E-92	1.11E-87	60.6	0.3652
FGF1	FGF1	31	fibroblast growth factor 1	protein_codin	-0.8037	0.0005	1	26.97	0.1470
DUSP1	DUSP1	31	dual specificity phosphatase 1	protein_codin	0.08354	0.124	1	60.6	0.1132
RIOK1	RIOK1	31	RIO kinase 1	protein_codin	0.2986	0.0754	1	41.83	0.06644
DTNBP1	DTNBP1	31	dystrobrevin binding protein 1	protein_codin	-0.3364	0.0262	1	8.51	0.0674
KDM1B	KDM1B	31	lysine demethylase 1B	protein_codin	0.2298	0.34	1	8.933	0.3432
SOX4	SOX4	31	SRY-box transcription factor 4	protein_codin	-0.2886	0.0306	1	60.54	0.07584
BTN2A2	BTN2A2	31	butyrophilin subfamily 2 member A2	protein_codin	0.1136	0.542	1	11.33	0.1321
TUBB	TUBB	31	tubulin beta class I	protein_codin	-0.04375	0.458	1	60.6	0.1408
HSPA1A	HSPA1A	31	heat shock protein family A (Hsp70) member 1A	protein_codin	0.5835	2.08E-08	0.00049	Inf	0.4560
HSPA1B	HSPA1B	31	heat shock protein family A (Hsp70) member 1B	protein_codin	0.7358	2.86E-12	6.8E-08	60.6	0.4981
RNF5	RNF5	31	ring finger protein 5	protein_codin	-0.06658	0.564	1	13.83	0.1012
TAPBP	TAPBP	31	TAP binding protein	protein_codin	0.07125	0.535	1	41.28	0.2412
CDKN1A	CDKN1A	31	cyclin dependent kinase inhibitor 1A	protein_codin	1.078	3.05E-14	7.27E-10	60.6	0.1617
AKIRIN2	AKIRIN2	31	akirin 2	protein_codin	0.387	0.0207	1	22.68	0.06679
TRAF3IP2-AS1	TRAF3IP2-AS1	31	TRAF3IP2 antisense RNA 1	antisense_RN	0.6444	0.00446	1	8.498	0.2808
NT5DC1	NT5DC1	31	5'-nucleotidase domain containing 1	protein_codin	-0.3153	0.00523	1	49.08	0.09932
HEY2	HEY2	31	hes related family bHLH transcription factor with YRP	protein_codin	-0.5217	1.61E-05	0.3703	33.03	0.1317
CTGF	CTGF	31	cellular communication network factor 2	protein_codin	-0.1651	0.035	1	NA	0.1519
SASH1	SASH1	31	SAM and SH3 domain containing 1	protein_codin	0.3372	0.0477	1	52.98	0.09912
FOXK1	FOXK1	31	forkhead box K1	protein_codin	0.2321	0.359	1	3.18	0.1742
IL6	IL6	31	interleukin 6	protein_codin	0.9313	0.086	1	40.88	0.6849
CYCS	CYCS	31	cytochrome c, somatic	protein_codin	0.4393	9.88E-05	1	60.6	0.4901
JAZF1	JAZF1	31	JAZF zinc finger 1	protein_codin	0.3975	0.0733	1	25.44	0.1416
H2AFV	H2AFV	31	H2A histone family member V	protein_codin	-0.215	0.0242	1	60.6	0.2205
TSC22D4	TSC22D4	31	TSC22 domain family member 4	protein_codin	0.297	0.122	1	6.555	0.06580
IFT22	IFT22	31	intraflagellar transport 22	protein_codin	0.09111	0.475	1	15.75	0.03218
CCDC71L	CCDC71L	31	coiled-coil domain containing 71 like	protein_codin	0.0985	0.479	1	60.58	0.3445
IMMP2L	IMMP2L	31	inner mitochondrial membrane peptidase subunit 2	protein_codin	0.1214	0.42	1	18.64	0.1324
HILPDA	HILPDA	31	hypoxia inducible lipid droplet associated	protein_codin	0.626	0.00300	1	59.63	0.1488
MKLN1	MKLN1	31	muskelin 1	protein_codin	0.001094	0.996	1	23.28	0.1702
TMUB1	TMUB1	31	transmembrane and ubiquitin like domain containing 1	protein_codin	-0.06352	0.754	1	6.702	0.128
CHPF2	CHPF2	31	chondroitin polymerizing factor 2	protein_codin	0.2788	0.202	1	9.734	0.1238
RHEB	RHEB	31	Ras homolog, mTORC1 binding	protein_codin	-0.01907	0.808	1	60.58	0.1026
WWC3	WWC3	31	WWC family member 3	protein_codin	0.1828	0.359	1	25.61	0.2131
ARHGAP6	ARHGAP6	31	Rho GTPase activating protein 6	protein_codin	0.1269	0.479	1	18.34	0.2598
HSD17B10	HSD17B10	31	hydroxysteroid 17-beta dehydrogenase 10	protein_codin	-0.3109	0.00147	1	22.66	0.1047
HUWE1	HUWE1	31	HECT, UBA and WWE domain containing E3 ubiquiti	protein_codin	0.306	0.0562	1	22.06	0.06784
SNX12	SNX12	31	sorting nexin 12	protein_codin	0.001476	0.999	1	2.966	0.2661
TCEAL7	TCEAL7	31	transcription elongation factor A like 7	protein_codin	0.9207	7.27E-06	0.1678	49.58	0.2161
PGRMC1	PGRMC1	31	progesterone receptor membrane component 1	protein_codin	-0.04585	0.472	1	51.99	0.1412
AGPAT5	AGPAT5	31	1-acylglycerol-3-phosphate O-acyltransferase 5	protein_codin	0.004132	0.985	1	8.333	0.2850
PLPBP	PLPBP	31	pyridoxal phosphate binding protein	protein_codin	0.09934	0.486	1	15.49	0.1396
CEBPD	CEBPD	31	CCAAT enhancer binding protein delta	protein_codin	1.472	4.67E-64	1.13E-59	60.6	0.2284
C8orf59	C8orf59	31	ribosomal biogenesis factor	protein_codin	-0.1598	0.0256	1	37.23	0.1352
YWHAZ	YWHAZ	31	tyrosine 3-monooxygenase/tryptophan 5-monooxyger	protein_codin	0.1747	0.00623	1	34.77	0.06641
KLF10	KLF10	31	Kruppel like factor 10	protein_codin	0.7538	6.55E-07	0.01527	29.01	0.169
MYC	MYC	31	MYC proto-oncogene, bHLH transcription factor	protein_codin	1.699	1.43E-23	3.44E-19	60.6	0.2914
FAM49B	FAM49B	31	family with sequence similarity 49 member B	protein_codin	-0.4694	0.0606	1	2.992	0.3909
NAPRT	NAPRT	31	nicotinate phosphoribosyltransferase	protein_codin	0.09920	0.626	1	6.235	0.1357
IFT74	IFT74	31	intraflagellar transport 74	protein_codin	0.1058	0.525	1	16.93	0.1002
RPP25L	RPP25L	31	ribonuclease P/MRP subunit p25 like	protein_codin	-0.05960	0.725	1	8.435	0.2750
HRCT1	HRCT1	31	histidine rich carboxyl terminus 1	protein_codin	-0.4706	0.00572	1	60.47	0.1198
RFK	RFK	31	riboflavin kinase	protein_codin	0.3007	0.0505	1	39.76	0.03244
PRUNE2	PRUNE2	31	prune homolog 2 with BCH domain	protein_codin	-1.572	2.6E-16	6.22E-12	36.88	0.3032
TLE1	TLE1	31	TLE family member 1, transcriptional corepressor	protein_codin	0.306	0.0766	1	22.55	0.1352
GADD45G	GADD45G	31	growth arrest and DNA damage inducible gamma	protein_codin	0.1854	0.271	1	60.6	0.1774
FAM120A	FAM120A	31	family with sequence similarity 120A	protein_codin	-0.06514	0.672	1	19.2	0.06799
ANP32B	ANP32B	31	acidic nuclear phosphoprotein 32 family member B	protein_codin	-0.4299	3.12E-10	7.38E-06	55.29	0.1012
MSANTD3	MSANTD3	31	Myb/SANT DNA binding domain containing 3	protein_codin	-0.283	0.135	1	52.8	0.06534
TMEM38B	TMEM38B	31	transmembrane protein 38B	protein_codin	0.406	0.0461	1	59.55	0.265
KLF4	KLF4	31	Kruppel like factor 4	protein_codin	2.678	1.59E-42	3.85E-38	59.34	0.06576
SLC25A25	SLC25A25	31	solute carrier family 25 member 25	protein_codin	0.2392	0.303	1	30.55	0.2482
TOR1A	TOR1A	31	torsin family 1 member A	protein_codin	-0.02283	0.882	1	8.419	0.1044
SNHG7	SNHG7	31	small nucleolar RNA host gene 7	antisense_RN	-0.04848	0.604	1	39.25	0.07342
SSNA1	SSNA1	31	SS nuclear autoantigen 1	protein_codin	-0.2749	0.00369	1	52.07	0.03222
ARRDC1-AS1	ARRDC1-AS1	31	ARRDC1 antisense RNA 1	antisense_RN	0.02261	0.934	1	2.904	0.352
DUSP8	DUSP8	31	dual specificity phosphatase 8	protein_codin	0.08029	0.735	1	32.97	0.2759
DENND5A	DENND5A	31	DENN domain containing 5A	protein_codin	0.4266	0.127	1	7.932	0.3361
USP47	USP47	31	ubiquitin specific peptidase 47	protein_codin	0.0753	0.616	1	16.95	0.2794
C11orf96	C11orf96	31	chromosome 11 open reading frame 96	protein_codin	0.2964	0.00050	1	Inf	0.1326
UBE2L6	UBE2L6	31	ubiquitin conjugating enzyme E2 L6	protein_codin	-0.06704	0.559	1	54.71	0.1677
ZFPL1	ZFPL1	31	zinc finger protein like 1	protein_codin	0.07472	0.624	1	48.46	0.2464
AP002884.3	AP002884.3	31	long intergenic non-protein coding RNA 2762	lincRNA	2.08E-05	0.994	1	21.93	0.1744
NNMT	NNMT	31	nicotinamide N-methyltransferase	protein_codin	0.08879	0.129	1	60.54	0.03345
UBE4A	UBE4A	31	ubiquitination factor E4A	protein_codin	0.4565	0.021	1	8.494	0.1407
HSPA8	HSPA8	31	heat shock protein family A (Hsp70) member 8	protein_codin	-0.00027	0.998	1	55.38	0.03454
SPA17	SPA17	31	sperm autoantigenic protein 17	protein_codin	0.04616	0.827	1	31.13	0.2067
KLF6	KLF6	31	Kruppel like factor 6	protein_codin	0.1258	0.129	1	60.54	0.03995
RBM17	RBM17	31	RNA binding motif protein 17	protein_codin	-0.1597	0.138	1	25.62	0.138
UPF2	UPF2	31	UPF2 regulator of nonsense mediated mRNA decay	protein_codin	-0.09077	0.539	1	14.02	0.2379
CREM	CREM	31	cAMP responsive element modulator	protein_codin	-0.1044	0.519	1	45.34	0.09037
CAMK2G	CAMK2G	31	calcium/calmodulin dependent protein kinase II gamma	protein_codin	-0.7227	7.56E-06	0.1744	16.33	0.1381
ZMIZ1	ZMIZ1	31	zinc finger MIZ-type containing 1	protein_codin	0.9616	1.63E-05	0.3749	24.2	0.2405
BMPR1A	BMPR1A	31	bone morphogenetic protein receptor type 1A	protein_codin	0.1913	0.316	1	8.642	0.1747
CH25H	CH25H	31	cholesterol 25-hydroxylase	protein_codin	1.921	0.00080	1	60.6	1.03
TRIM8	TRIM8	31	tripartite motif containing 8	protein_codin	0.6286	4.85E-05	1	60.6	0.1391
APOLD1	APOLD1	31	apolipoprotein L domain containing 1	protein_codin	2.269	2.27E-08	0.00052	55.38	0.7049
GPRC5A	GPRC5A	31	G protein-coupled receptor class C group 5 member A	protein_codin	1.677	1.91E-12	4.54E-08	39.38	0.2058
RERGL	RERGL	31	RERG like	protein_codin	1.145	5.08E-09	0.00012	60.6	0.3821
SOX5	SOX5	31	SRY-box transcription factor 5	protein_codin	-0.1797	0.352	1	26.46	0.4279
STK38L	STK38L	31	serine/threonine kinase 38 like	protein_codin	-0.3328	0.0238	1	60.58	0.1874
SENP1	SENP1	31	SUMO specific peptidase 1	protein_codin	0.2914	0.207	1	3.527	0.217
TUBA1B	TUBA1B	31	tubulin alpha 1b	protein_codin	-0.01086	0.82	1	60.6	0.1085
TUBA1A	TUBA1A	31	tubulin alpha 1a	protein_codin	0.2456	0.0003	1	60.6	0.1101
PRPH	PRPH	31	peripherin	protein_codin	2.121	3.55E-05	0.8129	60.6	0.7650
ATF1	ATF1	31	activating transcription factor 1	protein_codin	0.3306	0.0352	1	18.95	0.09855
NR4A1	NR4A1	31	nuclear receptor subfamily 4 group A member 1	protein_codin	2.234	2.28E-49	5.53E-45	60.6	0.2032
RARG	RARG	31	retinoic acid receptor gamma	protein_codin	0.9652	1.53E-06	0.03554	29.57	0.1668
CALCOCO1	CALCOCO1	31	calcium binding and coiled-coil domain 1	protein_codin	0.1998	0.191	1	32.35	0.06542
CS	CS	31	citrate synthase	protein_codin	0.0004761	0.999	1	2.886	0.3876
DCTN2	DCTN2	31	dynactin subunit 2	protein_codin	0.05352	0.498	1	21.69	0.03345
GNS	GNS	31	glucosamine (N-acetyl)-6-sulfatase	protein_codin	0.831	2.9E-06	0.06719	19.65	0.1010
CSRP2	CSRP2	31	cysteine and glycine rich protein 2	protein_codin	-0.5608	5.43E-09	0.00013	55.38	0.1412
PPP1R12A	PPP1R12A	31	protein phosphatase 1 regulatory subunit 12A	protein_codin	-0.2662	0.00010	1	42.86	0.03285
DUSP6	DUSP6	31	dual specificity phosphatase 6	protein_codin	-0.3316	0.0858	1	57.7	0.2097
C12orf75	C12orf75	31	chromosome 12 open reading frame 75	protein_codin	-0.7123	3.64E-22	8.76E-18	54.61	0.03438
CKAP4	CKAP4	31	cytoskeleton associated protein 4	protein_codin	0.3996	0.00441	1	30.69	0.07662
FICD	FICD	31	FIC domain containing	protein_codin	0.2214	0.396	1	10.06	0.3147
VSIG10	VSIG10	31	V-set and immunoglobulin domain containing 10	protein_codin	0.004837	0.981	1	3.356	0.1779
GTF2H3	GTF2H3	31	general transcription factor IIH subunit 3	protein_codin	0.3122	0.0669	1	23.82	0.2786
ANKLE2	ANKLE2	31	ankyrin repeat and LEM domain containing 2	protein_codin	0.0747	0.646	1	47.55	0.2024
HSPH1	HSPH1	31	heat shock protein family H (Hsp110) member 1	protein_codin	0.09872	0.552	1	41.95	0.2025
NUDT15	NUDT15	31	nudix hydrolase 15	protein_codin	0.1798	0.387	1	30.02	0.1706
STK24	STK24	31	serine/threonine kinase 24	protein_codin	0.4882	0.00288	1	7.222	0.2182
ANKRD10	ANKRD10	31	ankyrin repeat domain 10	protein_codin	0.1163	0.374	1	56.22	0.1376
C14orf119	C14orf119	31	chromosome 14 open reading frame 119	protein_codin	0.2814	0.0129	1	13.65	0.07147
EXOC5	EXOC5	31	exocyst complex component 5	protein_codin	0.301	0.115	1	25.62	0.1361
ZFP36L1	ZFP36L1	31	ZFP36 ring finger protein like 1	protein_codin	0.6318	2.81E-14	6.7E-10	54.68	0.1364
MED6	MED6	31	mediator complex subunit 6	protein_codin	0.1366	0.37	1	6.808	0.09857
FOS	FOS	31	Fos proto-oncogene, AP-1 transcription factor subunit	protein_codin	1.275	1.57E-87	3.82E-83	Inf	0.4750
SNW1	SNW1	31	SNW domain containing 1	protein_codin	0.1168	0.266	1	16.13	0.03220
CLMN	CLMN	31	calmin	protein_codin	0.8397	0.0002	1	59.87	0.3049
HSP90AA1	HSP90AA1	31	heat shock protein 90 alpha family class A member 1	protein_codin	0.02166	0.588	1	60.6	0.1391
THBS1	THBS1	31	thrombospondin 1	protein_codin	1.651	1.56E-12	3.71E-08	59.93	0.1332
MFAP1	MFAP1	31	microfibril associated protein 1	protein_codin	-0.05145	0.692	1	16.6	0.2064
HERC1	HERC1	31	HECT and RLD domain containing E3 ubiquitin prote	protein_codin	0.2988	0.179	1	7.065	0.2158
RBPMS2	RBPMS2	31	RNA binding protein, mRNA processing factor 2	protein_codin	1.593	2.62E-10	6.2E-06	41.75	0.07636
RASL12	RASL12	31	RAS like family 12	protein_codin	-0.2617	0.0857	1	45.77	0.1466
PDCD7	PDCD7	31	programmed cell death 7	protein_codin	-0.006662	0.958	1	11.84	0.2026
TRAF7	TRAF7	31	TNF receptor associated factor 7	protein_codin	0.6262	0.00241	1	32.79	0.1080
TNFRSF12A	TNFRSF12A	31	TNF receptor superfamily member 12A	protein_codin	-0.251	0.0216	1	55.33	0.07288
NDUFAB1	NDUFAB1	31	NADH:ubiquinone oxidoreductase subunit AB1	protein_codin	-0.1003	0.102	1	38.58	0.06558
YPEL3	YPEL3	31	yippee like 3	protein_codin	0.05106	0.486	1	35.05	0.03308
VPS35	VPS35	31	VPS35 retromer complex component	protein_codin	0.2337	0.0116	1	36.8	0.107
MT2A	MT2A	31	metallothionein 2A	protein_codin	0.1896	0.0441	1	Inf	0.4230
MT1E	MT1E	31	metallothionein 1E	protein_codin	0.1237	0.181	1	54.82	0.3348
MT1M	MT1M	31	metallothionein 1M	protein_codin	0.9913	8.44E-16	2.02E-12	60.6	0.4410
MT1A	MT1A	31	metallothionein 1A	protein_codin	2.111	8.42E-17	2.01E-12	55.3	0.501
MT1F	MT1F	31	metallothionein 1F	protein_codin	-0.2175	0.294	1	55.22	0.3
MT1G	MT1G	31	metallothionein 1G	protein_codin	1.183	0.0199	1	60.53	0.4250
MT1X	MT1X	31	metallothionein 1X	protein_codin	0.03497	0.7315	1	60.6	0.3812
CPNE2	CPNE2	31	copine 2	protein_codin	-0.3423	0.0822	1	28.72	0.2061
DOK4	DOK4	31	docking protein 4	protein_codin	-0.02133	0.92	1	8.606	0.3094
ATP6V0D1	ATP6V0D1	31	ATPase H+ transporting V0 subunit d1	protein_codin	-0.04725	0.709	1	35.2	0.1346
CFDP1	CFDP1	31	craniofacial development protein 1	protein_codin	0.1713	0.0836	1	28.22	0.3052
CRISPLD2	CRISPLD2	31	cysteine rich secretory protein LCCL domain containi	protein_codin	2.456	1.45E-19	3.48E-15	54.2	0.06786
FOXC2	FOXC2	31	forkhead box C2	protein_codin	-0.5749	5.87E-08	0.00138	59.88	0.1337
MIR22HG	MIR22HG	31	MIR22 host gene	lincRNA	0.9824	4.19E-10	9.91E-06	45.6	0.1067
PAFAH1B1	PAFAH1B1	31	platelet activating factor acetylhydrolase 1b regulatory	protein_codin	0.2002	0.0382	1	59.93	0.06849
NAA38	NAA38	31	N(alpha)-acetyltransferase 38, NatC auxiliary subunit	protein_codin	-0.07103	0.396	1	44.22	0.03219
PIGL	PIGL	31	phosphatidylinositol glycan anchor biosynthesis class	protein_codin	0.3604	0.193	1	3.21	0.2770
MPRIP	MPRIP	31	myosin phosphatase Rho interacting protein	protein_codin	0.06694	0.616	1	14.08	0.2520
NF1	NF1	31	neurofibromin 1	protein_codin	0.3267	0.0677	1	20.85	0.1332
PSMD11	PSMD11	31	proteasome 26S subunit, non-ATPase 11	protein_codin	-0.02519	0.831	1	13.73	0.03550
CISD3	CISD3	31	CDGSH iron sulfur domain 3	protein_codin	-0.312	0.00622	1	13.94	0.2730
ORMDL3	ORMDL3	31	ORMDL sphingolipid biosynthesis regulator 3	protein_codin	-0.4502	0.099	1	2.846	0.1757
TBX2-AS1	TBX2-AS1	31	TBX2 antisense RNA 1	antisense_RN	0.306	0.076	1	58.6	0.1830
CDC42EP4	CDC42EP4	31	CDC42 effector protein 4	protein_codin	0.432	0.0288	1	19.95	0.2082
SAP30BP	SAP30BP	31	SAP30 binding protein	protein_codin	0.3035	0.0242	1	15	0.1066
WBP2	WBP2	31	WW domain binding protein 2	protein_codin	-0.05526	0.624	1	14.26	0.2095
SOCS3	SOCS3	31	suppressor of cytokine signaling 3	protein_codin	0.8744	2.57E-16	6.15E-12	58.72	0.1372
TYMS	TYMS	31	thymidylate synthetase	protein_codin	-0.1423	0.569	1	60.56	0.282
MYOM1	MYOM1	31	myomesin 1	protein_codin	0.2371	0.302	1	53.86	0.2412
RPRD1A	RPRD1A	31	regulation of nuclear pre-mRNA domain containing 1A	protein_codin	-0.2893	0.135	1	3.256	0.236
MANBAL	MANBAL	31	mannosidase beta like	protein_codin	-0.1556	0.187	1	14.7	0.06559
TGM2	TGM2	31	transglutaminase 2	protein_codin	-1.376	9.12E-24	2.2E-19	60.6	0.06782
MTG2	MTG2	31	mitochondrial ribosome associated GTPase 2	protein_codin	0.3076	0.16	1	3.393	0.2837
PCMTD2	PCMTD2	31	protein-L-isoaspartate (D-aspartate) O-methyltransfer	protein_codin	0.2411	0.15	1	9.475	0.1788
TMEM259	TMEM259	31	transmembrane protein 259	protein_codin	0.3017	0.0359	1	16.08	0.1010
MIDN	MIDN	31	midnolin	protein_codin	0.6432	0.00018	1	53.77	0.1015
GADD45B	GADD45B	31	growth arrest and DNA damage inducible beta	protein_codin	1.307	7.27E-25	1.75E-20	Inf	0.268
NFIC	NFIC	31	nuclear factor I C	protein_codin	0.1549	0.0175	1	33.04	0.03223
EEF2	EEF2	31	eukaryotic translation elongation factor 2	protein_codin	-0.09097	0.056	1	55.3	0.0726
SNAPC2	SNAPC2	31	small nuclear RNA activating complex polypeptide 2	protein_codin	0.0492	0.775	1	21.4	0.2754
YIPF2	YIPF2	31	Yip1 domain family member 2	protein_codin	-0.2033	0.0805	1	24.35	0.03252
JUNB	JUNB	31	JunB proto-oncogene, AP-1 transcription factor subun	protein_codin	1.624	########	2.8E-97	60.6	0.3150
IER2	IER2	31	immediate early response 2	protein_codin	1.553	3.19E-70	7.75E-66	60	0.2726
DNAJB1	DNAJB1	31	DnaJ heat shock protein family (Hsp40) member B1	protein_codin	0.7792	1.21E-09	2.86E-05	60.58	0.3934
KLF2	KLF2	31	Kruppel like factor 2	protein_codin	1.398	3.56E-54	8.64E-50	60.6	0.2508
JUND	JUND	31	JunD proto-oncogene, AP-1 transcription factor subun	protein_codin	1.241	3.1E-101	7.54E-97	60.6	0.2268
GDF15	GDF15	31	growth differentiation factor 15	protein_codin	0.8913	0.00253	1	59.36	0.2726
WTIP	WTIP	31	WT1 interacting protein	protein_codin	0.3179	0.0201	1	60.58	0.04718
SDHAF1	SDHAF1	31	succinate dehydrogenase complex assembly factor 1	protein_codin	-0.1937	0.329	1	6.707	0.1989
ZFP36	ZFP36	31	ZFP36 ring finger protein	protein_codin	1.89	1.3E-103	3.16E-99	60.6	0.3902
SERTAD1	SERTAD1	31	SERTA domain containing 1	protein_codin	0.7511	3.49E-13	8.31E-09	46.08	0.06744
SERTAD3	SERTAD3	31	SERTA domain containing 3	protein_codin	0.3322	0.0286	1	53.43	0.1977
FOSB	FOSB	31	FosB proto-oncogene, AP-1 transcription factor subun	protein_codin	1.623	7.42E-36	1.8E-31	50.53	0.4529
SNRPD2	SNRPD2	31	small nuclear ribonucleoprotein D2 polypeptide	protein_codin	-0.266	1.58E-06	0.03669	60	0.09906
PPP1R15A	PPP1R15A	31	protein phosphatase 1 regulatory subunit 15A	protein_codin	1.055	3.2E-36	7.74E-32	60.54	0.06465
ZNF331	ZNF331	31	zinc finger protein 331	protein_codin	0.6072	0.00498	1	42.6	0.4530
MYADM	MYADM	31	myeloid associated differentiation marker	protein_codin	1.032	1.03E-29	2.49E-25	60	0.0402
SLC27A5	SLC27A5	31	solute carrier family 27 member 5	protein_codin	-0.05162	0.798	1	9.143	0.1411
KREMEN1	KREMEN1	31	kringle containing transmembrane protein 1	protein_codin	1.642	4.67E-09	0.00011	10.7	0.09802
MAFF	MAFF	31	MAF bZIP transcription factor F	protein_codin	1.59	7.83E-25	1.89E-20	40.88	0.03462
CYB5R3	CYB5R3	31	cytochrome b5 reductase 3	protein_codin	-0.04158	0.405	1	55.36	0.03394
APP	APP	31	amyloid beta precursor protein	protein_codin	1.005	6.48E-36	1.57E-31	55.32	0.03529
ADAMTS1	ADAMTS1	31	ADAM metallopeptidase with thrombospondin type 1	protein_codin	0.4065	0.0001	1	60.54	0.1981
LTN1	LTN1	31	listerin E3 ubiquitin protein ligase 1	protein_codin	-0.2042	0.161	1	15.96	0.07410
BACH1	BACH1	31	BTB domain and CNC homolog 1	protein_codin	0.1429	0.452	1	20.62	0.1962
CRYZL1	CRYZL1	31	crystallin zeta like 1	protein_codin	-0.128	0.391	1	17.78	0.2447
RCAN1	RCAN1	31	regulator of calcineurin 1	protein_codin	0.1453	0.417	1	42.8	0.4457
MCM3AP	MCM3AP	31	minichromosome maintenance complex component 3	protein_codin	0.09461	0.751	1	8.966	0.3918
