# Supplementary material for: Decoding the transcriptome of calcified atherosclerotic plaque at single-cell resolution
Source: Commun Biol. 2022 Oct 12;5:1084. doi: 10.1038/s42003-022-04056-7 (PMC9556750; doi:10.1038/s42003-022-04056-7)
Supplement: Supplementary file 15 — Supplementary Data 13 [file 42003_2022_4056_MOESM15_ESM.pdf]

STable20.EC.VSMC.Heatmap.Group.Abunds

		AC Abundance Ratic	Cell Count		
**EC Heatmap Groups**	Group1	0.084615385	390		
	Group2	0.675675676	259		
	Group3	0.967871486	249		
**VSMC Heatmap Groups**	Group1	0.007889546	507		
	Group2	0.413080895	581		
	Group3	0.717850288	521		
	Group4	0.722819594	837		
