# Supplementary material for: Decoding the transcriptome of calcified atherosclerotic plaque at single-cell resolution
Source: Commun Biol. 2022 Oct 12;5:1084. doi: 10.1038/s42003-022-04056-7 (PMC9556750; doi:10.1038/s42003-022-04056-7)
Supplement: Supplementary file 16 — Supplementary Data 14 [file 42003_2022_4056_MOESM16_ESM.pdf]

STable18.EC.Group.Regress

gene_short_nar	num_cells_express	status	estimate	std_err	test_val	p_value	normalized_effe	term	model_compone	q_value	Corrected NE
ITLN1	162	OK	6.6686822	0.8064015	8.2696796	1.34E-16	6.87632714	subgrp_cor	count	1.38E-12	6.87632714
OGN	137	OK	8.6115038	1.4766115	5.8219360	5.48E-09	6.6187938	subgrp_cor	count	5.57E-05	6.6187938
SULF1	234	OK	5.1209510	0.5078327	10.083932	6.51E-24	6.13724786	subgrp_cor	count	6.69E-20	6.13724786
FN1	242	OK	4.9834762	0.4699118	10.605131	2.82E-26	6.12542231	subgrp_cor	count	2.9E-22	6.12542231
BGN	269	OK	4.6654512	0.4009258	11.636693	2.68E-31	5.9430404	subgrp_cor	count	2.76E-27	5.9430404
COL8A1	152	OK	5.2392682	0.6787989	7.7184386	1.18E-14	5.74465014	subgrp_cor	count	1.21E-10	5.74465014
IGFBP3	135	OK	4.7686151	0.5864812	8.1308918	4.26E-16	5.49542229	subgrp_cor	count	4.37E-12	5.49542229
S100A4	134	OK	4.4090237	0.5436918	8.1094160	5.09E-16	5.22866354	subgrp_cor	count	5.21E-12	5.22866354
DCN	83	OK	4.829559	0.7905958	6.1087585	1E-09	5.17913924	subgrp_cor	count	1.02E-05	5.17913924
C1S	121	OK	6.2824800	1.1353537	5.5335002	3.14E-08	5.15119011	subgrp_cor	count	0.0003182	5.15119011
SERPINE1	124	OK	4.382582	0.5766229	7.60042	2.95E-14	5.12941362	subgrp_cor	count	3.02E-10	5.12941362
GJA5	98	OK	7.1081839	1.5186219	4.6806803	2.86E-06	5.05707466	subgrp_cor	count	0.0287497	5.05707466
TMEM120A	105	OK	4.9710035	0.805825	6.1688330	6.88E-10	5.04028672	subgrp_cor	count	7.01E-06	5.04028672
CP	112	OK	4.5103779	0.6444018	6.9993249	2.57E-12	4.887455	subgrp_cor	count	2.63E-08	4.887455
FBLIM1	105	OK	4.5752028	0.7190315	6.3630074	1.98E-10	4.85564677	subgrp_cor	count	2.02E-06	4.85564677
ANXA3	79	OK	4.8952671	0.8741948	5.5997437	2.15E-08	4.85319498	subgrp_cor	count	0.0002177	4.85319498
PROCR	244	OK	3.6790273	0.3400615	10.81871	2.81E-27	4.84062191	subgrp_cor	count	2.89E-23	4.84062191
MMP2	115	OK	6.5508558	1.1994542	5.4615303	4.72E-08	4.83816280	subgrp_cor	count	0.0004783	4.83816280
MPZL2	189	OK	3.7772623	0.4264616	8.8572155	8.2E-19	4.65505023	subgrp_cor	count	8.42E-15	4.65505023
ELN	156	OK	3.6281656	0.4026854	9.0099241	2.06E-19	4.64260676	subgrp_cor	count	2.12E-15	4.64260676
FAM107B	130	OK	3.7548379	0.4895834	7.6694544	1.73E-14	4.52121270	subgrp_cor	count	1.77E-10	4.52121270
PTGIS	107	OK	3.8049036	0.5394107	7.0538157	1.74E-12	4.41754582	subgrp_cor	count	1.78E-08	4.41754582
TMEM88	61	OK	4.1278983	0.7853593	5.2560630	1.47E-07	4.32131813	subgrp_cor	count	0.0014887	4.32131813
PMEPA1	77	OK	3.718100	0.6128724	6.0666781	1.31E-09	4.28717456	subgrp_cor	count	1.33E-05	4.28717456
EFEMP1	210	OK	3.1815653	0.2958149	10.755253	5.6E-27	4.27510396	subgrp_cor	count	5.76E-23	4.27510396
SELP	158	OK	3.3893446	0.4108753	8.2490825	1.6E-16	4.12780709	subgrp_cor	count	1.64E-12	4.12780709
BMX	192	OK	3.0496077	0.2966867	10.278880	8.77E-25	4.1071410	subgrp_cor	count	9.02E-21	4.1071410
THBS1	83	OK	3.4132055	0.554782	6.1523254	7.64E-10	4.05574276	subgrp_cor	count	7.77E-06	4.05574276
ALDH1A1	46	OK	3.9365742	0.8422233	4.6740262	2.95E-06	4.04837108	subgrp_cor	count	0.0296946	4.04837108
SMAD6	66	OK	4.4749341	0.9421055	4.7499288	2.03E-06	4.01961913	subgrp_cor	count	0.0204831	4.01961913
CDA	100	OK	3.3766867	0.5037932	6.7025248	2.05E-11	4.00888465	subgrp_cor	count	2.09E-07	4.00888465
TSTD1	45	OK	3.8108983	0.8264953	4.6109132	4.01E-06	3.85157177	subgrp_cor	count	0.0402547	3.85157177
EMP3	151	OK	2.9638474	0.3576139	8.2878400	1.15E-16	3.83532517	subgrp_cor	count	1.18E-12	3.83532517
HOMER3	59	OK	3.3142811	0.618732	5.3565698	8.48E-08	3.8141645	subgrp_cor	count	0.0008588	3.8141645
C1R	209	OK	2.7643217	0.2690925	10.272755	9.35E-25	3.72827396	subgrp_cor	count	9.62E-21	3.72827396
LTBP4	109	OK	3.3723136	0.5331658	6.3250743	2.53E-10	3.71489053	subgrp_cor	count	2.58E-06	3.71489053
TPM2	67	OK	3.103779	0.5687917	5.4567943	4.85E-08	3.61654262	subgrp_cor	count	0.0004912	3.61654262
CEBPB	105	OK	3.0554052	0.4674045	6.5369611	6.28E-11	3.60475420	subgrp_cor	count	6.4E-07	3.60475420
TNFRSF12A	54	OK	3.0502505	0.6384621	4.7776532	1.77E-06	3.4858360	subgrp_cor	count	0.0178594	3.4858360
CPE	146	OK	2.4635605	0.2825155	8.7200893	2.78E-18	3.35221880	subgrp_cor	count	2.85E-14	3.35221880
CYR61	237	OK	2.4281695	0.2398777	10.122529	4.39E-24	3.33820017	subgrp_cor	count	4.51E-20	3.33820017
ESM1	45	OK	3.0062622	0.6508952	4.6186575	3.86E-06	3.32573889	subgrp_cor	count	0.0387852	3.32573889
PLXNA2	62	OK	3.5720167	0.7364302	4.8504480	1.23E-06	3.23512617	subgrp_cor	count	0.0124156	3.23512617
EDN1	239	OK	2.3982044	0.2289539	10.474613	1.13E-25	3.30552860	subgrp_cor	count	1.16E-21	3.30552860
BMP4	110	OK	2.4971295	0.3496021	7.1427744	9.15E-13	3.28255220	subgrp_cor	count	9.35E-09	3.28255220
AAK1	89	OK	2.8677539	0.4933735	5.8125414	6.15E-09	3.2823423	subgrp_cor	count	6.25E-05	3.2823423
CCDC80	107	OK	2.6108513	0.3930701	6.6422022	3.09E-11	3.27911459	subgrp_cor	count	3.15E-07	3.27911459
PXDC1	90	OK	2.6136647	0.4292366	6.0891000	1.14E-09	3.27694209	subgrp_cor	count	1.16E-05	3.27694209
SKIL	192	OK	2.4821845	0.2909339	8.5317798	1.44E-17	3.24102309	subgrp_cor	count	1.48E-13	3.24102309
LIMCH1	109	OK	2.4498667	0.3561488	6.8787718	6.04E-12	3.1505809	subgrp_cor	count	6.16E-08	3.1505809
TAGLN	77	OK	2.4409199	0.4218021	5.7868832	7.17E-09	3.10436087	subgrp_cor	count	7.28E-05	3.10436087
LTBP3	62	OK	3.0426906	0.6607161	4.6051403	4.12E-06	3.07367701	subgrp_cor	count	0.0413795	3.07367701
CCSER2	77	OK	2.8266092	0.5524911	5.1161165	3.12E-07	3.04351829	subgrp_cor	count	0.0031529	3.04351829
SYNPO	95	OK	2.5312106	0.4234863	5.9773127	2.27E-09	3.04341985	subgrp_cor	count	2.31E-05	3.04341985
PTPRG	61	OK	2.9472446	0.6259773	4.7082288	2.5E-06	3.00795533	subgrp_cor	count	0.0251452	3.00795533
LEPR	84	OK	3.1950483	0.5696595	5.6104533	2.02E-08	2.92261266	subgrp_cor	count	0.0002047	2.92261266
GJA4	96	OK	2.2832575	0.3898047	5.8574389	4.7E-09	2.91293510	subgrp_cor	count	4.78E-05	2.91293510
MECOM	58	OK	2.8767389	0.6204540	4.6365058	3.54E-06	2.90948634	subgrp_cor	count	0.0356013	2.90948634
PTGS2	159	OK	2.1007369	0.2652487	7.9198743	2.38E-15	2.84908958	subgrp_cor	count	2.44E-11	2.84908958
DHRS3	65	OK	2.3959165	0.454002	5.2773260	1.31E-07	2.83987081	subgrp_cor	count	0.0013264	2.83987081
CYBA	132	OK	2.153866	0.3415054	6.3069768	2.85E-10	2.80332610	subgrp_cor	count	2.9E-06	2.80332610
C4orf48	74	OK	2.4001943	0.5151461	4.6592490	3.17E-06	2.7098693	subgrp_cor	count	0.0319015	2.7098693
CRIM1	245	OK	1.8736145	0.1972911	9.4966999	2.17E-21	2.5997316	subgrp_cor	count	2.23E-17	2.5997316
ITGA10	106	OK	2.1130326	0.3624108	5.830489	5.53E-09	2.59249221	subgrp_cor	count	5.62E-05	2.59249221
RBP1	73	OK	2.0400145	0.3897939	5.2335712	1.66E-07	2.58094180	subgrp_cor	count	0.0016812	2.58094180
SLPI	121	OK	1.9216695	0.280618	6.8827152	5.83E-12	2.56500910	subgrp_cor	count	5.95E-08	2.56500910
RGS3	99	OK	1.9516439	0.3728396	5.2345403	1.65E-07	2.52601231	subgrp_cor	count	0.0016726	2.52601231
CALU	117	OK	1.9824928	0.3504444	5.6570813	1.54E-08	2.49697251	subgrp_cor	count	0.0001562	2.49697251
LGALS1	304	OK	1.7813671	0.1939337	9.1854436	4.1E-20	2.47079594	subgrp_cor	count	4.21E-16	2.47079594
TSPAN3	97	OK	1.989756	0.3920469	5.0753001	3.87E-07	2.46509351	subgrp_cor	count	0.003908	2.46509351
ASS1	170	OK	1.8081195	0.2494576	7.2482015	4.22E-13	2.45116079	subgrp_cor	count	4.32E-09	2.45116079
SNRK	120	OK	1.8387478	0.3089334	5.9519223	2.65E-09	2.43262490	subgrp_cor	count	2.7E-05	2.43262490
BNIP3L	131	OK	1.8112968	0.2896771	6.2528116	4.03E-10	2.4326015	subgrp_cor	count	4.11E-06	2.4326015
ACVRL1	114	OK	1.9221683	0.3643272	5.2759383	1.32E-07	2.41817282	subgrp_cor	count	0.0013363	2.41817282
CYP1B1	175	OK	1.8701899	0.261366	7.158164	8.18E-13	2.40947461	subgrp_cor	count	8.36E-09	2.40947461
GMDS	83	OK	1.8929183	0.3755128	5.0408833	4.63E-07	2.39288772	subgrp_cor	count	0.0046791	2.39288772
MXD4	81	OK	2.0281829	0.4384466	4.6258372	3.73E-06	2.34935567	subgrp_cor	count	0.0374692	2.34935567
TTC3	92	OK	1.8593576	0.3774793	4.9257201	8.41E-07	2.33589696	subgrp_cor	count	0.0084806	2.33589696
HSD17B12	184	OK	1.6784638	0.2332676	7.1954430	6.23E-13	2.30915608	subgrp_cor	count	6.37E-09	2.30915608
HCFC1R1	83	OK	1.7797755	0.3621936	4.9089090	9.16E-07	2.29778086	subgrp_cor	count	0.0092381	2.29778086
PGRMC2	91	OK	1.8087620	0.3581206	5.0507055	4.4E-07	2.24844970	subgrp_cor	count	0.0044458	2.24844970
ITPR2	99	OK	1.8542371	0.3528785	5.2297561	1.61E-07	2.2185392	subgrp_cor	count	0.0016263	2.2185392
H2AFJ	112	OK	1.6540875	0.3158472	5.2368855	1.63E-07	2.17787664	subgrp_cor	count	0.0016508	2.17787664
TGFB1	102	OK	1.7465421	0.3640234	4.7978834	1.6E-06	2.14294429	subgrp_cor	count	0.0161489	2.14294429
RUNX1T1	72	OK	1.9939755	0.4221926	4.7229069	2.32E-06	2.10058578	subgrp_cor	count	0.0233987	2.10058578
CALD1	394	OK	1.4856231	0.1562567	9.5075752	1.95E-21	2.08704437	subgrp_cor	count	2.01E-17	2.08704437
TIMP2	143	OK	1.5598881	0.280243	5.5558324	2.76E-08	2.05486520	subgrp_cor	count	0.0002801	2.05486520
RBMS3	80	OK	1.6682320	0.3551892	4.6967412	2.64E-06	2.02633249	subgrp_cor	count	0.0265957	2.02633249
BMPR2	208	OK	1.4874826	0.2194364	6.7786489	1.21E-11	2.01640727	subgrp_cor	count	1.24E-07	2.01640727
RCN2	131	OK	1.5012148	0.2940365	5.1055380	3.3E-07	1.99675076	subgrp_cor	count	0.0033338	1.99675076
FXYD6	109	OK	1.6641455	0.336990	4.9386917	7.86E-07	1.98297511	subgrp_cor	count	0.0079387	1.98297511
METRNL	212	OK	1.3919024	0.2000187	6.9588593	3.43E-12	1.93380010	subgrp_cor	count	3.5E-08	1.93380010
CTSD	120	OK	1.4867765	0.3072256	4.8393665	1.3E-06	1.92250402	subgrp_cor	count	0.0131256	1.92250402
HTRA1	230	OK	1.4065081	0.2183007	6.4429837	1.17E-10	1.91803295	subgrp_cor	count	1.19E-06	1.91803295
CTGF	234	OK	1.3739004	0.1919889	7.1557697	8.32E-13	1.91436228	subgrp_cor	count	8.51E-09	1.91436228
NES	136	OK	1.418179	0.2878723	4.926415	8.38E-07	1.84654436	subgrp_cor	count	0.0084513	1.84654436
RHOB	330	OK	1.3165958	0.1630985	8.0723969	6.89E-16	1.83977304	subgrp_cor	count	7.06E-12	1.83977304
PTPRB	207	OK	1.3397722	0.2087015	6.4195602	1.37E-10	1.83485481	subgrp_cor	count	1.39E-06	1.83485481
TGM2	213	OK	1.3726408	0.2105328	6.5198430	7.04E-11	1.83450582	subgrp_cor	count	7.18E-07	1.83450582
TMED10	183	OK	1.3306089	0.2404801	5.5331349	3.15E-08	1.80612157	subgrp_cor	count	0.0003188	1.80612157
P4HB	204	OK	1.3437761	0.2315475	5.8034556	6.5E-09	1.79270431	subgrp_cor	count	6.6E-05	1.79270431
GJA1	140	OK	1.3848546	0.2724238	5.0834562	3.71E-07	1.7844362	subgrp_cor	count	0.0037448	1.7844362
LRPAP1	175	OK	1.3105468	0.2405393	5.4483672	5.08E-08	1.77269742	subgrp_cor	count	0.000515	1.77269742
CTNNAL1	143	OK	1.2841997	0.2516936	5.1022332	3.36E-07	1.74249756	subgrp_cor	count	0.0033922	1.74249756
PKIG	146	OK	1.2755137	0.2670743	4.7758756	1.79E-06	1.72948181	subgrp_cor	count	0.0180161	1.72948181
SRGN	416	OK	1.2209229	0.1446615	8.4398555	3.18E-17	1.71880943	subgrp_cor	count	3.26E-13	1.71880943
FAM198B	155	OK	1.2645128	0.2555579	4.9480470	7.5E-07	1.69668362	subgrp_cor	count	0.0075673	1.69668362
COMT	143	OK	1.2489789	0.2557242	4.8840847	1.04E-06	1.69564373	subgrp_cor	count	0.0104772	1.69564373
HSPG2	433	OK	1.1971612	0.139836	8.5611689	1.12E-17	1.68938338	subgrp_cor	count	1.15E-13	1.68938338
CFH	299	OK	1.2067628	0.1718269	7.0231307	2.17E-12	1.68276060	subgrp_cor	count	2.22E-08	1.68276060
CCND1	158	OK	1.2336260	0.2530013	4.8758660	1.08E-06	1.68081724	subgrp_cor	count	0.0109154	1.68081724
MYDGF	200	OK	1.2111916	0.2244405	5.3964930	6.8E-08	1.66521729	subgrp_cor	count	0.0006882	1.66521729
DNAJC3	164	OK	1.2182073	0.2467576	4.9372624	7.92E-07	1.64999439	subgrp_cor	count	0.0079955	1.64999439
C12orf57	297	OK	1.1629293	0.173943	6.6856729	2.3E-11	1.62119940	subgrp_cor	count	2.35E-07	1.62119940
WWTR1	310	OK	1.1410111	0.1639042	6.9614493	3.37E-12	1.59776584	subgrp_cor	count	3.44E-08	1.59776584
WSB1	306	OK	1.1332482	0.1668394	6.7924491	1.1E-11	1.57527427	subgrp_cor	count	1.13E-07	1.57527427
CXCL2	240	OK	1.105850	0.176031	6.2826752	3.33E-10	1.55665516	subgrp_cor	count	3.39E-06	1.55665516
GAS6	337	OK	1.0712058	0.1511618	7.0864847	1.38E-12	1.50912524	subgrp_cor	count	1.41E-08	1.50912524
PPP1CB	197	OK	1.0961133	0.2072981	5.2876166	1.24E-07	1.50688024	subgrp_cor	count	0.0012540	1.50688024
GADD45GIP1	236	OK	1.0721553	0.2016429	5.3170982	1.05E-07	1.46967463	subgrp_cor	count	0.0010673	1.46967463
PLPP1	170	OK	1.0862511	0.2345398	4.6314161	3.63E-06	1.46785432	subgrp_cor	count	0.0364807	1.46785432
COX7A1	184	OK	1.0832088	0.2274479	4.7624476	1.91E-06	1.46494572	subgrp_cor	count	0.0192559	1.46494572
NDUFB7	222	OK	1.0106353	0.2063643	4.8973356	9.71E-07	1.38953828	subgrp_cor	count	0.0097960	1.38953828
ATP5MC3	239	OK	0.982936	0.1921762	5.1147666	3.14E-07	1.36932700	subgrp_cor	count	0.0031752	1.36932700
DNAJB1	210	OK	0.9635696	0.2003972	4.8082977	1.52E-06	1.32966353	subgrp_cor	count	0.0153322	1.32966353
CRTAC1	269	OK	0.9505988	0.1645211	5.7779723	7.56E-09	1.32866849	subgrp_cor	count	7.68E-05	1.32866849
LTC4S	358	OK	0.940926	0.1421772	6.6180427	3.64E-11	1.32700692	subgrp_cor	count	3.71E-07	1.32700692
SSR3	227	OK	0.9532469	0.2034052	4.6864426	2.78E-06	1.31196094	subgrp_cor	count	0.0279634	1.31196094
NFIA	203	OK	0.9184144	0.1955065	4.6976151	2.63E-06	1.26761301	subgrp_cor	count	0.0264848	1.26761301
NDUFA1	250	OK	0.8865933	0.1904981	4.6540793	3.25E-06	1.22278209	subgrp_cor	count	0.0327091	1.22278209
CTSZ	253	OK	0.8441077	0.180221	4.6837132	2.82E-06	1.18233416	subgrp_cor	count	0.0283329	1.18233416
NFE2L2	237	OK	0.8505952	0.1846276	4.6070849	4.08E-06	1.18083598	subgrp_cor	count	0.0409986	1.18083598
NUCB2	354	OK	0.7857081	0.1480630	5.3065785	1.12E-07	1.10640641	subgrp_cor	count	0.0011306	1.10640641
PSAP	343	OK	0.7257625	0.1504731	4.8232036	1.41E-06	1.01747400	subgrp_cor	count	0.0142330	1.01747400
SAT1	425	OK	0.6336354	0.1364858	4.6424981	3.44E-06	0.89711349	subgrp_cor	count	0.0345874	0.89711349
SAMD11	2	OK	26.367955	84.916325	0.3105168	0.75617	2.49052421	subgrp_cor	count	1	0
NOC2L	5	OK	-1.537212	1.6487135	-0.932370	0.35115	-0.40044109	subgrp_cor	count	1	0
HES4	36	OK	0.3504160	0.4852934	0.7220703	0.47025	0.42010469	subgrp_cor	count	1	0
ISG15	142	OK	0.6680189	0.250329	2.6686003	0.00762	0.90047641	subgrp_cor	count	1	0
AGRN	23	OK	1.0135348	0.6292359	1.6107389	0.10724	1.02696710	subgrp_cor	count	1	0
TTLL10	1	OK	3.4598146	6.7714513	0.5109413	0.60939	0.00030179	subgrp_cor	count	1	0
TNFRSF4	22	OK	-0.279994	0.6755893	-0.414438	0.67855	-0.2826367	subgrp_cor	count	1	0
SDF4	122	OK	1.1647591	0.2987775	3.8984152	9.68E-05	1.45024963	subgrp_cor	count	0.9600127	0
B3GALT6	6	OK	0.9547241	1.5018923	0.6356808	0.52498	0.29618915	subgrp_cor	count	1	0
UBE2J2	22	OK	0.4026466	0.6075129	0.6627787	0.50747	0.39872702	subgrp_cor	count	1	0
PUSL1	1	OK	23.991896	84.899941	0.2825902	0.77749	1.65081709	subgrp_cor	count	1	0
INTS11	34	OK	-0.175832	0.5118574	-0.343517	0.73121	-0.20001502	subgrp_cor	count	1	0
CPTP	17	OK	-1.325110	0.7727768	-1.714739	0.08639	-1.21085824	subgrp_cor	count	1	0
DVL1	3	OK	-14.65800	45.690332	-0.320813	0.74835	-0.00067222	subgrp_cor	count	1	0
MXRA8	1	OK	1.5823999	2.7200188	0.5817606	0.56072	0.47433761	subgrp_cor	count	1	0
AURKAIP1	180	OK	0.1733152	0.2232205	0.7764304	0.43749	0.23585209	subgrp_cor	count	1	0
CCNL2	28	OK	0.2814510	0.5398337	0.5213661	0.60211	0.27864736	subgrp_cor	count	1	0
MRPL20	196	OK	0.4669707	0.2027392	2.303307	0.02126	0.64220390	subgrp_cor	count	1	0
ANKRD65	12	OK	-0.554136	0.890136	-0.625321	0.53159	-0.44732006	subgrp_cor	count	1	0
VWA1	202	OK	-0.592494	0.214031	-2.768262	0.00564	-0.79579779	subgrp_cor	count	1	0
ATAD3A	3	OK	2.4096337	2.4715209	0.9749599	0.32958	0.66517343	subgrp_cor	count	1	0
SSU72	104	OK	0.6170755	0.2919742	2.1134589	0.03456	0.81644072	subgrp_cor	count	1	0
MIB2	2	OK	21.105322	83.341271	0.2532397	0.80008	0.0003103	subgrp_cor	count	1	0
CDK11B	10	OK	0.0575443	0.9474873	0.0607336	0.95157	0.03724713	subgrp_cor	count	1	0
CDK11A	5	OK	-1.235285	1.2504678	-0.987858	0.32322	-0.00197223	subgrp_cor	count	1	0
NADK	5	OK	0.405043	2.1083409	0.1925883	0.84726	0.12643682	subgrp_cor	count	1	0
GNB1	108	OK	0.4044817	0.2990012	1.3527761	0.17612	0.44714321	subgrp_cor	count	1	0
TMEM52	1	OK	-15.99503	70.34942	-0.227365	0.82014	-0.00028474	subgrp_cor	count	1	0
GABRD	15	OK	0.6241605	1.0079417	0.6192427	0.53576	0.00114817	subgrp_cor	count	1	0
PRKCZ	2	OK	-1.295616	2.041425	-0.634662	0.52565	-0.00075754	subgrp_cor	count	1	0
FAAP20	70	OK	0.7269802	0.3790119	1.9180933	0.0551	0.92534017	subgrp_cor	count	1	0
SKI	25	OK	1.7549510	0.8367689	2.0972945	0.03597	1.06968465	subgrp_cor	count	1	0
RER1	85	OK	0.6247167	0.3523698	1.7729009	0.07625	0.78499556	subgrp_cor	count	1	0
PEX10	5	OK	-0.546377	1.297277	-0.421172	0.67362	-0.34177821	subgrp_cor	count	1	0
HES5	8	OK	-2.368351	1.3319601	-1.778098	0.07539	-1.15321820	subgrp_cor	count	1	0
AL139246.5	1	OK	1.5823999	2.7200188	0.5817606	0.56072	0.47433761	subgrp_cor	count	1	0
TNFRSF14	58	OK	-0.360479	0.3553389	-1.014465	0.31036	-0.45521069	subgrp_cor	count	1	0
FAM213B	4	OK	0.1681491	1.7253300	0.097459	0.92236	0.05201277	subgrp_cor	count	1	0
MMEL1	4	OK	-11.01941	43.249935	-0.254784	0.79889	-1.17482461	subgrp_cor	count	1	0
LINC00982	1	OK	18.759488	83.625179	0.2243282	0.8225	0.00028789	subgrp_cor	count	1	0
PRDM16	2	OK	4.2108437	4.2958243	0.9802178	0.32698	7.14E-14	subgrp_cor	count	1	0
MEGF6	8	OK	23.085900	54.063969	0.4270108	0.66937	1.62231891	subgrp_cor	count	1	0
TPRG1L	16	OK	0.2219909	0.8417237	0.2637337	0.79199	0.21963471	subgrp_cor	count	1	0
WRAP73	5	OK	-2.342222	1.7367717	-1.348606	0.17746	-0.53905893	subgrp_cor	count	1	0
LRRC47	3	OK	-10.58676	25.202461	-0.420068	0.67444	-0.00124361	subgrp_cor	count	1	0
CEP104	4	OK	-14.65800	45.690332	-0.320813	0.74835	-0.00067222	subgrp_cor	count	1	0
C1orf174	5	OK	2.4546467	1.6817506	1.4595782	0.14441	0.64777140	subgrp_cor	count	1	0
CHD5	1	OK	23.991896	84.899941	0.2825902	0.77749	1.65081709	subgrp_cor	count	1	0
RPL22	858	OK	-0.127374	0.0864517	-1.473355	0.14066	-0.18216315	subgrp_cor	count	1	0
ICMT	6	OK	9.2907794	6.6949586	1.3877276	0.16522	1.1756802	subgrp_cor	count	1	0
ACOT7	21	OK	3.1512652	1.1741648	2.6838354	0.00728	2.52579598	subgrp_cor	count	1	0
PLEKHG5	1	OK	1.5823999	2.7200188	0.5817606	0.56072	0.47433761	subgrp_cor	count	1	0
NOL9	8	OK	-0.010736	1.1438343	-0.009386	0.99251	-0.00889952	subgrp_cor	count	1	0
PHF13	6	OK	-0.133280	1.1707888	-0.113837	0.90937	-0.06670657	subgrp_cor	count	1	0
THAP3	1	OK	3.4598146	6.7714513	0.5109413	0.60939	0.00030179	subgrp_cor	count	1	0
DNAJC11	2	OK	-10.44227	41.465809	-0.251828	0.80117	-0.00045289	subgrp_cor	count	1	0
CAMTA1	205	OK	0.5157400	0.1966445	2.6227021	0.00872	0.71508243	subgrp_cor	count	1	0
VAMP3	143	OK	-0.300192	0.2310242	-1.299298	0.19381	-0.40295346	subgrp_cor	count	1	0
PER3	30	OK	-0.549050	0.5190384	-1.057822	0.29014	-0.62671149	subgrp_cor	count	1	0
PARK7	432	OK	0.3999158	0.1341940	2.9801308	0.00288	0.56529887	subgrp_cor	count	1	0
AL034417.4	1	OK	18.759488	83.625179	0.2243282	0.8225	0.00028789	subgrp_cor	count	1	0
ERRFI1	17	OK	2.7040227	1.1557136	2.3397082	0.0193	2.18353967	subgrp_cor	count	1	0
RERE	29	OK	-0.358386	0.5355772	-0.669158	0.50339	-0.37363700	subgrp_cor	count	1	0
ENO1	306	OK	0.4397310	0.166789	2.6364399	0.00838	0.61333355	subgrp_cor	count	1	0
GPR157	1	OK	-10.44227	41.465809	-0.251828	0.80117	-0.00045289	subgrp_cor	count	1	0
H6PD	8	OK	0.3156156	1.0425598	0.3024413	0.76232	0.30095134	subgrp_cor	count	1	0
SPSB1	7	OK	1.2504052	1.2754305	0.9803789	0.3269	0.62491299	subgrp_cor	count	1	0
SLC25A33	6	OK	-0.684924	1.3384235	-0.51174	0.60882	-0.19900120	subgrp_cor	count	1	0
CLSTN1	11	OK	1.1618235	0.9464243	1.227602	0.2196	0.94849485	subgrp_cor	count	1	0
CTNNBIP1	19	OK	0.7372871	0.7887085	0.9348030	0.34989	0.61212741	subgrp_cor	count	1	0
LZIC	23	OK	1.162738	0.6360342	1.8281063	0.06753	1.17091624	subgrp_cor	count	1	0
NMNAT1	1	OK	3.4598146	6.7714513	0.5109413	0.60939	0.00030179	subgrp_cor	count	1	0
UBE4B	1	OK	-10.44227	41.465809	-0.251828	0.80117	-0.00045289	subgrp_cor	count	1	0
KIF1B	12	OK	-1.596019	1.1606369	-1.37512	0.16909	-0.9937445	subgrp_cor	count	1	0
PGD	3	OK	2.0702523	1.7994826	1.1504708	0.24995	0.96210322	subgrp_cor	count	1	0
CENPS	3	OK	4.6613299	5.7683806	0.8080821	0.41904	0.00274918	subgrp_cor	count	1	0
DFFA	11	OK	0.6473303	1.1025466	0.5871229	0.55712	0.33099128	subgrp_cor	count	1	0
TARDBP	6	OK	0.0806078	1.0410433	0.0774298	0.93828	0.04093682	subgrp_cor	count	1	0
SRM	83	OK	0.3004049	0.3388939	0.8864275	0.37539	0.36904806	subgrp_cor	count	1	0
EXOSC10	6	OK	0.2156436	1.3069779	0.164841	0.86907	0.06761189	subgrp_cor	count	1	0
MTOR	2	OK	3.4598146	6.7714513	0.5109413	0.60939	0.00030179	subgrp_cor	count	1	0
UBIAD1	2	OK	1.9259645	2.5154879	0.7656425	0.44389	0.56022119	subgrp_cor	count	1	0
FBXO6	1	OK	1.5823999	2.7200188	0.5817606	0.56072	0.47433761	subgrp_cor	count	1	0
MAD2L2	17	OK	-0.123327	0.7132163	-0.172917	0.86272	-0.09200661	subgrp_cor	count	1	0
AGTRAP	85	OK	-0.110400	0.3228916	-0.341911	0.73242	-0.14111163	subgrp_cor	count	1	0
MTHFR	6	OK	-0.148549	1.2058097	-0.123194	0.90195	-0.00026149	subgrp_cor	count	1	0
KIAA2013	14	OK	0.7539123	0.9071496	0.8310782	0.40592	0.71385538	subgrp_cor	count	1	0
PLOD1	13	OK	2.6294861	1.1355581	2.3155892	0.02058	2.20320447	subgrp_cor	count	1	0
MFN2	1	OK	41.788303	174.29608	0.2397549	0.81052	0.00014488	subgrp_cor	count	1	0
MIIP	3	OK	-0.35720	1.5149899	-0.235803	0.81359	-0.22854173	subgrp_cor	count	1	0
TNFRSF1B	29	OK	-1.42532	0.588153	-2.423393	0.01538	-1.14874938	subgrp_cor	count	1	0
VPS13D	6	OK	4.3801518	3.436822	1.2744403	0.20251	0.00263449	subgrp_cor	count	1	0
PRDM2	23	OK	0.0921859	0.6045409	0.1524891	0.8788	0.09111615	subgrp_cor	count	1	0
KAZN	10	OK	0.3509452	1.2327587	0.2846828	0.77589	0.2366188	subgrp_cor	count	1	0
TMEM51	3	OK	-0.174796	1.820980	-0.095990	0.92352	-0.05309252	subgrp_cor	count	1	0
EFHD2	66	OK	-0.481297	0.3981132	-1.208945	0.22668	-0.58070951	subgrp_cor	count	1	0
CASP9	1	OK	1.5823999	2.7200188	0.5817606	0.56072	0.47433761	subgrp_cor	count	1	0
DNAJC16	3	OK	19.103032	50.772409	0.3762478	0.70672	0.00078880	subgrp_cor	count	1	0
DDI2	5	OK	-0.815226	2.2666336	-0.359664	0.7191	-4.1E-06	subgrp_cor	count	1	0
PLEKHM2	13	OK	2.8472102	1.3206243	2.1558577	0.03109	0.74209821	subgrp_cor	count	1	0
SLC25A34-AS1	4	OK	6.1490015	5.4123994	1.136095	0.25592	1.05209450	subgrp_cor	count	1	0
UQCRHL	2	OK	3.4598146	6.7714513	0.5109413	0.60939	0.00030179	subgrp_cor	count	1	0
AL450998.2	1	OK	3.4597879	4.7858528	0.7229198	0.46972	0.00081879	subgrp_cor	count	1	0
SPEN	21	OK	0.391805	0.6946418	0.5640392	0.57272	0.35133737	subgrp_cor	count	1	0
ZBTB17	2	OK	-10.44227	41.465809	-0.251828	0.80117	-0.00045289	subgrp_cor	count	1	0
EPHA2	2	OK	4.6613299	5.7683806	0.8080821	0.41904	0.00274918	subgrp_cor	count	1	0
ARHGEF19	1	OK	1.5823999	2.7200188	0.5817606	0.56072	0.47433761	subgrp_cor	count	1	0
SZRD1	25	OK	0.3665277	0.5516355	0.664428	0.50641	0.39681288	subgrp_cor	count	1	0
NECAP2	43	OK	0.4209825	0.4792042	0.8785034	0.37967	0.41682000	subgrp_cor	count	1	0
NBPF1	2	OK	-0.910223	2.1881125	-0.415985	0.67742	-0.25785735	subgrp_cor	count	1	0
CROCC	3	OK	-0.214966	1.5648178	-0.137374	0.89072	-0.06511276	subgrp_cor	count	1	0
BX284668.5	2	OK	1.9259645	2.5154879	0.7656425	0.44389	0.56022119	subgrp_cor	count	1	0
BX284668.6	2	OK	-0.910223	2.1881125	-0.415985	0.67742	-0.25785735	subgrp_cor	count	1	0
MFAP2	26	OK	2.4579895	0.7628751	3.2220076	0.00127	2.5040706	subgrp_cor	count	1	0
ATP13A2	1	OK	-10.44227	41.465809	-0.251828	0.80117	-0.00045289	subgrp_cor	count	1	0
SDHB	42	OK	0.6681251	0.5185807	1.2883723	0.19762	0.72158980	subgrp_cor	count	1	0
RCC2	7	OK	1.4140692	1.3954614	1.0133345	0.3109	0.4290282	subgrp_cor	count	1	0
IFFO2	3	OK	4.6613299	5.7683806	0.8080821	0.41904	0.00274918	subgrp_cor	count	1	0
UBR4	10	OK	0.829313	1.1353344	0.7304652	0.46507	0.26115515	subgrp_cor	count	1	0
AL035413.1	1	OK	3.4598146	6.7714513	0.5109413	0.60939	0.00030179	subgrp_cor	count	1	0
EMC1	2	OK	0.1681690	1.7253287	0.0974707	0.92235	0.05201879	subgrp_cor	count	1	0
MRTO4	21	OK	-0.951198	0.6874936	-1.383574	0.16649	-0.85373761	subgrp_cor	count	1	0
AKR7A2	55	OK	0.6039109	0.3866899	1.5617041	0.11836	0.76628132	subgrp_cor	count	1	0
CAPZB	293	OK	-0.360178	0.1633624	-2.204782	0.02747	-0.50221972	subgrp_cor	count	1	0
MINOS1	121	OK	0.2437191	0.2585293	0.9427135	0.34582	0.32239661	subgrp_cor	count	1	0
TMCO4	5	OK	0.2537029	1.2457212	0.2036595	0.83862	0.07870987	subgrp_cor	count	1	0
PLA2G2A	4	OK	4.9145501	2.8302550	1.7364336	0.08249	3.40096777	subgrp_cor	count	1	0
PLA2G5	19	OK	22.184387	32.569122	0.6811478	0.49578	2.33303475	subgrp_cor	count	1	0
UBXN10-AS1	1	OK	-11.01941	43.249935	-0.254784	0.79889	-1.17482461	subgrp_cor	count	1	0
UBXN10	1	OK	-12.14842	43.455309	-0.279561	0.77981	-1.91362624	subgrp_cor	count	1	0
CAMK2N1	2	OK	1.5823999	2.7200188	0.5817606	0.56072	0.47433761	subgrp_cor	count	1	0
PINK1	76	OK	1.3037067	0.3759151	3.4680880	0.00052	1.51915223	subgrp_cor	count	1	0
DDOST	101	OK	0.0624256	0.2865120	0.2178812	0.82752	0.0830994	subgrp_cor	count	1	0
KIF17	1	OK	3.4598146	6.7714513	0.5109413	0.60939	0.00030179	subgrp_cor	count	1	0
HP1BP3	186	OK	0.5411685	0.219420	2.4663512	0.01365	0.74006698	subgrp_cor	count	1	0
EIF4G3	40	OK	0.3275019	0.4641155	0.7056475	0.48041	0.33597859	subgrp_cor	count	1	0
ECE1	180	OK	0.7648205	0.2170996	3.5229006	0.00042	1.01837034	subgrp_cor	count	1	0
ALPL	14	OK	1.9324985	1.1684077	1.6539590	0.09814	0.00360789	subgrp_cor	count	1	0
USP48	30	OK	1.6989667	0.7051427	2.4093940	0.01598	1.43973608	subgrp_cor	count	1	0
CDC42	511	OK	-0.304085	0.1205653	-2.521999	0.01167	-0.43132378	subgrp_cor	count	1	0
ZBTB40	1	OK	17.747207	50.971927	0.3481761	0.72771	0.0007532	subgrp_cor	count	1	0
C1QA	2	OK	1.1469439	1.6004489	0.7166388	0.4736	0.37918447	subgrp_cor	count	1	0
C1QC	1	OK	18.759488	83.625179	0.2243282	0.8225	0.00028789	subgrp_cor	count	1	0
C1QB	3	OK	6.1490015	5.4123994	1.136095	0.25592	1.05209450	subgrp_cor	count	1	0
KDM1A	12	OK	0.4750378	0.9379506	0.5064625	0.61252	0.39599666	subgrp_cor	count	1	0
LUZP1	192	OK	-0.145860	0.223308	-0.653118	0.51175	-0.19629326	subgrp_cor	count	1	0
HNRNPR	158	OK	0.2075024	0.2435047	0.8521495	0.39412	0.2716924	subgrp_cor	count	1	0
ZNF436	3	OK	3.4598149	4.7858686	0.7229233	0.46972	0.00081935	subgrp_cor	count	1	0
TCEA3	13	OK	-1.952119	0.9578978	-2.037920	0.04156	-1.21032336	subgrp_cor	count	1	0
ASAP3	4	OK	-2.446592	1.7138131	-1.427572	0.15341	-0.00126286	subgrp_cor	count	1	0
ID3	722	OK	-0.120185	0.0966634	-1.243235	0.21376	-0.17143159	subgrp_cor	count	1	0
RPL11	890	OK	0.0167351	0.0816797	0.2048871	0.83766	0.02396905	subgrp_cor	count	1	0
ELOA	21	OK	1.6377336	0.8721310	1.8778526	0.0604	1.2935624	subgrp_cor	count	1	0
ELOA-AS1	1	OK	23.991896	84.899941	0.2825902	0.77749	1.65081709	subgrp_cor	count	1	0
PITHD1	33	OK	-0.836255	0.5289012	-1.581118	0.11385	-0.89485553	subgrp_cor	count	1	0
LYPLA2	17	OK	-2.005729	0.9211821	-2.177342	0.02945	-1.05396483	subgrp_cor	count	1	0
GALE	9	OK	-0.242347	0.9942402	-0.243751	0.80742	-0.17980642	subgrp_cor	count	1	0
HMGCL	12	OK	-0.086527	0.8918752	-0.097017	0.92271	-0.04362263	subgrp_cor	count	1	0
FUCA1	5	OK	8.7172801	8.5896888	1.0148528	0.31018	1.14378300	subgrp_cor	count	1	0
PNRC2	188	OK	-0.327660	0.2120273	-1.545368	0.12226	-0.45112233	subgrp_cor	count	1	0
SRSF10	159	OK	0.4310150	0.2283839	1.8872385	0.05912	0.57393648	subgrp_cor	count	1	0
MYOM3	1	OK	23.991896	84.899941	0.2825902	0.77749	1.65081709	subgrp_cor	count	1	0
STPG1	1	OK	3.4598146	6.7714513	0.5109413	0.60939	0.00030179	subgrp_cor	count	1	0
NIPAL3	5	OK	18.759488	83.625179	0.2243282	0.8225	0.00028789	subgrp_cor	count	1	0
RCAN3	1	OK	-15.99503	70.34942	-0.227365	0.82014	-0.00028474	subgrp_cor	count	1	0
SRRM1	356	OK	-0.223101	0.142219	-1.568714	0.11671	-0.31212227	subgrp_cor	count	1	0
CLIC4	130	OK	0.3277508	0.2695473	1.2159305	0.22401	0.41250527	subgrp_cor	count	1	0
SYF2	261	OK	-0.424749	0.1746691	-2.431739	0.01503	-0.58870120	subgrp_cor	count	1	0
RSRP1	207	OK	-0.168372	0.1904405	-0.884120	0.37662	-0.22860607	subgrp_cor	count	1	0
TMEM50A	344	OK	-0.209972	0.1495091	-1.404413	0.1602	-0.29503402	subgrp_cor	count	1	0
MACO1	19	OK	0.4201081	0.7434132	0.5651071	0.572	0.31697269	subgrp_cor	count	1	0
LDLRAP1	1	OK	18.759488	83.625179	0.2243282	0.8225	0.00028789	subgrp_cor	count	1	0
MAN1C1	9	OK	4.3552957	2.029228	2.1462817	0.03185	2.49379369	subgrp_cor	count	1	0
SELENON	38	OK	0.4258026	0.5537458	0.7689495	0.44192	0.38189174	subgrp_cor	count	1	0
MTFR1L	7	OK	1.5335582	1.1283736	1.3590873	0.17412	0.95339341	subgrp_cor	count	1	0
STMN1	146	OK	0.0779313	0.2363283	0.3297432	0.74159	0.10199600	subgrp_cor	count	1	0
ZNF593	52	OK	-0.222654	0.4090426	-0.544330	0.58621	-0.26797508	subgrp_cor	count	1	0
SH3BGRL3	714	OK	0.0501016	0.0983474	0.5094352	0.61045	0.07149653	subgrp_cor	count	1	0
DHDDS	3	OK	-0.150975	1.968815	-0.076683	0.93888	-9.78E-05	subgrp_cor	count	1	0
HMGN2	315	OK	0.1156482	0.1529357	0.7561884	0.44954	0.16184413	subgrp_cor	count	1	0

ARID1A	26	OK	0.76584396	0.6408916	1.19496645	0.2321	0.23884826	subgrp_cor	count		1	0
GPN2	4	OK	0.65325287	2.50012846	0.26128772	0.79387	0.00016297	subgrp_cor	count		1	0
NUDC	182	OK	-0.186903	0.2056896	-0.9086658	0.36352	-0.25510423	subgrp_cor	count		1	0
WDTC1	1	OK	-10.442278	41.4658099	-0.2518286	0.80117	-0.00045289	subgrp_cor	count		1	0
TMEM222	15	OK	0.13578887	0.71650479	0.18951565	0.84969	0.13429188	subgrp_cor	count		1	0
MAP3K6	7	OK	-11.147362	16.8475425	-0.661661	0.50819	-2.87886956	subgrp_cor	count		1	0
FCN3	8	OK	0.60014901	1.32210064	0.45393595	0.64987	0.00110630	subgrp_cor	count		1	0
WASF2	418	OK	-0.2806715	0.13173225	-2.1306214	0.03312	-0.39551720	subgrp_cor	count		1	0
AHDC1	1	OK	0.65325287	2.50012846	0.26128772	0.79387	0.00016297	subgrp_cor	count		1	0
FGR	10	OK	-0.2648028	0.9442005	-0.2804518	0.77912	-0.08012587	subgrp_cor	count		1	0
IFI6	222	OK	0.70319398	0.18931005	3.71450958	0.0002	0.9731288	subgrp_cor	count		1	0
FAM76A	7	OK	2.51290904	1.66457839	1.50963604	0.13114	1.65388372	subgrp_cor	count		1	0
STX12	39	OK	0.30209064	0.51564915	0.5858453	0.55798	0.2991531	subgrp_cor	count		1	0
RPA2	28	OK	-0.177282	0.5056820	-0.3505819	0.7259	-0.18053296	subgrp_cor	count		1	0
XKR8	3	OK	1.58227934	1.57050173	1.00756294	0.31366	0.97972300	subgrp_cor	count		1	0
EYA3	9	OK	1.2336159	1.11525794	1.106126	0.26867	0.61717700	subgrp_cor	count		1	0
PTAFR	1	OK	-15.995031	70.349421	-0.2273655	0.82014	-0.00028474	subgrp_cor	count		1	0
DNAJC8	222	OK	0.11173812	0.18376952	0.60803399	0.54316	0.1546660	subgrp_cor	count		1	0
ATP5IF1	289	OK	0.03428986	0.1658991	0.2066909	0.83625	0.04777384	subgrp_cor	count		1	0
SESN2	1	OK	-11.019418	43.2499352	-0.2547846	0.79889	-1.17482461	subgrp_cor	count		1	0
PHACTR4	59	OK	-0.0626043	0.3931636	-0.1592321	0.87349	-0.06392661	subgrp_cor	count		1	0
TRNAU1AP	14	OK	-0.8976539	0.8393473	-1.0694666	0.28486	-0.84599966	subgrp_cor	count		1	0
SNHG12	10	OK	-0.3197478	0.8706900	-0.3672349	0.71344	-0.09609344	subgrp_cor	count		1	0
TAF12	30	OK	-0.2185015	0.5518538	-0.3959409	0.69215	-0.2347157	subgrp_cor	count		1	0
GMEB1	2	OK	-10.442278	41.4658099	-0.2518286	0.80117	-0.00045289	subgrp_cor	count		1	0
YTHDF2	70	OK	-0.2868426	0.3559694	-0.8058068	0.42035	-0.35293195	subgrp_cor	count		1	0
EPB41	7	OK	0.77128352	1.03339568	0.74645514	0.45539	0.50030098	subgrp_cor	count		1	0
SRSF4	96	OK	-0.5831311	0.29412806	-1.9825754	0.04741	-0.70151467	subgrp_cor	count		1	0
MECR	3	OK	5.26127895	5.49058205	0.95823701	0.33794	0.00295034	subgrp_cor	count		1	0
LAPTM5	21	OK	2.76552264	1.04695702	2.64148629	0.00825	1.93589253	subgrp_cor	count		1	0
SDC3	4	OK	22.6877208	54.3157576	0.41770052	0.67617	1.6098774	subgrp_cor	count		1	0
PUM1	33	OK	0.56142977	0.51104088	1.09860042	0.27194	0.62021843	subgrp_cor	count		1	0
SNRNP40	33	OK	0.67253505	0.60813332	1.10590069	0.26877	0.66361189	subgrp_cor	count		1	0
ZCCHC17	65	OK	0.38291454	0.36617036	1.04572785	0.29569	0.47037288	subgrp_cor	count		1	0
SERINC2	6	OK	-0.2029407	1.65323657	-0.1227536	0.9023	-4.82E-05	subgrp_cor	count		1	0
TINAGL1	211	OK	0.24471129	0.19635107	1.24629463	0.21266	0.32937635	subgrp_cor	count		1	0
PEF1	57	OK	-0.2647777	0.3731303	-0.7096117	0.47794	-0.32579999	subgrp_cor	count		1	0
COL16A1	1	OK	18.7594894	83.6251792	0.22432824	0.8225	0.00028789	subgrp_cor	count		1	0
PTP4A2	125	OK	0.90468335	0.26844373	3.37010424	0.00075	1.16995755	subgrp_cor	count		1	0
KHDRBS1	224	OK	-0.5940824	0.1855936	-3.2009844	0.00137	-0.82015243	subgrp_cor	count		1	0
TMEM39B	13	OK	-0.0335329	0.83747759	-0.0400401	0.96806	-0.02777549	subgrp_cor	count		1	0
KPNA6	11	OK	0.23773116	0.92033938	0.25830815	0.79617	0.15457124	subgrp_cor	count		1	0
TXLNA	11	OK	2.76218781	1.50075337	1.84053414	0.06569	0.0050082	subgrp_cor	count		1	0
CCDC28B	7	OK	0.81196819	1.38206702	0.58750276	0.55687	0.41380800	subgrp_cor	count		1	0
TMEM234	5	OK	-0.4559703	1.26621233	-0.3601057	0.71877	-0.22432410	subgrp_cor	count		1	0
EIF3I	273	OK	-0.233917	0.1721559	-1.3587508	0.17422	-0.32517148	subgrp_cor	count		1	0
FAM167B	109	OK	-0.7748534	0.2925321	-2.6487802	0.00808	-1.00218236	subgrp_cor	count		1	0
HDAC1	49	OK	0.43352549	0.44068284	0.98378118	0.32522	0.47920399	subgrp_cor	count		1	0
BSDC1	4	OK	24.8460727	53.5664516	0.46383645	0.64276	2.92581147	subgrp_cor	count		1	0
ZBTB8OS	29	OK	0.5418359	0.56697775	0.95565637	0.33925	0.57221591	subgrp_cor	count		1	0
RBBP4	34	OK	0.05249413	0.54482253	0.09635098	0.92324	0.04358581	subgrp_cor	count		1	0
SYNC	7	OK	0.58218257	1.07028791	0.5438936	0.58651	0.1815886	subgrp_cor	count		1	0
YARS	4	OK	1.9258645	2.51548794	0.7656425	0.44389	0.56022119	subgrp_cor	count		1	0
S100PBP	4	OK	-0.6977694	1.69645387	-0.4113106	0.68084	-0.33591720	subgrp_cor	count		1	0
TMEM54	35	OK	-0.6609527	0.57537605	-1.1487317	0.25067	-0.57152960	subgrp_cor	count		1	0
RNF19B	3	OK	-1.5371297	1.64874199	-0.9323046	0.35119	-0.40043369	subgrp_cor	count		1	0
AK2	64	OK	-0.554778	0.3742823	-1.4822445	0.13828	-0.69792764	subgrp_cor	count		1	0
ZNF362	4	OK	0.45568755	1.46808638	0.3103956	0.75626	0.00030627	subgrp_cor	count		1	0
PHC2	33	OK	0.12075938	0.51450997	0.23470756	0.81444	0.11423734	subgrp_cor	count		1	0
SMIM12	25	OK	-0.3408286	0.67286682	-0.5065321	0.61248	-0.3326330	subgrp_cor	count		1	0
ZMYM6	3	OK	-10.475634	27.4919879	-0.3810432	0.70317	-1.13811785	subgrp_cor	count		1	0
ZMYM1	3	OK	-13.271979	28.0097917	-0.4738335	0.63562	-2.0156631	subgrp_cor	count		1	0
SFPQ	256	OK	-0.3170104	0.17584965	-1.8027354	0.07142	-0.43194446	subgrp_cor	count		1	0
ZMYM4	13	OK	-0.5120024	0.9202155	-0.556394	0.57794	-0.37370352	subgrp_cor	count		1	0
KIAA0319L	6	OK	-0.1902356	1.22474264	-0.155327	0.87656	-0.05772119	subgrp_cor	count		1	0
PSMB2	152	OK	-0.1777356	0.2373179	-0.7489345	0.4539	-0.24081532	subgrp_cor	count		1	0
CLSPN	1	OK	-11.019418	43.2499352	-0.2547846	0.79889	-1.17482461	subgrp_cor	count		1	0
AGO4	3	OK	-3.2376766	2.4624292	-1.3148303	0.18857	-1.30346697	subgrp_cor	count		1	0
AGO1	2	OK	3.45981497	4.78586864	0.72292309	0.46973	0.00081935	subgrp_cor	count		1	0
AGO3	19	OK	1.52488749	0.84682287	1.80071932	0.07175	0.94614632	subgrp_cor	count		1	0
ADPRHL2	4	OK	4.66122998	5.76838606	0.80808217	0.41906	0.00274918	subgrp_cor	count		1	0
COL8A2	1	OK	18.7594894	83.6251792	0.22432824	0.8225	0.00028789	subgrp_cor	count		1	0
TRAPPC3	55	OK	1.51394837	0.45856419	3.30149716	0.00096	1.74852273	subgrp_cor	count		1	0
MAP7D1	32	OK	0.62582628	0.56413098	1.10936337	0.26727	0.47136614	subgrp_cor	count		1	0
THRAP3	202	OK	0.31226957	0.1929951	1.61801808	0.10566	0.4307775	subgrp_cor	count		1	0
EVA1B	117	OK	0.89208438	0.29798673	2.99370489	0.00276	1.17237501	subgrp_cor	count		1	0
STK40	3	OK	-1.6462253	1.93519224	-0.8506776	0.39495	-0.7036061	subgrp_cor	count		1	0
LSM10.00	30	OK	0.71554709	0.55937725	1.27918517	0.20082	0.78835735	subgrp_cor	count		1	0
MRPS15	64	OK	0.4746795	0.3665018	1.29516279	0.19526	0.5828622	subgrp_cor	count		1	0
LINC01137	1	OK	-11.019418	43.2499352	-0.2547846	0.79889	-1.17482461	subgrp_cor	count		1	0
ZC3H12A	20	OK	-1.2897038	0.6707825	-1.9226856	0.05452	-1.17926813	subgrp_cor	count		1	0
MEAF6	63	OK	-0.4584177	0.3514586	-1.3043292	0.19212	-0.57532826	subgrp_cor	count		1	0
SNIP1	13	OK	0.3451859	0.93157672	0.37053942	0.71096	0.10720002	subgrp_cor	count		1	0
DNALI1	1	OK	1.58229999	2.72001883	0.58176068	0.56072	0.47433761	subgrp_cor	count		1	0
GNL2	13	OK	0.15741536	0.83654023	0.18817429	0.85074	0.15592848	subgrp_cor	count		1	0
YRDC	32	OK	-0.6048838	0.56597077	-1.0687545	0.28519	-0.64043168	subgrp_cor	count		1	0
C1orf122	109	OK	1.30056279	0.32294742	4.02716596	5.65E-05	1.67471710	subgrp_cor	count		0.56142767	0
MTF1	5	OK	3.69450387	2.48621439	1.48599569	0.13728	0.83624796	subgrp_cor	count		1	0
INPP5B	2	OK	0.16816909	1.72532872	0.09747075	0.92235	0.05201879	subgrp_cor	count		1	0
SF3A3	42	OK	-0.3089803	0.46646766	-0.6623831	0.50772	-0.3504555	subgrp_cor	count		1	0
FHL3	13	OK	1.24543493	1.02046359	1.22045993	0.22229	1.01191854	subgrp_cor	count		1	0
UTP11	51	OK	0.72452335	0.42148319	1.71898515	0.08562	0.88759931	subgrp_cor	count		1	0
RRAGC	13	OK	-0.0823207	0.83124358	-0.0990332	0.92111	-0.05300079	subgrp_cor	count		1	0
MYCBP	2	OK	2.40963371	2.47152069	0.97495997	0.32958	0.66517343	subgrp_cor	count		1	0
AKIRIN1	41	OK	-0.1251289	0.44504349	-0.2811607	0.77859	-0.13765295	subgrp_cor	count		1	0
NDUFS5	423	OK	0.4237568	0.1340726	3.16065178	0.00157	0.59925641	subgrp_cor	count		1	0
MACF1	203	OK	0.72012489	0.2148425	3.3518734	0.0008	1.00038521	subgrp_cor	count		1	0
PABPC4	34	OK	0.95069943	0.56447319	1.68422422	0.09214	0.89139789	subgrp_cor	count		1	0
PPIE	27	OK	-0.0718668	0.55002145	-0.1306615	0.89604	-0.07747746	subgrp_cor	count		1	0
TRIT1	2	OK	-2.1660098	2.70416007	-0.8009917	0.42314	-0.79067866	subgrp_cor	count		1	0
CAP1	176	OK	-0.0054795	0.21472866	-0.0255182	0.97964	-0.00732812	subgrp_cor	count		1	0
PPT1	30	OK	0.71642858	0.6623357	1.08166988	0.2794	0.59497816	subgrp_cor	count		1	0
RLF	11	OK	-0.4690224	0.94505283	-0.4962922	0.61969	-0.3425791	subgrp_cor	count		1	0
ZMPSTE24	8	OK	1.27165799	1.44550899	0.87973025	0.37901	0.64544097	subgrp_cor	count		1	0
COL9A2	1	OK	-11.019418	43.2499352	-0.2547846	0.79889	-1.17482461	subgrp_cor	count		1	0
SMAP2	3	OK	-1.2956167	2.0414257	-0.6346627	0.52565	-0.00075754	subgrp_cor	count		1	0
ZFP69	1	OK	3.45981463	6.7714513	0.51094137	0.60939	0.00030179	subgrp_cor	count		1	0
ZNF684	1	OK	18.7594894	83.6251792	0.22432824	0.8225	0.00028789	subgrp_cor	count		1	0
NFYC	9	OK	-2.2393055	1.28174479	-1.7470758	0.08062	-0.87347336	subgrp_cor	count		1	0
CITED4	24	OK	2.9307923	1.05927527	2.76679007	0.00566	2.41805085	subgrp_cor	count		1	0
CTPS1	4	OK	3.45981463	6.7714513	0.51094137	0.60939	0.00030179	subgrp_cor	count		1	0
HIVEP3	1	OK	3.45981463	6.7714513	0.51094137	0.60939	0.00030179	subgrp_cor	count		1	0
FOXJ3	31	OK	0.1301291	0.5054808	0.25743628	0.79684	0.14394472	subgrp_cor	count		1	0
RIMKLA	3	OK	-0.6348896	2.00896003	-0.3160275	0.75198	-0.000145	subgrp_cor	count		1	0
ZMYND12	1	OK	3.45981463	6.7714513	0.51094137	0.60939	0.00030179	subgrp_cor	count		1	0
PPCS	126	OK	-0.1849745	0.2518675	-0.7344117	0.4627	-0.24444097	subgrp_cor	count		1	0
PPIH	22	OK	-0.2518011	0.59974008	-0.4198504	0.67459	-0.26350831	subgrp_cor	count		1	0
YBX1	805	OK	-0.2248093	0.09255974	-2.428802	0.01515	-0.32111265	subgrp_cor	count		1	0
P3H1	5	OK	2.8951542	2.41327967	1.19967621	0.23027	0.75227995	subgrp_cor	count		1	0
SVBP	39	OK	-0.8063137	0.4683507	-1.7216025	0.08514	-0.96129268	subgrp_cor	count		1	0
SLC2A1	1	OK	23.9918963	84.8999419	0.28259026	0.77749	1.65081709	subgrp_cor	count		1	0
EBNA1BP2	41	OK	-0.3081826	0.50052279	-0.6157214	0.53808	-0.28810915	subgrp_cor	count		1	0
TIE1	85	OK	0.47503648	0.33013166	1.43893039	0.15017	0.58767811	subgrp_cor	count		1	0
ELOVL1	56	OK	0.02477259	0.42944688	0.05768487	0.954	0.03060427	subgrp_cor	count		1	0
MED8	11	OK	0.7364469	0.88402163	0.83306434	0.40481	0.61144280	subgrp_cor	count		1	0
HYI	37	OK	1.69808364	0.55651279	3.05129308	0.00228	1.91886929	subgrp_cor	count		1	0
PTPRF	2	OK	21.1053223	83.3412731	0.25323976	0.80008	0.0003103	subgrp_cor	count		1	0
DPH2	3	OK	1.77247723	1.84396277	0.96123265	0.33644	0.84969892	subgrp_cor	count		1	0
ATP6V0B	201	OK	0.68123771	0.2166785	3.14446396	0.00166	0.93254497	subgrp_cor	count		1	0
B4GALT2	4	OK	2.01764986	2.21208781	0.91210207	0.36171	0.00137857	subgrp_cor	count		1	0
DMAP1	11	OK	-0.5260986	0.83283704	-0.6316946	0.52759	-0.25731817	subgrp_cor	count		1	0
ERI3	18	OK	1.01583775	0.74624182	1.36127155	0.17342	0.95006949	subgrp_cor	count		1	0
RPS8	892	OK	0.01126809	0.08164721	0.13923386	0.88927	0.01627706	subgrp_cor	count		1	0
PLK3	20	OK	1.35784987	0.77180203	1.75932404	0.07852	1.17922642	subgrp_cor	count		1	0
BTBD19	1	OK	18.7594894	83.6251792	0.22432824	0.8225	0.00028789	subgrp_cor	count		1	0
EIF2B3	10	OK	0.72427479	1.04369781	0.69395066	0.48771	0.22576517	subgrp_cor	count		1	0
HECTD3	1	OK	3.45981463	6.7714513	0.51094137	0.60939	0.00030179	subgrp_cor	count		1	0
UROD	40	OK	0.37803438	0.46176234	0.8186773	0.41297	0.45720720	subgrp_cor	count		1	0
TOE1	2	OK	-0.1509758	1.968815	-0.0766836	0.93888	-9.78E-05	subgrp_cor	count		1	0
PRDX1	694	OK	0.00074873	0.10051452	0.00744898	0.99406	0.00106765	subgrp_cor	count		1	0
AKR1A1	120	OK	0.47639014	0.24957873	1.90877699	0.05629	0.65047022	subgrp_cor	count		1	0
NASP	244	OK	-0.2732432	0.17627293	-1.5501142	0.12111	-0.37516741	subgrp_cor	count		1	0
GPBP1L1	18	OK	1.42873941	0.86150379	1.65842498	0.09722	1.05164820	subgrp_cor	count		1	0
TMEM69	3	OK	-0.6348896	2.00896003	-0.3160275	0.75198	-0.000145	subgrp_cor	count		1	0
IPP	2	OK	21.642059	54.4868919	0.39719753	0.69122	1.57744735	subgrp_cor	count		1	0
MAST2	1	OK	-11.019418	43.2499352	-0.2547846	0.79889	-1.17482461	subgrp_cor	count		1	0
PIK3R3	41	OK	0.87108097	0.54268796	1.60512308	0.10847	0.65196617	subgrp_cor	count		1	0
TSPAN1	4	OK	-2.251343	2.141646	-1.0512209	0.29316	-1.08348305	subgrp_cor	count		1	0
POMGNT1	5	OK	-0.5463772	1.2972777	-0.4211721	0.67362	-0.34177821	subgrp_cor	count		1	0
LURAP1	2	OK	26.3679501	84.9163259	0.31051685	0.75617	2.49052421	subgrp_cor	count		1	0
LRRC41	4	OK	-0.8416402	1.46082315	-0.5761411	0.56452	-0.24034852	subgrp_cor	count		1	0
UQCRH	545	OK	-0.1605976	0.1170311	-1.3722668	0.16998	-0.22773842	subgrp_cor	count		1	0
NSUN4	3	OK	-0.910142	2.18812438	-0.4159466	0.67745	-0.25784050	subgrp_cor	count		1	0
MKNK1	4	OK	3.40162373	2.68088574	1.26884324	0.2045	0.9636701	subgrp_cor	count		1	0
MOB3C	2	OK	1.11124494	1.86858082	0.59469996	0.55204	0.34276981	subgrp_cor	count		1	0
ATPAF1	2	OK	21.1053223	83.3412731	0.25323976	0.80008	0.0003103	subgrp_cor	count		1	0
EFCAB14	81	OK	1.01236396	0.35649783	2.83974785	0.00451	1.2881379	subgrp_cor	count		1	0
CYP4X1	14	OK	23.8267197	32.9165826	0.72385156	0.46916	3.54150283	subgrp_cor	count		1	0
PDZK1IP1	8	OK	25.1387123	54.0636937	0.46498325	0.64194	2.93759967	subgrp_cor	count		1	0
TAL1	2	OK	21.1053223	83.3412731	0.25323976	0.80008	0.0003103	subgrp_cor	count		1	0
CMPK1	98	OK	-0.2786789	0.29162472	-0.9556078	0.33927	-0.35682756	subgrp_cor	count		1	0
TRABD2B	1	OK	-10.442278	41.4658099	-0.2518286	0.80117	-0.00045289	subgrp_cor	count		1	0
SPATA6	9	OK	0.35136761	0.98290673	0.35747808	0.72072	0.17965397	subgrp_cor	count		1	0
BEND5	9	OK	0.09267468	1.03246195	0.08976083	0.92848	0.07702634	subgrp_cor	count		1	0
FAF1	5	OK	-1.5608049	1.35047571	-1.1557446	0.24779	-0.87165418	subgrp_cor	count		1	0
CDKN2C	14	OK	5.56432483	2.79812345	1.98859162	0.04675	2.32480908	subgrp_cor	count		1	0
RNF11	56	OK	1.42369183	0.43463135	3.27563077	0.00105	1.66872849	subgrp_cor	count		1	0
EPS15	19	OK	1.45705479	0.8421979	1.7300621	0.08362	1.16731501	subgrp_cor	count		1	0
OSBPL9	24	OK	-0.049618	0.70105583	-0.0707761	0.94358	-0.03712321	subgrp_cor	count		1	0
NRDC	49	OK	0.17608954	0.42654053	0.41283183	0.67972	0.21109564	subgrp_cor	count		1	0
TXNDC12	111	OK	0.12431569	0.26899713	0.46214504	0.64398	0.16491701	subgrp_cor	count		1	0
KTI12	3	OK	4.21092061	4.28756165	0.9821248	0.32604	0.00257090	subgrp_cor	count		1	0
BTF3L4	66	OK	0.2669629	0.35540747	0.75114599	0.45256	0.32553440	subgrp_cor	count		1	0
ZFYVE9	2	OK	23.9918963	84.8999419	0.28259026	0.77749	1.65081709	subgrp_cor	count		1	0
PRPF38A	34	OK	0.17417443	0.52551884	0.33143262	0.74032	0.17852957	subgrp_cor	count		1	0
TUT4	63	OK	-0.0039911	0.3759023	-0.0106173	0.99153	-0.00481921	subgrp_cor	count		1	0
GPX7	9	OK	-0.5897972	1.18556066	-0.4974838	0.61885	-0.37748027	subgrp_cor	count		1	0
COA7	1	OK	23.9918963	84.8999419	0.28259026	0.77749	1.65081709	subgrp_cor	count		1	0
ZYG11B	6	OK	-0.1707575	1.26616852	-0.1348615	0.89272	-0.00011045	subgrp_cor	count		1	0
ECHDC2	5	OK	0.13080102	1.25921969	0.10387466	0.91727	0.0983791	subgrp_cor	count		1	0
SCP2	206	OK	-0.1497473	0.19811728	-0.7558518	0.44974	-0.20588865	subgrp_cor	count		1	0
CPT2	1	OK	3.45981463	6.7714513	0.51094137	0.60939	0.00030179	subgrp_cor	count		1	0
C1orf123	104	OK	-0.0421038	0.26683222	-0.1577911	0.87462	-0.05591683	subgrp_cor	count		1	0
MAGOH	120	OK	-0.5673723	0.26646634	-2.1292458	0.03323	-0.75583212	subgrp_cor	count		1	0
YIPF1	3	OK	-0.3495512	1.85447474	-0.1884907	0.85049	-0.17337783	subgrp_cor	count		1	0
HSPB11	34	OK	-0.0366516	0.54885883	-0.0667779	0.94676	-0.0395422	subgrp_cor	count		1	0
TMEM59	578	OK	0.04750119	0.11130215	0.42677693	0.66954	0.06758173	subgrp_cor	count		1	0
TCEANC2	2	OK	20.0743005	50.685294	0.39605769	0.69206	0.00081338	subgrp_cor	count		1	0
CYB5RL	1	OK	18.7594894	83.6251792	0.22432824	0.8225	0.00028789	subgrp_cor	count		1	0
MRPL37	21	OK	-1.2147578	0.75846809	-1.6015938	0.10925	-0.83011225	subgrp_cor	count		1	0
SSBP3	8	OK	-0.8240422	0.92876398	-0.8872461	0.37495	-0.23482530	subgrp_cor	count		1	0
ACOT11	2	OK	-0.2029407	1.65323657	-0.1227536	0.9023	-4.82E-05	subgrp_cor	count		1	0
TTC4	1	OK	18.7594894	83.6251792	0.22432824	0.8225	0.00028789	subgrp_cor	count		1	0
USP24	4	OK	-1.4072272	1.87423031	-0.7508294	0.45276	-0.35877740	subgrp_cor	count		1	0
PLPP3	56	OK	1.42534677	0.46162904	3.08764535	0.00202	1.68630916	subgrp_cor	count		1	0
PRKAA2	1	OK	-11.019418	43.2499352	-0.2547846	0.79889	-1.17482461	subgrp_cor	count		1	0
OMA1	1	OK	-10.442278	41.4658099	-0.2518286	0.80117	-0.00045289	subgrp_cor	count		1	0
TACSTD2	10	OK	-1.4719486	1.00569717	-1.4636102	0.1433	-1.25855173	subgrp_cor	count		1	0
MYSM1	7	OK	1.16974377	1.1885051	0.9842143	0.32501	0.77027054	subgrp_cor	count		1	0
JUN	714	OK	-0.20147	0.0956713	-2.1058555	0.03522	-0.28757229	subgrp_cor	count		1	0
FGGY	2	OK	-0.9102232	2.1881125	-0.4159856	0.67742	-0.25785735	subgrp_cor	count		1	0
NFIA-AS2	2	OK	1.9258645	2.51548794	0.7656425	0.44389	0.56022119	subgrp_cor	count		1	0
TM2D1	75	OK	1.09700604	0.34538366	3.1761955	0.00149	1.37520418	subgrp_cor	count		1	0
PATJ	1	OK	18.7594894	83.6251792	0.22432824	0.8225	0.00028789	subgrp_cor	count		1	0
USP1	44	OK	0.65018136	0.49886332	1.30332565	0.19246	0.70219301	subgrp_cor	count		1	0
ATG4C	4	OK	0.51030031	1.6034059	0.31826022	0.75029	0.15911328	subgrp_cor	count		1	0
ALG6	3	OK	0.51030031	1.6034059	0.31826022	0.75029	0.15911328	subgrp_cor	count		1	0
ITGB3BP	15	OK	-0.2371931	0.72797684	-0.3258251	0.74456	-0.19494072	subgrp_cor	count		1	0
PGM1	4	OK	-15.488617	42.8316448	-0.3616162	0.71764	-0.00073897	subgrp_cor	count		1	0
CACHD1	7	OK	-0.7743652	1.00436947	-0.7709962	0.44071	-0.22282340	subgrp_cor	count		1	0
JAK1	322	OK	-0.6566213	0.15704956	-4.1809815	2.9E-05	-0.91817422	subgrp_cor	count		0.28946875	0
AK4	12	OK	-2.9816188	1.24218666	-2.4002985	0.01638	-1.60514967	subgrp_cor	count		1	0
LEPROT	502	OK	-0.1456265	0.12068176	-1.2066982	0.22755	-0.20641284	subgrp_cor	count		1	0
PDE4B	16	OK	2.57952571	0.88060423	2.92926791	0.0034	2.59808575	subgrp_cor	count		1	0
SGIP1	1	OK	3.45981463	6.7714513	0.51094137	0.60939	0.00030179	subgrp_cor	count		1	0
TCTEX1D1	2	OK	-1.7585736	2.35544393	-0.7465986	0.45531	-0.00036089	subgrp_cor	count		1	0
WDR78	1	OK	-10.442278	41.4658099	-0.2518286	0.80117	-0.00045289	subgrp_cor	count		1	0
MIER1	139	OK	-0.137936	0.24332959	-0.5668692	0.5708	-0.1846061	subgrp_cor	count		1	0
SLC35D1	1	OK	18.7594894	83.6251792	0.22432824	0.8225	0.00028789	subgrp_cor	count		1	0
SERBP1	520	OK	-0.444174	0.11982488	-3.7068598	0.00021	-0.62842912	subgrp_cor	count		1	0
GADD45A	128	OK	-0.3465245	0.24961062	-1.3882614	0.16506	-0.4642867	subgrp_cor	count		1	0
GNG12	81	OK	0.38558108	0.34136105	1.12954022	0.25867	0.48757974	subgrp_cor	count		1	0
DIRAS3	2	OK	23.9918963	84.8999419	0.28259026	0.77749	1.65081709	subgrp_cor	count		1	0
WLS	46	OK	1.67861571	0.48535898	3.45850347	0.00054	1.96402183	subgrp_cor	count		1	0
LRRC40	1	OK	3.45981463	6.7714513	0.51094137	0.60939	0.00030179	subgrp_cor	count		1	0
SRSF11	291	OK	0.05832286	0.16220125	0.35957098	0.71917	0.08163391	subgrp_cor	count		1	0
ANKRD13C	6	OK	-10.406692	16.7648652	-0.6207436	0.53477	-2.19585791	subgrp_cor	count		1	0
HHLA3	4	OK	3.0502985	2.44043936	1.24989728	0.21134	1.24934625	subgrp_cor	count		1	0
ZRANB2	117	OK	-0.0455041	0.2583290	-0.1761477	0.86018	-0.06135114	subgrp_cor	count		1	0
NEGR1	5	OK	24.0001923	54.3454405	0.44162292	0.65876	2.40091057	subgrp_cor	count		1	0
FPGT	1	OK	-11.019418	43.2499352	-0.2547846	0.79889	-1.17482461	subgrp_cor	count		1	0
CRYZ	1	OK	3.45981463	6.7714513	0.51094137	0.60939	0.00030179	subgrp_cor	count		1	0
TYW3	49	OK	0.11194633	0.42573989	0.26294572	0.79259	0.12362520	subgrp_cor	count		1	0
ACADM	30	OK	0.37803459	0.5552163	0.68087803	0.49595	0.42574373	subgrp_cor	count		1	0
RABGGTB	19	OK	0.13120011	0.80978042	0.16201937	0.87129	0.06668946	subgrp_cor	count		1	0
ST6GALNAC3	6	OK	-1.0001779	1.19531481	-0.8367485	0.40272	-0.28026428	subgrp_cor	count		1	0
PIGK	19	OK	-0.7140677	0.7080386	-1.0085153	0.31321	-0.51308188	subgrp_cor	count		1	0
AC118549.1	11	OK	0.46017047	0.87500345	0.52590704	0.59895	0.23408988	subgrp_cor	count		1	0
USP33	13	OK	-0.2292723	0.84490973	-0.2713582	0.78612	-0.17033755	subgrp_cor	count		1	0
MIGA1	18	OK	1.29957348	0.73780169	1.76141294	0.07817	1.13272380	subgrp_cor	count		1	0
NEXN	14	OK	2.78908408	1.00403068	2.7778878	0.00547	2.62554324	subgrp_cor	count		1	0
FUBP1	30	OK	1.07434271	0.64642954	1.66196418	0.09652	0.79721511	subgrp_cor	count		1	0
DNAJB4	138	OK	0.13536029	0.24638294	0.54936757	0.58275	0.18216042	subgrp_cor	count		1	0
GIPC2	36	OK	-1.3148862	0.52135797	-2.522041	0.01167	-1.14152876	subgrp_cor	count		1	0
AC103591.3	2	OK	1.9258645	2.51548794	0.7656425	0.44389	0.56022119	subgrp_cor	count		1	0
IFI44L	141	OK	0.01354658	0.22386296	0.06050969	0.95175	0.01833240	subgrp_cor	count		1	0
IFI44	28	OK	0.79426368	0.51312464	1.54809109	0.1216	0.8738537	subgrp_cor	count		1	0
ADGRL2	26	OK	-0.5491576	0.65809334	-0.8344676	0.40402	-0.54927250	subgrp_cor	count		1	0
PRKACB	13	OK	2.39471136	1.17359931	2.04048463	0.0413	1.0708475	subgrp_cor	count		1	0
SAMD13	1	OK	3.45981463	6.7714513	0.51094137	0.60939	0.00030179	subgrp_cor	count		1	0
RPF1	8	OK	0.59320289	0.98395243	0.60287761	0.54659	0.18509337	subgrp_cor	count		1	0
GNG5	620	OK	0.10497487	0.10646473	0.98600606	0.32417	0.14951676	subgrp_cor	count		1	0
CTBS	50	OK	1.00126977	0.42769719	2.34107164	0.01923	1.25276752	subgrp_cor	count		1	0
SSX2IP	7	OK	21.1864031	33.1957895	0.63822561	0.52332	2.29602037	subgrp_cor	count		1	0
MCOLN3	1	OK	18.7594894	83.6251792	0.22432824	0.8225	0.00028789	subgrp_cor	count		1	0
C1orf52	38	OK	-0.347925	0.4901260	-0.7098684	0.47779	-0.38764899	subgrp_cor	count		1	0
BCL10	56	OK	0.113683	0.3791594	0.2998290	0.76431	0.14754489	subgrp_cor	count		1	0
ZNHIT6	24	OK	0.23569275	0.67814684	0.34755416	0.72818	0.2498688	subgrp_cor	count		1	0
ODF2L	131	OK	-0.0192815	0.24009023	-0.0803092	0.93599	-0.02561062	subgrp_cor	count		1	0
AL049597.2	1	OK	23.9918963	84.8999419	0.28259026	0.77749	1.65081709	subgrp_cor	count		1	0
SH3GLB1	281	OK	-0.0151375	0.16824297	-0.0899742	0.92831	-0.02111469	subgrp_cor	count		1	0
SELENOF	194	OK	0.33324243	0.2037404	1.63562275	0.10192	0.45716871	subgrp_cor	count		1	0
HS2ST1	1	OK	-11.019418	43.2499352	-0.2547846	0.79889	-1.17482461	subgrp_cor	count		1	0
LMO4	70	OK	-0.0835844	0.33379243	-0.2504082	0.80227	-0.10166619	subgrp_cor	count		1	0
PKN2	71	OK	0.34530588	0.39505062	0.87408007	0.38207	0.42201492	subgrp_cor	count		1	0
GTF2B	53	OK	-1.2762347	0.42025228	-3.0368299	0.00239	-1.4596568	subgrp_cor	count		1	0
KYAT3	2	OK	1.58229999	2.72001883	0.58176068	0.56072	0.47433761	subgrp_cor	count		1	0
RBMXL1	4	OK	-1.3967094	1.3668523	-1.0218437	0.30685	-0.37125920	subgrp_cor	count		1	0
GBP3	13	OK	0.31703751	0.70718955	0.44830627	0.65392	0.28425879	subgrp_cor	count		1	0
GBP1	112	OK	-1.1466899	0.26250081	-4.3611848	1.29E-05	-1.53819409	subgrp_cor	count		0.12962758	0
GBP7	2	OK	-11.019418	43.2499352	-0.2547846	0.79889	-1.17482461	subgrp_cor	count		1	0
GBP5	3	OK	-0.53361	1.64444679	-0.3244921	0.74557	-0.33080580	subgrp_cor	count		1	0
LRRC8B	2	OK	-11.189367	26.3349482	-0.4248866	0.67092	-2.27128013	subgrp_cor	count		1	0
LRRC8C-DT	2	OK	-15.488617	42.8316448	-0.3616162	0.71764	-0.00073897	subgrp_cor	count		1	0
LRRC8C	20	OK	1.12868743	0.79193721	1.42522337	0.15409	0.72149237	subgrp_cor	count		1	0
LRRC8D	2	OK	41.7883033	174.29608	0.23975469	0.81052	0.00014488	subgrp_cor	count		1	0
ZNF326	50	OK	1.41217435	0.49739136	2.83916143	0.00452	1.59781951	subgrp_cor	count		1	0
ZNF644	49	OK	0.1125434	0.41828146	0.2690614	0.78788	0.13203584	subgrp_cor	count		1	0
TGFBR3	117	OK	-0.5005229	0.24901259	-2.0100304	0.04442	-0.66730303	subgrp_cor	count		1	0
EPHX4	5	OK	2.33396234	2.24355	1.04029879	0.2982	0.64988602	subgrp_cor	count		1	0
RPAP2	20	OK	-0.3985916	0.64725728	-0.6157211	0.53809	-0.40147587	subgrp_cor	count		1	0
EVI5	63	OK	2.36886153	0.5471484	4.3294681	1.49E-05	2.47291504	subgrp_cor	count		0.14945497	0
RPL5	875	OK	-0.140712	0.0851849	-1.6518414	0.09857	-0.20134405	subgrp_cor	count		1	0
FAM69A	1	OK	1.58229999	2.72001883	0.58176068	0.56072	0.47433761	subgrp_cor	count		1	0
MTF2	18	OK	1.84454756	1.0828486	1.70342148	0.08849	0.0034537	subgrp_cor	count		1	0
TMED5	22	OK	1.23911938	0.71277132	1.73845286	0.08217	1.19800552	subgrp_cor	count		1	0
CCDC18	1	OK	-11.019418	43.2499352	-0.2547846	0.79889	-1.17482461	subgrp_cor	count		1	0
DR1	22	OK	0.32059512	0.62058419	0.51660214	0.60542	0.33886505	subgrp_cor	count		1	0
FNBP1L	94	OK	-0.6588619	0.30140402	-2.1859758	0.02882	-0.87024731	subgrp_cor	count		1	0
BCAR3	5	OK	-15.995031	70.349421	-0.2273655	0.82014	-0.00028474	subgrp_cor	count		1	0
CNTTIP2	117	OK	-0.2914485	0.26020276	-1.1200822	0.26269	-0.38766063	subgrp_cor	count		1	0
GCLM	12	OK	2.37849368	1.20062095	1.98105291	0.04759	1.56893861	subgrp_cor	count		1	0
ARHGAP29	386	OK	-0.108469	0.1393358	-0.7784651	0.43629	-0.15268731	subgrp_cor	count		1	0
ABCD3	2	OK	-0.3495512	1.85447474	-0.1884907	0.85049	-0.17337783	subgrp_cor	count		1	0
F3	1	OK	1.58229999	2.72001883	0.58176068	0.56072	0.47433761	subgrp_cor	count		1	0
SLC44A3	1	OK	1.58229999	2.72001883	0.58176068	0.56072	0.47433761	subgrp_cor	count		1	0
ALG14	7	OK	-2.0788657	1.39800223	-1.487026	0.13701	-0.49522376	subgrp_cor	count		1	0
PTBP2	7	OK	0.28837896	1.07055514	0.26937329	0.78764	0.00019219	subgrp_cor	count		1	0
DPYD	5	OK	-2.3422221	1.73677178	-1.3486067	0.17746	-0.53905893	subgrp_cor	count		1	0
SNX7	14	OK	-0.2237199	0.88817313	-0.2518878	0.80112	-0.16614060	subgrp_cor	count		1	0
PALMD	540	OK	0.02897028	0.11510526	0.2516855	0.80128	0.04108952	subgrp_cor	count		1	0
AGL	10	OK	0.92940289	0.96182445	0.9662916	0.3339	0.71909280	subgrp_cor	count		1	0
SLC35A3	10	OK	0.51027538	1.13121152	0.45108747	0.65193	0.26115712	subgrp_cor	count		1	0
SASS6	5	OK	-1.4843857	1.39837172	-1.0615101	0.28846	-0.6493907	subgrp_cor	count		1	0
TRMT13	6	OK	0.5102869	1.13123316	0.45108906	0.65193	0.26116205	subgrp_cor	count		1	0
DBT	4	OK	-11.448548	41.761485	-0.2741412	0.78398	-0.00049246	subgrp_cor	count		1	0
RTCA	15	OK	0.95530527	0.83094652	1.14965915	0.25028	0.71273426	subgrp_cor	count		1	0
CDC14A	4	OK	2.98494006	2.59888574	1.14854939	0.25074	1.23382860	subgrp_cor	count		1	0
VCAM1	195	OK	-0.638795	0.19689109	-3.2444077	0.00118	-0.8870860	subgrp_cor	count		1	0
EXTL2	2	OK	0.1681491	1.72533008	0.0974591	0.92236	0.05201277	subgrp_cor	count		1	0
AC104506.1	7	OK	-2.0999408	1.31769785	-1.5936437	0.11102	-1.69753133	subgrp_cor	count		1	0
SLC30A7	16	OK	-0.0944377	0.85997687	-0.1098142	0.91256	-0.04761448	subgrp_cor	count		1	0
DPH5	2	OK	21.1053223	83.3412731	0.25323976	0.80008	0.0003103	subgrp_cor	count		1	0
AC093157.1	5	OK	-1.3401314	1.68194103	-0.7967767	0.42558	-0.58807560	subgrp_cor	count		1	0
AL109741.1	10	OK	-2.9277763	1.31930347	-2.219185	0.02647	-1.29768001	subgrp_cor	count		1	0
S1PR1	90	OK	-0.6333933	0.31699817	-1.9980977	0.04571	-0.7825274	subgrp_cor	count		1	0
RNPC3	13	OK	-0.9886872	0.81816869	-1.2084149	0.22689	-0.95411103	subgrp_cor	count		1	0

PRMT6	1	OK	1.58229999	2.72001883	0.58176068	0.56072	0.47433761	subgrp_cor	count		1	0
VAV3	1	OK	3.45981463	6.77145131	0.51094137	0.60939	0.00030179	subgrp_cor	count		1	0
SLC25A24	21	OK	-0.3135878	0.69839319	-0.4490132	0.65342	-0.19943262	subgrp_cor	count		1	0
PRPF38B	91	OK	0.69936207	0.3410435	2.05065349	0.0403	0.85831497	subgrp_cor	count		1	0
STXBP3	20	OK	0.29692852	0.61870944	0.47991593	0.63129	0.30458963	subgrp_cor	count		1	0
CLCC1	3	OK	5.26127895	5.49058205	0.95823701	0.33794	0.00295034	subgrp_cor	count		1	0
WDR47	3	OK	3.45978634	3.90997673	0.88486111	0.37622	0.00081829	subgrp_cor	count		1	0
TAF13	12	OK	0.99747905	1.0625656	0.93874585	0.34786	0.00185672	subgrp_cor	count		1	0
TMEM167B	8	OK	0.98004711	1.0133837	0.96710372	0.33349	0.30380744	subgrp_cor	count		1	0
SARS	85	OK	0.17207265	0.32655498	0.52691703	0.59825	0.21409408	subgrp_cor	count		1	0
SORT1	6	OK	1.41194267	1.49593083	0.94385555	0.34524	0.69797651	subgrp_cor	count		1	0
PSMA5	93	OK	0.38725611	0.32609114	1.18757017	0.235	0.49600225	subgrp_cor	count		1	0
CYB561D1	1	OK	-12.148421	43.4553091	-0.2795612	0.77981	-1.91362624	subgrp_cor	count		1	0
GNAI3	85	OK	-0.4065263	0.33380937	-1.2178397	0.22329	-0.45959494	subgrp_cor	count		1	0
GSTM4	4	OK	2.8951547	2.41327967	1.19967621	0.23027	0.75227995	subgrp_cor	count		1	0
GSTM3	19	OK	1.39296596	0.80283147	1.73506647	0.08272	1.20694680	subgrp_cor	count		1	0
CSF1	11	OK	-0.2092285	0.8033421	-0.2604475	0.79452	-0.1047579	subgrp_cor	count		1	0
AHCYL1	23	OK	-0.3468118	0.69330734	-0.5002281	0.61691	-0.25562804	subgrp_cor	count		1	0
STRIP1	1	OK	3.45981463	6.77145131	0.51094137	0.60939	0.00030179	subgrp_cor	count		1	0
KCNC4	2	OK	0.58878195	1.7674148	0.33313173	0.73902	0.30122869	subgrp_cor	count		1	0
RBM15	1	OK	18.7594894	83.6251793	0.22432824	0.8225	0.00028789	subgrp_cor	count		1	0
SLC16A4	12	OK	4.48568825	2.40017267	1.86890231	0.06164	1.88818372	subgrp_cor	count		1	0
LAMTOR5	225	OK	0.70520177	0.20188793	3.49303575	0.00048	0.97545308	subgrp_cor	count		1	0
LRIF1	14	OK	-0.6012152	0.76858235	-0.7822391	0.43407	-0.52196727	subgrp_cor	count		1	0
DRAM2	12	OK	-1.7165084	0.91883163	-1.8681425	0.06174	-0.72607480	subgrp_cor	count		1	0
DENND2D	2	OK	1.17657919	2.3465057	0.50141757	0.61608	0.00080876	subgrp_cor	count		1	0
WDR77	4	OK	0.17216145	1.5344504	0.11219747	0.91067	0.00011395	subgrp_cor	count		1	0
ATP5PB	190	OK	0.21426932	0.2069805	1.035215	0.30057	0.29394566	subgrp_cor	count		1	0
RAP1A	146	OK	-0.2740334	0.23750734	-1.153789	0.24859	-0.35560284	subgrp_cor	count		1	0
DDX20	1	OK	18.7594894	83.6251793	0.22432824	0.8225	0.00028789	subgrp_cor	count		1	0
CTTNBP2NL	76	OK	0.90027991	0.38726622	2.3247055	0.02009	1.06110880	subgrp_cor	count		1	0
ST7L	3	OK	-10.475634	27.4919879	-0.3810432	0.70317	-1.13811785	subgrp_cor	count		1	0
CAPZA1	63	OK	-0.4717846	0.37693009	-1.2516496	0.2107	-0.55345745	subgrp_cor	count		1	0
MOV10	3	OK	3.50075187	2.83048881	1.23680117	0.21616	1.66526042	subgrp_cor	count		1	0
RHOC	571	OK	-0.380181	0.11522547	-3.2991662	0.00097	-0.53962138	subgrp_cor	count		1	0
SLC16A1	2	OK	4.66132998	5.76838606	0.80808217	0.41906	0.00274918	subgrp_cor	count		1	0
SLC16A1-AS1	3	OK	-2.023032	1.80712677	-1.1194741	0.26294	-0.00109400	subgrp_cor	count		1	0
MAGI3	2	OK	21.1053233	83.3412731	0.25323976	0.80008	0.0003103	subgrp_cor	count		1	0
PHTF1	3	OK	-0.0159578	1.81610225	-0.0087868	0.99299	-0.00486873	subgrp_cor	count		1	0
RSBN1	23	OK	0.62259771	0.66165299	0.94097317	0.34672	0.58790902	subgrp_cor	count		1	0
AP4B1-AS1	1	OK	1.58229999	2.72001883	0.58176068	0.56072	0.47433761	subgrp_cor	count		1	0
AP4B1	1	OK	23.9918968	84.8999419	0.28259026	0.77749	1.65081709	subgrp_cor	count		1	0
HIPK1	7	OK	6.87169477	5.08569886	1.35118014	0.17664	1.08844099	subgrp_cor	count		1	0
TRIM33	15	OK	0.56387119	0.93992166	0.59991297	0.54856	0.37240920	subgrp_cor	count		1	0
BCAS2	41	OK	-0.2934824	0.45497793	-0.6450476	0.5188	-0.33269855	subgrp_cor	count		1	0
DENND2C	1	OK	3.45981463	6.77145131	0.51094137	0.60939	0.00030179	subgrp_cor	count		1	0
NRAS	10	OK	-1.6108743	1.36602677	-1.1792406	0.2382	-0.41511206	subgrp_cor	count		1	0
CSDE1	278	OK	0.22479346	0.16263553	1.38213014	0.16692	0.31008453	subgrp_cor	count		1	0
SIKE1	17	OK	0.50322601	0.64470692	0.78055003	0.43507	0.4988867	subgrp_cor	count		1	0
TSPAN2	39	OK	3.38731996	0.82213873	4.12013193	3.79E-05	3.44056417	subgrp_cor	count		0.37729239	0
VANGL1	7	OK	0.97739537	1.47788083	0.66134925	0.50839	0.30300516	subgrp_cor	count		1	0
ATP1A1	255	OK	0.00702508	0.17394053	0.04044531	0.96774	0.00968450	subgrp_cor	count		1	0
CD58	29	OK	1.20336747	0.63846845	1.88477203	0.05946	1.11568264	subgrp_cor	count		1	0
PTGFRN	4	OK	19.107177	50.8032779	0.37610126	0.70684	0.00078799	subgrp_cor	count		1	0
TTF2	2	OK	18.7594894	83.6251793	0.22432824	0.8225	0.00028789	subgrp_cor	count		1	0
MAN1A2	43	OK	0.56049456	0.48902307	1.14613113	0.25174	0.64985839	subgrp_cor	count		1	0
TENT5C	2	OK	-0.2029407	1.65323657	-0.1227536	0.9022	-4.82E-05	subgrp_cor	count		1	0
GDAP2	2	OK	4.66132998	5.76838606	0.80808217	0.41906	0.00274918	subgrp_cor	count		1	0
WDR3	4	OK	-11.69486	27.7874484	-0.4208677	0.67385	-2.32043716	subgrp_cor	count		1	0
WARS2	2	OK	-0.3495512	1.85447474	-0.1884907	0.85049	-0.17337783	subgrp_cor	count		1	0
AL359915.2	1	OK	23.9918968	84.8999419	0.28259026	0.77749	1.65081709	subgrp_cor	count		1	0
SEC22B	44	OK	0.88249339	0.48318201	1.82639946	0.06779	0.94842064	subgrp_cor	count		1	0
SRGAP2C	4	OK	2.07025237	1.79948267	1.15047086	0.24995	0.96210332	subgrp_cor	count		1	0
AC239800.3	1	OK	-15.995031	70.349421	-0.2273655	0.82014	-0.00028474	subgrp_cor	count		1	0
AC245595.1	7	OK	-0.2610068	1.29170486	-0.2020638	0.83987	-0.16651949	subgrp_cor	count		1	0
SRGAP2B	7	OK	0.33615313	1.11153068	0.30242372	0.76232	0.10452415	subgrp_cor	count		1	0
AC245014.3	1	OK	-15.995031	70.349421	-0.2273655	0.82014	-0.00028474	subgrp_cor	count		1	0
RNF115	97	OK	-0.7483996	0.28403735	-2.6348634	0.00842	-0.9627607	subgrp_cor	count		1	0
POLR3C	2	OK	0.58878195	1.7674148	0.33313173	0.73902	0.30122869	subgrp_cor	count		1	0
PIAS3	2	OK	-1.2956167	2.0414257	-0.6346627	0.52565	-0.00075754	subgrp_cor	count		1	0
PEX11B	1	OK	-11.019418	43.2499352	-0.2547846	0.79889	-1.17482461	subgrp_cor	count		1	0
RBM8A	353	OK	-0.3368014	0.15240932	-2.2098481	0.02712	-0.47132651	subgrp_cor	count		1	0
LIX1L-AS1	1	OK	3.45981463	6.77145131	0.51094137	0.60939	0.00030179	subgrp_cor	count		1	0
LIX1L	31	OK	-0.3025624	0.55708083	-0.5431212	0.58705	-0.33776254	subgrp_cor	count		1	0
POLR3GL	121	OK	0.24771863	0.27253413	0.90894531	0.36338	0.32351522	subgrp_cor	count		1	0
CHD1L	4	OK	1.09958881	1.31889786	0.83371803	0.40444	0.55419909	subgrp_cor	count		1	0
NBPF14	1	OK	18.7594894	83.6251793	0.22432824	0.8225	0.00028789	subgrp_cor	count		1	0
PDE4DIP	3	OK	5.26127895	5.49058205	0.95823701	0.33794	0.00295034	subgrp_cor	count		1	0
AC245297.3	2	OK	21.642059	54.4868919	0.39719753	0.69122	1.57744735	subgrp_cor	count		1	0
HIST2H2AC	8	OK	1.33554099	1.78102864	0.74987059	0.45332	0.40720078	subgrp_cor	count		1	0
BOLA1	6	OK	0.64595447	1.23277164	0.52398551	0.60029	0.33028654	subgrp_cor	count		1	0
SV2A	1	OK	17.7472073	50.9719273	0.34817612	0.72771	0.0007532	subgrp_cor	count		1	0
SF3B4	17	OK	0.21678941	0.71992673	0.30112705	0.76332	0.19422915	subgrp_cor	count		1	0
MTMR11	17	OK	20.0698321	50.6521927	0.3962283	0.69194	0.00081457	subgrp_cor	count		1	0
OTUD7B	6	OK	1.74987168	1.37016143	1.27712812	0.20150	0.8186709	subgrp_cor	count		1	0
VPS45	2	OK	3.45981497	4.78586864	0.72292309	0.46972	0.00081935	subgrp_cor	count		1	0
PLEKHO1	42	OK	1.73397459	0.65904088	2.62105773	0.00851	1.62423196	subgrp_cor	count		1	0
ANP32E	47	OK	0.29092536	0.4307998	0.67531454	0.49948	0.3149540	subgrp_cor	count		1	0
APH1A	95	OK	0.5928258	0.29484055	2.01066575	0.04436	0.75552053	subgrp_cor	count		1	0
C1orf54	101	OK	0.71065447	0.34927267	2.03466954	0.04188	0.83187698	subgrp_cor	count		1	0
CIART	3	OK	5.80492735	5.66946625	1.0238931	0.30589	1.03285716	subgrp_cor	count		1	0
MRPS21	222	OK	-0.1218899	0.18403689	-0.6623124	0.50777	-0.1688399	subgrp_cor	count		1	0
PRPF3	4	OK	-2.7254339	1.88299805	-1.4472907	0.14779	-0.59041525	subgrp_cor	count		1	0
RPRD2	4	OK	0.17216145	1.5344504	0.11219747	0.91067	0.00011395	subgrp_cor	count		1	0
ECM1	6	OK	21.642059	54.4868919	0.39719753	0.69122	1.57744735	subgrp_cor	count		1	0
MCL1	291	OK	0.68912171	0.16493223	4.17821168	2.94E-05	0.95730928	subgrp_cor	count		0.29295809	0
ENSA	229	OK	-0.264219	0.18084336	-1.460988	0.14402	-0.36523592	subgrp_cor	count		1	0
GOLPH3L	4	OK	1.15146709	1.37134218	0.83966432	0.4011	0.00079060	subgrp_cor	count		1	0
CTSS	76	OK	0.77652289	0.34925658	2.22335946	0.02619	0.95716027	subgrp_cor	count		1	0
CTSK	38	OK	4.53425066	1.31775411	3.44089282	0.00058	3.45880859	subgrp_cor	count		1	0
ARNT	1	OK	3.45981463	6.77145131	0.51094137	0.60939	0.00030179	subgrp_cor	count		1	0
CERS2	44	OK	1.55820858	0.55579291	2.80357756	0.00505	1.41292137	subgrp_cor	count		1	0
C1orf56	20	OK	0.3133229	0.69316964	0.45201474	0.65126	0.20347106	subgrp_cor	count		1	0
CDC42SE1	37	OK	-0.1211839	0.48869347	-0.2479752	0.80415	-0.13052584	subgrp_cor	count		1	0
GABPB2	2	OK	4.66132998	5.76838606	0.80808217	0.41906	0.00274918	subgrp_cor	count		1	0
SCNM1	15	OK	0.29885215	0.73814585	0.40486872	0.68557	0.30600297	subgrp_cor	count		1	0
VPS72	9	OK	0.12739526	0.91404433	0.13937536	0.88915	0.0958103	subgrp_cor	count		1	0
PSMD4	145	OK	-0.0115697	0.23879404	-0.0484504	0.96136	-0.01570578	subgrp_cor	count		1	0
AL391069.2	1	OK	3.45981463	6.77145131	0.51094137	0.60939	0.00030179	subgrp_cor	count		1	0
RFX5	1	OK	-11.019418	43.2499352	-0.2547846	0.79889	-1.17482461	subgrp_cor	count		1	0
SELENBP1	8	OK	22.6877204	54.3157576	0.41770052	0.67617	1.6098774	subgrp_cor	count		1	0
PSMB4	183	OK	0.81700282	0.22395778	3.64802163	0.00026	1.11383670	subgrp_cor	count		1	0
POGZ	10	OK	-1.4474634	1.33574871	-1.0836345	0.27852	-0.98244622	subgrp_cor	count		1	0
SNX27	12	OK	1.64822215	0.9636546	1.71038685	0.08719	1.30069531	subgrp_cor	count		1	0
MRPL9	45	OK	0.16237181	0.41446003	0.39176712	0.69522	0.19054757	subgrp_cor	count		1	0
RORC	1	OK	18.7594894	83.6251793	0.22432824	0.8225	0.00028789	subgrp_cor	count		1	0
THEM4	6	OK	3.66421638	2.15550791	1.69993177	0.08914	2.10998156	subgrp_cor	count		1	0
S100A10	827	OK	-0.2837448	0.08914279	-3.1830368	0.00146	-0.40573128	subgrp_cor	count		1	0
S100A11	744	OK	-0.2289494	0.0966656	-2.3684681	0.01786	-0.32651755	subgrp_cor	count		1	0
S100A9	2	OK	-1.2956167	2.0414257	-0.6346627	0.52565	-0.00075754	subgrp_cor	count		1	0
S100A8	1	OK	-12.148421	43.4553091	-0.2795612	0.77981	-1.91362624	subgrp_cor	count		1	0
S100A6	846	OK	0.00636099	0.08663882	0.07341945	0.94147	0.00910045	subgrp_cor	count		1	0
S100A3	6	OK	4.62956245	2.54442552	1.30615312	0.1915	0.95266465	subgrp_cor	count		1	0
S100A16	361	OK	-0.0886363	0.15011108	-0.5904715	0.55487	-0.12446559	subgrp_cor	count		1	0
S100A13	487	OK	0.2914507	0.12314824	2.36666545	0.01795	0.41396304	subgrp_cor	count		1	0
CHTOP	25	OK	0.68942603	0.63370389	1.08793087	0.27662	0.6161841	subgrp_cor	count		1	0
SNAPIN	30	OK	-0.0690356	0.57050213	-0.1210085	0.90368	-0.07443265	subgrp_cor	count		1	0
ILF2	162	OK	0.43087765	0.23681981	1.81943248	0.06885	0.58520168	subgrp_cor	count		1	0
NPR1.00	20	OK	0.55099623	0.68738207	0.80157202	0.4228	0.45900421	subgrp_cor	count		1	0
INTS3	11	OK	-0.3075281	0.87079594	-0.3531575	0.72397	-0.19561760	subgrp_cor	count		1	0
SLC27A3	17	OK	1.81646923	0.9875659	1.83933976	0.06587	1.27535752	subgrp_cor	count		1	0
GATAD2B	10	OK	0.83091405	0.89896729	0.92429842	0.35532	0.61381172	subgrp_cor	count		1	0
DENND4B	1	OK	3.45981463	6.77145131	0.51094137	0.60939	0.00030179	subgrp_cor	count		1	0
CRTC2	2	OK	1.58240009	1.92403354	0.82243893	0.41082	0.77175617	subgrp_cor	count		1	0
SLC39A1	101	OK	-0.1360852	0.29107567	-0.4675252	0.64012	-0.17953514	subgrp_cor	count		1	0
AL358472.4	1	OK	-10.442278	41.4658099	-0.2518288	0.80117	-0.00045289	subgrp_cor	count		1	0
CREB3L4	3	OK	1.35697297	1.78752367	0.75913567	0.44777	0.41320240	subgrp_cor	count		1	0
JTB	201	OK	0.45954153	0.20625313	2.22804647	0.02588	0.62072692	subgrp_cor	count		1	0
RAB13	530	OK	-0.1036657	0.11904093	-0.8708411	0.38384	-0.14718108	subgrp_cor	count		1	0
RPS27	867	OK	-0.1209737	0.084958	-1.4239074	0.15447	-0.17295955	subgrp_cor	count		1	0
TPM3	637	OK	-0.300728	0.1063947	-2.8266348	0.0047	-0.4277839	subgrp_cor	count		1	0
C1orf43	149	OK	-0.0286824	0.23363163	-0.1227676	0.90229	-0.03852342	subgrp_cor	count		1	0
UBAP2L	7	OK	0.70079264	1.05078652	0.666922	0.50482	0.21849695	subgrp_cor	count		1	0
HAX1	118	OK	0.26951617	0.25736973	1.04719455	0.29501	0.36323652	subgrp_cor	count		1	0
ATP8B2	1	OK	3.45981463	6.77145131	0.51094137	0.60939	0.00030179	subgrp_cor	count		1	0
IL6R	1	OK	18.7594894	83.6251793	0.22432824	0.8225	0.00028789	subgrp_cor	count		1	0
SHE	34	OK	0.27832035	0.52372607	0.53143499	0.59512	0.23196862	subgrp_cor	count		1	0
TDRD10	2	OK	1.58229999	2.72001883	0.58176068	0.56072	0.47433761	subgrp_cor	count		1	0
UBE2Q1	22	OK	1.38526655	0.6735186	2.05676066	0.03971	1.42187093	subgrp_cor	count		1	0
ADAR	52	OK	0.50752809	0.39707751	1.27815873	0.20119	0.58006088	subgrp_cor	count		1	0
KCNN3	53	OK	0.47859009	0.43843906	1.0915771	0.27502	0.57350100	subgrp_cor	count		1	0
PMVK	23	OK	1.94852196	0.73725671	2.64293553	0.00822	1.79244502	subgrp_cor	count		1	0
PBXIP1	19	OK	1.52919767	0.81664373	1.87253956	0.06112	1.10146392	subgrp_cor	count		1	0
SHC1	51	OK	-0.2081296	0.47309817	-0.4399291	0.65999	-0.23649058	subgrp_cor	count		1	0
CKS1B	11	OK	0.93659265	0.92295793	1.01476203	0.31022	0.60412976	subgrp_cor	count		1	0
FLAD1	3	OK	4.63034423	3.5401477	1.30795227	0.19089	0.95270596	subgrp_cor	count		1	0
ZBTB7B	1	OK	-11.019418	43.2499352	-0.2547846	0.79889	-1.17482461	subgrp_cor	count		1	0
EFNA4	3	OK	21.642059	54.4868919	0.39719753	0.69122	1.57744735	subgrp_cor	count		1	0
EFNA3	1	OK	18.7594894	83.6251793	0.22432824	0.8225	0.00028789	subgrp_cor	count		1	0
EFNA1	175	OK	-0.2092346	0.20631475	-1.0141516	0.31051	-0.2884322	subgrp_cor	count		1	0
SLC50A1	15	OK	-0.0033156	0.74030454	-0.0044787	0.99642	-0.00274881	subgrp_cor	count		1	0
DPM3	54	OK	0.26465183	0.40761837	0.64926379	0.51617	0.31414778	subgrp_cor	count		1	0
KRTCAP2	191	OK	0.08424013	0.19692187	0.42778454	0.66881	0.11675602	subgrp_cor	count		1	0
THBS3	6	OK	0.12426577	1.55722389	0.07979956	0.9364	0.08171597	subgrp_cor	count		1	0
MTX1	22	OK	-0.0655249	0.67110028	-0.0976379	0.92222	-0.06689741	subgrp_cor	count		1	0
GBA	4	OK	-0.9727207	1.63750584	-0.5940258	0.55249	-0.27349270	subgrp_cor	count		1	0
FAM189B	1	OK	23.9918968	84.8999419	0.28259026	0.77749	1.65081709	subgrp_cor	count		1	0
SCAMP3	19	OK	0.16250688	0.7439508	0.21843767	0.82709	0.15382864	subgrp_cor	count		1	0
FDPS	161	OK	0.86224413	0.23625037	3.64970498	0.00026	1.16855187	subgrp_cor	count		1	0
RUSC1	2	OK	-10.442278	41.4658099	-0.2518288	0.80117	-0.00045289	subgrp_cor	count		1	0
ASH1L	62	OK	0.41788123	0.37906856	1.10238682	0.27029	0.49602596	subgrp_cor	count		1	0
YY1AP1	2	OK	-11.019418	43.2499352	-0.2547846	0.79889	-1.17482461	subgrp_cor	count		1	0
DAP3	24	OK	0.73098935	0.71814143	1.01789054	0.30873	0.60699354	subgrp_cor	count		1	0
GON4L	23	OK	0.67734058	0.63779925	1.06199651	0.28824	0.50989400	subgrp_cor	count		1	0
SYT11	13	OK	3.71878111	2.12922671	1.7465407	0.08072	0.86487762	subgrp_cor	count		1	0
RIT1	32	OK	1.22387298	0.61329681	1.9955629	0.04598	1.26653546	subgrp_cor	count		1	0
KHDC4	5	OK	0.73785345	1.30674578	0.56464958	0.57231	0.22996142	subgrp_cor	count		1	0
ARHGEF2	10	OK	2.93114443	1.70305898	1.72110564	0.08523	1.5782010	subgrp_cor	count		1	0
SSR2	309	OK	0.0281307	0.16158997	0.17407614	0.86181	0.03937222	subgrp_cor	count		1	0
UBQLN4	3	OK	0.3295369	1.40554809	0.23444726	0.81464	0.10244709	subgrp_cor	count		1	0
LAMTOR2	83	OK	-0.1750438	0.33247664	-0.5264848	0.59855	-0.21954944	subgrp_cor	count		1	0
LMNA	585	OK	-0.2401283	0.11163532	-2.1510067	0.03148	-0.34046491	subgrp_cor	count		1	0
SEMA4A	13	OK	0.28700179	0.84039549	0.34150801	0.73272	0.14639477	subgrp_cor	count		1	0
SLC25A44	3	OK	1.533588	2.24702247	0.68249481	0.49492	0.00105583	subgrp_cor	count		1	0
PMF1	33	OK	0.57510375	0.54674956	1.05185955	0.29286	0.60663351	subgrp_cor	count		1	0
GLMP	11	OK	-0.1487772	0.8415728	-0.1767847	0.85968	-0.11087238	subgrp_cor	count		1	0
CCT3	177	OK	-0.3119149	0.21811228	-1.4300658	0.1527	-0.41405493	subgrp_cor	count		1	0
MEF2D	4	OK	6.80913653	5.30501163	1.28352905	0.19931	1.696588	subgrp_cor	count		1	0
NAXE	56	OK	1.57359888	0.46503486	3.38382983	0.00071	1.88266279	subgrp_cor	count		1	0
GPATCH4	30	OK	-0.069246	0.62386758	-0.1109915	0.91162	-0.06523522	subgrp_cor	count		1	0
ISG20L2	8	OK	1.10069209	1.56872901	0.70163941	0.4829	0.55471951	subgrp_cor	count		1	0
RRNAD1	2	OK	-0.3495512	1.85447474	-0.1884907	0.85049	-0.17337783	subgrp_cor	count		1	0
MRPL24	29	OK	-0.2861212	0.60004413	-0.4768335	0.63349	-0.2798512	subgrp_cor	count		1	0
HDGF	67	OK	0.29824931	0.38149689	0.78178701	0.43434	0.35756127	subgrp_cor	count		1	0
PRCC	11	OK	-0.1230894	1.04246636	-0.1180752	0.90601	-0.00021701	subgrp_cor	count		1	0
PEAR1	4	OK	1.97379584	1.67711085	1.17690249	0.23923	1.19427293	subgrp_cor	count		1	0
ARHGEF11	2	OK	-11.019418	43.2499352	-0.2547846	0.79889	-1.17482461	subgrp_cor	count		1	0
ETV3	4	OK	0.51030031	1.6034059	0.31826022	0.75029	0.15911328	subgrp_cor	count		1	0
CD1D	3	OK	3.45981463	6.77145131	0.51094137	0.60939	0.00030179	subgrp_cor	count		1	0
SPTA1	1	OK	0.65325287	2.50012846	0.26128772	0.79387	0.00016297	subgrp_cor	count		1	0
MNDA	1	OK	3.45981463	6.77145131	0.51094137	0.60939	0.00030179	subgrp_cor	count		1	0
AIM2	6	OK	-1.8174474	1.45595424	-1.2482861	0.21192	-0.45400691	subgrp_cor	count		1	0
CADM3	7	OK	-1.0374932	1.18122493	-0.8783197	0.37977	-0.72293731	subgrp_cor	count		1	0
CADM3-AS1	17	OK	-4.5822423	1.63253677	-2.8068234	0.005	-3.20388575	subgrp_cor	count		1	0
TAGLN2	775	OK	-0.3591508	0.09425014	-3.8106134	0.00014	-0.51281192	subgrp_cor	count		1	0
LINC01133	27	OK	29.6416288	27.7082704	1.06977548	0.28472	2.61640946	subgrp_cor	count		1	0
PIGM	1	OK	18.7594894	83.6251793	0.22432824	0.8225	0.00028789	subgrp_cor	count		1	0
IGSF8	2	OK	21.642059	54.4868919	0.39719753	0.69122	1.57744735	subgrp_cor	count		1	0
PEA15	124	OK	0.2848236	0.26366987	1.08022811	0.28004	0.37997940	subgrp_cor	count		1	0
DCAF8	5	OK	-1.8923093	1.49482671	-1.2659055	0.20555	-1.15044693	subgrp_cor	count		1	0
PEX19	3	OK	0.8788176	1.64693117	0.53360919	0.59361	0.44699547	subgrp_cor	count		1	0
COPA	33	OK	0.65515743	0.56841061	1.15261334	0.24907	0.67253612	subgrp_cor	count		1	0
NCSTN	13	OK	0.55466434	0.90207304	0.61487742	0.53864	0.4126908	subgrp_cor	count		1	0
ITLN2	1	OK	18.7594894	83.6251793	0.22432824	0.8225	0.00028789	subgrp_cor	count		1	0
F11R	13	OK	0.8186329	0.8092912	1.01154307	0.31170	0.41713230	subgrp_cor	count		1	0
KLHDC9	2	OK	0.42513877	2.56228989	0.16592142	0.86822	0.1426475	subgrp_cor	count		1	0
PFDN2	226	OK	0.42052536	0.19083323	2.20362769	0.02755	0.58006625	subgrp_cor	count		1	0
NIT1	7	OK	-1.6917507	1.50105837	-1.1270370	0.25972	-0.71821867	subgrp_cor	count		1	0
DEDD	5	OK	0.65700982	1.41612318	0.46394963	0.64269	0.33589025	subgrp_cor	count		1	0
UFC1	318	OK	0.3256247	0.15759675	2.06618693	0.03881	0.45659852	subgrp_cor	count		1	0
PPOX	1	OK	18.7594894	83.6251793	0.22432824	0.8225	0.00028789	subgrp_cor	count		1	0
B4GALT3	4	OK	-1.8979105	1.60787469	-1.1803846	0.23785	-0.78103351	subgrp_cor	count		1	0
ADAMTS4	35	OK	-0.7329709	0.49581252	-1.4783229	0.13932	-0.85185771	subgrp_cor	count		1	0
NDUFS2	28	OK	-0.1641869	0.62027194	-0.2647015	0.79124	-0.12227247	subgrp_cor	count		1	0
FCER1G	1	OK	3.45981463	6.77145131	0.51094137	0.60939	0.00030179	subgrp_cor	count		1	0
SDHC	71	OK	0.09470517	0.34750396	0.27252976	0.78521	0.11909746	subgrp_cor	count		1	0
HSPA6	11	OK	6.29253557	3.73001277	1.68700108	0.0916	1.66214371	subgrp_cor	count		1	0
FCRLB	1	OK	1.58229999	2.72001883	0.58176068	0.56072	0.47433761	subgrp_cor	count		1	0
DUSP12	10	OK	3.81621809	2.20226994	1.73285664	0.08312	1.40013240	subgrp_cor	count		1	0
ATF6	32	OK	1.64072875	0.68060231	2.41070113	0.01592	1.01059724	subgrp_cor	count		1	0
OLFML2B	1	OK	17.7472073	50.9719273	0.34817612	0.72771	0.0007532	subgrp_cor	count		1	0
NOS1AP	1	OK	-11.019418	43.2499352	-0.2547846	0.79889	-1.17482461	subgrp_cor	count		1	0
UHMK1	23	OK	0.77329626	0.71341759	1.08407233	0.27832	0.69272264	subgrp_cor	count		1	0
UAP1	11	OK	0.24658297	1.49591083	0.16483798	0.86907	0.00044528	subgrp_cor	count		1	0
DDR2	3	OK	23.9918968	84.8999419	0.28259026	0.77749	1.65081709	subgrp_cor	count		1	0
HSD17B7	1	OK	3.45981463	6.77145131	0.51094137	0.60939	0.00030179	subgrp_cor	count		1	0
RGS5	10	OK	1.04736111	0.86673903	1.2083927	0.2269	0.91728656	subgrp_cor	count		1	0
PBX1	27	OK	0.13189747	0.50161265	0.26294685	0.79259	0.13039274	subgrp_cor	count		1	0
MGST3	547	OK	-0.1617485	0.11731283	-1.3787792	0.16796	-0.22955946	subgrp_cor	count		1	0
ALDH9A1	45	OK	-0.3422281	0.41856801	-0.8176145	0.41358	-0.35704259	subgrp_cor	count		1	0
TMCO1	165	OK	0.18704573	0.21482688	0.87068146	0.38393	0.25405362	subgrp_cor	count		1	0
UCK2	7	OK	-0.0059021	0.9864289	-0.0059833	0.99523	-0.00489279	subgrp_cor	count		1	0
POGK	4	OK	-0.2029407	1.16882308	-0.1736282	0.86216	-0.00013095	subgrp_cor	count		1	0
TADA1	1	OK	18.7594894	83.6251793	0.22432824	0.8225	0.00028789	subgrp_cor	count		1	0
POU2F1	3	OK	0.93967049	1.49854054	0.6270571	0.53062	0.29164784	subgrp_cor	count		1	0
CREG1	53	OK	0.46559375	0.39437655	1.18058173	0.23777	0.5322882	subgrp_cor	count		1	0
RCSD1	1	OK	3.45981463	6.77145131	0.51094137	0.60939	0.00030179	subgrp_cor	count		1	0
MPZL1	19	OK	0.47168117	0.76923625	0.61318115	0.53978	0.35584087	subgrp_cor	count		1	0
MPC2	113	OK	0.97892094	0.29450043	3.32400526	0.00089	1.30887798	subgrp_cor	count		1	0
DCAF6	13	OK	0.52958766	0.77325882	0.68487674	0.49342	0.4409374	subgrp_cor	count		1	0
GPR161	2	OK	18.7594894	83.6251793	0.22432824	0.8225	0.00028789	subgrp_cor	count		1	0
TIPRL	68	OK	0.70929173	0.36212029	1.95899472	0.05011	0.89922685	subgrp_cor	count		1	0
SFT2D2	24	OK	2.29160375	0.88814918	2.58020138	0.00987	1.94647798	subgrp_cor	count		1	0
ATP1B1	1	OK	23.9918968	84.8999419	0.28259026	0.77749	1.65081709	subgrp_cor	count		1	0
NME7	6	OK	-1.2520546	1.38784505	-0.9021572	0.36697	-0.56535377	subgrp_cor	count		1	0
BLZF1	12	OK	-2.3210076	1.07986068	-2.1493582	0.03161	-1.52661293	subgrp_cor	count		1	0
F5	79	OK	8.47289461	2.04812864	4.13689572	3.52E-05	5.05881280	subgrp_cor	count		0.35083907	0
METTL18	4	OK	0.84117939	1.40444781	0.59893958	0.54921	0.0005738	subgrp_cor	count		1	0
SCYL3	1	OK	-15.499043	42.9510917	-0.36085	0.71822	-0.00073756	subgrp_cor	count		1	0
KIFAP3	27	OK	-0.9516264	0.59452137	-1.6006597	0.10945	-1.00922204	subgrp_cor	count		1	0
FMO2	18	OK	3.8420357	1.18958873	3.22971767	0.00124	3.36306445	subgrp_cor	count		1	0
PRRC2C	326	OK	0.08754996	0.15218113	0.57530101	0.56509	0.12232258	subgrp_cor	count		1	0
VAMP4	7	OK	2.95348029	1.70042488	1.73690724	0.0824	1.55466382	subgrp_cor	count		1	0
METTL13	3	OK	-11.186792	26.3137535	-0.425133	0.67074	-2.27103070	subgrp_cor	count		1	0
DNM3	11	OK	-0.5884018	0.91103443	-0.6458612	0.51837	-0.42644171	subgrp_cor	count		1	0
DNM3OS	3	OK	-1.8538345	1.85439186	-0.9996995	0.31740	-0.46053279	subgrp_cor	count		1	0
PIGC	13	OK	-0.2664791	0.76439958	-0.3486121	0.72738	-0.27021265	subgrp_cor	count		1	0
SUCO	18	OK	0.68486679	0.62277521	1.09970141	0.27146	0.80184567	subgrp_cor	count		1	0
PRDX6	358	OK	0.31756288	0.14742759	2.15403612	0.03124	0.44637630	subgrp_cor	count		1	0
ANKRD45	1	OK	3.45978789	3.90172073	0.88673386	0.37522	0.00222136	subgrp_cor	count		1	0
KLHL20	11	OK	1.96309041	0.99829048	1.96645211	0.04925	1.45938132	subgrp_cor	count		1	0
CENPL	2	OK	-2.2785759	2.43693603	-0.9760518	0.32904	-6.09E-05	subgrp_cor	count		1	0
DARS2	2	OK	-11.019418	43.2499352	-0.2547846	0.79889	-1.17482461	subgrp_cor	count		1	0
ZBTB37	1	OK	18.7594894	83.6251793	0.22432824	0.8225	0.00028789	subgrp_cor	count		1	0
RC3H1	6	OK	0.26547321	1.21272933	0.21890558	0.82672	0.13768216	subgrp_cor	count		1	0
RABGAP1L	10	OK	0.06611886	0.97791995	0.06761173	0.94609	0.00011810	subgrp_cor	count		1	0
CACYBP	96	OK	-0.2280861	0.30156441	-0.7563429	0.44944	-0.29699374	subgrp_cor	count		1	0
MRPS14	15	OK	0.16326852	0.73366605	0.22267423	0.82379	0.15454264	subgrp_cor	count		1	0
KIAA0040	37	OK	0.43591763	0.43074104	1.01201788	0.31152	0.53144907	subgrp_cor	count		1	0
COP1	1	OK	3.45981463	6.77145131	0.51094137	0.60939	0.00030179	subgrp_cor	count		1	0
RASAL2	45	OK	-1.5636059	0.46599027	-3.3554476	0.00079	-1.70922919	subgrp_cor	count		1	0
RALGPS2	5	OK	2.19455215	1.45170837	1.51170319	0.13061	1.27593959	subgrp_cor	count		1	0
FAM20B	3	OK	4.66132998	5.76838606	0.80808217	0.41906	0.00274918	subgrp_cor	count		1	0
TOR3A	18	OK	-1.1475425	0.74290401	-1.5446714	0.12242	-1.1059177	subgrp_cor	count		1	0
ABL2	44	OK	2.87856888	0.93962413	3.06353229	0.00219	1.53132091	subgrp_cor	count		1	0
SOAT1	2	OK	21.642059	54.4868919	0.39719753	0.69122	1.57744735	subgrp_cor	count		1	0
TOR1AIP2	55	OK	1.12039253	0.46438425	2.41264113	0.01584	1.19448388	subgrp_cor	count		1	0
TOR1AIP1	23	OK	0.173907	0.61642362	0.28212255	0.77785	0.17201078	subgrp_cor	count		1	0
CEP350	39	OK	0.12969805	0.49819053	0.26033828	0.7946	0.11630126	subgrp_cor	count		1	0
QSOX1	20	OK	1.75987637	0.86977485	2.02337003	0.04304	1.23865087	subgrp_cor	count		1	0
ACBD6	41	OK	0.52023715	0.53689916	0.96896623	0.33256	0.56268446	subgrp_cor	count		1	0
XPR1	1	OK	23.9918968	84.8999419	0.28259026	0.77749	1.65081709	subgrp_cor	count		1	0
KIAA1614	1	OK	-11.019418	43.2499352	-0.2547846	0.79889	-1.17482461	subgrp_cor	count		1	0
STX6	10	OK	1.72758911	1.26375001	1.36703391	0.17161	1.05574644	subgrp_cor	count		1	0
IER5	82	OK	-0.3079688	0.31969873	-0.9633092	0.33539	-0.37313571	subgrp_cor	count		1	0
GLUL	124	OK	-1.074359	0.2570709	-4.1792327	2.92E-05	-1.40022902	subgrp_cor	count		0.29167527	0
LINC01686	1	OK	3.45978789	3.90172073	0.88673386	0.37522	0.00222136	subgrp_cor	count		1	0
DHX9	30	OK	0.46305549	0.57863755	0.80025137	0.42357	0.38603284	subgrp_cor	count		1	0
LAMC1	33	OK	-0.8847877	0.58047492	-1.5242479	0.12745	-0.9215641	subgrp_cor	count		1	0
SMG7	6	OK	-0.3611135	0.97283182	-0.3711982	0.71049	-0.10816307	subgrp_cor	count		1	0
ARPC5	348	OK	0.33119024	0.15352553	2.15722238	0.03099	0.46375767	subgrp_cor	count		1	0
RGL1	2	OK	1.58229999	2.72001883	0.58176068	0.56072	0.47433761	subgrp_cor	count		1	0
TSEN15	7	OK	0.14323966	1.10858465	0.12920949	0.89719	0.07290044	subgrp_cor	count		1	0
C1orf21	40	OK	-0.8530918	0.46296175	-1.8426832	0.06538	-0.9120036	subgrp_cor	count		1	0
EDEM3	8	OK	0.7789741	1.35318259	0.57566075	0.56484	0.51571745	subgrp_cor	count		1	0
FAM129A	15	OK	2.23584853	0.96708544	2.31194518	0.02078	1.82872764	subgrp_cor	count		1	0
RNF2	2	OK	-0.910142	2.18812438	-0.4159466	0.67745	-0.25784050	subgrp_cor	count		1	0
TRMT1L	2	OK	3.45981463	6.77145131	0.51094137	0.60939	0.00030179	subgrp_cor	count		1	0
SWT1	2	OK	-1.0311657	1.74795383	-0.5899272	0.55526	-0.00022765	subgrp_cor	count		1	0
IVNS1ABP	25	OK	0.66196354	0.69448171	0.95317638	0.3405	0.75663600	subgrp_cor	count		1	0
HMCN1	76	OK	28.1721568	18.16068	1.55127128	0.12084	5.64257129	subgrp_cor	count		1	0
PRG4	2	OK	4.66132998	5.76838606	0.80808217	0.41906	0.00274918	subgrp_cor	count		1	0
TPR	247	OK	0.41092405	0.18172288	2.26126752	0.02374	0.56651514	subgrp_cor	count		1	0
ODR4	19	OK	0.73936815	0.7276786	1.01606419	0.3096	0.81742085	subgrp_cor	count		1	0
PLA2G4A	24	OK	3.64970768	1.40380573	2.5998666	0.00932	2.01666995	subgrp_cor	count		1	0
RGS2	25	OK	0.57080148	0.64777709	0.8811696	0.37822	0.60241715	subgrp_cor	count		1	0
UCHL5	14	OK	-0.1955997	0.79600087	-0.245728	0.80589	-0.14553328	subgrp_cor	count		1	0

TROVE2	87	OK	0.3461773	0.3064081	1.1297915	0.25858	0.43232129	subgrp_con	count		1	0
GLRX2	20	OK	0.616929	0.6293957	0.9801933	0.32699	0.65010247	subgrp_con	count		1	0
CDC73	51	OK	0.2191126	0.4018959	0.5451974	0.58562	0.26716028	subgrp_con	count		1	0
KCNT2	19	OK	23.382146	32.93235	0.710005	0.4777	3.221789	subgrp_con	count		1	0
ASPM	1	OK	17.780255	15.808015	1.124762	0.26069	0.0069062	subgrp_con	count		1	0
ZBTB41	3	OK	3.4598146	6.771451	0.5109413	0.60939	0.00030179	subgrp_con	count		1	0
DENND1B	1	OK	-11.01941	43.249935	-0.254784	0.79889	-1.1748246	subgrp_con	count		1	0
NEK7	7	OK	0.9241711	1.201178	0.7693868	0.44168	0.0017165	subgrp_con	count		1	0
MIR181A1HG	1	OK	3.4598146	6.771451	0.5109413	0.60939	0.00030179	subgrp_con	count		1	0
NR5A2	15	OK	-0.627707	0.857789	-0.731773	0.46433	-0.5034191	subgrp_con	count		1	0
ZNF281	14	OK	0.4862261	0.985757	0.4932513	0.62186	0.3194642	subgrp_con	count		1	0
DDX59	10	OK	-1.739099	1.058741	-1.642610	0.10046	-1.2084944	subgrp_con	count		1	0
CAMSAP2	23	OK	0.0846323	0.693409	0.1220524	0.90286	0.05482705	subgrp_con	count		1	0
TMEM9	29	OK	1.7354716	0.595423	2.9146369	0.00356	1.98183254	subgrp_con	count		1	0
PHLDA3	27	OK	-0.321252	0.675947	-0.475263	0.6346	-0.1596660	subgrp_con	count		1	0
CSRP1	166	OK	0.9011488	0.222923	4.0424153	5.29E-05	1.24207815	subgrp_con	count		0.5262302	0
NAV1	52	OK	3.658417	0.858164	4.2630721	2.02E-05	3.21882509	subgrp_con	count		0.2014942	0
SHISA4	9	OK	1.4105850	1.100433	1.281845	0.1999	1.1338583	subgrp_con	count		1	0
LMOD1	5	OK	0.3531019	1.170937	0.3015549	0.76299	0.1805416	subgrp_con	count		1	0
TIMM17A	21	OK	-0.07519	0.699565	-0.107492	0.9144	-0.0622034	subgrp_con	count		1	0
RNPEP	7	OK	0.3361531	1.111530	0.3024237	0.76232	0.10452415	subgrp_con	count		1	0
ARL8A	26	OK	1.0235566	0.660754	1.5490723	0.12136	0.8413502	subgrp_con	count		1	0
KDM5B	15	OK	-2.142453	0.963246	-2.224201	0.02612	-0.8486810	subgrp_con	count		1	0
RABIF	7	OK	3.459816	3.026379	1.1432196	0.25295	0.0022229	subgrp_con	count		1	0
ADIPOR1	19	OK	-0.47687	0.632603	-0.75263	0.45167	-0.4419388	subgrp_con	count		1	0
CYB5R1	37	OK	0.5943223	0.455766	1.3040097	0.19222	0.6680107	subgrp_con	count		1	0
TMEM183A	27	OK	0.1752690	0.651456	0.2690418	0.7879	0.1319446	subgrp_con	count		1	0
CHI3L1	1	OK	18.759489	83.625179	0.2243282	0.8225	0.0002878	subgrp_con	count		1	0
BTG2	421	OK	0.003697	0.121312	0.0304799	0.97568	0.0052507	subgrp_con	count		1	0
FMOD	22	OK	24.577745	22.443697	0.757550	0.44872	3.5733650	subgrp_con	count		1	0
PRELP	48	OK	6.414406	7.333195	0.8747081	0.38173	6.4310669	subgrp_con	count		1	0
ATP2B4	57	OK	0.3450904	0.394962	0.8737291	0.38227	0.3886831	subgrp_con	count		1	0
SNRPE	160	OK	-0.112368	0.227845	-0.493176	0.62189	-0.1516296	subgrp_con	count		1	0
SOX13	2	OK	1.925964	2.515487	0.765642	0.44389	0.5602211	subgrp_con	count		1	0
PLEKHA6	2	OK	4.6613299	5.768386	0.8080821	0.41906	0.0027491	subgrp_con	count		1	0
PPP1R15B	40	OK	0.0854593	0.465960	0.1834045	0.85448	0.094347	subgrp_con	count		1	0
PIK3C2B	23	OK	0.806712	0.638938	1.262581	0.20674	0.5226957	subgrp_con	count		1	0
MDM4	24	OK	0.4241708	0.621303	0.6827116	0.49479	0.3821335	subgrp_con	count		1	0
LRRN2	1	OK	3.4598146	6.771451	0.5109413	0.60939	0.00030179	subgrp_con	count		1	0
RBBP5	5	OK	0.6851287	1.421670	0.4819179	0.62986	0.0012628	subgrp_con	count		1	0
DSTYK	10	OK	-0.45428	0.886647	-0.512472	0.60832	-0.2235742	subgrp_con	count		1	0
LEMD1	1	OK	1.5822999	2.720018	0.5817606	0.56072	0.4743376	subgrp_con	count		1	0
ELK4	47	OK	0.0907941	0.401965	0.2258754	0.8213	0.1020859	subgrp_con	count		1	0
NUCKS1	547	OK	0.285349	0.114016	2.5027041	0.01232	0.4055048	subgrp_con	count		1	0
RAB29	13	OK	1.0050522	0.888293	1.1301692	0.2584	0.5087885	subgrp_con	count		1	0
SLC41A1	3	OK	-11.19992	27.762997	-0.403412	0.68665	-1.8294085	subgrp_con	count		1	0
SRGAP2	12	OK	-0.60574	0.830718	-0.729186	0.46589	-0.3770780	subgrp_con	count		1	0
EIF2D	19	OK	1.4635408	0.732240	1.998715	0.04564	1.1740924	subgrp_con	count		1	0
AL591846.2	2	OK	-1.758573	2.355443	-0.746599	0.45531	-0.0003608	subgrp_con	count		1	0
DYRK3	2	OK	3.4598149	4.785868	0.7229230	0.46972	0.0008193	subgrp_con	count		1	0
MAPKAPK2	13	OK	0.3089195	0.859471	0.3594295	0.71927	0.2770037	subgrp_con	count		1	0
CD55	214	OK	-0.695664	0.191446	-3.633658	0.00028	-0.9616889	subgrp_con	count		1	0
CD46	137	OK	0.9300553	0.254770	3.6505577	0.00026	1.2363813	subgrp_con	count		1	0
MIR29B2CHG	1	OK	-10.44227	41.465809	-0.251828	0.80117	-0.0004528	subgrp_con	count		1	0
CD34	241	OK	0.473391	0.177094	2.673093	0.00752	0.6568667	subgrp_con	count		1	0
G0S2	6	OK	-1.945690	1.577988	-1.233011	0.21757	-1.4140761	subgrp_con	count		1	0
IRF6	24	OK	30.327809	29.703583	1.0210151	0.30725	4.2752436	subgrp_con	count		1	0
UTP25	2	OK	-1.758573	2.355443	-0.746599	0.45531	-0.0003608	subgrp_con	count		1	0
SERTAD4-AS1	63	OK	-0.158997	0.360025	-0.441627	0.65876	-0.1946309	subgrp_con	count		1	0
SERTAD4	3	OK	18.759489	83.625179	0.2243282	0.8225	0.0002878	subgrp_con	count		1	0
RCOR3	10	OK	0.0948233	0.914325	0.1037085	0.9174	0.0788925	subgrp_con	count		1	0
TRAF5	6	OK	-0.822793	1.179116	-0.706285	0.48001	-0.5077053	subgrp_con	count		1	0
LINC00467	3	OK	-0.545320	1.708550	-0.319171	0.7496	-0.000342	subgrp_con	count		1	0
SLC30A1	5	OK	-1.459665	1.668123	-0.875034	0.38156	-0.3998918	subgrp_con	count		1	0
LPGAT1	49	OK	-0.153170	0.443789	-0.345142	0.72998	-0.1845666	subgrp_con	count		1	0
PPP2R5A	34	OK	-2.349348	0.643776	-3.649322	0.00026	-2.341565	subgrp_con	count		1	0
TMEM206	1	OK	3.4598146	6.771451	0.5109413	0.60939	0.00030179	subgrp_con	count		1	0
NENF	177	OK	0.105553	0.204310	0.5166337	0.60541	0.1461042	subgrp_con	count		1	0
BATF3	9	OK	-0.946229	1.179088	-0.802509	0.42226	-0.2669034	subgrp_con	count		1	0
NSL1	45	OK	-0.692803	0.456787	-1.516687	0.12932	-0.7113280	subgrp_con	count		1	0
TATDN3	2	OK	3.4598146	6.771451	0.5109413	0.60939	0.00030179	subgrp_con	count		1	0
FLVCR1-DT	1	OK	3.4598146	6.771451	0.5109413	0.60939	0.00030179	subgrp_con	count		1	0
ANGEL2	4	OK	0.7689341	1.529886	0.5026054	0.61526	0.229548	subgrp_con	count		1	0
RPS6KC1	2	OK	21.64205	54.486891	0.3971975	0.69122	1.5774473	subgrp_con	count		1	0
PROX1	5	OK	0.1792095	1.147621	0.156157	0.87592	0.055457	subgrp_con	count		1	0
PTPN14	37	OK	1.18874	0.597214	1.990482	0.04654	0.757017	subgrp_con	count		1	0
CENPF	1	OK	-11.01941	43.249935	-0.254784	0.79889	-1.1748246	subgrp_con	count		1	0
KCTD3	6	OK	-1.265887	1.149582	-1.10116	0.27082	-0.570555	subgrp_con	count		1	0
GPATCH2	26	OK	0.1954252	0.599766	0.3258524	0.74454	0.1932659	subgrp_con	count		1	0
RRP15	33	OK	0.2400566	0.530962	0.4521163	0.65119	0.259826	subgrp_con	count		1	0
TGFB2	6	OK	25.138712	54.063693	0.4649832	0.64194	2.9375996	subgrp_con	count		1	0
LYPLAL1	25	OK	0.425493	0.636141	0.6688654	0.50358	0.4496931	subgrp_con	count		1	0
EPRS	77	OK	-0.043439	0.361411	-0.12019	0.90432	-0.0545674	subgrp_con	count		1	0
IARS2	44	OK	0.1688286	0.462981	0.3646770	0.71535	0.1957113	subgrp_con	count		1	0
RAB3GAP2	6	OK	0.5251098	1.138287	0.4613157	0.64457	0.4011620	subgrp_con	count		1	0
C1orf115	37	OK	-1.494549	0.505381	-2.957267	0.0031	-1.589555	subgrp_con	count		1	0
2-Mar	9	OK	-1.597404	1.068542	-1.494937	0.13492	-1.0426391	subgrp_con	count		1	0
HLX	10	OK	0.0814704	0.975437	0.0835219	0.93344	0.0612034	subgrp_con	count		1	0
MIA3	75	OK	0.9454128	0.362506	2.6008165	0.0093	1.1451200	subgrp_con	count		1	0
AIDA	41	OK	-0.128532	0.440690	-0.291661	0.77055	-0.1440105	subgrp_con	count		1	0
BROX	19	OK	0.5352269	0.656560	0.8150796	0.41503	0.4793631	subgrp_con	count		1	0
AL392172.1	3	OK	18.759489	83.625179	0.2243282	0.8225	0.0002878	subgrp_con	count		1	0
DISP1	2	OK	0.1681491	1.725330	0.097459	0.92236	0.0520127	subgrp_con	count		1	0
TLR5	2	OK	23.991896	84.899941	0.2825902	0.77749	1.6508170	subgrp_con	count		1	0
CAPN2	94	OK	-0.027277	0.291965	-0.093427	0.92556	-0.0347520	subgrp_con	count		1	0
TP53BP2	12	OK	-0.006659	0.822452	-0.008096	0.99354	-0.0043007	subgrp_con	count		1	0
DEGS1	47	OK	1.8002524	0.568803	3.1649830	0.00155	1.9636170	subgrp_con	count		1	0
NVL	5	OK	1.5162583	1.625265	0.9329294	0.35086	0.0010427	subgrp_con	count		1	0
CNIH4	82	OK	0.3574418	0.326489	1.0948126	0.2736	0.4560149	subgrp_con	count		1	0
WDR26	7	OK	1.5207849	1.392702	1.0919669	0.27485	0.4579806	subgrp_con	count		1	0
DNAH14	2	OK	-10.47563	27.491987	-0.381043	0.70317	-1.1381178	subgrp_con	count		1	0
LBR	18	OK	0.4300935	0.703357	0.6114864	0.54088	0.4263755	subgrp_con	count		1	0
ENAH	40	OK	-0.037774	0.456118	-0.082816	0.934	-0.032674	subgrp_con	count		1	0
SRP9	245	OK	-0.068285	0.177745	-0.384173	0.70085	-0.0945203	subgrp_con	count		1	0
PYCR2	16	OK	0.0422941	0.719975	0.0588828	0.95305	0.0318028	subgrp_con	count		1	0
LEFTY2	2	OK	1.34300	1.654457	0.8117507	0.41692	0.0009236	subgrp_con	count		1	0
SDE2	10	OK	0.0904937	1.082778	0.0835754	0.93339	0.0001619	subgrp_con	count		1	0
H3F3A	822	OK	-0.164872	0.090558	-1.820628	0.06866	-0.2354787	subgrp_con	count		1	0
ACBD3	101	OK	-0.311723	0.304567	-1.023497	0.30607	-0.4183567	subgrp_con	count		1	0
PARP1	61	OK	0.042690	0.378824	0.1126916	0.91027	0.0519957	subgrp_con	count		1	0
ITPKB	2	OK	4.6613299	5.768386	0.8080821	0.41906	0.0027491	subgrp_con	count		1	0
COQ8A	6	OK	-1.250701	1.360528	-0.919276	0.35795	-0.8445922	subgrp_con	count		1	0
CDC42BPA	35	OK	1.3942722	0.617556	2.257725	0.02396	1.0435516	subgrp_con	count		1	0
ZNF678	2	OK	2.4096233	2.471520	0.9749599	0.32958	0.6651734	subgrp_con	count		1	0
SNAP47	5	OK	0.5103003	1.603405	0.3182602	0.75029	0.1591132	subgrp_con	count		1	0
JMJD4	6	OK	0.8563093	1.378499	0.6212117	0.53446	0.5704827	subgrp_con	count		1	0
ARF1	414	OK	0.016898	0.134942	0.1252260	0.90034	0.0238417	subgrp_con	count		1	0
C1orf35	22	OK	-0.347535	0.620959	-0.559674	0.5757	-0.3068292	subgrp_con	count		1	0
MRPL55	41	OK	-0.132559	0.508236	-0.260823	0.79422	-0.1567513	subgrp_con	count		1	0
GUK1	745	OK	-0.295799	0.095573	-3.095006	0.00197	-0.422312	subgrp_con	count		1	0
GJC2	6	OK	-10.28366	16.527432	-0.622217	0.5338	-1.7497262	subgrp_con	count		1	0
IBA57	1	OK	-15.99503	70.34942	-0.227365	0.82014	-0.0002847	subgrp_con	count		1	0
TRIM11	1	OK	-11.01941	43.249935	-0.254784	0.79889	-1.1748246	subgrp_con	count		1	0
HIST3H2A	1	OK	3.4598146	6.771451	0.5109413	0.60939	0.00030179	subgrp_con	count		1	0
RNF187	37	OK	0.2094187	0.464001	0.4513321	0.65175	0.2356536	subgrp_con	count		1	0
RHOU	48	OK	-1.868906	0.485291	-3.851101	0.00012	-2.0205300	subgrp_con	count		1	0
RAB4A	43	OK	0.6917409	0.511177	1.3532301	0.17598	0.7283535	subgrp_con	count		1	0
ABCB10	1	OK	-11.01941	43.249935	-0.254784	0.79889	-1.1748246	subgrp_con	count		1	0
TAF5L	2	OK	19.10717	50.803277	0.3761012	0.70684	0.0007879	subgrp_con	count		1	0
GALNT2	19	OK	4.1608518	1.473805	2.8232034	0.00475	2.8853151	subgrp_con	count		1	0
COG2	3	OK	-1.646245	1.935192	-0.850688	0.39496	-0.7036029	subgrp_con	count		1	0
ARV1	5	OK	0.3531019	1.170937	0.3015549	0.76299	0.1805416	subgrp_con	count		1	0
FAM89A	34	OK	0.5635047	0.561449	1.0036609	0.31554	0.5944717	subgrp_con	count		1	0
C1orf131	40	OK	-0.947017	0.471321	-2.009280	0.04451	-0.8916353	subgrp_con	count		1	0
GNPAT	1	OK	-10.44227	41.465809	-0.251828	0.80117	-0.0004528	subgrp_con	count		1	0
EXOC8	4	OK	-1.295616	2.041425	-0.634662	0.52565	-0.0007575	subgrp_con	count		1	0
SPRTN	5	OK	0.1681593	1.219859	0.1378514	0.89036	0.0856494	subgrp_con	count		1	0
EGLN1	29	OK	-1.27725	0.624871	-2.044182	0.04094	-1.3811629	subgrp_con	count		1	0
AL445524.1	10	OK	-0.244130	1.036812	-0.235462	0.81385	-0.201221	subgrp_con	count		1	0
TSNAX	34	OK	0.5170101	0.517399	0.9992475	0.31767	0.5711486	subgrp_con	count		1	0
DISC1	1	OK	-11.01941	43.249935	-0.254784	0.79889	-1.1748246	subgrp_con	count		1	0
SIPA1L2	8	OK	4.6388072	3.003259	1.5445909	0.12245	0.9534050	subgrp_con	count		1	0
MAP10	1	OK	3.4598146	6.771451	0.5109413	0.60939	0.00030179	subgrp_con	count		1	0
NTPCR	36	OK	1.850840	0.616087	3.0041836	0.00266	2.0048041	subgrp_con	count		1	0
PCNX2	1	OK	17.747207	50.971927	0.3481761	0.72771	0.000753	subgrp_con	count		1	0
KCNK1	1	OK	3.4598146	6.771451	0.5109413	0.60939	0.00030179	subgrp_con	count		1	0
AL355472.1	1	OK	1.5822999	2.720018	0.5817606	0.56072	0.4743376	subgrp_con	count		1	0
COA6	32	OK	0.1697716	0.608638	0.2789365	0.78029	0.183654	subgrp_con	count		1	0
TARBP1	1	OK	18.759489	83.625179	0.2243282	0.8225	0.0002878	subgrp_con	count		1	0
LINC01354	2	OK	-10.47563	27.491987	-0.381043	0.70317	-1.1381178	subgrp_con	count		1	0
IRF2BP2	99	OK	-0.304280	0.281995	-1.079025	0.28058	-0.3957668	subgrp_con	count		1	0
LINC01132	1	OK	-10.44227	41.465809	-0.251828	0.80117	-0.0004528	subgrp_con	count		1	0
TOMM20	356	OK	0.1010735	0.148666	0.679868	0.49659	0.1416810	subgrp_con	count		1	0
ARID4B	166	OK	-0.022125	0.215196	-0.102816	0.91811	-0.0295877	subgrp_con	count		1	0
GGPS1	11	OK	0.2260810	0.828356	0.2729271	0.78492	0.1875318	subgrp_con	count		1	0
TBCE	1	OK	-10.44227	41.465809	-0.251828	0.80117	-0.0004528	subgrp_con	count		1	0
GNG4	2	OK	3.4598146	6.771451	0.5109413	0.60939	0.00030179	subgrp_con	count		1	0
LYST	97	OK	-0.621176	0.279586	-2.221690	0.0263	-0.8265874	subgrp_con	count		1	0
NID1	21	OK	0.4972068	0.788350	0.6306824	0.52826	0.4148946	subgrp_con	count		1	0
GPR137B	1	OK	3.4598146	6.771451	0.5109413	0.60939	0.00030179	subgrp_con	count		1	0
ERO1B	2	OK	-1.031165	1.747953	-0.589927	0.55524	-0.0002276	subgrp_con	count		1	0
LGALS8	18	OK	-1.011131	0.787350	-1.284220	0.19906	-0.8515855	subgrp_con	count		1	0
HEATR1	5	OK	-0.432431	1.097850	-0.393888	0.69366	-0.2131440	subgrp_con	count		1	0
MTR	18	OK	1.3781817	0.982016	1.4019926	0.16092	1.5925851	subgrp_con	count		1	0
RYR2	1	OK	3.4598146	6.771451	0.5109413	0.60939	0.00030179	subgrp_con	count		1	0
CHRM3	12	OK	5.5888872	3.153237	1.7724281	0.07632	1.0200104	subgrp_con	count		1	0
FH	11	OK	0.6961000	0.868039	0.8019221	0.4226	0.5236037	subgrp_con	count		1	0
OPN3	1	OK	-11.01941	43.249935	-0.254784	0.79889	-1.1748246	subgrp_con	count		1	0
CHML	2	OK	-1.758573	2.355443	-0.746599	0.45531	-0.0003608	subgrp_con	count		1	0
CEP170	25	OK	0.9113516	0.751260	1.2130967	0.22509	0.7502018	subgrp_con	count		1	0
SDCCAG8	40	OK	0.0191588	0.462740	0.0414031	0.96697	0.0201824	subgrp_con	count		1	0
AKT3	54	OK	-1.536294	0.433531	-3.543677	0.00039	-1.8896286	subgrp_con	count		1	0
ADSS	49	OK	1.4385086	0.50128	2.8692702	0.00411	1.6885606	subgrp_con	count		1	0
DESI2	29	OK	-0.219921	0.602670	-0.364911	0.71518	-0.1808813	subgrp_con	count		1	0
COX20	73	OK	-0.210767	0.355419	-0.593009	0.55317	-0.2577991	subgrp_con	count		1	0
HNRNPU	373	OK	-0.069273	0.138765	-0.499211	0.61762	-0.0972214	subgrp_con	count		1	0
EFCAB2	4	OK	0.5103003	1.603405	0.3182602	0.75029	0.1591132	subgrp_con	count		1	0
SMYD2	4	OK	-1.106879	1.712016	-0.646158	0.51818	-0.6550925	subgrp_con	count		1	0
TFB2M	3	OK	1.925964	2.515487	0.765642	0.44389	0.5602211	subgrp_con	count		1	0
CNST	15	OK	-0.201064	0.809316	-0.248437	0.8038	-0.1786230	subgrp_con	count		1	0
SCCPDH	22	OK	0.8136543	0.710715	1.144837	0.25228	0.6741107	subgrp_con	count		1	0
AHCTF1	24	OK	0.4549179	0.701999	0.6480312	0.51696	0.3814392	subgrp_con	count		1	0
ZNF669	1	OK	-15.99503	70.34942	-0.227365	0.82014	-0.0002847	subgrp_con	count		1	0
ZNF124	1	OK	0.6532528	2.500128	0.2612877	0.79387	0.0001629	subgrp_con	count		1	0
AL390728.6	2	OK	-15.99503	70.34942	-0.227365	0.82014	-0.0002847	subgrp_con	count		1	0
ZNF496	2	OK	21.105323	83.341273	0.2532397	0.80008	0.0003103	subgrp_con	count		1	0
ZNF672	1	OK	1.5822999	2.720018	0.5817606	0.56072	0.4743376	subgrp_con	count		1	0
ZNF692	1	OK	-10.44227	41.465809	-0.251828	0.80117	-0.0004528	subgrp_con	count		1	0
PGBD2	1	OK	-15.99503	70.34942	-0.227365	0.82014	-0.0002847	subgrp_con	count		1	0
SH3YL1	10	OK	-0.362735	1.002305	-0.361900	0.71742	-0.1797460	subgrp_con	count		1	0
ACP1	78	OK	-0.189446	0.322322	-0.587736	0.55671	-0.2460829	subgrp_con	count		1	0
ALKAL2	2	OK	-1.758573	2.355443	-0.746599	0.45531	-0.0003608	subgrp_con	count		1	0
TMEM18	79	OK	-0.420672	0.320930	-1.310791	0.18992	-0.5555629	subgrp_con	count		1	0
SNTG2	4	OK	-13.12169	27.86499	-0.470902	0.63771	-0.0015849	subgrp_con	count		1	0
TPO	4	OK	-2.725433	1.882998	-1.447390	0.14779	-0.5904152	subgrp_con	count		1	0
PXDN	21	OK	1.2617013	0.853244	1.4787098	0.13922	1.4735834	subgrp_con	count		1	0
TRAPPC12	5	OK	1.2304790	1.290432	0.9535397	0.34032	0.6157036	subgrp_con	count		1	0
ADI1	99	OK	1.3102493	0.299845	4.3697478	1.24E-05	1.7059048	subgrp_con	count		0.1244771	0
AC108488.1	1	OK	1.5822999	2.720018	0.5817606	0.56072	0.4743376	subgrp_con	count		1	0
RNASEH1	4	OK	1.4119426	1.495930	0.9438555	0.34526	0.6979765	subgrp_con	count		1	0
RNASEH1-AS1	1	OK	3.4598146	6.771451	0.5109413	0.60939	0.00030179	subgrp_con	count		1	0
RPS7	872	OK	-0.168643	0.085108	-1.981502	0.04752	-0.2412328	subgrp_con	count		1	0
CMPK2	3	OK	-0.102730	1.750918	-0.058672	0.95321	-0.0504952	subgrp_con	count		1	0
RSAD2	9	OK	-0.311902	0.949446	-0.32851	0.74252	-0.0937936	subgrp_con	count		1	0
RNF144A	1	OK	-10.44227	41.465809	-0.251828	0.80117	-0.0004528	subgrp_con	count		1	0
ID2	189	OK	0.486212	0.192918	2.5203017	0.01172	0.6713243	subgrp_con	count		1	0
KIDINS220	32	OK	0.789291	0.543241	1.4529311	0.14626	0.6546356	subgrp_con	count		1	0
MBOAT2	3	OK	-2.04066	2.108668	-0.96775	0.33317	-0.4726699	subgrp_con	count		1	0
ASAP2	34	OK	2.6343672	0.789908	3.3350286	0.00085	2.4342893	subgrp_con	count		1	0
ITGB1BP1	116	OK	-0.580967	0.272150	-2.134732	0.03278	-0.762109	subgrp_con	count		1	0
CPSF3	5	OK	0.7689341	1.529886	0.5026054	0.61526	0.229548	subgrp_con	count		1	0
IAH1	28	OK	0.8832314	0.602375	1.466241	0.14256	0.8989539	subgrp_con	count		1	0
ADAM17	26	OK	1.3732190	0.678113	2.0250586	0.04286	1.1868652	subgrp_con	count		1	0
YWHAQ	154	OK	-0.275786	0.234908	-1.174016	0.24039	-0.3633473	subgrp_con	count		1	0
TAF1B	13	OK	-1.275224	0.940179	-1.356362	0.17498	-1.1054482	subgrp_con	count		1	0
GRHL1	1	OK	-10.44227	41.465809	-0.251828	0.80117	-0.0004528	subgrp_con	count		1	0
AC104794.2	2	OK	2.4096233	2.471520	0.9749599	0.32958	0.6651734	subgrp_con	count		1	0
KLF11	21	OK	-2.318073	0.813215	-2.850502	0.00437	-1.3601504	subgrp_con	count		1	0
HPCAL1	46	OK	-0.73022	0.432468	-1.688516	0.09131	-0.7682599	subgrp_con	count		1	0
ODC1	92	OK	0.0067418	0.292672	0.0230354	0.98162	0.0087722	subgrp_con	count		1	0
NOL10	4	OK	-1.705348	1.618681	-1.053541	0.29209	-0.0003508	subgrp_con	count		1	0
ATP6V1C2	1	OK	41.788203	174.29608	0.2397549	0.81052	0.0001448	subgrp_con	count		1	0
PDIA6	338	OK	0.5394462	0.158228	3.4092821	0.00065	0.7562653	subgrp_con	count		1	0
PQLC3	19	OK	-1.554468	0.784383	-1.981607	0.04752	-1.3085741	subgrp_con	count		1	0
ROCK2	90	OK	0.5761628	0.345033	1.6698755	0.09494	0.649131	subgrp_con	count		1	0
E2F6	2	OK	18.759489	83.625179	0.2243282	0.8225	0.0002878	subgrp_con	count		1	0
LPIN1	4	OK	1.4985876	1.618142	0.9261160	0.35439	0.0010314	subgrp_con	count		1	0
TRIB2	14	OK	-0.727815	0.784241	-0.928050	0.35338	-0.4456827	subgrp_con	count		1	0
FAM84A	1	OK	-12.14842	43.45530	-0.279561	0.77981	-1.9136262	subgrp_con	count		1	0
NBAS	4	OK	-0.494583	1.440949	-0.343234	0.73142	-0.1464259	subgrp_con	count		1	0
DDX1	19	OK	0.6810675	0.744230	0.9151302	0.36012	0.5124590	subgrp_con	count		1	0
MYCN	2	OK	-11.01941	43.249935	-0.254784	0.79889	-1.1748246	subgrp_con	count		1	0
FAM49A	2	OK	1.5824000	1.924023	0.8224389	0.41082	0.7717561	subgrp_con	count		1	0
SMC6	22	OK	2.4428900	1.033056	2.3647198	0.01804	1.5985601	subgrp_con	count		1	0
GEN1	1	OK	18.759489	83.625179	0.2243282	0.8225	0.0002878	subgrp_con	count		1	0
RDH14	11	OK	-0.946821	0.985683	-0.960574	0.33677	-0.7405698	subgrp_con	count		1	0
TTC32	1	OK	3.4598146	6.771451	0.5109413	0.60939	0.00030179	subgrp_con	count		1	0
WDR35	1	OK	18.759489	83.625179	0.2243282	0.8225	0.0002878	subgrp_con	count		1	0
LAPTM4A	576	OK	-0.041262	0.111346	-0.370576	0.71095	-0.0587304	subgrp_con	count		1	0
PUM2	6	OK	-0.445599	1.189054	-0.374750	0.70785	-0.3898436	subgrp_con	count		1	0
GDF7	37	OK	8.394756	3.201862	2.6218350	0.00875	4.0764290	subgrp_con	count		1	0
AC018742.1	1	OK	0.6532528	2.500128	0.2612877	0.79387	0.0001629	subgrp_con	count		1	0
UBXN2A	17	OK	0.6660502	0.728809	0.913888	0.36078	0.5016847	subgrp_con	count		1	0
WDCP	1	OK	-11.01941	43.249935	-0.254784	0.79889	-1.1748246	subgrp_con	count		1	0
FKBP1B	11	OK	-0.228972	0.888263	-0.257776	0.79658	-0.1699976	subgrp_con	count		1	0
SF3B6	321	OK	0.1305815	0.159938	0.8164508	0.41426	0.1823521	subgrp_con	count		1	0
FAM228B	7	OK	1.648091	1.588193	1.0377146	0.2994	0.0030777	subgrp_con	count		1	0
TP53I3	37	OK	0.1858786	0.550029	0.3379370	0.73541	0.1547459	subgrp_con	count		1	0
ITSN2	69	OK	1.3218086	0.396482	3.3338388	0.00086	1.6499294	subgrp_con	count		1	0
NCOA1	14	OK	0.4989480	0.784079	0.6363485	0.52455	0.324972	subgrp_con	count		1	0
PTRHD1	36	OK	0.7962384	0.556598	1.4307242	0.15251	0.905754	subgrp_con	count		1	0
DNAJC27	1	OK	-11.01941	43.249935	-0.254784	0.79889	-1.1748246	subgrp_con	count		1	0
DNMT3A	5	OK	0.1681491	1.725330	0.097459	0.92236	0.0520127	subgrp_con	count		1	0
ASXL2	6	OK	0.1378315	1.178027	0.1170020	0.90686	0.0894239	subgrp_con	count		1	0
KIF3C	1	OK	-10.44227	41.465809	-0.251828	0.80117	-0.0004528	subgrp_con	count		1	0
RAB10	89	OK	-0.222296	0.308476	-0.720628	0.47114	-0.2858823	subgrp_con	count		1	0
HADHA	246	OK	-0.640317	0.183951	-3.480905	0.0005	-0.8844645	subgrp_con	count		1	0
HADHB	89	OK	-0.299895	0.304143	-0.986033	0.32412	-0.3661974	subgrp_con	count		1	0
SLC35F6	13	OK	0.6606612	0.925397	0.7139216	0.47526	0.5545899	subgrp_con	count		1	0
TMEM214	2	OK	-11.01941	43.249935	-0.254784	0.79889	-1.1748246	subgrp_con	count		1	0
AGBL5	2	OK	4.6613299	5.768386	0.8080821	0.41906	0.0027491	subgrp_con	count		1	0
OST4	487	OK	0.391977	0.127561	3.0728535	0.00212	0.553343	subgrp_con	count		1	0
EMILIN1	4	OK	4.1028912	2.741052	1.4968307	0.13444	2.3825605	subgrp_con	count		1	0
ATRAID	269	OK	0.1578376	0.169999	0.928458	0.35317	0.2200882	subgrp_con	count		1	0
CAD	2	OK	-11.01941	43.249935	-0.254784	0.79889	-1.1748246	subgrp_con	count		1	0
MPV17	28	OK	-0.117963	0.616757	-0.191264	0.84832	-0.1239115	subgrp_con	count		1	0
EIF2B4	8	OK	0.3541052	1.036099	0.3417674	0.73252	0.2952413	subgrp_con	count		1	0
SNX17	56	OK	0.5394037	0.402858	1.3389272	0.18167	0.6327165	subgrp_con	count		1	0
PPM1G	121	OK	0.0463843	0.260869	0.1778064	0.85887	0.0613939	subgrp_con	count		1	0
NRBP1	30	OK	-0.299481	0.541390	-0.553171	0.58015	-0.2928962	subgrp_con	count		1	0
FNDC4	9	OK	1.7611072	1.301052	1.353601	0.17586	1.2430564	subgrp_con	count		1	0
ZNF512	2	OK	-10.47563	27.491987	-0.381043	0.70317	-1.1381178	subgrp_con	count		1	0
CCDC121	3	OK	-11.19992	27.762997	-0.403412	0.68665	-1.8294085	subgrp_con	count		1	0
SUPT7L	2	OK	-10.44227	41.465809	-0.251828	0.80117	-0.0004528	subgrp_con	count		1	0
SLC4A1AP	18	OK	0.2332826	0.813273	0.2868439	0.77422	0.0723274	subgrp_con	count		1	0
MRPL33	205	OK	0.3783827	0.210983	1.7934271	0.0729	0.5139469	subgrp_con	count		1	0
RABAM2	17	OK	0.0946002	0.704271	0.1343235	0.89315	0.0710908	subgrp_con	count		1	0
FOSL2	66	OK	0.1526959	0.366694	0.4164088	0.67711	0.1887893	subgrp_con	count		1	0
AC092164.1	1	OK	1.5822999	2.720018	0.5817606	0.56072	0.4743376	subgrp_con	count		1	0
TRMT61B	3	OK	1.5822999	2.720018	0.5817606	0.56072	0.4743376	subgrp_con	count		1	0
WDR43	34	OK	-0.306681	0.531464	-0.577050	0.56391	-0.3105721	subgrp_con	count		1	0
YPEL5	86	OK	1.3223793	0.355538	3.71921	0.0002	1.6640761	subgrp_con	count		1	0
LBH	39	OK	0.7988283	0.548723	1.4557945	0.14545	0.9207628	subgrp_con	count		1	0
LCLAT1	1	OK	18.759489	83.625179	0.2243282	0.8225	0.0002878	subgrp_con	count		1	0
EHD3	1	OK	3.4598146	6.771451	0.5109413	0.60939	0.00030179	subgrp_con	count		1	0
DPY30	60	OK	0.3925196	0.400828	0.9792698	0.32745	0.4786115	subgrp_con	count		1	0
SPAST	3	OK	-10.47563	27.491987	-0.381043	0.70317	-1.1381178	subgrp_con	count		1	0
AL121658.1	1	OK	3.4598146	6.771451	0.5109413	0.60939	0.00030179	subgrp_con	count		1	0
YIPF4	28	OK	0.6665443	0.612765	1.0877649	0.2767	0.7667957	subgrp_con	count		1	0
BIRC6	20	OK	2.1499083	0.856127	2.5112011	0.01203	1.9447034	subgrp_con	count		1	0
TTC27	1	OK	-11.01941	43.249935	-0.254784	0.79889	-1.1748246	subgrp_con	count		1	0
LTBP1	3	OK	-1.853834	1.854391	-0.999699	0.31748	-0.4605327	subgrp_con	count		1	0
RASGRP3	22	OK	0.0924567	0.662178	0.1396250	0.88896	0.0469778	subgrp_con	count		1	0
FAM98A	27	OK	-0.163712	0.583724	-0.280460	0.77912	-0.1537966	subgrp_con	count		1	0
AC009414.2	29	OK	3.8094153	1.147657	3.3192971	0.0009	3.2150169	subgrp_con	count		1	0
FEZ2	129	OK	0.2758286	0.251524	1.0966258	0.2728	0.3739024	subgrp_con	count		1	0
STRN	14	OK	0.2883989	0.782825	0.3679374	0.71292	0.2403753	subgrp_con	count		1	0
GPATCH11	23	OK	0.0306002	0.575659	0.0531568	0.95761	0.0289114	subgrp_con	count		1	0
EIF2AK2	89	OK	0.4468567	0.308280	1.4495137	0.14719	0.5764133	subgrp_con	count		1	0
CEBPZOS	30	OK	0.7457285	0.553144	1.3481625	0.17761	0.7658017	subgrp_con	count		1	0
CEBPZ	74	OK	-0.767408	0.340092	-2.256470	0.02404	-0.9898188	subgrp_con	count		1	0
NDUFAF7	6	OK	-0.354830	1.335197	-0.265751	0.79042	-0.2201817	subgrp_con	count		1	0
PRKD3	22	OK	1.573668	0.711829	2.2107385	0.02705	1.3437386	subgrp_con	count		1	0
QPCT	14	OK	3.3320626	1.594964	2.089114	0.0367	1.6611418	subgrp_con	count		1	0
CDC42EP3	154	OK	-0.453744	0.232857	-1.948595	0.05134	-0.6072168	subgrp_con	count		1	0
CYP1B1-AS1	2	OK	-0.202946	1.653236	-0.122753	0.9022	-4.82E-05	subgrp_con	count		1	0
ATL2	11	OK	0.5838990	0.914883	0.6381524	0.52337	0.3800500	subgrp_con	count		1	0
HNRNPLL	4	OK	-1.089106	1.408619	-0.773173	0.43942	-0.6459026	subgrp_con	count		1	0
GALM	3	OK	0.111276	1.612038	0.0690285	0.94497	0.0565734	subgrp_con	count		1	0
SRSF7	282	OK	-0.135100	0.159922	-0.844788	0.39823	-0.1864031	subgrp_con	count		1	0
GEMIN6	5	OK	-1.254245	1.562761	-0.80258	0.42222	-0.7292682	subgrp_con	count		1	0
DHX57	1	OK	-11.01941	43.249935	-0.254784	0.79889	-1.1748246	subgrp_con	count		1	0
MORN2	14	OK	-0.266770	0.845501	-0.315517	0.75237	-0.1969270	subgrp_con	count		1	0
SOS1	29	OK	0.6169865	0.613668	1.0054055	0.3147	0.463976	subgrp_con	count		1	0
MAP4K3	9	OK	1.6542196	1.192915	1.3867076	0.16552	1.3047613	subgrp_con	count		1	0
AC007388.1	7	OK	-0.684821	1.338431	-0.511659	0.60889	-0.1989751	subgrp_con	count		1	0
TMEM178A	3	OK	3.4598155	3.909993	0.8848648	0.37622	0.0008187	subgrp_con	count		1	0

THUMPD2	7	OK	-0.667846	1.0177416	-0.656205	0.5116	-0.32245356	subgrp_con	count		1	0
SLC8A1	12	OK	-0.929150	0.8833067	-1.0519	0.2928	-0.82990122	subgrp_con	count		1	0
EML4	35	OK	-0.870277	0.5128431	-1.696965	0.0897	-0.85610946	subgrp_con	count		1	0
COX7A2L	228	OK	-0.265849	0.1822517	-1.459241	0.1445	-0.26708867	subgrp_con	count		1	0
MTA3	1	OK	3.4598146	6.7714513	0.5109413	0.6093	0.00030179	subgrp_con	count		1	0
THADA	3	OK	-12.14570	43.418889	-0.279733	0.7796	-1.91338276	subgrp_con	count		1	0
PLEKHH2	3	OK	20.818220	50.543461	0.411887	0.6804	0.00083506	subgrp_con	count		1	0
DYNC2LI1	3	OK	-0.174796	1.820980	-0.095990	0.9235	-0.05309252	subgrp_con	count		1	0
LRPPRC	10	OK	-1.622664	1.1165820	-1.453242	0.1461	-0.89842694	subgrp_con	count		1	0
PPM1B	3	OK	-14.65800	45.690332	-0.320813	0.7483	-0.00067223	subgrp_con	count		1	0
PREPL	11	OK	2.3144960	1.3497515	1.7147570	0.0863	1.04526964	subgrp_con	count		1	0
SRBD1	3	OK	-0.214985	1.5648174	-0.137287	0.8907	-0.06511836	subgrp_con	count		1	0
PRKCE	2	OK	4.6613299	5.7683860	0.8080821	0.4190	0.00274918	subgrp_con	count		1	0
EPAS1	348	OK	-0.197438	0.1420226	-1.390190	0.1644	-0.27711101	subgrp_con	count		1	0
RHOQ	12	OK	2.9373122	1.7705864	1.6589487	0.0971	1.54975084	subgrp_con	count		1	0
PIGF	14	OK	1.5279685	0.9252371	1.6514344	0.0986	0.95036071	subgrp_con	count		1	0
CRIPT	12	OK	0.3048756	0.8879829	0.3433350	0.7313	0.27333922	subgrp_con	count		1	0
SOCS5	7	OK	1.4079766	1.4864499	0.9472075	0.3435	0.0026313	subgrp_con	count		1	0
MCFD2	105	OK	0.7526822	0.2817779	2.6711894	0.0075	0.97513369	subgrp_con	count		1	0
CALM2	553	OK	-0.161735	0.1151142	-1.405002	0.1600	-0.22935156	subgrp_con	count		1	0
MSH2	3	OK	2.5552227	2.349844	1.0874007	0.2768	0.69313893	subgrp_con	count		1	0
MSH6	18	OK	-0.595409	0.7500206	-0.793857	0.4272	-0.42880063	subgrp_con	count		1	0
FBXO11	13	OK	0.9971794	0.8779628	1.1357877	0.2560	0.50469441	subgrp_con	count		1	0
FOXN2	19	OK	-0.118953	0.7212903	-0.164918	0.8690	-0.0764593	subgrp_con	count		1	0
PPP1R21	2	OK	-0.202940	1.6532236	-0.122753	0.9022	-4.82E-05	subgrp_con	count		1	0
STON1	6	OK	-0.834749	1.1107682	-0.751506	0.4523	-0.2361543	subgrp_con	count		1	0
ERLEC1	120	OK	0.9003842	0.2756256	3.2666821	0.0010	1.19936101	subgrp_con	count		1	0
PSME4	4	OK	-10.44227	41.465809	-0.251828	0.8011	-0.00045289	subgrp_con	count		1	0
ACYP2	5	OK	3.4598146	6.7714513	0.5109413	0.6093	0.00030179	subgrp_con	count		1	0
SPTBN1	419	OK	-0.400795	0.1274671	-3.14430	0.0016	-0.56604866	subgrp_con	count		1	0
RTN4	290	OK	0.0311634	0.1667443	0.1868933	0.8517	0.04322438	subgrp_con	count		1	0
RPS27A	888	OK	-0.002744	0.0817688	-0.033563	0.9732	-0.0039293	subgrp_con	count		1	0
MTIF2	17	OK	-0.787520	0.7997237	-0.984723	0.3247	-0.67504353	subgrp_con	count		1	0
CCDC88A	38	OK	0.9517673	0.5259249	1.8097017	0.0703	0.78481820	subgrp_con	count		1	0
CFAP36	99	OK	0.6821378	0.2950951	2.3115861	0.0208	0.88698541	subgrp_con	count		1	0
PPP4R3B	29	OK	0.7695852	0.5537535	1.3897613	0.1646	0.84665066	subgrp_con	count		1	0
PNPT1	14	OK	1.1515254	1.0130613	1.1366788	0.2556	0.57882480	subgrp_con	count		1	0
CCDC85A	3	OK	-1.031165	1.7479538	-0.589927	0.5552	-0.00022765	subgrp_con	count		1	0
VRK2	10	OK	-1.225449	1.0328309	-1.196177	0.2316	-0.84223391	subgrp_con	count		1	0
FANCL	12	OK	-0.502728	0.8750737	-0.574498	0.5656	-0.24637941	subgrp_con	count		1	0
PAPOLG	3	OK	0.1681491	1.7253300	0.097459	0.9223	0.05201277	subgrp_con	count		1	0
REL	59	OK	-0.363420	0.3993439	-0.910043	0.3628	-0.45190472	subgrp_con	count		1	0
PUS10	5	OK	-1.252086	1.3878362	-0.902185	0.3669	-0.56535851	subgrp_con	count		1	0
PEX13	6	OK	1.2785445	1.4228214	0.8979669	0.3692	0.39111381	subgrp_con	count		1	0
KIAA1841	1	OK	-10.44227	41.465809	-0.251828	0.8011	-0.00045289	subgrp_con	count		1	0
C2orf74	33	OK	0.4380835	0.4909016	0.8924058	0.3721	0.51932266	subgrp_con	count		1	0
USP34	64	OK	0.3878650	0.3578049	1.084012	0.2783	0.47328086	subgrp_con	count		1	0
XPO1	28	OK	0.4392768	0.5541565	0.7926944	0.4279	0.45101562	subgrp_con	count		1	0
FAM161A	5	OK	-1.878615	1.4424495	-1.302378	0.1927	-0.77540728	subgrp_con	count		1	0
CCT4	176	OK	-0.352581	0.2122130	-1.661452	0.0966	-0.47805488	subgrp_con	count		1	0
COMMD1	46	OK	0.5621786	0.4409644	1.2748842	0.2023	0.66660435	subgrp_con	count		1	0
B3GNT2	19	OK	-0.321239	0.7929055	-0.405268	0.6852	-0.23728627	subgrp_con	count		1	0
TMEM17	1	OK	-11.01941	43.249935	-0.254784	0.7988	-1.17482461	subgrp_con	count		1	0
EHBP1	4	OK	-1.295616	2.041425	-0.634662	0.5256	-0.00075754	subgrp_con	count		1	0
WDPCP	2	OK	0.5887819	1.7674148	0.3331317	0.7390	0.30122869	subgrp_con	count		1	0
MDH1	39	OK	0.0298674	0.461809	0.0646747	0.9484	0.03457818	subgrp_con	count		1	0
UGP2	102	OK	-0.193405	0.2727920	-0.708986	0.4783	-0.25656878	subgrp_con	count		1	0
VPS54	4	OK	7.4590921	7.6168296	0.9792909	0.3274	0.00128667	subgrp_con	count		1	0
PELI1	22	OK	-0.128353	0.6245522	-0.205512	0.8371	-0.12071810	subgrp_con	count		1	0
LGALSL	16	OK	-1.261720	0.858332	-1.469966	0.1415	-1.091525	subgrp_con	count		1	0
AFTPH	15	OK	0.1248491	0.8112934	0.153889	0.8777	0.06350334	subgrp_con	count		1	0
SERTAD2	13	OK	-0.863063	1.0629976	-0.811914	0.4168	-0.51444542	subgrp_con	count		1	0
SLC1A4	1	OK	3.4598146	6.7714513	0.5109413	0.6093	0.00030179	subgrp_con	count		1	0
CEP68	28	OK	-1.101319	0.5512923	-1.997704	0.0457	-1.13189360	subgrp_con	count		1	0
RAB1A	93	OK	0.1830253	0.3023311	0.6053803	0.5449	0.23528036	subgrp_con	count		1	0
ACTR2	163	OK	0.1726275	0.2268191	0.7610802	0.4466	0.22946616	subgrp_con	count		1	0
SPRED2	1	OK	18.759489	83.625179	0.2243282	0.8225	0.00028789	subgrp_con	count		1	0
ETAA1	5	OK	-0.024863	1.1958382	-0.020791	0.9834	-0.00762178	subgrp_con	count		1	0
C1D	39	OK	-0.434345	0.448560	-0.968308	0.3328	-0.47352292	subgrp_con	count		1	0
WDR92	1	OK	23.991896	84.899941	0.2825902	0.7774	1.65081709	subgrp_con	count		1	0
PNO1	19	OK	0.0708287	0.737310	0.0960636	0.9234	0.02181833	subgrp_con	count		1	0
PPP3R1	12	OK	0.1800918	0.8899687	0.2023574	0.8396	0.09175645	subgrp_con	count		1	0
AC017083.1	2	OK	-10.47563	27.491987	-0.381043	0.7031	-1.13811785	subgrp_con	count		1	0
CNRIP1	35	OK	-0.106896	0.525853	-0.203282	0.8389	-0.1051742	subgrp_con	count		1	0
FBXO48	2	OK	-1.295616	2.041425	-0.634662	0.5256	-0.00075754	subgrp_con	count		1	0
ARHGAP25	2	OK	-1.031165	1.7479538	-0.589927	0.5552	-0.00022765	subgrp_con	count		1	0
GFPT1	3	OK	2.0702522	1.7994826	1.1504708	0.2499	0.96210322	subgrp_con	count		1	0
NFU1	15	OK	2.7495958	1.2542847	2.1921624	0.0283	1.9979570	subgrp_con	count		1	0
ANXA4	63	OK	-0.331999	0.3566970	-0.930761	0.3519	-0.4174982	subgrp_con	count		1	0
GMCL1	11	OK	1.3685308	0.9421420	1.4525737	0.1463	0.86168409	subgrp_con	count		1	0
SNRNP27	25	OK	0.2770717	0.6526344	0.4245436	0.6711	0.26255403	subgrp_con	count		1	0
MXD1	3	OK	0.3472178	1.8140362	0.1914061	0.8482	0.00023212	subgrp_con	count		1	0
PCBP1	340	OK	-0.321754	0.1512760	-2.126937	0.0334	-0.44885953	subgrp_con	count		1	0
C2orf42	1	OK	-11.01941	43.249935	-0.254784	0.7988	-1.17482461	subgrp_con	count		1	0
TIA1	15	OK	-0.05856	0.8546778	-0.069663	0.9444	-0.04926540	subgrp_con	count		1	0
PCYOX1	15	OK	1.4051849	0.8693753	1.6163155	0.1060	1.21655576	subgrp_con	count		1	0
SNRPG	193	OK	0.3410431	0.2177294	1.5663621	0.1172	0.46018331	subgrp_con	count		1	0
FAM136A	8	OK	-0.568368	1.0683502	-0.532006	0.5947	-0.41315144	subgrp_con	count		1	0
VAX2	1	OK	23.991896	84.899941	0.2825902	0.7774	1.65081709	subgrp_con	count		1	0
TEX261	3	OK	0.878817	1.6469311	0.5336091	0.5936	0.44699547	subgrp_con	count		1	0
NAGK	28	OK	0.3216501	0.5768273	0.5576193	0.5771	0.33985070	subgrp_con	count		1	0
MCEE	3	OK	1.2533065	1.5409862	0.8133145	0.4160	0.62634666	subgrp_con	count		1	0
MPHOSPH10	64	OK	0.5210658	0.3638296	1.4321303	0.1521	0.64837049	subgrp_con	count		1	0
ZNF638	59	OK	0.9580233	0.4107224	2.3325566	0.0196	1.04939141	subgrp_con	count		1	0
DYSF	9	OK	1.0066889	1.2181133	0.8264329	0.4085	0.64741892	subgrp_con	count		1	0
EXOC6B	3	OK	-1.295616	2.041425	-0.634662	0.5256	-0.00075754	subgrp_con	count		1	0
SPR	14	OK	0.5218448	0.8226974	0.6343095	0.5258	0.4942387	subgrp_con	count		1	0
RAB11FIP5	10	OK	-1.900114	1.1303324	-1.681022	0.0927	-0.46867129	subgrp_con	count		1	0
PRADC1	18	OK	2.6231242	1.0172764	2.5785756	0.0099	2.24811523	subgrp_con	count		1	0
CCT7	130	OK	-0.128781	0.2566762	-0.501728	0.6158	-0.17182594	subgrp_con	count		1	0
EGR4	2	OK	-0.910223	2.1881125	-0.415985	0.6774	-0.25785735	subgrp_con	count		1	0
TPRKB	66	OK	-0.042515	0.3633514	-0.117009	0.9068	-0.0524832	subgrp_con	count		1	0
DUSP11	17	OK	-0.087828	0.6816656	-0.128843	0.8974	-0.07261850	subgrp_con	count		1	0
STAMBP	12	OK	-0.762661	0.9084185	-0.839548	0.4011	-0.47452422	subgrp_con	count		1	0
DGUOK	63	OK	0.8441859	0.3925010	2.1507865	0.0314	1.02451494	subgrp_con	count		1	0
BOLA3	34	OK	0.3018520	0.5275573	0.572169	0.5672	0.31884482	subgrp_con	count		1	0
BOLA3-AS1	1	OK	18.759489	83.625179	0.2243282	0.8225	0.00028789	subgrp_con	count		1	0
MOB1A	76	OK	0.1582925	0.3517241	0.4500475	0.6526	0.20270734	subgrp_con	count		1	0
MTHFD2	6	OK	0.3849845	1.3796765	0.2790397	0.7802	0.1198232	subgrp_con	count		1	0
DCTN1	14	OK	2.4148013	1.1589490	2.0836130	0.0372	1.36668469	subgrp_con	count		1	0
WDR54	25	OK	0.3336020	0.5842210	0.5710202	0.5679	0.3687996	subgrp_con	count		1	0
RTKN	3	OK	-0.841640	1.4608231	-0.576141	0.5645	-0.24034852	subgrp_con	count		1	0
MOGS	3	OK	21.642059	54.486891	0.3971975	0.6912	1.57744735	subgrp_con	count		1	0
MRPL53	1	OK	-11.01941	43.249935	-0.254784	0.7988	-1.17482461	subgrp_con	count		1	0
CCDC142	1	OK	-10.44227	41.465809	-0.251828	0.8011	-0.00045289	subgrp_con	count		1	0
AUP1	73	OK	0.4695904	0.3650661	1.2863160	0.1983	0.587566	subgrp_con	count		1	0
HTRA2	32	OK	0.3008522	0.5155292	0.5835794	0.5595	0.33262145	subgrp_con	count		1	0
DOK1	2	OK	3.4598146	6.7714513	0.5109413	0.6093	0.00030179	subgrp_con	count		1	0
TACR1	17	OK	-1.916540	0.7529802	-2.545273	0.0109	-1.44972998	subgrp_con	count		1	0
AC007681.1	1	OK	-11.01941	43.249935	-0.254784	0.7988	-1.17482461	subgrp_con	count		1	0
EVA1A	2	OK	23.991896	84.899941	0.2825902	0.7774	1.65081709	subgrp_con	count		1	0
MRPL19	26	OK	0.0852901	0.5434609	0.1569389	0.8752	0.09741999	subgrp_con	count		1	0
GCFC2	5	OK	0.1721614	1.534450	0.1121974	0.9106	0.00011395	subgrp_con	count		1	0
SUCLG1	58	OK	0.4446039	0.4063985	1.0940068	0.2739	0.52757584	subgrp_con	count		1	0
DNAH6	1	OK	-15.99503	70.34942	-0.227365	0.8201	-0.00028474	subgrp_con	count		1	0
TRABD2A	2	OK	3.4598146	6.7714513	0.5109413	0.6093	0.00030179	subgrp_con	count		1	0
TMSB10	878	OK	-0.120724	0.0841399	-1.434802	0.1513	-0.17276504	subgrp_con	count		1	0
KCMF1	23	OK	0.1635849	0.6229189	0.2626103	0.7928	0.14646214	subgrp_con	count		1	0
TCF7L1	10	OK	-0.406152	0.8475285	-0.479219	0.6317	-0.12119435	subgrp_con	count		1	0
TGOLN2	170	OK	0.8681563	0.2258636	3.8437184	0.0001	1.18314808	subgrp_con	count		1	0
RETSAT	2	OK	18.759489	83.625179	0.2243282	0.8225	0.00028789	subgrp_con	count		1	0
ELMOD3	1	OK	3.4598146	6.7714513	0.5109413	0.6093	0.00030179	subgrp_con	count		1	0
CAPG	10	OK	5.6831241	5.3331875	1.0656148	0.2866	0.00307912	subgrp_con	count		1	0
MAT2A	10	OK	-3.204390	1.6751715	-1.912873	0.0557	-1.61773675	subgrp_con	count		1	0
GGCX	9	OK	0.3913132	1.155381	0.3386875	0.7348	0.20016592	subgrp_con	count		1	0
VAMP8	91	OK	1.3559621	0.3238123	4.1874932	2.82E-05	1.77714472	subgrp_con	count		0.28137546	0
VAMP5	547	OK	-0.330544	0.116479	-2.837803	0.0045	-0.46833039	subgrp_con	count		1	0
RNF181	134	OK	0.0306446	0.2561143	0.1196520	0.9047	0.04105253	subgrp_con	count		1	0
TMEM150A	10	OK	0.364221	0.9299182	0.3916706	0.6952	0.32660282	subgrp_con	count		1	0
USP39	4	OK	0.5887819	1.7674148	0.3331317	0.7390	0.30122869	subgrp_con	count		1	0
GNLY	1	OK	3.4598146	6.7714513	0.5109413	0.6093	0.00030179	subgrp_con	count		1	0
ATOH8	6	OK	-0.599308	1.1521099	-0.520182	0.6029	-0.17565882	subgrp_con	count		1	0
PTCD3	1	OK	3.4598146	6.7714513	0.5109413	0.6093	0.00030179	subgrp_con	count		1	0
IMMT	24	OK	-0.195657	0.6473266	-0.302255	0.7624	-0.17350419	subgrp_con	count		1	0
MRPL35	20	OK	0.6247737	0.6316097	0.9891768	0.3225	0.51993137	subgrp_con	count		1	0
KDM3A	4	OK	5.2612789	5.4905820	0.9582370	0.3379	0.00295034	subgrp_con	count		1	0
CHMP3	140	OK	-0.201797	0.2425583	-0.831951	0.4054	-0.26046780	subgrp_con	count		1	0
RNF103	2	OK	5.8049273	5.6694662	1.023893	0.3058	1.03285716	subgrp_con	count		1	0
RMND5A	9	OK	2.5661893	1.365685	1.8790484	0.0602	1.12265528	subgrp_con	count		1	0
LINC01943	1	OK	3.4598146	6.7714513	0.5109413	0.6093	0.00030179	subgrp_con	count		1	0
CYTOR	33	OK	1.1728700	0.5631732	2.0826097	0.0372	1.21656210	subgrp_con	count		1	0
KRCC1	41	OK	0.1751665	0.4432267	0.3952075	0.6926	0.20305182	subgrp_con	count		1	0
RPIA	12	OK	0.1795193	0.9229082	0.1945148	0.8457	0.11658727	subgrp_con	count		1	0
IGKC	150	OK	0.9359628	0.3243280	2.8858521	0.0039	0.87804992	subgrp_con	count		1	0
MRPS5	43	OK	-0.138563	0.4462067	-0.310536	0.7561	-0.15519939	subgrp_con	count		1	0
ZNF514	1	OK	-11.01941	43.249935	-0.254784	0.7988	-1.17482461	subgrp_con	count		1	0
AC092835.1	1	OK	1.5823999	2.7200188	0.5817606	0.5607	0.47433761	subgrp_con	count		1	0
FAHD2A	1	OK	-10.44227	41.465809	-0.251828	0.8011	-0.00045289	subgrp_con	count		1	0
ANKRD36C	3	OK	-0.202940	1.6532236	-0.122753	0.9022	-4.82E-05	subgrp_con	count		1	0
DUSP2	21	OK	1.3967243	0.6359862	2.196155	0.0280	1.60315962	subgrp_con	count		1	0
STARD7	19	OK	0.2779085	0.708250	0.3923876	0.6947	0.2095292	subgrp_con	count		1	0
TMEM127	6	OK	2.8245297	2.2480480	1.2564365	0.2089	0.00186458	subgrp_con	count		1	0
CIAO1	17	OK	0.4076368	0.6882844	0.5922505	0.5536	0.20854850	subgrp_con	count		1	0
SNRNP200	17	OK	-0.731883	0.9219888	-0.793801	0.4273	-0.57728987	subgrp_con	count		1	0
ITPRIPL1	1	OK	-11.01941	43.249935	-0.254784	0.7988	-1.17482461	subgrp_con	count		1	0
ARID5A	91	OK	0.4702338	0.2854646	1.6472578	0.0995	0.60650134	subgrp_con	count		1	0
LMAN2L	1	OK	3.4598146	6.7714513	0.5109413	0.6093	0.00030179	subgrp_con	count		1	0
ANKRD39	5	OK	1.6052618	1.8644265	0.8609949	0.3892	0.99176187	subgrp_con	count		1	0
SEMA4C	3	OK	2.8951588	2.4132736	1.199681	0.2302	0.752269	subgrp_con	count		1	0
FAHD2B	2	OK	0.1681491	1.7253300	0.097459	0.9223	0.05201277	subgrp_con	count		1	0
ANKRD36	1	OK	18.759489	83.625179	0.2243282	0.8225	0.00028789	subgrp_con	count		1	0
AC092683.1	2	OK	-0.202940	1.6532236	-0.122753	0.9022	-4.82E-05	subgrp_con	count		1	0
ANKRD36B	2	OK	-10.44227	41.465809	-0.251828	0.8011	-0.00045289	subgrp_con	count		1	0
COX5B	517	OK	-0.122469	0.1205745	-1.015642	0.3098	-0.17372225	subgrp_con	count		1	0
ACTR1B	10	OK	-0.808803	0.9291428	-0.870482	0.3840	-0.23183765	subgrp_con	count		1	0
TMEM131	8	OK	-0.154085	0.9311237	-0.165481	0.8685	-0.11480148	subgrp_con	count		1	0
COA5	39	OK	-0.349576	0.4605964	-0.758964	0.4478	-0.41571824	subgrp_con	count		1	0
UNC50	41	OK	0.4055453	0.4600541	0.8815165	0.3780	0.49447517	subgrp_con	count		1	0
MGAT4A	61	OK	1.3199697	0.4366666	3.0228314	0.0025	1.54477534	subgrp_con	count		1	0
KIAA1211L	33	OK	1.2276214	0.6336938	1.9372469	0.0527	0.90328017	subgrp_con	count		1	0
C2orf15	1	OK	23.991896	84.899941	0.2825902	0.7774	1.65081709	subgrp_con	count		1	0
LIPT1	3	OK	0.878817	1.6469311	0.5336091	0.5936	0.44699547	subgrp_con	count		1	0
MITD1	26	OK	-0.793901	0.6061043	-1.309842	0.1902	-0.71883473	subgrp_con	count		1	0
MRPL30	1	OK	18.759489	83.625179	0.2243282	0.8225	0.00028789	subgrp_con	count		1	0
TXNDC9	42	OK	-0.296565	0.4556769	-0.650824	0.5151	-0.28995115	subgrp_con	count		1	0
EIF5B	250	OK	-0.061765	0.1780911	-0.346817	0.7287	-0.0851518	subgrp_con	count		1	0
REV1	25	OK	0.8445661	0.6047033	1.3966619	0.1625	0.78962625	subgrp_con	count		1	0
PDCL3	29	OK	0.533537	0.5957130	0.895628	0.3704	0.56305022	subgrp_con	count		1	0
RPL31	729	OK	-0.218687	0.0964982	-2.266232	0.0234	-0.3116904	subgrp_con	count		1	0
CNOT11	5	OK	-3.236473	2.1419797	-1.510972	0.1308	-1.05424964	subgrp_con	count		1	0
RNF149	11	OK	-0.482396	0.8894261	-0.542368	0.5875	-0.30404258	subgrp_con	count		1	0
MAP4K4	72	OK	0.4466610	0.3854100	1.1589240	0.2464	0.54513185	subgrp_con	count		1	0
IL1R2	1	OK	-10.44227	41.465809	-0.251828	0.8011	-0.00045289	subgrp_con	count		1	0
IL1R1	189	OK	-0.002282	0.1922409	-0.011871	0.9905	-0.00313164	subgrp_con	count		1	0
IL1RL1	21	OK	32.250566	42.405530	0.7605273	0.4469	0.00138821	subgrp_con	count		1	0
IL18R1	1	OK	-15.99503	70.34942	-0.227365	0.8201	-0.00028474	subgrp_con	count		1	0
MRPS9	10	OK	1.0110617	1.0187655	0.9924380	0.3209	0.74825460	subgrp_con	count		1	0
TGFBRAP1	1	OK	18.759489	83.625179	0.2243282	0.8225	0.00028789	subgrp_con	count		1	0
C2orf49	19	OK	0.0115845	0.7633059	0.0151767	0.9878	0.01278231	subgrp_con	count		1	0
FHL2	17	OK	1.1757261	0.823889	1.4270430	0.1535	1.21769059	subgrp_con	count		1	0
NCK2	18	OK	0.282649	0.7937209	0.3561072	0.7217	0.21311453	subgrp_con	count		1	0
C2orf40	18	OK	-1.716739	0.7602223	-2.258207	0.0239	-1.5634594	subgrp_con	count		1	0
UXS1	9	OK	0.7688825	1.079014	0.7125788	0.4761	0.39228530	subgrp_con	count		1	0
SULT1C2	3	OK	0.5103003	1.603405	0.3182602	0.7502	0.15911328	subgrp_con	count		1	0
GCC2	131	OK	0.0510185	0.2446006	0.2085789	0.8347	0.06802830	subgrp_con	count		1	0
LIMS1	282	OK	-0.09214	0.1671757	-0.551204	0.5814	-0.12934419	subgrp_con	count		1	0
RANBP2	23	OK	0.7515050	0.6418701	1.1708054	0.2416	0.48765669	subgrp_con	count		1	0
CCDC138	1	OK	-11.01941	43.249935	-0.254784	0.7988	-1.17482461	subgrp_con	count		1	0
SH3RF3	2	OK	-0.725185	1.8079191	-0.401116	0.6883	-0.20979917	subgrp_con	count		1	0
10-Sep	99	OK	-1.123556	0.2948892	-3.809967	0.0001	-1.43456007	subgrp_con	count		1	0
SOWAHC	2	OK	-0.725185	1.8079191	-0.401116	0.6883	-0.20979917	subgrp_con	count		1	0
AC074387.1	1	OK	3.4598146	6.7714513	0.5109413	0.6093	0.00030179	subgrp_con	count		1	0
AC013271.1	1	OK	3.4598146	6.7714513	0.5109413	0.6093	0.00030179	subgrp_con	count		1	0
MALL	135	OK	-0.332442	0.2440159	-1.362380	0.1730	-0.43769747	subgrp_con	count		1	0
NPHP1	4	OK	-11.19992	27.762997	-0.403412	0.6866	-1.82940850	subgrp_con	count		1	0
SMIM37	93	OK	-0.02408	0.3159920	-0.076207	0.9392	-0.03103147	subgrp_con	count		1	0
MIR4435-2HG	25	OK	0.862704	0.7502775	1.1498470	0.2502	0.76747030	subgrp_con	count		1	0
BCL2L11	5	OK	-0.860932	1.2926396	-0.666026	0.5053	-0.0014265	subgrp_con	count		1	0
ANAPC1	3	OK	1.2533065	1.5409862	0.8133145	0.4160	0.62634666	subgrp_con	count		1	0
MERTK	1	OK	18.759489	83.625179	0.2243282	0.8225	0.00028789	subgrp_con	count		1	0
TMEM87B	17	OK	0.4761051	0.7235331	0.6580280	0.5105	0.42113815	subgrp_con	count		1	0
FBLN7	8	OK	2.4141134	1.4781619	1.6331860	0.1024	1.58541476	subgrp_con	count		1	0
ZC3H8	21	OK	-0.602833	0.586400	-1.028023	0.3039	-0.52343005	subgrp_con	count		1	0
ZC3H6	20	OK	-0.206623	0.6746993	-0.306245	0.7594	-0.21005686	subgrp_con	count		1	0
TTL	5	OK	3.4598160	3.3871656	1.0214487	0.3070	0.00081829	subgrp_con	count		1	0
POLR1B	2	OK	-0.349551	1.8544747	-0.188490	0.8504	-0.17337783	subgrp_con	count		1	0
CHCHD5	52	OK	-0.105158	0.427660	-0.245893	0.8057	-0.11788483	subgrp_con	count		1	0
SLC20A1	54	OK	1.6069932	0.4920655	3.2657911	0.0010	1.67578653	subgrp_con	count		1	0
CBWD2	1	OK	-15.99503	70.34942	-0.227365	0.8201	-0.00028474	subgrp_con	count		1	0
SLC35F5	6	OK	0.6191907	1.1154500	0.5551039	0.5788	0.31669929	subgrp_con	count		1	0
ACTR3	109	OK	0.1133433	0.2876821	0.3939881	0.6935	0.14098128	subgrp_con	count		1	0
DDX18	151	OK	-0.437319	0.2322450	-1.883010	0.0597	-0.58849662	subgrp_con	count		1	0
CCDC93	8	OK	0.220228	1.2767573	0.1724907	0.8630	0.14314583	subgrp_con	count		1	0
INSIG2	27	OK	3.5757124	1.121952	3.1870458	0.0014	2.91257693	subgrp_con	count		1	0
C1QL2	3	OK	-1.705348	1.6186811	-1.053541	0.2920	-0.00035081	subgrp_con	count		1	0
STEAP3	3	OK	21.642059	54.486891	0.3971975	0.6912	1.57744735	subgrp_con	count		1	0
C2orf76	18	OK	0.2700371	0.7533036	0.3584705	0.7199	0.25587724	subgrp_con	count		1	0
DBI	250	OK	0.0097057	0.1810149	0.0536185	0.9572	0.01347787	subgrp_con	count		1	0
TMEM185B	1	OK	-15.99503	70.34942	-0.227365	0.8201	-0.00028474	subgrp_con	count		1	0
RALB	81	OK	0.0814884	0.3081664	0.2644300	0.7914	0.10765223	subgrp_con	count		1	0
INHBB	11	OK	-0.355663	0.8964221	-0.396758	0.6915	-0.22558643	subgrp_con	count		1	0
CLASP1	4	OK	1.1224437	1.4569312	0.7704163	0.4410	0.3460629	subgrp_con	count		1	0
NIFK-AS1	1	OK	-15.49804	42.951091	-0.36082	0.7182	-0.00073756	subgrp_con	count		1	0
NIFK	101	OK	-0.031161	0.2799636	-0.111305	0.9113	-0.04153413	subgrp_con	count		1	0
TSN	21	OK	0.9845872	0.7071228	1.3923849	0.1638	1.05267777	subgrp_con	count		1	0
GYPC	153	OK	-1.027604	0.2514232	-4.087149	4.37E-05	-1.3818504	subgrp_con	count		0.43487127	0
ERCC3	6	OK	0.0770030	1.0978566	0.0701394	0.9440	0.04987293	subgrp_con	count		1	0
MAP3K2	24	OK	1.3864104	0.7291437	1.9014225	0.0572	0.87772182	subgrp_con	count		1	0
IWS1	30	OK	-0.068915	0.5458199	-0.126260	0.8995	-0.07034340	subgrp_con	count		1	0
LIMS2	167	OK	0.1746551	0.2056162	0.8494226	0.3956	0.22978552	subgrp_con	count		1	0
WDR33	45	OK	0.7336787	0.5125805	1.4313153	0.1523	0.61015473	subgrp_con	count		1	0
SFT2D3	3	OK	5.2612789	5.4905820	0.9582370	0.3379	0.00295034	subgrp_con	count		1	0
POLR2D	6	OK	-0.256601	1.3412326	-0.191317	0.8482	-0.128091	subgrp_con	count		1	0
AC012306.2	7	OK	0.8200785	1.1232657	0.7300841	0.4653	0.531140	subgrp_con	count		1	0
UGGT1	1	OK	0.6532528	2.5001284	0.2612877	0.7938	0.00016297	subgrp_con	count		1	0
HS6ST1	3	OK	0.6103599	1.4407638	0.4236363	0.6718	0.3122109	subgrp_con	count		1	0
MZT2B	379	OK	0.2164277	0.1423262	1.5206447	0.1283	0.30458957	subgrp_con	count		1	0
CCDC115	32	OK	0.1252292	0.5087008	0.2461746	0.8055	0.143096	subgrp_con	count		1	0
IMP4	38	OK	-0.725576	0.5070704	-1.430919	0.1524	-0.74361839	subgrp_con	count		1	0
PTPN18	10	OK	-1.959486	1.1347387	-1.726817	0.0842	-1.35856022	subgrp_con	count		1	0
FAM168B	5	OK	-0.209243	1.136738	-0.184073	0.8539	-0.06340479	subgrp_con	count		1	0
PLEKHB2	19	OK	0.1584564	0.7242310	0.2187926	0.8268	0.11924910	subgrp_con	count		1	0
MZT2A	73	OK	0.6986795	0.3640775	1.9190406	0.0549	0.89657934	subgrp_con	count		1	0
LYPD1	4	OK	21.105323	83.341273	0.2532397	0.8000	0.0003103	subgrp_con	count		1	0
NCKAP5	5	OK	22.971512	51.721583	0.444051	0.6570	2.36236892	subgrp_con	count		1	0
MGAT5	1	OK	-11.01941	43.249935	-0.254784	0.7988	-1.17482461	subgrp_con	count		1	0
CCNT2	4	OK	-1.314262	1.5485585	-0.848700	0.3960	-0.76471835	subgrp_con	count		1	0
RAB3GAP1	18	OK	-0.512507	0.6567115	-0.780414	0.4351	-0.474044	subgrp_con	count		1	0
R3HDM1	11	OK	0.2066654	0.7983862	0.258850	0.7957	0.17210261	subgrp_con	count		1	0
UBXN4	342	OK	0.2602985	0.147955	1.759302	0.0785	0.36645671	subgrp_con	count		1	0
DARS	33	OK	-0.230520	0.5088222	-0.453047	0.6505	-0.25768879	subgrp_con	count		1	0
CXCR4	5	OK	0.2757225	1.3361644	0.2063537	0.8365	0.14079937	subgrp_con	count		1	0
THSD7B	1	OK	18.759489	83.625179	0.2243282	0.8225	0.00028789	subgrp_con	count		1	0
SPOPL	2	OK	-0.202940	1.6532236	-0.122753	0.9022	-4.82E-05	subgrp_con	count		1	0
KYNU	5	OK	0.2466666	1.4959047	0.164894	0.8690	0.00044539	subgrp_con	count		1	0
GTDC1	3	OK	-11.19992	27.762997	-0.403412	0.6866	-1.82940850	subgrp_con	count		1	0
ZEB2	6	OK	0.7689292	1.5298876	0.5026017	0.6152	0.23855204	subgrp_con	count		1	0
ACVR2A	2	OK	-10.47563	27.491987	-0.381043	0.7031	-1.13811785	subgrp_con	count		1	0
ORC4	3	OK	-10.47563	27.491987	-0.381043	0.7031	-1.13811785	subgrp_con	count		1	0
MBD5	5	OK	-1.610874	1.3660267	-1.179240	0.2382	-0.41511206	subgrp_con	count		1	0
EPC2	14	OK	-1.458954	0.9762743	-1.49441	0.1350	-1.17161624	subgrp_con	count		1	0
LYPD6	1	OK	1.5823999	2.7200188	0.5817606	0.5607	0.47433761	subgrp_con	count		1	0
MMADHC	60	OK	0.9566058	0.4213125	2.2705376	0.0231	1.16723642	subgrp_con	count		1	0
AC007364.1	1	OK	18.759489	83.625179	0.2243282	0.8225	0.00028789	subgrp_con	count		1	0
RND3	78	OK	1.7807016	0.4209410	4.2302874	2.33E-05	2.14481162	subgrp_con	count		0.23306615	0
RBM43	26	OK	-0.636793	0.5429144	-1.172916	0.2408	-0.67574781	subgrp_con	count		1	0
NMI	35	OK	-1.227780	0.5427703	-2.262063	0.0236	-1.45029220	subgrp_con	count		1	0
RIF1	20	OK	0.1466429	0.6874931	0.2133009	0.8310	0.13904893	subgrp_con	count		1	0
ARL5A	2	OK	18.759489	83.625179	0.2243282	0.8225	0.00028789	subgrp_con	count		1	0
STAM2	7	OK	-0.470717	0.9424969	-0.499436	0.6174	-0.13966414	subgrp_con	count		1	0
FMNL2	7	OK	-0.136434	1.1444334	-0.119215	0.9051	-0.0874707	subgrp_con	count		1	0
PRPF40A	118	OK	-0.046609	0.2585949	-0.180239	0.8569	-0.06189858	subgrp_con	count		1	0
ARL6IP6	15	OK	0.1901594	0.7276649	0.2613283	0.7938	0.15828338	subgrp_con	count		1	0
NR4A2	52	OK	1.2962221	0.4750247	2.7287466	0.0063	1.59545633	subgrp_con	count		1	0
[illegible]	[illegible]	OK	3.4598146	6.7714513	0.5109413	0.6093	0.00030179	subgrp_con	count		1	0

SCN9A	5	OK	-0.630652	1.2356596	-0.5103768	0.60979	-0.18426540	subgrp_con	count		1	0
CERS6	2	OK	21.1053223	83.341273	0.2532397	0.80008	0.0003103	subgrp_con	count		1	0
PPIG	248	OK	-0.1438356	0.1754077	-0.8200071	0.41221	-0.20002029	subgrp_con	count		1	0
KLHL23	1	OK	-10.442278	41.4658099	-0.2518286	0.80117	-0.00045289	subgrp_con	count		1	0
SSB	309	OK	-0.1388129	0.1598291	-0.868508	0.38512	-0.19389827	subgrp_con	count		1	0
METTL5	115	OK	-0.2680573	0.2701470	-0.9922645	0.32107	-0.36174522	subgrp_con	count		1	0
GORASP2	17	OK	0.0043668	0.7496156	0.0058254	0.99535	0.00327320	subgrp_con	count		1	0
TLK1	58	OK	0.8616943	0.4026537	2.1400382	0.03235	1.01723614	subgrp_con	count		1	0
METTL8	5	OK	-1.5382228	1.4082018	-1.0923212	0.27469	-0.8157862	subgrp_con	count		1	0
DCAF17	1	OK	-11.019418	43.2499352	-0.2547846	0.79889	-1.17482461	subgrp_con	count		1	0
CYBRD1	30	OK	4.1252837	1.1987505	3.4414031	0.00058	3.23663026	subgrp_con	count		1	0
DYNC1I2	152	OK	-0.1059685	0.2296836	-0.4613672	0.64454	-0.14264215	subgrp_con	count		1	0
SLC25A12	3	OK	4.6613299	5.7683860	0.8080821	0.41906	0.00274918	subgrp_con	count		1	0
HAT1	25	OK	0.0571601	0.5692384	0.1004150	0.92001	0.05850172	subgrp_con	count		1	0
METAP1D	1	OK	18.7594894	83.625179	0.2243282	0.8225	0.00028789	subgrp_con	count		1	0
AC078883.3	2	OK	4.6613299	5.7683860	0.8080821	0.41906	0.00274918	subgrp_con	count		1	0
RAPGEF4	14	OK	0.9759744	0.8287992	1.1775763	0.23897	0.92332609	subgrp_con	count		1	0
MAP3K20	11	OK	-0.0219473	0.9076138	-0.0241812	0.98071	-0.01643807	subgrp_con	count		1	0
SP3	14	OK	0.6716978	0.9006028	0.7458214	0.45577	0.43662549	subgrp_con	count		1	0
OLA1	67	OK	-0.2623446	0.3467460	-0.7565895	0.4492	-0.32682300	subgrp_con	count		1	0
CIR1	118	OK	-0.3940866	0.2562555	-1.5378654	0.12408	-0.52357233	subgrp_con	count		1	0
SCRN3	5	OK	0.5524930	1.3480003	0.4098611	0.68190	0.17231298	subgrp_con	count		1	0
GPR155	5	OK	-11.788041	17.656216	-0.6676425	0.50436	-1.22788846	subgrp_con	count		1	0
WIPF1	5	OK	0.6217488	1.5248630	0.4077407	0.68346	0.6253064	subgrp_con	count		1	0
CHN1	9	OK	-1.3657028	1.1341509	-1.2041631	0.22852	-0.36460482	subgrp_con	count		1	0
ATF2	5	OK	-1.434092	1.5260949	-0.9397141	0.34736	-0.63181067	subgrp_con	count		1	0
LNPK	3	OK	-10.475634	27.491987	-0.3810432	0.70317	-1.13811785	subgrp_con	count		1	0
HOXD-AS2	3	OK	0.1112767	1.6120387	0.0690285	0.94497	0.05657342	subgrp_con	count		1	0
HOXD9	2	OK	-10.475634	27.491987	-0.3810432	0.70317	-1.13811785	subgrp_con	count		1	0
HOXD8	27	OK	-1.7198329	0.6630207	-2.5939352	0.00949	-1.6529252	subgrp_con	count		1	0
HAGLR	1	OK	0.6532528	2.5001286	0.2612877	0.79387	0.00016297	subgrp_con	count		1	0
HAGLROS	11	OK	0.1510817	1.0600396	0.1425246	0.88667	0.09805314	subgrp_con	count		1	0
HOXD1	2	OK	-11.448548	41.761485	-0.2741412	0.78398	-0.00049246	subgrp_con	count		1	0
MTX2	27	OK	0.7038783	0.6586670	1.0686405	0.28522	0.75996827	subgrp_con	count		1	0
LINC01116	23	OK	-1.8628752	0.6991596	-2.6644606	0.00771	-1.6661782	subgrp_con	count		1	0
LINC01117	2	OK	-1.0311657	1.7479538	-0.5899272	0.55526	-0.00022765	subgrp_con	count		1	0
HNRNPA3	337	OK	-0.0213994	0.1511013	-0.1416226	0.88738	-0.02980063	subgrp_con	count		1	0
AC079305.1	1	OK	23.9918966	84.899941	0.2825902	0.77749	1.65081709	subgrp_con	count		1	0
AGPS	6	OK	-0.0755604	1.2476219	-0.0605635	0.95171	-0.04866258	subgrp_con	count		1	0
PDE11A	1	OK	-10.442278	41.4658099	-0.2518286	0.80117	-0.00045289	subgrp_con	count		1	0
OSBPL6	5	OK	22.6877204	54.315757	0.4177005	0.67617	1.6098774	subgrp_con	count		1	0
AC009948.1	1	OK	-11.019418	43.2499352	-0.2547846	0.79889	-1.17482461	subgrp_con	count		1	0
PRKRA	27	OK	0.0859864	0.5276266	0.1629684	0.87054	0.09294621	subgrp_con	count		1	0
FKBP7	11	OK	2.3445219	1.3310420	1.7614184	0.07817	1.33862113	subgrp_con	count		1	0
PLEKHA3	71	OK	0.9695228	0.3494874	2.7741280	0.00553	1.22435817	subgrp_con	count		1	0
TTN-AS1	1	OK	-11.019418	43.2499352	-0.2547846	0.79889	-1.17482461	subgrp_con	count		1	0
SESTD1	16	OK	5.2612938	2.7484956	1.9142449	0.05559	0.00810907	subgrp_con	count		1	0
CWC22	28	OK	-0.0802357	0.5318024	-0.1508751	0.88007	-0.0714396	subgrp_con	count		1	0
UBE2E3	111	OK	0.0455176	0.2801834	0.1624565	0.87095	0.05923961	subgrp_con	count		1	0
ITGA4	1	OK	17.7472079	50.971927	0.3481761	0.72771	0.0007532	subgrp_con	count		1	0
SSFA2	146	OK	0.8568035	0.2351750	3.6432591	0.00027	1.15619561	subgrp_con	count		1	0
PDE1A	2	OK	1.9259646	2.5154879	0.7656425	0.44389	0.56022119	subgrp_con	count		1	0
DNAJC10	49	OK	1.0375649	0.5006385	2.0724833	0.03822	1.06204668	subgrp_con	count		1	0
FRZB	2	OK	0.5887819	1.767414	0.3331317	0.73903	0.30122869	subgrp_con	count		1	0
NCKAP1	58	OK	1.422021	0.4873359	2.9284693	0.0032	1.57214461	subgrp_con	count		1	0
DUSP19	3	OK	0.5103003	1.603405	0.3182602	0.75029	0.15811328	subgrp_con	count		1	0
NUP35	3	OK	-0.357207	1.5148489	-0.2358037	0.81359	-0.22654173	subgrp_con	count		1	0
FSIP2	1	OK	18.7594894	83.625179	0.2243282	0.8225	0.00028789	subgrp_con	count		1	0
ZC3H15	230	OK	-0.4083469	0.186197	-2.193086	0.0282	-0.56554919	subgrp_con	count		1	0
ITGAV	42	OK	12.5261589	4.2614913	2.9393840	0.00329	3.04939886	subgrp_con	count		1	0
FAM171B	25	OK	0.7511279	0.7623890	0.9839386	0.32515	0.38468012	subgrp_con	count		1	0
CALCRL	585	OK	0.3356830	0.1104533	3.0391396	0.00237	0.47638559	subgrp_con	count		1	0
GULP1	15	OK	0.4103620	0.7247647	0.5662003	0.57126	0.38827794	subgrp_con	count		1	0
COL3A1	54	OK	4.9570849	1.1850012	4.1831895	2.87E-05	3.22508967	subgrp_con	count		0.2867289	0
COL5A2	4	OK	11.5419164	4.7775515	2.4158643	0.0157	0.00521789	subgrp_con	count		1	0
WDR75	3	OK	-11.199925	27.762997	-0.403412	0.68665	-1.82940850	subgrp_con	count		1	0
SLC40A1	33	OK	-0.931892	0.4875153	-1.9111032	0.05599	-1.04587201	subgrp_con	count		1	0
ASNSD1	17	OK	0.5213646	0.6892734	0.7563974	0.44941	0.43446104	subgrp_con	count		1	0
ANKAR	7	OK	-1.1817077	1.2059343	-0.9799104	0.32712	-0.32350761	subgrp_con	count		1	0
ORMDL1	65	OK	0.0333288	0.3447846	0.096684	0.92297	0.04211662	subgrp_con	count		1	0
PMS1	7	OK	-0.3655229	1.159098	-0.3152509	0.7525	-0.10944449	subgrp_con	count		1	0
C2orf88	2	OK	-0.2029407	1.6532365	-0.1227536	0.9023	-4.82E-05	subgrp_con	count		1	0
HIBCH	17	OK	0.5254766	0.7086670	0.7415000	0.45839	0.49698918	subgrp_con	count		1	0
INPP1	95	OK	-0.431792	0.2877458	-1.5006022	0.13346	-0.56067441	subgrp_con	count		1	0
MFSD6	7	OK	1.0268744	1.3898596	0.7388331	0.46001	0.52373824	subgrp_con	count		1	0
NAB1	9	OK	1.2376430	1.0461443	1.1830518	0.23679	0.00231491	subgrp_con	count		1	0
GLS	151	OK	-0.0029132	0.2202909	-0.0132244	0.98945	-0.00400773	subgrp_con	count		1	0
STAT1	56	OK	0.7198380	0.3715160	1.9375690	0.05268	0.91320425	subgrp_con	count		1	0
MYO1B	10	OK	1.7147351	1.1317962	1.5150564	0.12976	1.21555919	subgrp_con	count		1	0
NABP1	5	OK	-0.9206809	1.1213217	-0.8210676	0.41161	-0.26049779	subgrp_con	count		1	0
CAVIN2	430	OK	-0.2033967	0.1247876	-1.6299425	0.10311	-0.28776088	subgrp_con	count		1	0
SLC39A10	35	OK	0.2572952	0.5206946	0.4941384	0.62121	0.28948011	subgrp_con	count		1	0
STK17B	2	OK	3.4598146	6.7714513	0.5109413	0.60939	0.00030179	subgrp_con	count		1	0
AC114760.2	1	OK	3.4597879	4.7858528	0.7229198	0.46972	0.00081879	subgrp_con	count		1	0
HECW2	21	OK	-0.396086	0.7122944	-0.5560678	0.57816	-0.32127618	subgrp_con	count		1	0
GTF3C3	6	OK	7.8870122	3.7267296	2.1163360	0.03432	0.00405462	subgrp_con	count		1	0
ANKRD44	1	OK	18.7594894	83.625179	0.2243282	0.8225	0.00028789	subgrp_con	count		1	0
SF3B1	172	OK	-0.0963698	0.2066050	-0.4664445	0.6409	-0.12860939	subgrp_con	count		1	0
COQ10B	44	OK	-0.710481	0.4563948	-1.5567249	0.11954	-0.76535289	subgrp_con	count		1	0
HSPE1	285	OK	0.0103075	0.1679281	0.0613806	0.95106	0.01436479	subgrp_con	count		1	0
MOB4	37	OK	-0.2575836	0.4808649	-0.5356671	0.59219	-0.21149925	subgrp_con	count		1	0
RFTN2	3	OK	3.3648273	2.5447810	1.3222463	0.18609	1.31777762	subgrp_con	count		1	0
PLCL1	1	OK	-10.442278	41.4658099	-0.2518286	0.80117	-0.00045289	subgrp_con	count		1	0
C2orf69	4	OK	5.0027563	4.2286829	1.1830530	0.23679	0.98115823	subgrp_con	count		1	0
MAIP1	7	OK	3.6034358	2.1080764	1.7093476	0.08739	1.36341139	subgrp_con	count		1	0
SPATS2L	76	OK	0.4348906	0.3704073	1.1740874	0.24036	0.52605889	subgrp_con	count		1	0
SGO2	8	OK	-1.2730247	0.9993884	-1.2738037	0.20272	-0.57324908	subgrp_con	count		1	0
BZW1	338	OK	0.5877160	0.1568225	3.7476497	0.00018	0.824223	subgrp_con	count		1	0
CLK1	73	OK	0.4057524	0.3385308	1.1985685	0.2307	0.4485454	subgrp_con	count		1	0
PPIL3	3	OK	2.9849400	2.5988857	1.1485460	0.25074	1.23382860	subgrp_con	count		1	0
NIF3L1	1	OK	-11.019418	43.2499352	-0.2547846	0.79889	-1.17482461	subgrp_con	count		1	0
ORC2	7	OK	1.6315080	1.3708437	1.1901487	0.23399	0.48716736	subgrp_con	count		1	0
FAM126B	7	OK	1.5146440	1.1992448	1.2629982	0.20659	0.4563386	subgrp_con	count		1	0
NDUFB3	102	OK	0.5878834	0.3007836	1.9545057	0.05064	0.76030101	subgrp_con	count		1	0
CFLAR	186	OK	0.0544517	0.1906461	0.2856168	0.77517	0.07325998	subgrp_con	count		1	0
CASP10	8	OK	-0.2149664	1.1047585	-0.1945822	0.84572	-0.10759279	subgrp_con	count		1	0
CASP8	2	OK	-12.145707	43.418869	-0.2797334	0.77968	-1.91338276	subgrp_con	count		1	0
TRAK2	11	OK	2.7193762	1.3595097	2.0002623	0.04547	1.16580822	subgrp_con	count		1	0
TMEM237	1	OK	-10.442278	41.4658099	-0.2518286	0.80117	-0.00045289	subgrp_con	count		1	0
ALS2	1	OK	18.7594894	83.625179	0.2243282	0.8225	0.00028789	subgrp_con	count		1	0
SUMO1	237	OK	-0.4756255	0.1861872	-2.5545549	0.01063	-0.6569225	subgrp_con	count		1	0
NOP58	40	OK	-0.7325139	0.4650557	-1.5751097	0.11523	-0.83005876	subgrp_con	count		1	0
AC064836.3	1	OK	-10.442278	41.4658099	-0.2518286	0.80117	-0.00045289	subgrp_con	count		1	0
ICA1L	1	OK	-10.442278	41.4658099	-0.2518286	0.80117	-0.00045289	subgrp_con	count		1	0
WDR12	5	OK	0.0064164	1.2548604	0.0051132	0.99592	0.00324896	subgrp_con	count		1	0
NBEAL1	28	OK	-0.6751283	0.6415073	-1.0524090	0.29261	-0.00310309	subgrp_con	count		1	0
CYP20A1	31	OK	0.1712532	0.4978108	0.3442136	0.73069	0.19852551	subgrp_con	count		1	0
ABI2	21	OK	0.7944439	0.8069265	0.9845307	0.32485	0.40507326	subgrp_con	count		1	0
RAPH1	25	OK	-0.9233555	0.6422350	-1.4354869	0.15115	-0.87098350	subgrp_con	count		1	0
PARD3B	1	OK	18.7594894	83.625179	0.2243282	0.8225	0.00028789	subgrp_con	count		1	0
NRP2	93	OK	-0.4720029	0.3017859	-1.564032	0.11781	-0.58202156	subgrp_con	count		1	0
INO80D	14	OK	0.5147698	0.7540826	0.6826438	0.49482	0.3920727	subgrp_con	count		1	0
NDUFS1	18	OK	0.1226168	0.6952892	0.1763538	0.86002	0.10196757	subgrp_con	count		1	0
EEF1B2	795	OK	-0.1937649	0.0923747	-2.0975964	0.03594	-0.27677951	subgrp_con	count		1	0
ADAM23	3	OK	-10.442278	41.4658099	-0.2518286	0.80117	-0.00045289	subgrp_con	count		1	0
FASTKD2	6	OK	-0.2931643	1.0236494	-0.2863914	0.77458	-0.21692785	subgrp_con	count		1	0
KLF7	37	OK	-0.8856888	0.4624797	-1.9150864	0.05548	-0.94510869	subgrp_con	count		1	0
CREB1	26	OK	0.6132567	0.573242	1.0698023	0.28471	0.58154307	subgrp_con	count		1	0
METTL21A	13	OK	-0.181758	0.7795721	-0.2331522	0.81564	-0.17064535	subgrp_con	count		1	0
CCNYL1	8	OK	-0.484872	1.0072	-0.481358	0.63026	-0.00083107	subgrp_con	count		1	0
FZD5	1	OK	-10.442278	41.4658099	-0.2518286	0.80117	-0.00045289	subgrp_con	count		1	0
IDH1	12	OK	-0.3247247	0.8038095	-0.4039823	0.68623	-0.23985882	subgrp_con	count		1	0
PIKFYVE	2	OK	-11.019418	43.2499352	-0.2547846	0.79889	-1.17482461	subgrp_con	count		1	0
MAP2	5	OK	19.1030323	50.772489	0.3762478	0.70672	0.00078880	subgrp_con	count		1	0
RPE	3	OK	-0.6348896	2.0089600	-0.3160275	0.75198	-0.000145	subgrp_con	count		1	0
KANSL1L	3	OK	-0.2029407	1.6532365	-0.1227536	0.9022	-4.82E-05	subgrp_con	count		1	0
LANCL1	6	OK	-1.9381096	1.4629785	-1.3247697	0.18525	-1.28771449	subgrp_con	count		1	0
SPAG16	11	OK	1.0119052	0.9299922	1.0880790	0.27656	0.31334602	subgrp_con	count		1	0
BARD1	12	OK	-1.069286	0.9268810	-1.153639	0.24865	-0.82654706	subgrp_con	count		1	0
ATIC	1	OK	-15.995032	70.349421	-0.2273655	0.82014	-0.00028474	subgrp_con	count		1	0
XRCC5	248	OK	0.0873803	0.1834734	0.476256	0.63389	0.12016463	subgrp_con	count		1	0
SMARCAL1	3	OK	-14.042889	27.411700	-0.5122954	0.60844	-0.00174737	subgrp_con	count		1	0
RPL37A	822	OK	-0.1994024	0.0901849	-2.2110391	0.02703	-0.28479255	subgrp_con	count		1	0
IGFBP2	257	OK	0.0495877	0.1688182	0.2937343	0.76896	0.06929817	subgrp_con	count		1	0
IGFBP5	70	OK	1.3538644	0.4219788	3.2012599	0.00137	1.52927124	subgrp_con	count		1	0
TNS1	8	OK	0.4585666	1.0368652	0.4422625	0.6582	0.38521972	subgrp_con	count		1	0
ARPC2	491	OK	0.2235277	0.125159	1.7858399	0.07411	0.31647034	subgrp_con	count		1	0
AAMP	32	OK	0.046564	0.5133153	0.0907126	0.92772	0.04598949	subgrp_con	count		1	0
PNKD	88	OK	0.7534440	0.3235634	2.3285821	0.01988	0.97302408	subgrp_con	count		1	0
TMBIM1	86	OK	0.8256274	0.3152281	2.6191424	0.00882	1.00992943	subgrp_con	count		1	0
CTDSP1	54	OK	0.8160674	0.4100185	1.9903183	0.04656	0.91381232	subgrp_con	count		1	0
USP37	2	OK	-0.3495512	1.8544747	-0.1884907	0.85049	-0.17337783	subgrp_con	count		1	0
CNOT9	6	OK	-1.2520546	1.3878450	-0.9021572	0.36697	-0.56535277	subgrp_con	count		1	0
PLCD4	1	OK	18.7594894	83.625179	0.2243282	0.8225	0.00028789	subgrp_con	count		1	0
BCS1L	3	OK	3.4598146	6.7714513	0.5109413	0.60939	0.00030179	subgrp_con	count		1	0
RNF25	2	OK	-13.04562	27.674761	-0.4713905	0.63736	-1.3178643	subgrp_con	count		1	0
CYP27A1	8	OK	0.8357807	1.1349618	0.7363955	0.46149	0.55426136	subgrp_con	count		1	0
CNPPD1	7	OK	1.3462853	1.2660218	1.0633980	0.2876	0.66862932	subgrp_con	count		1	0
RETREG2	14	OK	3.3059243	1.7475615	1.8917355	0.05852	1.65439004	subgrp_con	count		1	0
ZFAND2B	24	OK	0.6101654	0.600115	1.0167473	0.30927	0.62490420	subgrp_con	count		1	0
ANKZF1	2	OK	0.1681491	1.7253300	0.097459	0.92236	0.05201277	subgrp_con	count		1	0
STK16	3	OK	3.3648273	2.5447810	1.3222463	0.18609	1.31777762	subgrp_con	count		1	0
TUBA4A	69	OK	-0.1331947	0.3445025	-0.386629	0.69903	-0.16055348	subgrp_con	count		1	0
DNAJB2	6	OK	0.6210801	1.2477937	0.4977426	0.61867	0.31766016	subgrp_con	count		1	0
DNPEP	26	OK	-0.0442578	0.5856587	-0.0755690	0.93976	-0.04361213	subgrp_con	count		1	0
GMPPA	4	OK	4.3938286	2.8675507	1.5322583	0.12546	1.94227546	subgrp_con	count		1	0
CHPF	1	OK	23.9918966	84.899941	0.2825902	0.77749	1.65081709	subgrp_con	count		1	0
OBSL1	2	OK	-0.9102232	2.1881125	-0.4159856	0.67742	-0.25785735	subgrp_con	count		1	0
EPHA4	26	OK	-0.0537499	0.6680122	-0.0804625	0.93587	-0.04789021	subgrp_con	count		1	0
FARSB	1	OK	1.5823999	2.7200188	0.5817606	0.56072	0.47433761	subgrp_con	count		1	0
ACSL3	23	OK	-0.336758	0.6109010	-0.5512482	0.58146	-0.29642971	subgrp_con	count		1	0
WDFY1	9	OK	-1.8540209	1.3189071	-1.4057252	0.15981	-0.76811798	subgrp_con	count		1	0
MRPL44	9	OK	-0.2478955	0.9656512	-0.2567132	0.7974	-0.15827420	subgrp_con	count		1	0
SERPINE2	50	OK	12.2721963	3.0811802	3.9829530	6.81E-05	3.80506732	subgrp_con	count		0.6764221	0
FAM124B	4	OK	-0.4954258	1.5039132	-0.3294245	0.74183	-0.00031231	subgrp_con	count		1	0
CUL3	15	OK	0.150973	0.7442342	0.2028575	0.83925	0.12551323	subgrp_con	count		1	0
DOCK10	1	OK	3.4598146	6.7714513	0.5109413	0.60939	0.00030179	subgrp_con	count		1	0
RHBDD1	1	OK	18.7594894	83.625179	0.2243282	0.8225	0.00028789	subgrp_con	count		1	0
MFF	29	OK	0.9720299	0.6043989	1.608274	0.10779	0.95171795	subgrp_con	count		1	0
AGFG1	23	OK	1.3971478	0.766343	1.8231342	0.06829	1.354038	subgrp_con	count		1	0
TRIP12	39	OK	0.5783404	0.4742257	1.2195466	0.22264	0.66232906	subgrp_con	count		1	0
FBXO36	1	OK	-15.995032	70.349421	-0.2273655	0.82014	-0.00028474	subgrp_con	count		1	0
SLC16A14	1	OK	3.4598146	6.7714513	0.5109413	0.60939	0.00030179	subgrp_con	count		1	0
SP110	104	OK	-0.7034332	0.2784092	-2.526616	0.01152	-0.91371263	subgrp_con	count		1	0
SP140L	13	OK	-0.32452	0.7830644	-0.4144232	0.67856	-0.28618098	subgrp_con	count		1	0
SP100	281	OK	-0.3941015	0.1639983	-2.4030822	0.01626	-0.5485534	subgrp_con	count		1	0
CAB39	7	OK	0.9801526	1.3385170	0.7322675	0.46401	0.49669676	subgrp_con	count		1	0
ITM2C	1	OK	23.9918966	84.899941	0.2825902	0.77749	1.65081709	subgrp_con	count		1	0
PSMD1	46	OK	0.9341697	0.4832389	1.9331423	0.05322	0.91588022	subgrp_con	count		1	0
HTR2B	3	OK	19.1030323	50.772489	0.3762478	0.70672	0.00078880	subgrp_con	count		1	0
NCL	551	OK	-0.2129024	0.1125606	-1.8914456	0.05856	-0.30256815	subgrp_con	count		1	0
PTMA	885	OK	-0.1093292	0.0836958	-1.3062678	0.19146	-0.15644245	subgrp_con	count		1	0
PDE6D	6	OK	3.3934792	2.1394596	1.5861384	0.11271	1.32352972	subgrp_con	count		1	0
COPS7B	2	OK	1.5823999	2.7200188	0.5817606	0.56072	0.47433761	subgrp_con	count		1	0
NPPC	6	OK	1.2785256	1.4238165	0.8979567	0.36921	0.39110799	subgrp_con	count		1	0
DIS3L2	4	OK	-0.712926	1.3887485	-0.5133571	0.6077	-0.00044093	subgrp_con	count		1	0
TIGD1	1	OK	-10.442278	41.4658099	-0.2518286	0.80117	-0.00045289	subgrp_con	count		1	0
EIF4E2	38	OK	0.8377798	0.5479276	1.5289970	0.12627	0.87924629	subgrp_con	count		1	0
GIGYF2	13	OK	-0.19021	0.8654936	-0.2197706	0.82605	-0.09534502	subgrp_con	count		1	0
NGEF	1	OK	18.7594894	83.625179	0.2243282	0.8225	0.00028789	subgrp_con	count		1	0
INPP5D	6	OK	-0.6905869	1.3657653	-0.505641	0.61311	-0.19746312	subgrp_con	count		1	0
DGKD	2	OK	2.4096337	2.4715206	0.9749599	0.32958	0.66517342	subgrp_con	count		1	0
USP40	1	OK	-10.442278	41.4658099	-0.2518286	0.80117	-0.00045289	subgrp_con	count		1	0
ARL4C	20	OK	0.6783965	0.7753866	0.8749128	0.38162	0.51047675	subgrp_con	count		1	0
SH3BP4	21	OK	-1.0193133	0.7888313	-1.2921815	0.19629	-0.85260577	subgrp_con	count		1	0
AGAP1	3	OK	-2.023032	1.8071267	-1.1194743	0.26294	-0.00109400	subgrp_con	count		1	0
IQCA1	10	OK	1.3741333	1.1340198	1.2117365	0.22561	1.09918086	subgrp_con	count		1	0
ACKR3	69	OK	1.5233058	0.4020035	3.7892845	0.00015	1.91429172	subgrp_con	count		1	0
COPS8	55	OK	0.2521011	0.4125028	0.6111500	0.5411	0.29237268	subgrp_con	count		1	0
MLPH	30	OK	-3.553715	0.9100036	-3.9051658	9.42E-05	-2.26339284	subgrp_con	count		0.9238869	0
RAB17	1	OK	23.9918966	84.899941	0.2825902	0.77749	1.65081709	subgrp_con	count		1	0
LRRFIP1	596	OK	-0.3226176	0.1077660	-2.9936835	0.00276	-0.45888495	subgrp_con	count		1	0
RAMP1	2	OK	5.8049273	5.6694662	1.023893	0.30589	1.03285716	subgrp_con	count		1	0
UBE2F	11	OK	-0.64630	1.075837	-0.6007449	0.54801	-0.00109241	subgrp_con	count		1	0
SCLY	1	OK	-10.442278	41.4658099	-0.2518286	0.80117	-0.00045289	subgrp_con	count		1	0
ILKAP	6	OK	1.3170372	1.1963862	1.1008461	0.27096	0.83352147	subgrp_con	count		1	0
HES6	2	OK	-1.7585735	2.3554439	-0.7465996	0.45531	-0.00036089	subgrp_con	count		1	0
PER2	15	OK	-2.1119525	0.9470933	-2.2299306	0.02575	-1.56057374	subgrp_con	count		1	0
TRAF3IP1	4	OK	-2.1660098	2.7041600	-0.8009917	0.42314	-0.79067866	subgrp_con	count		1	0
TWIST2	2	OK	18.7594894	83.625179	0.2243282	0.8225	0.00028789	subgrp_con	count		1	0
NDUFA10	118	OK	0.0668016	0.2588873	0.2580335	0.79638	0.08936706	subgrp_con	count		1	0
COPS9	207	OK	0.1142728	0.1994377	0.5729750	0.56666	0.15628349	subgrp_con	count		1	0
GPC1	2	OK	3.4598149	4.7858686	0.7229230	0.46972	0.00081925	subgrp_con	count		1	0
DUSP28	1	OK	3.4597878	3.9017207	0.8867338	0.37522	0.00222136	subgrp_con	count		1	0
RNPEPL1	16	OK	-0.8439868	0.8904979	-0.9477695	0.34325	-0.6030075	subgrp_con	count		1	0
SNED1	2	OK	-14.65800	45.690332	-0.3208138	0.74835	-0.00067223	subgrp_con	count		1	0
MTERF4	8	OK	1.6303893	1.1609139	1.4044015	0.1602	1.16447637	subgrp_con	count		1	0
PASK	1	OK	3.4598146	6.7714513	0.5109413	0.60939	0.00030179	subgrp_con	count		1	0
PPP1R7	45	OK	0.0740531	0.4685890	0.1580343	0.87442	0.08003472	subgrp_con	count		1	0
HDLBP	109	OK	0.7605054	0.3000675	2.5344474	0.01126	0.9985465	subgrp_con	count		1	0
2-Sep	221	OK	0.3739488	0.1922377	1.9452418	0.05175	0.50842274	subgrp_con	count		1	0
STK25	54	OK	0.1387086	0.4454653	0.3113792	0.75551	0.13125959	subgrp_con	count		1	0
BOK	4	OK	11.5072273	4.8024160	2.3961329	0.01657	0.00201746	subgrp_con	count		1	0
THAP4	8	OK	-0.1902356	1.2247426	-0.155327	0.87656	-0.05772119	subgrp_con	count		1	0
ATG4B	6	OK	2.6265438	2.0961357	1.2530409	0.21019	0.0017549	subgrp_con	count		1	0
DTYMK	19	OK	0.1742534	0.8005282	0.2176730	0.82768	0.11292127	subgrp_con	count		1	0
ING5	12	OK	1.373220	0.9901089	1.3869413	0.16546	1.17919697	subgrp_con	count		1	0
CNTN4	1	OK	18.7594894	83.625179	0.2243282	0.8225	0.00028789	subgrp_con	count		1	0
TRNT1	13	OK	0.873264	0.8638555	1.0108918	0.31207	0.72210615	subgrp_con	count		1	0
CRBN	125	OK	-0.0943732	0.2521558	-0.3742656	0.70821	-0.12594662	subgrp_con	count		1	0
SUMF1	8	OK	0.0516335	1.2085531	0.0427234	0.96592	9.21E-05	subgrp_con	count		1	0
LRRN1	1	OK	3.4598146	6.7714513	0.5109413	0.60939	0.00030179	subgrp_con	count		1	0
ITPR1	28	OK	0.3462661	0.5846748	0.5922371	0.55369	0.31174176	subgrp_con	count		1	0
EGOT	2	OK	-1.2956167	2.041425	-0.6346627	0.52565	-0.00075754	subgrp_con	count		1	0
BHLHE40	131	OK	-0.1044652	0.2500780	-0.4177320	0.67614	-0.13993547	subgrp_con	count		1	0
ARL8B	31	OK	1.1000387	0.5832657	1.8856759	0.05934	1.1992671	subgrp_con	count		1	0
EDEM1	4	OK	18.7594894	83.625179	0.2243282	0.8225	0.00028789	subgrp_con	count		1	0
SSUH2	5	OK	0.7003107	1.7308960	0.4045943	0.68579	0.00047551	subgrp_con	count		1	0
OXTR	2	OK	1.1765791	2.346505	0.5014175	0.61608	0.00080876	subgrp_con	count		1	0
RAD18	3	OK	0.1681491	1.7253300	0.097459	0.92236	0.05201277	subgrp_con	count		1	0
THUMPD3-AS1	35	OK	-1.8938409	0.5506140	-3.4395072	0.00058	-1.81111882	subgrp_con	count		1	0
THUMPD3	14	OK	0.0429843	0.7372935	0.0583001	0.95351	0.04529838	subgrp_con	count		1	0
SETD5	21	OK	1.0983222	0.7613121	1.4426831	0.14911	1.06226316	subgrp_con	count		1	0
MTMR14	3	OK	-1.363526	1.7092410	-0.7977378	0.42502	-0.57591456	subgrp_con	count		1	0
OGG1	4	OK	0.2606514	1.6620474	0.1568255	0.87538	0.08088269	subgrp_con	count		1	0
CAMK1	12	OK	0.2089977	0.7997806	0.2613188	0.79385	0.19794192	subgrp_con	count		1	0
TADA3	20	OK	1.9338428	0.8870903	2.1799841	0.02926	1.69973847	subgrp_con	count		1	0
ARPC4	111	OK	0.0401933	0.2640448	0.1522217	0.87901	0.05052757	subgrp_con	count		1	0
JAGN1	41	OK	0.1445669	0.464155	0.311462	0.75545	0.16262981	subgrp_con	count		1	0
IL17RC	3	OK	0.1112767	1.6120387	0.0690285	0.94497	0.05657342	subgrp_con	count		1	0
CRELD1	5	OK	-1.4966957	1.5631792	-0.957468	0.33832	-0.00085699	subgrp_con	count		1	0
PRRT3	2	OK	-0.9102232	2.1881125	-0.4159856	0.67742	-0.25785735	subgrp_con	count		1	0
EMC3	65	OK	-0.0072377	0.3740632	-0.019349	0.98456	-0.00873918	subgrp_con	count		1	0
FANCD2	1	OK	-15.995032	70.349421	-0.2273655	0.82014	-0.00028474	subgrp_con	count		1	0
BRK1	357	OK	0.0591645	0.147176	0.4019973	0.68769	0.0828917	subgrp_con	count		1	0
VHL	2	OK	-10.442278	41.4658099	-0.2518286	0.80117	-0.00045289	subgrp_con	count		1	0
SEC13	32	OK	0.5103133	0.5672222	0.899670	0.3682	0.50495227	subgrp_con	count		1	0
SLC6A1	4	OK	24.0001923	54.345440	0.4416229	0.65876	2.40091057	subgrp_con	count		1	0
HRH1	24	OK	-0.015582	0.6777508	-0.0229906	0.98166	-0.01167203	subgrp_con	count		1	0
ATG7	3	OK	2.4096337	2.4715206	0.9749599	0.32958	0.66517342	subgrp_con	count		1	0
VGLL4	136	OK	0.0009305	0.2536409	0.0036686	0.99707	0.0012233	subgrp_con	count		1	0
TAMM41	3	OK	-0.8152267	2.2666336	-0.3596641	0.7191	-4.1E-06	subgrp_con	count		1	0
TIMP4	4	OK	-1.851627	1.447537	-1.2791560	0.20086	-0.00102086	subgrp_con	count		1	0
PPARG	43	OK	-0.2435327	0.4686434	-0.5196546	0.6033	-0.2672146	subgrp_con	count		1	0
MKRN2	4	OK	0.9396704	1.4985405	0.627057	0.53062	0.29164784	subgrp_con	count		1	0
RAF1	10	OK	-0.1649746	1.0645653	-0.1549688	0.87685	-0.0002899	subgrp_con	count		1	0
RPL32	886	OK	-0.0431398	0.0829981	-0.5197678	0.60322	-0.06175898	subgrp_con	count		1	0
IQSEC1	5	OK	2.4096254	1.7474468	1.3789406	0.16791	1.07550689	subgrp_con	count		1	0
FBLN2	141	OK	-0.4012848	0.2242739	-1.7892622	0.07357	-0.53734086	subgrp_con	count		1	0
CHCHD4	4	OK	0.1104031	1.7448244	0.0632746	0.94955	0.05700340	subgrp_con	count		1	0
TMEM43	46	OK	0.7847328	0.4335510	1.8100150	0.07029	0.93734329	subgrp_con	count		1	0
XPC	30	OK	0.2829321	0.5207711	0.5432946	0.58692	0.26814743	subgrp_con	count		1	0
LSM3.00	141	OK	0.8097168	0.2396584	3.3786285	0.00073	1.0930677	subgrp_con	count		1	0
SLC6A6	36	OK	3.5902818	1.2872764	2.7890528	0.00529	0.01737100	subgrp_con	count		1	0
GRIP2	3	OK	21.1053223	83.341273	0.2532397	0.80008	0.0003103	subgrp_con	count		1	0
CCDC174	34	OK	1.0219104	0.5365665	1.9045361	0.05684	1.09344130	subgrp_con	count		1	0
FGD5	26	OK	-1.6190504	0.7074103	-2.2887003	0.0221	-1.17512001	subgrp_con	count		1	0
FGD5-AS1	48	OK	0.4022898	0.4402972	0.9136051	0.36077	0.4529353	subgrp_con	count		1	0
NR2C2	5	OK	20.817553	50.538527	0.4119145	0.6804	0.00083571	subgrp_con	count		1	0
MRPS25	20	OK	0.0716217	0.6168042	0.1161174	0.90756	0.07331699	subgrp_con	count		1	0
RBSN	2	OK	3.4598146	6.7714513	0.5109413	0.60939	0.00030179	subgrp_con	count		1	0
CAPN7	3	OK	-10.967865	27.153658	-0.4039185	0.68627	-1.17125107	subgrp_con	count		1	0
METTL6	3	OK	-0.6348896	2.0089600	-0.3160275	0.75198	-0.000145	subgrp_con	count		1	0
EAF1	4	OK	-0.9602659	1.5876113	-0.6048484	0.54529	-0.29064357	subgrp_con	count		1	0
HACL1	3	OK	-10.967865	27.153658	-0.4039185	0.68627	-1.17125107	subgrp_con	count		1	0
BTD	4	OK	-0.5911893	1.4254704	-0.4147327	0.67834	-0.36846215	subgrp_con	count		1	0
ANKRD28	69	OK	0.2250738	0.4103386	0.5485077	0.58334	0.25332875	subgrp_con	count		1	0
GALNT15	16	OK	-0.574508	0.7298814	-0.7871251	0.43121	-0.55439042	subgrp_con	count		1	0
DPH3	28	OK	1.1317095	0.5909069	1.9152076	0.05547	1.20599988	subgrp_con	count		1	0
OXNAD1	2	OK	3.4598149	4.7858686	0.7229230	0.46972	0.00081925	subgrp_con	count		1	0
RFTN1	9	OK	-1.4897976	1.0430230	-1.4283458	0.15319	-0.65125968	subgrp_con	count		1	0
TBC1D5	33	OK	0.9680718	0.5793734	1.6708944	0.09474	0.90710253	subgrp_con	count		1	0
SATB1	2	OK	-0.2029407	1.6532365	-0.1227536	0.9022	-4.82E-05	subgrp_con	count		1	0
RAB5A	63	OK	-0.0870735	0.3469670	-0.2509561	0.80185	-0.09764490	subgrp_con	count		1	0
KAT2B	5	OK	0.9396704	1.4985405	0.627057	0.53062	0.29164784	subgrp_con	count		1	0
UBE2E2	7	OK	-0.2681983	1.2291585	-0.2181967	0.82728	-0.13377831	subgrp_con	count		1	0
UBE2E1	25	OK	-0.2366506	0.5772214	-0.4099824	0.68182	-0.23190914	subgrp_con	count		1	0
NKIRAS1	1	OK	17.7472079	50.971927	0.3481761	0.72771	0.0007532	subgrp_con	count		1	0
RPL15	861	OK	-0.1331834	0.0862802	-1.543615	0.12268	-0.19046256	subgrp_con	count		1	0
NR1D2	34	OK	0.1952551	0.5301603	0.3682946	0.71265	0.18501525	subgrp_con	count		1	0
RARB	3	OK	-0.6154176	1.4920971	-0.4124514	0.68001	-0.29861013	subgrp_con	count		1	0
TOP2B	11	OK	2.6822781	1.4903138	1.7998075	0.07189	0.00487777	subgrp_con	count		1	0
NGLY1	21	OK	-0.8882578	0.6836871	-1.2992167	0.19387	-0.80125364	subgrp_con	count		1	0
OXSM	2	OK	1.5824000	1.9240335	0.8224389	0.41082	0.77175617	subgrp_con	count		1	0
SLC4A7	14	OK	1.8269273	1.0656048	1.7144511	0.08645	1.52969768	subgrp_con	count		1	0
CMC1	39	OK	0.4176940	0.5000064	0.835377	0.40351	0.47025299	subgrp_con	count		1	0
AZI2	21	OK	0.2272840	0.6337945	0.3587662	0.71977	0.22504430	subgrp_con	count		1	0

RBMS3-AS3	4	OK	1.1034521	1.6520588	0.6679250	0.50419	0.00075806	subgrp_con	count	1	0
LINC01985	9	OK	-2.5589912	1.2966509	-1.973539	0.04842	-1.62312009	subgrp_con	count	1	0
STT3B	9	OK	1.6396497	1.3469685	1.2172888	0.22349	0.48929643	subgrp_con	count	1	0
OSBPL10	11	OK	0.5187505	0.9878792	0.5251153	0.5995	0.33783520	subgrp_con	count	1	0
GPD1L	1	OK	1.5822999	2.7200188	0.5817606	0.56072	0.47433761	subgrp_con	count	1	0
CMTM8	46	OK	0.2270327	0.4287350	0.5295410	0.59642	0.25860934	subgrp_con	count	1	0
CMTM7	9	OK	2.6572087	1.965893	1.3516545	0.17649	0.7117613	subgrp_con	count	1	0
CMTM6	53	OK	1.0869154	0.4444813	2.4453565	0.01447	1.31204539	subgrp_con	count	1	0
DYNC1LI1	45	OK	0.2321589	0.4274520	0.5431227	0.58705	0.25661488	subgrp_con	count	1	0
CNOT10	3	OK	-11.019418	43.249935	-0.254784	0.79889	-1.17482461	subgrp_con	count	1	0
GLB1	6	OK	-1.379179	1.6976076	-0.812425	0.41655	-1.01347372	subgrp_con	count	1	0
CRTAP	125	OK	-0.471096	0.2398895	-1.963726	0.04956	-0.63030433	subgrp_con	count	1	0
UBP1	9	OK	-0.193819	1.1688759	-0.165816	0.8682	-0.00012516	subgrp_con	count	1	0
PDCD6IP	62	OK	-0.254728	0.3733917	-0.682202	0.49511	-0.30358574	subgrp_con	count	1	0
TRANK1	1	OK	-11.019418	43.249935	-0.254784	0.79889	-1.17482461	subgrp_con	count	1	0
EPM2AIP1	8	OK	-0.634045	1.0346179	-0.612830	0.53996	-0.1877592	subgrp_con	count	1	0
MLH1	2	OK	18.759489	83.625179	0.2243282	0.8225	0.00028789	subgrp_con	count	1	0
LRRFIP2	79	OK	-0.104644	0.3214291	-0.325559	0.74476	-0.13520261	subgrp_con	count	1	0
GOLGA4	172	OK	0.9694534	0.2303717	4.2082131	2.57E-05	1.28586823	subgrp_con	count	0.2568317	0
ITGA9	7	OK	32.562672	44.560900	0.7310735	0.46472	1.93304344	subgrp_con	count	1	0
CTDSPL	7	OK	0.0147770	1.4255587	0.0103657	0.99172	0.00748501	subgrp_con	count	1	0
VILL	1	OK	-10.442278	41.465809	-0.251828	0.80117	-0.00045289	subgrp_con	count	1	0
PLCD1	2	OK	-11.448548	41.76148	-0.274141	0.78398	-0.00049246	subgrp_con	count	1	0
ACAA1	35	OK	2.1119407	0.6223515	3.3880412	0.0007	2.114234	subgrp_con	count	1	0
MYD88	6	OK	0.1112766	1.1395450	0.097650	0.92221	0.08365687	subgrp_con	count	1	0
OXSR1	4	OK	-0.494490	1.4409485	-0.343169	0.73147	-0.14639957	subgrp_con	count	1	0
EXOG	1	OK	1.5822999	2.7200188	0.5817606	0.56072	0.47433761	subgrp_con	count	1	0
WDR48	7	OK	0.7712835	1.0333956	0.7464551	0.45539	0.50030098	subgrp_con	count	1	0
GORASP1	2	OK	-0.202940	1.6532236	-0.122753	0.9022	-4.82E-05	subgrp_con	count	1	0
CSRNP1	147	OK	-0.374307	0.2207910	-1.695302	0.09002	-0.48910629	subgrp_con	count	1	0
SLC25A38	4	OK	21.642059	54.486891	0.3971975	0.69122	1.57744735	subgrp_con	count	1	0
RPSA	794	OK	-0.210744	0.0925534	-2.277001	0.02279	-0.30083394	subgrp_con	count	1	0
MYRIP	18	OK	-1.358648	0.7577768	-1.792940	0.07298	-0.9127105	subgrp_con	count	1	0
EIF1B	229	OK	0.1062541	0.1821768	0.5832472	0.55972	0.1479055	subgrp_con	count	1	0
RPL14	867	OK	-0.187047	0.0857611	-2.181027	0.02918	-0.26763938	subgrp_con	count	1	0
ZNF621	6	OK	1.339557	1.2142667	1.1031818	0.26995	0.40833625	subgrp_con	count	1	0
CTNNB1	182	OK	0.6777434	0.2131671	3.1793993	0.00148	0.90145198	subgrp_con	count	1	0
ULK4	1	OK	3.4597879	4.7858528	0.7229198	0.46972	0.00081879	subgrp_con	count	1	0
TRAK1	3	OK	5.8049273	5.6694662	1.023892	0.30589	1.03285716	subgrp_con	count	1	0
VIPR1	2	OK	21.105322	83.341273	0.2532397	0.80009	0.0003103	subgrp_con	count	1	0
SEC22C	17	OK	1.7275190	0.8924091	1.9357926	0.05289	1.4591854	subgrp_con	count	1	0
SS18L2	39	OK	0.4767736	0.5139927	0.9275882	0.35362	0.51582737	subgrp_con	count	1	0
NKTR	175	OK	0.0530131	0.2117363	0.2503735	0.8022	0.07002572	subgrp_con	count	1	0
HIGD1A	25	OK	0.2291720	0.6004435	0.3816712	0.70271	0.22681693	subgrp_con	count	1	0
ANO10	5	OK	3.2995982	2.4741640	1.3336214	0.18232	1.56134392	subgrp_con	count	1	0
ABHD5	45	OK	0.8552435	0.4655925	1.836892	0.06622	0.95784219	subgrp_con	count	1	0
TCAIM	8	OK	-2.434995	1.4794582	-1.645869	0.09979	-0.92047446	subgrp_con	count	1	0
ZNF445	3	OK	-1.340131	1.6819410	-0.796776	0.42558	-0.59807560	subgrp_con	count	1	0
ZNF197	2	OK	-0.910223	2.1881125	-0.415985	0.67742	-0.25785735	subgrp_con	count	1	0
ZNF35	2	OK	-1.758573	2.3554439	-0.746599	0.45531	-0.00036089	subgrp_con	count	1	0
KIAA1143	33	OK	-0.070727	0.4873253	-0.145134	0.8846	-0.08176215	subgrp_con	count	1	0
TMEM42	5	OK	1.6575270	1.457682	1.1370974	0.2555	0.80310915	subgrp_con	count	1	0
ZDHHC3	31	OK	0.345419	0.5568003	0.6203658	0.53502	0.30979953	subgrp_con	count	1	0
EXOSC7	17	OK	-1.616757	0.8114527	-1.992423	0.04632	-1.27439223	subgrp_con	count	1	0
CLEC3B	14	OK	2.3077297	1.2747903	1.8102817	0.07025	0.64461778	subgrp_con	count	1	0
TMEM158	1	OK	1.5822999	2.7200188	0.5817606	0.56072	0.47433761	subgrp_con	count	1	0
LIMD1	1	OK	3.4598146	6.7714513	0.5109413	0.60939	0.00030179	subgrp_con	count	1	0
SACM1L	6	OK	0.3692481	1.5490836	0.2383655	0.8116	0.11310683	subgrp_con	count	1	0
LZTFL1	3	OK	0.3472110	1.814026	0.1914583	0.84817	0.00023216	subgrp_con	count	1	0
CCRL2	7	OK	3.618481	2.2215615	1.6288010	0.10336	1.36614183	subgrp_con	count	1	0
LTF	1	OK	18.759489	83.625179	0.2243282	0.8225	0.00028789	subgrp_con	count	1	0
LRRC2	1	OK	3.4598146	6.7714513	0.5109413	0.60939	0.00030179	subgrp_con	count	1	0
TMIE	1	OK	3.4598146	6.7714513	0.5109413	0.60939	0.00030179	subgrp_con	count	1	0
CCDC12	69	OK	-0.256728	0.3510876	-0.731238	0.46462	-0.31985718	subgrp_con	count	1	0
NBEAL2	1	OK	-15.99503	70.34942	-0.227365	0.82014	-0.00028474	subgrp_con	count	1	0
SETD2	30	OK	0.6799251	0.565353	1.2026733	0.2291	0.60782139	subgrp_con	count	1	0
KIF9	30	OK	1.0862446	0.7194129	1.509904	0.13107	0.88971058	subgrp_con	count	1	0
KLHL18	2	OK	-0.910142	2.1881243	-0.415946	0.67745	-0.25784050	subgrp_con	count	1	0
AC099778.1	1	OK	18.759489	83.625179	0.2243282	0.8225	0.00028789	subgrp_con	count	1	0
SCAP	2	OK	3.4598146	6.7714513	0.5109413	0.60939	0.00030179	subgrp_con	count	1	0
ELP6	24	OK	1.1291290	0.5925079	1.905677	0.05669	1.20337512	subgrp_con	count	1	0
SMARCC1	10	OK	-0.308565	1.0312508	-0.299214	0.76478	-0.1524939	subgrp_con	count	1	0
DHX30	11	OK	1.0528057	0.9312047	1.1305846	0.25822	0.32548791	subgrp_con	count	1	0
MAP4	157	OK	0.1600493	0.2289021	0.6992041	0.48442	0.2123426	subgrp_con	count	1	0
CAMP	3	OK	23.991896	84.899941	0.2825902	0.77749	1.65081709	subgrp_con	count	1	0
NME6	3	OK	2.1611158	2.3677943	0.9127126	0.36139	0.61359306	subgrp_con	count	1	0
CCDC51	2	OK	0.1681690	1.7253287	0.0974707	0.92235	0.05201879	subgrp_con	count	1	0
TMA7	566	OK	-0.059305	0.1140311	-0.520083	0.60301	-0.08422804	subgrp_con	count	1	0
SHISA5	97	OK	0.225021	0.2897182	0.7766820	0.43734	0.28930114	subgrp_con	count	1	0
UQCRC1	29	OK	1.992885	0.7993799	2.4930395	0.01267	1.74191991	subgrp_con	count	1	0
NCKIPSD	4	OK	-0.725215	1.278201	-0.567372	0.57046	-0.34817812	subgrp_con	count	1	0
IP6K2	27	OK	0.0781386	0.5742973	0.1360595	0.89177	0.06884764	subgrp_con	count	1	0
PRKAR2A	19	OK	0.04135	0.6622791	0.0624419	0.95021	0.0310321	subgrp_con	count	1	0
ARIH2OS	2	OK	0.6532528	2.5001284	0.2612877	0.79387	0.00016297	subgrp_con	count	1	0
ARIH2	19	OK	0.0049252	0.6437026	0.007667	0.99388	0.00440472	subgrp_con	count	1	0
P4HTM	12	OK	2.5933285	1.3085805	1.981795	0.0475	0.70018216	subgrp_con	count	1	0
DALRD3	5	OK	0.5103003	1.603405	0.3182602	0.75029	0.15911328	subgrp_con	count	1	0
NDUFAF3	215	OK	0.4412613	0.1951275	2.2613991	0.02373	0.6061002	subgrp_con	count	1	0
IMPDH2	78	OK	-0.817527	0.333999	-2.447691	0.01438	-0.97549735	subgrp_con	count	1	0
QARS	20	OK	-0.153052	0.7127522	-0.214734	0.82997	-0.12624030	subgrp_con	count	1	0
LAMB2	23	OK	0.1419981	0.554458	0.2561023	0.79787	0.16237512	subgrp_con	count	1	0
KLHDC8B	9	OK	-2.371251	1.2449401	-1.904711	0.05682	-1.5478657	subgrp_con	count	1	0
C3orf62	2	OK	3.4598146	6.7714513	0.5109413	0.60939	0.00030179	subgrp_con	count	1	0
USP4	8	OK	0.510280	1.1312213	0.451088	0.65192	0.26115827	subgrp_con	count	1	0
RHOA	650	OK	-0.232964	0.1052001	-2.214488	0.0268	-0.33133174	subgrp_con	count	1	0
TCTA	6	OK	1.1224437	1.4569313	0.7704163	0.44105	0.3460629	subgrp_con	count	1	0
DAG1	14	OK	0.4165442	0.8189093	0.5086573	0.61099	0.31428394	subgrp_con	count	1	0
APEH	2	OK	3.4598146	6.7714513	0.5109413	0.60939	0.00030179	subgrp_con	count	1	0
GMPPB	3	OK	-13.04562	27.67476	-0.471390	0.63736	-1.3178641	subgrp_con	count	1	0
IP6K1	5	OK	3.4598155	3.9099931	0.8848648	0.37622	0.00081877	subgrp_con	count	1	0
INKA1	6	OK	3.3860344	2.2519862	1.5035768	0.13269	1.32204360	subgrp_con	count	1	0
UBA7	4	OK	0.1112627	1.6120392	0.0690198	0.94497	0.0565662	subgrp_con	count	1	0
MON1A	1	OK	3.4598146	6.7714513	0.5109413	0.60939	0.00030179	subgrp_con	count	1	0
RBM6	36	OK	0.7159705	0.5751776	1.2447815	0.21321	0.77988907	subgrp_con	count	1	0
RBM5	18	OK	0.2480719	0.7214487	0.343852	0.73096	0.1267010	subgrp_con	count	1	0
SEMA3F	38	OK	5.1614789	1.515667	3.4054154	0.00066	2.94552448	subgrp_con	count	1	0
GNAI2	533	OK	-0.109422	0.1155909	-0.946633	0.34382	-0.1549128	subgrp_con	count	1	0
IFRD2	18	OK	0.3283749	0.7034765	0.4667886	0.64065	0.29443623	subgrp_con	count	1	0
HYAL1	7	OK	1.6019796	1.3172991	1.2161092	0.22396	0.00299417	subgrp_con	count	1	0
HYAL2	478	OK	0.1216120	0.1245123	0.9767060	0.32871	0.17203049	subgrp_con	count	1	0
TUSC2	23	OK	1.4004065	0.6826388	2.0514603	0.04022	1.34277160	subgrp_con	count	1	0
RASSF1	21	OK	-0.009996	0.6123143	-0.016325	0.98697	-0.00891822	subgrp_con	count	1	0
NPRL2	2	OK	-12.145707	43.418869	-0.279733	0.77968	-1.91338276	subgrp_con	count	1	0
CYB561D2	6	OK	4.8875530	3.3795676	1.446206	0.14812	0.97277335	subgrp_con	count	1	0
TMEM115	18	OK	0.9398781	0.7416202	1.2673307	0.20506	0.83385901	subgrp_con	count	1	0
HEMK1	1	OK	18.759489	83.625179	0.2243282	0.8225	0.00028789	subgrp_con	count	1	0
MANF	55	OK	0.5929367	0.4134786	1.4340203	0.15157	0.70286337	subgrp_con	count	1	0
TEX264	95	OK	0.4917799	0.3218539	1.5279597	0.12652	0.63834241	subgrp_con	count	1	0
RRP9	3	OK	-11.653559	26.358195	-0.442122	0.6584	-2.65715881	subgrp_con	count	1	0
PCBP4	1	OK	18.759489	83.625179	0.2243282	0.8225	0.00028789	subgrp_con	count	1	0
ABHD14B	33	OK	-0.243037	0.5146641	-0.472225	0.63677	-0.2716233	subgrp_con	count	1	0
ABHD14A	10	OK	0.1303176	0.920413	0.1415859	0.88741	0.11658475	subgrp_con	count	1	0
RPL29	880	OK	-0.066127	0.0841970	-0.785391	0.43222	-0.09464843	subgrp_con	count	1	0
DUSP7	6	OK	0.2465829	1.4959108	0.1648379	0.86907	0.00044528	subgrp_con	count	1	0
ALAS1	12	OK	-0.065687	1.1258878	-0.058343	0.95348	-0.04232233	subgrp_con	count	1	0
TWF2	57	OK	-0.296470	0.4285132	-0.691878	0.48901	-0.34943466	subgrp_con	count	1	0
PPM1M	5	OK	0.8036947	1.1338078	0.7088456	0.47842	0.52078526	subgrp_con	count	1	0
WDR82	19	OK	1.8091696	0.985339	1.8360883	0.06634	1.52238572	subgrp_con	count	1	0
BAP1	1	OK	-10.442278	41.465809	-0.251828	0.80117	-0.00045289	subgrp_con	count	1	0
PHF7	1	OK	3.4598146	6.7714513	0.5109413	0.60939	0.00030179	subgrp_con	count	1	0
SEMA3G	9	OK	2.9276042	1.8224246	1.6064942	0.10814	1.25684687	subgrp_con	count	1	0
NISCH	3	OK	0.1681491	1.7253300	0.097459	0.92236	0.05201277	subgrp_con	count	1	0
STAB1	53	OK	4.1167443	0.9170672	4.4890322	7.15E-06	3.65650872	subgrp_con	count	0.0717191	0
SMIM4	31	OK	0.465933	0.5754482	0.8096885	0.41812	0.4179064	subgrp_con	count	1	0
PBRM1	35	OK	-0.278428	0.5112334	-0.544620	0.58601	-0.28241443	subgrp_con	count	1	0
GNL3	25	OK	0.2543496	0.6130644	0.4148823	0.67822	0.25177931	subgrp_con	count	1	0
GLT8D1	25	OK	2.1965352	0.9489526	2.3146943	0.02063	1.27682344	subgrp_con	count	1	0
SPCS1	423	OK	0.2864402	0.1345193	2.1293612	0.03322	0.40530272	subgrp_con	count	1	0
NEK4	2	OK	7.1529884	9.4615387	0.7560068	0.44965	0.00046099	subgrp_con	count	1	0
SFMBT1	1	OK	3.4598146	6.7714513	0.5109413	0.60939	0.00030179	subgrp_con	count	1	0
TKT	135	OK	1.1520303	0.2768742	4.160843	3.17E-05	1.52959337	subgrp_con	count	0.3160588	0
DCP1A	8	OK	1.601163	1.4825787	1.0799854	0.28015	1.0132741	subgrp_con	count	1	0
ACTR8	1	OK	-12.148421	43.455309	-0.279561	0.77981	-1.91362624	subgrp_con	count	1	0
SELENOK	249	OK	0.6940972	0.1835704	3.7810958	0.00016	0.96726586	subgrp_con	count	1	0
CCDC66	47	OK	0.4102228	0.4575744	0.8965403	0.36996	0.50006017	subgrp_con	count	1	0
FAM208A	35	OK	-0.516110	0.4854675	-1.063121	0.28773	-0.41767522	subgrp_con	count	1	0
ARHGEF3	34	OK	-1.792912	0.5425257	-3.304750	0.00095	-1.62022319	subgrp_con	count	1	0
IL17RD	1	OK	-10.442278	41.465809	-0.251828	0.80117	-0.00045289	subgrp_con	count	1	0
APPL1	137	OK	-0.032938	0.2361490	-0.139479	0.88907	-0.04404401	subgrp_con	count	1	0
PDE12	4	OK	0.5103003	1.603405	0.3182602	0.75029	0.15911328	subgrp_con	count	1	0
ARF4	185	OK	0.4647947	0.2193921	2.1185569	0.03412	0.63183797	subgrp_con	count	1	0
DENND6A	6	OK	-0.042035	1.1592036	-0.036262	0.97107	-0.02123820	subgrp_con	count	1	0
SLMAP	13	OK	0.5020142	0.8943609	0.5613107	0.57459	0.00091875	subgrp_con	count	1	0
FLNB	68	OK	0.8363754	0.3989614	2.0963815	0.03605	1.05883285	subgrp_con	count	1	0
DNASE1L3	117	OK	-0.919104	0.2700208	-3.403825	0.00066	-1.19698498	subgrp_con	count	1	0
ABHD6	12	OK	-2.018589	1.0057016	-2.007142	0.04473	-1.76718696	subgrp_con	count	1	0
RPP14	4	OK	2.0702523	1.7994826	1.1504708	0.24995	0.96210332	subgrp_con	count	1	0
PXK	11	OK	0.3920637	0.8691428	0.451092	0.65192	0.29580674	subgrp_con	count	1	0
PDHB	56	OK	0.2680067	0.42372	0.6325089	0.52705	0.32417470	subgrp_con	count	1	0
KCTD6	3	OK	1.791902	2.2833539	0.7847677	0.43259	0.00045246	subgrp_con	count	1	0
FAM107A	102	OK	0.6492409	0.2881849	2.2528623	0.02427	0.80991575	subgrp_con	count	1	0
C3orf14	10	OK	1.2095313	1.1301840	1.0702074	0.28452	0.60597100	subgrp_con	count	1	0
THOC7	124	OK	-0.352539	0.2539613	-1.388162	0.16509	-0.46598843	subgrp_con	count	1	0
PSMD6	49	OK	0.8830905	0.4315166	2.0464809	0.04071	1.05253368	subgrp_con	count	1	0
PRICKLE2	2	OK	-1.295616	2.041425	-0.634662	0.52565	-0.00075754	subgrp_con	count	1	0
ADAMTS9	66	OK	-0.851470	0.3960278	-2.150026	0.03155	-0.9593011	subgrp_con	count	1	0
MAGI1	13	OK	-0.396504	0.8307767	-0.477268	0.63317	-0.34784171	subgrp_con	count	1	0
SLC25A26	6	OK	-0.190177	1.2247469	-0.15527	0.8766	-0.05770397	subgrp_con	count	1	0
LRIG1	27	OK	1.6238064	0.7045270	2.304817	0.02118	1.53615498	subgrp_con	count	1	0
SUCLG2	24	OK	-0.628207	0.591413	-1.062213	0.28814	-0.5771141	subgrp_con	count	1	0
EOGT	4	OK	1.5659305	1.5666919	0.9995140	0.31755	0.97089418	subgrp_con	count	1	0
TMF1	82	OK	0.697400	0.3566889	1.9552067	0.05056	0.80645976	subgrp_con	count	1	0
UBA3	18	OK	0.9646554	0.843756	1.1432863	0.25292	0.79493700	subgrp_con	count	1	0
ARL6IP5	486	OK	0.2875160	0.1227423	2.3424358	0.01916	0.40702863	subgrp_con	count	1	0
FRMD4B	172	OK	0.867242	0.216722	4.0016250	6.29E-05	1.181884	subgrp_con	count	0.625377	0
FOXP1	292	OK	0.1691279	0.1604504	1.0540819	0.29185	0.23444016	subgrp_con	count	1	0
EIF4E3	3	OK	-1.340131	1.6819410	-0.796776	0.42558	-0.59807560	subgrp_con	count	1	0
RYBP	29	OK	-0.513513	0.5483641	-0.936445	0.34904	-0.4969014	subgrp_con	count	1	0
SHQ1	1	OK	-11.019418	43.249935	-0.254784	0.79889	-1.17482461	subgrp_con	count	1	0
GXYLT2	8	OK	21.981615	33.054715	0.6650069	0.50605	2.81118157	subgrp_con	count	1	0
PPP4R2	53	OK	0.8486040	0.4340315	1.9551667	0.05056	0.99117899	subgrp_con	count	1	0
ROBO1	5	OK	1.7966220	2.1603505	0.831634	0.40562	0.0012336	subgrp_con	count	1	0
GBE1	8	OK	-0.181478	1.2658618	-0.143363	0.886	-0.00011729	subgrp_con	count	1	0
CADM2	3	OK	-12.145707	43.418869	-0.279733	0.77968	-1.91338276	subgrp_con	count	1	0
VGLL3	1	OK	18.759489	83.625179	0.2243282	0.8225	0.00028789	subgrp_con	count	1	0
CHMP2B	82	OK	0.1686737	0.3254854	0.518222	0.6043	0.20855819	subgrp_con	count	1	0
CGGBP1	49	OK	0.664647	0.4591898	1.4474345	0.14779	0.83029626	subgrp_con	count	1	0
ZNF654	3	OK	0.6393470	1.4406245	0.4437985	0.65719	0.00043313	subgrp_con	count	1	0
C3orf38	8	OK	0.2171419	0.900452	0.2411475	0.80944	0.16358366	subgrp_con	count	1	0
PROS1	50	OK	0.1407104	0.4271388	0.3294254	0.74182	0.17155580	subgrp_con	count	1	0
ARL13B	25	OK	-0.493178	0.6276548	-0.785747	0.43202	-0.39992090	subgrp_con	count	1	0
NSUN3	2	OK	0.1681491	1.7253300	0.097459	0.92236	0.05201277	subgrp_con	count	1	0
MTRNR2L12	556	OK	-0.429662	0.1117391	-3.845227	0.00012	-0.60933489	subgrp_con	count	1	0
ARL6.00	1	OK	3.4598146	6.7714513	0.5109413	0.60939	0.00030179	subgrp_con	count	1	0
CRYBG3	54	OK	-0.862468	0.4404112	-1.958325	0.05019	-0.99395576	subgrp_con	count	1	0
RIOX2	4	OK	-2.342222	1.7367717	-1.348606	0.17746	-0.53905893	subgrp_con	count	1	0
CLDND1	49	OK	0.265603	0.4128386	0.6433592	0.51999	0.32388139	subgrp_con	count	1	0
ST3GAL6	11	OK	-0.749570	1.0425785	-0.718957	0.47217	-0.00125739	subgrp_con	count	1	0
CMSS1	27	OK	-0.150704	0.6981665	-0.215857	0.8291	-0.13390331	subgrp_con	count	1	0
FILIP1L	215	OK	-0.560055	0.1891499	-2.960913	0.00307	-0.78245222	subgrp_con	count	1	0
TBC1D23	10	OK	-0.226098	0.8799661	-0.256940	0.79722	-0.11308646	subgrp_con	count	1	0
NIT2	16	OK	0.1408979	0.8235662	0.1710827	0.86416	0.10595239	subgrp_con	count	1	0
TOMM70	16	OK	-0.144866	0.8614848	-0.168158	0.86646	-0.11952662	subgrp_con	count	1	0
LNP1	1	OK	23.991896	84.899941	0.2825902	0.77749	1.65081709	subgrp_con	count	1	0
TMEM45A	5	OK	21.642059	54.486891	0.3971975	0.69122	1.57744735	subgrp_con	count	1	0
TFG	93	OK	0.0888586	0.3243944	0.2739215	0.78414	0.10643074	subgrp_con	count	1	0
ABI3BP	35	OK	5.0185696	1.7428147	2.8795772	0.00398	2.2713935	subgrp_con	count	1	0
IMPG2	1	OK	41.788303	174.29608	0.2397546	0.81052	0.00014488	subgrp_con	count	1	0
SENP7	19	OK	0.1970945	0.6216605	0.3170452	0.75121	0.14861616	subgrp_con	count	1	0
TRMT10C	40	OK	0.3090118	0.4833280	0.6393418	0.5226	0.36275932	subgrp_con	count	1	0
PCNP	109	OK	0.3825197	0.2747765	1.3921117	0.16389	0.49344286	subgrp_con	count	1	0
ZBTB11	11	OK	0.7689192	1.0790763	0.7125716	0.47611	0.3923047	subgrp_con	count	1	0
RPL24	867	OK	-0.120531	0.0860272	-1.401087	0.16119	-0.17239248	subgrp_con	count	1	0
CEP97	2	OK	-1.295616	2.041425	-0.634662	0.52565	-0.00075754	subgrp_con	count	1	0
NXPE3	10	OK	-0.531081	0.9809599	-0.541388	0.58824	-0.33261298	subgrp_con	count	1	0
NFKBIZ	147	OK	-0.646485	0.2236851	-2.890157	0.00385	-0.88027098	subgrp_con	count	1	0
ALCAM	28	OK	3.1709218	1.0595840	2.9925818	0.00277	2.08532922	subgrp_con	count	1	0
CBLB	10	OK	-2.389794	1.2284751	-1.945333	0.05172	-0.54398599	subgrp_con	count	1	0
LINC00882	1	OK	0.6532528	2.5001284	0.2612877	0.79387	0.00016297	subgrp_con	count	1	0
DUBR	1	OK	-10.442278	41.465809	-0.251828	0.80117	-0.00045289	subgrp_con	count	1	0
BBX	87	OK	0.024536	0.299995	0.0817885	0.93481	0.03173486	subgrp_con	count	1	0
LINC00636	1	OK	-10.442278	41.465809	-0.251828	0.80117	-0.00045289	subgrp_con	count	1	0
CD47	94	OK	0.2433157	0.2886633	0.842905	0.39928	0.32088134	subgrp_con	count	1	0
IFT57	52	OK	-0.110576	0.4263751	-0.259340	0.79537	-0.13080574	subgrp_con	count	1	0
DZIP3	4	OK	5.2970356	4.0741023	1.3001724	0.19354	1.0014446	subgrp_con	count	1	0
NECTIN3	3	OK	-1.776530	2.1943298	-0.809600	0.41817	-0.00098664	subgrp_con	count	1	0
PHLDB2	15	OK	0.2589006	0.8805283	0.2940287	0.76874	0.13216568	subgrp_con	count	1	0
ABHD10	3	OK	0.1721614	1.534450	0.1121974	0.91067	0.00011395	subgrp_con	count	1	0
CD200	74	OK	-0.207898	0.3796432	-0.547616	0.58396	-0.24550636	subgrp_con	count	1	0
ATG3	68	OK	0.0131223	0.3734965	0.0351337	0.97197	0.01620800	subgrp_con	count	1	0
SLC35A5	1	OK	18.759489	83.625179	0.2243282	0.8225	0.00028789	subgrp_con	count	1	0
GTPBP8	12	OK	-0.355936	0.7983585	-0.445834	0.65572	-0.33185875	subgrp_con	count	1	0
NEPRO	4	OK	5.2970356	4.0741023	1.3001724	0.19354	1.0014446	subgrp_con	count	1	0
BOC	2	OK	-1.439802	2.5201708	-0.571311	0.56779	-4.13E-05	subgrp_con	count	1	0
USF3	7	OK	1.1243469	1.3040772	0.8621781	0.38859	0.56596321	subgrp_con	count	1	0
NAA50	20	OK	0.2612710	0.698789	0.3738924	0.70848	0.21771346	subgrp_con	count	1	0
ATP6V1A	7	OK	4.6613686	4.0891078	1.1399475	0.25431	0.00274223	subgrp_con	count	1	0
ZDHHC23	1	OK	18.759489	83.625179	0.2243282	0.8225	0.00028789	subgrp_con	count	1	0
CCDC191	7	OK	0.227717	0.9203400	0.2582931	0.79618	0.15456250	subgrp_con	count	1	0
QTRT2	5	OK	-0.545320	1.708550	-0.319171	0.7496	-0.0003423	subgrp_con	count	1	0
ZBTB20	190	OK	0.5382948	0.1937564	2.7782031	0.00547	0.73304399	subgrp_con	count	1	0
LSAMP	7	OK	4.1203745	2.5258514	1.6312814	0.10282	1.44689194	subgrp_con	count	1	0
AC092691.1	1	OK	18.759489	83.625179	0.2243282	0.8225	0.00028789	subgrp_con	count	1	0
B4GALT4	2	OK	-10.442278	41.465809	-0.251828	0.80117	-0.00045289	subgrp_con	count	1	0
ARHGAP31	19	OK	-0.606415	0.6774297	-0.89475	0.37092	-0.43849853	subgrp_con	count	1	0
POGLUT1	15	OK	0.9952057	0.7834350	1.2703105	0.20397	1.00837861	subgrp_con	count	1	0
TIMMDC1	88	OK	-0.062042	0.3108183	-0.199610	0.84179	-0.08019368	subgrp_con	count	1	0
ADPRH	4	OK	-1.119738	1.4934041	-0.749789	0.45338	-0.30904529	subgrp_con	count	1	0
PLA1A	6	OK	-3.457949	1.8257462	-1.893992	0.05822	-1.42115681	subgrp_con	count	1	0
POPDC2	1	OK	18.759489	83.625179	0.2243282	0.8225	0.00028789	subgrp_con	count	1	0
COX17	150	OK	0.6089114	0.2435884	2.4997554	0.01243	0.82105474	subgrp_con	count	1	0
GSK3B	9	OK	0.9338618	1.2033443	0.7760554	0.43772	0.00173491	subgrp_con	count	1	0
LRRC58	6	OK	4.3479796	2.3220574	1.8724685	0.06114	2.83809267	subgrp_con	count	1	0
FSTL1	53	OK	3.2435915	0.7666478	4.2308753	2.33E-05	3.14409180	subgrp_con	count	0.2324810	0
NDUFB4	331	OK	-0.027996	0.1537636	-0.182075	0.85552	-0.03925372	subgrp_con	count	1	0
RABL3	3	OK	0.4061000	2.1083412	0.1926158	0.84726	0.12645366	subgrp_con	count	1	0
GTF2E1	1	OK	18.759489	83.625179	0.2243282	0.8225	0.00028789	subgrp_con	count	1	0
HCLS1	8	OK	-0.389696	1.3046085	-0.298707	0.76516	-0.00067293	subgrp_con	count	1	0
GOLGB1	177	OK	0.4782744	0.214866	2.2259132	0.02602	0.62606782	subgrp_con	count	1	0
IQCB1	9	OK	0.2035398	0.9396108	0.2166214	0.8285	0.15369129	subgrp_con	count	1	0
EAF2	1	OK	-11.019418	43.249935	-0.254784	0.79889	-1.17482461	subgrp_con	count	1	0
CCDC58	4	OK	1.0791023	1.401781	0.7698075	0.44141	0.69158497	subgrp_con	count	1	0
FAM162A	83	OK	0.2536567	0.3161624	0.8022988	0.42238	0.32075737	subgrp_con	count	1	0
KPNA1	5	OK	0.4304565	1.1045519	0.3897114	0.69675	0.28038964	subgrp_con	count	1	0
PARP9	33	OK	0.1883422	0.4913085	0.3833481	0.70146	0.19304225	subgrp_con	count	1	0
DTX3L	17	OK	-0.176060	0.6703267	-0.262648	0.79282	-0.15624266	subgrp_con	count	1	0
PARP14	136	OK	-0.151077	0.2365134	-0.638768	0.52297	-0.20471132	subgrp_con	count	1	0
DIRC2	1	OK	23.991896	84.899941	0.2825902	0.77749	1.65081709	subgrp_con	count	1	0
SEC22A	2	OK	-12.145707	43.418869	-0.279733	0.77968	-1.91338276	subgrp_con	count	1	0
HACD2	5	OK	-0.163777	1.3379262	-0.122411	0.90257	-0.0497823	subgrp_con	count	1	0
MYLK	20	OK	-2.025322	0.8052313	-2.515206	0.0119	-2.0043363	subgrp_con	count	1	0
CCDC14	11	OK	-0.103344	0.9311813	-0.110982	0.91163	-0.06647424	subgrp_con	count	1	0
KALRN	1	OK	-11.019418	43.249935	-0.254784	0.79889	-1.17482461	subgrp_con	count	1	0
UMPS	2	OK	1.5822999	2.7200188	0.5817606	0.56072	0.47433761	subgrp_con	count	1	0
ITGB5	8	OK	4.7249608	2.7101381	1.7434391	0.08126	1.96006978	subgrp_con	count	1	0
HEG1	200	OK	0.7282101	0.2031610	3.5843975	0.00034	0.98673453	subgrp_con	count	1	0
ZNF148	41	OK	0.478037	0.4789518	0.9980916	0.31822	0.50476830	subgrp_con	count	1	0
SNX4	10	OK	1.7428585	1.1049759	1.5772819	0.11472	1.0635464	subgrp_con	count	1	0
OSBPL11	2	OK	-0.910223	2.1881125	-0.415985	0.67742	-0.25785735	subgrp_con	count	1	0
SLC41A3	58	OK	0.5848603	0.3737582	1.5648090	0.11762	0.70025470	subgrp_con	count	1	0
KLF15	2	OK	23.991896	84.899941	0.2825902	0.77749	1.65081709	subgrp_con	count	1	0
ZXDC	2	OK	-0.202940	1.6532236	-0.122753	0.9022	-4.82E-05	subgrp_con	count	1	0
CHCHD6	2	OK	-11.019418	43.249935	-0.254784	0.79889	-1.17482461	subgrp_con	count	1	0
TPRA1	4	OK	-2.222919	1.7760535	-1.251606	0.21071	-0.8693730	subgrp_con	count	1	0
PODXL2	1	OK	18.759489	83.625179	0.2243282	0.8225	0.00028789	subgrp_con	count	1	0
ABTB1	18	OK	0.3357557	0.6261538	0.536219	0.59181	0.27992432	subgrp_con	count	1	0
SEC61A1	17	OK	2.8918677	1.2371447	2.3375339	0.01941	1.76590697	subgrp_con	count	1	0
RUVBL1	30	OK	-1.840632	0.6962225	-2.643741	0.0082	-1.5020489	subgrp_con	count	1	0
GATA2	40	OK	2.7120958	0.766828	3.5367716	0.00041	2.18810934	subgrp_con	count	1	0
GATA2-AS1	31	OK	2.2668470	0.6862437	3.303267	0.00096	2.46257400	subgrp_con	count	1	0
RPN1	53	OK	0.4730922	0.415265	1.1392531	0.2546	0.57183547	subgrp_con	count	1	0
RAB7A	176	OK	0.5769222	0.2184089	2.6414776	0.00825	0.77706058	subgrp_con	count	1	0
ACAD9	3	OK	5.2612789	5.4905820	0.9582370	0.33794	0.00295034	subgrp_con	count	1	0
ISY1	16	OK	-0.804197	0.7443893	-1.080344	0.27999	-0.63504312	subgrp_con	count	1	0
CNBP	445	OK	0.0161764	0.1288026	0.1255912	0.90006	0.02288061	subgrp_con	count	1	0
COPG1	4	OK	1.0791232	1.4017658	0.7698313	0.4414	0.69160196	subgrp_con	count	1	0
HMCES	8	OK	0.9622198	0.9971456	0.9649741	0.33456	0.62001722	subgrp_con	count	1	0
H1FX	264	OK	-0.106046	0.1696152	-0.625219	0.53182	-0.14818499	subgrp_con	count	1	0
MBD4	43	OK	-0.326263	0.4361936	-0.747979	0.45447	-0.39493532	subgrp_con	count	1	0
IFT122	2	OK	1.791902	2.2833539	0.7847677	0.43259	0.00045246	subgrp_con	count	1	0
PLXND1	84	OK	-0.381783	0.3107178	-1.228713	0.21918	-0.4685842	subgrp_con	count	1	0
TMCC1	5	OK	-0.684924	1.3384235	-0.51174	0.60882	-0.19900120	subgrp_con	count	1	0
ATP2C1	12	OK	0.8262848	0.9828956	0.8406656	0.40049	0.42099794	subgrp_con	count	1	0
NUDT16	35	OK	0.7821654	0.5324901	1.4688824	0.14186	0.87723665	subgrp_con	count	1	0
MRPL3	35	OK	0.1007540	0.5091798	0.1978751	0.84314	0.11672877	subgrp_con	count	1	0
DNAJC13	6	OK	1.2769755	1.3468569	0.9481151	0.34307	0.00238546	subgrp_con	count	1	0
ACKR4	7	OK	-2.417098	1.5752746	-1.534300	0.12496	-1.39660844	subgrp_con	count	1	0
UBA5	12	OK	0.6035756	0.909571	0.6635823	0.50696	0.39276467	subgrp_con	count	1	0
NPHP3	5	OK	-0.465329	1.3093717	-0.355391	0.7222	-0.13813694	subgrp_con	count	1	0
CDV3	227	OK	0.5206490	0.1902285	2.7369653	0.0062	0.72248617	subgrp_con	count	1	0
TOPBP1	12	OK	-1.825388	0.9345639	-1.953198	0.0508	-1.25898698	subgrp_con	count	1	0
SRPRB	26	OK	1.3049442	0.6912963	1.8876771	0.05907	1.30946880	subgrp_con	count	1	0
SLCO2A1	111	OK	-0.531785	0.2760280	-1.926562	0.05403	-0.64577558	subgrp_con	count	1	0
RYK	21	OK	0.1597498	0.7078661	0.2256780	0.82145	0.10370072	subgrp_con	count	1	0
AMOTL2	9	OK	4.0468789	2.3342969	1.7336607	0.08298	1.43614321	subgrp_con	count	1	0
ANAPC13	48	OK	0.4314275	0.4296498	1.0041374	0.31531	0.52598438	subgrp_con	count	1	0
CEP63	18	OK	-0.689297	0.7522080	-0.916365	0.35948	-0.62948953	subgrp_con	count	1	0

PPP2R3A	2	OK	1.58229999	2.7200188	0.58176068	0.56072	0.47433761	subgrp_cor	count		1	0
MSL2	5	OK	0.78489939	1.18353473	0.66318238	0.50721	0.41411647	subgrp_cor	count		1	0
STAG1	12	OK	0.89772584	0.97677412	0.91908233	0.35805	0.45633126	subgrp_cor	count		1	0
SLC35G2	12	OK	2.56142962	1.3209942	1.93901655	0.0525	1.65093322	subgrp_cor	count		1	0
NCK1-DT	2	OK	-10.588084	25.214284	-0.419926	0.67454	-0.00124279	subgrp_cor	count		1	0
NCK1	62	OK	-1.3611564	0.3856652	-3.5293732	0.00042	-1.54631372	subgrp_cor	count		1	0
DBR1	2	OK	-1.6133232	2.8064683	-0.5748588	0.56539	0	subgrp_cor	count		1	0
ARMC8	8	OK	-0.7466774	1.11830748	-0.6676852	0.50432	-0.45141444	subgrp_cor	count		1	0
MRAS	30	OK	0.63084896	0.57792505	1.09157573	0.27502	0.5953109	subgrp_cor	count		1	0
CEP70	2	OK	1.58229999	2.7200188	0.58176068	0.56072	0.47433761	subgrp_cor	count		1	0
FAIM	2	OK	-0.9102232	2.1881125	-0.4159856	0.67742	-0.25785735	subgrp_cor	count		1	0
PIK3CB	3	OK	0.90334498	1.46049118	0.61852136	0.53622	0.5834292	subgrp_cor	count		1	0
MRPS22	22	OK	-0.2753287	0.6010001	-0.4581174	0.64687	-0.29529001	subgrp_cor	count		1	0
COPB2	68	OK	0.12459727	0.3769361	0.3305527	0.74098	0.14238128	subgrp_cor	count		1	0
NMNAT3	1	OK	1.58229999	2.7200188	0.58176068	0.56072	0.47433761	subgrp_cor	count		1	0
SLC25A36	63	OK	0.384084	0.3740196	1.02690883	0.30446	0.46829760	subgrp_cor	count		1	0
ZBTB38	115	OK	0.04185274	0.27070835	0.15460454	0.87712	0.05528223	subgrp_cor	count		1	0
RASA2	7	OK	-3.8466456	1.85227627	-2.0767127	0.03782	-1.76218693	subgrp_cor	count		1	0
RNF7	266	OK	0.31130237	0.17225243	1.80724532	0.07072	0.43580462	subgrp_cor	count		1	0
ATP1B3	172	OK	0.19550268	0.24055849	0.81270748	0.41638	0.25836380	subgrp_cor	count		1	0
TFDP2	34	OK	1.24672504	0.5555781	2.24401397	0.02482	1.40033253	subgrp_cor	count		1	0
XRN1	34	OK	0.13751417	0.48492552	0.28357792	0.77672	0.13016981	subgrp_cor	count		1	0
PCOLCE2	5	OK	21.4247248	50.4712	0.42449314	0.67121	0.00085385	subgrp_cor	count		1	0
U2SURP	168	OK	-0.0587963	0.21582076	-0.272432	0.78529	-0.08042966	subgrp_cor	count		1	0
AC026204.1	1	OK	-15.995031	70.349421	-0.2272655	0.82014	-0.00028474	subgrp_cor	count		1	0
SLC9A9	2	OK	3.45981462	6.7714513	0.51094137	0.60939	0.00030179	subgrp_cor	count		1	0
C3orf58	25	OK	0.55683589	0.61832482	0.90055568	0.36782	0.52717555	subgrp_cor	count		1	0
PLOD2	15	OK	0.96499252	0.95089616	1.01482429	0.31019	0.71967099	subgrp_cor	count		1	0
PLSCR4	50	OK	-0.194834	0.39214826	-0.4968377	0.6192	-0.24002117	subgrp_cor	count		1	0
PLSCR1	333	OK	-0.4434377	0.15143534	-2.9282315	0.00341	-0.62253020	subgrp_cor	count		1	0
GYG1	30	OK	1.44846956	0.68379029	2.11829501	0.03415	1.55458575	subgrp_cor	count		1	0
HLTF	22	OK	0.48204987	0.61858732	0.77927539	0.43582	0.43210212	subgrp_cor	count		1	0
TM4SF18	126	OK	-0.3352278	0.269693	-1.2429975	0.21387	-0.44488691	subgrp_cor	count		1	0
TM4SF1	732	OK	-0.0724661	0.09627896	-0.7526682	0.45165	-0.10351947	subgrp_cor	count		1	0
TM4SF1-AS1	1	OK	3.45981462	6.7714513	0.51094137	0.60939	0.00030179	subgrp_cor	count		1	0
COMMD2	27	OK	1.30954925	0.70669058	1.85307302	0.06387	1.26680982	subgrp_cor	count		1	0
RNF13	126	OK	0.00645488	0.25611408	0.025203	0.97989	0.0085748	subgrp_cor	count		1	0
PFN2	7	OK	0.9136678	1.01156546	0.90322163	0.36641	0.75135128	subgrp_cor	count		1	0
TSC22D2	45	OK	0.49492783	0.49034556	1.00934498	0.31281	0.48631819	subgrp_cor	count		1	0
SERP1	357	OK	0.00056694	0.14686225	0.00386014	0.99692	0.00079738	subgrp_cor	count		1	0
EIF2A	130	OK	0.02012953	0.27061852	0.0743834	0.94071	0.02626754	subgrp_cor	count		1	0
SELENOT	92	OK	0.63617008	0.31420864	2.02467407	0.0429	0.81378985	subgrp_cor	count		1	0
ERICH6-AS1	2	OK	-0.9102232	2.1881125	-0.4159856	0.67742	-0.25785735	subgrp_cor	count		1	0
SIAH2	9	OK	0.5817263	0.86029366	0.67619503	0.49892	0.4844236	subgrp_cor	count		1	0
SUCNR1	2	OK	23.9918968	84.8999419	0.28259026	0.77749	1.65081709	subgrp_cor	count		1	0
MBNL1	219	OK	0.15535433	0.17526014	0.88642133	0.37539	0.21353364	subgrp_cor	count		1	0
MBNL1-AS1	7	OK	-1.2944068	1.14540536	-1.1300862	0.25844	-0.97691260	subgrp_cor	count		1	0
P2RY1	1	OK	3.45981462	6.7714513	0.51094137	0.60939	0.00030179	subgrp_cor	count		1	0
RAP2B	12	OK	1.26928361	1.25967158	1.00763059	0.31363	0.63359868	subgrp_cor	count		1	0
DHX36	119	OK	0.22648941	0.28468677	0.79557404	0.42628	0.29870715	subgrp_cor	count		1	0
MME	4	OK	0.27574647	1.33616325	0.20637184	0.8365	0.14081207	subgrp_cor	count		1	0
C3orf33	1	OK	-10.442278	41.4658099	-0.2518286	0.80117	-0.00045289	subgrp_cor	count		1	0
GMPS	14	OK	-0.9214185	0.80186497	-1.1490944	0.25052	-0.55669042	subgrp_cor	count		1	0
TIPARP-AS1	1	OK	-10.442278	41.4658099	-0.2518286	0.80117	-0.00045289	subgrp_cor	count		1	0
TIPARP	93	OK	0.27231528	0.2821778	0.96504854	0.33452	0.33206889	subgrp_cor	count		1	0
CCNL1	275	OK	-0.3931457	0.16372079	-2.4013179	0.01634	-0.5472258	subgrp_cor	count		1	0
VEPH1	2	OK	3.45981462	6.7714513	0.51094137	0.60939	0.00030179	subgrp_cor	count		1	0
PTX3	4	OK	1.58240009	1.92403354	0.82243893	0.41082	0.77175617	subgrp_cor	count		1	0
RSRC1	42	OK	0.40605629	0.4588593	0.88492547	0.3762	0.41637728	subgrp_cor	count		1	0
MLF1.00	44	OK	-0.6386739	0.45818653	-1.3939168	0.16334	-0.65765037	subgrp_cor	count		1	0
GFM1	6	OK	2.10295729	1.68778516	1.24598636	0.21277	0.60080772	subgrp_cor	count		1	0
LXN	122	OK	0.46245629	0.24812734	1.86378609	0.06235	0.63518819	subgrp_cor	count		1	0
RARRES1	6	OK	1.45223197	1.3125850	1.10639079	0.26856	0.90869407	subgrp_cor	count		1	0
MFSD1	13	OK	0.12701001	0.73733937	0.17225447	0.86324	0.1136499	subgrp_cor	count		1	0
AC080013.1	1	OK	-11.019418	43.2499352	-0.2547846	0.79889	-1.17482461	subgrp_cor	count		1	0
SMC4	38	OK	0.82713768	0.54544609	1.51644248	0.12941	0.77871310	subgrp_cor	count		1	0
KPNA4	55	OK	0.9970765	0.46463757	2.14592311	0.03188	1.14514794	subgrp_cor	count		1	0
AC069224.1	1	OK	18.7594894	83.6251792	0.22432824	0.8225	0.00028789	subgrp_cor	count		1	0
PPM1L	7	OK	6.01469026	5.22348967	1.15146973	0.24954	0.0031765	subgrp_cor	count		1	0
B3GALNT1	14	OK	8.45501791	4.97240338	1.70038836	0.08906	1.78226228	subgrp_cor	count		1	0
NMD3	19	OK	3.51092413	1.3957102	2.51551083	0.01189	2.20160731	subgrp_cor	count		1	0
PDCD10	99	OK	-0.5305231	0.30580106	-1.7348635	0.08276	-0.67629849	subgrp_cor	count		1	0
SERPINI1	8	OK	-0.094928	0.94865691	-0.1000772	0.92028	-0.04784253	subgrp_cor	count		1	0
GOLIM4	98	OK	1.13369677	0.30407648	3.72832776	0.00019	1.47738988	subgrp_cor	count		1	0
MYNN	6	OK	-0.7106474	1.23769091	-0.5741719	0.56585	-0.43832273	subgrp_cor	count		1	0
LRRC34	1	OK	-11.019418	43.2499352	-0.2547846	0.79889	-1.17482461	subgrp_cor	count		1	0
SEC62	627	OK	-0.067708	0.10364869	-0.6532455	0.5130	-0.0965204	subgrp_cor	count		1	0
GPR160	2	OK	1.9258645	2.51548794	0.76564251	0.44389	0.56022119	subgrp_cor	count		1	0
PHC3	8	OK	-10.810449	16.6333369	-0.6499267	0.51574	-2.23452070	subgrp_cor	count		1	0
PRKCI	5	OK	-0.712926	1.38874852	-0.5133257	0.6077	-0.00044093	subgrp_cor	count		1	0
CLDN11	3	OK	-0.9602659	1.58761136	-0.6048494	0.54528	-0.29064257	subgrp_cor	count		1	0
RPL22L1	156	OK	-0.2981869	0.24097553	-1.2374158	0.21592	-0.39971276	subgrp_cor	count		1	0
EIF5A2	5	OK	-0.910142	2.18812438	-0.4159466	0.67745	-0.25784050	subgrp_cor	count		1	0
TNIK	1	OK	22.9715125	51.7315836	0.4440519	0.65701	2.26236892	subgrp_cor	count		1	0
PLD1	2	OK	5.80492735	5.66946625	1.0238932	0.30589	1.03285716	subgrp_cor	count		1	0
FNDC3B	30	OK	1.18570173	0.67294645	1.75934123	0.07852	0.96671632	subgrp_cor	count		1	0
NCEH1	6	OK	-0.256601	1.34123268	-0.1913174	0.84829	-0.128091	subgrp_cor	count		1	0
ECT2	3	OK	4.21092061	4.28756165	0.9821248	0.32604	0.00257090	subgrp_cor	count		1	0
NLGN1	5	OK	3.25174846	2.39836786	1.35581723	0.17516	0.80586482	subgrp_cor	count		1	0
NAALADL2	3	OK	21.1053238	83.341273	0.25323976	0.80008	0.0003103	subgrp_cor	count		1	0
TBL1XR1	47	OK	0.57294459	0.44150848	1.29769781	0.19439	0.65501571	subgrp_cor	count		1	0
ZMAT3	15	OK	1.67598152	1.14097765	1.46889951	0.14186	0.49863643	subgrp_cor	count		1	0
PIK3CA	15	OK	1.13132532	0.89703836	1.26117831	0.20724	0.72307813	subgrp_cor	count		1	0
KCNMB3	1	OK	-10.442278	41.4658099	-0.2518286	0.80117	-0.00045289	subgrp_cor	count		1	0
ZNF639	11	OK	0.11860188	1.02365606	0.11586107	0.90776	0.06031330	subgrp_cor	count		1	0
MFN1	6	OK	0.05163359	1.20855314	0.04272348	0.96592	9.21E-05	subgrp_cor	count		1	0
GNB4	49	OK	-0.1437718	0.39090345	-0.3677935	0.71302	-0.17327413	subgrp_cor	count		1	0
ACTL6A	10	OK	-0.1362473	0.9765202	-0.1395232	0.88904	-0.10158965	subgrp_cor	count		1	0
MRPL47	34	OK	1.30023031	0.66826803	1.94567187	0.05169	1.25515141	subgrp_cor	count		1	0
NDUFB5	193	OK	0.08899013	0.20098568	0.44276874	0.65792	0.12252775	subgrp_cor	count		1	0
USP13	7	OK	1.43688628	1.26029671	1.14011742	0.25424	0.70898343	subgrp_cor	count		1	0
TTC14	15	OK	0.67643603	0.94665343	0.71455509	0.47488	0.44280396	subgrp_cor	count		1	0
FXR1	131	OK	0.07026313	0.24466062	0.2871863	0.77397	0.09300970	subgrp_cor	count		1	0
DNAJC19	82	OK	1.15671211	0.3403498	3.39859789	0.00068	1.46169083	subgrp_cor	count		1	0
ATP11B	7	OK	3.15630613	2.25990861	1.39665213	0.16252	0.00204500	subgrp_cor	count		1	0
DCUN1D1	9	OK	0.5141295	1.14466925	0.44915114	0.65332	0.42722559	subgrp_cor	count		1	0
MCCC1	2	OK	3.45981462	6.7714513	0.51094137	0.60939	0.00030179	subgrp_cor	count		1	0
B3GNT5	4	OK	-2.2911888	2.05792275	-1.1132501	0.26556	-0.53157637	subgrp_cor	count		1	0
KLHL24	26	OK	-0.112661	0.61703983	-0.1825831	0.85512	-0.09304061	subgrp_cor	count		1	0
YEATS2	3	OK	18.5273313	30.5501885	0.60645555	0.54421	0.00212367	subgrp_cor	count		1	0
PARL	18	OK	1.38095462	0.79843161	1.72958411	0.0837	1.11232398	subgrp_cor	count		1	0
EIF2B5	7	OK	-1.394118	1.39765735	-0.9974677	0.31854	-0.3903728	subgrp_cor	count		1	0
DVL3	5	OK	1.15819855	1.65445052	0.70005028	0.4839	0.00079558	subgrp_cor	count		1	0
AP2M1	221	OK	0.1712595	0.18916879	0.90532643	0.36529	0.23493141	subgrp_cor	count		1	0
ABCF3	3	OK	0.16816909	1.72532872	0.09747075	0.92235	0.05201879	subgrp_cor	count		1	0
ALG3	12	OK	-0.6137925	0.93429455	-0.6569582	0.51121	-0.44463180	subgrp_cor	count		1	0
EEF1AKMT4	1	OK	-11.019418	43.2499352	-0.2547846	0.79889	-1.17482461	subgrp_cor	count		1	0
PSMD2	48	OK	0.70860607	0.47540326	1.49053684	0.13606	0.86874682	subgrp_cor	count		1	0
EIF4G1	46	OK	0.1058415	0.43396216	0.24412612	0.80712	0.11174542	subgrp_cor	count		1	0
FAM131A	2	OK	-11.448548	41.761485	-0.2741412	0.78398	-0.00049246	subgrp_cor	count		1	0
POLR2H	65	OK	0.22590156	0.3773621	0.5986324	0.54942	0.28268199	subgrp_cor	count		1	0
MAGEF1	3	OK	1.58229999	2.7200188	0.58176068	0.56072	0.47433761	subgrp_cor	count		1	0
C3orf70	2	OK	-10.475634	27.4919879	-0.3810432	0.70317	-1.13811785	subgrp_cor	count		1	0
EHHADH	4	OK	-11.86868	17.8081448	-0.6664754	0.50511	-1.88863766	subgrp_cor	count		1	0
MAP3K13	69	OK	0.79329688	0.37361935	2.11551369	0.03439	0.98023258	subgrp_cor	count		1	0
TMEM41A	4	OK	-1.537212	1.64871356	-0.9323706	0.35115	-0.40044109	subgrp_cor	count		1	0
SENP2	7	OK	-0.3768204	1.23104768	-0.3060974	0.75952	-0.23889864	subgrp_cor	count		1	0
IGF2BP2	2	OK	0.16816909	1.72532872	0.09747075	0.92235	0.05201879	subgrp_cor	count		1	0
TRA2B	111	OK	-0.19219	0.27155979	-0.7077261	0.47912	-0.23969019	subgrp_cor	count		1	0
ETV5	1	OK	-11.019418	43.2499352	-0.2547846	0.79889	-1.17482461	subgrp_cor	count		1	0
TBCCD1	1	OK	18.7594894	83.6251792	0.22432824	0.8225	0.00028789	subgrp_cor	count		1	0
DNAJB11	26	OK	0.28472477	0.63782551	0.44639915	0.65531	0.21468329	subgrp_cor	count		1	0
EIF4A2	274	OK	-0.3311406	0.16345964	-2.0258247	0.04278	-0.45294773	subgrp_cor	count		1	0
RFC4	3	OK	-1.1068798	1.71301689	-0.6461581	0.51818	-0.65509255	subgrp_cor	count		1	0
ST6GAL1	40	OK	1.51085017	0.5119987	2.95088	0.00317	1.69053631	subgrp_cor	count		1	0
MASP1	1	OK	-11.019418	43.2499352	-0.2547846	0.79889	-1.17482461	subgrp_cor	count		1	0
RTP4	6	OK	0.54583421	1.18972732	0.45878933	0.64639	0.35540674	subgrp_cor	count		1	0
BCL6	7	OK	-0.838295	1.08022719	-0.7760358	0.43772	-0.00139238	subgrp_cor	count		1	0
LPP	106	OK	0.57794447	0.31200184	1.84645713	0.06482	0.7140228	subgrp_cor	count		1	0
P3H2	8	OK	0.16911166	1.14127116	0.14817833	0.8822	0.08636387	subgrp_cor	count		1	0
CCDC50	180	OK	0.30828847	0.21854565	1.41063648	0.15835	0.42404825	subgrp_cor	count		1	0
MB21D2	2	OK	18.7594894	83.6251792	0.22432824	0.8225	0.00028789	subgrp_cor	count		1	0
OPA1	5	OK	-2.8771992	1.86158275	-1.5455661	0.12221	-1.31136528	subgrp_cor	count		1	0
HES1	264	OK	-0.259968	0.16953206	-1.5334442	0.12517	-0.36375755	subgrp_cor	count		1	0
ATP13A3	59	OK	3.91905875	0.99564503	3.93620088	8.28E-05	1.79404751	subgrp_cor	count		0.8215256	0
TMEM44	11	OK	2.49556508	1.51890805	1.64299942	0.10038	0.68187277	subgrp_cor	count		1	0
LSG1	15	OK	0.06688668	0.85074522	0.07862128	0.93732	0.05558962	subgrp_cor	count		1	0
FAM43A	39	OK	0.21403908	0.48883568	0.43785478	0.66149	0.24464472	subgrp_cor	count		1	0
XXYLT1-AS2	1	OK	1.58229999	2.7200188	0.58176068	0.56072	0.47433761	subgrp_cor	count		1	0
ACAP2	92	OK	-0.3452924	0.29591506	-1.1668632	0.24327	-0.43996172	subgrp_cor	count		1	0
PPP1R2	112	OK	0.17854799	0.28270125	0.63157835	0.52766	0.23435134	subgrp_cor	count		1	0
APOD	54	OK	1.13529067	0.41573326	2.73081512	0.00632	1.47287643	subgrp_cor	count		1	0
MUC20-OT1	12	OK	1.07832615	0.82869637	1.30123189	0.19318	0.54406270	subgrp_cor	count		1	0
TNK2	1	OK	3.45981462	6.7714513	0.51094137	0.60939	0.00030179	subgrp_cor	count		1	0
TFRC	2	OK	3.45981462	6.7714513	0.51094137	0.60939	0.00030179	subgrp_cor	count		1	0
PCYT1A	7	OK	1.34136848	1.44971127	0.92526595	0.35482	1.26504468	subgrp_cor	count		1	0
TCTEX1D2	6	OK	2.55522274	2.3498446	1.08740073	0.27686	0.69313893	subgrp_cor	count		1	0
TM4SF19-AS1	1	OK	-10.442278	41.4658099	-0.2518286	0.80117	-0.00045289	subgrp_cor	count		1	0
UBXN7	5	OK	-1.8978878	1.607882	-1.180365	0.23786	-0.78103666	subgrp_cor	count		1	0
RNF168	35	OK	1.56645729	0.61471223	2.5482774	0.01082	1.72820583	subgrp_cor	count		1	0
FBXO45	3	OK	0.1112767	1.61203873	0.06902855	0.94497	0.05657342	subgrp_cor	count		1	0
PIGX	5	OK	-0.2681983	1.22915854	-0.2181967	0.82728	-0.13377831	subgrp_cor	count		1	0
CEP19	4	OK	-0.9072285	1.64418474	-0.5517802	0.5811	-0.52553220	subgrp_cor	count		1	0
PAK2	221	OK	-0.1708954	0.18556352	-0.9209538	0.35707	-0.23677861	subgrp_cor	count		1	0
SENP5	11	OK	-0.4605365	0.90991465	-0.5061316	0.61276	-0.28998186	subgrp_cor	count		1	0
NCBP2	27	OK	-0.141564	0.53100785	-0.2665951	0.78978	-0.15857719	subgrp_cor	count		1	0
NCBP2-AS2	31	OK	0.54768715	0.5321442	1.02920818	0.30338	0.63450680	subgrp_cor	count		1	0
PIGZ	3	OK	-13.271979	28.0097917	-0.4738335	0.63562	-2.0156633	subgrp_cor	count		1	0
DLG1	11	OK	-0.2318086	0.83587724	-0.2773237	0.78152	-0.20471265	subgrp_cor	count		1	0
FYTTD1	63	OK	0.93526217	0.39409489	2.37319029	0.01764	1.0611303	subgrp_cor	count		1	0
LRCH3	7	OK	-0.2352951	1.04124601	-0.2259745	0.82122	-0.11761641	subgrp_cor	count		1	0
IQCG	4	OK	0.51030031	1.6034059	0.31826032	0.75029	0.15911328	subgrp_cor	count		1	0
RPL35A	874	OK	-0.1083481	0.08416797	-1.2872845	0.198	-0.15504980	subgrp_cor	count		1	0
ZNF141	2	OK	0.03920501	2.28606678	0.01714955	0.98632	0.01985577	subgrp_cor	count		1	0
ZNF721	12	OK	0.17036275	1.14708762	0.14851765	0.88192	0.05270200	subgrp_cor	count		1	0
PIGG	3	OK	4.09418501	2.86684182	1.42811682	0.15326	1.94504264	subgrp_cor	count		1	0
ATP5ME	211	OK	0.19110195	0.19576818	0.97616451	0.32898	0.2609741	subgrp_cor	count		1	0
SLC49A3	2	OK	-11.448548	41.761485	-0.2741412	0.78398	-0.00049246	subgrp_cor	count		1	0
PCGF3	6	OK	1.27697551	1.34685698	0.94811515	0.34307	0.00238546	subgrp_cor	count		1	0
AC139887.2	1	OK	-10.442278	41.4658099	-0.2518286	0.80117	-0.00045289	subgrp_cor	count		1	0
CPLX1	38	OK	-1.057328	0.49135538	-2.1518601	0.03141	-0.88551120	subgrp_cor	count		1	0
TMEM175	4	OK	-2.3422221	1.73677178	-1.3486067	0.17746	-0.53905893	subgrp_cor	count		1	0
FGFRL1	6	OK	21.642059	54.4868919	0.39719753	0.69122	1.57744735	subgrp_cor	count		1	0
CTBP1	8	OK	-2.192906	1.37394017	-1.5960695	0.11047	-1.09788300	subgrp_cor	count		1	0
MAEA	4	OK	-11.18473	26.2967873	-0.4253269	0.6706	-2.27083089	subgrp_cor	count		1	0
UVSSA	3	OK	3.52482161	3.04884185	1.1561215	0.24762	1.19374976	subgrp_cor	count		1	0
AC147067.1	2	OK	3.45981462	6.7714513	0.51094137	0.60939	0.00030179	subgrp_cor	count		1	0
SLBP	32	OK	0.32480667	0.54237618	0.59885896	0.54927	0.35165118	subgrp_cor	count		1	0
TMEM129	6	OK	-0.9102232	1.54642098	-0.5885996	0.55612	-0.42849043	subgrp_cor	count		1	0
LETM1	7	OK	1.3602522	1.2636872	1.07641527	0.28174	0.67765103	subgrp_cor	count		1	0
NSD2	3	OK	-1.8538345	1.85439186	-0.9996995	0.31746	-0.46053279	subgrp_cor	count		1	0
HAUS3	6	OK	1.1560669	1.58798091	0.72800913	0.46661	0.38296539	subgrp_cor	count		1	0
ZFYVE28	3	OK	3.45981559	3.90999315	0.88486487	0.37622	0.00081877	subgrp_cor	count		1	0
RNF4	6	OK	0.98204847	1.23185942	0.79720824	0.42532	0.63226296	subgrp_cor	count		1	0
FAM193A	3	OK	0.93961099	1.49856075	0.62700893	0.53068	0.2916352	subgrp_cor	count		1	0
TNIP2	45	OK	-0.1367409	0.46104778	-0.2965872	0.76679	-0.16332517	subgrp_cor	count		1	0
SH3BP2	21	OK	0.91401928	0.71058906	1.28626576	0.19835	0.6850063	subgrp_cor	count		1	0
ADD1	79	OK	0.52498198	0.3475580	1.51048727	0.13092	0.63494339	subgrp_cor	count		1	0
MFSD10	63	OK	0.12812784	0.3732009	0.34332135	0.73136	0.16116201	subgrp_cor	count		1	0
NOP14	6	OK	-2.9323615	1.71291879	-1.7119093	0.08691	-0.61160745	subgrp_cor	count		1	0
HTT	3	OK	0.90334498	1.46049118	0.61852136	0.53622	0.5834292	subgrp_cor	count		1	0
RGS12	10	OK	2.70561682	1.61807247	1.67212339	0.0945	0.70455449	subgrp_cor	count		1	0
DOK7	1	OK	18.7594894	83.6251792	0.22432824	0.8225	0.00028789	subgrp_cor	count		1	0
ADRA2C	3	OK	1.49544582	1.91900207	0.77928307	0.43581	5.08E-05	subgrp_cor	count		1	0
TMEM128	4	OK	1.39610123	1.41763559	0.98480967	0.32472	0.42407085	subgrp_cor	count		1	0
LYAR	40	OK	0.30585181	0.51466856	0.59428946	0.55232	0.3230721	subgrp_cor	count		1	0
STX18	5	OK	-10.442278	41.4658099	-0.2518286	0.80117	-0.00045289	subgrp_cor	count		1	0
MSX1	32	OK	0.03459177	0.55816305	0.06197423	0.95058	0.03267336	subgrp_cor	count		1	0
CYTL1	110	OK	-0.6340571	0.2707296	-2.3420308	0.01918	-0.86876711	subgrp_cor	count		1	0
WFS1	5	OK	1.79662209	2.16035059	0.83163453	0.40562	0.0012336	subgrp_cor	count		1	0
MAN2B2	2	OK	18.7594894	83.6251792	0.22432824	0.8225	0.00028789	subgrp_cor	count		1	0
MRFAP1	264	OK	0.52352005	0.17503431	2.99095673	0.00278	0.72159191	subgrp_cor	count		1	0
LINC02482	1	OK	1.58229999	2.7200188	0.58176068	0.56072	0.47433761	subgrp_cor	count		1	0
AC093323.1	1	OK	-10.442278	41.4658099	-0.2518286	0.80117	-0.00045289	subgrp_cor	count		1	0
MRFAP1L1	8	OK	-1.1912924	1.01177105	-1.1774328	0.23902	-0.69802693	subgrp_cor	count		1	0
BLOC1S4	17	OK	0.41086343	0.78545007	0.523093	0.60091	0.43468045	subgrp_cor	count		1	0
KIAA0232	18	OK	1.55235742	0.8721584	1.77990308	0.07509	0.75693856	subgrp_cor	count		1	0
CCDC96	1	OK	-10.442278	41.4658099	-0.2518286	0.80117	-0.00045289	subgrp_cor	count		1	0
GRPEL1	48	OK	-0.137222	0.41680289	-0.3292275	0.74198	-0.1653988	subgrp_cor	count		1	0
LINC02447	1	OK	3.45981462	6.7714513	0.51094137	0.60939	0.00030179	subgrp_cor	count		1	0
AFAP1	17	OK	0.28945613	0.66053466	0.43821492	0.66122	0.28702463	subgrp_cor	count		1	0
ABLIM2	2	OK	-15.995031	70.349421	-0.2272655	0.82014	-0.00028474	subgrp_cor	count		1	0
SH3TC1	6	OK	41.2857975	66.2929997	0.62277763	0.53342	0.00100678	subgrp_cor	count		1	0
HTRA3	1	OK	3.45981462	6.7714513	0.51094137	0.60939	0.00030179	subgrp_cor	count		1	0
WDR1	50	OK	0.24864492	0.4500367	0.5524992	0.58061	0.20716613	subgrp_cor	count		1	0
ZNF518B	3	OK	5.26127895	5.49058205	0.95823701	0.33794	0.00295034	subgrp_cor	count		1	0
HS3ST1	28	OK	-0.4756329	0.56207592	-0.8462075	0.39744	-0.49222199	subgrp_cor	count		1	0
RAB28	5	OK	0.60173508	1.116688	0.5388564	0.58990	0.45339585	subgrp_cor	count		1	0
BOD1L1	171	OK	-0.0129365	0.21237642	-0.0606275	0.95166	-0.01773819	subgrp_cor	count		1	0
CC2D2A	4	OK	-0.4944595	1.44095493	-0.3431471	0.73149	-0.14639255	subgrp_cor	count		1	0
FBXL5	19	OK	-1.4931774	0.87433892	-1.7077787	0.08768	-1.3261624	subgrp_cor	count		1	0
FAM200B	38	OK	0.72810023	0.48548739	1.49973047	0.13368	0.86189381	subgrp_cor	count		1	0
BST1	1	OK	-10.442278	41.4658099	-0.2518286	0.80117	-0.00045289	subgrp_cor	count		1	0
PROM1	1	OK	18.7594894	83.6251792	0.22432824	0.8225	0.00028789	subgrp_cor	count		1	0
TAPT1	1	OK	23.9918968	84.8999419	0.28259026	0.77749	1.65081709	subgrp_cor	count		1	0
LDB2	302	OK	-0.5438298	0.15880194	-3.4245792	0.00062	-0.75035486	subgrp_cor	count		1	0
QDPR	3	OK	0.90334498	1.46049118	0.61852136	0.53622	0.5834292	subgrp_cor	count		1	0
LAP3	292	OK	-0.4113665	0.15801883	-2.6032587	0.00923	-0.57653613	subgrp_cor	count		1	0
MED28	43	OK	-0.1681764	0.44260447	-0.37997	0.70397	-0.18827765	subgrp_cor	count		1	0
DCAF16	1	OK	3.45981462	6.7714513	0.51094137	0.60939	0.00030179	subgrp_cor	count		1	0
LCORL	4	OK	0.32954695	1.40554589	0.23446189	0.81462	0.10245319	subgrp_cor	count		1	0
SLIT2	1	OK	18.7594894	83.6251792	0.22432824	0.8225	0.00028789	subgrp_cor	count		1	0
DHX15	13	OK	-3.4143792	1.27706133	-2.673622	0.0075	-1.10025160	subgrp_cor	count		1	0
SOD3	4	OK	0.51031165	1.60340574	0.31826732	0.75029	0.15911454	subgrp_cor	count		1	0
CCDC149	3	OK	-13.335695	29.1122333	-0.4580787	0.6469	-1.33917170	subgrp_cor	count		1	0
LGI2	2	OK	-15.995031	70.349421	-0.2272655	0.82014	-0.00028474	subgrp_cor	count		1	0
SEPSECS-AS1	1	OK	3.45981462	6.7714513	0.51094137	0.60939	0.00030179	subgrp_cor	count		1	0
ANAPC4	1	OK	3.45981462	6.7714513	0.51094137	0.60939	0.00030179	subgrp_cor	count		1	0
SEL1L3	5	OK	3.45981462	6.7714513	0.51094137	0.60939	0.00030179	subgrp_cor	count		1	0
SMIM20	14	OK	0.51723703	0.82256215	0.62883366	0.52939	0.4899913	subgrp_cor	count		1	0
RBPJ	36	OK	0.43555287	0.49010936	0.88868496	0.37417	0.47143772	subgrp_cor	count		1	0
STIM2	45	OK	-0.5182376	0.44358899	-1.168257	0.2427	-0.59982631	subgrp_cor	count		1	0
AC106047.1	1	OK	3.45981462	6.7714513	0.51094137	0.60939	0.00030179	subgrp_cor	count		1	0
PCDH7	45	OK	3.99120388	0.98790932	4.04015203	5.34E-05	2.97644469	subgrp_cor	count		0.53128113	0
ARAP2	8	OK	-0.9228443	1.04121073	-0.8863185	0.37545	-0.7252301	subgrp_cor	count		1	0
C4orf19	1	OK	3.45981462	6.7714513	0.51094137	0.60939	0.00030179	subgrp_cor	count		1	0
RELL1	16	OK	0.07627813	0.8591263	0.08878572	0.92925	0.06336966	subgrp_cor	count		1	0
PGM2	31	OK	1.9408868	0.74243034	2.61423153	0.00894	1.85669672	subgrp_cor	count		1	0
TBC1D1	44	OK	0.56388281	0.52819047	1.06757475	0.28571	0.55920154	subgrp_cor	count		1	0
KLF3	197	OK	0.50050229	0.19950522	2.50871777	0.01212	0.68910599	subgrp_cor	count		1	0
FAM114A1	69	OK	1.73081588	0.42790632	4.04484767	5.24E-05	2.1138074	subgrp_cor	count		0.520849	0
KLHL5	17	OK	-0.5511538	0.86993144	-0.63356	0.52637	-0.44801752	subgrp_cor	count		1	0
RFC1	135	OK	0.13732208	0.23252637	0.59056572	0.55481	0.1845533	subgrp_cor	count		1	0
RPL9	866	OK	-0.0982254	0.08523158	-1.1524526	0.24914	-0.14049933	subgrp_cor	count		1	0
LIAS	4	OK	-2.2229067	1.77603141	-1.2516145	0.21071	-0.86934838	subgrp_cor	count		1	0
UGDH	2	OK	4.66132998	5.76838606	0.80808217	0.41904	0.00274918	subgrp_cor	count		1	0
SMIM14	81	OK	0.7174396	0.34706436	2.0670996	0.03872	0.91356733	subgrp_cor	count		1	0
UBE2K	44	OK	0.18061406	0.46593269	0.38763981	0.69828	0.21197581	subgrp_cor	count		1	0
PDS5A	13	OK	-0.2719398	0.71685582	-0.3793508	0.70442	-0.08202754	subgrp_cor	count		1	0
N4BP2	3	OK	4.66132998	5.76838606	0.80808217	0.41904	0.00274918	subgrp_cor	count		1	0
APBB2	11	OK	1.38486608	1.0684071	1.296197	0.19491	0.42096116	subgrp_cor	count		1	0
UCHL1	3	OK	24.0045017	54.379622	0.44142458	0.65891	2.40107252	subgrp_cor	count		1	0
TMEM33	13	OK	-0.0963084	0.77147448	-0.1248368	0.90065	-0.06195277	subgrp_cor	count		1	0
SLC30A9	16	OK	-0.0245439	0.75335909	-0.0325793	0.97401	-0.0241873	subgrp_cor	count		1	0
SHISA3	1	OK	23.9918968	84.8999419	0.28259026	0.77749	1.65081709	subgrp_cor	count		1	0
ATP8A1	3	OK	-0.2381161	1.56462708	-0.1521871	0.87904	-0.07272580	subgrp_cor	count		1	0
GUF1	1	OK	-11.019418	43.2499352	-0.2547846	0.79889	-1.17482461	subgrp_cor	count		1	0
GNPDA2	9	OK	-1.2352853	1.25046783	-0.9878585	0.32322	-0.00197323	subgrp_cor	count		1	0
GABRA2	7	OK	-3.4380385	1.81428769	-1.8949798	0.0581	-2.05968180	subgrp_cor	count		1	0
COMMD8	27	OK	-0.2446035	0.6799002	-0.3597638	0.71902	-0.23930539	subgrp_cor	count		1	0
ATP10D	24	OK	1.55418603	0.66664283	2.33837778	0.01937	1.40968755	subgrp_cor	count		1	0
NFXL1	1	OK	18.7594894	83.6251792	0.22432824	0.8225	0.00028789	subgrp_cor	count		1	0
TEC	1	OK	0.65325287	2.50012846	0.26128772	0.79387	0.00016297	subgrp_cor	count		1	0
SLAIN2	20	OK	0.10219465	0.61885954	0.16513384	0.86884	0.10098951	subgrp_cor	count		1	0
FRYL	47	OK	0.27141562	0.49226417	0.55136171	0.58139	0.31172412	subgrp_cor	count		1	0
OCIAD1	350	OK	0.04274941	0.14532703	0.29416004	0.76864	0.06017400	subgrp_cor	count		1	0
OCIAD2	187	OK	0.58755768	0.20999763	2.79792511	0.00514	0.810165	subgrp_cor	count		1	0
DCUN1D4	4	OK	0.04190965	1.30404483	0.0321382	0.97436	0.01289204	subgrp_cor	count		1	0
SGCB	18	OK	1.51128083	0.8162988	1.85150441	0.0641	1.3750567	subgrp_cor	count		1	0
USP46	7	OK	2.75578992	2.1436892	1.28553613	0.1986	0.72904048	subgrp_cor	count		1	0
FIP1L1	20	OK	-0.8474	0.70711481	-1.198395	0.23070	-0.51585374	subgrp_cor	count		1	0
LNX1	4	OK	-1.8979105	1.60787469	-1.1803846	0.23785	-0.78103351	subgrp_cor	count		1	0
CHIC2	51	OK	-1.236178	0.42298002	-2.9235078	0.00346	-1.40035633	subgrp_cor	count		1	0
PDGFRA	2	OK	-0.9102232	2.1881125	-0.4159856	0.67742	-0.25785735	subgrp_cor	count		1	0
KIT	2	OK	1.58229999	2.7200188	0.58176068	0.56072	0.47433761	subgrp_cor	count		1	0
KDR	51	OK	-0.0191225	0.45502764	-0.0420249	0.96648	-0.02108862	subgrp_cor	count		1	0
SRD5A3	15	OK	0.15566545	0.90215463	0.17254853	0.86301	0.11714220	subgrp_cor	count		1	0
TMEM165	157	OK	0.23423745	0.23595791	0.99270858	0.32085	0.3129957	subgrp_cor	count		1	0
CLOCK	13	OK	0.58448339	0.84779324	0.68941737	0.49056	0.53693126	subgrp_cor	count		1	0
EXOC1	24	OK	-0.0512991	0.61613772	-0.0832581	0.93365	-0.05053516	subgrp_cor	count		1	0
CEP135	3	OK	-15.498043	42.9510917	-0.36082	0.71822	-0.00073756	subgrp_cor	count		1	0
AASDH	7	OK	-2.8680849	1.6560513	-1.7318817	0.08329	-1.01107936	subgrp_cor	count		1	0
PPAT	4	OK	-0.6508873	1.2753209	-0.5103714	0.60979	-0.00040481	subgrp_cor	count		1	0
PAICS	16	OK	-1.0277626	0.79476558	-1.2931645	0.19595	-0.91656772	subgrp_cor	count		1	0
SRP72	179	OK	-0.2703019	0.21199118	-1.275062	0.20229	-0.36709976	subgrp_cor	count		1	0
ARL9.00	1	OK	3.45981462	6.7714513	0.51094137	0.60939	0.00030179	subgrp_cor	count		1	0
HOPX	5	OK	-1.3107744	1.6383999	-0.8000332	0.42369	-0.35262481	subgrp_cor	count		1	0
REST	72	OK	0.40634641	0.35402303	1.14779658	0.25105	0.46463804	subgrp_cor	count		1	0
NOA1	11	OK	0.29954691	1.00422126	0.29828776	0.76548	0.19473179	subgrp_cor	count		1	0
POLR2B	35	OK	0.31566355	0.51508442	0.61283847	0.53998	0.32382471	subgrp_cor	count		1	0
IGFBP7	944	OK	-0.1334961	0.08578936	-1.5560911	0.11969	-0.19104703	subgrp_cor	count		1	0
CENPC	57	OK	-0.6047137	0.36692111	-1.6480755	0.09934	-0.75197473	subgrp_cor	count		1	0
UBA6	14	OK	-0.5292886	0.7667666	-0.6902865	0.49003	-0.49511116	subgrp_cor	count		1	0
YTHDC1	88	OK	-0.4618442	0.2995762	-1.5416584	0.12316	-0.58216315	subgrp_cor	count		1	0
SULT1B1	5	OK	19.1030323	50.7724696	0.37624795	0.70672	0.00078880	subgrp_cor	count		1	0
SULT1E1	1	OK	18.7594894	83.6251792	0.22432824	0.8225	0.00028789	subgrp_cor	count		1	0
JCHAIN	2	OK	3.45981497	4.78586864	0.72292309	0.46972	0.00081935	subgrp_cor	count		1	0

UTP3	13	OK	0.2588905	0.8805251	0.2940183	0.7687	0.13216056	subgrp_cor	count		1	0
RUFY3	5	OK	-1.6793547	1.5548187	-1.0800966	0.2801	-0.69151270	subgrp_cor	count		1	0
GRSF1	23	OK	0.3283329	0.6198080	0.5297332	0.5962	0.32510760	subgrp_cor	count		1	0
MOB1B	2	OK	0.1681491	1.7253300	0.097459	0.9223	0.05201277	subgrp_cor	count		1	0
DCK	3	OK	-11.448548	41.761485	-0.2741412	0.7839	-0.00049246	subgrp_cor	count		1	0
SLC4A4	3	OK	18.759489	83.625179	0.2243282	0.8225	0.00028789	subgrp_cor	count		1	0
ADAMTS3	1	OK	1.5823999	2.7200188	0.5817606	0.5607	0.47433761	subgrp_cor	count		1	0
COX18	1	OK	18.759489	83.625179	0.2243282	0.8225	0.00028789	subgrp_cor	count		1	0
ANKRD17	22	OK	0.4558978	0.7168714	0.6359547	0.5248	0.29865504	subgrp_cor	count		1	0
CXCL8	19	OK	0.3997601	0.6261400	0.6384516	0.5231	0.42222919	subgrp_cor	count		1	0
CXCL1	15	OK	1.3994000	0.8781419	1.5935821	0.1110	1.28009844	subgrp_cor	count		1	0
CXCL3	64	OK	0.6258464	0.3343669	1.8717356	0.0613	0.8281756	subgrp_cor	count		1	0
MTHFD2L	4	OK	3.7563488	2.4593475	1.5273761	0.1266	1.75931225	subgrp_cor	count		1	0
AREG	10	OK	0.7054197	0.9658203	0.7303840	0.4651	0.6340514	subgrp_cor	count		1	0
BTC	1	OK	23.991896	84.899941	0.2825902	0.7774	1.65081709	subgrp_cor	count		1	0
RCHY1	5	OK	-2.7471059	1.7708095	-1.5512276	0.1208	-0.00050543	subgrp_cor	count		1	0
THAP6	3	OK	-10.586763	25.202461	-0.4200686	0.6744	-0.00124361	subgrp_cor	count		1	0
G3BP2	101	OK	0.295546	0.2975039	0.9934187	0.3205	0.38799905	subgrp_cor	count		1	0
USO1	26	OK	-0.7797712	0.6523588	-1.1952103	0.2319	-0.78961192	subgrp_cor	count		1	0
NAAA	7	OK	0.5457685	1.1897347	0.4587312	0.6464	0.35536169	subgrp_cor	count		1	0
SDAD1	45	OK	-0.6595734	0.4604554	-1.4324371	0.1520	-0.78408246	subgrp_cor	count		1	0
CXCL9	8	OK	-1.9908403	1.2209146	-1.6306138	0.1029	-1.67862869	subgrp_cor	count		1	0
CXCL10	26	OK	-2.59752	0.7983043	-3.2537967	0.0011	-2.99130505	subgrp_cor	count		1	0
CXCL11	14	OK	-2.0442797	0.9058711	-2.2567002	0.0240	-1.71440738	subgrp_cor	count		1	0
NUP54	20	OK	-1.0046872	0.6645836	-1.5117542	0.1306	-0.78154909	subgrp_cor	count		1	0
SCARB2	85	OK	0.9626238	0.3413147	2.8203406	0.0048	1.21004845	subgrp_cor	count		1	0
FAM47E	2	OK	-11.448548	41.761485	-0.2741412	0.7839	-0.00049246	subgrp_cor	count		1	0
SHROOM3	2	OK	18.759489	83.625179	0.2243282	0.8225	0.00028789	subgrp_cor	count		1	0
11-Sep	83	OK	1.2560438	0.3328124	3.774029	0.0001	1.62857191	subgrp_cor	count		1	0
CCNI	592	OK	-0.0444186	0.1094479	-0.4058427	0.6848	-0.06311838	subgrp_cor	count		1	0
CCNG2	21	OK	-0.7145439	0.6698633	-1.066703	0.2861	-0.74956237	subgrp_cor	count		1	0
CNOT6L	63	OK	-0.060978	0.3539028	-0.1723016	0.8632	-0.07573440	subgrp_cor	count		1	0
MRPL1	21	OK	-0.4697283	0.7846867	-0.5986111	0.5494	-0.4345178	subgrp_cor	count		1	0
AC098818.2	1	OK	18.759489	83.625179	0.2243282	0.8225	0.00028789	subgrp_cor	count		1	0
BMP2K	4	OK	-2.8119193	1.8396743	-1.5284875	0.1263	-0.00139885	subgrp_cor	count		1	0
ANTXR2	4	OK	18.759489	83.625179	0.2243282	0.8225	0.00028789	subgrp_cor	count		1	0
RASGEF1B	17	OK	1.3047151	0.9296104	1.4035074	0.1604	0.64980049	subgrp_cor	count		1	0
HNRNPD	246	OK	-0.1673616	0.1805066	-0.9271771	0.3538	-0.23049895	subgrp_cor	count		1	0
HNRNPDL	412	OK	-0.1920259	0.1337367	-1.4358492	0.1510	-0.27011752	subgrp_cor	count		1	0
ENOPH1	13	OK	6.6862228	3.0548615	2.1887155	0.0286	2.70841718	subgrp_cor	count		1	0
TMEM150C	15	OK	1.6597573	0.8651395	1.9184852	0.0550	1.18241986	subgrp_cor	count		1	0
SCD5	4	OK	3.4598160	3.3871656	1.0214487	0.3070	0.00081839	subgrp_cor	count		1	0
SEC31A	72	OK	0.6968861	0.3462922	2.0124223	0.0441	0.82502218	subgrp_cor	count		1	0
THAP9-AS1	22	OK	-0.398816	0.7050105	-0.565688	0.5716	-0.29328712	subgrp_cor	count		1	0
COPS4	23	OK	1.7249673	0.7894147	2.1851218	0.0288	1.54407039	subgrp_cor	count		1	0
COQ2	1	OK	3.4598146	6.7714513	0.5109413	0.6093	0.00030179	subgrp_cor	count		1	0
HELQ	4	OK	-0.9102232	2.1881125	-0.4159856	0.6774	-0.25785735	subgrp_cor	count		1	0
MRPS18C	121	OK	0.1563513	0.2416799	0.6469354	0.5176	0.21013684	subgrp_cor	count		1	0
ABRAXAS1	16	OK	-0.4526986	0.7119123	-0.6358909	0.5248	-0.39658323	subgrp_cor	count		1	0
GPAT3	5	OK	-3.026378	1.8954835	-1.5966256	0.1103	-1.01528925	subgrp_cor	count		1	0
WDFY3	4	OK	0.4061000	2.1083412	0.1926158	0.8472	0.12645366	subgrp_cor	count		1	0
SLC10A6	3	OK	1.3569729	1.7875236	0.7591356	0.4477	0.41320240	subgrp_cor	count		1	0
AC093827.4	3	OK	-11.199925	27.762997	-0.403412	0.6866	-1.82940850	subgrp_cor	count		1	0
AFF1	41	OK	-0.0308996	0.4826712	-0.0640116	0.9489	-0.0352308	subgrp_cor	count		1	0
KLHL8	3	OK	-10.475634	27.491987	-0.3810432	0.7031	-1.13811785	subgrp_cor	count		1	0
NUDT9	18	OK	-0.0959667	0.706264	-0.1358792	0.8919	-0.07932485	subgrp_cor	count		1	0
SPP1	24	OK	2.7486669	0.8588747	3.2003117	0.0013	2.69584974	subgrp_cor	count		1	0
PKD2	9	OK	2.6063789	1.2954307	2.0119786	0.0442	1.87148306	subgrp_cor	count		1	0
ABCG2	41	OK	-2.4820674	0.5666563	-4.3801989	1.19E-05	-2.21255253	subgrp_cor	count		0.1186777	0
PPM1K	11	OK	-0.3245182	0.8445317	-0.3842581	0.7007	-0.20624424	subgrp_cor	count		1	0
HERC6	3	OK	0.5103116	1.6034057	0.3182673	0.7502	0.15911454	subgrp_cor	count		1	0
HERC5	28	OK	-0.671117	0.5175709	-1.296667	0.1947	-0.73856216	subgrp_cor	count		1	0
PYURF	210	OK	0.0195134	0.1884945	0.1035227	0.9175	0.02705340	subgrp_cor	count		1	0
HERC3	2	OK	-11.019418	43.249935	-0.2547846	0.7988	-1.17482461	subgrp_cor	count		1	0
NAP1L5	2	OK	-15.99503	70.34942	-0.2273655	0.8201	-0.00028474	subgrp_cor	count		1	0
FAM13A	5	OK	0.4556875	1.4680863	0.3103956	0.7562	0.00030627	subgrp_cor	count		1	0
GPRIN3	4	OK	1.5824001	1.5705105	1.0075705	0.3136	0.97973527	subgrp_cor	count		1	0
SNCA	20	OK	-0.3332523	0.631925	-0.5273602	0.5979	-0.36457095	subgrp_cor	count		1	0
CCSER1	1	OK	0.6532528	2.5001284	0.2612877	0.7938	0.00016297	subgrp_cor	count		1	0
SMARCAD1	10	OK	-0.1854299	1.0152215	-0.1826592	0.8550	-0.0929800	subgrp_cor	count		1	0
PDLIM5	143	OK	0.1900739	0.2453500	0.7747049	0.4385	0.25171197	subgrp_cor	count		1	0
RAP1GDS1	6	OK	-2.2229579	1.7760683	-1.2516172	0.2107	-0.86936325	subgrp_cor	count		1	0
TSPAN5	24	OK	0.9795164	0.660677	1.4825934	0.1381	0.86637137	subgrp_cor	count		1	0
EIF4E	25	OK	-0.3183403	0.6078053	-0.5237537	0.6004	-0.34880713	subgrp_cor	count		1	0
METAP1	5	OK	0.449533	1.0943930	0.4107601	0.6812	0.14007308	subgrp_cor	count		1	0
ADH5	92	OK	0.1264810	0.3062831	0.4129547	0.6796	0.16513355	subgrp_cor	count		1	0
TRMT10A	3	OK	4.2109206	4.2875616	0.9821248	0.3260	0.00257090	subgrp_cor	count		1	0
LAMTOR3	11	OK	3.9550138	1.9464312	2.0319309	0.0421	1.41248942	subgrp_cor	count		1	0
DNAJB14	70	OK	-0.0456044	0.3336857	-0.1366687	0.8912	-0.05728682	subgrp_cor	count		1	0
H2AFZ	425	OK	-0.0456945	0.1346879	-0.3392624	0.7344	-0.06451448	subgrp_cor	count		1	0
PPP3CA	160	OK	-0.3882755	0.228383	-1.7001061	0.0891	-0.52920058	subgrp_cor	count		1	0
AP001816.1	19	OK	-1.4532976	0.7332909	-1.9818841	0.0474	-1.44987637	subgrp_cor	count		1	0
NFKB1	24	OK	-0.838604	0.6753936	-1.2416522	0.2143	-0.79511657	subgrp_cor	count		1	0
UBE2D3	330	OK	-0.248587	0.1525824	-1.6291982	0.1032	-0.34719496	subgrp_cor	count		1	0
AC018797.2	1	OK	-15.99503	70.34942	-0.2273655	0.8201	-0.00028474	subgrp_cor	count		1	0
CISD2	21	OK	1.8609113	0.8835274	2.1062292	0.0351	1.64652640	subgrp_cor	count		1	0
SLC9B1	1	OK	1.5823999	2.7200188	0.5817606	0.5607	0.47433761	subgrp_cor	count		1	0
SLC9B2	4	OK	-0.6508873	1.2753209	-0.5103714	0.6097	-0.00040481	subgrp_cor	count		1	0
BDH2	57	OK	0.6388623	0.3777523	1.6912201	0.0907	0.79824387	subgrp_cor	count		1	0
TET2	10	OK	-0.0299772	0.981936	-0.0305286	0.9756	-0.01515447	subgrp_cor	count		1	0
PPA2	18	OK	0.8669265	0.8377565	1.0348191	0.3007	0.64896615	subgrp_cor	count		1	0
INTS12	8	OK	-0.3714082	1.2493673	-0.297277	0.7662	-0.11115247	subgrp_cor	count		1	0
NPNT	1	OK	-15.498043	42.951091	-0.36082	0.7182	-0.00073756	subgrp_cor	count		1	0
TBCK	1	OK	-10.442278	41.465809	-0.2518286	0.8011	-0.00045289	subgrp_cor	count		1	0
AIMP1	89	OK	0.0926575	0.3191828	0.2902961	0.7715	0.1198851	subgrp_cor	count		1	0
DKK2.00	109	OK	11.875846	2.7302975	4.3496527	1.36E-05	5.88808531	subgrp_cor	count		0.1364078	0
PAPSS1	59	OK	2.6109309	0.5874789	4.4442974	8.82E-06	2.86655194	subgrp_cor	count		0.0882382	0
SGMS2	3	OK	4.6613299	5.7683860	0.8080821	0.4190	0.00274918	subgrp_cor	count		1	0
CYP2U1	4	OK	1.1389358	1.3676122	0.8327915	0.4049	0.00078202	subgrp_cor	count		1	0
HADH	4	OK	0.8664780	1.2872623	0.673116	0.5008	0.26944509	subgrp_cor	count		1	0
LEF1	9	OK	-0.0882709	1.2951000	-0.0681576	0.9456	-0.02696008	subgrp_cor	count		1	0
RPL34	884	OK	-0.1361872	0.0842437	-1.6165851	0.1059	-0.19489458	subgrp_cor	count		1	0
OSTC	248	OK	0.3214516	0.1802707	1.7831609	0.0745	0.44811522	subgrp_cor	count		1	0
SEC24B-AS1	1	OK	3.4598146	6.7714513	0.5109413	0.6093	0.00030179	subgrp_cor	count		1	0
SEC24B	7	OK	-1.3568516	1.2681728	-1.0699264	0.2846	-0.7841671	subgrp_cor	count		1	0
MCUB	52	OK	-2.410192	0.5294318	-4.5524125	5.3E-06	-2.50789445	subgrp_cor	count		0.0532145	0
CASP6	4	OK	-0.9101889	1.5464254	-0.588578	0.5561	-0.42847801	subgrp_cor	count		1	0
PLA2G12A	1	OK	-10.442278	41.465809	-0.2518286	0.8011	-0.00045289	subgrp_cor	count		1	0
CFI	105	OK	0.1586292	0.2803469	0.5658321	0.5715	0.20584392	subgrp_cor	count		1	0
GAR1	20	OK	0.5600288	0.7106436	0.7880586	0.4306	0.53017689	subgrp_cor	count		1	0
FAM241A	117	OK	-1.162182	0.262801	-4.4222894	9.77E-06	-1.54460172	subgrp_cor	count		0.0977972	0
AP1AR	5	OK	-0.9512394	1.4452003	-0.6582058	0.5104	-7.77E-05	subgrp_cor	count		1	0
TIFA	46	OK	-1.1996017	0.4533137	-2.6462947	0.0081	-1.39323268	subgrp_cor	count		1	0
ALPK1	5	OK	-0.5452686	1.7085577	-0.3191397	0.7496	-0.00034230	subgrp_cor	count		1	0
ZGRF1	2	OK	-1.096672	2.6557302	-0.4129456	0.6796	-0.28735259	subgrp_cor	count		1	0
LARP7	126	OK	0.2286068	0.2456224	0.9307245	0.352	0.30547017	subgrp_cor	count		1	0
ANK2	1	OK	18.759489	83.625179	0.2243282	0.8225	0.00028789	subgrp_cor	count		1	0
CAMK2D	28	OK	0.4006037	0.6465628	0.6195897	0.5355	0.35922270	subgrp_cor	count		1	0
SNHG8	271	OK	-0.3164917	0.1691803	-1.870736	0.0613	-0.43857142	subgrp_cor	count		1	0
METTL14	10	OK	-0.7379943	1.0174129	-0.7253636	0.4682	-0.35133165	subgrp_cor	count		1	0
SEC24D	6	OK	2.1931013	1.3907135	1.5769612	0.1148	1.00493965	subgrp_cor	count		1	0
SYNPO2	1	OK	18.759489	83.625179	0.2243282	0.8225	0.00028789	subgrp_cor	count		1	0
MYOZ2	10	OK	20.048637	32.869799	0.6099409	0.5419	1.52868947	subgrp_cor	count		1	0
USP53	37	OK	1.3196959	0.5047930	2.6143306	0.0089	1.27458167	subgrp_cor	count		1	0
C4orf3	320	OK	0.1166192	0.1535821	0.7593278	0.4476	0.16328410	subgrp_cor	count		1	0
PDE5A	2	OK	0.5887819	1.767414	0.3331317	0.7390	0.30122869	subgrp_cor	count		1	0
PRDM5	1	OK	1.5823999	2.7200188	0.5817606	0.5607	0.47433761	subgrp_cor	count		1	0
ANXA5	435	OK	0.1801918	0.1330775	1.3540359	0.1757	0.25443732	subgrp_cor	count		1	0
EXOSC9	17	OK	0.397676	0.7361091	0.5402410	0.5890	0.35701110	subgrp_cor	count		1	0
BBS7	3	OK	-0.9102232	2.1881125	-0.4159856	0.6774	-0.25785735	subgrp_cor	count		1	0
KIAA1109	32	OK	0.855620	0.5374927	1.5918735	0.1114	0.80575147	subgrp_cor	count		1	0
FGF2	29	OK	1.6837540	0.608339	2.7677863	0.0056	1.74201274	subgrp_cor	count		1	0
NUDT6	8	OK	-1.3641912	1.2120061	-1.1255646	0.2603	-1.17262152	subgrp_cor	count		1	0
SPATA5	2	OK	-15.99503	70.34942	-0.2273655	0.8201	-0.00028474	subgrp_cor	count		1	0
ANKRD50	6	OK	5.6831241	5.3321875	1.0656148	0.2866	0.00307912	subgrp_cor	count		1	0
FAT4	16	OK	7.4940081	3.1428968	2.3844270	0.0171	1.11652839	subgrp_cor	count		1	0
INTU	7	OK	-0.585090	1.1193505	-0.5227055	0.6011	-0.2846892	subgrp_cor	count		1	0
HSPA4L	3	OK	3.7524367	2.7019469	1.3887899	0.1649	1.03336371	subgrp_cor	count		1	0
MFSD8	1	OK	-10.442278	41.465809	-0.2518286	0.8011	-0.00045289	subgrp_cor	count		1	0
LARP1B	4	OK	2.4096337	2.4715206	0.9749599	0.3295	0.66517342	subgrp_cor	count		1	0
JADE1	9	OK	1.3462960	1.2660257	1.0634033	0.2876	0.66864089	subgrp_cor	count		1	0
SCLT1	9	OK	-1.9058769	1.2144965	-1.5692732	0.1165	-1.30704584	subgrp_cor	count		1	0
C4orf33	5	OK	-2.5935646	1.7016536	-1.5241436	0.1274	-0.95570064	subgrp_cor	count		1	0
AC105383.1	4	OK	21.105323	83.341273	0.2532397	0.8000	0.0003103	subgrp_cor	count		1	0
PCDH10	49	OK	30.425446	29.500363	1.0313583	0.3023	4.27953585	subgrp_cor	count		1	0
NOCT	2	OK	-10.475634	27.491987	-0.3810432	0.7031	-1.13811785	subgrp_cor	count		1	0
ELF2	103	OK	-0.2312604	0.2672842	-0.865222	0.3869	-0.30415584	subgrp_cor	count		1	0
MGARP	1	OK	3.4598146	6.7714513	0.5109413	0.6093	0.00030179	subgrp_cor	count		1	0
NDUFC1	256	OK	0.4763260	0.1768889	2.6927976	0.0070	0.66370680	subgrp_cor	count		1	0
NAA15	12	OK	-0.2204173	0.903479	-0.243965	0.8072	-0.14094693	subgrp_cor	count		1	0
AC097376.2	3	OK	-2.023032	1.8071267	-1.1194743	0.2629	-0.00109400	subgrp_cor	count		1	0
RAB33B	9	OK	1.1374782	1.0216341	1.1133910	0.2655	0.5527578	subgrp_cor	count		1	0
SETD7	12	OK	-0.0116657	0.8176857	-0.0142667	0.9886	-0.00357862	subgrp_cor	count		1	0
MGST2	438	OK	-0.0447873	0.1283500	-0.3489465	0.7271	-0.06344846	subgrp_cor	count		1	0
MAML3	3	OK	-1.8538345	1.8543918	-0.9996995	0.3174	-0.46053279	subgrp_cor	count		1	0
SCOC	77	OK	0.4127352	0.3579888	1.1529277	0.2489	0.51938765	subgrp_cor	count		1	0
ELMOD2	6	OK	2.5016136	1.7866259	1.4001887	0.1614	1.40015402	subgrp_cor	count		1	0
TBC1D9	8	OK	0.7526486	1.0919369	0.6892784	0.4906	0.38413322	subgrp_cor	count		1	0
ZNF330	12	OK	-1.3939634	0.8708758	-1.6006453	0.1094	-1.04009356	subgrp_cor	count		1	0
IL15	8	OK	-0.1948826	0.9899096	-0.1968691	0.8439	-0.16073994	subgrp_cor	count		1	0
INPP4B	1	OK	22.971512	51.731583	0.4440519	0.6570	2.36236892	subgrp_cor	count		1	0
USP38	3	OK	-11.186792	26.313753	-0.425132	0.6707	-2.27103070	subgrp_cor	count		1	0
GAB1	5	OK	-1.2029145	1.3773214	-0.8733724	0.3824	-0.52919171	subgrp_cor	count		1	0
SMARCA5	84	OK	0.2733891	0.3218480	0.8494356	0.3956	0.33338235	subgrp_cor	count		1	0
HHIP-AS1	16	OK	3.510013	1.3056450	2.6883366	0.0071	2.90297918	subgrp_cor	count		1	0
HHIP	26	OK	4.6332650	1.548362	2.9923649	0.0027	3.27654193	subgrp_cor	count		1	0
ANAPC10	18	OK	-1.5287726	0.7707327	-1.9835314	0.0473	-1.52363666	subgrp_cor	count		1	0
ABCE1	51	OK	-0.7948748	0.449296	-1.7691545	0.0768	-0.91045304	subgrp_cor	count		1	0
OTUD4	6	OK	-2.0841657	1.8296746	-1.1390900	0.2546	-1.07774579	subgrp_cor	count		1	0
SMAD1	55	OK	-1.6049852	0.4383368	-3.6615337	0.0002	-1.88490701	subgrp_cor	count		1	0
MMAA	1	OK	-11.019418	43.249935	-0.2547846	0.7988	-1.17482461	subgrp_cor	count		1	0
LSM6.00	64	OK	-0.3122636	0.3882813	-0.80422	0.4212	-0.38388371	subgrp_cor	count		1	0
TMEM184C	1	OK	26.075713	51.462821	0.5066903	0.6123	3.63710153	subgrp_cor	count		1	0
PRMT9	2	OK	-10.442278	41.465809	-0.2518286	0.8011	-0.00045289	subgrp_cor	count		1	0
ARHGAP10	7	OK	-1.5807265	1.2206833	-1.2949522	0.1953	-0.91479087	subgrp_cor	count		1	0
NR3C2	1	OK	-11.019418	43.249935	-0.2547846	0.7988	-1.17482461	subgrp_cor	count		1	0
DCLK2	1	OK	3.4598146	6.7714513	0.5109413	0.6093	0.00030179	subgrp_cor	count		1	0
LRBA	4	OK	4.6613686	4.0891078	1.1399475	0.2543	0.00274223	subgrp_cor	count		1	0
RPS3A	875	OK	-0.115194	0.0842695	-1.3669707	0.1716	-0.16479426	subgrp_cor	count		1	0
SH3D19	12	OK	0.7417000	0.8934693	0.8301348	0.4064	0.48141104	subgrp_cor	count		1	0
GATB	1	OK	-11.019418	43.249935	-0.2547846	0.7988	-1.17482461	subgrp_cor	count		1	0
FBXW7	20	OK	-0.2423784	0.7519307	-0.3223413	0.7471	-0.17982210	subgrp_cor	count		1	0
ARFIP1	17	OK	0.6980195	0.6694666	1.0426501	0.2971	0.58006017	subgrp_cor	count		1	0
TRIM2	5	OK	-1.6462456	1.9351929	-0.8506881	0.3949	-0.70360391	subgrp_cor	count		1	0
TMEM131L	1	OK	1.5823776	1.9240213	0.8224325	0.4108	0.77174550	subgrp_cor	count		1	0
TLR2	1	OK	18.759489	83.625179	0.2243282	0.8225	0.00028789	subgrp_cor	count		1	0
SFRP2	1	OK	1.582381	1.2168202	1.3004230	0.1934	1.25554822	subgrp_cor	count		1	0
PLRG1	20	OK	0.8803385	0.6561381	1.341697	0.1796	0.86460799	subgrp_cor	count		1	0
GUCY1A1	20	OK	1.1633180	0.6728635	1.7289063	0.0838	1.20996054	subgrp_cor	count		1	0
GUCY1B1	1	OK	1.5823999	2.7200188	0.5817606	0.5607	0.47433761	subgrp_cor	count		1	0
CTSO	20	OK	-0.5586486	0.6104738	-0.9151066	0.3601	-0.48633523	subgrp_cor	count		1	0
PDGFC	4	OK	24.000192	54.345440	0.4416229	0.6587	2.40091057	subgrp_cor	count		1	0
FAM198B-AS1	2	OK	18.759489	83.625179	0.2243282	0.8225	0.00028789	subgrp_cor	count		1	0
TMEM144	1	OK	18.759489	83.625179	0.2243282	0.8225	0.00028789	subgrp_cor	count		1	0
ETFDH	3	OK	0.1721614	1.534450	0.1121974	0.9106	0.00011395	subgrp_cor	count		1	0
PPID	12	OK	-1.8538602	1.0715562	-1.7300633	0.0836	-0.99269273	subgrp_cor	count		1	0
FNIP2	84	OK	0.7865900	0.3106030	2.5324609	0.0113	1.01117042	subgrp_cor	count		1	0
C4orf45	1	OK	3.4598146	6.7714513	0.5109413	0.6093	0.00030179	subgrp_cor	count		1	0
RAPGEF2	9	OK	0.2953152	1.0926390	0.2702770	0.7869	0.09156996	subgrp_cor	count		1	0
NPY1R	1	OK	23.991896	84.899941	0.2825902	0.7774	1.65081709	subgrp_cor	count		1	0
TMA16	20	OK	-0.3656427	0.6786852	-0.5387436	0.5900	-0.32179904	subgrp_cor	count		1	0
TMEM192	6	OK	2.1412669	1.5249372	1.4041672	0.1602	1.05850244	subgrp_cor	count		1	0
KLHL2	1	OK	-11.019418	43.249935	-0.2547846	0.7988	-1.17482461	subgrp_cor	count		1	0
MSMO1	12	OK	-0.2204194	0.9034796	-0.2439672	0.8072	-0.14094834	subgrp_cor	count		1	0
TLL1	8	OK	-1.4564595	1.149722	-1.2667918	0.2052	-1.23773237	subgrp_cor	count		1	0
DDX60	13	OK	0.1733058	0.7767035	0.2231299	0.8234	0.1307448	subgrp_cor	count		1	0
DDX60L	13	OK	-1.5695228	0.871594	-1.8007495	0.0717	-1.14634779	subgrp_cor	count		1	0
PALLD	24	OK	0.9781292	0.7631105	1.2817662	0.1999	0.79898216	subgrp_cor	count		1	0
CBR4	5	OK	0.329526	1.4055480	0.2344472	0.8146	0.10244709	subgrp_cor	count		1	0
SH3RF1	3	OK	21.64205	54.486891	0.3971975	0.6912	1.57744735	subgrp_cor	count		1	0
NEK1	15	OK	4.106609	2.0167617	2.0362391	0.0417	1.47118925	subgrp_cor	count		1	0
CLCN3	38	OK	0.8275696	0.4795312	1.7257889	0.0843	0.90964895	subgrp_cor	count		1	0
HPF1	47	OK	-0.2732559	0.4263990	-0.6408454	0.5216	-0.33862984	subgrp_cor	count		1	0
GALNT7	6	OK	-0.4403523	1.1421467	-0.3855470	0.6998	-0.27767988	subgrp_cor	count		1	0
AC097534.2	7	OK	-1.148757	1.0748536	-1.0687567	0.2851	-0.86326149	subgrp_cor	count		1	0
SAP30	77	OK	-1.5413126	0.3471570	-4.4398138	9E-06	-1.91352606	subgrp_cor	count		0.0901898	0
FBXO8	7	OK	0.6139864	1.3007398	0.4720286	0.6369	0.57888619	subgrp_cor	count		1	0
CEP44	5	OK	0.3531019	1.1709373	0.3015569	0.7629	0.18054163	subgrp_cor	count		1	0
GPM6A	31	OK	-0.8931774	0.507946	-1.7584172	0.0786	-0.90693469	subgrp_cor	count		1	0
SPCS3	168	OK	0.3018784	0.228269	1.3224672	0.1860	0.4100541	subgrp_cor	count		1	0
VEGFC	50	OK	-0.2551038	0.4543253	-0.5614984	0.5744	-0.31165460	subgrp_cor	count		1	0
AGA	9	OK	1.0349415	1.0446771	0.9906807	0.3218	0.52322654	subgrp_cor	count		1	0
DCTD	18	OK	0.3072936	0.8194882	0.3749823	0.7076	0.15689141	subgrp_cor	count		1	0
WWC2	15	OK	1.7702612	1.2359651	1.4322905	0.1520	0.86010694	subgrp_cor	count		1	0
CDKN2AIP	24	OK	0.2998941	0.5789684	0.517953	0.6044	0.29386822	subgrp_cor	count		1	0
ING2	41	OK	1.827602	0.6152199	2.9706495	0.0029	1.91573680	subgrp_cor	count		1	0
RWDD4	38	OK	-0.6422395	0.4800638	-1.337822	0.1809	-0.69412891	subgrp_cor	count		1	0
TRAPPC11	2	OK	0.1681491	1.7253300	0.097459	0.9223	0.05201277	subgrp_cor	count		1	0
STOX2	6	OK	-1.897298	1.4010550	-1.3541924	0.1756	-0.76229785	subgrp_cor	count		1	0
IRF2	54	OK	0.127869	0.390509	0.3276722	0.7431	0.16093389	subgrp_cor	count		1	0
CASP3	26	OK	0.2586401	0.6177597	0.4186742	0.6754	0.25560464	subgrp_cor	count		1	0
PRIMPOL	2	OK	-0.2029407	1.6532265	-0.1227536	0.9022	-4.82E-05	subgrp_cor	count		1	0
CENPU	1	OK	-10.442278	41.465809	-0.2518286	0.8011	-0.00045289	subgrp_cor	count		1	0
SLC25A4	24	OK	4.3036270	1.369866	3.1416391	0.0016	3.29866230	subgrp_cor	count		1	0
CFAP97	130	OK	0.1162175	0.2484516	0.4677674	0.6399	0.15573567	subgrp_cor	count		1	0
SNX25	1	OK	0.6532528	2.5001284	0.2612877	0.7938	0.00016297	subgrp_cor	count		1	0
ANKRD37	24	OK	1.3390710	0.6374389	2.1007047	0.0356	1.51314608	subgrp_cor	count		1	0
UFSP2	15	OK	1.7695089	0.9861835	1.7942997	0.0727	1.24801018	subgrp_cor	count		1	0
PDLIM3	112	OK	-1.26679	0.29174	-4.3421288	1.41E-05	-1.6840782	subgrp_cor	count		0.1411088	0
SORBS2	46	OK	1.0535636	0.5898614	1.7861205	0.0740	0.81165619	subgrp_cor	count		1	0
TLR3	4	OK	0.2606514	1.6620474	0.1568255	0.8753	0.08088269	subgrp_cor	count		1	0
CYP4V2	5	OK	1.5206865	1.3920887	1.091899	0.2748	0.45797223	subgrp_cor	count		1	0
FRG1	78	OK	0.2844709	0.3260347	0.8725172	0.3829	0.36438312	subgrp_cor	count		1	0
CCDC127	6	OK	0.0751322	1.3781715	0.054515	0.9565	0.03814830	subgrp_cor	count		1	0
SDHA	8	OK	0.6302261	1.1043464	0.570776	0.5681	0.47473440	subgrp_cor	count		1	0
PDCD6	242	OK	0.0569956	0.1846581	0.3086548	0.7575	0.07905872	subgrp_cor	count		1	0
EXOC3-AS1	2	OK	5.8049273	5.6694662	1.023893	0.3058	1.03285716	subgrp_cor	count		1	0
EXOC3	1	OK	-11.019418	43.249935	-0.2547846	0.7988	-1.17482461	subgrp_cor	count		1	0
SLC9A3	1	OK	18.759489	83.625179	0.2243282	0.8225	0.00028789	subgrp_cor	count		1	0
TPPP	1	OK	3.4598146	6.7714513	0.5109413	0.6093	0.00030179	subgrp_cor	count		1	0
BRD9	9	OK	-0.2533849	1.0875558	-0.2329854	0.8157	-0.0761972	subgrp_cor	count		1	0
SLC12A7	5	OK	-10.406683	16.764866	-0.6207436	0.5347	-2.19585791	subgrp_cor	count		1	0
CLPTM1L	16	OK	1.0972895	0.8874857	1.2364023	0.2163	0.89881580	subgrp_cor	count		1	0
SLC6A3	3	OK	18.759489	83.625179	0.2243282	0.8225	0.00028789	subgrp_cor	count		1	0
LPCAT1	7	OK	-1.3967094	1.366852	-1.0218437	0.3068	-0.37125920	subgrp_cor	count		1	0
MRPL36	54	OK	0.2380006	0.4207346	0.565678	0.5716	0.29788751	subgrp_cor	count		1	0
NDUFS6	167	OK	0.7243298	0.2467128	2.9359631	0.0033	0.9556293	subgrp_cor	count		1	0
ICE1	28	OK	0.3822669	0.559475	0.6832600	0.4944	0.39238170	subgrp_cor	count		1	0
MED10	85	OK	-0.1757705	0.3027908	-0.5805015	0.5615	-0.22768183	subgrp_cor	count		1	0
NSUN2	5	OK	-3.25655	2.3988764	-1.3575313	0.1746	-0.64364427	subgrp_cor	count		1	0
MIR4458HG	4	OK	-1.1068798	1.7130168	-0.6461581	0.5181	-0.65509255	subgrp_cor	count		1	0
SEMA5A	13	OK	22.375167	33.053129	0.6769455	0.4984	2.82684843	subgrp_cor	count		1	0
SNHG18	4	OK	22.221790	54.522322	0.4075655	0.6835	1.5953836	subgrp_cor	count		1	0
FAM173B	4	OK	-3.7608267	2.122701	-1.7717172	0.0764	-0.69498291	subgrp_cor	count		1	0
CCT5	121	OK	0.293607	0.2768886	1.0603814	0.2889	0.38239108	subgrp_cor	count		1	0
CMBL	14	OK	1.4111345	0.8485891	1.662918	0.0963	1.13425631	subgrp_cor	count		1	0
AC012640.2	1	OK	-11.019418	43.249935	-0.2547846	0.7988	-1.17482461	subgrp_cor	count		1	0
6-Mar	39	OK	-0.2329176	0.4643185	-0.5016332	0.6159	-0.24994976	subgrp_cor	count		1	0
ROPN1L	2	OK	-0.9102232	2.1881125	-0.4159856	0.6774	-0.25785735	subgrp_cor	count		1	0
DAP	84	OK	1.096420	0.3340669	3.2820388	0.0010	1.38734932	subgrp_cor	count		1	0
TRIO	2	OK	-13.04562	27.67476	-0.4713905	0.6373	-1.3178641	subgrp_cor	count		1	0
OTULINL	12	OK	-11.868132	16.711042	-0.710197	0.4775	-3.85918791	subgrp_cor	count		1	0
OTULIN	5	OK	5.0027563	4.2286829	1.1830530	0.2367	0.98115823	subgrp_cor	count		1	0
ANKH	8	OK	1.1581985	1.6544505	0.7000502	0.4839	0.00079558	subgrp_cor	count		1	0
ZNF622	6	OK	-1.3222082	1.2976654	-1.018912	0.3082	-0.58149545	subgrp_cor	count		1	0
RETREG1	4	OK	-1.0379627	1.4280017	-0.7268637	0.4673	-0.00062210	subgrp_cor	count		1	0
MYO10	19	OK	-0.019959	0.8385713	-0.0238011	0.9810	-0.01288709	subgrp_cor	count		1	0
BASP1	1	OK	3.4598146	6.7714513	0.5109413	0.6093	0.00030179	subgrp_cor	count		1	0
GOLPH3	20	OK	0.5390164	0.6417643	0.8398978	0.4009	0.44909165	subgrp_cor	count		1	0
AC025181.2	1	OK	-11.019418	43.249935	-0.2547846	0.7988	-1.17482461	subgrp_cor	count		1	0
MTMR12	9	OK	1.2384611	1.019532	1.2147340	0.2244	1.00666617	subgrp_cor	count		1	0
ZFR	68	OK	0.374163	0.3542978	1.0560706	0.2909	0.47728619	subgrp_cor	count		1	0
SUB1	357	OK	0.5161353	0.1531650	3.3697980	0.0007	0.72468032	subgrp_cor	count		1	0
NPR3.00	2	OK	-1.0311657	1.7479538	-0.5899271	0.5552	-0.00022765	subgrp_cor	count		1	0
TARS	16	OK	-0.6890249	0.696615	-0.9891038	0.3226	-0.68582280	subgrp_cor	count		1	0
RAI14	67	OK	0.4858593	0.3897417	1.2466186	0.2125	0.54728977	subgrp_cor	count		1	0
RAD1	9	OK	0.5989814	1.1732274	0.5105415	0.6096	0.18683341	subgrp_cor	count		1	0
BRIX1	44	OK	-0.2856292	0.4372563	-0.6530788	0.5137	-0.32873013	subgrp_cor	count		1	0
DNAJC21	122	OK	-0.0799959	0.2596162	-0.3081312	0.7579	-0.1060132	subgrp_cor	count		1	0
SPEF2	2	OK	0.1681690	1.7253287	0.0974707	0.9223	0.05201879	subgrp_cor	count		1	0
IL7R	3	OK	-0.5453205	1.708550	-0.3191715	0.7496	-0.0003423	subgrp_cor	count		1	0
NADK2	2	OK	-0.3495512	1.8544747	-0.1884907	0.8504	-0.17237782	subgrp_cor	count		1	0
NIPBL	58	OK	0.422443	0.3714419	1.1373056	0.2554	0.46697185	subgrp_cor	count		1	0
CPLANE1	4	OK	-2.4465924	1.7138131	-1.4275724	0.1534	-0.00126286	subgrp_cor	count		1	0
NUP155	3	OK	-1.0379618	1.4280127	-0.7268575	0.4673	-0.00062229	subgrp_cor	count		1	0
WDR70	9	OK	-0.9477928	1.2366561	-0.7664158	0.4434	-0.43818333	subgrp_cor	count		1	0
EGFLAM	1	OK	18.759489	83.625179	0.2243282	0.8225	0.00028789	subgrp_cor	count		1	0
LIFR	145	OK	-0.7584697	0.2248878	-3.3726578	0.0007	-1.03183256	subgrp_cor	count		1	0
LIFR-AS1	3	OK	5.0027563	4.2286829	1.1830530	0.2367	0.98115823	subgrp_cor	count		1	0
OSMR	10	OK	0.643106	0.9357673	0.6872506	0.4919	0.32884969	subgrp_cor	count		1	0
RICTOR	9	OK	0.3131020	1.1290566	0.2773130	0.7815	0.20379679	subgrp_cor	count		1	0
DAB2	94	OK	-0.288384	0.3054936	-0.9439947	0.3451	-0.35697964	subgrp_cor	count		1	0
TTC33	7	OK	-1.0325478	1.0653474	-0.9692122	0.3324	-0.71986706	subgrp_cor	count		1	0
PTGER4	9	OK	-1.8538705	1.0715842	-1.7300116	0.0836	-0.99268922	subgrp_cor	count		1	0
PRKAA1	9	OK	0.0234720	0.903828	0.0259696	0.9792	0.0151754	subgrp_cor	count		1	0
RPL37	866	OK	-0.170480	0.0866574	-1.9672961	0.0491	-0.24385157	subgrp_cor	count		1	0
CARD6	28	OK	0.5096003	0.6427085	0.7928949	0.4278	0.5660365	subgrp_cor	count		1	0
C7	86	OK	-0.2013763	0.3285527	-0.6129184	0.5399	-0.22527160	subgrp_cor	count		1	0
PLCXD3	37	OK	0.1392257	0.4334144	0.3212301	0.7480	0.14690247	subgrp_cor	count		1	0
OXCT1	6	OK	0.7449229	1.2009177	0.6202947	0.5350	0.55868246	subgrp_cor	count		1	0
C5orf51	3	OK	0.5103003	1.6034059	0.3182603	0.7502	0.15911328	subgrp_cor	count		1	0
FBXO4	9	OK	-1.1912739	1.0117445	-1.1774453	0.2390	-0.69802322	subgrp_cor	count		1	0
CCDC152	7	OK	7.7555284	4.917793	1.5770342	0.1147	2.18633627	subgrp_cor	count		1	0
SELENOP	276	OK	-0.2109722	0.1627994	-1.2959027	0.1950	-0.29212487	subgrp_cor	count		1	0
ANXA2R	27	OK	-0.0445078	0.5320350	-0.0836557	0.9333	-0.04386109	subgrp_cor	count		1	0
ZNF131	22	OK	0.7851844	0.7450784	1.0538279	0.2919	0.65109665	subgrp_cor	count		1	0
HMGCS1	6	OK	0.9801526	1.3385170	0.7322675	0.4640	0.49669576	subgrp_cor	count		1	0

CCL28	3	OK	22.2230389	54.5331384	0.40751436	0.68362	1.59542235	subgrp_con	count	1	0
TMEM267	1	OK	18.7594894	83.6251792	0.2243282	0.8225	0.00028789	subgrp_con	count	1	0
C5orf34	1	OK	-11.019418	43.2499352	-0.2547846	0.79889	-1.17482461	subgrp_con	count	1	0
PAIP1	68	OK	0.0862332	0.3513271	0.24545	0.80611	0.10724225	subgrp_con	count	1	0
NNT-AS1	19	OK	-0.858838	0.81026505	-1.0599472	0.28917	-0.60918234	subgrp_con	count	1	0
NNT	2	OK	-15.488617	42.8316449	-0.3616162	0.71764	-0.00073897	subgrp_con	count	1	0
MRPS30	13	OK	-0.407932	0.8127546	-0.501912	0.61573	-0.29981979	subgrp_con	count	1	0
PARP8	1	OK	-10.442278	41.4658099	-0.2518286	0.80117	-0.00045289	subgrp_con	count	1	0
ITGA1	9	OK	-1.3768869	1.0718637	-1.2845724	0.19894	-0.77537476	subgrp_con	count	1	0
PELO	26	OK	-0.753960	0.6346082	-1.1880722	0.2348	-0.79174858	subgrp_con	count	1	0
ITGA2	27	OK	-0.028714	0.5451637	-0.0526711	0.95799	-0.03164866	subgrp_con	count	1	0
MOCS2	53	OK	0.2997726	0.3647186	0.8219286	0.41112	0.37531977	subgrp_con	count	1	0
AC008966.1	1	OK	3.4598146	6.7714513	0.5109413	0.60930	0.00030179	subgrp_con	count	1	0
NDUFS4	80	OK	0.7885393	0.3491250	2.2586155	0.02391	0.95793082	subgrp_con	count	1	0
ARL15.00	23	OK	1.8071282	0.9195758	1.9651757	0.04939	0.53155849	subgrp_con	count	1	0
SNX18	19	OK	6.8014435	3.510504	1.9374547	0.05268	1.08516455	subgrp_con	count	1	0
GPX8	24	OK	1.2782862	0.6958704	1.8371038	0.06619	1.23440637	subgrp_con	count	1	0
DHX29	60	OK	0.1816140	0.3754072	0.4837788	0.62854	0.22458644	subgrp_con	count	1	0
MTREX	11	OK	1.0588648	0.9516085	1.1127105	0.26583	0.53473891	subgrp_con	count	1	0
IL6ST	497	OK	-0.170025	0.1170133	-1.4530409	0.14621	-0.24054038	subgrp_con	count	1	0
AC016596.1	1	OK	1.5822999	2.7200188	0.5817606	0.56072	0.47433761	subgrp_con	count	1	0
MAP3K1	14	OK	-2.295234	0.9716440	-2.3622169	0.01817	-2.00368100	subgrp_con	count	1	0
SETD9	6	OK	-0.223118	1.1048737	-0.2019400	0.83996	-0.11161655	subgrp_con	count	1	0
GPBP1	158	OK	-0.238733	0.2137055	-1.1171145	0.26395	-0.32146754	subgrp_con	count	1	0
PLK2	81	OK	-0.830058	0.3422188	-2.4255095	0.01529	-1.04554761	subgrp_con	count	1	0
RAB3C	26	OK	-0.265392	0.6373393	-0.416406	0.67711	-0.24831517	subgrp_con	count	1	0
PDE4D	6	OK	2.9836403	1.8750428	1.5912384	0.11150	1.75079475	subgrp_con	count	1	0
PART1	1	OK	18.7594894	83.6251792	0.2243282	0.8225	0.00028789	subgrp_con	count	1	0
ELOVL7	9	OK	-11.573144	16.777382	-0.6898063	0.49032	-3.5289439	subgrp_con	count	1	0
NDUFAF2	50	OK	-0.146613	0.4234325	-0.3462505	0.72915	-0.16687884	subgrp_con	count	1	0
SMIM15	12	OK	0.6596009	0.8467382	0.7789904	0.43599	0.54856979	subgrp_con	count	1	0
ZSWIM6	6	OK	-1.606445	1.6185685	-0.99251	0.32095	-0.411208	subgrp_con	count	1	0
KIF2A	53	OK	-0.155221	0.3963201	-0.3916567	0.69531	-0.1957071	subgrp_con	count	1	0
DIMT1	22	OK	0.587650	0.7543483	0.7790175	0.43597	0.44286950	subgrp_con	count	1	0
IPO11	87	OK	0.2477606	0.3256994	0.7607034	0.44682	0.31966328	subgrp_con	count	1	0
RNF180	7	OK	10.004463	7.1213630	1.4048523	0.16007	2.26813971	subgrp_con	count	1	0
RGS7BP	11	OK	2.4982532	1.3746851	1.8173276	0.06917	1.29886662	subgrp_con	count	1	0
SREK1IP1	92	OK	-0.285997	0.3037584	-0.9415302	0.34642	-0.36992314	subgrp_con	count	1	0
CWC27	20	OK	0.0985650	0.6886966	0.1431182	0.8862	0.0638793	subgrp_con	count	1	0
ADAMTS6	16	OK	1.1703047	1.0777080	1.0859199	0.27751	0.36002075	subgrp_con	count	1	0
CENPK	3	OK	-2.023032	1.8071267	-1.1194743	0.26294	-0.00109400	subgrp_con	count	1	0
PPWD1	10	OK	-1.098253	0.8466989	-1.297100	0.1946	-0.65064292	subgrp_con	count	1	0
TRIM23	3	OK	-10.475634	27.491987	-0.3810432	0.70317	-1.13811785	subgrp_con	count	1	0
TRAPPC13	4	OK	-0.150975	1.968815	-0.0766836	0.93888	-9.78E-05	subgrp_con	count	1	0
SGTB	6	OK	-1.998204	1.5544495	-1.2841876	0.19908	-1.26285654	subgrp_con	count	1	0
NLN	1	OK	-10.442278	41.4658099	-0.2518286	0.80117	-0.00045289	subgrp_con	count	1	0
ERBIN	29	OK	-1.401579	0.6300948	-2.2242947	0.02612	-1.20504412	subgrp_con	count	1	0
SREK1	52	OK	0.6890281	0.4553019	1.5133433	0.13019	0.74346839	subgrp_con	count	1	0
MAST4	46	OK	1.3995018	0.513824	2.7236946	0.00646	1.43733924	subgrp_con	count	1	0
PIK3R1	13	OK	0.6442162	0.8405225	0.7664473	0.44341	0.48889432	subgrp_con	count	1	0
SLC30A5	5	OK	0.9396109	1.4985607	0.6270089	0.53065	0.2916352	subgrp_con	count	1	0
CENPH	2	OK	3.4598146	6.7714513	0.5109413	0.60930	0.00030179	subgrp_con	count	1	0
MRPS36	64	OK	0.0203224	0.3790274	0.0536172	0.95724	0.02540703	subgrp_con	count	1	0
CDK7	1	OK	-11.019418	43.2499352	-0.2547846	0.79889	-1.17482461	subgrp_con	count	1	0
CCDC125	4	OK	0.5103003	1.6034059	0.3182602	0.75029	0.15911328	subgrp_con	count	1	0
AK6	37	OK	0.8713116	0.5085187	1.7134305	0.08663	0.91348704	subgrp_con	count	1	0
TAF9	50	OK	0.0974773	0.397342	0.2453234	0.80621	0.11966303	subgrp_con	count	1	0
RAD17	6	OK	0.1703627	1.1470876	0.1485176	0.88193	0.05270200	subgrp_con	count	1	0
MARVELD2	1	OK	-11.019418	43.2499352	-0.2547846	0.79889	-1.17482461	subgrp_con	count	1	0
GTF2H2C	2	OK	-0.150975	1.968815	-0.0766836	0.93888	-9.78E-05	subgrp_con	count	1	0
SMN1	1	OK	-10.442278	41.4658099	-0.2518286	0.80117	-0.00045289	subgrp_con	count	1	0
NAIP	3	OK	-0.725215	1.8079140	-0.4011341	0.68832	-0.20880483	subgrp_con	count	1	0
GTF2H2	1	OK	18.7594894	83.6251792	0.2243282	0.8225	0.00028789	subgrp_con	count	1	0
LINC02197	2	OK	-11.186792	26.313753	-0.425131	0.67074	-2.27103070	subgrp_con	count	1	0
BDP1	84	OK	-0.002222	0.3201452	-0.0069437	0.99446	-0.00282052	subgrp_con	count	1	0
MCCC2	2	OK	0.6532528	2.5001284	0.2612877	0.79387	0.00016297	subgrp_con	count	1	0
MAP1B	59	OK	1.9652255	0.6194449	3.1725586	0.00151	1.72227933	subgrp_con	count	1	0
MRPS27	9	OK	-0.838295	1.0802271	-0.7760358	0.43772	-0.00139238	subgrp_con	count	1	0
ZNF366	3	OK	-0.174796	1.820980	-0.0959904	0.92353	-0.05309252	subgrp_con	count	1	0
TNPO1	40	OK	0.2345975	0.4906458	0.4781401	0.63250	0.23264433	subgrp_con	count	1	0
FCHO2	55	OK	-0.944330	0.3972761	-2.377012	0.01745	-1.07391638	subgrp_con	count	1	0
BTF3	802	OK	-0.310205	0.0927451	-3.3447048	0.00082	-0.44280555	subgrp_con	count	1	0
UTP15	3	OK	-11.199925	27.762997	-0.403412	0.68665	-1.82940850	subgrp_con	count	1	0
ENC1	2	OK	18.7594894	83.6251792	0.2243282	0.8225	0.00028789	subgrp_con	count	1	0
HEXB	49	OK	1.3343015	0.4635048	2.8787218	0.00399	1.44034484	subgrp_con	count	1	0
GFM2	1	OK	-11.019418	43.2499352	-0.2547846	0.79889	-1.17482461	subgrp_con	count	1	0
NSA2	262	OK	0.1868771	0.1714326	1.0900906	0.27567	0.25887791	subgrp_con	count	1	0
GCNT4	1	OK	3.4598146	6.7714513	0.5109413	0.60930	0.00030179	subgrp_con	count	1	0
ANKRD31	1	OK	3.4598146	6.7714513	0.5109413	0.60930	0.00030179	subgrp_con	count	1	0
HMGCR	2	OK	18.7594894	83.6251792	0.2243282	0.8225	0.00028789	subgrp_con	count	1	0
COL4A3BP	59	OK	-0.981196	0.3717617	-2.6393164	0.00831	-1.06224284	subgrp_con	count	1	0
POLK	31	OK	-0.126235	0.5759901	-0.2191631	0.82652	-0.1286539	subgrp_con	count	1	0
POC5	2	OK	-0.910223	2.1881125	-0.4159856	0.67742	-0.25785735	subgrp_con	count	1	0
F2R	62	OK	-0.317227	0.4072332	-0.778982	0.43599	-0.3595951	subgrp_con	count	1	0
CRHBP	38	OK	-0.650241	0.4787383	-1.3582405	0.17439	-0.68693927	subgrp_con	count	1	0
AGGF1	2	OK	0.5887819	1.7674148	0.3331317	0.73903	0.30122869	subgrp_con	count	1	0
ZBED3	5	OK	1.5207849	1.3927023	1.0919669	0.27485	0.45798065	subgrp_con	count	1	0
ZBED3-AS1	1	OK	3.4598146	6.7714513	0.5109413	0.60930	0.00030179	subgrp_con	count	1	0
WDR41	5	OK	-1.484432	1.3983751	-1.0615412	0.28844	-0.64939975	subgrp_con	count	1	0
TBCA	302	OK	0.1050493	0.1653808	0.6351966	0.5252	0.14622813	subgrp_con	count	1	0
AP3B1	30	OK	0.9714849	0.5779843	1.6807862	0.0928	0.91017952	subgrp_con	count	1	0
SCAMP1-AS1	5	OK	-1.926410	1.4185129	-1.3580494	0.17445	-0.00105388	subgrp_con	count	1	0
SCAMP1	23	OK	0.2403079	0.6405964	0.3751315	0.70756	0.2276595	subgrp_con	count	1	0
LHFPL2	2	OK	3.4598146	6.7714513	0.5109413	0.60930	0.00030179	subgrp_con	count	1	0
BHMT2	1	OK	-15.995031	70.349421	-0.2273655	0.82014	-0.00028474	subgrp_con	count	1	0
JMY	9	OK	-1.057388	1.0437082	-1.0131074	0.31101	-0.61917335	subgrp_con	count	1	0
TENT2	4	OK	-0.147078	1.8100873	-0.081255	0.93524	-0.04445379	subgrp_con	count	1	0
SERINC5	1	OK	-10.442278	41.4658099	-0.2518286	0.80117	-0.00045289	subgrp_con	count	1	0
ZFYVE16	25	OK	0.4845433	0.6875325	0.7047569	0.48096	0.46367128	subgrp_con	count	1	0
AC008771.1	1	OK	3.4598146	6.7714513	0.5109413	0.60930	0.00030179	subgrp_con	count	1	0
DHFR	2	OK	-10.968199	27.210083	-0.4030932	0.68688	-1.17122783	subgrp_con	count	1	0
MSH3	8	OK	-0.502963	1.0633263	-0.4730096	0.63621	-0.00086075	subgrp_con	count	1	0
RASGRF2	9	OK	4.4078724	3.1185113	1.4134540	0.15752	0.93395207	subgrp_con	count	1	0
CKMT2-AS1	1	OK	-10.442278	41.4658099	-0.2518286	0.80117	-0.00045289	subgrp_con	count	1	0
ZCCHC9	25	OK	0.836217	0.6367479	1.3132632	0.18909	0.85664392	subgrp_con	count	1	0
SSBP2	15	OK	-1.884168	0.8606429	-2.189257	0.02858	-1.43553291	subgrp_con	count	1	0
RPS23	885	OK	-0.070145	0.0831223	-0.843879	0.39874	-0.10040676	subgrp_con	count	1	0
ATP6AP1L	1	OK	-11.019418	43.2499352	-0.2547846	0.79889	-1.17482461	subgrp_con	count	1	0
TMEM167A	53	OK	0.2482814	0.417637	0.5947303	0.55202	0.29159453	subgrp_con	count	1	0
XRCC4	5	OK	-0.819246	1.7280912	-0.4740762	0.63545	-0.24845814	subgrp_con	count	1	0
VCAN	33	OK	-0.310377	0.5436698	-0.5708935	0.56807	-0.28956974	subgrp_con	count	1	0
HAPLN1	1	OK	3.4598146	6.7714513	0.5109413	0.60930	0.00030179	subgrp_con	count	1	0
EDIL3	24	OK	2.6462089	1.0534198	2.5120172	0.012	1.45320023	subgrp_con	count	1	0
COX7C	647	OK	0.0267062	0.106013	0.2519129	0.80111	0.0379635	subgrp_con	count	1	0
RASA1	6	OK	1.5334351	1.7222502	0.8898505	0.37355	0.46136546	subgrp_con	count	1	0
CCNH	32	OK	1.3974508	0.573256	2.4377413	0.01478	1.58221266	subgrp_con	count	1	0
TMEM161B-AS1	3	OK	-0.697769	1.6964538	-0.4113106	0.68084	-0.33591720	subgrp_con	count	1	0
MEF2C-AS1	4	OK	-0.910223	1.5464209	-0.5885998	0.55617	-0.42849043	subgrp_con	count	1	0
CETN3	4	OK	1.791902	2.2832539	0.7847677	0.43259	0.00045246	subgrp_con	count	1	0
MBLAC2	2	OK	1.925866	2.5154879	0.7656425	0.44389	0.56022119	subgrp_con	count	1	0
POLR3G	1	OK	3.4598146	6.7714513	0.5109413	0.60930	0.00030179	subgrp_con	count	1	0
LYSMD3	6	OK	1.1224427	1.4569313	0.7704163	0.44105	0.3460629	subgrp_con	count	1	0
ARRDC3	74	OK	-0.121825	0.339070	-0.3592927	0.71938	-0.15965074	subgrp_con	count	1	0
NR2F1-AS1	4	OK	-0.546377	1.297277	-0.4211723	0.67362	-0.34177821	subgrp_con	count	1	0
NR2F1	17	OK	2.1010178	0.962586	2.1826790	0.02906	1.58686194	subgrp_con	count	1	0
FAM172A	21	OK	0.2102817	0.6571271	0.320001	0.74897	0.18827278	subgrp_con	count	1	0
KIAA0825	1	OK	18.7594894	83.6251792	0.2243282	0.8225	0.00028789	subgrp_con	count	1	0
SLF1	4	OK	0.111276	1.6120387	0.0690285	0.94497	0.05657342	subgrp_con	count	1	0
MCTP1	145	OK	0.2188933	0.2550802	0.8581352	0.39082	0.28750711	subgrp_con	count	1	0
TTC37	31	OK	1.1624376	0.6004231	1.9360307	0.05286	1.01871857	subgrp_con	count	1	0
ARSK	2	OK	18.7594894	83.6251792	0.2243282	0.8225	0.00028789	subgrp_con	count	1	0
RHOBTB3	5	OK	0.4061000	2.1083412	0.1926158	0.84726	0.12645366	subgrp_con	count	1	0
GLRX	59	OK	-0.218189	0.3806709	-0.573171	0.56652	-0.25471543	subgrp_con	count	1	0
ELL2	6	OK	1.1034521	1.6520598	0.6679250	0.50418	0.00075806	subgrp_con	count	1	0
PCSK1	1	OK	23.991896	84.899941	0.2825902	0.77749	1.65081709	subgrp_con	count	1	0
CAST	556	OK	-0.334165	0.114415	-2.9206238	0.00349	-0.47439322	subgrp_con	count	1	0
ERAP1	2	OK	-1.295616	2.041425	-0.6346627	0.52565	-0.00075754	subgrp_con	count	1	0
ERAP2	24	OK	0.0182227	0.617966	0.0294881	0.97648	0.01799548	subgrp_con	count	1	0
LNPEP	23	OK	0.5095100	0.6353932	0.8018815	0.42262	0.45661701	subgrp_con	count	1	0
RIOK2	4	OK	-0.972720	1.6375058	-0.5940258	0.55249	-0.27349270	subgrp_con	count	1	0
RGMB	1	OK	-10.442278	41.4658099	-0.2518286	0.80117	-0.00045289	subgrp_con	count	1	0
RGMB-AS1	1	OK	18.7594894	83.6251792	0.2243282	0.8225	0.00028789	subgrp_con	count	1	0
CHD1	97	OK	-0.786498	0.3019200	-2.6049892	0.00919	-1.00401265	subgrp_con	count	1	0
FAM174A	32	OK	0.6507488	0.5514082	1.1801580	0.23796	0.7624026	subgrp_con	count	1	0
ST8SIA4	9	OK	-1.622893	1.1165788	-1.4532086	0.14615	-0.89843220	subgrp_con	count	1	0
PAM	27	OK	3.8527641	1.1093285	3.4730595	0.00051	3.49866274	subgrp_con	count	1	0
GIN1	1	OK	-15.995031	70.349421	-0.2273655	0.82014	-0.00028474	subgrp_con	count	1	0
PPIP5K2	24	OK	-0.409649	0.6110672	-0.6703834	0.50262	-0.35962728	subgrp_con	count	1	0
NUDT12	1	OK	18.7594894	83.6251792	0.2243282	0.8225	0.00028789	subgrp_con	count	1	0
FBXL17	4	OK	-0.202940	1.6532365	-0.1227536	0.9022	-4.82E-05	subgrp_con	count	1	0
FER	9	OK	-0.406686	0.8901014	-0.4568995	0.64774	-0.33123386	subgrp_con	count	1	0
PJA2	113	OK	0.0911451	0.2786742	0.3270668	0.74362	0.11235425	subgrp_con	count	1	0
MAN2A1	2	OK	5.8032311	5.6696270	1.0235648	0.30606	1.03280544	subgrp_con	count	1	0
SLC25A46	4	OK	5.8049273	5.6694662	1.023893	0.30589	1.03285716	subgrp_con	count	1	0
TSLP	3	OK	2.4096337	2.4715206	0.9749599	0.32958	0.66517343	subgrp_con	count	1	0
WDR36	5	OK	0.1681491	1.7253300	0.097459	0.92236	0.05201277	subgrp_con	count	1	0
NREP	19	OK	0.9622775	0.7697108	1.2501804	0.21122	0.85276426	subgrp_con	count	1	0
EPB41L4A	21	OK	0.422944	0.5986948	0.7221465	0.46958	0.36097411	subgrp_con	count	1	0
EPB41L4A-AS1	42	OK	-0.784430	0.446242	-1.757858	0.07877	-0.88855705	subgrp_con	count	1	0
EPB41L4A-DT	2	OK	-11.019418	43.2499352	-0.2547846	0.79889	-1.17482461	subgrp_con	count	1	0
APC	31	OK	-0.252514	0.5383170	-0.4690809	0.63901	-0.25655968	subgrp_con	count	1	0
SRP19	56	OK	-0.113449	0.3719266	-0.3050317	0.76034	-0.13792594	subgrp_con	count	1	0
REEP5	248	OK	0.5685947	0.1838126	3.0933389	0.00198	0.79381172	subgrp_con	count	1	0
DCP2	24	OK	0.563223	0.6506817	0.8655763	0.38672	0.42311038	subgrp_con	count	1	0
MCC	3	OK	-0.634886	2.0089600	-0.3160275	0.75198	-0.000145	subgrp_con	count	1	0
YTHDC2	12	OK	-0.764358	0.9767714	-0.7825351	0.4339	-0.47020233	subgrp_con	count	1	0
KCNN2	1	OK	3.4598146	6.7714513	0.5109413	0.60930	0.00030179	subgrp_con	count	1	0
PGGT1B	11	OK	0.7706202	0.8563263	0.8999141	0.36817	0.57859649	subgrp_con	count	1	0
CCDC112	20	OK	0.7030600	0.740904	0.9489207	0.34266	0.4570438	subgrp_con	count	1	0
FEM1C	17	OK	0.8182773	0.8227839	0.9934369	0.3205	0.5300679	subgrp_con	count	1	0
AC010226.1	2	OK	-10.475634	27.491987	-0.3810432	0.70317	-1.13811785	subgrp_con	count	1	0
TMED7	19	OK	0.7596577	0.6523396	1.1645128	0.24422	0.5705351	subgrp_con	count	1	0
CDO1	24	OK	1.5968262	0.6118205	2.6099584	0.00906	1.83644249	subgrp_con	count	1	0
ATG12	58	OK	0.4578745	0.3916611	1.169058	0.24238	0.56612485	subgrp_con	count	1	0
AP3S1	63	OK	0.4985396	0.3952057	1.2614688	0.20714	0.60268732	subgrp_con	count	1	0
COMMD10	23	OK	0.2462466	0.6764597	0.3640225	0.71584	0.2051625	subgrp_con	count	1	0
LINC02147	2	OK	1.1112449	1.8685808	0.5946999	0.55206	0.34276981	subgrp_con	count	1	0
DMXL1	14	OK	0.860915	0.8226735	1.0464849	0.29536	0.55686201	subgrp_con	count	1	0
TNFAIP8	9	OK	0.408653	1.0571107	0.3865759	0.69907	0.20906690	subgrp_con	count	1	0
HSD17B4	20	OK	0.7048858	0.6674867	1.0560298	0.29095	0.71811835	subgrp_con	count	1	0
SRFBP1	46	OK	0.2572393	0.4551608	0.5653812	0.57181	0.28970338	subgrp_con	count	1	0
LOX	15	OK	2.6510635	1.4259353	1.8591750	0.063	1.68873634	subgrp_con	count	1	0
SNCAIP	16	OK	1.1457984	0.8444943	1.3579705	0.17447	1.00813634	subgrp_con	count	1	0
SNX2	90	OK	0.3609454	0.3031452	1.1906682	0.23379	0.47005566	subgrp_con	count	1	0
SNX24	6	OK	20.817553	50.528527	0.4119145	0.6804	0.00083571	subgrp_con	count	1	0
PPIC	230	OK	0.471321	0.1901997	2.4780337	0.01321	0.6493484	subgrp_con	count	1	0
PRDM6	3	OK	21.642059	54.486891	0.3971975	0.69122	1.57744735	subgrp_con	count	1	0
CEP120	1	OK	-10.442278	41.4658099	-0.2518286	0.80117	-0.00045289	subgrp_con	count	1	0
CSNK1G3	31	OK	0.165633	0.5955338	0.2781255	0.78092	0.17951736	subgrp_con	count	1	0
ZNF608	22	OK	-1.504388	0.6941937	-2.1671015	0.03022	-1.34100116	subgrp_con	count	1	0
GRAMD2B	3	OK	3.4598149	4.7858686	0.7229230	0.46972	0.00081935	subgrp_con	count	1	0
ALDH7A1	6	OK	1.6688807	1.5674495	1.0647110	0.28701	0.83065212	subgrp_con	count	1	0
PHAX	44	OK	0.4168156	0.4573774	0.911316	0.36212	0.48929497	subgrp_con	count	1	0
LMNB1	2	OK	-11.448548	41.761485	-0.2741411	0.78398	-0.00049246	subgrp_con	count	1	0
2-Mar	8	OK	-0.371408	1.2493673	-0.297277	0.76626	-0.11115247	subgrp_con	count	1	0
C5orf63	1	OK	-11.019418	43.2499352	-0.2547846	0.79889	-1.17482461	subgrp_con	count	1	0
PRRC1	12	OK	1.8641658	1.1565326	1.6118575	0.10699	1.12302179	subgrp_con	count	1	0
LINC01184	14	OK	1.6694246	1.0467379	1.5948830	0.11076	0.80806426	subgrp_con	count	1	0
SLC12A2	36	OK	0.0103223	0.5443975	0.0189636	0.98487	0.00317344	subgrp_con	count	1	0
ISOC1	1	OK	18.7594894	83.6251792	0.2243282	0.8225	0.00028789	subgrp_con	count	1	0
HINT1	727	OK	-0.256779	0.098523	-2.6062857	0.00915	-0.36626980	subgrp_con	count	1	0
LYRM7	7	OK	1.010366	1.260543	0.8015325	0.42282	0.31288283	subgrp_con	count	1	0
CDC42SE2	16	OK	-0.269199	0.9567424	-0.2813712	0.77842	-0.13426903	subgrp_con	count	1	0
RAPGEF6	1	OK	-15.498043	42.951091	-0.36082	0.71822	-0.00073756	subgrp_con	count	1	0
FNIP1	14	OK	0.1362295	0.8079760	0.1687295	0.86601	0.11340082	subgrp_con	count	1	0
P4HA2	6	OK	3.4016237	2.6808857	1.2688422	0.2049	0.9636701	subgrp_con	count	1	0
PDLIM4	88	OK	1.7510830	0.387317	4.5210554	6.15E-06	2.17159392	subgrp_con	count	0.0616981	0
SLC22A4	3	OK	18.7594894	83.6251792	0.2243282	0.8225	0.00028789	subgrp_con	count	1	0
SLC22A5	1	OK	18.7594894	83.6251792	0.2243282	0.8225	0.00028789	subgrp_con	count	1	0
AC116366.1	4	OK	-0.605635	1.1833363	-0.5118036	0.60879	-0.00037816	subgrp_con	count	1	0
KIF3A	9	OK	-0.031285	1.0587072	-0.0295508	0.97642	-0.01581500	subgrp_con	count	1	0
8-Sep	4	OK	0.6128592	2.3301820	0.2630091	0.79254	5.79E-05	subgrp_con	count	1	0
UQCRQ	262	OK	0.8394516	0.1839499	4.5634782	5.03E-06	1.16674681	subgrp_con	count	0.0504941	0
AFF4	63	OK	0.3378139	0.3789223	0.8915124	0.37265	0.40499033	subgrp_con	count	1	0
ZCCHC10	27	OK	-0.038296	0.5730866	-0.0668242	0.94672	-0.03171752	subgrp_con	count	1	0
HSPA4	23	OK	0.6125008	0.6906580	0.8868365	0.37517	0.31330463	subgrp_con	count	1	0
C5orf15	9	OK	0.4432217	0.9153548	0.4842076	0.62826	0.39738678	subgrp_con	count	1	0
VDAC1	113	OK	0.7159526	0.2956722	2.4214460	0.01546	0.92186409	subgrp_con	count	1	0
TCF7	2	OK	41.285797	66.292999	0.6227776	0.53342	0.00100678	subgrp_con	count	1	0
SKP1	594	OK	0.1675710	0.1105812	1.5153659	0.12968	0.2383883	subgrp_con	count	1	0
PPP2CA	34	OK	-0.778290	0.4730661	-1.6452045	0.09992	-0.77080367	subgrp_con	count	1	0
CDKL3	1	OK	-10.442278	41.4658099	-0.2518286	0.80117	-0.00045289	subgrp_con	count	1	0
UBE2B	294	OK	-0.02229	0.1614098	-0.1380959	0.89016	-0.03106148	subgrp_con	count	1	0
CDKN2AIPNL	10	OK	2.4446888	1.2475543	1.9595850	0.05004	1.77438501	subgrp_con	count	1	0
JADE2	2	OK	-11.448548	41.761485	-0.2741412	0.78398	-0.00049246	subgrp_con	count	1	0
SAR1B	29	OK	1.2595412	0.5988374	2.1033107	0.03544	1.33486152	subgrp_con	count	1	0
SEC24A	6	OK	1.6575270	1.457682	1.1370974	0.2555	0.80310915	subgrp_con	count	1	0
CAMLG	139	OK	-0.017449	0.2367426	-0.0736682	0.94127	-0.02292623	subgrp_con	count	1	0
DDX46	164	OK	-0.428713	0.2260123	-1.8968586	0.05785	-0.57696974	subgrp_con	count	1	0
C5orf24	54	OK	0.3392974	0.4300547	0.788963	0.43012	0.40681677	subgrp_con	count	1	0
TXNDC15	37	OK	0.987005	0.5184162	1.9038856	0.05692	1.09980354	subgrp_con	count	1	0
PCBD2	3	OK	1.2533065	1.5409862	0.8133145	0.41604	0.62624666	subgrp_con	count	1	0
H2AFY	76	OK	-0.196248	0.3528114	-0.5562416	0.57805	-0.23828038	subgrp_con	count	1	0
CXCL14	1	OK	1.582381	1.2168202	1.3004230	0.19346	1.25554822	subgrp_con	count	1	0
SMAD5	49	OK	-0.612252	0.4487828	-1.3642518	0.17249	-0.6867978	subgrp_con	count	1	0
KLHL3	10	OK	-0.373370	1.0363189	-0.360285	0.71863	-0.23548914	subgrp_con	count	1	0
HNRNPA0	183	OK	0.0649061	0.210805	0.3078965	0.75816	0.08732875	subgrp_con	count	1	0
AC106791.1	1	OK	-11.019418	43.2499352	-0.2547846	0.79889	-1.17482461	subgrp_con	count	1	0
FAM13B	7	OK	0.8411792	1.4044478	0.5989395	0.54921	0.0005738	subgrp_con	count	1	0
NME5	5	OK	-0.105387	1.465562	-0.0719094	0.94267	-0.08681449	subgrp_con	count	1	0
BRD8	18	OK	2.0650418	0.8903617	2.3193289	0.02038	1.88071060	subgrp_con	count	1	0
CDC23	4	OK	0.0506299	1.8128827	0.0279278	0.97772	0.03279048	subgrp_con	count	1	0
FAM53C	4	OK	-2.251343	2.141646	-1.0512209	0.29316	-1.08348305	subgrp_con	count	1	0
KDM3B	8	OK	-0.163702	0.9894241	-0.1654525	0.86859	-0.1347680	subgrp_con	count	1	0
EGR1	454	OK	-0.204854	0.1195807	-1.7129609	0.08672	-0.29122315	subgrp_con	count	1	0
ETF1	28	OK	0.3790704	0.5817797	0.651570	0.51468	0.3401018	subgrp_con	count	1	0
HSPA9	55	OK	0.5279624	0.3927490	1.3442742	0.17886	0.60297879	subgrp_con	count	1	0
CTNNA1	125	OK	0.4177872	0.2679954	1.5589341	0.11901	0.55316474	subgrp_con	count	1	0
SIL1	18	OK	1.2420609	0.8686148	1.4299328	0.15274	1.20164128	subgrp_con	count	1	0
PAIP2	147	OK	0.2489819	0.2410331	1.0329779	0.30161	0.33421129	subgrp_con	count	1	0
SPATA24	1	OK	-10.442278	41.4658099	-0.2518286	0.80117	-0.00045289	subgrp_con	count	1	0
DNAJC18	6	OK	-2.593606	1.7016757	-1.5241487	0.12747	-0.95569460	subgrp_con	count	1	0
ECSCR	666	OK	-0.111406	0.1028265	-1.083445	0.27861	-0.15870410	subgrp_con	count	1	0
TMEM173	162	OK	-0.609646	0.2208550	-2.760392	0.00577	-0.81967260	subgrp_con	count	1	0
UBE2D2	243	OK	0.021921	0.1798553	0.1218812	0.90299	0.03042926	subgrp_con	count	1	0
CXXC5	35	OK	1.7799370	0.615381	2.8924108	0.00382	1.82926665	subgrp_con	count	1	0
PURA	97	OK	-0.181806	0.2949336	-0.6164316	0.53761	-0.23749147	subgrp_con	count	1	0
CYSTM1	233	OK	-0.399824	0.1803629	-2.2167768	0.02664	-0.55401104	subgrp_con	count	1	0
PFDN1	101	OK	-0.293807	0.2980253	-0.985848	0.32422	-0.36971248	subgrp_con	count	1	0
HBEGF	72	OK	0.0547351	0.3350408	0.1633689	0.87022	0.07102815	subgrp_con	count	1	0
SRA1	50	OK	0.4568084	0.4360955	1.0474961	0.29487	0.54207356	subgrp_con	count	1	0
SLC35A4	2	OK	-10.442278	41.4658099	-0.2518286	0.80117	-0.00045289	subgrp_con	count	1	0
CD14	50	OK	-0.871724	0.3999576	-2.1795427	0.02929	-0.99492091	subgrp_con	count	1	0
NDUFA2	135	OK	0.2739265	0.2489715	1.1002321	0.27122	0.36716767	subgrp_con	count	1	0
TMCO6	1	OK	-11.019418	43.2499352	-0.2547846	0.79889	-1.17482461	subgrp_con	count	1	0
IK	185	OK	-0.051823	0.1951249	-0.2655915	0.79055	-0.07072437	subgrp_con	count	1	0
WDR55	4	OK	-0.841640	1.4608231	-0.5761411	0.56452	-0.24034852	subgrp_con	count	1	0
HARS	24	OK	0.0221456	0.6287553	0.0352213	0.9719	0.02265021	subgrp_con	count	1	0
HARS2	3	OK	2.1611158	2.3677943	0.9127126	0.36139	0.61359306	subgrp_con	count	1	0
ZMAT2	45	OK	0.0418659	0.4526402	0.0924929	0.92631	0.04522323	subgrp_con	count	1	0
PCDHB2	1	OK	23.991896	84.899941	0.2825902	0.77749	1.65081709	subgrp_con	count	1	0
PCDHB4	8	OK	0.4189281	0.9819488	0.4266292	0.66965	0.27516072	subgrp_con	count	1	0
PCDHB6	1	OK	-10.442278	41.4658099	-0.2518286	0.80117	-0.00045289	subgrp_con	count	1	0
PCDHB16	3	OK	21.642059	54.486891	0.3971975	0.69122	1.57744735	subgrp_con	count	1	0
PCDHB9	1	OK	18.7594894	83.6251792	0.2243282	0.8225	0.00028789	subgrp_con	count	1	0
PCDHB10	2	OK	-10.442278	41.4658099	-0.2518286	0.80117	-0.00045289	subgrp_con	count	1	0
PCDHB11	2	OK	-10.442278	41.4658099	-0.2518286	0.80117	-0.00045289	subgrp_con	count	1	0
PCDHB13	1	OK	-10.442278	41.4658099	-0.2518286	0.80117	-0.00045289	subgrp_con	count	1	0
PCDHB15	2	OK	-15.995031	70.349421	-0.2273655	0.82014	-0.00028474	subgrp_con	count	1	0
TAF7	84	OK	-0.547369	0.293922	-1.8622905	0.06256	-0.66821956	subgrp_con	count	1	0
PCDHGA8	1	OK	-10.442278	41.4658099	-0.2518286	0.80117	-0.00045289	subgrp_con	count	1	0
PCDHGA9	2	OK	-13.04562	27.674761	-0.4713905	0.63736	-1.3178643	subgrp_con	count	1	0
PCDHGA10	1	OK	22.971512	51.731593	0.444051	0.65701	2.38236892	subgrp_con	count	1	0
PCDHGB7	2	OK	1.5822999	2.7200188	0.5817606	0.56072	0.47433761	subgrp_con	count	1	0
PCDHGA12	2	OK	-0.349548	1.3110379	-0.2666190	0.78976	-0.25782434	subgrp_con	count	1	0
DIAPH1	26	OK	-0.049340	0.5856782	-0.0842452	0.93286	-0.04860946	subgrp_con	count	1	0
HDAC3	12	OK	0.8899544	1.0063706	0.8843207	0.37652	0.57506753	subgrp_con	count	1	0
ARAP3	9	OK	1.0138128	1.3748066	0.737422	0.46087	0.00188658	subgrp_con	count	1	0
PCDH1	14	OK	-0.653998	0.9971983	-0.6558356	0.51192	-0.40543068	subgrp_con	count	1	0
DELE1	18	OK	-2.380835	0.9463339	-2.5158514	0.01187	-1.3834480	subgrp_con	count	1	0
PCDH12	17	OK	0.5614955	0.9467579	0.5930713	0.55313	0.42329724	subgrp_con	count	1	0
RNF14	3	OK	4.6613299	5.7683860	0.8080821	0.41906	0.00274918	subgrp_con	count	1	0
GNPDA1	14	OK	2.1436197	0.9914065	2.1622004	0.0306	1.61151221	subgrp_con	count	1	0
NDFIP1	129	OK	0.2434450	0.2564889	0.9491444	0.34255	0.32421507	subgrp_con	count	1	0
SPRY4	10	OK	-0.371408	1.2493673	-0.297277	0.76626	-0.11115247	subgrp_con	count	1	0
ARHGAP26	30	OK	-0.493263	0.519290	-0.949881	0.34217	-0.49762525	subgrp_con	count	1	0
NR3C1	102	OK	0.5346942	0.2821586	1.8950126	0.05809	0.69785797	subgrp_con	count	1	0
YIPF5	71	OK	0.3445961	0.3319680	1.0380400	0.29925	0.42362048	subgrp_con	count	1	0
PRELID2	2	OK	0.5887819	1.7674148	0.3331317	0.73902	0.30122869	subgrp_con	count	1	0
SH3RF2	1	OK	1.5822999	2.7200188	0.5817606	0.56072	0.47433761	subgrp_con	count	1	0
LARS	73	OK	-0.960614	0.3656010	-2.6274928	0.0086	-1.22430300	subgrp_con	count	1	0
RBM27	1	OK	-15.995031	70.349421	-0.2273655	0.82014	-0.00028474	subgrp_con	count	1	0
TCERG1	27	OK	0.031084	0.5634546	0.0551671	0.95601	0.03358069	subgrp_con	count	1	0
DPYSL3	54	OK	0.6983138	0.4912424	1.4215258	0.15516	0.83614437	subgrp_con	count	1	0
SPINK5	2	OK	18.7594894	83.6251792	0.2243282	0.8225	0.00028789	subgrp_con	count	1	0
FBXO38	4	OK	20.069832	50.652192	0.396228	0.69196	0.00081457	subgrp_con	count	1	0
ADRB2	36	OK	1.2500085	0.5364123	2.3303129	0.01979	0.79369908	subgrp_con	count	1	0
ABLIM3	5	OK	-0.920753	1.1212962	-0.8211507	0.41156	-0.26051363	subgrp_con	count	1	0
AFAP1L1	21	OK	-0.364466	0.7327035	-0.497427	0.61889	-0.33861786	subgrp_con	count	1	0
GRPEL2	5	OK	3.4051463	2.6022549	1.3085367	0.19069	0.91619239	subgrp_con	count	1	0
CARMN	3	OK	4.6613299	5.7683860	0.8080821	0.41906	0.00274918	subgrp_con	count	1	0
CSNK1A1	366	OK	0.355386	0.1482556	2.3971051	0.01652	0.49713154	subgrp_con	count	1	0
ARHGEF37	3	OK	0.329536	1.4055480	0.2344472	0.81464	0.10244709	subgrp_con	count	1	0
SLC26A2	9	OK	2.4226821	1.2967951	1.8682072	0.06173	1.62925574	subgrp_con	count	1	0
HMGXB3	19	OK	2.5008865	1.2373565	2.0211527	0.04326	0.00461474	subgrp_con	count	1	0
PDGFRB	2	OK	18.7594894	83.6251792	0.2243282	0.8225	0.00028789	subgrp_con	count	1	0
TCOF1	1	OK	-10.442278	41.4658099	-0.2518286	0.80117	-0.00045289	subgrp_con	count	1	0

CD74	798	OK	-0.2365805	0.0873656	-2.707936	0.00677	-0.33849210	subgrp_con	count		1	0
RPS14	884	OK	-0.099204	0.0831844	-1.1925804	0.23302	-0.14200413	subgrp_con	count		1	0
NDST1	3	OK	-0.2427562	2.0774687	-0.1168519	0.90698	-0.12394189	subgrp_con	count		1	0
RBM22	71	OK	-0.7332426	0.3551626	-2.0645265	0.03897	-0.88483209	subgrp_con	count		1	0
DCTN4	1	OK	18.7594894	83.625179	0.2243282	0.8225	0.00028789	subgrp_con	count		1	0
GPX3	21	OK	2.0456693	0.765750	2.6714581	0.00755	1.21069706	subgrp_con	count		1	0
TNIP1	26	OK	-0.1032655	0.5905067	-0.174876	0.86118	-0.09716857	subgrp_con	count		1	0
ANXA6	12	OK	-0.6451309	0.7820293	-0.8249446	0.4094	-0.18821781	subgrp_con	count		1	0
CCDC69	62	OK	-1.520522	0.4216426	-3.606189	0.00031	-1.88758530	subgrp_con	count		1	0
GM2A	1	OK	1.5822999	2.7200188	0.5817606	0.56072	0.47433761	subgrp_con	count		1	0
SPARC	305	OK	0.3276257	0.1676256	1.9545090	0.05064	0.45118953	subgrp_con	count		1	0
ATOX1	207	OK	-0.2207738	0.2026384	-1.0894962	0.27594	-0.29835016	subgrp_con	count		1	0
G3BP1	74	OK	-0.2621427	0.3613130	-0.7255279	0.46812	-0.32991912	subgrp_con	count		1	0
FAM114A2	6	OK	0.1432295	1.1085806	0.1292008	0.8972	0.07289541	subgrp_con	count		1	0
MFAP3	2	OK	-1.2956167	2.041425	-0.6346627	0.52565	-0.00075754	subgrp_con	count		1	0
GALNT10	5	OK	2.0176498	2.2120878	0.9121020	0.36171	0.00137857	subgrp_con	count		1	0
SAP30L	16	OK	1.2629468	0.9485266	1.3314826	0.18302	0.80092225	subgrp_con	count		1	0
LARP1	24	OK	0.4400457	0.6389633	0.6886868	0.49102	0.3330103	subgrp_con	count		1	0
FAXDC2	13	OK	0.7985400	0.7660807	1.0423706	0.29726	0.75837747	subgrp_con	count		1	0
CNOT8	11	OK	0.9622198	0.9971456	0.9649741	0.33458	0.62001722	subgrp_con	count		1	0
GEMIN5	4	OK	-1.2956167	2.041425	-0.6346627	0.52565	-0.00075754	subgrp_con	count		1	0
MRPL22	52	OK	0.6794624	0.4217498	1.6110556	0.10717	0.81263843	subgrp_con	count		1	0
SGCD	4	OK	2.1611158	2.3677943	0.9127126	0.36139	0.61359306	subgrp_con	count		1	0
MED7	4	OK	-1.0891069	1.4086192	-0.7731734	0.43942	-0.64590266	subgrp_con	count		1	0
CYFIP2	1	OK	23.991896	84.899941	0.2825902	0.77749	1.65081709	subgrp_con	count		1	0
ADAM19	1	OK	1.5822999	2.7200188	0.5817606	0.56072	0.47433761	subgrp_con	count		1	0
THG1L	6	OK	-0.828539	1.3183665	-0.628458	0.5297	-0.69818302	subgrp_con	count		1	0
CLINT1	66	OK	0.501805	0.3583946	1.4001477	0.16147	0.60131655	subgrp_con	count		1	0
EBF1	10	OK	-2.419858	1.136520	-2.129181	0.03324	-1.57349158	subgrp_con	count		1	0
RNF145	20	OK	1.0096211	0.8266010	1.2214128	0.22192	0.89846954	subgrp_con	count		1	0
UBLCP1	10	OK	-0.363981	0.9371073	-0.388409	0.69771	-0.23073456	subgrp_con	count		1	0
TTC1	81	OK	-0.224239	0.3075537	-0.729106	0.46594	-0.28733489	subgrp_con	count		1	0
PWWP2A	10	OK	0.5328431	0.9836562	0.5416965	0.58803	0.27993304	subgrp_con	count		1	0
C1QTNF2	3	OK	-0.1747967	1.820980	-0.095990	0.92352	-0.05309252	subgrp_con	count		1	0
SLU7	58	OK	-0.039785	0.3639469	-0.1093157	0.91295	-0.04971149	subgrp_con	count		1	0
PTTG1	1	OK	-15.99503	70.34942	-0.227365	0.82014	-0.00028474	subgrp_con	count		1	0
CCNG1	25	OK	0.4632251	0.7399577	0.6260157	0.5312	0.51983952	subgrp_con	count		1	0
NUDCD2	53	OK	1.0633455	0.4379853	2.4278107	0.01519	1.28438471	subgrp_con	count		1	0
MAT2B	9	OK	-0.018566	0.9649125	-0.019241	0.98465	-0.01656001	subgrp_con	count		1	0
RARS	13	OK	-0.32488	0.8146223	-0.398811	0.69003	-0.20646961	subgrp_con	count		1	0
PANK3	8	OK	3.3837495	2.512641	1.3466902	0.17808	0.0007883	subgrp_con	count		1	0
SLIT3	4	OK	20.916475	23.252354	0.6290223	0.52932	2.28603486	subgrp_con	count		1	0
SPDL1	2	OK	4.6613299	5.7683860	0.8080821	0.41904	0.00274918	subgrp_con	count		1	0
C5orf58	1	OK	23.991896	84.899941	0.2825902	0.77749	1.65081709	subgrp_con	count		1	0
KCNMB1	2	OK	26.367955	84.916325	0.3105168	0.75617	2.49052421	subgrp_con	count		1	0
NPM1	712	OK	-0.253176	0.1000351	-2.530874	0.01138	-0.36088438	subgrp_con	count		1	0
FGF18	61	OK	5.9509157	1.5141876	3.9301045	8.49E-05	4.18756908	subgrp_con	count		0.8425514	0
FBXW11	22	OK	1.4011426	0.6802402	2.0597761	0.03942	1.39487076	subgrp_con	count		1	0
STK10	2	OK	5.8049273	5.6694662	1.023892	0.30589	1.03285716	subgrp_con	count		1	0
UBTD2	7	OK	2.0080883	1.4564896	1.3787178	0.16798	1.19254312	subgrp_con	count		1	0
SH3PXD2B	3	OK	3.4598146	6.7714513	0.5109413	0.60939	0.00030179	subgrp_con	count		1	0
NEURL1B	26	OK	-1.640369	0.6296426	-2.605238	0.00918	-1.56276740	subgrp_con	count		1	0
DUSP1	726	OK	-0.105130	0.0966621	-1.087609	0.27677	-0.14990146	subgrp_con	count		1	0
ERGIC1	15	OK	2.3374011	1.1569231	2.0203599	0.04335	1.05266271	subgrp_con	count		1	0
RPL26L1	80	OK	0.551034	0.3538783	1.5571298	0.11944	0.67631761	subgrp_con	count		1	0
ATP6V0E1	495	OK	0.2368506	0.1239285	1.9111872	0.05598	0.33575727	subgrp_con	count		1	0
CREBRF	55	OK	-0.024713	0.3752880	-0.065851	0.9475	-0.02774320	subgrp_con	count		1	0
BNIP1	2	OK	-10.44227	41.465809	-0.251828	0.80117	-0.00045289	subgrp_con	count		1	0
STC2	4	OK	-14.04288	27.411700	-0.512295	0.60844	-0.00174737	subgrp_con	count		1	0
BOD1	49	OK	0.3138935	0.4297031	0.7304893	0.46509	0.37628656	subgrp_con	count		1	0
CPEB4	48	OK	0.8522243	0.4882993	1.7452908	0.08092	0.90312905	subgrp_con	count		1	0
SFXN1	6	OK	1.4522319	1.3125850	1.1063907	0.26856	0.90869407	subgrp_con	count		1	0
THOC3	5	OK	1.2268690	1.3580315	0.9034172	0.3662	0.77980226	subgrp_con	count		1	0
KIAA1191	12	OK	0.8549650	0.9495584	0.9003817	0.36792	0.26593385	subgrp_con	count		1	0
NOP16	17	OK	-0.003594	0.8411639	-0.004273	0.99659	-0.00269373	subgrp_con	count		1	0
HIGD2A	224	OK	-0.18928	0.1896489	-0.998602	0.31799	-0.26237298	subgrp_con	count		1	0
CLTB	204	OK	1.0303308	0.231222	4.4560069	8.35E-06	1.38631476	subgrp_con	count		0.0836940	0
FAF2	22	OK	-0.733934	0.6544376	-1.121473	0.26209	-0.70151206	subgrp_con	count		1	0
RNF44	2	OK	0.1681690	1.7253287	0.0974707	0.92235	0.05201879	subgrp_con	count		1	0
TSPAN17	3	OK	-0.910223	2.1881125	-0.415985	0.67742	-0.25785735	subgrp_con	count		1	0
UIMC1	9	OK	-0.478563	0.9682791	-0.494241	0.62114	-0.23500933	subgrp_con	count		1	0
NSD1	40	OK	0.2903174	0.5082444	0.5712161	0.56785	0.31569408	subgrp_con	count		1	0
PRELID1	277	OK	0.1634286	0.1732479	0.9433224	0.34552	0.22802690	subgrp_con	count		1	0
LMAN2	152	OK	0.3030601	0.2414473	1.2551810	0.20941	0.40430869	subgrp_con	count		1	0
RGS14	3	OK	-1.698643	1.9355446	-0.877605	0.38016	-1.09785319	subgrp_con	count		1	0
F12	1	OK	3.4598146	6.7714513	0.5109413	0.60939	0.00030179	subgrp_con	count		1	0
GRK6	2	OK	21.105322	83.341273	0.2532397	0.80008	0.0003103	subgrp_con	count		1	0
PRR7	5	OK	-1.496695	1.5631792	-0.957469	0.33832	-0.00085699	subgrp_con	count		1	0
DBN1	32	OK	1.4545345	0.6588615	2.2076482	0.02727	1.25502282	subgrp_con	count		1	0
PDLIM7	50	OK	1.4181755	0.571693	2.4806588	0.01311	1.409218	subgrp_con	count		1	0
DDX41	5	OK	0.1255948	1.4500075	0.086616	0.93098	0.03878308	subgrp_con	count		1	0
TMED9	170	OK	1.0573352	0.234305	4.5126273	6.4E-06	1.43859870	subgrp_con	count		0.0641959	0
B4GALT7	4	OK	18.7594894	83.625179	0.2243282	0.8225	0.00028789	subgrp_con	count		1	0
N4BP3	2	OK	3.4598146	6.7714513	0.5109413	0.60939	0.00030179	subgrp_con	count		1	0
RMND5B	2	OK	26.367955	84.916325	0.3105168	0.75617	2.49052421	subgrp_con	count		1	0
NHP2	185	OK	-0.116591	0.2079903	-0.560561	0.5751	-0.1602266	subgrp_con	count		1	0
HNRNPAB	176	OK	0.0306009	0.2240725	0.1365673	0.89137	0.0413220	subgrp_con	count		1	0
PHYKPL	16	OK	3.7148582	1.8120450	2.050091	0.04036	1.38311791	subgrp_con	count		1	0
COL23A1	6	OK	18.7594894	83.625179	0.2243282	0.8225	0.00028789	subgrp_con	count		1	0
CLK4	4	OK	-0.841640	1.4608231	-0.576141	0.56452	-0.24034852	subgrp_con	count		1	0
ZNF354A	3	OK	-0.907228	1.6441847	-0.551780	0.5811	-0.52553220	subgrp_con	count		1	0
ZNF354B	3	OK	-11.19992	27.762997	-0.403412	0.68665	-1.82940850	subgrp_con	count		1	0
ZNF354C	1	OK	3.4598146	6.7714513	0.5109413	0.60939	0.00030179	subgrp_con	count		1	0
RUFY1	12	OK	1.1285698	1.0603432	1.0643438	0.28717	0.72141834	subgrp_con	count		1	0
HNRNPH1	275	OK	-0.462090	0.1597552	-2.892203	0.00382	-0.64152294	subgrp_con	count		1	0
CANX	280	OK	0.6682969	0.1721429	3.8828008	0.0001	0.92378362	subgrp_con	count		1	0
MGAT4B	45	OK	1.218297	0.5313858	2.2926359	0.02187	1.32251186	subgrp_con	count		1	0
SQSTM1	441	OK	-0.0591887	0.1285622	-0.460389	0.64524	-0.08357493	subgrp_con	count		1	0
MRNIP	5	OK	0.1084560	1.0696415	0.1013947	0.91924	0.03346609	subgrp_con	count		1	0
TBC1D9B	5	OK	5.6831241	5.3331875	1.0656148	0.2866	0.00307912	subgrp_con	count		1	0
RNF130	52	OK	0.7622363	0.4088524	1.8643311	0.06228	0.92635905	subgrp_con	count		1	0
MAPK9	5	OK	1.3360870	1.3423686	0.9953206	0.31958	0.84317478	subgrp_con	count		1	0
CNOT6	3	OK	5.0027563	4.2286829	1.1830530	0.23679	0.98115823	subgrp_con	count		1	0
SCGB3A1	3	OK	-0.634889	2.0089600	-0.316027	0.75198	-0.000145	subgrp_con	count		1	0
FLT4	3	OK	0.6532528	2.5001284	0.2612877	0.79387	0.00016297	subgrp_con	count		1	0
MGAT1	76	OK	0.0884152	0.3372501	0.262165	0.79319	0.10589928	subgrp_con	count		1	0
ZFP62	3	OK	-1.537212	1.6487135	-0.932370	0.35115	-0.40044109	subgrp_con	count		1	0
BTNL9	11	OK	0.0950428	0.8944540	0.1062579	0.91538	0.00017006	subgrp_con	count		1	0
TRIM41	1	OK	0.6532528	2.5001284	0.2612877	0.79387	0.00016297	subgrp_con	count		1	0
RACK1	870	OK	-0.100397	0.0849516	-1.181815	0.23728	-0.14367762	subgrp_con	count		1	0
TRIM52	4	OK	-0.841532	1.4608505	-0.576056	0.56458	-0.24032510	subgrp_con	count		1	0
DUSP22	18	OK	-0.730594	0.7508156	-0.973068	0.33052	-0.21057446	subgrp_con	count		1	0
EXOC2	2	OK	5.8049273	5.6694662	1.023892	0.30589	1.03285716	subgrp_con	count		1	0
AL357054.4	5	OK	2.4096254	1.7474468	1.3789406	0.16791	1.07550689	subgrp_con	count		1	0
FOXC1	254	OK	0.6046716	0.1699305	3.5583449	0.00037	0.84231868	subgrp_con	count		1	0
GMDS-DT	2	OK	21.105322	83.341273	0.2532397	0.80008	0.0003103	subgrp_con	count		1	0
WRNIP1	6	OK	3.2756645	2.2671140	1.4448609	0.1485	0.80917957	subgrp_con	count		1	0
SERPINB1	298	OK	0.1575660	0.1510580	1.0430829	0.29691	0.22088314	subgrp_con	count		1	0
SERPINB9P1	3	OK	-0.590592	1.845458	-0.320024	0.74895	-0.36470820	subgrp_con	count		1	0
SERPINB9	22	OK	1.2735293	0.6568649	1.9387993	0.05252	1.03269048	subgrp_con	count		1	0
SERPINB6	280	OK	-0.123780	0.1678727	-0.737346	0.46091	-0.17130183	subgrp_con	count		1	0
NQO2	13	OK	-0.163767	0.9450840	-0.173283	0.86242	-0.08221776	subgrp_con	count		1	0
RIPK1	13	OK	-2.543104	1.1097784	-2.291542	0.02193	-1.22320467	subgrp_con	count		1	0
BPHL	2	OK	1.925866	2.5154879	0.7656425	0.44389	0.56022119	subgrp_con	count		1	0
TUBB2A	23	OK	0.8590617	0.8232689	1.0434764	0.29672	0.26718393	subgrp_con	count		1	0
PSMG4	33	OK	0.5026675	0.5530126	0.9089621	0.36337	0.45051117	subgrp_con	count		1	0
FAM50B	4	OK	0.1681593	1.2198591	0.1378514	0.89036	0.08564646	subgrp_con	count		1	0
PRPF4B	181	OK	-0.119658	0.1950999	-0.613318	0.53967	-0.16483537	subgrp_con	count		1	0
ECI2	67	OK	-0.476650	0.3517797	-1.354968	0.17542	-0.60828256	subgrp_con	count		1	0
CDYL	6	OK	0.9064719	1.293807	0.7006233	0.48354	0.43726195	subgrp_con	count		1	0
RPP40	4	OK	-0.910142	2.1881243	-0.415946	0.67745	-0.25784050	subgrp_con	count		1	0
LYRM4	8	OK	3.3995765	2.0417980	1.6649915	0.09591	1.32474321	subgrp_con	count		1	0
FARS2	2	OK	1.925866	2.5154879	0.7656425	0.44389	0.56022119	subgrp_con	count		1	0
RREB1	4	OK	1.1364931	1.8169267	0.6255029	0.53164	0.35017300	subgrp_con	count		1	0
SSR1	45	OK	0.5805774	0.4855649	1.1956740	0.23182	0.69776144	subgrp_con	count		1	0
RIOK1	24	OK	-0.247277	0.5675392	-0.435700	0.66305	-0.25115827	subgrp_con	count		1	0
DSP	4	OK	-11.18472	26.296787	-0.425326	0.6706	-2.27083089	subgrp_con	count		1	0
SNRNP48	9	OK	1.5157683	1.3318027	1.1381327	0.25507	1.10726701	subgrp_con	count		1	0
BMP6	47	OK	23.315088	19.347466	1.205071	0.22818	4.45553270	subgrp_con	count		1	0
BLOC1S5	20	OK	-0.157081	0.6524040	-0.240773	0.80972	-0.15999288	subgrp_con	count		1	0
EEF1E1	35	OK	0.3374765	0.553429	0.6097916	0.542	0.3653692	subgrp_con	count		1	0
SLC35B3	4	OK	-0.910223	2.1881125	-0.415985	0.67742	-0.25785735	subgrp_con	count		1	0
TFAP2A	6	OK	2.7999747	1.4786808	1.893562	0.05828	1.94140532	subgrp_con	count		1	0
GCNT2	7	OK	-0.36547	1.1590985	-0.315311	0.75252	-0.1094311	subgrp_con	count		1	0
PAK1IP1	35	OK	-0.274409	0.4997051	-0.549143	0.58291	-0.31143917	subgrp_con	count		1	0
TMEM14C	345	OK	-0.147231	0.1510400	-0.974782	0.32967	-0.20698704	subgrp_con	count		1	0
TMEM14B	169	OK	-0.260000	0.2217597	-1.172441	0.24102	-0.35441279	subgrp_con	count		1	0
SMIM13	2	OK	-0.910223	2.1881125	-0.415985	0.67742	-0.25785735	subgrp_con	count		1	0
NEDD9	117	OK	-0.016430	0.2741397	-0.059935	0.95221	-0.01901012	subgrp_con	count		1	0
HIVEP1	13	OK	0.5668603	0.9930589	0.5708224	0.56812	0.42896347	subgrp_con	count		1	0
PHACTR1	5	OK	0.6393470	1.4406245	0.4437985	0.65719	0.00043313	subgrp_con	count		1	0
TBC1D7	6	OK	1.6656161	1.427239	1.1670194	0.2432	0.80646122	subgrp_con	count		1	0
GFOD1	36	OK	-0.078743	0.466954	-0.168632	0.86609	-0.07417534	subgrp_con	count		1	0
SIRT5	6	OK	-1.322208	1.2976654	-1.018912	0.30824	-0.58149545	subgrp_con	count		1	0
NOL7	277	OK	-0.000136	0.1700687	-0.000802	0.99936	-0.00019006	subgrp_con	count		1	0
RANBP9	12	OK	0.7241977	1.0437127	0.6938669	0.48777	0.22574304	subgrp_con	count		1	0
MCUR1	57	OK	-0.879779	0.3792517	-2.319776	0.02035	-1.12834349	subgrp_con	count		1	0
CD83	14	OK	-1.456565	0.8331442	-1.748275	0.08042	-1.36508183	subgrp_con	count		1	0
JARID2	1	OK	3.4598146	6.7714513	0.5109413	0.60939	0.00030179	subgrp_con	count		1	0
DTNBP1	20	OK	0.082216	0.6495858	0.1265671	0.89928	0.07350687	subgrp_con	count		1	0
MYLIP	18	OK	0.2364246	0.6934764	0.3409267	0.73316	0.07330920	subgrp_con	count		1	0
GMPR	12	OK	2.0557680	1.0358853	1.9845517	0.04719	1.6835588	subgrp_con	count		1	0
ATXN1	7	OK	1.7779881	1.5612795	1.1388019	0.25479	0.0033153	subgrp_con	count		1	0
FAM8A1	7	OK	-0.523218	1.4597376	-0.358433	0.72002	-0.37039835	subgrp_con	count		1	0
NUP153	7	OK	-1.731287	1.1641332	-1.487190	0.13696	-0.00262919	subgrp_con	count		1	0
AL138724.1	3	OK	-1.353265	1.7824963	-0.759196	0.44772	-0.34509379	subgrp_con	count		1	0
KIF13A	19	OK	3.546752	1.3887942	2.5538360	0.01065	2.39763852	subgrp_con	count		1	0
TPMT	2	OK	18.7594894	83.625179	0.2243282	0.8225	0.00028789	subgrp_con	count		1	0
DEK	325	OK	-0.318476	0.1553027	-2.050680	0.0403	-0.44571675	subgrp_con	count		1	0
RNF144B	3	OK	5.8049273	5.6694662	1.023892	0.30589	1.03285716	subgrp_con	count		1	0
ID4	13	OK	2.9562824	1.1426693	2.5871722	0.00968	2.53538704	subgrp_con	count		1	0
E2F3	6	OK	0.6191907	1.1154500	0.5551039	0.57882	0.31669928	subgrp_con	count		1	0
CDKAL1	3	OK	4.6613299	5.7683860	0.8080821	0.41904	0.00274918	subgrp_con	count		1	0
SOX4	264	OK	0.669167	0.1790869	3.7365509	0.00019	0.91229430	subgrp_con	count		1	0
CASC15	11	OK	-0.769949	1.171340	-0.657322	0.51097	-0.22166315	subgrp_con	count		1	0
MRS2	4	OK	0.1721614	1.534450	0.1121974	0.91067	0.00011395	subgrp_con	count		1	0
ALDH5A1	1	OK	-10.44227	41.465809	-0.251828	0.80117	-0.00045289	subgrp_con	count		1	0
TDP2	2	OK	0.1681491	1.7253300	0.097459	0.92236	0.05201277	subgrp_con	count		1	0
ACOT13	25	OK	-0.342965	0.649340	-0.528175	0.59738	-0.31993425	subgrp_con	count		1	0
C6orf62	51	OK	0.1026015	0.3991633	0.2570415	0.79715	0.10507721	subgrp_con	count		1	0
GMNN	11	OK	-0.157901	0.9452366	-0.167050	0.86732	-0.07929933	subgrp_con	count		1	0
RIPOR2	4	OK	-2.068972	1.6710595	-1.238120	0.21567	-0.48734237	subgrp_con	count		1	0
CARMIL1	13	OK	2.2937022	1.1987841	1.9133572	0.0557	1.31783005	subgrp_con	count		1	0
TRIM38	40	OK	0.2348315	0.4291217	0.547237	0.58422	0.24082158	subgrp_con	count		1	0
HIST1H1A	2	OK	1.925866	2.5154879	0.7656425	0.44389	0.56022119	subgrp_con	count		1	0
HIST1H1C	11	OK	0.1926354	0.8542700	0.2254971	0.82159	0.14506374	subgrp_con	count		1	0
HFE	1	OK	-15.99503	70.34942	-0.227365	0.82014	-0.00028474	subgrp_con	count		1	0
HIST1H4C	190	OK	0.0282356	0.2050239	0.1382065	0.89008	0.03893641	subgrp_con	count		1	0
HIST1H2AC	10	OK	-0.861297	0.9361761	-0.920016	0.35756	-0.67888900	subgrp_con	count		1	0
HIST1H1E	16	OK	-0.282921	0.8186475	-0.345596	0.72965	-0.2767554	subgrp_con	count		1	0
HIST1H4E	1	OK	-11.019418	43.249935	-0.254784	0.79889	-1.17482461	subgrp_con	count		1	0
HIST1H1D	1	OK	3.4598146	6.7714513	0.5109413	0.60939	0.00030179	subgrp_con	count		1	0
BTN2A2	7	OK	-0.164974	1.0645653	-0.154968	0.87685	-0.0002899	subgrp_con	count		1	0
BTN3A1	4	OK	-1.754582	1.6323569	-1.074876	0.28242	-0.95331468	subgrp_con	count		1	0
BTN3A3	5	OK	-11.32529	26.916785	-0.420752	0.67394	-1.19509663	subgrp_con	count		1	0
BTN2A1	5	OK	6.4385882	6.8672152	0.9375835	0.34846	0.00043628	subgrp_con	count		1	0
HCG11	8	OK	-1.531011	1.0701549	-1.430644	0.15252	-0.83274665	subgrp_con	count		1	0
HMGN4	1	OK	-10.44227	41.465809	-0.251828	0.80117	-0.00045289	subgrp_con	count		1	0
ABT1	13	OK	1.7967311	1.0197706	1.7618973	0.07809	1.39931706	subgrp_con	count		1	0
ZNF322	16	OK	-1.193043	0.8822768	-1.352232	0.1762	-0.70636946	subgrp_con	count		1	0
ZNF391	1	OK	18.7594894	83.625179	0.2243282	0.8225	0.00028789	subgrp_con	count		1	0
ZSCAN16-AS1	8	OK	0.3912848	1.1553860	0.3386615	0.73486	0.20015046	subgrp_con	count		1	0
NKAPL	5	OK	-11.65026	26.331208	-0.442450	0.65816	-2.6568200	subgrp_con	count		1	0
ZSCAN26	2	OK	-10.47563	27.491987	-0.381043	0.70317	-1.13811785	subgrp_con	count		1	0
ZSCAN31	3	OK	-0.357207	1.5148489	-0.235803	0.81359	-0.22654173	subgrp_con	count		1	0
TRIM27	15	OK	0.5002911	0.769042	0.6506676	0.51526	0.4484790	subgrp_con	count		1	0
GABBR1	4	OK	-1.295616	1.4442412	-0.897091	0.36967	-0.00075720	subgrp_con	count		1	0
HLA-A	859	OK	-0.230138	0.0855871	-2.688936	0.00717	-0.32917154	subgrp_con	count		1	0
ZNRD1	32	OK	-0.153831	0.5425593	-0.283528	0.77677	-0.17226247	subgrp_con	count		1	0
PPP1R11	37	OK	0.2475056	0.4861200	0.5091451	0.61065	0.27835193	subgrp_con	count		1	0
TRIM26	2	OK	-12.14570	43.418869	-0.279733	0.77968	-1.91338276	subgrp_con	count		1	0
HCG18	5	OK	-0.841640	1.4608231	-0.576141	0.56452	-0.24034852	subgrp_con	count		1	0
HLA-E	856	OK	-0.182653	0.0866873	-2.107038	0.03511	-0.26116966	subgrp_con	count		1	0
GNL1	39	OK	-0.376310	0.4649500	-0.809356	0.41832	-0.42576631	subgrp_con	count		1	0
ABCF1	53	OK	0.6938461	0.4167958	1.6647147	0.09597	0.84378164	subgrp_con	count		1	0
PPP1R10	192	OK	-0.337352	0.1967271	-1.714825	0.08638	-0.45138479	subgrp_con	count		1	0
MRPS18B	55	OK	-0.986236	0.417932	-2.359796	0.01829	-1.15632159	subgrp_con	count		1	0
ATAT1	3	OK	3.050298	2.4404393	1.2498972	0.21134	1.24934625	subgrp_con	count		1	0
C6orf136	3	OK	4.6613299	5.7683860	0.8080821	0.41904	0.00274918	subgrp_con	count		1	0
DHX16	6	OK	-2.725433	1.8829980	-1.447390	0.14779	-0.58041525	subgrp_con	count		1	0
PPP1R18	41	OK	-0.050105	0.4871512	-0.102853	0.91808	-0.05404434	subgrp_con	count		1	0
NRM	3	OK	-1.646225	1.9351922	-0.850677	0.39495	-0.7036061	subgrp_con	count		1	0
TUBB	471	OK	-0.365964	0.1253876	-2.918665	0.00352	-0.51732038	subgrp_con	count		1	0
FLOT1	258	OK	-0.420593	0.1751552	-2.401263	0.01634	-0.58057188	subgrp_con	count		1	0
IER3	487	OK	0.1019406	0.1175601	0.8671362	0.38587	0.14499357	subgrp_con	count		1	0
HCG21	1	OK	3.4598146	6.7714513	0.5109413	0.60939	0.00030179	subgrp_con	count		1	0
PSORS1C1	3	OK	32.638224	45.170896	0.7225498	0.46996	2.73354919	subgrp_con	count		1	0
CCHCR1	1	OK	23.991896	84.899941	0.2825902	0.77749	1.65081709	subgrp_con	count		1	0
AL662844.4	1	OK	-11.019418	43.249935	-0.254784	0.79889	-1.17482461	subgrp_con	count		1	0
HLA-C	836	OK	-0.203374	0.089306	-2.277275	0.02277	-0.29062750	subgrp_con	count		1	0
HLA-B	864	OK	-0.217669	0.0852957	-2.551937	0.01071	-0.31139460	subgrp_con	count		1	0
MICA	13	OK	-0.394926	0.9026670	-0.437511	0.66174	-0.34947261	subgrp_con	count		1	0
DDX39B	14	OK	-1.142829	0.9175848	-1.245475	0.21296	-0.7873299	subgrp_con	count		1	0
NFKBIL1	11	OK	1.3895652	1.1881883	1.1694822	0.24221	1.01155523	subgrp_con	count		1	0
LTB	1	OK	3.4598146	6.7714513	0.5109413	0.60939	0.00030179	subgrp_con	count		1	0
AIF1	1	OK	-15.99503	70.34942	-0.227365	0.82014	-0.00028474	subgrp_con	count		1	0
PRRC2A	8	OK	1.0501293	1.0078014	1.0420001	0.29741	0.32472266	subgrp_con	count		1	0
BAG6	15	OK	0.9328958	0.8601147	1.0846179	0.27809	0.83815471	subgrp_con	count		1	0
C6orf47	5	OK	3.1002558	2.3039102	1.3456496	0.17842	1.26089264	subgrp_con	count		1	0
GPANK1	12	OK	-1.290756	0.859841	-1.501156	0.13332	-0.8742376	subgrp_con	count		1	0
CSNK2B	222	OK	0.0808511	0.1836639	0.4402124	0.65978	0.11254005	subgrp_con	count		1	0
LY6G5C	1	OK	23.991896	84.899941	0.2825902	0.77749	1.65081709	subgrp_con	count		1	0
DDAH2	157	OK	0.3692337	0.233339	1.5823911	0.11356	0.49561187	subgrp_con	count		1	0
CLIC1	607	OK	-0.254620	0.1113907	-2.285834	0.02226	-0.362118	subgrp_con	count		1	0
VARS	2	OK	3.4598146	6.7714513	0.5109413	0.60939	0.00030179	subgrp_con	count		1	0
LSM2.00	195	OK	-0.487152	0.1968054	-2.475297	0.01331	-0.67518687	subgrp_con	count		1	0
HSPA1A	441	OK	0.3896764	0.1264865	3.0807747	0.00206	0.55050164	subgrp_con	count		1	0
HSPA1B	287	OK	0.7358343	0.1636351	4.4967987	6.9E-06	1.02460796	subgrp_con	count		0.0691566	0
C6orf48	80	OK	1.1520142	0.3467085	3.3227167	0.00089	1.36353251	subgrp_con	count		1	0
NEU1	21	OK	0.8243427	0.7942833	1.0378447	0.29934	0.73422738	subgrp_con	count		1	0
EHMT2	5	OK	-1.693796	1.4305819	-1.183991	0.23642	-0.00096024	subgrp_con	count		1	0
CFB	1	OK	3.4598146	6.7714513	0.5109413	0.60939	0.00030179	subgrp_con	count		1	0
NELFE	45	OK	-1.196827	0.4652098	-2.572662	0.01009	-1.34209874	subgrp_con	count		1	0
DXO	5	OK	1.4119426	1.4959308	0.9438555	0.34526	0.69797651	subgrp_con	count		1	0
STK19	16	OK	-0.045967	0.6417597	-0.071627	0.9429	-0.04957544	subgrp_con	count		1	0
TNXB	53	OK	0.6466131	0.4341056	1.4895294	0.13635	0.66527885	subgrp_con	count		1	0
ATF6B	11	OK	0.2279322	0.8743943	0.2606745	0.79434	0.11763694	subgrp_con	count		1	0
FKBPL	16	OK	-0.890347	0.7494200	-1.188049	0.23481	-0.42007301	subgrp_con	count		1	0
AL662884.4	1	OK	-11.019418	43.249935	-0.254784	0.79889	-1.17482461	subgrp_con	count		1	0
PPT2	3	OK	4.6613299	5.7683860	0.8080821	0.41904	0.00274918	subgrp_con	count		1	0
AGPAT1	17	OK	1.2730905	0.9872589	1.2895191	0.19722	0.38956606	subgrp_con	count		1	0
RNF5	36	OK	0.1621796	0.4860139	0.3336934	0.73861	0.17116961	subgrp_con	count		1	0
PBX2	4	OK	5.8049273	5.6694662	1.023892	0.30589	1.03285716	subgrp_con	count		1	0
GPSM3	5	OK	0.2914024	1.4447553	0.2016967	0.84015	0.18962305	subgrp_con	count		1	0
NOTCH4	24	OK	0.6807051	0.6716690	1.0134532	0.31084	0.44240510	subgrp_con	count		1	0
HLA-DRB1	796	OK	-0.385540	0.0904047	-4.264606	2E-05	-0.55111162	subgrp_con	count		0.2001347	0
HLA-DQA2	24	OK	-1.758573	0.6801866	-2.585428	0.00972	-0.00267815	subgrp_con	count		1	0
TAP2	13	OK	-1.266352	0.7922813	-1.598362	0.10996	-0.86107909	subgrp_con	count		1	0
PSMB8-AS1	11	OK	-2.043114	1.0430441	-1.958799	0.05014	-1.06322085	subgrp_con	count		1	0
TAP1	54	OK	-1.434709	0.4205774	-3.411284	0.00065	-1.53330819	subgrp_con	count		1	0
HLA-DMB	37	OK	-1.392713	0.4829929	-2.883507	0.00393	-1.6555670	subgrp_con	count		1	0
BRD2	112	OK	0.313460	0.2662089	1.1774977	0.239	0.41152840	subgrp_con	count		1	0
HLA-DOA	13	OK	-1.837439	0.8574537	-2.142901	0.03212	-1.43103926	subgrp_con	count		1	0
SLC39A7	49	OK	1.4926099	0.4856693	3.0733049	0.00212	1.68239410	subgrp_con	count		1	0
HSD17B8	6	OK	1.007862	1.1696602	0.8616712	0.38887	0.51014631	subgrp_con	count		1	0
RING1	9	OK	1.8883003	1.2298349	1.5354095	0.12468	1.13578327	subgrp_con	count		1	0
VPS52	3	OK	-0.615396	1.492102	-0.412435	0.68002	-0.29859809	subgrp_con	count		1	0
RPS18	879	OK	-0.109086	0.0844869	-1.291167	0.19665	-0.15611225	subgrp_con	count		1	0
B3GALT4	1	OK	41.788203	174.29608	0.2397549	0.81052	0.00014488	subgrp_con	count		1	0
WDR46	9	OK	0.7007926	1.0507865	0.666922	0.50482	0.21849695	subgrp_con	count		1	0
PFDN6	16	OK	0.6512328	0.8233347	0.7909697	0.42896	0.49029348	subgrp_con	count		1	0
RGL2	47	OK	-0.353756	0.4295185	-0.823611	0.41016	-0.38674586	subgrp_con	count		1	0
TAPBP	135	OK	-0.163751	0.2366794	-0.691870	0.48902	-0.21686605	subgrp_con	count		1	0
ZBTB22	2	OK	-0.150975	1.968815	-0.076682	0.93888	-9.78E-05	subgrp_con	count		1	0
DAXX	8	OK	3.6371462	2.3560678	1.5437357	0.12265	1.36949466	subgrp_con	count		1	0
PHF1	9	OK	-0.148789	0.8415739	-0.176798	0.85967	-0.11088112	subgrp_con	count		1	0
CUTA	203	OK	0.3664756	0.2035125	1.8007518	0.07174	0.50274899	subgrp_con	count		1	0
SYNGAP1	2	OK	-0.202940	1.6532365	-0.122753	0.9023	-4.82E-05	subgrp_con	count		1	0
BAK1	1	OK	-10.44227	41.465809	-0.251828	0.80117	-0.00045289	subgrp_con	count		1	0
ITPR3	9	OK	4.3801518	3.436822	1.2744400	0.20251	0.00263449	subgrp_con	count		1	0
UQCC2	43	OK	1.6574593	0.589592	2.8111833	0.00494	1.56113367	subgrp_con	count		1	0
LEMD2	2	OK	-1.031165	1.7479538	-0.589927	0.55524	-0.00022765	subgrp_con	count		1	0
HMGA1	8	OK	-0.74881	1.1063965	-0.676804	0.49852	-0.21428950	subgrp_con	count		1	0
SMIM29	19	OK	0.422327	0.7724751	0.5467195	0.58457	0.43411527	subgrp_con	count		1	0
NUDT3	25	OK	-0.343191	0.6471607	-0.530303	0.5959	-0.31979603	subgrp_con	count		1	0
RPS10	427	OK	0.0259927	0.1380302	0.1883120	0.85062	0.0364712	subgrp_con	count		1	0
C6orf106	8	OK	1.3315811	1.0413296	1.2787316	0.20099	0.84060079	subgrp_con	count		1	0
AL451165.2	2	OK	0.5887819	1.767414	0.3331317	0.73903	0.30122869	subgrp_con	count		1	0
SNRPC	81	OK	0.0407667	0.3259291	0.1250786	0.90046	0.05174162	subgrp_con	count		1	0
UHRF1BP1	2	OK	-0.202940	1.6532365	-0.122753	0.9022	-4.82E-05	subgrp_con	count		1	0
TAF11	29	OK	0.0737519	0.5347609	0.1379157	0.89031	0.06971595	subgrp_con	count		1	0
ANKS1A	8	OK	-3.485885	1.6670733	-2.091020	0.03652	-1.88583834	subgrp_con	count		1	0
SCUBE3	3	OK	2.9849400	2.5988857	1.1485460	0.25074	1.23382860	subgrp_con	count		1	0
ZNF76	3	OK	-0.725185	1.8079191	-0.401116	0.68832	-0.20979917	subgrp_con	count		1	0
RPL10A	850	OK	-0.252219	0.0884832	-2.850477	0.00437	-0.36052563	subgrp_con	count		1	0
FKBP5	74	OK	1.402997	0.3732910	3.7584545	0.00017	1.02034806	subgrp_con	count		1	0
SRPK1	35	OK	-0.035834	0.5376584	-0.066649	0.94686	-0.0394908	subgrp_con	count		1	0
MAPK14	5	OK	1.2304790	1.2904328	0.9535397	0.34032	0.61570362	subgrp_con	count		1	0
ETV7	1	OK	-10.44227	41.465809	-0.251828	0.80117	-0.00045289	subgrp_con	count		1	0
KCTD20	23	OK	1.2363248	0.8413061	1.4695302	0.14169	0.90920501	subgrp_con	count		1	0
STK38	4	OK	2.5552227	2.349844	1.0874007	0.27686	0.69313892	subgrp_con	count		1	0
SRSF3	305	OK	0.0504646	0.1566644	0.3221195	0.74736	0.07013804	subgrp_con	count		1	0
CDKN1A	209	OK	-0.850088	0.1908272	-4.454756	8.4E-06	-1.168089	subgrp_con	count		0.0841647	0
CPNE5	4	OK	2.9849400	2.5988857	1.1485460	0.25074	1.23382860	subgrp_con	count		1	0
PPIL1	1	OK	18.7594894	83.625179	0.2243282	0.8225	0.00028789	subgrp_con	count		1	0
C6orf89	31	OK	0.2327108	0.508415	0.4577181	0.64715	0.25722552	subgrp_con	count		1	0
PI16	21	OK	4.6358895	2.5568829	1.813102	0.06982	1.51358985	subgrp_con	count		1	0

MTCH1	195	OK	0.27036609	0.20063499	1.347552	0.1779	0.36687906	subgrp_cor	count		1	0
PIM1	13	OK	-0.8687473	0.821504	-1.0575082	0.29028	-0.52771002	subgrp_cor	count		1	0
TBC1D22B	1	OK	-11.019418	43.2499352	-0.2547846	0.79889	-1.17482461	subgrp_cor	count		1	0
RNF8	20	OK	1.05696957	0.80619386	1.3110613	0.18984	0.93229898	subgrp_cor	count		1	0
CMTR1	4	OK	-11.019418	43.2499352	-0.2547846	0.79889	-1.17482461	subgrp_cor	count		1	0
CCDC167	16	OK	3.5571363	1.7140817	2.0752431	0.03796	1.35498690	subgrp_cor	count		1	0
MDGA1	2	OK	21.1053223	83.341273	0.2532397	0.80008	0.0003103	subgrp_cor	count		1	0
ZFAND3	10	OK	1.4412185	1.0658198	1.3522159	0.17631	0.43645732	subgrp_cor	count		1	0
GLO1	54	OK	1.0422911	0.4207856	2.4770123	0.01325	1.23759577	subgrp_cor	count		1	0
SAYSD1	6	OK	1.1517809	1.4373287	0.8013344	0.42294	0.35462896	subgrp_cor	count		1	0
OARD1	21	OK	1.1821325	0.7985953	1.4802647	0.1388	0.87215129	subgrp_cor	count		1	0
MDFI	6	OK	0.9819801	1.2318749	0.7971427	0.42537	0.63221222	subgrp_cor	count		1	0
TFEB	6	OK	-1.722465	1.3369256	-1.2883777	0.19761	-0.00261489	subgrp_cor	count		1	0
CCND3	59	OK	-0.8632703	0.3600972	-2.3972256	0.01652	-1.06528607	subgrp_cor	count		1	0
TAF8	5	OK	1.1224686	1.4569290	0.7704346	0.44106	0.34606345	subgrp_cor	count		1	0
MRPS10	39	OK	0.4421714	0.5120062	0.8636055	0.3879	0.46837478	subgrp_cor	count		1	0
UBR2	16	OK	1.0517343	0.8835973	1.1902869	0.23392	0.67993641	subgrp_cor	count		1	0
TBCC	7	OK	-0.1081063	1.1457882	-0.094351	0.92482	-0.06942081	subgrp_cor	count		1	0
RPL7L1	100	OK	0.0831395	0.2970803	0.2798554	0.77959	0.10600479	subgrp_cor	count		1	0
C6orf226	5	OK	2.9849402	1.8374842	1.6244712	0.10428	1.81651269	subgrp_cor	count		1	0
CNPY3	30	OK	0.8210013	0.6371748	1.2885025	0.19757	0.8077280	subgrp_cor	count		1	0
PEX6	3	OK	-2.040668	2.1086684	-0.967752	0.33317	-0.47266997	subgrp_cor	count		1	0
MEA1	56	OK	0.9713473	0.3944221	2.4627097	0.01379	1.21461341	subgrp_cor	count		1	0
KLHDC3	3	OK	-1.2956167	2.0414257	-0.6346627	0.52565	-0.00075754	subgrp_cor	count		1	0
RRP36	23	OK	0.5475104	0.6734177	0.8130324	0.4162	0.54155181	subgrp_cor	count		1	0
MRPL2	18	OK	-0.926069	0.7070469	-1.3097703	0.19027	-0.78451853	subgrp_cor	count		1	0
PTK7	1	OK	-12.148421	43.455309	-0.2795612	0.77981	-1.91362624	subgrp_cor	count		1	0
SRF	1	OK	-10.442278	41.4658099	-0.2518286	0.80117	-0.00045289	subgrp_cor	count		1	0
DNPH1	16	OK	1.4017626	0.7780546	1.8016249	0.0716	1.44121492	subgrp_cor	count		1	0
ABCC10	1	OK	3.4598146	6.7714513	0.5109413	0.60939	0.00030179	subgrp_cor	count		1	0
TJAP1	1	OK	3.4598146	6.7714513	0.5109413	0.60939	0.00030179	subgrp_cor	count		1	0
POLR1C	5	OK	0.6851084	1.4216744	0.4819024	0.62988	0.0012628	subgrp_cor	count		1	0
YIPF3	129	OK	0.3324566	0.2548935	1.3042957	0.19217	0.45113018	subgrp_cor	count		1	0
XPO5	1	OK	18.7594889	83.6251792	0.2243282	0.8225	0.00028789	subgrp_cor	count		1	0
POLH	1	OK	-15.99503	70.34942	-0.2273655	0.82014	-0.00028474	subgrp_cor	count		1	0
MAD2L1BP	3	OK	3.4598146	6.7714513	0.5109413	0.60939	0.00030179	subgrp_cor	count		1	0
MRPS18A	14	OK	1.4231740	0.8796375	1.6179096	0.10568	1.30255101	subgrp_cor	count		1	0
VEGFA	3	OK	-1.2956167	2.0414257	-0.6346627	0.52565	-0.00075754	subgrp_cor	count		1	0
MRPL14	60	OK	0.8780490	0.4203906	2.0886506	0.03674	1.07305813	subgrp_cor	count		1	0
SLC29A1	49	OK	-0.2507445	0.4157513	-0.6031116	0.54642	-0.28882866	subgrp_cor	count		1	0
HSP90AB1	845	OK	-0.2294201	0.0875021	-2.6218792	0.00874	-0.32792936	subgrp_cor	count		1	0
SLC35B2	2	OK	-12.148421	43.455309	-0.2795612	0.77981	-1.91362624	subgrp_cor	count		1	0
NFKBIE	6	OK	0.7689927	1.5299032	0.5026414	0.61522	0.23956880	subgrp_cor	count		1	0
CDC5L	67	OK	0.2105173	0.3618121	0.5818415	0.56067	0.25666748	subgrp_cor	count		1	0
SUPT3H	1	OK	3.4598146	6.7714513	0.5109413	0.60939	0.00030179	subgrp_cor	count		1	0
CLIC5	11	OK	0.8288893	1.0279103	0.8063828	0.42002	0.25796544	subgrp_cor	count		1	0
ENPP4	8	OK	0.4172160	1.2213267	0.3416907	0.73258	0.00076344	subgrp_cor	count		1	0
RCAN2	23	OK	-0.004648	0.7374982	-0.0063038	0.99497	-0.00300265	subgrp_cor	count		1	0
ADGRF5	75	OK	-0.1973443	0.3573195	-0.5522906	0.58075	-0.23763530	subgrp_cor	count		1	0
TNFRSF21	9	OK	20.0499960	32.8203073	0.6107158	0.54139	1.52874421	subgrp_cor	count		1	0
CD2AP	55	OK	0.6955957	0.4085304	1.7026764	0.08863	0.85858024	subgrp_cor	count		1	0
PTCHD4	2	OK	3.4598149	4.7858686	0.7229230	0.46972	0.00081935	subgrp_cor	count		1	0
MUT	3	OK	19.1030323	50.7724696	0.3762478	0.70672	0.00078880	subgrp_cor	count		1	0
CENPQ	1	OK	-11.019418	43.2499352	-0.2547846	0.79889	-1.17482461	subgrp_cor	count		1	0
C6orf141	8	OK	-1.4768749	1.0529638	-1.4025885	0.16074	-1.09110057	subgrp_cor	count		1	0
MCM3	15	OK	-0.5297930	0.8514721	-0.6222093	0.5338	-0.33183954	subgrp_cor	count		1	0
EFHC1	7	OK	0.4495331	1.0943930	0.4107601	0.68125	0.14007308	subgrp_cor	count		1	0
TRAM2-AS1	5	OK	-12.01282	26.372569	-0.455504	0.64875	-2.97079701	subgrp_cor	count		1	0
TMEM14A	21	OK	0.5990117	0.7074930	0.8466679	0.39719	0.6618874	subgrp_cor	count		1	0
GSTA4	9	OK	0.7280654	1.4056971	0.5179390	0.6045	0.00134439	subgrp_cor	count		1	0
ICK	3	OK	3.4598149	4.7858686	0.7229230	0.46972	0.00081935	subgrp_cor	count		1	0
FBXO9	37	OK	1.0980111	0.5104531	2.1510516	0.03147	1.27330565	subgrp_cor	count		1	0
ELOVL5	19	OK	0.6137995	0.7691807	0.7979913	0.42488	0.39936274	subgrp_cor	count		1	0
LRRC1	16	OK	-2.142165	0.8516970	-2.5151727	0.0119	-1.45349375	subgrp_cor	count		1	0
MLIP	2	OK	0.5887819	1.767414	0.3331317	0.73903	0.30122869	subgrp_cor	count		1	0
HMGCLL1	2	OK	21.642059	54.4868919	0.3971975	0.69122	1.57744735	subgrp_cor	count		1	0
BMP5	1	OK	18.7594889	83.6251792	0.2243282	0.8225	0.00028789	subgrp_cor	count		1	0
COL21A1	16	OK	2.9821596	1.5725801	1.8963482	0.05792	0.76610794	subgrp_cor	count		1	0
DST	82	OK	1.5673757	0.3817986	4.1052416	4.04E-05	1.98309507	subgrp_cor	count		0.40225771	0
KIAA1586	16	OK	0.2442310	0.6897993	0.3540614	0.72329	0.2418202	subgrp_cor	count		1	0
ZNF451	17	OK	-0.4718470	0.7986354	-0.5908176	0.55464	-0.13929818	subgrp_cor	count		1	0
PRIM2	3	OK	-0.5453205	1.7085504	-0.3191715	0.7496	-0.0003423	subgrp_cor	count		1	0
PTP4A1	9	OK	1.6656161	1.4272294	1.1670294	0.2432	0.80646122	subgrp_cor	count		1	0
PHF3	95	OK	0.1530339	0.2951035	0.5185770	0.60400	0.19742583	subgrp_cor	count		1	0
LMBRD1	23	OK	-0.6709742	0.6030512	-1.1126321	0.26587	-0.61446679	subgrp_cor	count		1	0
FAM135A	1	OK	18.7594889	83.6251792	0.2243282	0.8225	0.00028789	subgrp_cor	count		1	0
SDHAF4	1	OK	-11.019418	43.2499352	-0.2547846	0.79889	-1.17482461	subgrp_cor	count		1	0
SMAP1	53	OK	-0.1258086	0.4155990	-0.3027162	0.76211	-0.15291092	subgrp_cor	count		1	0
OGFRL1	3	OK	-2.426	1.9736479	-1.2342627	0.21711	-0.00126331	subgrp_cor	count		1	0
DDX43	2	OK	-13.04562	27.67476	-0.4713905	0.63736	-1.3178643	subgrp_cor	count		1	0
CGAS	10	OK	-1.3298136	1.0920233	-1.2269092	0.21986	-1.00599002	subgrp_cor	count		1	0
MTO1	2	OK	-10.475634	27.4919879	-0.3810432	0.70317	-1.13811785	subgrp_cor	count		1	0
EEF1A1	893	OK	0.0025503	0.0818227	0.0311688	0.97513	0.00365204	subgrp_cor	count		1	0
SLC17A5	4	OK	-10.967865	27.1536587	-0.4039185	0.68627	-1.17125107	subgrp_cor	count		1	0
CD109	43	OK	-1.1059848	0.5013613	-2.2059636	0.02739	-1.10318656	subgrp_cor	count		1	0
COL12A1	6	OK	1.9803390	1.4600074	1.3563897	0.17498	1.40877414	subgrp_cor	count		1	0
COX7A2	428	OK	0.4950533	0.1374022	3.6029483	0.00031	0.69861122	subgrp_cor	count		1	0
TMEM30A	85	OK	0.9259959	0.3223335	2.8727883	0.00407	1.12196643	subgrp_cor	count		1	0
FILIP1	117	OK	1.1237472	0.2859786	3.9294789	8.51E-05	1.50825864	subgrp_cor	count		0.84456107	0
SENP6	53	OK	0.5025232	0.4206042	1.1947649	0.23219	0.62461577	subgrp_cor	count		1	0
MYO6	37	OK	0.0300508	0.5197970	0.0578129	0.9539	0.02254302	subgrp_cor	count		1	0
IRAK1BP1	4	OK	-1.1257225	1.7984653	-0.6259353	0.53136	-0.58135011	subgrp_cor	count		1	0
PHIP	79	OK	0.8687822	0.3305137	2.6285808	0.00857	0.91191139	subgrp_cor	count		1	0
HMGN3	356	OK	-0.0942639	0.1437938	-0.6555487	0.51211	-0.1326309	subgrp_cor	count		1	0
HMGN3-AS1	2	OK	-1.7585735	2.3554439	-0.7465998	0.45532	-0.00036089	subgrp_cor	count		1	0
LCA5	11	OK	-0.714472	0.8227837	-0.8683584	0.3852	-0.51335258	subgrp_cor	count		1	0
BCKDHB	5	OK	-12.029447	17.823513	-0.6749201	0.49972	-2.35325944	subgrp_cor	count		1	0
TENT5A	47	OK	2.6232450	0.6030884	4.3496855	1.36E-05	2.24823779	subgrp_cor	count		0.13640103	0
IBTK	10	OK	1.1899035	1.0256166	1.1601825	0.24597	0.59681527	subgrp_cor	count		1	0
TPBG	22	OK	1.1781254	0.7436895	1.5841629	0.11316	1.20574337	subgrp_cor	count		1	0
DOPEY1	4	OK	2.8951588	2.4132736	1.199681	0.23026	0.752269	subgrp_cor	count		1	0
PGM3	13	OK	1.2807818	0.9088946	1.4091642	0.15879	0.93927539	subgrp_cor	count		1	0
RIPPLY2	1	OK	-11.019418	43.2499352	-0.2547846	0.79889	-1.17482461	subgrp_cor	count		1	0
CYB5R4	4	OK	-1.6462456	1.9351929	-0.8506881	0.39494	-0.70360391	subgrp_cor	count		1	0
MRAP2	2	OK	26.3679560	84.9163259	0.3105168	0.75617	2.49052421	subgrp_cor	count		1	0
CEP162	6	OK	-0.4559601	1.2662142	-0.3600971	0.71877	-0.22431830	subgrp_cor	count		1	0
NT5E	28	OK	2.9349272	0.8232111	3.565218	0.00036	2.94931158	subgrp_cor	count		1	0
SNX14	13	OK	1.3846664	1.0935188	1.2660655	0.20549	0.68578934	subgrp_cor	count		1	0
SYNCRIP	126	OK	0.0763410	0.2473673	0.3086142	0.75762	0.10105780	subgrp_cor	count		1	0
AL139274.2	3	OK	-0.214986	1.5648174	-0.1373872	0.89072	-0.06511836	subgrp_cor	count		1	0
ZNF292	29	OK	0.5899218	0.6166738	0.9566189	0.33876	0.49313396	subgrp_cor	count		1	0
SMIM8	2	OK	3.4598146	6.7714513	0.5109413	0.60939	0.00030179	subgrp_cor	count		1	0
SLC35A1	3	OK	0.8788174	1.6469311	0.5336091	0.59361	0.44699547	subgrp_cor	count		1	0
RARS2	16	OK	-0.2278645	0.7620295	-0.2986312	0.76522	-0.20180474	subgrp_cor	count		1	0
ORC3	7	OK	0.6191907	1.1154500	0.5551039	0.57882	0.31669928	subgrp_cor	count		1	0
AKIRIN2	40	OK	-1.064132	0.4966689	-2.142538	0.03215	-1.06686922	subgrp_cor	count		1	0
CNR1	2	OK	18.7594889	83.6251792	0.2243282	0.8225	0.00028789	subgrp_cor	count		1	0
RNGTT	2	OK	1.0504293	2.0348410	0.5162217	0.6057	0.47406546	subgrp_cor	count		1	0
PNRC1	699	OK	-0.2249082	0.0989121	-2.2738174	0.02298	-0.32083323	subgrp_cor	count		1	0
UBE2J1	68	OK	0.1853903	0.3888670	0.4767602	0.63352	0.20862382	subgrp_cor	count		1	0
LYRM2	53	OK	0.6193104	0.4043624	1.5315725	0.12562	0.79237911	subgrp_cor	count		1	0
MDN1	6	OK	-1.5836592	1.6674753	-0.9497347	0.34225	-0.43611199	subgrp_cor	count		1	0
CASP8AP2	18	OK	0.3908626	0.6789442	0.5756917	0.56482	0.40102483	subgrp_cor	count		1	0
MAP3K7	4	OK	0.1681690	1.7253287	0.0974707	0.92235	0.05201879	subgrp_cor	count		1	0
MANEA	5	OK	-0.4944902	1.4409495	-0.3431697	0.73147	-0.14639957	subgrp_cor	count		1	0
UFL1	38	OK	0.3197772	0.4523159	0.7069777	0.47958	0.3754514	subgrp_cor	count		1	0
NDUFAF4	16	OK	-0.6535846	0.777776	-0.8403224	0.40072	-0.56484570	subgrp_cor	count		1	0
FBXL4	3	OK	0.1721614	1.534450	0.1121974	0.91067	0.00011395	subgrp_cor	count		1	0
PNISR	193	OK	-0.0483624	0.1957934	-0.247007	0.8049	-0.06594684	subgrp_cor	count		1	0
USP45	2	OK	-1.2956167	2.0414257	-0.6346627	0.52565	-0.00075754	subgrp_cor	count		1	0
CCNC	19	OK	0.6859788	0.7140713	0.9606587	0.33672	0.3505531	subgrp_cor	count		1	0
ASCC3	14	OK	1.3533620	1.0213144	1.3251178	0.18513	0.85305481	subgrp_cor	count		1	0
HACE1	3	OK	-0.214986	1.5648174	-0.1373872	0.89072	-0.06511836	subgrp_cor	count		1	0
ATG5	17	OK	0.1188813	0.7429702	0.1600082	0.87287	0.09885377	subgrp_cor	count		1	0
PRDM1	8	OK	0.9631393	1.3030450	0.7391450	0.45982	0.29876821	subgrp_cor	count		1	0
CRYBG1	6	OK	-1.7177116	1.3707138	-1.2531512	0.21015	-0.88431591	subgrp_cor	count		1	0
QRSL1	4	OK	1.4118376	1.4959328	0.9437840	0.34528	0.69790486	subgrp_cor	count		1	0
CD24	1	OK	1.5823999	2.7200188	0.5817606	0.56072	0.47433761	subgrp_cor	count		1	0
C6orf203	2	OK	1.1112446	1.8685808	0.5946999	0.55204	0.34276981	subgrp_cor	count		1	0
PDSS2	1	OK	1.5823999	2.7200188	0.5817606	0.56072	0.47433761	subgrp_cor	count		1	0
SOBP	5	OK	3.3648273	2.5447810	1.3222446	0.18609	1.31777762	subgrp_cor	count		1	0
SEC63	54	OK	0.9800666	0.4793985	2.0443670	0.04092	0.95917612	subgrp_cor	count		1	0
AL024507.2	1	OK	18.7594889	83.6251792	0.2243282	0.8225	0.00028789	subgrp_cor	count		1	0
OSTM1	15	OK	0.5685781	1.1156641	0.5096319	0.61032	0.36926581	subgrp_cor	count		1	0
SNX3	484	OK	-0.2389078	0.1268604	-1.8832325	0.05967	-0.33830052	subgrp_cor	count		1	0
FOXO3	24	OK	0.0775959	0.5497630	0.1411440	0.88776	0.06446883	subgrp_cor	count		1	0
SESN1	3	OK	21.642059	54.4868919	0.3971975	0.69122	1.57744735	subgrp_cor	count		1	0
CEP57L1	6	OK	0.9780701	1.5523686	0.6296441	0.52893	0.29428065	subgrp_cor	count		1	0
CD164	77	OK	1.8815187	0.4512255	4.1697967	3.05E-05	2.17753851	subgrp_cor	count		0.30395675	0
ZBTB24	7	OK	-0.0669504	1.2468371	-0.0536962	0.95718	-0.05028806	subgrp_cor	count		1	0
AK9	8	OK	0.6922667	1.1526604	0.6005816	0.54812	0.44981832	subgrp_cor	count		1	0
CDC40	34	OK	0.1585801	0.5114564	0.3100560	0.75652	0.16733182	subgrp_cor	count		1	0
CDK19	2	OK	4.6613299	5.7683860	0.8080821	0.41906	0.00274918	subgrp_cor	count		1	0
AMD1.00	98	OK	-0.6140899	0.2926328	-2.0984997	0.03586	-0.70868452	subgrp_cor	count		1	0
GTF3C6	92	OK	0.4415596	0.3229477	1.3672791	0.17154	0.57655737	subgrp_cor	count		1	0
RPF2	44	OK	-0.5629689	0.4072435	-1.3823876	0.16685	-0.66561933	subgrp_cor	count		1	0
MFSD4B	1	OK	-10.442278	41.4658099	-0.2518286	0.80117	-0.00045289	subgrp_cor	count		1	0
REV3L	46	OK	0.1918146	0.4681673	0.4097138	0.68202	0.19662832	subgrp_cor	count		1	0
TRAF3IP2-AS1	1	OK	18.7594889	83.6251792	0.2243282	0.8225	0.00028789	subgrp_cor	count		1	0
TRAF3IP2	10	OK	-1.4999259	0.8950147	-1.6758672	0.09376	-0.39285834	subgrp_cor	count		1	0
FYN	29	OK	-0.8842698	0.5826110	-1.5177705	0.12907	-0.8365308	subgrp_cor	count		1	0
TUBE1	9	OK	1.9495399	1.3799572	1.4127538	0.15772	0.91797958	subgrp_cor	count		1	0
FAM229B	16	OK	2.2207857	0.9894201	2.2445225	0.0248	1.65515836	subgrp_cor	count		1	0
LAMA4	118	OK	0.4259236	0.2809042	1.5162590	0.12945	0.56398514	subgrp_cor	count		1	0
MARCKS	252	OK	-0.620400	0.1845201	-3.3622841	0.00077	-0.85056914	subgrp_cor	count		1	0
HDAC2	31	OK	0.8947407	0.6348556	1.4093608	0.15873	0.73996509	subgrp_cor	count		1	0
NT5DC1	26	OK	-0.6221438	0.5471413	-1.1370805	0.2555	-0.71775746	subgrp_cor	count		1	0
TSPYL4	1	OK	24.3602009	51.7229675	0.4709745	0.63766	2.90627168	subgrp_cor	count		1	0
DSE	9	OK	6.4280329	5.2748401	1.2186213	0.22299	1.06669891	subgrp_cor	count		1	0
TSPYL1	35	OK	1.0922896	0.5237027	2.0857055	0.03701	1.10341102	subgrp_cor	count		1	0
TRAPPC3L	2	OK	21.642059	54.4868919	0.3971975	0.69122	1.57744735	subgrp_cor	count		1	0
CALHM5	9	OK	4.1203745	2.5258514	1.6312814	0.10282	1.44689194	subgrp_cor	count		1	0
CALHM4	2	OK	26.3679560	84.9163259	0.3105168	0.75617	2.49052421	subgrp_cor	count		1	0
RWDD1	347	OK	0.2240159	0.1507327	1.4861799	0.13722	0.31526972	subgrp_cor	count		1	0
RSPH4A	4	OK	3.4598160	3.3871656	1.0214487	0.30706	0.00081839	subgrp_cor	count		1	0
ZUP1	1	OK	-15.99503	70.34942	-0.2273655	0.82014	-0.00028474	subgrp_cor	count		1	0
KPNA5	5	OK	-0.239524	1.4877131	-0.1610015	0.87209	-0.11969813	subgrp_cor	count		1	0
DCBLD1	7	OK	-0.5828276	1.3029660	-0.4473082	0.65465	-0.00099041	subgrp_cor	count		1	0
GOPC	51	OK	0.4588960	0.391691	1.1715740	0.24137	0.56738495	subgrp_cor	count		1	0
NUS1	5	OK	-0.920753	1.1212962	-0.8211507	0.41156	-0.26051363	subgrp_cor	count		1	0
CEP85L	19	OK	-0.4227899	0.6972640	-0.6063555	0.54426	-0.31044582	subgrp_cor	count		1	0
PLN	1	OK	18.7594889	83.6251792	0.2243282	0.8225	0.00028789	subgrp_cor	count		1	0
MCM9	1	OK	-10.442278	41.4658099	-0.2518286	0.80117	-0.00045289	subgrp_cor	count		1	0
ASF1A	19	OK	1.4735583	0.7718309	1.9091725	0.05624	1.34415258	subgrp_cor	count		1	0
FAM184A	2	OK	-0.7251858	1.8079191	-0.4011162	0.68832	-0.20979917	subgrp_cor	count		1	0
MAN1A1	43	OK	-0.5528272	0.4361731	-1.267449	0.20496	-0.61145354	subgrp_cor	count		1	0
HSF2	5	OK	0.6851084	1.4216744	0.4819024	0.62988	0.0012628	subgrp_cor	count		1	0
SERINC1	141	OK	0.3826745	0.2514968	1.5215875	0.12811	0.51518929	subgrp_cor	count		1	0
PKIB	1	OK	-15.99503	70.34942	-0.2273655	0.82014	-0.00028474	subgrp_cor	count		1	0
RNF217	7	OK	-0.8227509	1.0560139	-0.7791090	0.43592	-0.23546273	subgrp_cor	count		1	0
HDDC2	14	OK	-0.9725207	0.7772744	-1.2511934	0.21086	-0.68221542	subgrp_cor	count		1	0
HEY2	29	OK	10.805209	4.0223904	2.6865979	0.00724	4.22146561	subgrp_cor	count		1	0
NCOA7	220	OK	-0.0945132	0.1823237	-0.5183814	0.60419	-0.13167920	subgrp_cor	count		1	0
HINT3	6	OK	-0.6508873	1.2753209	-0.5103714	0.60979	-0.00040481	subgrp_cor	count		1	0
TRMT11	6	OK	0.7378357	1.306749	0.5646358	0.57232	0.22995700	subgrp_cor	count		1	0
CENPW	5	OK	7.7419315	5.9792459	1.2948006	0.19539	1.71690540	subgrp_cor	count		1	0
RSPO3	33	OK	-0.5346064	0.4738536	-1.1282086	0.25923	-0.3896310	subgrp_cor	count		1	0
RNF146	9	OK	-0.0798786	1.0590647	-0.0754237	0.93988	-0.05159030	subgrp_cor	count		1	0
ECHDC1	24	OK	-0.3035914	0.6284578	-0.4830736	0.62904	-0.29671815	subgrp_cor	count		1	0
PTPRK	11	OK	0.3226585	0.9575341	0.3369681	0.73614	0.21003964	subgrp_cor	count		1	0
ARHGAP18	103	OK	1.2825825	0.3018171	4.2495354	2.14E-05	1.71076112	subgrp_cor	count		0.21402152	0
L3MBTL3	5	OK	-0.5905927	1.845458	-0.3200249	0.74895	-0.36470820	subgrp_cor	count		1	0
EPB41L2	21	OK	1.5383292	0.7223848	2.1265711	0.03346	1.52626621	subgrp_cor	count		1	0
AKAP7	8	OK	0.3295372	0.9941318	0.3314824	0.74028	0.16843740	subgrp_cor	count		1	0
MED23	5	OK	1.0371349	1.5664808	0.6620795	0.50792	0.33268875	subgrp_cor	count		1	0
LINC01013	11	OK	1.8603487	1.0458502	1.7787907	0.07527	1.666103	subgrp_cor	count		1	0
STX7	40	OK	0.7385366	0.4826762	1.5269234	0.12679	0.75442268	subgrp_cor	count		1	0
RPS12	889	OK	-0.0313129	0.0822758	-0.3805847	0.70352	-0.04483223	subgrp_cor	count		1	0
TBPL1	16	OK	-0.687068	0.748004	-0.9185346	0.35834	-0.42468619	subgrp_cor	count		1	0
SGK1	121	OK	-0.1614374	0.2830364	-0.5703768	0.56842	-0.17369999	subgrp_cor	count		1	0
AL078590.3	1	OK	3.4598146	6.7714513	0.5109413	0.60939	0.00030179	subgrp_cor	count		1	0
HBS1L	11	OK	-1.3616163	1.0437718	-1.3045152	0.19206	-0.60591219	subgrp_cor	count		1	0
AHI1	43	OK	-0.3430228	0.4304318	-0.7969271	0.42549	-0.41853981	subgrp_cor	count		1	0
LINC00271	1	OK	1.5823999	2.7200188	0.5817606	0.56072	0.47433761	subgrp_cor	count		1	0
BCLAF1	142	OK	0.0960294	0.2298625	0.4177691	0.67612	0.12827882	subgrp_cor	count		1	0
MAP7	2	OK	4.6613299	5.7683860	0.8080821	0.41906	0.00274918	subgrp_cor	count		1	0
MAP3K5	4	OK	21.1053223	83.341273	0.2532397	0.80008	0.0003103	subgrp_cor	count		1	0
PEX7	1	OK	23.9918965	84.8999418	0.2825902	0.77749	1.65081709	subgrp_cor	count		1	0
IFNGR1	183	OK	-0.0393509	0.1972880	-0.1994575	0.8419	-0.05398850	subgrp_cor	count		1	0
AL357060.1	1	OK	-10.442278	41.4658099	-0.2518286	0.80117	-0.00045289	subgrp_cor	count		1	0
TNFAIP3	58	OK	0.1246720	0.4128412	0.3019877	0.76266	0.15421646	subgrp_cor	count		1	0
ARFGEF3	3	OK	6.6626465	6.9572133	0.9576349	0.33825	0.00121744	subgrp_cor	count		1	0
HEBP2	220	OK	0.0710398	0.1960221	0.3624069	0.71705	0.09904621	subgrp_cor	count		1	0
NHSL1	3	OK	3.4598149	4.7858686	0.7229230	0.46972	0.00081935	subgrp_cor	count		1	0
CCDC28A	5	OK	2.8951588	2.4132736	1.199681	0.23026	0.752269	subgrp_cor	count		1	0
REPS1	1	OK	-11.019418	43.2499352	-0.2547846	0.79889	-1.17482461	subgrp_cor	count		1	0
ABRACL	64	OK	0.4226887	0.3908728	1.0813970	0.27952	0.54707919	subgrp_cor	count		1	0
HECA	10	OK	0.1739952	0.8354967	0.2082536	0.83503	0.11298578	subgrp_cor	count		1	0
TXLNB	1	OK	3.4597878	3.9017207	0.8867338	0.37522	0.00222136	subgrp_cor	count		1	0
CITED2	67	OK	1.3502812	0.3629917	3.7198678	0.0002	1.71885839	subgrp_cor	count		1	0
AL355596.1	12	OK	24.0442849	32.848004	0.7319861	0.46419	3.55072445	subgrp_cor	count		1	0
VTA1	15	OK	2.0681200	1.1427652	1.8097506	0.07033	0.96134452	subgrp_cor	count		1	0
ADGRG6	14	OK	-1.3456086	0.8007500	-1.6804352	0.09287	-1.00210671	subgrp_cor	count		1	0
HIVEP2	3	OK	-0.2029437	1.6532265	-0.1227534	0.9023	-4.82E-05	subgrp_cor	count		1	0
AIG1	88	OK	0.7834738	0.3105497	2.5228610	0.01164	1.03910183	subgrp_cor	count		1	0
PEX3	12	OK	0.8835992	1.1057308	0.7991087	0.42422	0.44965980	subgrp_cor	count		1	0
FUCA2	22	OK	1.2431905	0.7001274	1.7756631	0.07579	1.20266789	subgrp_cor	count		1	0
PHACTR2	215	OK	0.6123778	0.1860097	3.2921812	0.00099	0.85092283	subgrp_cor	count		1	0
LTV1	12	OK	-0.4008214	0.7962825	-0.5033657	0.61471	-0.25345732	subgrp_cor	count		1	0
PLAGL1	1	OK	-10.442278	41.4658099	-0.2518286	0.80117	-0.00045289	subgrp_cor	count		1	0
SF3B5	204	OK	0.4638164	0.2066164	2.2448188	0.02478	0.63619595	subgrp_cor	count		1	0
STX11	5	OK	-0.256601	1.3412326	-0.1913174	0.84828	-0.128091	subgrp_cor	count		1	0
UTRN	166	OK	-0.0216176	0.2345677	-0.0921580	0.92657	-0.02854401	subgrp_cor	count		1	0
AL356599.1	2	OK	-10.442278	41.4658099	-0.2518286	0.80117	-0.00045289	subgrp_cor	count		1	0
SHPRH	20	OK	0.3602495	0.7320110	0.4914647	0.6231	0.23458882	subgrp_cor	count		1	0
RAB32	44	OK	1.9324717	0.6078286	3.1793034	0.00148	2.15658044	subgrp_cor	count		1	0
STXBP5-AS1	1	OK	-15.99503	70.34942	-0.2273655	0.82014	-0.00028474	subgrp_cor	count		1	0
SAMD5	1	OK	3.4598146	6.7714513	0.5109413	0.60939	0.00030179	subgrp_cor	count		1	0
SASH1	86	OK	-0.9584852	0.3104334	-3.0875702	0.00202	-1.23714818	subgrp_cor	count		1	0
TAB2	5	OK	0.0206923	1.5208729	0.0136055	0.98914	0.01048895	subgrp_cor	count		1	0
PPIL4	77	OK	0.3547621	0.3150854	1.1259235	0.2602	0.44861367	subgrp_cor	count		1	0
GINM1	204	OK	0.3743753	0.1956059	1.9139307	0.05563	0.51427408	subgrp_cor	count		1	0
KATNA1	9	OK	-0.235295	1.0412460	-0.2259745	0.82122	-0.11761641	subgrp_cor	count		1	0
LATS1	5	OK	0.9732317	1.6704237	0.5826256	0.56015	0.00180907	subgrp_cor	count		1	0
PCMT1	89	OK	0.0502200	0.30092	0.1668860	0.86746	0.06571863	subgrp_cor	count		1	0
LRP11	8	OK	-0.362491	0.9123613	-0.3973109	0.69114	-0.17962594	subgrp_cor	count		1	0
AL355312.4	3	OK	18.7594889	83.6251792	0.2243282	0.8225	0.00028789	subgrp_cor	count		1	0
PLEKHG1	14	OK	-0.7894383	0.9932207	-0.7948066	0.42667	-0.37773658	subgrp_cor	count		1	0
MTHFD1L	9	OK	7.0747884	4.6860163	1.5097669	0.1311	1.68815612	subgrp_cor	count		1	0
AKAP12	67	OK	-1.3730176	0.3665919	-3.745357	0.00018	-1.58390468	subgrp_cor	count		1	0
ZBTB2	2	OK	-11.448548	41.76148	-0.2741412	0.78398	-0.00049246	subgrp_cor	count		1	0
RMND1	12	OK	-0.6800252	0.8227288	-0.8265485	0.40849	-0.53886375	subgrp_cor	count		1	0
ARMT1	9	OK	-0.681752	0.8872174	-0.768417	0.44224	-0.42160148	subgrp_cor	count		1	0
SYNE1	56	OK	0.1600855	0.3920160	0.4083647	0.68301	0.18010803	subgrp_cor	count		1	0
MYCT1	156	OK	-0.9620615	0.2262380	-4.2524312	2.11E-05	-1.29933164	subgrp_cor	count		0.21129313	0
MTRF1L	39	OK	0.6824892	0.4786939	1.4257317	0.15395	0.79895985	subgrp_cor	count		1	0
RGS17	4	OK	-3.1382695	1.9572121	-1.6034386	0.10884	-0.63773439	subgrp_cor	count		1	0
CNKSR3	38	OK	0.5995330	0.6038017	0.9929302	0.32074	0.45395627	subgrp_cor	count		1	0
SCAF8	6	OK	0.6393479	1.4406134	0.4438025	0.65719	0.00043305	subgrp_cor	count		1	0
TFB1M	3	OK	-13.121696	27.864993	-0.4709025	0.63771	-0.00158491	subgrp_cor	count		1	0
ARID1B	39	OK	1.1131321	0.5277615	2.1091575	0.03493	1.03176365	subgrp_cor	count		1	0
TMEM242	32	OK	-0.178096	0.5232872	-0.3403466	0.7336	-0.20265823	subgrp_cor	count		1	0
ZDHHC14	7	OK	0.2883798	1.0705617	0.2693713	0.78764	0.00019216	subgrp_cor	count		1	0
SNX9	50	OK	-0.2802416	0.4292608	-0.6528472	0.51385	-0.29275703	subgrp_cor	count		1	0
SYNJ2	13	OK	1.5257625	1.0983922	1.3890871	0.16481	0.74764319	subgrp_cor	count		1	0
GTF2H5	90	OK	0.4949550	0.2997663	1.6512696	0.09868	0.63613968	subgrp_cor	count		1	0
TULP4	21	OK	1.2061536	0.7565109	1.5943637	0.11085	0.88862092	subgrp_cor	count		1	0
TMEM181	8	OK	1.5238343	1.2673346	1.2023893	0.22921	1.10050474	subgrp_cor	count		1	0
DYNLT1	172	OK	-0.0415382	0.2255143	-0.1841932	0.85386	-0.05673678	subgrp_cor	count		1	0
EZR	19	OK	1.7228094	1.0286631	1.6748043	0.09397	1.29943035	subgrp_cor	count		1	0
RSPH3	2	OK	-0.3495512	1.8544747	-0.1884907	0.85049	-0.17337783	subgrp_cor	count		1	0
TAGAP	8	OK	-12.398979	17.8914229	-0.6930124	0.4883	-3.01205145	subgrp_cor	count		1	0
SOD2	235	OK	-0.1911083	0.1769403	-1.0800719	0.28011	-0.26097204	subgrp_cor	count		1	0
WTAP	86	OK	0.2799921	0.2961929	0.9453033	0.3445	0.36125897	subgrp_cor	count		1	0
ACAT2	5	OK	-0.9607313	1.395414	-0.6884910	0.49114	-0.00157680	subgrp_cor	count		1	0
TCP1	96	OK	0.2329479	0.2933697	0.7940421	0.42717	0.3033718	subgrp_cor	count		1	0
MRPL18	92	OK	0.2754704	0.3173682	0.8679838	0.3854	0.35290320	subgrp_cor	count		1	0
IGF2R	13	OK	-1.0056082	0.8988293	-1.1187977	0.26322	-0.70304229	subgrp_cor	count		1	0
MAP3K4	9	OK	-0.9398129	1.1256898	-0.8348774	0.40379	-0.56666572	subgrp_cor	count		1	0
QKI	195	OK	0.1620755	0.2059357	0.7870199	0.43127	0.2226299	subgrp_cor	count		1	0
PDE10A	4	OK	-1.3107746	1.6383999	-0.8000332	0.42369	-0.35262481	subgrp_cor	count		1	0
SFT2D1	15	OK	-0.9813463	0.8790125	-1.1164116	0.26425	-0.83081822	subgrp_cor	count		1	0
MPC1	47	OK	1.5513560	0.4774585	3.2491950	0.00116	1.8411832	subgrp_cor	count		1	0
RPS6KA2	23	OK	0.5232867	0.7443473	0.7030141	0.48205	0.34078041	subgrp_cor	count		1	0
RNASET2	10	OK	2.1751906	1.2345703	1.7619009	0.07809	1.47000312	subgrp_cor	count		1	0
LINC01558	2	OK	3.4598146	6.7714513	0.5109413	0.60939	0.00030179	subgrp_cor	count		1	0
AFDN-DT	1	OK	3.4598146	6.7714513	0.5109413	0.60939	0.00030179	subgrp_cor	count		1	0
AFDN	107	OK	0.3074141	0.2903526	1.0587612	0.28971	0.40167392	subgrp_cor	count		1	0
SMOC2	1	OK	3.4598146	6.7714513	0.5109413	0.60939	0.00030179	subgrp_cor	count		1	0
LINC01615	2	OK	-0.9102232	2.1881125	-0.4159856	0.67742	-0.25785735	subgrp_cor	count		1	0
THBS2	1	OK	3.4598146	6.7714513	0.5109413	0.60939	0.00030179	subgrp_cor	count		1	0
WDR27	1	OK	0.6532528	2.5001284	0.2612877	0.79387	0.00016297	subgrp_cor	count		1	0
C6orf120	4	OK	1.0667075	1.3819375	0.7718927	0.44018	0.87247101	subgrp_cor	count		1	0
PHF10	13	OK	-0.561479	0.7557984	-0.7428963	0.45754	-0.35080153	subgrp_cor	count		1	0
ERMARD	1	OK	-11.019418	43.2499352	-0.2547846	0.79889	-1.17482461	subgrp_cor	count		1	0
DLL1	9	OK	-1.5404839	0.9185163	-1.6771437	0.09352	-0.00239318	subgrp_cor	count		1	0
FAM120B	2	OK	-1.2956167	2.0414257	-0.6346627	0.52565	-0.00075754	subgrp_cor	count		1	0
PSMB1	439	OK	0.1575240	0.1318606	1.1946252	0.23222	0.22266610	subgrp_cor	count		1	0
TBP	1	OK	-10.442278	41.4658099	-0.2518286	0.80117	-0.00045289	subgrp_cor	count		1	0
PDCD2	55	OK	0.1385048	0.4038515	0.3429603	0.73163	0.16881563	subgrp_cor	count		1	0
PDGFA	52	OK	1.4361486	0.4684737	3.0655802	0.00217	1.72790754	subgrp_cor	count		1	0
PRKAR1B	12	OK	-0.9746008	0.8869520	-1.0988201	0.27185	-0.87303780	subgrp_cor	count		1	0
AC147651.4	1	OK	3.4598146	6.7714513	0.5109413	0.60939	0.00030179	subgrp_cor	count		1	0
SUN1	46	OK	-1.2301927	0.5044036	-2.438905	0.01473	-1.36610670	subgrp_cor	count		1	0
COX19	3	OK	-1.2956167	2.0414257	-0.6346627	0.52565	-0.00075754	subgrp_cor	count		1	0
C7orf50	88	OK	1.6505891	0.3796242	4.3479545	1.37E-05	2.04493224	subgrp_cor	count		0.13745429	0
GPR146	89	OK	-0.5069875	0.2965516	-1.7096082	0.08734	-0.6593274	subgrp_cor	count		1	0

ZFAND2A	9	OK	-0.5850907	1.1193505	-0.5227055	0.60119	-0.2846892	subgrp_cor	count		1	0
INTS1	4	OK	2.0176498	2.2120878	0.9121020	0.36171	0.00137857	subgrp_cor	count		1	0
MAFK	3	OK	21.642059	54.486891	0.3971975	0.69122	1.57744725	subgrp_cor	count		1	0
PSMG3	16	OK	-0.1739764	0.7898526	-0.2202641	0.82567	-0.15440537	subgrp_cor	count		1	0
ELFN1	1	OK	23.991896	84.899941	0.2825902	0.77749	1.65081709	subgrp_cor	count		1	0
MAD1L1	1	OK	-10.44227	41.465809	-0.2518286	0.80117	-0.00045289	subgrp_cor	count		1	0
MRM2	4	OK	1.3361195	1.3423634	0.9953485	0.31957	0.84319757	subgrp_cor	count		1	0
NUDT1	4	OK	1.1367732	1.8168872	0.6256707	0.53152	0.3502482	subgrp_cor	count		1	0
SNX8	3	OK	-11.019418	43.249935	-0.2547846	0.79889	-1.17482461	subgrp_cor	count		1	0
EIF3B	22	OK	-0.395450	0.682651	-0.579285	0.5624	-0.24973714	subgrp_cor	count		1	0
CHST12	30	OK	-0.4985307	0.618797	-0.805644	0.42045	-0.51641435	subgrp_cor	count		1	0
LFNG	35	OK	-1.361574	0.5057747	-2.692056	0.0071	-1.58166571	subgrp_cor	count		1	0
BRAT1	7	OK	-0.4403878	1.1421408	-0.385558	0.69981	-0.27770276	subgrp_cor	count		1	0
IQCE	4	OK	-0.1509758	1.968815	-0.076683	0.93888	-9.78E-05	subgrp_cor	count		1	0
TTYH3	3	OK	4.6613299	5.7683860	0.8080821	0.41904	0.00274918	subgrp_cor	count		1	0
GNA12	6	OK	-0.4944902	1.4409495	-0.343169	0.72147	-0.14639957	subgrp_cor	count		1	0
FOXK1	5	OK	-1.2352853	1.2504678	-0.987858	0.32322	-0.00197223	subgrp_cor	count		1	0
RBAK-RBAKDN	5	OK	6.0171118	5.6425028	1.0663905	0.28625	0.00015459	subgrp_cor	count		1	0
WIPI2	27	OK	-0.4819813	0.580177	-0.830748	0.40612	-0.46736839	subgrp_cor	count		1	0
TNRC18	4	OK	0.1721614	1.534450	0.1121974	0.91067	0.00011395	subgrp_cor	count		1	0
FBXL18	1	OK	3.4598146	6.7714513	0.5109413	0.60939	0.00030179	subgrp_cor	count		1	0
ACTB	859	OK	-0.2406259	0.0871218	-2.761946	0.00575	-0.34404112	subgrp_cor	count		1	0
FSCN1	65	OK	-0.3646297	0.3882044	-0.939272	0.34759	-0.42903082	subgrp_cor	count		1	0
RNF216	10	OK	-0.6204027	1.004379	-0.617697	0.53677	-0.29808147	subgrp_cor	count		1	0
CCZ1	10	OK	0.8455396	1.2043961	0.7020444	0.48265	0.43177897	subgrp_cor	count		1	0
AIMP2	10	OK	0.3338555	0.9931695	0.3361516	0.73676	0.25183029	subgrp_cor	count		1	0
EIF2AK1	22	OK	0.4108383	0.6884989	0.5967159	0.5507	0.21019121	subgrp_cor	count		1	0
USP42	3	OK	2.0136551	1.3033100	1.5450315	0.12234	0.00138768	subgrp_cor	count		1	0
RAC1	733	OK	-0.2290556	0.0973322	-2.353236	0.01861	-0.32655055	subgrp_cor	count		1	0
KDELR2	187	OK	0.8293690	0.2126825	3.8995626	9.64E-05	1.13745052	subgrp_cor	count		0.95557131	0
ZDHHC4	6	OK	2.704828	1.7963230	1.5057581	0.13212	1.47366775	subgrp_cor	count		1	0
ZNF316	1	OK	-11.019418	43.249935	-0.2547846	0.79889	-1.17482461	subgrp_cor	count		1	0
CCZ1B	1	OK	18.759489	83.625179	0.2243282	0.8225	0.00028789	subgrp_cor	count		1	0
C1GALT1	6	OK	2.3339623	2.2435	1.0402987	0.2982	0.64998602	subgrp_cor	count		1	0
MIOS	3	OK	4.6613299	5.7683860	0.8080821	0.41904	0.00274918	subgrp_cor	count		1	0
RPA3	25	OK	0.8814156	0.6316849	1.3953185	0.16292	0.9683855	subgrp_cor	count		1	0
UMAD1	1	OK	-10.44227	41.465809	-0.2518286	0.80117	-0.00045289	subgrp_cor	count		1	0
ICA1	51	OK	-0.4453764	0.4140499	-1.075658	0.28208	-0.52834540	subgrp_cor	count		1	0
NDUFA4	585	OK	0.1503780	0.1127591	1.3336219	0.18232	0.21365277	subgrp_cor	count		1	0
PHF14	110	OK	0.2160022	0.2797848	0.7720296	0.4401	0.28210821	subgrp_cor	count		1	0
THSD7A	34	OK	0.3414319	0.571290	0.5976499	0.55007	0.30614681	subgrp_cor	count		1	0
TMEM106B	35	OK	0.9267218	0.5516305	1.6799682	0.09296	1.06609594	subgrp_cor	count		1	0
ARL4A	358	OK	-0.6141006	0.1452504	-4.227874	2.36E-05	-0.86254768	subgrp_cor	count		0.23555581	0
ETV1	9	OK	-1.2866995	1.0818823	-1.189315	0.23432	-0.87049629	subgrp_cor	count		1	0
MEOX2	54	OK	-2.1164449	0.4753293	-4.452588	8.48E-06	-2.5154185	subgrp_cor	count		0.08501195	0
ISPD	1	OK	-10.44227	41.465809	-0.2518286	0.80117	-0.00045289	subgrp_cor	count		1	0
SOSTDC1	2	OK	-1.0311657	1.7479538	-0.589927	0.55534	-0.00022765	subgrp_cor	count		1	0
ANKMY2	1	OK	3.4598146	6.7714513	0.5109413	0.60939	0.00030179	subgrp_cor	count		1	0
BZW2	37	OK	-0.7200669	0.5000650	-1.439945	0.14988	-0.77450147	subgrp_cor	count		1	0
TSPAN13	35	OK	-1.0054912	0.5499762	-1.828244	0.06751	-0.70281040	subgrp_cor	count		1	0
AHR	47	OK	0.796948	0.4409364	1.8073989	0.0707	0.83956978	subgrp_cor	count		1	0
AC073332.1	1	OK	18.759489	83.625179	0.2243282	0.8225	0.00028789	subgrp_cor	count		1	0
SNX13	11	OK	0.0500646	0.9413665	0.0531829	0.95759	0.0154067	subgrp_cor	count		1	0
HDAC9	2	OK	3.4598146	6.7714513	0.5109413	0.60939	0.00030179	subgrp_cor	count		1	0
TWISTNB	35	OK	0.3883051	0.4661676	0.832972	0.40486	0.47349770	subgrp_cor	count		1	0
SP4	1	OK	3.4598146	6.7714513	0.5109413	0.60939	0.00030179	subgrp_cor	count		1	0
CDCA7L	1	OK	18.759489	83.625179	0.2243282	0.8225	0.00028789	subgrp_cor	count		1	0
RAPGEF5	84	OK	0.5306084	0.327138	1.6219682	0.10481	0.66365642	subgrp_cor	count		1	0
AC073072.1	2	OK	-13.04562	27.67476	-0.4713905	0.63736	-1.3178641	subgrp_cor	count		1	0
TOMM7	621	OK	0.1210067	0.1055575	1.1463582	0.25165	0.17168063	subgrp_cor	count		1	0
KLHL7	3	OK	0.9033449	1.4604911	0.6185213	0.53622	0.5834292	subgrp_cor	count		1	0
NUPL2	10	OK	-0.4833587	0.8543478	-0.565763	0.57155	-0.35245230	subgrp_cor	count		1	0
GPNMB	1	OK	-10.44227	41.465809	-0.2518286	0.80117	-0.00045289	subgrp_cor	count		1	0
MALSU1	28	OK	-0.014115	0.5078690	-0.027792	0.97782	-0.01653600	subgrp_cor	count		1	0
TRA2A	55	OK	-0.2138818	0.3848583	-0.555741	0.57839	-0.25512127	subgrp_cor	count		1	0
CCDC126	3	OK	-2.2911888	2.0579227	-1.113350	0.26556	-0.53157637	subgrp_cor	count		1	0
MPP6	1	OK	3.4598146	6.7714513	0.5109413	0.60939	0.00030179	subgrp_cor	count		1	0
CYCS	142	OK	-0.312505	0.2451139	-1.274937	0.20232	-0.40967211	subgrp_cor	count		1	0
NFE2L3	17	OK	3.2805531	1.4498240	2.262725	0.02365	1.95849034	subgrp_cor	count		1	0
HNRNPA2B1	665	OK	-0.1124688	0.1013801	-1.109375	0.26727	-0.16003667	subgrp_cor	count		1	0
CBX3	230	OK	-0.144653	0.191749	-0.754387	0.45062	-0.19794638	subgrp_cor	count		1	0
SNX10	2	OK	-12.14842	43.455309	-0.279561	0.77981	-1.91362624	subgrp_cor	count		1	0
AC004540.2	47	OK	6.5305749	1.5400352	4.2405360	2.23E-05	5.2690886	subgrp_cor	count		0.22276362	0
AC004540.1	3	OK	3.4598146	6.7714513	0.5109413	0.60939	0.00030179	subgrp_cor	count		1	0
SKAP2	31	OK	-1.4898072	0.5695515	-2.615754	0.0089	-1.33455425	subgrp_cor	count		1	0
HOXA1	2	OK	-0.3495512	1.8544747	-0.188490	0.85049	-0.17337783	subgrp_cor	count		1	0
HOTAIRM1	29	OK	-1.145697	0.5485870	-2.088452	0.03676	-0.95120620	subgrp_cor	count		1	0
HOXA2	2	OK	21.105322	83.341273	0.2532397	0.80008	0.0003103	subgrp_cor	count		1	0
HOXA3	1	OK	-15.99503	70.34942	-0.227365	0.82014	-0.00028474	subgrp_cor	count		1	0
HOXA-AS2	4	OK	-10.72179	16.814827	-0.637639	0.52371	-2.56187940	subgrp_cor	count		1	0
HOXA4	4	OK	-0.841532	1.4608505	-0.576056	0.56458	-0.24032510	subgrp_cor	count		1	0
HOXA5	1	OK	-15.49804	42.951091	-0.36082	0.71822	-0.00073756	subgrp_cor	count		1	0
HOXA7	1	OK	23.991896	84.899941	0.2825902	0.77749	1.65081709	subgrp_cor	count		1	0
HIBADH	7	OK	-1.1568462	1.1654364	-0.992629	0.32089	-0.68065351	subgrp_cor	count		1	0
TAX1BP1	242	OK	0.2785881	0.1798506	1.5489995	0.12138	0.38668184	subgrp_cor	count		1	0
JAZF1	7	OK	1.1242277	1.3040779	0.8621629	0.3886	0.56595077	subgrp_cor	count		1	0
CREB5	17	OK	3.2905343	1.4046376	2.3426214	0.01915	2.12158420	subgrp_cor	count		1	0
CPVL	21	OK	-2.6410496	0.8926882	-2.958535	0.00309	-2.49304461	subgrp_cor	count		1	0
PRR15	5	OK	3.2517484	2.3983678	1.3558172	0.17516	0.80586482	subgrp_cor	count		1	0
WIPF3	12	OK	23.70217	32.908410	0.7202467	0.47137	3.53622515	subgrp_cor	count		1	0
FKBP14	2	OK	3.4598146	6.7714513	0.5109413	0.60939	0.00030179	subgrp_cor	count		1	0
MTURN	35	OK	0.1020983	0.523141	0.195164	0.84526	0.11038390	subgrp_cor	count		1	0
GGCT	49	OK	-0.213102	0.4251075	-0.501290	0.61617	-0.24880335	subgrp_cor	count		1	0
GARS	14	OK	2.2447473	1.2873502	1.7436958	0.08121	0.63150201	subgrp_cor	count		1	0
INMT	57	OK	1.5043955	0.4121752	3.6498911	0.00026	1.60981133	subgrp_cor	count		1	0
MINDY4	9	OK	-1.1537827	1.0822298	-1.066116	0.28637	-0.94954489	subgrp_cor	count		1	0
LSM5.86	93	OK	-0.1568377	0.2990595	-0.524770	0.59974	-0.20608008	subgrp_cor	count		1	0
KBTBD2	51	OK	-0.4799227	0.4208149	-1.140460	0.25409	-0.56286578	subgrp_cor	count		1	0
FKBP9	62	OK	-0.946403	0.3841352	-2.463723	0.01375	-1.08960835	subgrp_cor	count		1	0
NTSC3A	25	OK	-1.1875566	0.603327	-1.968345	0.04903	-0.9068229	subgrp_cor	count		1	0
RP9	34	OK	-0.720288	0.5303876	-1.358231	0.17439	-0.77408542	subgrp_cor	count		1	0
BBS9	4	OK	0.8788176	1.6469311	0.5336091	0.59361	0.44699547	subgrp_cor	count		1	0
BMPER	8	OK	22.221790	54.523227	0.4075655	0.68359	1.5953836	subgrp_cor	count		1	0
DPY19L1	5	OK	2.9646162	2.2923517	1.2932641	0.19592	0.76336654	subgrp_cor	count		1	0
HERPUD2	11	OK	0.4638373	0.8310364	0.5581431	0.57675	0.30213355	subgrp_cor	count		1	0
7-Sep	550	OK	-0.2586776	0.1151573	-2.246297	0.02468	-0.36694904	subgrp_cor	count		1	0
EEPD1	1	OK	-15.99503	70.34942	-0.227365	0.82014	-0.00028474	subgrp_cor	count		1	0
ELMO1	23	OK	-0.059992	0.5903745	-0.101618	0.91906	-0.05344633	subgrp_cor	count		1	0
EPDR1	3	OK	-0.3495512	1.8544747	-0.188490	0.85049	-0.17337783	subgrp_cor	count		1	0
SFRP4	2	OK	26.367955	84.916325	0.3105168	0.75617	2.49052421	subgrp_cor	count		1	0
STARD3NL	30	OK	0.4778793	0.5345857	0.893907	0.37137	0.54629118	subgrp_cor	count		1	0
TRGC2	1	OK	23.991896	84.899941	0.2825902	0.77749	1.65081709	subgrp_cor	count		1	0
VPS41	16	OK	0.1351831	0.851740	0.1587139	0.87389	0.06878263	subgrp_cor	count		1	0
YAE1D1	2	OK	1.5824000	1.9240335	0.8224389	0.41082	0.77175617	subgrp_cor	count		1	0
RALA	87	OK	0.6567246	0.3303449	1.9879964	0.04681	0.85650728	subgrp_cor	count		1	0
AC072061.1	1	OK	-11.019418	43.249935	-0.2547846	0.79889	-1.17482461	subgrp_cor	count		1	0
CDK13	23	OK	0.0952499	0.5619447	0.169500	0.8654	0.09753953	subgrp_cor	count		1	0
MPLKIP	52	OK	0.0432282	0.4583585	0.087765	0.93006	0.05000807	subgrp_cor	count		1	0
INHBA	2	OK	18.759489	83.625179	0.2243282	0.8225	0.00028789	subgrp_cor	count		1	0
PSMA2	54	OK	0.6512886	0.4200202	1.5507316	0.12097	0.80918738	subgrp_cor	count		1	0
MRPL32	102	OK	-0.2161145	0.2916951	-0.740891	0.45876	-0.27355837	subgrp_cor	count		1	0
STK17A	29	OK	0.8054207	0.5705456	1.4116675	0.15805	0.90210005	subgrp_cor	count		1	0
COA1	8	OK	-0.4701574	0.9593202	-0.490094	0.62407	-0.0002970	subgrp_cor	count		1	0
BLVRA	27	OK	0.444426	0.5208122	0.8533322	0.39347	0.50018900	subgrp_cor	count		1	0
URGCP	1	OK	-10.44227	41.465809	-0.2518286	0.80117	-0.00045289	subgrp_cor	count		1	0
UBE2D4	8	OK	-1.3921003	1.1325247	-1.229200	0.219	-0.93133974	subgrp_cor	count		1	0
AC004951.1	1	OK	-10.44227	41.465809	-0.2518286	0.80117	-0.00045289	subgrp_cor	count		1	0
DBNL	32	OK	-0.0402378	0.4837619	-0.083176	0.93371	-0.04341229	subgrp_cor	count		1	0
POLM	2	OK	-10.44227	41.465809	-0.2518286	0.80117	-0.00045289	subgrp_cor	count		1	0
AEBP1	13	OK	1.2334199	1.2311566	1.001838	0.31642	0.37823729	subgrp_cor	count		1	0
POLD2	16	OK	0.2297258	0.7298057	0.3147767	0.75293	0.19136229	subgrp_cor	count		1	0
YKT6	4	OK	0.6247355	1.8560295	0.3365978	0.73642	0.20899665	subgrp_cor	count		1	0
NUDCD3	7	OK	0.1681559	0.9961478	0.1688062	0.86595	0.10917917	subgrp_cor	count		1	0
DDX56	8	OK	0.171002	0.9190410	0.1860663	0.85239	0.05290121	subgrp_cor	count		1	0
TMED4	37	OK	1.1386475	0.5014553	2.2706857	0.02317	1.30222646	subgrp_cor	count		1	0
OGDH	5	OK	3.4598149	4.7858686	0.7229230	0.46972	0.00081935	subgrp_cor	count		1	0
ZMIZ2	2	OK	3.4598146	6.7714513	0.5109413	0.60939	0.00030179	subgrp_cor	count		1	0
PPIA	743	OK	-0.2306762	0.0976828	-2.361480	0.0182	-0.32914549	subgrp_cor	count		1	0
H2AFV	111	OK	0.8256195	0.2804573	2.9438325	0.00324	1.07804096	subgrp_cor	count		1	0
PURB	29	OK	-0.6355748	0.5618348	-1.131248	0.25795	-0.6111880	subgrp_cor	count		1	0
SNHG15	34	OK	0.0334950	0.5673948	0.0590330	0.95293	0.0361801	subgrp_cor	count		1	0
CCM2	30	OK	0.2487633	0.5973493	0.4164454	0.67709	0.27493051	subgrp_cor	count		1	0
TNS3	4	OK	19.103032	50.772469	0.3762478	0.70672	0.00078880	subgrp_cor	count		1	0
HUS1	8	OK	1.1594686	1.2988961	0.892656	0.37206	0.7270112	subgrp_cor	count		1	0
UPP1	116	OK	-1.068978	0.296001	-3.543834	0.00039	-1.33351710	subgrp_cor	count		1	0
FIGNL1	1	OK	1.5822999	2.7200188	0.5817606	0.56072	0.47433761	subgrp_cor	count		1	0
GRB10	26	OK	-0.8238531	0.5568906	-1.479380	0.13904	-0.78205659	subgrp_cor	count		1	0
SEC61G	252	OK	0.7469794	0.1905630	3.9198536	8.86E-05	1.02990586	subgrp_cor	count		0.8789396	0
VOPP1	17	OK	1.3953493	0.8214164	1.6987111	0.08937	1.11466765	subgrp_cor	count		1	0
NIPSNAP2	42	OK	-0.3287122	0.4707668	-0.698248	0.48502	-0.35191292	subgrp_cor	count		1	0
PSPH	1	OK	1.5822999	2.7200188	0.5817606	0.56072	0.47433761	subgrp_cor	count		1	0
CCT6A	214	OK	-0.4565213	0.1979525	-2.306215	0.0211	-0.62073879	subgrp_cor	count		1	0
SUMF2	67	OK	1.3183400	0.3890128	3.3889365	0.0007	1.63457036	subgrp_cor	count		1	0
PHKG1	9	OK	1.3812324	1.1121465	1.2419518	0.21425	0.6843026	subgrp_cor	count		1	0
CHCHD2	599	OK	-0.0266343	0.1116139	-0.238628	0.81139	-0.03782340	subgrp_cor	count		1	0
ZNF138	3	OK	-13.335695	29.112233	-0.458078	0.6469	-1.33917170	subgrp_cor	count		1	0
ZNF273	1	OK	3.4598146	6.7714513	0.5109413	0.60939	0.00030179	subgrp_cor	count		1	0
ZNF117	1	OK	18.759489	83.625179	0.2243282	0.8225	0.00028789	subgrp_cor	count		1	0
ERV3-1	2	OK	3.4598149	4.7858686	0.7229230	0.46972	0.00081935	subgrp_cor	count		1	0
ZNF92	4	OK	0.1721614	1.534450	0.1121974	0.91067	0.00011395	subgrp_cor	count		1	0
VKORC1L1	9	OK	1.6982137	1.5207309	1.1167746	0.26409	1.04069886	subgrp_cor	count		1	0
GUSB	41	OK	1.6567515	0.5396820	3.0698658	0.00214	1.79189944	subgrp_cor	count		1	0
ASL	7	OK	0.4806421	1.0841576	0.4433223	0.65752	0.14982334	subgrp_cor	count		1	0
CRCP	18	OK	0.7098659	0.7722073	0.9180796	0.35858	0.53379454	subgrp_cor	count		1	0
TPST1	4	OK	0.1255948	1.4500075	0.086616	0.93098	0.03878308	subgrp_cor	count		1	0
AC008267.5	1	OK	0.6532528	2.5001284	0.2612877	0.79387	0.00016297	subgrp_cor	count		1	0
RABGEF1	1	OK	18.759489	83.625179	0.2243282	0.8225	0.00028789	subgrp_cor	count		1	0
AC027644.3	5	OK	-1.4516747	1.5498004	-0.936684	0.34892	-0.97674657	subgrp_cor	count		1	0
TMEM248	41	OK	1.4344094	0.519943	2.7587804	0.0058	1.42534525	subgrp_cor	count		1	0
SBDS	400	OK	0.1077581	0.1380117	0.7807899	0.43492	0.15166705	subgrp_cor	count		1	0
AUTS2	1	OK	-11.019418	43.249935	-0.2547846	0.79889	-1.17482461	subgrp_cor	count		1	0
NSUN5	2	OK	0.4251287	2.5622898	0.1659214	0.86822	0.1426475	subgrp_cor	count		1	0
BAZ1B	32	OK	0.4386186	0.5020625	0.8736334	0.38232	0.39324370	subgrp_cor	count		1	0
BCL7B	32	OK	0.1452490	0.5224398	0.2780205	0.781	0.15708772	subgrp_cor	count		1	0
TBL2	3	OK	0.6393470	1.4406245	0.4437985	0.65719	0.00043312	subgrp_cor	count		1	0
DNAJC30	4	OK	-13.335695	29.112233	-0.458078	0.6469	-1.33917170	subgrp_cor	count		1	0
BUD23	72	OK	0.0838725	0.3453585	0.2428563	0.80812	0.10648146	subgrp_cor	count		1	0
ABHD11	1	OK	18.759489	83.625179	0.2243282	0.8225	0.00028789	subgrp_cor	count		1	0
EIF4H	98	OK	0.2392431	0.2890928	0.8275650	0.40792	0.29588318	subgrp_cor	count		1	0
RFC2	4	OK	-1.2520544	1.3878450	-0.902157	0.36697	-0.56535377	subgrp_cor	count		1	0
GTF2I	102	OK	1.0401004	0.3271256	3.1795135	0.00148	1.31407856	subgrp_cor	count		1	0
GTF2IRD2	1	OK	18.759489	83.625179	0.2243282	0.8225	0.00028789	subgrp_cor	count		1	0
RCC1L	5	OK	0.1084560	1.0696415	0.1013947	0.91924	0.03346608	subgrp_cor	count		1	0
POM121C	6	OK	0.7446742	1.0909250	0.6826080	0.49485	0.38012890	subgrp_cor	count		1	0
HIP1	40	OK	-0.2542179	0.4841054	-0.525129	0.59949	-0.26601725	subgrp_cor	count		1	0
RHBDD2	18	OK	-0.0737253	0.734766	-0.100338	0.92008	-0.05511055	subgrp_cor	count		1	0
POR	14	OK	4.6299696	2.2184585	2.0870210	0.03689	1.90904526	subgrp_cor	count		1	0
STYXL1	3	OK	-0.2029407	1.6532365	-0.122753	0.9023	-4.82E-05	subgrp_cor	count		1	0
MDH2	179	OK	-0.0239649	0.2123385	-0.112285	0.91052	-0.03300579	subgrp_cor	count		1	0
HSPB1	772	OK	-0.0908539	0.0920097	-0.987437	0.32342	-0.12978820	subgrp_cor	count		1	0
YWHAG	66	OK	0.340950	0.4173040	0.8170306	0.41391	0.37695644	subgrp_cor	count		1	0
ZP3	1	OK	-10.44227	41.465809	-0.2518286	0.80117	-0.00045289	subgrp_cor	count		1	0
POMZP3	1	OK	-11.019418	43.249935	-0.2547846	0.79889	-1.17482461	subgrp_cor	count		1	0
CCDC146	3	OK	-13.271979	28.009791	-0.473833	0.63562	-2.0156632	subgrp_cor	count		1	0
FGL2	46	OK	3.5755614	0.8759643	4.0818571	4.47E-05	3.02100634	subgrp_cor	count		0.44485141	0
PTPN12	108	OK	-0.1680973	0.2844178	-0.591022	0.55451	-0.22170669	subgrp_cor	count		1	0
RSBN1L	59	OK	-0.3675822	0.3914033	-0.939139	0.34766	-0.45138589	subgrp_cor	count		1	0
TMEM60	6	OK	1.1643006	1.3515277	0.8614700	0.38896	0.00217239	subgrp_cor	count		1	0
PHTF2	1	OK	-11.019418	43.249935	-0.2547846	0.79889	-1.17482461	subgrp_cor	count		1	0
GNAI1	7	OK	-2.2525449	1.3511253	-1.667162	0.09548	-1.13466663	subgrp_cor	count		1	0
CD36	40	OK	-1.849538	0.5184153	-3.567676	0.00036	-2.0772215	subgrp_cor	count		1	0
SEMA3C	5	OK	25.72320	53.189585	0.4836170	0.62866	3.32915949	subgrp_cor	count		1	0
HGF	8	OK	-1.2456629	0.9804887	-1.270450	0.20392	-0.84819107	subgrp_cor	count		1	0
CACNA2D1	3	OK	4.6613299	5.7683860	0.8080821	0.41904	0.00274918	subgrp_cor	count		1	0
PCLO	1	OK	23.991896	84.899941	0.2825902	0.77749	1.65081709	subgrp_cor	count		1	0
SEMA3E	7	OK	-12.279116	17.914721	-0.685420	0.49308	-2.7217341	subgrp_cor	count		1	0
SEMA3A	2	OK	-11.448548	41.761485	-0.274141	0.78398	-0.00049246	subgrp_cor	count		1	0
SEMA3D	3	OK	-10.58676	25.202461	-0.420068	0.67444	-0.00124361	subgrp_cor	count		1	0
KIAA1324L	1	OK	-11.019418	43.249935	-0.2547846	0.79889	-1.17482461	subgrp_cor	count		1	0
AC005076.1	1	OK	3.4598146	6.7714513	0.5109413	0.60939	0.00030179	subgrp_cor	count		1	0
DMTF1	2	OK	0.1681491	1.7253300	0.097459	0.92236	0.05201277	subgrp_cor	count		1	0
TMEM243	29	OK	-0.2213776	0.565221	-0.391665	0.69531	-0.26411721	subgrp_cor	count		1	0
TP53TG1	27	OK	0.5310530	0.6423933	0.8266789	0.40842	0.52559210	subgrp_cor	count		1	0
CROT	4	OK	-0.4654296	1.3093639	-0.355462	0.72224	-0.13816105	subgrp_cor	count		1	0
ABCB1	12	OK	0.3744964	0.9742210	0.3844060	0.70068	0.00068100	subgrp_cor	count		1	0
RUNDC3B	10	OK	-0.972964	0.9757734	-0.997120	0.31871	-0.27355356	subgrp_cor	count		1	0
DBF4	8	OK	1.0721274	1.0548028	1.0164244	0.30942	0.87928172	subgrp_cor	count		1	0
ADAM22	1	OK	3.4598146	6.7714513	0.5109413	0.60939	0.00030179	subgrp_cor	count		1	0
SRI	221	OK	0.0680298	0.1872578	0.3632948	0.71639	0.09436641	subgrp_cor	count		1	0
STEAP2	2	OK	21.105322	83.341273	0.2532397	0.80008	0.0003103	subgrp_cor	count		1	0
GTPBP10	2	OK	-15.48861	42.831644	-0.361616	0.71764	-0.00073897	subgrp_cor	count		1	0
MTERF1	1	OK	19.111790	50.837591	0.3759381	0.70696	0.00078702	subgrp_cor	count		1	0
AKAP9	147	OK	0.1262232	0.2300573	0.5486599	0.58324	0.16604332	subgrp_cor	count		1	0
KRIT1	40	OK	0.9550945	0.4907208	1.9463091	0.05162	0.99892824	subgrp_cor	count		1	0
ANKIB1	12	OK	2.1882890	1.3037672	1.6784353	0.09326	0.61946804	subgrp_cor	count		1	0
GATAD1	44	OK	-0.7971709	0.4454303	-1.789664	0.07351	-0.95962244	subgrp_cor	count		1	0
PEX1	9	OK	-0.9640805	1.0269917	-0.938742	0.34786	-0.76732384	subgrp_cor	count		1	0
RBM48	2	OK	-13.27240	28.013322	-0.473789	0.63565	-2.01570217	subgrp_cor	count		1	0
FAM133B	170	OK	0.0330481	0.2228135	0.1483221	0.88209	0.04462709	subgrp_cor	count		1	0
CDK6	8	OK	1.6963364	1.3031108	1.3017591	0.192	0.00316910	subgrp_cor	count		1	0
SAMD9	28	OK	-1.5478339	0.6376241	-2.427501	0.0152	-1.31067680	subgrp_cor	count		1	0
SAMD9L	34	OK	-0.1932169	0.5052527	-0.382416	0.70215	-0.19655244	subgrp_cor	count		1	0
TFPI2	3	OK	-0.3495512	1.8544747	-0.188490	0.85049	-0.17337783	subgrp_cor	count		1	0
BET1	5	OK	0.8120212	1.3820622	0.587542	0.55684	0.4139407	subgrp_cor	count		1	0
COL1A2	24	OK	24.901896	22.503269	0.7661351	0.4436	3.8300218	subgrp_cor	count		1	0
CASD1	11	OK	-0.1989312	0.9997841	-0.198974	0.84229	-0.20223217	subgrp_cor	count		1	0
SGCE	30	OK	2.9035134	0.7145737	4.0632802	4.84E-05	3.2254617	subgrp_cor	count		0.48165289	0
PPP1R9A	1	OK	-12.14842	43.455309	-0.279561	0.77981	-1.91362624	subgrp_cor	count		1	0
PON2	88	OK	0.8611177	0.3310406	2.6012446	0.00929	1.10689064	subgrp_cor	count		1	0
ASB4	1	OK	-10.44227	41.465809	-0.2518286	0.80117	-0.00045289	subgrp_cor	count		1	0
DYNC1I1	1	OK	1.5822999	2.7200188	0.5817606	0.56072	0.47433761	subgrp_cor	count		1	0
SLC25A13	10	OK	-0.283344	1.0037239	-0.282293	0.77772	-0.24989528	subgrp_cor	count		1	0
SEM1	386	OK	-0.1791965	0.1442768	-1.242032	0.21422	-0.25176627	subgrp_cor	count		1	0
DLX6-AS1	2	OK	0.5887819	1.767414	0.3331317	0.73903	0.30122869	subgrp_cor	count		1	0
SDHAF3	1	OK	-11.019418	43.249935	-0.2547846	0.79889	-1.17482461	subgrp_cor	count		1	0
TECPR1	6	OK	0.1681559	1.2198581	0.1378514	0.89036	0.08564646	subgrp_cor	count		1	0
BRI3	496	OK	-0.173168	0.1217966	-1.421780	0.15509	-0.24563447	subgrp_cor	count		1	0
NPTX2	4	OK	-0.256612	1.3412322	-0.191325	0.84827	-0.12809702	subgrp_cor	count		1	0
TRRAP	2	OK	-10.44227	41.465809	-0.2518286	0.80117	-0.00045289	subgrp_cor	count		1	0
SMURF1	1	OK	0.6532528	2.5001284	0.2612877	0.79387	0.00016297	subgrp_cor	count		1	0
ARPC1A	193	OK	0.0932360	0.2006037	0.4647769	0.64209	0.12866469	subgrp_cor	count		1	0
ARPC1B	342	OK	-0.0335766	0.1515531	-0.221550	0.82466	-0.04708704	subgrp_cor	count		1	0
PDAP1	136	OK	0.7798637	0.2835290	2.7505600	0.00595	1.01596548	subgrp_cor	count		1	0
BUD31	122	OK	-0.0664163	0.2683687	-0.247481	0.80454	-0.08785106	subgrp_cor	count		1	0
CPSF4	3	OK	2.5552227	2.349844	1.0874007	0.27686	0.69313893	subgrp_cor	count		1	0
ATP5MF	94	OK	0.5877290	0.3251045	1.8078156	0.07064	0.7600937	subgrp_cor	count		1	0
ZNF789	3	OK	-1.2956167	2.041425	-0.634662	0.52565	-0.00075754	subgrp_cor	count		1	0
ZNF394	8	OK	0.0097397	1.133294	0.0085941	0.99314	0.00483242	subgrp_cor	count		1	0
ZKSCAN5	1	OK	-11.019418	43.249935	-0.2547846	0.79889	-1.17482461	subgrp_cor	count		1	0
FAM200A	3	OK	-11.18473	26.296787	-0.425326	0.6706	-2.27083089	subgrp_cor	count		1	0
ZNF655	2	OK	-0.910223	2.1881125	-0.415985	0.67742	-0.25785735	subgrp_cor	count		1	0
TRIM4	5	OK	0.3473110	1.814020	0.1914583	0.84817	0.00022216	subgrp_cor	count		1	0
GJC3	1	OK	-11.019418	43.249935	-0.2547846	0.79889	-1.17482461	subgrp_cor	count		1	0
ZKSCAN1	25	OK	0.4379426	0.6133011	0.7140743	0.47519	0.28526898	subgrp_cor	count		1	0
ZSCAN21	1	OK	-10.44227	41.465809	-0.2518286	0.80117	-0.00045289	subgrp_cor	count		1	0
ZNF3	2	OK	4.6613299	5.7683860	0.8080821	0.41904	0.00274918	subgrp_cor	count		1	0
COPS6	125	OK	0.685012	0.2736263	2.5034587	0.0122	0.9059128	subgrp_cor	count		1	0
MCM7	3	OK	-11.019418	43.249935	-0.2547846	0.79889	-1.17482461	subgrp_cor	count		1	0
AP4M1	3	OK	0.1681690	1.7253287	0.0974707	0.92235	0.05201879	subgrp_cor	count		1	0
TAF6	9	OK	-0.412299	0.8807500	-0.468122	0.6397	-0.12290274	subgrp_cor	count		1	0
CNPY4	7	OK	0.0203365	1.1688642	0.0173985	0.98612	0.00624904	subgrp_cor	count		1	0
LAMTOR4	272	OK	0.2132212	0.1713164	1.2446047	0.21329	0.29834226	subgrp_cor	count		1	0
PILRB	2	OK	3.2106545	2.8728928	1.1175684	0.26375	1.53266440	subgrp_cor	count		1	0
ZCWPW1	1	OK	-15.99503	70.34942	-0.227365	0.82014	-0.00028474	subgrp_cor	count		1	0
MEPCE	3	OK	-10.44227	41.465809	-0.2518286	0.80117	-0.00045289	subgrp_cor	count		1	0
PPP1R35	24	OK	0.1966730	0.6150566	0.3197641	0.74915	0.21282286	subgrp_cor	count		1	0
TSC22D4	12	OK	-0.500200	0.904473	-0.553029	0.58024	-0.40521438	subgrp_cor	count		1	0
AGFG2	1	OK	-10.44227	41.465809	-0.2518286	0.80117	-0.00045289	subgrp_cor	count		1	0
LRCH4	1	OK	-11.019418	43.249935	-0.2547846	0.79889	-1.17482461	subgrp_cor	count		1	0
MOSPD3	13	OK	-0.54397	0.8708289	-0.624662	0.53219	-0.39612841	subgrp_cor	count		1	0
GNB2	201	OK	0.138046	0.2028230	0.6806227	0.49611	0.18818427	subgrp_cor	count		1	0
GIGYF1	5	OK	-0.7152769	1.1983617	-0.596878	0.55059	-0.34909885	subgrp_cor	count		1	0
POP7	26	OK	-0.089932	0.6412357	-0.140248	0.88846	-0.09178625	subgrp_cor	count		1	0
EPHB4	52	OK	0.1977857	0.482427	0.4099800	0.68182	0.17767870	subgrp_cor	count		1	0
SLC12A9	5	OK	-11.19992	27.762997	-0.403412	0.68665	-1.82940850	subgrp_cor	count		1	0
TRIP6	26	OK	1.9875046	0.7448731	2.6682457	0.00762	1.89359578	subgrp_cor	count		1	0
SRRT	10	OK	0.7003324	1.0357981	0.6761283	0.49896	0.00129195	subgrp_cor	count		1	0
TRIM56	47	OK	1.0609131	0.5031824	2.1084065	0.035	1.02432080	subgrp_cor	count		1	0
AP1S1	34	OK	0.3458477	0.5605586	0.6169697	0.53725	0.36560791	subgrp_cor	count		1	0
PLOD3	21	OK	-0.825235	0.6552775	-1.259368	0.2079	-0.7026738	subgrp_cor	count		1	0
ZNHIT1	350	OK	-0.0924424	0.1514049	-0.610563	0.54149	-0.12985920	subgrp_cor	count		1	0
CLDN15	31	OK	1.0192286	0.6069612	1.6792317	0.09311	1.11486259	subgrp_cor	count		1	0
FIS1	505	OK	-0.0314469	0.1218757	-0.258024	0.79639	-0.04459553	subgrp_cor	count		1	0
IFT22	17	OK	0.8341865	1.038789	0.8030369	0.42195	0.42076275	subgrp_cor	count		1	0
CUX1	23	OK	0.3208708	0.6485323	0.4947647	0.62077	0.28798399	subgrp_cor	count		1	0
SH2B2	2	OK	-0.910223	2.1881125	-0.415985	0.67742	-0.25785735	subgrp_cor	count		1	0
PRKRIP1	13	OK	-0.4576523	1.2259536	-0.373303	0.70892	-0.28822727	subgrp_cor	count		1	0
ORAI2	15	OK	0.7659737	1.1388567	0.6725812	0.50121	0.2386403	subgrp_cor	count		1	0
ALKBH4	2	OK	-0.2029407	1.6532365	-0.122753	0.9022	-4.82E-05	subgrp_cor	count		1	0
LRWD1	2	OK	4.6613299	5.7683860	0.8080821	0.41904	0.00274918	subgrp_cor	count		1	0
POLR2J	126	OK	1.0838939	0.2950055	3.674148	0.00024	1.40997929	subgrp_cor	count		1	0
POLR2J3	27	OK	0.0504113	0.5664264	0.0889989	0.92908	0.03262140	subgrp_cor	count		1	0
FAM185A	1	OK	18.759489	83.625179	0.2243282	0.8225	0.00028789	subgrp_cor	count		1	0
LRRC17	5	OK	3.2517626	2.3983585	1.3558283	0.17515	0.80585438	subgrp_cor	count		1	0
ARMC10	20	OK	-0.0179617	0.6966612	-0.025782	0.97943	-0.01694535	subgrp_cor	count		1	0
PMPCB	44	OK	0.413479	0.4170554	0.9914256	0.32148	0.49075183	subgrp_cor	count		1	0
DNAJC2	44	OK	0.3694738	0.4590907	0.8047948	0.42094	0.40847894	subgrp_cor	count		1	0
PSMC2	42	OK	0.463300	0.4718044	0.9819755	0.32611	0.53708421	subgrp_cor	count		1	0
ORC5	3	OK	21.642059	54.486891	0.3971975	0.69122	1.57744725	subgrp_cor	count		1	0
KMT2E-AS1	1	OK	0.6532528	2.5001284	0.2612877	0.79387	0.00016297	subgrp_cor	count		1	0
KMT2E	186	OK	-0.175831	0.1997373	-0.880812	0.37842	-0.23974043	subgrp_cor	count		1	0
SRPK2	49	OK	-0.9644953	0.4535387	-2.126599	0.03345	-1.12141020	subgrp_cor	count		1	0
PUS7	6	OK	-3.3132418	1.7468637	-1.896680	0.05787	-0.68069515	subgrp_cor	count		1	0
ATXN7L1	1	OK	-11.019418	43.249935	-0.2547846	0.79889	-1.17482461	subgrp_cor	count		1	0
SYPL1	246	OK	-0.3048599	0.1767334	-1.72497	0.08452	-0.41878767	subgrp_cor	count		1	0
NAMPT	245	OK	-0.0205657	0.1740333	-0.118170	0.90592	-0.02791675	subgrp_cor	count		1	0
AC007032.1	1	OK	3.4598146	6.7714513	0.5109413	0.60939	0.00030179	subgrp_cor	count		1	0

CCDC71L	59	OK	0.30737002	0.38106889	0.80659962	0.4199	0.26088302	subgrp_cor	count	1	0
PRKAR2B	6	OK	1.58240031	1.21682751	1.30043107	0.19345	1.2555626	subgrp_cor	count	1	0
HBP1	31	OK	-0.235258	0.50954329	-0.4618999	0.64415	-0.19343280	subgrp_cor	count	1	0
COG5	1	OK	3.45981462	6.77145131	0.51094137	0.60939	0.00030179	subgrp_cor	count	1	0
DUS4L	5	OK	-1.144924	1.33271434	-0.8590919	0.39029	-0.00067934	subgrp_cor	count	1	0
BCAP29	258	OK	0.7074303	0.17623654	4.01409559	5.97E-05	0.9878865	subgrp_cor	count	0.59324025	0
SLC26A4-AS1	2	OK	2.98494006	2.59888574	1.14854609	0.25074	1.23382860	subgrp_cor	count	1	0
SLC26A4	4	OK	4.04715036	2.63721613	1.53462976	0.12487	1.81582407	subgrp_cor	count	1	0
CBLL1	5	OK	0.34721101	1.814029	0.19145836	0.84817	0.00023216	subgrp_cor	count	1	0
DLD	18	OK	0.56243497	0.68504392	0.8210203	0.41162	0.46847856	subgrp_cor	count	1	0
LAMB1	89	OK	1.65549284	0.41384848	4.00021485	6.33E-05	2.0216339	subgrp_cor	count	0.62905292	0
NRCAM	2	OK	1.58240009	1.92403354	0.82243893	0.41082	0.77175617	subgrp_cor	count	1	0
PNPLA8	47	OK	-0.3779167	0.45584739	-0.8290422	0.40709	-0.36824828	subgrp_cor	count	1	0
THAP5	19	OK	0.42139565	0.60225768	0.69969328	0.48412	0.43223521	subgrp_cor	count	1	0
DNAJB9	48	OK	0.08329725	0.37743434	0.2209583	0.82512	0.09206827	subgrp_cor	count	1	0
IMMP2L	22	OK	2.49376508	0.87328482	2.85528781	0.0043	2.17750857	subgrp_cor	count	1	0
DOCK4	26	OK	-1.5350213	0.66957213	-2.2925407	0.02187	-1.12290787	subgrp_cor	count	1	0
ZNF277	9	OK	2.31209575	1.39620363	1.65598749	0.09772	1.32538316	subgrp_cor	count	1	0
IFRD1	68	OK	-0.6881254	0.3485006	-1.9745314	0.04832	-0.7567070	subgrp_cor	count	1	0
TMEM168	6	OK	-1.729293	1.51458687	-1.1417589	0.25355	-0.42952234	subgrp_cor	count	1	0
SMIM30	30	OK	-0.4402553	0.61672281	-0.7138625	0.47531	-0.35799054	subgrp_cor	count	1	0
MDFIC	28	OK	-0.3409287	0.55255675	-0.6170021	0.53722	-0.27866270	subgrp_cor	count	1	0
TES	13	OK	-0.4078351	0.92799346	-0.4394806	0.66033	-0.3322464	subgrp_cor	count	1	0
CAV2	327	OK	0.40813978	0.14968407	2.72649267	0.0064	0.57311894	subgrp_cor	count	1	0
CAV1	776	OK	-0.3310245	0.09238381	-3.5831438	0.00034	-0.47309100	subgrp_cor	count	1	0
MET	30	OK	0.51769989	0.541002	0.95692787	0.3386	0.51228712	subgrp_cor	count	1	0
CAPZA2	298	OK	0.38461429	0.16028655	2.39954195	0.01642	0.5361480	subgrp_cor	count	1	0
ST7	2	OK	1.7919026	2.28235398	0.78476776	0.43259	0.00045246	subgrp_cor	count	1	0
CTTNBP2	3	OK	-2.023032	1.80712677	-1.1194743	0.26294	-0.00109400	subgrp_cor	count	1	0
LSM8.00	124	OK	-0.0086051	0.24968193	-0.0344642	0.97251	-0.01150802	subgrp_cor	count	1	0
TSPAN12	32	OK	-2.556178	0.7172059	-3.5640785	0.00037	-2.5322502	subgrp_cor	count	1	0
ING3	10	OK	-1.4298085	1.03162145	-1.3859818	0.16575	-1.13389930	subgrp_cor	count	1	0
FAM3C	96	OK	-0.305531	0.29712946	-1.0282758	0.30382	-0.38792727	subgrp_cor	count	1	0
AASS	2	OK	-1.7585725	2.35544393	-0.7465996	0.45531	-0.00036089	subgrp_cor	count	1	0
CADPS2	27	OK	-0.8973099	0.62774811	-1.4294107	0.15289	-0.63414258	subgrp_cor	count	1	0
RNF148	1	OK	-11.019418	43.2499352	-0.2547846	0.79889	-1.17482461	subgrp_cor	count	1	0
NDUFA5	124	OK	0.73459839	0.26309191	2.79217404	0.00524	0.96475921	subgrp_cor	count	1	0
WASL	90	OK	0.23868777	0.31452339	0.75888719	0.44792	0.30687129	subgrp_cor	count	1	0
POT1	3	OK	-1.8538345	1.85439186	-0.9996995	0.31746	-0.46053279	subgrp_cor	count	1	0
ZNF800	25	OK	0.20199623	0.60919417	0.33157942	0.74021	0.18093403	subgrp_cor	count	1	0
GCC1	1	OK	18.7594894	83.6251792	0.22432824	0.8225	0.00028789	subgrp_cor	count	1	0
ARF5	110	OK	-0.5938433	0.27940936	-2.1253521	0.03356	-0.77882444	subgrp_cor	count	1	0
SND1	10	OK	0.31786779	0.94048736	0.33798199	0.72536	0.20920326	subgrp_cor	count	1	0
RBM28	15	OK	-0.064327	0.74782599	-0.0860189	0.93145	-0.05322990	subgrp_cor	count	1	0
IMPDH1	12	OK	0.87780826	0.98247858	0.89346311	0.37161	0.00162924	subgrp_cor	count	1	0
HILPDA	11	OK	-0.2508664	0.78410065	-0.3199418	0.74901	-0.20604840	subgrp_cor	count	1	0
METTL2B	4	OK	0.16816905	1.21985865	0.13785946	0.89035	0.08565132	subgrp_cor	count	1	0
AC018638.7	1	OK	18.7594894	83.6251792	0.22432824	0.8225	0.00028789	subgrp_cor	count	1	0
FLNC	9	OK	-0.3516244	1.28014306	-0.2746758	0.78357	-0.17354887	subgrp_cor	count	1	0
ATP6V1F	258	OK	-0.1558176	0.17171956	-0.9079782	0.36389	-0.21631223	subgrp_cor	count	1	0
TNPO3	1	OK	18.7594894	83.6251792	0.22432824	0.8225	0.00028789	subgrp_cor	count	1	0
NRF1	1	OK	-10.442278	41.4658099	-0.2518288	0.80117	-0.00045289	subgrp_cor	count	1	0
UBE2H	46	OK	0.80786604	0.49719146	1.62485905	0.10419	0.919288	subgrp_cor	count	1	0
ZC3HC1	3	OK	-14.65800	45.6903329	-0.3208138	0.74835	-0.00067223	subgrp_cor	count	1	0
KLHDC10	9	OK	-0.6763363	1.28587443	-0.5259738	0.59891	-0.4049758	subgrp_cor	count	1	0
TMEM209	2	OK	1.9259645	2.51548794	0.76564251	0.44389	0.56022119	subgrp_cor	count	1	0
MEST	1	OK	-10.442278	41.4658099	-0.2518288	0.80117	-0.00045289	subgrp_cor	count	1	0
COPG2	2	OK	18.7594894	83.6251792	0.22432824	0.8225	0.00028789	subgrp_cor	count	1	0
AC016831.7	10	OK	-1.7465899	0.96406331	-1.8116962	0.07003	-0.42984672	subgrp_cor	count	1	0
LINC00513	1	OK	1.58239999	2.72001883	0.58176068	0.56072	0.47433761	subgrp_cor	count	1	0
AC016831.1	1	OK	-10.442278	41.4658099	-0.2518288	0.80117	-0.00045289	subgrp_cor	count	1	0
AC016831.5	11	OK	-1.3846999	0.90854842	-1.5240793	0.12749	-0.79184168	subgrp_cor	count	1	0
AC058791.1	36	OK	-0.578072	0.44275172	-1.3056368	0.19168	-0.62659098	subgrp_cor	count	1	0
MKLN1	16	OK	0.21012768	0.74307857	0.28277989	0.77735	0.15895953	subgrp_cor	count	1	0
AC008264.2	2	OK	-0.2029407	1.65322657	-0.1227536	0.9023	-4.82E-05	subgrp_cor	count	1	0
PODXL	156	OK	0.87685412	0.24666722	3.55480599	0.00038	1.14742299	subgrp_cor	count	1	0
PLXNA4	21	OK	25.4708359	32.6019233	0.78126789	0.43464	4.24444086	subgrp_cor	count	1	0
CHCHD3	29	OK	-1.0063243	0.61691586	-1.6312181	0.10284	-1.03997021	subgrp_cor	count	1	0
EXOC4	9	OK	0.7432149	1.00583234	0.72890535	0.45996	0.54991519	subgrp_cor	count	1	0
SLC35B4	5	OK	0.06141457	1.2093467	0.05078326	0.9595	0.00010965	subgrp_cor	count	1	0
AKR1B1	20	OK	1.52997027	0.73299665	2.0872814	0.03686	1.59841959	subgrp_cor	count	1	0
BPGM	12	OK	1.41194262	1.49593083	0.94385555	0.34526	0.69797651	subgrp_cor	count	1	0
AGBL3	2	OK	1.9259645	2.51548794	0.76564251	0.44389	0.56022119	subgrp_cor	count	1	0
CYREN	6	OK	-0.8463989	1.53706861	-0.5506578	0.58187	-0.38748378	subgrp_cor	count	1	0
TMEM140	36	OK	-0.0636339	0.43474415	-0.1463709	0.88363	-0.07449486	subgrp_cor	count	1	0
CNOT4	32	OK	-0.0215184	0.53404845	-0.0402929	0.96786	-0.02265234	subgrp_cor	count	1	0
STMP1	68	OK	0.91006759	0.39513684	2.30317067	0.02127	1.14520065	subgrp_cor	count	1	0
MTPN	228	OK	0.1789419	0.18812017	0.95121061	0.3415	0.24865442	subgrp_cor	count	1	0
PTN	5	OK	21.642059	54.4868919	0.39719753	0.69122	1.57744735	subgrp_cor	count	1	0
DGKI	1	OK	0.65325287	2.50012846	0.26128772	0.79387	0.00016297	subgrp_cor	count	1	0
CREB3L2	29	OK	3.64469813	1.07315198	3.39625534	0.00068	2.93658397	subgrp_cor	count	1	0
TRIM24	6	OK	0.73785346	1.30674578	0.56464958	0.57231	0.22996142	subgrp_cor	count	1	0
ZC3HAV1	66	OK	-0.0063435	0.33993594	-0.0186607	0.98511	-0.00566001	subgrp_cor	count	1	0
UBN2	3	OK	-2.023032	1.80712677	-1.1194743	0.26294	-0.00109400	subgrp_cor	count	1	0
FMC1	6	OK	3.7563655	2.45935196	1.5273803	0.12667	1.75930479	subgrp_cor	count	1	0
LUC7L2	22	OK	0.68872701	0.6524851	1.05522098	0.29132	0.5698062	subgrp_cor	count	1	0
HIPK2	21	OK	1.7347913	0.77626753	2.23478534	0.02543	1.55589241	subgrp_cor	count	1	0
PARP12	10	OK	1.14631663	1.16258145	0.98600974	0.32412	0.38415001	subgrp_cor	count	1	0
KDM7A	4	OK	3.45981462	6.77145131	0.51094137	0.60939	0.00030179	subgrp_cor	count	1	0
MKRN1	9	OK	0.75167129	1.25702954	0.59797424	0.54986	0.00138724	subgrp_cor	count	1	0
ADCK2	1	OK	3.45981462	6.77145131	0.51094137	0.60939	0.00030179	subgrp_cor	count	1	0
NDUFB2	322	OK	0.46612966	0.16470397	2.83010579	0.00465	0.65149343	subgrp_cor	count	1	0
BRAF	6	OK	-1.5256412	1.34685813	-1.1327408	0.25732	-0.00086992	subgrp_cor	count	1	0
MRPS33	36	OK	0.18809408	0.53664511	0.35049986	0.72596	0.19878324	subgrp_cor	count	1	0
AGK	2	OK	23.9918968	84.8999419	0.28259026	0.77749	1.65081709	subgrp_cor	count	1	0
KIAA1147	4	OK	19.1030323	50.7724696	0.37624785	0.70672	0.00078880	subgrp_cor	count	1	0
SSBP1	246	OK	0.05972848	0.18232469	0.32758377	0.74322	0.08277901	subgrp_cor	count	1	0
PRSS1	1	OK	1.58239999	2.72001883	0.58176068	0.56072	0.47433761	subgrp_cor	count	1	0
PRSS2	5	OK	2.90706132	1.62938877	1.78414224	0.0744	2.35087600	subgrp_cor	count	1	0
GSTK1	279	OK	-0.5663724	0.16588486	-3.4142503	0.00064	-0.79349338	subgrp_cor	count	1	0
CASP2	2	OK	-0.2029407	1.65322657	-0.1227536	0.9023	-4.82E-05	subgrp_cor	count	1	0
AC093673.1	4	OK	-0.3495512	1.85447474	-0.1884907	0.85049	-0.17337783	subgrp_cor	count	1	0
ZYX	39	OK	2.66328283	0.70610287	3.77194873	0.00016	3.16536057	subgrp_cor	count	1	0
TCAF1	15	OK	0.47951024	0.71407223	0.67151494	0.50189	0.24567099	subgrp_cor	count	1	0
TPK1	4	OK	-3.5229022	2.14366662	-1.6434002	0.1003	-0.67130448	subgrp_cor	count	1	0
CUL1	6	OK	-0.181478	1.26586186	-0.1433632	0.886	-0.00011729	subgrp_cor	count	1	0
PDIA4	40	OK	1.55490791	0.55473995	2.80294923	0.00506	1.70144614	subgrp_cor	count	1	0
ZNF425	1	OK	-15.99503	70.349421	-0.2273655	0.82014	-0.00028474	subgrp_cor	count	1	0
ZNF282	1	OK	-10.442278	41.4658099	-0.2518288	0.80117	-0.00045289	subgrp_cor	count	1	0
ZNF746	1	OK	18.7594894	83.6251792	0.22432824	0.8225	0.00028789	subgrp_cor	count	1	0
ZNF467	20	OK	-0.4566702	0.67528873	-0.6762592	0.49888	-0.33420901	subgrp_cor	count	1	0
ZNF862	1	OK	3.45981462	6.77145131	0.51094137	0.60939	0.00030179	subgrp_cor	count	1	0
ATP6V0E2	2	OK	18.7594894	83.6251792	0.22432824	0.8225	0.00028789	subgrp_cor	count	1	0
RARRES2	24	OK	2.24691211	0.95136041	2.36178959	0.01819	1.50587046	subgrp_cor	count	1	0
REPIN1	8	OK	0.69220624	1.15267181	0.60052327	0.54816	0.44977596	subgrp_cor	count	1	0
ZNF775	1	OK	-15.99503	70.349421	-0.2273655	0.82014	-0.00028474	subgrp_cor	count	1	0
GIMAP8	66	OK	-0.6874914	0.3285354	-2.0925945	0.03639	-0.86185802	subgrp_cor	count	1	0
GIMAP2	16	OK	-0.8340747	0.74011409	-1.1269542	0.25976	-0.66113856	subgrp_cor	count	1	0
GIMAP5	1	OK	-10.442278	41.4658099	-0.2518288	0.80117	-0.00045289	subgrp_cor	count	1	0
TMEM176B	7	OK	-3.6201645	1.81792403	-1.9914824	0.04642	-1.72842488	subgrp_cor	count	1	0
TMEM176A	7	OK	-1.8862408	1.32533598	-1.4232171	0.15467	-1.32119055	subgrp_cor	count	1	0
NOS3	43	OK	2.44257783	0.73425758	3.3265954	0.00088	2.03566840	subgrp_cor	count	1	0
ABCB8	1	OK	-11.019418	43.2499352	-0.2547846	0.79889	-1.17482461	subgrp_cor	count	1	0
CDK5	3	OK	-1.7585725	2.35544393	-0.7465996	0.45531	-0.00036089	subgrp_cor	count	1	0
SLC4A2	4	OK	0.17216145	1.5344504	0.11219747	0.91067	0.00011395	subgrp_cor	count	1	0
FASTK	8	OK	0.96593117	1.300792	0.74257782	0.45774	0.64203860	subgrp_cor	count	1	0
TMUB1	16	OK	0.70984775	0.7732003	0.91806452	0.35859	0.53278364	subgrp_cor	count	1	0
AGAP3	5	OK	0.17216145	1.5344504	0.11219747	0.91067	0.00011395	subgrp_cor	count	1	0
ABCF2	5	OK	-0.9607313	1.395414	-0.6884919	0.49114	-0.00157680	subgrp_cor	count	1	0
NUB1	82	OK	-0.8557307	0.30555728	-2.8005574	0.0051	-1.07740096	subgrp_cor	count	1	0
RHEB	188	OK	0.33034578	0.21213301	1.54995104	0.12115	0.4507765	subgrp_cor	count	1	0
PRKAG2	15	OK	-0.9059573	0.8480959	-1.068225	0.28542	-0.54820163	subgrp_cor	count	1	0
PRKAG2-AS1	1	OK	-11.019418	43.2499352	-0.2547846	0.79889	-1.17482461	subgrp_cor	count	1	0
GALNT11	13	OK	0.82007571	0.91097806	0.90021456	0.36801	0.41785847	subgrp_cor	count	1	0
KMT2C	36	OK	0.50931196	0.48105056	1.05874932	0.28971	0.38417824	subgrp_cor	count	1	0
LINC01003	10	OK	0.19301132	0.88258485	0.21868899	0.8268	0.16070115	subgrp_cor	count	1	0
DPP6	1	OK	18.7594894	83.6251792	0.22432824	0.8225	0.00028789	subgrp_cor	count	1	0
PAXIP1-AS2	2	OK	18.7594894	83.6251792	0.22432824	0.8225	0.00028789	subgrp_cor	count	1	0
PAXIP1-AS1	3	OK	4.21092061	4.28756165	0.9821248	0.32604	0.00257090	subgrp_cor	count	1	0
INSIG1	66	OK	0.87266001	0.37588873	2.32152959	0.02026	1.09443579	subgrp_cor	count	1	0
RBM33	10	OK	-0.1987744	0.92987332	-0.213765	0.83072	-0.14789221	subgrp_cor	count	1	0
LINC01006	3	OK	1.40004994	2.08531454	0.6713855	0.50197	0.87174583	subgrp_cor	count	1	0
RNF32	2	OK	21.642059	54.4868919	0.39719753	0.69122	1.57744735	subgrp_cor	count	1	0
LMBR1	3	OK	-1.2956167	2.0414257	-0.6346627	0.52565	-0.00075754	subgrp_cor	count	1	0
NOM1	1	OK	-10.442278	41.4658099	-0.2518288	0.80117	-0.00045289	subgrp_cor	count	1	0
UBE3C	3	OK	-13.271979	28.0097917	-0.4738335	0.63562	-2.0156631	subgrp_cor	count	1	0
DNAJB6	125	OK	-0.2484404	0.26673414	-0.9314157	0.35164	-0.31701858	subgrp_cor	count	1	0
PTPRN2	18	OK	-0.8936648	0.72082224	-1.2397853	0.21505	-0.84483659	subgrp_cor	count	1	0
ESYT2	23	OK	-0.5523754	0.66197558	-0.8344348	0.40404	-0.53438320	subgrp_cor	count	1	0
WDR60	47	OK	-0.2233707	0.40504162	-0.551478	0.58131	-0.27100384	subgrp_cor	count	1	0
GTPBP6	22	OK	0.05431456	0.63525165	0.08550086	0.93186	0.03515287	subgrp_cor	count	1	0
LINC00685	1	OK	-10.442278	41.4658099	-0.2518288	0.80117	-0.00045289	subgrp_cor	count	1	0
SLC25A6	596	OK	-0.1938562	0.11226346	-1.7267968	0.0842	-0.27545939	subgrp_cor	count	1	0
ASMTL	19	OK	-1.0728144	0.69064425	-1.5533532	0.12034	-0.89694557	subgrp_cor	count	1	0
AKAP17A	19	OK	-0.3781303	0.77363016	-0.4887739	0.625	-0.23768465	subgrp_cor	count	1	0
DHRSX	8	OK	1.16060945	1.18256385	0.98143492	0.32638	0.57202220	subgrp_cor	count	1	0
ZBED1	11	OK	0.39371469	1.12185156	0.35095078	0.72562	0.19732012	subgrp_cor	count	1	0
CD99	661	OK	-0.0246928	0.10342456	-0.2387521	0.8112	-0.03518946	subgrp_cor	count	1	0
ARSD	6	OK	0.44916523	1.25221424	0.35869679	0.71982	0.14267849	subgrp_cor	count	1	0
ARSE	1	OK	1.58239999	2.72001883	0.58176068	0.56072	0.47433761	subgrp_cor	count	1	0
MXRA5	2	OK	3.45981497	4.78586864	0.72292309	0.46972	0.00081925	subgrp_cor	count	1	0
PRKX	2	OK	0.65325287	2.50012846	0.26128772	0.79387	0.00016297	subgrp_cor	count	1	0
BX890604.1	1	OK	23.9918968	84.8999419	0.28259026	0.77749	1.65081709	subgrp_cor	count	1	0
STS	1	OK	-11.019418	43.2499352	-0.2547846	0.79889	-1.17482461	subgrp_cor	count	1	0
PNPLA4	3	OK	0.83595158	1.92553229	0.43414049	0.66419	0.26012312	subgrp_cor	count	1	0
TBL1X	3	OK	2.39922841	2.24960179	1.0665125	0.28619	0.00059488	subgrp_cor	count	1	0
SHROOM2	1	OK	3.45981462	6.77145131	0.51094137	0.60939	0.00030179	subgrp_cor	count	1	0
WWC3	12	OK	3.58188421	1.62296462	2.20564177	0.02741	1.72137819	subgrp_cor	count	1	0
CLCN4	1	OK	23.9918968	84.8999419	0.28259026	0.77749	1.65081709	subgrp_cor	count	1	0
MID1	8	OK	-1.7368823	1.08859999	-1.5955282	0.1106	-0.94612280	subgrp_cor	count	1	0
HCCS	11	OK	-0.5731349	0.93670229	-0.6118645	0.54062	-0.27917049	subgrp_cor	count	1	0
ARHGAP6	5	OK	1.15288977	1.51684659	0.76005693	0.44722	0.33628872	subgrp_cor	count	1	0
MSL3	33	OK	0.72044385	0.57558938	1.25166286	0.21069	0.64346470	subgrp_cor	count	1	0
PRPS2	2	OK	0.40610002	2.10834129	0.19261588	0.84726	0.12645366	subgrp_cor	count	1	0
TMSB4X	886	OK	-0.099418	0.08350252	-1.190575	0.23382	-0.14229793	subgrp_cor	count	1	0
RAB9A	12	OK	-0.0197185	0.85129055	-0.023163	0.98152	-0.01476841	subgrp_cor	count	1	0
TRAPPC2	6	OK	0.38987436	1.06866912	0.36482233	0.71524	0.29415334	subgrp_cor	count	1	0
OFD1	18	OK	-1.3084936	0.71795391	-1.8225315	0.06837	-1.14028809	subgrp_cor	count	1	0
GPM6B	2	OK	1.58240009	1.92403354	0.82243893	0.41082	0.77175617	subgrp_cor	count	1	0
GEMIN8	1	OK	3.45981462	6.77145131	0.51094137	0.60939	0.00030179	subgrp_cor	count	1	0
MOSPD2	4	OK	-1.6462456	1.93519292	-0.8506881	0.39494	-0.70360391	subgrp_cor	count	1	0
ASB9	17	OK	-2.5183352	0.91103197	-2.7642666	0.00571	-2.07721864	subgrp_cor	count	1	0
PIR	44	OK	0.56443168	0.40284493	1.40111393	0.16118	0.70582479	subgrp_cor	count	1	0
CA5B	6	OK	1.41245154	1.42415025	0.99178548	0.3212	0.73805866	subgrp_cor	count	1	0
ZRSR2	8	OK	-1.7312876	1.16413322	-1.4871903	0.13696	-0.00262919	subgrp_cor	count	1	0
AP1S2	108	OK	-0.7449223	0.28120593	-2.6490276	0.00807	-0.97630551	subgrp_cor	count	1	0
SYAP1	52	OK	0.49242147	0.43323237	1.13635976	0.25581	0.58979649	subgrp_cor	count	1	0
TXLNG	9	OK	0.72515827	0.88989915	0.81487693	0.41514	0.60223799	subgrp_cor	count	1	0
RBBP7	44	OK	-0.091938	0.44739149	-0.2054970	0.83718	-0.10475753	subgrp_cor	count	1	0
SCML1	20	OK	-0.3072719	0.67643084	-0.4542547	0.64965	-0.30029125	subgrp_cor	count	1	0
RAI2	7	OK	-1.2352853	1.25046783	-0.9878585	0.32322	-0.00197223	subgrp_cor	count	1	0
SCML2	1	OK	3.45981462	6.77145131	0.51094137	0.60939	0.00030179	subgrp_cor	count	1	0
CDKL5	8	OK	2.21320771	1.29726707	1.70605404	0.088	0.00150828	subgrp_cor	count	1	0
PHKA2	2	OK	-10.475634	27.4918879	-0.3810432	0.70317	-1.13811785	subgrp_cor	count	1	0
PDHA1	16	OK	0.94354323	0.82363655	1.14558203	0.25197	0.83699347	subgrp_cor	count	1	0
SH3KBP1	13	OK	1.4195078	0.91305416	1.55468083	0.12002	0.70134300	subgrp_cor	count	1	0
BCLAF3	3	OK	0.16814912	1.72533008	0.0974591	0.92236	0.05201277	subgrp_cor	count	1	0
EIF1AX	182	OK	0.20795133	0.21241903	0.97896751	0.3276	0.28187725	subgrp_cor	count	1	0
RPS6KA3	36	OK	-0.1970688	0.4901103	-0.4020490	0.68765	-0.1849241	subgrp_cor	count	1	0
SMS	9	OK	0.84821096	1.36279487	0.62247883	0.53362	0.4318898	subgrp_cor	count	1	0
PRDX4	222	OK	-0.2631907	0.18410445	-1.4295728	0.15284	-0.36815322	subgrp_cor	count	1	0
ACOT9	23	OK	0.76632596	0.71215508	1.07606613	0.2819	0.49700541	subgrp_cor	count	1	0
APOO	2	OK	-11.448548	41.761485	-0.2741412	0.78398	-0.00049246	subgrp_cor	count	1	0
EIF2S3	93	OK	-0.9084386	0.28670701	-3.1685258	0.00153	-1.13669855	subgrp_cor	count	1	0
ZFX	13	OK	0.03432919	0.75271676	0.04560705	0.96362	0.02220329	subgrp_cor	count	1	0
TMEM47	32	OK	2.25837319	0.65449381	3.45056465	0.00056	2.22017078	subgrp_cor	count	1	0
PRRG1	16	OK	-1.9151496	0.92595308	-2.068305	0.03861	-1.45312682	subgrp_cor	count	1	0
LANCL3	3	OK	2.16111585	2.36779439	0.91271263	0.36139	0.61359306	subgrp_cor	count	1	0
DYNLT3	50	OK	0.70658366	0.47845326	1.47680813	0.13972	0.80561250	subgrp_cor	count	1	0
SRPX	67	OK	1.40144048	0.37985199	3.68943833	0.00022	1.74212884	subgrp_cor	count	1	0
RPGR	75	OK	0.52843505	0.32948434	1.60382446	0.10875	0.63300925	subgrp_cor	count	1	0
OTC	27	OK	4.87251223	1.47965074	3.2930151	0.00099	3.20353021	subgrp_cor	count	1	0
MID1IP1	24	OK	0.94937258	0.58902912	1.6117583	0.10701	0.9302797	subgrp_cor	count	1	0
BCOR	1	OK	23.9918968	84.8999419	0.28259026	0.77749	1.65081709	subgrp_cor	count	1	0
ATP6AP2	184	OK	0.42828888	0.20988296	2.04108438	0.04124	0.58449756	subgrp_cor	count	1	0
CXorf38	5	OK	-2.0242839	1.42191547	-1.4236318	0.15455	-0.8034941	subgrp_cor	count	1	0
MED14OS	3	OK	0.1112767	1.61203872	0.06902855	0.94497	0.05657342	subgrp_cor	count	1	0
USP9X	17	OK	2.98496096	1.17857634	2.5326826	0.01132	2.04450781	subgrp_cor	count	1	0
DDX3X	280	OK	0.21532826	0.16615303	1.29596353	0.19499	0.29862341	subgrp_cor	count	1	0
CASK	5	OK	-0.691827	1.06525457	-0.6494475	0.51605	-0.00042873	subgrp_cor	count	1	0
MAOA	31	OK	-2.3352245	0.63494624	-3.6778303	0.00024	-2.05233683	subgrp_cor	count	1	0
MAOB	6	OK	-2.9288941	1.63528697	-1.7909350	0.0733	-0.61462545	subgrp_cor	count	1	0
FUNDC1	6	OK	4.38015185	3.4369227	1.27444003	0.20251	0.00263449	subgrp_cor	count	1	0
MIR222HG	22	OK	0.71410597	0.6358631	1.12304986	0.26142	0.82815670	subgrp_cor	count	1	0
KRBOX4	6	OK	-2.0841657	1.82967462	-1.1390909	0.25467	-1.07774579	subgrp_cor	count	1	0
CHST7	15	OK	-2.5458942	0.98327699	-2.5891932	0.00962	-1.44158006	subgrp_cor	count	1	0
SLC9A7	3	OK	0.83961098	1.49856075	0.62700893	0.53065	0.2916352	subgrp_cor	count	1	0
RP2	1	OK	-10.442278	41.4658099	-0.2518288	0.80117	-0.00045289	subgrp_cor	count	1	0
RGN	3	OK	19.1030323	50.7724696	0.37624785	0.70672	0.00078880	subgrp_cor	count	1	0
NDUFB11	313	OK	0.52409602	0.16126947	3.24981557	0.00115	0.73443255	subgrp_cor	count	1	0
RBM10	5	OK	-1.0379618	1.42801275	-0.7268575	0.46731	-0.00062229	subgrp_cor	count	1	0
UBA1	7	OK	6.14900159	5.41239945	1.1360953	0.25592	1.05209450	subgrp_cor	count	1	0
CDK16	2	OK	0.16814912	1.72533008	0.0974591	0.92236	0.05201277	subgrp_cor	count	1	0
USP11	6	OK	0.64395055	1.14383456	0.56297525	0.57345	0.48511172	subgrp_cor	count	1	0
ZNF41	1	OK	23.9918968	84.8999419	0.28259026	0.77749	1.65081709	subgrp_cor	count	1	0
LINC01560	1	OK	3.45978799	4.78585287	0.72291982	0.46972	0.00081879	subgrp_cor	count	1	0
ARAF	7	OK	-1.7224651	1.3369256	-1.2883777	0.19761	-0.00261489	subgrp_cor	count	1	0
TIMP1	749	OK	0.24714608	0.0929792	2.65810734	0.00786	0.35324471	subgrp_cor	count	1	0
CFP	2	OK	-0.9102232	2.18811251	-0.4159856	0.67742	-0.25785735	subgrp_cor	count	1	0
ELK1	4	OK	3.45981497	4.78586864	0.72292309	0.46972	0.00081925	subgrp_cor	count	1	0
UXT	280	OK	0.06544315	0.166346	0.39341585	0.69401	0.09085233	subgrp_cor	count	1	0
ZNF630	1	OK	-11.019418	43.2499352	-0.2547846	0.79889	-1.17482461	subgrp_cor	count	1	0
EBP	8	OK	0.49100923	1.141223	0.43021138	0.66704	0.37038455	subgrp_cor	count	1	0
RBM3	311	OK	0.0215502	0.16065803	0.13413706	0.89329	0.02983757	subgrp_cor	count	1	0
WDR13	41	OK	0.59050511	0.48571688	1.21573931	0.22408	0.5588067	subgrp_cor	count	1	0
HDAC6	3	OK	-1.2956167	2.0414257	-0.6346627	0.52565	-0.00075754	subgrp_cor	count	1	0
TIMM17B	11	OK	1.14138917	0.86316443	1.3223311	0.18606	1.06152146	subgrp_cor	count	1	0
PQBP1	56	OK	0.03262821	0.37377085	0.0872947	0.93044	0.0423256	subgrp_cor	count	1	0
SLC35A2	8	OK	0.20056699	0.98275878	0.20408568	0.83829	0.13031528	subgrp_cor	count	1	0
PIM2	2	OK	-11.448548	41.761485	-0.2741412	0.78398	-0.00049246	subgrp_cor	count	1	0
OTUD5	8	OK	-1.7465736	1.53002912	-1.1415296	0.25365	-0.7126714	subgrp_cor	count	1	0
GRIPAP1	10	OK	0.10931171	0.89111171	0.12266891	0.90237	0.07086594	subgrp_cor	count	1	0
TFE3	2	OK	-0.2029407	1.65322657	-0.1227536	0.9023	-4.82E-05	subgrp_cor	count	1	0
PRAF2	37	OK	-0.4622812	0.45936812	-1.0063415	0.31425	-0.42874819	subgrp_cor	count	1	0
WDR45	31	OK	0.2488053	0.52461057	0.47426656	0.63531	0.28010640	subgrp_cor	count	1	0
GPKOW	3	OK	3.45978799	4.78585287	0.72291982	0.46972	0.00081879	subgrp_cor	count	1	0
MAGIX	1	OK	-15.99503	70.349421	-0.2273655	0.82014	-0.00028474	subgrp_cor	count	1	0
PLP2	85	OK	-0.1700468	0.30627336	-0.5552126	0.57875	-0.22850259	subgrp_cor	count	1	0
SYP	1	OK	23.9918968	84.8999419	0.28259026	0.77749	1.65081709	subgrp_cor	count	1	0
PPP1R3F	1	OK	3.45981462	6.77145131	0.51094137	0.60939	0.00030179	subgrp_cor	count	1	0
USP27X	3	OK	21.642059	54.4868919	0.39719753	0.69122	1.57744735	subgrp_cor	count	1	0
SHROOM4	31	OK	0.59962539	0.65169843	0.92009642	0.35752	0.6013654	subgrp_cor	count	1	0
MAGED1	9	OK	4.09321054	2.2536942	1.81622269	0.06934	1.82420569	subgrp_cor	count	1	0
GPR173	1	OK	-11.019418	43.2499352	-0.2547846	0.79889	-1.17482461	subgrp_cor	count	1	0
TSPYL2	16	OK	0.3498252	0.87324438	0.40060401	0.68871	0.28989707	subgrp_cor	count	1	0
KANTR	1	OK	-11.019418	43.2499352	-0.2547846	0.79889	-1.17482461	subgrp_cor	count	1	0
IQSEC2	3	OK	-1.8538345	1.85439186	-0.9996995	0.31746	-0.46053279	subgrp_cor	count	1	0
SMC1A	22	OK	0.74176275	0.78177396	0.94881998	0.34271	0.55328070	subgrp_cor	count	1	0
HSD17B10	40	OK	0.27555889	0.50535045	0.54528277	0.58556	0.29120252	subgrp_cor	count	1	0
HUWE1	26	OK	0.01879598	0.54807256	0.03429458	0.97264	0.01774792	subgrp_cor	count	1	0
PHF8	1	OK	3.45981462	6.77145131	0.51094137	0.60939	0.00030179	subgrp_cor	count	1	0
WNK3	2	OK	1.9259645	2.51548794	0.76564251	0.44389	0.56022119	subgrp_cor	count	1	0
TSR2	43	OK	-0.1002323	0.41223302	-0.2431448	0.80789	-0.11727289	subgrp_cor	count	1	0
GNL3L	4	OK	1.5231809	1.72315008	0.88975471	0.3736	0.46131746	subgrp_cor	count	1	0
MAGED2	129	OK	0.54870995	0.25727396	2.13195845	0.03301	0.71803960	subgrp_cor	count	1	0
APEX2	3	OK	-10.475634	27.4918879	-0.3810432	0.70317	-1.13811785	subgrp_cor	count	1	0
FAM104B	16	OK	1.63432942	0.87868419	1.85997363	0.06289	1.291229	subgrp_cor	count	1	0
MAGEH1	24	OK	0.56645737	0.66190206	0.85580239	0.39211	0.57985420	subgrp_cor	count	1	0
USP51	2	OK	21.1053238	83.3412731	0.25323976	0.80008	0.0003103	subgrp_cor	count	1	0
RRAGB	7	OK	0.9577923	1.21984272	0.78517688	0.43235	0.72589091	subgrp_cor	count	1	0
AL445472.1	1	OK	23.9918968	84.8999419	0.28259026	0.77749	1.65081709	subgrp_cor	count	1	0
KLF8	2	OK	5.68306046	5.33217267	1.06580594	0.2866	0.00307231	subgrp_cor	count	1	0
UBQLN2	3	OK	0.16814912	1.72533008	0.0974591	0.92236	0.05201277	subgrp_cor	count	1	0
NBDY	153	OK	0.34721135	0.23323855	1.48865332	0.13658	0.47207859	subgrp_cor	count	1	0
SPIN2B	2	OK	18.7594894	83.6251792	0.22432824	0.8225	0.00028789	subgrp_cor	count	1	0
ZXDA	1	OK	18.7594894	83.6251792	0.22432824	0.8225	0.00028789	subgrp_cor	count	1	0
ARHGEF9	5	OK	6.66246465	6.95721339	0.95763408	0.33825	0.00121744	subgrp_cor	count	1	0
ZC4H2	2	OK	21.642059	54.4868919	0.39719753	0.69122	1.57744735	subgrp_cor	count	1	0
LAS1L	4	OK	1.12250508	1.45693183	0.7704582	0.44103	0.34607098	subgrp_cor	count	1	0
MSN	222	OK	0.26097043	0.20152449	1.29498124	0.19532	0.3551658	subgrp_cor	count	1	0
AR	18	OK	6.54565748	3.07214508	2.13064726	0.03312	3.28724705	subgrp_cor	count	1	0
OPHN1	2	OK	4.21084377	4.29582435	0.98021786	0.32698	7.14E-14	subgrp_cor	count	1	0
YIPF6	24	OK	1.48688479	0.67906429	2.18960829	0.02855	1.42603569	subgrp_cor	count	1	0
STARD8	5	OK	-0.2565733	1.3412338	-0.1912964	0.84829	-0.12807708	subgrp_cor	count	1	0
EFNB1	5	OK	0.70021075	1.73088606	0.40454632	0.68579	0.00047551	subgrp_cor	count	1	0
IGBP1	99	OK	-0.1620535	0.29494209	-0.5494418	0.5827	-0.21278687	subgrp_cor	count	1	0
PDZD11	34	OK	0.6445482	0.5087676	1.2668814	0.2052	0.74590829	subgrp_cor	count	1	0
SNX12	6	OK	1.12244377	1.45693139	0.77041635	0.44105	0.3460629	subgrp_cor	count	1	0
FOXO4	1	OK	18.7594894	83.6251792	0.22432824	0.8225	0.00028789	subgrp_cor	count	1	0
IL2RG	1	OK	-11.019418	43.2499352	-0.2547846	0.79889	-1.17482461	subgrp_cor	count	1	0
MED12	1	OK	18.7594894	83.6251792	0.22432824	0.8225	0.00028789	subgrp_cor	count	1	0
ZMYM3	1	OK	41.7883033	174.29608	0.23975469	0.81052	0.00014488	subgrp_cor	count	1	0
NONO	60	OK	0.63178227	0.4013526	1.57413274	0.11546	0.73973986	subgrp_cor	count	1	0
TAF1	7	OK	0.21616589	1.19733271	0.18053953	0.85672	0.11023974	subgrp_cor	count	1	0
OGT	8	OK	0.27655792	0.98157191	0.28175003	0.77814	0.23048401	subgrp_cor	count	1	0
NHSL2	27	OK	-0.1888188	0.60651884	-0.3113151	0.75556	-0.19213962	subgrp_cor	count	1	0
PIN4	53	OK	0.04000235	0.39992494	0.10002465	0.92032	0.04567137	subgrp_cor	count	1	0
RPS4X	880	OK	-0.1845088	0.08461436	-2.1805858	0.02921	-0.26395401	subgrp_cor	count	1	0
HDAC8	3	OK	23.9918968	84.8999419	0.28259026	0.77749	1.65081709	subgrp_cor	count	1	0
CHIC1	1	OK	23.9918968	84.8999419	0.28259026	0.77749	1.65081709	subgrp_cor	count	1	0
JPX	25	OK	0.18231458	0.63232321	0.28832499	0.7731	0.15172532	subgrp_cor	count	1	0
FTX	33	OK	0.25562158	0.50164091	0.50957083	0.61035	0.30321044	subgrp_cor	count	1	0
SLC16A2	1	OK	18.7594894	83.6251792	0.22432824	0.8225	0.00028789	subgrp_cor	count	1	0
RLIM	11	OK	-0.7098325	1.4197009	-0.4999871	0.61708	-0.5229127	subgrp_cor	count	1	0
ABCB7	7	OK	-1.5478218	1.26151866	-1.2269514	0.21984	-0.64688814	subgrp_cor	count	1	0
UPRT	1	OK	3.45981462	6.77145131	0.51094137	0.60939	0.00030179	subgrp_cor	count	1	0
ZDHHC15	1	OK	3.45981462	6.77145131	0.51094137	0.60939	0.00030179	subgrp_cor	count	1	0

PBDC1	32	OK	-0.058809	0.5278516	-0.1114122	0.91129	-0.06885265	subgrp_con	count	1	0
MAGEE1	1	OK	23.991896	84.899941	0.2825802	0.77749	1.65081709	subgrp_con	count	1	0
ATRX	249	OK	0.2499392	0.171540	1.4570311	0.14511	0.34440428	subgrp_con	count	1	0
MAGT1	41	OK	1.0161203	0.473362	2.1465985	0.03182	1.16885548	subgrp_con	count	1	0
COX7B	254	OK	0.3604926	0.1876420	1.9211719	0.05471	0.49552969	subgrp_con	count	1	0
ATP7A	1	OK	23.991896	84.899941	0.2825802	0.77749	1.65081709	subgrp_con	count	1	0
PGK1.00	298	OK	-0.162891	0.1653057	-0.985396	0.32442	-0.22625097	subgrp_con	count	1	0
TAF9B	4	OK	21.744260	23.025778	0.6584026	0.51028	2.80170450	subgrp_con	count	1	0
CYSLTR1	47	OK	-1.740276	0.4634701	-3.754883	0.00017	-2.09058441	subgrp_con	count	1	0
BRWD3	4	OK	1.4118376	1.4959328	0.9437840	0.34526	0.69790486	subgrp_con	count	1	0
HMGN5	19	OK	0.5041539	0.654895	0.7700589	0.44126	0.47802629	subgrp_con	count	1	0
SH3BGRL	295	OK	-0.078937	0.1594276	-0.495127	0.62052	-0.11034129	subgrp_con	count	1	0
HDX	3	OK	4.2109206	4.2875616	0.982124	0.32606	0.00257090	subgrp_con	count	1	0
APOOL	9	OK	-3.143003	1.7900557	-1.755813	0.07912	-1.93779720	subgrp_con	count	1	0
POF1B	3	OK	21.105322	83.341273	0.2532397	0.80008	0.0003103	subgrp_con	count	1	0
CHM	22	OK	-0.130806	0.7053760	-0.185442	0.85288	-0.10798500	subgrp_con	count	1	0
KLHL4	3	OK	3.3463743	2.0762529	1.611737	0.10702	2.36911986	subgrp_con	count	1	0
PCDH11X	2	OK	21.105322	83.341273	0.2532397	0.80008	0.0003103	subgrp_con	count	1	0
DIAPH2	30	OK	-1.454338	0.6648002	-2.187631	0.0287	-1.51348998	subgrp_con	count	1	0
PCDH19	6	OK	-11.20641	16.843031	-0.665344	0.50582	-3.11522939	subgrp_con	count	1	0
TNMD	1	OK	-10.44227	41.465809	-0.251828	0.80117	-0.00045289	subgrp_con	count	1	0
TSPAN6	44	OK	2.53864	0.5781555	4.3909359	1.13E-05	2.86338729	subgrp_con	count	0.1129879	0
SRPX2	5	OK	4.0471503	2.6372161	1.5346297	0.12487	1.81592407	subgrp_con	count	1	0
SYTL4	2	OK	3.4598146	6.7714513	0.5109413	0.60939	0.00030179	subgrp_con	count	1	0
TRMT2B	1	OK	17.747207	50.971927	0.3481761	0.72771	0.0007532	subgrp_con	count	1	0
RPL36A	214	OK	-0.045398	0.1939508	-0.234074	0.81492	-0.06135561	subgrp_con	count	1	0
GLA	6	OK	5.3102004	3.3807821	1.5707313	0.11625	1.58327458	subgrp_con	count	1	0
HNRNPH2	30	OK	0.037688	0.5152626	0.0731434	0.94169	0.03128061	subgrp_con	count	1	0
ARMCX4	1	OK	23.991896	84.899941	0.2825802	0.77749	1.65081709	subgrp_con	count	1	0
ARMCX1	15	OK	-1.788774	0.8980964	-1.991728	0.0464	-1.26970120	subgrp_con	count	1	0
ARMCX6	28	OK	1.2224892	0.6148132	1.9883910	0.04677	1.12265389	subgrp_con	count	1	0
ARMCX3	64	OK	0.3981762	0.3792058	1.0500267	0.29371	0.43104611	subgrp_con	count	1	0
ARMCX2	10	OK	1.5257815	1.0983760	1.3891249	0.16479	0.74764155	subgrp_con	count	1	0
ZMAT1	10	OK	1.1849225	1.0605240	1.1172990	0.26387	0.7550348	subgrp_con	count	1	0
TCEAL2	12	OK	4.0805451	1.5382169	2.6527761	0.00798	3.11629198	subgrp_con	count	1	0
BEX5	14	OK	0.9808827	0.7824691	1.2535737	0.21	0.72102645	subgrp_con	count	1	0
BEX1	5	OK	22.576026	22.795857	0.6883783	0.49121	3.19841235	subgrp_con	count	1	0
BEX4	86	OK	0.3912638	0.2923937	1.3381402	0.18085	0.51352655	subgrp_con	count	1	0
TCEAL8	82	OK	0.7372093	0.3425403	2.1521822	0.03138	0.91494653	subgrp_con	count	1	0
BEX2	4	OK	1.1224437	1.4569313	0.7704163	0.44105	0.3460629	subgrp_con	count	1	0
TCEAL9	131	OK	1.217652	0.2729403	4.4612387	8.15E-06	1.61503273	subgrp_con	count	0.0816746	0
BEX3	220	OK	0.6826321	0.1909316	3.5752690	0.00035	0.95101600	subgrp_con	count	1	0
TCEAL4	301	OK	0.5442199	0.1672041	3.2548232	0.00113	0.75885802	subgrp_con	count	1	0
TCEAL3	95	OK	0.3852833	0.2854133	1.3502640	0.17692	0.49884702	subgrp_con	count	1	0
TCEAL1	20	OK	2.1556571	0.7851256	2.7456208	0.00604	2.08893107	subgrp_con	count	1	0
MORF4L2	85	OK	0.2227916	0.3142551	0.7089515	0.47835	0.28171154	subgrp_con	count	1	0
FAM199X	8	OK	1.6319859	1.5121304	1.079262	0.28047	0.52772204	subgrp_con	count	1	0
MUM1L1	2	OK	21.105322	83.341273	0.2532397	0.80008	0.0003103	subgrp_con	count	1	0
TBC1D8B	4	OK	3.2016089	2.1673531	1.4771976	0.13962	1.72003022	subgrp_con	count	1	0
MORC4	2	OK	23.991896	84.899941	0.2825802	0.77749	1.65081709	subgrp_con	count	1	0
RBM41	3	OK	3.4598149	4.7858686	0.7229230	0.46972	0.00081935	subgrp_con	count	1	0
PRPS1	6	OK	4.6304640	3.5442653	1.3064665	0.19139	0.95271434	subgrp_con	count	1	0
TSC22D3	326	OK	-0.130699	0.1410882	-0.926365	0.35426	-0.18134286	subgrp_con	count	1	0
PSMD10	5	OK	2.7048028	1.7963237	1.5057317	0.13214	1.47364766	subgrp_con	count	1	0
ATG4A	8	OK	2.4666989	2.1392766	1.1530528	0.24889	0.67632650	subgrp_con	count	1	0
ACSL4	12	OK	0.4069426	0.9068526	0.4487417	0.65362	0.0007412	subgrp_con	count	1	0
TMEM164	2	OK	3.4598146	6.7714513	0.5109413	0.60939	0.00030179	subgrp_con	count	1	0
ALG13	37	OK	0.8437400	0.4962327	1.7002909	0.08908	1.00650217	subgrp_con	count	1	0
IL13RA2	9	OK	1.4193525	1.0558988	1.344212	0.17888	1.0310178	subgrp_con	count	1	0
LRCH2	5	OK	-10.98256	16.856594	-0.651528	0.5147	-2.86146472	subgrp_con	count	1	0
PLS3	126	OK	0.4628682	0.2742908	1.6875091	0.09151	0.59700549	subgrp_con	count	1	0
KLHL13	4	OK	21.64205	54.486891	0.3971975	0.69122	1.57744725	subgrp_con	count	1	0
WDR44	1	OK	-15.99503	70.34942	-0.227365	0.82014	-0.00028474	subgrp_con	count	1	0
DOCK11	11	OK	-2.988814	1.2745568	-2.344982	0.01902	-2.06273245	subgrp_con	count	1	0
IL13RA1	73	OK	0.5072024	0.3294706	1.5394466	0.1237	0.60168672	subgrp_con	count	1	0
LONRF3	1	OK	-11.01941	43.249935	-0.254784	0.79889	-1.17482461	subgrp_con	count	1	0
PGRMC1	135	OK	0.5044521	0.2322634	2.1718964	0.02986	0.67786549	subgrp_con	count	1	0
SLC25A43	1	OK	-10.44227	41.465809	-0.251828	0.80117	-0.00045289	subgrp_con	count	1	0
SLC25A5	389	OK	-0.177209	0.1429653	-1.239528	0.21515	-0.24920082	subgrp_con	count	1	0
CXorf56	1	OK	18.759489	83.625179	0.2243282	0.8225	0.00028789	subgrp_con	count	1	0
UBE2A	41	OK	0.2602642	0.467077	0.5572180	0.57738	0.30181257	subgrp_con	count	1	0
NKRF	5	OK	0.329526	1.4055480	0.2344472	0.81464	0.10244709	subgrp_con	count	1	0
6-Sep	24	OK	-1.010629	0.6025417	-1.677277	0.09349	-0.78572051	subgrp_con	count	1	0
RPL39	861	OK	-0.180324	0.0866820	-2.080292	0.0375	-0.25789258	subgrp_con	count	1	0
UPF3B	25	OK	-0.558974	0.618977	-0.903061	0.36649	-0.54050997	subgrp_con	count	1	0
RNF113A	8	OK	-1.035477	0.9767412	-1.060134	0.28908	-0.72169004	subgrp_con	count	1	0
NKAP	62	OK	0.1974821	0.3702424	0.5333860	0.59377	0.22223913	subgrp_con	count	1	0
ZBTB33	3	OK	-0.202940	1.6532365	-0.122753	0.9022	-4.82E-05	subgrp_con	count	1	0
LAMP2	61	OK	0.9330260	0.4066272	2.2945486	0.02176	1.17367394	subgrp_con	count	1	0
CUL4B	7	OK	0.8664780	1.2872623	0.673116	0.50087	0.26844509	subgrp_con	count	1	0
MCTS1	64	OK	1.0926430	0.4385845	2.4912937	0.01272	1.26779176	subgrp_con	count	1	0
C1GALT1C1	29	OK	0.3641525	0.588191	0.6191052	0.53585	0.4102315	subgrp_con	count	1	0
GRIA3	1	OK	18.759489	83.625179	0.2243282	0.8225	0.00028789	subgrp_con	count	1	0
THOC2	41	OK	0.308507	0.4727387	0.6525985	0.51402	0.30553656	subgrp_con	count	1	0
XIAP	26	OK	0.718727	0.5839784	1.2307425	0.21842	0.73449455	subgrp_con	count	1	0
STAG2	49	OK	0.3012988	0.4193077	0.7185620	0.47241	0.35760074	subgrp_con	count	1	0
SMARCA1	13	OK	-0.140561	0.7841780	-0.179246	0.85774	-0.09025480	subgrp_con	count	1	0
OCRL	2	OK	3.4598146	6.7714513	0.5109413	0.60939	0.00030179	subgrp_con	count	1	0
APLN	9	OK	1.6829366	1.3298617	1.265497	0.20568	1.19645557	subgrp_con	count	1	0
ZDHHC9	3	OK	4.6613299	5.7683860	0.8080821	0.41906	0.00274918	subgrp_con	count	1	0
UTP14A	9	OK	-0.052460	0.921466	-0.056931	0.9546	-0.0467478	subgrp_con	count	1	0
ELF4	2	OK	-0.910223	2.1881125	-0.415985	0.67742	-0.25785735	subgrp_con	count	1	0
AIFM1	3	OK	-0.545320	1.708550	-0.319171	0.7496	-0.0003423	subgrp_con	count	1	0
ZNF280C	1	OK	-11.01941	43.249935	-0.254784	0.79889	-1.17482461	subgrp_con	count	1	0
RBMX2	26	OK	0.1212200	0.5415013	0.2238592	0.82287	0.13106834	subgrp_con	count	1	0
ENOX2	3	OK	4.2109206	4.2875616	0.982124	0.32606	0.00257090	subgrp_con	count	1	0
PHF6	15	OK	-0.253656	0.962489	-0.263542	0.79212	-0.18917577	subgrp_con	count	1	0
HPRT1	35	OK	-0.109781	0.5026587	-0.218402	0.82712	-0.11826116	subgrp_con	count	1	0
MIR503HG	1	OK	3.4598146	6.7714513	0.5109413	0.60939	0.00030179	subgrp_con	count	1	0
MOSPD1	7	OK	-1.339557	1.432781	-0.934932	0.34982	-0.77052088	subgrp_con	count	1	0
SMIM10	21	OK	-1.724900	0.6874194	-2.509240	0.0121	-1.23483708	subgrp_con	count	1	0
RTL8B	7	OK	-2.142510	1.3658173	-1.568665	0.11673	-0.50889916	subgrp_con	count	1	0
RTL8C	68	OK	0.2025501	0.348300	0.5815390	0.56088	0.24495581	subgrp_con	count	1	0
RTL8A	53	OK	-0.120362	0.4050922	-0.297124	0.76637	-0.14631661	subgrp_con	count	1	0
ZNF75D	1	OK	-10.44227	41.465809	-0.251828	0.80117	-0.00045289	subgrp_con	count	1	0
MMGT1	14	OK	-1.778977	0.9057117	-1.964175	0.04951	-1.46593110	subgrp_con	count	1	0
FHL1	64	OK	1.5801845	0.4133596	3.8227837	0.00013	1.95910791	subgrp_con	count	1	0
MAP7D3	21	OK	0.1982811	0.7415087	0.2675372	0.78906	0.19629111	subgrp_con	count	1	0
HTATSF1	23	OK	0.7312590	0.6410156	1.1407818	0.25396	0.69017751	subgrp_con	count	1	0
ARHGEF6	2	OK	4.6613299	5.7683860	0.8080821	0.41906	0.00274918	subgrp_con	count	1	0
RBMX	125	OK	-0.514528	0.2548402	-2.019023	0.04348	-0.68249663	subgrp_con	count	1	0
MCF2.00	1	OK	18.759489	83.625179	0.2243282	0.8225	0.00028789	subgrp_con	count	1	0
ATP11C	40	OK	0.1842169	0.4855336	0.3796173	0.70422	0.165163	subgrp_con	count	1	0
LDOC1	6	OK	3.2074606	1.7415957	1.8416791	0.06552	1.89100619	subgrp_con	count	1	0
FMR1	6	OK	-1.649434	1.3358386	-1.234756	0.21692	-1.01589916	subgrp_con	count	1	0
IDS	39	OK	2.3904018	0.6511017	3.6713184	0.00024	2.48487884	subgrp_con	count	1	0
TMEM185A	2	OK	1.5823776	1.9240213	0.822422	0.41082	0.77174550	subgrp_con	count	1	0
CXorf40B	1	OK	3.4598146	6.7714513	0.5109413	0.60939	0.00030179	subgrp_con	count	1	0
MTM1	2	OK	-1.295616	2.041425	-0.634662	0.52565	-0.00075754	subgrp_con	count	1	0
CD99L2	4	OK	1.1225052	1.4569318	0.770458	0.44102	0.34607098	subgrp_con	count	1	0
HMGB3	5	OK	4.0129835	3.5901511	1.1177756	0.26366	0.0024854	subgrp_con	count	1	0
VMA21	18	OK	-0.033990	0.720884	-0.047151	0.96239	-0.02544516	subgrp_con	count	1	0
GABRE	1	OK	41.147020	64.794804	0.6350358	0.52541	0.00104799	subgrp_con	count	1	0
CETN2	25	OK	-0.580948	0.5839831	-0.994802	0.31982	-0.56040470	subgrp_con	count	1	0
NSDHL	1	OK	-11.45102	41.79736	-0.273965	0.78411	-0.00049189	subgrp_con	count	1	0
CCNQ	12	OK	2.1037558	1.0278727	2.0467084	0.04069	1.71433999	subgrp_con	count	1	0
BCAP31	280	OK	-0.197911	0.1688154	-1.172353	0.24106	-0.27647060	subgrp_con	count	1	0
ABCD1	1	OK	17.747207	50.971927	0.3481761	0.72771	0.0007532	subgrp_con	count	1	0
IDH3G	34	OK	0.2889155	0.5389964	0.5360250	0.59194	0.29636518	subgrp_con	count	1	0
SSR4	541	OK	0.5232393	0.1187502	4.4062155	1.05E-05	0.7432518	subgrp_con	count	0.1053185	0
L1CAM	2	OK	-12.14570	43.418869	-0.279733	0.77968	-1.91338276	subgrp_con	count	1	0
AVPR2	31	OK	-2.678474	0.7337843	-3.650220	0.00026	-2.7751554	subgrp_con	count	1	0
ARHGAP4	6	OK	32.156634	42.147544	0.7629539	0.44549	0.00142193	subgrp_con	count	1	0
NAA10	242	OK	0.2296906	0.1863939	1.2322858	0.21786	0.31854161	subgrp_con	count	1	0
RENBP	1	OK	-11.01941	43.249935	-0.254784	0.79889	-1.17482461	subgrp_con	count	1	0
TMEM187	4	OK	3.1002559	2.3039102	1.3456496	0.17842	1.26089264	subgrp_con	count	1	0
IRAK1	7	OK	21.105322	83.341273	0.2532397	0.80008	0.0003103	subgrp_con	count	1	0
MECP2	12	OK	-0.363131	0.7865895	-0.461653	0.64432	-0.00062878	subgrp_con	count	1	0
FLNA	133	OK	0.6626090	0.2637359	2.5123857	0.01199	0.85349061	subgrp_con	count	1	0
EMD	30	OK	-0.06865	0.5574007	-0.123162	0.90198	-0.07704169	subgrp_con	count	1	0
RPL10	890	OK	-0.031516	0.0822660	-0.383108	0.70164	-0.045124	subgrp_con	count	1	0
DNASE1L1	18	OK	1.3132301	0.8402746	1.5628582	0.11809	0.96102924	subgrp_con	count	1	0
TAZ	2	OK	-0.150975	1.968815	-0.076683	0.93888	-9.78E-05	subgrp_con	count	1	0
AC244090.1	5	OK	1.4119426	1.4959308	0.9438555	0.34524	0.69797651	subgrp_con	count	1	0
ATP6AP1	36	OK	1.4825087	0.6174085	2.4011794	0.01634	1.35148870	subgrp_con	count	1	0
GDI1	8	OK	14.666007	10.099021	1.452220	0.14644	1.3563655	subgrp_con	count	1	0
FAM50A	41	OK	-0.381657	0.5011344	-0.761587	0.44631	-0.43192272	subgrp_con	count	1	0
LAGE3	49	OK	0.4390407	0.4210882	1.0426311	0.29712	0.54627020	subgrp_con	count	1	0
UBL4A	10	OK	0.6088753	1.0621111	0.5732688	0.56640	0.18991916	subgrp_con	count	1	0
SLC10A3	10	OK	0.9434985	0.9407764	1.0028934	0.31591	0.83695387	subgrp_con	count	1	0
FAM3A	11	OK	-0.576019	0.8617067	-0.668463	0.50386	-0.41847290	subgrp_con	count	1	0
G6PD	6	OK	1.8342002	1.6829423	1.0898770	0.27577	0.53818379	subgrp_con	count	1	0
IKBKG	6	OK	-0.181478	1.2658618	-0.143363	0.886	-0.00011729	subgrp_con	count	1	0
GAB3	1	OK	-10.44227	41.465809	-0.251828	0.80117	-0.00045289	subgrp_con	count	1	0
DKC1	11	OK	-1.672077	1.2694971	-1.317117	0.1878	-1.22950736	subgrp_con	count	1	0
MPP1	10	OK	-1.520516	1.1025525	-1.379087	0.16787	-0.85387132	subgrp_con	count	1	0
F8	30	OK	-1.728562	0.5808437	-2.97595	0.00292	-1.56575894	subgrp_con	count	1	0
F8A1	1	OK	3.4598146	6.7714513	0.5109413	0.60939	0.00030179	subgrp_con	count	1	0
FUNDC2	137	OK	-0.226257	0.2453034	-0.922356	0.35634	-0.29759098	subgrp_con	count	1	0
BRCC3	7	OK	-0.465407	1.3093679	-0.355444	0.72226	-0.13815566	subgrp_con	count	1	0
VBP1	34	OK	0.5129007	0.5526637	0.9280520	0.35338	0.54138019	subgrp_con	count	1	0
RAB39B	1	OK	1.5823999	2.7200188	0.5817606	0.56072	0.47433761	subgrp_con	count	1	0
CLIC2	73	OK	-1.046918	0.3453268	-3.031672	0.00243	-1.29713258	subgrp_con	count	1	0
VAMP7	7	OK	-0.024939	1.1958373	-0.02085	0.98336	-0.00764495	subgrp_con	count	1	0
FBXO25	7	OK	0.8411793	1.4044478	0.5989395	0.54921	0.0005738	subgrp_con	count	1	0
TDRP	3	OK	-0.907228	1.6441847	-0.551780	0.5811	-0.52553220	subgrp_con	count	1	0
ERICH1	79	OK	-1.357989	0.3345677	-4.058936	4.93E-05	-1.74485845	subgrp_con	count	0.4906012	0
CLN8	6	OK	-0.517197	1.1290765	-0.458071	0.6469	-0.00082999	subgrp_con	count	1	0
AC100810.1	1	OK	18.759489	83.625179	0.2243282	0.8225	0.00028789	subgrp_con	count	1	0
ARHGEF10	2	OK	1.5823999	2.7200188	0.5817606	0.56072	0.47433761	subgrp_con	count	1	0
AC016065.1	1	OK	3.4598146	6.7714513	0.5109413	0.60939	0.00030179	subgrp_con	count	1	0
MCPH1	5	OK	0.5458342	1.1897273	0.4587893	0.64639	0.35540674	subgrp_con	count	1	0
ANGPT2	50	OK	1.3117029	0.4405761	2.9772445	0.00291	1.71557773	subgrp_con	count	1	0
AGPAT5	19	OK	1.0614794	0.6781534	1.5652496	0.11752	0.99467249	subgrp_con	count	1	0
CLDN23	1	OK	3.4598146	6.7714513	0.5109413	0.60939	0.00030179	subgrp_con	count	1	0
MFHAS1	4	OK	-1.853834	1.8543918	-0.999699	0.31749	-0.46053279	subgrp_con	count	1	0
ERI1	2	OK	-14.65800	45.690332	-0.320813	0.74835	-0.00067223	subgrp_con	count	1	0
PPP1R3B	3	OK	-0.202940	1.6532365	-0.122753	0.9022	-4.82E-05	subgrp_con	count	1	0
AC022784.1	1	OK	3.4598146	6.7714513	0.5109413	0.60939	0.00030179	subgrp_con	count	1	0
TNKS	3	OK	-0.495425	1.5039132	-0.329424	0.74182	-0.00031231	subgrp_con	count	1	0
MSRA	31	OK	0.0534445	0.539025	0.0991503	0.92102	0.05277149	subgrp_con	count	1	0
SOX7	45	OK	-0.672875	0.4589621	-1.466081	0.14262	-0.78481206	subgrp_con	count	1	0
PINX1	7	OK	0.7540371	1.191782	0.6326971	0.52692	0.57006282	subgrp_con	count	1	0
MTMR9	8	OK	-0.227836	1.0793571	-0.211084	0.83282	-0.14563108	subgrp_con	count	1	0
NEIL2	2	OK	1.5823999	2.7200188	0.5817606	0.56072	0.47433761	subgrp_con	count	1	0
FDFT1	13	OK	0.9754567	1.1085784	0.8799168	0.3789	0.30242875	subgrp_con	count	1	0
CTSB	109	OK	0.6829419	0.2913582	2.3439862	0.01908	0.90131719	subgrp_con	count	1	0
LONRF1	4	OK	-2.342099	1.7367233	-1.348573	0.17747	-0.53907020	subgrp_con	count	1	0
DLC1	99	OK	-0.499427	0.2901892	-1.721041	0.08524	-0.61917936	subgrp_con	count	1	0
TUSC3	19	OK	1.6781485	0.6886663	2.436809	0.01482	1.87296665	subgrp_con	count	1	0
MICU3	4	OK	-1.758572	2.3554439	-0.746599	0.45531	-0.00036089	subgrp_con	count	1	0
ZDHHC2	20	OK	24.591137	22.753879	0.7507854	0.45279	3.81663043	subgrp_con	count	1	0
CNOT7	32	OK	0.5718639	0.5361891	1.0665340	0.28618	0.60323784	subgrp_con	count	1	0
VPS37A	11	OK	-0.75055	0.9259062	-0.810614	0.41759	-0.59712457	subgrp_con	count	1	0
SLC7A2	36	OK	29.422457	26.579457	1.1069623	0.26833	1.82708825	subgrp_con	count	1	0
PDGFRL	6	OK	4.2598113	2.6316753	1.6186690	0.10552	1.85273386	subgrp_con	count	1	0
MTUS1	464	OK	0.5015399	0.1187430	4.2237408	2.4E-05	0.71148360	subgrp_con	count	0.2398238	0
PCM1	130	OK	0.6490709	0.2604220	2.4923810	0.01269	0.85356761	subgrp_con	count	1	0
ASAH1	55	OK	0.6438143	0.3641057	1.7682071	0.07702	0.76242879	subgrp_con	count	1	0
SH2D4A	3	OK	4.2109206	4.2875616	0.982124	0.32606	0.00257090	subgrp_con	count	1	0
CSGALNACT1	71	OK	1.2518841	0.3781518	3.3105332	0.00093	1.47691590	subgrp_con	count	1	0
INTS10	22	OK	-0.574613	0.6772564	-0.848218	0.39636	-0.52960090	subgrp_con	count	1	0
LPL	2	OK	0.6532529	1.7687836	0.3693232	0.71189	0.0001629	subgrp_con	count	1	0
ATP6V1B2	2	OK	1.791902	2.2833539	0.7847677	0.43259	0.00045246	subgrp_con	count	1	0
XPO7	4	OK	1.4118376	1.4959328	0.9437840	0.34526	0.69790486	subgrp_con	count	1	0
FAM160B2	2	OK	3.4598146	6.7714513	0.5109413	0.60939	0.00030179	subgrp_con	count	1	0
NUDT18	1	OK	3.4598146	6.7714513	0.5109413	0.60939	0.00030179	subgrp_con	count	1	0
REEP4	1	OK	-11.01941	43.249935	-0.254784	0.79889	-1.17482461	subgrp_con	count	1	0
BMP1	3	OK	0.1721614	1.534450	0.1121974	0.91067	0.00011395	subgrp_con	count	1	0
POLR3D	5	OK	-1.897887	1.607882	-1.18036	0.23786	-0.78103666	subgrp_con	count	1	0
SLC39A14	9	OK	4.3801518	3.436922	1.2744400	0.20252	0.00263449	subgrp_con	count	1	0
PPP3CC	4	OK	-10.96786	27.152658	-0.403918	0.68627	-1.17125107	subgrp_con	count	1	0
SORBS3	26	OK	0.3030553	0.5566165	0.5444598	0.58612	0.2872116	subgrp_con	count	1	0
PDLIM2	81	OK	0.9209819	0.338068	2.7242425	0.00644	1.20413992	subgrp_con	count	1	0
C8orf58	3	OK	3.4598146	6.7714513	0.5109413	0.60939	0.00030179	subgrp_con	count	1	0
CCAR2	3	OK	18.759489	83.625179	0.2243282	0.8225	0.00028789	subgrp_con	count	1	0
BIN3	2	OK	18.759489	83.625179	0.2243282	0.8225	0.00028789	subgrp_con	count	1	0
EGR3	26	OK	1.3400654	0.6618817	2.0246296	0.04291	1.49413728	subgrp_con	count	1	0
RHOBTB2	1	OK	18.759489	83.625179	0.2243282	0.8225	0.00028789	subgrp_con	count	1	0
TNFRSF10B	30	OK	0.4050850	0.691415	0.5858775	0.55796	0.36323839	subgrp_con	count	1	0
AC107959.4	2	OK	3.4598146	6.7714513	0.5109413	0.60939	0.00030179	subgrp_con	count	1	0
TNFRSF10D	42	OK	-0.132240	0.4314590	-0.306495	0.75922	-0.13885870	subgrp_con	count	1	0
TNFRSF10A	5	OK	-1.825974	1.6282295	-1.12137	0.26212	-0.50172544	subgrp_con	count	1	0
CHMP7	2	OK	-2.166009	2.7041600	-0.800991	0.42314	-0.79067868	subgrp_con	count	1	0
R3HCC1	11	OK	0.7926934	1.0244354	0.773785	0.43906	0.51382088	subgrp_con	count	1	0
LOXL2	9	OK	-0.242355	0.9942429	-0.243758	0.80742	-0.17981221	subgrp_con	count	1	0
ENTPD4	2	OK	0.5887819	1.767414	0.3331317	0.73903	0.30122869	subgrp_con	count	1	0
SLC25A37	119	OK	0.485283	0.2492072	1.9473082	0.0515	0.64247209	subgrp_con	count	1	0
NKX3-1	1	OK	3.4598146	6.7714513	0.5109413	0.60939	0.00030179	subgrp_con	count	1	0
KCTD9	9	OK	0.5102685	1.1312005	0.4510858	0.65192	0.2611530	subgrp_con	count	1	0
PPP2R2A	109	OK	-0.607150	0.2886337	-2.103532	0.03542	-0.78583938	subgrp_con	count	1	0
DPYSL2	143	OK	-0.646421	0.240705	-2.685534	0.00724	-0.82505811	subgrp_con	count	1	0
EPHX2	4	OK	-0.024863	1.1958382	-0.020791	0.98341	-0.00762178	subgrp_con	count	1	0
CLU	724	OK	-0.009372	0.0884637	-0.105950	0.91562	-0.01341338	subgrp_con	count	1	0
SCARA3	7	OK	1.411023	1.2245040	1.1523222	0.24920	0.00265275	subgrp_con	count	1	0
CCDC25	98	OK	0.0831494	0.2825268	0.2943062	0.76852	0.11106791	subgrp_con	count	1	0
ELP3	1	OK	-10.44227	41.465809	-0.251828	0.80117	-0.00045289	subgrp_con	count	1	0
ZNF395	1	OK	-10.44227	41.465809	-0.251828	0.80117	-0.00045289	subgrp_con	count	1	0
HMBOX1	17	OK	0.3025048	0.6677484	0.4530221	0.65052	0.19687530	subgrp_con	count	1	0
KIF13B	25	OK	1.5934633	0.6750919	2.3603647	0.01826	1.56579270	subgrp_con	count	1	0
DUSP4	15	OK	0.1264774	0.7798674	0.1621781	0.87117	0.10519163	subgrp_con	count	1	0
SARAF	274	OK	0.5040445	0.1715702	2.937831	0.00331	0.69477986	subgrp_con	count	1	0
AC044849.1	4	OK	1.7966220	2.1603505	0.831634	0.40562	0.0012336	subgrp_con	count	1	0
LEPROTL1	32	OK	0.2650656	0.5774145	0.4590560	0.64619	0.28695045	subgrp_con	count	1	0
DCTN6	51	OK	-0.172777	0.3876899	-0.445658	0.65584	-0.21145451	subgrp_con	count	1	0
RBPMS	120	OK	-0.404672	0.2639196	-1.533317	0.1252	-0.54335747	subgrp_con	count	1	0
GTF2E2	10	OK	0.5194599	1.0366477	0.5010959	0.6162	0.39176786	subgrp_con	count	1	0
UBXN8	4	OK	-0.697762	1.6964571	-0.411305	0.68085	-0.33591144	subgrp_con	count	1	0
PPP2CB	52	OK	-0.106376	0.4408448	-0.241301	0.80932	-0.10850434	subgrp_con	count	1	0
WRN	6	OK	-0.778415	1.3420495	-0.580019	0.5619	-0.47709280	subgrp_con	count	1	0
NRG1	30	OK	25.842478	22.547822	0.7929846	0.4272	4.42230872	subgrp_con	count	1	0
AC090204.1	1	OK	-11.01941	43.249935	-0.254784	0.79889	-1.17482461	subgrp_con	count	1	0
FUT10	2	OK	21.64205	54.486891	0.3971975	0.69122	1.57744725	subgrp_con	count	1	0
TTI2	2	OK	-0.910223	2.1881125	-0.415985	0.67742	-0.25785735	subgrp_con	count	1	0
MAK16	4	OK	-1.310774	1.638399	-0.800033	0.42369	-0.35262481	subgrp_con	count	1	0
RNF122	3	OK	-11.44854	41.76168	-0.274141	0.78398	-0.00049246	subgrp_con	count	1	0
DUSP26	1	OK	18.759489	83.625179	0.2243282	0.8225	0.00028789	subgrp_con	count	1	0
ZNF703	1	OK	19.111790	50.827591	0.3759381	0.70696	0.00078703	subgrp_con	count	1	0
ERLIN2	3	OK	3.4598155	3.9099931	0.8848648	0.37622	0.00081877	subgrp_con	count	1	0
PLPBP	25	OK	-0.154376	0.5931110	-0.260282	0.79465	-0.16612536	subgrp_con	count	1	0
ADGRA2	14	OK	-3.007096	1.1377383	-2.643047	0.00822	-2.41322458	subgrp_con	count	1	0
BRF2	1	OK	23.991896	84.899941	0.2825802	0.77749	1.65081709	subgrp_con	count	1	0
RAB11FIP1	44	OK	0.5907427	0.4233887	1.3952725	0.16292	0.7358867	subgrp_con	count	1	0
EIF4EBP1	40	OK	1.3360732	0.5313657	2.5144135	0.01192	1.5555052	subgrp_con	count	1	0
ASH2L	4	OK	0.8411793	1.4044478	0.5989395	0.54921	0.0005738	subgrp_con	count	1	0
LSM1.00	36	OK	0.2639168	0.5316122	0.4964461	0.61958	0.27874084	subgrp_con	count	1	0
BAG4	10	OK	6.1492695	3.1325221	1.9630410	0.04964	2.07241822	subgrp_con	count	1	0
DDHD2	3	OK	4.6613299	5.7683860	0.8080821	0.41906	0.00274918	subgrp_con	count	1	0
PLPP5	34	OK	1.5846761	0.6568710	2.4124614	0.01585	1.43412562	subgrp_con	count	1	0
NSD3	120	OK	1.2242002	0.2921634	4.1901208	2.79E-05	1.63669706	subgrp_con	count	0.2781647	0
AC087623.3	1	OK	3.4598146	6.7714513	0.5109413	0.60939	0.00030179	subgrp_con	count	1	0
FGFR1	17	OK	2.2547816	1.072267	2.1028169	0.03548	1.28071392	subgrp_con	count	1	0
TACC1	385	OK	-0.234859	0.1315731	-1.785012	0.07426	-0.33090284	subgrp_con	count	1	0
TM2D2	50	OK	0.1480315	0.3991080	0.3709060	0.71071	0.18043681	subgrp_con	count	1	0
ADAM9	15	OK	4.3669542	1.7878485	2.4425750	0.01458	2.79205432	subgrp_con	count	1	0
TCIM	99	OK	0.631746	0.3422243	1.8460002	0.06489	0.74847345	subgrp_con	count	1	0
SFRP1	31	OK	4.8446631	1.2823988	3.7778100	0.00016	3.9520437	subgrp_con	count	1	0
GOLGA7	81	OK	0.479536	0.3261352	1.4703605	0.14146	0.62053379	subgrp_con	count	1	0
GINS4	1	OK	18.759489	83.625179	0.2243282	0.8225	0.00028789	subgrp_con	count	1	0
GPAT4	2	OK	18.759489	83.625179	0.2243282	0.8225	0.00028789	subgrp_con	count	1	0
ANK1	3	OK	18.759489	83.625179	0.2243282	0.8225	0.00028789	subgrp_con	count	1	0
KAT6A	22	OK	-1.744248	0.7143713	-2.441651	0.01462	-1.51429147	subgrp_con	count	1	0
AP3M2	2	OK	-10.44227	41.465809	-0.251828	0.80117	-0.00045289	subgrp_con	count	1	0
IKBKB	4	OK	-1.119738	1.4934041	-0.749788	0.45338	-0.30904529	subgrp_con	count	1	0
POLB	14	OK	-2.136443	0.9455292	-2.259521	0.02385	-1.77364212	subgrp_con	count	1	0
DKK4.00	1	OK	-11.01941	43.249935	-0.254784	0.79889	-1.17482461	subgrp_con	count	1	0
VDAC3	73	OK	-0.318561	0.3310913	-0.962156	0.33597	-0.40914106	subgrp_con	count	1	0
SMIM19	36	OK	-0.097239	0.4590567	-0.21182	0.83226	-0.11236185	subgrp_con	count	1	0
THAP1	10	OK	-0.523184	0.9625825	-0.543522	0.58677	-0.38156201	subgrp_con	count	1	0
RNF170	7	OK	0.3530920	1.1709379	0.3015453	0.763	0.18053741	subgrp_con	count	1	0
HOOK3	56	OK	0.7825996	0.4151356	1.8851657	0.05941	0.84329120	subgrp_con	count	1	0
FNTA	61	OK	-0.665819	0.3635608	-1.831383	0.06704	-0.82182258	subgrp_con	count	1	0
HGSNAT	11	OK	0.0037160	1.0304823	0.0036061	0.99712	6.61E-06	subgrp_con	count	1	0
SPIDR	4	OK	-2.453925	1.9317402	-1.270318	0.20397	-1.36997073	subgrp_con	count	1	0
CEBPD	591	OK	-0.309225	0.1056780	-2.926076	0.00343	-0.44029730	subgrp_con	count	1	0
PRKDC	100	OK	0.5872257	0.314054	1.8698222	0.06151	0.76411355	subgrp_con	count	1	0
UBE2V2	44	OK	-0.186444	0.4543436	-0.410360	0.68154	-0.22809650	subgrp_con	count	1	0
SNAI2	3	OK	-10.47563	27.491987	-0.381043	0.70317	-1.13811785	subgrp_con	count	1	0
PCMTD1	213	OK	0.508849	0.1939205	2.6240114	0.00869	0.69883829	subgrp_con	count	1	0
RB1CC1	79	OK	1.4670780	0.3939119	3.7243805	0.0002	1.68682905	subgrp_con	count	1	0
ATP6V1H	10	OK	0.5582932	1.1125272	0.5018243	0.61579	0.17362150	subgrp_con	count	1	0
TCEA1	175	OK	0.360513	0.2131463	1.691389	0.09076	0.49234209	subgrp_con	count	1	0
LYPLA1	75	OK	0.7289574	0.3427393	2.1268568	0.03342	0.92382662	subgrp_con	count	1	0
MRPL15	19	OK	-0.205850	0.8258597	-0.249277	0.80307	-0.16949896	subgrp_con	count	1	0
SOX17	121	OK	0.7263188	0.2767742	2.6242288	0.00868	0.94671194	subgrp_con	count	1	0
TMEM68	4	OK	2.164878	1.7332019	1.2489908	0.21167	0.99526412	subgrp_con	count	1	0
TGS1	29	OK	-0.219253	0.5240932	-0.418348	0.67569	-0.21497138	subgrp_con	count	1	0
LYN	12	OK	1.7837258	1.4840782	1.2019082	0.2294	0.00332683	subgrp_con	count	1	0
RPS20	743	OK	-0.158742	0.0954552	-1.663001	0.09631	-0.22609228	subgrp_con	count	1	0
CHCHD7	7	OK	0.4517720	1.0153651	0.4449355	0.65637	0.37664578	subgrp_con	count	1	0
IMPAD1	13	OK	3.7317025	1.7374641	2.1477867	0.03172	2.0917586	subgrp_con	count	1	0
FAM110B	1	OK	1.5823999	2.7200188	0.5817606	0.56072	0.47433761	subgrp_con	count	1	0
SDCBP	526	OK	-0.478003	0.1173963	-4.071703	4.67E-05	-0.67823643	subgrp_con	count	0.4646051	0
NSMAF	2	OK	-0.910223	2.1881125	-0.415985	0.67742	-0.25785735	subgrp_con	count	1	0
CA8	5	OK	-0.860932	1.2926396	-0.666026	0.50539	-0.0014265	subgrp_con	count	1	0
RAB2A	268	OK	0.4631565	0.178004	2.6019387	0.00927	0.64250850	subgrp_con	count	1	0
CHD7	8	OK	-0.151570	1.1073556	-0.136876	0.89112	-0.04610916	subgrp_con	count	1	0

ASPH	159	OK	-0.4824113	0.2260747	-2.133858	0.03285	-0.66218738	subgrp_con	count	1	0
GGH	1	OK	-15.99503	70.34942	-0.2273655	0.82014	-0.00028474	subgrp_con	count	1	0
YTHDF3-AS1	1	OK	18.759489	83.625179	0.2243282	0.8225	0.00028789	subgrp_con	count	1	0
YTHDF3	5	OK	4.6613299	5.7683860	0.8080821	0.41906	0.00274918	subgrp_con	count	1	0
CYP7B1	1	OK	3.4598146	6.7714513	0.5109413	0.60939	0.00030179	subgrp_con	count	1	0
ARMC1	15	OK	0.5681212	0.758116	0.7493855	0.45362	0.474807	subgrp_con	count	1	0
PDE7A	1	OK	-10.99763	25.231832	-0.4358635	0.66294	-0.00129008	subgrp_con	count	1	0
RRS1	3	OK	-10.44227	41.465809	-0.2518286	0.80117	-0.00045289	subgrp_con	count	1	0
MYBL1	1	OK	23.991896	84.899941	0.2825902	0.77749	1.65081709	subgrp_con	count	1	0
VCPIP1	3	OK	-11.01941	43.249935	-0.2547846	0.79889	-1.17482461	subgrp_con	count	1	0
SGK3	11	OK	-0.576944	0.9300542	-0.6203234	0.53506	-0.46458248	subgrp_con	count	1	0
C8orf44	1	OK	3.4598146	6.7714513	0.5109413	0.60939	0.00030179	subgrp_con	count	1	0
COPS5	73	OK	0.0722576	0.3449363	0.2097709	0.83385	0.09185622	subgrp_con	count	1	0
CSPP1	9	OK	0.1422849	1.1599746	0.1226620	0.90237	0.07227324	subgrp_con	count	1	0
ARFGEF1	25	OK	0.4391718	0.6601039	0.6653070	0.50585	0.41478646	subgrp_con	count	1	0
PREX2	62	OK	-0.845402	0.3758629	-2.2492298	0.0245	-1.01598693	subgrp_con	count	1	0
AC083967.1	4	OK	21.105322	83.341273	0.2532397	0.80008	0.0003103	subgrp_con	count	1	0
NCOA2	2	OK	0.1681491	1.7253300	0.097459	0.92236	0.05201277	subgrp_con	count	1	0
TRAM1	203	OK	0.1949921	0.1970912	0.9893495	0.32249	0.2691272	subgrp_con	count	1	0
LACTB2	13	OK	-0.724528	0.9525685	-0.759806	0.44737	-0.44119188	subgrp_con	count	1	0
MSC	1	OK	-10.44227	41.465809	-0.2518286	0.80117	-0.00045289	subgrp_con	count	1	0
TERF1	26	OK	1.3618881	0.7048400	1.9321945	0.05334	1.27090864	subgrp_con	count	1	0
RPL7	826	OK	-0.162087	0.0897682	-1.805626	0.07098	-0.23155109	subgrp_con	count	1	0
STAU2	10	OK	0.980127	1.0133543	0.9672109	0.33344	0.30382676	subgrp_con	count	1	0
UBE2W	22	OK	1.1047258	0.6892200	1.6028636	0.10896	0.82160116	subgrp_con	count	1	0
ELOC	271	OK	0.0527897	0.1697220	0.3110363	0.75577	0.07339729	subgrp_con	count	1	0
TMEM70	103	OK	-0.819712	0.3006703	-2.7262865	0.00641	-1.04549414	subgrp_con	count	1	0
LY96	100	OK	-0.607491	0.2995019	-2.0283382	0.04253	-0.77883557	subgrp_con	count	1	0
AC087672.2	1	OK	1.5823999	2.7200188	0.5817606	0.56072	0.47433761	subgrp_con	count	1	0
PI15	2	OK	23.991896	84.899941	0.2825902	0.77749	1.65081709	subgrp_con	count	1	0
CRISPLD1	6	OK	8.4703615	8.5047516	0.9959563	0.31927	1.13069500	subgrp_con	count	1	0
PEX2	20	OK	-0.040787	0.8453686	-0.048247	0.96152	-0.03634445	subgrp_con	count	1	0
PKIA	3	OK	3.2254063	2.3053320	1.3991070	0.16178	1.50796693	subgrp_con	count	1	0
ZC2HC1A	19	OK	2.0694254	0.996090	2.0775474	0.03775	1.56832414	subgrp_con	count	1	0
IL7	4	OK	3.0404222	1.990914	1.5271485	0.12672	1.79478554	subgrp_con	count	1	0
HEY1	19	OK	1.0612798	0.847838	1.2517470	0.21066	0.53581460	subgrp_con	count	1	0
MRPS28	9	OK	0.6289205	0.9433122	0.6667151	0.50495	0.32164192	subgrp_con	count	1	0
TPD52	8	OK	0.3920617	0.8691430	0.4510899	0.65192	0.295805	subgrp_con	count	1	0
AC009812.1	2	OK	-1.758573	2.3554439	-0.7465996	0.45531	-0.00036089	subgrp_con	count	1	0
ZBTB10	18	OK	-0.640207	0.7994081	-0.8008522	0.42322	-0.61256749	subgrp_con	count	1	0
ZNF704	7	OK	0.9134545	0.9879926	0.924556	0.3552	0.00062485	subgrp_con	count	1	0
IMPA1	10	OK	1.0791153	0.9918611	1.0879701	0.27661	0.95177642	subgrp_con	count	1	0
ZFAND1	61	OK	-0.916104	0.3863597	-2.371117	0.01772	-1.15232929	subgrp_con	count	1	0
SNX16	7	OK	1.3961960	1.4175964	0.9849037	0.32467	0.42408480	subgrp_con	count	1	0
LRRCC1	17	OK	-0.045032	0.6699263	-0.067287	0.94635	-0.04013765	subgrp_con	count	1	0
C8orf59	236	OK	0.2068154	0.1848471	1.1188457	0.26321	0.28656415	subgrp_con	count	1	0
CA13	1	OK	-10.44227	41.465809	-0.2518286	0.80117	-0.00045289	subgrp_con	count	1	0
CA2	7	OK	0.4209682	1.490919	0.2823547	0.77767	0.21538577	subgrp_con	count	1	0
WWP1	46	OK	0.3673792	0.4335132	0.8474462	0.39675	0.44830284	subgrp_con	count	1	0
RMDN1	15	OK	0.0060423	0.8570600	0.0070512	0.99437	0.00539255	subgrp_con	count	1	0
CPNE3	147	OK	-0.118846	0.2278832	-0.521524	0.602	-0.16055463	subgrp_con	count	1	0
AF117829.1	1	OK	1.5823793	1.5705017	1.0075629	0.31366	0.97972300	subgrp_con	count	1	0
RIPK2	8	OK	-10.77433	16.712840	-0.64467	0.51914	-2.83962193	subgrp_con	count	1	0
OSGIN2	8	OK	1.2533065	1.0898857	1.1499324	0.25017	0.92072565	subgrp_con	count	1	0
NBN	33	OK	0.1532287	0.5031896	0.3045148	0.76074	0.17758699	subgrp_con	count	1	0
DECR1	134	OK	0.4560822	0.251598	1.8127387	0.06987	0.60931529	subgrp_con	count	1	0
TMEM64	5	OK	4.2109206	4.2875616	0.982124	0.32606	0.00257090	subgrp_con	count	1	0
PIP4P2	10	OK	0.2500698	0.8530144	0.2931601	0.7694	0.22411919	subgrp_con	count	1	0
OTUD6B-AS1	88	OK	-0.003197	0.3196827	-0.0100017	0.99202	-0.0040380	subgrp_con	count	1	0
OTUD6B	3	OK	0.1681491	1.7253300	0.097459	0.92236	0.05201277	subgrp_con	count	1	0
SLC26A7	1	OK	18.759489	83.625179	0.2243282	0.8225	0.00028789	subgrp_con	count	1	0
TRIQK	14	OK	-0.668491	0.8290252	-0.806358	0.42006	-0.48297900	subgrp_con	count	1	0
FAM92A	18	OK	0.9265815	0.7893628	1.1738348	0.24048	0.6920976	subgrp_con	count	1	0
RBM12B	3	OK	-0.815226	2.2666336	-0.359664	0.7191	-4.1E-06	subgrp_con	count	1	0
TMEM67	3	OK	-0.150975	1.96881	-0.076683	0.93888	-9.78E-05	subgrp_con	count	1	0
PDP1	1	OK	-10.44227	41.465809	-0.2518286	0.80117	-0.00045289	subgrp_con	count	1	0
GEM	4	OK	1.1006820	1.5687290	0.7016394	0.4829	0.55471951	subgrp_con	count	1	0
VIRMA	20	OK	-0.465784	0.7039072	-0.6617126	0.50810	-0.46910283	subgrp_con	count	1	0
DPY19L4	7	OK	2.0955149	1.5313606	1.3684008	0.17119	0.0038826	subgrp_con	count	1	0
INTS8	3	OK	-1.646245	1.9351929	-0.850688	0.39494	-0.70360391	subgrp_con	count	1	0
CCNE2	2	OK	1.925964	2.5154879	0.7656425	0.44389	0.56022119	subgrp_con	count	1	0
NDUFAF6	3	OK	-1.340131	1.6819410	-0.796776	0.42558	-0.58807560	subgrp_con	count	1	0
PLEKHF2	8	OK	1.3811968	1.1121530	1.2419125	0.21427	0.68430100	subgrp_con	count	1	0
C8orf37	1	OK	18.759489	83.625179	0.2243282	0.8225	0.00028789	subgrp_con	count	1	0
GDF6	3	OK	24.346015	51.616027	0.4716755	0.63716	2.905701	subgrp_con	count	1	0
UQCRB	661	OK	-0.067719	0.1036261	-0.653494	0.51346	-0.09639453	subgrp_con	count	1	0
MTERF3	4	OK	2.1611158	2.3677943	0.9127126	0.36139	0.61359306	subgrp_con	count	1	0
PTDSS1	1	OK	1.5823999	2.7200188	0.5817606	0.56072	0.47433761	subgrp_con	count	1	0
SDC2	1	OK	18.759489	83.625179	0.2243282	0.8225	0.00028789	subgrp_con	count	1	0
CPQ	42	OK	2.061499	0.5550267	3.7142344	0.0002	2.33432784	subgrp_con	count	1	0
MTDH	347	OK	-0.230328	0.149224	-1.5435030	0.12271	-0.32206132	subgrp_con	count	1	0
LAPTM4B	81	OK	-0.473430	0.3314365	-1.428418	0.15317	-0.59657756	subgrp_con	count	1	0
MATN2	23	OK	2.3655053	1.255316	1.8843896	0.05952	0.00437018	subgrp_con	count	1	0
RPL30	879	OK	-0.132100	0.0847262	-1.559146	0.11896	-0.18899922	subgrp_con	count	1	0
RIDA	9	OK	0.712914	1.2965792	0.5506137	0.5819	0.46367215	subgrp_con	count	1	0
NIPAL2	1	OK	-11.01941	43.249935	-0.2547846	0.79889	-1.17482461	subgrp_con	count	1	0
STK3	10	OK	0.6157140	0.9776126	0.6298139	0.52882	0.19205108	subgrp_con	count	1	0
AC104986.2	1	OK	-15.99503	70.34942	-0.2273655	0.82014	-0.00028474	subgrp_con	count	1	0
VPS13B	3	OK	-0.615396	1.492102	-0.412435	0.68002	-0.29859809	subgrp_con	count	1	0
COX6C	500	OK	0.1182837	0.1245427	0.9497441	0.34224	0.16732192	subgrp_con	count	1	0
POLR2K	132	OK	0.7802103	0.2558447	3.0499369	0.00229	1.0469138	subgrp_con	count	1	0
SPAG1	11	OK	-1.428348	0.9190181	-1.554211	0.12012	-0.95126849	subgrp_con	count	1	0
RNF19A	50	OK	-0.679058	0.4352713	-1.56008	0.11874	-0.81373627	subgrp_con	count	1	0
ANKRD46	4	OK	-0.087672	1.574177	-0.055694	0.95559	-5.71E-05	subgrp_con	count	1	0
PABPC1	483	OK	-0.423381	0.1222231	-3.42777	0.00061	-0.58605573	subgrp_con	count	1	0
YWHAZ	236	OK	0.1945506	0.1893779	1.027314	0.30427	0.26631240	subgrp_con	count	1	0
ZNF706	203	OK	-0.052662	0.1990954	-0.264507	0.79139	-0.0723903	subgrp_con	count	1	0
RRM2B	9	OK	0.0064664	1.2548604	0.0051517	0.99589	0.00327341	subgrp_con	count	1	0
UBR5	10	OK	1.1376388	1.2382587	0.9187406	0.35823	0.35050309	subgrp_con	count	1	0
KLF10	115	OK	-0.632375	0.2639826	-2.395520	0.0166	-0.82878248	subgrp_con	count	1	0
GASAL1	1	OK	-15.99503	70.34942	-0.2273655	0.82014	-0.00028474	subgrp_con	count	1	0
AZIN1	18	OK	0.4950021	0.7508580	0.659248	0.50974	0.32240699	subgrp_con	count	1	0
ATP6V1C1	7	OK	-1.396709	1.366852	-1.021843	0.30685	-0.37125920	subgrp_con	count	1	0
BAALC	14	OK	-2.243012	1.0367180	-2.163568	0.0305	-1.92034724	subgrp_con	count	1	0
FZD6	44	OK	0.3471028	0.4430938	0.7833618	0.43341	0.40254277	subgrp_con	count	1	0
CTHRC1	85	OK	-0.041929	0.3249662	-0.129026	0.89734	-0.05535512	subgrp_con	count	1	0
SLC25A32	12	OK	-0.762817	0.8775494	-0.869253	0.38471	-0.36483968	subgrp_con	count	1	0
DCAF13	30	OK	-0.486885	0.577561	-0.843002	0.39923	-0.45090711	subgrp_con	count	1	0
LRP12	1	OK	23.991896	84.899941	0.2825902	0.77749	1.65081709	subgrp_con	count	1	0
ZFPM2	5	OK	1.5207849	1.3927023	1.0919669	0.27485	0.45798365	subgrp_con	count	1	0
AC027031.2	3	OK	-13.45074	28.143897	-0.477927	0.6327	-2.49443659	subgrp_con	count	1	0
OXR1	43	OK	0.1534827	0.448513	0.3422034	0.7322	0.17781988	subgrp_con	count	1	0
EIF3E	527	OK	-0.455252	0.1213291	-3.752213	0.00018	-0.64420483	subgrp_con	count	1	0
EMC2	76	OK	0.1700024	0.351013	0.4843190	0.62816	0.20878151	subgrp_con	count	1	0
NUDCD1	13	OK	-1.669901	0.9706957	-1.720314	0.08538	-1.38861643	subgrp_con	count	1	0
ENY2	364	OK	-0.37408	0.1472519	-2.540428	0.01107	-0.52437005	subgrp_con	count	1	0
PKHD1L1	26	OK	2.498815	0.72428	3.4500589	0.00056	2.48092276	subgrp_con	count	1	0
EBAG9	49	OK	-1.139710	0.4439694	-2.567092	0.01026	-1.28262719	subgrp_con	count	1	0
SYBU	10	OK	-2.023352	0.9954324	-2.032636	0.04209	-0.48961782	subgrp_con	count	1	0
EIF3H	412	OK	-0.111523	0.1375412	-0.810835	0.41748	-0.15718031	subgrp_con	count	1	0
UTP23	49	OK	-0.475562	0.4183319	-1.136806	0.25562	-0.59634059	subgrp_con	count	1	0
RAD21	96	OK	-0.165218	0.2809599	-0.588049	0.5565	-0.19905156	subgrp_con	count	1	0
MED30	35	OK	0.4560903	0.4806711	0.9488614	0.34269	0.52874760	subgrp_con	count	1	0
EXT1	26	OK	4.2717319	1.4511237	2.9437405	0.00324	2.95713519	subgrp_con	count	1	0
SAMD12	5	OK	0.9719089	1.2305288	0.7898303	0.42962	0.6260073	subgrp_con	count	1	0
TNFRSF11B	3	OK	0.8359515	1.9255322	0.4341404	0.66419	0.26012312	subgrp_con	count	1	0
NOV	3	OK	2.0176498	2.2120878	0.9121020	0.36171	0.00137857	subgrp_con	count	1	0
ENPP2	26	OK	-0.020945	0.5220696	-0.040120	0.968	-0.02453596	subgrp_con	count	1	0
TAF2	3	OK	6.8091365	5.3050116	1.2835290	0.19932	1.696588	subgrp_con	count	1	0
DEPTOR	7	OK	0.4920367	1.2873872	0.3821979	0.70232	0.00089920	subgrp_con	count	1	0
COL14A1	3	OK	0.9396704	1.4985405	0.627057	0.53062	0.29164784	subgrp_con	count	1	0
MRPL13	54	OK	-0.354315	0.3942739	-0.898652	0.36884	-0.43220191	subgrp_con	count	1	0
MTBP	1	OK	-10.44227	41.465809	-0.2518286	0.80117	-0.00045289	subgrp_con	count	1	0
HAS2	21	OK	2.5269074	0.9920474	2.5471639	0.01086	1.63545311	subgrp_con	count	1	0
AC016405.3	1	OK	-11.01941	43.249935	-0.2547846	0.79889	-1.17482461	subgrp_con	count	1	0
DERL1	22	OK	0.0654234	0.6628087	0.0987062	0.92137	0.05433887	subgrp_con	count	1	0
C8orf76	1	OK	-10.44227	41.465809	-0.2518286	0.80117	-0.00045289	subgrp_con	count	1	0
ZHX1	22	OK	0.2550828	0.5616860	0.4541377	0.64973	0.24146811	subgrp_con	count	1	0
ATAD2	2	OK	3.5007518	2.8304888	1.2368011	0.21616	1.66526042	subgrp_con	count	1	0
WDYHV1	1	OK	-11.01941	43.249935	-0.2547846	0.79889	-1.17482461	subgrp_con	count	1	0
FBXO32	2	OK	-12.14570	43.418869	-0.279733	0.77968	-1.91338276	subgrp_con	count	1	0
FAM91A1	3	OK	0.0535803	1.8127528	0.0295574	0.97642	0.04448980	subgrp_con	count	1	0
TMEM65	2	OK	0.1681491	1.7253300	0.097459	0.92236	0.05201277	subgrp_con	count	1	0
TRMT12	3	OK	-1.758573	2.3554439	-0.7465996	0.45531	-0.00036089	subgrp_con	count	1	0
RNF139	10	OK	-1.218385	1.0293097	-1.183691	0.23654	-0.69978603	subgrp_con	count	1	0
TATDN1	43	OK	0.8512206	0.4393273	1.9377379	0.05266	1.04020007	subgrp_con	count	1	0
NDUFB9	242	OK	0.3170465	0.1783539	1.7776237	0.07547	0.44290768	subgrp_con	count	1	0
MTSS1	17	OK	-0.237718	0.9498917	-0.250258	0.80229	-0.19486509	subgrp_con	count	1	0
SQLE	7	OK	4.7890907	2.7126659	1.7646097	0.07763	1.53092668	subgrp_con	count	1	0
WASHC5	2	OK	23.991896	84.899941	0.2825902	0.77749	1.65081709	subgrp_con	count	1	0
NSMCE2	13	OK	0.6262165	0.850251	0.7365071	0.46142	0.40736996	subgrp_con	count	1	0
TRIB1	34	OK	-1.06050	0.5320655	-1.993190	0.04624	-1.22787311	subgrp_con	count	1	0
FAM84B	17	OK	-1.954678	0.763374	-2.560576	0.01045	-1.21248628	subgrp_con	count	1	0
PCAT1	2	OK	21.64205	54.486891	0.3971975	0.69122	1.57744735	subgrp_con	count	1	0
MYC	112	OK	-0.35904	0.267999	-1.33972	0.18032	-0.46801671	subgrp_con	count	1	0
FAM49B	7	OK	0.2161201	1.1973328	0.1805013	0.85676	0.11021650	subgrp_con	count	1	0
ASAP1	128	OK	-0.247700	0.2557006	-0.968713	0.33268	-0.32644344	subgrp_con	count	1	0
EFR3A	13	OK	0.450774	0.7442806	0.605651	0.54475	0.40414464	subgrp_con	count	1	0
LRRC6	4	OK	0.5103003	1.603405	0.3182602	0.75029	0.15811328	subgrp_con	count	1	0
PHF20L1	66	OK	0.0736607	0.3921405	0.1878427	0.852	0.08638179	subgrp_con	count	1	0
NDRG1	124	OK	-0.494769	0.2649576	-1.867351	0.06185	-0.63363571	subgrp_con	count	1	0
ST3GAL1	32	OK	0.7765566	0.4891742	1.5874847	0.1124	0.58297068	subgrp_con	count	1	0
AC103706.1	1	OK	-15.99503	70.34942	-0.2273655	0.82014	-0.00028474	subgrp_con	count	1	0
AC083843.3	1	OK	-10.44227	41.465809	-0.2518286	0.80117	-0.00045289	subgrp_con	count	1	0
KHDRBS3	3	OK	-0.214986	1.5648174	-0.137387	0.89072	-0.06511836	subgrp_con	count	1	0
TRAPPC9	2	OK	-1.295616	2.041425	-0.634662	0.52565	-0.00075754	subgrp_con	count	1	0
CHRAC1	30	OK	1.3342302	0.6261988	2.1306779	0.03312	1.37324165	subgrp_con	count	1	0
AGO2	8	OK	-1.583558	1.1799341	-1.342072	0.17957	-0.00264108	subgrp_con	count	1	0
PTK2	50	OK	1.2429928	0.4847998	2.5639299	0.01035	1.34806607	subgrp_con	count	1	0
DENND3	7	OK	1.4368675	1.2903060	1.1400941	0.25425	0.70898876	subgrp_con	count	1	0
SLC45A4	1	OK	18.759489	83.625179	0.2243282	0.8225	0.00028789	subgrp_con	count	1	0
PTP4A3	56	OK	-0.895001	0.4095242	-2.18546	0.02885	-0.9326763	subgrp_con	count	1	0
ARC	3	OK	2.0702522	1.7994826	1.1504708	0.24995	0.96210332	subgrp_con	count	1	0
LY6K	2	OK	23.991896	84.899941	0.2825902	0.77749	1.65081709	subgrp_con	count	1	0
THEM6	21	OK	3.0112867	1.0798772	2.7885452	0.00529	1.92583339	subgrp_con	count	1	0
LYPD2	16	OK	23.446713	32.275694	0.726451	0.46756	2.86963898	subgrp_con	count	1	0
LY6E-DT	1	OK	1.5823999	2.7200188	0.5817606	0.56072	0.47433761	subgrp_con	count	1	0
LY6H	12	OK	2.5288329	1.1792389	2.1444618	0.032	2.53667691	subgrp_con	count	1	0
GPIHBP1	22	OK	0.2474092	0.6416206	0.3856005	0.69979	0.25373880	subgrp_con	count	1	0
GLI4	1	OK	-11.01941	43.249935	-0.2547846	0.79889	-1.17482461	subgrp_con	count	1	0
MINCR	2	OK	3.4598149	4.7858686	0.7229230	0.46972	0.00081935	subgrp_con	count	1	0
TOP1MT	6	OK	1.8344122	1.6316521	1.1242667	0.2609	0.53824028	subgrp_con	count	1	0
GSDMD	96	OK	-0.299785	0.294263	-1.018763	0.30832	-0.38373429	subgrp_con	count	1	0
NAPRT	35	OK	0.207248	0.5434400	0.381363	0.70292	0.18566095	subgrp_con	count	1	0
EEF1D	765	OK	-0.171659	0.0941585	-1.823088	0.06829	-0.24517001	subgrp_con	count	1	0
TSTA3	35	OK	0.0575720	0.546431	0.1053600	0.91609	0.05892170	subgrp_con	count	1	0
PUF60	72	OK	0.0781873	0.3510351	0.2227337	0.82374	0.10115764	subgrp_con	count	1	0
NRBP2	1	OK	18.759489	83.625179	0.2243282	0.8225	0.00028789	subgrp_con	count	1	0
PLEC	95	OK	0.4615995	0.3305435	1.3964863	0.16257	0.58357158	subgrp_con	count	1	0
PARP10	2	OK	-11.01941	43.249935	-0.2547846	0.79889	-1.17482461	subgrp_con	count	1	0
GRINA	12	OK	0.073119	0.968300	0.0755120	0.93981	0.03712376	subgrp_con	count	1	0
EXOSC4	11	OK	3.5639495	1.6515547	2.1579360	0.03093	2.21762200	subgrp_con	count	1	0
GPAA1	41	OK	1.2530900	0.5145395	2.4353610	0.01488	1.29511481	subgrp_con	count	1	0
CYC1	78	OK	0.2262525	0.3524699	0.641905	0.52092	0.2860898	subgrp_con	count	1	0
SHARPIN	37	OK	0.6801751	0.5470567	1.2433356	0.21374	0.73455683	subgrp_con	count	1	0
MAF1.00	72	OK	-0.374211	0.3609364	-1.036780	0.29984	-0.45604762	subgrp_con	count	1	0
HGH1	2	OK	18.759489	83.625179	0.2243282	0.8225	0.00028789	subgrp_con	count	1	0
BOP1.00	1	OK	3.4598146	6.7714513	0.5109413	0.60939	0.00030179	subgrp_con	count	1	0
HSF1	31	OK	-0.115064	0.4986550	-0.230749	0.81752	-0.12087453	subgrp_con	count	1	0
DGAT1	8	OK	0.8569919	1.0670059	0.8031744	0.42187	0.43620956	subgrp_con	count	1	0
SLC52A2	10	OK	-1.817447	1.4559542	-1.248286	0.21192	-0.45400691	subgrp_con	count	1	0
FBXL6	2	OK	3.4598146	6.7714513	0.5109413	0.60939	0.00030179	subgrp_con	count	1	0
CPSF1	1	OK	-11.01941	43.249935	-0.2547846	0.79889	-1.17482461	subgrp_con	count	1	0
SLC39A4	4	OK	1.0647369	1.2800063	0.831821	0.40551	0.79046237	subgrp_con	count	1	0
VPS28	385	OK	0.1674661	0.1405484	1.1915189	0.23345	0.23594206	subgrp_con	count	1	0
CYHR1	20	OK	0.5557319	0.685943	0.8101711	0.41786	0.52613695	subgrp_con	count	1	0
PPP1R16A	3	OK	-11.44854	41.76168	-0.274141	0.78398	-0.00049246	subgrp_con	count	1	0
MFSD3	2	OK	3.4598146	6.7714513	0.5109413	0.60939	0.00030179	subgrp_con	count	1	0
LRRC14	2	OK	3.4597863	3.9099767	0.8848611	0.37622	0.00081828	subgrp_con	count	1	0
C8orf82	8	OK	1.5972904	1.3306339	1.2004732	0.22996	1.36338373	subgrp_con	count	1	0
ARHGAP39	1	OK	3.4598146	6.7714513	0.5109413	0.60939	0.00030179	subgrp_con	count	1	0
ZNF251	1	OK	3.4598146	6.7714513	0.5109413	0.60939	0.00030179	subgrp_con	count	1	0
ZNF34	2	OK	-1.295616	2.041425	-0.634662	0.52565	-0.00075754	subgrp_con	count	1	0
RPL8	882	OK	-0.057589	0.0834153	-0.690359	0.48997	-0.08242950	subgrp_con	count	1	0
ZNF7	4	OK	0.9339908	1.3622225	0.6856374	0.49294	0.28911211	subgrp_con	count	1	0
COMMD5	12	OK	0.3481969	0.8550384	0.4072295	0.68384	0.26341794	subgrp_con	count	1	0
ZNF250	1	OK	23.991896	84.899941	0.2825902	0.77749	1.65081709	subgrp_con	count	1	0
C8orf33	42	OK	0.8004745	0.5141426	1.5569113	0.11949	0.78798114	subgrp_con	count	1	0
WASHC1	3	OK	-0.202946	1.6532265	-0.122753	0.9022	-4.82E-05	subgrp_con	count	1	0
CBWD1	5	OK	23.616643	53.90594	0.4381083	0.66132	2.38851165	subgrp_con	count	1	0
C9orf66	1	OK	3.4598146	6.7714513	0.5109413	0.60939	0.00030179	subgrp_con	count	1	0
DOCK8	2	OK	3.4598149	4.7858686	0.7229230	0.46972	0.00081935	subgrp_con	count	1	0
KANK1	9	OK	20.358018	32.658351	0.6233633	0.53305	1.53810498	subgrp_con	count	1	0
SMARCA2	45	OK	0.3417471	0.4663155	0.7328668	0.46364	0.41935606	subgrp_con	count	1	0
PUM3	33	OK	-1.201900	0.5336549	-2.252221	0.02431	-0.99230507	subgrp_con	count	1	0
RFX3	2	OK	0.5887819	1.767414	0.3331317	0.73903	0.30122869	subgrp_con	count	1	0
GLIS3	18	OK	3.5547431	1.3492614	2.6345844	0.00842	2.36664486	subgrp_con	count	1	0
CDC37L1-DT	1	OK	3.4598146	6.7714513	0.5109413	0.60939	0.00030179	subgrp_con	count	1	0
CDC37L1	9	OK	-0.99605	1.0601365	-0.939555	0.34745	-0.60821812	subgrp_con	count	1	0
AK3	35	OK	1.1729666	0.5162432	2.2721203	0.02308	1.27520674	subgrp_con	count	1	0
RCL1	4	OK	-1.119738	1.4934041	-0.749789	0.45338	-0.30904529	subgrp_con	count	1	0
JAK2	1	OK	-11.18836	26.334948	-0.424858	0.67092	-2.27128013	subgrp_con	count	1	0
RLN2	1	OK	23.991896	84.899941	0.2825902	0.77749	1.65081709	subgrp_con	count	1	0
PLGRKT	11	OK	-0.681756	0.8872210	-0.768418	0.44224	-0.42160246	subgrp_con	count	1	0
CD274	1	OK	0.6532528	2.5001284	0.2612877	0.79387	0.00016297	subgrp_con	count	1	0
KIAA2026	24	OK	0.4626233	0.6389031	0.7241056	0.469	0.34901838	subgrp_con	count	1	0
RANBP6	4	OK	-0.465429	1.3093839	-0.355462	0.72224	-0.13816105	subgrp_con	count	1	0
IL33	315	OK	-0.251313	0.1465679	-1.714654	0.08641	-0.35229695	subgrp_con	count	1	0
UHRF2	16	OK	-0.212161	0.7190854	-0.295043	0.76796	-0.17456257	subgrp_con	count	1	0
DMAC1	64	OK	0.2520951	0.3602522	0.6997739	0.48407	0.31553666	subgrp_con	count	1	0
MPDZ	43	OK	-0.571384	0.4843330	-1.179735	0.23811	-0.64994533	subgrp_con	count	1	0
LINC01235	21	OK	0.5793573	0.8892721	0.6514961	0.51472	0.18067228	subgrp_con	count	1	0
AL583785.1	24	OK	-0.579454	0.6823262	-0.849248	0.39574	-0.46665810	subgrp_con	count	1	0
NFIB	328	OK	0.0902464	0.1458706	0.6186743	0.53612	0.12676683	subgrp_con	count	1	0
ZDHHC21	4	OK	3.4016237	2.6808857	1.2688432	0.2045	0.9636701	subgrp_con	count	1	0
TTC39B	2	OK	0.1681491	1.7253300	0.097459	0.92236	0.05201277	subgrp_con	count	1	0
SNAPC3	5	OK	-1.396709	1.366852	-1.021843	0.30685	-0.37125920	subgrp_con	count	1	0
PSIP1	229	OK	0.2347103	0.1802026	1.3024812	0.19275	0.32640650	subgrp_con	count	1	0
CCDC171	4	OK	0.1255848	1.4500080	0.0866097	0.93098	0.0387800	subgrp_con	count	1	0
BNC2	4	OK	-2.436168	1.9735948	-1.234381	0.21706	-0.00126210	subgrp_con	count	1	0
CNTLN	24	OK	-0.044625	0.5316	-0.083936	0.93311	-0.03977226	subgrp_con	count	1	0
ADAMTSL1	6	OK	1.7966220	2.1603505	0.831634	0.40562	0.0012336	subgrp_con	count	1	0
RRAGA	98	OK	-0.962175	0.2965437	-3.244633	0.00118	-1.20175541	subgrp_con	count	1	0
HAUS6	6	OK	-0.79443	1.2718958	-0.624608	0.53222	-0.00132644	subgrp_con	count	1	0
PLIN2	72	OK	-1.050061	0.3749889	-2.800247	0.00511	-1.17083913	subgrp_con	count	1	0
DENND4C	9	OK	-2.020771	1.3143911	-1.537420	0.12419	-1.38892821	subgrp_con	count	1	0
RPS6	874	OK	-0.102908	0.0839971	-1.225143	0.22052	-0.14725569	subgrp_con	count	1	0
MLLT3	8	OK	1.1849225	1.0605240	1.1172990	0.26387	0.7550349	subgrp_con	count	1	0
FOCAD	2	OK	-11.01941	43.249935	-0.2547846	0.79889	-1.17482461	subgrp_con	count	1	0
KLHL9	2	OK	23.991896	84.899941	0.2825902	0.77749	1.65081709	subgrp_con	count	1	0
MTAP	13	OK	-0.861959	0.8856545	-0.973246	0.33042	-0.40795077	subgrp_con	count	1	0
TUSC1	30	OK	0.6185037	0.5345429	1.1570702	0.24726	0.65044219	subgrp_con	count	1	0
CAAP1	13	OK	0.5396809	0.7796100	0.6922447	0.48878	0.44964212	subgrp_con	count	1	0
IFT74	22	OK	0.2924324	0.621341	0.4706470	0.63789	0.30015655	subgrp_con	count	1	0
TEK	53	OK	-0.146257	0.4114014	-0.355510	0.72221	-0.1607918	subgrp_con	count	1	0
MOB3B	1	OK	-11.01941	43.249935	-0.2547846	0.79889	-1.17482461	subgrp_con	count	1	0
C9orf72	2	OK	-11.01941	43.249935	-0.2547846	0.79889	-1.17482461	subgrp_con	count	1	0
ACO1	10	OK	-3.035798	1.6421262	-1.848699	0.0645	-1.3487944	subgrp_con	count	1	0
DDX58	20	OK	-1.466683	0.7106698	-2.063804	0.03904	-1.3694308	subgrp_con	count	1	0
TOPORS	50	OK	-0.194845	0.4223244	-0.461364	0.64454	-0.19839011	subgrp_con	count	1	0
SMIM27	4	OK	-0.801898	1.530316	-0.524008	0.60027	-0.0004920	subgrp_con	count	1	0
NDUFB6	109	OK	0.7762928	0.2919321	2.6591545	0.00783	1.01109268	subgrp_con	count	1	0
APTX	9	OK	0.4304669	1.1045510	0.3897211	0.69674	0.28039717	subgrp_con	count	1	0
DNAJA1	550	OK	-0.458404	0.1156048	-3.965267	7.33E-05	-0.64994219	subgrp_con	count	0.7282240	0
SMU1	20	OK	-0.122614	0.6071732	-0.201943	0.83996	-0.1153417	subgrp_con	count	1	0
B4GALT1	56	OK	1.3203592	0.4359254	3.0288648	0.00245	1.53733453	subgrp_con	count	1	0
BAG1	151	OK	0.3927416	0.2327524	1.6873795	0.09152	0.53396089	subgrp_con	count	1	0
CHMP5	280	OK	-0.122390	0.1635061	-0.748536	0.45414	-0.17119576	subgrp_con	count	1	0
NFX1	4	OK	-13.33569	29.112233	-0.458078	0.6469	-1.33917170	subgrp_con	count	1	0
AQP7	7	OK	0.1037581	1.1862982	0.087463	0.9302	0.0001857	subgrp_con	count	1	0
AQP3	32	OK	-0.543238	0.5346955	-1.015976	0.30964	-0.52470053	subgrp_con	count	1	0
UBE2R2	96	OK	-0.056890	0.3055378	-0.186196	0.85229	-0.0721469	subgrp_con	count	1	0
UBAP2	9	OK	-0.910223	1.5464209	-0.588599	0.55617	-0.42849043	subgrp_con	count	1	0
DCAF12	3	OK	1.925964	2.5154879	0.7656425	0.44389	0.56022119	subgrp_con	count	1	0
UBAP1	3	OK	5.8049273	5.6694662	1.023893	0.30589	1.03285716	subgrp_con	count	1	0
NUDT2	21	OK	0.2214780	0.7348208	0.3014041	0.76311	0.11296539	subgrp_con	count	1	0
MYORG	1	OK	18.759489	83.625179	0.2243282	0.8225	0.00028789	subgrp_con	count	1	0
FAM219A	2	OK	-0.725185	1.8079191	-0.401116	0.68832	-0.20979917	subgrp_con	count	1	0
RPP25L	10	OK	-0.507756	0.9435807	-0.538116	0.5905	-0.31572694	subgrp_con	count	1	0
DCTN3	162	OK	0.7190688	0.2371091	3.0326493	0.00242	0.96776524	subgrp_con	count	1	0
SIGMAR1	5	OK	1.5824000	1.9240335	0.8224389	0.41082	0.77175617	subgrp_con	count	1	0
GALT	8	OK	0.2581669	1.0384353	0.2486114	0.80366	0.08010574	subgrp_con	count	1	0
IL11RA	24	OK	26.637220	32.242047	0.8261640	0.40871	4.96761854	subgrp_con	count	1	0
AL162231.1	1	OK	3.4598146	6.7714513	0.5109413	0.60939	0.00030179	subgrp_con	count	1	0
AL162231.2	2	OK	-10.47563	27.491987	-0.381043	0.70317	-1.13811785	subgrp_con	count	1	0
CCL21	9	OK	2.1100601	1.3710039	1.5390620	0.12379	0.97622193	subgrp_con	count	1	0
VCP	101	OK	1.2742231	0.3450976	3.6924502	0.00022	1.46934515	subgrp_con	count	1	0
STOML2	57	OK	0.1664907	0.3953176	0.4211569	0.67364	0.20446137	subgrp_con	count	1	0
FAM214B	7	OK	-1.037962	1.4280017	-0.726863	0.46731	-0.00062210	subgrp_con	count	1	0
UNC13B	2	OK	3.4598146	6.7714513	0.5109413	0.60939	0.00030179	subgrp_con	count	1	0
TESK1	2	OK	3.4598146	6.7714513	0.5109413	0.60939	0.00030179	subgrp_con	count	1	0
CCDC107	51	OK	1.1053479	0.4703003	2.3503022	0.01876	1.22750744	subgrp_con	count	1	0
TLN1	215	OK	0.3726386	0.1917382	1.9434767	0.05196	0.51677692	subgrp_con	count	1	0
CREB3	20	OK	2.4196656	0.9674115	2.5011751	0.01238	1.34206143	subgrp_con	count	1	0
GBA2	3	OK	1.791902	2.2833539	0.7847677	0.43259	0.00045246	subgrp_con	count	1	0
RGP1	4	OK	23.622321	53.950611	0.4378508	0.66149	2.38672454	subgrp_con	count	1	0
MSMP	7	OK	2.2469801	1.2896131	1.7416989	0.08157	1.66916971	subgrp_con	count	1	0
NPR2.00	1	OK	18.759489	83.625179	0.2243282	0.8225	0.00028789	subgrp_con	count	1	0
HINT2	54	OK	1.3027052	0.4300032	3.0295244	0.00245	1.62455416	subgrp_con	count	1	0
TMEM8B	1	OK	3.4598146	6.7714513	0.5109413	0.60939	0.00030179	subgrp_con	count	1	0
HRCT1	84	OK	1.2062615	0.3055968	3.9472316	7.91E-05	1.5776658	subgrp_con	count	0.7850662	0
SPAAR	2	OK	18.759489	83.625179	0.2243282	0.8225	0.00028789	subgrp_con	count	1	0
GLIPR2	15	OK	2.9721053	1.9786122	1.5021160	0.13307	0.00530394	subgrp_con	count	1	0
CLTA	400	OK	0.1414304	0.1391077	1.0166969	0.3092	0.19840464	subgrp_con	count	1	0
GNE	2	OK	-1.096672	2.6557302	-0.412945	0.67965	-0.28735258	subgrp_con	count	1	0
RNF38	3	OK	-0.910223	2.1881125	-0.415985	0.67742	-0.25785735	subgrp_con	count	1	0
EBLN2P	19	OK	0.3780280	0.7203512	0.5247829	0.59977	0.28520792	subgrp_con	count	1	0

ZCCHC7	19	OK	-0.9440639	0.66517169	-1.4192785	0.15582	-0.79947721	subgrp_cor	count		1	0
GRHPR	81	OK	-0.3061548	0.33891088	-0.9033491	0.36636	-0.37885285	subgrp_cor	count		1	0
POLR1E	8	OK	-1.4882516	1.17402733	-1.2676464	0.20492	-1.10183009	subgrp_cor	count		1	0
TOMM5	23	OK	0.8322913	0.76934323	1.08182054	0.27932	0.79118333	subgrp_cor	count		1	0
TRMT10B	6	OK	-1.825889	1.40925302	-1.2956434	0.1951	-1.00924292	subgrp_cor	count		1	0
EXOSC3	2	OK	3.4598146	6.7714513	0.51094137	0.60939	0.00030179	subgrp_cor	count		1	0
DCAF10	6	OK	-0.5672087	1.39570102	-0.406397	0.68445	-0.40583734	subgrp_cor	count		1	0
SLC25A51	2	OK	-0.3495512	1.85447474	-0.1884907	0.85049	-0.17337783	subgrp_cor	count		1	0
SHB	5	OK	0.5103003	1.6034059	0.31826022	0.75029	0.15911328	subgrp_cor	count		1	0
CNTNAP3	7	OK	-1.313542	1.031222	-1.2737722	0.20276	-0.35323457	subgrp_cor	count		1	0
CNTNAP3B	81	OK	0.1133092	0.31521148	0.35947043	0.71926	0.14708860	subgrp_cor	count		1	0
FAM27C	1	OK	18.759489	83.6251793	0.22432824	0.8225	0.00028789	subgrp_cor	count		1	0
CBWD5	6	OK	-0.910223	2.1881125	-0.4159856	0.67742	-0.25785735	subgrp_cor	count		1	0
ZNF658	3	OK	4.6613299	5.7683860	0.80808217	0.41906	0.00274918	subgrp_cor	count		1	0
CBWD3	1	OK	-10.44227	41.4658099	-0.2518286	0.80117	-0.00045289	subgrp_cor	count		1	0
PGM5	46	OK	-1.4696708	0.4473847	-3.2850268	0.00102	-1.63711728	subgrp_cor	count		1	0
PGM5-AS1	11	OK	-12.04943	10.8068828	-1.1149781	0.26486	-2.97473221	subgrp_cor	count		1	0
FAM122A	3	OK	-13.04562	27.674761	-0.4713905	0.63736	-1.3178643	subgrp_cor	count		1	0
FXN	8	OK	-1.950109	1.21132637	-1.6098956	0.10742	-1.20945043	subgrp_cor	count		1	0
FAM189A2	3	OK	2.8951543	2.41227967	1.1996762	0.23027	0.75227995	subgrp_cor	count		1	0
PTAR1	7	OK	0.5820988	1.07041274	0.54380789	0.58657	0.1815643	subgrp_cor	count		1	0
SMC5	22	OK	-0.0602729	0.58647546	-0.1027713	0.91814	-0.04507741	subgrp_cor	count		1	0
KLF9	160	OK	-0.958116	0.2214989	-4.3256048	1.52E-05	-1.22149048	subgrp_cor	count		0.15208343	0
TRPM3	1	OK	22.971512	51.7215836	0.4440519	0.65708	2.36236892	subgrp_cor	count		1	0
CEMIP2	96	OK	-0.577985	0.32551285	-1.7756138	0.0758	-0.72931204	subgrp_cor	count		1	0
ABHD17B	2	OK	-1.7585735	2.35544393	-0.7465996	0.45533	-0.00036089	subgrp_cor	count		1	0
C9orf85	9	OK	-0.4727607	0.9507355	-0.4972579	0.61902	-0.29741074	subgrp_cor	count		1	0
ZFAND5	152	OK	-0.6507113	0.21937	-2.9662732	0.00301	-0.85625768	subgrp_cor	count		1	0
ANXA1	652	OK	-0.2531698	0.10438296	-2.4253941	0.01529	-0.3606576	subgrp_cor	count		1	0
TRPM6	1	OK	-11.019418	43.2499352	-0.2547846	0.79889	-1.17482461	subgrp_cor	count		1	0
C9orf40	9	OK	-1.489915	1.0430045	-1.4284838	0.15315	-0.65129533	subgrp_cor	count		1	0
CARNMT1	3	OK	-2.023032	1.80712677	-1.1194743	0.26294	-0.00109400	subgrp_cor	count		1	0
NMRK1	43	OK	-0.7219062	0.45103415	-1.6005578	0.10947	-0.80600393	subgrp_cor	count		1	0
OSTF1	68	OK	-0.3988849	0.36501017	-1.0928049	0.27448	-0.48946001	subgrp_cor	count		1	0
PCSK5	4	OK	1.1112449	1.8685808	0.59469996	0.55206	0.34276981	subgrp_cor	count		1	0
RFK	92	OK	1.1154203	0.31119894	3.58426786	0.00034	1.49521767	subgrp_cor	count		1	0
GCNT1	1	OK	18.759489	83.6251793	0.22432824	0.8225	0.00028789	subgrp_cor	count		1	0
PRUNE2	3	OK	0.9241545	1.69936285	0.5438241	0.58656	0.00063197	subgrp_cor	count		1	0
VPS13A	18	OK	0.4061817	0.63739606	0.63725179	0.52396	0.33867747	subgrp_cor	count		1	0
GNA14	3	OK	-10.44227	41.4658099	-0.2518286	0.80117	-0.00045289	subgrp_cor	count		1	0
GNAQ	70	OK	0.7102736	0.35155136	2.0206824	0.04331	0.87646372	subgrp_cor	count		1	0
CEP78	4	OK	-10.283873	16.5503053	-0.6213706	0.53436	-1.74969161	subgrp_cor	count		1	0
TLE4	38	OK	-0.4968027	0.46258888	-1.0739382	0.28285	-0.51520720	subgrp_cor	count		1	0
TLE1	15	OK	1.3061177	0.82147028	1.58997565	0.11186	0.81681728	subgrp_cor	count		1	0
FRMD3	3	OK	-0.5453205	1.7085506	-0.3191715	0.7496	-0.0003423	subgrp_cor	count		1	0
IDNK	6	OK	-0.416767	1.01311203	-0.411372	0.6808	-0.26324822	subgrp_cor	count		1	0
UBQLN1	11	OK	0.1198232	1.06051877	0.11298547	0.91006	0.07819044	subgrp_cor	count		1	0
GKAP1	10	OK	-1.0393413	0.85383583	-1.2172612	0.2235	-0.72408479	subgrp_cor	count		1	0
KIF27	5	OK	1.5207849	1.39270225	1.09196694	0.27485	0.45798065	subgrp_cor	count		1	0
HNRNPK	356	OK	-0.4008112	0.1454511	-2.7556412	0.00586	-0.56152368	subgrp_cor	count		1	0
RMI1	1	OK	-10.44227	41.4658099	-0.2518286	0.80117	-0.00045289	subgrp_cor	count		1	0
NTRK2	1	OK	-15.498043	42.9510917	-0.36082	0.71822	-0.00073756	subgrp_cor	count		1	0
AGTPBP1	5	OK	-0.9101889	1.54642544	-0.588576	0.55615	-0.42847801	subgrp_cor	count		1	0
NAA35	5	OK	-1.4437862	1.27558598	-1.1318611	0.25769	-0.38122243	subgrp_cor	count		1	0
GOLM1	29	OK	1.4398594	0.70799504	2.03371407	0.04198	1.15666049	subgrp_cor	count		1	0
ISCA1	30	OK	-0.346677	0.5115987	-0.6776347	0.498	-0.27104868	subgrp_cor	count		1	0
TUT7	12	OK	-0.749613	0.86413688	-0.8674702	0.38568	-0.46069091	subgrp_cor	count		1	0
DAPK1	3	OK	-1.3401314	1.68194103	-0.7967767	0.42558	-0.59807560	subgrp_cor	count		1	0
CTSL	116	OK	-0.3307826	0.2669782	-1.2389872	0.21535	-0.43819409	subgrp_cor	count		1	0
SPIN1	8	OK	-2.058018	1.39346893	-1.4769022	0.1397	-0.82612912	subgrp_cor	count		1	0
CKS2	10	OK	0.3849845	1.37967654	0.27903972	0.78021	0.1198232	subgrp_cor	count		1	0
SECISBP2	41	OK	-0.6064315	0.4596044	-1.3194641	0.18701	-0.70738625	subgrp_cor	count		1	0
SEMA4D	1	OK	18.759489	83.6251793	0.22432824	0.8225	0.00028789	subgrp_cor	count		1	0
GADD45G	32	OK	0.0333406	0.51164153	0.06516405	0.94804	0.03414306	subgrp_cor	count		1	0
AUH	2	OK	-10.44227	41.4658099	-0.2518286	0.80117	-0.00045289	subgrp_cor	count		1	0
NFIL3	52	OK	-0.6222169	0.41016974	-1.5169742	0.12927	-0.62168702	subgrp_cor	count		1	0
SPTLC1	29	OK	1.2265514	0.6201632	1.97778822	0.04795	1.22990594	subgrp_cor	count		1	0
IARS	7	OK	-0.8515402	1.1638723	-0.7316434	0.46439	-0.24045461	subgrp_cor	count		1	0
NOL8	27	OK	-0.8110487	0.60223457	-1.3467322	0.17807	-0.86743189	subgrp_cor	count		1	0
OMD	157	OK	14.308518	3.42146863	4.18199138	2.89E-05	7.02038594	subgrp_cor	count		0.28822829	0
ASPN	1	OK	-11.019418	43.2499352	-0.2547846	0.79889	-1.17482461	subgrp_cor	count		1	0
ECM2	13	OK	24.594688	32.7972338	0.74990741	0.45331	3.81678417	subgrp_cor	count		1	0
BICD2	2	OK	1.0504292	2.03484104	0.51622177	0.6057	0.47406546	subgrp_cor	count		1	0
ZNF484	3	OK	-13.271979	28.0097917	-0.4738335	0.63562	-2.0156633	subgrp_cor	count		1	0
CARD19	56	OK	-0.070526	0.40282777	-0.1750776	0.86102	-0.08702938	subgrp_cor	count		1	0
NINJ1	33	OK	0.9035787	0.52206102	1.7307914	0.08349	1.07648665	subgrp_cor	count		1	0
FAM120AOS	43	OK	1.4725412	0.49454465	2.97758993	0.00291	1.72237086	subgrp_cor	count		1	0
FAM120A	29	OK	1.8539832	0.70711322	2.62190439	0.00874	1.86870084	subgrp_cor	count		1	0
PHF2	5	OK	-1.0890608	1.40863724	-0.7731307	0.43945	-0.64587094	subgrp_cor	count		1	0
MFSD14B	3	OK	1.5823999	2.72001883	0.58176068	0.56072	0.47433761	subgrp_cor	count		1	0
FBP2	5	OK	3.6609756	2.52606659	1.44927917	0.14726	1.37370436	subgrp_cor	count		1	0
FBP1	1	OK	18.759489	83.6251793	0.22432824	0.8225	0.00028789	subgrp_cor	count		1	0
C9orf3	83	OK	1.1267401	0.37382372	3.01409487	0.00258	1.27089710	subgrp_cor	count		1	0
PTCH1	1	OK	-10.44227	41.4658099	-0.2518286	0.80117	-0.00045289	subgrp_cor	count		1	0
LINC00476	3	OK	0.9241545	1.69936285	0.5438241	0.58656	0.00063197	subgrp_cor	count		1	0
ERCC6L2	12	OK	6.507689	3.37642479	1.9273905	0.05392	0.00153757	subgrp_cor	count		1	0
LINC00092	1	OK	18.759489	83.6251793	0.22432824	0.8225	0.00028789	subgrp_cor	count		1	0
SLC35D2	8	OK	-12.073089	17.7762624	-0.6791691	0.49703	-1.90688357	subgrp_cor	count		1	0
ZNF367	1	OK	3.4598146	6.7714513	0.51094137	0.60939	0.00030179	subgrp_cor	count		1	0
HABP4	22	OK	0.2545481	0.69109665	0.36832493	0.71263	0.07897047	subgrp_cor	count		1	0
CDC14B	5	OK	0.4367464	1.76872585	0.24692717	0.80496	0.00029324	subgrp_cor	count		1	0
AAED1	89	OK	-0.6962559	0.33170243	-2.0990377	0.03581	-0.88418953	subgrp_cor	count		1	0
MFSD14C	2	OK	-11.448548	41.761485	-0.2741412	0.78398	-0.00049246	subgrp_cor	count		1	0
TDRD7	2	OK	-1.2956167	2.0414257	-0.6346627	0.52565	-0.00075754	subgrp_cor	count		1	0
TMOD1	26	OK	1.3271481	0.5687411	2.35106652	0.01872	1.41595283	subgrp_cor	count		1	0
NCBP1	2	OK	3.4598146	6.7714513	0.51094137	0.60939	0.00030179	subgrp_cor	count		1	0
XPA	34	OK	0.1026931	0.49370528	0.20800497	0.83522	0.12603602	subgrp_cor	count		1	0
ANP32B	369	OK	-0.6614282	0.14531728	-4.5516142	5.32E-06	-0.92821314	subgrp_cor	count		0.05340623	0
NANS	84	OK	-0.4630787	0.32611355	-1.4199922	0.15561	-0.58326440	subgrp_cor	count		1	0
ANKS6	2	OK	0.4061000	2.10834129	0.19261588	0.84726	0.12645366	subgrp_cor	count		1	0
TGFBR1	3	OK	3.4598155	3.90999315	0.88486487	0.37622	0.00081877	subgrp_cor	count		1	0
ALG2	9	OK	1.2143251	1.177928	1.03089928	0.30259	0.77241374	subgrp_cor	count		1	0
SEC61B	430	OK	0.3878068	0.13340654	2.90695534	0.00365	0.54883084	subgrp_cor	count		1	0
NR4A3	1	OK	1.5823999	2.72001883	0.58176068	0.56072	0.47433761	subgrp_cor	count		1	0
STX17-AS1	1	OK	3.4598146	6.7714513	0.51094137	0.60939	0.00030179	subgrp_cor	count		1	0
STX17	19	OK	-1.288823	0.74949646	-1.7195852	0.08551	-1.22704301	subgrp_cor	count		1	0
ERP44	82	OK	0.3736276	0.31818086	1.17426198	0.24029	0.48793385	subgrp_cor	count		1	0
TEX10	1	OK	-11.019418	43.2499352	-0.2547846	0.79889	-1.17482461	subgrp_cor	count		1	0
MSANTD3	13	OK	2.1752565	1.23455375	1.76197802	0.07807	1.47007129	subgrp_cor	count		1	0
MRPL50	22	OK	0.2415303	0.68198399	0.35415835	0.72322	0.24770082	subgrp_cor	count		1	0
ZNF189	2	OK	-11.019418	43.2499352	-0.2547846	0.79889	-1.17482461	subgrp_cor	count		1	0
TMEM246	1	OK	-11.019418	43.2499352	-0.2547846	0.79889	-1.17482461	subgrp_cor	count		1	0
RNF20	6	OK	2.1388646	1.43143338	1.49421183	0.13512	1.33472288	subgrp_cor	count		1	0
SMC2	26	OK	0.1504882	0.57582995	0.26134145	0.79382	0.16625377	subgrp_cor	count		1	0
NIPSNAP3A	9	OK	-0.036478	0.93874823	-0.0388585	0.969	-0.03021248	subgrp_cor	count		1	0
ABCA1	4	OK	22.687720	54.3157576	0.41770052	0.67617	1.6098774	subgrp_cor	count		1	0
SLC44A1	22	OK	0.7748275	0.66706807	1.16154023	0.24542	0.39536151	subgrp_cor	count		1	0
FKTN	4	OK	1.1006820	1.5687290	0.7016394	0.4829	0.55471951	subgrp_cor	count		1	0
TMEM38B	2	OK	-11.019418	43.2499352	-0.2547846	0.79889	-1.17482461	subgrp_cor	count		1	0
ZNF462	10	OK	-1.986916	1.1967990	-1.660192	0.09688	-1.44902388	subgrp_cor	count		1	0
RAD23B	35	OK	-0.2789274	0.5424654	-0.5141847	0.60712	-0.20655179	subgrp_cor	count		1	0
KLF4	366	OK	-0.4774542	0.14092729	-3.3879472	0.0007	-0.67043294	subgrp_cor	count		1	0
FAM206A	1	OK	3.4598146	6.7714513	0.51094137	0.60939	0.00030179	subgrp_cor	count		1	0
TMEM245	23	OK	1.6582206	0.74012885	2.24058396	0.02505	1.76136754	subgrp_cor	count		1	0
AKAP2	2	OK	-1.7585735	2.35544393	-0.7465996	0.45533	-0.00036089	subgrp_cor	count		1	0
TXN	555	OK	-0.1046119	0.11604794	-0.9014541	0.36735	-0.14856441	subgrp_cor	count		1	0
SVEP1	5	OK	1.0226767	1.39333713	0.73397651	0.46296	0.76927284	subgrp_cor	count		1	0
ECPAS	11	OK	1.0339216	1.07279216	0.96376694	0.33516	0.94451020	subgrp_cor	count		1	0
PTGR1	15	OK	5.2064087	2.37807457	2.1893379	0.02857	1.98131256	subgrp_cor	count		1	0
GNG10	1	OK	-10.44227	41.4658099	-0.2518286	0.80117	-0.00045289	subgrp_cor	count		1	0
UGCG	105	OK	-0.7366827	0.28918691	-2.5474275	0.01085	-0.96568287	subgrp_cor	count		1	0
PTBP3	38	OK	-0.9681372	0.50671086	-1.9106304	0.05605	-0.81636769	subgrp_cor	count		1	0
HSDL2	18	OK	0.1013700	0.67941881	0.14920119	0.88139	0.09586950	subgrp_cor	count		1	0
INIP	5	OK	-1.2520544	1.38784505	-0.9021572	0.36697	-0.56535377	subgrp_cor	count		1	0
SNX30	1	OK	-10.44227	41.4658099	-0.2518286	0.80117	-0.00045289	subgrp_cor	count		1	0
ZFP37	1	OK	-15.99503	70.349421	-0.2273655	0.82014	-0.00029474	subgrp_cor	count		1	0
SLC31A1	4	OK	23.991896	84.8998419	0.28259026	0.77749	1.65281709	subgrp_cor	count		1	0
CDC26	86	OK	0.4244217	0.31564813	1.34460394	0.17875	0.55266683	subgrp_cor	count		1	0
PRPF4	3	OK	3.4598146	6.7714513	0.51094137	0.60939	0.00030179	subgrp_cor	count		1	0
HDHD3	1	OK	3.4598146	6.7714513	0.51094137	0.60939	0.00030179	subgrp_cor	count		1	0
ALAD	1	OK	3.4598146	6.7714513	0.51094137	0.60939	0.00030179	subgrp_cor	count		1	0
POLE3	20	OK	0.7823297	0.64797059	1.20735379	0.2272	0.82188545	subgrp_cor	count		1	0
COL27A1	1	OK	1.5823999	2.72001883	0.58176068	0.56072	0.47433761	subgrp_cor	count		1	0
AKNA	3	OK	18.759489	83.6251793	0.22432824	0.8225	0.00028789	subgrp_cor	count		1	0
ATP6V1G1	415	OK	0.0332013	0.13374207	0.2489968	0.80336	0.04708841	subgrp_cor	count		1	0
TLR4	21	OK	-0.6574332	0.6316418	-1.0408324	0.29795	-0.69630992	subgrp_cor	count		1	0
CDK5RAP2	4	OK	-0.2029407	1.65322657	-0.1227536	0.9022	-4.82E-05	subgrp_cor	count		1	0
MEGF9	1	OK	18.759489	83.6251793	0.22432824	0.8225	0.00028789	subgrp_cor	count		1	0
FBXW2	9	OK	1.1224437	1.45693139	0.77041635	0.44105	0.3460629	subgrp_cor	count		1	0
PSMD5	3	OK	-1.096672	2.65573025	-0.4129456	0.67965	-0.28735358	subgrp_cor	count		1	0
PHF19	6	OK	20.917329	23.1820625	0.62038063	0.52845	2.28610964	subgrp_cor	count		1	0
CNTRL	12	OK	-0.126651	0.833560	-0.1519389	0.87922	-0.06374702	subgrp_cor	count		1	0
RAB14	156	OK	0.5612849	0.23639018	2.37440043	0.01758	0.7540513	subgrp_cor	count		1	0
GSN	716	OK	-0.4099775	0.09703563	-4.2250198	2.39E-05	-0.5848779	subgrp_cor	count		0.23851365	0
STOM	294	OK	-0.005752	0.13849739	-0.0415318	0.96687	-0.00804293	subgrp_cor	count		1	0
DAB2IP	3	OK	0.6393470	1.44062452	0.44379853	0.65719	0.00043313	subgrp_cor	count		1	0
NDUFA8	112	OK	-0.2315473	0.28391595	-0.8155489	0.41476	-0.30585028	subgrp_cor	count		1	0
LHX6	7	OK	-13.327064	27.7366216	-0.4804862	0.63088	-0.00160251	subgrp_cor	count		1	0
RBM18	10	OK	-0.4088015	0.87068615	-0.4695165	0.6387	-0.25787538	subgrp_cor	count		1	0
MRRF	6	OK	0.4531379	1.62838623	0.27827427	0.7808	0.22734854	subgrp_cor	count		1	0
PTGS1	2	OK	23.622321	53.9506118	0.43785085	0.66149	2.38672454	subgrp_cor	count		1	0
PDCL	46	OK	0.8134454	0.45379536	1.79253802	0.07305	0.95056008	subgrp_cor	count		1	0
RC3H2	11	OK	-0.7467069	0.9000451	-0.8296328	0.40675	-0.70513261	subgrp_cor	count		1	0
RABGAP1	10	OK	-1.7992389	1.15635954	-1.5559511	0.11972	-0.74364890	subgrp_cor	count		1	0
STRBP	7	OK	0.7623427	1.70127539	0.44810072	0.65408	0.0005186	subgrp_cor	count		1	0
DENND1A	1	OK	-10.44227	41.4658099	-0.2518286	0.80117	-0.00045289	subgrp_cor	count		1	0
PSMB7	97	OK	0.2336620	0.30502823	0.76603936	0.44366	0.30515224	subgrp_cor	count		1	0
OLFML2A	8	OK	0.0050675	1.09458655	0.00462963	0.99631	0.0033199	subgrp_cor	count		1	0
RPL35	812	OK	-0.2210133	0.09099146	-2.428945	0.01514	-0.31574099	subgrp_cor	count		1	0
ARPC5L	145	OK	0.8448181	0.25132864	3.36141079	0.00078	1.15249759	subgrp_cor	count		1	0
GOLGA1	9	OK	-1.0325383	1.06534803	-0.9692029	0.33244	-0.71985702	subgrp_cor	count		1	0
SCAI	1	OK	-11.019418	43.2499352	-0.2547846	0.79889	-1.17482461	subgrp_cor	count		1	0
PPP6C	23	OK	0.2095826	0.66440564	0.3154438	0.75242	0.19849686	subgrp_cor	count		1	0
RABEPK	5	OK	2.704828	1.79632305	1.50575817	0.13212	1.47366775	subgrp_cor	count		1	0
HSPA5	314	OK	0.5497261	0.16162027	3.40134397	0.00067	0.76427420	subgrp_cor	count		1	0
GAPVD1	12	OK	0.205909	1.39153645	0.1479727	0.88236	0.15156229	subgrp_cor	count		1	0
MAPKAP1	15	OK	1.0274788	0.84874249	1.21058964	0.22605	0.66016041	subgrp_cor	count		1	0
PBX3	3	OK	19.103032	50.7724696	0.37624785	0.70672	0.00078880	subgrp_cor	count		1	0
ZBTB43	4	OK	0.5103003	1.6034059	0.31826022	0.75029	0.15911328	subgrp_cor	count		1	0
RALGPS1	1	OK	-11.019418	43.2499352	-0.2547846	0.79889	-1.17482461	subgrp_cor	count		1	0
ANGPTL2	19	OK	1.9170622	0.99907317	1.91884072	0.055	1.60845862	subgrp_cor	count		1	0
SLC2A8	2	OK	5.8049273	5.66946625	1.0238931	0.30589	1.03285716	subgrp_cor	count		1	0
RPL12	878	OK	-0.057804	0.08372736	-0.6903839	0.48995	-0.08274490	subgrp_cor	count		1	0
LRSAM1	2	OK	18.759489	83.6251793	0.22432824	0.8225	0.00028789	subgrp_cor	count		1	0
FAM129B	17	OK	0.952525	0.86105839	1.10622603	0.26862	0.6140133	subgrp_cor	count		1	0
STXBP1	9	OK	-0.4605365	0.90991465	-0.5061316	0.61276	-0.28998186	subgrp_cor	count		1	0
PTRH1	9	OK	2.0275897	1.3573128	1.49382647	0.13522	0.94674418	subgrp_cor	count		1	0
SH2D3C	29	OK	-0.228866	0.5368938	-0.4262793	0.6699	-0.18807291	subgrp_cor	count		1	0
CDK9	11	OK	-1.1782336	0.89538575	-1.3158802	0.18821	-0.53710953	subgrp_cor	count		1	0
ENG	477	OK	0.2977904	0.12593569	2.36462337	0.01805	0.41988160	subgrp_cor	count		1	0
AK1	12	OK	0.3471661	0.86407599	0.4017773	0.68785	0.00063048	subgrp_cor	count		1	0
ST6GALNAC6	1	OK	1.5823776	1.92402136	0.8224325	0.41082	0.77174550	subgrp_cor	count		1	0
ST6GALNAC4	18	OK	1.857555	0.91091051	2.03922953	0.04142	1.29895767	subgrp_cor	count		1	0
DPM2	30	OK	-0.3669696	0.5447586	-0.6736359	0.50054	-0.32294556	subgrp_cor	count		1	0
FAM102A	2	OK	3.4598149	4.78586864	0.72292339	0.46972	0.00081925	subgrp_cor	count		1	0
SLC25A25	32	OK	-0.8083362	0.5071843	-1.593772	0.11099	-0.88458090	subgrp_cor	count		1	0
SLC25A25-AS1	1	OK	0.6532528	2.50012846	0.26128772	0.79387	0.00016297	subgrp_cor	count		1	0
PTGES2	12	OK	0.0318800	1.04093323	0.03062637	0.97557	0.02645597	subgrp_cor	count		1	0
C9orf16	362	OK	-0.0082708	0.1484742	-0.0557052	0.95558	-0.01165032	subgrp_cor	count		1	0
CIZ1	1	OK	-10.44227	41.4658099	-0.2518286	0.80117	-0.00045289	subgrp_cor	count		1	0
GOLGA2	36	OK	1.4540941	0.55039012	2.64193396	0.00824	1.56026410	subgrp_cor	count		1	0
SWI5	32	OK	-0.133714	0.51677318	-0.2587482	0.79582	-0.15636038	subgrp_cor	count		1	0
TRUB2	7	OK	0.980104	1.33852566	0.73222676	0.46402	0.49666511	subgrp_cor	count		1	0
COQ4	15	OK	-0.1024622	0.86099331	-0.1190047	0.90527	-0.06586737	subgrp_cor	count		1	0
URM1	64	OK	0.0242181	0.3560657	0.06801606	0.94577	0.02898979	subgrp_cor	count		1	0
CERCAM	4	OK	5.2970356	4.07410236	1.30017245	0.19354	1.0014446	subgrp_cor	count		1	0
ODF2	5	OK	-0.920753	1.12129622	-0.8211507	0.41156	-0.26051363	subgrp_cor	count		1	0
AL356481.1	2	OK	-13.04562	27.674761	-0.4713905	0.63736	-1.3178643	subgrp_cor	count		1	0
SPTAN1	120	OK	0.7346528	0.25943333	2.82175987	0.00462	0.96704686	subgrp_cor	count		1	0
WDR34	3	OK	-10.44227	41.4658099	-0.2518286	0.80117	-0.00045289	subgrp_cor	count		1	0
SET	472	OK	-0.252288	0.12595886	-2.0029414	0.04518	-0.35560120	subgrp_cor	count		1	0
PKN3	9	OK	0.7108667	1.15748593	0.61414723	0.53912	0.22161777	subgrp_cor	count		1	0
ZDHHC12	5	OK	1.1006820	1.5687290	0.7016394	0.4829	0.55471951	subgrp_cor	count		1	0
AL441992.1	2	OK	-0.910223	2.1881125	-0.4159856	0.67742	-0.25785735	subgrp_cor	count		1	0
ZER1	1	OK	3.4598146	6.7714513	0.51094137	0.60939	0.00030179	subgrp_cor	count		1	0
TBC1D13	1	OK	3.4598146	6.7714513	0.51094137	0.60939	0.00030179	subgrp_cor	count		1	0
ENDOG	10	OK	-0.029972	0.98193654	-0.0305238	0.97565	-0.01515206	subgrp_cor	count		1	0
SPOUT1	2	OK	-0.3495512	1.85447474	-0.1884907	0.85049	-0.17337783	subgrp_cor	count		1	0
LRRC8A	29	OK	1.1455229	0.73463209	1.55931513	0.11892	0.57598502	subgrp_cor	count		1	0
PHYHD1	2	OK	1.925966	2.51548794	0.7656425	0.44388	0.56022119	subgrp_cor	count		1	0
NUP188	2	OK	4.6613299	5.7683860	0.80808217	0.41906	0.00274918	subgrp_cor	count		1	0
SH3GLB2	5	OK	0.7378534	1.30674578	0.56464958	0.57231	0.22996142	subgrp_cor	count		1	0
DOLPP1	1	OK	18.759489	83.6251793	0.22432824	0.8225	0.00028789	subgrp_cor	count		1	0
CRAT	2	OK	3.4598146	6.7714513	0.51094137	0.60939	0.00030179	subgrp_cor	count		1	0
PTPA	12	OK	1.3921652	0.89194777	1.56081478	0.11857	1.01325993	subgrp_cor	count		1	0
IER5L	106	OK	0.3544250	0.29476506	1.20238854	0.22921	0.46515487	subgrp_cor	count		1	0
NTMT1	12	OK	1.5016764	0.97467865	1.54068874	0.12339	1.1990722	subgrp_cor	count		1	0
ASB6	1	OK	18.759489	83.6251793	0.22432824	0.8225	0.00028789	subgrp_cor	count		1	0
PRRX2	3	OK	23.616643	53.905947	0.4381083	0.66131	2.38651165	subgrp_cor	count		1	0
TOR1B	2	OK	3.4598146	6.7714513	0.51094137	0.60939	0.00030179	subgrp_cor	count		1	0
TOR1A	11	OK	1.0275787	0.96957974	1.05981872	0.28922	0.84450877	subgrp_cor	count		1	0
C9orf78	106	OK	0.72103	0.28845366	2.49963899	0.01242	0.95327946	subgrp_cor	count		1	0
FNBP1	16	OK	0.7569213	0.76827685	0.98521947	0.32452	0.56852059	subgrp_cor	count		1	0
GPR107	7	OK	2.0726658	1.58260543	1.30966688	0.19031	1.23791122	subgrp_cor	count		1	0
HMCN2	6	OK	1.1389358	1.3676122	0.83279154	0.40496	0.00078202	subgrp_cor	count		1	0
FUBP3	5	OK	-0.6508873	1.2753209	-0.5103714	0.60979	-0.00049481	subgrp_cor	count		1	0
ABL1	8	OK	4.2598113	2.63167534	1.61866902	0.10552	1.85273386	subgrp_cor	count		1	0
AIF1L	20	OK	2.5546368	1.06001538	2.40999983	0.01595	1.97709296	subgrp_cor	count		1	0
NUP214	7	OK	-0.3681577	1.19085117	-0.3091551	0.7572	-0.00063706	subgrp_cor	count		1	0
AL157938.3	4	OK	0.5524930	1.34800031	0.40986119	0.68191	0.17231298	subgrp_cor	count		1	0
PRRC2B	30	OK	0.8547988	0.68260145	1.25226646	0.21047	0.64978308	subgrp_cor	count		1	0
UCK1	2	OK	-0.2029407	1.65322657	-0.1227536	0.9022	-4.82E-05	subgrp_cor	count		1	0
RAPGEF1	11	OK	0.234009	0.87808108	0.26650056	0.78985	0.17703817	subgrp_cor	count		1	0
MED27	10	OK	1.2095496	1.13017839	1.07022806	0.28452	0.60597204	subgrp_cor	count		1	0
SETX	28	OK	0.0725704	0.59525975	0.12191397	0.90297	0.07842102	subgrp_cor	count		1	0
TTF1	30	OK	-1.1060696	0.5222166	-2.118028	0.03417	-1.18850211	subgrp_cor	count		1	0

RNH1	281	OK	0.38073681	0.17337856	2.19587308	0.02809	0.53107952	subgrp_con	count		1	0
HRAS	24	OK	0.48093312	0.64907177	0.74095523	0.45872	0.52041170	subgrp_con	count		1	0
RASSF7	4	OK	-0.024939	1.19583735	-0.020855	0.98336	-0.00764495	subgrp_con	count		1	0
IRF7	18	OK	1.08011305	0.81198662	1.33021041	0.18345	0.80126817	subgrp_con	count		1	0
DEAF1	16	OK	0.58194509	0.81750963	0.71185102	0.47656	0.48378534	subgrp_con	count		1	0
TMEM80	9	OK	-0.7658105	0.94218868	-0.811928	0.41682	-0.54781236	subgrp_con	count		1	0
TALDO1	172	OK	0.96432435	0.23464195	4.10976967	3.96E-05	1.32128317	subgrp_con	count		0.3945885	0
RPLP2	864	OK	-0.0748485	0.08564922	-0.873896	0.38217	-0.1070549	subgrp_con	count		1	0
PNPLA2	77	OK	-0.726099	0.3265493	-2.2235522	0.02618	-0.9214065	subgrp_con	count		1	0
CRACR2B	32	OK	-1.303186	0.52357979	-2.488988	0.01281	-1.47034128	subgrp_con	count		1	0
CD151	582	OK	0.11125305	0.10947284	1.0162618	0.3095	0.15818639	subgrp_con	count		1	0
POLR2L	420	OK	0.20481485	0.13279767	1.54230764	0.123	0.28861700	subgrp_con	count		1	0
TSPAN4	243	OK	0.00551891	0.176872	0.03120281	0.97511	0.00771400	subgrp_con	count		1	0
CHID1	38	OK	2.01401423	0.57889634	3.47905849	0.0005	2.16436856	subgrp_con	count		1	0
AP2A2	13	OK	-0.1014743	0.7516996	-0.1349931	0.89262	-0.05111837	subgrp_con	count		1	0
TOLLIP	10	OK	1.09077757	1.121882	0.97227565	0.33094	0.80874434	subgrp_con	count		1	0
TOLLIP-AS1	1	OK	-11.019418	43.2499352	-0.2547846	0.79889	-1.17482461	subgrp_con	count		1	0
MOB2	103	OK	-0.2592674	0.2760963	-0.9390466	0.34771	-0.34236542	subgrp_con	count		1	0
DUSP8	7	OK	-0.0358088	1.32787954	-0.0270422	0.97842	-0.02313129	subgrp_con	count		1	0
MRPL23	18	OK	0.04025026	0.7365829	0.0629279	0.94982	0.03478855	subgrp_con	count		1	0
IGF2	7	OK	-1.775478	1.4131647	-1.2562844	0.20898	-0.93372295	subgrp_con	count		1	0
CD81	784	OK	-0.0258289	0.09261405	-0.2788877	0.78032	-0.03689463	subgrp_con	count		1	0
TSSC4	36	OK	0.3532409	0.5262523	0.671238	0.50207	0.3623823	subgrp_con	count		1	0
KCNQ1	2	OK	3.45981462	6.7714513	0.51094137	0.60939	0.00030179	subgrp_con	count		1	0
KCNQ1OT1	30	OK	0.29678279	0.49621318	0.59808534	0.54978	0.2238012	subgrp_con	count		1	0
CDKN1C	126	OK	0.78412772	0.2427968	3.22956327	0.00124	1.06110655	subgrp_con	count		1	0
SLC22A18AS	1	OK	1.58237769	1.92402136	0.8224325	0.41082	0.77174550	subgrp_con	count		1	0
PHLDA2	12	OK	-0.17682	1.05818663	-0.1670971	0.86729	-0.13167290	subgrp_con	count		1	0
NAP1L4	55	OK	0.4258494	0.39668731	1.07376613	0.28292	0.49970997	subgrp_con	count		1	0
CARS	9	OK	-0.2870624	1.11257749	-0.2580157	0.79639	-0.18286044	subgrp_con	count		1	0
OSBPL5	2	OK	-11.448548	41.761485	-0.2741412	0.78398	-0.00049246	subgrp_con	count		1	0
ZNF195	6	OK	0.13783153	1.17802725	0.11700203	0.90686	0.08942392	subgrp_con	count		1	0
NUP98	2	OK	-10.442278	41.4658099	-0.2518286	0.80117	-0.00045289	subgrp_con	count		1	0
PGAP2	4	OK	-0.98441	1.3645039	-0.7214416	0.47064	-0.27638727	subgrp_con	count		1	0
RHOG	36	OK	0.0004559	0.5034555	0.00090534	0.99928	0.00052747	subgrp_con	count		1	0
STIM1	2	OK	-15.99503	70.349421	-0.2273655	0.82014	-0.00028474	subgrp_con	count		1	0
RRM1	4	OK	-0.1747011	1.82098429	-0.0959377	0.92357	-0.05306391	subgrp_con	count		1	0
TRIM21	2	OK	-11.448548	41.761485	-0.2741412	0.78398	-0.00049246	subgrp_con	count		1	0
OR51E1	3	OK	1.17657919	2.3465057	0.50141757	0.61608	0.00080876	subgrp_con	count		1	0
HBB	2	OK	-13.04562	27.674761	-0.4713905	0.63736	-1.3178641	subgrp_con	count		1	0
TRIM5	9	OK	22.6877208	54.3157576	0.41770052	0.67617	1.6098774	subgrp_con	count		1	0
TRIM22	45	OK	0.18341962	0.41619674	0.44070413	0.65942	0.20268235	subgrp_con	count		1	0
FAM160A2	4	OK	-1.1197385	1.49340414	-0.7497892	0.45338	-0.30904529	subgrp_con	count		1	0
CAVIN3	226	OK	-0.3905024	0.19873504	-1.9649399	0.04942	-0.52760948	subgrp_con	count		1	0
SMPD1	5	OK	1.39610128	1.41703559	0.98480967	0.32472	0.42407085	subgrp_con	count		1	0
APBB1	4	OK	6.42803292	5.27484014	1.21862137	0.22299	1.06669691	subgrp_con	count		1	0
ARFIP2	6	OK	1.33602055	1.34240069	0.99524722	0.31962	0.84312343	subgrp_con	count		1	0
TIMM10B	9	OK	-0.0908395	1.20647722	-0.0752907	0.93998	-0.06785652	subgrp_con	count		1	0
RRP8	4	OK	-0.1380197	1.39391242	-0.099018	0.92112	-8.95E-05	subgrp_con	count		1	0
ILK	46	OK	1.42037973	0.50961626	2.78715544	0.00532	1.60649771	subgrp_con	count		1	0
TAF10	182	OK	-0.4291205	0.21029127	-2.0406007	0.04129	-0.5865107	subgrp_con	count		1	0
TPP1	19	OK	0.00404509	0.73497958	0.00550363	0.99561	0.00413567	subgrp_con	count		1	0
DCHS1	9	OK	4.0758934	1.6667007	2.445486	0.01447	3.49916620	subgrp_con	count		1	0
MRPL17	122	OK	1.15761681	0.30496343	3.79592009	0.00015	1.52846656	subgrp_con	count		1	0
PPFIBP2	1	OK	3.45981462	6.7714513	0.51094137	0.60939	0.00030179	subgrp_con	count		1	0
EIF3F	469	OK	-0.0623946	0.12680452	-0.4920531	0.62268	-0.08816473	subgrp_con	count		1	0
STK33	1	OK	-11.189367	26.3349482	-0.4248866	0.67092	-2.27128013	subgrp_con	count		1	0
RPL27A	686	OK	-0.075893	0.1009746	-0.7516056	0.45229	-0.10795139	subgrp_con	count		1	0
ST5	2	OK	3.45981462	6.7714513	0.51094137	0.60939	0.00030179	subgrp_con	count		1	0
AKIP1	16	OK	0.59389488	0.75973728	0.78171081	0.43438	0.49894602	subgrp_con	count		1	0
TMEM9B	64	OK	-0.0476016	0.3500219	-0.135996	0.89182	-0.06037270	subgrp_con	count		1	0
DENND5A	7	OK	0.79511567	1.13287854	0.7018543	0.48277	0.51535561	subgrp_con	count		1	0
IPO7	24	OK	0.91007068	0.64404349	1.4130578	0.15764	0.88894582	subgrp_con	count		1	0
ZNF143	9	OK	0.84132773	0.89673308	0.93821422	0.34813	0.74896526	subgrp_con	count		1	0
WEE1	10	OK	2.09330751	1.42276194	1.4712985	0.14125	0.61574650	subgrp_con	count		1	0
SWAP70	126	OK	-0.2900359	0.25203986	-1.1507542	0.24982	-0.37393856	subgrp_con	count		1	0
SBF2	3	OK	2.55520307	2.34984433	1.08739249	0.27686	0.69311810	subgrp_con	count		1	0
ADM	61	OK	0.99081703	0.45292297	2.18760604	0.0287	1.13740263	subgrp_con	count		1	0
MTRNR2L8	174	OK	-0.9656335	0.21726369	-4.4445228	8.81E-06	-1.29053288	subgrp_con	count		0.0882544	0
RNF141	4	OK	4.21092061	4.28756165	0.9821248	0.32606	0.00257090	subgrp_con	count		1	0
LYVE1	11	OK	1.39937513	1.14053204	1.22694942	0.21984	1.02136318	subgrp_con	count		1	0
CTR9	16	OK	-0.8194104	0.80048043	-1.0236483	0.306	-0.74490758	subgrp_con	count		1	0
EIF4G2	304	OK	0.50641361	0.16339144	3.09938896	0.00194	0.70331650	subgrp_con	count		1	0
ZBED5	11	OK	-0.6650699	1.11571058	-0.5960954	0.55111	-0.41056757	subgrp_con	count		1	0
ZBED5-AS1	2	OK	-10.442278	41.4658099	-0.2518286	0.80117	-0.00045289	subgrp_con	count		1	0
GALNT18	11	OK	-0.305864	1.0932292	-0.2797803	0.77965	-0.1521786	subgrp_con	count		1	0
AC104031.1	1	OK	18.7594894	83.6251793	0.22432824	0.8225	0.00028789	subgrp_con	count		1	0
USP47	10	OK	1.54081171	1.04268821	1.47771588	0.13948	1.10879478	subgrp_con	count		1	0
DKK3.00	33	OK	1.96769267	0.61272927	3.21135742	0.00132	1.62681234	subgrp_con	count		1	0
MICAL2	8	OK	0.79511567	1.13287854	0.7018543	0.48277	0.51535561	subgrp_con	count		1	0
PARVA	33	OK	3.00472752	0.95210535	3.15587715	0.0016	2.3341225	subgrp_con	count		1	0
TEAD1	22	OK	1.78337055	0.74223806	2.4026935	0.01627	1.58859050	subgrp_con	count		1	0
BTBD10	13	OK	-0.3069389	1.04101657	-0.2948454	0.76811	-0.09182532	subgrp_con	count		1	0
FAR1	12	OK	0.79269347	1.02443541	0.7737857	0.43906	0.51382088	subgrp_con	count		1	0
SPON1	2	OK	3.45978638	3.90997672	0.88486111	0.37622	0.00081828	subgrp_con	count		1	0
RRAS2	11	OK	2.90901901	1.42257126	2.04490214	0.04086	1.78916176	subgrp_con	count		1	0
COPB1	63	OK	-0.2212916	0.37717147	-0.5867136	0.5574	-0.2514675	subgrp_con	count		1	0
PSMA1	232	OK	-0.0095783	0.18879447	-0.0507343	0.95954	-0.01314279	subgrp_con	count		1	0
C11orf58	399	OK	0.23242709	0.14012203	1.65874764	0.09717	0.32790128	subgrp_con	count		1	0
RPS13	879	OK	-0.1527475	0.08503965	-1.7961918	0.07246	-0.21853951	subgrp_con	count		1	0
PIK3C2A	121	OK	-0.1851565	0.27284213	-0.6786214	0.49738	-0.22299651	subgrp_con	count		1	0
SERGEF	6	OK	-0.0248099	1.36386208	-0.0181909	0.98549	-0.01601159	subgrp_con	count		1	0
SAAL1	4	OK	-1.2956167	2.0414257	-0.6346627	0.52565	-0.00075754	subgrp_con	count		1	0
HPS5	5	OK	1.56593057	1.56669195	0.99951402	0.31755	0.97089418	subgrp_con	count		1	0
GTF2H1	8	OK	0.56583588	1.0707885	0.52842917	0.5972	0.36836572	subgrp_con	count		1	0
LDHA	602	OK	-0.3382609	0.10920537	-3.097475	0.00195	-0.48060350	subgrp_con	count		1	0
TSG101	79	OK	0.56627298	0.32676855	1.73294809	0.0831	0.70406421	subgrp_con	count		1	0
UEVLD	1	OK	18.7594894	83.6251793	0.22432824	0.8225	0.00028789	subgrp_con	count		1	0
SPTY2D1	11	OK	-0.5258524	0.91626995	-0.5739055	0.56603	-0.42130664	subgrp_con	count		1	0
TMEM86A	1	OK	1.58229999	2.72001883	0.58176068	0.56073	0.47433761	subgrp_con	count		1	0
PTPN5	4	OK	-11.543108	27.6317575	-0.4177479	0.67613	-1.85965091	subgrp_con	count		1	0
E2F8	1	OK	3.45981462	6.7714513	0.51094137	0.60939	0.00030179	subgrp_con	count		1	0
NAV2	4	OK	0.31027286	1.97731199	0.15691649	0.87531	0.1666194	subgrp_con	count		1	0
HTATIP2	32	OK	0.17701236	0.57575163	0.3074457	0.7585	0.17510995	subgrp_con	count		1	0
FANCF	2	OK	-11.186792	26.3137535	-0.425131	0.67076	-2.27103070	subgrp_con	count		1	0
SVIP	108	OK	0.64373449	0.28093731	2.29138127	0.02194	0.87091212	subgrp_con	count		1	0
FIBIN	16	OK	21.3267171	32.0107174	0.66623678	0.50526	1.56773798	subgrp_con	count		1	0
CCDC34	13	OK	1.32603411	0.93871942	1.412586	0.15777	0.83742131	subgrp_con	count		1	0
LIN7C	10	OK	0.47918929	1.0264321	0.46684948	0.64061	0.31438942	subgrp_con	count		1	0
METTL15	4	OK	0.93961099	1.49856075	0.62700893	0.53065	0.2916352	subgrp_con	count		1	0
ARL14EP	17	OK	-0.291082	0.80103041	-0.3633857	0.71632	-0.18537705	subgrp_con	count		1	0
DNAJC24	3	OK	3.56535443	2.36687343	1.5063562	0.13198	2.38350095	subgrp_con	count		1	0
IMMP1L	8	OK	0.21292165	1.12955578	0.18850034	0.85048	0.06596965	subgrp_con	count		1	0
ELP4	5	OK	-3.1389321	1.95696544	-1.6039793	0.10872	-0.63775877	subgrp_con	count		1	0
RCN1	129	OK	-0.3868103	0.24220035	-1.5970673	0.11025	-0.51649984	subgrp_con	count		1	0
EIF3M	188	OK	-0.1073282	0.21643295	-0.4958957	0.61997	-0.14548160	subgrp_con	count		1	0
PRRG4	8	OK	-0.0423525	0.93896761	-0.0451054	0.96402	-0.03507115	subgrp_con	count		1	0
QSER1	10	OK	0.92154485	0.92793201	0.99311678	0.32065	0.59477234	subgrp_con	count		1	0
TCP11L1	2	OK	3.45981462	6.7714513	0.51094137	0.60939	0.00030179	subgrp_con	count		1	0
CSTF3	3	OK	21.1053239	83.3412731	0.25323976	0.80008	0.0003103	subgrp_con	count		1	0
HIPK3	119	OK	-1.0471568	0.26552083	-3.9437842	8.02E-05	-1.35223962	subgrp_con	count		0.7962033	0
CD59	690	OK	-0.0799763	0.10067816	-0.7943758	0.42698	-0.11390900	subgrp_con	count		1	0
FBXO3	17	OK	0.68107751	0.74423225	0.91514108	0.36012	0.51246568	subgrp_con	count		1	0
LMO2	467	OK	0.40362915	0.1220845	3.30614569	0.00095	0.57244692	subgrp_con	count		1	0
CAPRIN1	31	OK	0.4798545	0.5779746	0.8302346	0.40641	0.36245620	subgrp_con	count		1	0
NAT10	1	OK	3.45981462	6.7714513	0.51094137	0.60939	0.00030179	subgrp_con	count		1	0
CAT	35	OK	-0.417482	0.4710382	-0.8863018	0.37545	-0.4062466	subgrp_con	count		1	0
APIP	16	OK	0.59566317	0.75347186	0.79055795	0.4292	0.30473671	subgrp_con	count		1	0
CD44	5	OK	18.7594894	83.6251793	0.22432824	0.8225	0.00028789	subgrp_con	count		1	0
FJX1	7	OK	0.18374725	1.15428996	0.15918646	0.87352	0.11934421	subgrp_con	count		1	0
TRIM44	56	OK	-0.3478195	0.38331788	-0.9073918	0.3642	-0.42724383	subgrp_con	count		1	0
LDLRAD3	5	OK	-0.075569	1.2476219	-0.0605635	0.95171	-0.04866258	subgrp_con	count		1	0
COMMD9	14	OK	0.43604129	0.72927321	0.59791212	0.5499	0.36355213	subgrp_con	count		1	0
C11orf74	17	OK	-1.3546049	0.73832474	-1.8347006	0.06655	-1.17124349	subgrp_con	count		1	0
API5	8	OK	0.56583588	1.0707885	0.52842917	0.5972	0.36836572	subgrp_con	count		1	0
TTC17	11	OK	1.10525779	1.11832536	0.98831505	0.323	0.71501889	subgrp_con	count		1	0
ALKBH3	3	OK	21.642059	54.4868919	0.39719753	0.69122	1.57744735	subgrp_con	count		1	0
C11orf96	158	OK	-0.5748773	0.22458744	-2.5597035	0.01048	-0.77232577	subgrp_con	count		1	0
EXT2	5	OK	0.51030031	1.6034059	0.31826022	0.75029	0.15911328	subgrp_con	count		1	0
CD82	5	OK	2.50161364	1.78662599	1.40018877	0.16146	1.40015402	subgrp_con	count		1	0
TSPAN18	73	OK	-0.211812	0.34853496	-0.607721	0.54337	-0.24419959	subgrp_con	count		1	0
TP53I11	17	OK	0.92060253	0.79922904	1.15186322	0.24938	0.86283181	subgrp_con	count		1	0
CHST1	8	OK	0.52597899	1.35580619	0.38794552	0.69806	0.34252675	subgrp_con	count		1	0
SLC35C1	4	OK	-0.2681983	1.22915854	-0.2181967	0.82728	-0.13377831	subgrp_con	count		1	0
CRY2	2	OK	18.7594894	83.6251793	0.22432824	0.8225	0.00028789	subgrp_con	count		1	0
PEX16	15	OK	0.1570537	0.74174409	0.21173569	0.83231	0.13068805	subgrp_con	count		1	0
PHF21A	3	OK	-0.3379094	1.94220883	-0.173982	0.86188	-0.00021590	subgrp_con	count		1	0
CREB3L1	12	OK	-1.1698008	0.81019682	-1.4438477	0.14879	-0.32075316	subgrp_con	count		1	0
DGKZ	8	OK	1.79662209	2.16035059	0.8316345	0.40562	0.0012236	subgrp_con	count		1	0
MDK	272	OK	0.19643641	0.1609656	1.22036271	0.22232	0.27544247	subgrp_con	count		1	0
ATG13	2	OK	0.65325287	2.50012866	0.26128772	0.79387	0.00016297	subgrp_con	count		1	0
ARHGAP1	6	OK	-0.2415775	1.33818825	-0.1805258	0.85674	-0.17661359	subgrp_con	count		1	0
ZNF408	1	OK	18.7594894	83.6251793	0.22432824	0.8225	0.00028789	subgrp_con	count		1	0
CKAP5	9	OK	1.765883	1.3982139	1.26295634	0.2066	0.54029512	subgrp_con	count		1	0
C11orf49	2	OK	18.7594894	83.6251793	0.22432824	0.8225	0.00028789	subgrp_con	count		1	0
ARFGAP2	9	OK	1.86310705	1.40385015	1.32714098	0.18446	0.88515937	subgrp_con	count		1	0
PACSIN3	1	OK	18.7594894	83.6251793	0.22432824	0.8225	0.00028789	subgrp_con	count		1	0
DDB2	16	OK	1.99401655	1.05132591	1.89666832	0.05787	1.52229751	subgrp_con	count		1	0
ACP2	6	OK	3.97588323	2.42850823	1.63717099	0.10159	1.80276123	subgrp_con	count		1	0
NR1H3	2	OK	1.58240009	1.92403354	0.82243893	0.41082	0.77175617	subgrp_con	count		1	0
SLC39A13	7	OK	0.12502745	0.90948557	0.13747053	0.89066	0.0635936	subgrp_con	count		1	0
PSMC3	107	OK	0.40315143	0.28358983	1.42160062	0.15514	0.52646038	subgrp_con	count		1	0
CELF1	14	OK	1.40490957	0.9100899	1.54370416	0.12266	1.135785	subgrp_con	count		1	0
NDUFS3	53	OK	0.15173479	0.39621477	0.38296096	0.70175	0.18985314	subgrp_con	count		1	0
PTPMT1	8	OK	-0.2958832	1.28260451	-0.2306884	0.81756	-0.24235932	subgrp_con	count		1	0
KBTBD4	1	OK	-12.148421	43.455309	-0.2795613	0.77981	-1.91362624	subgrp_con	count		1	0
C1QTNF4	1	OK	23.9918963	84.8999419	0.28259026	0.77749	1.65081709	subgrp_con	count		1	0
MTCH2	20	OK	0.82945025	0.84055006	0.98679458	0.32374	0.73866140	subgrp_con	count		1	0
FNBP4	59	OK	0.5296096	0.38322158	1.40810893	0.1591	0.59574335	subgrp_con	count		1	0
NUP160	7	OK	2.19068155	1.86208867	1.17646468	0.23941	0.00151552	subgrp_con	count		1	0
PTPRJ	33	OK	38.1757033	234.792085	0.16258366	0.87084	3.85822674	subgrp_con	count		1	0
FOLH1	1	OK	3.45981462	6.7714513	0.51094137	0.60939	0.00030179	subgrp_con	count		1	0
APLNR	53	OK	-0.6984515	0.47064443	-1.4840322	0.1378	-0.77960736	subgrp_con	count		1	0
TNKS1BP1	9	OK	0.30412117	0.94620205	0.3214125	0.7479	0.22786067	subgrp_con	count		1	0
SSRP1	12	OK	-0.6852793	0.99279554	-0.6902522	0.49004	-0.32391573	subgrp_con	count		1	0
SLC43A3	8	OK	1.69741737	1.22645874	1.38399875	0.16636	0.81947827	subgrp_con	count		1	0
TIMM10	62	OK	-0.123162	0.36283736	-0.3394415	0.73428	-0.14025575	subgrp_con	count		1	0
UBE2L6	125	OK	-0.4115109	0.24507959	-1.6790909	0.09312	-0.5484544	subgrp_con	count		1	0
SERPING1	249	OK	-0.3526232	0.16471689	-2.1407834	0.03229	-0.49559076	subgrp_con	count		1	0
CLP1	5	OK	-0.7251858	1.80791919	-0.4011162	0.68832	-0.20979917	subgrp_con	count		1	0
ZDHHC5	12	OK	3.50742719	1.57609882	2.22538533	0.02606	1.97921317	subgrp_con	count		1	0
MED19	46	OK	-0.4448101	0.41794403	-1.0642816	0.2872	-0.50224898	subgrp_con	count		1	0
TMX2	21	OK	1.14034008	0.6710516	1.69933286	0.08926	1.06059937	subgrp_con	count		1	0
SELENOH	286	OK	-0.0646967	0.16805847	-0.3849653	0.70026	-0.09048651	subgrp_con	count		1	0
CTNND1	36	OK	0.13964575	0.56099306	0.24892599	0.80342	0.13215397	subgrp_con	count		1	0
ZFP91	5	OK	1.01381281	1.37480646	0.7374222	0.46087	0.00188658	subgrp_con	count		1	0
GLYATL2	2	OK	3.45981462	6.7714513	0.51094137	0.60939	0.00030179	subgrp_con	count		1	0
FAM111A	25	OK	0.25823013	0.64254839	0.40141527	0.68811	0.25596374	subgrp_con	count		1	0
OSBP	9	OK	-0.4728818	1.00069853	-0.4725517	0.63652	-0.38179188	subgrp_con	count		1	0
PATL1	5	OK	-0.8416402	1.46082315	-0.5761411	0.56452	-0.24034852	subgrp_con	count		1	0
STX3	4	OK	18.7594894	83.6251793	0.22432824	0.8225	0.00028789	subgrp_con	count		1	0
MRPL16	58	OK	0.60731273	0.35720784	1.70016642	0.0891	0.77388567	subgrp_con	count		1	0
MS4A6A	2	OK	1.9259645	2.51548794	0.7656425	0.44389	0.56022119	subgrp_con	count		1	0
CCDC86	1	OK	3.45981462	6.7714513	0.51094137	0.60939	0.00030179	subgrp_con	count		1	0
PRPF19	6	OK	2.4487393	1.9385827	1.32186247	0.18292	1.02563747	subgrp_con	count		1	0
TMEM109	160	OK	-0.3141769	0.22180833	-1.4164342	0.15665	-0.42478416	subgrp_con	count		1	0
SLC15A3	3	OK	-0.7252159	1.80791401	-0.4011341	0.68832	-0.20980483	subgrp_con	count		1	0
VPS37C	1	OK	3.45981462	6.7714513	0.51094137	0.60939	0.00030179	subgrp_con	count		1	0
VWCE	1	OK	0.65325287	2.50012866	0.26128772	0.79387	0.00016297	subgrp_con	count		1	0
DDB1	14	OK	0.37248005	0.82215106	0.45305548	0.65051	0.28101780	subgrp_con	count		1	0
TKFC	1	OK	1.58229999	2.72001883	0.58176068	0.56073	0.47433761	subgrp_con	count		1	0
CYB561A3	5	OK	26.3679501	84.9163259	0.31051685	0.75617	2.49052421	subgrp_con	count		1	0
TMEM138	4	OK	4.21092061	4.28756165	0.9821248	0.32606	0.00257090	subgrp_con	count		1	0
TMEM216	6	OK	0.4831432	1.27696596	0.37835245	0.70517	0.2472630	subgrp_con	count		1	0
CPSF7	2	OK	3.45981462	6.7714513	0.51094137	0.60939	0.00030179	subgrp_con	count		1	0
SDHAF2	29	OK	0.72008908	0.57902069	1.24363267	0.21362	0.85832221	subgrp_con	count		1	0
TMEM258	288	OK	0.61484894	0.16940487	3.62946447	0.00028	0.85034738	subgrp_con	count		1	0
FADS1	6	OK	4.22637529	2.98885092	1.41404687	0.15735	1.90865498	subgrp_con	count		1	0
FADS3	26	OK	2.59399259	0.93412385	2.77692576	0.00549	2.23393517	subgrp_con	count		1	0
RAB3IL1	6	OK	-0.5561292	1.05719913	-0.5260401	0.59886	-0.27129238	subgrp_con	count		1	0
BEST1	1	OK	18.7594894	83.6251793	0.22432824	0.8225	0.00028789	subgrp_con	count		1	0
FTH1	888	OK	-0.0564823	0.08243129	-0.6852045	0.49322	-0.08085559	subgrp_con	count		1	0
ASRGL1	16	OK	0.41605531	0.90648442	0.45897679	0.64625	0.21286900	subgrp_con	count		1	0
TUT1	4	OK	-0.4654295	1.30936391	-0.3554622	0.72224	-0.13816105	subgrp_con	count		1	0
MTA2	3	OK	-2.4361688	1.97359482	-1.2343815	0.21706	-0.00126310	subgrp_con	count		1	0
EML3	3	OK	3.45981462	6.7714513	0.51094137	0.60939	0.00030179	subgrp_con	count		1	0
ROM1	19	OK	2.10379083	0.85028131	2.47422918	0.01335	1.89459954	subgrp_con	count		1	0
B3GAT3	16	OK	2.42051943	0.95229298	2.54178018	0.01103	2.28646942	subgrp_con	count		1	0
GANAB	28	OK	1.43279133	0.62827388	2.28052036	0.02258	1.31289269	subgrp_con	count		1	0
CSKMT	5	OK	-0.0420939	1.15920361	-0.0363271	0.97102	-0.02124088	subgrp_con	count		1	0
UQCC3	37	OK	0.43289620	0.49150293	0.88076027	0.37845	0.50824964	subgrp_con	count		1	0
UBXN1	321	OK	-0.6114684	0.1571309	-3.8914587	9.96E-05	-0.85234932	subgrp_con	count		0.9878640	0
HNRNPUL2	4	OK	5.68312411	5.33318758	1.06561489	0.2866	0.00307912	subgrp_con	count		1	0
TTC9C	9	OK	-0.3796101	1.36222755	-0.2786686	0.78051	-0.11352900	subgrp_con	count		1	0
POLR2G	95	OK	0.55365677	0.31950587	1.73285322	0.08312	0.69240499	subgrp_con	count		1	0
TMEM223	36	OK	0.27972323	0.52076563	0.5371386	0.59117	0.30277149	subgrp_con	count		1	0
TMEM179B	209	OK	-0.1781256	0.19376253	-0.9192970	0.35794	-0.24720130	subgrp_con	count		1	0
NXF1	3	OK	1.7919026	2.28325398	0.78476776	0.43259	0.00045246	subgrp_con	count		1	0
STX5	17	OK	-0.0114756	0.70454032	-0.0162881	0.987	-0.01023765	subgrp_con	count		1	0
AP001160.1	5	OK	0.98655309	1.35858221	0.7261637	0.46774	0.50589436	subgrp_con	count		1	0
WDR74	13	OK	-0.9596599	0.82586353	-1.1620079	0.24522	-0.57740547	subgrp_con	count		1	0
SLC3A2	273	OK	-0.1885534	0.16755052	-1.1253525	0.26044	-0.26123196	subgrp_con	count		1	0
RARRES3	138	OK	-0.6217887	0.22282559	-2.7904733	0.00526	-0.84470842	subgrp_con	count		1	0
PLA2G16	177	OK	0.24582943	0.21329972	1.15255394	0.24909	0.33282960	subgrp_con	count		1	0
ATL3	47	OK	-0.3434375	0.44067815	-0.7793386	0.43578	-0.36752216	subgrp_con	count		1	0
RTN3	96	OK	-0.0337826	0.28398869	-0.1189577	0.90531	-0.04440406	subgrp_con	count		1	0
C11orf95	2	OK	-10.442278	41.4658099	-0.2518286	0.80117	-0.00045289	subgrp_con	count		1	0
MARK2	4	OK	3.45981497	4.78586864	0.72292309	0.46972	0.00081935	subgrp_con	count		1	0
COX8A	326	OK	0.68110538	0.1612256	4.22454853	2.39E-05	0.95477719	subgrp_con	count		0.2389889	0
OTUB1	28	OK	-0.8706703	0.57756699	-1.5074792	0.13169	-0.9300308	subgrp_con	count		1	0
MACROD1	6	OK	0.35309207	1.1709379	0.30154636	0.763	0.18053741	subgrp_con	count		1	0
STIP1	19	OK	1.27612389	0.93349859	1.36703359	0.17161	0.80428660	subgrp_con	count		1	0
FERMT3	7	OK	21.1053239	83.3412731	0.25323976	0.80008	0.0003103	subgrp_con	count		1	0
TRPT1	11	OK	1.54756095	1.0288705	1.50413579	0.13258	1.11304232	subgrp_con	count		1	0
NUDT22	14	OK	-0.2063097	0.79098227	-0.2608273	0.79422	-0.19352590	subgrp_con	count		1	0
DNAJC4	65	OK	0.43999327	0.36931315	1.19138263	0.2335	0.55353463	subgrp_con	count		1	0
VEGFB	73	OK	0.85569547	0.34138877	2.50651325	0.01219	1.11681496	subgrp_con	count		1	0
FKBP2	319	OK	0.63386221	0.15661709	4.04720975	5.18E-05	0.89097339	subgrp_con	count		0.5157264	0
PPP1R14B	332	OK	0.31892784	0.15392938	2.07191013	0.03827	0.44906895	subgrp_con	count		1	0
AP001453.2	1	OK	3.45981462	6.7714513	0.51094137	0.60939	0.00030179	subgrp_con	count		1	0
PLCB3	2	OK	4.66132993	5.76838606	0.80808217	0.41904	0.00274918	subgrp_con	count		1	0
BAD	94	OK	0.65515613	0.30737503	2.13145541	0.03305	0.85447234	subgrp_con	count		1	0
GPR137	12	OK	0.40812063	0.94463923	0.43203862	0.66571	0.2658290	subgrp_con	count		1	0
ESRRA	3	OK	4.66132993	5.76838606	0.80808217	0.41904	0.00274918	subgrp_con	count		1	0
TRMT112	423	OK	0.16499564	0.13392381	1.23201102	0.21794	0.23279558	subgrp_con	count		1	0
PRDX5	320	OK	0.50949133	0.15888607	3.2066457	0.00134	0.71492052	subgrp_con	count		1	0
RPS6KA4	1	OK	18.7594894	83.6251793	0.22432824	0.8225	0.00028789	subgrp_con	count		1	0
SF1	155	OK	0.12041405	0.22857104	0.52681236	0.59832	0.1613623	subgrp_con	count		1	0
MEN1	1	OK	3.45981462	6.7714513	0.51094137	0.60939	0.00030179	subgrp_con	count		1	0
EHD1	6	OK	7.24684899	6.17288547	1.17398079	0.2404	0.00128168	subgrp_con	count		1	0
ATG2A	4	OK	21.1053239	83.3412731	0.25323976	0.80008	0.0003103	subgrp_con	count		1	0
PPP2R5B	8	OK	6.66246465	6.95721239	0.95763408	0.33825	0.00121744	subgrp_con	count		1	0
BATF2	1	OK	18.7594894	83.6251793	0.22432824	0.8225	0.00028789	subgrp_con	count		1	0
ARL2.00	353	OK	-0.2220213	0.1474132	-1.5061155	0.13204	-0.31217688	subgrp_con	count		1	0
SAC3D1	4	OK	1.10069613	1.56872329	0.70165092	0.4829	0.55472334	subgrp_con	count		1	0
NAALADL1	2	OK	18.7594894	83.6251793	0.22432824	0.8225	0.00028789	subgrp_con	count		1	0
ZFPL1	15	OK	1.35007459	0.91382677	1.47738568	0.13957	1.08971334	subgrp_con	count		1	0
VPS51	56	OK	0.40468917	0.39125734	1.03432994	0.30098	0.46278114	subgrp_con	count		1	0
TM7SF2	1	OK	3.45981462	6.7714513	0.51094137	0.60939	0.00030179	subgrp_con	count		1	0
ZNHIT2	2	OK	-2.1660098	2.70416007	-0.8009917	0.42314	-0.79067866	subgrp_con	count		1	0
FAU	874	OK	-0.1459086	0.08540947	-1.7083418	0.08757	-0.20870436	subgrp_con	count		1	0
MRPL49	8	OK	-0.1902356	1.22474264	-0.155327	0.87656	-0.05772119	subgrp_con	count		1	0
CAPN1	17	OK	1.34417349	0.97145974	1.38366361	0.16646	0.40961982	subgrp_con	count		1	0
CDC42EP2	32	OK	1.04228567	0.53086384	1.96356493	0.04958	1.11464355	subgrp_con	count		1	0
DPF2	5	OK	4.21092061	4.28756165	0.9821248	0.32606	0.00257090	subgrp_con	count		1	0
FRMD8	4	OK	0.30934059	1.30434383	0.23716182	0.81253	0.00020636	subgrp_con	count		1	0
NEAT1	669	OK	0.27595754	0.0989094	2.79000332	0.00527	0.39305051	subgrp_con	count		1	0
MALAT1	891	OK	0.1703123	0.08279265	2.05709441	0.03968	0.24392149	subgrp_con	count		1	0
SCYL1	6	OK	0.12559487	1.45000757	0.0866167	0.93098	0.03878308	subgrp_con	count		1	0
SSSCA1	44	OK	-0.1664919	0.46521412	-0.3578822	0.72042	-0.19876582	subgrp_con	count		1	0
FAM89B	164	OK	0.15830261	0.23711199	0.66762803	0.50437	0.21276028	subgrp_con	count		1	0
EHBP1L1	15	OK	-0.792094	0.86179479	-0.9191218	0.35803	-0.6741234	subgrp_con	count		1	0
MAP3K11	63	OK	0.77087659	0.41418332	1.86119652	0.06272	0.83063804	subgrp_con	count		1	0
SIPA1	5	OK	1.58229999	2.72001883	0.58176068	0.56073	0.47433761	subgrp_con	count		1	0
RELA	16	OK	0.04098817	0.81694566	0.05017246	0.95998	0.02651869	subgrp_con	count		1	0
KAT5	3	OK	3.45981462	6.7714513	0.51094137	0.60939	0.00030179	subgrp_con	count		1	0
RNASEH2C	155	OK	0.85941883	0.25329922	3.39289964	0.00069	1.15227690	subgrp_con	count		1	0
CFL1	702	OK	-0.1270424	0.10020183	-1.2678655	0.20485	-0.180910	subgrp_con	count		1	0
EFEMP2	28	OK	4.70779321	1.6825646	2.7979866	0.00514	3.0164630	subgrp_con	count		1	0
FIBP	73	OK	0.93451926	0.378518	2.46888791	0.01355	1.17531383	subgrp_con	count		1	0
CCDC85B	683	OK	0.0579234	0.10289985	0.56291051	0.5735	0.0825928	subgrp_con	count		1	0
FOSL1	8	OK	-0.2829187	1.15823604	-0.2442668	0.80702	-0.20946244	subgrp_con	count		1	0
C11orf68	4	OK	-1.3965204	1.36691908	-1.0216555	0.30694	-0.37123200	subgrp_con	count		1	0
DRAP1	77	OK	0.74047882	0.36839115	2.01003422	0.04442	0.94211656	subgrp_con	count		1	0
SART1	3	OK	-10.124821	16.771925	-0.6036767	0.54606	-2.16898761	subgrp_con	count		1	0
EIF1AD	2	OK	21.642059	54.4868919	0.39719753	0.69122	1.57744735	subgrp_con	count		1	0
BANF1	208	OK	-0.192072	0.19594648	-0.9802271	0.32697	-0.26548185	subgrp_con	count		1	0
CST6	2	OK	26.3679501	84.9163259	0.31051685	0.75617	2.49052421	subgrp_con	count		1	0
SF3B2	149	OK	-0.2921025	0.23301389	-1.2535842	0.20999	-0.39157924	subgrp_con	count		1	0
PACS1	6	OK	-0.432431	1.09785088	-0.3938887	0.69366	-0.21314406	subgrp_con	count		1	0
RAB1B	27	OK	0.60695659	0.56844503	1.06774892	0.28563	0.50621156	subgrp_con	count		1	0
YIF1A	72	OK	0.692747	0.3517109	1.9696492	0.04888	0.88565727	subgrp_con	count		1	0
RIN1	2	OK	3.45981462	6.7714513	0.51094137	0.60939	0.00030179	subgrp_con	count		1	0
BRMS1	18	OK	-0.6163125	0.71295615	-0.8644466	0.38734	-0.65173122	subgrp_con	count		1	0
B4GAT1	7	OK	0.58209889	1.07041274	0.54380789	0.58657	0.1815643	subgrp_con	count		1	0
AP001107.9	1	OK	-10.442278	41.4658099	-0.2518286	0.80117	-0.00045289	subgrp_con	count		1	0
MRPL11	78	OK	0.69686347	0.34507499	2.01945695	0.04344	0.91561189	subgrp_con	count		1	0
PELI3	2	OK	0.16816909	1.72532873	0.09747075	0.92235	0.05201879	subgrp_con	count		1	0
AP002748.3	1	OK	3.45981462	6.7714513	0.51094137	0.60939	0.00030179	subgrp_con	count		1	0
DPP3	3	OK	-11.019418	43.2499352	-0.2547846	0.79889	-1.17482461	subgrp_con	count		1	0
ZDHHC24	24	OK	0.07095620	0.62990496	0.11264692	0.91031	0.07832188	subgrp_con	count		1	0
CTSF	54	OK	1.64610677	0.45867889	3.58879995	0.00032	2.0053713	subgrp_con	count		1	0
CCS	26	OK	0.41566345	0.58409526	0.71163641	0.47669	0.4110250	subgrp_con	count		1	0
RBM4	5	OK	-2.4465924	1.71381312	-1.4275724	0.15341	-0.00126286	subgrp_con	count		1	0
AP001157.1	1	OK	-11.019418	43.2499352	-0.2547846	0.79889	-1.17482461	subgrp_con	count		1	0
RCE1	4	OK	1.21307139	1.77813069	0.68221731	0.4951	0.54679985	subgrp_con	count		1	0
LRFN4	1	OK	18.7594894	83.6251793	0.22432824	0.8225	0.00028789	subgrp_con	count		1	0

RHOD	6	OK	2.66728225	2.24180553	1.18979203	0.23412	0.71356325	subgrp_con	count		1	0
KDM2A	27	OK	-0.2186195	0.63707186	-0.343162	0.72148	-0.19358467	subgrp_con	count		1	0
GRK2	6	OK	-0.3631316	1.11178674	-0.3266198	0.74396	-0.0006286	subgrp_con	count		1	0
ANKRD13D	6	OK	-0.717499	1.10151536	-0.6513746	0.5148	-0.2077451	subgrp_con	count		1	0
RAD9A	3	OK	-1.8538345	1.85439186	-0.9996995	0.31748	-0.46053279	subgrp_con	count		1	0
CLCF1	2	OK	-10.442278	41.4658099	-0.2518286	0.80117	-0.00045289	subgrp_con	count		1	0
PPP1CA	78	OK	0.69329196	0.35009899	1.98055971	0.04764	0.88290161	subgrp_con	count		1	0
RPS6KB2	4	OK	-0.214986	1.56481749	-0.1373872	0.89072	-0.06511836	subgrp_con	count		1	0
CORO1B	58	OK	-0.2137766	0.38716131	-0.5521642	0.58086	-0.26327656	subgrp_con	count		1	0
TMEM134	27	OK	-0.7310505	0.58860538	-1.2420044	0.21422	-0.78672727	subgrp_con	count		1	0
AIP	80	OK	-0.8284548	0.34151487	-2.4258234	0.01527	-1.04831253	subgrp_con	count		1	0
CDK2AP2	49	OK	1.76594413	0.51426656	3.43390813	0.00059	1.8882055	subgrp_con	count		1	0
GSTP1	676	OK	0.11608956	0.1011456	1.14774706	0.25107	0.16558503	subgrp_con	count		1	0
NDUFV1	48	OK	0.2343028	0.452872	0.5173709	0.6049	0.27810839	subgrp_con	count		1	0
NUDT8	7	OK	0.20324053	1.07690436	0.18872663	0.85031	0.1692424	subgrp_con	count		1	0
UNC93B1	4	OK	0.81196819	1.38206702	0.58750276	0.55687	0.41380800	subgrp_con	count		1	0
NDUFS8	124	OK	0.35751213	0.24703063	1.44723827	0.14782	0.48560883	subgrp_con	count		1	0
TCIRG1	6	OK	-1.3221817	1.29766572	-1.0188924	0.30825	-0.59149214	subgrp_con	count		1	0
CHKA	2	OK	3.45981497	4.78586864	0.72292309	0.46972	0.00081935	subgrp_con	count		1	0
KMT5B	20	OK	0.66747368	0.76049131	0.87768738	0.38011	0.55503111	subgrp_con	count		1	0
C11orf24	1	OK	23.9918963	84.8999419	0.28259026	0.77749	1.65081709	subgrp_con	count		1	0
LRP5	12	OK	-0.479265	0.8041381	-0.5959989	0.55118	-0.30135610	subgrp_con	count		1	0
PPP6R3	17	OK	-1.005526	0.7993642	-1.2579074	0.20842	-0.46820460	subgrp_con	count		1	0
GAL	1	OK	1.58229999	2.72001883	0.58176068	0.56072	0.47433761	subgrp_con	count		1	0
CPT1A	17	OK	0.79689767	0.71241081	1.11859289	0.26333	0.66055774	subgrp_con	count		1	0
MRPL21	82	OK	-0.1739372	0.31460816	-0.5528694	0.58035	-0.22597457	subgrp_con	count		1	0
FADD	5	OK	3.45981497	4.78586864	0.72292309	0.46972	0.00081935	subgrp_con	count		1	0
PPFIA1	20	OK	-0.0253123	0.64362875	-0.0393275	0.96863	-0.026642	subgrp_con	count		1	0
CTTN	94	OK	1.13178621	0.33614226	3.36688575	0.00076	1.37515114	subgrp_con	count		1	0
DHCR7	1	OK	3.45981463	6.77145131	0.51094137	0.60939	0.00030179	subgrp_con	count		1	0
NADSYN1	3	OK	0.01477708	1.4255587	0.01036578	0.99173	0.00748501	subgrp_con	count		1	0
FAM86C1	1	OK	-10.442278	41.4658099	-0.2518286	0.80117	-0.00045289	subgrp_con	count		1	0
RNF121	5	OK	0.51030031	1.6034059	0.31826022	0.75029	0.15911328	subgrp_con	count		1	0
IL18BP	21	OK	-0.0210109	0.57445053	-0.0365756	0.97082	-0.02071596	subgrp_con	count		1	0
NUMA1	30	OK	-0.0779739	0.53044649	-0.1469967	0.88313	-0.09127264	subgrp_con	count		1	0
LAMTOR1	260	OK	0.12613737	0.17388787	0.72539485	0.46821	0.17576582	subgrp_con	count		1	0
ANAPC15	10	OK	3.62895633	1.71679056	2.1138026	0.03452	2.91751131	subgrp_con	count		1	0
CLPB	1	OK	-10.442278	41.4658099	-0.2518286	0.80117	-0.00045289	subgrp_con	count		1	0
PDE2A	20	OK	-3.6849889	1.14271611	-3.2235142	0.00122	-3.00705858	subgrp_con	count		1	0
STARD10	5	OK	0.60175573	1.11668489	0.53887698	0.58997	0.45341194	subgrp_con	count		1	0
ATG16L2	3	OK	4.66132998	5.76838606	0.80808217	0.41906	0.00274918	subgrp_con	count		1	0
FCHSD2	26	OK	1.4495068	0.67909956	2.13445405	0.03281	1.16190693	subgrp_con	count		1	0
P2RY6	12	OK	1.44656743	1.35328596	1.06892964	0.2851	0.43792209	subgrp_con	count		1	0
FAM168A	1	OK	-15.498043	42.9510917	-0.36082	0.71822	-0.00073756	subgrp_con	count		1	0
RAB6A	40	OK	0.10198023	0.49049886	0.20791141	0.8352	0.10078674	subgrp_con	count		1	0
MRPL48	6	OK	0.31310209	1.12905662	0.27731301	0.78154	0.20379678	subgrp_con	count		1	0
COA4	78	OK	0.29029069	0.33412569	0.86880685	0.38495	0.37323446	subgrp_con	count		1	0
PAAF1	3	OK	0.51031165	1.60340574	0.31826732	0.75029	0.15911454	subgrp_con	count		1	0
C2CD3	3	OK	2.40962373	2.47152069	0.97495997	0.32958	0.66517342	subgrp_con	count		1	0
PPME1	4	OK	0.63934704	1.44062452	0.44379853	0.65719	0.00043312	subgrp_con	count		1	0
P4HA3	3	OK	2.55522271	2.3498446	1.08740073	0.27686	0.69313893	subgrp_con	count		1	0
PGM2L1	49	OK	1.71502021	0.58724457	2.9204696	0.0035	1.85467910	subgrp_con	count		1	0
KCNE3	1	OK	1.58229999	2.72001883	0.58176068	0.56072	0.47433761	subgrp_con	count		1	0
POLD3	9	OK	-1.2765853	1.0590989	-1.2053504	0.22807	-0.74019055	subgrp_con	count		1	0
RNF169	5	OK	-12.167015	17.8102076	-0.6831484	0.49451	-2.71013712	subgrp_con	count		1	0
XRRA1	2	OK	-12.145707	43.4188694	-0.2797334	0.77968	-1.91338276	subgrp_con	count		1	0
SPCS2	349	OK	0.3862568	0.14823696	2.6056713	0.00917	0.54444467	subgrp_con	count		1	0
ARRB1	8	OK	-2.9509266	1.81194574	-1.6285955	0.1034	-1.02658816	subgrp_con	count		1	0
RPS3	875	OK	-0.1745063	0.08450178	-2.06512	0.03892	-0.24968851	subgrp_con	count		1	0
GDPD5	3	OK	8.75501389	7.58596821	1.15410633	0.24840	1.76119183	subgrp_con	count		1	0
SERPINH1	141	OK	-0.0399519	0.24977389	-0.1599522	0.87292	-0.05238486	subgrp_con	count		1	0
UVRAG	47	OK	0.6112446	0.44581763	1.37106422	0.17035	0.73818244	subgrp_con	count		1	0
THAP12	31	OK	-1.3488122	0.57091744	-2.3625345	0.01815	-1.42553588	subgrp_con	count		1	0
AP002360.1	2	OK	-1.2725896	2.31041901	-0.5508034	0.58177	-0.74406113	subgrp_con	count		1	0
EMSY	4	OK	-1.5256412	1.34685813	-1.1327406	0.25732	-0.00086992	subgrp_con	count		1	0
LRRC32	40	OK	-1.4101259	0.47479167	-2.9699886	0.00298	-1.53552826	subgrp_con	count		1	0
ACER3	5	OK	0.16814912	1.72533008	0.0974591	0.92236	0.05201277	subgrp_con	count		1	0
PAK1	1	OK	3.45981463	6.77145131	0.51094137	0.60939	0.00030179	subgrp_con	count		1	0
CLNS1A	80	OK	-0.0620149	0.32725896	-0.1894998	0.84975	-0.0804092	subgrp_con	count		1	0
RSF1	161	OK	-0.3439713	0.22806776	-1.5081978	0.1315	-0.45951424	subgrp_con	count		1	0
AAMDC	106	OK	1.03312982	0.28239254	3.65848834	0.00025	1.37849570	subgrp_con	count		1	0
KCTD14	1	OK	23.9918963	84.8999419	0.28259026	0.77749	1.65081709	subgrp_con	count		1	0
NDUFC2	409	OK	0.45432223	0.13663257	3.32521189	0.00088	0.64254975	subgrp_con	count		1	0
ALG8	5	OK	-1.3967094	1.3668523	-1.0218437	0.30685	-0.37125920	subgrp_con	count		1	0
KCTD21	1	OK	-15.995032	70.349423	-0.2273655	0.82014	-0.00028474	subgrp_con	count		1	0
RAB30	1	OK	23.9918963	84.8999419	0.28259026	0.77749	1.65081709	subgrp_con	count		1	0
RAB30-AS1	5	OK	1.92315949	1.66311474	1.15636008	0.24752	0.00132266	subgrp_con	count		1	0
PCF11	48	OK	-0.8074414	0.3928763	-2.0552052	0.03986	-0.63938495	subgrp_con	count		1	0
ANKRD42	4	OK	21.1053223	83.3412731	0.25323976	0.80008	0.0003103	subgrp_con	count		1	0
CCDC90B	87	OK	-0.0927422	0.31666309	-0.2928734	0.76962	-0.11984186	subgrp_con	count		1	0
DLG2	1	OK	-10.442278	41.4658099	-0.2518286	0.80117	-0.00045289	subgrp_con	count		1	0
TMEM126B	42	OK	0.79467819	0.48210794	1.64492886	0.09998	0.91474304	subgrp_con	count		1	0
TMEM126A	24	OK	-1.3049311	0.67334212	-1.9379912	0.05262	-1.19146549	subgrp_con	count		1	0
CREBZF	8	OK	-3.9814606	1.88468565	-2.1123088	0.03466	-0.71273858	subgrp_con	count		1	0
PICALM	51	OK	0.17637056	0.44322275	0.3979276	0.69068	0.2044573	subgrp_con	count		1	0
EED	3	OK	-2.023032	1.80712677	-1.1194742	0.26294	-0.00109400	subgrp_con	count		1	0
HIKESHI	55	OK	0.60049629	0.42738236	1.40505634	0.16	0.71886289	subgrp_con	count		1	0
ME3	2	OK	3.45981463	6.77145131	0.51094137	0.60939	0.00030179	subgrp_con	count		1	0
PRSS23	170	OK	0.15850713	0.2273774	0.69711053	0.48573	0.2135887	subgrp_con	count		1	0
AP001528.2	14	OK	-1.8924088	0.88646168	-2.1347891	0.03278	-1.4391861	subgrp_con	count		1	0
FZD4	39	OK	-0.784882	0.45299417	-1.7288349	0.08384	-0.87376823	subgrp_con	count		1	0
RAB38	8	OK	-0.3536322	0.86149511	-0.4104866	0.68145	-0.22432747	subgrp_con	count		1	0
CHORDC1	35	OK	-0.0577375	0.48787566	-0.1183448	0.90579	-0.05145108	subgrp_con	count		1	0
SLC36A4	4	OK	24.0001923	54.3454405	0.44162292	0.65876	2.40091057	subgrp_con	count		1	0
SMCO4	10	OK	-0.9503536	1.31210675	-0.724298	0.46888	-0.44534842	subgrp_con	count		1	0
CEP295	9	OK	1.20332880	1.01512077	1.1854038	0.23586	0.60309968	subgrp_con	count		1	0
TAF1D	174	OK	-0.0639022	0.21553582	-0.2964805	0.76686	-0.08625318	subgrp_con	count		1	0
C11orf54	9	OK	0.84125923	0.94032088	0.89465122	0.37097	0.54449870	subgrp_con	count		1	0
MED17	2	OK	0.16814912	1.72533008	0.0974591	0.92236	0.05201277	subgrp_con	count		1	0
MRE11	13	OK	-0.086505	0.89187426	-0.0969924	0.92273	-0.04361120	subgrp_con	count		1	0
ANKRD49	2	OK	35.3043164	67.4881674	0.52311861	0.60089	0.00071431	subgrp_con	count		1	0
AMOTL1	8	OK	-0.036682	0.97355624	-0.0376782	0.96994	-6.51E-05	subgrp_con	count		1	0
CWC15	82	OK	0.07266688	0.30885942	0.23527486	0.814	0.0903622	subgrp_con	count		1	0
SRSF8	46	OK	-0.140206	0.38828236	-0.361092	0.71803	-0.15960826	subgrp_con	count		1	0
ENDOD1	44	OK	-1.1201038	0.44869309	-2.4963697	0.01255	-1.34044057	subgrp_con	count		1	0
SESN3	27	OK	-1.1756003	0.58037815	-2.0255764	0.04281	-1.3398523	subgrp_con	count		1	0
FAM76B	16	OK	-0.7358552	0.68469191	-1.0747246	0.2825	-0.53052578	subgrp_con	count		1	0
CEP57	56	OK	-0.6186947	0.39679095	-1.559248	0.11894	-0.76006623	subgrp_con	count		1	0
MTMR2	9	OK	1.59826166	1.18622981	1.34733436	0.17787	0.47850310	subgrp_con	count		1	0
MAML2	4	OK	0.76892923	1.52989765	0.50260178	0.61524	0.23955204	subgrp_con	count		1	0
CCDC82	80	OK	0.07016759	0.32261403	0.21749703	0.82782	0.08775721	subgrp_con	count		1	0
JRKL	1	OK	-11.019418	43.2499352	-0.2547846	0.79889	-1.17482461	subgrp_con	count		1	0
CEP126	6	OK	-0.2093075	1.13673479	-0.1841305	0.85391	-0.06342379	subgrp_con	count		1	0
AP001527.2	1	OK	-11.019418	43.2499352	-0.2547846	0.79889	-1.17482461	subgrp_con	count		1	0
YAP1	12	OK	1.97266843	1.10852092	1.77955002	0.07515	1.50680670	subgrp_con	count		1	0
BIRC3	59	OK	-0.785182	0.35508176	-2.2112715	0.02702	-0.89974048	subgrp_con	count		1	0
BIRC2	30	OK	1.17094617	0.73452148	1.59416193	0.1109	0.36103538	subgrp_con	count		1	0
TMEM123	150	OK	-0.0533218	0.23009559	-0.2317374	0.81674	-0.07214154	subgrp_con	count		1	0
AP001830.1	2	OK	4.66132998	5.76838606	0.80808217	0.41906	0.00274918	subgrp_con	count		1	0
DCUN1D5	15	OK	4.52037077	1.81071868	2.49645114	0.01254	2.98119897	subgrp_con	count		1	0
DYNC2H1	9	OK	0.26139003	1.13121669	0.23106982	0.81726	0.22866074	subgrp_con	count		1	0
PDGFD	42	OK	4.61372143	1.14617785	4.02531894	5.69E-05	3.56371850	subgrp_con	count		0.56579617	0
CASP4	149	OK	-0.1110052	0.22719498	-0.48859	0.62512	-0.15105193	subgrp_con	count		1	0
CASP1	28	OK	0.12975067	0.51174419	0.25354596	0.79985	0.15035126	subgrp_con	count		1	0
CARD16	112	OK	0.18998809	0.25457056	0.74630818	0.45548	0.25805104	subgrp_con	count		1	0
MSANTD4	2	OK	-1.7585735	2.35544393	-0.7465996	0.45533	-0.00036089	subgrp_con	count		1	0
KBTBD3	5	OK	0.00115825	1.85957505	0.00062286	0.9995	7.58E-07	subgrp_con	count		1	0
AASDHPPT	21	OK	0.51964365	0.6333093	0.82052111	0.41192	0.46565402	subgrp_con	count		1	0
CWF19L2	43	OK	-0.1562116	0.465427	-0.3356306	0.73715	-0.19136047	subgrp_con	count		1	0
ALKBH8	1	OK	3.45981463	6.77145131	0.51094137	0.60939	0.00030179	subgrp_con	count		1	0
SLC35F2	8	OK	3.38603443	2.25198624	1.50357687	0.13269	1.32204360	subgrp_con	count		1	0
CUL5	39	OK	-0.3035319	0.47767786	-0.6354322	0.52515	-0.34391330	subgrp_con	count		1	0
ACAT1	74	OK	-0.4100014	0.32778577	-1.2508213	0.211	-0.47633319	subgrp_con	count		1	0
NPAT	11	OK	-1.8103785	1.32847349	-1.362752	0.17296	-1.14553212	subgrp_con	count		1	0
ATM	12	OK	-0.7571789	0.93383068	-0.8108312	0.41746	-0.46149888	subgrp_con	count		1	0
KDELC2	3	OK	21.642059	54.4868919	0.39719753	0.69122	1.57744735	subgrp_con	count		1	0
DDX10	8	OK	-1.8035614	1.078799	-1.6718358	0.09456	-0.75296570	subgrp_con	count		1	0
RDX	379	OK	-0.1881807	0.14163309	-1.3286494	0.18396	-0.26302881	subgrp_con	count		1	0
ZC3H12C	3	OK	1.25576317	2.03774815	0.61625043	0.53772	0.00031670	subgrp_con	count		1	0
FDX1	35	OK	0.76572705	0.50640313	1.5120899	0.13051	0.85807947	subgrp_con	count		1	0
ARHGAP20	14	OK	2.02321383	1.17835259	1.71698508	0.08598	1.37093735	subgrp_con	count		1	0
LAYN	16	OK	-0.2900636	0.80573145	-0.3600004	0.71885	-0.23779536	subgrp_con	count		1	0
SIK2	6	OK	0.70779233	1.76425942	0.40118382	0.68829	0.37107769	subgrp_con	count		1	0
PPP2R1B	1	OK	3.45981463	6.77145131	0.51094137	0.60939	0.00030179	subgrp_con	count		1	0
C11orf1	21	OK	-0.2210899	0.70633853	-0.3130084	0.75427	-0.21682107	subgrp_con	count		1	0
CRYAB	17	OK	3.7217906	1.41344741	2.63312987	0.00846	2.45252307	subgrp_con	count		1	0
DIXDC1	57	OK	1.40057275	0.43135824	3.2468825	0.00117	1.67455847	subgrp_con	count		1	0
DLAT	2	OK	22.2230369	54.5331384	0.40751436	0.68362	1.59542235	subgrp_con	count		1	0
PIH1D2	1	OK	3.45981463	6.77145131	0.51094137	0.60939	0.00030179	subgrp_con	count		1	0
NKAPD1	25	OK	0.1091555	0.59854492	0.18236809	0.85529	0.11181695	subgrp_con	count		1	0
TIMM8B	77	OK	0.684043	0.38139409	1.79353388	0.07289	0.80996443	subgrp_con	count		1	0
SDHD	157	OK	-0.1706414	0.2239383	-0.7620018	0.44606	-0.23487566	subgrp_con	count		1	0
IL18	3	OK	20.0721963	50.669702	0.39613804	0.692	0.00081401	subgrp_con	count		1	0
BCO2	17	OK	22.1777677	31.2519264	0.70964482	0.47792	1.59402044	subgrp_con	count		1	0
PTS	41	OK	1.03166987	0.5674759	1.81799898	0.06906	1.167292	subgrp_con	count		1	0
AP002884.1	4	OK	1.56593057	1.56669195	0.99951402	0.31755	0.97089418	subgrp_con	count		1	0
NCAM1	5	OK	22.2217907	54.5232277	0.40756558	0.68359	1.5953836	subgrp_con	count		1	0
USP28	3	OK	-1.2956167	2.0414257	-0.6346627	0.52565	-0.00075754	subgrp_con	count		1	0
ZBTB16	33	OK	0.08359355	0.43168456	0.19364498	0.84645	0.06280189	subgrp_con	count		1	0
NNMT	560	OK	0.25046594	0.1109186	2.25810584	0.02394	0.35586535	subgrp_con	count		1	0
C11orf71	4	OK	-0.9102232	1.54642098	-0.5885996	0.55612	-0.42849043	subgrp_con	count		1	0
RBM7	25	OK	-1.1950808	0.5641978	-2.1181946	0.03416	-1.04978719	subgrp_con	count		1	0
REXO2	213	OK	0.65303956	0.20934455	3.11946843	0.00181	0.89815407	subgrp_con	count		1	0
ZPR1	14	OK	-0.6566761	0.7542579	-0.8706254	0.38396	-0.56799783	subgrp_con	count		1	0
APOA1	1	OK	23.9918963	84.8999419	0.28259026	0.77749	1.65081709	subgrp_con	count		1	0
SIK3	6	OK	-1.8174474	1.45595424	-1.2482861	0.21192	-0.45400691	subgrp_con	count		1	0
PAFAH1B2	33	OK	1.63215173	0.63128185	2.58545647	0.00973	1.5407662	subgrp_con	count		1	0
SIDT2	2	OK	5.80492735	5.66946625	1.0238931	0.30589	1.03285716	subgrp_con	count		1	0
PCSK7	10	OK	1.33962787	1.21424681	1.1032581	0.26992	0.40834447	subgrp_con	count		1	0
RNF214	11	OK	-3.0426392	1.3924813	-2.1850485	0.02889	-1.91880046	subgrp_con	count		1	0
BACE1	3	OK	-10.442278	41.4658099	-0.2518286	0.80117	-0.00045289	subgrp_con	count		1	0
CEP164	9	OK	-0.3749047	1.28930321	-0.2907806	0.77122	-0.00064838	subgrp_con	count		1	0
JAML	1	OK	18.7594894	83.6251792	0.22432824	0.8225	0.00028789	subgrp_con	count		1	0
MPZL3	1	OK	-10.442278	41.4658099	-0.2518286	0.80117	-0.00045289	subgrp_con	count		1	0
UBE4A	15	OK	0.46229508	0.80526217	0.57409263	0.56592	0.2365861	subgrp_con	count		1	0
ATP5MG	479	OK	-0.0213123	0.12640779	-0.1685992	0.86611	-0.03014011	subgrp_con	count		1	0
KMT2A	116	OK	-0.3673229	0.26817128	-1.3697324	0.17077	-0.48548984	subgrp_con	count		1	0
TMEM25	4	OK	1.099586	1.31889629	0.8337168	0.40444	0.55419423	subgrp_con	count		1	0
IFT46	2	OK	-10.442278	41.4658099	-0.2518286	0.80117	-0.00045289	subgrp_con	count		1	0
ARCN1	50	OK	0.78395296	0.47051449	1.66616115	0.09568	0.9166584	subgrp_con	count		1	0
PHLDB1	13	OK	5.03322653	2.55903386	1.96684639	0.0492	1.68392474	subgrp_con	count		1	0
DDX6	73	OK	0.73567777	0.37121415	1.98181501	0.0475	0.89434524	subgrp_con	count		1	0
AP004609.3	1	OK	22.9715125	51.7315836	0.4440519	0.65701	2.36236892	subgrp_con	count		1	0
BCL9L	7	OK	0.25309207	1.1709379	0.30154636	0.762	0.18053741	subgrp_con	count		1	0
CCDC84	6	OK	-2.0581009	1.39347207	-1.4769589	0.13969	-0.82613993	subgrp_con	count		1	0
RPS25	869	OK	-0.2089328	0.0855987	-2.4408409	0.01465	-0.29883532	subgrp_con	count		1	0
TRAPPC4	56	OK	0.16312121	0.3871297	0.42138643	0.67347	0.16135364	subgrp_con	count		1	0
VPS11	2	OK	-0.5232457	2.06622527	-0.2532375	0.80008	-0.24602840	subgrp_con	count		1	0
HMBS	1	OK	3.45978793	4.78585287	0.72291983	0.46972	0.00081879	subgrp_con	count		1	0
H2AFX	35	OK	-0.7641315	0.46649294	-1.6380345	0.10141	-0.8519414	subgrp_con	count		1	0
DPAGT1	4	OK	-0.8009709	1.39742131	-0.5731778	0.56652	-0.38156508	subgrp_con	count		1	0
HINFP	1	OK	1.58229999	2.72001883	0.58176068	0.56072	0.47433761	subgrp_con	count		1	0
NLRX1	1	OK	18.7594894	83.6251792	0.22432824	0.8225	0.00028789	subgrp_con	count		1	0
CBL	9	OK	1.14837186	1.22428999	0.93799007	0.34825	0.35364207	subgrp_con	count		1	0
MCAM	112	OK	0.72448849	0.28793947	2.51611384	0.01187	0.94974429	subgrp_con	count		1	0
THY1	24	OK	1.26925303	0.77104871	1.64613861	0.09974	1.10831365	subgrp_con	count		1	0
OAF	18	OK	0.07904609	0.81071633	0.09750154	0.92233	0.04014187	subgrp_con	count		1	0
TMEM136	3	OK	1.58229999	2.72001883	0.58176068	0.56072	0.47433761	subgrp_con	count		1	0
ARHGEF12	43	OK	0.11453423	0.45234864	0.25319913	0.80011	0.14251603	subgrp_con	count		1	0
SCSD	14	OK	21.8282101	32.8168469	0.66515257	0.50596	2.31980174	subgrp_con	count		1	0
JHY	1	OK	-10.442278	41.4658099	-0.2518286	0.80117	-0.00045289	subgrp_con	count		1	0
HSPA8	676	OK	-0.0822847	0.1030287	-0.7986582	0.42449	-0.11719132	subgrp_con	count		1	0
TBRG1	35	OK	1.254238	0.53128609	2.36075859	0.01824	1.32957154	subgrp_con	count		1	0
SIAE	2	OK	1.58229999	2.72001883	0.58176068	0.56072	0.47433761	subgrp_con	count		1	0
SPA17	5	OK	0.13783159	1.17802725	0.11700203	0.90686	0.08942392	subgrp_con	count		1	0
NRGN	60	OK	1.000106	0.46424	2.15428656	0.03122	1.14890157	subgrp_con	count		1	0
ESAM	312	OK	-0.4601271	0.16185314	-2.8428678	0.00447	-0.64143148	subgrp_con	count		1	0
MSANTD2	2	OK	4.66132998	5.76838606	0.80808217	0.41906	0.00274918	subgrp_con	count		1	0
ROBO3	10	OK	1.65755209	1.15998469	1.42894307	0.15302	0.48008128	subgrp_con	count		1	0
ROBO4	64	OK	0.11417677	0.37313838	0.30598946	0.75961	0.14498148	subgrp_con	count		1	0
TMEM218	5	OK	-0.2681877	1.22915915	-0.2181879	0.82728	-0.13377334	subgrp_con	count		1	0
FEZ1	46	OK	24.1970267	19.4440544	1.24444348	0.21334	5.14845578	subgrp_con	count		1	0
EI24	71	OK	-0.088556	0.36501275	-0.2426107	0.80831	-0.10585275	subgrp_con	count		1	0
STT3A	10	OK	1.22686905	1.35803153	0.90341723	0.3662	0.77980226	subgrp_con	count		1	0
CHEK1	1	OK	3.45981463	6.77145131	0.51094137	0.60939	0.00030179	subgrp_con	count		1	0
HYLS1	1	OK	1.58229999	2.72001883	0.58176068	0.56072	0.47433761	subgrp_con	count		1	0
PUS3	4	OK	-0.6348894	2.00896003	-0.3160275	0.75198	-0.0001452	subgrp_con	count		1	0
CDON	2	OK	-0.2029407	1.65323657	-0.1227536	0.9022	-4.82E-05	subgrp_con	count		1	0
RPUSD4	4	OK	-0.0755904	1.2476219	-0.0605625	0.95171	-0.04866258	subgrp_con	count		1	0
FAM118B	1	OK	41.7882039	174.29908	0.23975469	0.81052	0.00014889	subgrp_con	count		1	0
SRPRA	85	OK	0.67948189	0.32080051	2.11801638	0.03417	0.88853927	subgrp_con	count		1	0
DCPS	8	OK	0.72051061	1.14043687	0.63178474	0.52752	0.46788710	subgrp_con	count		1	0
ST3GAL4	7	OK	2.57427143	1.73496196	1.48376247	0.13787	1.42721216	subgrp_con	count		1	0
ETS1	119	OK	-0.4288262	0.27005367	-1.5879294	0.1123	-0.54772941	subgrp_con	count		1	0
FLI1	29	OK	-1.9896647	0.67209801	-2.9603788	0.00307	-1.75667788	subgrp_con	count		1	0
SENCR	15	OK	-0.1611862	0.75505776	-0.2134752	0.83096	-0.14312165	subgrp_con	count		1	0
ARHGAP32	1	OK	18.7594894	83.6251792	0.22432824	0.8225	0.00028789	subgrp_con	count		1	0
NFRKB	1	OK	-12.148421	43.455309	-0.2795612	0.77981	-1.91362624	subgrp_con	count		1	0
APLP2	209	OK	0.83406089	0.2036996	4.09456334	4.23E-05	1.14461409	subgrp_con	count		0.42127299	0
ZBTB44	9	OK	0.11864029	1.02366821	0.11589721	0.90773	0.06033287	subgrp_con	count		1	0
JAM3	11	OK	0.7695688	0.89323495	0.86155249	0.38892	0.23974616	subgrp_con	count		1	0
NCAPD3	2	OK	-11.019418	43.2499352	-0.2547846	0.79889	-1.17482461	subgrp_con	count		1	0
VPS26B	1	OK	-10.442278	41.4658099	-0.2518286	0.80117	-0.00045289	subgrp_con	count		1	0
THYN1	69	OK	-0.336237	0.35315382	-0.9520979	0.34105	-0.42482531	subgrp_con	count		1	0
ACAD8	3	OK	-0.9102232	2.18811251	-0.4159856	0.67742	-0.25785735	subgrp_con	count		1	0
ZMYND11	29	OK	0.35353542	0.56832918	0.622061	0.5339	0.31701645	subgrp_con	count		1	0
DIP2C	1	OK	18.7594894	83.6251792	0.22432824	0.8225	0.00028789	subgrp_con	count		1	0
LARP4B	6	OK	-0.6106617	1.21896463	-0.5009675	0.61639	-0.2909350	subgrp_con	count		1	0
GTPBP4	28	OK	-1.0402518	0.65239249	-1.5945184	0.11082	-0.92690098	subgrp_con	count		1	0
IDI1	18	OK	-0.4008597	0.74803353	-0.5358848	0.59206	-0.11966922	subgrp_con	count		1	0
WDR37	2	OK	-0.0826528	11.1675942	-0.0074011	0.99409	-0.04381574	subgrp_con	count		1	0
PFKP	16	OK	1.84702099	0.9751089	1.89416889	0.0582	1.11565069	subgrp_con	count		1	0
PITRM1	2	OK	-10.442278	41.4658099	-0.2518286	0.80117	-0.00045289	subgrp_con	count		1	0
KLF6	470	OK	-0.2410036	0.12665653	-1.9028125	0.05707	-0.34012030	subgrp_con	count		1	0
MANCR	2	OK	0.65325287	2.50012866	0.26128772	0.79387	0.00035297	subgrp_con	count		1	0
AKR1C1	60	OK	-1.4008087	0.40523208	-3.456806	0.00055	-1.77032783	subgrp_con	count		1	0
AKR1C2	28	OK	-1.7604425	0.59107878	-2.9783551	0.0029	-1.97797429	subgrp_con	count		1	0
AKR1C3	265	OK	-0.2987582	0.16605823	-1.7991064	0.072	-0.41604695	subgrp_con	count		1	0
NET1	71	OK	-0.7438469	0.31924003	-2.3300549	0.0198	-0.85391068	subgrp_con	count		1	0
FAM208B	16	OK	0.01422865	0.70371256	0.02021912	0.98387	0.00929611	subgrp_con	count		1	0
GDI2	175	OK	-0.2405546	0.21479125	-1.1199461	0.26276	-0.32431513	subgrp_con	count		1	0
ANKRD16	2	OK	-0.3495512	1.85447474	-0.1884907	0.85049	-0.17337783	subgrp_con	count		1	0
FBH1	6	OK	4.66126865	4.08910788	1.13994758	0.25433	0.00274223	subgrp_con	count		1	0
AL137186.2	2	OK	-15.995031	70.349421	-0.2273655	0.82014	-0.00028474	subgrp_con	count		1	0
IL15RA	27	OK	0.5019378	0.53665403	0.93530988	0.34962	0.47547325	subgrp_con	count		1	0
RBM17	117	OK	-0.256082	0.26691297	-0.9594251	0.33734	-0.33333945	subgrp_con	count		1	0
PFKFB3	6	OK	-1.1197385	1.49340414	-0.7497892	0.45338	-0.30904529	subgrp_con	count		1	0
SFMBT2	1	OK	18.7594894	83.6251792	0.22432824	0.8225	0.00028789	subgrp_con	count		1	0
ITIH5	6	OK	3.38578395	2.25197881	1.50347061	0.13272	1.321999	subgrp_con	count		1	0
KIN	32	OK	-0.3799038	0.51744411	-0.7341929	0.46282	-0.42965694	subgrp_con	count		1	0
ATP5F1C	324	OK	-0.0451147	0.15513236	-0.2908139	0.77119	-0.06339369	subgrp_con	count		1	0
TAF3	16	OK	0.81199707	0.77470356	1.04813908	0.29457	0.41245080	subgrp_con	count		1	0
CELF2	17	OK	2.35938581	1.12145926	2.10385335	0.03539	1.72124690	subgrp_con	count		1	0
USP6NL	4	OK	-13.231989	27.8280289	-0.4754914	0.63444	-0.00159191	subgrp_con	count		1	0
PROSER2	1	OK	3.45981463	6.77145131	0.51094137	0.60939	0.00030179	subgrp_con	count		1	0
UPF2	67	OK	-0.7400613	0.36429519	-2.031488	0.04221	-0.93072568	subgrp_con	count		1	0
NUDT5	42	OK	0.1916058	0.46512661	0.41194332	0.68038	0.22738392	subgrp_con	count		1	0
CDC123	75	OK	0.41407746	0.38390895	1.07858246	0.28077	0.48608526	subgrp_con	count		1	0
CAMK1D	4	OK	-0.197292	1.53061314	-0.1288974	0.89744	-0.00012738	subgrp_con	count		1	0
CCDC3	52	OK	0.79157028	0.45635273	1.734558	0.08282	0.80767075	subgrp_con	count		1	0
OPTN	88	OK	0.04322417	0.30427229	0.14238618	0.88677	0.05565767	subgrp_con	count		1	0
PHYH	23	OK	-0.8377583	0.55064399	-1.5214155	0.12816	-0.75835312	subgrp_con	count		1	0
SEPHS1	12	OK	2.71397956	1.54636778	1.75506733	0.07925	1.16431535	subgrp_con	count		1	0
BEND7	17	OK	0.28210435	0.67605177	0.41728217	0.67647	0.26733031	subgrp_con	count		1	0
PRPF18	4	OK	0.45568755	1.46808638	0.3103956	0.75626	0.00030627	subgrp_con	count		1	0
FRMD4A	9	OK	0.37257973	1.02344065	0.36404626	0.71582	0.2426267	subgrp_con	count		1	0
[illegible]	[illegible]	[illegible]	[illegible]	[illegible]	[illegible]	[illegible]	[illegible]	[illegible]	[illegible]		[illegible]	[illegible]

BAMBI	16	OK	5.4718113	2.0473746	2.6725889	0.00752	3.66237874	subgrp_con	count		1	0
SVIL	63	OK	-0.5156144	0.4048450	-1.2736091	0.2028	-0.63113933	subgrp_con	count		1	0
JCAD	36	OK	1.6506798	0.5876622	2.8088919	0.00497	1.61284696	subgrp_con	count		1	0
MTPAP	4	OK	-0.910142	2.1881243	-0.4159466	0.67745	-0.25784050	subgrp_con	count		1	0
ZNF438	1	OK	-12.14842	43.455309	-0.2795612	0.77981	-1.91362624	subgrp_con	count		1	0
ZEB1-AS1	3	OK	4.6613299	5.7683860	0.8080821	0.41906	0.00274918	subgrp_con	count		1	0
ZEB1	110	OK	-0.046583	0.2752936	-0.1692124	0.86562	-0.06040916	subgrp_con	count		1	0
ARHGAP12	9	OK	-0.2877317	1.0842204	-0.2653811	0.79072	-0.23852515	subgrp_con	count		1	0
KIF5B	190	OK	0.3344142	0.2103034	1.5901508	0.1118	0.45232077	subgrp_con	count		1	0
EPC1	73	OK	0.6564989	0.3376387	1.9443827	0.05185	0.83262630	subgrp_con	count		1	0
ITGB1	427	OK	0.3849528	0.1354490	2.8420489	0.00448	0.54299457	subgrp_con	count		1	0
NRP1	134	OK	0.0470263	0.2443826	0.1924291	0.84741	0.06224388	subgrp_con	count		1	0
PARD3	4	OK	5.2612789	5.4905820	0.9582370	0.33794	0.00295034	subgrp_con	count		1	0
CUL2	17	OK	0.0342178	0.6904539	0.0497032	0.96036	0.03241451	subgrp_con	count		1	0
CREM	38	OK	0.2654099	0.4812499	0.5515012	0.58129	0.32370402	subgrp_con	count		1	0
CCNY	42	OK	0.4050992	0.4425718	0.9153298	0.36002	0.36325708	subgrp_con	count		1	0
FZD8	11	OK	2.8157696	1.2564076	2.2411274	0.02502	2.10772403	subgrp_con	count		1	0
ZNF248	2	OK	3.4598149	4.7858686	0.7229230	0.46972	0.00081935	subgrp_con	count		1	0
ZNF33A	19	OK	1.1110581	0.7214177	1.5401037	0.12354	0.97845874	subgrp_con	count		1	0
ZNF37A	11	OK	-0.1943923	1.0050183	-0.1934217	0.84662	-0.12490747	subgrp_con	count		1	0
ZNF33B	7	OK	-0.3689299	1.0858849	-0.3397505	0.73406	-0.11043304	subgrp_con	count		1	0
BMS1	10	OK	0.8680319	0.9581566	0.9059394	0.36497	0.7720750	subgrp_con	count		1	0
CSGALNACT2	6	OK	-3.5874562	2.2336039	-1.6061291	0.10825	-0.00061220	subgrp_con	count		1	0
HNRNPF	247	OK	-0.491312	0.175176	-2.8046772	0.00504	-0.67883453	subgrp_con	count		1	0
AL450326.1	1	OK	3.4597879	4.7858528	0.7229198	0.46972	0.00081879	subgrp_con	count		1	0
ZNF239	1	OK	18.759489	83.625179	0.2243282	0.8225	0.00028789	subgrp_con	count		1	0
ZNF32	30	OK	1.0247767	0.6386511	1.6045955	0.10858	1.00134357	subgrp_con	count		1	0
CXCL12	172	OK	-0.5500642	0.2178014	-2.5255307	0.01155	-0.7060693	subgrp_con	count		1	0
RASSF4	1	OK	3.4598146	6.7714513	0.5109413	0.60939	0.00030179	subgrp_con	count		1	0
DEPP1	203	OK	0.4495289	0.1758341	2.5565508	0.01057	0.62335375	subgrp_con	count		1	0
C10orf25	1	OK	18.759489	83.625179	0.2243282	0.8225	0.00028789	subgrp_con	count		1	0
ZNF22	83	OK	-0.1758974	0.3263239	-0.5384141	0.59029	-0.22251868	subgrp_con	count		1	0
8-Mar	3	OK	18.759489	83.625179	0.2243282	0.8225	0.00028789	subgrp_con	count		1	0
WASHC2C	5	OK	1.6870461	1.4126881	1.1942098	0.2324	0.76488159	subgrp_con	count		1	0
TIMM23	8	OK	-0.0248099	1.3638620	-0.0181909	0.98549	-0.01601158	subgrp_con	count		1	0
NCOA4	54	OK	0.5619085	0.4412440	1.2734640	0.20285	0.55584684	subgrp_con	count		1	0
LINC00842	1	OK	-15.99503	70.34942	-0.2273655	0.82014	-0.00028474	subgrp_con	count		1	0
SYT15	10	OK	-0.1368807	0.8926909	-0.1533349	0.87812	-0.06884897	subgrp_con	count		1	0
MAPK8	3	OK	3.4597863	3.9099767	0.8848611	0.37622	0.00081828	subgrp_con	count		1	0
VSTM4	1	OK	18.759489	83.625179	0.2243282	0.8225	0.00028789	subgrp_con	count		1	0
ERCC6	4	OK	6.4280329	5.2748401	1.2186213	0.22299	1.06669691	subgrp_con	count		1	0
PARG	3	OK	-0.5443403	1.6805338	-0.3239091	0.74601	-0.00034258	subgrp_con	count		1	0
WASHC2A	2	OK	-0.7252159	1.8079140	-0.4011341	0.68832	-0.20980483	subgrp_con	count		1	0
SGMS1	8	OK	0.0299067	1.278882	0.0233886	0.98134	0.01516999	subgrp_con	count		1	0
PRKG1	3	OK	19.102032	50.772469	0.3762479	0.70672	0.00078880	subgrp_con	count		1	0
CSTF2T	3	OK	1.7075095	1.7381067	0.9823962	0.3259	0.50667270	subgrp_con	count		1	0
PCDH15	1	OK	18.759489	83.625179	0.2243282	0.8225	0.00028789	subgrp_con	count		1	0
IPMK	2	OK	-0.9102232	2.1881125	-0.4159856	0.67742	-0.25785735	subgrp_con	count		1	0
CISD1	89	OK	0.8496990	0.3744898	2.2689509	0.02327	1.00327906	subgrp_con	count		1	0
UBE2D1	37	OK	0.0102468	0.4961979	0.0206507	0.98352	0.01106407	subgrp_con	count		1	0
TFAM	60	OK	0.0685304	0.3918438	0.1748923	0.86116	0.08469121	subgrp_con	count		1	0
BICC1	8	OK	21.981615	33.054715	0.6650069	0.50605	2.81118157	subgrp_con	count		1	0
LINC00844	1	OK	3.4598146	6.7714513	0.5109413	0.60939	0.00030179	subgrp_con	count		1	0
FAM13C	28	OK	-2.040148	0.6359469	-3.2080479	0.00134	-1.79062125	subgrp_con	count		1	0
CCDC6	20	OK	-0.0168409	0.678005	-0.0248389	0.98018	-0.01395727	subgrp_con	count		1	0
ANK3	1	OK	18.759489	83.625179	0.2243282	0.8225	0.00028789	subgrp_con	count		1	0
CDK1	2	OK	4.6613299	5.7683860	0.8080821	0.41906	0.00274918	subgrp_con	count		1	0
RHOBTB1	2	OK	0.6532528	2.5001284	0.2612877	0.79387	0.00016297	subgrp_con	count		1	0
ARID5B	344	OK	-0.393082	0.1474816	-2.665295	0.00769	-0.55174649	subgrp_con	count		1	0
RTKN2	2	OK	23.991896	84.899941	0.2825902	0.77749	1.65081709	subgrp_con	count		1	0
ADO	2	OK	4.0104108	3.3070236	1.2126949	0.22525	1.2141728	subgrp_con	count		1	0
EGR2	17	OK	0.4381229	0.7310945	0.5992698	0.54899	0.49192434	subgrp_con	count		1	0
NRBF2	41	OK	0.3441022	0.4620979	0.7446523	0.45648	0.40857389	subgrp_con	count		1	0
JMJD1C	204	OK	-0.131459	0.1951865	-0.6735048	0.50062	-0.17923605	subgrp_con	count		1	0
REEP3	57	OK	0.721693	0.4145888	1.7407458	0.08172	0.88418099	subgrp_con	count		1	0
DNAJC12	3	OK	-1.340131	1.6819410	-0.7967767	0.42558	-0.59807560	subgrp_con	count		1	0
SIRT1	6	OK	1.5336804	1.2416095	1.2352356	0.21674	0.75104284	subgrp_con	count		1	0
HERC4	3	OK	-10.47563	27.491987	-0.3810432	0.70317	-1.13811785	subgrp_con	count		1	0
HNRNPH3	137	OK	-0.192405	0.235878	-0.8156988	0.41467	-0.25424418	subgrp_con	count		1	0
RUFY2	12	OK	0.112467	0.7787967	0.1444121	0.88518	0.09478357	subgrp_con	count		1	0
TET1	4	OK	3.4598149	4.7858686	0.7229230	0.46972	0.00081935	subgrp_con	count		1	0
CCAR1	46	OK	-0.1209975	0.4135110	-0.2926101	0.76982	-0.13312151	subgrp_con	count		1	0
DDX50	24	OK	-0.6593372	0.6040984	-1.09144	0.27508	-0.71198752	subgrp_con	count		1	0
DDX21	245	OK	0.0492324	0.1862771	0.2642968	0.79155	0.06852405	subgrp_con	count		1	0
KIF1BP	13	OK	-0.4803986	0.8255036	-0.5819461	0.5606	-0.42018918	subgrp_con	count		1	0
VPS26A	39	OK	-0.2639489	0.4604332	-0.5732621	0.56647	-0.29469285	subgrp_con	count		1	0
SUPV3L1	3	OK	4.2109206	4.2875616	0.9821248	0.32606	0.00257090	subgrp_con	count		1	0
HK1	15	OK	1.1312787	0.9417048	1.2013092	0.22962	0.56925492	subgrp_con	count		1	0
TSPAN15	20	OK	0.7550338	0.7898370	0.9559360	0.3391	0.77163149	subgrp_con	count		1	0
COL13A1	5	OK	2.472958	1.8823568	1.3137565	0.18892	1.1452953	subgrp_con	count		1	0
TYSND1	2	OK	-12.145707	43.418869	-0.2797334	0.77968	-1.91338276	subgrp_con	count		1	0
SAR1A	83	OK	0.8867701	0.3194112	2.7762645	0.0055	1.13534733	subgrp_con	count		1	0
PPA1	221	OK	-0.1445194	0.1917137	-0.7538292	0.45095	-0.1971390	subgrp_con	count		1	0
EIF4EBP2	14	OK	2.4496385	1.088014	2.2514758	0.02436	1.7694534	subgrp_con	count		1	0
PALD1	8	OK	-2.4371764	1.3588453	-1.7935642	0.07288	-1.1742432	subgrp_con	count		1	0
PCBD1	72	OK	0.7559940	0.3141813	2.4062345	0.01612	1.00837193	subgrp_con	count		1	0
UNC5B	14	OK	1.5434940	1.2251034	1.2598886	0.20771	0.7552310	subgrp_con	count		1	0
CDH23	6	OK	0.2883786	1.0705617	0.2693713	0.78764	0.00019216	subgrp_con	count		1	0
VSIR	58	OK	-0.4393016	0.3810939	-1.1527384	0.24902	-0.50975164	subgrp_con	count		1	0
CHST3	3	OK	21.642059	54.486891	0.3971975	0.69122	1.57744735	subgrp_con	count		1	0
ASCC1	3	OK	-0.2029437	1.6532236	-0.1227536	0.9023	-4.82E-05	subgrp_con	count		1	0
ANAPC16	328	OK	0.0842068	0.1532399	0.5489381	0.58305	0.11816345	subgrp_con	count		1	0
DDIT4	244	OK	0.2524540	0.1808716	1.3957631	0.16279	0.32970114	subgrp_con	count		1	0
DNAJB12	25	OK	0.3655408	0.6096927	0.5995492	0.54881	0.36310007	subgrp_con	count		1	0
MICU1	9	OK	-0.6678418	1.0177303	-0.656201	0.51169	-0.32245221	subgrp_con	count		1	0
MCU	2	OK	-11.019418	43.249935	-0.2547846	0.79889	-1.17482461	subgrp_con	count		1	0
OIT3	2	OK	3.4598146	6.7714513	0.5109413	0.60939	0.00030179	subgrp_con	count		1	0
P4HA1	18	OK	0.8676213	0.7134641	1.2160686	0.22396	0.81665680	subgrp_con	count		1	0
ECD	3	OK	-0.150994	1.968814	-0.0766929	0.93887	-9.78E-05	subgrp_con	count		1	0
DNAJC9	9	OK	0.2005598	0.9827569	0.2040787	0.83829	0.13031079	subgrp_con	count		1	0
MRPS16	50	OK	0.2641701	0.433804	0.6089613	0.54255	0.31954534	subgrp_con	count		1	0
ANXA7	149	OK	0.733387	0.2472282	2.9664368	0.00301	0.96539095	subgrp_con	count		1	0
PPP3CB	10	OK	-0.0950013	0.948659	-0.1001427	0.92022	-0.047873	subgrp_con	count		1	0
SEC24C	2	OK	1.9258645	2.5154879	0.7656425	0.44389	0.56022119	subgrp_con	count		1	0
FUT11	10	OK	0.3467781	0.9135602	0.3795897	0.70425	0.26292503	subgrp_con	count		1	0
CHCHD1	37	OK	0.2909663	0.5705494	0.5099756	0.61007	0.27573477	subgrp_con	count		1	0
ZSWIM8	1	OK	23.991896	84.899941	0.2825902	0.77749	1.65081709	subgrp_con	count		1	0
CAMK2G	2	OK	21.105322	83.341273	0.2532397	0.80008	0.0003103	subgrp_con	count		1	0
PLAU	3	OK	2.1611158	2.3677943	0.9127126	0.36139	0.61359306	subgrp_con	count		1	0
VCL	38	OK	2.0477243	0.6794332	3.0138712	0.00258	1.94228651	subgrp_con	count		1	0
AP3M1	5	OK	4.6295624	3.5444255	1.3061531	0.1915	0.95266465	subgrp_con	count		1	0
ADK	2	OK	-0.9102232	2.1881125	-0.4159856	0.67742	-0.25785735	subgrp_con	count		1	0
KAT6B	13	OK	0.0234870	0.9038293	0.0259861	0.97927	0.01518512	subgrp_con	count		1	0
SAMD8	8	OK	0.980104	1.3385256	0.7322267	0.46403	0.49666511	subgrp_con	count		1	0
VDAC2	257	OK	0.6159009	0.1847756	3.3332371	0.00086	0.85445696	subgrp_con	count		1	0
COMTD1	9	OK	0.8396963	1.0595494	0.7925032	0.42807	0.62915557	subgrp_con	count		1	0
ZNF503	25	OK	-0.695448	0.5994205	-1.1602004	0.24597	-0.6002806	subgrp_con	count		1	0
LRMDA	43	OK	0.818808	0.5091521	1.6081799	0.1079	0.9453795	subgrp_con	count		1	0
POLR3A	2	OK	3.4598146	6.7714513	0.5109413	0.60939	0.00030179	subgrp_con	count		1	0
RPS24	886	OK	-0.061132	0.0834421	-0.7326361	0.46379	-0.08749197	subgrp_con	count		1	0
ZMIZ1	23	OK	0.0083899	0.5758849	0.0145688	0.98838	0.0069596	subgrp_con	count		1	0
ZCCHC24	20	OK	1.8978221	0.7593905	2.4991386	0.01245	1.75002210	subgrp_con	count		1	0
NUTM2B-AS1	17	OK	-0.698950	0.7751468	-0.9017002	0.36722	-0.50897622	subgrp_con	count		1	0
AL135925.1	6	OK	4.0129835	3.5901511	1.1177756	0.26366	0.0024854	subgrp_con	count		1	0
SFTPD-AS1	1	OK	24.360201	51.722967	0.4709745	0.63766	2.90627168	subgrp_con	count		1	0
ANXA11	134	OK	-0.3809197	0.2350629	-1.6205008	0.10512	-0.51685741	subgrp_con	count		1	0
FAM213A	224	OK	-0.4874929	0.1760808	-2.7685737	0.00563	-0.68170503	subgrp_con	count		1	0
TSPAN14	50	OK	-0.6295255	0.3934323	-1.6000857	0.10958	-0.72588497	subgrp_con	count		1	0
GHITM	147	OK	0.5886530	0.2399111	2.4536294	0.01414	0.77735921	subgrp_con	count		1	0
WAPL	23	OK	0.1150845	0.7430552	0.1548802	0.87692	0.09597225	subgrp_con	count		1	0
AL731569.1	1	OK	-10.44227	41.465809	-0.2518286	0.80117	-0.00045289	subgrp_con	count		1	0
MMRN2	225	OK	0.2220709	0.1747571	1.2707403	0.20382	0.30102109	subgrp_con	count		1	0
GLUD1	44	OK	0.1825651	0.4666402	0.3912331	0.69562	0.20873372	subgrp_con	count		1	0
SHLD2	6	OK	-2.2229579	1.7760683	-1.2516172	0.21071	-0.86936325	subgrp_con	count		1	0
NUTM2A-AS1	13	OK	-0.286008	0.7912424	-0.3614657	0.71775	-0.27974210	subgrp_con	count		1	0
MINPP1	17	OK	2.6805434	0.9758175	2.7469718	0.00601	2.04195043	subgrp_con	count		1	0
PAPSS2	32	OK	6.0122171	2.1352316	2.8157212	0.00487	3.67514062	subgrp_con	count		1	0
ATAD1	13	OK	1.9733610	1.1920520	1.6554319	0.09784	0.92686835	subgrp_con	count		1	0
PTEN	54	OK	0.9459073	0.4272130	2.2141349	0.02682	1.10230218	subgrp_con	count		1	0
ACTA2	7	OK	1.8194202	1.3578133	1.3399633	0.18026	0.53457130	subgrp_con	count		1	0
FAS	1	OK	18.759489	83.625179	0.2243282	0.8225	0.00028789	subgrp_con	count		1	0
CH25H	13	OK	0.3835278	0.8849757	0.4333766	0.66476	0.24978128	subgrp_con	count		1	0
LIPA	27	OK	-1.7691417	0.665782	-2.6572386	0.00788	-1.6588159	subgrp_con	count		1	0
IFIT2	47	OK	-1.8043908	0.4536409	-3.9775748	6.96E-05	-2.01153301	subgrp_con	count		0.6916918	0
IFIT3	109	OK	-0.6744356	0.2730149	-2.4703251	0.0135	-0.91205267	subgrp_con	count		1	0
IFIT1	58	OK	-0.990076	0.3724975	-2.6579386	0.00786	-1.1619930	subgrp_con	count		1	0
IFIT5	4	OK	-1.4966957	1.5621792	-0.957488	0.33832	-0.00085699	subgrp_con	count		1	0
KIF20B	3	OK	-0.5453205	1.708550	-0.3191715	0.7496	-0.0003423	subgrp_con	count		1	0
RPP30	15	OK	0.4349307	0.8335247	0.5217970	0.60181	0.3633034	subgrp_con	count		1	0
ANKRD1	6	OK	23.616643	53.905947	0.4381083	0.66131	2.38651165	subgrp_con	count		1	0
PCGF5	21	OK	2.2123218	0.8631898	2.5629725	0.01038	1.8914054	subgrp_con	count		1	0
HECTD2	4	OK	1.3961012	1.4176355	0.9848096	0.32472	0.42407085	subgrp_con	count		1	0
PPP1R3C	1	OK	-11.451025	41.797363	-0.2739652	0.78411	-0.00049189	subgrp_con	count		1	0
TNKS2	14	OK	0.3793687	0.8336867	0.4550495	0.64907	0.31759601	subgrp_con	count		1	0
BTAF1	7	OK	-1.757021	1.4927361	-1.1770472	0.23918	-0.70790546	subgrp_con	count		1	0
CPEB3	1	OK	18.759489	83.625179	0.2243282	0.8225	0.00028789	subgrp_con	count		1	0
5-Mar	16	OK	2.1634906	0.9917022	2.1815928	0.02914	2.12588805	subgrp_con	count		1	0
HHEX	69	OK	0.4520580	0.3521651	1.2800189	0.20054	0.5810637	subgrp_con	count		1	0
EXOC6	25	OK	1.801812	0.6287484	2.8657122	0.00416	2.13782985	subgrp_con	count		1	0
MYOF	68	OK	-0.2039632	0.3909479	-0.5217146	0.60187	-0.23814923	subgrp_con	count		1	0
FRA10AC1	46	OK	0.4036762	0.3918646	1.0301420	0.30294	0.49921504	subgrp_con	count		1	0
NOC3L	44	OK	-0.0822109	0.4764902	-0.1725342	0.86302	-0.10162066	subgrp_con	count		1	0
TBC1D12	10	OK	0.5591067	0.9874736	0.5661991	0.57126	0.46572105	subgrp_con	count		1	0
HELLS	5	OK	-1.2956167	2.041425	-0.6346627	0.52565	-0.00075754	subgrp_con	count		1	0
PDLIM1	606	OK	-0.344472	0.1087545	-3.1674252	0.00154	-0.48939152	subgrp_con	count		1	0
SORBS1	1	OK	3.4598146	6.7714513	0.5109413	0.60939	0.00030179	subgrp_con	count		1	0
TCTN3	11	OK	-0.7217954	1.1396158	-0.6333667	0.52649	-0.56564915	subgrp_con	count		1	0
ENTPD1	71	OK	-0.8085396	0.3507683	-2.3050525	0.02116	-0.97288118	subgrp_con	count		1	0
ZNF518A	10	OK	0.0820934	0.9296173	0.0883088	0.92963	0.02530156	subgrp_con	count		1	0
TLL2	1	OK	1.5823999	2.7200188	0.5817606	0.56073	0.47433761	subgrp_con	count		1	0
TM9SF3	133	OK	0.990611	0.2630578	3.7657559	0.00017	1.29713758	subgrp_con	count		1	0
LCOR	10	OK	0.5463626	0.7805602	0.6999622	0.48395	0.17039620	subgrp_con	count		1	0
FRAT1	1	OK	3.4598146	6.7714513	0.5109413	0.60939	0.00030179	subgrp_con	count		1	0
FRAT2	3	OK	-1.2956167	2.041425	-0.6346627	0.52565	-0.00075754	subgrp_con	count		1	0
PGAM1	181	OK	0.4507362	0.2260442	1.9940181	0.04615	0.61274639	subgrp_con	count		1	0
EXOSC1	17	OK	1.4858146	0.8042731	1.8474002	0.06469	0.92729243	subgrp_con	count		1	0
MMS19	1	OK	-12.14842	43.455309	-0.2795612	0.77981	-1.91362624	subgrp_con	count		1	0
UBTD1	25	OK	0.0621317	0.5904673	0.1052246	0.9162	0.07096072	subgrp_con	count		1	0
AVPI1	2	OK	2.9849400	2.5988857	1.1485460	0.25074	1.23382860	subgrp_con	count		1	0
MARVELD1	12	OK	2.751782	1.3373579	2.0576257	0.03962	1.17452033	subgrp_con	count		1	0
SFRP5	3	OK	18.759489	83.625179	0.2243282	0.8225	0.00028789	subgrp_con	count		1	0
GOLGA7B	2	OK	-15.48861	42.831644	-0.3616162	0.71764	-0.00073897	subgrp_con	count		1	0
R3HCC1L	2	OK	3.4598149	4.7858686	0.7229230	0.46972	0.00081935	subgrp_con	count		1	0
PYROXD2	1	OK	-10.44227	41.465809	-0.2518286	0.80117	-0.00045289	subgrp_con	count		1	0
HPS1	5	OK	5.2612789	5.4905820	0.9582370	0.33794	0.00295034	subgrp_con	count		1	0
GOT1	7	OK	5.4777193	3.2813212	1.6693639	0.09505	1.59830643	subgrp_con	count		1	0
SLC25A28	20	OK	-1.126691	0.6521669	-1.7276117	0.08406	-0.66527496	subgrp_con	count		1	0
CUTC	12	OK	0.6309098	0.9359325	0.6740976	0.50025	0.47516124	subgrp_con	count		1	0
COX15	7	OK	-0.711650	1.004366	-0.7085565	0.4786	-0.20551762	subgrp_con	count		1	0
DNMBP	2	OK	18.759489	83.625179	0.2243282	0.8225	0.00028789	subgrp_con	count		1	0
ERLIN1	13	OK	0.0832298	0.8311242	0.1001412	0.92022	0.06252800	subgrp_con	count		1	0
CHUK	1	OK	3.4598146	6.7714513	0.5109413	0.60939	0.00030179	subgrp_con	count		1	0
CWF19L1	2	OK	-11.019418	43.249935	-0.2547846	0.79889	-1.17482461	subgrp_con	count		1	0
BLOC1S2	26	OK	0.5579220	0.609226	0.9157883	0.35979	0.58531440	subgrp_con	count		1	0
NDUFB8	248	OK	0.4190828	0.1796582	2.3326592	0.01967	0.58277379	subgrp_con	count		1	0
SLF2	12	OK	-0.4470078	0.9268420	-0.4822912	0.6296	-0.32339925	subgrp_con	count		1	0
MRPL43	123	OK	0.7212285	0.2726355	2.6453946	0.00816	0.97421657	subgrp_con	count		1	0
TWNK	1	OK	3.4598146	6.7714513	0.5109413	0.60939	0.00030179	subgrp_con	count		1	0
LZTS2	9	OK	0.3338050	0.9931456	0.3361088	0.73679	0.25179185	subgrp_con	count		1	0
SFXN3	11	OK	0.1703592	0.8967290	0.1899784	0.84932	0.11051941	subgrp_con	count		1	0
KAZALD1	10	OK	22.548414	33.0322	0.682619	0.49485	2.83374644	subgrp_con	count		1	0
BTRC	3	OK	-0.3495512	1.8544747	-0.1884907	0.85049	-0.17337783	subgrp_con	count		1	0
DPCD	7	OK	1.0103298	1.260556	0.8014952	0.42284	0.31287661	subgrp_con	count		1	0
POLL	2	OK	0.6532528	2.5001284	0.2612877	0.79387	0.00016297	subgrp_con	count		1	0
NPM3	2	OK	-10.47563	27.491987	-0.3810432	0.70317	-1.13811785	subgrp_con	count		1	0
OGA	35	OK	0.2473445	0.5126638	0.4816237	0.63007	0.27449328	subgrp_con	count		1	0
PPRC1	1	OK	3.4598146	6.7714513	0.5109413	0.60939	0.00030179	subgrp_con	count		1	0
NOLC1	38	OK	-0.7790318	0.4544650	-1.7141732	0.0865	-0.81736035	subgrp_con	count		1	0
ELOVL3	1	OK	3.4598146	6.7714513	0.5109413	0.60939	0.00030179	subgrp_con	count		1	0
NFKB2	9	OK	-0.4785897	0.9682931	-0.4942612	0.62112	-0.23502059	subgrp_con	count		1	0
FBXL15	27	OK	0.6367524	0.5815711	1.0948831	0.27357	0.74480499	subgrp_con	count		1	0
CUEDC2	71	OK	1.2014377	0.3901025	3.0797994	0.00207	1.49434111	subgrp_con	count		1	0
ACTR1A	33	OK	-0.0575443	0.5529952	-0.1040589	0.91712	-0.06051780	subgrp_con	count		1	0
SUFU	1	OK	3.4597879	4.7858528	0.7229198	0.46972	0.00081879	subgrp_con	count		1	0
TRIM8	55	OK	0.1306271	0.4123486	0.3167883	0.7514	0.1603462	subgrp_con	count		1	0
AL391121.1	2	OK	-12.145707	43.418869	-0.2797334	0.77968	-1.91338276	subgrp_con	count		1	0
ARL3.00	55	OK	0.8171781	0.4373093	1.8686500	0.06167	0.95466098	subgrp_con	count		1	0
WBP1L	10	OK	8.5504519	4.9412887	1.7304092	0.08356	1.77807496	subgrp_con	count		1	0
BORCS7	96	OK	0.0438088	0.2833742	0.1545969	0.87714	0.05628044	subgrp_con	count		1	0
CNNM2	1	OK	18.759489	83.625179	0.2243282	0.8225	0.00028789	subgrp_con	count		1	0
PCGF6	2	OK	18.759489	83.625179	0.2243282	0.8225	0.00028789	subgrp_con	count		1	0
ATP5MD	246	OK	-0.1735684	0.1824058	-0.9515507	0.34132	-0.23956412	subgrp_con	count		1	0
CALHM2	25	OK	-0.4230362	0.5745492	-0.7362924	0.46155	-0.39313401	subgrp_con	count		1	0
SH3PXD2A	7	OK	0.3393435	1.0955305	0.3097526	0.75675	0.30513588	subgrp_con	count		1	0
STN1.00	14	OK	0.6387493	0.8740777	0.7307694	0.46492	0.60359479	subgrp_con	count		1	0
SLK	121	OK	0.1354152	0.2883343	0.4696466	0.63862	0.17035154	subgrp_con	count		1	0
COL17A1	2	OK	-10.44227	41.465809	-0.2518286	0.80117	-0.00045289	subgrp_con	count		1	0
SFR1	5	OK	0.9396109	1.4985607	0.6270088	0.53065	0.2916352	subgrp_con	count		1	0
GSTO1	272	OK	0.6760183	0.1823014	3.7082447	0.00021	0.93880588	subgrp_con	count		1	0
GSTO2	2	OK	-11.448548	41.761485	-0.2741412	0.78398	-0.00049246	subgrp_con	count		1	0
ITPRIP	18	OK	0.6773492	0.874729	0.7743528	0.43872	0.00124935	subgrp_con	count		1	0
XPNPEP1	5	OK	1.1581985	1.6544505	0.7000502	0.4839	0.00079558	subgrp_con	count		1	0
ADD3	13	OK	-1.0228533	0.9154235	-1.1173652	0.26384	-0.61118045	subgrp_con	count		1	0
MX1	46	OK	-1.2167739	0.4536749	-2.6820392	0.00732	-1.34287724	subgrp_con	count		1	0
SMNDC1	41	OK	-0.1526355	0.4019480	-0.3797385	0.70414	-0.17093834	subgrp_con	count		1	0
DUSP5	143	OK	-0.0616148	0.2380739	-0.2588052	0.79579	-0.08237666	subgrp_con	count		1	0
SMC3	97	OK	0.4586722	0.2943478	1.5582662	0.11917	0.59559254	subgrp_con	count		1	0
PDCD4	120	OK	0.4907592	0.2606433	1.8828768	0.05972	0.65098086	subgrp_con	count		1	0
BBIP1	19	OK	0.1394907	0.6829418	0.2042497	0.83816	0.12485913	subgrp_con	count		1	0
SHOC2	39	OK	-0.3790089	0.4558179	-0.8314910	0.4057	-0.39478605	subgrp_con	count		1	0
GPAM	2	OK	0.5887819	1.7674148	0.3331317	0.73903	0.30122869	subgrp_con	count		1	0
ACSL5	10	OK	0.9812119	0.9647751	1.0170369	0.30914	0.49721511	subgrp_con	count		1	0
ZDHHC6	4	OK	1.9928384	1.7182483	1.1598081	0.24612	1.36009744	subgrp_con	count		1	0
VTI1A	2	OK	-1.2956167	2.041425	-0.6346627	0.52565	-0.00075754	subgrp_con	count		1	0
TCF7L2	26	OK	0.0745562	0.558391	0.1335195	0.89379	0.05599902	subgrp_con	count		1	0
CASP7	15	OK	-1.7152132	0.7474905	-2.2946286	0.02175	-0.00264026	subgrp_con	count		1	0
DCLRE1A	1	OK	-11.019418	43.249935	-0.2547846	0.79889	-1.17482461	subgrp_con	count		1	0
NHLRC2	13	OK	-0.0648284	0.8217548	-0.0788900	0.93712	-0.04845762	subgrp_con	count		1	0
CCDC186	51	OK	-0.087543	0.4637202	-0.1887844	0.85026	-0.10351617	subgrp_con	count		1	0
AFAP1L2	6	OK	-1.263047	1.0736979	-1.1763522	0.23945	-0.3420207	subgrp_con	count		1	0
ABLIM1	113	OK	0.6782584	0.2557676	2.6518540	0.00801	0.90049072	subgrp_con	count		1	0
FAM160B1	9	OK	2.1769082	1.3503500	1.6121065	0.10694	0.99943356	subgrp_con	count		1	0
SHTN1	1	OK	-15.99503	70.34942	-0.2273655	0.82014	-0.00028474	subgrp_con	count		1	0
PDZD8	11	OK	0.1037555	0.9928179	0.1045061	0.91677	0.06722208	subgrp_con	count		1	0
EMX2OS	1	OK	18.759489	83.625179	0.2243282	0.8225	0.00028789	subgrp_con	count		1	0
EMX2	6	OK	21.981615	33.054715	0.6650069	0.50605	2.81118157	subgrp_con	count		1	0
RAB11FIP2	4	OK	1.3355409	1.7810286	0.7498705	0.45332	0.40720078	subgrp_con	count		1	0
CASC2	1	OK	3.4598146	6.7714513	0.5109413	0.60939	0.00030179	subgrp_con	count		1	0
FAM204A	132	OK	-0.0204097	0.2475854	-0.0824351	0.9343	-0.02720498	subgrp_con	count		1	0
CACUL1	20	OK	1.6884512	0.7074514	2.3866673	0.017	1.70049604	subgrp_con	count		1	0
NANOS1	1	OK	1.5823999	2.7200188	0.5817606	0.56073	0.47433761	subgrp_con	count		1	0
EIF3A	138	OK	0.0788655	0.2672311	0.2951210	0.7679	0.10583235	subgrp_con	count		1	0
FAM45A	28	OK	0.8979807	0.5084426	1.7661398	0.07737	1.03432305	subgrp_con	count		1	0
SFXN4	2	OK	-14.65800	45.690332	-0.3208138	0.74835	-0.00067223	subgrp_con	count		1	0
PRDX3	131	OK	0.5001988	0.2582444	1.9369203	0.05276	0.66816158	subgrp_con	count		1	0
GRK5	5	OK	2.202634	1.6454796	1.3385914	0.1807	0.62256279	subgrp_con	count		1	0
RGS10	14	OK	4.4210418	1.7634409	2.5070540	0.01217	2.82319562	subgrp_con	count		1	0
TIAL1	64	OK	0.4647493	0.3601938	1.2902756	0.19695	0.55147377	subgrp_con	count		1	0
BAG3	76	OK	0.0404595	0.3432865	0.1178594	0.90618	0.04964616	subgrp_con	count		1	0
INPP5F	7	OK	-1.7779396	1.3192990	-1.3476396	0.17777	-0.44681836	subgrp_con	count		1	0
MCMBP	12	OK	0.0362489	0.9212415	0.0393479	0.96861	0.01837780	subgrp_con	count		1	0
SEC23IP	4	OK	-10.96786	27.153658	-0.4039185	0.68627	-1.17125107	subgrp_con	count		1	0
WDR11	3	OK	0.5887819	1.7674148	0.3331317	0.73903	0.30122869	subgrp_con	count		1	0
ATE1	13	OK	0.9967819	0.9465744	1.0530413	0.29232	1.0126672	subgrp_con	count		1	0
NSMCE4A	39	OK	0.0396872	0.5071719	0.0782520	0.93763	0.04060538	subgrp_con	count		1	0
TACC2	8	OK	0.029293	1.1693581	0.0250509	0.98001	0.00900545	subgrp_con	count		1	0
PLEKHA1	53	OK	0.5458843	0.4335830	1.2590075	0.20802	0.67020944	subgrp_con	count		1	0
C10orf88	2	OK	-0.3495512	1.8544747	-0.1884907	0.85049	-0.17337783	subgrp_con	count		1	0
PSTK	1	OK	-15.99503	70.34942	-0.2273655	0.82014	-0.00028474	subgrp_con	count		1	0
IKZF5	1	OK	18.759489	83.625179	0.2243282	0.8225	0.00028789	subgrp_con	count		1	0
BUB3	57	OK	-0.3469229	0.394454	-0.8795016	0.37912	-0.38654441	subgrp_con	count		1	0
CPXM2	103	OK	0.9267938	0.2997804	3.0915749	0.00199	1.18587489	subgrp_con	count		1	0
CHST15	6	OK	0.2466666	1.4959047	0.1648946	0.86903	0.00044539	subgrp_con	count		1	0
OAT	8	OK	2.0012984	1.5562192	1.2860003	0.19844	0.00371568	subgrp_con	count		1	0
LHPP	2	OK	23.991896	84.899941	0.2825902	0.77749	1.65081709	subgrp_con	count		1	0
FAM53B	2	OK	23.991896	84.899941	0.2825902	0.77749	1.65081709	subgrp_con	count		1	0
EEF1AKMT2	10	OK	0.4464022	0.9392057	0.4752977	0.63457	0.326314	subgrp_con	count		1	0
ABRAXAS2	6	OK	-11.78804	17.656216	-0.6676425	0.50436	-1.22788846	subgrp_con	count		1	0
ZRANB1	5	OK	-1.6692539	1.3317404	-1.2534379	0.21005	-0.000936	subgrp_con	count		1	0
CTBP2	39	OK	1.0193322	0.5277054	1.9316317	0.0534	1.03308186	subgrp_con	count		1	0
EDRF1	4	OK	-1.5883495	1.4907273	-1.0654862	0.28666	-0.41069514	subgrp_con	count		1	0
UROS	7	OK	-0.3715185	1.2493608	-0.2973660	0.76619	-0.11118403	subgrp_con	count		1	0
BCCIP	21	OK	-0.1734588	0.6345549	-0.2733552	0.78458	-0.15394891	subgrp_con	count		1	0
DHX32	4	OK	-1.3634593	1.5336512	-0.8890282	0.37399	-0.62860673	subgrp_con	count		1	0
DOCK1	22	OK	0.1381936	0.701629	0.1969613	0.84386	0.11517734	subgrp_con	count		1	0
PTPRE	57	OK	0.5480668	0.4325524	1.2670527	0.20514	0.69209641	subgrp_con	count		1	0
MGMT	169	OK	0.0891070	0.2296413	0.3880270	0.698	0.12075553	subgrp_con	count		1	0
EBF3	11	OK	-2.4226679	1.0982019	-2.2060312	0.02738	-1.39860150	subgrp_con	count		1	0
GLRX3	38	OK	1.0166962	0.5954605	1.7074116	0.08775	0.65455979	subgrp_con	count		1	0
PPP2R2D	10	OK	0.3902484	1.0139508	0.3848790	0.70032	0.1214831	subgrp_con	count		1	0
BNIP3	70	OK	0.4275210	0.3334956	1.2819387	0.19986	0.55332350	subgrp_con	count		1	0
STK32C	3	OK	-11.542805	27.656566	-0.4173622	0.67641	-1.85958926	subgrp_con	count		1	0
PWWP2B	3	OK	-1.8538345	1.8543918	-0.9996995	0.31746	-0.46053279	subgrp_con	count		1	0
INPP5A	2	OK	3.4597863	3.9099767	0.8848611	0.37622	0.00081828	subgrp_con	count		1	0
TUBGCP2	18	OK	0.9700438	0.8578182	1.1308267	0.25812	0.62485067	subgrp_con	count		1	0
ZNF511	10	OK	0.1474529	0.9718997	0.1517162	0.87941	0.11094327	subgrp_con	count		1	0
FUOM	7	OK	0.7765549	1.1241030	0.6908218	0.48968	0.6443666	subgrp_con	count		1	0
ECHS1	104	OK	0.9560999	0.2869002	3.3325173	0.00086	1.27279175	subgrp_con	count		1	0
IQSEC3	2	OK	3.4598146	6.7714513	0.5109413	0.60939	0.00030179	subgrp_con	count		1	0
KDM5A	30	OK	1.5608091	0.6477426	2.4096133	0.01597	1.4966714	subgrp_con	count		1	0
CCDC77	2	OK	2.4096337	2.4715209	0.9749599	0.32958	0.66517343	subgrp_con	count		1	0
NINJ2	4	OK	-13.27197	28.009791	-0.4738335	0.63562	-2.0156632	subgrp_con	count		1	0
AC021054.1	1	OK	-10.44227	41.465809	-0.2518286	0.80117	-0.00045289	subgrp_con	count		1	0
ERC1	15	OK	1.8251795	1.1604948	1.5727597	0.11577	1.14343954	subgrp_con	count		1	0
ADIPOR2	13	OK	-0.2169682	0.776800	-0.2793102	0.78001	-0.10858122	subgrp_con	count		1	0
DCP1B	1	OK	-15.99503	70.34942	-0.2273655	0.82014	-0.00028474	subgrp_con	count		1	0
CACNA1C	3	OK	3.4597795	3.3818931	1.0230304	0.30629	0.00222221	subgrp_con	count		1	0

FKBP4	10	OK	0.90660139	1.02002472	0.88880335	0.37411	0.28164283	subgrp_cor	count		1	0
RHNO1	4	OK	-1.7545825	1.63223695	-1.0748767	0.28242	-0.95331468	subgrp_cor	count		1	0
TULP3	7	OK	-1.5885338	1.49071057	-1.0656219	0.28659	-0.41070797	subgrp_cor	count		1	0
TEAD4	12	OK	-0.1514263	0.96548482	-0.1568396	0.87537	-0.04606565	subgrp_cor	count		1	0
AC125807.2	1	OK	-11.019418	43.2499352	-0.2547846	0.79889	-1.17482461	subgrp_cor	count		1	0
TSPAN9	29	OK	0.45397957	0.6046377	0.75082909	0.45276	0.44940118	subgrp_cor	count		1	0
CCND2	29	OK	0.96527263	0.63551237	1.51888882	0.12879	1.15624935	subgrp_cor	count		1	0
C12orf4	4	OK	2.89515881	2.41327366	1.1996811	0.23026	0.752289	subgrp_cor	count		1	0
RAD51AP1	2	OK	5.80492735	5.66946625	1.0238921	0.30589	1.03285716	subgrp_cor	count		1	0
DYRK4	27	OK	1.83615573	0.62958022	2.9164762	0.00354	2.03405960	subgrp_cor	count		1	0
NDUFA9	27	OK	-0.4043765	0.5800098	-0.6971891	0.48568	-0.39344447	subgrp_cor	count		1	0
AC005833.1	1	OK	1.58229999	2.72001883	0.58176068	0.56072	0.47433761	subgrp_cor	count		1	0
KCNA5	1	OK	-10.442278	41.4658099	-0.2518286	0.80117	-0.00045289	subgrp_cor	count		1	0
VWF	608	OK	0.17830086	0.10120153	1.76183946	0.0781	0.25442207	subgrp_cor	count		1	0
CD9	654	OK	0.18204612	0.10290501	1.7690696	0.07688	0.25944241	subgrp_cor	count		1	0
TNFRSF1A	228	OK	-0.2406586	0.18035855	-1.3343341	0.18209	-0.32836466	subgrp_cor	count		1	0
LTBR	38	OK	-1.1337772	0.49174195	-2.3056346	0.02112	-1.21227612	subgrp_cor	count		1	0
TAPBPL	8	OK	0.61051273	1.07102135	0.57002853	0.56866	0.00122210	subgrp_cor	count		1	0
MRPL51	225	OK	0.45521241	0.19492622	2.33530626	0.01952	0.63122952	subgrp_cor	count		1	0
NCAPD2	2	OK	3.45979833	3.90997673	0.8848611	0.37622	0.00081828	subgrp_cor	count		1	0
GAPDH	872	OK	-0.0801703	0.08467699	-0.9490196	0.34261	-0.11472875	subgrp_cor	count		1	0
IFFO1	1	OK	-11.019418	43.2499352	-0.2547846	0.79889	-1.17482461	subgrp_cor	count		1	0
CHD4	76	OK	0.73021091	0.34523163	2.11513337	0.03442	0.872867	subgrp_cor	count		1	0
ING4	9	OK	0.7008639	1.05076724	0.66700205	0.50477	0.21851659	subgrp_cor	count		1	0
ZNF384	2	OK	3.45981497	4.78586864	0.72292309	0.46972	0.00081935	subgrp_cor	count		1	0
COPS7A	27	OK	2.38178693	0.8022952	2.9687164	0.00299	2.1840023	subgrp_cor	count		1	0
MLF2.00	75	OK	0.07700386	0.31538516	0.24415815	0.80711	0.09993835	subgrp_cor	count		1	0
PTMS	622	OK	-0.0903615	0.10603483	-0.8521871	0.39411	-0.12863276	subgrp_cor	count		1	0
LAG3	2	OK	-12.145707	43.4188694	-0.2797334	0.77968	-1.91338276	subgrp_cor	count		1	0
P3H3	3	OK	0.17216145	1.5344504	0.11219747	0.91067	0.00011395	subgrp_cor	count		1	0
TPI1	426	OK	-0.043036	0.13505613	-0.3186533	0.74999	-0.06075363	subgrp_cor	count		1	0
LRRC23	4	OK	-0.4654295	1.3093639	-0.3554622	0.72224	-0.13816105	subgrp_cor	count		1	0
ENO2	1	OK	23.9918963	84.8999419	0.28259026	0.77749	1.65081709	subgrp_cor	count		1	0
ATN1	5	OK	3.14495939	2.48058509	1.26782456	0.20486	0.86187553	subgrp_cor	count		1	0
PHB2	208	OK	-0.3047817	0.1953646	-1.5600661	0.11874	-0.41841377	subgrp_cor	count		1	0
EMG1	10	OK	0.5487273	0.89168863	0.6153798	0.5382	0.41372733	subgrp_cor	count		1	0
LPCAT3	5	OK	0.5753754	1.1859972	0.4851406	0.62758	0.17946481	subgrp_cor	count		1	0
C1RL	10	OK	0.49345311	1.05558713	0.4674679	0.64017	0.25842149	subgrp_cor	count		1	0
RBP5	14	OK	-1.9247675	0.88494388	-2.1750165	0.02963	-1.19812279	subgrp_cor	count		1	0
CLSTN3	3	OK	10.1741528	10.4794248	0.97086935	0.33168	1.19375212	subgrp_cor	count		1	0
GDF3	1	OK	1.58229999	2.72001883	0.58176068	0.56072	0.47433761	subgrp_cor	count		1	0
FOXJ2	2	OK	0.65325287	2.50012846	0.26128772	0.79387	0.00016297	subgrp_cor	count		1	0
NECAP1	6	OK	1.33962787	1.2142468	1.1032583	0.26992	0.40834447	subgrp_cor	count		1	0
MFAP5	8	OK	4.01298359	3.59015119	1.11777565	0.26366	0.0024854	subgrp_cor	count		1	0
RIMKLB	3	OK	18.7594894	83.6251792	0.22432824	0.8225	0.00028789	subgrp_cor	count		1	0
PHC1	1	OK	-11.019418	43.2499352	-0.2547846	0.79889	-1.17482461	subgrp_cor	count		1	0
M6PR	40	OK	1.08992389	0.54440499	2.00204618	0.04528	1.16335901	subgrp_cor	count		1	0
KLRG1	3	OK	-0.1747011	1.82098429	-0.0959377	0.92357	-0.05306391	subgrp_cor	count		1	0
LINC00987	4	OK	-2.342223	1.73680068	-1.348585	0.17747	-0.53905944	subgrp_cor	count		1	0
CLEC2D	1	OK	0.65325287	2.50012846	0.26128772	0.79387	0.00016297	subgrp_cor	count		1	0
CLEC12A	1	OK	-15.995031	70.349421	-0.2273655	0.82014	-0.00028474	subgrp_cor	count		1	0
CLEC1A	7	OK	-1.537212	1.16640209	-1.3179092	0.18752	-0.66746902	subgrp_cor	count		1	0
GABARAPL1	66	OK	0.57075268	0.35576862	1.60428047	0.10865	0.72099202	subgrp_cor	count		1	0
KLRD1	1	OK	-10.442278	41.4658099	-0.2518286	0.80117	-0.00045289	subgrp_cor	count		1	0
MAGOHB	19	OK	0.18810409	0.83083931	0.22640246	0.82089	0.15671640	subgrp_cor	count		1	0
YBX3	709	OK	-0.2378289	0.0985779	-2.412596	0.01584	-0.33938077	subgrp_cor	count		1	0
PRH1	5	OK	0.28837869	1.07056173	0.26937138	0.78764	0.00019216	subgrp_cor	count		1	0
TAS2R14	1	OK	-10.442278	41.4658099	-0.2518286	0.80117	-0.00045289	subgrp_cor	count		1	0
SMIM10L1	60	OK	0.15178935	0.40416246	0.37556518	0.70726	0.1927521	subgrp_cor	count		1	0
LINC01252	2	OK	2.40963373	2.47152069	0.97495997	0.32958	0.66517343	subgrp_cor	count		1	0
ETV6	17	OK	0.94068179	0.76323813	1.23248795	0.21777	0.77592746	subgrp_cor	count		1	0
LRP6	6	OK	0.93961099	1.49856075	0.62700893	0.53065	0.2916352	subgrp_cor	count		1	0
MANSC1	26	OK	1.33397379	0.65026844	2.05142014	0.04022	1.33376726	subgrp_cor	count		1	0
LOH12CR2	2	OK	4.66132993	5.76838606	0.80808217	0.41906	0.00274918	subgrp_cor	count		1	0
CREBL2	2	OK	2.40963373	2.47152069	0.97495997	0.32958	0.66517343	subgrp_cor	count		1	0
CDKN1B	42	OK	-0.3406406	0.44424085	-0.7667925	0.4432	-0.37960519	subgrp_cor	count		1	0
APOLD1	94	OK	-0.7264867	0.2984055	-2.4345621	0.01490	-0.74447842	subgrp_cor	count		1	0
GPRC5A	37	OK	2.12590201	0.59683189	3.5619779	0.00037	2.46940498	subgrp_cor	count		1	0
GPRC5D-AS1	3	OK	2.98494009	2.59888574	1.14854609	0.25076	1.22382860	subgrp_cor	count		1	0
HEBP1	172	OK	0.66365261	0.22194141	2.9902154	0.00279	0.91484541	subgrp_cor	count		1	0
EMP1	377	OK	-0.1563463	0.14249346	-1.0972174	0.27258	-0.21837710	subgrp_cor	count		1	0
ATF7IP	27	OK	-0.3801245	0.57759365	-0.6581175	0.51048	-0.38360357	subgrp_cor	count		1	0
PLBD1	11	OK	0.22043899	0.78968212	0.27914902	0.78012	0.14328317	subgrp_cor	count		1	0
WBP11	40	OK	-0.0310167	0.4646509	-0.0667527	0.94678	-0.03222130	subgrp_cor	count		1	0
ART4	11	OK	20.817553	50.5285273	0.4119145	0.6804	0.00083571	subgrp_cor	count		1	0
MGP	725	OK	0.31257263	0.09087673	3.43952327	0.00058	0.44716977	subgrp_cor	count		1	0
ARHGDIB	294	OK	-0.332525	0.1641233	-2.026067	0.04276	-0.46284060	subgrp_cor	count		1	0
STRAP	98	OK	0.34002664	0.30142704	1.12805619	0.2592	0.43195692	subgrp_cor	count		1	0
DERA	26	OK	0.80518328	0.56294194	1.43031311	0.15263	0.91634964	subgrp_cor	count		1	0
MGST1	4	OK	-1.8979105	1.60787469	-1.1803846	0.23785	-0.78103351	subgrp_cor	count		1	0
PLEKHA5	6	OK	0.14853379	1.61597268	0.09191598	0.92676	0.09694857	subgrp_cor	count		1	0
AEBP2	11	OK	-0.545035	0.885874	-0.6152512	0.53836	-0.16017107	subgrp_cor	count		1	0
PDE3A	26	OK	23.2850707	19.5721198	1.18970612	0.23416	4.706208	subgrp_cor	count		1	0
PYROXD1	10	OK	0.88995441	1.00637065	0.88432071	0.37652	0.57506753	subgrp_cor	count		1	0
RECQL	43	OK	-0.9794954	0.47755553	-2.0510607	0.04026	-1.01454007	subgrp_cor	count		1	0
GOLT1B	7	OK	0.30422099	1.00694478	0.3021228	0.76256	0.22942550	subgrp_cor	count		1	0
LDHB	290	OK	0.28526628	0.16827176	1.69527101	0.09003	0.39839951	subgrp_cor	count		1	0
CMAS	17	OK	-0.7420527	0.8513616	-0.8716069	0.38342	-0.53191566	subgrp_cor	count		1	0
C2CD5	2	OK	-0.2029437	1.65223657	-0.1227536	0.9022	-4.82E-05	subgrp_cor	count		1	0
ETNK1	18	OK	0.35129549	0.80669697	0.43559784	0.66312	0.29298003	subgrp_cor	count		1	0
SOX5	1	OK	18.7594894	83.6251792	0.22432824	0.8225	0.00028789	subgrp_cor	count		1	0
BCAT1	3	OK	21.1053239	83.3412731	0.25323976	0.80008	0.0003103	subgrp_cor	count		1	0
LRMP	2	OK	-12.145707	43.4188694	-0.2797334	0.77968	-1.91338276	subgrp_cor	count		1	0
ETFRF1	48	OK	0.16476695	0.40868073	0.40316789	0.68682	0.1933612	subgrp_cor	count		1	0
KRAS	119	OK	0.3260227	0.284094	1.18278702	0.23689	0.42687210	subgrp_cor	count		1	0
RASSF8-AS1	6	OK	0.17044573	1.14708404	0.14859045	0.88188	0.05272766	subgrp_cor	count		1	0
RASSF8	26	OK	0.60449513	0.61457434	0.9835997	0.32531	0.65328437	subgrp_cor	count		1	0
BHLHE41	2	OK	21.642059	54.4868919	0.39719753	0.69122	1.57744735	subgrp_cor	count		1	0
SSPN	7	OK	-0.0085248	0.91675946	-0.0092989	0.99258	-0.00550512	subgrp_cor	count		1	0
INTS13	3	OK	19.1030323	50.7724696	0.37624785	0.70672	0.00078880	subgrp_cor	count		1	0
FGFR1OP2	99	OK	0.45603339	0.28664433	1.5909381	0.11162	0.60482402	subgrp_cor	count		1	0
TM7SF3	22	OK	0.98639938	0.6502513	1.51695095	0.12929	0.8735397	subgrp_cor	count		1	0
MED21	23	OK	1.18010713	0.6560842	1.79871293	0.07206	1.03561100	subgrp_cor	count		1	0
STK38L	31	OK	2.37246945	0.78509249	3.02189805	0.00251	2.09217277	subgrp_cor	count		1	0
ARNTL2	6	OK	0.51030031	1.6034059	0.31826022	0.75029	0.15911328	subgrp_cor	count		1	0
PPFIBP1	124	OK	-0.5379298	0.26532293	-2.0274525	0.04262	-0.69919455	subgrp_cor	count		1	0
MRPS35	25	OK	-0.774229	0.62549346	-1.2377882	0.21579	-0.78716016	subgrp_cor	count		1	0
KLHL42	2	OK	18.7594894	83.6251792	0.22432824	0.8225	0.00028789	subgrp_cor	count		1	0
PTHLH	8	OK	22.2217907	54.5232277	0.40756559	0.68359	1.5953836	subgrp_cor	count		1	0
CCDC91	25	OK	1.20430785	0.69822594	1.72481113	0.08456	1.05529099	subgrp_cor	count		1	0
FAR2	6	OK	-1.0540603	1.2346784	-0.8537124	0.39326	-0.48795849	subgrp_cor	count		1	0
ERGIC2	53	OK	0.72181709	0.41477372	1.74026713	0.08181	0.87059455	subgrp_cor	count		1	0
TMTC1	47	OK	-0.8529997	0.40888875	-2.0861412	0.03697	-0.96094335	subgrp_cor	count		1	0
IPO8	3	OK	0.93961099	1.49856075	0.62700893	0.53065	0.2916352	subgrp_cor	count		1	0
CAPRIN2	8	OK	0.61035997	1.44076382	0.42363638	0.67182	0.3122109	subgrp_cor	count		1	0
LINC00941	1	OK	-11.019418	43.2499352	-0.2547846	0.79889	-1.17482461	subgrp_cor	count		1	0
TSPAN11	1	OK	18.7594894	83.6251792	0.22432824	0.8225	0.00028789	subgrp_cor	count		1	0
DDX11	1	OK	3.45981462	6.7714513	0.51094137	0.60936	0.00030179	subgrp_cor	count		1	0
SINHCAF	43	OK	-1.0215563	0.4841736	-2.1098967	0.03487	-1.05581740	subgrp_cor	count		1	0
DENND5B	3	OK	3.71904477	2.27618501	1.63389386	0.10228	2.45754779	subgrp_cor	count		1	0
ETFBKMT	1	OK	-10.442278	41.4658099	-0.2518286	0.80117	-0.00045289	subgrp_cor	count		1	0
AMN1	10	OK	0.41275889	0.86272871	0.4784341	0.63234	0.31142822	subgrp_cor	count		1	0
AC023157.3	20	OK	-0.424346	0.6744586	-0.6291651	0.52926	-0.20879365	subgrp_cor	count		1	0
KIAA1551	59	OK	-0.5814635	0.38937243	-1.4933351	0.13535	-0.70437426	subgrp_cor	count		1	0
BICD1	11	OK	0.26424623	0.95560462	0.27652256	0.78215	0.2374911	subgrp_cor	count		1	0
FGD4	11	OK	-1.3469093	0.8531916	-1.5786716	0.11441	-0.77402040	subgrp_cor	count		1	0
DNM1L	23	OK	-0.3095456	0.62405358	-0.4960237	0.61988	-0.2287558	subgrp_cor	count		1	0
YARS2	2	OK	18.7594894	83.6251792	0.22432824	0.8225	0.00028789	subgrp_cor	count		1	0
ALG10B	2	OK	-12.145707	43.4188694	-0.2797334	0.77968	-1.91338276	subgrp_cor	count		1	0
CPNE8	70	OK	-1.1419934	0.35087483	-3.2547032	0.00114	-1.38496083	subgrp_cor	count		1	0
KIF21A	4	OK	0.87880152	1.64693725	0.53359745	0.59362	0.44698448	subgrp_cor	count		1	0
SLC2A13	1	OK	18.7594894	83.6251792	0.22432824	0.8225	0.00028789	subgrp_cor	count		1	0
LRRK2	3	OK	1.27602469	1.68001949	0.75952967	0.44754	0.44280304	subgrp_cor	count		1	0
PDZRN4	2	OK	21.1053239	83.3412731	0.25323976	0.80008	0.0003103	subgrp_cor	count		1	0
GXYLT1	12	OK	1.46036403	0.99407564	1.46906729	0.14181	0.71929340	subgrp_cor	count		1	0
YAF2	16	OK	0.94927409	0.89412599	1.06167816	0.28838	0.48164121	subgrp_cor	count		1	0
PPHLN1	34	OK	-0.4482147	0.57143123	-0.7843721	0.43282	-0.4658087	subgrp_cor	count		1	0
ZCRB1	121	OK	0.81971299	0.26425995	3.10191884	0.00192	1.0994262	subgrp_cor	count		1	0
PRICKLE1	4	OK	5.26127895	5.49058205	0.95823701	0.33796	0.00295034	subgrp_cor	count		1	0
PUS7L	18	OK	0.37091473	0.68508435	0.54141476	0.58822	0.30926825	subgrp_cor	count		1	0
IRAK4	17	OK	0.60605275	0.73517114	0.82436962	0.40972	0.50450313	subgrp_cor	count		1	0
TWF1	22	OK	0.59661413	0.64573698	0.92392751	0.35552	0.53412266	subgrp_cor	count		1	0
ANO6	30	OK	1.82990064	0.69207612	2.64407422	0.00819	1.52958060	subgrp_cor	count		1	0
AC008124.1	5	OK	-0.5453205	1.7085504	-0.3191715	0.7496	-0.0003423	subgrp_cor	count		1	0
ARID2	3	OK	0.93961099	1.49856075	0.62700893	0.53065	0.2916352	subgrp_cor	count		1	0
SCAF11	193	OK	-0.3049394	0.1962835	-1.5535658	0.12029	-0.41038439	subgrp_cor	count		1	0
SLC38A1	3	OK	-1.8538345	1.85439186	-0.9996995	0.31748	-0.46053279	subgrp_cor	count		1	0
SLC38A2	212	OK	0.57808045	0.20563707	2.81116853	0.00494	0.77862612	subgrp_cor	count		1	0
AMIGO2	9	OK	-1.4792302	1.00858467	-1.4666396	0.14247	-0.64760440	subgrp_cor	count		1	0
PCED1B	1	OK	3.45981462	6.7714513	0.51094137	0.60936	0.00030179	subgrp_cor	count		1	0
RPAP3	11	OK	-1.6621947	0.97244944	-1.7092865	0.0874	-1.40565454	subgrp_cor	count		1	0
RAPGEF3	27	OK	-1.4900205	0.59308883	-2.5123058	0.01199	-1.26986705	subgrp_cor	count		1	0
SLC48A1	18	OK	-0.085839	0.64588702	-0.1330558	0.89415	-0.0808748	subgrp_cor	count		1	0
HDAC7	51	OK	-0.2139183	0.39892711	-0.536234	0.5918	-0.23916908	subgrp_cor	count		1	0
TMEM106C	15	OK	24.5117626	32.6109289	0.75164258	0.45227	4.02086865	subgrp_cor	count		1	0
SENP1	2	OK	3.45981462	6.7714513	0.51094137	0.60936	0.00030179	subgrp_cor	count		1	0
PFKM	3	OK	18.7594894	83.6251792	0.22432824	0.8225	0.00028789	subgrp_cor	count		1	0
ASB8	9	OK	0.15053949	0.93368747	0.16123114	0.87192	0.09770006	subgrp_cor	count		1	0
CCDC184	1	OK	0.65325287	2.50012846	0.26128772	0.79387	0.00016297	subgrp_cor	count		1	0
KANSL2	1	OK	-11.019418	43.2499352	-0.2547846	0.79889	-1.17482461	subgrp_cor	count		1	0
CCNT1	7	OK	5.53677109	3.9708163	1.39436596	0.16322	1.01678103	subgrp_cor	count		1	0
ADCY6	2	OK	18.7594894	83.6251792	0.22432824	0.8225	0.00028789	subgrp_cor	count		1	0
DDX23	12	OK	-0.8842151	0.82386262	-1.0852934	0.27779	-0.5417592	subgrp_cor	count		1	0
RND1	33	OK	-0.2694458	0.56058594	-0.4806418	0.63077	-0.3092838	subgrp_cor	count		1	0
FKBP11	67	OK	-0.49868	0.34499429	-1.4454733	0.14832	-0.6145155	subgrp_cor	count		1	0
ARF3	18	OK	0.86987983	0.76110395	1.14291856	0.25307	0.44258430	subgrp_cor	count		1	0
PRKAG1	6	OK	2.66728235	2.24180553	1.18979203	0.23412	0.71356325	subgrp_cor	count		1	0
KMT2D	3	OK	-0.5453205	1.7085504	-0.3191715	0.7496	-0.0003423	subgrp_cor	count		1	0
DHH	5	OK	18.7594894	83.6251792	0.22432824	0.8225	0.00028789	subgrp_cor	count		1	0
LMBR1L	3	OK	37.9504577	40.8758203	0.92843282	0.35318	0.00237266	subgrp_cor	count		1	0
TUBA1B	627	OK	-0.3103313	0.10616103	-2.9232131	0.00346	-0.44069773	subgrp_cor	count		1	0
TUBA1A	329	OK	0.0981243	0.16157417	0.6073019	0.54365	0.13658708	subgrp_cor	count		1	0
TUBA1C	130	OK	1.14367057	0.29567196	3.86803864	0.00011	1.4901205	subgrp_cor	count		1	0
SPATS2	29	OK	-0.4542235	0.61425557	-0.7394686	0.45962	-0.45782713	subgrp_cor	count		1	0
MCRS1	6	OK	1.23047661	1.29043287	0.95353787	0.34032	0.61569752	subgrp_cor	count		1	0
FMNL3	4	OK	0.84117939	1.40444781	0.59893958	0.54921	0.0005738	subgrp_cor	count		1	0
TMBIM6	526	OK	-0.0144859	0.11754798	-0.123234	0.90192	-0.02057174	subgrp_cor	count		1	0
NCKAP5L	1	OK	-15.995031	70.349421	-0.2273655	0.82014	-0.00028474	subgrp_cor	count		1	0
BCDIN3D	2	OK	-10.475634	27.4919879	-0.3810432	0.70317	-1.13811785	subgrp_cor	count		1	0
AQP5	3	OK	24.3460159	51.6160277	0.4716755	0.63716	2.905701	subgrp_cor	count		1	0
RACGAP1	1	OK	18.7594894	83.6251792	0.22432824	0.8225	0.00028789	subgrp_cor	count		1	0
SMARCD1	17	OK	0.94265455	0.72202347	1.30557329	0.1917	0.88411688	subgrp_cor	count		1	0
COX14	99	OK	0.93506215	0.3300009	2.83351397	0.0046	1.19168148	subgrp_cor	count		1	0
AC074032.1	1	OK	18.7594894	83.6251792	0.22432824	0.8225	0.00028789	subgrp_cor	count		1	0
CERS5	12	OK	2.07409729	1.1177346	1.8556259	0.06351	1.57107225	subgrp_cor	count		1	0
LIMA1	328	OK	0.42683877	0.14897786	2.86511545	0.00417	0.59821825	subgrp_cor	count		1	0
LARP4	23	OK	-0.027287	0.63063281	-0.0432692	0.96549	-0.02788224	subgrp_cor	count		1	0
DIP2B	1	OK	18.7594894	83.6251792	0.22432824	0.8225	0.00028789	subgrp_cor	count		1	0
ATF1	14	OK	0.00562723	0.97129615	0.00579353	0.99538	0.00366299	subgrp_cor	count		1	0
METTL7A	34	OK	-0.787346	0.48166852	-1.6346222	0.10212	-0.80251854	subgrp_cor	count		1	0
SLC11A2	4	OK	1.79190275	1.61417387	1.11010516	0.26696	0.00122917	subgrp_cor	count		1	0
LETMD1	33	OK	-0.0530703	0.46880097	-0.1132043	0.90987	-0.05228838	subgrp_cor	count		1	0
DAZAP2	104	OK	0.19232261	0.2844794	0.6760511	0.49902	0.24631353	subgrp_cor	count		1	0
SMAGP	64	OK	-1.7496147	0.39453926	-4.4345769	9.23E-06	-2.06616452	subgrp_cor	count		0.09229169	0
SCN8A	6	OK	20.0998321	50.6521927	0.3962283	0.69194	0.00081457	subgrp_cor	count		1	0
ACVR1B	2	OK	18.7594894	83.6251792	0.22432824	0.8225	0.00028789	subgrp_cor	count		1	0
GRASP	271	OK	0.39357671	0.17140999	2.29611297	0.02167	0.53791129	subgrp_cor	count		1	0
NR4A1	207	OK	0.75999351	0.19200321	3.95782117	7.56E-05	1.05916514	subgrp_cor	count		0.75122222	0
ATG101	28	OK	0.37875689	0.59778847	0.6335969	0.52636	0.37396627	subgrp_cor	count		1	0
KRT7	8	OK	0.16816909	0.99614733	0.16881945	0.86594	0.10918750	subgrp_cor	count		1	0
KRT8	15	OK	-0.4617406	0.855513	-0.5397222	0.58930	-0.22705996	subgrp_cor	count		1	0
KRT18	69	OK	0.05721064	0.35196391	0.1625469	0.87088	0.07400988	subgrp_cor	count		1	0
EIF4B	347	OK	-0.28848	0.15079492	-1.9195605	0.05492	-0.40212971	subgrp_cor	count		1	0
AC068888.1	12	OK	0.43198282	0.75308733	0.57361584	0.56622	0.40743025	subgrp_cor	count		1	0
TNS2	70	OK	0.11894869	0.34105849	0.34876318	0.72727	0.14609809	subgrp_cor	count		1	0
SPRYD3	7	OK	0.16815931	1.21985913	0.13785142	0.89036	0.08564646	subgrp_cor	count		1	0
IGFBP6	48	OK	1.24014213	0.438480	2.82827464	0.00468	1.47627944	subgrp_cor	count		1	0
CSAD	10	OK	0.9546585	1.11661587	0.85495695	0.39257	0.29616963	subgrp_cor	count		1	0
RARG	4	OK	0.83595159	1.92553229	0.43414049	0.66419	0.26012312	subgrp_cor	count		1	0
MFSD5	2	OK	-12.145707	43.4188694	-0.2797334	0.77968	-1.91338276	subgrp_cor	count		1	0
PFDN5	790	OK	-0.1591907	0.09316483	-1.7087	0.08752	-0.22722845	subgrp_cor	count		1	0
C12orf10	73	OK	0.08884413	0.32305994	0.27500821	0.78331	0.11494845	subgrp_cor	count		1	0
AAAS	1	OK	3.45981462	6.7714513	0.51094137	0.60936	0.00030179	subgrp_cor	count		1	0
SP1	4	OK	0.16814912	1.72533008	0.0974591	0.92236	0.05201277	subgrp_cor	count		1	0
PRR13	122	OK	-0.2275772	0.25014475	-0.9097819	0.36294	-0.3064720	subgrp_cor	count		1	0
PCBP2	389	OK	-0.2269589	0.13998655	-1.6212904	0.10496	-0.31911463	subgrp_cor	count		1	0
MAP3K12	14	OK	0.09040135	1.04826412	0.0862391	0.93128	0.06792820	subgrp_cor	count		1	0
AC023509.4	1	OK	18.7594894	83.6251792	0.22432824	0.8225	0.00028789	subgrp_cor	count		1	0
TARBP2	2	OK	26.3679503	84.9163259	0.31051685	0.75617	2.49052421	subgrp_cor	count		1	0
ATF7	10	OK	2.09834649	1.43300955	1.46289773	0.1435	1.119790	subgrp_cor	count		1	0
ATP5MC2	680	OK	-0.2747236	0.10135245	-2.7105771	0.00672	-0.39188101	subgrp_cor	count		1	0
CALCOCO1	13	OK	-0.197008	0.67710734	-0.2909551	0.77109	-0.1746824	subgrp_cor	count		1	0
SMUG1	3	OK	-2.1660098	2.70416007	-0.8009917	0.42314	-0.79067868	subgrp_cor	count		1	0
CBX5	41	OK	0.87821055	0.50660626	1.733517	0.083	1.01259956	subgrp_cor	count		1	0
HNRNPA1	521	OK	-0.242142	0.11939103	-2.0281421	0.04255	-0.34243372	subgrp_cor	count		1	0
COPZ1	73	OK	0.65909169	0.37069742	1.77797755	0.07541	0.83183366	subgrp_cor	count		1	0
ITGA5	75	OK	0.80634395	0.36437697	2.21293885	0.0269	0.97131468	subgrp_cor	count		1	0
BLOC1S1	119	OK	0.06102109	0.25832702	0.2362163	0.81326	0.08093132	subgrp_cor	count		1	0
CD63	755	OK	-0.0062681	0.09510279	-0.0659082	0.94745	-0.00894494	subgrp_cor	count		1	0
GDF11	3	OK	2.40963373	2.47152069	0.97495997	0.32958	0.66517343	subgrp_cor	count		1	0
ORMDL2	32	OK	0.41625097	0.54227182	0.76619283	0.44356	0.42696813	subgrp_cor	count		1	0
DNAJC14	2	OK	18.7594894	83.6251792	0.22432824	0.8225	0.00028789	subgrp_cor	count		1	0
PYM1	7	OK	1.10508753	1.2025228	0.91897429	0.35811	0.70729781	subgrp_cor	count		1	0
DGKA	4	OK	-2.9438786	2.40065873	-1.2262795	0.22009	-0.589171	subgrp_cor	count		1	0
RAB5B	3	OK	6.80913659	5.30501163	1.28352905	0.19932	1.696588	subgrp_cor	count		1	0
SUOX	2	OK	-1.2956167	2.041425	-0.6346627	0.52568	-0.00075754	subgrp_cor	count		1	0
IKZF4	1	OK	23.9918963	84.8999419	0.28259026	0.77749	1.65081709	subgrp_cor	count		1	0
RPS26	853	OK	-0.1066327	0.08727576	-1.2217911	0.22179	-0.15248374	subgrp_cor	count		1	0
PA2G4	230	OK	-0.1196503	0.18189268	-0.6578064	0.51068	-0.16535093	subgrp_cor	count		1	0
RPL41	882	OK	-0.1288194	0.08449151	-1.5246433	0.12735	-0.18434197	subgrp_cor	count		1	0
ESYT1	14	OK	1.10867135	0.88260318	1.25613795	0.20907	0.90762216	subgrp_cor	count		1	0
MYL6B	63	OK	1.12270949	0.38284649	2.93253178	0.00336	1.4418277	subgrp_cor	count		1	0
MYL6	839	OK	-0.1597359	0.08865006	-1.8018686	0.07157	-0.22829300	subgrp_cor	count		1	0
SMARCC2	48	OK	-0.14298	0.40004012	-0.3574143	0.72078	-0.17231912	subgrp_cor	count		1	0
RNF41	9	OK	1.69741737	1.22645874	1.38398875	0.16638	0.81947827	subgrp_cor	count		1	0
NABP2	13	OK	1.17816913	0.93451805	1.26072384	0.20741	1.09879332	subgrp_cor	count		1	0
ANKRD52	1	OK	-12.148421	43.4553091	-0.2795612	0.77981	-1.91362624	subgrp_cor	count		1	0
COQ10A	2	OK	3.45981462	6.7714513	0.51094137	0.60936	0.00030179	subgrp_cor	count		1	0
CS	2	OK	-11.186792	26.3137535	-0.425133	0.67074	-2.27103070	subgrp_cor	count		1	0
CNPY2	106	OK	0.90399567	0.30110369	3.00227362	0.00268	1.19779409	subgrp_cor	count		1	0
PAN2	1	OK	-10.442278	41.4658099	-0.2518286	0.80117	-0.00045289	subgrp_cor	count		1	0
STAT2	19	OK	0.72381432	0.62464652	1.15875827	0.24655	0.47026213	subgrp_cor	count		1	0
SPRYD4	1	OK	23.9918963	84.8999419	0.28259026	0.77749	1.65081709	subgrp_cor	count		1	0
RBMS2	20	OK	0.26656135	0.69992328	0.38084368	0.70332	0.25257872	subgrp_cor	count		1	0
BAZ2A	22	OK	1.57979532	0.70551685	2.2392028	0.02514	1.43434103	subgrp_cor	count		1	0
ATP5F1B	215	OK	0.27247283	0.20034699	1.3600046	0.17382	0.36854403	subgrp_cor	count		1	0
PTGES3	376	OK	-0.492785	0.14353499	-3.4332052	0.0006	-0.69021870	subgrp_cor	count		1	0
NACA	866	OK	-0.1946548	0.0862577	-2.2566662	0.02403	-0.27842297	subgrp_cor	count		1	0
PRIM1	5	OK	-0.8009852	1.39742091	-0.5731882	0.56652	-0.38157488	subgrp_cor	count		1	0
HSD17B6	1	OK	3.45981462	6.7714513	0.51094137	0.60936	0.00030179	subgrp_cor	count		1	0
GPR182	2	OK	3.45981497	4.78586864	0.72292309	0.46972	0.00081935	subgrp_cor	count		1	0
NAB2	4	OK	0.27572253	1.33616448	0.20635377	0.83651	0.14079937	subgrp_cor	count		1	0
STAT6	5	OK	-13.335695	29.1122333	-0.4580787	0.6469	-1.33917170	subgrp_cor	count		1	0
LRP1	2	OK	18.7594894	83.6251792	0.22432824	0.8225	0.00028789	subgrp_cor	count		1	0
SHMT2	4	OK	0.51031165	1.60340574	0.31826732	0.75028	0.15911454	subgrp_cor	count		1	0
NDUFA4L2	1	OK	-11.019418	43.2499352	-0.2547846	0.79889	-1.17482461	subgrp_cor	count		1	0
R3HDM2	45	OK	-0.5847904	0.44673716	-1.3314103	0.18305	-0.65695703	subgrp_cor	count		1	0
MARS	2	OK	1.58229999	2.72001883	0.58176068	0.56072	0.47433761	subgrp_cor	count		1	0
DDIT3	52	OK	-0.8226728	0.38509664	-2.1362761	0.03266	-0.96308111	subgrp_cor	count		1	0
MBD6	1	OK	-15.995031	70.349421	-0.2273655	0.82014	-0.00028474	subgrp_cor	count		1	0
DCTN2	96	OK	-0.2776723	0.3041415	-0.9129706	0.36126	-0.3415857	subgrp_cor	count		1	0
OS9	144	OK	0.82697069	0.24380539	3.3919295	0.00069	1.05965831	subgrp_cor	count		1	0
AGAP2-AS1	10	OK	0.41619777	0.98371879	0.42308607	0.67222	0.3476906	subgrp_cor	count		1	0
TSPAN31	17	OK	0.93987813	0.74162022	1.26733079	0.20506	0.83385901	subgrp_cor	count		1	0
CDK4	48	OK	-0.6198063	0.436726	-1.419211	0.15584	-0.75070660	subgrp_cor	count		1	0
9-Mar	3	OK	0.8788176	1.64693117	0.53360919	0.59361	0.44699547	subgrp_cor	count		1	0
METTL1	1	OK	18.7594894	83.6251792	0.22432824	0.8225	0.00028789	subgrp_cor	count		1	0
TSFM	11	OK	-0.2135574	1.15191393	-0.1853936	0.85292	-0.13661164	subgrp_cor	count		1	0
CTDSP2	21	OK	-0.1664946	0.64871663	-0.2566522	0.79745	-0.14780618	subgrp_cor	count		1	0
AC084033.3	7	OK	-0.7174013	1.10153987	-0.6512713	0.51487	-0.20772194	subgrp_cor	count		1	0
ATP23	6	OK	-0.2280882	1.04106915	-0.2190903	0.82658	-0.11406564	subgrp_cor	count		1	0
LRIG3	21	OK	0.09781819	0.61777179	0.15834034	0.87419	0.10999221	subgrp_cor	count		1	0
SLC16A7	12	OK	-2.769985	1.22403251	-2.2629995	0.02364	-1.50439025	subgrp_cor	count		1	0
USP15	41	OK	-0.386005	0.46016704	-0.8388368	0.40156	-0.44862925	subgrp_cor	count		1	0
MON2	6	OK	-2.7205282	1.55727218	-1.7469831	0.08064	-0.97255287	subgrp_cor	count		1	0
RXYLT1	14	OK	1.01255273	0.81078165	1.24885994	0.21172	0.89572418	subgrp_cor	count		1	0
SRGAP1	16	OK	0.61809199	0.96775337	0.6386875	0.52303	0.40212911	subgrp_cor	count		1	0
XPOT	4	OK	0.32948919	1.40555397	0.23441945	0.81466	0.10243567	subgrp_cor	count		1	0
TBK1	12	OK	4.28269917	1.67825186	2.55188108	0.01071	3.96375306	subgrp_cor	count		1	0
RASSF3	8	OK	-0.3009637	1.00293662	-0.3000824	0.76411	-0.2465979	subgrp_cor	count		1	0
GNS	35	OK	-0.6475162	0.51219524	-1.2641979	0.20616	-0.72516845	subgrp_cor	count		1	0
TBC1D30	2	OK	-13.04562	27.674761	-0.4712905	0.63736	-1.3178643	subgrp_cor	count		1	0
LEMD3	3	OK	1.7919026	2.28235398	0.78476776	0.43259	0.00045246	subgrp_cor	count		1	0
MSRB3	75	OK	0.10341189	0.32588682	0.31732454	0.752	0.12499245	subgrp_cor	count		1	0
LLPH	25	OK	-1.4261299	0.62718491	-2.2738588	0.02297	-1.38719703	subgrp_cor	count		1	0
TMBIM4	384	OK	-0.0877062	0.1391962	-0.6300906	0.52864	-0.12365431	subgrp_cor	count		1	0
IRAK3	30	OK	0.06355367	0.50554367	0.12571351	0.89996	0.06278033	subgrp_cor	count		1	0
HELB	2	OK	-0.3495512	1.85447474	-0.1884907	0.85049	-0.17337783	subgrp_cor	count		1	0
CAND1	17	OK	-0.2251938	0.67879279	-0.3317561	0.74007	-0.14396340	subgrp_cor	count		1	0
DYRK2	4	OK	-1.8538345	1.85439186	-0.9996995	0.31748	-0.46053279	subgrp_cor	count		1	0
MDM1	1	OK	3.45981462	6.7714513	0.51094137	0.60936	0.00030179	subgrp_cor	count		1	0
LINC02384	1	OK	-11.019418	43.2499352	-0.2547846	0.79889	-1.17482461	subgrp_cor	count		1	0
RAP1B	279	OK	-0.4200695	0.16491012	-2.5472631	0.01086	-0.58461207	subgrp_cor	count		1	0
NUP107	6	OK	1.12244377	1.45693139	0.77041635	0.44105	0.3460629	subgrp_cor	count		1	0
SLC35E3	7	OK	-0.5311004	0.98097028	-0.5414032	0.58822	-0.33262271	subgrp_cor	count		1	0
MDM2	25	OK	1.83672507	1.08077374	1.69945383	0.08922	0.00344018	subgrp_cor	count		1	0
CPSF6	9	OK	2.12692365	1.32026355	1.61098416	0.10718	0.60610494	subgrp_cor	count		1	0
LYZ	2	OK	0.00115825	1.85956448	0.00062286	0.9995	7.58E-07	subgrp_cor	count		1	0
YEATS4	13	OK	-0.0118971	0.83875663	-0.0141842	0.98868	-0.0112256	subgrp_cor	count		1	0
FRS2	3	OK	19.1030323	50.7724696	0.37624785	0.70672	0.00078880	subgrp_cor	count		1	0
CCT2	152	OK	-0.1265483	0.24208938	-0.522734	0.60116	-0.16989992	subgrp_cor	count		1	0
RAB3IP	1	OK	-11.019418	43.2499352	-0.2547846	0.79889	-1.17482461	subgrp_cor	count		1	0
AC025159.1	1	OK	-10.442278	41.4658099	-0.2518286	0.80117	-0.00045289	subgrp_cor	count		1	0
CNOT2	55	OK	-0.1959342	0.39465526	-0.4964692	0.61958	-0.23145141	subgrp_cor	count		1	0
KCNMB4	17	OK	23.5044346	32.8721166	0.71502651	0.47459	3.23685695	subgrp_cor	count		1	0
PTPRR	4	OK	21.642059	54.4868919	0.39719753	0.69122	1.57744735	subgrp_cor	count		1	0
ZFC3H1	31	OK	1.03893091	0.59094132	1.75809488	0.07873	0.97073451	subgrp_cor	count		1	0

THAP2	5	OK	-0.256601	1.3412326	-0.191317	0.8482	-0.128091	subgrp_con	count		1	0
TMEM19	4	OK	0.5103003	1.603405	0.3182602	0.7502	0.1591132	subgrp_con	count		1	0
RAB21	58	OK	0.2295388	0.3970669	0.5780861	0.5632	0.2662118	subgrp_con	count		1	0
TBC1D15	67	OK	-0.651362	0.3592283	-1.81322	0.0698	-0.8175285	subgrp_con	count		1	0
ATXN7L3B	35	OK	0.7213100	0.5198572	1.387515	0.1652	0.8225594	subgrp_con	count		1	0
GLIPR1	20	OK	5.1002880	1.9856688	2.568549	0.0102	3.2492284	subgrp_con	count		1	0
KRR1	85	OK	-0.133474	0.3052426	-0.437272	0.6619	-0.1683433	subgrp_con	count		1	0
PHLDA1	32	OK	1.368996	0.6395056	2.140709	0.0322	1.478703	subgrp_con	count		1	0
NAP1L1	386	OK	0.2650766	0.1414839	1.873549	0.0609	0.3705139	subgrp_con	count		1	0
OSBPL8	56	OK	0.675585	0.403457	1.674516	0.0940	0.8439738	subgrp_con	count		1	0
ZDHHC17	9	OK	-2.326809	1.1341685	-2.051555	0.0402	-0.5368109	subgrp_con	count		1	0
NAV3	6	OK	-0.371518	1.2493608	-0.297366	0.7661	-0.1111840	subgrp_con	count		1	0
SYT1	2	OK	1.5823999	2.7200188	0.5817606	0.5607	0.4743376	subgrp_con	count		1	0
PAWR	45	OK	1.7304164	0.4947818	3.497332	0.0004	2.1632043	subgrp_con	count		1	0
PPP1R12A	112	OK	-0.056405	0.2671203	-0.211162	0.8327	-0.0735669	subgrp_con	count		1	0
CCDC59	88	OK	-0.586162	0.3040964	-1.927555	0.0539	-0.7251156	subgrp_con	count		1	0
METTL25	9	OK	0.2244599	1.0079581	0.2226877	0.8237	0.1144945	subgrp_con	count		1	0
TMTC2	9	OK	20.355739	32.679991	0.6228808	0.5333	1.5380279	subgrp_con	count		1	0
LRRIQ1	1	OK	3.4598146	6.7714513	0.5109413	0.6092	0.0003017	subgrp_con	count		1	0
NTS	8	OK	-0.358101	0.9368739	-0.382230	0.7022	-0.2270967	subgrp_con	count		1	0
C12orf29	9	OK	0.1681593	0.8627485	0.1949111	0.8454	0.1265752	subgrp_con	count		1	0
CEP290	43	OK	-0.143415	0.4328261	-0.330506	0.7410	-0.1543726	subgrp_con	count		1	0
TMTC3	10	OK	1.5422438	1.2091085	1.275521	0.2021	0.8868878	subgrp_con	count		1	0
KITLG	10	OK	-0.508569	0.8895132	-0.571738	0.5675	-0.1503718	subgrp_con	count		1	0
DUSP6	185	OK	0.3770604	0.2246780	1.678225	0.0932	0.4896041	subgrp_con	count		1	0
AC024909.2	1	OK	3.4598146	6.7714513	0.5109413	0.6092	0.0003017	subgrp_con	count		1	0
POC1B	5	OK	21.105323	83.341273	0.2532397	0.8000	0.0003103	subgrp_con	count		1	0
POC1B-AS1	1	OK	-10.44227	41.465809	-0.251828	0.8011	-0.0004528	subgrp_con	count		1	0
ATP2B1	135	OK	-0.330637	0.2540713	-1.301355	0.1931	-0.4343618	subgrp_con	count		1	0
ATP2B1-AS1	15	OK	0.9859419	0.847798	1.162944	0.2448	0.9238320	subgrp_con	count		1	0
LUM	7	OK	22.777241	32.908149	0.6921459	0.4888	3.2067512	subgrp_con	count		1	0
BTG1	534	OK	0.1137664	0.1151845	0.9874291	0.3234	0.1614392	subgrp_con	count		1	0
AC025164.1	5	OK	0.6851084	1.4216744	0.4819024	0.6298	0.0012628	subgrp_con	count		1	0
EEA1	143	OK	0.1755050	0.2470447	0.7104179	0.4774	0.2314269	subgrp_con	count		1	0
NUDT4	65	OK	2.5085464	0.5732195	4.376239	1.21E-05	2.7123589	subgrp_con	count		0.12084067	0
UBE2N	91	OK	-0.162950	0.3136968	-0.519451	0.6034	-0.2044136	subgrp_con	count		1	0
MRPL42	41	OK	0.5468212	0.5290395	1.0336111	0.3013	0.5408457	subgrp_con	count		1	0
SOCS2	172	OK	0.0609668	0.2039229	0.2989780	0.7649	0.0835584	subgrp_con	count		1	0
TMCC3	12	OK	1.029488	0.950354	1.0832677	0.2786	0.7566322	subgrp_con	count		1	0
NDUFA12	257	OK	0.1652703	0.1743561	0.9478892	0.3431	0.2299099	subgrp_con	count		1	0
NR2C1	6	OK	1.4119426	1.4959308	0.9438555	0.3452	0.6979765	subgrp_con	count		1	0
FGD6	12	OK	-0.272114	0.9528988	-0.28556	0.7752	-0.1733930	subgrp_con	count		1	0
VEZT	33	OK	0.2765218	0.5464293	0.5060523	0.6128	0.2996276	subgrp_con	count		1	0
METAP2	134	OK	-0.35778	0.2454361	-1.45776	0.1449	-0.4763651	subgrp_con	count		1	0
NTN4	12	OK	0.0304173	0.8092205	0.0375884	0.9700	0.0228179	subgrp_con	count		1	0
SNRPF	166	OK	-0.271985	0.2224682	-1.222579	0.2214	-0.3678677	subgrp_con	count		1	0
LTA4H	21	OK	-1.029964	0.7114406	-1.447688	0.1477	-0.8631836	subgrp_con	count		1	0
ELK3	246	OK	-0.704646	0.1850623	-3.807618	0.0001	-0.9603456	subgrp_con	count		1	0
CDK17	22	OK	0.9221404	0.7364983	1.2520604	0.2105	0.8162967	subgrp_con	count		1	0
NEDD1	2	OK	-10.44227	41.465809	-0.251828	0.8011	-0.0004528	subgrp_con	count		1	0
TMPO	26	OK	-0.580134	0.6057350	-0.957736	0.3382	-0.5043537	subgrp_con	count		1	0
SLC25A3	539	OK	-0.159691	0.1158075	-1.378937	0.1679	-0.2263988	subgrp_con	count		1	0
IKBIP	114	OK	-0.381168	0.270982	-1.406619	0.1595	-0.5097727	subgrp_con	count		1	0
APAF1	5	OK	0.6351719	1.2743206	0.4984399	0.6181	0.4194298	subgrp_con	count		1	0
UHRF1BP1L	6	OK	1.726175	1.3776028	1.2530285	0.2102	0.5113905	subgrp_con	count		1	0
ACTR6	21	OK	1.0549948	0.671741	1.5705229	0.1162	0.9818042	subgrp_con	count		1	0
DEPDC4	1	OK	-15.99503	70.34942	-0.227365	0.8201	-0.0002847	subgrp_con	count		1	0
SCYL2	7	OK	2.7684922	1.4524499	1.9060844	0.0566	2.3456583	subgrp_con	count		1	0
UTP20	3	OK	-0.545320	1.708550	-0.319171	0.7496	-0.0003423	subgrp_con	count		1	0
ARL1.00	83	OK	0.9658815	0.3390585	2.8487162	0.0043	1.2438832	subgrp_con	count		1	0
CHPT1	10	OK	-1.178201	0.8954070	-1.315827	0.1882	-0.5370962	subgrp_con	count		1	0
GNPTAB	11	OK	1.2445084	1.1895369	1.0462125	0.2954	1.0217946	subgrp_con	count		1	0
DRAM1	13	OK	1.8300619	1.1327976	1.6155241	0.1062	1.2832119	subgrp_con	count		1	0
WASHC3	53	OK	0.9773013	0.4587512	2.1303513	0.0331	1.0698708	subgrp_con	count		1	0
NUP37	6	OK	1.6688807	1.5674495	1.0647110	0.2870	0.8306521	subgrp_con	count		1	0
PARPBP	1	OK	-10.44227	41.465809	-0.251828	0.8011	-0.0004528	subgrp_con	count		1	0
STAB2	1	OK	3.4598146	6.7714513	0.5109413	0.6092	0.0003017	subgrp_con	count		1	0
HSP90B1	651	OK	0.2788246	0.1053262	2.6472464	0.0081	0.3971516	subgrp_con	count		1	0
C12orf73	4	OK	-13.45038	28.110956	-0.478474	0.6323	-2.4943965	subgrp_con	count		1	0
TDG	22	OK	1.1969246	0.8804912	1.3593821	0.1740	0.8881528	subgrp_con	count		1	0
GLT8D2	18	OK	2.9227600	1.1731574	2.4913621	0.0127	1.794248	subgrp_con	count		1	0
HCFC2	5	OK	1.6556946	1.8916818	0.8752500	0.3814	0.0011289	subgrp_con	count		1	0
NFYB	19	OK	0.1188785	0.6466207	0.1838459	0.8541	0.0770885	subgrp_con	count		1	0
TXNRD1	42	OK	1.4960328	0.5156556	2.9012244	0.0037	1.6626048	subgrp_con	count		1	0
EID3	1	OK	-10.44227	41.465809	-0.251828	0.8011	-0.0004528	subgrp_con	count		1	0
CHST11	2	OK	-1.295616	2.041425	-0.634662	0.5256	-0.0007575	subgrp_con	count		1	0
SLC41A2	1	OK	1.5823999	2.7200188	0.5817606	0.5607	0.4743376	subgrp_con	count		1	0
C12orf45	15	OK	-0.670176	0.9144999	-0.732833	0.4636	-0.5789656	subgrp_con	count		1	0
WASHC4	17	OK	3.0553030	1.2970923	2.355501	0.0185	1.6216500	subgrp_con	count		1	0
APPL2	2	OK	-0.523245	2.0662252	-0.253237	0.8000	-0.2460284	subgrp_con	count		1	0
C12orf75	6	OK	-0.675640	1.0414804	-0.648730	0.5165	-0.1964965	subgrp_con	count		1	0
NUAK1	107	OK	0.2824695	0.2806661	1.0064254	0.3142	0.3515419	subgrp_con	count		1	0
CKAP4	15	OK	3.9865481	1.9841182	2.0092290	0.0445	1.8048430	subgrp_con	count		1	0
TCP11L2	14	OK	-1.648727	0.8511383	-1.937084	0.0527	-1.5684293	subgrp_con	count		1	0
RIC8B	3	OK	-11.01941	43.249935	-0.254784	0.7988	-1.1748246	subgrp_con	count		1	0
AC007541.1	1	OK	-10.44227	41.465809	-0.251828	0.8011	-0.0004528	subgrp_con	count		1	0
TMEM263	18	OK	1.2186390	0.8497307	1.4341472	0.1515	0.8971542	subgrp_con	count		1	0
MTERF2	3	OK	-1.853834	1.8543918	-0.999699	0.3174	-0.4605327	subgrp_con	count		1	0
CRY1	1	OK	1.5823999	2.7200188	0.5817606	0.5607	0.4743376	subgrp_con	count		1	0
PWP1	32	OK	0.349865	0.5140208	0.6806449	0.4961	0.3589311	subgrp_con	count		1	0
PRDM4	4	OK	4.6613026	4.0890786	1.139929	0.2543	0.0027372	subgrp_con	count		1	0
CMKLR1	4	OK	-0.841640	1.4608231	-0.576141	0.5645	-0.2403485	subgrp_con	count		1	0
FICD	3	OK	-0.725185	1.8079191	-0.401116	0.6883	-0.2097991	subgrp_con	count		1	0
SART3	5	OK	-2.834116	2.0003677	-1.416797	0.1565	-0.9566137	subgrp_con	count		1	0
ISCU	289	OK	0.2023593	0.164602	1.2293843	0.2189	0.2836212	subgrp_con	count		1	0
CORO1C	17	OK	-2.105182	0.9932500	-2.119489	0.0340	-1.5563334	subgrp_con	count		1	0
SSH1	10	OK	-1.942547	1.2971622	-1.497536	0.1342	-0.4760054	subgrp_con	count		1	0
ALKBH2	13	OK	-0.325349	0.7541428	-0.431283	0.6662	-0.2402391	subgrp_con	count		1	0
UNG	4	OK	-0.495425	1.5039132	-0.329424	0.7418	-0.0003123	subgrp_con	count		1	0
ACACB	7	OK	-3.24158	1.7269142	-1.877096	0.0605	-0.6484415	subgrp_con	count		1	0
KCTD10	12	OK	1.2683317	1.1103820	1.1422480	0.2533	0.8124737	subgrp_con	count		1	0
UBE3B	2	OK	0.1681491	1.7253300	0.097459	0.9223	0.0520127	subgrp_con	count		1	0
MMAB	13	OK	0.720155	0.9194984	0.7832047	0.4335	0.5414033	subgrp_con	count		1	0
GLTP	62	OK	0.79852	0.4544860	1.7570065	0.0789	0.9089082	subgrp_con	count		1	0
TCHP	7	OK	0.4886593	1.2435071	0.3929687	0.6943	0.3785615	subgrp_con	count		1	0
GIT2	2	OK	18.759488	83.625179	0.2243282	0.8225	0.0002878	subgrp_con	count		1	0
ANKRD13A	3	OK	0.9396704	1.4985405	0.627057	0.5306	0.2916478	subgrp_con	count		1	0
C12orf76	14	OK	-0.084215	0.8102006	-0.103944	0.9172	-0.0829090	subgrp_con	count		1	0
IFT81	3	OK	-14.15184	27.792762	-0.509191	0.6106	-0.0017462	subgrp_con	count		1	0
ATP2A2	16	OK	0.4519307	0.8572787	0.5271689	0.5980	0.3000550	subgrp_con	count		1	0
ANAPC7	2	OK	1.925966	2.5154879	0.7656425	0.4438	0.5602211	subgrp_con	count		1	0
ARPC3	544	OK	-0.012565	0.116228	-0.108113	0.9139	-0.0178565	subgrp_con	count		1	0
GPN3	22	OK	0.0764842	0.6720861	0.113801	0.9094	0.0723321	subgrp_con	count		1	0
FAM216A	4	OK	1.1006820	1.5687290	0.7016394	0.4829	0.5547195	subgrp_con	count		1	0
VPS29	183	OK	0.6410822	0.2184501	2.9346843	0.0033	0.8717703	subgrp_con	count		1	0
PPTC7	1	OK	18.759488	83.625179	0.2243282	0.8225	0.0002878	subgrp_con	count		1	0
TCTN1	5	OK	1.1367732	1.8168873	0.6256707	0.5315	0.3502482	subgrp_con	count		1	0
HVCN1	2	OK	-1.295616	2.041425	-0.634662	0.5256	-0.0007575	subgrp_con	count		1	0
PPP1CC	69	OK	-0.283228	0.3556678	-0.796329	0.4258	-0.3161646	subgrp_con	count		1	0
PHETA1	1	OK	18.759488	83.625179	0.2243282	0.8225	0.0002878	subgrp_con	count		1	0
SH2B3	12	OK	1.0044184	1.3570834	0.740130	0.4592	0.3107329	subgrp_con	count		1	0
ATXN2	31	OK	0.4523796	0.5697257	0.7940305	0.4271	0.4058851	subgrp_con	count		1	0
BRAP	5	OK	-13.0456	27.67476	-0.471390	0.6373	-1.317864	subgrp_con	count		1	0
ALDH2	138	OK	1.1172850	0.2637384	4.2367169	2.27E-05	1.4132330	subgrp_con	count		0.22656263	0
MAPKAPK5-AS1	18	OK	0.2564500	0.7102560	0.3610670	0.7180	0.2707965	subgrp_con	count		1	0
MAPKAPK5	3	OK	-1.295616	2.041425	-0.634662	0.5256	-0.0007575	subgrp_con	count		1	0
ERP29	301	OK	-0.225161	0.1598102	-1.408928	0.1588	-0.3154533	subgrp_con	count		1	0
NAA25	2	OK	-11.01941	43.249935	-0.254784	0.7988	-1.1748246	subgrp_con	count		1	0
TRAFD1	2	OK	0.1681690	1.7253287	0.0974707	0.9223	0.0520187	subgrp_con	count		1	0
HECTD4	1	OK	18.759488	83.625179	0.2243282	0.8225	0.0002878	subgrp_con	count		1	0
RPL6	880	OK	-0.139596	0.0846889	-1.648340	0.0992	-0.1997259	subgrp_con	count		1	0
PTPN11	42	OK	0.2214058	0.517759	0.4276230	0.6689	0.2095764	subgrp_con	count		1	0
OAS1	33	OK	-0.964221	0.4902629	-1.966743	0.0492	-1.0631664	subgrp_con	count		1	0
OAS3	5	OK	0.5724548	1.5699850	0.3646243	0.7152	0.1785511	subgrp_con	count		1	0
OAS2	8	OK	-0.63193	1.0529245	-0.600175	0.5482	-0.1846158	subgrp_con	count		1	0
DDX54	6	OK	0.1084054	1.0696446	0.1013471	0.9192	0.0334504	subgrp_con	count		1	0
RITA1	3	OK	-1.295616	2.041425	-0.634662	0.5256	-0.0007575	subgrp_con	count		1	0
TPCN1	1	OK	-10.44227	41.465809	-0.251828	0.8011	-0.0004528	subgrp_con	count		1	0
SLC8B1	4	OK	0.1721614	1.534450	0.1121974	0.9106	0.0001139	subgrp_con	count		1	0
PLBD2	3	OK	0.1681491	1.7253300	0.097459	0.9223	0.0520127	subgrp_con	count		1	0
RBM19	1	OK	3.4598146	6.7714513	0.5109413	0.6092	0.0003017	subgrp_con	count		1	0
TBX3	10	OK	1.7944002	1.5678850	1.1444718	0.2524	0.0023448	subgrp_con	count		1	0
MED13L	28	OK	0.0216654	0.5889376	0.0367873	0.9706	0.0162737	subgrp_con	count		1	0
C12orf49	7	OK	0.2068195	1.7742016	0.116570	0.9072	0.0645036	subgrp_con	count		1	0
FBXO21	34	OK	0.5711251	0.4918897	1.161083	0.2456	0.6825169	subgrp_con	count		1	0
WSB2	11	OK	1.701946	1.2082035	1.408658	0.1589	0.8213215	subgrp_con	count		1	0
VSIG10	3	OK	1.2533065	1.5409862	0.8133145	0.4160	0.6262466	subgrp_con	count		1	0
PEBP1	424	OK	0.3544712	0.1314518	2.6965855	0.0070	0.5016096	subgrp_con	count		1	0
TAOK3	24	OK	0.7892026	0.725352	1.0880262	0.2765	0.7036429	subgrp_con	count		1	0
SUDS3	14	OK	0.26779	0.7712433	0.3472276	0.7284	0.2400410	subgrp_con	count		1	0
HSPB8	8	OK	-1.339811	1.09202	-1.226906	0.2198	-1.0059829	subgrp_con	count		1	0
TMEM233	8	OK	1.3761794	1.0257653	1.341612	0.1797	1.1937272	subgrp_con	count		1	0
RAB35	4	OK	-2.023738	1.5852411	-1.276612	0.2017	-0.4896968	subgrp_con	count		1	0
GCN1	1	OK	18.759488	83.625179	0.2243282	0.8225	0.0002878	subgrp_con	count		1	0
RPLP0	850	OK	-0.196137	0.0876794	-2.236987	0.0252	-0.2804759	subgrp_con	count		1	0
PXN	17	OK	-1.231280	0.772871	-1.59312	0.1111	-1.0619715	subgrp_con	count		1	0
COX6A1	483	OK	0.0730198	0.1238154	0.5897474	0.5553	0.1035290	subgrp_con	count		1	0
TRIAP1	25	OK	-0.719888	0.6839723	-1.052508	0.2925	-0.7575418	subgrp_con	count		1	0
GATC	1	OK	23.991896	84.899941	0.2825902	0.7774	1.6508170	subgrp_con	count		1	0
SRSF9	265	OK	-0.140183	0.1727157	-0.811641	0.417	-0.1953429	subgrp_con	count		1	0
DYNLL1	682	OK	0.2015358	0.1016051	1.9835202	0.0473	0.2870604	subgrp_con	count		1	0
COQ5	3	OK	5.2612789	5.4905820	0.9582370	0.3379	0.0029503	subgrp_con	count		1	0
RNF10	41	OK	2.4809851	0.6923974	3.5831805	0.0003	2.2524385	subgrp_con	count		1	0
POP5	21	OK	-0.487301	0.7127753	-0.683667	0.4941	-0.4719220	subgrp_con	count		1	0
MLEC	79	OK	1.0578922	0.4106382	2.576214	0.0099	1.1958020	subgrp_con	count		1	0
ACADS	3	OK	0.111276	1.6120387	0.0690285	0.9449	0.0565734	subgrp_con	count		1	0
SPPL3	12	OK	2.1881468	1.3037829	1.6783061	0.0932	0.6194797	subgrp_con	count		1	0
C12orf43	12	OK	-0.568577	0.8491618	-0.669575	0.5031	-0.4585267	subgrp_con	count		1	0
OASL	5	OK	-1.610874	1.3660267	-1.179240	0.2382	-0.4151120	subgrp_con	count		1	0
P2RX4	5	OK	1.4985876	1.6181423	0.9261159	0.3543	0.0010314	subgrp_con	count		1	0
CAMKK2	8	OK	-0.256586	0.9478485	-0.270703	0.7866	-0.1902313	subgrp_con	count		1	0
ANAPC5	68	OK	0.3352385	0.353079	0.9494699	0.3423	0.3980397	subgrp_con	count		1	0
RNF34	3	OK	21.105323	83.341273	0.2532397	0.8000	0.0003103	subgrp_con	count		1	0
ORAI1	66	OK	-0.547265	0.3741192	-1.462811	0.1435	-0.6690962	subgrp_con	count		1	0
SETD1B	1	OK	-12.14842	43.455309	-0.279561	0.7798	-1.9136262	subgrp_con	count		1	0
PSMD9	3	OK	21.64205	54.486891	0.3971975	0.6912	1.5774473	subgrp_con	count		1	0
MLXIP	14	OK	0.380022	0.8855581	0.4291334	0.6678	0.1182772	subgrp_con	count		1	0
VPS33A	1	OK	-10.44227	41.465809	-0.251828	0.8011	-0.0004528	subgrp_con	count		1	0
CLIP1	47	OK	0.6127200	0.4616530	1.3272305	0.1844	0.6620658	subgrp_con	count		1	0
ZCCHC8	6	OK	0.4160004	1.217848	0.3415864	0.7326	0.2709653	subgrp_con	count		1	0
RSRC2	206	OK	0.1335877	0.1969047	0.678428	0.4974	0.1806572	subgrp_con	count		1	0
DENR	37	OK	-0.212672	0.5283204	-0.402544	0.6872	-0.2378429	subgrp_con	count		1	0
HIP1R	10	OK	1.6019796	1.3172991	1.2161092	0.2239	0.0029941	subgrp_con	count		1	0
VPS37B	11	OK	-0.917376	1.0146163	-0.904160	0.3659	-0.5602357	subgrp_con	count		1	0
ARL6IP4	394	OK	0.1016854	0.1384299	0.734509	0.4626	0.1433831	subgrp_con	count		1	0
C12orf65	25	OK	-0.795499	0.6047401	-1.315438	0.1883	-0.8517496	subgrp_con	count		1	0
CDK2AP1	2	OK	1.5823776	1.9240213	0.822432	0.4108	0.7717455	subgrp_con	count		1	0
SBNO1	13	OK	-0.318015	0.9314068	-0.341436	0.7327	-0.1580923	subgrp_con	count		1	0
AC137767.1	4	OK	-1.722571	1.4606253	-1.179338	0.2382	-0.0009622	subgrp_con	count		1	0
KMT5A	39	OK	0.8801319	0.5197441	1.6933947	0.0903	0.9214587	subgrp_con	count		1	0
RILPL2	138	OK	0.5642193	0.2505288	2.2521135	0.0243	0.7546753	subgrp_con	count		1	0
SNRNP35	16	OK	-1.864644	0.8982095	-2.075957	0.0379	-1.5202297	subgrp_con	count		1	0
RILPL1	14	OK	-0.318894	0.907512	-0.35139	0.7252	-0.0958434	subgrp_con	count		1	0
TMED2	99	OK	0.7126515	0.2930429	2.4319015	0.0150	0.9143813	subgrp_con	count		1	0
DDX55	5	OK	-0.44492	1.2070041	-0.368616	0.7124	-0.1323161	subgrp_con	count		1	0
EIF2B1	19	OK	-0.778222	0.6785490	-1.146892	0.2514	-0.6165533	subgrp_con	count		1	0
GTF2H3	7	OK	-0.910223	2.1881125	-0.415985	0.6774	-0.2578573	subgrp_con	count		1	0
ATP6V0A2	3	OK	-0.910223	2.1881125	-0.415985	0.6774	-0.2578573	subgrp_con	count		1	0
CCDC92	7	OK	-0.209243	1.136738	-0.184073	0.8539	-0.0634047	subgrp_con	count		1	0
ZNF664	1	OK	23.991896	84.899941	0.2825902	0.7774	1.6508170	subgrp_con	count		1	0
RFLNA	1	OK	-10.44227	41.465809	-0.251828	0.8011	-0.0004528	subgrp_con	count		1	0
NCOR2	13	OK	1.2252219	0.8601234	1.4244721	0.1543	0.6071731	subgrp_con	count		1	0
SCARB1	16	OK	-4.106042	1.6127425	-2.546	0.0109	-3.3116193	subgrp_con	count		1	0
UBC	825	OK	-0.245102	0.0889225	-2.756353	0.0058	-0.3503226	subgrp_con	count		1	0
BRI3BP	2	OK	-11.44854	41.76148	-0.274141	0.7839	-0.0004924	subgrp_con	count		1	0
STX2	22	OK	1.5864779	0.8600601	1.8446126	0.0650	1.1343890	subgrp_con	count		1	0
RAN	411	OK	-0.060749	0.1357253	-0.447556	0.6544	-0.0857223	subgrp_con	count		1	0
SFSWAP	20	OK	0.957782	0.7639322	1.2537526	0.2099	0.6258605	subgrp_con	count		1	0
ULK1	2	OK	-1.295616	2.041425	-0.634662	0.5256	-0.0007575	subgrp_con	count		1	0
PUS1	1	OK	-10.44227	41.465809	-0.251828	0.8011	-0.0004528	subgrp_con	count		1	0
EP400	1	OK	-10.44227	41.465809	-0.251828	0.8011	-0.0004528	subgrp_con	count		1	0
DDX51	1	OK	-10.44227	41.465809	-0.251828	0.8011	-0.0004528	subgrp_con	count		1	0
NOC4L	2	OK	1.5823999	2.7200188	0.5817606	0.5607	0.4743376	subgrp_con	count		1	0
FBRSL1	2	OK	-10.44227	41.465809	-0.251828	0.8011	-0.0004528	subgrp_con	count		1	0
PXMP2	10	OK	3.8992628	3.1583425	1.2345914	0.2169	0.0024342	subgrp_con	count		1	0
ANKLE2	27	OK	-0.007075	0.5488597	-0.012892	0.9897	-0.0063123	subgrp_con	count		1	0
GOLGA3	13	OK	1.2527756	0.929412	1.3479226	0.1776	0.6260171	subgrp_con	count		1	0
ZNF26	1	OK	-10.44227	41.465809	-0.251828	0.8011	-0.0004528	subgrp_con	count		1	0
ZNF84	6	OK	-0.190235	1.2247426	-0.155327	0.8765	-0.0577211	subgrp_con	count		1	0
ZNF891	2	OK	-10.44227	41.465809	-0.251828	0.8011	-0.0004528	subgrp_con	count		1	0
ZNF268	2	OK	-1.295616	2.041425	-0.634662	0.5256	-0.0007575	subgrp_con	count		1	0
MPHOSPH8	242	OK	-0.466635	0.1750704	-2.665417	0.0076	-0.6515985	subgrp_con	count		1	0
PSPC1	5	OK	-0.44492	1.2070041	-0.368616	0.7124	-0.1323161	subgrp_con	count		1	0
ZMYM5	10	OK	1.6031882	1.0273943	1.5604410	0.1186	1.1477257	subgrp_con	count		1	0
AL355001.2	2	OK	-0.523245	2.0662252	-0.253237	0.8000	-0.2460284	subgrp_con	count		1	0
ZMYM2	10	OK	1.5541975	1.0254720	1.5155822	0.1296	1.3456652	subgrp_con	count		1	0
GJB2	1	OK	-10.44227	41.465809	-0.251828	0.8011	-0.0004528	subgrp_con	count		1	0
CRYL1	39	OK	0.4708932	0.4631176	1.0167898	0.3092	0.5587450	subgrp_con	count		1	0
IFT88	15	OK	-1.082394	0.7881768	-1.373289	0.1696	-0.9020450	subgrp_con	count		1	0
AL161772.1	1	OK	1.5823999	2.7200188	0.5817606	0.5607	0.4743376	subgrp_con	count		1	0
EEF1AKMT1	5	OK	-1.642345	1.7455082	-0.940897	0.3467	-0.8720342	subgrp_con	count		1	0
LATS2	10	OK	0.7302666	1.0005401	0.7298724	0.4654	0.3729829	subgrp_con	count		1	0
SAP18	452	OK	-0.093599	0.1302351	-0.718692	0.4723	-0.1320520	subgrp_con	count		1	0
MRPL57	182	OK	0.5194928	0.2193285	2.3685599	0.0178	0.7134358	subgrp_con	count		1	0
ZDHHC20	13	OK	-0.871869	1.054167	-0.827068	0.4082	-0.5247879	subgrp_con	count		1	0
MICU2	16	OK	0.6337968	0.8052766	0.7870547	0.4312	0.1976826	subgrp_con	count		1	0
SGCG	2	OK	23.991896	84.899941	0.2825902	0.7774	1.6508170	subgrp_con	count		1	0
SACS	3	OK	18.759488	83.625179	0.2243282	0.8225	0.0002878	subgrp_con	count		1	0
MIPEP	1	OK	3.4598146	6.7714513	0.5109413	0.6092	0.0003017	subgrp_con	count		1	0
SPATA13	3	OK	-0.615417	1.4920971	-0.412451	0.6800	-0.2986101	subgrp_con	count		1	0
C1QTNF9	5	OK	-10.48270	16.745283	-0.626008	0.5313	-2.5376455	subgrp_con	count		1	0
PARP4	10	OK	-0.792104	0.8780192	-0.902149	0.3669	-0.3753363	subgrp_con	count		1	0
CENPJ	2	OK	-12.14570	43.418869	-0.279733	0.7796	-1.9133827	subgrp_con	count		1	0
MTMR6	8	OK	4.2109206	4.2875616	0.982124	0.3260	0.0025709	subgrp_con	count		1	0
NUP58	5	OK	-2.342222	1.7367717	-1.348606	0.1774	-0.5390589	subgrp_con	count		1	0
RNF6	9	OK	-0.235333	1.0412448	-0.226012	0.8211	-0.1176352	subgrp_con	count		1	0
AL138966.2	1	OK	-10.44227	41.465809	-0.251828	0.8011	-0.0004528	subgrp_con	count		1	0
WASF3	5	OK	-12.80545	17.467058	-0.733120	0.4634	-1.3003720	subgrp_con	count		1	0
USP12	11	OK	1.7014875	1.2813926	1.3278424	0.1842	0.0031795	subgrp_con	count		1	0
RPL21	864	OK	-0.100988	0.0856245	-1.179433	0.2382	-0.1444624	subgrp_con	count		1	0
RASL11A	4	OK	0.4209530	1.4909211	0.2823442	0.7776	0.2153794	subgrp_con	count		1	0
GTF3A	127	OK	-0.081006	0.2532136	-0.319912	0.7490	-0.1071388	subgrp_con	count		1	0
MTIF3	143	OK	-0.814710	0.2442179	-3.335996	0.0008	-1.0984264	subgrp_con	count		1	0
LNX2	3	OK	23.991896	84.899941	0.2825902	0.7774	1.6508170	subgrp_con	count		1	0
POLR1D	159	OK	-0.26226	0.2338261	-1.121588	0.2620	-0.3483305	subgrp_con	count		1	0
PAN3	4	OK	-0.214966	1.5648178	-0.137374	0.8907	-0.0651127	subgrp_con	count		1	0
FLT1	206	OK	-0.16179	0.1921961	-0.841801	0.3999	-0.2215414	subgrp_con	count		1	0
POMP	400	OK	0.2561401	0.1413670	1.8118803	0.07	0.3606021	subgrp_con	count		1	0
SLC7A1	2	OK	18.759488	83.625179	0.2243282	0.8225	0.0002878	subgrp_con	count		1	0
UBL3	27	OK	1.7886688	0.6551053	2.7306584	0.0063	1.7394898	subgrp_con	count		1	0
HMGB1	799	OK	-0.107671	0.0909514	-1.18383	0.2364	-0.1537516	subgrp_con	count		1	0
MEDAG	33	OK	3.3935863	0.817333	4.1520221	3.3E-05	3.6042154	subgrp_con	count		0.32846286	0
HSPH1	71	OK	0.4511821	0.3863165	1.1679078	0.2428	0.5077204	subgrp_con	count		1	0
FRY	31	OK	-1.604867	0.5864247	-2.736697	0.0062	-1.5777652	subgrp_con	count		1	0
BRCA2	2	OK	-15.99503	70.34942	-0.227365	0.8201	-0.0002847	subgrp_con	count		1	0
N4BP2L1	10	OK	-1.97926	1.054068	-1.877774	0.0604	-1.4877944	subgrp_con	count		1	0
N4BP2L2	284	OK	0.0097671	0.1597740	0.06113	0.9512	0.0135885	subgrp_con	count		1	0
PDS5B	18	OK	-0.714524	0.6629855	-1.077737	0.2811	-0.4407357	subgrp_con	count		1	0
KL	1	OK	-11.45102	41.79736	-0.273965	0.7841	-0.0004918	subgrp_con	count		1	0
STARD13	2	OK	1.1765791	2.346505	0.5014175	0.6160	0.0008087	subgrp_con	count		1	0
RFC3	3	OK	-0.459144	2.2050407	-0.208224	0.8350	-0.1332891	subgrp_con	count		1	0
DCLK1	1	OK	3.4598146	6.7714513	0.5109413	0.6092	0.0003017	subgrp_con	count		1	0
SPART	45	OK	1.2238068	0.485839	2.5184393	0.0117	1.2990931	subgrp_con	count		1	0
RFXAP	3	OK	-2.905766	2.0547455	-1.414173	0.1573	-0.0005300	subgrp_con	count		1	0
SMAD9	25	OK	0.4024523	0.5787039	0.6954373	0.4867	0.3608794	subgrp_con	count		1	0
ALG5	43	OK	-0.526680	0.5067944	-1.039239	0.2986	-0.4873041	subgrp_con	count		1	0
EXOSC8	44	OK	-0.212181	0.4278001	-0.495982	0.6199	-0.2372968	subgrp_con	count		1	0
SUPT20H	7	OK	-0.365666	0.9535164	-0.383492	0.7013	-0.1811593	subgrp_con	count		1	0
TRPC4	1	OK	-10.44227	41.465809	-0.251828	0.8011	-0.0004528	subgrp_con	count		1	0
UFM1	80	OK	-0.167830	0.3221468	-0.520975	0.6023	-0.2125761	subgrp_con	count		1	0
NHLRC3	7	OK	-0.979666	1.133505	-0.864280	0.3874	-0.6706788	subgrp_con	count		1	0
LHFPL6	125	OK	-0.512311	0.2506488	-2.043942	0.0409	-0.6870166	subgrp_con	count		1	0
COG6	5	OK	1.1581985	1.6544505	0.7000502	0.4839	0.0007955	subgrp_con	count		1	0
FOXO1	51	OK	-0.569853	0.4000481	-1.424461	0.1543	-0.6500605	subgrp_con	count		1	0
MRPS31	38	OK	-0.636597	0.4663946	-1.364933	0.1722	-0.7137763	subgrp_con	count		1	0
ELF1	218	OK	-0.052754	0.1900873	-0.277527	0.7813	-0.0723749	subgrp_con	count		1	0
WBP4	66	OK	-0.062879	0.3572918	-0.175995	0.8602	-0.0793610	subgrp_con	count		1	0
KBTBD6	1	OK	-10.44227	41.465809	-0.251828	0.8011	-0.0004528	subgrp_con	count		1	0
KBTBD7	2	OK	0.6071996	2.0068355	0.3025657	0.7622	0.2053010	subgrp_con	count		1	0
MTRF1	1	OK	23.991896	84.899941	0.2825902	0.7774	1.6508170	subgrp_con	count		1	0
NAA16	4	OK	-3.450147	2.1893454	-1.575880	0.1150	-1.0973991	subgrp_con	count		1	0
RGCC	125	OK	0.1252511	0.2736064	0.4581442	0.6468	0.1611019	subgrp_con	count		1	0
DGKH	7	OK	-1.054093	1.234678	-0.853712	0.3932	-0.4879584	subgrp_con	count		1	0
AKAP11	11	OK	-0.653998	0.9971983	-0.655835	0.5119	-0.4054306	subgrp_con	count		1	0
EPSTI1	25	OK	-0.640832	0.5582524	-1.147925	0.252	-0.6926075	subgrp_con	count		1	0
DNAJC15	206	OK	0.3470248	0.2018465	1.7192505	0.0855	0.4824185	subgrp_con	count		1	0
CCDC122	8	OK	-1.514069	1.0628962	-1.424456	0.1543	-0.8510052	subgrp_con	count		1	0
LACC1	3	OK	3.4598146	6.7714513	0.5109413	0.6092	0.0003017	subgrp_con	count		1	0
TSC22D1	301	OK	0.0170064	0.1620558	0.104942	0.9164	0.0235353	subgrp_con	count		1	0
NUFIP1	23	OK	-0.304676	0.5812297	-0.524192	0.6001	-0.2689308	subgrp_con	count		1	0
GPALPP1	15	OK	-0.792758	0.8238662	-0.962242	0.3359	-0.7209390	subgrp_con	count		1	0
GTF2F2	15	OK	-0.512995	0.6623617	-0.774494	0.4386	-0.4478425	subgrp_con	count		1	0
TPT1	887	OK	-0.086591	0.0833547	-1.038826	0.2988	-0.1239565	subgrp_con	count		1	0
COG3	4	OK	0.878817	1.6469311	0.5336091	0.5936	0.4469954	subgrp_con	count		1	0
ZC3H13	100	OK	-0.215120	0.2819681	-0.762924	0.4455	-0.2649209	subgrp_con	count		1	0
LRCH1	8	OK	-2.991796	1.4456718	-2.069485	0.0385	-1.8749948	subgrp_con	count		1	0
ESD	154	OK	-0.322255	0.2229429	-1.445463	0.1483	-0.4370503	subgrp_con	count		1	0
SUCLA2	11	OK	1.1849334	1.0605217	1.1173117	0.2638	0.7550376	subgrp_con	count		1	0
NUDT15	9	OK	0.0261246	0.8873208	0.0294422	0.9765	0.0246714	subgrp_con	count		1	0
MED4	81	OK	-0.457175	0.3301701	-1.384667	0.1661	-0.5472722	subgrp_con	count		1	0
ITM2B	872	OK	-0.130641	0.0850150	-1.536691	0.1243	-0.1868960	subgrp_con	count		1	0
RB1	30	OK	0.0860240	0.6330447	0.1358893	0.8919	0.0951297	subgrp_con	count		1	0
RCBTB2	11	OK	-0.88126	0.9909480	-0.889318	0.3738	-0.4162068	subgrp_con	count		1	0
AL161421.1	1	OK	1.5823999	2.7200188	0.5817606	0.5607	0.4743376	subgrp_con	count		1	0
FNDC3A	55	OK	0.4207844	0.4036537	1.0424391	0.2972	0.5131057	subgrp_con	count		1	0
CDADC1	4	OK	0.5103116	1.6034057	0.3182673	0.7502	0.1591145	subgrp_con	count		1	0
CAB39L	1	OK	-10.44227	41.465809	-0.251828	0.8011	-0.0004528	subgrp_con	count		1	0

PHF11	25	OK	-0.478300	0.6131681	-0.780047	0.43536	-0.41830188	subgrp_cor	count	1	0
RCBTB1	1	OK	18.759489	83.625179	0.2243282	0.8225	0.00028789	subgrp_cor	count	1	0
ARL11.00	1	OK	3.4598146	6.7714513	0.5109413	0.60939	0.00030179	subgrp_cor	count	1	0
EBPL	147	OK	-0.853165	0.2381538	-3.582414	0.00034	-1.1427285	subgrp_cor	count	1	0
KPNA3	31	OK	-1.127531	0.5474041	-2.059779	0.03942	-1.23158254	subgrp_cor	count	1	0
SPRYD7	14	OK	-0.810393	0.7738954	-1.047161	0.29502	-0.49514237	subgrp_cor	count	1	0
DLEU2	5	OK	0.8120212	1.3820622	0.587543	0.55686	0.4138407	subgrp_cor	count	1	0
TRIM13	28	OK	1.1702964	0.6820040	1.7158669	0.08617	0.95455545	subgrp_cor	count	1	0
RNASEH2B	5	OK	-0.455839	1.2662125	-0.360081	0.71879	-0.22431057	subgrp_cor	count	1	0
INTS6	99	OK	-0.640290	0.2811589	-2.277324	0.02277	-0.69208489	subgrp_cor	count	1	0
WDFY2	4	OK	1.53358	2.2470324	0.6824968	0.49492	0.00105583	subgrp_cor	count	1	0
DHRS12	2	OK	-0.202940	1.6532236	-0.122753	0.9022	-4.82E-05	subgrp_cor	count	1	0
AL162377.1	1	OK	-15.99503	70.34942	-0.227365	0.82014	-0.00028474	subgrp_cor	count	1	0
ALG11	1	OK	-10.44227	41.465809	-0.251828	0.80117	-0.00045289	subgrp_cor	count	1	0
NEK3	27	OK	-0.79692	0.5397945	-1.47635	0.13985	-0.83543447	subgrp_cor	count	1	0
THSD1	9	OK	0.6156961	0.9776126	0.6297957	0.52882	0.19204548	subgrp_cor	count	1	0
VPS36	55	OK	0.6885514	0.4271893	1.6118180	0.107	0.82341847	subgrp_cor	count	1	0
CKAP2	6	OK	2.6370529	1.687012	1.563149	0.11802	1.44989409	subgrp_cor	count	1	0
SUGT1	151	OK	0.0292468	0.2280988	0.1282201	0.89797	0.03934518	subgrp_cor	count	1	0
PCDH17	89	OK	1.5828529	0.364170	4.346458	1.38E-05	2.00310227	subgrp_cor	count	0.1382808	0
TDRD3	5	OK	-1.000177	1.1953148	-0.836748	0.40272	-0.28026428	subgrp_cor	count	1	0
PCDH9	9	OK	27.637969	51.108329	0.5407723	0.58866	4.33977476	subgrp_cor	count	1	0
DACH1	2	OK	4.6613299	5.7683860	0.8080821	0.41904	0.00274918	subgrp_cor	count	1	0
MZT1	8	OK	1.6542239	1.1929544	1.3866614	0.16555	1.3047560	subgrp_cor	count	1	0
DIS3	34	OK	0.7616708	0.5263503	1.4470794	0.14787	0.67931109	subgrp_cor	count	1	0
PIBF1	28	OK	1.2031320	0.7628332	1.5771887	0.11475	0.8030038	subgrp_cor	count	1	0
KLF5	3	OK	18.759489	83.625179	0.2243282	0.8225	0.00028789	subgrp_cor	count	1	0
KLF12	2	OK	-10.58808	25.214284	-0.41992	0.67454	-0.00124279	subgrp_cor	count	1	0
TBC1D4	5	OK	3.4598155	3.9099931	0.8848648	0.37622	0.00081877	subgrp_cor	count	1	0
COMMD6	584	OK	-0.227829	0.1124475	-2.026099	0.04275	-0.22392476	subgrp_cor	count	1	0
UCHL3	25	OK	1.1546207	0.8063989	1.4318232	0.15219	1.07313448	subgrp_cor	count	1	0
LMO7	2	OK	4.6613299	5.7683860	0.8080821	0.41904	0.00274918	subgrp_cor	count	1	0
KCTD12	128	OK	0.0682540	0.2630291	0.2598724	0.79496	0.08870575	subgrp_cor	count	1	0
CLN5	28	OK	1.0094763	0.629658	1.6032132	0.10889	1.08426001	subgrp_cor	count	1	0
FBXL3	15	OK	0.1137405	0.7401311	0.1536762	0.87787	0.07374626	subgrp_cor	count	1	0
MYCBP2	153	OK	0.7111804	0.2265323	3.139421	0.00169	0.95464375	subgrp_cor	count	1	0
EDNRB	22	OK	-1.208004	0.6428499	-1.879139	0.06022	-0.0053181	subgrp_cor	count	1	0
POU4F1	10	OK	4.4829392	2.4862214	1.8031134	0.07137	0.95035104	subgrp_cor	count	1	0
RNF219	13	OK	0.7470097	1.0420737	0.7168492	0.47347	0.23555011	subgrp_cor	count	1	0
RBM26	32	OK	0.2787141	0.520183	0.5358	0.5921	0.27622626	subgrp_cor	count	1	0
NDFIP2	4	OK	1.9259644	1.7780488	1.0831899	0.27872	0.90913919	subgrp_cor	count	1	0
SPRY2	7	OK	-2.303261	1.4487624	-1.589812	0.11188	-1.12488064	subgrp_cor	count	1	0
TGDS	6	OK	0.3938977	1.1961016	0.3293179	0.74192	0.29889558	subgrp_cor	count	1	0
GPR180	3	OK	1.925964	2.5154879	0.7656425	0.44389	0.56022119	subgrp_cor	count	1	0
ABCC4	9	OK	2.3060879	1.5805597	1.459022	0.14456	1.18912257	subgrp_cor	count	1	0
CLDN10	13	OK	3.8313502	1.7418428	2.199595	0.02784	2.29179662	subgrp_cor	count	1	0
DNAJC3-DT	1	OK	-11.01941	43.249935	-0.254784	0.79889	-1.17482461	subgrp_cor	count	1	0
UGGT2	6	OK	2.2272870	1.6062600	1.3866291	0.16555	0.5833736	subgrp_cor	count	1	0
MBNL2	63	OK	-0.624769	0.3515641	-1.777112	0.07555	-0.72065893	subgrp_cor	count	1	0
RAP2A	6	OK	-0.465429	1.3093639	-0.355462	0.72224	-0.13816105	subgrp_cor	count	1	0
IPO5	21	OK	-0.254665	0.6136377	-0.415010	0.67812	-0.16253293	subgrp_cor	count	1	0
FARP1	14	OK	1.8512007	1.0735662	1.7243470	0.08465	1.11771069	subgrp_cor	count	1	0
STK24	15	OK	0.6716985	0.9006001	0.7458344	0.45571	0.43662712	subgrp_cor	count	1	0
DOCK9	46	OK	0.4172471	0.5008375	0.8330988	0.40479	0.44451489	subgrp_cor	count	1	0
UBAC2	17	OK	0.9492740	0.8941259	1.0616781	0.28838	0.48164121	subgrp_cor	count	1	0
TM9SF2	101	OK	1.2251073	0.3120543	3.925941	8.64E-05	1.58938037	subgrp_cor	count	0.8570856	0
ZIC2	1	OK	18.759489	83.625179	0.2243282	0.8225	0.00028789	subgrp_cor	count	1	0
AL355338.1	2	OK	-0.202940	1.6532236	-0.122753	0.9022	-4.82E-05	subgrp_cor	count	1	0
PCCA	4	OK	-2.023032	1.8071267	-1.119474	0.26294	-0.00109400	subgrp_cor	count	1	0
TMTC4	1	OK	3.4598146	6.7714513	0.5109413	0.60939	0.00030179	subgrp_cor	count	1	0
ITGBL1	7	OK	2.2496224	1.4202102	1.5840065	0.11319	1.29940684	subgrp_cor	count	1	0
TPP2	6	OK	1.8928767	1.4767540	1.2817819	0.19992	0.85393147	subgrp_cor	count	1	0
TEX30	1	OK	-11.01941	43.249935	-0.254784	0.79889	-1.17482461	subgrp_cor	count	1	0
ERCC5	1	OK	23.991896	84.899941	0.2825802	0.77749	1.65081709	subgrp_cor	count	1	0
EFNB2	155	OK	0.3809958	0.2308100	1.6506898	0.0988	0.49150257	subgrp_cor	count	1	0
AL138689.2	1	OK	18.759489	83.625179	0.2243282	0.8225	0.00028789	subgrp_cor	count	1	0
ARGLU1	426	OK	-0.218263	0.128265	-1.701654	0.08882	-0.30758348	subgrp_cor	count	1	0
FAM155A	1	OK	-10.44227	41.465809	-0.251828	0.80117	-0.00045289	subgrp_cor	count	1	0
LIG4	3	OK	21.105322	83.341273	0.2532397	0.80009	0.0003103	subgrp_cor	count	1	0
ABHD13	5	OK	0.5103003	1.603405	0.3182603	0.75029	0.15911329	subgrp_cor	count	1	0
IRS2	1	OK	-10.44227	41.465809	-0.251828	0.80117	-0.00045289	subgrp_cor	count	1	0
COL4A1	135	OK	1.0492912	0.3190883	3.288403	0.00101	1.25731180	subgrp_cor	count	1	0
COL4A2	114	OK	1.4835866	0.3519919	4.2148312	2.5E-05	1.85817456	subgrp_cor	count	0.249465	0
NAXD	2	OK	-0.349551	1.8544747	-0.188490	0.85049	-0.17337783	subgrp_cor	count	1	0
CARS2	4	OK	-0.465429	1.3093639	-0.355462	0.72224	-0.13816105	subgrp_cor	count	1	0
ING1	33	OK	-0.886688	0.5344461	-1.659078	0.0971	-0.79993710	subgrp_cor	count	1	0
ANKRD10	54	OK	0.2656115	0.3998055	0.6643518	0.50647	0.32629386	subgrp_cor	count	1	0
ARHGEF7	4	OK	-1.310774	1.638399	-0.800032	0.42369	-0.35262481	subgrp_cor	count	1	0
TUBGCP3	6	OK	0.5524930	1.3480003	0.4098611	0.68191	0.17231298	subgrp_cor	count	1	0
ATP11A	3	OK	-1.646245	1.9351929	-0.850688	0.39494	-0.70360391	subgrp_cor	count	1	0
MCF2L	3	OK	-1.340131	1.6819410	-0.796776	0.42558	-0.58807560	subgrp_cor	count	1	0
PCID2	19	OK	1.0668185	0.8025108	1.3293509	0.18372	0.94146905	subgrp_cor	count	1	0
CUL4A	5	OK	-0.35720	1.5148489	-0.235803	0.81359	-0.22654173	subgrp_cor	count	1	0
LAMP1	159	OK	0.4201901	0.2196809	1.912728	0.05579	0.55749452	subgrp_cor	count	1	0
TMCO3	13	OK	0.0893140	0.7910712	0.1129026	0.91011	0.08445017	subgrp_cor	count	1	0
TFDP1	2	OK	1.5823999	2.7200188	0.5817606	0.56072	0.47433761	subgrp_cor	count	1	0
TMEM255B	67	OK	-0.770846	0.385517	-1.999512	0.04555	-0.96405777	subgrp_cor	count	1	0
RASA3	1	OK	3.4598146	6.7714513	0.5109413	0.60939	0.00030179	subgrp_cor	count	1	0
CDC16	9	OK	0.176282	1.0929171	0.1612953	0.87186	0.08980462	subgrp_cor	count	1	0
UPF3A	117	OK	0.2399559	0.2485074	0.9655884	0.33425	0.32583994	subgrp_cor	count	1	0
CHAMP1	3	OK	-1.853784	1.8543592	-0.99969	0.31746	-0.46052349	subgrp_cor	count	1	0
TTC5	6	OK	1.5659305	1.5666919	0.9995140	0.31755	0.97089418	subgrp_cor	count	1	0
CCNB1IP1	23	OK	1.2098510	0.6550158	1.8470561	0.06474	1.0600101	subgrp_cor	count	1	0
PARP2	3	OK	-1.295616	2.041425	-0.634662	0.52565	-0.00075754	subgrp_cor	count	1	0
OSGEP	15	OK	1.8312812	0.9204402	1.9895709	0.04664	1.62625905	subgrp_cor	count	1	0
APEX1	139	OK	0.1354403	0.2495326	0.5427761	0.58729	0.18123181	subgrp_cor	count	1	0
PIP4P1	17	OK	-0.283289	0.8101585	-0.349671	0.72659	-0.28746640	subgrp_cor	count	1	0
PNP	164	OK	0.0467362	0.2299688	0.203228	0.83896	0.06352022	subgrp_cor	count	1	0
ANG	2	OK	2.4096337	2.4715206	0.9749599	0.32958	0.66517343	subgrp_cor	count	1	0
RNASE1	779	OK	-0.105529	0.0917014	-1.150793	0.24982	-0.15078548	subgrp_cor	count	1	0
METTL17	2	OK	-0.202940	1.6532236	-0.122753	0.9022	-4.82E-05	subgrp_cor	count	1	0
NDRG2	13	OK	-1.610624	0.8500992	-1.894631	0.05814	-1.0475735	subgrp_cor	count	1	0
ARHGEF40	2	OK	1.5823776	1.9240213	0.822422	0.41082	0.77174550	subgrp_cor	count	1	0
ZNF219	2	OK	-10.44227	41.465809	-0.251828	0.80117	-0.00045289	subgrp_cor	count	1	0
HNRNPC	228	OK	0.2548851	0.1896264	1.3441439	0.1789	0.35161456	subgrp_cor	count	1	0
SUPT16H	44	OK	-0.397540	0.4581656	-0.867678	0.38557	-0.44201466	subgrp_cor	count	1	0
CHD8	9	OK	-0.725185	1.0438424	-0.694727	0.48722	-0.4466955	subgrp_cor	count	1	0
RAB2B	9	OK	-0.256418	0.9722946	-0.263725	0.79199	-0.00044774	subgrp_cor	count	1	0
TOX4	101	OK	-0.246619	0.2763643	-0.892371	0.37219	-0.31916942	subgrp_cor	count	1	0
METTL3	3	OK	-1.295616	2.041425	-0.634662	0.52565	-0.00075754	subgrp_cor	count	1	0
TRDC	3	OK	3.7994304	2.6548369	1.4311351	0.15239	1.76818710	subgrp_cor	count	1	0
TRAC	1	OK	-15.99503	70.34942	-0.227365	0.82014	-0.00028474	subgrp_cor	count	1	0
DAD1	524	OK	0.2798268	0.1208685	2.3151338	0.02061	0.39739991	subgrp_cor	count	1	0
OXA1L	32	OK	0.1238040	0.514734	0.2405201	0.80992	0.13065081	subgrp_cor	count	1	0
SLC7A7	3	OK	2.0702523	1.7994826	1.1504708	0.24995	0.96210332	subgrp_cor	count	1	0
MRPL52	122	OK	0.4143276	0.2737246	1.5136657	0.13011	0.54246850	subgrp_cor	count	1	0
MMP14	6	OK	0.0011582	1.8595750	0.0006228	0.9995	7.58E-07	subgrp_cor	count	1	0
LRP10	80	OK	0.9498513	0.3342304	2.8410555	0.0045	1.18821366	subgrp_cor	count	1	0
RBM23	13	OK	-0.856500	0.7580175	-1.129922	0.25851	-0.52091440	subgrp_cor	count	1	0
PRMT5	4	OK	2.704828	1.7963230	1.5057581	0.13212	1.47366775	subgrp_cor	count	1	0
HAUS4	1	OK	3.4598146	6.7714513	0.5109413	0.60939	0.00030179	subgrp_cor	count	1	0
C14orf93	1	OK	-11.01941	43.249935	-0.254784	0.79889	-1.17482461	subgrp_cor	count	1	0
PSMB5	265	OK	0.5361775	0.182905	2.931447	0.00337	0.74573333	subgrp_cor	count	1	0
ACIN1	34	OK	0.2167741	0.5454208	0.3974437	0.69104	0.18057218	subgrp_cor	count	1	0
C14orf119	49	OK	-0.026797	0.4282103	-0.062580	0.9501	-0.03261558	subgrp_cor	count	1	0
SLC7A8	5	OK	7.6236562	4.9764408	1.5319495	0.12553	2.17816624	subgrp_cor	count	1	0
RNF212B	3	OK	26.367950	84.916325	0.3105168	0.75617	2.49052421	subgrp_cor	count	1	0
HOMEZ	2	OK	0.4061000	2.1083412	0.1926158	0.84726	0.12645366	subgrp_cor	count	1	0
PPP1R3E	1	OK	-11.01941	43.249935	-0.254784	0.79889	-1.17482461	subgrp_cor	count	1	0
BCL2L2	3	OK	0.6103599	1.4407638	0.4236363	0.67182	0.3122109	subgrp_cor	count	1	0
PABPN1	20	OK	-0.169649	0.6963347	-0.243631	0.80752	-0.05154698	subgrp_cor	count	1	0
SLC22A17	7	OK	0.4304669	1.1045510	0.3897211	0.69674	0.28039717	subgrp_cor	count	1	0
NGDN	22	OK	-1.604654	0.764878	-2.097922	0.03591	-1.53074879	subgrp_cor	count	1	0
ZFHX2	1	OK	18.759489	83.625179	0.2243282	0.8225	0.00028789	subgrp_cor	count	1	0
THTPA	3	OK	3.3648273	2.5447810	1.322246	0.18609	1.31777762	subgrp_cor	count	1	0
JPH4	1	OK	18.759489	83.625179	0.2243282	0.8225	0.00028789	subgrp_cor	count	1	0
DHRS4-AS1	4	OK	0.8412799	1.2679586	0.6634910	0.50702	0.54450918	subgrp_cor	count	1	0
DHRS4	5	OK	-2.951075	1.8120570	-1.628577	0.1034	-1.02658532	subgrp_cor	count	1	0
DHRS4L2	31	OK	-0.297085	0.5325975	-0.557804	0.57698	-0.29045315	subgrp_cor	count	1	0
PCK2	1	OK	23.991896	84.899941	0.2825802	0.77749	1.65081709	subgrp_cor	count	1	0
DCAF11	6	OK	0.5073705	1.2646970	0.4011795	0.68829	0.00092844	subgrp_cor	count	1	0
PSME1	548	OK	-0.35475	0.1143838	-3.101400	0.00193	-0.5038890	subgrp_cor	count	1	0
EMC9	1	OK	3.4598146	6.7714513	0.5109413	0.60939	0.00030179	subgrp_cor	count	1	0
PSME2	314	OK	-0.29817	0.1559366	-1.912167	0.05585	-0.41791760	subgrp_cor	count	1	0
AL136295.5	1	OK	-10.44227	41.465809	-0.251828	0.80117	-0.00045289	subgrp_cor	count	1	0
IRF9	21	OK	-0.806180	0.6722099	-1.199299	0.23041	-0.38313313	subgrp_cor	count	1	0
IPO4	1	OK	3.4598146	6.7714513	0.5109413	0.60939	0.00030179	subgrp_cor	count	1	0
CHMP4A	68	OK	0.4363261	0.3503874	1.2452677	0.21302	0.54602292	subgrp_cor	count	1	0
MDP1	1	OK	3.4598146	6.7714513	0.5109413	0.60939	0.00030179	subgrp_cor	count	1	0
NEDD8	421	OK	0.1454424	0.1344434	1.0818110	0.27934	0.20528666	subgrp_cor	count	1	0
GMPR2	22	OK	0.1525163	0.7169507	0.2127291	0.83154	0.14435255	subgrp_cor	count	1	0
TINF2	14	OK	-0.262033	0.7648124	-0.342611	0.73189	-0.24527614	subgrp_cor	count	1	0
RABGGTA	1	OK	-15.99503	70.34942	-0.227365	0.82014	-0.00028474	subgrp_cor	count	1	0
DHRS1	4	OK	3.1002559	2.3039102	1.3456496	0.17842	1.26089264	subgrp_cor	count	1	0
CIDEB	3	OK	-0.725185	1.8079191	-0.401116	0.68832	-0.20979917	subgrp_cor	count	1	0
LTB4R2	1	OK	-10.44227	41.465809	-0.251828	0.80117	-0.00045289	subgrp_cor	count	1	0
LTB4R	2	OK	3.4598146	6.7714513	0.5109413	0.60939	0.00030179	subgrp_cor	count	1	0
ADCY4	24	OK	-0.564236	0.6286861	-0.897484	0.36948	-0.27470705	subgrp_cor	count	1	0
SDR39U1	11	OK	0.9514789	0.9471172	1.0046050	0.31509	0.78768274	subgrp_cor	count	1	0
CTSG	1	OK	23.991896	84.899941	0.2825802	0.77749	1.65081709	subgrp_cor	count	1	0
NOVA1	14	OK	1.4779616	0.8837222	1.6724278	0.09444	1.2706159	subgrp_cor	count	1	0
G2E3	9	OK	-0.017076	1.1551516	-0.014782	0.98821	-0.01680109	subgrp_cor	count	1	0
SCFD1	19	OK	1.8127235	0.9077257	1.9970056	0.04582	1.27320714	subgrp_cor	count	1	0
STRN3	40	OK	0.1845046	0.4712589	0.3915144	0.69542	0.20761057	subgrp_cor	count	1	0
AP4S1	1	OK	18.759489	83.625179	0.2243282	0.8225	0.00028789	subgrp_cor	count	1	0
HECTD1	45	OK	0.703782	0.4899880	1.4363260	0.15091	0.74217045	subgrp_cor	count	1	0
AL139353.1	1	OK	-15.99503	70.34942	-0.227365	0.82014	-0.00028474	subgrp_cor	count	1	0
NUBPL	2	OK	-10.44227	41.465809	-0.251828	0.80117	-0.00045289	subgrp_cor	count	1	0
ARHGAP5	14	OK	-0.761362	0.909132	-0.837460	0.40232	-0.64528013	subgrp_cor	count	1	0
AKAP6	1	OK	18.759489	83.625179	0.2243282	0.8225	0.00028789	subgrp_cor	count	1	0
EGLN3	33	OK	-1.292082	0.5333759	-2.422461	0.01542	-1.49201496	subgrp_cor	count	1	0
SPTSSA	35	OK	1.4354338	0.5425211	2.6458580	0.00815	1.5716504	subgrp_cor	count	1	0
EAPP	102	OK	-0.102147	0.2778142	-0.367682	0.71311	-0.13585492	subgrp_cor	count	1	0
SNX6	232	OK	-0.21255	0.1886644	-1.126619	0.2599	-0.29376197	subgrp_cor	count	1	0
CFL2	25	OK	0.9101169	0.6213598	1.4647180	0.143	0.8545794	subgrp_cor	count	1	0
BAZ1A	68	OK	-1.570219	0.4052656	-3.874543	0.00011	-1.8842193	subgrp_cor	count	1	0
AL121603.2	5	OK	-2.022361	1.7907921	-1.129311	0.25877	-1.39520054	subgrp_cor	count	1	0
SRP54	27	OK	-0.559217	0.6052531	-0.923940	0.35552	-0.59320637	subgrp_cor	count	1	0
FAM177A1	155	OK	-0.508286	0.2284778	-2.224664	0.0261	-0.69423763	subgrp_cor	count	1	0
PPP2R3C	24	OK	0.8803487	0.6561391	1.3417104	0.17969	0.86461783	subgrp_cor	count	1	0
PSMA6	8	OK	-1.47207	1.1724345	-1.255567	0.20927	-0.97501891	subgrp_cor	count	1	0
NFKBIA	670	OK	-0.243600	0.0979786	-2.486261	0.01291	-0.34739298	subgrp_cor	count	1	0
RALGAPA1	15	OK	0.3338972	0.7817384	0.4271214	0.66929	0.21782065	subgrp_cor	count	1	0
BRMS1L	4	OK	-1.497488	2.0936499	-0.715252	0.47445	-0.61992563	subgrp_cor	count	1	0
MBIP	7	OK	0.565823	1.07297	0.5273405	0.59796	0.36835291	subgrp_cor	count	1	0
SLC25A21-AS1	1	OK	18.759489	83.625179	0.2243282	0.8225	0.00028789	subgrp_cor	count	1	0
MIPOL1	4	OK	6.1490015	5.4123994	1.136095	0.25592	1.05209450	subgrp_cor	count	1	0
SSTR1	6	OK	25.138712	54.063693	0.4649832	0.64194	2.93759967	subgrp_cor	count	1	0
CLEC14A	658	OK	0.0298626	0.1022240	0.2921296	0.77019	0.04257875	subgrp_cor	count	1	0
SEC23A	13	OK	2.6505063	1.5748647	1.6830056	0.09237	1.14675213	subgrp_cor	count	1	0
GEMIN2	2	OK	-10.47563	27.491987	-0.381043	0.70317	-1.13811785	subgrp_cor	count	1	0
TRAPPC6B	7	OK	-1.926410	1.4185129	-1.358048	0.17445	-0.00105388	subgrp_cor	count	1	0
AL132639.3	1	OK	3.4598146	6.7714513	0.5109413	0.60939	0.00030179	subgrp_cor	count	1	0
PNN	175	OK	0.0465229	0.2205399	0.2109501	0.83292	0.0625069	subgrp_cor	count	1	0
MIA2	18	OK	-0.414997	0.6592773	-0.629473	0.52904	-0.38413844	subgrp_cor	count	1	0
FBXO33	6	OK	-0.495425	1.5039132	-0.329424	0.74182	-0.00031231	subgrp_cor	count	1	0
KLHL28	9	OK	0.0511076	1.0660907	0.0479393	0.96176	0.04529510	subgrp_cor	count	1	0
TOGARAM1	12	OK	0.4137284	0.9708733	0.426140	0.67001	0.21386022	subgrp_cor	count	1	0
PRPF39	3	OK	0.5103116	1.6034057	0.3182673	0.75028	0.15911454	subgrp_cor	count	1	0
FKBP3	71	OK	0.6441242	0.379710	1.6963544	0.08982	0.83450602	subgrp_cor	count	1	0
MIS18BP1	52	OK	-0.420179	0.4068770	-1.03269	0.30175	-0.49841510	subgrp_cor	count	1	0
RPS29	772	OK	-0.156016	0.0942179	-1.655914	0.09774	-0.22256338	subgrp_cor	count	1	0
LRR1	6	OK	-0.078139	1.0826627	-0.072173	0.94246	-0.03940996	subgrp_cor	count	1	0
RPL36AL	711	OK	-0.229440	0.0985872	-2.327280	0.01995	-0.3268652	subgrp_cor	count	1	0
MGAT2	10	OK	-0.362680	1.0022825	-0.361854	0.71746	-0.17972068	subgrp_cor	count	1	0
AL139099.1	1	OK	18.759489	83.625179	0.2243282	0.8225	0.00028789	subgrp_cor	count	1	0
DNAAF2	6	OK	-0.455970	1.2662122	-0.360105	0.71877	-0.22432410	subgrp_cor	count	1	0
KLHDC1	2	OK	21.64205	54.486891	0.3971975	0.69122	1.57744725	subgrp_cor	count	1	0
KLHDC2	28	OK	-0.409736	0.5586859	-0.733280	0.46332	-0.44654770	subgrp_cor	count	1	0
NEMF	48	OK	0.8996067	0.4601390	1.955076	0.05057	1.06192366	subgrp_cor	count	1	0
ARF6	68	OK	-0.167689	0.3588267	-0.467326	0.64027	-0.18429571	subgrp_cor	count	1	0
VCPKMT	2	OK	-1.295616	2.041425	-0.634662	0.52565	-0.00075754	subgrp_cor	count	1	0
SOS2	15	OK	0.1545276	0.8214069	0.1881255	0.85079	0.11628333	subgrp_cor	count	1	0
L2HGDH	1	OK	-11.01941	43.249935	-0.254784	0.79889	-1.17482461	subgrp_cor	count	1	0
ATP5S	18	OK	-0.546506	0.6937097	-0.787802	0.43081	-0.50324291	subgrp_cor	count	1	0
AL359397.2	1	OK	3.4598146	6.7714513	0.5109413	0.60939	0.00030179	subgrp_cor	count	1	0
CDKL1	2	OK	-1.758573	2.3554439	-0.746599	0.45531	-0.00036089	subgrp_cor	count	1	0
MAP4K5	29	OK	-0.585391	0.5472849	-1.087534	0.2768	-0.51694027	subgrp_cor	count	1	0
ATL1	3	OK	-0.150975	1.96881	-0.076683	0.93888	-9.78E-05	subgrp_cor	count	1	0
SAV1	52	OK	-1.123681	0.435422	-2.580670	0.00986	-1.22762322	subgrp_cor	count	1	0
NIN	8	OK	0.9772933	1.4778829	0.6613469	0.50839	0.30301174	subgrp_cor	count	1	0
PYGL	2	OK	5.8049273	5.6694662	1.023893	0.30589	1.03285716	subgrp_cor	count	1	0
TMX1	33	OK	0.9725933	0.5908576	1.6460705	0.09975	1.0432048	subgrp_cor	count	1	0
FRMD6	5	OK	4.4086152	2.1183580	1.413761	0.15742	0.93399720	subgrp_cor	count	1	0
GNG2	2	OK	3.4598146	6.7714513	0.5109413	0.60939	0.00030179	subgrp_cor	count	1	0
RTRAF	324	OK	-0.085197	0.1559380	-0.546355	0.58482	-0.11921412	subgrp_cor	count	1	0
NID2	6	OK	1.2692227	1.5445810	0.8217327	0.41123	0.63357232	subgrp_cor	count	1	0
TXNDC16	3	OK	2.4096168	2.4715214	0.9749528	0.32958	0.66515773	subgrp_cor	count	1	0
ERO1A	14	OK	-0.755608	0.7900679	-0.956384	0.33888	-0.46411398	subgrp_cor	count	1	0
AL133453.1	2	OK	5.8049273	5.6694662	1.023893	0.30589	1.03285716	subgrp_cor	count	1	0
PSMC6	60	OK	0.2658819	0.4071375	0.653051	0.51372	0.29924878	subgrp_cor	count	1	0
STYX	5	OK	-0.581189	1.4254706	-0.414732	0.67834	-0.36846215	subgrp_cor	count	1	0
GNPNAT1	6	OK	2.5552227	2.349844	1.0874007	0.27686	0.69313893	subgrp_cor	count	1	0
FERMT2	180	OK	0.0480273	0.2113172	0.2272758	0.82021	0.06556751	subgrp_cor	count	1	0
DDHD1	7	OK	2.9646238	2.292357	1.2932641	0.19592	0.76335144	subgrp_cor	count	1	0
CDKN3	6	OK	-0.480995	1.3823581	-0.347952	0.72788	-0.2361515	subgrp_cor	count	1	0
CNIH1	119	OK	-0.396830	0.2690665	-1.474841	0.14026	-0.53554644	subgrp_cor	count	1	0
GMFB	2	OK	-0.910223	2.1881125	-0.415985	0.67742	-0.25785735	subgrp_cor	count	1	0
CGRRF1	10	OK	0.7026272	0.954660	0.7359972	0.46173	0.65758057	subgrp_cor	count	1	0
SAMD4A	4	OK	-12.14570	43.418869	-0.279733	0.77968	-1.91338276	subgrp_cor	count	1	0
GCH1	6	OK	-0.712934	1.3887467	-0.513365	0.6077	-0.00044093	subgrp_cor	count	1	0
SOCS4	15	OK	0.3837505	0.7843926	0.4892326	0.62466	0.2895297	subgrp_cor	count	1	0
MAPK1IP1L	64	OK	-0.213463	0.3652426	-0.584443	0.55892	-0.2645757	subgrp_cor	count	1	0
FBXO34	30	OK	0.1192698	0.6496988	0.1835773	0.85435	0.10674821	subgrp_cor	count	1	0
ATG14	7	OK	-1.185382	1.2798142	-0.926214	0.35432	-0.31926640	subgrp_cor	count	1	0
KTN1	560	OK	-0.129632	0.1130393	-1.137945	0.25514	-0.18262133	subgrp_cor	count	1	0
AL138995.1	1	OK	18.759489	83.625179	0.2243282	0.8225	0.00028789	subgrp_cor	count	1	0
PELI2	2	OK	21.105322	83.341273	0.2532397	0.80009	0.0003103	subgrp_cor	count	1	0
TMEM260	2	OK	-15.99503	70.34942	-0.227365	0.82014	-0.00028474	subgrp_cor	count	1	0
EXOC5	18	OK	0.198365	0.6797244	0.2918323	0.77041	0.12887921	subgrp_cor	count	1	0
AP5M1	2	OK	-0.202940	1.6532236	-0.122753	0.9022	-4.82E-05	subgrp_cor	count	1	0
NAA30	5	OK	-2.222919	1.7760535	-1.251606	0.21071	-0.8693730	subgrp_cor	count	1	0
ARMH4	8	OK	-2.926706	1.4205361	-2.060283	0.03937	-1.54576271	subgrp_cor	count	1	0
ACTR10	84	OK	-0.368107	0.3012373	-1.221580	0.22187	-0.47068302	subgrp_cor	count	1	0
PSMA3	206	OK	-0.229779	0.1926614	-1.192661	0.233	-0.31689259	subgrp_cor	count	1	0
PSMA3-AS1	29	OK	-0.48106	0.5365540	-0.896582	0.36994	-0.44572274	subgrp_cor	count	1	0
ARID4A	76	OK	-0.266092	0.3399029	-0.782848	0.43372	-0.33495879	subgrp_cor	count	1	0
TIMM9	24	OK	0.479189	0.6581718	0.728061	0.46658	0.31195226	subgrp_cor	count	1	0
KIAA0586	6	OK	-1.000177	1.1953148	-0.836748	0.40272	-0.28026428	subgrp_cor	count	1	0
L3HYPDH	4	OK	0.4209530	1.4909211	0.2823442	0.77768	0.21537946	subgrp_cor	count	1	0
JKAMP	23	OK	0.2533976	0.6373319	0.3975912	0.69092	0.28011613	subgrp_cor	count	1	0
PCNX4	11	OK	-0.792351	0.9466766	-0.836982	0.4026	-0.00132231	subgrp_cor	count	1	0
DHRS7	143	OK	-0.472821	0.2372535	-1.992895	0.04627	-0.6396769	subgrp_cor	count	1	0
PPM1A	6	OK	0.6270896	1.3968900	0.4489183	0.65349	0.00115334	subgrp_cor	count	1	0
SIX1	2	OK	21.105322	83.341273	0.2532397	0.80009	0.0003103	subgrp_cor	count	1	0
MNAT1	34	OK	0.7916968	0.5665481	1.3974043	0.16229	0.87106877	subgrp_cor	count	1	0
TRMT5	4	OK	2.1946847	2.1400930	1.0255090	0.30512	0.00149216	subgrp_cor	count	1	0
SLC38A6	2	OK	5.8049273	5.6694662	1.023893	0.30589	1.03285716	subgrp_cor	count	1	0
PRKCH	27	OK	-0.697570	0.6135021	-1.137029	0.25552	-0.62876535	subgrp_cor	count	1	0
AL359220.1	7	OK	0.5562488	1.3975796	0.3980089	0.69062	0.29160630	subgrp_cor	count	1	0
HIF1A	96	OK	0.1985170	0.3106637	0.6390094	0.52282	0.25695354	subgrp_cor	count	1	0
SNAPC1	30	OK	-0.03182	0.5293743	-0.060123	0.95206	-0.03434216	subgrp_cor	count	1	0
RHOJ	66	OK	0.0319775	0.4086407	0.0782534	0.93762	0.03748281	subgrp_cor	count	1	0
PPP2R5E	37	OK	1.4631479	0.5817143	2.5152346	0.0119	1.33692214	subgrp_cor	count	1	0
AL136038.3	2	OK	-0.725185	1.8079191	-0.401116	0.68832	-0.20979917	subgrp_cor	count	1	0
MTHFD1	4	OK	-0.465429	1.3093639	-0.355462	0.72224	-0.13816105	subgrp_cor	count	1	0
ZBTB25	2	OK	-0.349551	1.8544747	-0.188490	0.85049	-0.17337783	subgrp_cor	count	1	0
ZBTB1	12	OK	-0.839339	0.9952777	-0.843322	0.39905	-0.39587549	subgrp_cor	count	1	0
HSPA2	37	OK	-1.154475	0.4769871	-2.420349	0.01551	-0.88465032	subgrp_cor	count	1	0
CHURC1	157	OK	0.1670840	0.2201844	0.7588369	0.44795	0.22570994	subgrp_cor	count	1	0
RAB15	1	OK	1.5823999	2.7200188	0.5817606	0.56072	0.47433761	subgrp_cor	count	1	0
MAX	36	OK	0.8561134	0.5556486	1.5407459	0.12338	0.87194541	subgrp_cor	count	1	0
FUT8	25	OK	8.1993562	4.6430181	1.7659539	0.0774	1.1458093	subgrp_cor	count	1	0
MPP5	4	OK	1.1224437	1.4569313	0.7704163	0.44105	0.3460629	subgrp_cor	count	1	0
ATP6V1D	32	OK	-0.036452	0.5502222	-0.066250	0.94718	-0.04017170	subgrp_cor	count	1	0
EIF2S1	48	OK	-0.308447	0.4264996	-0.723207	0.46955	-0.35471321	subgrp_cor	count	1	0
PLEK2	1	OK	1.5823999	2.7200188	0.5817606	0.56072	0.47433761	subgrp_cor	count	1	0
PIGH	6	OK	-0.174751	1.2871310	-0.13576	0.892	-0.08767490	subgrp_cor	count	1	0
ARG2	3	OK	1.925964	2.5154879	0.7656425	0.44389	0.56022119	subgrp_cor	count	1	0
VTI1B	57	OK	-0.314782	0.4159139	-0.756845	0.44914	-0.37466309	subgrp_cor	count	1	0
RDH11	18	OK	-0.317725	0.6730178	-0.472091	0.63686	-0.29766741	subgrp_cor	count	1	0
ZFP36L1	380	OK	-0.624746	0.1423344	-4.389281	1.14E-05	-0.87691985	subgrp_cor	count	0.1138394	0
ACTN1	264	OK	-0.079467	0.1696483	-0.468421	0.63948	-0.10936020	subgrp_cor	count	1	0
DCAF5	8	OK	4.1656877	2.4138723	1.725728	0.0844	1.83688686	subgrp_cor	count	1	0
EXD2	2	OK	-0.202940	1.6532236	-0.122753	0.9022	-4.82E-05	subgrp_cor	count	1	0
ERH	332	OK	-0.112371	0.1517391	-0.740555	0.45896	-0.15765807	subgrp_cor	count	1	0
SLC39A9	5	OK	20.099832	50.652192	0.396228	0.69194	0.00081457	subgrp_cor	count	1	0
SUSD6	10	OK	0.3307878	0.9649121	0.3428165	0.73174	0.10328587	subgrp_cor	count	1	0
SRSF5	315	OK	-0.435253	0.1546634	-2.814196	0.00489	-0.60647205	subgrp_cor	count	1	0
SMOC1	3	OK	2.4096337	2.4715206	0.9749599	0.32958	0.66517343	subgrp_cor	count	1	0
COX16	1	OK	-10.44227	41.465809	-0.251828	0.80117	-0.00045289	subgrp_cor	count	1	0
SYNJ2BP	37	OK	-0.252195	0.4648139	-0.542572	0.58742	-0.29054692	subgrp_cor	count	1	0
MED6	15	OK	-0.064337	0.7478261	-0.086032	0.93144	-0.05323852	subgrp_cor	count	1	0
TTC9	3	OK	5.2612789	5.4905820	0.9582370	0.33794	0.00295034	subgrp_cor	count	1	0
PCNX1	10	OK	0.7264722	1.3048429	0.5567507	0.5777	0.22644375	subgrp_cor	count	1	0
SIPA1L1	4	OK	1.1389358	1.367612	0.8327915	0.40496	0.00078202	subgrp_cor	count	1	0
DCAF4	2	OK	0.5887819	1.7674148	0.3331317	0.73902	0.30122869	subgrp_cor	count	1	0
RBM25	272	OK	0.1668679	0.1698759	0.9822929	0.32596	0.2306630	subgrp_cor	count	1	0
PSEN1	7	OK	-2.07534	1.3994087	-1.483016	0.13807	-0.00111619	subgrp_cor	count	1	0
NUMB	25	OK	0.8246460	0.7801475	1.0570385	0.29049	0.77685791	subgrp_cor	count	1	0
PNMA1	5	OK	4.6613299	5.7683860	0.8080821	0.41904	0.00274918	subgrp_cor	count	1	0
ELMSAN1	40	OK	-1.010516	0.4936789	-2.046910	0.04067	-0.98370357	subgrp_cor	count	1	0
PTGR2	2	OK	18.759489	83.625179	0.2243282	0.8225	0.00028789	subgrp_cor	count	1	0
COQ6	3	OK	0.9033449	1.4604911	0.6185213	0.53622	0.5834292	subgrp_cor	count	1	0
ENTPD5	2	OK	-10.47563	27.491987	-0.381043	0.70317	-1.13811785	subgrp_cor	count	1	0
ALDH6A1	13	OK	0.5361981	0.7470422	0.7177614	0.4729	0.44675682	subgrp_cor	count	1	0
ABCD4	47	OK	-0.972933	0.474771	-2.049267	0.04044	-1.00503866	subgrp_cor	count	1	0

NPC2	571	OK	-0.001488	0.1111388	-0.013389	0.98932	-0.00211657	subgrp_con	count		1	0
ISCA2	10	OK	0.2024240	0.8922917	0.2268585	0.82052	0.19171094	subgrp_con	count		1	0
LTBP2	68	OK	10.806348	3.3228166	3.2511869	0.00115	5.33910981	subgrp_con	count		1	0
FCF1	1	OK	-11.18936	26.334948	-0.424886	0.67092	-2.27128013	subgrp_con	count		1	0
YLPM1	8	OK	0.5848714	1.0377261	0.5636089	0.57302	0.42883027	subgrp_con	count		1	0
DLST	5	OK	0.1112767	1.6120387	0.0690285	0.94497	0.05657342	subgrp_con	count		1	0
PGF	48	OK	1.2139151	0.4788758	2.534927	0.01125	1.44810688	subgrp_con	count		1	0
EIF2B2	20	OK	0.9043022	0.6597869	1.3705976	0.1705	1.0427609	subgrp_con	count		1	0
MLH3	6	OK	-0.368151	1.1908516	-0.309149	0.75721	-0.00063705	subgrp_con	count		1	0
ACYP1	7	OK	-0.602821	1.0319416	-0.584162	0.55911	-0.48498581	subgrp_con	count		1	0
NEK9	2	OK	19.111790	50.837581	0.3759381	0.70696	0.00078703	subgrp_con	count		1	0
FOS	818	OK	-0.155057	0.0870020	-1.782228	0.07471	-0.22179767	subgrp_con	count		1	0
JDP2	34	OK	0.6989622	0.5823550	1.2002340	0.23005	0.65948935	subgrp_con	count		1	0
ERG28	26	OK	1.5451040	0.6584972	2.346409	0.01896	1.57107315	subgrp_con	count		1	0
IFT43	43	OK	0.4219507	0.4630550	0.9112322	0.36217	0.48826633	subgrp_con	count		1	0
TGFB3	1	OK	0.6532528	2.5001286	0.2612877	0.79387	0.00016297	subgrp_con	count		1	0
GPATCH2L	13	OK	1.1698153	1.0258203	1.1403705	0.25412	0.35086748	subgrp_con	count		1	0
VASH1	7	OK	1.7724772	1.8439627	0.9612326	0.33644	0.84969892	subgrp_con	count		1	0
IRF2BPL	19	OK	-1.036850	0.6408573	-1.617911	0.10568	-0.28921492	subgrp_con	count		1	0
GSTZ1	6	OK	-0.822804	1.1791166	-0.706295	0.48	-0.50770942	subgrp_con	count		1	0
TMED8	1	OK	23.991896	84.899941	0.2825902	0.77749	1.65081709	subgrp_con	count		1	0
AHSA1	27	OK	0.1492467	0.69908	0.2134902	0.83094	0.11229712	subgrp_con	count		1	0
SPTLC2	54	OK	-0.499497	0.403147	-1.238995	0.21535	-0.62936360	subgrp_con	count		1	0
ALKBH1	2	OK	0.1681491	1.7253300	0.097459	0.92236	0.05201277	subgrp_con	count		1	0
SLIRP	91	OK	0.2746923	0.3198425	0.8588360	0.39042	0.32250384	subgrp_con	count		1	0
SNW1	89	OK	-0.083736	0.3064006	-0.273290	0.78462	-0.1051467	subgrp_con	count		1	0
GTF2A1	7	OK	5.6061539	4.6319498	1.2103226	0.22616	0.00040456	subgrp_con	count		1	0
SEL1L	22	OK	0.4929131	0.6588718	0.7481168	0.45439	0.48780913	subgrp_con	count		1	0
FLRT2	19	OK	0.1254715	0.8397498	0.1494153	0.88122	0.03876580	subgrp_con	count		1	0
GALC	2	OK	-0.150975	1.968815	-0.076683	0.93888	-9.78E-05	subgrp_con	count		1	0
SPATA7	5	OK	-0.465429	1.3093639	-0.355462	0.72224	-0.13816105	subgrp_con	count		1	0
ZC3H14	18	OK	0.3902217	0.7166429	0.5445134	0.58609	0.19960751	subgrp_con	count		1	0
FOXN3	19	OK	-1.05482	0.7066375	-1.492721	0.13551	-0.98498591	subgrp_con	count		1	0
EFCAB11	1	OK	-11.01941	43.249935	-0.254784	0.79889	-1.17482461	subgrp_con	count		1	0
PSMC1	97	OK	0.306877	0.2943544	1.0425437	0.29716	0.38807080	subgrp_con	count		1	0
NRDE2	3	OK	3.4016237	2.6808857	1.2688432	0.2045	0.9636701	subgrp_con	count		1	0
CALM1	815	OK	-0.056955	0.0897940	-0.634286	0.52589	-0.08138944	subgrp_con	count		1	0
TTC7B	1	OK	-11.01941	43.249935	-0.254784	0.79889	-1.17482461	subgrp_con	count		1	0
RPS6KA5	2	OK	23.991896	84.899941	0.2825902	0.77749	1.65081709	subgrp_con	count		1	0
DGLUCY	10	OK	3.5172255	2.0394317	1.7246106	0.0846	0.84081643	subgrp_con	count		1	0
CCDC88C	5	OK	1.8340679	1.6828964	1.0898281	0.27579	0.53818925	subgrp_con	count		1	0
PPP4R3A	14	OK	-0.412314	0.8807501	-0.468140	0.63969	-0.12296818	subgrp_con	count		1	0
TC2N	1	OK	23.991896	84.899941	0.2825902	0.77749	1.65081709	subgrp_con	count		1	0
FBLN5	27	OK	8.4146337	2.8352045	2.9679106	0.003	3.84385360	subgrp_con	count		1	0
TRIP11	88	OK	0.7927529	0.331193	2.393626	0.01668	1.01864927	subgrp_con	count		1	0
ATXN3	36	OK	-1.495820	0.5502853	-2.718444	0.00656	-1.54762665	subgrp_con	count		1	0
NDUFB1	174	OK	0.6156754	0.228829	2.690576	0.00713	0.83651912	subgrp_con	count		1	0
CPSF2	4	OK	-0.545320	1.708550	-0.319171	0.7496	-0.0003423	subgrp_con	count		1	0
RIN3	4	OK	-0.495425	1.5039132	-0.329424	0.74182	-0.00031231	subgrp_con	count		1	0
LGMN	18	OK	0.7138348	0.6943861	1.0280085	0.30395	0.75100933	subgrp_con	count		1	0
GOLGA5	6	OK	-1.223218	1.3768776	-0.895663	0.37042	-0.33530171	subgrp_con	count		1	0
ITPK1	4	OK	1.791902	2.2823539	0.7847677	0.43259	0.00045246	subgrp_con	count		1	0
MOAP1	5	OK	0.2530274	1.185319	0.2134677	0.83096	0.16258771	subgrp_con	count		1	0
TMEM251	4	OK	2.4165575	1.843624	1.3107647	0.18994	1.36735324	subgrp_con	count		1	0
GON7	4	OK	0.1255948	1.4500075	0.086616	0.93098	0.03878308	subgrp_con	count		1	0
UBR7	5	OK	1.4119426	1.4959308	0.9438555	0.34524	0.69797651	subgrp_con	count		1	0
BTBD7	45	OK	0.0522678	0.4592437	0.1138129	0.90939	0.05767955	subgrp_con	count		1	0
DDX24	368	OK	0.5760361	0.1476397	3.9016320	9.55E-05	0.80886414	subgrp_con	count		0.9475327	0
IFI27L1	8	OK	2.5967910	1.6108020	1.6121105	0.10694	1.13154824	subgrp_con	count		1	0
IFI27	790	OK	-0.249547	0.091902	-2.715257	0.00662	-0.3563413	subgrp_con	count		1	0
IFI27L2	51	OK	2.0512436	0.5756843	3.5630779	0.00037	2.15752891	subgrp_con	count		1	0
SERPINA3	1	OK	18.759489	83.625179	0.2243282	0.8225	0.00028789	subgrp_con	count		1	0
DICER1	15	OK	0.4221056	0.7942394	0.5314589	0.5951	0.27494618	subgrp_con	count		1	0
SYNE3	3	OK	18.759489	83.625179	0.2243282	0.8225	0.00028789	subgrp_con	count		1	0
AL133467.1	1	OK	3.4598146	6.7714513	0.5109413	0.60939	0.00030179	subgrp_con	count		1	0
SNHG10	1	OK	3.4597879	4.7858528	0.7229198	0.46972	0.00081879	subgrp_con	count		1	0
GLRX5	79	OK	0.4824517	0.3318572	1.4537929	0.146	0.62582637	subgrp_con	count		1	0
C14orf132	6	OK	-1.777839	1.3192990	-1.347639	0.17777	-0.44681836	subgrp_con	count		1	0
BDKRB2	3	OK	2.1611158	2.3677943	0.9127126	0.36139	0.61359306	subgrp_con	count		1	0
ATG2B	4	OK	-1.340131	1.6819410	-0.796776	0.42558	-0.59807560	subgrp_con	count		1	0
GSKIP	5	OK	2.895154	2.4132796	1.1996762	0.23027	0.75227995	subgrp_con	count		1	0
AK7	1	OK	3.4598146	6.7714513	0.5109413	0.60939	0.00030179	subgrp_con	count		1	0
PAPOLA	129	OK	-0.503943	0.262312	-1.921153	0.05471	-0.64517197	subgrp_con	count		1	0
VRK1	8	OK	-1.650498	1.0425244	-1.583175	0.11338	-0.42283623	subgrp_con	count		1	0
SETD3	17	OK	0.2876486	0.7244914	0.3970352	0.69134	0.27259197	subgrp_con	count		1	0
CCNK	17	OK	0.4928419	0.8984397	0.5485531	0.58331	0.46887604	subgrp_con	count		1	0
CYP46A1	3	OK	-1.295616	2.041425	-0.634662	0.52565	-0.00075754	subgrp_con	count		1	0
EML1	9	OK	0.1432229	1.1085781	0.1291951	0.8972	0.07289211	subgrp_con	count		1	0
EVL	24	OK	1.7111188	0.6944331	2.4641507	0.01373	1.96620351	subgrp_con	count		1	0
YY1	232	OK	-0.068993	0.1846922	-0.373561	0.70873	-0.09495078	subgrp_con	count		1	0
SLC25A29	26	OK	-0.362277	0.5375008	-0.674003	0.50031	-0.33907499	subgrp_con	count		1	0
WARS	103	OK	-0.637912	0.2721334	-2.335534	0.01952	-0.82739577	subgrp_con	count		1	0
WDR25	1	OK	3.4598146	6.7714513	0.5109413	0.60939	0.00030179	subgrp_con	count		1	0
DIO3OS	1	OK	18.759489	83.625179	0.2243282	0.8225	0.00028789	subgrp_con	count		1	0
PPP2R5C	29	OK	-0.201128	0.5188506	-0.387642	0.69829	-0.2108025	subgrp_con	count		1	0
DYNC1H1	89	OK	0.5762900	0.3510519	1.6418938	0.10061	0.60798118	subgrp_con	count		1	0
HSP90AA1	700	OK	0.0923602	0.0985476	0.9372145	0.34865	0.1316690	subgrp_con	count		1	0
WDR20	3	OK	5.0027561	4.2286829	1.1830530	0.23679	0.98115823	subgrp_con	count		1	0
MOK	1	OK	-11.01941	43.249935	-0.254784	0.79889	-1.17482461	subgrp_con	count		1	0
CINP	12	OK	0.2626418	1.0391193	0.2527542	0.80046	0.17083800	subgrp_con	count		1	0
TECPR2	2	OK	18.759489	83.625179	0.2243282	0.8225	0.00028789	subgrp_con	count		1	0
ANKRD9	7	OK	-2.427826	1.5276322	-1.589274	0.112	-0.00126226	subgrp_con	count		1	0
RCOR1	10	OK	2.8148250	1.3537073	2.079352	0.03758	1.51070586	subgrp_con	count		1	0
TRAF3	3	OK	-11.01941	43.249935	-0.254784	0.79889	-1.17482461	subgrp_con	count		1	0
CDC42BPB	44	OK	0.048499	0.4827889	0.100456	0.91998	0.05240938	subgrp_con	count		1	0
TNFAIP2	16	OK	0.1779533	0.7413052	0.2400540	0.81029	0.13420077	subgrp_con	count		1	0
LINC00605	1	OK	-15.99503	70.34942	-0.227365	0.82014	-0.00028474	subgrp_con	count		1	0
EIF5	247	OK	0.0930297	0.1766865	0.5264943	0.59854	0.12702101	subgrp_con	count		1	0
MARK3	25	OK	0.0405599	0.6318037	0.0641971	0.94881	0.02056747	subgrp_con	count		1	0
CKB	10	OK	20.635628	32.492427	0.6350903	0.52537	1.54656705	subgrp_con	count		1	0
TRMT61A	4	OK	-1.106879	1.7130168	-0.646158	0.51818	-0.65509255	subgrp_con	count		1	0
BAG5	61	OK	0.8097644	0.406078	1.9941090	0.04614	0.96650979	subgrp_con	count		1	0
KLC1	17	OK	0.290830	0.7580892	0.3836366	0.70125	0.1485591	subgrp_con	count		1	0
APOPT1	37	OK	-0.303310	0.5110646	-0.593487	0.55285	-0.33177536	subgrp_con	count		1	0
AL049840.1	2	OK	3.4598146	6.7714513	0.5109413	0.60939	0.00030179	subgrp_con	count		1	0
ZFYVE21	129	OK	-0.473136	0.2453671	-1.928281	0.05382	-0.64435130	subgrp_con	count		1	0
PPP1R13B	16	OK	0.0289012	0.7051315	0.040987	0.96731	0.0216869	subgrp_con	count		1	0
ATP5MPL	192	OK	0.281782	0.2119516	1.3294638	0.18369	0.3782450	subgrp_con	count		1	0
TDRD9	1	OK	23.991896	84.899941	0.2825902	0.77749	1.65081709	subgrp_con	count		1	0
KIF26A	9	OK	0.2550225	0.9085094	0.2807043	0.77894	0.07912237	subgrp_con	count		1	0
INF2	7	OK	-0.065689	1.1258878	-0.058344	0.95347	-0.0423235	subgrp_con	count		1	0
SIVA1	207	OK	0.5848135	0.1999460	2.9248561	0.00345	0.81000747	subgrp_con	count		1	0
AKT1	38	OK	1.330501	0.574847	2.3145281	0.02064	1.28101316	subgrp_con	count		1	0
AHNAK2	56	OK	1.3605642	0.4016129	3.3877500	0.0007	1.64002938	subgrp_con	count		1	0
JAG2	12	OK	1.3764456	1.4646408	0.9397837	0.34732	0.4191910	subgrp_con	count		1	0
NUDT14	79	OK	0.7572942	0.388956	1.9468895	0.05154	0.91290090	subgrp_con	count		1	0
BRF1	2	OK	4.6613299	5.7683860	0.8080821	0.41904	0.00274918	subgrp_con	count		1	0
BTBD6	35	OK	0.8212458	0.5055078	1.6245956	0.10425	0.88391228	subgrp_con	count		1	0
PACS2	8	OK	-1.000177	1.1953148	-0.836748	0.40272	-0.28026428	subgrp_con	count		1	0
MTA1	10	OK	0.5239310	0.9239202	0.5664964	0.57108	0.16303320	subgrp_con	count		1	0
CRIP2	717	OK	-0.199832	0.0972976	-2.051712	0.0402	-0.28510915	subgrp_con	count		1	0
CRIP1	79	OK	-1.08118	0.3623514	-2.983807	0.00285	-1.28625498	subgrp_con	count		1	0
TMEM121	10	OK	1.9822165	1.2355680	1.6042957	0.10865	1.26849636	subgrp_con	count		1	0
IGHA1	21	OK	-0.202793	0.7944191	-0.255272	0.79851	-0.00035559	subgrp_con	count		1	0
IGHG1	3	OK	3.4598155	3.9099931	0.8848648	0.37622	0.00081877	subgrp_con	count		1	0
IGHG3	2	OK	3.4598149	4.7858686	0.7229230	0.46972	0.00081935	subgrp_con	count		1	0
IGHD	1	OK	3.4598146	6.7714513	0.5109413	0.60939	0.00030179	subgrp_con	count		1	0
NIPA2	10	OK	0.5608770	1.0214413	0.5491035	0.58292	0.28701064	subgrp_con	count		1	0
CYFIP1	33	OK	-0.812536	0.548853	-1.480425	0.13876	-0.86857588	subgrp_con	count		1	0
TUBGCP5	5	OK	1.1560646	1.5879809	0.7280091	0.46661	0.38296539	subgrp_con	count		1	0
NDN	37	OK	0.2863538	0.4617841	0.6201032	0.53519	0.22750000	subgrp_con	count		1	0
SNRPN	92	OK	1.1138381	0.3118945	3.5712006	0.00036	1.47663702	subgrp_con	count		1	0
UBE3A	44	OK	0.6387674	0.5170324	1.2354493	0.21666	0.68999455	subgrp_con	count		1	0
ATP10A	2	OK	4.6613299	5.7683860	0.8080821	0.41904	0.00274918	subgrp_con	count		1	0
HERC2	59	OK	0.9331586	0.4060441	2.2981705	0.02155	1.11107789	subgrp_con	count		1	0
NSMCE3	16	OK	0.5462134	0.6525317	0.8370679	0.40255	0.41184281	subgrp_con	count		1	0
TJP1	142	OK	-0.426047	0.237087	-1.797002	0.07234	-0.56950062	subgrp_con	count		1	0
FAN1	2	OK	-12.14842	43.455309	-0.279561	0.77981	-1.91362624	subgrp_con	count		1	0
MTMR10	3	OK	-1.037962	1.4280017	-0.726863	0.46731	-0.00062210	subgrp_con	count		1	0
KLF13	108	OK	-0.063770	0.2768266	-0.230362	0.81781	-0.0851220	subgrp_con	count		1	0
LINC02256	2	OK	1.5823999	2.7200188	0.5817606	0.56072	0.47433761	subgrp_con	count		1	0
AC055874.1	3	OK	1.2533065	1.5409862	0.8133145	0.41604	0.62624666	subgrp_con	count		1	0
RYR3	7	OK	-2.681998	1.6192685	-1.656301	0.09766	-1.93155583	subgrp_con	count		1	0
AVEN	10	OK	1.1618487	0.9464317	1.2276097	0.21959	0.94850962	subgrp_con	count		1	0
EMC7	75	OK	1.1256788	0.3787431	2.9721427	0.00296	1.37770229	subgrp_con	count		1	0
KATNBL1	45	OK	0.1264940	0.4575749	0.2764443	0.78221	0.14227625	subgrp_con	count		1	0
EMC4	133	OK	0.4109263	0.2533337	1.6220751	0.10479	0.55153888	subgrp_con	count		1	0
SLC12A6	9	OK	-2.252544	1.3511253	-1.667162	0.09548	-1.13466663	subgrp_con	count		1	0
NOP10	307	OK	0.0903560	0.1630516	0.5541559	0.57947	0.12638002	subgrp_con	count		1	0
LPCAT4	36	OK	-1.381	0.5155289	-2.679965	0.00736	-1.6030335	subgrp_con	count		1	0
GOLGA8A	5	OK	0.4209530	1.4909211	0.2823442	0.77768	0.21537946	subgrp_con	count		1	0
GOLGA8B	2	OK	21.105322	83.341273	0.2532397	0.80008	0.0003103	subgrp_con	count		1	0
ACTC1	1	OK	23.991896	84.899941	0.2825902	0.77749	1.65081709	subgrp_con	count		1	0
AQR	3	OK	-0.725185	1.8079191	-0.401116	0.68832	-0.20979917	subgrp_con	count		1	0
ZNF770	17	OK	0.7507912	0.8203012	0.9152629	0.36005	0.23570909	subgrp_con	count		1	0
DPH6	4	OK	-1.853834	1.8543918	-0.999699	0.31746	-0.46053279	subgrp_con	count		1	0
SPRED1	3	OK	3.4598155	3.9099931	0.8848648	0.37622	0.00081877	subgrp_con	count		1	0
FAM98B	5	OK	0.1703627	1.1470876	0.1485176	0.88193	0.05270200	subgrp_con	count		1	0
AC023908.3	1	OK	-11.01941	43.249935	-0.254784	0.79889	-1.17482461	subgrp_con	count		1	0
GPR176	2	OK	5.8049273	5.6694662	1.023893	0.30589	1.03285716	subgrp_con	count		1	0
EIF2AK4	60	OK	-0.530556	0.4131875	-1.284056	0.19912	-0.62652602	subgrp_con	count		1	0
SRP14	835	OK	-0.176618	0.0889672	-1.985204	0.04712	-0.25245193	subgrp_con	count		1	0
INAFM2	1	OK	18.759489	83.625179	0.2243282	0.8225	0.00028789	subgrp_con	count		1	0
IVD	9	OK	1.2051016	1.1763685	1.0244252	0.30563	0.7669766	subgrp_con	count		1	0
BAHD1	3	OK	6.6844809	6.7550163	0.9895580	0.32239	4.48E-1	subgrp_con	count		1	0
CCDC32	12	OK	-0.633939	0.7631219	-0.830718	0.40612	-0.3936811	subgrp_con	count		1	0
RMDN3	8	OK	-13.32706	27.726621	-0.480486	0.63088	-0.00160251	subgrp_con	count		1	0
GCHFR	60	OK	0.4938535	0.3519485	1.4031979	0.16056	0.64956574	subgrp_con	count		1	0
DNAJC17	15	OK	-0.780074	0.7520947	-1.037202	0.29964	-0.47802559	subgrp_con	count		1	0
ZFYVE19	2	OK	-0.349551	1.8564747	-0.188490	0.85049	-0.17237782	subgrp_con	count		1	0
DLL4	13	OK	-0.437941	0.8293898	-0.528028	0.59749	-0.32125744	subgrp_con	count		1	0
INO80	14	OK	-0.231839	0.8725925	-0.265690	0.79049	-0.19058721	subgrp_con	count		1	0
CHP1	61	OK	0.4301204	0.3649352	1.1786213	0.23855	0.52439368	subgrp_con	count		1	0
RTF1	159	OK	0.6413475	0.2405405	2.6662757	0.00767	0.85613548	subgrp_con	count		1	0
MGA	4	OK	1.5823999	2.7200188	0.5817606	0.56072	0.47433761	subgrp_con	count		1	0
MAPKBP1	1	OK	-11.45102	41.797363	-0.273965	0.78411	-0.00049189	subgrp_con	count		1	0
EHD4	97	OK	-0.825005	0.3096738	-2.664111	0.00772	-0.97531604	subgrp_con	count		1	0
VPS39	2	OK	3.4598146	6.7714513	0.5109413	0.60939	0.00030179	subgrp_con	count		1	0
TMEM87A	45	OK	-0.041149	0.4430924	-0.092869	0.92601	-0.04055523	subgrp_con	count		1	0
GANC	2	OK	21.105322	83.341273	0.2532397	0.80008	0.0003103	subgrp_con	count		1	0
ZNF106	85	OK	0.0827979	0.3190736	0.2594948	0.79525	0.10463326	subgrp_con	count		1	0
SNAP23	83	OK	0.3397898	0.3247044	1.0464588	0.29535	0.438407	subgrp_con	count		1	0
LRRC57	5	OK	-0.444980	1.2069935	-0.368668	0.71237	-0.13233229	subgrp_con	count		1	0
HAUS2	13	OK	-0.130564	0.7375898	-0.177014	0.8595	-0.12837375	subgrp_con	count		1	0
STARD9	5	OK	-10.72144	16.830118	-0.63703	0.5241	-2.56179814	subgrp_con	count		1	0
TTBK2	7	OK	1.0171822	1.0833239	0.9389455	0.34776	0.65386087	subgrp_con	count		1	0
UBR1	10	OK	1.7954086	1.0402338	1.725966	0.08435	1.10107010	subgrp_con	count		1	0
CCNDBP1	50	OK	0.3347846	0.3659922	0.9147314	0.36032	0.42712001	subgrp_con	count		1	0
ADAL	4	OK	1.2532170	1.5410077	0.8132451	0.41608	0.62618913	subgrp_con	count		1	0
TUBGCP4	2	OK	-10.44227	41.465809	-0.251828	0.80117	-0.00045289	subgrp_con	count		1	0
TP53BP1	8	OK	0.4159896	1.2178490	0.3415773	0.73267	0.27095870	subgrp_con	count		1	0
MAP1A	1	OK	41.788303	174.29608	0.2397546	0.81052	0.00014488	subgrp_con	count		1	0
PDIA3	453	OK	0.4310134	0.1303664	3.3061699	0.00095	0.60866547	subgrp_con	count		1	0
SERF2	830	OK	-0.127377	0.089307	-1.426291	0.15379	-0.18206142	subgrp_con	count		1	0
HYPK	1	OK	-15.99503	70.34942	-0.227365	0.82014	-0.00028474	subgrp_con	count		1	0
MFAP1	32	OK	-0.450428	0.5155700	-0.873650	0.38231	-0.45365720	subgrp_con	count		1	0
CASC4	41	OK	0.9653043	0.4822621	2.0016175	0.04532	1.0768192	subgrp_con	count		1	0
CTDSPL2	13	OK	0.2463871	0.8284852	0.2973947	0.76617	0.20527990	subgrp_con	count		1	0
EIF3J-DT	9	OK	-1.610162	1.1824191	-1.361752	0.17328	-0.89306169	subgrp_con	count		1	0
EIF3J	127	OK	0.1017086	0.2588633	0.3929048	0.69439	0.1366737	subgrp_con	count		1	0
SPG11	5	OK	-2.593606	1.7016757	-1.524148	0.12747	-0.95568460	subgrp_con	count		1	0
B2M	896	OK	0.0985768	0.0807120	1.2213395	0.22196	0.14120538	subgrp_con	count		1	0
TRIM69	18	OK	-1.391546	0.7975787	-1.744712	0.08103	-0.61865864	subgrp_con	count		1	0
SHF	4	OK	26.367950	84.916325	0.3105168	0.75617	2.49052421	subgrp_con	count		1	0
GATM	2	OK	-14.6580	45.690332	-0.320813	0.74835	-0.00067223	subgrp_con	count		1	0
SLC30A4	2	OK	-10.47563	27.491987	-0.381043	0.70317	-1.13811785	subgrp_con	count		1	0
BLOC1S6	31	OK	0.3617842	0.5815895	0.6220504	0.53391	0.38330968	subgrp_con	count		1	0
SQOR	31	OK	0.206224	0.5045297	0.408746	0.68273	0.22317340	subgrp_con	count		1	0
SEMA6D	5	OK	-0.960731	1.395414	-0.688491	0.49114	-0.00157680	subgrp_con	count		1	0
MYEF2	4	OK	0.0711722	1.3601836	0.0523255	0.95827	4.68E-05	subgrp_con	count		1	0
DUT	307	OK	0.0776424	0.1546517	0.5020471	0.61563	0.10922066	subgrp_con	count		1	0
FBN1	39	OK	0.5629196	0.5902250	0.9537373	0.34022	0.50646190	subgrp_con	count		1	0
EID1	672	OK	0.1203397	0.1016538	1.1838188	0.23649	0.17164768	subgrp_con	count		1	0
SECISBP2L	31	OK	0.6906628	0.5129495	1.346453	0.17816	0.76195226	subgrp_con	count		1	0
COPS2	90	OK	-0.327300	0.2884466	-1.134699	0.2565	-0.42901097	subgrp_con	count		1	0
GALK2	3	OK	6.4386681	6.8673603	0.937575	0.34846	0.00043590	subgrp_con	count		1	0
FAM227B	1	OK	-11.01941	43.249935	-0.254784	0.79889	-1.17482461	subgrp_con	count		1	0
DTWD1	24	OK	0.0565891	0.6136816	0.0922125	0.92653	0.05791471	subgrp_con	count		1	0
GABPB1	9	OK	-0.441068	0.9954556	-0.443081	0.65771	-0.22073585	subgrp_con	count		1	0
GABPB1-AS1	16	OK	0.0505857	0.863700	0.0585688	0.9532	0.05327727	subgrp_con	count		1	0
USP8	61	OK	0.6535622	0.4083496	1.6004969	0.10949	0.80575486	subgrp_con	count		1	0
TRPM7	9	OK	1.6315080	1.3708437	1.1901487	0.23399	0.48716726	subgrp_con	count		1	0
SPPL2A	70	OK	0.670312	0.380768	1.7604204	0.07834	0.76469354	subgrp_con	count		1	0
TNFAIP8L3	6	OK	-1.963664	1.4246309	-1.378366	0.16800	-1.03430556	subgrp_con	count		1	0
SCG3	3	OK	-1.499244	1.6042469	-0.934547	0.35002	-0.00085793	subgrp_con	count		1	0
LYSMD2	63	OK	-0.722782	0.3787720	-1.908225	0.05636	-0.91347958	subgrp_con	count		1	0
TMOD2	9	OK	-0.856443	1.0631059	-0.805604	0.42047	-0.67279495	subgrp_con	count		1	0
TMOD3	358	OK	-0.634935	0.1481357	-4.286170	1.82E-05	-0.8889965	subgrp_con	count		0.1817067	0
LEO1	26	OK	-0.341054	0.6056428	-0.563128	0.57335	-0.33285064	subgrp_con	count		1	0
MAPK6	5	OK	-1.606445	1.618566	-0.992511	0.32095	-0.41120881	subgrp_con	count		1	0
GNB5	6	OK	-0.268198	1.2291585	-0.218196	0.82728	-0.13377831	subgrp_con	count		1	0
MYO5C	19	OK	-1.944748	0.8254452	-2.355999	0.01847	-1.6516253	subgrp_con	count		1	0
MYO5A	8	OK	5.2970356	4.0741023	1.3001724	0.19354	1.0014446	subgrp_con	count		1	0
ARPP19	37	OK	0.8672689	0.5437892	1.5950466	0.1107	0.90957254	subgrp_con	count		1	0
FAM214A	6	OK	1.6609888	1.3318866	1.2470941	0.21236	0.82226607	subgrp_con	count		1	0
RSL24D1	204	OK	-0.423539	0.1971785	-2.148001	0.03171	-0.58016336	subgrp_con	count		1	0
RAB27A	34	OK	-0.512209	0.5169448	-0.990839	0.32176	-0.54508353	subgrp_con	count		1	0
PIGBOS1	14	OK	-0.268119	0.9016737	-0.297357	0.76619	-0.19866338	subgrp_con	count		1	0
CCPG1	222	OK	0.8694681	0.1916300	4.5372220	5.7E-06	1.19663412	subgrp_con	count		0.0571653	0
DNAAF4	10	OK	0.1026664	0.9797538	0.1047880	0.91654	0.07726742	subgrp_con	count		1	0
NEDD4	39	OK	0.5676105	0.5577642	1.017653	0.30884	0.56035076	subgrp_con	count		1	0
RFX7	2	OK	-1.272589	2.3104190	-0.550803	0.58177	-0.74406113	subgrp_con	count		1	0
TEX9	1	OK	3.4598146	6.7714513	0.5109413	0.60939	0.00030179	subgrp_con	count		1	0
MNS1	2	OK	-11.01941	43.249935	-0.254784	0.79889	-1.17482461	subgrp_con	count		1	0
ZNF280D	9	OK	-0.167450	0.9199452	-0.182022	0.85557	-0.05088650	subgrp_con	count		1	0
TCF12	14	OK	1.2102035	0.9272878	1.3051002	0.19186	0.60630131	subgrp_con	count		1	0
CGNL1	2	OK	21.105322	83.341273	0.2532397	0.80008	0.0003103	subgrp_con	count		1	0
POLR2M	2	OK	-1.295616	2.041425	-0.634662	0.52565	-0.00075754	subgrp_con	count		1	0
ALDH1A2	29	OK	-0.126835	0.5163747	-0.245627	0.80597	-0.11929773	subgrp_con	count		1	0
ADAM10	51	OK	0.1486281	0.409827	0.3626600	0.71686	0.17440356	subgrp_con	count		1	0
MINDY2	27	OK	1.076782	0.6160175	1.7479742	0.08047	1.05196504	subgrp_con	count		1	0
RNF111	7	OK	2.887049	1.676733	1.7218301	0.0851	1.67492432	subgrp_con	count		1	0
SLTM	153	OK	0.1180097	0.2228009	0.5296646	0.59634	0.16151879	subgrp_con	count		1	0
MYO1E	17	OK	0.6272909	0.749155	0.8374638	0.40232	0.56140302	subgrp_con	count		1	0
GTF2A2	175	OK	0.3447383	0.2120188	1.6259798	0.10395	0.47475195	subgrp_con	count		1	0
BNIP2	146	OK	-0.09372	0.2362377	-0.396719	0.69157	-0.12582981	subgrp_con	count		1	0
ANXA2	724	OK	-0.182156	0.098863	-1.842506	0.0654	-0.25971545	subgrp_con	count		1	0
ICE2	12	OK	-0.264884	0.8621141	-0.307249	0.75865	-0.23279200	subgrp_con	count		1	0
RORA	34	OK	-1.171348	0.5447530	-2.150238	0.03154	-1.03124455	subgrp_con	count		1	0
VPS13C	81	OK	-0.122686	0.3077586	-0.398644	0.69016	-0.15397354	subgrp_con	count		1	0
TLN2	1	OK	-10.44227	41.465809	-0.251828	0.80117	-0.00045289	subgrp_con	count		1	0
TPM1	150	OK	-0.092641	0.2330934	-0.397445	0.69104	-0.12471594	subgrp_con	count		1	0
TPM1-AS	1	OK	-10.44227	41.465809	-0.251828	0.80117	-0.00045289	subgrp_con	count		1	0
LACTB	37	OK	-0.104554	0.496333	-0.210652	0.83316	-0.0780490	subgrp_con	count		1	0
RPS27L	396	OK	0.3328397	0.1441386	2.309166	0.02093	0.46858012	subgrp_con	count		1	0
RAB8B	35	OK	0.0293452	0.4879531	0.0601393	0.95204	0.03573720	subgrp_con	count		1	0
APH1B	7	OK	-1.233312	1.3768454	-0.895752	0.37038	-0.33531194	subgrp_con	count		1	0
USP3	14	OK	1.4815960	0.926111	1.5998032	0.10964	0.92496689	subgrp_con	count		1	0
HERC1	10	OK	2.7288739	1.6699498	1.6341053	0.10224	1.168370	subgrp_con	count		1	0
FAM96A	25	OK	-0.940019	0.6953895	-1.351769	0.17645	-0.91678926	subgrp_con	count		1	0
SNX1	17	OK	0.5103143	0.7174463	0.7112927	0.4769	0.42529458	subgrp_con	count		1	0
PPIB	628	OK	0.1076452	0.1082898	0.9940471	0.3202	0.15316514	subgrp_con	count		1	0
TRIP4	3	OK	-0.910223	2.1881125	-0.415985	0.67742	-0.25785735	subgrp_con	count		1	0
ZNF609	6	OK	1.010366	1.260543	0.8015325	0.42282	0.31288283	subgrp_con	count		1	0
OAZ2	38	OK	0.5855080	0.5328544	1.0988143	0.27185	0.6175269	subgrp_con	count		1	0
PIF1	1	OK	17.780255	15.808015	1.1247620	0.26069	0.00690633	subgrp_con	count		1	0
PLEKHO2	12	OK	-0.012031	0.9600735	-0.012531	0.99	-0.00905166	subgrp_con	count		1	0
SPG21	21	OK	0.5003915	0.6578895	0.7606011	0.4469	0.52823305	subgrp_con	count		1	0
MTFMT	1	OK	3.4598146	6.7714513	0.5109413	0.60939	0.00030179	subgrp_con	count		1	0
PDCD7	54	OK	0.4204326	0.3853665	1.0909940	0.27528	0.51260118	subgrp_con	count		1	0
CLPX	7	OK	0.0605254	1.1297061	0.0535763	0.95727	0.04523458	subgrp_con	count		1	0
DPP8	12	OK	-1.888500	1.2700757	-1.486919	0.13704	-0.76832180	subgrp_con	count		1	0
HACD3	77	OK	-0.225982	0.3415200	-0.661696	0.50817	-0.29250995	subgrp_con	count		1	0
INTS14	4	OK	0.329539	1.4055480	0.2344472	0.81464	0.10244709	subgrp_con	count		1	0
DENND4A	5	OK	1.1006820	1.5687290	0.7016394	0.4829	0.55471951	subgrp_con	count		1	0
RAB11A	368	OK	-0.524759	0.1453977	-3.609133	0.00031	-0.73682738	subgrp_con	count		1	0
TIPIN	6	OK	1.1006820	1.5687290	0.7016394	0.4829	0.55471951	subgrp_con	count		1	0
MAP2K1	2	OK	5.8049273	5.6694662	1.023893	0.30589	1.03285716	subgrp_con	count		1	0
SNAPC5	12	OK	-0.009978	0.8656791	-0.011526	0.9908	-0.00644335	subgrp_con	count		1	0
RPL4	655	OK	-0.211293	0.1045577	-2.020830	0.0433	-0.29991268	subgrp_con	count		1	0
SMAD3	4	OK	-12.14842	43.455309	-0.279561	0.77981	-1.91362624	subgrp_con	count		1	0
AAGAB	6	OK	-0.796035	1.1365339	-0.700406	0.48367	-0.37941249	subgrp_con	count		1	0
C15orf61	16	OK	-0.527568	0.7444479	-0.708670	0.47852	-0.4865373	subgrp_con	count		1	0
PIAS1	7	OK	-4.43E-06	1.119316	-3.96E-06	1	-7.88E-0	subgrp_con	count		1	0
FEM1B	5	OK	-15.99503	70.34942	-0.227365	0.82014	-0.00028474	subgrp_con	count		1	0
ANP32A	87	OK	-0.053720	0.3213836	-0.167152	0.86725	-0.06896292	subgrp_con	count		1	0
SPESP1	1	OK	3.4598146	6.7714513	0.5109413	0.60939	0.00030179	subgrp_con	count		1	0
NOX5	1	OK	0.6532528	2.5001286	0.2612877	0.79387	0.00016297	subgrp_con	count		1	0
EWSAT1	1	OK	3.4598146	6.7714513	0.5109413	0.60939	0.00030179	subgrp_con	count		1	0
GLCE	10	OK	0.5102625	1.1311913	0.4510842	0.65193	0.26115165	subgrp_con	count		1	0
RPLP1	889	OK	0.0091870	0.0820122	0.1120208	0.91081	0.01315156	subgrp_con	count		1	0
TLE3	6	OK	4.2109206	4.2875616	0.982124	0.32604	0.00257090	subgrp_con	count		1	0
UACA	159	OK	-0.305736	0.2287562	-1.336516	0.18138	-0.40982374	subgrp_con	count		1	0
LARP6	30	OK	-0.575386	0.5487543	-1.048531	0.29430	-0.6357722	subgrp_con	count		1	0
LRRC49	4	OK	0.1681491	1.2198596	0.1378430	0.89036	0.08564139	subgrp_con	count		1	0
THAP10	1	OK	18.759489	83.625179	0.2243282	0.8225	0.00028789	subgrp_con	count		1	0
THSD4	6	OK	4.1203745	2.5258514	1.6312814	0.10282	1.44689194	subgrp_con	count		1	0
MYO9A	24	OK	-0.479590	0.5801207	-0.826707	0.4084	-0.49777109	subgrp_con	count		1	0
PKM	259	OK	-0.288027	0.1751872	-1.644114	0.10015	-0.39730655	subgrp_con	count		1	0
PARP6	1	OK	3.4598146	6.7714513	0.5109413	0.60939	0.00030179	subgrp_con	count		1	0
HEXA	75	OK	-0.084216	0.3121129	-0.269826	0.78729	-0.10883326	subgrp_con	count		1	0
ARIH1	30	OK	-0.664938	0.5219259	-1.274009	0.20266	-0.47972856	subgrp_con	count		1	0
BBS4	6	OK	1.3477282	1.5268314	0.8826961	0.3774	0.44189371	subgrp_con	count		1	0
ADPGK	1	OK	3.4598146	6.7714513	0.5109413	0.60939	0.00030179	subgrp_con	count		1	0
NPTN	48	OK	0.8822539	0.4589678	1.9222564	0.05457	1.00301129	subgrp_con	count		1	0
CD276	7	OK	1.9780750	1.6528914	1.1967361	0.23141	0.57244123	subgrp_con	count		1	0
STOML1	3	OK	4.6613299	5.7683860	0.8080821	0.41904	0.00274918	subgrp_con	count		1	0

PML	35	OK	-1.5251038	0.5440099	-2.8034482	0.00506	-1.58376852	subgrp_con	count	1	0
ISLR	6	OK	0.17452762	1.11176869	0.15698195	0.87526	0.08795558	subgrp_con	count	1	0
SEMA7A	4	OK	24.0001923	54.3454405	0.44162292	0.65870	2.40091057	subgrp_con	count	1	0
UBL7	17	OK	1.28995515	0.87628647	1.47190217	0.14105	1.14043913	subgrp_con	count	1	0
UBL7-AS1	3	OK	4.66132998	5.76838606	0.80808217	0.41906	0.00274918	subgrp_con	count	1	0
CLK3	13	OK	-1.289712	0.9465132	-1.3625929	0.17301	-0.87363421	subgrp_con	count	1	0
CSK	6	OK	0.93967049	1.49854054	0.6270571	0.53062	0.29164784	subgrp_con	count	1	0
ULK3	2	OK	-11.019418	43.2499352	-0.2547846	0.79889	-1.17482461	subgrp_con	count	1	0
SCAMP2	45	OK	0.4003621	0.44379808	0.9021267	0.36699	0.51833834	subgrp_con	count	1	0
MPI	2	OK	18.7594898	83.6251793	0.22432824	0.8225	0.00028789	subgrp_con	count	1	0
COX5A	270	OK	0.4931967	0.18278613	2.69821736	0.00697	0.68719552	subgrp_con	count	1	0
SCAMP5	3	OK	1.9258645	2.51548794	0.7656425	0.44389	0.56022119	subgrp_con	count	1	0
PPCDC	2	OK	23.9918968	84.8999419	0.28259026	0.77749	1.65081709	subgrp_con	count	1	0
COMMD4	40	OK	0.14993895	0.5378606	0.27876916	0.78042	0.16218343	subgrp_con	count	1	0
NEIL1	13	OK	-0.2204194	0.9034796	-0.2439672	0.80726	-0.14094834	subgrp_con	count	1	0
MAN2C1	1	OK	23.9918968	84.8999419	0.28259026	0.77749	1.65081709	subgrp_con	count	1	0
SIN3A	12	OK	-0.5597229	0.90176156	-0.6207005	0.5348	-0.47780785	subgrp_con	count	1	0
PTPN9	1	OK	18.7594898	83.6251793	0.22432824	0.8225	0.00028789	subgrp_con	count	1	0
SNUPN	8	OK	0.82013909	1.12225096	0.73014768	0.4652	0.53118321	subgrp_con	count	1	0
IMP3	155	OK	-0.3404537	0.2282857	-1.4913487	0.13587	-0.46757122	subgrp_con	count	1	0
UBE2Q2	53	OK	-0.059243	0.3961654	-0.1495413	0.88112	-0.07311259	subgrp_con	count	1	0
FBXO22	12	OK	0.17215509	0.8859863	0.19430897	0.84593	0.00030951	subgrp_con	count	1	0
ETFA	45	OK	1.00278826	0.4923858	2.03659073	0.04169	1.12559872	subgrp_con	count	1	0
SCAPER	11	OK	-0.4123149	0.88075018	-0.4681405	0.63968	-0.12296818	subgrp_con	count	1	0
PEAK1	8	OK	-2.9861246	1.67561223	-1.7821095	0.07472	-1.29979885	subgrp_con	count	1	0
HMG20A	9	OK	0.52638409	0.96309118	0.54655686	0.58468	0.49410232	subgrp_con	count	1	0
TBC1D2B	9	OK	2.44069513	1.54928953	1.57536413	0.11517	1.42568840	subgrp_con	count	1	0
CIB2	1	OK	23.9918968	84.8999419	0.28259026	0.77749	1.65081709	subgrp_con	count	1	0
IDH3A	4	OK	-10.967865	27.1536587	-0.4039185	0.68627	-1.17125107	subgrp_con	count	1	0
DNAJA4	1	OK	-15.99503	70.34942	-0.2272655	0.82014	-0.00028474	subgrp_con	count	1	0
WDR61	53	OK	0.3775421	0.41698815	0.9054025	0.36525	0.42496774	subgrp_con	count	1	0
IREB2	5	OK	1.69632904	1.67450798	1.01303132	0.31105	0.50382681	subgrp_con	count	1	0
HYKK	1	OK	-10.442278	41.4658099	-0.2518286	0.80117	-0.00045289	subgrp_con	count	1	0
PSMA4	189	OK	-0.1334148	0.21444797	-0.6221314	0.53386	-0.18000681	subgrp_con	count	1	0
ADAMTS7	3	OK	1.58240009	1.92403354	0.82243893	0.41082	0.77175617	subgrp_con	count	1	0
MORF4L1	512	OK	-0.018913	0.11894353	-0.159009	0.87366	-0.02681716	subgrp_con	count	1	0
TMED3	33	OK	1.93036964	0.554198	3.4831762	0.0005	2.3054029	subgrp_con	count	1	0
MTHFS	1	OK	1.58229999	2.72001883	0.58176068	0.56072	0.47433761	subgrp_con	count	1	0
ZFAND6	71	OK	0.6850259	0.37282302	1.83740234	0.06615	0.82663431	subgrp_con	count	1	0
ABHD17C	7	OK	1.68293668	1.32986173	1.2654975	0.20568	1.19645557	subgrp_con	count	1	0
CEMIP	5	OK	2.07025237	1.79948267	1.15047086	0.24995	0.96210332	subgrp_con	count	1	0
MESD	149	OK	0.6442897	0.24503877	2.62933809	0.00856	0.86520939	subgrp_con	count	1	0
TLNRD1	46	OK	0.48442275	0.45653865	1.06107723	0.28865	0.51283326	subgrp_con	count	1	0
SAXO2	1	OK	-11.019418	43.2499352	-0.2547846	0.79889	-1.17482461	subgrp_con	count	1	0
RPS17	39	OK	0.3006433	0.51084886	0.5885170	0.55619	0.25065404	subgrp_con	count	1	0
SNHG21	1	OK	3.4598146	6.7714513	0.51094137	0.60930	0.00030179	subgrp_con	count	1	0
WHAMM	16	OK	0.8817229	0.90465814	0.97464872	0.32972	0.64844084	subgrp_con	count	1	0
RAMMET	15	OK	-0.3591032	0.7629177	-0.4706958	0.63788	-0.33474183	subgrp_con	count	1	0
C15orf40	24	OK	0.57264553	0.58957196	0.97129029	0.3314	0.61931469	subgrp_con	count	1	0
BTBD1	12	OK	-0.0387652	1.05855672	-0.0366208	0.97079	-0.01958909	subgrp_con	count	1	0
TM6SF1	20	OK	2.09789113	0.9773092	2.1465992	0.03182	1.82348276	subgrp_con	count	1	0
HDGFL3	252	OK	-0.1734356	0.16974884	-1.021719	0.30691	-0.24132715	subgrp_con	count	1	0
ADAMTSL3	2	OK	18.7594898	83.6251793	0.22432824	0.8225	0.00028789	subgrp_con	count	1	0
AC068282.5	1	OK	1.58229999	2.72001883	0.58176068	0.56072	0.47433761	subgrp_con	count	1	0
WDR73	2	OK	-14.65800	45.6903329	-0.3208138	0.74835	-0.00067223	subgrp_con	count	1	0
NMB	2	OK	1.9258645	2.51548794	0.7656425	0.44389	0.56022119	subgrp_con	count	1	0
SEC11A	519	OK	-0.2094079	0.12090377	-1.7320213	0.08327	-0.29708687	subgrp_con	count	1	0
ALPK3	3	OK	4.66132998	5.76838606	0.80808217	0.41906	0.00274918	subgrp_con	count	1	0
PDE8A	11	OK	1.16713436	0.92094749	1.26731912	0.20506	0.8619232	subgrp_con	count	1	0
AKAP13	170	OK	-0.0816746	0.2178332	-0.3749413	0.7077	-0.11048584	subgrp_con	count	1	0
MRPL46	13	OK	0.7182669	0.92284725	0.7784244	0.43632	0.4693632	subgrp_con	count	1	0
MRPS11	24	OK	1.21439731	0.70977016	1.71097262	0.08709	1.21988967	subgrp_con	count	1	0
ISG20	28	OK	0.5289731	0.5724244	0.92409259	0.35544	0.54216518	subgrp_con	count	1	0
HAPLN3	115	OK	-0.3446064	0.25113247	-1.3722096	0.17	-0.45045470	subgrp_con	count	1	0
MFGE8	49	OK	0.93666883	0.45002628	2.08136475	0.0374	0.91823782	subgrp_con	count	1	0
ABHD2	31	OK	4.60750245	1.50002291	3.07162139	0.00213	3.00053555	subgrp_con	count	1	0
PEX11A	3	OK	1.533588	2.24703247	0.68249481	0.49492	0.00105583	subgrp_con	count	1	0
ARPIN	12	OK	-0.5645106	0.92085996	-0.6130254	0.53986	-0.35260596	subgrp_con	count	1	0
ZNF710	2	OK	5.80492735	5.66946625	1.0238931	0.30589	1.03285716	subgrp_con	count	1	0
IDH2	14	OK	0.08781007	0.89068095	0.09858751	0.92147	0.05699644	subgrp_con	count	1	0
CIB1	136	OK	1.00776931	0.27584024	3.65345287	0.00026	1.32821552	subgrp_con	count	1	0
NGRN	1	OK	17.7472079	50.9719273	0.34817612	0.72771	0.0007532	subgrp_con	count	1	0
IQGAP1	163	OK	0.10242957	0.22959248	0.44617978	0.65547	0.13853394	subgrp_con	count	1	0
CRTC3	4	OK	1.77247723	1.84296277	0.96123265	0.33644	0.84969892	subgrp_con	count	1	0
FURIN	1	OK	3.4598146	6.7714513	0.51094137	0.60930	0.00030179	subgrp_con	count	1	0
FES	7	OK	-0.5561292	1.05719913	-0.5260401	0.59886	-0.27129238	subgrp_con	count	1	0
HDDC3	9	OK	0.82211238	0.93301579	0.88113444	0.37825	0.68094617	subgrp_con	count	1	0
UNC45A	6	OK	-1.3401314	1.68194103	-0.7967767	0.42558	-0.59807560	subgrp_con	count	1	0
VPS33B	1	OK	-11.019418	43.2499352	-0.2547846	0.79889	-1.17482461	subgrp_con	count	1	0
SV2B	1	OK	-11.019418	43.2499352	-0.2547846	0.79889	-1.17482461	subgrp_con	count	1	0
SLCO3A1	28	OK	-1.3806576	0.64100349	-2.1539002	0.03125	-1.10616268	subgrp_con	count	1	0
CHD2	24	OK	1.08017519	0.68928617	1.56709249	0.11709	0.88894083	subgrp_con	count	1	0
MCTP2	2	OK	35.3043164	67.4881674	0.5231186	0.60089	0.00071431	subgrp_con	count	1	0
LINC01197	12	OK	-0.6935696	0.83787759	-0.8277696	0.4079	-0.42845300	subgrp_con	count	1	0
IGF1R	22	OK	1.81930105	0.7837905	2.32115728	0.02028	1.10198107	subgrp_con	count	1	0
SYNM	8	OK	1.63097677	1.37045834	1.19009585	0.23401	0.49536710	subgrp_con	count	1	0
TTC23	2	OK	-0.9102232	2.1881125	-0.4159856	0.67742	-0.25785735	subgrp_con	count	1	0
LRRC28	4	OK	1.11124491	1.86858082	0.59469996	0.55206	0.34276981	subgrp_con	count	1	0
MEF2A	111	OK	0.76668007	0.30303223	2.5300281	0.01141	0.96195168	subgrp_con	count	1	0
LYSMD4	1	OK	3.45978793	4.78585287	0.72292983	0.46972	0.00081879	subgrp_con	count	1	0
LINS1	3	OK	-0.9727207	1.63750584	-0.5940258	0.55249	-0.27349270	subgrp_con	count	1	0
ALDH1A3	17	OK	0.72622257	0.6880515	1.05547705	0.29121	0.74203567	subgrp_con	count	1	0
LRRK1	3	OK	-12.145707	43.4188694	-0.2797334	0.77968	-1.91338276	subgrp_con	count	1	0
CHSY1	50	OK	1.69679929	0.59156156	2.86833935	0.00413	1.33346222	subgrp_con	count	1	0
SELENOS	218	OK	0.6299725	0.19748311	3.19000597	0.00142	0.86852677	subgrp_con	count	1	0
SNRPA1	19	OK	2.03782349	0.82953004	2.45659999	0.01403	1.9328239	subgrp_con	count	1	0
TM2D3	35	OK	0.09401011	0.48415458	0.19417376	0.84606	0.10572418	subgrp_con	count	1	0
TARSL2	4	OK	5.26127895	5.49058205	0.9582370	0.33794	0.00295034	subgrp_con	count	1	0
POLR3K	19	OK	0.87597623	0.7272146	1.20439795	0.22844	0.82345843	subgrp_con	count	1	0
SNRNP25	12	OK	0.58308223	0.77370369	0.75362485	0.45107	0.55183480	subgrp_con	count	1	0
RHBDF1	2	OK	5.80492735	5.66946625	1.0238931	0.30589	1.03285716	subgrp_con	count	1	0
MPG	97	OK	0.52029546	0.32430374	1.60434616	0.10864	0.67726237	subgrp_con	count	1	0
NPRL3	5	OK	1.36704329	1.74186477	0.78481597	0.43256	0.41600004	subgrp_con	count	1	0
LUC7L	29	OK	0.50768288	0.62489604	0.81242774	0.41655	0.50405689	subgrp_con	count	1	0
FAM234A	10	OK	0.01007329	0.94172432	0.01069665	0.99147	0.00755187	subgrp_con	count	1	0
RGS11	1	OK	18.7594898	83.6251793	0.22432824	0.8225	0.00028789	subgrp_con	count	1	0
ARHGDIG	1	OK	23.9918968	84.8999419	0.28259026	0.77749	1.65081709	subgrp_con	count	1	0
AXIN1	2	OK	2.40963373	2.47152069	0.97495997	0.32958	0.66517343	subgrp_con	count	1	0
MRPL28	36	OK	0.30828923	0.48673268	0.63359055	0.52635	0.3576165	subgrp_con	count	1	0
TMEM8A	6	OK	3.4598146	6.7714513	0.51094137	0.60930	0.00030179	subgrp_con	count	1	0
NME4	48	OK	1.24480555	0.46596092	2.67148061	0.00755	1.49570114	subgrp_con	count	1	0
DECR2	3	OK	0.87880157	1.64693725	0.53359745	0.59362	0.44698448	subgrp_con	count	1	0
RAB11FIP3	2	OK	3.4598146	6.7714513	0.51094137	0.60930	0.00030179	subgrp_con	count	1	0
CAPN15	2	OK	-12.148421	43.455309	-0.2795613	0.77981	-1.91362624	subgrp_con	count	1	0
PIGQ	4	OK	0.07441875	1.68072532	0.04427777	0.96468	0.02306319	subgrp_con	count	1	0
NHLRC4	1	OK	0.65325287	2.50012846	0.26128772	0.79387	0.00016297	subgrp_con	count	1	0
METTL26	132	OK	0.57256713	0.25396679	2.25449605	0.02416	0.77732165	subgrp_con	count	1	0
MCRIP2	6	OK	1.28047999	1.51293457	0.84635517	0.39735	0.63872727	subgrp_con	count	1	0
RHOT2	3	OK	0.65325287	2.50012846	0.26128772	0.79387	0.00016297	subgrp_con	count	1	0
STUB1	137	OK	0.57554609	0.24244532	2.37392116	0.0176	0.78056421	subgrp_con	count	1	0
JMJD8	22	OK	0.53047908	0.60614682	0.87516599	0.38148	0.54369991	subgrp_con	count	1	0
METRN	42	OK	-0.1784959	0.47990674	-0.3719387	0.70994	-0.1997837	subgrp_con	count	1	0
FAM173A	27	OK	0.18962285	0.57781713	0.32817104	0.74278	0.20964980	subgrp_con	count	1	0
NARFL	2	OK	-0.3495512	1.85447474	-0.1884907	0.85049	-0.17337783	subgrp_con	count	1	0
RPUSD1	1	OK	23.9918968	84.8999419	0.28259026	0.77749	1.65081709	subgrp_con	count	1	0
AC009041.2	2	OK	21.642059	54.4868919	0.39719753	0.69122	1.57744735	subgrp_con	count	1	0
UBE2I	127	OK	0.25807438	0.26380955	0.97828006	0.32795	0.34108122	subgrp_con	count	1	0
TSR3	37	OK	0.09049169	0.50734431	0.17836345	0.85844	0.10174236	subgrp_con	count	1	0
GNPTG	54	OK	0.91466069	0.39053341	2.34208037	0.01918	1.17481849	subgrp_con	count	1	0
UNKL	1	OK	1.58229999	2.72001883	0.58176068	0.56072	0.47433761	subgrp_con	count	1	0
C16orf91	12	OK	0.51673169	0.87033309	0.59371715	0.5527	0.43061747	subgrp_con	count	1	0
TMEM204	124	OK	0.62674365	0.26623683	2.35408325	0.01857	0.81976334	subgrp_con	count	1	0
CRAMP1	1	OK	23.9918968	84.8999419	0.28259026	0.77749	1.65081709	subgrp_con	count	1	0
AL031708.1	1	OK	-11.019418	43.2499352	-0.2547846	0.79889	-1.17482461	subgrp_con	count	1	0
JPT2	1	OK	-11.019418	43.2499352	-0.2547846	0.79889	-1.17482461	subgrp_con	count	1	0
MAPK8IP3	2	OK	23.9918968	84.8999419	0.28259026	0.77749	1.65081709	subgrp_con	count	1	0
NME3	231	OK	0.52815375	0.18342389	2.8794164	0.00398	0.72561157	subgrp_con	count	1	0
MRPS34	102	OK	0.08754348	0.28792304	0.30405153	0.76109	0.11589232	subgrp_con	count	1	0
SPSB3	40	OK	-0.8249897	0.49153423	-1.678397	0.09327	-0.90104420	subgrp_con	count	1	0
NUBP2	26	OK	0.38927453	0.58700878	0.66314942	0.50722	0.39945023	subgrp_con	count	1	0
HAGH	40	OK	1.48902205	0.5251764	2.83527978	0.00458	1.62580702	subgrp_con	count	1	0
FAHD1	11	OK	-0.1833169	0.86212377	-0.212634	0.83162	-0.16263771	subgrp_con	count	1	0
MSRB1	5	OK	3.37674291	2.38312277	1.41694039	0.1565	1.32018257	subgrp_con	count	1	0
NDUFB10	337	OK	0.37939534	0.15291939	2.48489637	0.01371	0.52375344	subgrp_con	count	1	0
RPS2	884	OK	-0.0197556	0.08267578	-0.2389499	0.81114	-0.02828837	subgrp_con	count	1	0
SNHG9	15	OK	1.29794473	0.99071767	1.31010561	0.19016	1.31863863	subgrp_con	count	1	0
TBL3	1	OK	-15.99503	70.34942	-0.2272655	0.82014	-0.00028474	subgrp_con	count	1	0
GFER	13	OK	-1.1871092	0.93877986	-1.2645235	0.20606	-0.81303913	subgrp_con	count	1	0
ZNF598	9	OK	0.16934047	0.83535871	0.20271587	0.83936	0.10995132	subgrp_con	count	1	0
NPW	2	OK	21.642059	54.4868919	0.39719753	0.69122	1.57744735	subgrp_con	count	1	0
SLC9A3R2	536	OK	-0.0329746	0.11288958	-0.2920942	0.77021	-0.04682979	subgrp_con	count	1	0
NTHL1	54	OK	-0.476785	0.37380273	-1.2754994	0.20212	-0.5914241	subgrp_con	count	1	0
TSC2	4	OK	18.7594898	83.6251793	0.22432824	0.8225	0.00028789	subgrp_con	count	1	0
PKD1	6	OK	32.4037175	43.7444212	0.74075086	0.45886	1.92760869	subgrp_con	count	1	0
TRAF7	11	OK	1.98068681	1.32233199	1.49787408	0.13417	0.57303448	subgrp_con	count	1	0
MLST8	5	OK	3.0502985	2.44043936	1.24989728	0.21134	1.24934625	subgrp_con	count	1	0
PGP	27	OK	1.08578473	0.64846404	1.67439457	0.09405	1.01243921	subgrp_con	count	1	0
ECI1	40	OK	0.43979534	0.51489201	0.85415063	0.39302	0.46444120	subgrp_con	count	1	0
RNPS1	102	OK	-0.1717202	0.27013422	-0.6356846	0.52498	-0.22491284	subgrp_con	count	1	0
AMDHD2	4	OK	4.21092061	4.28756165	0.9821248	0.32606	0.00257090	subgrp_con	count	1	0
PDPK1	3	OK	0.8788174	1.64693117	0.53360919	0.59361	0.44699547	subgrp_con	count	1	0
PRSS27	1	OK	-15.99503	70.34942	-0.2272655	0.82014	-0.00028474	subgrp_con	count	1	0
SRRM2	414	OK	0.11328696	0.13305393	0.85143641	0.39452	0.16054342	subgrp_con	count	1	0
ELOB	501	OK	0.31339277	0.12253793	2.55751664	0.01054	0.44437416	subgrp_con	count	1	0
FLYWCH2	55	OK	-0.1199685	0.40347313	-0.2973396	0.76621	-0.1366258	subgrp_con	count	1	0
FLYWCH1	1	OK	18.7594898	83.6251793	0.22432824	0.8225	0.00028789	subgrp_con	count	1	0
PAQR4	3	OK	5.26127895	5.49058205	0.9582370	0.33794	0.00295034	subgrp_con	count	1	0
THOC6	2	OK	-0.3495512	1.85447474	-0.1884907	0.85049	-0.17337783	subgrp_con	count	1	0
MMP25-AS1	1	OK	18.7594898	83.6251793	0.22432824	0.8225	0.00028789	subgrp_con	count	1	0
IL32	127	OK	0.68172527	0.28270894	2.41140332	0.01589	0.88117771	subgrp_con	count	1	0
AC108134.3	4	OK	-1.8537846	1.85435929	-0.99969	0.31748	-0.46052349	subgrp_con	count	1	0
ZNF205	3	OK	-1.6462456	1.93519292	-0.8506881	0.39494	-0.70360391	subgrp_con	count	1	0
AC108134.2	2	OK	-10.475634	27.4919879	-0.3810432	0.70317	-1.13811795	subgrp_con	count	1	0
ZNF200	2	OK	-0.910142	2.18812438	-0.4159464	0.67745	-0.25784050	subgrp_con	count	1	0
ZNF263	2	OK	0.65325287	2.50012846	0.26128772	0.79387	0.00016297	subgrp_con	count	1	0
ZNF75A	2	OK	-11.448548	41.761485	-0.2741412	0.78398	-0.00049246	subgrp_con	count	1	0
ZSCAN32	1	OK	-11.019418	43.2499352	-0.2547846	0.79889	-1.17482461	subgrp_con	count	1	0
AC025283.2	1	OK	-11.019418	43.2499352	-0.2547846	0.79889	-1.17482461	subgrp_con	count	1	0
ZNF174	4	OK	-1.2520862	1.38783627	-0.9021858	0.36696	-0.56535851	subgrp_con	count	1	0
ZNF597	1	OK	1.58229999	2.72001883	0.58176068	0.56072	0.47433761	subgrp_con	count	1	0
NAA60	9	OK	0.14745295	0.97189979	0.1517162	0.87941	0.11094327	subgrp_con	count	1	0
CLUAP1	13	OK	-0.0828112	0.72613908	-0.1140432	0.9092	-0.06848228	subgrp_con	count	1	0
DNASE1	1	OK	41.7883033	174.29608	0.23975499	0.81052	0.00014488	subgrp_con	count	1	0
TRAP1	11	OK	-1.157114	0.98665594	-1.1727634	0.24089	-0.79590086	subgrp_con	count	1	0
CREBBP	13	OK	0.87687171	0.97851841	0.89612183	0.37019	0.58107860	subgrp_con	count	1	0
ADCY9	1	OK	-11.019418	43.2499352	-0.2547846	0.79889	-1.17482461	subgrp_con	count	1	0
GLIS2	4	OK	2.96462385	2.2923576	1.29326413	0.19592	0.76335144	subgrp_con	count	1	0
PAM16	1	OK	3.4598146	6.7714513	0.51094137	0.60930	0.00030179	subgrp_con	count	1	0
CORO7	1	OK	18.7594898	83.6251793	0.22432824	0.8225	0.00028789	subgrp_con	count	1	0
VASN	4	OK	21.1053223	83.341273	0.25323976	0.80008	0.0003103	subgrp_con	count	1	0
NMRAL1	12	OK	0.65699825	1.41612758	0.46392891	0.6427	0.33587741	subgrp_con	count	1	0
HMOX2	42	OK	2.43325249	0.75906132	3.20560727	0.00135	2.36294375	subgrp_con	count	1	0
CDIP1	11	OK	-0.7106237	0.87459825	-0.8125145	0.4165	-0.61232238	subgrp_con	count	1	0
UBALD1	1	OK	-10.442278	41.4658099	-0.2518286	0.80117	-0.00045289	subgrp_con	count	1	0
NUDT16L1	6	OK	1.41194268	1.49593083	0.94385555	0.34524	0.69797651	subgrp_con	count	1	0
ROGDI	3	OK	-0.6977694	1.69645387	-0.4113106	0.68084	-0.33591720	subgrp_con	count	1	0
GLYR1	9	OK	0.71990447	1.15885432	0.62122085	0.53445	0.22441317	subgrp_con	count	1	0
UBN1	52	OK	-0.4002206	0.44147974	-0.9065435	0.36465	-0.44508732	subgrp_con	count	1	0
PPL	7	OK	-1.4690786	1.15727729	-1.2694266	0.20429	-0.00084048	subgrp_con	count	1	0
NAGPA	3	OK	1.58229999	2.72001883	0.58176068	0.56072	0.47433761	subgrp_con	count	1	0
ALG1	1	OK	23.9918968	84.8999419	0.28259026	0.77749	1.65081709	subgrp_con	count	1	0
EEF2KMT	1	OK	-10.442278	41.4658099	-0.2518286	0.80117	-0.00045289	subgrp_con	count	1	0
METTL22	2	OK	-1.7585726	2.35544393	-0.7465996	0.45533	-0.00036089	subgrp_con	count	1	0
PMM2	5	OK	1.0103665	1.2605423	0.80153256	0.42282	0.31288283	subgrp_con	count	1	0
CARHSP1	151	OK	-0.5705747	0.23967047	-2.3806632	0.01728	-0.77106524	subgrp_con	count	1	0
USP7	24	OK	0.10228288	0.5971392	0.17145561	0.86387	0.07695379	subgrp_con	count	1	0
C16orf72	19	OK	2.0617629	1.0439358	1.97499108	0.04827	0.59359657	subgrp_con	count	1	0
ATF7IP2	3	OK	1.58240009	1.92403354	0.82243893	0.41082	0.77175617	subgrp_con	count	1	0
LINC01290	2	OK	-0.910142	2.18812438	-0.4159464	0.67745	-0.25784050	subgrp_con	count	1	0
EMP2	410	OK	-0.086123	0.1305207	-0.659845	0.50935	-0.12201115	subgrp_con	count	1	0
AC027277.1	1	OK	-15.99503	70.34942	-0.2272655	0.82014	-0.00028474	subgrp_con	count	1	0
NUBP1	12	OK	-1.284638	0.99222784	-1.2947007	0.19542	-0.87072713	subgrp_con	count	1	0
CIITA	7	OK	-0.8934795	1.17388291	-0.7611217	0.44658	-0.24979193	subgrp_con	count	1	0
DEXI	23	OK	1.47500066	0.67578115	2.18268026	0.02906	1.32899516	subgrp_con	count	1	0
SOCS1	10	OK	1.29156338	1.02429389	1.26093042	0.20732	0.79358154	subgrp_con	count	1	0
LITAF	53	OK	1.24588988	0.45546555	2.73421995	0.00625	1.49695245	subgrp_con	count	1	0
SNN	5	OK	-11.325296	26.9167859	-0.4207522	0.67394	-1.19569663	subgrp_con	count	1	0
TXNDC11	1	OK	-10.442278	41.4658099	-0.2518286	0.80117	-0.00045289	subgrp_con	count	1	0
ZC3H7A	19	OK	0.34699143	0.72050041	0.48155053	0.63012	0.32904959	subgrp_con	count	1	0
GSPT1	83	OK	0.05039691	0.32806137	0.15362568	0.87790	0.06420888	subgrp_con	count	1	0
SNX29	5	OK	5.00275631	4.22868299	1.18305305	0.23679	0.98115823	subgrp_con	count	1	0
CPPED1	14	OK	0.39665209	0.87919714	0.45115262	0.65188	0.12349128	subgrp_con	count	1	0
ERCC4	10	OK	0.02717521	0.89047591	0.03051763	0.97565	0.00839500	subgrp_con	count	1	0
LINC02185	75	OK	-0.9838618	0.32002501	-3.074328	0.00211	-1.26443590	subgrp_con	count	1	0
MKL2	126	OK	-1.167473	0.25682851	-4.545725	5.47E-06	-1.55363141	subgrp_con	count	0.0549094	0
PARN	3	OK	0.16814913	1.72533008	0.0974591	0.92236	0.05201277	subgrp_con	count	1	0
BFAR	42	OK	-0.1587529	0.42601197	-0.372649	0.70941	-0.18065889	subgrp_con	count	1	0
NOMO1	1	OK	18.7594898	83.6251793	0.22432824	0.8225	0.00028789	subgrp_con	count	1	0
PDXDC1	9	OK	-0.1158957	0.83645475	-0.1385550	0.8898	-0.03533727	subgrp_con	count	1	0
NTAN1	107	OK	0.16227361	0.29444971	0.55110805	0.58156	0.21397214	subgrp_con	count	1	0
RRN3	2	OK	-0.9102232	2.1881125	-0.4159856	0.67742	-0.25785735	subgrp_con	count	1	0
C16orf45	30	OK	-1.1455437	0.56188877	-2.0387012	0.04148	-1.17332387	subgrp_con	count	1	0
MARF1	8	OK	-1.6871368	1.02988334	-1.6381665	0.10139	-0.71675485	subgrp_con	count	1	0
NDE1	4	OK	1.099586	1.31888629	0.8337168	0.40444	0.55419423	subgrp_con	count	1	0
MYH11	9	OK	1.07430079	0.92761773	1.15812878	0.24681	0.94587791	subgrp_con	count	1	0
FOPNL	9	OK	2.50786971	1.41656384	1.77038947	0.07666	1.62759021	subgrp_con	count	1	0
ABCC1	3	OK	18.7594898	83.6251793	0.22432824	0.8225	0.00028789	subgrp_con	count	1	0
XYLT1	1	OK	18.7594898	83.6251793	0.22432824	0.8225	0.00028789	subgrp_con	count	1	0
RPS15A	872	OK	-0.1179977	0.08520463	-1.3848744	0.16609	-0.16881119	subgrp_con	count	1	0
ARL6IP1	169	OK	-0.3689828	0.20523651	-1.7978418	0.0722	-0.50898740	subgrp_con	count	1	0
SMG1	30	OK	0.07195219	0.55446591	0.12976842	0.89675	0.07106818	subgrp_con	count	1	0
TMC7	6	OK	2.45104359	1.52685244	1.60529173	0.10842	1.60305062	subgrp_con	count	1	0
COQ7	5	OK	-1.4345025	1.36672583	-1.0495905	0.29391	-0.95463182	subgrp_con	count	1	0
ITPRIPL2	43	OK	1.034778	0.5117585	2.02200472	0.04318	1.20528215	subgrp_con	count	1	0
GDE1	14	OK	0.28714829	0.93178643	0.30816983	0.75795	0.14669835	subgrp_con	count	1	0
CCP110	3	OK	-12.145707	43.4188694	-0.2797334	0.77968	-1.91338276	subgrp_con	count	1	0
VPS35L	8	OK	2.62654389	2.09613573	1.25304096	0.21019	0.0017549	subgrp_con	count	1	0
KNOP1	41	OK	0.02802165	0.46851298	0.05980976	0.95231	0.0309132	subgrp_con	count	1	0
IQCK	37	OK	0.45790047	0.47406205	0.96590831	0.33409	0.38170360	subgrp_con	count	1	0
GPRC5B	27	OK	0.51794043	0.55547675	0.93242503	0.35112	0.51267586	subgrp_con	count	1	0
ACSM3	7	OK	3.99180469	2.42006106	1.64946443	0.09905	0.89447291	subgrp_con	count	1	0
THUMPD1	82	OK	0.42500719	0.32215917	1.31924691	0.18709	0.53452016	subgrp_con	count	1	0
ERI2	1	OK	3.4598146	6.7714513	0.51094137	0.60930	0.00030179	subgrp_con	count	1	0
DCUN1D3	10	OK	2.43996065	1.21706182	2.00479598	0.04498	1.59720637	subgrp_con	count	1	0
LYRM1	45	OK	-1.0216547	0.440192	-2.3209246	0.02029	-1.15742471	subgrp_con	count	1	0
TMEM159	14	OK	-0.4728757	0.74344376	-0.6360612	0.52474	-0.43852313	subgrp_con	count	1	0
METTL9	103	OK	0.0766195	0.29650624	0.25840773	0.79609	0.09808105	subgrp_con	count	1	0
IGSF6	2	OK	0.16814913	1.72533008	0.0974591	0.92236	0.05201277	subgrp_con	count	1	0
OTOA	1	OK	-10.442278	41.4658099	-0.2518286	0.80117	-0.00045289	subgrp_con	count	1	0
UQCRC2	129	OK	0.23347573	0.24031116	0.9715561	0.33127	0.31339327	subgrp_con	count	1	0
MOSMO	16	OK	0.12898832	0.8134224	0.15857483	0.874	0.12203092	subgrp_con	count	1	0
SDR42E2	1	OK	1.58229999	2.72001883	0.58176068	0.56072	0.47433761	subgrp_con	count	1	0
EEF2K	6	OK	0.3002409	1.19206856	0.25186521	0.80115	0.22905584	subgrp_con	count	1	0
POLR3E	2	OK	-11.019418	43.2499352	-0.2547846	0.79889	-1.17482461	subgrp_con	count	1	0
CDR2	5	OK	0.63934773	1.01915039	0.62733405	0.53044	0.00043355	subgrp_con	count	1	0
NPIPB5	5	OK	5.68312411	5.33318758	1.06561489	0.2866	0.00307912	subgrp_con	count	1	0
HS2ST2	1	OK	1.58229999	2.72001883	0.58176068	0.56072	0.47433761	subgrp_con	count	1	0
SCNN1B	2	OK	3.45981497	4.78586864	0.72292309	0.46972	0.00081935	subgrp_con	count	1	0
COG7	1	OK	3.4598146	6.7714513	0.51094137	0.60930	0.00030179	subgrp_con	count	1	0
GGA2	3	OK	0.51031165	1.60346574	0.31826732	0.75028	0.15811454	subgrp_con	count	1	0
UBFD1	3	OK	0.17216145	1.5344504	0.11219747	0.91067	0.00011395	subgrp_con	count	1	0
NDUFAB1	200	OK	0.49305879	0.20664825	2.38597946	0.01703	0.68003161	subgrp_con	count	1	0
DCTN5	6	OK	0.18621725	1.20493826	0.15454505	0.87718	0.0575300	subgrp_con	count	1	0
PRKCB	1	OK	18.7594898	83.6251793	0.22432824	0.8225	0.00028789	subgrp_con	count	1	0
RBBP6	67	OK	0.37828696	0.37031343	1.02180189	0.30687	0.46130923	subgrp_con	count	1	0
TNRC6A	18	OK	1.62674341	0.8942513	1.81911215	0.06889	1.18139744	subgrp_con	count	1	0
ARHGAP17	4	OK	-0.4654295	1.30938391	-0.3554622	0.72224	-0.13816105	subgrp_con	count	1	0
LCMT1	10	OK	-11.325296	26.9167859	-0.4207522	0.67394	-1.19569663	subgrp_con	count	1	0
NSMCE1	43	OK	-0.2359471	0.42320923	-0.5575188	0.57717	-0.26371776	subgrp_con	count	1	0
IL4R	6	OK	-0.2029407	1.65323657	-0.1227536	0.9023	-4.82E-05	subgrp_con	count	1	0
GTF3C1	5	OK	1.25330657	1.54098629	0.81331452	0.41604	0.62624666	subgrp_con	count	1	0
GSG1L	3	OK	24.3460159	51.6160273	0.4716755	0.63716	2.905701	subgrp_con	count	1	0
XPO6	1	OK	41.7883033	174.29608	0.23975499	0.81052	0.00014488	subgrp_con	count	1	0
NUPR1	180	OK	0.58404846	0.19264326	3.03176175	0.00242	0.81491621	subgrp_con	count	1	0
AC020765.2	1	OK	1.58237769	1.92402136	0.8224325	0.41082	0.77174550	subgrp_con	count	1	0
SGF29	2	OK	0.65325287	2.50012846	0.26128772	0.79387	0.00016297	subgrp_con	count	1	0
SULT1A1	1	OK	-11.019418	43.2499352	-0.2547846	0.79889	-1.17482461	subgrp_con	count	1	0
EIF3C	1	OK	3.4598146	6.7714513	0.51094137	0.60930	0.00030179	subgrp_con	count	1	0
ATXN2L	5	OK	-0.0876726	1.5741778	-0.0556942	0.95559	-5.71E-05	subgrp_con	count	1	0
TUFM	185	OK	0.14131017	0.2079788	0.67944506	0.49686	0.19210603	subgrp_con	count	1	0
SH2B1	2	OK	3.4598146	6.7714513	0.51094137	0.60930	0.00030179	subgrp_con	count	1	0
NFATC2IP	8	OK	1.40820523	1.21848398	1.15570273	0.2478	0.71815557	subgrp_con	count	1	0
SPNS1	2	OK	2.40963373	2.47152069	0.97495997	0.32958	0.66517343	subgrp_con	count	1	0
SPN	1	OK	-15.99503	70.34942	-0.2272655	0.82014	-0.00028474	subgrp_con	count	1	0
KIF22	17	OK	-1.1001038	0.74603865	-1.4734152	0.14064	-1.09988013	subgrp_con	count	1	0
MAZ	31	OK	0.57184719	0.63510828	0.9003932	0.36791	0.37225557	subgrp_con	count	1	0
MVP	55	OK	-1.0164824	0.39349603	-2.5832088	0.00979	-1.15073835	subgrp_con	count	1	0
CDIPT	60	OK	0.66403965	0.38605455	1.72006702	0.08542	0.77783587	subgrp_con	count	1	0
SEZ6L2	5	OK	5.80493005	4.01081887	1.44731793	0.14781	1.62552891	subgrp_con	count	1	0
ASPHD1	1	OK	1.58229999	2.72001883	0.58176068	0.56072	0.47433761	subgrp_con	count	1	0
TMEM219	362	OK	0.00592033	0.14242387	0.04156839	0.96684	0.00836445	subgrp_con	count	1	0
TAOK2	11	OK	0.29276543	0.95001859	0.30816770	0.75795	0.22473350	subgrp_con	count	1	0
HIRIP3	12	OK	0.0657277	0.85438697	0.07692878	0.93868	0.05456697	subgrp_con	count	1	0
INO80E	4	OK	-0.2727703	1.36508978	-0.1998185	0.84162	-0.17377179	subgrp_con	count	1	0
ALDOA	3	OK	1.9258645	2.51548794	0.7656425	0.44389	0.56022119	subgrp_con	count	1	0
PPP4C	80	OK	-0.2856072	0.35295259	-0.8069082	0.41972	-0.35761738	subgrp_con	count	1	0
YPEL3	157	OK	0.51454565	0.23158153	2.22178096	0.0262	0.71162032	subgrp_con	count	1	0
MAPK3	84	OK	0.60694821	0.33554011	1.80889934	0.07047	0.77343476	subgrp_con	count	1	0
SULT1A3	1	OK	-10.442278	41.4658099	-0.2518286	0.80117	-0.00045289	subgrp_con	count	1	0
CD2BP2	37	OK	0.66129030	0.58113169	1.13792548	0.25515	0.59137202	subgrp_con	count	1	0
ZNF771	5	OK	0.98198018	1.23187493	0.79714272	0.42537	0.63221222	subgrp_con	count	1	0
DCTPP1	41	OK	-0.1869669	0.50374163	-0.3711564	0.71052	-0.21845287	subgrp_con	count	1	0
SEPHS2	67	OK	-0.253517	0.34845699	-0.7275416	0.46689	-0.30492854	subgrp_con	count	1	0
ZNF768	1	OK	-11.019418	43.2499352	-0.2547846	0.79889	-1.17482461	subgrp_con	count	1	0
ZNF747	1	OK	-10.442278	41.4658099	-0.2518286	0.80117	-0.00045289	subgrp_con	count	1	0
ZNF688	8	OK	-0.2645831	1.07252575	-0.2466916	0.80515	-0.19645124	subgrp_con	count	1	0
ZNF785	2	OK	0.65325287	2.50012846	0.26128772	0.79387	0.00016297	subgrp_con	count	1	0

PRR14	2	OK	1.58239999	2.72001883	0.58176068	0.56072	0.47433761	subgrp_cor	count	1	0
FBRS	2	OK	0.16816909	1.72532872	0.09747075	0.92235	0.05201879	subgrp_cor	count	1	0
PHKG2	4	OK	4.21092061	4.28756165	0.9821248	0.32606	0.00257090	subgrp_cor	count	1	0
RNF40	2	OK	4.66132998	5.76838606	0.80808217	0.41906	0.00274918	subgrp_cor	count	1	0
ZNF629	1	OK	23.9918963	84.8999419	0.28259026	0.77749	1.65081709	subgrp_cor	count	1	0
BCL7C	145	OK	0.16925955	0.23958235	0.70647753	0.47988	0.23113866	subgrp_cor	count	1	0
MIR762HG	1	OK	1.58239999	2.72001883	0.58176068	0.56072	0.47433761	subgrp_cor	count	1	0
ORAI3	14	OK	0.30813316	0.75150654	0.4100206	0.68179	0.29202929	subgrp_cor	count	1	0
HSD3B7	6	OK	-3.2416204	1.72691551	-1.8771158	0.0605	-0.64844543	subgrp_cor	count	1	0
STX4	19	OK	1.46269821	0.83729806	1.74692655	0.08065	1.17135437	subgrp_cor	count	1	0
VKORC1	172	OK	0.46906507	0.21370244	2.19494483	0.02817	0.64338833	subgrp_cor	count	1	0
BCKDK	16	OK	-0.47198	0.80247171	-0.5881578	0.55642	-0.45789734	subgrp_cor	count	1	0
KAT8	12	OK	0.47442667	0.82783167	0.57309559	0.56658	0.14787474	subgrp_cor	count	1	0
FUS	315	OK	-0.5719242	0.15453135	-3.7010236	0.00021	-0.79081535	subgrp_cor	count	1	0
PYCARD	66	OK	0.44895465	0.36736207	1.22210398	0.22167	0.55511887	subgrp_cor	count	1	0
ARMC5	3	OK	-1.537212	1.64871355	-0.9323706	0.35115	-0.40044109	subgrp_cor	count	1	0
TGFB1I1	41	OK	0.91522757	0.48306824	1.8946123	0.05814	1.11762314	subgrp_cor	count	1	0
ZNF720	11	OK	0.2171461	0.90045323	0.241152	0.80944	0.16358704	subgrp_cor	count	1	0
ZNF267	20	OK	-0.8679724	0.66413982	-1.3069122	0.19126	-0.85234799	subgrp_cor	count	1	0
VPS35	89	OK	-0.2222294	0.30531624	-0.727898	0.46668	-0.28675096	subgrp_cor	count	1	0
C16orf87	16	OK	-1.4871324	0.90968358	-1.6347798	0.1021	-0.83892056	subgrp_cor	count	1	0
DNAJA2	56	OK	0.20588462	0.40233945	0.5117187	0.60885	0.21737710	subgrp_cor	count	1	0
ITFG1	20	OK	1.61281695	0.86679588	1.86066521	0.06279	1.00316055	subgrp_cor	count	1	0
PHKB	31	OK	0.07901056	0.51111548	0.15458456	0.87715	0.07804579	subgrp_cor	count	1	0
LONP2	15	OK	0.43460462	0.81407262	0.53386468	0.59344	0.32790586	subgrp_cor	count	1	0
SIAH1	11	OK	-2.0785573	1.11250915	-1.8682508	0.06171	-1.07579463	subgrp_cor	count	1	0
N4BP1	21	OK	-1.3911312	0.70662937	-1.9686858	0.04899	-1.25329665	subgrp_cor	count	1	0
ZNF423	2	OK	19.107177	50.8032779	0.37610126	0.70686	0.00078799	subgrp_cor	count	1	0
CNEP1R1	1	OK	1.58237769	1.92402136	0.8224325	0.41082	0.77174550	subgrp_cor	count	1	0
TENT4B	2	OK	4.21084377	4.29582435	0.98021786	0.32698	7.14E-1	subgrp_cor	count	1	0
BRD7	78	OK	-0.1968855	0.31681959	-0.6214435	0.53433	-0.25237138	subgrp_cor	count	1	0
CYLD	16	OK	0.12979821	0.71988538	0.18030399	0.85690	0.1079546	subgrp_cor	count	1	0
AC007906.2	1	OK	-11.019418	43.2499352	-0.2547846	0.79889	-1.17482461	subgrp_cor	count	1	0
CHD9	189	OK	-0.4038305	0.19885979	-2.0307298	0.04228	-0.55206246	subgrp_cor	count	1	0
RBL2	18	OK	0.02781334	0.68088381	0.04084888	0.96742	0.02843684	subgrp_cor	count	1	0
AKTIP	12	OK	-0.5487619	0.8311813	-0.6602193	0.50911	-0.50645236	subgrp_cor	count	1	0
RPGRIP1L	1	OK	0.65325287	2.50012846	0.26128772	0.79387	0.00016297	subgrp_cor	count	1	0
FTO	2	OK	18.7594884	83.6251792	0.2243282	0.8225	0.00028789	subgrp_cor	count	1	0
IRX3	1	OK	3.45981463	6.77145131	0.51094137	0.60939	0.00030179	subgrp_cor	count	1	0
CRNDE	19	OK	0.45602491	0.68426763	0.66644233	0.50512	0.34404849	subgrp_cor	count	1	0
IRX6	1	OK	0.65325287	2.50012846	0.26128772	0.79387	0.00016297	subgrp_cor	count	1	0
LPCAT2	52	OK	2.29021738	0.62982045	3.63630206	0.00028	2.25539166	subgrp_cor	count	1	0
CES1	3	OK	26.3679501	84.9163259	0.31051685	0.75617	2.49052421	subgrp_cor	count	1	0
AMFR	11	OK	2.05047165	1.02164794	2.00702372	0.04475	1.40119388	subgrp_cor	count	1	0
NUDT21	18	OK	0.80192889	0.68583309	1.16929504	0.24228	0.84143070	subgrp_cor	count	1	0
OGFOD1	6	OK	0.8788176	1.64693117	0.53360919	0.59361	0.44699547	subgrp_cor	count	1	0
BBS2	1	OK	22.9715125	51.7315836	0.4440519	0.65701	2.36236892	subgrp_cor	count	1	0
MT2A	242	OK	0.00266563	0.18237072	0.01461654	0.98834	0.003681	subgrp_cor	count	1	0
MT1E	15	OK	1.41700857	0.79686776	1.77822298	0.07537	1.13850353	subgrp_cor	count	1	0
MT1M	10	OK	0.01097406	1.02140639	0.01074407	0.99142	0.00909765	subgrp_cor	count	1	0
MT1X	41	OK	-0.3426637	0.43012349	-0.7966449	0.42566	-0.33440307	subgrp_cor	count	1	0
NUP93	3	OK	-2.532961	2.47632114	-1.0228726	0.30637	-0.88788594	subgrp_cor	count	1	0
HERPUD1	272	OK	-0.3128309	0.17011928	-1.8388917	0.06592	-0.43049735	subgrp_cor	count	1	0
CETP	2	OK	-11.200751	27.7703836	-0.4033241	0.6867	-1.82948107	subgrp_cor	count	1	0
CPNE2	61	OK	-0.4777181	0.36074324	-1.3242608	0.18542	-0.58514052	subgrp_cor	count	1	0
FAM192A	24	OK	0.97371928	0.64651663	1.50610085	0.13206	0.72691565	subgrp_cor	count	1	0
RSPRY1	5	OK	0.51030031	1.6034059	0.31826022	0.75029	0.15911328	subgrp_cor	count	1	0
ARL2BP	131	OK	0.90149102	0.25740332	3.50225092	0.00046	1.21149820	subgrp_cor	count	1	0
PLLP	28	OK	2.61818936	0.81716581	3.20398788	0.00136	2.41965057	subgrp_cor	count	1	0
CX3CL1	24	OK	0.00040287	0.54081536	0.00074493	0.99941	0.00042431	subgrp_cor	count	1	0
CIAPIN1	10	OK	0.97747629	1.47789286	0.66139861	0.50836	0.30303051	subgrp_cor	count	1	0
COQ9	5	OK	-0.3681514	1.19085163	-0.3091497	0.75721	-0.00063705	subgrp_cor	count	1	0
POLR2C	34	OK	0.18694004	0.52170526	0.358325	0.7201	0.19163743	subgrp_cor	count	1	0
DOK4	3	OK	18.7594884	83.6251792	0.2243282	0.8225	0.00028789	subgrp_cor	count	1	0
CCDC102A	2	OK	3.45981463	6.77145131	0.51094137	0.60939	0.00030179	subgrp_cor	count	1	0
ADGRG1	32	OK	1.6167139	0.7415867	2.18004459	0.02925	0.78672157	subgrp_cor	count	1	0
KIFC3	17	OK	-1.3093812	0.7703227	-1.6998846	0.08942	-0.75558785	subgrp_cor	count	1	0
USB1	12	OK	0.42775056	0.88476566	0.48346198	0.62877	0.32320288	subgrp_cor	count	1	0
MMP15	3	OK	-0.1747967	1.8209803	-0.0959904	0.92352	-0.05309252	subgrp_cor	count	1	0
CFAP20	44	OK	0.50546605	0.48226375	1.04811122	0.29459	0.56865785	subgrp_cor	count	1	0
CSNK2A2	44	OK	1.28943381	0.55511732	2.32281315	0.02019	1.33524517	subgrp_cor	count	1	0
CCDC113	2	OK	4.66132998	5.76838606	0.80808217	0.41906	0.00274918	subgrp_cor	count	1	0
GINS3	1	OK	-11.019418	43.2499352	-0.2547846	0.79889	-1.17482461	subgrp_cor	count	1	0
AC009118.2	1	OK	1.58237769	1.92402136	0.8224325	0.41082	0.77174550	subgrp_cor	count	1	0
NDRG4	11	OK	5.05377783	2.73821389	1.84564757	0.06494	2.27609324	subgrp_cor	count	1	0
SETD6	2	OK	0.16814912	1.72533008	0.0974591	0.92236	0.05201277	subgrp_cor	count	1	0
CNOT1	8	OK	4.66132998	5.76838606	0.80808217	0.41906	0.00274918	subgrp_cor	count	1	0
GOT2	7	OK	0.55249302	1.34800031	0.40986119	0.68192	0.17231298	subgrp_cor	count	1	0
CDH11	67	OK	6.02788058	6.59364407	0.91419563	0.36061	5.91668540	subgrp_cor	count	1	0
CDH5	140	OK	0.29353723	0.24956214	1.17620899	0.23951	0.39026276	subgrp_cor	count	1	0
LINC00920	2	OK	-0.3495512	1.85447474	-0.1884907	0.85049	-0.17237783	subgrp_cor	count	1	0
TK2	3	OK	4.66132998	5.76838606	0.80808217	0.41906	0.00274918	subgrp_cor	count	1	0
CKLF	36	OK	1.60306743	0.58353732	2.74715494	0.00601	1.89151801	subgrp_cor	count	1	0
CMTM3	28	OK	-0.0528657	0.63440107	-0.0833317	0.93359	-0.05396800	subgrp_cor	count	1	0
DYNC1LI2	131	OK	0.03896281	0.25009461	0.15579229	0.8762	0.04966470	subgrp_cor	count	1	0
NAE1	18	OK	-1.3675341	0.81983485	-1.6680605	0.0952	-1.24105990	subgrp_cor	count	1	0
RRAD	3	OK	1.25320657	1.54098629	0.81331455	0.41604	0.62624666	subgrp_cor	count	1	0
FAM96B	224	OK	0.43045592	0.19645424	2.19112562	0.02844	0.59789776	subgrp_cor	count	1	0
CES2	1	OK	18.7594884	83.6251792	0.2243282	0.8225	0.00028789	subgrp_cor	count	1	0
CBFB	7	OK	-3.1389321	1.95696544	-1.6039792	0.10872	-0.63775877	subgrp_cor	count	1	0
B3GNT9	3	OK	21.642059	54.4868919	0.39719753	0.69122	1.57744725	subgrp_cor	count	1	0
TRADD	21	OK	-0.4255542	0.70808569	-0.6009925	0.54784	-0.34632452	subgrp_cor	count	1	0
FBXL8	1	OK	-15.498043	42.9510917	-0.36082	0.71822	-0.00073756	subgrp_cor	count	1	0
NOL3	6	OK	1.23047157	1.29043195	0.95353464	0.34032	0.61568944	subgrp_cor	count	1	0
EXOC3L1	1	OK	-15.995021	70.349421	-0.2272655	0.82014	-0.00028474	subgrp_cor	count	1	0
E2F4	20	OK	1.37353621	0.87406888	1.57142788	0.11608	1.10298608	subgrp_cor	count	1	0
TMEM208	68	OK	0.835345	0.38795233	2.15321582	0.0312	1.01418295	subgrp_cor	count	1	0
FHOD1	5	OK	-15.498043	42.9510917	-0.36082	0.71822	-0.00073756	subgrp_cor	count	1	0
TPPP3	13	OK	1.12238825	1.02724997	1.09261454	0.27456	0.56505669	subgrp_cor	count	1	0
ZDHHC1	2	OK	0.58878195	1.7674148	0.33313173	0.73903	0.30122869	subgrp_cor	count	1	0
ATP6V0D1	29	OK	1.06691467	0.68104387	1.5665873	0.11721	0.87523332	subgrp_cor	count	1	0
AC027682.6	1	OK	23.9918963	84.8999419	0.28259026	0.77749	1.65081709	subgrp_cor	count	1	0
RIPOR1	20	OK	-0.977445	0.84338741	-1.1589518	0.24648	-0.58850647	subgrp_cor	count	1	0
CTCF	29	OK	0.19264723	0.52458194	0.36723954	0.71344	0.2130577	subgrp_cor	count	1	0
ACD	4	OK	-0.2565731	1.3412338	-0.1912964	0.84829	-0.12807708	subgrp_cor	count	1	0
PARD6A	5	OK	1.91553346	1.48845431	1.28692796	0.19812	1.14884144	subgrp_cor	count	1	0
GFOD2	25	OK	-1.5415285	0.69925751	-2.2045219	0.02749	-1.11970288	subgrp_cor	count	1	0
RANBP10	2	OK	23.9918963	84.8999419	0.28259026	0.77749	1.65081709	subgrp_cor	count	1	0
CENPT	4	OK	0.1112767	1.61203872	0.06902852	0.94497	0.05657342	subgrp_cor	count	1	0
THAP11	15	OK	-0.2761991	0.67841891	-0.4071229	0.68392	-0.27024846	subgrp_cor	count	1	0
NUTF2	78	OK	0.0307125	0.32961534	0.09317679	0.92576	0.03984954	subgrp_cor	count	1	0
PSKH1	1	OK	-11.019418	43.2499352	-0.2547846	0.79889	-1.17482461	subgrp_cor	count	1	0
PSMB10	20	OK	-0.3544762	0.61615256	-0.5753058	0.56508	-0.32956440	subgrp_cor	count	1	0
SLC12A4	2	OK	35.5507685	69.3238151	0.51274791	0.60812	2.03654130	subgrp_cor	count	1	0
DPEP2	2	OK	2.98494006	2.59888574	1.14854609	0.25074	1.23382860	subgrp_cor	count	1	0
NFATC3	13	OK	-0.4208346	0.70048103	-0.6007794	0.54799	-0.12541740	subgrp_cor	count	1	0
PLA2G15	1	OK	1.58239999	2.72001883	0.58176068	0.56072	0.47433761	subgrp_cor	count	1	0
SLC7A6	3	OK	21.1053233	83.3412731	0.25323976	0.80008	0.0003103	subgrp_cor	count	1	0
SLC7A6OS	4	OK	-2.2229576	1.77606832	-1.2516172	0.21071	-0.86936325	subgrp_cor	count	1	0
ZFP90	10	OK	0.09594759	0.84523883	0.11351032	0.90962	0.06217789	subgrp_cor	count	1	0
HAS3	2	OK	26.3679501	84.9163259	0.31051685	0.75617	2.49052421	subgrp_cor	count	1	0
CHTF8	10	OK	0.75409345	0.94288776	0.79977011	0.42386	0.62581458	subgrp_cor	count	1	0
UTP4	2	OK	-10.475634	27.4919879	-0.3810432	0.70317	-1.13811785	subgrp_cor	count	1	0
SNTB2	203	OK	0.21035321	0.20134564	1.04473696	0.29614	0.28914795	subgrp_cor	count	1	0
VPS4A	26	OK	-0.0066804	0.61869755	-0.0107975	0.99138	-0.00703410	subgrp_cor	count	1	0
COG8	2	OK	-0.9102232	2.18811251	-0.4159856	0.67742	-0.25785735	subgrp_cor	count	1	0
NIP7	10	OK	-1.276548	1.05904866	-1.2053705	0.22806	-0.74017684	subgrp_cor	count	1	0
TERF2	10	OK	0.09268547	1.03245839	0.08977163	0.92847	0.0770353	subgrp_cor	count	1	0
CYB5B	35	OK	0.033849	0.51240058	0.06625501	0.94717	0.03351253	subgrp_cor	count	1	0
NFAT5	41	OK	0.08902342	0.47301274	0.18820512	0.85072	0.06810001	subgrp_cor	count	1	0
NQO1	59	OK	1.86320875	0.47157554	3.95102928	7.78E-05	2.17919122	subgrp_cor	count	0.77278875	0
NOB1	40	OK	-1.0118969	0.48253771	-2.0970318	0.03599	-1.16113524	subgrp_cor	count	1	0
PDPR	1	OK	18.7594884	83.6251792	0.2243282	0.8225	0.00028789	subgrp_cor	count	1	0
EXOSC6	30	OK	0.94708962	0.58499981	1.61895714	0.10546	0.99080608	subgrp_cor	count	1	0
AARS	5	OK	0.21082055	1.18900263	0.17731715	0.85926	0.00013987	subgrp_cor	count	1	0
DDX19B	5	OK	-1.3967094	1.3668523	-1.0218437	0.30685	-0.37125920	subgrp_cor	count	1	0
DDX19A	3	OK	1.58239999	2.72001883	0.58176068	0.56072	0.47433761	subgrp_cor	count	1	0
ST3GAL2	7	OK	-0.0950471	1.44529459	-0.0657631	0.94757	-0.04775710	subgrp_cor	count	1	0
FUK	4	OK	0.406043	2.10834695	0.19258832	0.84729	0.12643682	subgrp_cor	count	1	0
COG4	3	OK	0.51031165	1.60340574	0.31826732	0.75028	0.15911454	subgrp_cor	count	1	0
SF3B3	5	OK	-1.1016806	1.28067604	-0.8602336	0.38966	-0.30477216	subgrp_cor	count	1	0
IL34	9	OK	2.78717855	1.82653833	1.52593485	0.12703	0.73439054	subgrp_cor	count	1	0
CMTR2	4	OK	-1.3967094	1.3668523	-1.0218437	0.30685	-0.37125920	subgrp_cor	count	1	0
PHLPP2	1	OK	3.45981463	6.77145131	0.51094137	0.60939	0.00030179	subgrp_cor	count	1	0
AP1G1	2	OK	0.16814912	1.72533008	0.0974591	0.92236	0.05201277	subgrp_cor	count	1	0
IST1	19	OK	0.28737029	0.72560597	0.39604193	0.69207	0.14678249	subgrp_cor	count	1	0
DHX38	6	OK	-0.0587026	1.02372164	-0.0573423	0.95427	-0.01796061	subgrp_cor	count	1	0
ZFHX3	9	OK	2.66728235	2.24180553	1.18979203	0.23412	0.71356325	subgrp_cor	count	1	0
PSMD7	122	OK	0.59974666	0.26895302	2.22993096	0.02575	0.79515921	subgrp_cor	count	1	0
GLG1	64	OK	2.91256099	0.639321	4.55570986	5.22E-06	2.68577212	subgrp_cor	count	0.05229189	0
RFWD3	1	OK	18.7594884	83.6251792	0.2243282	0.8225	0.00028789	subgrp_cor	count	1	0
MLKL	18	OK	0.25930144	0.7788349	0.33293505	0.73919	0.16865633	subgrp_cor	count	1	0
ZNRF1	47	OK	0.86189142	0.4793701	1.79796656	0.07218	0.99358223	subgrp_cor	count	1	0
BCAR1	28	OK	-0.535532	0.59063192	-0.9067110	0.36456	-0.49457634	subgrp_cor	count	1	0
CFDP1	106	OK	-0.3239762	0.27481661	-1.1788812	0.23845	-0.42360272	subgrp_cor	count	1	0
TMEM170A	28	OK	-0.7254498	0.56240267	-1.2899117	0.19708	-0.72052688	subgrp_cor	count	1	0
TMEM231	1	OK	18.7594884	83.6251792	0.2243282	0.8225	0.00028789	subgrp_cor	count	1	0
GABARAPL2	514	OK	0.42154375	0.1205732	3.49616459	0.00047	0.59719070	subgrp_cor	count	1	0
KARS	16	OK	-1.2244927	0.86437128	-1.4166282	0.15659	-0.83582858	subgrp_cor	count	1	0
TERF2IP	213	OK	0.15766798	0.19019803	0.82896716	0.40712	0.21825587	subgrp_cor	count	1	0
MON1B	7	OK	0.33615318	1.11153068	0.30242372	0.76232	0.10452415	subgrp_cor	count	1	0
SYCE1L	8	OK	1.30364828	1.15502717	1.12867326	0.25904	0.82452316	subgrp_cor	count	1	0
ADAMTS18	5	OK	2.4598163	3.02637821	1.14321969	0.25295	0.00222292	subgrp_cor	count	1	0
NUDT7	7	OK	-12.844241	17.6051335	-0.7295736	0.46565	-2.43380993	subgrp_cor	count	1	0
WWOX	7	OK	0.16298123	1.13073297	0.1441377	0.88539	0.12268049	subgrp_cor	count	1	0
MAF	6	OK	0.26075945	1.66204	0.1568912	0.87532	0.08091627	subgrp_cor	count	1	0
CDYL2	1	OK	19.1117901	50.8375913	0.37593815	0.70696	0.00078703	subgrp_cor	count	1	0
CMC2	21	OK	0.36713086	0.70997112	0.51710675	0.60508	0.34798011	subgrp_cor	count	1	0
ATMIN	10	OK	1.12238825	1.02724997	1.09261454	0.27456	0.56505669	subgrp_cor	count	1	0
GCSH	29	OK	1.93129687	0.65638803	2.94231873	0.00326	1.90843268	subgrp_cor	count	1	0
GAN	3	OK	18.7594884	83.6251792	0.2243282	0.8225	0.00028789	subgrp_cor	count	1	0
CMIP	86	OK	-0.533837	0.32740003	-1.6305344	0.10299	-0.67757395	subgrp_cor	count	1	0
MPHOSPH6	5	OK	1.19649991	1.18944638	1.0059301	0.31445	0.7618921	subgrp_cor	count	1	0
CDH13	66	OK	1.24088011	0.39418406	3.14797135	0.00164	1.58636035	subgrp_cor	count	1	0
HSBP1	292	OK	0.22339366	0.16430461	1.35963112	0.17395	0.31273425	subgrp_cor	count	1	0
MLYCD	4	OK	-0.8416402	1.46082315	-0.5761411	0.56452	-0.24034852	subgrp_cor	count	1	0
MBTPS1	31	OK	2.01715381	0.69101715	2.91910819	0.00351	1.91468663	subgrp_cor	count	1	0
HSDL1	3	OK	0.16814912	1.72533008	0.0974591	0.92236	0.05201277	subgrp_cor	count	1	0
TAF1C	2	OK	23.9918963	84.8999419	0.28259026	0.77749	1.65081709	subgrp_cor	count	1	0
WFDC1	1	OK	18.7594884	83.6251792	0.2243282	0.8225	0.00028789	subgrp_cor	count	1	0
TLDC1	3	OK	-10.475634	27.4919879	-0.3810432	0.70317	-1.13811785	subgrp_cor	count	1	0
KLHL36	7	OK	-0.2352535	1.04124707	-0.2259344	0.82125	-0.11759627	subgrp_cor	count	1	0
USP10	7	OK	1.01381281	1.37480665	0.7374222	0.46087	0.00188658	subgrp_cor	count	1	0
ZDHHC7	3	OK	-11.019418	43.2499352	-0.2547846	0.79889	-1.17482461	subgrp_cor	count	1	0
GSE1	8	OK	1.58287906	1.27662795	1.23989064	0.21502	1.04111925	subgrp_cor	count	1	0
EMC8	16	OK	0.56759931	0.83503943	0.67972756	0.49668	0.50835094	subgrp_cor	count	1	0
COX4I1	724	OK	-0.1526399	0.098936	-1.5428147	0.12288	-0.21770195	subgrp_cor	count	1	0
IRF8	1	OK	0.65325287	2.50012846	0.26128772	0.79387	0.00016297	subgrp_cor	count	1	0
FOXC2	44	OK	2.95860546	0.72945569	4.05590849	4.99E-05	3.10249999	subgrp_cor	count	0.4969512	0
FBXO31	4	OK	-1.8537846	1.85435929	-0.99969	0.31746	-0.46052349	subgrp_cor	count	1	0
ZCCHC14	5	OK	1.37534679	1.64644137	0.83534514	0.40352	0.48312098	subgrp_cor	count	1	0
BANP	1	OK	18.7594884	83.6251792	0.2243282	0.8225	0.00028789	subgrp_cor	count	1	0
ZFPM1	5	OK	0.55249302	1.34800031	0.40986119	0.68192	0.17231298	subgrp_cor	count	1	0
ZC3H18	8	OK	-0.3639812	0.93710737	-0.3884093	0.69771	-0.23073456	subgrp_cor	count	1	0
MVD	2	OK	18.7594884	83.6251792	0.2243282	0.8225	0.00028789	subgrp_cor	count	1	0
RNF166	6	OK	1.6575426	1.45768333	1.13710747	0.25549	0.80313281	subgrp_cor	count	1	0
CTU2	2	OK	2.98494006	2.59888574	1.14854609	0.25074	1.23382860	subgrp_cor	count	1	0
PIEZO1	8	OK	-0.8009674	0.98694704	-0.8115607	0.41704	-0.57116958	subgrp_cor	count	1	0
APRT	297	OK	0.11221489	0.16591709	0.67633112	0.49882	0.15699882	subgrp_cor	count	1	0
TRAPPC2L	101	OK	0.8581587	0.3120069	2.75044782	0.00595	1.11378350	subgrp_cor	count	1	0
CBFA2T3	2	OK	-10.475634	27.4919879	-0.3810432	0.70317	-1.13811785	subgrp_cor	count	1	0
AC092384.1	4	OK	-2.4465924	1.71381313	-1.4275724	0.15341	-0.00126286	subgrp_cor	count	1	0
ANKRD11	115	OK	0.58672071	0.27857271	2.10616723	0.03519	0.72922717	subgrp_cor	count	1	0
SPG7	10	OK	-0.0811959	0.89195427	-0.0910314	0.92747	-0.04094567	subgrp_cor	count	1	0
RPL13	890	OK	-0.000511	0.08202638	-0.0062290	0.99503	-0.00073183	subgrp_cor	count	1	0
CHMP1A	16	OK	0.3361476	0.78551897	0.42793059	0.6687	0.17183582	subgrp_cor	count	1	0
SPATA33	6	OK	-1.6108743	1.36602677	-1.1792406	0.2382	-0.41511206	subgrp_cor	count	1	0
CDK10	5	OK	-0.6508873	1.2753209	-0.5103714	0.60979	-0.00040481	subgrp_cor	count	1	0
SPATA2L	1	OK	1.58239999	2.72001883	0.58176068	0.56072	0.47433761	subgrp_cor	count	1	0
ZNF276	4	OK	-1.0379618	1.42801275	-0.7268575	0.46731	-0.00062229	subgrp_cor	count	1	0
TCF25	166	OK	0.21985375	0.22544613	0.97519414	0.32946	0.29627955	subgrp_cor	count	1	0
DEF8	18	OK	0.93728048	0.94946657	0.98716529	0.32356	0.77307470	subgrp_cor	count	1	0
GAS8	2	OK	-1.2956167	2.0414257	-0.6346627	0.52565	-0.00075754	subgrp_cor	count	1	0
DOC2B	36	OK	-2.0502066	0.56671213	-3.6177214	0.0003	-1.79869023	subgrp_cor	count	1	0
LINC02091	2	OK	3.45981463	6.77145131	0.51094137	0.60939	0.00030179	subgrp_cor	count	1	0
RPH3AL	9	OK	-3.0930081	1.52844927	-2.023625	0.04301	-1.60513300	subgrp_cor	count	1	0
AC129507.4	1	OK	3.45981463	6.77145131	0.51094137	0.60939	0.00030179	subgrp_cor	count	1	0
C17orf97	1	OK	18.7594884	83.6251792	0.2243282	0.8225	0.00028789	subgrp_cor	count	1	0
RFLNB	53	OK	0.60566399	0.44943374	1.34761573	0.17779	0.71785283	subgrp_cor	count	1	0
VPS53	6	OK	-0.8416402	1.46082315	-0.5761411	0.56452	-0.24034852	subgrp_cor	count	1	0
GLOD4	33	OK	-0.2684374	0.54432987	-0.4931431	0.62191	-0.26265665	subgrp_cor	count	1	0
MRM3	2	OK	-12.145707	43.4188694	-0.2797334	0.77968	-1.91338276	subgrp_cor	count	1	0
NXN	12	OK	-1.9535832	1.01251179	-1.9294426	0.05368	-1.21096930	subgrp_cor	count	1	0
TIMM22	8	OK	1.25154888	1.21888115	1.02679298	0.30452	0.38342687	subgrp_cor	count	1	0
ABR	5	OK	1.9259645	2.51548794	0.7656425	0.44389	0.56022119	subgrp_cor	count	1	0
YWHAE	461	OK	-0.1959998	0.12736863	-1.5388312	0.12385	-0.27653065	subgrp_cor	count	1	0
CRK	56	OK	0.66422408	0.44588888	1.48966282	0.13631	0.49995296	subgrp_cor	count	1	0
MYO1C	49	OK	0.24661632	0.44652567	0.55230042	0.58074	0.27757929	subgrp_cor	count	1	0
INPP5K	6	OK	-0.1902356	1.22474264	-0.155327	0.87656	-0.05772119	subgrp_cor	count	1	0
PITPNA-AS1	7	OK	0.68544664	1.04563654	0.6555305	0.51212	0.44544667	subgrp_cor	count	1	0
PITPNA	33	OK	0.83083246	0.55589948	1.49457319	0.13503	0.84679195	subgrp_cor	count	1	0
SLC43A2	1	OK	3.45981463	6.77145131	0.51094137	0.60939	0.00030179	subgrp_cor	count	1	0
SCARF1	36	OK	0.43161587	0.5112214	0.84428366	0.39851	0.38718742	subgrp_cor	count	1	0
RILP	16	OK	0.62736781	0.74915193	0.83743737	0.40235	0.56137921	subgrp_cor	count	1	0
PRPF8	25	OK	-0.7458589	0.62848968	-1.186748	0.23532	-0.73696178	subgrp_cor	count	1	0
TLCD2	11	OK	1.13125775	0.94167997	1.20131871	0.22962	0.56924800	subgrp_cor	count	1	0
MIR22HG	115	OK	-0.2325809	0.26005909	-0.8943385	0.37114	-0.29909919	subgrp_cor	count	1	0
SMYD4	1	OK	-11.019418	43.2499352	-0.2547846	0.79889	-1.17482461	subgrp_cor	count	1	0
RPA1	4	OK	0.62072077	1.75640758	0.3534036	0.72379	0.46831355	subgrp_cor	count	1	0
HIC1	4	OK	0.12559487	1.45000757	0.0866167	0.93098	0.03878308	subgrp_cor	count	1	0
SMG6	16	OK	2.32970349	1.03735194	2.24581784	0.02472	1.37336526	subgrp_cor	count	1	0
SRR	2	OK	3.79943049	2.65483698	1.43113514	0.15239	1.76818710	subgrp_cor	count	1	0
TSR1	4	OK	-10.475634	27.4919879	-0.3810432	0.70317	-1.13811785	subgrp_cor	count	1	0
SGSM2	1	OK	18.7594884	83.6251792	0.2243282	0.8225	0.00028789	subgrp_cor	count	1	0
MNT	4	OK	-1.2956167	2.0414257	-0.6346627	0.52565	-0.00075754	subgrp_cor	count	1	0
METTL16	2	OK	0.58878195	1.7674148	0.33313173	0.73903	0.30122869	subgrp_cor	count	1	0
PAFAH1B1	82	OK	0.74414168	0.3438554	2.16411223	0.03046	0.93462234	subgrp_cor	count	1	0
TAX1BP3	35	OK	1.66714268	0.64256025	2.5904997	0.00958	1.41536092	subgrp_cor	count	1	0
EMC6	55	OK	1.13650738	0.42276586	2.68826666	0.00718	1.40816119	subgrp_cor	count	1	0
ITGAE	36	OK	-0.2987724	0.47357706	-0.6308845	0.52812	-0.3557563	subgrp_cor	count	1	0
NCBP3	25	OK	-0.9021847	0.64984142	-1.3883151	0.16504	-0.81267935	subgrp_cor	count	1	0
ATP2A3	7	OK	5.29562619	4.07422745	1.29978659	0.19367	1.00139069	subgrp_cor	count	1	0
ZZEF1	6	OK	-0.7696064	1.48221063	-0.5192288	0.6036	-0.45705798	subgrp_cor	count	1	0
CYB5D2	23	OK	1.38241255	0.66574781	2.07648079	0.03785	1.37654066	subgrp_cor	count	1	0
ANKFY1	5	OK	4.66132998	5.76838606	0.80808217	0.41906	0.00274918	subgrp_cor	count	1	0
UBE2G1	12	OK	-1.2001722	0.95292518	-1.2594611	0.20786	-0.70247228	subgrp_cor	count	1	0
SPNS2	52	OK	-0.7672175	0.43738479	-1.7541019	0.07941	-0.82406763	subgrp_cor	count	1	0
MYBBP1A	6	OK	-1.8174474	1.45595424	-1.2482861	0.21193	-0.45400691	subgrp_cor	count	1	0
PELP1	4	OK	-1.2520862	1.38783627	-0.9021858	0.36696	-0.56535851	subgrp_cor	count	1	0
MED11	5	OK	1.91578079	1.48838833	1.28715117	0.19804	1.14901874	subgrp_cor	count	1	0
CXCL16	4	OK	1.77247729	1.84396277	0.96122365	0.33644	0.84969892	subgrp_cor	count	1	0
VMO1	2	OK	0.65325298	1.76878362	0.36932325	0.71189	0.0001629	subgrp_cor	count	1	0
PSMB6	252	OK	0.82680861	0.19311441	4.28144438	1.86E-05	1.14355676	subgrp_cor	count	0.1855913	0
PLD2	1	OK	3.45981463	6.77145131	0.51094137	0.60939	0.00030179	subgrp_cor	count	1	0
MINK1	1	OK	1.58239999	2.72001883	0.58176068	0.56072	0.47433761	subgrp_cor	count	1	0
SLC25A11	32	OK	0.91019696	0.53153251	1.71240132	0.08682	1.01689382	subgrp_cor	count	1	0
RNF167	30	OK	0.58846733	0.52714389	1.1163316	0.26429	0.6360968	subgrp_cor	count	1	0
PFN1	634	OK	-0.1127713	0.10630603	-1.0608179	0.28877	-0.16064057	subgrp_cor	count	1	0
SPAG7	49	OK	-0.5095026	0.4305407	-1.1834017	0.23665	-0.6190763	subgrp_cor	count	1	0
CAMTA2	1	OK	3.45981463	6.77145131	0.51094137	0.60939	0.00030179	subgrp_cor	count	1	0
KIF1C	19	OK	2.69320691	1.19856405	2.24702794	0.02464	1.70597261	subgrp_cor	count	1	0
RABEP1	25	OK	0.56343485	0.5774271	0.97576793	0.32918	0.55719606	subgrp_cor	count	1	0
RPAIN	60	OK	0.88448329	0.4013616	2.20370679	0.02754	1.1076790	subgrp_cor	count	1	0
AC004148.2	2	OK	0.16814912	1.72533008	0.0974591	0.92236	0.05201277	subgrp_cor	count	1	0
C1QBP	182	OK	0.08800099	0.21666377	0.40616384	0.68462	0.12070010	subgrp_cor	count	1	0
DHX33	3	OK	1.9259645	2.51548794	0.7656425	0.44389	0.56022119	subgrp_cor	count	1	0
DERL2	28	OK	1.10587535	0.73401229	1.50661694	0.13191	1.03022485	subgrp_cor	count	1	0
MIS12	2	OK	18.7594884	83.6251792	0.2243282	0.8225	0.00028789	subgrp_cor	count	1	0
NLRP1	9	OK	-0.8524611	0.96944966	-0.8793247	0.37923	-0.24313552	subgrp_cor	count	1	0
WSCD1	2	OK	-0.7251858	1.80791919	-0.4011162	0.68832	-0.20979917	subgrp_cor	count	1	0
PITPNM3	1	OK	18.7594884	83.6251792	0.2243282	0.8225	0.00028789	subgrp_cor	count	1	0
KIAA0753	1	OK	3.45981463	6.77145131	0.51094137	0.60939	0.00030179	subgrp_cor	count	1	0
TXNDC17	144	OK	0.49358737	0.25502619	1.80603947	0.07092	0.61217033	subgrp_cor	count	1	0
MED31	26	OK	0.0407444	0.5829301	0.06989585	0.94428	0.04495645	subgrp_cor	count	1	0
XAF1	87	OK	-0.2990015	0.29708987	-1.0064344	0.31421	-0.36509341	subgrp_cor	count	1	0
RNASEK	28	OK	-0.8744429	0.58365271	-1.4982247	0.13407	-0.93368645	subgrp_cor	count	1	0
BCL6B	30	OK	0.24763192	0.59886692	0.41350074	0.67924	0.25369631	subgrp_cor	count	1	0
ASGR1	10	OK	-0.2898596	0.9222174	-0.3142071	0.75329	-0.29421723	subgrp_cor	count	1	0
ACADVL	53	OK	1.6365882	0.50071482	3.2685036	0.00108	1.83894434	subgrp_cor	count	1	0
[illegible]											

TMEM107	17	OK	-1.0929366	0.7341189	-1.4887732	0.13655	-0.91603649	subgrp_cor	count		1	0
BORCS6	10	OK	-1.0093742	0.9555082	-1.0563741	0.2908	-0.78484177	subgrp_cor	count		1	0
LINC00324	6	OK	-12.55615	26.348029	-0.47655	0.63368	-3.4628712	subgrp_cor	count		1	0
CTC1	2	OK	-11.20075	27.770283	-0.4033234	0.6867	-1.82948107	subgrp_cor	count		1	0
PFAS	1	OK	18.759489	83.625179	0.2243282	0.8225	0.00028789	subgrp_cor	count		1	0
SLC25A35	1	OK	-11.01941	43.249935	-0.254784	0.79889	-1.17482461	subgrp_cor	count		1	0
RANGRF	15	OK	0.5138477	0.734413	0.6996705	0.48412	0.4604863	subgrp_cor	count		1	0
ARHGEF15	17	OK	-0.238981	0.720001	-0.331918	0.73995	-0.21155325	subgrp_cor	count		1	0
KRBA2	3	OK	0.111276	1.6120387	0.0690285	0.94497	0.05657342	subgrp_cor	count		1	0
RPL26	867	OK	-0.214060	0.086503	-2.474577	0.01334	-0.30613047	subgrp_cor	count		1	0
NDEL1	7	OK	-0.943293	1.0217259	-0.92322	0.35588	-0.2661694	subgrp_cor	count		1	0
MYH10	18	OK	0.3904402	0.7797098	0.5007506	0.61655	0.39993124	subgrp_cor	count		1	0
NTN1	5	OK	32.558212	45.670899	0.7128874	0.47592	1.93289083	subgrp_cor	count		1	0
STX8	15	OK	0.4560905	0.773206	0.589869	0.55528	0.34409894	subgrp_cor	count		1	0
SCO1	2	OK	23.991896	84.899941	0.2825902	0.77749	1.65081709	subgrp_cor	count		1	0
TMEM220	7	OK	0.6192086	1.115446	0.5551219	0.57881	0.31670700	subgrp_cor	count		1	0
SHISA6	1	OK	18.759489	83.625179	0.2243282	0.8225	0.00028789	subgrp_cor	count		1	0
MAP2K4	2	OK	0.1681690	1.7253287	0.0974707	0.92235	0.05201879	subgrp_cor	count		1	0
ARHGAP44	1	OK	17.747207	50.971927	0.3481761	0.72771	0.0007532	subgrp_cor	count		1	0
ELAC2	3	OK	-0.202940	1.6532236	-0.122753	0.9022	-4.82E-05	subgrp_cor	count		1	0
COX10	1	OK	3.4598146	6.7714513	0.5109413	0.60939	0.00030179	subgrp_cor	count		1	0
TRIM16	3	OK	-1.853834	1.8543918	-0.999699	0.31748	-0.46053279	subgrp_cor	count		1	0
ZSWIM7	29	OK	0.5547224	0.5599785	0.9906137	0.32187	0.64282001	subgrp_cor	count		1	0
TTC19	30	OK	-0.754702	0.5249806	-1.437581	0.15055	-0.74740692	subgrp_cor	count		1	0
NCOR1	120	OK	0.1429091	0.2673034	0.5346327	0.5929	0.18552513	subgrp_cor	count		1	0
PIGL	1	OK	18.759489	83.625179	0.2243282	0.8225	0.00028789	subgrp_cor	count		1	0
UBB	705	OK	0.0620673	0.0988236	0.6280613	0.52996	0.08855502	subgrp_cor	count		1	0
LRRC75A	52	OK	0.6005222	0.4151174	1.446632	0.148	0.68489599	subgrp_cor	count		1	0
ZNF287	1	OK	3.4598146	6.7714513	0.5109413	0.60939	0.00030179	subgrp_cor	count		1	0
MPRIP	28	OK	0.3777656	0.5829626	0.6480100	0.51698	0.38681250	subgrp_cor	count		1	0
MPRIP-AS1	1	OK	3.4598146	6.7714513	0.5109413	0.60939	0.00030179	subgrp_cor	count		1	0
FLCN	2	OK	18.759489	83.625179	0.2243282	0.8225	0.00028789	subgrp_cor	count		1	0
COPS3	19	OK	-0.548877	0.6723078	-0.816407	0.41427	-0.47812195	subgrp_cor	count		1	0
NT5M	1	OK	-10.44227	41.465809	-0.251828	0.80117	-0.00045289	subgrp_cor	count		1	0
MED9	1	OK	18.759489	83.625179	0.2243282	0.8225	0.00028789	subgrp_cor	count		1	0
RASD1	13	OK	2.2152404	0.9772039	2.2668171	0.0234	1.90305585	subgrp_cor	count		1	0
PEMT	14	OK	0.4651244	0.962369	0.4833117	0.62887	0.23803556	subgrp_cor	count		1	0
RAI1	1	OK	3.4598146	6.7714513	0.5109413	0.60939	0.00030179	subgrp_cor	count		1	0
SREBF1	2	OK	3.4598146	6.7714513	0.5109413	0.60939	0.00030179	subgrp_cor	count		1	0
TOM1L2	1	OK	23.991896	84.899941	0.2825902	0.77749	1.65081709	subgrp_cor	count		1	0
DRC3	1	OK	-11.01941	43.249935	-0.254784	0.79889	-1.17482461	subgrp_cor	count		1	0
ATPAF2	1	OK	3.4598146	6.7714513	0.5109413	0.60939	0.00030179	subgrp_cor	count		1	0
GID4	4	OK	-0.349551	1.311049	-0.266619	0.78978	-0.25782604	subgrp_cor	count		1	0
DRG2	9	OK	-1.332397	1.0040631	-1.32700	0.18451	-0.35544891	subgrp_cor	count		1	0
ALKBH5	7	OK	1.6639852	1.4590722	1.1404405	0.2541	0.52318246	subgrp_cor	count		1	0
FLII	17	OK	-0.058045	0.7573757	-0.076640	0.93891	-0.04341525	subgrp_cor	count		1	0
SMCR8	2	OK	0.6532528	2.5001284	0.2612877	0.79387	0.00016297	subgrp_cor	count		1	0
TVP23B	1	OK	23.991896	84.899941	0.2825902	0.77749	1.65081709	subgrp_cor	count		1	0
PRPSAP2	4	OK	2.5552227	2.349844	1.0874007	0.27686	0.69313893	subgrp_cor	count		1	0
GRAP	3	OK	3.4598149	4.7858686	0.7229230	0.46972	0.00081925	subgrp_cor	count		1	0
AC007952.4	3	OK	-0.015957	1.8161022	-0.008786	0.99299	-0.00486873	subgrp_cor	count		1	0
GRAPL	1	OK	-11.01941	43.249935	-0.254784	0.79889	-1.17482461	subgrp_cor	count		1	0
EPN2	27	OK	-0.481598	0.6230858	-0.772925	0.43957	-0.48511250	subgrp_cor	count		1	0
B9D1	3	OK	6.8091365	5.3050116	1.2835290	0.19931	1.696588	subgrp_cor	count		1	0
MAPK7	3	OK	0.3472176	1.8140362	0.1914061	0.84821	0.00023212	subgrp_cor	count		1	0
SLC47A1	1	OK	18.759489	83.625179	0.2243282	0.8225	0.00028789	subgrp_cor	count		1	0
ALDH3A2	28	OK	2.5702619	0.8160583	3.1496017	0.00163	2.30787312	subgrp_cor	count		1	0
USP22	35	OK	0.6594694	0.5124202	1.2869699	0.1981	0.69504113	subgrp_cor	count		1	0
DHRS7B	26	OK	0.2464755	0.6528341	0.3775469	0.70577	0.20535391	subgrp_cor	count		1	0
TMEM11	15	OK	-0.291839	0.7906061	-0.369134	0.71203	-0.21596299	subgrp_cor	count		1	0
MAP2K3	14	OK	1.3610072	0.7842944	1.7353267	0.08268	1.25056811	subgrp_cor	count		1	0
KCNJ12	1	OK	18.759489	83.625179	0.2243282	0.8225	0.00028789	subgrp_cor	count		1	0
C17orf51	1	OK	-11.01941	43.249935	-0.254784	0.79889	-1.17482461	subgrp_cor	count		1	0
KSR1	2	OK	-1.295616	2.041425	-0.634662	0.52565	-0.00075754	subgrp_cor	count		1	0
LGALS9	70	OK	0.2477607	0.3895283	0.6360530	0.52474	0.31010930	subgrp_cor	count		1	0
LYRM9	2	OK	-11.18679	26.313753	-0.42513	0.67074	-2.27103070	subgrp_cor	count		1	0
NLK	1	OK	18.759489	83.625179	0.2243282	0.8225	0.00028789	subgrp_cor	count		1	0
TMEM97	2	OK	-13.0456	27.67476	-0.471390	0.63736	-1.3178643	subgrp_cor	count		1	0
IFT20	52	OK	0.4262146	0.437947	0.9732090	0.33045	0.49418461	subgrp_cor	count		1	0
TNFAIP1	41	OK	0.0739816	0.4672001	0.1583511	0.87418	0.06613534	subgrp_cor	count		1	0
POLDIP2	7	OK	1.0331191	1.324192	0.7801883	0.43528	0.3424257	subgrp_cor	count		1	0
TMEM199	3	OK	-0.725185	1.8079191	-0.401116	0.68832	-0.20979917	subgrp_cor	count		1	0
UNC119	4	OK	1.7138889	1.5246445	1.1241236	0.26096	1.04872804	subgrp_cor	count		1	0
PIGS	5	OK	0.9772933	1.4778829	0.6613469	0.50839	0.30301174	subgrp_cor	count		1	0
KIAA0100	7	OK	-3.236473	2.1419797	-1.510972	0.1308	-1.05424964	subgrp_cor	count		1	0
SDF2	71	OK	0.3005775	0.3647710	0.8240170	0.40993	0.37623121	subgrp_cor	count		1	0
SUPT6H	11	OK	2.7133284	1.3719325	1.9777491	0.04796	1.71409530	subgrp_cor	count		1	0
RAB34	42	OK	2.8047074	0.634582	4.4197645	9.88E-06	3.22841160	subgrp_cor	count		0.09893696	0
RPL23A	845	OK	-0.069952	0.0868987	-0.804990	0.42083	-0.10004476	subgrp_cor	count		1	0
TRAF4	7	OK	1.9182525	1.2811619	1.4972756	0.13432	1.33314744	subgrp_cor	count		1	0
FAM222B	5	OK	6.8091365	5.3050116	1.2835290	0.19931	1.696588	subgrp_cor	count		1	0
ERAL1	2	OK	-0.910223	2.1881125	-0.415985	0.67742	-0.25785735	subgrp_cor	count		1	0
FLOT2	15	OK	0.9081953	0.7653639	1.186619	0.23538	0.75478405	subgrp_cor	count		1	0
PHF12	1	OK	-11.01941	43.249935	-0.254784	0.79889	-1.17482461	subgrp_cor	count		1	0
MYO18A	6	OK	-0.690393	1.3210380	-0.522614	0.60124	-0.32534467	subgrp_cor	count		1	0
NUFIP2	81	OK	0.9438409	0.3632031	2.5986583	0.00936	1.21717432	subgrp_cor	count		1	0
TAOK1	33	OK	0.4714887	0.5712563	0.8253540	0.40917	0.39334161	subgrp_cor	count		1	0
ABHD15	1	OK	3.4598146	6.7714513	0.5109413	0.60939	0.00030179	subgrp_cor	count		1	0
TP53I13	10	OK	1.949678	1.3799767	1.4128341	0.1577	0.91803278	subgrp_cor	count		1	0
GIT1	15	OK	0.4990408	0.8944699	0.5579099	0.57691	0.44983914	subgrp_cor	count		1	0
SSH2	3	OK	-0.214966	1.5648178	-0.137374	0.89073	-0.06511276	subgrp_cor	count		1	0
NSRP1	180	OK	-0.422775	0.2097710	-2.015414	0.04386	-0.57743015	subgrp_cor	count		1	0
SLC6A4	1	OK	23.991896	84.899941	0.2825902	0.77749	1.65081709	subgrp_cor	count		1	0
BLMH	13	OK	-0.055040	0.8763565	-0.062806	0.94992	-0.0411647	subgrp_cor	count		1	0
CPD	29	OK	0.851918	0.5698165	1.4950747	0.13489	0.97549172	subgrp_cor	count		1	0
GOSR1	42	OK	-0.919794	0.5109762	-1.800072	0.07185	-1.11904596	subgrp_cor	count		1	0
CRLF3	1	OK	-11.01941	43.249935	-0.254784	0.79889	-1.17482461	subgrp_cor	count		1	0
TEFM	6	OK	-1.674711	1.334501	-1.254933	0.2095	-0.71277837	subgrp_cor	count		1	0
RNF135	6	OK	0.5658288	1.0707832	0.5284252	0.5972	0.36836042	subgrp_cor	count		1	0
NF1	16	OK	2.3428964	1.0369022	2.2595151	0.02385	1.33799568	subgrp_cor	count		1	0
COPRS	86	OK	1.0220328	0.3147825	3.2467897	0.00117	1.34083210	subgrp_cor	count		1	0
UTP6	20	OK	-0.359262	0.645237	-0.556790	0.57767	-0.33345109	subgrp_cor	count		1	0
SUZ12	44	OK	0.6622642	0.4786690	1.3835534	0.1665	0.71510352	subgrp_cor	count		1	0
RHOT1	6	OK	1.5700459	1.3578364	1.1562849	0.24756	1.12712103	subgrp_cor	count		1	0
C17orf75	4	OK	-0.024863	1.1958382	-0.020791	0.98341	-0.00762178	subgrp_cor	count		1	0
ZNF207	64	OK	0.3641917	0.3724591	0.9778021	0.32817	0.40263964	subgrp_cor	count		1	0
AC005899.5	1	OK	-10.44227	41.465809	-0.251828	0.80117	-0.00045289	subgrp_cor	count		1	0
PSMD11	52	OK	0.6313558	0.4492912	1.4052263	0.15996	0.68205518	subgrp_cor	count		1	0
MYO1D	15	OK	2.1931013	1.3907135	1.5769612	0.1148	1.00493965	subgrp_cor	count		1	0
CCL2	220	OK	0.6192722	0.1780182	3.4786810	0.0005	0.87303765	subgrp_cor	count		1	0
CCL8	4	OK	0.5524930	1.3480003	0.4098611	0.68191	0.17231298	subgrp_cor	count		1	0
CCT6B	1	OK	-11.45102	41.79736	-0.273965	0.78411	-0.00049189	subgrp_cor	count		1	0
ZNF830	10	OK	-1.82514	1.0834093	-1.684626	0.09206	-1.28915590	subgrp_cor	count		1	0
LIG3	1	OK	-15.99503	70.34942	-0.227365	0.82014	-0.00028474	subgrp_cor	count		1	0
RFFL	1	OK	-11.01941	43.249935	-0.254784	0.79889	-1.17482461	subgrp_cor	count		1	0
RAD51D	1	OK	-11.01941	43.249935	-0.254784	0.79889	-1.17482461	subgrp_cor	count		1	0
NLE1	1	OK	-11.01941	43.249935	-0.254784	0.79889	-1.17482461	subgrp_cor	count		1	0
SLFN5	112	OK	-0.570834	0.2741416	-2.082260	0.03732	-0.74336722	subgrp_cor	count		1	0
AC022706.1	5	OK	-2.464013	1.6248819	-1.51642	0.12941	-0.54978098	subgrp_cor	count		1	0
SLFN11	9	OK	-0.800974	0.9869609	-0.811558	0.41705	-0.57117605	subgrp_cor	count		1	0
SLFN12	11	OK	2.0850638	1.5954907	1.3068479	0.19126	0.00384883	subgrp_cor	count		1	0
LINC02001	9	OK	0.208842	1.0275939	0.2032342	0.83895	0.1573110	subgrp_cor	count		1	0
AP2B1	44	OK	0.747891	0.4446436	1.6820025	0.09257	0.91823789	subgrp_cor	count		1	0
TAF15	60	OK	-0.006373	0.3549132	-0.017958	0.98567	-0.00796661	subgrp_cor	count		1	0
MMP28	61	OK	-0.142916	0.3432838	-0.416321	0.67717	-0.1889507	subgrp_cor	count		1	0
CCL5	3	OK	4.2109206	4.2875616	0.982124	0.32606	0.00257090	subgrp_cor	count		1	0
CCL3	3	OK	19.103032	50.772469	0.3762478	0.70672	0.00078880	subgrp_cor	count		1	0
CCL4	8	OK	2.0731662	1.6554499	1.2523279	0.21045	1.70903430	subgrp_cor	count		1	0
CCL4L2	4	OK	3.3648273	2.5447810	1.322246	0.18609	1.31777762	subgrp_cor	count		1	0
ZNHIT3	34	OK	1.0293589	0.5623020	1.8306159	0.06716	1.14603950	subgrp_cor	count		1	0
PIGW	1	OK	3.4598146	6.7714513	0.5109413	0.60939	0.00030179	subgrp_cor	count		1	0
GGNBP2	100	OK	-0.618267	0.2936737	-2.105287	0.03527	-0.78007745	subgrp_cor	count		1	0
DHRS11	4	OK	-11.8686	17.808144	-0.666475	0.50511	-1.88863766	subgrp_cor	count		1	0
AATF	17	OK	-0.277174	0.7018631	-0.39491	0.69291	-0.17666682	subgrp_cor	count		1	0
TADA2A	2	OK	5.0026588	4.2286244	1.1830463	0.23679	0.98115258	subgrp_cor	count		1	0
DUSP14	15	OK	1.2488164	1.112267	1.1227668	0.26154	0.62417554	subgrp_cor	count		1	0
SYNRG	10	OK	0.5609257	1.0214983	0.5491205	0.58292	0.29703291	subgrp_cor	count		1	0
DDX52	30	OK	0.1485792	0.5836065	0.2545879	0.79904	0.14059005	subgrp_cor	count		1	0
MRPL45	20	OK	0.4231498	0.6554374	0.6456151	0.5187	0.41049529	subgrp_cor	count		1	0
ARHGAP23	9	OK	-0.049105	1.2213978	-0.040204	0.96793	-0.03166016	subgrp_cor	count		1	0
EPOP	2	OK	-1.295616	2.041425	-0.634662	0.52565	-0.00075754	subgrp_cor	count		1	0
AC006449.2	2	OK	0.1681491	1.7253300	0.097459	0.92236	0.05201277	subgrp_cor	count		1	0
MLLT6	21	OK	-0.92185	0.6665778	-1.382960	0.16668	-0.93425254	subgrp_cor	count		1	0
CISD3	38	OK	-0.251871	0.4572193	-0.550876	0.58172	-0.29714485	subgrp_cor	count		1	0
PCGF2	52	OK	0.4785877	0.3896129	1.2283671	0.21931	0.59167403	subgrp_cor	count		1	0
PSMB3	323	OK	0.1496341	0.1574617	0.9502885	0.34197	0.2095677	subgrp_cor	count		1	0
PIP4K2B	3	OK	0.5887819	1.767414	0.3331317	0.73903	0.30122869	subgrp_cor	count		1	0
CWC25	37	OK	0.041650	0.4858173	0.0857322	0.93168	0.04755428	subgrp_cor	count		1	0
RPL23	746	OK	-0.153910	0.095846	-1.605835	0.10831	-0.21945896	subgrp_cor	count		1	0
LASP1	16	OK	0.2075228	0.8189255	0.2534087	0.79995	0.06428472	subgrp_cor	count		1	0
PLXDC1	2	OK	-0.725215	1.807914	-0.401134	0.68832	-0.20980483	subgrp_cor	count		1	0
CACNB1	1	OK	-15.99503	70.34942	-0.227365	0.82014	-0.00028474	subgrp_cor	count		1	0
RPL19	882	OK	-0.150726	0.0845487	-1.782716	0.07463	-0.21567579	subgrp_cor	count		1	0
FBXL20	2	OK	18.759489	83.625179	0.2243282	0.8225	0.00028789	subgrp_cor	count		1	0
MED1	7	OK	-1.038591	1.167183	-0.889827	0.37356	-0.84325998	subgrp_cor	count		1	0
CDK12	22	OK	-0.329241	0.7774607	-0.42348	0.67194	-0.32117964	subgrp_cor	count		1	0
STARD3	25	OK	-1.299216	0.5834405	-2.226819	0.02596	-1.12981258	subgrp_cor	count		1	0
PNMT	4	OK	-1.754582	1.6323569	-1.074876	0.28242	-0.95331468	subgrp_cor	count		1	0
PGAP3	2	OK	-0.202940	1.6532236	-0.122753	0.9022	-4.82E-05	subgrp_cor	count		1	0
MIEN1	67	OK	0.5805752	0.383422	1.5141835	0.12998	0.71245955	subgrp_cor	count		1	0
ORMDL3	1	OK	18.759489	83.625179	0.2243282	0.8225	0.00028789	subgrp_cor	count		1	0
PSMD3	25	OK	0.0823450	0.788108	0.1044843	0.91679	0.0418080	subgrp_cor	count		1	0
CSF3	25	OK	-0.477469	0.6180005	-0.772602	0.43976	-0.49562231	subgrp_cor	count		1	0
MED24	3	OK	-0.841640	1.4608231	-0.576141	0.56452	-0.24034852	subgrp_cor	count		1	0
THRA	12	OK	0.0298214	0.8704550	0.0342599	0.97267	0.03049657	subgrp_cor	count		1	0
NR1D1	10	OK	-0.872088	0.9793677	-0.89046	0.37322	-0.68216476	subgrp_cor	count		1	0
MSL1	12	OK	0.8505302	0.8176520	1.0402104	0.29826	0.70384747	subgrp_cor	count		1	0
CASC3	11	OK	0.0267449	0.8948410	0.0298879	0.97616	0.01734991	subgrp_cor	count		1	0
WIPF2	12	OK	0.0682413	0.9683165	0.0704742	0.94382	0.0512543	subgrp_cor	count		1	0
RARA	16	OK	-0.38948	0.722722	-0.538905	0.58995	-0.38229906	subgrp_cor	count		1	0
RARA-AS1	10	OK	1.4291570	1.0901671	1.3109521	0.18987	1.14727574	subgrp_cor	count		1	0
TOP2A	1	OK	0.6532528	2.5001284	0.2612877	0.79387	0.00016297	subgrp_cor	count		1	0
IGFBP4	745	OK	-0.437352	0.0960832	-4.551808	5.32E-06	-0.62432020	subgrp_cor	count		0.0523862	0
SMARCE1	41	OK	-0.009737	0.4540126	-0.021447	0.98289	-0.01025247	subgrp_cor	count		1	0
AC073508.3	1	OK	-10.44227	41.465809	-0.251828	0.80117	-0.00045289	subgrp_cor	count		1	0
KRT222	16	OK	-0.798053	0.7467384	-1.068719	0.2852	-0.38029966	subgrp_cor	count		1	0
KRT27	3	OK	-11.44854	41.76148	-0.274141	0.78398	-0.00049246	subgrp_cor	count		1	0
KRT10	352	OK	0.4272506	0.149877	2.8513368	0.00435	0.60088122	subgrp_cor	count		1	0
TMEM99	7	OK	-1.484432	1.3983751	-1.061541	0.28844	-0.64939975	subgrp_cor	count		1	0
KRTAP4-11	1	OK	1.5822999	2.7200188	0.5817608	0.56072	0.47433761	subgrp_cor	count		1	0
EIF1	863	OK	-0.202469	0.0865884	-2.338297	0.01937	-0.28956960	subgrp_cor	count		1	0
JUP	22	OK	1.8859792	0.8218485	2.2948013	0.02174	1.96177800	subgrp_cor	count		1	0
P3H4	2	OK	1.1112449	1.8685808	0.5946999	0.55204	0.34276981	subgrp_cor	count		1	0
FKBP10	26	OK	1.2416400	0.7162201	1.7336011	0.08299	0.9128089	subgrp_cor	count		1	0
NT5C3B	30	OK	1.0420671	0.6218303	1.6758062	0.09378	0.92065369	subgrp_cor	count		1	0
ACLY	9	OK	-2.400317	1.567376	-1.531423	0.12566	-1.42693734	subgrp_cor	count		1	0
CNP	16	OK	-1.13660	0.796329	-1.427309	0.15349	-0.78718626	subgrp_cor	count		1	0
DNAJC7	92	OK	0.1355401	0.3112642	0.4354501	0.66324	0.17048947	subgrp_cor	count		1	0
NKIRAS2	18	OK	0.3074733	0.7143082	0.4304491	0.66687	0.20012019	subgrp_cor	count		1	0
DHX58	4	OK	1.09958	1.3188962	0.833716	0.40446	0.55419423	subgrp_cor	count		1	0
KAT2A	2	OK	-10.44227	41.465809	-0.251828	0.80117	-0.00045289	subgrp_cor	count		1	0
RAB5C	214	OK	-0.10035	0.1967439	-0.510094	0.60990	-0.13692975	subgrp_cor	count		1	0
AC099811.3	1	OK	-11.01941	43.249935	-0.254784	0.79889	-1.17482461	subgrp_cor	count		1	0
GHDC	8	OK	1.7938809	1.4194299	1.2638037	0.2062	0.55322908	subgrp_cor	count		1	0
STAT5B	9	OK	0.9932482	1.3624169	0.7290319	0.46732	0.49792729	subgrp_cor	count		1	0
STAT5A	9	OK	2.3719910	1.351467	1.7551230	0.07924	1.22216585	subgrp_cor	count		1	0
STAT3	249	OK	-0.098358	0.1680016	-0.585460	0.55824	-0.13675050	subgrp_cor	count		1	0
CAVIN1	497	OK	0.0305786	0.1163104	0.2629055	0.79262	0.04335331	subgrp_cor	count		1	0
ATP6V0A1	6	OK	2.8248494	1.6725554	1.6879323	0.09142	0.76067192	subgrp_cor	count		1	0
NAGLU	4	OK	19.103032	50.772469	0.3762478	0.70672	0.00078880	subgrp_cor	count		1	0
COASY	4	OK	2.5552030	2.3498443	1.0873924	0.27686	0.69311810	subgrp_cor	count		1	0
MLX	23	OK	-0.808567	0.6304603	-1.282503	0.19967	-0.79893516	subgrp_cor	count		1	0
TUBG1	11	OK	1.2504052	1.2754305	0.980378	0.3269	0.62491299	subgrp_cor	count		1	0
PLEKHH3	9	OK	7.351849	5.0116738	1.4669449	0.14239	1.72950772	subgrp_cor	count		1	0
AC100793.2	1	OK	18.759489	83.625179	0.2243282	0.8225	0.00028789	subgrp_cor	count		1	0
EZH1	14	OK	0.6352158	0.749225	0.8478297	0.39652	0.47836971	subgrp_cor	count		1	0
RAMP2-AS1	2	OK	-0.202940	1.6532236	-0.122753	0.9022	-4.82E-05	subgrp_cor	count		1	0
RAMP2	778	OK	-0.315849	0.0930232	-3.395282	0.00069	-0.45115833	subgrp_cor	count		1	0
VPS25	23	OK	1.0385666	0.7342414	1.4144756	0.15722	0.97780822	subgrp_cor	count		1	0
COA3	84	OK	0.6810271	0.3370818	2.0203616	0.04335	0.88299190	subgrp_cor	count		1	0
BECN1	27	OK	-0.981725	0.6322275	-1.550351	0.12106	-0.98893987	subgrp_cor	count		1	0
PSME3	6	OK	-0.615396	1.492102	-0.412435	0.68002	-0.29859809	subgrp_cor	count		1	0
AOC3	17	OK	-1.756580	0.8245671	-2.130306	0.03315	-1.36069188	subgrp_cor	count		1	0
RUNDC1	1	OK	18.759489	83.625179	0.2243282	0.8225	0.00028789	subgrp_cor	count		1	0
RPL27	749	OK	-0.031795	0.0961744	-0.330606	0.74094	-0.04530609	subgrp_cor	count		1	0
IFI35	60	OK	0.1546197	0.3842744	0.4023681	0.68741	0.17920668	subgrp_cor	count		1	0
VAT1	48	OK	-0.579876	0.4316818	-1.343296	0.17918	-0.65098320	subgrp_cor	count		1	0
BRCA1	1	OK	3.4598146	6.7714513	0.5109413	0.60939	0.00030179	subgrp_cor	count		1	0
NBR1	22	OK	0.2524319	0.643051	0.3925533	0.69465	0.22652984	subgrp_cor	count		1	0
TMEM106A	1	OK	-10.44227	41.465809	-0.251828	0.80117	-0.00045289	subgrp_cor	count		1	0
ARL4D	40	OK	6.1883686	1.8938558	3.2676027	0.00108	3.87089643	subgrp_cor	count		1	0
DHX8	9	OK	0.9255608	1.1718178	0.7898504	0.42962	0.47001577	subgrp_cor	count		1	0
MEOX1	33	OK	-2.207823	0.6455216	-3.420215	0.00062	-1.89884485	subgrp_cor	count		1	0
SOST	1	OK	-11.01941	43.249935	-0.254784	0.79889	-1.17482461	subgrp_cor	count		1	0
DUSP3	18	OK	1.5336833	0.8174229	1.8762421	0.06062	1.10429943	subgrp_cor	count		1	0
MPP3	2	OK	21.105323	83.341273	0.2532397	0.80008	0.0003103	subgrp_cor	count		1	0
CD300LG	4	OK	-2.342222	1.7367717	-1.348606	0.17748	-0.53905893	subgrp_cor	count		1	0
NAGS	2	OK	1.925964	2.5154879	0.7656425	0.44389	0.56022119	subgrp_cor	count		1	0
TMEM101	34	OK	0.6110070	0.5250325	1.163750	0.24452	0.67410450	subgrp_cor	count		1	0
LSM12.00	46	OK	0.4234561	0.4422661	0.957468	0.33832	0.5025638	subgrp_cor	count		1	0
G6PC3	8	OK	-0.107220	1.031342	-0.103961	0.9172	-0.08004306	subgrp_cor	count		1	0
HDAC5	13	OK	0.6337141	0.917471	0.6907184	0.48974	0.47724472	subgrp_cor	count		1	0
TMUB2	10	OK	0.548734	0.8916922	0.615385	0.5382	0.41373457	subgrp_cor	count		1	0
ATXN7L3	3	OK	21.64205	54.486891	0.3971975	0.69122	1.57744735	subgrp_cor	count		1	0
UBTF	43	OK	1.1764585	0.48746	2.4134067	0.0158	1.27771520	subgrp_cor	count		1	0
SLC25A39	27	OK	0.098319	0.5979420	0.1644298	0.86939	0.08793196	subgrp_cor	count		1	0
GRN	216	OK	0.4751463	0.1888005	2.516658	0.01185	0.65639300	subgrp_cor	count		1	0
GPATCH8	27	OK	3.6036310	1.2522778	2.8776609	0.00401	1.71579349	subgrp_cor	count		1	0
CCDC43	33	OK	0.638206	0.5655363	1.1284972	0.25911	0.6695920	subgrp_cor	count		1	0
EFTUD2	4	OK	0.1721614	1.534450	0.1121974	0.91067	0.00011395	subgrp_cor	count		1	0
DCAKD	1	OK	23.991896	84.899941	0.2825902	0.77749	1.65081709	subgrp_cor	count		1	0
NMT1	22	OK	0.1754070	0.7363283	0.2382152	0.81171	0.14600461	subgrp_cor	count		1	0
PLCD3	5	OK	2.3992284	2.2496017	1.066512	0.28619	0.00059488	subgrp_cor	count		1	0
AC142472.1	1	OK	3.4598146	6.7714513	0.5109413	0.60939	0.00030179	subgrp_cor	count		1	0
HEXIM1	20	OK	0.7610350	0.8043874	0.9461051	0.34409	0.62209847	subgrp_cor	count		1	0
HEXIM2	1	OK	-11.01941	43.249935	-0.254784	0.79889	-1.17482461	subgrp_cor	count		1	0
PLEKHM1	2	OK	0.1681491	1.7253300	0.097459	0.92236	0.05201277	subgrp_cor	count		1	0
AC091132.5	1	OK	-11.01941	43.249935	-0.254784	0.79889	-1.17482461	subgrp_cor	count		1	0
KANSL1	40	OK	0.7157211	0.578930	1.2362995	0.21635	0.64163093	subgrp_cor	count		1	0
KANSL1-AS1	9	OK	2.2235183	1.3578791	1.7111377	0.08706	0.64009590	subgrp_cor	count		1	0
FAM215B	1	OK	18.759489	83.625179	0.2243282	0.8225	0.00028789	subgrp_cor	count		1	0
NSF	4	OK	-15.99503	70.34942	-0.227365	0.82014	-0.00028474	subgrp_cor	count		1	0
WNT9B	2	OK	-10.44227	41.465809	-0.251828	0.80117	-0.00045289	subgrp_cor	count		1	0
GOSR2	19	OK	0.5396825	0.7796104	0.6922463	0.48878	0.4496439	subgrp_cor	count		1	0
CDC27	65	OK	0.6514130	0.3562823	1.8283616	0.0675	0.79876563	subgrp_cor	count		1	0
MYL4	1	OK	41.788303	174.29608	0.2397549	0.81052	0.00014488	subgrp_cor	count		1	0
ITGB3	1	OK	18.759489	83.625179	0.2243282	0.8225	0.00028789	subgrp_cor	count		1	0
EFCAB13	5	OK	0.2537540	1.2457170	0.2037012	0.83859	0.07872548	subgrp_cor	count		1	0
NPEPPS	40	OK	0.652203	0.5136190	1.2700148	0.20409	0.78896594	subgrp_cor	count		1	0
KPNB1	68	OK	-0.157598	0.3577238	-0.440558	0.65952	-0.18628467	subgrp_cor	count		1	0
TBKBP1	3	OK	0.5103116	1.6034057	0.3182673	0.75028	0.15911454	subgrp_cor	count		1	0
MRPL10	11	OK	0.0991450	0.9969795	0.099445	0.92079	0.06425633	subgrp_cor	count		1	0
SCRN2	9	OK	2.3120807	1.3961963	1.6559853	0.09772	1.32539092	subgrp_cor	count		1	0
SP2	3	OK	-0.725215	1.807914	-0.401134	0.68832	-0.20980483	subgrp_cor	count		1	0
PNPO	5	OK	-0.024863	1.1958382	-0.020791	0.98341	-0.00762178	subgrp_cor	count		1	0
CDK5RAP3	17	OK	1.7891645	0.9441168	1.8950668	0.05808	1.39245067	subgrp_cor	count		1	0
COPZ2	39	OK	1.6279936	0.5545855	2.9355140	0.00333	1.76567520	subgrp_cor	count		1	0
NFE2L1	73	OK	-0.729755	0.3279653	-2.22509	0.02607	-0.90850014	subgrp_cor	count		1	0
CBX1	37	OK	1.3048818	0.5658935	2.3058787	0.02112	1.41048252	subgrp_cor	count		1	0
SNX11	1	OK	-11.01941	43.249935	-0.254784	0.79889	-1.17482461	subgrp_cor	count		1	0
HOXB2	13	OK	-3.365481	1.521858	-2.211428	0.02701	-1.88045023	subgrp_cor	count		1	0
HOXB3	2	OK	-11.44854	41.76148	-0.274141	0.78398	-0.00049246	subgrp_cor	count		1	0
HOXB4	19	OK	-1.594129	0.7540694	-2.114035	0.03452	-1.72118012	subgrp_cor	count		1	0
HOXB5	3	OK	1.5824001	1.5705105	1.0075705	0.31366	0.97973537	subgrp_cor	count		1	0
CALCOCO2	237	OK	-0.800447	0.1758700	-4.551256	5.33E-06	-1.10392680	subgrp_cor	count		0.05346643	0
ATP5MC1	161	OK	0.2079256	0.2268298	0.9166185	0.35934	0.27868497	subgrp_cor	count		1	0
UBE2Z	22	OK	0.6565503	0.8716213	0.7532517	0.4513	0.33566277	subgrp_cor	count		1	0
SNF8	93	OK	0.6976411	0.3155253	2.2110463	0.02702	0.89187091	subgrp_cor	count		1	0
GNGT2	1	OK	-15.99503	70.34942	-0.227365	0.82014	-0.00028474	subgrp_cor	count		1	0
ABI3	55	OK	-1.25898	0.4299364	-2.928314	0.00341	-1.44142083	subgrp_cor	count		1	0
ZNF652	18	OK	0.5597084	0.661106	0.8466243	0.3972	0.5298756	subgrp_cor	count		1	0
PHB	125	OK	0.0848749	0.2530543	0.3354019	0.73732	0.1143517	subgrp_cor	count		1	0
SPOP	48	OK	1.3289975	0.4789270	2.774947	0.00552	1.48716072	subgrp_cor	count		1	0
SLC35B1	23	OK	0.2643852	0.6192703	0.4269304	0.66942	0.25051327	subgrp_cor	count		1	0
FAM117A	6	OK	-0.688176	1.1979986	-0.574438	0.56567	-0.49554981	subgrp_cor	count		1	0
KAT7	8	OK	1.5337779	1.0191056	1.5050235	0.13232	0.95351203	subgrp_cor	count		1	0
ITGA3	10	OK	0.4044904	0.8630720	0.4686636	0.63931	0.20683060	subgrp_cor	count		1	0
PDK2	7	OK	0.7755380	1.2121452	0.6398061	0.5222	0.39560908	subgrp_cor	count		1	0
SAMD14	1	OK	18.759489	83.625179	0.2243282	0.8225	0.00028789	subgrp_cor	count		1	0
PPP1R9B	6	OK	-0.465429	1.309363	-0.355462	0.72226	-0.13816105	subgrp_cor	count		1	0
TMEM92	1	OK	23.991896	84.899941	0.2825902	0.77749	1.65081709	subgrp_cor	count		1	0
MRPL27	51	OK	0.3350443	0.4320761	0.7754288	0.43809	0.40167324	subgrp_cor	count		1	0
LRRC59	16	OK	0.0349460	0.8700948	0.0401635	0.96796	0.01771632	subgrp_cor	count		1	0
ACSF2	2	OK	-11.01941	43.249935	-0.254784	0.79889	-1.17482461	subgrp_cor	count		1	0
RSAD1	2	OK	2.8849400	2.5988857	1.1485460	0.25074	1.23382860	subgrp_cor	count		1	0
SPATA20	7	OK	5.6831241	5.3331875	1.0656148	0.2866	0.00307912	subgrp_cor	count		1	0
ABCC3	6	OK	20.917329	33.182062	0.6303806	0.52845	2.28610964	subgrp_cor	count		1	0
ANKRD40	16	OK	-0.223463	0.7675863	-0.291121	0.77096	-0.14287113	subgrp_cor	count		1	0
AC005921.2	11	OK	0.8609042	0.8226665	1.0464801	0.29536	0.55685498	subgrp_cor	count		1	0
LUC7L3	107	OK	0.8866959	0.3058167	2.8994359	0.00376	1.13463451	subgrp_cor	count		1	0
TOB1	48	OK	-0.121073	0.424559	-0.285174	0.77551	-0.13575396	subgrp_cor	count		1	0
SPAG9	217	OK	0.0147229	0.1937760	0.075979	0.93944	0.02009783	subgrp_cor	count		1	0
NME1	80	OK	0.7533420	0.3417418	2.2044186	0.02749	0.95436754	subgrp_cor	count		1	0
NME2	1	OK	-10.44227	41.465809	-0.251828	0.80117	-0.00045289	subgrp_cor	count		1	0
UTP18	14	OK	-0.01994	0.8098982	-0.024620	0.98036	-0.01493422	subgrp_cor	count		1	0
COX11	10	OK	0.8649534	1.3849582	0.6245334	0.53228	0.00160356	subgrp_cor	count		1	0
HLF	1	OK	-10.44227	41.465809	-0.251828	0.80117	-0.00045289	subgrp_cor	count		1	0
MMD	1	OK	3.4598146	6.7714513	0.5109413	0.60939	0.00030179	subgrp_cor	count		1	0
TMEM100	3	OK	4.2109206	4.2875616	0.982124	0.32606	0.00257090	subgrp_cor	count		1	0
PCTP	2	OK	-15.99503	70.34942	-0.227365	0.82014	-0.00028474	subgrp_cor	count		1	0
NOG	7	OK	22.221790	54.523227	0.4075655	0.68359	1.5953836	subgrp_cor	count		1	0
C17orf67	3	OK	4.4237837	2.6360667	1.6781759	0.09331	2.97812552	subgrp_cor	count		1	0
DGKE	17	OK	0.2422800	0.7702316	0.3146847	0.753	0.22845920	subgrp_cor	count		1	0
TRIM25	14	OK	-0.976591	0.8123960	-1.202112	0.22932	-0.76172586	subgrp_cor	count		1	0
COIL	15	OK	1.0158160	0.7462362	1.3612526	0.17342	0.95004613	subgrp_cor	count		1	0
SCPEP1	20	OK	0.8514465	0.6540730	1.3017606	0.193	0.7045820	subgrp_cor	count		1	0
AC007114.1	2	OK	-0.910223	2.1881125	-0.415985	0.67742	-0.25785735	subgrp_cor	count		1	0

AKAP1	3	OK	-12.145707	43.4188694	-0.2797334	0.77968	-1.91338276	subgrp_cor	count		1	0
MSI2	12	OK	0.28837869	1.07056173	0.26937138	0.78764	0.00019216	subgrp_cor	count		1	0
MRPS23	27	OK	-0.1028764	0.62429418	-0.1647884	0.86911	-0.11322808	subgrp_cor	count		1	0
VEZF1	33	OK	0.49390134	0.52080725	0.94833808	0.34296	0.52140903	subgrp_cor	count		1	0
SRSF1	26	OK	-0.7938997	0.59632165	-1.3312279	0.18330	-0.48608121	subgrp_cor	count		1	0
DYNLL2	14	OK	0.74600307	0.9399867	0.79363151	0.42741	0.38962621	subgrp_cor	count		1	0
MKS1	1	OK	3.45981462	6.77145131	0.51094137	0.60939	0.00030179	subgrp_cor	count		1	0
SUPT4H1	60	OK	-0.2986048	0.3589824	-0.831809	0.40552	-0.38223055	subgrp_cor	count		1	0
MTMR4	4	OK	-2.2156624	1.60719433	-1.3785903	0.16802	-0.00117967	subgrp_cor	count		1	0
4-Sep	8	OK	1.33158906	1.04132663	1.27874292	0.20090	0.84059799	subgrp_cor	count		1	0
RAD51C	9	OK	-1.0398125	1.08041621	-0.9624185	0.33584	-0.86698295	subgrp_cor	count		1	0
TRIM37	3	OK	-1.5371297	1.64874199	-0.9323046	0.35119	-0.40043369	subgrp_cor	count		1	0
SKA2	12	OK	0.71223674	0.86372583	0.82460975	0.40959	0.53554662	subgrp_cor	count		1	0
YPEL2	113	OK	-0.5528527	0.28025609	-1.9726686	0.04852	-0.72202782	subgrp_cor	count		1	0
DHX40	8	OK	-0.6608909	1.01722656	-0.6496988	0.51589	-0.21931085	subgrp_cor	count		1	0
AC091271.1	4	OK	-1.6462456	1.93519292	-0.8506881	0.39494	-0.70360391	subgrp_cor	count		1	0
CLTC	53	OK	0.68789548	0.4535789	1.5165949	0.12937	0.79540430	subgrp_cor	count		1	0
PTRH2	17	OK	1.44558299	0.90132356	1.60384467	0.10875	1.15908482	subgrp_cor	count		1	0
VMP1	77	OK	0.71858346	0.37456719	1.91843676	0.05506	0.8805912	subgrp_cor	count		1	0
TUBD1	1	OK	23.9918963	84.8999419	0.28259026	0.77749	1.65081709	subgrp_cor	count		1	0
RPS6KB1	4	OK	0.26065145	1.66204742	0.15682552	0.87538	0.08088269	subgrp_cor	count		1	0
RNFT1	1	OK	3.45981462	6.77145131	0.51094137	0.60939	0.00030179	subgrp_cor	count		1	0
HEATR6	1	OK	1.58239999	2.72001883	0.58176068	0.56072	0.47433761	subgrp_cor	count		1	0
CA4	6	OK	-10.908865	16.7918392	-0.6496528	0.51592	-2.58086635	subgrp_cor	count		1	0
USP32	2	OK	-12.148421	43.4553091	-0.2795613	0.77981	-1.91362624	subgrp_cor	count		1	0
APPBP2	11	OK	-0.5877102	0.80352987	-0.7314106	0.46452	-0.36639980	subgrp_cor	count		1	0
PPM1D	24	OK	-1.1688728	0.60273586	-1.9392786	0.05247	-0.96884632	subgrp_cor	count		1	0
BCAS3	2	OK	3.45981462	6.77145131	0.51094137	0.60939	0.00030179	subgrp_cor	count		1	0
MED13	27	OK	0.94754929	0.62901016	1.50641332	0.13196	0.89064729	subgrp_cor	count		1	0
METTL2A	4	OK	-0.6154176	1.49209719	-0.4124514	0.68001	-0.29861013	subgrp_cor	count		1	0
TLK2	8	OK	-0.1854396	1.01522159	-0.1826592	0.85507	-0.0929800	subgrp_cor	count		1	0
MRC2	23	OK	25.9248923	32.4922202	0.79788	0.42494	4.57122531	subgrp_cor	count		1	0
TANC2	7	OK	2.03643721	1.39222417	1.4627222	0.14354	0.94995223	subgrp_cor	count		1	0
CYB561	9	OK	1.88346237	1.04715461	1.79864783	0.07207	1.5827402	subgrp_cor	count		1	0
ACE	24	OK	0.00428129	0.68458294	0.00625385	0.99501	0.00320905	subgrp_cor	count		1	0
DCAF7	15	OK	2.13427112	1.00015301	2.1339446	0.03285	1.62989550	subgrp_cor	count		1	0
TACO1	5	OK	2.20606141	1.55467262	1.41898775	0.1559	1.3026459	subgrp_cor	count		1	0
MAP3K3	22	OK	0.35367977	0.64999981	0.54412288	0.58636	0.2668131	subgrp_cor	count		1	0
STRADA	1	OK	3.45981462	6.77145131	0.51094137	0.60939	0.00030179	subgrp_cor	count		1	0
LIMD2	10	OK	0.08787787	0.90719161	0.09686803	0.92282	0.07302557	subgrp_cor	count		1	0
CCDC47	174	OK	0.49151008	0.21783515	2.25633942	0.02405	0.6716354	subgrp_cor	count		1	0
DDX42	52	OK	0.84624752	0.4628375	1.82839015	0.06749	1.09558964	subgrp_cor	count		1	0
FTSJ3	2	OK	3.45981462	6.77145131	0.51094137	0.60939	0.00030179	subgrp_cor	count		1	0
PSMC5	208	OK	0.69225913	0.2068353	3.34691017	0.00082	0.95134424	subgrp_cor	count		1	0
SMARCD2	8	OK	0.17216144	1.08468911	0.15871962	0.87389	0.0003095	subgrp_cor	count		1	0
CD79B	71	OK	-1.0506533	0.36630828	-2.8682214	0.00412	-1.1880726	subgrp_cor	count		1	0
SCN4A	1	OK	0.65325287	2.50012846	0.26128772	0.79387	0.00016297	subgrp_cor	count		1	0
ICAM2	199	OK	0.73621364	0.22128335	3.32701786	0.00088	0.98807606	subgrp_cor	count		1	0
ERN1.00	10	OK	-0.4251942	1.15482629	-0.3681888	0.71272	-0.34788604	subgrp_cor	count		1	0
PECAM1	714	OK	-0.1082101	0.09499777	-1.1390803	0.25467	-0.15449898	subgrp_cor	count		1	0
POLG2	1	OK	1.58239999	2.72001883	0.58176068	0.56072	0.47433761	subgrp_cor	count		1	0
DDX5	680	OK	-0.0191798	0.09994436	-0.1919052	0.84782	-0.02728237	subgrp_cor	count		1	0
CEP95	5	OK	1.69821373	1.52073093	1.11677463	0.26409	1.04069886	subgrp_cor	count		1	0
SMURF2	27	OK	1.60981732	0.68087264	2.36434427	0.01806	1.27042487	subgrp_cor	count		1	0
LRRC37A3	1	OK	-10.442278	41.4658099	-0.2518286	0.80117	-0.00045289	subgrp_cor	count		1	0
GNA13	7	OK	1.04216691	1.28431867	0.81145511	0.4171	0.50439027	subgrp_cor	count		1	0
RGS9	1	OK	1.58239999	2.72001883	0.58176068	0.56072	0.47433761	subgrp_cor	count		1	0
CEP112	29	OK	0.112487	0.49336421	0.22799992	0.81965	0.11865820	subgrp_cor	count		1	0
PRKCA	1	OK	18.7594894	83.6251792	0.22432824	0.8225	0.00028789	subgrp_cor	count		1	0
HELZ	6	OK	6.42803292	5.27484014	1.21862137	0.22299	1.06669691	subgrp_cor	count		1	0
PSMD12	70	OK	-0.2095098	0.36241624	-0.5780915	0.5632	-0.24740066	subgrp_cor	count		1	0
PITPNC1	26	OK	-1.7776386	0.69677588	-2.5512342	0.01073	-1.54317927	subgrp_cor	count		1	0
NOL11	12	OK	0.54198904	1.12288469	0.4826756	0.62932	0.27736238	subgrp_cor	count		1	0
BPTF	140	OK	-0.1043566	0.22709569	-0.459527	0.64586	-0.13709565	subgrp_cor	count		1	0
C17orf58	11	OK	1.77265503	0.9983894	1.77551457	0.07581	1.46127868	subgrp_cor	count		1	0
KPNA2	16	OK	0.01369139	0.90757264	0.01508572	0.98796	0.01135771	subgrp_cor	count		1	0
AC005332.7	2	OK	4.01287811	3.59010066	1.11776201	0.26367	0.00248239	subgrp_cor	count		1	0
AC005332.4	3	OK	3.45981462	6.77145131	0.51094137	0.60939	0.00030179	subgrp_cor	count		1	0
AMZ2	3	OK	4.66132999	5.76838606	0.80808217	0.41904	0.00274918	subgrp_cor	count		1	0
WIPI1	3	OK	2.55522271	2.3498646	1.08740073	0.27686	0.69313893	subgrp_cor	count		1	0
PRKAR1A	71	OK	0.80929331	0.38779791	2.08689451	0.0369	0.92094584	subgrp_cor	count		1	0
FAM20A	1	OK	-10.442278	41.4658099	-0.2518286	0.80117	-0.00045289	subgrp_cor	count		1	0
ABCA8	8	OK	25.1387123	54.0636937	0.46498325	0.64194	2.93759967	subgrp_cor	count		1	0
ABCA9	1	OK	1.58239999	2.72001883	0.58176068	0.56072	0.47433761	subgrp_cor	count		1	0
ABCA6	2	OK	26.3679501	84.9163259	0.31051685	0.75617	2.49052421	subgrp_cor	count		1	0
ABCA5	6	OK	-0.1817875	1.24407214	-0.146122	0.88382	-0.14860488	subgrp_cor	count		1	0
MAP2K6	3	OK	-1.8538345	1.85439186	-0.9996995	0.31740	-0.46053279	subgrp_cor	count		1	0
LINC01497	1	OK	18.7594894	83.6251792	0.22432824	0.8225	0.00028789	subgrp_cor	count		1	0
KCNJ2-AS1	1	OK	1.58237769	1.92402136	0.8224325	0.41082	0.77174550	subgrp_cor	count		1	0
KCNJ2	19	OK	1.76780613	0.7706145	2.29402136	0.02179	1.94765595	subgrp_cor	count		1	0
SLC39A11	2	OK	0.58878195	1.7674148	0.33313173	0.73903	0.30122869	subgrp_cor	count		1	0
FAM104A	9	OK	-0.8163116	0.96433512	-0.846502	0.39727	-0.6920222	subgrp_cor	count		1	0
C17orf80	5	OK	1.84906689	1.53934796	1.20120137	0.22967	0.91442635	subgrp_cor	count		1	0
CDC42EP4	29	OK	-0.9489472	0.53097254	-1.7871869	0.07391	-0.57161550	subgrp_cor	count		1	0
RPL38	713	OK	-0.2048724	0.09965442	-2.0558287	0.0398	-0.29154254	subgrp_cor	count		1	0
GPRC5C	21	OK	-0.2879984	0.61257476	-0.470144	0.63825	-0.21320591	subgrp_cor	count		1	0
SLC9A3R1	1	OK	3.45981462	6.77145131	0.51094137	0.60939	0.00030179	subgrp_cor	count		1	0
NAT9	4	OK	-0.9727207	1.63750584	-0.5940258	0.55249	-0.27349270	subgrp_cor	count		1	0
TMEM104	1	OK	3.45981462	6.77145131	0.51094137	0.60939	0.00030179	subgrp_cor	count		1	0
FDXR	2	OK	3.45981462	6.77145131	0.51094137	0.60939	0.00030179	subgrp_cor	count		1	0
HID1	3	OK	-13.335695	29.1122331	-0.4580787	0.6469	-1.33917170	subgrp_cor	count		1	0
HID1-AS1	3	OK	-10.968199	27.2100839	-0.4030932	0.68688	-1.17122783	subgrp_cor	count		1	0
CDR2L	4	OK	1.58965014	1.67297115	0.95019579	0.34201	0.00109513	subgrp_cor	count		1	0
MRPL58	6	OK	-1.5504866	1.41942443	-1.0923347	0.27469	-0.00088235	subgrp_cor	count		1	0
KCTD2	1	OK	23.9918963	84.8999419	0.28259026	0.77749	1.65081709	subgrp_cor	count		1	0
ATP5PD	299	OK	0.30002239	0.16368881	1.83288272	0.06682	0.41969516	subgrp_cor	count		1	0
SLC16A5	13	OK	19.7150485	30.415247	0.64819623	0.51686	0.00221832	subgrp_cor	count		1	0
ARMC7	5	OK	1.39610124	1.41763559	0.98480967	0.32472	0.42407085	subgrp_cor	count		1	0
NT5C	15	OK	0.52877253	0.90655191	0.58327882	0.55971	0.56964757	subgrp_cor	count		1	0
JPT1	72	OK	1.27784449	0.41349343	3.09036229	0.002	1.40948306	subgrp_cor	count		1	0
SUMO2	719	OK	-0.1735651	0.09873659	-1.7578586	0.07877	-0.24736463	subgrp_cor	count		1	0
NUP85	1	OK	3.45981462	6.77145131	0.51094137	0.60939	0.00030179	subgrp_cor	count		1	0
MRPS7	45	OK	0.10134067	0.45991464	0.2203467	0.8256	0.11395338	subgrp_cor	count		1	0
MIF4GD	14	OK	0.35579437	0.83152699	0.42788072	0.66874	0.36402421	subgrp_cor	count		1	0
GRB2	100	OK	-0.1527712	0.3002754	-0.5087702	0.61091	-0.19063473	subgrp_cor	count		1	0
TMEM94	2	OK	-1.7585735	2.35544393	-0.7465996	0.45531	-0.00036089	subgrp_cor	count		1	0
CASKIN2	12	OK	1.72033592	1.13163445	1.5202223	0.12846	0.00322026	subgrp_cor	count		1	0
TSEN54	4	OK	-0.1747967	1.8209803	-0.0959904	0.92352	-0.05309252	subgrp_cor	count		1	0
LLGL2	3	OK	-10.967865	27.1536587	-0.4039185	0.68627	-1.17125107	subgrp_cor	count		1	0
MYO15B	1	OK	17.7472079	50.9719273	0.34817612	0.72771	0.0007532	subgrp_cor	count		1	0
SMIM6	2	OK	23.9918963	84.8999419	0.28259026	0.77749	1.65081709	subgrp_cor	count		1	0
SAP30BP	35	OK	0.81883741	0.52971971	1.54578375	0.12215	0.83483276	subgrp_cor	count		1	0
ITGB4	106	OK	-0.9287495	0.27510972	-3.3759241	0.00074	-1.20274436	subgrp_cor	count		1	0
GALK1	12	OK	-0.3252293	0.7541413	-0.4312578	0.66628	-0.24022495	subgrp_cor	count		1	0
H3F3B	858	OK	-0.2268586	0.08709463	-2.6047372	0.00919	-0.32440278	subgrp_cor	count		1	0
UNK	1	OK	3.45981462	6.77145131	0.51094137	0.60939	0.00030179	subgrp_cor	count		1	0
WBP2	61	OK	0.32250416	0.36751063	0.87753743	0.38019	0.36885708	subgrp_cor	count		1	0
TRIM47	60	OK	-0.2662063	0.39626757	-0.6717842	0.50172	-0.32009647	subgrp_cor	count		1	0
ACOX1	4	OK	0.3295369	1.40554809	0.23444726	0.81464	0.10244709	subgrp_cor	count		1	0
TEN1	11	OK	4.24656361	2.24021648	1.89560413	0.05801	1.85054516	subgrp_cor	count		1	0
SRP68	10	OK	7.10967254	3.38264665	2.10180764	0.03557	0.00369984	subgrp_cor	count		1	0
EXOC7	9	OK	-0.1947311	0.98428978	-0.1978392	0.84317	-0.05906690	subgrp_cor	count		1	0
RNF157	1	OK	-11.019418	43.2499352	-0.2547846	0.79889	-1.17482461	subgrp_cor	count		1	0
UBALD2	78	OK	-0.3711629	0.33956975	-1.0930386	0.27438	-0.4587083	subgrp_cor	count		1	0
PRPSAP1	70	OK	-1.4277986	0.36183483	-3.9459946	7.95E-05	-1.73944154	subgrp_cor	count		0.78905069	0
UBE2O	2	OK	-0.7251858	1.80791919	-0.4011162	0.68832	-0.20979917	subgrp_cor	count		1	0
RHBDF2	1	OK	0.65325287	2.50012846	0.26128772	0.79387	0.00016297	subgrp_cor	count		1	0
CYGB	15	OK	-4.3897318	1.66840173	-2.6311	0.00851	-2.7485076	subgrp_cor	count		1	0
PRCD	1	OK	-11.019418	43.2499352	-0.2547846	0.79889	-1.17482461	subgrp_cor	count		1	0
ST6GALNAC1	5	OK	0.03048815	1.30363393	0.02338705	0.98134	0.00937328	subgrp_cor	count		1	0
AC005837.1	1	OK	-11.019418	43.2499352	-0.2547846	0.79889	-1.17482461	subgrp_cor	count		1	0
MXRA7	54	OK	-0.6110331	0.39753169	-1.5370675	0.12428	-0.75520447	subgrp_cor	count		1	0
JMJD6	11	OK	-0.8156504	0.88335982	-0.9233502	0.35582	-0.64602363	subgrp_cor	count		1	0
METTL23	35	OK	0.67278987	0.52941913	1.27080762	0.2038	0.75541019	subgrp_cor	count		1	0
SRSF2	161	OK	-0.262202	0.22735246	-1.1532887	0.24879	-0.33702932	subgrp_cor	count		1	0
MFSD11	12	OK	0.95881766	1.04958629	0.91351091	0.36097	0.4863054	subgrp_cor	count		1	0
SEC14L1	133	OK	0.15331841	0.25314661	0.60565065	0.54475	0.20661620	subgrp_cor	count		1	0
9-Sep	26	OK	-1.0559084	0.61157885	-1.7265286	0.08425	-0.93332922	subgrp_cor	count		1	0
TNRC6C	7	OK	0.84117929	1.40344781	0.59939958	0.54921	0.0005738	subgrp_cor	count		1	0
TMC6	4	OK	3.22540632	2.30532205	1.39910705	0.16176	1.50796693	subgrp_cor	count		1	0
SYNGR2	159	OK	-0.4369681	0.21178394	-2.0632741	0.03909	-0.5942634	subgrp_cor	count		1	0
AFMID	9	OK	-0.245884	1.01751929	-0.2416506	0.80905	-0.15700843	subgrp_cor	count		1	0
AC087645.2	1	OK	-10.442278	41.4658099	-0.2518286	0.80117	-0.00045289	subgrp_cor	count		1	0
SOCS3	569	OK	-0.376985	0.11026715	-3.4188344	0.00063	-0.53609856	subgrp_cor	count		1	0
PGS1	2	OK	3.45981497	4.78586864	0.72292309	0.46972	0.00081935	subgrp_cor	count		1	0
CYTH1	21	OK	-2.2900438	0.81139505	-2.8222536	0.00477	-1.14676705	subgrp_cor	count		1	0
USP36	2	OK	-10.442278	41.4658099	-0.2518286	0.80117	-0.00045289	subgrp_cor	count		1	0
CANT1	2	OK	-11.019418	43.2499352	-0.2547846	0.79889	-1.17482461	subgrp_cor	count		1	0
C1QTNF1-AS1	1	OK	18.7594894	83.6251792	0.22432824	0.8225	0.00028789	subgrp_cor	count		1	0
C1QTNF1	35	OK	-0.4224687	0.50837762	-0.8310136	0.40597	-0.39235185	subgrp_cor	count		1	0
CBX4	10	OK	-0.6380537	1.01901321	-0.6261486	0.53122	-0.40169802	subgrp_cor	count		1	0
TBC1D16	3	OK	1.9259645	2.51548794	0.7656425	0.44389	0.56022119	subgrp_cor	count		1	0
GAA	16	OK	2.40949336	1.16966554	2.05998431	0.0394	1.83714359	subgrp_cor	count		1	0
EIF4A3	62	OK	0.249597	0.37595546	0.66390045	0.50675	0.31062135	subgrp_cor	count		1	0
SGSH	2	OK	-0.2029407	1.65323657	-0.1227536	0.9022	-4.82E-05	subgrp_cor	count		1	0
SLC26A11	2	OK	4.66132999	5.76838606	0.80808217	0.41904	0.00274918	subgrp_cor	count		1	0
RNF213	77	OK	-0.1643727	0.32244232	-0.5097726	0.61021	-0.19800540	subgrp_cor	count		1	0
ENDOV	5	OK	-0.1901836	1.22474529	-0.1552842	0.8766	-0.05770574	subgrp_cor	count		1	0
CHMP6	9	OK	-0.3119276	0.94944186	-0.3285376	0.7425	-0.09380085	subgrp_cor	count		1	0
BAIAP2-DT	2	OK	21.1053233	83.3412731	0.25323976	0.80008	0.0003103	subgrp_cor	count		1	0
BAIAP2	11	OK	-0.6560036	0.88869208	-0.7381675	0.46041	-0.70568723	subgrp_cor	count		1	0
CEP131	3	OK	-0.7251858	1.80791919	-0.4011162	0.68832	-0.20979917	subgrp_cor	count		1	0
TEPSIN	1	OK	-11.019418	43.2499352	-0.2547846	0.79889	-1.17482461	subgrp_cor	count		1	0
NDUFAF8	40	OK	1.56141867	0.63316966	2.46603521	0.01366	1.62812105	subgrp_cor	count		1	0
SLC38A10	10	OK	0.50192508	0.99340195	0.5052568	0.61338	0.15648906	subgrp_cor	count		1	0
ACTG1	849	OK	-0.1644564	0.08715313	-1.8869825	0.05916	-0.23511351	subgrp_cor	count		1	0
FAAP100	1	OK	-11.019418	43.2499352	-0.2547846	0.79889	-1.17482461	subgrp_cor	count		1	0
NPLOC4	11	OK	20.9172295	33.1820625	0.63038063	0.52845	2.28610964	subgrp_cor	count		1	0
OXLD1	17	OK	-1.0380812	0.72390689	-1.4339982	0.15157	-0.92856320	subgrp_cor	count		1	0
CCDC137	15	OK	-0.1762078	0.8402174	-0.209717	0.83389	-0.13111252	subgrp_cor	count		1	0
ARL16.00	26	OK	0.34019488	0.62865365	0.54114828	0.58841	0.17391425	subgrp_cor	count		1	0
HGS	4	OK	1.06473698	1.28000634	0.8318216	0.40551	0.79046237	subgrp_cor	count		1	0
MRPL12	41	OK	-0.1284625	0.47815443	-0.2686632	0.78819	-0.15486074	subgrp_cor	count		1	0
MCRIP1	81	OK	0.85453673	0.33835656	2.52555098	0.01155	1.09038602	subgrp_cor	count		1	0
ARHGDIA	98	OK	0.21525636	0.28912876	0.74449998	0.45657	0.27950156	subgrp_cor	count		1	0
ALYREF	54	OK	0.8443359	0.41137831	2.05245604	0.04012	1.03248270	subgrp_cor	count		1	0
ANAPC11	219	OK	0.6009334	0.20582421	2.91964389	0.0035	0.82990002	subgrp_cor	count		1	0
PCYT2	3	OK	1.9259645	2.51548794	0.7656425	0.44389	0.56022119	subgrp_cor	count		1	0
SIRT7	1	OK	3.45981462	6.77145131	0.51094137	0.60939	0.00030179	subgrp_cor	count		1	0
MAFG	23	OK	0.09218783	0.61872401	0.14899678	0.88156	0.09109482	subgrp_cor	count		1	0
ASPSCR1	6	OK	4.07007701	2.38949665	1.70331982	0.08851	1.84522350	subgrp_cor	count		1	0
CENPX	81	OK	0.73867089	0.33141332	2.22885096	0.02582	0.96269943	subgrp_cor	count		1	0
DCXR	32	OK	0.93959713	0.51292271	1.83184935	0.06697	1.09447751	subgrp_cor	count		1	0
RFNG	9	OK	0.91496356	1.01797619	0.89880644	0.36876	0.75684532	subgrp_cor	count		1	0
GPS1	9	OK	0.60233837	0.97691201	0.61657382	0.53752	0.39196561	subgrp_cor	count		1	0
DUS1L	7	OK	-0.1122634	1.22847011	-0.0913848	0.92719	-0.00019808	subgrp_cor	count		1	0
SLC16A3	5	OK	-2.2911888	2.05792275	-1.1133503	0.26556	-0.53157637	subgrp_cor	count		1	0
CSNK1D	24	OK	0.28674843	0.69101921	0.41496449	0.67817	0.27132144	subgrp_cor	count		1	0
SECTM1	1	OK	-11.019418	43.2499352	-0.2547846	0.79889	-1.17482461	subgrp_cor	count		1	0
OGFOD3	9	OK	-0.4471311	1.17381209	-0.3809222	0.70326	-0.2201322	subgrp_cor	count		1	0
HEXDC	4	OK	-2.0238546	1.58519258	-1.2767247	0.2017	-0.4897003	subgrp_cor	count		1	0
CYBC1	24	OK	0.83856411	0.65353445	1.28312151	0.19945	0.69421214	subgrp_cor	count		1	0
NARF	19	OK	-0.8971573	0.8213218	-1.0923335	0.27469	-0.80857698	subgrp_cor	count		1	0
FOXK2	5	OK	-0.8862995	1.25712583	-0.7050206	0.4808	-0.25179390	subgrp_cor	count		1	0
WDR45B	22	OK	0.76895225	0.76534014	1.00471961	0.31503	0.57737346	subgrp_cor	count		1	0
RAB40B	2	OK	3.45981497	4.78586864	0.72292309	0.46972	0.00081935	subgrp_cor	count		1	0
FN3KRP	1	OK	-11.019418	43.2499352	-0.2547846	0.79889	-1.17482461	subgrp_cor	count		1	0
TBCD	6	OK	0.03517585	1.23613074	0.02845642	0.9772	0.01789198	subgrp_cor	count		1	0
AC144831.1	1	OK	23.9918963	84.8999419	0.28259026	0.77749	1.65081709	subgrp_cor	count		1	0
USP14	39	OK	-0.5763403	0.5341787	-1.0789279	0.28062	-0.5957244	subgrp_cor	count		1	0
THOC1	2	OK	-12.148421	43.4553091	-0.2795613	0.77981	-1.91362624	subgrp_cor	count		1	0
COLEC12	1	OK	18.7594894	83.6251792	0.22432824	0.8225	0.00028789	subgrp_cor	count		1	0
TYMS	3	OK	0.8788176	1.64693117	0.53360919	0.59361	0.44699547	subgrp_cor	count		1	0
ENOSF1	6	OK	1.22341999	1.23115663	1.0018284	0.31642	0.37823729	subgrp_cor	count		1	0
YES1	81	OK	0.30203126	0.33290877	0.90724935	0.36427	0.38194250	subgrp_cor	count		1	0
METTL4	4	OK	2.55522271	2.3498646	1.08740073	0.27686	0.69313893	subgrp_cor	count		1	0
SMCHD1	48	OK	-0.6811529	0.41712423	-1.6329736	0.10247	-0.82342263	subgrp_cor	count		1	0
LPIN2	3	OK	1.25330657	1.54098629	0.81331455	0.41604	0.62624666	subgrp_cor	count		1	0
MYL12A	810	OK	-0.3064646	0.09189153	-3.3350692	0.00085	-0.43781472	subgrp_cor	count		1	0
MYL12B	766	OK	0.10334956	0.09494934	1.08847059	0.27639	0.14748222	subgrp_cor	count		1	0
TGIF1	37	OK	-0.6041003	0.50043864	-1.2071412	0.22738	-0.60429038	subgrp_cor	count		1	0
DLGAP1	1	OK	18.7594894	83.6251792	0.22432824	0.8225	0.00028789	subgrp_cor	count		1	0
DLGAP1-AS1	1	OK	1.58239999	2.72001883	0.58176068	0.56072	0.47433761	subgrp_cor	count		1	0
LINC00526	1	OK	-11.019418	43.2499352	-0.2547846	0.79889	-1.17482461	subgrp_cor	count		1	0
LINC00667	18	OK	-0.1101846	0.72524887	-0.1519267	0.87924	-0.09800974	subgrp_cor	count		1	0
EPB41L3	52	OK	-0.4371367	0.39932362	-1.0946928	0.27365	-0.56006878	subgrp_cor	count		1	0
PTPRM	40	OK	0.14493937	0.47419696	0.30565227	0.75987	0.12974387	subgrp_cor	count		1	0
RAB12	12	OK	-2.3025637	1.1524821	-1.9979171	0.04572	-1.51863585	subgrp_cor	count		1	0
NDUFV2	225	OK	-0.012812	0.18825434	-0.0680568	0.94574	-0.01784228	subgrp_cor	count		1	0
NDUFV2-AS1	2	OK	0.16814912	1.72533008	0.0974591	0.92238	0.05201277	subgrp_cor	count		1	0
ANKRD12	270	OK	0.23893589	0.16204193	1.47453159	0.14034	0.3336927	subgrp_cor	count		1	0
TWSG1	2	OK	21.1053233	83.3412731	0.25323976	0.80008	0.0003103	subgrp_cor	count		1	0
RALBP1	148	OK	0.27292682	0.23347359	1.1689837	0.24241	0.37035428	subgrp_cor	count		1	0
PPP4R1	19	OK	-1.5680677	0.97780172	-1.6036664	0.10879	-1.24327975	subgrp_cor	count		1	0
RAB31	12	OK	2.5952593	1.55811134	1.66564432	0.09578	0.70054924	subgrp_cor	count		1	0
VAPA	322	OK	0.26057796	0.15948049	1.63391752	0.10228	0.36442123	subgrp_cor	count		1	0
NAPG	22	OK	-0.0078671	0.65310982	-0.0120457	0.99039	-0.00828239	subgrp_cor	count		1	0
PIEZO2	4	OK	3.4597796	3.38189313	1.02303043	0.30629	0.00222231	subgrp_cor	count		1	0
GNAL	5	OK	-0.8416402	1.46082315	-0.5761411	0.56452	-0.24034852	subgrp_cor	count		1	0
CHMP1B	47	OK	1.40743329	0.53454897	2.63293612	0.00847	1.48072745	subgrp_cor	count		1	0
MPPE1	8	OK	0.58209889	1.07041274	0.54380789	0.58657	0.1815643	subgrp_cor	count		1	0
IMPA2	1	OK	18.7594894	83.6251792	0.22432824	0.8225	0.00028789	subgrp_cor	count		1	0
TUBB6	156	OK	-0.7132755	0.24342282	-2.9301915	0.00339	-0.94641456	subgrp_cor	count		1	0
AFG3L2	12	OK	-0.3639662	0.93710153	-0.3883957	0.69772	-0.23072627	subgrp_cor	count		1	0
SPIRE1	5	OK	1.6611503	1.68196075	0.98762721	0.32334	0.92687993	subgrp_cor	count		1	0
PSMG2	129	OK	-0.1769631	0.2453933	-0.7211408	0.47082	-0.24001746	subgrp_cor	count		1	0
PTPN2	31	OK	-1.296722	0.56591139	-2.2914029	0.02194	-1.40149588	subgrp_cor	count		1	0
CEP192	5	OK	1.39610124	1.41763559	0.98480967	0.32472	0.42407085	subgrp_cor	count		1	0
LDLRAD4	8	OK	0.85702485	1.06707913	0.80315023	0.42189	0.43621861	subgrp_cor	count		1	0
FAM210A	6	OK	-0.778415	1.34204959	-0.5800195	0.5619	-0.47708280	subgrp_cor	count		1	0
RNMT	70	OK	-0.6278365	0.34031229	-1.8448834	0.06505	-0.78030543	subgrp_cor	count		1	0
ROCK1	132	OK	0.28765425	0.2421333	1.18799952	0.23482	0.38095946	subgrp_cor	count		1	0
ESCO1	17	OK	0.37425498	0.65171321	0.5742633	0.56579	0.33560235	subgrp_cor	count		1	0
SNRPD1	151	OK	-0.2657742	0.23988753	-1.1079115	0.2679	-0.35637043	subgrp_cor	count		1	0
MIB1	12	OK	0.83734071	1.09702628	0.76328226	0.4452	0.26421442	subgrp_cor	count		1	0
GATA6-AS1	5	OK	4.31521111	2.4808281	1.73942367	0.08196	2.54729950	subgrp_cor	count		1	0
GATA6	50	OK	1.81341696	0.45994739	3.94266173	8.06E-05	2.10678377	subgrp_cor	count		0.79985967	0
RBBP8	10	OK	-1.7717794	1.23890758	-1.4301142	0.15268	-1.10249259	subgrp_cor	count		1	0
RIOK3	69	OK	1.00227425	0.39473606	2.53909979	0.01111	1.22173410	subgrp_cor	count		1	0
LAMA3	1	OK	-11.019418	43.2499352	-0.2547846	0.79889	-1.17482461	subgrp_cor	count		1	0
TTC39C	3	OK	-11.18472	26.2967872	-0.4253269	0.6706	-2.27083089	subgrp_cor	count		1	0
OSBPL1A	30	OK	-1.1655883	0.5296063	-2.2008581	0.02775	-1.24630869	subgrp_cor	count		1	0
IMPACT	16	OK	-0.6913704	0.97888553	-0.7062832	0.48001	-0.59655664	subgrp_cor	count		1	0
ZNF521	35	OK	0.21526388	0.50382982	0.42725506	0.66919	0.2329234	subgrp_cor	count		1	0
SS18	10	OK	-1.2765819	1.05910782	-1.2053371	0.22807	-0.74019561	subgrp_cor	count		1	0
KCTD1	2	OK	0.40610007	2.10834129	0.19261588	0.84726	0.12645366	subgrp_cor	count		1	0
CDH2	2	OK	3.45981497	4.78586864	0.72292309	0.46972	0.00081935	subgrp_cor	count		1	0
DSG2	10	OK	-1.5188076	1.08305103	-1.4023417	0.16081	-1.11372216	subgrp_cor	count		1	0
TRAPPC8	4	OK	-0.0248099	1.36386208	-0.0181909	0.98549	-0.01601159	subgrp_cor	count		1	0
RNF125	19	OK	-2.3491797	0.8870523	-2.6482989	0.00809	-2.18032737	subgrp_cor	count		1	0
RNF138	12	OK	-2.2453066	1.01855416	-2.2044057	0.0275	-0.87498807	subgrp_cor	count		1	0
CCDC178	3	OK	-1.1068798	1.71301689	-0.6461581	0.51818	-0.65509255	subgrp_cor	count		1	0
ASXL3	1	OK	23.9918963	84.8999419	0.28259026	0.77749	1.65081709	subgrp_cor	count		1	0
MAPRE2	9	OK	5.26129471	3.88861499	1.35299965	0.17606	0.00295028	subgrp_cor	count		1	0
ZNF397	9	OK	0.98123699	0.96477011	1.01706819	0.30912	0.49722350	subgrp_cor	count		1	0
ZSCAN30	6	OK	0.17517579	1.57806869	0.11100637	0.91161	0.00011650	subgrp_cor	count		1	0
ZNF24	33	OK	-0.0145047	0.5310015	-0.0273157	0.97821	-0.01628555	subgrp_cor	count		1	0
AC007998.3	3	OK	1.9259645	2.51548794	0.7656425	0.44389	0.56022119	subgrp_cor	count		1	0
INO80C	23	OK	[illegible]	[illegible]	[illegible]	[illegible]	[illegible]	subgrp_cor	count		1	0

KIAA1468	2	OK	3.4598146	6.7714513	0.5109413	0.60929	0.00030179	subgrp_con	count		1	0
ZCCHC2	11	OK	-1.3288417	1.0238728	-1.297858	0.19436	-0.8959284	subgrp_con	count		1	0
BCL2	1	OK	3.4598146	6.7714513	0.5109413	0.60929	0.00030179	subgrp_con	count		1	0
KDSR	47	OK	0.7268353	0.4488792	1.641500	0.10069	0.87177697	subgrp_con	count		1	0
VPS4B	47	OK	-0.2463766	0.480397	-0.512860	0.60805	-0.27962720	subgrp_con	count		1	0
SERPINB8	2	OK	21.642059	54.486891	0.3971975	0.69122	1.57744735	subgrp_con	count		1	0
DSEL	11	OK	-0.3489828	0.9318606	-0.374501	0.70802	-0.22097631	subgrp_con	count		1	0
TMX3	32	OK	1.1595808	0.6179361	1.8765382	0.06058	0.85659972	subgrp_con	count		1	0
CCDC102B	3	OK	0.1681690	1.7253287	0.0974707	0.92235	0.05201879	subgrp_con	count		1	0
SOCS6	1	OK	23.991896	84.899941	0.2825902	0.77749	1.65081709	subgrp_con	count		1	0
TIMM21	3	OK	0.3472110	1.814029	0.1914583	0.84817	0.00023216	subgrp_con	count		1	0
CYB5A	204	OK	0.4466544	0.1948010	2.2928748	0.02186	0.61826757	subgrp_con	count		1	0
CNDP2	32	OK	-0.4043659	0.5765132	-0.701399	0.48305	-0.32948675	subgrp_con	count		1	0
LINC00909	2	OK	5.8049273	5.6694662	1.023893	0.30589	1.03285716	subgrp_con	count		1	0
ZNF407	5	OK	-0.0853996	1.3967657	-0.061141	0.95125	-0.06406928	subgrp_con	count		1	0
AC015819.2	1	OK	3.4598146	6.7714513	0.5109413	0.60929	0.00030179	subgrp_con	count		1	0
ZADH2	6	OK	-0.665045	1.5499432	-0.429077	0.66787	-0.454282	subgrp_con	count		1	0
TSHZ1	7	OK	-2.1263403	1.2694054	-1.675067	0.09392	-0.84442518	subgrp_con	count		1	0
ZNF516	1	OK	0.6532528	2.5001284	0.2612877	0.79387	0.00016297	subgrp_con	count		1	0
ZNF236-DT	1	OK	-15.99503	70.34942	-0.227365	0.82014	-0.00028474	subgrp_con	count		1	0
ZNF236	1	OK	3.4598146	6.7714513	0.5109413	0.60929	0.00030179	subgrp_con	count		1	0
MBP	2	OK	-10.47563	27.491987	-0.381043	0.70317	-1.13811785	subgrp_con	count		1	0
ATP9B	3	OK	0.6102599	1.4407638	0.4236363	0.67182	0.3122109	subgrp_con	count		1	0
NFATC1	8	OK	2.4678965	1.6875105	1.4624480	0.14362	1.0933980	subgrp_con	count		1	0
PQLC1	32	OK	-0.800811	0.5114517	-1.565762	0.1174	-0.72715426	subgrp_con	count		1	0
HSBP1L1	14	OK	-0.776136	0.9212870	-0.842448	0.39954	-0.47772245	subgrp_con	count		1	0
TXNL4A	102	OK	0.5950833	0.3229613	1.8425839	0.06539	0.75513296	subgrp_con	count		1	0
RBFA	14	OK	-0.70764	0.7403166	-0.955869	0.33914	-0.60988968	subgrp_con	count		1	0
ZCCHC3	6	OK	0.6428183	1.2485503	0.514851	0.60666	0.00043555	subgrp_con	count		1	0
NRSN2-AS1	1	OK	18.759489	83.625179	0.2243282	0.8225	0.00028789	subgrp_con	count		1	0
SOX12	1	OK	3.4598146	6.7714513	0.5109413	0.60929	0.00030179	subgrp_con	count		1	0
TRIB3	2	OK	3.4598149	4.7858686	0.7229230	0.46972	0.00081935	subgrp_con	count		1	0
RBCK1	71	OK	-0.147536	0.3178992	-0.464097	0.64258	-0.18773435	subgrp_con	count		1	0
TBC1D20	6	OK	-0.684924	1.3384235	-0.51174	0.60882	-0.19900120	subgrp_con	count		1	0
CSNK2A1	48	OK	0.5870347	0.4433389	1.3241217	0.18546	0.71016410	subgrp_con	count		1	0
TCF15	4	OK	0.1781001	1.2200630	0.1459762	0.88394	0.09073525	subgrp_con	count		1	0
FAM110A	1	OK	3.4598146	6.7714513	0.5109413	0.60929	0.00030179	subgrp_con	count		1	0
PSMF1	67	OK	0.5197391	0.3676680	1.4136098	0.15749	0.62822480	subgrp_con	count		1	0
TMEM74B	1	OK	3.4598146	6.7714513	0.5109413	0.60929	0.00030179	subgrp_con	count		1	0
SDCBP2	5	OK	4.8875530	3.3795676	1.4462066	0.14812	0.97277335	subgrp_con	count		1	0
FKBP1A	717	OK	-0.151952	0.0993831	-1.528952	0.12628	-0.21665883	subgrp_con	count		1	0
NSFL1C	30	OK	0.5103917	0.5623845	0.9075493	0.36412	0.5741760	subgrp_con	count		1	0
SIRPB2	1	OK	18.759489	83.625179	0.2243282	0.8225	0.00028789	subgrp_con	count		1	0
SIRPA	4	OK	-2.023032	1.8071267	-1.119474	0.26294	-0.00109400	subgrp_con	count		1	0
STK35	1	OK	3.4598146	6.7714513	0.5109413	0.60929	0.00030179	subgrp_con	count		1	0
AL121899.1	1	OK	3.4598146	6.7714513	0.5109413	0.60929	0.00030179	subgrp_con	count		1	0
SNRPB	222	OK	-0.2647107	0.1964439	-1.347512	0.17782	-0.36112372	subgrp_con	count		1	0
NOP56	42	OK	-0.619540	0.4928733	-1.256997	0.20875	-0.49954725	subgrp_con	count		1	0
IDH3B	24	OK	-1.09893	0.6550009	-1.677762	0.09339	-0.91651875	subgrp_con	count		1	0
PCED1A	3	OK	3.4598149	4.7858686	0.7229230	0.46972	0.00081935	subgrp_con	count		1	0
VPS16	2	OK	4.6613299	5.7683860	0.8080821	0.41904	0.00274918	subgrp_con	count		1	0
PTPRA	74	OK	-0.007434	0.368170	-0.020193	0.98389	-0.00880801	subgrp_con	count		1	0
MRPS26	41	OK	0.5448246	0.4333754	1.2571653	0.20869	0.67755077	subgrp_con	count		1	0
FASTKD5	2	OK	19.111790	50.837591	0.3759381	0.70696	0.00078702	subgrp_con	count		1	0
DDRGK1	84	OK	-0.065215	0.3035623	-0.214834	0.8299	-0.08626280	subgrp_con	count		1	0
ITPA	18	OK	-0.373083	0.8979317	-0.415491	0.67779	-0.11163814	subgrp_con	count		1	0
C20orf194	2	OK	-10.47563	27.491987	-0.381043	0.70317	-1.13811785	subgrp_con	count		1	0
ATRN	2	OK	-10.44227	41.465809	-0.251828	0.80117	-0.00045289	subgrp_con	count		1	0
HSPA12B	29	OK	-0.626678	0.5962917	-1.050958	0.29328	-0.60197274	subgrp_con	count		1	0
C20orf27	9	OK	0.6854383	1.0456263	0.6555289	0.51212	0.44543979	subgrp_con	count		1	0
CENPB	14	OK	-0.105419	1.029130	-0.102435	0.91841	-0.08706544	subgrp_con	count		1	0
CDC25B	17	OK	-1.337103	0.7933835	-1.685318	0.09192	-1.19267455	subgrp_con	count		1	0
APSS1	3	OK	0.9396109	1.4985607	0.6270089	0.53065	0.2916352	subgrp_con	count		1	0
AL353194.1	1	OK	3.4598146	6.7714513	0.5109413	0.60929	0.00030179	subgrp_con	count		1	0
PANK2	11	OK	0.8283487	0.8765385	0.945022	0.34465	0.53636207	subgrp_con	count		1	0
RNF24	2	OK	-1.295616	2.041425	-0.634662	0.52565	-0.00075754	subgrp_con	count		1	0
SMOX	7	OK	5.6831241	5.3331875	1.0656148	0.2866	0.00307912	subgrp_con	count		1	0
PRNP	27	OK	1.5246585	0.6652569	2.2918340	0.02192	1.593369	subgrp_con	count		1	0
RASSF2	3	OK	-1.106879	1.7130168	-0.646158	0.51819	-0.65509255	subgrp_con	count		1	0
SLC23A2	2	OK	11.471804	4.8277143	2.3762392	0.01749	0.00081379	subgrp_con	count		1	0
TMEM230	184	OK	0.362734	0.2160503	1.6789343	0.09316	0.49828827	subgrp_con	count		1	0
PCNA	22	OK	-0.751403	0.6127126	-1.226355	0.22007	-0.71487313	subgrp_con	count		1	0
CDS2	11	OK	1.9689333	1.1707806	1.6817268	0.09262	0.92522663	subgrp_con	count		1	0
GPCPD1	30	OK	-1.244617	0.5530970	-2.25027	0.02442	-1.3817243	subgrp_con	count		1	0
SHLD1	1	OK	1.5822999	2.7200188	0.5817606	0.56072	0.47433761	subgrp_con	count		1	0
TRMT6	14	OK	0.6542295	0.711535	0.9194617	0.35785	0.68930872	subgrp_con	count		1	0
CRLS1	10	OK	0.6151046	1.090722	0.5639426	0.57279	0.50692553	subgrp_con	count		1	0
BMP2	37	OK	2.1636266	0.6181697	3.5000524	0.00047	2.47503774	subgrp_con	count		1	0
TMX4	87	OK	1.2098288	0.3318702	3.6454878	0.00027	1.58533680	subgrp_con	count		1	0
PLCB1	45	OK	-0.094128	0.4750864	-0.198128	0.84294	-0.07031290	subgrp_con	count		1	0
PLCB4	19	OK	-1.684097	0.7654303	-2.200196	0.02779	-1.68318456	subgrp_con	count		1	0
SNAP25	1	OK	3.4598146	6.7714513	0.5109413	0.60929	0.00030179	subgrp_con	count		1	0
MKKS	1	OK	3.4598146	6.7714513	0.5109413	0.60929	0.00030179	subgrp_con	count		1	0
SLX4IP	1	OK	18.759489	83.625179	0.2243282	0.8225	0.00028789	subgrp_con	count		1	0
JAG1	58	OK	2.8948385	0.7097589	4.0786218	4.53E-05	1.98156643	subgrp_con	count		0.4510411	0
BTBD3	14	OK	0.8283487	0.8765385	0.945022	0.34465	0.53636207	subgrp_con	count		1	0
AL035448.1	1	OK	0.6532528	2.5001284	0.2612877	0.79387	0.00016297	subgrp_con	count		1	0
ISM1	1	OK	3.4598146	6.7714513	0.5109413	0.60929	0.00030179	subgrp_con	count		1	0
TASP1	4	OK	-0.087672	1.574177	-0.055694	0.95559	-5.71E-05	subgrp_con	count		1	0
ESF1	77	OK	0.2815912	0.3372594	0.834939	0.40375	0.34593114	subgrp_con	count		1	0
NDUFAF5	1	OK	18.759489	83.625179	0.2243282	0.8225	0.00028789	subgrp_con	count		1	0
SNRPB2	150	OK	0.2601281	0.2378494	1.093709	0.27409	0.34967538	subgrp_con	count		1	0
BFSP1	1	OK	1.5822999	2.7200188	0.5817606	0.56072	0.47433761	subgrp_con	count		1	0
DSTN	669	OK	0.13992	0.1005399	1.3917149	0.16401	0.19937480	subgrp_con	count		1	0
RRBP1	198	OK	-0.012876	0.2027871	-0.06349	0.94937	-0.01742467	subgrp_con	count		1	0
SNX5	50	OK	0.3576157	0.398681	0.8969856	0.36972	0.40900617	subgrp_con	count		1	0
MGME1	4	OK	0.1721614	1.534450	0.1121974	0.91067	0.00011395	subgrp_con	count		1	0
PET117	1	OK	18.759489	83.625179	0.2243282	0.8225	0.00028789	subgrp_con	count		1	0
POLR3F	1	OK	18.759489	83.625179	0.2243282	0.8225	0.00028789	subgrp_con	count		1	0
RBBP9	2	OK	21.642059	54.486891	0.3971975	0.69122	1.57744735	subgrp_con	count		1	0
SEC23B	3	OK	-11.44854	41.76148	-0.274141	0.78398	-0.00049246	subgrp_con	count		1	0
SMIM26	119	OK	0.1446880	0.2654572	0.5450520	0.58572	0.18839043	subgrp_con	count		1	0
DTD1	30	OK	0.3353755	0.5962792	0.5624470	0.57381	0.1714386	subgrp_con	count		1	0
RIN2	50	OK	-0.89831	0.4004044	-2.243526	0.02486	-1.04793286	subgrp_con	count		1	0
NAA20	130	OK	0.0927739	0.2599842	0.3568445	0.72121	0.12515323	subgrp_con	count		1	0
CRNKL1	13	OK	0.6158080	1.2454837	0.494432	0.621	0.19819232	subgrp_con	count		1	0
RALGAPA2	50	OK	-0.901924	0.4024853	-2.240888	0.02503	-1.01384310	subgrp_con	count		1	0
KIZ	3	OK	-0.357207	1.5148489	-0.235803	0.81359	-0.22654173	subgrp_con	count		1	0
AL117332.1	1	OK	1.5822999	2.7200188	0.5817606	0.56072	0.47433761	subgrp_con	count		1	0
XRN2	172	OK	-0.435635	0.2206366	-1.974445	0.04832	-0.59245772	subgrp_con	count		1	0
THBD	353	OK	-0.231557	0.1408572	-1.643916	0.10019	-0.32352068	subgrp_con	count		1	0
CD93	172	OK	-0.689667	0.2336439	-2.951787	0.00316	-0.94377364	subgrp_con	count		1	0
NXT1	105	OK	-0.417846	0.2685531	-1.55592	0.11972	-0.54268840	subgrp_con	count		1	0
GZF1	1	OK	-15.99503	70.34942	-0.227365	0.82014	-0.00028474	subgrp_con	count		1	0
CST3	795	OK	-0.308120	0.091474	-3.368365	0.00076	-0.44043083	subgrp_con	count		1	0
SYNDIG1	2	OK	18.759489	83.625179	0.2243282	0.8225	0.00028789	subgrp_con	count		1	0
APMAP	22	OK	0.8772166	0.7149471	1.2269671	0.21982	0.7800041	subgrp_con	count		1	0
ACSS1	1	OK	3.4598146	6.7714513	0.5109413	0.60929	0.00030179	subgrp_con	count		1	0
PYGB	3	OK	0.1721614	1.534450	0.1121974	0.91067	0.00011395	subgrp_con	count		1	0
ABHD12	3	OK	2.895154	2.4132796	1.1996762	0.23027	0.75227995	subgrp_con	count		1	0
ZNF337	1	OK	-11.01941	43.249935	-0.254784	0.79889	-1.17482461	subgrp_con	count		1	0
HM13	96	OK	0.3852579	0.3060086	1.2593040	0.20792	0.49160539	subgrp_con	count		1	0
ID1	582	OK	0.3637295	0.1087725	3.3439454	0.00083	0.51598900	subgrp_con	count		1	0
BCL2L1	18	OK	1.1245249	0.8688816	1.2942210	0.19559	0.56024701	subgrp_con	count		1	0
PDRG1	6	OK	-2.164436	1.4858669	-1.456682	0.1452	-0.00115540	subgrp_con	count		1	0
CCM2L	19	OK	-0.502468	0.6440436	-0.780178	0.43529	-0.36538491	subgrp_con	count		1	0
TM9SF4	4	OK	3.3648273	2.5447810	1.3222463	0.18609	1.31777762	subgrp_con	count		1	0
POFUT1	2	OK	5.8049273	5.6694662	1.023893	0.30589	1.03285716	subgrp_con	count		1	0
KIF3B	12	OK	1.100682	1.1101727	0.9914512	0.32147	0.8156701	subgrp_con	count		1	0
ASXL1	43	OK	0.0667091	0.4772927	0.1397657	0.88885	0.05540994	subgrp_con	count		1	0
NOL4L	10	OK	2.0511147	1.2422728	1.6510984	0.09872	1.40560749	subgrp_con	count		1	0
COMMD7	62	OK	-0.116756	0.366164	-0.318862	0.74982	-0.14399924	subgrp_con	count		1	0
MAPRE1	31	OK	0.1279545	0.6199707	0.2063880	0.83649	0.12650872	subgrp_con	count		1	0
CDK5RAP1	1	OK	0.6532528	2.5001284	0.2612877	0.79387	0.00016297	subgrp_con	count		1	0
SNTA1	1	OK	1.5822999	2.7200188	0.5817606	0.56072	0.47433761	subgrp_con	count		1	0
CBFA2T2	2	OK	0.8091085	2.1695665	0.372935	0.7092	0.29050946	subgrp_con	count		1	0
E2F1	1	OK	-11.01941	43.249935	-0.254784	0.79889	-1.17482461	subgrp_con	count		1	0
PXMP4	1	OK	18.759489	83.625179	0.2243282	0.8225	0.00028789	subgrp_con	count		1	0
ZNF341-AS1	2	OK	0.4061000	2.1083412	0.1926158	0.84726	0.12645366	subgrp_con	count		1	0
CHMP4B	36	OK	-0.720469	0.5086279	-1.416495	0.15662	-0.7910438	subgrp_con	count		1	0
RALY	119	OK	-0.046096	0.2597970	-0.177431	0.85917	-0.06132838	subgrp_con	count		1	0
EIF2S2	322	OK	-0.175477	0.1571095	-1.116912	0.26403	-0.24565667	subgrp_con	count		1	0
AHCY	17	OK	0.2834153	0.7647270	0.3706098	0.71092	0.21369305	subgrp_con	count		1	0
ITCH	6	OK	0.9772933	1.4778829	0.6613469	0.50839	0.30301174	subgrp_con	count		1	0
DYNLRB1	324	OK	-0.010875	0.1526088	-0.071264	0.94319	-0.01524561	subgrp_con	count		1	0
MAP1LC3A	56	OK	-0.353299	0.395441	-0.893430	0.37162	-0.44184133	subgrp_con	count		1	0
PIGU	2	OK	3.4598146	6.7714513	0.5109413	0.60929	0.00030179	subgrp_con	count		1	0
NCOA6	6	OK	-1.124193	1.1736798	-0.957836	0.33815	-0.00181598	subgrp_con	count		1	0
ACSS2	3	OK	-10.48367	16.750523	-0.625871	0.5314	-2.53774386	subgrp_con	count		1	0
GSS	13	OK	0.4442548	0.8515309	0.5218305	0.60179	0.22738763	subgrp_con	count		1	0
TRPC4AP	3	OK	0.1681491	1.7253300	0.097459	0.92236	0.05201277	subgrp_con	count		1	0
EDEM2	16	OK	1.7136627	1.0210686	1.6783031	0.09329	1.21491809	subgrp_con	count		1	0
MMP24OS	68	OK	0.6248274	0.3540971	1.764564	0.07764	0.78514242	subgrp_con	count		1	0
MMP24	1	OK	0.6532528	2.5001284	0.2612877	0.79387	0.00016297	subgrp_con	count		1	0
EIF6	97	OK	0.4476668	0.2980684	1.5018925	0.13312	0.59026149	subgrp_con	count		1	0
ERGIC3	77	OK	0.728713	0.3640670	2.001591	0.04532	0.93129304	subgrp_con	count		1	0
SPAG4	2	OK	23.991896	84.899941	0.2825902	0.77749	1.65081709	subgrp_con	count		1	0
CPNE1	33	OK	0.168725	0.4939842	0.3415608	0.73268	0.14043536	subgrp_con	count		1	0
RBM12	4	OK	-10.47563	27.491987	-0.381043	0.70317	-1.13811785	subgrp_con	count		1	0
NFS1	3	OK	4.6613299	5.7683860	0.8080821	0.41904	0.00274918	subgrp_con	count		1	0
ROMO1	139	OK	0.581797	0.2595450	2.2416066	0.02499	0.77930578	subgrp_con	count		1	0
RBM39	341	OK	0.0745478	0.1480482	0.5035371	0.61459	0.10349382	subgrp_con	count		1	0
PHF20	40	OK	-0.57270	0.489108	-1.170907	0.24164	-0.58167644	subgrp_con	count		1	0
SCAND1	217	OK	0.4283078	0.1988448	2.1539802	0.03124	0.59342739	subgrp_con	count		1	0
CNBD2	1	OK	1.5822999	2.7200188	0.5817606	0.56072	0.47433761	subgrp_con	count		1	0
NORAD	198	OK	0.837015	0.2025660	4.132062	3.6E-05	1.15412750	subgrp_con	count		0.3582638	0
EPB41L1	2	OK	23.991896	84.899941	0.2825902	0.77749	1.65081709	subgrp_con	count		1	0
AAR2	2	OK	1.925864	2.5154879	0.765642	0.44389	0.56022119	subgrp_con	count		1	0
DLGAP4	22	OK	0.4737191	0.6943837	0.6822152	0.4951	0.30856620	subgrp_con	count		1	0
MYL9	126	OK	0.8121914	0.2461479	3.2996053	0.00097	1.10538868	subgrp_con	count		1	0
TGIF2	3	OK	-11.01941	43.249935	-0.254784	0.79889	-1.17482461	subgrp_con	count		1	0
RABSIF	38	OK	1.0869613	0.5734828	1.8953684	0.05804	1.12910037	subgrp_con	count		1	0
NDRG3	4	OK	-0.494490	1.4409495	-0.343169	0.73147	-0.14639957	subgrp_con	count		1	0
DSN1	3	OK	1.925864	2.5154879	0.765642	0.44389	0.56022119	subgrp_con	count		1	0
SOGA1	1	OK	-11.01941	43.249935	-0.254784	0.79889	-1.17482461	subgrp_con	count		1	0
SAMHD1	57	OK	-1.53686	0.4091738	-3.756018	0.00017	-1.78207588	subgrp_con	count		1	0
MROH8	1	OK	-10.44227	41.465809	-0.251828	0.80117	-0.00045289	subgrp_con	count		1	0
RPN2	145	OK	0.9603858	0.2595106	3.7007573	0.00021	1.26917702	subgrp_con	count		1	0
MANBAL	13	OK	1.2275076	0.8035397	1.5276251	0.12661	1.1366078	subgrp_con	count		1	0
BLCAP	31	OK	0.1215155	0.5581735	0.2177021	0.82766	0.09137637	subgrp_con	count		1	0
CTNNBL1	16	OK	0.3186459	0.7459648	0.4271594	0.66926	0.26563730	subgrp_con	count		1	0
TTI1	2	OK	0.4061000	2.1083412	0.1926158	0.84726	0.12645366	subgrp_con	count		1	0
RPRD1B	5	OK	6.1490015	5.4123994	1.136095	0.25592	1.05209450	subgrp_con	count		1	0
RALGAPB	5	OK	21.422762	50.402002	0.4250379	0.67081	0.00085383	subgrp_con	count		1	0
PPP1R16B	2	OK	-0.725185	1.8079191	-0.401116	0.68832	-0.20979917	subgrp_con	count		1	0
MAFB	97	OK	-0.007018	0.2863279	-0.024512	0.98044	-0.0091817	subgrp_con	count		1	0
TOP1.00	152	OK	0.4658621	0.2367341	1.9678704	0.04908	0.63068889	subgrp_con	count		1	0
PLCG1	4	OK	0.329526	1.4055480	0.2344472	0.81464	0.10244709	subgrp_con	count		1	0
ZHX3	3	OK	-11.44854	41.76148	-0.274141	0.78398	-0.00049246	subgrp_con	count		1	0
CHD6	25	OK	0.4268454	0.584067	0.7308154	0.46489	0.3819027	subgrp_con	count		1	0
SRSF6	38	OK	-1.039899	0.4956911	-2.097872	0.03592	-0.62018094	subgrp_con	count		1	0
IFT52	16	OK	1.9332257	1.0359395	1.8662534	0.06201	0.91196455	subgrp_con	count		1	0
TOX2	6	OK	1.1367637	1.4604746	0.7783522	0.43636	0.35024770	subgrp_con	count		1	0
OSER1	20	OK	0.7716913	0.6363109	1.2127582	0.22522	0.72761639	subgrp_con	count		1	0
TTPAL	1	OK	18.759489	83.625179	0.2243282	0.8225	0.00028789	subgrp_con	count		1	0
SERINC3	122	OK	0.5819010	0.2579824	2.2555840	0.0241	0.76324755	subgrp_con	count		1	0
ADA	3	OK	0.2914024	1.4447553	0.2016967	0.84015	0.18962305	subgrp_con	count		1	0
KCNK15	24	OK	24.685219	32.741172	0.7539503	0.45088	3.82068669	subgrp_con	count		1	0
RIMS4	3	OK	18.759489	83.625179	0.2243282	0.8225	0.00028789	subgrp_con	count		1	0
YWHAB	591	OK	-0.155411	0.1106486	-1.404546	0.16016	-0.22085833	subgrp_con	count		1	0
TOMM34	2	OK	3.4598149	4.7858686	0.7229230	0.46972	0.00081935	subgrp_con	count		1	0
STK4	20	OK	1.245161	0.7551644	1.6488613	0.09919	0.91520645	subgrp_con	count		1	0
SDC4	5	OK	22.221790	54.523227	0.4075655	0.68359	1.5953836	subgrp_con	count		1	0
SYS1	28	OK	1.1784906	0.6075542	1.9397289	0.05241	1.25344315	subgrp_con	count		1	0
PIGT	59	OK	0.8471722	0.3812321	2.2221950	0.02627	1.05588363	subgrp_con	count		1	0
WFDC2	1	OK	23.991896	84.899941	0.2825902	0.77749	1.65081709	subgrp_con	count		1	0
DNTTIP1	9	OK	0.5949231	1.0475352	0.5679266	0.57008	0.56356680	subgrp_con	count		1	0
SNX21	1	OK	18.759489	83.625179	0.2243282	0.8225	0.00028789	subgrp_con	count		1	0
NEURL2	1	OK	-15.99503	70.34942	-0.227365	0.82014	-0.00028474	subgrp_con	count		1	0
CTSA	50	OK	0.7442534	0.4680716	1.5902555	0.11179	0.8196503	subgrp_con	count		1	0
PLTP	48	OK	4.3836046	1.0680873	4.1041629	4.06E-05	2.79471463	subgrp_con	count		0.4041987	0
PCIF1	5	OK	0.1721614	1.534450	0.1121974	0.91067	0.00011395	subgrp_con	count		1	0
SLC12A5	1	OK	-11.01941	43.249935	-0.254784	0.79889	-1.17482461	subgrp_con	count		1	0
CD40	147	OK	-0.952304	0.2391192	-3.982551	6.82E-05	-1.29550191	subgrp_con	count		0.6774994	0
SLC35C2	4	OK	1.925864	2.5154879	0.765642	0.44389	0.56022119	subgrp_con	count		1	0
ZNF334	1	OK	3.4597879	4.7858528	0.7229198	0.46972	0.00081879	subgrp_con	count		1	0
TP53RK	9	OK	1.3832536	1.0267001	1.3472810	0.17789	1.199312	subgrp_con	count		1	0
ZMYND8	14	OK	1.290973	0.9263971	1.3935421	0.16346	1.03505521	subgrp_con	count		1	0
NCOA3	27	OK	0.9962957	0.650886	1.5308277	0.12581	1.00795920	subgrp_con	count		1	0
SULF2	101	OK	0.6076432	0.2992807	2.0296771	0.04239	0.78035160	subgrp_con	count		1	0
PREX1	8	OK	0.2865178	1.3546823	0.2115018	0.8325	0.00019144	subgrp_con	count		1	0
ARFGEF2	8	OK	5.4808811	2.9176359	1.8785349	0.06031	1.56328035	subgrp_con	count		1	0
CSE1L	5	OK	-0.480995	1.3823581	-0.347952	0.72788	-0.2361515	subgrp_con	count		1	0
STAU1	176	OK	-0.140420	0.2076302	-0.676290	0.49885	-0.19138866	subgrp_con	count		1	0
DDX27	24	OK	-0.168189	0.5350031	-0.314365	0.75324	-0.18090202	subgrp_con	count		1	0
ZNFX1	18	OK	0.9445241	0.6640802	1.4223041	0.15494	0.96014681	subgrp_con	count		1	0
ZFAS1	302	OK	-0.401565	0.1608869	-2.495950	0.01256	-0.55794696	subgrp_con	count		1	0
KCNB1	2	OK	-0.910223	2.1881125	-0.415985	0.67742	-0.25785735	subgrp_con	count		1	0
B4GALT5	8	OK	0.3131020	1.1290566	0.2773130	0.78154	0.20379679	subgrp_con	count		1	0
RNF114	34	OK	-0.811022	0.5135274	-1.579316	0.11426	-0.77216335	subgrp_con	count		1	0
SNAI1	38	OK	-1.821148	0.5482232	-3.321911	0.00089	-1.64050170	subgrp_con	count		1	0
UBE2V1	7	OK	-12.06780	17.054961	-0.707582	0.4792	-2.9766998	subgrp_con	count		1	0
TMEM189	7	OK	2.9646238	2.2923576	1.2932641	0.19592	0.76335144	subgrp_con	count		1	0
PTPN1	33	OK	-0.189934	0.634055	-0.299555	0.76452	-0.17828575	subgrp_con	count		1	0
ADNP	31	OK	-0.25453	0.5078827	-0.501162	0.61626	-0.23819473	subgrp_con	count		1	0
DPM1	26	OK	-0.342171	0.579684	-0.590272	0.55501	-0.34635267	subgrp_con	count		1	0
ZNF217	8	OK	0.2537029	1.2457212	0.2036595	0.83862	0.07870987	subgrp_con	count		1	0
PFDN4	45	OK	0.941680	0.4831438	1.9490686	0.05129	1.05116672	subgrp_con	count		1	0
DOK5	9	OK	3.1888290	1.8141633	1.7577408	0.07879	1.88504826	subgrp_con	count		1	0
FAM210B	42	OK	-0.912331	0.4553167	-2.003728	0.0451	-1.09289957	subgrp_con	count		1	0
CSTF1	5	OK	1.657542	1.4576833	1.1371074	0.25549	0.80313281	subgrp_con	count		1	0
RTF2	134	OK	0.1632493	0.2343806	0.6965140	0.48611	0.21998968	subgrp_con	count		1	0
RAE1	5	OK	0.7689292	1.5298976	0.5026017	0.61524	0.23955204	subgrp_con	count		1	0
RBM38	5	OK	0.4831635	1.2769626	0.3783693	0.70516	0.24727463	subgrp_con	count		1	0
RAB22A	28	OK	0.6478751	0.5617611	1.153292	0.24879	0.66355524	subgrp_con	count		1	0
VAPB	13	OK	-0.07832	0.7713330	-0.101542	0.91912	-0.05043115	subgrp_con	count		1	0
STX16	10	OK	0.0920861	0.9523123	0.0966974	0.92297	0.02844361	subgrp_con	count		1	0
NPEPL1	5	OK	-0.214966	1.5648178	-0.137374	0.89072	-0.06511276	subgrp_con	count		1	0
GNAS	818	OK	-0.203180	0.0910185	-2.232302	0.02559	-0.29022649	subgrp_con	count		1	0
NELFCD	10	OK	-0.063573	1.0515834	-0.060454	0.95179	-0.04755152	subgrp_con	count		1	0
ATP5F1E	760	OK	0.0248153	0.0955420	0.2597320	0.79507	0.03541390	subgrp_con	count		1	0
PRELID3B	18	OK	-0.103812	0.7833410	-0.132524	0.89457	-0.10586439	subgrp_con	count		1	0
EDN3	1	OK	3.4598146	6.7714513	0.5109413	0.60929	0.00030179	subgrp_con	count		1	0
SYCP2	1	OK	3.4598146	6.7714513	0.5109413	0.60929	0.00030179	subgrp_con	count		1	0
FAM217B	7	OK	-0.660255	1.1511583	-0.573557	0.56627	-0.40908608	subgrp_con	count		1	0
PPP1R3D	3	OK	-14.6580	45.690332	-0.320813	0.74835	-0.00067223	subgrp_con	count		1	0
CDH26	2	OK	-0.910223	2.1881125	-0.415985	0.67742	-0.25785735	subgrp_con	count		1	0
LSM14B	3	OK	2.4096168	2.4715214	0.9749528	0.32959	0.66515773	subgrp_con	count		1	0
PSMA7	621	OK	-0.090485	0.1067082	-0.847974	0.39645	-0.12874673	subgrp_con	count		1	0
MTG2	3	OK	-10.44227	41.465809	-0.251828	0.80117	-0.00045289	subgrp_con	count		1	0
OSBPL2	4	OK	-2.626438	1.9322051	-1.359295	0.17405	-0.96366359	subgrp_con	count		1	0
ADRM1	148	OK	0.4972486	0.249553	1.9929573	0.04627	0.66831707	subgrp_con	count		1	0
LAMA5	15	OK	-0.553412	0.8110611	-0.682330	0.49502	-0.40163791	subgrp_con	count		1	0
RPS21	775	OK	-0.164945	0.0938871	-1.756843	0.07894	-0.23535956	subgrp_con	count		1	0
GATA5	3	OK	21.105323	83.341273	0.2532397	0.80009	0.0003103	subgrp_con	count		1	0
AL357033.1	1	OK	-11.01941	43.249935	-0.254784	0.79889	-1.17482461	subgrp_con	count		1	0
SLCO4A1	14	OK	-1.350374	0.7931992	-1.702440	0.08867	-0.90806966	subgrp_con	count		1	0
MRGBP	4	OK	-0.174796	1.820980	-0.095990	0.92352	-0.05309252	subgrp_con	count		1	0
OGFR	12	OK	-2.002999	0.9601991	-2.086024	0.03698	-1.50185811	subgrp_con	count		1	0
COL9A3	1	OK	-10.44227	41.465809	-0.251828	0.80117	-0.00045289	subgrp_con	count		1	0
TCFL5	5	OK	-12.5999	17.388327	-0.724622	0.46868	-1.28553328	subgrp_con	count		1	0
DIDO1	6	OK	-1.300942	1.2162501	-1.069633	0.28478	-0.00075964	subgrp_con	count		1	0
AL117379.1	1	OK	0.6532528	2.5001284	0.2612877	0.79387	0.00016297	subgrp_con	count		1	0
GID8	31	OK	-0.333670	0.508158	-0.656628	0.51142	-0.378059	subgrp_con	count		1	0
YTHDF1	9	OK	-0.069958	0.9992382	-0.070012	0.94418	-0.03529888	subgrp_con	count		1	0
EEF1A2	1	OK	18.759489	83.625179	0.2243282	0.8225	0.00028789	subgrp_con	count		1	0
PPDPF	392	OK	0.1726975	0.1390018	1.2424120	0.21409	0.24403191	subgrp_con	count		1	0
HELZ2	10	OK	-1.420582	0.9796035	-1.450160	0.14701	-0.00223085	subgrp_con	count		1	0
STMN3	21	OK	7.0323179	3.5684145	1.9707121	0.04876	2.13943837	subgrp_con	count		1	0
ARFRP1	24	OK	-0.628235	0.6387206	-0.983583	0.32532	-0.54445011	subgrp_con	count		1	0
SLC2A4RG	65	OK	1.7268496	0.4143408	4.1677028	3.08E-05	2.13271784	subgrp_con	count		0.3067309	0
ZBTB46	2	OK	-13.27340	28.013322	-0.47378	0.63565	-2.01570217	subgrp_con	count		1	0
TPD52L2	25	OK	1.1938570	0.6504007	1.8355715	0.06642	1.15783062	subgrp_con	count		1	0
DNAJC5	5	OK	0.9820484	1.2318594	0.7972082	0.42532	0.63226296	subgrp_con	count		1	0
UCKL1	6	OK	-2.502839	1.7300341	-1.446697	0.14796	-1.21160770	subgrp_con	count		1	0
ZNF512B	2	OK	1.5824000	1.9240335	0.8224389	0.41082	0.77175617	subgrp_con	count		1	0
SAMD10	1	OK	-10.44227	41.465809	-0.251828	0.80117	-0.00045289	subgrp_con	count		1	0
PRPF6	84	OK	-0.001086	0.3082291	-0.003523	0.99719	-0.00128695	subgrp_con	count		1	0
C20orf204	8	OK	2.0912030	1.3934877	1.5006971	0.13342	1.23046554	subgrp_con	count		1	0
SOX18	478	OK	-0.165930	0.1260716	-1.316162	0.18812	-0.23528659	subgrp_con	count		1	0
TCEA2	30	OK	0.8182937	0.5587005	1.4648163	0.14297	0.8319924	subgrp_con	count		1	0
RGS19	8	OK	-0.427152	1.0700230	-0.399199	0.68975	-0.26961075	subgrp_con	count		1	0
PCMTD2	19	OK	-0.341868	0.6224990	-0.549187	0.58288	-0.31892632	subgrp_con	count		1	0
PLPP2	14	OK	-0.859802	0.9326411	-0.921900	0.35658	-0.24502115	subgrp_con	count		1	0
MIER2	3	OK	-0.174796	1.820980	-0.095990	0.92352	-0.05309252	subgrp_con	count		1	0
C2CD4C	2	OK	-14.6580	45.690332	-0.320813	0.74835	-0.00067223	subgrp_con	count		1	0
SHC2	5	OK	-0.660255	1.1511577	-0.57355	0.56627	-0.409087	subgrp_con	count		1	0
MADCAM1	1	OK	3.4598146	6.7714513	0.5109413	0.60929	0.00030179	subgrp_con	count		1	0
TPGS1	45	OK	0.5937184	0.506335	1.1725782	0.24096	0.71730288	subgrp_con	count		1	0
CDC34	26	OK	-0.012228	0.6090985	-0.020076	0.98398	-0.01249920	subgrp_con	count		1	0
BSG	297	OK	0.6290511	0.1666772	3.7740681	0.00016	0.87734197	subgrp_con	count		1	0
RNF126	26	OK	-0.041104	0.565940	-0.072630	0.9421	-0.04051066	subgrp_con	count		1	0
FSTL3	1	OK	18.759489	83.625179	0.2243282	0.8225	0.00028789	subgrp_con	count		1	0
PALM	13	OK	-2.465076	0.9893454	-2.491624	0.01272	-1.20056767	subgrp_con	count		1	0
PTBP1	25	OK	1.0403019	0.7566295	1.3749158	0.16916	0.32165827	subgrp_con	count		1	0
CFD	1	OK	1.582381	1.2168202	1.3004230	0.19340	1.25554822	subgrp_con	count		1	0
MED16	2	OK	21.105323	83.341273	0.2532397	0.80009	0.0003103	subgrp_con	count		1	0
R3HDM4	14	OK	-0.047046	0.847583	-0.055506	0.95573	-0.03895053	subgrp_con	count		1	0
WDR18	7	OK	0.7378094	1.3067546	0.5646121	0.57234	0.22995124	subgrp_con	count		1	0
TMEM259	50	OK	-0.242570	0.4309738	-0.562841	0.57354	-0.27107402	subgrp_con	count		1	0
CNN2	1	OK	1.5822999	2.7200188	0.5817606	0.56072	0.47433761	subgrp_con	count		1	0
POLR2E	104	OK	0.2172981	0.285771	0.7603822	0.44702	0.2793620	subgrp_con	count		1	0
GPX4	469	OK	0.3546284	0.126663	2.799779	0.00511	0.50283972	subgrp_con	count		1	0
SBNO2	18	OK	1.8277841	0.8954048	2.0412935	0.04122	1.1061517	subgrp_con	count		1	0
STK11	7	OK	4.6613299	5.7683860	0.8080821	0.41904	0.00274918	subgrp_con	count		1	0
ATP5F1D	383	OK	-0.052988	0.1408231	-0.376278	0.70671	-0.0749258	subgrp_con	count		1	0
MIDN	152	OK	0.3959692	0.231533	1.7102012	0.08722	0.52328618	subgrp_con	count		1	0

CIRBP	527	OK	-0.2877848	0.1183915	-2.4307889	0.01507	-0.4071095	subgrp_con	count		1	0
C19orf24	61	OK	0.2790763	0.4077238	0.6844739	0.49368	0.34732177	subgrp_con	count		1	0
MUM1	11	OK	4.6152962	2.2724898	2.0309865	0.04226	2.21218499	subgrp_con	count		1	0
NDUFS7	96	OK	1.048954	0.3119584	3.3624798	0.00077	1.38168586	subgrp_con	count		1	0
GAMT	2	OK	3.4598146	6.7714513	0.5109413	0.60939	0.00030179	subgrp_con	count		1	0
DAZAP1	29	OK	0.8688124	0.6298630	1.3793672	0.16779	0.77256742	subgrp_con	count		1	0
RPS15	878	OK	-0.155234	0.0844814	-1.8374996	0.06614	-0.22211373	subgrp_con	count		1	0
AC027307.2	1	OK	18.759489	83.625179	0.2243282	0.8225	0.00028789	subgrp_con	count		1	0
C19orf25	8	OK	-1.392112	1.1325222	-1.2292145	0.21890	-0.93134463	subgrp_con	count		1	0
MEX3D	7	OK	4.2109206	4.2875616	0.982124	0.32604	0.00257090	subgrp_con	count		1	0
MBD3	5	OK	0.9396109	1.4985607	0.6270089	0.53065	0.2916252	subgrp_con	count		1	0
UQCR11	254	OK	0.684896	0.1889549	3.6246578	0.00029	0.94507574	subgrp_con	count		1	0
TCF3	5	OK	0.4209530	1.4909211	0.2823442	0.77768	0.21537946	subgrp_con	count		1	0
REXO1	3	OK	3.4598146	6.7714513	0.5109413	0.60939	0.00030179	subgrp_con	count		1	0
KLF16	8	OK	-1.374361	1.0613833	-1.294877	0.19536	-0.78699171	subgrp_con	count		1	0
AC012615.1	2	OK	1.5822999	2.7200188	0.5817606	0.56072	0.47433761	subgrp_con	count		1	0
ABHD17A	152	OK	0.2095931	0.2386891	0.8781009	0.37989	0.28132803	subgrp_con	count		1	0
SCAMP4	6	OK	2.0210287	1.4780632	1.3673493	0.17152	0.60537032	subgrp_con	count		1	0
ADAT3	1	OK	-15.99503	70.34942	-0.2273655	0.82014	-0.00028474	subgrp_con	count		1	0
CSNK1G2	13	OK	-0.31978	0.8706787	-0.3672778	0.71341	-0.0961027	subgrp_con	count		1	0
BTBD2	5	OK	1.5207849	1.3927023	1.0919669	0.27485	0.45798065	subgrp_con	count		1	0
MKNK2	83	OK	0.0329697	0.2867190	0.1149896	0.90845	0.04264476	subgrp_con	count		1	0
MOB3A	1	OK	-11.019418	43.249935	-0.2547846	0.79889	-1.17482461	subgrp_con	count		1	0
AP3D1	14	OK	0.7516912	0.8329322	0.9024639	0.36682	0.48777592	subgrp_con	count		1	0
DOT1L	1	OK	18.759489	83.625179	0.2243282	0.8225	0.00028789	subgrp_con	count		1	0
PLEKHJ1	7	OK	-0.684834	1.338424	-0.5116722	0.60888	-0.19897682	subgrp_con	count		1	0
OAZ1	610	OK	0.4208991	0.1089744	3.8623641	0.00011	0.59907826	subgrp_con	count		1	0
LSM7.00	184	OK	0.1888443	0.2179368	0.8665097	0.38621	0.25745390	subgrp_con	count		1	0
SPPL2B	4	OK	9.3507449	9.9226405	0.9423645	0.34600	1.36E-0	subgrp_con	count		1	0
TIMM13	105	OK	0.5812334	0.3025040	1.9217378	0.05464	0.75422490	subgrp_con	count		1	0
LMNB2	1	OK	-10.442278	41.465809	-0.2518286	0.80117	-0.00045289	subgrp_con	count		1	0
GADD45B	500	OK	-0.2419427	0.115191	-2.1003589	0.0357	-0.34386208	subgrp_con	count		1	0
GNG7	3	OK	0.1681491	1.7253300	0.097459	0.92236	0.05201277	subgrp_con	count		1	0
SLC39A3	7	OK	-0.979640	1.046110	-0.9364589	0.34904	-0.45753534	subgrp_con	count		1	0
SGTA	11	OK	-0.965872	0.8822182	-1.0948228	0.27359	-0.27179504	subgrp_con	count		1	0
ZNF554	1	OK	3.4598146	6.7714513	0.5109413	0.60939	0.00030179	subgrp_con	count		1	0
TLE2	9	OK	-0.209212	1.1367401	-0.1840464	0.85398	-0.06339579	subgrp_con	count		1	0
AES	254	OK	0.6126937	0.1779664	3.4427479	0.00058	0.84883112	subgrp_con	count		1	0
GNA11	17	OK	0.9945498	0.8848256	1.1240065	0.26101	0.65378385	subgrp_con	count		1	0
S1PR4	2	OK	-10.47563	27.491987	-0.3810432	0.70317	-1.13811785	subgrp_con	count		1	0
NCLN	3	OK	1.7724772	1.8439627	0.9612326	0.33644	0.84969892	subgrp_con	count		1	0
NFIC	190	OK	0.8907557	0.2262712	3.9366724	8.26E-05	1.21674244	subgrp_con	count		0.81999613	0
DOHH	4	OK	0.3473110	1.814029	0.1914583	0.84817	0.00022216	subgrp_con	count		1	0
FZR1	2	OK	1.5822999	2.7200188	0.5817606	0.56072	0.47433761	subgrp_con	count		1	0
MFSD12	3	OK	-11.019418	43.249935	-0.2547846	0.79889	-1.17482461	subgrp_con	count		1	0
HMG20B	41	OK	-0.058944	0.4780282	-0.123308	0.90186	-0.06978776	subgrp_con	count		1	0
GIPC3	2	OK	3.4598146	6.7714513	0.5109413	0.60939	0.00030179	subgrp_con	count		1	0
TBXA2R	6	OK	-0.379610	1.3622755	-0.278658	0.78052	-0.11352900	subgrp_con	count		1	0
PIP5K1C	10	OK	-1.864356	1.2441477	-1.498500	0.134	-1.04298228	subgrp_con	count		1	0
APBA3	1	OK	1.5822999	2.7200188	0.5817606	0.56072	0.47433761	subgrp_con	count		1	0
MRPL54	96	OK	0.7057810	0.3078989	2.2922642	0.02189	0.92257389	subgrp_con	count		1	0
DAPK3	11	OK	2.0067443	1.2733819	1.5759170	0.11504	0.00373478	subgrp_con	count		1	0
EEF2	550	OK	-0.072076	0.1136471	-0.634213	0.52594	-0.10306679	subgrp_con	count		1	0
PIAS4	2	OK	18.759489	83.625179	0.2243282	0.8225	0.00028789	subgrp_con	count		1	0
ZBTB7A	77	OK	-0.556153	0.3365433	-1.652547	0.09842	-0.70575809	subgrp_con	count		1	0
MAP2K2	134	OK	0.2015332	0.2589108	0.7783886	0.43634	0.26970750	subgrp_con	count		1	0
SIRT6	3	OK	1.925964	2.5154879	0.7656425	0.44389	0.56022119	subgrp_con	count		1	0
YJU2	3	OK	0.878817	1.6469311	0.5336091	0.59361	0.44699547	subgrp_con	count		1	0
STAP2	2	OK	-0.910223	2.1881125	-0.4159856	0.67742	-0.25785735	subgrp_con	count		1	0
SH3GL1	14	OK	-1.185753	0.8218024	-1.442868	0.14906	-0.69595546	subgrp_con	count		1	0
UBXN6	17	OK	-0.643027	0.7774875	-0.827058	0.4082	-0.51569743	subgrp_con	count		1	0
HDGFL2	13	OK	-0.963175	0.93229	-1.033017	0.3016	-0.57232782	subgrp_con	count		1	0
PLIN5	1	OK	0.6532528	2.5001284	0.2612877	0.79387	0.00016297	subgrp_con	count		1	0
LRG1	42	OK	-1.027054	0.4366818	-2.3519522	0.01868	-1.27347723	subgrp_con	count		1	0
SEMA6B	20	OK	1.3744443	0.7754864	1.7723640	0.07632	1.3710994	subgrp_con	count		1	0
TNFAIP8L1	37	OK	0.254393	0.5008697	0.5079041	0.61152	0.26864309	subgrp_con	count		1	0
DPP9	2	OK	21.105322	83.341273	0.2532397	0.80008	0.0003103	subgrp_con	count		1	0
FEM1A	2	OK	2.4096337	2.4715206	0.9749599	0.32958	0.66517343	subgrp_con	count		1	0
PLIN3	23	OK	0.9974355	0.7413370	1.3454548	0.17846	0.74282399	subgrp_con	count		1	0
PTPRS	1	OK	18.759489	83.625179	0.2243282	0.8225	0.00028789	subgrp_con	count		1	0
SAFB2	22	OK	-0.856123	0.5754322	-1.487791	0.13681	-0.52070621	subgrp_con	count		1	0
SAFB	21	OK	-0.754387	0.6567042	-1.148747	0.25066	-0.71830953	subgrp_con	count		1	0
RPL36	826	OK	-0.165614	0.0885893	-1.869463	0.06156	-0.23674263	subgrp_con	count		1	0
C19orf70	207	OK	0.2322433	0.2009688	1.1556127	0.24786	0.31963990	subgrp_con	count		1	0
HSD11B1L	1	OK	-10.442278	41.465809	-0.2518286	0.80117	-0.00045289	subgrp_con	count		1	0
LONP1	2	OK	-1.295616	2.041425	-0.634662	0.52565	-0.00075754	subgrp_con	count		1	0
NDUFA11	231	OK	0.4877870	0.1865620	2.6146101	0.00893	0.6777342	subgrp_con	count		1	0
CAPS	2	OK	18.759489	83.625179	0.2243282	0.8225	0.00028789	subgrp_con	count		1	0
RANBP3	2	OK	-1.758573	2.3554439	-0.7465996	0.45531	-0.00036089	subgrp_con	count		1	0
MLLT1	14	OK	0.386798	0.9760162	0.3963029	0.69188	0.29688276	subgrp_con	count		1	0
CLPP	68	OK	0.5909447	0.3588755	1.6466565	0.09962	0.76170615	subgrp_con	count		1	0
ALKBH7	202	OK	0.5435172	0.2068636	2.6274183	0.0086	0.74911653	subgrp_con	count		1	0
GTF2F1	58	OK	-0.139382	0.3813240	-0.365523	0.71472	-0.17295491	subgrp_con	count		1	0
KHSRP	5	OK	-0.465339	1.3093717	-0.355291	0.7222	-0.13813694	subgrp_con	count		1	0
SLC25A23	2	OK	0.1681491	1.7253300	0.097459	0.92236	0.05201277	subgrp_con	count		1	0
TNFSF9	31	OK	-1.494381	0.5579612	-2.678288	0.0074	-1.80357727	subgrp_con	count		1	0
C3	3	OK	22.221790	54.523227	0.4075655	0.68359	1.5953836	subgrp_con	count		1	0
GPR108	52	OK	0.1601956	0.3841001	0.4170674	0.67662	0.19930648	subgrp_con	count		1	0
TRIP10	20	OK	0.0356414	0.6622219	0.0538210	0.95708	0.02674077	subgrp_con	count		1	0
ZNF557	1	OK	-10.442278	41.465809	-0.2518286	0.80117	-0.00045289	subgrp_con	count		1	0
INSR	49	OK	-0.396111	0.4362337	-0.908025	0.36387	-0.4545091	subgrp_con	count		1	0
ZNF358	40	OK	-1.20417	0.4978697	-2.418654	0.01558	-1.36703889	subgrp_con	count		1	0
MCOLN1	5	OK	1.2784729	1.4238208	0.897917	0.36922	0.39110554	subgrp_con	count		1	0
PNPLA6	1	OK	3.4598146	6.7714513	0.5109413	0.60939	0.00030179	subgrp_con	count		1	0
PET100	49	OK	0.6571110	0.4469686	1.4701501	0.14152	0.70959791	subgrp_con	count		1	0
STXBP2	1	OK	1.5822999	2.7200188	0.5817606	0.56072	0.47433761	subgrp_con	count		1	0
MAP2K7	4	OK	-10.442278	41.465809	-0.2518286	0.80117	-0.00045289	subgrp_con	count		1	0
SNAPC2	15	OK	-0.433787	0.9891195	-0.438559	0.66098	-0.21379049	subgrp_con	count		1	0
TIMM44	5	OK	-0.862817	1.2168600	-0.709052	0.47829	-0.4083153	subgrp_con	count		1	0
ELAVL1	27	OK	0.9466536	0.6371452	1.4857736	0.13734	0.88938071	subgrp_con	count		1	0
NDUFA7	1	OK	3.4598146	6.7714513	0.5109413	0.60939	0.00030179	subgrp_con	count		1	0
RPS28	876	OK	-0.074537	0.0840086	-0.88726	0.37494	-0.10666640	subgrp_con	count		1	0
ANGPTL4	23	OK	1.629147	0.7810299	2.0858964	0.03699	1.47689483	subgrp_con	count		1	0
RAB11B-AS1	9	OK	0.6016934	1.1167009	0.5388133	0.59002	0.45336317	subgrp_con	count		1	0
RAB11B	56	OK	0.0463780	0.3917522	0.1183861	0.90576	0.05602445	subgrp_con	count		1	0
2-Mar	99	OK	0.1864056	0.2913618	0.6397735	0.52232	0.23451969	subgrp_con	count		1	0
HNRNPM	187	OK	-0.0653	0.2139464	-0.304936	0.76041	-0.08835791	subgrp_con	count		1	0
ZNF414	4	OK	6.1490015	5.4123994	1.136095	0.25592	1.05209450	subgrp_con	count		1	0
ADAMTS10	1	OK	3.4598146	6.7714513	0.5109413	0.60939	0.00030179	subgrp_con	count		1	0
ZNF558	1	OK	-15.99503	70.34942	-0.2273655	0.82014	-0.00028474	subgrp_con	count		1	0
ZNF699	2	OK	-1.295616	2.041425	-0.634662	0.52565	-0.00075754	subgrp_con	count		1	0
ZNF559	1	OK	18.759489	83.625179	0.2243282	0.8225	0.00028789	subgrp_con	count		1	0
ZNF426	7	OK	2.0703214	1.318256	1.5703787	0.11633	0.57840517	subgrp_con	count		1	0
ZNF121	5	OK	-1.254245	1.5627617	-0.802582	0.42222	-0.72926821	subgrp_con	count		1	0
ZNF561	1	OK	-11.019418	43.249935	-0.2547846	0.79889	-1.17482461	subgrp_con	count		1	0
ZNF562	2	OK	2.4096337	2.4715206	0.9749599	0.32958	0.66517343	subgrp_con	count		1	0
FBXL12	4	OK	2.164878	1.7323019	1.2489908	0.21167	0.99526412	subgrp_con	count		1	0
UBL5	368	OK	0.5655260	0.1494125	3.7849979	0.00015	0.79279022	subgrp_con	count		1	0
PIN1	82	OK	0.7839796	0.3302082	2.3741975	0.01759	1.02403193	subgrp_con	count		1	0
C19orf66	35	OK	-0.180088	0.4859229	-0.370606	0.71092	-0.20477341	subgrp_con	count		1	0
PPAN	1	OK	3.4598146	6.7714513	0.5109413	0.60939	0.00030179	subgrp_con	count		1	0
EIF3G	372	OK	-0.067849	0.1454589	-0.466458	0.64089	-0.09543876	subgrp_con	count		1	0
DNMT1	30	OK	-0.37485	0.5225604	-0.717335	0.47317	-0.3903682	subgrp_con	count		1	0
MRPL4	43	OK	-0.050218	0.4541784	-0.110570	0.91196	-0.05807387	subgrp_con	count		1	0
ICAM4	10	OK	-0.955777	0.9160560	-1.043361	0.29679	-0.74695819	subgrp_con	count		1	0
ICAM5	2	OK	-10.47563	27.491987	-0.3810432	0.70317	-1.13811785	subgrp_con	count		1	0
ICAM3	9	OK	0.5723336	1.0339828	0.553522	0.57992	0.47992556	subgrp_con	count		1	0
TYK2	1	OK	-10.442278	41.465809	-0.2518286	0.80117	-0.00045289	subgrp_con	count		1	0
CDC37	388	OK	-0.35877	0.1414670	-2.53613	0.01121	-0.50413997	subgrp_con	count		1	0
KEAP1	8	OK	-1.157129	1.0858175	-1.065675	0.28657	-0.31780893	subgrp_con	count		1	0
CDKN2D	56	OK	-0.864068	0.418768	-2.063255	0.03908	-0.94175649	subgrp_con	count		1	0
SLC44A2	33	OK	0.8136135	0.5769562	1.410182	0.15849	0.76835816	subgrp_con	count		1	0
ILF3-DT	28	OK	-0.841393	0.5369974	-1.566848	0.11715	-0.90041617	subgrp_con	count		1	0
ILF3	68	OK	0.8164272	0.3988273	2.0470692	0.04065	0.91472814	subgrp_con	count		1	0
QTRT1	16	OK	0.2172055	0.7083953	0.3066163	0.75914	0.16381265	subgrp_con	count		1	0
DNM2	12	OK	0.2134942	0.8423775	0.2534420	0.80016	0.1088716	subgrp_con	count		1	0
TMED1	24	OK	1.2112878	0.6797548	1.7819481	0.07476	1.2170495	subgrp_con	count		1	0
CARM1	3	OK	-0.523245	2.0662252	-0.253237	0.80008	-0.24602840	subgrp_con	count		1	0
YIPF2	19	OK	0.7772281	0.6613303	1.175249	0.23989	0.64461296	subgrp_con	count		1	0
TIMM29	2	OK	-0.202940	1.6532365	-0.122753	0.9023	-4.82E-0	subgrp_con	count		1	0
SMARCA4	21	OK	0.6085267	0.7372650	0.8253839	0.40915	0.51020879	subgrp_con	count		1	0
LDLR	29	OK	-0.591669	0.5767534	-1.025862	0.30496	-0.62698098	subgrp_con	count		1	0
KANK2	6	OK	0.4830730	1.0016093	0.4822968	0.6296	0.15058388	subgrp_con	count		1	0
DOCK6	7	OK	0.6459443	1.3674778	0.4723618	0.63667	0.20013519	subgrp_con	count		1	0
TMEM205	107	OK	1.1122815	0.2893499	3.8440741	0.00012	1.47769209	subgrp_con	count		1	0
PLPPR2	2	OK	21.642059	54.486891	0.3971975	0.69122	1.57744735	subgrp_con	count		1	0
SWSAP1	2	OK	18.759489	83.625179	0.2243282	0.8225	0.00028789	subgrp_con	count		1	0
EPOR	3	OK	1.925964	2.5154879	0.7656425	0.44389	0.56022119	subgrp_con	count		1	0
PRKCSH	42	OK	1.355970	0.5319468	2.549071	0.0108	1.24668722	subgrp_con	count		1	0
ECSIT	9	OK	-1.681215	1.2409218	-1.354811	0.17548	-0.71485776	subgrp_con	count		1	0
CNN1	1	OK	-10.442278	41.465809	-0.2518286	0.80117	-0.00045289	subgrp_con	count		1	0
ELOF1	8	OK	1.2972240	1.3309556	0.974656	0.32977	0.00242401	subgrp_con	count		1	0
ACP5	3	OK	-12.145707	43.418869	-0.279733	0.77968	-1.91338276	subgrp_con	count		1	0
AC008543.1	1	OK	-10.442278	41.465809	-0.2518286	0.80117	-0.00045289	subgrp_con	count		1	0
ZNF69	1	OK	3.4598146	6.7714513	0.5109413	0.60939	0.00030179	subgrp_con	count		1	0
ZNF763	1	OK	-10.442278	41.465809	-0.2518286	0.80117	-0.00045289	subgrp_con	count		1	0
ZNF433	1	OK	-10.442278	41.465809	-0.2518286	0.80117	-0.00045289	subgrp_con	count		1	0
ZNF844	1	OK	22.971512	51.721583	0.444051	0.65701	2.36236892	subgrp_con	count		1	0
ZNF136	3	OK	-0.841640	1.4608231	-0.576141	0.56452	-0.24034852	subgrp_con	count		1	0
ZNF44	2	OK	18.759489	83.625179	0.2243282	0.8225	0.00028789	subgrp_con	count		1	0
ZNF563	1	OK	-10.442278	41.465809	-0.2518286	0.80117	-0.00045289	subgrp_con	count		1	0
ZNF799	2	OK	-1.295616	2.041425	-0.634662	0.52565	-0.00075754	subgrp_con	count		1	0
ZNF443	1	OK	1.5822999	2.7200188	0.5817606	0.56072	0.47433761	subgrp_con	count		1	0
ZNF791	34	OK	-1.092384	0.4810707	-2.270736	0.02316	-0.91162104	subgrp_con	count		1	0
WDR83	3	OK	2.5552030	2.3498443	1.0873924	0.27686	0.69311810	subgrp_con	count		1	0
WDR83OS	479	OK	0.0613149	0.1241206	0.4939949	0.62133	0.0870074	subgrp_con	count		1	0
DHPS	31	OK	-0.825551	0.5610550	-1.471426	0.14118	-0.88167929	subgrp_con	count		1	0
TRIR	390	OK	0.114145	0.1430363	0.7980148	0.42486	0.16027810	subgrp_con	count		1	0
ASNA1	39	OK	0.0140470	0.5156134	0.0272434	0.97827	0.01577899	subgrp_con	count		1	0
HOOK2	6	OK	-2.058333	1.3934720	-1.476958	0.13969	-0.82613993	subgrp_con	count		1	0
JUNB	790	OK	-0.208720	0.0918392	-2.272675	0.02305	-0.29825818	subgrp_con	count		1	0
PRDX2	321	OK	0.6230121	0.1590981	3.9158986	9.01E-05	0.87384568	subgrp_con	count		0.89338697	0
DNASE2	12	OK	0.9908091	0.8470193	1.1697597	0.2421	0.81560285	subgrp_con	count		1	0
GCDH	5	OK	5.6831241	5.3321875	1.0656148	0.2866	0.00307912	subgrp_con	count		1	0
FARSA	6	OK	0.483143	1.2769659	0.3783524	0.70517	0.2472630	subgrp_con	count		1	0
CALR	447	OK	0.450354	0.1324951	3.3990280	0.00068	0.63309769	subgrp_con	count		1	0
AC092069.1	5	OK	1.3961960	1.4175864	0.9849037	0.32467	0.42408480	subgrp_con	count		1	0
RAD23A	150	OK	0.1319154	0.2231514	0.5911475	0.55442	0.17949845	subgrp_con	count		1	0
NFIX	17	OK	3.2480364	1.3516865	2.4029508	0.01626	1.63914957	subgrp_con	count		1	0
LYL1	93	OK	1.1219877	0.3253015	3.4490692	0.00056	1.43052505	subgrp_con	count		1	0
TRMT1	2	OK	-0.910223	2.1881125	-0.4159856	0.67742	-0.25785735	subgrp_con	count		1	0
NACC1	2	OK	1.5822999	2.7200188	0.5817606	0.56072	0.47433761	subgrp_con	count		1	0
STX10	33	OK	0.1265017	0.5676279	0.2228603	0.82364	0.12959657	subgrp_con	count		1	0
IER2	638	OK	-0.276490	0.1030385	-2.683365	0.00729	-0.39399403	subgrp_con	count		1	0
CCDC130	10	OK	-0.507206	0.9072458	-0.559061	0.57612	-0.3196940	subgrp_con	count		1	0
MRI1	2	OK	21.105322	83.341273	0.2532397	0.80008	0.0003103	subgrp_con	count		1	0
C19orf53	369	OK	0.1216396	0.1458429	0.8340455	0.40426	0.17114972	subgrp_con	count		1	0
ZSWIM4	2	OK	-0.202940	1.6532365	-0.122753	0.9022	-4.82E-0	subgrp_con	count		1	0
AC020916.1	27	OK	0.3673399	0.5326136	0.6896916	0.49039	0.41333427	subgrp_con	count		1	0
NANOS3	1	OK	0.6532528	2.5001284	0.2612877	0.79387	0.00016297	subgrp_con	count		1	0
CC2D1A	2	OK	0.6532528	2.5001284	0.2612877	0.79387	0.00016297	subgrp_con	count		1	0
DCAF15	1	OK	-12.14842	43.455309	-0.279561	0.77981	-1.91362624	subgrp_con	count		1	0
RFX1	3	OK	-0.910223	2.1881125	-0.4159856	0.67742	-0.25785735	subgrp_con	count		1	0
IL27RA	7	OK	-2.094069	1.2694471	-1.649584	0.09903	-1.24801645	subgrp_con	count		1	0
SAMD1	12	OK	-0.086527	0.8918752	-0.097017	0.92271	-0.04362263	subgrp_con	count		1	0
PRKACA	6	OK	-1.385027	1.2210385	-1.134303	0.25667	-0.61434341	subgrp_con	count		1	0
DDX39A	25	OK	0.0533853	0.6119019	0.0872449	0.93048	0.05044412	subgrp_con	count		1	0
PKN1	45	OK	-0.518962	0.4878046	-1.063874	0.28739	-0.56405257	subgrp_con	count		1	0
GIPC1	19	OK	2.1004896	0.9861307	2.1300316	0.03317	1.58653096	subgrp_con	count		1	0
TECR	66	OK	-0.463849	0.3781619	-1.226590	0.21998	-0.58729477	subgrp_con	count		1	0
ZNF333	1	OK	-10.442278	41.465809	-0.2518286	0.80117	-0.00045289	subgrp_con	count		1	0
ILVBL	4	OK	-1.573752	1.5163546	-1.037852	0.29934	-0.84374156	subgrp_con	count		1	0
BRD4	57	OK	-0.294026	0.3654113	-0.804646	0.42102	-0.36797637	subgrp_con	count		1	0
AKAP8	4	OK	-1.295603	1.4442432	-0.897081	0.36968	-0.00075721	subgrp_con	count		1	0
AKAP8L	7	OK	0.4806421	1.084157	0.4433323	0.65752	0.14982334	subgrp_con	count		1	0
WIZ	1	OK	-12.14842	43.455309	-0.279561	0.77981	-1.91362624	subgrp_con	count		1	0
TPM4	515	OK	-0.398528	0.1217608	-3.273041	0.00106	-0.56376311	subgrp_con	count		1	0
RAB8A	31	OK	0.3561329	0.5230629	0.6808604	0.49596	0.39373792	subgrp_con	count		1	0
FAM32A	59	OK	0.2821977	0.4145187	0.6807841	0.49602	0.34413391	subgrp_con	count		1	0
AP1M1	5	OK	0.7026314	1.4772493	0.4756349	0.63432	0.46608579	subgrp_con	count		1	0
AC020911.2	1	OK	23.991896	84.899941	0.2825902	0.77749	1.65081709	subgrp_con	count		1	0
KLF2	762	OK	-0.321319	0.0939940	-3.418510	0.00063	-0.45856694	subgrp_con	count		1	0
EPS15L1	2	OK	18.759489	83.625179	0.2243282	0.8225	0.00028789	subgrp_con	count		1	0
CHERP	1	OK	1.5822999	2.7200188	0.5817606	0.56072	0.47433761	subgrp_con	count		1	0
SLC35E1	4	OK	-2.446592	1.7138131	-1.427572	0.15341	-0.00126286	subgrp_con	count		1	0
MED26	1	OK	3.4598146	6.7714513	0.5109413	0.60939	0.00030179	subgrp_con	count		1	0
SMIM7	80	OK	1.3264097	0.3710203	3.5750162	0.00035	1.63475712	subgrp_con	count		1	0
TMEM38A	2	OK	21.105322	83.341273	0.2532397	0.80008	0.0003103	subgrp_con	count		1	0
SIN3B	12	OK	1.4442885	0.9079722	1.5906747	0.11168	1.15816354	subgrp_con	count		1	0
CPAMD8	14	OK	1.891118	0.9415817	2.0084276	0.0446	0.00356951	subgrp_con	count		1	0
MYO9B	8	OK	2.2060903	1.3956270	1.5807162	0.11394	1.00936506	subgrp_con	count		1	0
USE1	29	OK	0.5591788	0.5457637	1.0245803	0.30556	0.61676912	subgrp_con	count		1	0
OCEL1	5	OK	-0.432460	1.0978471	-0.393916	0.69364	-0.21315736	subgrp_con	count		1	0
NR2F6	17	OK	1.9496305	0.973226	2.0032657	0.04515	1.35062248	subgrp_con	count		1	0
USHBP1	5	OK	0.3093405	1.3043438	0.2371618	0.81253	0.00020636	subgrp_con	count		1	0
BABAM1	38	OK	0.721252	0.4772533	1.5322103	0.12547	0.81981801	subgrp_con	count		1	0
MRPL34	108	OK	1.0977531	0.3032235	3.6201364	0.00029	1.45076566	subgrp_con	count		1	0
DDA1	5	OK	5.002756	4.2286829	1.1830530	0.23679	0.98115823	subgrp_con	count		1	0
GTPBP3	1	OK	-10.442278	41.465809	-0.2518286	0.80117	-0.00045289	subgrp_con	count		1	0
BST2	511	OK	0.5032716	0.1195646	4.209201	2.56E-05	0.71568013	subgrp_con	count		0.25573682	0
MVB12A	66	OK	0.1837776	0.3721448	0.4925102	0.62236	0.22571252	subgrp_con	count		1	0
SLC27A1	3	OK	21.642059	54.486891	0.3971975	0.69122	1.57744735	subgrp_con	count		1	0
PGLS	181	OK	0.4481687	0.223418	2.0059581	0.04486	0.61146274	subgrp_con	count		1	0
COLGALT1	5	OK	3.459816	3.0263792	1.1432199	0.25295	0.00222292	subgrp_con	count		1	0
MAP1S	5	OK	-0.305257	1.5883470	-0.192185	0.8476	-0.19325804	subgrp_con	count		1	0
FCHO1	2	OK	-12.145707	43.418869	-0.279733	0.77968	-1.91338276	subgrp_con	count		1	0
RPL18A	872	OK	-0.077058	0.0845817	-0.911058	0.36226	-0.11029159	subgrp_con	count		1	0
CCDC124	88	OK	0.1016523	0.311587	0.3262405	0.74424	0.13270405	subgrp_con	count		1	0
ARRDC2	16	OK	-0.290309	0.8116108	-0.357695	0.72057	-0.14459542	subgrp_con	count		1	0
MPV17L2	2	OK	-11.019418	43.249935	-0.2547846	0.79889	-1.17482461	subgrp_con	count		1	0
RAB3A	1	OK	-10.442278	41.465809	-0.2518286	0.80117	-0.00045289	subgrp_con	count		1	0
JUND	741	OK	-0.29130	0.0959583	-3.035701	0.0024	-0.41581978	subgrp_con	count		1	0
LSM4.00	81	OK	-0.155554	0.3269521	-0.47577	0.63426	-0.19791921	subgrp_con	count		1	0
PGPEP1	4	OK	3.3648273	2.5447810	1.322246	0.18609	1.31777762	subgrp_con	count		1	0
GDF15	30	OK	3.2887154	0.9447172	3.4811637	0.0005	3.24106282	subgrp_con	count		1	0
SSBP4	78	OK	1.3165788	0.3847642	3.4217804	0.00062	1.68352403	subgrp_con	count		1	0
ISYNA1	16	OK	1.2305281	0.784570	1.5684091	0.11679	1.23538239	subgrp_con	count		1	0
FKBP8	111	OK	0.6117771	0.2702119	2.2640637	0.02357	0.80225852	subgrp_con	count		1	0
KXD1	52	OK	0.4971440	0.4182758	1.1885554	0.23461	0.58233952	subgrp_con	count		1	0
UBA52	827	OK	-0.122909	0.0898072	-1.368593	0.17112	-0.17564223	subgrp_con	count		1	0
REX1BD	210	OK	0.3401176	0.1951738	1.742629	0.0814	0.46866270	subgrp_con	count		1	0
KLHL26	1	OK	0.6532528	2.5001284	0.2612877	0.79387	0.00016297	subgrp_con	count		1	0
UPF1	5	OK	-0.176520	1.7800985	-0.099163	0.92101	-0.08923128	subgrp_con	count		1	0
COPE	184	OK	0.9549425	0.2260079	4.2252609	2.39E-05	1.29938762	subgrp_con	count		0.23828219	0
DDX49	2	OK	3.4598149	4.7858686	0.7229230	0.46972	0.00081935	subgrp_con	count		1	0
SUGP2	7	OK	0.5118995	1.7578682	0.291204	0.77089	0.17008884	subgrp_con	count		1	0
TMEM161A	3	OK	-0.91014	2.1881243	-0.415946	0.67745	-0.25784050	subgrp_con	count		1	0
BORCS8	12	OK	-0.27748	0.8897971	-0.311853	0.75515	-0.08366573	subgrp_con	count		1	0
RFXANK	11	OK	-0.42428	0.9419696	-0.450423	0.65241	-0.20926519	subgrp_con	count		1	0
NR2C2AP	11	OK	-2.112653	1.0384972	-2.034237	0.04192	-1.43303158	subgrp_con	count		1	0
MAU2	3	OK	0.1681690	1.7253287	0.0974707	0.92235	0.05201879	subgrp_con	count		1	0
GATAD2A	14	OK	2.0613223	1.1791160	1.7481936	0.08042	1.2169787	subgrp_con	count		1	0
NDUFA13	41	OK	1.6878733	0.5607519	3.0100176	0.00261	1.85351411	subgrp_con	count		1	0
ATP13A1	2	OK	-11.451032	41.79736	-0.273965	0.78411	-0.00049189	subgrp_con	count		1	0
ZNF101	1	OK	-12.14842	43.455309	-0.279561	0.77981	-1.91362624	subgrp_con	count		1	0
ZNF506	4	OK	-0.268171	1.2291601	-0.218174	0.82729	-0.13376592	subgrp_con	count		1	0
ZNF253	1	OK	-10.442278	41.465809	-0.2518286	0.80117	-0.00045289	subgrp_con	count		1	0
ZNF93	2	OK	-13.0456	27.67476	-0.471390	0.63736	-1.3178643	subgrp_con	count		1	0
ZNF90	1	OK	18.759489	83.625179	0.2243282	0.8225	0.00028789	subgrp_con	count		1	0
AC011447.3	1	OK	3.4598146	6.7714513	0.5109413	0.60939	0.00030179	subgrp_con	count		1	0
ZNF486	2	OK	1.925964	2.5154879	0.7656425	0.44389	0.56022119	subgrp_con	count		1	0
ZNF626	4	OK	1.925964	2.5154879	0.7656425	0.44389	0.56022119	subgrp_con	count		1	0
ZNF85	3	OK	-1.853834	1.8543918	-0.999699	0.31740	-0.46053279	subgrp_con	count		1	0
ZNF430	4	OK	0.0147770	1.425558	0.0103657	0.99172	0.00748501	subgrp_con	count		1	0
ZNF431	7	OK	-1.16497	1.2427412	-0.937422	0.34854	-0.54417528	subgrp_con	count		1	0
ZNF708	4	OK	-2.350809	2.3603730	-0.995948	0.31929	-0.51168042	subgrp_con	count		1	0
ZNF738	2	OK	-11.019418	43.249935	-0.2547846	0.79889	-1.17482461	subgrp_con	count		1	0
ZNF493	4	OK	0.5524576	1.3480048	0.4098335	0.68193	0.17230335	subgrp_con	count		1	0
ZNF429	1	OK	-11.019418	43.249935	-0.2547846	0.79889	-1.17482461	subgrp_con	count		1	0
ZNF100	3	OK	1.5659305	1.5666919	0.9995140	0.31755	0.97089418	subgrp_con	count		1	0
ZNF43	13	OK	-1.255921	0.8693458	-1.444673	0.14855	-0.72009720	subgrp_con	count		1	0
ZNF208	2	OK	-1.295616	2.041425	-0.634662	0.52565	-0.00075754	subgrp_con	count		1	0
ZNF257	1	OK	-10.442278	41.465809	-0.2518286	0.80117	-0.00045289	subgrp_con	count		1	0
ZNF676	4	OK	1.4119335	1.4959362	0.9438469	0.34525	0.6979627	subgrp_con	count		1	0
ZNF91	11	OK	-0.446723	0.9925427	-0.450080	0.65265	-0.32983802	subgrp_con	count		1	0
ZNF675	5	OK	0.7689927	1.5299032	0.5026414	0.61522	0.23956880	subgrp_con	count		1	0
ZNF681	2	OK	0.5887819	1.767414	0.3331317	0.73903	0.30122869	subgrp_con	count		1	0
ZNF726	1	OK	1.5822999	2.7200188	0.5817606	0.56072	0.47433761	subgrp_con	count		1	0
ZNF254	4	OK	2.2572088	1.7549585	1.2861893	0.19838	1.32689219	subgrp_con	count		1	0
LINC00662	6	OK	0.7754798	1.2826867	0.6045746	0.54540	0.23507395	subgrp_con	count		1	0
UQCRFS1	60	OK	-0.074546	0.3944835	-0.188972	0.85011	-0.08496696	subgrp_con	count		1	0
POP4	19	OK	1.0071908	0.7847059	1.2835264	0.19931	0.94231277	subgrp_con	count		1	0
PLEKHF1	5	OK	0.6569825	1.4161275	0.4639289	0.6427	0.33587741	subgrp_con	count		1	0
C19orf12	2	OK	0.40604	2.1083469	0.1925883	0.84728	0.12643683	subgrp_con	count		1	0
URI1	42	OK	-1.016726	0.4878393	-2.084141	0.03715	-1.16652048	subgrp_con	count		1	0
TSHZ3	5	OK	4.2109206	4.2875616	0.982124	0.32604	0.00257090	subgrp_con	count		1	0
DPY19L3	4	OK	0.0801233	1.6024050	0.0500019	0.96012	0.04092734	subgrp_con	count		1	0
PDCD5	187	OK	0.0815595	0.2067557	0.3944728	0.69322	0.11215844	subgrp_con	count		1	0
CEP89	5	OK	-2.674942	1.6743951	-1.597558	0.11014	-0.58410445	subgrp_con	count		1	0
CEBPG	28	OK	-0.07441	0.5550818	-0.134063	0.89335	-0.0732803	subgrp_con	count		1	0
PEPD	97	OK	0.5410084	0.3040028	1.7796161	0.07514	0.68362659	subgrp_con	count		1	0
KCTD15	2	OK	1.1112449	1.8685808	0.5946999	0.55204	0.34276981	subgrp_con	count		1	0
LSM14A	51	OK	0.0260556	0.4115666	0.0633083	0.94952	0.02814074	subgrp_con	count		1	0
KIAA0355	52	OK	0.9632655	0.4664196	2.0654478	0.03888	0.97788748	subgrp_con	count		1	0
GPI	23	OK	0.8255527	0.6759599	1.2213042	0.22197	0.77627542	subgrp_con	count		1	0
PDCD2L	1	OK	-11.019418	43.249935	-0.2547846	0.79889	-1.17482461	subgrp_con	count		1	0
UBA2	45	OK	0.7382272	0.44173	1.6712068	0.09468	0.82795881	subgrp_con	count		1	0
WTIP	5	OK	1.0315898	1.620334	0.6366522	0.52435	0.0019189	subgrp_con	count		1	0
ZNF302	15	OK	1.0819021	0.7101703	1.5234402	0.12765	1.12643054	subgrp_con	count		1	0
AC020910.4	1	OK	1.5822999	2.7200188	0.5817606	0.56072	0.47433761	subgrp_con	count		1	0
ZNF181	6	OK	-0.492370	1.1826952	-0.416311	0.67719	-0.30761332	subgrp_con	count		1	0
ZNF599	1	OK	-11.019418	43.249935	-0.2547846	0.79889	-1.17482461	subgrp_con	count		1	0
AC008555.5	1	OK	-11.019418	43.249935	-0.2547846	0.79889	-1.17482461	subgrp_con	count		1	0
ZNF792	2	OK	1.925964	2.5154879	0.7656425	0.44389	0.56022119	subgrp_con	count		1	0
GRAMD1A	49	OK	0.2700900	0.44337	0.6091670	0.54241	0.30407523	subgrp_con	count		1	0
SCN1B	45	OK	-0.591866	0.4068534	-1.454741	0.14574	-0.70567908	subgrp_con	count		1	0
LGI4	1	OK	-10.442278	41.465809	-0.2518286	0.80117	-0.00045289	subgrp_con	count		1	0

FXYD1	3	OK	0.16816909	1.72532872	0.09747075	0.92235	0.05201879	subgrp_cor	count	1	0
FXYD5	672	OK	-0.07826	0.10156149	-0.7705678	0.44096	-0.11159089	subgrp_cor	count	1	0
LSR	31	OK	1.84316663	0.64235873	2.86937274	0.00411	1.87213266	subgrp_cor	count	1	0
USF2	46	OK	0.27011291	0.45674102	0.59139183	0.55426	0.29243958	subgrp_cor	count	1	0
MAG	2	OK	18.7594894	83.6251792	0.22432824	0.8225	0.00028789	subgrp_cor	count	1	0
DMKN	12	OK	2.22082757	0.98946307	2.24447747	0.0248	1.65516807	subgrp_cor	count	1	0
TMEM147	122	OK	0.30291341	0.25918204	1.16872841	0.24251	0.40875553	subgrp_cor	count	1	0
RBM42	34	OK	-0.2666179	0.4909743	-0.5430384	0.5871	-0.27882189	subgrp_cor	count	1	0
COX6B1	432	OK	0.37325846	0.13214201	2.8034611	0.00506	0.527742	subgrp_cor	count	1	0
KMT2B	10	OK	0.38046742	0.90017385	0.42265994	0.67254	0.37642434	subgrp_cor	count	1	0
IGFLR1	1	OK	-10.442278	41.4658099	-0.2518286	0.80117	-0.00045289	subgrp_cor	count	1	0
U2AF1L4	3	OK	5.80492723	5.66946625	1.0238921	0.30589	1.03285716	subgrp_cor	count	1	0
PSENEN	88	OK	0.21056584	0.30587148	0.6884128	0.49119	0.27705252	subgrp_cor	count	1	0
HSPB6	1	OK	3.45981462	6.7714513	0.51094137	0.60929	0.00030179	subgrp_cor	count	1	0
PROSER3	1	OK	18.7594894	83.6251792	0.22432824	0.8225	0.00028789	subgrp_cor	count	1	0
APLP1	1	OK	23.9918963	84.8999419	0.28259026	0.77749	1.65081709	subgrp_cor	count	1	0
NFKBID	9	OK	-2.3712651	1.24494135	-1.9047203	0.05682	-1.54785899	subgrp_cor	count	1	0
SDHAF1	3	OK	4.66132998	5.76838606	0.80808217	0.41904	0.00274918	subgrp_cor	count	1	0
POLR2I	120	OK	0.40074604	0.26169841	1.53132773	0.12569	0.52332219	subgrp_cor	count	1	0
TBCB	159	OK	0.69124411	0.2360203	2.92874855	0.0034	0.94605925	subgrp_cor	count	1	0
CAPNS1	261	OK	-0.0011179	0.16827542	-0.0066432	0.9947	-0.00155040	subgrp_cor	count	1	0
ZNF146	4	OK	-2.4465924	1.71381312	-1.4275724	0.15341	-0.00126286	subgrp_cor	count	1	0
ZFP14	4	OK	1.5231809	1.72215008	0.8897547	0.3736	0.46131746	subgrp_cor	count	1	0
ZFP82	1	OK	-11.019418	43.2499352	-0.2547846	0.79889	-1.17482461	subgrp_cor	count	1	0
ZNF566	1	OK	-10.442278	41.4658099	-0.2518286	0.80117	-0.00045289	subgrp_cor	count	1	0
ZNF529	1	OK	23.9918963	84.8999419	0.28259026	0.77749	1.65081709	subgrp_cor	count	1	0
LINC01534	2	OK	-12.145707	43.4188694	-0.2797324	0.77968	-1.91338276	subgrp_cor	count	1	0
ZNF567	9	OK	0.68285176	1.03495205	0.65979072	0.50939	0.51665748	subgrp_cor	count	1	0
ZNF790	1	OK	1.58239999	2.72001883	0.58176068	0.56072	0.47433761	subgrp_cor	count	1	0
ZNF345	1	OK	3.45981462	6.7714513	0.51094137	0.60929	0.00030179	subgrp_cor	count	1	0
ZNF829	1	OK	-10.442278	41.4658099	-0.2518286	0.80117	-0.00045289	subgrp_cor	count	1	0
ZNF420	2	OK	-0.9102232	2.1881125	-0.4159856	0.67742	-0.25785735	subgrp_cor	count	1	0
ZNF383	3	OK	6.14900159	5.41239945	1.1360953	0.25592	1.05209450	subgrp_cor	count	1	0
HKR1	2	OK	23.9918963	84.8999419	0.28259026	0.77749	1.65081709	subgrp_cor	count	1	0
ZNF569	2	OK	-11.448548	41.761485	-0.2741412	0.78398	-0.00049246	subgrp_cor	count	1	0
ZNF570	4	OK	-0.7252159	1.807914	-0.4011341	0.68832	-0.20980483	subgrp_cor	count	1	0
ZNF793	1	OK	-11.019418	43.2499352	-0.2547846	0.79889	-1.17482461	subgrp_cor	count	1	0
ZNF571	1	OK	-11.019418	43.2499352	-0.2547846	0.79889	-1.17482461	subgrp_cor	count	1	0
ZNF781	3	OK	0.8788176	1.64693117	0.53360919	0.59361	0.44699547	subgrp_cor	count	1	0
SPINT2	57	OK	5.45424106	1.25594953	4.342723	1.41E-05	4.53371695	subgrp_cor	count	0.14074153	0
PPP1R14A	30	OK	2.8121214	0.84619582	3.32325136	0.00089	2.60619345	subgrp_cor	count	1	0
C19orf33	26	OK	3.64414749	1.23638073	2.9474215	0.0032	2.7280265	subgrp_cor	count	1	0
YIF1B	11	OK	1.12604937	1.06416531	1.05815263	0.28999	1.01093954	subgrp_cor	count	1	0
KCNK6	3	OK	2.55522271	2.3498446	1.08740073	0.27686	0.69313893	subgrp_cor	count	1	0
PSMD8	249	OK	0.65662259	0.18836869	3.48583709	0.00049	0.91083377	subgrp_cor	count	1	0
FAM98C	13	OK	0.0034429	0.84007259	0.00409952	0.99673	0.0028563	subgrp_cor	count	1	0
EIF3K	637	OK	-0.1277771	0.10576911	-1.2080756	0.22702	-0.18208888	subgrp_cor	count	1	0
ACTN4	198	OK	0.28563857	0.20705625	1.37952068	0.16773	0.38678396	subgrp_cor	count	1	0
ECH1	150	OK	0.07509215	0.23515068	0.3192938	0.7495	0.10283074	subgrp_cor	count	1	0
HNRNPL	26	OK	-0.1992936	0.63875709	-0.3120022	0.75504	-0.16406736	subgrp_cor	count	1	0
RINL	1	OK	23.9918963	84.8999419	0.28259026	0.77749	1.65081709	subgrp_cor	count	1	0
SIRT2	11	OK	0.85703442	1.06709804	0.80314497	0.42189	0.43622194	subgrp_cor	count	1	0
NFKBIB	5	OK	0.45568755	1.46808638	0.3103956	0.75626	0.00030627	subgrp_cor	count	1	0
MRPS12	46	OK	0.71300075	0.47764003	1.49275752	0.1355	0.81284382	subgrp_cor	count	1	0
FBXO17	3	OK	5.26127895	5.49058205	0.95823701	0.33794	0.00295034	subgrp_cor	count	1	0
PAK4	3	OK	0.07117225	1.36018365	0.05232554	0.95827	4.68E-05	subgrp_cor	count	1	0
SAMD4B	5	OK	-1.434093	1.52609497	-0.9397141	0.34736	-0.63181067	subgrp_cor	count	1	0
PAF1	14	OK	-0.3940447	0.767652	-0.513311	0.60772	-0.38368844	subgrp_cor	count	1	0
MED29	23	OK	0.79111918	0.63750389	1.24096368	0.21462	0.86920125	subgrp_cor	count	1	0
ZFP36	721	OK	-0.4315391	0.0953225	-4.5271485	5.98E-06	-0.61668142	subgrp_cor	count	0.05995245	0
RPS16	829	OK	-0.1046045	0.08965259	-1.1667757	0.2433	-0.14943736	subgrp_cor	count	1	0
SUPT5H	12	OK	0.95509169	1.10520859	0.86417324	0.38749	0.29251256	subgrp_cor	count	1	0
TIMM50	6	OK	-0.0420409	1.15920361	-0.0362671	0.97107	-0.0212408	subgrp_cor	count	1	0
EID2B	3	OK	-0.714306	1.96229939	-0.3638304	0.71598	-6.05E-05	subgrp_cor	count	1	0
EID2	16	OK	-0.3599009	0.82488529	-0.4363042	0.66262	-0.3168369	subgrp_cor	count	1	0
DYRK1B	3	OK	1.533588	2.24703247	0.68249481	0.49493	0.00105583	subgrp_cor	count	1	0
FBL	67	OK	-0.5381768	0.35199379	-1.5289382	0.12629	-0.63667131	subgrp_cor	count	1	0
PSMC4	68	OK	0.23578966	0.3715657	0.63458403	0.5257	0.28748334	subgrp_cor	count	1	0
ZNF546	1	OK	-11.019418	43.2499352	-0.2547846	0.79889	-1.17482461	subgrp_cor	count	1	0
ZNF780B	1	OK	3.45981462	6.7714513	0.51094137	0.60929	0.00030179	subgrp_cor	count	1	0
ZNF780A	5	OK	-0.6602558	1.15115834	-0.5735578	0.56627	-0.40908608	subgrp_cor	count	1	0
PLD3	47	OK	0.7364163	0.42023294	1.75240022	0.07971	0.88801436	subgrp_cor	count	1	0
SERTAD1	306	OK	-0.1759846	0.15679475	-1.1223881	0.2617	-0.24670595	subgrp_cor	count	1	0
SERTAD3	91	OK	-0.5858805	0.28910463	-2.0265346	0.04271	-0.74880546	subgrp_cor	count	1	0
BLVRB	72	OK	0.55472671	0.33949388	1.63400395	0.10226	0.71758272	subgrp_cor	count	1	0
SHKBP1	21	OK	0.59916613	0.85527771	0.70055162	0.48358	0.49882421	subgrp_cor	count	1	0
COQ8B	2	OK	18.7594894	83.6251792	0.22432824	0.8225	0.00028789	subgrp_cor	count	1	0
ITPKC	41	OK	-1.5519028	0.48452094	-3.2029634	0.00136	-1.61123198	subgrp_cor	count	1	0
SNRPA	21	OK	-1.8104455	0.76330927	-2.3718374	0.0177	-1.48608985	subgrp_cor	count	1	0
AXL	1	OK	18.7594894	83.6251792	0.22432824	0.8225	0.00028789	subgrp_cor	count	1	0
HNRNPUL1	30	OK	-1.1412337	0.62268813	-1.8327532	0.06684	-0.87380047	subgrp_cor	count	1	0
CCDC97	1	OK	18.7594894	83.6251792	0.22432824	0.8225	0.00028789	subgrp_cor	count	1	0
TMEM91	13	OK	0.55686873	0.79659717	0.69905939	0.48451	0.49880881	subgrp_cor	count	1	0
B9D2	6	OK	1.96313791	1.58118064	1.24156476	0.2144	0.90660126	subgrp_cor	count	1	0
EXOSC5	7	OK	-1.6916763	1.50108089	-1.1269721	0.25975	-0.71820096	subgrp_cor	count	1	0
DMAC2	9	OK	-0.5427675	0.8779628	-0.6182124	0.53644	-0.15996718	subgrp_cor	count	1	0
PCAT19	33	OK	-1.7029959	0.56019496	-3.0400057	0.00237	-1.32770523	subgrp_cor	count	1	0
RPS19	872	OK	-0.1528362	0.08518855	-1.7940937	0.0728	-0.21865831	subgrp_cor	count	1	0
ARHGEF1	13	OK	-0.9117545	0.80068907	-1.1387122	0.25482	-0.25824475	subgrp_cor	count	1	0
RABAC1	498	OK	0.07685866	0.12300793	0.62482685	0.53208	0.10911304	subgrp_cor	count	1	0
POU2F2	1	OK	18.7594894	83.6251792	0.22432824	0.8225	0.00028789	subgrp_cor	count	1	0
DEDD2	2	OK	-10.442278	41.4658099	-0.2518286	0.80117	-0.00045289	subgrp_cor	count	1	0
ZNF526	1	OK	18.7594894	83.6251792	0.22432824	0.8225	0.00028789	subgrp_cor	count	1	0
GSK3A	5	OK	0.55249302	1.34800031	0.40986119	0.68192	0.17231298	subgrp_cor	count	1	0
ERF	25	OK	0.71086449	0.57061096	1.24576033	0.21285	0.67121606	subgrp_cor	count	1	0
CIC	5	OK	1.34301765	1.65446373	0.81175406	0.41692	0.00092378	subgrp_cor	count	1	0
PAFAH1B3	29	OK	-0.9227286	0.57469864	-1.6055875	0.10836	-0.96041345	subgrp_cor	count	1	0
MEGF8	1	OK	3.45981462	6.7714513	0.51094137	0.60929	0.00030179	subgrp_cor	count	1	0
CNFN	1	OK	-10.442278	41.4658099	-0.2518286	0.80117	-0.00045289	subgrp_cor	count	1	0
ETHE1	112	OK	0.76389538	0.27842543	2.74362653	0.00608	1.03034012	subgrp_cor	count	1	0
ZNF575	1	OK	-11.019418	43.2499352	-0.2547846	0.79889	-1.17482461	subgrp_cor	count	1	0
XRCC1	3	OK	-1.2956167	2.0414257	-0.6346627	0.52565	-0.00075754	subgrp_cor	count	1	0
PINLYP	1	OK	-11.019418	43.2499352	-0.2547846	0.79889	-1.17482461	subgrp_cor	count	1	0
ZNF576	3	OK	1.9258645	2.51548794	0.76564251	0.44389	0.56022119	subgrp_cor	count	1	0
ZNF428	80	OK	1.43767173	0.40822388	3.52177272	0.00043	1.76542732	subgrp_cor	count	1	0
PLAUR	44	OK	0.88528428	0.48197943	1.83676768	0.06624	1.07369810	subgrp_cor	count	1	0
KCNN4	15	OK	5.11851214	2.05503623	2.49071627	0.01275	2.53907604	subgrp_cor	count	1	0
LYPD5	1	OK	3.45981462	6.7714513	0.51094137	0.60929	0.00030179	subgrp_cor	count	1	0
ZNF223	1	OK	-11.019418	43.2499352	-0.2547846	0.79889	-1.17482461	subgrp_cor	count	1	0
ZNF224	8	OK	0.96498589	0.95089273	1.01482096	0.31019	0.71966427	subgrp_cor	count	1	0
ZNF234	1	OK	-11.019418	43.2499352	-0.2547846	0.79889	-1.17482461	subgrp_cor	count	1	0
ZNF226	6	OK	1.10069614	1.56872329	0.70165092	0.4829	0.55472334	subgrp_cor	count	1	0
ZNF227	1	OK	3.45978793	4.78585287	0.72291983	0.46972	0.00081879	subgrp_cor	count	1	0
ZNF112	1	OK	26.1931853	26.8565524	0.97529962	0.32941	5.48034831	subgrp_cor	count	1	0
IGSF23	1	OK	23.9918963	84.8999419	0.28259026	0.77749	1.65081709	subgrp_cor	count	1	0
PVR	7	OK	7.01053936	5.16881947	1.35631345	0.175	1.70916359	subgrp_cor	count	1	0
CEACAM19	1	OK	1.58239999	2.72001883	0.58176068	0.56072	0.47433761	subgrp_cor	count	1	0
BCL3	62	OK	-0.4689434	0.34901236	-1.3436296	0.17907	-0.58616920	subgrp_cor	count	1	0
NECTIN2	163	OK	-0.876198	0.24150864	-3.6280193	0.00029	-1.16653742	subgrp_cor	count	1	0
TOMM40	12	OK	-0.0802376	0.97801533	-0.0820411	0.93461	-0.05166423	subgrp_cor	count	1	0
APOE	6	OK	22.6877204	54.3157576	0.41770052	0.67617	1.6098774	subgrp_cor	count	1	0
CLPTM1	15	OK	1.36363014	0.92259805	1.47803276	0.1394	0.67643728	subgrp_cor	count	1	0
RELB	37	OK	-0.620462	0.5382873	-1.1526614	0.24905	-0.5702737	subgrp_cor	count	1	0
CLASRP	1	OK	-11.019418	43.2499352	-0.2547846	0.79889	-1.17482461	subgrp_cor	count	1	0
ZNF296	2	OK	-1.0311657	1.74795383	-0.5899273	0.55524	-0.00022765	subgrp_cor	count	1	0
GEMIN7	3	OK	1.58239999	2.72001883	0.58176068	0.56072	0.47433761	subgrp_cor	count	1	0
MARK4	2	OK	-0.7251858	1.80791919	-0.4011162	0.68832	-0.20979917	subgrp_cor	count	1	0
PPP1R37	2	OK	-1.0311657	1.74795383	-0.5899273	0.55524	-0.00022765	subgrp_cor	count	1	0
TRAPPC6A	39	OK	-0.6321666	0.46078915	-1.3719216	0.17009	-0.72898155	subgrp_cor	count	1	0
EXOC3L2	17	OK	0.62825577	0.93498652	0.67194099	0.50162	0.0011559	subgrp_cor	count	1	0
PPP1R13L	2	OK	18.7594894	83.6251792	0.22432824	0.8225	0.00028789	subgrp_cor	count	1	0
CD3EAP	1	OK	3.45981462	6.7714513	0.51094137	0.60929	0.00030179	subgrp_cor	count	1	0
ERCC1	26	OK	0.99971735	0.65490874	1.52649873	0.12689	0.93559739	subgrp_cor	count	1	0
FOSB	377	OK	-0.0479786	0.13708788	-0.3499842	0.72635	-0.06757870	subgrp_cor	count	1	0
RTN2	4	OK	-1.6462456	1.93519293	-0.8506881	0.39494	-0.70360391	subgrp_cor	count	1	0
PPM1N	1	OK	-11.019418	43.2499352	-0.2547846	0.79889	-1.17482461	subgrp_cor	count	1	0
VASP	62	OK	0.43929696	0.40118778	1.09499086	0.27352	0.52651690	subgrp_cor	count	1	0
OPA3	7	OK	0.887566	1.02615928	0.86493979	0.38707	0.27586715	subgrp_cor	count	1	0
GPR4	2	OK	1.58239999	2.72001883	0.58176068	0.56072	0.47433761	subgrp_cor	count	1	0
EML2	5	OK	1.83420028	1.68294236	1.08987706	0.27577	0.53818379	subgrp_cor	count	1	0
SNRPD2	379	OK	-0.0724792	0.14208187	-0.5101227	0.60997	-0.10200129	subgrp_cor	count	1	0
FBXO46	1	OK	18.7594894	83.6251792	0.22432824	0.8225	0.00028789	subgrp_cor	count	1	0
DMWD	5	OK	1.15815841	1.65445535	0.70002399	0.48392	0.00079560	subgrp_cor	count	1	0
IRF2BP1	1	OK	3.45981462	6.7714513	0.51094137	0.60929	0.00030179	subgrp_cor	count	1	0
MYPOP	1	OK	-10.442278	41.4658099	-0.2518286	0.80117	-0.00045289	subgrp_cor	count	1	0
NOVA2	13	OK	-0.6176348	0.85505463	-0.7223337	0.47009	-0.18069849	subgrp_cor	count	1	0
CCDC61	4	OK	0.1112767	1.61203873	0.06902855	0.94497	0.05657342	subgrp_cor	count	1	0
HIF3A	11	OK	0.23782691	0.83074114	0.2862828	0.77466	0.00043010	subgrp_cor	count	1	0
PPP5C	1	OK	0.65325287	2.50012846	0.26128772	0.79387	0.00016297	subgrp_cor	count	1	0
PPP5D1	1	OK	-10.442278	41.4658099	-0.2518286	0.80117	-0.00045289	subgrp_cor	count	1	0
PNMA8B	2	OK	-0.1993711	1.9964692	-0.0998618	0.92045	-0.06364451	subgrp_cor	count	1	0
CALM3	118	OK	0.03035056	0.27546455	0.11017964	0.91227	0.03981304	subgrp_cor	count	1	0
PRKD2	13	OK	-1.5961268	0.92446489	-1.7265412	0.08425	-1.04015347	subgrp_cor	count	1	0
STRN4	6	OK	1.99874575	2.07992047	0.9609722	0.33657	0.0013669	subgrp_cor	count	1	0
SLC1A5	13	OK	-2.4650815	0.98939494	-2.491506	0.01272	-1.20056916	subgrp_cor	count	1	0
AP2S1	224	OK	0.38800881	0.1968767	1.9708215	0.04874	0.53523432	subgrp_cor	count	1	0
ARHGAP35	4	OK	1.66308861	1.58721948	1.04780002	0.29472	0.93975760	subgrp_cor	count	1	0
TMEM160	86	OK	0.80472411	0.32604566	2.4681332	0.01358	1.04509720	subgrp_cor	count	1	0
ZC3H4	4	OK	-0.0249392	1.19583735	-0.020855	0.98336	-0.00764495	subgrp_cor	count	1	0
SAE1	11	OK	0.53506345	1.03011375	0.51942172	0.60347	0.27382809	subgrp_cor	count	1	0
BBC3	17	OK	-0.9051887	0.9191847	-0.9847734	0.32476	-0.42636847	subgrp_cor	count	1	0
CCDC9	1	OK	3.45981462	6.7714513	0.51094137	0.60929	0.00030179	subgrp_cor	count	1	0
INAFM1	1	OK	-10.442278	41.4658099	-0.2518286	0.80117	-0.00045289	subgrp_cor	count	1	0
C5AR2	1	OK	-11.189367	26.3349482	-0.4248866	0.67092	-2.27128013	subgrp_cor	count	1	0
NAPA-AS1	1	OK	3.45981462	6.7714513	0.51094137	0.60929	0.00030179	subgrp_cor	count	1	0
NAPA	62	OK	0.26994243	0.36941347	0.73073248	0.46494	0.31304174	subgrp_cor	count	1	0
EHD2	41	OK	-0.1368261	0.47711847	-0.2867759	0.77428	-0.14365740	subgrp_cor	count	1	0
SELENOW	792	OK	-0.2761855	0.09357059	-2.9516268	0.00316	-0.39430878	subgrp_cor	count	1	0
PLA2G4C	5	OK	0.93967049	1.49854054	0.6270571	0.53062	0.29164784	subgrp_cor	count	1	0
ZSWIM9	3	OK	21.642059	54.4868919	0.39719753	0.69122	1.57744735	subgrp_cor	count	1	0
CARD8	43	OK	-0.3119317	0.45629634	-0.6836165	0.49422	-0.38087112	subgrp_cor	count	1	0
CARD8-AS1	18	OK	-1.8125148	0.8320369	-2.1784068	0.02938	-1.63829440	subgrp_cor	count	1	0
KDELR1	352	OK	0.31100559	0.14521988	2.14161854	0.03222	0.4393246	subgrp_cor	count	1	0
CYTH2	41	OK	0.24851996	0.47776489	0.52017209	0.60294	0.2880674	subgrp_cor	count	1	0
RPL18	872	OK	-0.2066479	0.08590552	-2.4055254	0.01615	-0.29561372	subgrp_cor	count	1	0
SPHK2	2	OK	3.45981462	6.7714513	0.51094137	0.60929	0.00030179	subgrp_cor	count	1	0
DBP	9	OK	0.34345945	0.89920013	0.38196108	0.70249	0.22362382	subgrp_cor	count	1	0
CA11	3	OK	1.14694397	1.60044893	0.71663888	0.4736	0.37918447	subgrp_cor	count	1	0
RASIP1	112	OK	-0.3750564	0.28042383	-1.3374628	0.18107	-0.46344066	subgrp_cor	count	1	0
BCAT2	4	OK	0.62108147	1.24779259	0.49774415	0.61866	0.31765969	subgrp_cor	count	1	0
HSD17B14	7	OK	1.91578073	1.48838833	1.28715117	0.19806	1.14901874	subgrp_cor	count	1	0
PLEKHA4	9	OK	22.3754543	50.1688854	0.44600173	0.6556	0.00088526	subgrp_cor	count	1	0
PPP1R15A	492	OK	-0.0543815	0.12290124	-0.4424811	0.65814	-0.07695907	subgrp_cor	count	1	0
NUCB1	229	OK	-0.0168594	0.17602279	-0.0963476	0.92324	-0.0233844	subgrp_cor	count	1	0
BAX	137	OK	1.25204587	0.3061372	4.08981947	4.32E-05	1.63610862	subgrp_cor	count	0.42993807	0
FTL	875	OK	-0.0564775	0.08394385	-0.6728004	0.50107	-0.08082264	subgrp_cor	count	1	0
RUVBL2	9	OK	0.82013909	1.12325096	0.73014768	0.4652	0.53118321	subgrp_cor	count	1	0
SNRNP70	49	OK	-0.1153134	0.4414212	-0.2612321	0.79392	-0.12924460	subgrp_cor	count	1	0
LIN7B	1	OK	18.7594894	83.6251792	0.22432824	0.8225	0.00028789	subgrp_cor	count	1	0
PPFIA3	27	OK	-0.2478702	0.52568373	-0.4715106	0.63728	-0.25942674	subgrp_cor	count	1	0
CD37	2	OK	-1.2956167	2.0414257	-0.6346627	0.52565	-0.00075754	subgrp_cor	count	1	0
TEAD2	16	OK	-0.2249953	0.68930998	-0.3264064	0.74412	-0.16707714	subgrp_cor	count	1	0
PIH1D1	30	OK	1.05209708	0.61724853	1.70449511	0.08829	1.12528866	subgrp_cor	count	1	0
ALDH16A1	8	OK	-2.0533747	1.28662699	-1.5959363	0.1105	-1.06687712	subgrp_cor	count	1	0
FLT3LG	2	OK	0.16814911	1.72532009	0.0974591	0.92236	0.05201277	subgrp_cor	count	1	0
RPL13A	854	OK	-0.1189902	0.08626546	-1.3793494	0.16779	-0.17017069	subgrp_cor	count	1	0
RPS11	805	OK	-0.1261094	0.09181586	-1.3734929	0.1696	-0.18001863	subgrp_cor	count	1	0
FCGRT	528	OK	-0.0452103	0.115934	-0.3899655	0.69656	-0.06434962	subgrp_cor	count	1	0
RCN3	19	OK	8.98862098	4.53450622	1.98227117	0.04745	3.11371381	subgrp_cor	count	1	0
NOSIP	81	OK	0.32240268	0.310704	1.03765216	0.29942	0.4286107	subgrp_cor	count	1	0
PRRG2	4	OK	-2.2229579	1.77606832	-1.2516173	0.21071	-0.86936325	subgrp_cor	count	1	0
RRAS	191	OK	0.87929283	0.20755681	4.23639597	2.27E-05	1.21211898	subgrp_cor	count	0.22686352	0
SCAF1	3	OK	4.66132998	5.76838606	0.80808217	0.41904	0.00274918	subgrp_cor	count	1	0
IRF3	25	OK	0.68394249	0.63566724	1.0759442	0.28195	0.72043553	subgrp_cor	count	1	0
BCL2L12	5	OK	-2.2911888	2.05792275	-1.1133503	0.26556	-0.53157637	subgrp_cor	count	1	0
PRMT1	213	OK	-0.2820936	0.1976652	-1.427129	0.15354	-0.38827260	subgrp_cor	count	1	0
AP2A1	6	OK	-1.2658877	1.14958263	-1.101162	0.27082	-0.5705552	subgrp_cor	count	1	0
FUZ	1	OK	-11.019418	43.2499352	-0.2547846	0.79889	-1.17482461	subgrp_cor	count	1	0
PTOV1	24	OK	1.08795321	0.63315987	1.71829149	0.08574	1.0603028	subgrp_cor	count	1	0
PNKP	10	OK	1.25350588	1.18457136	1.05819361	0.28997	0.61186944	subgrp_cor	count	1	0
AKT1S1	16	OK	1.69255197	0.89996967	1.88067668	0.06002	1.33325546	subgrp_cor	count	1	0
TBC1D17	3	OK	3.45981462	6.7714513	0.51094137	0.60929	0.00030179	subgrp_cor	count	1	0
NUP62	5	OK	0.54229585	1.36387325	0.39761463	0.69092	0.3616530	subgrp_cor	count	1	0
ATF5	17	OK	0.01709866	0.7635404	0.02239392	0.98213	0.01418388	subgrp_cor	count	1	0
VRK3	5	OK	-2.626438	1.93220513	-1.3592957	0.17405	-0.96366359	subgrp_cor	count	1	0
NR1H2	34	OK	-0.1398355	0.52736973	-0.2651564	0.79089	-0.12425867	subgrp_cor	count	1	0
EMC10	28	OK	0.8396628	0.58601159	1.43284334	0.1519	0.88480140	subgrp_cor	count	1	0
JOSD2	27	OK	1.20454855	0.6400758	1.88188423	0.05985	1.28006846	subgrp_cor	count	1	0
CLEC11A	3	OK	2.40963371	2.47152069	0.97495997	0.32958	0.66517343	subgrp_cor	count	1	0
C19orf48	4	OK	1.533588	2.24703247	0.68249481	0.49493	0.00105583	subgrp_cor	count	1	0
CTU1	3	OK	1.58239999	2.72001883	0.58176068	0.56072	0.47433761	subgrp_cor	count	1	0
ETFB	152	OK	0.13506767	0.22929733	0.58905035	0.55582	0.18598652	subgrp_cor	count	1	0
ZNF175	1	OK	3.45981462	6.7714513	0.51094137	0.60929	0.00030179	subgrp_cor	count	1	0
ZNF577	2	OK	1.58239999	2.72001883	0.58176068	0.56072	0.47433761	subgrp_cor	count	1	0
ZNF614	1	OK	-15.995031	70.349421	-0.2273655	0.82014	-0.00028474	subgrp_cor	count	1	0
ZNF432	3	OK	-1.2956167	2.0414257	-0.6346627	0.52565	-0.00075754	subgrp_cor	count	1	0
ZNF841	1	OK	18.7594894	83.6251792	0.22432824	0.8225	0.00028789	subgrp_cor	count	1	0
ZNF616	3	OK	4.66132998	5.76838606	0.80808217	0.41904	0.00274918	subgrp_cor	count	1	0
ZNF836	5	OK	-1.434093	1.52609497	-0.9397141	0.34736	-0.63181067	subgrp_cor	count	1	0
AC010320.1	1	OK	-12.148421	43.455309	-0.2795612	0.77981	-1.91362624	subgrp_cor	count	1	0
PPP2R1A	50	OK	-0.1613648	0.40362541	-0.3997392	0.68935	-0.19069210	subgrp_cor	count	1	0
ZNF766	4	OK	-10.124769	16.10465	-0.6286861	0.52955	-1.11477900	subgrp_cor	count	1	0
ZNF480	8	OK	-0.5072308	1.01197016	-0.501231	0.61621	-0.3173818	subgrp_cor	count	1	0
ZNF880	5	OK	2.16111585	2.36779439	0.91271263	0.36139	0.61359306	subgrp_cor	count	1	0
ZNF528	1	OK	18.7594894	83.6251792	0.22432824	0.8225	0.00028789	subgrp_cor	count	1	0
ZNF578	1	OK	-10.442278	41.4658099	-0.2518286	0.80117	-0.00045289	subgrp_cor	count	1	0
ZNF701	1	OK	-11.019418	43.2499352	-0.2547846	0.79889	-1.17482461	subgrp_cor	count	1	0
ZNF83	12	OK	-0.361028	0.83679714	-0.4314534	0.66614	-0.29489490	subgrp_cor	count	1	0
ZNF611	2	OK	-11.448548	41.761485	-0.2741412	0.78398	-0.00049246	subgrp_cor	count	1	0
ZNF468	2	OK	-11.019418	43.2499352	-0.2547846	0.79889	-1.17482461	subgrp_cor	count	1	0
ZNF160	6	OK	0.35156559	1.62408617	0.21646977	0.82862	0.00023572	subgrp_cor	count	1	0
ZNF415	3	OK	-2.2201404	1.93161127	-1.1493722	0.2504	-0.83118514	subgrp_cor	count	1	0
ZNF347	6	OK	0.44491013	1.39321023	0.31934175	0.74947	0.14208579	subgrp_cor	count	1	0
ZNF665	1	OK	-15.995031	70.349421	-0.2273655	0.82014	-0.00028474	subgrp_cor	count	1	0
ZNF677	16	OK	0.68018135	0.77320529	0.87969051	0.37903	0.67140902	subgrp_cor	count	1	0
ZNF845	1	OK	-11.019418	43.2499352	-0.2547846	0.79889	-1.17482461	subgrp_cor	count	1	0
ZNF765	1	OK	3.45981462	6.7714513	0.51094137	0.60929	0.00030179	subgrp_cor	count	1	0
ZNF761	1	OK	-15.995031	70.349421	-0.2273655	0.82014	-0.00028474	subgrp_cor	count	1	0
ZNF331	5	OK	-0.437932	1.17318375	-0.3732858	0.70894	-0.21576258	subgrp_cor	count	1	0
MYADM	169	OK	-0.2863943	0.21795603	-1.3140004	0.18885	-0.38772971	subgrp_cor	count	1	0
CACNG8	2	OK	1.58237769	1.92402136	0.8224325	0.41082	0.77174550	subgrp_cor	count	1	0
NDUFA3	90	OK	0.63243667	0.3118912	2.02774773	0.04259	0.8312526	subgrp_cor	count	1	0
TFPT	26	OK	0.62664965	0.64022074	0.97880248	0.32768	0.66053624	subgrp_cor	count	1	0
PRPF31	43	OK	0.43159707	0.44363225	0.97287105	0.33062	0.51276800	subgrp_cor	count	1	0
CNOT3	3	OK	3.45981497	4.78586864	0.72292309	0.46972	0.00081935	subgrp_cor	count	1	0
LENG1	27	OK	0.63876373	0.56260636	1.13536529	0.25622	0.65374521	subgrp_cor	count	1	0
MBOAT7	7	OK	18.7594894	83.6251792	0.22432824	0.8225	0.00028789	subgrp_cor	count	1	0
TSEN34	34	OK	1.61196123	0.5705907	2.82507448	0.00472	1.74928051	subgrp_cor	count	1	0
RPS9	859	OK	-0.2499844	0.08758552	-2.8541744	0.00432	-0.3573793	subgrp_cor	count	1	0
LENG8	3	OK	-0.8416402	1.46082315	-0.5761411	0.56452	-0.24034852	subgrp_cor	count	1	0
LENG9	5	OK	-1.6462456	1.93519293	-0.8506881	0.39494	-0.70360391	subgrp_cor	count	1	0
CDC42EP5	103	OK	0.76028799	0.30510916	2.49185566	0.01271	0.98786112	subgrp_cor	count	1	0
RDH13	5	OK	0.3849922	1.3796747	0.27904562	0.78021	0.11983507	subgrp_cor	count	1	0
EPS8L1	3	OK	3.45981462	6.7714513	0.51094137	0.60929	0.00030179	subgrp_cor	count	1	0
PPP1R12C	1	OK	-11.019418	43.2499352	-0.2547846	0.79889	-1.17482461	subgrp_cor	count	1	0
PPP6R1	3	OK	1.58239999	2.72001883	0.58176068	0.56072	0.47433761	subgrp_cor	count	1	0
HSPBP1	6	OK	-0.8328049	1.17911664	-0.7062956	0.48	-0.50770942	subgrp_cor	count	1	0
KMT5C	1	OK	3.45981462	6.7714513	0.51094137	0.60929	0.00030179	subgrp_cor	count	1	0
TMEM238	4	OK	1.39619609	1.41759646	0.98490376	0.32467	0.42408480	subgrp_cor	count	1	0
RPL28	878	OK	-0.0677542	0.08406604	-0.805966	0.42028	-0.09697039	subgrp_cor	count	1	0
UBE2S	67	OK	1.00437923	0.37582438	2.67246957	0.00753	1.27850313	subgrp_cor	count	1	0
ISOC2	22	OK	0.99778239	0.63452756	1.57248076	0.11584	1.13110518	subgrp_cor	count	1	0
NAT14	3	OK	23.9918963	84.8999419	0.28259026	0.77749	1.65081709	subgrp_cor	count	1	0
ZNF579	1	OK	24.3602015	51.7229675	0.47097456	0.63766	2.90627168	subgrp_cor	count	1	0
ZNF524	45	OK	-0.6130846	0.45054054	-1.3607756	0.17358	-0.70749178	subgrp_cor	count	1	0
ZNF580	27	OK	-0.4272421	0.60513528	-0.7060275	0.48017	-0.46589950	subgrp_cor	count	1	0
ZNF581	10	OK	-1.7668902	1.14765726	-1.5395626	0.12367	-1.35908282	subgrp_cor	count	1	0
CCDC106	4	OK	1.25321704	1.54100777	0.81324511	0.41608	0.62618913	subgrp_cor	count	1	0
U2AF2	5	OK	0.45568755	1.46808638	0.3103956	0.75626	0.00030627	subgrp_cor	count	1	0
EPN1	31	OK	1.28229627	0.71360998	1.79691472	0.07235	0.94010420	subgrp_cor	count	1	0
ZNF787	17	OK	-0.1838949	0.71290465	-0.2579516	0.79644	-0.1729018	subgrp_cor	count	1	0
ZNF444	2	OK	1.58239999	2.72001883	0.58176068	0.56072	0.47433761	subgrp_cor	count	1	0
ZNF582	3	OK	-10.442278	41.4658099	-0.2518286	0.80117	-0.00045289	subgrp_cor	count	1	0
ZNF582-AS1	4	OK	2.55522271	2.3498446	1.08740073	0.27686	0.69313893	subgrp_cor	count	1	0
ZNF583	4	OK	-10.967865	27.1536587	-0.4039185	0.68627	-1.17125107	subgrp_cor	count	1	0
ZNF667-AS1	36	OK	-0.486866	0.50232737	-0.9692205	0.33246	-0.47199194	subgrp_cor	count	1	0
ZNF264	9	OK	0.35762204	0.89186977	0.40098011	0.68843	0.11125375	subgrp_cor	count	1	0
ZNF460	1	OK	-12.148421	43.455309	-0.2795612	0.77981	-1.91362624	subgrp_cor	count	1	0
AC005261.1	7	OK	-0.2029437	1.65223657	-0.1227536	0.9022	-4.82E-05	subgrp_cor	count	1	0
ZNF543	1	OK	-11.019418	43.2499352	-0.2547846	0.79889	-1.17482461	subgrp_cor	count	1	0
TRAPPC2B	28	OK	-0.9937582	0.58616379	-1.6953582	0.09001	-1.0290193	subgrp_cor	count	1	0
ZNF548	2	OK	-0.7251858	1.80791919	-0.4011162	0.68832	-0.20979917	subgrp_cor	count	1	0
ZNF772	1	OK	-11.019418	43.2499352	-0.2547846	0.79889	-1.17482461	subgrp_cor	count	1	0
ZNF416	1	OK	-10.442278	41.4658099	-0.2518286	0.80117	-0.00045289	subgrp_cor	count	1	0
ZNF530	1	OK	-10.442278	41.4658099	-0.2518286	0.80117	-0.00045289	subgrp_cor	count	1	0
ZNF587B	1	OK	3.45981462	6.7714513	0.51094137	0.60929	0.00030179	subgrp_cor	count	1	0
ZNF814	3	OK	-2.9057663	2.05474551	-1.4141723	0.15731	-0.00053009	subgrp_cor	count	1	0
ZSCAN18	23	OK	1.73209661	0.94287151	1.83768053	0.06611	0.8238037	subgrp_cor	count	1	0
ZNF274	7	OK	-0.0107357	1.14383436	-0.0093866	0.99251	-0.00889952	subgrp_cor	count	1	0
AC020915.3	1	OK	-11.019418	43.2499352	-0.2547846	0.79889	-1.17482461	subgrp_cor	count	1	0
ZNF8	1	OK	-11.019418	43.2499352	-0.2547846	0.79889	-1.17482461	subgrp_cor	count	1	0
ERVK3-1	1	OK	3.45981462	6.7714513	0.51094137	0.60929	0.00030179	subgrp_cor	count	1	0
AC010642.2	21	OK	0.50306144	0.70323309	0.71535519	0.47429	0.45086198	subgrp_cor	count	1	0
A1BG	3	OK	3.45981559	3.90999315	0.88486487	0.37622	0.00081877	subgrp_cor	count	1	0
RPS5	852	OK	-0.1953216	0.08704097	-2.2440187	0.02482	-0.27930295	subgrp_cor	count	1	0
SLC27A5	13	OK	1.20511345	1.17636768	1.02443604	0.30562	0.76698011	subgrp_cor	count	1	0
TRIM28	27	OK	0.91073957	0.63779747	1.42794479	0.15331	0.92951307	subgrp_cor	count	1	0
CHMP2A	312	OK	0.26596413	0.15910336	1.67164401	0.09459	0.37260355	subgrp_cor	count	1	0
UBE2M	129	OK	0.23995807	0.26501436	0.90545289	0.36522	0.32209624	subgrp_cor	count	1	0
MZF1-AS1	1	OK	3.45981462	6.7714513	0.51094137	0.60929	0.00030179	subgrp_cor	count	1	0
MZF1	1	OK	18.7594894	83.6251792	0.22432824	0.8225	0.00028789	subgrp_cor	count	1	0
RPS4Y1	645	OK	-0.4009	0.10532953	-3.8057884	0.00014	-0.57056821	subgrp_cor	count	1	0
ZFY	1	OK	0.65325287	2.50012846	0.26128772	0.79387	0.00016297	subgrp_cor	count	1	0
ZFY-AS1	1	OK	18.7594894	83.6251792	0.22432824	0.8225	0.00028789	subgrp_cor	count	1	0
TTTY15	1	OK	1.58239999	2.72001883	0.58176068	0.56072	0.47433761	subgrp_cor	count	1	0
DDX3Y	79	OK	-0.4532653	0.32819435	-1.3810870	0.16725	-0.54264883	subgrp_cor	count	1	0
UTY	6	OK	-0.3645825	1.02487382	-0.355734	0.72206	-0.10917092	subgrp_cor	count	1	0

KDM5D	1	OK	3.4598146	6.7714513	0.5109413	0.6093	0.00030179	subgrp_cor	count	1	0
EIF1AY	50	OK	0.98740799	0.4171043	2.3672927	0.01792	1.2343118	subgrp_cor	count	1	0
IL17RA	1	OK	18.759489	83.625179	0.2243282	0.8225	0.00028789	subgrp_cor	count	1	0
HDHD5	3	OK	0.3295269	1.4055480	0.2344472	0.81464	0.10244709	subgrp_cor	count	1	0
CECR2	2	OK	-10.475634	27.491987	-0.381043	0.70317	-1.13811785	subgrp_cor	count	1	0
ATP6V1E1	72	OK	0.7292028	0.3558503	2.0491842	0.04044	0.89942525	subgrp_cor	count	1	0
BCL2L13	4	OK	-0.256573	1.341233	-0.191296	0.84829	-0.12807708	subgrp_cor	count	1	0
BID	22	OK	-0.848329	0.7020344	-1.208387	0.2269	-0.8046046	subgrp_cor	count	1	0
MICAL3	5	OK	3.4598155	3.9099931	0.8848648	0.37622	0.00081877	subgrp_cor	count	1	0
PEX26	4	OK	-0.214986	1.5648174	-0.137387	0.89072	-0.06511836	subgrp_cor	count	1	0
USP18	3	OK	-0.5453205	1.708550	-0.319171	0.7496	-0.0003423	subgrp_cor	count	1	0
DGCR2	3	OK	-1.853784	1.8542582	-0.99969	0.31746	-0.46052349	subgrp_cor	count	1	0
ESS2	1	OK	-10.44227	41.465809	-0.251828	0.80117	-0.00045289	subgrp_cor	count	1	0
SLC25A1	11	OK	1.0459855	0.9830539	1.0640164	0.28732	0.67146344	subgrp_cor	count	1	0
C22orf39	15	OK	-0.934728	0.8090054	-1.155417	0.24792	-0.73408542	subgrp_cor	count	1	0
MRPL40	66	OK	1.2071663	0.4135557	2.9189933	0.00351	1.52984422	subgrp_cor	count	1	0
UFD1	124	OK	1.2316721	0.3074189	4.0065199	6.16E-05	1.61745753	subgrp_cor	count	0.61262609	0
CDC45	1	OK	23.991896	84.899941	0.2825902	0.77749	1.65081709	subgrp_cor	count	1	0
CLDN5	360	OK	0.1122654	0.1374108	0.8170054	0.41392	0.1590258	subgrp_cor	count	1	0
TBX1	31	OK	6.9999925	2.636403	2.655124	0.00792	3.47950129	subgrp_cor	count	1	0
RTL10	8	OK	2.2565289	1.3693670	1.6478627	0.09938	1.02628821	subgrp_cor	count	1	0
TXNRD2	10	OK	23.085901	54.06396	0.4270108	0.66937	1.62231891	subgrp_cor	count	1	0
ARVCF	1	OK	18.759489	83.625179	0.2243282	0.8225	0.00028789	subgrp_cor	count	1	0
RANBP1	153	OK	0.2046325	0.2382260	0.858984	0.39035	0.27794738	subgrp_cor	count	1	0
ZDHHC8	2	OK	-10.475634	27.491987	-0.381043	0.70317	-1.13811785	subgrp_cor	count	1	0
DGCR6L	36	OK	0.2589116	0.4603681	0.5624013	0.57384	0.31038407	subgrp_cor	count	1	0
ZNF74	1	OK	3.4598146	6.7714513	0.5109413	0.6093	0.00030179	subgrp_cor	count	1	0
KLHL22	1	OK	23.991896	84.899941	0.2825902	0.77749	1.65081709	subgrp_cor	count	1	0
PI4KA	2	OK	3.4598146	6.7714513	0.5109413	0.6093	0.00030179	subgrp_cor	count	1	0
SNAP29	14	OK	-0.514340	0.8006248	-0.642423	0.5206	-0.41628951	subgrp_cor	count	1	0
CRKL	2	OK	21.105322	83.341273	0.2532397	0.80009	0.0003103	subgrp_cor	count	1	0
LZTR1	1	OK	3.4598146	6.7714513	0.5109413	0.6093	0.00030179	subgrp_cor	count	1	0
THAP7	7	OK	-0.429708	1.0483136	-0.409904	0.68188	-0.3153867	subgrp_cor	count	1	0
UBE2L3	155	OK	0.1977717	0.2367843	0.835229	0.40358	0.26337255	subgrp_cor	count	1	0
YDJC	2	OK	-10.44227	41.465809	-0.251828	0.80117	-0.00045289	subgrp_cor	count	1	0
SDF2L1	44	OK	0.8079759	0.5039825	1.6031825	0.10889	0.90491233	subgrp_cor	count	1	0
PPIL2	2	OK	3.4598146	6.7714513	0.5109413	0.6093	0.00030179	subgrp_cor	count	1	0
YPEL1	2	OK	3.4598149	4.7858686	0.7229230	0.46973	0.00081935	subgrp_cor	count	1	0
MAPK1	13	OK	1.4854041	0.9446646	1.5724142	0.11585	1.07362533	subgrp_cor	count	1	0
PPM1F	13	OK	0.7102809	1.2254296	0.5796179	0.56217	0.00131063	subgrp_cor	count	1	0
IGLC2	75	OK	1.0285037	0.5078329	2.0252797	0.04284	0.00192167	subgrp_cor	count	1	0
IGLC3	13	OK	1.4665354	1.4087906	1.0409889	0.29789	3.75E-05	subgrp_cor	count	1	0
BCR	25	OK	-0.670527	0.6820027	-0.983174	0.32552	-0.57775993	subgrp_cor	count	1	0
CHCHD10	50	OK	3.6945553	0.8327786	4.4364194	9.15E-06	3.71356143	subgrp_cor	count	0.09161383	0
SMARCB1	30	OK	0.2065721	0.5901067	0.3500589	0.7263	0.22832627	subgrp_cor	count	1	0
SLC2A11	1	OK	3.4598146	6.7714513	0.5109413	0.6093	0.00030179	subgrp_cor	count	1	0
MIF	191	OK	-0.029634	0.2131296	-0.139043	0.88942	-0.04078923	subgrp_cor	count	1	0
GSTT2B	20	OK	1.8202265	0.7743691	2.350592	0.01874	1.81554143	subgrp_cor	count	1	0
DDTL	1	OK	-10.44227	41.465809	-0.251828	0.80117	-0.00045289	subgrp_cor	count	1	0
DDT	209	OK	-0.013545	0.2024361	-0.066910	0.94665	-0.01875232	subgrp_cor	count	1	0
CABIN1	4	OK	-0.615417	1.4920971	-0.412451	0.68001	-0.29861013	subgrp_cor	count	1	0
GGT5	19	OK	0.810219	0.7262445	1.1156288	0.26458	0.72532021	subgrp_cor	count	1	0
GUCD1	8	OK	-2.928894	1.6352869	-1.790925	0.0732	-0.61462545	subgrp_cor	count	1	0
SNRPD3	191	OK	0.3623661	0.2147684	1.6872414	0.09156	0.49445914	subgrp_cor	count	1	0
SGSM1	1	OK	41.788303	174.2960	0.2397546	0.81052	0.00014488	subgrp_cor	count	1	0
KIAA1671	12	OK	-1.714941	1.0632039	-1.612994	0.10675	-1.0823653	subgrp_cor	count	1	0
HPS4	6	OK	-0.097120	1.0546481	-0.092088	0.92662	-0.06268831	subgrp_cor	count	1	0
SRRD	6	OK	0.974608	1.1459232	0.8505009	0.39505	0.72654740	subgrp_cor	count	1	0
TPST2	54	OK	-0.562642	0.3804531	-1.478874	0.13917	-0.67728603	subgrp_cor	count	1	0
MIAT	6	OK	19.103032	50.772469	0.3762478	0.70672	0.00078880	subgrp_cor	count	1	0
PITPNB	74	OK	-0.189731	0.3607941	-0.525872	0.59898	-0.23214195	subgrp_cor	count	1	0
TTC28	6	OK	-1.000177	1.1953148	-0.836748	0.40272	-0.28026428	subgrp_cor	count	1	0
HSCB	19	OK	-0.105102	0.6912883	-0.152038	0.87916	-0.09892075	subgrp_cor	count	1	0
CCDC117	3	OK	-13.33569	29.112233	-0.458078	0.6469	-1.33917170	subgrp_cor	count	1	0
XBP1	77	OK	0.6755460	0.3512143	1.9234581	0.05442	0.86033013	subgrp_cor	count	1	0
Z93930.2	1	OK	22.971512	51.721583	0.444051	0.65701	2.36236892	subgrp_cor	count	1	0
KREMEN1	2	OK	-0.202940	1.6532365	-0.122753	0.9022	-4.82E-05	subgrp_cor	count	1	0
RHBDD3	2	OK	-11.01941	43.249935	-0.254784	0.79889	-1.17482461	subgrp_cor	count	1	0
EWSR1	57	OK	0.5056835	0.398164	1.270027	0.20407	0.58735782	subgrp_cor	count	1	0
GAS2L1	26	OK	0.1987898	0.5517939	0.3602619	0.71865	0.16552581	subgrp_cor	count	1	0
RASL10A	10	OK	0.1585854	0.8991977	0.1763632	0.86001	0.08074857	subgrp_cor	count	1	0
AP1B1	4	OK	-0.597961	1.3409867	-0.445911	0.65566	-0.30195179	subgrp_cor	count	1	0
THOC5	1	OK	3.4598146	6.7714513	0.5109413	0.6093	0.00030179	subgrp_cor	count	1	0
NIPSNAP1	2	OK	21.64205	54.486891	0.3971975	0.69122	1.57744735	subgrp_cor	count	1	0
ZMAT5	14	OK	0.8484752	0.8078346	1.0503080	0.29358	0.79829186	subgrp_cor	count	1	0
UQCR10	145	OK	0.641797	0.2551702	2.5151737	0.0119	0.86187430	subgrp_cor	count	1	0
ASCC2	4	OK	2.5552227	2.349844	1.0874007	0.27686	0.69313893	subgrp_cor	count	1	0
MTMR3	2	OK	-2.378575	2.4369360	-0.976051	0.32906	-6.09E-05	subgrp_cor	count	1	0
TBC1D10A	7	OK	0.7544778	1.139986	0.661832	0.50808	0.00139472	subgrp_cor	count	1	0
SF3A1	12	OK	-0.882349	0.908099	-0.972744	0.33068	-0.69726907	subgrp_cor	count	1	0
CCDC157	1	OK	-10.44227	41.465809	-0.251828	0.80117	-0.00045289	subgrp_cor	count	1	0
SEC14L2	1	OK	0.6532528	2.5001284	0.2612877	0.79387	0.00016297	subgrp_cor	count	1	0
PES1.00	6	OK	-0.342273	1.4045035	-0.243697	0.80747	-0.00059237	subgrp_cor	count	1	0
TCN2	99	OK	1.0685138	0.3129258	3.4145912	0.00064	1.38682592	subgrp_cor	count	1	0
SLC35E4	6	OK	2.6672823	2.2418055	1.1897920	0.23412	0.71356325	subgrp_cor	count	1	0
DUSP18	5	OK	0.1681593	1.2198591	0.1378514	0.89036	0.08564646	subgrp_cor	count	1	0
MORC2-AS1	1	OK	-11.01941	43.249935	-0.254784	0.79889	-1.17482461	subgrp_cor	count	1	0
SMTN	4	OK	-0.5453205	1.708550	-0.319171	0.7496	-0.0003423	subgrp_cor	count	1	0
SELENOM	502	OK	0.4652163	0.1169419	3.9790372	6.92E-05	0.66190548	subgrp_cor	count	0.68752061	0
LIMK2	10	OK	0.8876562	1.0261343	0.8650487	0.38701	0.27589058	subgrp_cor	count	1	0
PIK3IP1	21	OK	-0.556724	0.5799010	-0.960033	0.33706	-0.57561187	subgrp_cor	count	1	0
PATZ1	1	OK	41.788303	174.2960	0.2297546	0.81052	0.00014488	subgrp_cor	count	1	0
DRG1	9	OK	-0.256599	0.9478544	-0.270716	0.78661	-0.19024146	subgrp_cor	count	1	0
EIF4ENIF1	2	OK	-12.14842	43.455309	-0.279561	0.77981	-1.91362624	subgrp_cor	count	1	0
PISD	1	OK	3.4598146	6.7714513	0.5109413	0.6093	0.00030179	subgrp_cor	count	1	0
PRR14L	11	OK	1.5037442	1.1936551	1.2597812	0.20775	0.43040637	subgrp_cor	count	1	0
YWHAH	198	OK	0.4314032	0.210933	2.0452117	0.04082	0.57899825	subgrp_cor	count	1	0
RTCB	40	OK	-0.195208	0.4735210	-0.412248	0.68016	-0.19155030	subgrp_cor	count	1	0
FBXO7	44	OK	0.1995410	0.4208692	0.4741164	0.63542	0.23414928	subgrp_cor	count	1	0
HMGXB4	28	OK	-0.077586	0.5888200	-0.131765	0.89517	-0.08701208	subgrp_cor	count	1	0
TOM1	1	OK	-10.44227	41.465809	-0.251828	0.80117	-0.00045289	subgrp_cor	count	1	0
HMOX1	16	OK	2.1391900	1.0981500	1.9479943	0.05142	1.45988481	subgrp_cor	count	1	0
APOL6	13	OK	-1.488546	0.9175500	-1.622305	0.10476	-0.9943349	subgrp_cor	count	1	0
RBFOX2	27	OK	0.3736982	0.6098636	0.6127570	0.54006	0.35449879	subgrp_cor	count	1	0
APOL3	25	OK	1.6448917	0.6827128	2.4093463	0.01598	1.15826698	subgrp_cor	count	1	0
APOL4	3	OK	3.4597918	2.5591854	1.3519113	0.1764	0.00221855	subgrp_cor	count	1	0
APOL2	15	OK	-0.295010	0.760489	-0.387923	0.69807	-0.1468903	subgrp_cor	count	1	0
APOL1	47	OK	0.7880556	0.4282957	1.8399798	0.06577	0.65339582	subgrp_cor	count	1	0
MYH9	320	OK	-0.139336	0.1502096	-0.927615	0.35361	-0.19451929	subgrp_cor	count	1	0
TXN2	105	OK	-0.009957	0.2881768	-0.034553	0.97244	-0.01309033	subgrp_cor	count	1	0
EIF3D	182	OK	-0.288121	0.2237001	-1.287981	0.19775	-0.38186036	subgrp_cor	count	1	0
IFT27	15	OK	1.0837822	0.8285971	1.3079724	0.19088	1.05554763	subgrp_cor	count	1	0
CSF2RB	22	OK	-0.030170	0.678405	-0.044472	0.96452	-0.02971833	subgrp_cor	count	1	0
TST	17	OK	0.9015477	0.8051122	1.1197789	0.26281	0.00167744	subgrp_cor	count	1	0
MPST	48	OK	0.8511850	0.4226144	2.0140935	0.044	1.0546191	subgrp_cor	count	1	0
KCTD17	3	OK	-0.150975	1.96881	-0.076683	0.93888	-9.78E-05	subgrp_cor	count	1	0
RAC2	4	OK	2.4096237	2.4715206	0.9749599	0.32958	0.66517343	subgrp_cor	count	1	0
MFNG	57	OK	-0.407959	0.3762217	-1.084359	0.27821	-0.5170336	subgrp_cor	count	1	0
CARD10	10	OK	-1.440334	1.033156	-1.394111	0.16328	-0.38049042	subgrp_cor	count	1	0
CDC42EP1	27	OK	0.1874106	0.6736167	0.2782155	0.78085	0.17733112	subgrp_cor	count	1	0
GGA1	3	OK	3.4598155	3.9099931	0.8848648	0.37622	0.00081877	subgrp_cor	count	1	0
NOL12	1	OK	-10.44227	41.465809	-0.251828	0.80117	-0.00045289	subgrp_cor	count	1	0
TRIOBP	78	OK	-0.639225	0.3356401	-1.904496	0.05685	-0.75366972	subgrp_cor	count	1	0
H1F0	49	OK	0.0854324	0.4100048	0.2083692	0.83494	0.10623879	subgrp_cor	count	1	0
GCAT	4	OK	0.1037183	1.5399871	0.0673501	0.9462	0.08473990	subgrp_cor	count	1	0
ANKRD54	5	OK	-13.33569	29.112233	-0.458078	0.6469	-1.33917170	subgrp_cor	count	1	0
EIF3L	252	OK	-0.204600	0.1736426	-1.178286	0.23868	-0.27875933	subgrp_cor	count	1	0
POLR2F	70	OK	0.0700828	0.3603841	0.1944671	0.84581	0.08468579	subgrp_cor	count	1	0
PICK1	1	OK	-10.44227	41.465809	-0.251828	0.80117	-0.00045289	subgrp_cor	count	1	0
MAFF	135	OK	-0.698770	0.2460558	-2.839885	0.00451	-0.92579695	subgrp_cor	count	1	0
TMEM184B	7	OK	4.6612686	4.0891078	1.1399475	0.25433	0.00274223	subgrp_cor	count	1	0
CSNK1E	11	OK	-1.681165	1.2409215	-1.354772	0.17549	-0.71485242	subgrp_cor	count	1	0
KDELR3	21	OK	1.8992945	0.8192639	2.318292	0.02043	1.57962857	subgrp_cor	count	1	0
DDX17	269	OK	0.4072888	0.1798405	2.2647216	0.02353	0.56559334	subgrp_cor	count	1	0
CBY1	16	OK	0.3348891	0.7415850	0.4515794	0.65157	0.27920085	subgrp_cor	count	1	0
TOMM22	94	OK	0.2875435	0.306101	0.9393740	0.34754	0.37223170	subgrp_cor	count	1	0
JOSD1	21	OK	1.2967709	0.7757519	1.6716309	0.0946	1.12467409	subgrp_cor	count	1	0
GTPBP1	1	OK	-10.44227	41.465809	-0.251828	0.80117	-0.00045289	subgrp_cor	count	1	0
SUN2	19	OK	2.5403310	0.9695765	2.6200418	0.00879	1.818842	subgrp_cor	count	1	0
DNAL4	4	OK	-1.698643	1.9355446	-0.877605	0.38016	-1.09785319	subgrp_cor	count	1	0
NPTXR	1	OK	0.6532528	2.5001284	0.2612877	0.79387	0.00016297	subgrp_cor	count	1	0
CBX6	20	OK	2.1491821	0.8590218	2.5018945	0.01235	1.47352632	subgrp_cor	count	1	0
APOBEC3C	3	OK	18.759489	83.625179	0.2243282	0.8225	0.00028789	subgrp_cor	count	1	0
APOBEC3D	1	OK	-10.44227	41.465809	-0.251828	0.80117	-0.00045289	subgrp_cor	count	1	0
APOBEC3G	4	OK	-2.436168	1.9725948	-1.234281	0.21709	-0.00126310	subgrp_cor	count	1	0
CBX7	5	OK	3.2756868	2.2671298	1.444860	0.1485	0.80916594	subgrp_cor	count	1	0
PDGFB	40	OK	0.7318064	0.5147724	1.4216117	0.15514	0.74765659	subgrp_cor	count	1	0
RPL3	877	OK	-0.175518	0.085089	-2.062763	0.03914	-0.25109052	subgrp_cor	count	1	0
TAB1	3	OK	0.5887819	1.767414	0.3331317	0.73903	0.30122869	subgrp_cor	count	1	0
MGAT3	1	OK	3.4598146	6.7714513	0.5109413	0.6093	0.00030179	subgrp_cor	count	1	0
MIEF1	3	OK	-0.615417	1.4920971	-0.412451	0.68001	-0.29861013	subgrp_cor	count	1	0
ATF4	172	OK	-0.614624	0.2144979	-2.865408	0.00416	-0.81546575	subgrp_cor	count	1	0
RPS19BP1	156	OK	-0.078996	0.2303770	-0.342901	0.73167	-0.10788256	subgrp_cor	count	1	0
TNRC6B	124	OK	-0.363354	0.2501693	-1.452434	0.14638	-0.46634946	subgrp_cor	count	1	0
ADSL	2	OK	1.5822999	2.7200188	0.5817606	0.56072	0.47433761	subgrp_cor	count	1	0
MKL1	2	OK	23.991896	84.899941	0.2825902	0.77749	1.65081709	subgrp_cor	count	1	0
SLC25A17	2	OK	-0.725185	1.8079191	-0.401116	0.68832	-0.20979917	subgrp_cor	count	1	0
ST13	429	OK	-0.104388	0.1296267	-0.805296	0.42065	-0.14728968	subgrp_cor	count	1	0
XPNPEP3	4	OK	-1.340131	1.6819410	-0.796776	0.42558	-0.59807560	subgrp_cor	count	1	0
RBX1	253	OK	-0.139605	0.1785462	-0.781899	0.43427	-0.19392409	subgrp_cor	count	1	0
EP300	12	OK	-1.396709	0.966840	-1.444611	0.14857	-0.61853489	subgrp_cor	count	1	0
L3MBTL2	4	OK	-0.800985	1.3974209	-0.573188	0.56652	-0.38157489	subgrp_cor	count	1	0
CHADL	1	OK	18.759489	83.625179	0.2243282	0.8225	0.00028789	subgrp_cor	count	1	0
RANGAP1	5	OK	32.04112	42.148112	0.7602030	0.44713	0.00151978	subgrp_cor	count	1	0
ZC3H7B	4	OK	1.8342002	1.6829423	1.0898770	0.27577	0.53818379	subgrp_cor	count	1	0
TEF	3	OK	0.1721614	1.534450	0.1121974	0.91067	0.00011395	subgrp_cor	count	1	0
TOB2	24	OK	-0.110714	0.6641566	-0.166699	0.86761	-0.09143973	subgrp_cor	count	1	0
PHF5A	46	OK	-0.341210	0.4268139	-0.799436	0.42406	-0.4017230	subgrp_cor	count	1	0
ACO2	10	OK	-0.238898	0.8831747	-0.270500	0.78679	-0.19565098	subgrp_cor	count	1	0
POLR3H	5	OK	1.1581985	1.6544505	0.7000502	0.4839	0.00079558	subgrp_cor	count	1	0
DESI1	5	OK	4.6612686	4.0891078	1.1399475	0.25433	0.00274223	subgrp_cor	count	1	0
XRCC6	141	OK	0.4150548	0.2523946	1.6444680	0.10008	0.54730883	subgrp_cor	count	1	0
SNU13	358	OK	0.0602051	0.1493847	0.4030209	0.68692	0.08434994	subgrp_cor	count	1	0
C22orf46	1	OK	3.4598146	6.7714513	0.5109413	0.6093	0.00030179	subgrp_cor	count	1	0
AL021453.1	2	OK	-10.44227	41.465809	-0.251828	0.80117	-0.00045289	subgrp_cor	count	1	0
SREBF2	2	OK	3.4598149	4.7858686	0.7229230	0.46973	0.00081935	subgrp_cor	count	1	0
NAGA	3	OK	4.2108437	4.2958243	0.9802178	0.32698	7.14E-14	subgrp_cor	count	1	0
PHETA2	1	OK	18.759489	83.625179	0.2243282	0.8225	0.00028789	subgrp_cor	count	1	0
SMDT1	200	OK	0.0477702	0.1965285	0.2430703	0.80795	0.06580555	subgrp_cor	count	1	0
NDUFA6	98	OK	0.8522368	0.3192504	2.669493	0.0076	1.09560504	subgrp_cor	count	1	0
RRP7A	30	OK	0.2043424	0.485647	0.4207628	0.67392	0.25097360	subgrp_cor	count	1	0
POLDIP3	3	OK	3.4598149	4.7858686	0.7229230	0.46973	0.00081935	subgrp_cor	count	1	0
Z93241.1	2	OK	-10.475634	27.491987	-0.381043	0.70317	-1.13811785	subgrp_cor	count	1	0
CYB5R3	339	OK	0.5152951	0.1451875	3.549166	0.00039	0.72459436	subgrp_cor	count	1	0
A4GALT	33	OK	-0.875662	0.4772299	-1.834882	0.06652	-0.93491863	subgrp_cor	count	1	0
ARFGAP3	56	OK	1.1339994	0.4426083	2.5620833	0.0104	1.34210333	subgrp_cor	count	1	0
PACSIN2	8	OK	-0.796004	1.1365422	-0.700373	0.48369	-0.37940097	subgrp_cor	count	1	0
TTLL1	2	OK	1.925864	2.5154879	0.765642	0.44389	0.56022119	subgrp_cor	count	1	0
BIK	1	OK	-11.01941	43.249935	-0.254784	0.79889	-1.17482461	subgrp_cor	count	1	0
MCAT	1	OK	1.5822999	2.7200188	0.5817606	0.56072	0.47433761	subgrp_cor	count	1	0
TSPO	565	OK	0.1594287	0.1132911	1.4073360	0.15932	0.22686629	subgrp_cor	count	1	0
SAMM50	22	OK	1.0179061	0.7801177	1.3048108	0.19196	0.51501068	subgrp_cor	count	1	0
PARVB	92	OK	0.041938	0.2936489	0.1428167	0.88642	0.054418	subgrp_cor	count	1	0
PRR5	8	OK	-2.647708	1.4047637	-1.884807	0.05946	-1.45232929	subgrp_cor	count	1	0
NUP50-DT	1	OK	-11.01941	43.249935	-0.254784	0.79889	-1.17482461	subgrp_cor	count	1	0
NUP50	13	OK	0.0445667	0.8637065	0.0515994	0.95885	0.02885602	subgrp_cor	count	1	0
KIAA0930	4	OK	21.105322	83.341273	0.2532397	0.80009	0.0003103	subgrp_cor	count	1	0
FAM118A	32	OK	1.3377980	0.6416470	2.0849437	0.03707	1.4109360	subgrp_cor	count	1	0
FBLN1	1	OK	1.582381	1.2168202	1.3004230	0.19346	1.25554822	subgrp_cor	count	1	0
ATXN10	46	OK	0.7935344	0.4342137	1.8275202	0.06762	0.97117927	subgrp_cor	count	1	0
PRR34-AS1	2	OK	-2.920220	2.7480114	-1.062666	0.28792	-0.00053078	subgrp_cor	count	1	0
MIRLET7BHG	3	OK	-0.174796	1.820880	-0.095990	0.92352	-0.05309252	subgrp_cor	count	1	0
PPARA	5	OK	0.8665002	1.2872569	0.6731369	0.50086	0.26945087	subgrp_cor	count	1	0
CDPF1	2	OK	23.622221	53.950611	0.4378508	0.66149	2.38672454	subgrp_cor	count	1	0
TTC38	2	OK	3.4598146	6.7714513	0.5109413	0.6093	0.00030179	subgrp_cor	count	1	0
TRMU	2	OK	0.1681491	1.7253200	0.097459	0.92236	0.05201277	subgrp_cor	count	1	0
CELSR1	1	OK	3.4598146	6.7714513	0.5109413	0.6093	0.00030179	subgrp_cor	count	1	0
CERK	7	OK	1.010366	1.260543	0.8015325	0.42282	0.31288283	subgrp_cor	count	1	0
AL118516.1	27	OK	-0.686679	0.5765598	-1.190994	0.23366	-0.76893098	subgrp_cor	count	1	0
TBC1D22A	5	OK	1.4119426	1.4959308	0.9438555	0.34524	0.69797651	subgrp_cor	count	1	0
FAM19A5	2	OK	3.4598146	6.7714513	0.5109413	0.6093	0.00030179	subgrp_cor	count	1	0
BRD1	10	OK	-0.008524	0.9167594	-0.009298	0.99258	-0.00550512	subgrp_cor	count	1	0
ALG12	5	OK	6.4280329	5.2748401	1.2186213	0.2229	1.06669691	subgrp_cor	count	1	0
CRELD2	40	OK	1.7753699	0.6059740	2.9297776	0.00339	1.90567504	subgrp_cor	count	1	0
MOV10L1	2	OK	23.991896	84.899941	0.2825902	0.77749	1.65081709	subgrp_cor	count	1	0
TRABD	7	OK	-12.85333	17.046726	-0.754006	0.45085	-3.06078019	subgrp_cor	count	1	0
AL022328.4	1	OK	-15.99503	70.34942	-0.227365	0.82014	-0.00028474	subgrp_cor	count	1	0
SELENOO	2	OK	3.4598146	6.7714513	0.5109413	0.6093	0.00030179	subgrp_cor	count	1	0
MAPK12	12	OK	-0.039634	0.9807307	-0.040412	0.96776	-0.03283288	subgrp_cor	count	1	0
MAPK11	25	OK	-1.001390	0.5826551	-1.718667	0.08567	-1.0042418	subgrp_cor	count	1	0
PLXNB2	2	OK	18.759489	83.625179	0.2243282	0.8225	0.00028789	subgrp_cor	count	1	0
DENND6B	1	OK	3.4598146	6.7714513	0.5109413	0.6093	0.00030179	subgrp_cor	count	1	0
PPP6R2	3	OK	4.2109206	4.2875616	0.982124	0.32604	0.00257090	subgrp_cor	count	1	0
LMF2	21	OK	0.4242289	0.6210678	0.6830637	0.49457	0.40323996	subgrp_cor	count	1	0
NCAPH2	1	OK	1.5822999	2.7200188	0.5817606	0.56072	0.47433761	subgrp_cor	count	1	0
TYMP	59	OK	-1.236990	0.3973063	-3.113442	0.00185	-1.44636486	subgrp_cor	count	1	0
ODF3B	65	OK	-1.160838	0.3660210	-3.171508	0.00152	-1.48982424	subgrp_cor	count	1	0
ARSA	7	OK	1.523692	1.2416064	1.235248	0.21674	0.75103270	subgrp_cor	count	1	0
SHANK3	14	OK	1.6546296	1.028233	1.6091960	0.10757	1.881508	subgrp_cor	count	1	0
RABL2B	1	OK	3.4597878	3.9017207	0.8867338	0.37522	0.00222136	subgrp_cor	count	1	0
RBM11	1	OK	-10.44227	41.465809	-0.251828	0.80117	-0.00045289	subgrp_cor	count	1	0
HSPA13	3	OK	3.4598146	6.7714513	0.5109413	0.6093	0.00030179	subgrp_cor	count	1	0
NRIP1	12	OK	0.7921319	1.0372008	0.7637209	0.44503	0.41090267	subgrp_cor	count	1	0
USP25	8	OK	-0.209125	1.0874174	-0.192313	0.8475	-0.10467736	subgrp_cor	count	1	0
MIR99AHG	41	OK	3.6836094	0.9067590	4.0623905	4.86E-05	3.38619164	subgrp_cor	count	0.48344443	0
BTG3	76	OK	-0.01434	0.2223629	-0.064508	0.9645	-0.01793245	subgrp_cor	count	1	0
CHODL	2	OK	21.105322	83.341273	0.2532397	0.80009	0.0003103	subgrp_cor	count	1	0
MIR155HG	8	OK	-0.127802	0.8902644	-0.143556	0.88585	-0.08211282	subgrp_cor	count	1	0
MRPL39	8	OK	-1.556364	1.2121941	-1.283923	0.19917	-0.6729377	subgrp_cor	count	1	0
ATP5PF	522	OK	0.0999568	0.1187946	0.8414262	0.40011	0.14182448	subgrp_cor	count	1	0
GABPA	20	OK	0.7079210	0.7097476	0.9974263	0.31856	0.36163180	subgrp_cor	count	1	0
APP	345	OK	0.203838	0.1484783	1.372849	0.1698	0.28483224	subgrp_cor	count	1	0
ADAMTS1	321	OK	0.2251490	0.1482171	1.5190488	0.12875	0.3164153	subgrp_cor	count	1	0
ADAMTS5	7	OK	2.1611157	1.6737947	1.2911474	0.19665	0.99299457	subgrp_cor	count	1	0
N6AMT1	2	OK	4.6613299	5.7683860	0.8080821	0.41904	0.00274918	subgrp_cor	count	1	0
LTN1	13	OK	0.5654248	0.8895323	0.6356428	0.52501	0.28932796	subgrp_cor	count	1	0
RWDD2B	5	OK	-2.680455	2.2252050	-1.204588	0.22836	-0.92874357	subgrp_cor	count	1	0
USP16	54	OK	0.4995943	0.3959469	1.2617707	0.20702	0.59868185	subgrp_cor	count	1	0
CCT8	154	OK	0.3891512	0.2399197	1.6220059	0.1048	0.52632748	subgrp_cor	count	1	0
MAP3K7CL	1	OK	3.4598146	6.7714513	0.5109413	0.6093	0.00030179	subgrp_cor	count	1	0
BACH1	14	OK	1.2896929	0.9777042	1.3191034	0.18712	0.9029309	subgrp_cor	count	1	0
TIAM1	2	OK	-1.758573	2.3554439	-0.746599	0.45533	-0.00036089	subgrp_cor	count	1	0
SOD1	426	OK	-0.001740	0.129002	-0.013490	0.98924	-0.00246372	subgrp_cor	count	1	0
SCAF4	2	OK	4.6613299	5.7683860	0.8080821	0.41904	0.00274918	subgrp_cor	count	1	0
MIS18A	3	OK	-0.972752	1.6375112	-0.594043	0.55248	-0.27350085	subgrp_cor	count	1	0
URB1	1	OK	-10.44227	41.465809	-0.251828	0.80117	-0.00045289	subgrp_cor	count	1	0
URB1-AS1	1	OK	-10.44227	41.465809	-0.251828	0.80117	-0.00045289	subgrp_cor	count	1	0
EVA1C	82	OK	0.3156161	0.321804	0.9807708	0.32671	0.39033782	subgrp_cor	count	1	0
CFAP298	40	OK	0.0740616	0.468595	0.1580502	0.87442	0.08004383	subgrp_cor	count	1	0
SYNJ1	1	OK	3.4598146	6.7714513	0.5109413	0.6093	0.00030179	subgrp_cor	count	1	0
PAXBP1	17	OK	-0.225851	0.7886497	-0.286504	0.77449	-0.16697886	subgrp_cor	count	1	0
C21orf62-AS1	1	OK	18.759489	83.625179	0.2243282	0.8225	0.00028789	subgrp_cor	count	1	0
IFNAR2	15	OK	-0.409863	0.8483057	-0.483155	0.62899	-0.30073061	subgrp_cor	count	1	0
IFNAR1	85	OK	0.4546855	0.329853	1.3784481	0.16806	0.59027881	subgrp_cor	count	1	0
IFNGR2	39	OK	0.2568244	0.4705452	0.5458018	0.5852	0.28290798	subgrp_cor	count	1	0
TMEM50B	59	OK	0.0594304	0.3749323	0.1585097	0.87406	0.07046010	subgrp_cor	count	1	0
DNAJC28	1	OK	18.759489	83.625179	0.2243282	0.8225	0.00028789	subgrp_cor	count	1	0
GART	6	OK	2.4666988	2.1392766	1.1530528	0.24889	0.67632650	subgrp_cor	count	1	0
SON	428	OK	0.186065	0.1311848	1.4183489	0.15609	0.2617666	subgrp_cor	count	1	0
CRYZL1	12	OK	-0.0051	0.8167856	-0.006244	0.99502	-0.00329489	subgrp_cor	count	1	0
ITSN1	18	OK	0.1360671	0.6905821	0.1970325	0.8438	0.11318192	subgrp_cor	count	1	0
ATP5PO	222	OK	-0.028383	0.1535195	-0.184887	0.85332	-0.03971198	subgrp_cor	count	1	0
MRPS6	52	OK	2.1204787	0.492731	4.3035141	1.68E-05	2.58462489	subgrp_cor	count	0.16805964	0
SLC5A3	6	OK	2.3215290	1.8148839	1.2791540	0.20084	1.55838210	subgrp_cor	count	1	0
RCAN1	82	OK	-0.752649	0.3492453	-2.155074	0.03116	-0.97349985	subgrp_cor	count	1	0
SETD4	2	OK	1.925864	2.5154879	0.765642	0.44389	0.56022119	subgrp_cor	count	1	0
CBR1	14	OK	0.3026995	0.7349250	0.4118780	0.68042	0.27138515	subgrp_cor	count	1	0
CBR3	46	OK	-2.001452	0.5045682	-3.966663	7.29E-05	-2.3841997	subgrp_cor	count	0.72404675	0
DOPEY2	2	OK	3.4598149	4.7858686	0.7229230	0.46973	0.00081935	subgrp_cor	count	1	0
AP000692.2	2	OK	-1.031165	1.7479538	-0.589927	0.55524	-0.00022765	subgrp_cor	count	1	0
MORC3	13	OK	-1.277403	0.8985851	-1.421572	0.15515	-0.57489007	subgrp_cor	count	1	0
CHAF1B	2	OK	-10.44227	41.465809	-0.251828	0.80117	-0.00045289	subgrp_cor	count	1	0
PIGP	43	OK	1.0597725	0.4643089	2.2824728	0.02246	1.25825496	subgrp_cor	count	1	0
VPS26C	4	OK	-0.910188	1.5464254	-0.588576	0.55615	-0.42847801	subgrp_cor	count	1	0
AP001437.1	1	OK	-15.99503	70.34942	-0.227365	0.82014	-0.00028474	subgrp_cor	count	1	0
DYRK1A	8	OK	-0.841599	1.033349	-0.814438	0.41539	-0.39919144	subgrp_cor	count	1	0
KCNJ15	1	OK	-15.99503	70.34942	-0.227365	0.82014	-0.00028474	subgrp_cor	count	1	0
ERG	141	OK	-0.15133	0.2283886	-0.662616	0.50758	-0.19918933	subgrp_cor	count	1	0
AP001043.1	1	OK	-11.01941	43.249935	-0.254784	0.79889	-1.17482461	subgrp_cor	count	1	0
PSMG1	14	OK	-0.067247	0.8587519	-0.078308	0.93758	-0.0205648	subgrp_cor	count	1	0
BRWD1	15	OK	-1.070263	0.9938030	-1.076937	0.28151	-0.62458210	subgrp_cor	count	1	0
HMGN1	305	OK	0.3992910	0.1618848	2.4665127	0.01364	0.55823117	subgrp_cor	count	1	0
WRB	25	OK	0.2837890	0.5807043	0.488698	0.62506	0.32912957	subgrp_cor	count	1	0
LCA5L	1	OK	0.6532528	2.5001284	0.2612877	0.79387	0.00016297	subgrp_cor	count	1	0
SH3BGR	1	OK	23.991896	84.899941	0.2825902	0.77749	1.65081709	subgrp_cor	count	1	0
LINC00323	1	OK	18.759489	83.625179	0.2243282	0.8225	0.00028789	subgrp_cor	count	1	0
BACE2	154	OK	0.3163391	0.2315761	1.3660262	0.17192	0.43202605	subgrp_cor	count	1	0
MX2	9	OK	0.4461548	1.0511044	0.4244629	0.67123	0.13978012	subgrp_cor	count	1	0
MX1	106	OK	0.1048233	0.2653898	0.3950186	0.69282	0.13789471	subgrp_cor	count	1	0
C2CD2	4	OK	-0.465429	1.3093639	-0.355462	0.72224	-0.13816105	subgrp_cor	count	1	0
ZBTB21	8	OK	-1.549088	1.158653	-1.336972	0.18123	-0.65489782	subgrp_cor	count	1	0
ABCG1	24	OK	-0.716865	0.606744	-1.181495	0.23741	-0.61742290	subgrp_cor	count	1	0
TFF3	40	OK	-0.846461	0.5092965	-1.662021	0.09651	-0.83452240	subgrp_cor	count	1	0
PDE9A	5	OK	-0.078158	1.0826623	-0.072190	0.94245	-0.03941927	subgrp_cor	count	1	0
AP001627.1	2	OK	-10.44227	41.465809	-0.251828	0.80117	-0.00045289	subgrp_cor	count	1	0
WDR4	2	OK	-0.910223	2.1881125	-0.415985	0.67742	-0.25785735	subgrp_cor	count	1	0
NDUFV3	28	OK	-0.366286	0.5921489	-0.618572	0.5362	-0.35709458	subgrp_cor	count	1	0
PKNOX1	6	OK	1.2769755	1.3468568	0.9481151	0.34307	0.00238546	subgrp_cor	count	1	0
HSF2BP	1	OK	23.991896	84.899941	0.2825902	0.77749	1.65081709	subgrp_cor	count	1	0
RRP1B	12	OK	-1.08662	0.9509391	-1.142689	0.25317	-0.64452234	subgrp_cor	count	1	0
PDXK	9	OK	1.460357	1.0992855	1.3284604	0.18402	0.71928944	subgrp_cor	count	1	0
CSTB	351	OK	0.2938314	0.1446475	2.0313614	0.04222	0.41469824	subgrp_cor	count	1	0
RRP1	6	OK	0.4209530	1.4909211	0.2823442	0.77768	0.21537946	subgrp_cor	count	1	0
AGPAT3	4	OK	-1.980263	1.9519158	-1.014522	0.31032	-1.0869269	subgrp_cor	count	1	0
TRAPPC10	6	OK	-2.524252	1.5508636	-1.627644	0.1036	-1.19462406	subgrp_cor	count	1	0
PFKL	12	OK	-0.393271	1.063950	-0.369633	0.71166	-0.24588554	subgrp_cor	count	1	0
C21orf2	7	OK	2.5971200	1.6109336	1.6121831	0.10692	1.13159859	subgrp_cor	count	1	0

UBE2G2	47	OK	0.87070069	0.47915716	1.81715052	0.06919	0.97375486	subgrp_con	count		1	0
SUMO3	114	OK	-0.0346283	0.27492813	-0.1259541	0.89977	-0.0454063	subgrp_con	count		1	0
PTTG1IP	410	OK	0.43143733	0.13440539	3.20997093	0.00132	0.61024596	subgrp_con	count		1	0
FAM207A	5	OK	3.45981558	3.90999315	0.88486487	0.37622	0.00081877	subgrp_con	count		1	0
ADARB1	5	OK	0.1112767	1.61203873	0.06902855	0.94497	0.05657342	subgrp_con	count		1	0
POFUT2	1	OK	0.65325287	2.50012846	0.26128772	0.79387	0.00016297	subgrp_con	count		1	0
COL18A1	139	OK	0.41165004	0.27609537	1.49097048	0.13597	0.52287369	subgrp_con	count		1	0
COL6A1	3	OK	4.66132998	5.76838606	0.80808217	0.41906	0.00274918	subgrp_con	count		1	0
COL6A2	33	OK	1.19843781	0.73870106	1.62235832	0.10472	0.60088737	subgrp_con	count		1	0
SPATC1L	10	OK	0.22568249	0.90344453	0.24980227	0.80274	0.20221589	subgrp_con	count		1	0
MCM3AP	3	OK	5.80492726	5.66946625	1.0238931	0.30589	1.03285716	subgrp_con	count		1	0
YBEY	15	OK	-0.4889748	0.74432764	-0.6569349	0.51122	-0.4274780	subgrp_con	count		1	0
PCNT	3	OK	3.45981462	6.77145131	0.51094137	0.60939	0.00030179	subgrp_con	count		1	0
DIP2A	9	OK	-0.2629062	0.90578018	-0.2902538	0.77162	-0.19485264	subgrp_con	count		1	0
S100B	20	OK	25.4805017	32.6285193	0.78092731	0.43485	4.24487018	subgrp_con	count		1	0
PRMT2	102	OK	0.84824815	0.28572153	2.96914324	0.00299	1.12853106	subgrp_con	count		1	0
MT-ND1	854	OK	-0.0619334	0.08492193	-0.7292983	0.46582	-0.08861414	subgrp_con	count		1	0
MT-ND2	862	OK	-0.1026697	0.0853839	-1.2024475	0.22919	-0.14683524	subgrp_con	count		1	0
MT-CO1	885	OK	-0.0734792	0.08357228	-0.8792295	0.37928	-0.10514259	subgrp_con	count		1	0
MT-CO2	882	OK	-0.04259	0.08375	-0.5085372	0.61108	-0.06094939	subgrp_con	count		1	0
MT-ATP8	28	OK	-0.5644081	0.5886748	-0.9587774	0.33767	-0.41001565	subgrp_con	count		1	0
MT-ATP6	878	OK	-0.0685974	0.08372907	-0.8192782	0.41262	-0.09814486	subgrp_con	count		1	0
MT-CO3	887	OK	-0.0450284	0.0828686	-0.5432708	0.58687	-0.06444773	subgrp_con	count		1	0
MT-ND3	826	OK	-0.2039019	0.0868253	-2.3484154	0.01885	-0.29140827	subgrp_con	count		1	0
MT-ND4L	386	OK	-0.3402729	0.1327236	-2.5637706	0.01035	-0.47724592	subgrp_con	count		1	0
MT-ND4	880	OK	-0.0808789	0.08350808	-0.9685163	0.33279	-0.11573661	subgrp_con	count		1	0
MT-ND5	806	OK	-0.1860537	0.0886433	-2.0989034	0.03582	-0.26589291	subgrp_con	count		1	0
MT-ND6	18	OK	0.74586789	0.75137667	0.99266842	0.32087	0.48406624	subgrp_con	count		1	0
MT-CYB	887	OK	-0.0465182	0.0831548	-0.5594175	0.57588	-0.06658345	subgrp_con	count		1	0
SPARCL1	789	OK	-0.4407485	0.089652	-4.9161597	8.83E-07	-0.62994790	subgrp_con	count		0.00890439	-0.6299479
ADIRF	715	OK	-0.4694007	0.09579716	-4.899944	9.59E-07	-0.66944284	subgrp_con	count		0.00966783	-0.66944284
LGALS3	750	OK	-0.5431225	0.0976263	-5.5632796	2.65E-08	-0.77469484	subgrp_con	count		0.00026845	-0.77469484
NOPS3	480	OK	-0.5708218	0.12401544	-4.6028282	4.17E-06	-0.8060374	subgrp_con	count		0.04183755	-0.80603741
TNFSF10	531	OK	-0.652931	0.11395499	-5.7297282	1.01E-08	-0.92874940	subgrp_con	count		0.0001021	-0.92874941
LMCD1	379	OK	-0.6674158	0.14051626	-4.7497405	2.04E-06	-0.94061166	subgrp_con	count		0.02050013	-0.94061166
TGFBR2	581	OK	-0.6647028	0.11121983	-5.9764775	2.28E-09	-0.94531856	subgrp_con	count		2.32E-05	-0.94531857
DUSP23	362	OK	-0.6907192	0.14735903	-4.6873221	2.77E-06	-0.97323519	subgrp_con	count		0.0278464	-0.9732352
ZFP36L2	565	OK	-0.685962	0.11150104	-6.1520686	7.65E-10	-0.9755370	subgrp_con	count		7.78E-06	-0.97553709
IFI16	360	OK	-0.713282	0.14766663	-4.8310236	1.36E-06	-0.99961806	subgrp_con	count		0.01368652	-0.99961807
HSPD1	352	OK	-0.7152582	0.14881377	-4.8063978	1.54E-06	-1.00380490	subgrp_con	count		0.01547806	-1.0038049
HLA-DRB5	649	OK	-0.7271003	0.10432197	-6.9697712	3.17E-12	-1.03628998	subgrp_con	count		3.24E-08	-1.03628999
MAP1LC3B	372	OK	-0.7551749	0.14851654	-5.0847867	3.68E-07	-1.06106097	subgrp_con	count		0.00371904	-1.06106097
ETS2	476	OK	-0.7570193	0.12873364	-5.8805084	4.09E-09	-1.06777089	subgrp_con	count		4.16E-05	-1.0677709
AHNAK	341	OK	-0.7676257	0.14368614	-5.3423782	9.17E-08	-1.07900758	subgrp_con	count		0.00092883	-1.07900758
RBP7	301	OK	-0.7870633	0.16134565	-4.8781188	1.07E-06	-1.08857275	subgrp_con	count		0.01079807	-1.08857275
LY6E	418	OK	-0.7701328	0.13257752	-5.8089244	6.29E-09	-1.08983723	subgrp_con	count		6.39E-05	-1.08983724
PSMB8	327	OK	-0.7833348	0.1597759	-4.9027099	9.45E-07	-1.09665613	subgrp_con	count		0.00953366	-1.09665614
GIMAP7	662	OK	-0.7721074	0.10424107	-7.4069404	1.29E-13	-1.09984053	subgrp_con	count		1.32E-09	-1.09984053
RSL1D1	261	OK	-0.8295568	0.17795368	-4.6616446	3.14E-06	-1.15173828	subgrp_con	count		0.03153552	-1.15173828
TXNIP	634	OK	-0.810287	0.10453239	-7.7515405	9.08E-15	-1.15307805	subgrp_con	count		9.3E-11	-1.15307806
MARCKSL1	402	OK	-0.8222847	0.14181538	-5.7982759	6.7E-09	-1.15897560	subgrp_con	count		6.8E-05	-1.15897561
TFPI	408	OK	-0.8229662	0.13371169	-6.1547807	7.52E-10	-1.1664167	subgrp_con	count		7.65E-06	-1.1664167
PLVAP	569	OK	-0.8331736	0.11696882	-7.1230402	1.06E-12	-1.17769572	subgrp_con	count		1.08E-08	-1.17769572
AQP1	556	OK	-0.8330119	0.11442199	-7.2801732	3.33E-13	-1.18069023	subgrp_con	count		3.41E-09	-1.18069024
EMCN	355	OK	-0.8780275	0.15073496	-5.8249759	5.71E-09	-1.22915219	subgrp_con	count		5.8E-05	-1.2291522
NRN1	312	OK	-0.879855	0.15795858	-5.5701302	2.55E-08	-1.23686494	subgrp_con	count		0.0002581	-1.23686494
MEF2C	218	OK	-0.9048462	0.19526417	-4.6339581	3.59E-06	-1.23877725	subgrp_con	count		0.0360388	-1.23877726
HLA-DRA	662	OK	-0.8717627	0.10285092	-8.4759835	2.33E-17	-1.24267227	subgrp_con	count		2.39E-13	-1.24267228
SLC2A3	254	OK	-0.9219423	0.18246493	-5.0527094	4.36E-07	-1.24837268	subgrp_con	count		0.00429986	-1.24837269
ADGRL4	355	OK	-0.9214885	0.15268071	-6.0353962	1.59E-09	-1.28493943	subgrp_con	count		1.61E-05	-1.28493944
IRF1	320	OK	-0.9273876	0.15146719	-6.122696	9.2E-10	-1.30305355	subgrp_con	count		9.36E-06	-1.30305355
CD320	284	OK	-0.945224	0.1685905	-5.6066273	2.06E-08	-1.32073440	subgrp_con	count		0.00020929	-1.32073441
GIMAP1	275	OK	-0.959966	0.16803325	-5.7129533	1.11E-08	-1.32856145	subgrp_con	count		0.0001127	-1.32856145
JAM2	350	OK	-0.9492829	0.15196486	-6.2467264	4.19E-10	-1.3340369	subgrp_con	count		4.27E-06	-1.3340369
POSTN	213	OK	-1.0027689	0.19239344	-5.1851238	2.16E-07	-1.33745640	subgrp_con	count		0.00218268	-1.33745641
NR2F2	326	OK	-0.9872184	0.16036246	-6.156169	7.45E-10	-1.37538401	subgrp_con	count		7.59E-06	-1.37538401
POLE4	217	OK	-1.051179	0.19835651	-5.299439	1.16E-07	-1.4388562	subgrp_con	count		0.00117565	-1.4388562
TSPAN7	414	OK	-1.0200802	0.135299	-7.5343283	4.91E-14	-1.44067778	subgrp_con	count		5.02E-10	-1.44067778
FABP5	271	OK	-1.0396852	0.17300101	-6.0097058	1.86E-09	-1.45564254	subgrp_con	count		1.89E-05	-1.45564254
ITM2A	403	OK	-1.0369273	0.13977858	-7.4182033	1.19E-13	-1.45827023	subgrp_con	count		1.21E-09	-1.45827024
PLCG2	211	OK	-1.0506059	0.19722413	-5.3269648	9.99E-08	-1.46352364	subgrp_con	count		0.00101105	-1.46352364
LPAR6	210	OK	-1.0820386	0.19784881	-5.4740723	4.4E-08	-1.4844237	subgrp_con	count		0.0004457	-1.4844237
IL3RA	159	OK	-1.113046	0.24045356	-4.6289356	3.68E-06	-1.49001066	subgrp_con	count		0.03691677	-1.49001066
HLA-F	260	OK	-1.0999164	0.17848123	-6.1626447	7.15E-10	-1.52693480	subgrp_con	count		7.28E-06	-1.52693481
TPD52L1	138	OK	-1.1497747	0.24535696	-4.6861302	2.78E-06	-1.53071207	subgrp_con	count		0.0280034	-1.53071208
COL15A1	141	OK	-1.2292656	0.25869041	-4.7518794	2.02E-06	-1.54689706	subgrp_con	count		0.02028849	-1.54689706
MMRN1	207	OK	-1.1343751	0.19477326	-5.8240804	5.74E-09	-1.55623343	subgrp_con	count		5.83E-05	-1.55623344
RAMP3	530	OK	-1.111393	0.12077724	-9.2020075	3.51E-20	-1.57469154	subgrp_con	count		3.61E-16	-1.57469154
FABP4	286	OK	-1.1508472	0.17332972	-6.6396411	3.14E-11	-1.57666215	subgrp_con	count		3.21E-07	-1.57666215
WNK1	89	OK	-1.3968068	0.30037927	-4.6501438	3.32E-06	-1.58548599	subgrp_con	count		0.03323627	-1.58548599
KANK3	176	OK	-1.1685413	0.22577387	-5.1757154	2.27E-07	-1.59006658	subgrp_con	count		0.00229537	-1.59006659
COTL1	144	OK	-1.1932383	0.2568752	-4.6452065	3.4E-06	-1.60096645	subgrp_con	count		0.03414012	-1.60096646
CNN3	361	OK	-1.1468687	0.15335804	-7.4914145	6.81E-14	-1.60915006	subgrp_con	count		6.97E-10	-1.60915007
BCAM	339	OK	-1.1580576	0.15577893	-7.4339809	1.05E-13	-1.62157610	subgrp_con	count		1.08E-09	-1.62157611
EPHX1	123	OK	-1.2397999	0.27133334	-4.5692871	4.89E-06	-1.62530949	subgrp_con	count		0.04911967	-1.62530949
CYYR1	244	OK	-1.2226703	0.19225833	-6.3594847	2.02E-10	-1.68121551	subgrp_con	count		2.06E-06	-1.68121552
A2M	452	OK	-1.1973549	0.1330671	-8.998126	2.3E-19	-1.68814738	subgrp_con	count		2.36E-15	-1.68814739
ICAM1	196	OK	-1.2410329	0.20910737	-5.9349074	2.94E-09	-1.70429061	subgrp_con	count		2.99E-05	-1.70429061
PSMB9	345	OK	-1.2204583	0.15653337	-7.796792	6.35E-15	-1.70827854	subgrp_con	count		6.5E-11	-1.70827855
ADAM15	218	OK	-1.2804826	0.20465899	-6.2566642	3.93E-10	-1.70847795	subgrp_con	count		4.01E-06	-1.70847796
SNCG	452	OK	-1.2242516	0.1352202	-9.053762	1.38E-19	-1.7312879	subgrp_con	count		1.42E-15	-1.73128798
PRCP	419	OK	-1.2362832	0.14473925	-8.5414515	1.33E-17	-1.73178839	subgrp_con	count		1.36E-13	-1.7317884
GBP4	120	OK	-1.2972486	0.26970641	-4.8098545	1.51E-06	-1.76100929	subgrp_con	count		0.01521579	-1.7610093
HLA-DPA1	598	OK	-1.256284	0.11499321	-10.924854	8.77E-28	-1.78583940	subgrp_con	count		9.02E-24	-1.7858394
GNG11	609	OK	-1.2741362	0.11434611	-11.142807	7.78E-29	-1.80993573	subgrp_con	count		7.99E-25	-1.80993574
GIMAP4	384	OK	-1.29157	0.14583188	-8.8565683	8.25E-19	-1.81171678	subgrp_con	count		8.47E-15	-1.81171678
CXorf36	122	OK	-1.4358887	0.28353707	-5.064209	4.1E-07	-1.81824427	subgrp_con	count		0.004143	-1.81824428
HSD17B11	146	OK	-1.3593957	0.24342337	-5.5844914	2.34E-08	-1.81899145	subgrp_con	count		0.00023771	-1.81899146
FAM110D	162	OK	-1.3680327	0.24323736	-5.6242705	1.86E-08	-1.8258542	subgrp_con	count		0.0001890	-1.82585424
GIMAP6	79	OK	-1.6053516	0.34291689	-4.6814586	2.85E-06	-1.88487614	subgrp_con	count		0.02864346	-1.88487614
GMFG	204	OK	-1.372267	0.21961325	-6.2485622	4.14E-10	-1.88610091	subgrp_con	count		4.22E-06	-1.88610092
MAP3K8	105	OK	-1.5026032	0.30432094	-4.9375617	7.91E-07	-1.90370279	subgrp_con	count		0.00798413	-1.90370279
PIM3	357	OK	-1.3608087	0.15993833	-8.5083336	1.76E-17	-1.90999641	subgrp_con	count		1.81E-13	-1.90999642
HMGB2	156	OK	-1.4088454	0.23827432	-5.9127034	3.37E-09	-1.9191227	subgrp_con	count		3.42E-05	-1.91912278
SELE	134	OK	-1.4626615	0.26971343	-5.4230209	5.86E-08	-1.97278830	subgrp_con	count		0.00059355	-1.97278831
EPS8	106	OK	-1.5694124	0.31095903	-5.0470077	4.49E-07	-2.01698454	subgrp_con	count		0.00453227	-2.01698455
GBP2	106	OK	-1.5749888	0.2899682	-5.4315991	5.59E-08	-2.01855718	subgrp_con	count		0.0005659	-2.01855719
SH3BP5	304	OK	-1.4564299	0.17346478	-8.3961127	4.62E-17	-2.01908194	subgrp_con	count		4.73E-13	-2.01908195
PLAC9	107	OK	-1.5924582	0.28983445	-5.4943716	3.92E-08	-2.049772	subgrp_con	count		0.0003974	-2.0497729
SYNE2	252	OK	-1.5086598	0.18603397	-8.1095951	5.08E-16	-2.0686962	subgrp_con	count		5.2E-12	-2.06869622
PMP22	177	OK	-1.5515156	0.23389035	-6.6338003	3.27E-11	-2.08742343	subgrp_con	count		3.34E-07	-2.08742344
C2CD4B	224	OK	-1.4984893	0.20539839	-7.2955262	2.97E-13	-2.09409105	subgrp_con	count		3.04E-09	-2.09409106
SPRY1	384	OK	-1.527472	0.15375787	-9.9342698	2.95E-23	-2.1167033	subgrp_con	count		3.03E-19	-2.11670315
CLEC2B	218	OK	-1.5625288	0.21195147	-7.3721065	1.68E-13	-2.12989633	subgrp_con	count		1.72E-09	-2.12989633
ATF3	273	OK	-1.5659857	0.18505582	-8.4622347	2.62E-17	-2.16746126	subgrp_con	count		2.69E-13	-2.16746127
MGLL	224	OK	-1.6039713	0.21085302	-7.6070585	2.8E-14	-2.22602908	subgrp_con	count		2.87E-10	-2.22602909
RASSF9	101	OK	-1.687070	0.314134	-5.3705429	7.85E-08	-2.24259940	subgrp_con	count		0.00079497	-2.2425994
CTSC	364	OK	-1.5971742	0.15960885	-10.006803	1.42E-23	-2.24289489	subgrp_con	count		1.46E-19	-2.24289489
ANKRD29	79	OK	-1.7992881	0.36051879	-4.9908302	6.01E-07	-2.27737879	subgrp_con	count		0.00606975	-2.2773788
ZNF385D	154	OK	-1.7546495	0.25985713	-6.7523627	1.45E-11	-2.29076597	subgrp_con	count		1.48E-07	-2.29076598
TMEM88	102	OK	-1.7775046	0.31254479	-5.6871997	1.29E-08	-2.30999614	subgrp_con	count		0.00013106	-2.30999615
PERP	137	OK	-1.7068806	0.26527492	-6.4343838	1.24E-10	-2.32224973	subgrp_con	count		1.26E-06	-2.32224973
CCL14	247	OK	-1.7142189	0.19728284	-8.6891438	3.65E-18	-2.34811343	subgrp_con	count		3.75E-14	-2.34811344
RASA4	107	OK	-1.7974967	0.30479861	-5.8973258	3.69E-09	-2.41025910	subgrp_con	count		3.76E-05	-2.41025911
IL6	80	OK	-1.8625005	0.37006064	-5.0329602	4.83E-07	-2.41722471	subgrp_con	count		0.00487648	-2.41722472
PLAT	324	OK	-1.7555286	0.17255744	-10.17359	2.6E-24	-2.43135051	subgrp_con	count		2.67E-20	-2.43135052
PDK4	157	OK	-1.975627	0.25000314	-7.9024089	2.74E-15	-2.45444213	subgrp_con	count		2.8E-11	-2.45444214
HLA-DPB1	459	OK	-1.7447628	0.14474364	-12.054156	1.84E-33	-2.46011322	subgrp_con	count		1.9E-29	-2.46011322
RGS16	288	OK	-1.7775859	0.1848916	-9.6246175	6.29E-22	-2.47242314	subgrp_con	count		6.47E-18	-2.47242314
ITGA6	194	OK	-1.8338786	0.23528319	-7.7943465	6.47E-15	-2.48760338	subgrp_con	count		6.63E-11	-2.48760339
OLFM1	254	OK	-1.8171557	0.2041072	-8.9029474	5.44E-19	-2.50175523	subgrp_con	count		5.58E-15	-2.50175524
SEMA6A	60	OK	-2.3155945	0.4711499	-4.9147723	8.89E-07	-2.59870159	subgrp_con	count		0.00896679	-2.59870159
HLA-DMA	292	OK	-1.9076936	0.19137639	-9.9682807	2.1E-23	-2.65172390	subgrp_con	count		2.16E-19	-2.6517239
TSHZ2	119	OK	-2.0639009	0.30211579	-6.8089523	9.83E-12	-2.67391898	subgrp_con	count		1E-07	-2.67391899
TIMP3	311	OK	-1.970705	0.18230988	-10.809645	3.1E-27	-2.7247118	subgrp_con	count		3.19E-23	-2.7247118
IFITM1	235	OK	-1.9805848	0.2204255	-8.9852796	2.58E-19	-2.73467490	subgrp_con	count		2.65E-15	-2.7346749
CSRP2	96	OK	-2.2055668	0.37061705	-5.9510666	2.66E-09	-2.7492900	subgrp_con	count		2.71E-05	-2.74929005
SPHK1	80	OK	-2.3317253	0.40613404	-5.7412703	9.4E-09	-2.79907439	subgrp_con	count		9.54E-05	-2.7990744
C21orf91	88	OK	-2.2702158	0.38796719	-5.8515664	4.87E-09	-2.88598105	subgrp_con	count		4.95E-05	-2.88598106
HLA-DQB1	217	OK	-2.1186703	0.22818009	-9.2850801	1.62E-20	-2.89346270	subgrp_con	count		1.66E-16	-2.89346271
BTN3A2	68	OK	-2.5199312	0.44267893	-5.6796278	1.35E-08	-2.92086913	subgrp_con	count		0.00013699	-2.92086914
PDIA5	51	OK	-2.6680213	0.54735037	-4.8744308	1.09E-06	-2.92306151	subgrp_con	count		0.01099955	-2.92306152
DAAM1	184	OK	-2.171446	0.2540002	-8.5489937	1.24E-17	-2.92630538	subgrp_con	count		1.27E-13	-2.92630538
CCL23	67	OK	-2.3763329	0.45078822	-5.2715062	1.35E-07	-2.95768618	subgrp_con	count		0.00126892	-2.95768619
ACKR1	466	OK	-2.124455	0.1517302	-14.001533	1.53E-44	-2.99352988	subgrp_con	count		1.57E-40	-2.9935299
TESC	127	OK	-2.2467306	0.31257761	-7.1648312	7.79E-13	-2.99793994	subgrp_con	count		7.96E-09	-2.99793995
NBL1	118	OK	-2.3679798	0.34857599	-6.7932958	1.1E-11	-3.00777991	subgrp_con	count		1.12E-07	-3.00777992
ADM5	98	OK	-2.341003	0.365442	-6.4059497	1.49E-10	-3.04078876	subgrp_con	count		1.52E-06	-3.04078877
NOSTRIN	178	OK	-2.3161526	0.26338603	-8.7934226	1.45E-18	-3.10056638	subgrp_con	count		1.49E-14	-3.10056639
TMEM273	60	OK	-2.8666574	0.5155070	-5.560842	2.68E-08	-3.10487432	subgrp_con	count		0.00027221	-3.10487432
STC1	243	OK	-2.2855694	0.22452597	-10.179535	2.45E-24	-3.13022854	subgrp_con	count		2.52E-20	-3.13022855
SH3BGRL2	46	OK	-3.5423234	0.73099670	-4.8458811	1.26E-06	-3.15794279	subgrp_con	count		0.01270242	-3.1579428
CTSH	216	OK	-2.5140652	0.25074008	-10.026576	1.16E-23	-3.36659620	subgrp_con	count		1.2E-19	-3.36659621
HLA-DQA1	227	OK	-3.5562251	0.34282832	-10.373164	3.28E-25	-4.6612873	subgrp_con	count		3.38E-21	-4.66128736
