# Supplementary material for: Decoding the transcriptome of calcified atherosclerotic plaque at single-cell resolution
Source: Commun Biol. 2022 Oct 12;5:1084. doi: 10.1038/s42003-022-04056-7 (PMC9556750; doi:10.1038/s42003-022-04056-7)
Supplement: Supplementary file 17 — Supplementary Data 15 [file 42003_2022_4056_MOESM17_ESM.pdf]

gene_short_name	num_cells_expressed	status	term	estimate	std_err	test_val	p_value	normalized_effect	model_component	q_value	Corrected NE
VEGFC	27	FAIL	subgrp_cont	-49.58313775	8.997166326	-5.510972672	3.57E-08	-51.22005055	count	0.000814845	-51.220051
CFD	564	OK	subgrp_cont	-10.64254054	0.209308313	-50.84623909	0	-13.93484881	count	0	-13.934849
APOD	491	OK	subgrp_cont	-8.480395165	0.236279743	-35.89133403	4.17E-282	-11.48818872	count	1.01E-277	-11.488189
CXCL14	274	OK	subgrp_cont	-8.009029729	0.347042551	-23.07794737	7.71E-118	-10.1545977	count	1.87E-113	-10.154598
PLA2G2A	386	OK	subgrp_cont	-9.089615308	0.301146354	-30.18338161	3.91E-200	-9.76845838	count	9.50E-196	-9.7684584
PI16	459	OK	subgrp_cont	-8.546690383	0.255677776	-33.42758415	5.45E-245	-9.269911527	count	1.32E-240	-9.2699115
MFAP5	495	OK	subgrp_cont	-7.401288084	0.190099792	-38.93369905	0	-9.257865641	count	0	-9.2578656
C3	486	OK	subgrp_cont	-8.616328157	0.256901545	-33.53941746	1.28E-246	-8.803008658	count	3.12E-242	-8.8030087
SLPI	521	OK	subgrp_cont	-5.830079922	0.249483188	-23.36862843	8.91E-121	-8.258481162	count	2.16E-116	-8.2584812
SFRP2	764	OK	subgrp_cont	-5.296024734	0.197245061	-26.84997387	8.44E-159	-7.555682777	count	2.05E-154	-7.5556828
IGF1	335	OK	subgrp_cont	-6.720508093	0.268850376	-24.99720544	6.56E-138	-7.494874076	count	1.59E-133	-7.4948741
LEPR	425	OK	subgrp_cont	-6.416465504	0.240727341	-26.65449416	1.59E-156	-7.469635316	count	3.85E-152	-7.4696353
ACKR3	686	OK	subgrp_cont	-5.484178854	0.145986899	-37.5662399	0	-7.414244937	count	0	-7.4142449
CHRDL1	444	OK	subgrp_cont	-7.072515483	0.263499002	-26.84076759	1.08E-158	-7.337753501	count	2.62E-154	-7.3377535
SCARA5	402	OK	subgrp_cont	-6.217794213	0.23443655	-26.52229015	5.36E-155	-7.326715848	count	1.30E-150	-7.3267158
MGST1	552	OK	subgrp_cont	-5.412217582	0.15241485	-35.50977862	3.47E-276	-7.090040438	count	8.44E-272	-7.0900404
LRRN4CL	355	OK	subgrp_cont	-7.308934383	0.355845671	-20.53961867	9.53E-94	-7.007924653	count	2.31E-89	-7.0079247
PRG4	100	OK	subgrp_cont	-10.41837383	0.81305569	-12.81385022	1.37E-37	-6.952454368	count	3.29E-33	-6.9524544
SERPINF1	807	OK	subgrp_cont	-4.962893758	0.138145204	-35.92519774	1.24E-282	-6.929821086	count	3.00E-278	-6.9298211
PLAT	535	OK	subgrp_cont	-5.361188417	0.177294168	-30.23894406	7.29E-201	-6.921206434	count	1.77E-196	-6.9212064
GAS1	513	OK	subgrp_cont	-5.555386557	0.188429619	-29.48255473	4.82E-191	-6.835655913	count	1.17E-186	-6.8356559
FBLN1	1014	OK	subgrp_cont	-4.831983812	0.123042661	-39.27080056	0	-6.829284984	count	0	-6.829285
DCN	1010	OK	subgrp_cont	-4.752971234	0.167870076	-28.3133918	2.36E-176	-6.813039115	count	5.74E-172	-6.8130391
SFRP1	478	OK	subgrp_cont	-4.662098782	0.182912935	-25.48807598	2.67E-143	-6.491696645	count	6.48E-139	-6.4916966
TMEM176B	324	OK	subgrp_cont	-6.162537594	0.278771411	-22.1060602	2.76E-108	-6.454304377	count	6.69E-104	-6.4543044
TSHZ2	337	OK	subgrp_cont	-5.862986358	0.266783825	-21.97654355	4.83E-107	-6.348148541	count	1.17E-102	-6.3481485
ITM2A	659	OK	subgrp_cont	-4.65995233	0.135651317	-34.35242977	1.30E-258	-6.331836326	count	3.15E-254	-6.3318363
CPVL	235	OK	subgrp_cont	-6.19347146	0.360857658	-17.16319807	5.01E-66	-6.296414541	count	1.21E-61	-6.2964145
TMEM176A	290	OK	subgrp_cont	-5.763315935	0.277098978	-20.79876289	4.44E-96	-6.190504946	count	1.07E-91	-6.1905049
VIT	229	OK	subgrp_cont	-10.02595754	0.970830885	-10.32719262	5.31E-25	-6.108594495	count	1.26E-20	-6.1085945
C16orf89	247	OK	subgrp_cont	-5.400960552	0.300407947	-17.97875392	2.86E-72	-6.09626166	count	6.90E-68	-6.0962617
TNXB	653	OK	subgrp_cont	-4.465758736	0.147569107	-30.26215195	3.61E-201	-6.0583683	count	8.77E-197	-6.0583683
SEMA3C	360	OK	subgrp_cont	-5.373125719	0.239124874	-22.46995736	8.17E-112	-5.929124905	count	1.98E-107	-5.9291249
FBLN2	684	OK	subgrp_cont	-4.346363764	0.124494368	-34.9121317	4.87E-267	-5.906589275	count	1.18E-262	-5.9065893
CD34	482	OK	subgrp_cont	-4.477996279	0.174797621	-25.61817635	9.57E-145	-5.838572181	count	2.32E-140	-5.8385722
